# Epigenetic re-wiring of breast cancer by pharmacological targeting of C-terminal binding protein

**DOI:** 10.1038/s41419-019-1892-7

**Published:** 2019-09-18

**Authors:** Jung S. Byun, Samson Park, Dae Ik Yi, Jee-Hye Shin, Sara Gil Hernandez, Stephen M. Hewitt, Marc C. Nicklaus, Megan L. Peach, Laura Guasch, Binwu Tang, Lalage M. Wakefield, Tingfen Yan, Ambar Caban, Alana Jones, Mohamed Kabbout, Nasreen Vohra, Anna María Nápoles, Sandeep Singhal, Ryan Yancey, Adriana De Siervi, Kevin Gardner

**Affiliations:** 1 National Institute on Minority Health and Health Disparities, Bethesda, MD 20892 USA; 2 Genetics Branch, National Cancer Institute, Bethesda, MD 20892 USA; 3 Laboratory of Cancer Biology and Genetics, National Cancer Institute, Bethesda, MD 20892 USA; 4 Laboratory of Pathology, National Cancer Institute, Bethesda, MD 20892 USA; 5 Chemical Biology Laboratory, Center for Cancer Research, National Cancer Institute, Frederick, MD 20892 USA; 6 Basic Science Program, Chemical Biology Laboratory, Leidos Biomedical Research Inc., Frederick National Laboratory for Cancer Research, Frederick, MD 21702 USA; 7 National Human Genome Institute, Bethesda, MD 20892 USA; 8 Brody School of Medicine at East Carolina University, Greenville, NC 27834 USA; 9 Department of Pathology and Cell Biology, Columbia University Medical Center, New York, NY 10032 USA; 10 Laboratorio de Oncologıa Molecular y Nuevos Blancos Terapeuticos, Instituto de Biologıa y Medicina Experimental (IBYME), CONICET, Buenos Aires, Argentina

**Keywords:** Breast cancer, Target validation

## Abstract

The C-terminal binding protein (CtBP) is an NADH-dependent dimeric family of nuclear proteins that scaffold interactions between transcriptional regulators and chromatin-modifying complexes. Its association with poor survival in several cancers implicates CtBP as a promising target for pharmacological intervention. We employed computer-assisted drug design to search for CtBP inhibitors, using quantitative structure-activity relationship (QSAR) modeling and docking. Functional screening of these drugs identified 4 compounds with low toxicity and high water solubility. Micro molar concentrations of these CtBP inhibitors produces significant de-repression of epigenetically silenced pro-epithelial genes, preferentially in the triple-negative breast cancer cell line MDA-MB-231. This epigenetic reprogramming occurs through eviction of CtBP from gene promoters; disrupted recruitment of chromatin-modifying protein complexes containing LSD1, and HDAC1; and re-wiring of activating histone marks at targeted genes. In functional assays, CtBP inhibition disrupts CtBP dimerization, decreases cell migration, abolishes cellular invasion, and improves DNA repair. Combinatorial use of CtBP inhibitors with the LSD1 inhibitor pargyline has synergistic influence. Finally, integrated correlation of gene expression in breast cancer patients with nuclear levels of CtBP1 and LSD1, reveals new potential therapeutic vulnerabilities. These findings implicate a broad role for this class of compounds in strategies for epigenetically targeted therapeutic intervention.

## Introduction

The C-terminal binding protein (CtBP) was first described as a phosphoprotein that binds specifically to the C-terminal end of the E1a adenovirus oncogene^1–3^. These proteins were later found to represent a dimeric family of proteins, composed of CtBP1 and CtBP2, that can homodimerize or heterodimerize in the nucleus to influence multiple different epigenetic nuclear events by recruiting a diverse array of chromatin-modifying complexes^2,4^. Binding partners for CtBP include histone deacetylases, histone methyltransferases, and histone demethylases in addition to several different classes of sequence-specific DNA binding proteins and chromatin-associated complexes^2,3^. Therefore, in its dimeric form, CtBP has the broad potential of re-shaping the landscape of epigenetic regulation throughout the nucleus^5,6^. CtBP belongs to a family of NAD-dependent D-2-hydroxy acid dehydrogenases including *E. Coli* D-3-phosphoglycerate dehydrogenase, bacterial D-lactate dehydrogenase (D-LDH) and D-hydroxyisocaproate dehydrogenase^7^. Though the actual substrate for CtBP remains unclear^8–10^, its ability to dimerize and form higher order oligomers is positively regulated by NADH/NAD+^7,11^. The ability of CtBP to bind and undergo redox cycles with NADH/NAD+ and substrate implicates a substantial role for CtBP in the regulation of genomic responses to changes in cellular metabolism^9,12^.

CtBP levels are elevated in multiple different cancers to profoundly influence cellular phenotypic plasticity by promoting pathways linked to epithelial-to-mesenchymal transition, cell migration, decreased genome stability and the acquisition of stem cell self-renewal features^13–16^. The increasing role of epigenetic regulation in tumor heterogeneity, cellular plasticity and the acquisition of drug resistance^17^ suggests a significant potential function for CtBP as a major determinant in the epigenetic control of cancer. These dramatic properties implicate CtBP as a promising candidate for targeted disruption by small molecule inhibitors as a therapeutic approach against cancer^18–23^. The first proof of this principle was provided by the discovery that 2-Keto-4-methylthiobutyrate (MTOB), an intermediate in methionine metabolism, is a selective inhibitor of CtBP activity capable of disrupting tumor growth in murine models^10,18^. However, MTOB requires 10 mM concentration to be effective and is therefore considered impractical as a therapeutic agent^10^. Recently, the crystallographic structure of the dehydrogenase domains of both CtBP2 and CtBP1 in complex with MTOB and NAD+ has been resolved^20^. This advance provided a framework through which more effective CtBP inhibitors were designed through computational methods^20–22^. Using a similar approach, 24 commercially available compounds with potential as CtBP inhibitors were identified. Four lead compounds were selected from these candidates based on their solubility, low cytotoxicity and ability to reverse transcriptional repression by CtBP. Further characterization of these compounds indicates that they have potent activity against CtBP at low micromolar concentrations to induce significant alterations in epigenetic transcriptional programming in breast cancer.

## Results

### Identification of small molecular inhibitors of CtBP

**Fig. 1:**
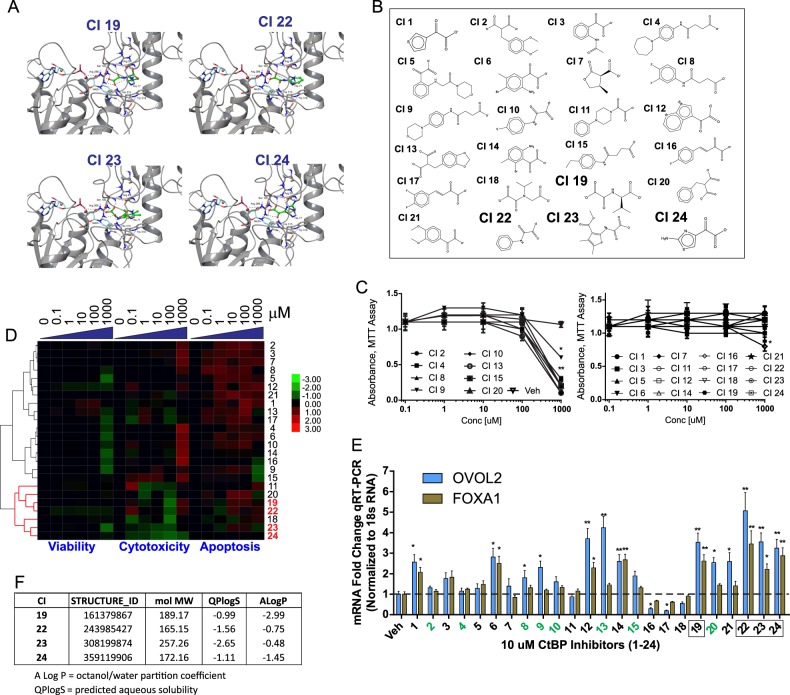
Identification and validation of small molecule CtBP inhibitors by computer-assisted drug design using QSAR-based modeling. **a** Representative docked structures of four small molecular inhibitors in the active site of CtBP. Four lead compounds (CI19, CI22, CI23, and CI24) are shown in green in the CtBP substrate binding site. The NAD+ cofactor is colored in light blue. Hydrogen bonds are indicated with dashed black lines. **b** Structures of 24 commercially available predicted inhibitors of CtBP screened based on best QSAR predicted activities and highest docking scores. **c** Proliferation and viability screening of MDA-MB-231 cells treated 24 h with 10 μM of the 24 predicted CtBP inhibitors as determined by MTT assay. **d** Viability, cytotoxicity, and apoptosis screen of MDA-MB-231 cells treated 24 h with 10 μM of the 24 predicted CtBP inhibitors as assessed by the ApoTox-Glo™ Triplex Assay. Samples were normalized and subject to hierarchical clustering using a correlation matrix and centroid linkage. Dendrogram highlighted in red contains the 4 lead compounds with the lowest cytotoxicity and highest de-repression of CtBP target genes. **e** qRT-PCR profiling of mRNA abundance of CtBP-repressed genes, *FOXA1* and *OVOL2*, in MDA-MB-231 cells after treatment for 24 h with 10 μM of the indicated CtBP inhibitors. **f** Table showing the relative water solubility of the 4 lead compounds based on AlogP (octanol/water partition coefficient) and QPlogS (predicted aqueous solubility). All experiments represent the average and standard error of at least two biological replicates. The error bars represent the s.d. of the mean from at least two independent experiments. *Indicates *p*-values < 0.05 and **indicated *p*-value < 0.001

We exploited the observation that most dehydrogenases have strict substrate specificities and the recent publication of the crystallographic structure of MTOB in complex with CtBP^20^ to conduct virtual screening of the ChemNavigator iResearch Library from Sigma Aldrich^24^ to select molecules that showed favorable interactions with three residues (His315, Glu295, Arg 266) demonstrated to function as a catalytic triad in the active site of CtBP^8^. This computational screen identified 31 compounds of which 24 were commercially available. The docked structures of four representative compounds are shown in Fig. 1a and the structures of the 24 compounds identified are shown in Fig. 1b. These 24 compounds were then experimentally screened for influence on viability and proliferation by MTT assay (Fig. 1c) and combined viability, cytotoxicity and apoptosis assay (Fig. 1d).

CtBP functions as both an activator and repressor of transcription although most interactions described thus far are repressive^2^. The transcriptionally repressive activity of CtBP is ascribed to its ability to bind and recruit a variety of chromatin-modifying enzymes that remove activating chromatin modifications including histone 3 and 4 lysine acetylation and trimethylation of histone 3 at lysine 4^2,3^. However, the function of CtBP is highly cell-type specific, in part due to the differential expression of various chromatin regulatory complexes and the differential abundance of proteins that destabilize and, therefore, downregulate CtBP activity including: APC, HIPK2, AMPK, and JNK1^2,3^. In breast cancer, over-expression of CtBP is associated with downregulation of a variety of pro-epithelial genes in ER+ tumors but has little effect on the expression of these genes in breast cancer cell types with more mesenchymal or stromal features^14^. Downregulation or depletion of CtBP by RNAi-mediated gene depletion results in upregulation of pro-epithelial genes in the mesenchymal breast cancer cell line MDA-MB-231^14^. We applied a functional screen for the loss of CtBP transcriptional repressive activity by screening the 24 compounds for the ability to upregulate *FOXA1* and *OVOL2*, two genes that are intimately involved in maintaining the epithelial phenotype^25–28^. This functional screen identified several compounds that substantially (greater than two-fold) upregulate *FOXA1* and/or *OVOL2* expression (Fig. 1e). From this screen, 4 compounds (CI19, CI22, CI23, and CI24) showing both the lowest cytotoxicity, and upregulated *FOXA1* and *OVOL2* expression in MDA-MB-231, were selected (Fig. 1e). All four of these compounds have molecular weights less than 300 Da and are water-soluble (Fig. 1f).

### CtBP inhibitors disrupt CtBP dimerization in vivo

**Fig. 2:**
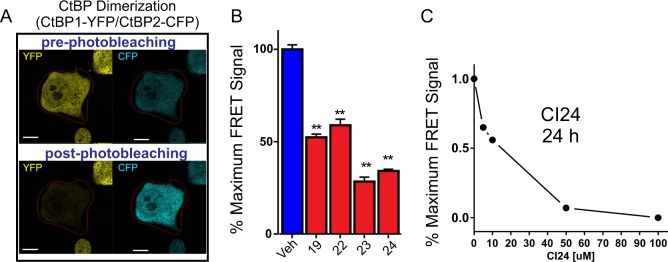
Pharmacological Inhibition of CtBP1/CtBP2 heterodimer formation by CtBP inhibitors. **a** CtBP1-YFP and CtBP2-CFP heterodimerization was measured by fluorescence resonance energy transfer (FRET) acceptor photobleaching (see Materials and methods). **b** Inhibition of CtBP dimerization FRET signal by the CtBP inhibitors CI19, CI22, CI23, and CI24 at 10 μM concentrations. **c** Dose-response curve of inhibition of CtBP dimerization FRET signal by increased concentrations of CI24 CtBP inhibitor. The error bars represent the s.d. of the mean from at least two independent experiments. *Indicates *P* < 0.05 and **indicates *P* < 0.01. Error bars in **c** are smaller than the size of the symbols

CtBP activity is dependent on its ability to form dimers and higher order oligomers. This property enables CtBP to recruit chromatin-modifying complexes to specific chromatin locations and stabilizes CtBP against nuclear export and degradation^2,3^. As shown in Fig. 2, assessment of CtBP1/CtBP2 dimerization by fluorescent resonance energy transfer (FRET) acceptor photobleaching^29^ reveals a significant decrease in the paired CtBP1-YFP/CtBP2-CFP FRET signal, after incubation with CI19, CI22, CI23 or CI24 (Fig. 2a, b). This is consistent with the observation that CI19, CI22, CI23, and CI24 are also able to induce the release of CtBP2 from immuno-precipitated CtBP1 complexes in vitro (Supplementary Fig. 2). Finally, the dose-response for CI24 suggests the IC50 for CtBP dimerization lies between 10 and 20 μM (Fig. 2c).

### Disruption of epigenetic control of transcription by CtBP small-molecule inhibition

**Fig. 3:**
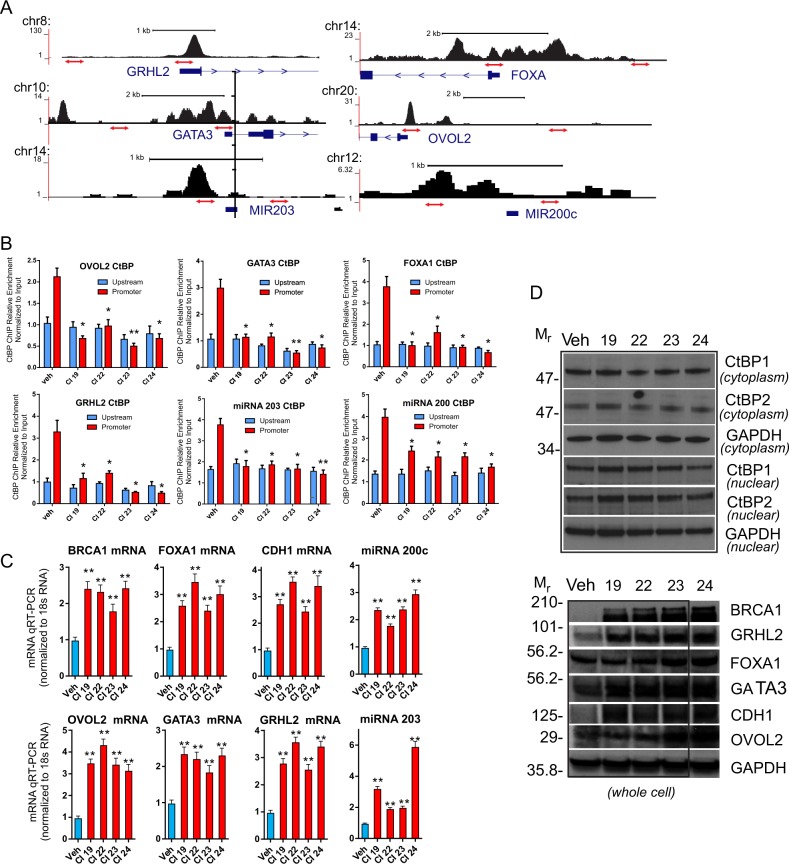
Epigenetic reactivation of pro-epithelial gene expression in MDA-MB-231 cells following treatment with CtBP inhibitors. **a** Diagram of CtBP ChIP-seq profile^14^ used to select amplicon regions for detecting CtBP occupancy at the proximal promoter and upstream regions of the *GRHL2*, *FOXA1*, *GATA3*, *OVOL2*, miRNA 203, and miRNA 200c genes. **b** qChIP profile of CtBP binding to the promoter and upstream regions of *OVOL2*, *GATA3*, *FOXA1*, *GRHL2*, miRNA 203, and miRNA 200c following treatment of MDA-MB-231 cells with the vehicle and 10 μM CI19, CI22, CI23, and CI24 inhibitors for 24 h prior to ChIP. **c** qRT-PCR profiles of *BRCA1*, *FOXA1*, *CDH1*, *OVOL2*, *GATA3*, and *GRHL2* mRNA and miRNA 200c and miRNA 203 abundance in MDA-MB-231 cells treated 24 h with 10 μM CtBP inhibitors as indicated. **d** (Top), immuno-blot profiling of CtBP1 and CtBP2 in nuclear and cytoplasmic extracts of MDA-MB-231 treated 24 h with vehicle or 10 μM of the indicated CtBP inhibitors. (Bottom), immuno-blot profiling of BRCA1, GRHL2, FOXA1, GATA3, CDH1, OVOL2, and GAPDH protein expression in whole-cell lysates of MDA-MB-231 cells, treated as described above. The error bars represent the s.d. of the mean from at least two independent experiments. *P*-values are calculated from the Students to *T*-Test relative to the vehicle control. *Indicates *P* < 0.05 and **indicates *P* < 0.01

These four lead compounds were then screened for their specific ability to target and de-repress pro-epithelial genes that are known to be transcriptionally silenced in the mesenchymal triple negative breast cancer cell line MDA-MB-231^30,31^. As shown in Fig. 3a, b, analysis by CtBP chromatin immunoprecipitation, using an antibody that recognizes both CtBP1 and CtBP2^13,14^, demonstrates that treatment of MDA-MB-231 cells with 10 μM of the CtBP inhibitor results in eviction of CtBP from the promoter regions of the master epithelial regulatory genes *OVOL2*, *GATA3*, *FOXA1*, and *GRHL2*^14^, in addition to the pro-epithelial micro-RNAs, miR200c and miR203. This CtBP promoter eviction is associated with significant upregulation of the RNAs for *OVOL2*, *GATA3*, *FOXA1*, *GRHL2*, miRNA *miR200c*, *miR203*, and the well-known CtBP-repressed genes, *E-Cadherin* and *BRCA1*^13,32^ (Fig. 3c). This gene reactivation in MDA-MB-231 is reflected by an increased level of protein expression (Fig. 3d, bottom). Notably, as shown in Fig. 3d, this drug-induced eviction occurs in the absence of appreciable loss or shift in neither total CtBP1 nor CtBP2 protein levels from the cytosolic and nuclear compartments (Fig. 3d, top). The relatively modest increase in both OVOL2 and FOXA1 protein levels likely reflects differential influences of both post-transcriptional (RNA stability and decay) and post-translational steps in OVOL2 and FOXA1 regulation.

**Fig. 4:**
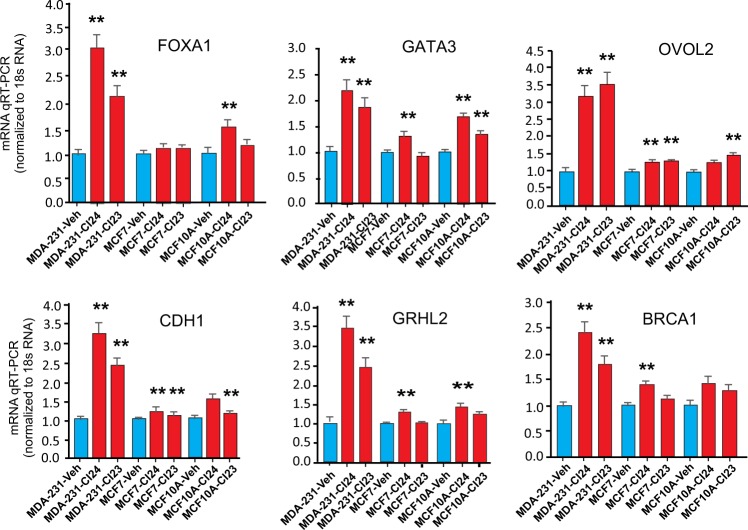
The magnitude of de-repression of pro-epithelial genes by CtBP inhibitors is cell-type specific. qRT-PCR profiles of *FOXA1*, *GATA3*, *OVOL2*, *CDH1*, *GRHL2*, and *BRCA1* mRNA in the presence of the 10 μM CtBP inhibitors CI23, and CI24, in MDA-MB-231, MCF-7, and MCF10A cell lines. The vehicle (Veh) used for all cell lines was phosphate-buffered saline. Results show the average and standard error of the mean (SEM) for three biological replicates. *P*-values are calculated from the Student’s T-Test relative to the vehicle control. *Indicates *P* < 0.05 and **indicates *P* < 0.01

A survey comparing the CtBP inhibitor-induced transcriptional de-repression of the pro-epithelial genes in cell lines characterized by higher levels of epithelial differentiation, including the estrogen-receptor positive MCF-7 cell line, and the non-transformed mammary epithelial cell line MCF-10A is shown in Fig. 4. For each compound, this survey demonstrates similar trends but lower levels of transcriptional reactivation by the CtBP inhibitor compounds in both MCF-7 and MCF-10A (Fig. 4). These findings indicate a cell-specific dose-sensitivity to CtBP inhibition.

### CtBP inhibitors disrupt recruitment of histone modification machinery

**Fig. 5:**
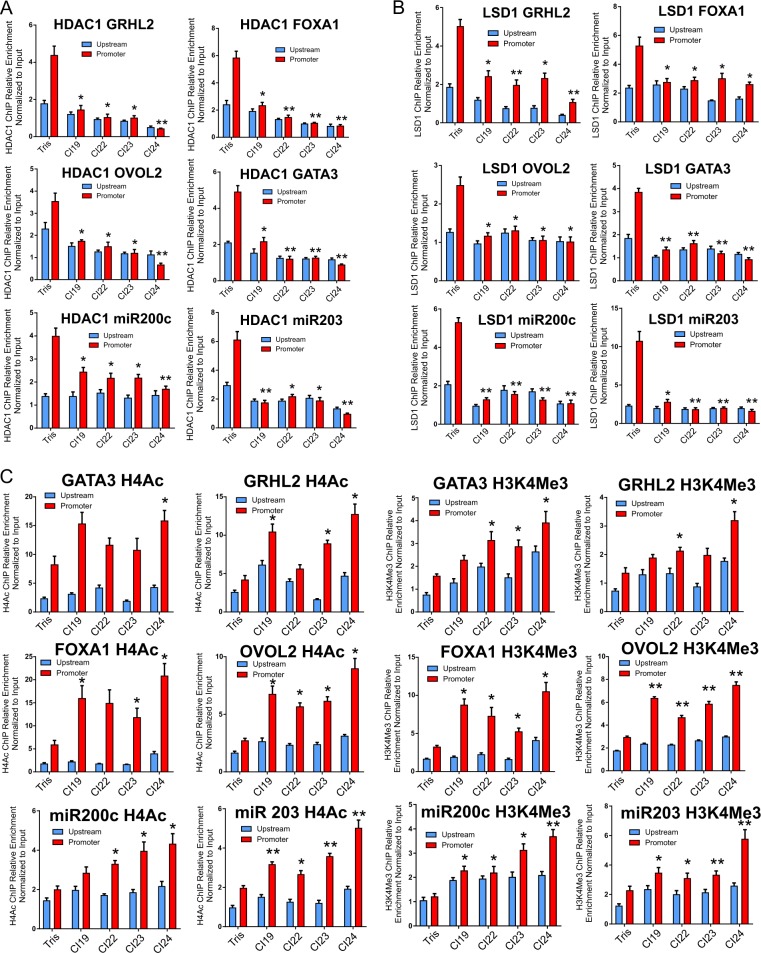
Treatment with CtBP inhibitors is associated with LSD1 and HDAC1 eviction and increased deposition of activating chromatin marks at the promoter-proximal regions of pro-epithelial genes in MDA-MB-231. **a** qChIP profiles in MDA-MB-231 cells of HDAC1 eviction from the *GRHL2, FOXA1, OVOL2, GATA3*, miRNA 200c, and miRNA 203 proximal promoter regions following 24-h incubation with vehicle or 10 μM CtBP inhibitors. **b** qChIP profiles of LSD1 eviction from the *GRHL2, FOXA1, OVOL2, GATA3*, miRNA 200c, and miRNA 203 proximal promoter regions following 24-h incubation with vehicle or 10 μM CtBP inhibitors. **c** qChIP profiles of changes in histone 4 (K5,8,12,16) pan-acetylation (left) and histone 3 lysine 4 trimethylation (right) at the promoters and upstream regions of *GATA3, GRHL2, FOXA1, OVOL2*, miRNA 203, and miRNA 200c following 24-h incubation of MDA-MB-231 cells with 10 μM CtBP inhibitors. The error bars represent the s.d. of the mean from at least two independent experiments. *P*-values are calculated from the Student’s *T*-Test relative to the vehicle control. *Indicates *P* < 0.05 and **indicates *P* < 0.01

Multiple studies have shown that CtBP ferries different chromatin-modifying complexes to chromatin depending on the promoter or enhancer context^13–15,33^. These recruited assemblies then affect changes in the local epigenetic marks specific to the composition of the assembled complexes. Two classes of chromatin modifiers that have been commonly shown to associate with CtBP are the histone deacetylases HDAC1 and HDAC2 and the H3K4Me2/1 demethylase, LSD1^6,34,35^. LSD1 (*KDM1A*) is a monoamine oxidase that catalyzes the removal of H3K4Me2 and K3K4Me1 activation marks by demethylation, thus disrupting the ultimate accumulation of the active H3K4Me3 modification to play a pivotal role in modulating epithelial-to-mesenchymal transition^36^. By ChIP analysis, the addition of the CtBP inhibitors results in significant eviction of HDAC1 from the *OVOL2*, *FOXA1*, *GRHL2*, *GATA3*, *miRNA 200c*, and *miRNA 203* promoters (Fig. 5a) with a similar pattern for LSD1 ChIP (Fig. 5b). Accordingly, loss of LSD1 and HDAC1 from these respective promoter regions is associated with significant increases in both histone 4 acetylation and the deposition of H3K4Me3 marks at the promoter regions of *GATA3*, *GRHL2*, *FOXA1*, *OVOL2*, *miRNA 200c*, and *miRNA 203* (Fig. 5c).

### CtBP inhibition increases DNA repair and blocks cell migration and invasion

**Fig. 6:**
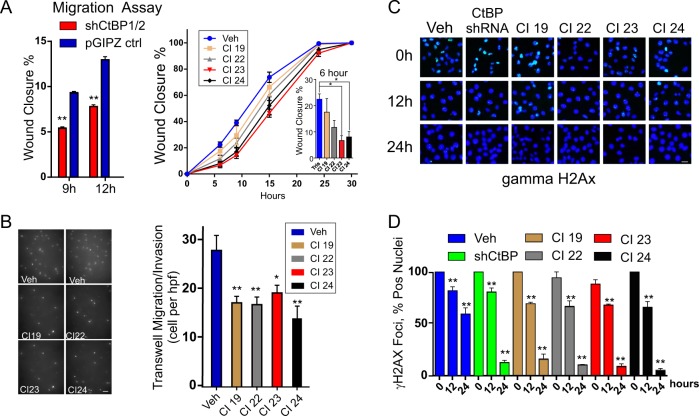
Pharmacologic inhibition of CtBP decreases cellular invasion and migration and increases DNA repair in MDA-MB-231. **a** (Left), Relative migration at 9 and 12 h of MDA-MB-231 cells transduced with doxycycline-inducible (96 h) lentiviral vector expressing a shRNA that targets both CtBP1 and CtBP2^14^. (Right), Wound closure assay of MDA-MB-231 cells pre-treated 24 h with 10 μM CtBP inhibitors. The inset shows relative migration at 6 h of incubation. (*indicates *P* < 0.05). **b** Fluorescent images (left) and graph (right) of invading and migrating MDA-MB-231 cells on DAPI stained membranes following 24-h treatment of cells with vehicle or 10 μM CtBP inhibitors during invasion and migration in Corning® Biocoat™ Matrigel® Invasion Chambers. All experiments represent the average and standard error of at least two biological replicates with each experiment performed at least twice. The error bars represent the s.d. of the mean from at least two independent experiments. *P*-values are calculated from the Student’s *T*-Test relative to the vehicle control. *Indicates *P* < 0.05 and **indicates *P* < 0.01. **c** (Top) Phospho-gamma H2AX foci profile of cells following ionizing radiation (5 Gy) and recovery at 0, 12 and 24 h. **d** Relative rate of DNA repair is expressed as total percent of nuclei containing two or more phospho-gamma H2AX foci per high power field. The error bars represent the s.d. of the mean from at least two independent experiments. *P*-values are calculated from the Students *T*-Test relative to the vehicle control. *Indicates *P* < 0.05 and **indicates *P* < 0.01

One of the most characterized influences of CtBP on cellular phenotype is its role in promoting cellular migration, a key gain of function in cells undergoing epithelial-to-mesenchymal transition and a central feature during tumor metastasis^32^. As shown in Fig. 6a, RNAi-mediated depletion of CtBP in MDA-MB-231 leads to a demonstrable decrease in cell migration, compared to empty vector control (pGIPz) as measured by a wound closure assay (right). Incubation of wild type MDA-MB-231 cells with 10 μM CtBP inhibitors decreases cell migration throughout the time course of the wound closure assay with greater influences caused by compounds CI23 and CI24 (Fig. 6a, right). Similarly, at 10 micromolar concentration, all 4 compounds inhibit both invasion and migration as demonstrated in Matrigel® invasion chamber assays (Fig. 6b).

CtBP forms complexes at the promoters of numerous genes involved in DNA repair, including the *BRCA1* promoter and numerous members of the Fanconi Anemia complementation group^5,13,14,23^. Prior studies have shown that depletion of CtBP results in increased DNA repair detectable in both comet and gamma H2A.X foci formation assays^14,37^. In gamma H2A.X-labeled DNA repair foci assays, the addition of either CtBP inhibitors CI19, CI22, CI23, or CI24 at 10 micromolar concentrations causes increases in DNA repair as demonstrated by the significant decrease in remaining repair foci 24 h following exposure to ionizing radiation (Fig. 6c, d). Notably, the increase is comparable and, in some cases, exceeds that caused by RNAi-mediated CtBP gene depletion (Fig. 6d)^14^.

### Synergistic pharmacological targeting of LSD1 in combination with CtBP

**Fig. 7:**
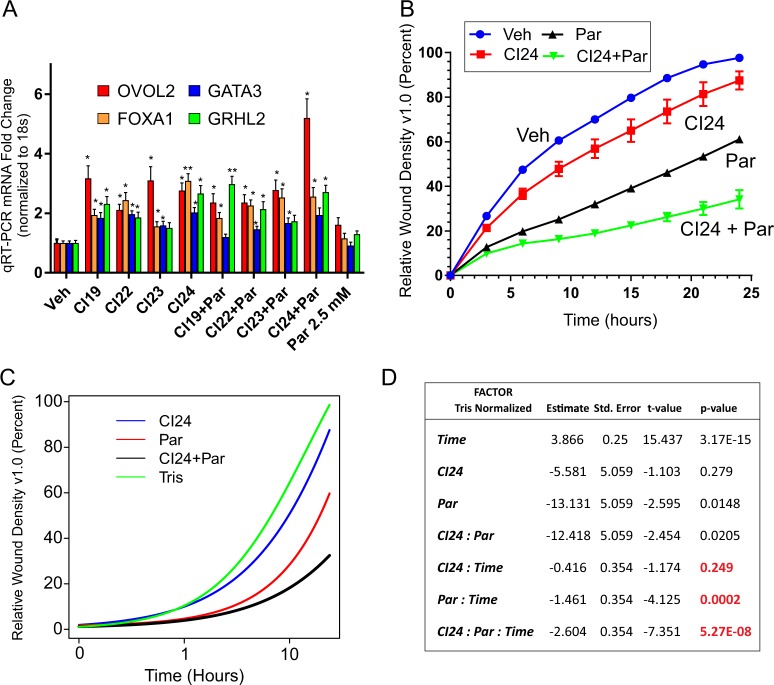
Pharmacologic inhibition of CtBP potentiates LSD1 inhibition to depress pro-epithelial gene expression and decrease breast cancer cell migration. **a** Relative expression of the pro-epithelial genes *OVOL2*, *GATA3, FOXA1*, and *GRHL2* in MDA-MB-231 cells treated with vehicle alone, 2.5 mM pargyline alone, CI24 inhibitor alone or in combination with 2.5 mM pargyline and 10 μM CtBP inhibitors. The error bars represent the s.d. of the mean derived from at least two independent experiments. *P*-values are calculated from the Student’s *T*-Test relative to the vehicle control. *Indicates *P* < 0.05 and **indicates *P* < 0.01. **b** IncuCyte ZOOM™ assay of MDA-MB-231 cell migration in cells treated with either vehicle, 10 μM CI24 CtBP inhibitor, 2.5 mM pargyline, or 2.5 mM pargyline with 10 μM CI24. The error bars represent the s.d. of the mean derived from at least two independent experiments *indicates *P* < 0.05 and **indicates *P* < 0.01. **c** Logistic regression modeling of the dependence of cell mobility (wound density) on time and added drug(s) based on data plotted in (**b**). Hours are shown in log scale. **d** Tabulation of values of the multivariate analysis based on logistic analysis of the single and combined influence of time, CI24, pargyline, and the combination of pargyline and CI24 on wound density shown in (**c**). *P*-values indicate the significance of the strength of interaction of the combined conditions with respect to their influence on cell mobility

LSD1 was one of the first chromatin regulatory complexes to be found in association with CtBP^6^. Several recent studies have suggested the value of targeting LSD1 in therapeutic strategies to epigenetically treat breast cancer alone or in combination with other epigenetic disruptors^38–43^. The LSD1 inhibitor, pargyline has been used in the past alone or in combination with HDAC inhibitors to induce growth inhibition and apoptosis in TNBC cells^39,42,44^. Notably, the combination of these drugs were found to be more than additive for TNBCs and additive or competitive in non-TNBC cell lines^39^. Because CtBP is a well-characterized HDAC associated component of chromatin-modifying complexes, we explored the effectiveness of combining LSD1 inhibition with CtBP inhibition as a surrogate for HDAC disruption. As shown in Fig. 7a, while the addition of pargyline alone minimally reactivates pro-epithelial gene expression in MDA-MB-231, its combination with CtBP inhibitors show additive to synergistic responses in the de-repression of pro-epithelial genes, particularly with respect to CI24. A comparison of the functional influences of pargyline and CI24 alone and in combination on cellular migration was profiled over time (24 h) using the IncuCyte ZOOM™ assay. As shown in Fig. 7b, while both CI24 and pargyline repress migration in this assay, their combination produces greater than additive effects. Logistic regression reveals that the combination of CI24 and pargyline have their greatest influence over time in the migration assay (Fig. 7c, d). Using the T-statistic, the combination of Pargyline and CI24 have significantly greater than additive influence on TNBC cell migration: (*p*-value = 5.27 E−08) compared to CI24 (*p*-value = 0.249) or Pargyline (*p*-value = 0.0002) alone.

### Nuclear CtBP and LSD1 associated pathways in breast cancer patients

**Fig. 8:**
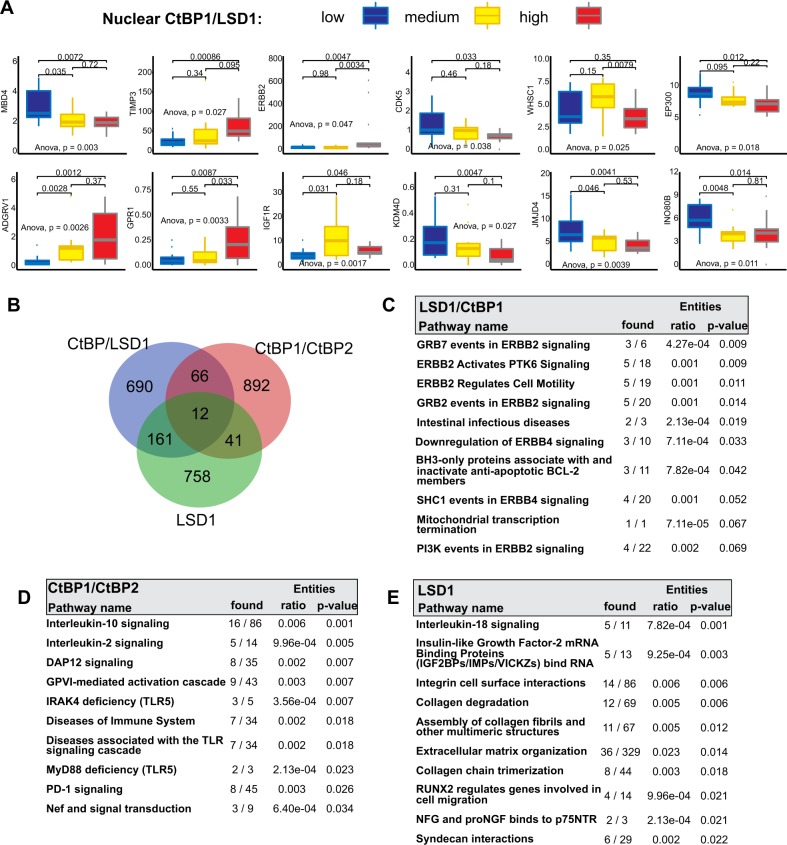
Inferred gene targets and pathway vulnerabilities associated with combined nuclear enrichment of CtBP1 and LSD1 in breast cancer patients. **a** Boxplot displays representative of genes that showed significant differential expression by ANOVA analysis (*p*-value < 0.05) across the three breast cancer patient categories stratified by low, medium, and high nuclear co-expression of CtBP1 and LSD1 using a quantitative immune-histochemical analysis of nuclear staining for CtBP1, and LSD1 based on analysis of the RNA-seq profiles from each patient in each class (see materials and methods). ANOVA test was applied to identify which genes differed significantly across the three categories. (*p*-values for paired categories are also shown). **b** Venn diagram showing the overlap between genes there were significantly differentially expressed in the categories: CtBP1/LSD1, CtBP1/CtBP2, and LSD1. **c** Pathway analysis showing significantly enriched pathway genes that are differentially expressed in association with CtBP1/LSD1 nuclear expression. **d** Pathway analysis showing significantly enriched pathway genes that are differentially expressed in association with CtBP1/CtBP2 nuclear expression. **e** Pathway analysis showing significantly enriched pathway genes that are differentially expressed in association with LSD1 nuclear expression alone

Previous studies have shown that patient breast cancer samples that co-express high levels of LSD1 and histone deacetylases (SIRT1, HDAC2) have decreased survival^45^. To evaluate the possible significance of high nuclear levels of CtBP and LSD1 in breast cancer survival, we utilized a unique resource of patient samples in which nuclear, CtBP1, CtBP2, and LSD1 have been quantitatively determined and compared to paired gene expression data based on RNA-seq analysis (Material and methods, also see Supplementary Fig. 3). Using this available data, 126 breast cancer patients were quantitively stratified according to CtBP1, CtBP2, and LSD1 nuclear expression into low, medium and high terciles of expression (see Materials and methods). Notably, within this cohort, the level of CtBP1 or CtBP2 did not differ greatly in patients between breast cancer subtypes or estrogen receptor status. In fact, slightly higher levels were demonstrated in estrogen-receptor-positive subtypes as opposed to estrogen receptor-negative subtypes (Supplementary Fig. 3). These categories were then used to group patients, according to their CtBP1, CtBP2 and LSD1 nuclear expression, in to three categories: (I) CtBP1/LSD1 = *Low:Low* = low; (II) CtBP1:LSD1, *medium:medium* *=* medium; and (III) CtBP1/LSD1 = *high:high* = high (Fig. 8a). The gene expression of the patients in these three categories were then assessed by analysis of variance (ANOVA) (Fig. 8a) to identify genes that were differentially expressed between the three groups. Examples of differentially expressed genes are shown by boxplot in Fig. 8a. Notably, many genes linked to chromatin modification and cell signaling are enriched in the genes differentially expressed in these categories (Fig. 8a, and Supplementary Tables 1–4). Similarly, genes that are differentially expressed based on the stratification of CtBP1/CtBP2 or LSD1, respectively, were identified (Supplementary Table 1). A Venn diagram of the genes common to all three stratifications is shown in Fig. 8b and demonstrates very little overlap. Moreover, the gene pathways enriched in the genes differentially expressed by ANOVA of the three stratifications show significant differences (Fig. 8c–e). CtBP1/LSD1 differentially expressed genes show significant enrichment for growth factor signaling, CtBP1/CtBP2 differential expressed genes are enriched for immune response genes including immune checkpoint regulators (Fig. 8d), and LSD1 only differentially expressed genes are enriched with genes associated with regulation of the extracellular matrix (Fig. 8e). Unique genes identified by ANOVA profiling of differentially expressed genes across 5 categories of CtBP1:LSD1 combinations (including CtBP1:LSD1 = *Low/High* and CtBP1:LSD1 = *High/Low*) was not additionally informative (Fig. S4 and Supplementary Tables 1 and 5).

## Discussion

In summary, using computer-assisted drug design to screen for possible inhibitors of CtBP function, we have identified four lead compounds that, through disruption of CtBP dimerization, reactivate CtBP-repressed genes. The mechanisms underlying this inhibitory activity are associated with the ability of this class of compounds to disrupt the CtBP-mediated recruitment of multiple different chromatin-modifying complexes to targeted genes. These include the histone deacetylase, HDAC1; and the histone demethylase, LSD1. These findings not only implicate an important application of these compounds in strategies for therapeutic intervention but also demonstrate their utility as investigative tools to define epigenetic gene regulatory pathways and mechanisms linked to CtBP. The application of these compounds as investigative tools also provides a proof of principle for the combinatorial use of CtBP inhibitors with other epigenetically targeted compounds or therapeutic strategies. The combined use of LSD1 and CtBP inhibitors offers a new concept in this therapeutic approach.

The current rapid expansion of novel strategies to target both epigenetic regulatory enzymes and epigenetic regulatory complexes^46–48^ suggests a prominent emerging new role for these compounds and other recently characterized CtBP inhibitors^21,22^ in combinatorial strategies for epigenetic intervention. Although these compounds bind with modest affinity (in the micromolar range), they serve as prototypes from which to bootstrap the design of novel molecules with higher binding affinity and activity toward CtBP-regulated pathways. Similarly, although pargyline shows measurable synergy with CtBP inhibitors, its primary use in clinical trials has been associated with treatments for hypertension. Nonetheless, there is renewed interest in therapeutic targeting of LSD1 as several new compounds that disrupt LSD1 function including: GSK2879552, ORY-100, 4SC-202, IMG-7289, INCB-59872, and tranylcypromine derivatives, are being assessed in clinical trials for activity against leukemia^43^. This is particularly relevant given the recent finding that loss of LSD1 is associated with an enhanced anti-tumor response through upregulation of double-stranded RNA stress response secondary to lost repression of endogenous retroviral elements^49^. Furthermore, analysis of TCGA data show an inverse relationship between CD8+ T-cell infiltration and the RNA levels of LSD1^49^. It is not clear what underlies the cell-type specific dose sensitivity we have observed in the current study, however, this has been observed with other CtBP inhibitors^10^. Whether or not it reflects sensitivity thresholds based on the level of epithelial differentiation in breast cancer will require extensive investigation beyond the scope of this initial proof-of-principle report. Such issues may become better resolved with the development of 2nd or 3rd generation CtBP inhibitors that act with higher affinity. Currently, the most potent compound appears to be CI24 which shows the highest uniform correlation in potency of transcriptional de-repression, with eviction of chromatin modifiers, influences on cell migration/invasion, and increase in DNA repair. The provocative finding that differential enrichment of CtBP1/LSD1 shows selective influence on ERRB2/4 signaling (Fig. 8c) suggests a broader application of this targeting strategy beyond TNBC subtypes. Moreover, it suggests additional opportunities for combined therapy. An important aspect of this current study is the correlation of nuclear levels of CtBP1 and LSD1 with gene expression patterns in patient samples. Although cell lines are an easily tractable system that led to the cell-type specific identification of *GATA3*, *FOXA1*, and *OVOL2* as major regulatory targets of CtBP, these genes do not show up as top targets in patient samples (Fig. 8). This reflects the innate heterogeneity and plasticity of patient breast cancer tissues and also is consistent with the significant cell type-specificity of the influence of CtBP inhibition, mentioned previously (Fig. 4), and observed in CtBP-depleted cell lines^14^. Other notable correlations found in patient samples is the high significance of the DNA repair genes *ATM*, *LIG4*, *PARP3*, *SETX*, and *GRF2H4* genes revealed by ANOVA analysis of the CtBP1/CTBP2 high category (see Supplementary Table 1). In summary, the findings in this study provide a new and novel conceptual groundwork for the development of more effective treatment strategies and introduce a novel class of compounds to be explored in combinatorial strategies to exploit vulnerabilities in the epigenetic regulation of breast cancer.

## Materials and methods

### Cell lines and constructs

MDA-MB-231 and MCF-7 cells were purchased from ATCC. All starting cultures were screened for mycoplasma contamination approximately 4–5 months prior to use, and experiments were conducted on cell lines grown for no more than 13–15 passages. Short hairpin sequences for RNAi depletion of CtBP1 and CtBP2 were cloned into the doxycycline-inducible (Tet-ON) pINDUCER lentiviral vector system that produces a miR30-based short hairpin that targets both CtBP1 and CtBP2^13,50^. CFP-CTBP1 and YFP-CTBP2 plasmids were provided by Dr. Jermey P. Blaydes^51^.

### Cell culture and tissues

MDA-MB-231 and MCF-7 cells were maintained in regular DMEM supplemented with 10% (v/v) FBS, penicillin-streptomycin (Invitrogen) and insulin as previously described^14^.

### Computer-assisted drug design

ChemNavigator iResearch Library from Sigma Aldrich^24^ was searched for molecules containing oxaldehydic acid structures similar to MTOB or structurally similar propanedioic, 3-oxobutanoic, and 4-oxopropanoic acid. These moieties can form favorable interactions with three residues (His315, Glu295, Arg 266) demonstrated to function as a catalytic triad in the active site of CtBP in addition to other essential residues at the catalytic site, including Arg266 and Arg67^8^. Quantitative structure-activity relationship (QSAR) models were generated using MTOB and a series of 11 alpha-keto acids with measured kinetic properties for the catalytic domain of CtBP. The best model obtained was then applied against the set of selected acidic compounds from ChemNavigator, and compounds with high predicted substrate activity values were filtered for drug-like properties using the Lipinski Rule^52^. Filtered candidates selected by the QSAR modeling were then further evaluated by docking into the published crystallographic structure of the CtBP dehydrogenase core with NAD+ and acetic acid^8^. Docking was done using Glide (27) and the docked compound structures were required to be stabilized by at least two hydrogen bonds to Arg266, Gly101 or His315 in the CtBP active site. This screen identified 31 compounds of which 24 were commercially available. (See virtual screening methods in Supplementary materials and methods)

### Acceptor photobleaching FRET

Expression vectors for CFP-CtBP1 (pSCFP3A) and YFP-CtBP2 (pSYFP2) were co-transfected into MDA-MB-231 wild-type cells using lipofectamine 2000 reagent (Thermo Fischer Scientific). 2 × 10^4^ cells/well were seeded into Nunc Lab-Tek 8 chambered coverglass (Thermo Fischer Scientific). Cells were then treated with either 100 μM of Tris control vehicle or 10 μM of the four different CtBP Inhibitors (CI19, CI22, CI23, and CI24). We used a plasmid encoding a CFP-YFP fusion protein with a 2-aa spacer between CFP and YFP as a positive control for FRET^29^. Co-transfection with CFP-CtBP1 (pSCFP3A) and unconjugated YFP (pCMV6AC-mYFP) plasmids were used as negative control. Cells were imaged with a 60 × 1.4 NA Zeiss immersion objective and ×2 zoom using 514 nm laser line of argon laser (25 mW) and 405 nm laser line of Diode laser (30 mW) on LSM780 confocal microscope (Carl Zeiss, Inc, Thornwood, NY, USA) in the Optical Microscopy Core (NCI/CCR/LRBGE). YFP was photobleached by scanning the whole cell 10 times using the 514 laser line of an argon laser at 100% intensity. The efficiency of Fluorescence Resonance Energy Transfer (FRET) E_F_ was measured by acceptor photobleaching method as described^29^ based on the equation: *E*_*F*=_ (I_CFP, after_ − I_CFP, before_)/I_CFP, after_, where I_CFP_, _after_ and I_CFP_, _before_ referring to CFP intensities after and before YFP photobleaching, respectively. Each sample was scored for greater than 15 nuclei in order to achieve statistical significance. 15 regions of interest were randomly measured within each nucleus. A FRET signal was considered be positive if the FRET efficiency values (*E*_*F*_) obtained in the experiment exceeded those of random FRET in the negative control.

### Chromatin immunoprecipitation and immunoprecipitation

ChIP was performed as previously described^53^ and the list of primers and antibodies used in the study are provided in the Supplementary materials and methods.

### Western blotting RNA isolation, and RT-qPCR

RNA isolation from cell lysates and RT-qPCR were performed as described previously^53^ and normalized to 18S rRNA. The total RNA was prepared using the RNAeasy kit (Qiagen) following the manufacturer’s protocol. The RNA was quantified and 1 μg of total RNA was used for each reverse transcription. Reverse transcription was carried out by following the QuantiTect® Reverse Transcription procedure (Qiagen). For western blotting, the cells were collected and resuspended in RIPA lysis buffer (50 mM Tris pH 7.5, 1 mM EDTA, 150 mM NaCl, 0.1% SDS, 1% TritonX-100, 1% sodium deoxycholate and freshly added proteinase inhibitor cocktail) for 30 min on ice. The lysates were centrifuged for 20 min at 12,000 rpm and the supernatants were used for quantitation and western blotting. Nuclear extracts were prepared and analyzed as described previously^53^.

### Invasion and migration assays

Invasion assay was performed using MDA-MB-231 cells with the Corning® Biocoat™ Matrigel® Invasion Chambers (cat. 354480). Replicates were carried out according to the manufacturer’s recommendation after 24 h of treatment of Tris and 10 μM CtBP inhibitors (CI19, CI22, CI23, and CI24). After 24 h of the invasion, the membranes were washed in 1X PBS, fixated in methanol, stained with DAPI, and visualized. Wound closure assay was performed using the Radius™ assay from Cell Biolabs, Inc. Cells were incubated 24 h with 10 μM CtBP inhibitors prior to initiation of the migration assay. For assays comparing cellular migration in response to combined addition of drugs, cellular migration was measured using the IncuCyte® ZOOM System, real-time, a quantitative live-cell analysis system for high definition phase contrast images to monitor wound closer over time as directed by the manufacturer’s instructions.

### Gamma H2A.X foci formation assay

MDA-MB-231 wild type and CtBP1/2 knockdown cells were seeded in 8 chamber slides at a density of 10,000 cells/well. MDA-MB-231 wild type cells were treated with the CtBP inhibitors (CI19, CI22, CI23, and CI24) and Tris Control for 24 h prior to Gamma Irradiation. MDA-MB-231 wild type and CtBP1/2 knockdown cells were exposed to 5 Gy of Gamma irradiation. After 0, 12, and 24 h post-Gamma irradiation, cells were washed three times with 1× PBS, fixed with 3.5% paraformaldehyde for 20 min followed by 70% cold ethanol overnight. Cells were then washed three times with 1× PBS, stained with anti-phospho-Histone H2A.X antibody (Ser139) (catalog # 05-636, Millipore) at 1/1000 primary antibody dilution for 1 h, washed three times with 1× PBS, followed by staining with secondary Alexa Fluor 488 goat anti-mouse antibody (Catalog # A11001, Life Technologies) at 1/2000 dilution for one hour. Cells were then washed three times with 1× PBS followed by staining with 1/5000 DAPI dilution. Cells were then mounted with anti-fade mounting media and covered with coverslips. Different fluorescent images were taken with Axiovert 200 M and only cells with 3 plus phospho-H2A foci were scored.

### Methods for immunohistochemistry

#### Tissue microarray construction and scoring

Following IRB approval from East Carolina University and the National Institutes of Health Intramural research program, approximately 180 de-identified formalin-fixed and paraffin-embedded tissue from patients who were diagnosed and underwent surgery for Stage 0 to Stage IV breast cancer between 2001 and 2010 at Pitt County Memorial Hospital (now Vidant Medical Center), Greenville, NC. Following a review of hematoxylin and eosin-stained sections, regions of interest were outlined, and 1 mm cores were removed from corresponding blocks using a Pathology Devices TMArrayer (Westminster, MD). All arrays contained appropriately chosen positive and negative control tissue. Digital image analysis and scoring of IHC staining was performed using Leica Aperio digital analysis platforms, in which three representative regions of tumor were outlined on each core by a pathologist and scored digitally using the Nuclear v9 algorithm (CtBP1, CtBP2, and LSD1) to generate a histo-score (H-Score; 0–300) based on the percent of positive cells with assigned intensity thresholds of negative (0), low (1), moderate (2) or high (3) as previously described^54^.

Breast tumor tissue microarrays were stained, using monoclonal primary antibodies, CTBP1 and CTBP2 (33871, 39008 Millipore) at 1:100,000 overnight with high pH (Catalog No. M361201-2, DAKO). LSD1 (Abcam, ab129195) staining was done at 1:2000 overnight with low pH (Catalog No. MA5-13191, DAKO). Using the assigned scale of 1–300, patients were then classified into terciles based on the H-Scores for CtBP1, CtBP2, and LSD1, respectively. The data was then categorized into terciles based on the divided ordered distribution into three parts, each containing a third, i.e. 33.33% of a given population. The first tercile (low) represents the lowest 33.33% of the data (1–33.33%); the second tercile represents the medium 33.33% value (33.34–66.66%) and third highest 33.33 value (66.34–100%). Based on those protein values, the patients were classified into these three categories and defined them as low, medium and high. Finally, patients were segregated into combined CtBP1: LSD1 categories of Low: Low; Medium: Medium; and High: High and used for comparison of their gene expression patterns.

#### RNA-seq

Following a review of H&E stained slides areas of tumor with >80% nuclei were circled, and 2.5 × 2–3 mm tissue cores were extracted from the corresponding regions of FFPE tissue blocks for *N* = 126 patients. Sample cores were shipped to BGI Beijing Genome Institute (BGI) for further processing as previously described^55,56^. Briefly, the total RNA samples were first treated with DNase I, followed by an mRNA enrichment step enriched by using the oligo (dT) coupled magnetic beads. Following fragmentation, the first strand of cDNA was synthesized by using random hexamer-primers. Buffer, dNTPs, RNase H and DNA polymerase I were added to synthesize the second strand. The double-strand cDNA was purified with magnetic beads and end reparation and 3’-end single nucleotide A (adenine) addition was then performed. Finally, sequencing adaptors were ligated to the fragments. The fragments were enriched by PCR amplification. During the QC step, Agilent 2100 Bioanalyzer and ABI StepOnePlus Real-Time PCR System were used to qualify and quantify the sample library. The library was sequenced (60 M paired-end read per sample) on an Illumina HiSeqTM4000.

#### Sequence data analysis

After sequencing, the raw reads were filtered (BGI). Data filtering included removing adapter sequences, contamination and low-quality read from raw reads. The cleaned reads (fastQ) were mapped to the reference sequence using HISAT^57^. Raw reads and RPKM for each sample were calculated using HOMER^58^. Differential gene expression was performed using EdgeR^59^.

### Statistical analysis

A Spearman rank correlation test was performed to test the relation between its protein H-score and gene expression (RPKM value) values^60^. A completely unsupervised hierarchical clustering approach was performed on all 486 breast samples of the protein data set. Complete linkage and distance correlations were used for clustering protein data with bootstrap resampling techniques. The stability of the clustering was estimated with the ‘pvclust’ R package^61^ available on CRAN http://cran.rproject.org/web/packages/ pvclust/). A two-sided *t*-test was employed to test the null hypothesis (H0) assumption of equality of the protein values in two defined groups of data and demonstrated by violin plots using R software and ggplot2 package^62^. ANOVA and logistic regression analysis were conducted using R statistical analysis software. A *p*-value thresholds 0.05 was applied to select the top significant genes (Supplementary Table 1). A reactome open-source browser (version 3.6), with curated and peer-reviewed pathway database (reactome release 67) was used for pathway analysis of the top genes identified (https://reactome.org/PathwayBrowser/).

## Supplementary information

Supplementary Figures.

Supplementary Table 1.

Supplementary Table 2.

Supplementary Table 3.

Supplementary Table 4.

Supplementary Table 5.

Supplementary Material and Methods.
