# Supplementary material for: Epigenetic re-wiring of breast cancer by pharmacological targeting of C-terminal binding protein
Source: Cell Death Dis. 2019 Sep 18;10(10):689. doi: 10.1038/s41419-019-1892-7 (PMC6751206; doi:10.1038/s41419-019-1892-7)
Supplement: Supplementary file 1 — Supplementary Figures. [file 41419_2019_1892_MOESM1_ESM.pdf]

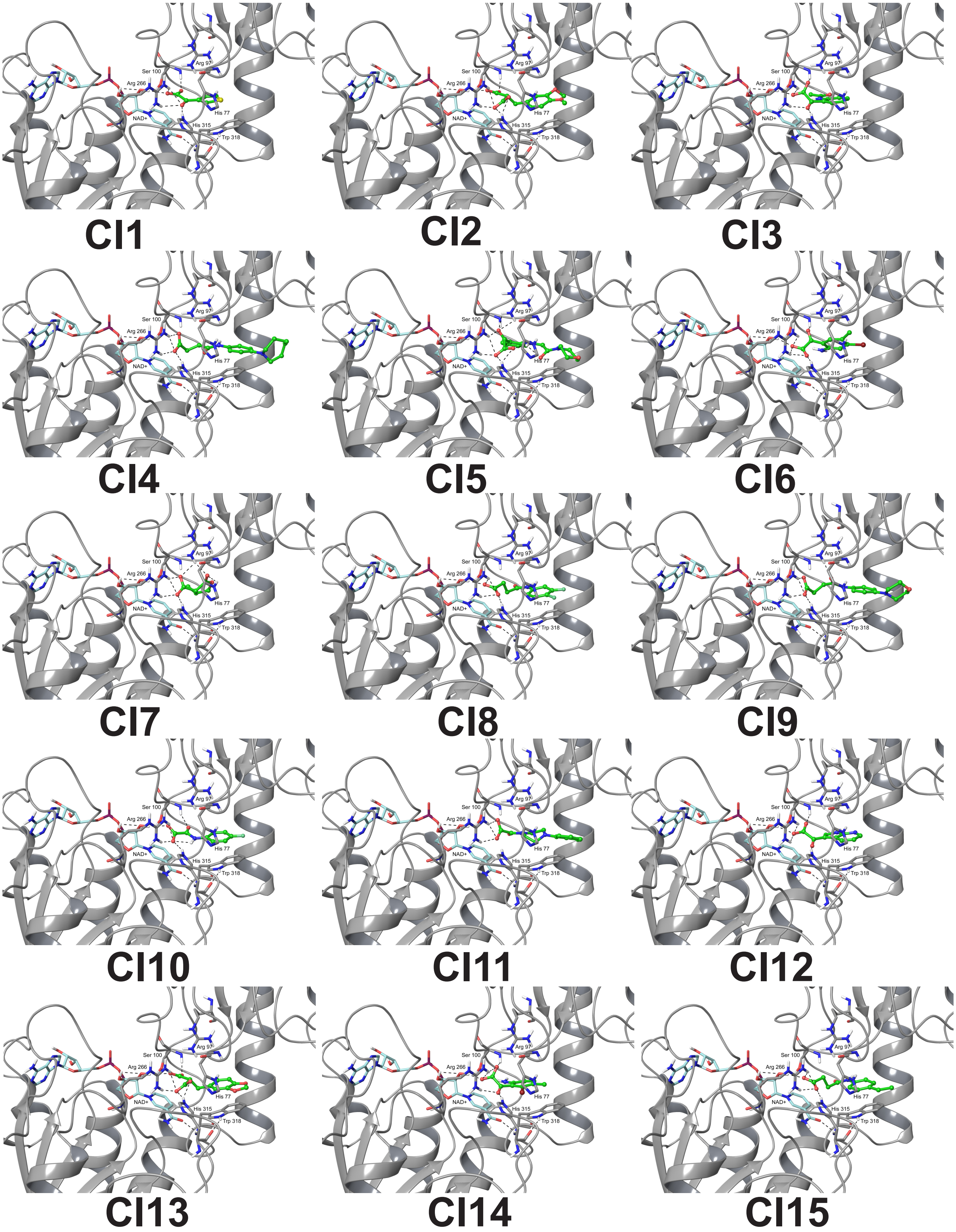

Supplementary Figure 1

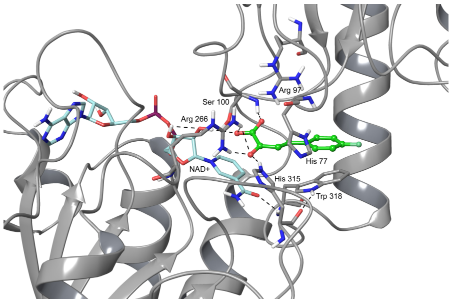

CI16

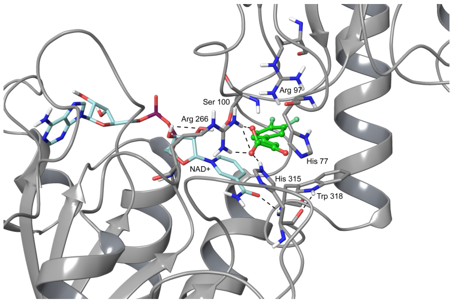

CI17

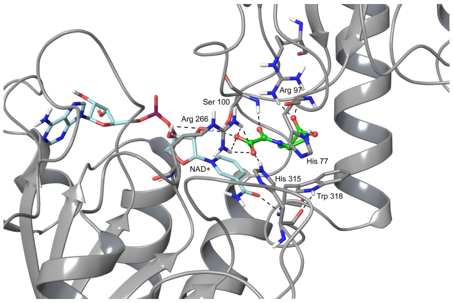

CI18

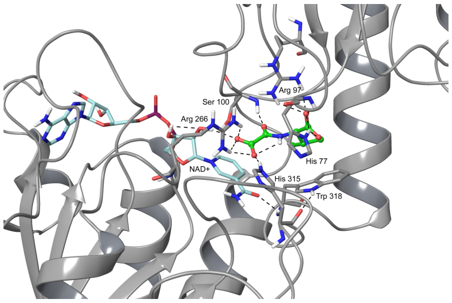

CI19

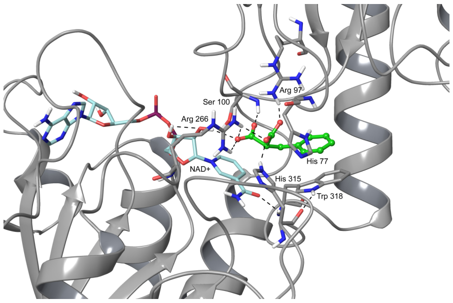

CI20

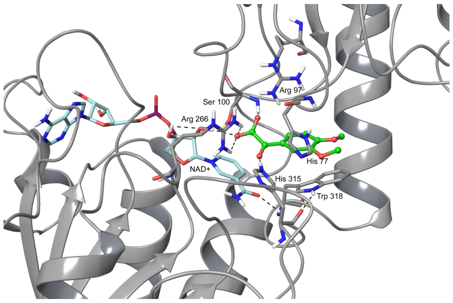

CI21

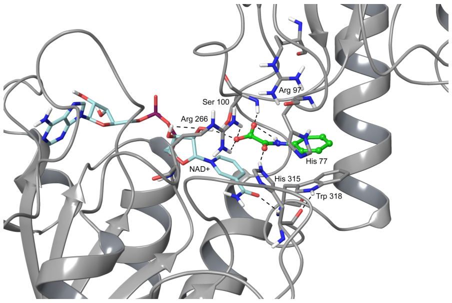

CI22

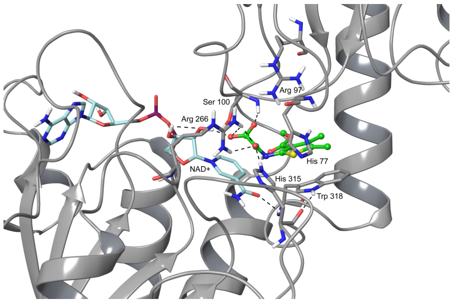

CI23

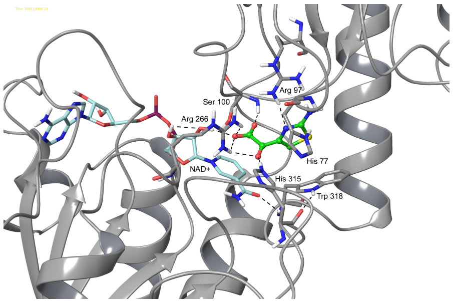

CI24

Supplementary Figure 1. Docking structure of the 24 small molecules screened in this study. Compounds are shown in green in the CtBP substrate binding site. The NAD+ cofactor is colored in light blue. Hydrogen bonds are indicated with dashed black lines.

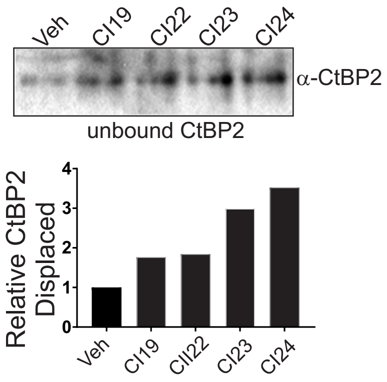

Supplementary Figure 2. Drug displacement of CtBP2 from immunoprecipated CtBP1. Whole cell lysates from 5 x 10^7 MCF-7 cells overexpressing flag epitope tagged CtBP1 (fCtBP1) were immunopreciptated with 10 ug anti-flag antibody coupled to magnetic beads (Sigma aldrich). Washed immunopreciptates were divided and then incubated 4h at 4C° with vehicle or 10 uM CI19, CI22, CI23, or CI24. Eluted protein was analyzed for released endogenous CtBP2 by western blot. Densitometer reading of relative displacement is shown below.

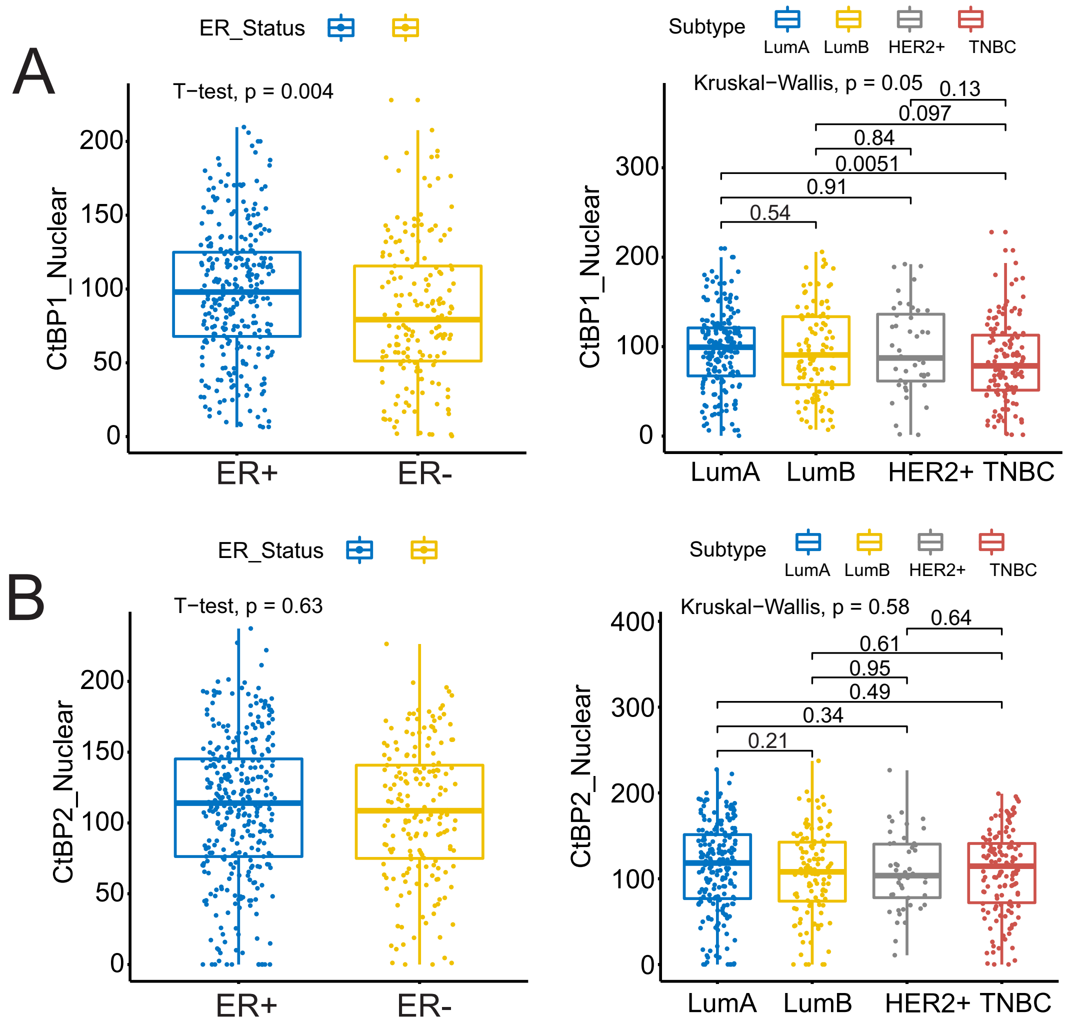

Supplementary Figure 3. Analysis of the relative expression of nuclear CtBP1 and CtBP2 in the breast cancer cohort characterized in Figure 8. **A,** Boxplot analysis demonstrating the relative nuclear expression of CtBP1 in ER+ compared to ER- breast cancer patients (left) including a comparison by intrinsic breast cancer subtypes (right) in the patient cohort used for the RNA-seq analysis shown in Figure 8. **B**, Boxplot analysis demonstrating the relative nuclear expression of CtBP2 in ER+ compared to ER- breast cancer (left) and also compared by intrinsic breast cancer subtype (right) in the patient cohort used for the RNA-seq analysis shown in (Figure 8) (N=126 of 555 patients samples were available for RNA-seq analysis)

A

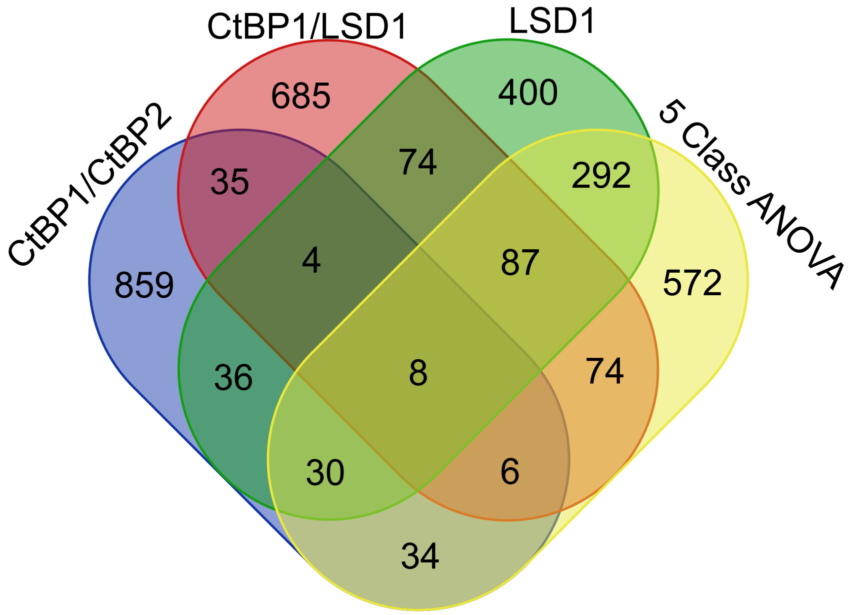

B

Pathway name	Entities			
	found	ratio	p-value	FDR*
**Defective SFTPA2 causes idiopathic pulmonary fibrosis (IPF)**	1 / 1	7.11e-05	0.042	0.78
**Defective CSF2RA causes pulmonary surfactant metabolism dysfunction 4 (SMDP4)**	2 / 8	5.69e-04	0.047	0.78
**Defective CSF2RB causes pulmonary surfactant metabolism dysfunction 5 (SMDP5)**	2 / 8	5.69e-04	0.047	0.78

C

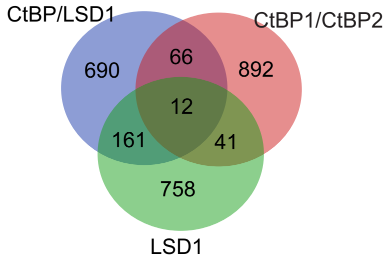

(From Figure 8)

Supplementary Figure 4 A, Venn diagram of overlap between genes strata CtBP1:LSD1; CtBP1:CtBP2; LSD1 alone and the 5 class ANOVA strata analysis comparing the following 5 classes: I) CtBP1/LSD1= *low:low*; II) CtBP1:LSD1, *medium:medium*; III) CtBP1/LSD1 = *high:high* ; IV) (CtBP CtBP1/LSD1= *high:low*; and V) CtBP1:LSD1, = *low:high*. (See supplemental table 5). **B,** Pathway enrichment analysis from genes unique to those enriched in 5 Class ANOVA analysis. **C,** Venn diagram from Figure 8 shown as reference.
