# Supplementary material for: Epigenetic re-wiring of breast cancer by pharmacological targeting of C-terminal binding protein
Source: Cell Death Dis. 2019 Sep 18;10(10):689. doi: 10.1038/s41419-019-1892-7 (PMC6751206; doi:10.1038/s41419-019-1892-7)
Supplement: Supplementary file 7 — Supplementary Material and Methods. [file 41419_2019_1892_MOESM7_ESM.doc]

**Supplemental Materials and Methods**

**Virtual Screening Methods**

*Database Filtering.*

The ChemNavigator iResearch Library (iRL) from Sigma-Aldrich (1) was used as the main library for our virtual screening. The iRL is a collection of over 91.5 million chemical samples (representing over 55 million unique compounds) aggregated from more than 300 chemistry suppliers world-wide. In preliminary filtering using the program Pipeline Pilot (2), we removed any salts and solvents, keeping only the largest fragment in each molecular record, and filtered out compounds containing inorganic atoms (other than H, C, N, O, P, S, F, Cl, Br, and I) and compounds with a molecular weight less than 100. Any duplicate structures were eliminated.

Because most dehydrogenases have strict substrate specificities, and the structure of one potential substrate of CtBP (2-keto-4-methylthiobutyrate, MTOB) is known, we then filtered the database to select molecules containing oxaldehydic acid (which is present in the MTOB structure) or structurally similar propanedioic acid, 3-oxobutanoic acid or 4-oxopropanoic acid. These moieties allow good interactions with the catalytic triad residues of CtBP (His315, Glu295, Arg266) and other key residues in the active site such as Arg266 and Arg67 (3).

The drug-like properties of the selected compounds were evaluated using the Lipinski rule of five (4), to eliminate compounds with more than 5 hydrogen bond donors, more than 10 hydrogen bond acceptors, a molecular weight over 500, or an octanol-water partition coefficient log P greater than 5. Additionally, the water solubility of the remaining compounds was predicted using Qikprop (5), and compounds with QPlogS greater than 0.5 and less than -6.0 were filtered out.

*QSAR Modeling.*

A series of 11 α-keto acids with measured kinetic properties for the catalytic domain of CtBP1 (6) was used to generate quantitative structure-activity relationships (QSAR) models. This set of potential substrates for CtBP1 includes 2-keto-4-methylthiobutyrate (MTOB) which is currently the most potent known substrate for CtBP. The catalytic efficiencies expressed as *k_cat_/K_M_* (s^-1^/M^-1^) were transformed to –log (*k_cat_/K_M_*). 3D structures were built for the 11 compounds with Maestro 9.2 (7), and minimized with LigPrep (8), using the OPLS 2005 force ﬁeld at pH 7.0. This series of compounds was also used for docking validation (see below).

QSAR models were constructed using the GUSAR program (9). We obtained consensus models using QNA and MNA descriptors with additional variables (*i.e.* length, volume, lipophilicity). All compounds were used for the training set due to the limited number of available molecules in the series. The performance of the QSAR models was evaluated by measuring the accuracy of the predictions. The statistical parameters that were used to evaluate the predictions were as follows: R^2^ (determination coefficient), SD (standard deviation) and Q^2^ (determination coefficient calculated for leave-one-out cross validation procedure). The best model obtained was applied against our pool of pre-filtered compounds, selecting for compounds with high predicted substrate activity values using a cut-off score of 2.8.

*Docking.*

The compounds selected by the QSAR models were docked using Glide (10, 11) into the crystal structure (PDB ID: 1MX3 (3)) of CtBP dehydrogenase core with NAD^+^ and acetic acid solved at 1.95Å. The crystal structure (PDB ID: 1DXY (12)) of D-2-hydroxyisocaproate dehydrogenase complexed with NAD^+^ and 2-oxoisocaproate at 1.9Å resolution was also used for structural analysis. Both crystal structures were downloaded from the RCBS Protein Data Bank ([http://www.pdb.org](http://www.pdb.org/)) (13) and from PDBePISA (14), which provided the dimer assemblies for both structures. The coordinates of the PDB complexes were superimposed into the same relative orientation.

An initial docking run was performed with the series of 11 α-keto acid substrates (6) to establish parameters and a GlideScore fitness threshold for defining good poses. The protein preparation wizard in Maestro (7) was used to optimize and minimize the protein by deleting the crystal waters and adding explicit hydrogens. The binding site was defined using the Receptor Grid Generation panel with default options. Standard-precision (SP) docking was selected for screening the ligands. We selected the flexible docking mode, meaning that Glide internally generated the conformations during the docking process. Docking constraints were applied in order to be sure that compounds were placed properly in the binding site due to their small size. Compounds were required to have at least two hydrogen bonds with Arg266, Gly101 or His315 of the CtBP binding domain. The 10 highest-scoring poses were saved for each compound, and poses with a GlideScore fitness of less than 4.0 were rejected. Maestro (7) was used for analyzing and visually investigating the ligand-protein interactions of the saved poses.

Based on these results, a set of 31 compounds were selected as hits from the virtual screening. Of these, 24 were immediately commercially available and were selected for purchase and experimental assaying.

|  | **CNC_ID** | **Structure_ID** | | **Supplier** | **CHEM_NAME** |  |  |  |  |  |
| --- | --- | --- | --- | --- | --- | --- | --- | --- | --- | --- |
| **1** | 2464139 | | 28667305 | Specs | 2-oxo-2-(thiophen-3-yl)acetate | |  |  |  |  |
| **2** | 5840690 | | 27195152 | ChemBridge Corporation | 2-[(3,4-dimethoxyphenyl)methyl]propanedioic acid | | | |  |  |
| **3** | 12291930 | | 34405495 | Labotest | potassium 2-(2-acetamidophenyl)-2-oxoacetate | | | |  |  |
| **4** | 17927669 | | 30375639 | ChemBridge Corporation | 3-{[4-(azepan-1-yl)phenyl]carbamoyl}propanoic acid | | | |  |  |
| **5** | 19322461 | | 37183068 | Otava | potassium 2-(2-{[2-(morpholin-4-yl)-2-oxoethyl]amino}phenyl)-2-oxoacetate | | | | | |
| **6** | 150553063 | | 150553018 | Apollo Scientific Ltd | sodium 2-(2-amino-4-bromo-5-methylphenyl)-2-oxoacetate | | | | |  |
| **7** | 183174394 | | 28724388 | Vitas-M Laboratory, Ltd. | 4-methyl-2-oxooxolane-3-carboxylic acid | | |  |  |  |
| **8** | 187734488 | | 29573563 | Vitas-M Laboratory, Ltd. | 3-[(3,4-difluorophenyl)carbamoyl]propanoic acid | | | |  |  |
| **9** | 187753384 | | 30375640 | Vitas-M Laboratory, Ltd. | 3-{[4-(morpholin-4-yl)phenyl]carbamoyl}propanoic acid | | | |  |  |
| **10** | 188380200 | | 37576185 | Vitas-M Laboratory, Ltd. | [(4-fluorophenyl)carbamoyl]formic acid | | |  |  |  |
| **11** | 189573355 | | 75809820 | Life Chemicals Inc. | 2-oxo-2-(4-phenylpiperazin-1-yl)acetate | | |  |  |  |
| **12** | 234543335 | | 48698131 | Sigma-Aldrich | potassium 2-oxo-2-{1H-pyrrolo[2,3-b]pyridin-3-yl}acetate | | | |  |  |
| **13** | 234559327 | | 234559040 | Sigma-Aldrich | 2-(2H-1,3-benzodioxol-5-ylmethyl)propanedioic acid | | | |  |  |
| **14** | 249393301 | | 27646592 | Vitas-M Laboratory, Ltd. | 2-(6-amino-2-bromo-3-methylphenyl)-2-oxoacetate | | | |  |  |
| **15** | 314152780 | | 28147989 | Sigma-Aldrich | 3-[(4-ethylphenyl)carbamoyl]propanoic acid | | |  |  |  |
| **16** | 333196555 | | 161229083 | Sigma-Aldrich | potassium (3E)-4-(4-fluorophenyl)-2-oxobut-3-enoate | | | |  |  |
| **17** | 333196608 | | 161231173 | Sigma-Aldrich | potassium (3E)-4-(3,4-difluorophenyl)-2-oxobut-3-enoate | | | |  |  |
| **18** | 333200838 | | 161374374 | Sigma-Aldrich | disodium 2-[1-carboxylato-N-(propan-2-yl)formamido]acetate | | | | |  |
| **19** | 333201007 | | 161379867 | Sigma-Aldrich | disodium 2-(carboxylatoformamido)-3-methylbutanoate | | | |  |  |
| **20** | 333884125 | | 28116018 | Vitas-M Laboratory, Ltd. | 2-benzylpropanedioic acid | |  |  |  |  |
| **21** | 366259061 | | 48648177 | Sigma-Aldrich | sodium 2-(3,4-dimethoxyphenyl)-2-oxoacetate | | |  |  |  |
| **22** | 362924866 | | 243985427 | FCH | potassium (phenylcarbamoyl)formate | | |  |  |  |
| **23** | 364193097 | | 308199874 | Tim Tec, Inc. | sodium {[3-(methoxycarbonyl)-4,5-dimethylthiophen-2-yl]carbamoyl}formate | | | | | |
| **24** | 362835349 | | 359119906 | FCH Group | sodium (2-amino-1,3-thiazol-4-yl)(oxo)acetate | | |  |  |  |

N-Methyl-N-propargylbenzylamine was from Sigma-Aldrich,

**Antibodies**

CTBP1 612042 BD Biosciences

CTBP2 624088 BD Biosciences

GAPDH 6C5 Santa Cruz

BRCA1 Purified in lab

FOXA1 ab23738 Abcam

GATA3 HG3-31 Santa Cruz

CDH1 24E10 Cell Signaling

OVOL2 HPA038531 Sigma-Aldrich

HDAC1 PA1-860 Thermo scientific

LSD1 ab17721 Abcam

H4Ac 06-598 Millipore

H3K4Me3 07-473 Millipore

Phosphor-Histone H2A.X (Ser139) 05-636 Millipore

Alexa Fluo488 goat anti-mouse A11001 Life technologies

LSD1 ab129195 Abcam

**Primers**

**Gene expression primers**

OVOL2 F TTCTTCAGGTGGGACTCCAG

OVOL2 R TTCAACGACACCTTCGACCT

FOXA1 F GCCTGAGTTCATGTTGCTGA

FOXA1 R CTGTGAAGATGGAAGGGCAT

GRHL2 F ACTTCCAGGCTTCATCCTCA

GRHL2 R CATGTCACAAGAGTCGGACAA

GATA3 F TCTGACAGTTCGCACAGGAC

GATA3 R AAAATGAACGGACAGAACCG

BRCA1 F GAAGGCCCTTTCTTCTGGTT

BRCA1 R AGAGTGTCCCATCTGTCTGGA

CDH1 F GACCGGTGCAATCTTCAAA

CDH1 R TTGACGCCGAGAGCTACAC

ABCB1 F TCAGCCTCACCACAGATGAC

ABCB1 R TCACTTCAGGAAGCAACCAG

ABCC2 F GGGATCTCTTCCACACTGGAT

ABCC2 R CATACAGGCCCTGAAGAGGA

18S F GCCCGAAGCGTTTACTTTGA

18S R TCCATTATTCCTAGCTGCGGTATC

miR203a Hs03302931_pri TaqMan Pri-miRNA Assays

miR200c Hs03303157_pri TaqMan Pri-miRNA Assays

RNU48 TM 001006 control miRNA

**ChIP primers**

GRHL2 upstream F CCTGGGCAACAGAGAGAGAC

GRHL2upstreamR CCCTGAAAACAATTCCTCCA

GRHL2promoterF ATTCAGCTCCTTGCGAGAAA

GRHL2promoterR TAGCCGGAAAGGTGGGATA

OVOL2upstreamF GATCGTGCCATTGTACTCCA

OVOL2upstreamR TGGAGCACACAGCAATAAGG

OVOL2promoterF CGAGCTTGTTGACACCGTTA

OVOL2promoterR GGCGACACCTATGCCTTAAA

FOXA1upstreamF AACTCCCAGACAGCAGCATT

FOXA1upstreamR AGCCAGTCACTTTCCCTGAG

FOXA1promoterF CAGCTCTGGGCTTCCTCTT

FOXA1promoterR TTTCATGCCCTTCCATCTTC

GATA3upstreamF GTGACGTCAAACCCAGTGTG

GATA3upstreamR CTTGGTCTGGGTGGTTTGAG

GATA3promoterF CGCGGATGACACTAGAACCT

GATA3promoterR TACCAACCTGGGTAGCGAAG

miR203controlF CAGGGCCTGGCTGGCTCCAC

miR203controlR CCCACCCAGCGGTTCCCACA

miR203promoterF CAGAACCCGGGAGGCCAGGTG

miR203promoterR GGCCCATAGGTCCCGCGACTG

miR200ccontrolF ACAAGGGGTGGTTCTTGTTG

miR200ccontrolR CGCTCTTCCTCCTTCCTTCT

miR200cpromoterF GGGAAGGGGTTAAGGCAGT

miR200cpromoterR CGCTCTTCCTCCTTCCTTCT

ABCB1upstreamF AAACTTGGCTAAAATGACATAAAAGAA

ABCB1upstreamR GGGCAGAGATGTAAACTGACAA

ABCB1promoterF GCTTTGGAGCCATAGTCATGT

ABCB1promoterR TCCCAGTACCAGAGGAGGAG

ABCC2upstreamF GCTTGCTGAGGAAAAGTTGG

ABCC2upstreamR CAAACAAAAGCCCTACAACCA

ABCC2promoterF TTCTGCGCCAGTAAATTGTG

ABCC2promoterR GCAAGTAAGGAGCCAGGAAG

**References**

1. ChemNavigator iResearch Library, version 2012 Q2; Sigma-Aldrich Co. LLC: St. Louis, MO, 2012.

2. Pipeline Pilot, version 5.0; SciTegic, Inc.: San Diego, CA, 2005.

3. Kumar V, Carlson JE, Ohgi KA, Edwards TA, Rose DW, *et al*. Transcription corepressor CtBP is an NAD(+)-regulated dehydrogenase. *Mol Cell* 2002; 10: 857–869.

4. Lipinski CA, Lombardo F, Dominy BW, Feeney PJ. Experimental and computational approaches to estimate solubility and permeability in drug discovery and development settings. *Adv Drug Deliv Rev* 2001; 46: 3–26.

5. QikProp, version 3.4; Schrödinger LLC.: New York, NY, 2012.

6. Achouri Y, Noël G, Van Schaftingen E. 2-Keto-4-methylthiobutyrate, an intermediate in the methionine salvage pathway, is a good substrate for CtBP1. *Biochem Biophys Res Commun* 2007; 352: 903–906.

7. Maestro, version 9.2; Schrödinger LLC.: New York, NY, 2012.

8. LigPrep, version 2.5; Schrödinger LLC.: New York, NY, 2012.

9. Filimonov DA, Zakharov AV, Lagunin AA, Poroikov VV. QNA-based “Star Track” QSAR approach. *SAR QSAR Environ Res* 2009; 20: 679–709.

10. Friesner RA, Banks JL, Murphy RB, Halgren TA, Klicic JJ, Mainz DT *et al*. Glide: A new approach for rapid, accurate docking and scoring. 1. Method and assessment of docking accuracy. *J Med Chem* 2004; 47: 1739–1749.

11. Glide, version 5.7; Schrödinger LLC.: New York, NY, 2012.

12. Dengler U, Niefind K, Kiess M, Schomburg D. Crystal structure of a ternary complex of D-2-hydroxyisocaproate dehydrogenase from Lactobacillus casei, NAD+ and 2-oxoisocaproate at 1.9 A resolution. *J Mol Biol* 1997; 267: 640–660.

13. Berman HM, Westbrook J, Feng Z, Gilliland G, Bhat TN, *et al*. The Protein Data Bank. *Nucl Acids Res* 2000; 28: 235–242.

14. Krissinel E, Henrick K (2007) Inference of macromolecular assemblies from crystalline state. *J Mol Biol* 2007; 372: 774–797.
