# Supplementary material for: Epigenetic re-wiring of breast cancer by pharmacological targeting of C-terminal binding protein
Source: Cell Death Dis. 2019 Sep 18;10(10):689. doi: 10.1038/s41419-019-1892-7 (PMC6751206; doi:10.1038/s41419-019-1892-7)
Supplement: Supplementary file 3 — Supplementary Table 2. [file 41419_2019_1892_MOESM3_ESM.pdf]

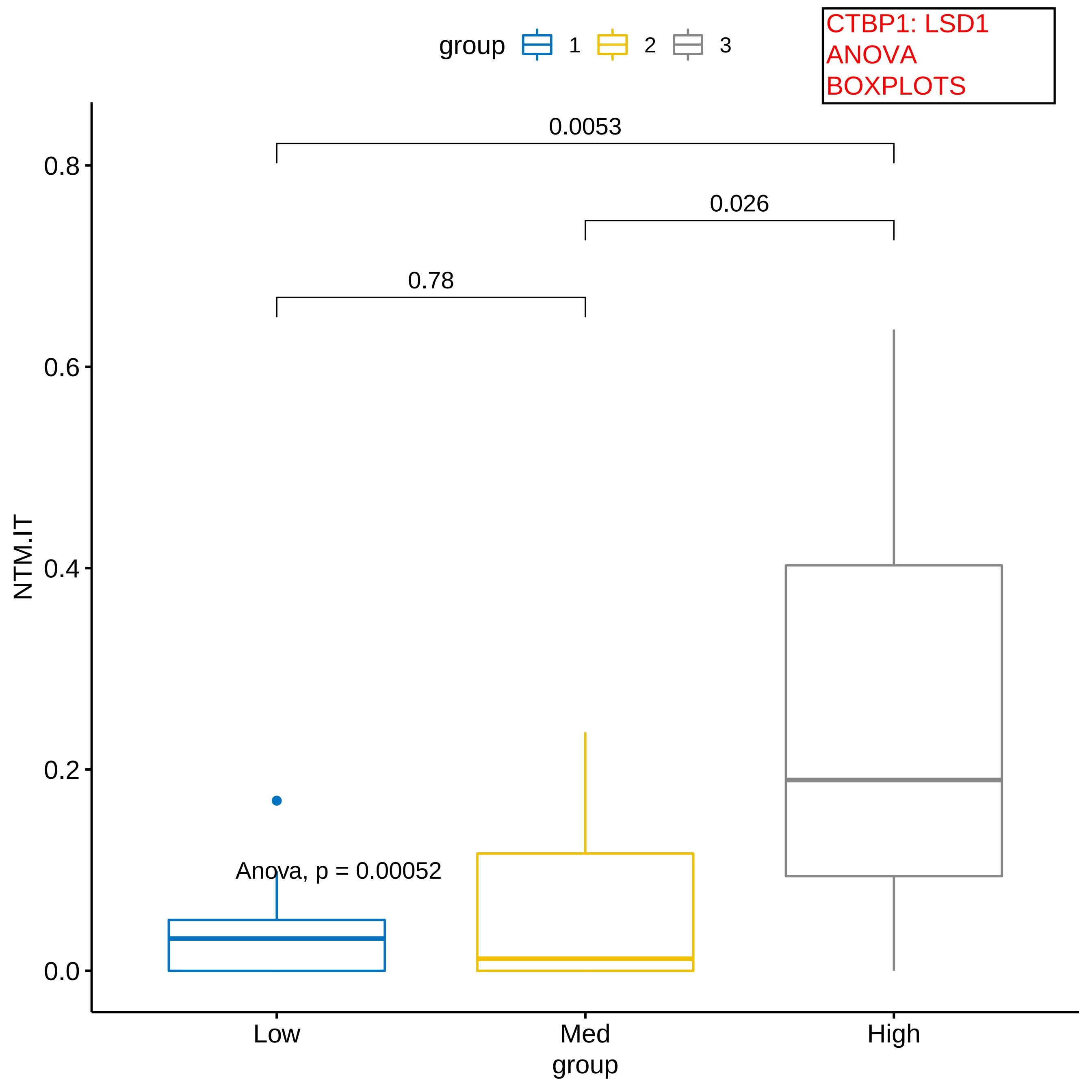

CTBP1: LSD1
ANOVA
BOXPLOTS
group 1 2 3
0.0053
0.026
0.78
Anova, p = 0.00052
NTM.IT
0.0
0.2
0.4
0.6
0.8
Low
Med
High
group

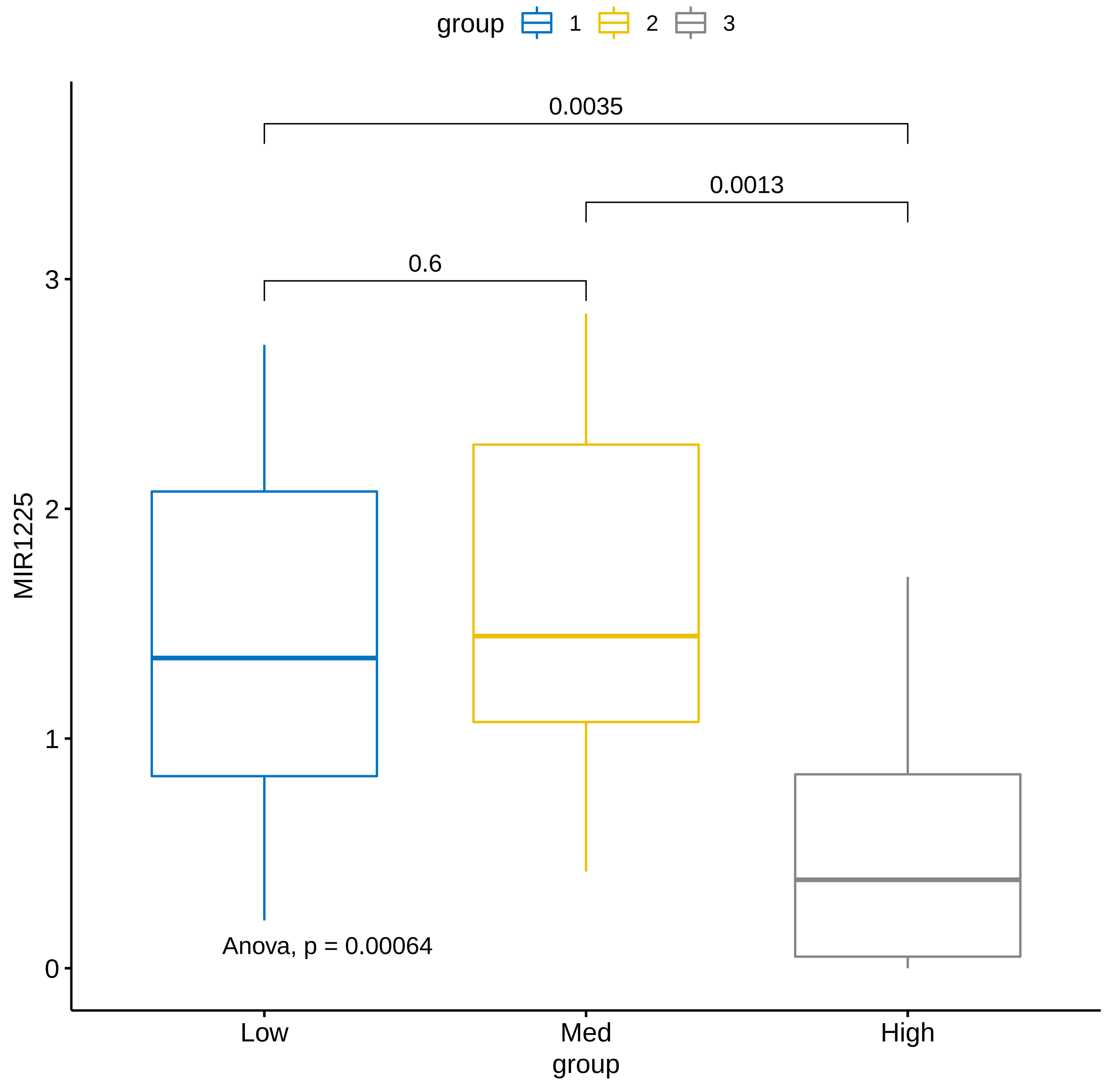
group
1
2
3
0.0035
0.0013
0.6
3
2
1
0
MIR1225
Anova, p = 0.00064
Low
Med
High
group

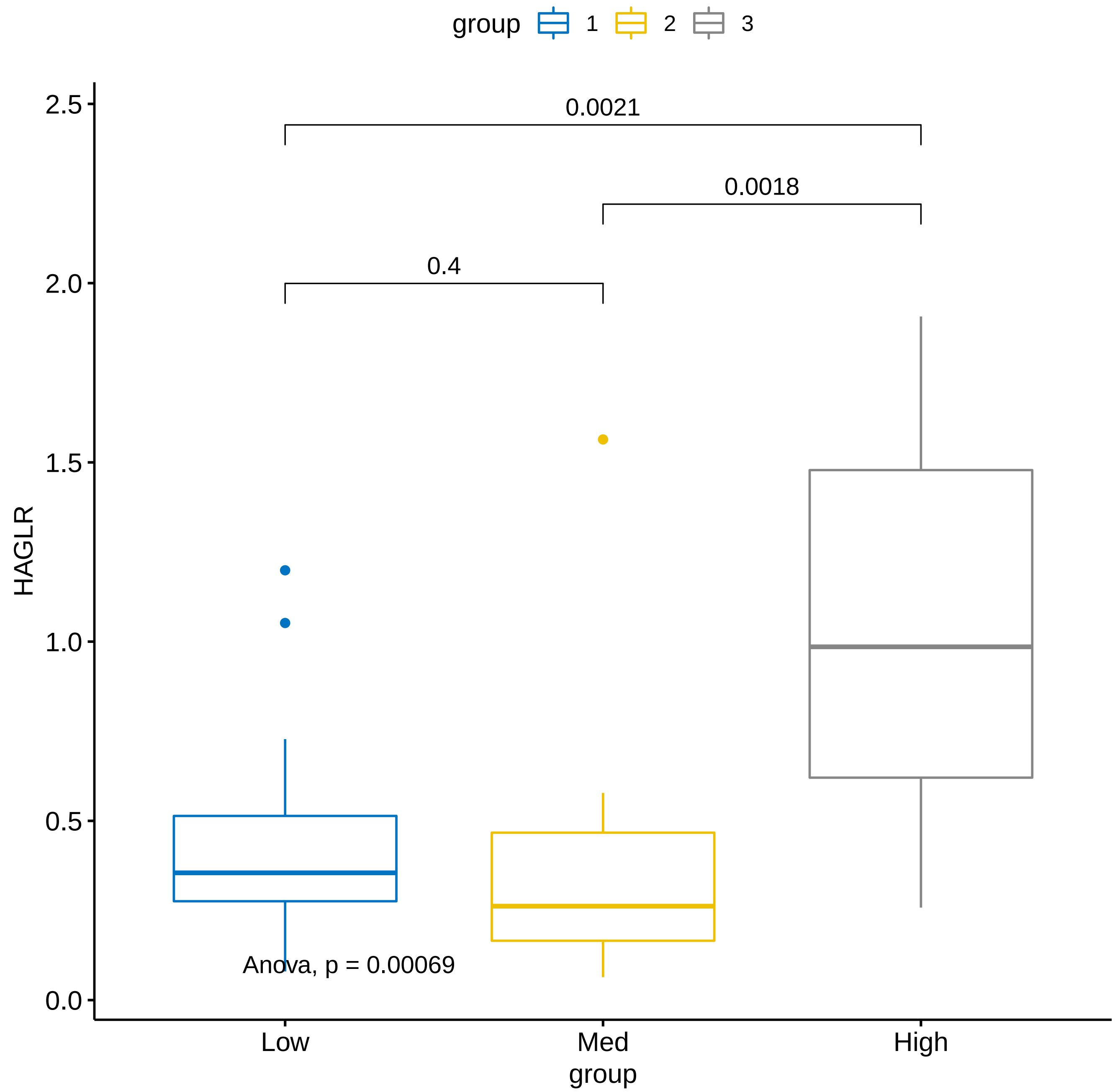

group 1 2 3
0.0021
0.0018
0.4
2.5
2.0
1.5
1.0
0.5
0.0
HAGLR
Anova, p = 0.00069
Low
Med
High
group

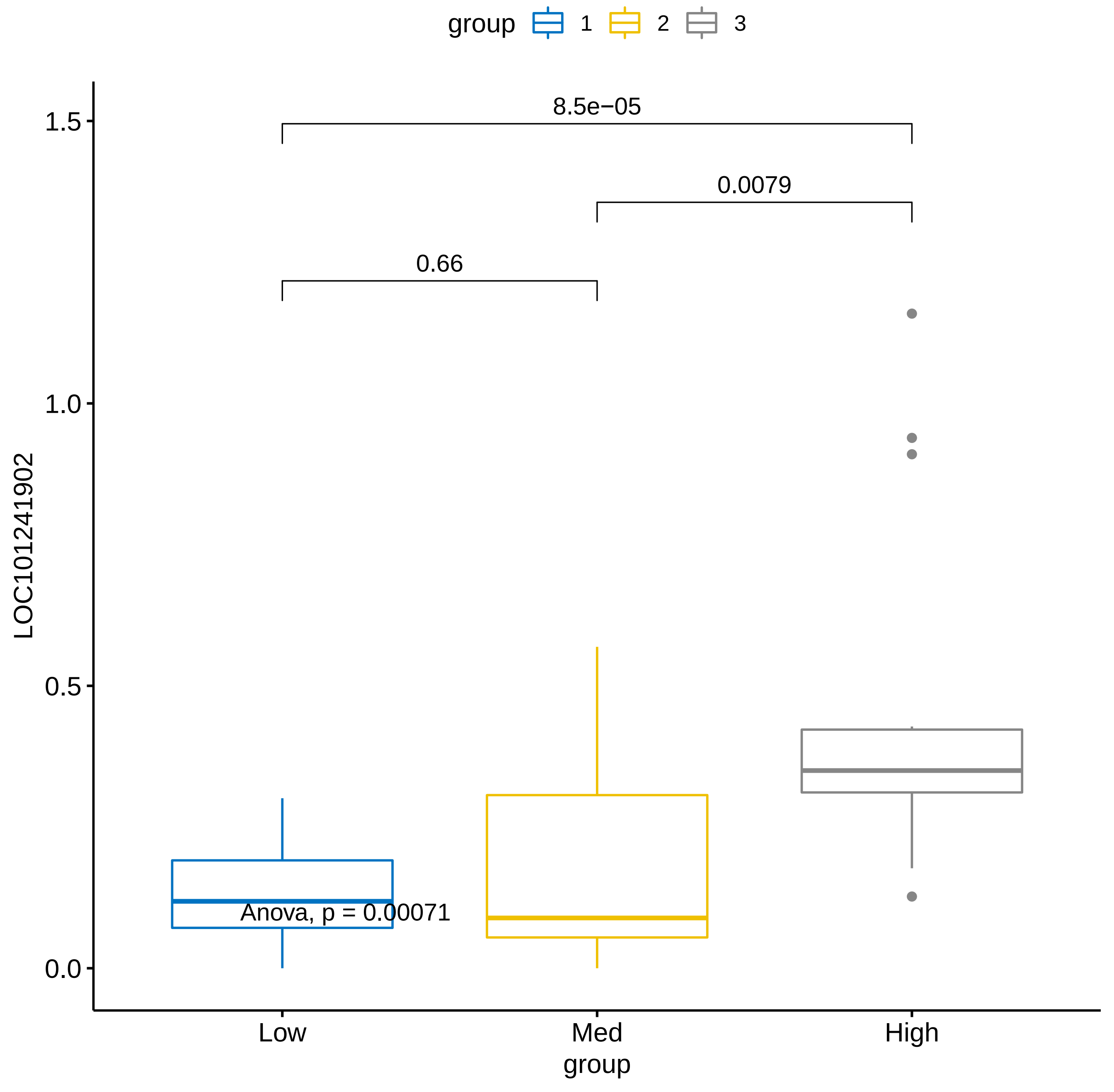
group
1
2
3
8.5e−05
0.0079
0.66
1.5
1.0
0.5
0.0
LOC101241902
Anova, p = 0.00071
Low
Med
High
group

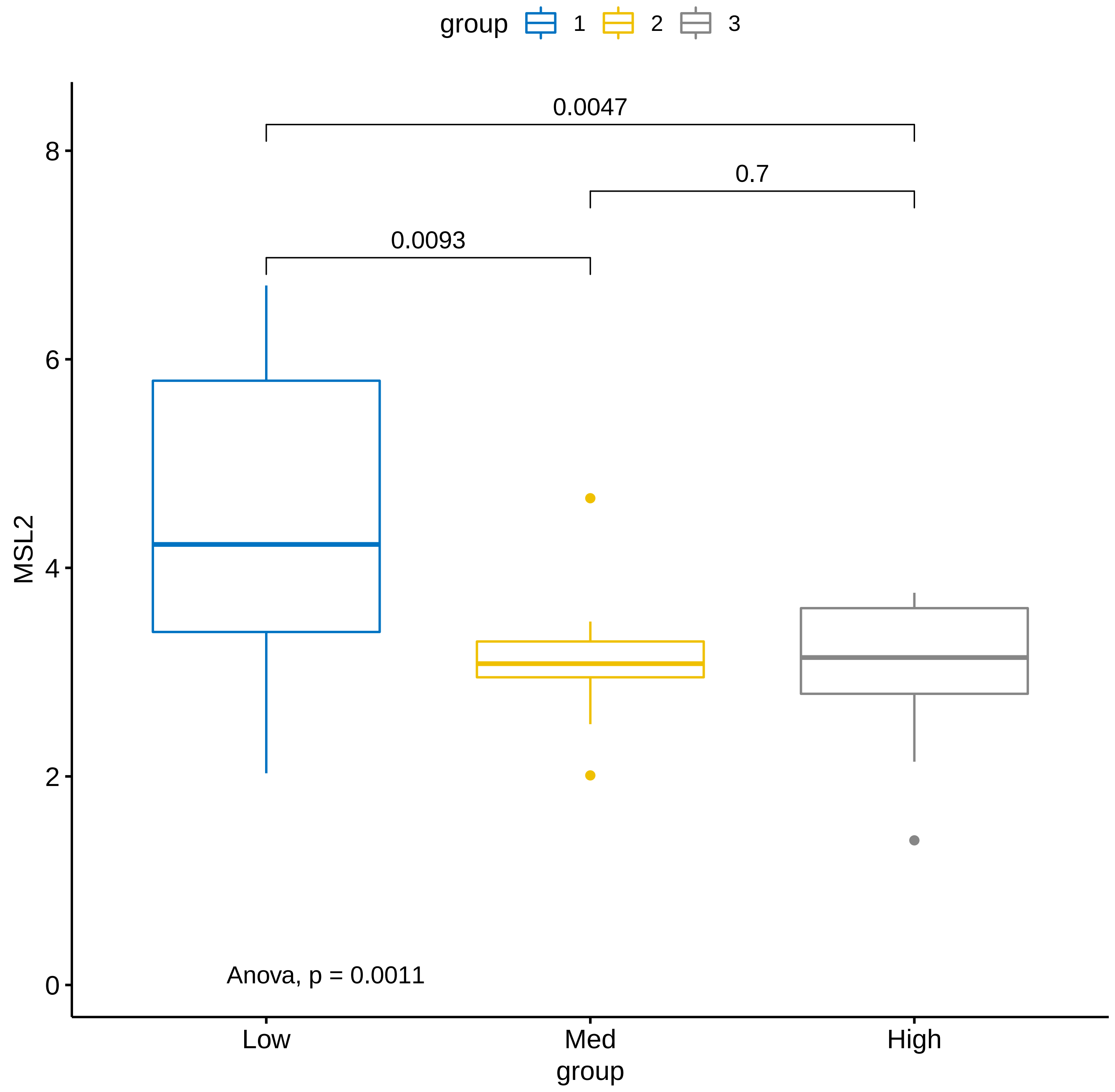

group
1
2
3
0.0047
0.7
0.0093
MSL2
8
6
4
2
0
Anova, p = 0.0011
Low
Med
High
group

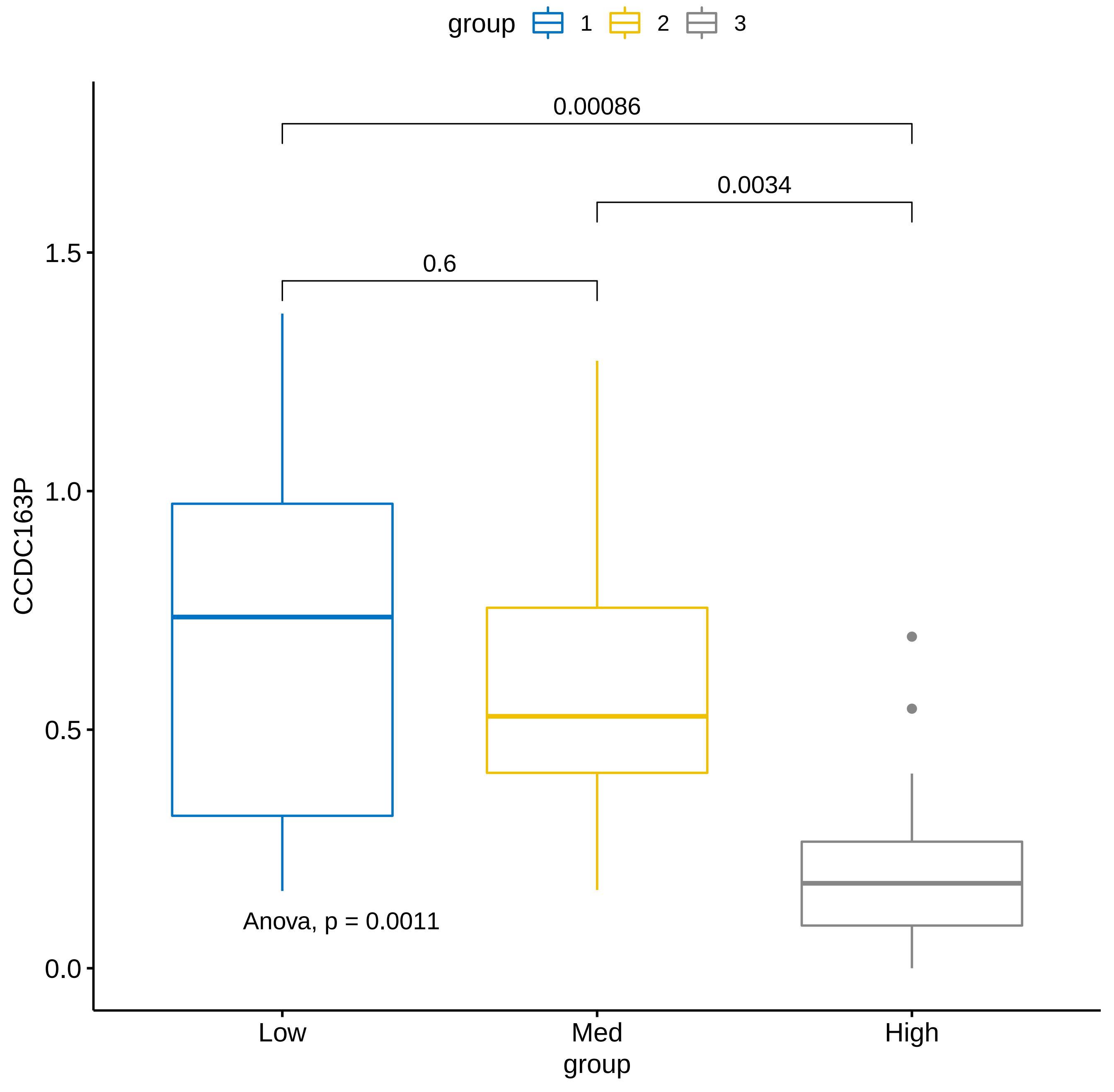

group
1
2
3
0.00086
0.0034
0.6
1.5
1.0
0.5
0.0
CCDC163P
Anova, p = 0.0011
Low
Med
High
group

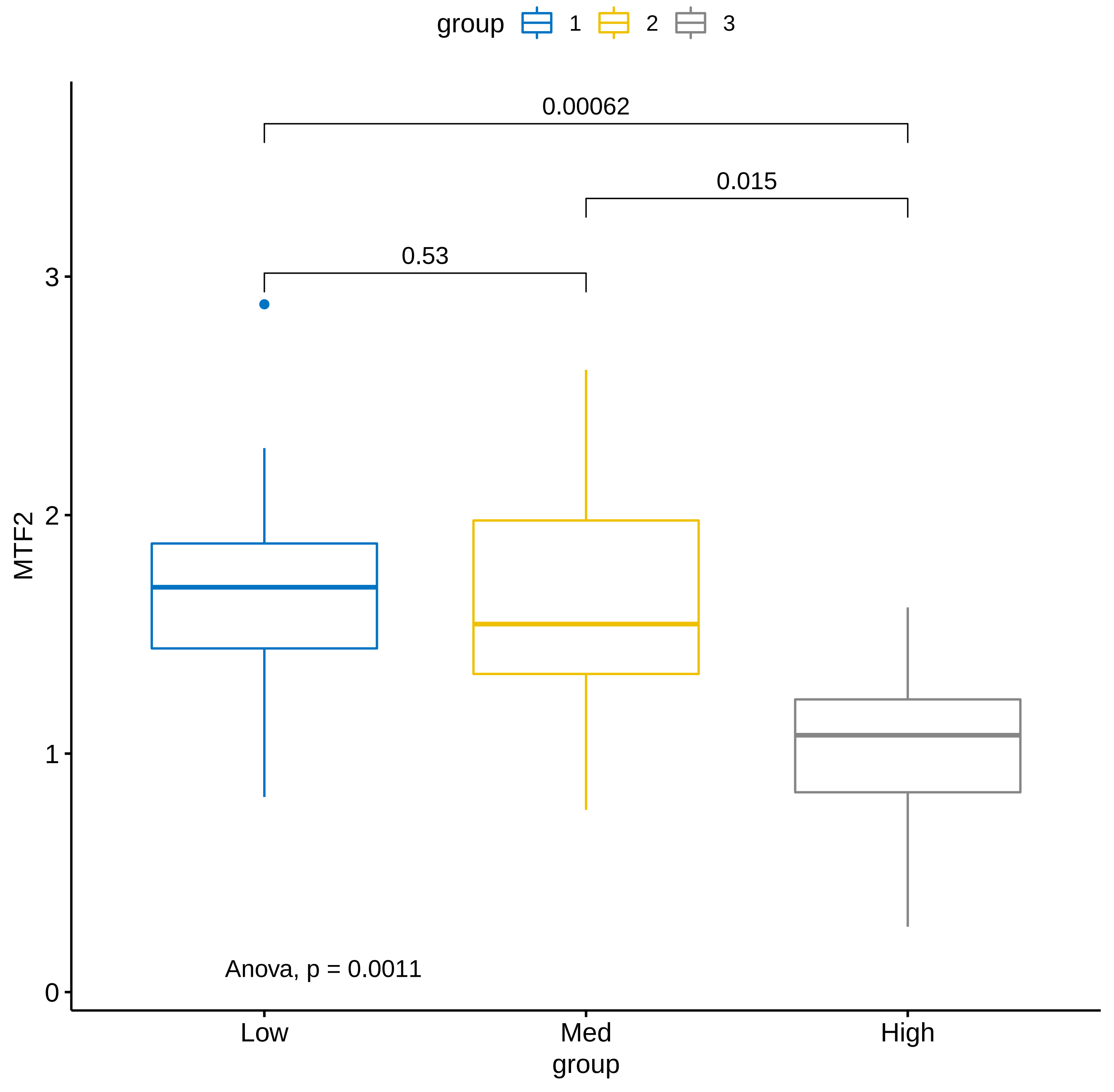

group 1 2 3
0.00062
0.015
0.53
3
2
1
0
MTF2
Anova, p = 0.0011
Low
Med
High
group

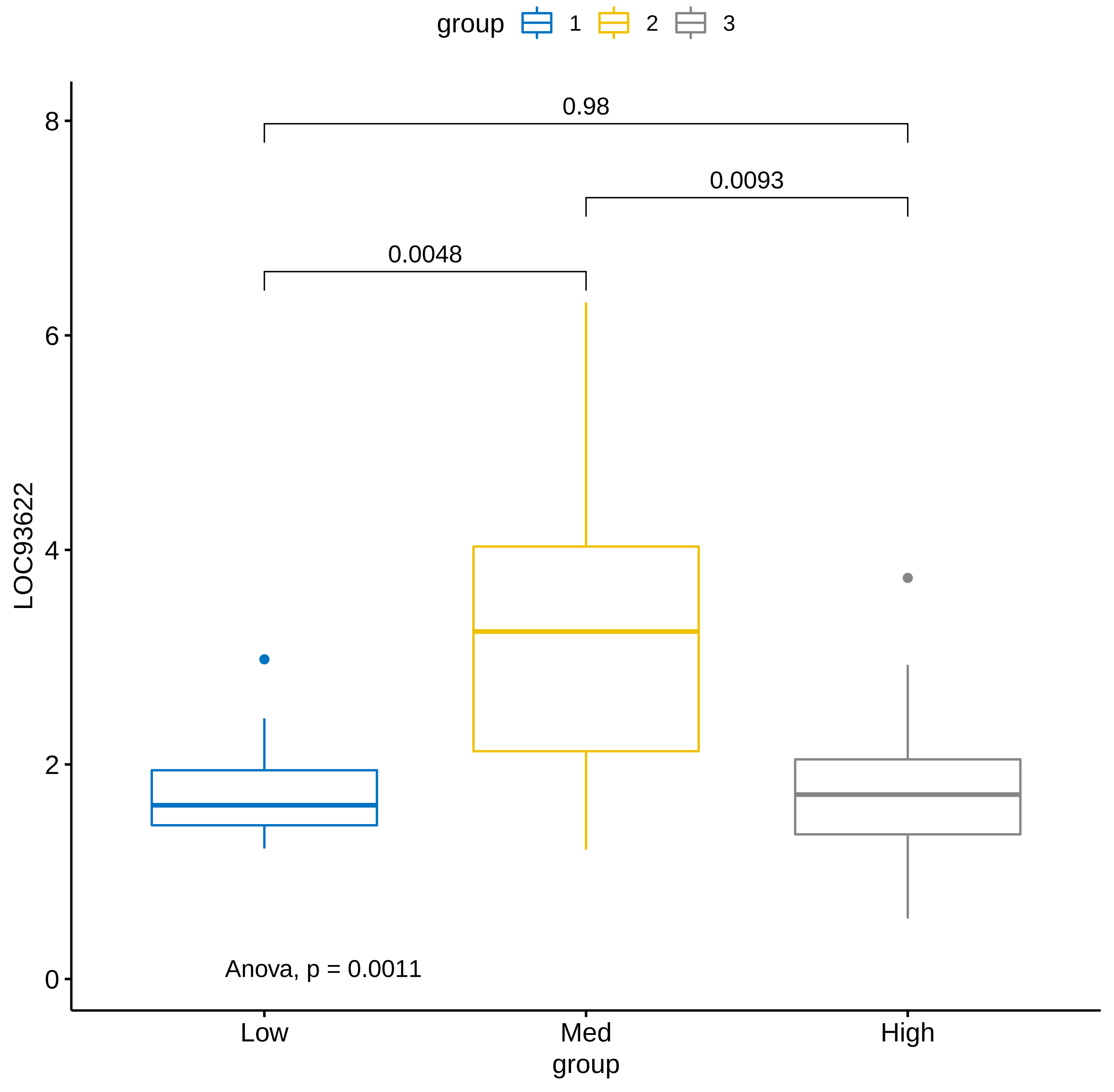
group
1
2
3
0.98
0.0093
0.0048
8
6
4
2
0
LOC93622
Anova, p = 0.0011
Low
Med
High
group

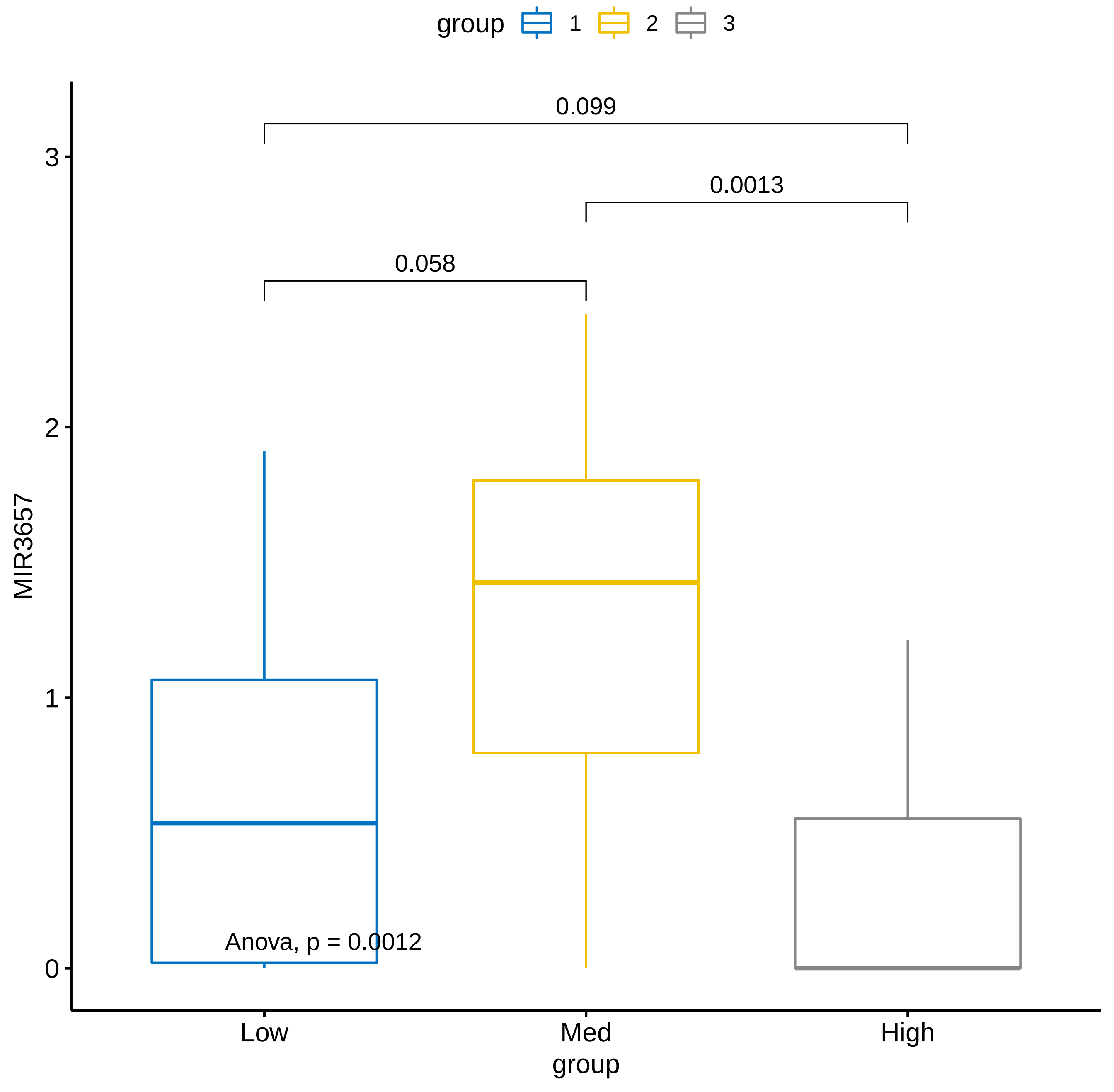
group
1
2
3
0.099
0.0013
0.058
Anova, p = 0.0012
MIR3657
0
1
2
3
Low
Med
High
group

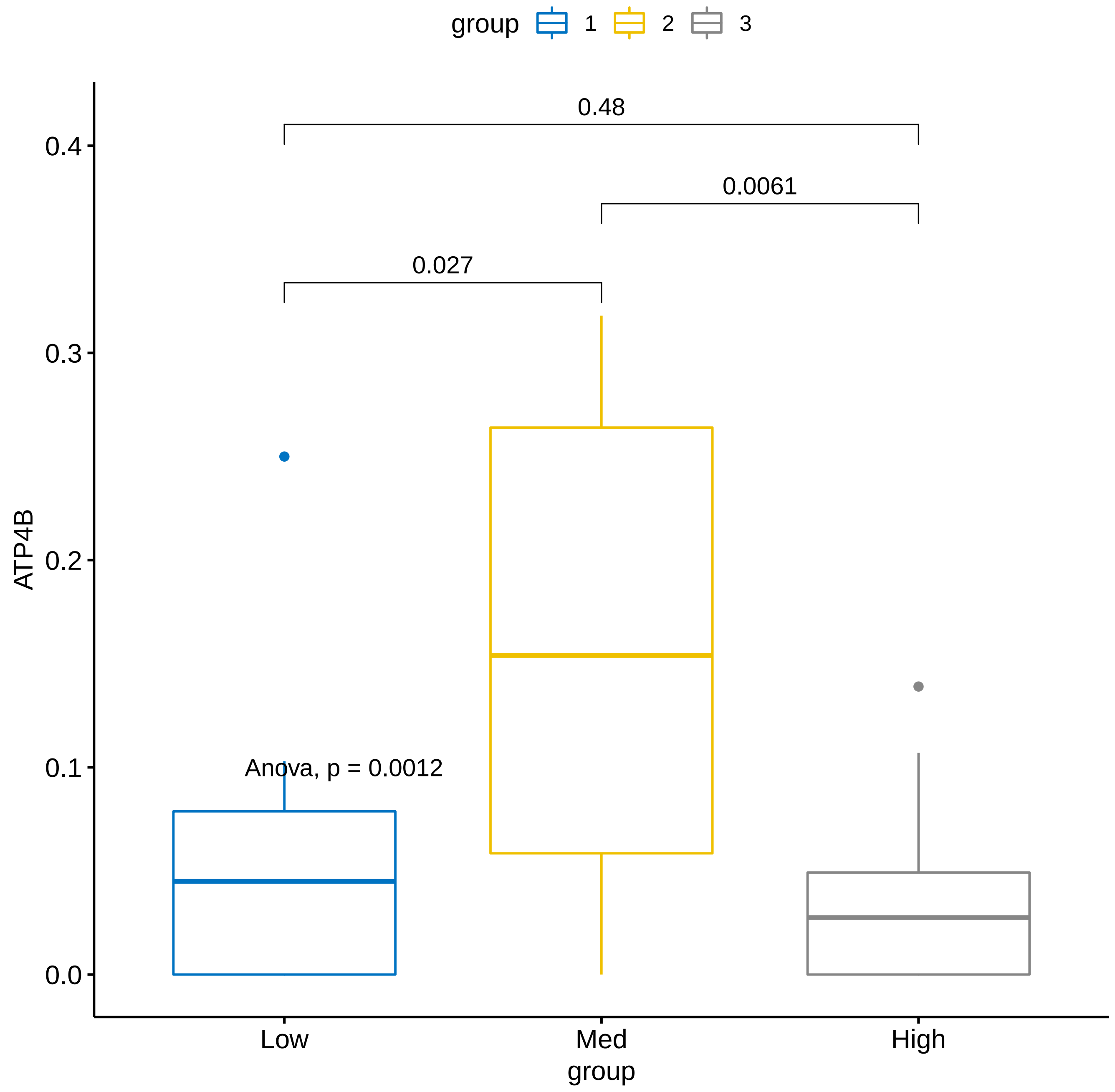
group
1
2
3
0.48
0.0061
0.027
Anova, p = 0.0012
0.4
0.3
0.2
0.1
0.0
ATP4B
Low
Med
High
group

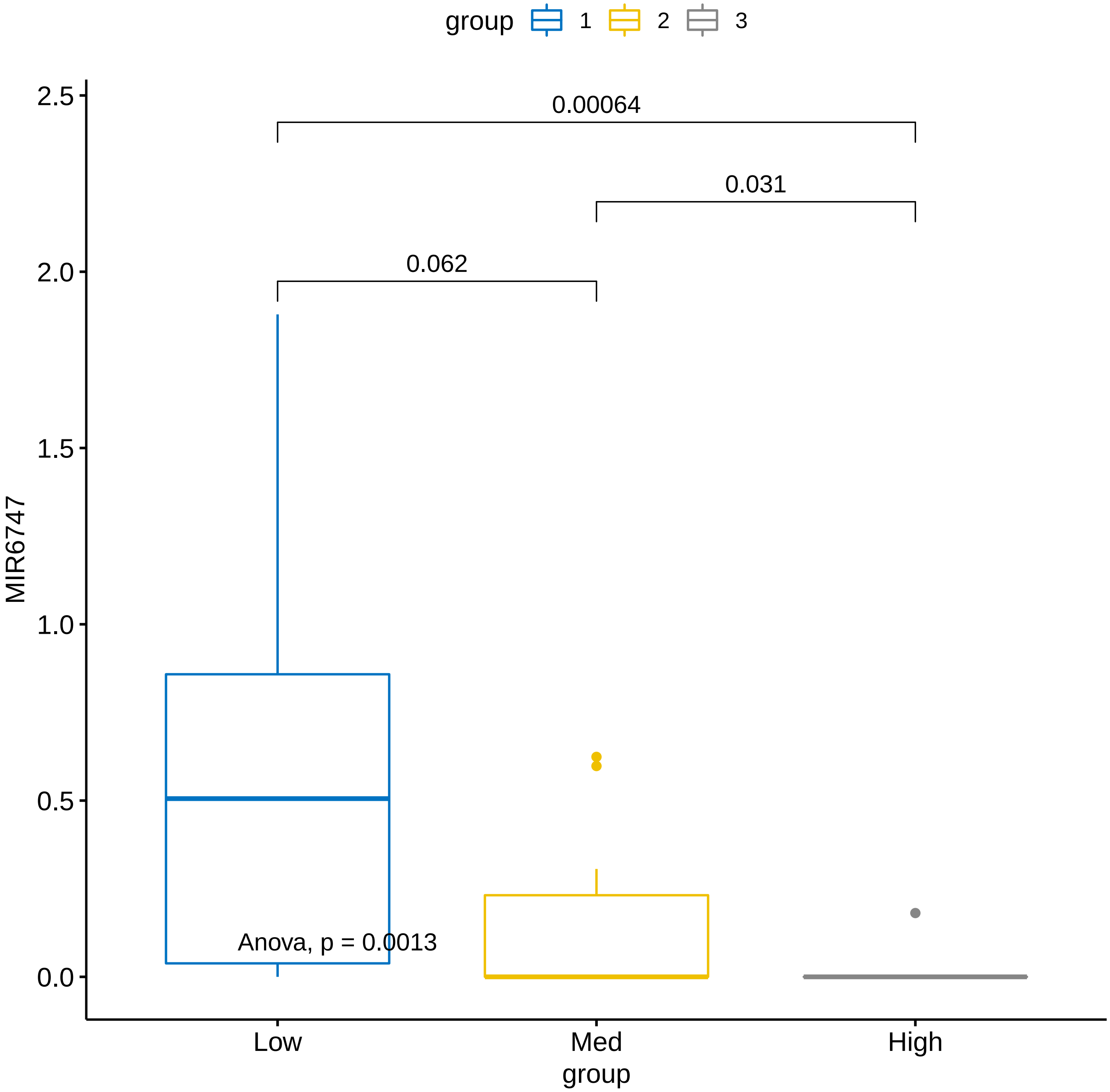
group
1
2
3
2.5
2.0
1.5
1.0
0.5
0.0
MIR6747
0.00064
0.031
0.062
Anova, p = 0.0013
Low
Med
High
group

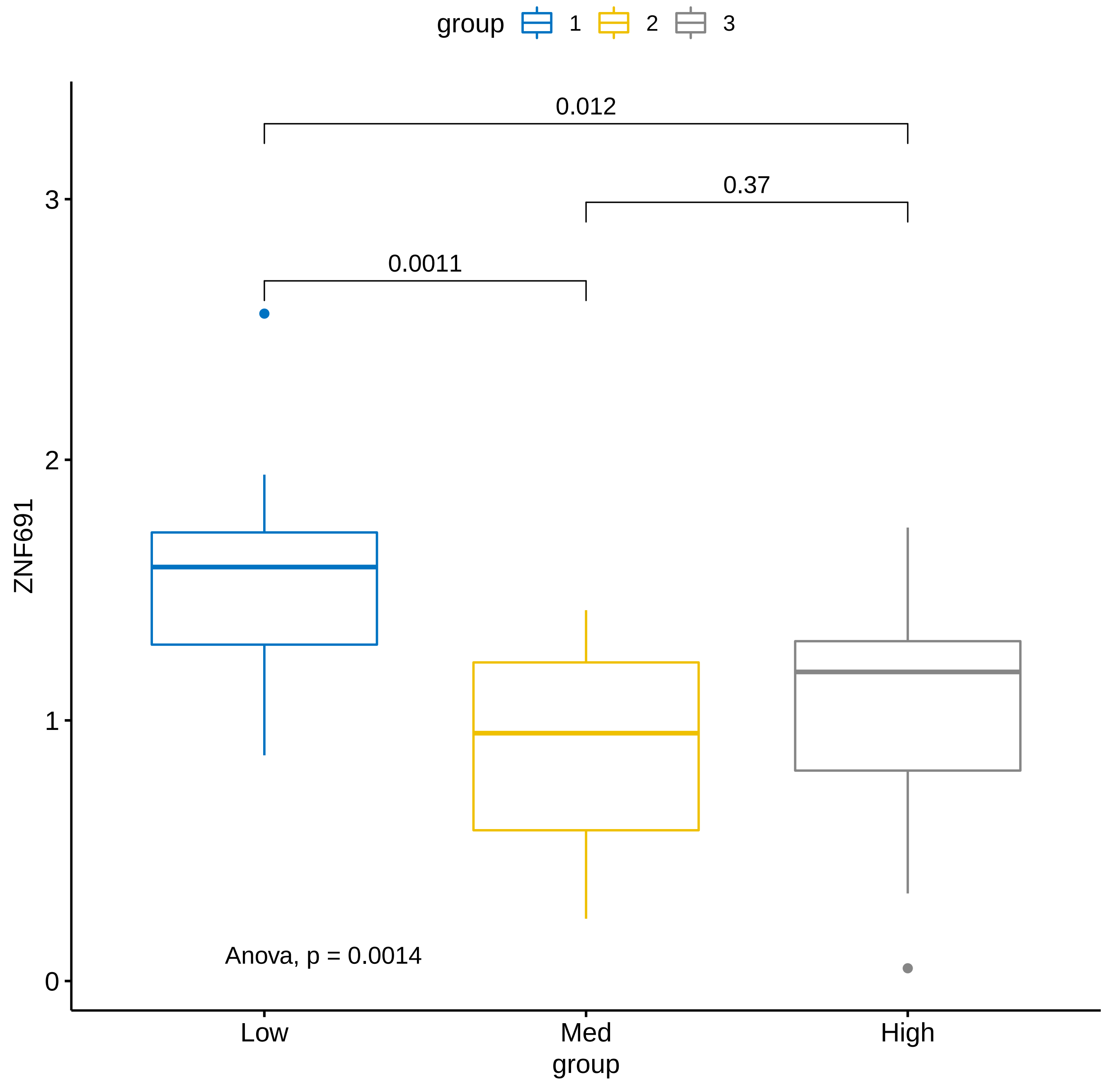

group
1
2
3
0.012
0.37
0.0011
3
2
1
0
ZNF691
Anova, p = 0.0014
Low
Med
High
group

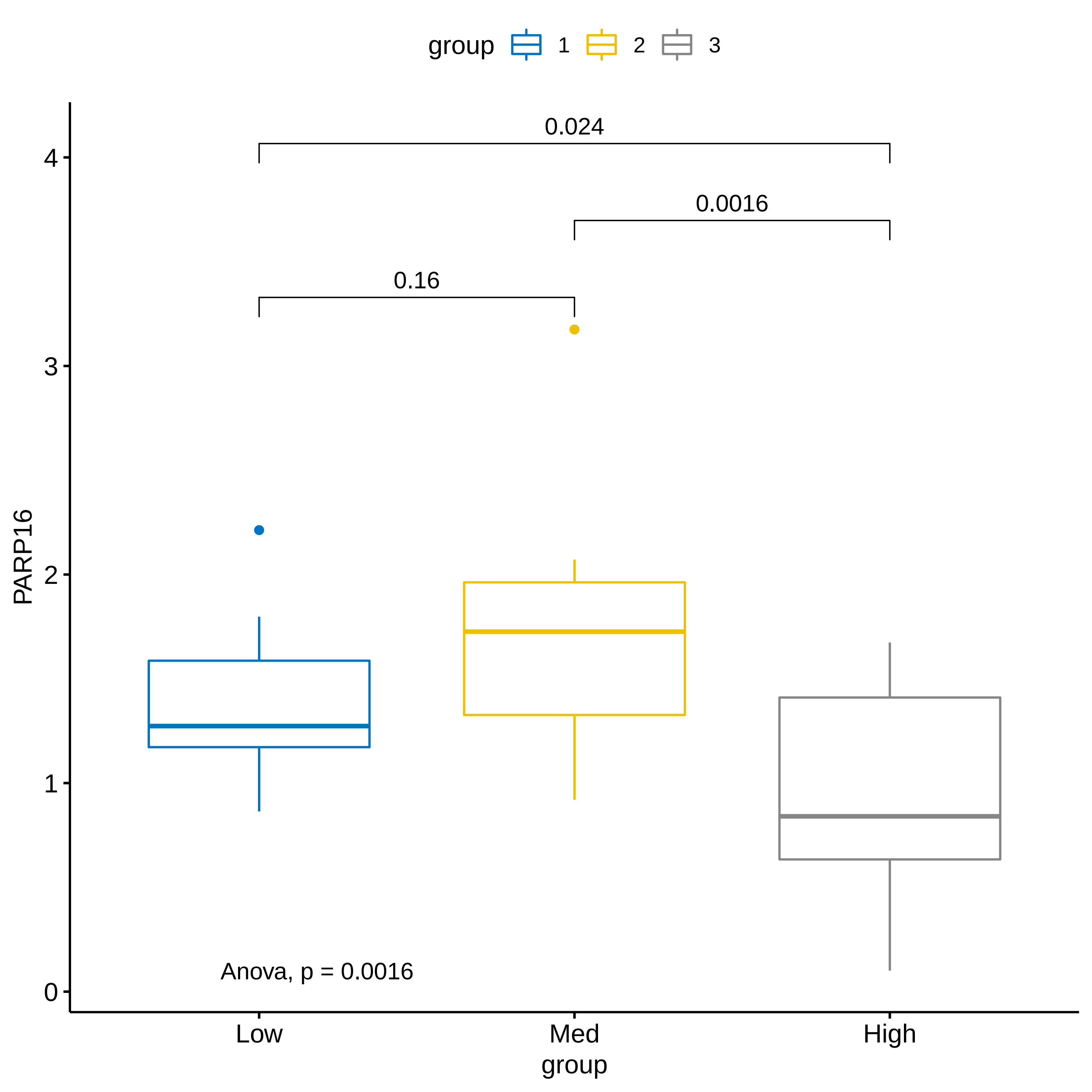

group 1 2 3
0.024
0.0016
0.16
PARP16
4
3
2
1
0
Anova, p = 0.0016
Low
Med
High
group

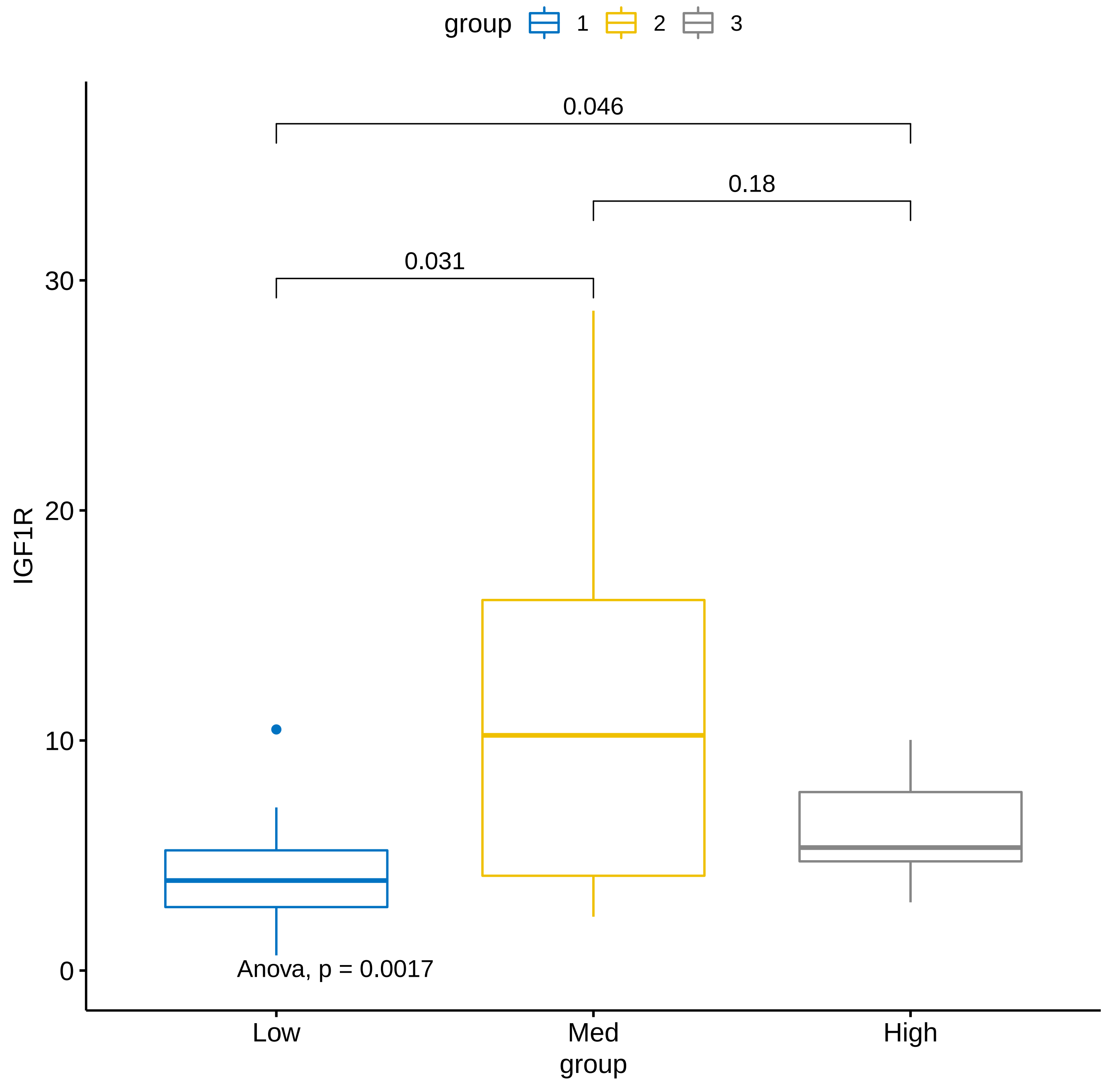

group 1 2 3
0.046
0.18
0.031
IGF1R
30
20
10
0
Anova, p = 0.0017
Low
Med
High
group

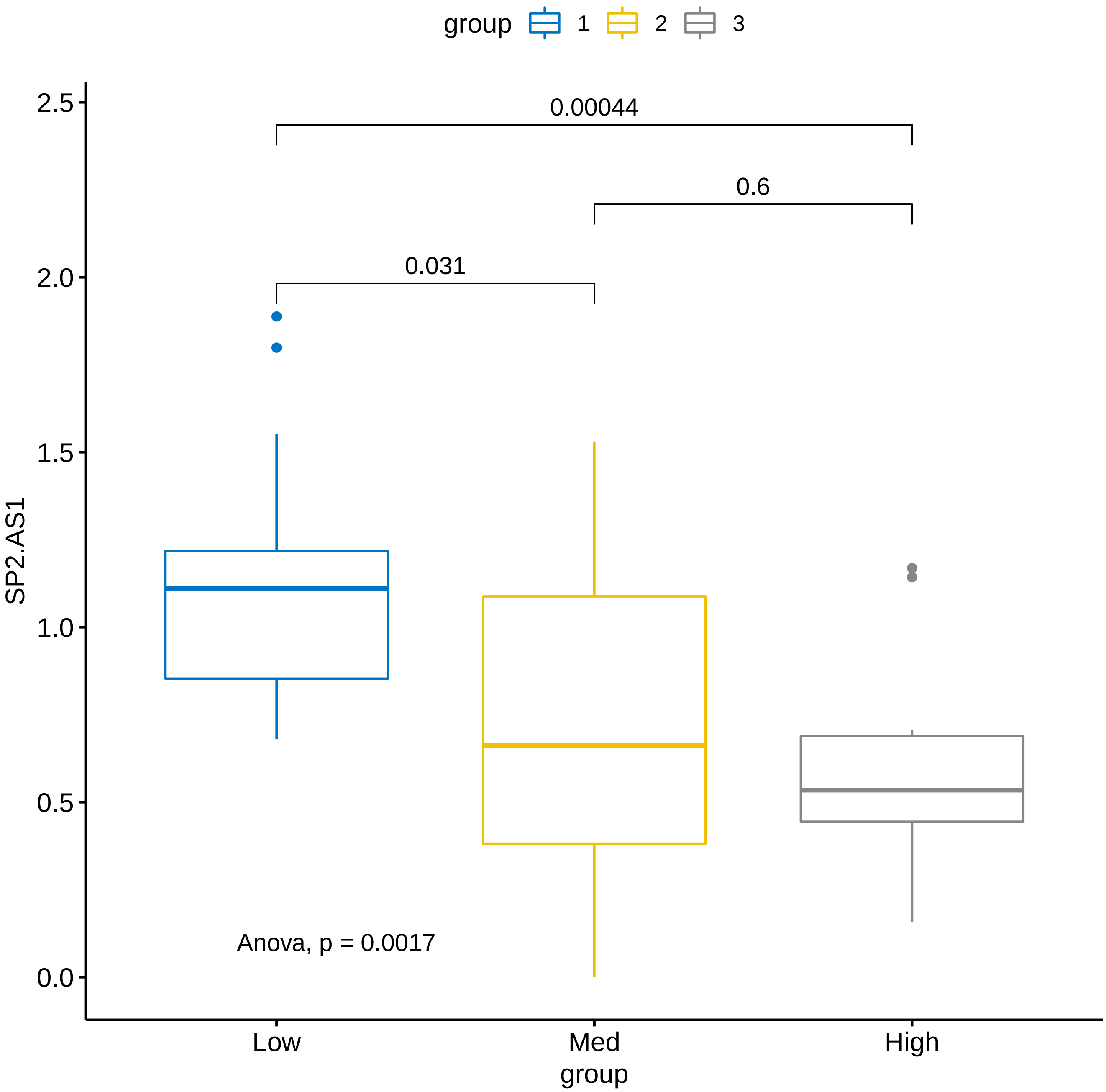
group
1
2
3
0.00044
0.6
0.031
2.5
2.0
1.5
1.0
0.5
0.0
SP2.AS1
Anova, p = 0.0017
Low
Med
High
group

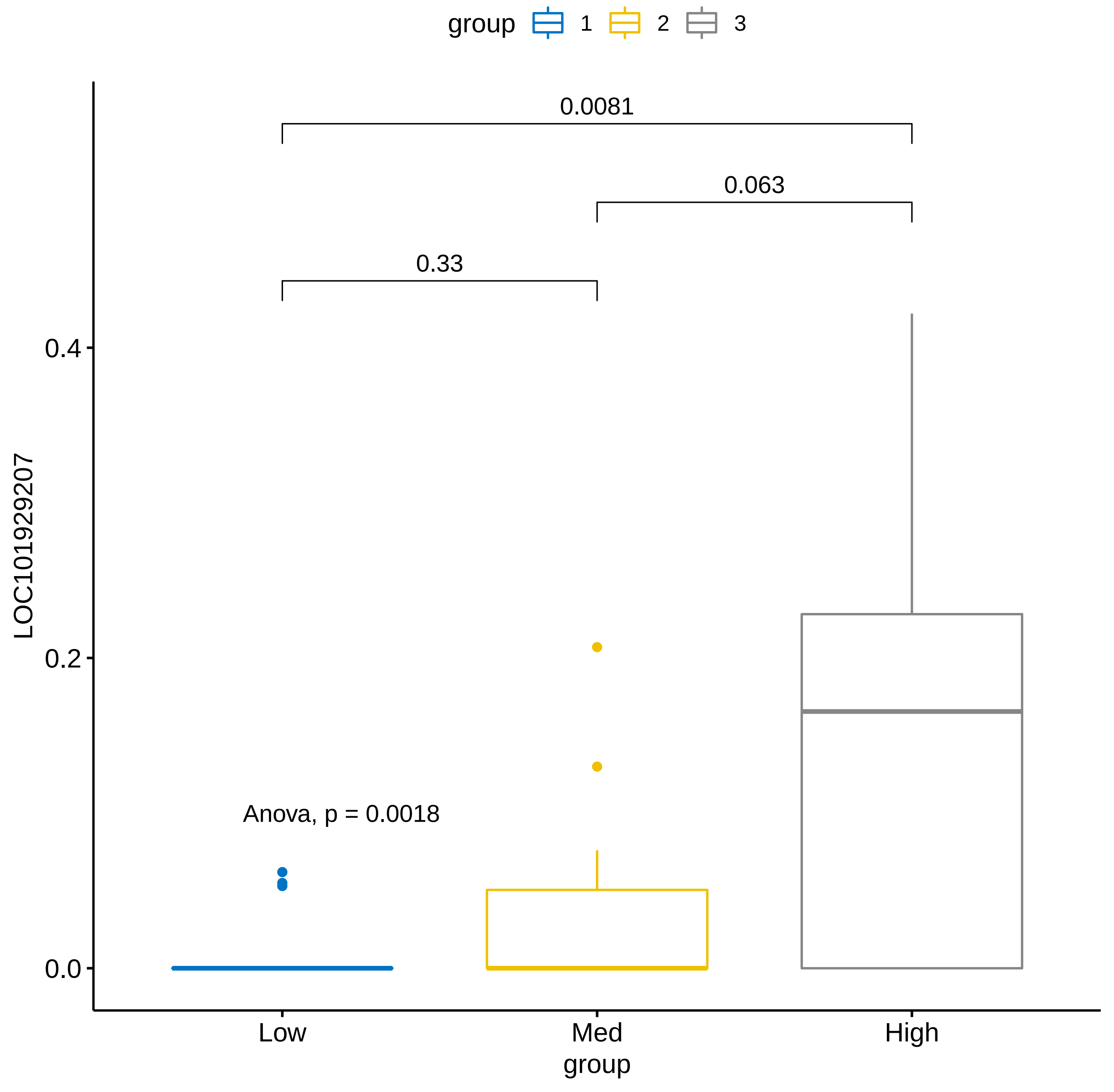

group 1 2 3
0.0081
0.063
0.33
Anova, p = 0.0018
LOC101929207
0.4
0.2
0.0
Low
Med
High
group

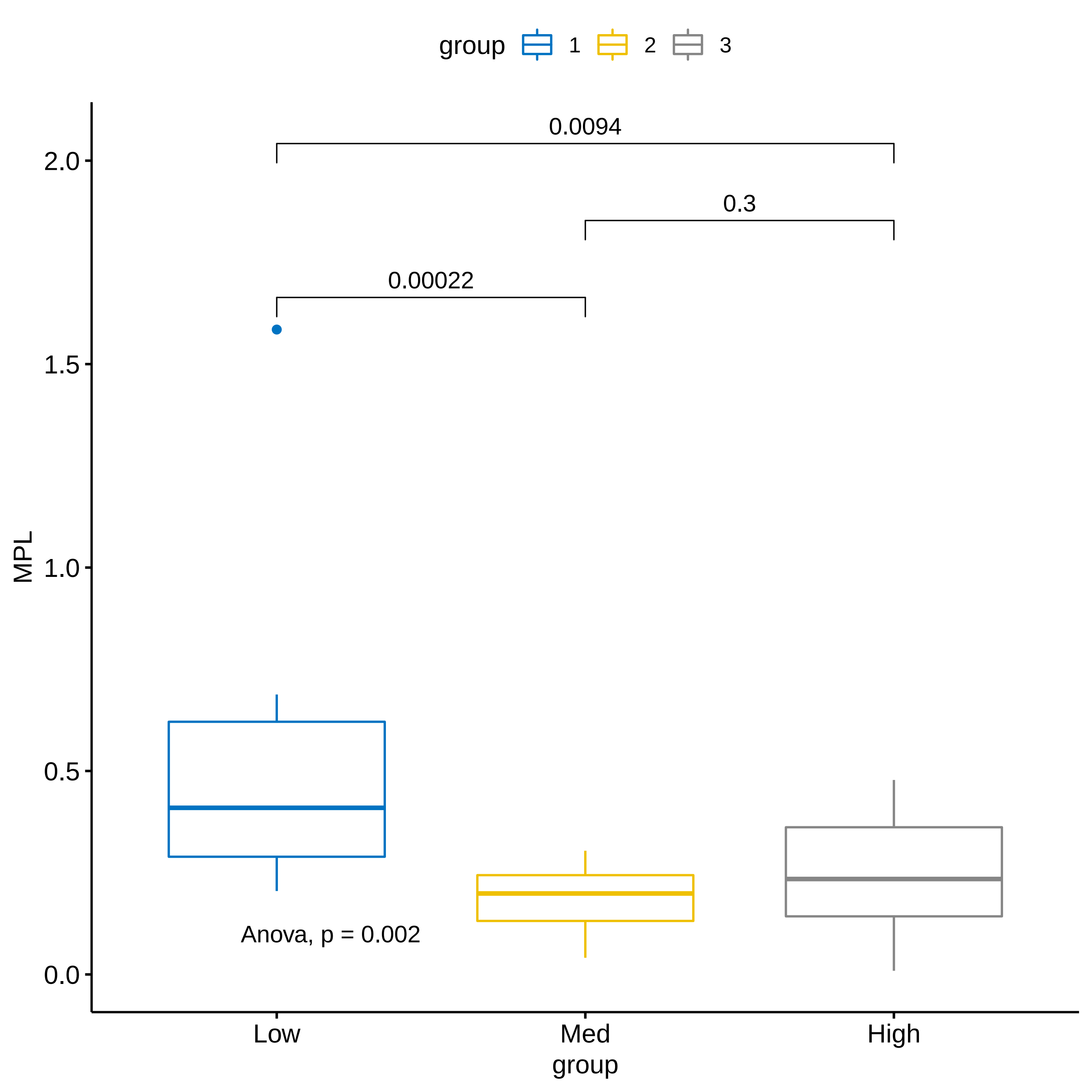
group
1
2
3
0.0094
0.3
0.00022
2.0
1.5
1.0
0.5
0.0
MPL
Anova, p = 0.002
Low
Med
High
group

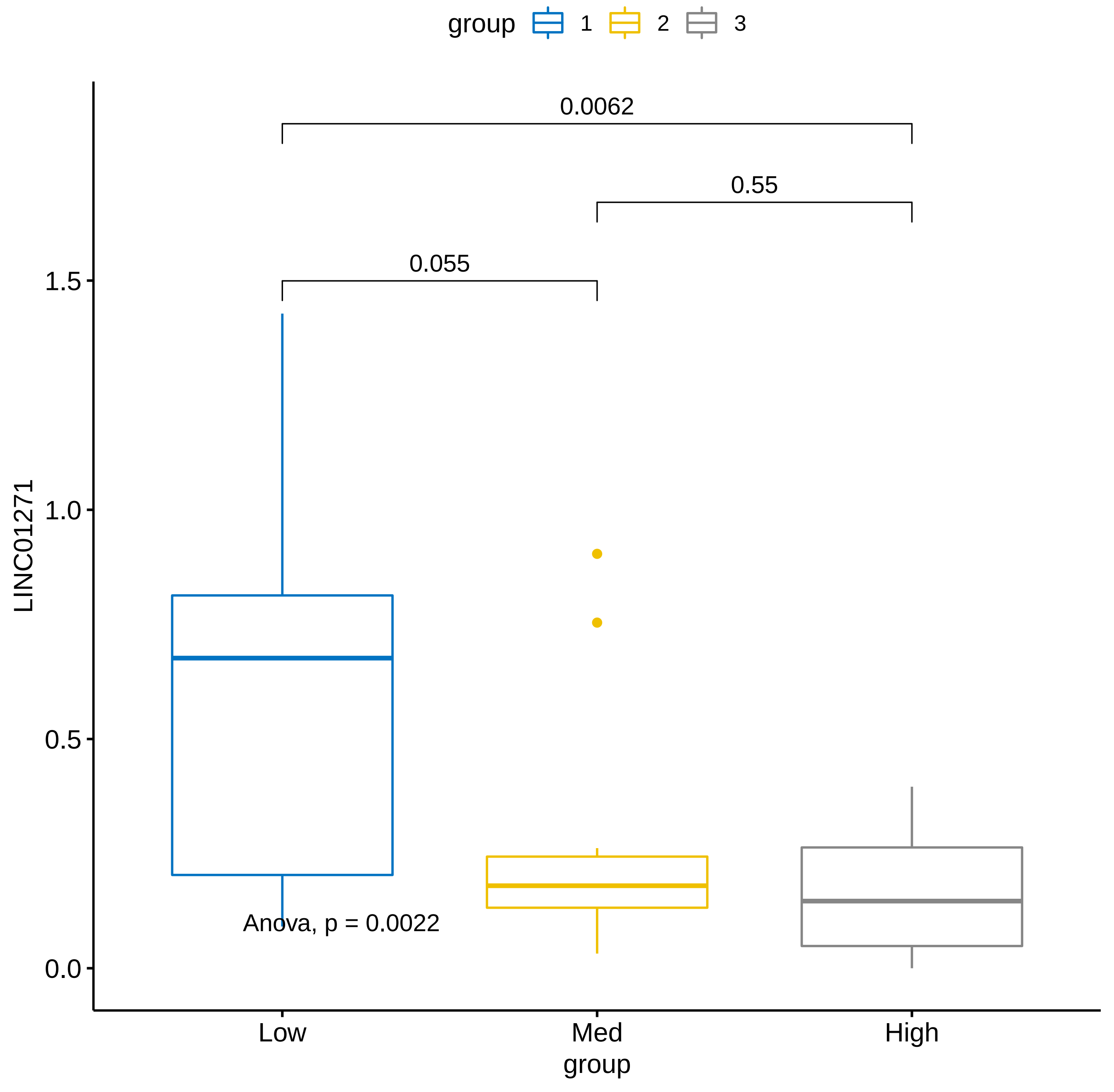
group
1
2
3
0.0062
0.55
0.055
1.5
1.0
0.5
0.0
LINC01271
Anova, p = 0.0022
Low
Med
High
group

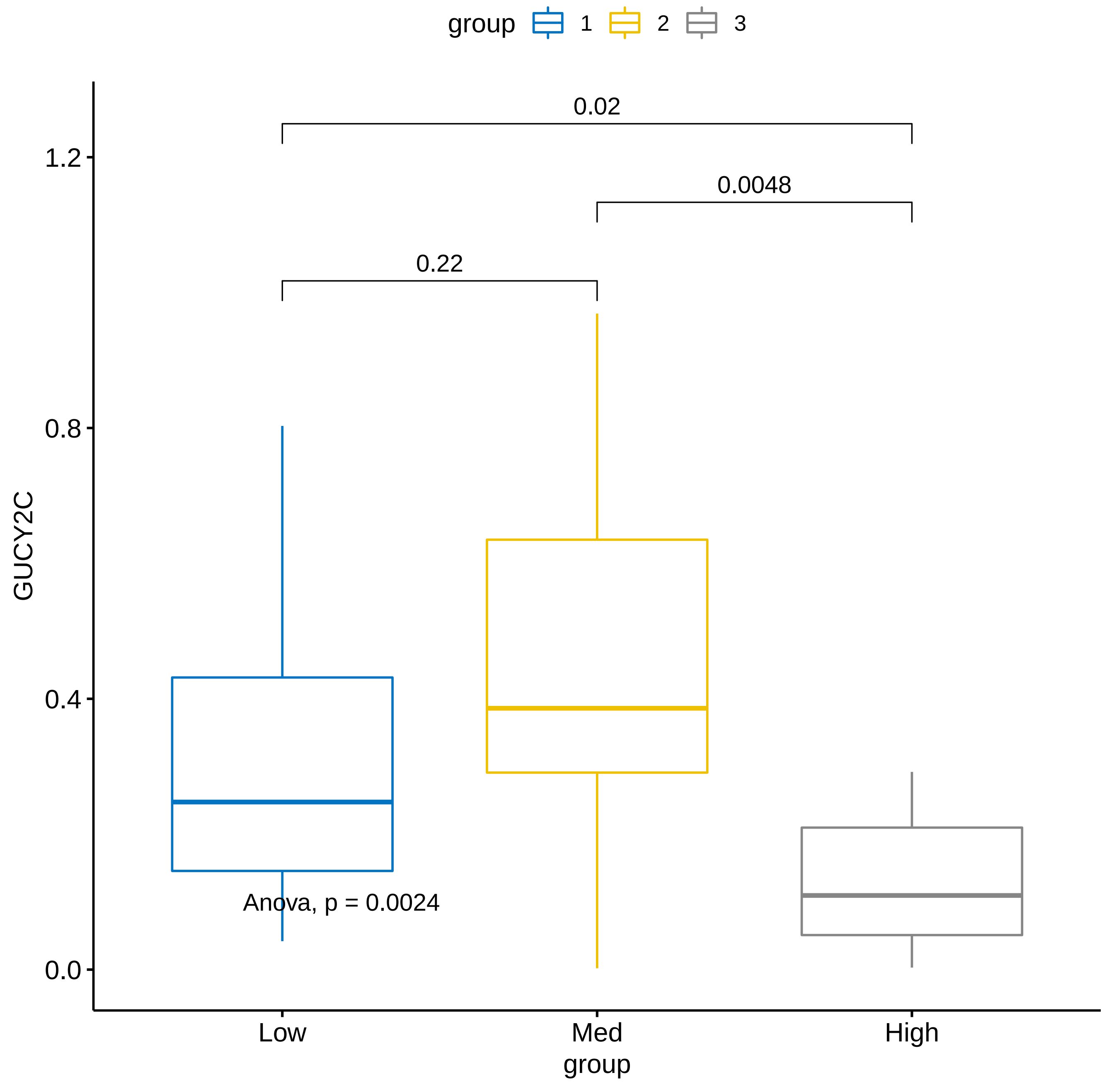

group
1
2
3
0.02
0.0048
0.22
1.2
0.8
0.4
0.0
GUCY2C
Anova, p = 0.0024
Low
Med
High
group

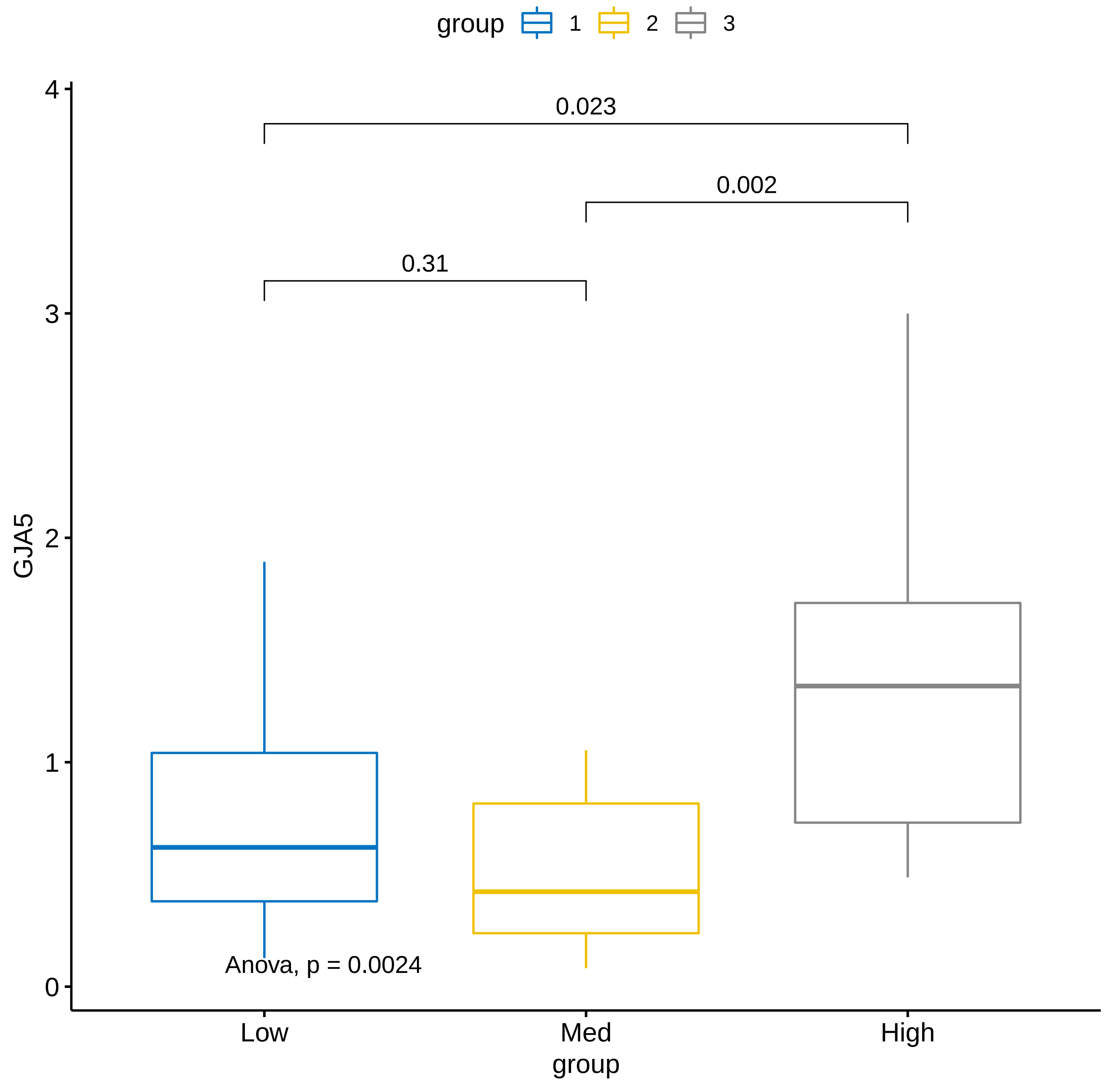

group 1 2 3
0.023
0.002
0.31
GJA5
4
3
2
1
0
Anova, p = 0.0024
Low
Med
High
group

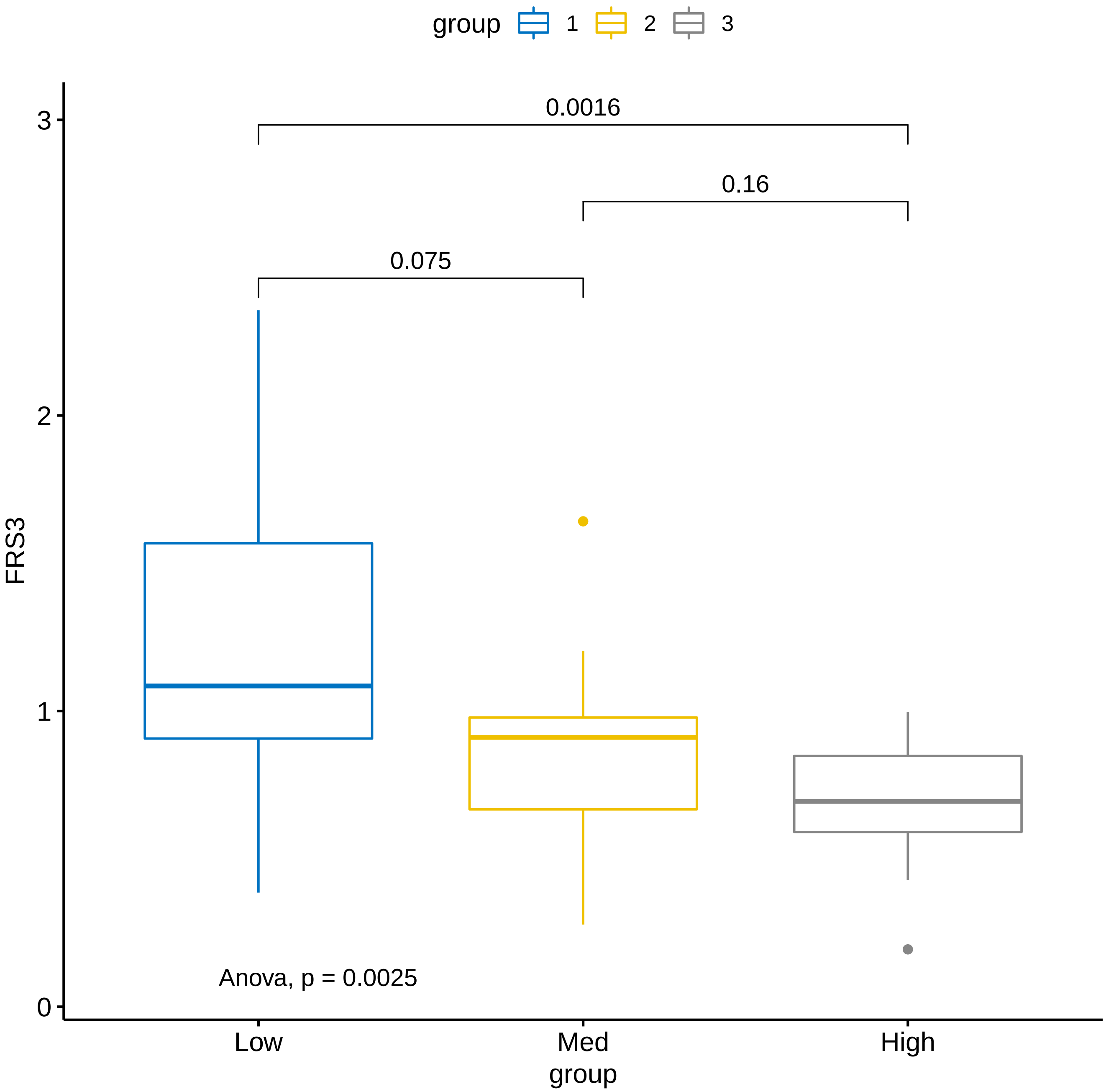

group 1 2 3
0.0016
0.16
0.075
FRS3
3
2
1
0
Anova, p = 0.0025
Low
Med
High
group

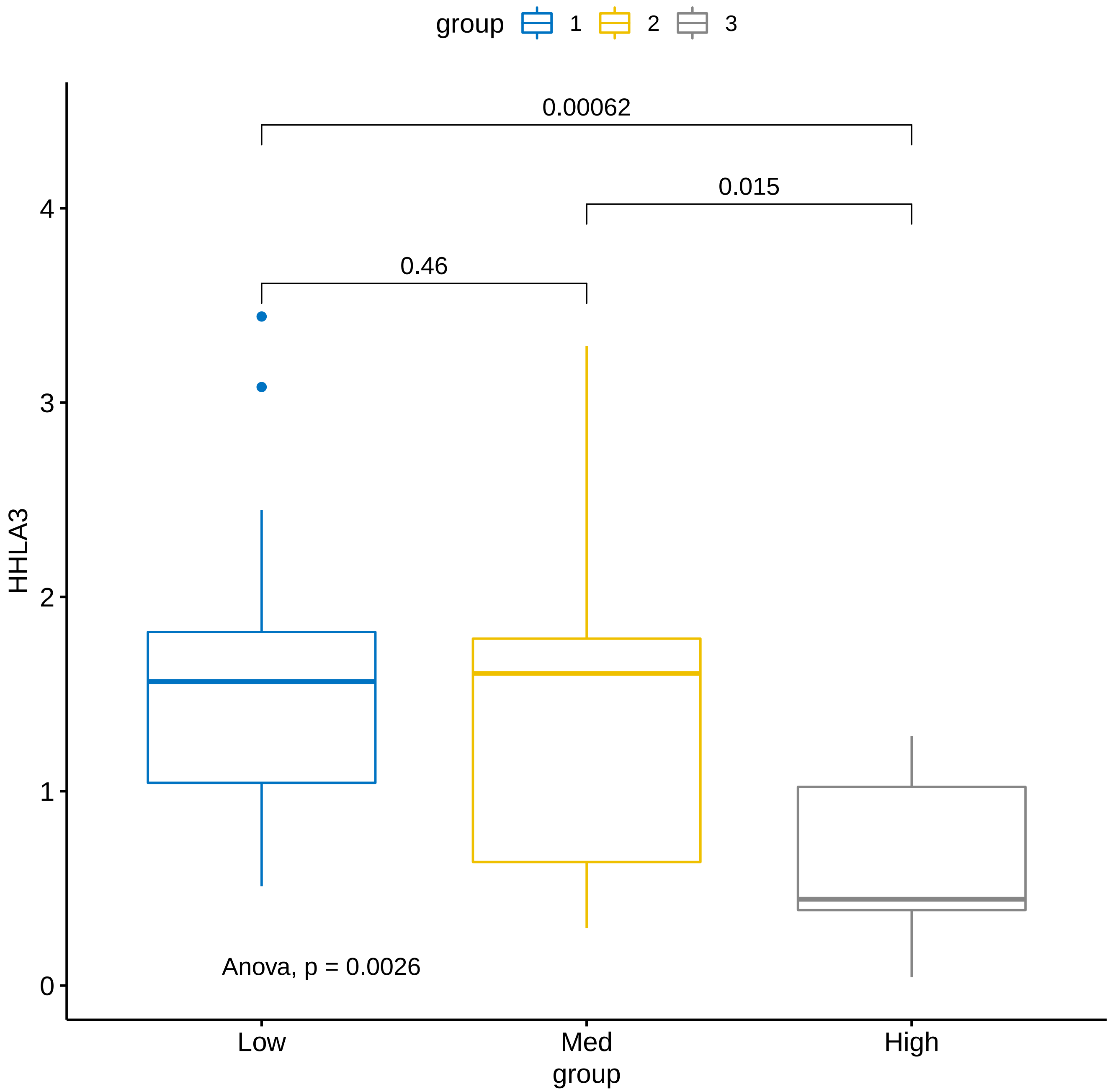

group 1 2 3
0.00062
0.015
0.46
4
3
2
1
0
HHLA3
Anova, p = 0.0026
Low
Med
High
group

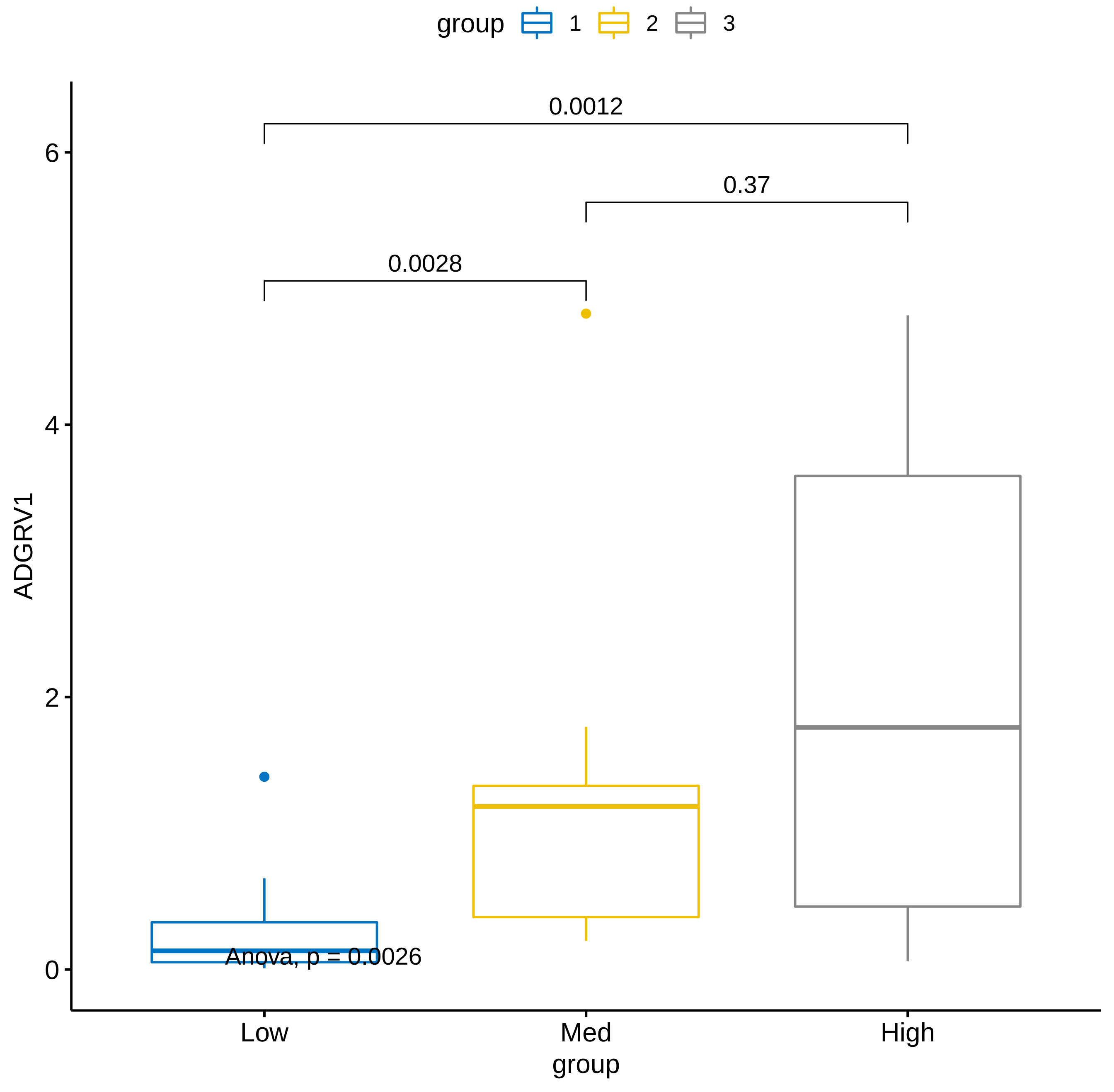

group
1
2
3
0.0012
0.37
0.0028
6
4
2
0
ADGRV1
Anova, p = 0.0026
Low
Med
High
group

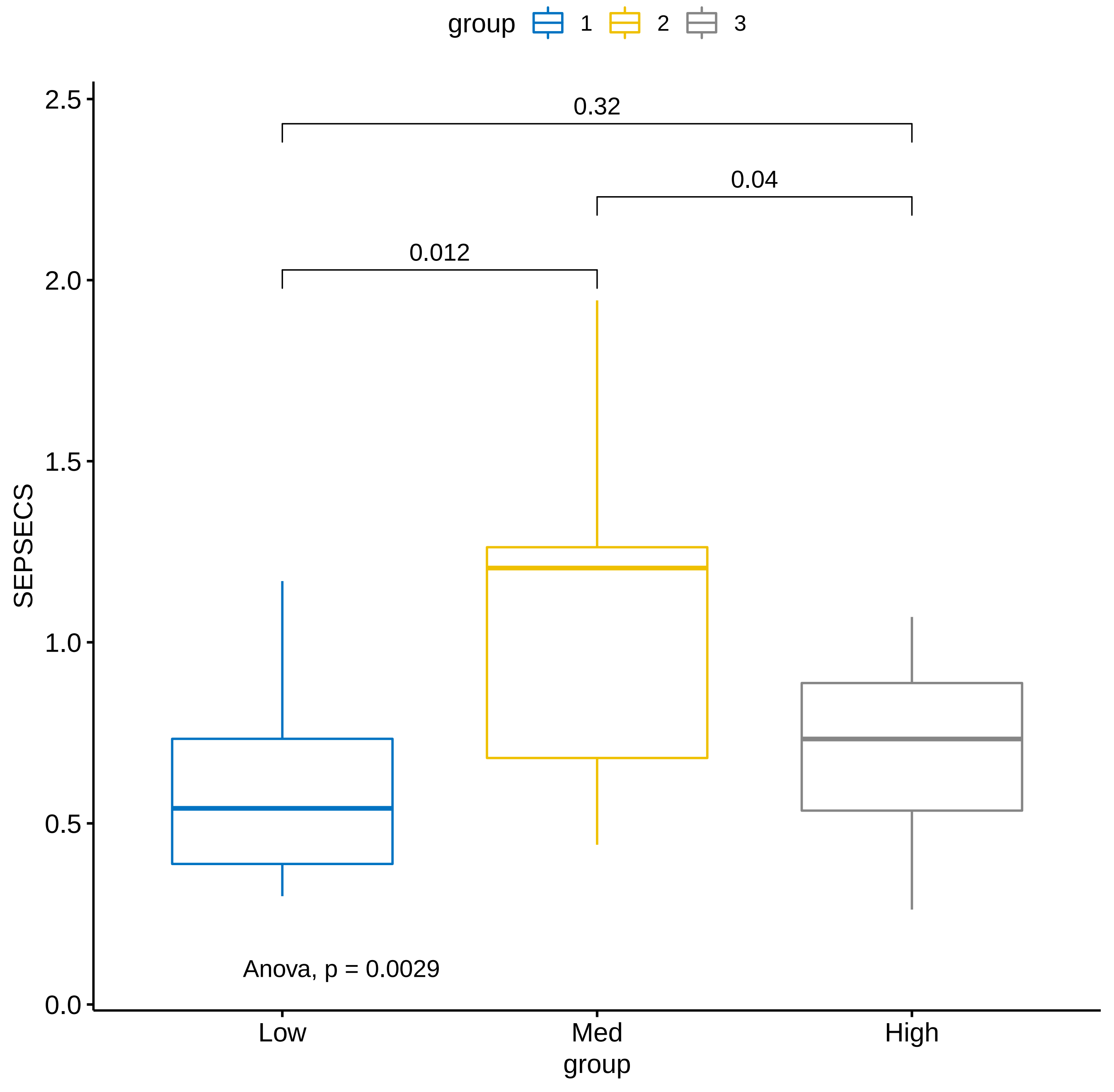
group 1 2 3
0.32
0.04
0.012
2.5
2.0
1.5
1.0
0.5
0.0
SEPSECS
Anova, p = 0.0029
Low
Med
High
group

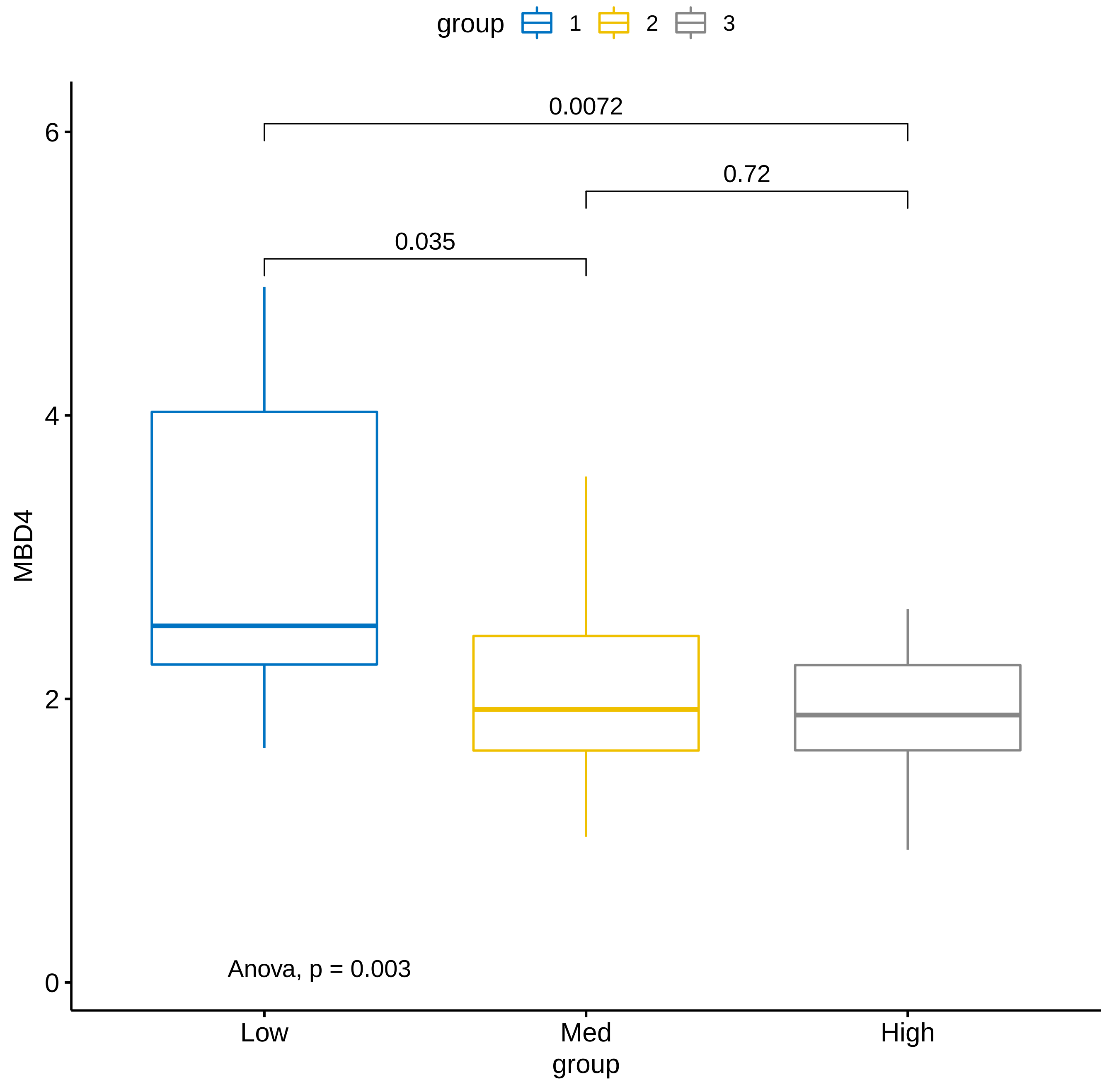

group 1 2 3
0.0072
0.72
0.035
6
4
2
0
MBD4
Anova, p = 0.003
Low
Med
High
group

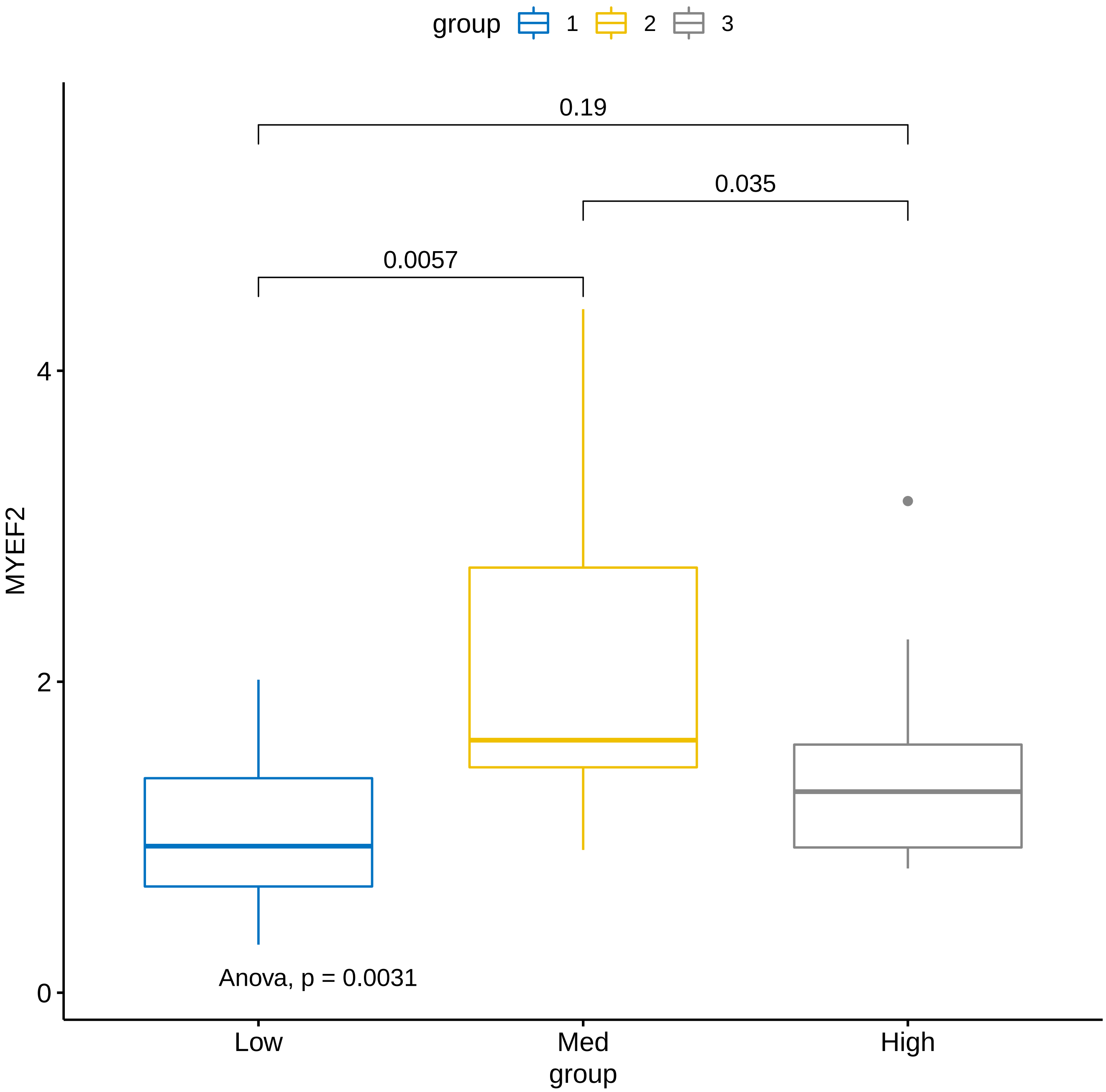
group 1 2 3
0.19
0.035
0.0057
4
2
0
MYEF2
Anova, p = 0.0031
Low
Med
High
group

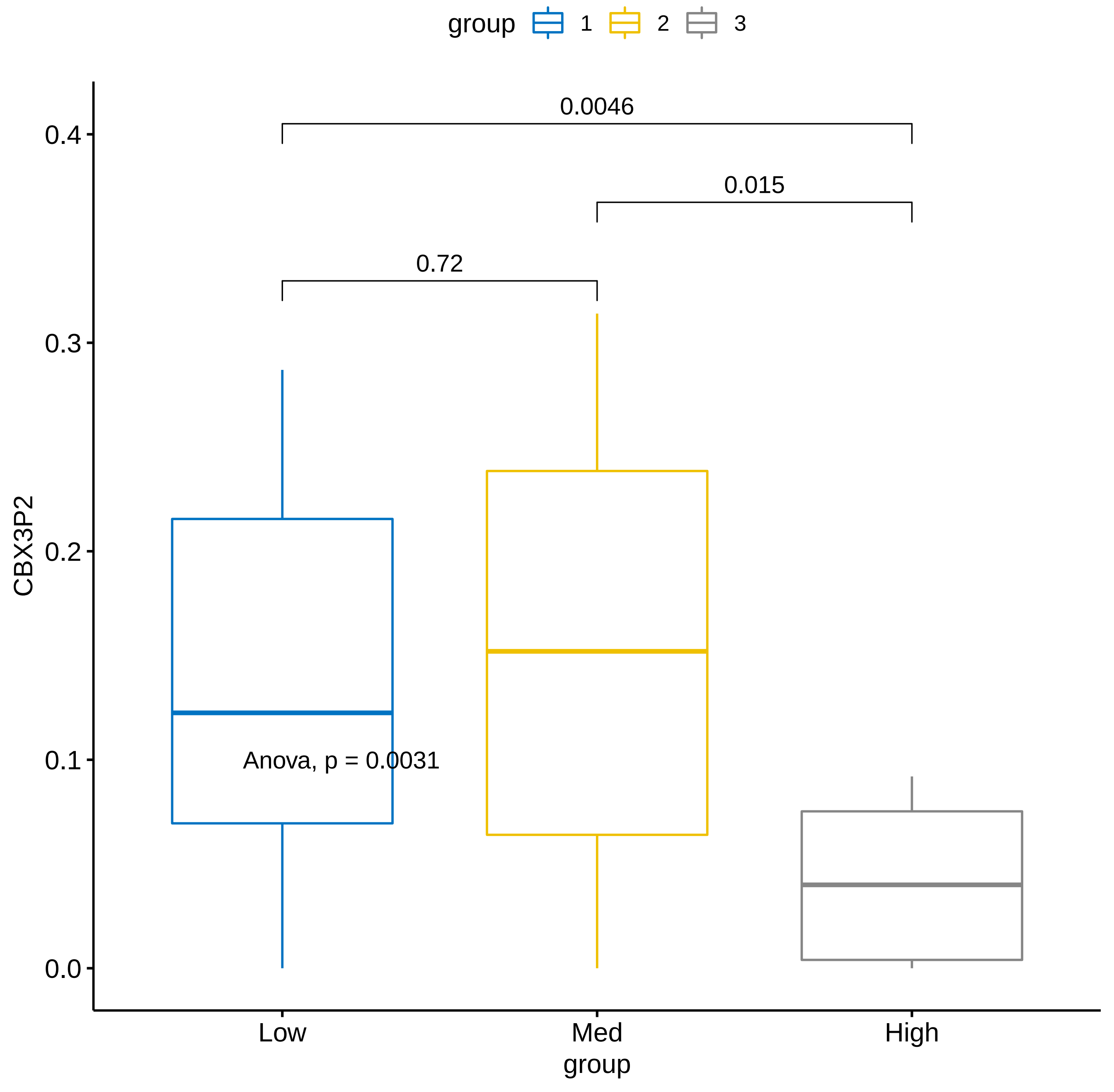
group 1 2 3
0.0046
0.015
0.72
CBX3P2
0.4
0.3
0.2
0.1
0.0
Anova, p = 0.0031
Low
Med
High
group

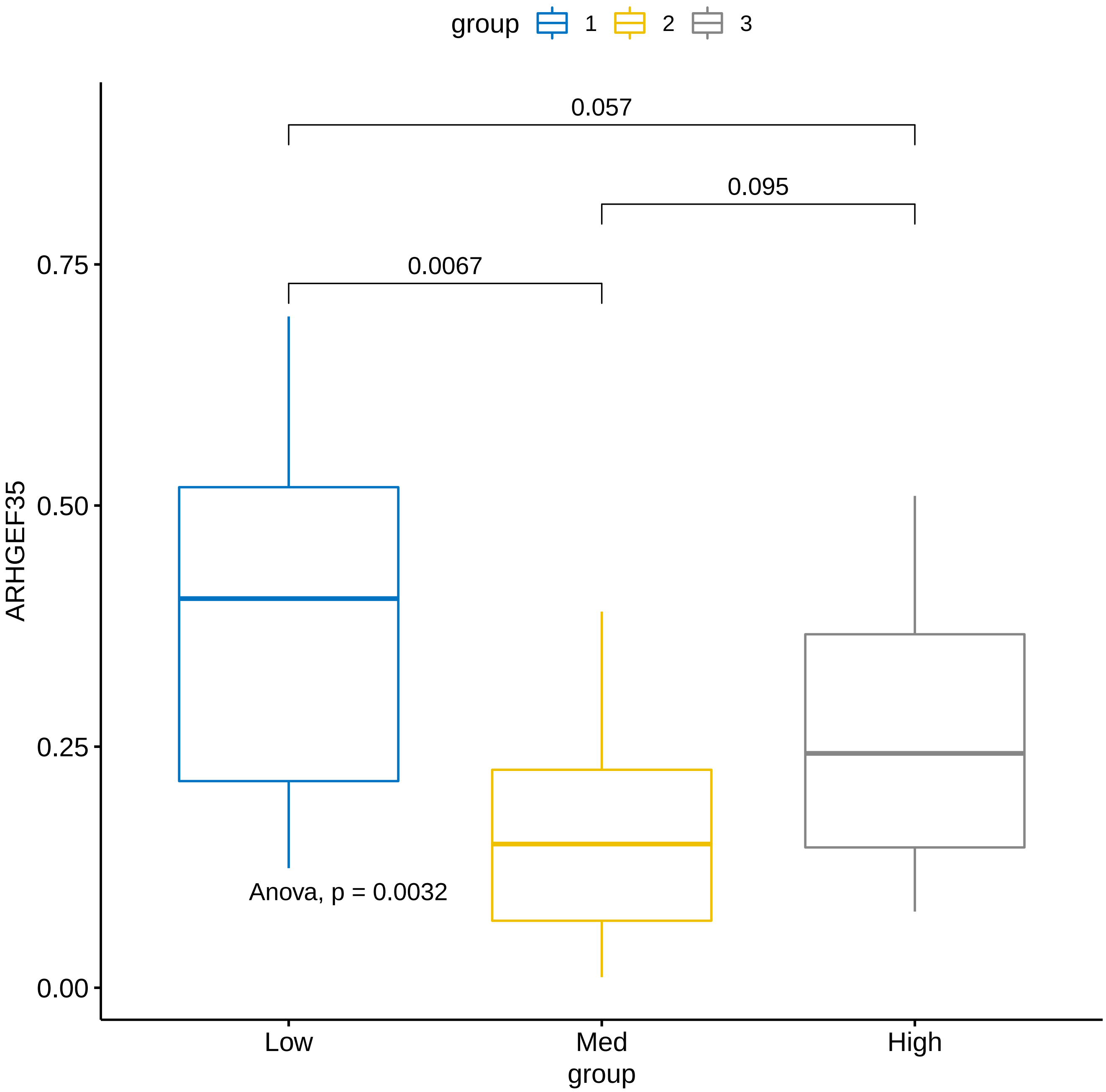

group
1
2
3
0.057
0.095
0.0067
0.75
0.50
0.25
0.00
ARHGEF35
Anova, p = 0.0032
Low
Med
High
group

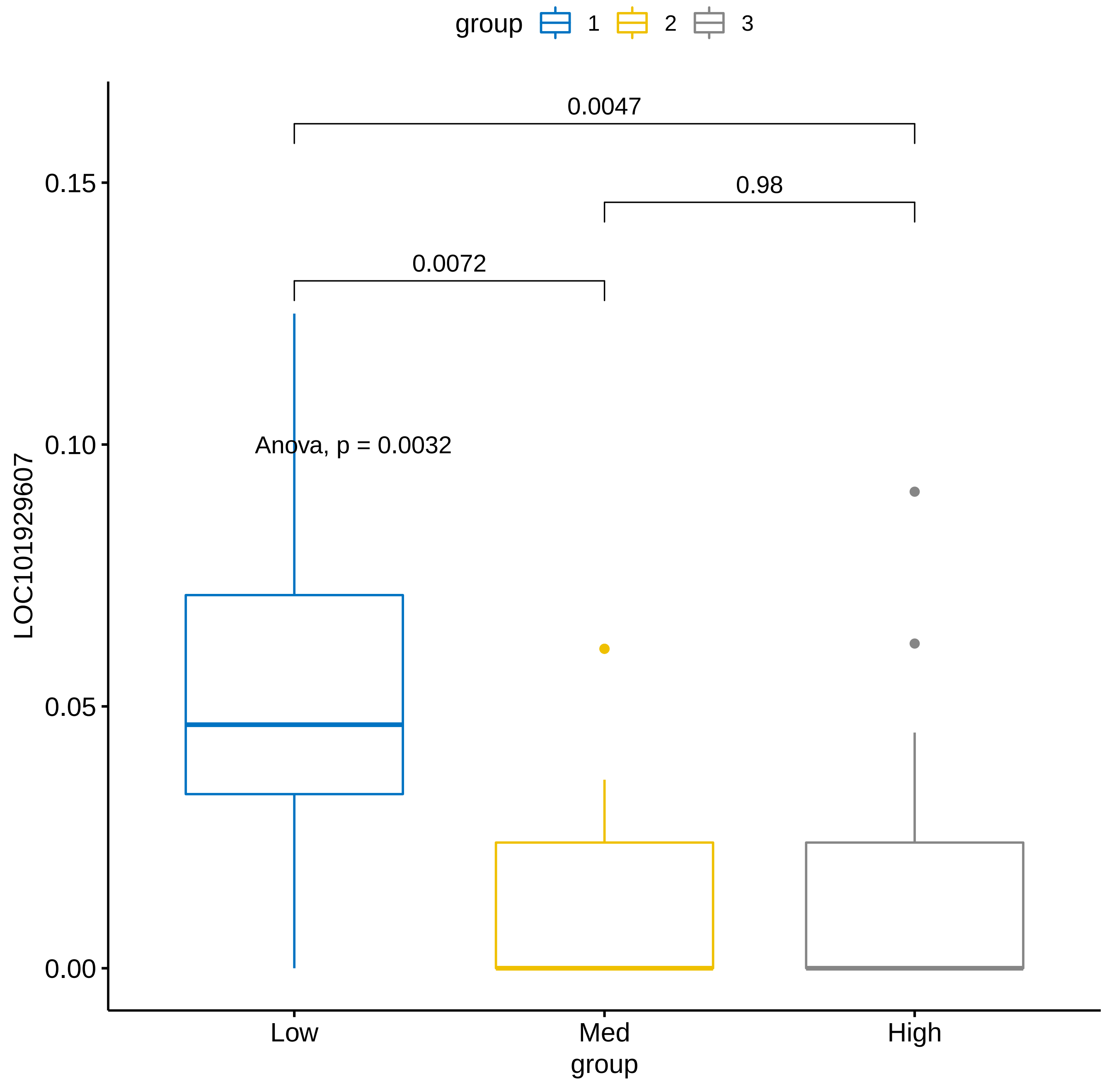

group 1 2 3
0.0047
0.98
0.0072
Anova, p = 0.0032
0.15
0.10
0.05
0.00
LOC101929607
Low
Med
High
group

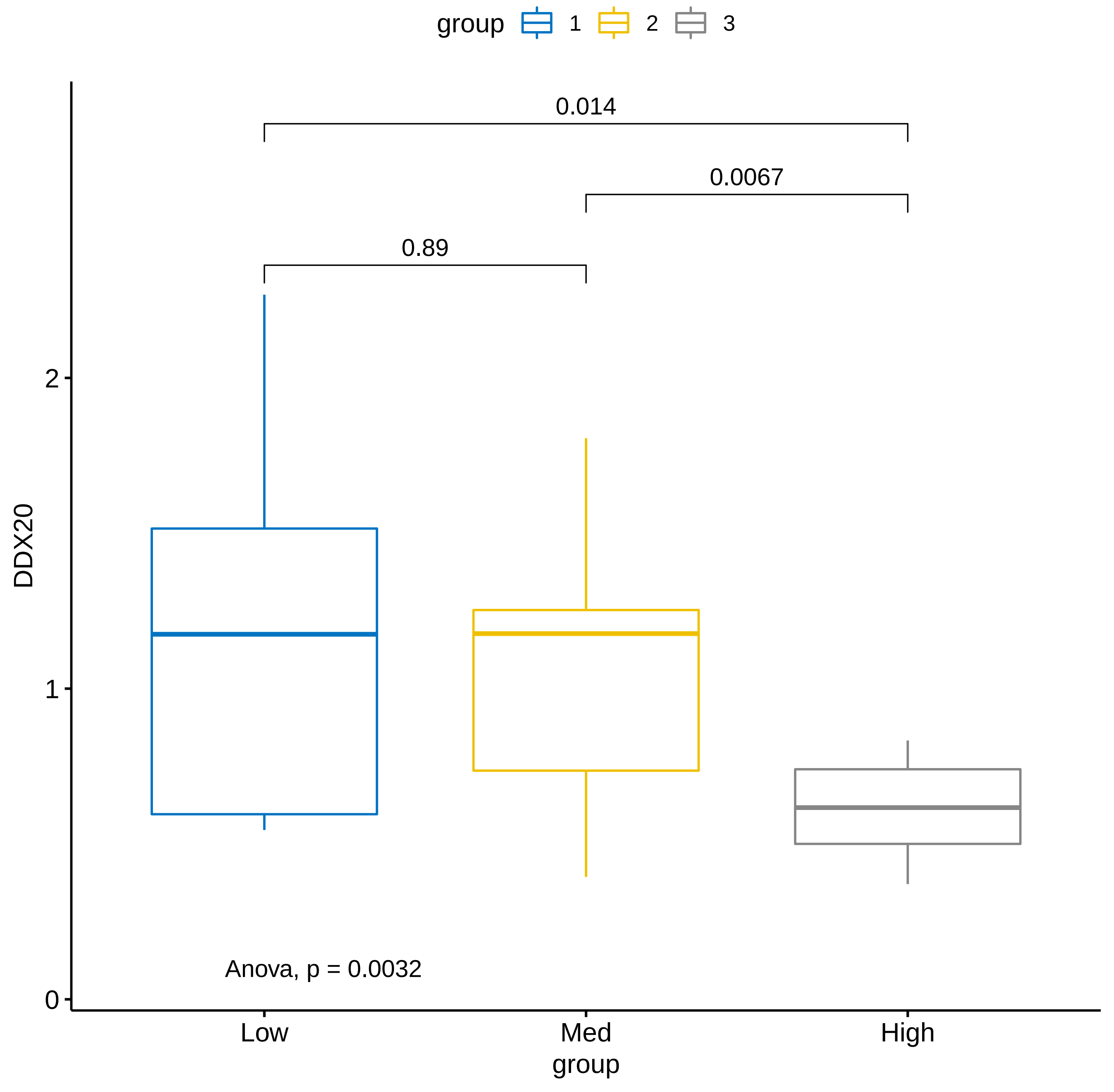

group
1
2
3
0.014
0.0067
0.89
DDX20
2
1
0
Anova, p = 0.0032
Low
Med
High
group

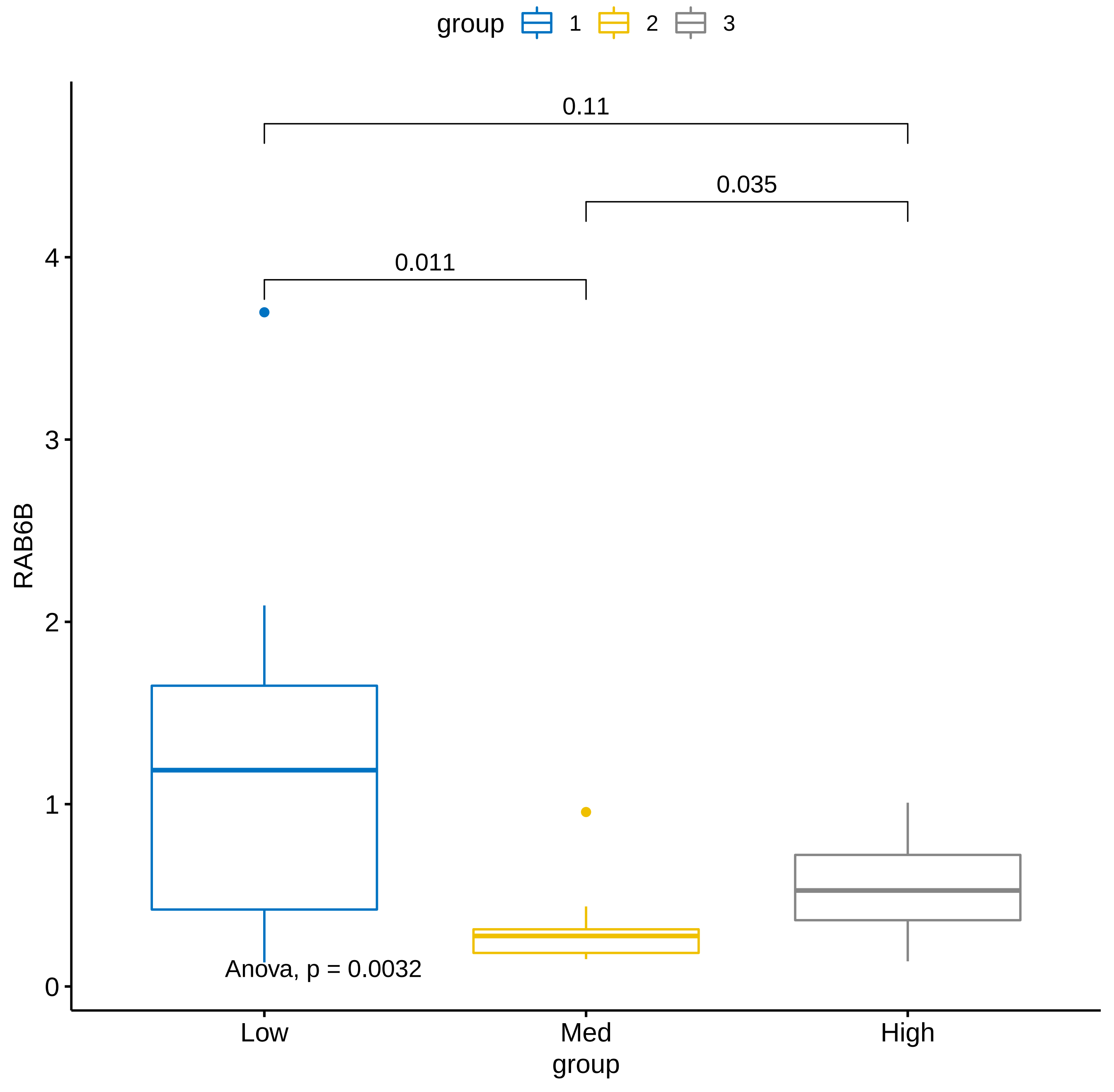
group
1
2
3
0.11
0.035
0.011
RAB6B
4
3
2
1
0
Anova, p = 0.0032
Low
Med
High
group

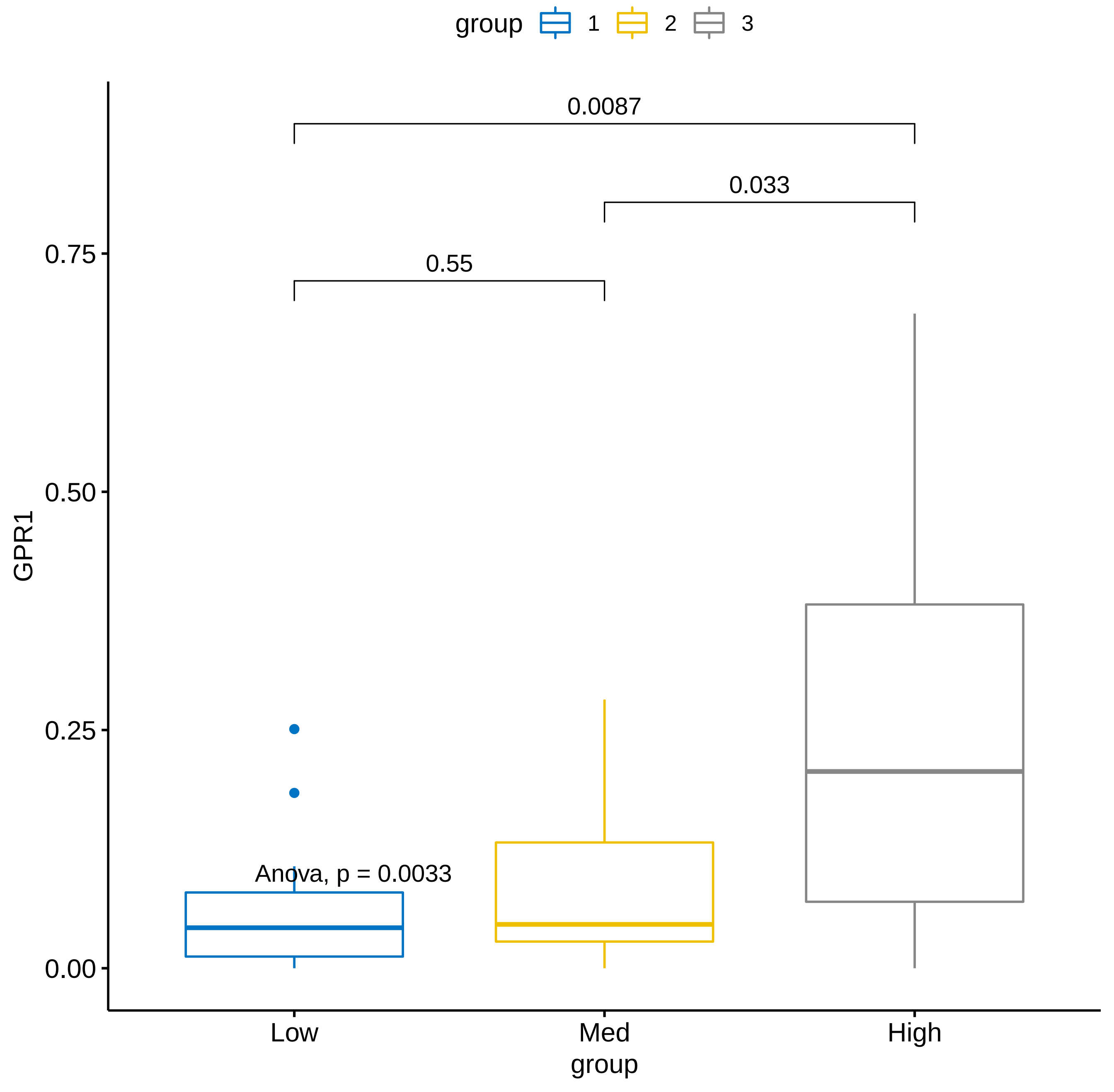

group 1 2 3
0.0087
0.033
0.55
0.75
0.50
0.25
0.00
GPR1
Anova, p = 0.0033
Low
Med
High
group

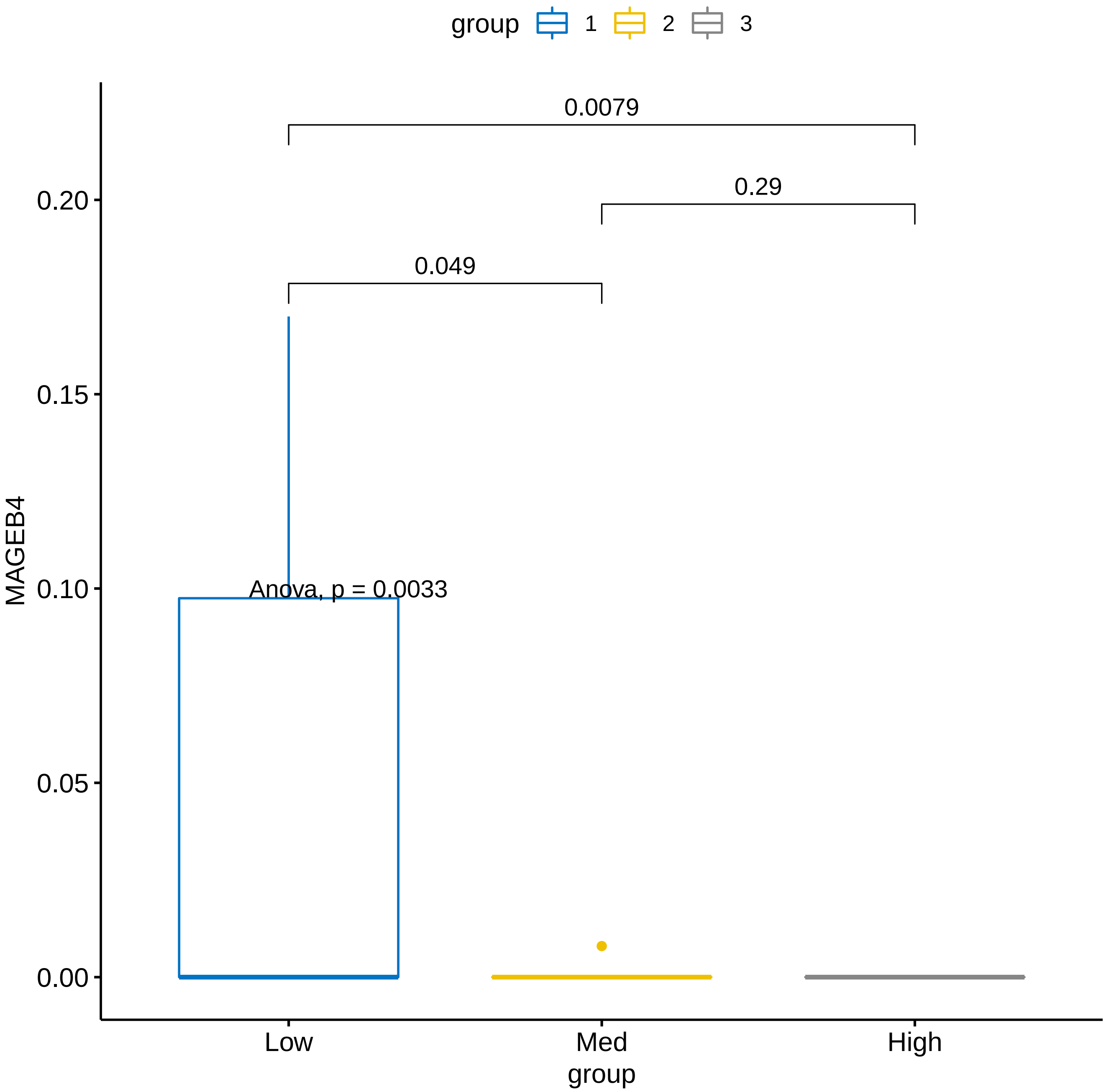
group
1
2
3
0.0079
0.29
0.049
Anova, p = 0.0033
MAGEB4
0.20
0.15
0.10
0.05
0.00
Low
Med
High
group

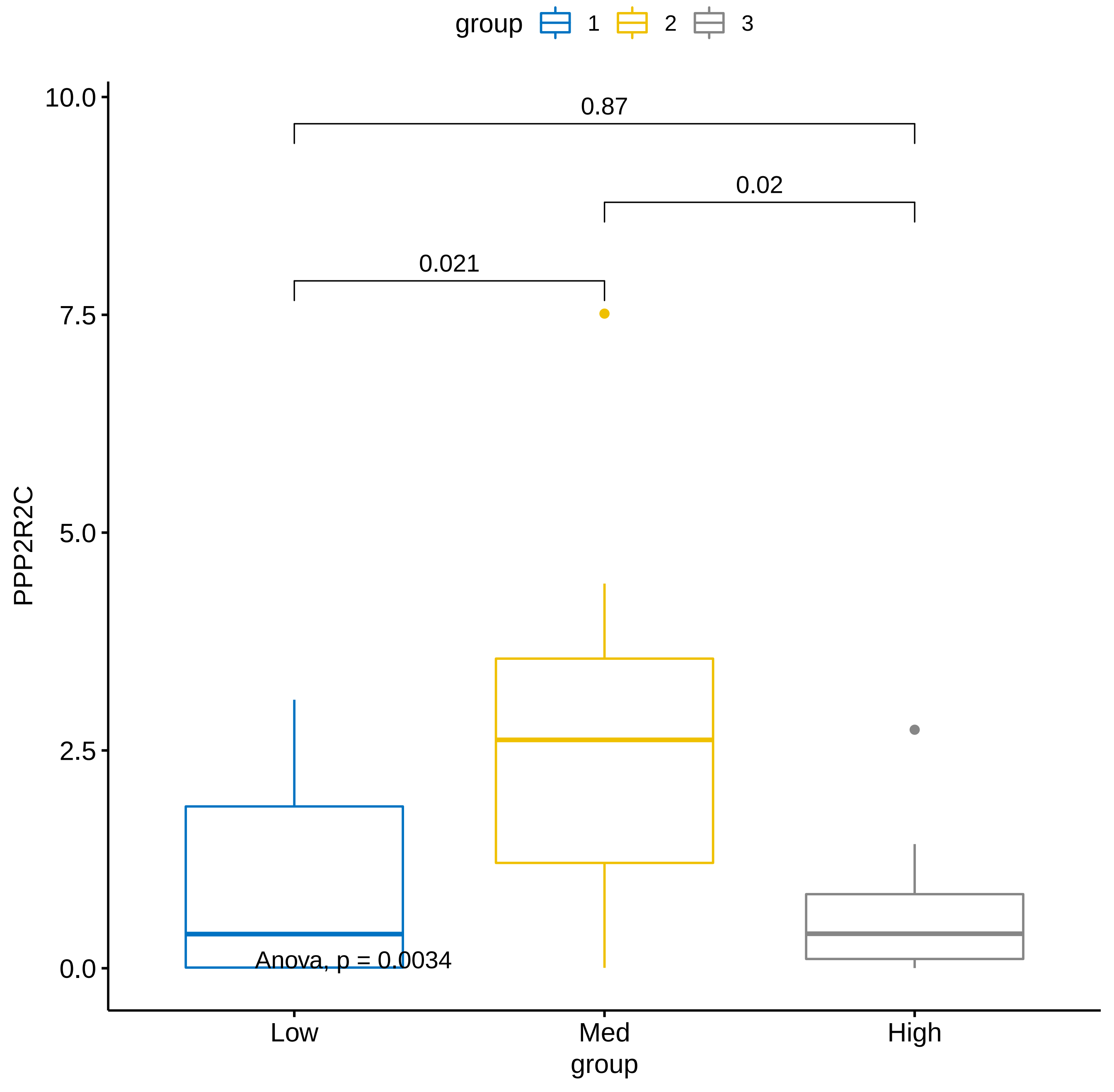

group 1 2 3
0.87
0.02
0.021
10.0
7.5
5.0
2.5
0.0
PPP2R2C
Anova, p = 0.0034
Low
Med
High
group

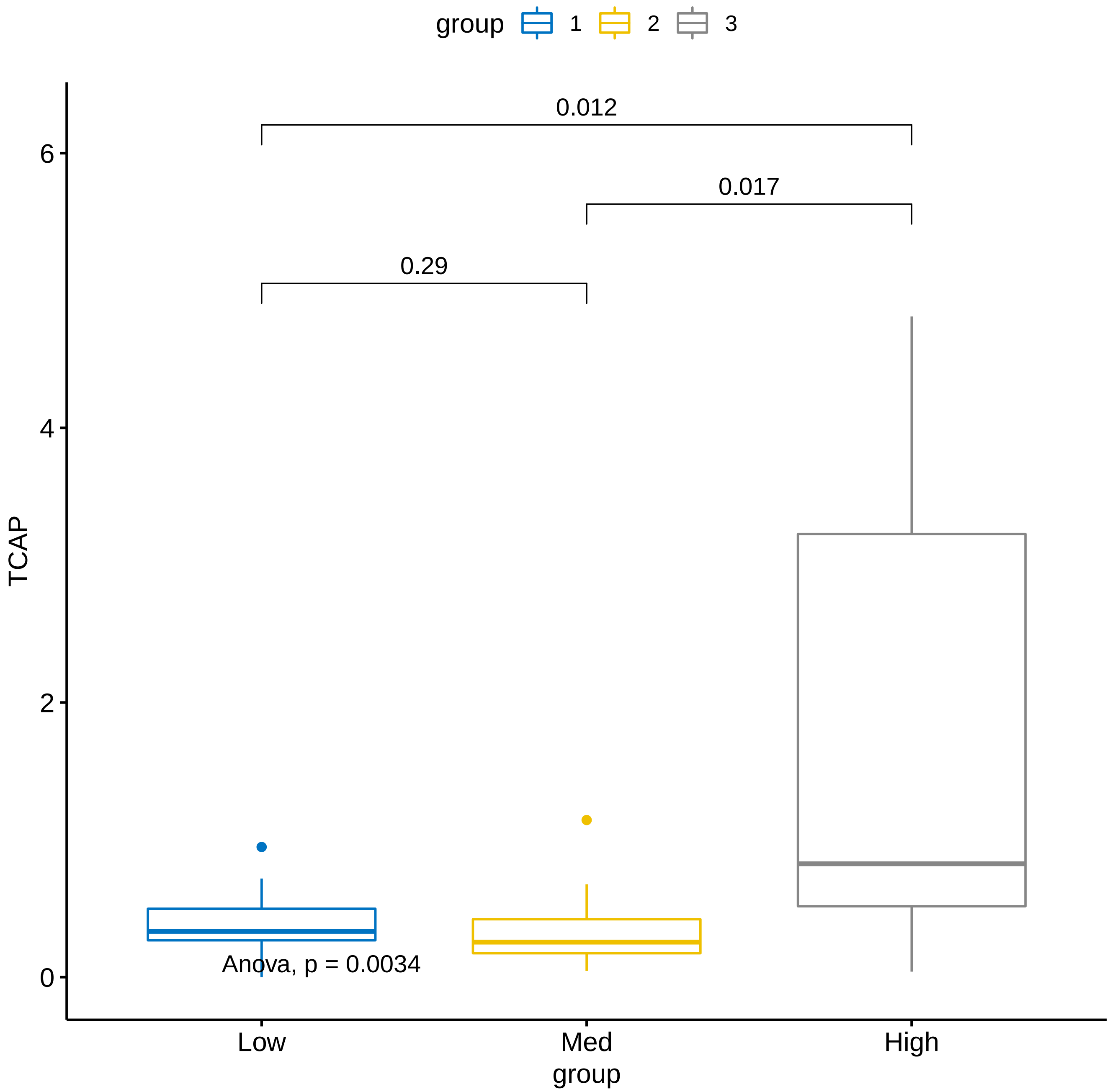
group
1
2
3
0.012
0.017
0.29
6
4
2
0
TCAP
Anova, p = 0.0034
Low
Med
High
group

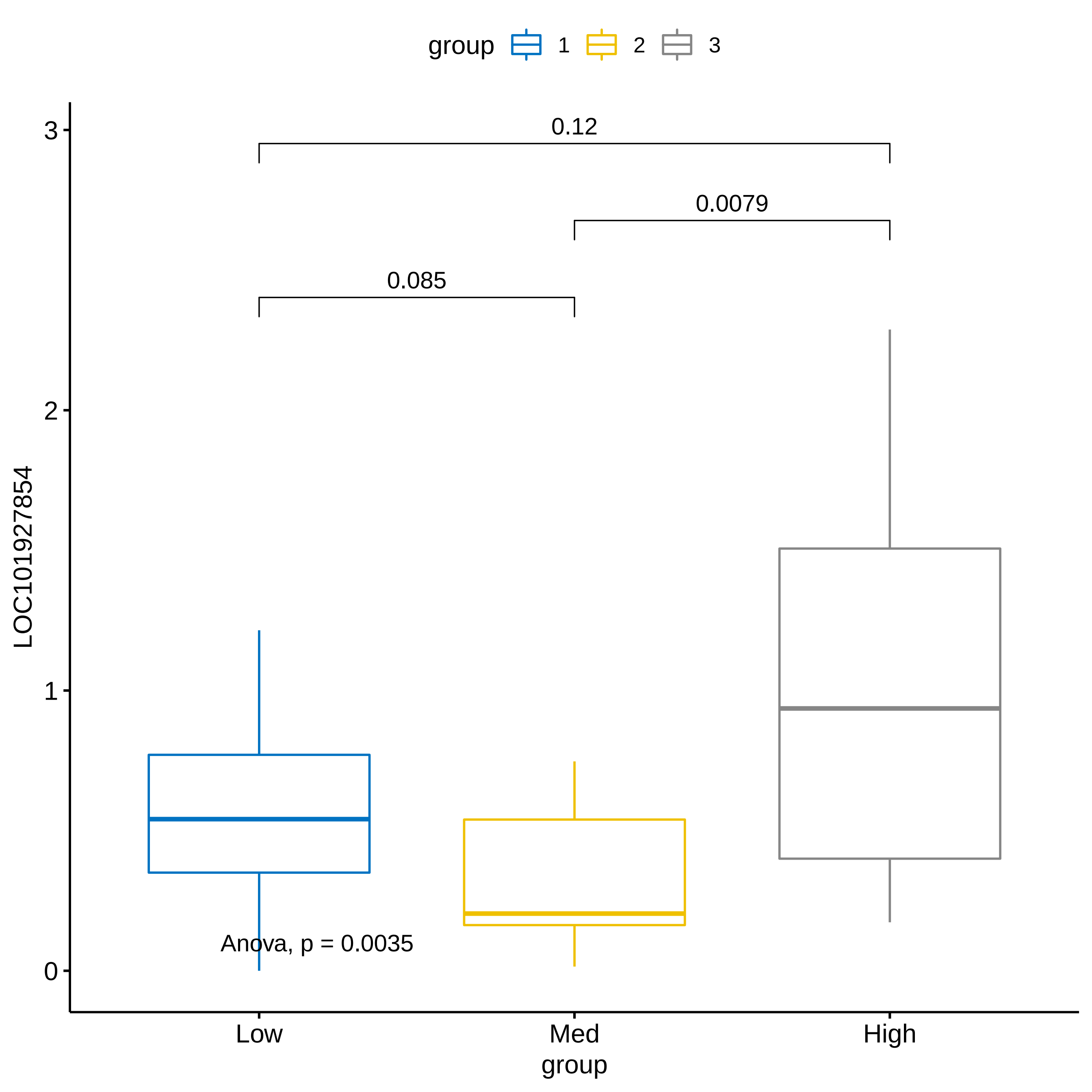
group
1
2
3
0.12
0.0079
0.085
Anova, p = 0.0035
LOC101927854
3
2
1
0
Low
Med
High
group

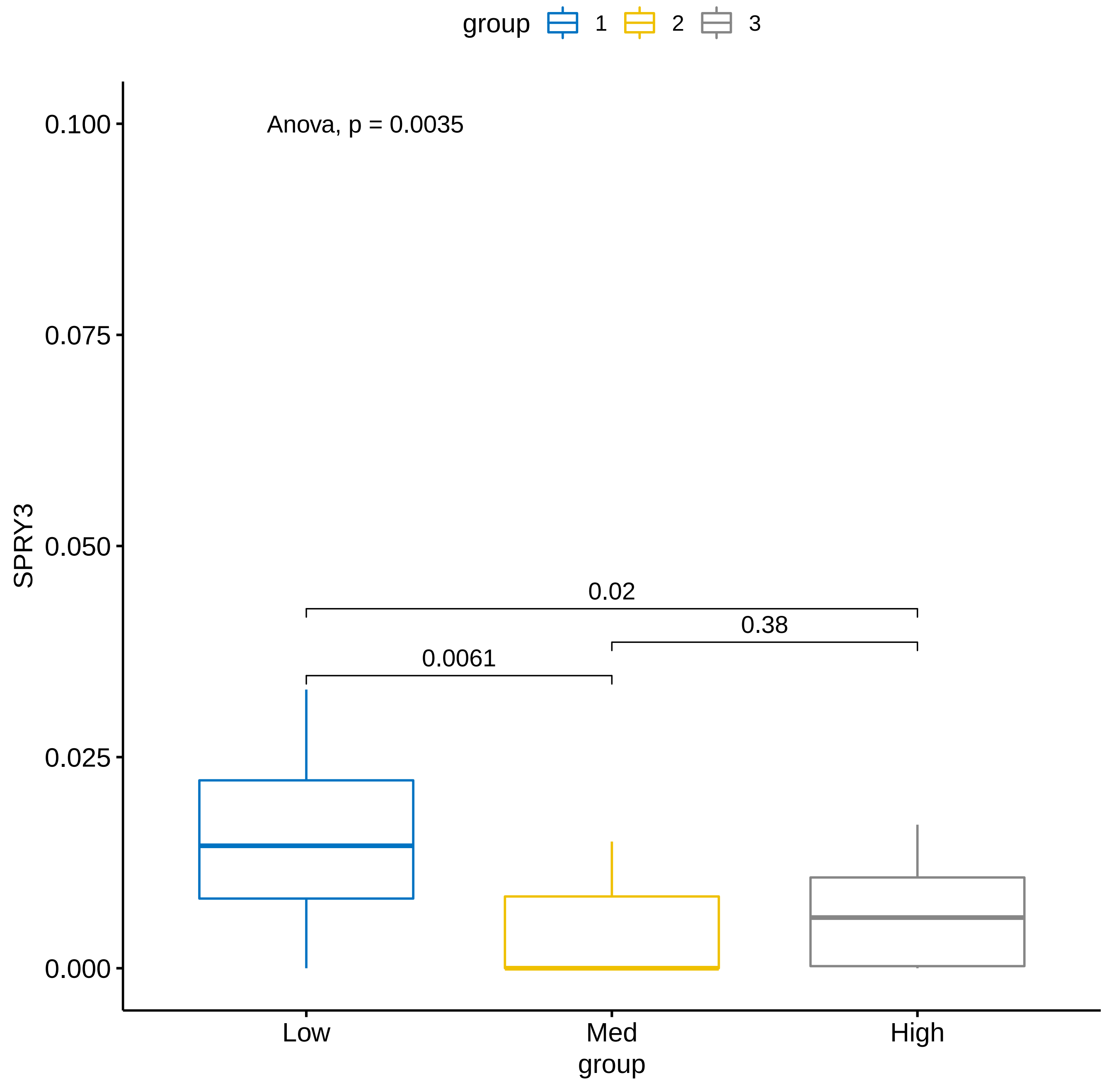
group
1
2
3
Anova, p = 0.0035
0.100
0.075
0.050
0.025
0.000
SPRY3
0.02
0.38
0.0061
Low
Med
High
group

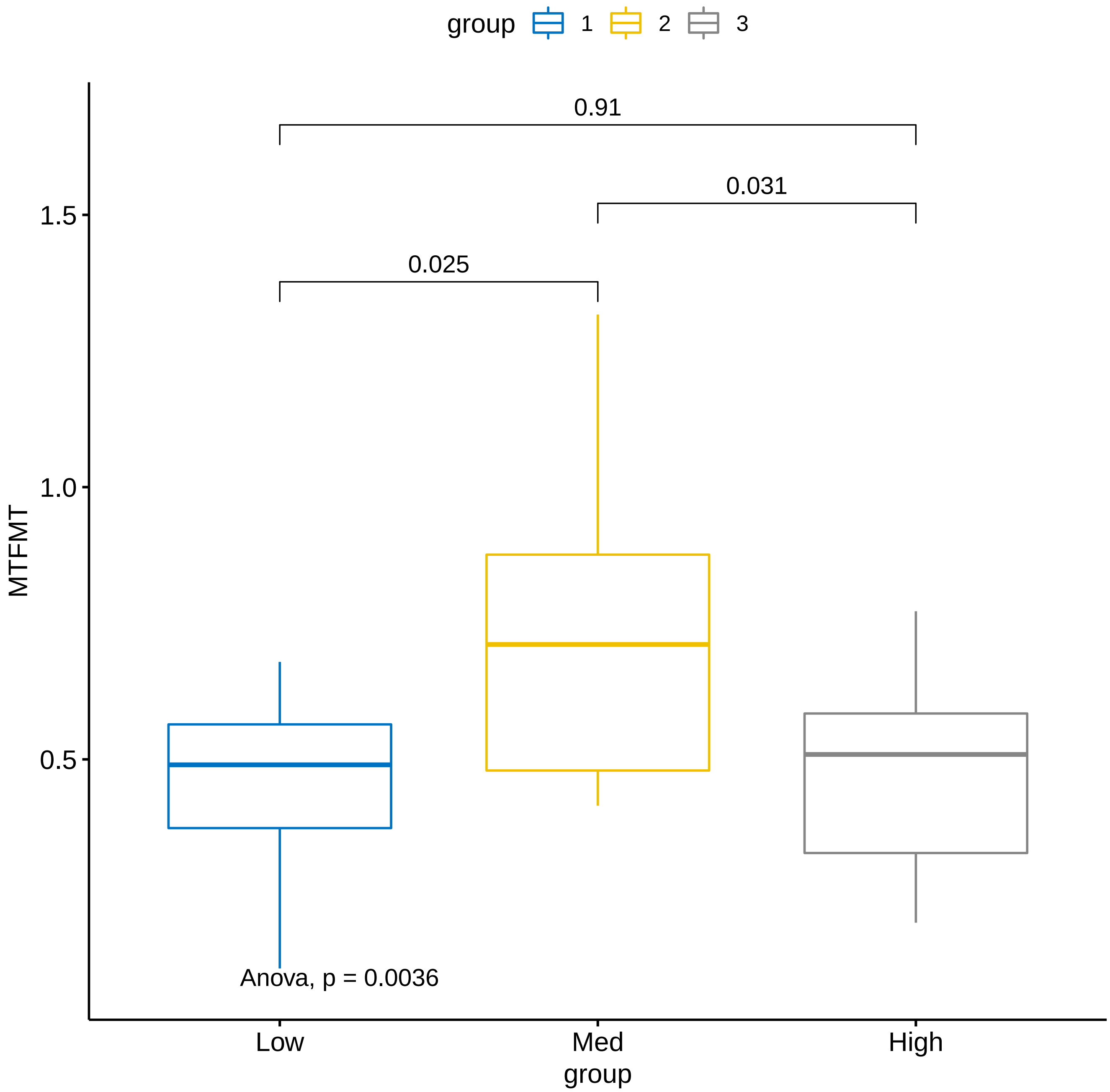
group 1 2 3
0.91
0.031
0.025
1.5
1.0
0.5
MTFMT
Anova, p = 0.0036
Low
Med
High
group

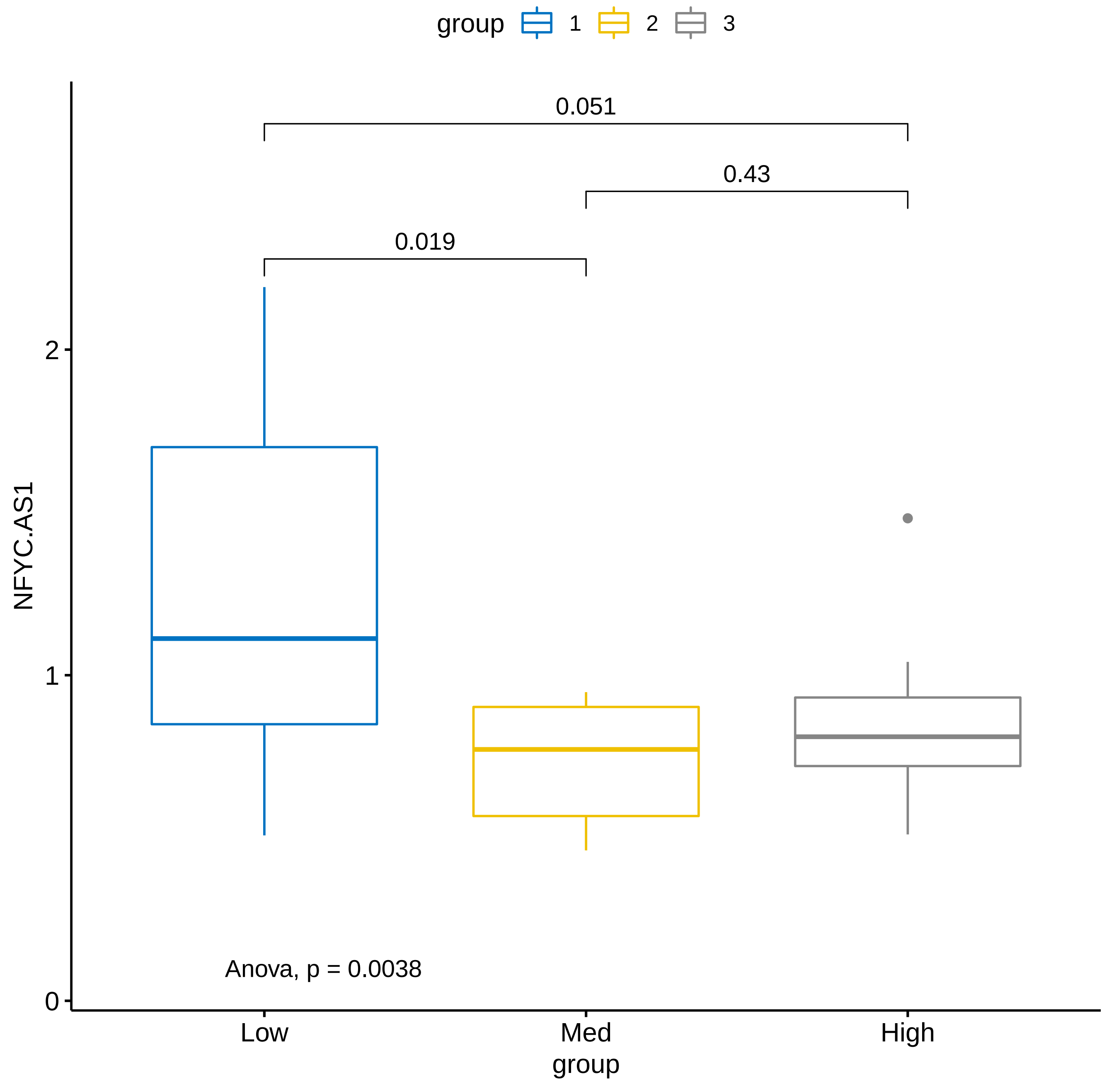
group
1
2
3
0.051
0.43
0.019
NFYC.AS1
2
1
0
Anova, p = 0.0038
Low
Med
High
group

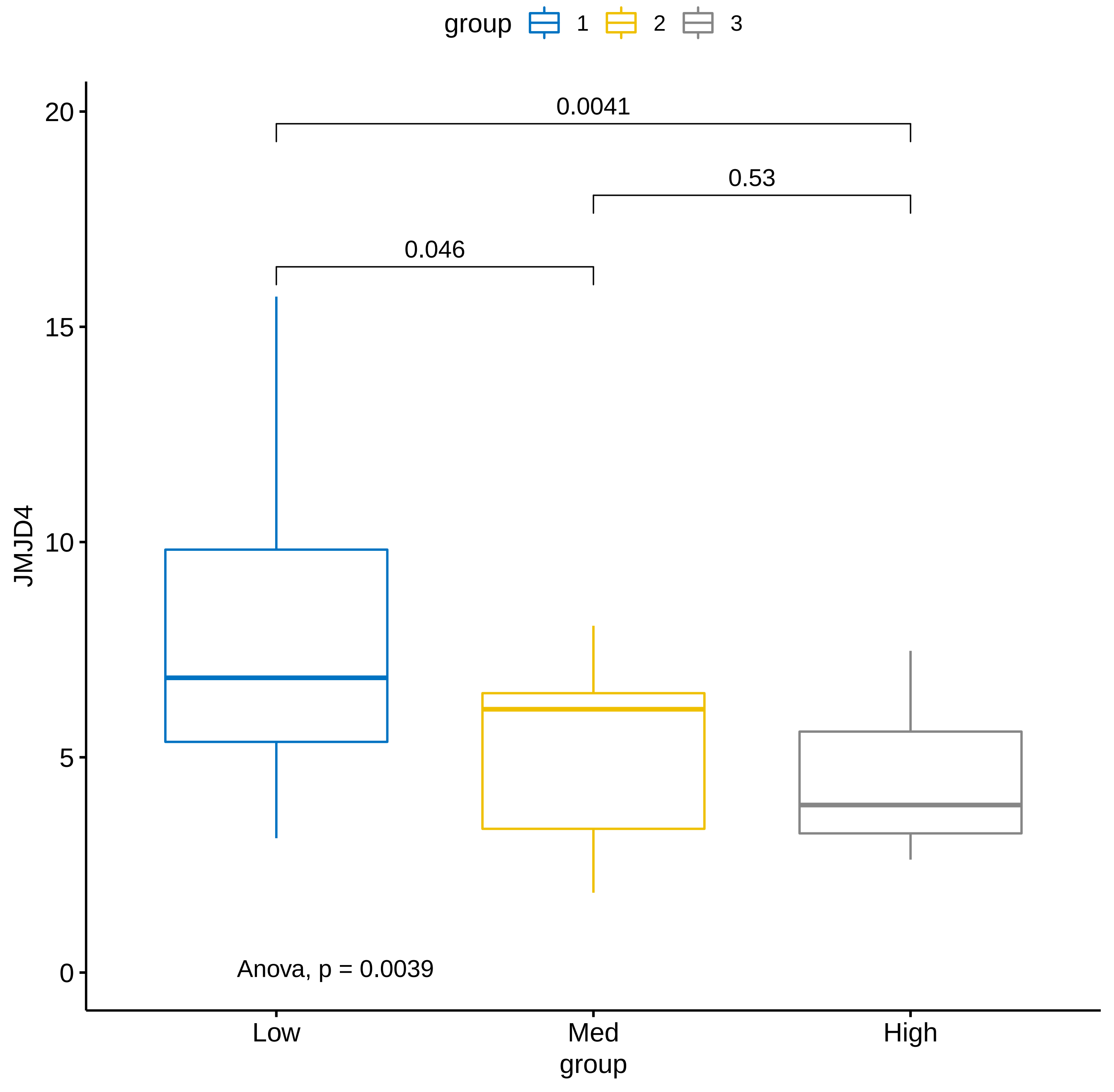
group 1 2 3
0.0041
0.53
0.046
JMJD4
20
15
10
5
0
Anova, p = 0.0039
Low
Med
High
group

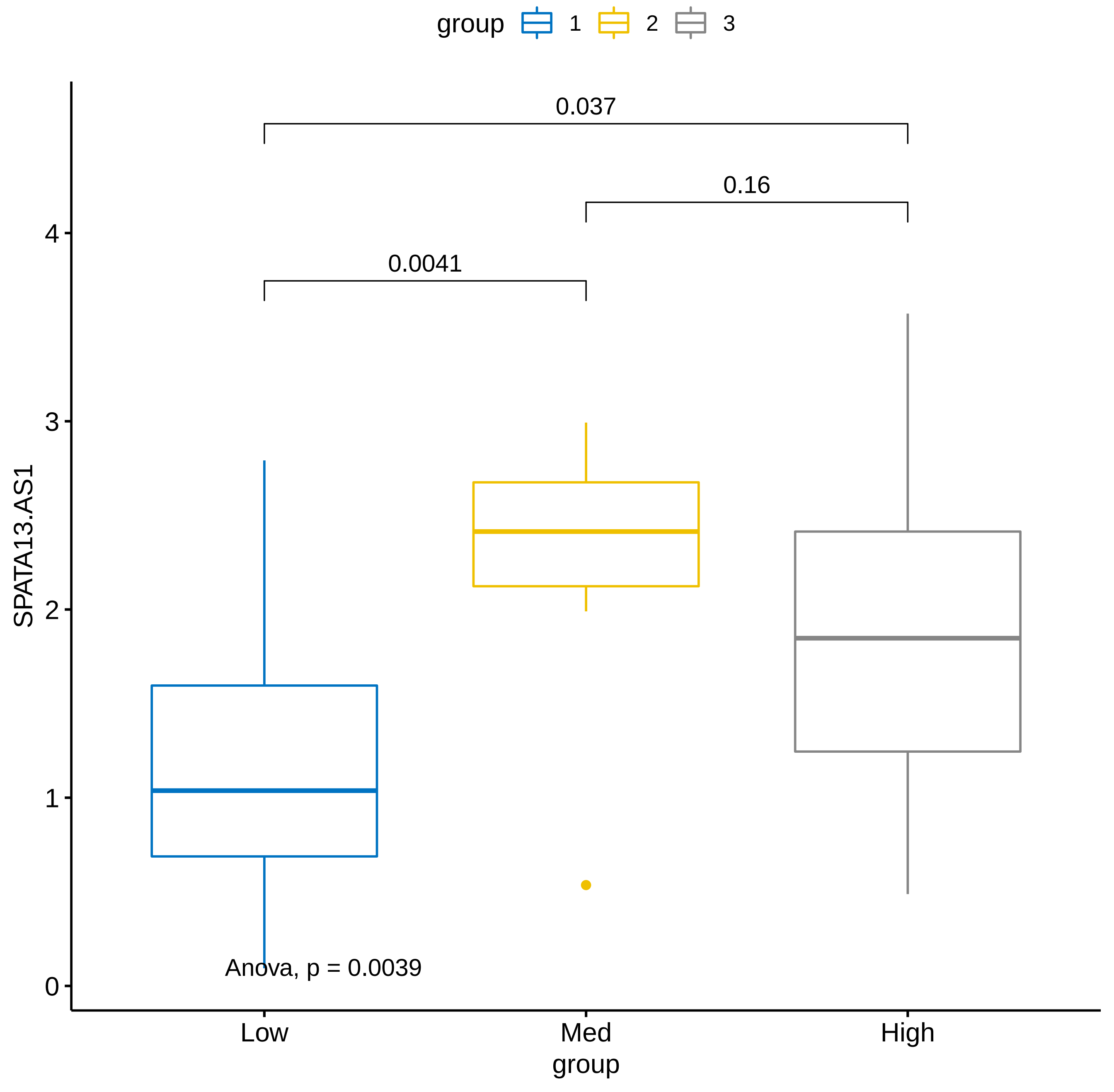
group
1
2
3
0.037
0.16
0.0041
SPATA13.AS1
4
3
2
1
0
Anova, p = 0.0039
Low
Med
High
group

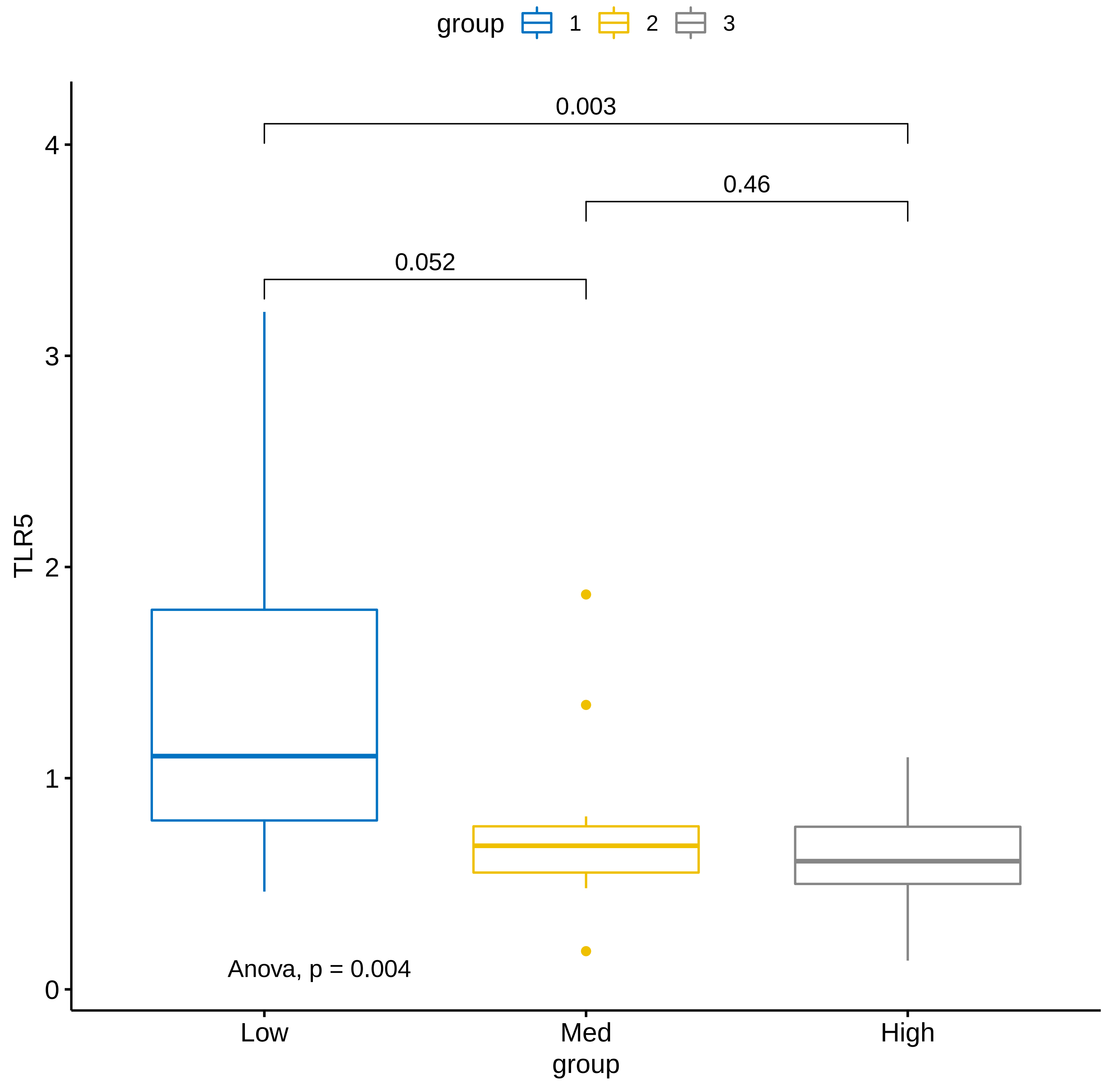
group
1
2
3
0.003
0.46
0.052
TLR5
4
3
2
1
0
Anova, p = 0.004
Low
Med
High
group

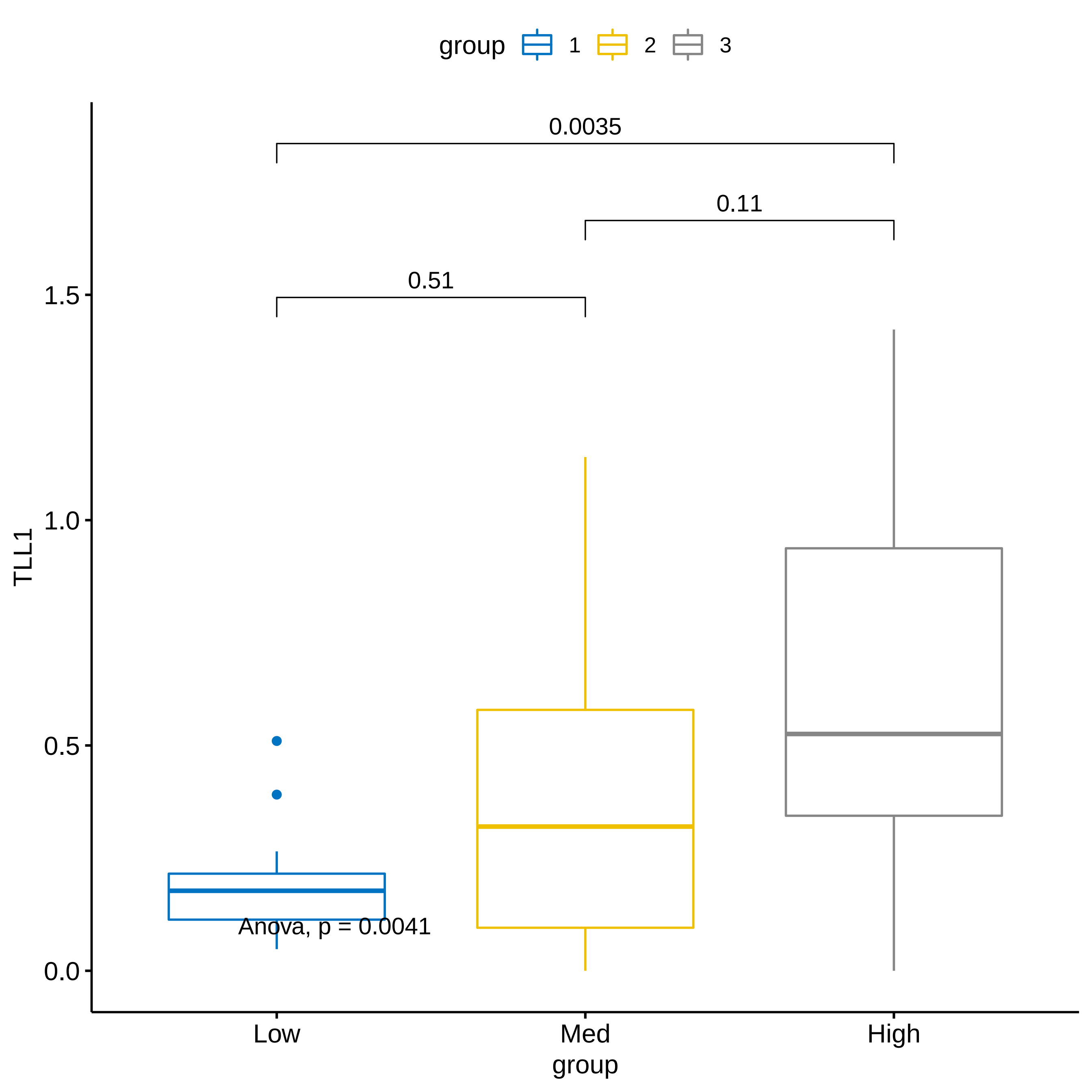
group
1
2
3
0.0035
0.11
0.51
Anova, p = 0.0041
TLL1
1.5
1.0
0.5
0.0
Low
Med
High
group

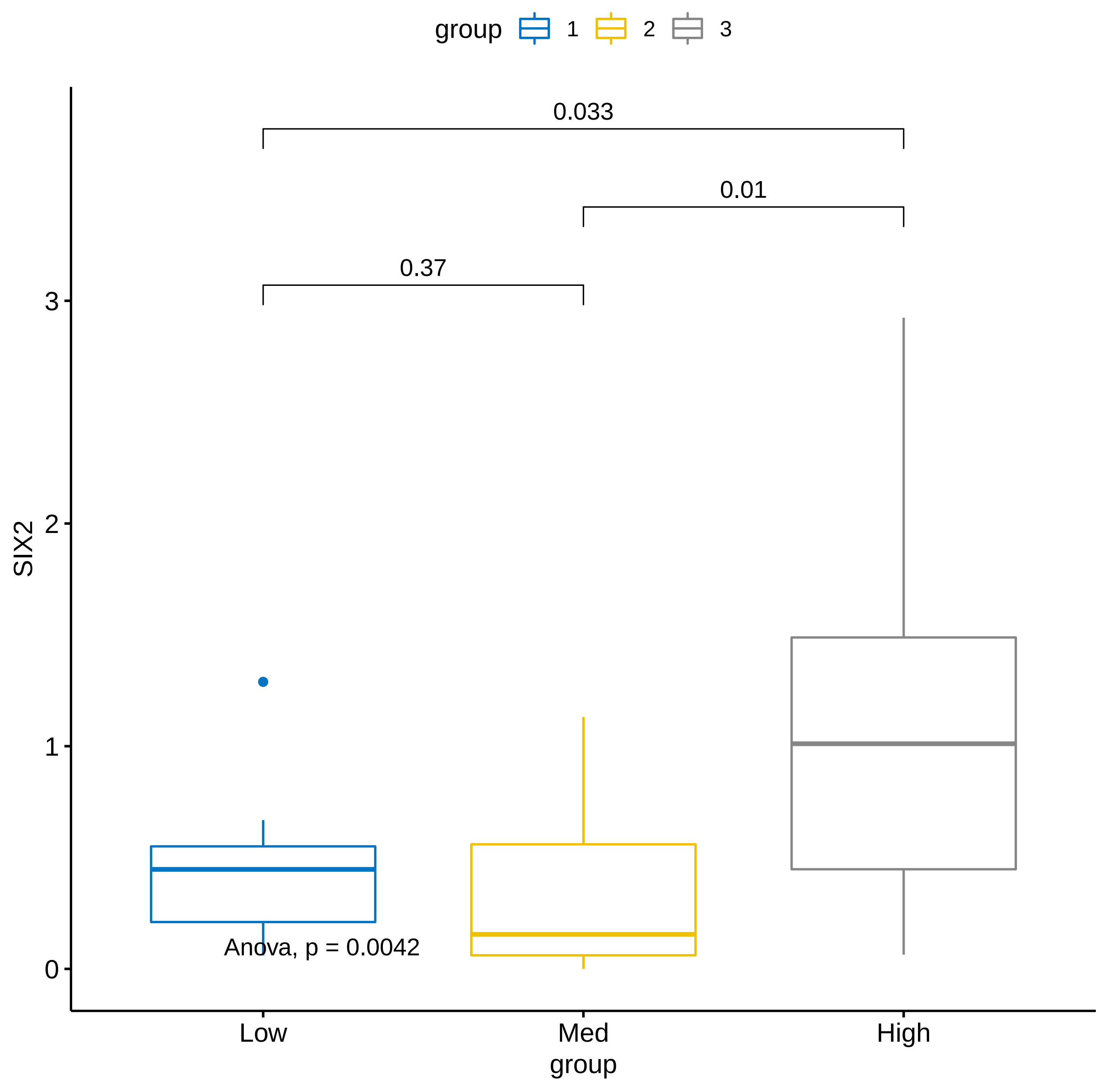

group
1
2
3
0.033
0.01
0.37
SIX2
3
2
1
0
Anova, p = 0.0042
Low
Med
High
group

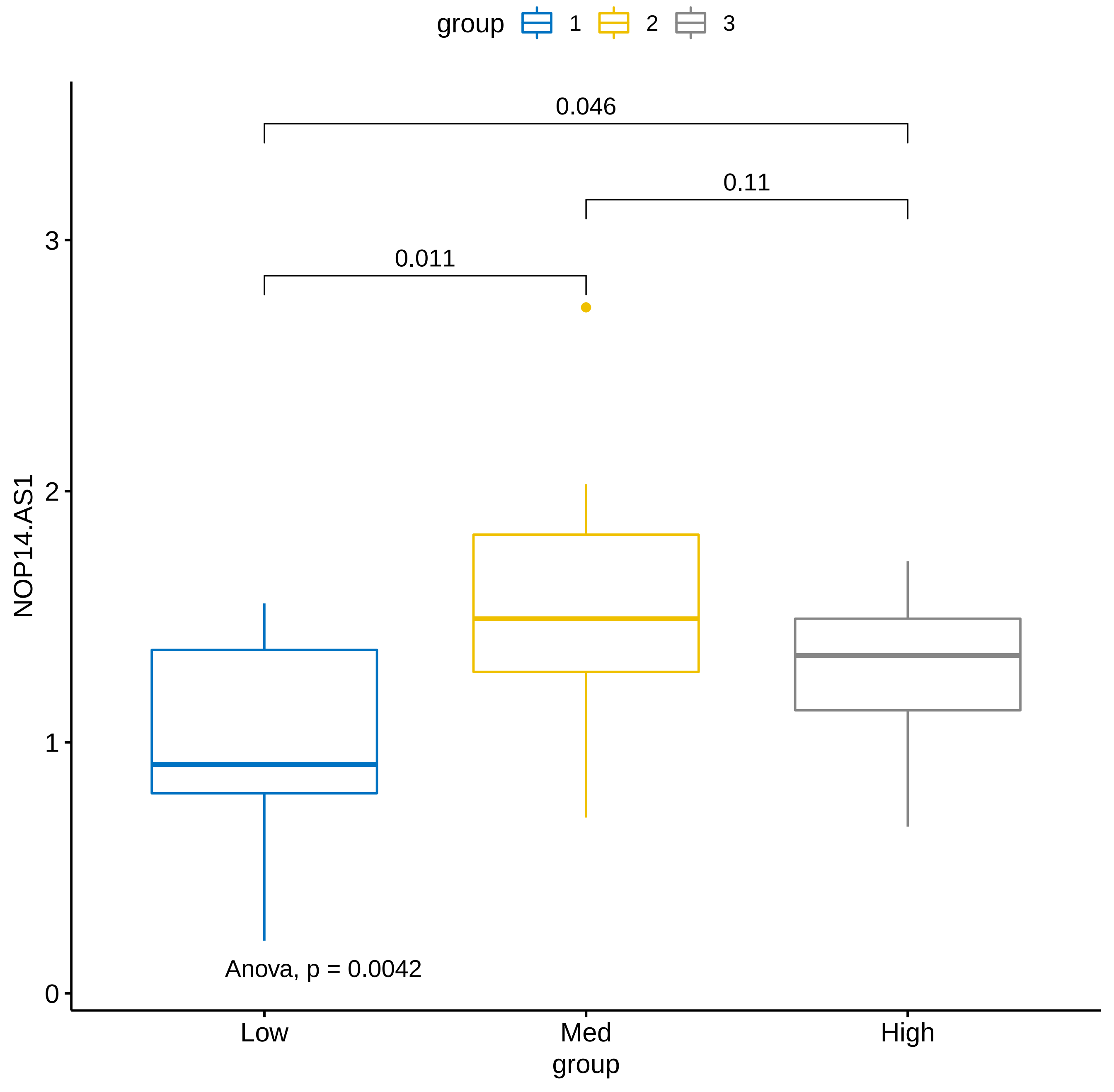
group
1
2
3
0.046
0.11
0.011
3
2
1
0
NOP14.AS1
Anova, p = 0.0042
Low
Med
High
group

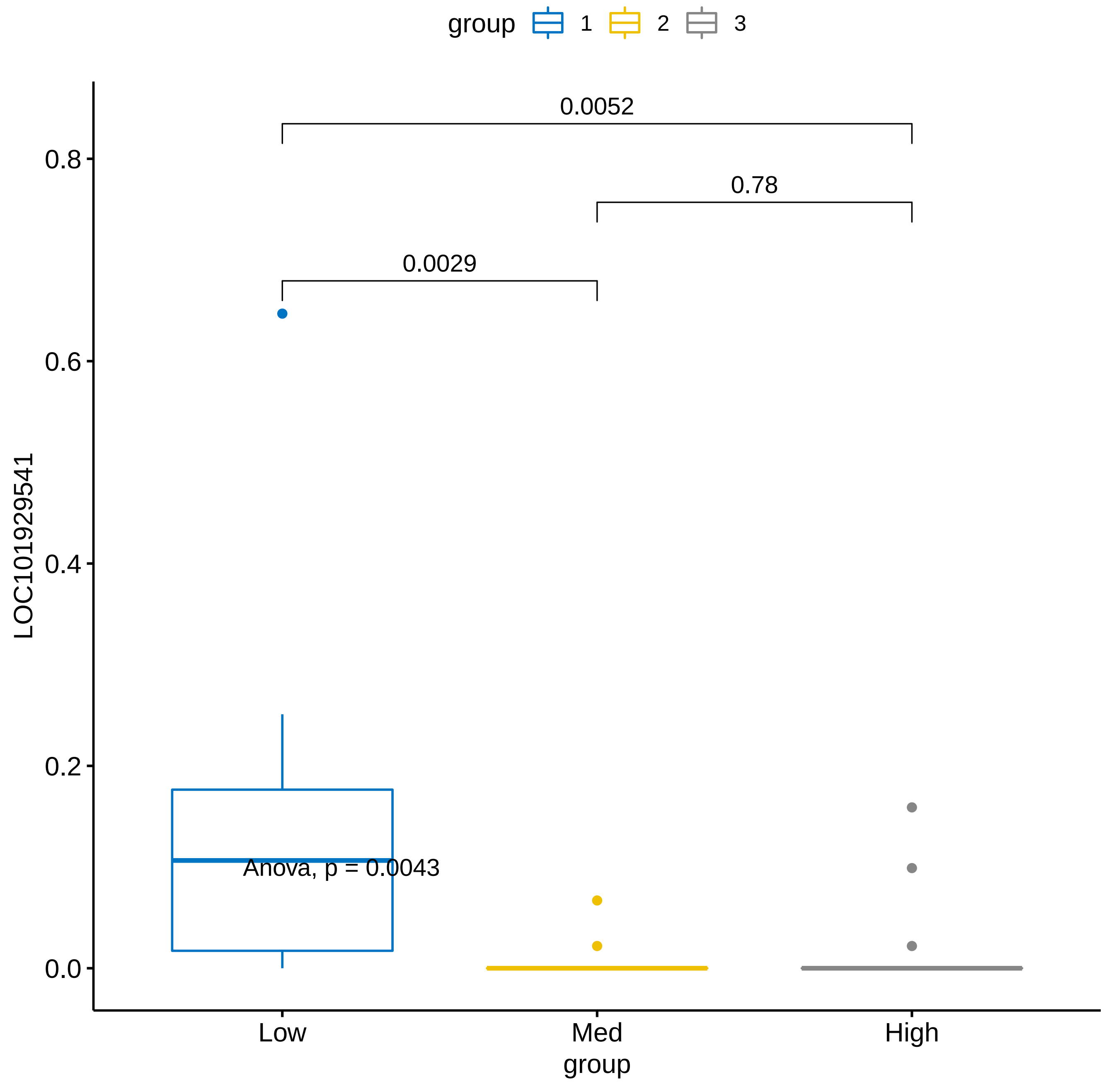

group
1
2
3
0.0052
0.78
0.0029
Anova, p = 0.0043
0.8
0.6
0.4
0.2
0.0
LOC101929541
Low
Med
High
group

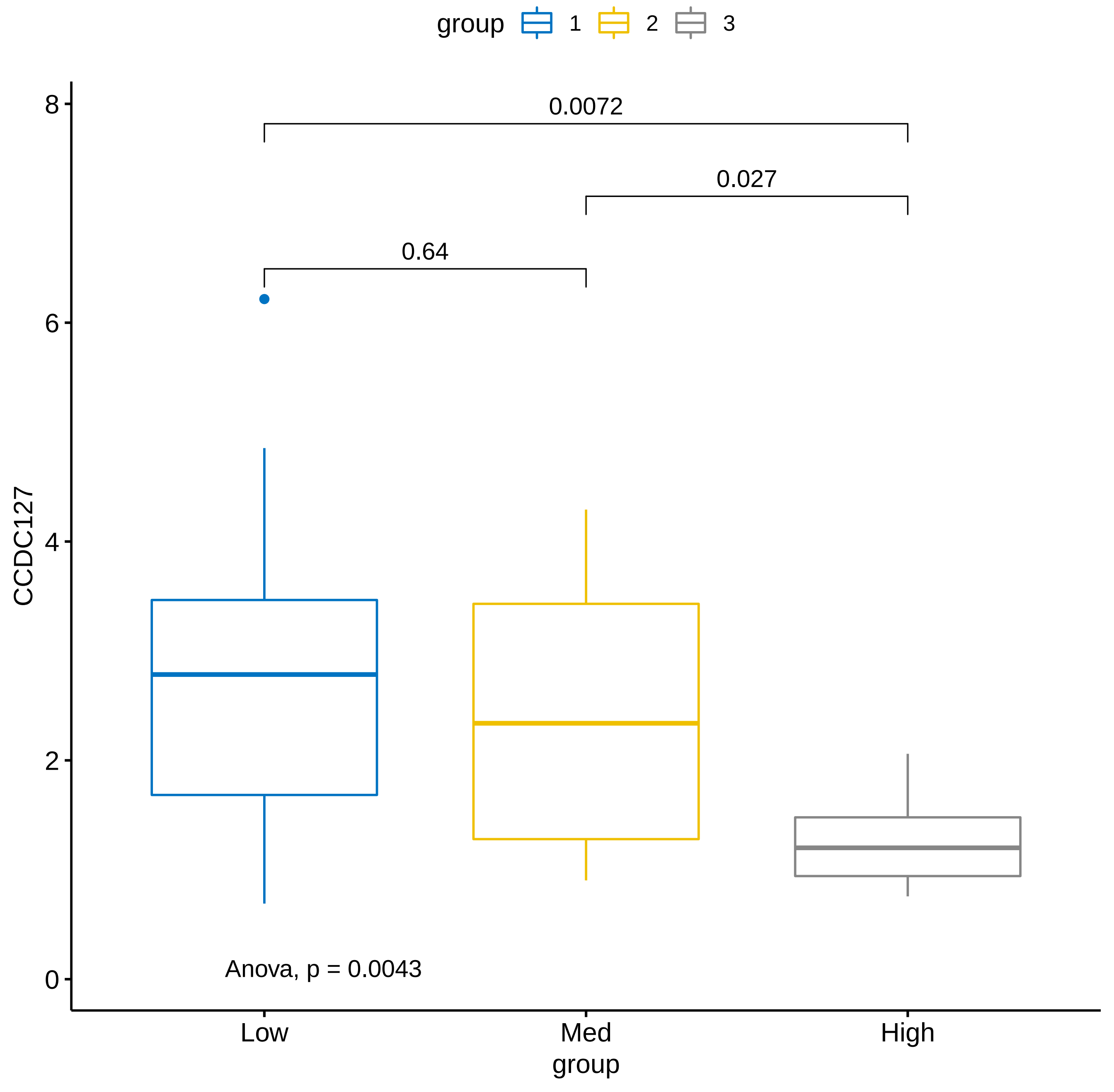
group
1
2
3
0.0072
0.027
0.64
8
6
4
2
0
CCDC127
Anova, p = 0.0043
Low
Med
High
group

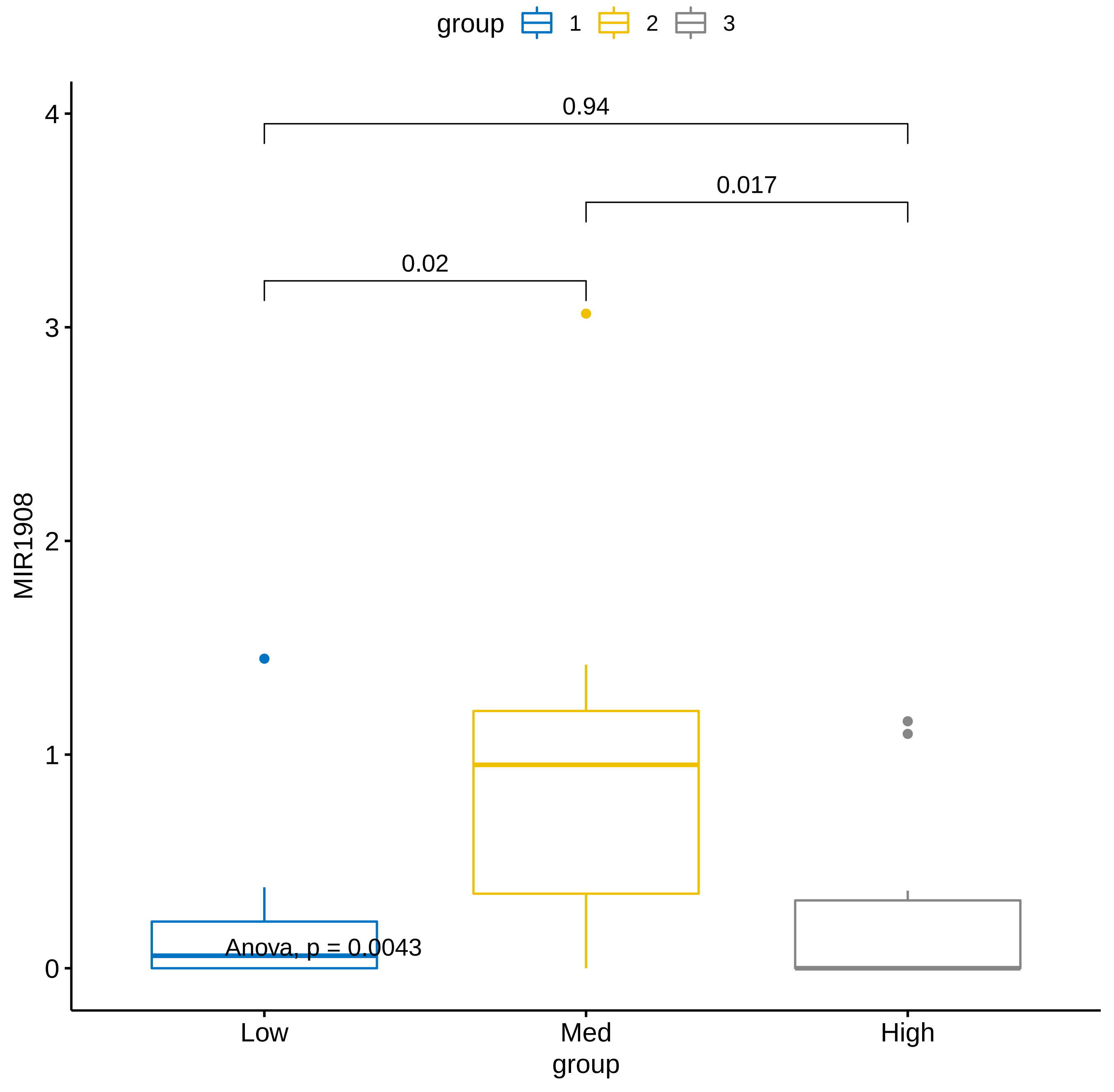
group
1
2
3
0.94
0.017
0.02
4
3
2
1
0
MIR1908
Anova, p = 0.0043
Low
Med
High
group

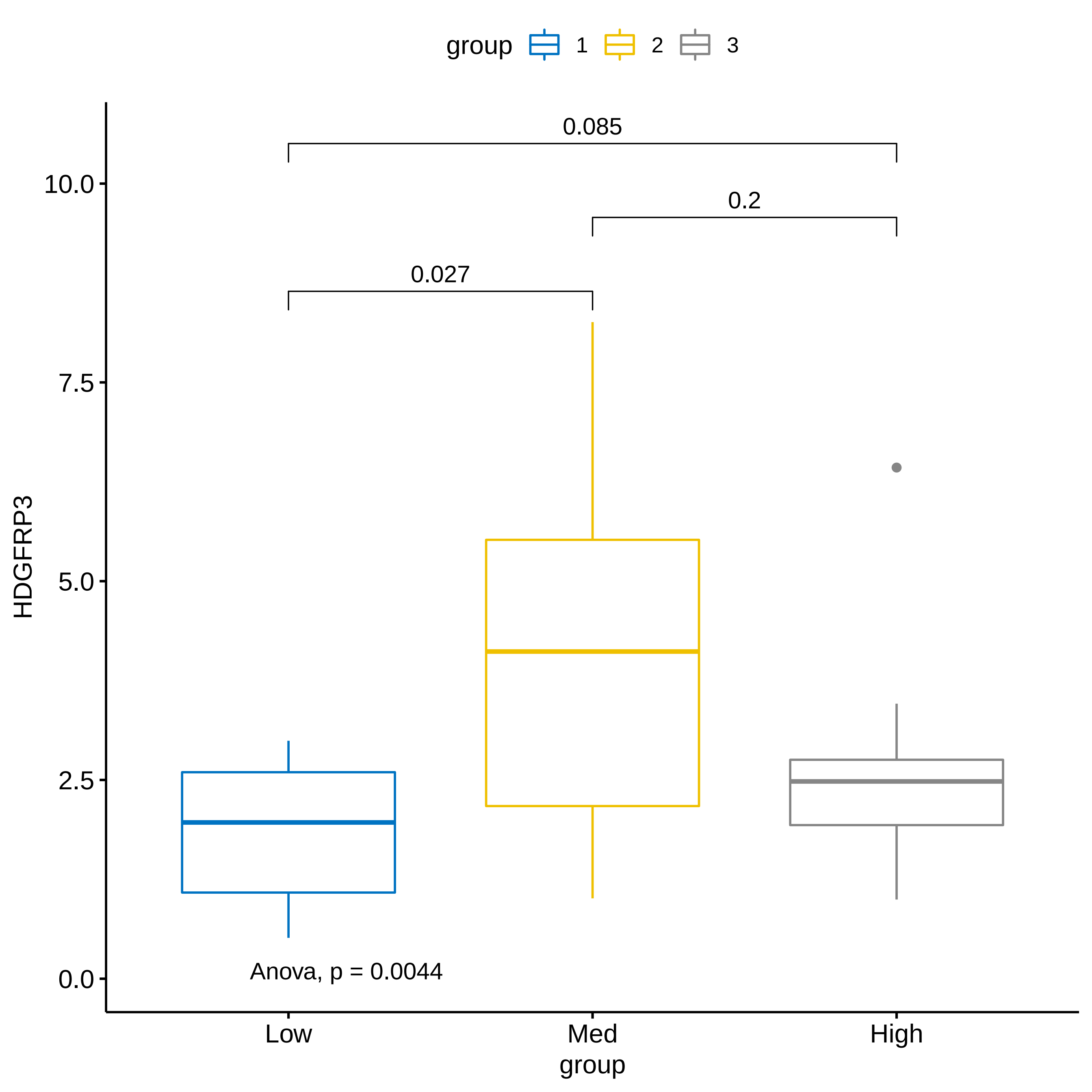

group 1 2 3
0.085
0.2
0.027
10.0
7.5
5.0
2.5
0.0
HDGFRP3
Anova, p = 0.0044
Low
Med
High
group

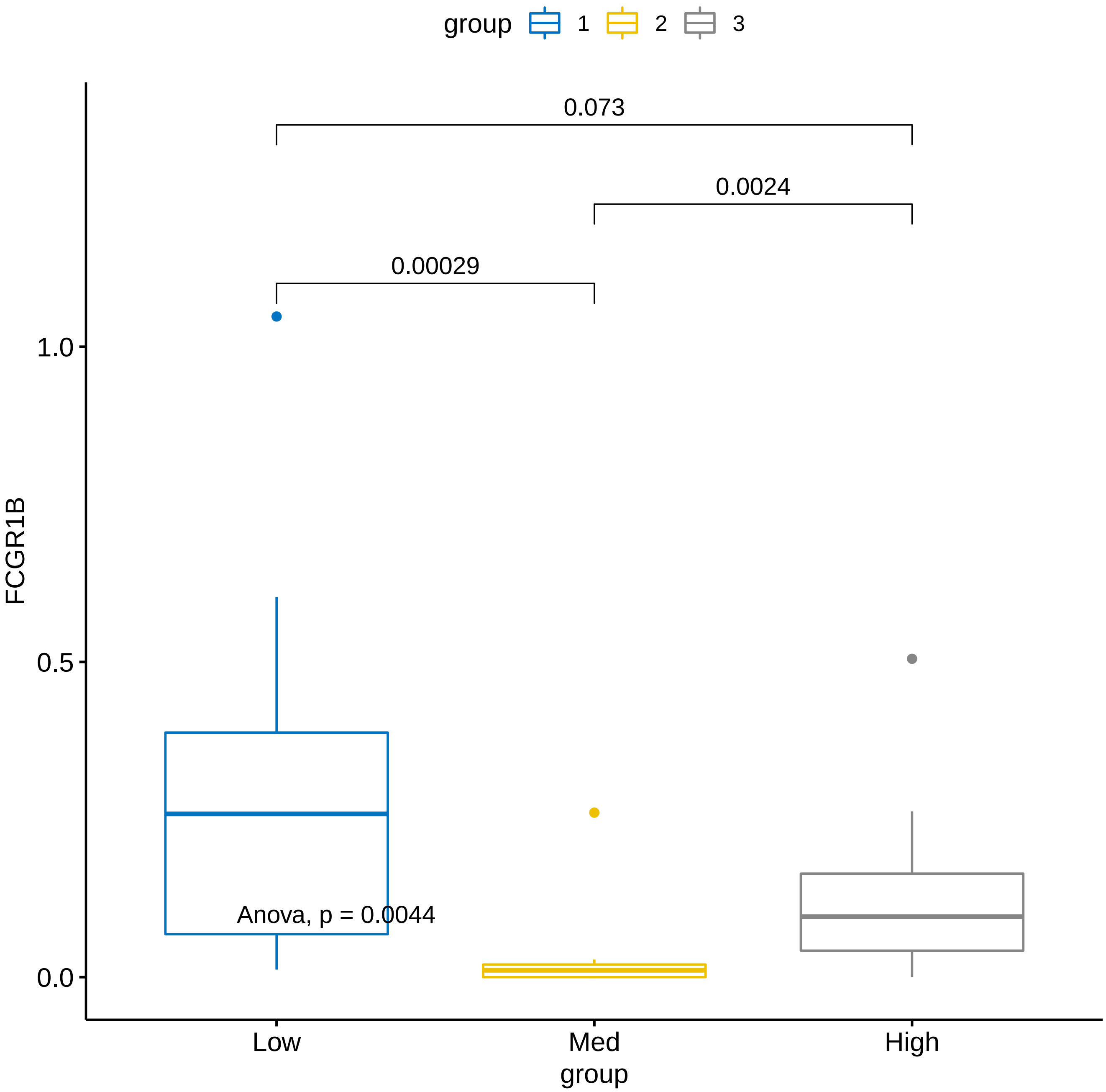

group 1 2 3
0.073
0.0024
0.00029
FCGR1B
1.0
0.5
0.0
Anova, p = 0.0044
Low
Med
High
group

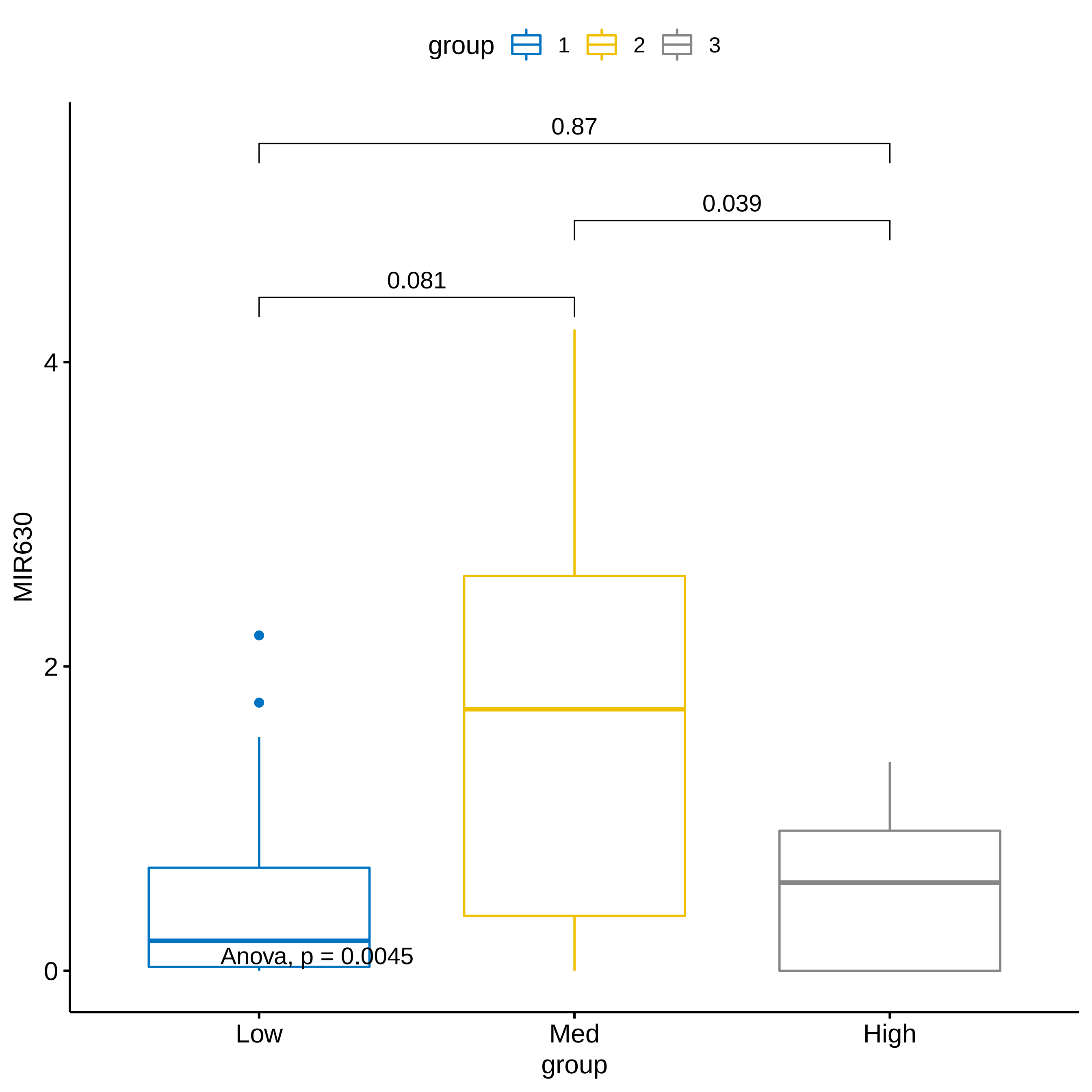

group
1
2
3
0.87
0.039
0.081
4
2
0
MIR630
Anova, p = 0.0045
Low
Med
High
group

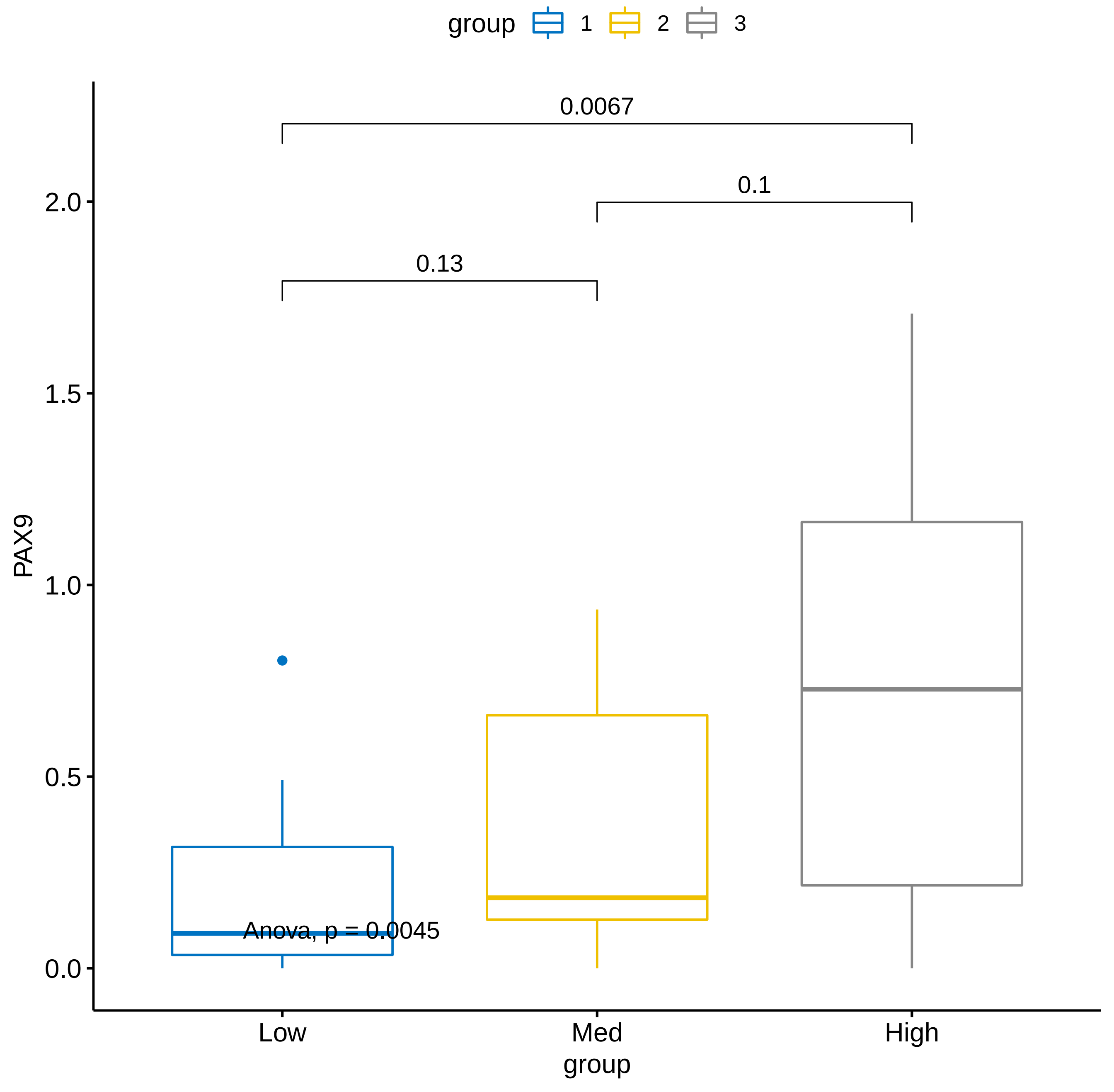

group 1 2 3
0.0067
0.1
0.13
Anova, p = 0.0045
PAX9
2.0
1.5
1.0
0.5
0.0
Low
Med
High
group

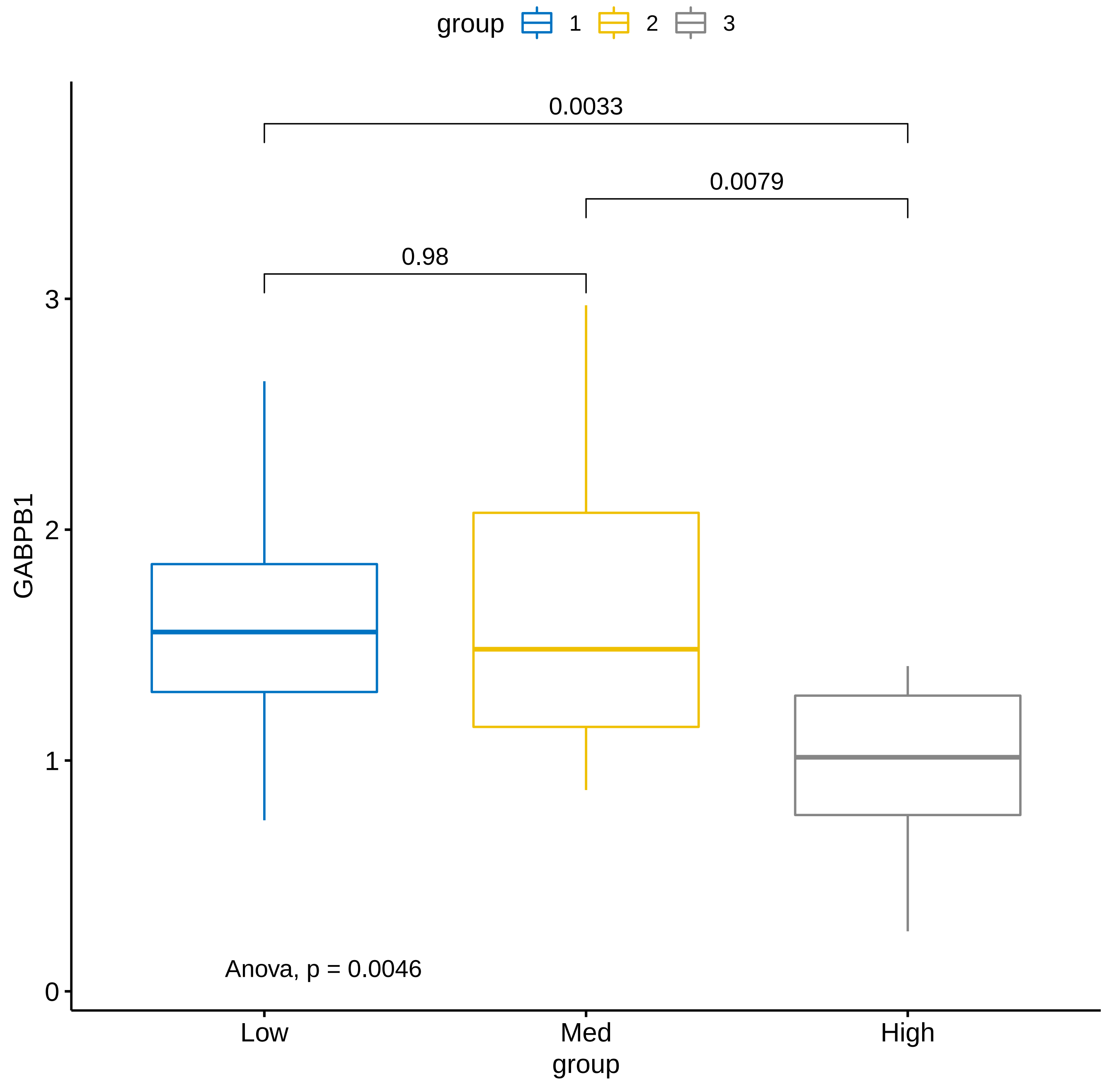

group 1 2 3
0.0033
0.0079
0.98
GABPB1
3
2
1
0
Anova, p = 0.0046
Low
Med
High
group

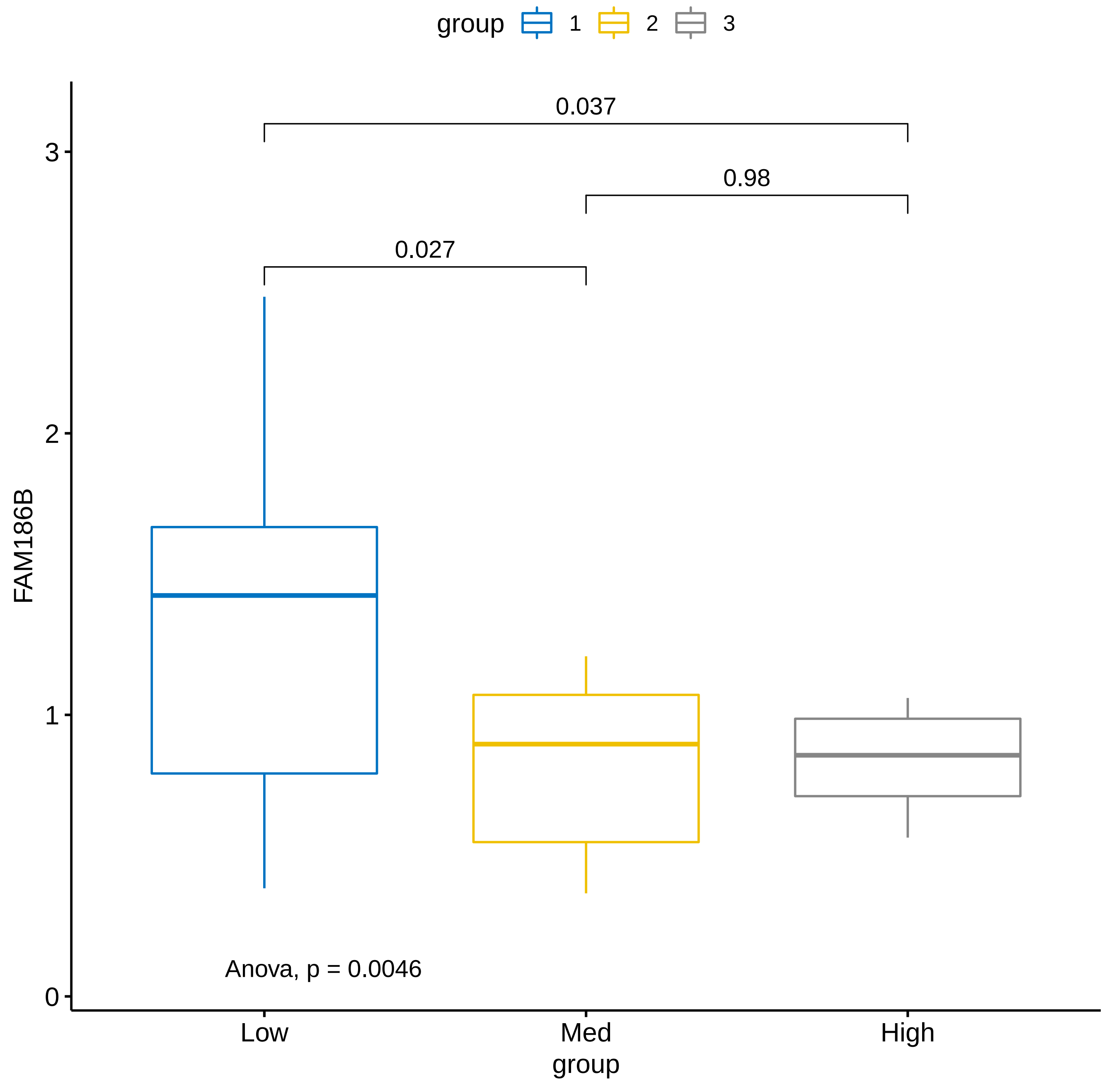
group
1
2
3
0.037
0.98
0.027
FAM186B
3
2
1
0
Anova, p = 0.0046
Low
Med
High
group

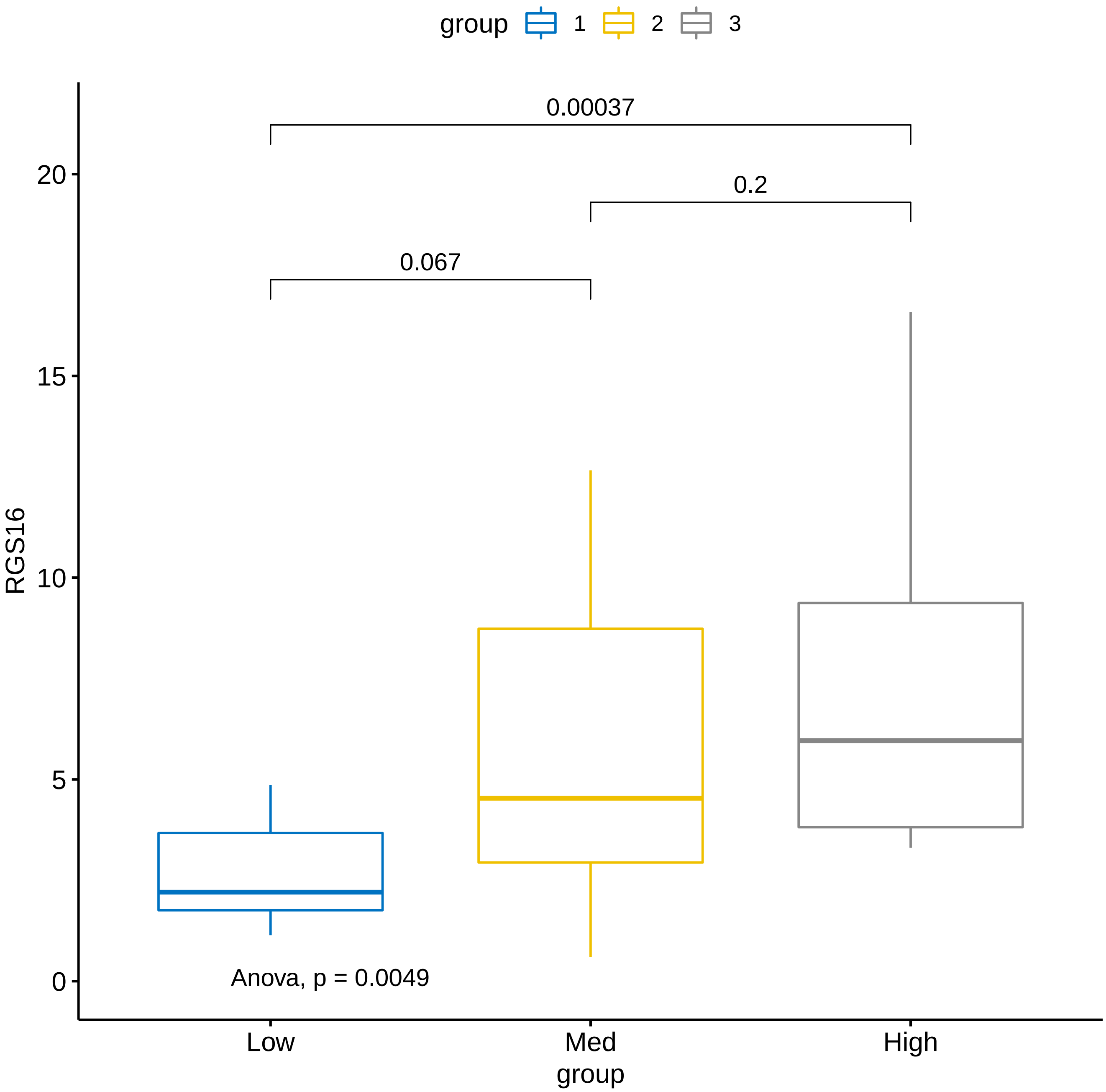
group 1 2 3
0.00037
0.2
0.067
20
15
10
5
0
RGS16
Anova, p = 0.0049
Low
Med
High
group

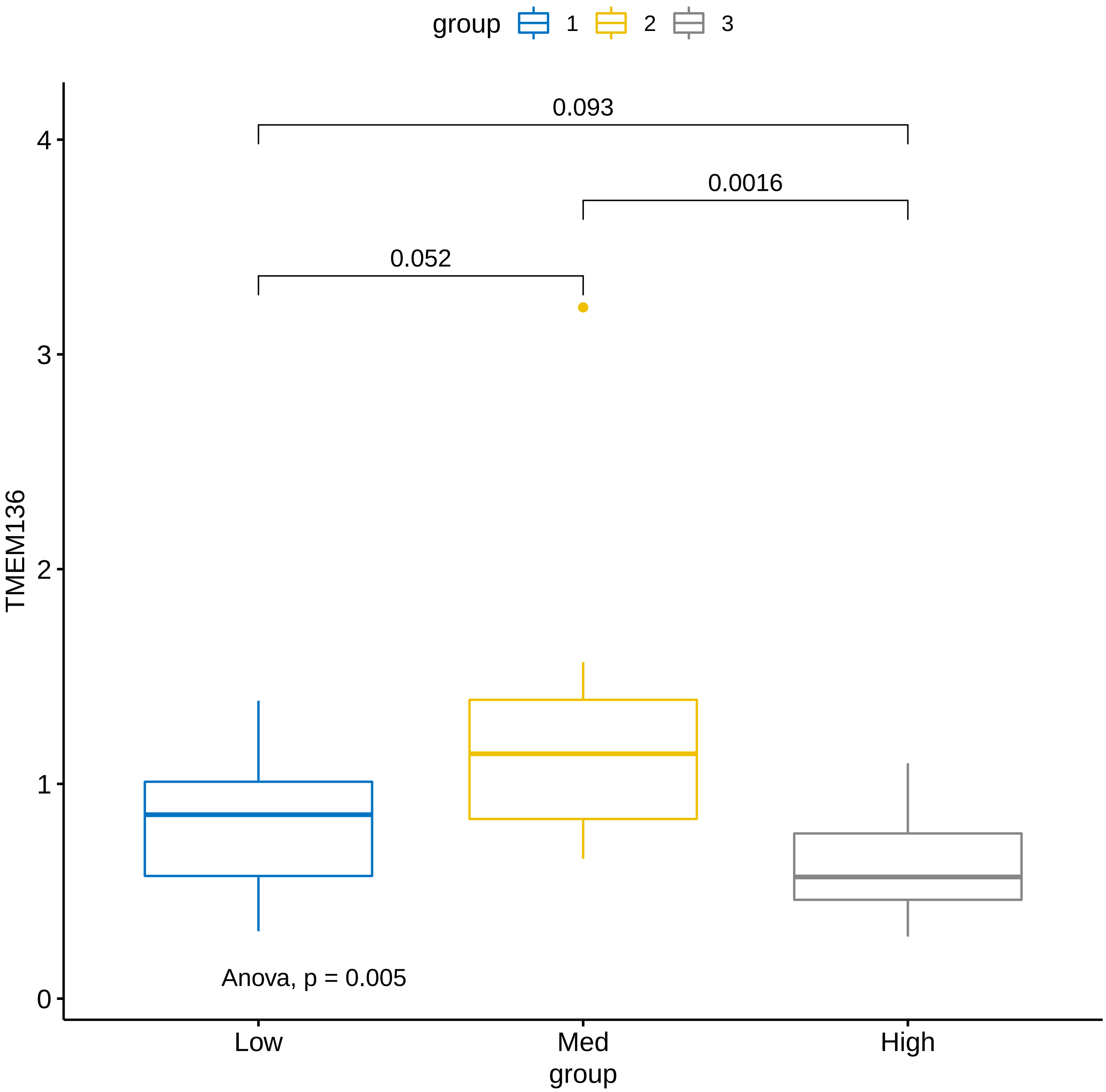

group
1
2
3
0.093
0.0016
0.052
TMEM136
4
3
2
1
0
Anova, p = 0.005
Low
Med
High
group

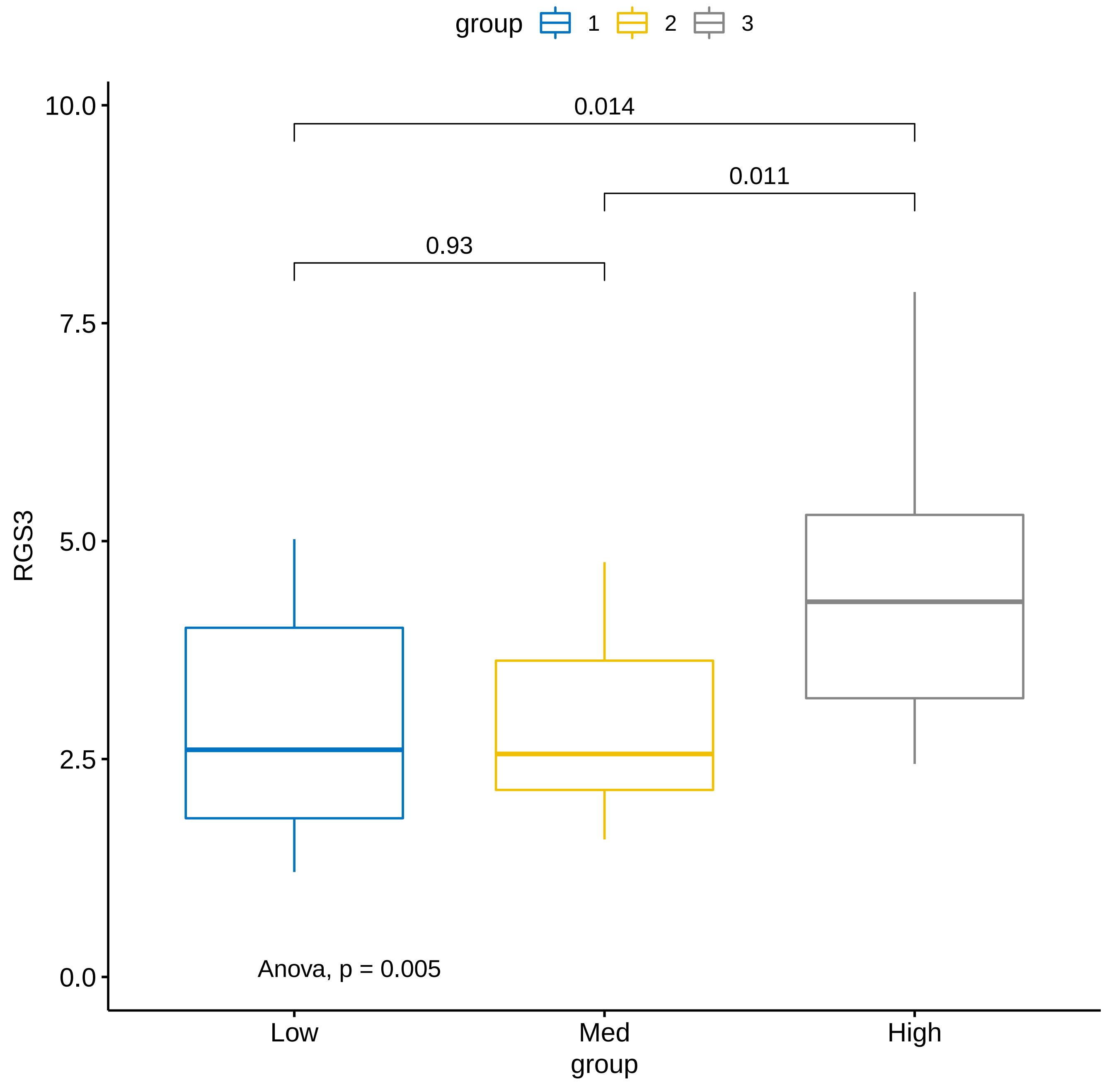

group 1 2 3
0.014
0.011
0.93
10.0
7.5
5.0
2.5
0.0
RGS3
Anova, p = 0.005
Low
Med
High
group

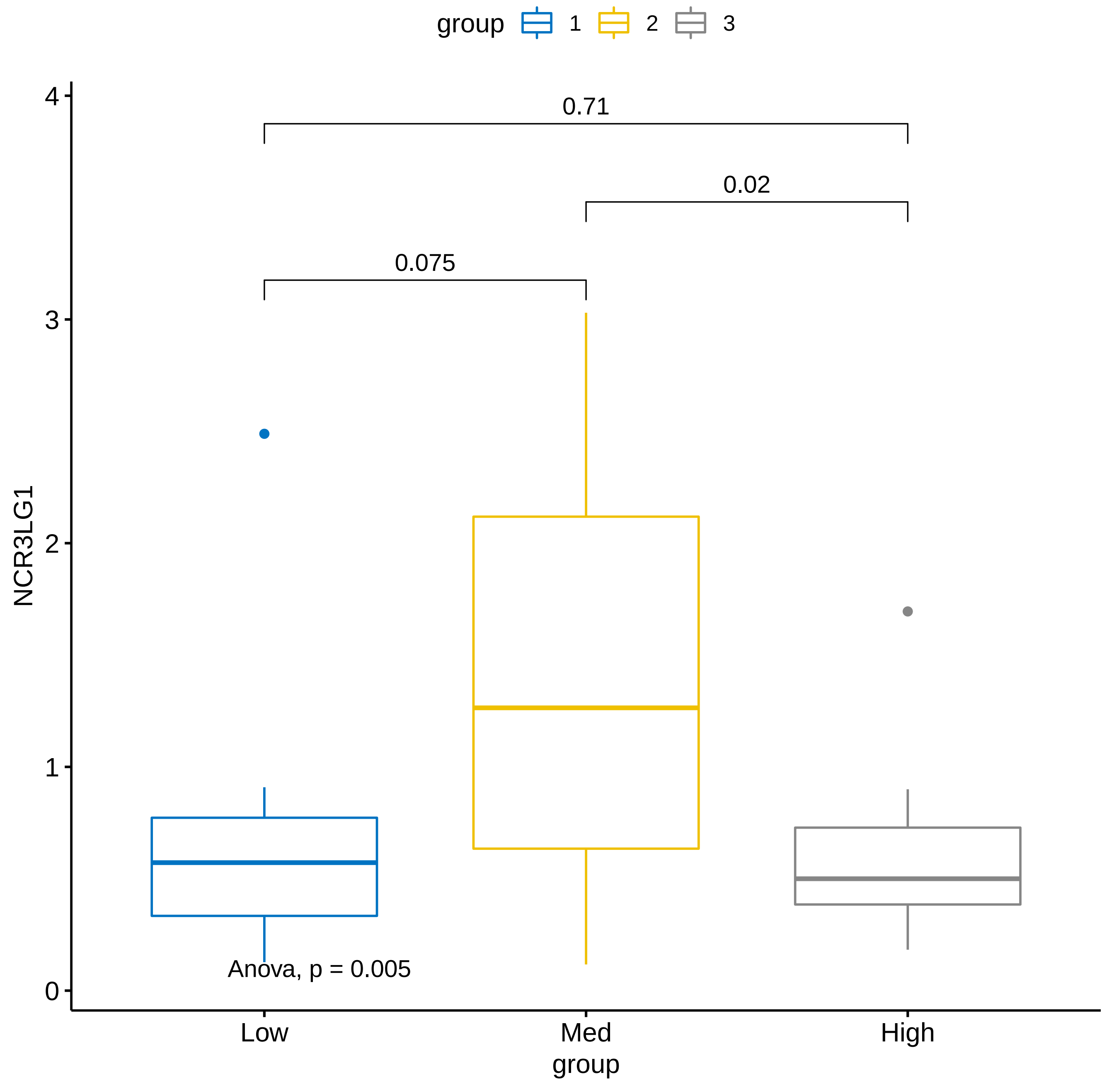
group 1 2 3
0.71
0.02
0.075
NCR3LG1
4
3
2
1
0
Anova, p = 0.005
Low
Med
High
group

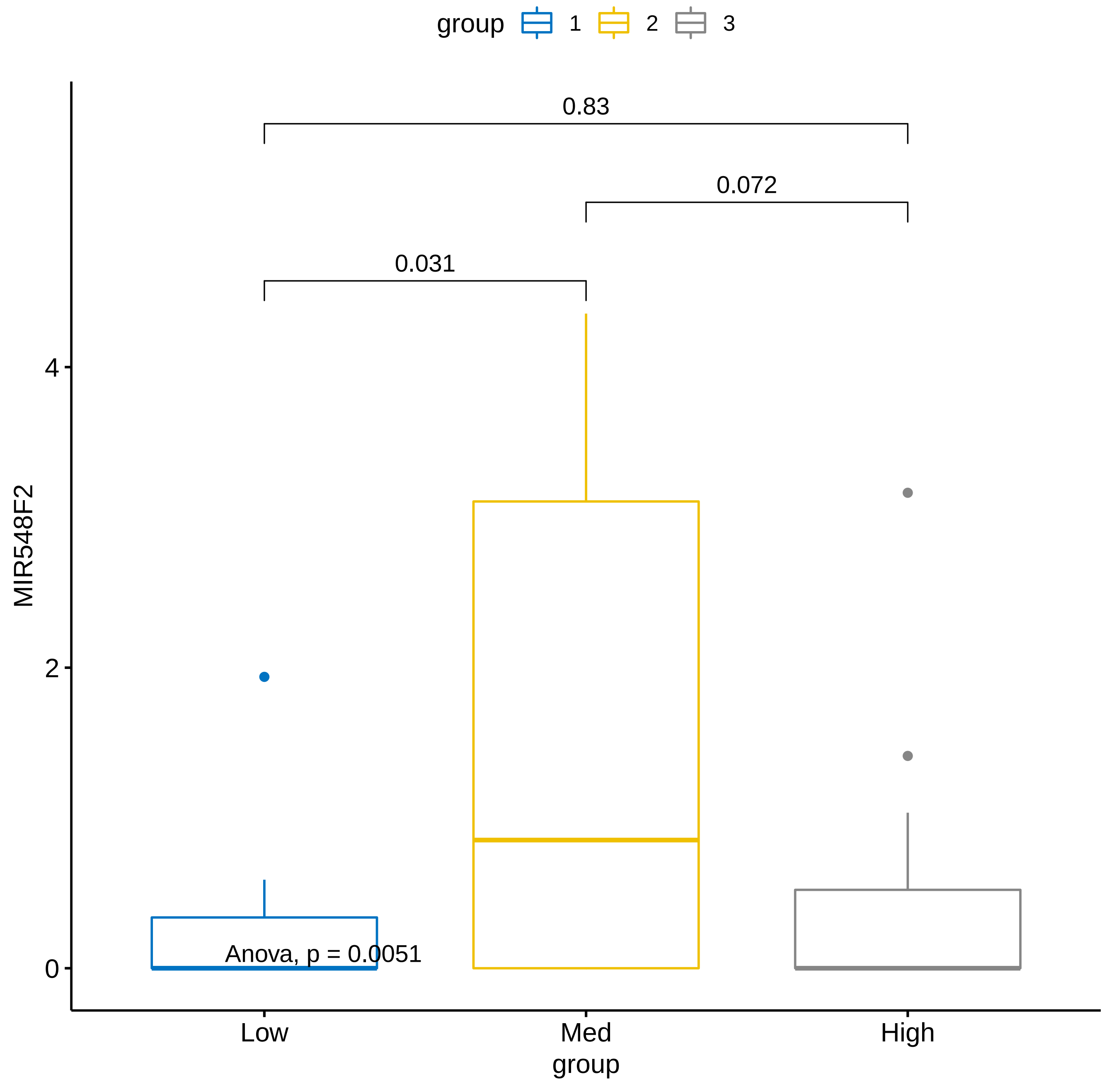
group
1
2
3
0.83
0.072
0.031
4
2
0
MIR548F2
Anova, p = 0.0051
Low
Med
High
group

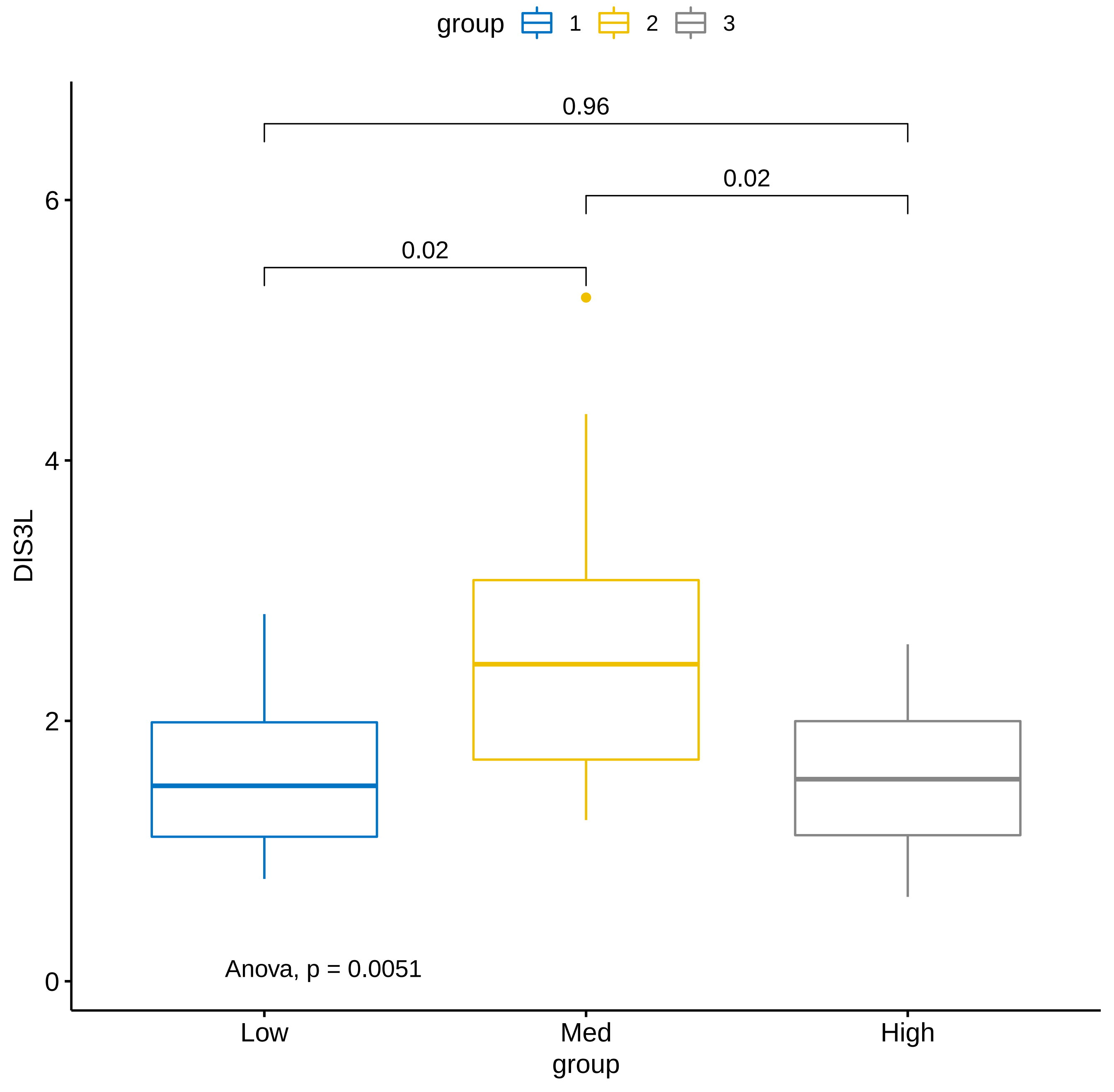

group
1
2
3
0.96
0.02
0.02
6
4
2
0
DIS3L
Anova, p = 0.0051
Low
Med
High
group

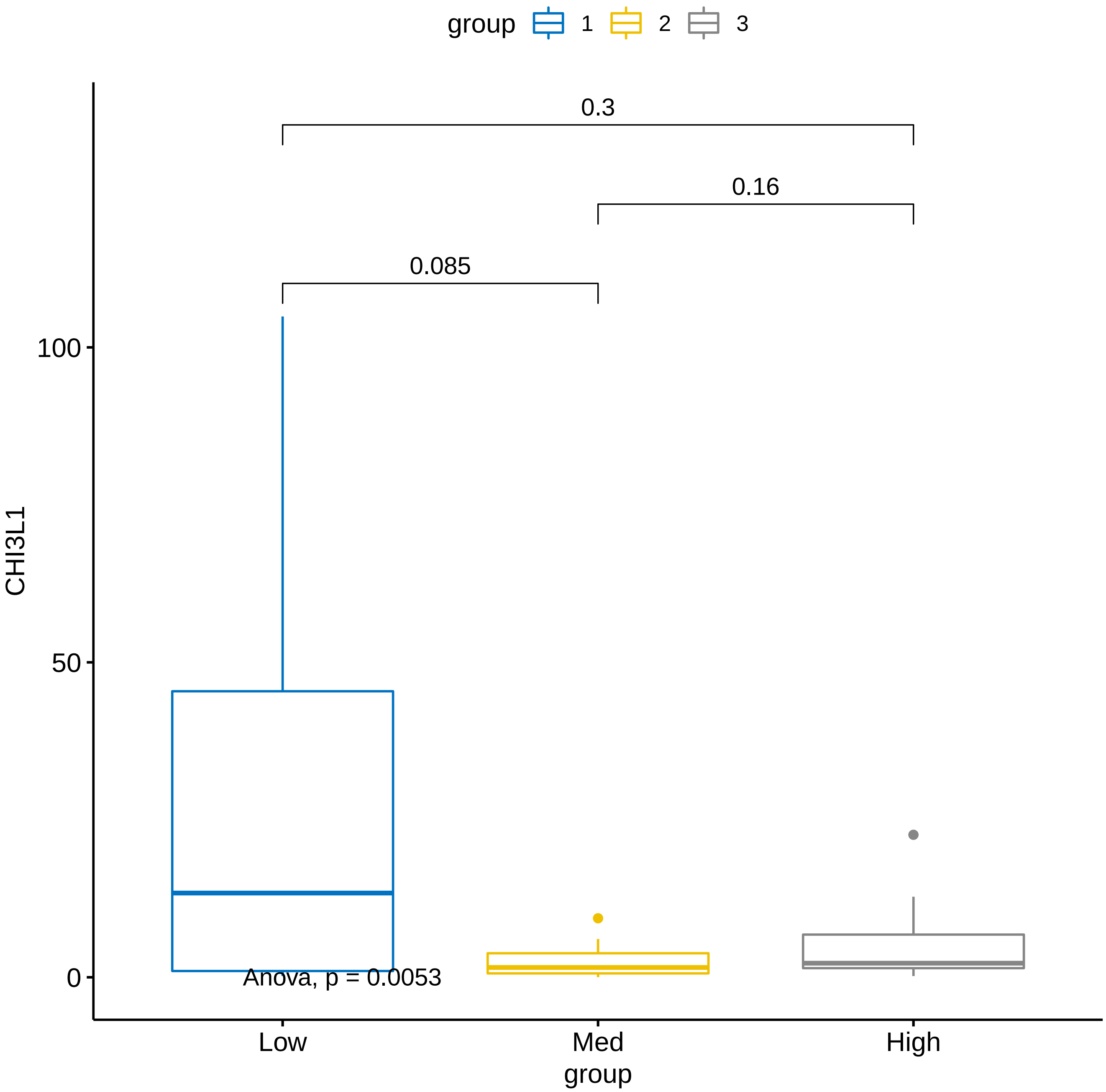
group
1
2
3
0.3
0.16
0.085
100
50
0
CHI3L1
Anova, p = 0.0053
Low
Med
High
group

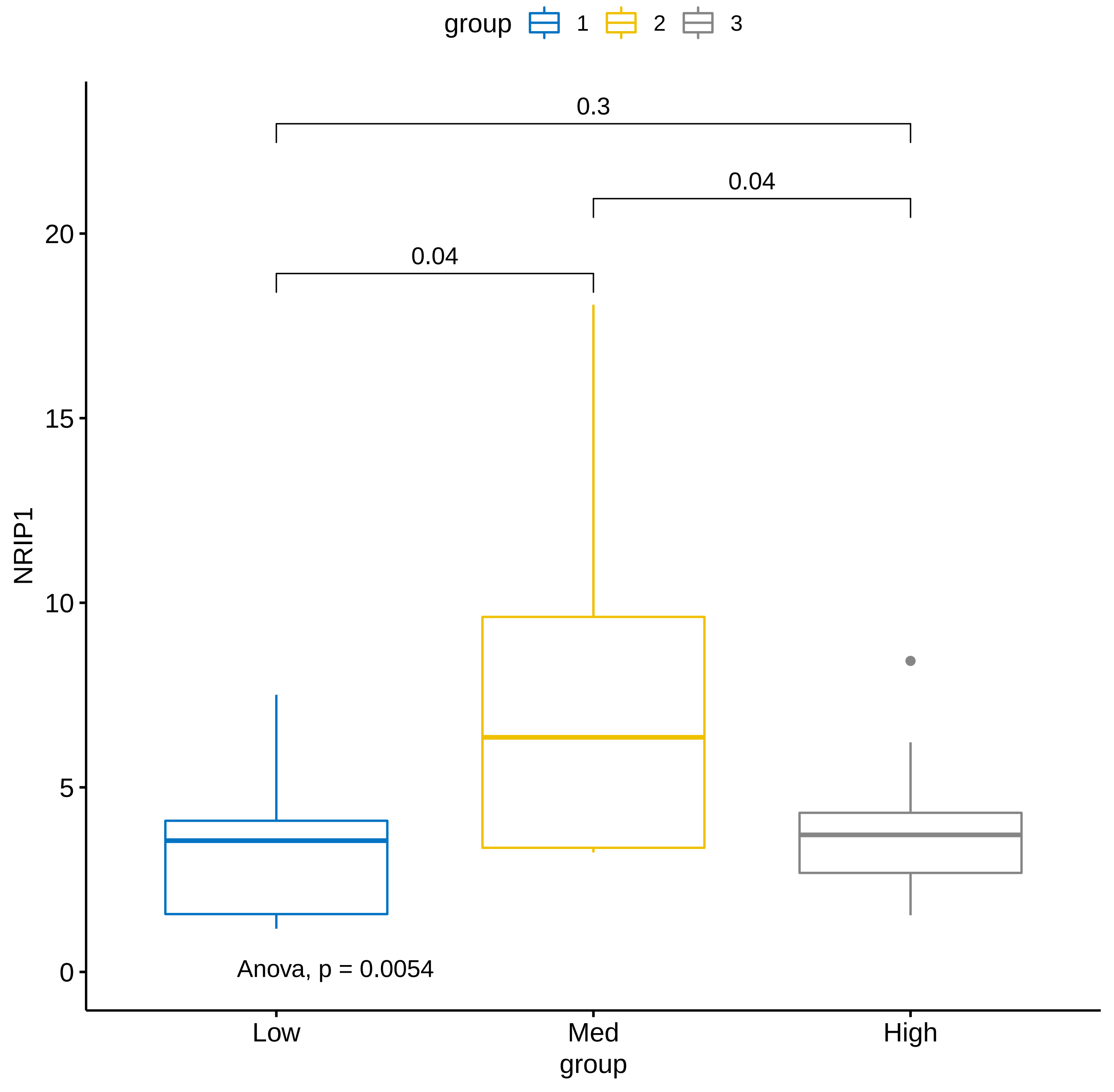
group 1 2 3
0.3
0.04
0.04
NRIP1
20
15
10
5
0
Anova, p = 0.0054
Low
Med
High
group

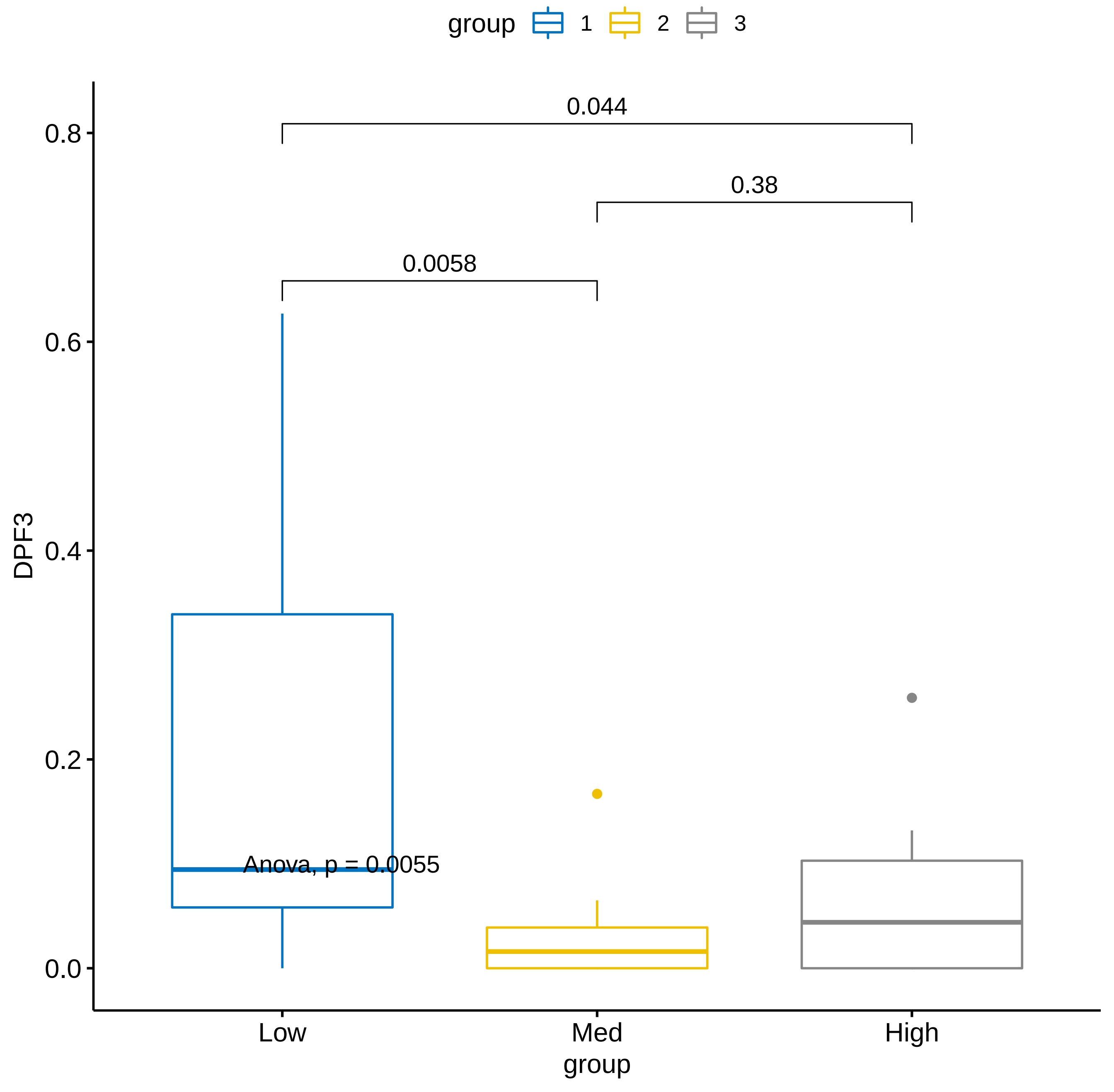

group
1
2
3
0.044
0.38
0.0058
Anova, p = 0.0055
DPF3
0.8
0.6
0.4
0.2
0.0
Low
Med
High
group

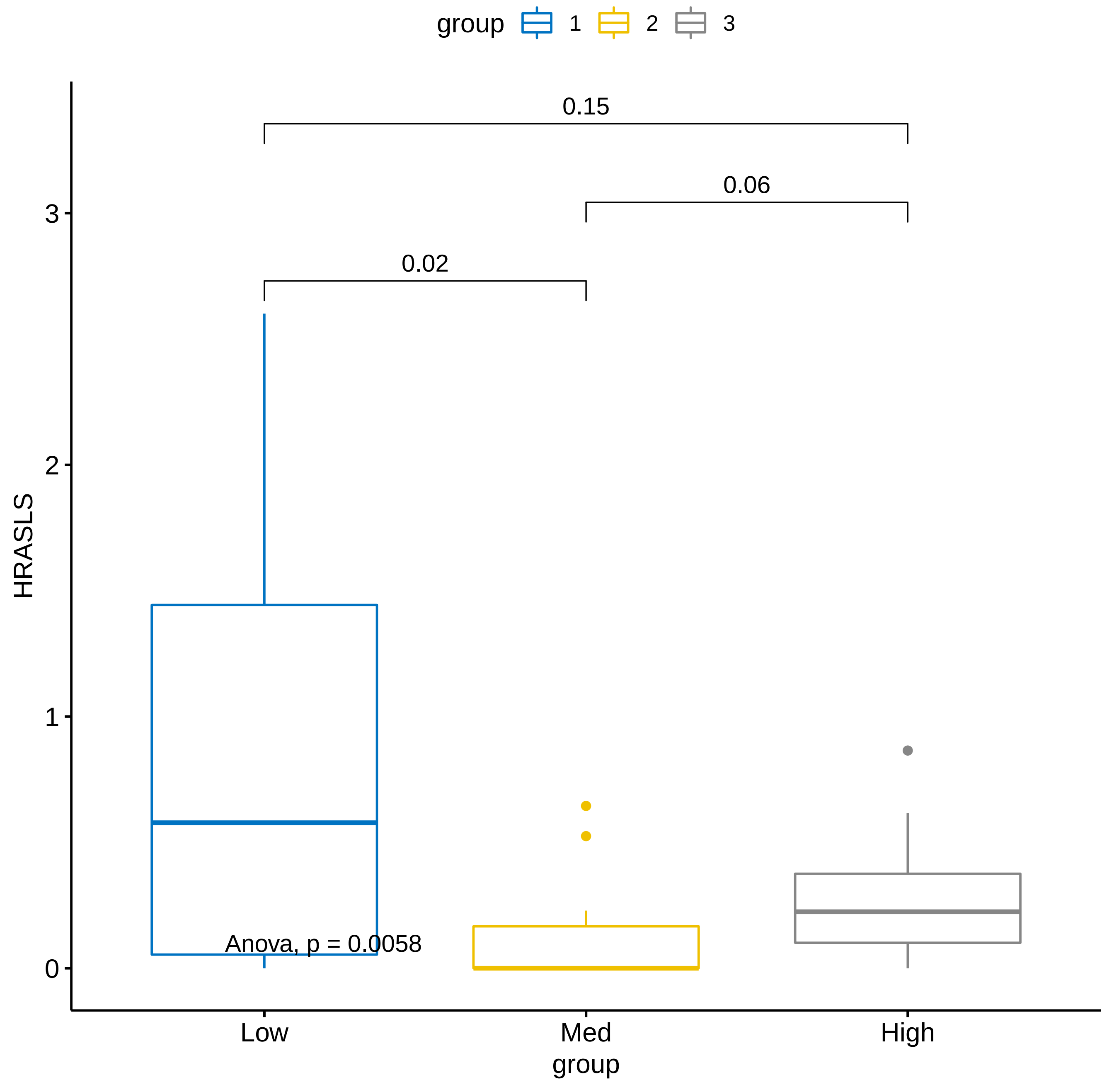
group
1
2
3
0.15
0.06
0.02
3
2
1
0
HRASLS
Anova, p = 0.0058
Low
Med
High
group

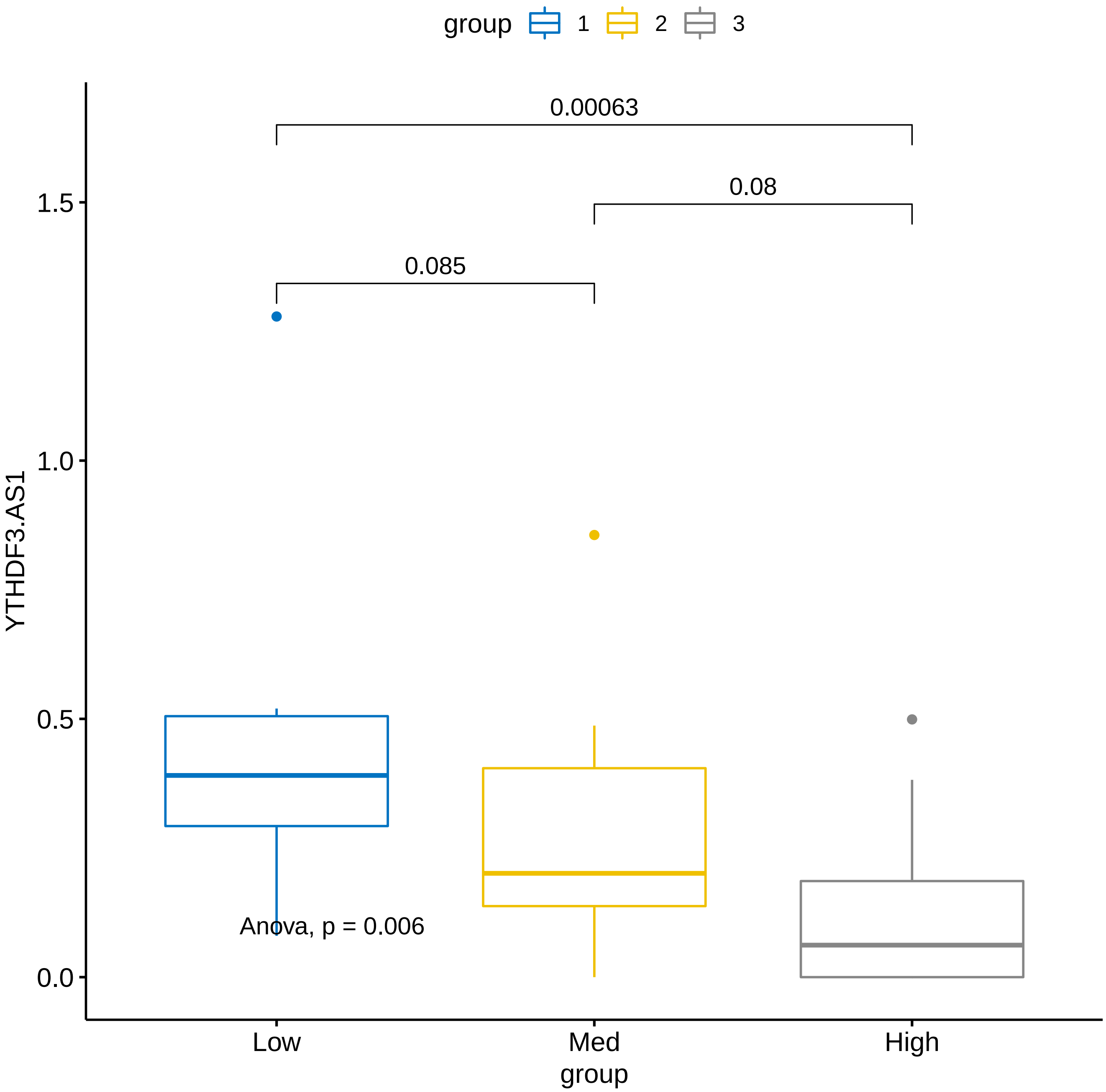

group
1
2
3
0.00063
0.08
0.085
1.5
1.0
0.5
0.0
YTHDF3.AS1
Anova, p = 0.006
Low
Med
High
group

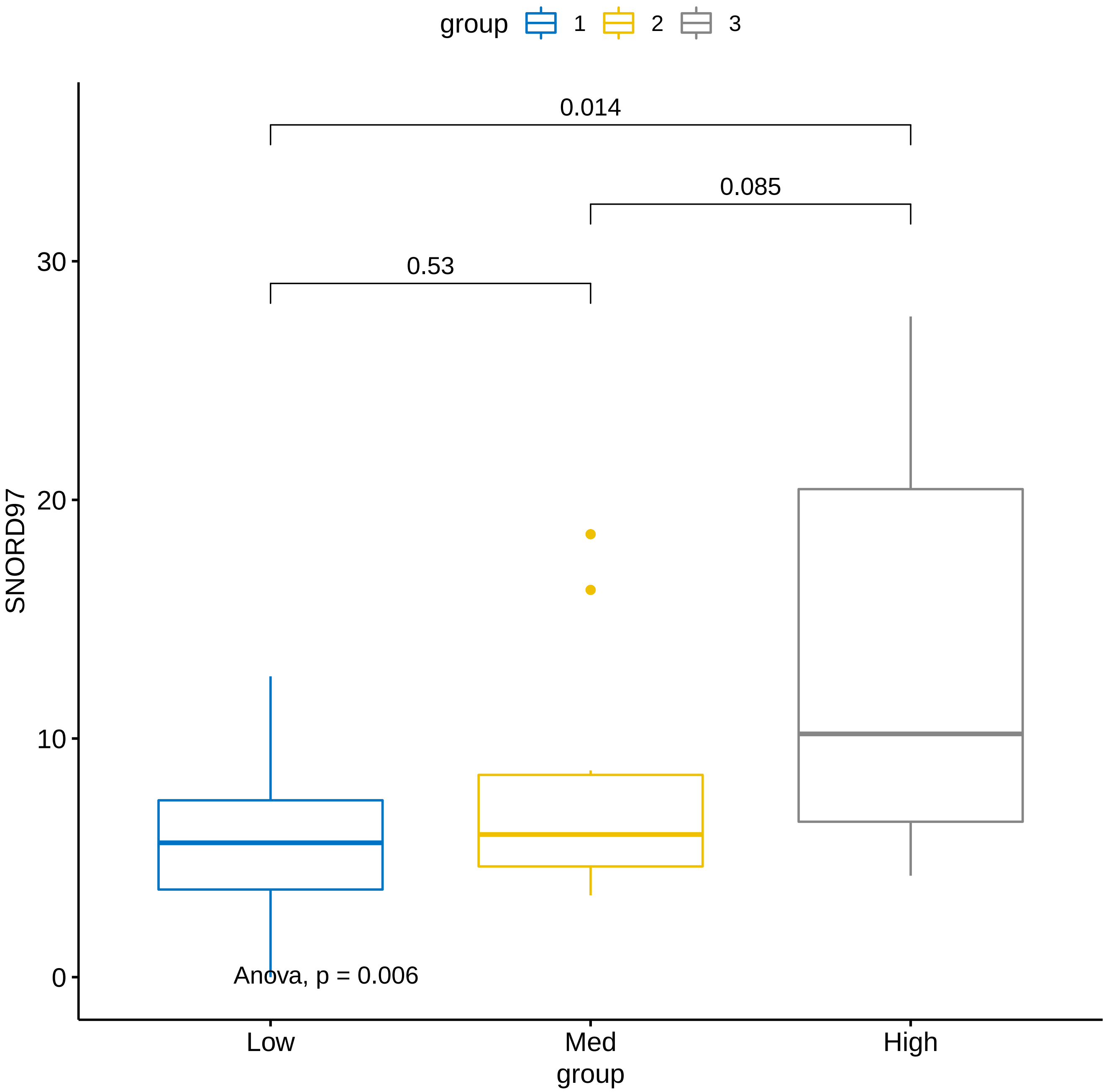
group 1 2 3
0.014
0.085
0.53
30
20
10
0
SNORD97
Anova, p = 0.006
Low
Med
High
group

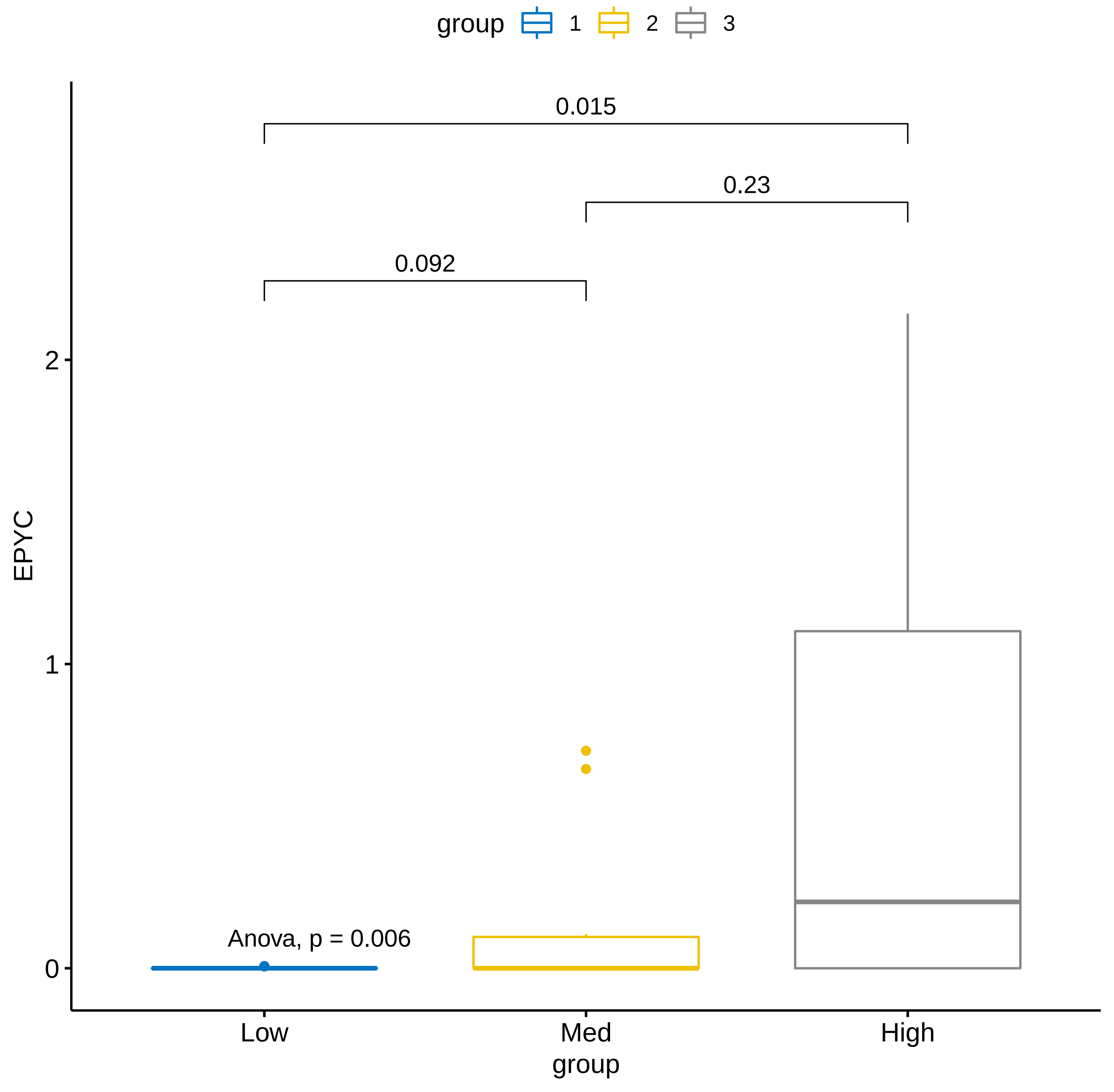
group
1
2
3
0.015
0.23
0.092
2
1
0
EPYC
Anova, p = 0.006
Low
Med
High
group

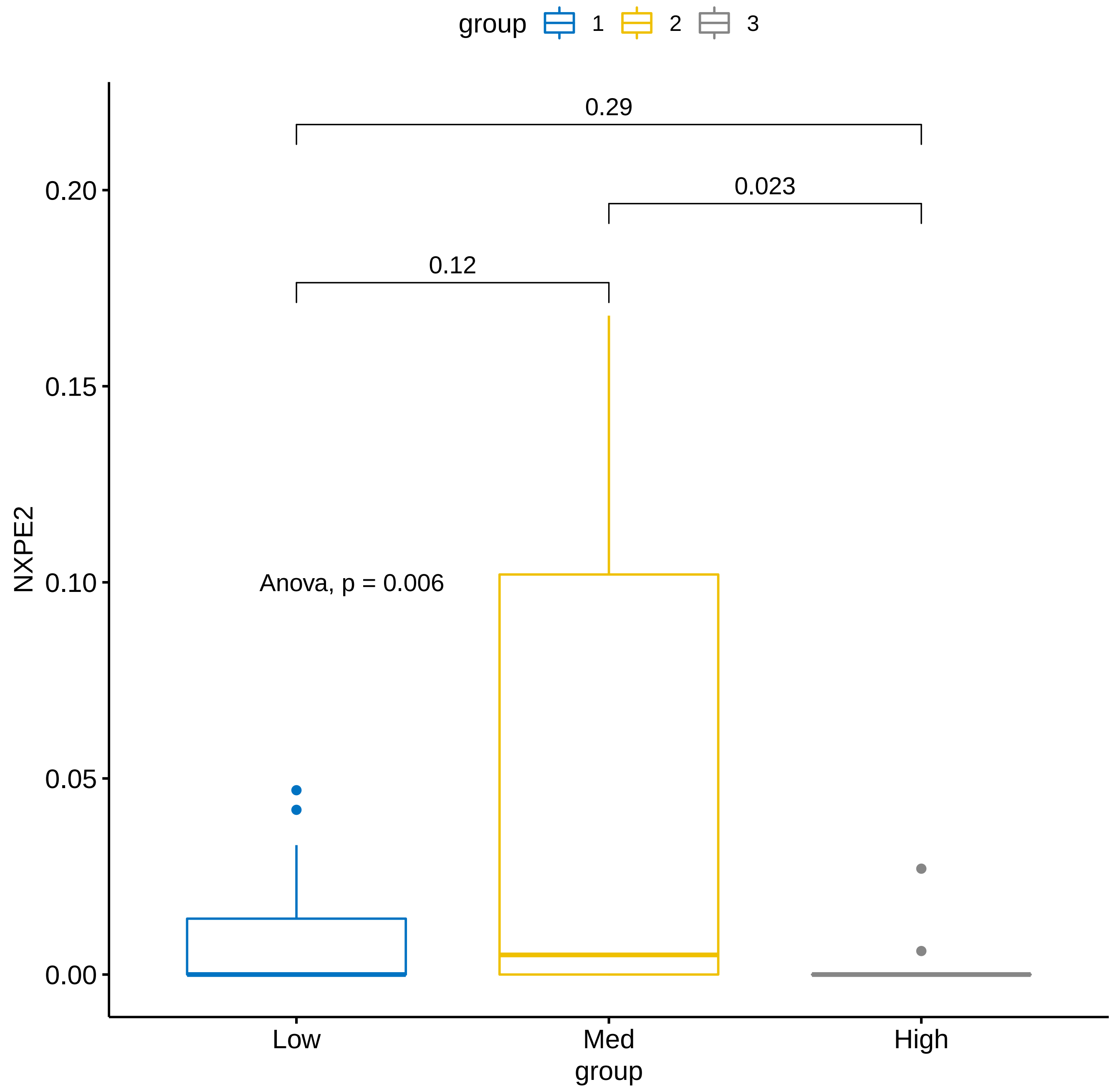
group
1
2
3
0.29
0.023
0.12
Anova, p = 0.006
NXPE2
0.20
0.15
0.10
0.05
0.00
Low
Med
High
group

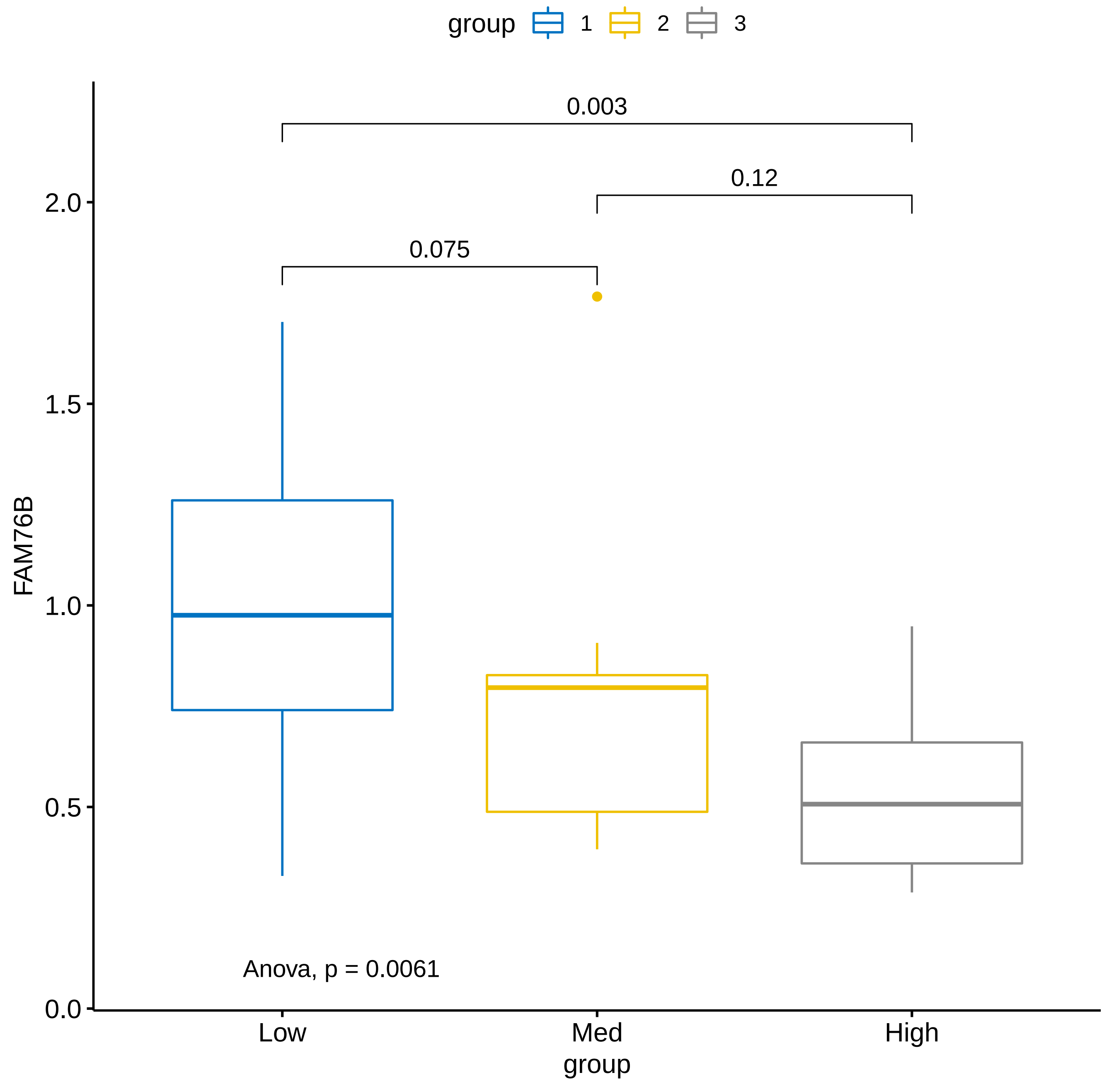
group
1
2
3
0.003
0.12
0.075
2.0
1.5
1.0
0.5
0.0
FAM76B
Anova, p = 0.0061
Low
Med
High
group

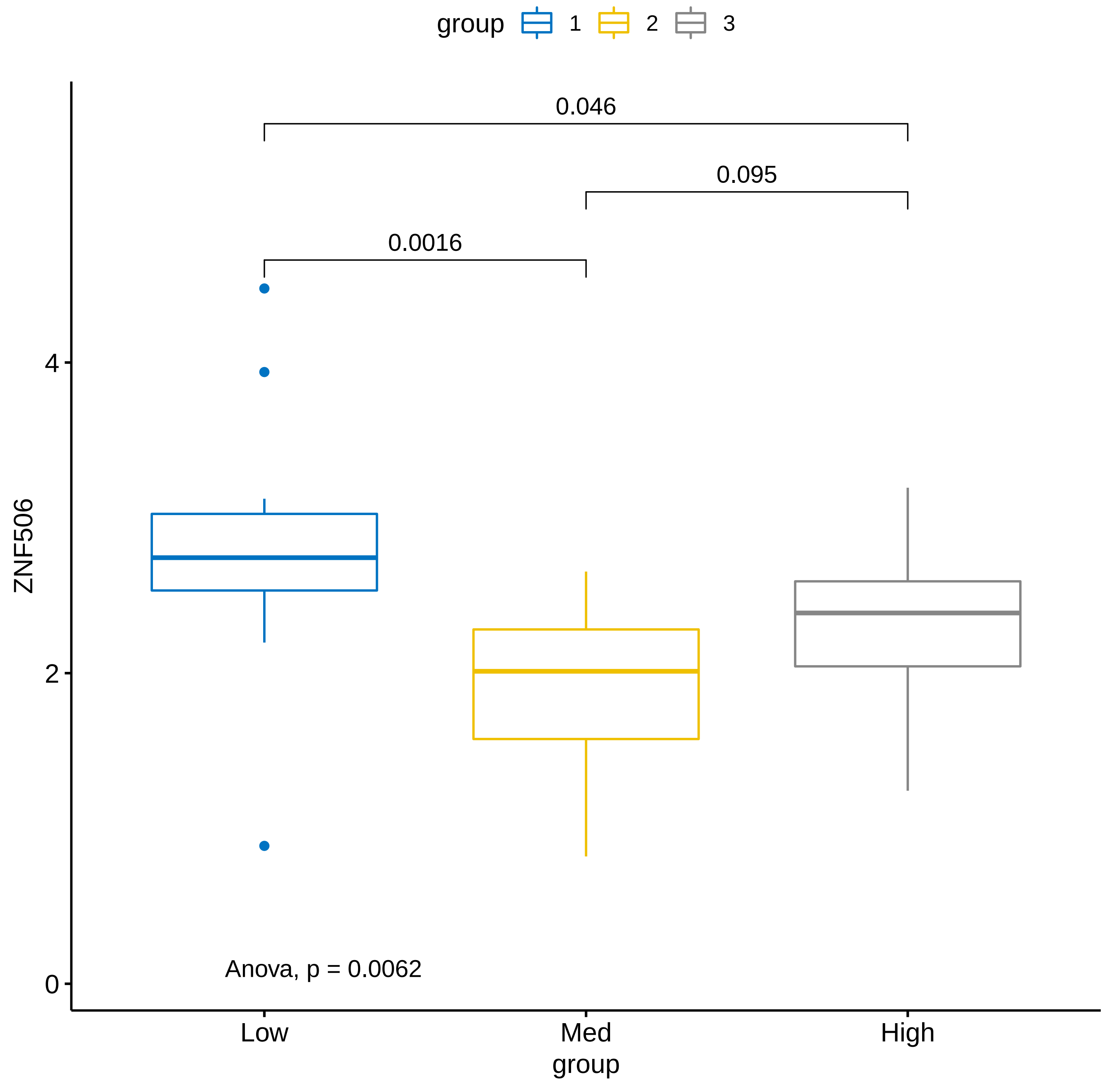
group 1 2 3
0.046
0.095
0.0016
ZNF506
4
2
0
Anova, p = 0.0062
Low
Med
High
group

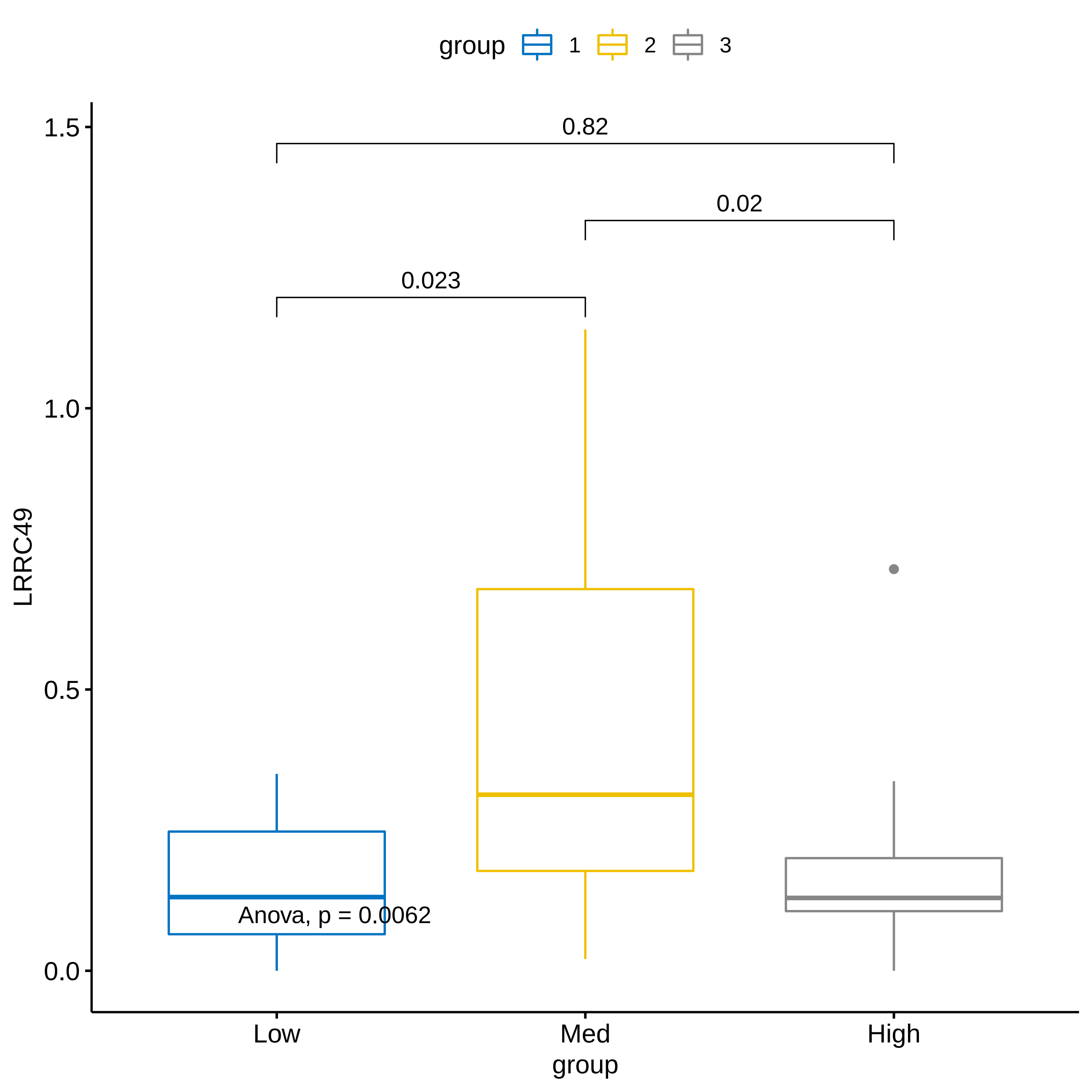

group
1
2
3
0.82
0.02
0.023
1.5
1.0
0.5
0.0
LRRC49
Anova, p = 0.0062
Low
Med
High
group

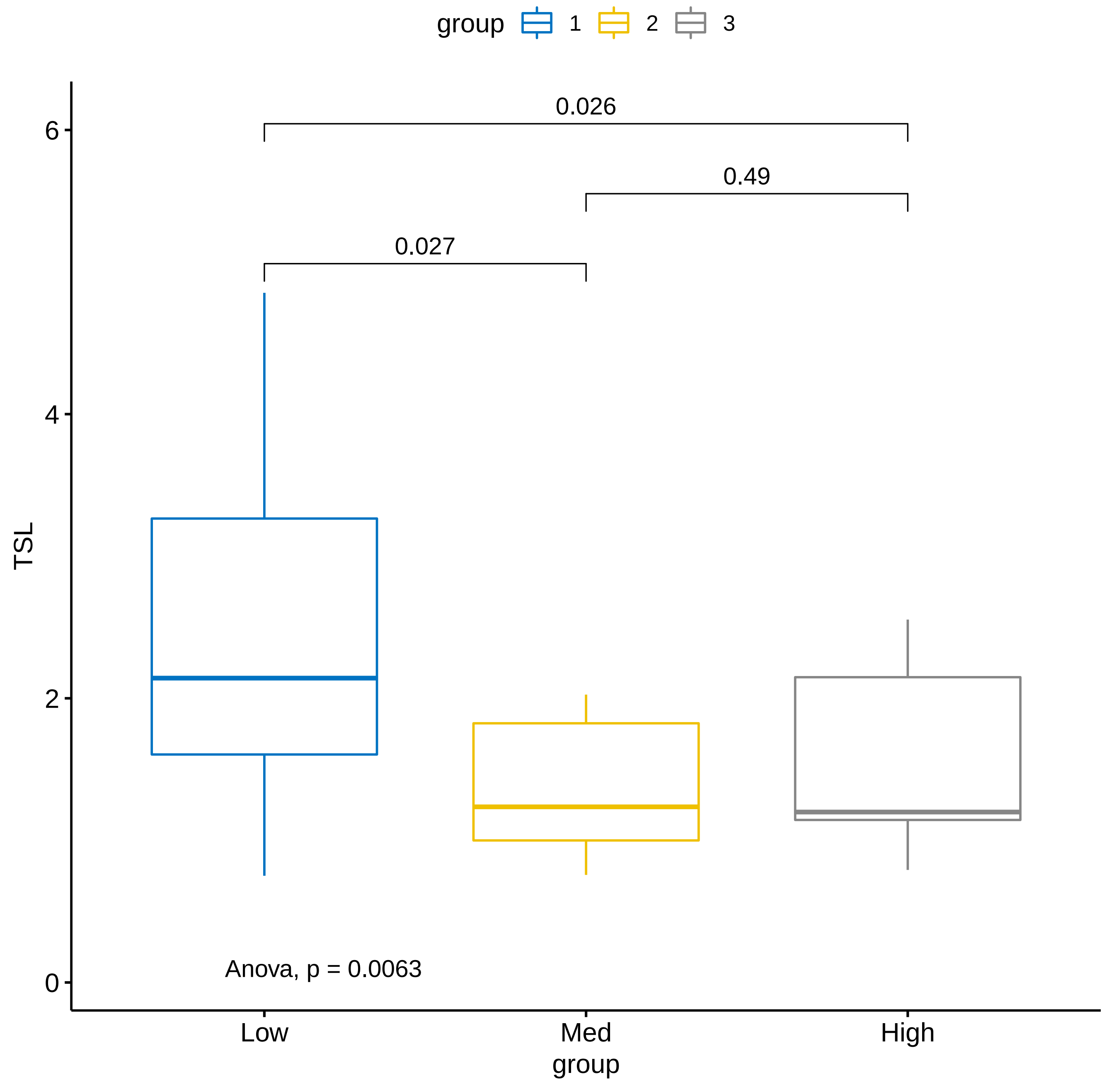
group 1 2 3
0.026
0.49
0.027
TSL
6
4
2
0
Anova, p = 0.0063
Low
Med
High
group

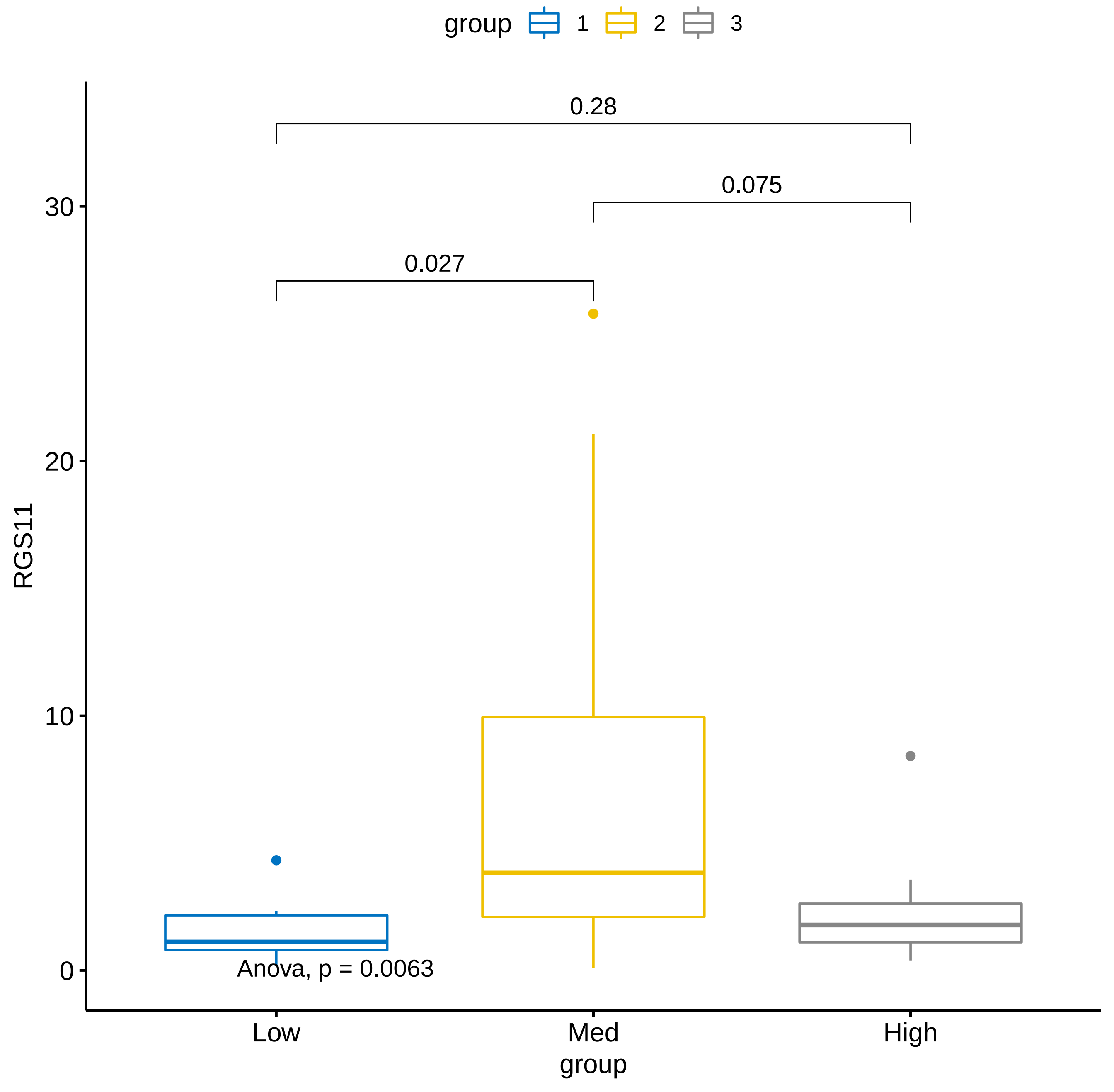

group 1 2 3
0.28
0.075
0.027
30
20
10
0
RGS11
Anova, p = 0.0063
Low
Med
High
group

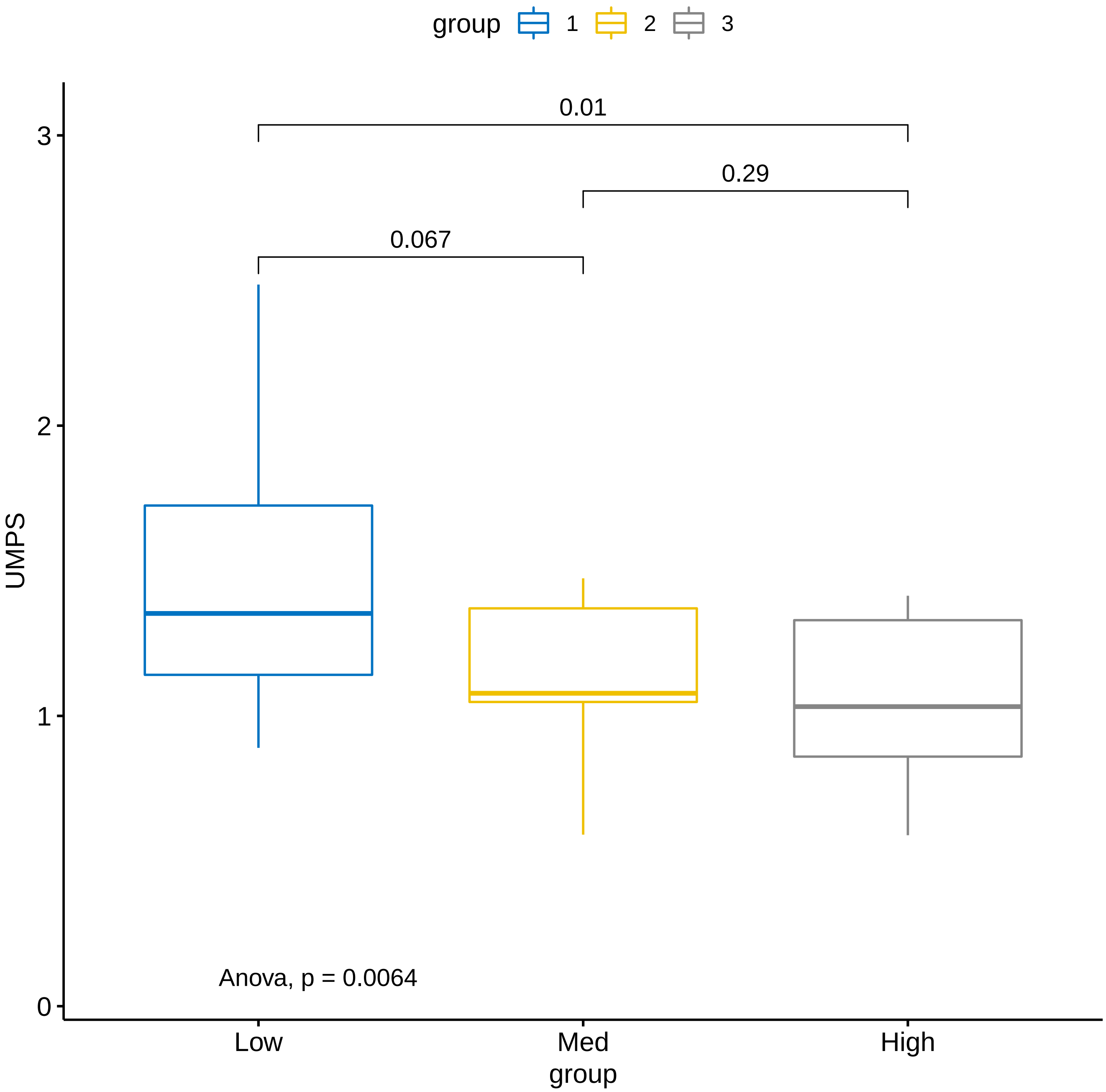
group 1 2 3
0.01
0.29
0.067
3
2
1
0
UMPS
Anova, p = 0.0064
Low
Med
High
group

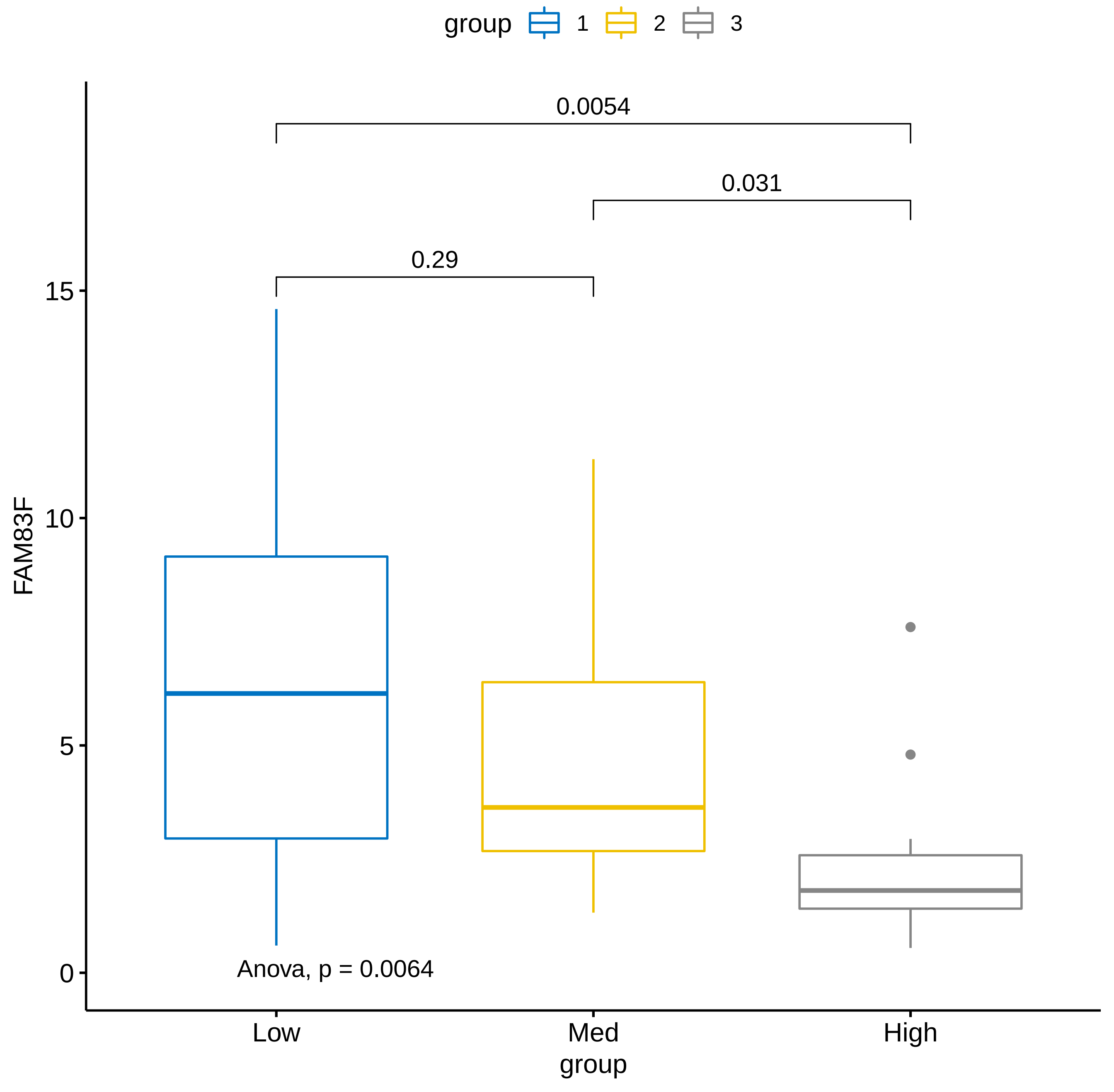

group
1
2
3
0.0054
0.031
0.29
15
10
5
0
FAM83F
Anova, p = 0.0064
Low
Med
High
group

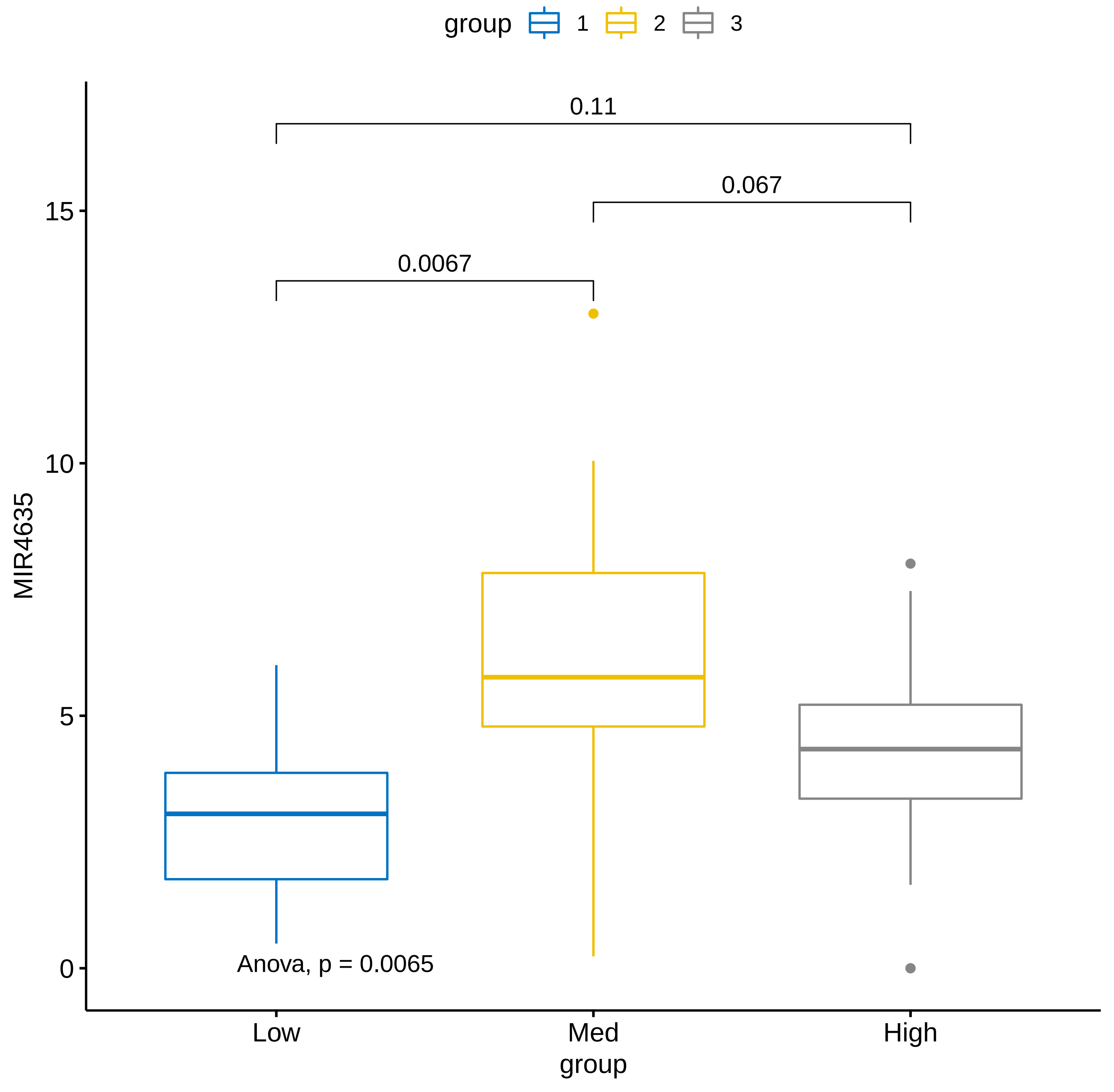
group
1
2
3
0.11
0.067
0.0067
15
10
5
0
MIR4635
Anova, p = 0.0065
Low
Med
High
group

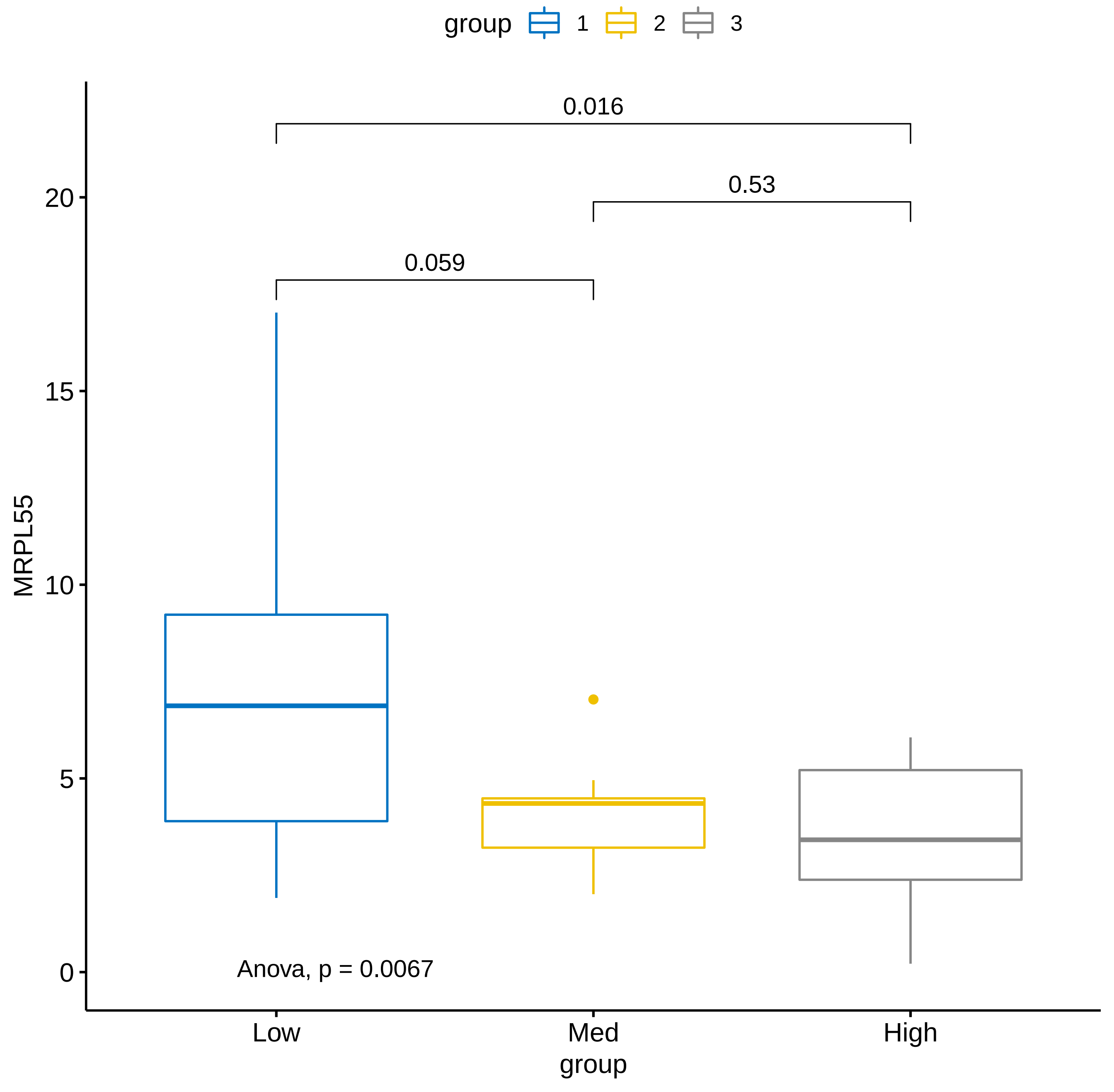
group
1
2
3
0.016
0.53
0.059
MRPL55
20
15
10
5
0
Anova, p = 0.0067
Low
Med
High
group

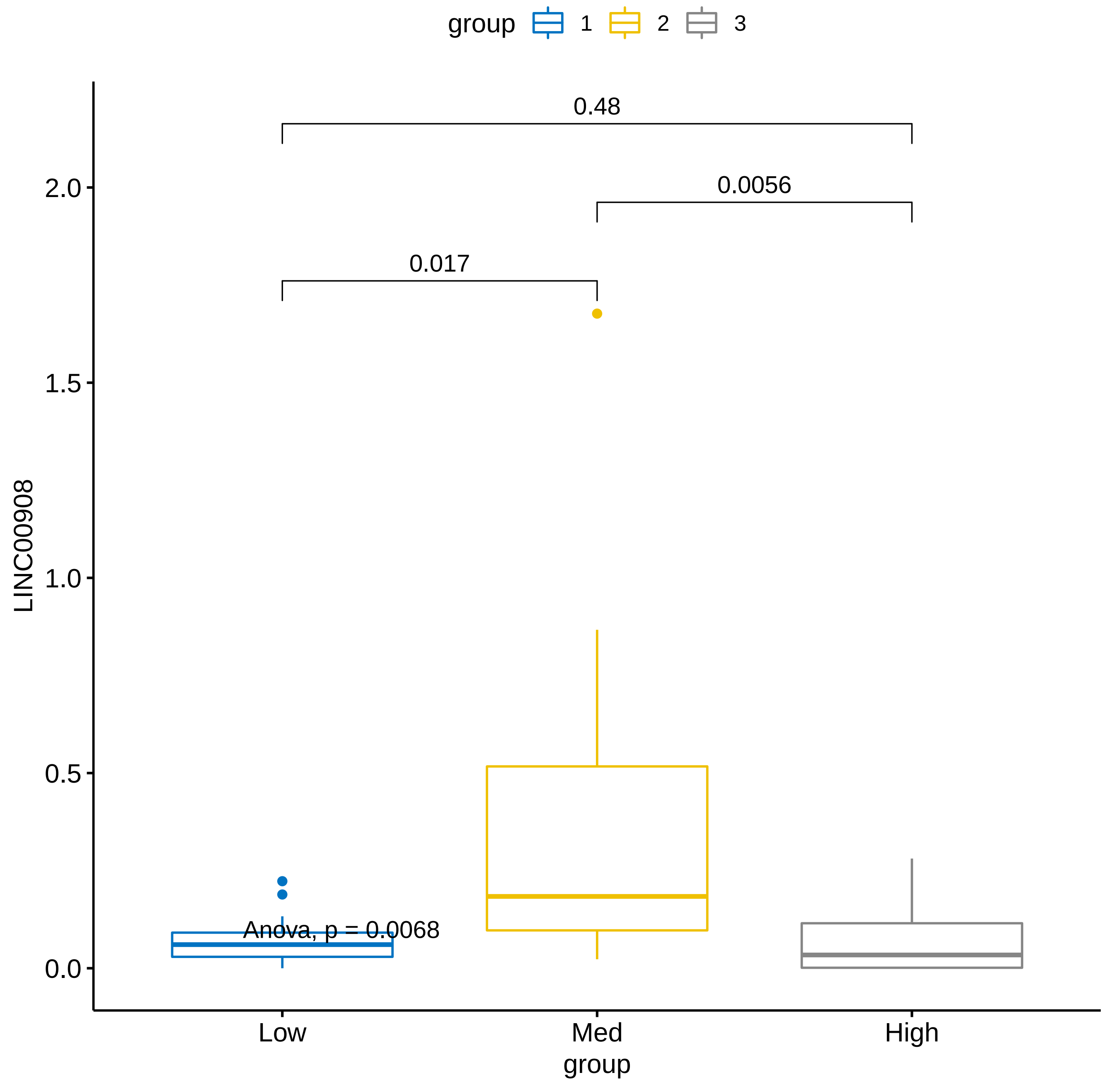
group
1
2
3
0.48
0.0056
0.017
Anova, p = 0.0068
2.0
1.5
1.0
0.5
0.0
LINC00908
Low
Med
High
group

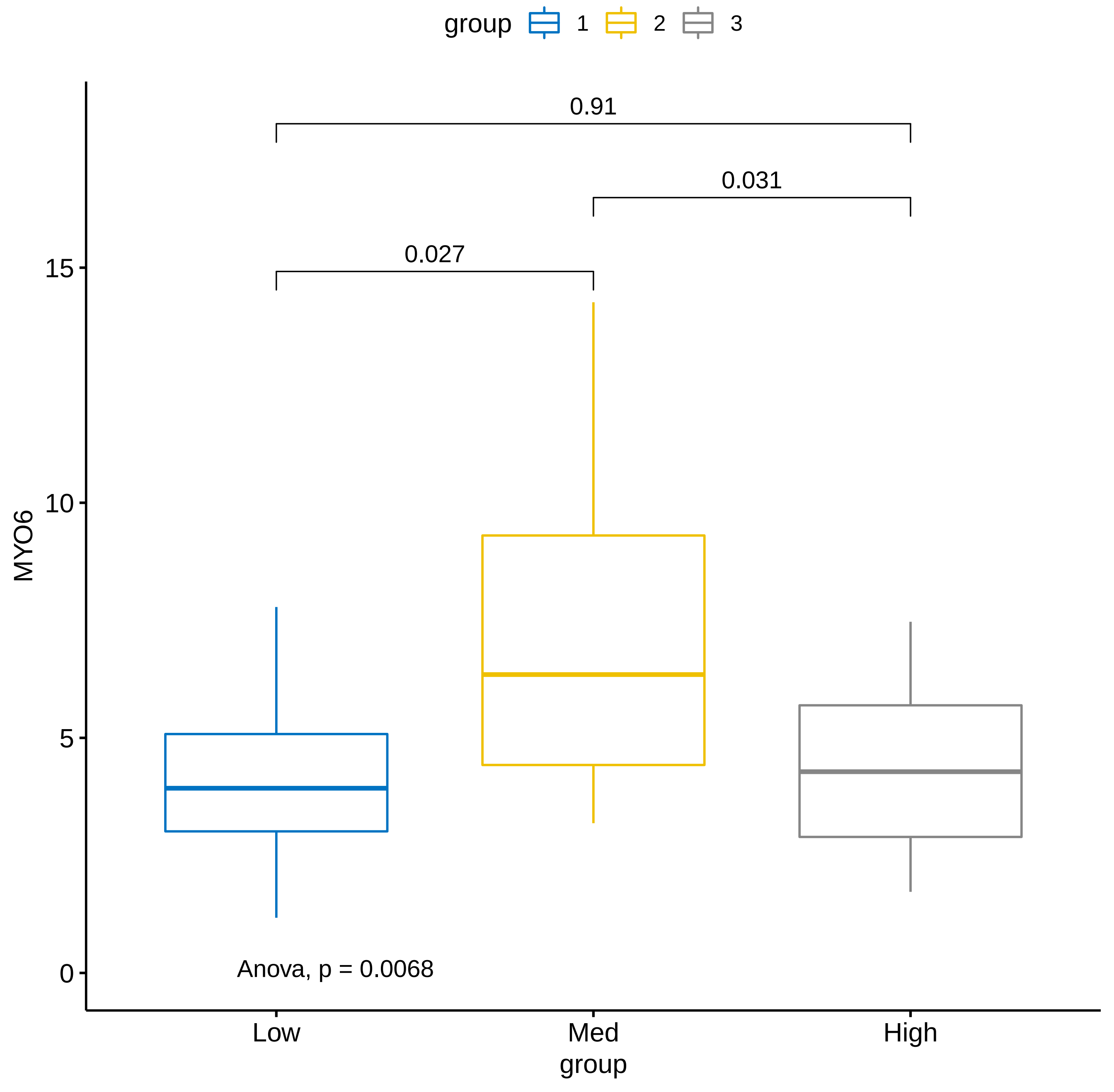
group
1
2
3
0.91
0.031
0.027
15
10
5
0
MYO6
Anova, p = 0.0068
Low
Med
High
group

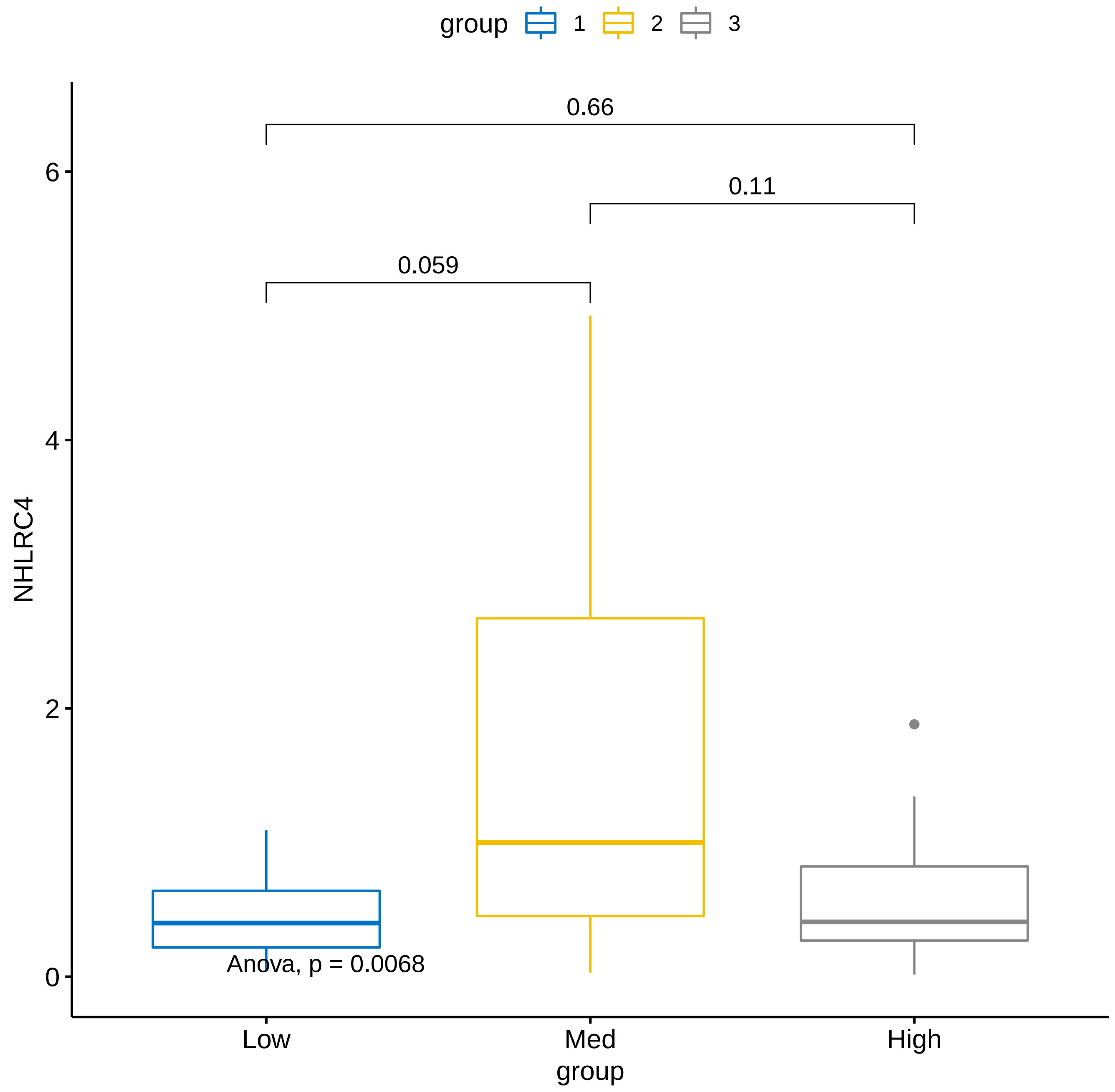
group
1
2
3
0.66
0.11
0.059
NHLRC4
6
4
2
0
Anova, p = 0.0068
Low
Med
High
group

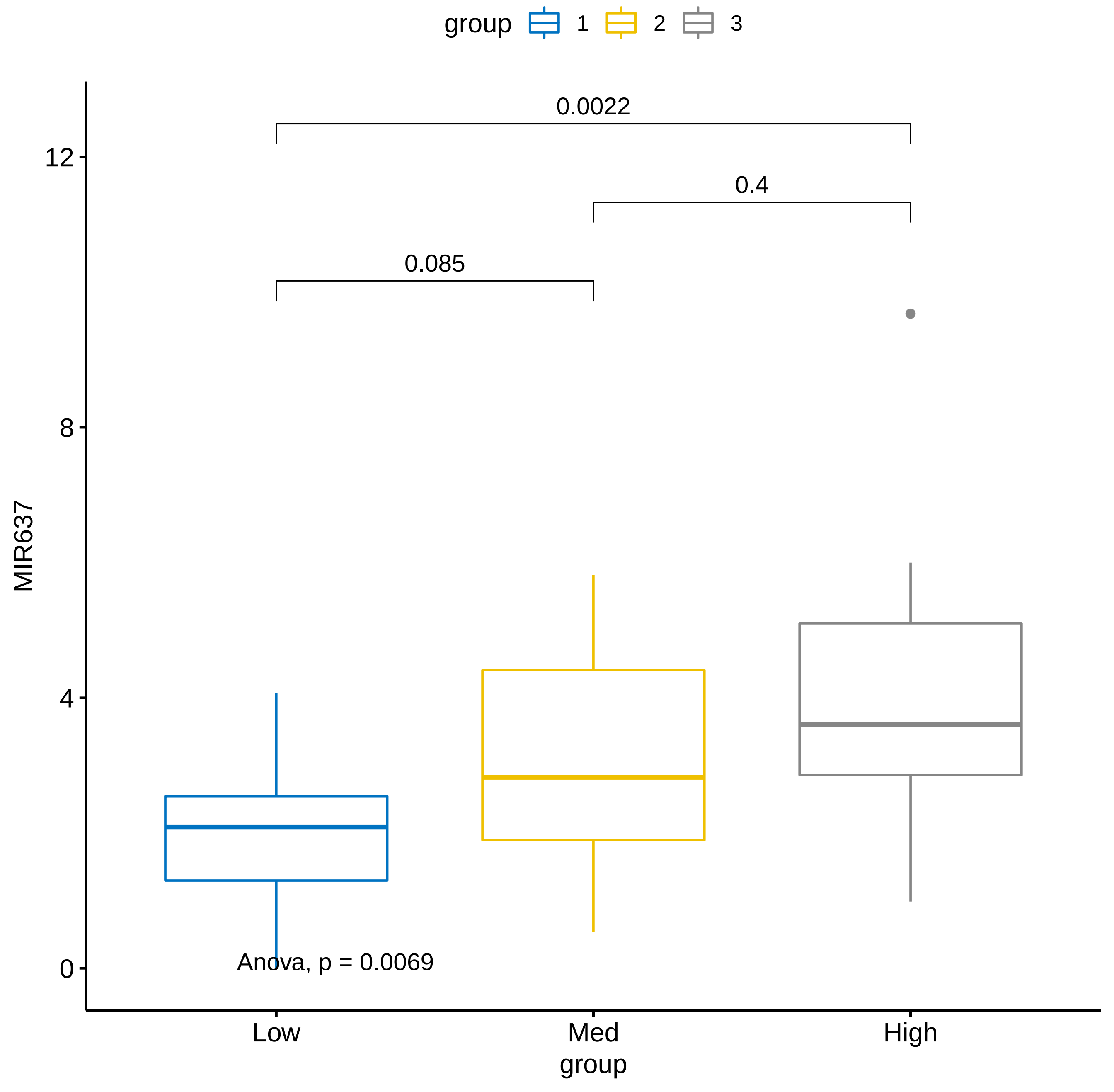
group
1
2
3
0.0022
0.4
0.085
12
8
4
0
MIR637
Anova, p = 0.0069
Low
Med
High
group

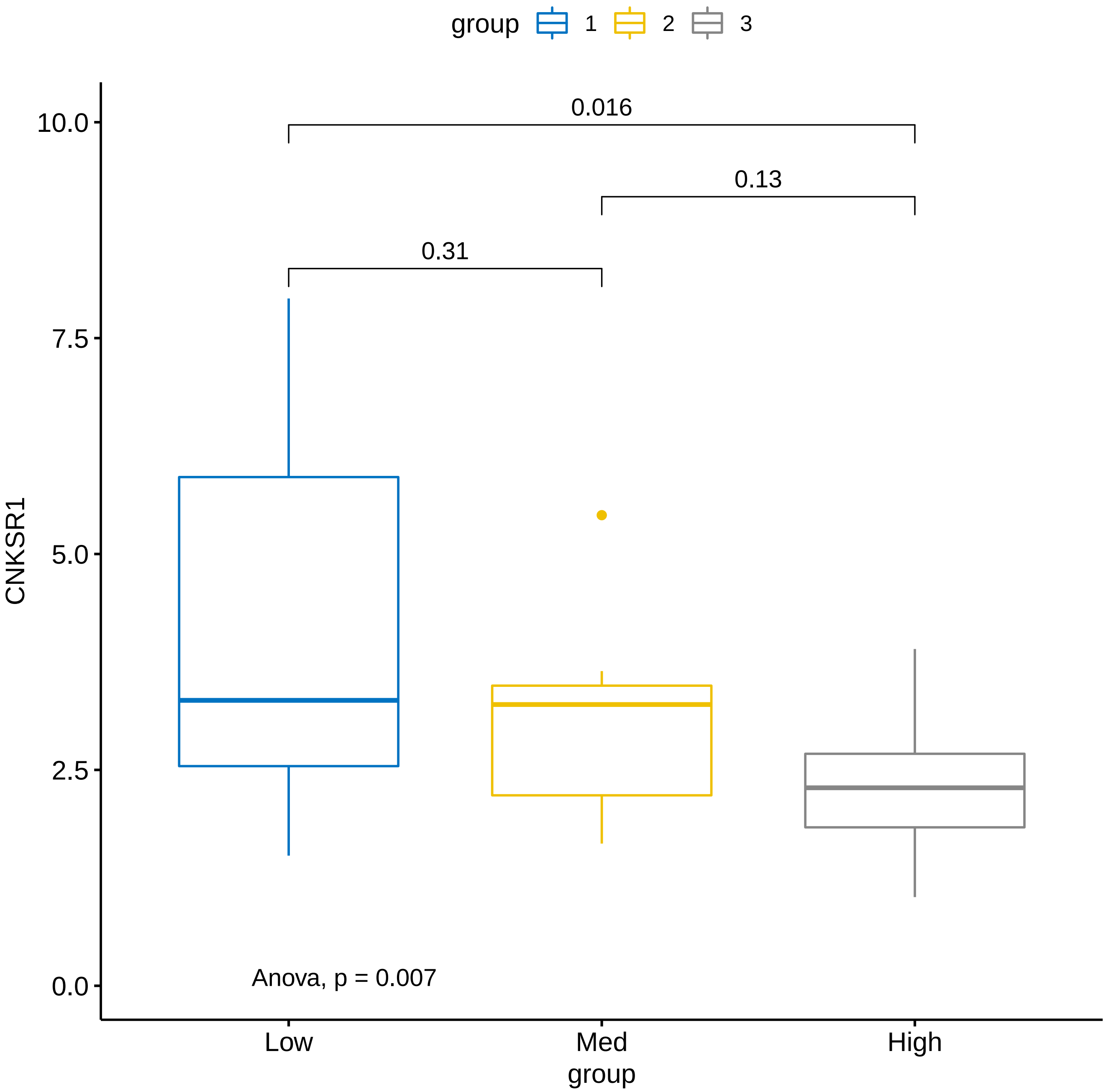

group
1
2
3
0.016
0.13
0.31
10.0
7.5
5.0
2.5
0.0
CNKSR1
Anova, p = 0.007
Low
Med
High
group

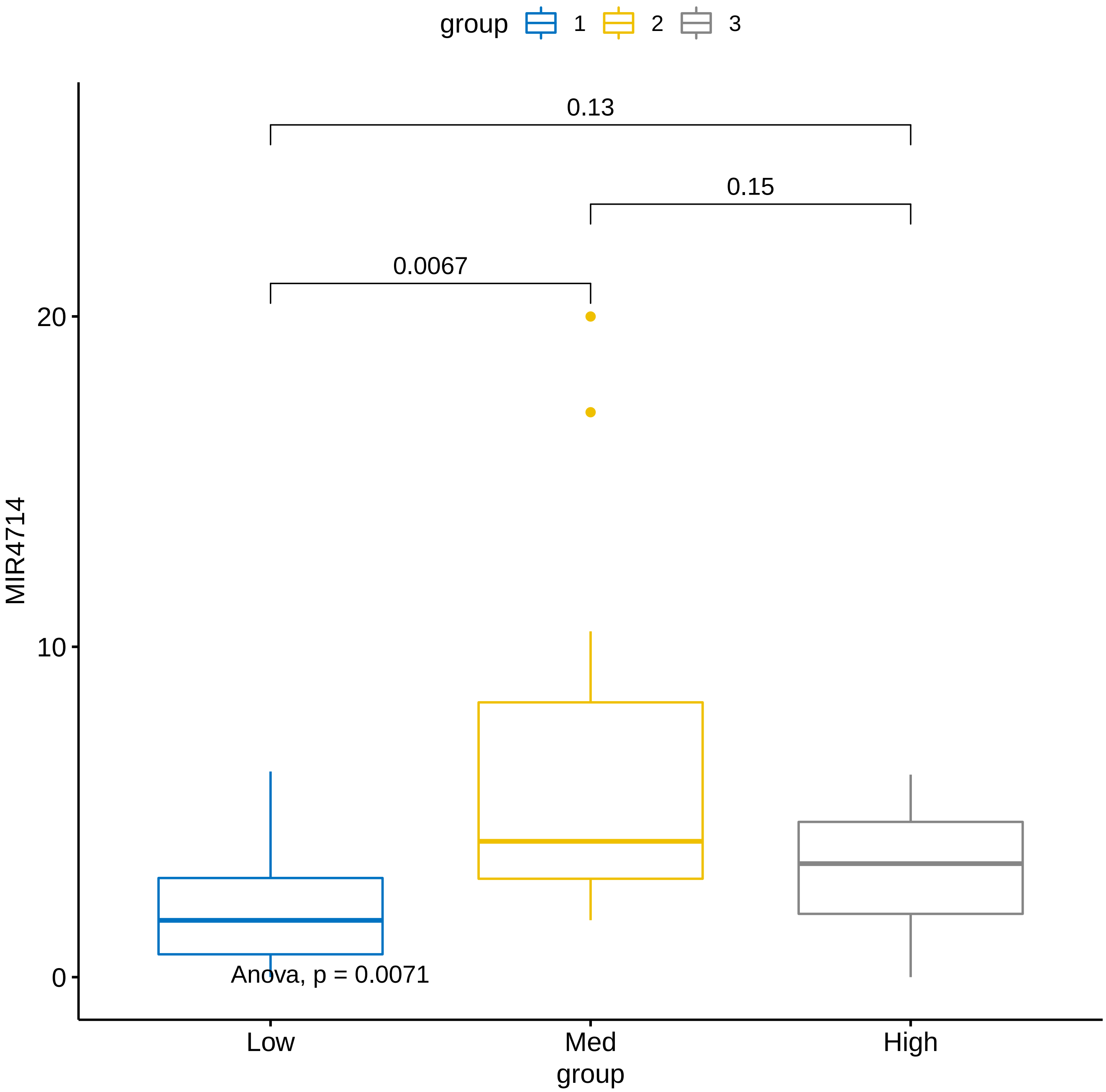
group
1
2
3
0.13
0.15
0.0067
20
10
0
MIR4714
Anova, p = 0.0071
Low
Med
High
group

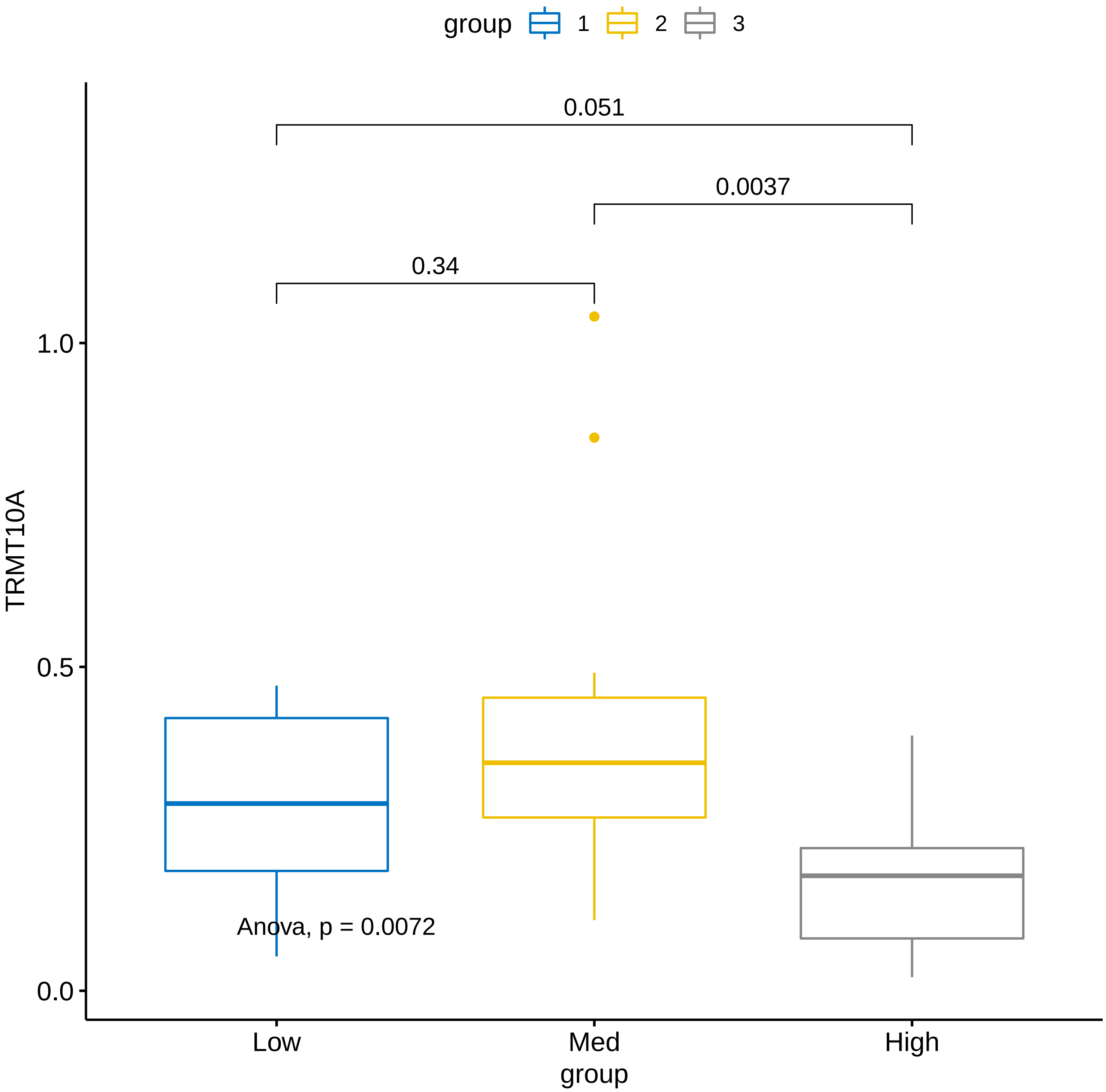
group 1 2 3
0.051
0.0037
0.34
TRMT10A
1.0
0.5
0.0
Anova, p = 0.0072
Low
Med
High
group

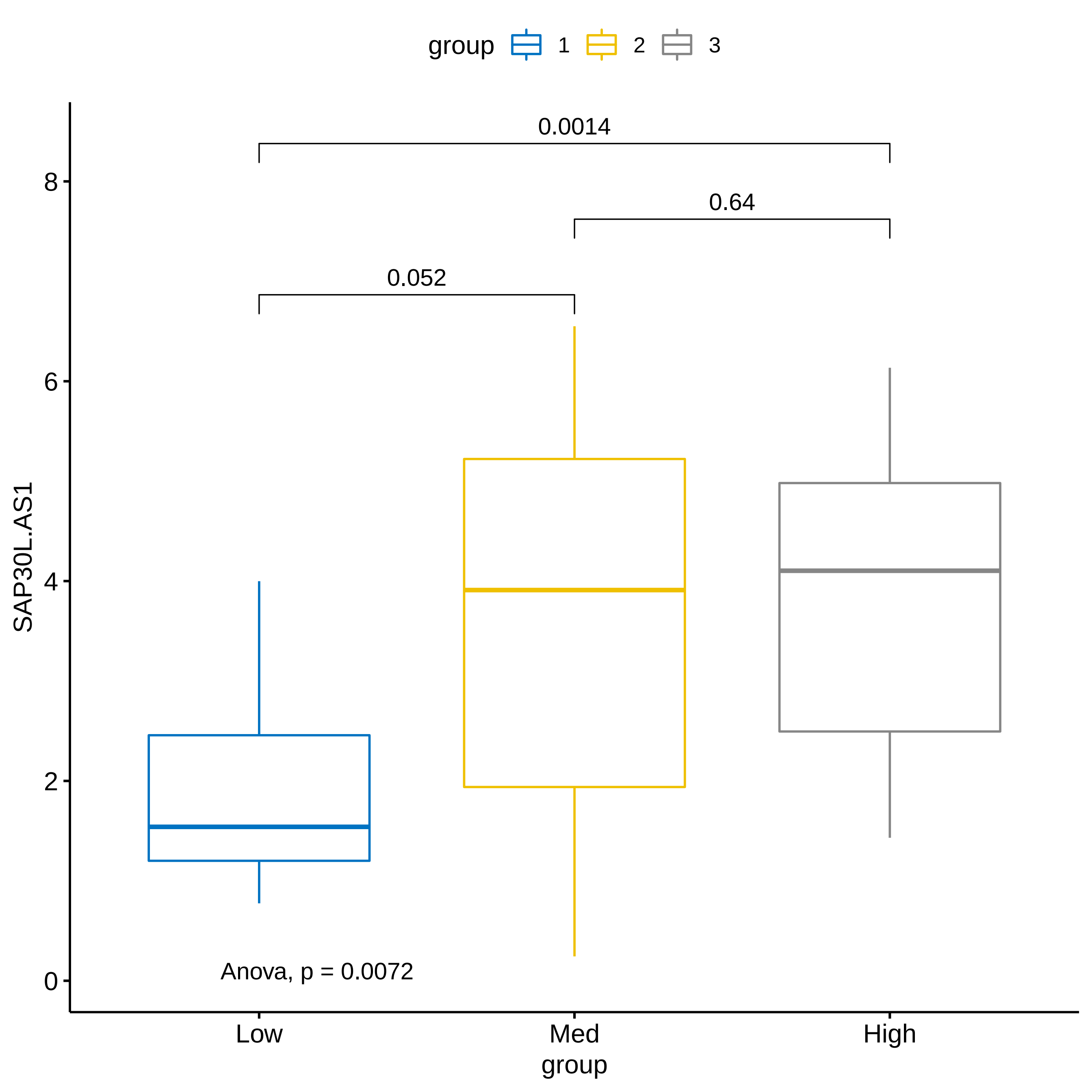

group
1
2
3
0.0014
0.64
0.052
Anova, p = 0.0072
SAP30L.AS1
8
6
4
2
0
Low
Med
High
group

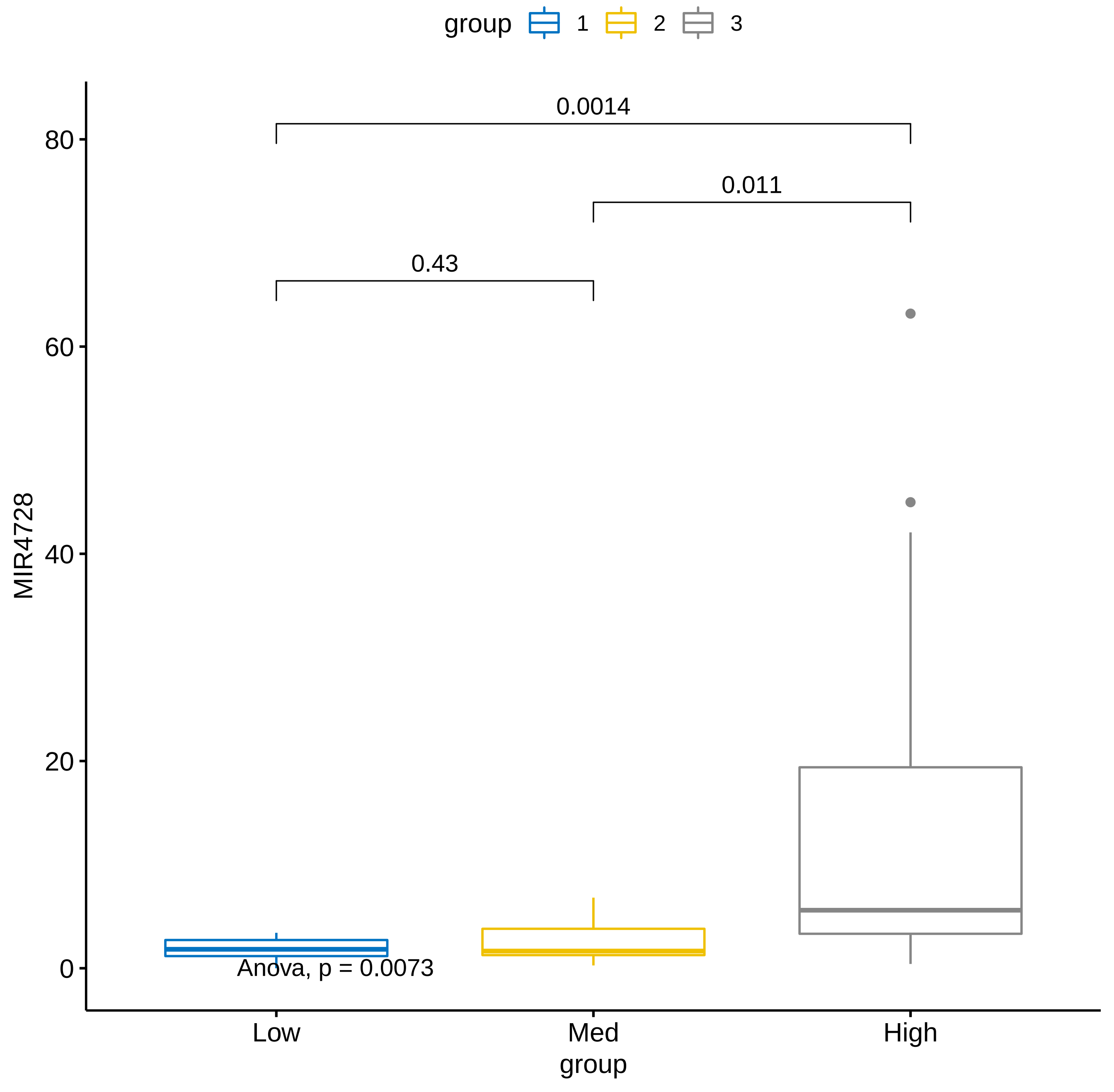

group
1
2
3
0.0014
0.011
0.43
80
60
40
20
0
MIR4728
Anova, p = 0.0073
Low
Med
High
group

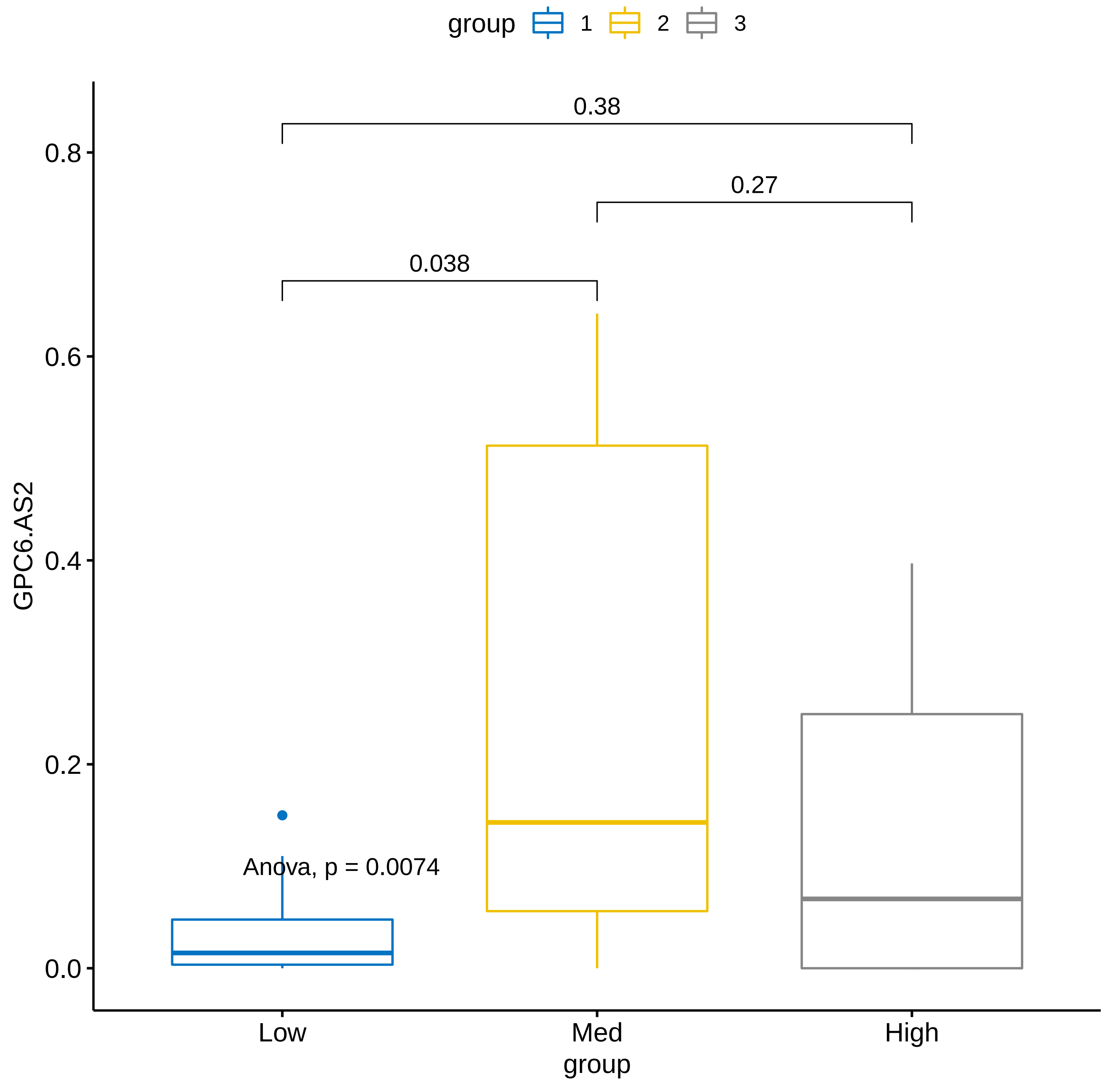
group 1 2 3
0.38
0.27
0.038
0.8
0.6
0.4
0.2
0.0
GPC6.AS2
Anova, p = 0.0074
Low
Med
High
group

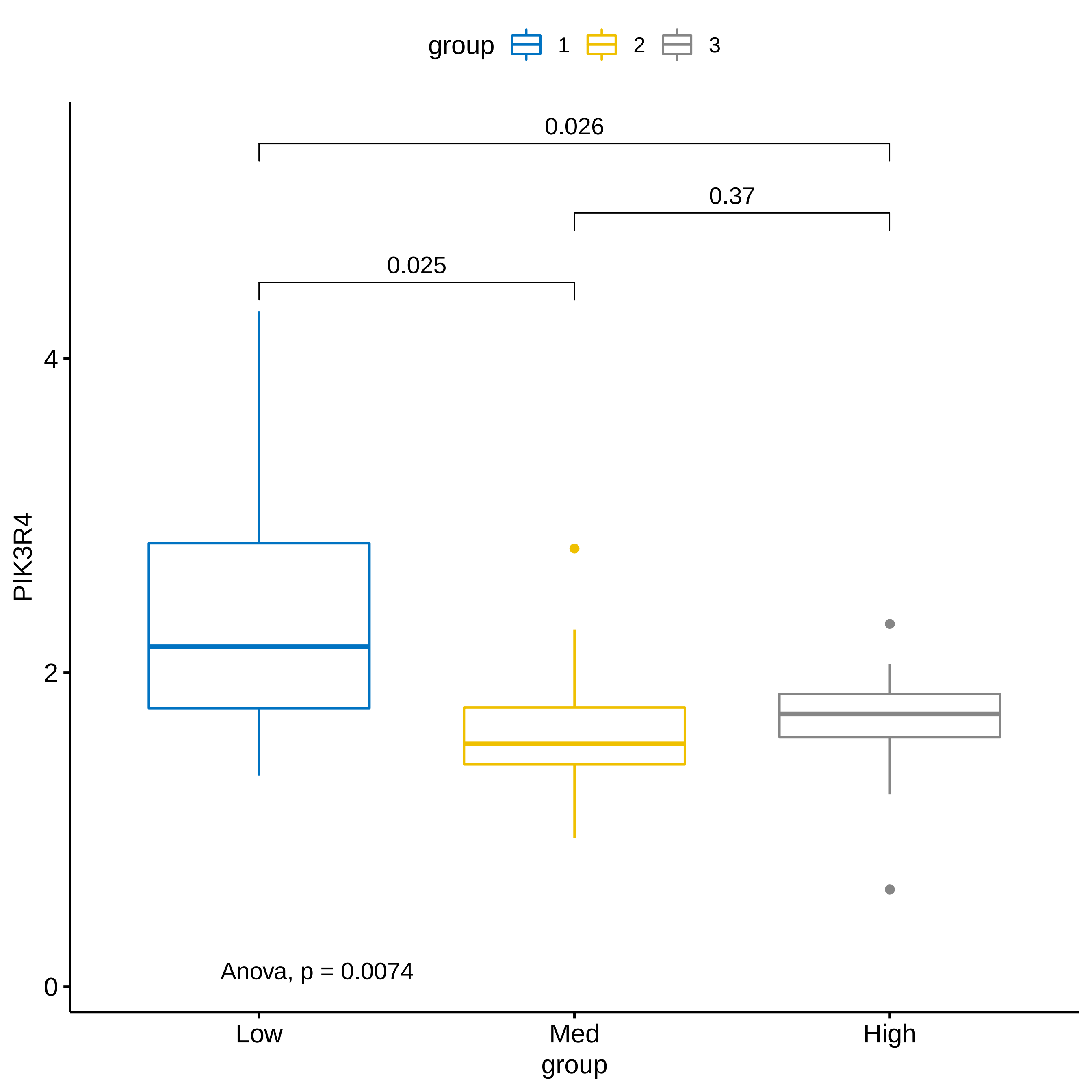

group 1 2 3
0.026
0.37
0.025
PIK3R4
4
2
0
Anova, p = 0.0074
Low
Med
High
group

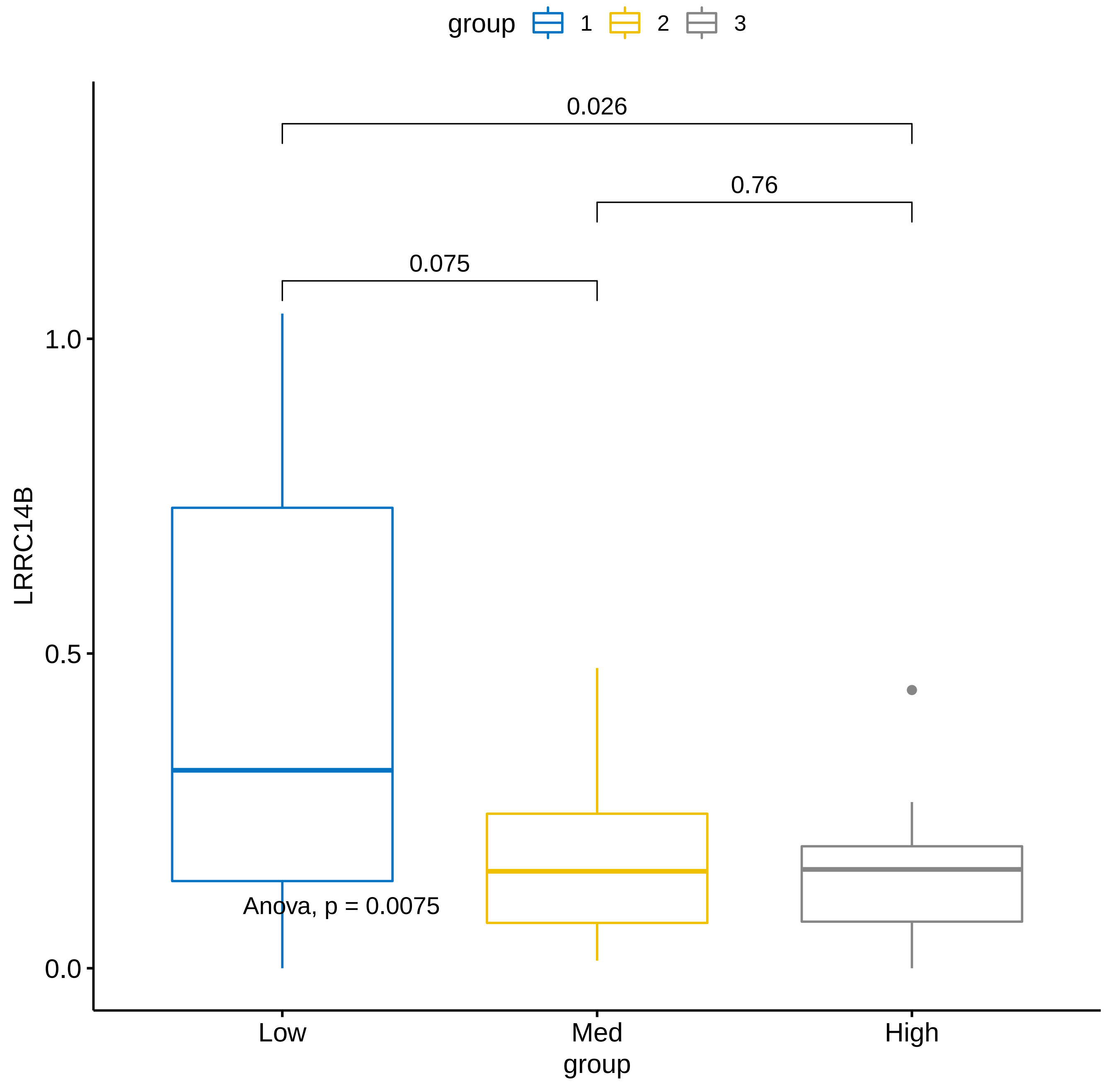
group
1
2
3
0.026
0.76
0.075
LRRC14B
1.0
0.5
0.0
Anova, p = 0.0075
Low
Med
High
group

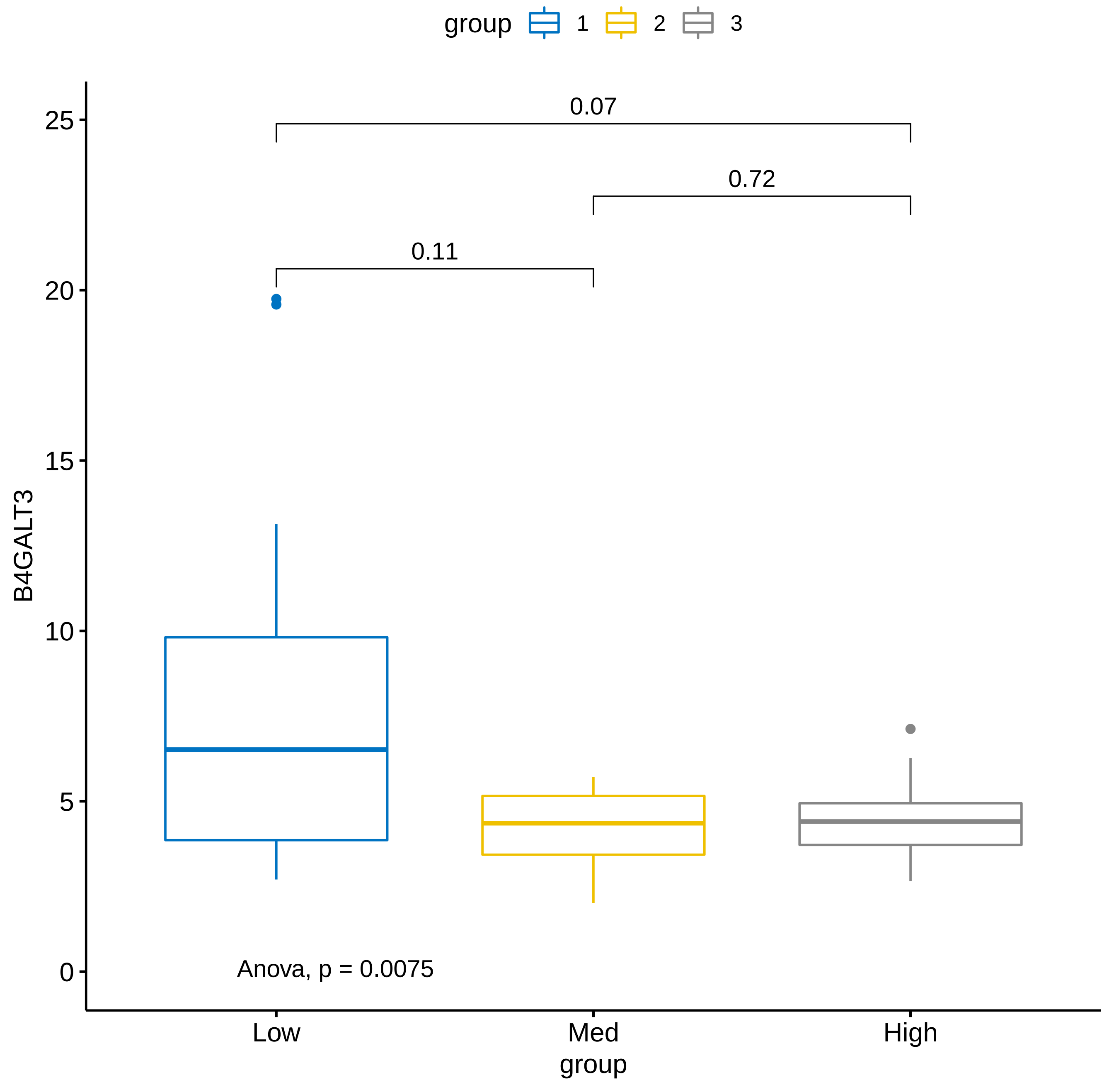
group 1 2 3
0.07
0.72
0.11
25
20
15
10
5
0
B4GALT3
Anova, p = 0.0075
Low
Med
High
group

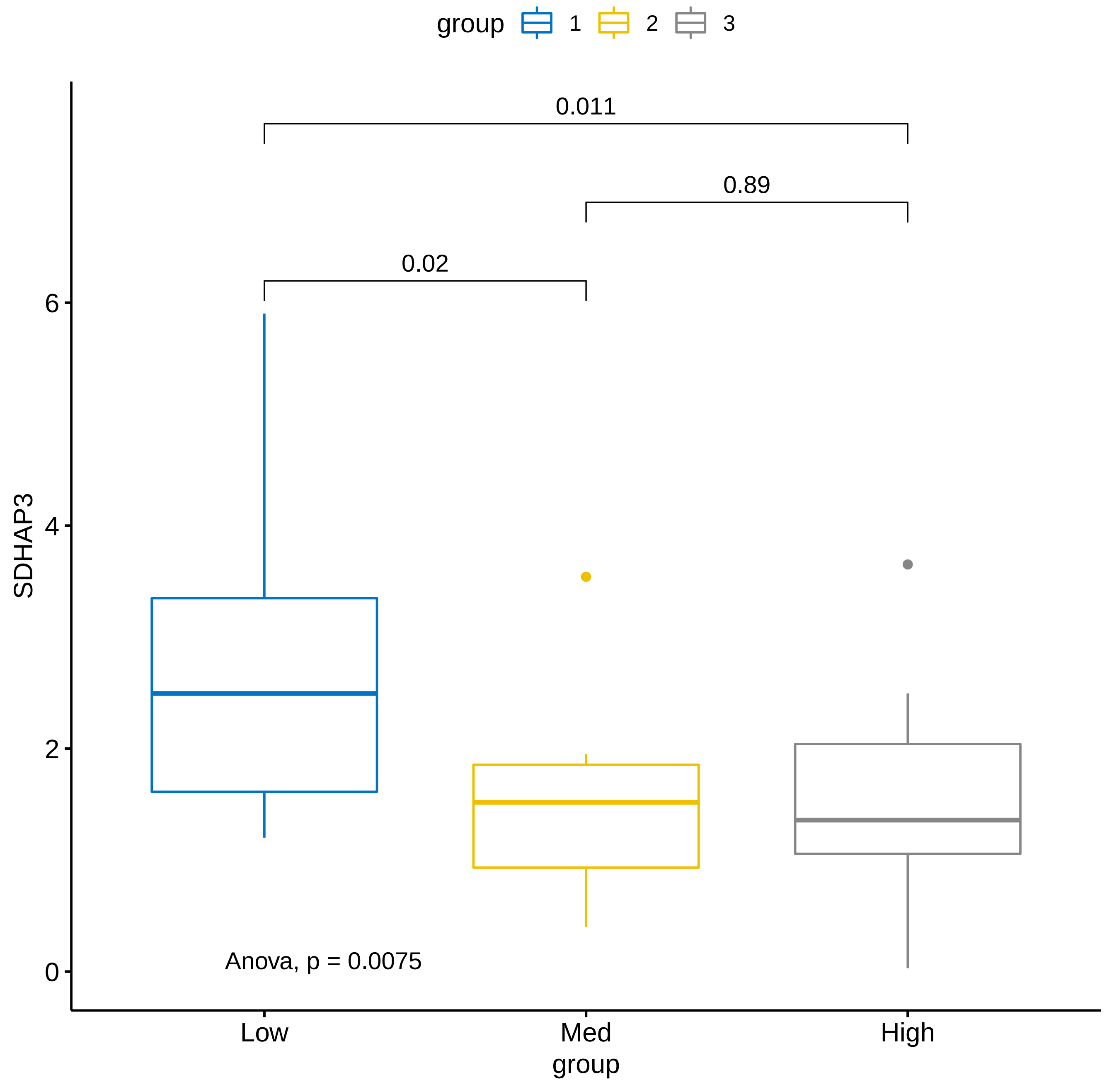
group 1 2 3
0.011
0.89
0.02
6
4
2
0
SDHAP3
Anova, p = 0.0075
Low
Med
High
group

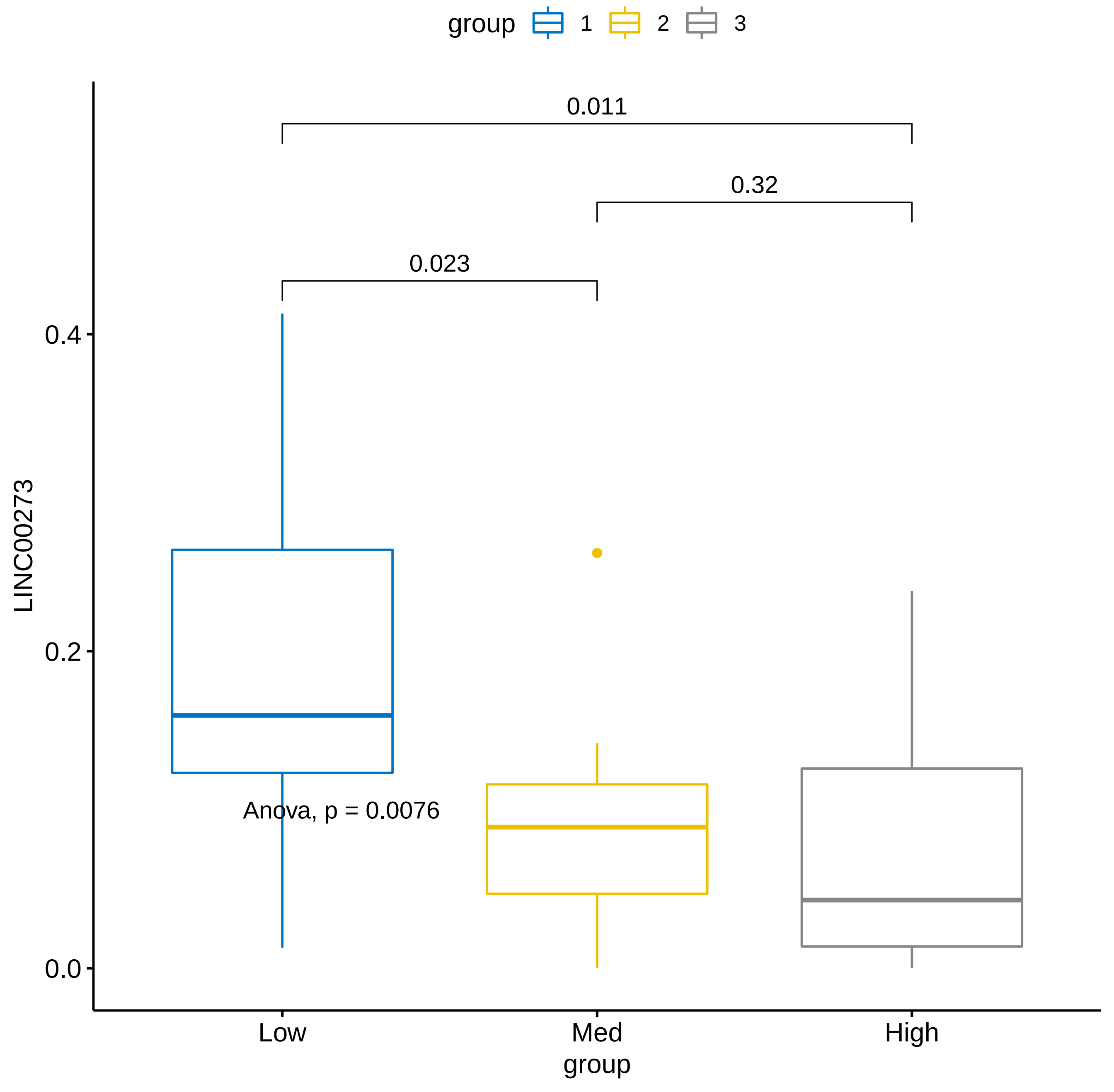
group 1 2 3
0.011
0.32
0.023
0.4
0.2
0.0
LINC00273
Anova, p = 0.0076
Low
Med
High
group

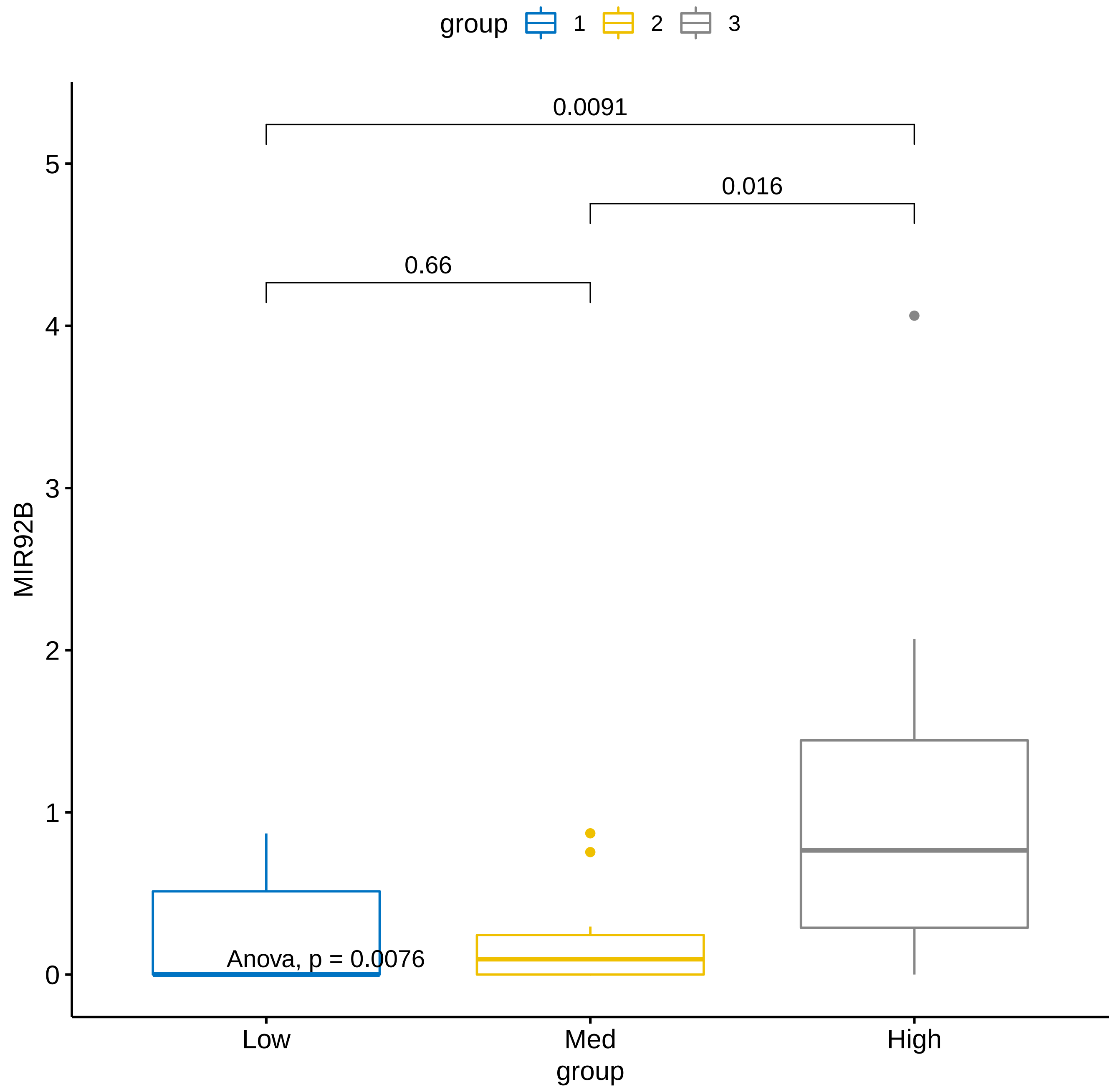
group 1 2 3
0.0091
0.016
0.66
MIR92B
5
4
3
2
1
0
Anova, p = 0.0076
Low
Med
High
group

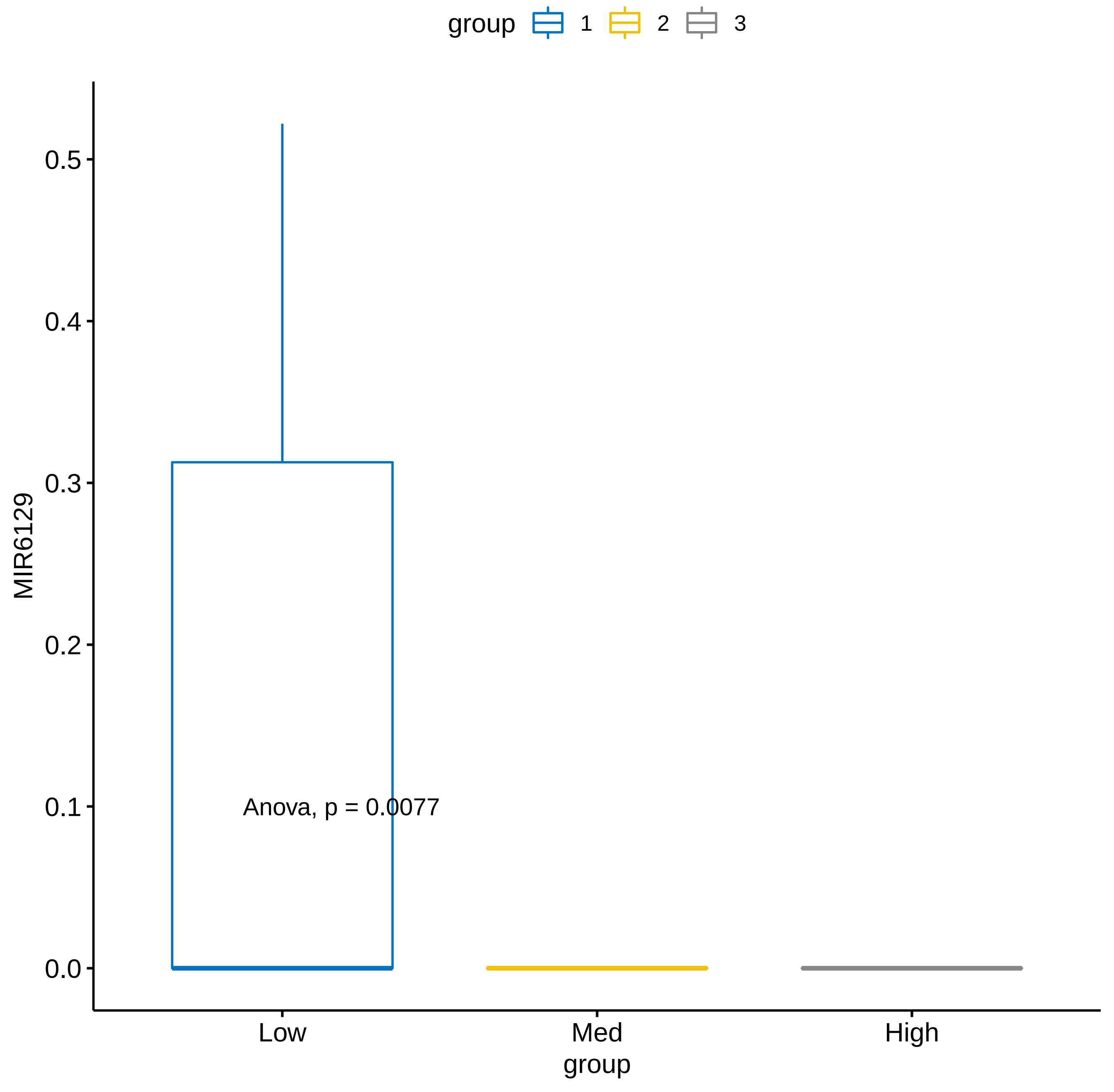

group
1
2
3
MIR6129
0.5
0.4
0.3
0.2
0.1
0.0
Anova, p = 0.0077
Low
Med
High
group

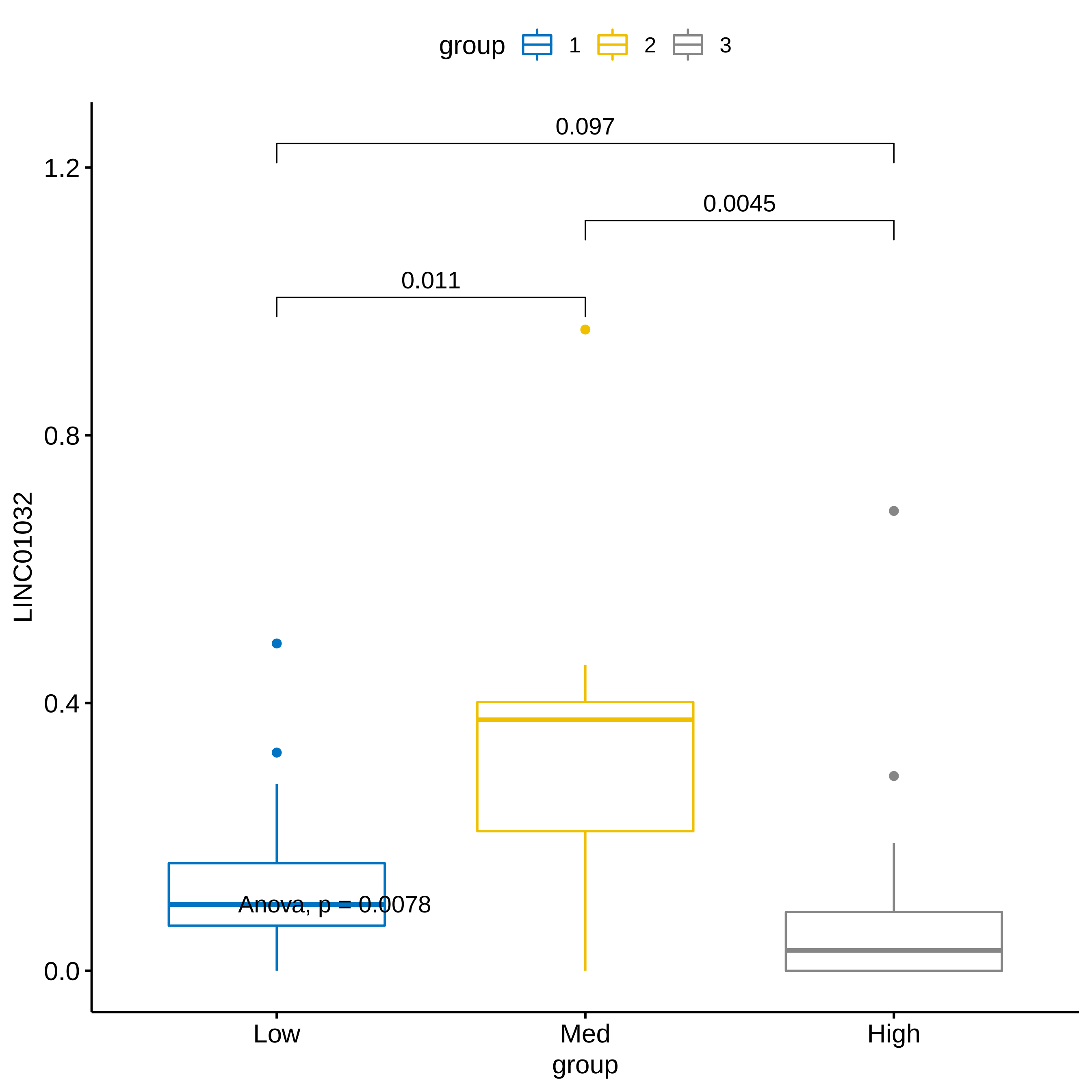
group
1
2
3
0.097
0.0045
0.011
1.2
0.8
0.4
0.0
LINC01032
Anova, p = 0.0078
Low
Med
High
group

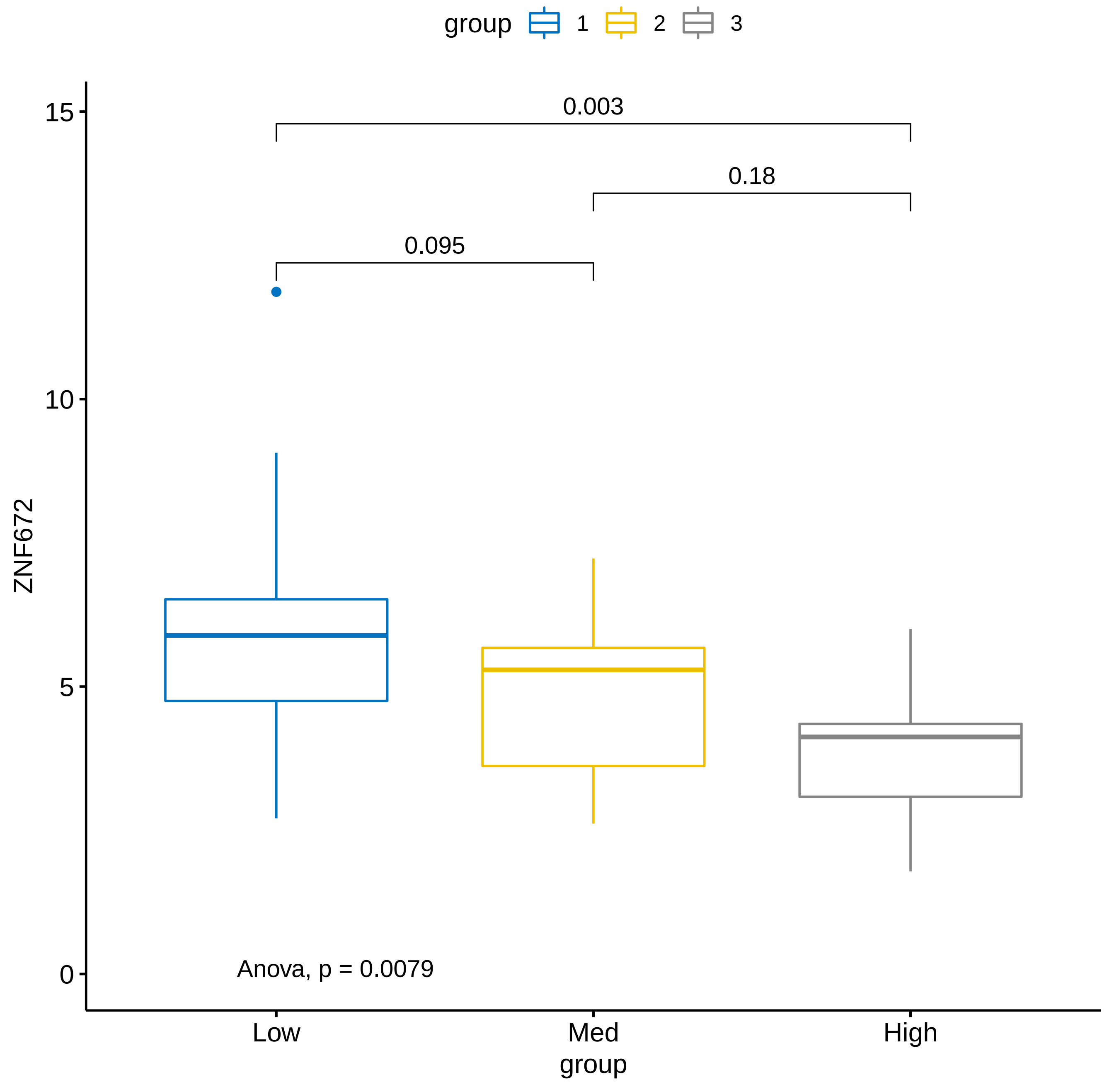
group
1
2
3
0.003
0.18
0.095
15
10
5
0
ZNF672
Anova, p = 0.0079
Low
Med
High
group

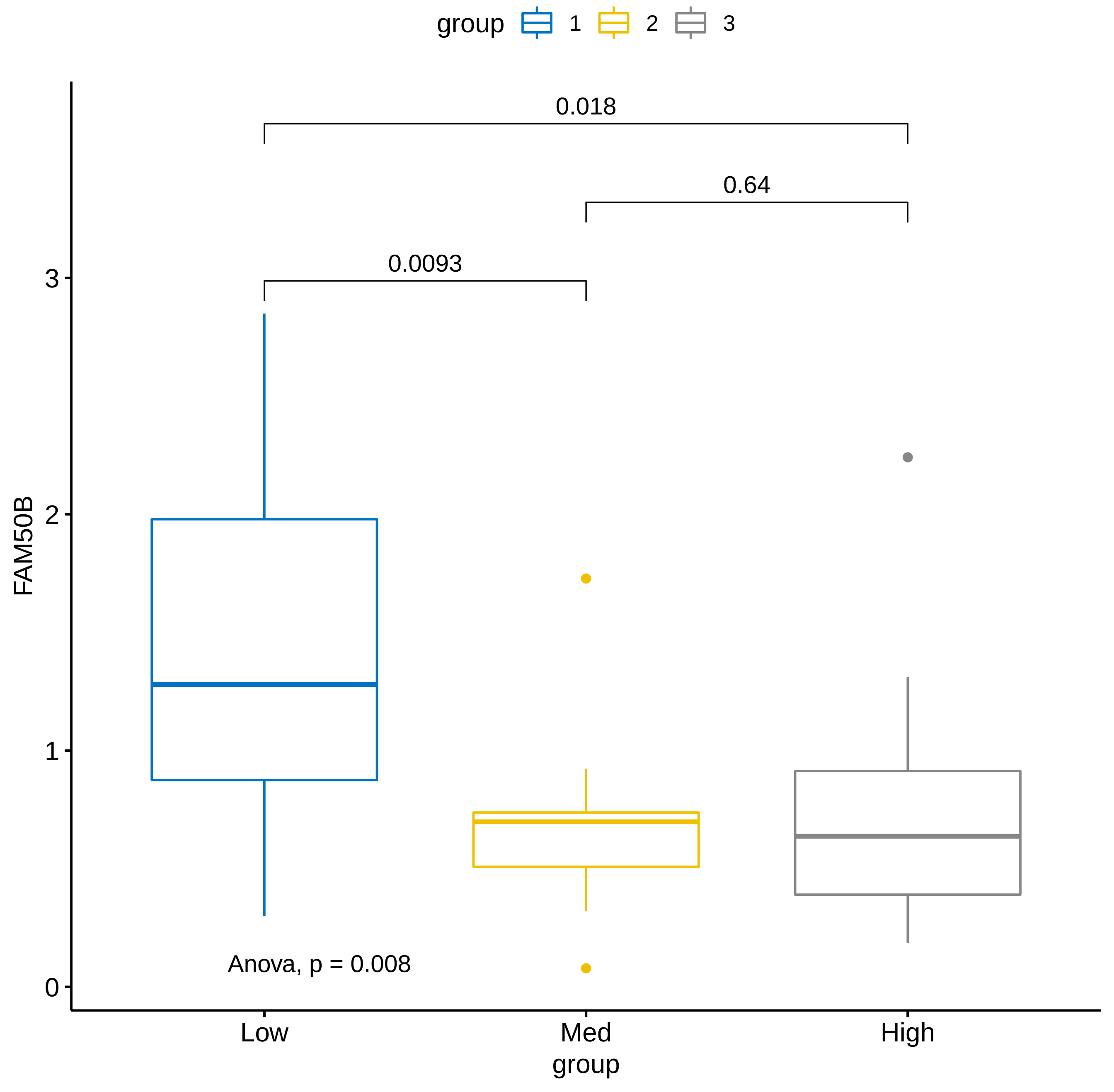
group
1
2
3
0.018
0.64
0.0093
3
2
1
0
FAM50B
Anova, p = 0.008
Low
Med
High
group

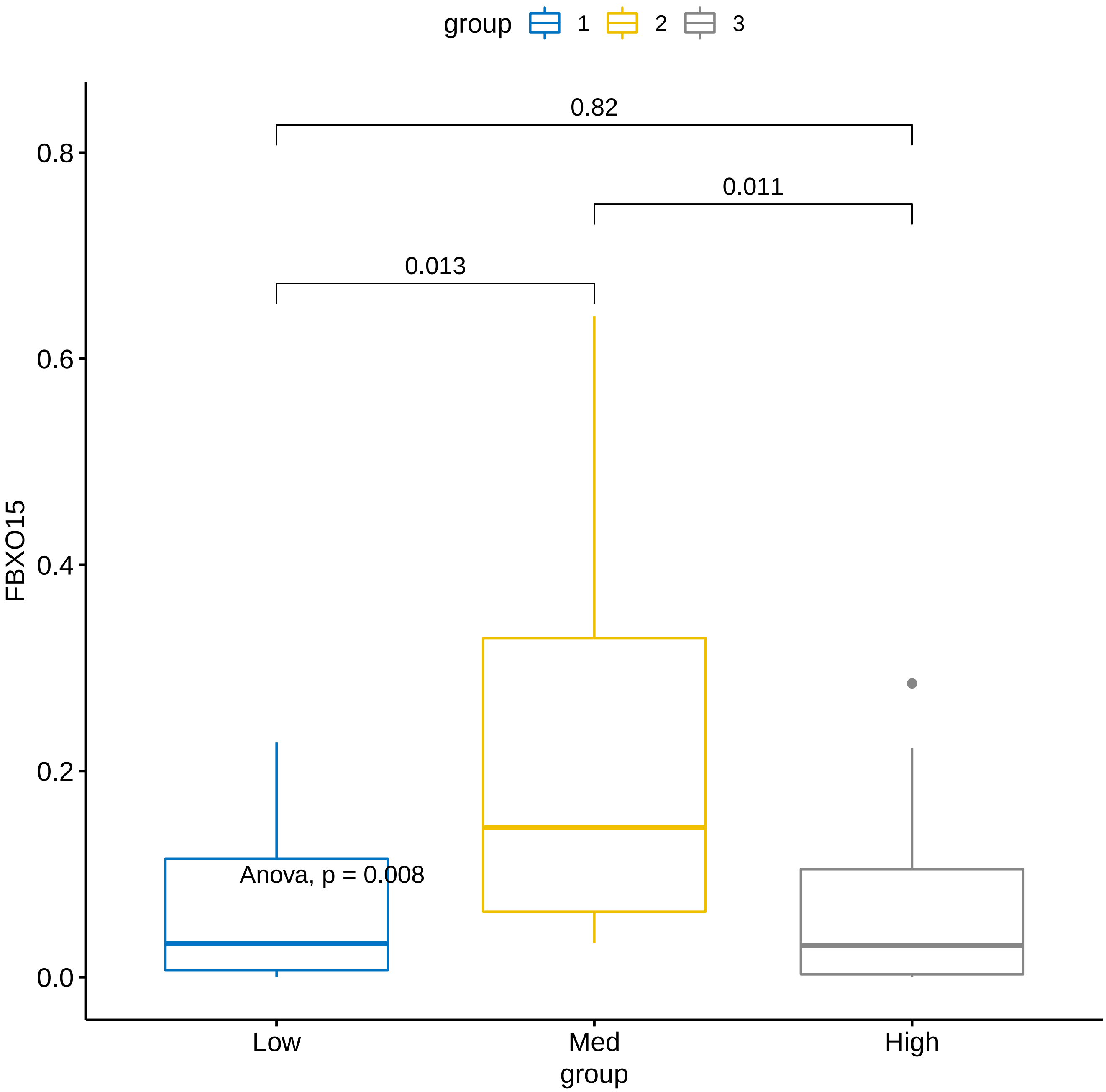
group
1
2
3
0.82
0.011
0.013
Anova, p = 0.008
FBXO15
0.8
0.6
0.4
0.2
0.0
Low
Med
High
group

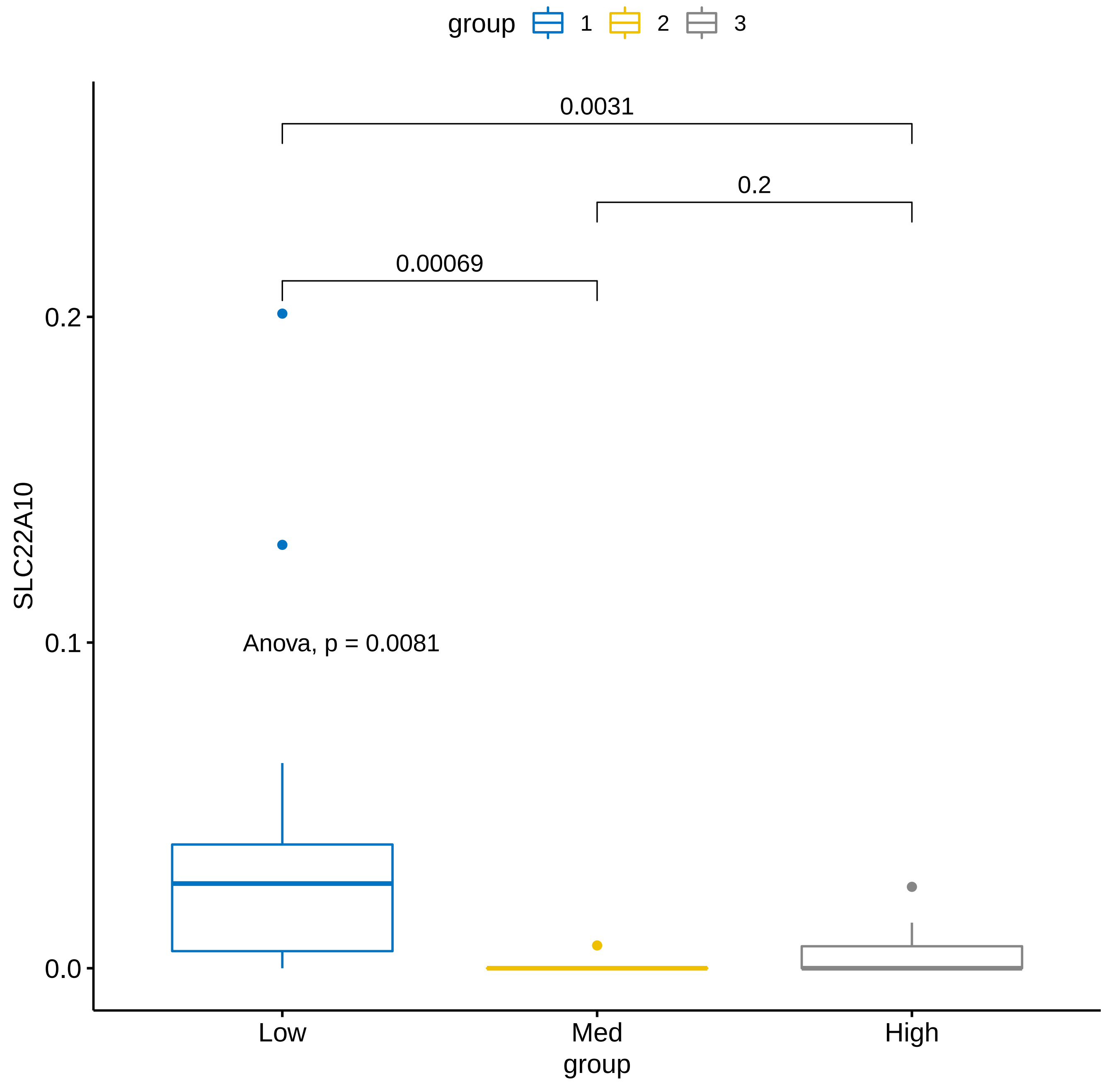

group
1
2
3
0.0031
0.2
0.00069
0.2
0.1
0.0
SLC22A10
Anova, p = 0.0081
Low
Med
High
group

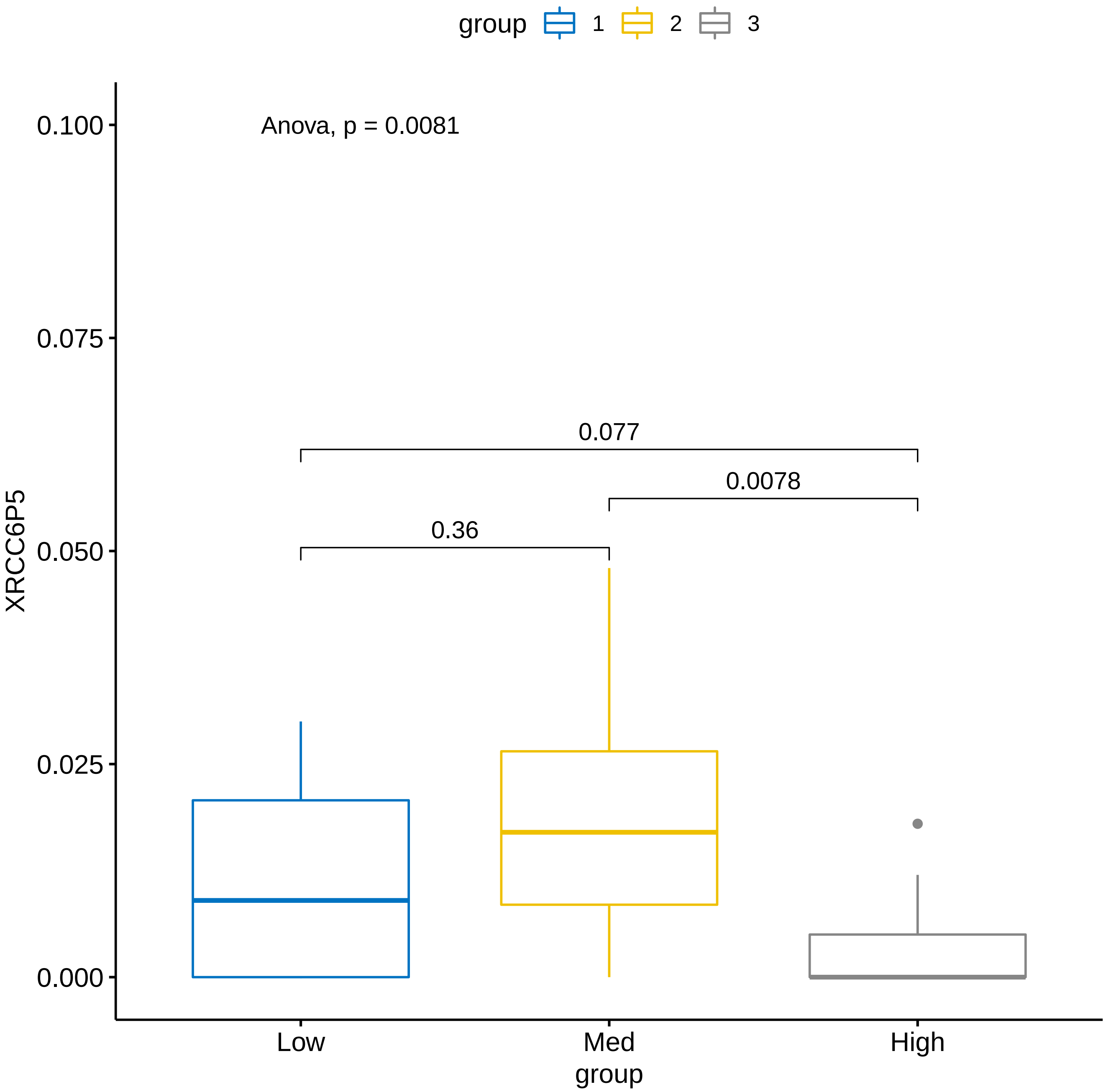

group 1 2 3
Anova, p = 0.0081
0.077
0.0078
0.36
0.100
0.075
0.050
0.025
0.000
XRCC6P5
Low
Med
High
group

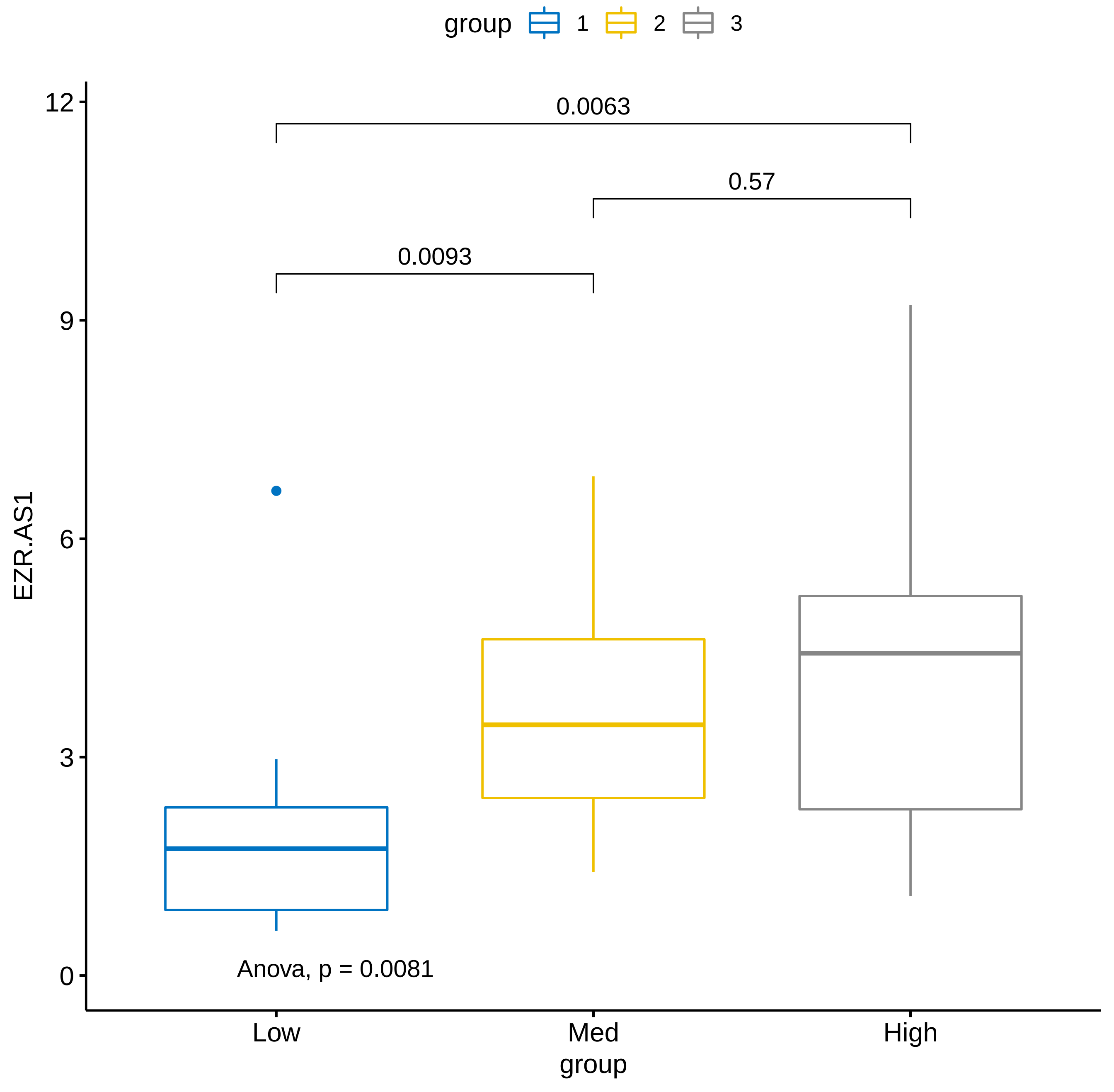

group
1
2
3
12
9
6
3
0
EZR.AS1
0.0063
0.57
0.0093
Anova, p = 0.0081
Low
Med
High
group

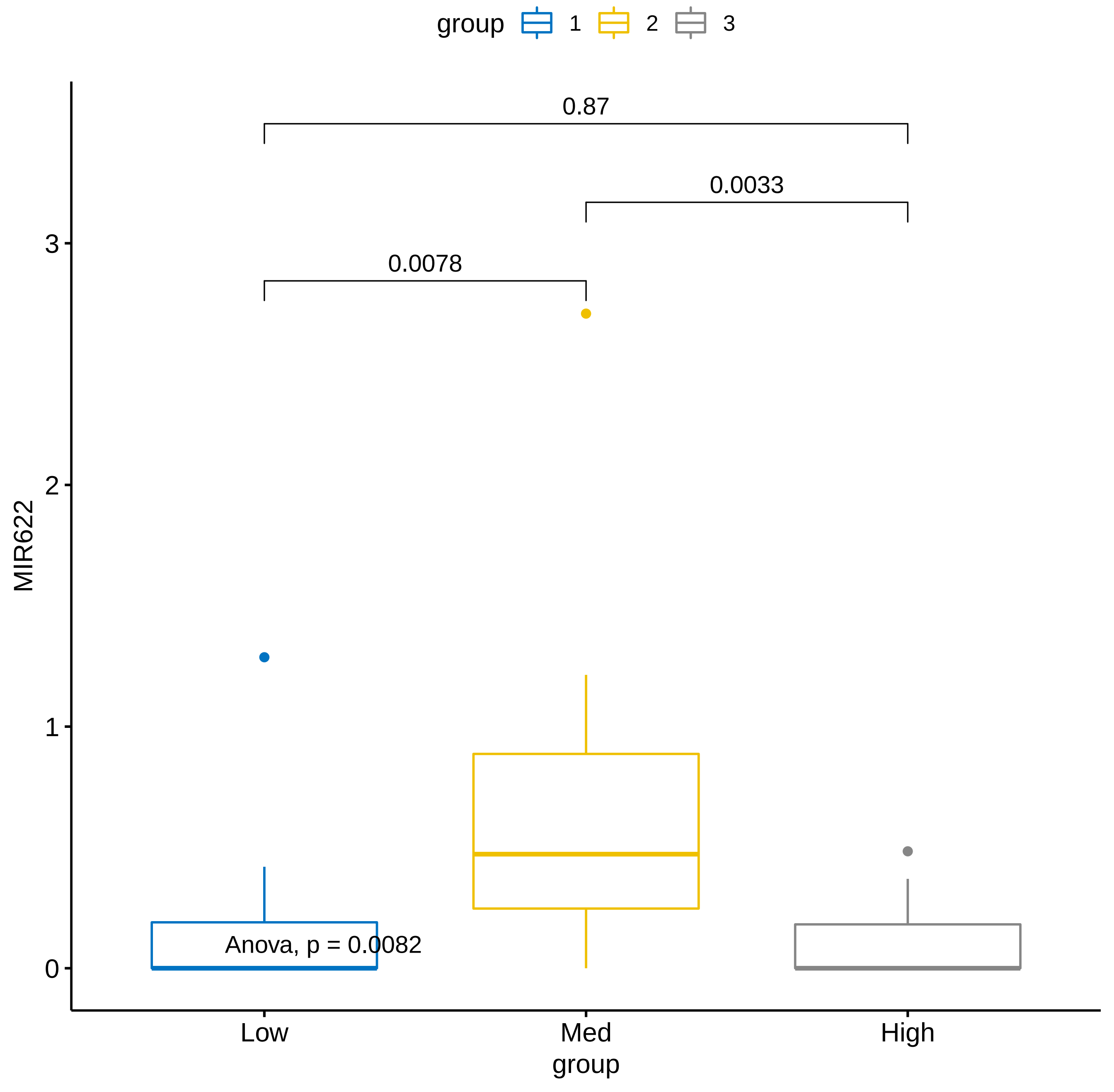
group 1 2 3
0.87
0.0033
0.0078
Anova, p = 0.0082
MIR622
3
2
1
0
Low
Med
High
group

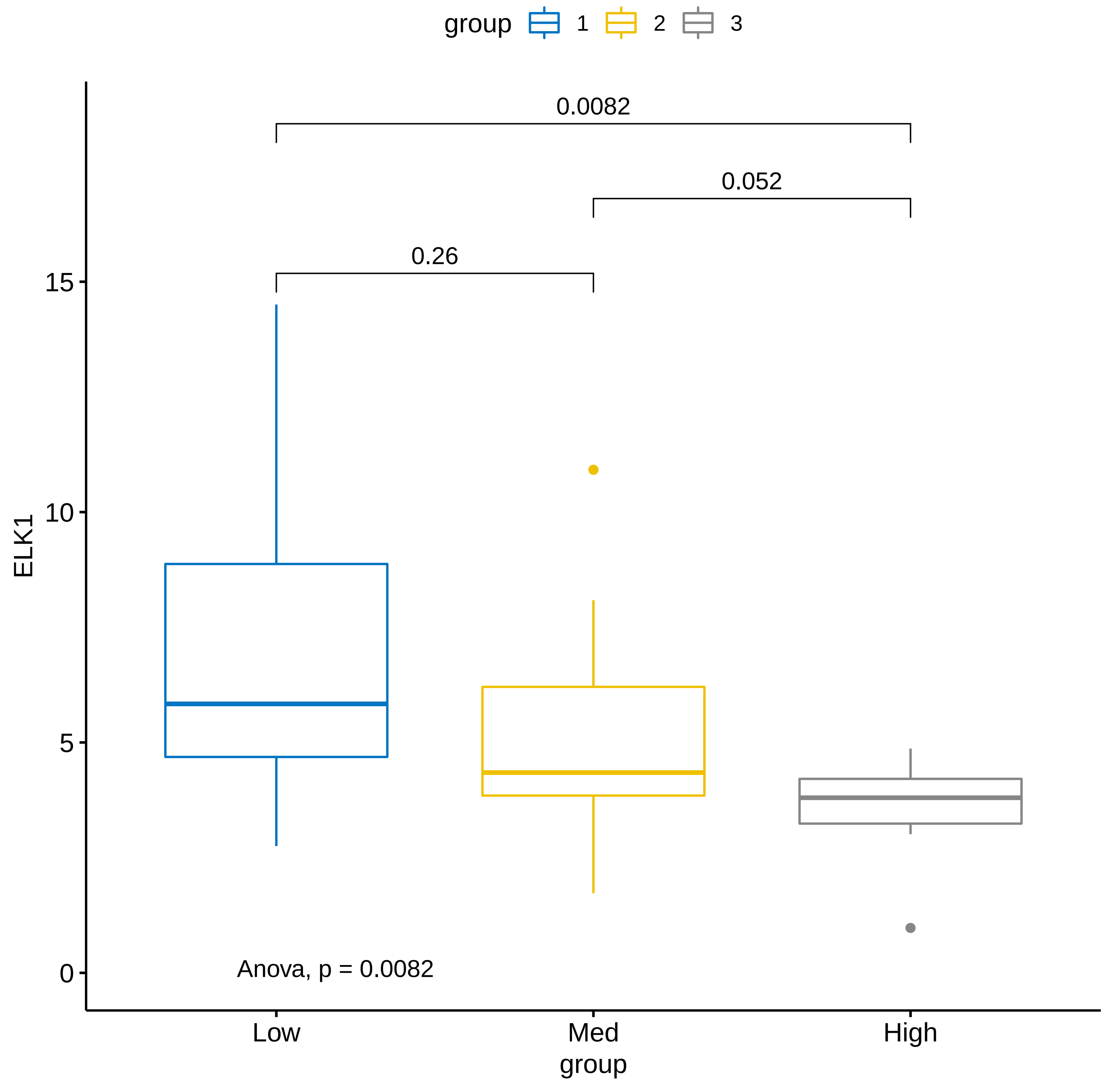

group
1
2
3
0.0082
0.052
0.26
15
10
5
0
ELK1
Anova, p = 0.0082
Low
Med
High
group

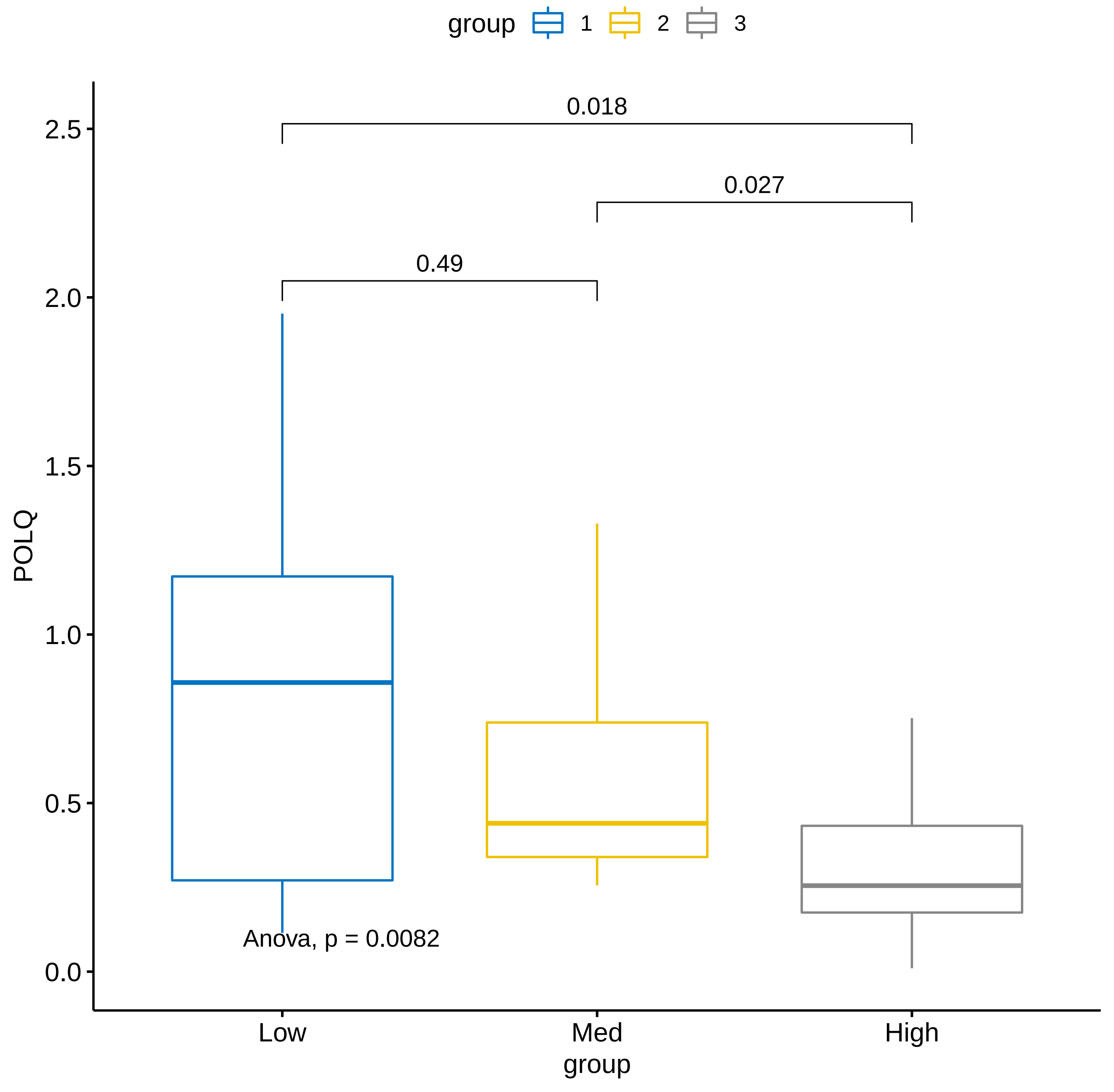

group
1
2
3
0.018
0.027
0.49
2.5
2.0
1.5
1.0
0.5
0.0
POLQ
Anova, p = 0.0082
Low
Med
High
group

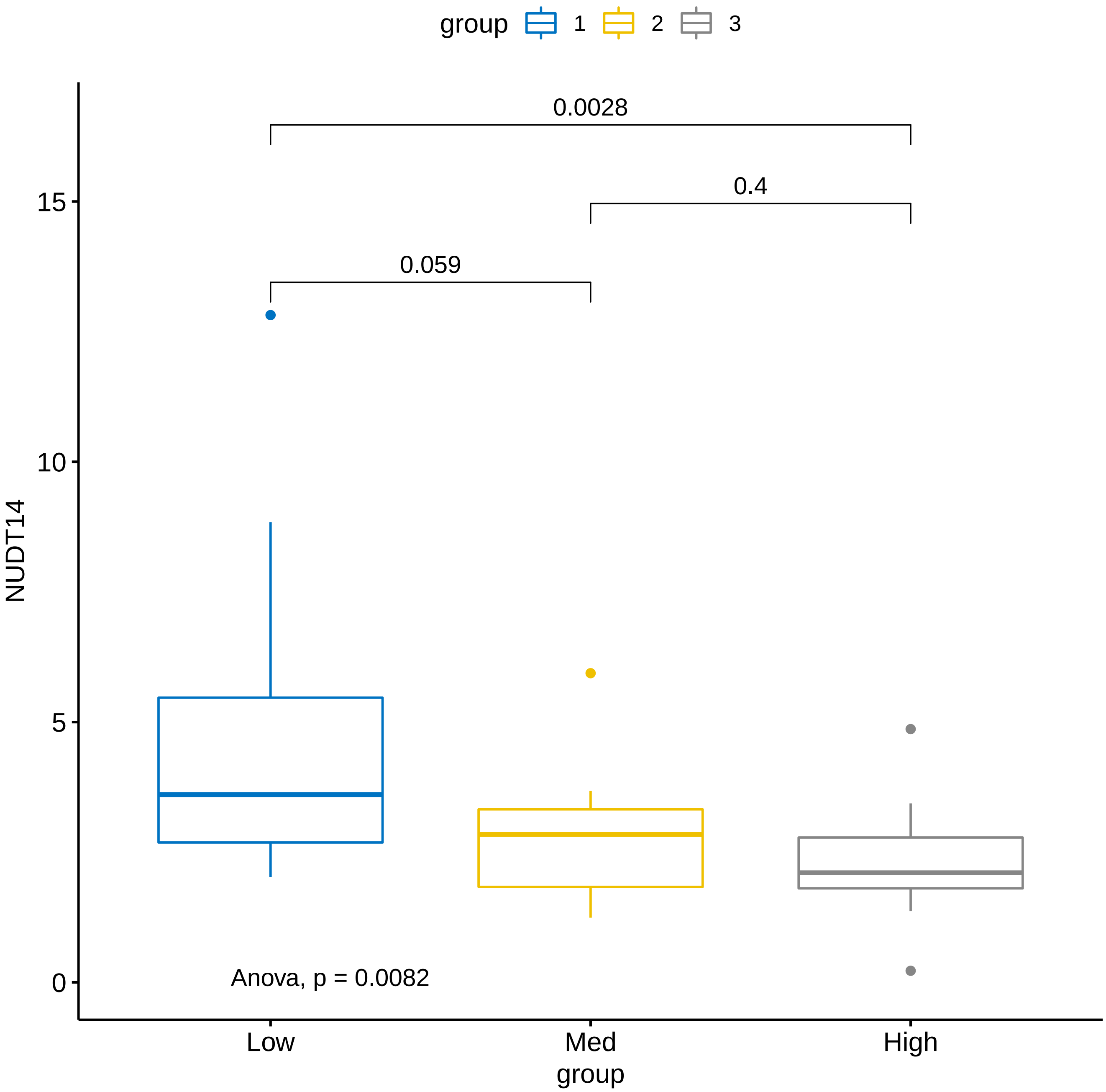
group
1
2
3
0.0028
0.4
0.059
15
10
5
0
NUDT14
Anova, p = 0.0082
Low
Med
High
group

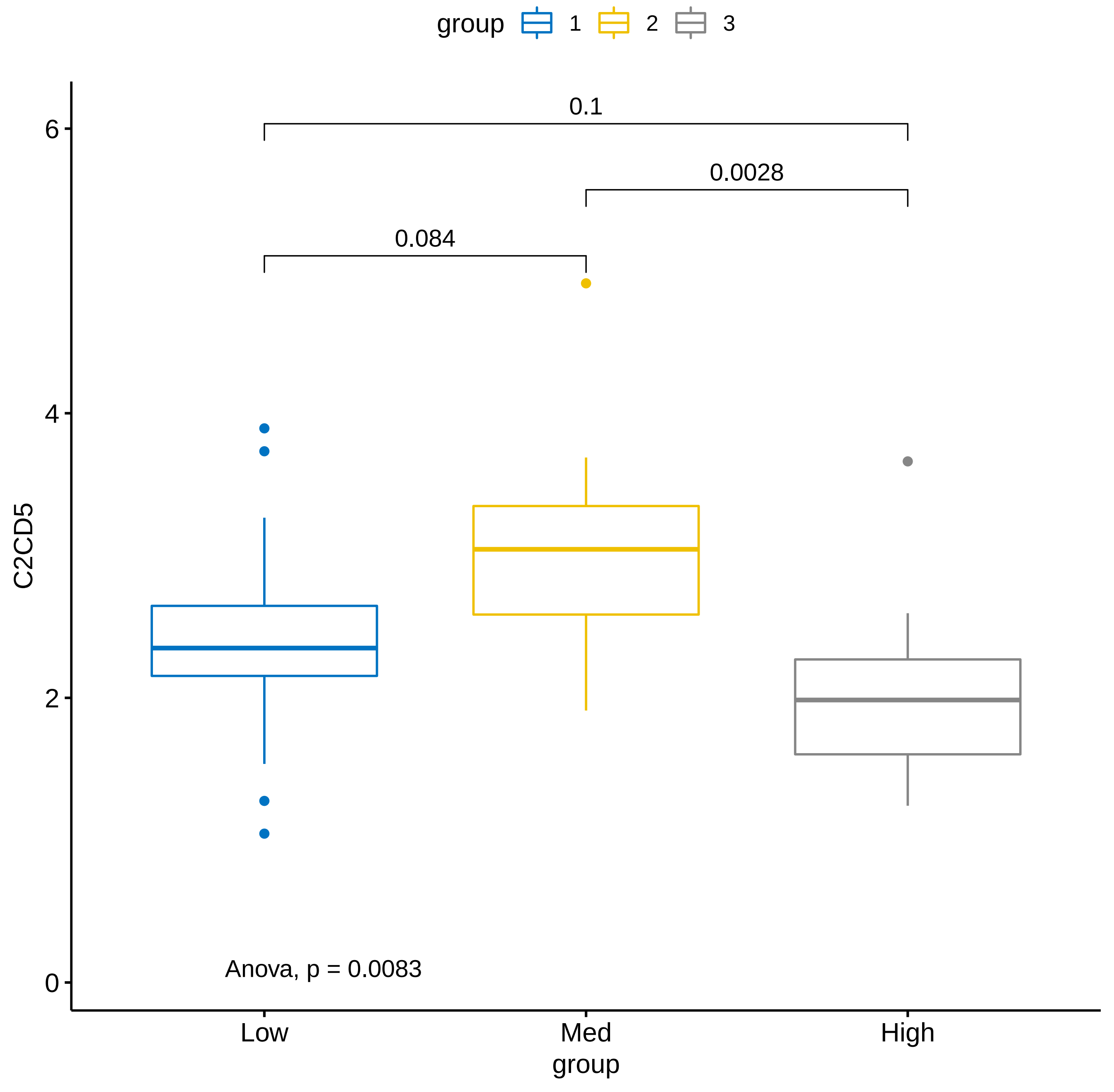
group 1 2 3
0.1
0.0028
0.084
C2CD5
6
4
2
0
Anova, p = 0.0083
Low
Med
High
group

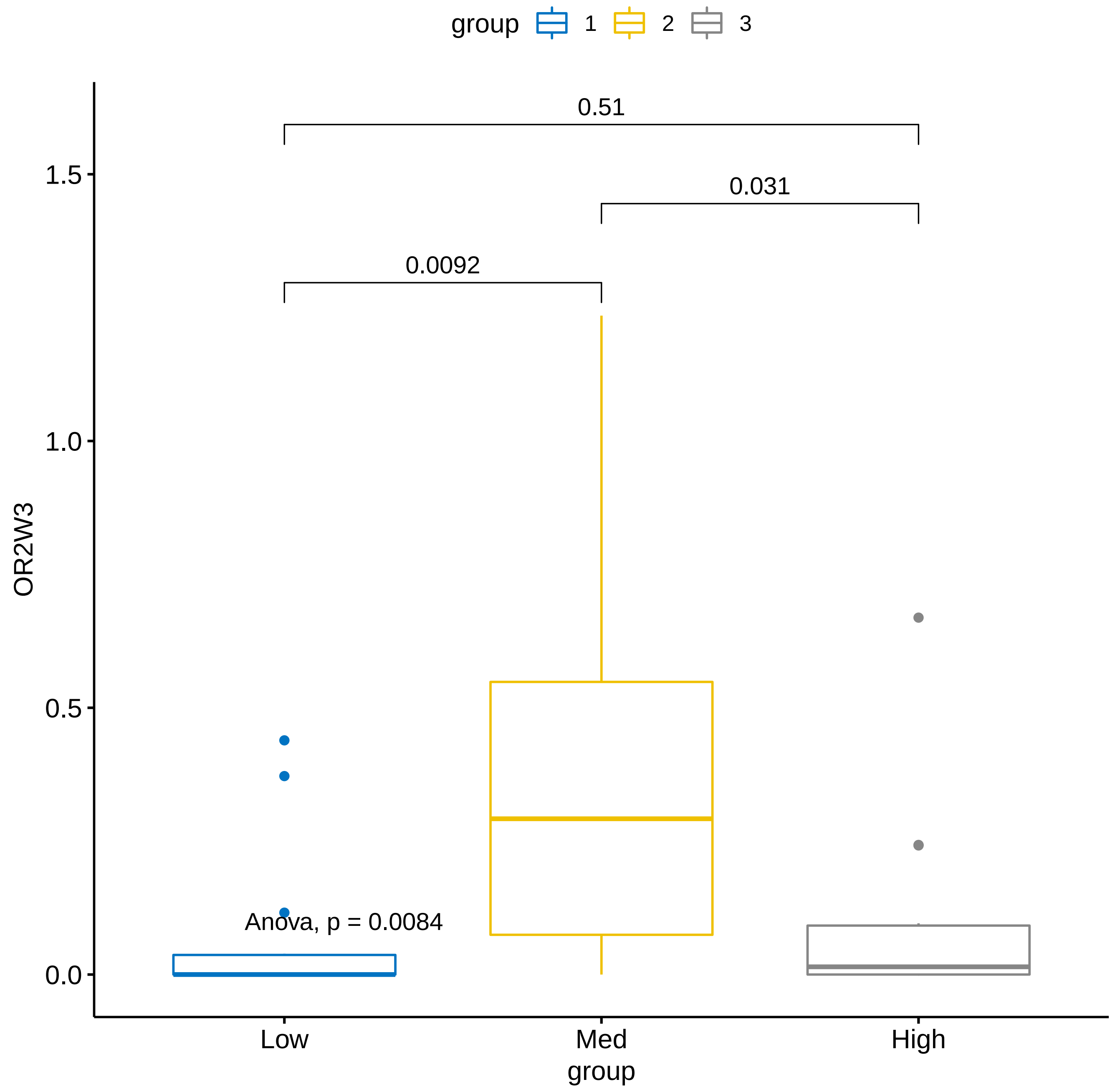
group 1 2 3
0.51
0.031
0.0092
1.5
1.0
0.5
0.0
OR2W3
Anova, p = 0.0084
Low
Med
High
group

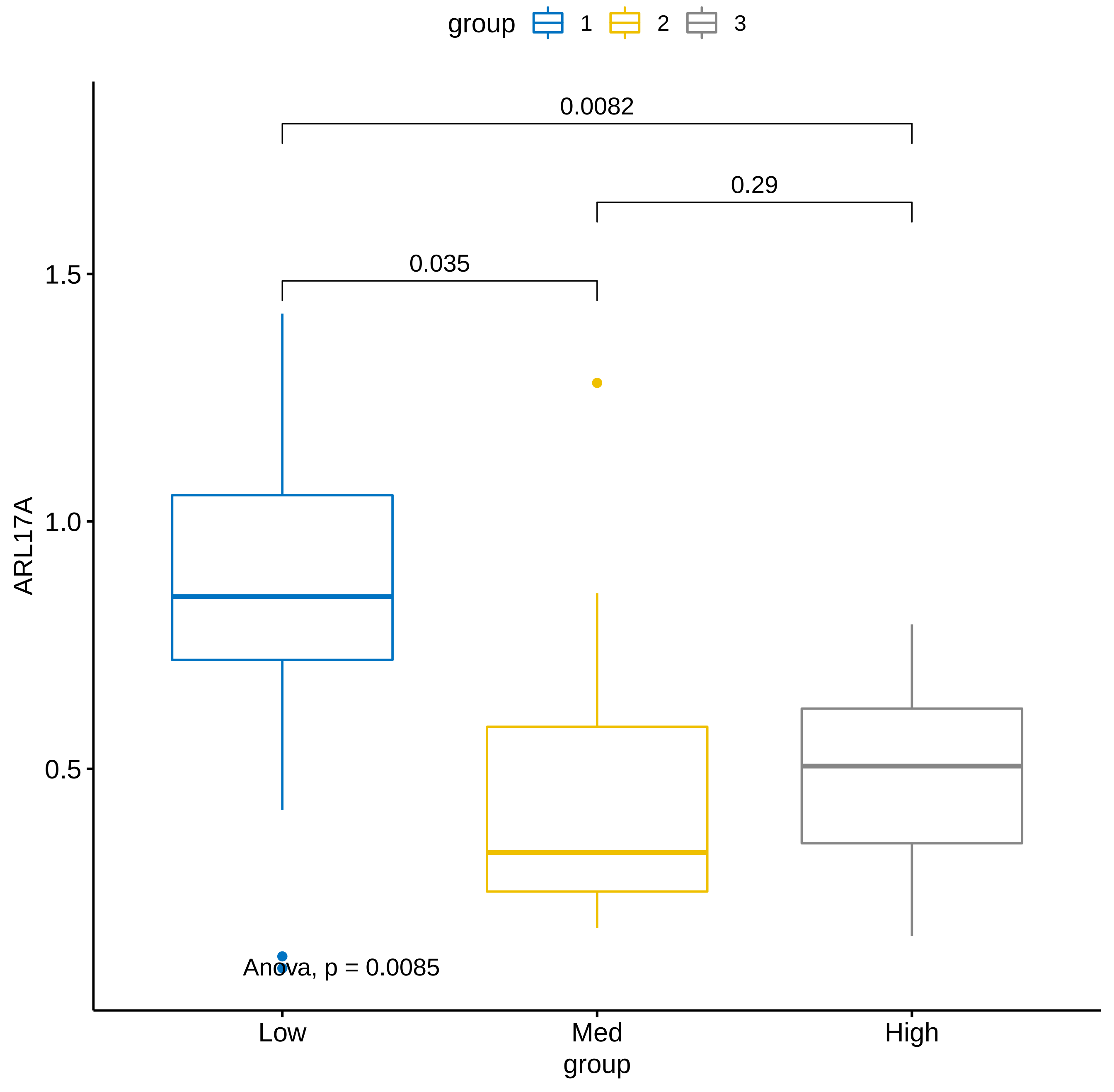
group
1
2
3
0.0082
0.29
0.035
1.5
1.0
0.5
ARL17A
Anova, p = 0.0085
Low
Med
High
group

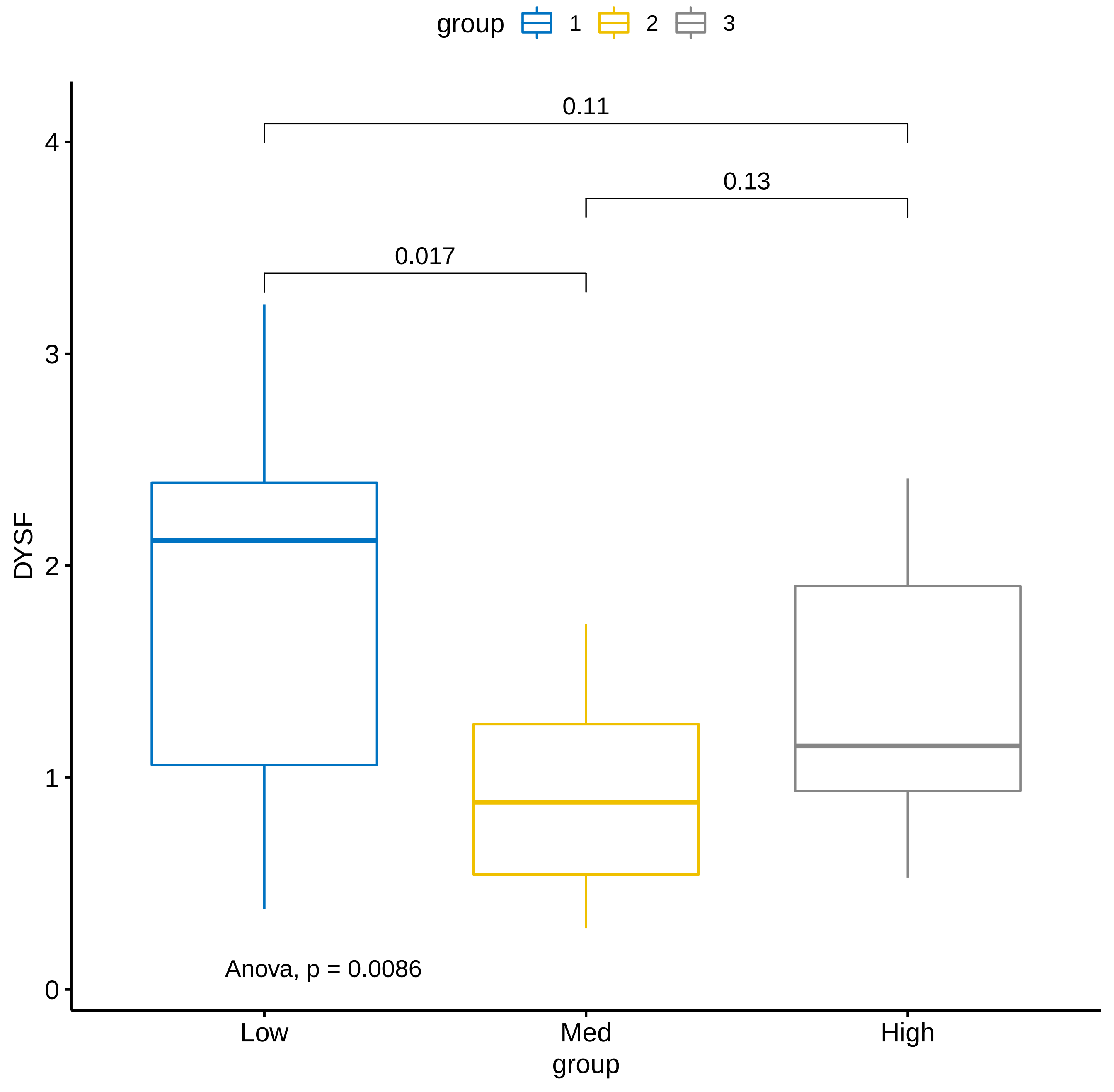

group 1 2 3
0.11
0.13
0.017
DYSF
4
3
2
1
0
Anova, p = 0.0086
Low
Med
High
group

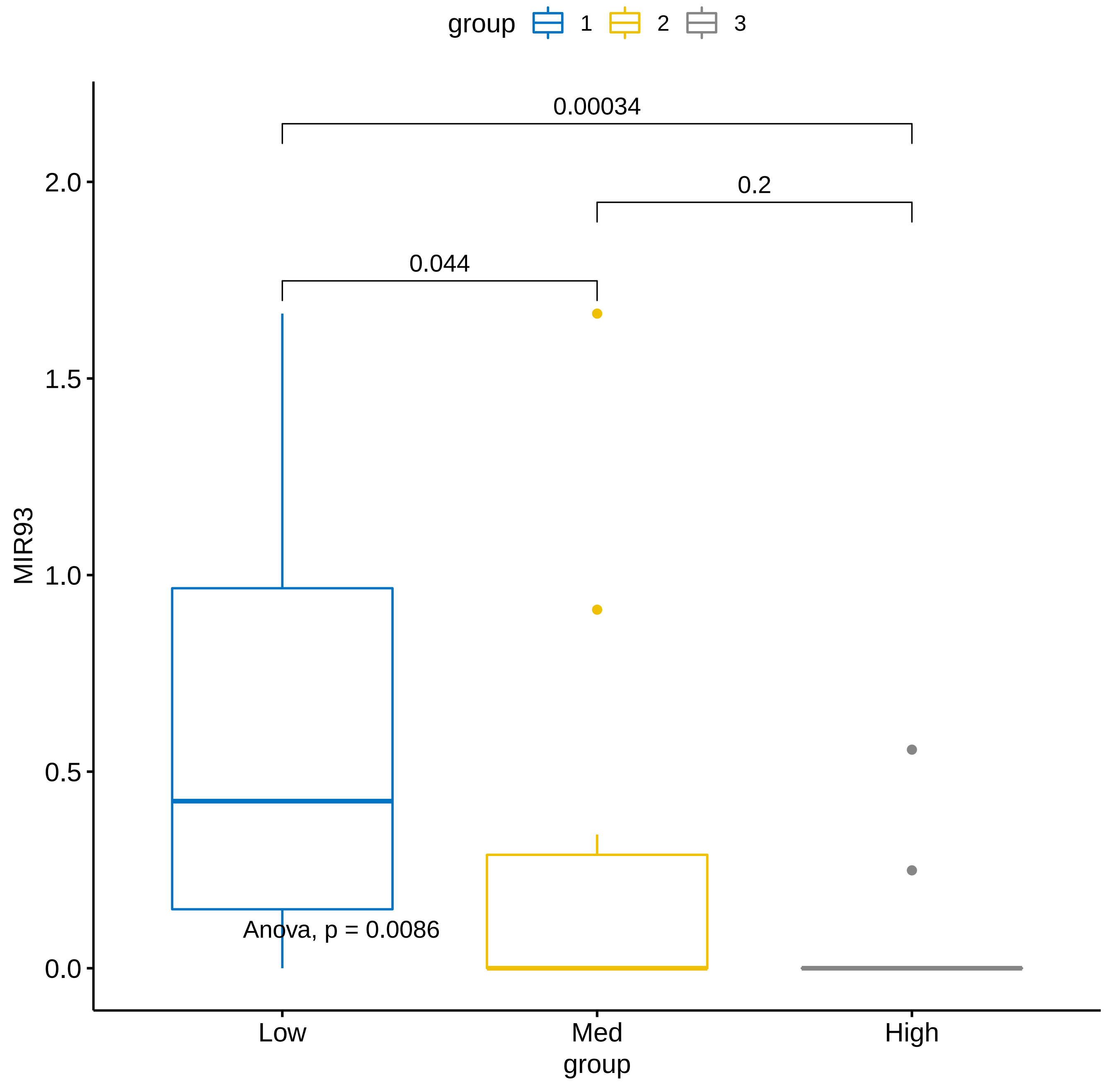
group
1
2
3
0.00034
0.2
0.044
2.0
1.5
1.0
0.5
0.0
MIR93
Anova, p = 0.0086
Low
Med
High
group

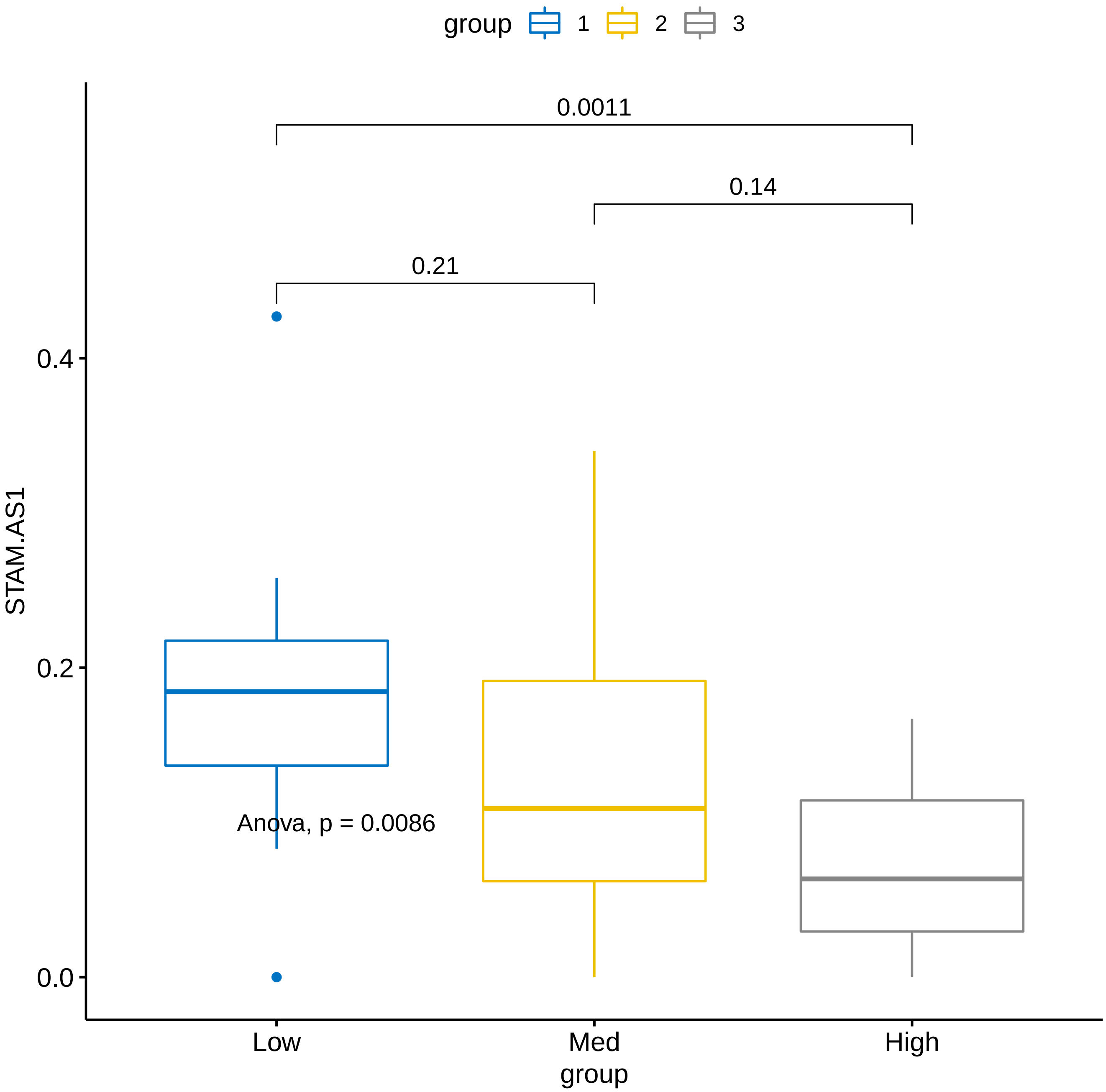
group
1
2
3
0.0011
0.14
0.21
Anova, p = 0.0086
STAM.AS1
0.4
0.2
0.0
Low
Med
High
group

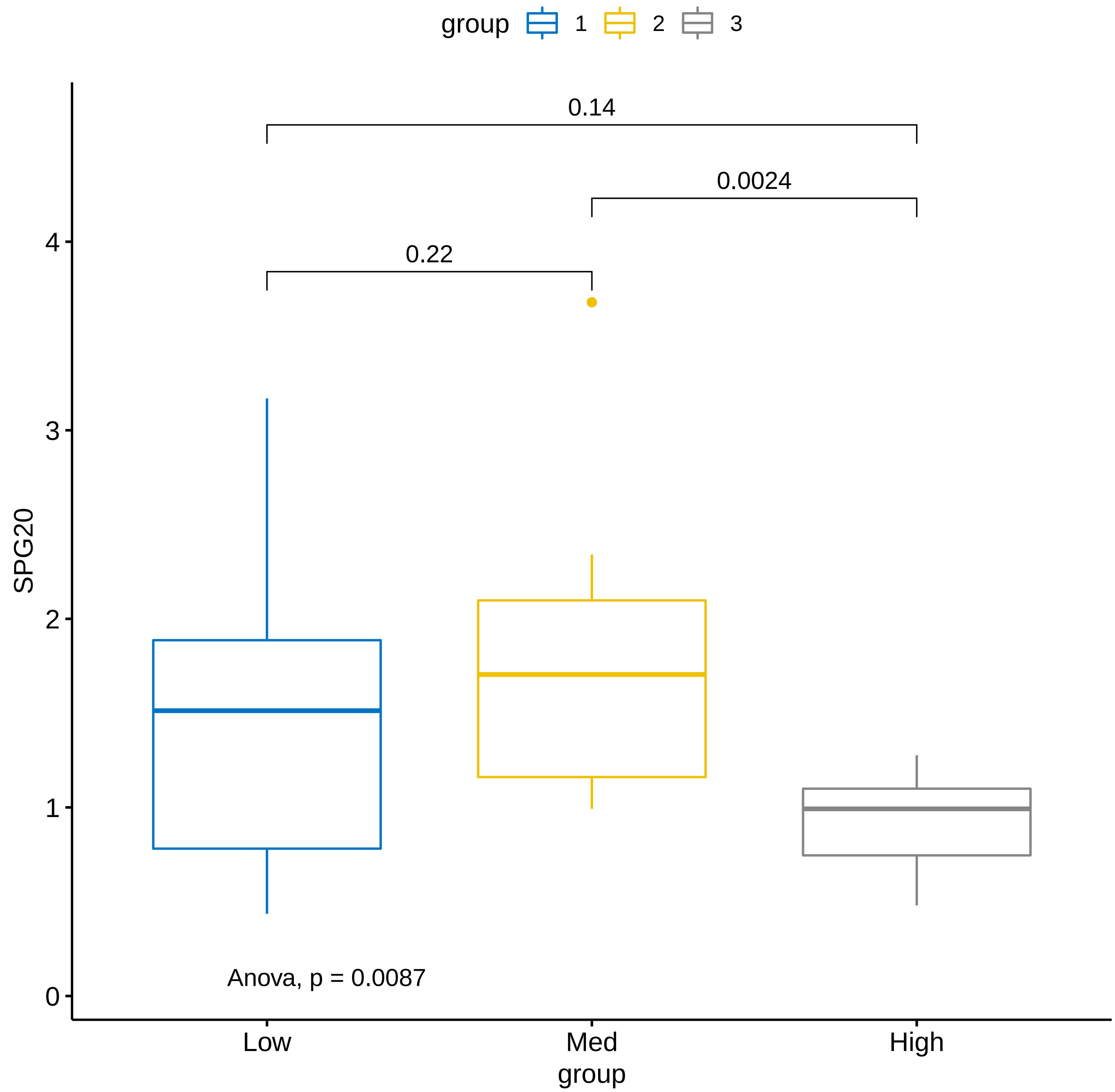
group
1
2
3
0.14
0.0024
0.22
4
3
2
1
0
SPG20
Anova, p = 0.0087
Low
Med
High
group

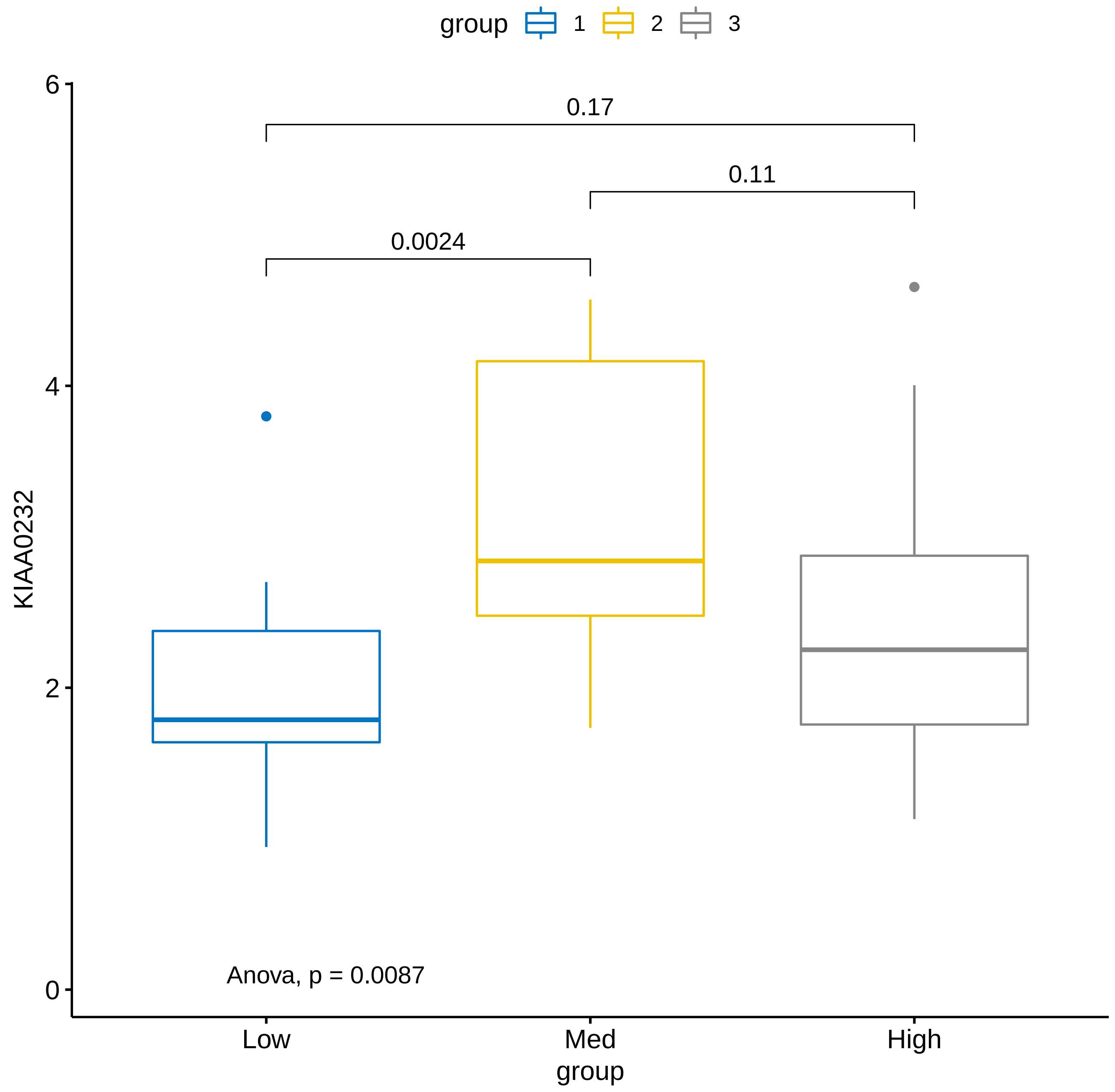
group
1
2
3
6
4
2
0
0.17
0.11
0.0024
KIAA0232
Anova, p = 0.0087
Low
Med
High
group

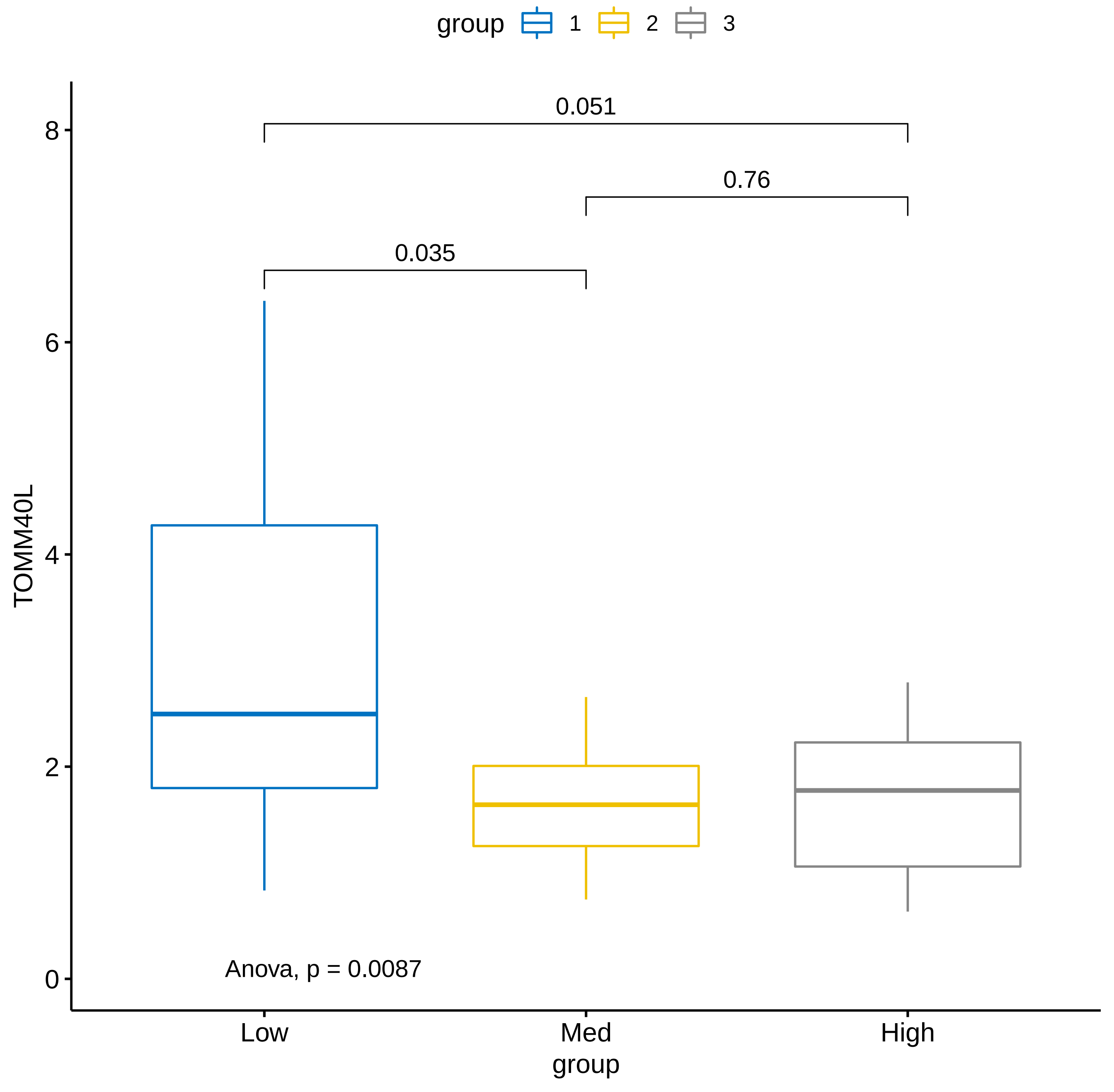

group
1
2
3
0.051
0.76
0.035
8
6
4
2
0
TOMM40L
Anova, p = 0.0087
Low
Med
High
group

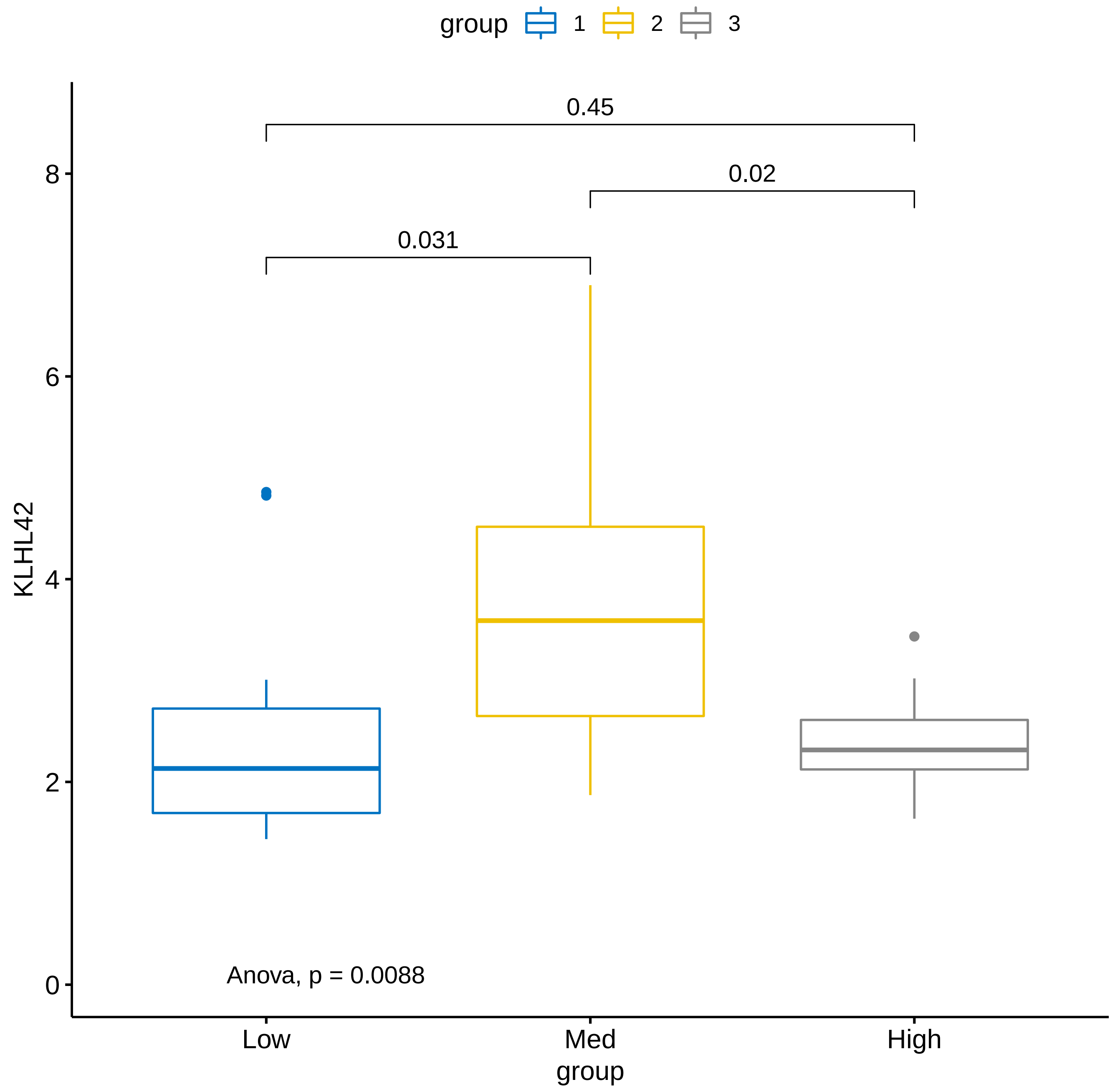
group
1
2
3
0.45
0.02
0.031
8
6
4
2
0
KLHL42
Anova, p = 0.0088
Low
Med
High
group

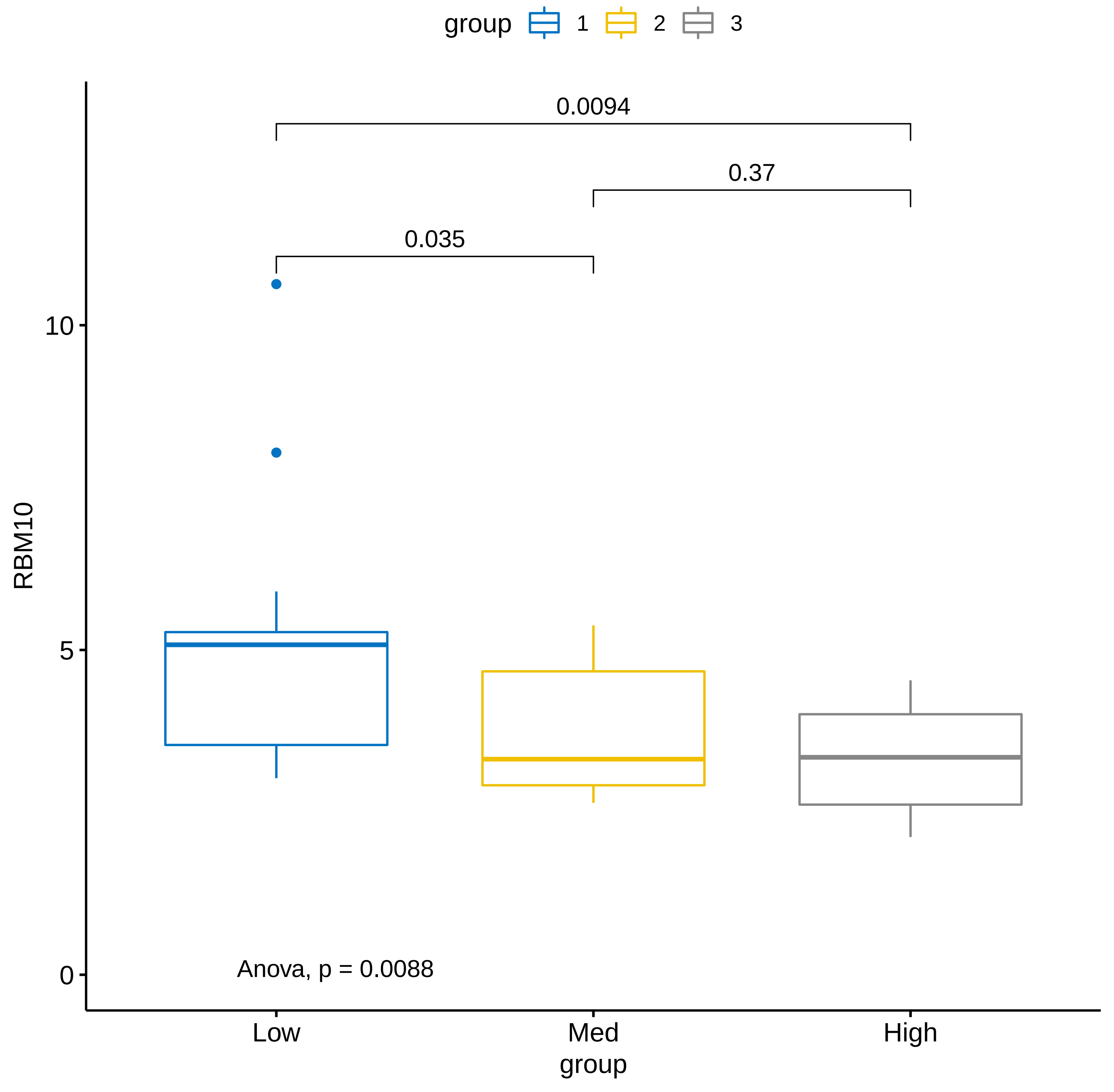
group
1
2
3
0.0094
0.37
0.035
10
5
0
RBM10
Anova, p = 0.0088
Low
Med
High
group

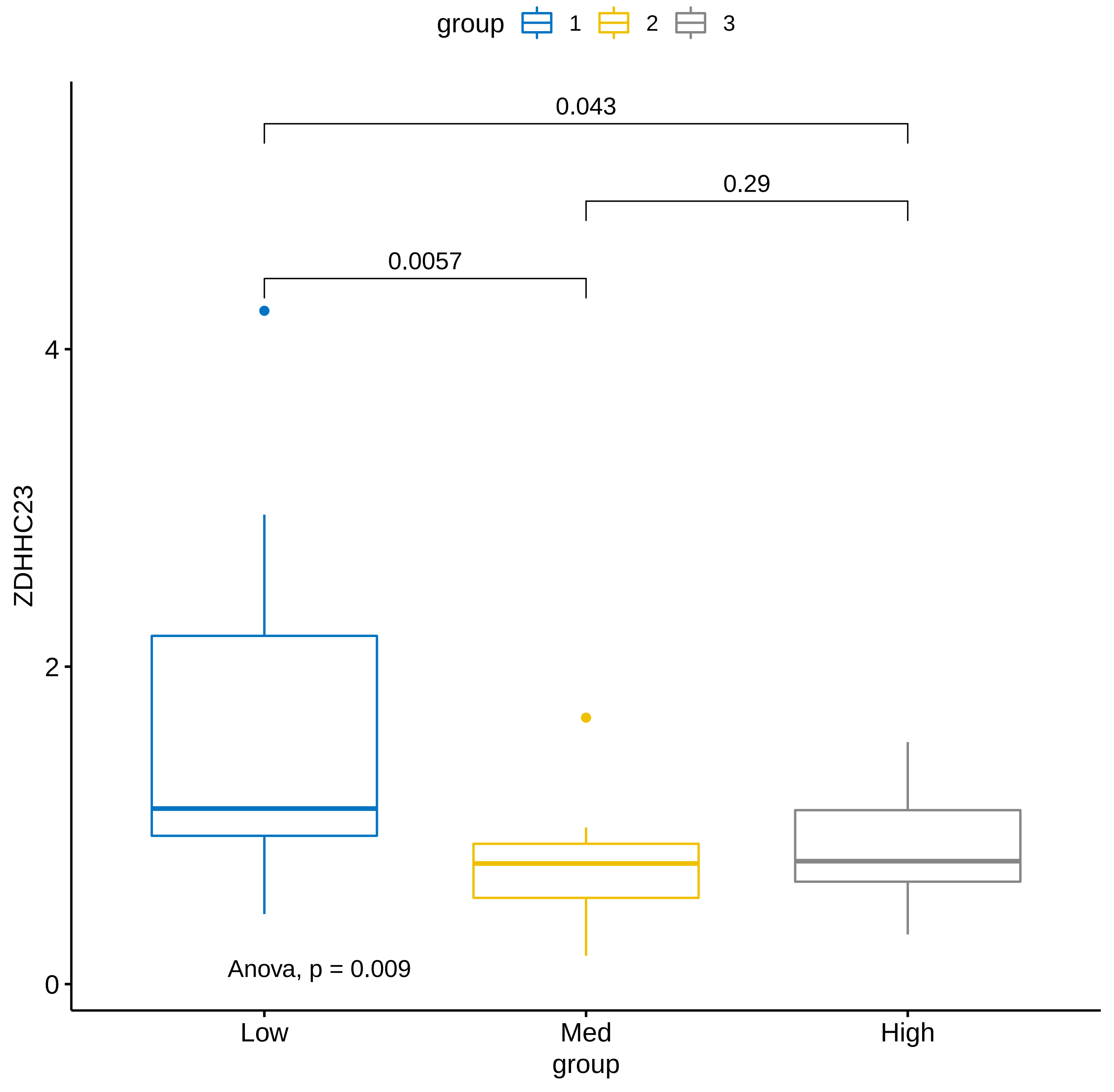
group
1
2
3
0.043
0.29
0.0057
ZDHHC23
4
2
0
Anova, p = 0.009
Low
Med
High
group

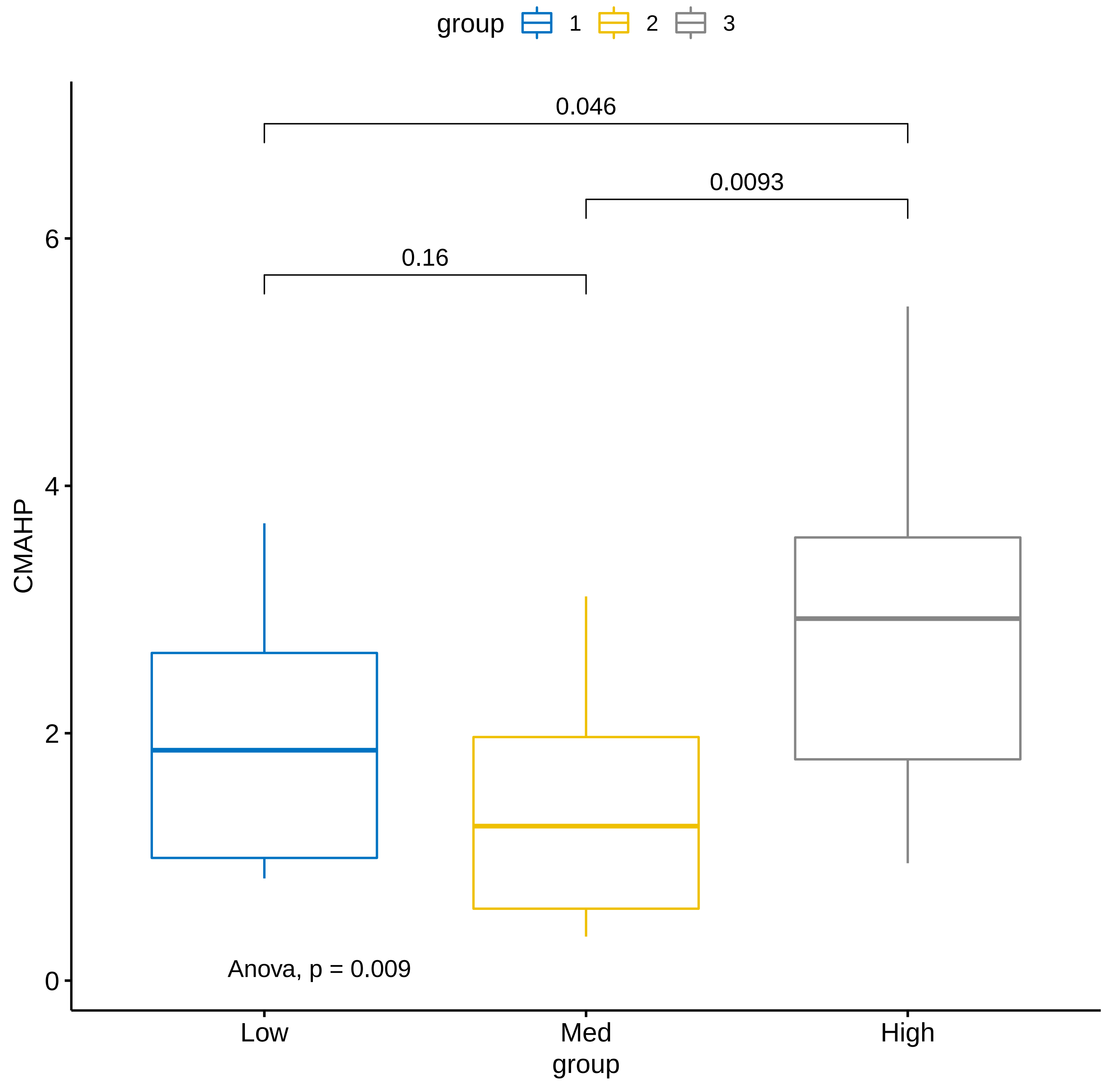
group
1
2
3
0.046
0.0093
0.16
6
4
2
0
CMAHP
Anova, p = 0.009
Low
Med
High
group

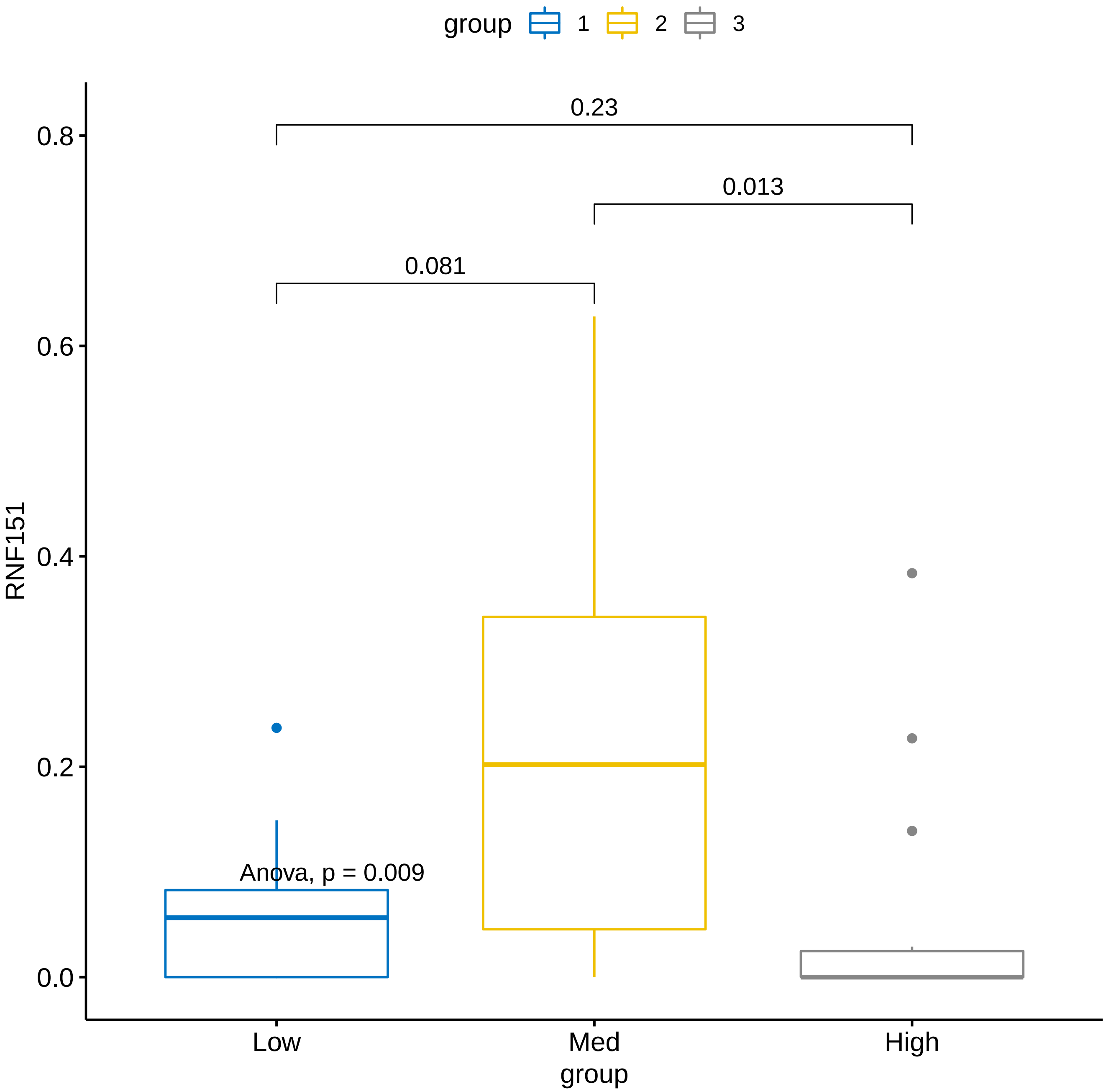
group
1
2
3
0.23
0.013
0.081
Anova, p = 0.009
RNF151
0.8
0.6
0.4
0.2
0.0
Low
Med
High
group

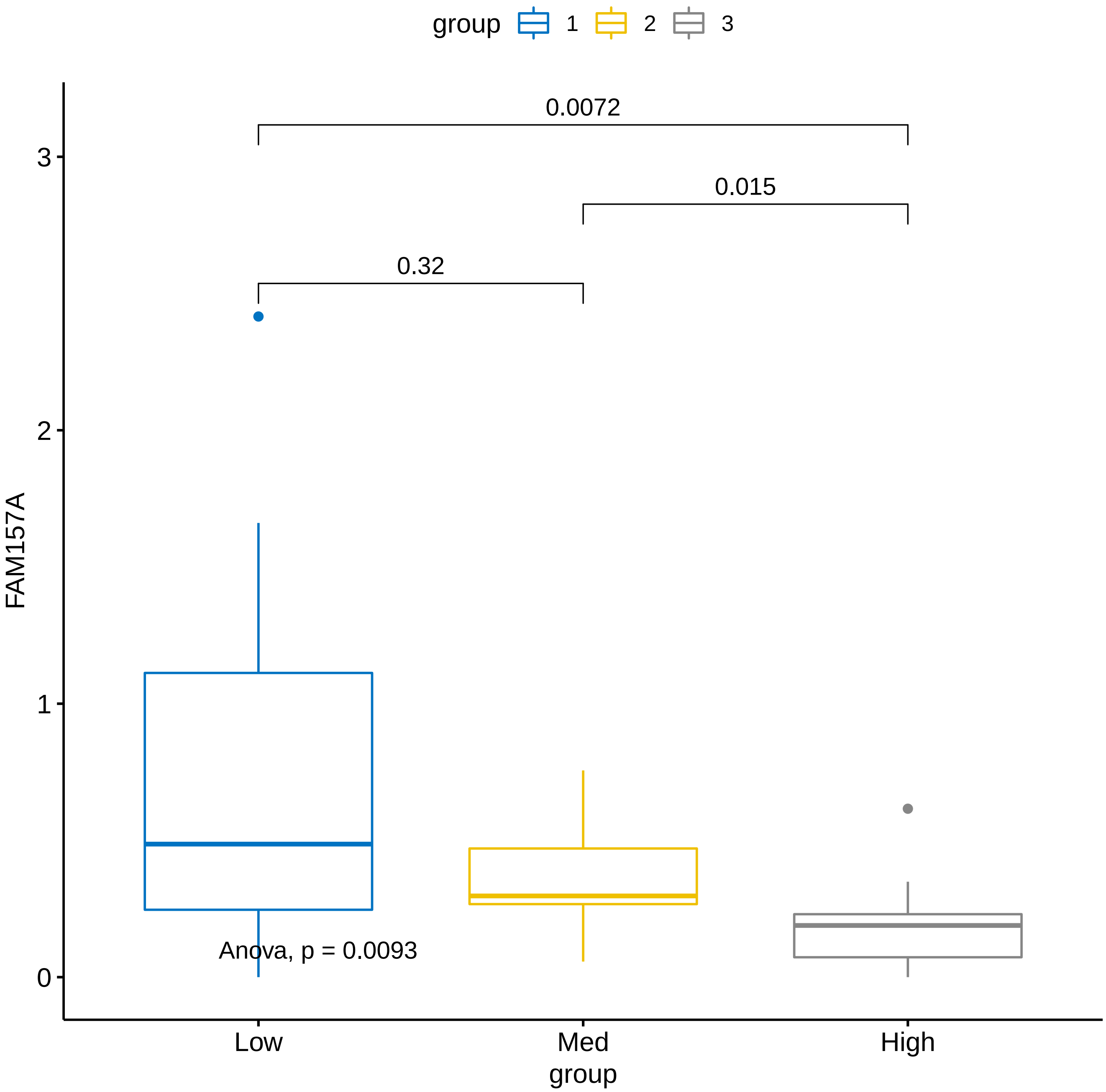

group
1
2
3
0.0072
0.015
0.32
Anova, p = 0.0093
3
2
1
0
FAM157A
Low
Med
High
group

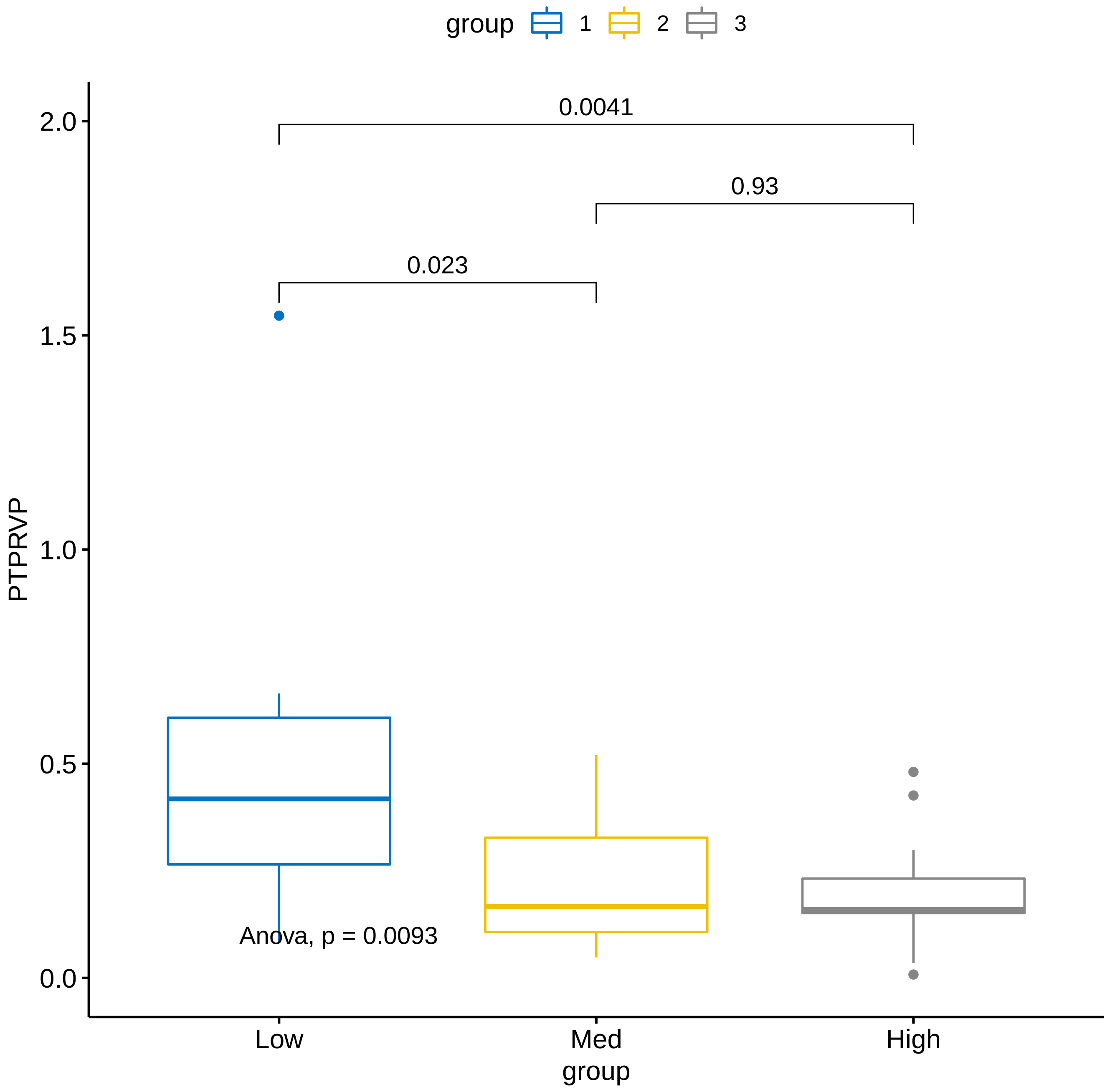
group
1
2
3
0.0041
0.93
0.023
2.0
1.5
1.0
0.5
0.0
PTPRVP
Anova, p = 0.0093
Low
Med
High
group

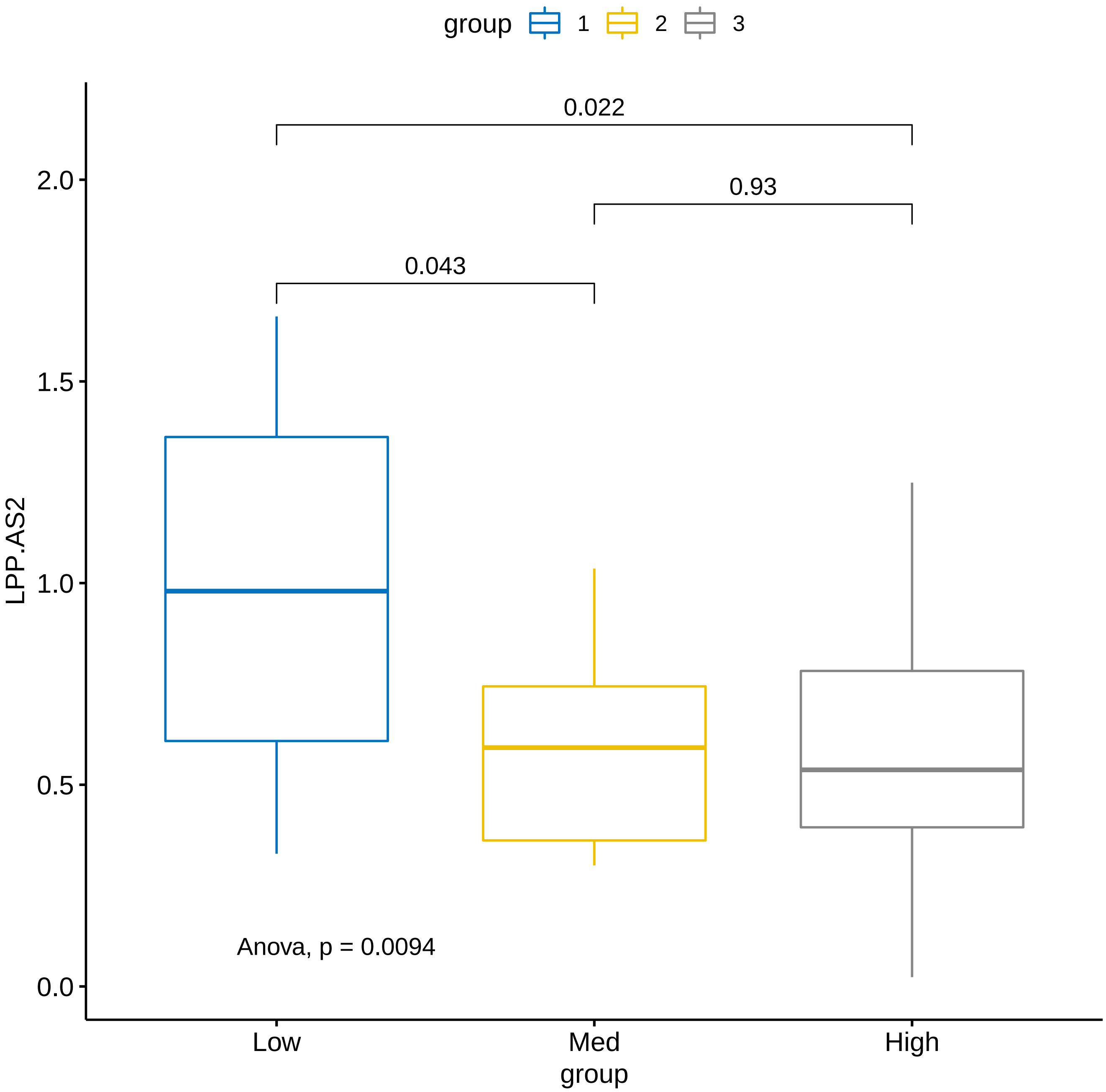

group 1 2 3
0.022
0.93
0.043
2.0
1.5
1.0
0.5
0.0
LPP.AS2
Anova, p = 0.0094
Low
Med
High
group

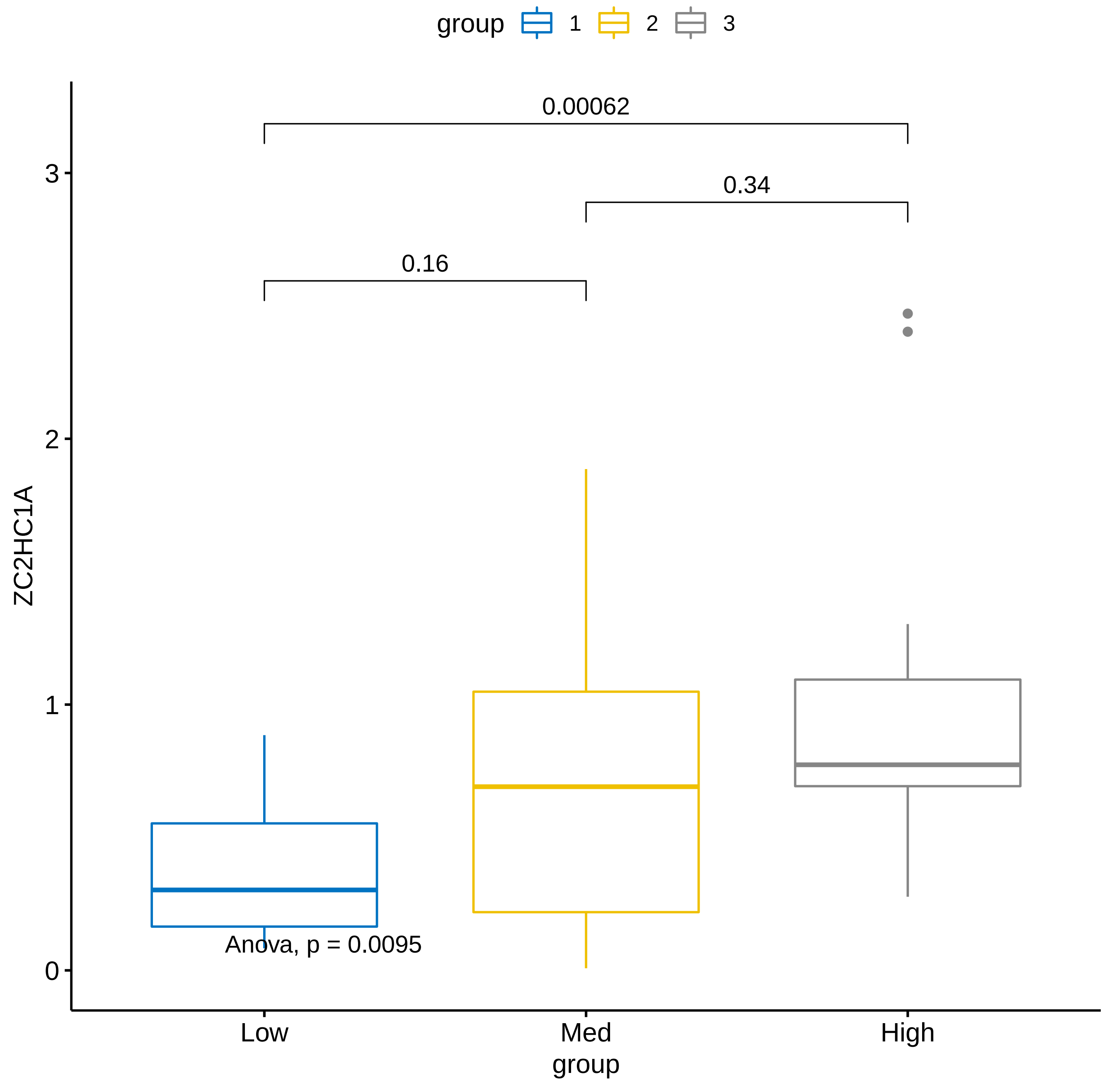

group 1 2 3
0.00062
0.34
0.16
3
2
1
0
ZC2HC1A
Anova, p = 0.0095
Low
Med
High
group

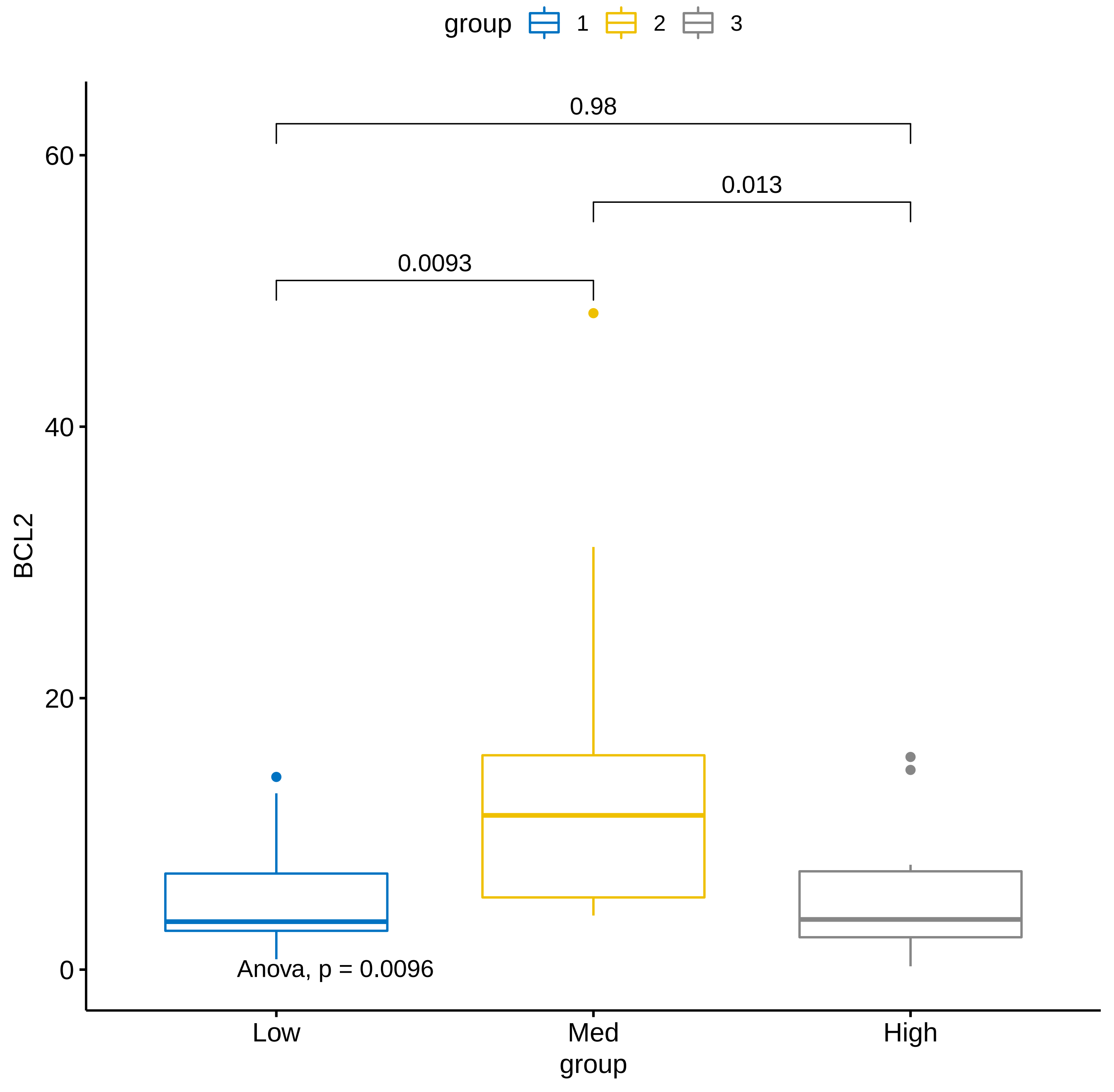
group
1
2
3
0.98
0.013
0.0093
60
40
20
0
BCL2
Anova, p = 0.0096
Low
Med
High
group

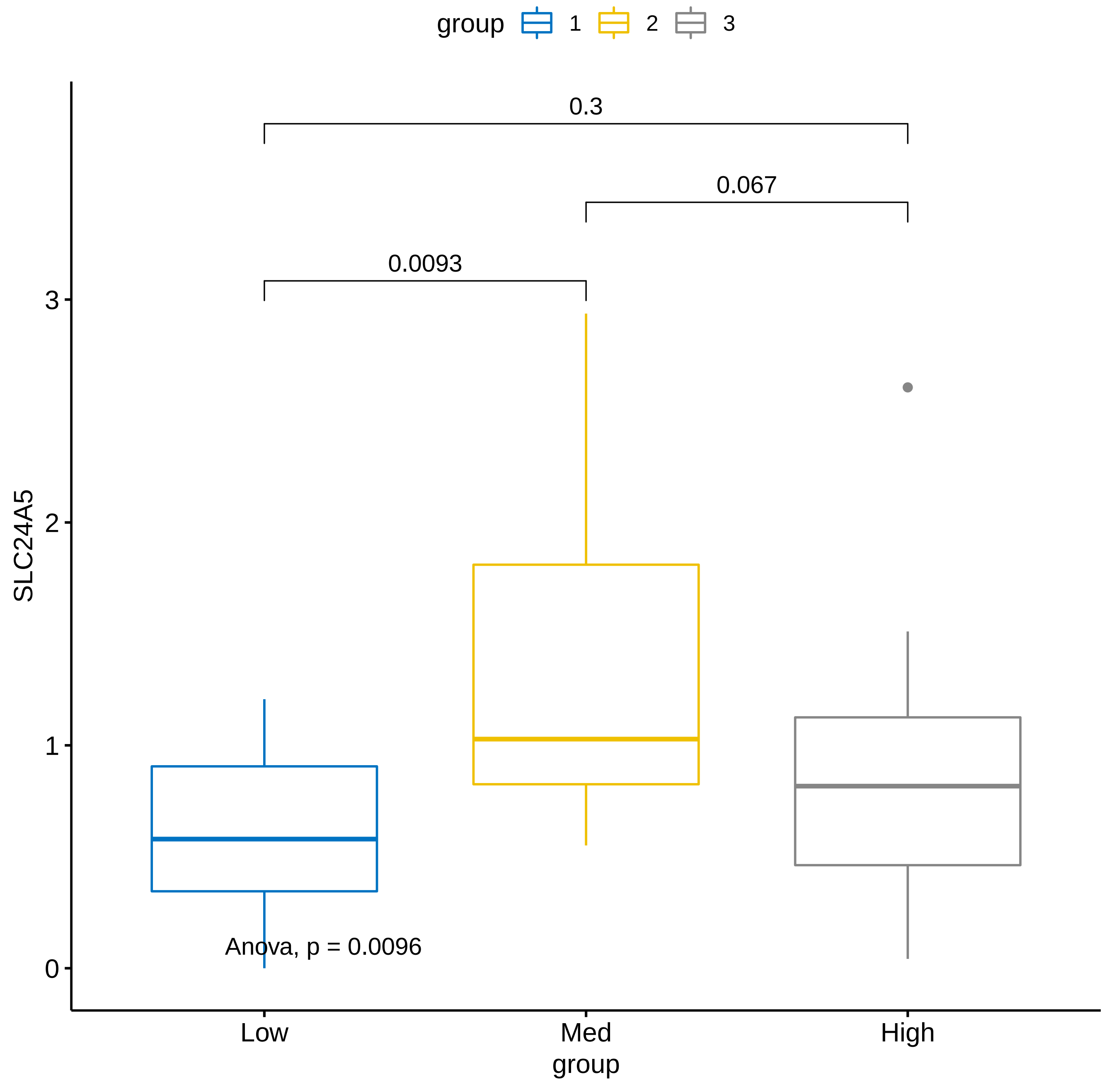
group
1
2
3
0.3
0.067
0.0093
SLC24A5
3
2
1
0
Anova, p = 0.0096
Low
Med
High
group

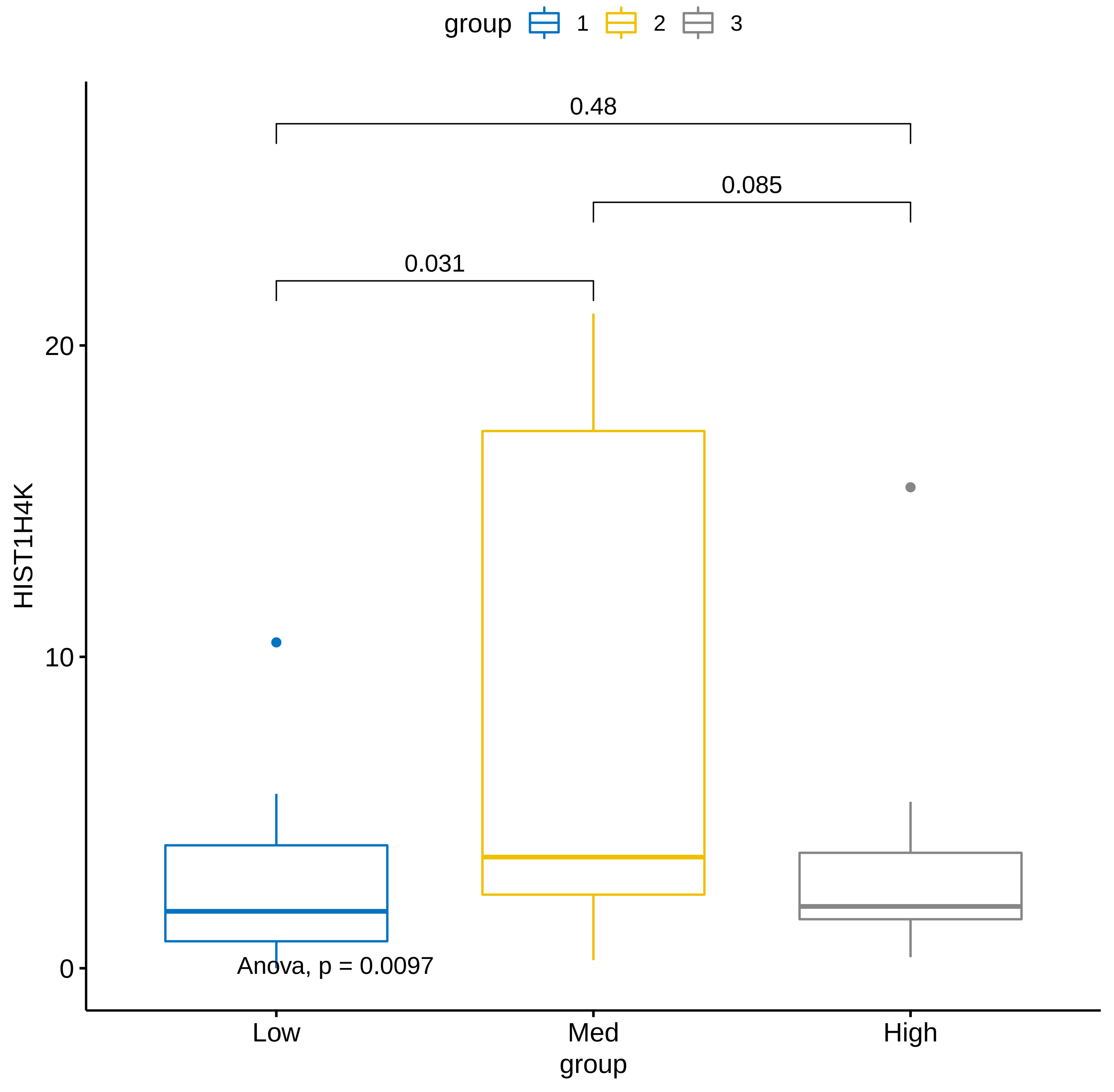
group 1 2 3
0.48
0.085
0.031
20
10
0
HIST1H4K
Anova, p = 0.0097
Low
Med
High
group

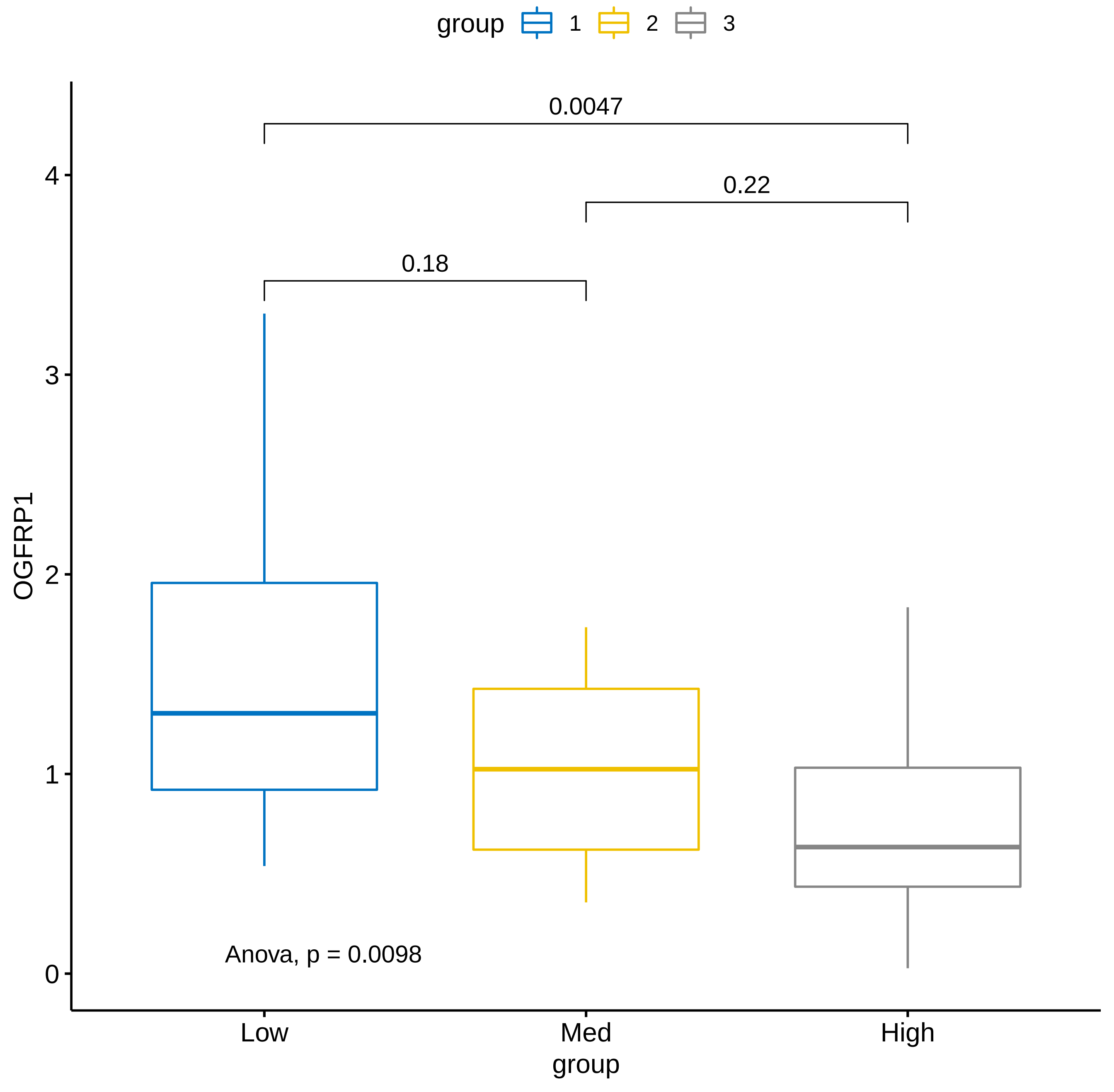
group
1
2
3
0.0047
0.22
0.18
4
3
2
1
0
OGFRP1
Anova, p = 0.0098
Low
Med
High
group

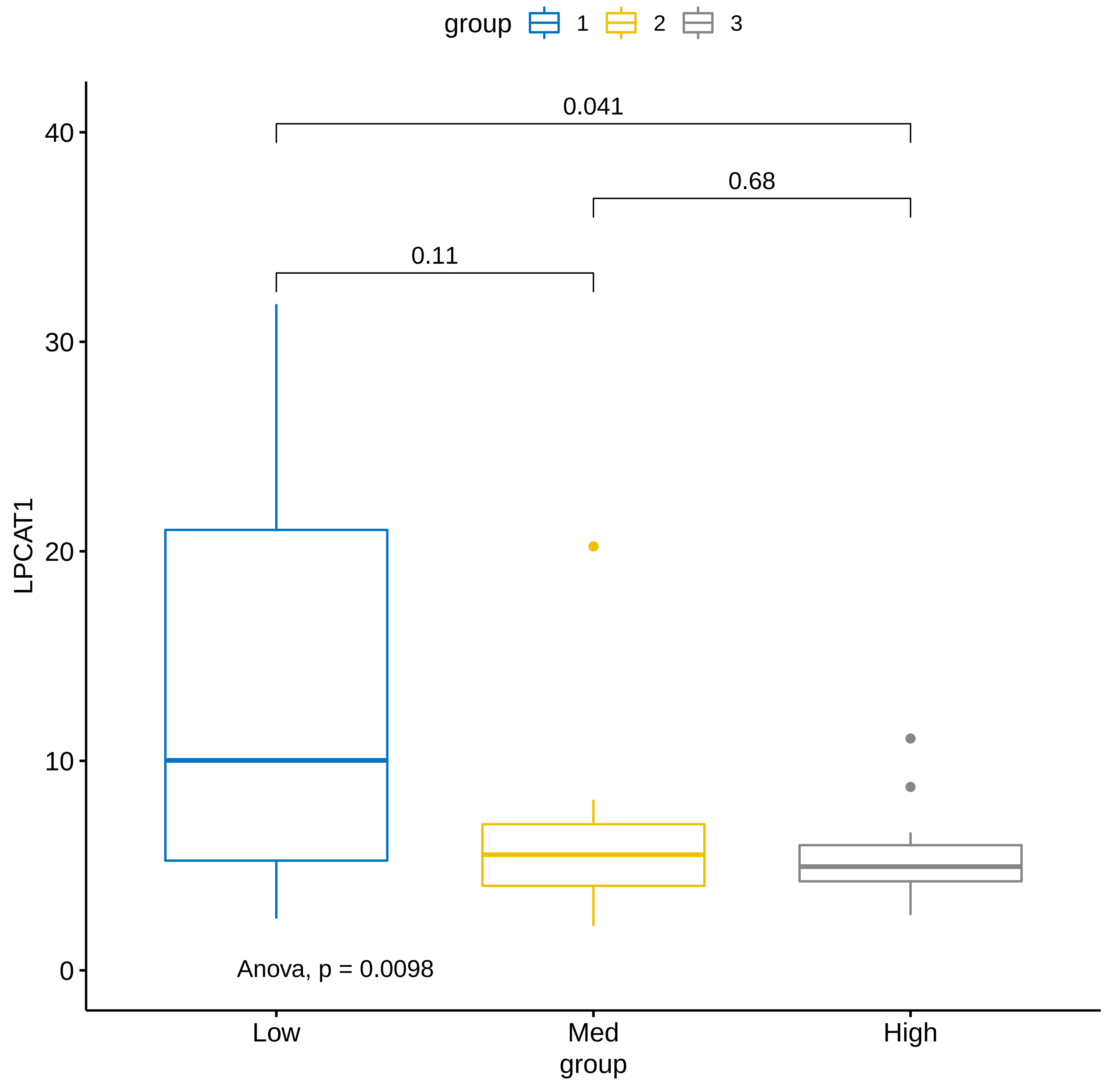
group
1
2
3
0.041
0.68
0.11
40
30
20
10
0
LPCAT1
Anova, p = 0.0098
Low
Med
High
group

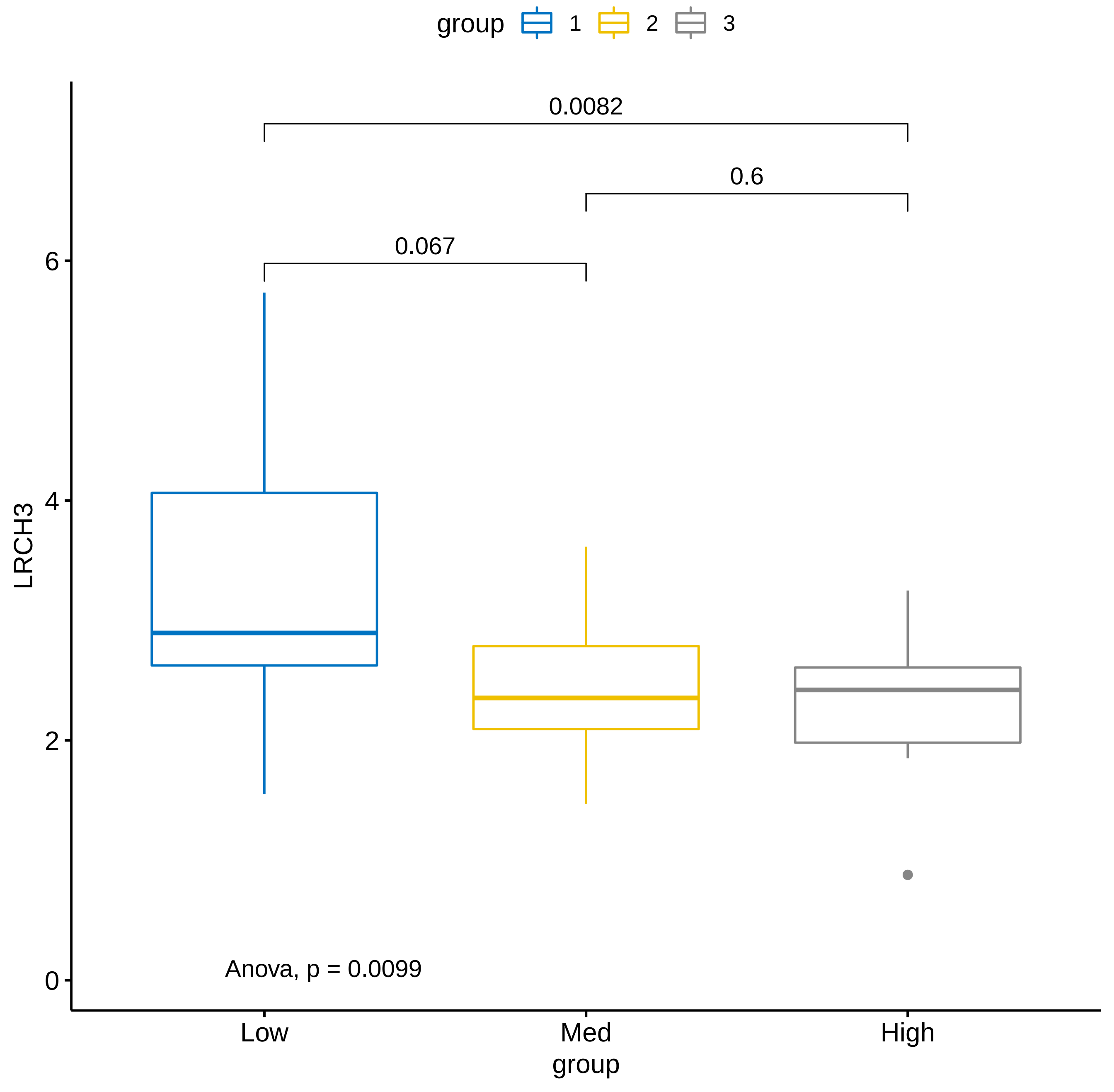

group
1
2
3
0.0082
0.6
0.067
LRCH3
6
4
2
0
Anova, p = 0.0099
Low
Med
High
group

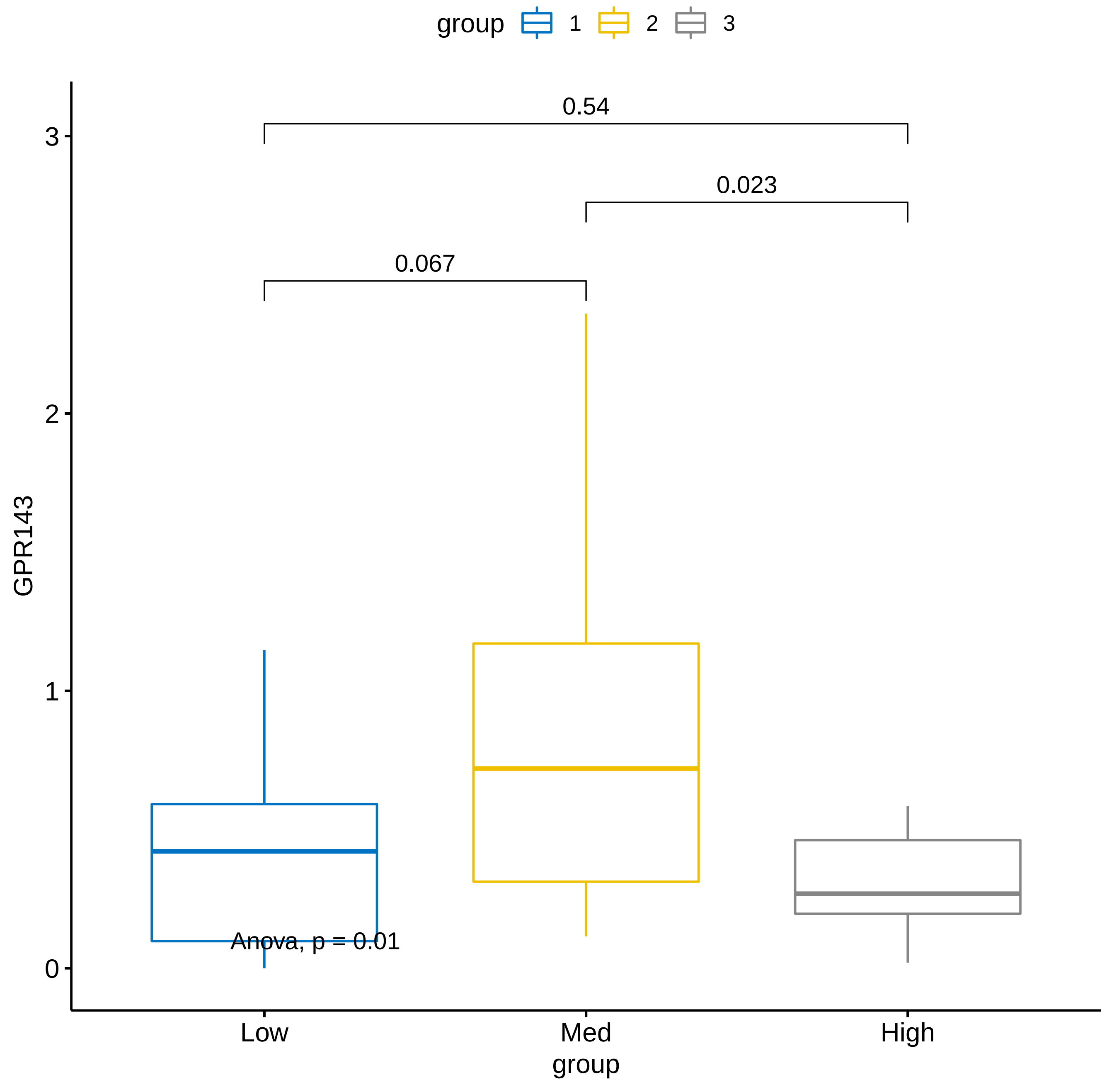

group
1
2
3
0.54
0.023
0.067
3
2
1
0
GPR143
Anova, p = 0.01
Low
Med
High
group

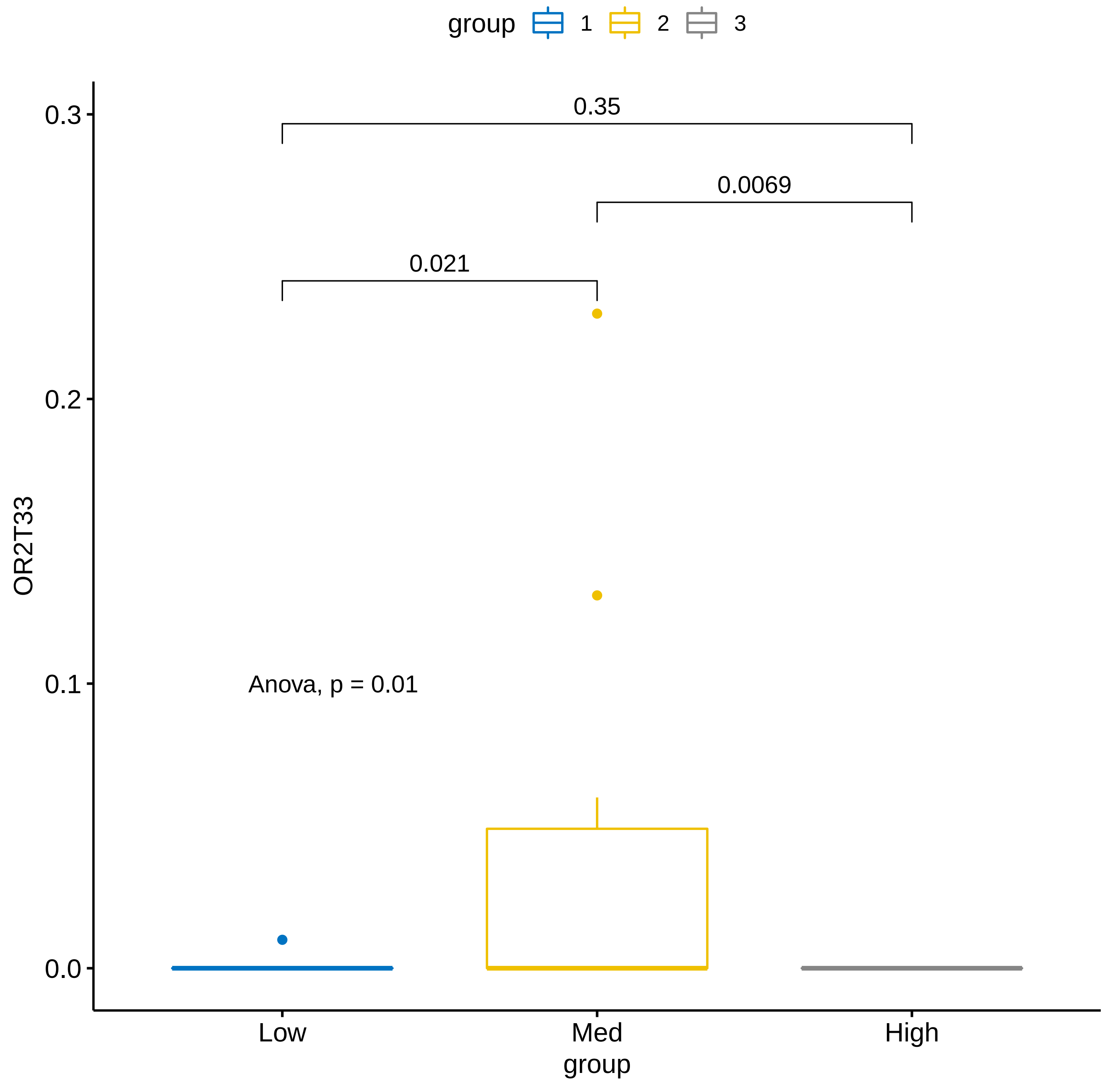
group
1
2
3
0.35
0.0069
0.021
Anova, p = 0.01
0.3
0.2
0.1
0.0
OR2T33
Low
Med
High
group

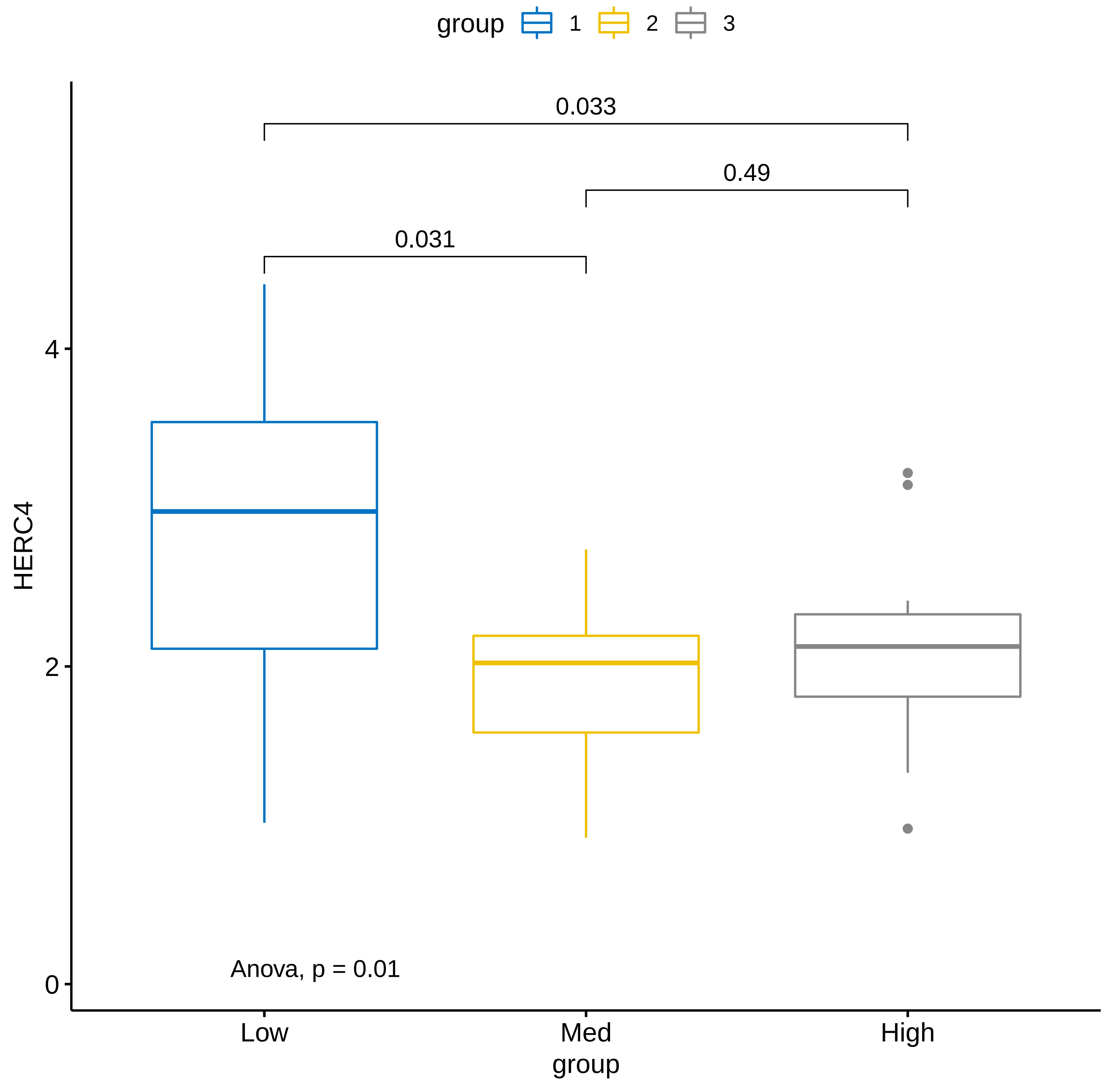
group 1 2 3
0.033
0.49
0.031
HERC4
4
2
0
Anova, p = 0.01
Low
Med
High
group

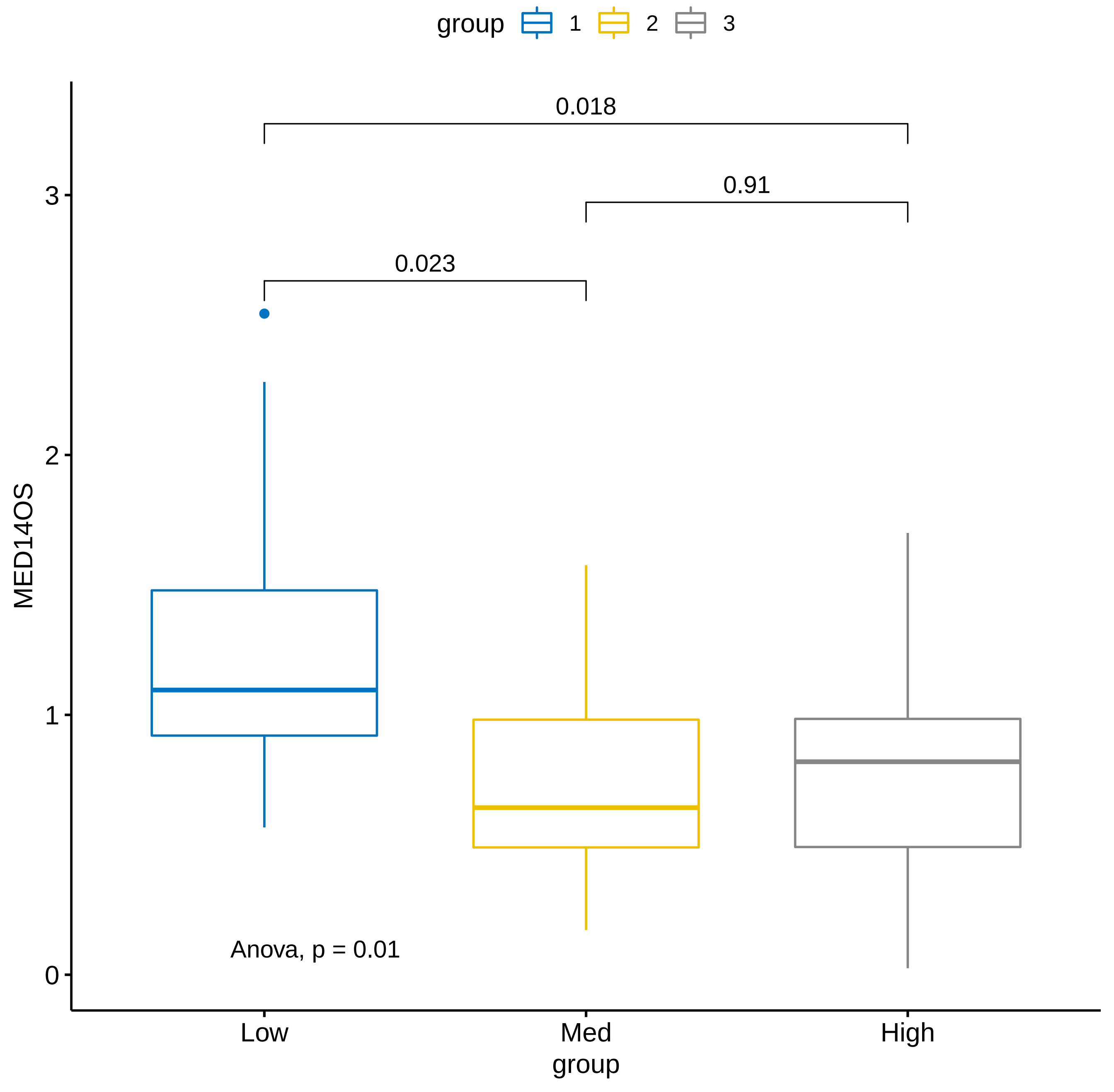
group
1
2
3
0.018
0.91
0.023
Anova, p = 0.01
MED14OS
3
2
1
0
Low
Med
High
group

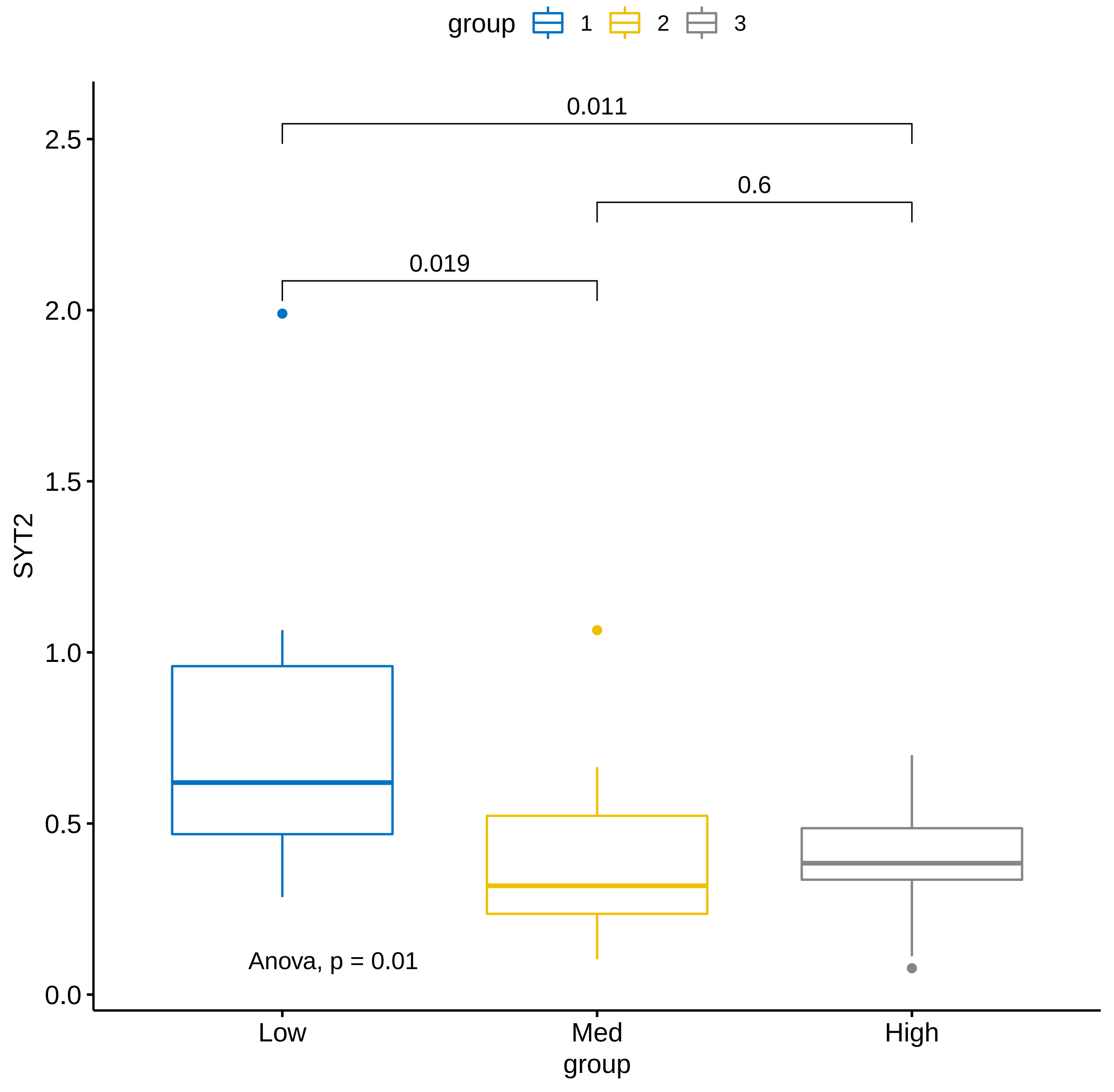
group
1
2
3
0.011
0.6
0.019
2.5
2.0
1.5
1.0
0.5
0.0
SYT2
Anova, p = 0.01
Low
Med
High
group

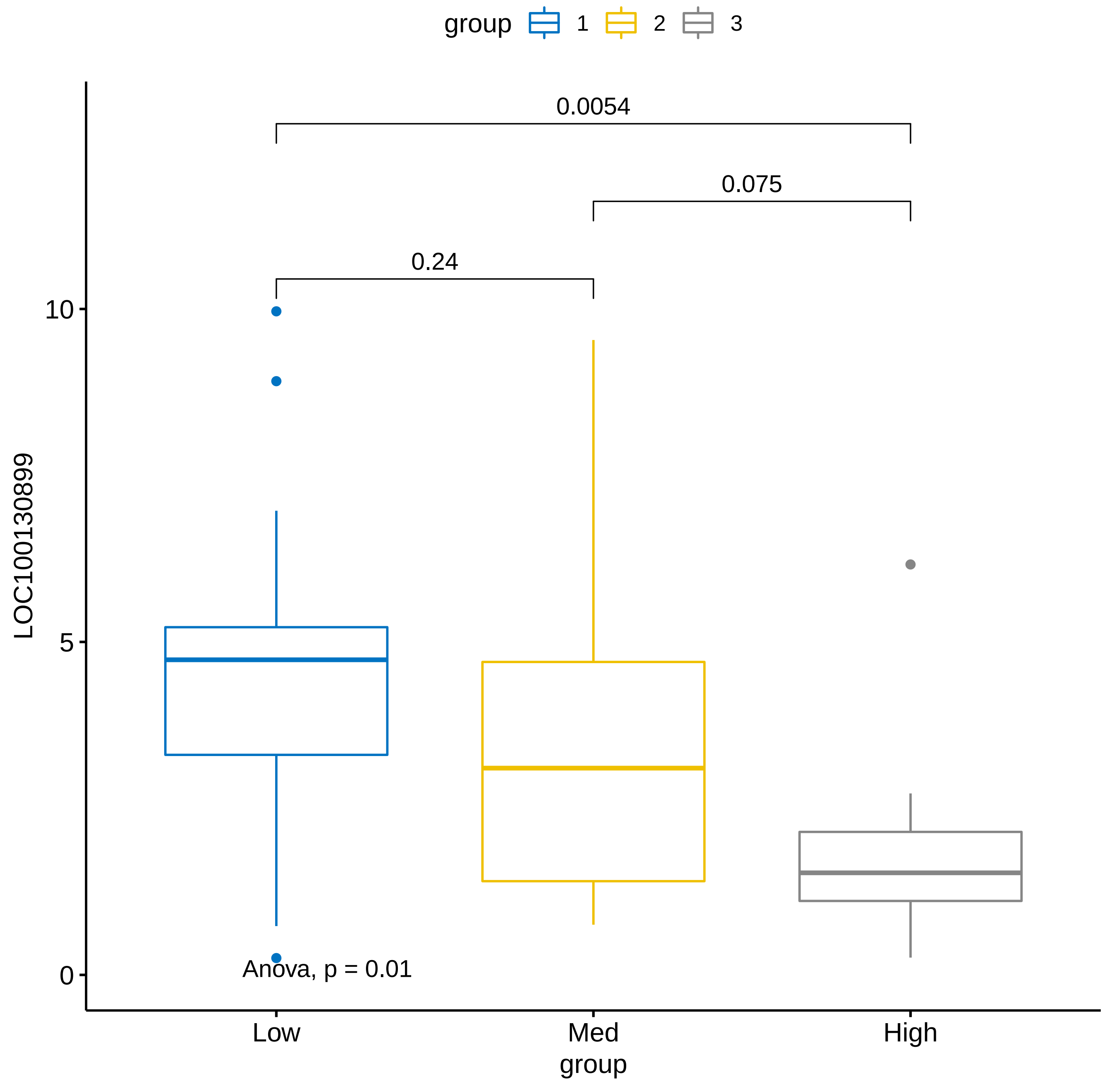
group 1 2 3
0.0054
0.075
0.24
LOC100130899
10
5
0
Anova, p = 0.01
Low
Med
High
group

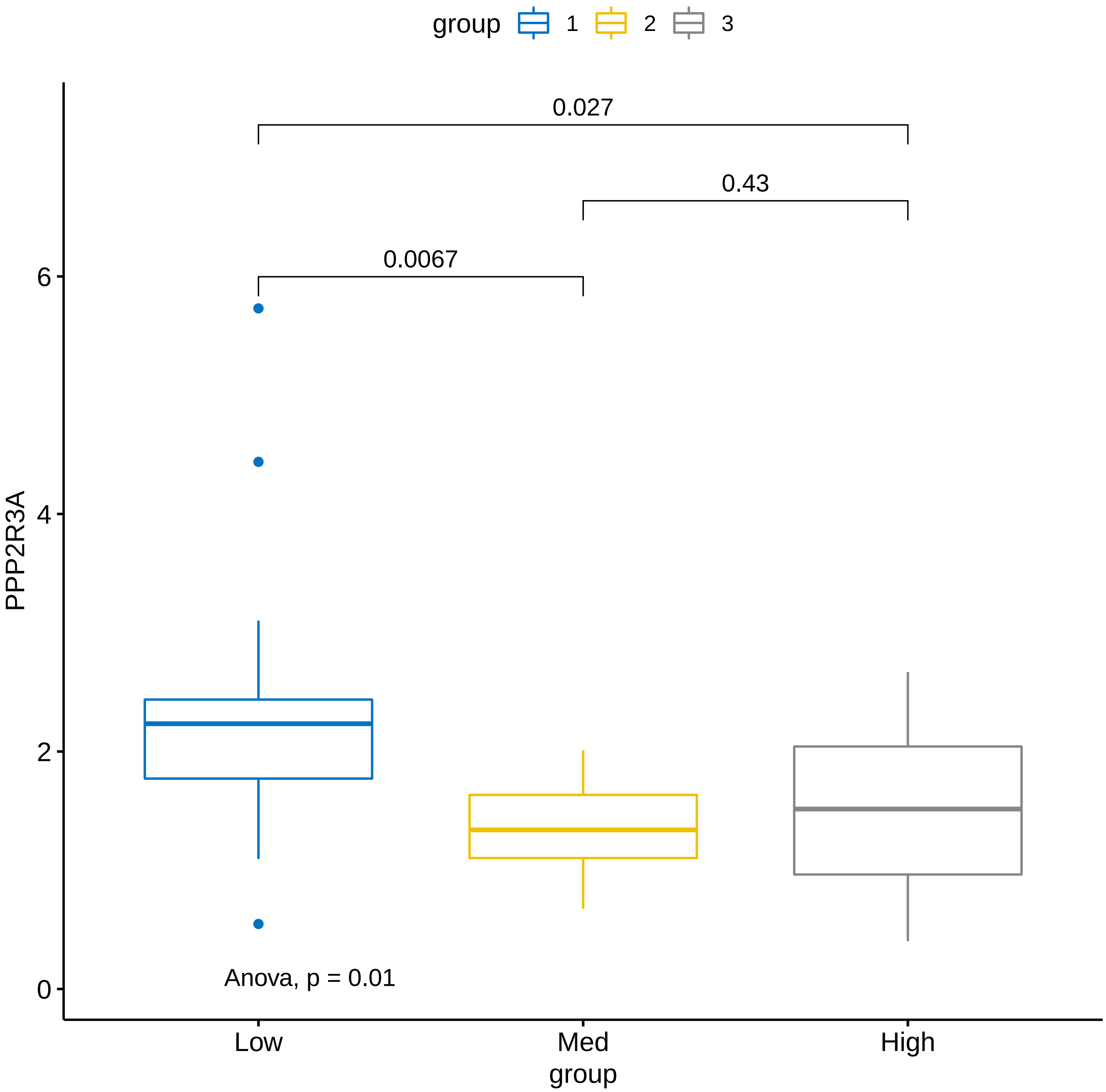
group
1
2
3
0.027
0.43
0.0067
PPP2R3A
6
4
2
0
Anova, p = 0.01
Low
Med
High
group

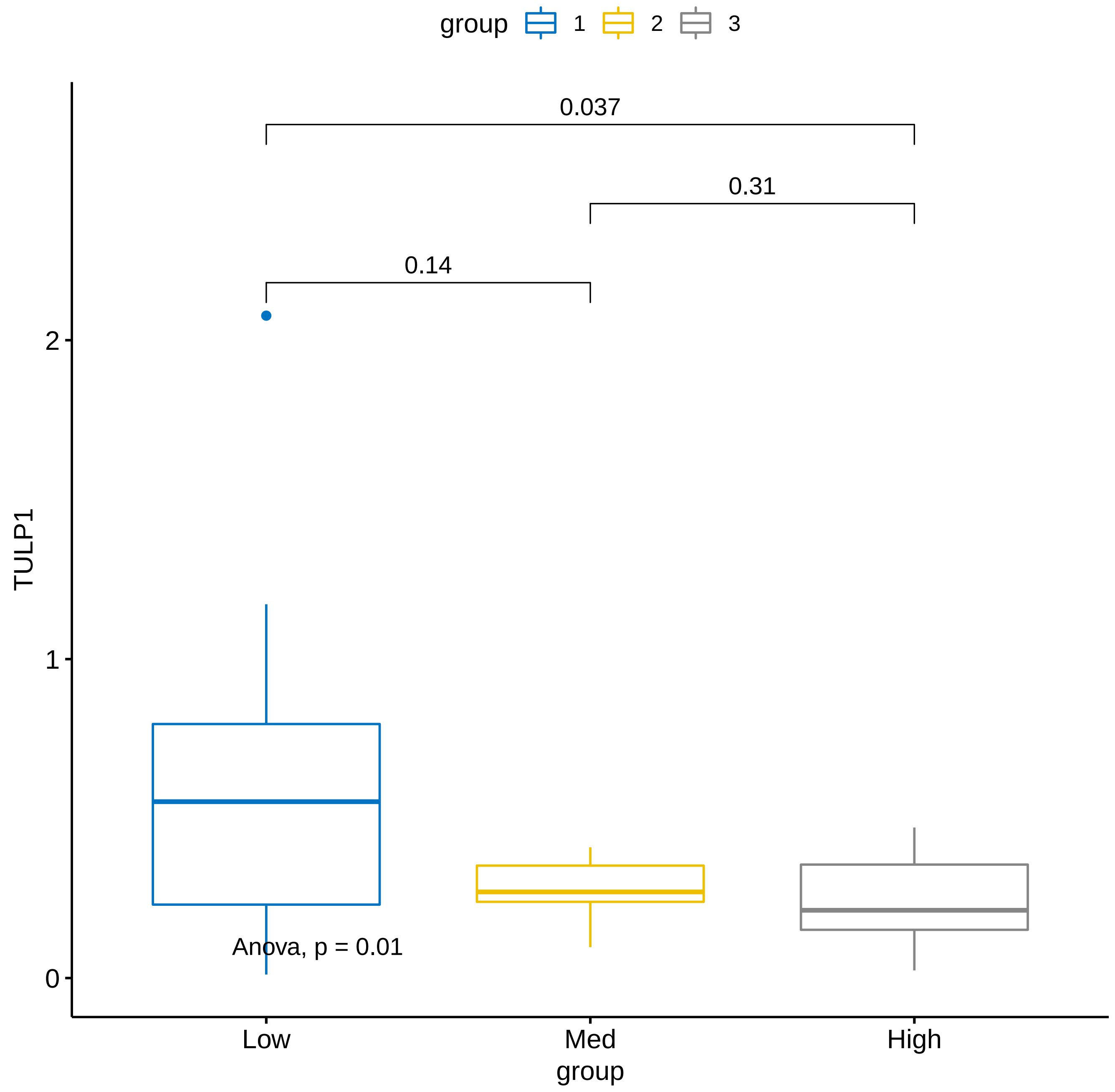
group
1
2
3
0.037
0.31
0.14
2
1
0
TULP1
Anova, p = 0.01
Low
Med
High
group

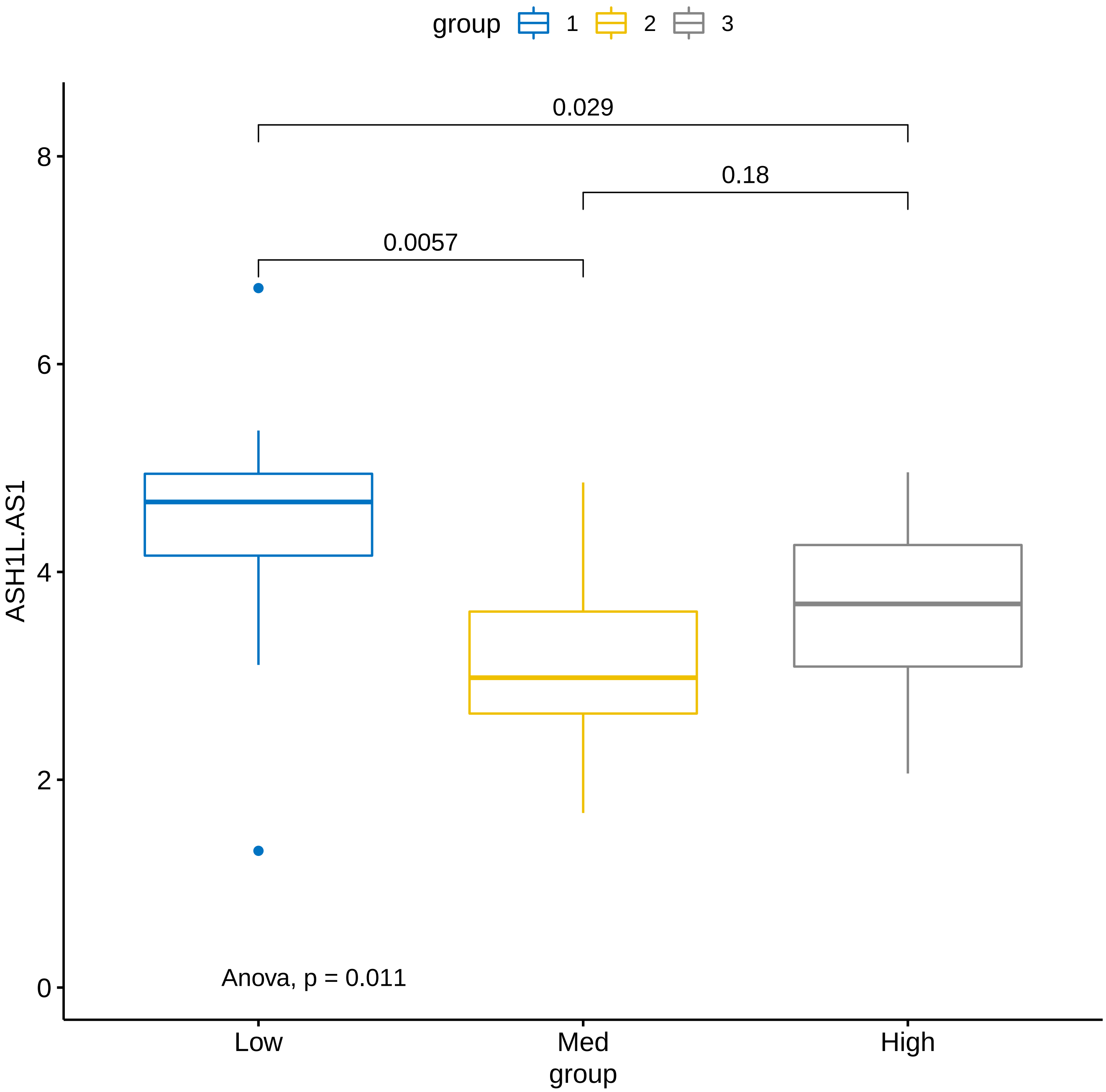
group 1 2 3
0.029
0.18
0.0057
8
6
4
2
0
ASH1L.AS1
Anova, p = 0.011
Low
Med
High
group

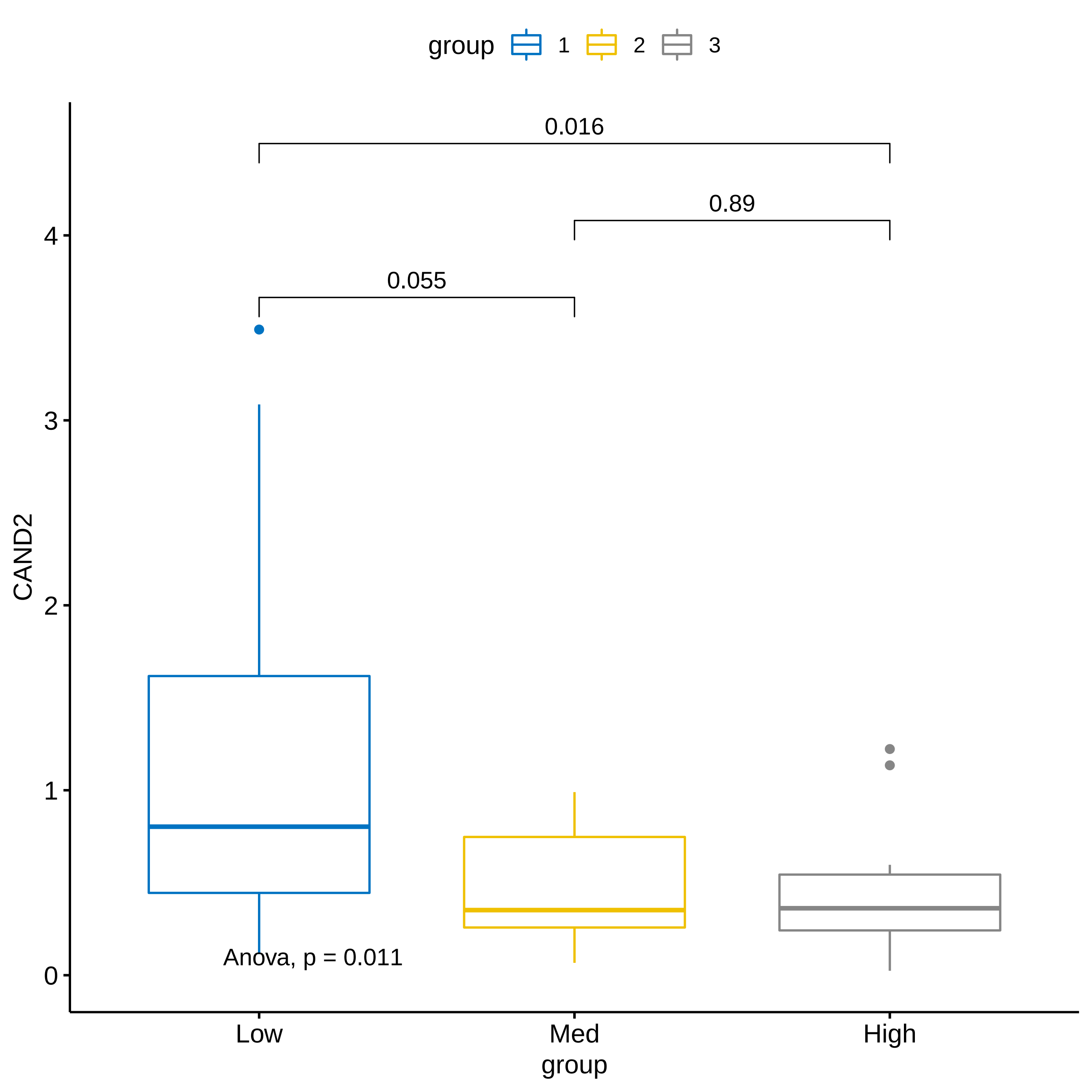

group 1 2 3
0.016
0.89
0.055
CAND2
4
3
2
1
0
Anova, p = 0.011
Low
Med
High
group

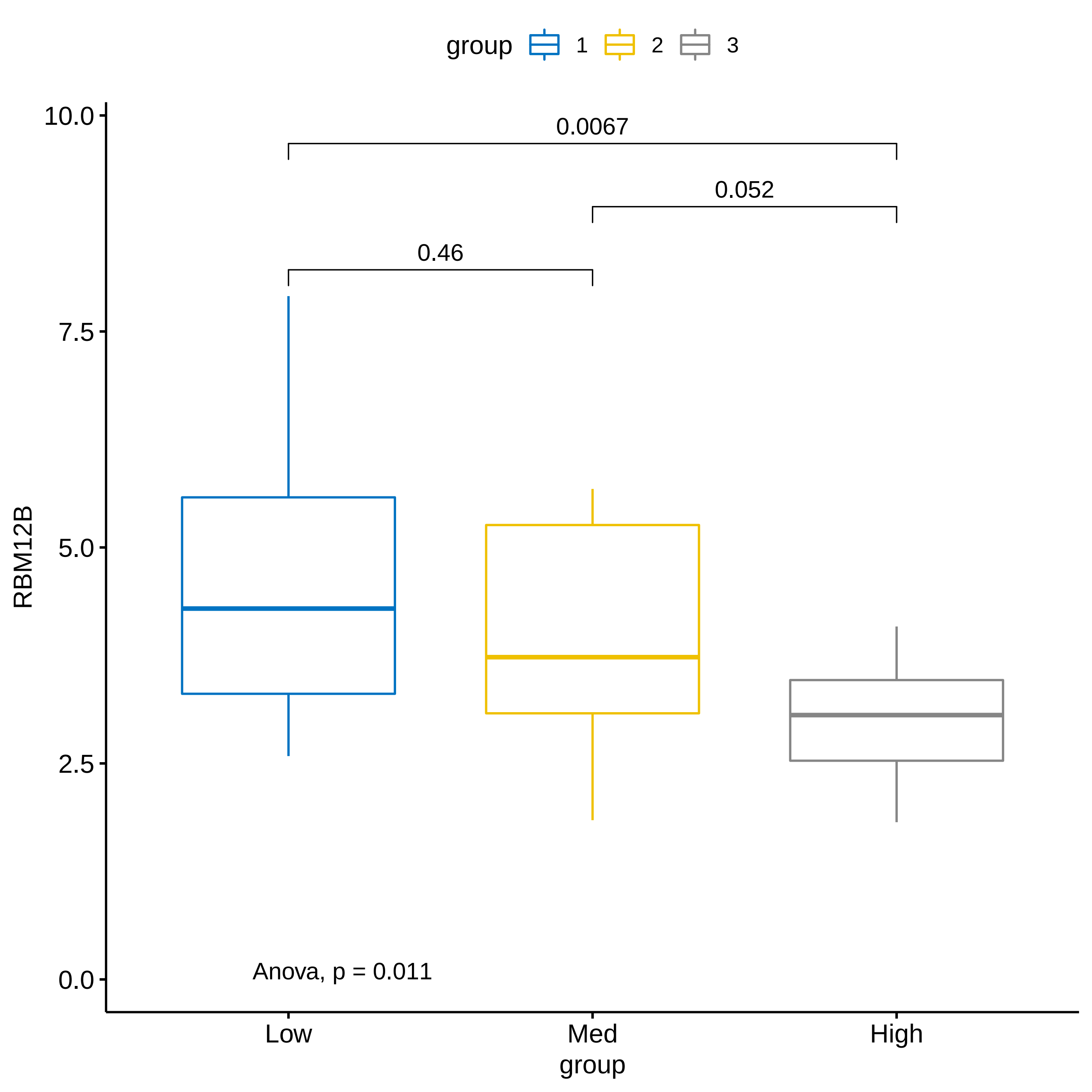

group
1
2
3
10.0
7.5
5.0
2.5
0.0
RBM12B
0.0067
0.052
0.46
Anova, p = 0.011
Low
Med
High
group

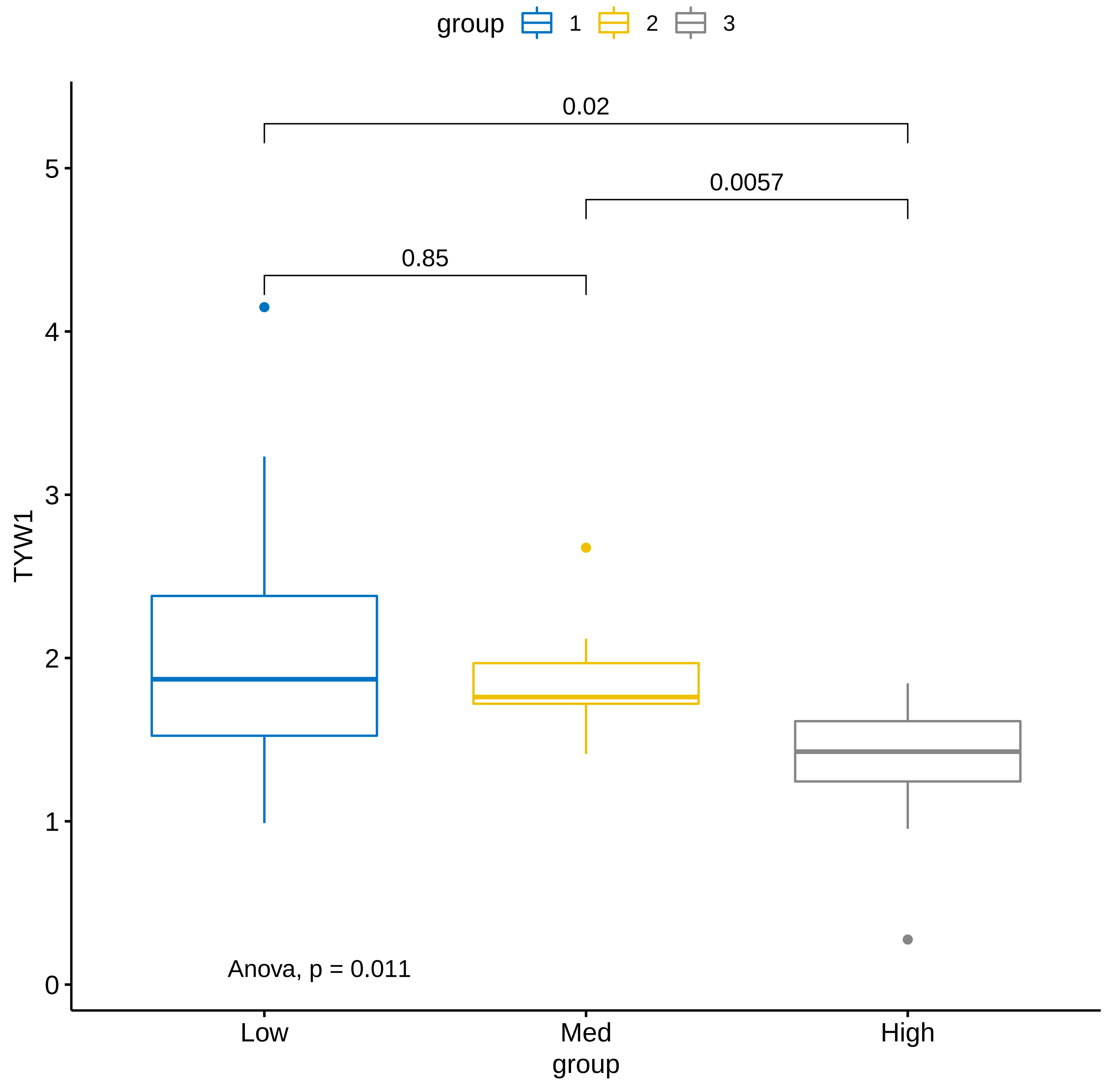

group 1 2 3
0.02
0.0057
0.85
5
4
3
2
1
0
TYW1
Anova, p = 0.011
Low
Med
High
group

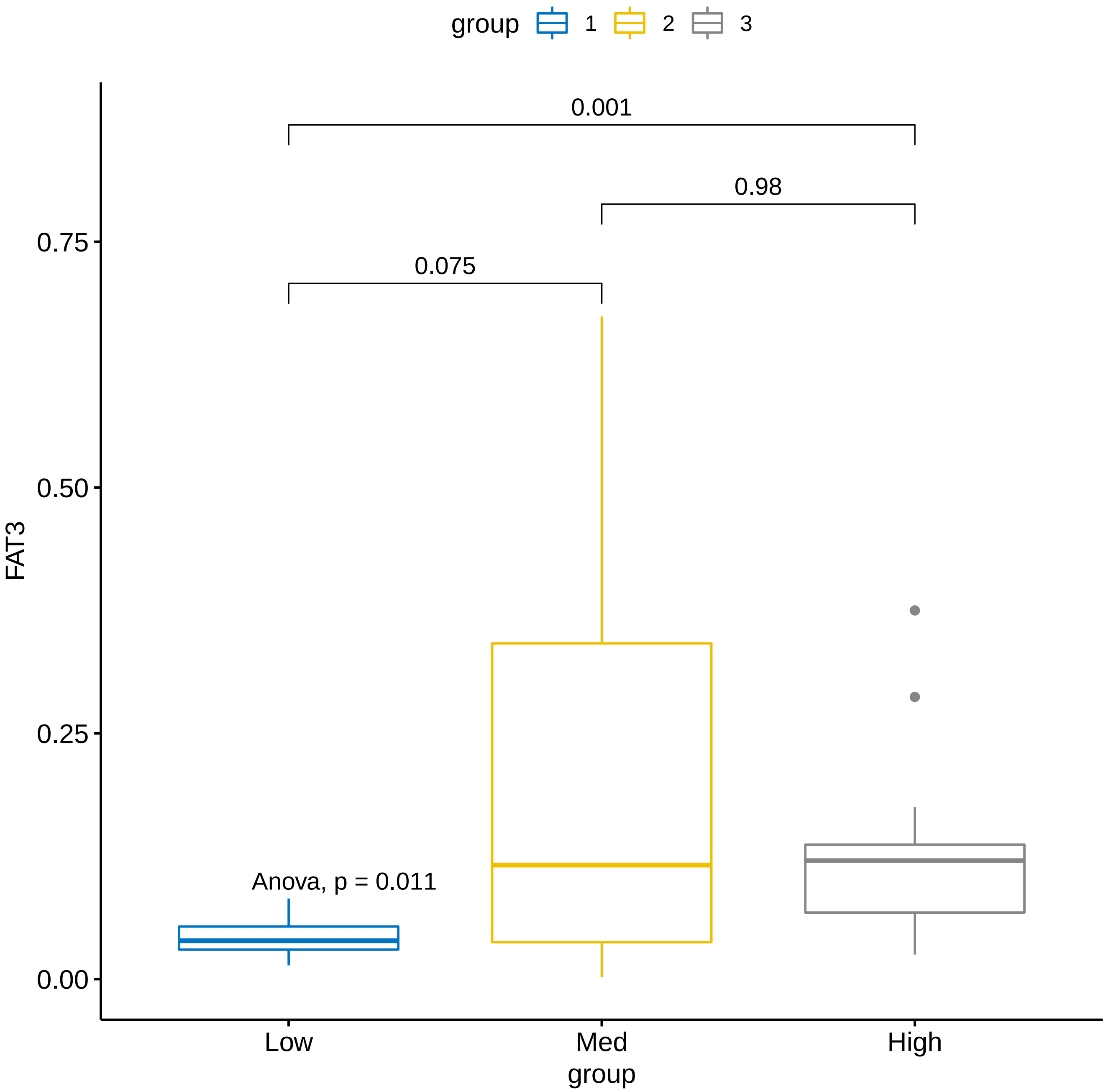
group
1
2
3
0.001
0.98
0.075
0.75
0.50
0.25
0.00
FAT3
Anova, p = 0.011
Low
Med
High
group

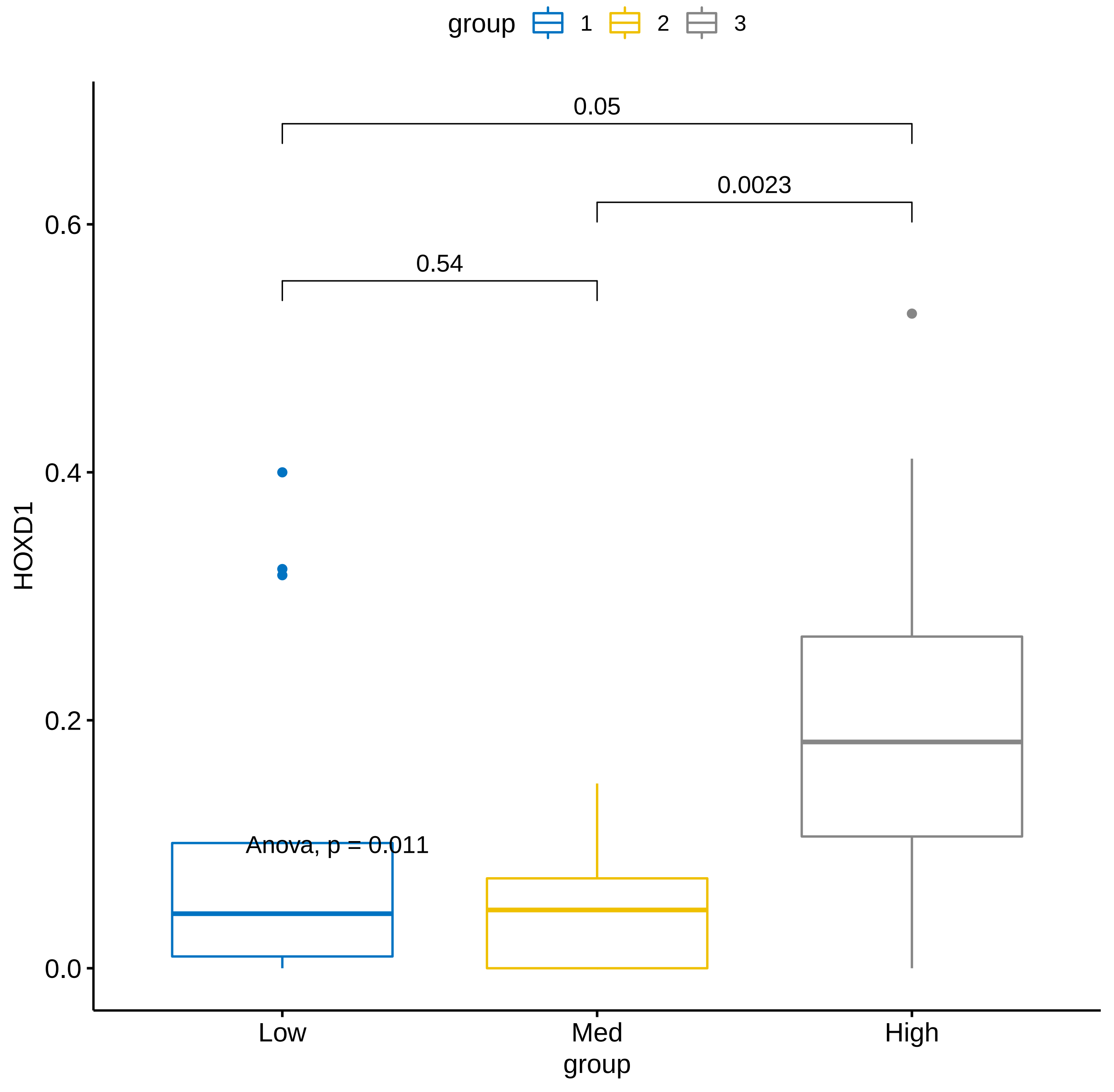

group 1 2 3
0.05
0.0023
0.54
Anova, p = 0.011
HOXD1
0.0
0.2
0.4
0.6
Low
Med
High
group

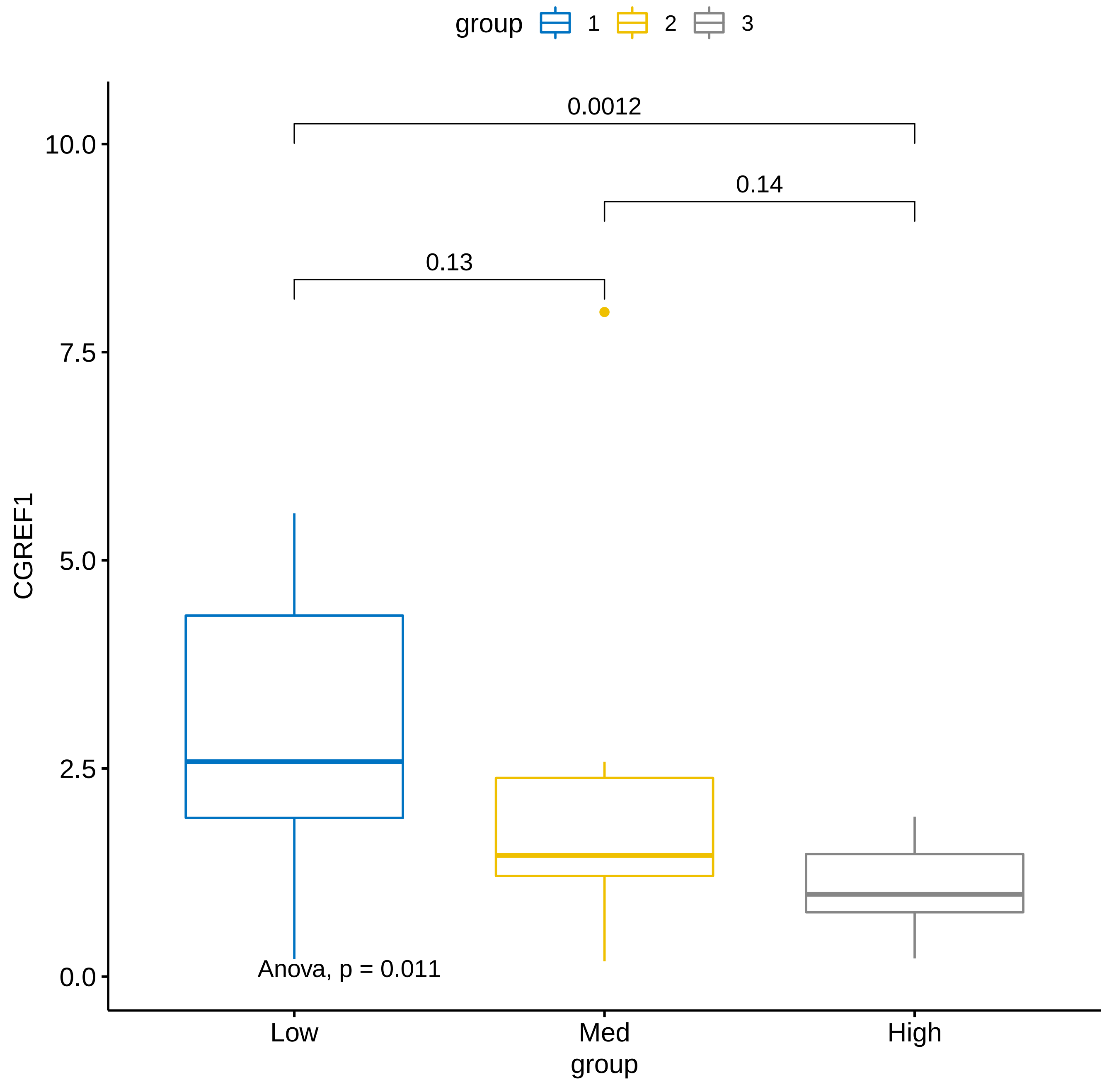
group
1
2
3
0.0012
0.14
0.13
10.0
7.5
5.0
2.5
0.0
CGREF1
Anova, p = 0.011
Low
Med
High
group

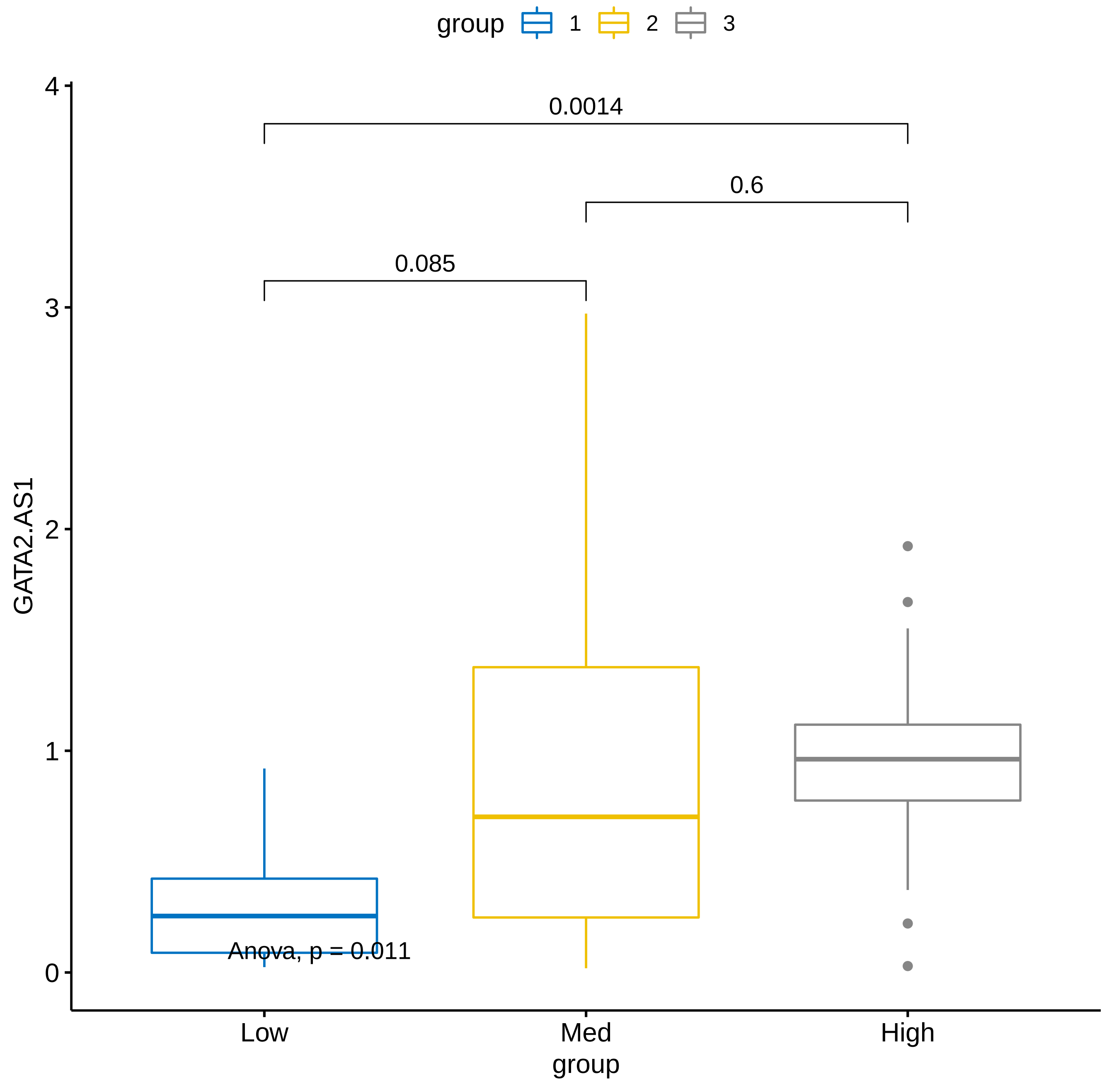
group 1 2 3
0.0014
0.6
0.085
4
3
2
1
0
GATA2.AS1
Anova, p = 0.011
Low
Med
High
group

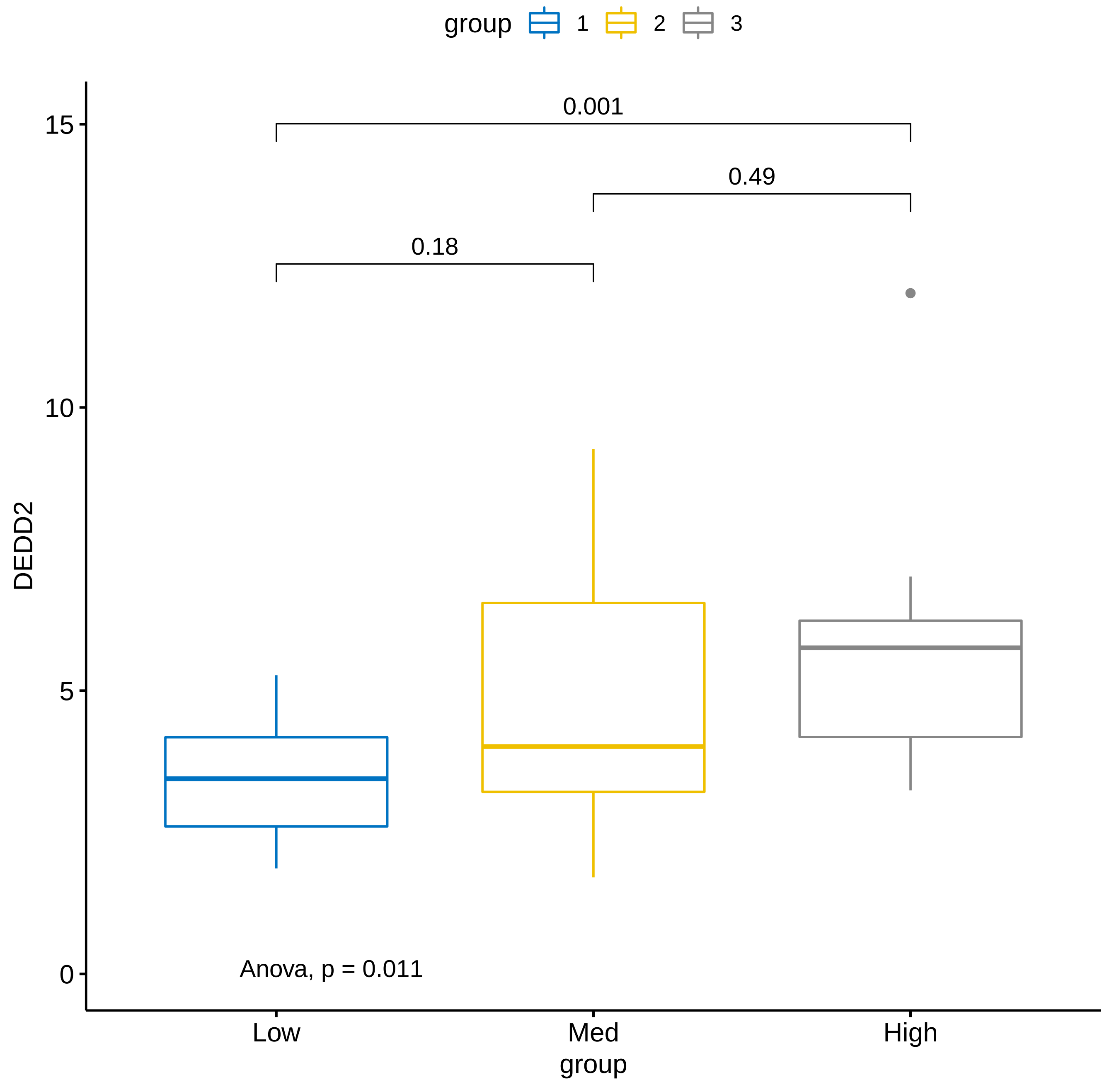
group
1
2
3
0.001
0.49
0.18
15
10
5
0
DEDD2
Anova, p = 0.011
Low
Med
High
group

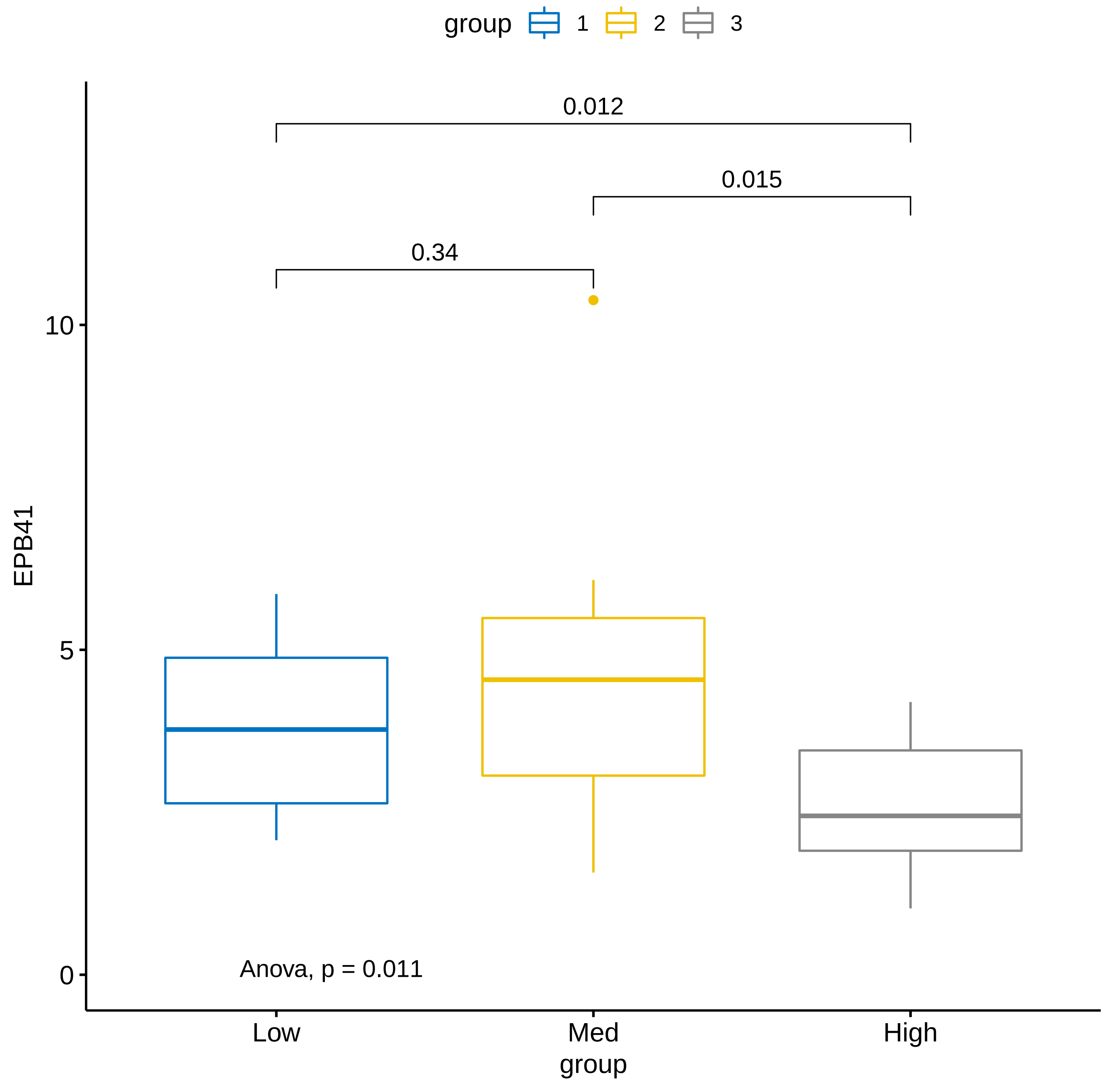

group 1 2 3
0.012
0.015
0.34
10
5
0
EPB41
Anova, p = 0.011
Low
Med
High
group

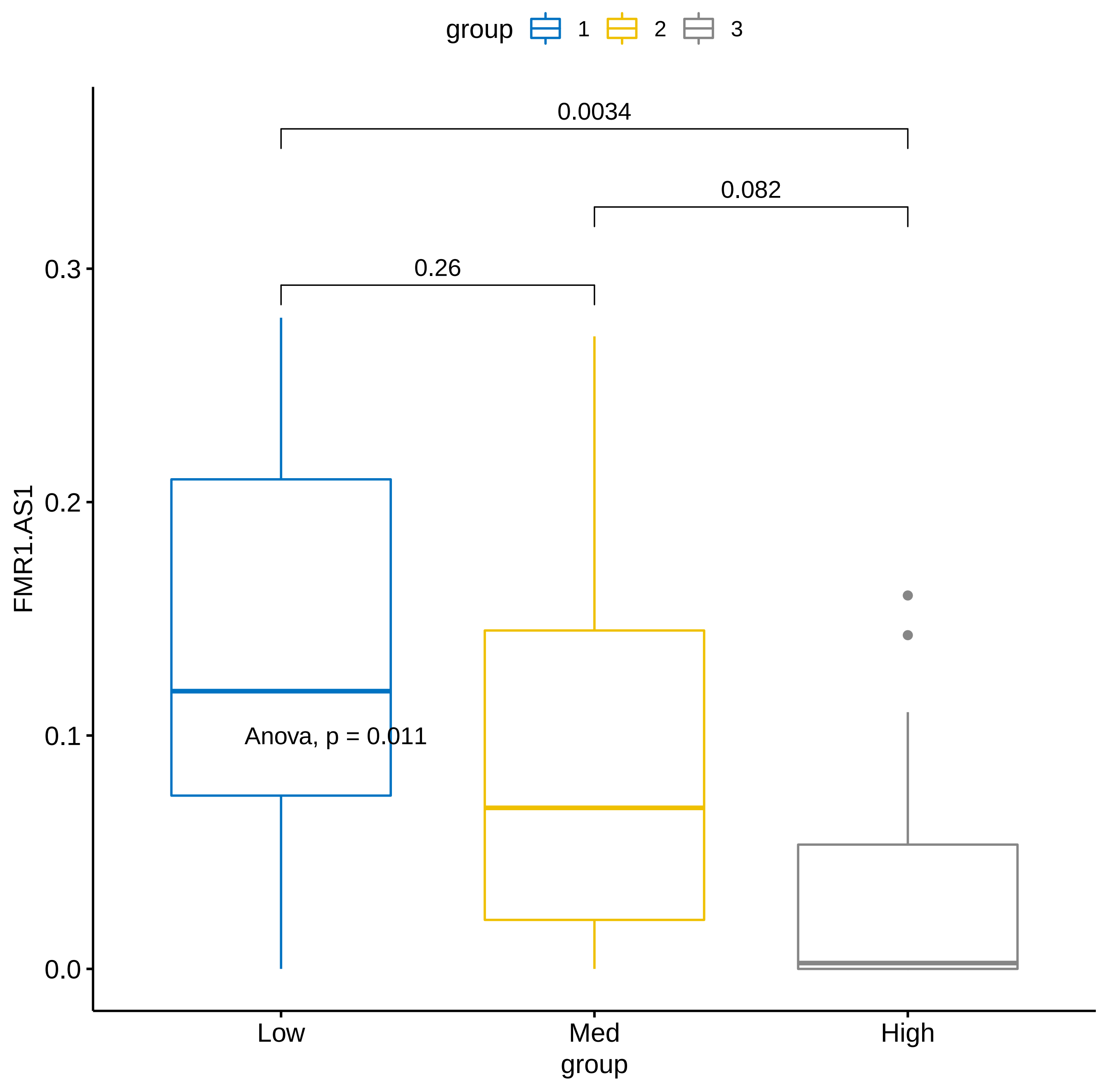
group
1
2
3
0.0034
0.082
0.26
Anova, p = 0.011
FMR1.AS1
0.3
0.2
0.1
0.0
Low
Med
High
group

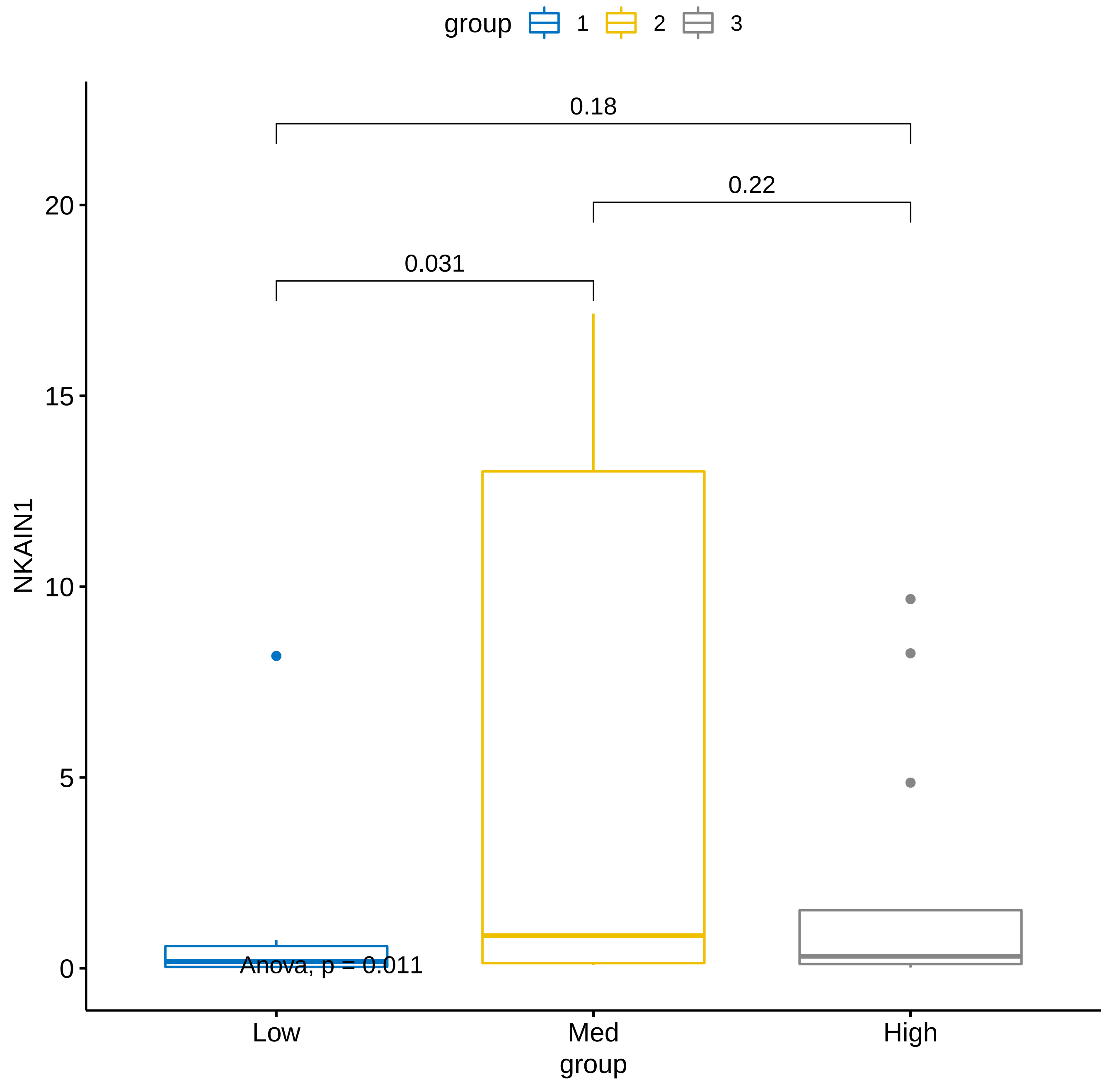

group
1
2
3
0.18
0.22
0.031
20
15
10
5
0
NKAIN1
Anova, p = 0.011
Low
Med
High
group

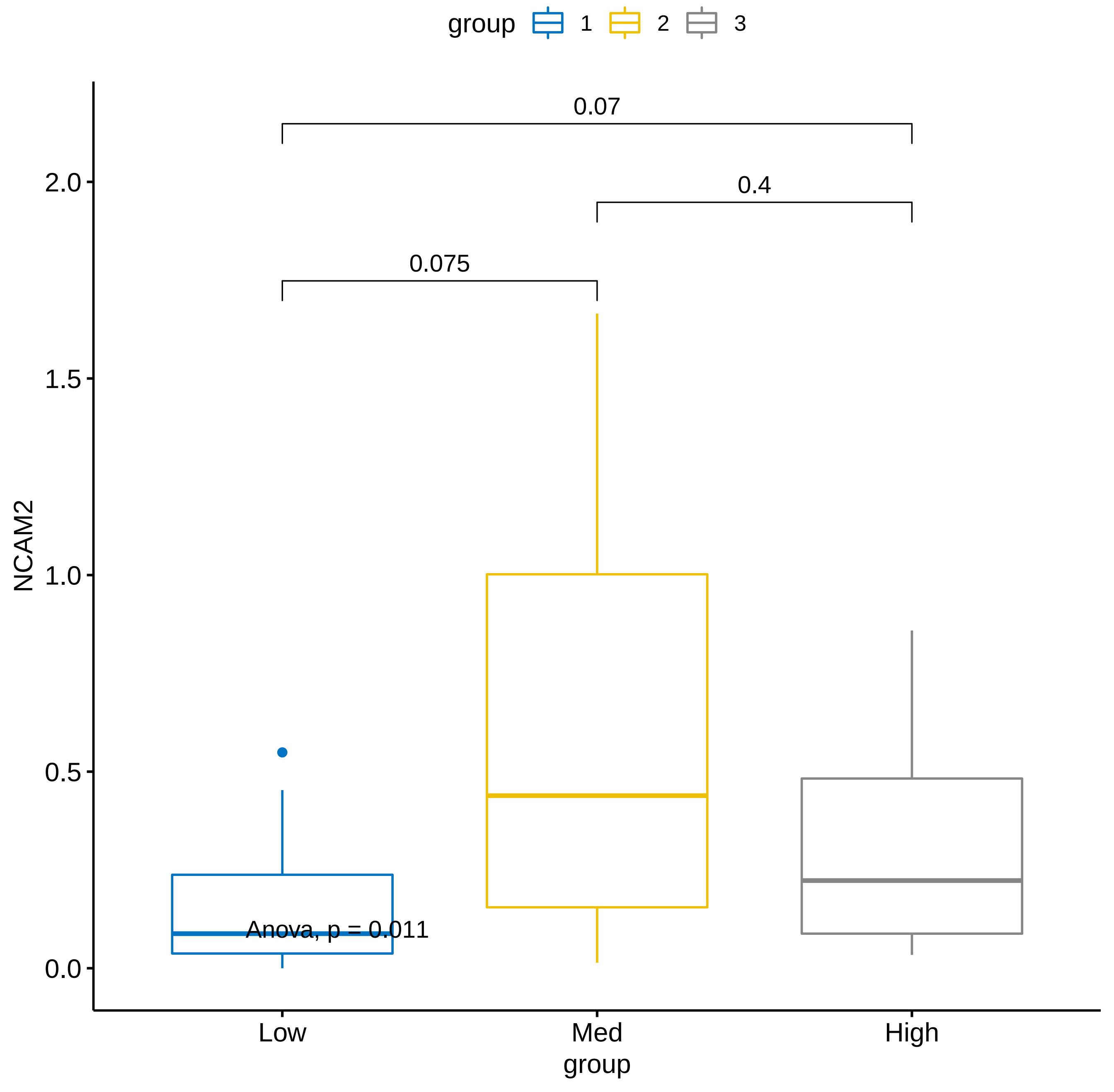
group 1 2 3
0.07
0.4
0.075
2.0
1.5
1.0
0.5
0.0
NCAM2
Anova, p = 0.011
Low
Med
High
group

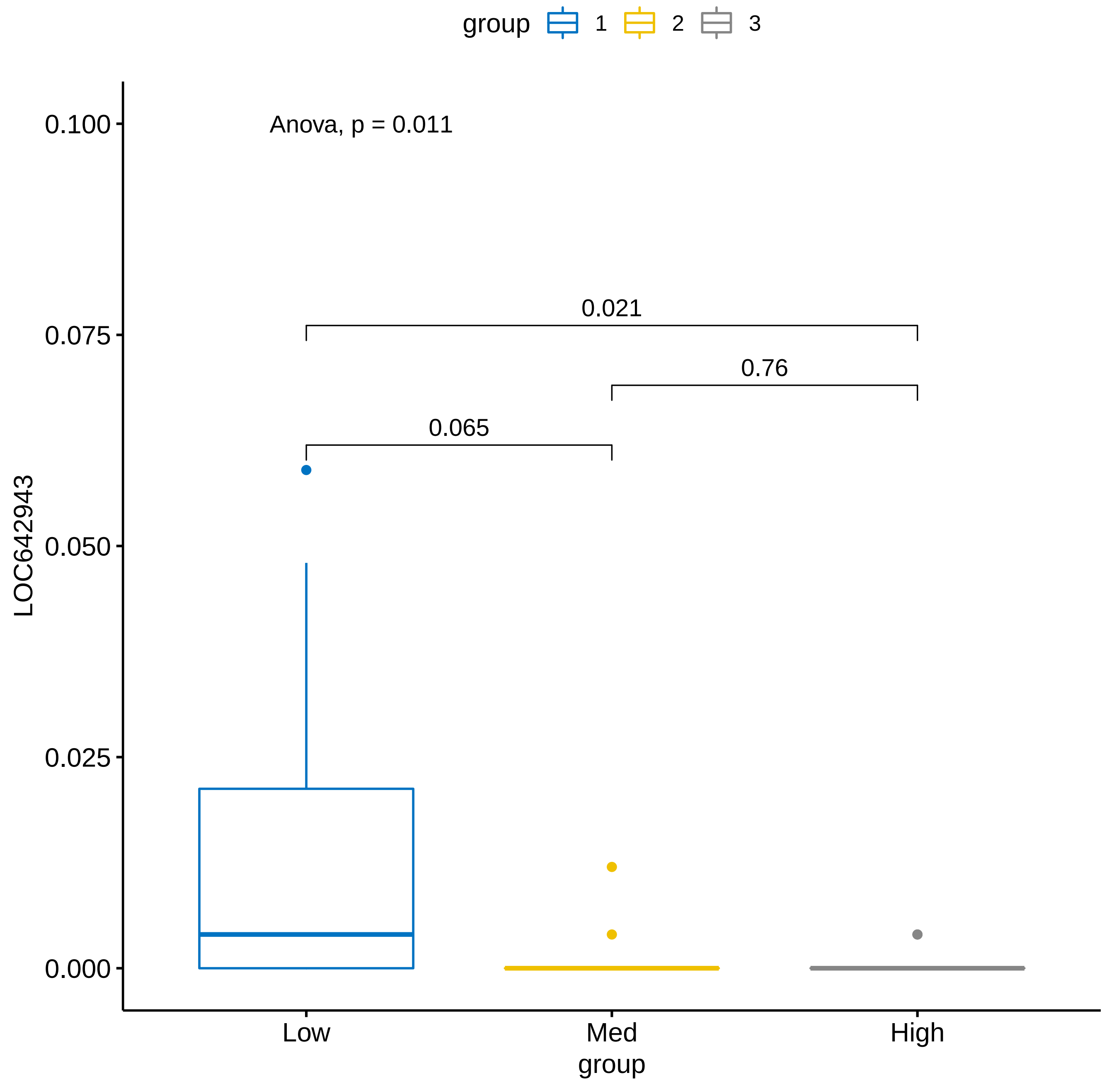

group 1 2 3
Anova, p = 0.011
0.021
0.76
0.065
0.100
0.075
0.050
0.025
0.000
LOC642943
Low
Med
High
group

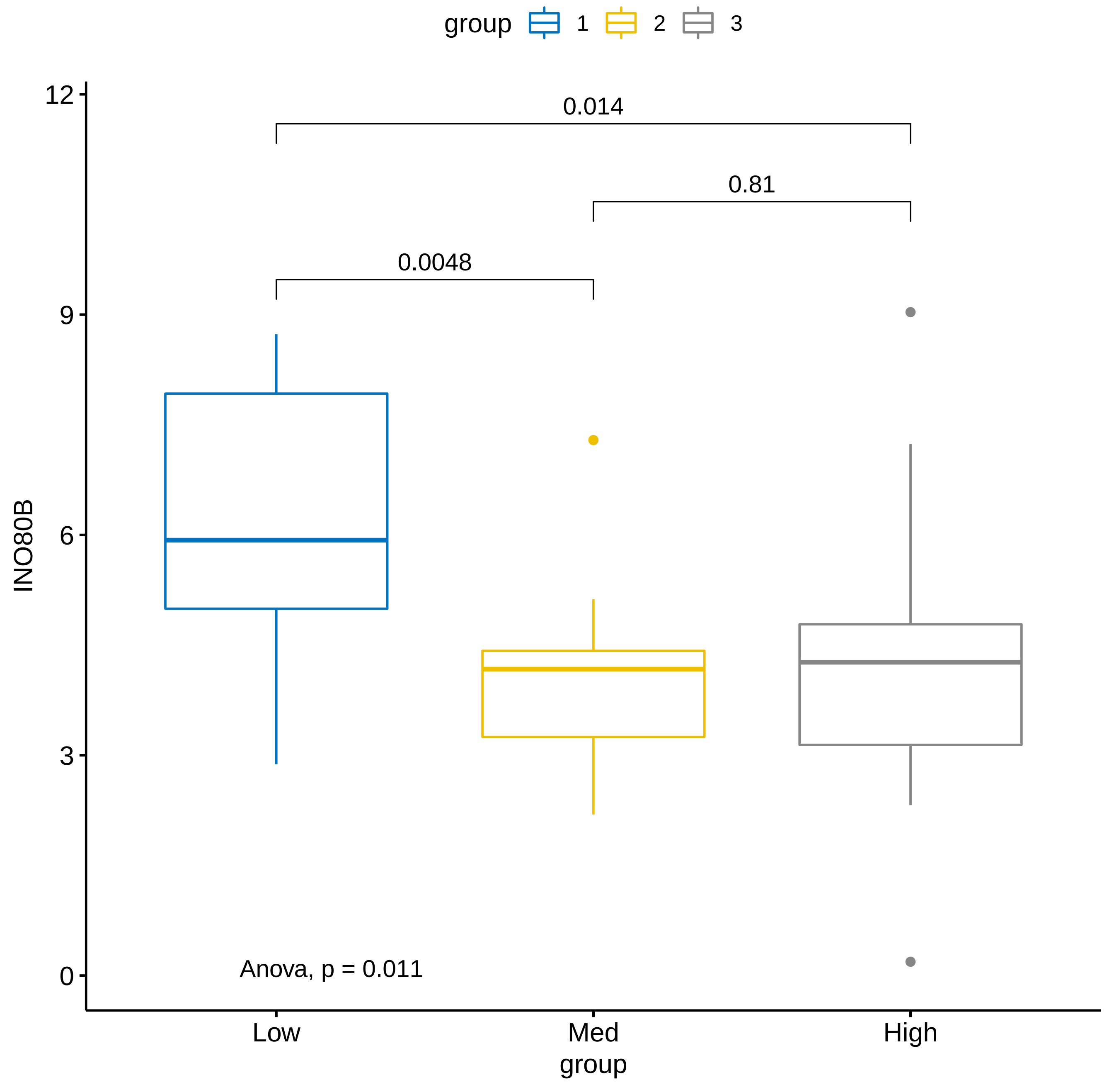
group
1
2
3
0.014
0.81
0.0048
INO80B
12
9
6
3
0
Anova, p = 0.011
Low
Med
High
group

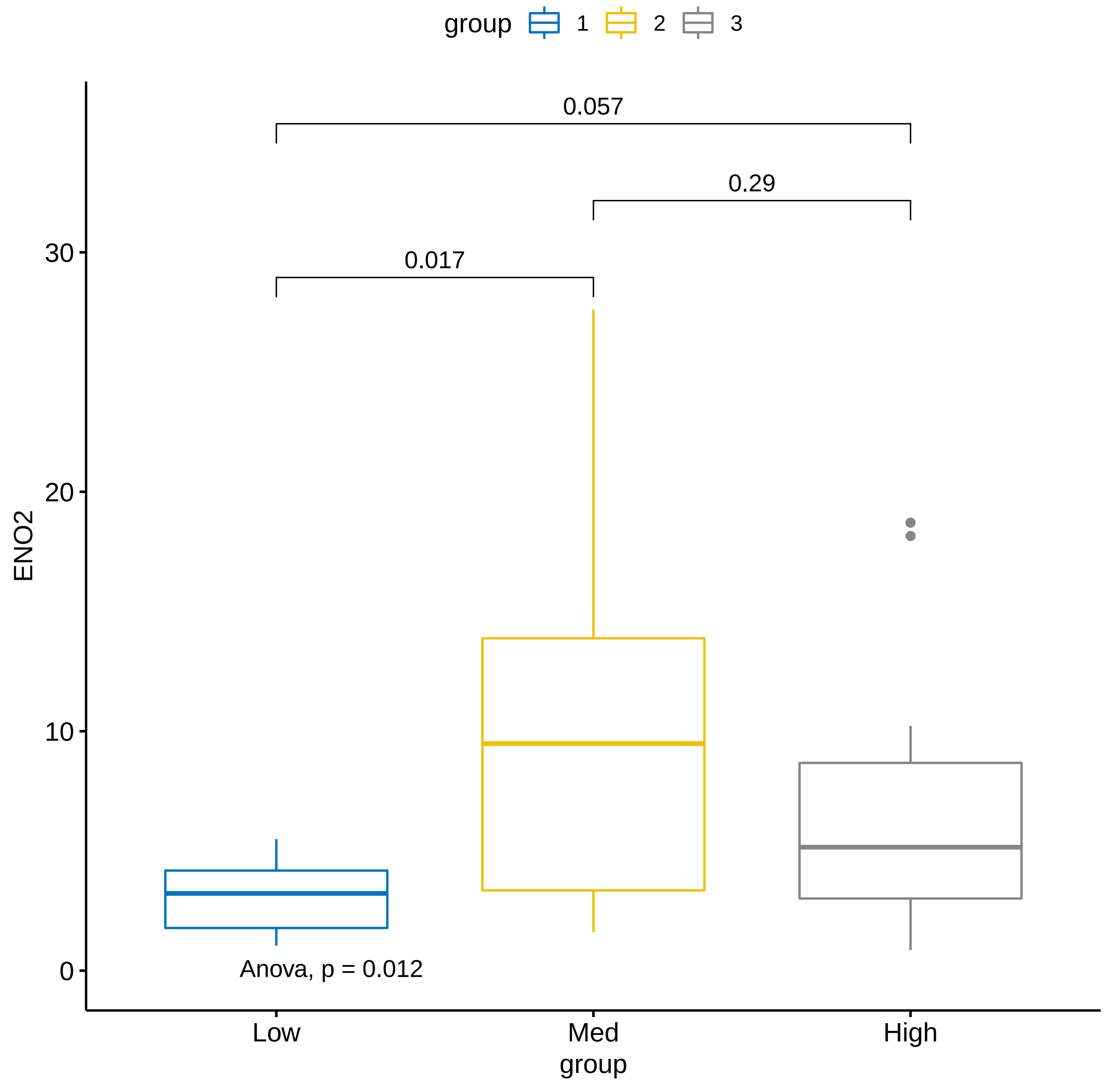

group
1
2
3
0.057
0.29
0.017
30
20
10
0
ENO2
Anova, p = 0.012
Low
Med
High
group

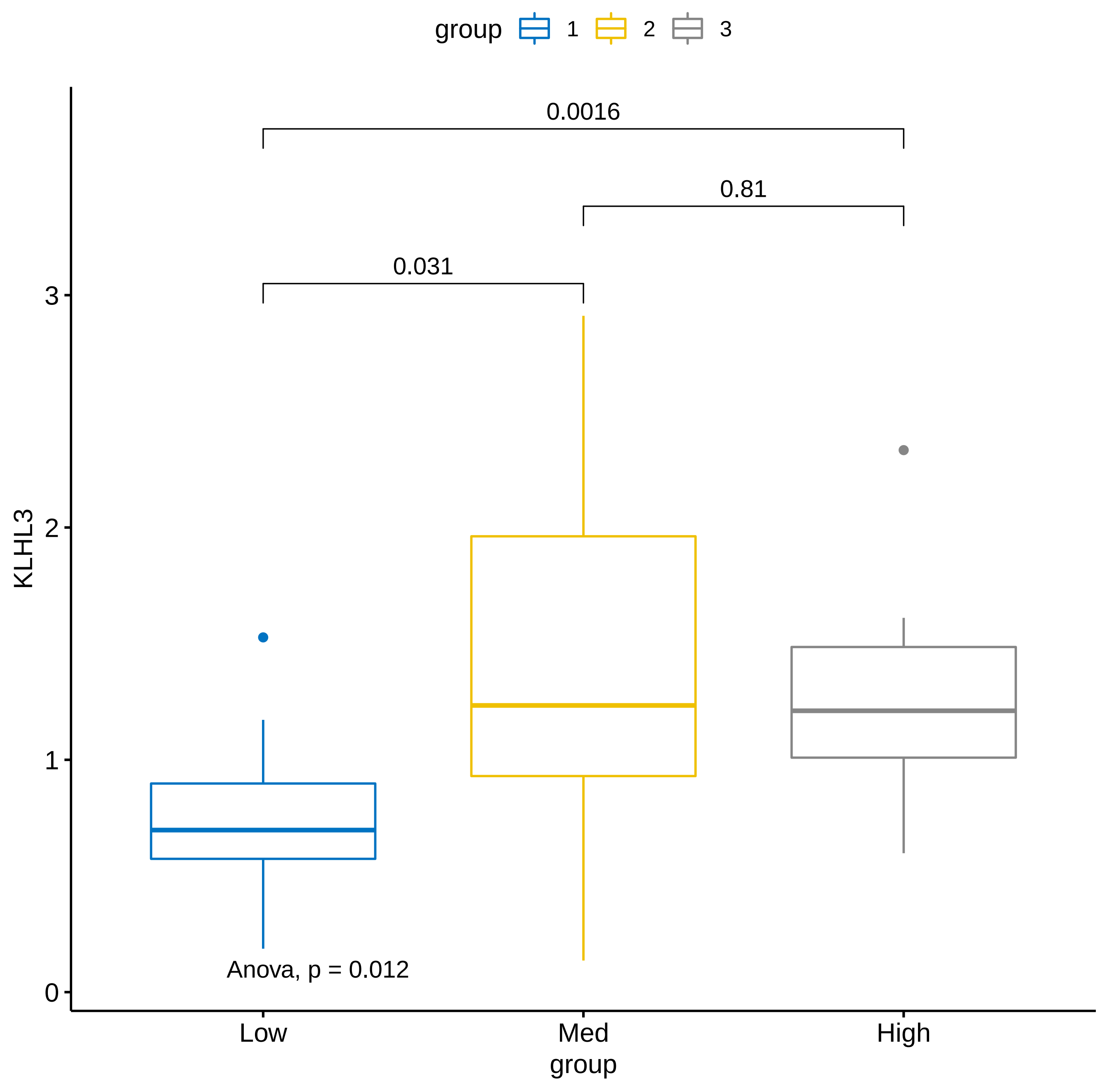
group 1 2 3
0.0016
0.81
0.031
3
2
1
0
KLHL3
Anova, p = 0.012
Low
Med
High
group

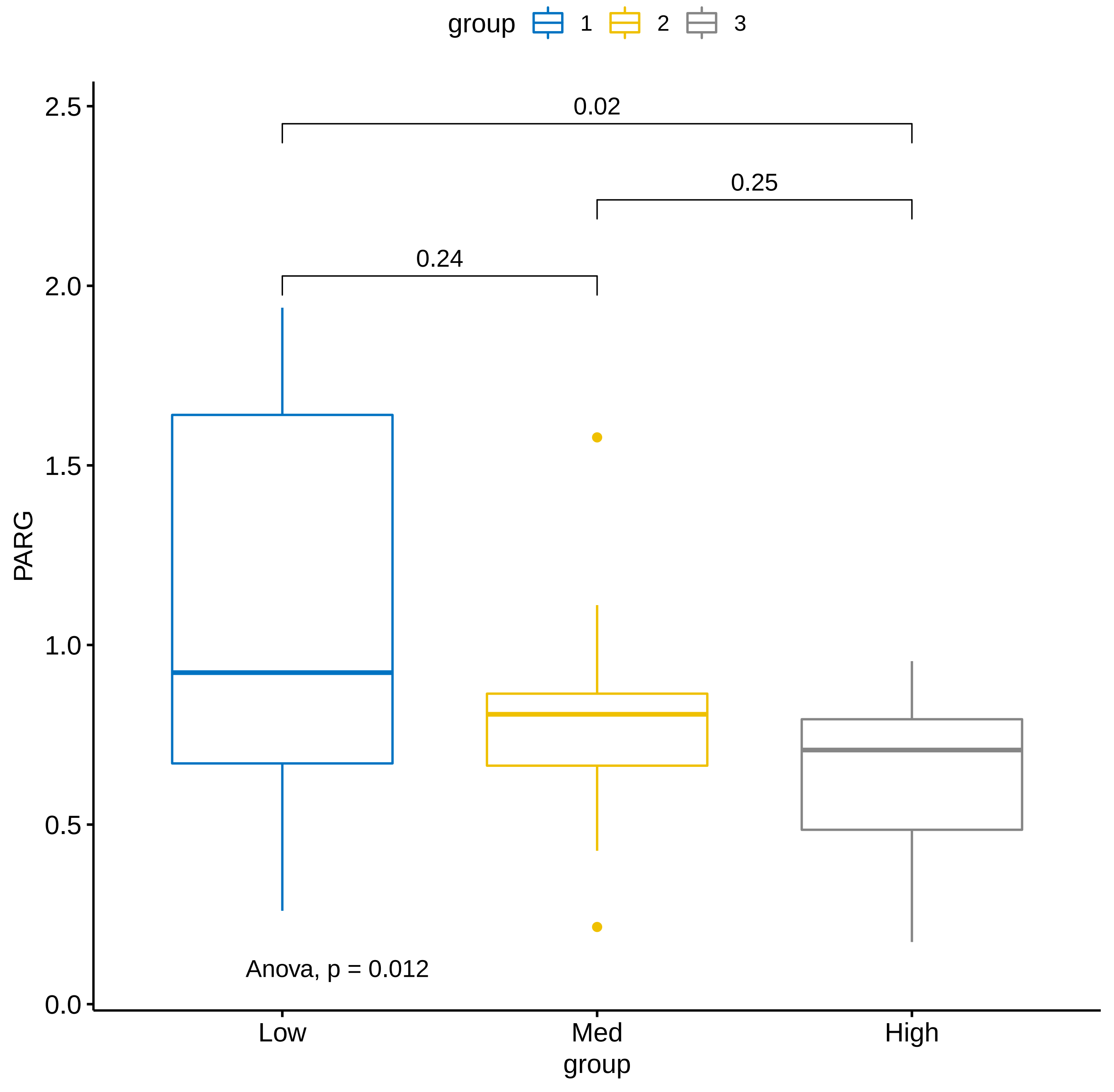

group 1 2 3
0.02
0.25
0.24
PARG
2.5
2.0
1.5
1.0
0.5
0.0
Anova, p = 0.012
Low
Med
High
group

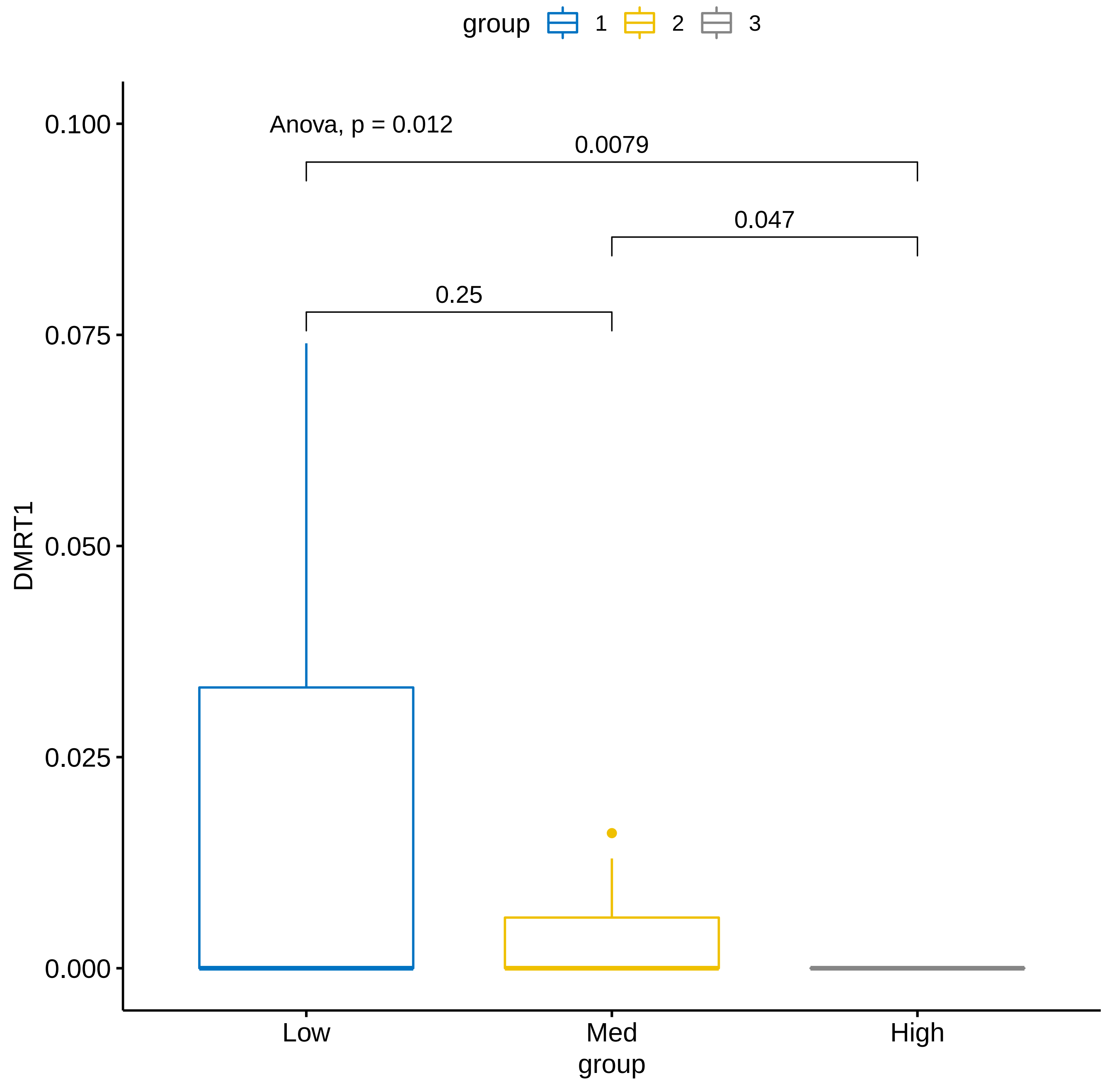
group
1
2
3
Anova, p = 0.012
0.0079
0.047
0.25
0.100
0.075
0.050
0.025
0.000
DMRT1
Low
Med
High
group

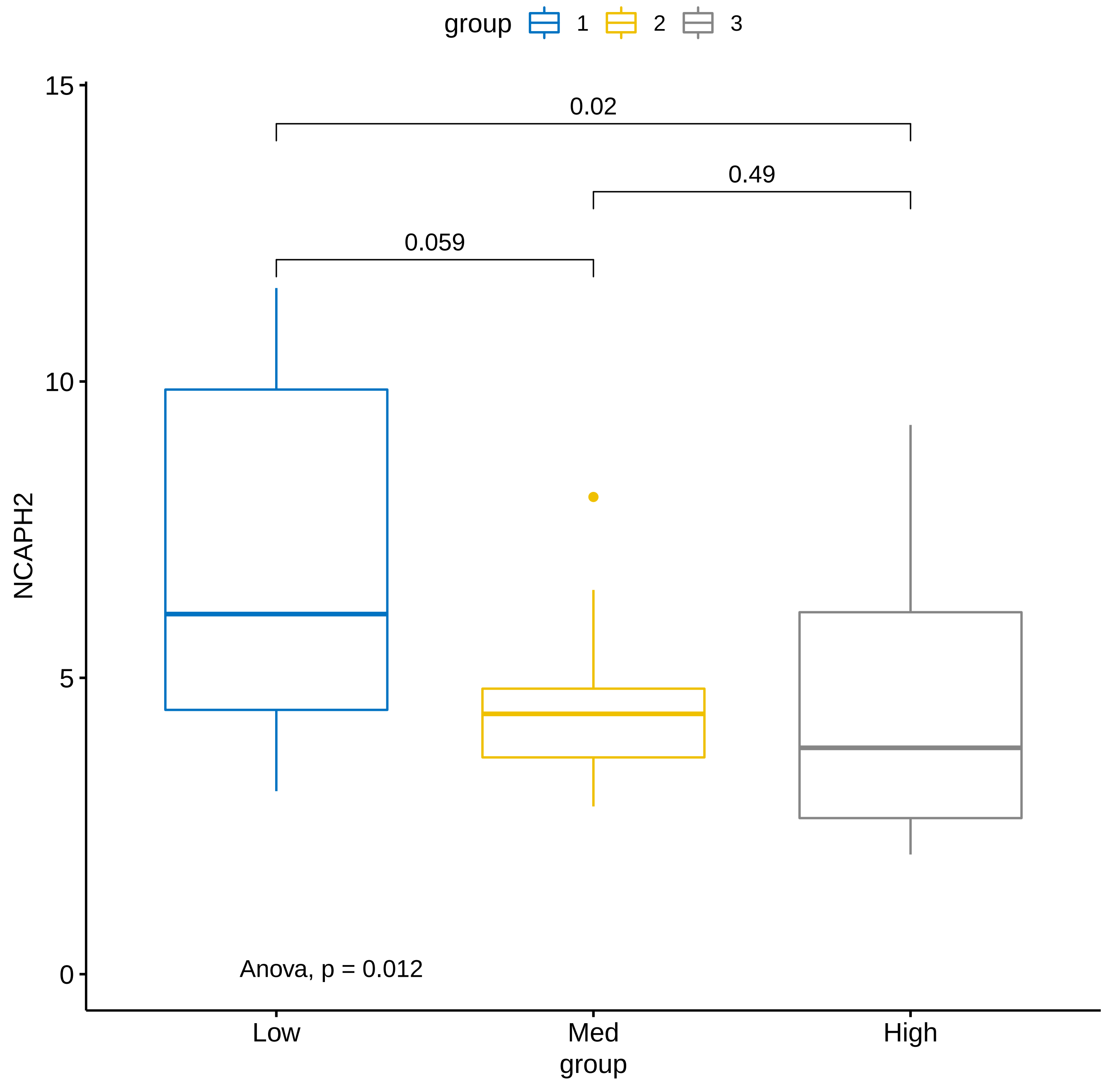

group 1 2 3
15
10
5
0
NCAPH2
0.02
0.49
0.059
Anova, p = 0.012
Low
Med
High
group

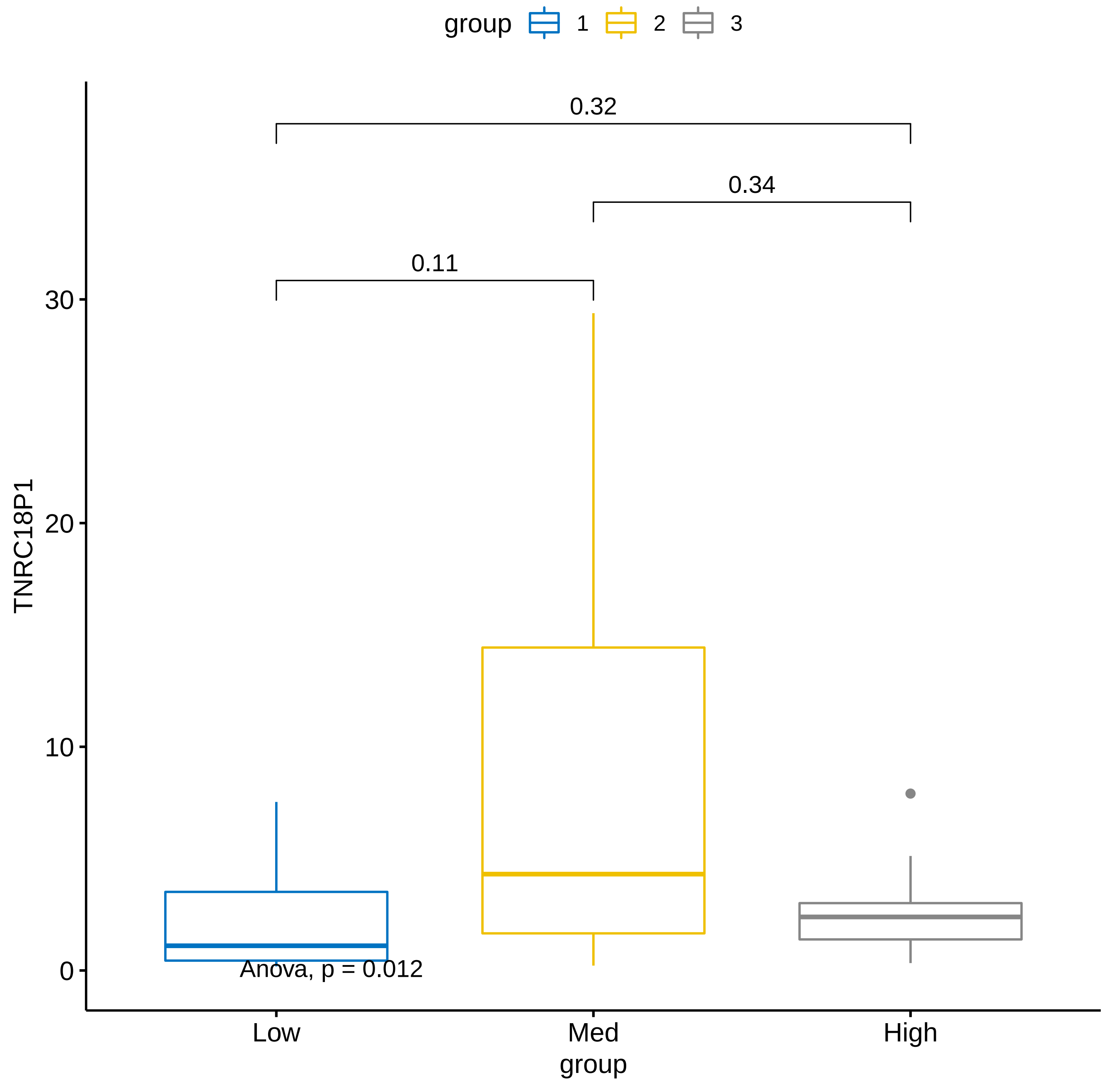
group
1
2
3
0.32
0.34
0.11
30
20
10
0
TNRC18P1
Anova, p = 0.012
Low
Med
High
group

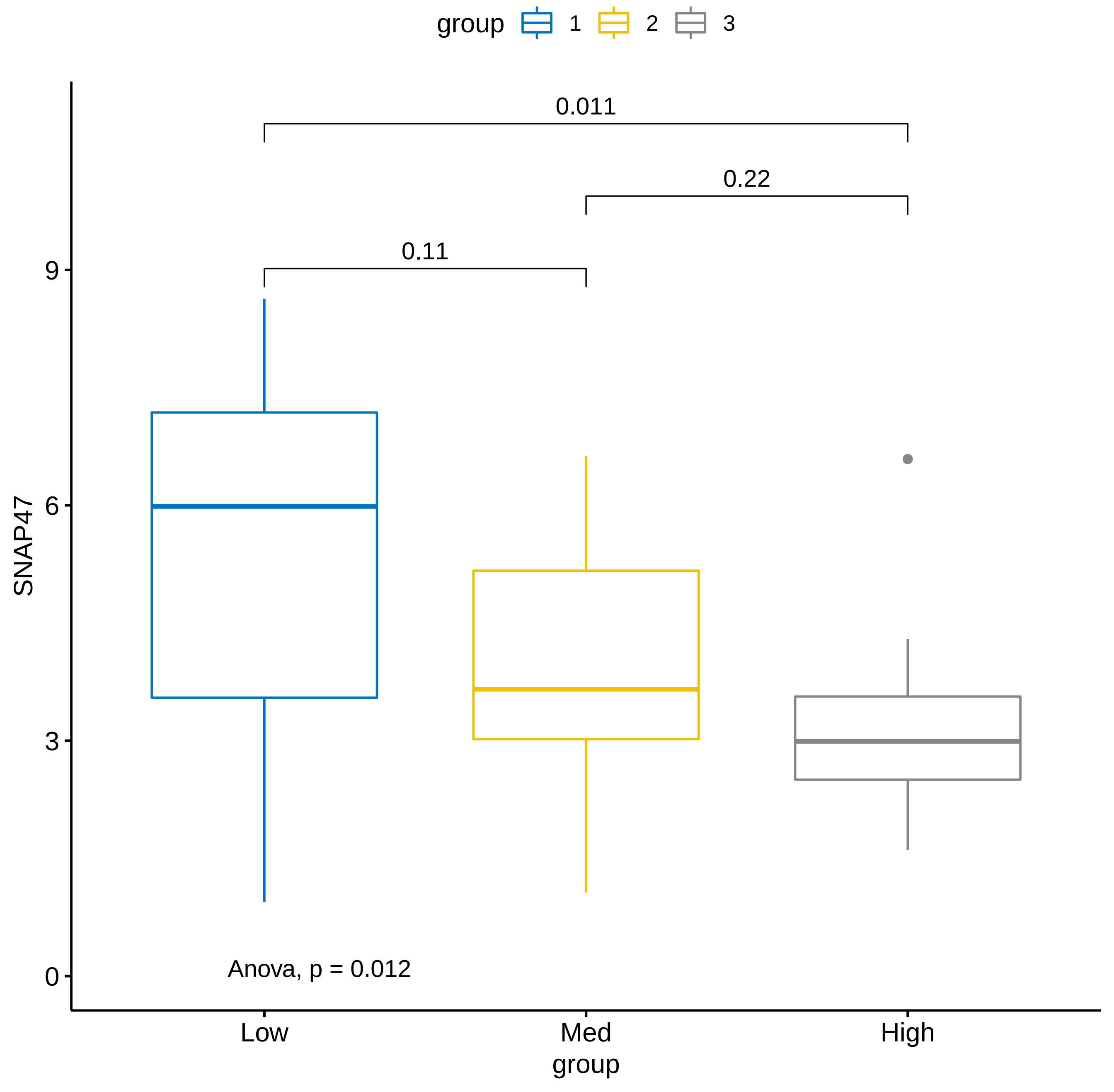
group 1 2 3
0.011
0.22
0.11
9
6
3
0
SNAP47
Anova, p = 0.012
Low
Med
High
group

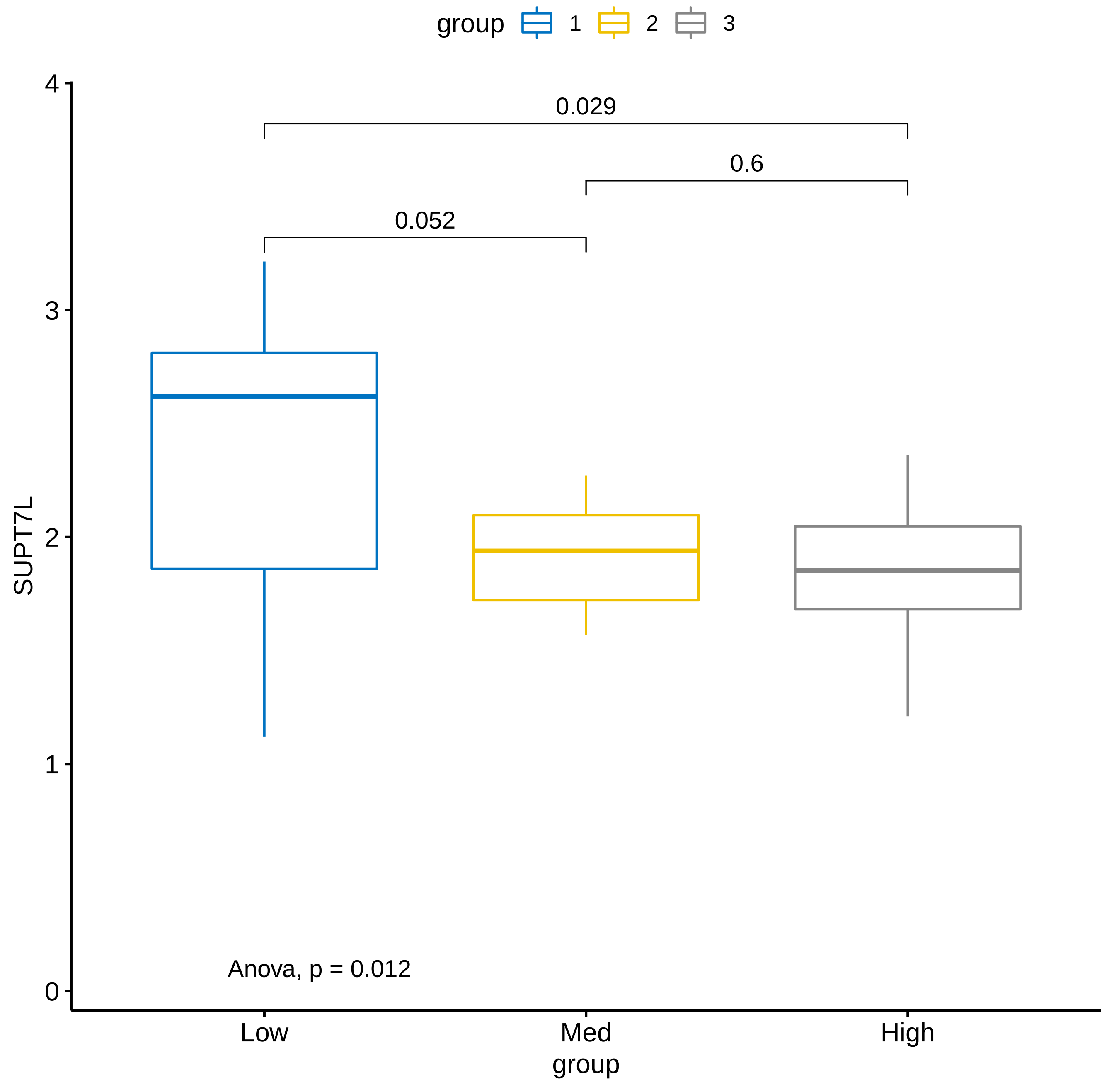
group
1
2
3
4
3
2
1
0
SUPT7L
0.029
0.6
0.052
Anova, p = 0.012
Low
Med
High
group

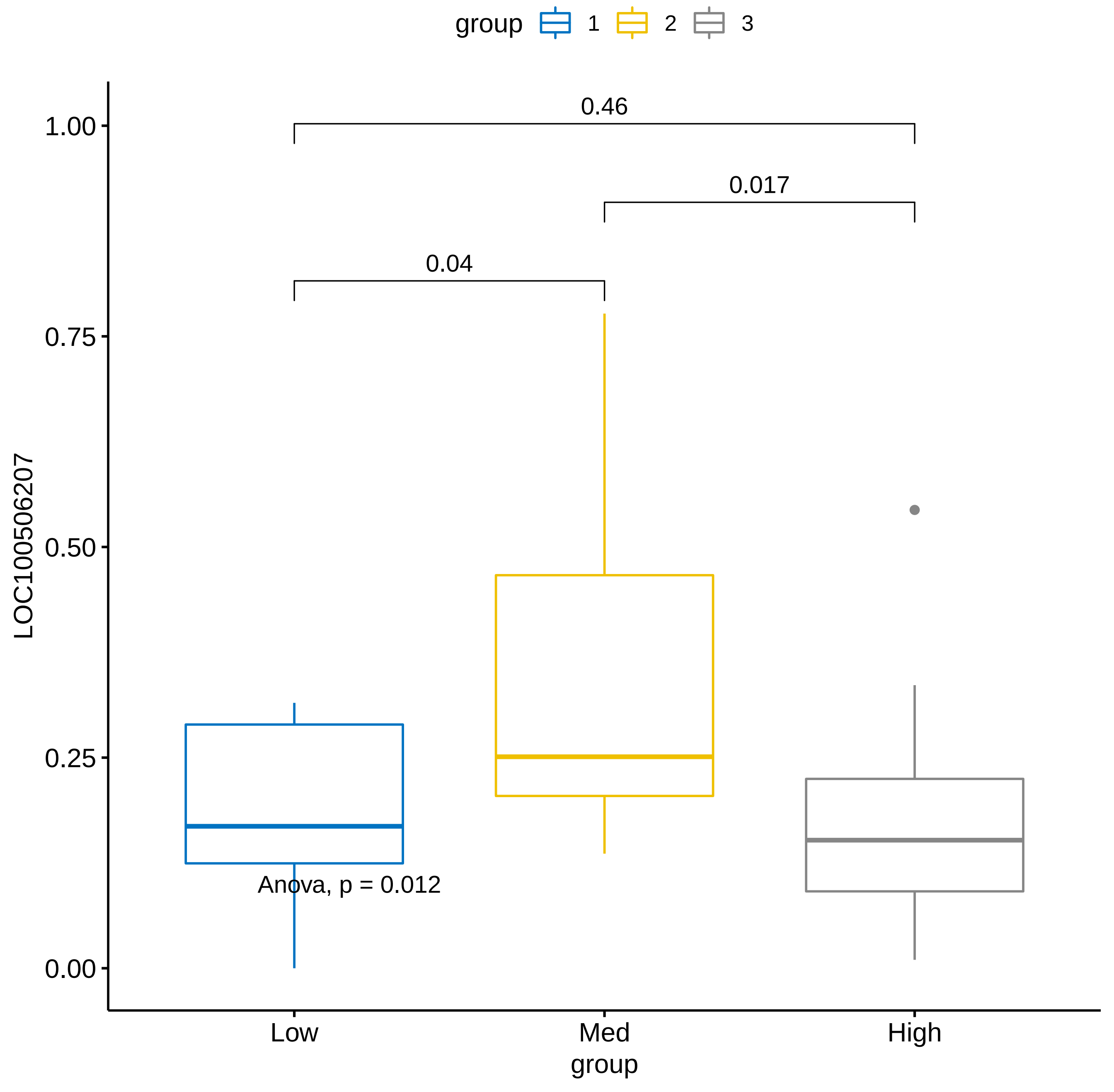
group
1
2
3
0.46
0.017
0.04
1.00
0.75
0.50
0.25
0.00
LOC100506207
Anova, p = 0.012
Low
Med
High
group

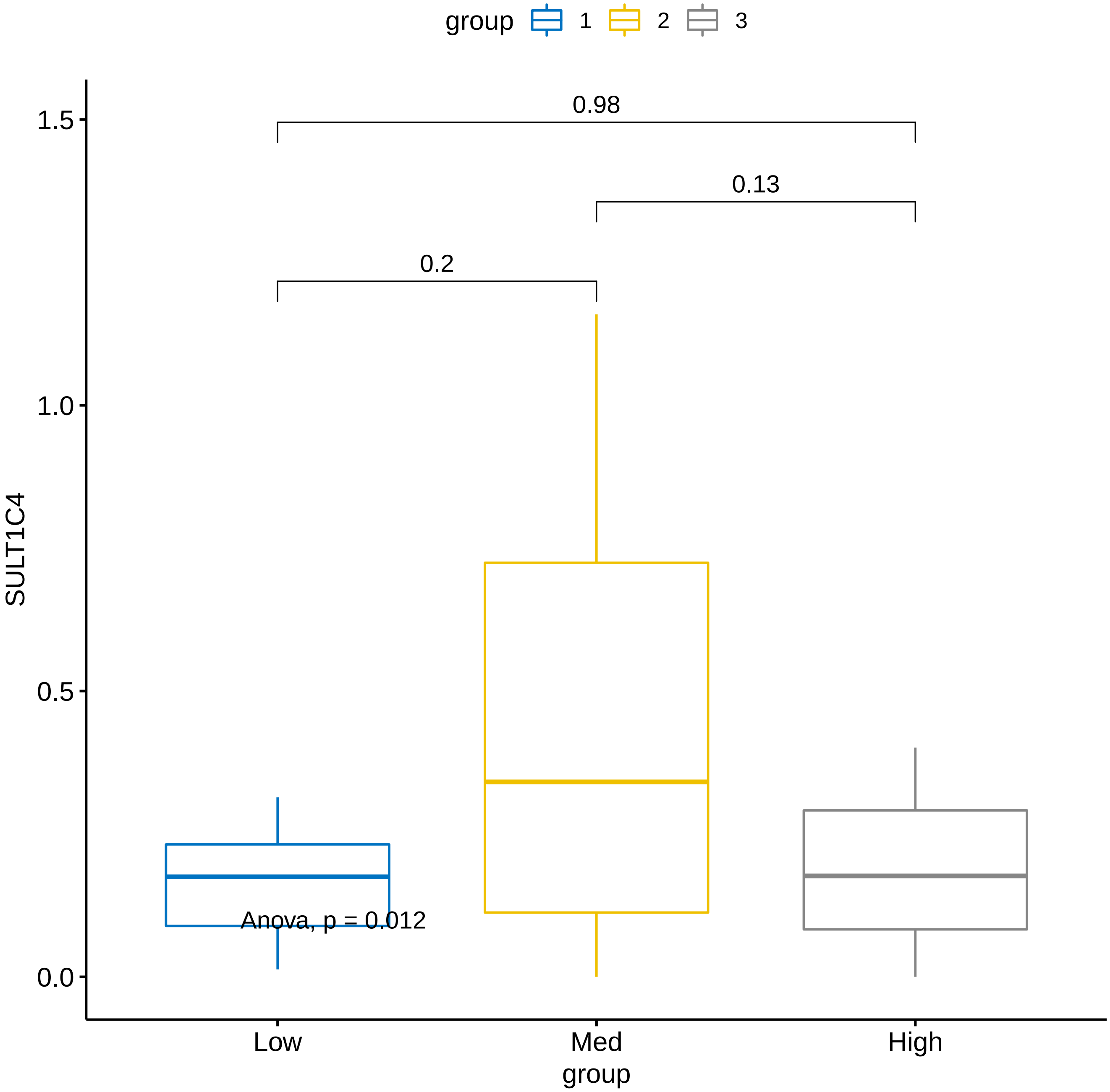
group
1
2
3
0.98
0.13
0.2
Anova, p = 0.012
SULT1C4
1.5
1.0
0.5
0.0
Low
Med
High
group

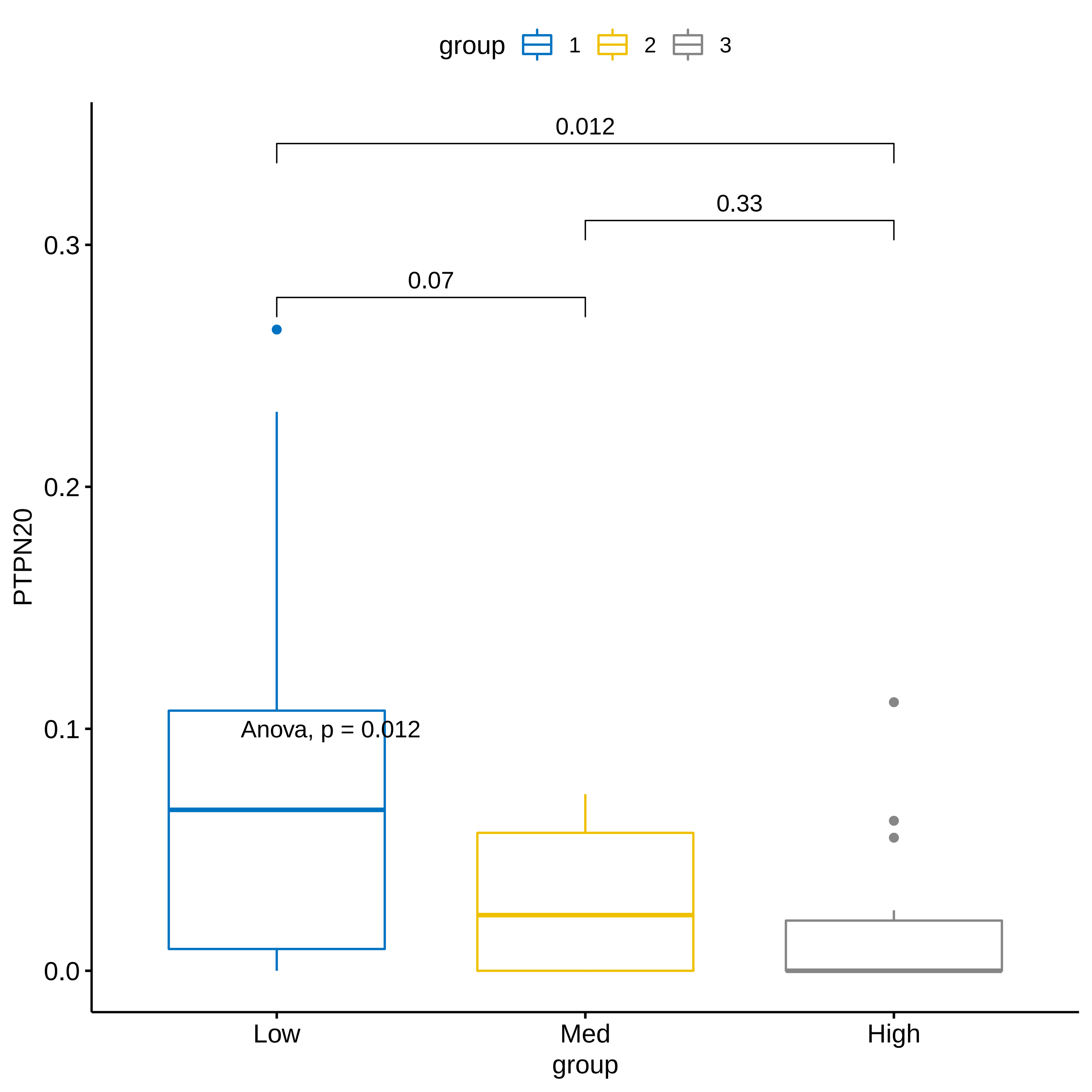
group
1
2
3
0.012
0.33
0.07
Anova, p = 0.012
PTPN20
0.3
0.2
0.1
0.0
Low
Med
High
group

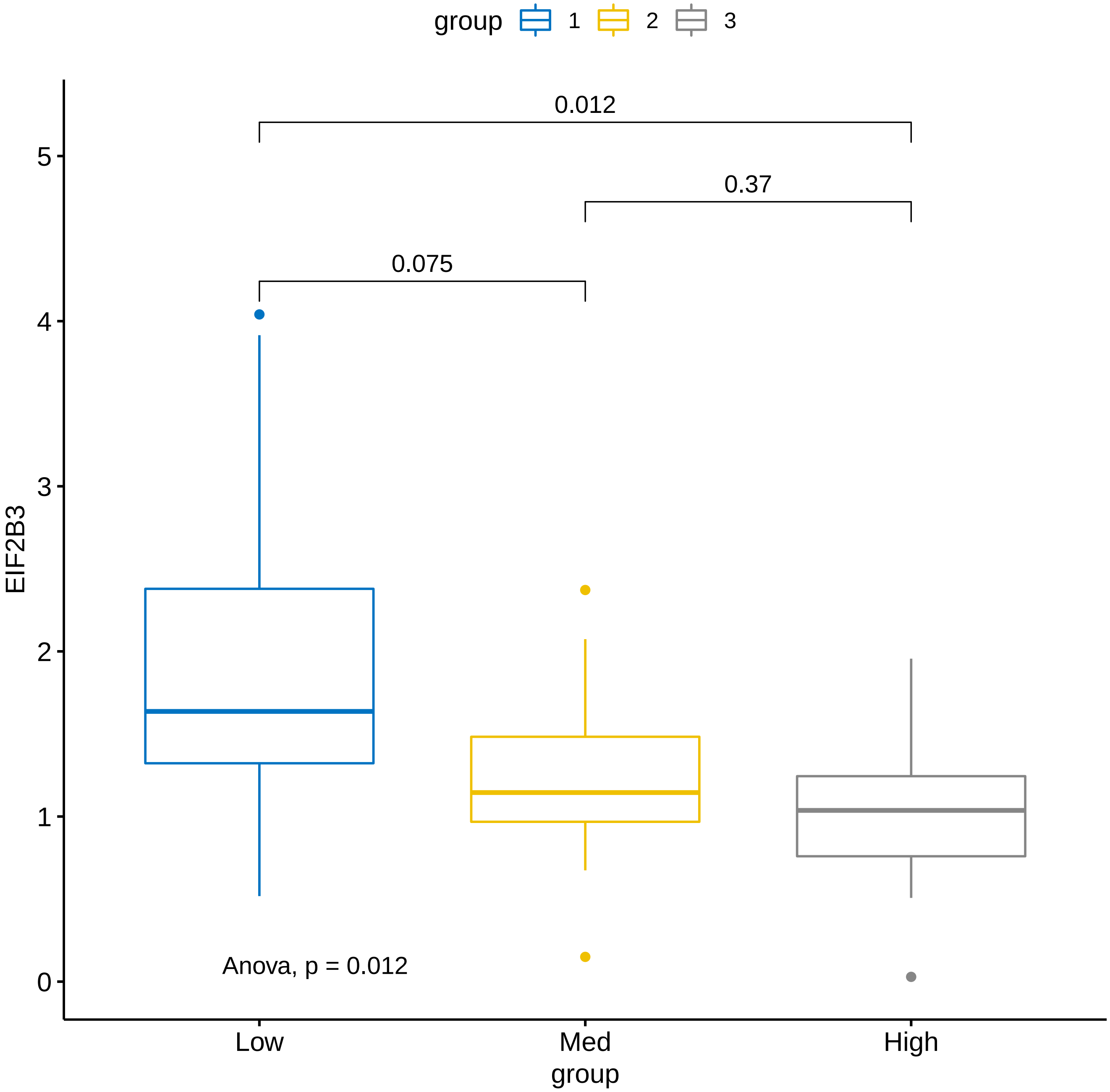

group
1
2
3
0.012
0.37
0.075
5
4
3
2
1
0
EIF2B3
Anova, p = 0.012
Low
Med
High
group

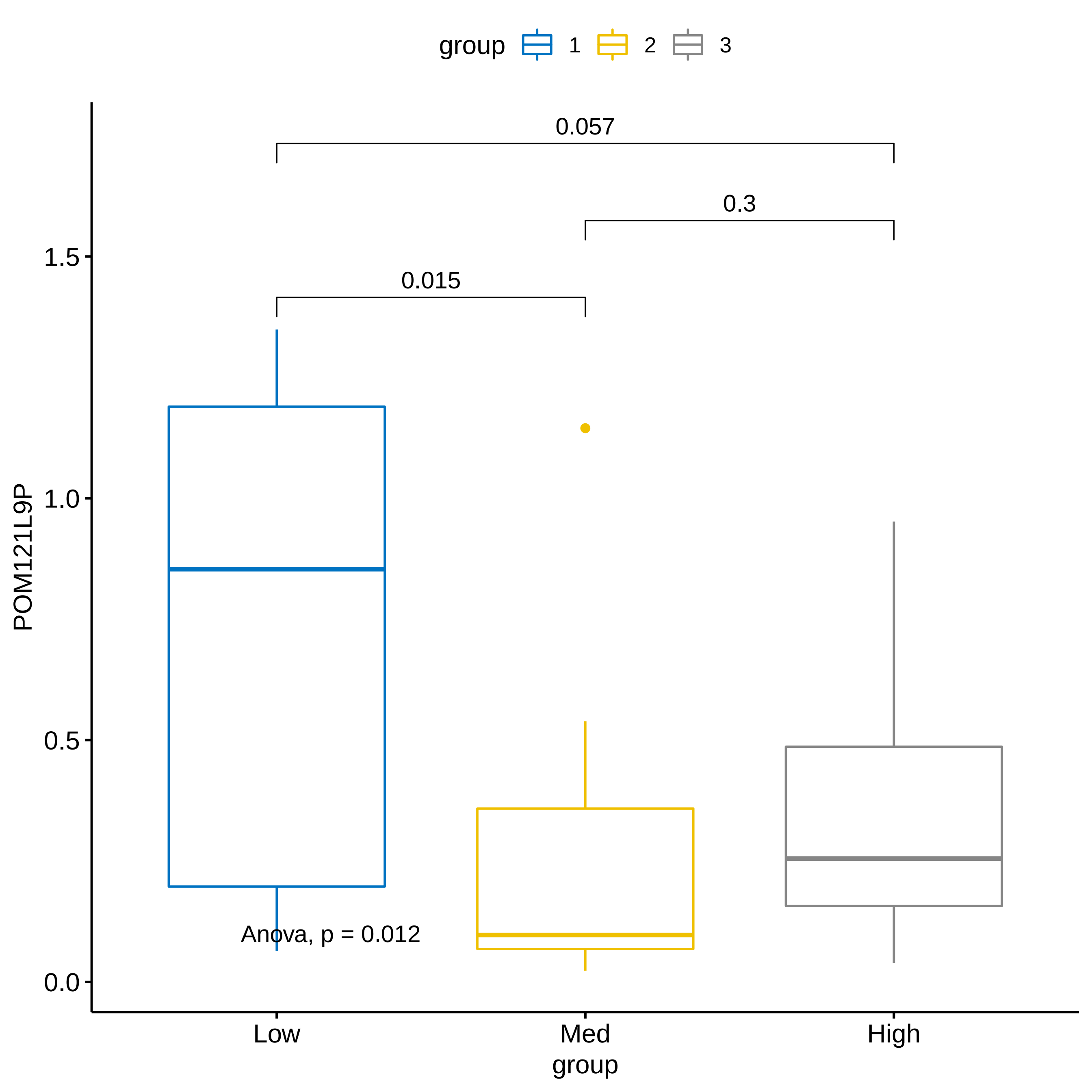
group 1 2 3
0.057
0.3
0.015
1.5
1.0
0.5
0.0
POM121L9P
Anova, p = 0.012
Low
Med
High
group

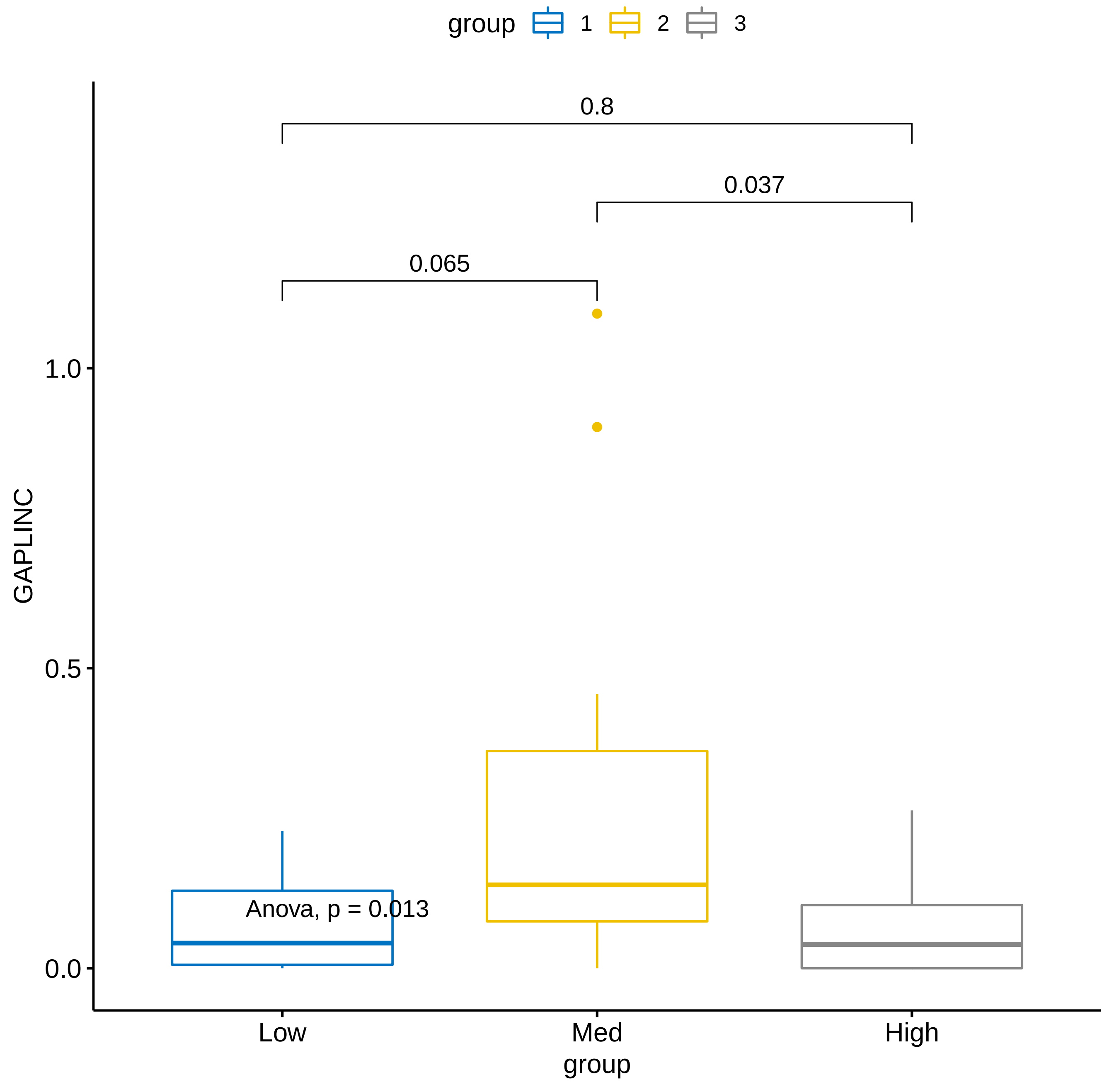
group
1
2
3
0.8
0.037
0.065
1.0
0.5
0.0
GAPLINC
Anova, p = 0.013
Low
Med
High
group

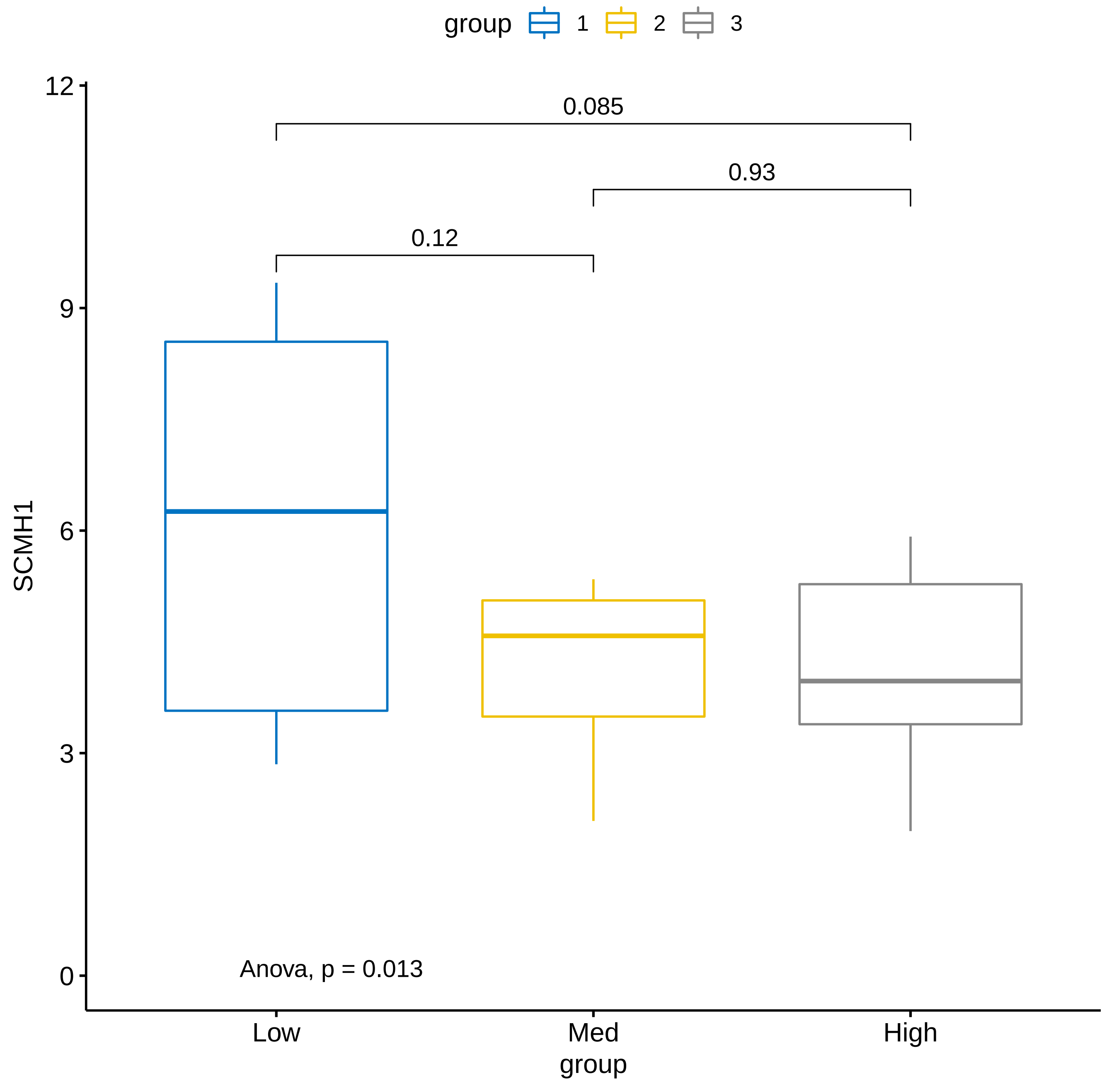
group
1
2
3
12
9
6
3
0
SCMH1
0.085
0.93
0.12
Anova, p = 0.013
Low
Med
High
group

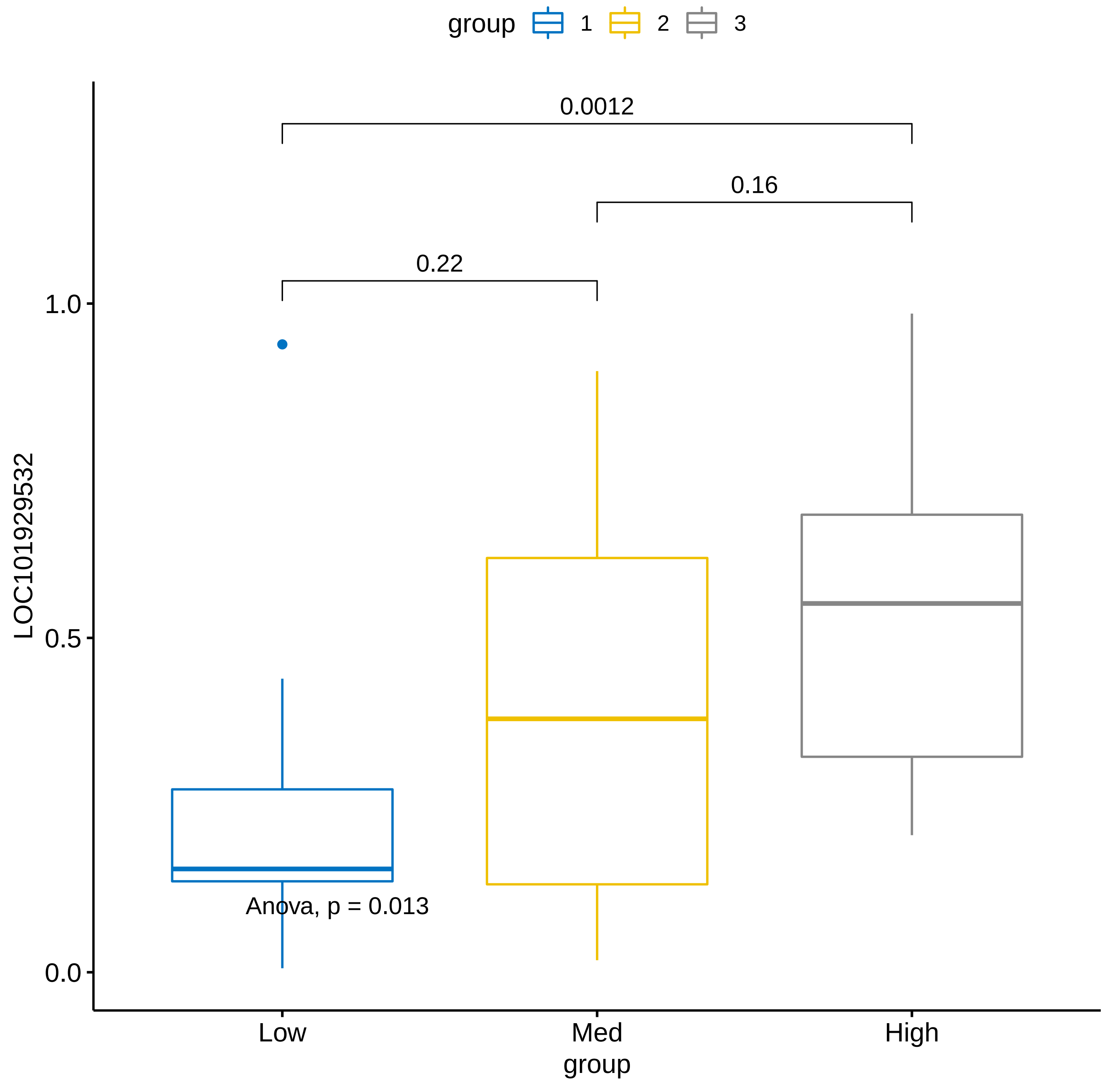
group
1
2
3
0.0012
0.16
0.22
1.0
0.5
0.0
LOC101929532
Anova, p = 0.013
Low
Med
High
group

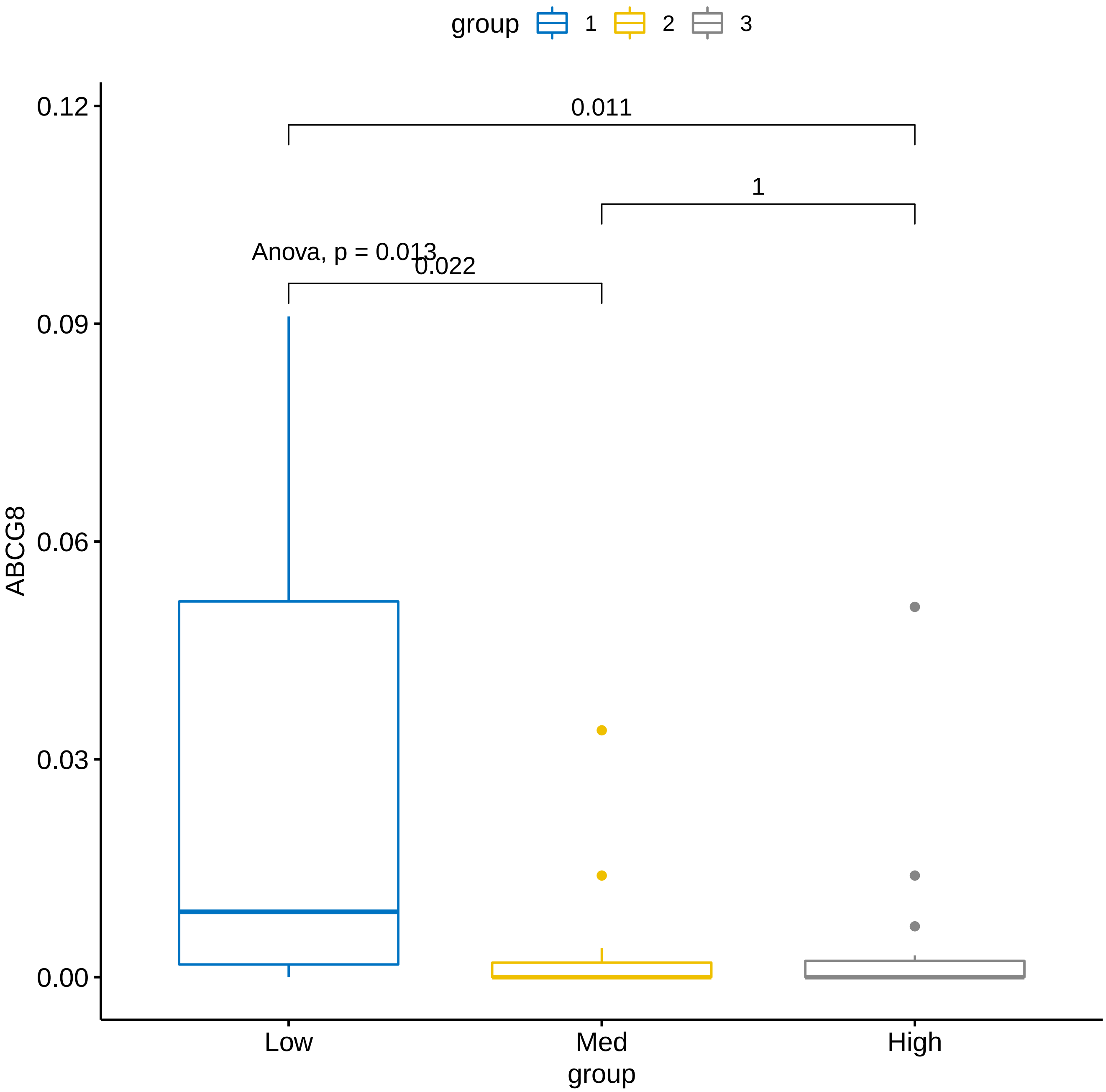
group
1
2
3
0.011
1
Anova, p = 0.013
0.022
0.12
0.09
0.06
0.03
0.00
ABCG8
Low
Med
High
group

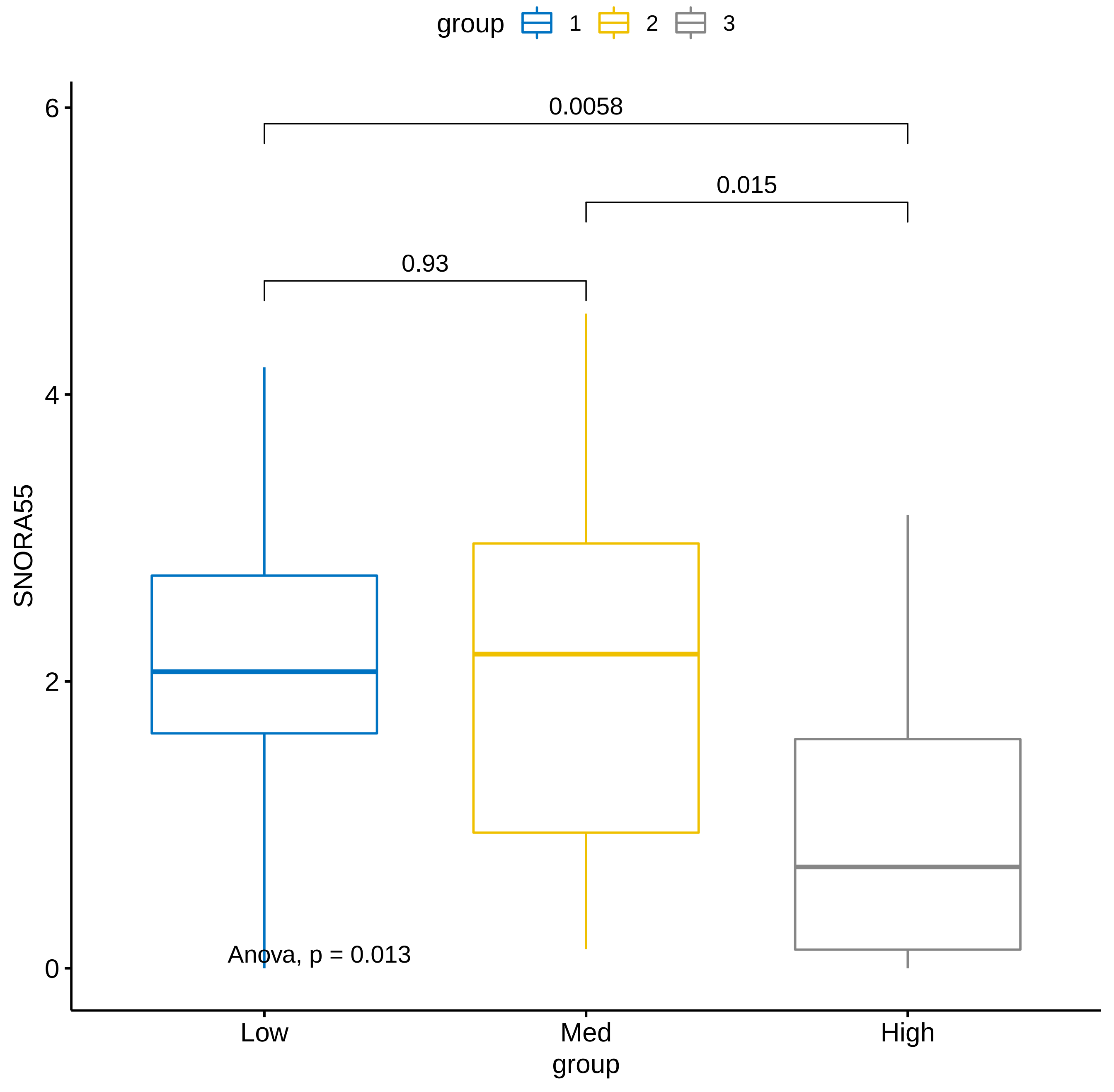
group 1 2 3
0.0058
0.015
0.93
6
4
2
0
SNORA55
Anova, p = 0.013
Low
Med
High
group

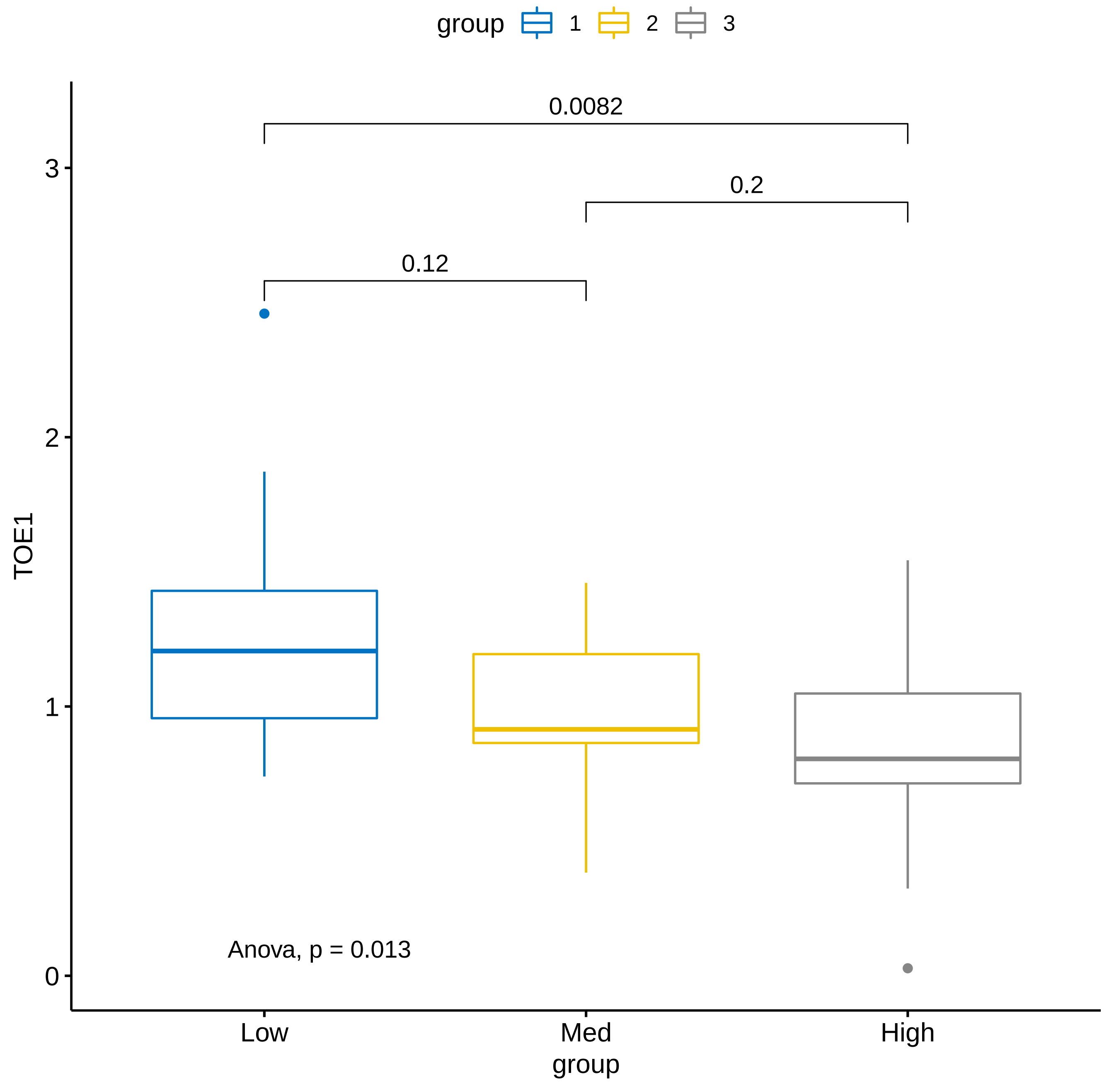

group 1 2 3
0.0082
0.2
0.12
3
2
1
0
TOE1
Anova, p = 0.013
Low
Med
High
group

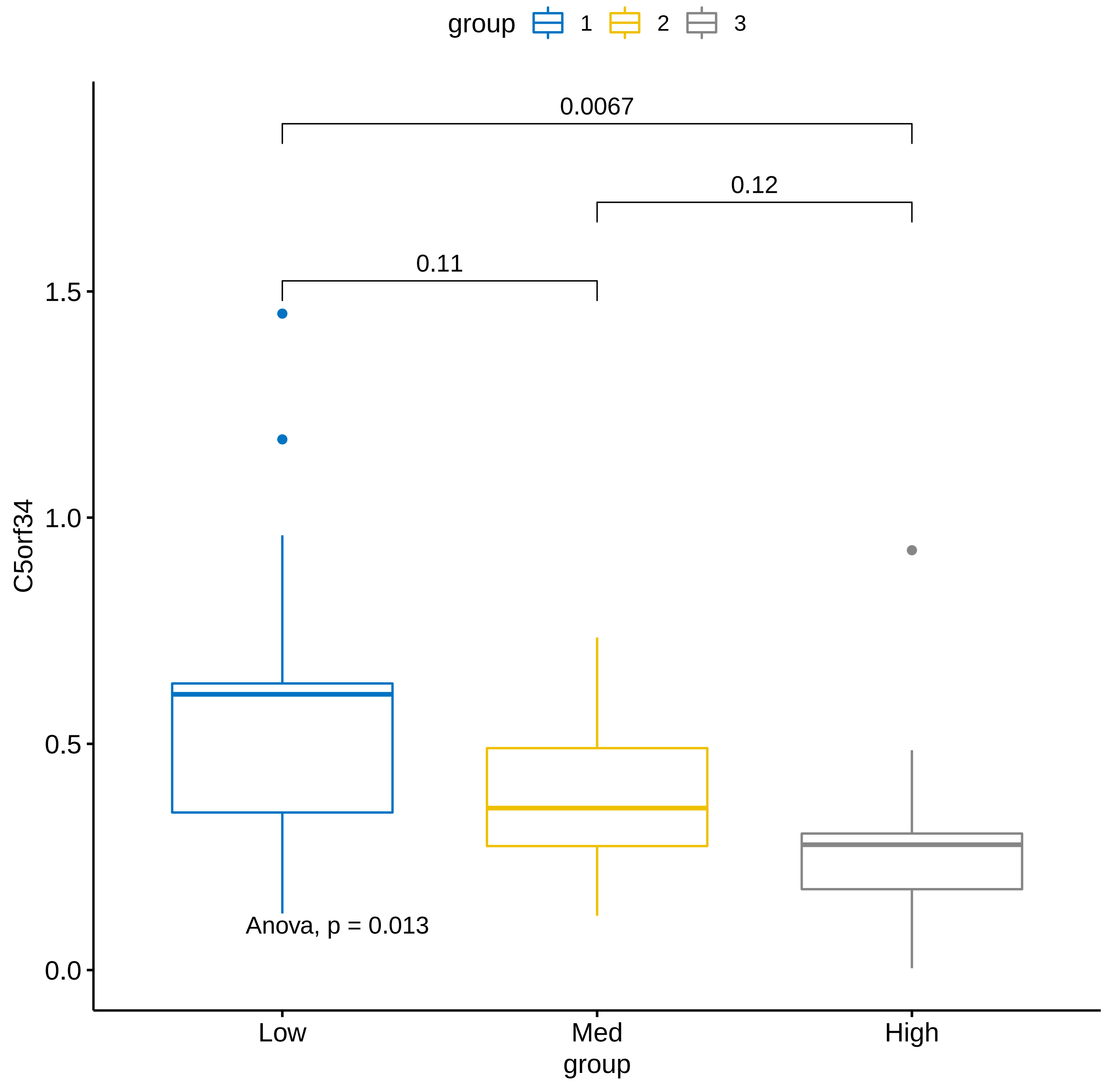
group
1
2
3
0.0067
0.12
0.11
1.5
1.0
0.5
0.0
C5orf34
Anova, p = 0.013
Low
Med
High
group

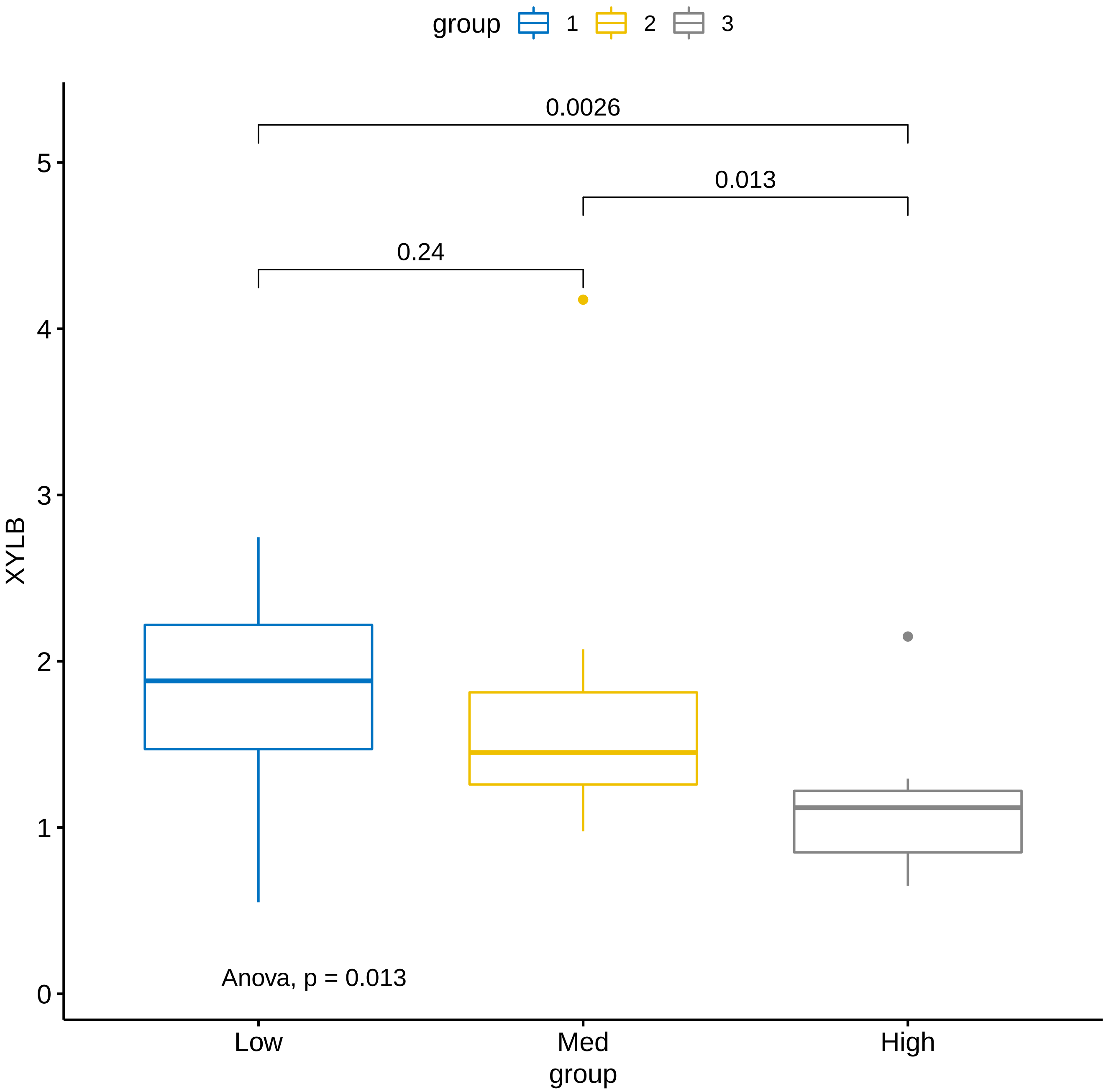
group
1
2
3
0.0026
0.013
0.24
XYLB
5
4
3
2
1
0
Anova, p = 0.013
Low
Med
High
group

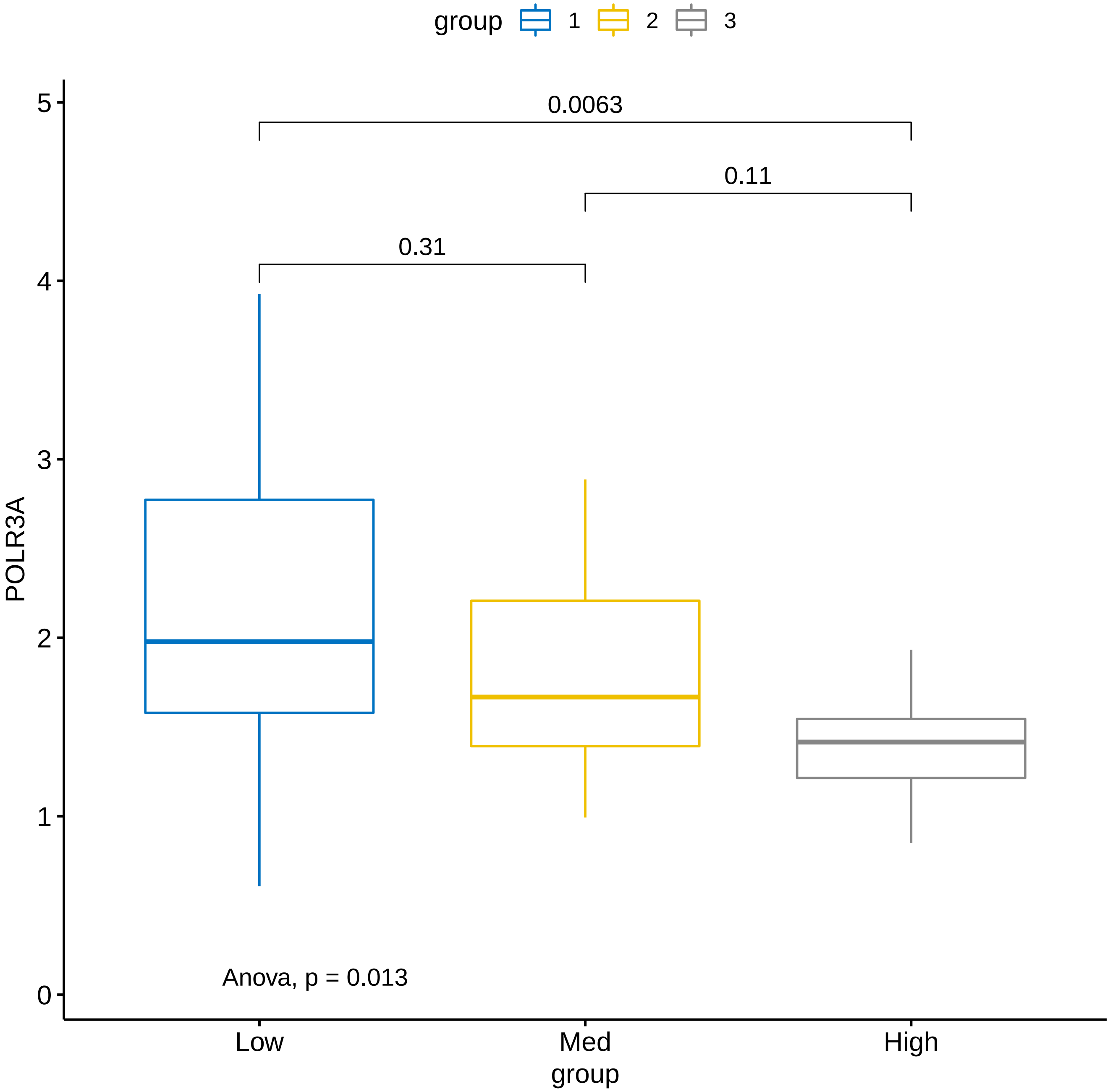
group
1
2
3
0.0063
0.11
0.31
5
4
3
2
1
0
POLR3A
Anova, p = 0.013
Low
Med
High
group

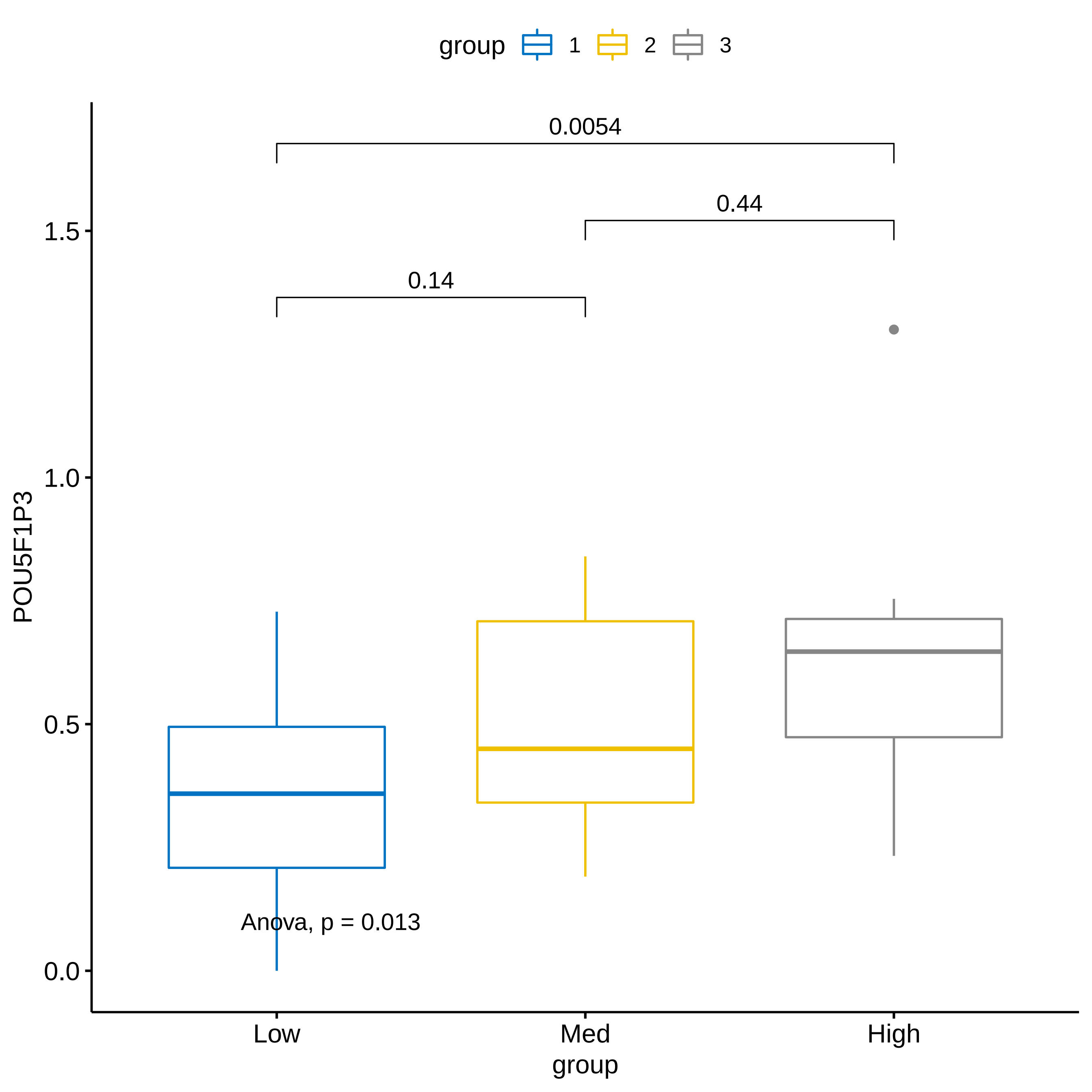
group
1
2
3
0.0054
0.44
0.14
1.5
1.0
0.5
0.0
POU5F1P3
Anova, p = 0.013
Low
Med
High
group

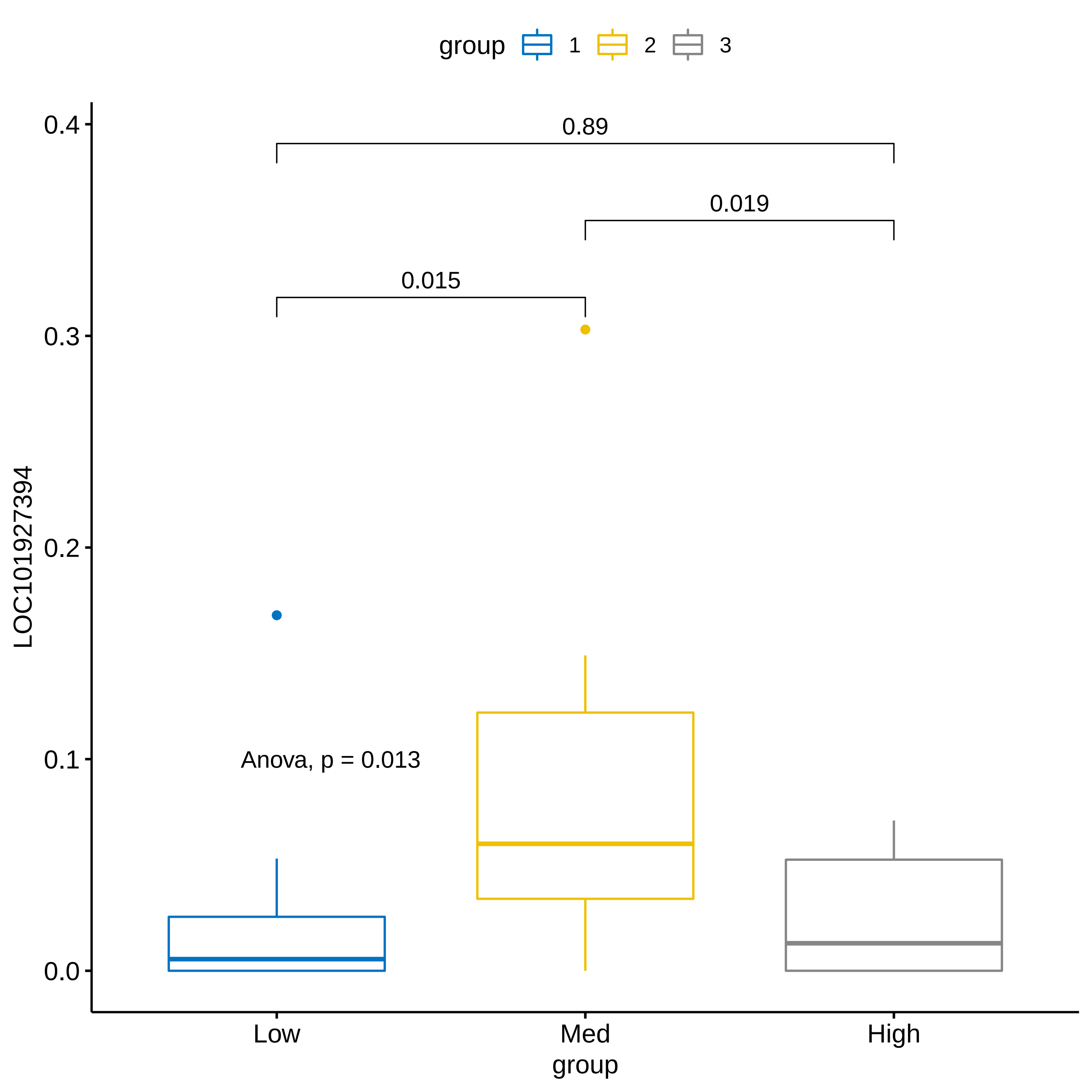
group
1
2
3
0.89
0.019
0.015
0.4
0.3
0.2
0.1
0.0
LOC101927394
Anova, p = 0.013
Low
Med
High
group

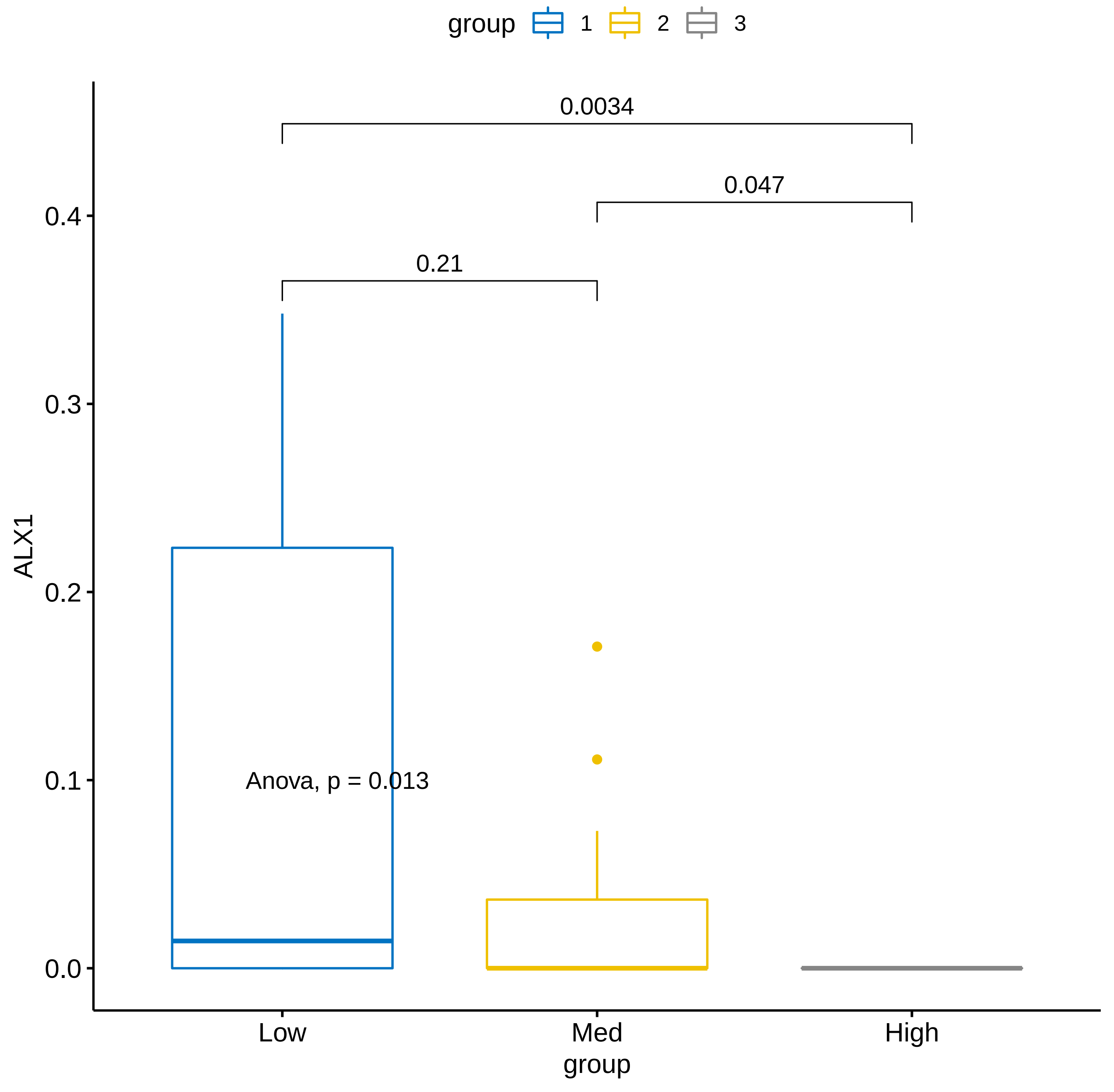

group 1 2 3
0.0034
0.047
0.21
Anova, p = 0.013
ALX1
0.4
0.3
0.2
0.1
0.0
Low
Med
High
group

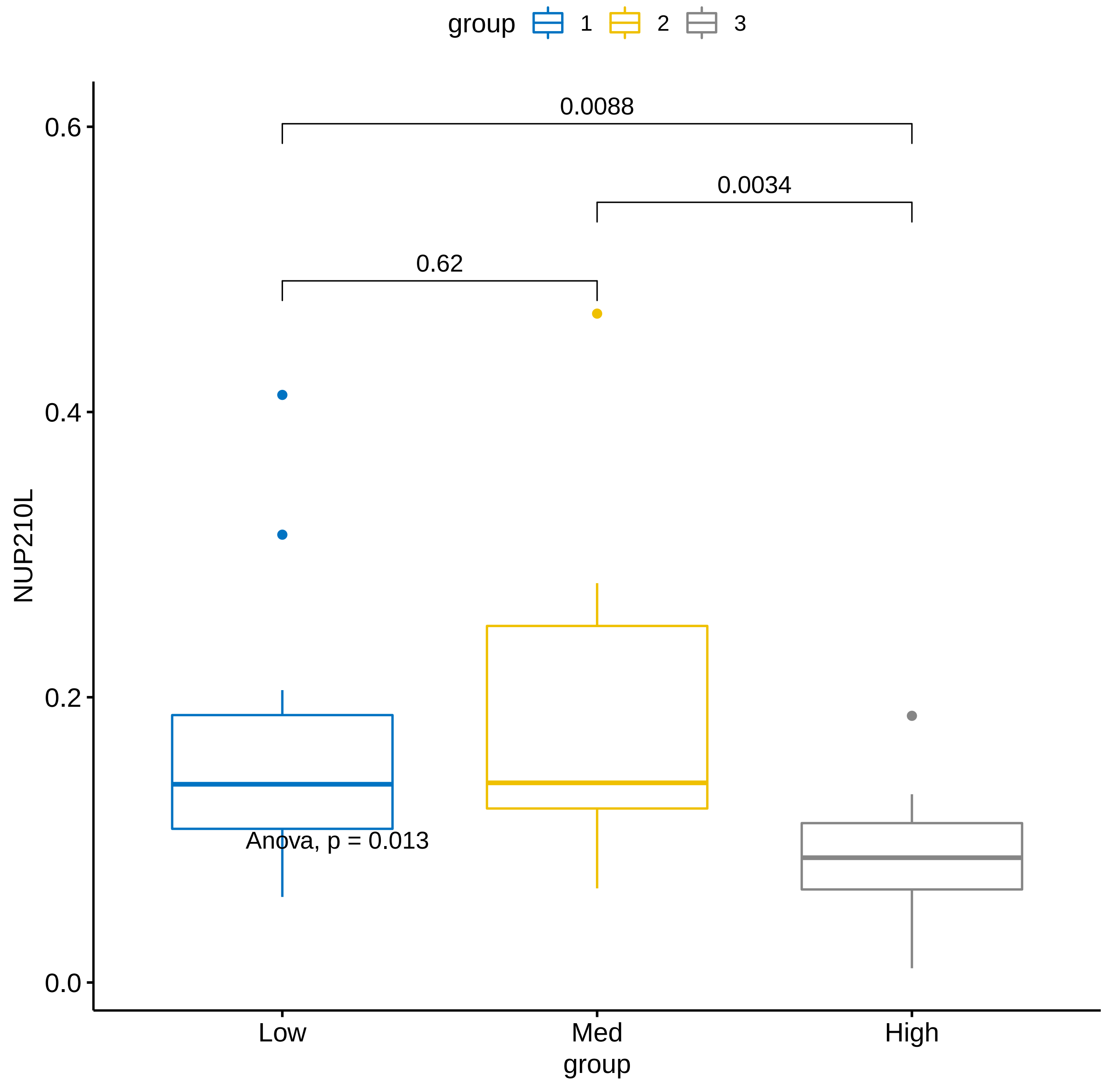
group
1
2
3
0.0088
0.0034
0.62
0.6
0.4
0.2
0.0
NUP210L
Anova, p = 0.013
Low
Med
High
group

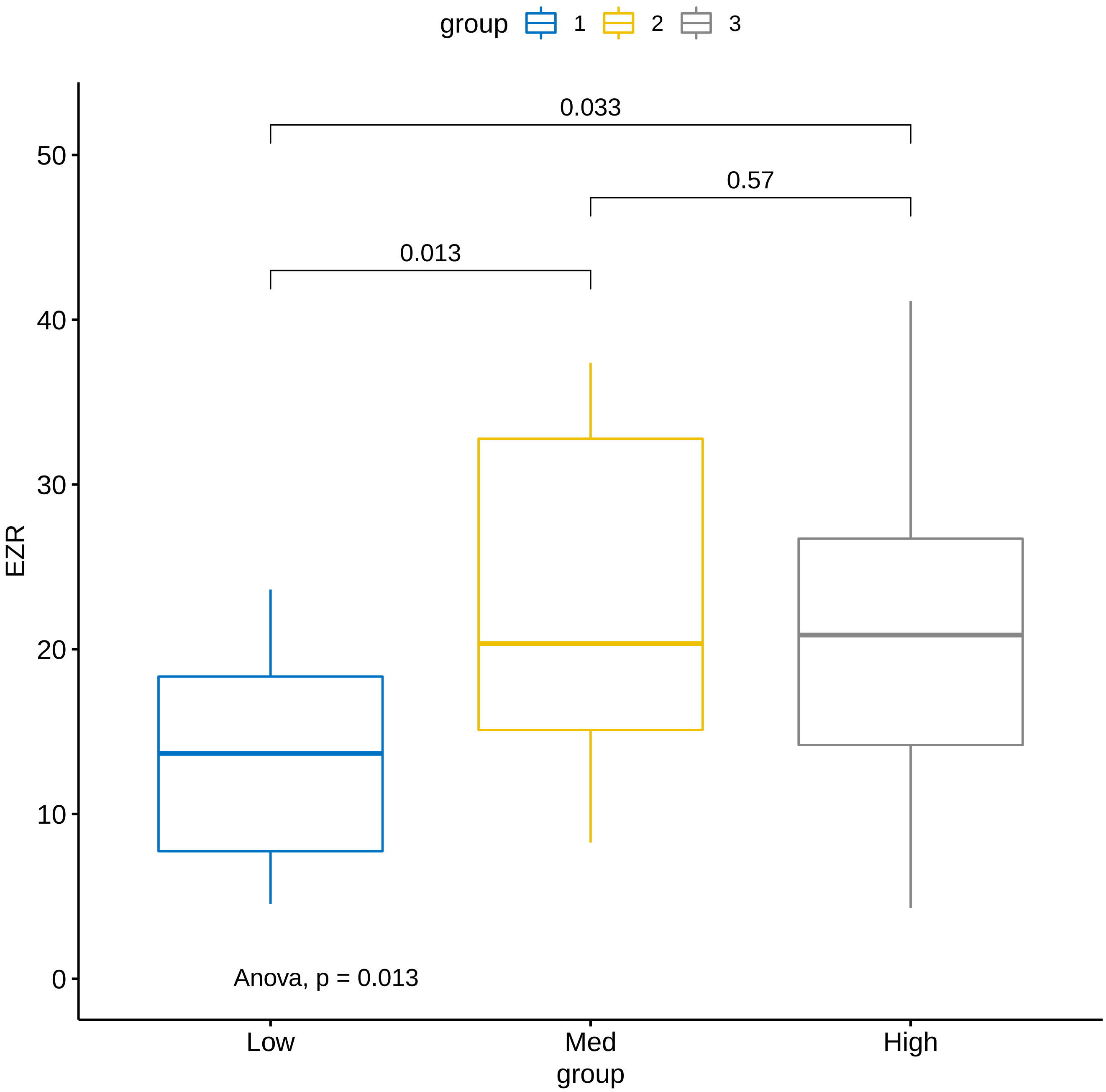
group
1
2
3
0.033
0.57
0.013
EZR
50
40
30
20
10
0
Anova, p = 0.013
Low
Med
High
group

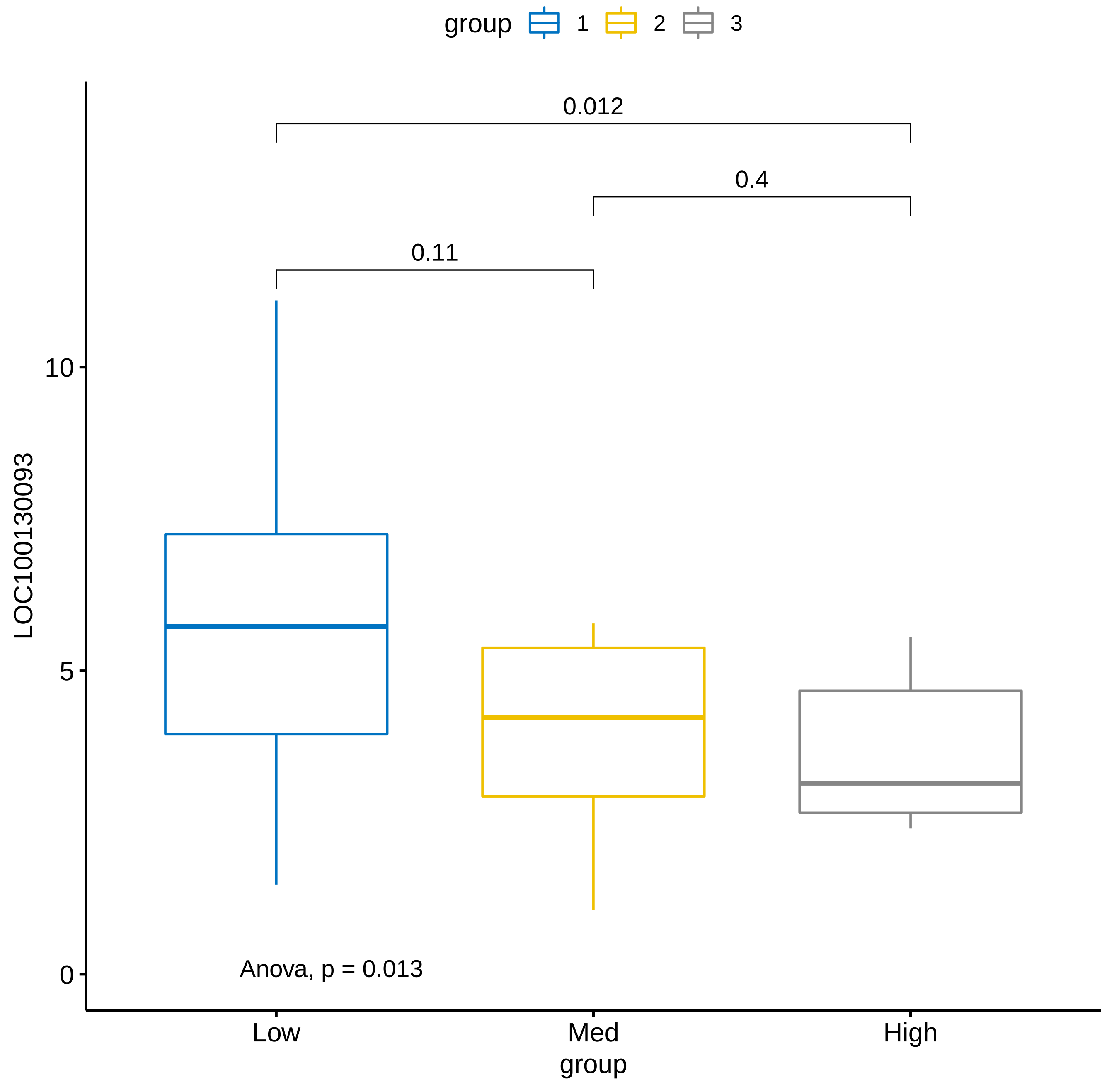

group
1
2
3
0.012
0.4
0.11
10
5
0
LOC100130093
Anova, p = 0.013
Low
Med
High
group

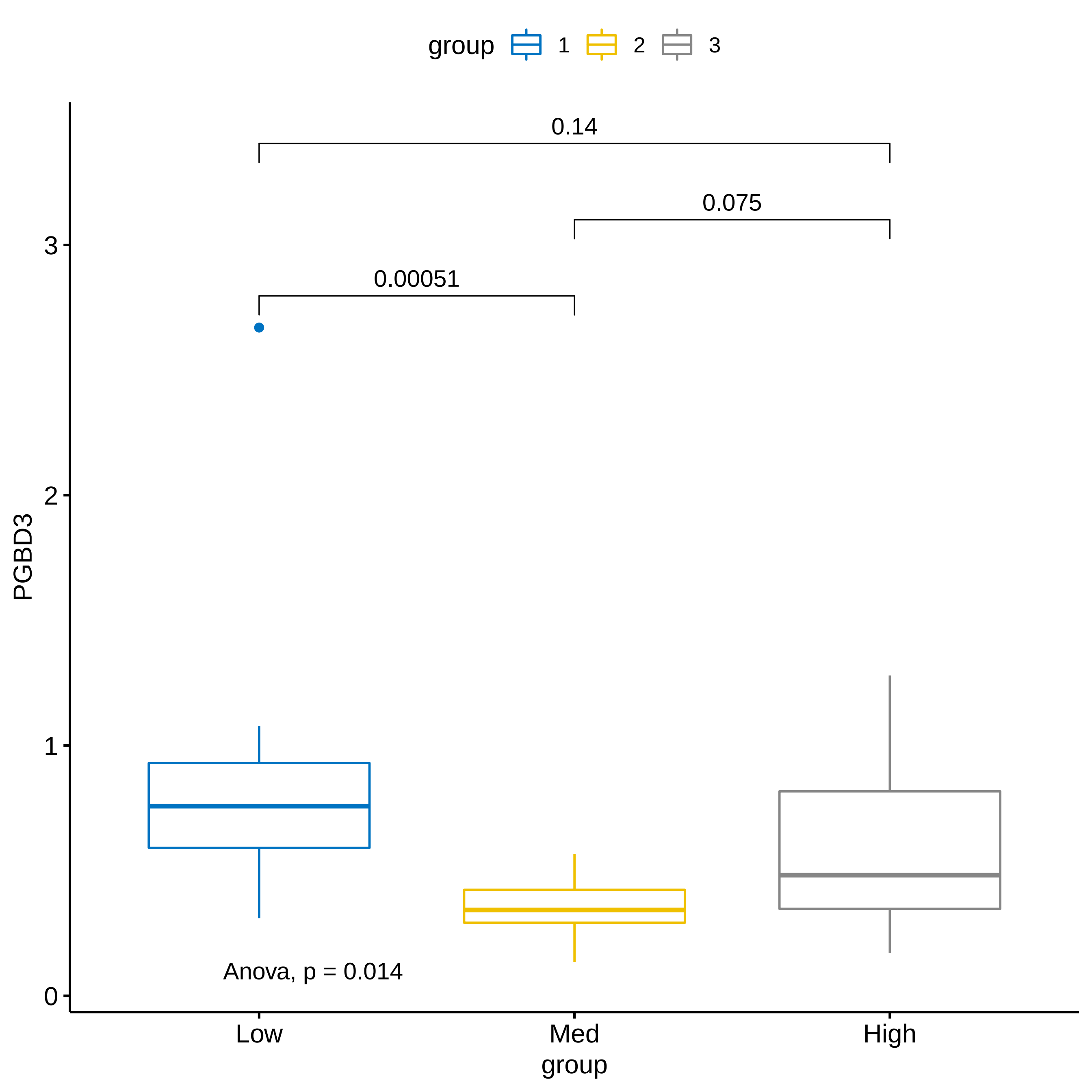

group
1
2
3
0.14
0.075
0.00051
3
2
1
0
PGBD3
Anova, p = 0.014
Low
Med
High
group

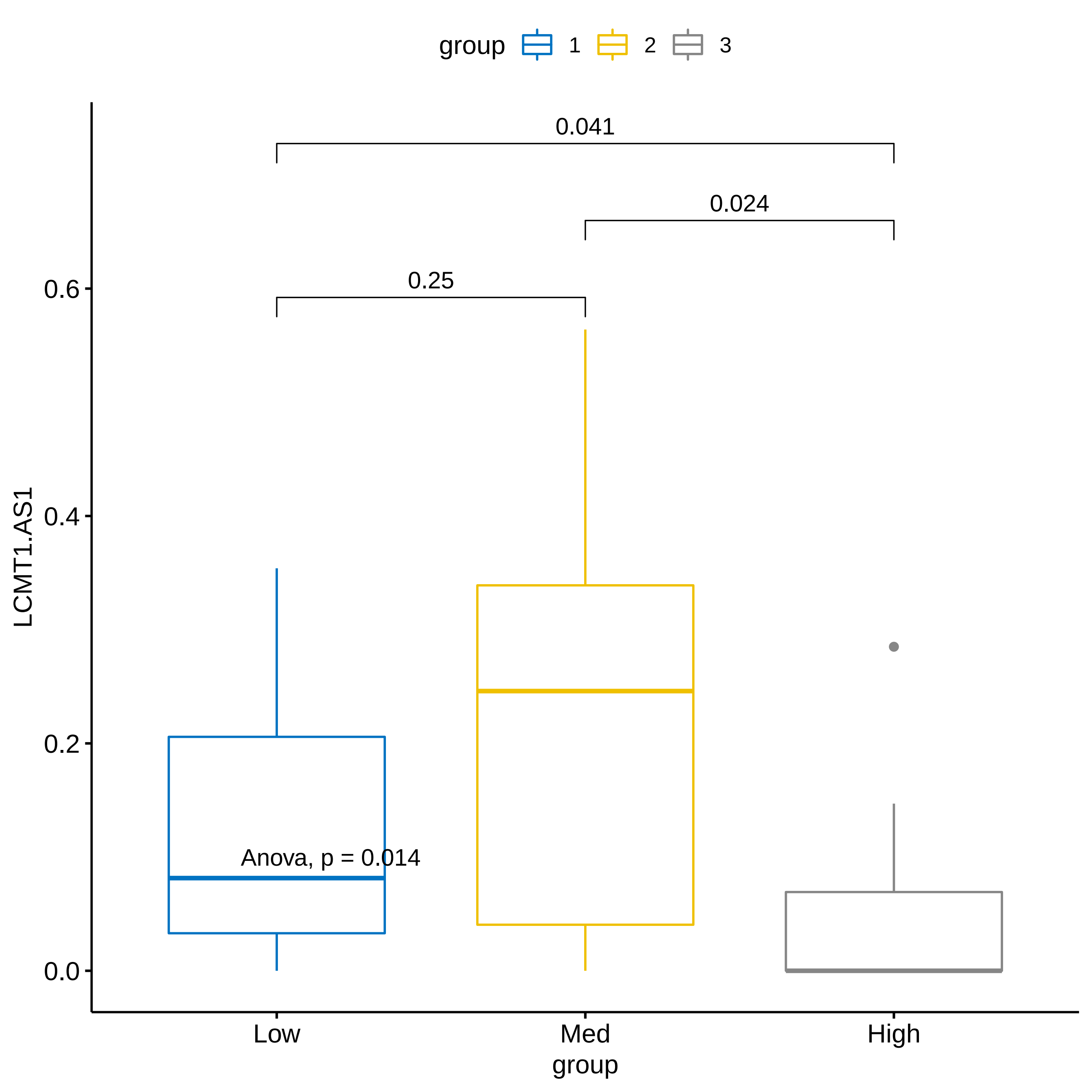
group
1
2
3
0.041
0.024
0.25
0.6
0.4
0.2
0.0
LCMT1.AS1
Anova, p = 0.014
Low
Med
High
group

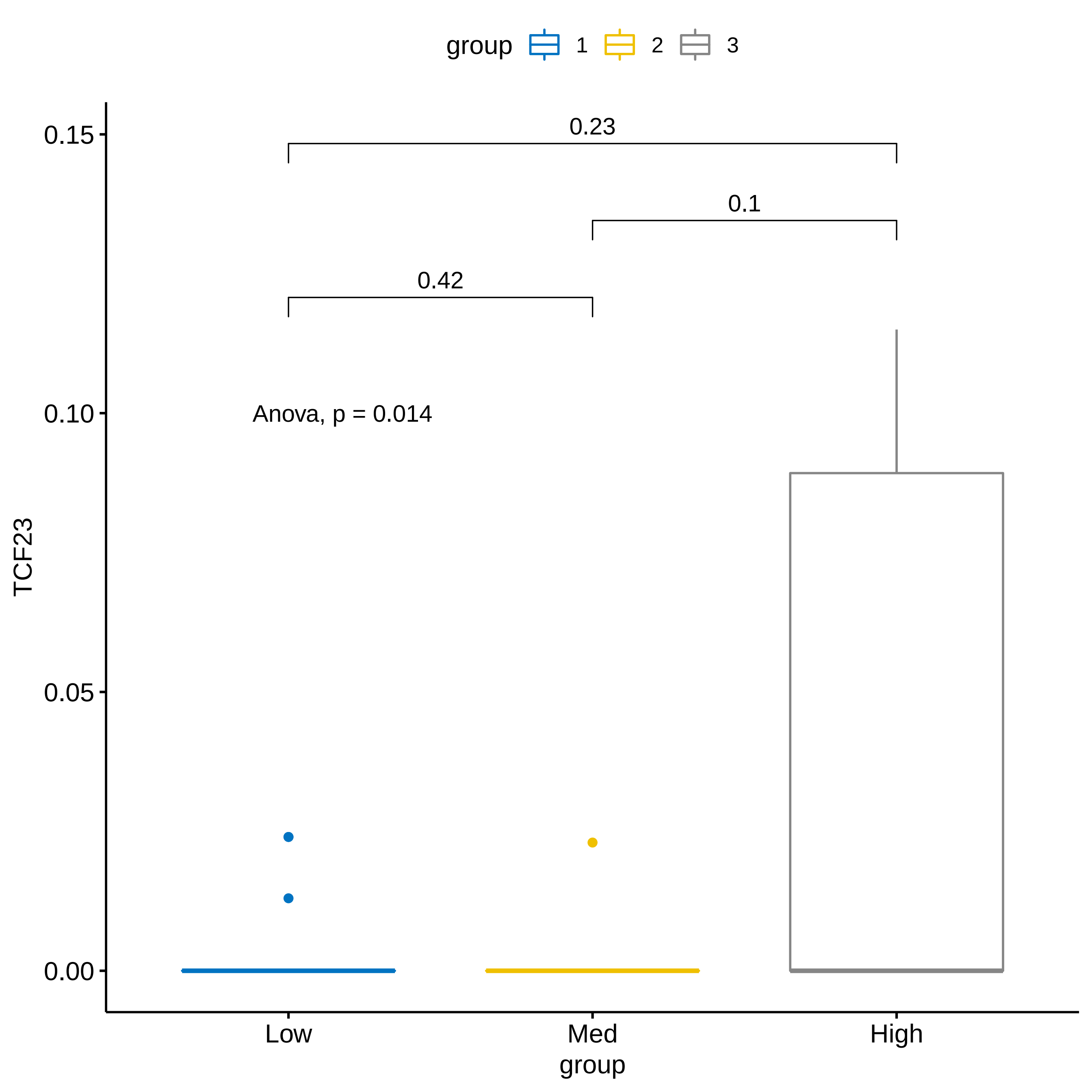

group 1 2 3
0.23
0.1
0.42
Anova, p = 0.014
TCF23
0.15
0.10
0.05
0.00
Low
Med
High
group

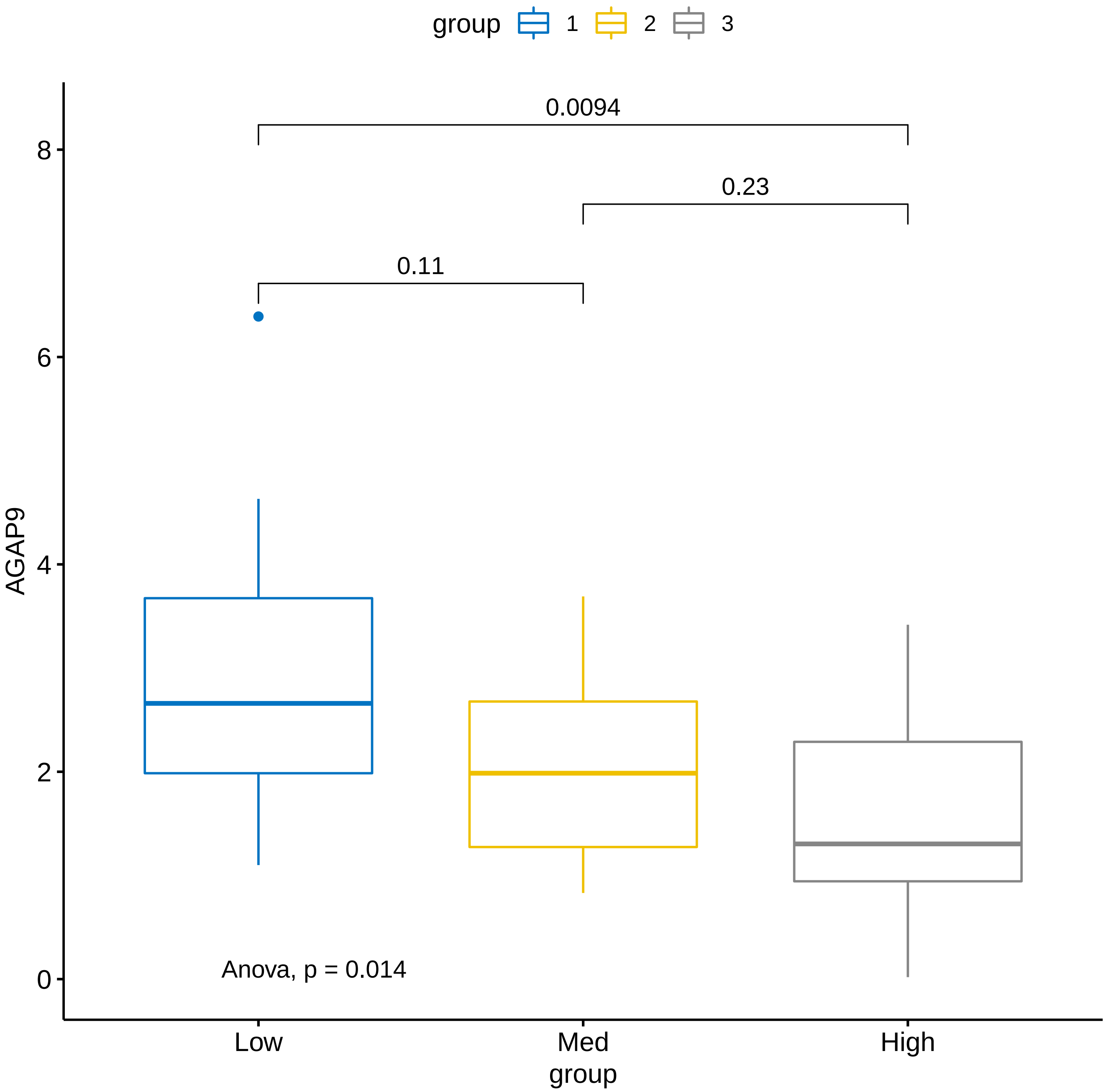

group
1
2
3
0.0094
0.23
0.11
8
6
4
2
0
AGAP9
Anova, p = 0.014
Low
Med
High
group

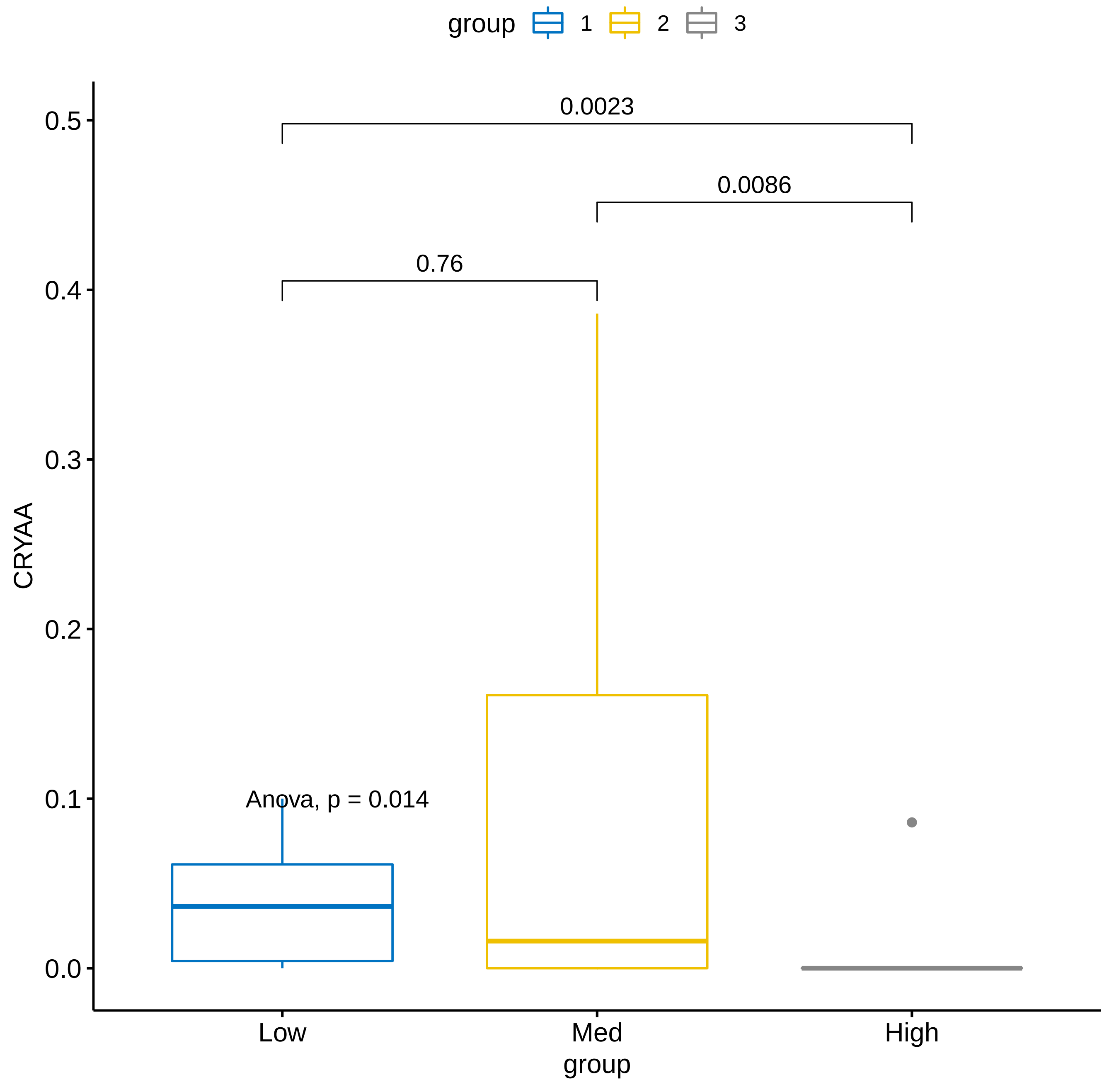
group
1
2
3
0.0023
0.0086
0.76
Anova, p = 0.014
0.5
0.4
0.3
0.2
0.1
0.0
CRYAA
Low
Med
High
group

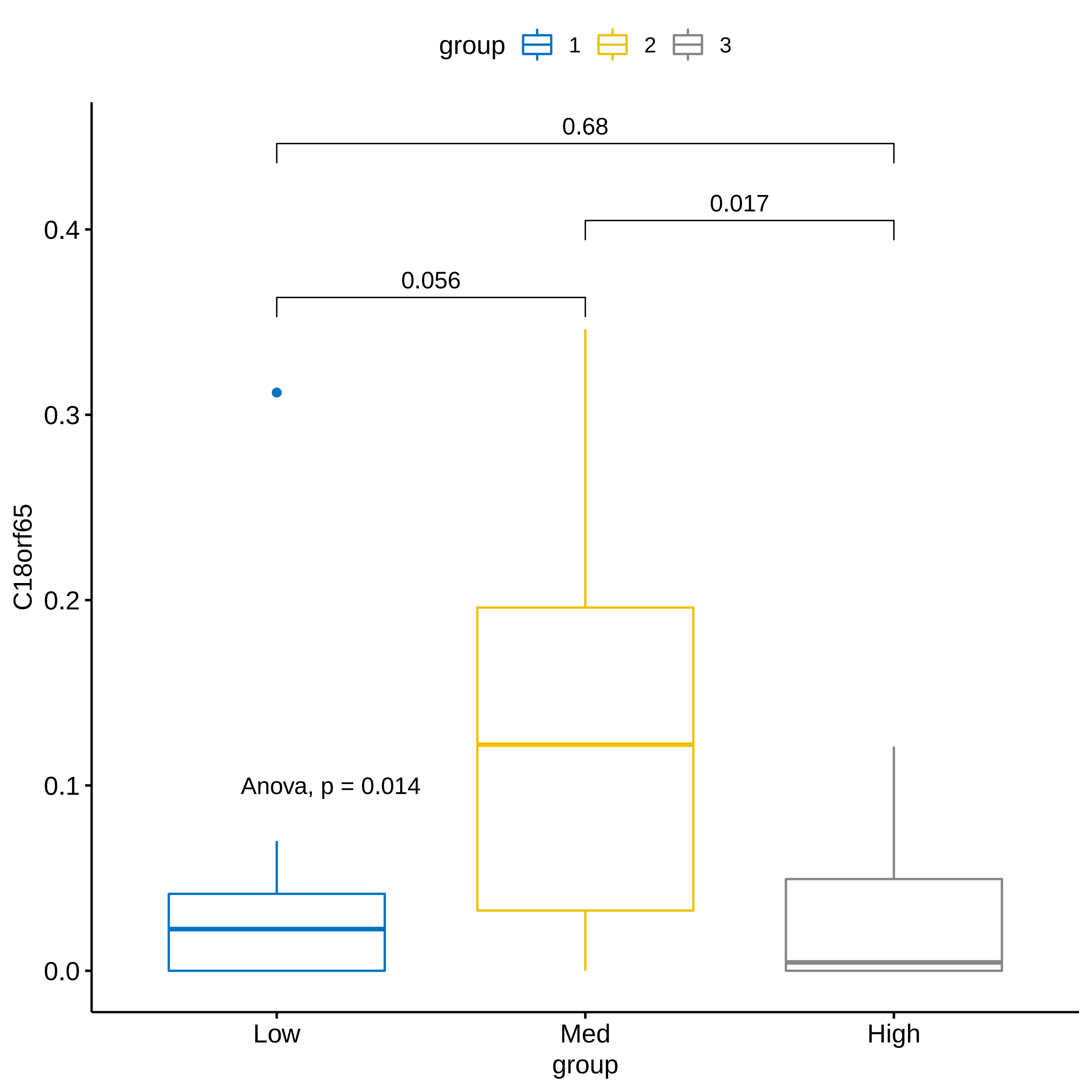

group 1 2 3
0.68
0.017
0.056
Anova, p = 0.014
0.4
0.3
0.2
0.1
0.0
C18orf65
Low
Med
High
group

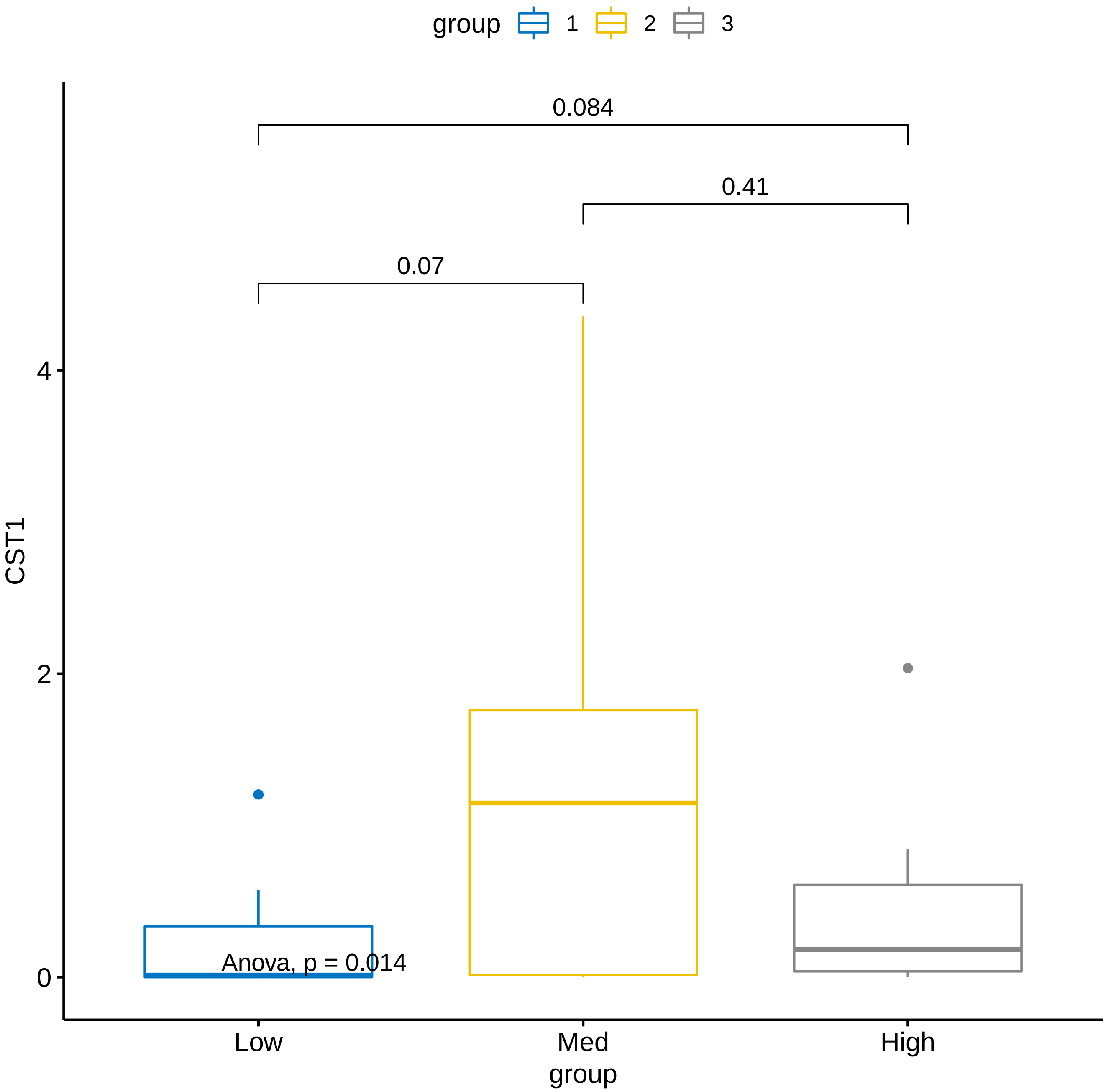
group 1 2 3
0.084
0.41
0.07
4
2
0
CST1
Anova, p = 0.014
Low
Med
High
group

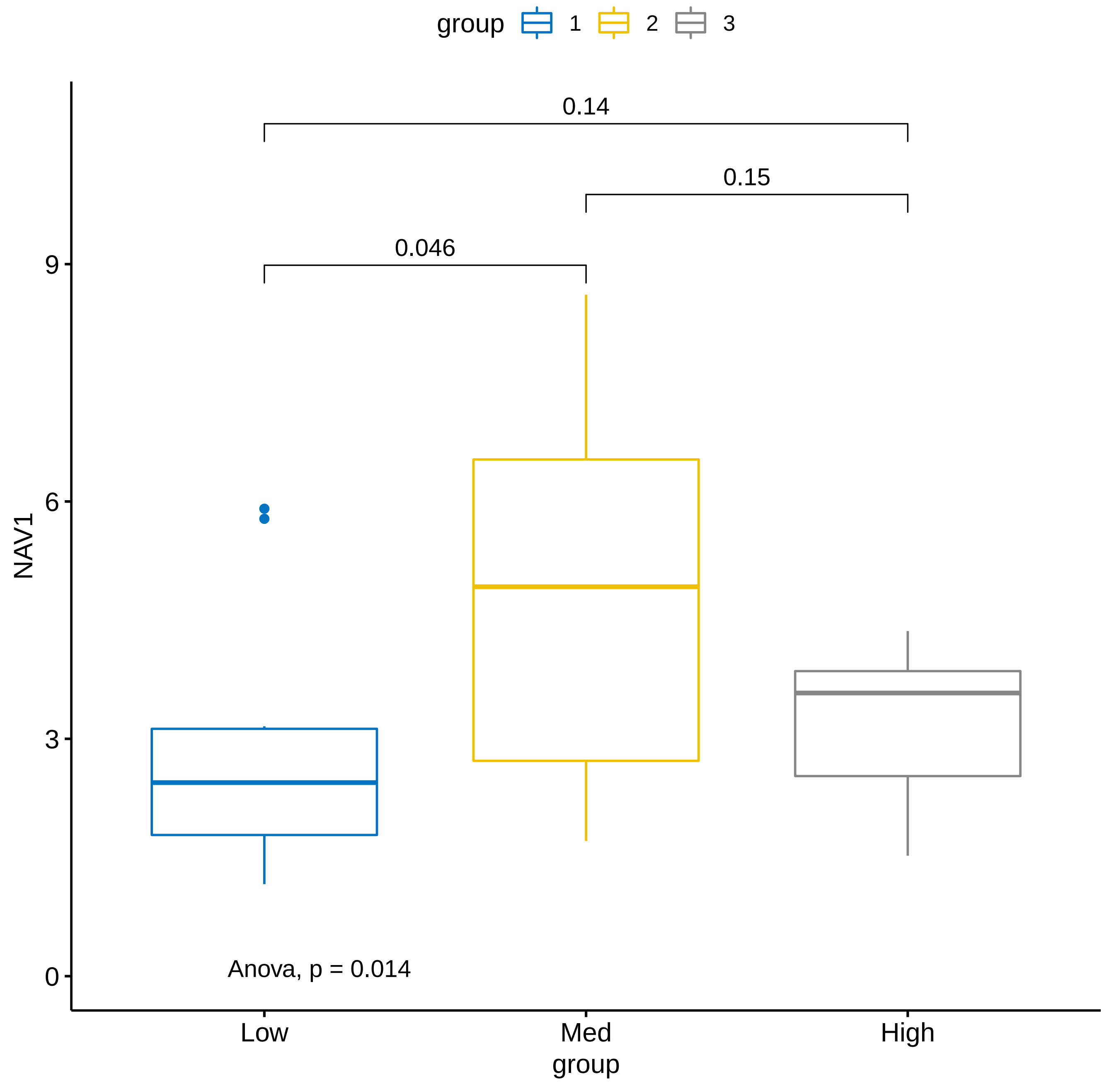

group
1
2
3
0.14
0.15
0.046
9
6
3
0
NAV1
Anova, p = 0.014
Low
Med
High
group

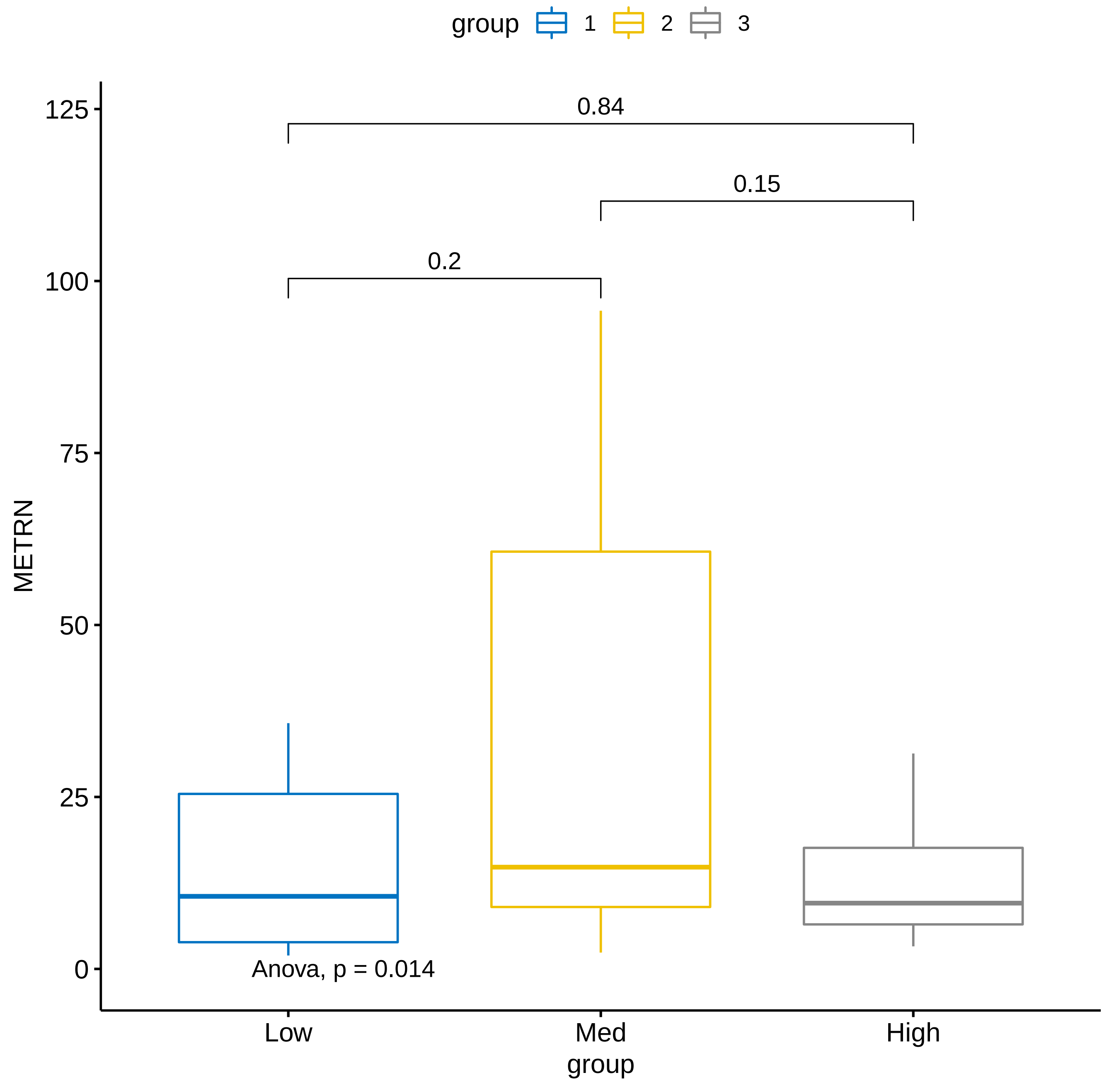

group 1 2 3
0.84
0.15
0.2
125
100
75
50
25
0
METRN
Anova, p = 0.014
Low
Med
High
group

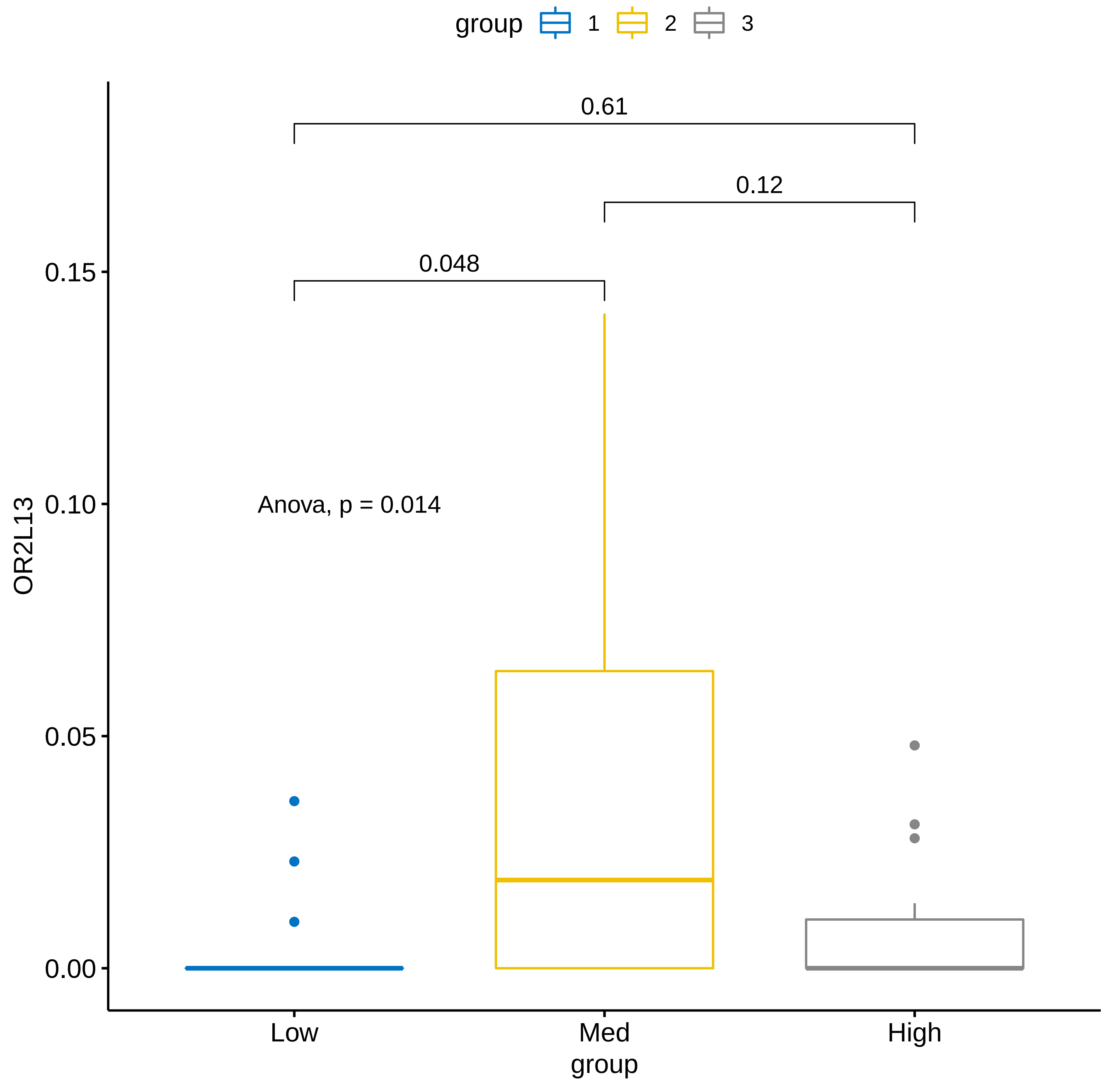

group
1
2
3
0.61
0.12
0.048
Anova, p = 0.014
0.15
0.10
0.05
0.00
OR2L13
Low
Med
High
group

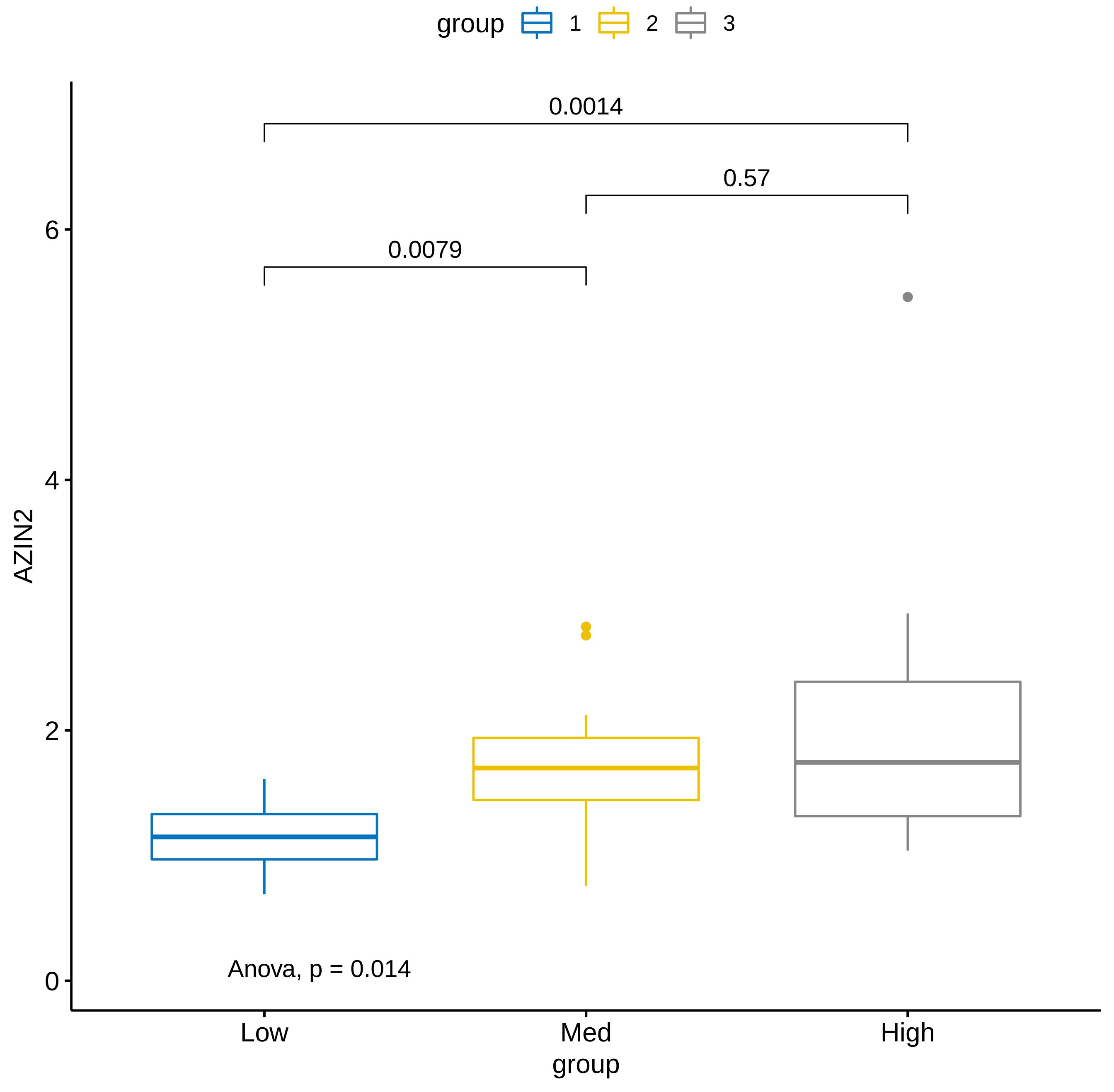

group
1
2
3
0.0014
0.57
0.0079
AZIN2
6
4
2
0
Anova, p = 0.014
Low
Med
High
group

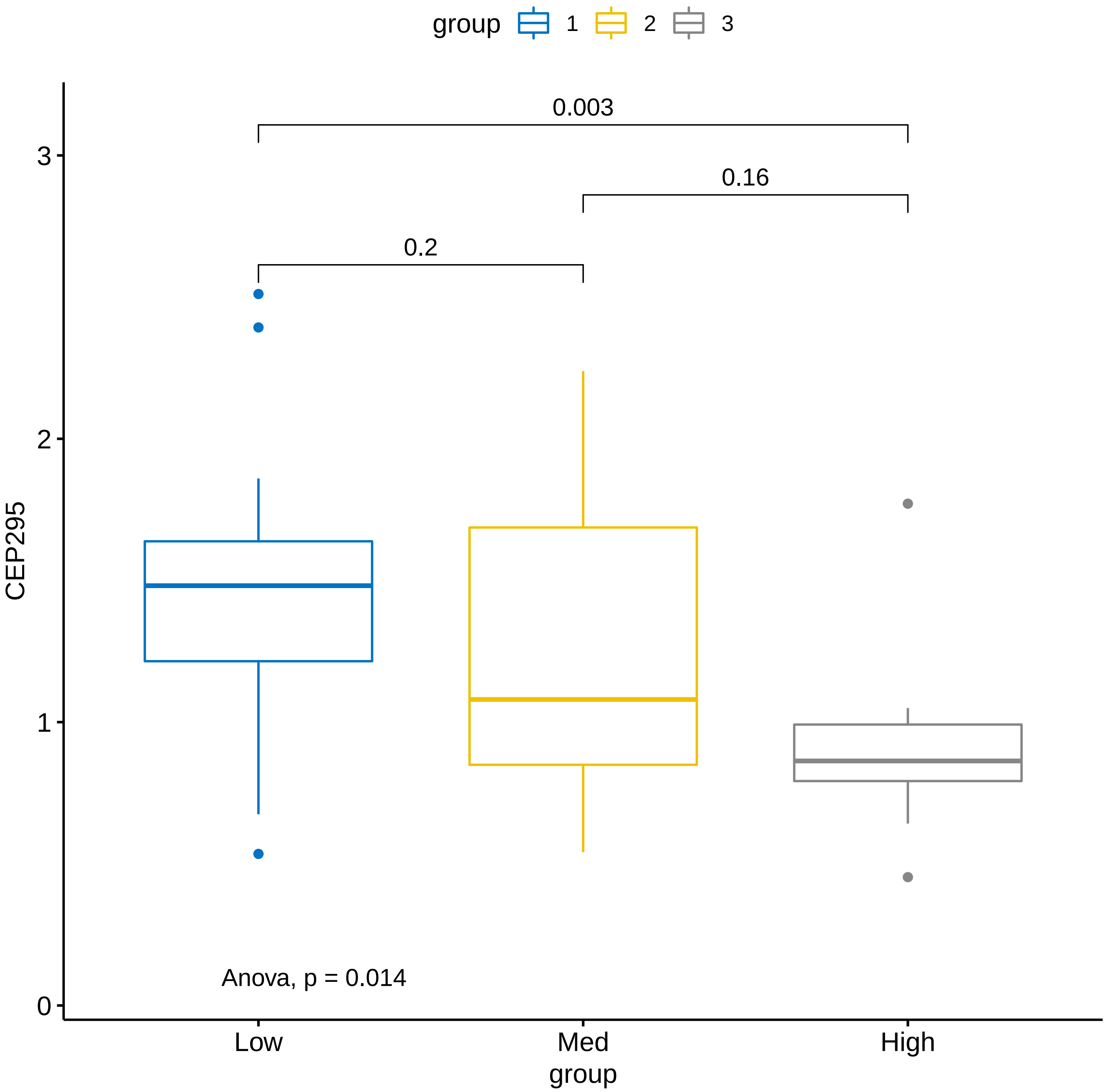

group 1 2 3
0.003
0.16
0.2
CEP295
3
2
1
0
Anova, p = 0.014
Low
Med
High
group

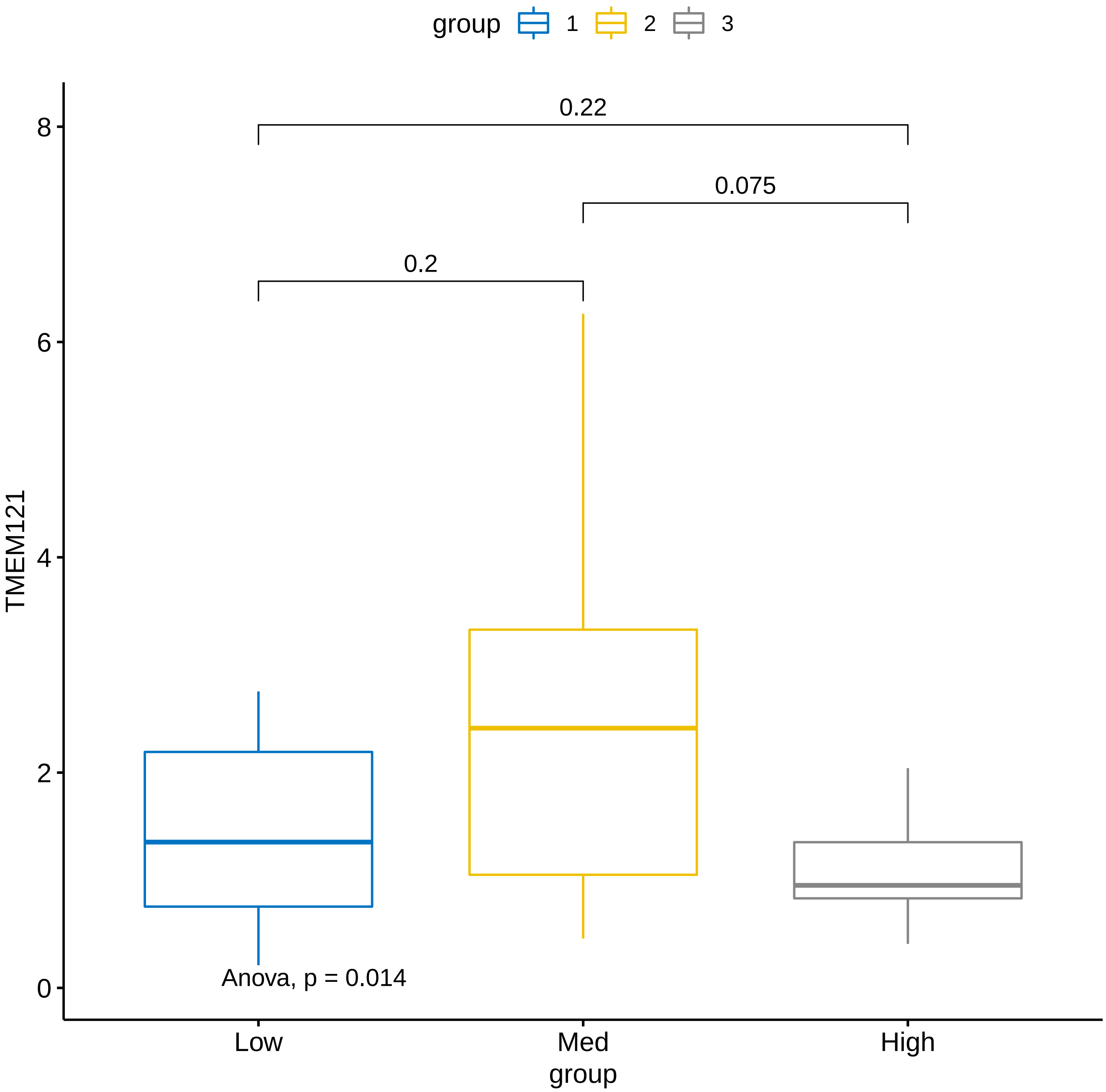
group
1
2
3
0.22
0.075
0.2
8
6
4
2
0
TMEM121
Anova, p = 0.014
Low
Med
High
group

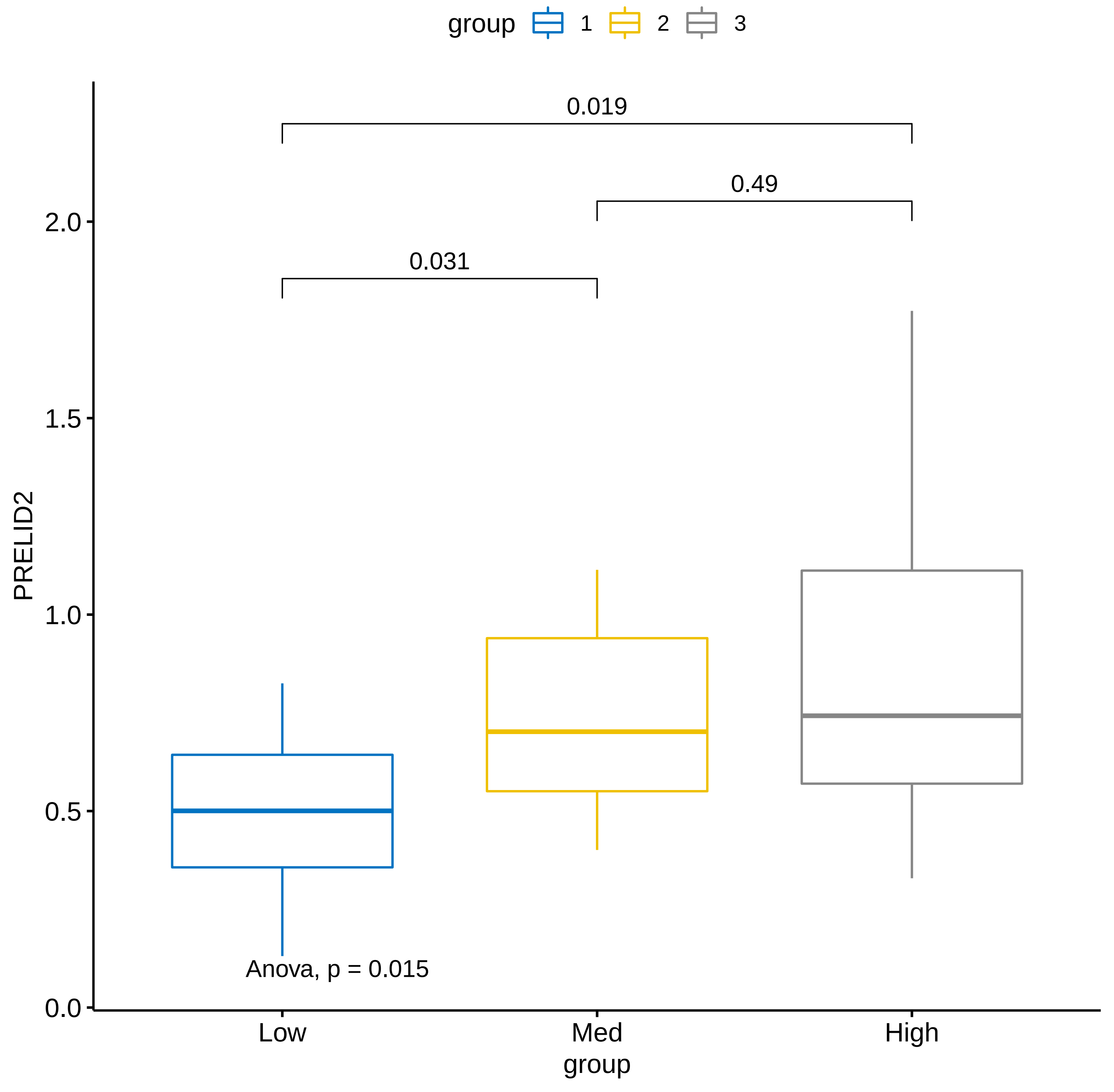

group 1 2 3
0.019
0.49
0.031
2.0
1.5
1.0
0.5
0.0
PRELID2
Anova, p = 0.015
Low
Med
High
group

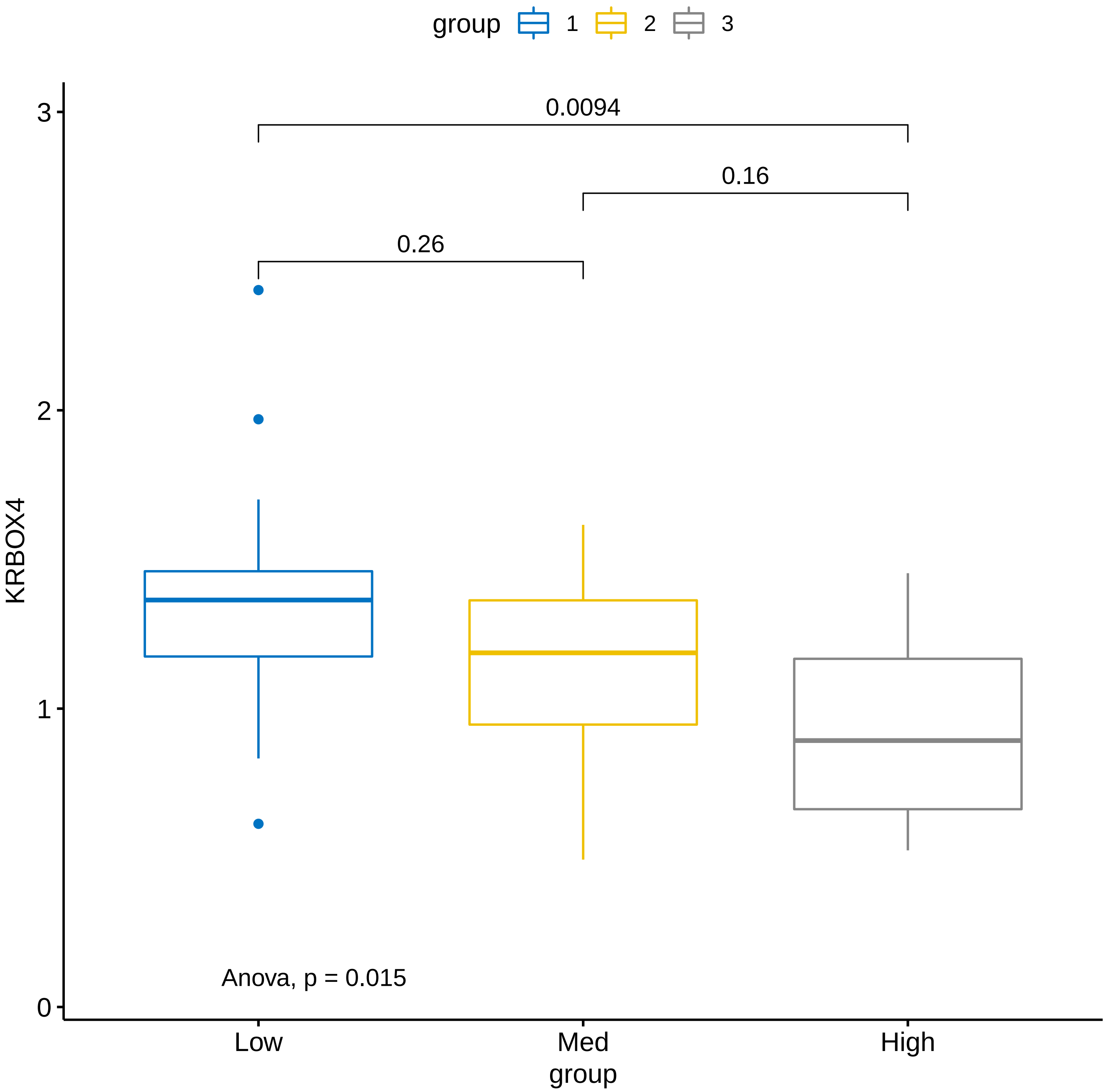

group 1 2 3
0.0094
0.16
0.26
KRBOX4
3
2
1
0
Anova, p = 0.015
Low
Med
High
group

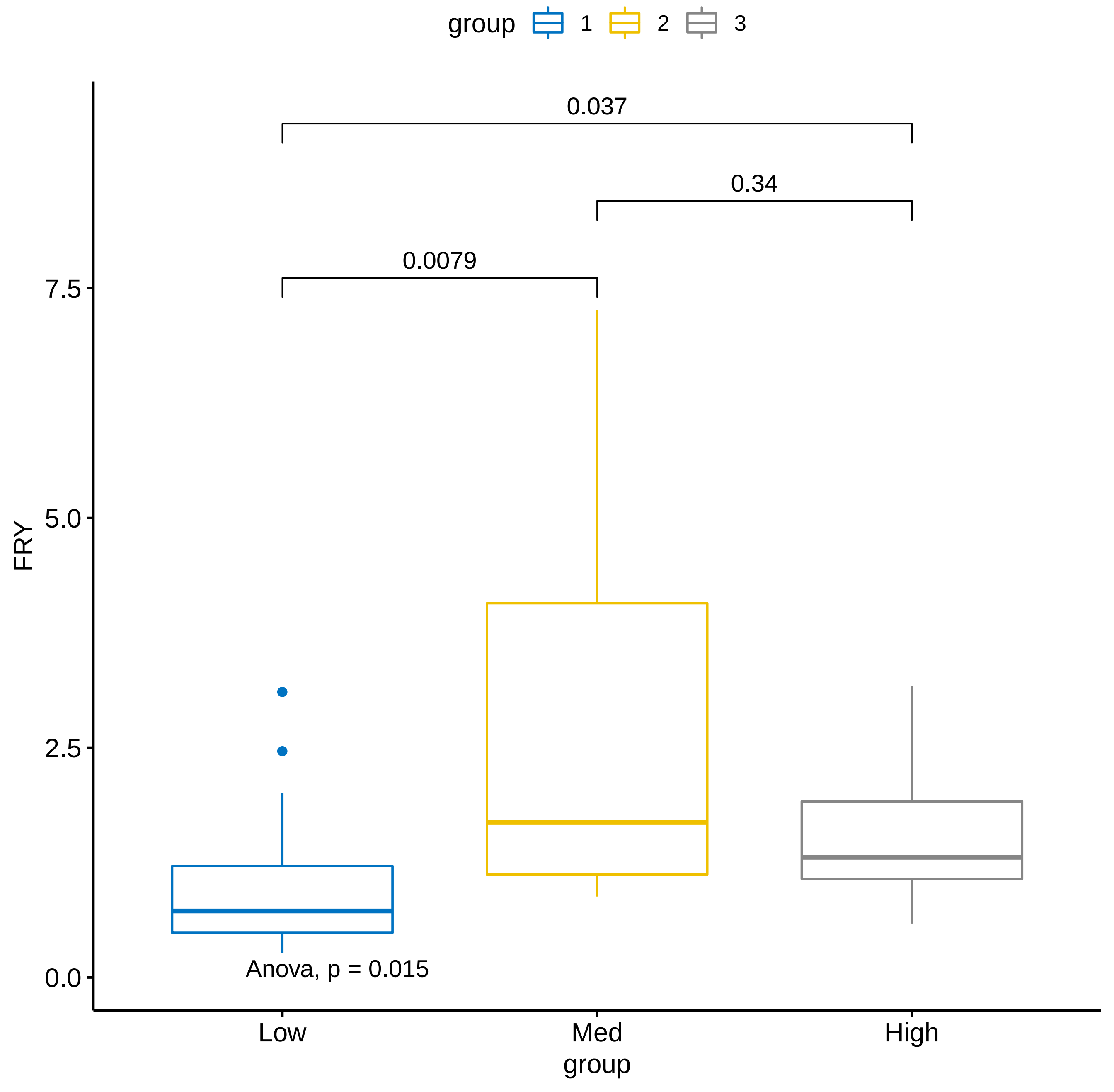

group 1 2 3
0.037
0.34
0.0079
FRY
7.5
5.0
2.5
0.0
Anova, p = 0.015
Low
Med
High
group

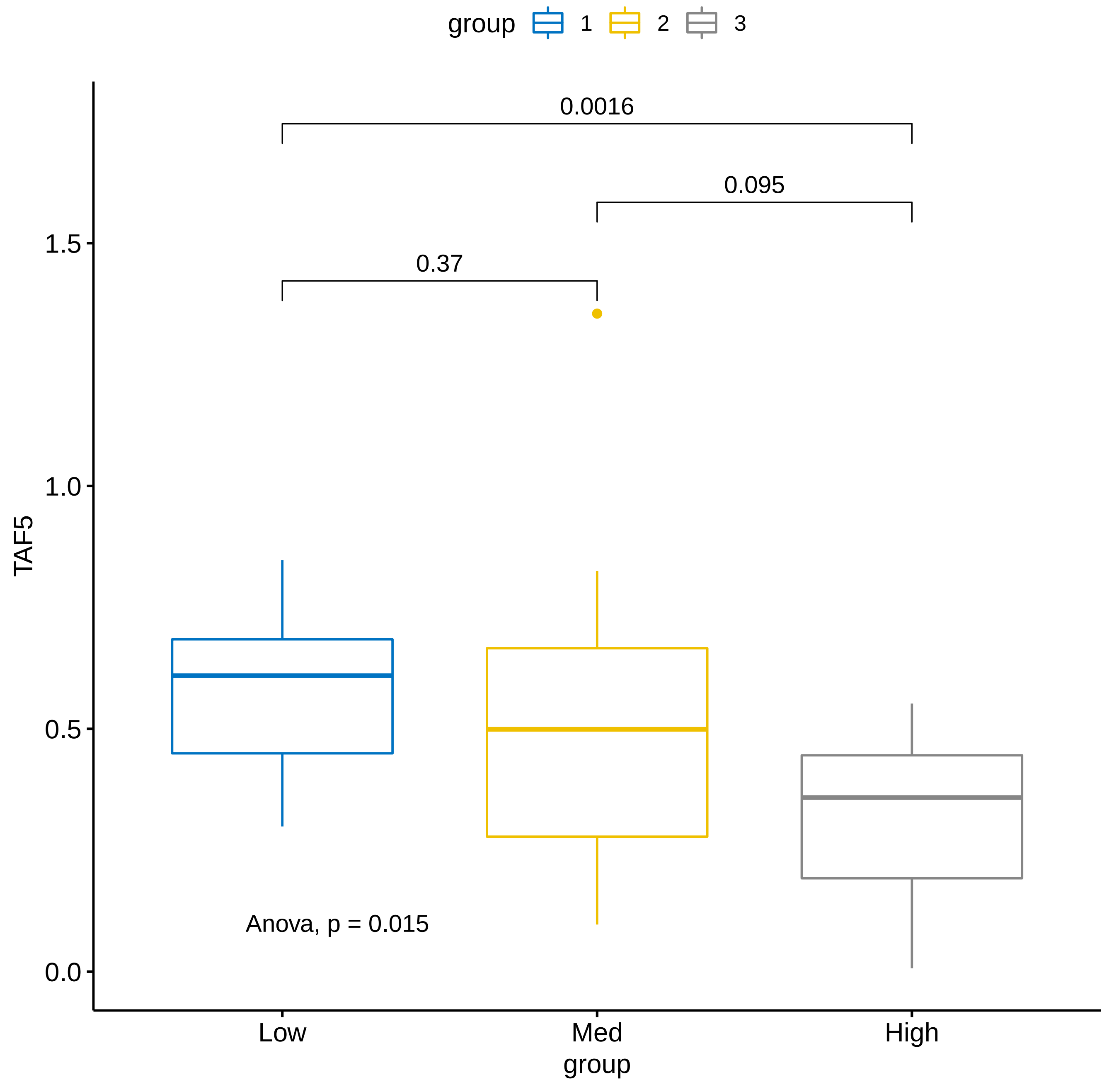
group 1 2 3
0.0016
0.095
0.37
1.5
1.0
0.5
0.0
TAF5
Anova, p = 0.015
Low
Med
High
group

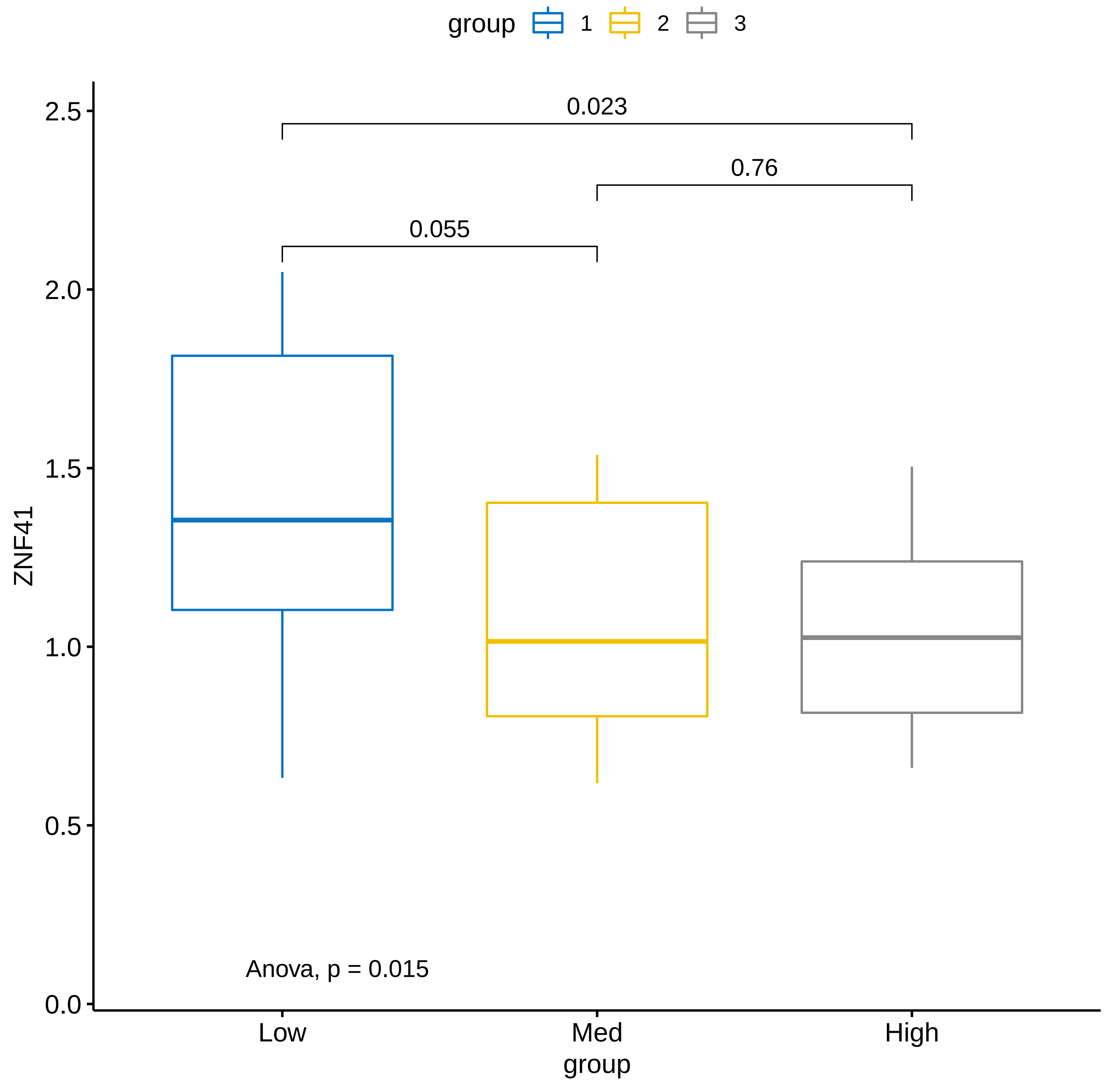
group
1
2
3
0.023
0.76
0.055
2.5
2.0
1.5
1.0
0.5
0.0
ZNF41
Anova, p = 0.015
Low
Med
High
group

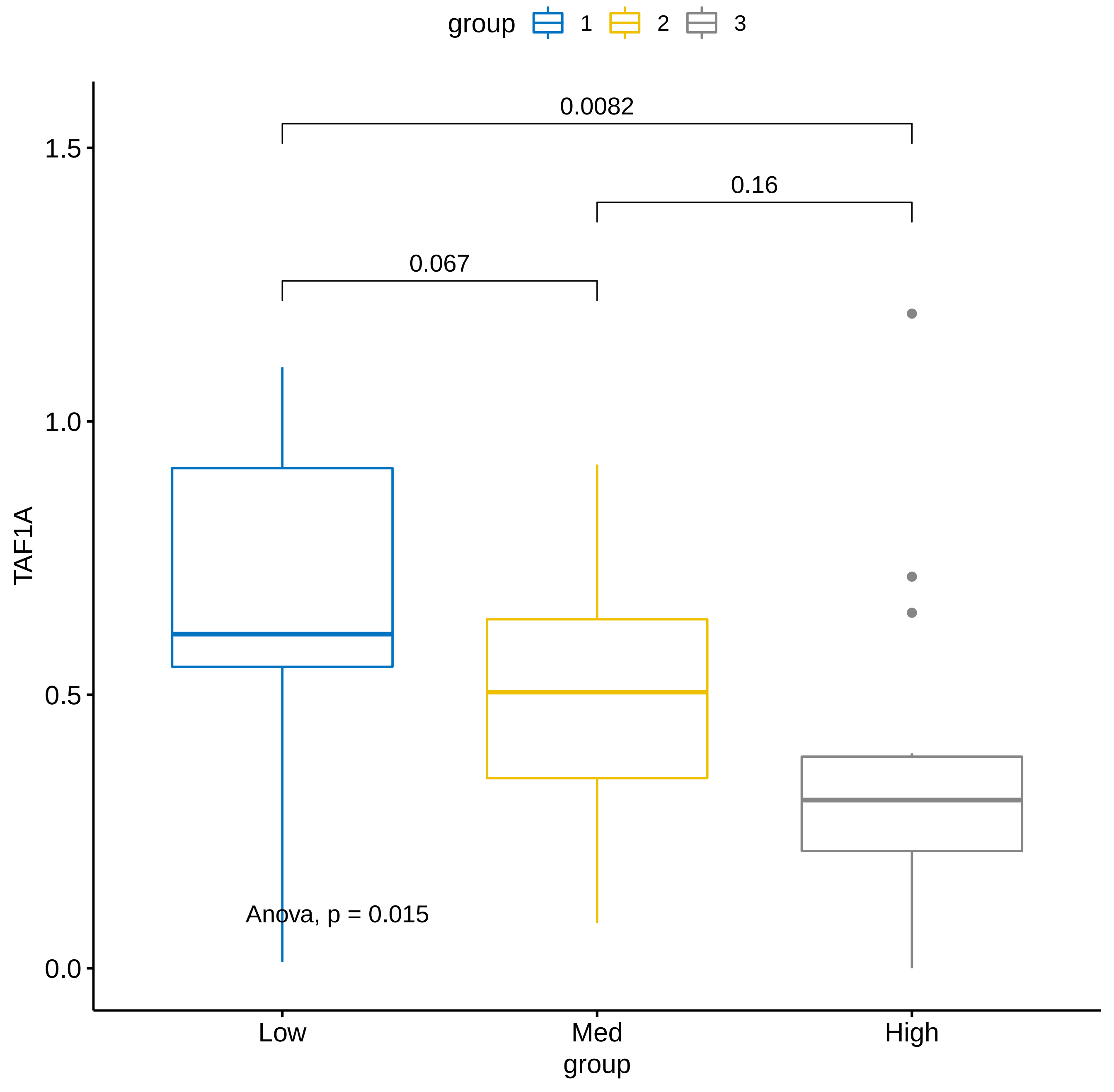

group
1
2
3
0.0082
0.16
0.067
1.5
1.0
0.5
0.0
TAF1A
Anova, p = 0.015
Low
Med
High
group

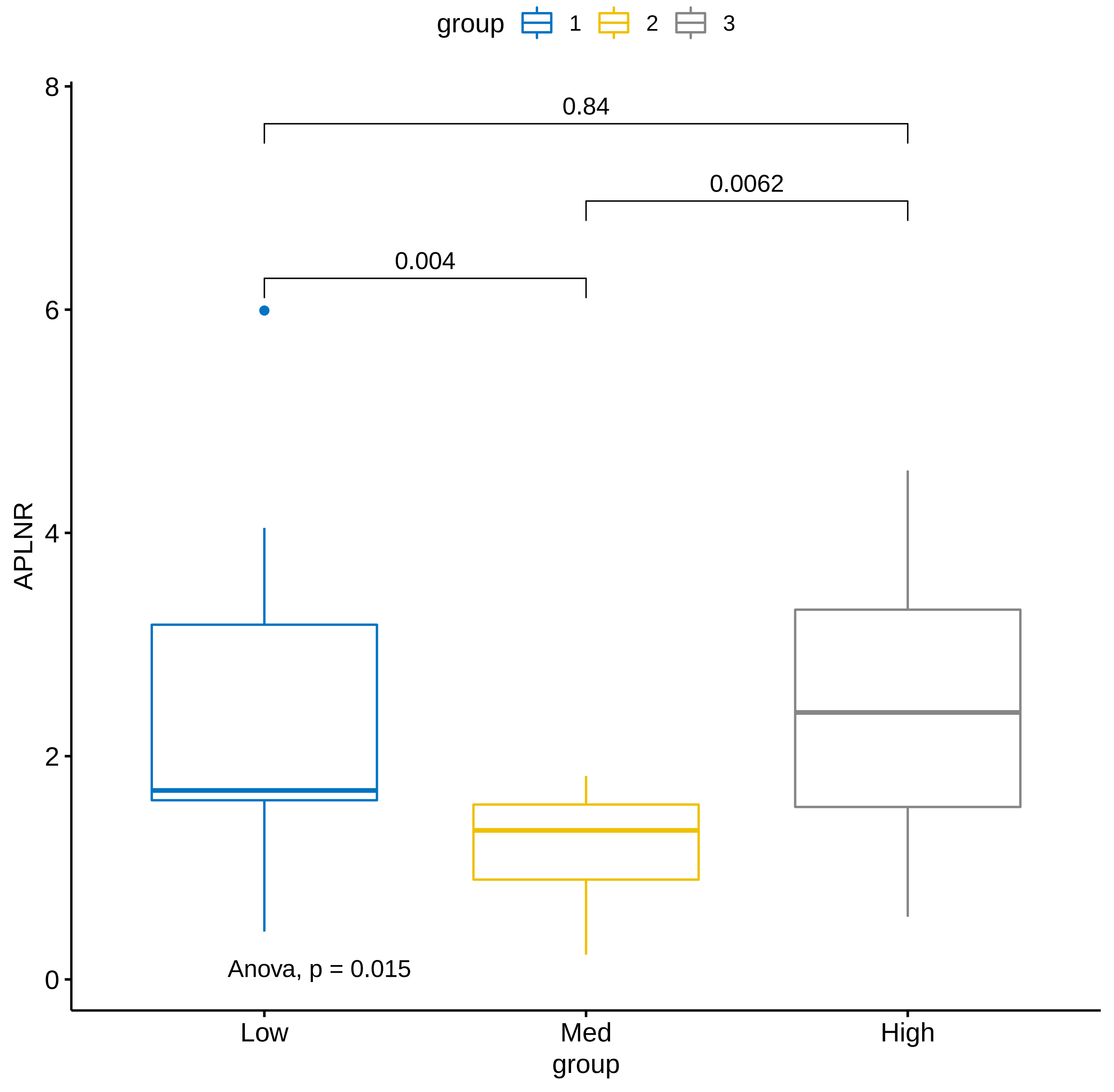
group 1 2 3
0.84
0.0062
0.004
Anova, p = 0.015
APLNR
8
6
4
2
0
Low
Med
High
group

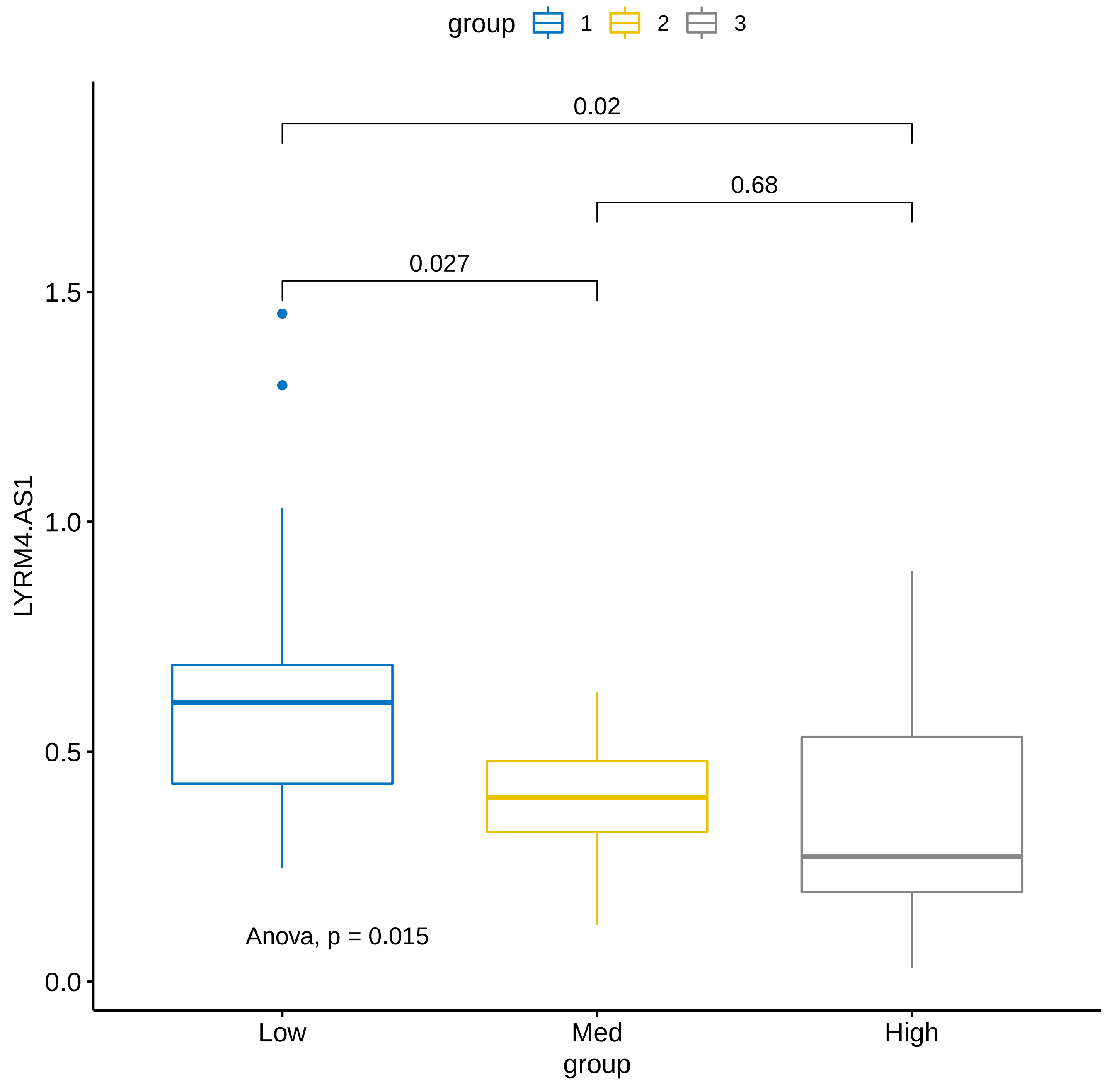
group
1
2
3
0.02
0.68
0.027
1.5
1.0
0.5
0.0
LYRM4.AS1
Anova, p = 0.015
Low
Med
High
group

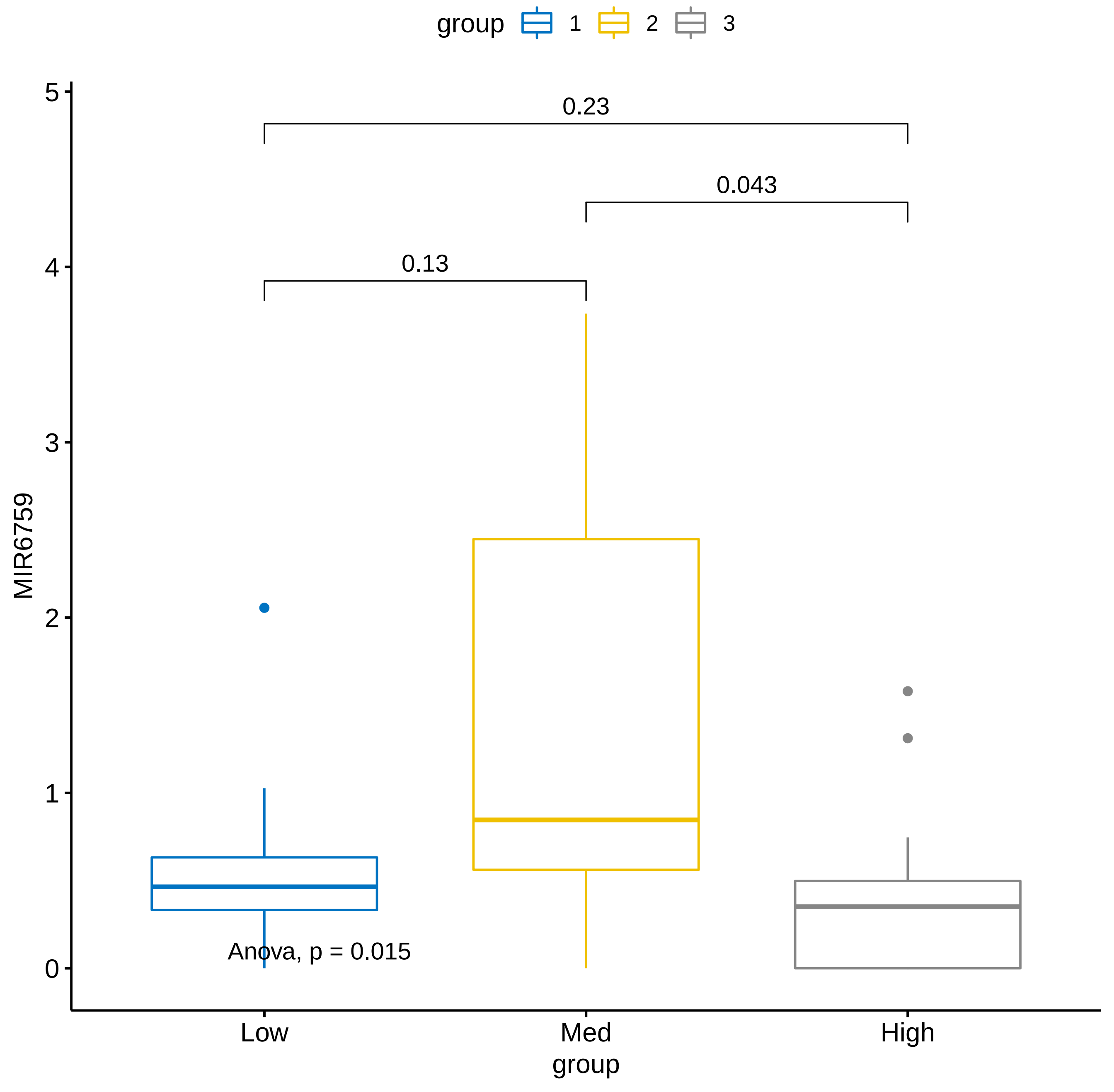

group 1 2 3
0.23
0.043
0.13
5
4
3
2
1
0
MIR6759
Anova, p = 0.015
Low
Med
High
group

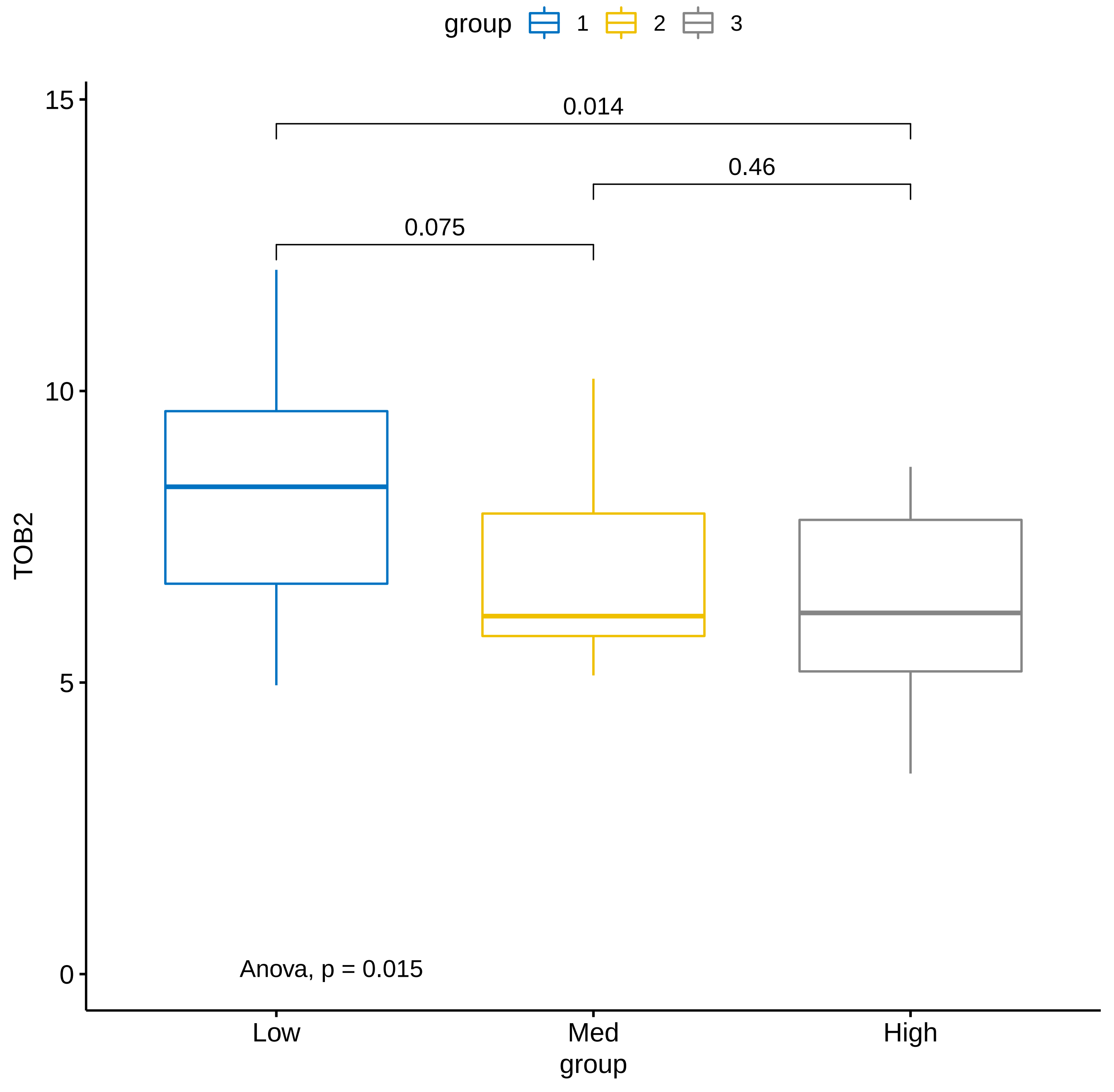
group 1 2 3
15
10
5
0
TOB2
0.014
0.46
0.075
Anova, p = 0.015
Low
Med
High
group

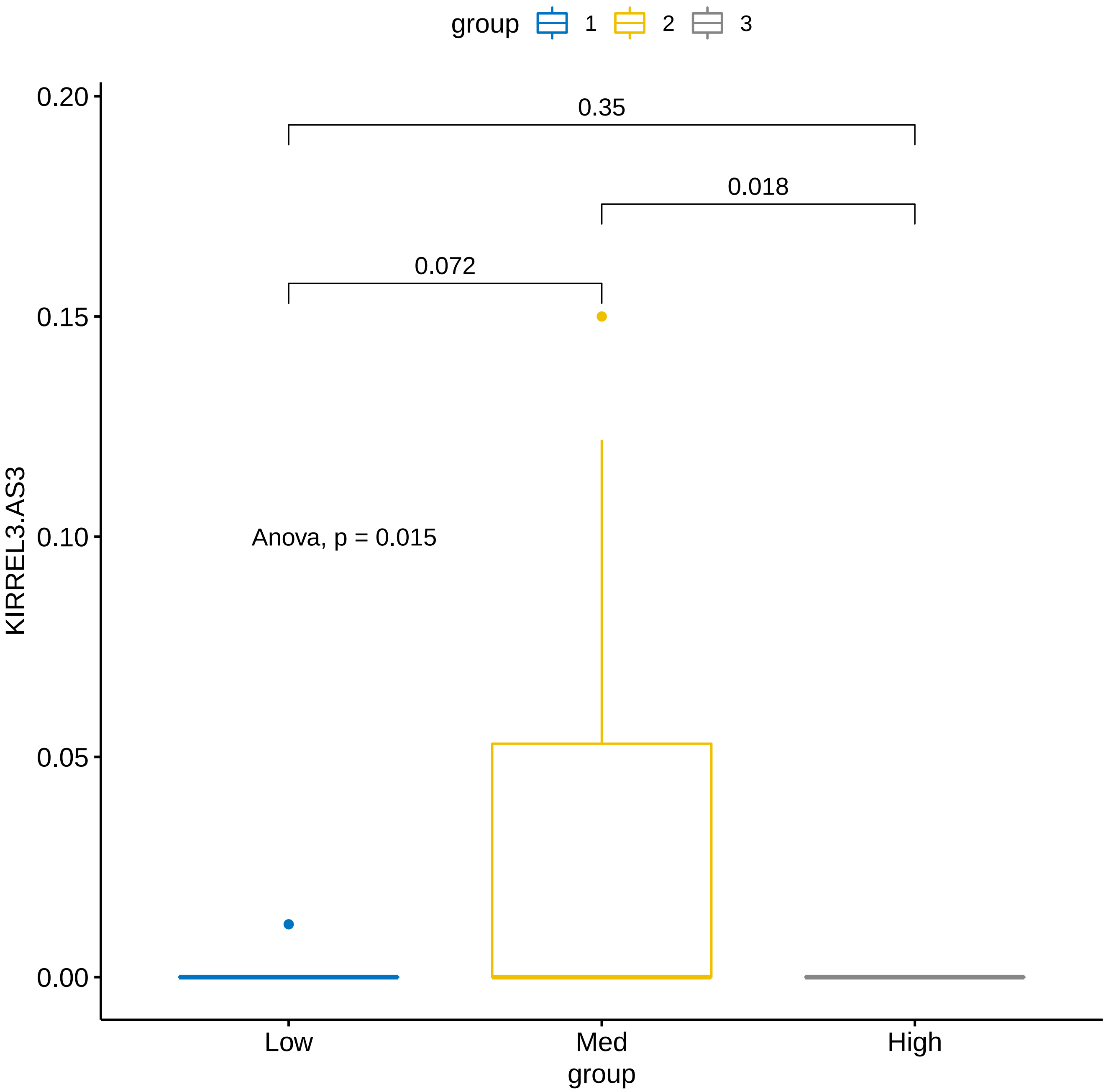
group
1
2
3
0.35
0.018
0.072
Anova, p = 0.015
KIRREL3.AS3
0.20
0.15
0.10
0.05
0.00
Low
Med
High
group

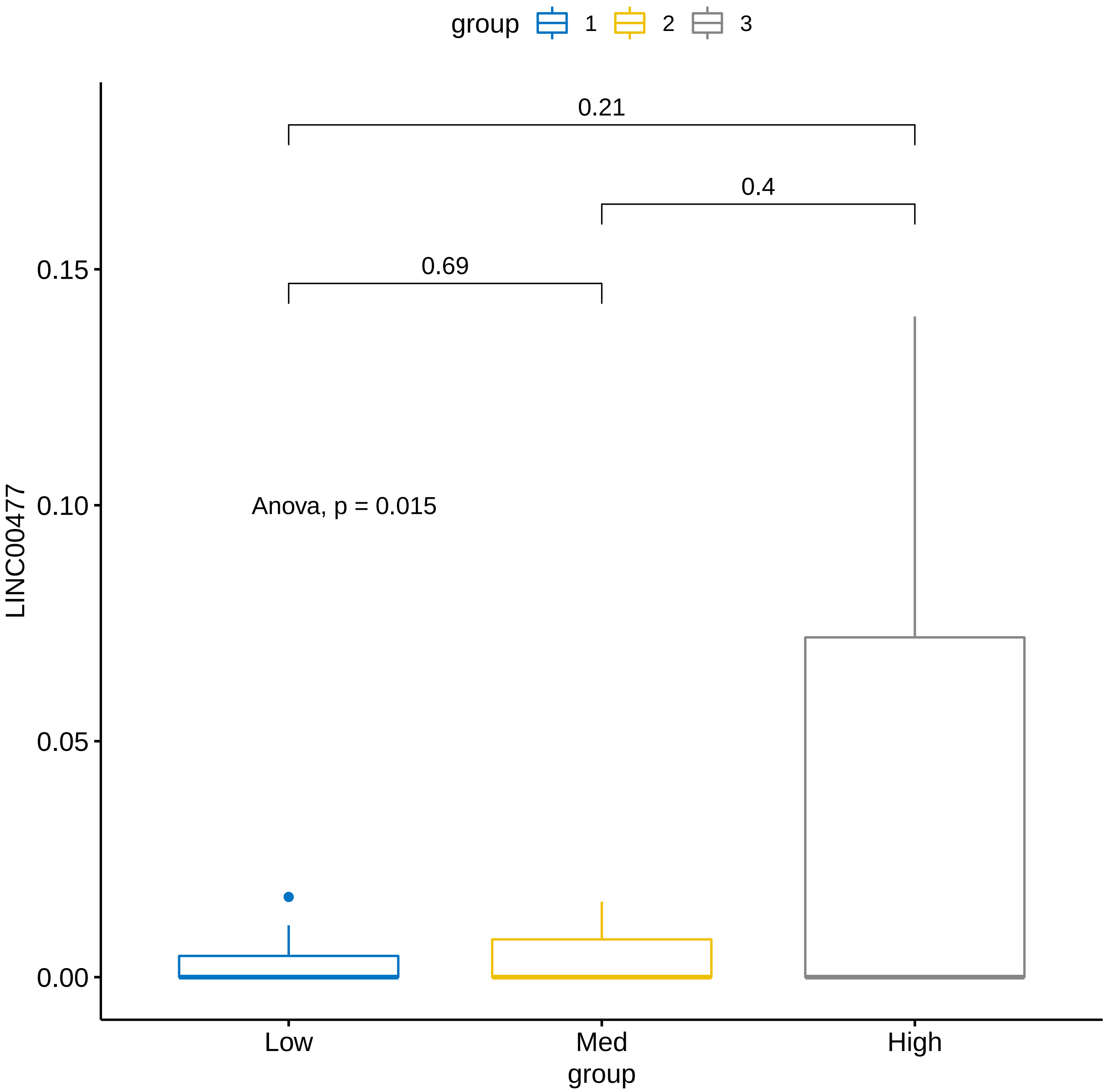

group
1
2
3
0.21
0.4
0.69
Anova, p = 0.015
LINC00477
0.15
0.10
0.05
0.00
Low
Med
High
group

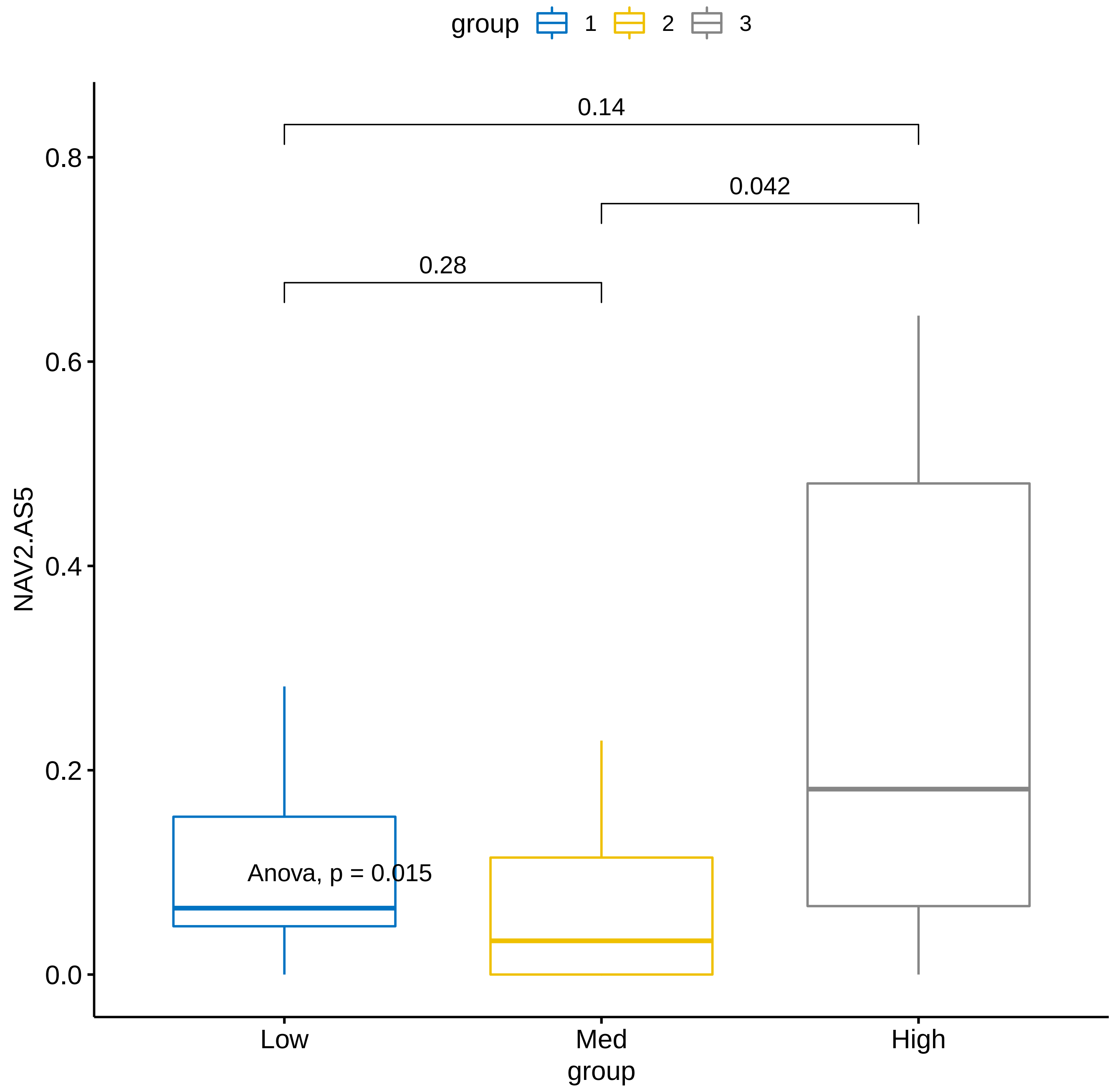
group
1
2
3
0.14
0.042
0.28
0.8
0.6
0.4
0.2
0.0
NAV2.AS5
Anova, p = 0.015
Low
Med
High
group

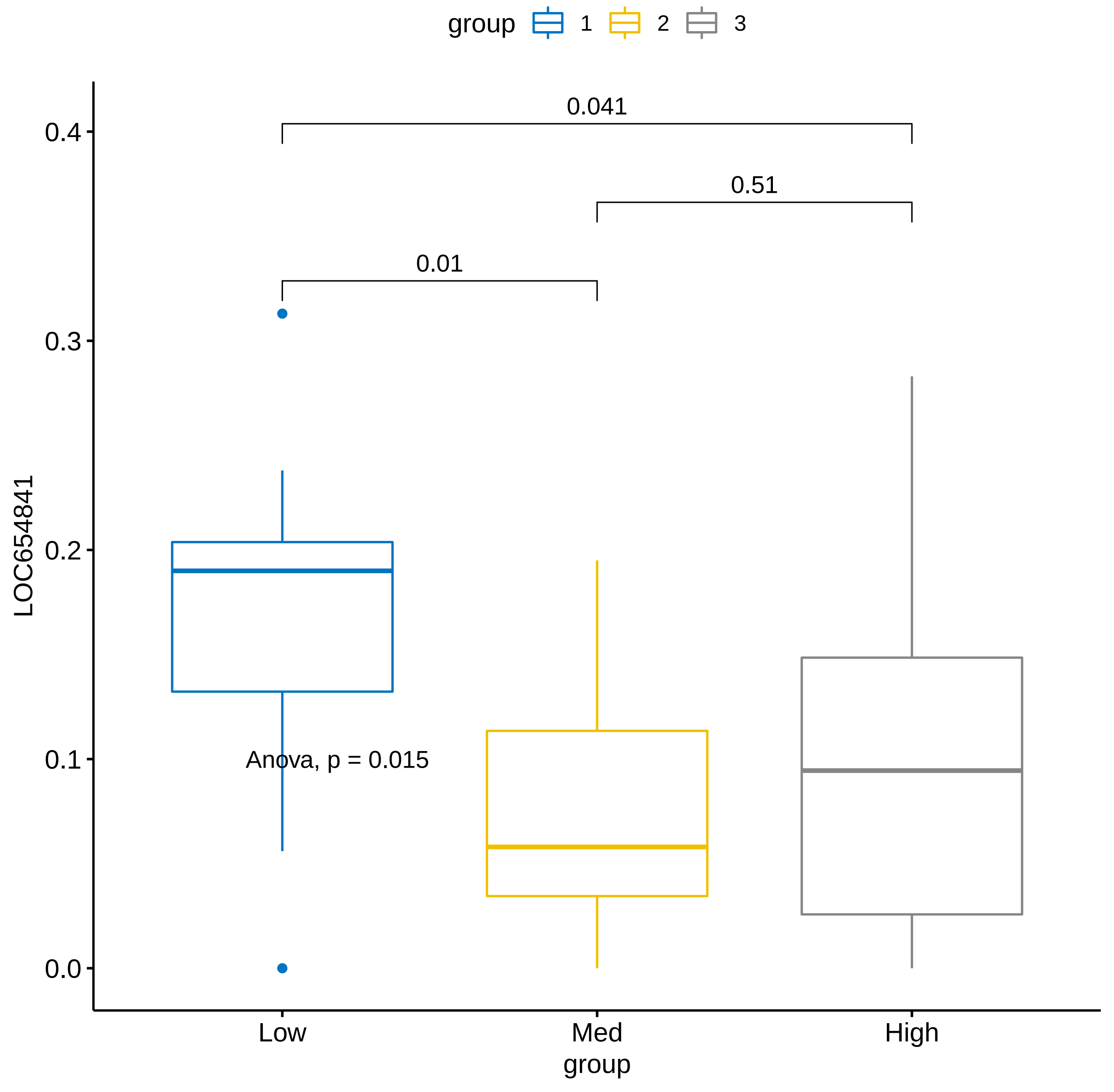
group
1
2
3
0.041
0.51
0.01
0.4
0.3
0.2
0.1
0.0
LOC654841
Anova, p = 0.015
Low
Med
High
group

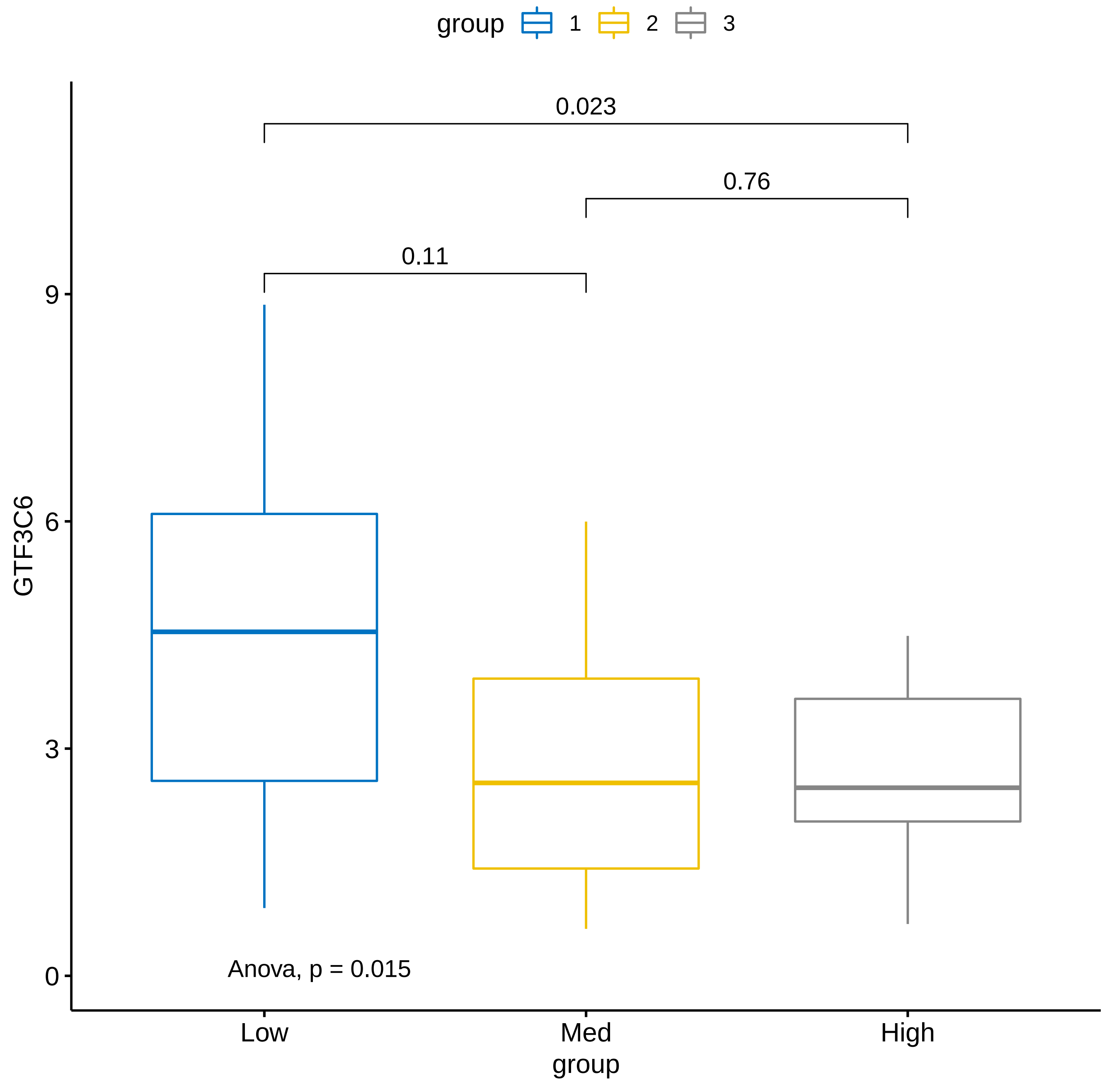
group 1 2 3
0.023
0.76
0.11
9
6
3
0
GTF3C6
Anova, p = 0.015
Low
Med
High
group

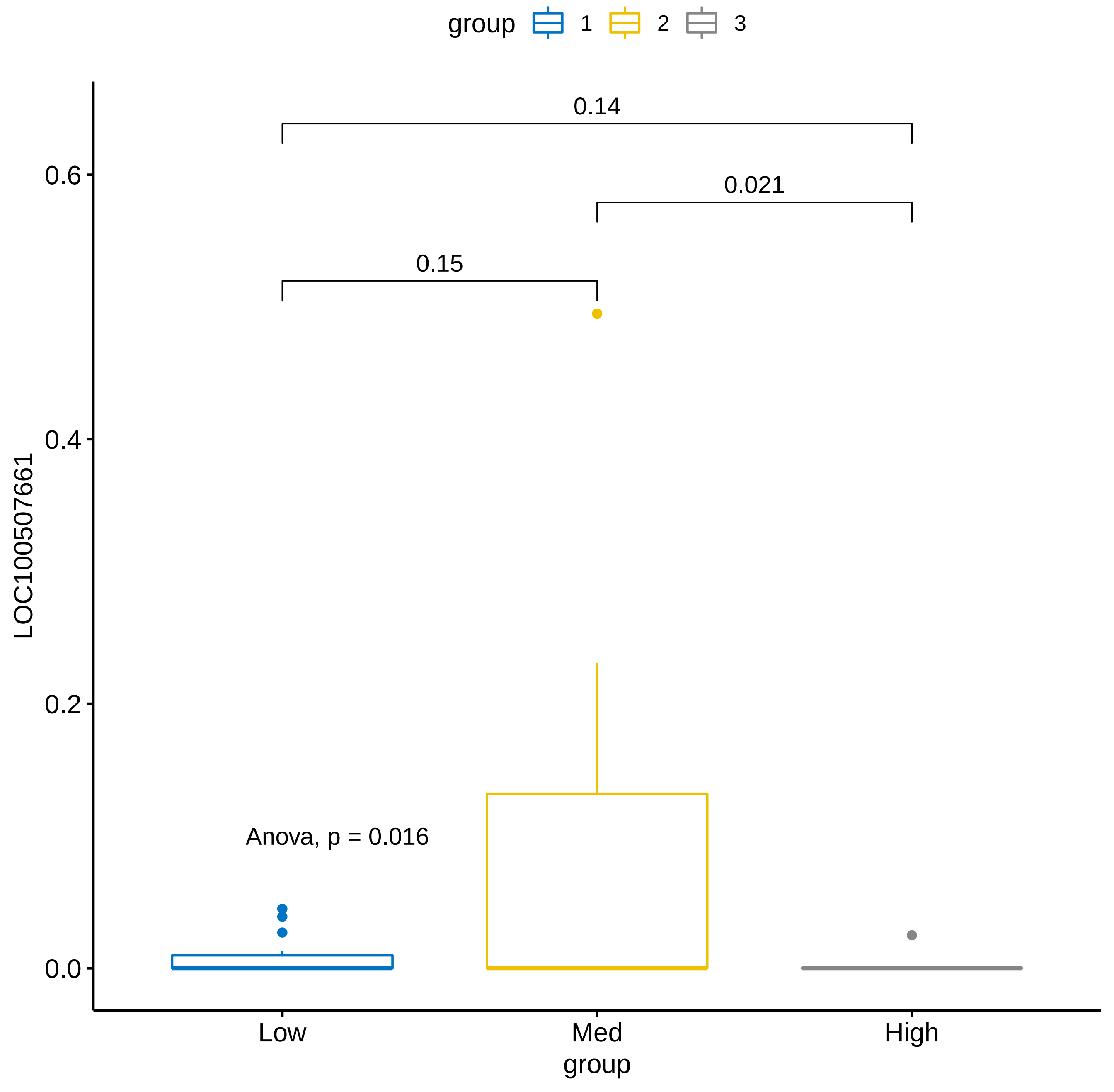

group 1 2 3
0.14
0.021
0.15
Anova, p = 0.016
0.6
0.4
0.2
0.0
LOC100507661
Low
Med
High
group

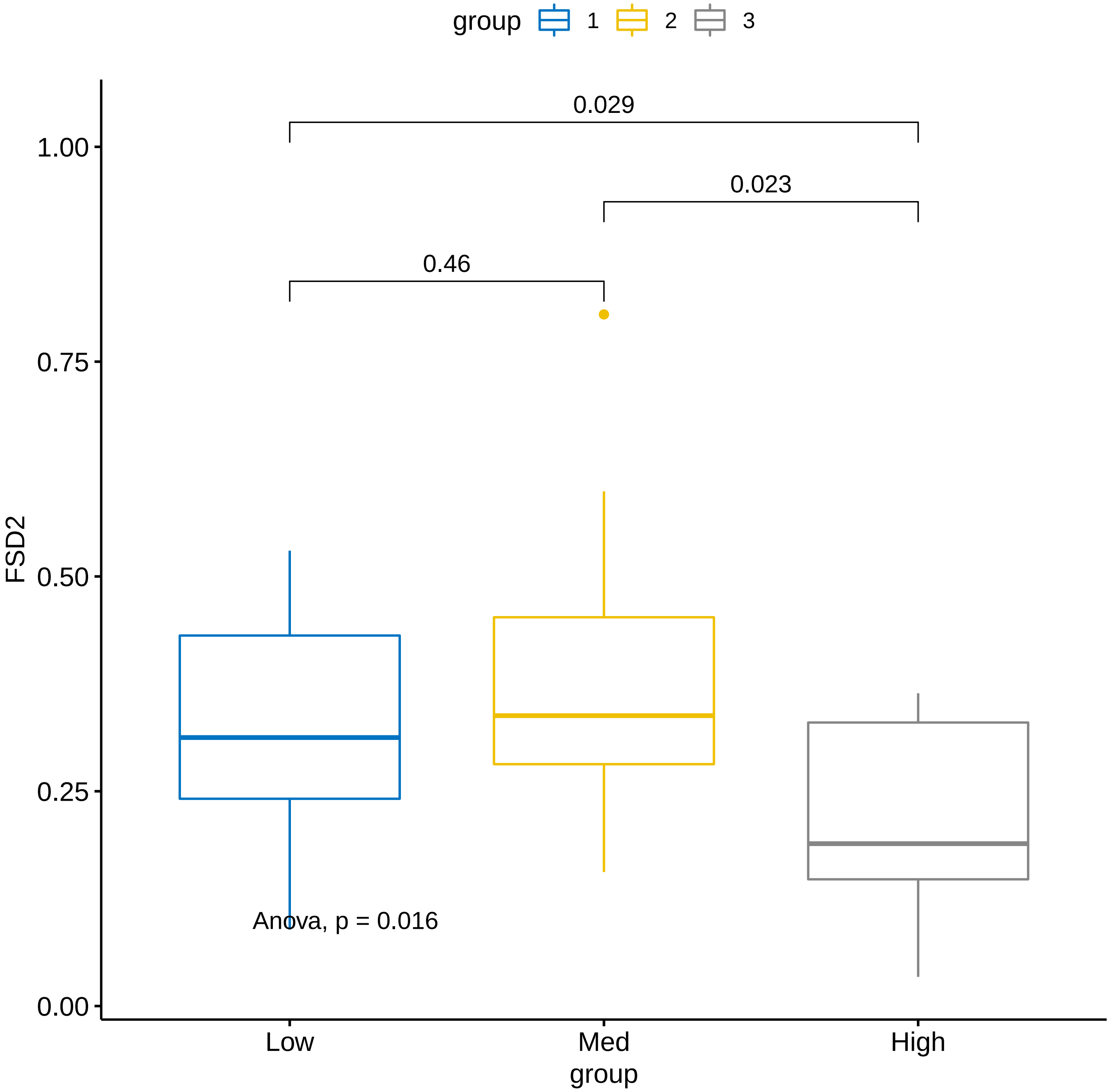
group
1
2
3
0.029
0.023
0.46
1.00
0.75
0.50
0.25
0.00
FSD2
Anova, p = 0.016
Low
Med
High
group

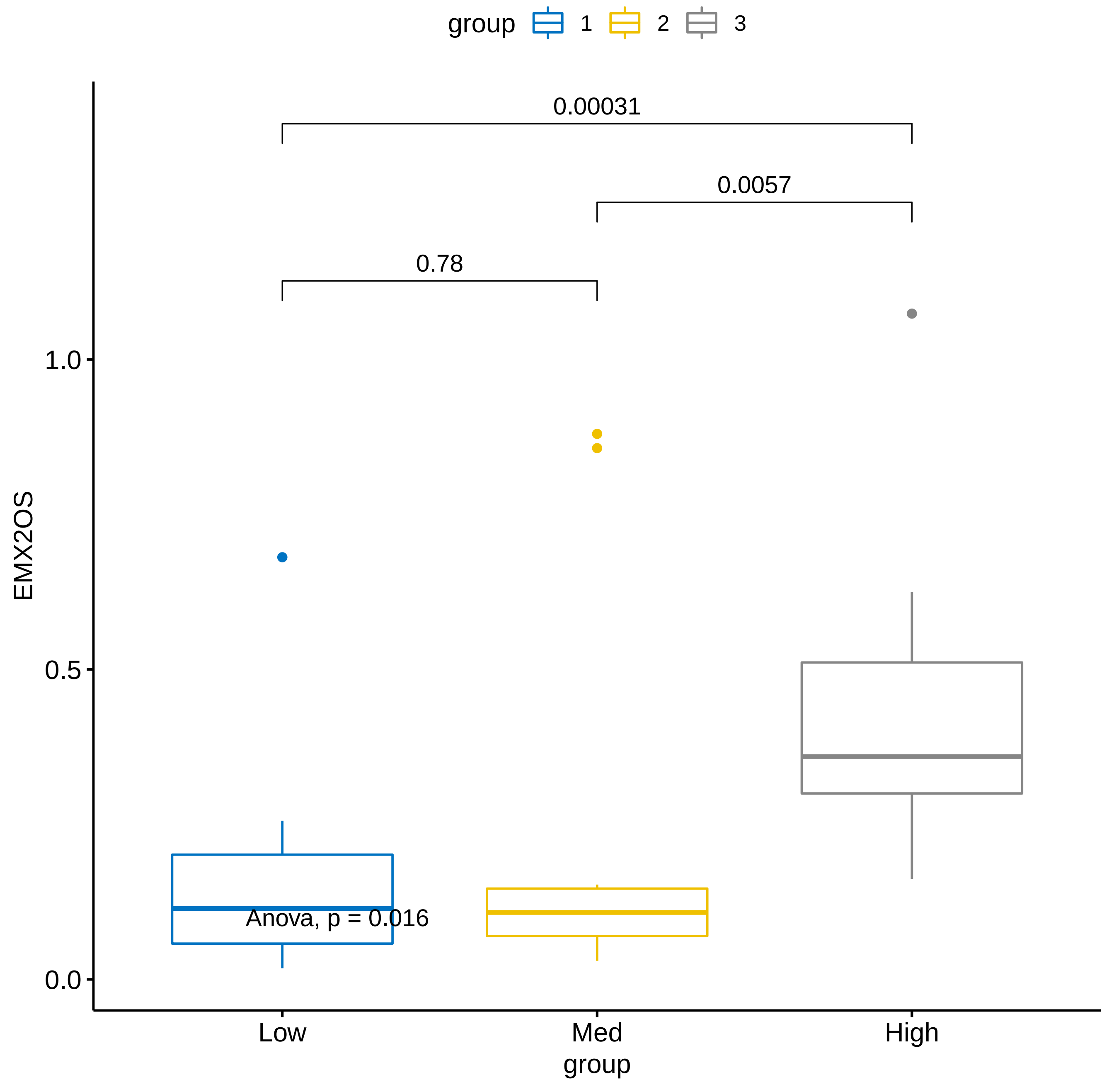
group
1
2
3
0.00031
0.0057
0.78
1.0
0.5
0.0
EMX2OS
Anova, p = 0.016
Low
Med
High
group

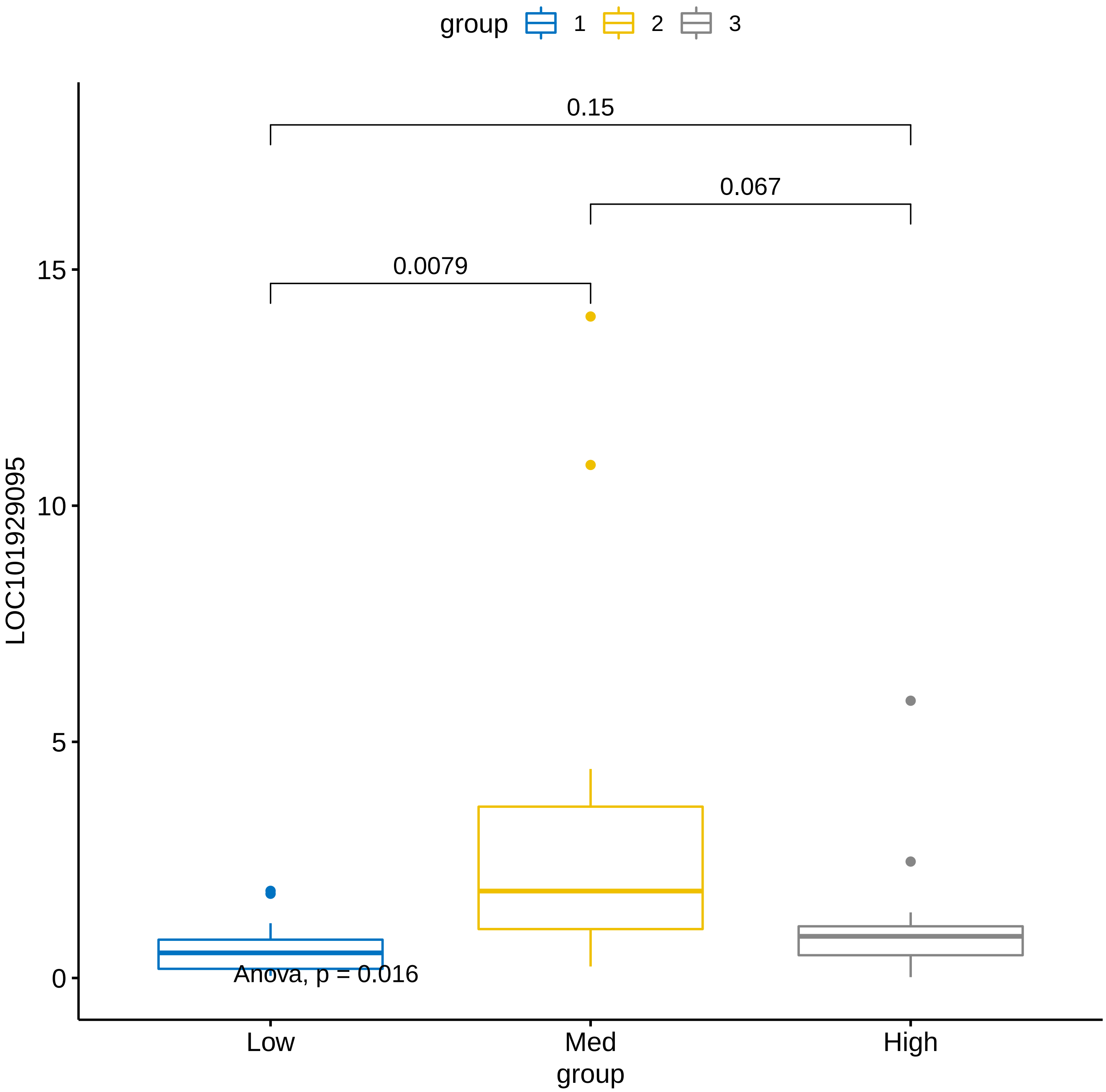
group 1 2 3
0.15
0.067
0.0079
15
10
5
0
LOC101929095
Anova, p = 0.016
Low
Med
High
group

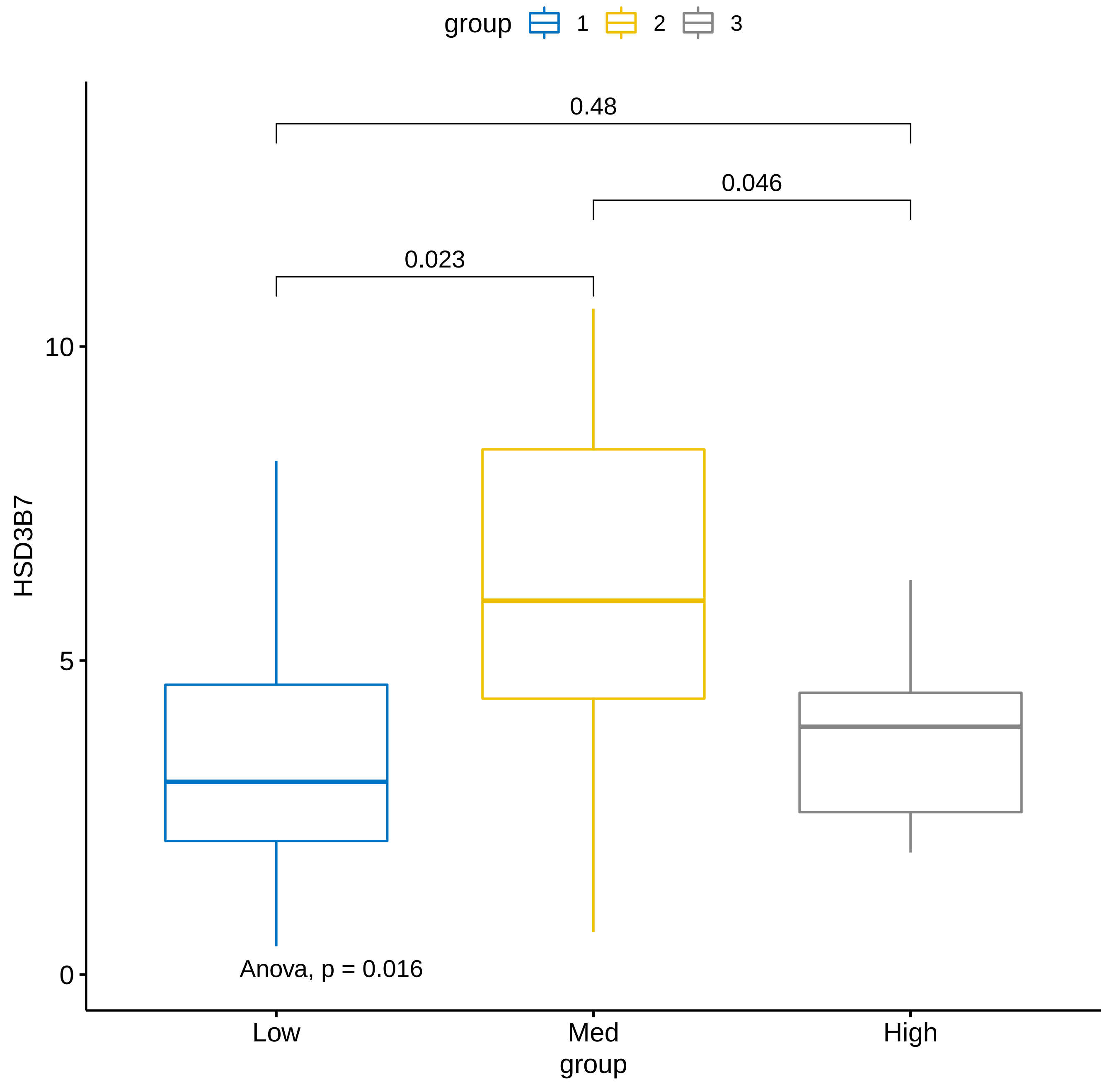
group 1 2 3
0.48
0.046
0.023
10
5
0
HSD3B7
Anova, p = 0.016
Low
Med
High
group

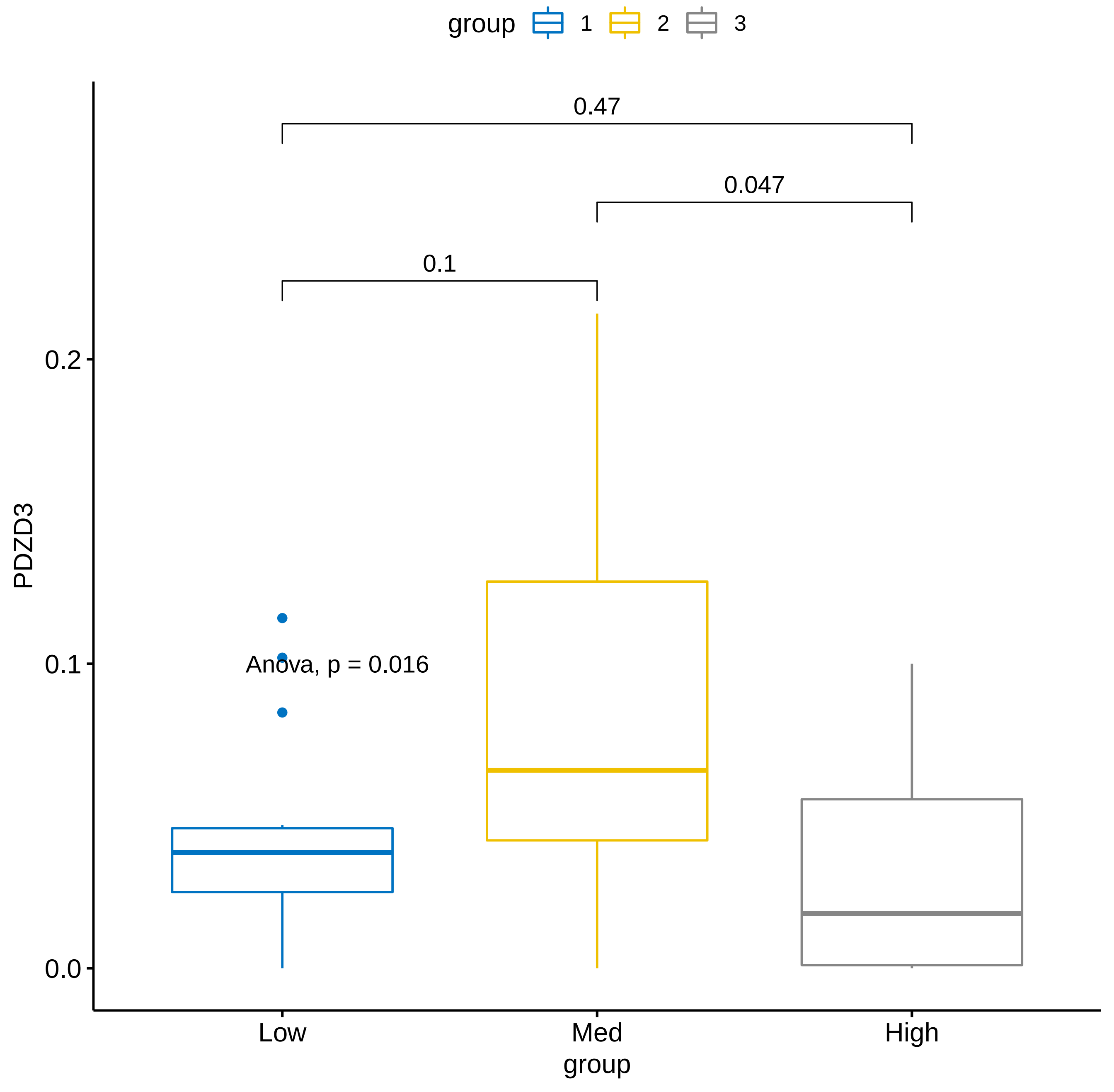

group 1 2 3
0.47
0.047
0.1
Anova, p = 0.016
PDZD3
0.2
0.1
0.0
Low
Med
High
group

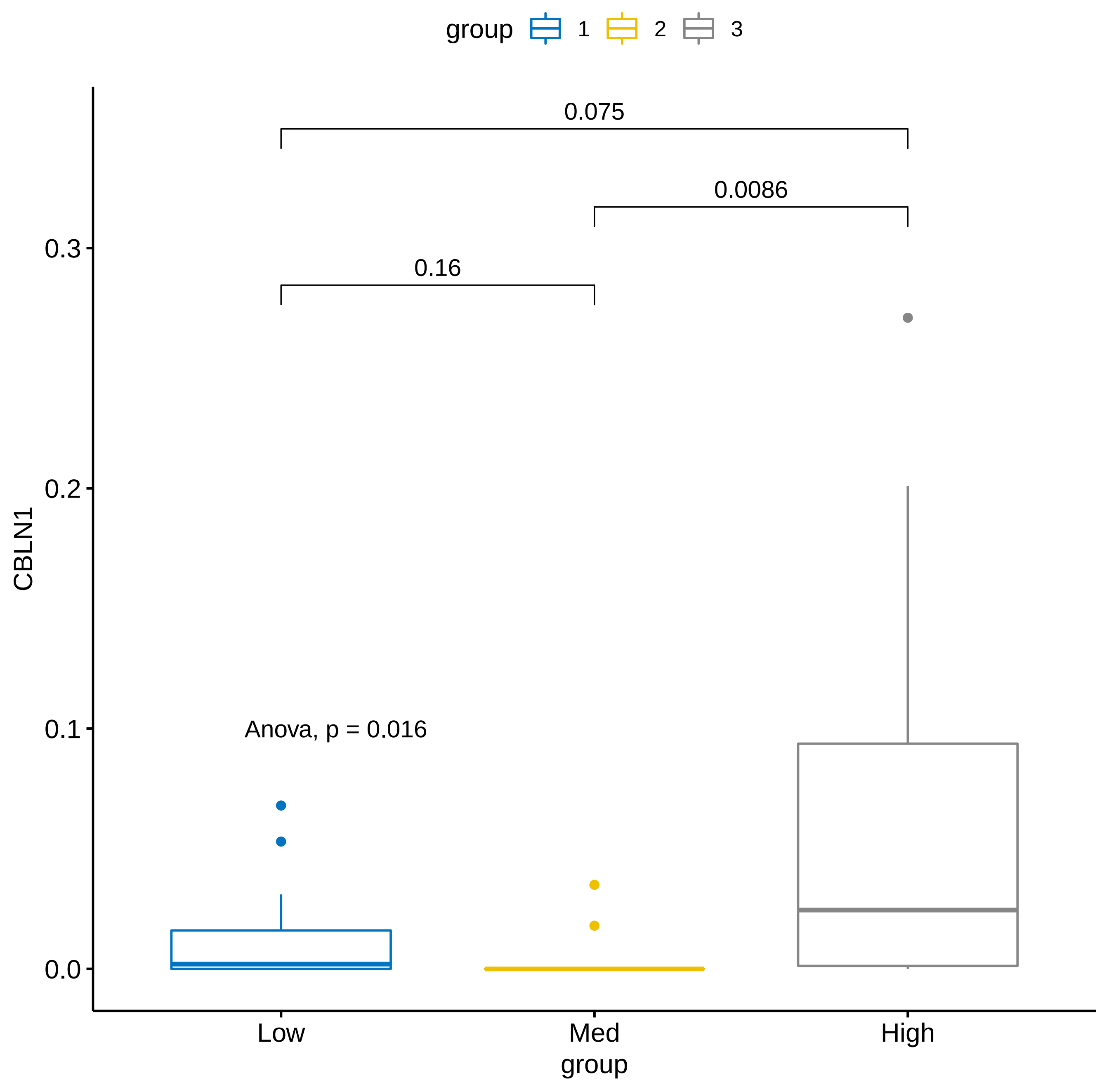
group
1
2
3
0.075
0.0086
0.16
Anova, p = 0.016
0.3
0.2
0.1
0.0
CBLN1
Low
Med
High
group

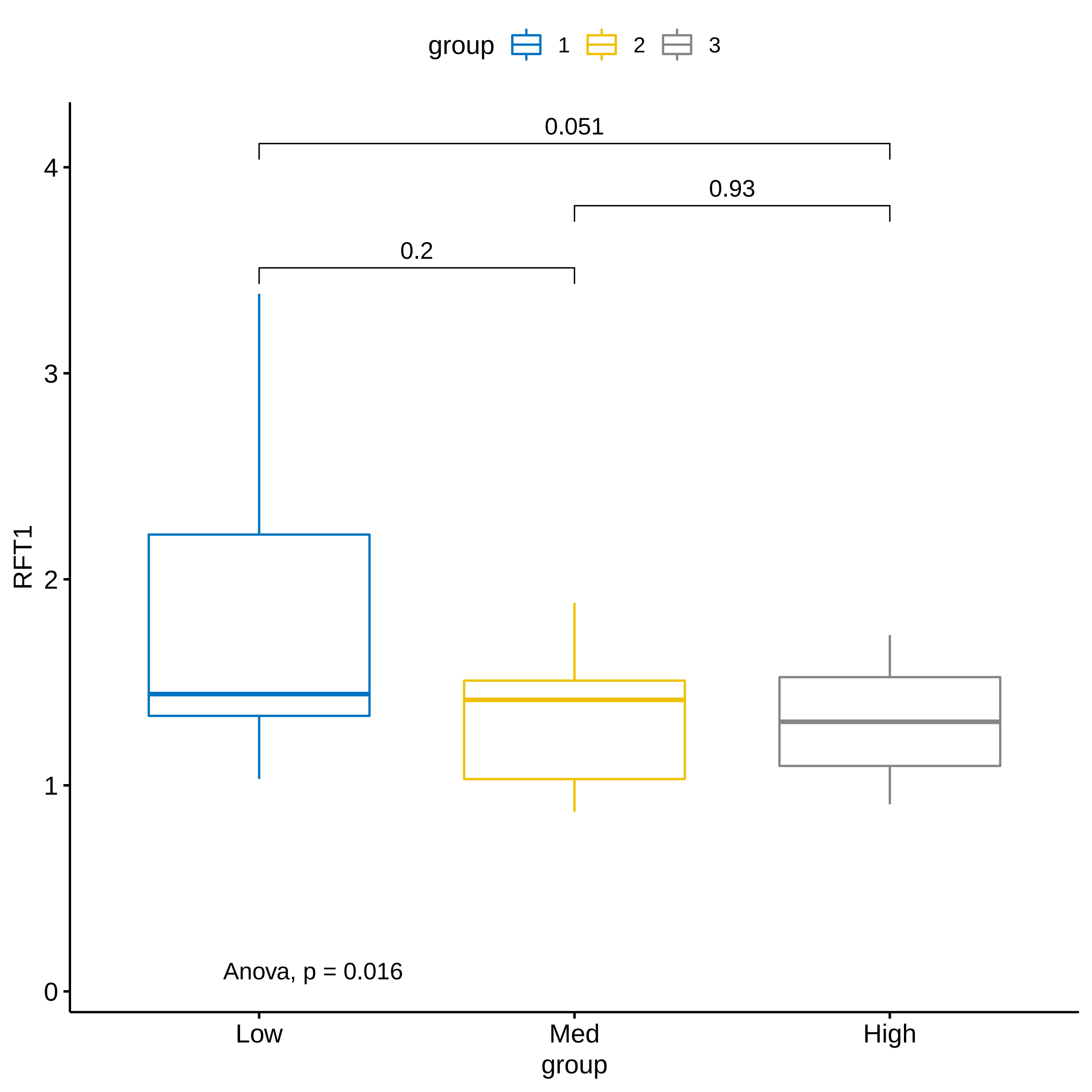

group
1
2
3
0.051
0.93
0.2
RFT1
4
3
2
1
0
Anova, p = 0.016
Low
Med
High
group

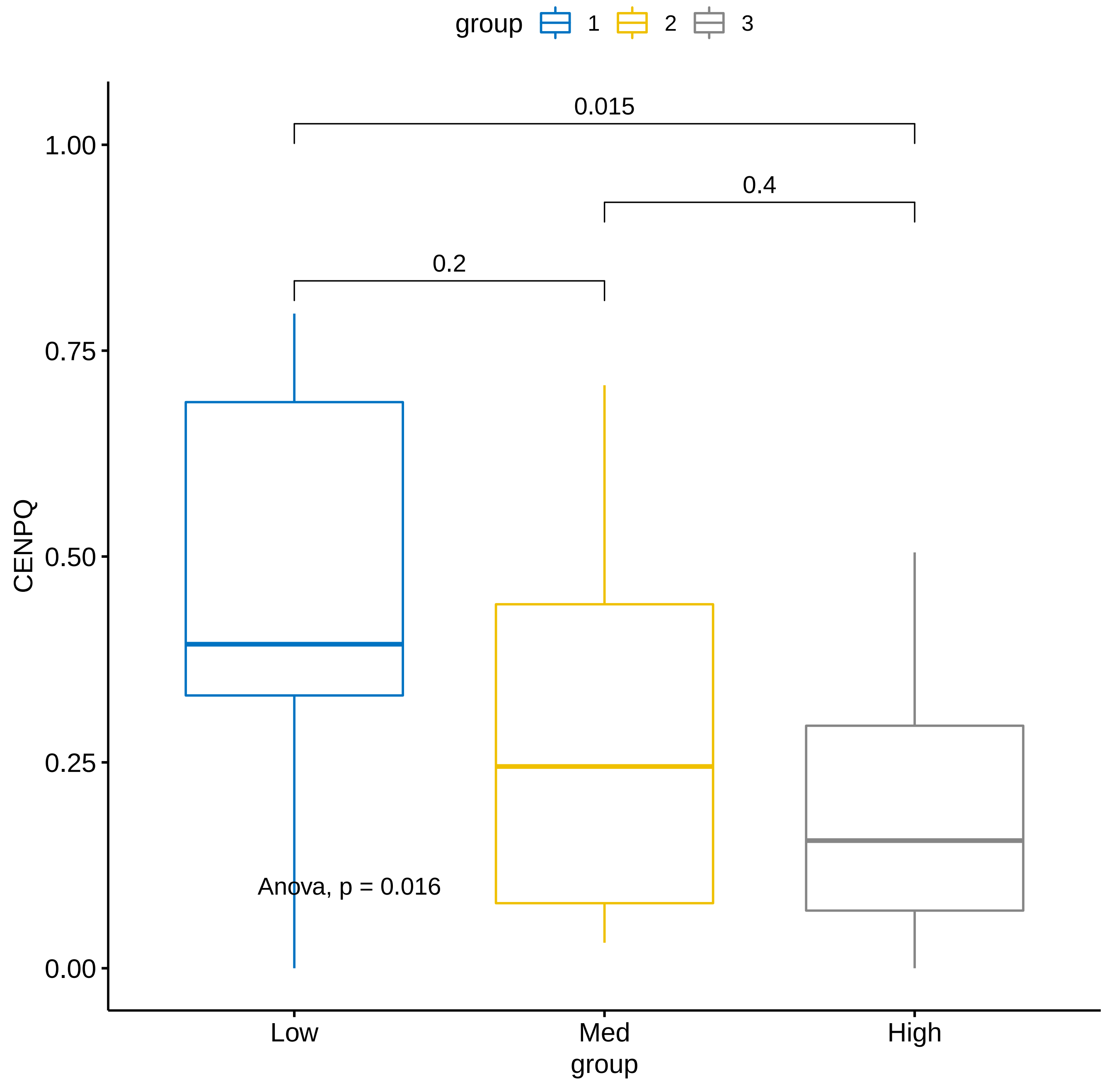

group 1 2 3
0.015
0.4
0.2
1.00
0.75
0.50
0.25
0.00
CENPQ
Anova, p = 0.016
Low
Med
High
group

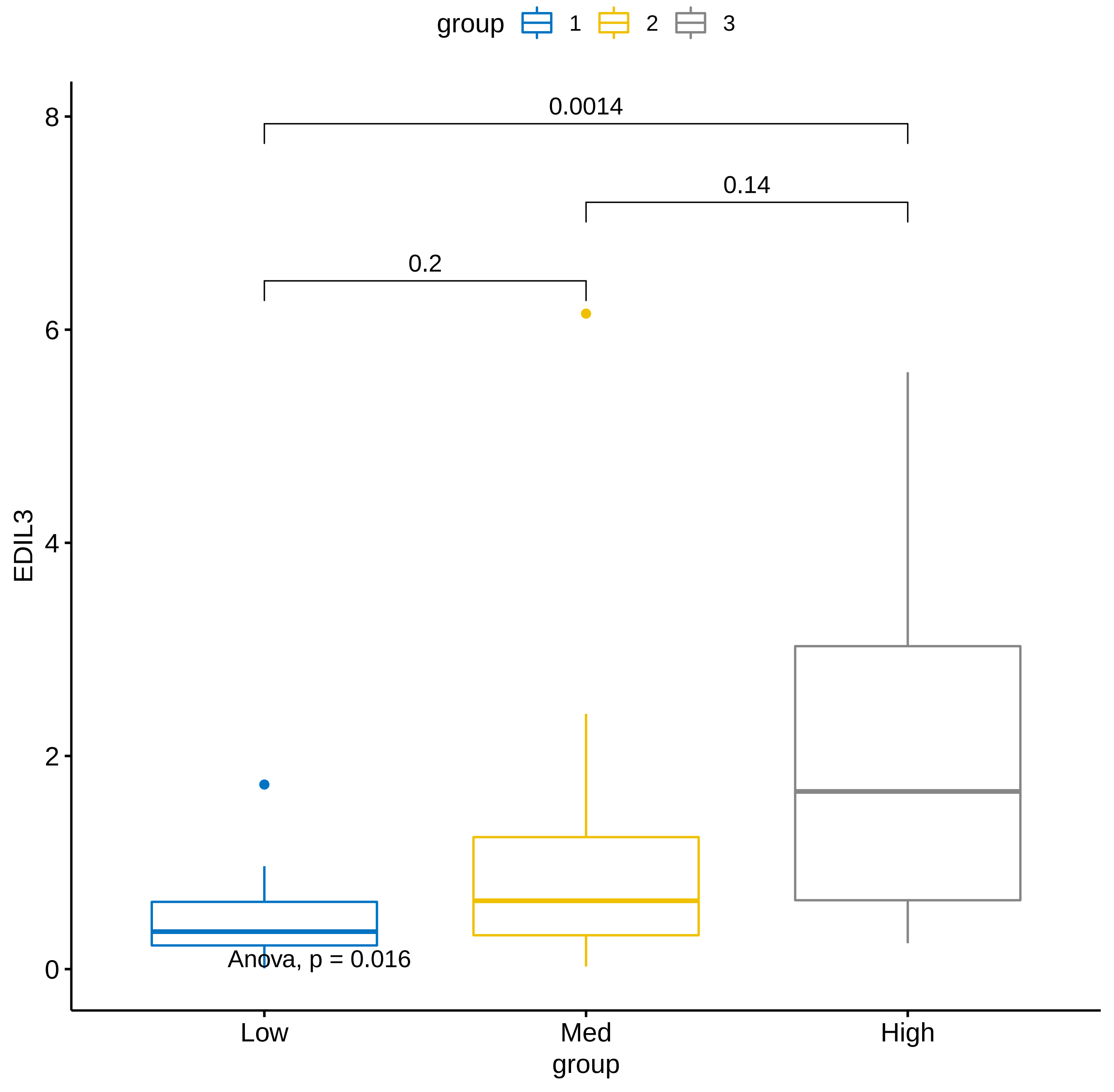
group
1
2
3
0.0014
0.14
0.2
8
6
4
2
0
EDIL3
Anova, p = 0.016
Low
Med
High
group

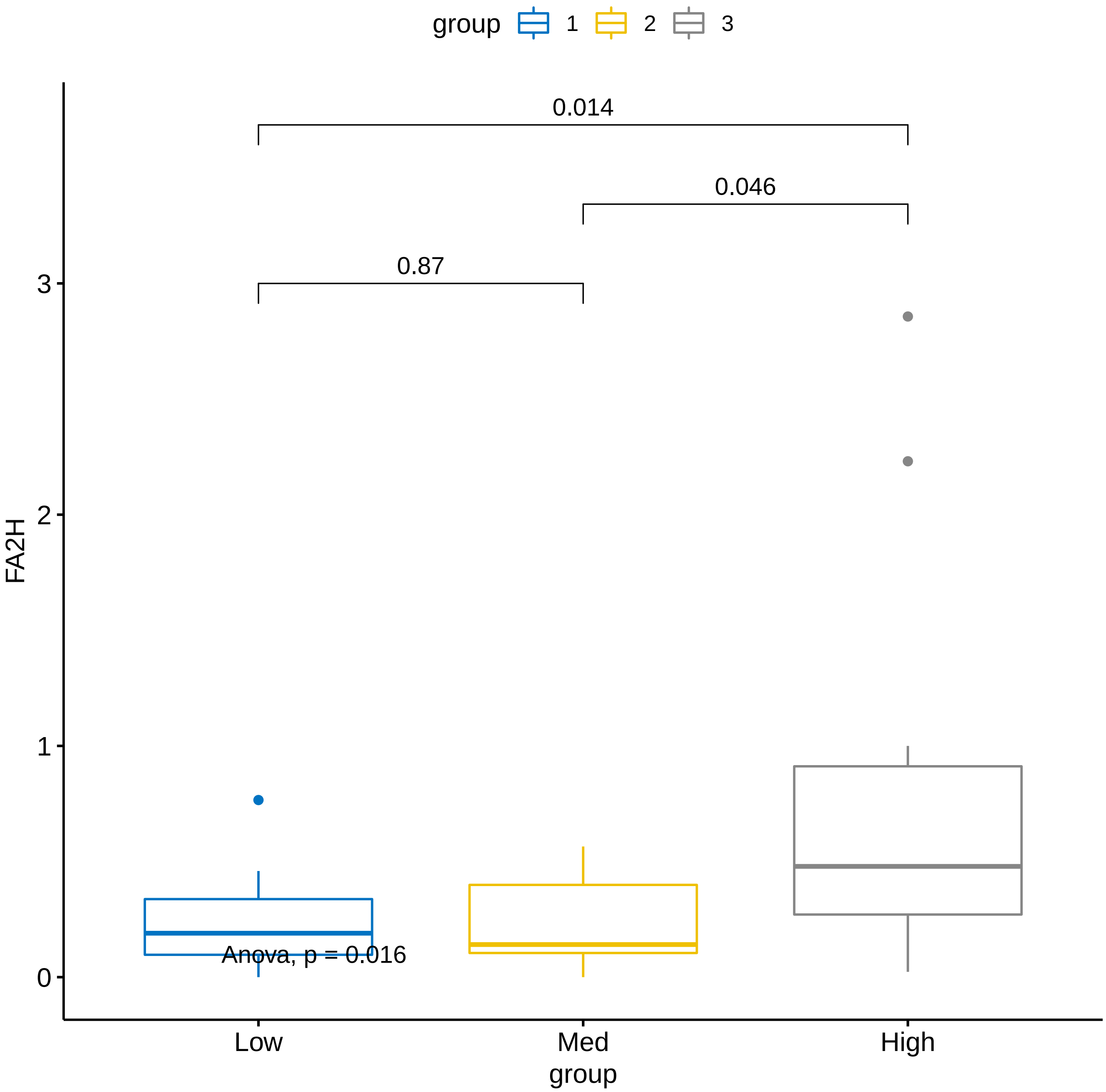

group
1
2
3
0.014
0.046
0.87
FA2H
3
2
1
0
Anova, p = 0.016
Low
Med
High
group

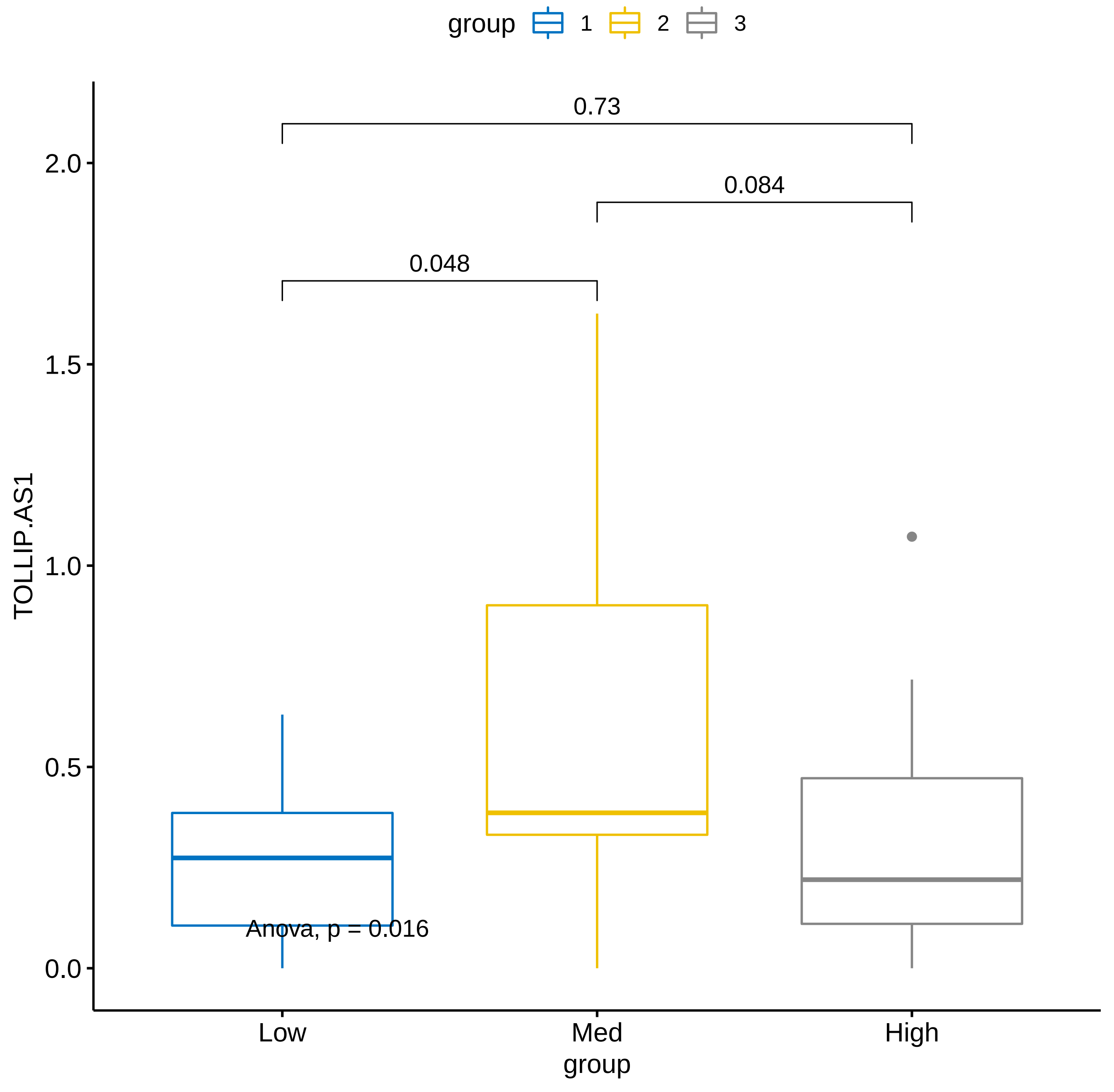

group
1
2
3
0.73
0.084
0.048
2.0
1.5
1.0
0.5
0.0
TOLLIP.AS1
Anova, p = 0.016
Low
Med
High
group

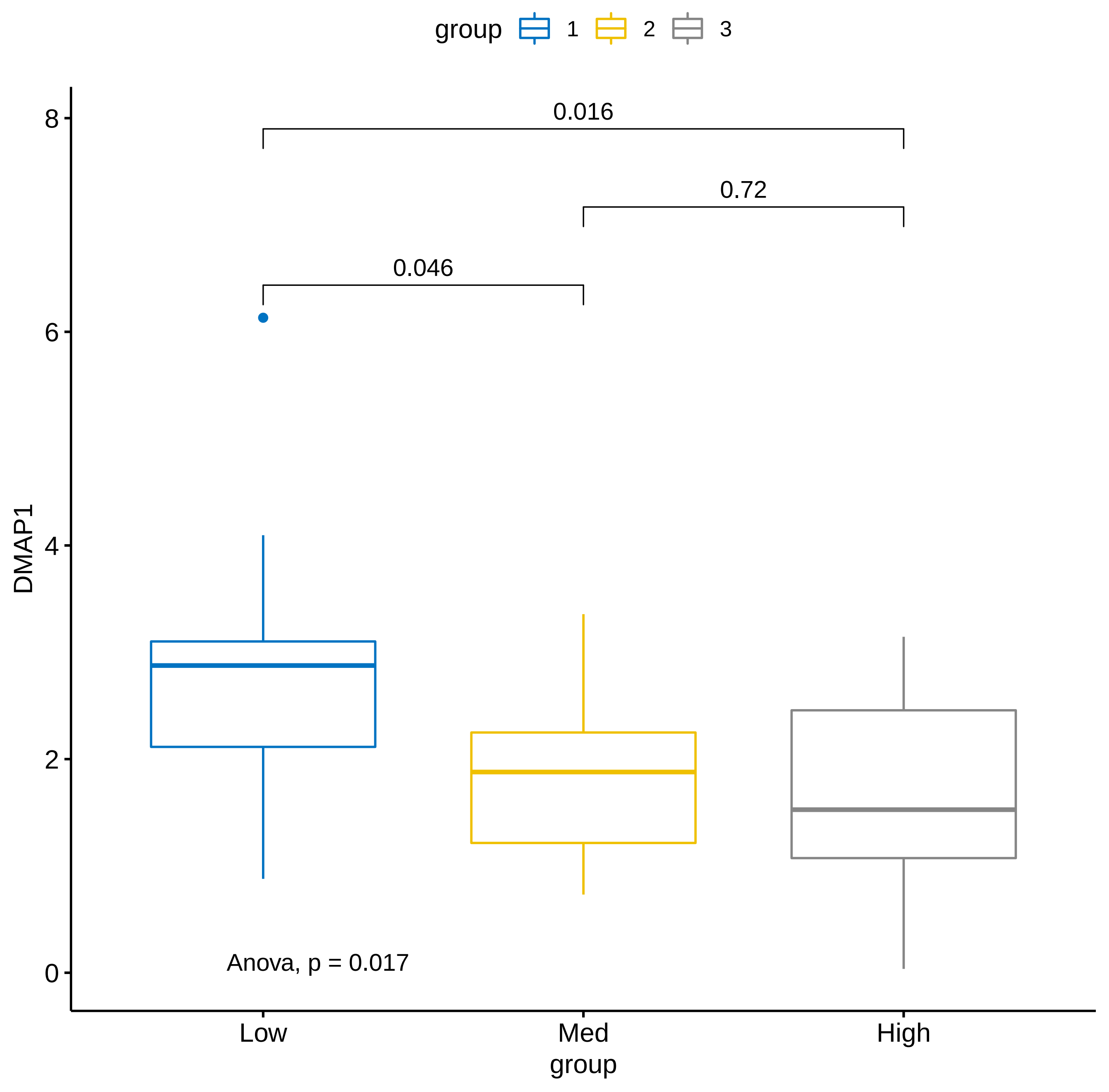

group 1 2 3
0.016
0.72
0.046
DMAP1
8
6
4
2
0
Anova, p = 0.017
Low
Med
High
group

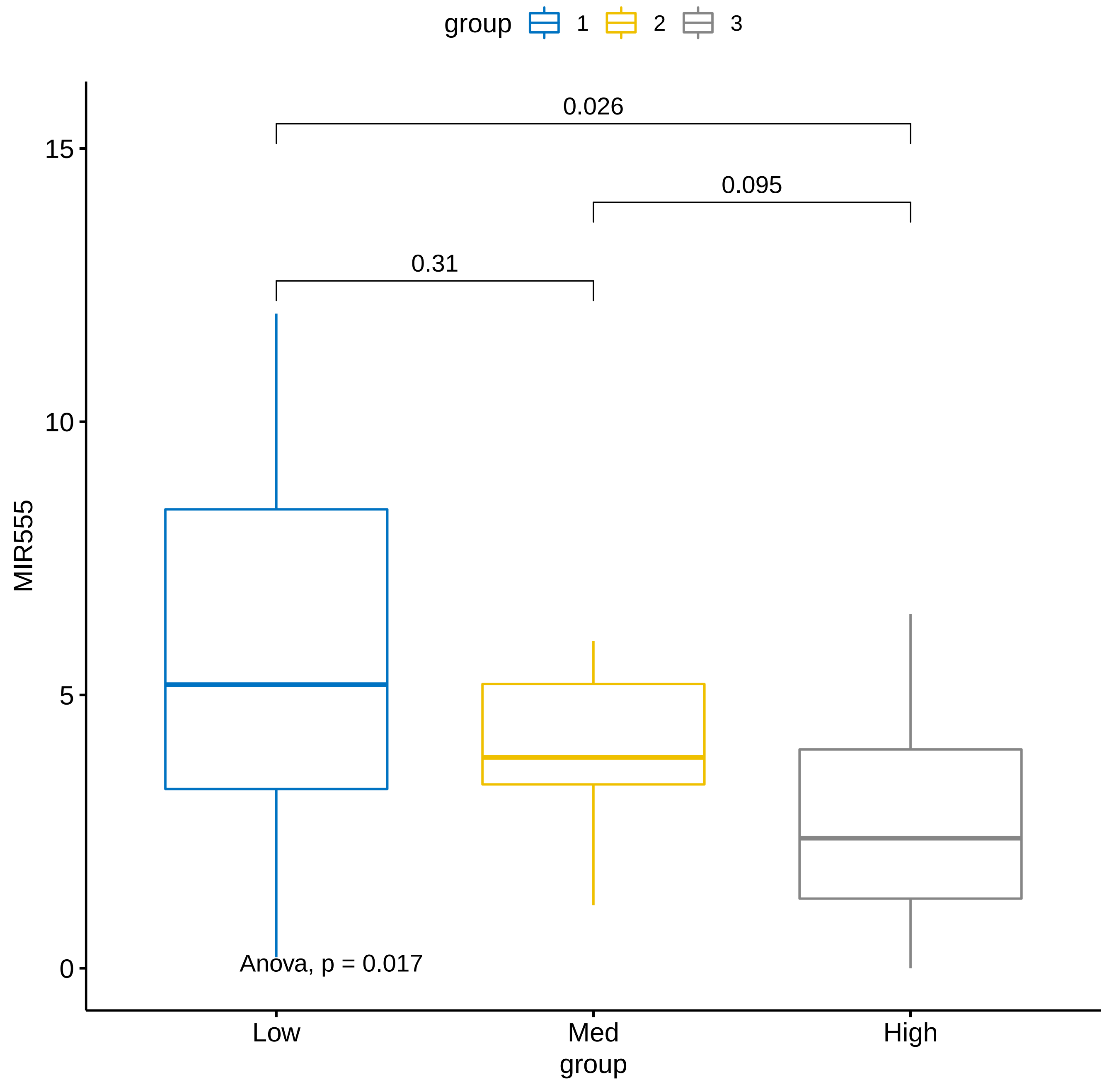

group 1 2 3
0.026
0.095
0.31
15
10
5
0
MIR555
Anova, p = 0.017
Low
Med
High
group

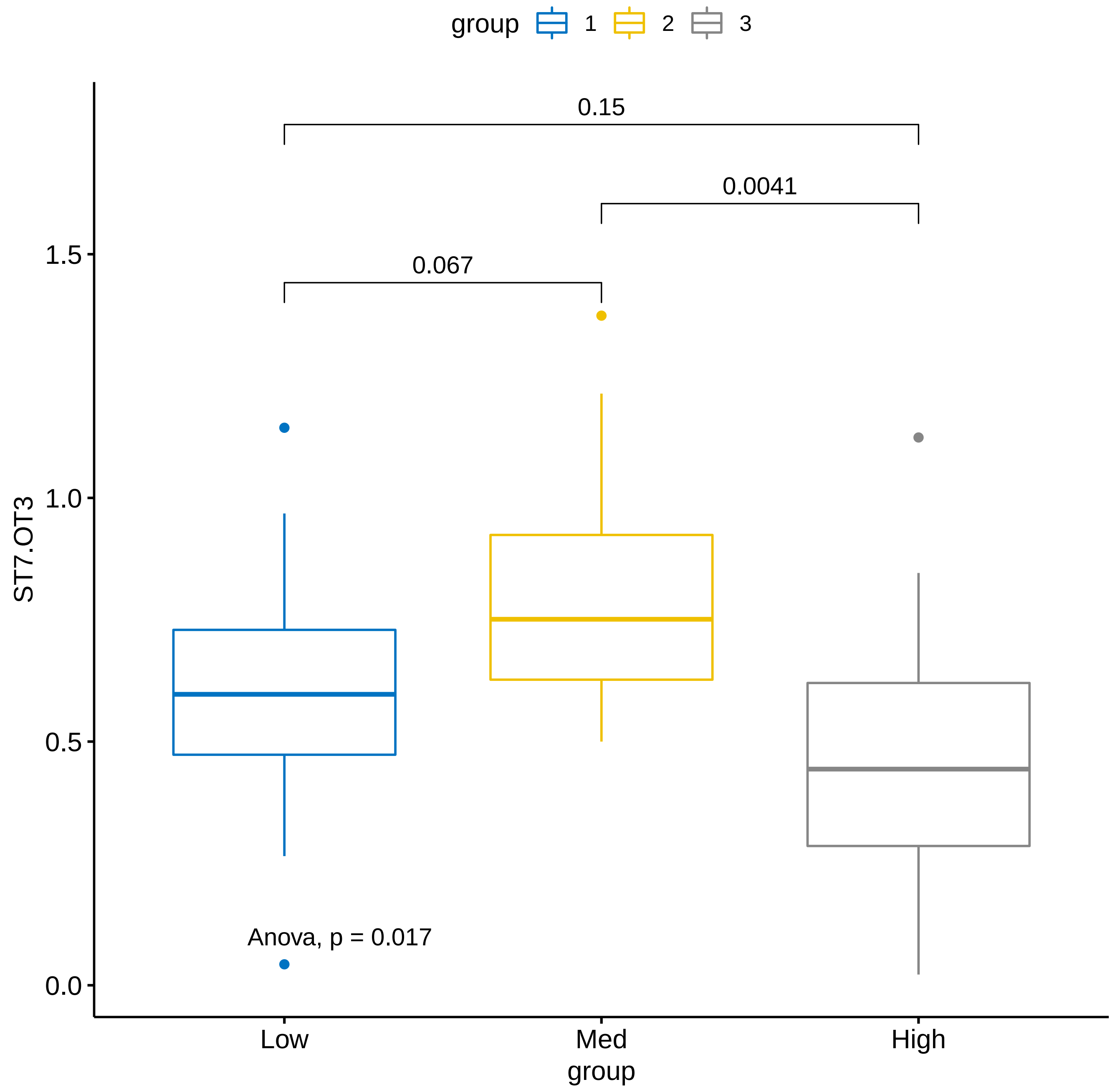
group 1 2 3
0.15
0.0041
0.067
1.5
1.0
0.5
0.0
ST7.OT3
Anova, p = 0.017
Low
Med
High
group

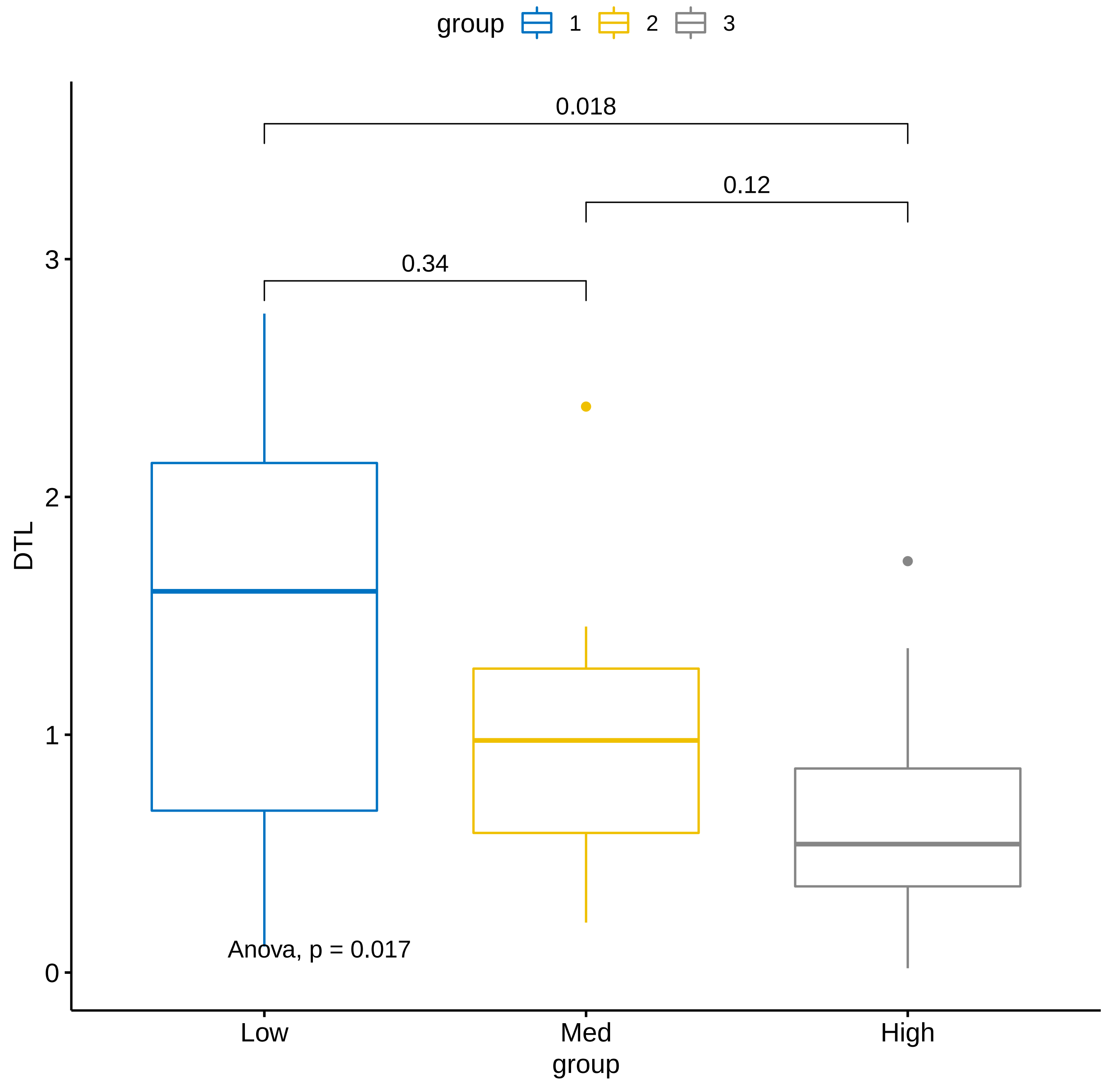
group
1
2
3
0.018
0.12
0.34
3
2
1
0
DTL
Anova, p = 0.017
Low
Med
High
group

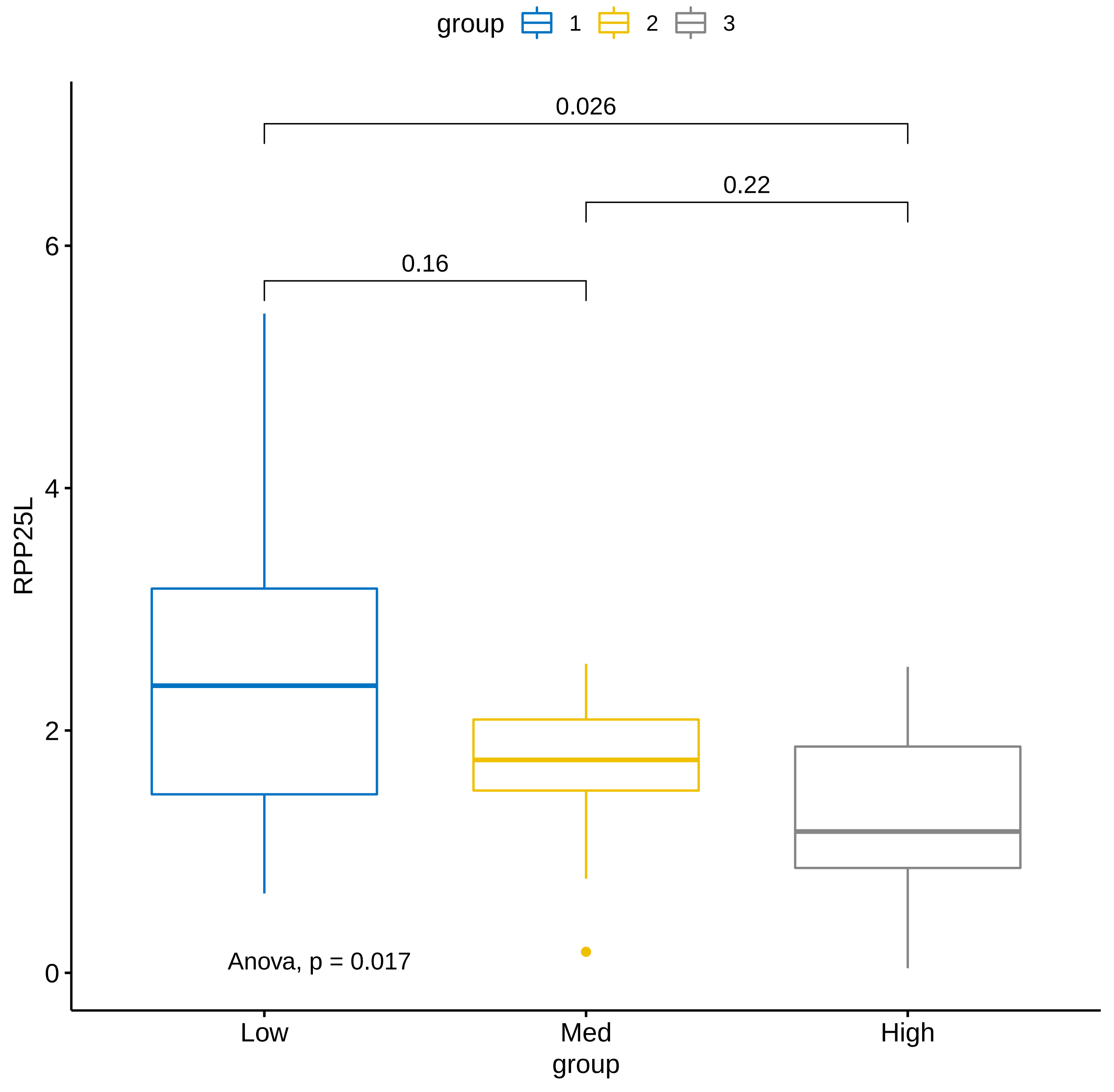
group
1
2
3
0.026
0.22
0.16
RPP25L
6
4
2
0
Anova, p = 0.017
Low
Med
High
group

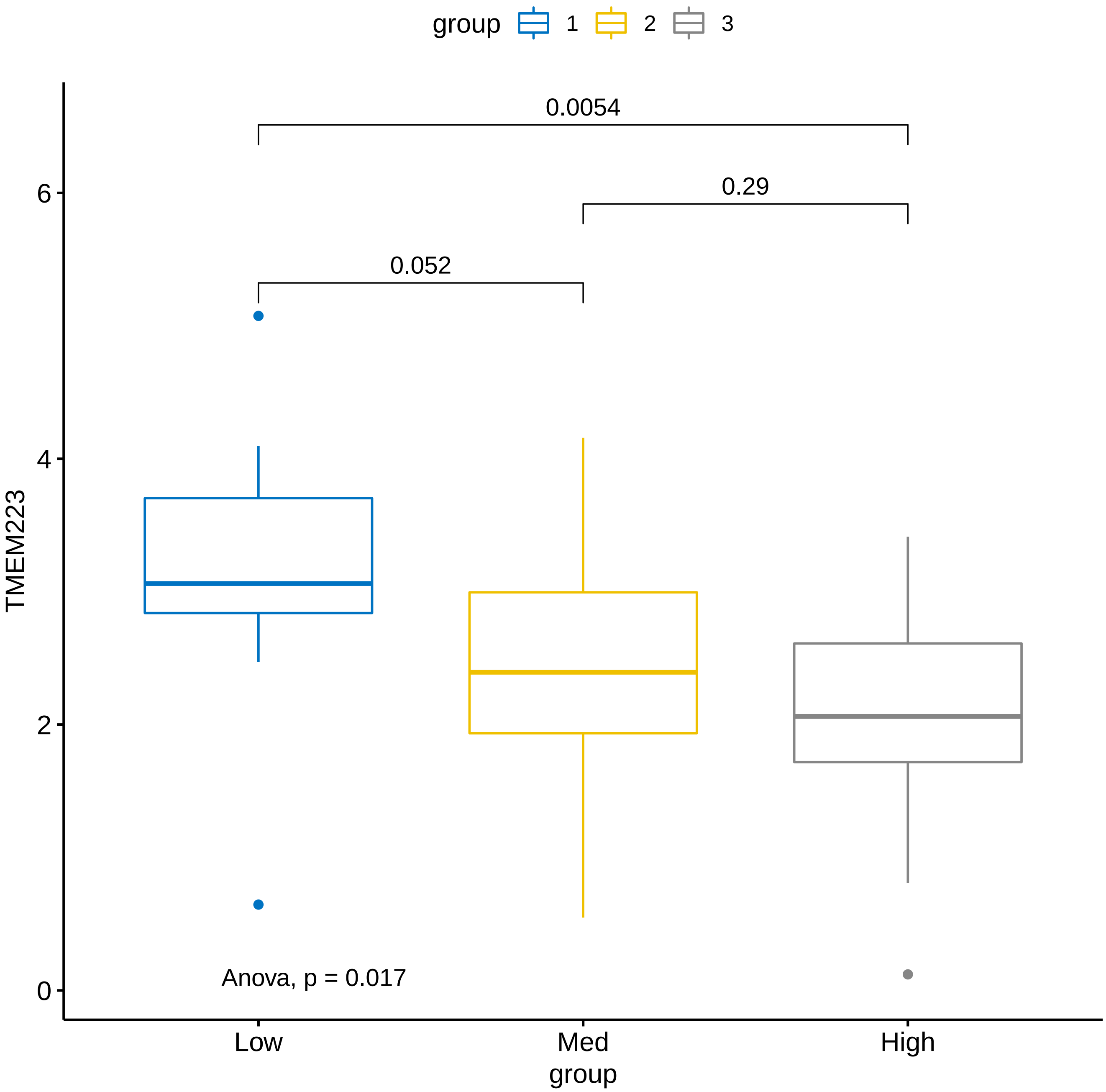
group
1
2
3
0.0054
0.29
0.052
TMEM223
6
4
2
0
Anova, p = 0.017
Low
Med
High
group

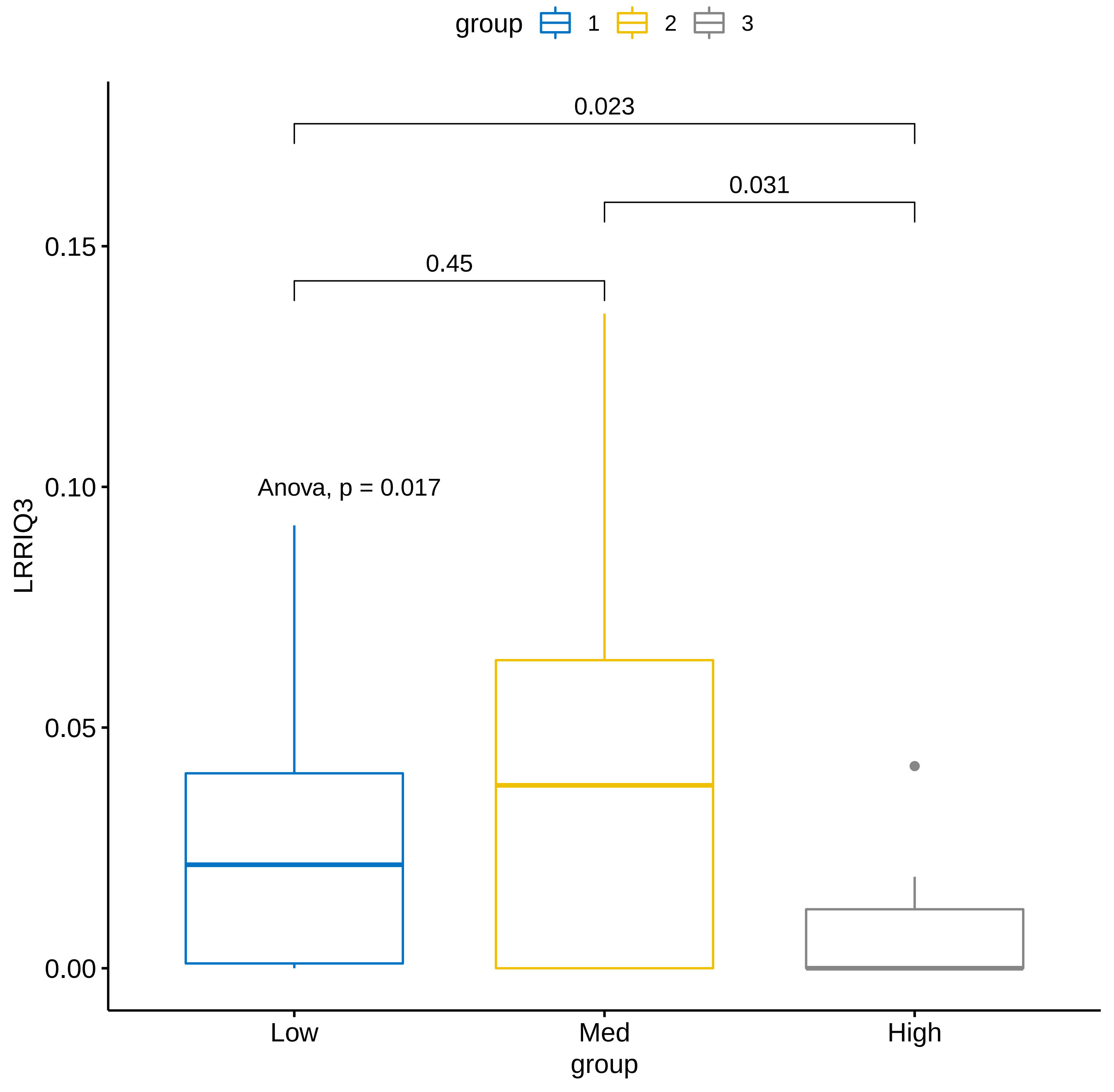

group
1
2
3
0.023
0.031
0.45
Anova, p = 0.017
LRRIQ3
0.15
0.10
0.05
0.00
Low
Med
High
group

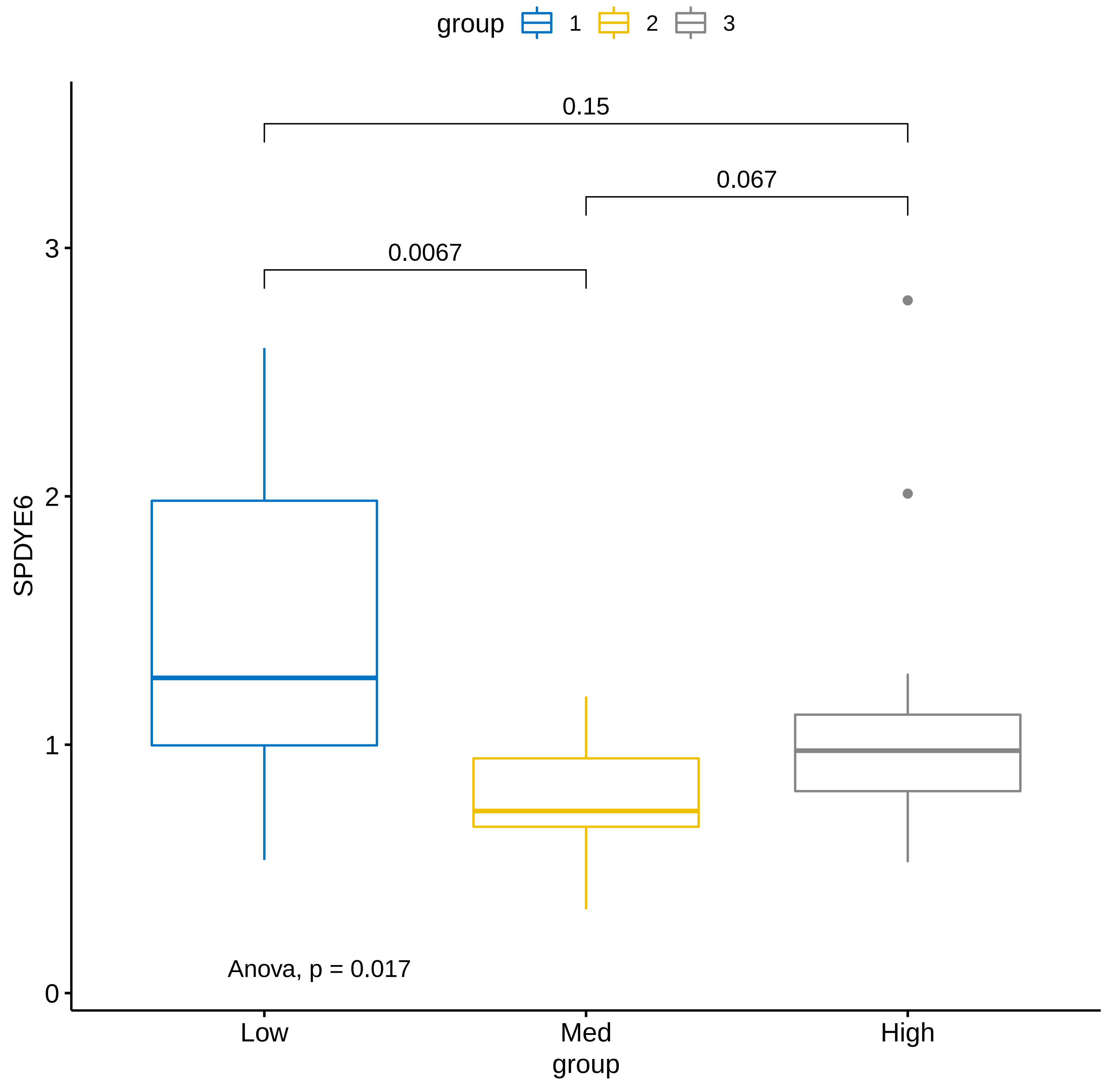

group 1 2 3
0.15
0.067
0.0067
3
2
1
0
SPDYE6
Anova, p = 0.017
Low
Med
High
group

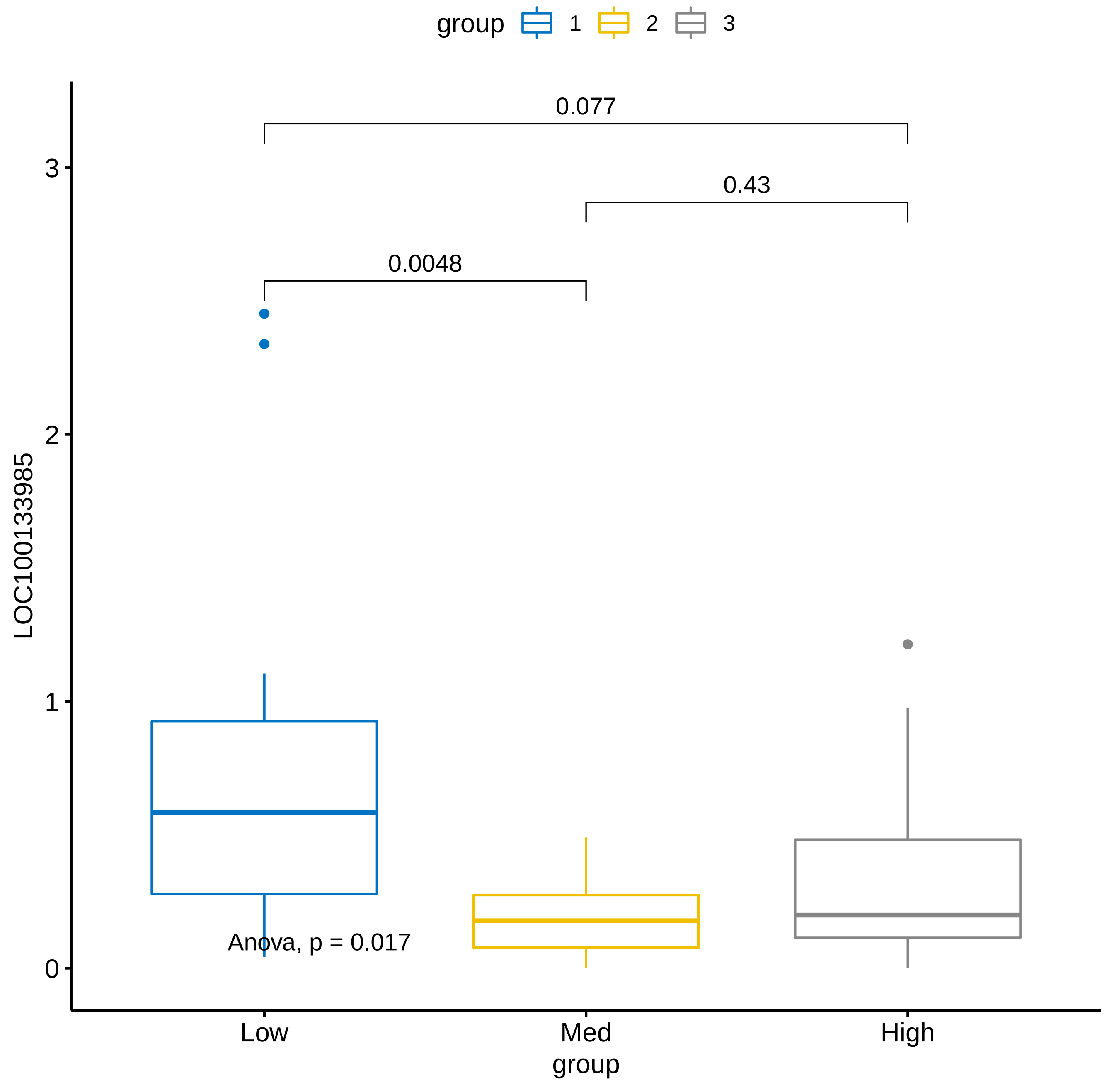

group 1 2 3
0.077
0.43
0.0048
LOC100133985
3
2
1
0
Anova, p = 0.017
Low
Med
High
group

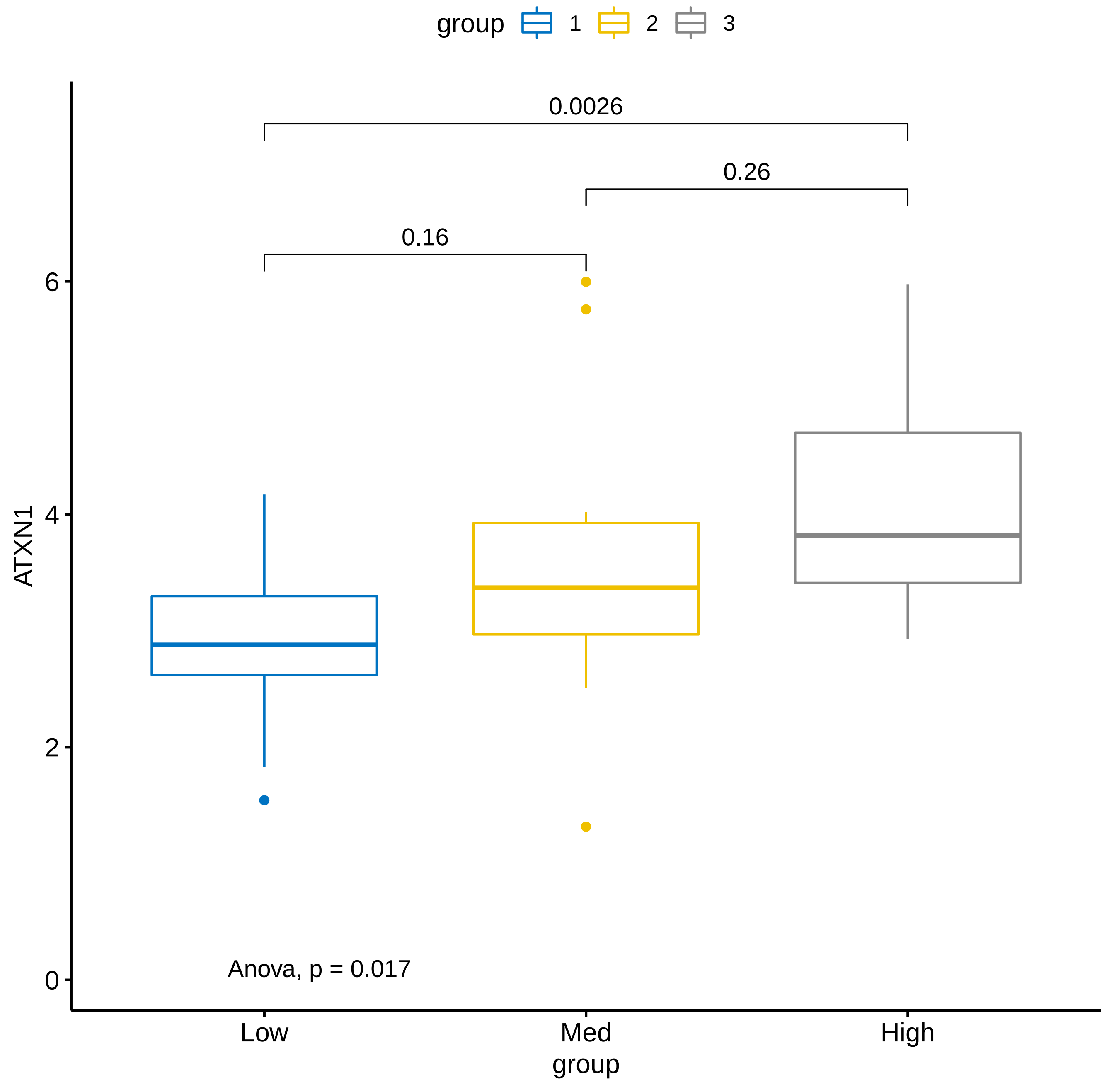

group 1 2 3
0.0026
0.26
0.16
ATXN1
6
4
2
0
Anova, p = 0.017
Low
Med
High
group

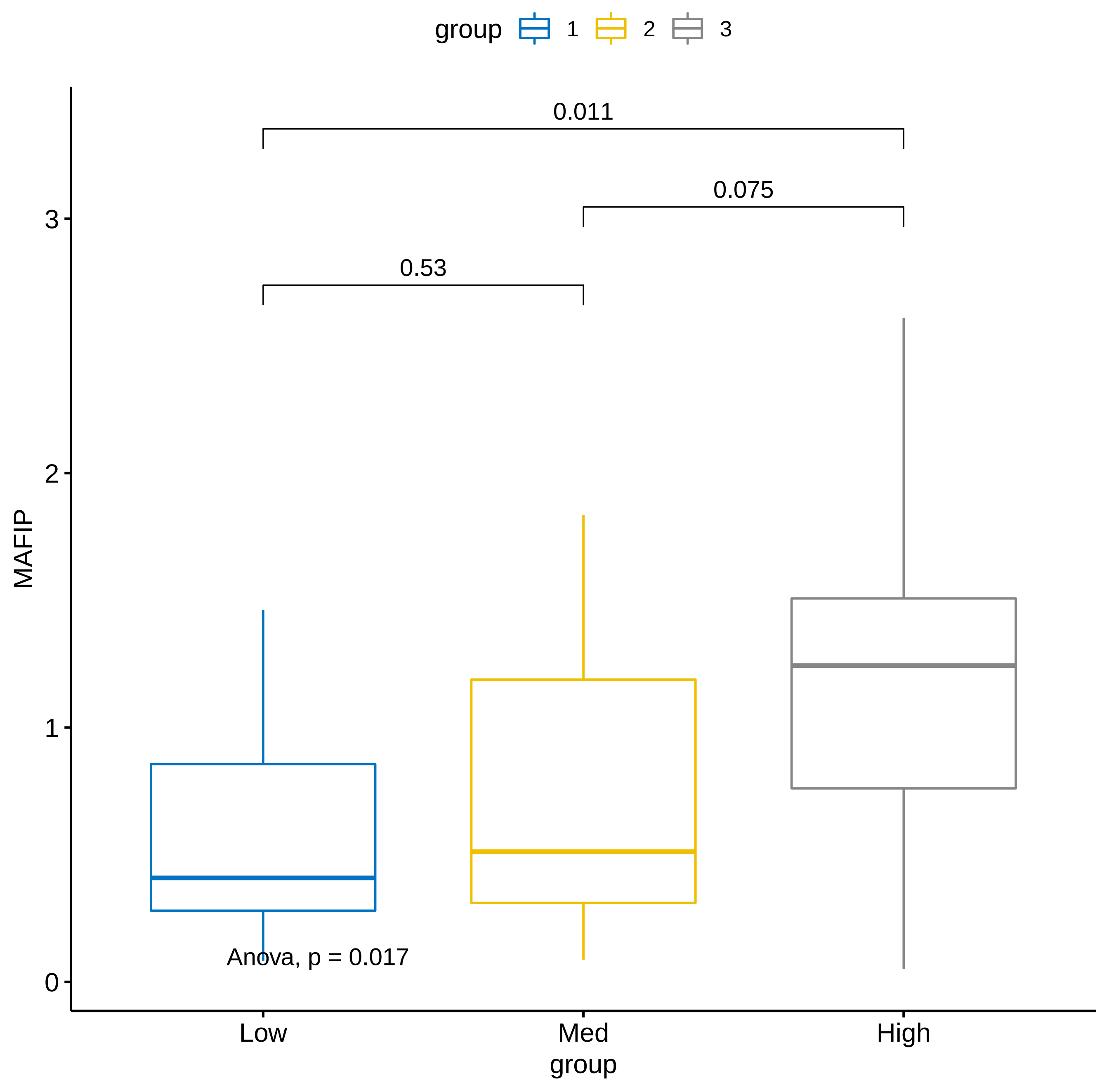
group
1
2
3
0.011
0.075
0.53
3
2
1
0
MAFIP
Anova, p = 0.017
Low
Med
High
group

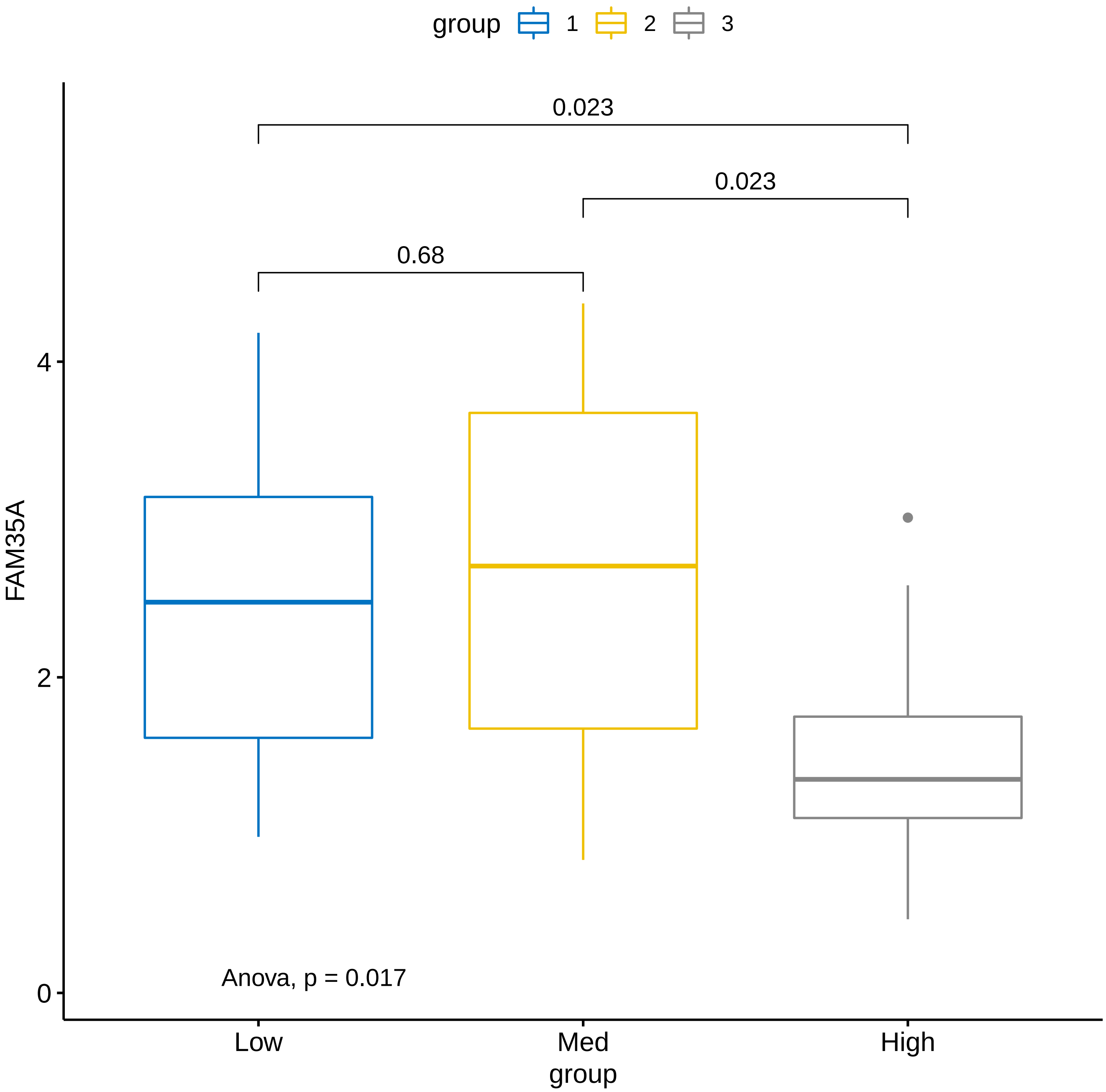

group
1
2
3
0.023
0.023
0.68
FAM35A
4
2
0
Anova, p = 0.017
Low
Med
High
group

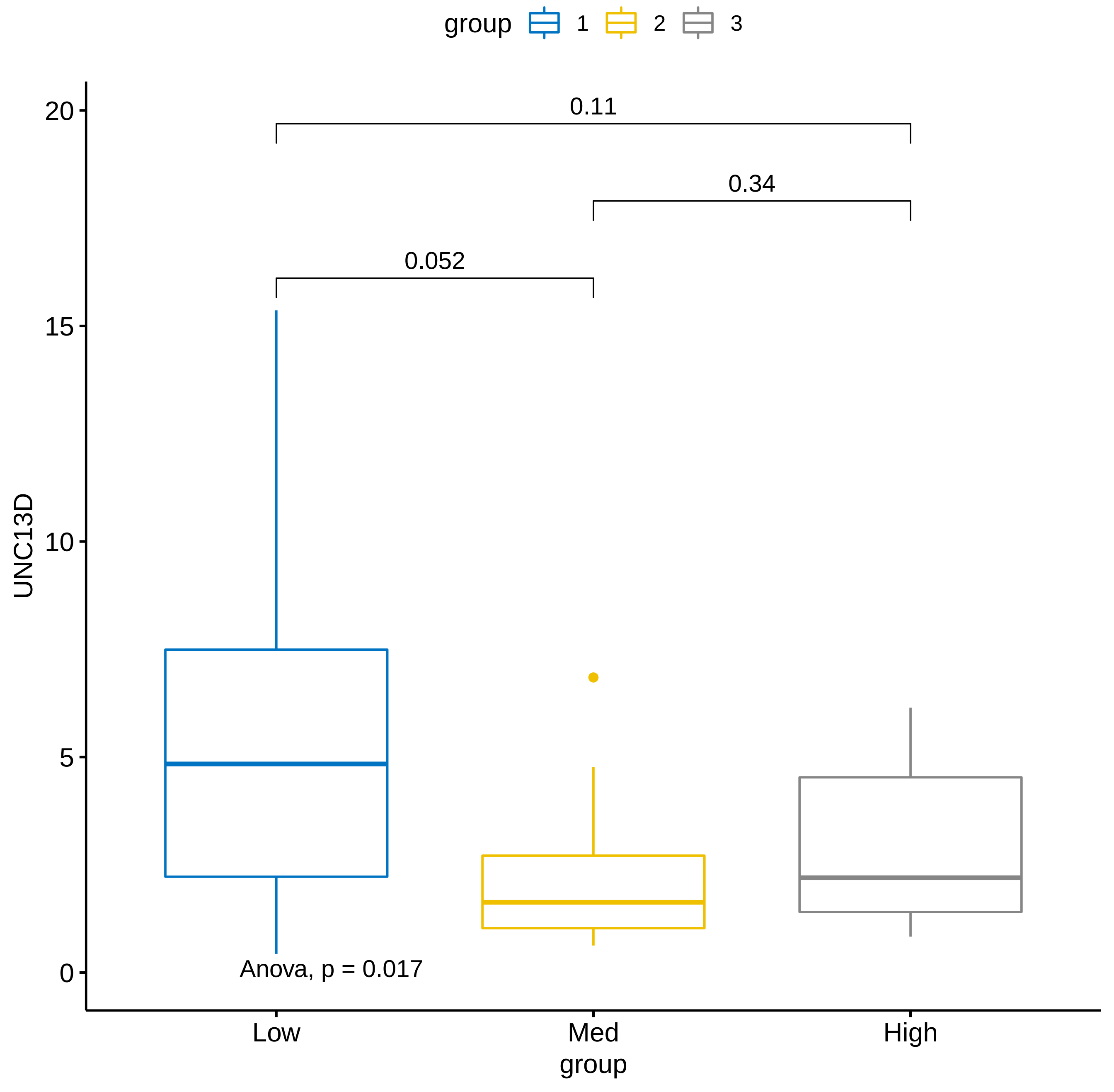

group
1
2
3
0.11
0.34
0.052
20
15
10
5
0
UNC13D
Anova, p = 0.017
Low
Med
High
group

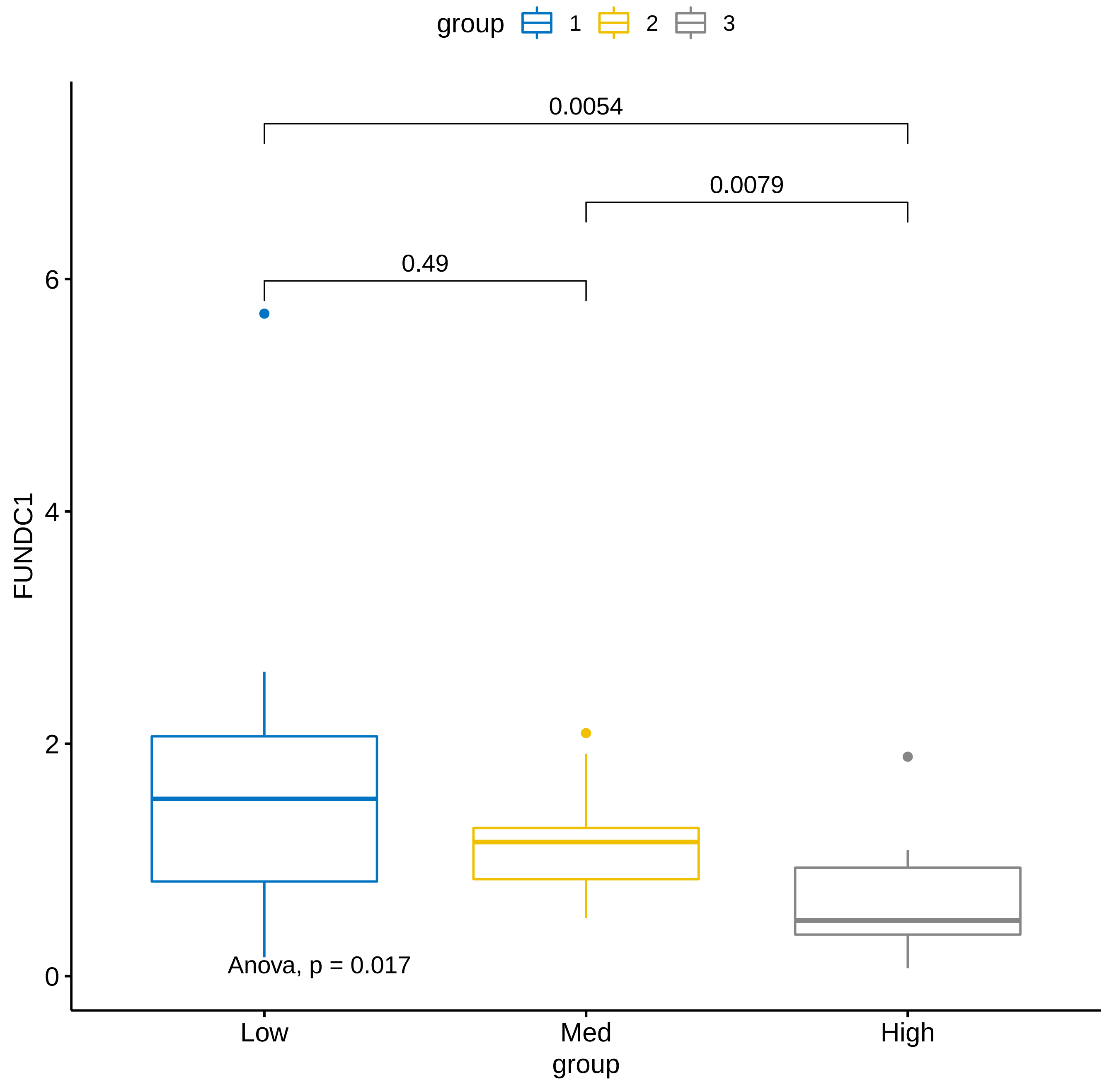

group 1 2 3
0.0054
0.0079
0.49
FUNDC1
6
4
2
0
Anova, p = 0.017
Low
Med
High
group

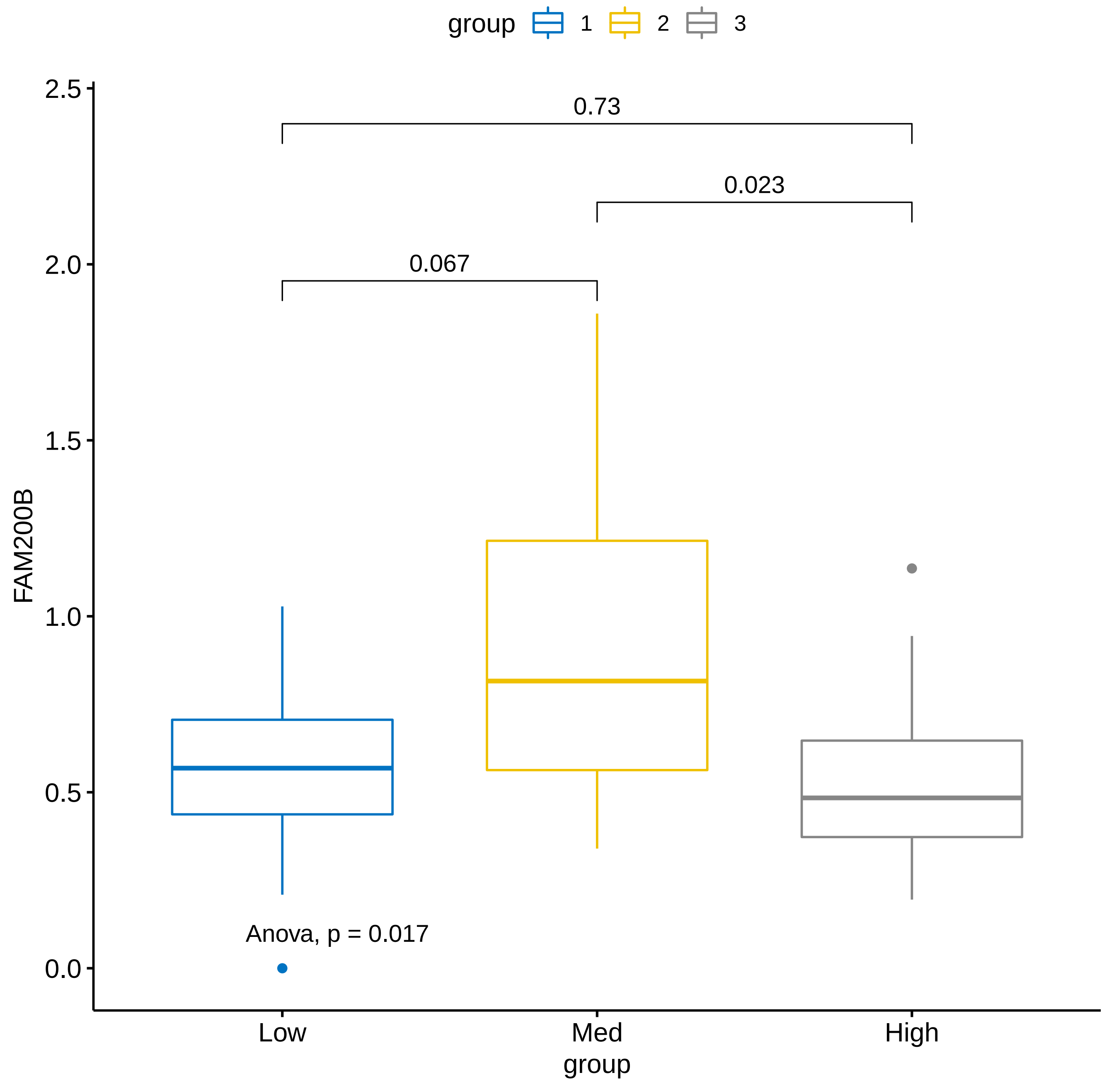

group
1
2
3
2.5
2.0
1.5
1.0
0.5
0.0
FAM200B
0.73
0.023
0.067
Anova, p = 0.017
Low
Med
High
group

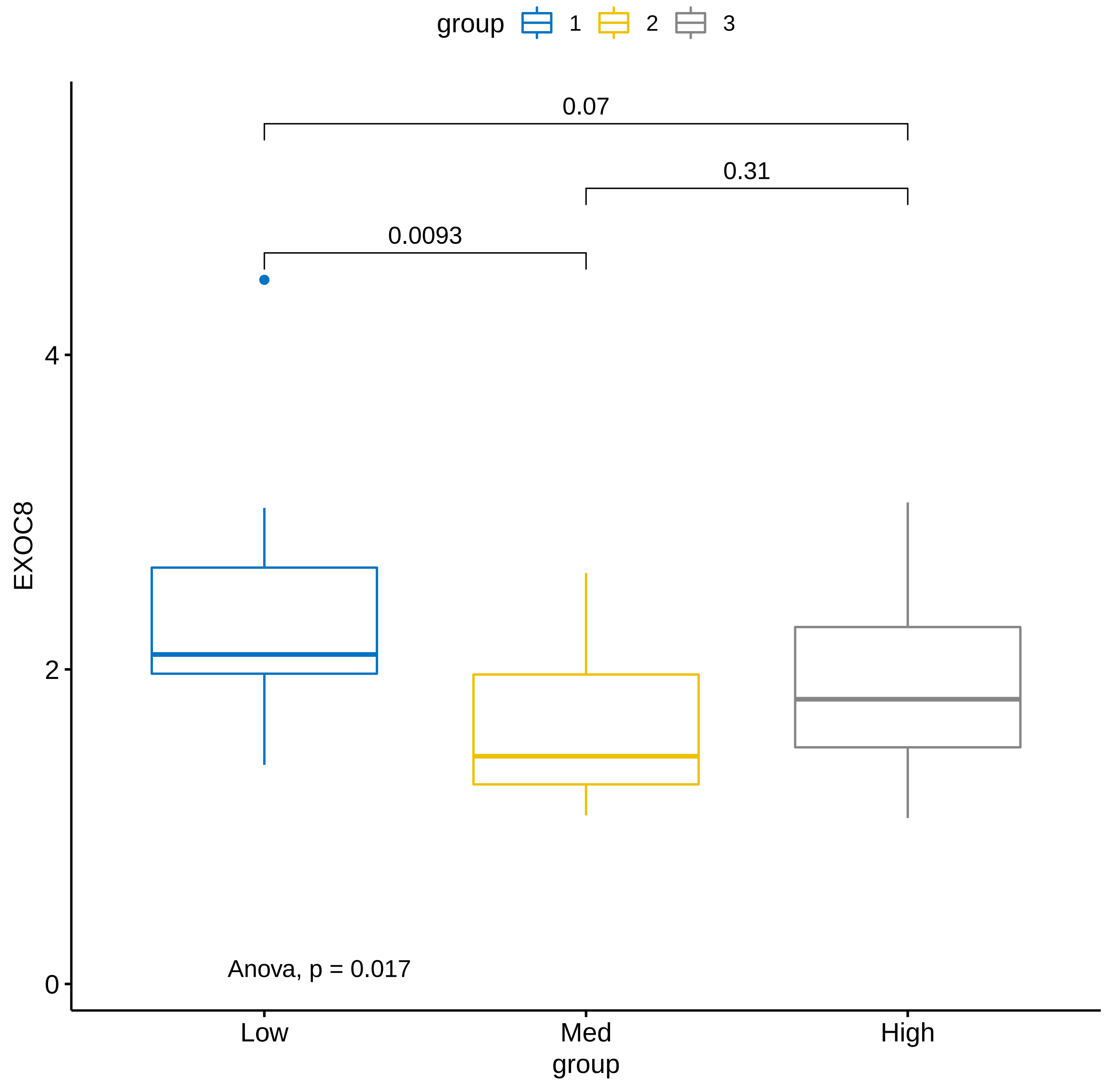
group
1
2
3
0.07
0.31
0.0093
EXOC8
4
2
0
Anova, p = 0.017
Low
Med
High
group

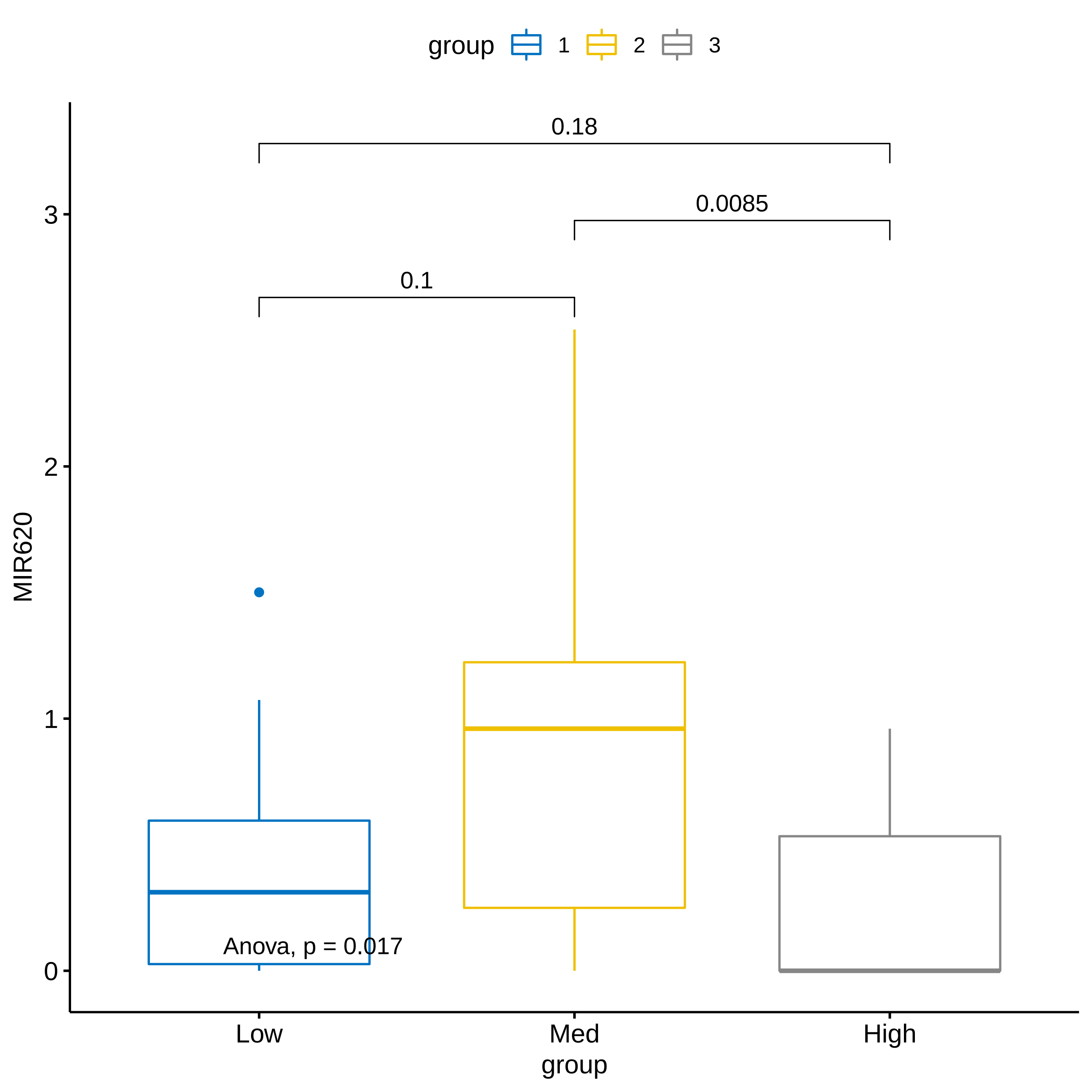
group
1
2
3
0.18
0.0085
0.1
3
2
1
0
MIR620
Anova, p = 0.017
Low
Med
High
group

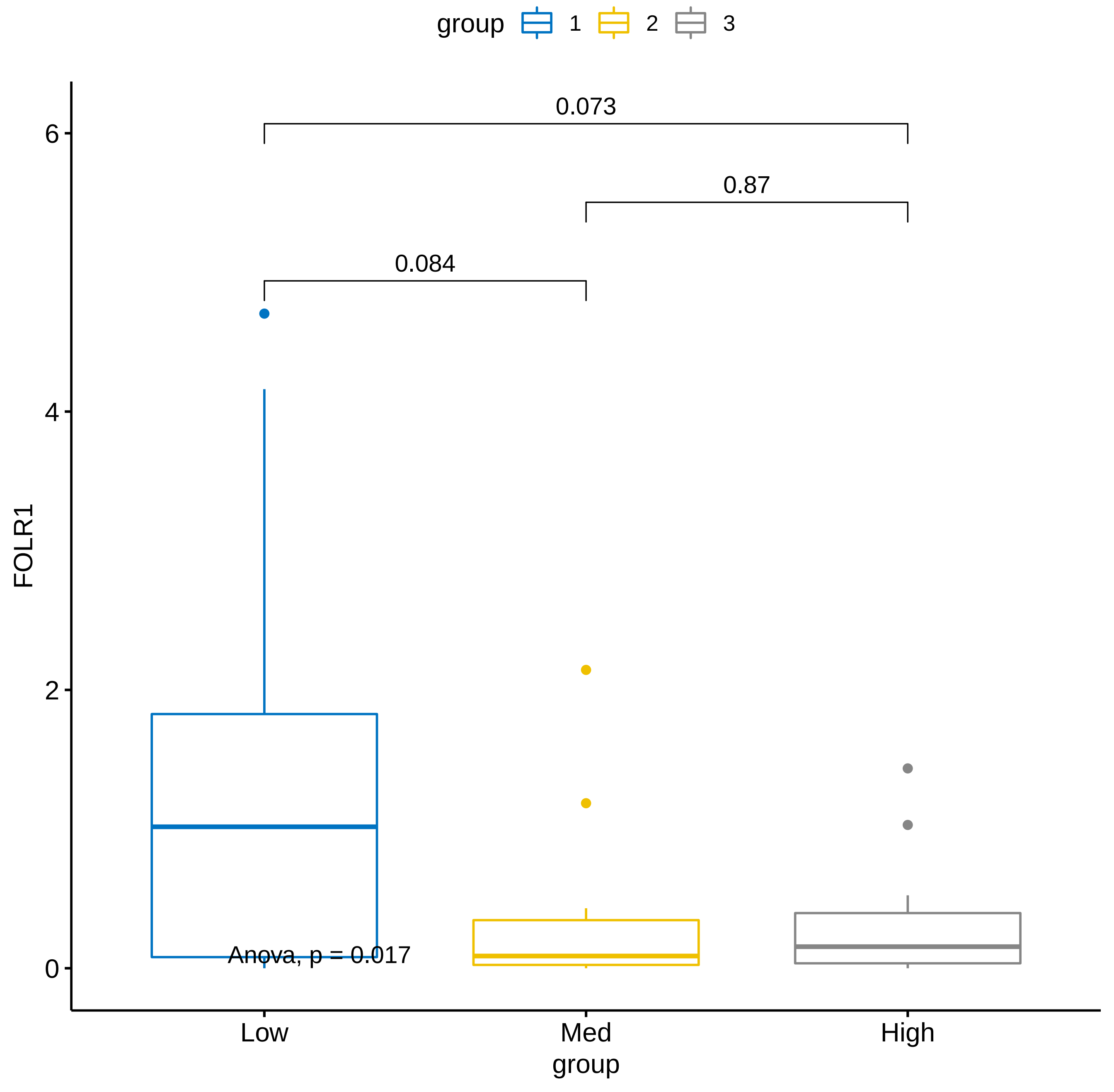
group
1
2
3
0.073
0.87
0.084
6
4
2
0
FOLR1
Anova, p = 0.017
Low
Med
High
group

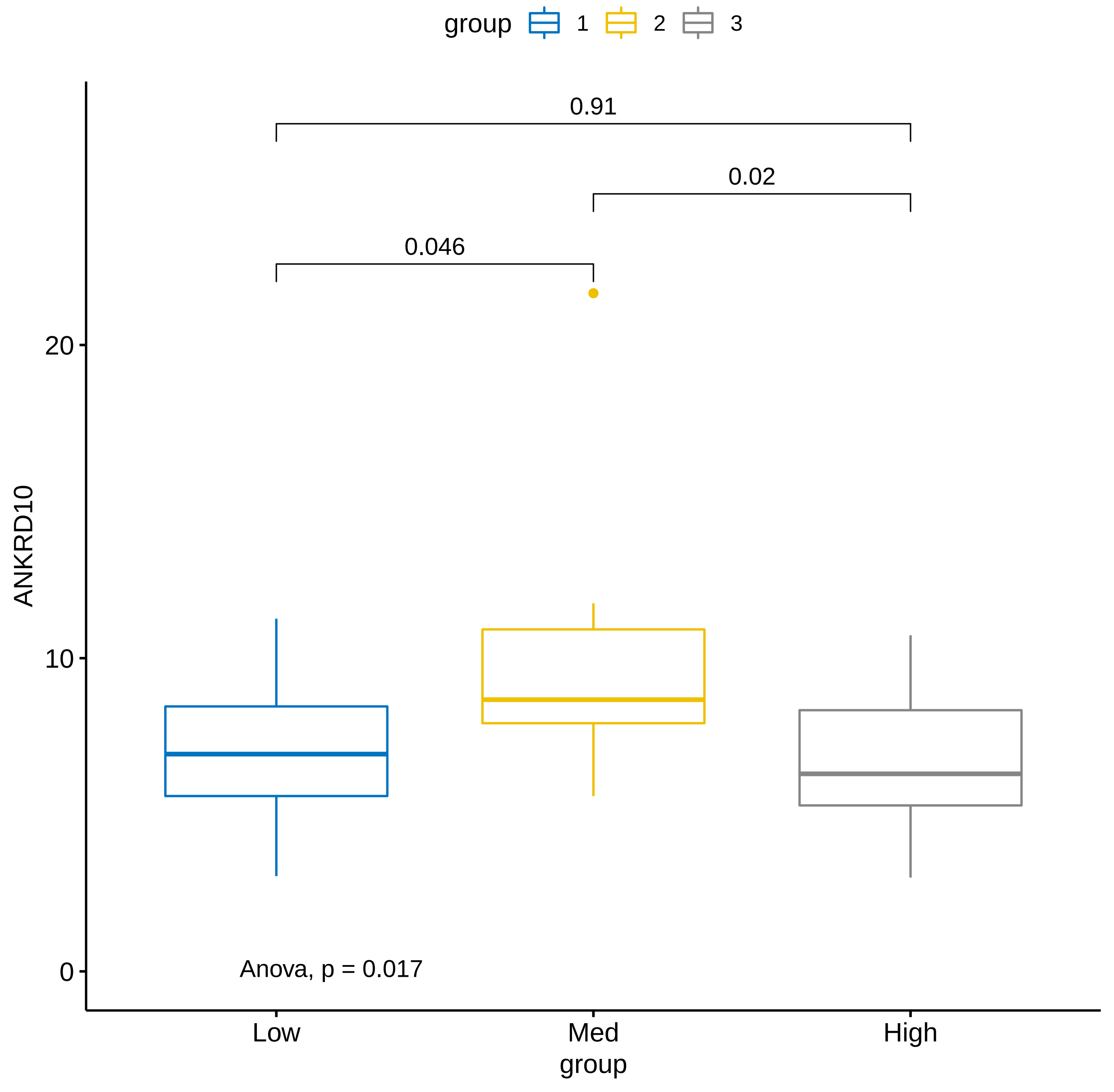

group 1 2 3
0.91
0.02
0.046
20
10
0
ANKRD10
Anova, p = 0.017
Low
Med
High
group

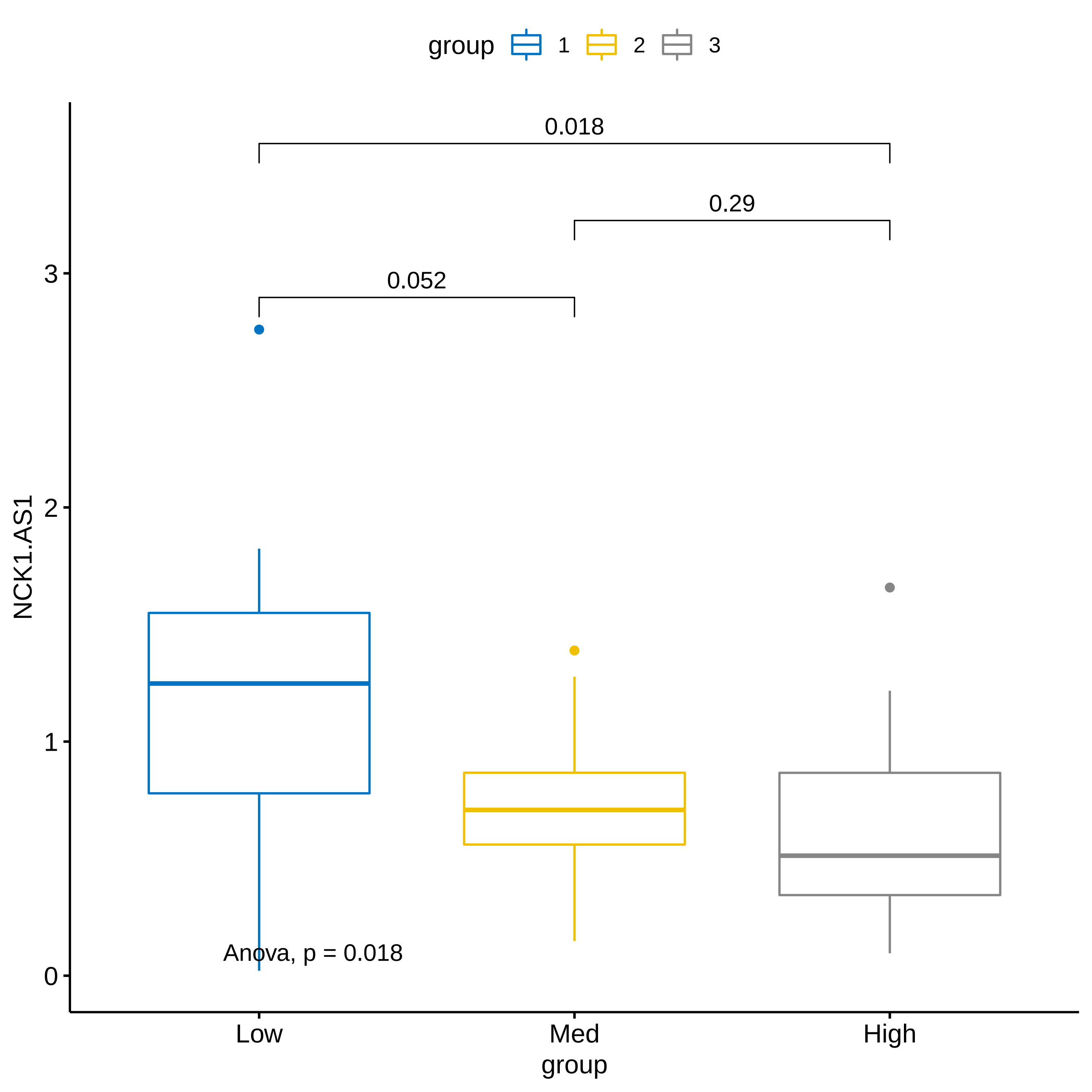
group 1 2 3
0.018
0.29
0.052
NCK1.AS1
3
2
1
0
Anova, p = 0.018
Low
Med
High
group

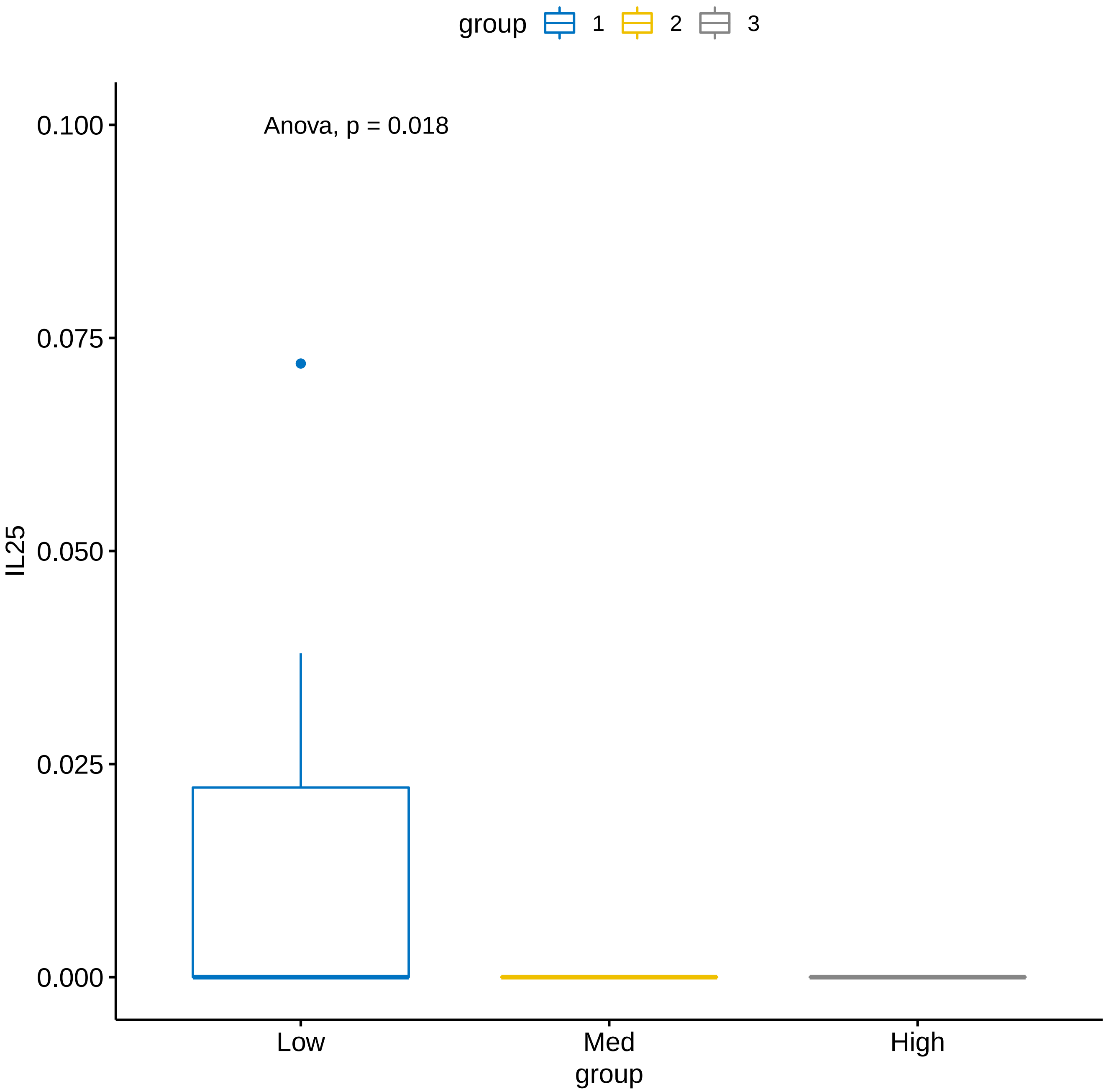

group
1
2
3
Anova, p = 0.018
IL25
0.100
0.075
0.050
0.025
0.000
Low
Med
High
group

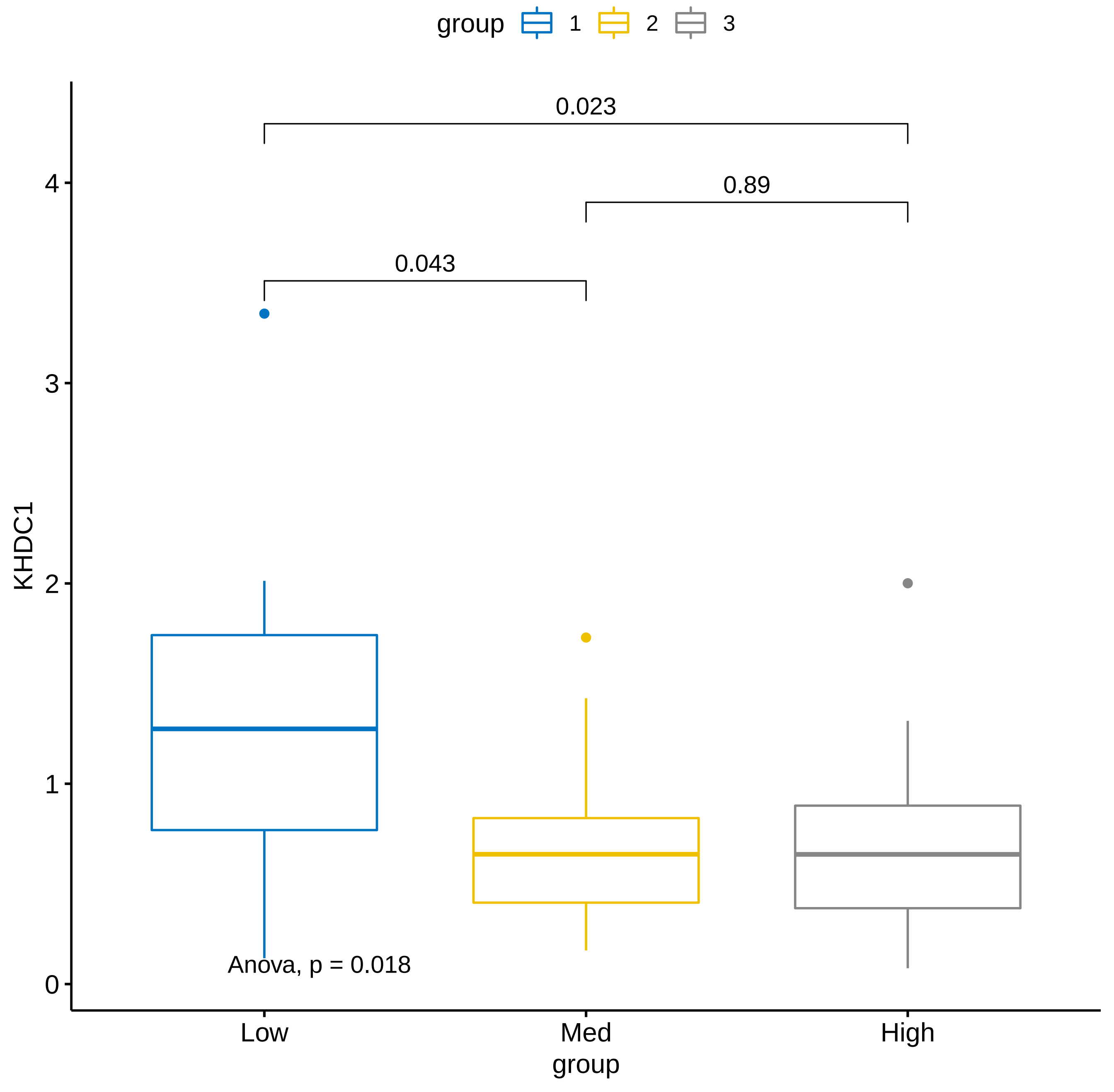

group 1 2 3
0.023
0.89
0.043
KHDC1
4
3
2
1
0
Anova, p = 0.018
Low
Med
High
group

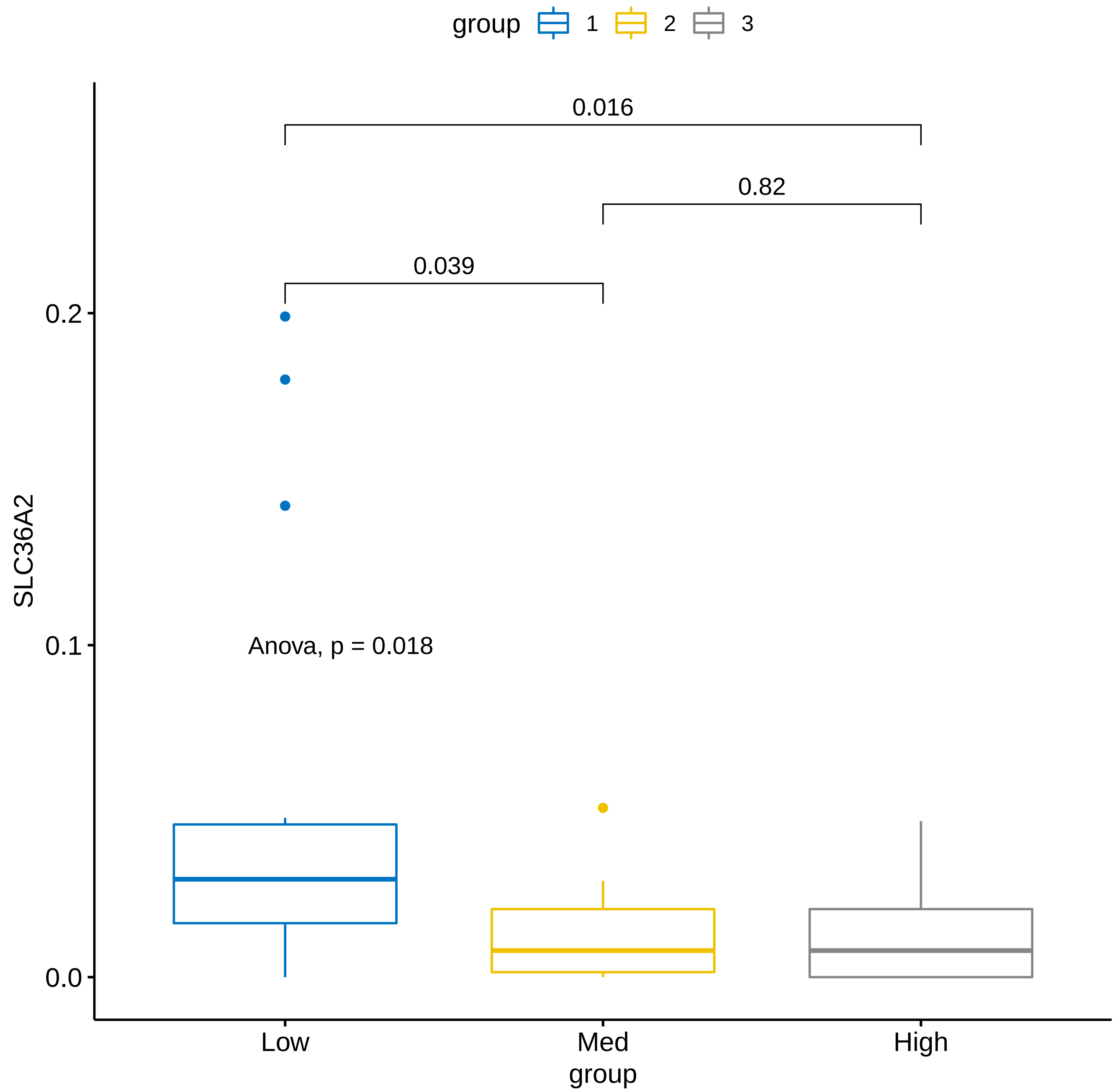
group
1
2
3
0.016
0.82
0.039
0.2
0.1
0.0
SLC36A2
Anova, p = 0.018
Low
Med
High
group

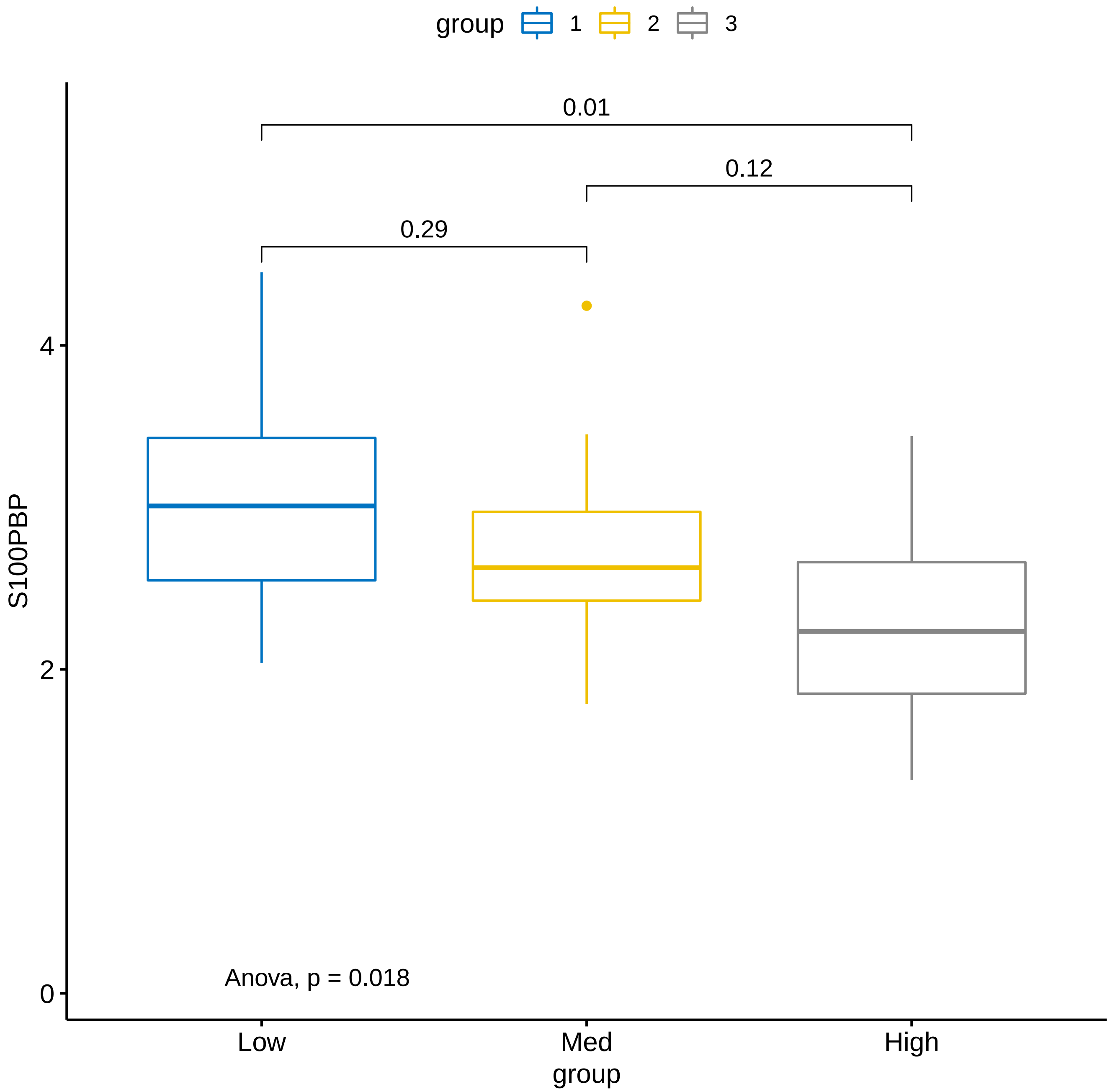
group 1 2 3
0.01
0.12
0.29
S100PBP
4
2
0
Anova, p = 0.018
Low
Med
High
group

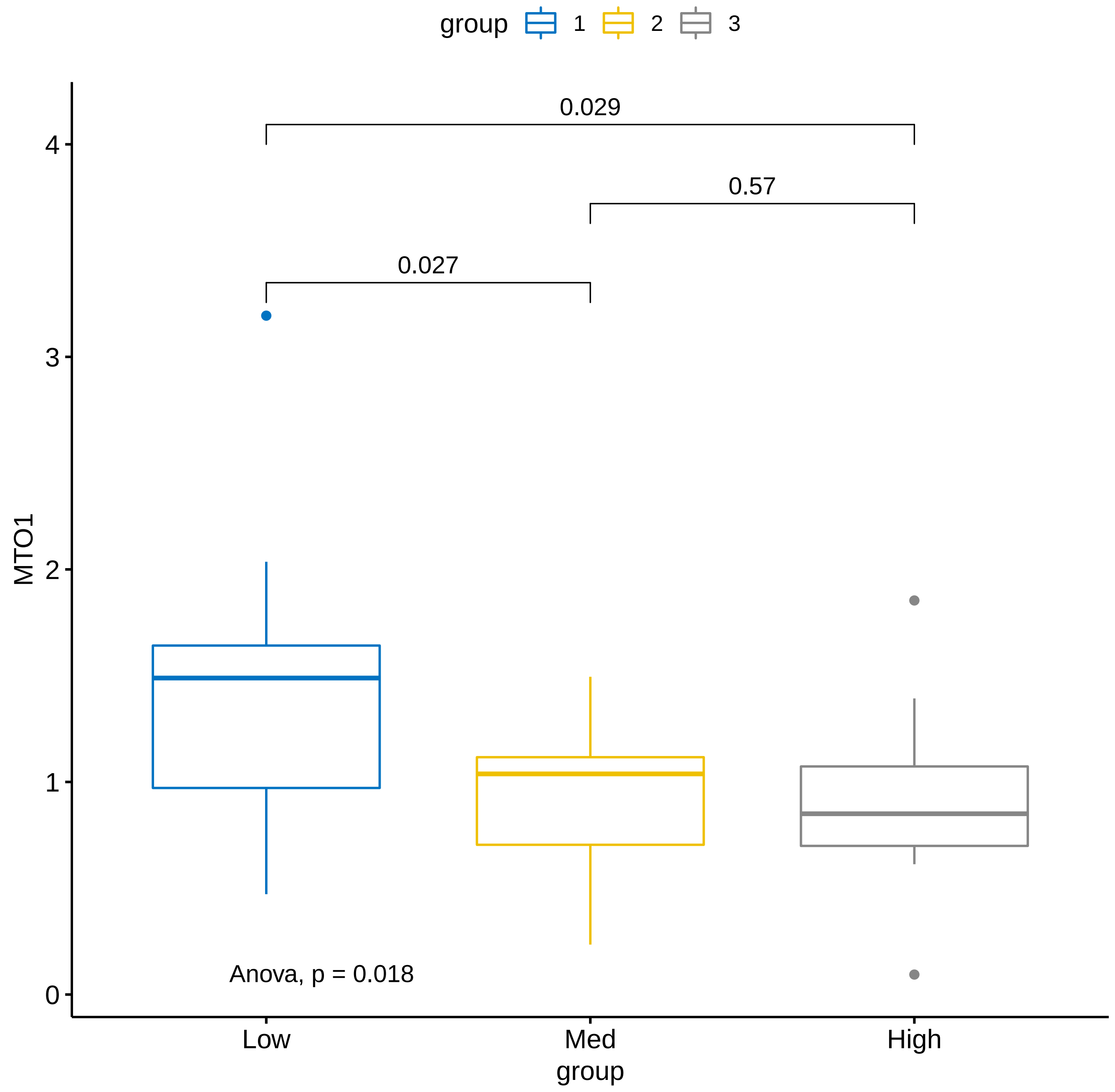
group
1
2
3
0.029
0.57
0.027
MTO1
4
3
2
1
0
Anova, p = 0.018
Low
Med
High
group

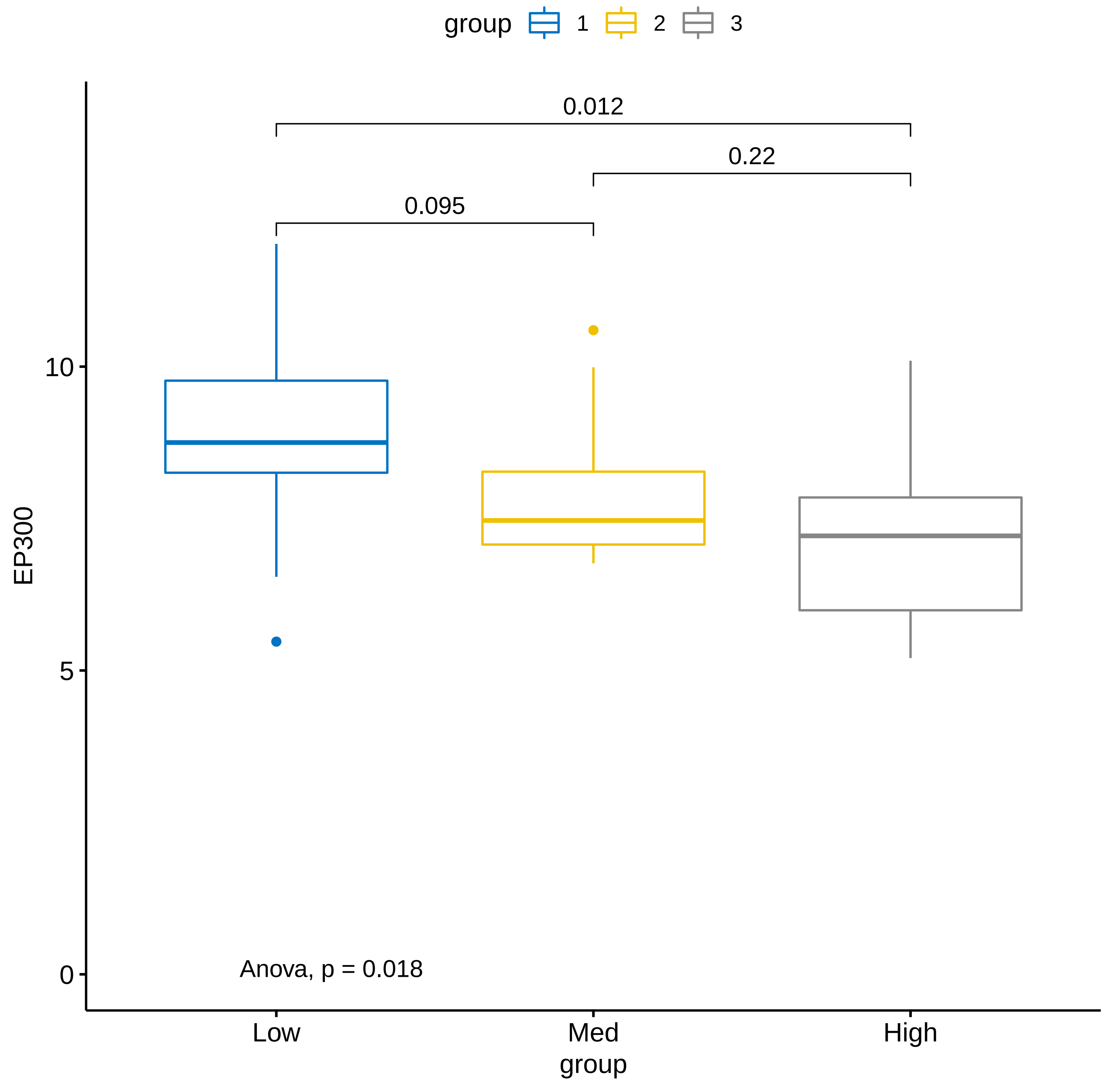
group
1
2
3
0.012
0.22
0.095
10
5
0
EP300
Anova, p = 0.018
Low
Med
High
group

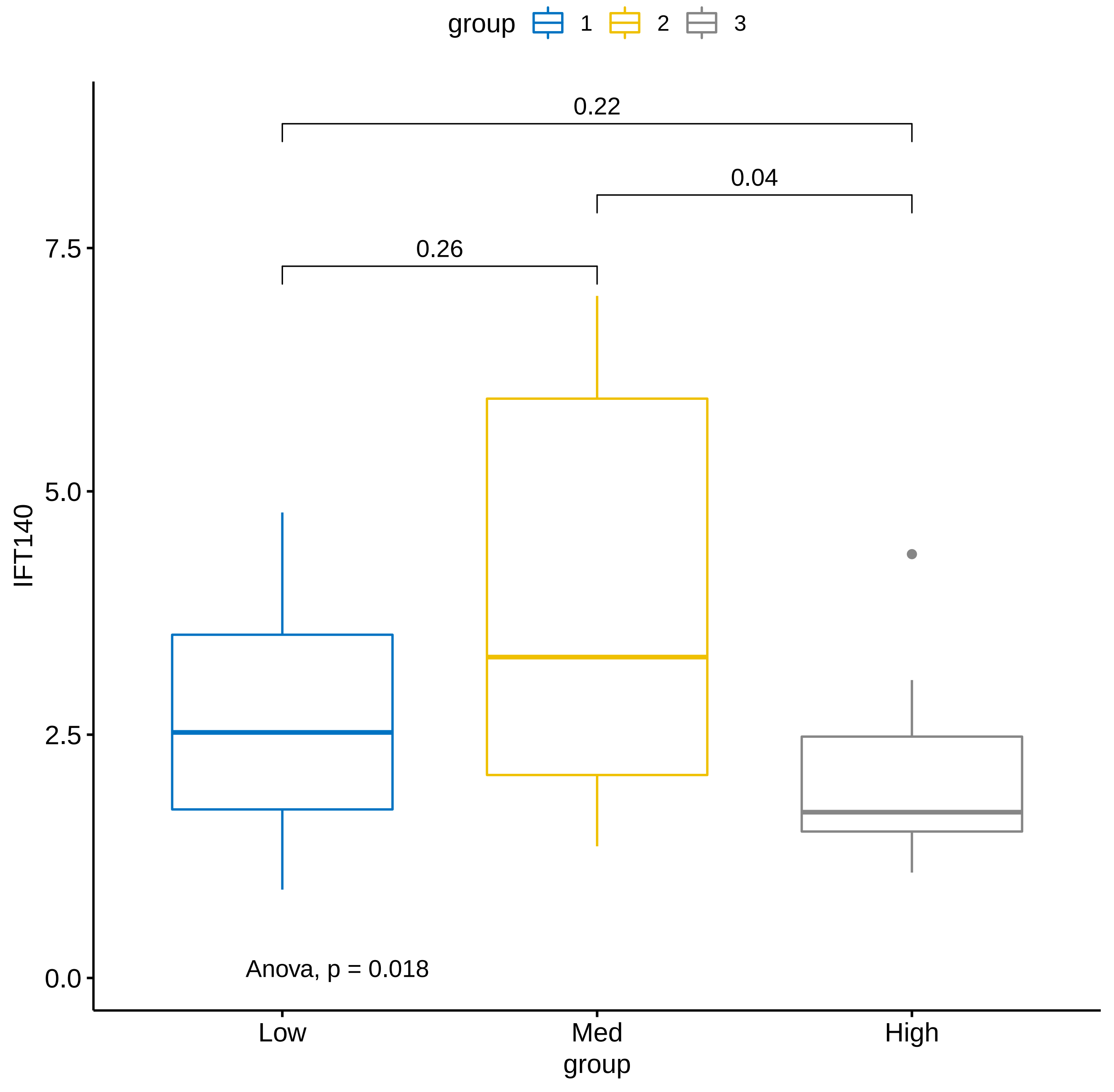
group
1
2
3
0.22
0.04
0.26
7.5
5.0
2.5
0.0
IFT140
Anova, p = 0.018
Low
Med
High
group

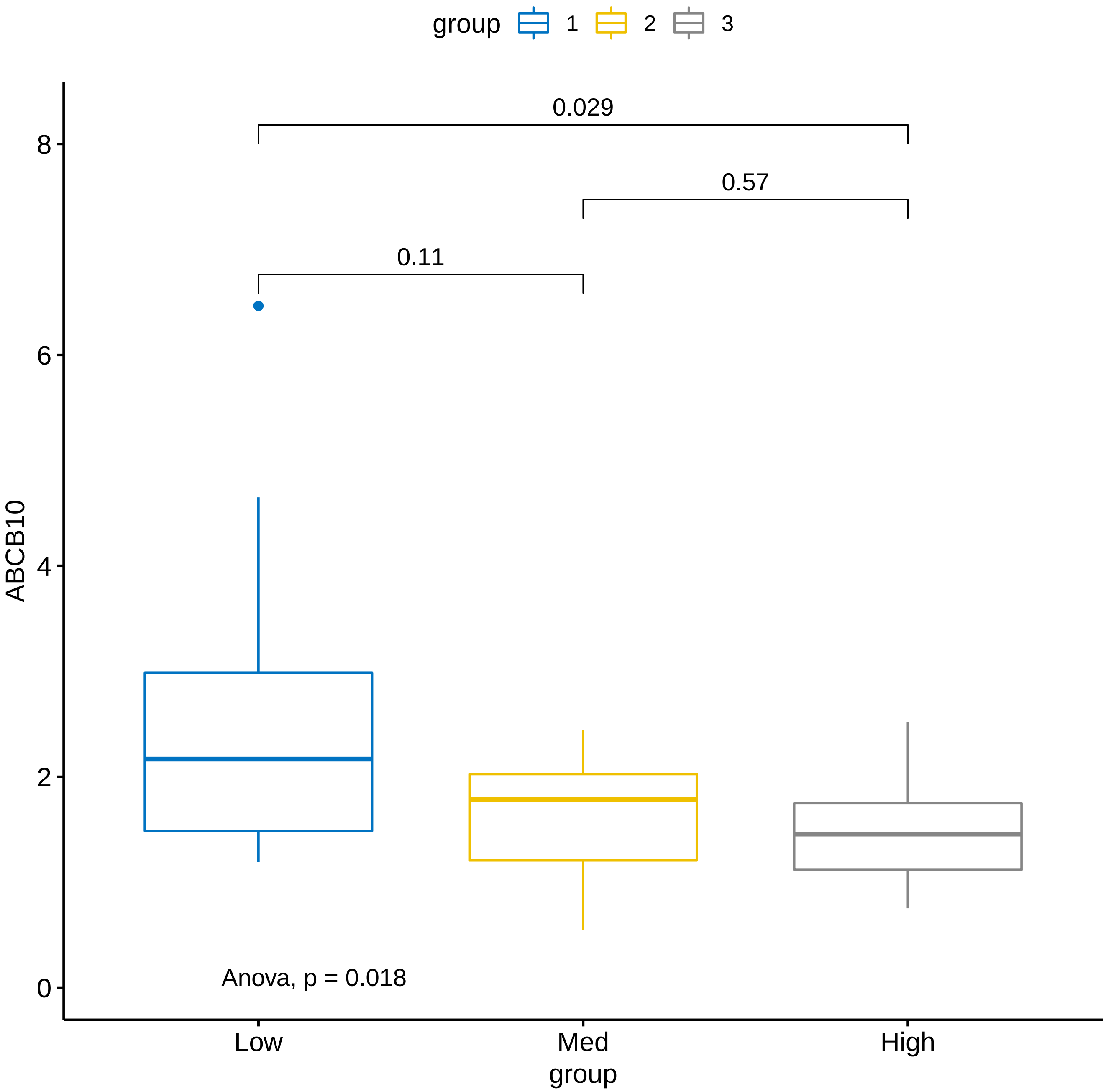
group
1
2
3
0.029
0.57
0.11
ABCB10
8
6
4
2
0
Anova, p = 0.018
Low
Med
High
group

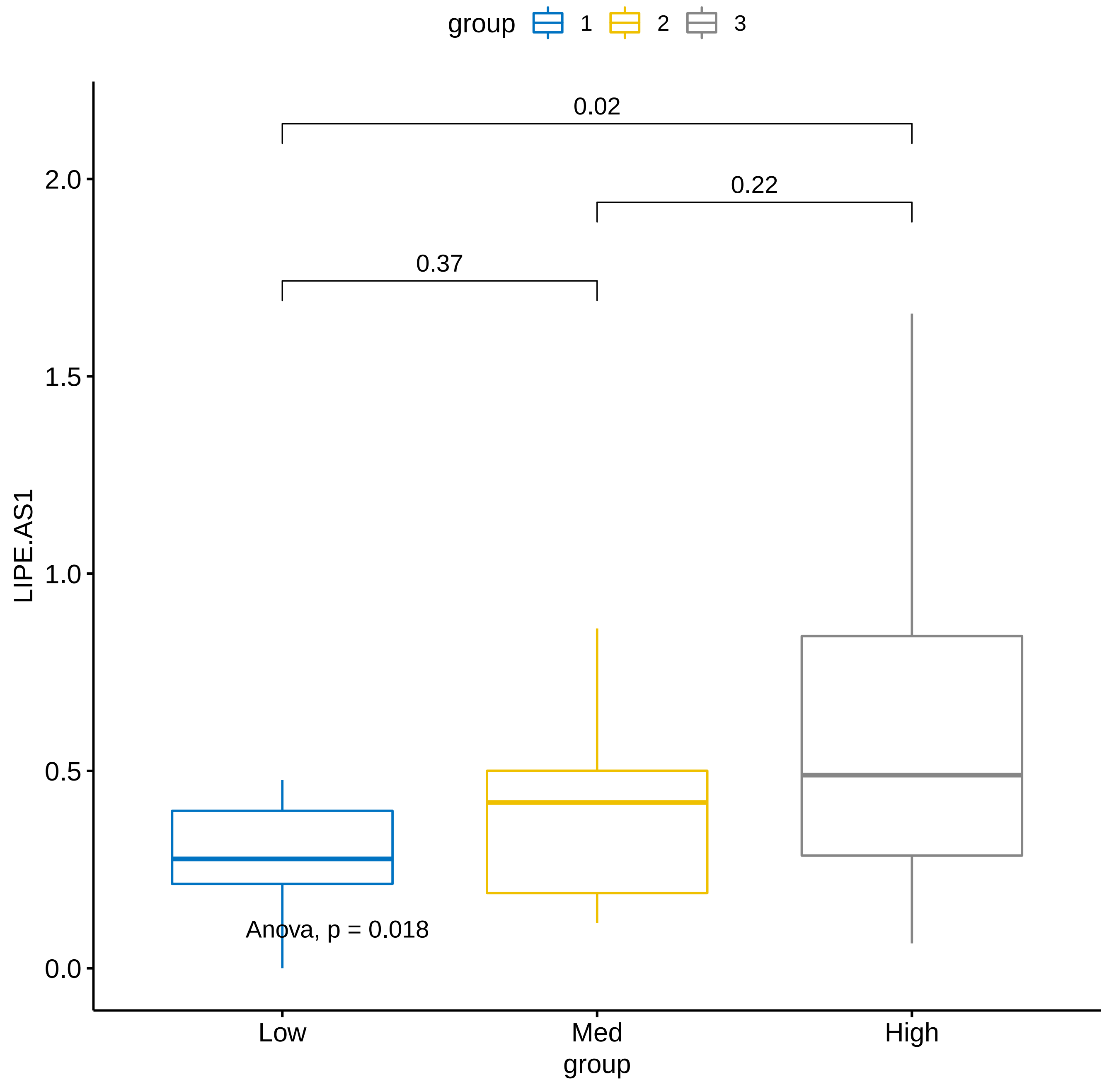
group 1 2 3
0.02
0.22
0.37
2.0
1.5
1.0
0.5
0.0
LIPE.AS1
Anova, p = 0.018
Low
Med
High
group

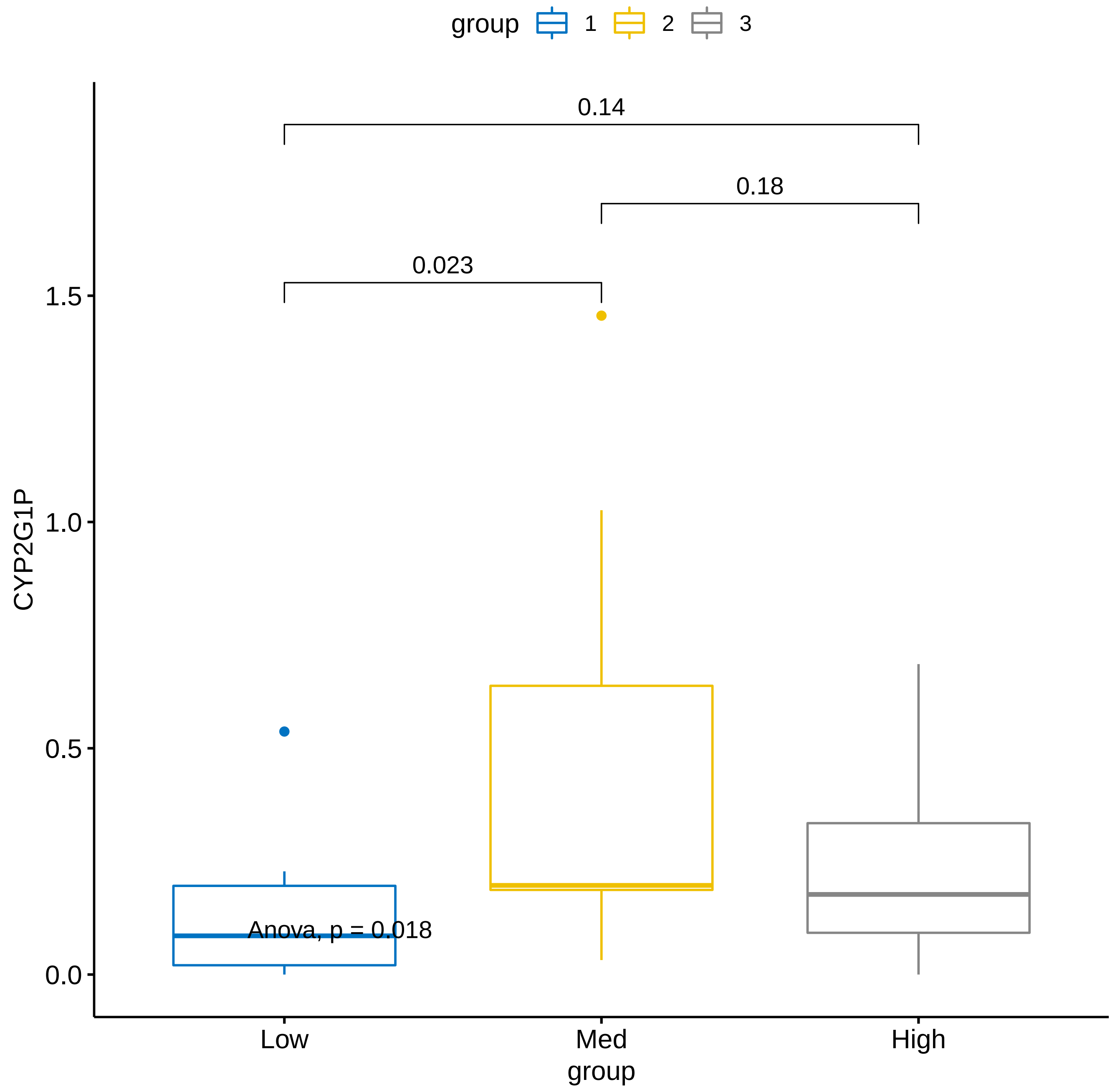

group 1 2 3
0.14
0.18
0.023
1.5
1.0
0.5
0.0
CYP2G1P
Anova, p = 0.018
Low
Med
High
group

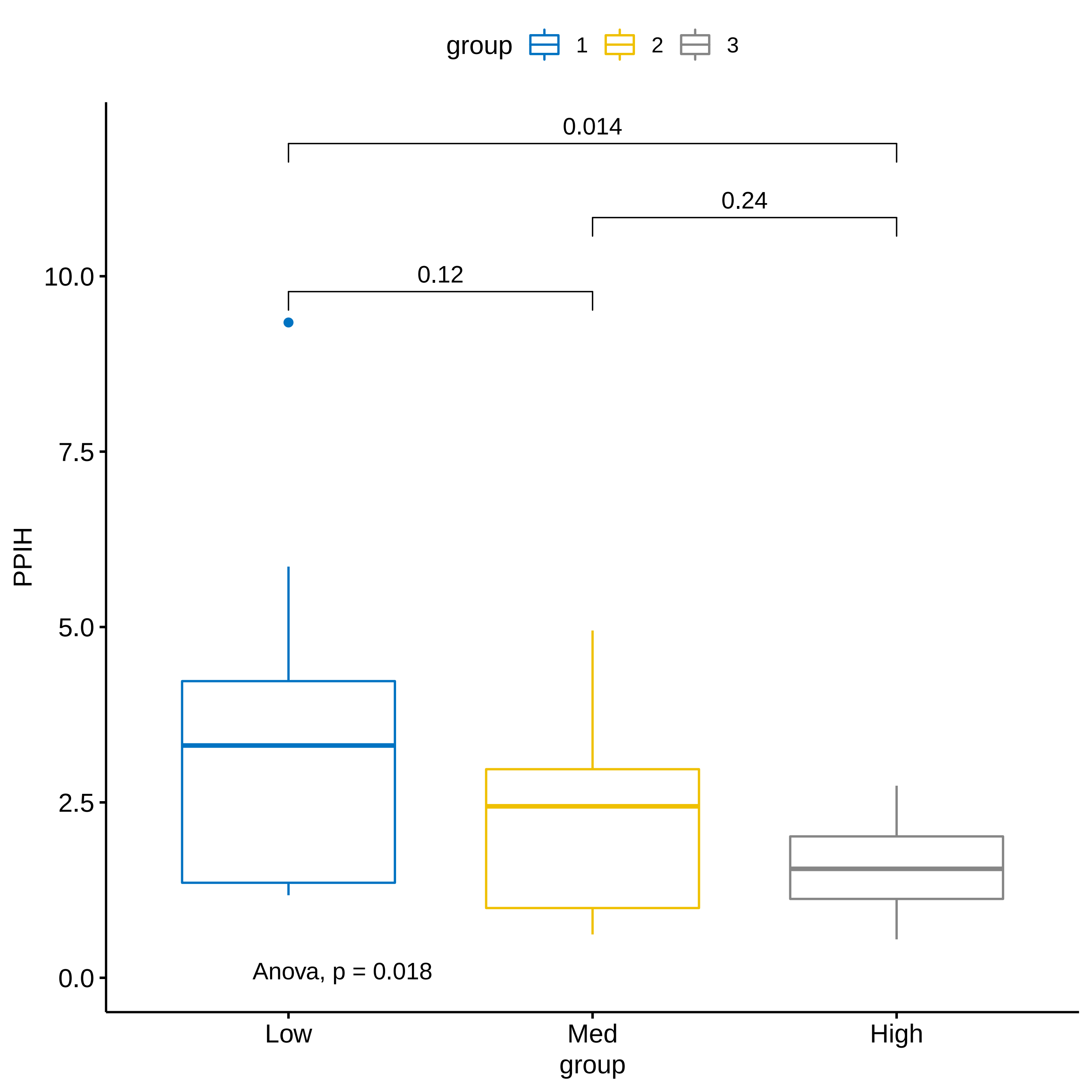
group
1
2
3
0.014
0.24
0.12
10.0
7.5
5.0
2.5
0.0
PPIH
Anova, p = 0.018
Low
Med
High
group

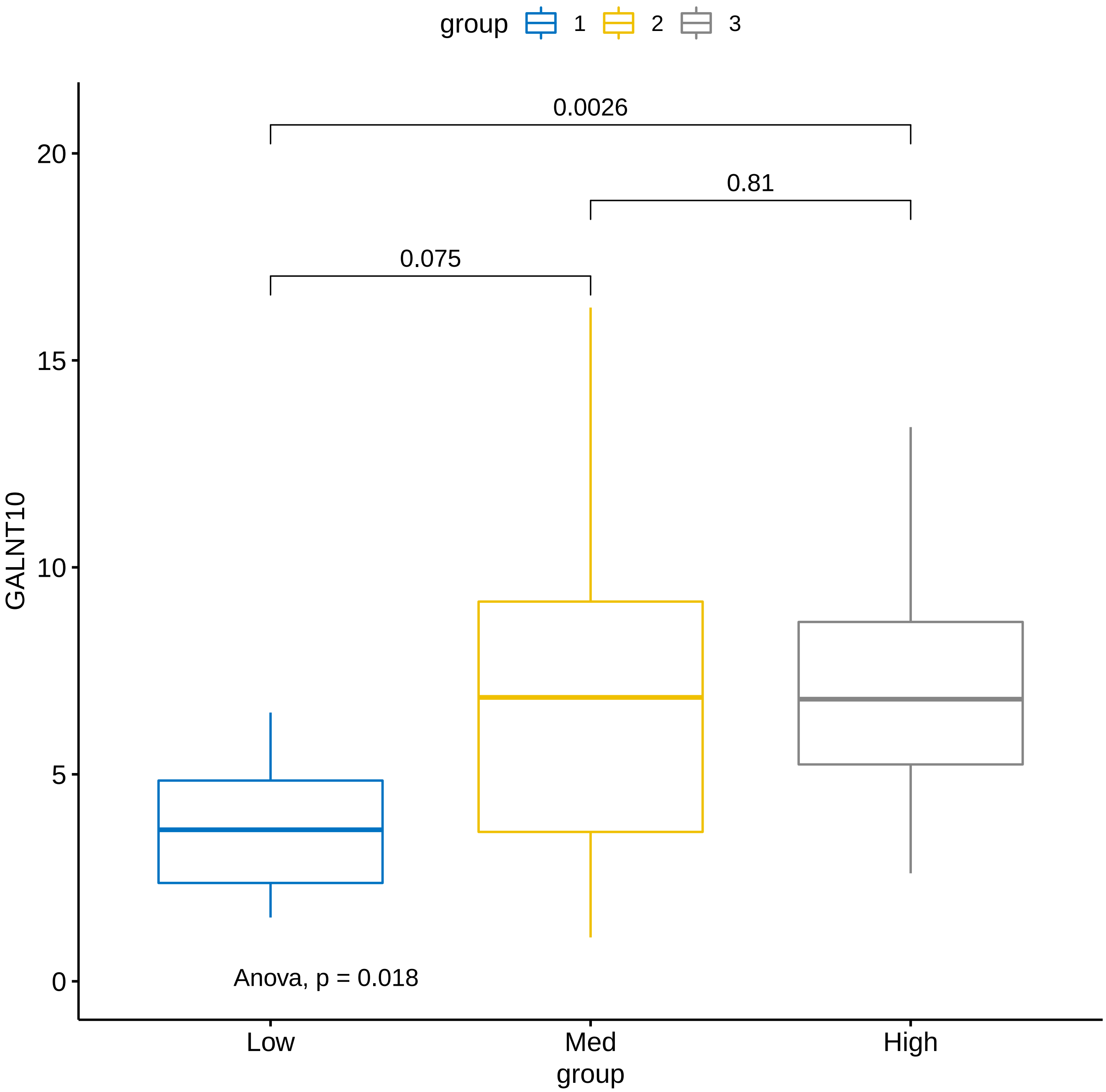
group 1 2 3
0.0026
0.81
0.075
20
15
10
5
0
GALNT10
Anova, p = 0.018
Low
Med
High
group

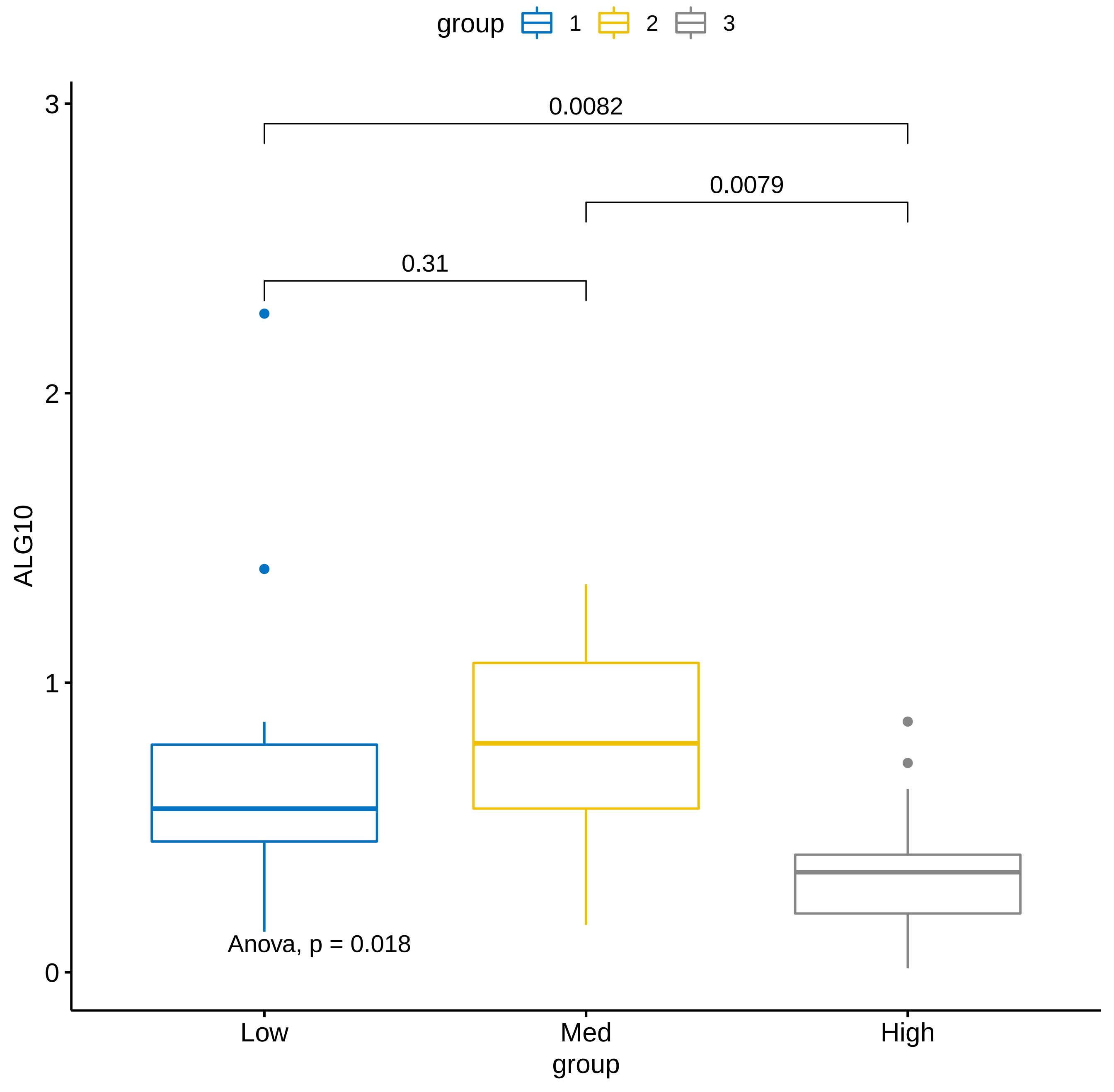

group 1 2 3
0.0082
0.0079
0.31
ALG10
3
2
1
0
Anova, p = 0.018
Low
Med
High
group

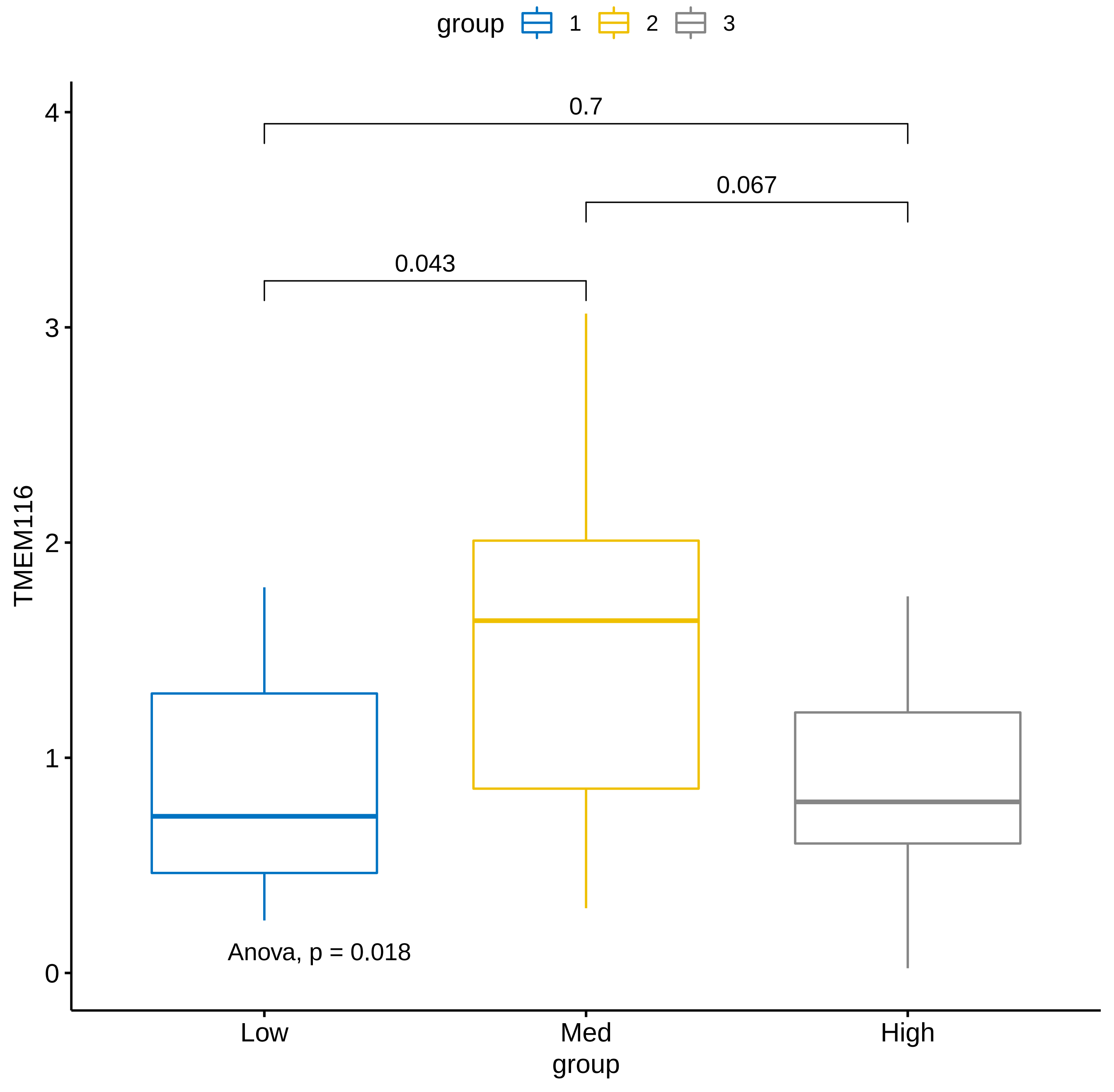
group 1 2 3
0.7
0.067
0.043
TMEM116
4
3
2
1
0
Anova, p = 0.018
Low
Med
High
group

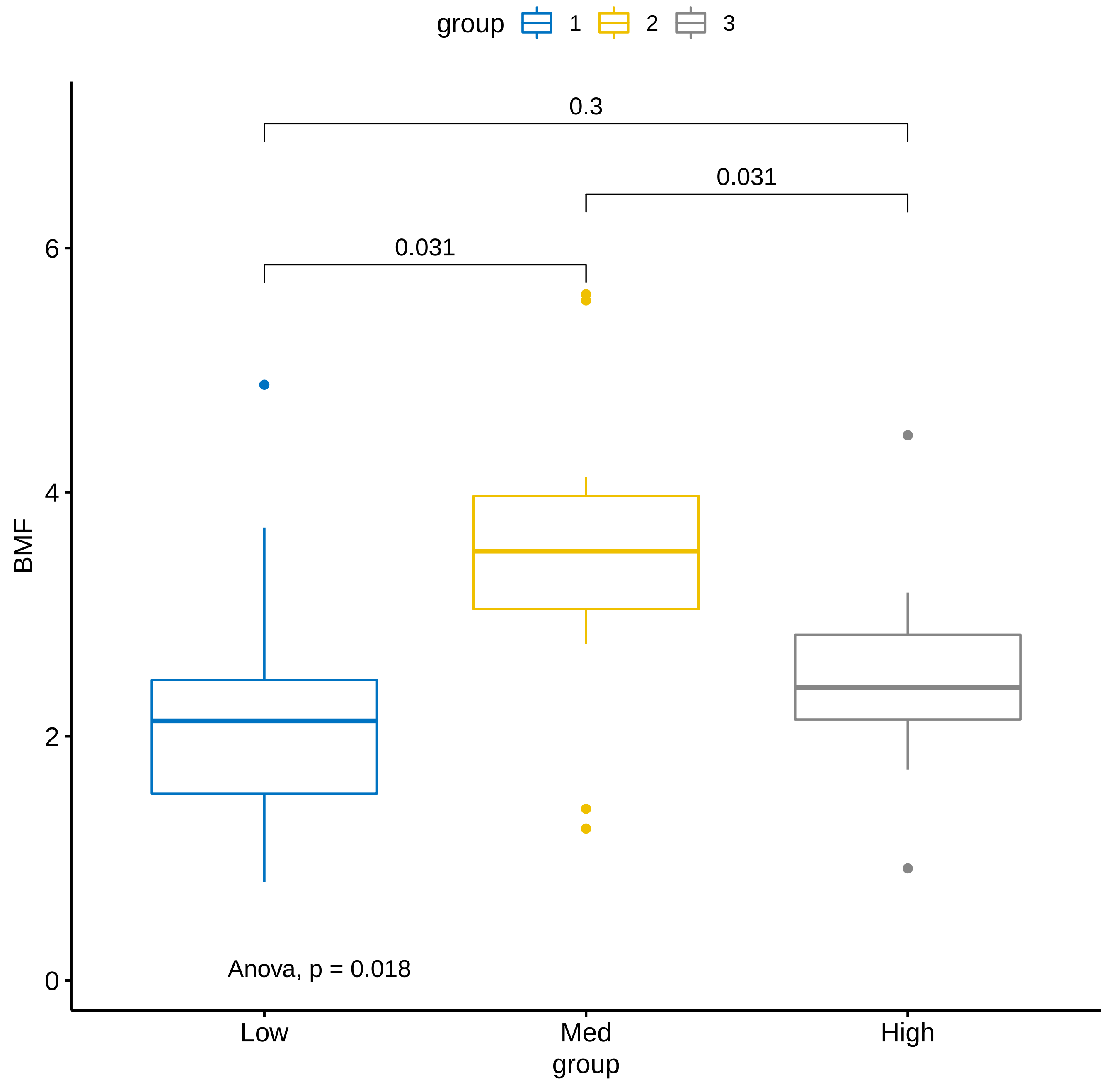
group
1
2
3
0.3
0.031
0.031
6
4
2
0
BMF
Anova, p = 0.018
Low
Med
High
group

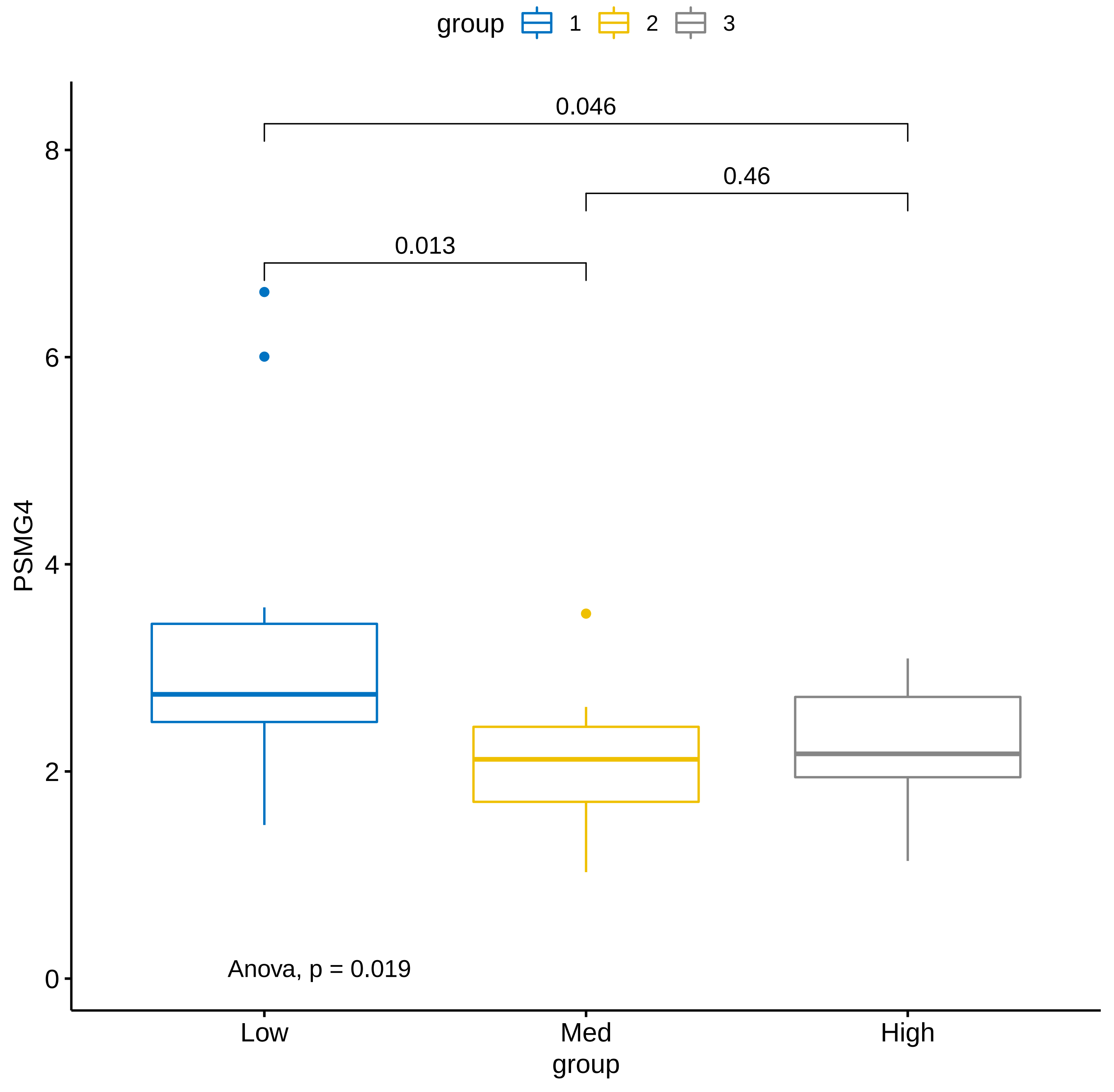

group 1 2 3
0.046
0.46
0.013
8
6
4
2
0
PSMG4
Anova, p = 0.019
Low
Med
High
group

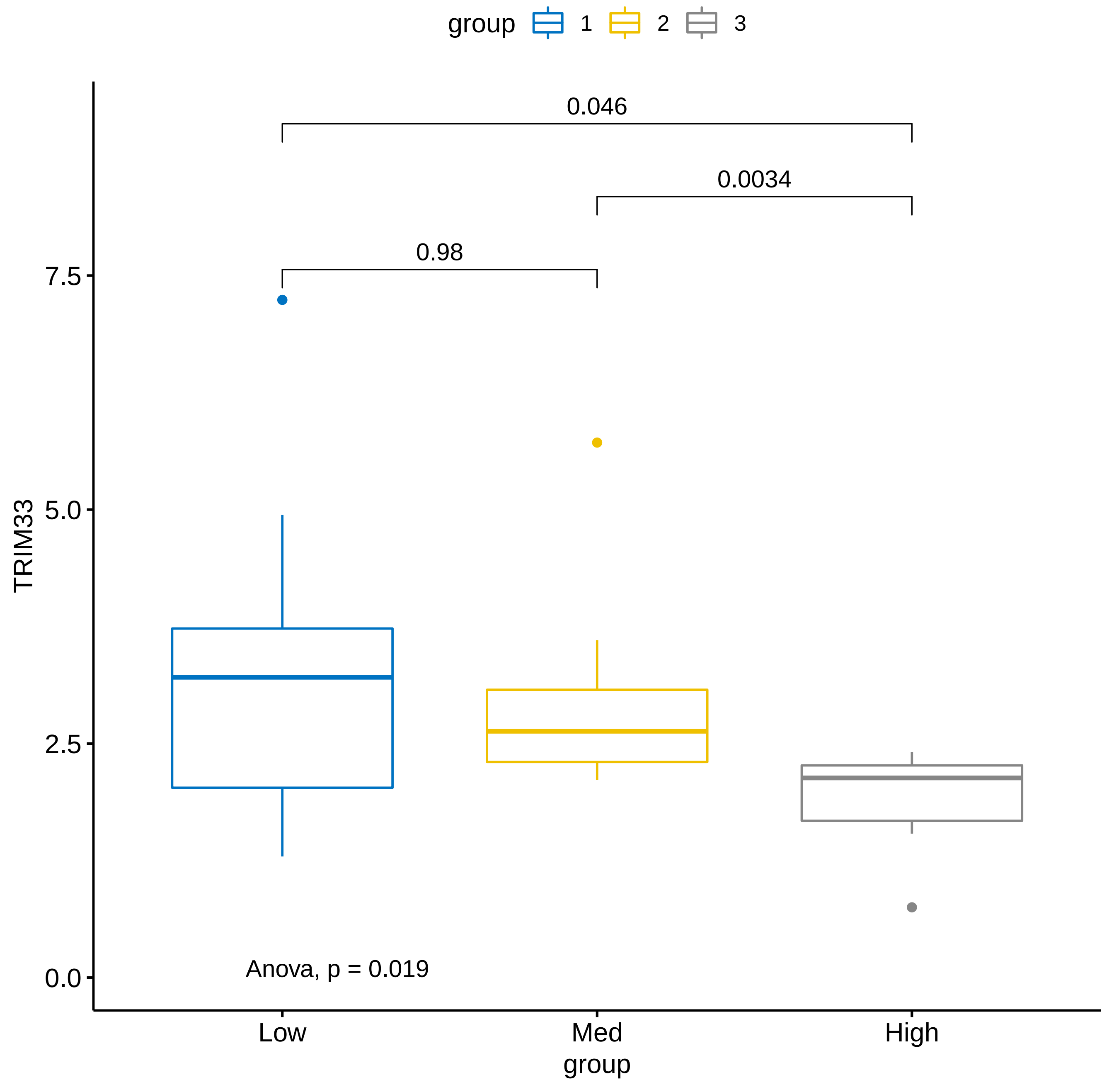
group
1
2
3
0.046
0.0034
0.98
7.5
5.0
2.5
0.0
TRIM33
Anova, p = 0.019
Low
Med
High
group

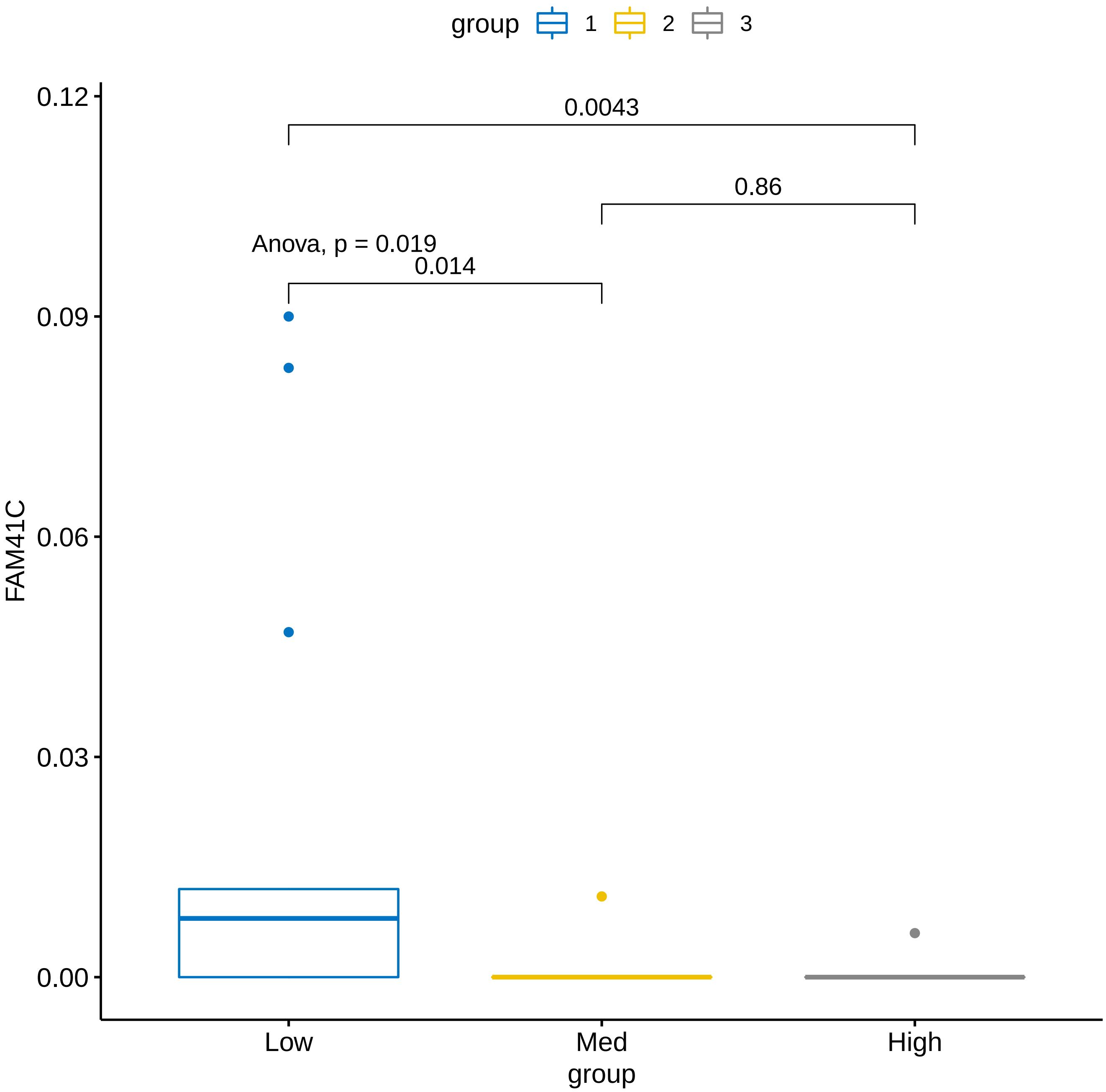

group
1
2
3
0.0043
0.86
Anova, p = 0.019
0.014
0.12
0.09
0.06
0.03
0.00
FAM41C
Low
Med
High
group

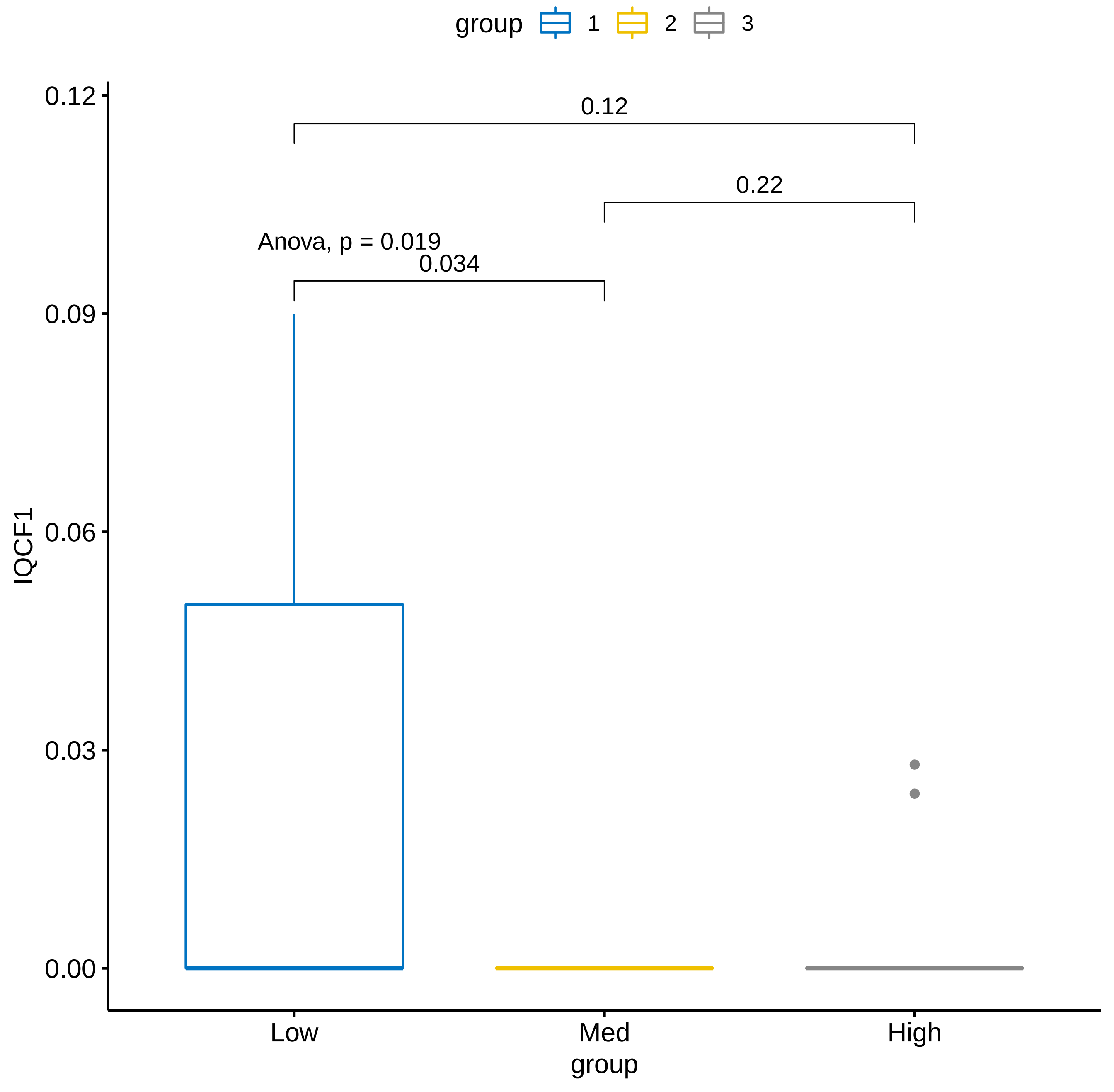
group 1 2 3
0.12
0.22
Anova, p = 0.019
0.034
0.12
0.09
0.06
0.03
0.00
IQCF1
Low
Med
High
group

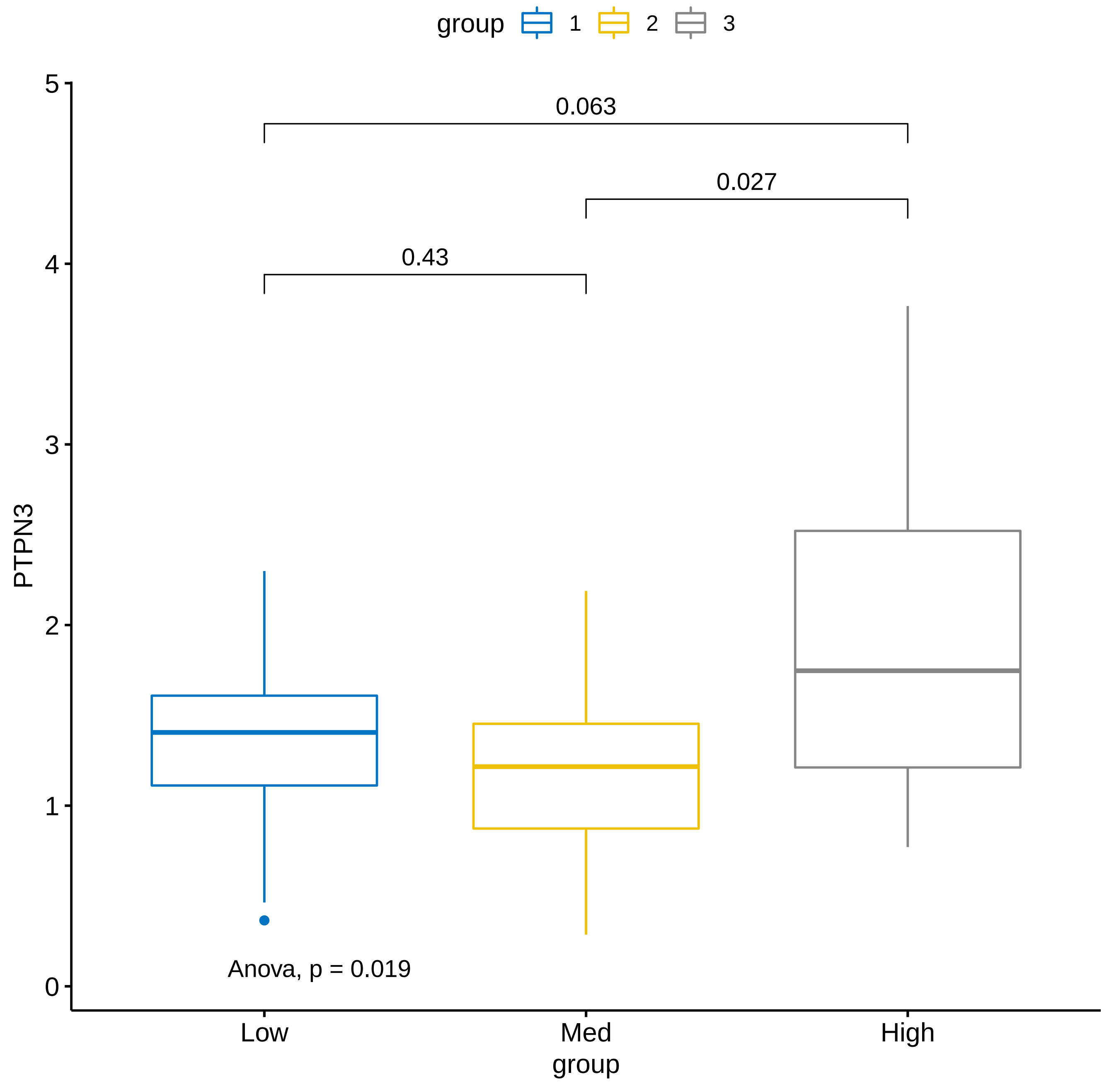
group 1 2 3
0.063
0.027
0.43
PTPN3
5
4
3
2
1
0
Anova, p = 0.019
Low
Med
High
group

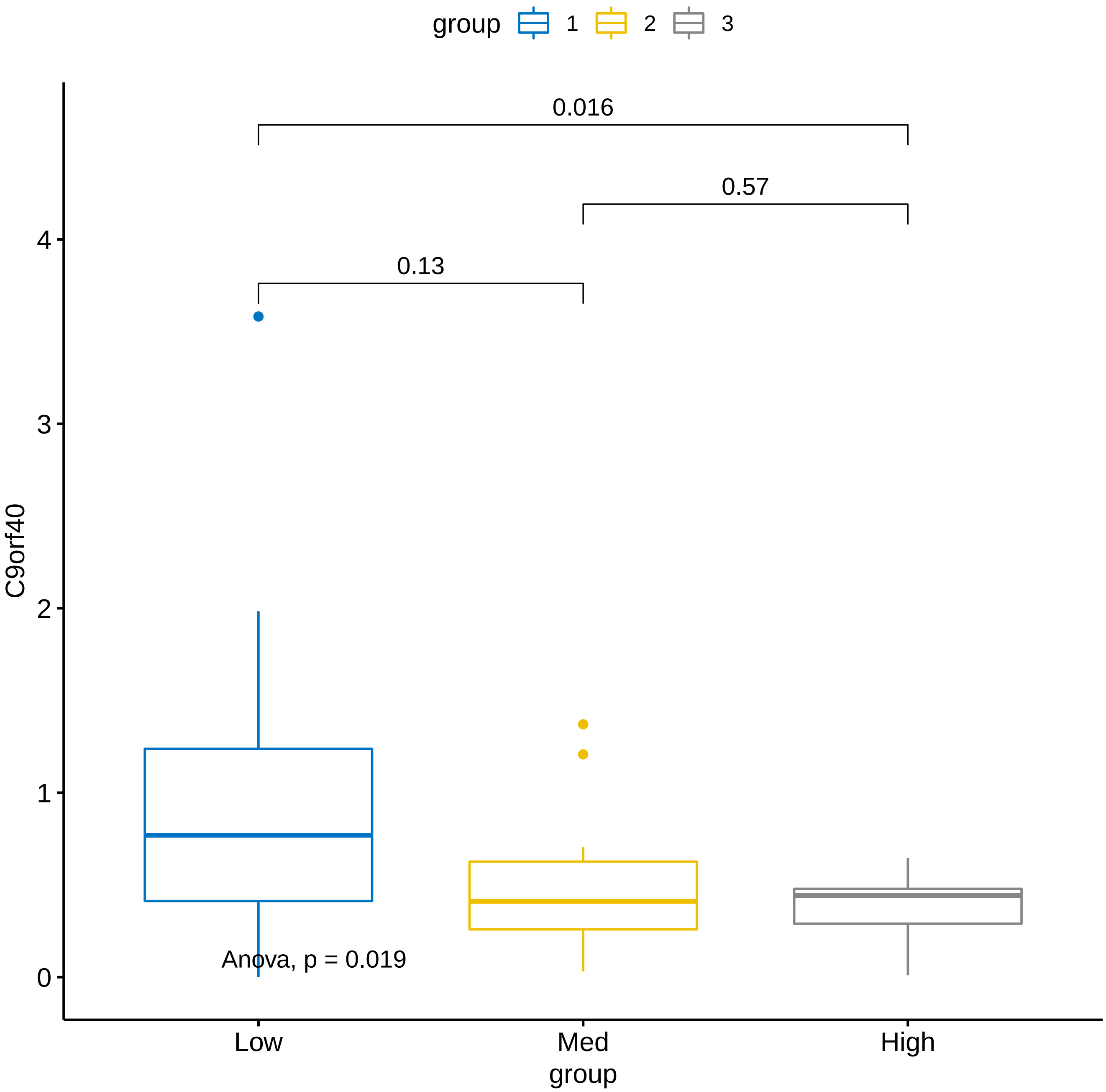
group
1
2
3
0.016
0.57
0.13
C9orf40
4
3
2
1
0
Anova, p = 0.019
Low
Med
High
group

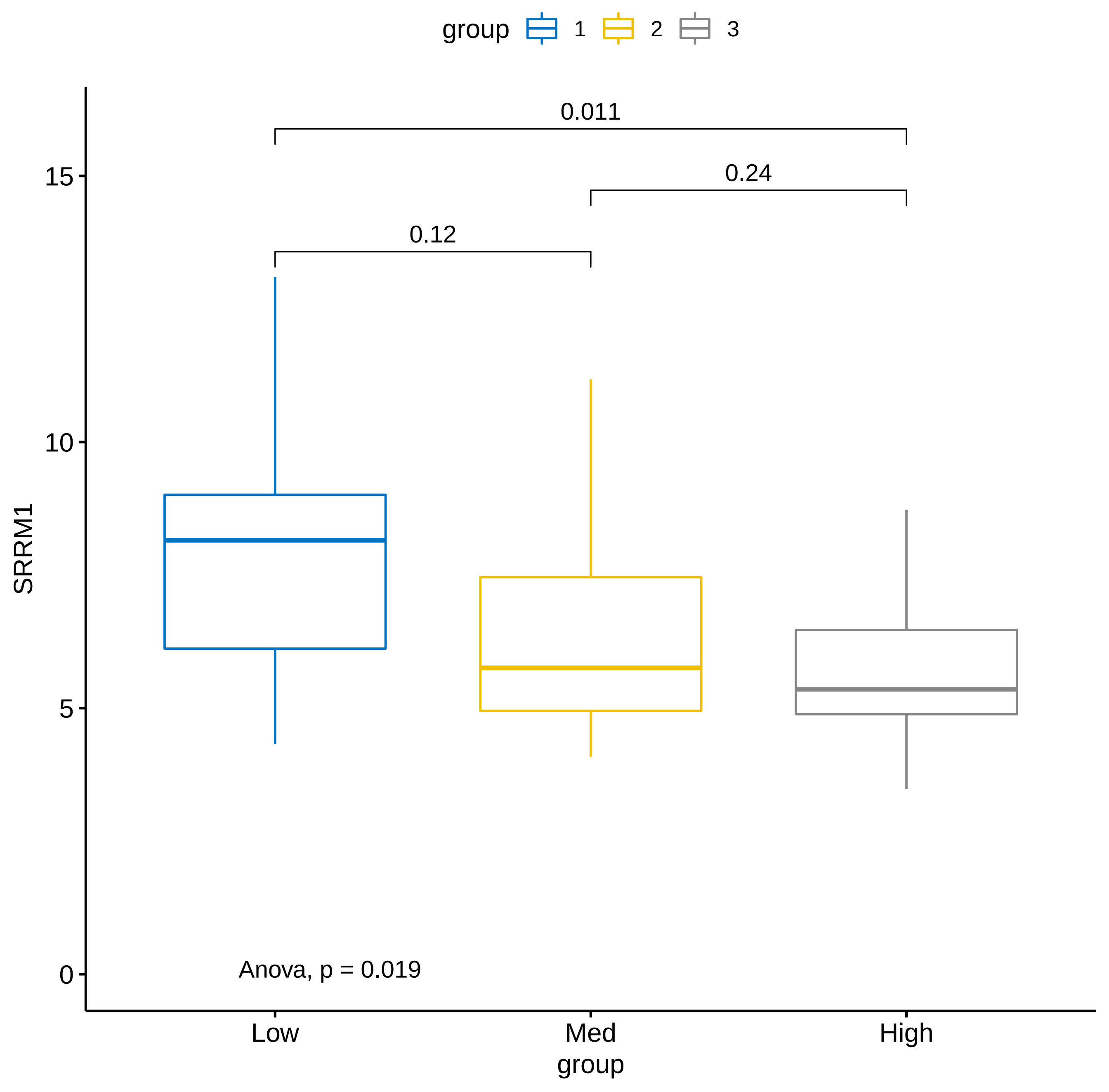

group
1
2
3
0.011
0.24
0.12
15
10
5
0
SRRM1
Anova, p = 0.019
Low
Med
High
group

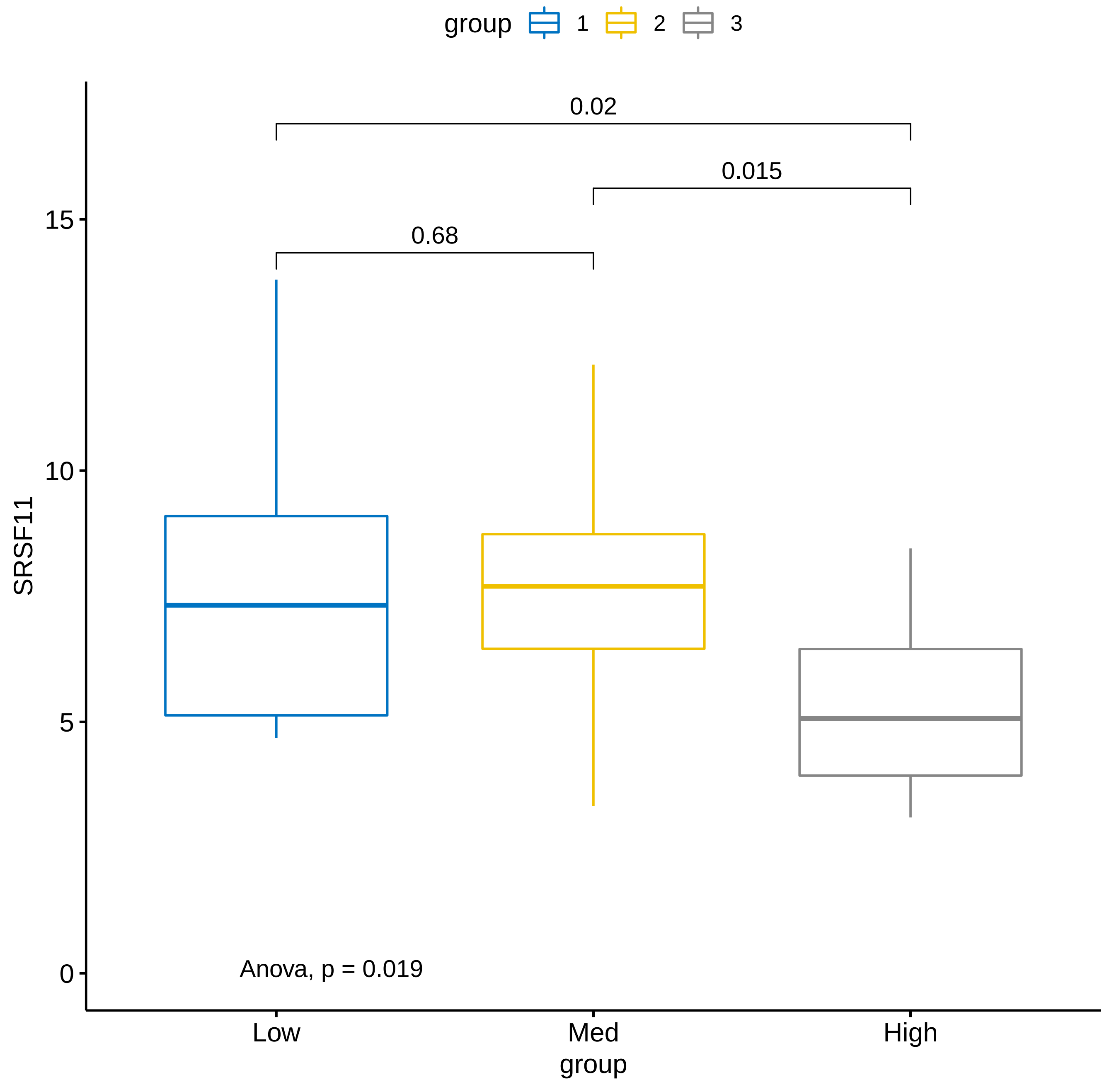

group
1
2
3
0.02
0.015
0.68
15
10
5
0
SRSF11
Anova, p = 0.019
Low
Med
High
group

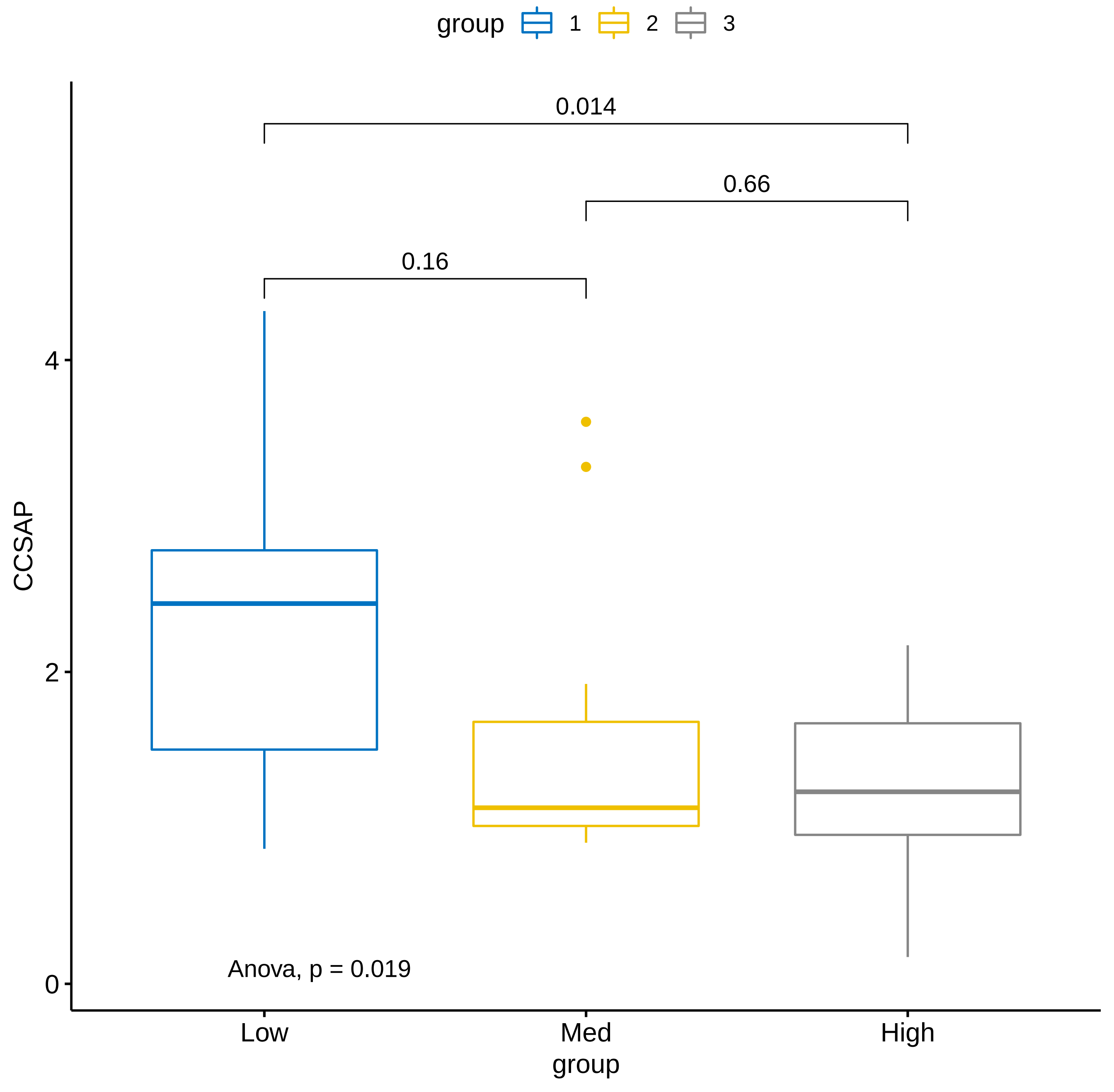

group 1 2 3
0.014
0.66
0.16
4
2
0
CCSAP
Anova, p = 0.019
Low
Med
High
group

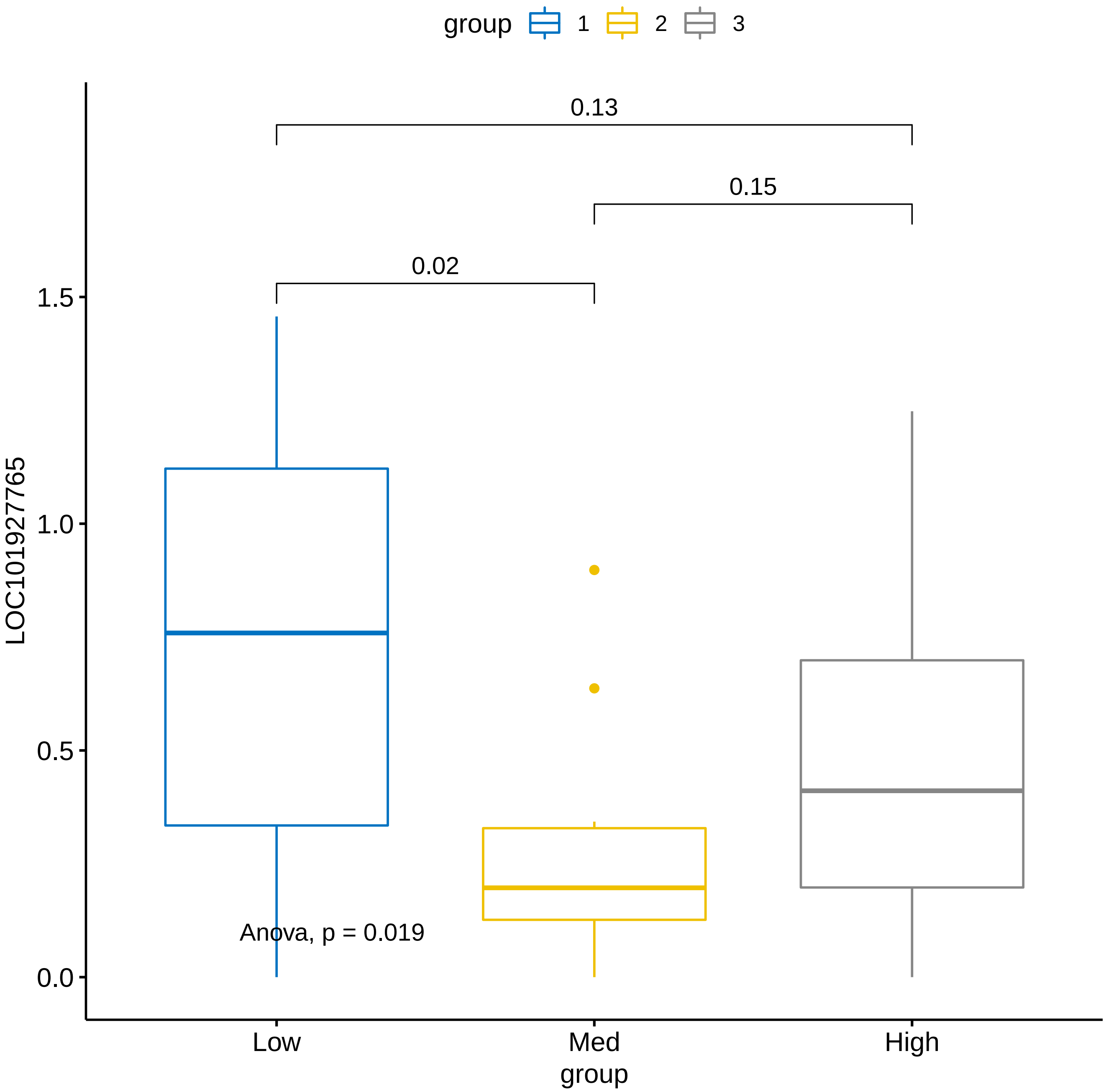
group
1
2
3
0.13
0.15
0.02
1.5
1.0
0.5
0.0
LOC101927765
Anova, p = 0.019
Low
Med
High
group

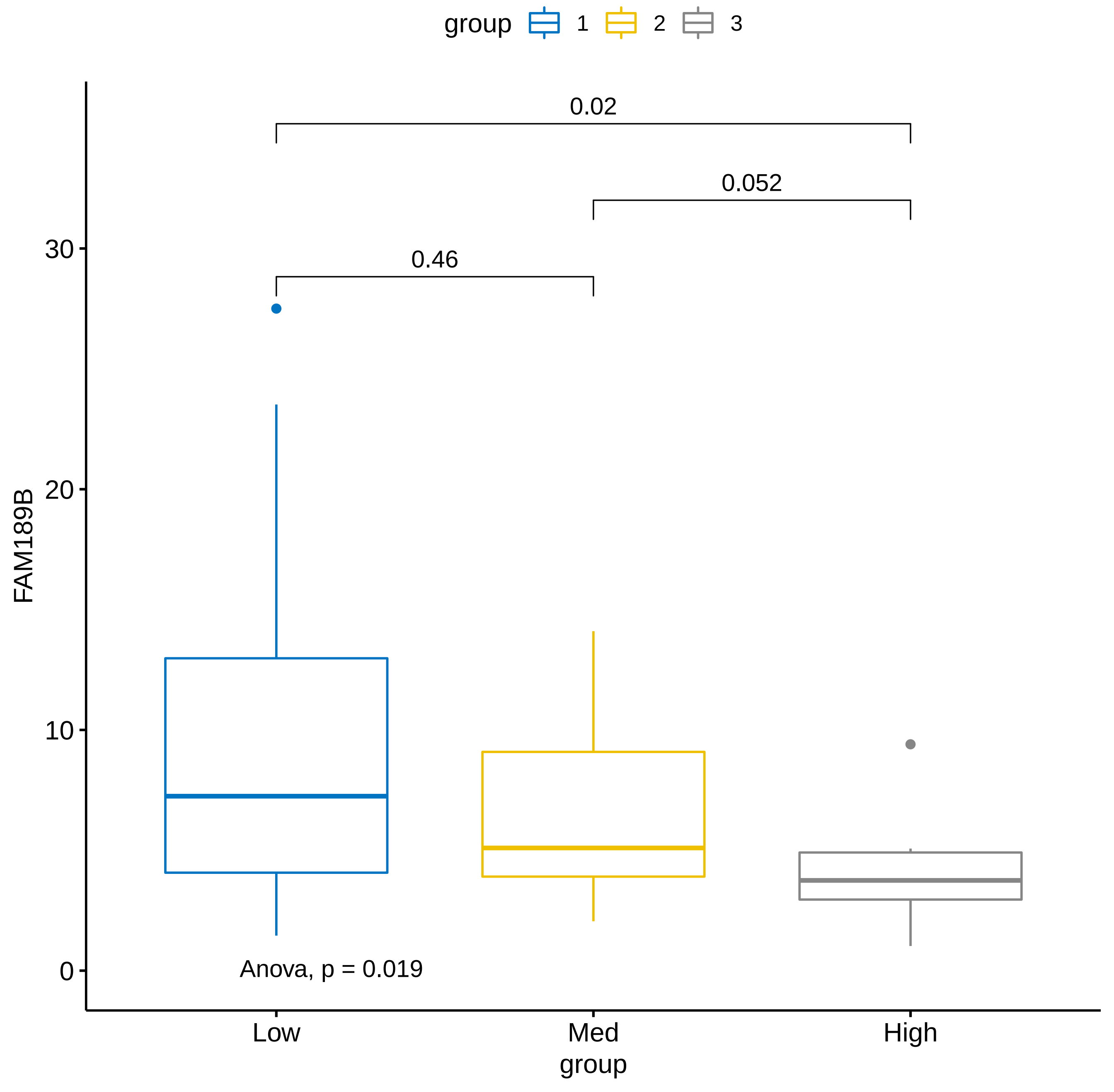

group
1
2
3
0.02
0.052
0.46
30
20
10
0
FAM189B
Anova, p = 0.019
Low
Med
High
group

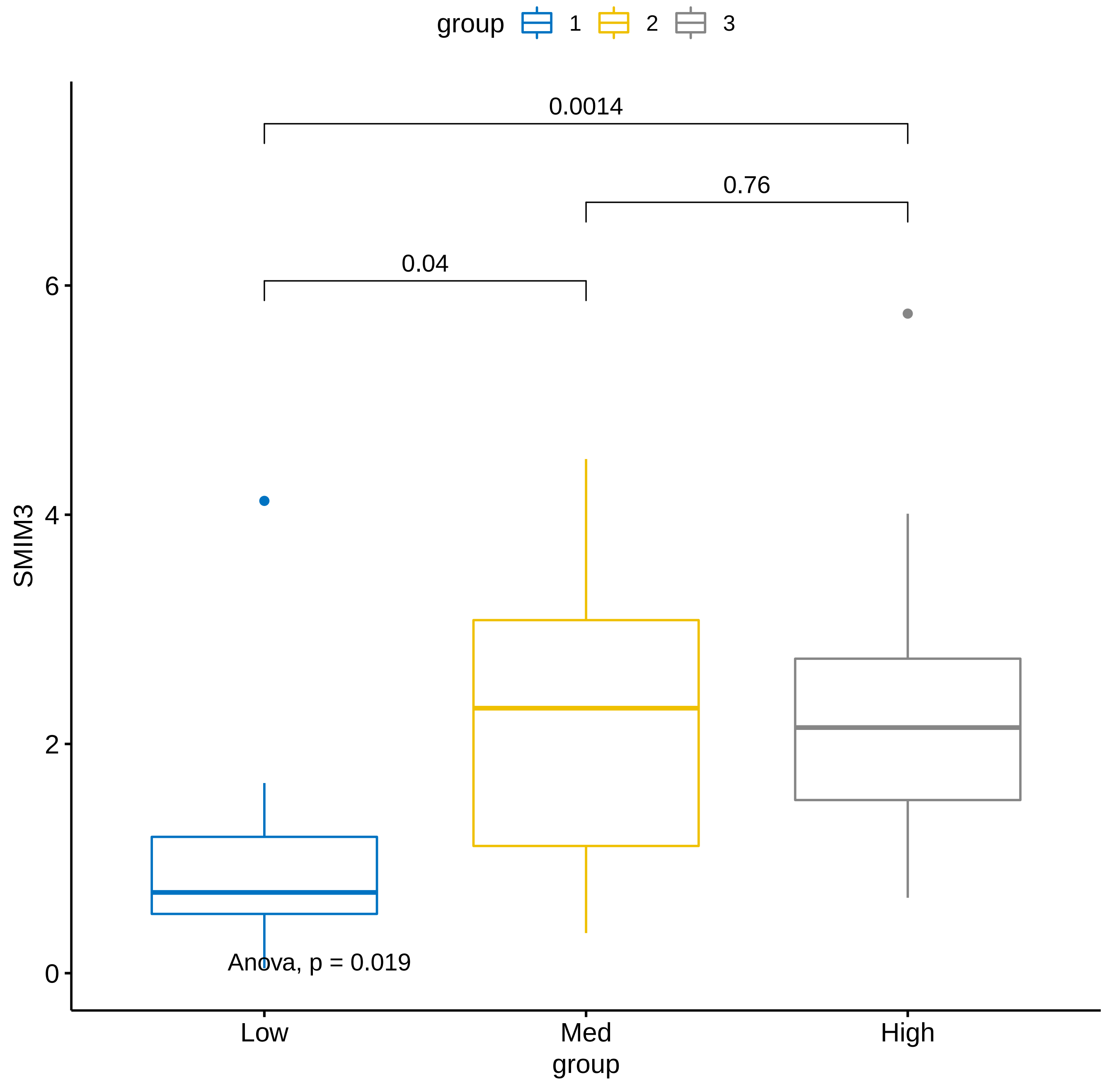
group
1
2
3
0.0014
0.76
0.04
6
4
2
0
SMIM3
Anova, p = 0.019
Low
Med
High
group

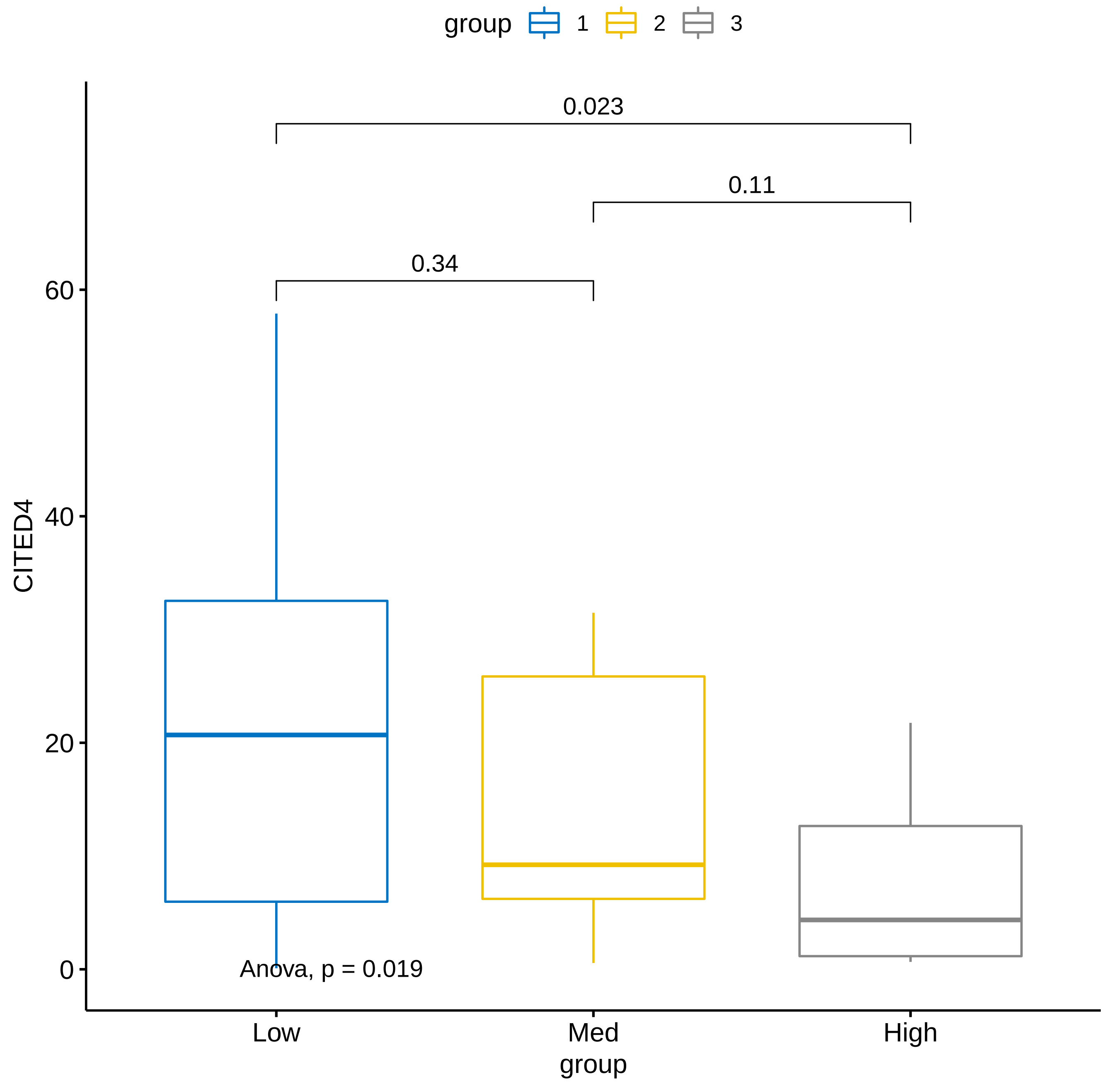
group
1
2
3
0.023
0.11
0.34
60
40
20
0
CITED4
Anova, p = 0.019
Low
Med
High
group

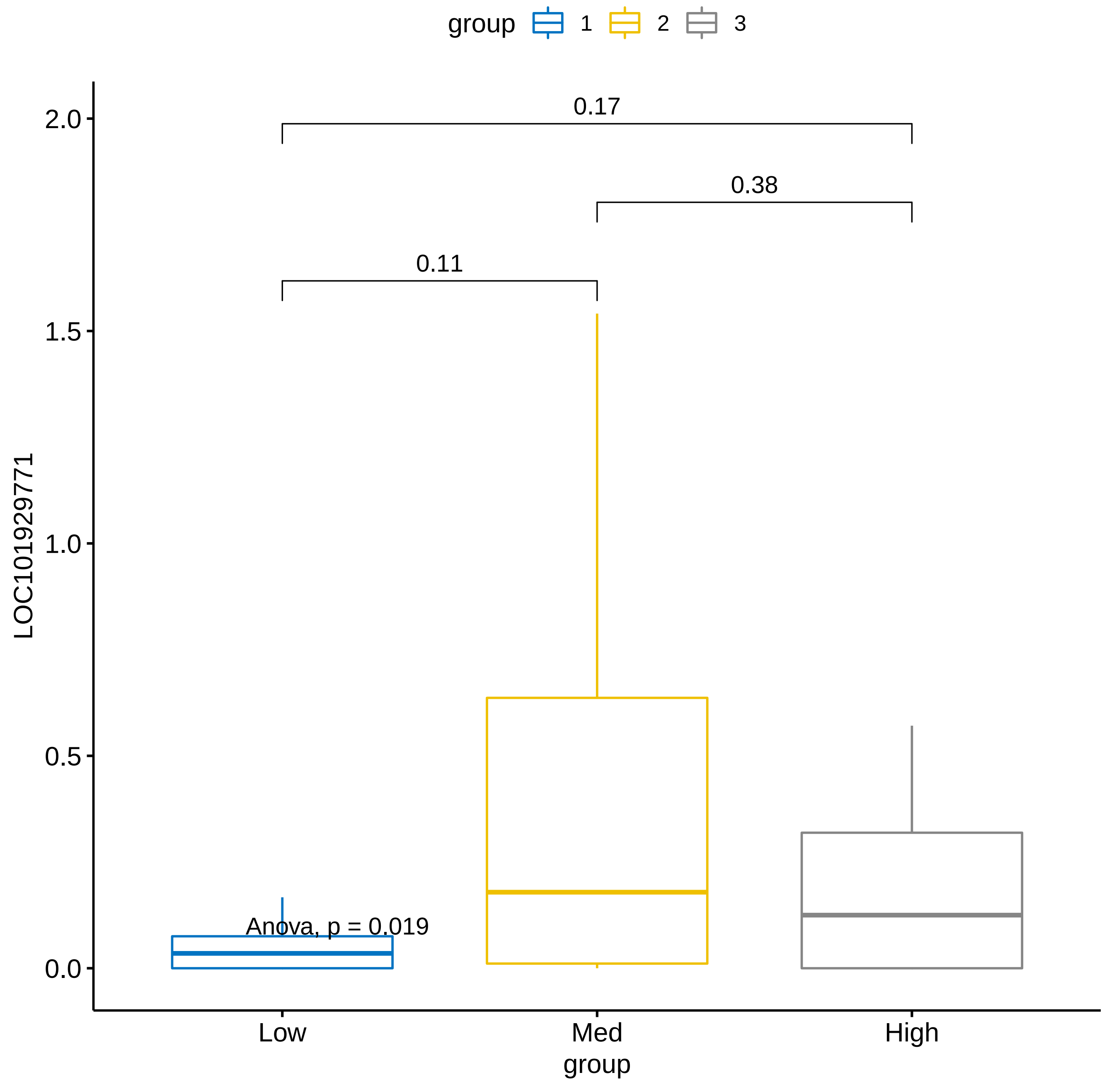
group 1 2 3
0.17
0.38
0.11
2.0
1.5
1.0
0.5
0.0
LOC101929771
Anova, p = 0.019
Low
Med
High
group

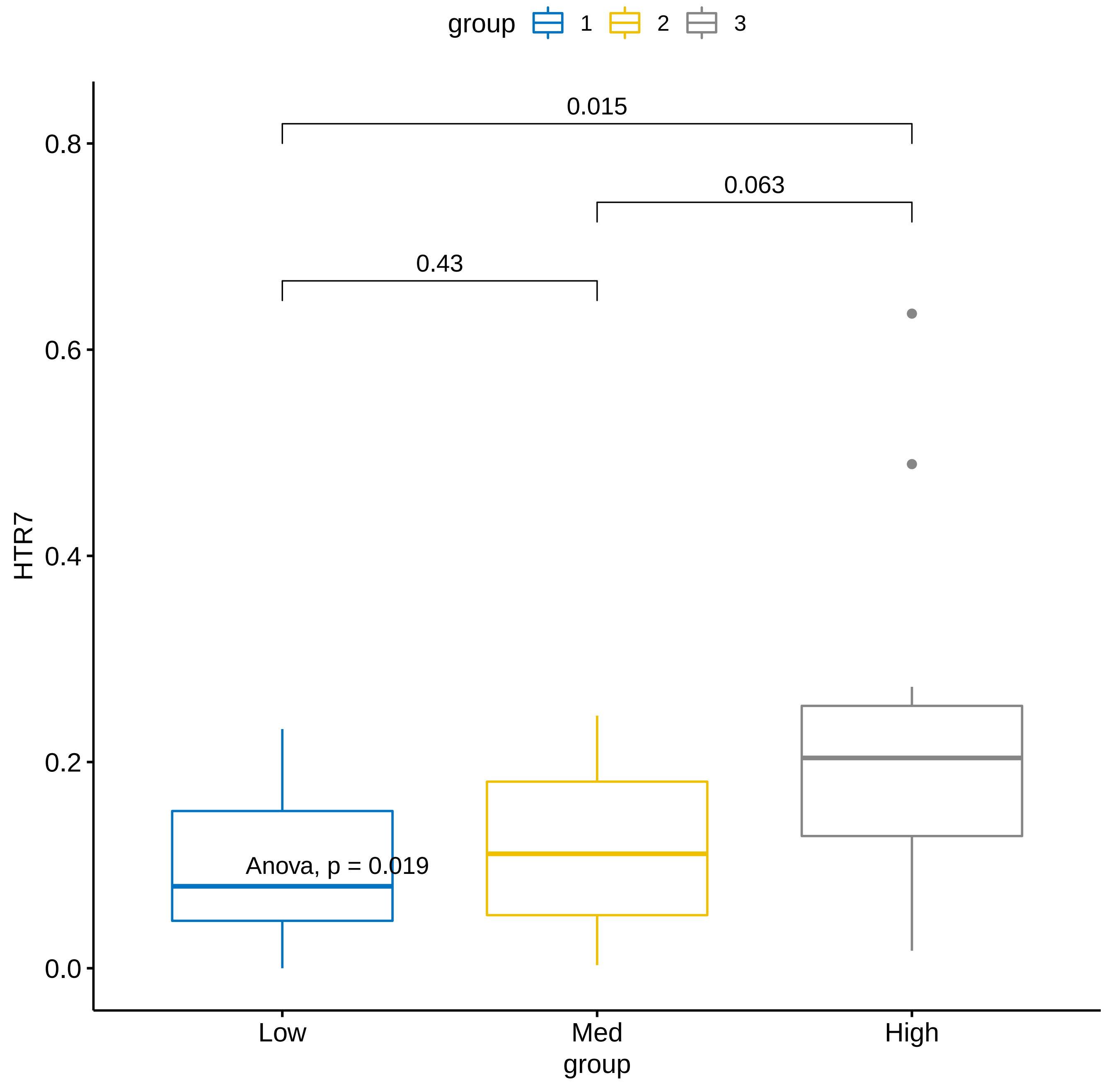

group
1
2
3
0.015
0.063
0.43
Anova, p = 0.019
HTR7
0.0
0.2
0.4
0.6
0.8
Low
Med
High
group

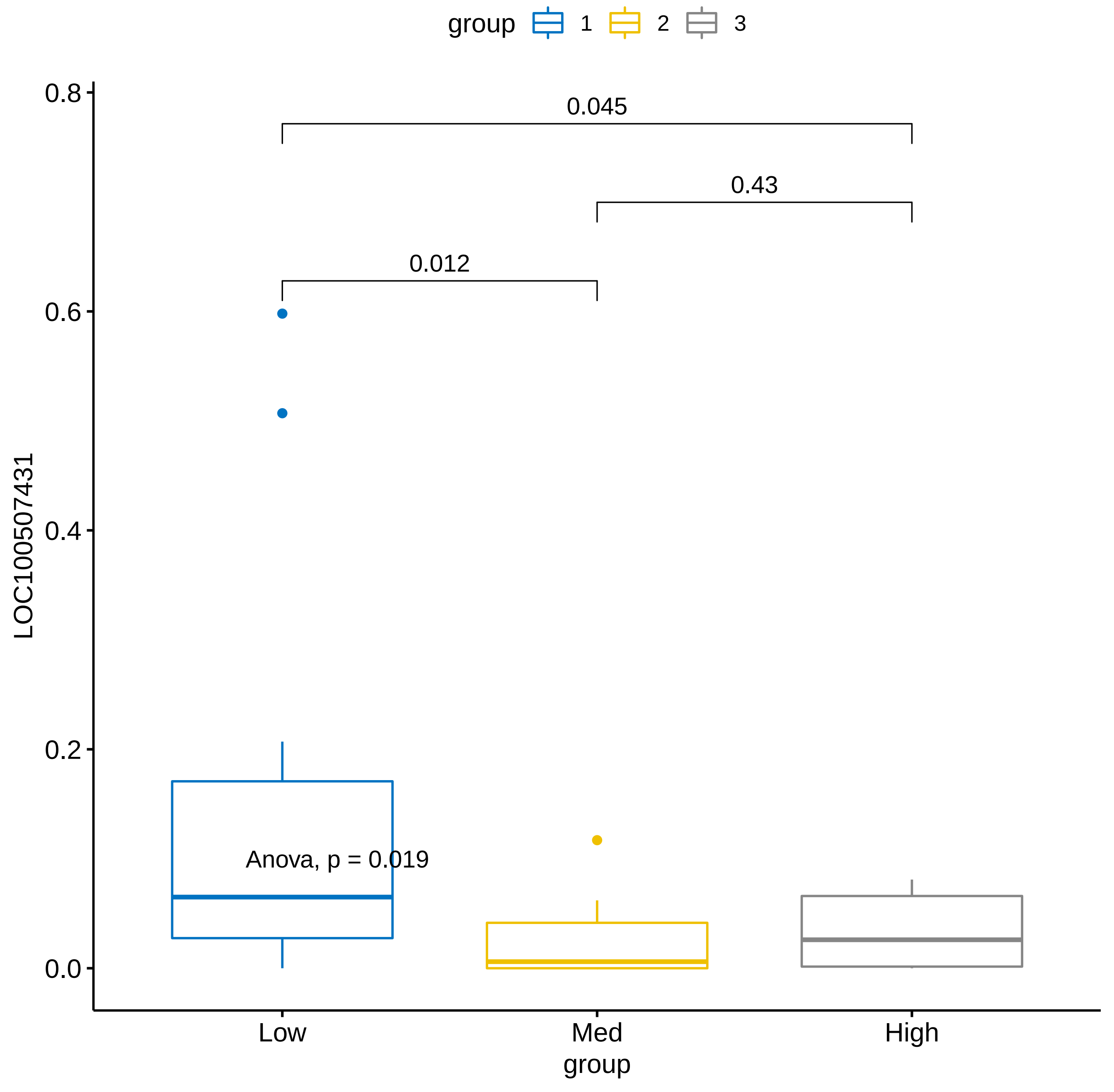
group
1
2
3
0.8
0.6
0.4
0.2
0.0
LOC100507431
0.045
0.43
0.012
Anova, p = 0.019
Low
Med
High
group

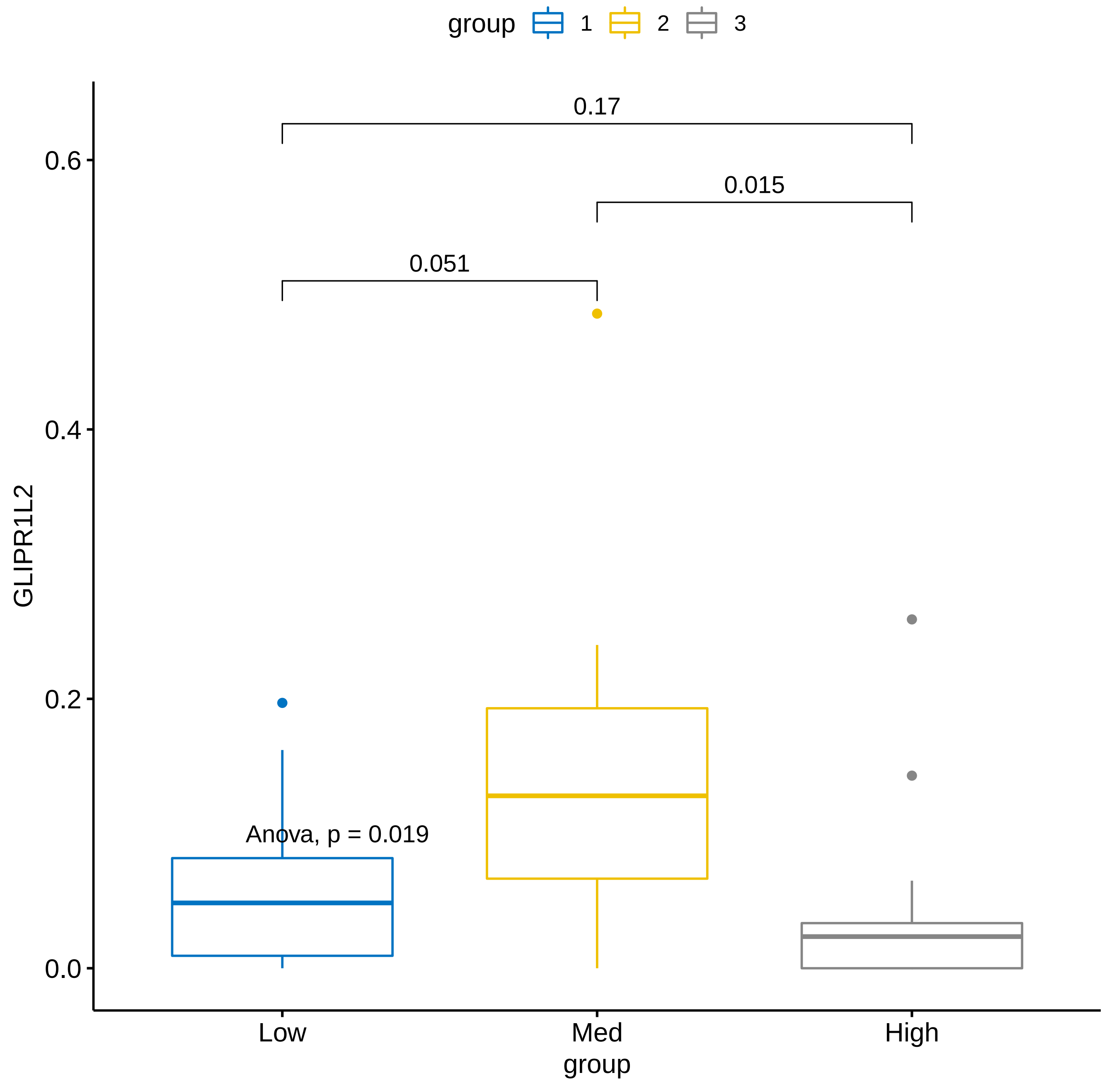

group 1 2 3
0.17
0.015
0.051
Anova, p = 0.019
0.6
0.4
0.2
0.0
GLIPR1L2
Low
Med
High
group

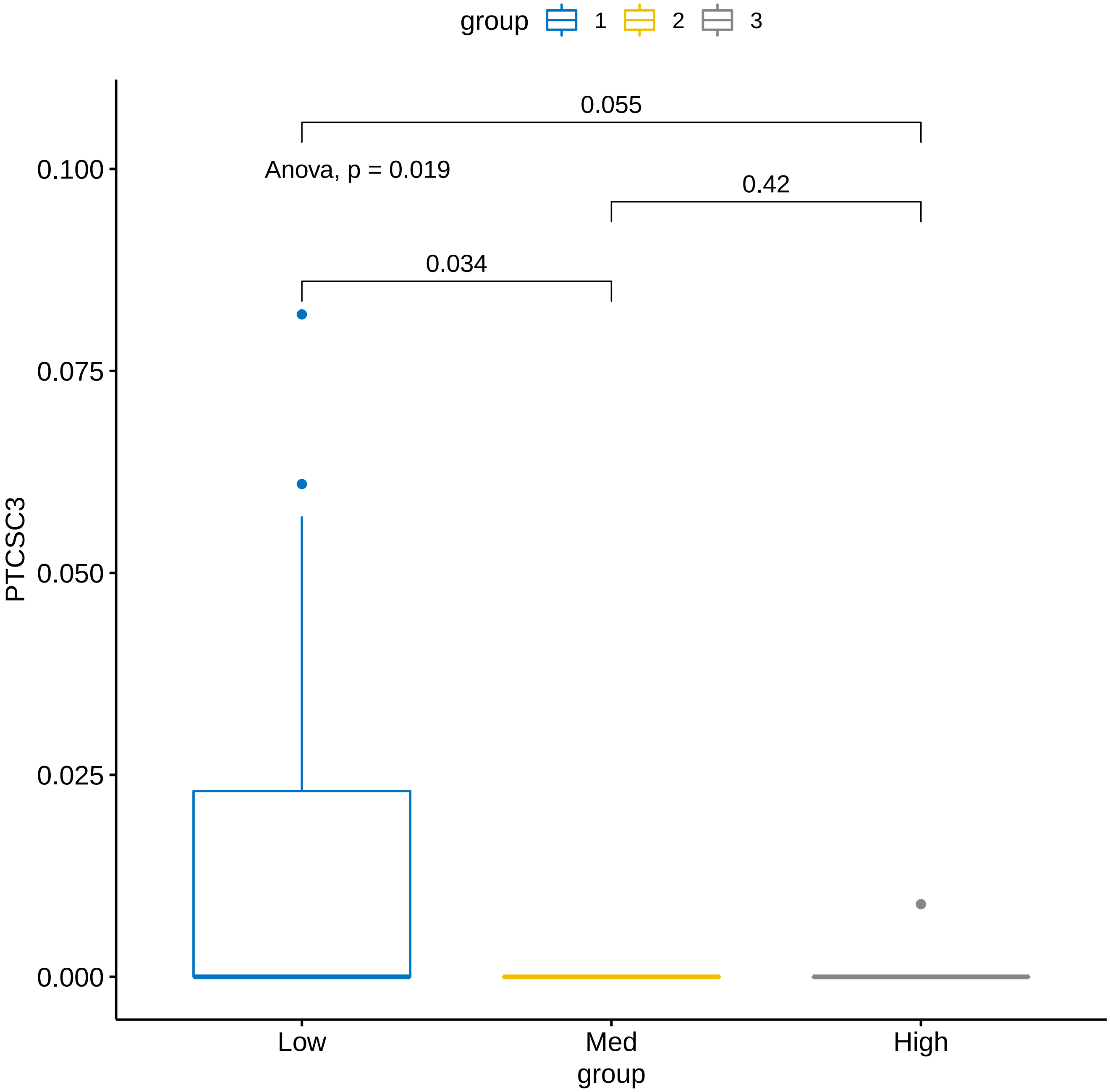

group
1
2
3
0.055
Anova, p = 0.019
0.42
0.034
0.100
0.075
0.050
0.025
0.000
PTCSC3
Low
Med
High
group

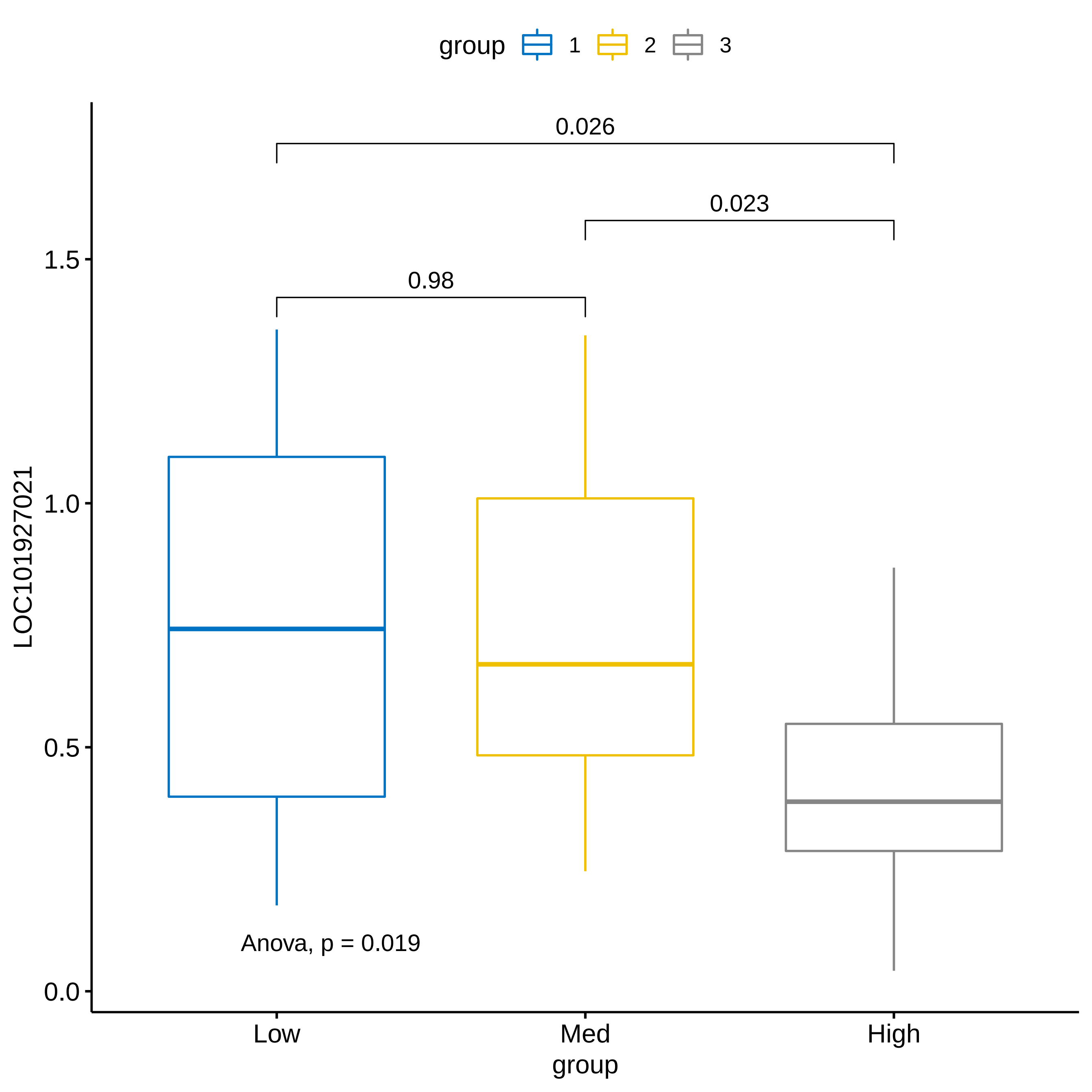
group
1
2
3
0.026
0.023
0.98
1.5
1.0
0.5
0.0
LOC101927021
Anova, p = 0.019
Low
Med
High
group

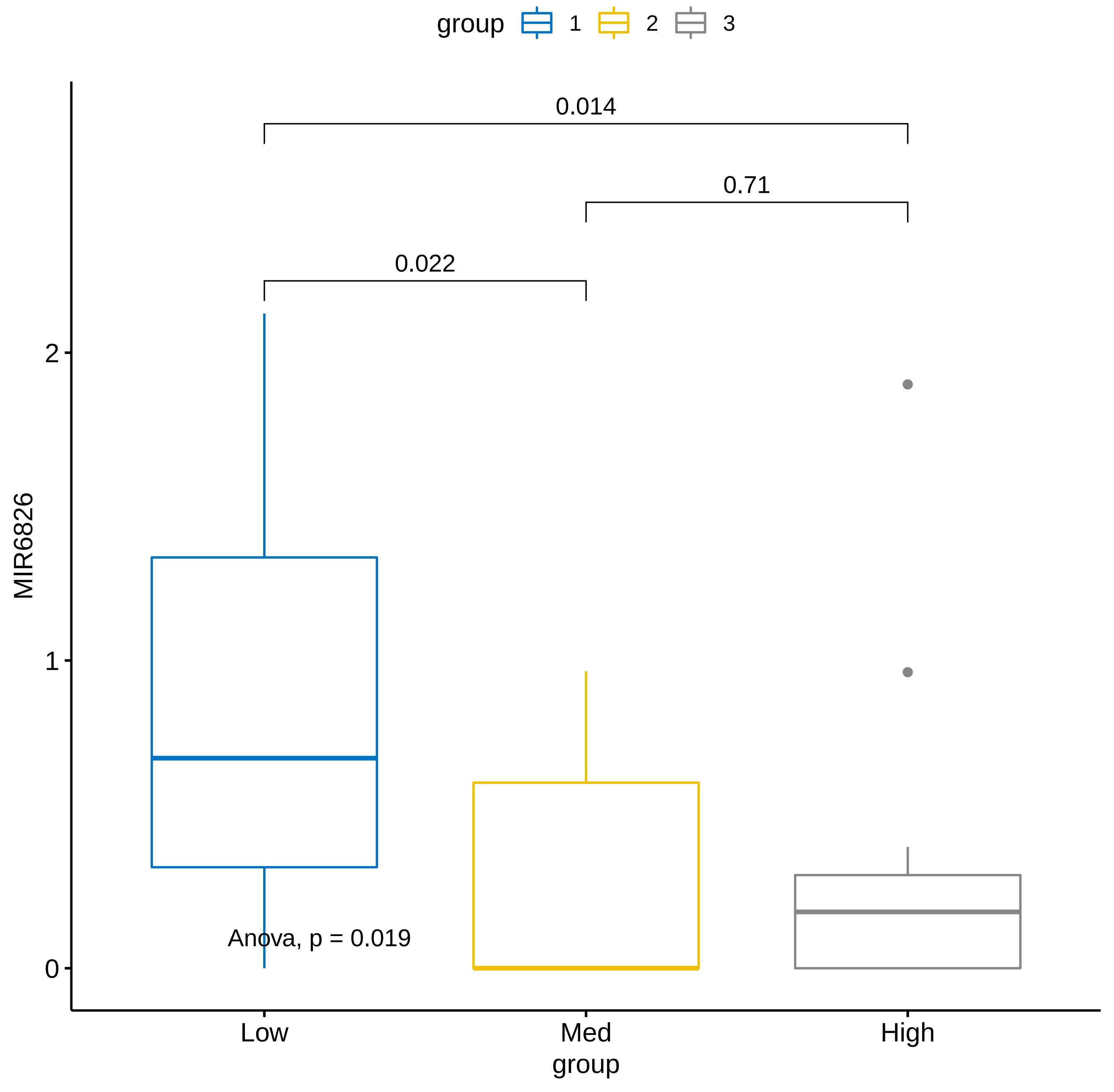
group
1
2
3
0.014
0.71
0.022
2
1
0
MIR6826
Anova, p = 0.019
Low
Med
High
group

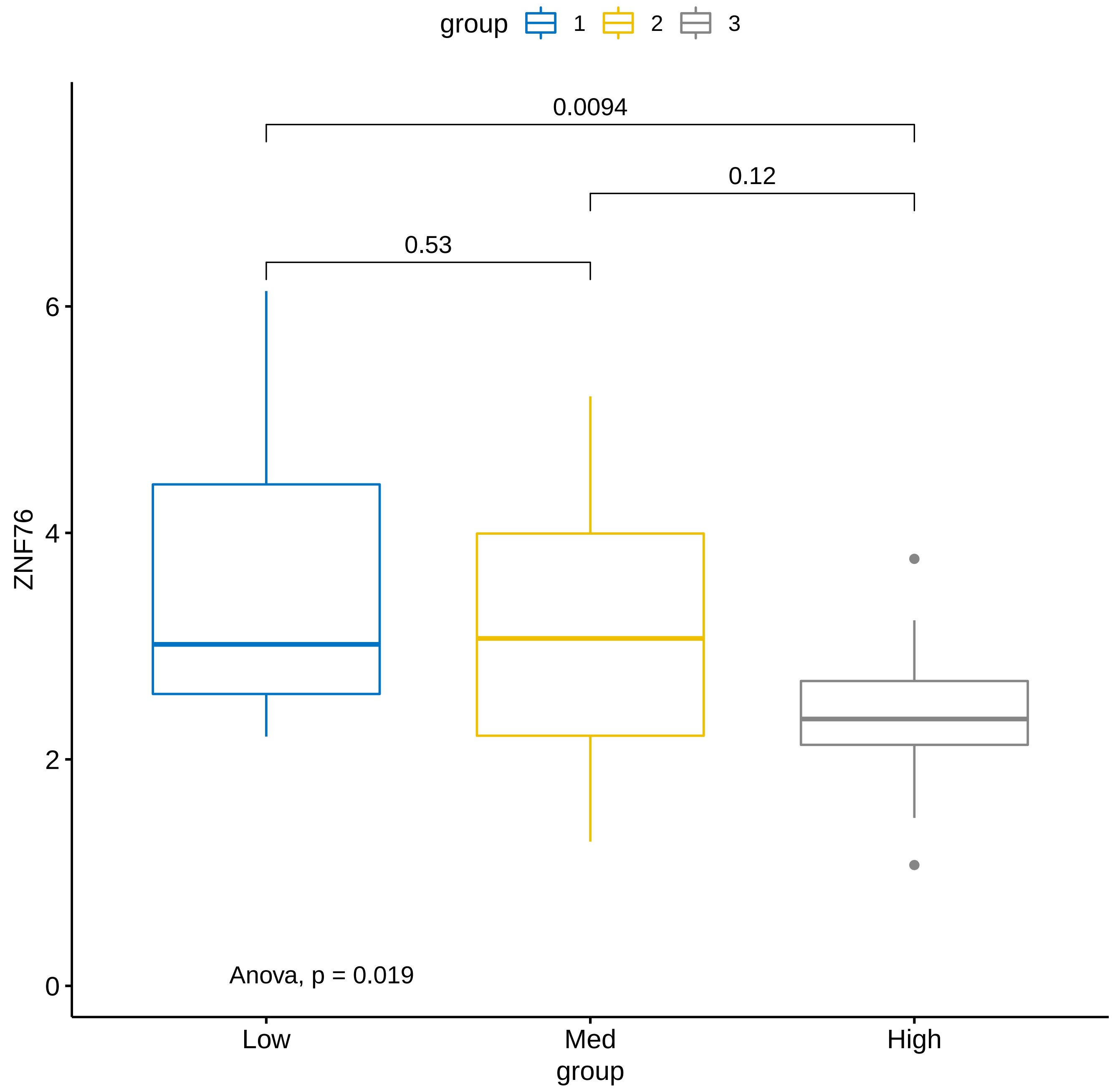

group
1
2
3
0.0094
0.12
0.53
ZNF76
6
4
2
0
Anova, p = 0.019
Low
Med
High
group

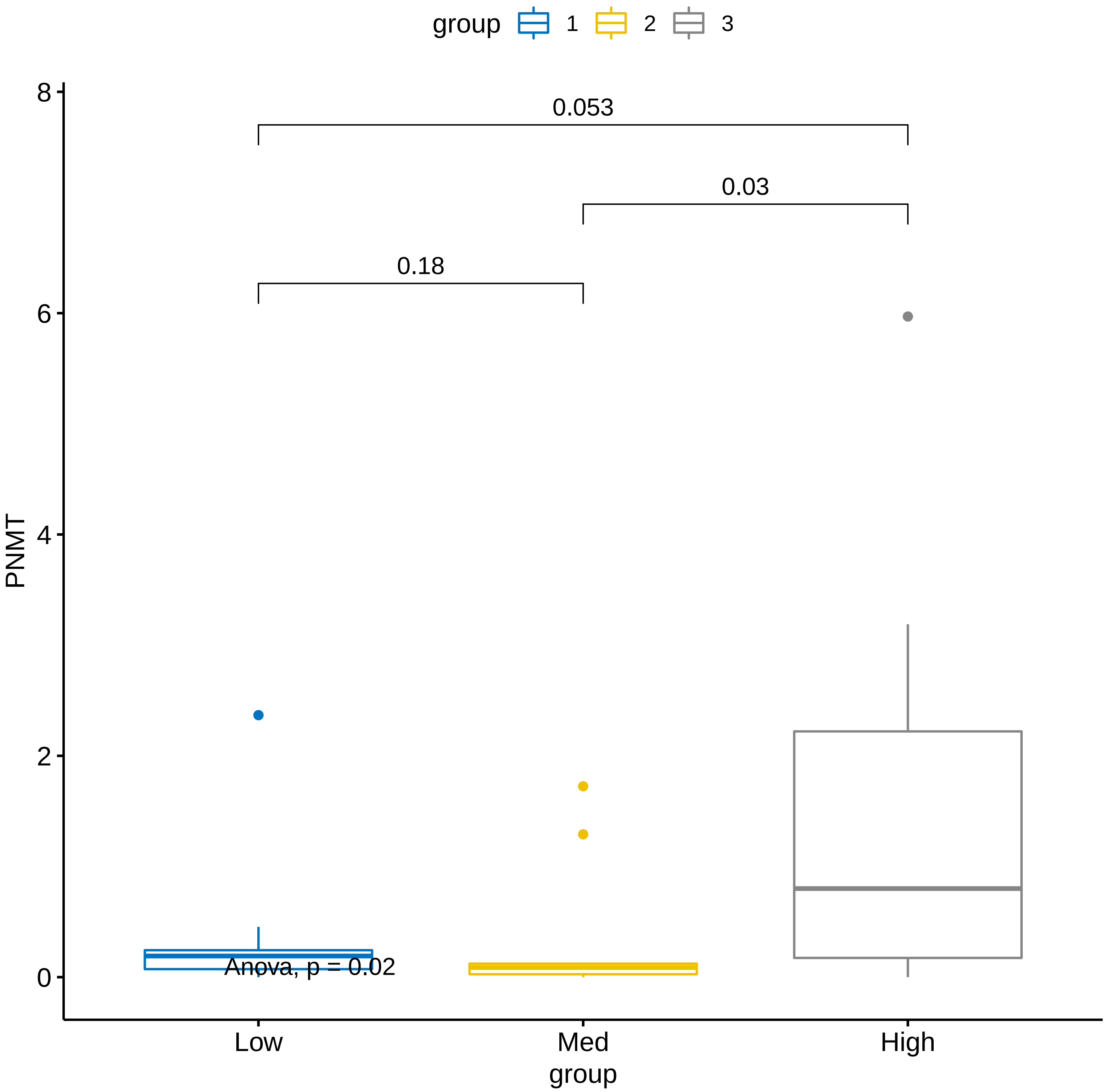
group
1
2
3
0.053
0.03
0.18
8
6
4
2
0
PNMT
Anova, p = 0.02
Low
Med
High
group

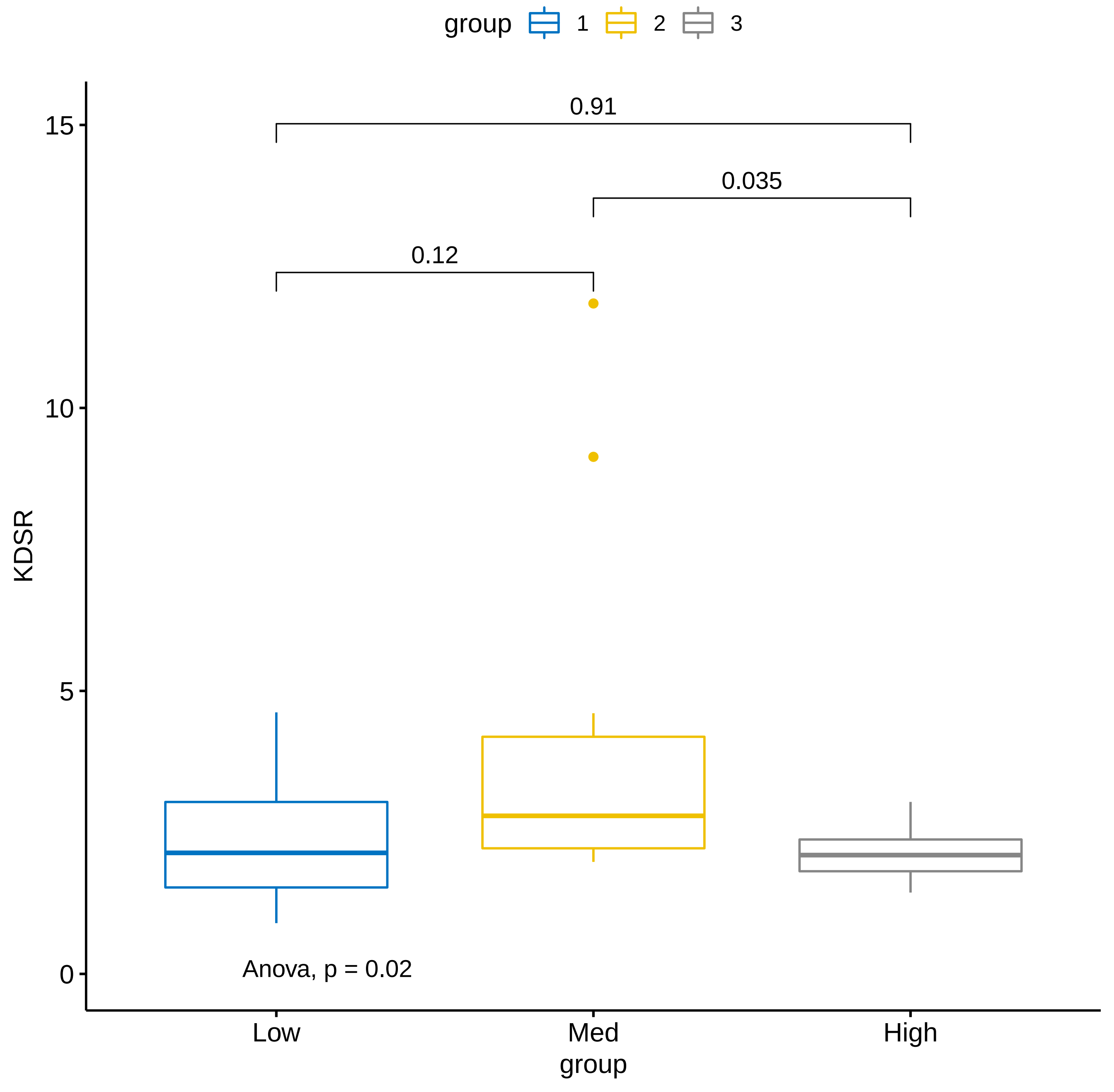
group 1 2 3
0.91
0.035
0.12
15
10
5
0
KDSR
Anova, p = 0.02
Low
Med
High
group

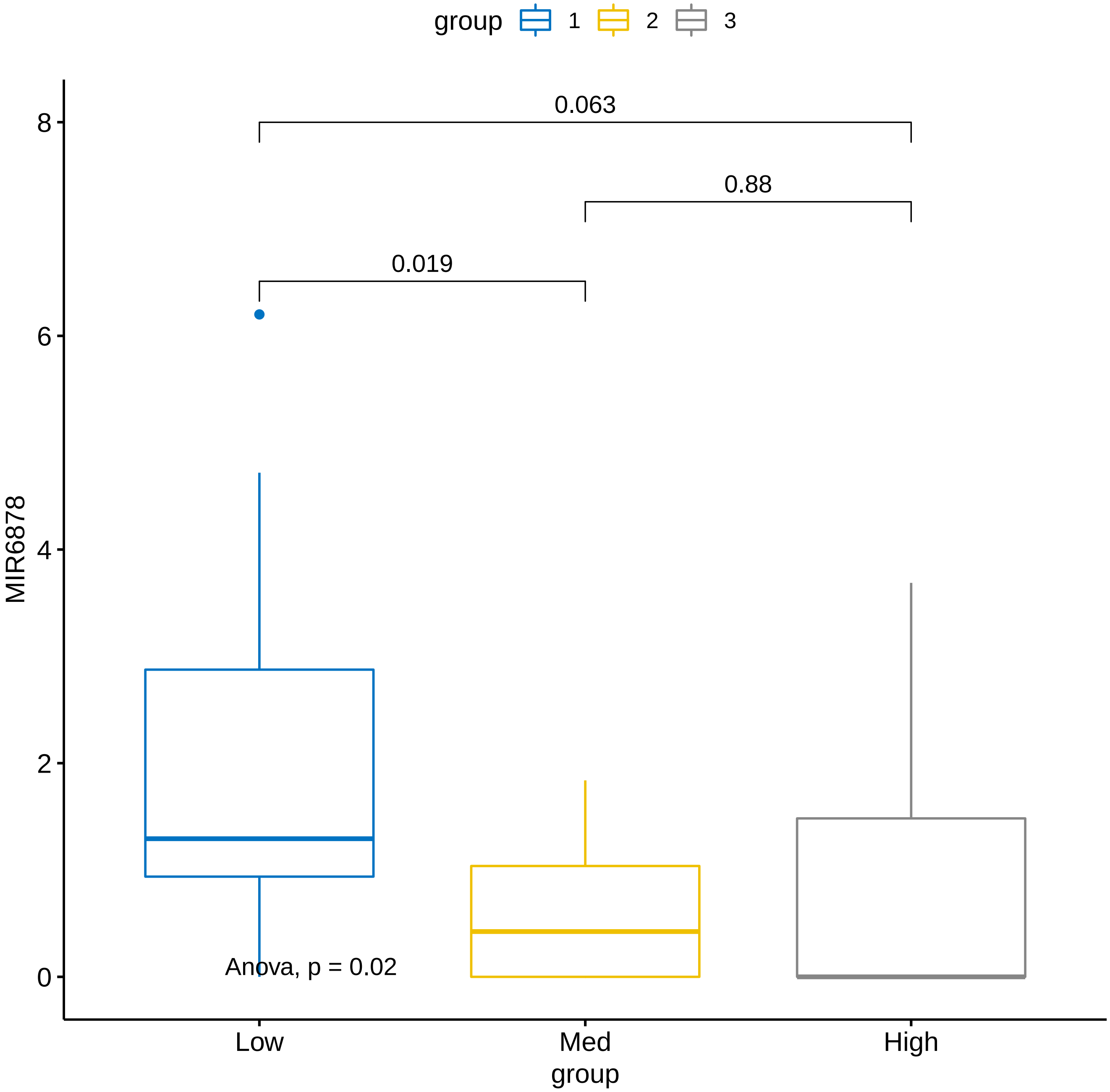
group
1
2
3
0.063
0.88
0.019
8
6
4
2
0
MIR6878
Anova, p = 0.02
Low
Med
High
group

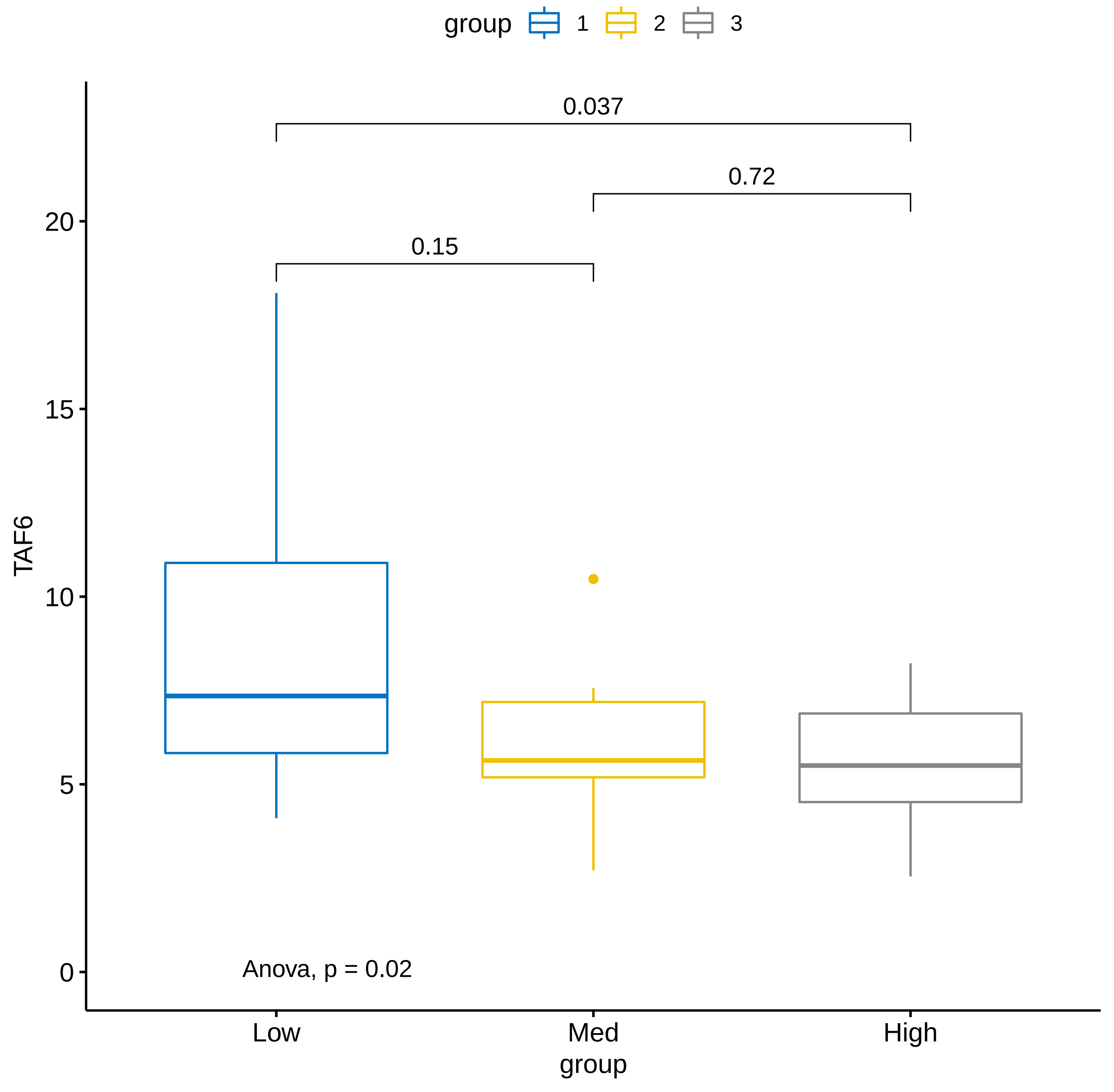
group 1 2 3
0.037
0.72
0.15
20
15
10
5
0
TAF6
Anova, p = 0.02
Low
Med
High
group

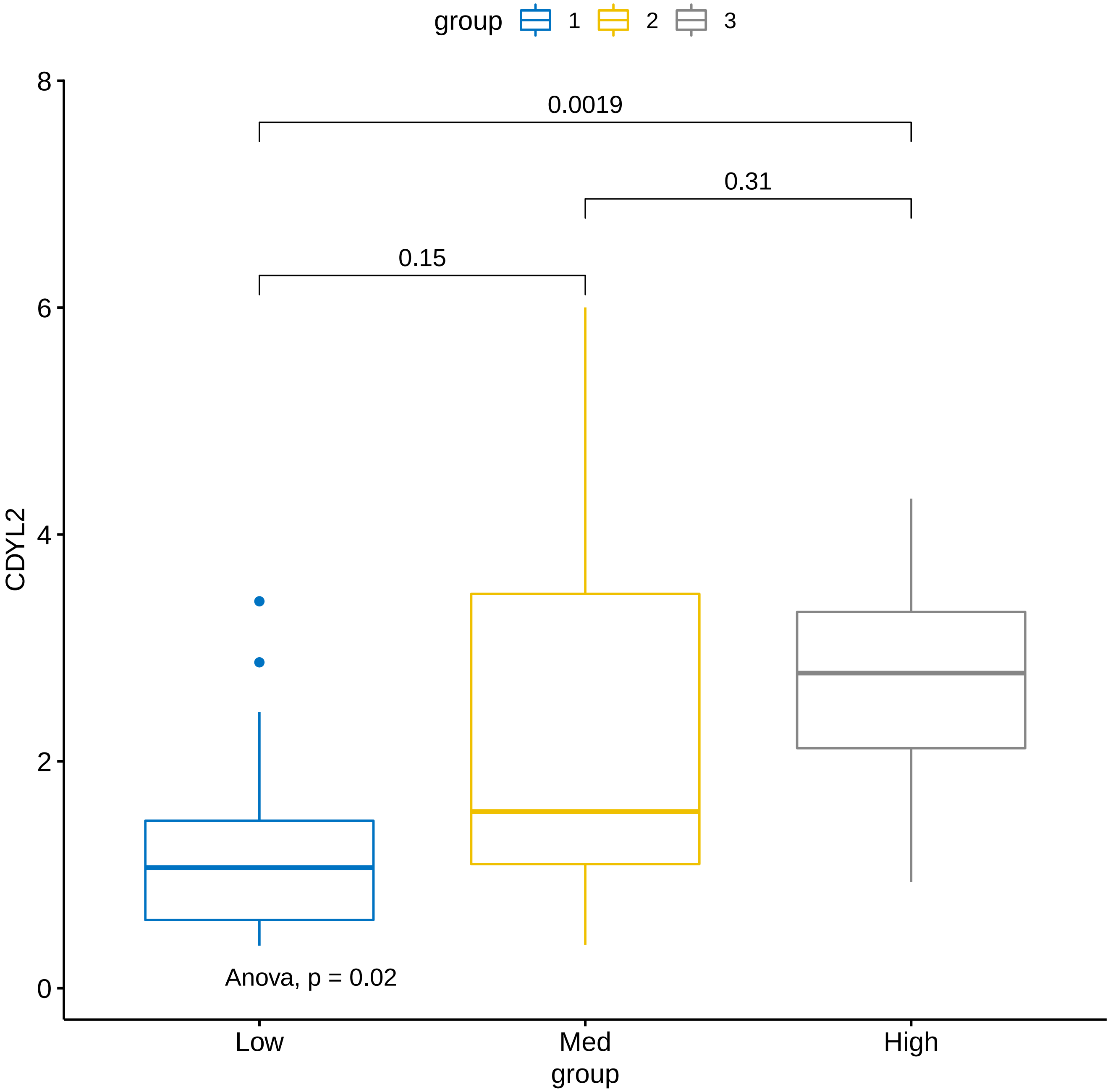

group 1 2 3
0.0019
0.31
0.15
8
6
4
2
0
CDYL2
Anova, p = 0.02
Low
Med
High
group

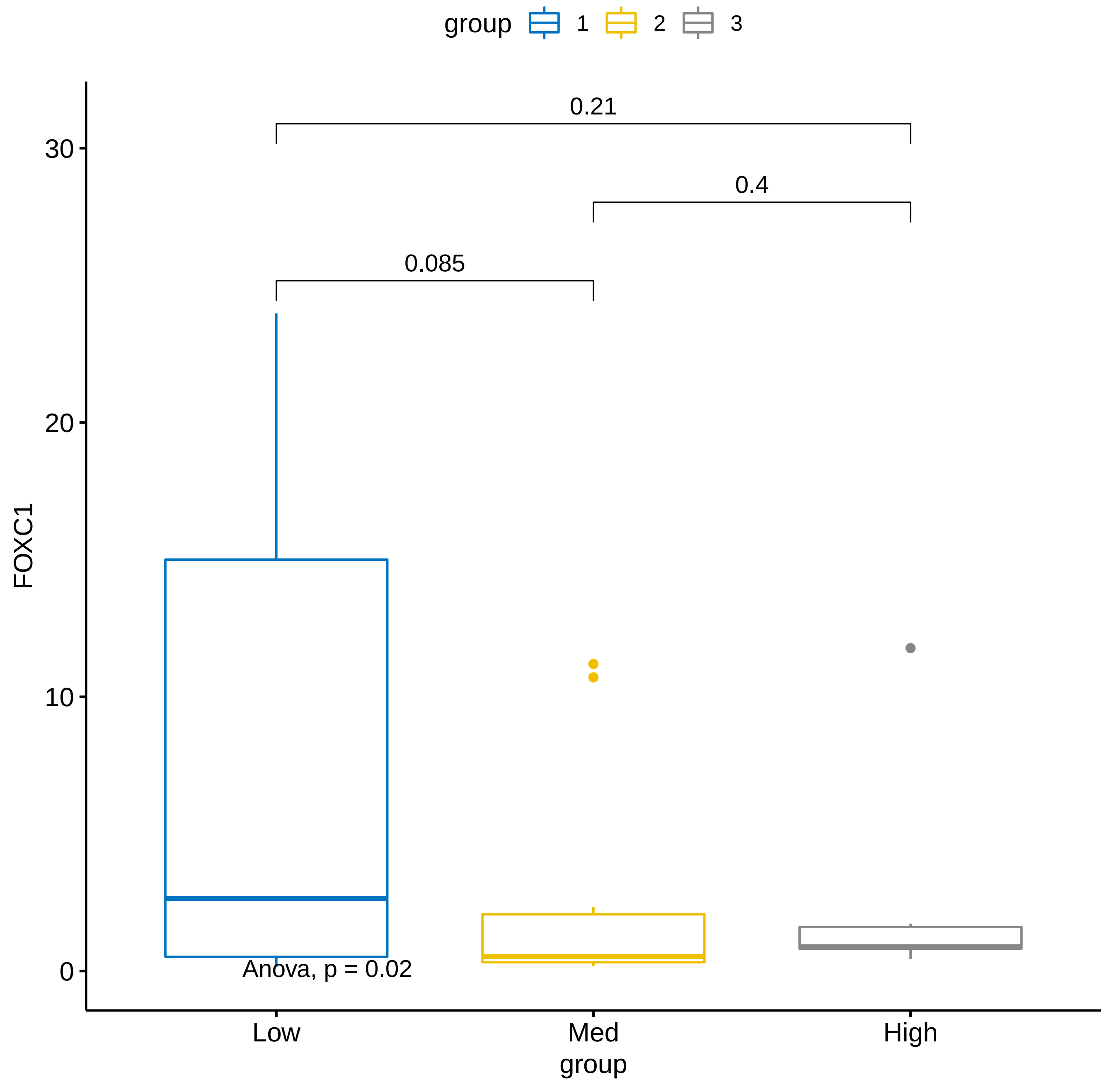
group 1 2 3
0.21
0.4
0.085
30
20
10
0
FOXC1
Anova, p = 0.02
Low
Med
High
group

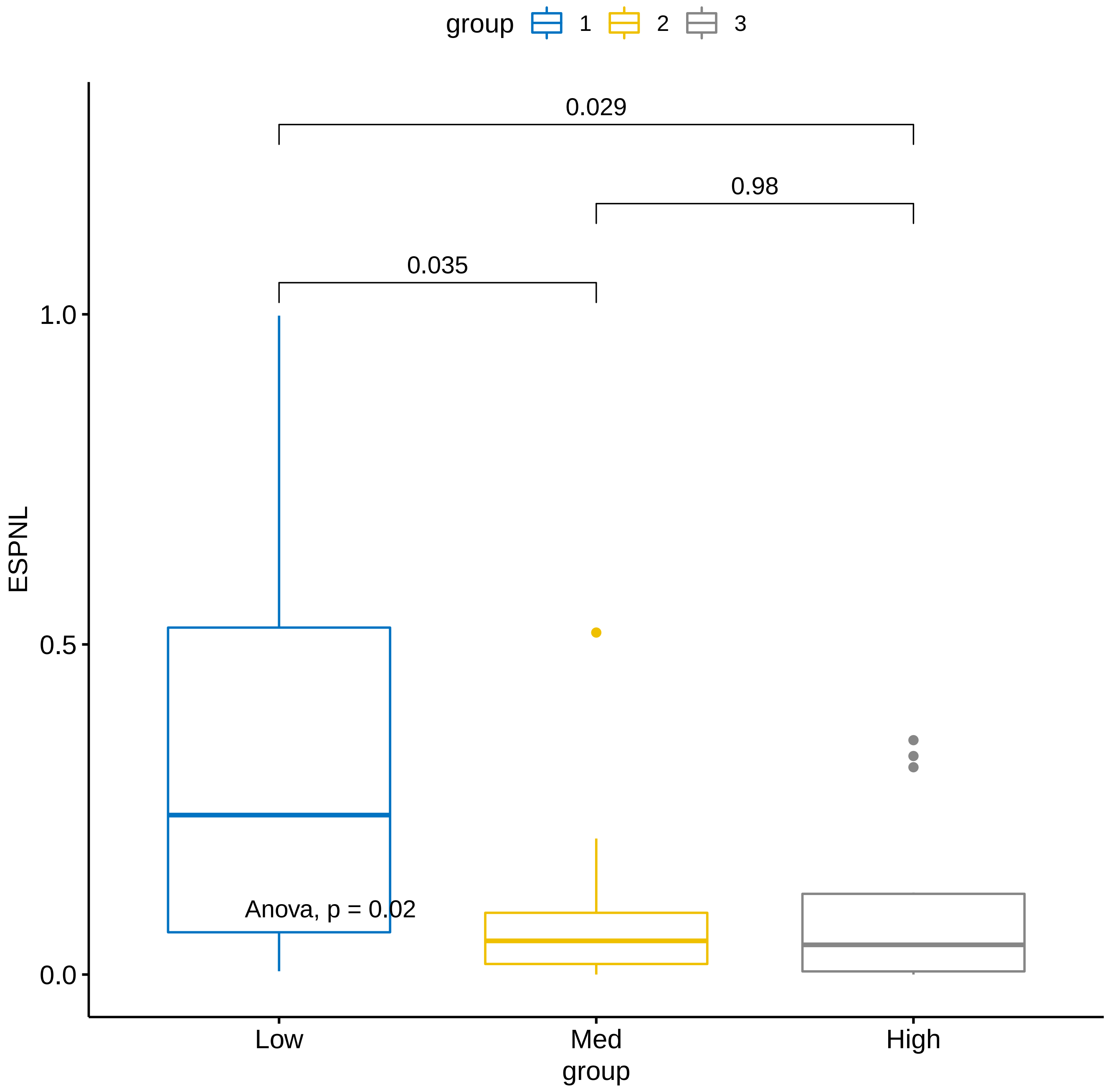
group
1
2
3
0.029
0.98
0.035
1.0
0.5
0.0
ESPNL
Anova, p = 0.02
Low
Med
High
group

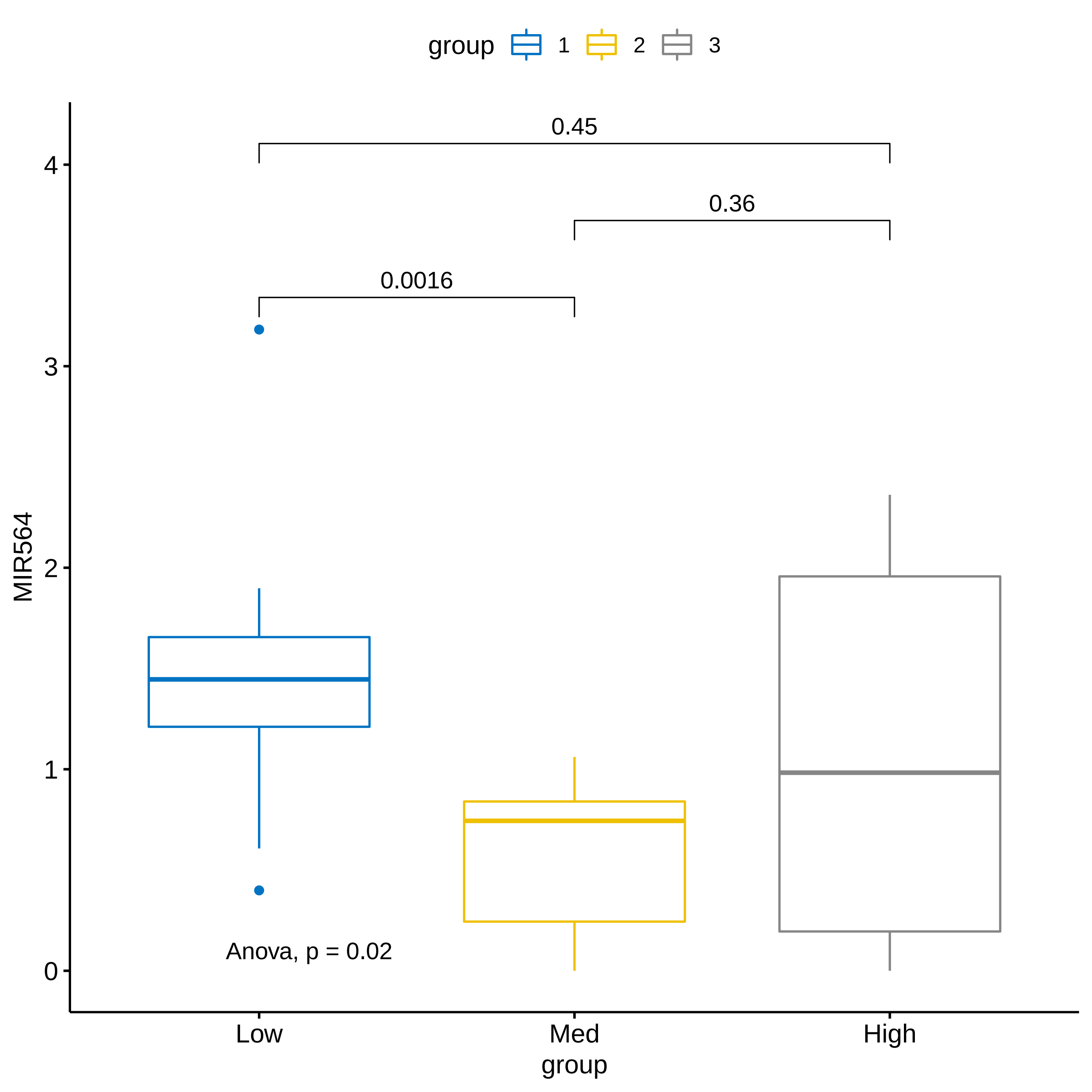

group 1 2 3
0.45
0.36
0.0016
4
3
2
1
0
MIR564
Anova, p = 0.02
Low
Med
High
group

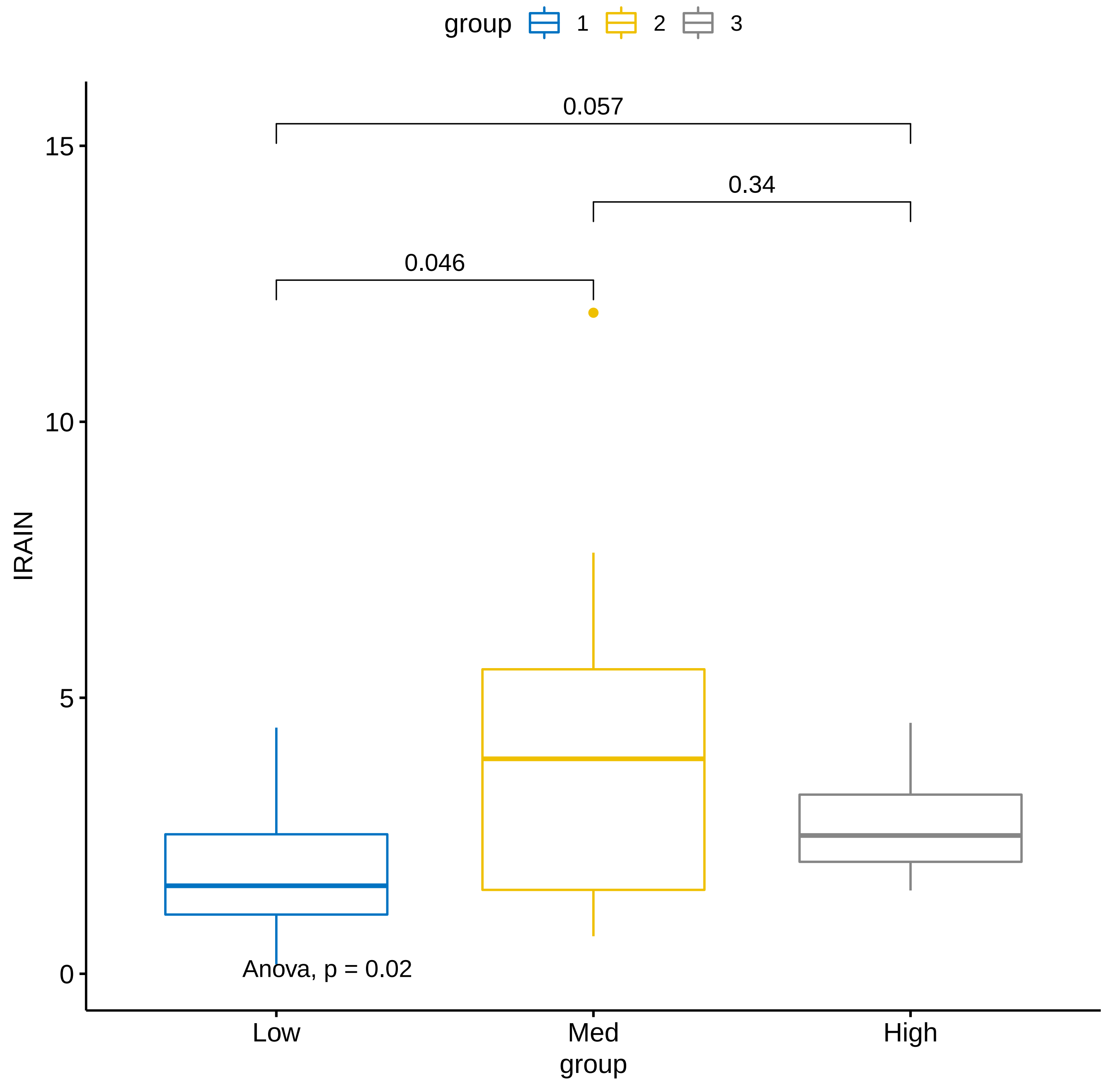
group
1
2
3
0.057
0.34
0.046
15
10
5
0
IRAIN
Anova, p = 0.02
Low
Med
High
group

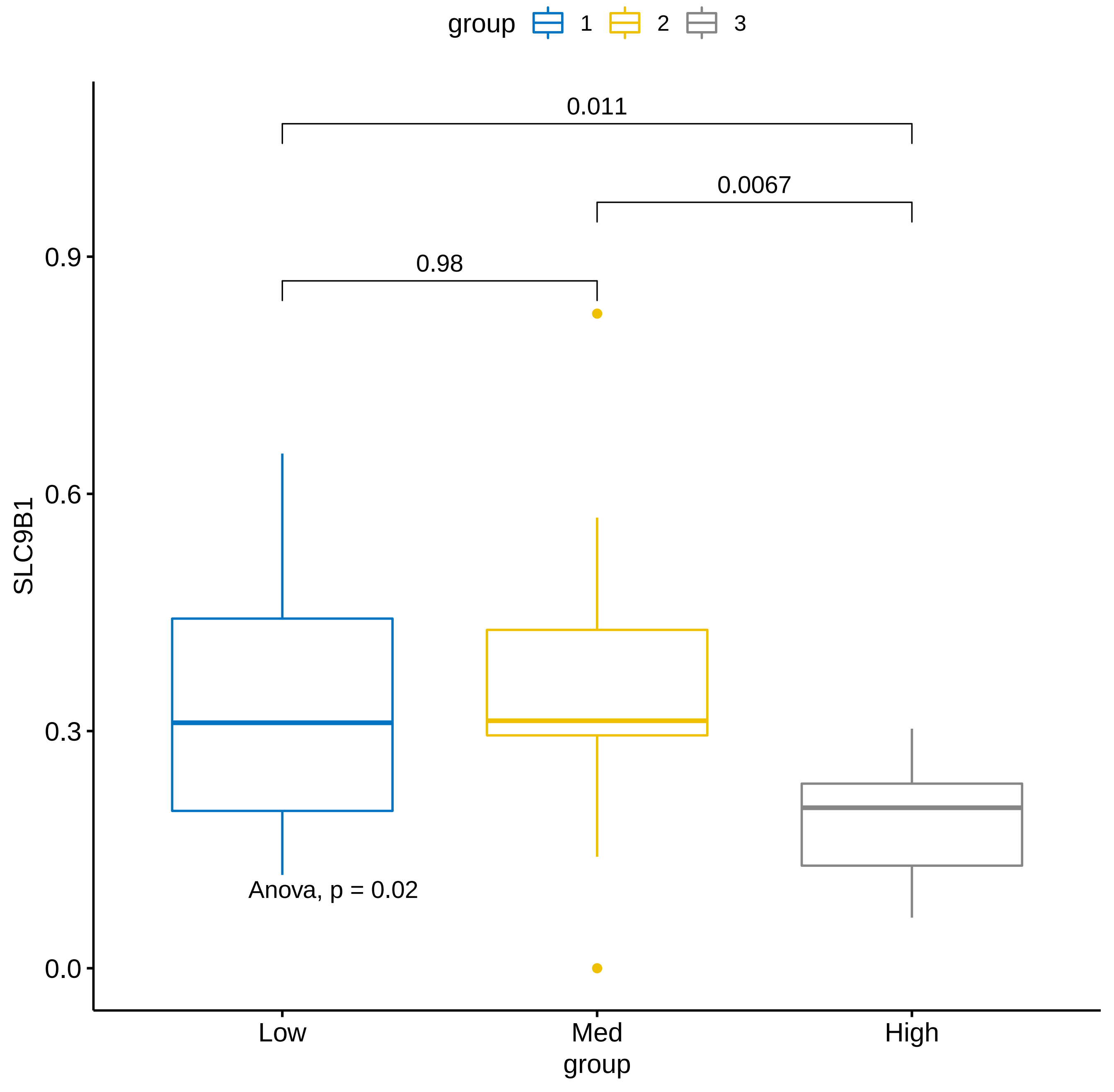

group
1
2
3
0.011
0.0067
0.98
0.9
0.6
0.3
0.0
SLC9B1
Anova, p = 0.02
Low
Med
High
group

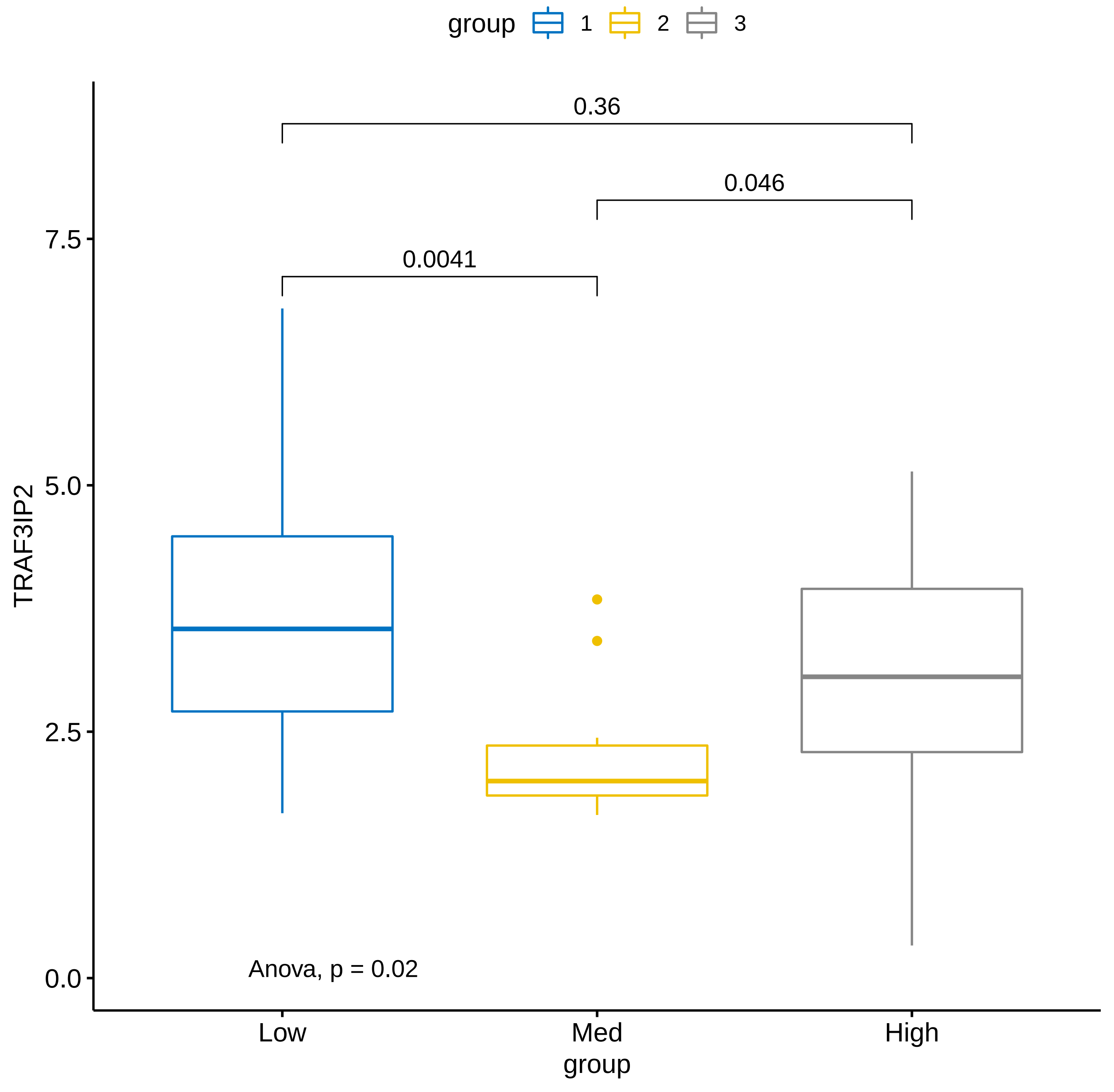

group
1
2
3
0.36
0.046
0.0041
TRAF3IP2
7.5
5.0
2.5
0.0
Anova, p = 0.02
Low
Med
High
group

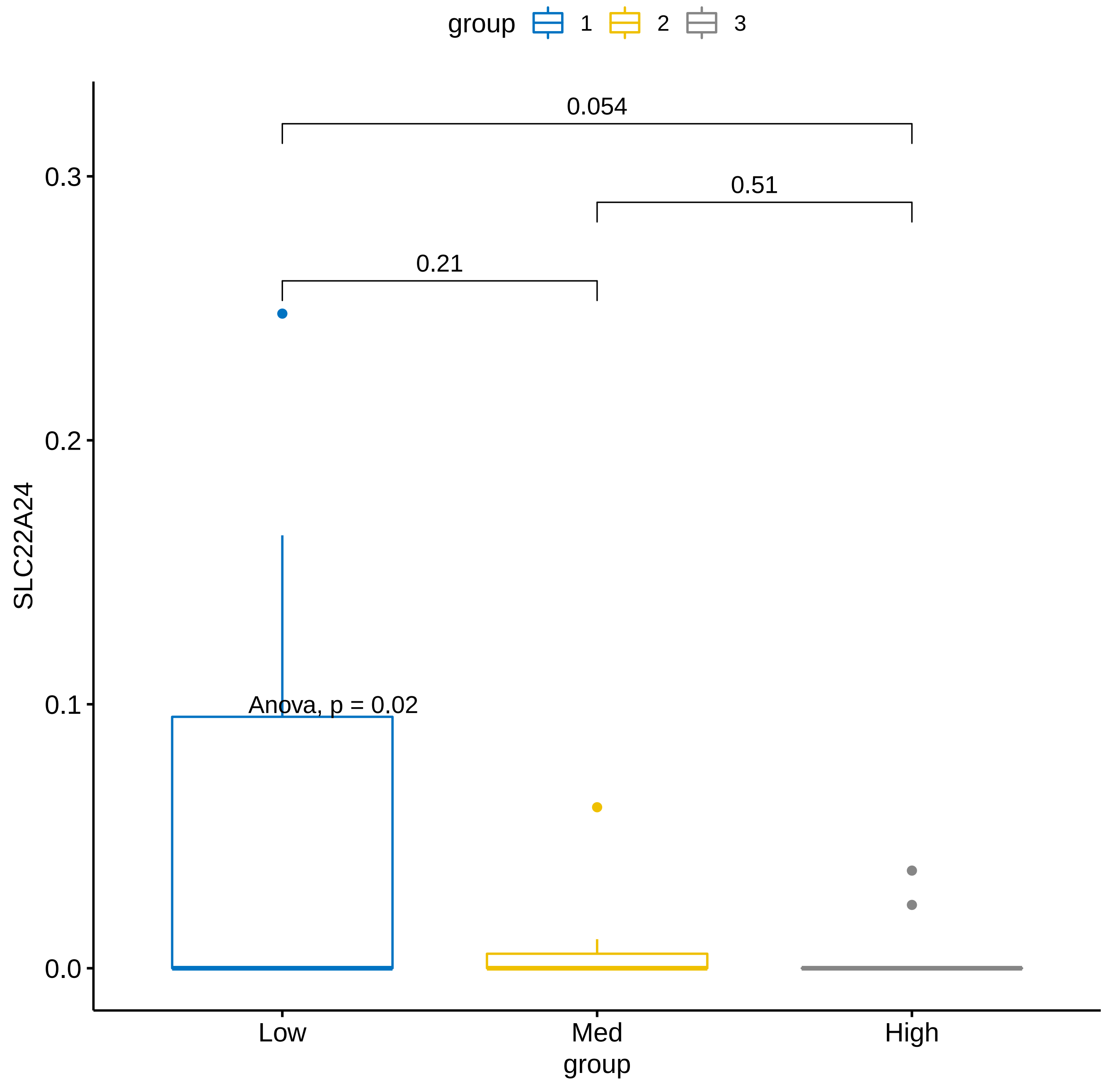
group
1
2
3
0.054
0.51
0.21
Anova, p = 0.02
0.3
0.2
0.1
0.0
SLC22A24
Low
Med
High
group

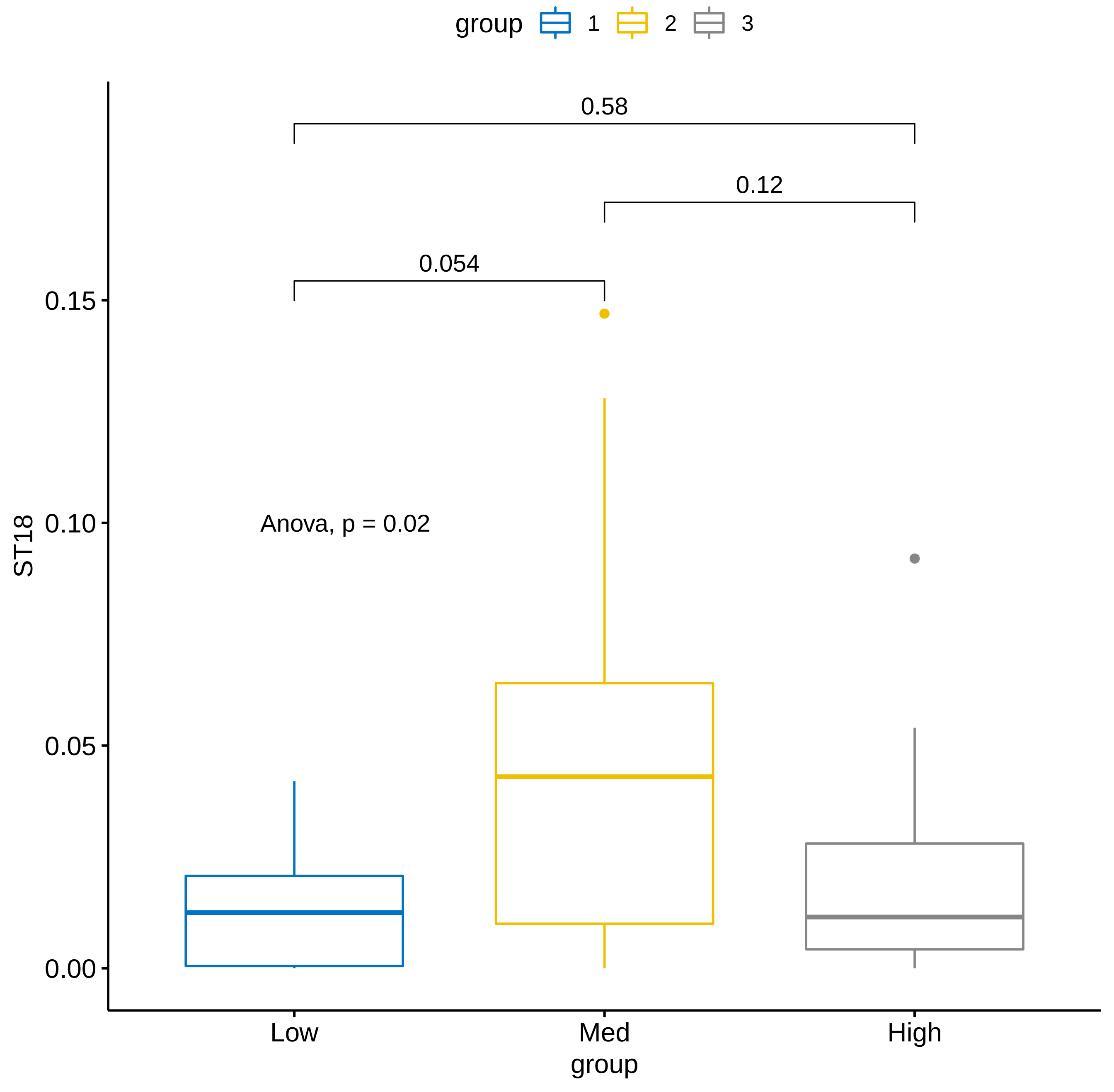

group 1 2 3
0.58
0.12
0.054
Anova, p = 0.02
ST18
0.15
0.10
0.05
0.00
Low
Med
High
group

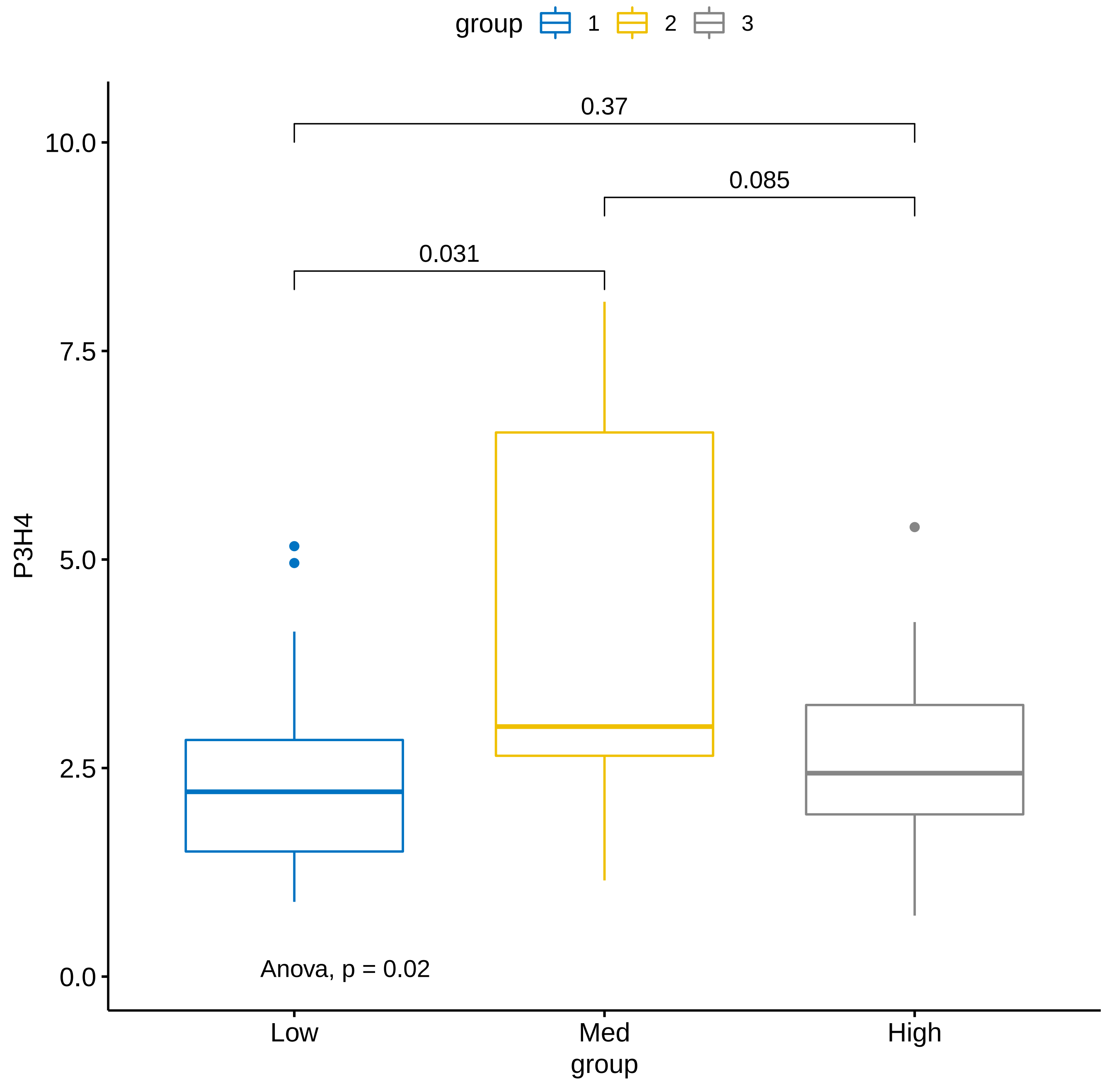
group
1
2
3
0.37
0.085
0.031
10.0
7.5
5.0
2.5
0.0
P3H4
Anova, p = 0.02
Low
Med
High
group

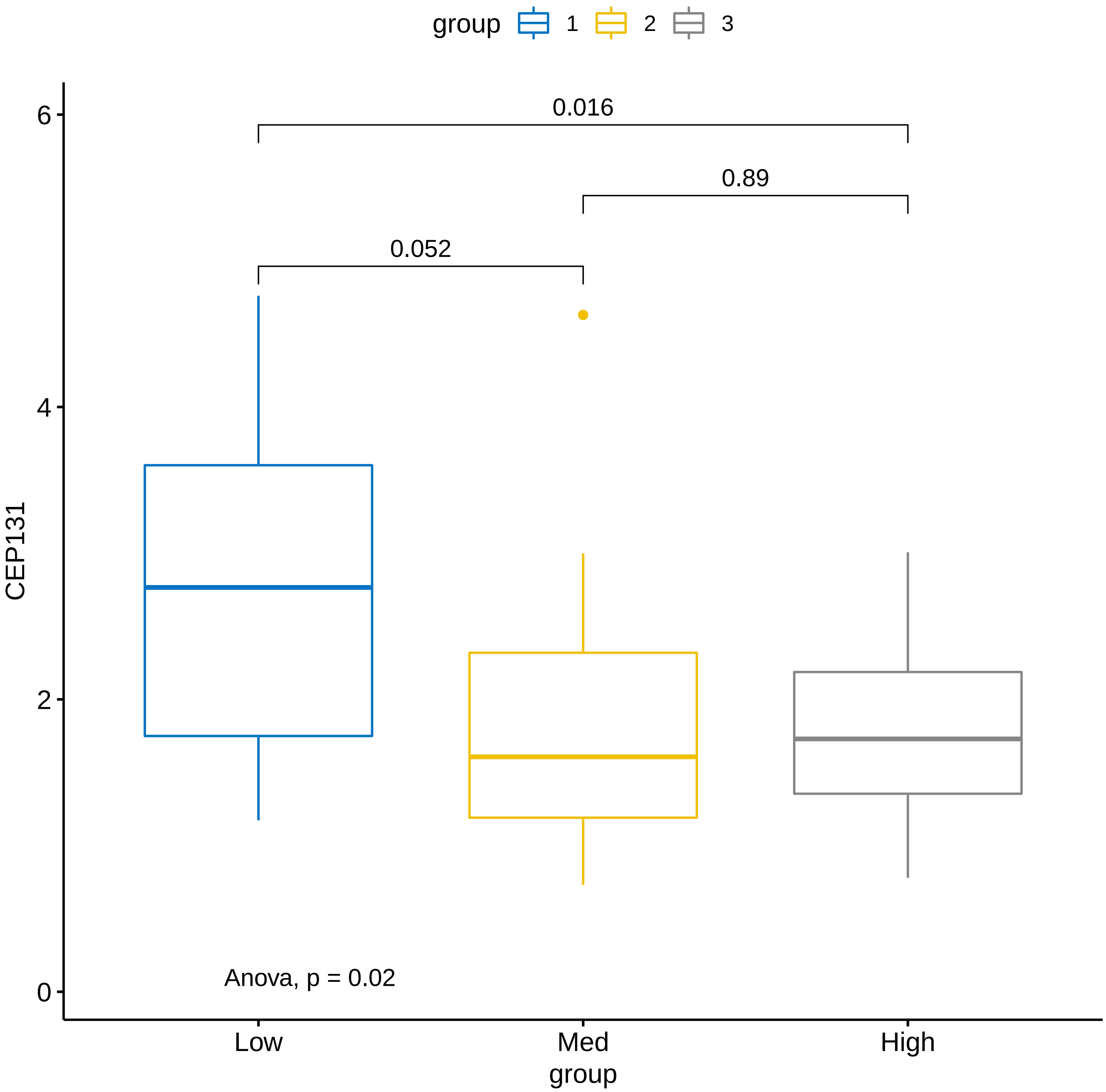

group 1 2 3
0.016
0.89
0.052
6
4
2
0
CEP131
Anova, p = 0.02
Low
Med
High
group

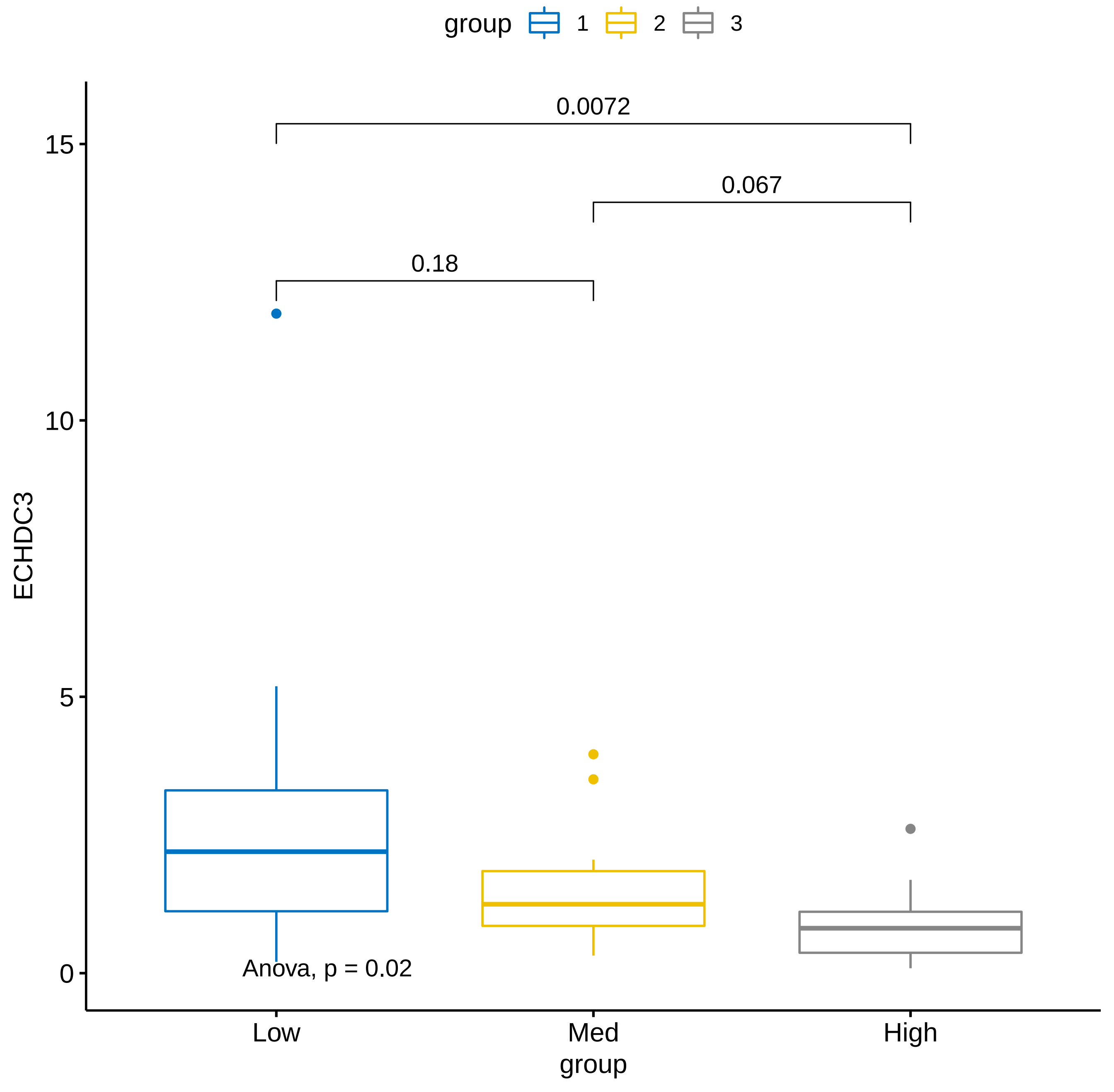
group
1
2
3
0.0072
0.067
0.18
15
10
5
0
ECHDC3
Anova, p = 0.02
Low
Med
High
group

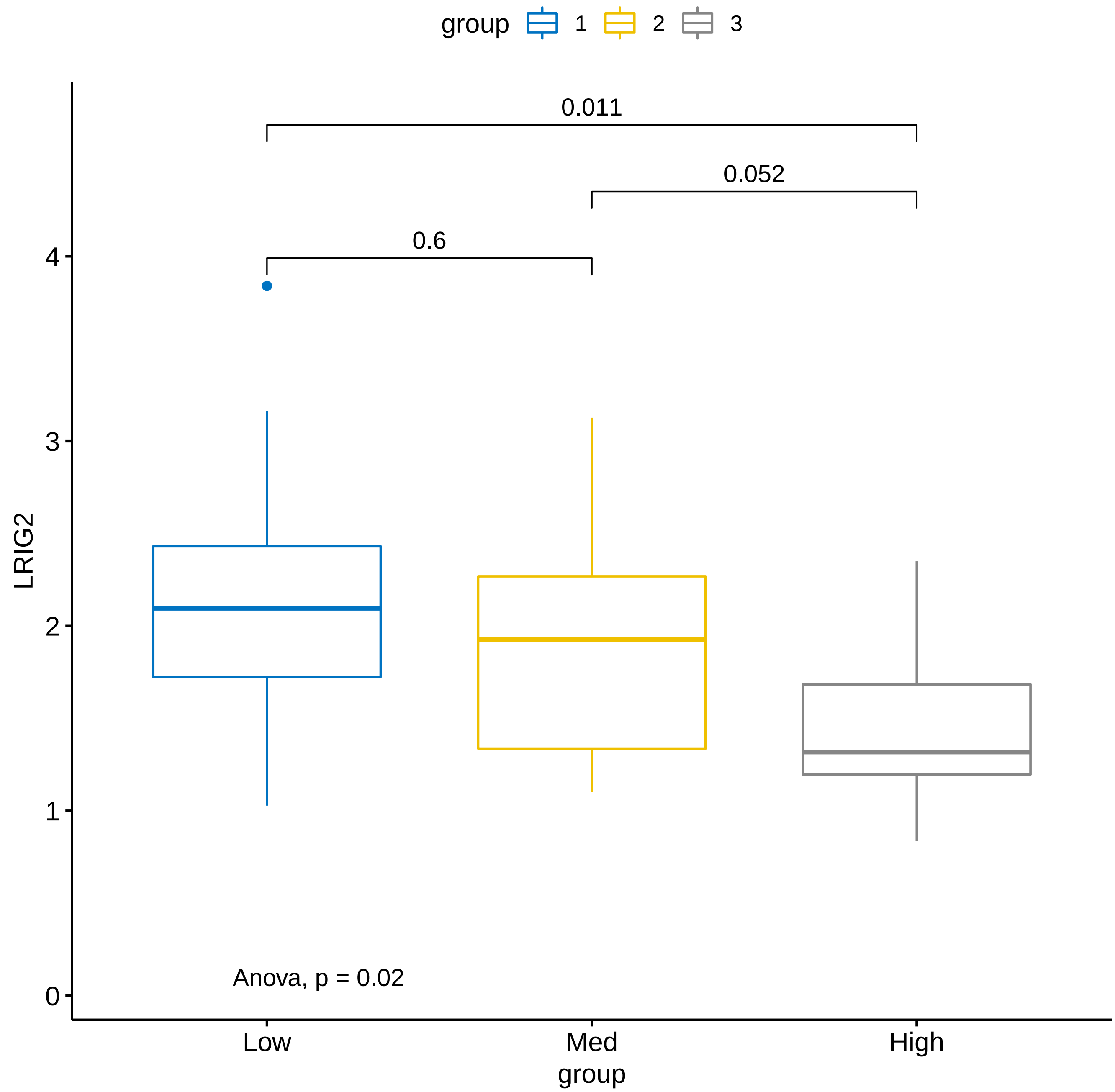
group
1
2
3
0.011
0.052
0.6
LRIG2
4
3
2
1
0
Anova, p = 0.02
Low
Med
High
group

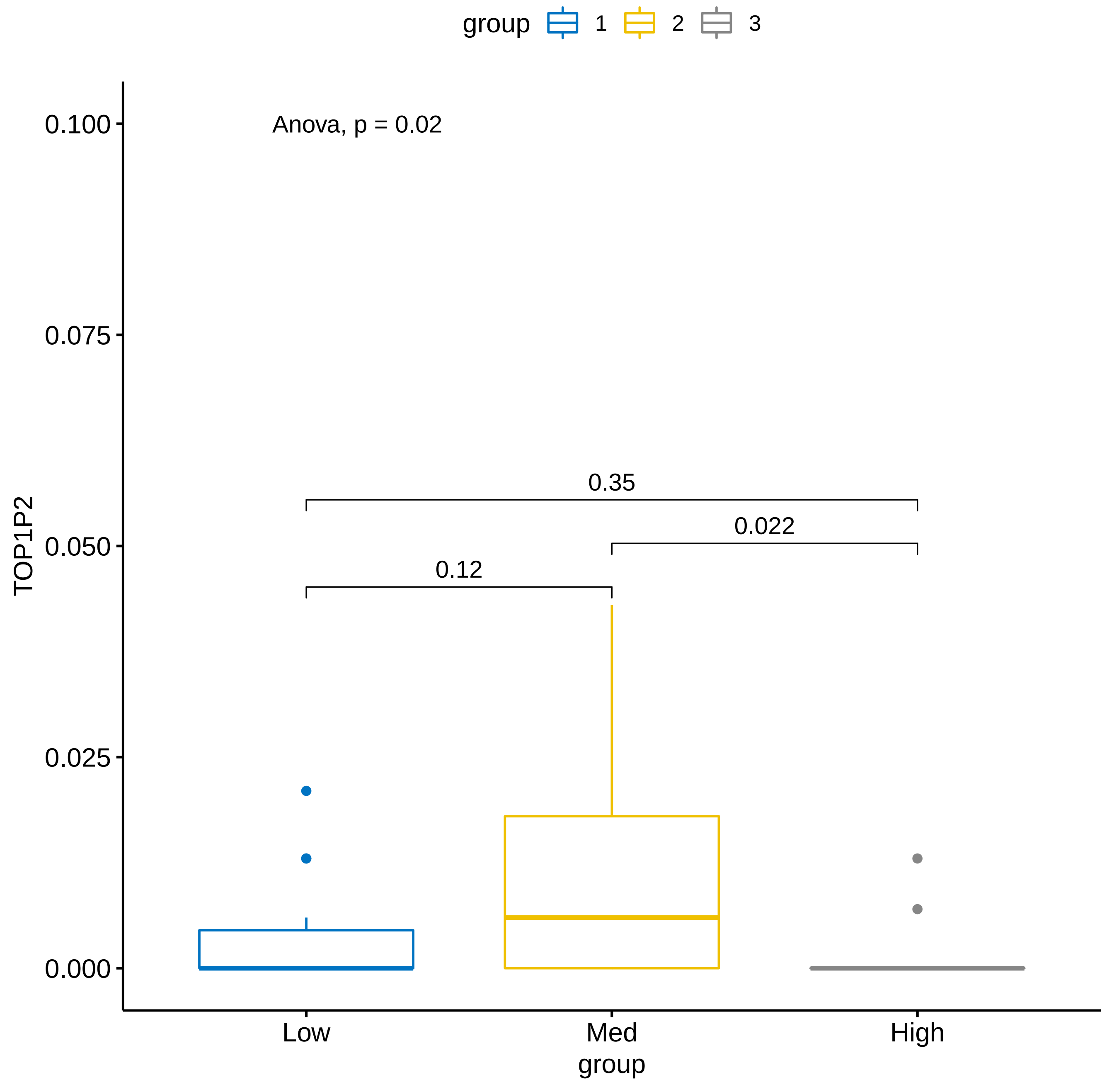

group 1 2 3
Anova, p = 0.02
0.35
0.022
0.12
TOP1P2
0.100
0.075
0.050
0.025
0.000
Low
Med
High
group

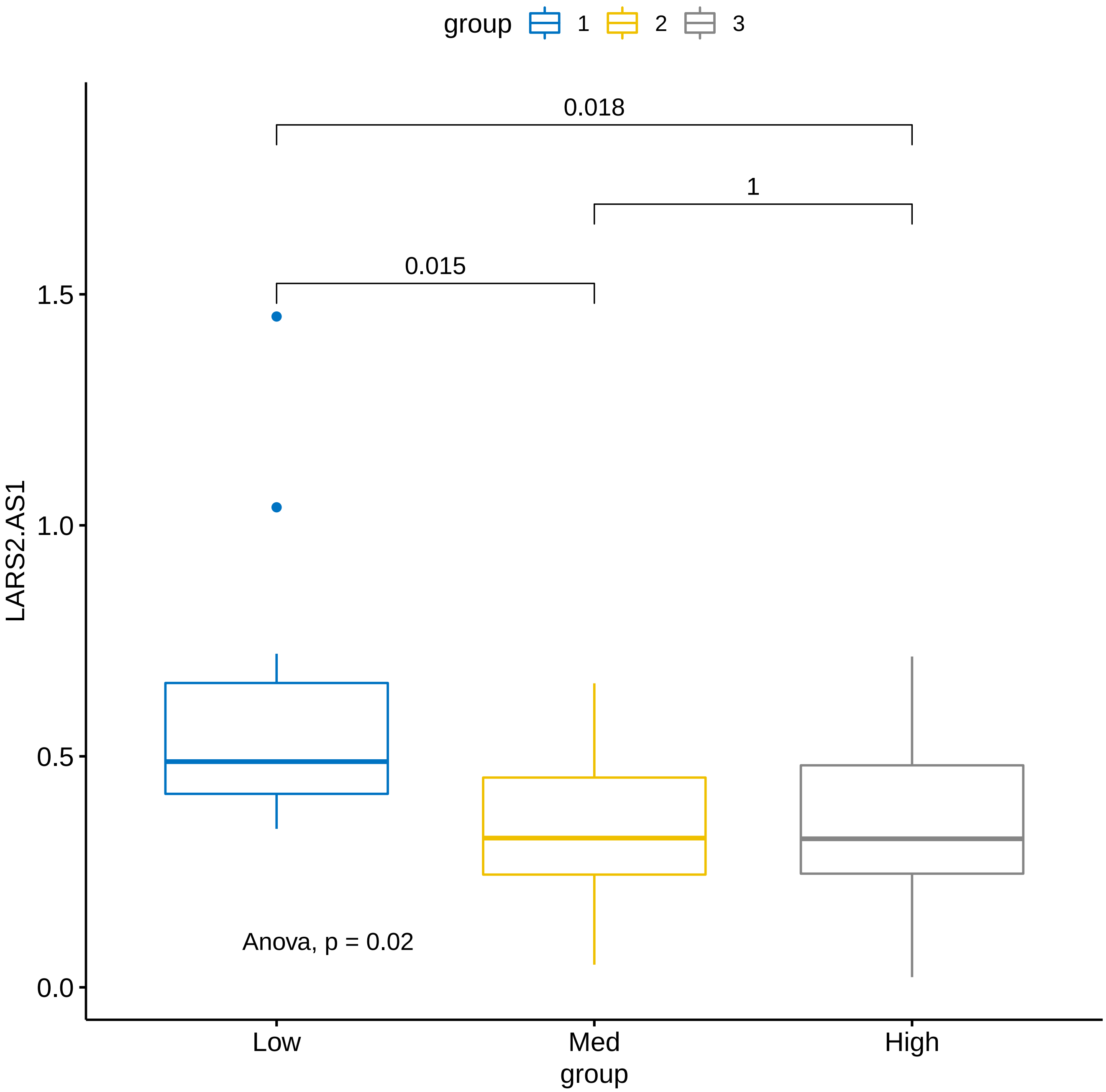
group
1
2
3
0.018
1
0.015
1.5
1.0
0.5
0.0
LARS2.AS1
Anova, p = 0.02
Low
Med
High
group

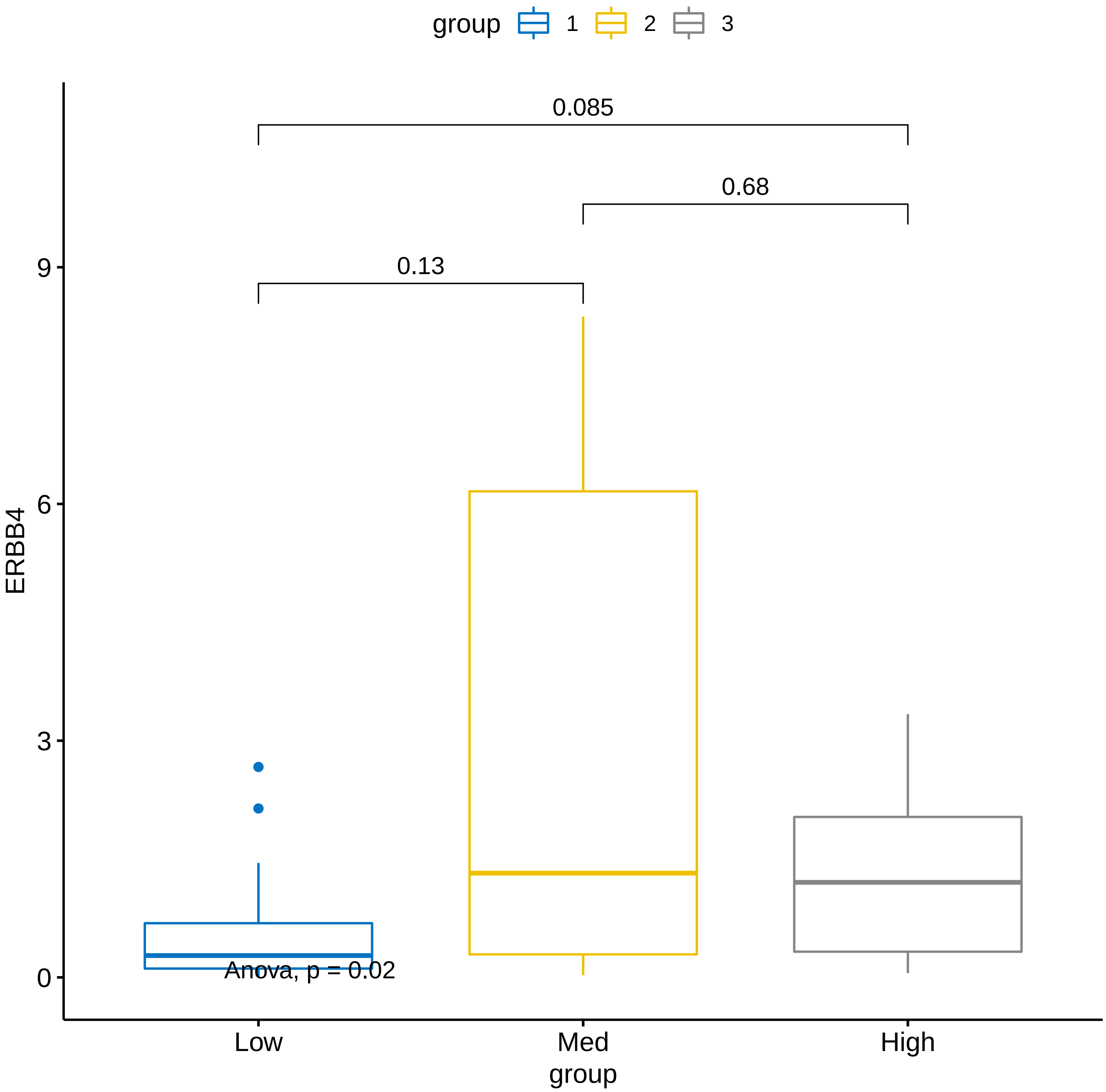
group
1
2
3
0.085
0.68
0.13
9
6
3
0
ERBB4
Anova, p = 0.02
Low
Med
High
group

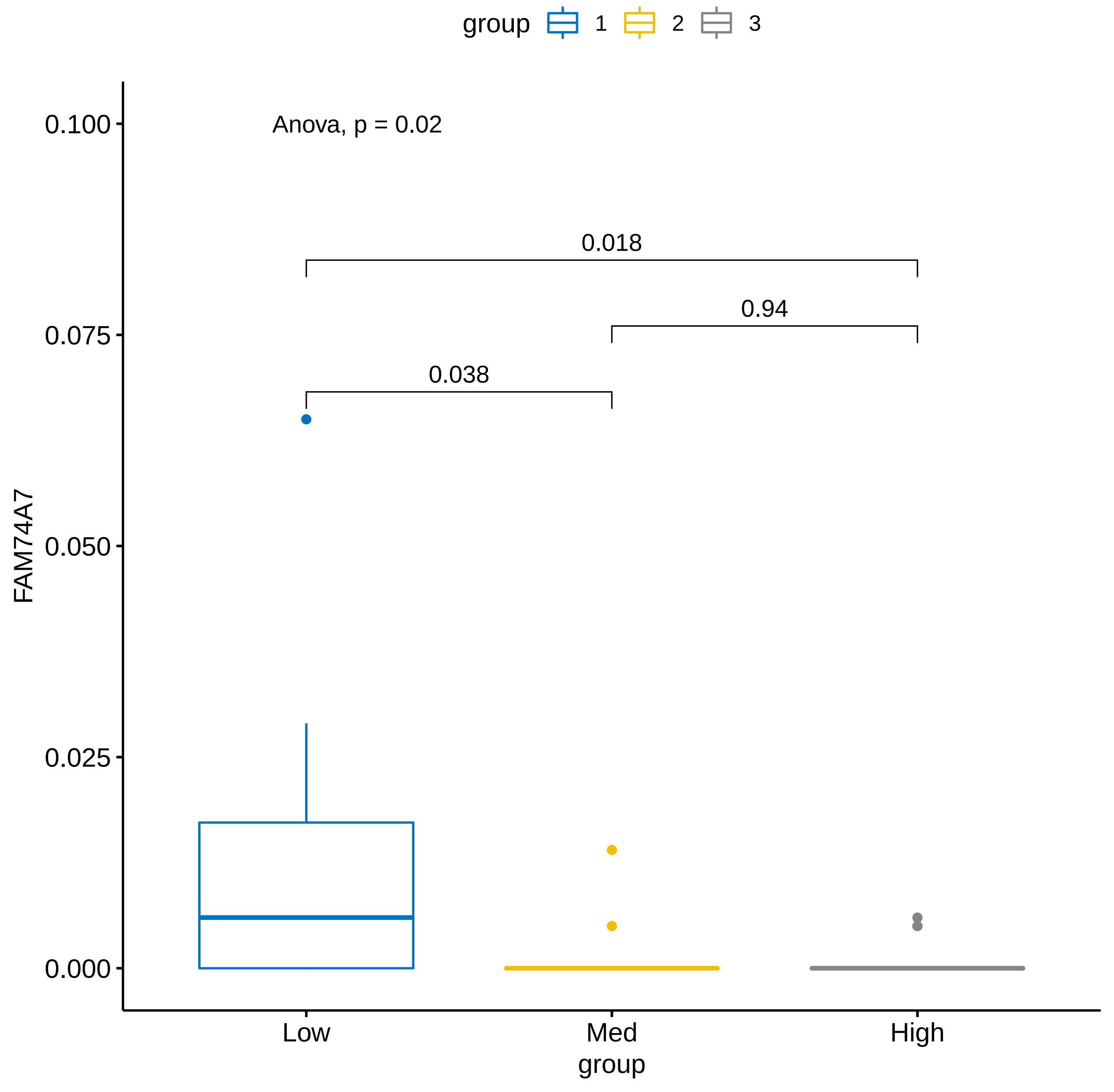
group
1
2
3
Anova, p = 0.02
0.018
0.94
0.038
0.100
0.075
0.050
0.025
0.000
FAM74A7
Low
Med
High
group

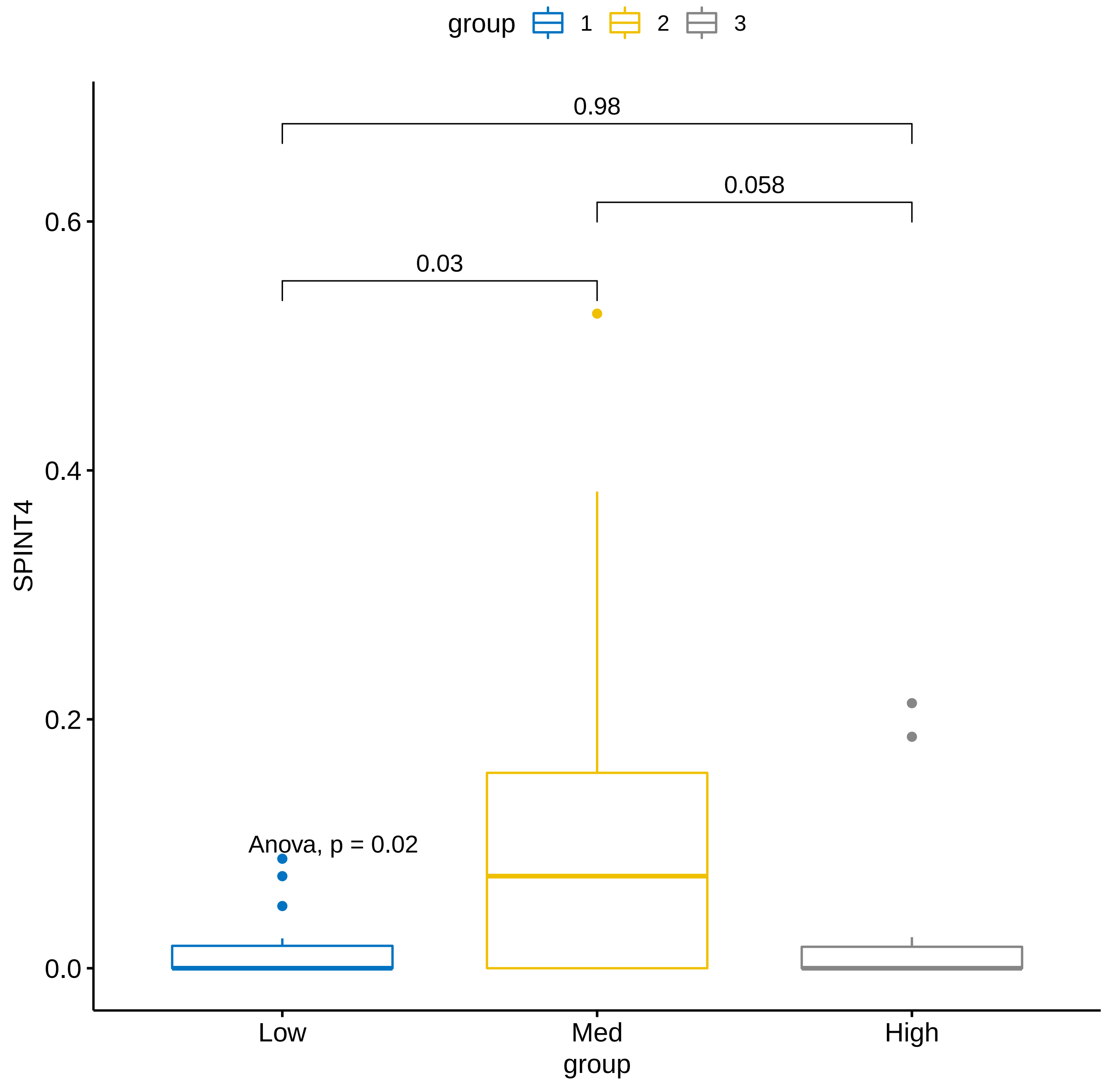
group
1
2
3
0.98
0.058
0.03
Anova, p = 0.02
0.6
0.4
0.2
0.0
SPINT4
Low
Med
High
group

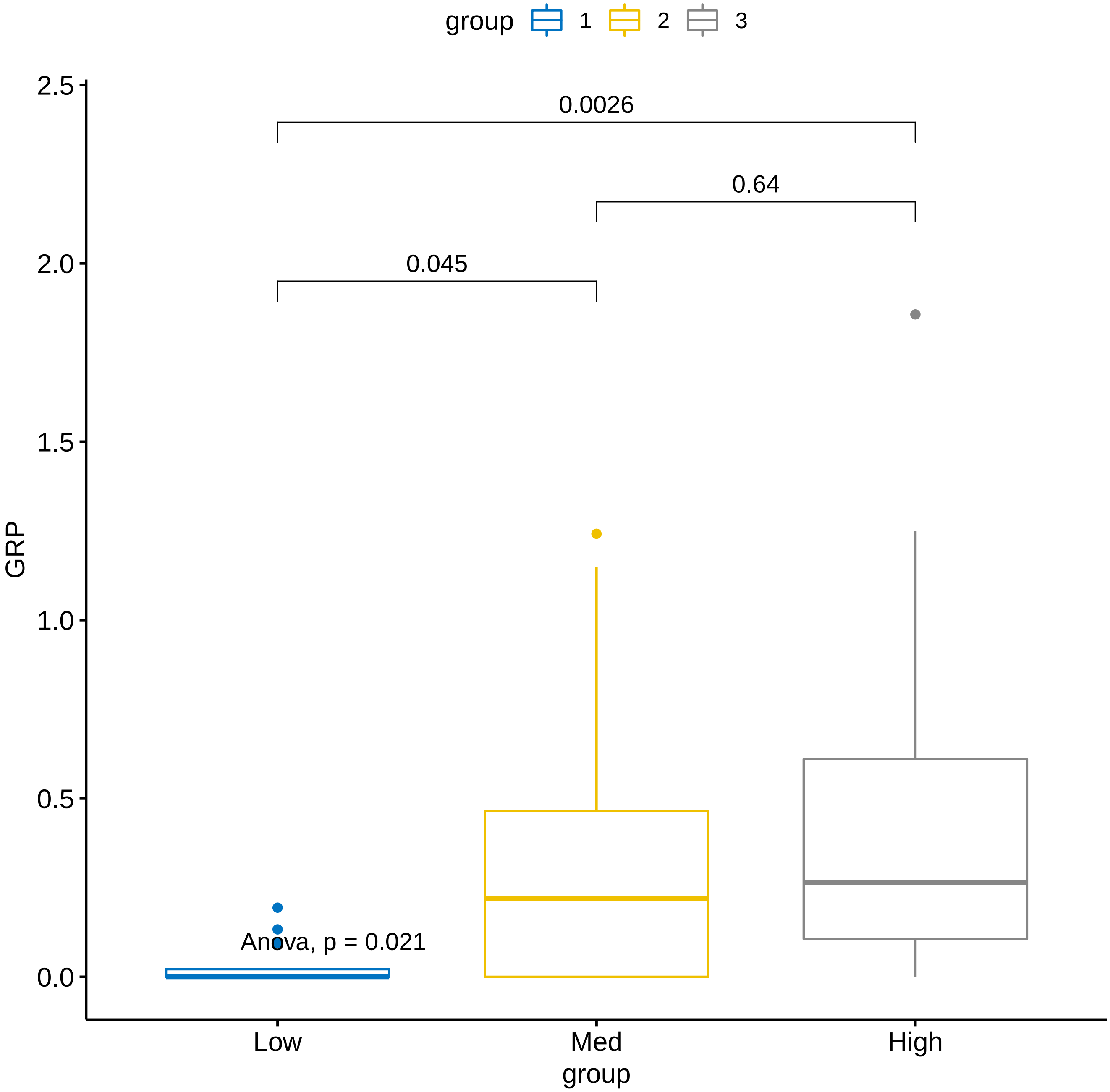

group 1 2 3
2.5
2.0
1.5
1.0
0.5
0.0
GRP
0.0026
0.64
0.045
Anova, p = 0.021
Low
Med
High
group

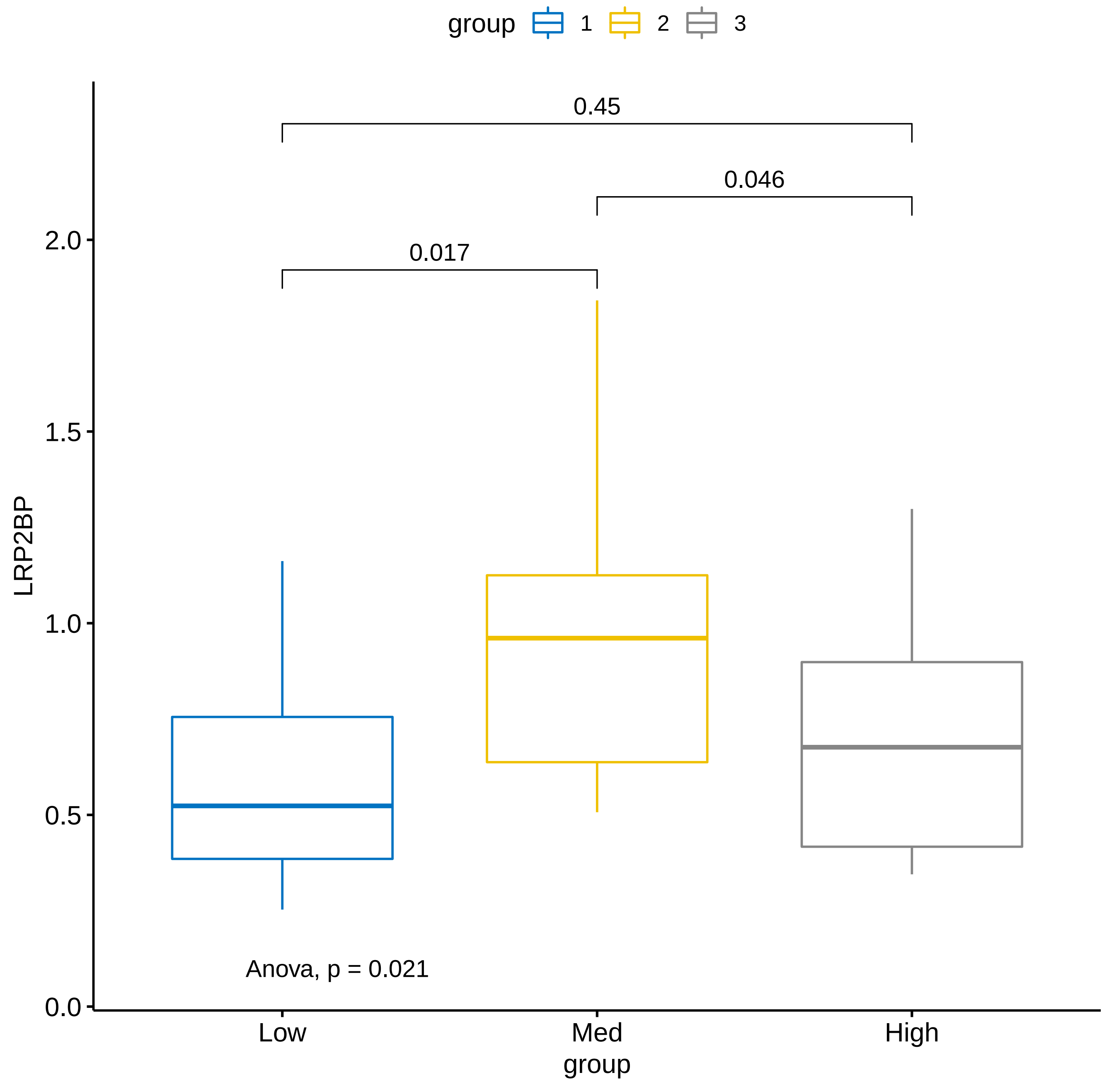
group
1
2
3
0.45
0.046
0.017
2.0
1.5
1.0
0.5
0.0
LRP2BP
Anova, p = 0.021
Low
Med
High
group

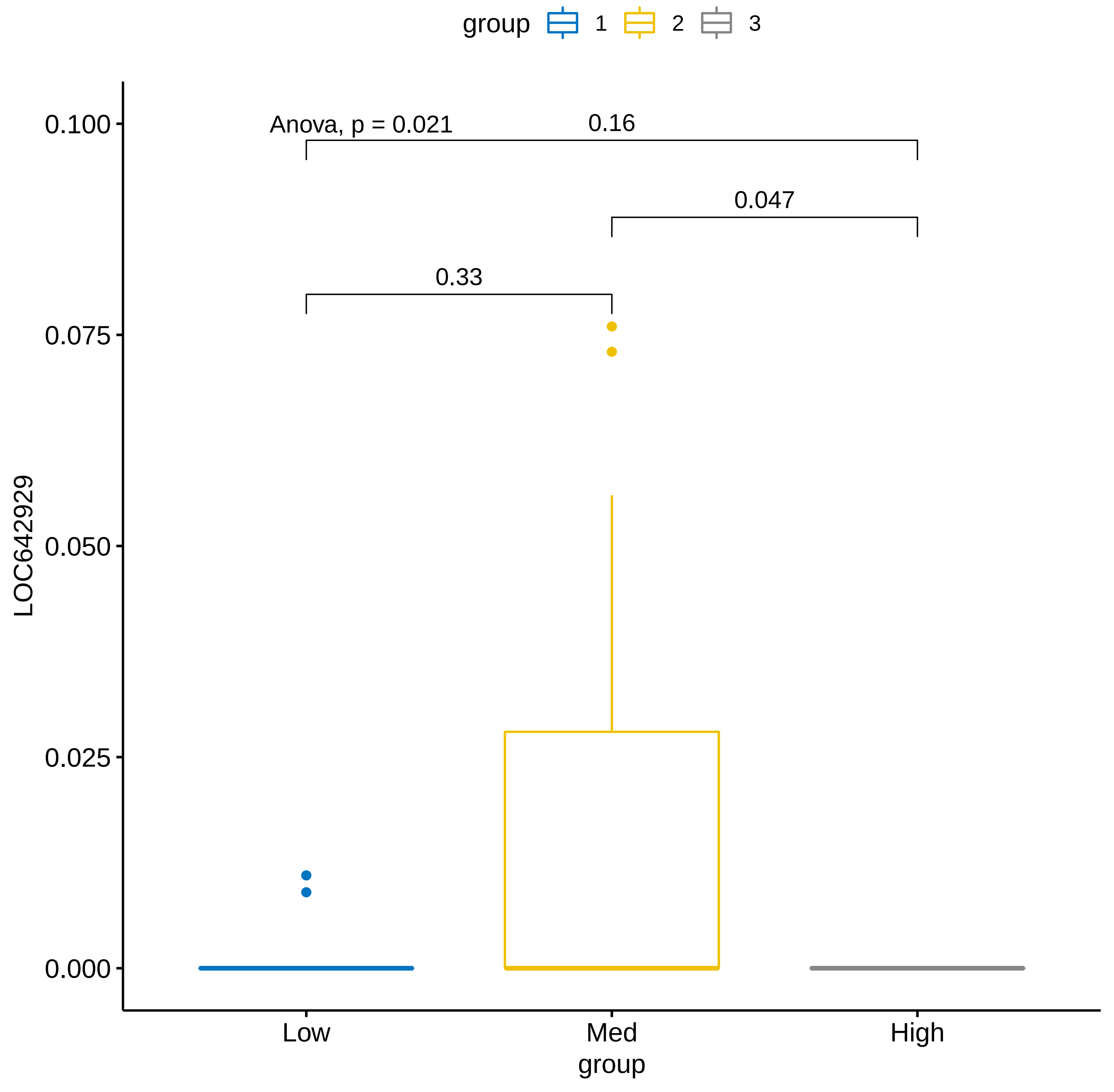

group 1 2 3
Anova, p = 0.021
0.16
0.047
0.33
0.100
0.075
0.050
0.025
0.000
LOC642929
Low
Med
High
group

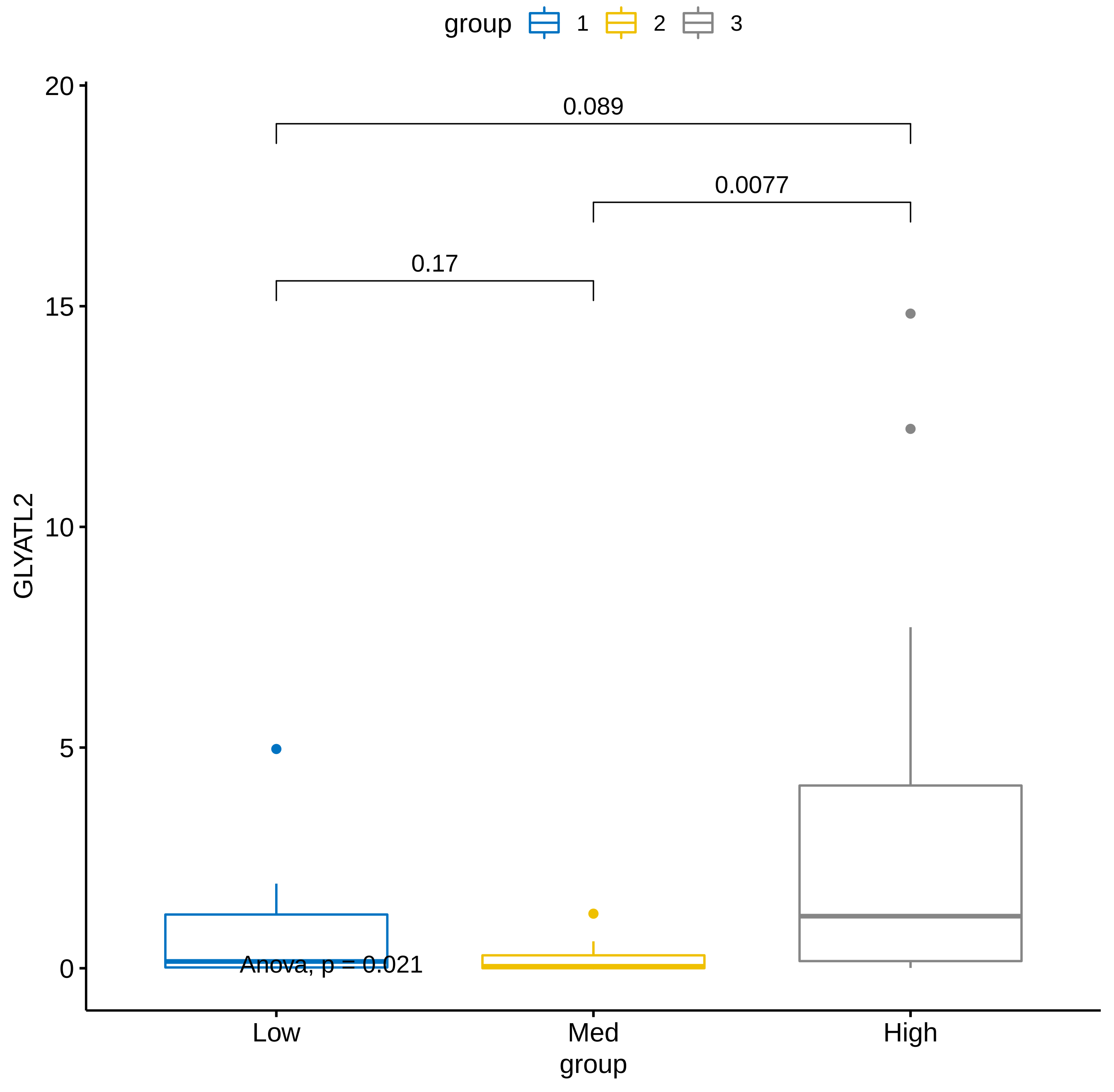

group
1
2
3
20
15
10
5
0
GLYATL2
0.089
0.0077
0.17
Anova, p = 0.021
Low
Med
High
group

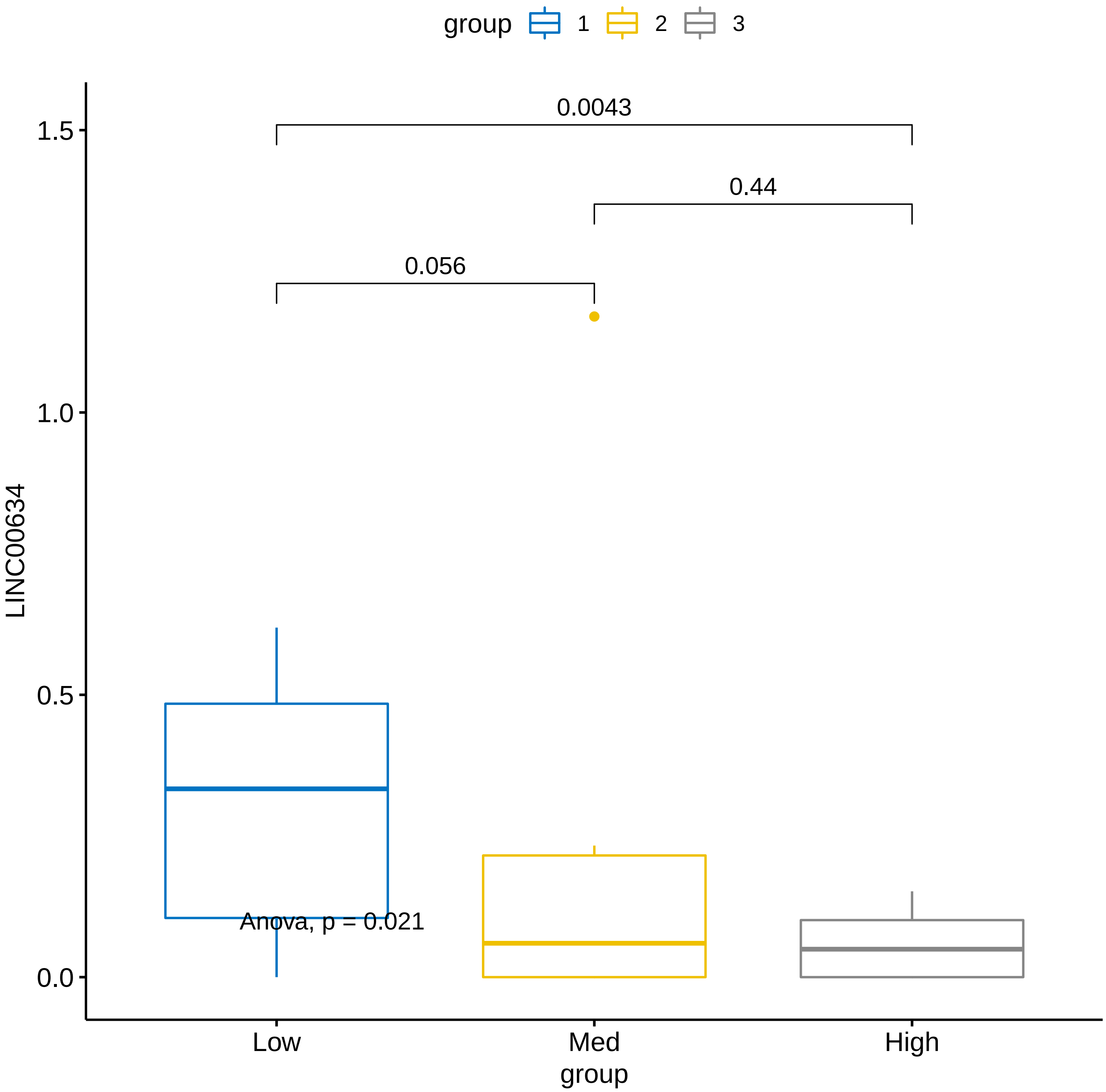

group 1 2 3
0.0043
0.44
0.056
Anova, p = 0.021
1.5
1.0
0.5
0.0
LINC00634
Low
Med
High
group

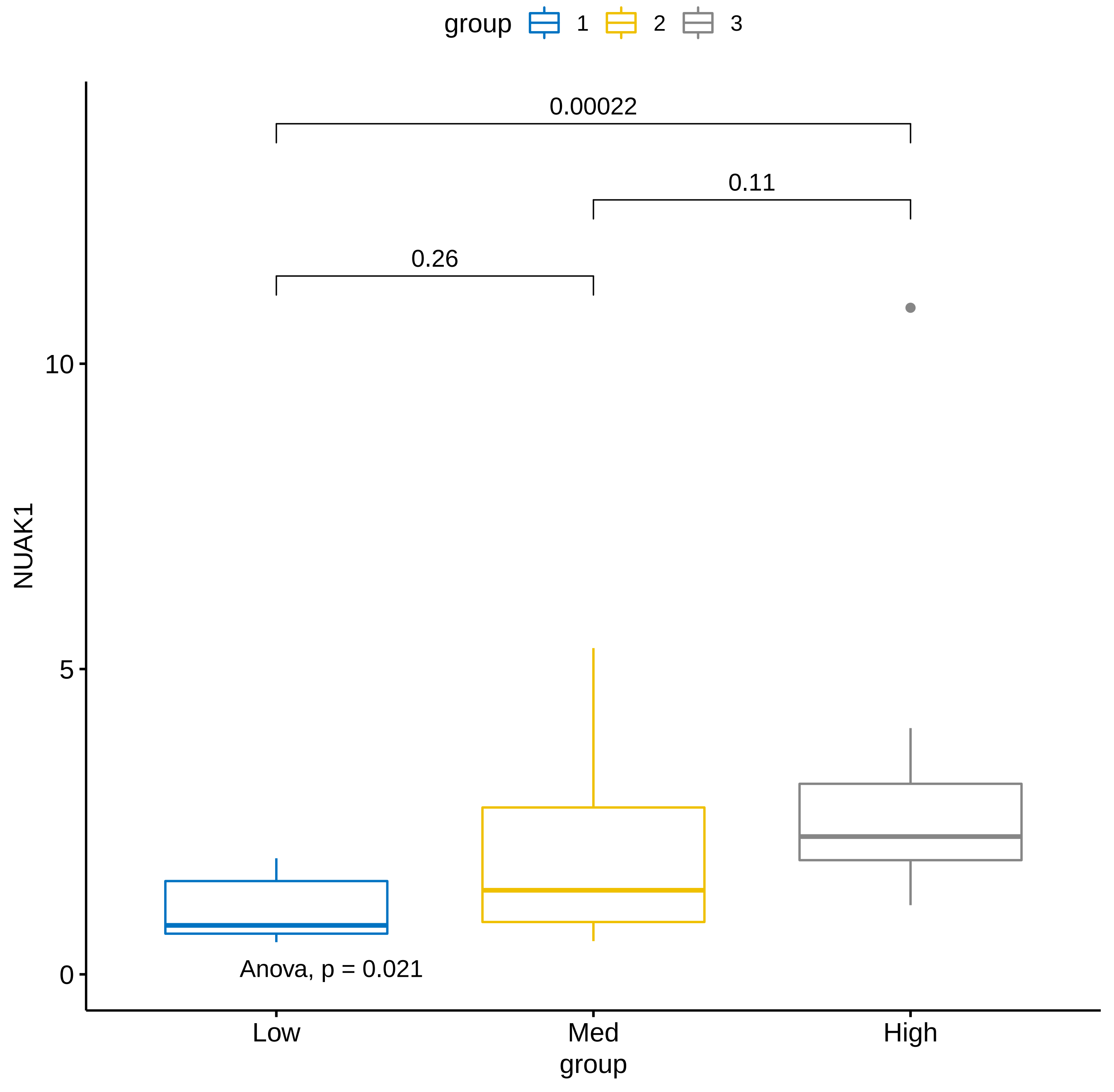

group
1
2
3
0.00022
0.11
0.26
10
5
0
NUAK1
Anova, p = 0.021
Low
Med
High
group

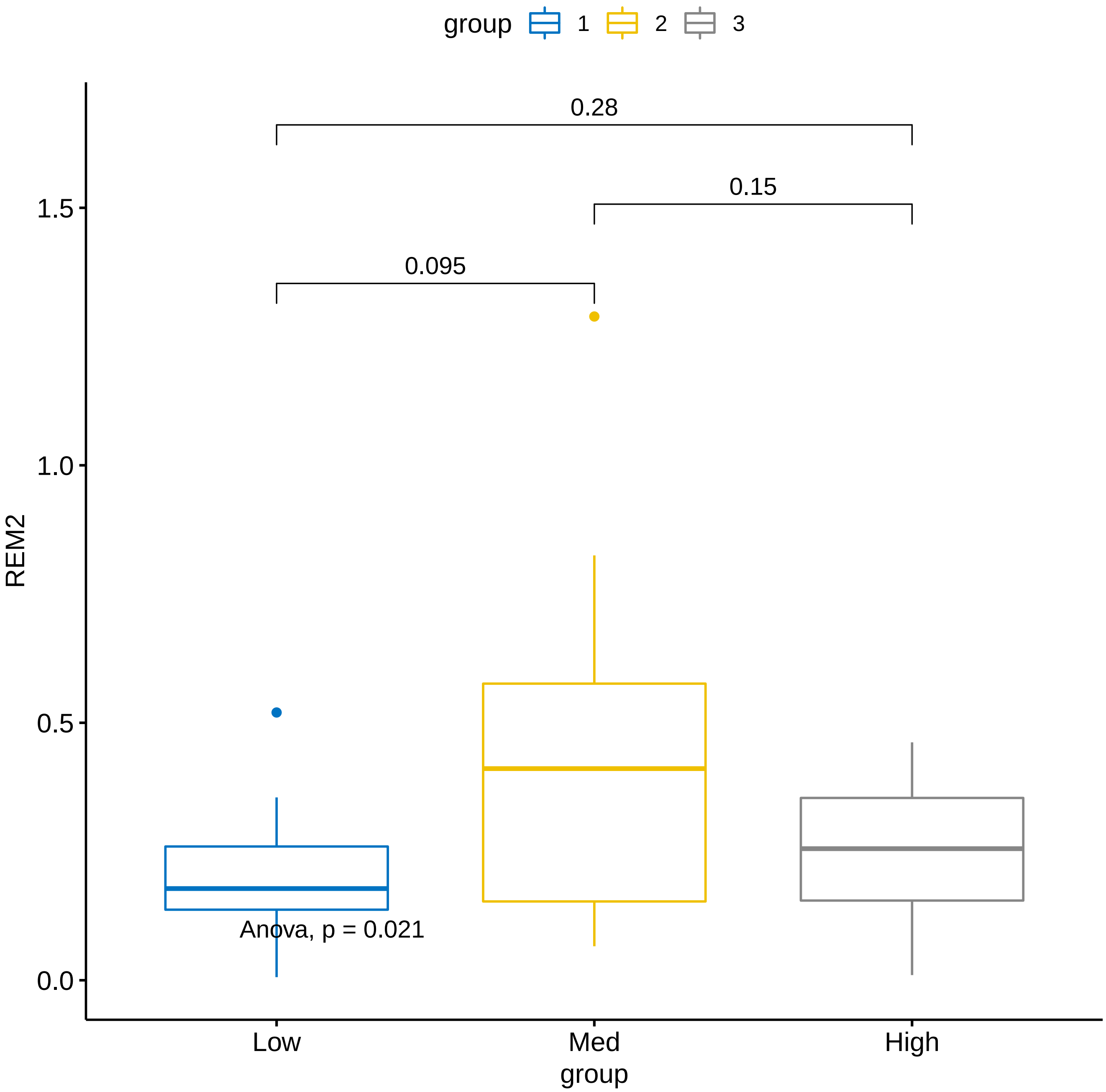
group
1
2
3
0.28
0.15
0.095
1.5
1.0
0.5
0.0
REM2
Anova, p = 0.021
Low
Med
High
group

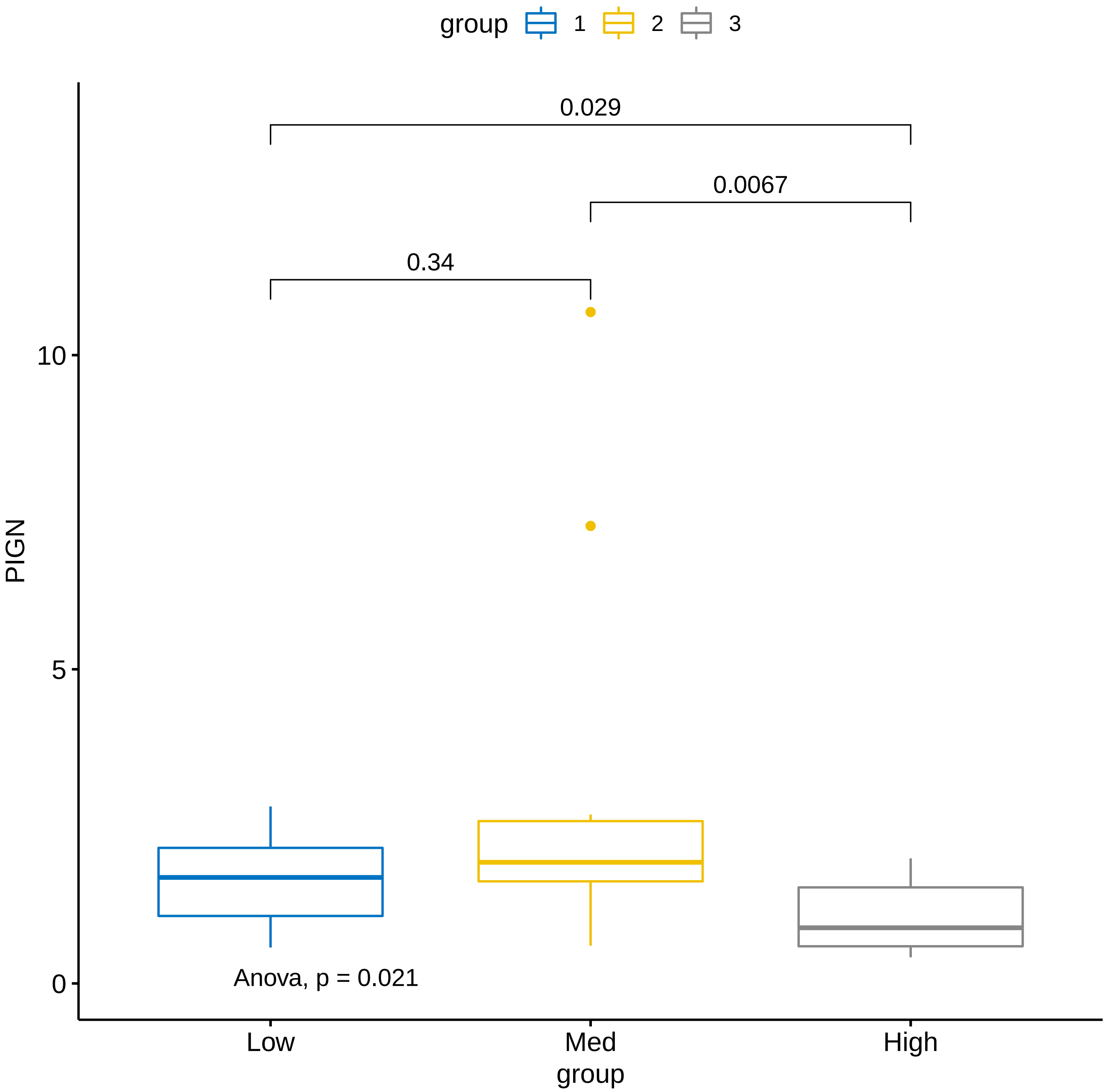
group
1
2
3
0.029
0.0067
0.34
10
5
0
PIGN
Anova, p = 0.021
Low
Med
High
group

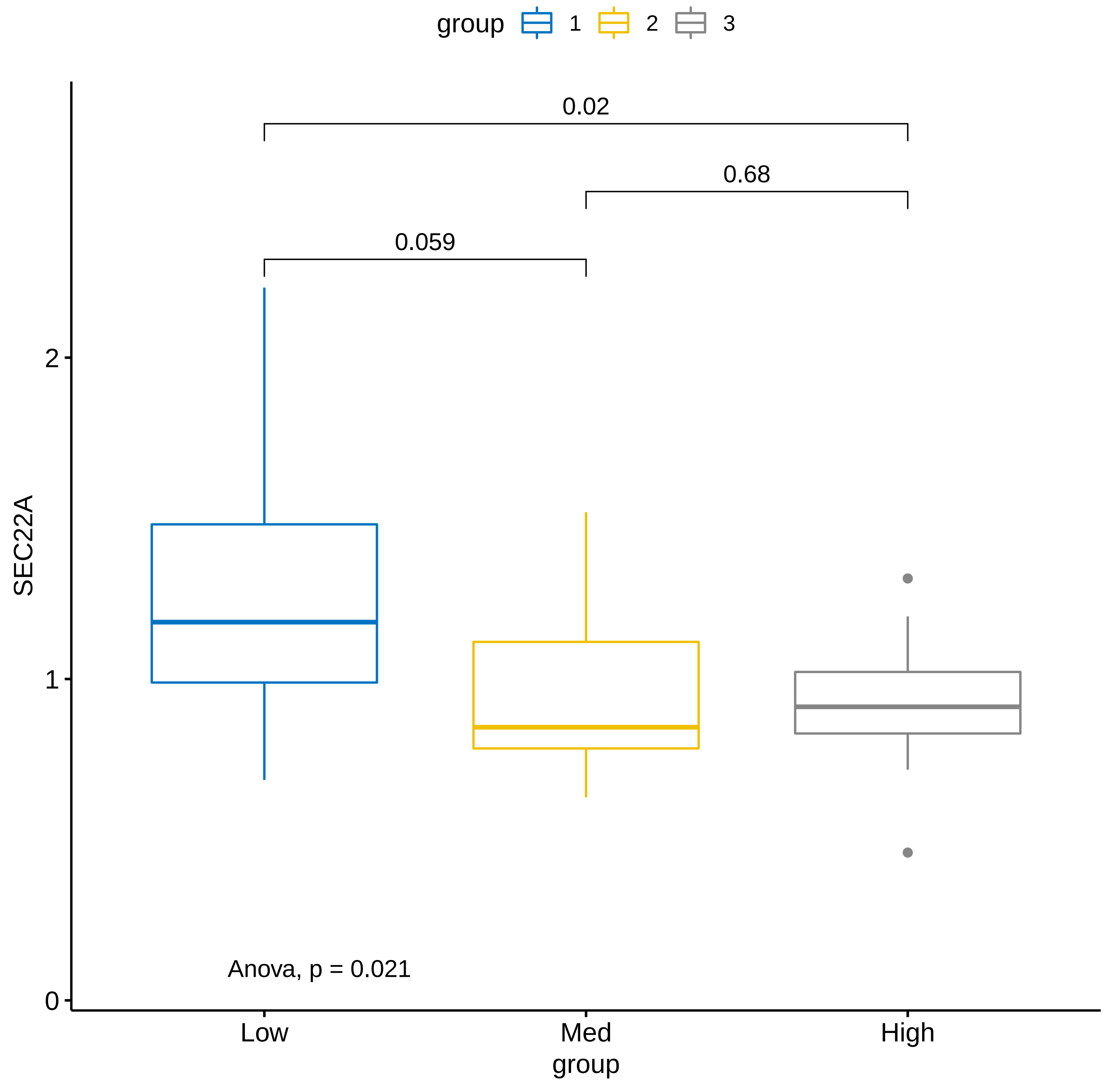

group
1
2
3
0.02
0.68
0.059
SEC22A
2
1
0
Anova, p = 0.021
Low
Med
High
group

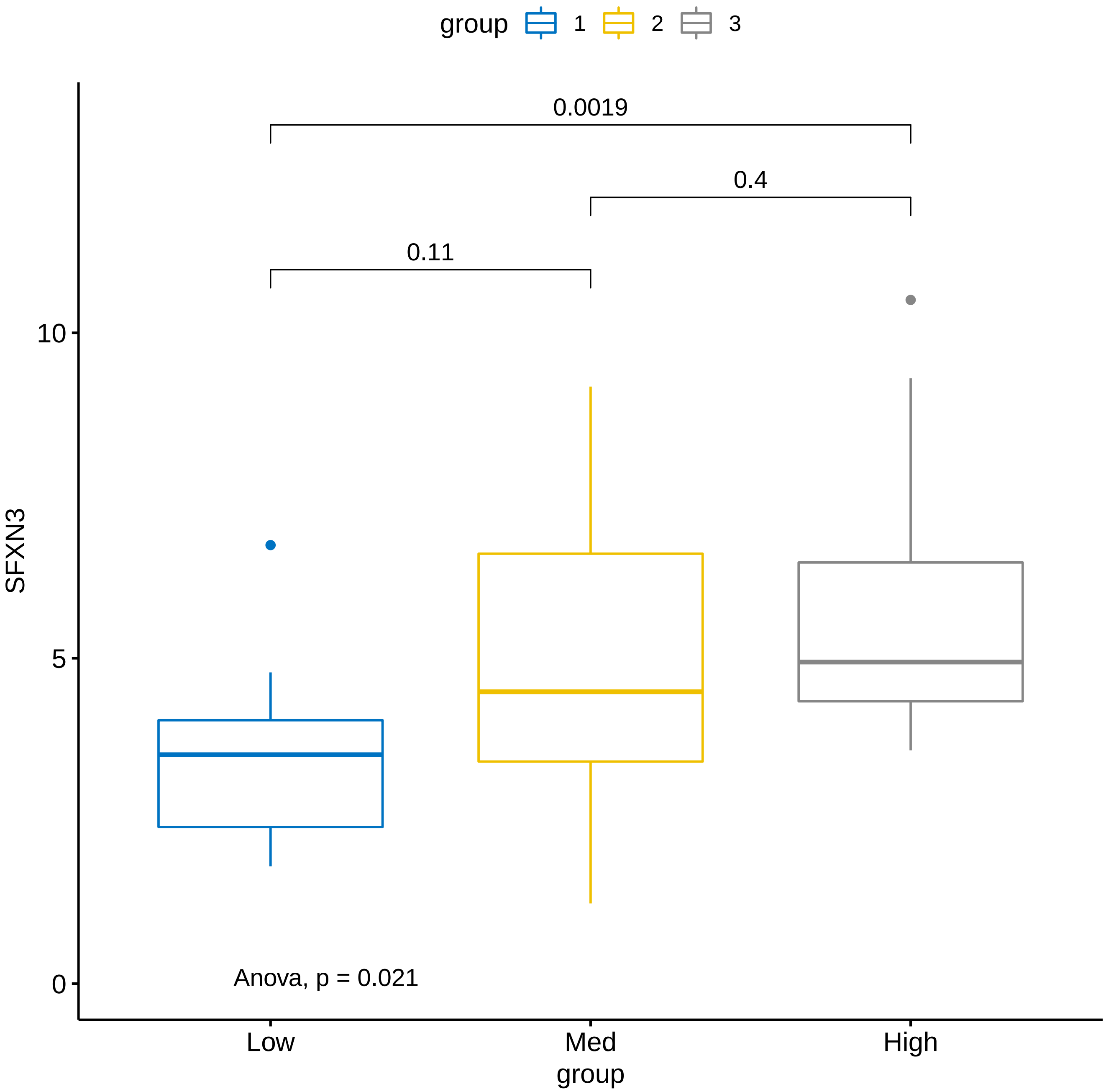

group
1
2
3
0.0019
0.4
0.11
10
5
0
SFXN3
Anova, p = 0.021
Low
Med
High
group

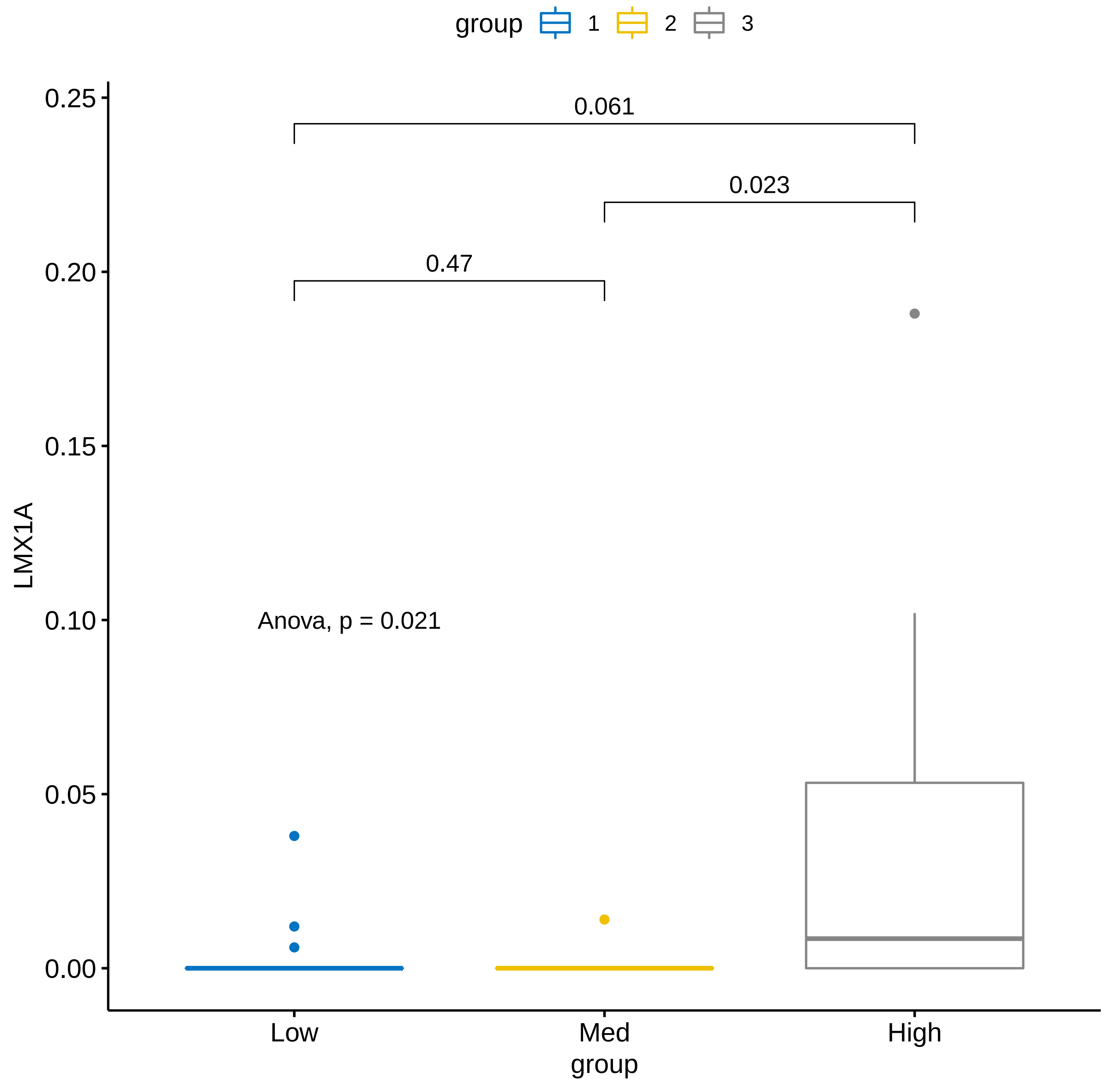

group 1 2 3
0.061
0.023
0.47
Anova, p = 0.021
0.25
0.20
0.15
0.10
0.05
0.00
LMX1A
Low
Med
High
group

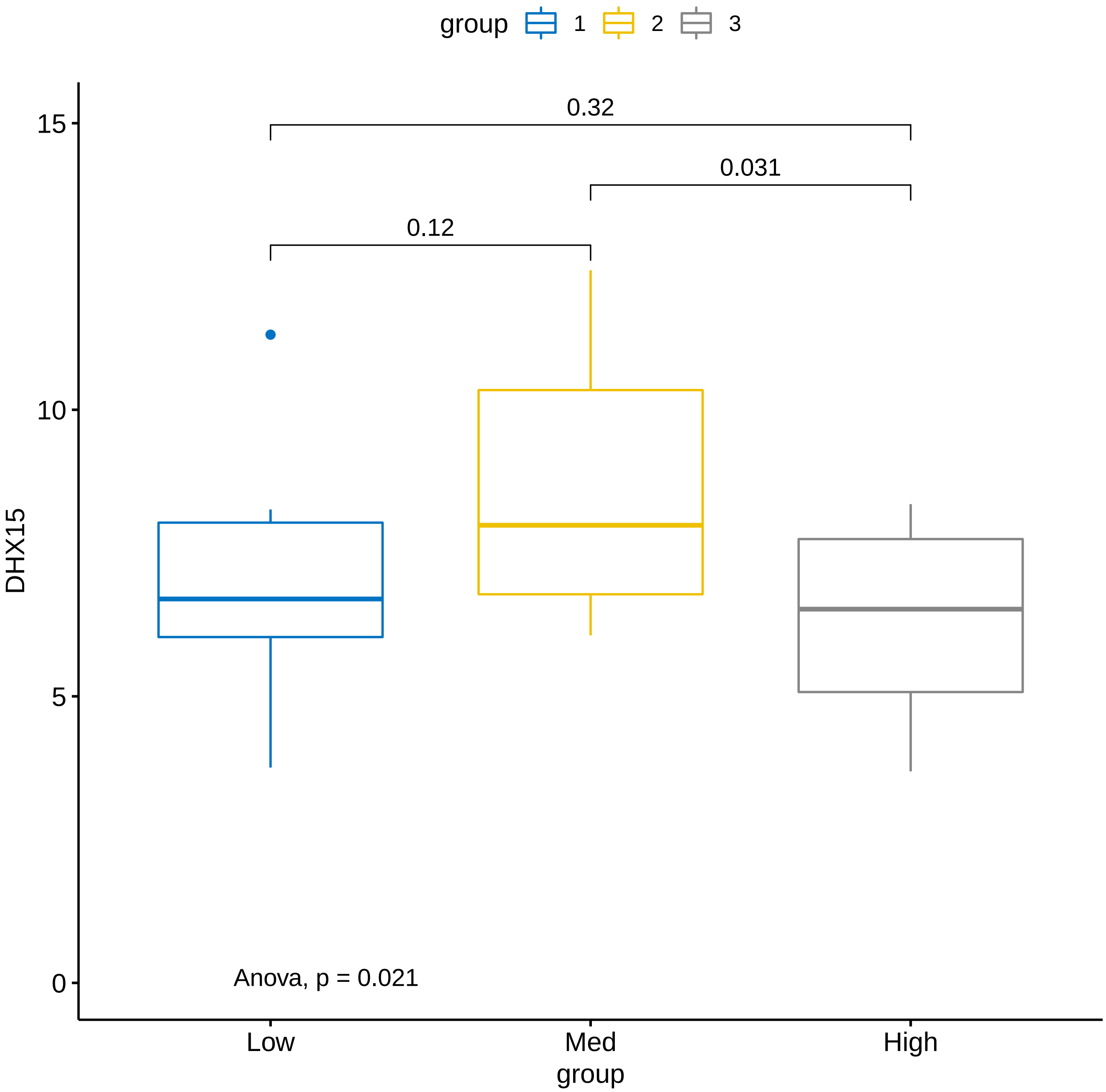
group 1 2 3
0.32
0.031
0.12
15
10
5
0
DHX15
Anova, p = 0.021
Low
Med
High
group

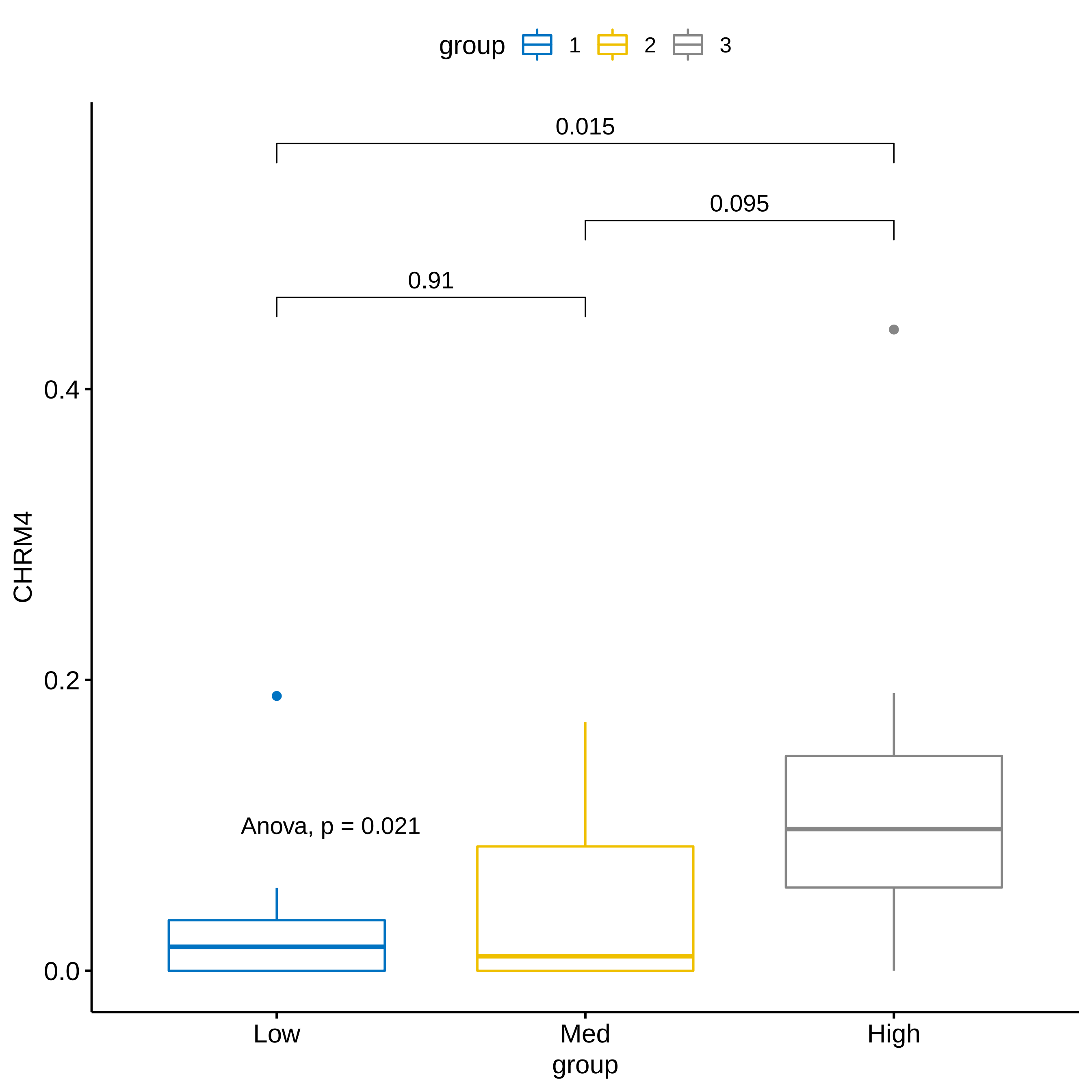
group
1
2
3
0.015
0.095
0.91
0.4
0.2
0.0
CHRM4
Anova, p = 0.021
Low
Med
High
group

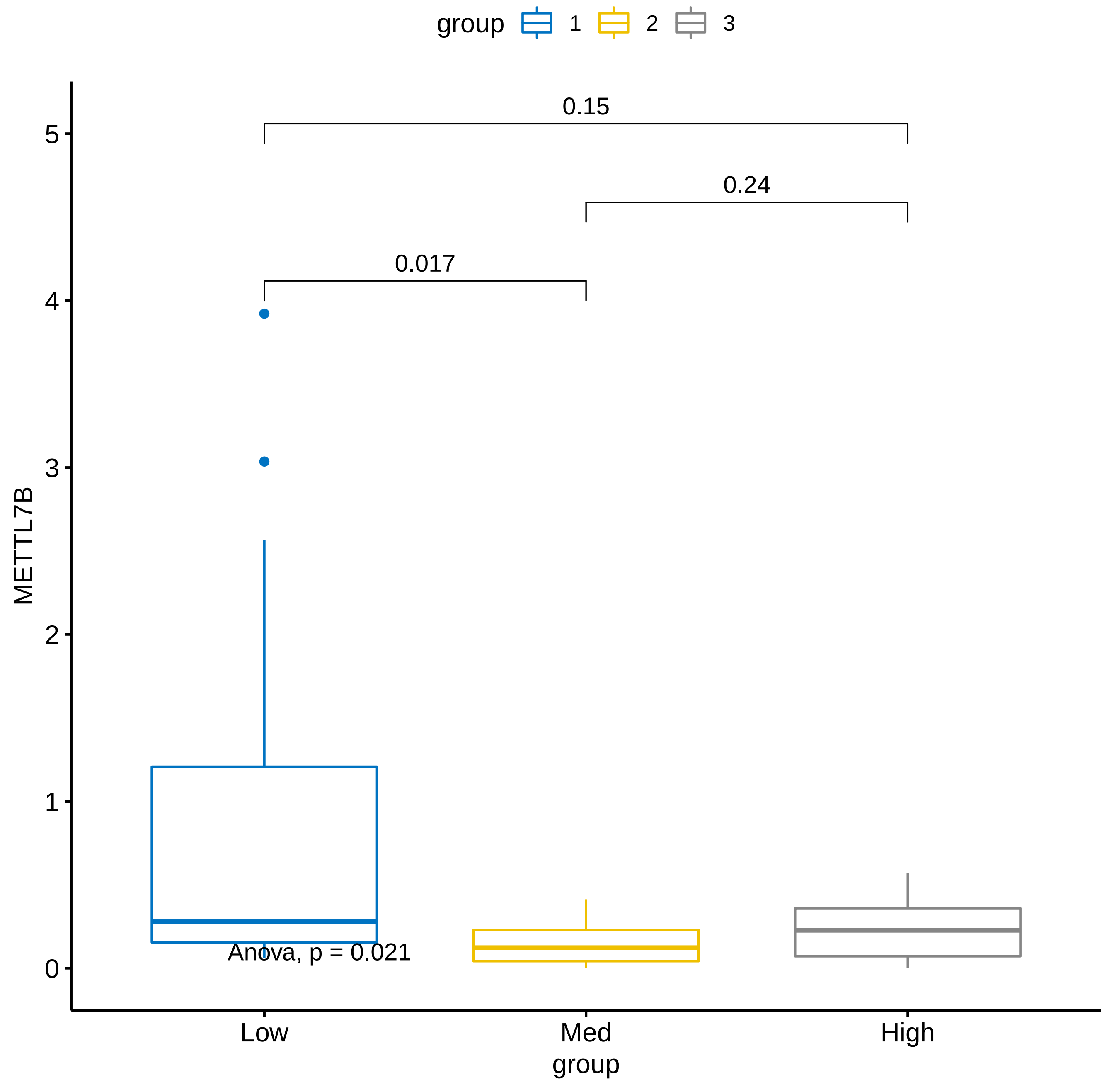

group
1
2
3
0.15
0.24
0.017
5
4
3
2
1
0
METTL7B
Anova, p = 0.021
Low
Med
High
group

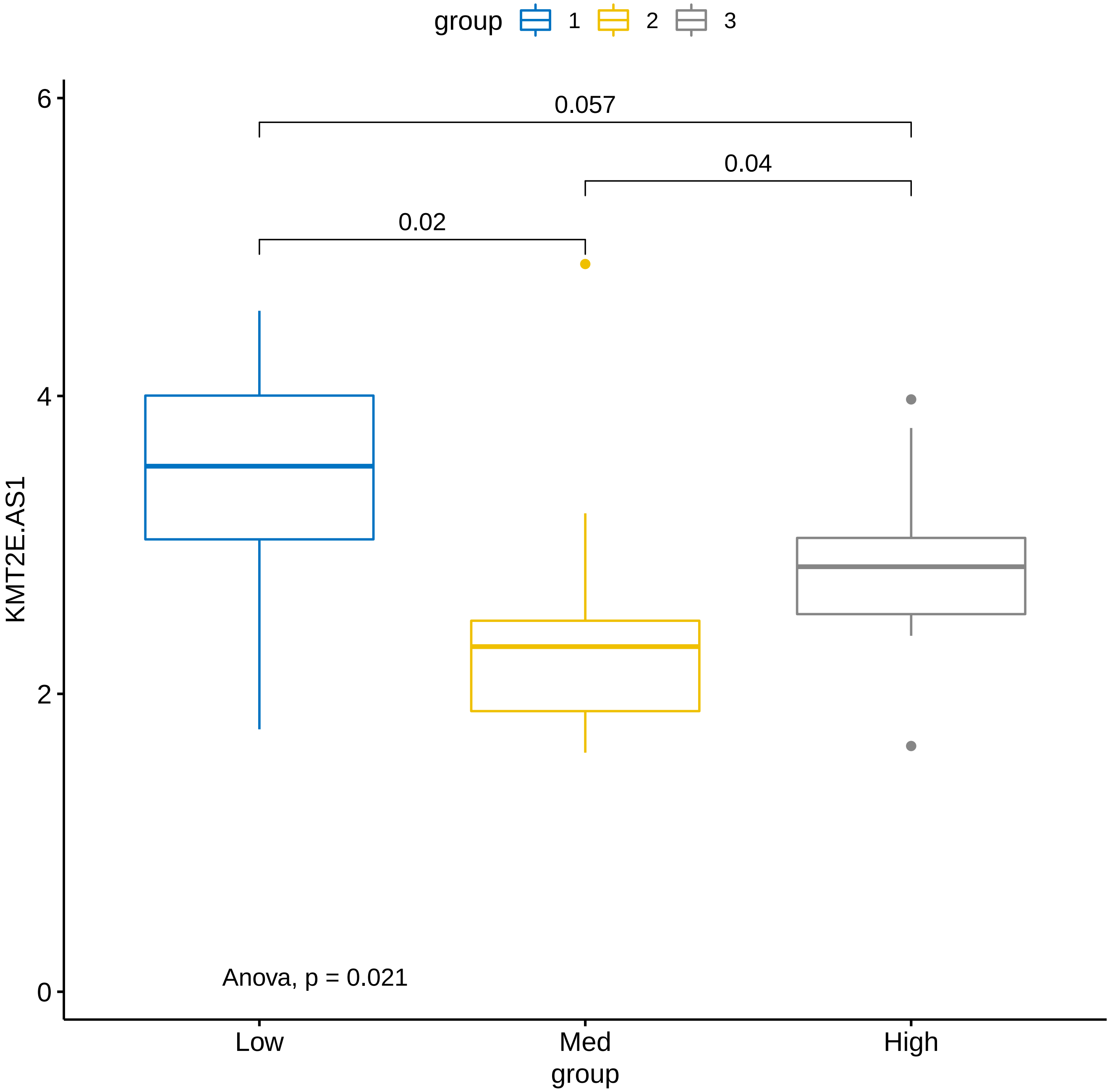

group 1 2 3
0.057
0.04
0.02
KMT2E.AS1
6
4
2
0
Anova, p = 0.021
Low
Med
High
group

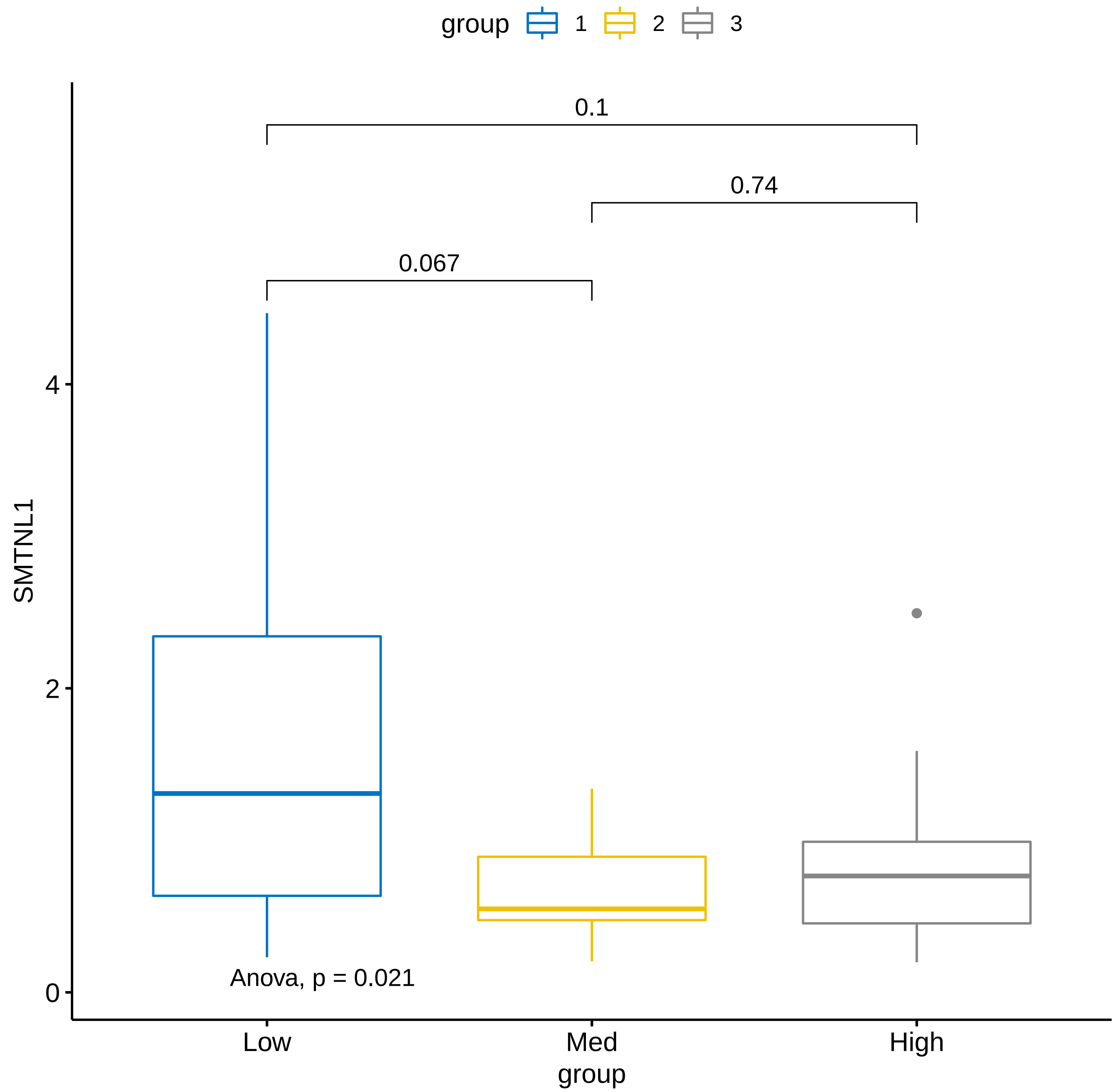
group
1
2
3
0.1
0.74
0.067
SMTNL1
4
2
0
Anova, p = 0.021
Low
Med
High
group

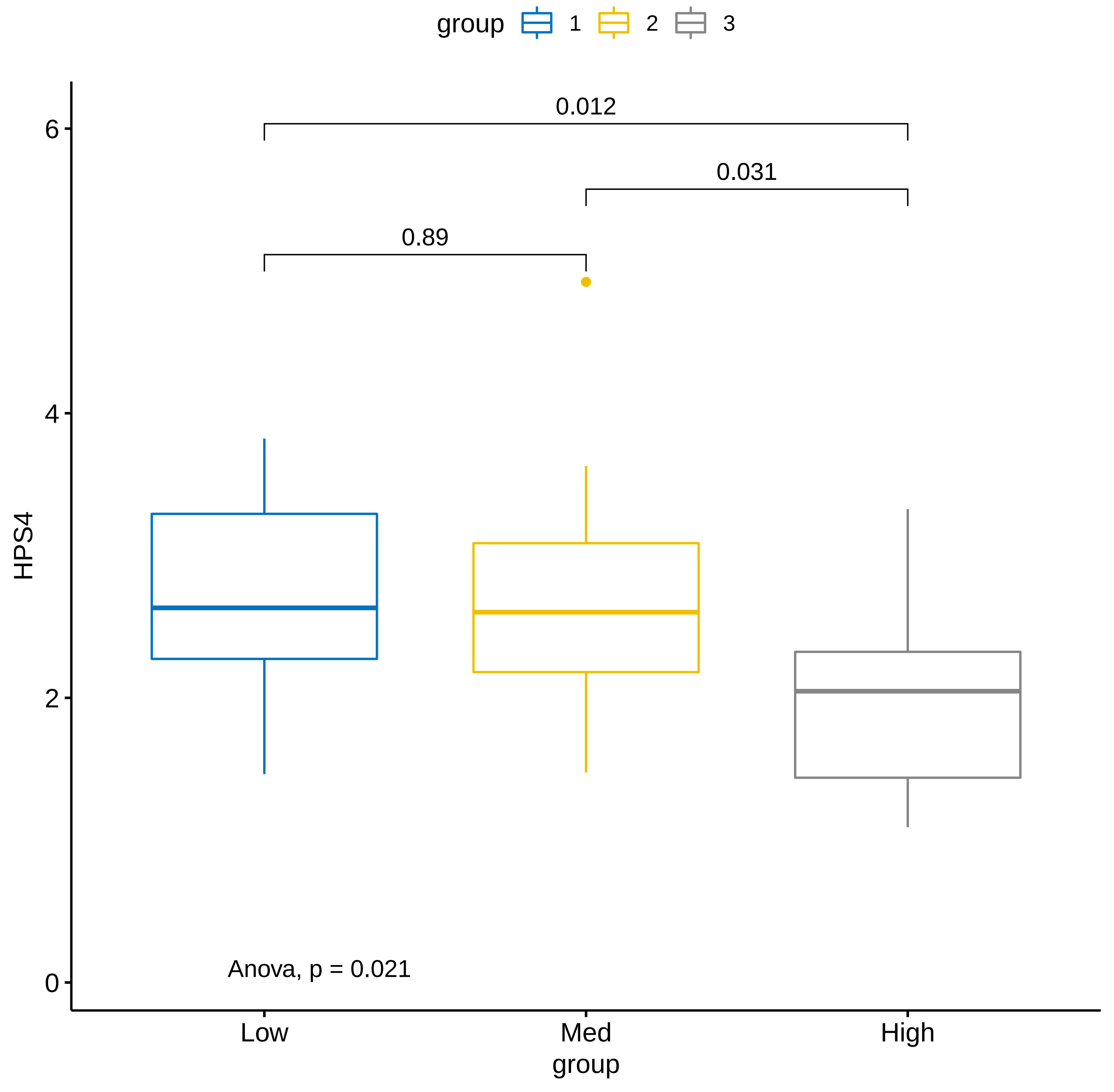
group
1
2
3
0.012
0.031
0.89
6
4
2
0
HPS4
Anova, p = 0.021
Low
Med
High
group

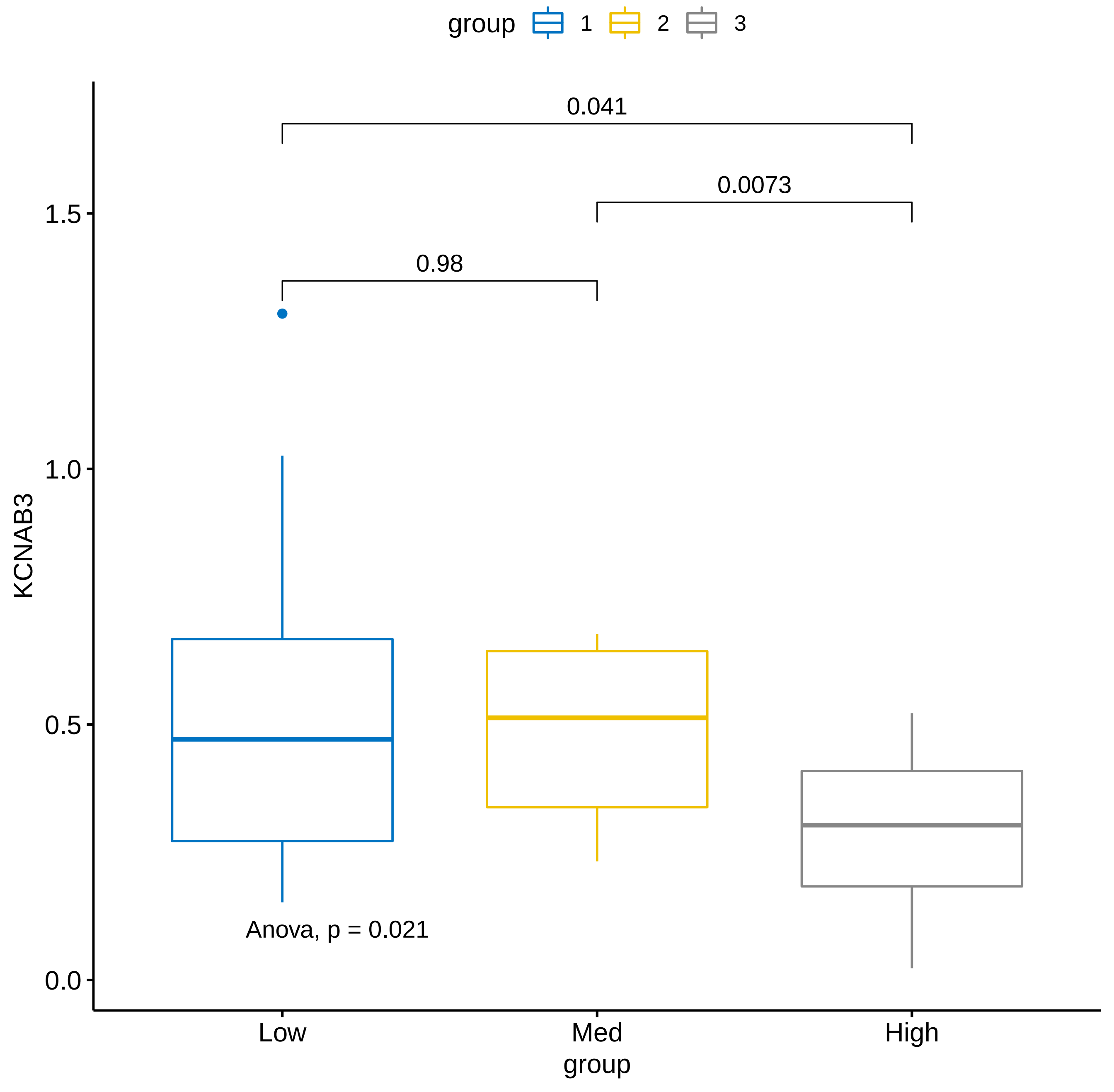
group
1
2
3
0.041
0.0073
0.98
1.5
1.0
0.5
0.0
KCNAB3
Anova, p = 0.021
Low
Med
High
group

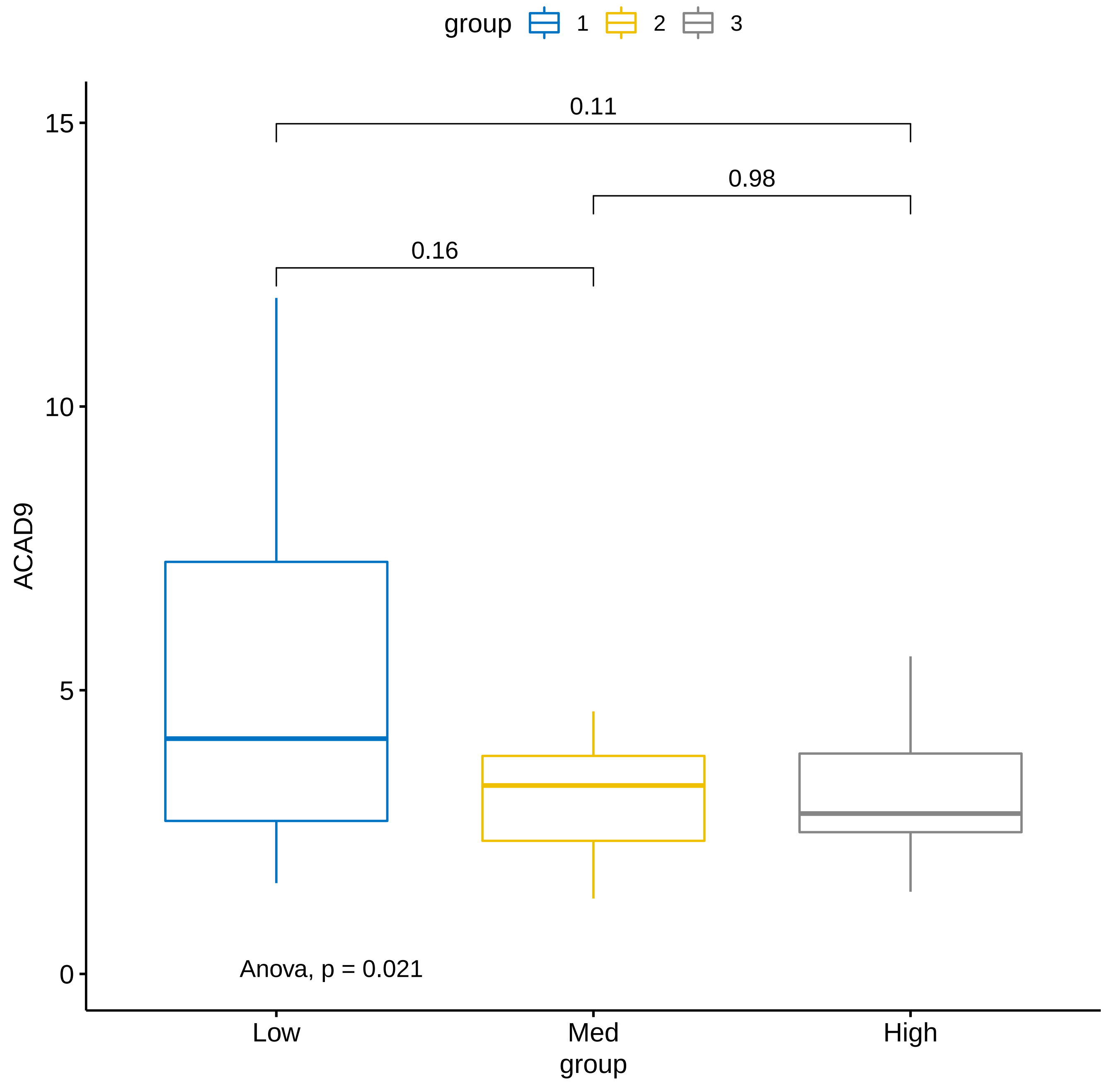
group
1
2
3
0.11
0.98
0.16
15
10
5
0
ACAD9
Anova, p = 0.021
Low
Med
High
group

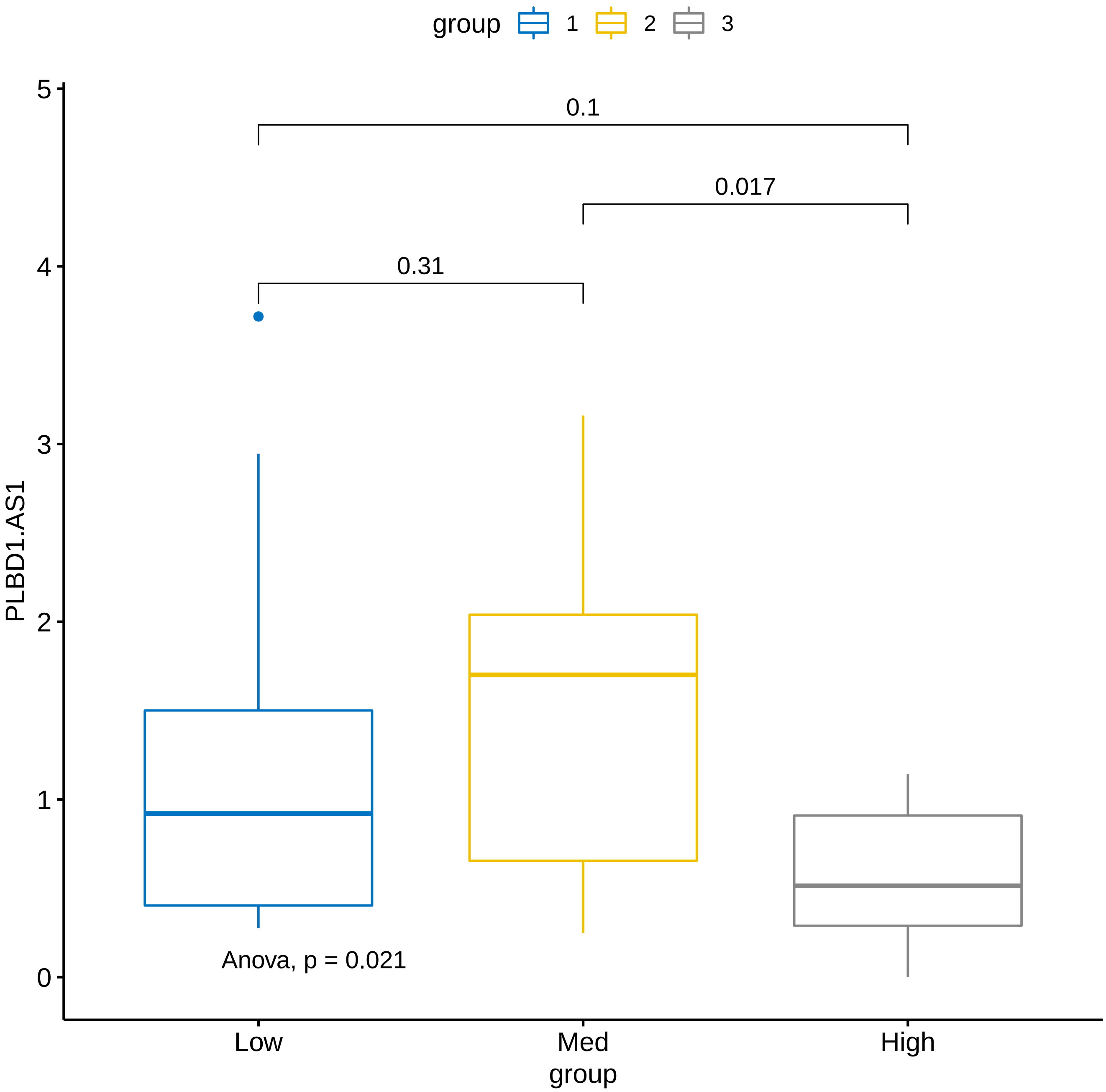

group 1 2 3
0.1
0.017
0.31
PLBD1.AS1
5
4
3
2
1
0
Anova, p = 0.021
Low
Med
High
group

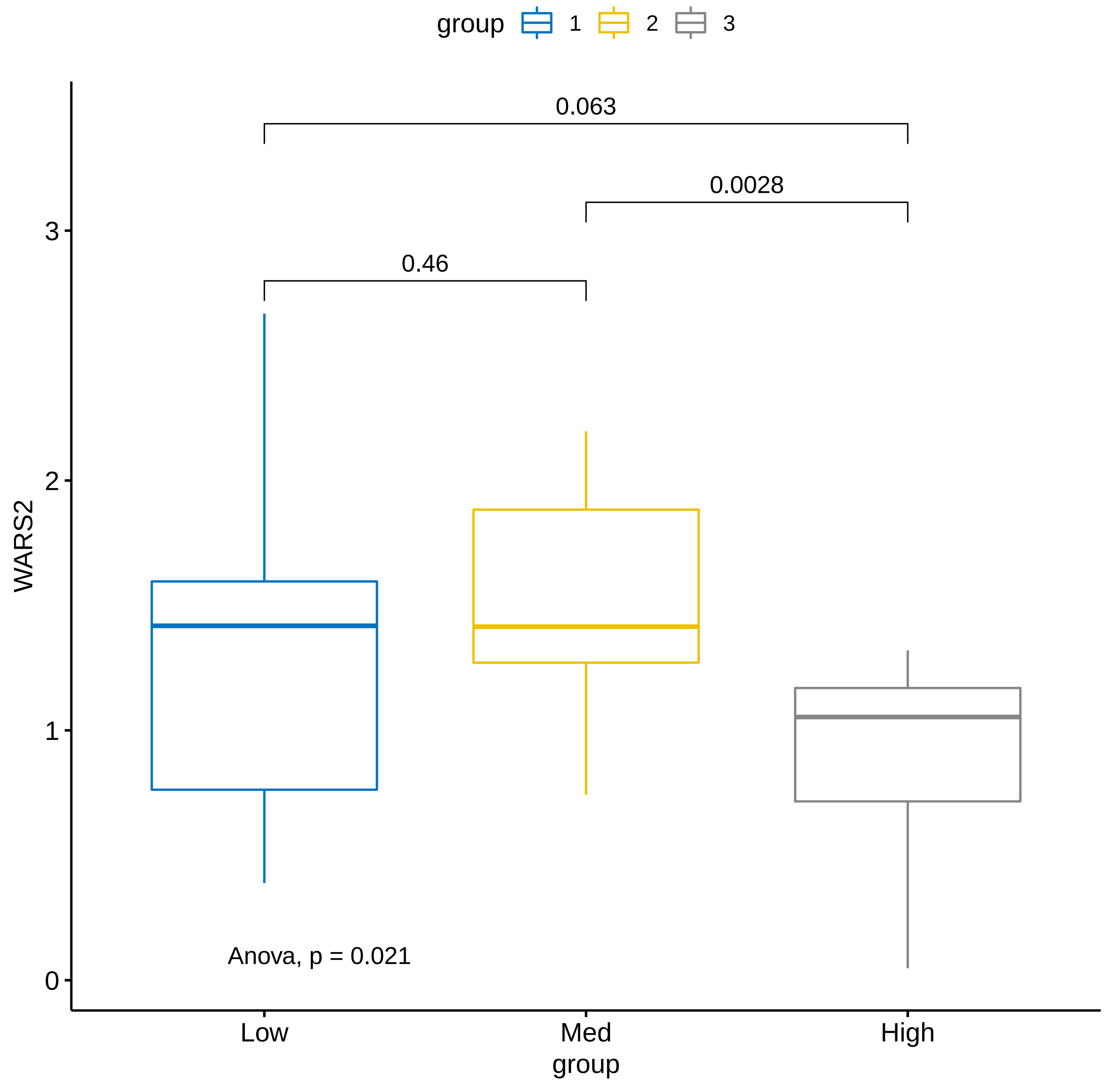
group 1 2 3
0.063
0.0028
0.46
Anova, p = 0.021
WARS2
3
2
1
0
Low
Med
High
group

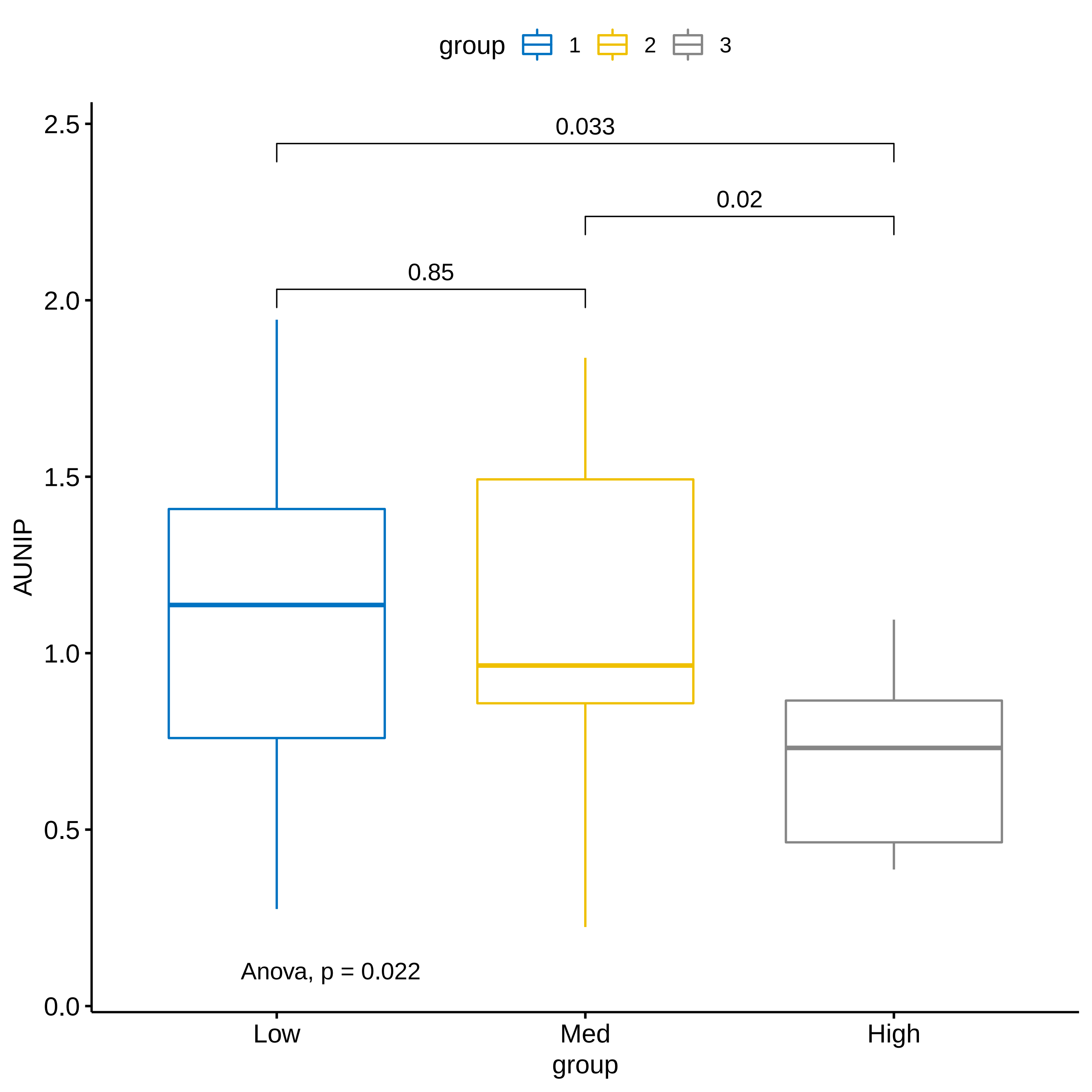

group 1 2 3
0.033
0.02
0.85
2.5
2.0
1.5
1.0
0.5
0.0
AUNIP
Anova, p = 0.022
Low
Med
High
group

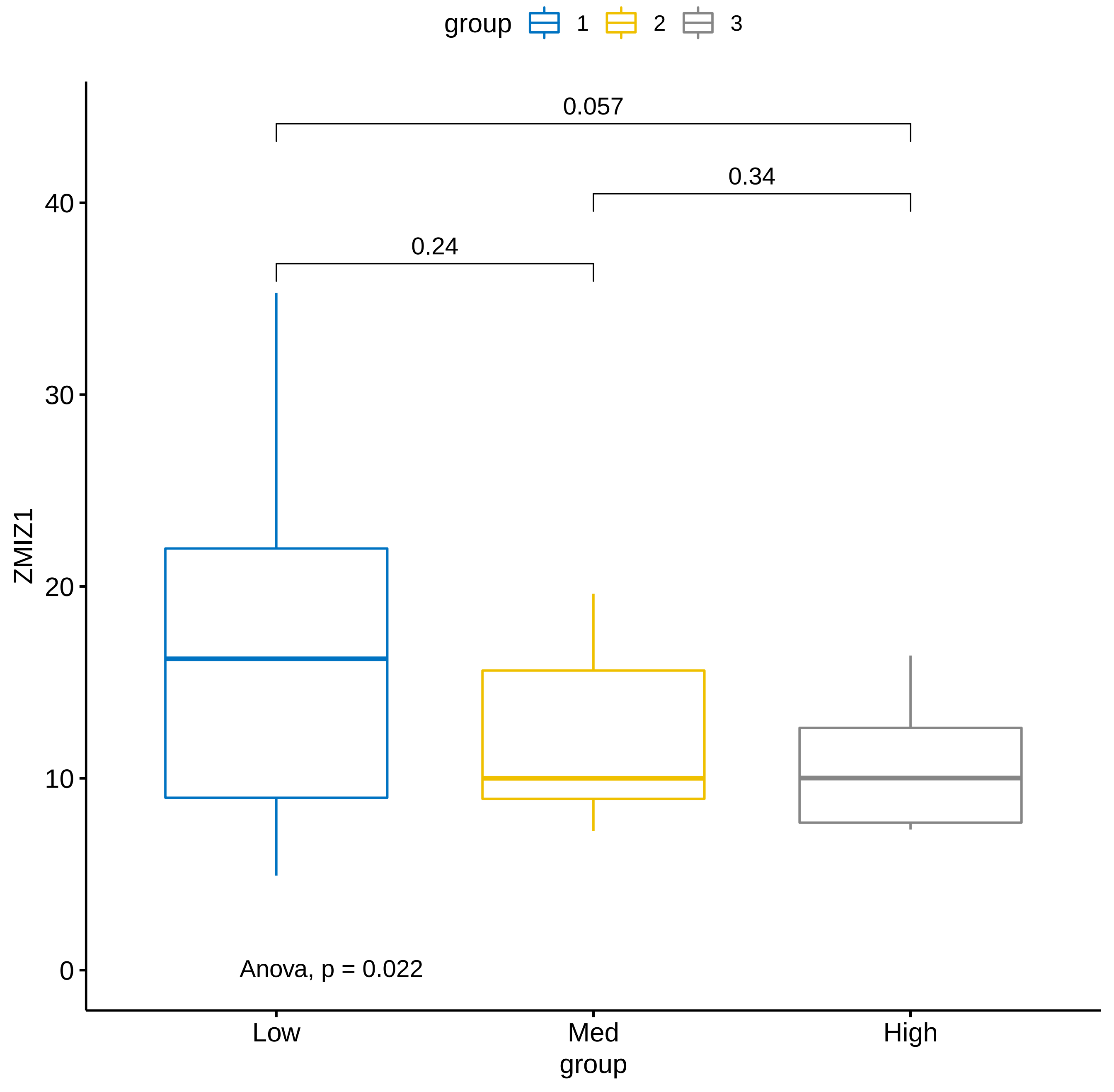

group
1
2
3
0.057
0.34
0.24
40
30
20
10
0
ZMIZ1
Anova, p = 0.022
Low
Med
High
group

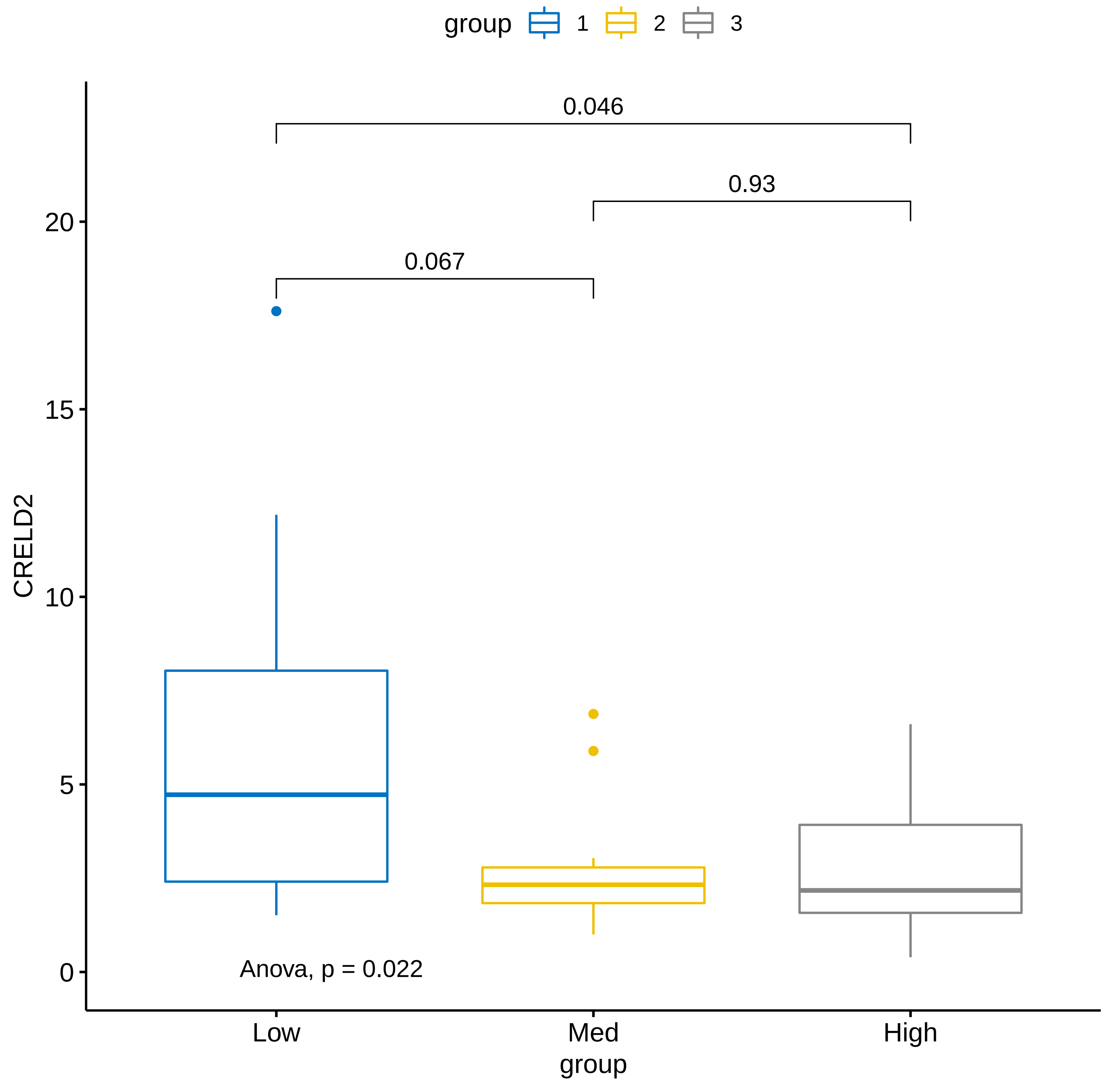
group
1
2
3
0.046
0.93
0.067
20
15
10
5
0
CRELD2
Anova, p = 0.022
Low
Med
High
group

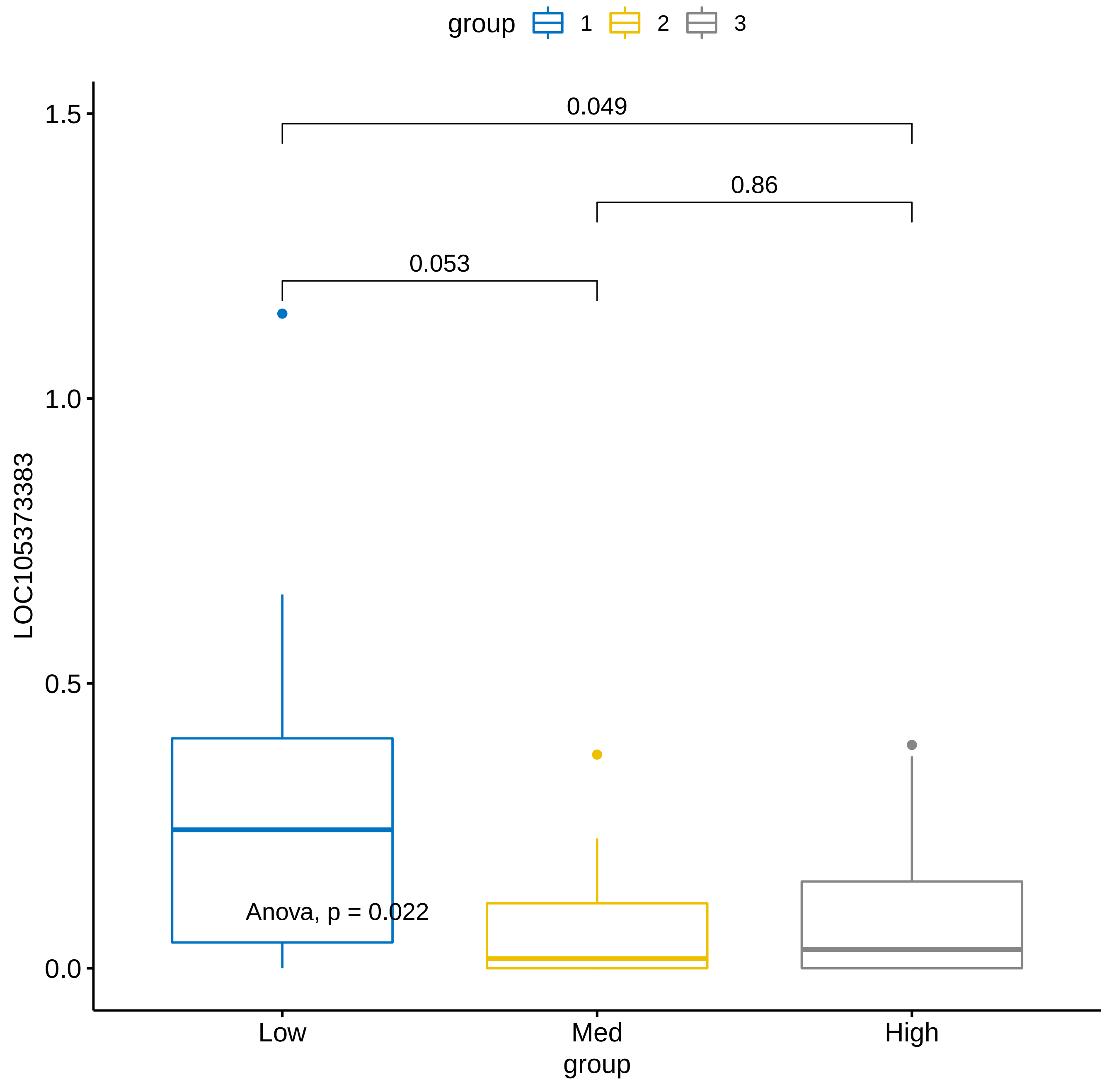

group
1
2
3
0.049
0.86
0.053
Anova, p = 0.022
1.5
1.0
0.5
0.0
LOC105373383
Low
Med
High
group

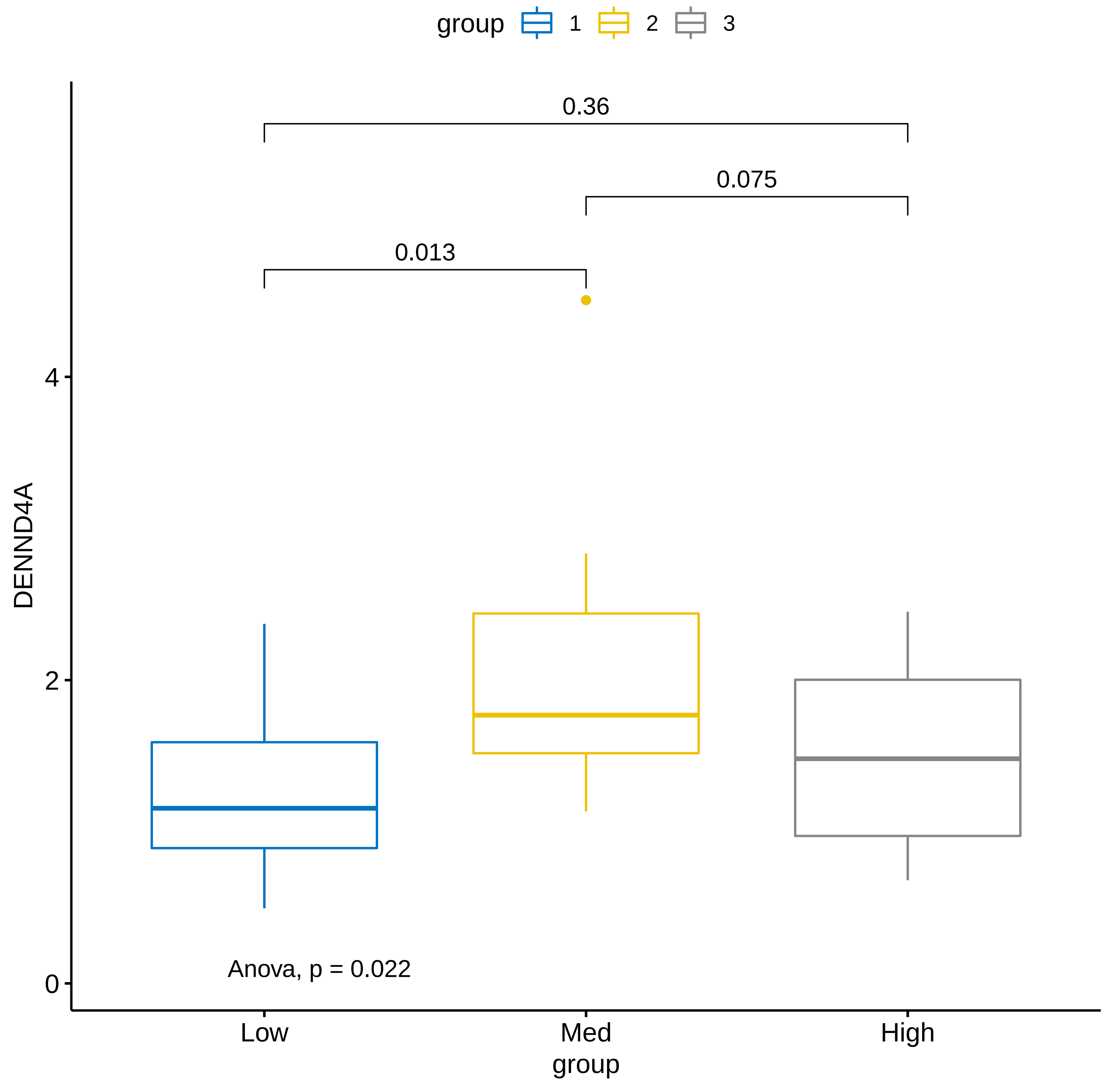
group
1
2
3
0.36
0.075
0.013
DENND4A
4
2
0
Anova, p = 0.022
Low
Med
High
group

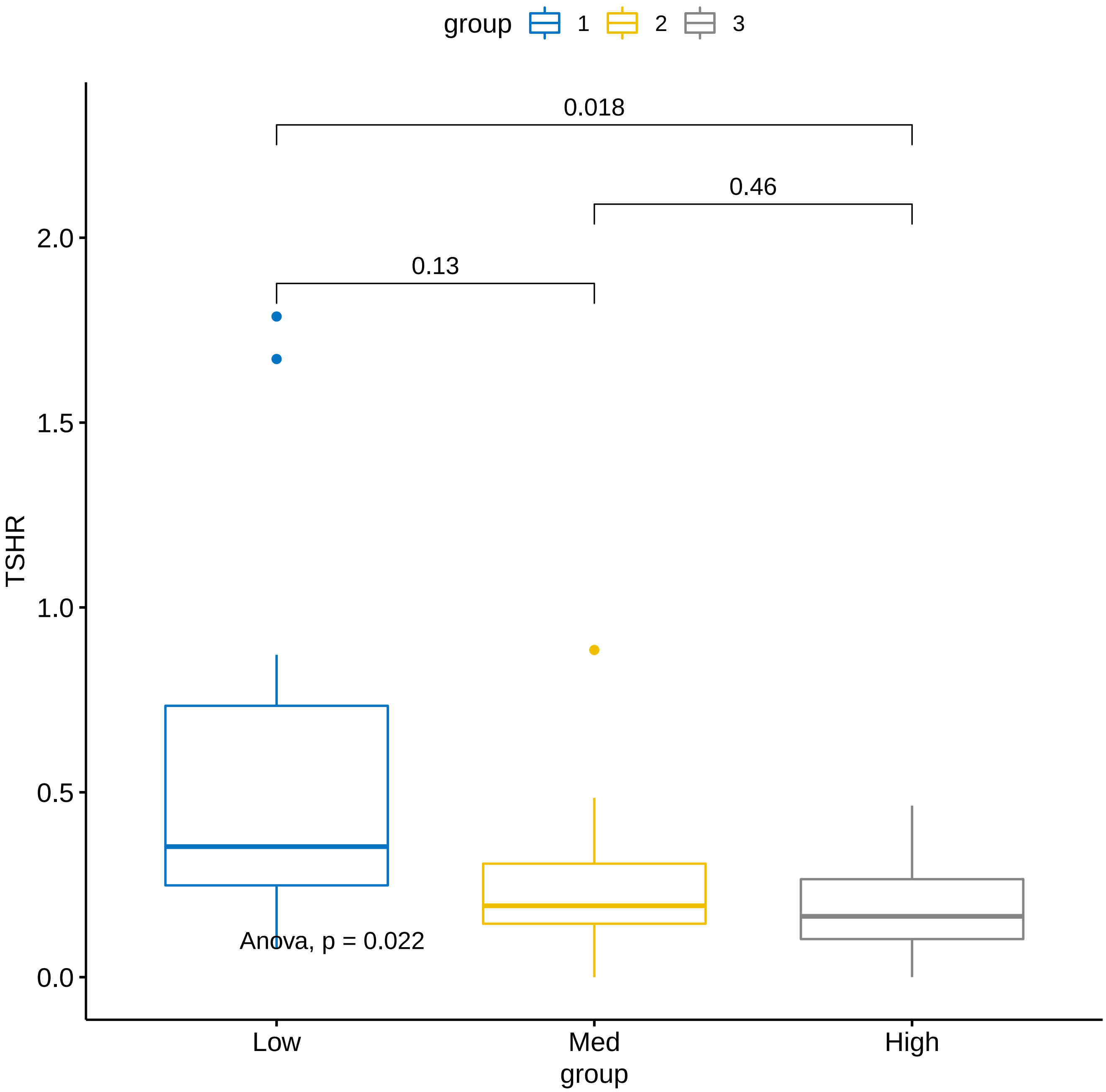
group 1 2 3
0.018
0.46
0.13
2.0
1.5
1.0
0.5
0.0
TSHR
Anova, p = 0.022
Low
Med
High
group

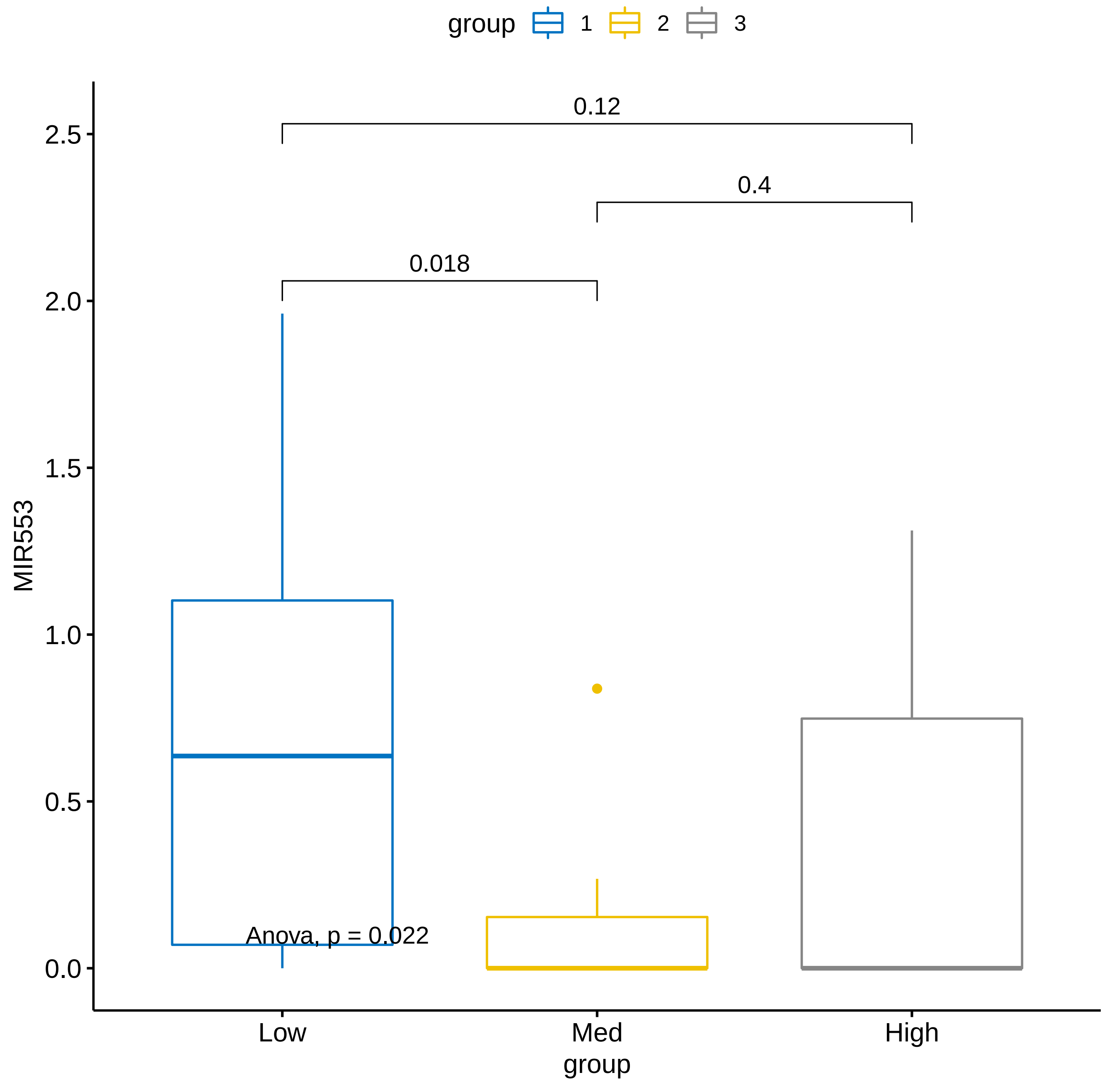

group 1 2 3
0.12
0.4
0.018
2.5
2.0
1.5
1.0
0.5
0.0
MIR553
Anova, p = 0.022
Low
Med
High
group

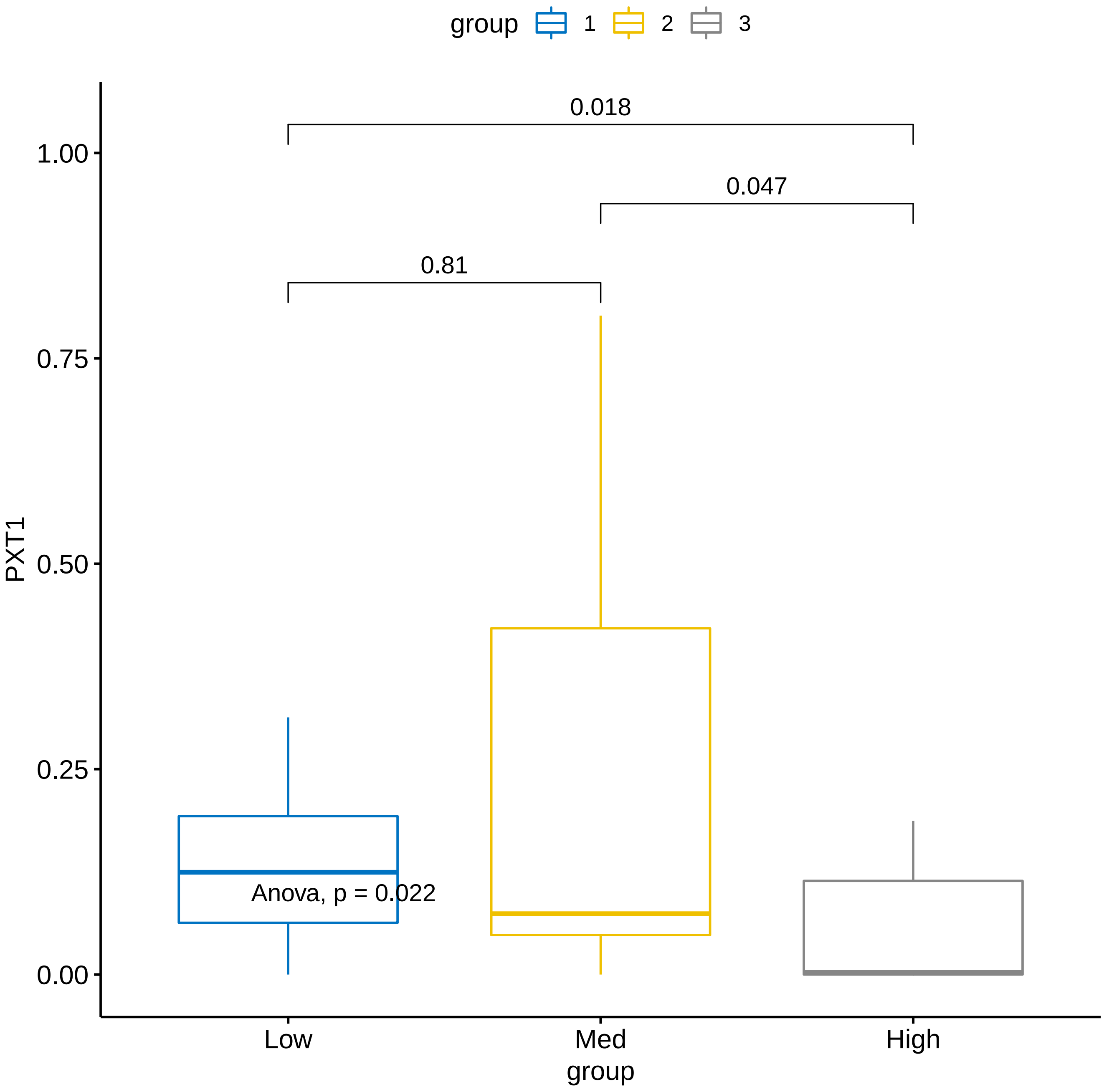
group
1
2
3
0.018
0.047
0.81
1.00
0.75
0.50
0.25
0.00
PXT1
Anova, p = 0.022
Low
Med
High
group

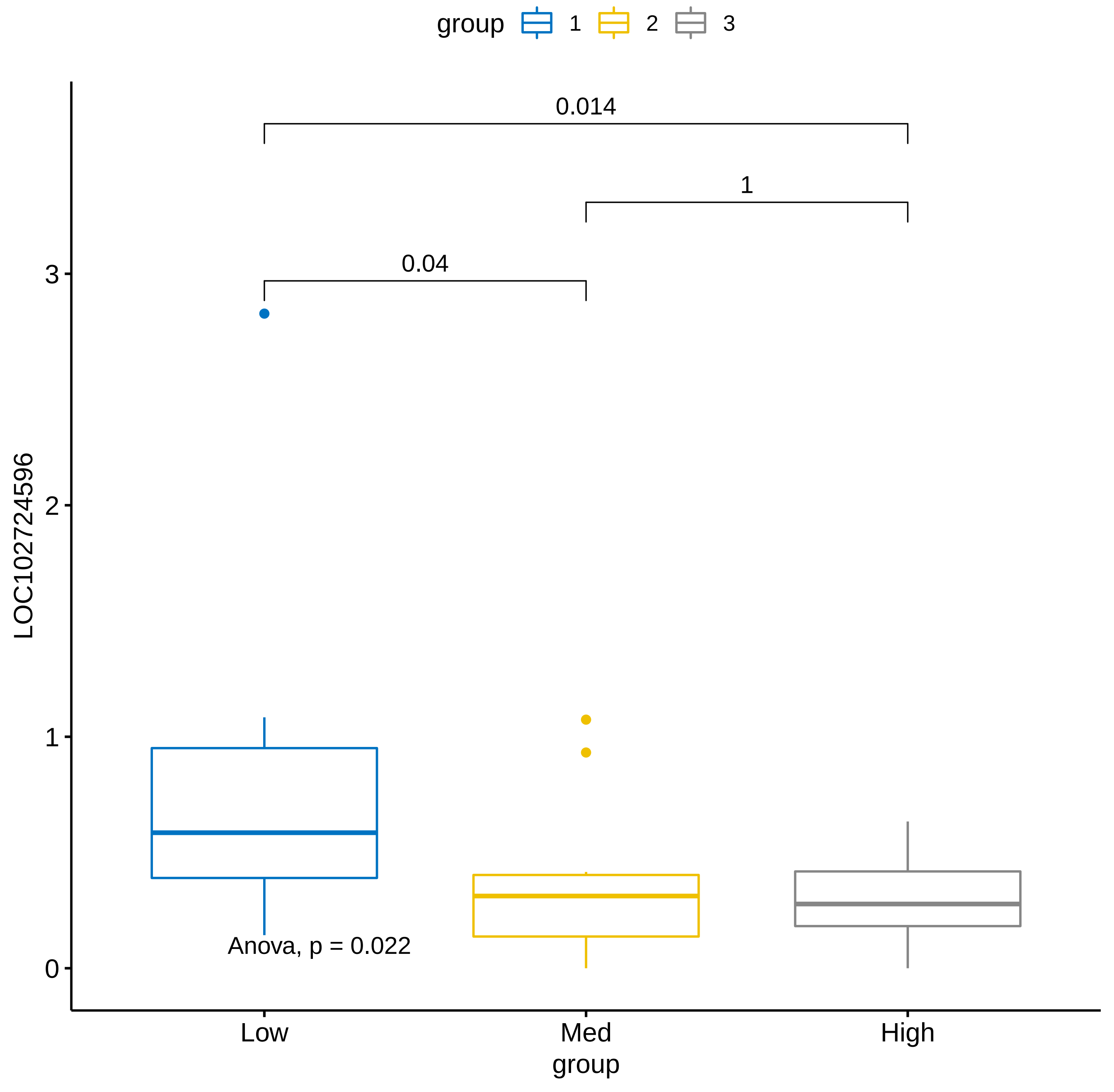
group
1
2
3
0.014
1
0.04
3
2
1
0
LOC102724596
Anova, p = 0.022
Low
Med
High
group

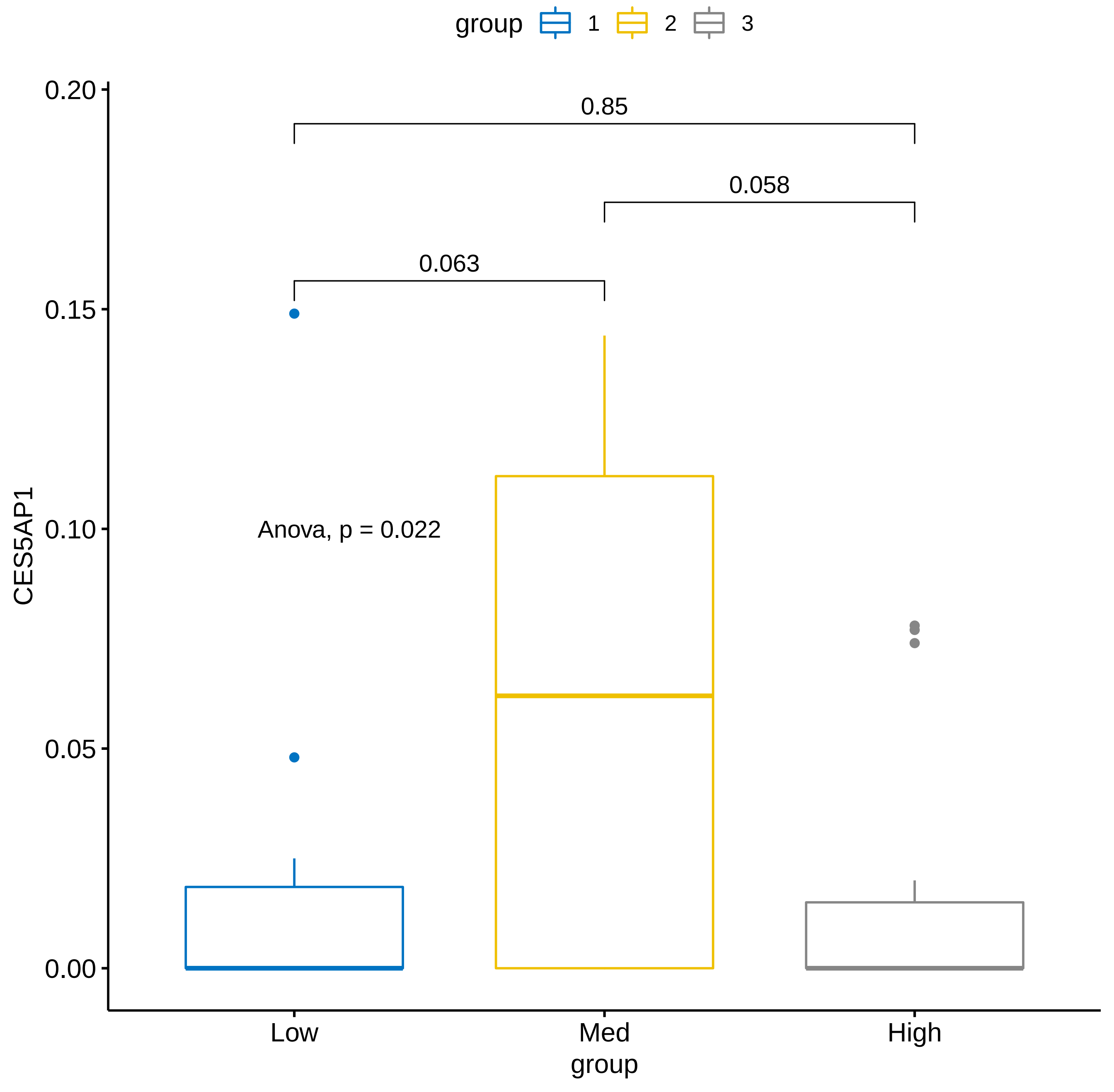
group
1
2
3
0.20
0.15
0.10
0.05
0.00
CES5AP1
0.85
0.058
0.063
Anova, p = 0.022
Low
Med
High
group

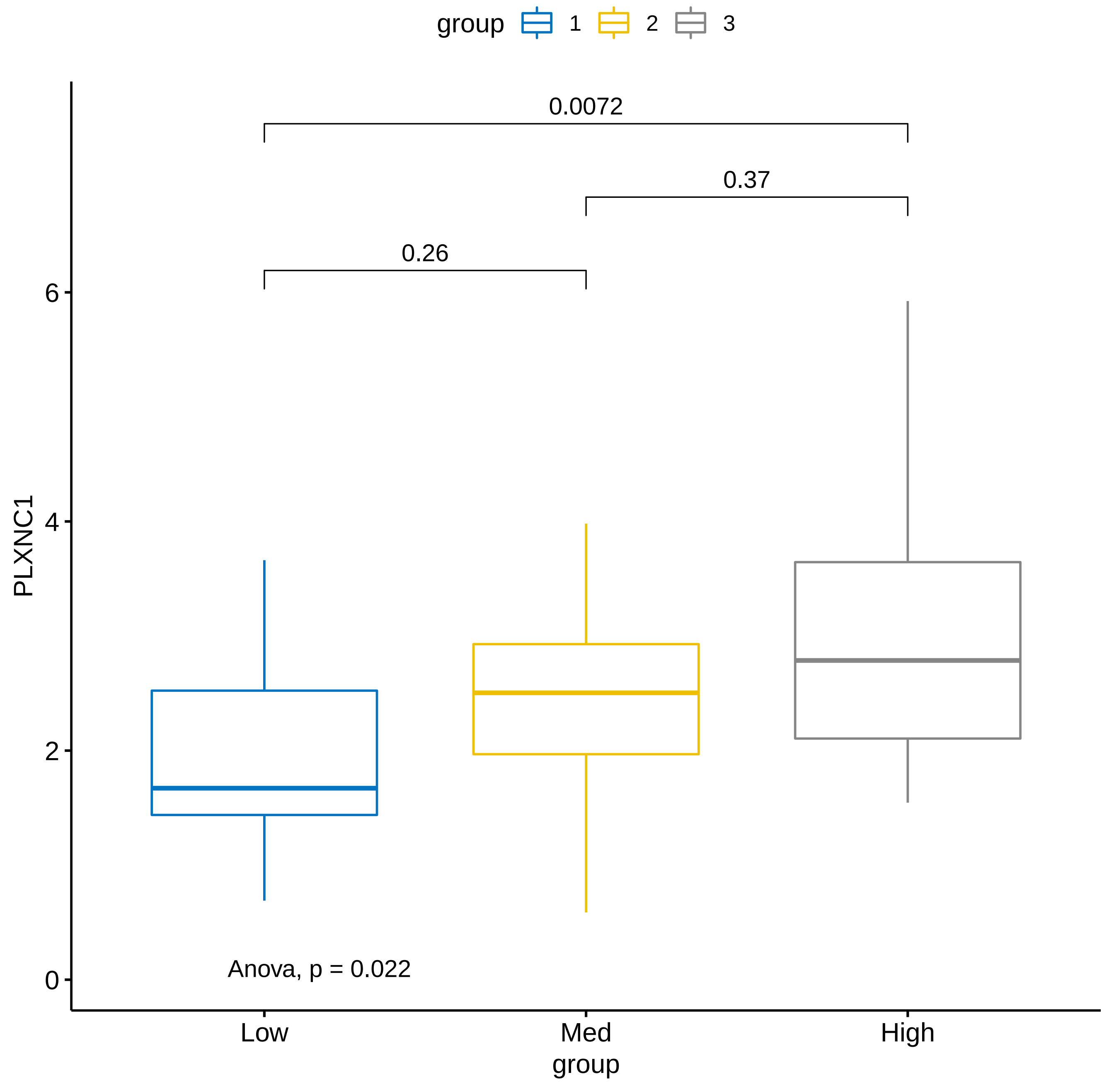

group
1
2
3
0.0072
0.37
0.26
6
4
2
0
PLXNC1
Anova, p = 0.022
Low
Med
High
group

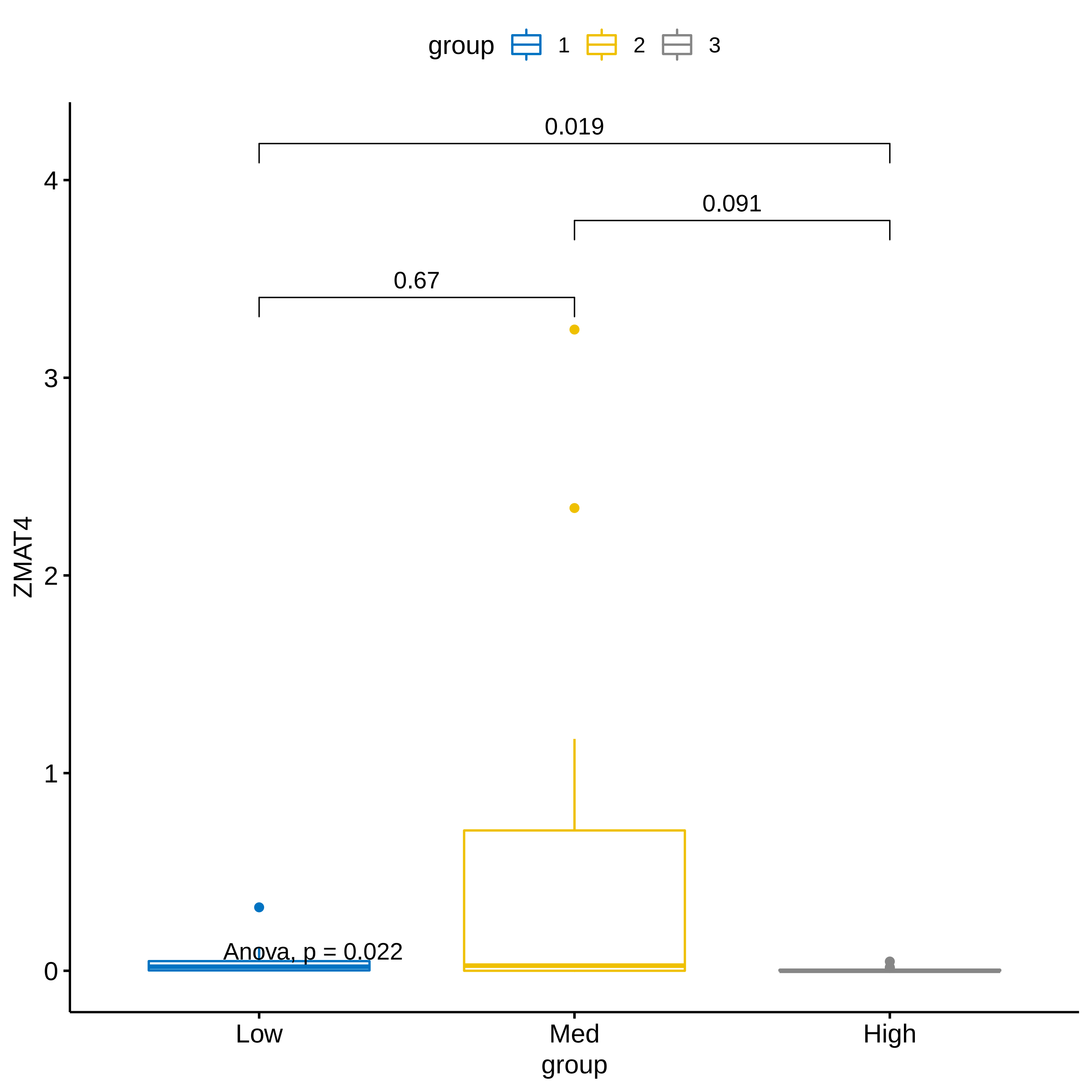

group 1 2 3
0.019
0.091
0.67
Anova, p = 0.022
ZMAT4
0
1
2
3
4
Low
Med
High
group

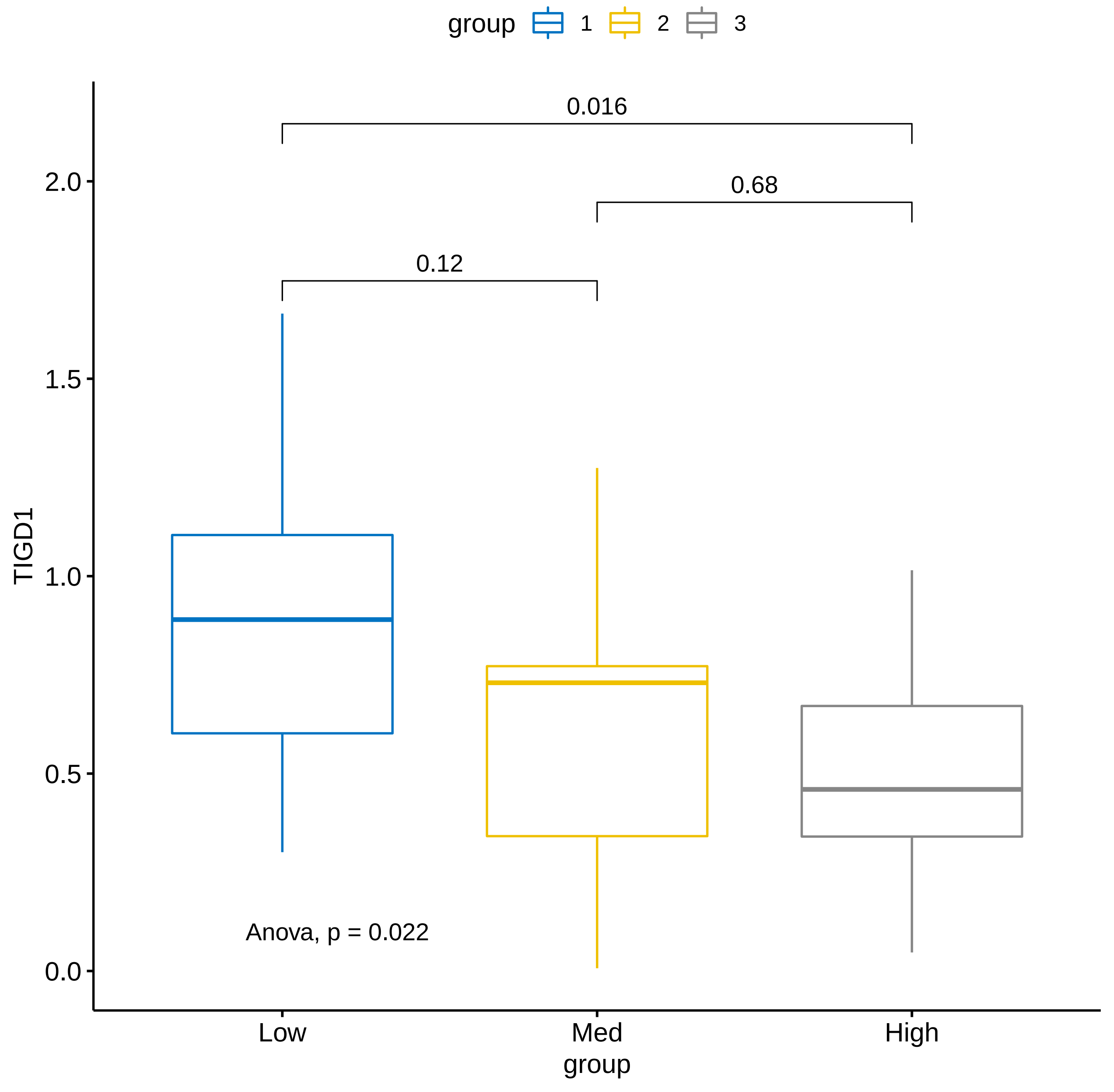
group
1
2
3
0.016
0.68
0.12
2.0
1.5
1.0
0.5
0.0
TIGD1
Anova, p = 0.022
Low
Med
High
group

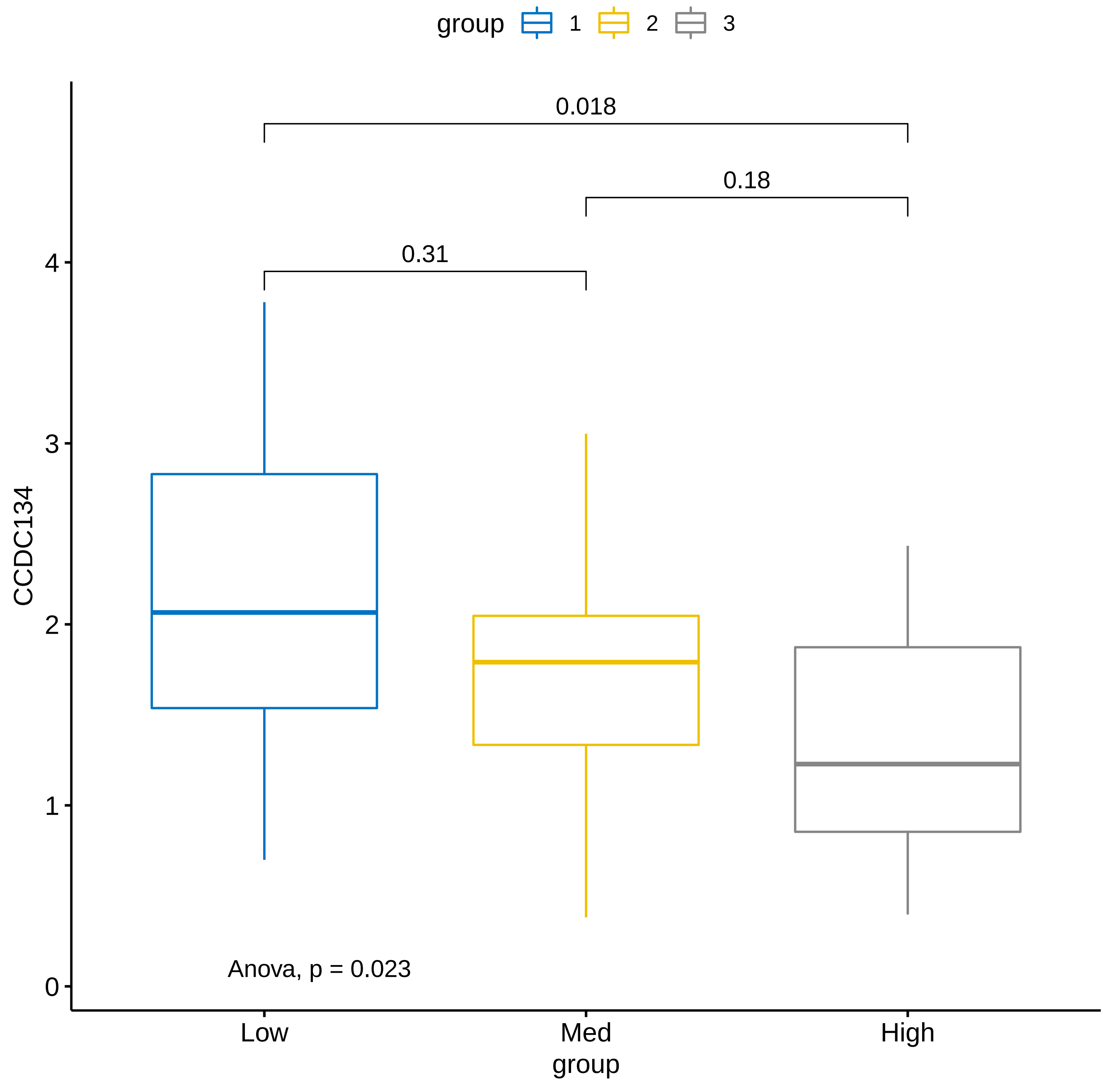
group
1
2
3
0.018
0.18
0.31
4
3
2
1
0
CCDC134
Anova, p = 0.023
Low
Med
High
group

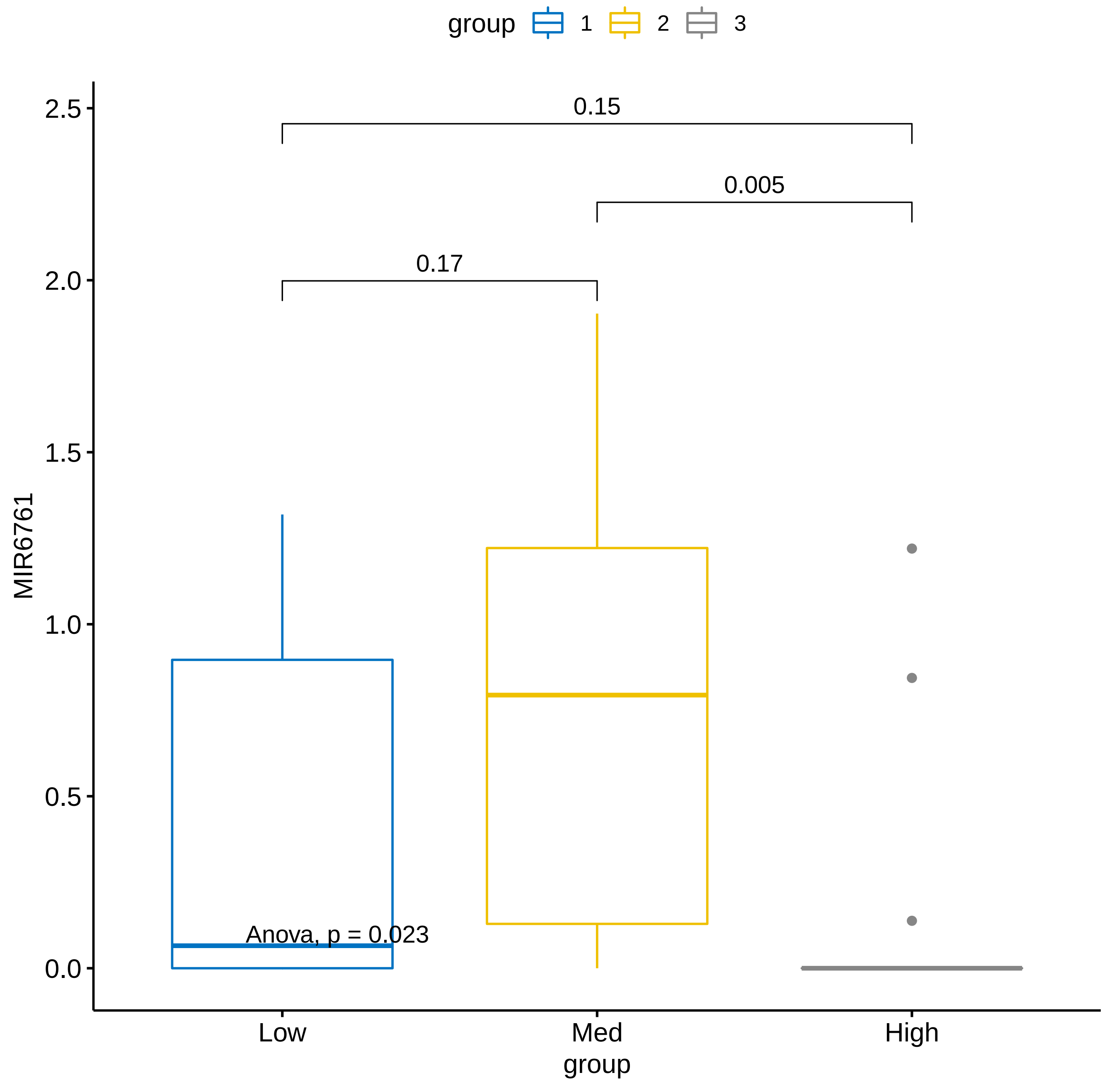

group
1
2
3
2.5
2.0
1.5
1.0
0.5
0.0
MIR6761
0.15
0.005
0.17
Anova, p = 0.023
Low
Med
High
group

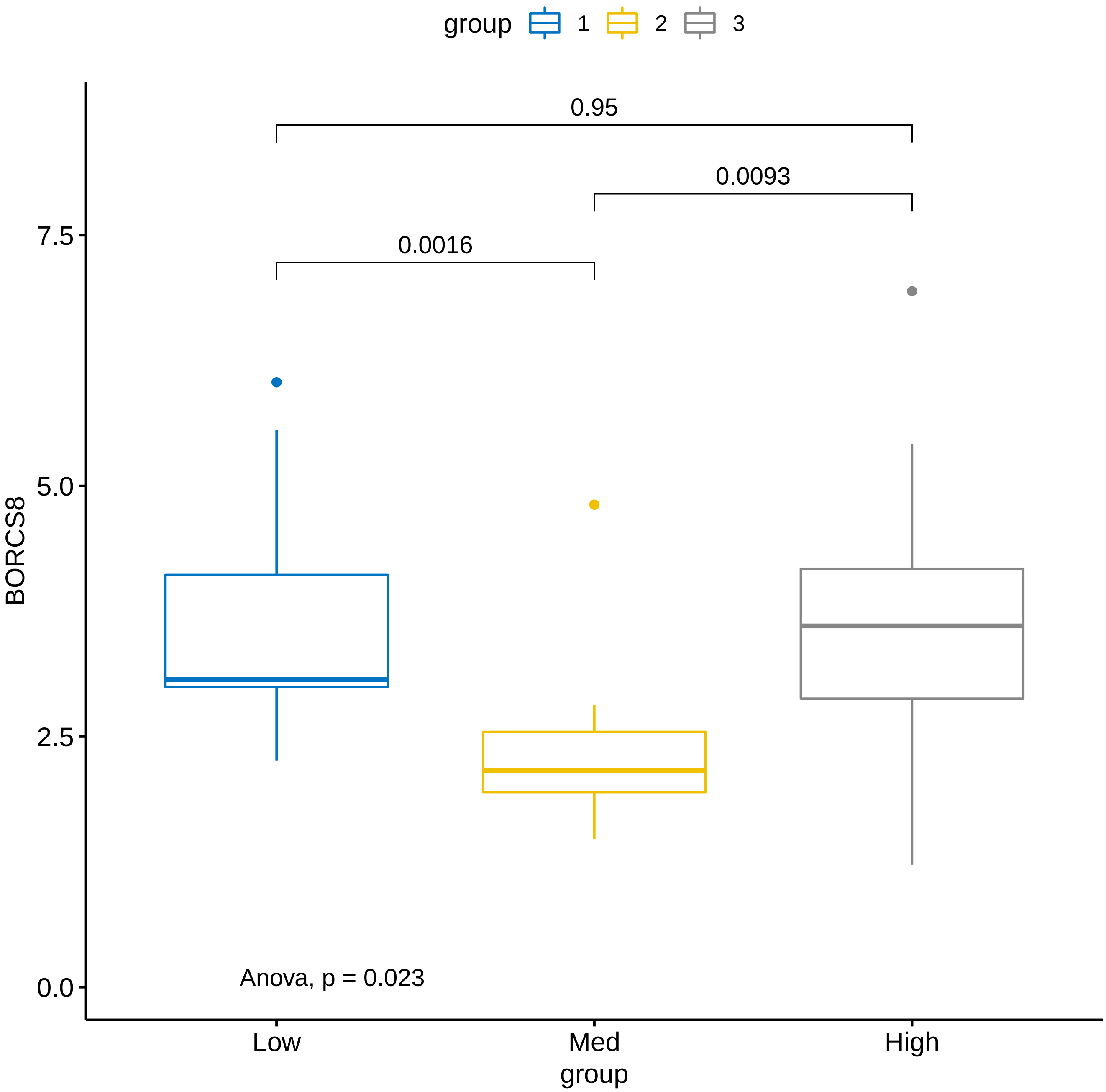

group
1
2
3
0.95
0.0093
0.0016
7.5
5.0
2.5
0.0
BORCS8
Anova, p = 0.023
Low
Med
High
group

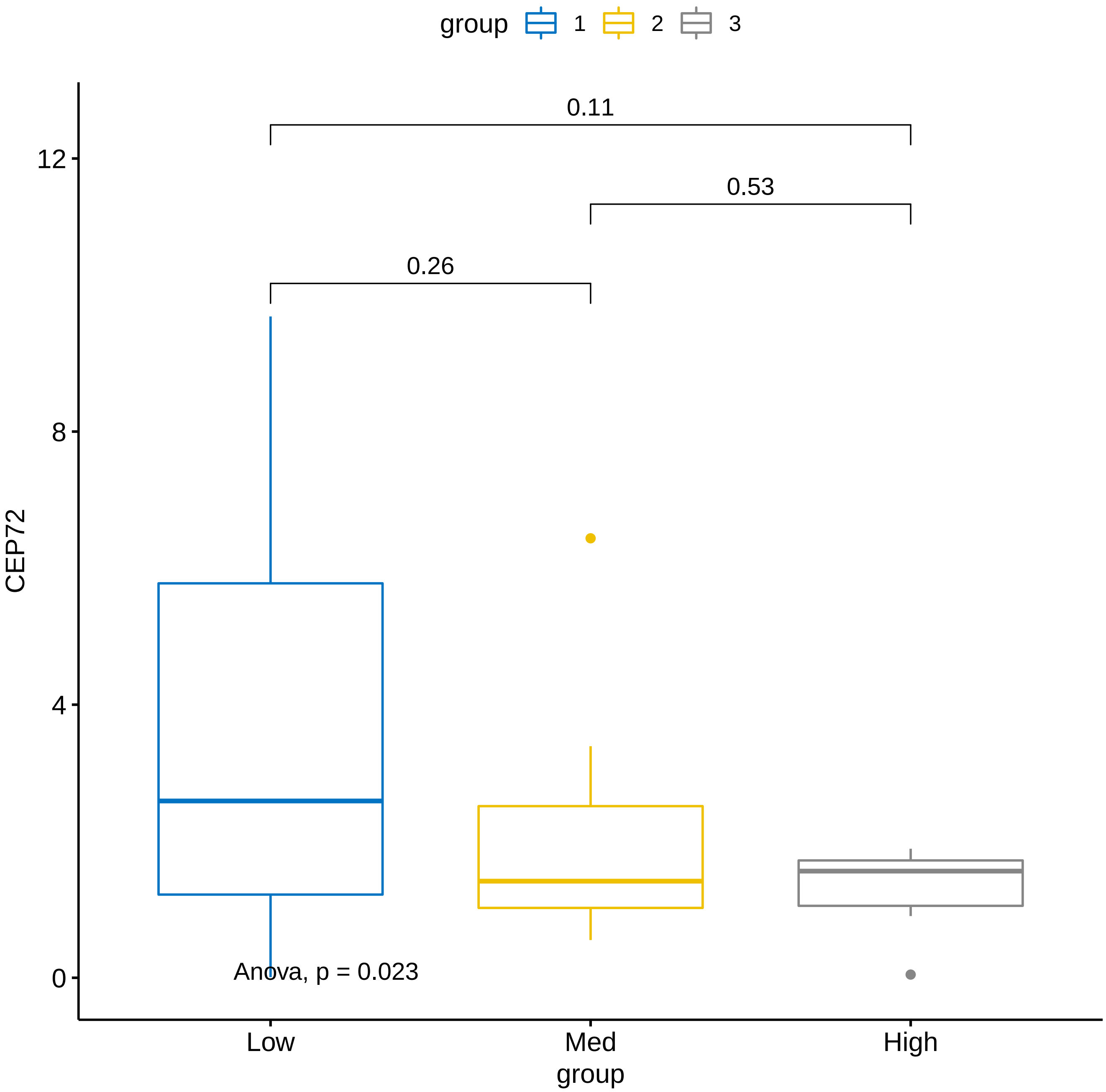
group
1
2
3
0.11
0.53
0.26
12
8
4
0
CEP72
Anova, p = 0.023
Low
Med
High
group

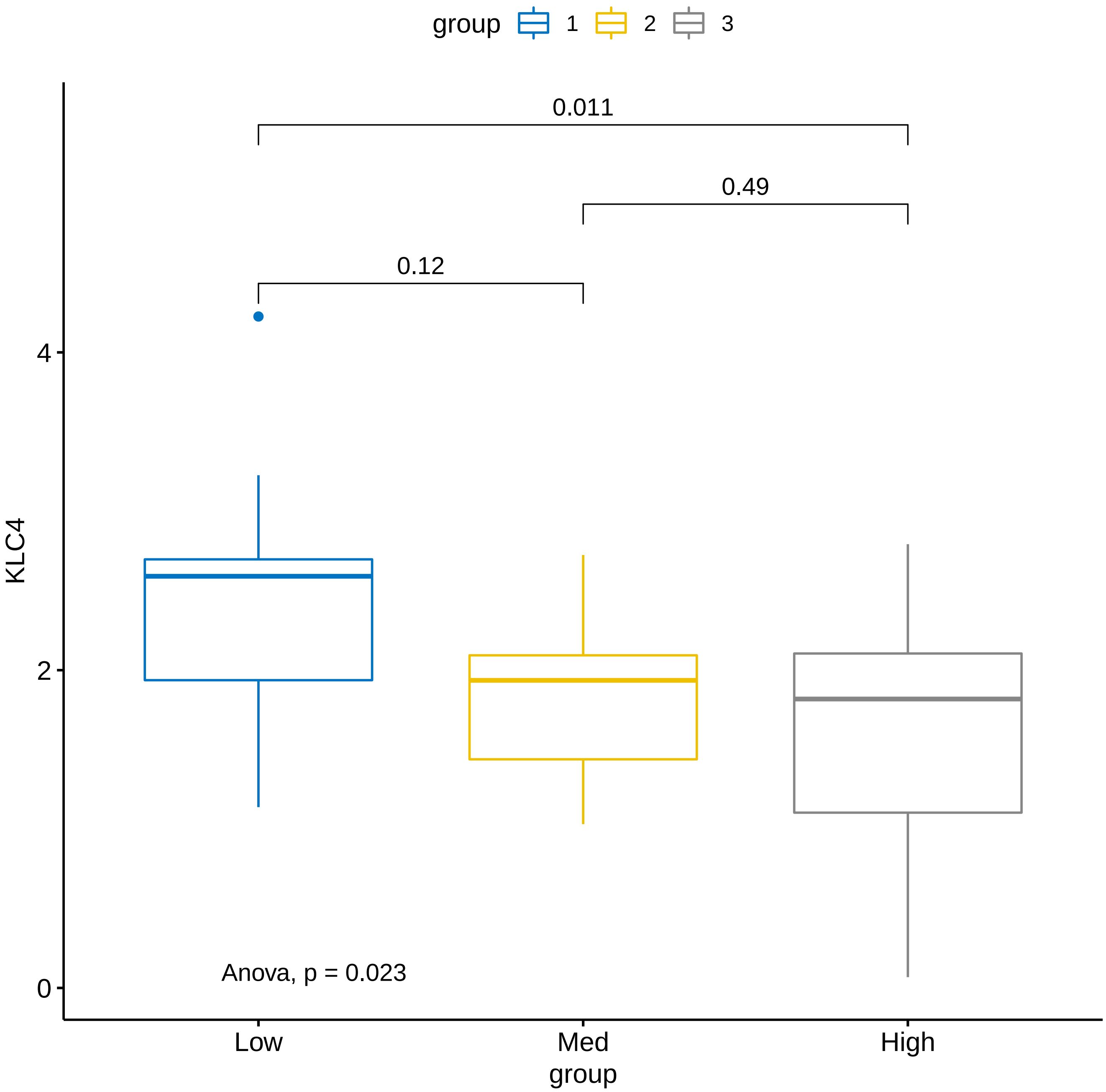
group
1
2
3
0.011
0.49
0.12
KLC4
4
2
0
Anova, p = 0.023
Low
Med
High
group

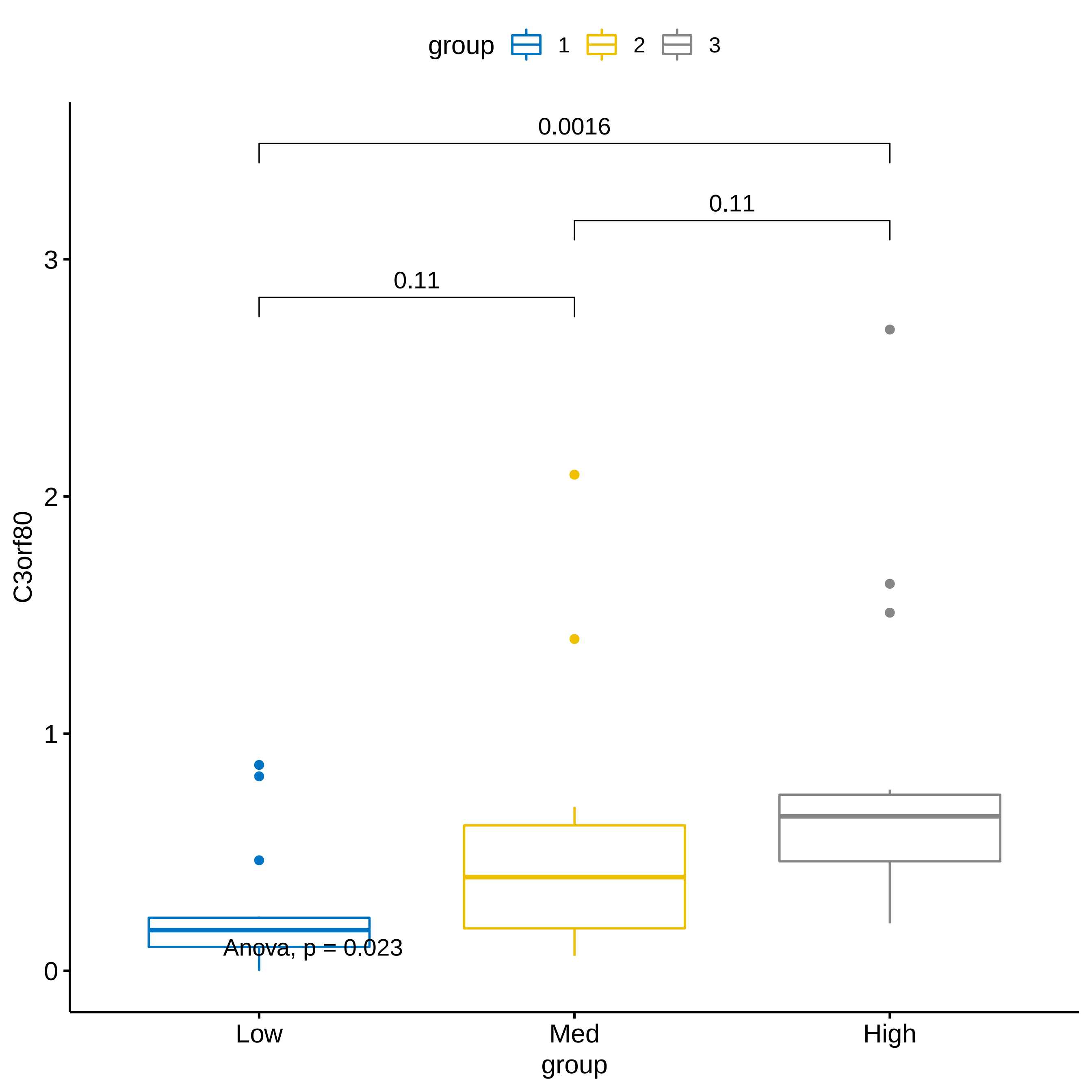
group
1
2
3
0.0016
0.11
0.11
3
2
1
0
C3orf80
Anova, p = 0.023
Low
Med
High
group

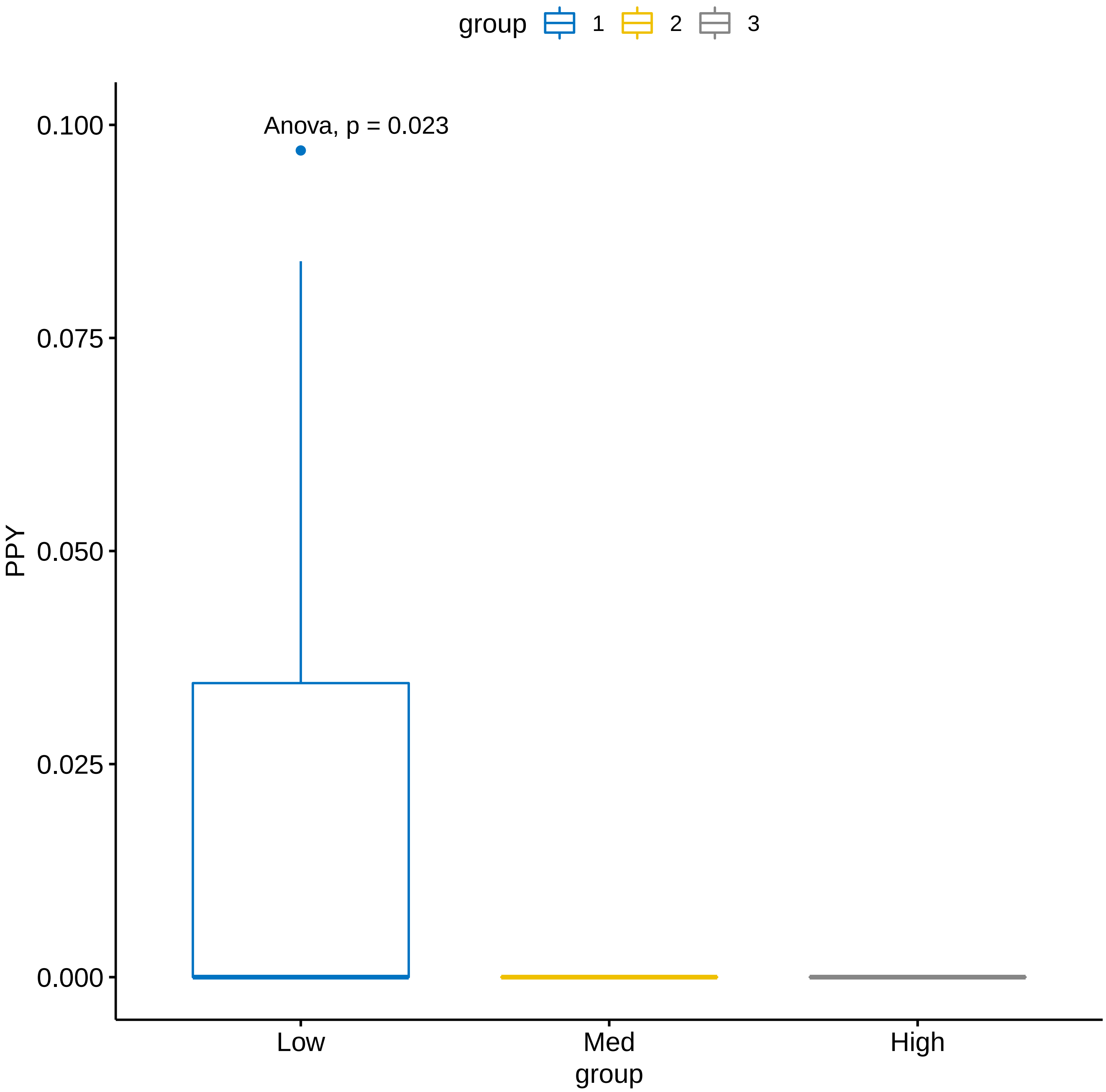
group 1 2 3
Anova, p = 0.023
PPY
0.100
0.075
0.050
0.025
0.000
Low
Med
High
group

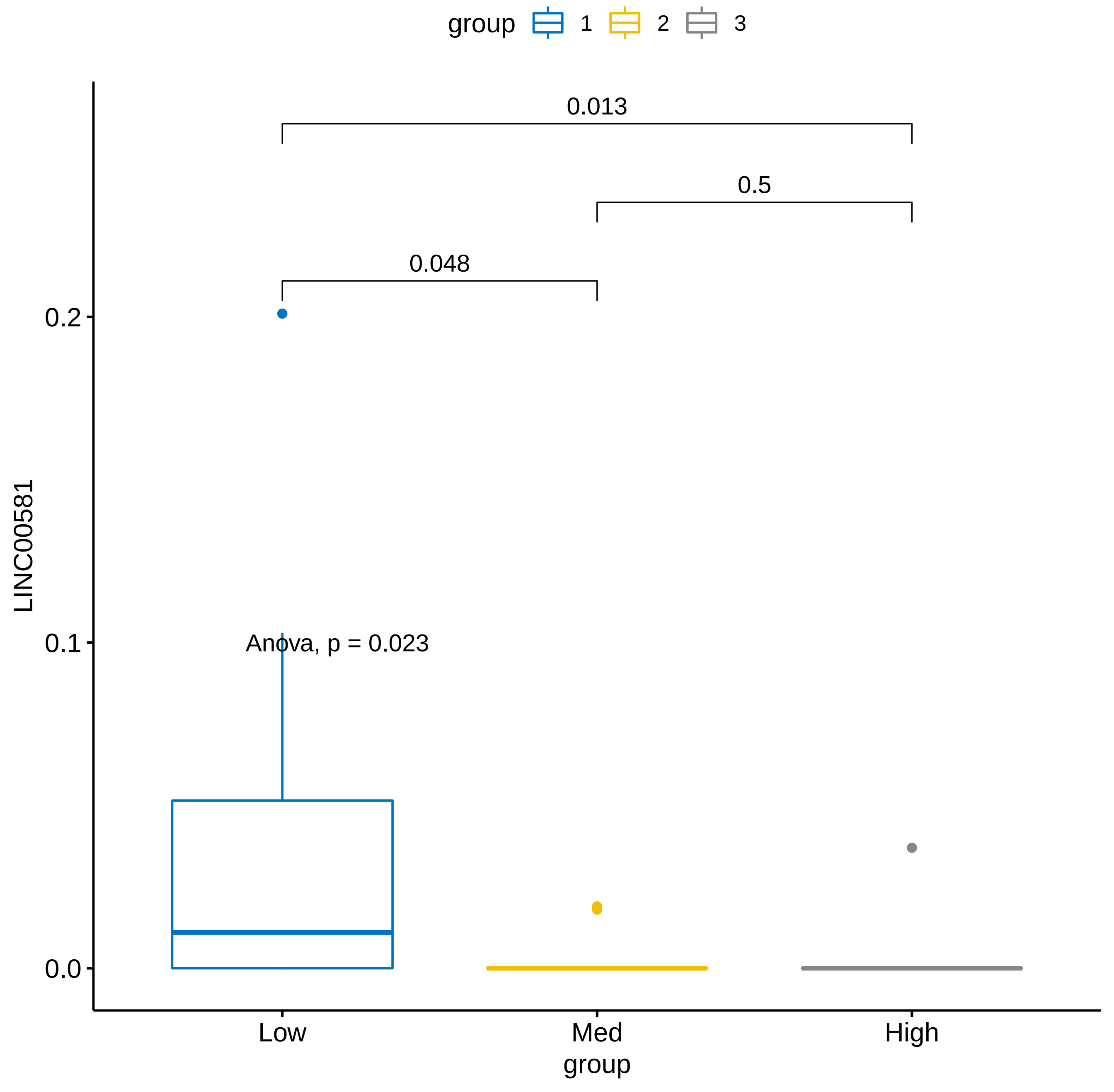
group 1 2 3
0.013
0.5
0.048
0.2
0.1
0.0
LINC00581
Anova, p = 0.023
Low
Med
High
group

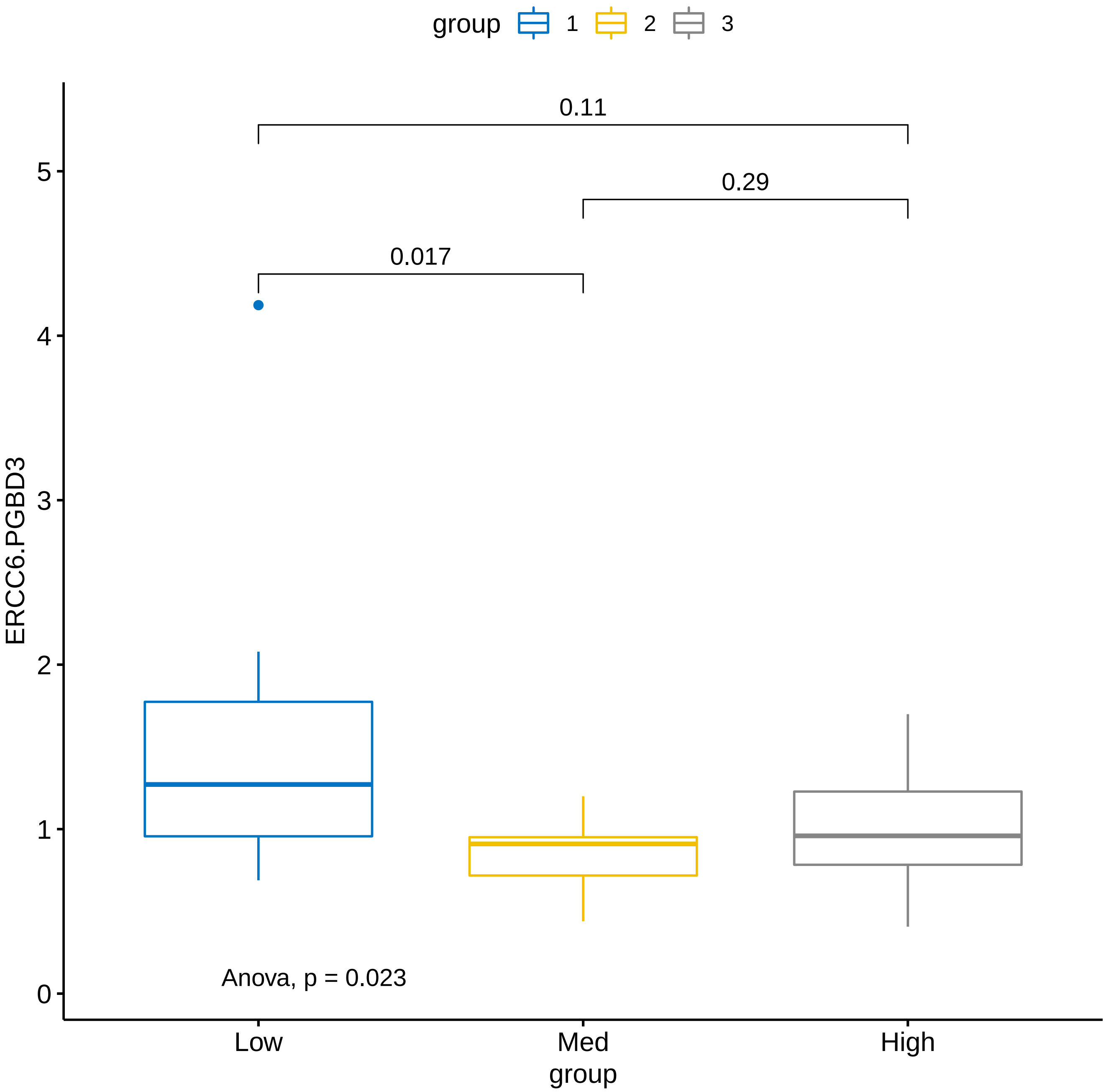
group
1
2
3
0.11
0.29
0.017
5
4
3
2
1
0
ERCC6.PGBD3
Anova, p = 0.023
Low
Med
High
group

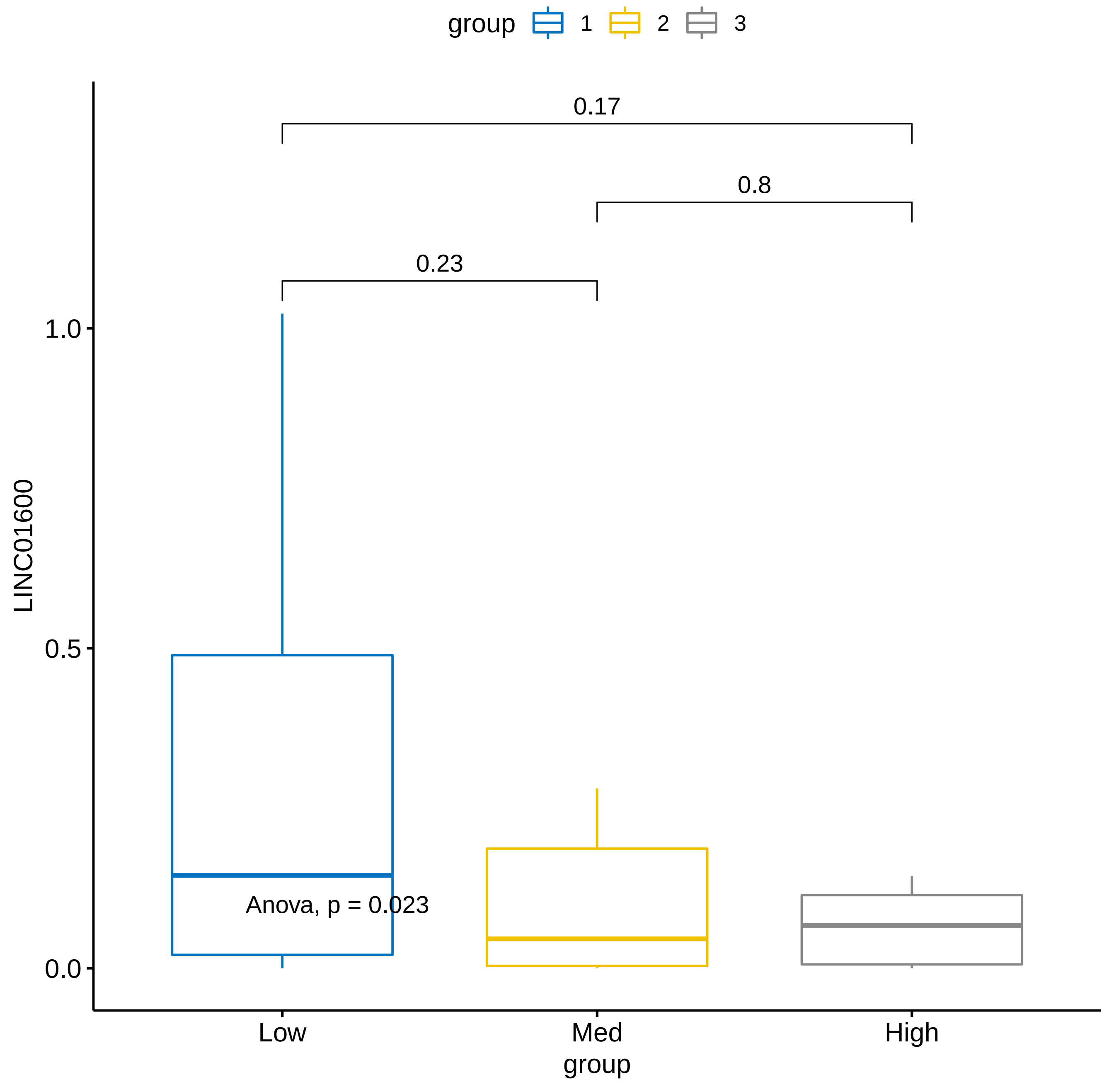

group
1
2
3
0.17
0.8
0.23
1.0
0.5
0.0
LINC01600
Anova, p = 0.023
Low
Med
High
group

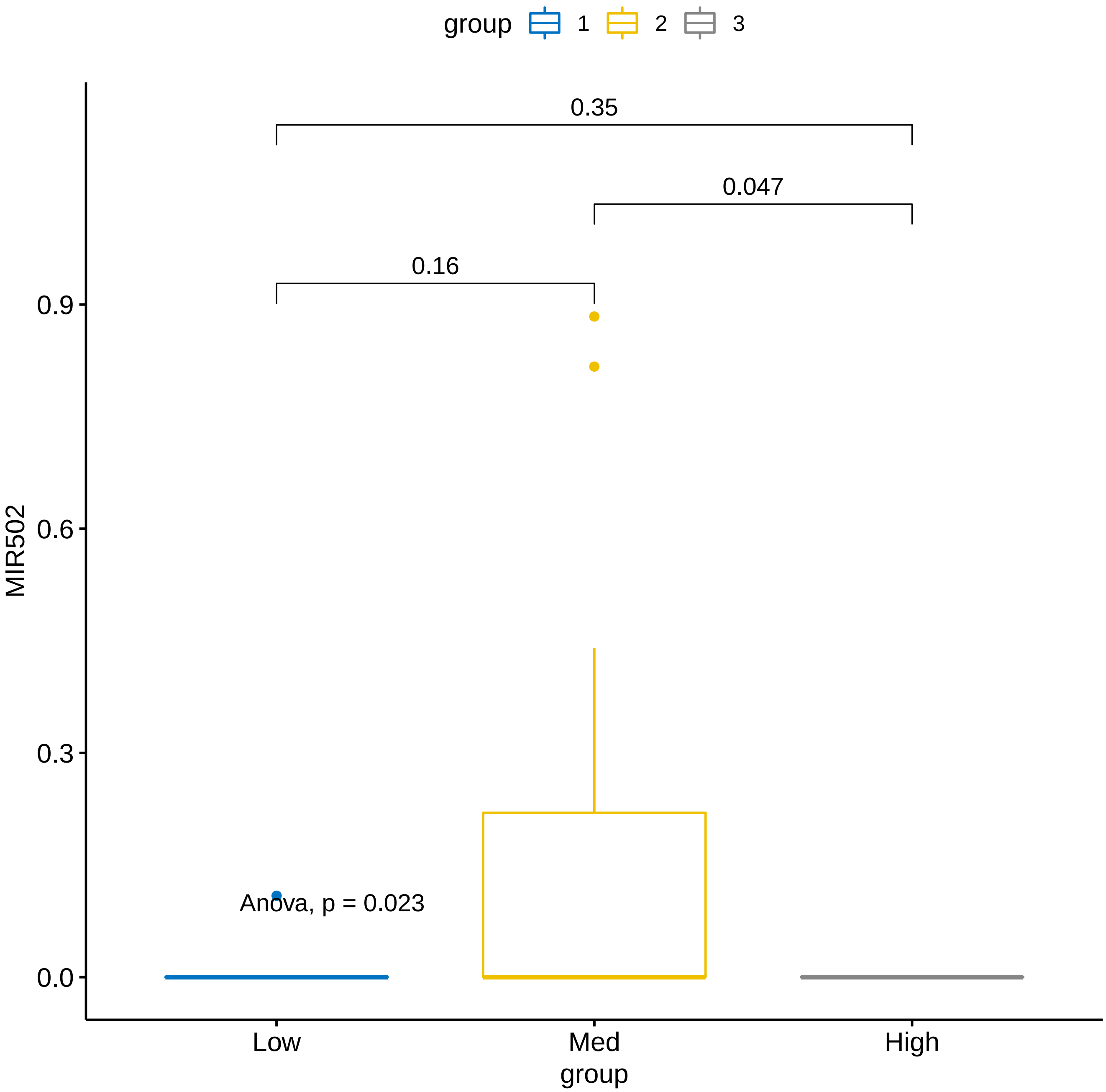

group
1
2
3
0.35
0.047
0.16
Anova, p = 0.023
0.9
0.6
0.3
0.0
MIR502
Low
Med
High
group

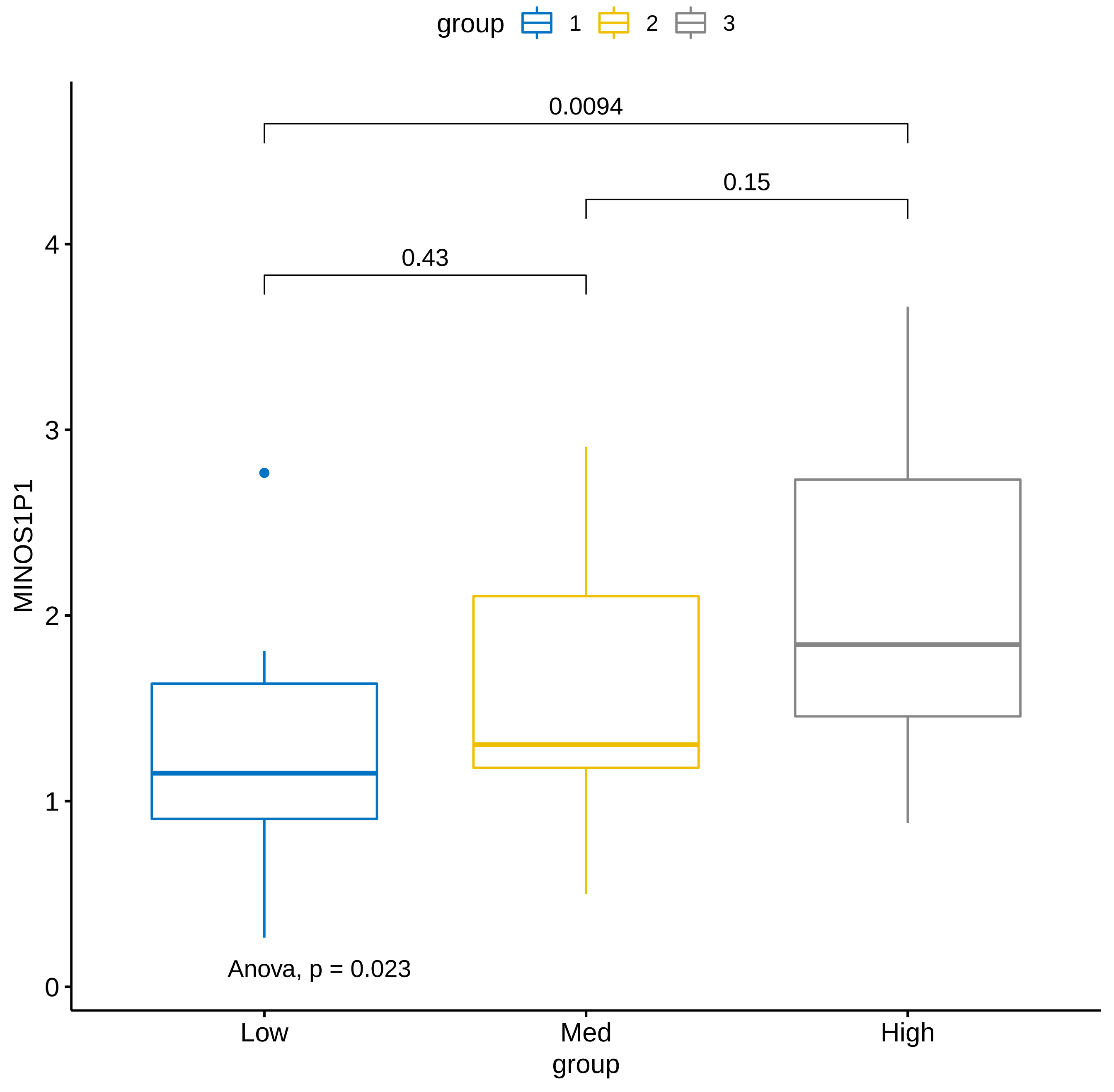

group 1 2 3
0.0094
0.15
0.43
MINOS1P1
4
3
2
1
0
Anova, p = 0.023
Low
Med
High
group

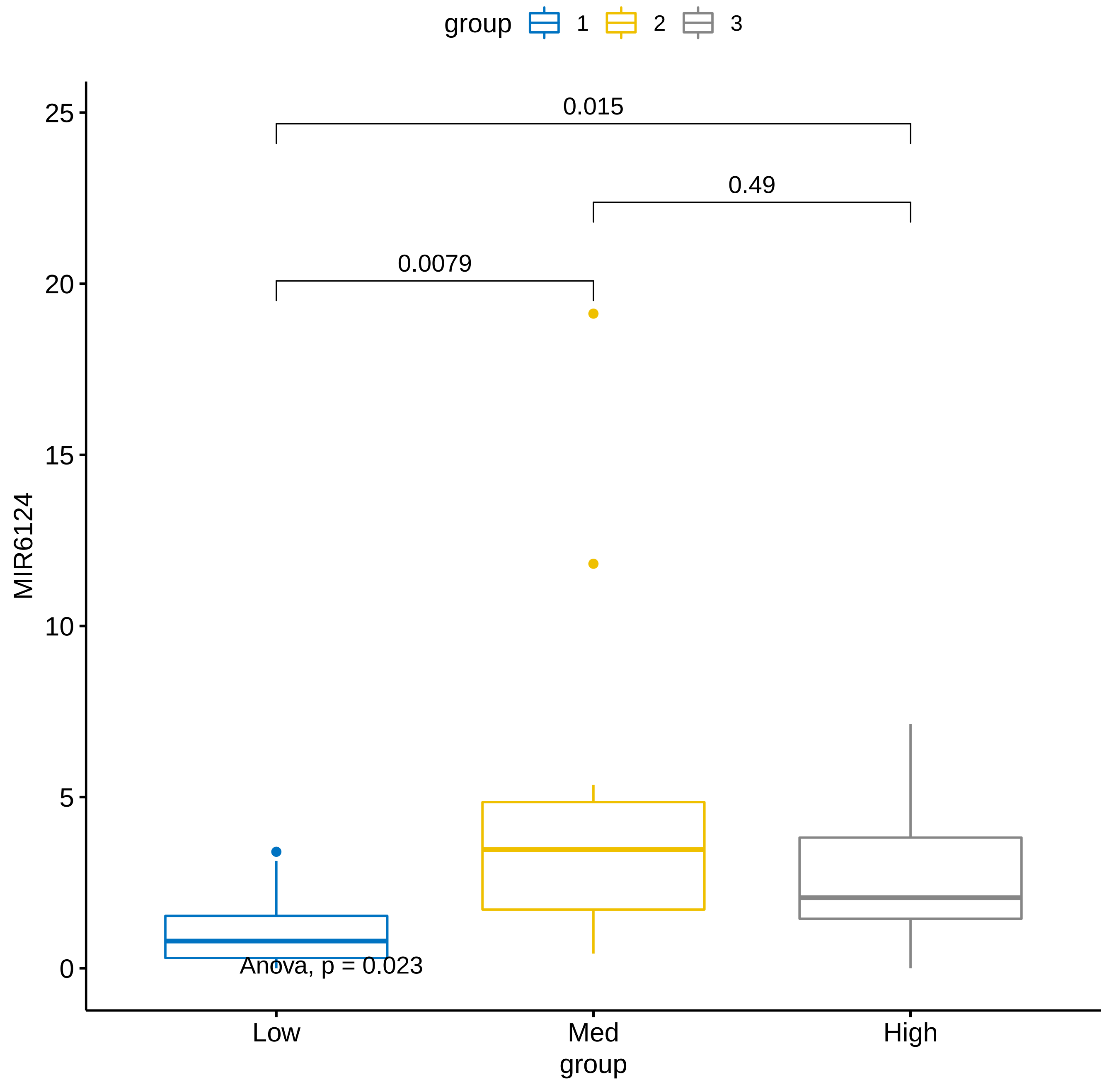
group 1 2 3
0.015
0.49
0.0079
25
20
15
10
5
0
MIR6124
Anova, p = 0.023
Low
Med
High
group

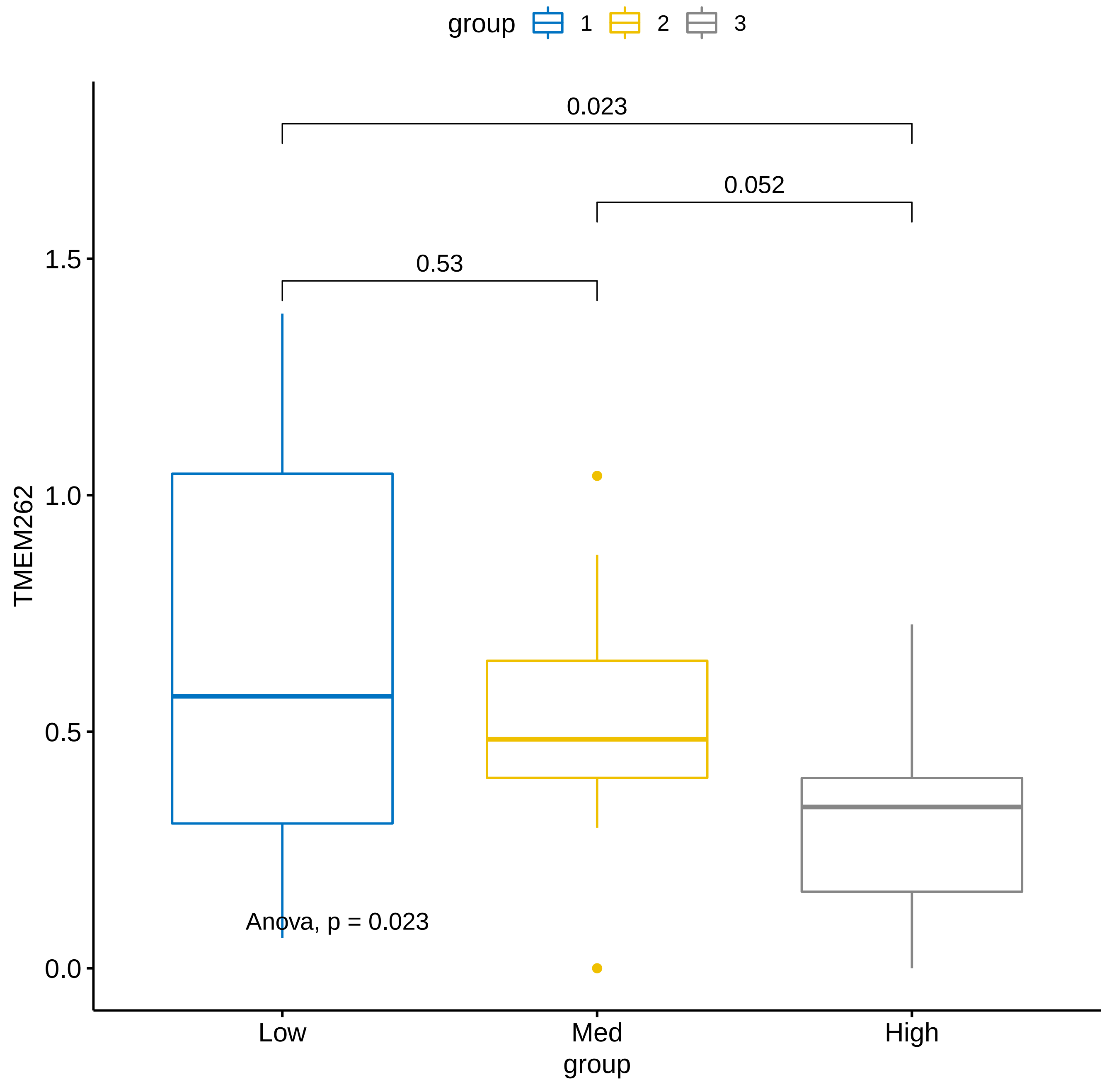

group
1
2
3
0.023
0.052
0.53
1.5
1.0
0.5
0.0
TMEM262
Anova, p = 0.023
Low
Med
High
group

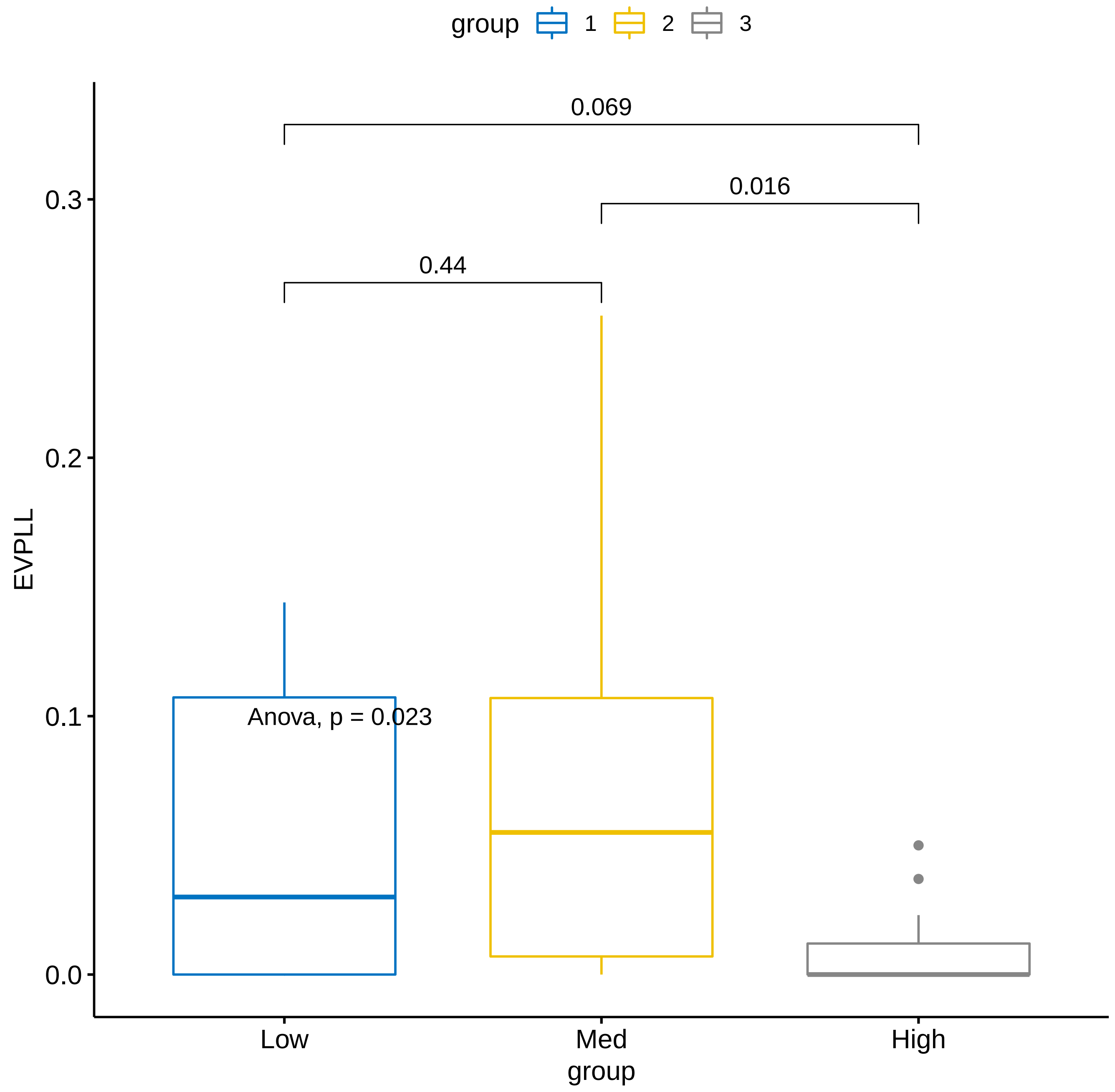

group
1
2
3
0.069
0.016
0.44
Anova, p = 0.023
0.3
0.2
0.1
0.0
EVPLL
Low
Med
High
group

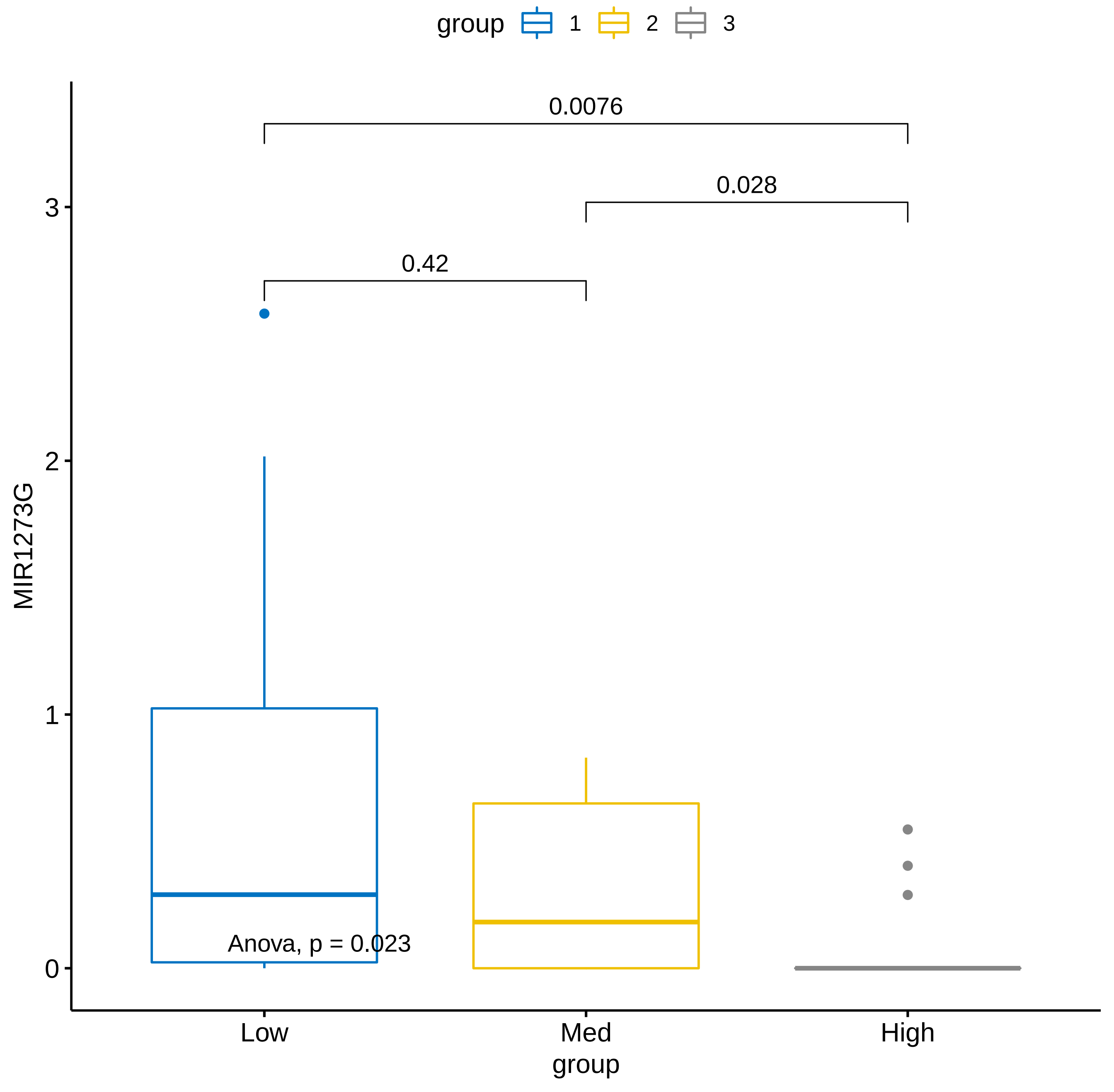
group 1 2 3
0.0076
0.028
0.42
3
2
1
0
MIR1273G
Anova, p = 0.023
Low
Med
High
group

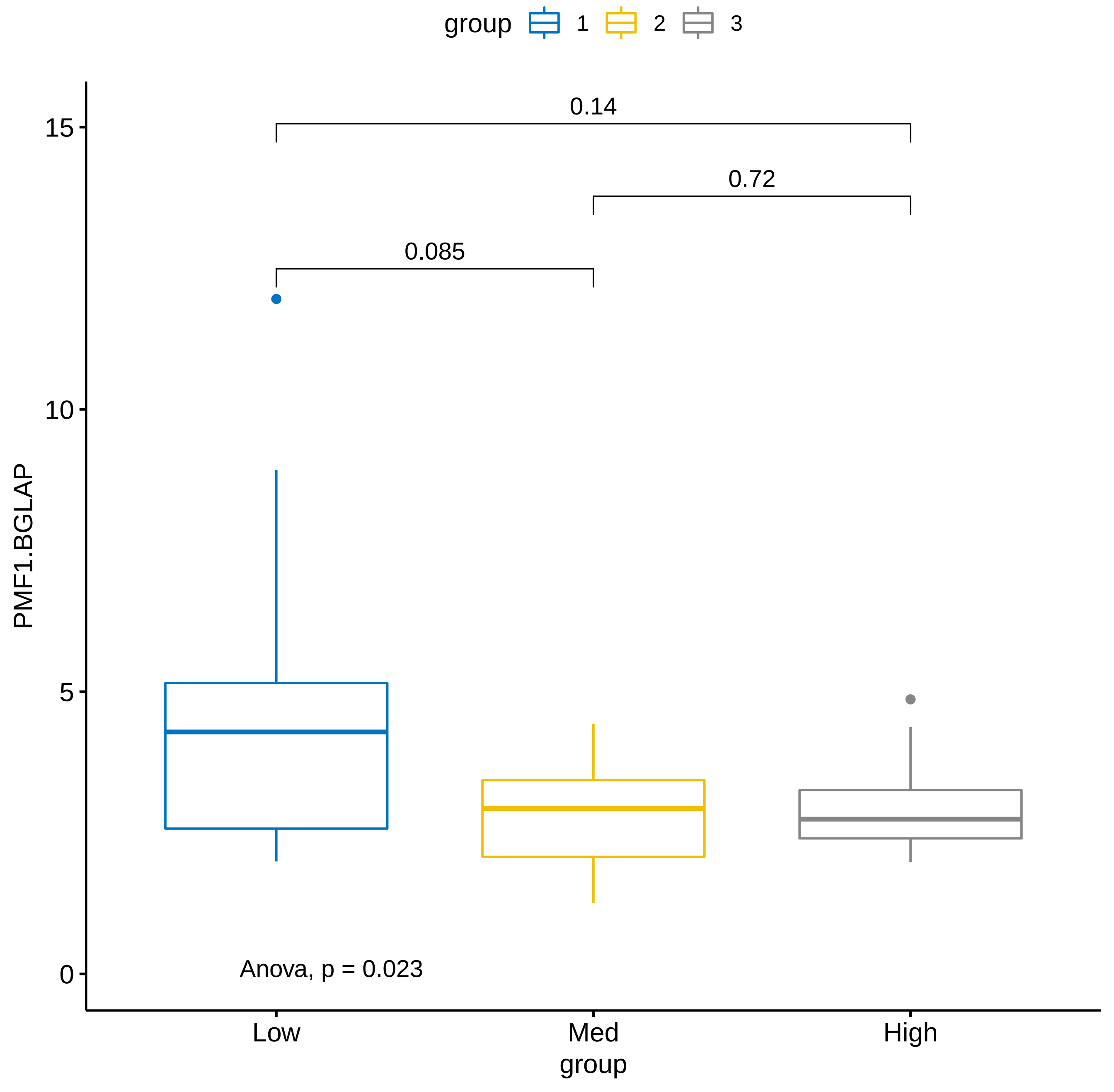
group 1 2 3
0.14
0.72
0.085
15
10
5
0
PMF1.BGLAP
Anova, p = 0.023
Low
Med
High
group

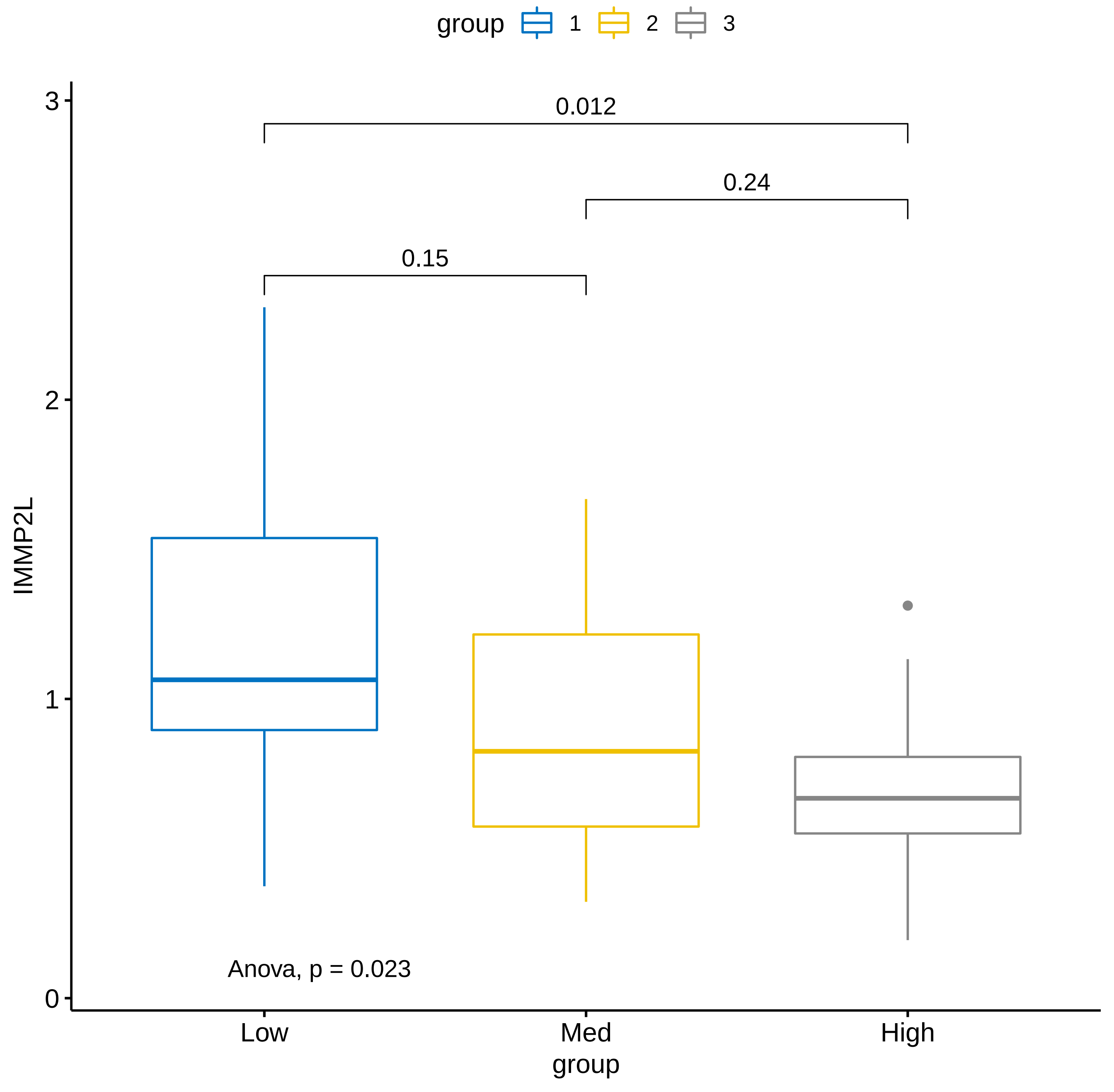
group
1
2
3
0.012
0.24
0.15
IMMP2L
3
2
1
0
Anova, p = 0.023
Low
Med
High
group

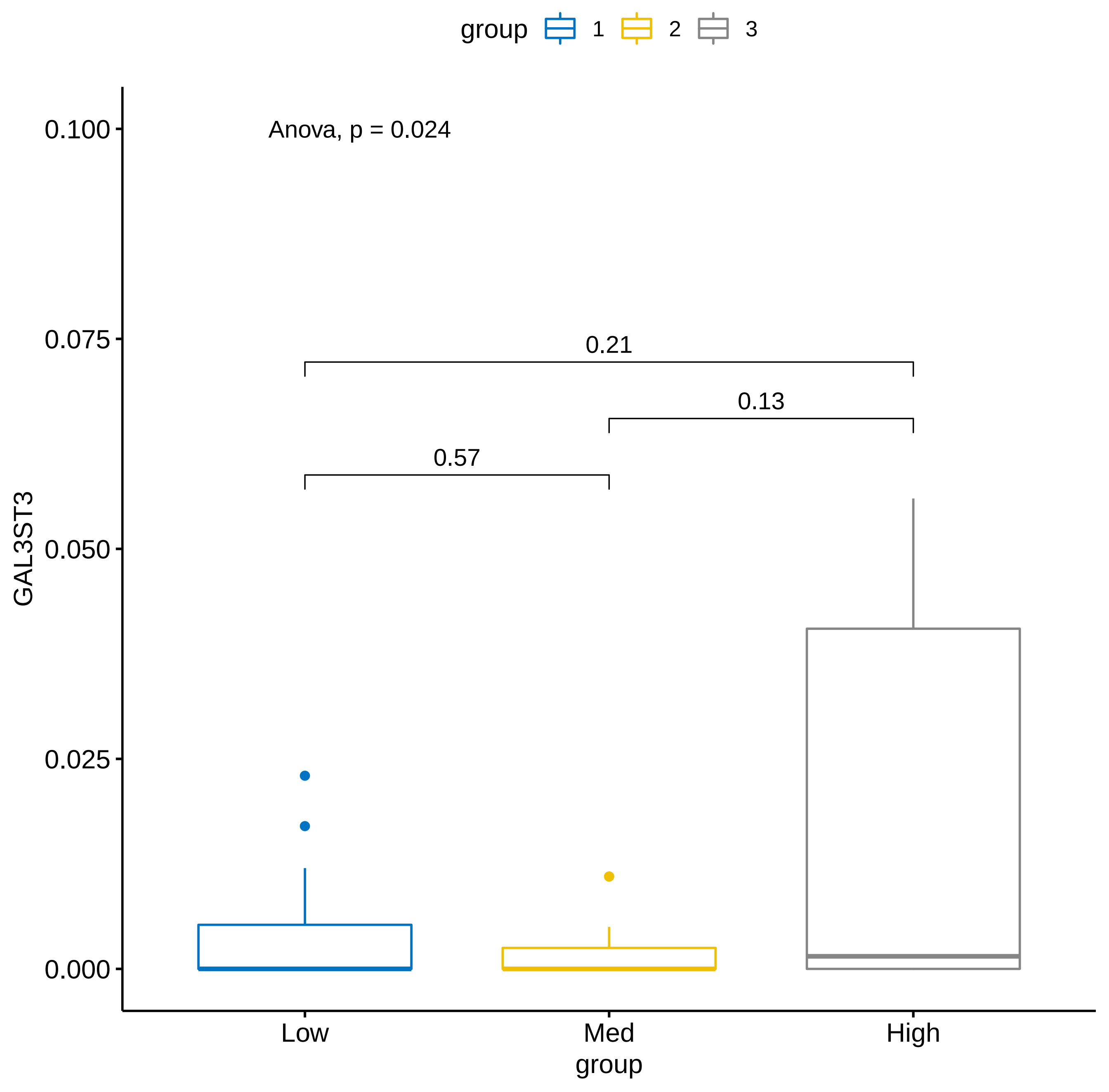
group 1 2 3
Anova, p = 0.024
0.21
0.13
0.57
GAL3ST3
0.100
0.075
0.050
0.025
0.000
Low
Med
High
group

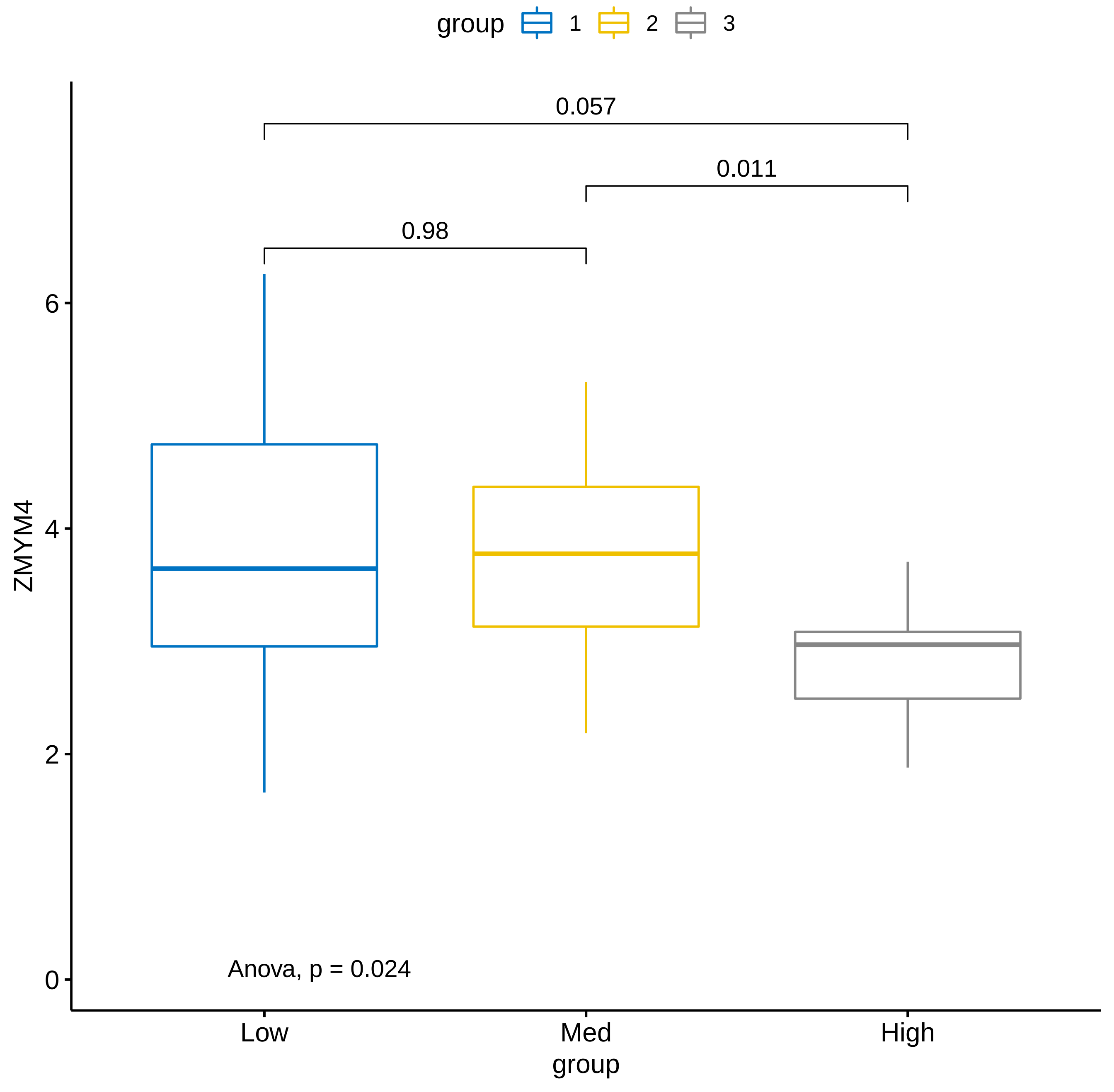
group
1
2
3
0.057
0.011
0.98
6
4
2
0
ZMYM4
Anova, p = 0.024
Low
Med
High
group

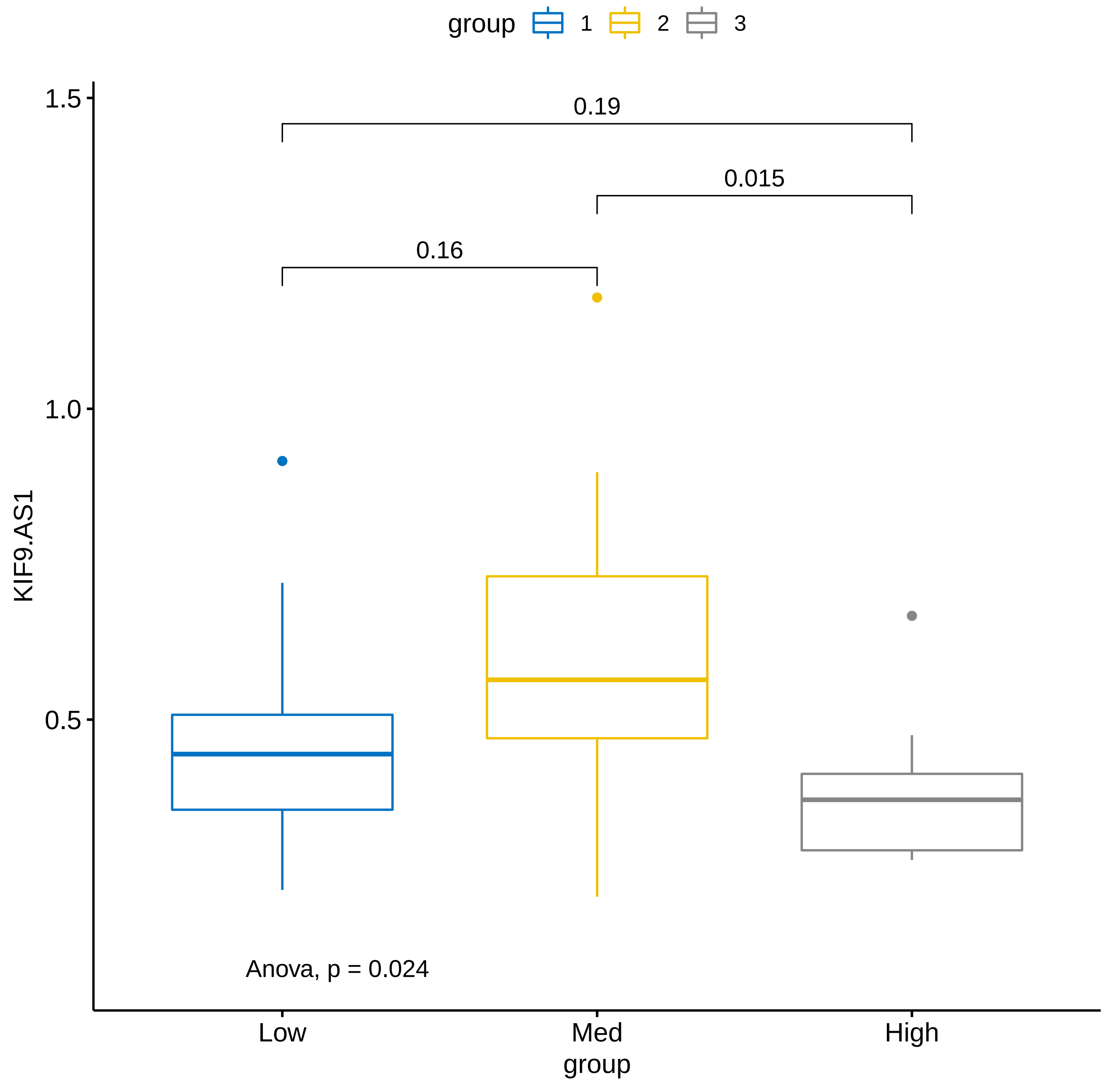

group
1
2
3
0.19
0.015
0.16
1.5
1.0
0.5
KIF9.AS1
Anova, p = 0.024
Low
Med
High
group

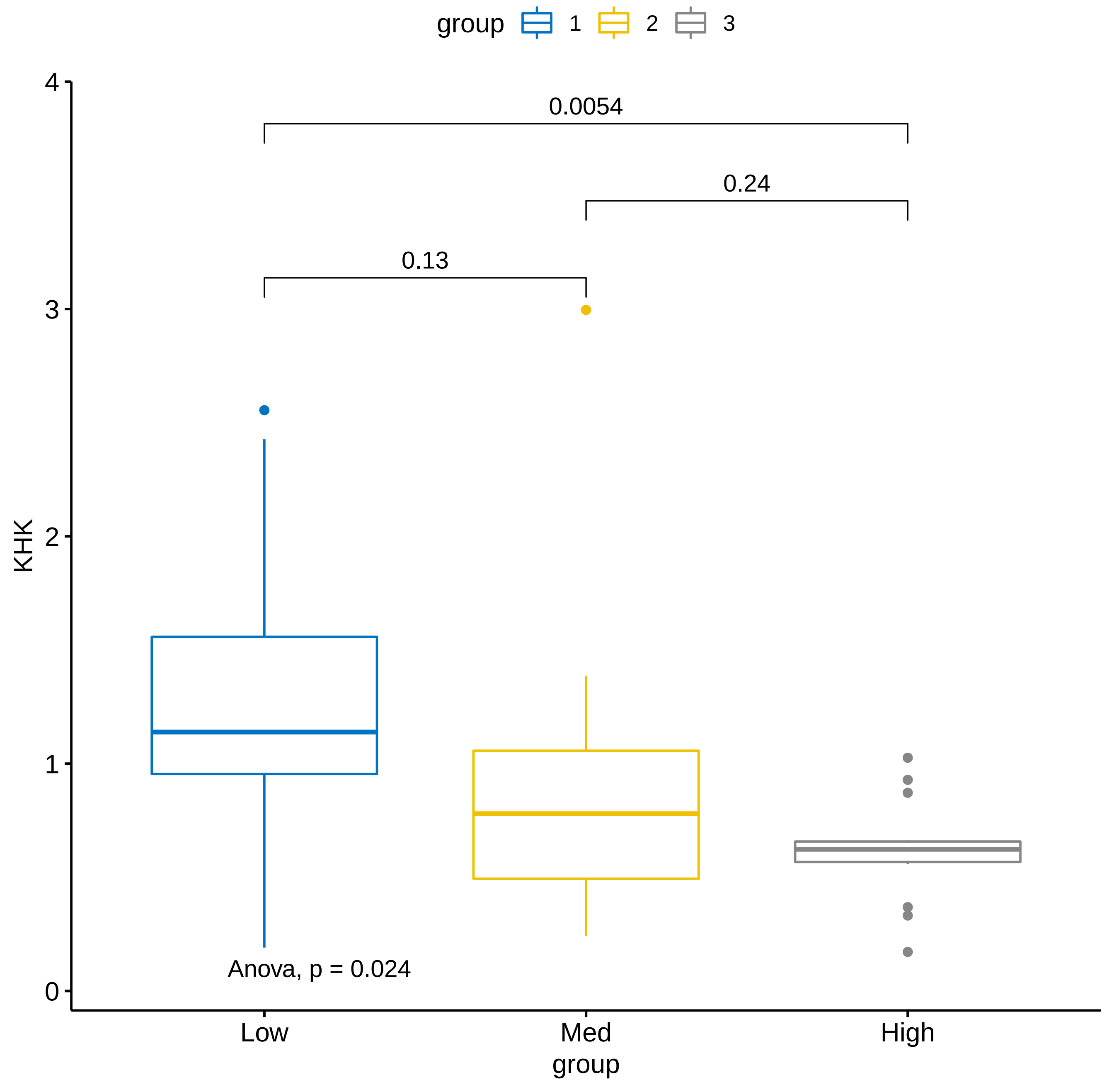

group
1
2
3
0.0054
0.24
0.13
KHK
4
3
2
1
0
Anova, p = 0.024
Low
Med
High
group

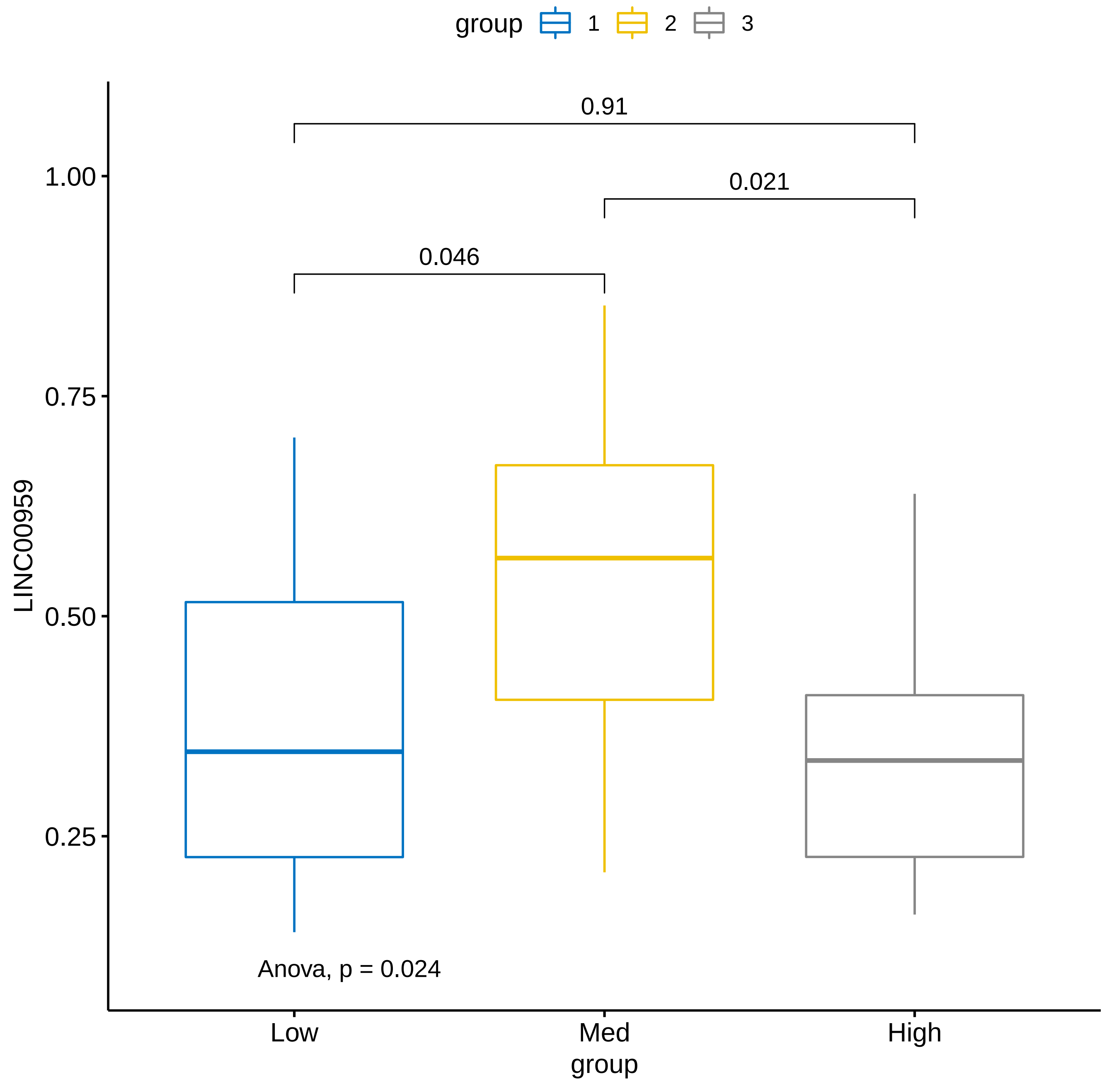

group
1
2
3
0.91
0.021
0.046
1.00
0.75
0.50
0.25
LINC00959
Anova, p = 0.024
Low
Med
High
group

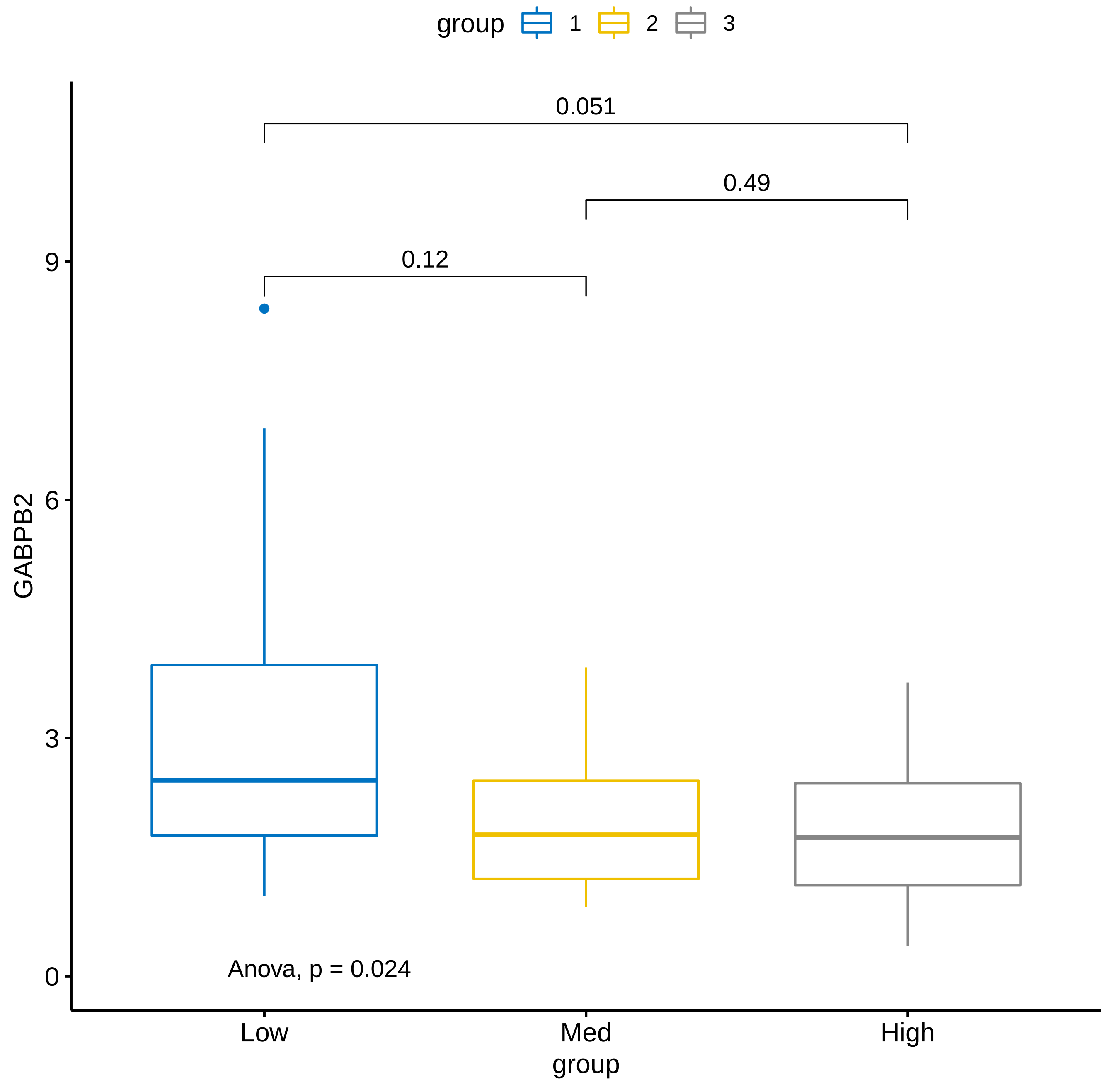
group
1
2
3
0.051
0.49
0.12
9
6
3
0
GABPB2
Anova, p = 0.024
Low
Med
High
group

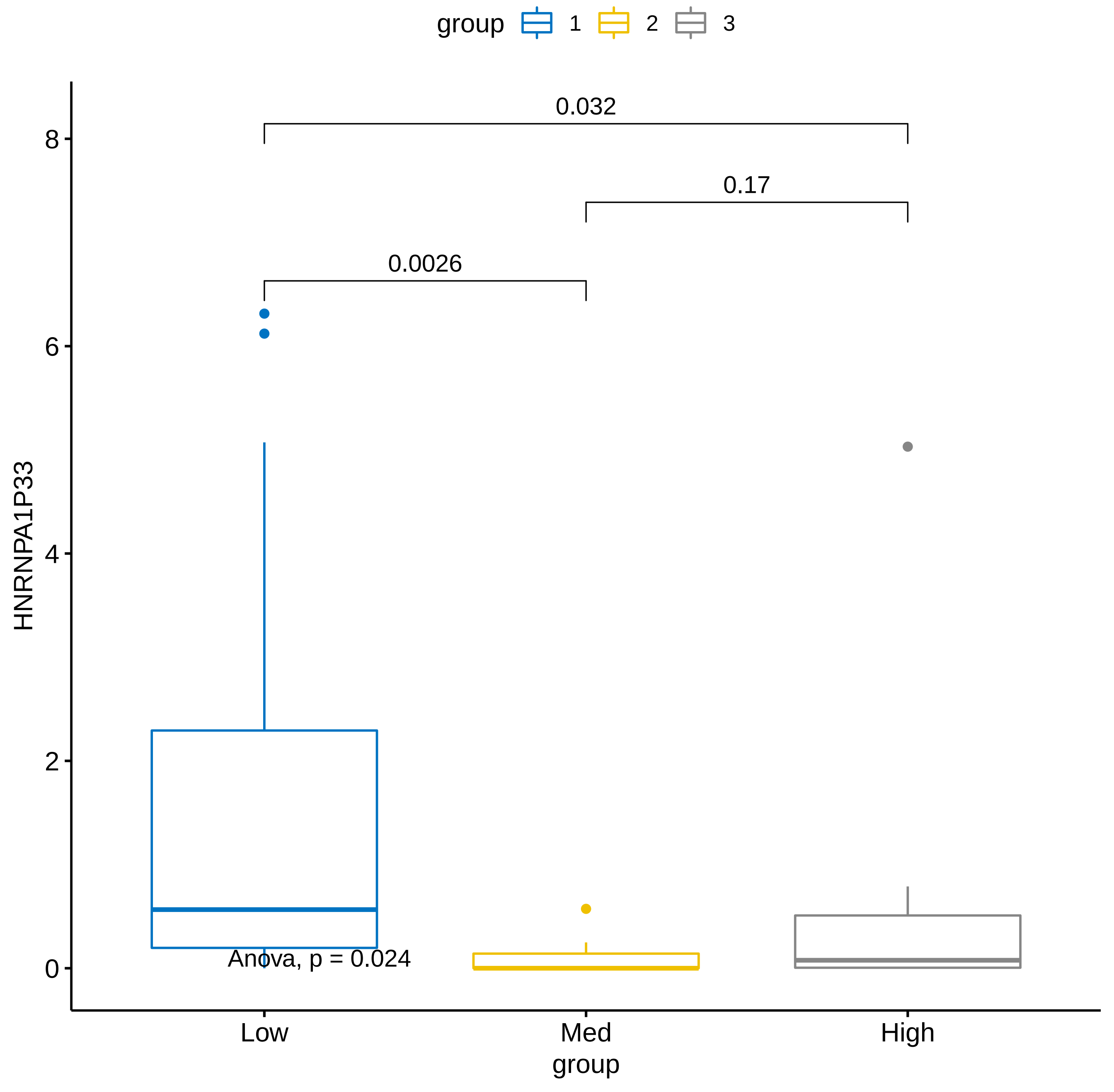
group 1 2 3
0.032
0.17
0.0026
HNRNPA1P33
8
6
4
2
0
Anova, p = 0.024
Low
Med
High
group

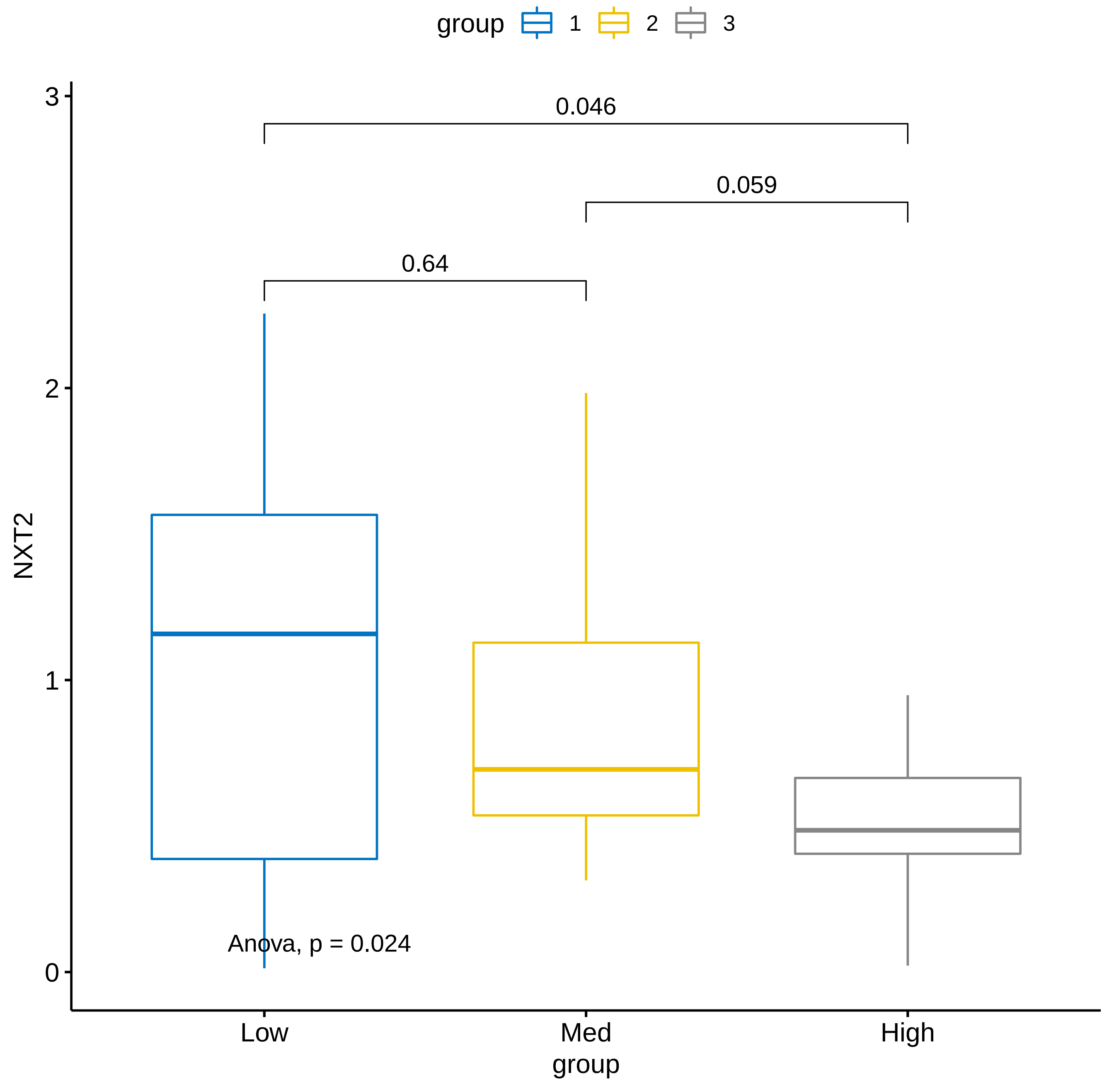

group
1
2
3
0.046
0.059
0.64
Anova, p = 0.024
NXT2
0
1
2
3
Low
Med
High
group

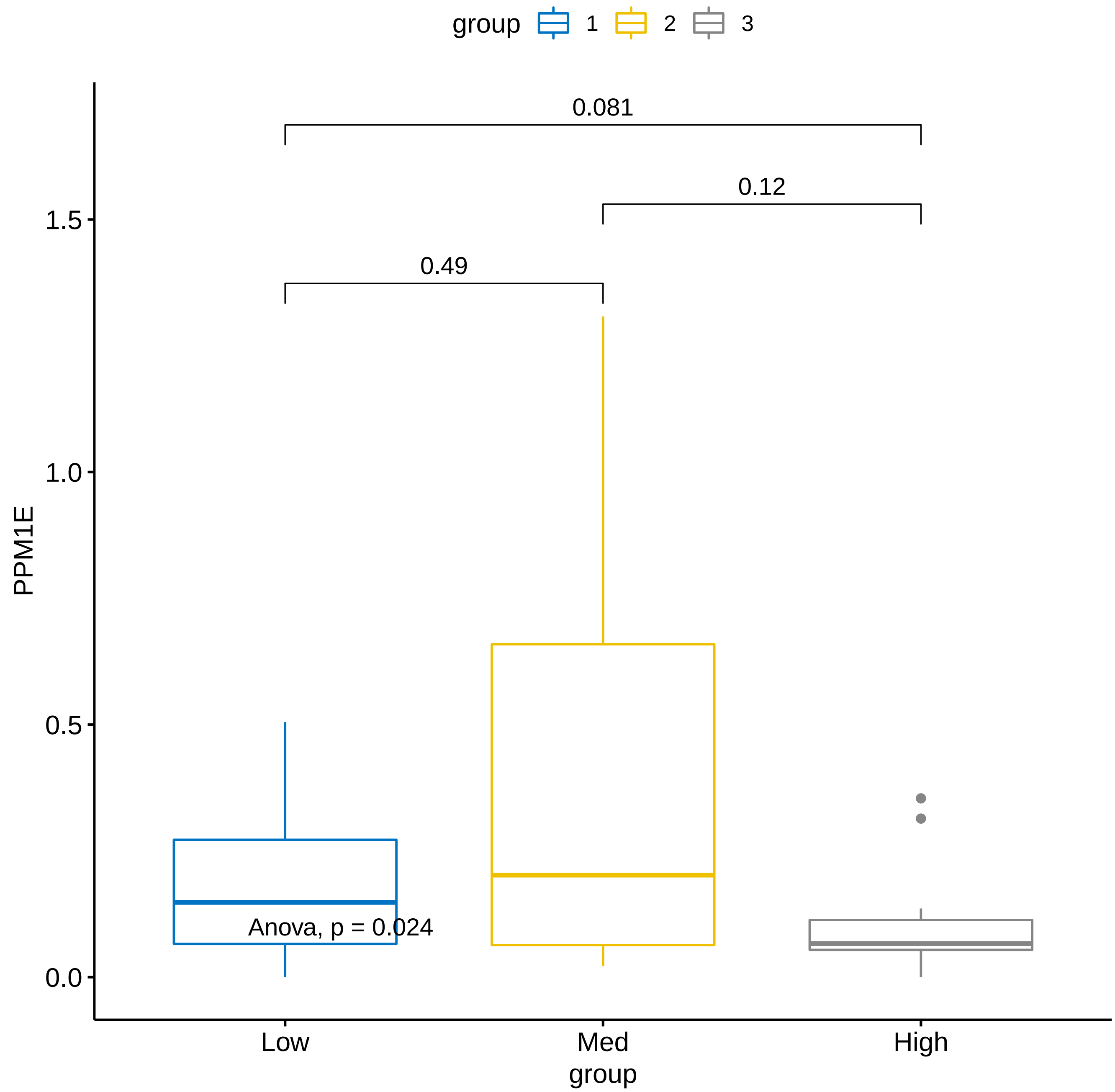
group
1
2
3
0.081
0.12
0.49
1.5
1.0
0.5
0.0
PPM1E
Anova, p = 0.024
Low
Med
High
group

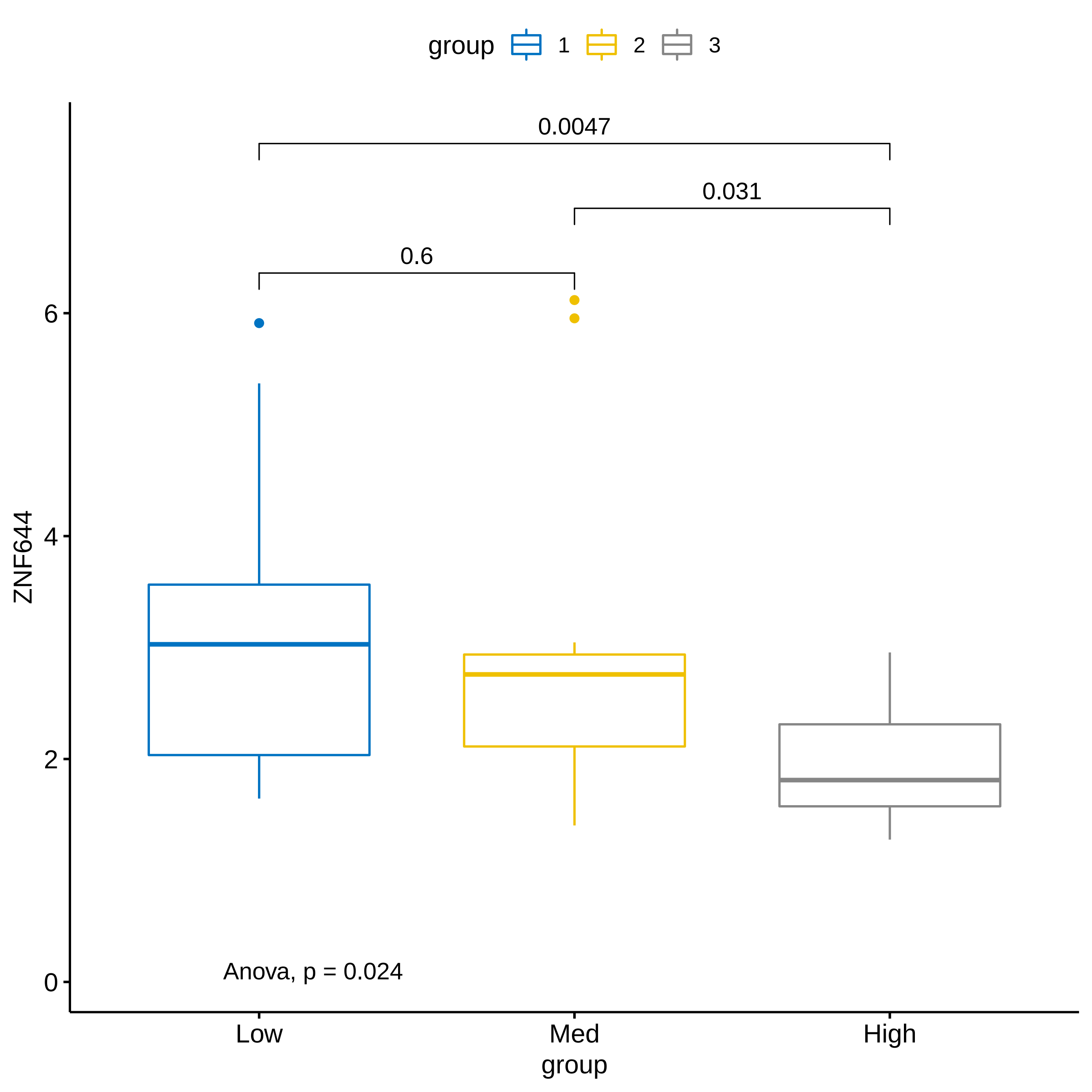

group 1 2 3
0.0047
0.031
0.6
6
4
2
0
ZNF644
Anova, p = 0.024
Low
Med
High
group

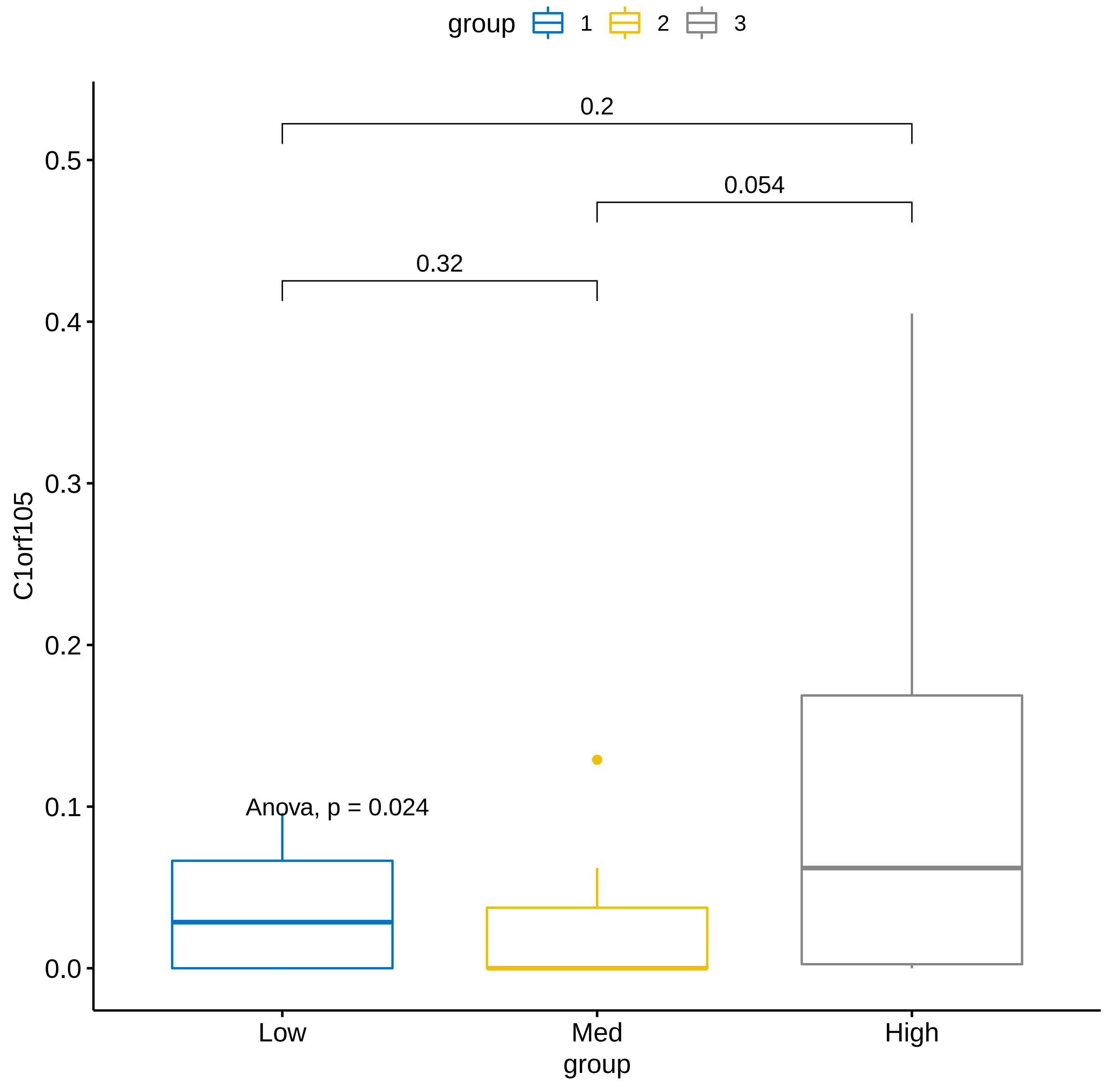

group 1 2 3
0.2
0.054
0.32
0.5
0.4
0.3
0.2
0.1
0.0
C1orf105
Anova, p = 0.024
Low
Med
High
group

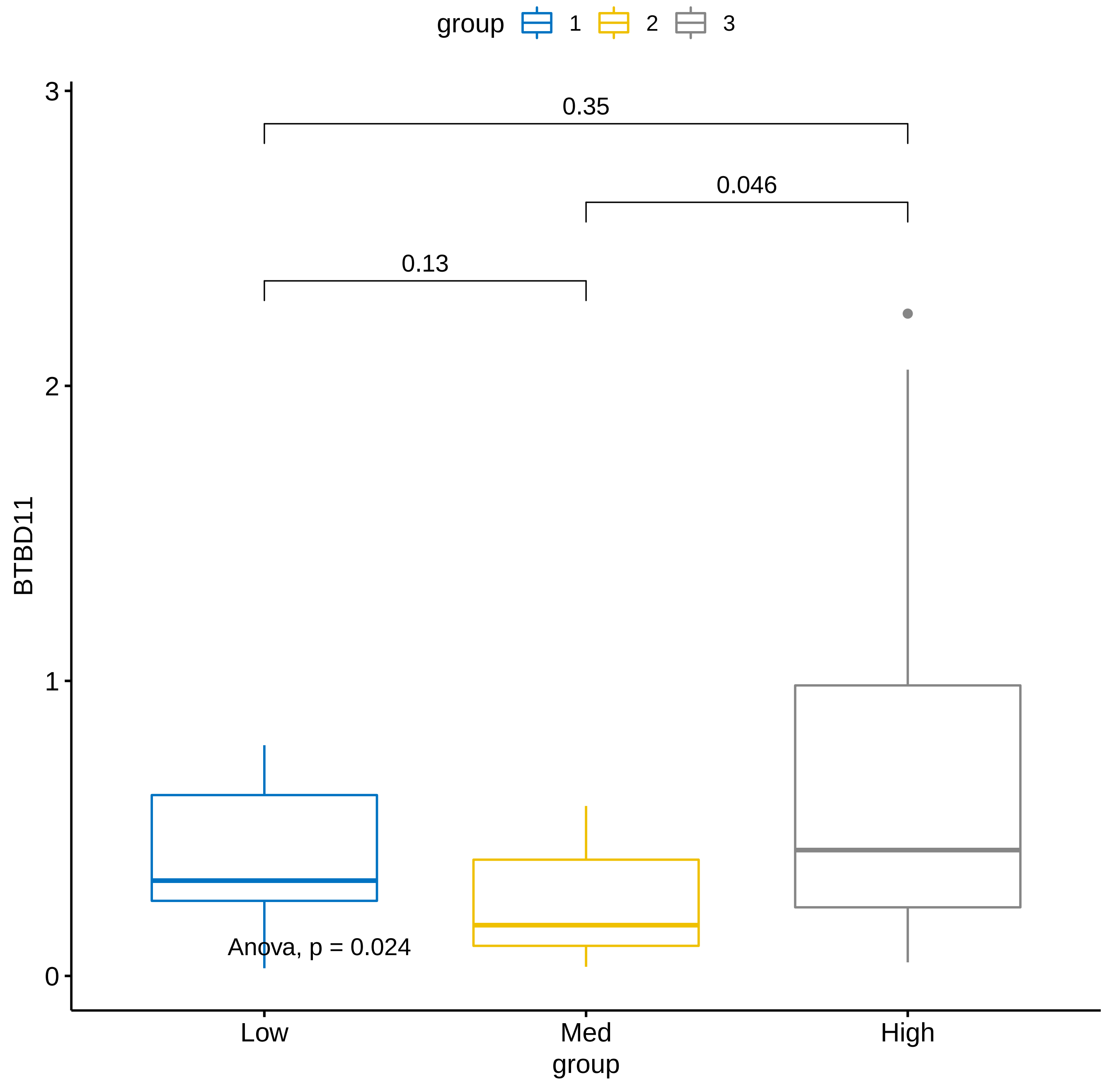
group
1
2
3
0.35
0.046
0.13
Anova, p = 0.024
3
2
1
0
BTBD11
Low
Med
High
group

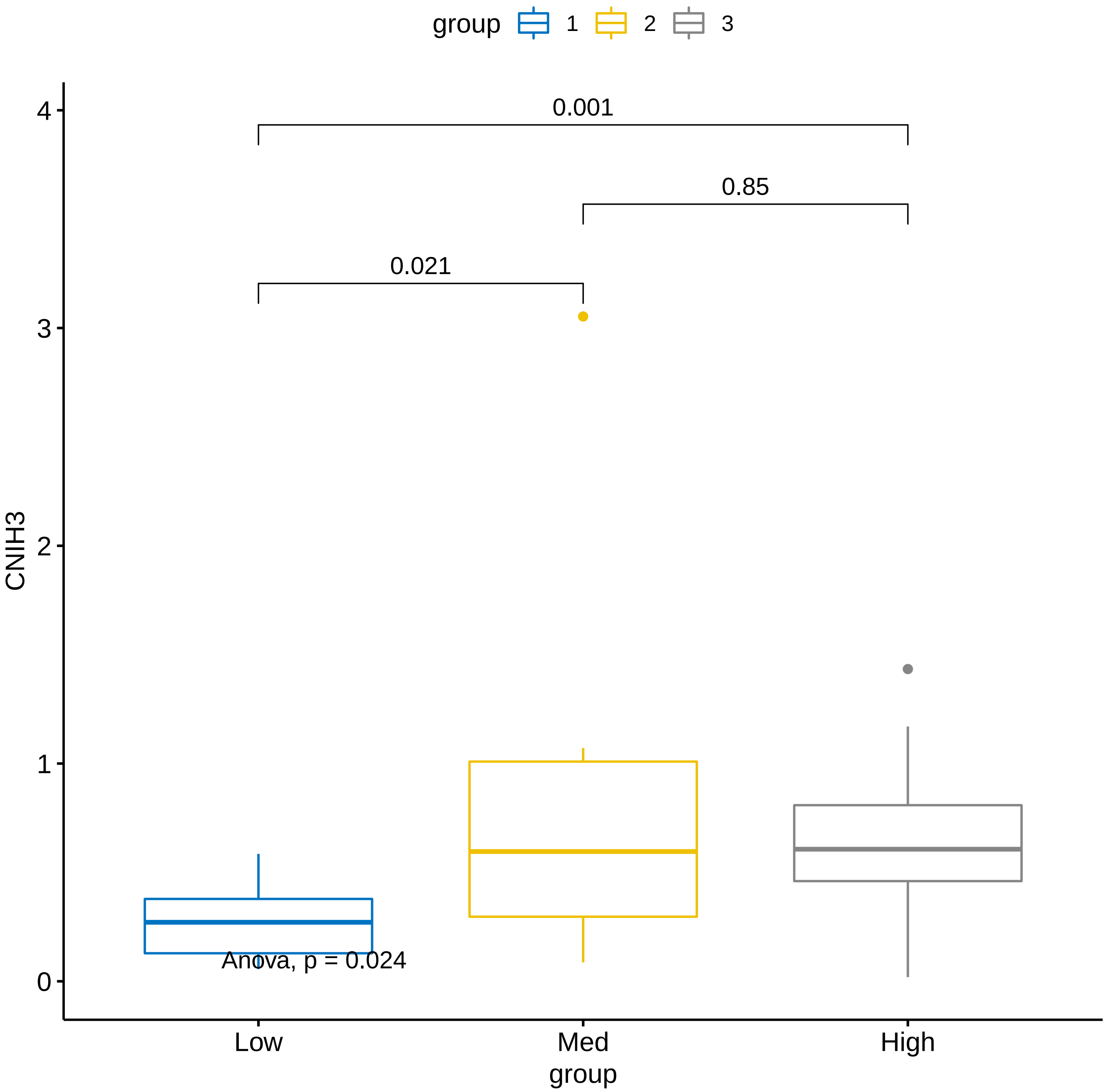

group
1
2
3
0.001
0.85
0.021
Anova, p = 0.024
CNIH3
4
3
2
1
0
Low
Med
High
group

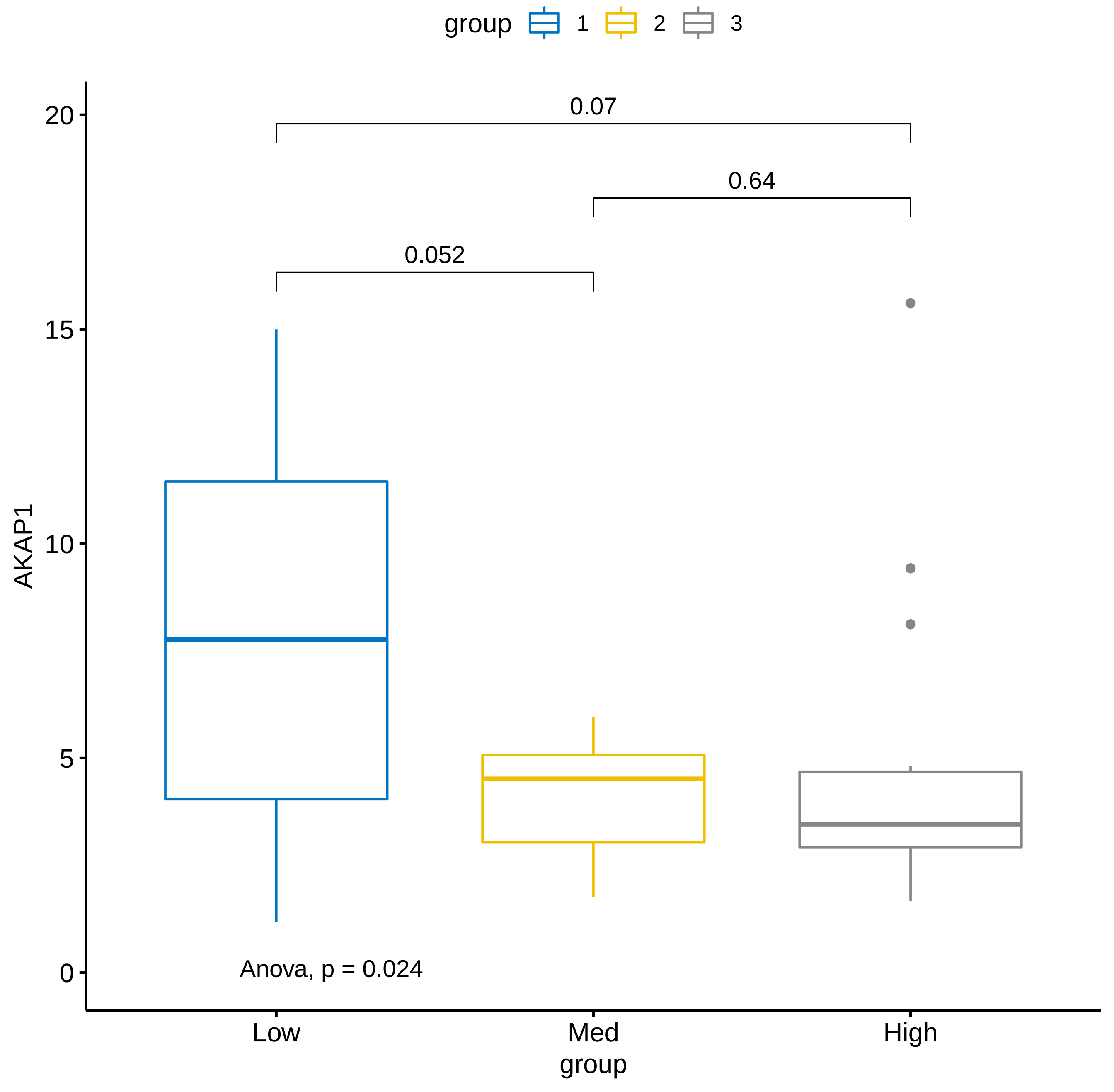

group
1
2
3
0.07
0.64
0.052
20
15
10
5
0
AKAP1
Anova, p = 0.024
Low
Med
High
group

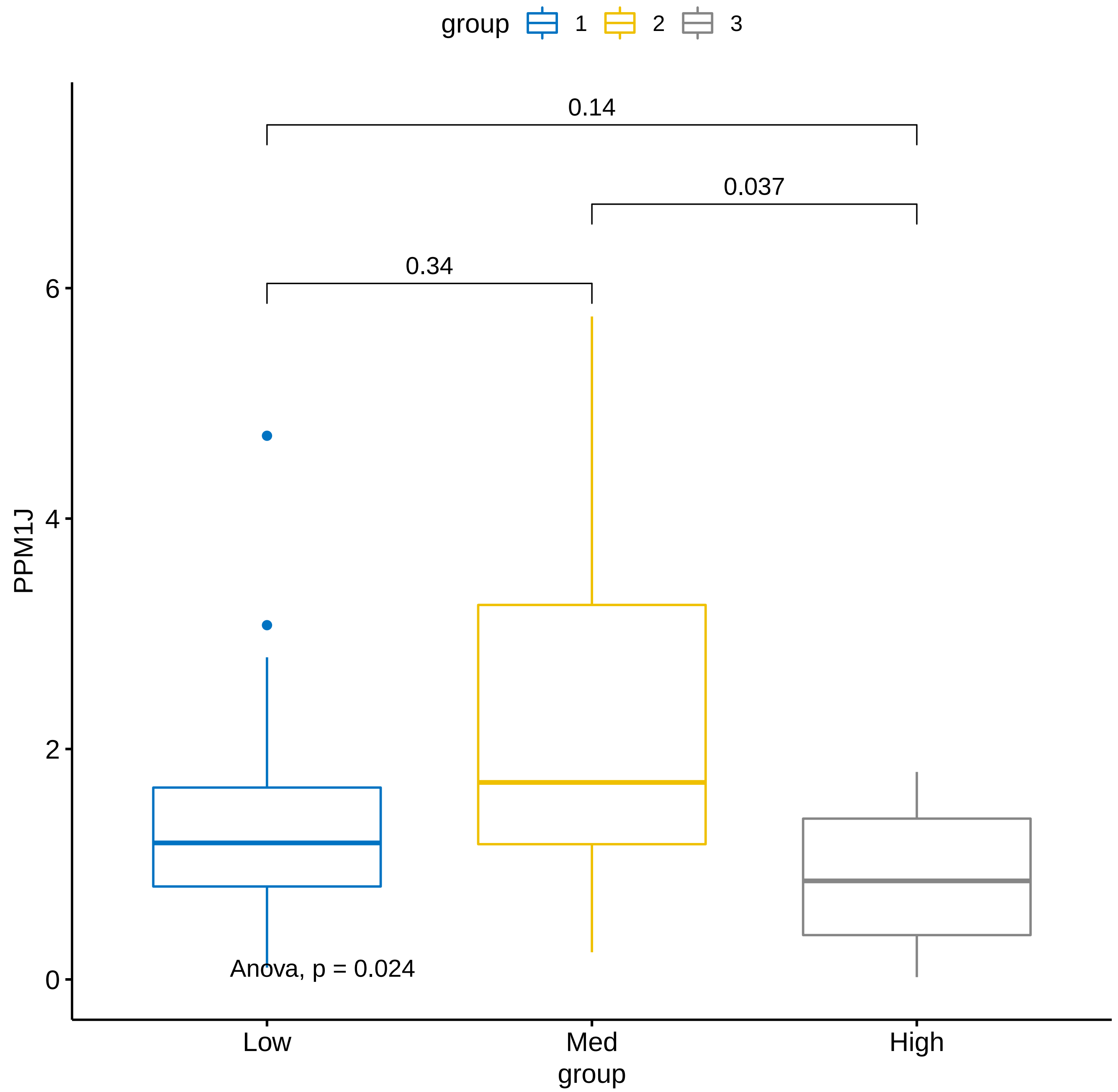
group
1
2
3
0.14
0.037
0.34
6
4
2
0
PPM1J
Anova, p = 0.024
Low
Med
High
group

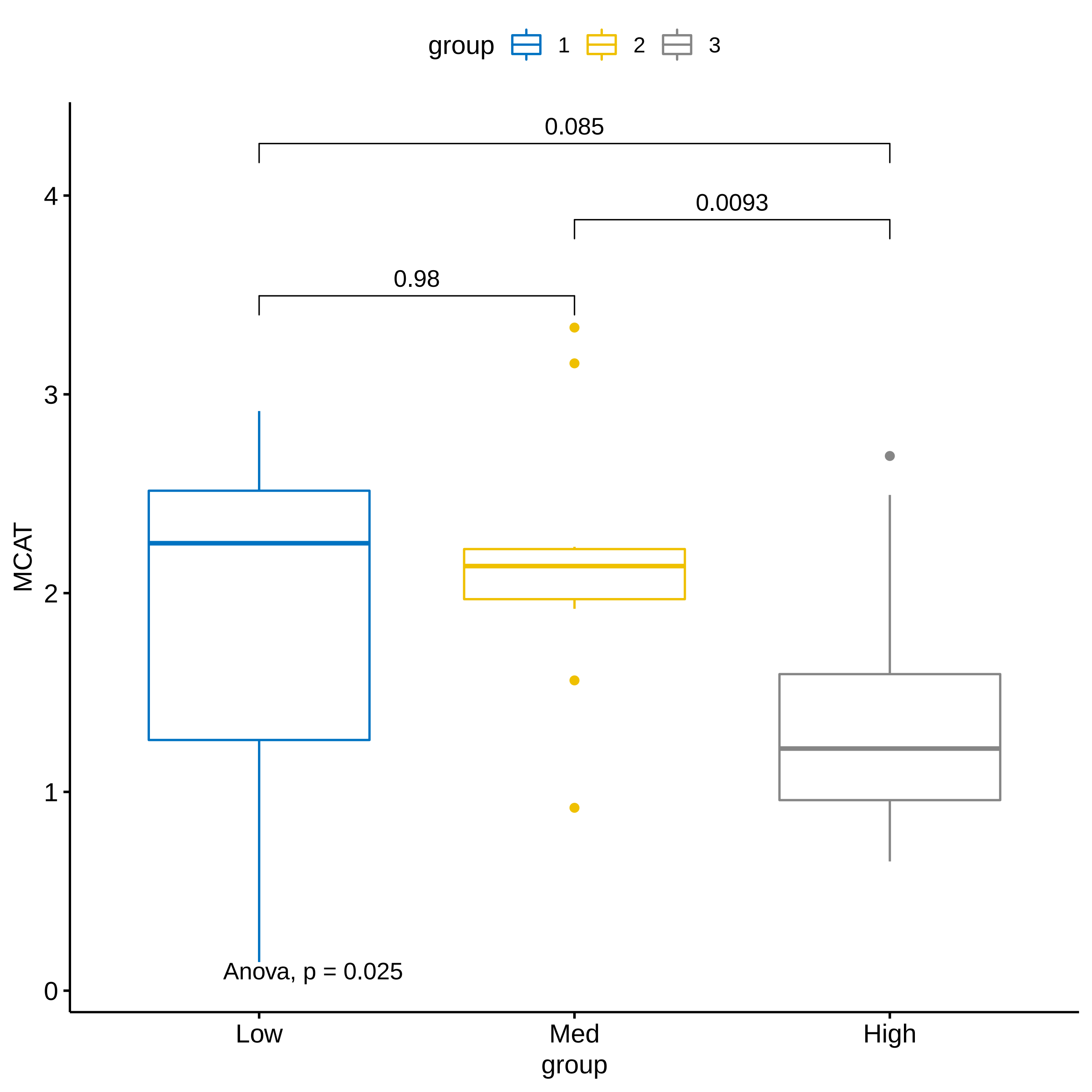
group
1
2
3
0.085
0.0093
0.98
4
3
2
1
0
MCAT
Anova, p = 0.025
Low
Med
High
group

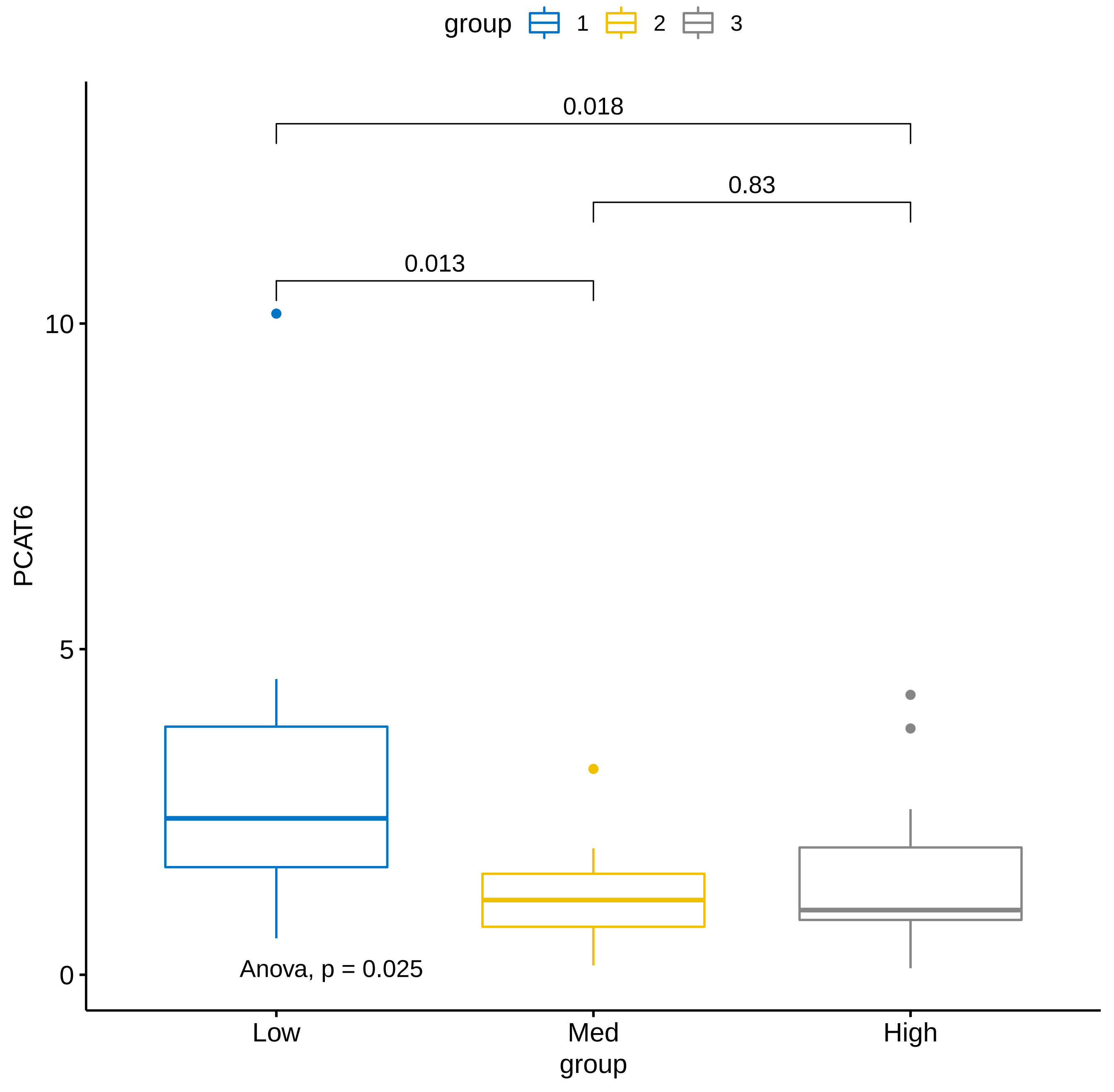

group
1
2
3
0.018
0.83
0.013
10
5
0
PCAT6
Anova, p = 0.025
Low
Med
High
group

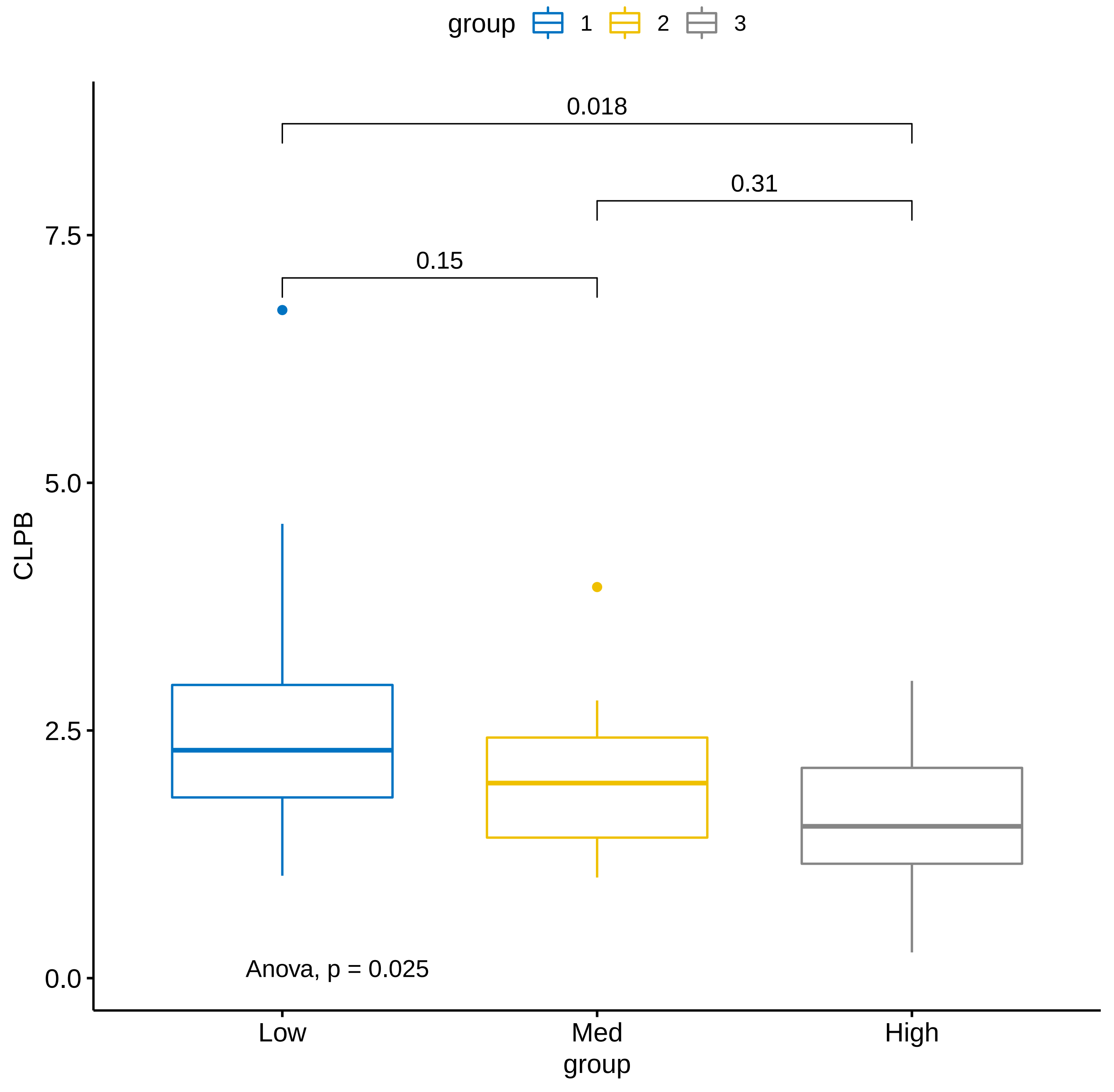
group
1
2
3
0.018
0.31
0.15
7.5
5.0
2.5
0.0
CLPB
Anova, p = 0.025
Low
Med
High
group

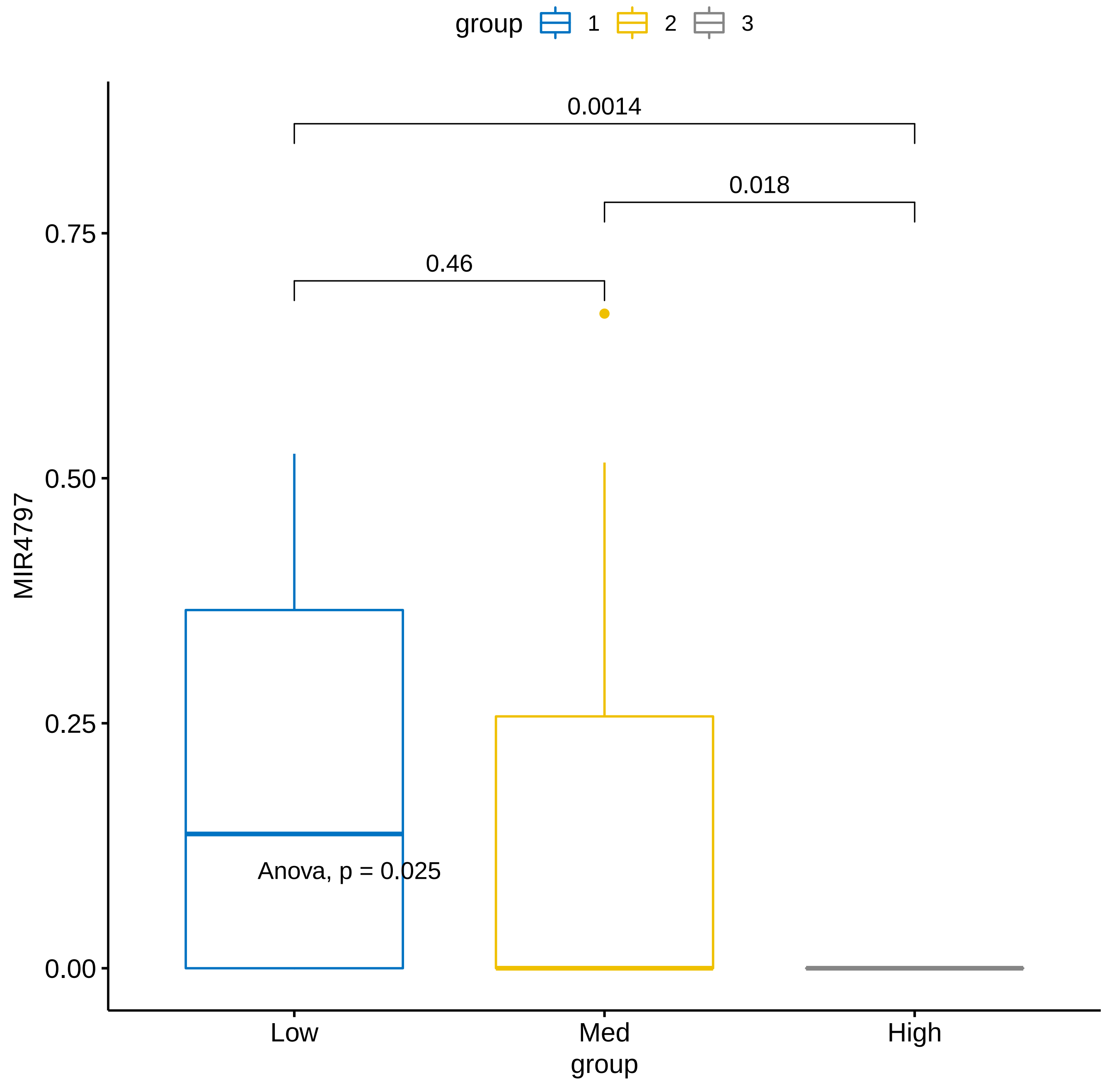
group
1
2
3
0.0014
0.018
0.46
0.75
0.50
0.25
0.00
MIR4797
Anova, p = 0.025
Low
Med
High
group

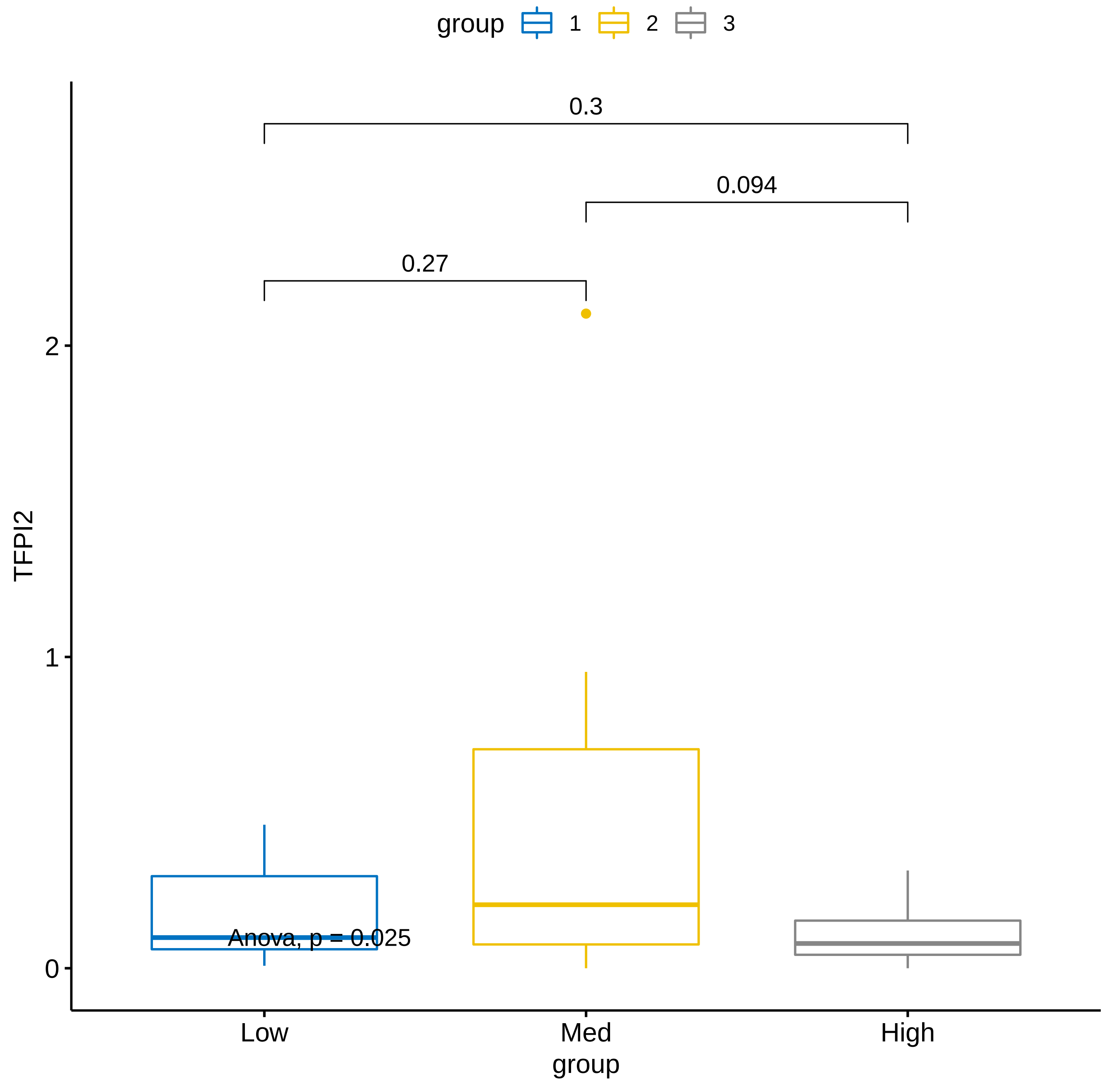
group
1
2
3
0.3
0.094
0.27
TFPI2
2
1
0
Anova, p = 0.025
Low
Med
High
group

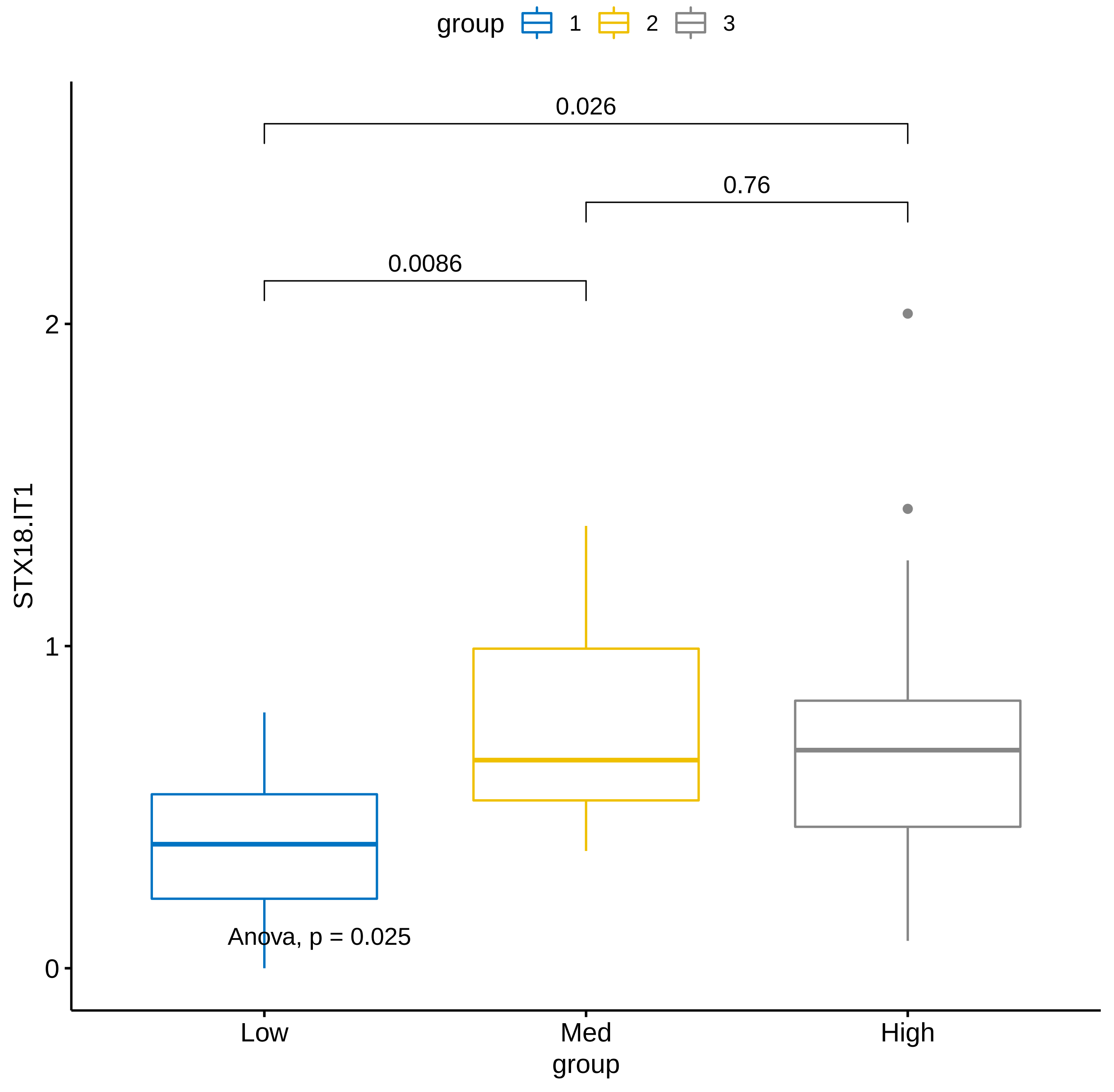

group 1 2 3
0.026
0.76
0.0086
STX18.IT1
2
1
0
Anova, p = 0.025
Low
Med
High
group

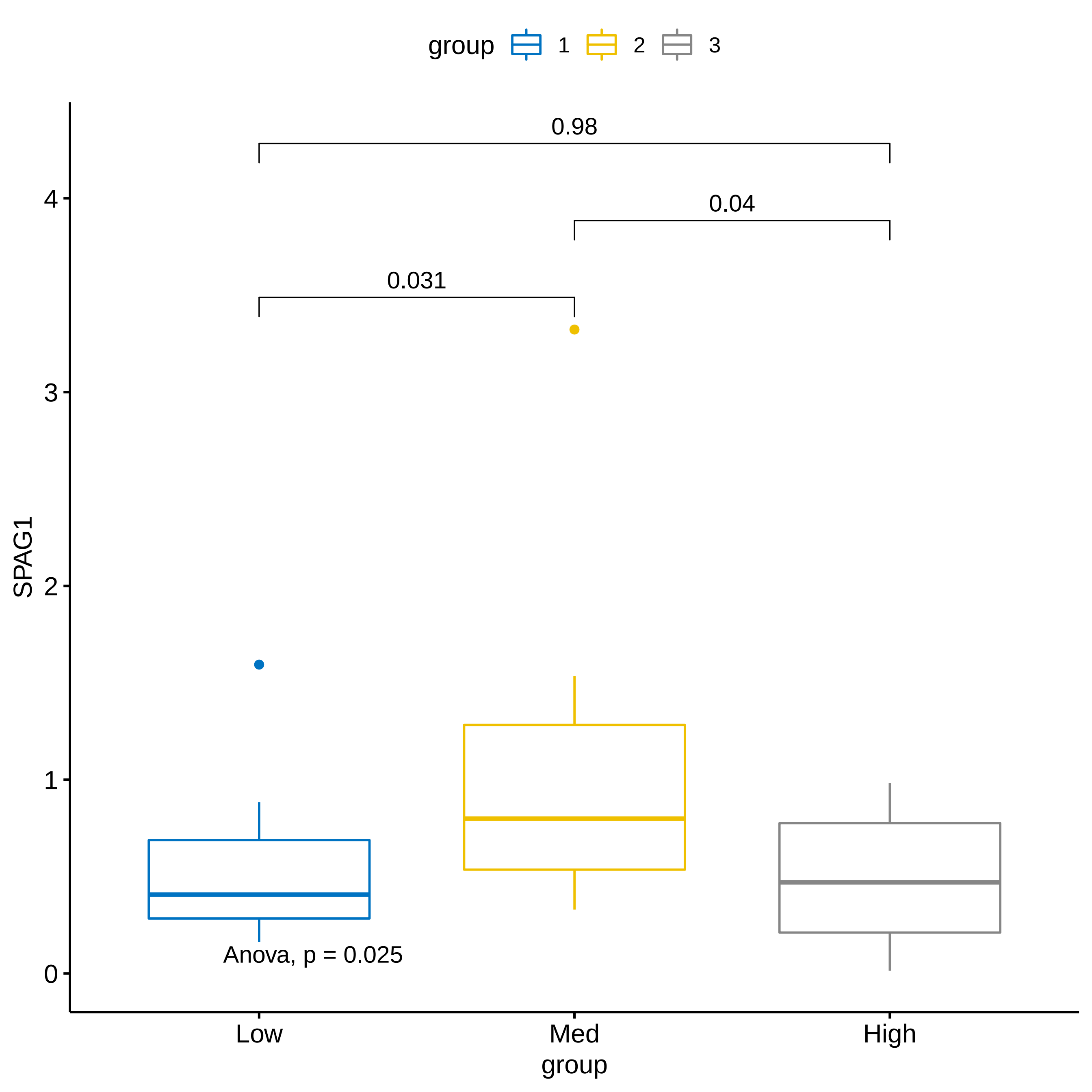

group 1 2 3
0.98
0.04
0.031
SPAG1
4
3
2
1
0
Anova, p = 0.025
Low
Med
High
group

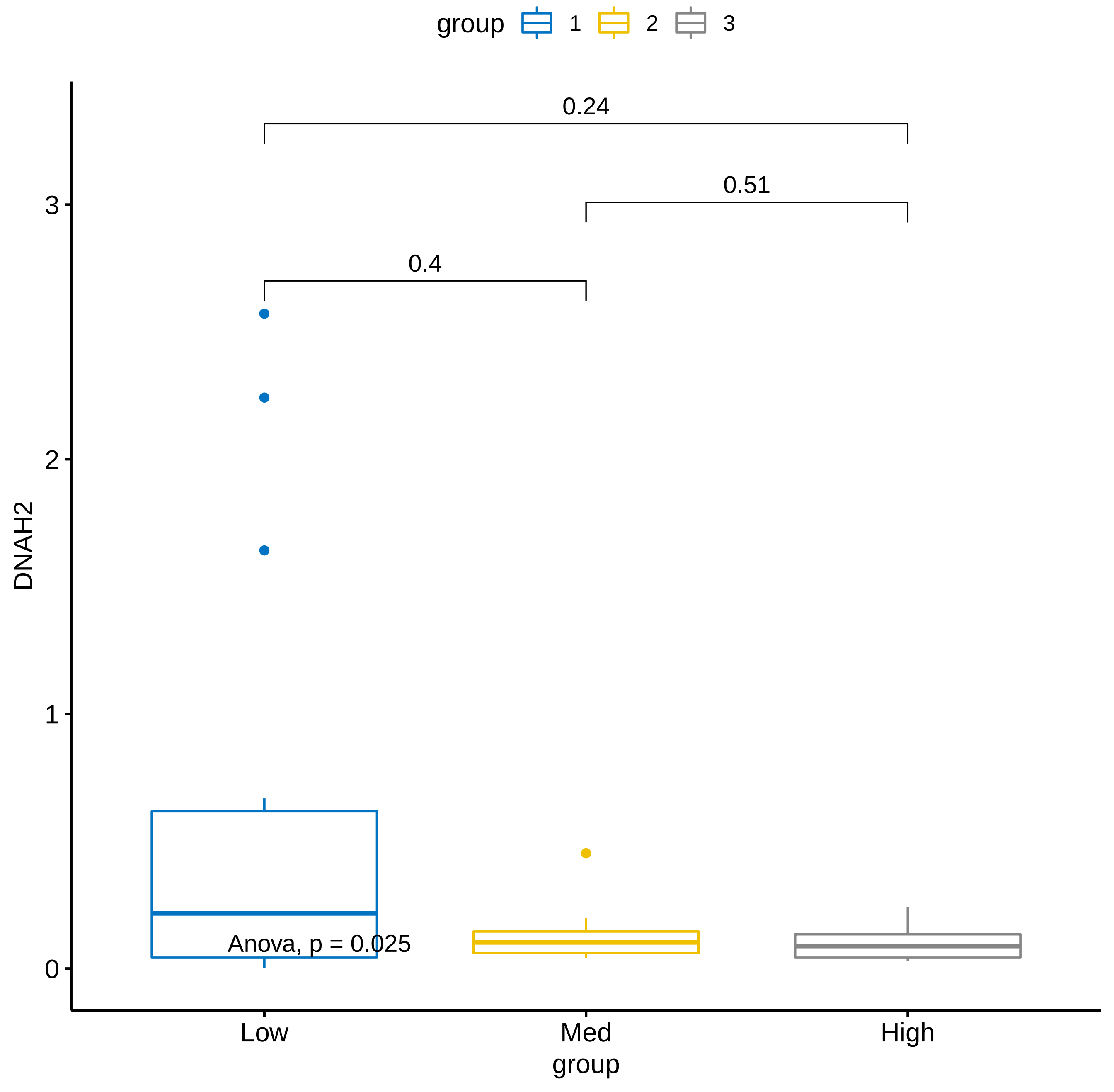

group
1
2
3
0.24
0.51
0.4
3
2
1
0
DNAH2
Anova, p = 0.025
Low
Med
High
group

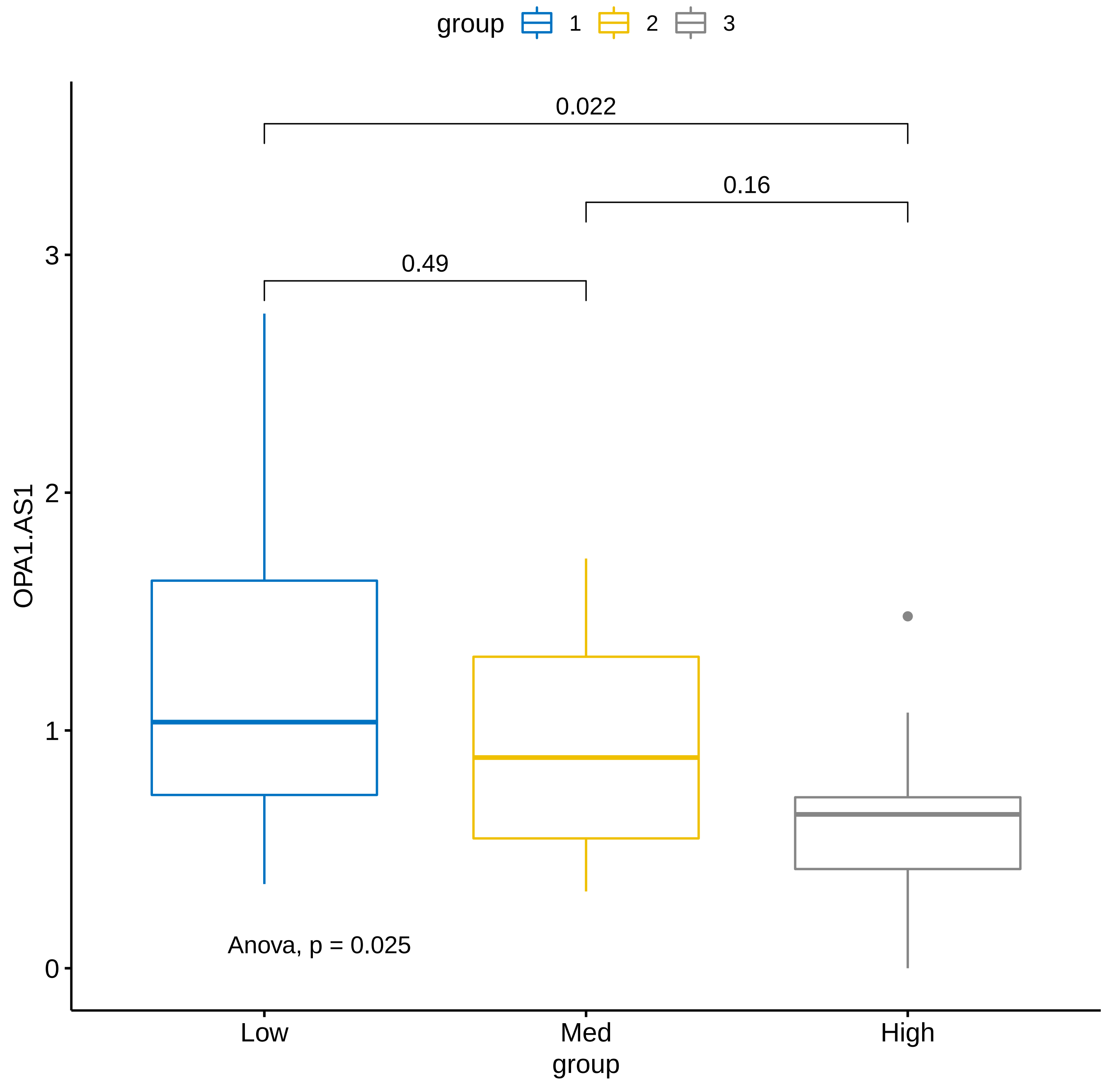
group
1
2
3
0.022
0.16
0.49
3
2
1
0
OPA1.AS1
Anova, p = 0.025
Low
Med
High
group

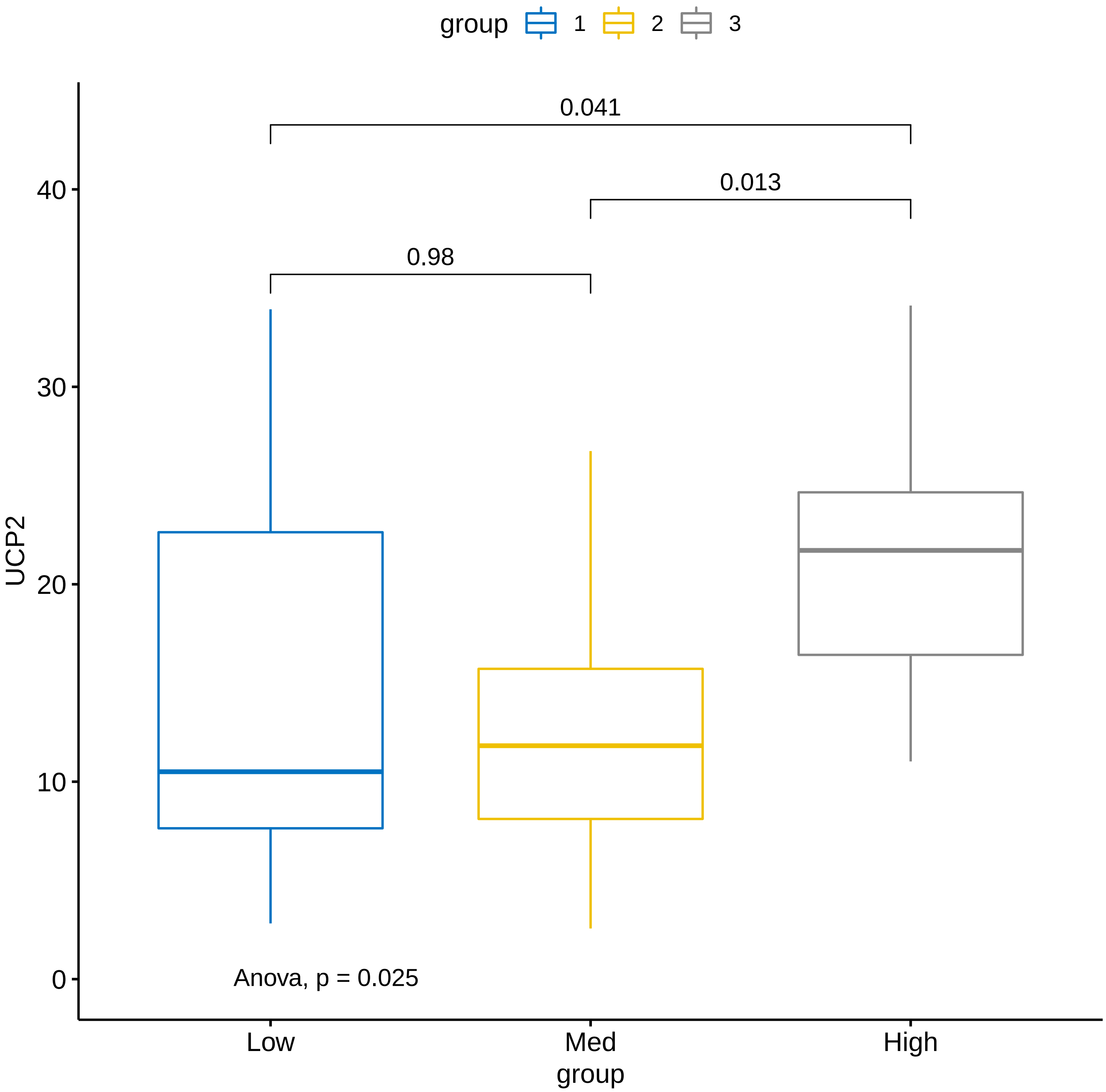
group
1
2
3
0.041
0.013
0.98
40
30
20
10
0
UCP2
Anova, p = 0.025
Low
Med
High
group

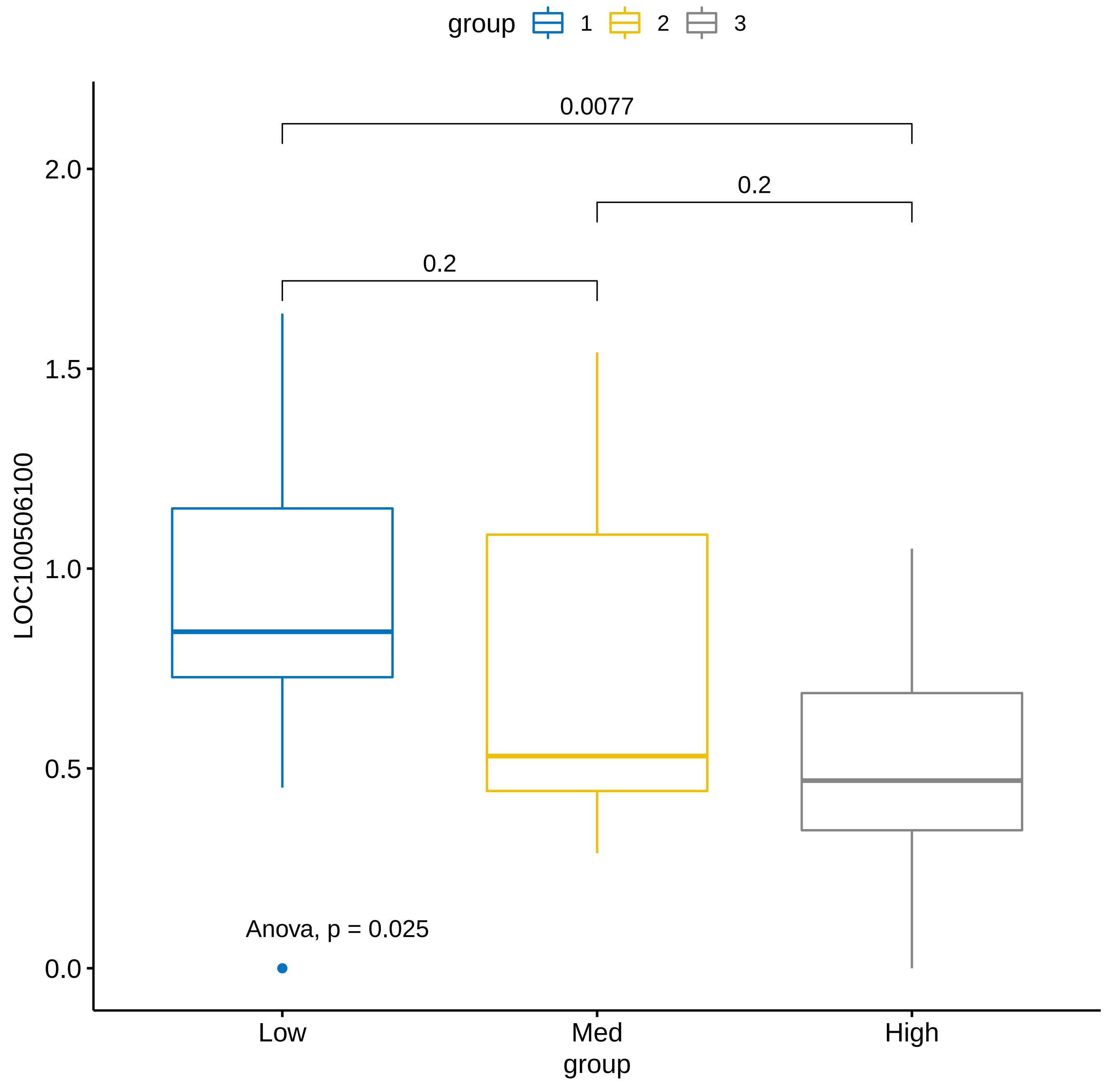
group
1
2
3
0.0077
0.2
0.2
2.0
1.5
1.0
0.5
0.0
LOC100506100
Anova, p = 0.025
Low
Med
High
group

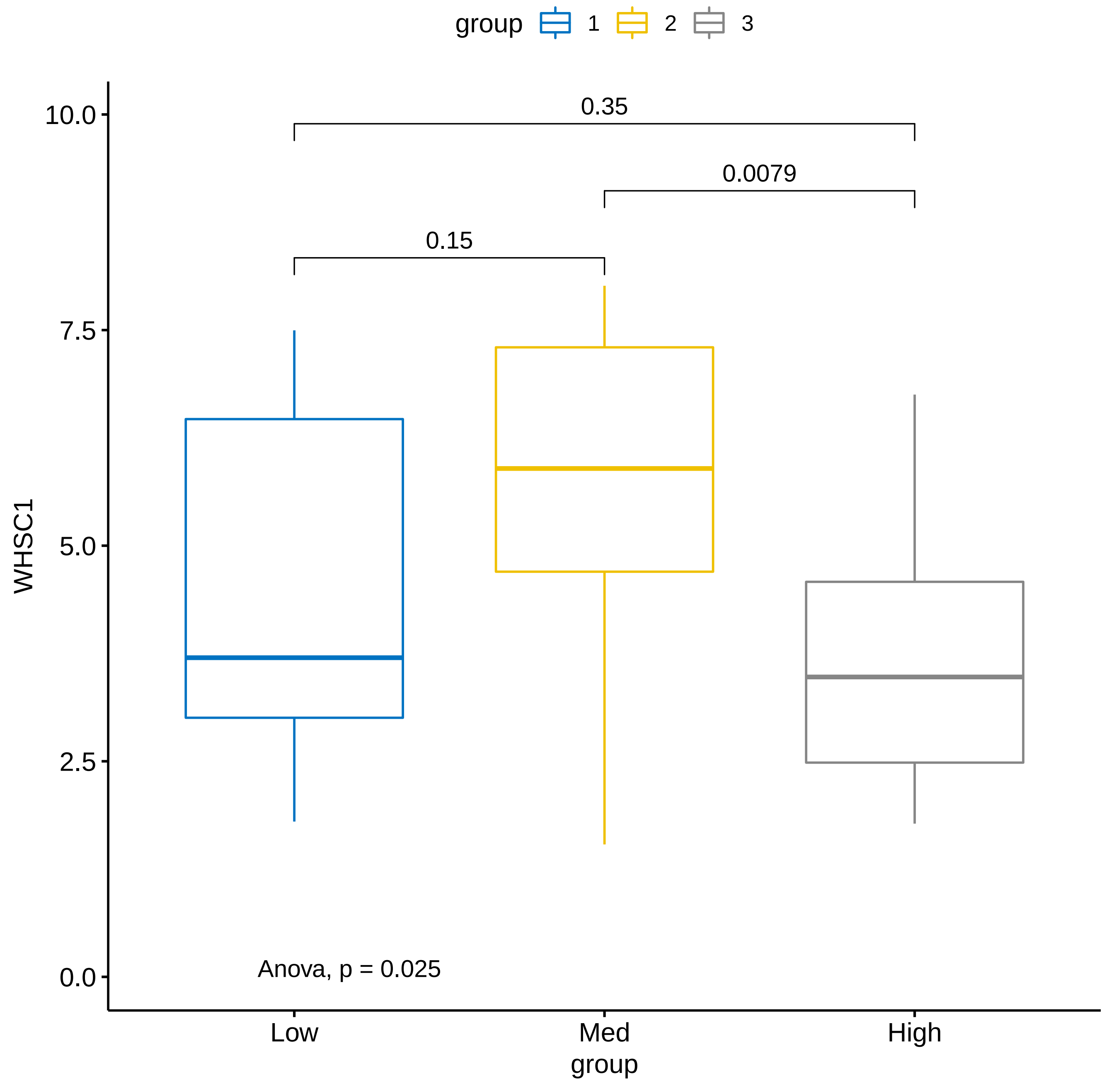

group
1
2
3
0.35
0.0079
0.15
10.0
7.5
5.0
2.5
0.0
WHSC1
Anova, p = 0.025
Low
Med
High
group

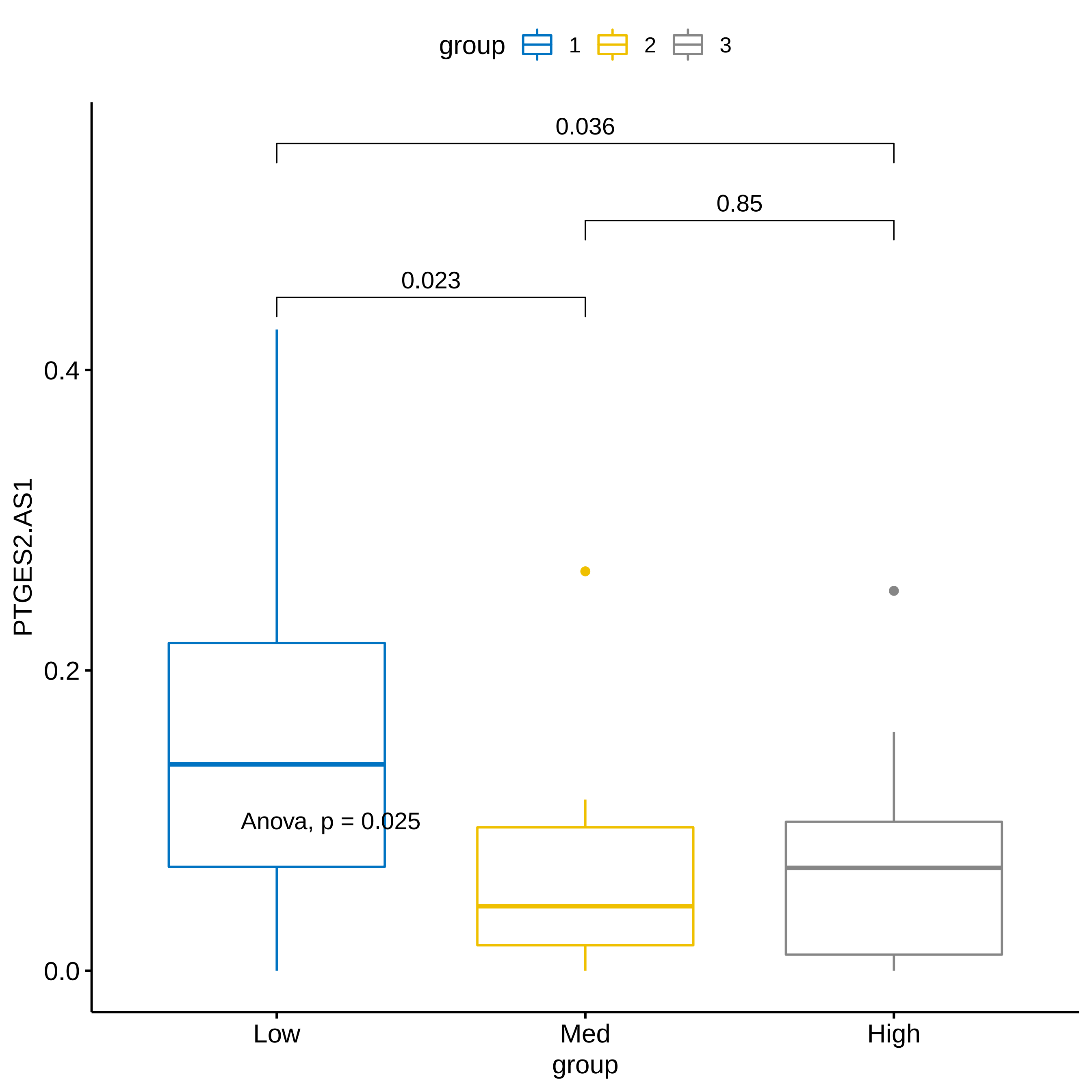

group 1 2 3
0.036
0.85
0.023
0.4
0.2
0.0
PTGES2.AS1
Anova, p = 0.025
Low
Med
High
group

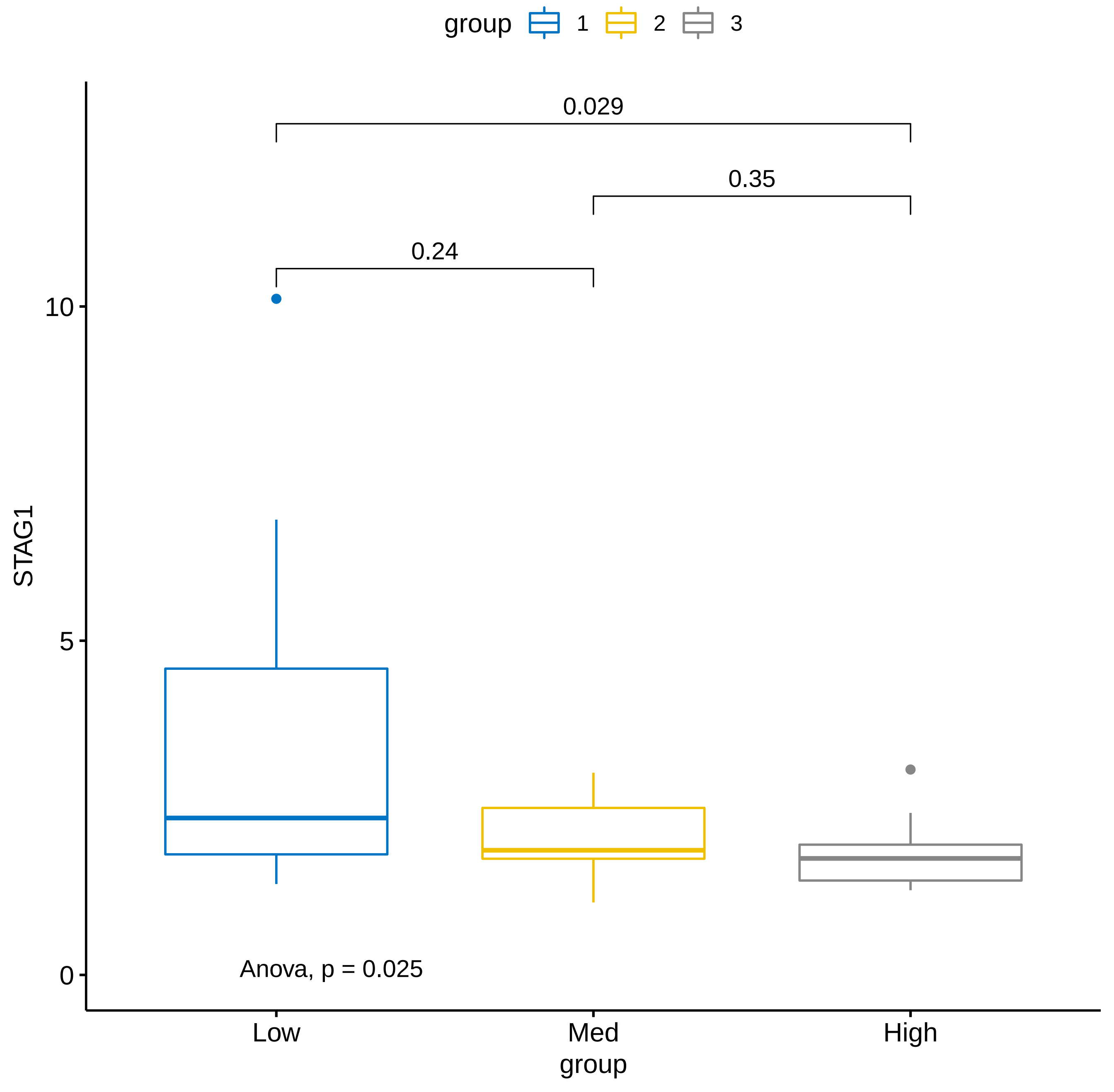

group 1 2 3
0.029
0.35
0.24
10
5
0
STAG1
Anova, p = 0.025
Low
Med
High
group

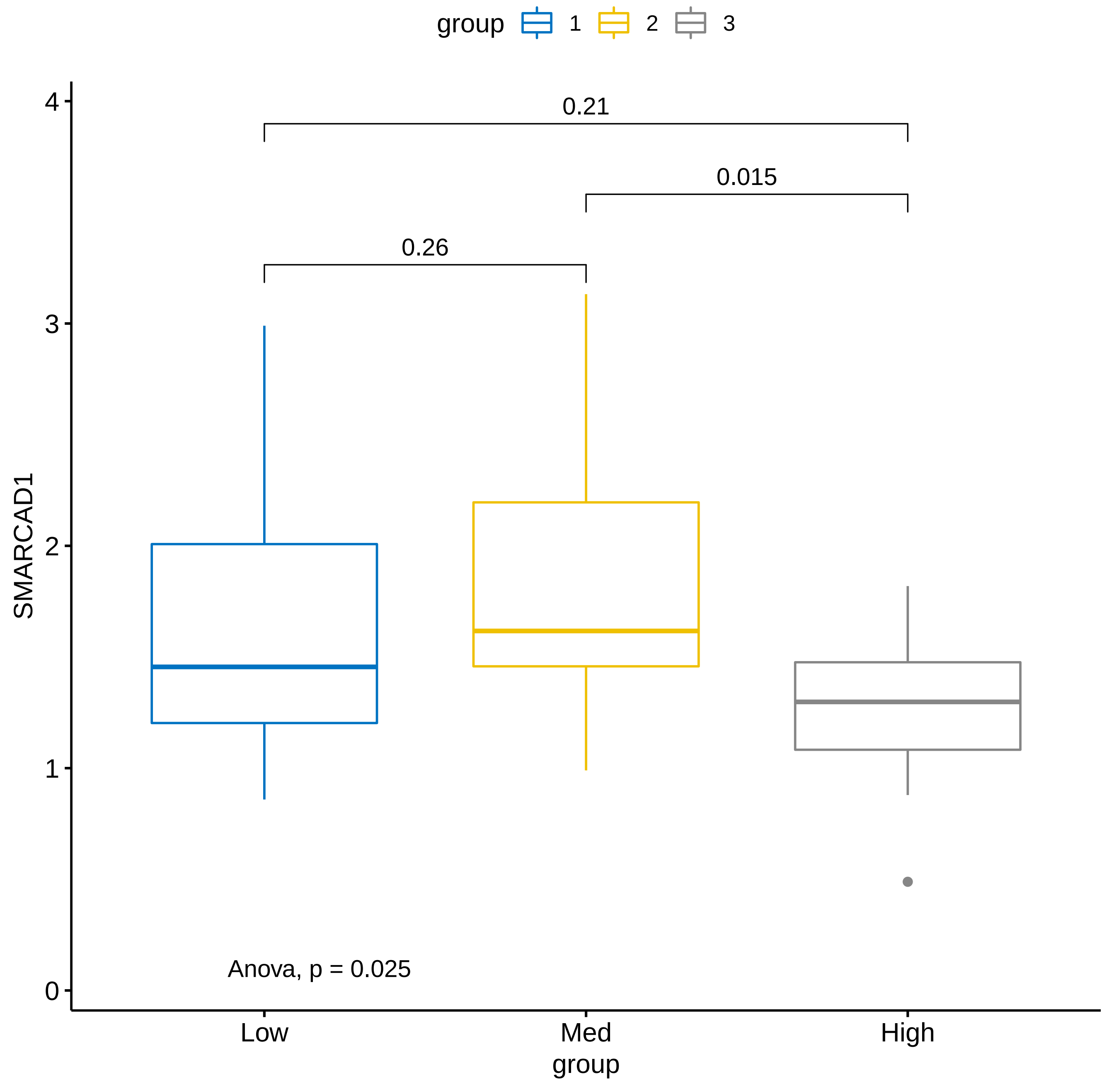
group
1
2
3
0.21
0.015
0.26
SMARCAD1
4
3
2
1
0
Anova, p = 0.025
Low
Med
High
group

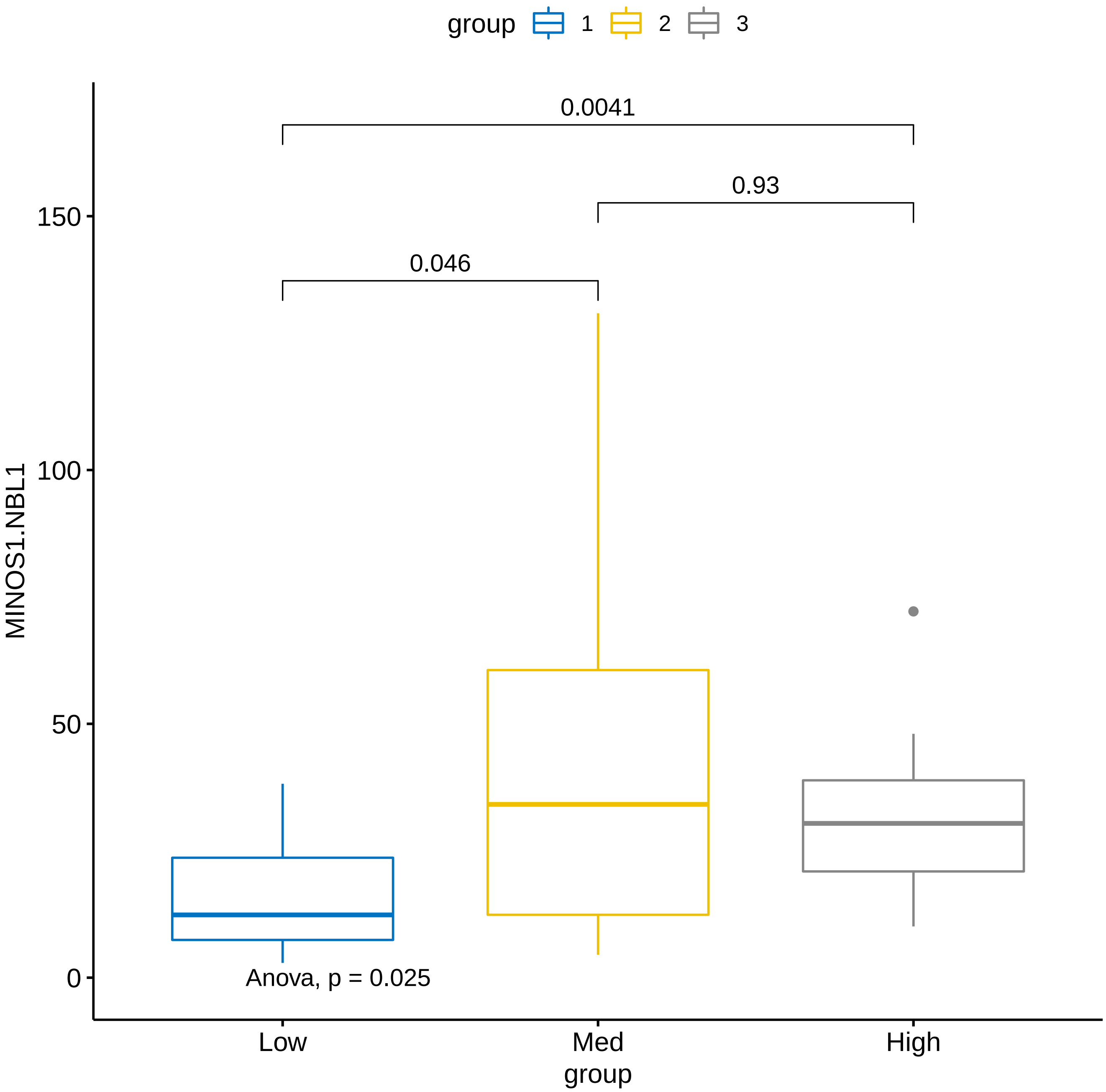
group 1 2 3
0.0041
0.93
0.046
150
100
50
0
MINOS1.NBL1
Anova, p = 0.025
Low
Med
High
group

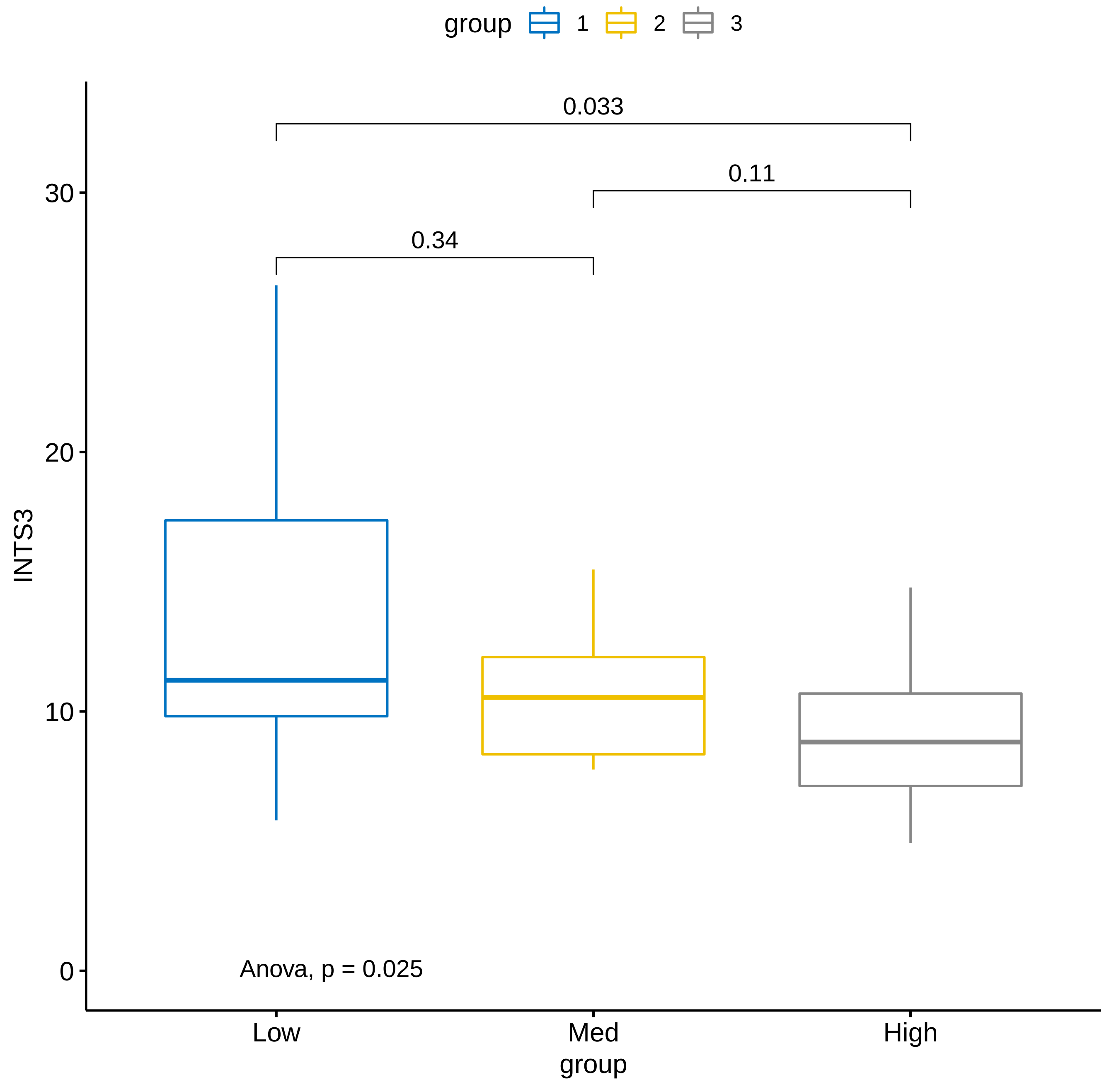
group
1
2
3
0.033
0.11
0.34
30
20
10
0
INTS3
Anova, p = 0.025
Low
Med
High
group

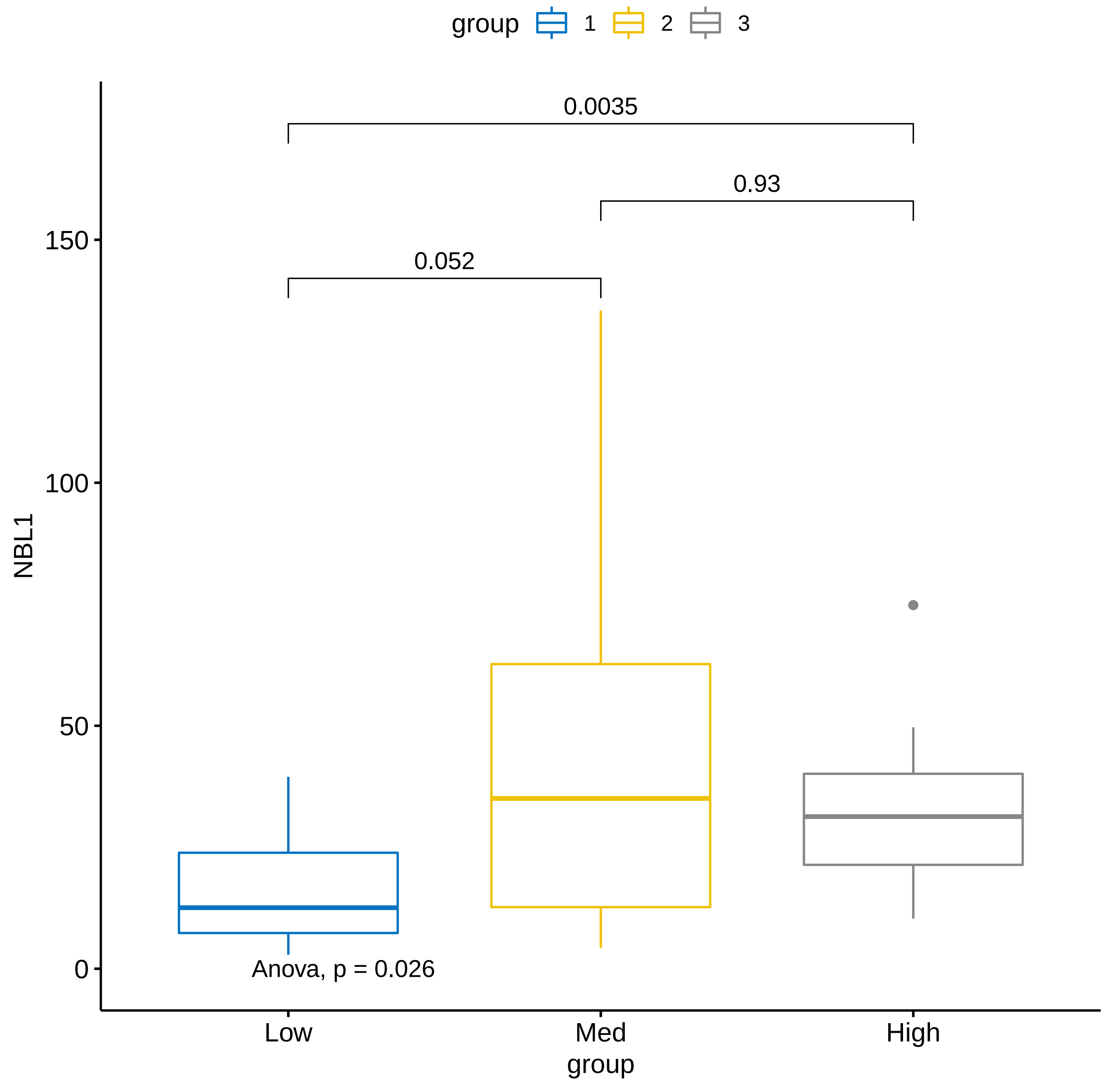

group 1 2 3
0.0035
0.93
0.052
150
100
50
0
NBL1
Anova, p = 0.026
Low
Med
High
group

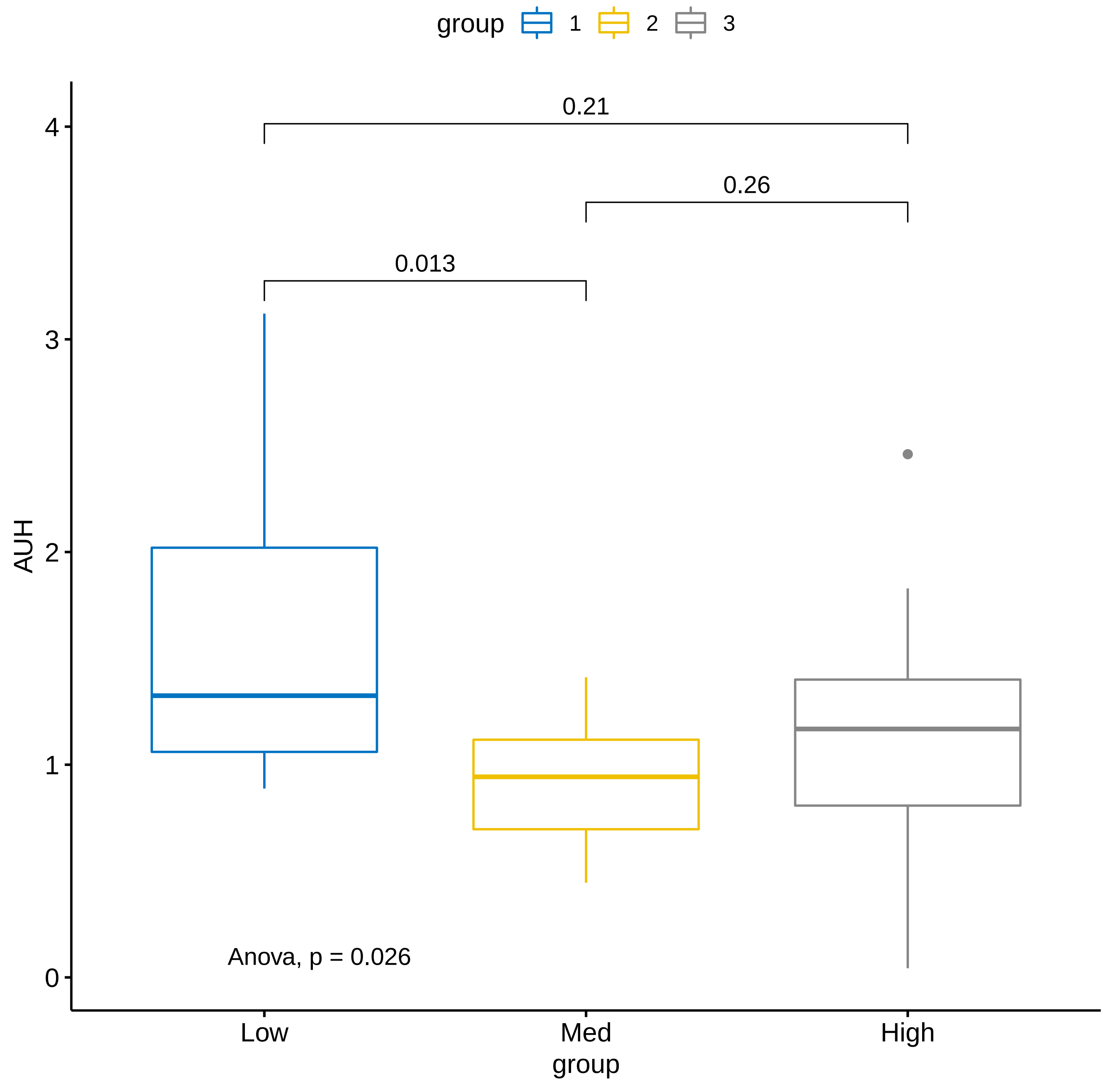
group
1
2
3
0.21
0.26
0.013
AUH
4
3
2
1
0
Anova, p = 0.026
Low
Med
High
group

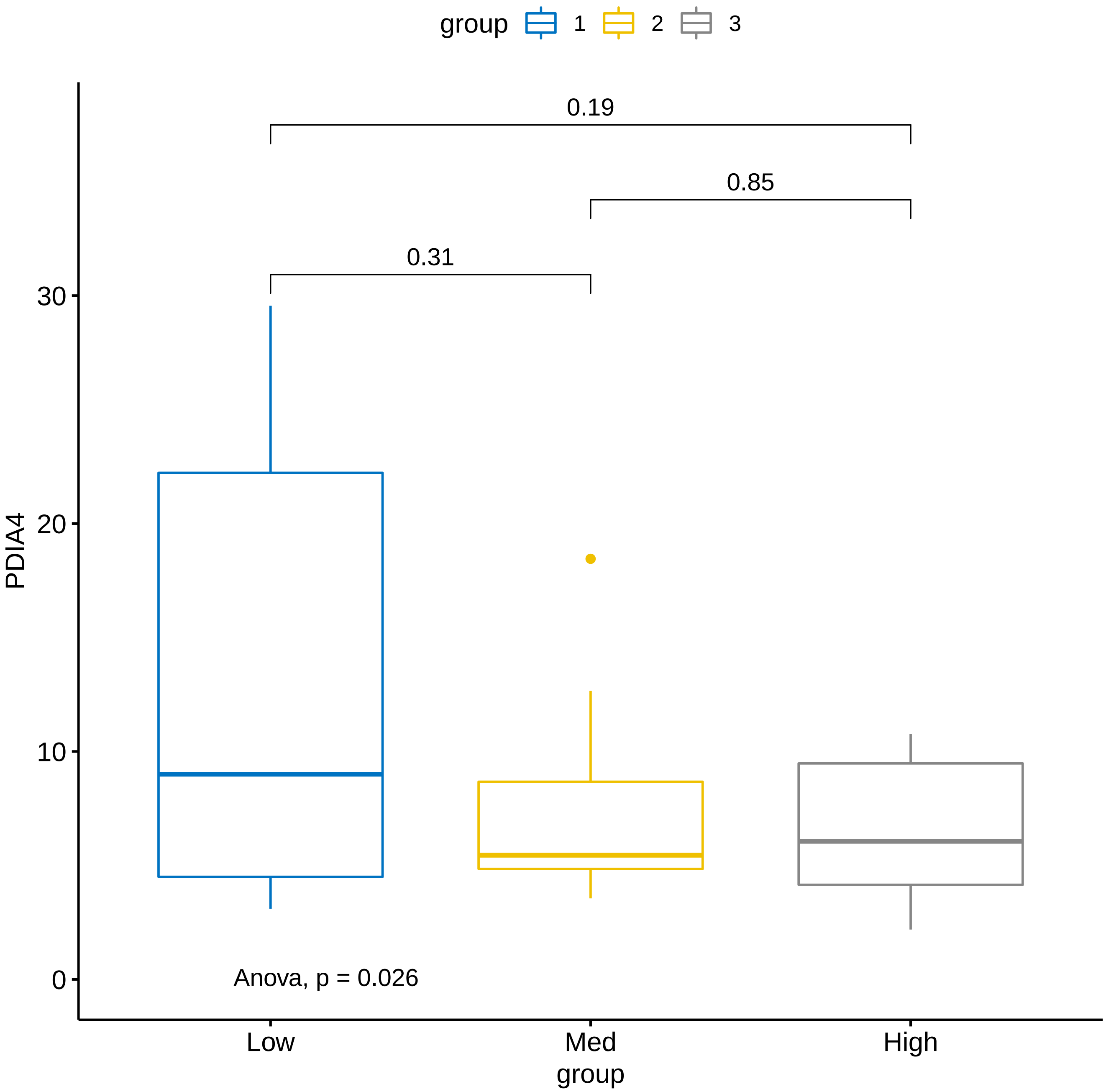
group
1
2
3
0.19
0.85
0.31
30
20
10
0
PDIA4
Anova, p = 0.026
Low
Med
High
group

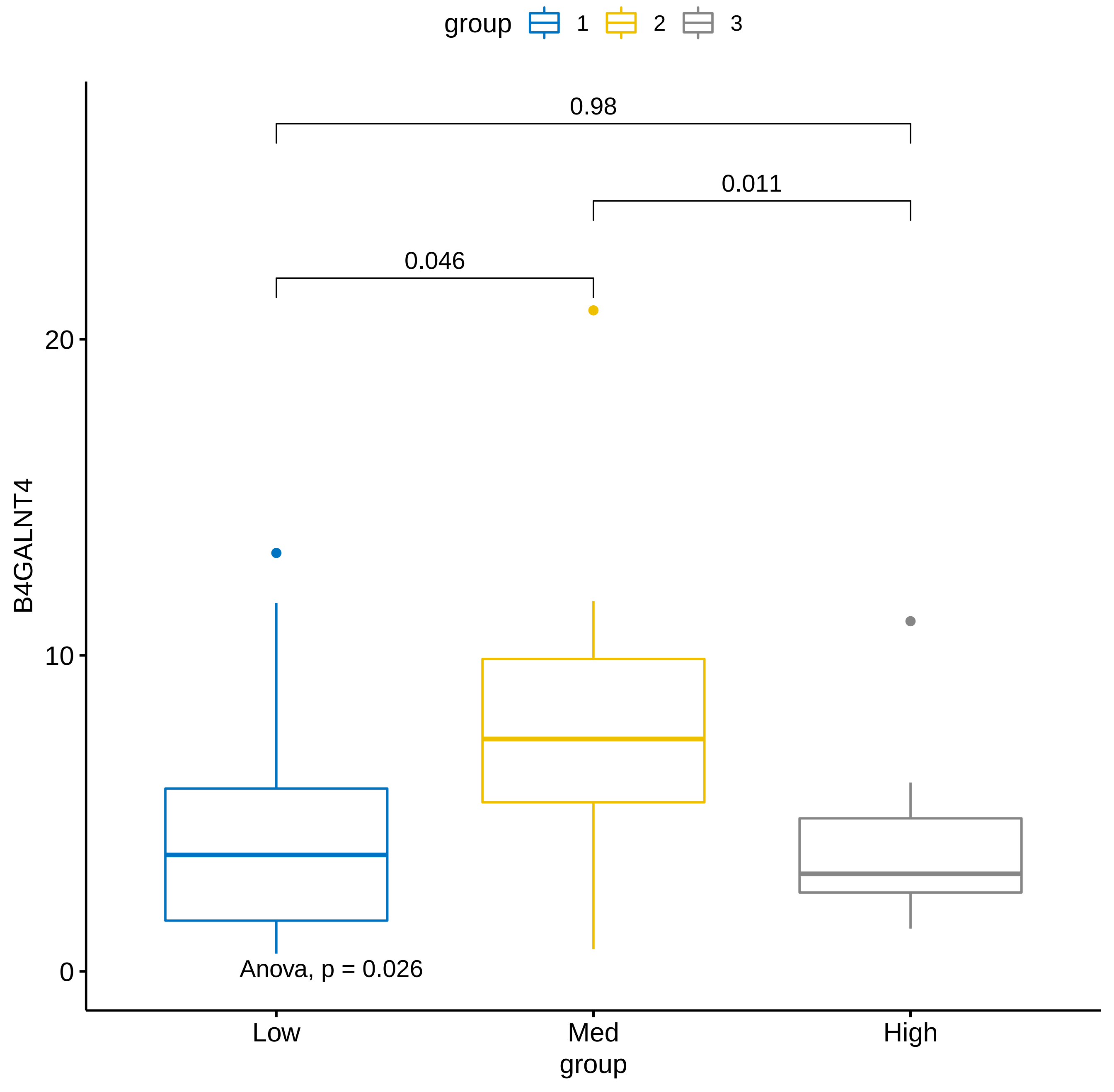
group
1
2
3
0.98
0.011
0.046
20
10
0
B4GALNT4
Anova, p = 0.026
Low
Med
High
group

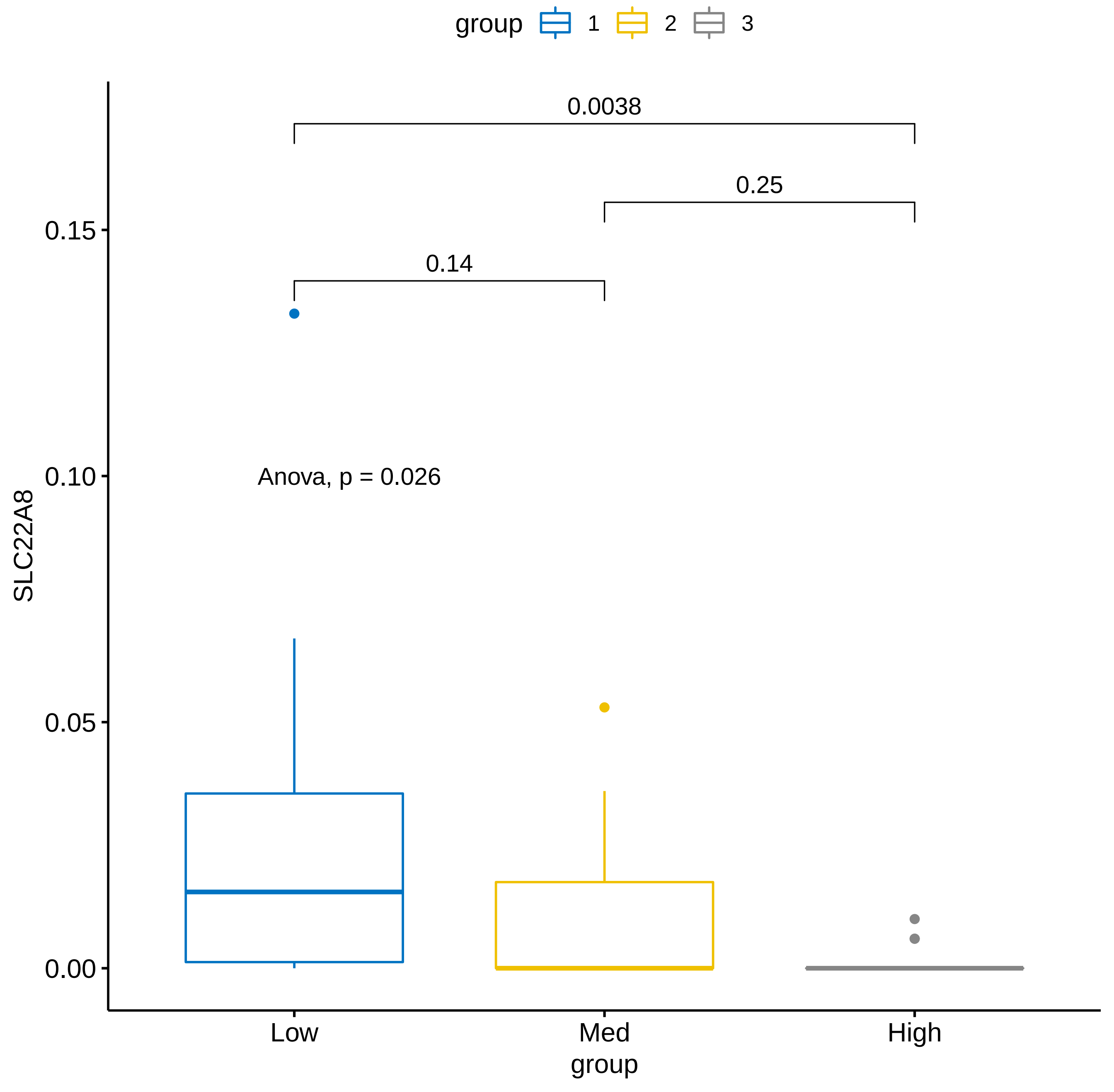
group
1
2
3
0.0038
0.25
0.14
Anova, p = 0.026
SLC22A8
0.15
0.10
0.05
0.00
Low
Med
High
group

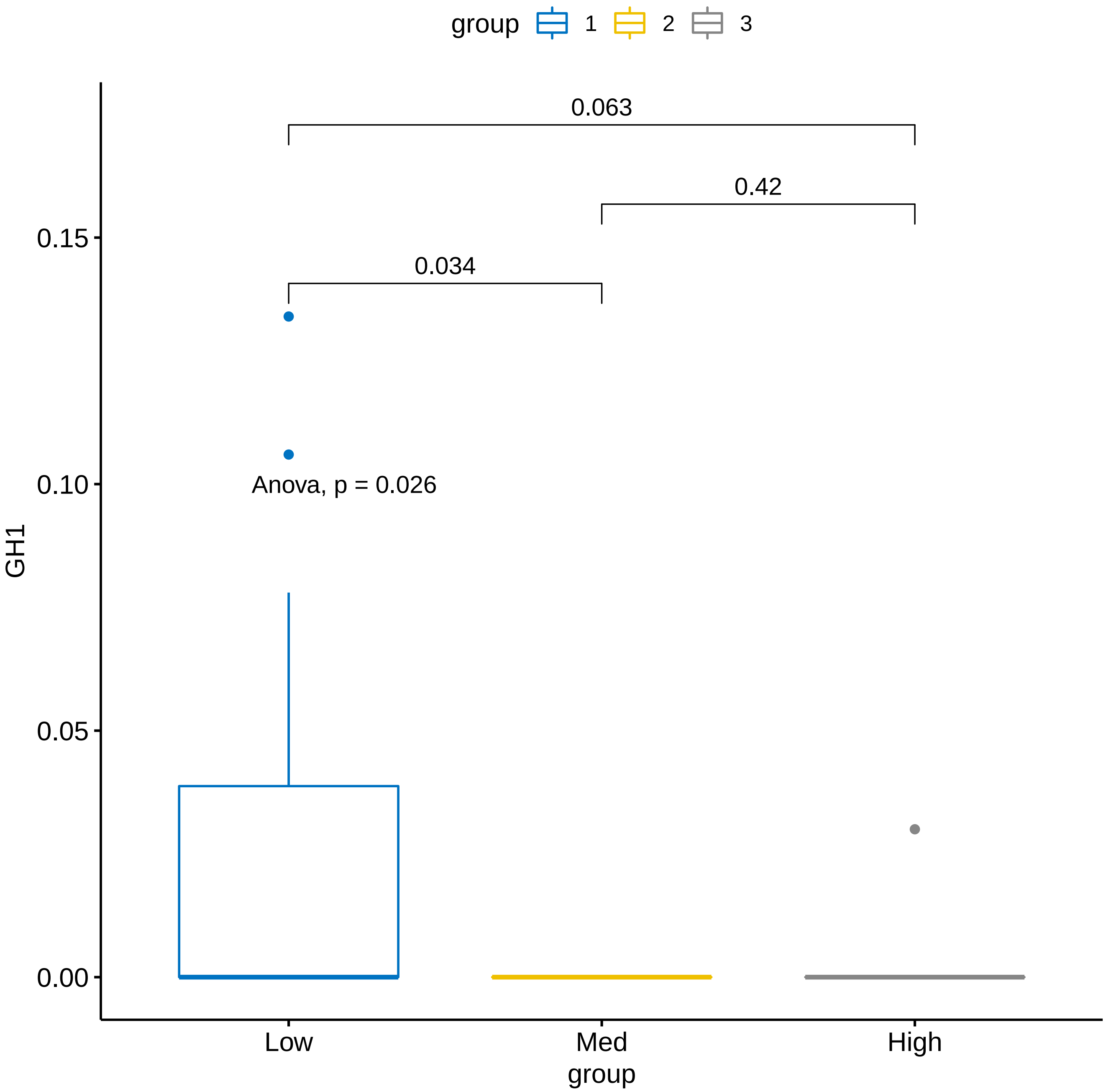
group
1
2
3
0.063
0.42
0.034
Anova, p = 0.026
0.15
0.10
0.05
0.00
GH1
Low
Med
High
group

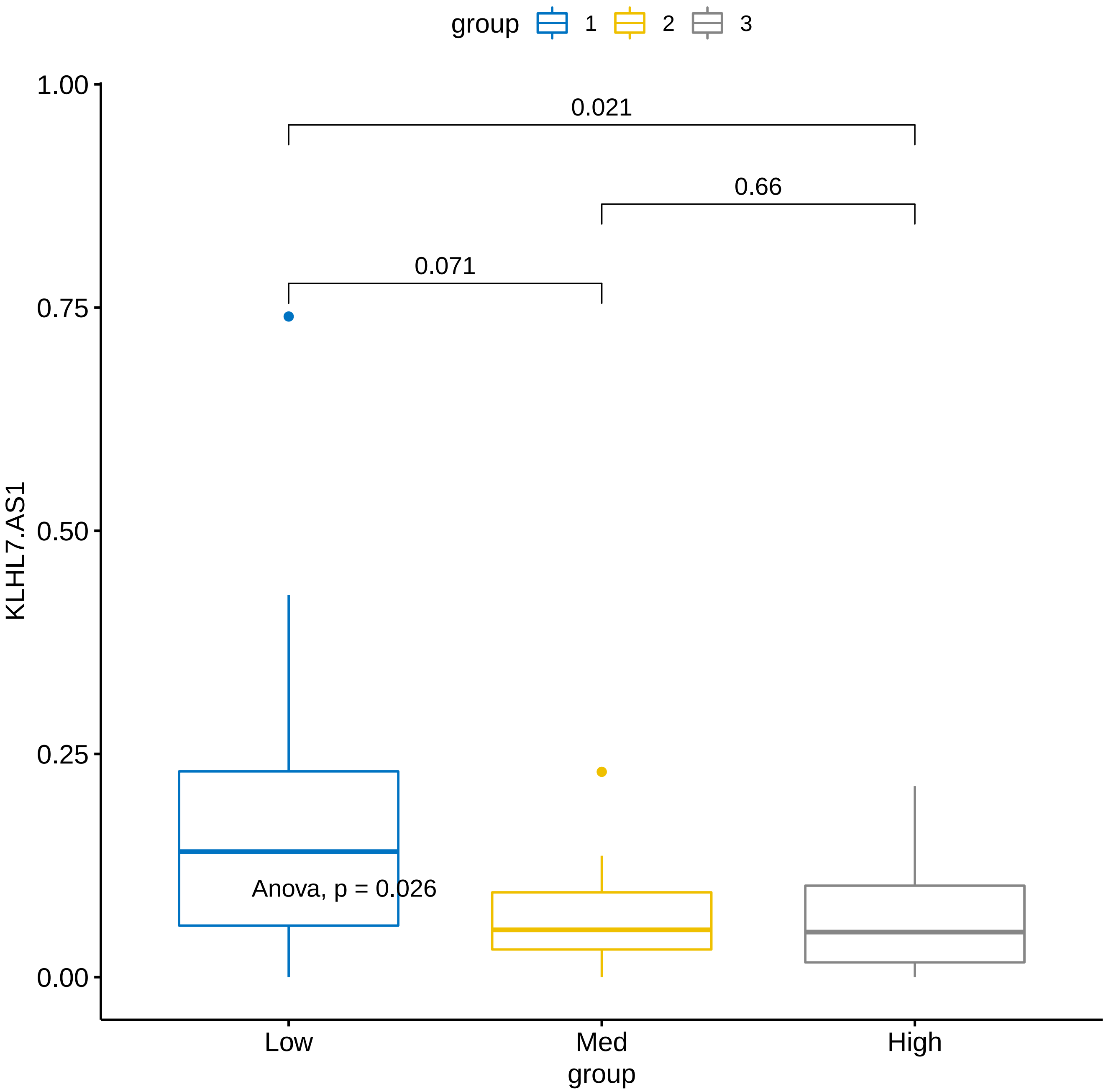

group 1 2 3
1.00
0.75
0.50
0.25
0.00
KLHL7.AS1
0.021
0.66
0.071
Anova, p = 0.026
Low
Med
High
group

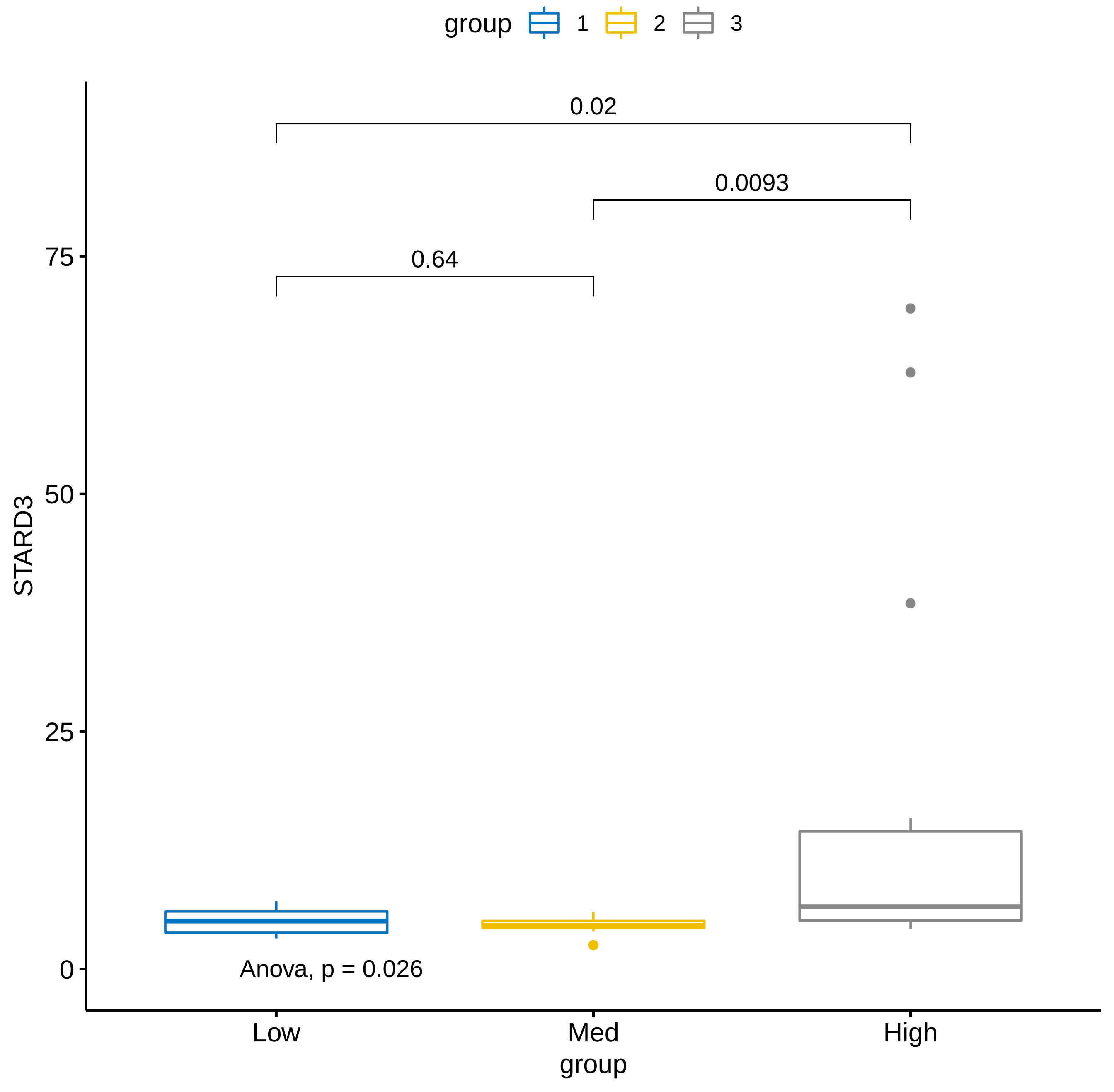
group
1
2
3
0.02
0.0093
0.64
STARD3
75
50
25
0
Anova, p = 0.026
Low
Med
High
group

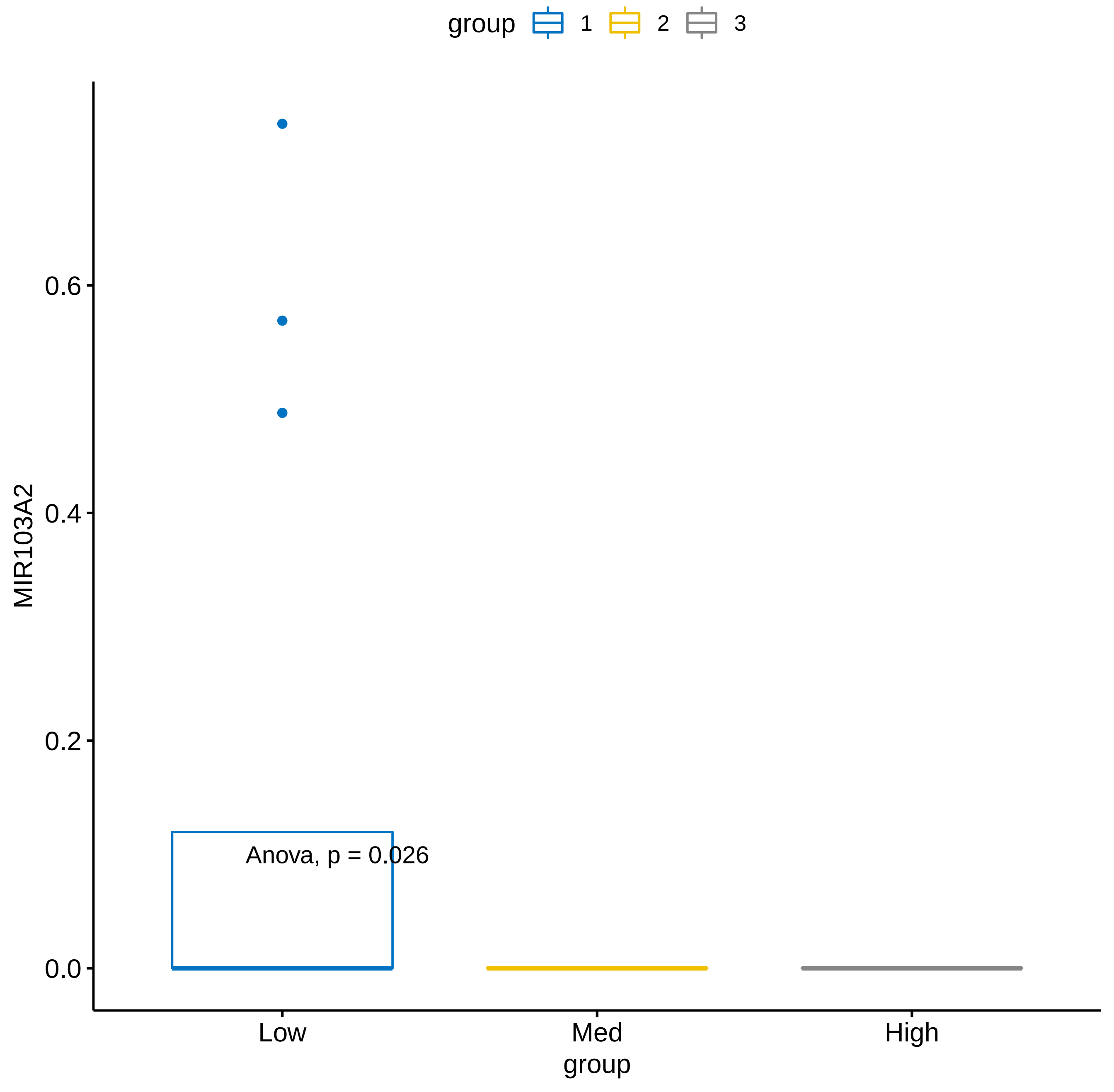

group 1 2 3
MIR103A2
0.6
0.4
0.2
0.0
Anova, p = 0.026
Low
Med
High
group

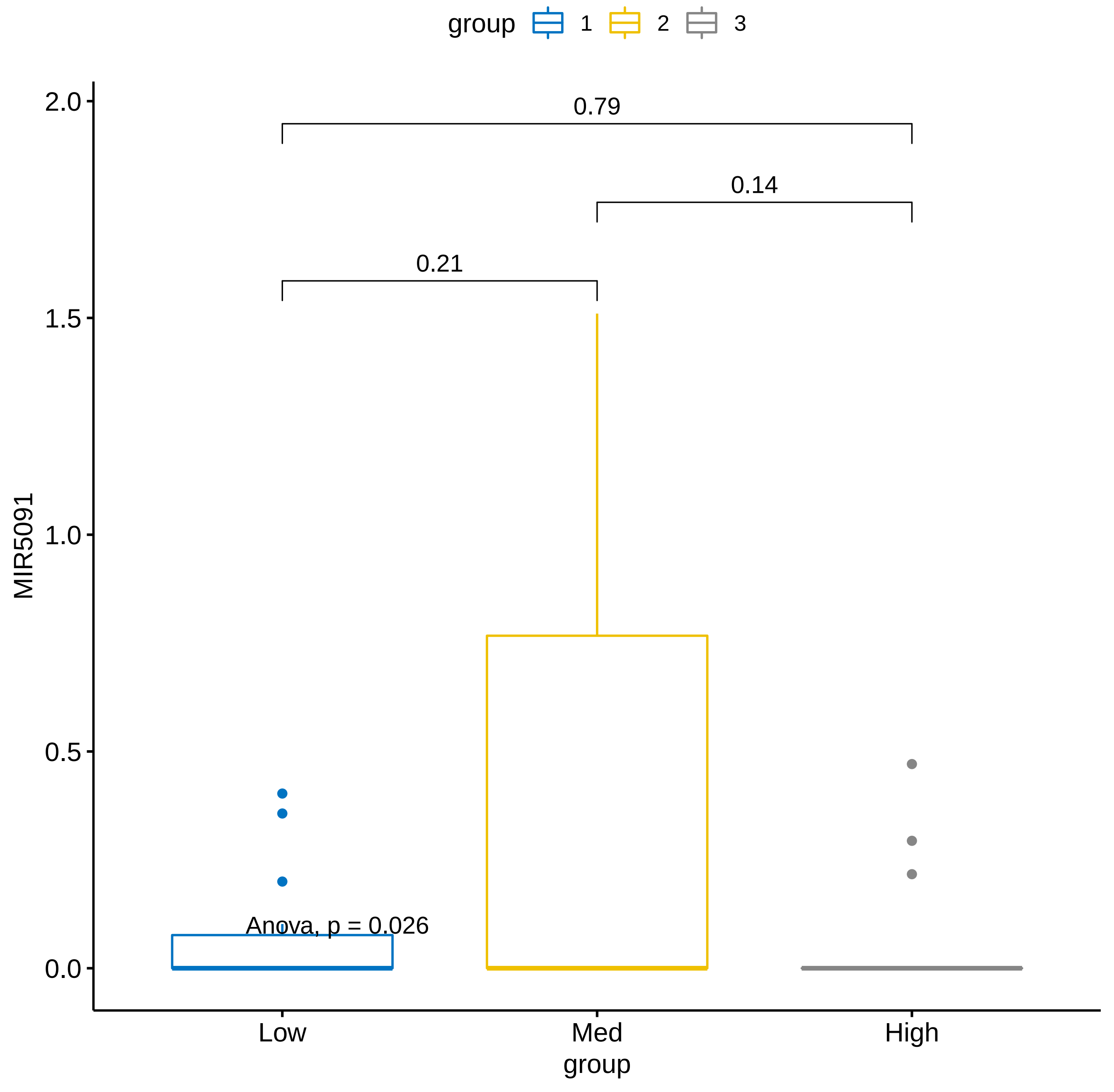
group
1
2
3
2.0
1.5
1.0
0.5
0.0
MIR5091
0.79
0.14
0.21
Anova, p = 0.026
Low
Med
High
group

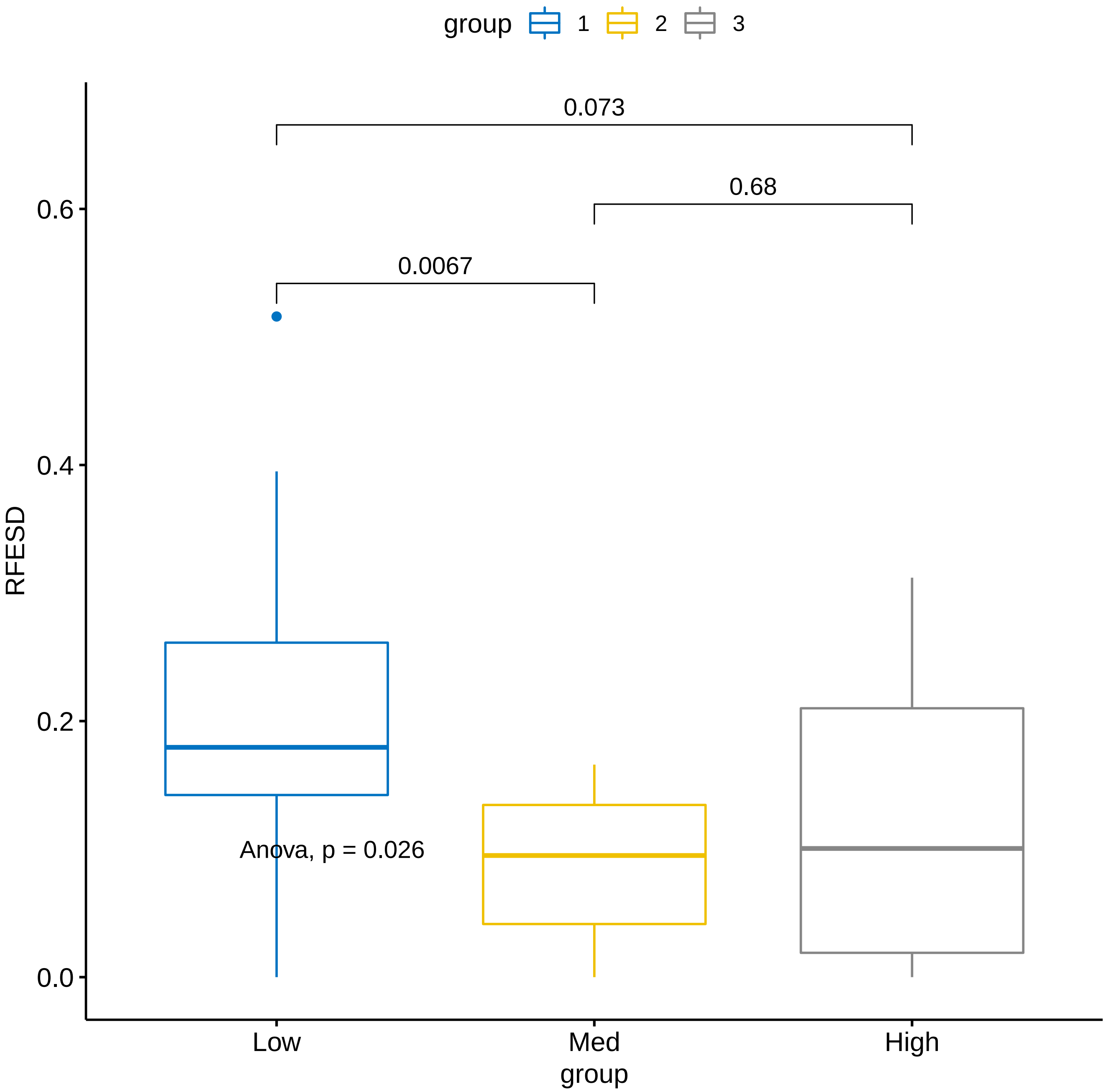
group
1
2
3
0.073
0.68
0.0067
0.6
0.4
0.2
0.0
RFESD
Anova, p = 0.026
Low
Med
High
group

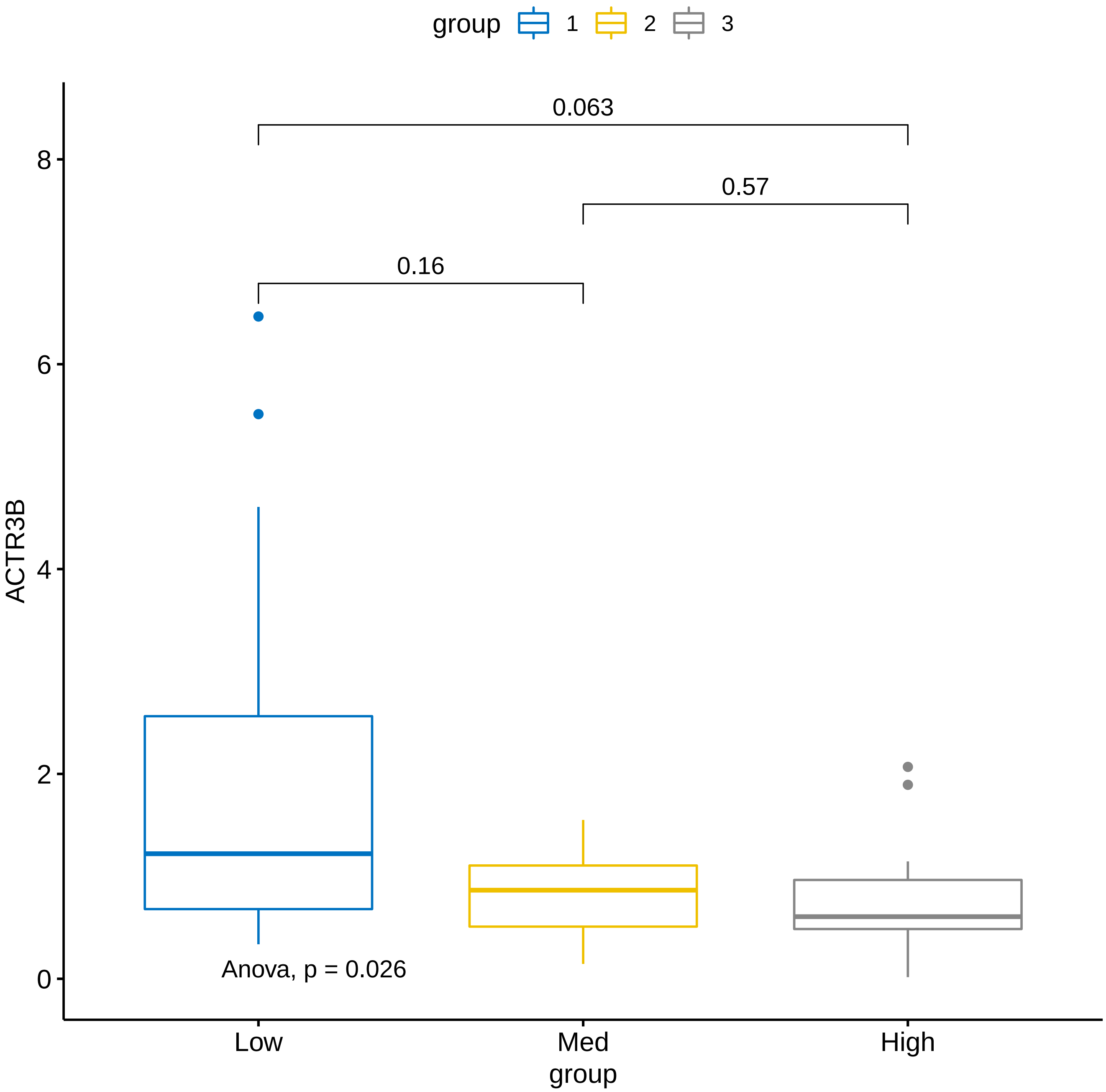
group
1
2
3
0.063
0.57
0.16
ACTR3B
8
6
4
2
0
Anova, p = 0.026
Low
Med
High
group

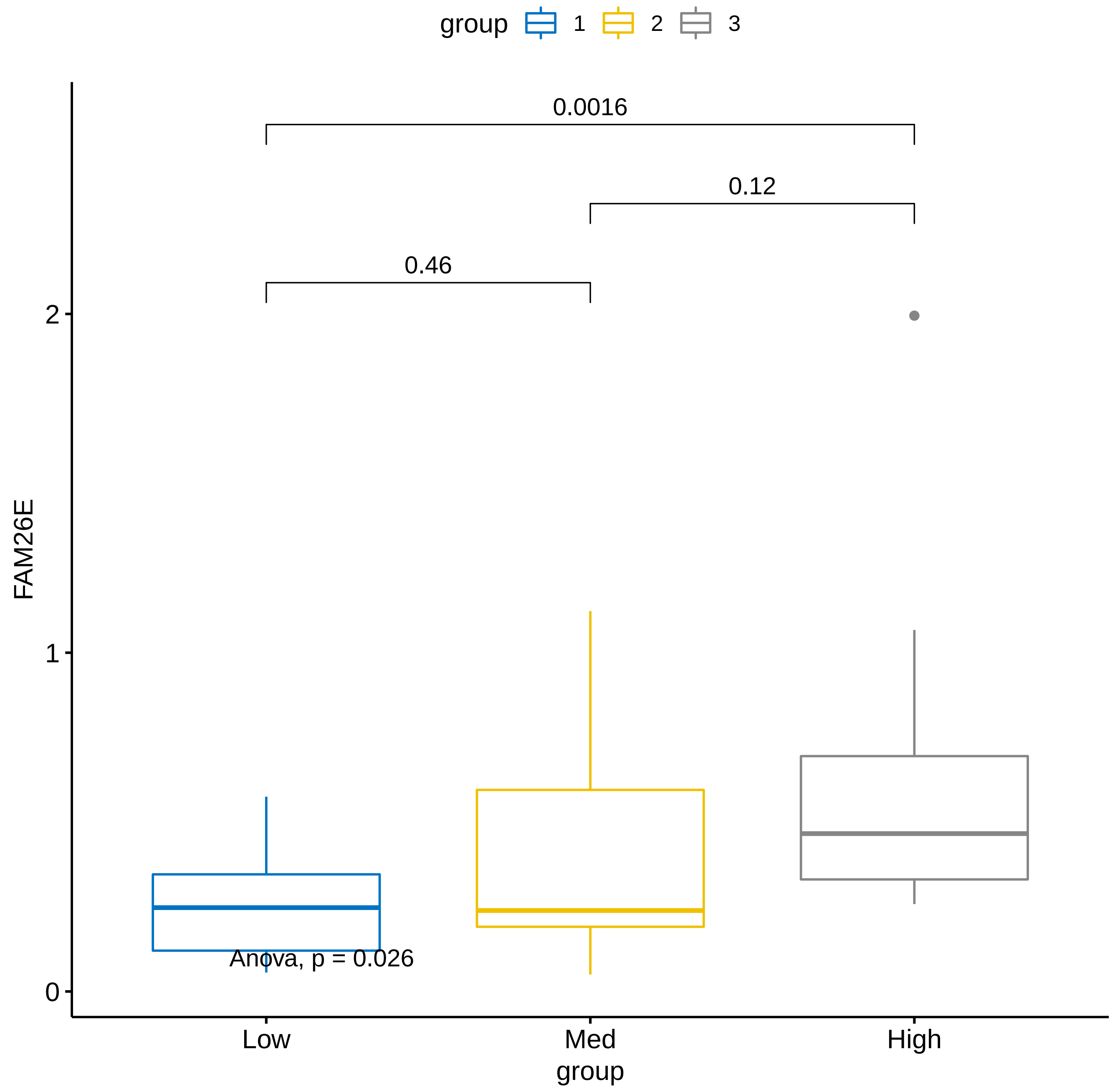

group 1 2 3
0.0016
0.12
0.46
FAM26E
2
1
0
Anova, p = 0.026
Low
Med
High
group

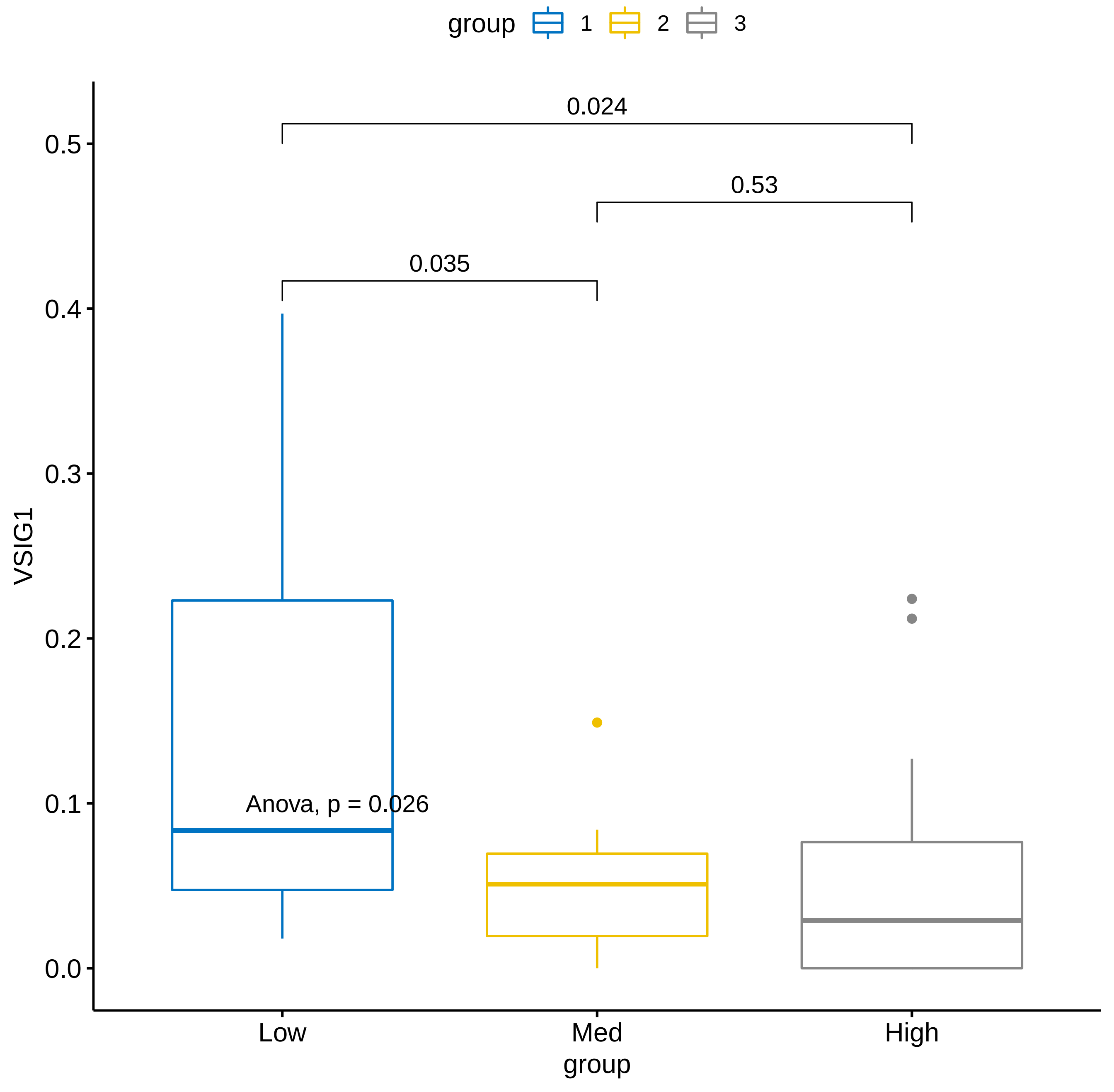
group
1
2
3
0.024
0.53
0.035
Anova, p = 0.026
VSIG1
0.0
0.1
0.2
0.3
0.4
0.5
Low
Med
High
group

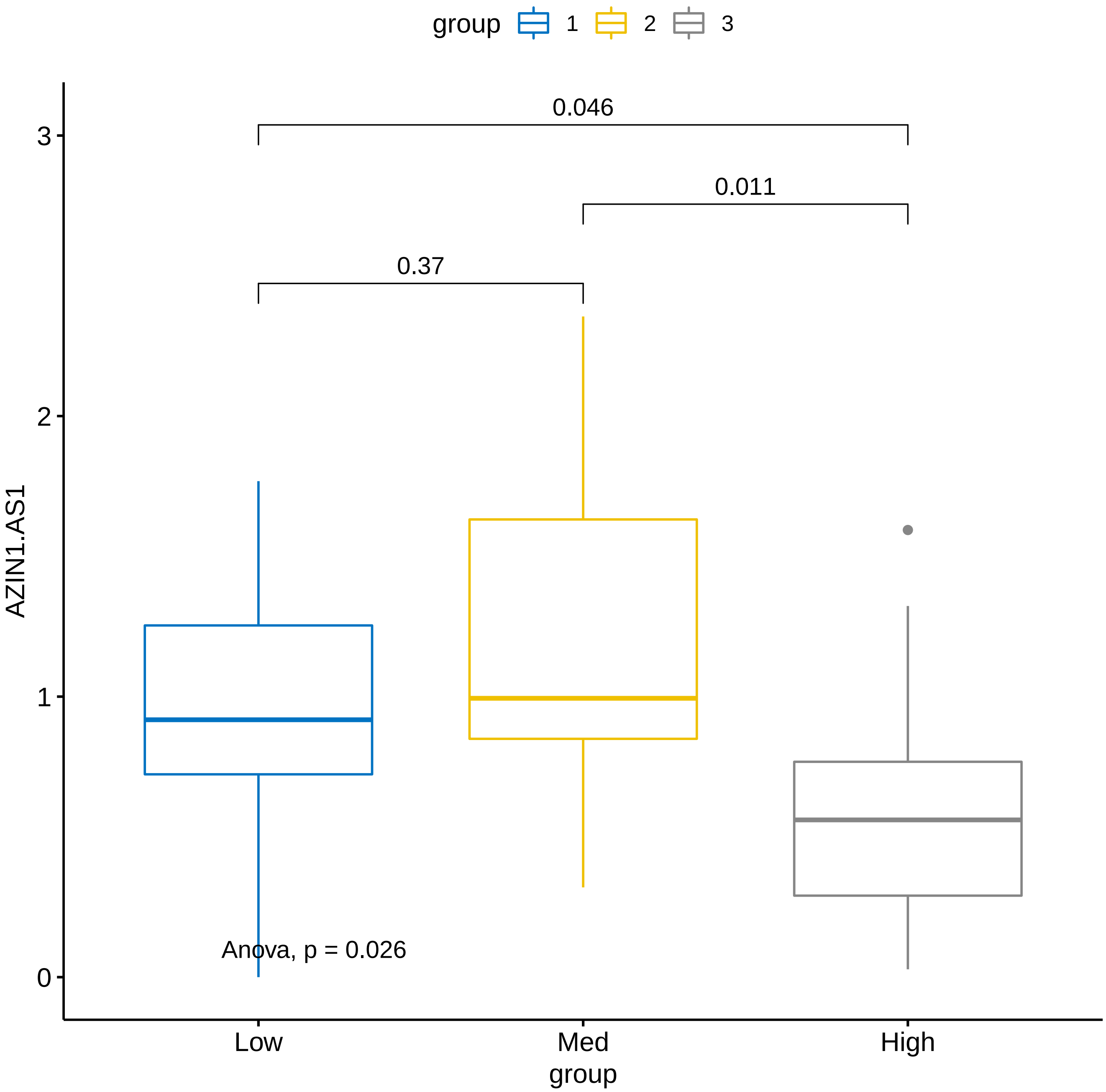

group 1 2 3
0.046
0.011
0.37
AZIN1.AS1
3
2
1
0
Anova, p = 0.026
Low
Med
High
group

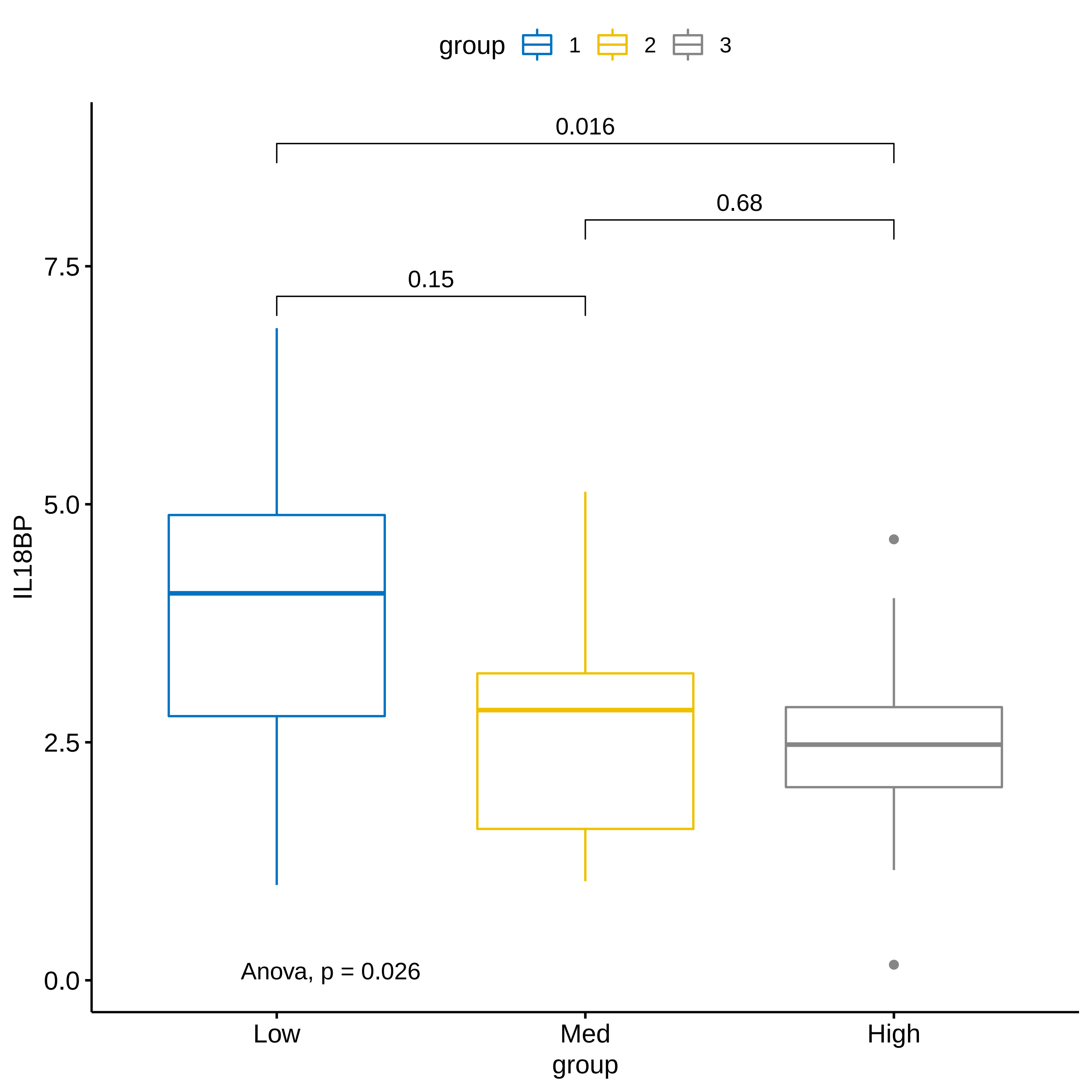
group
1
2
3
0.016
0.68
0.15
7.5
5.0
2.5
0.0
IL18BP
Anova, p = 0.026
Low
Med
High
group

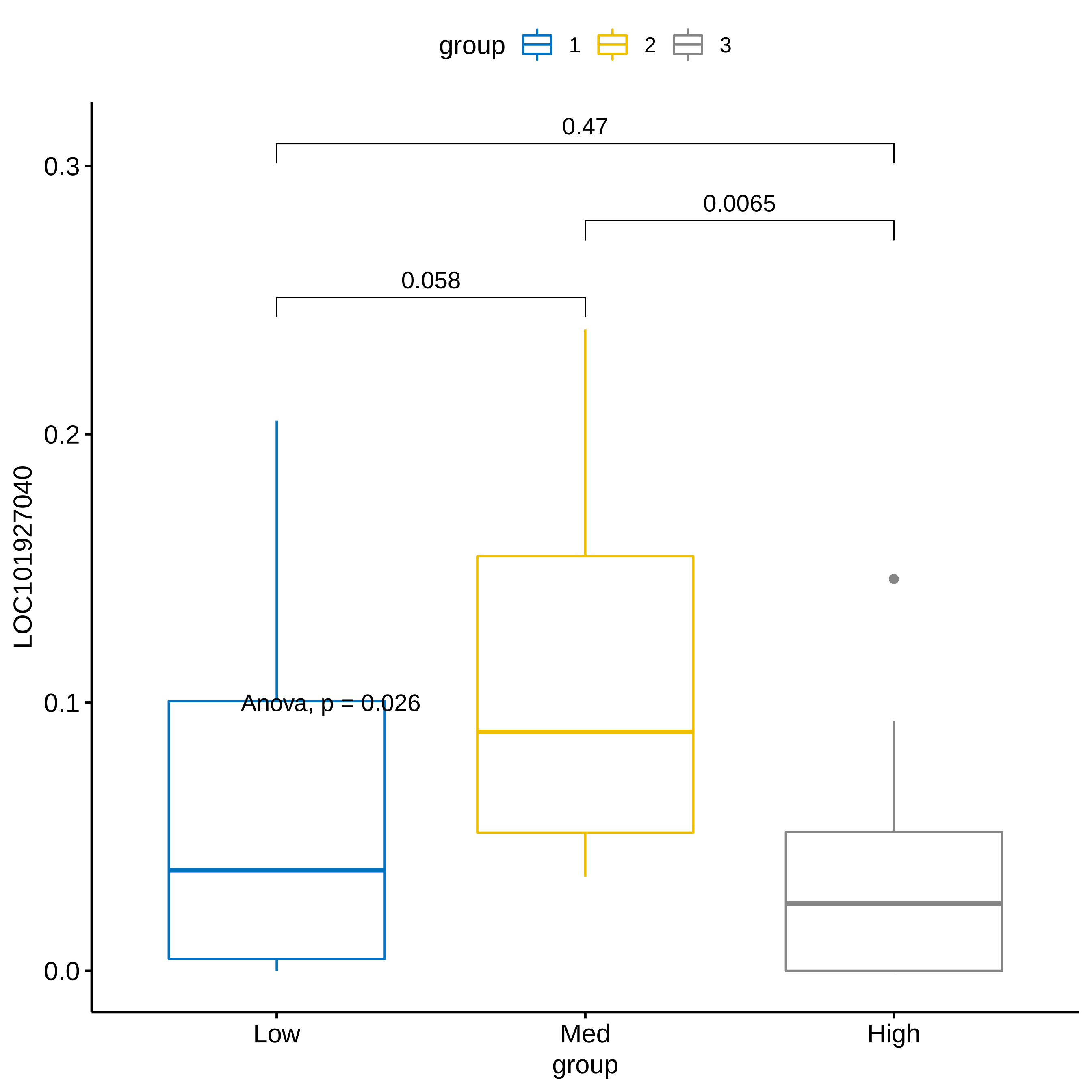
group
1
2
3
0.47
0.0065
0.058
Anova, p = 0.026
0.3
0.2
0.1
0.0
LOC101927040
Low
Med
High
group

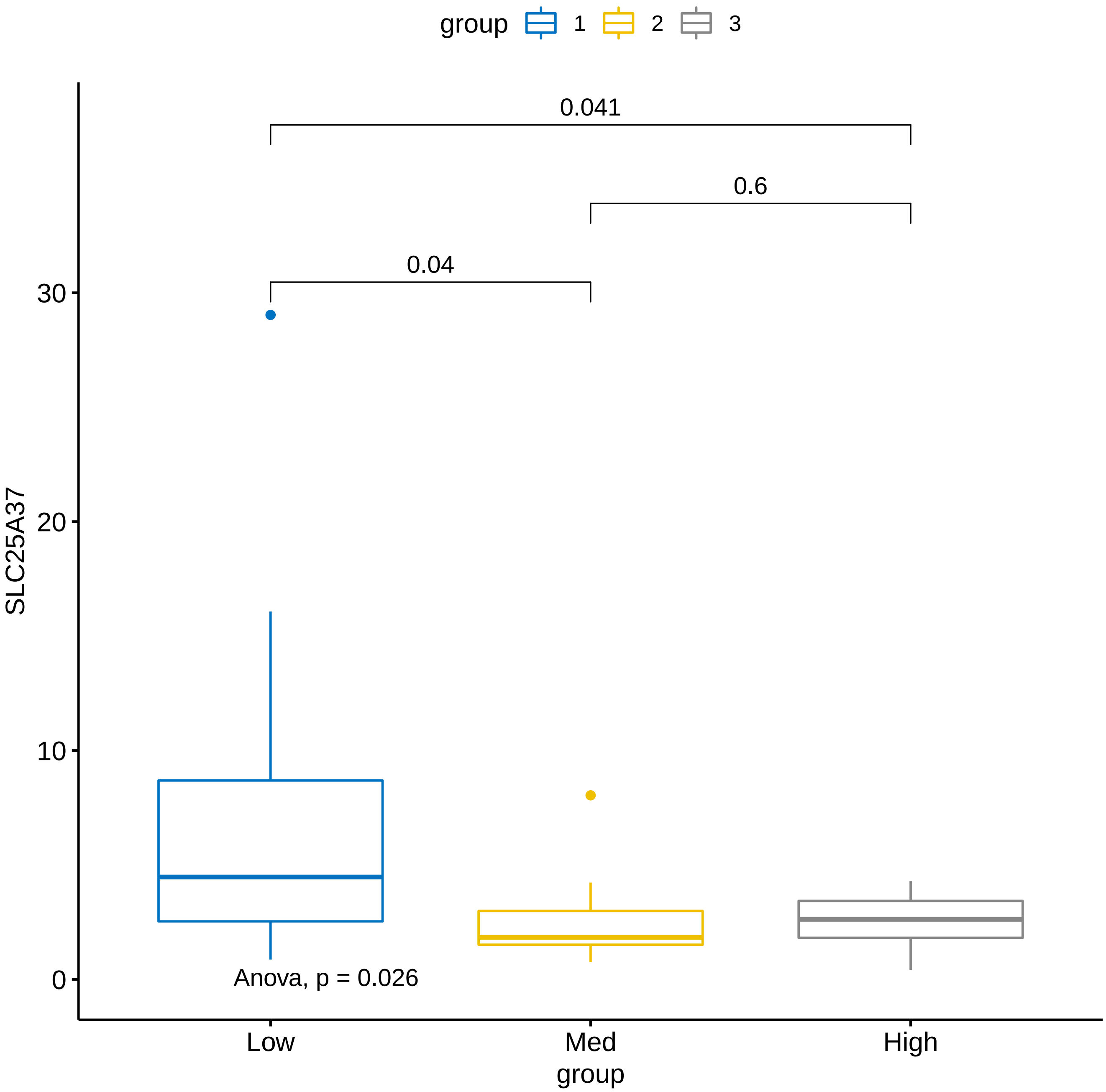

group
1
2
3
0.041
0.6
0.04
30
20
10
0
SLC25A37
Anova, p = 0.026
Low
Med
High
group

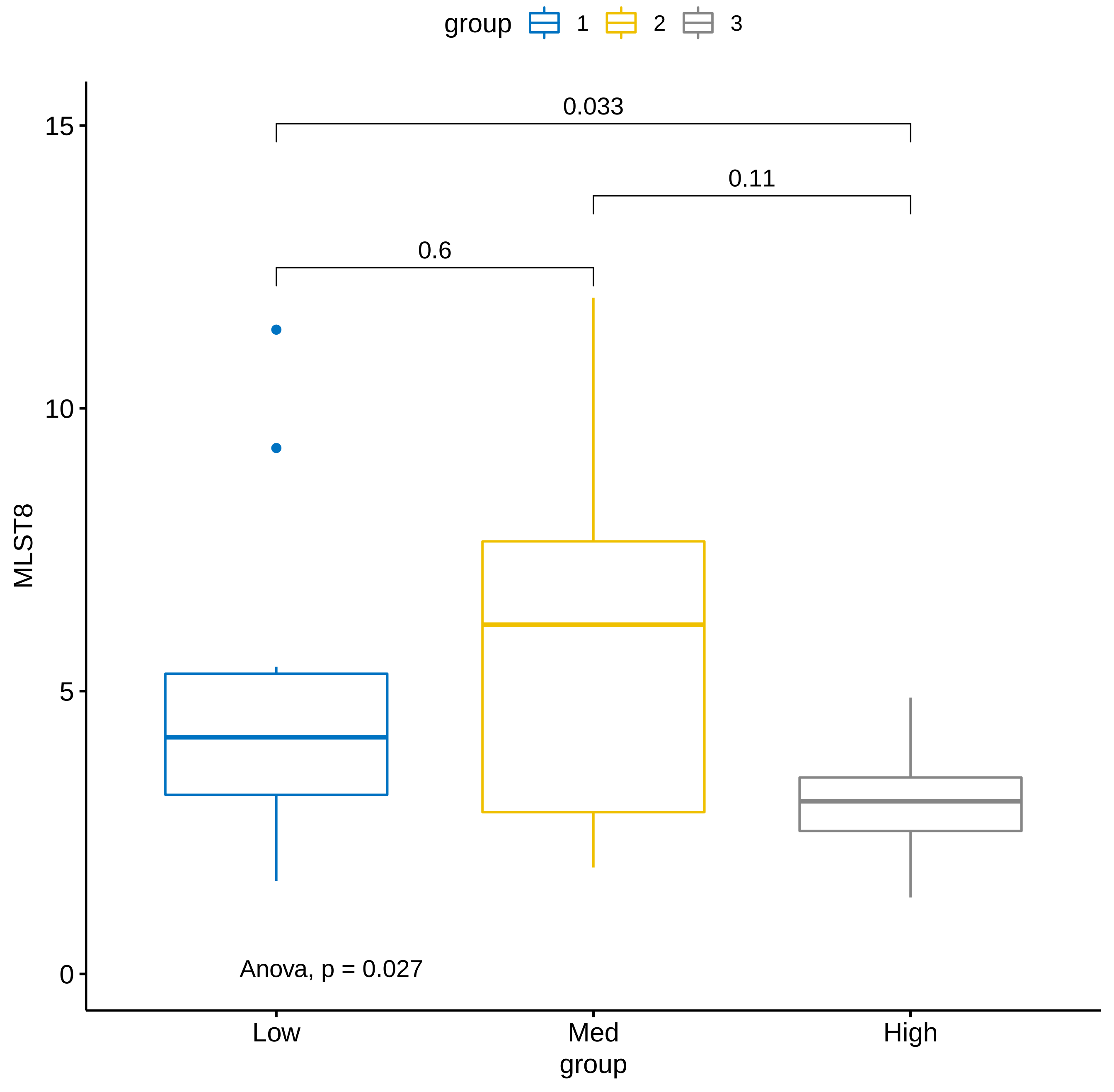
group
1
2
3
0.033
0.11
0.6
15
10
5
0
MLST8
Anova, p = 0.027
Low
Med
High
group

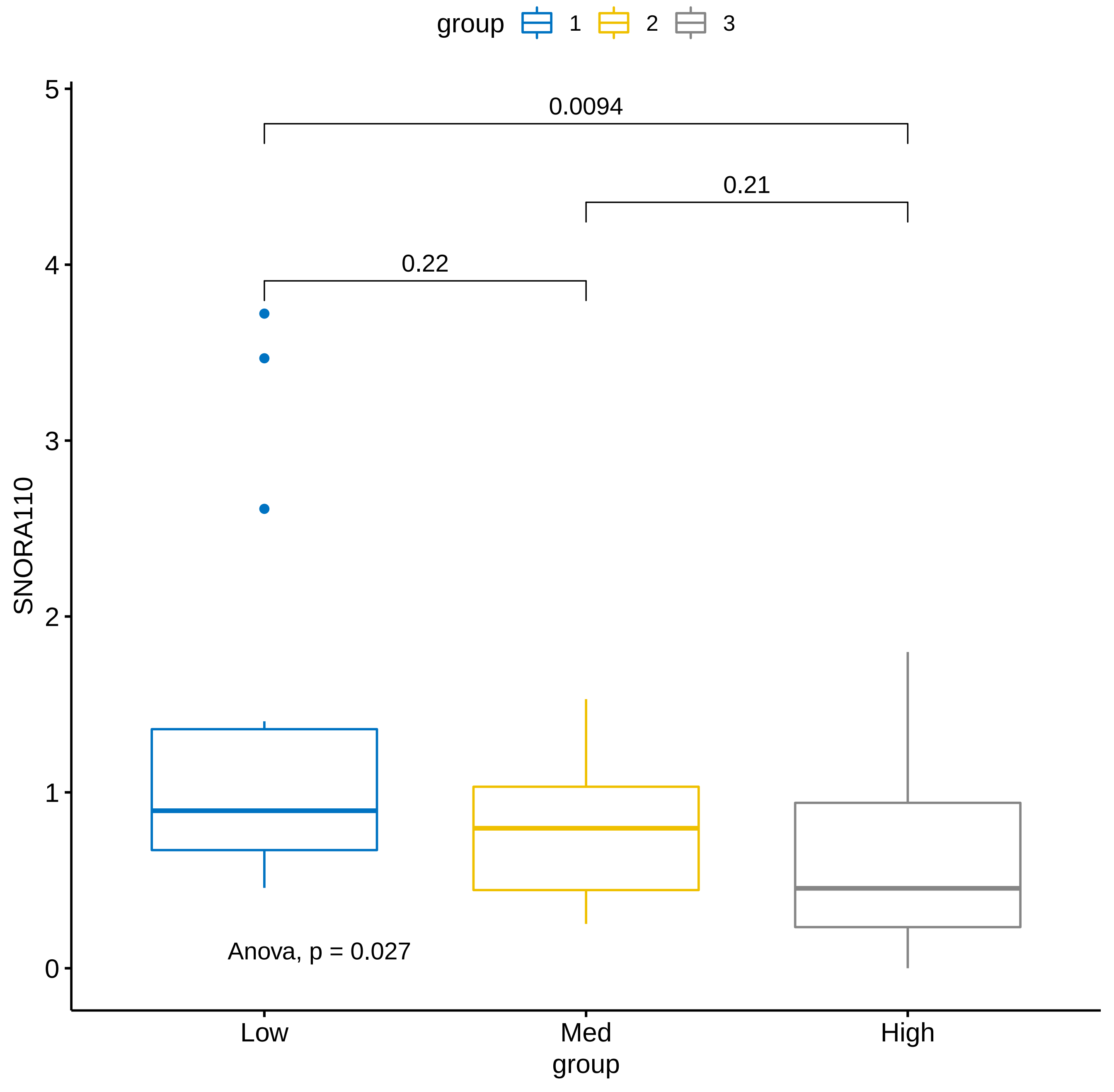
group 1 2 3
0.0094
0.21
0.22
5
4
3
2
1
0
SNORA110
Anova, p = 0.027
Low
Med
High
group

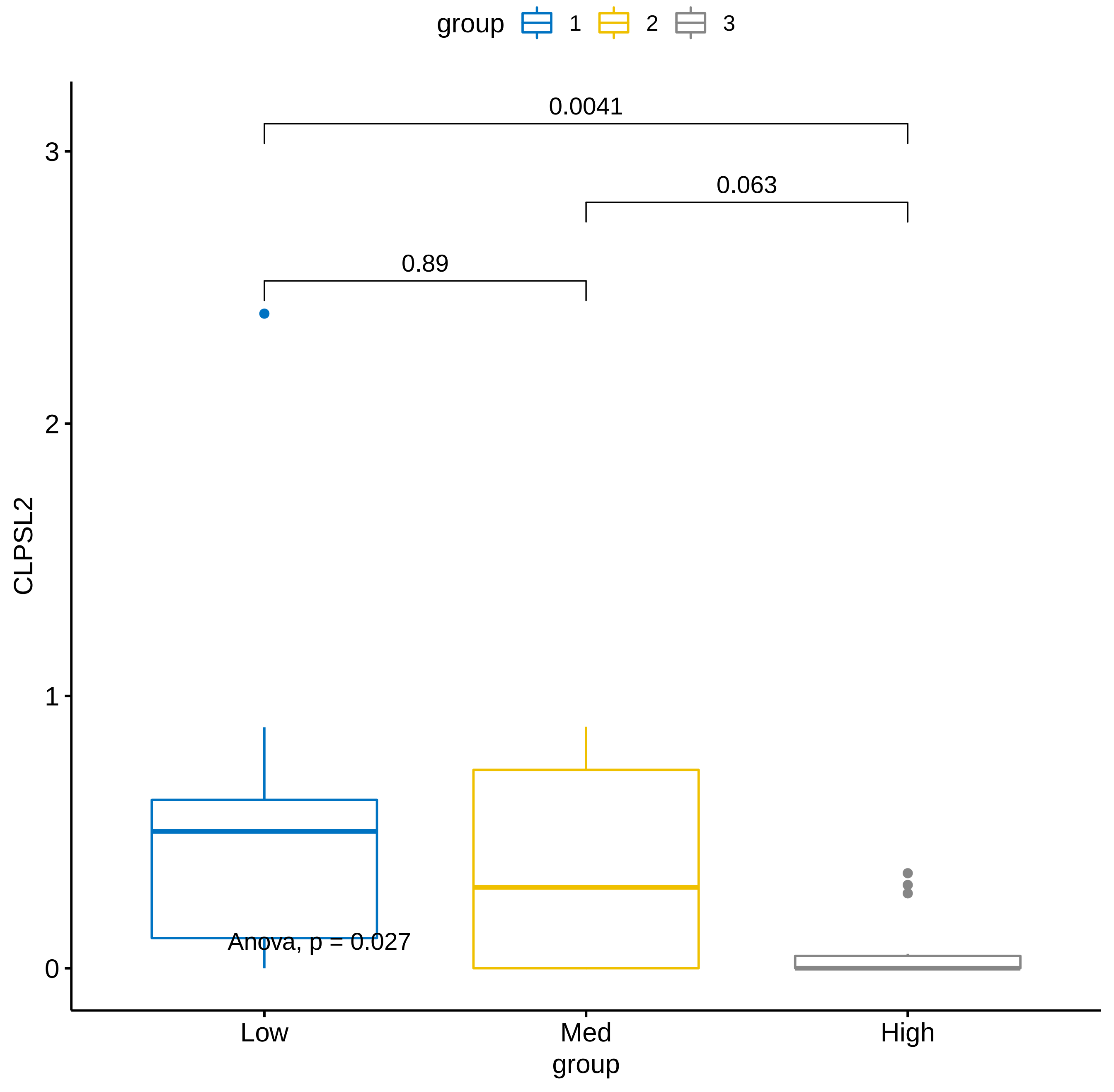
group
1
2
3
0.0041
0.063
0.89
3
2
1
0
CLPSL2
Anova, p = 0.027
Low
Med
High
group

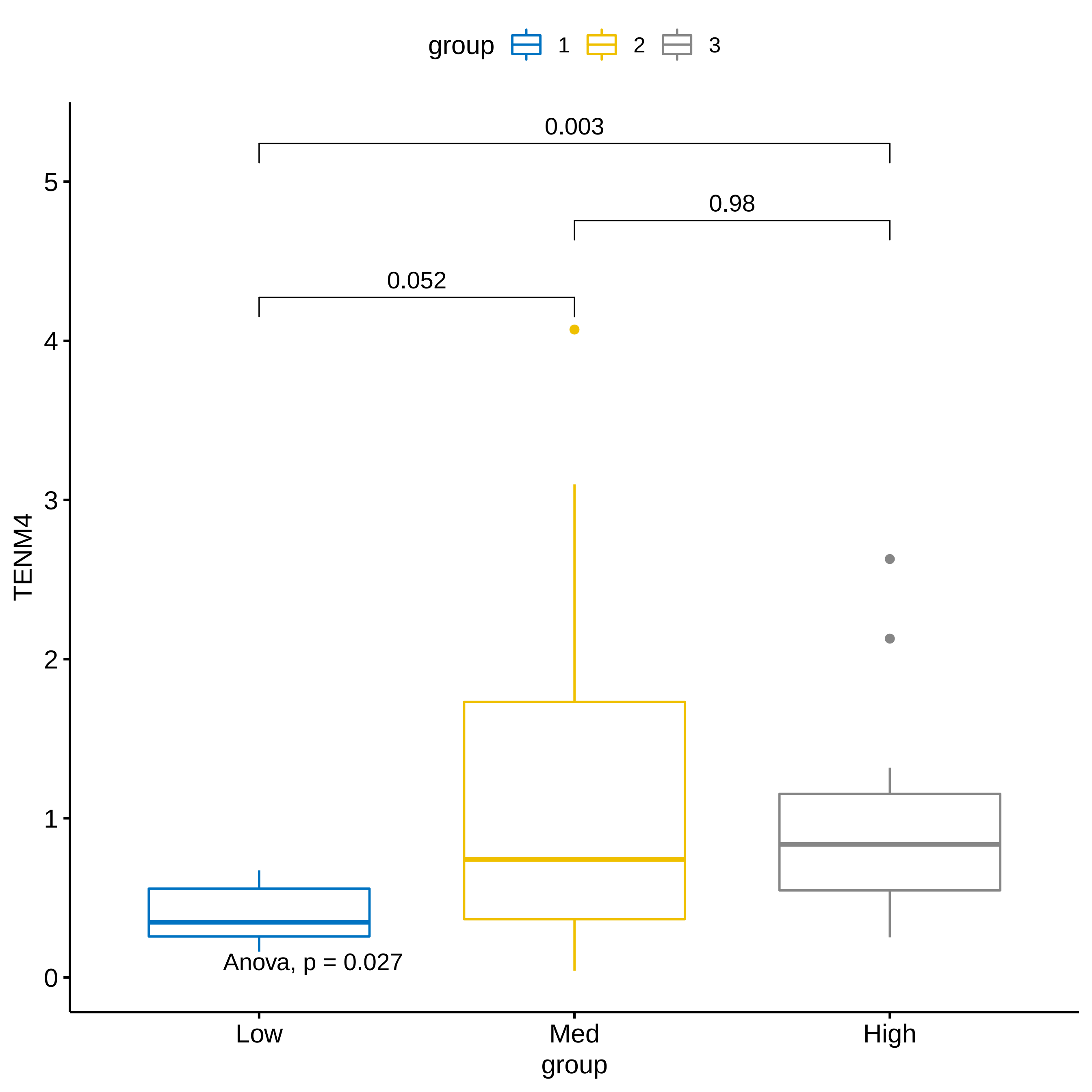
group
1
2
3
0.003
0.98
0.052
5
4
3
2
1
0
TENM4
Anova, p = 0.027
Low
Med
High
group

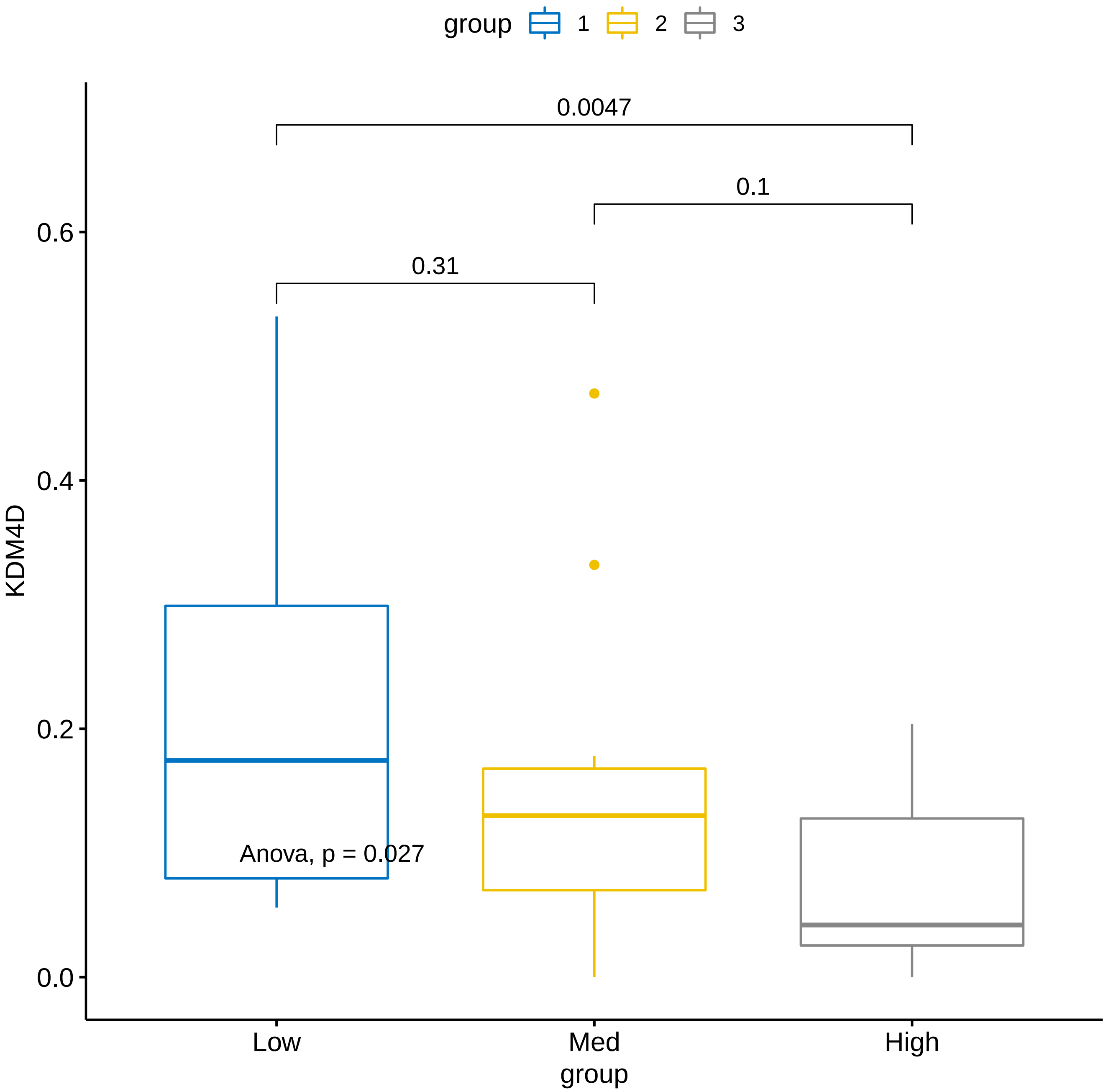

group 1 2 3
0.0047
0.1
0.31
Anova, p = 0.027
KDM4D
0.6
0.4
0.2
0.0
Low
Med
High
group

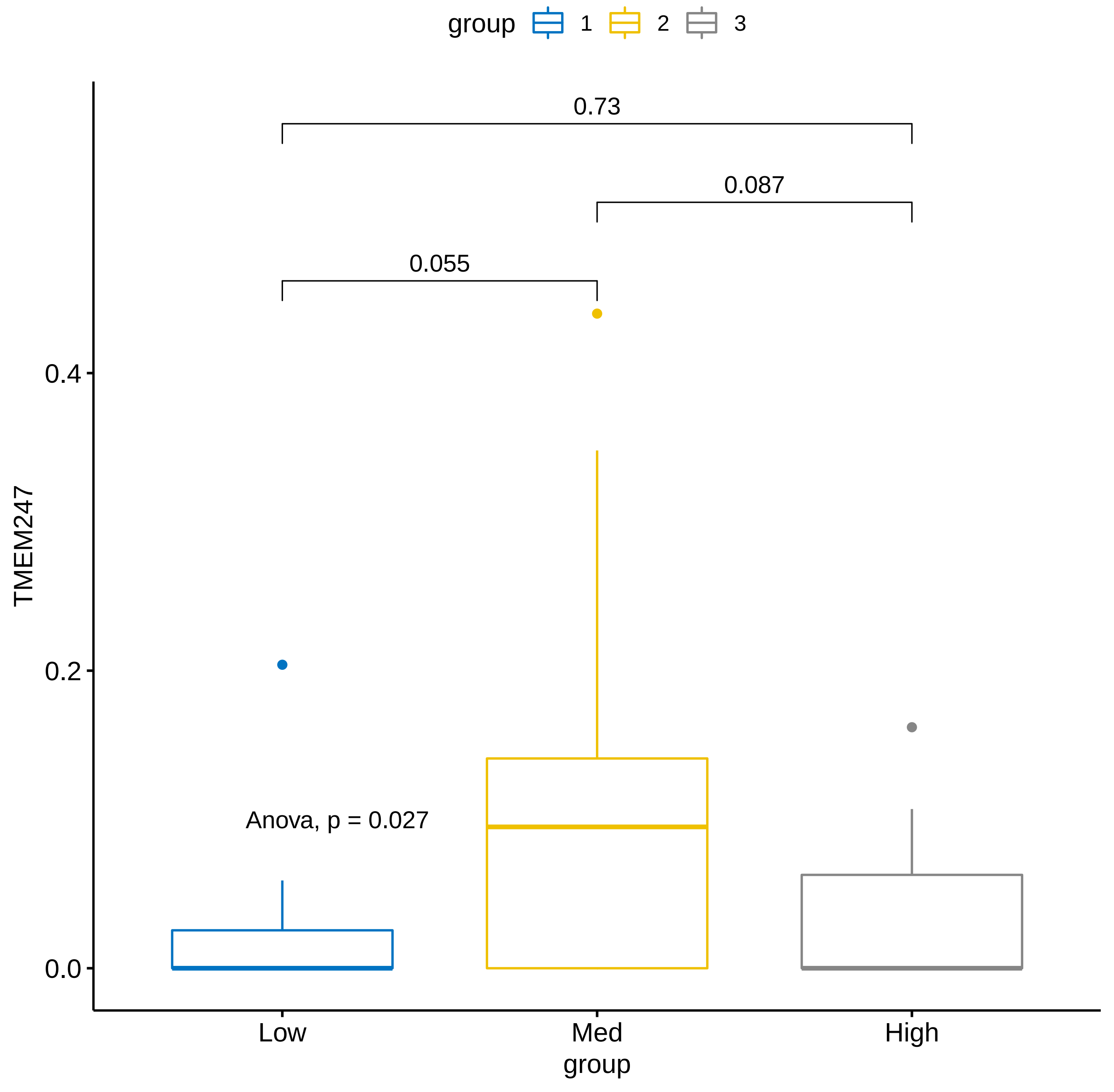
group
1
2
3
0.73
0.087
0.055
0.4
0.2
0.0
TMEM247
Anova, p = 0.027
Low
Med
High
group

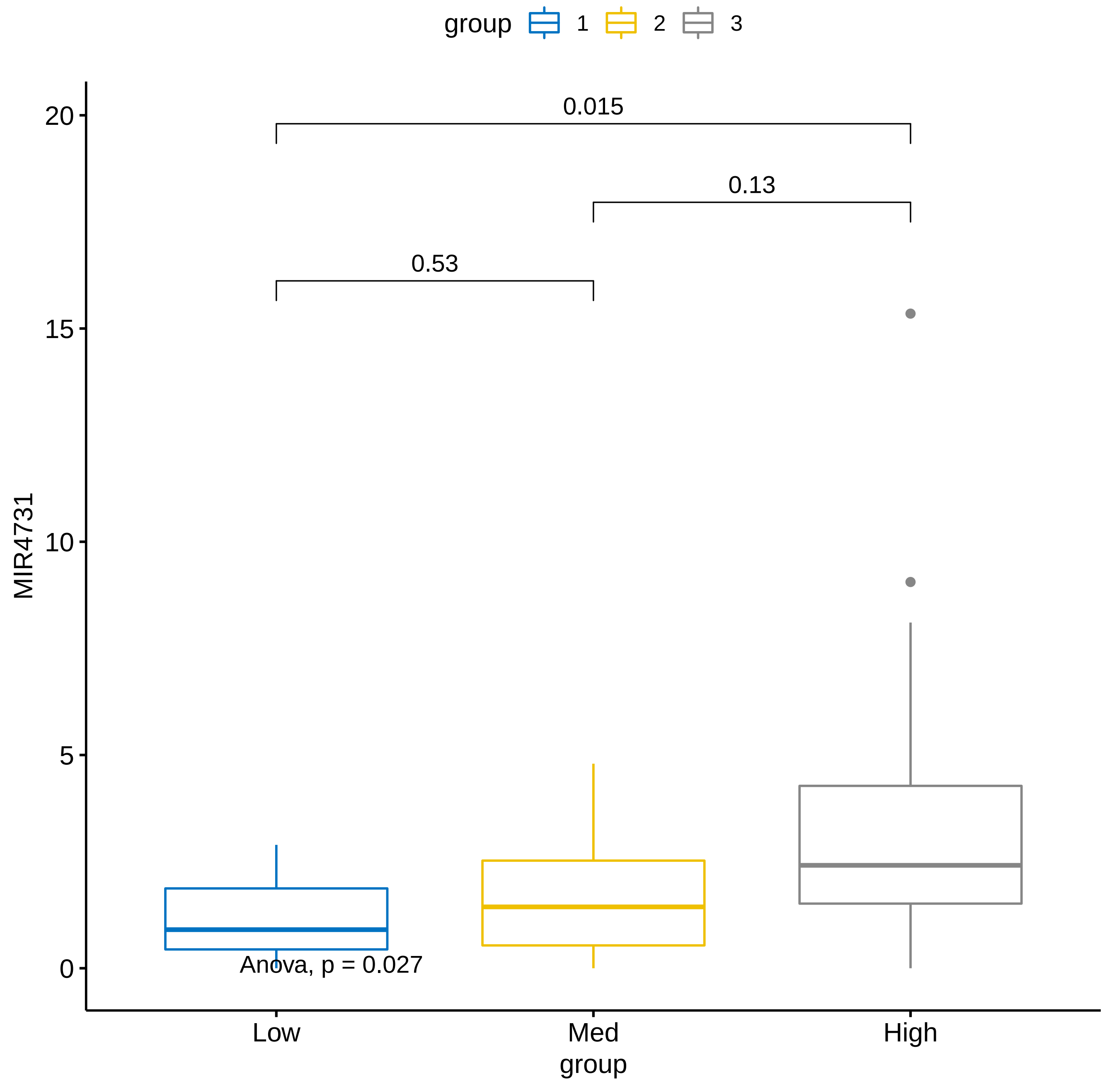

group 1 2 3
0.015
0.13
0.53
MIR4731
20
15
10
5
0
Anova, p = 0.027
Low
Med
High
group

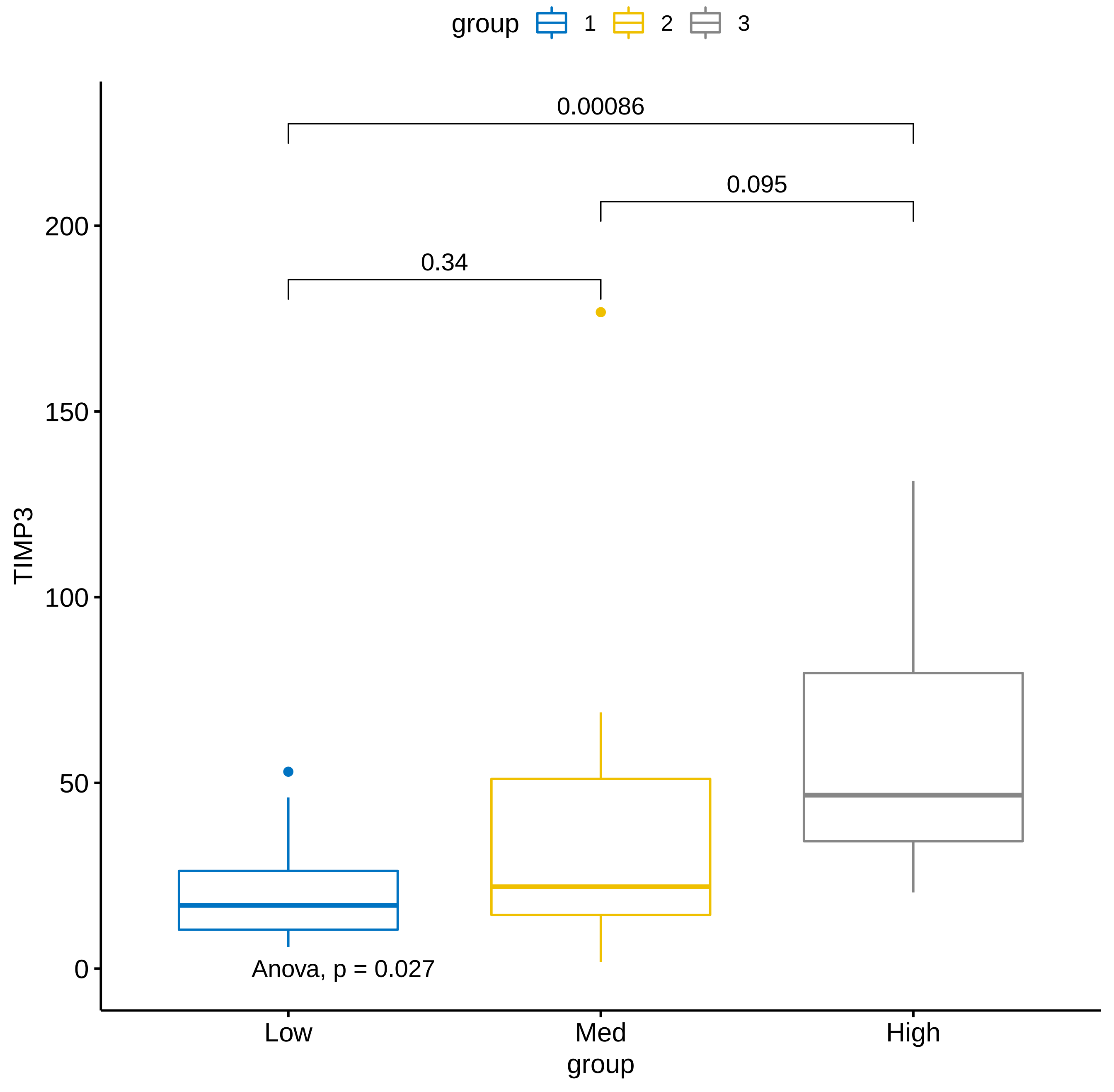
group
1
2
3
0.00086
0.095
0.34
TIMP3
200
150
100
50
0
Anova, p = 0.027
Low
Med
High
group

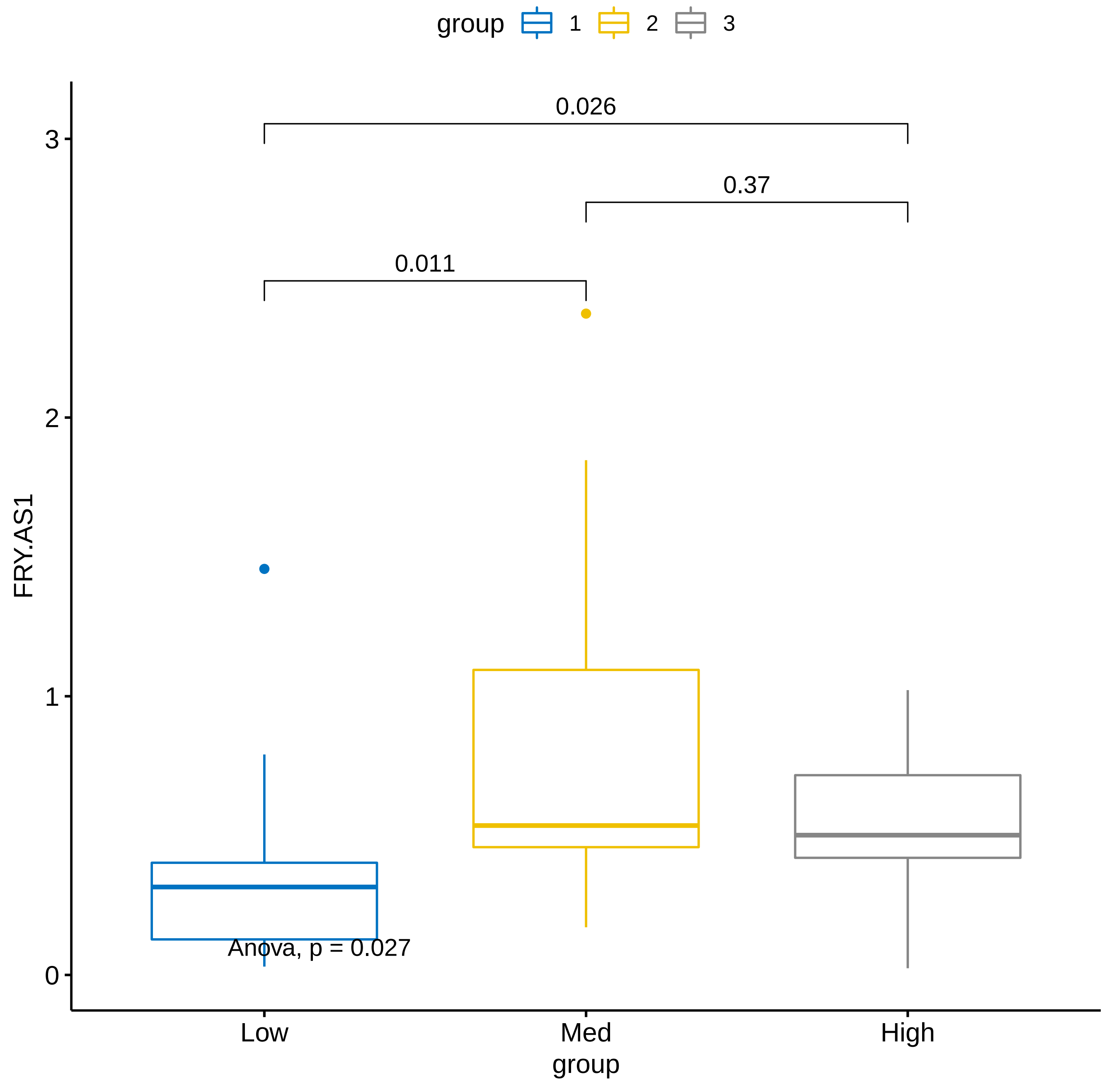
group 1 2 3
0.026
0.37
0.011
3
2
1
0
FRY.AS1
Anova, p = 0.027
Low
Med
High
group

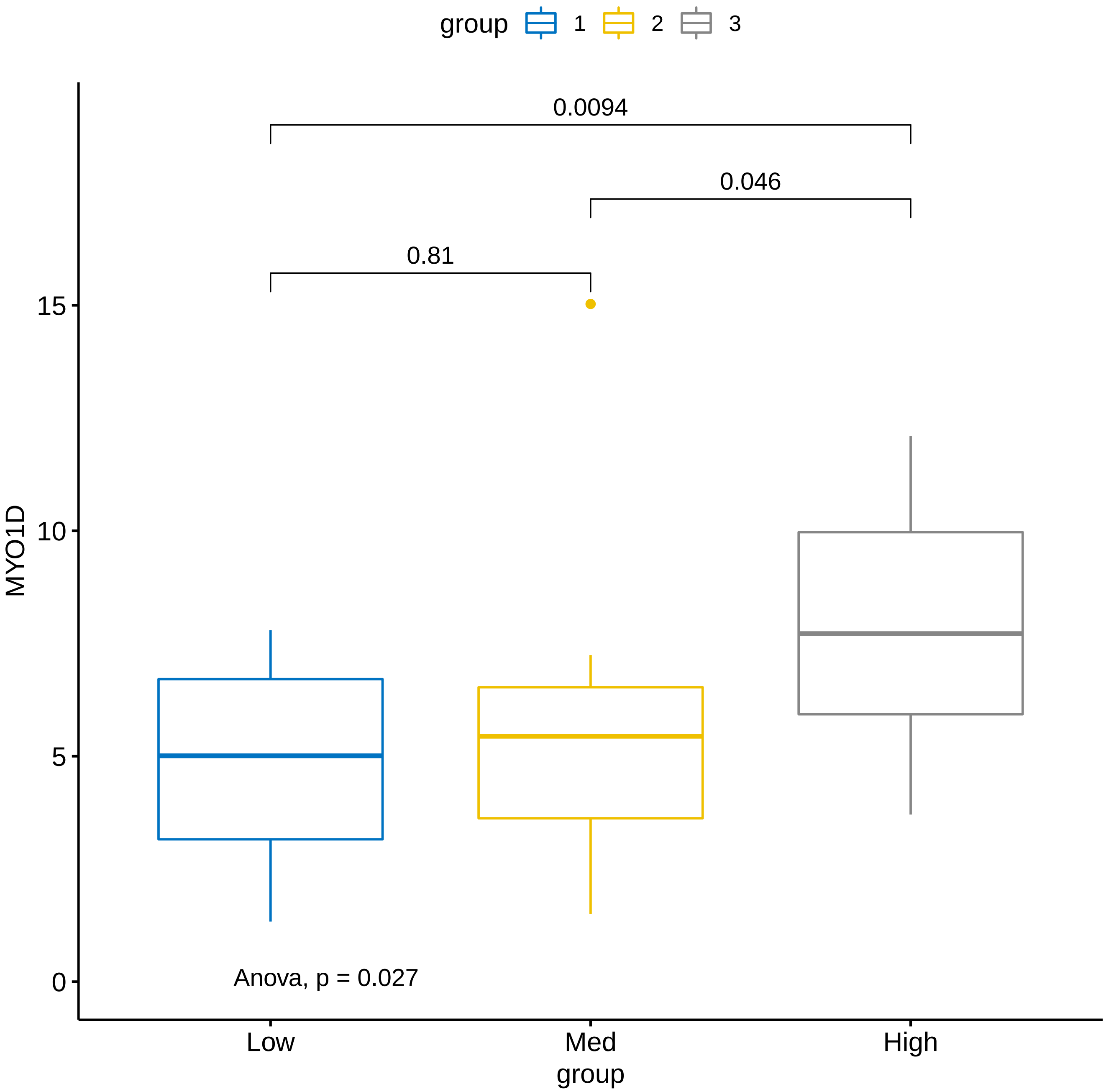
group
1
2
3
0.0094
0.046
0.81
15
10
5
0
MYO1D
Anova, p = 0.027
Low
Med
High
group

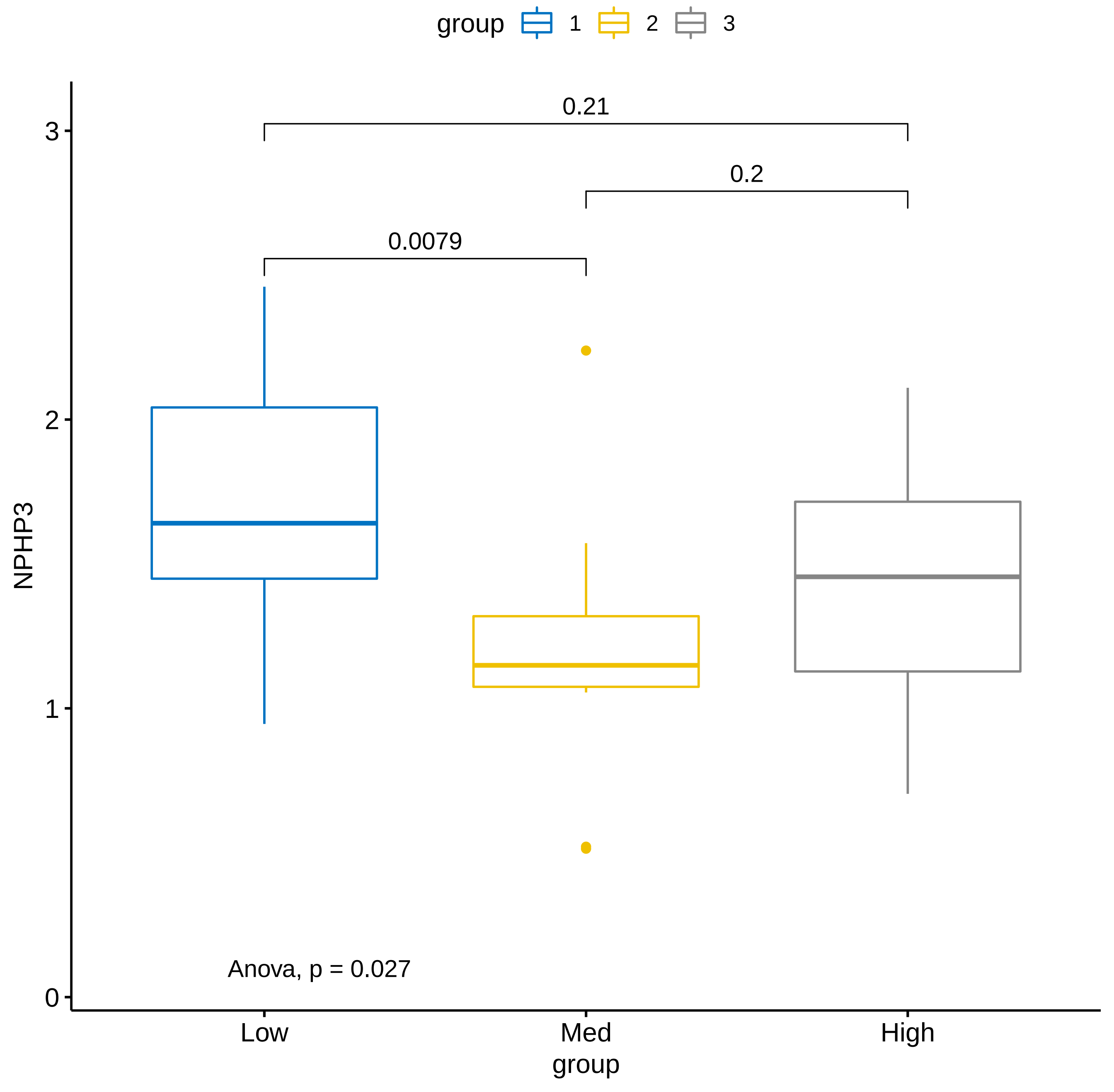

group
1
2
3
0.21
0.2
0.0079
3
2
1
0
NPHP3
Anova, p = 0.027
Low
Med
High
group

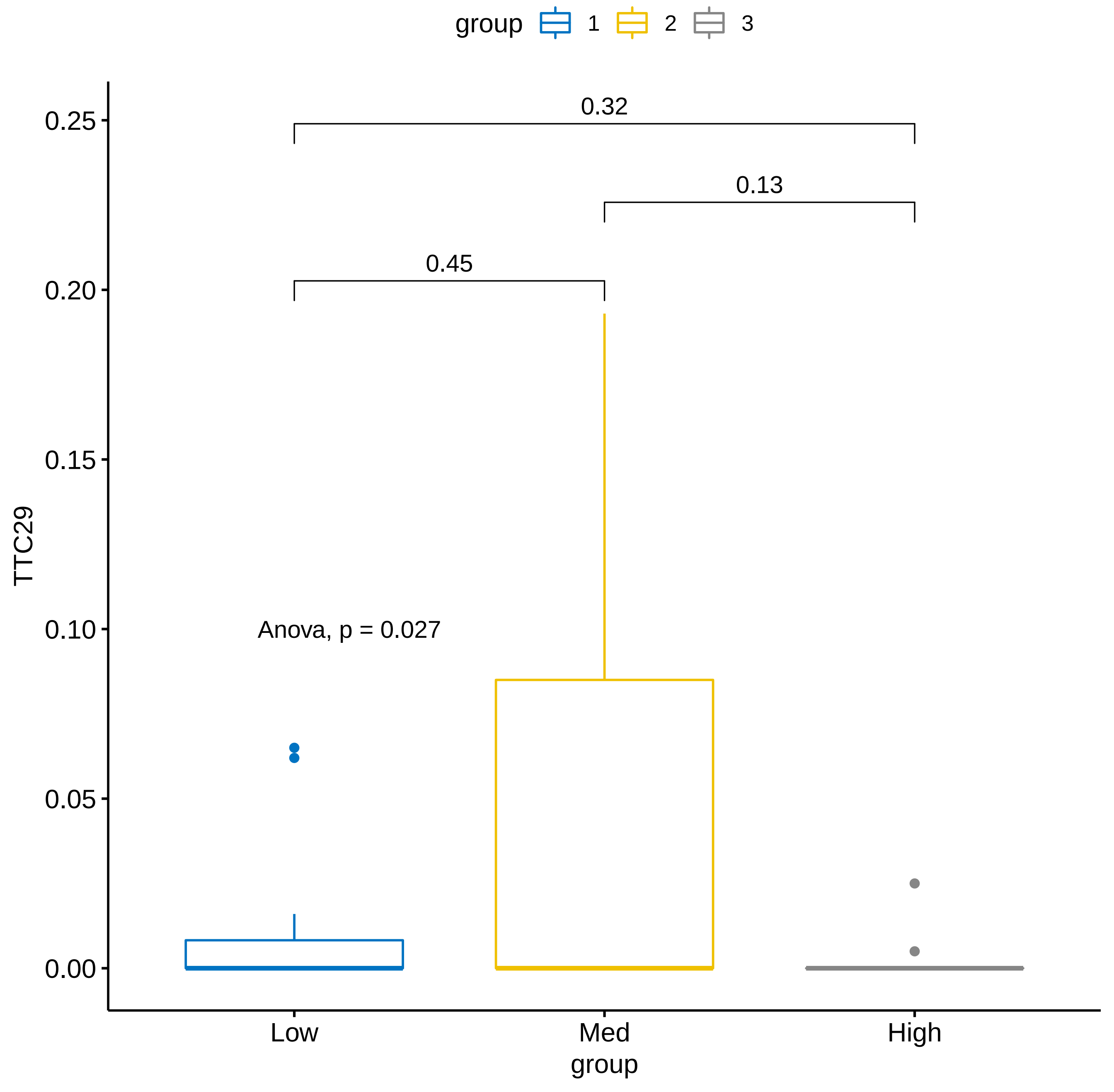
group
1
2
3
0.32
0.13
0.45
0.25
0.20
0.15
0.10
0.05
0.00
TTC29
Anova, p = 0.027
Low
Med
High
group

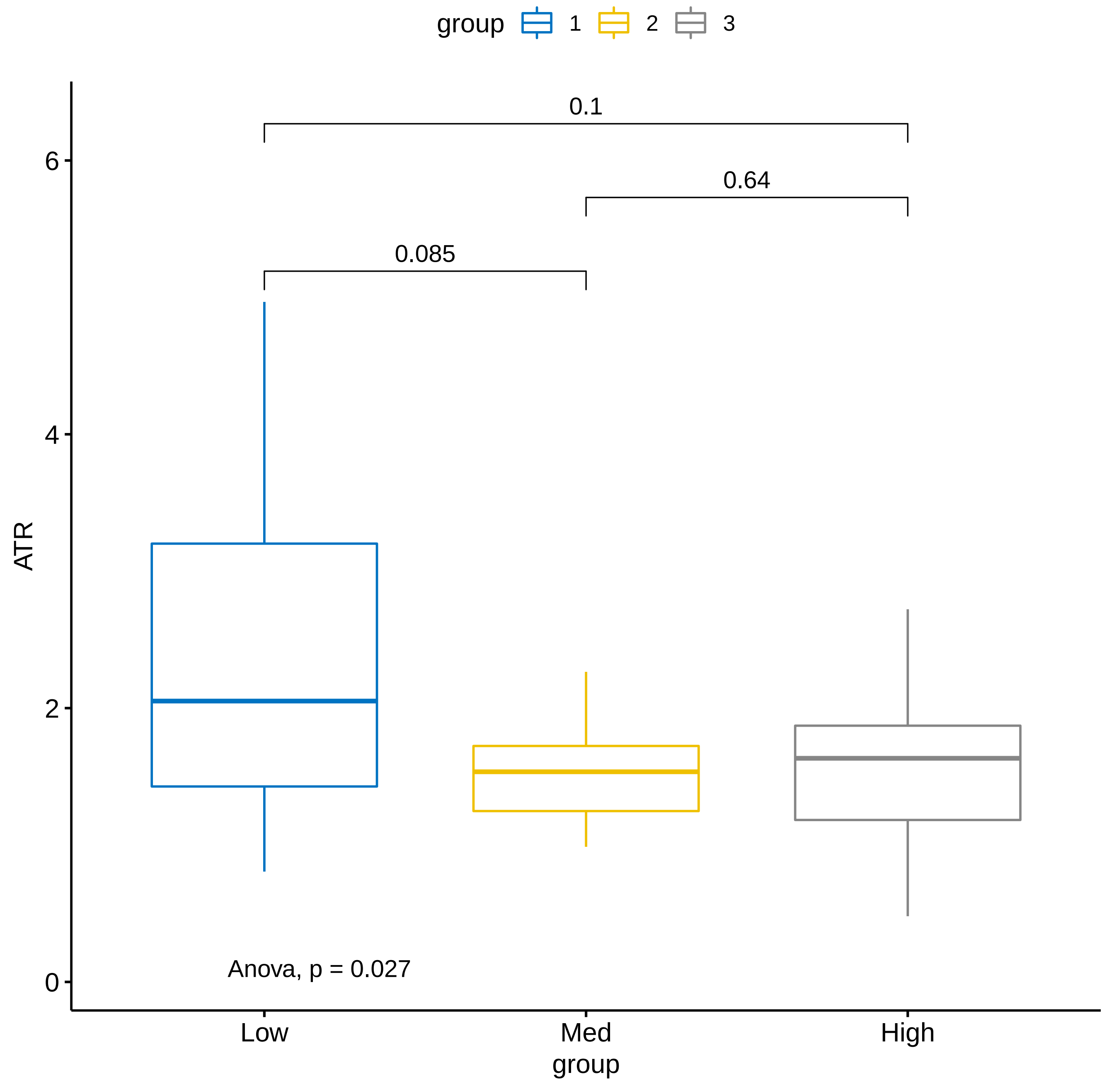
group
1
2
3
0.1
0.64
0.085
6
4
2
0
ATR
Anova, p = 0.027
Low
Med
High
group

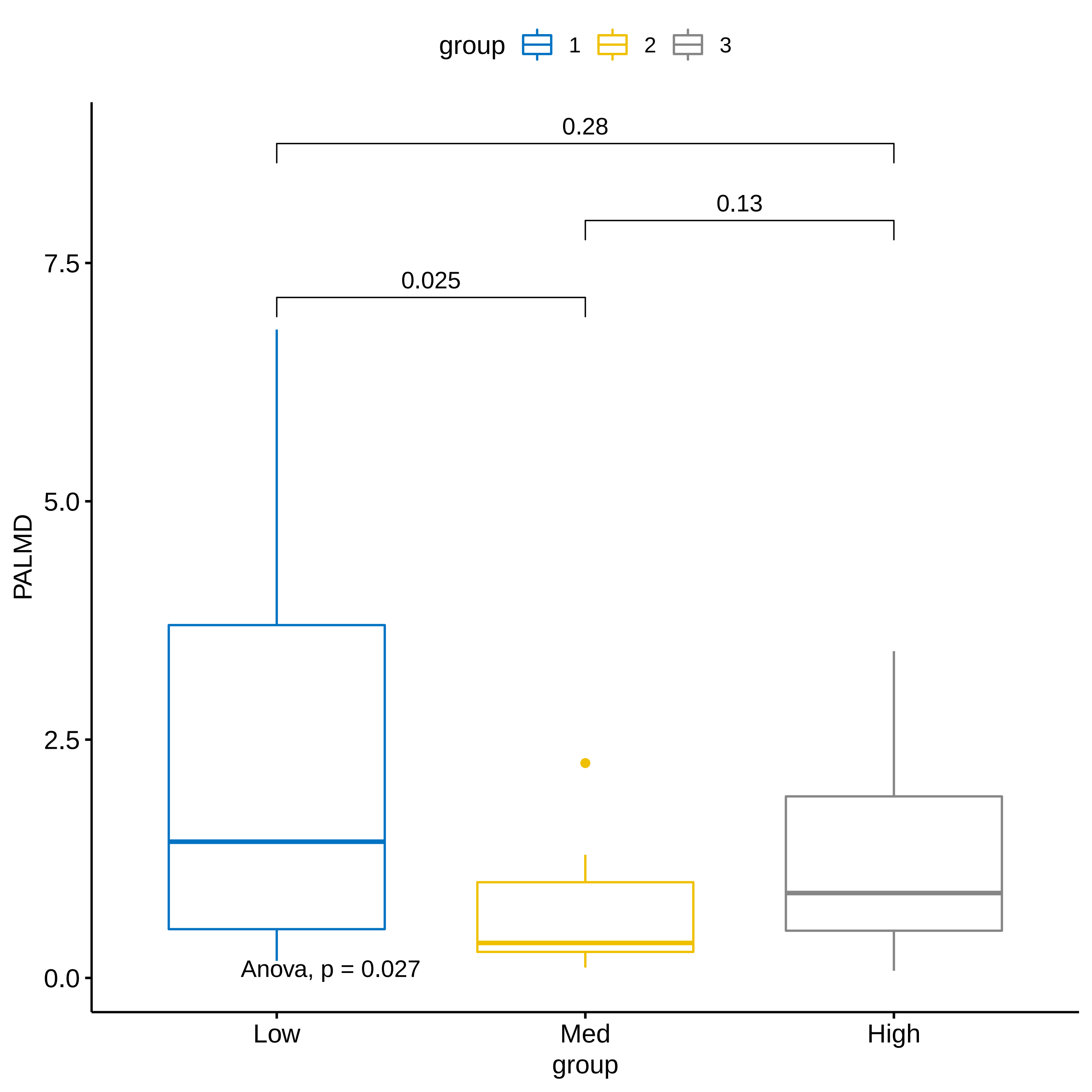
group
1
2
3
0.28
0.13
0.025
7.5
5.0
2.5
0.0
PALMD
Anova, p = 0.027
Low
Med
High
group

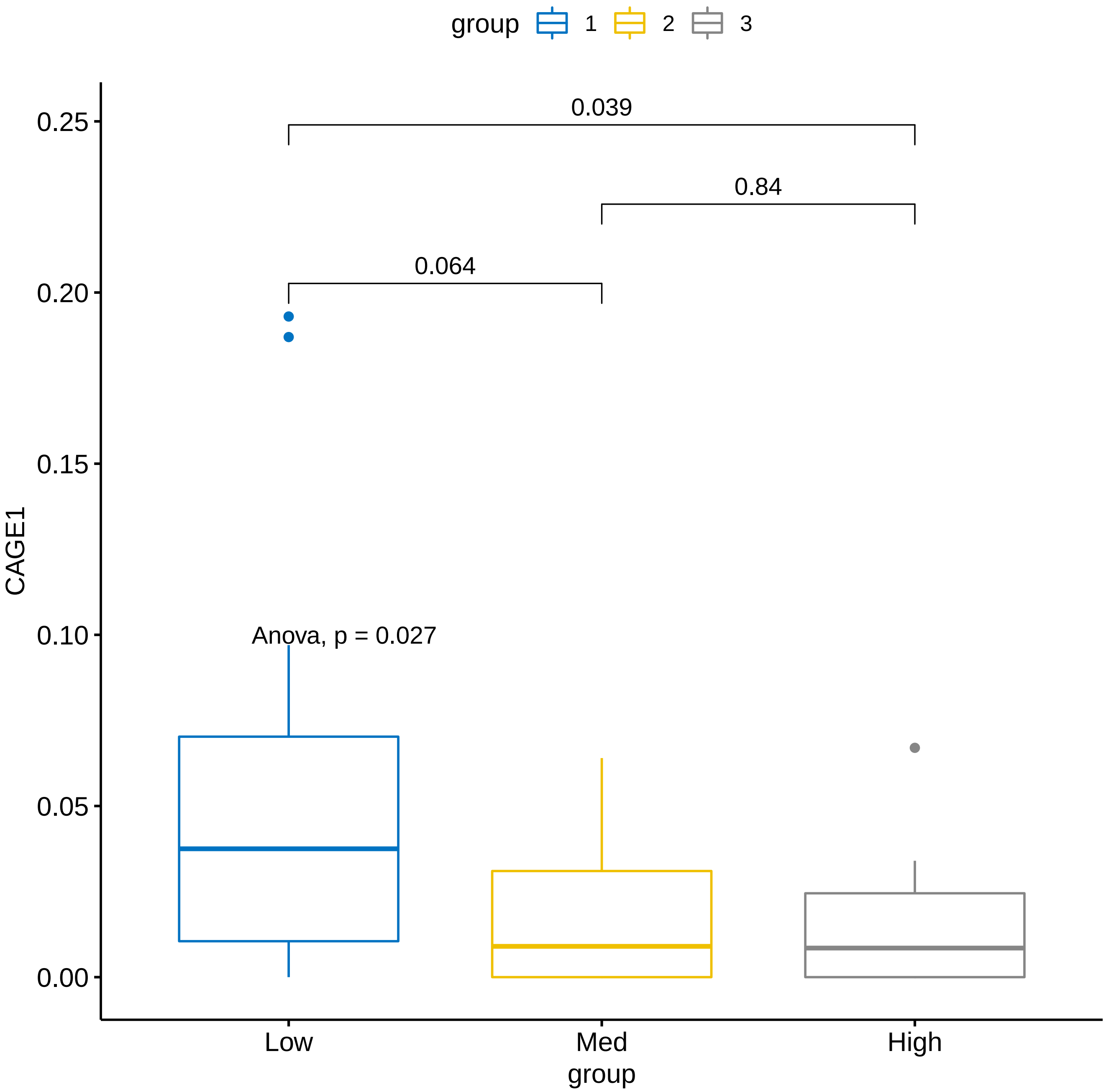
group
1
2
3
0.039
0.84
0.064
Anova, p = 0.027
CAGE1
0.25
0.20
0.15
0.10
0.05
0.00
Low
Med
High
group

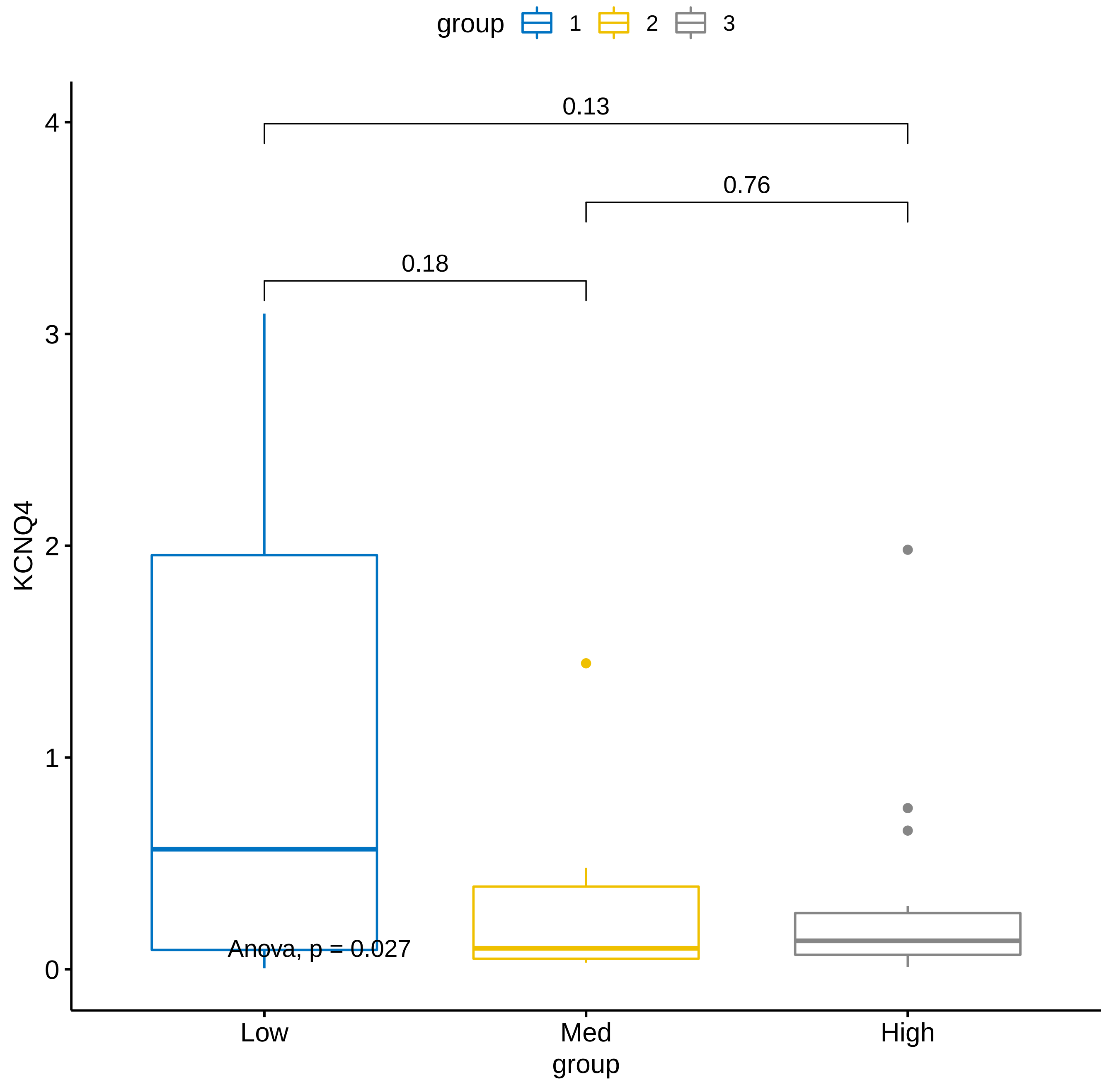

group
1
2
3
0.13
0.76
0.18
KCNQ4
4
3
2
1
0
Anova, p = 0.027
Low
Med
High
group

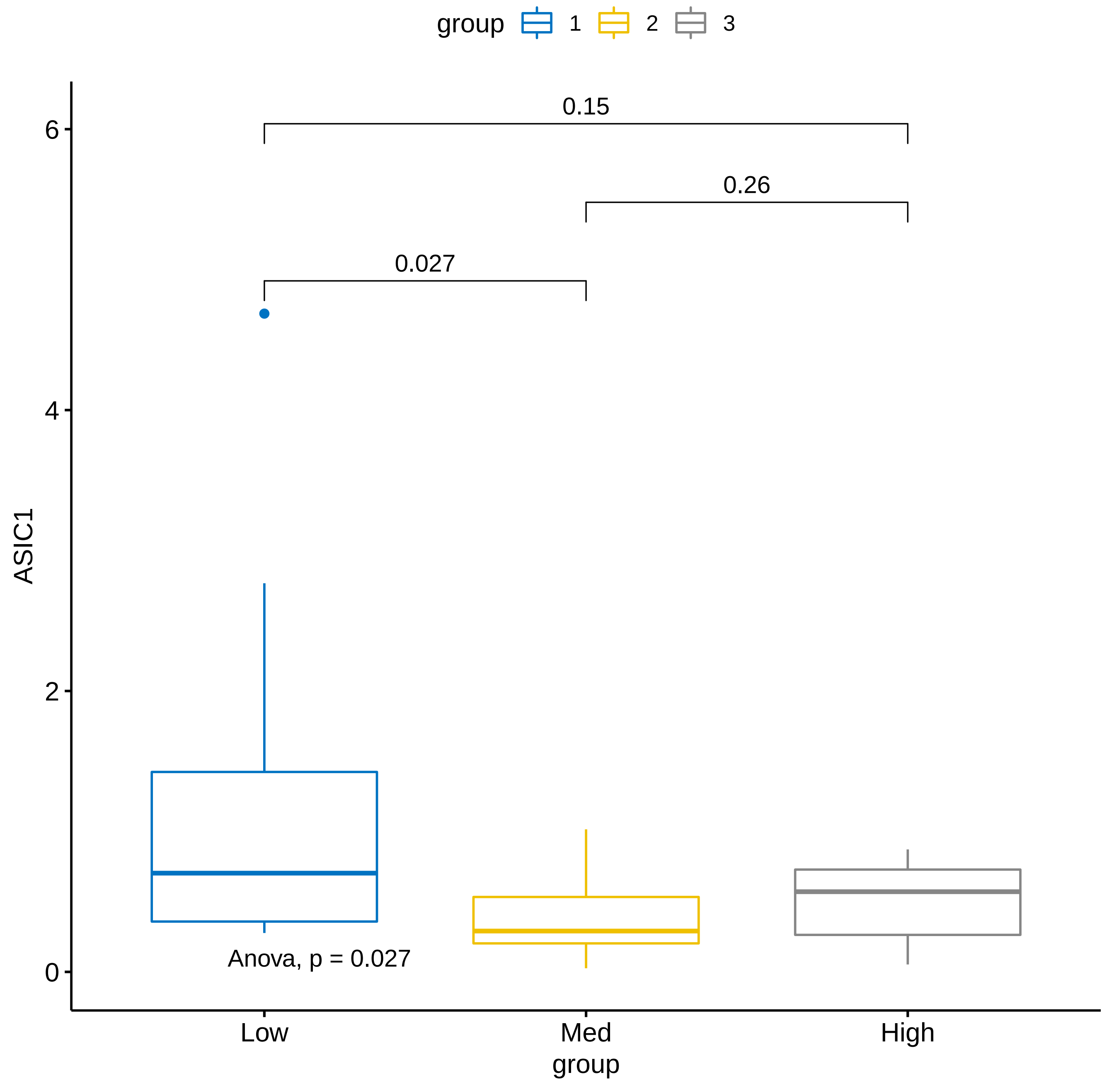
group 1 2 3
0.15
0.26
0.027
ASIC1
6
4
2
0
Anova, p = 0.027
Low
Med
High
group

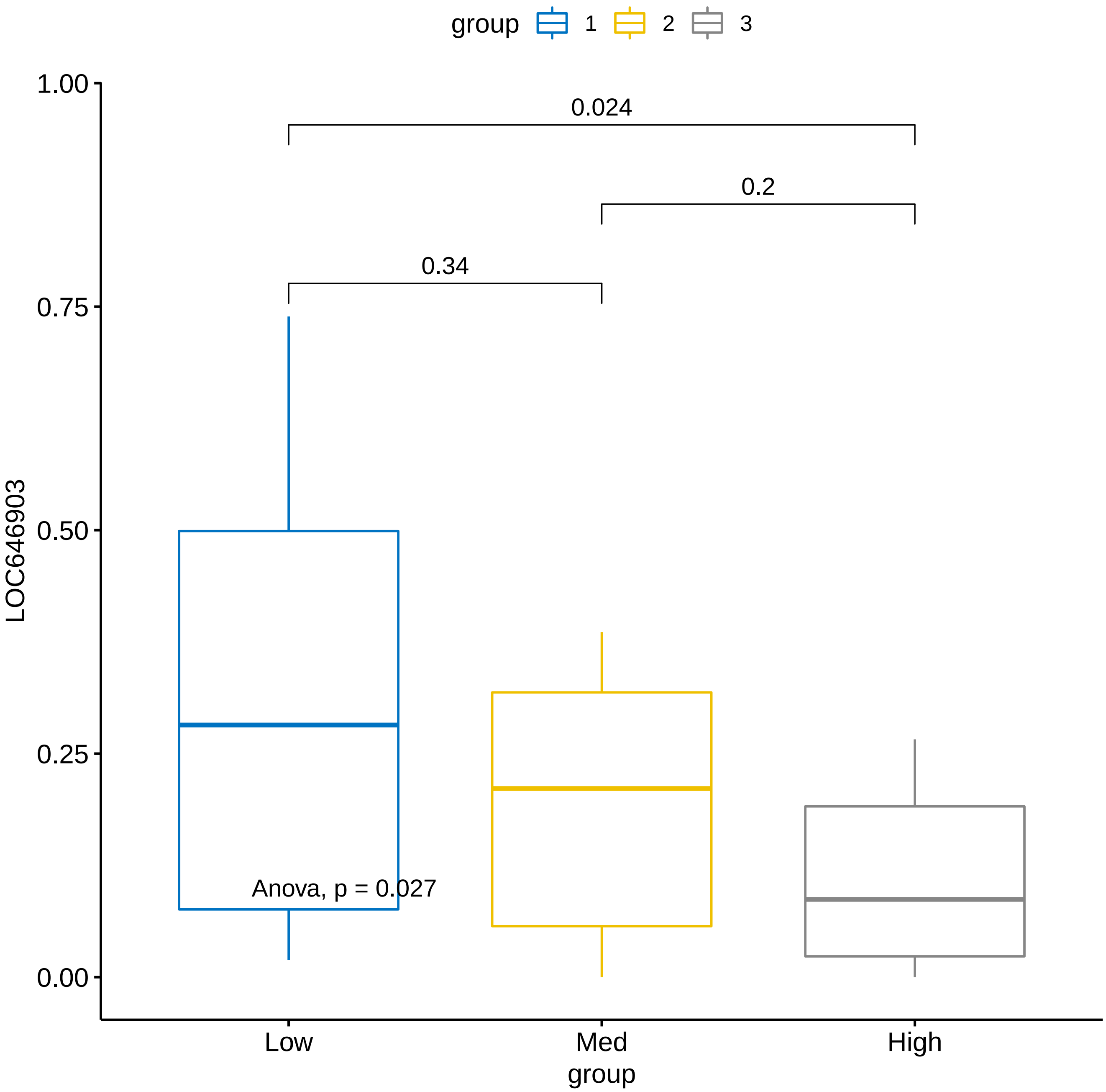
group
1
2
3
1.00
0.75
0.50
0.25
0.00
LOC646903
0.024
0.2
0.34
Anova, p = 0.027
Low
Med
High
group

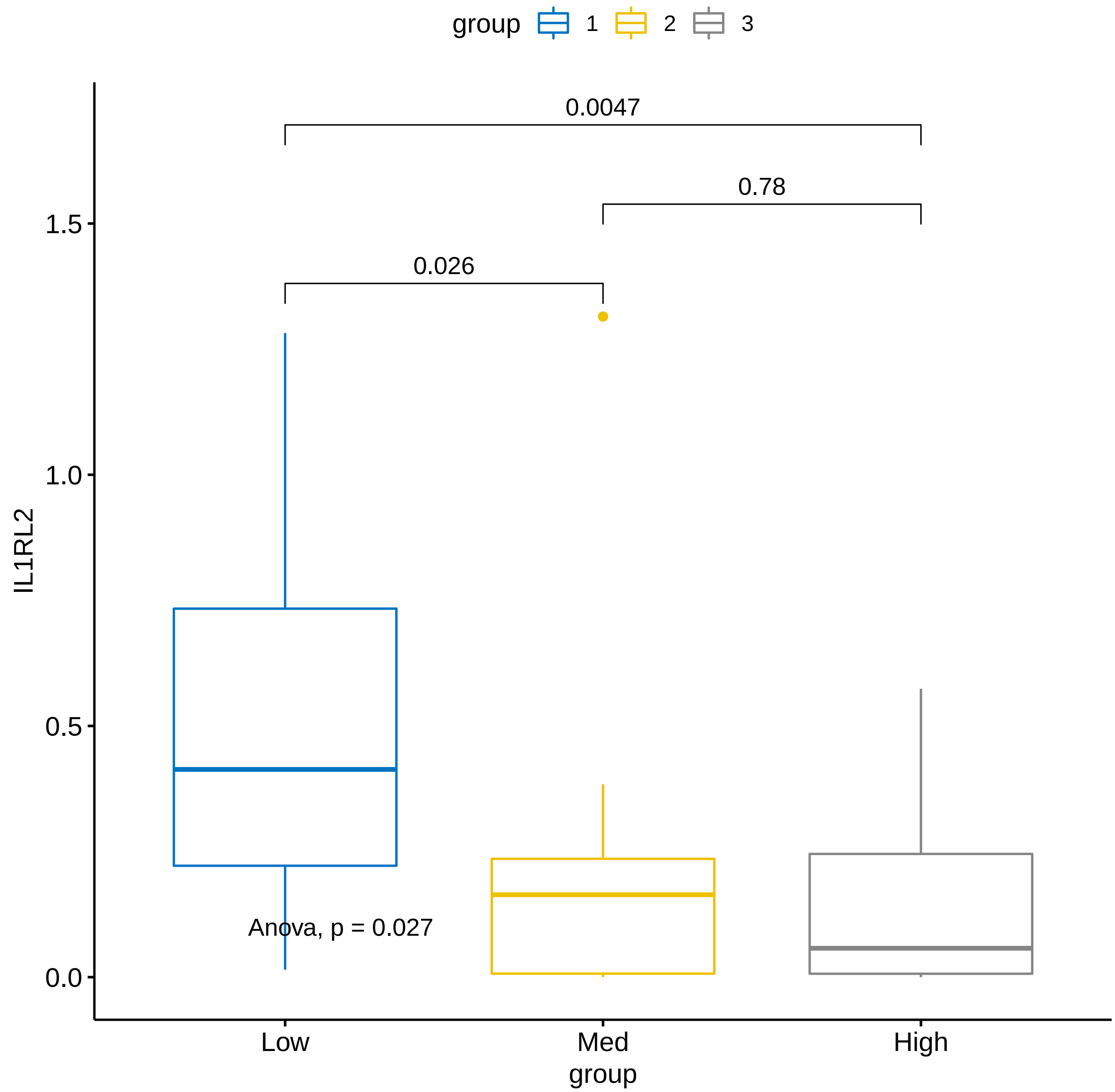

group
1
2
3
0.0047
0.78
0.026
1.5
1.0
0.5
0.0
IL1RL2
Anova, p = 0.027
Low
Med
High
group

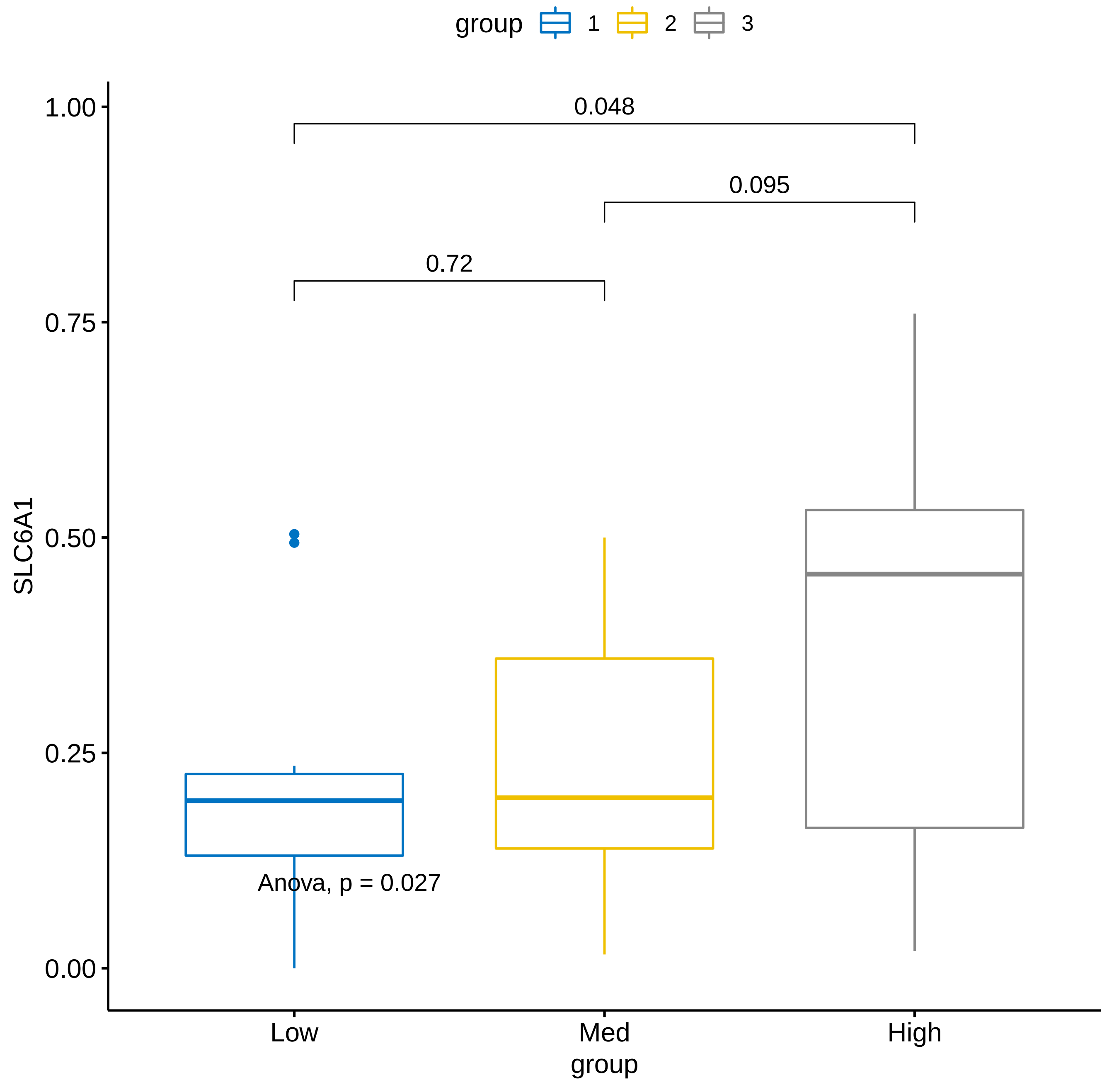
group 1 2 3
1.00
0.75
0.50
0.25
0.00
SLC6A1
0.048
0.095
0.72
Anova, p = 0.027
Low
Med
High
group

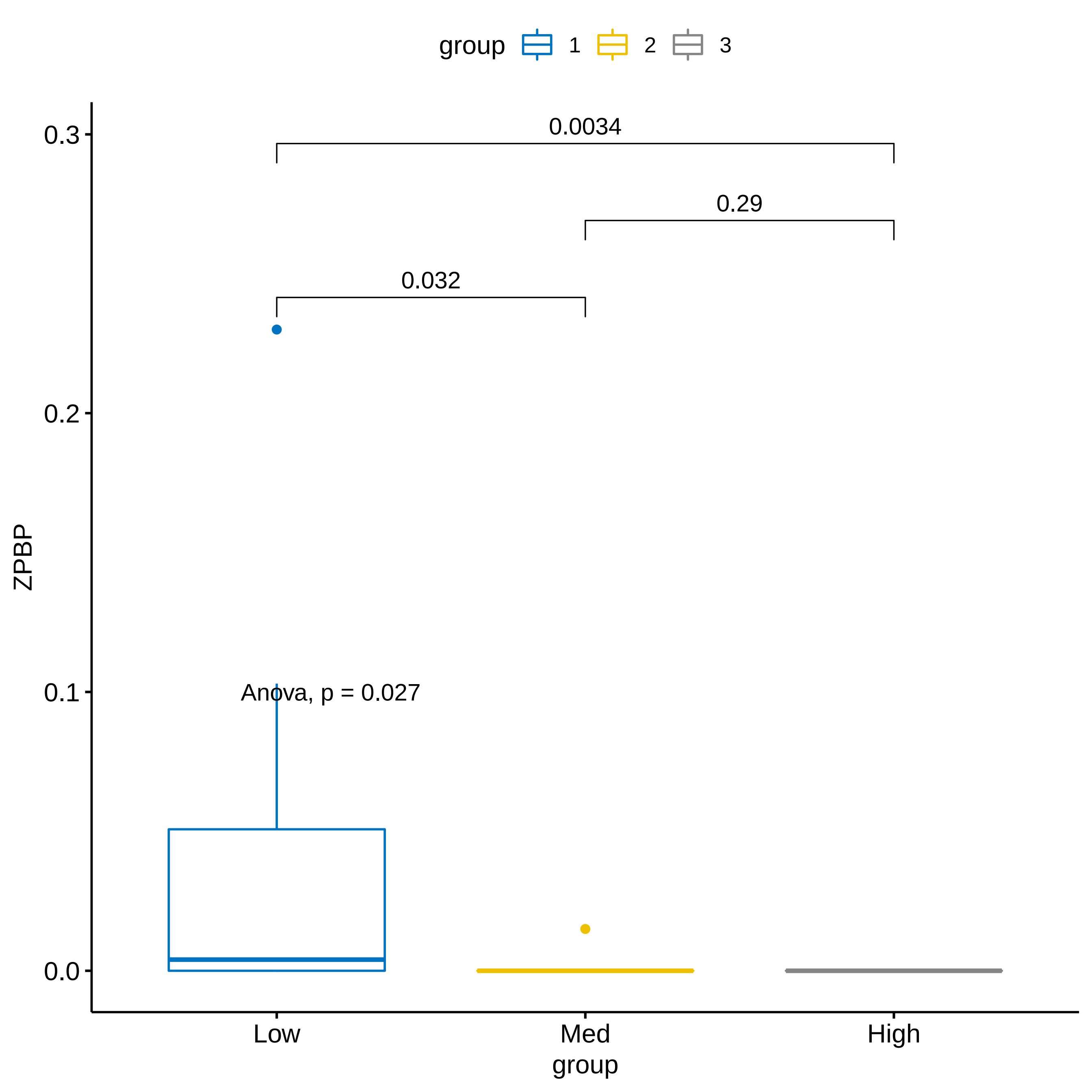
group
1
2
3
0.0034
0.29
0.032
Anova, p = 0.027
0.3
0.2
0.1
0.0
ZPBP
Low
Med
High
group

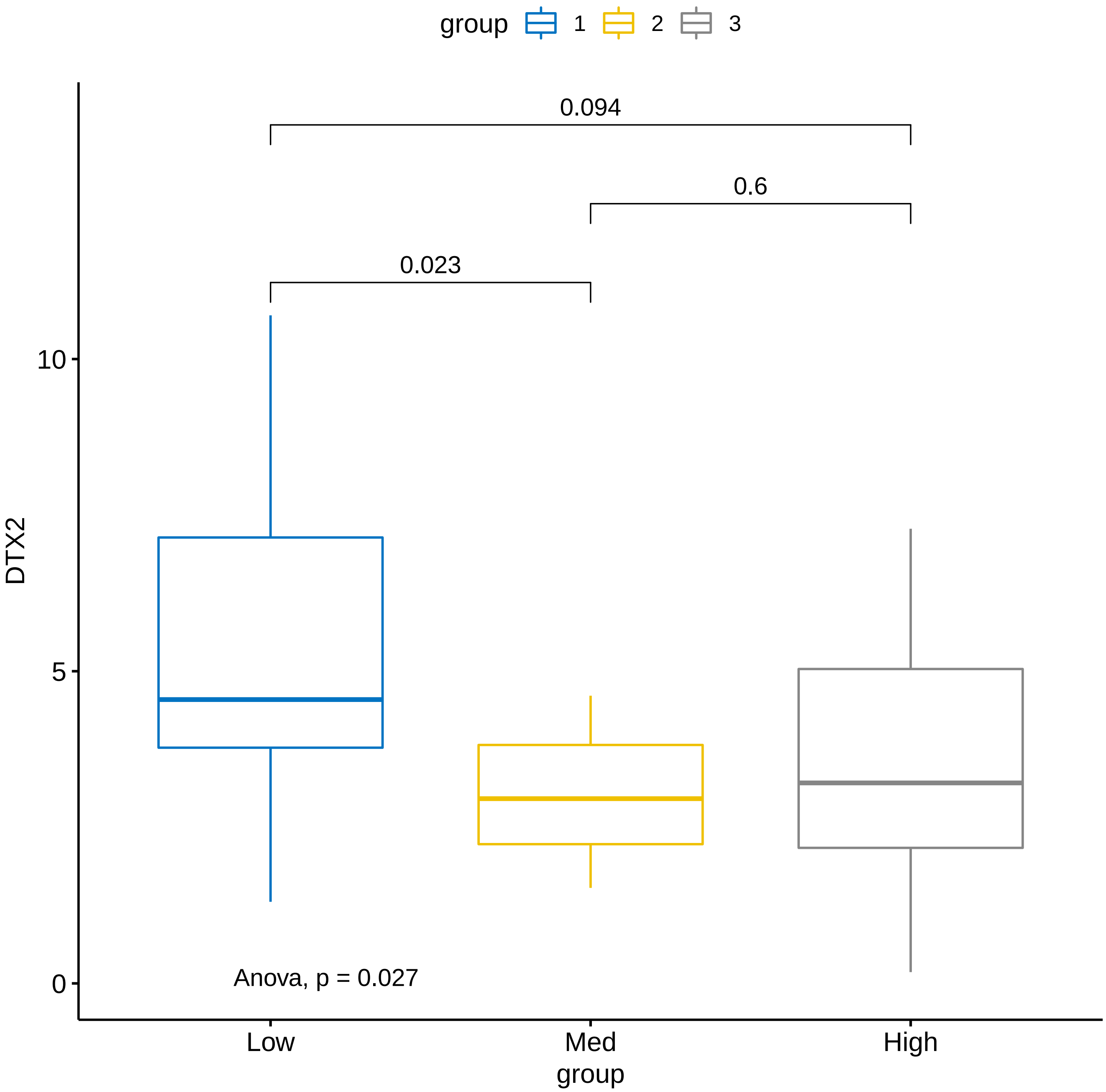
group
1
2
3
0.094
0.6
0.023
10
5
0
DTX2
Anova, p = 0.027
Low
Med
High
group

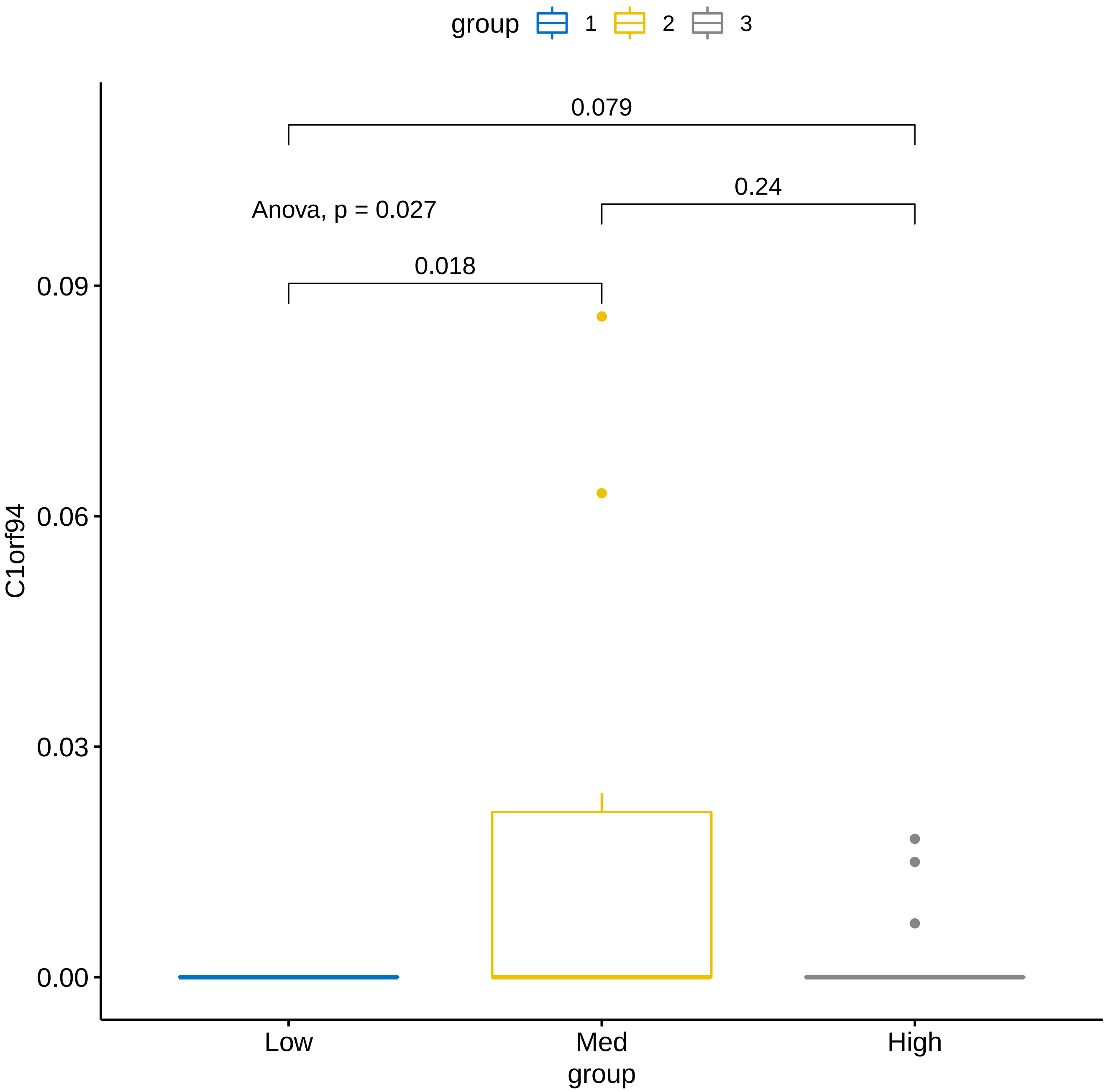

group 1 2 3
0.079
0.24
Anova, p = 0.027
0.018
0.09
0.06
0.03
0.00
C1orf94
Low
Med
High
group

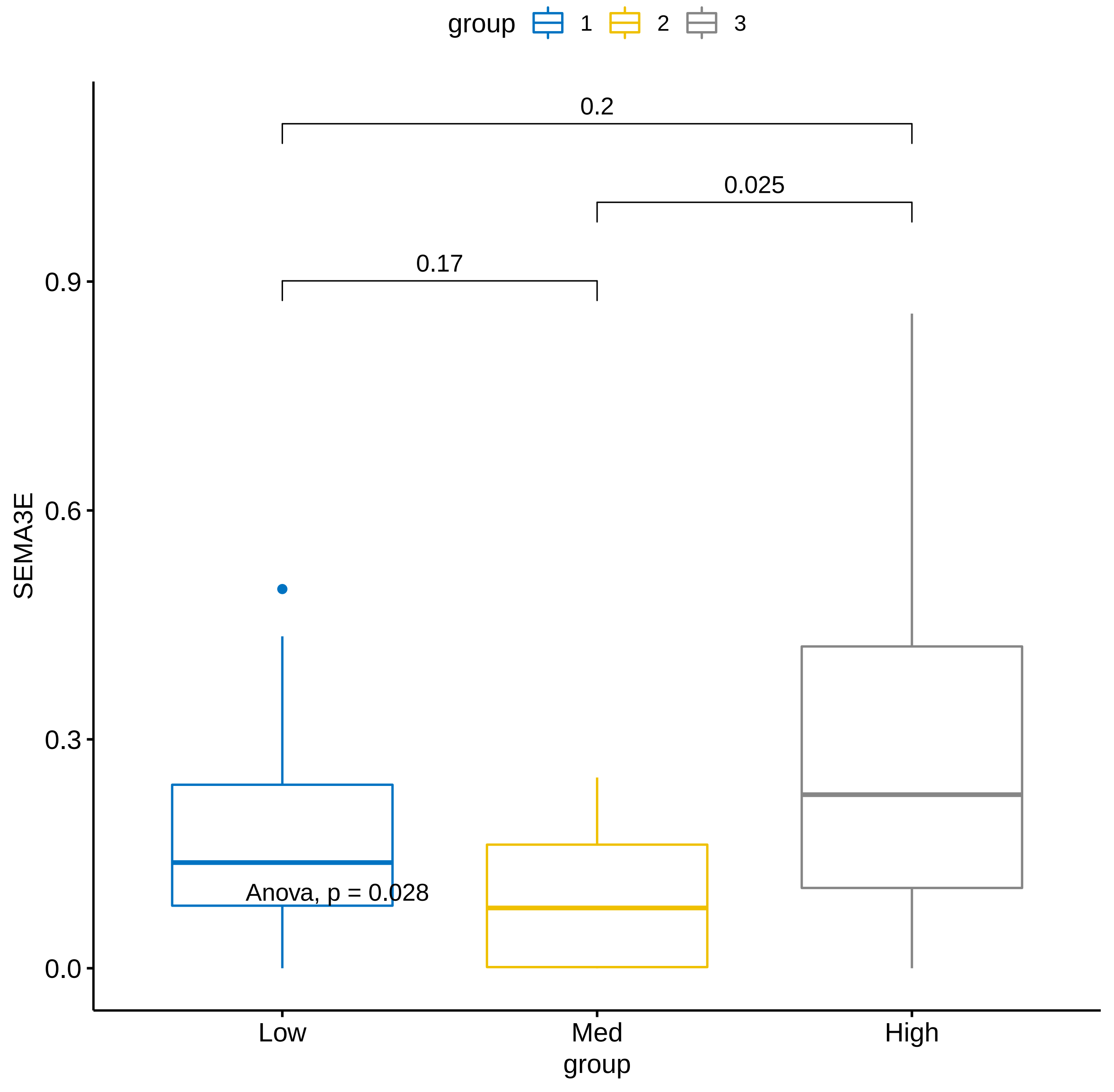
group
1
2
3
0.2
0.025
0.17
Anova, p = 0.028
SEMA3E
0.9
0.6
0.3
0.0
Low
Med
High
group

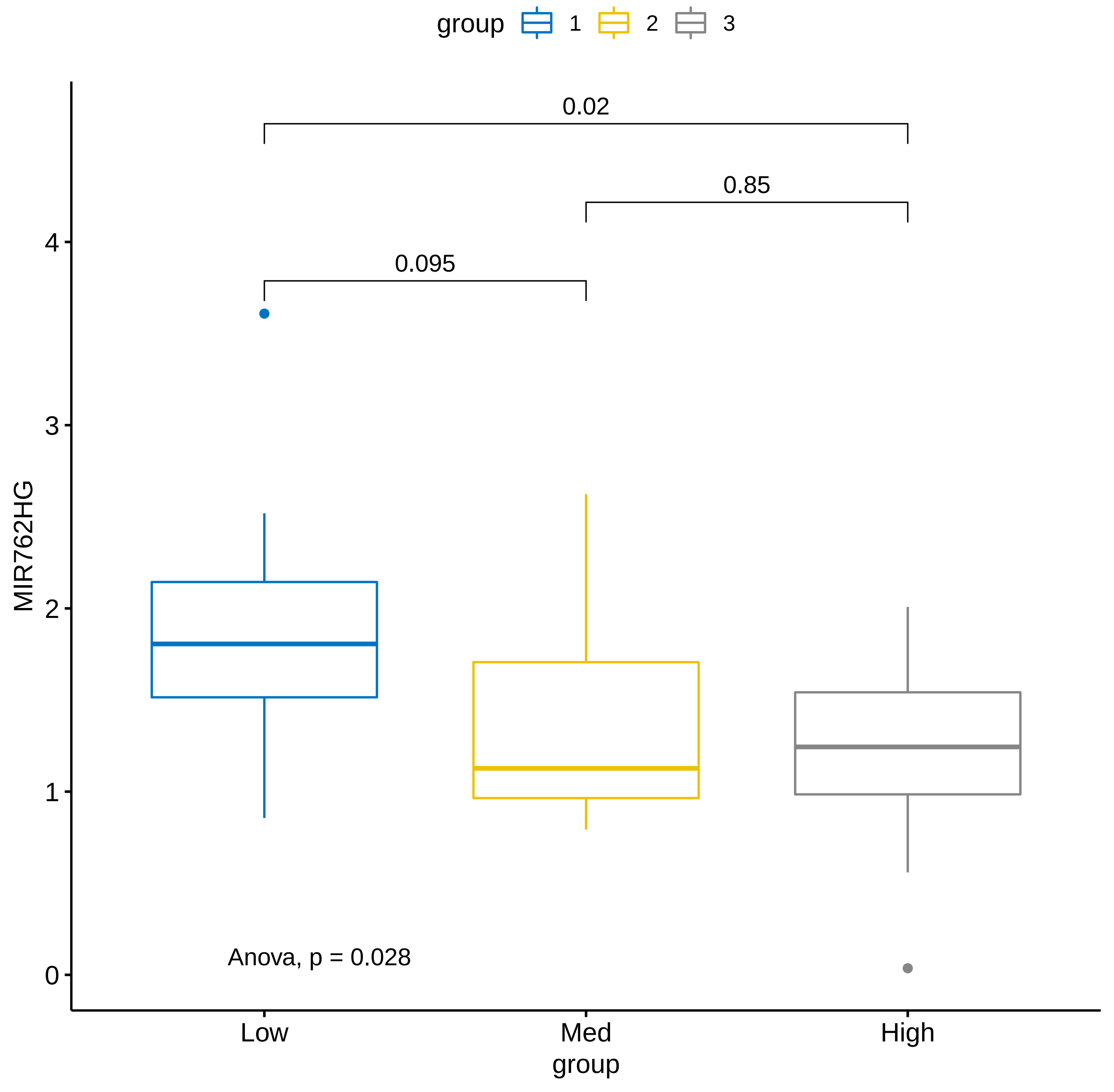
group 1 2 3
0.02
0.85
0.095
4
3
2
1
0
MIR762HG
Anova, p = 0.028
Low
Med
High
group

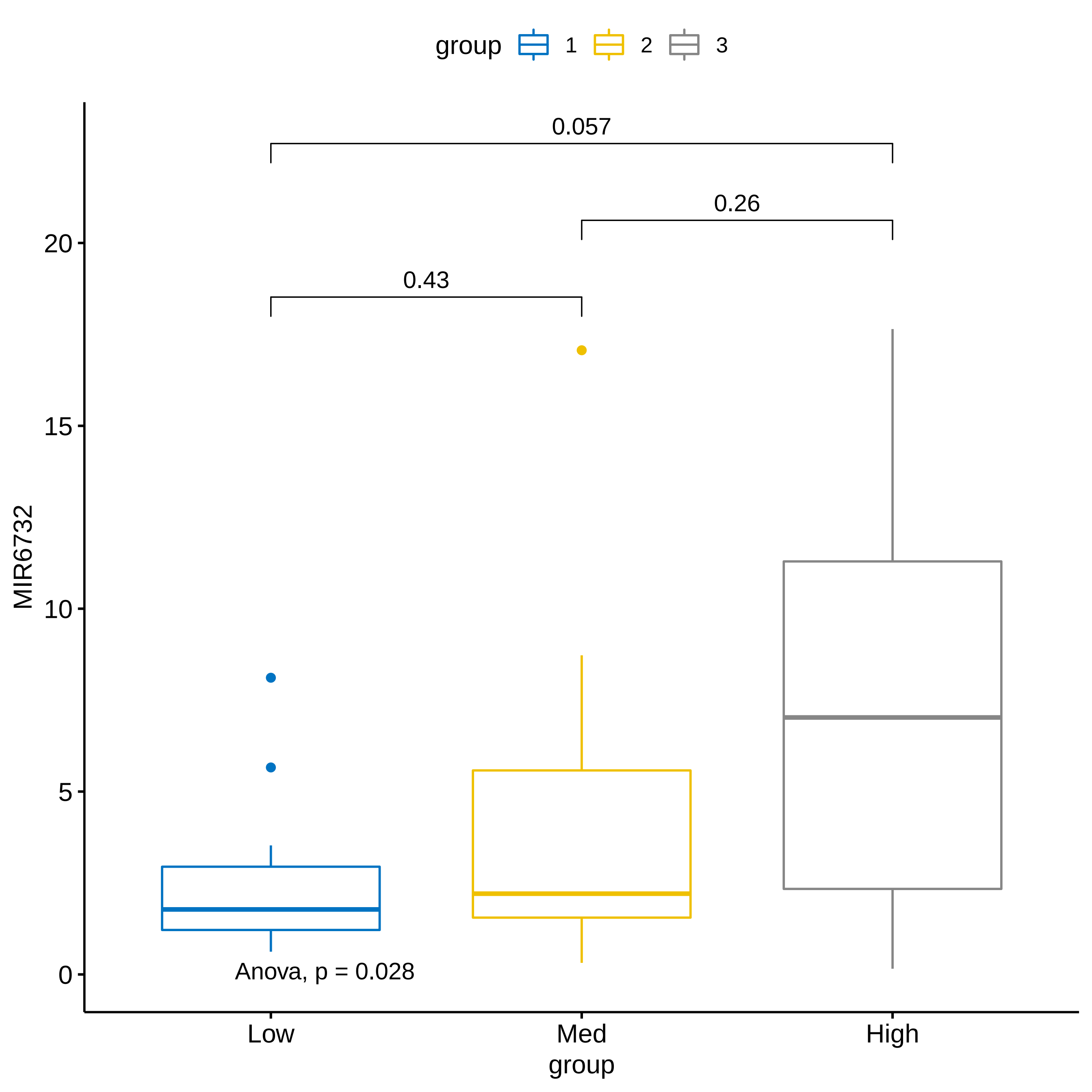

group 1 2 3
0.057
0.26
0.43
20
15
10
5
0
MIR6732
Anova, p = 0.028
Low
Med
High
group

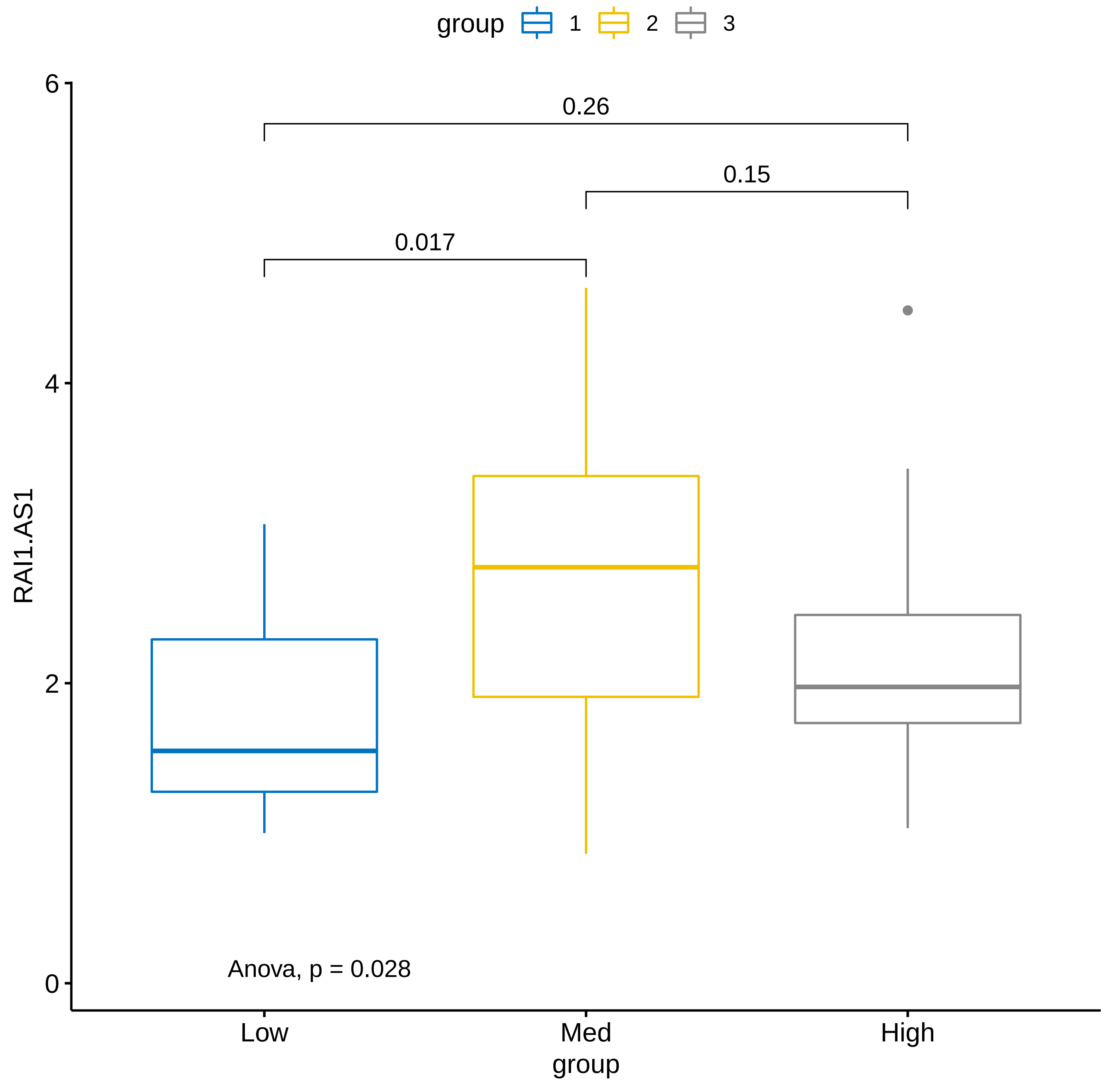
group
1
2
3
6
4
2
0
RAI1.AS1
0.26
0.15
0.017
Anova, p = 0.028
Low
Med
High
group

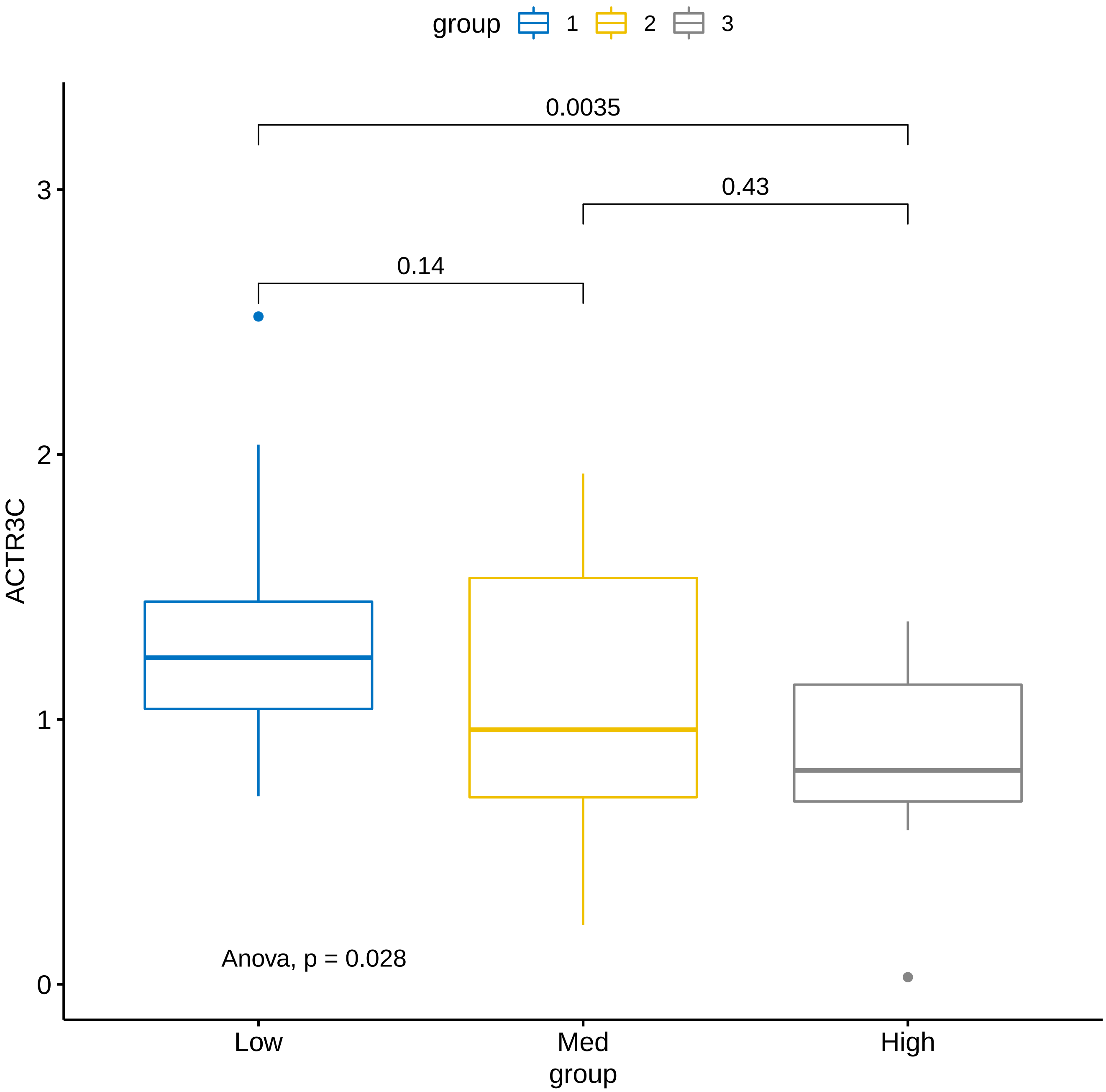

group
1
2
3
0.0035
0.43
0.14
ACTR3C
3
2
1
0
Anova, p = 0.028
Low
Med
High
group

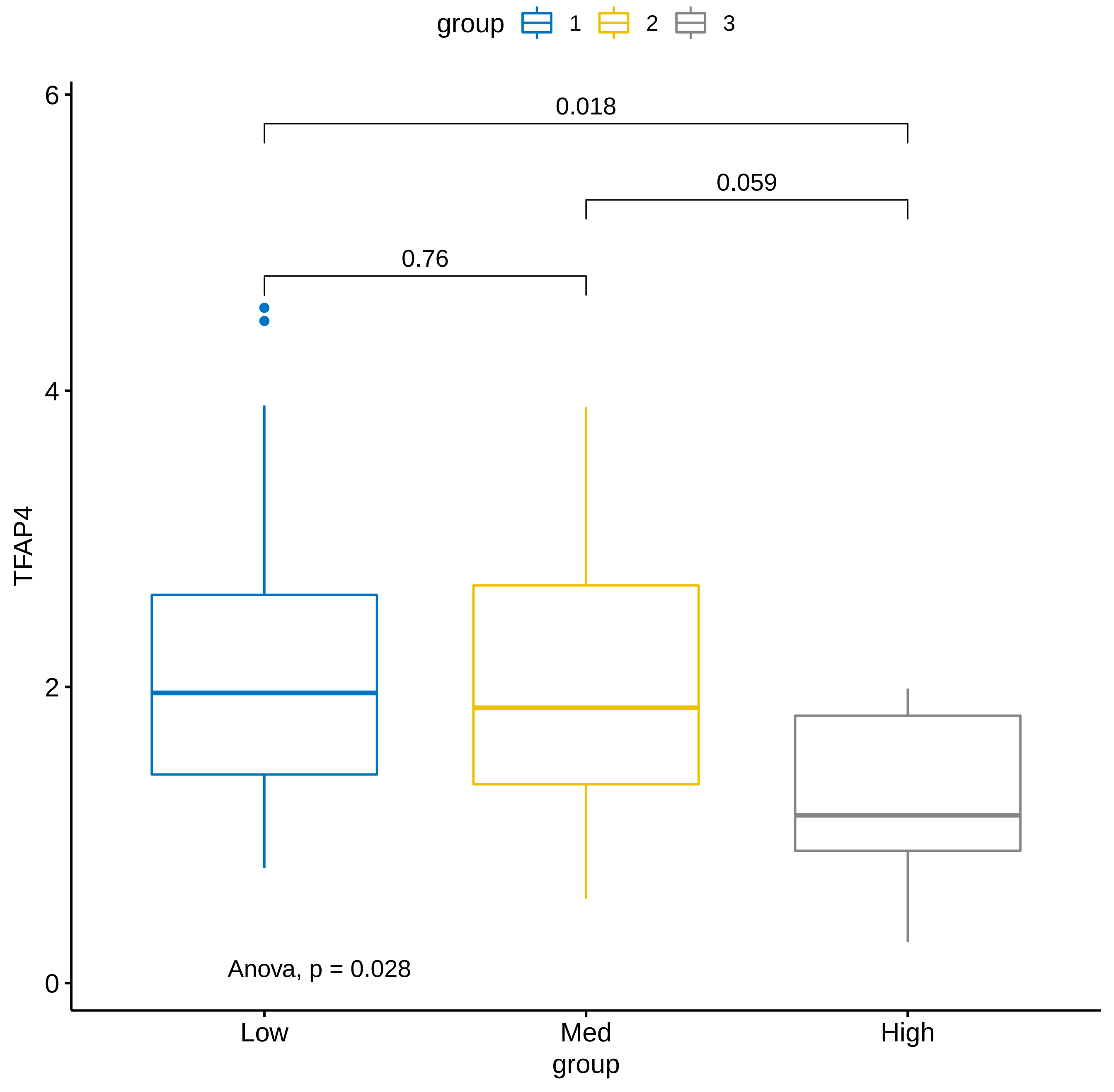

group 1 2 3
0.018
0.059
0.76
TFAP4
6
4
2
0
Anova, p = 0.028
Low
Med
High
group

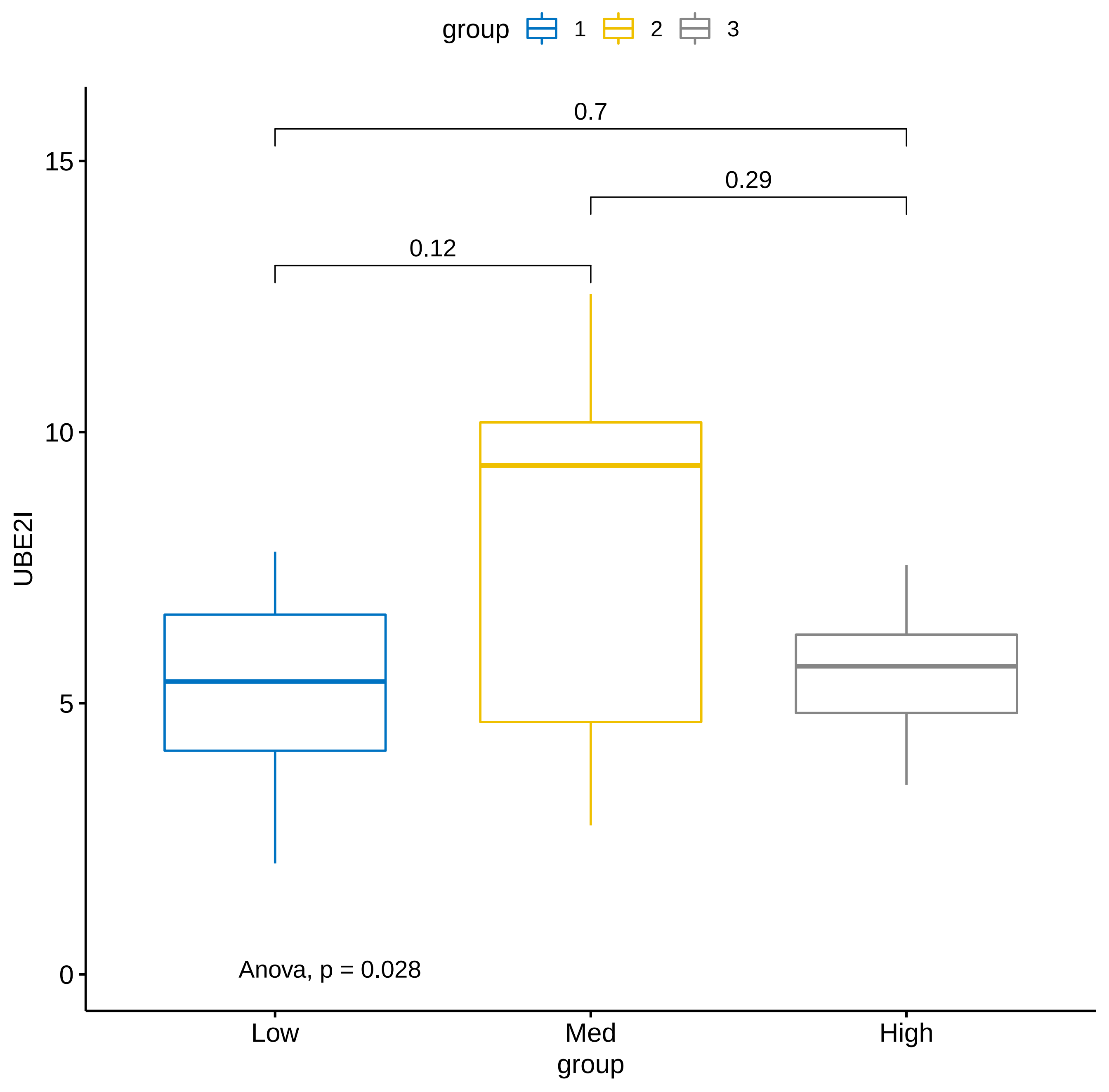
group
1
2
3
0.7
0.29
0.12
15
10
5
0
UBE2I
Anova, p = 0.028
Low
Med
High
group

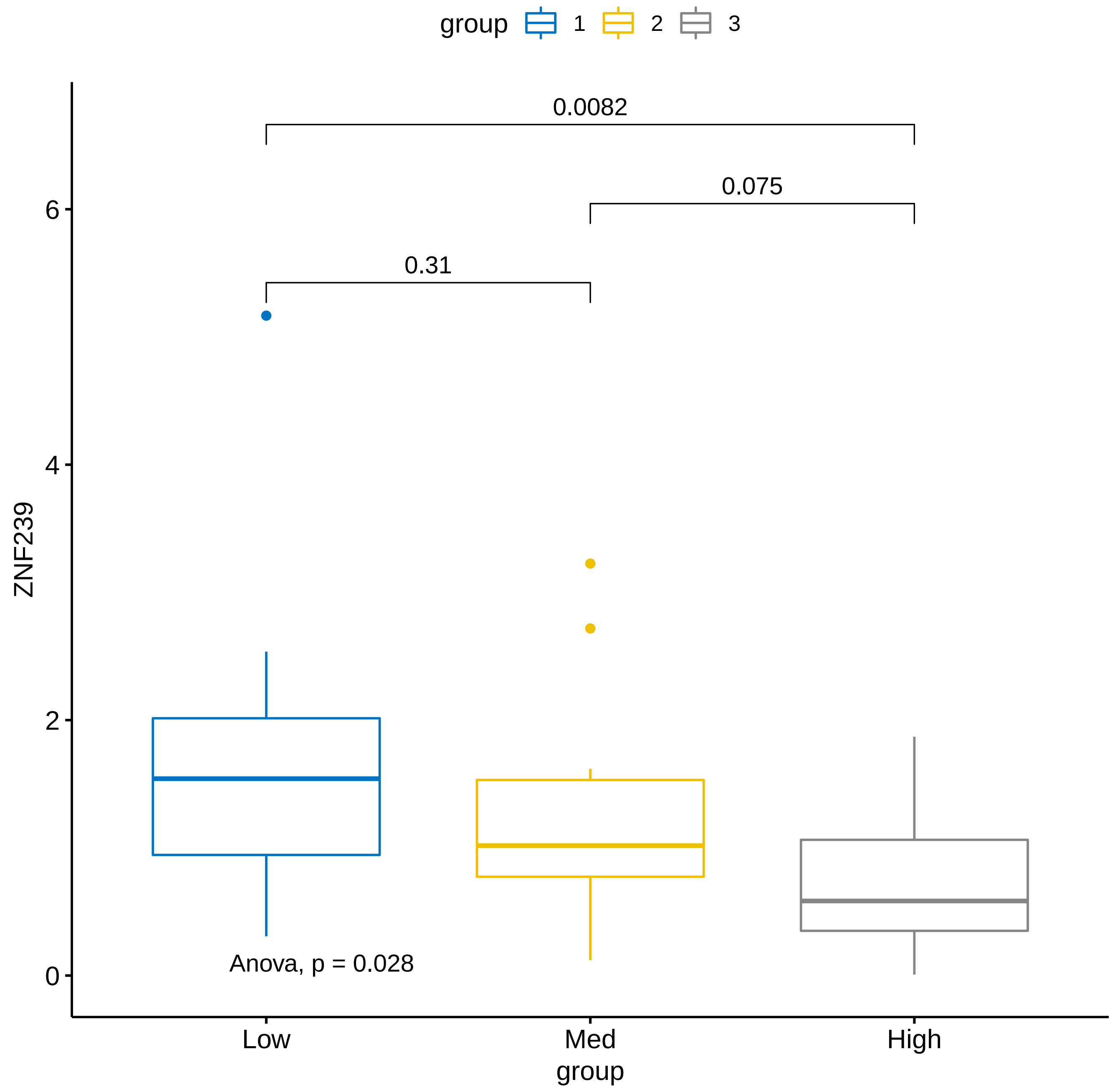

group 1 2 3
0.0082
0.075
0.31
ZNF239
6
4
2
0
Anova, p = 0.028
Low
Med
High
group

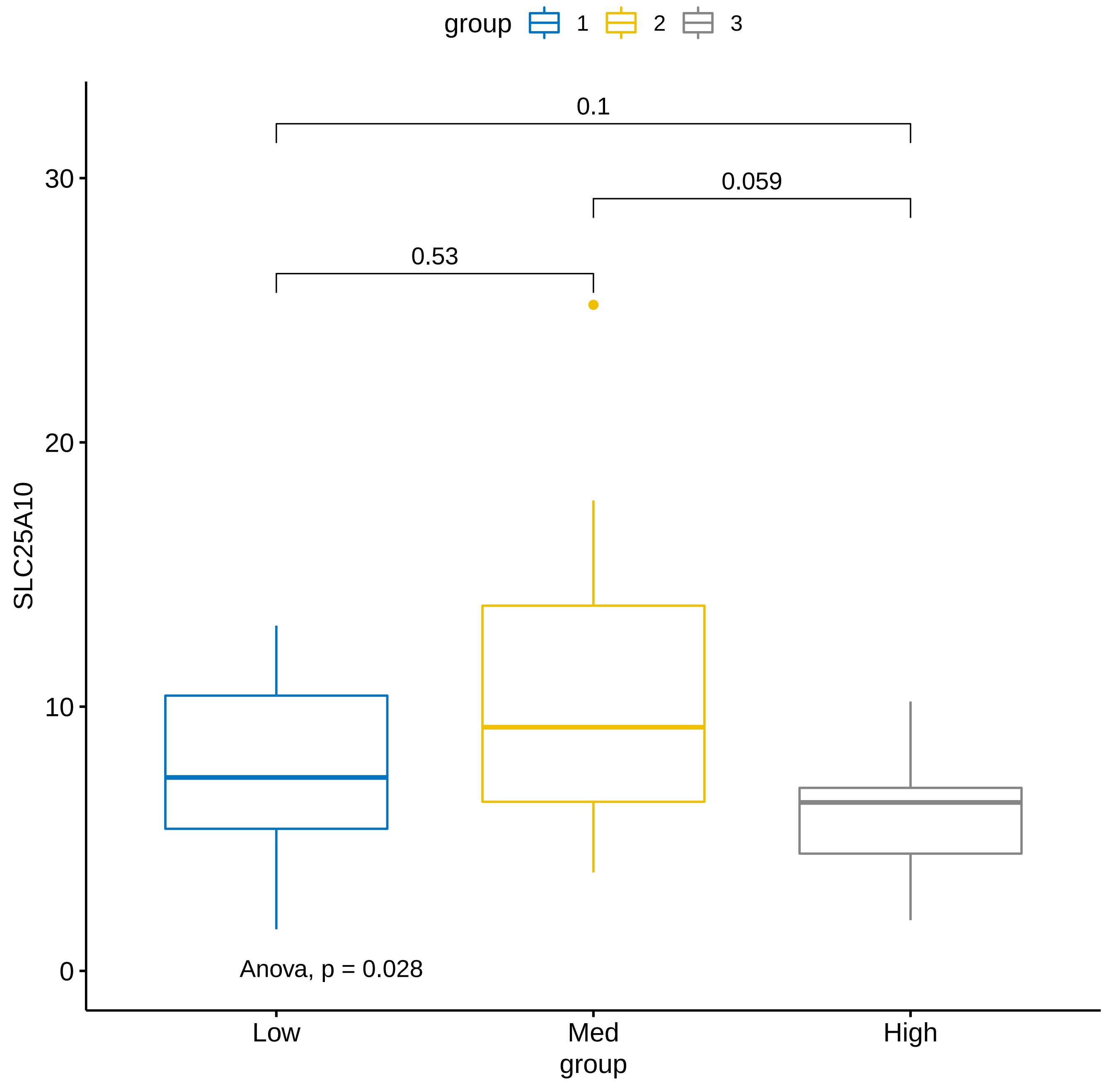

group
1
2
3
0.1
0.059
0.53
30
20
10
0
SLC25A10
Anova, p = 0.028
Low
Med
High
group

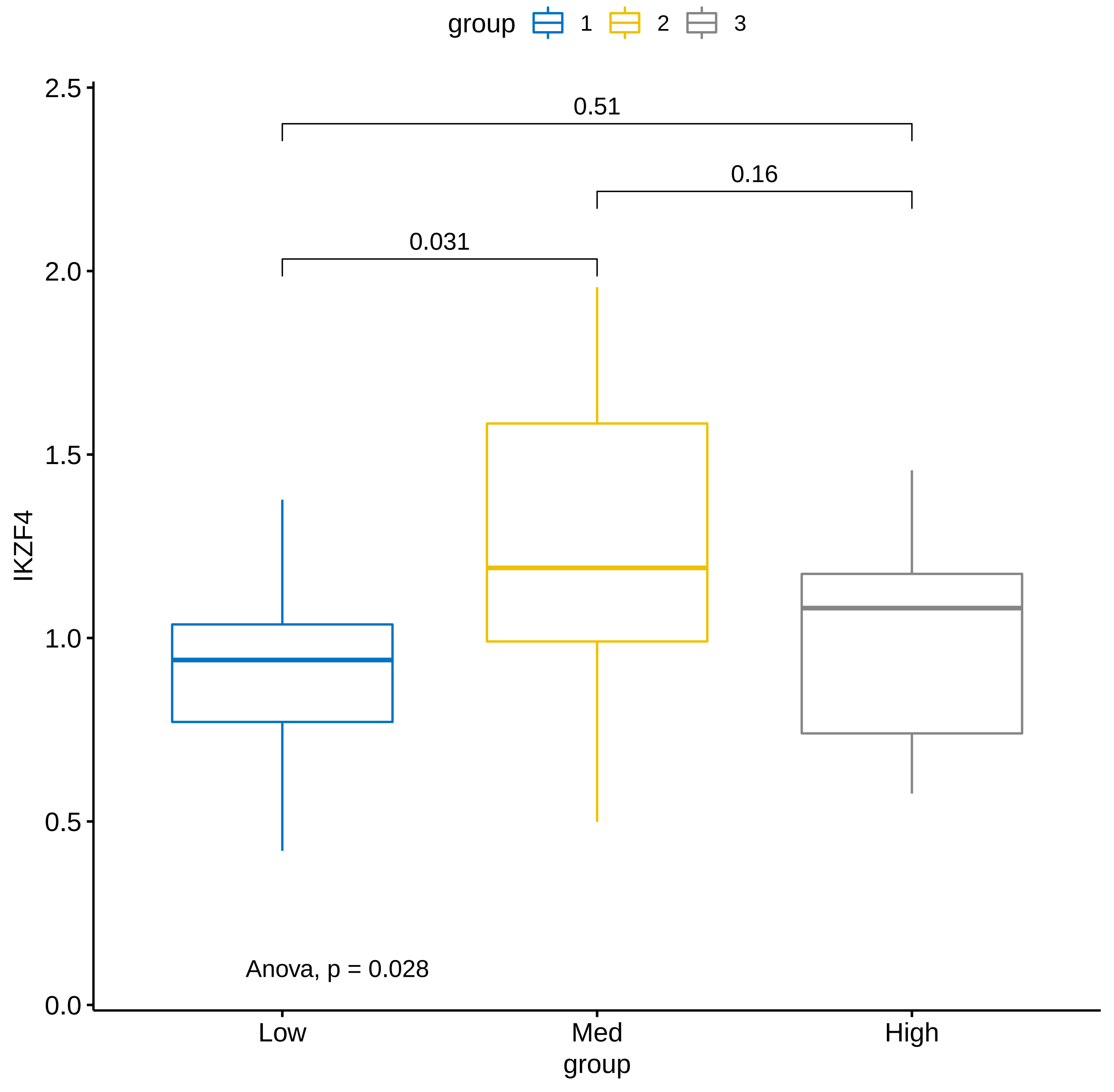
group
1
2
3
2.5
2.0
1.5
1.0
0.5
0.0
IKZF4
0.51
0.16
0.031
Anova, p = 0.028
Low
Med
High
group

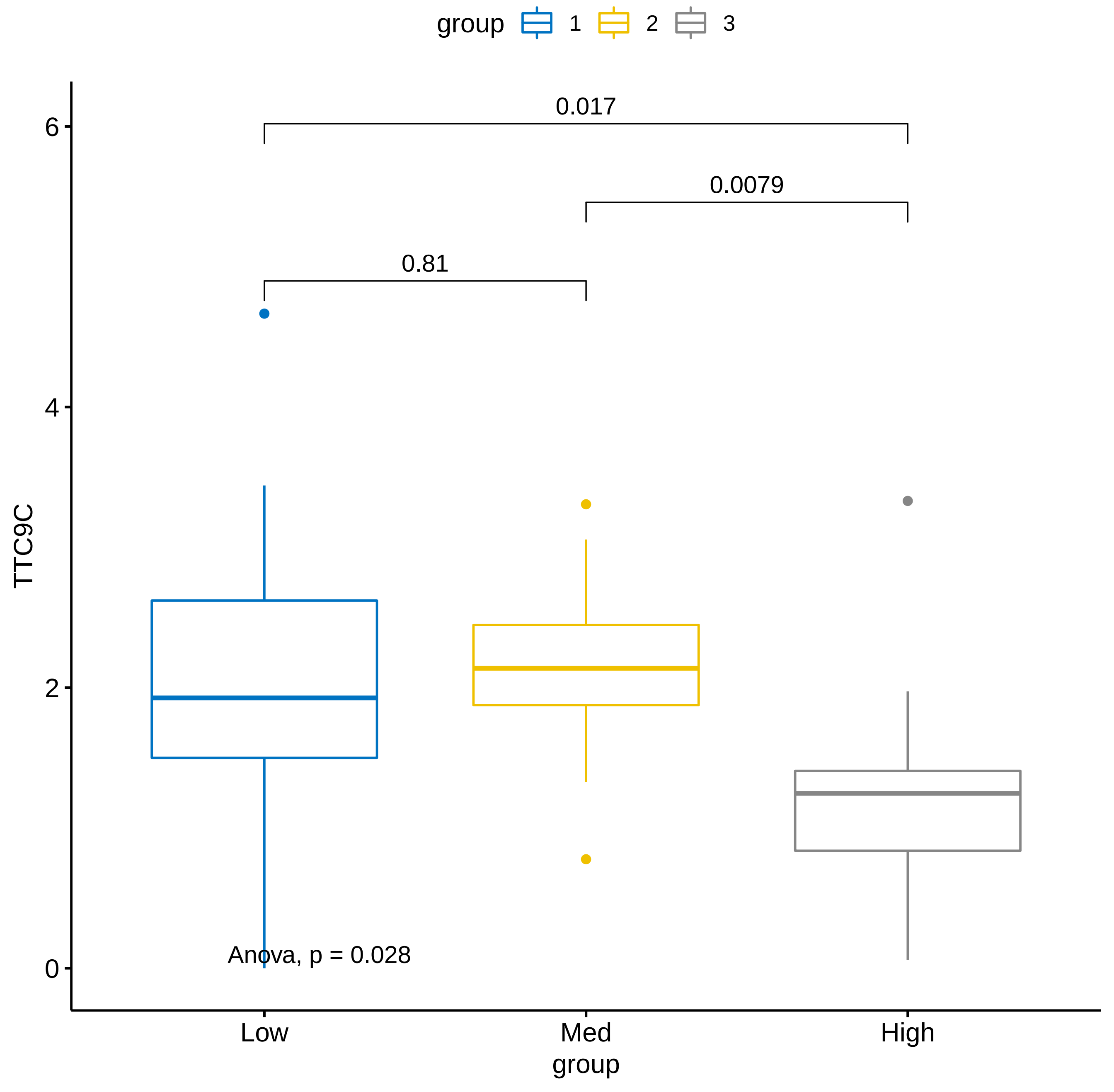
group 1 2 3
0.017
0.0079
0.81
6
4
2
0
TTC9C
Anova, p = 0.028
Low
Med
High
group

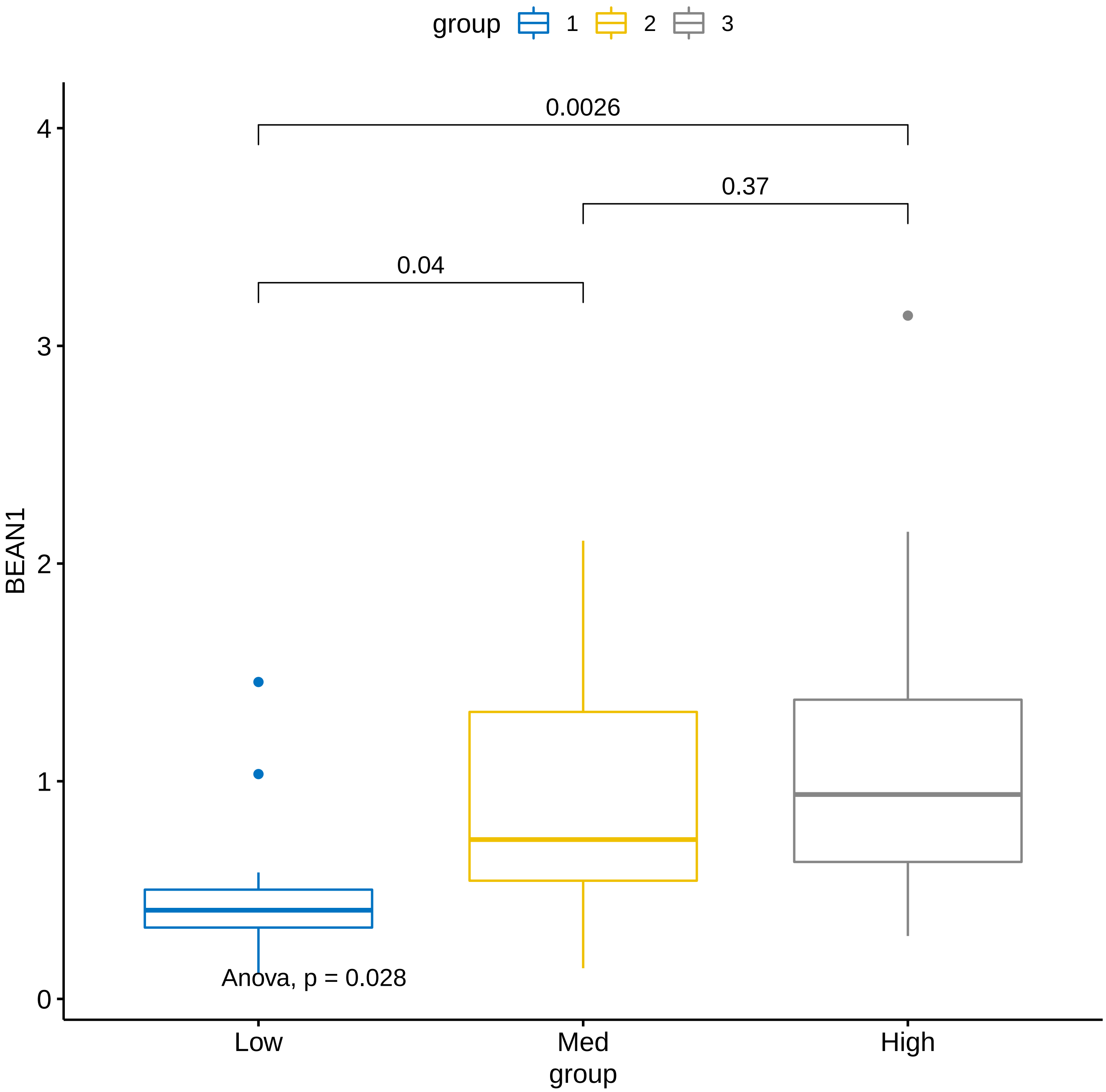
group
1
2
3
0.0026
0.37
0.04
4
3
2
1
0
BEAN1
Anova, p = 0.028
Low
Med
High
group

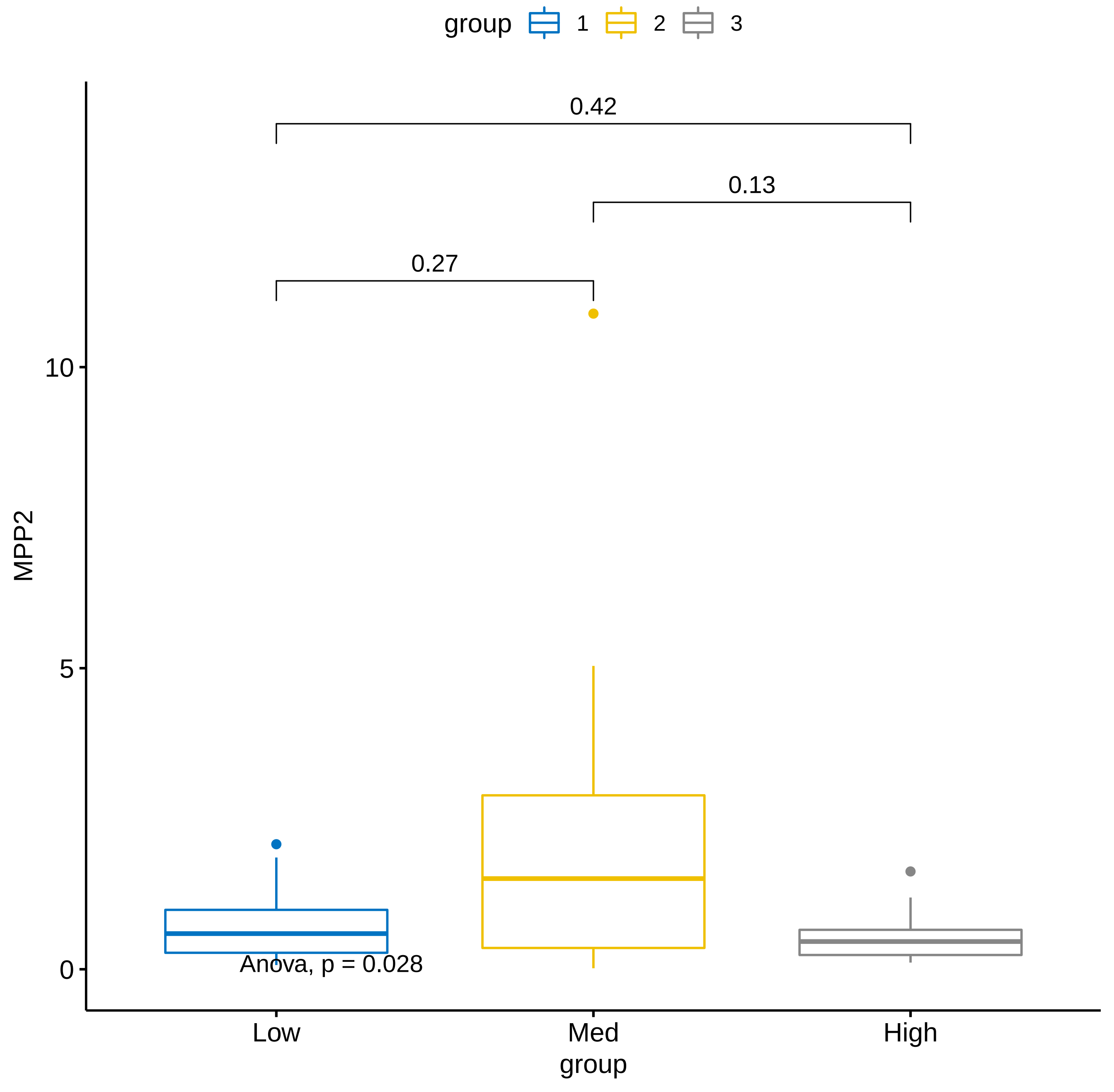

group 1 2 3
0.42
0.13
0.27
MPP2
10
5
0
Anova, p = 0.028
Low
Med
High
group

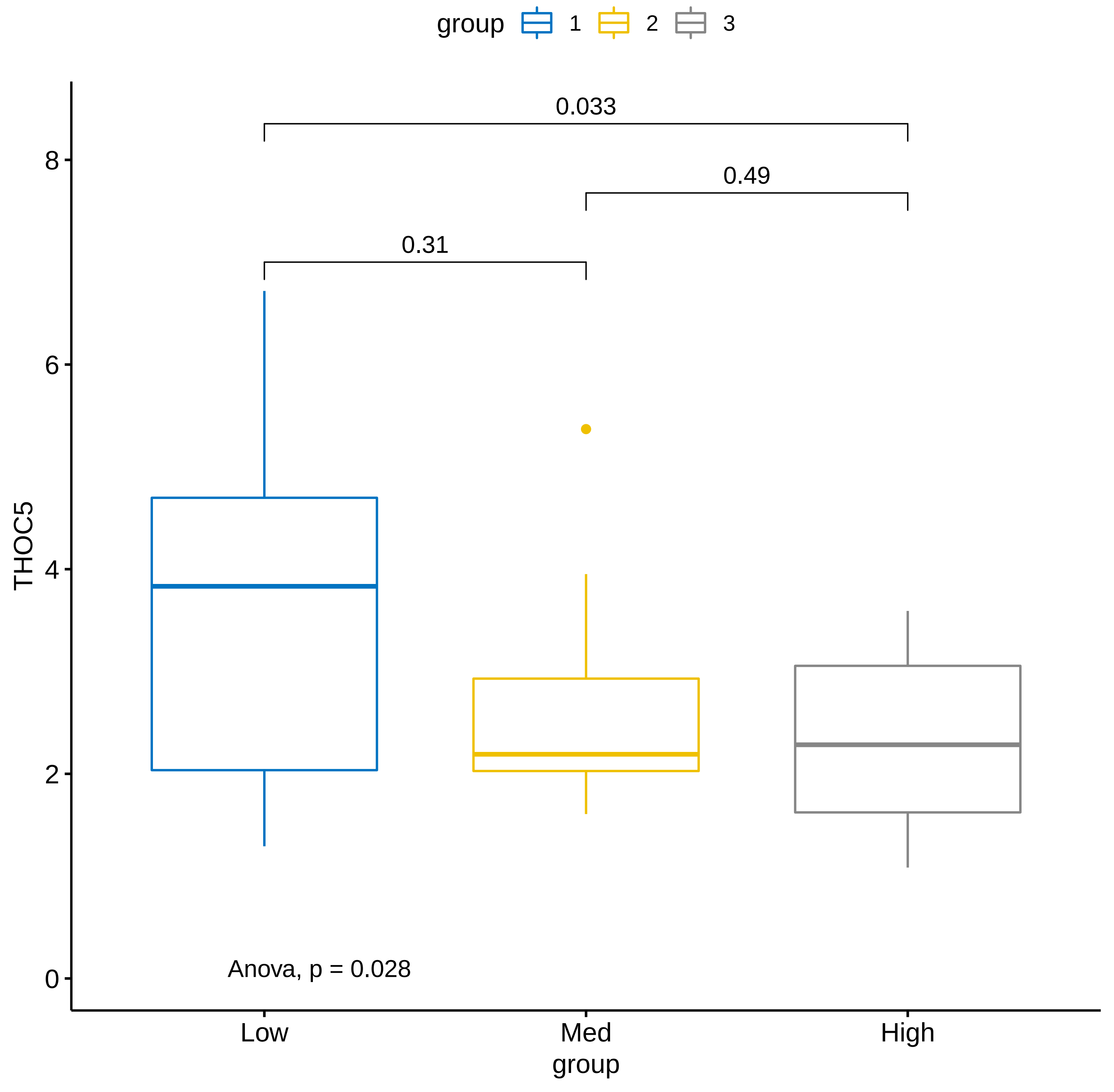
group
1
2
3
0.033
0.49
0.31
8
6
4
2
0
THOC5
Anova, p = 0.028
Low
Med
High
group

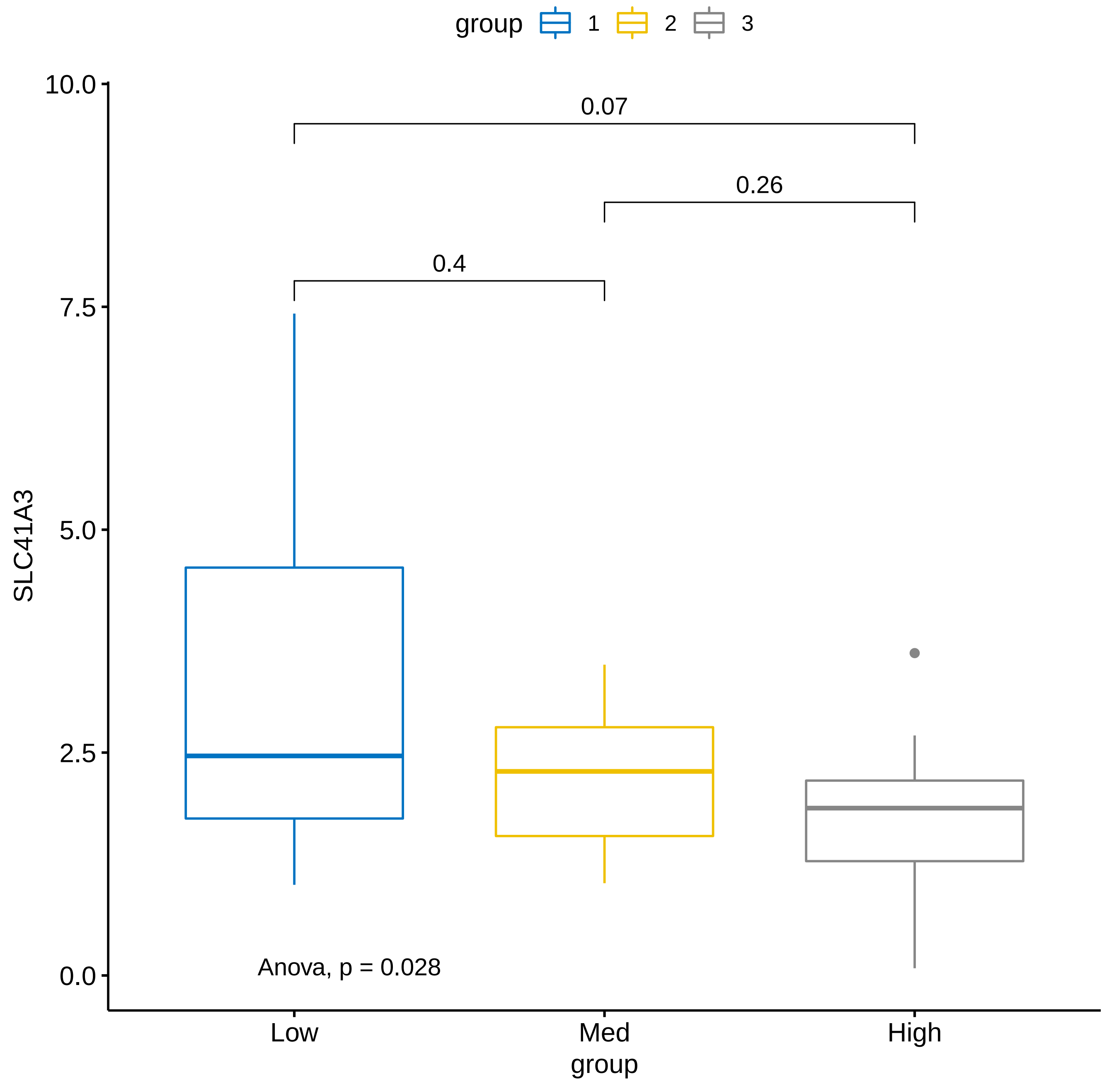
group
1
2
3
10.0
7.5
5.0
2.5
0.0
SLC41A3
0.07
0.26
0.4
Anova, p = 0.028
Low
Med
High
group

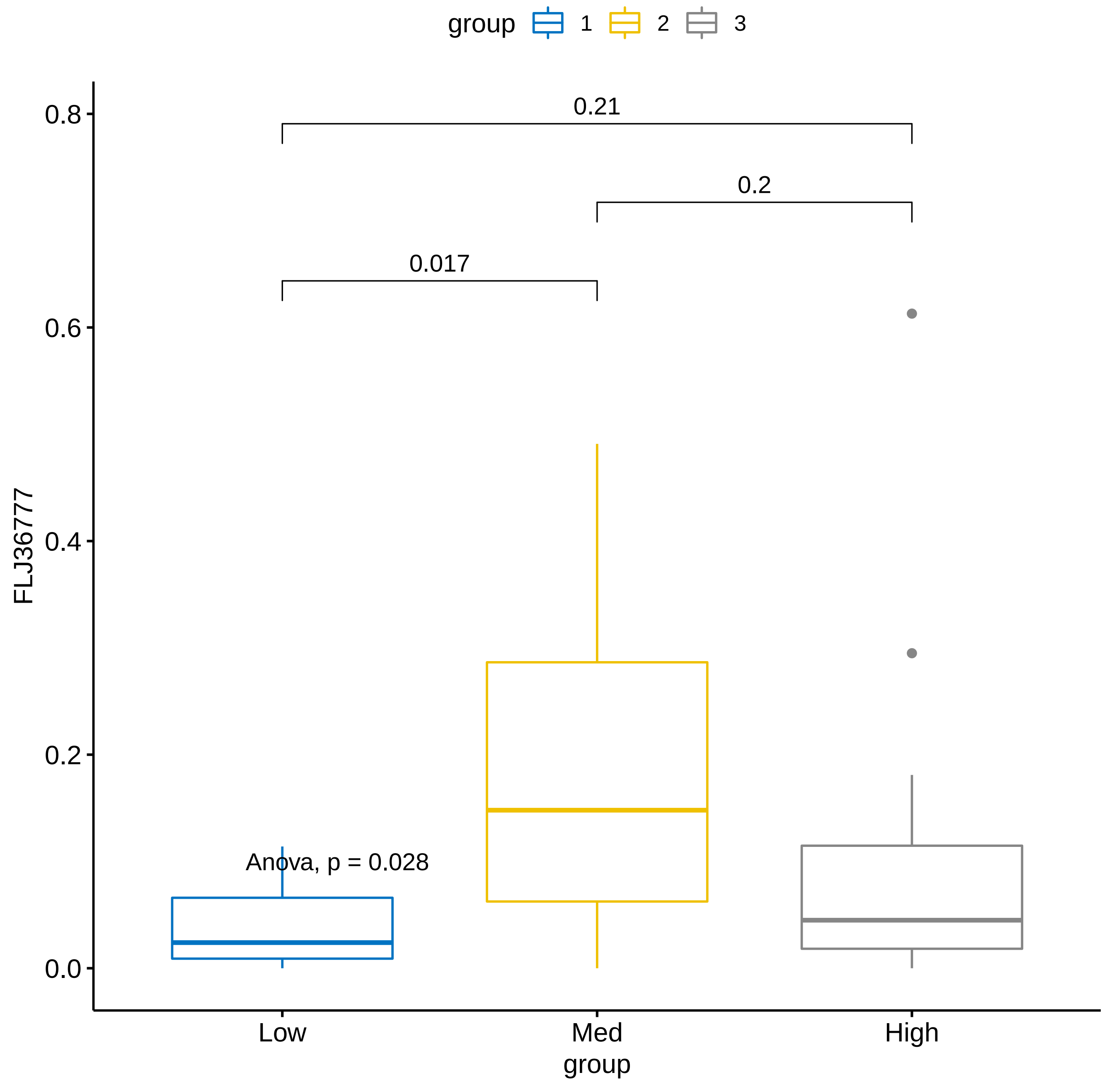
group 1 2 3
0.21
0.2
0.017
Anova, p = 0.028
FLJ36777
0.8
0.6
0.4
0.2
0.0
Low
Med
High
group

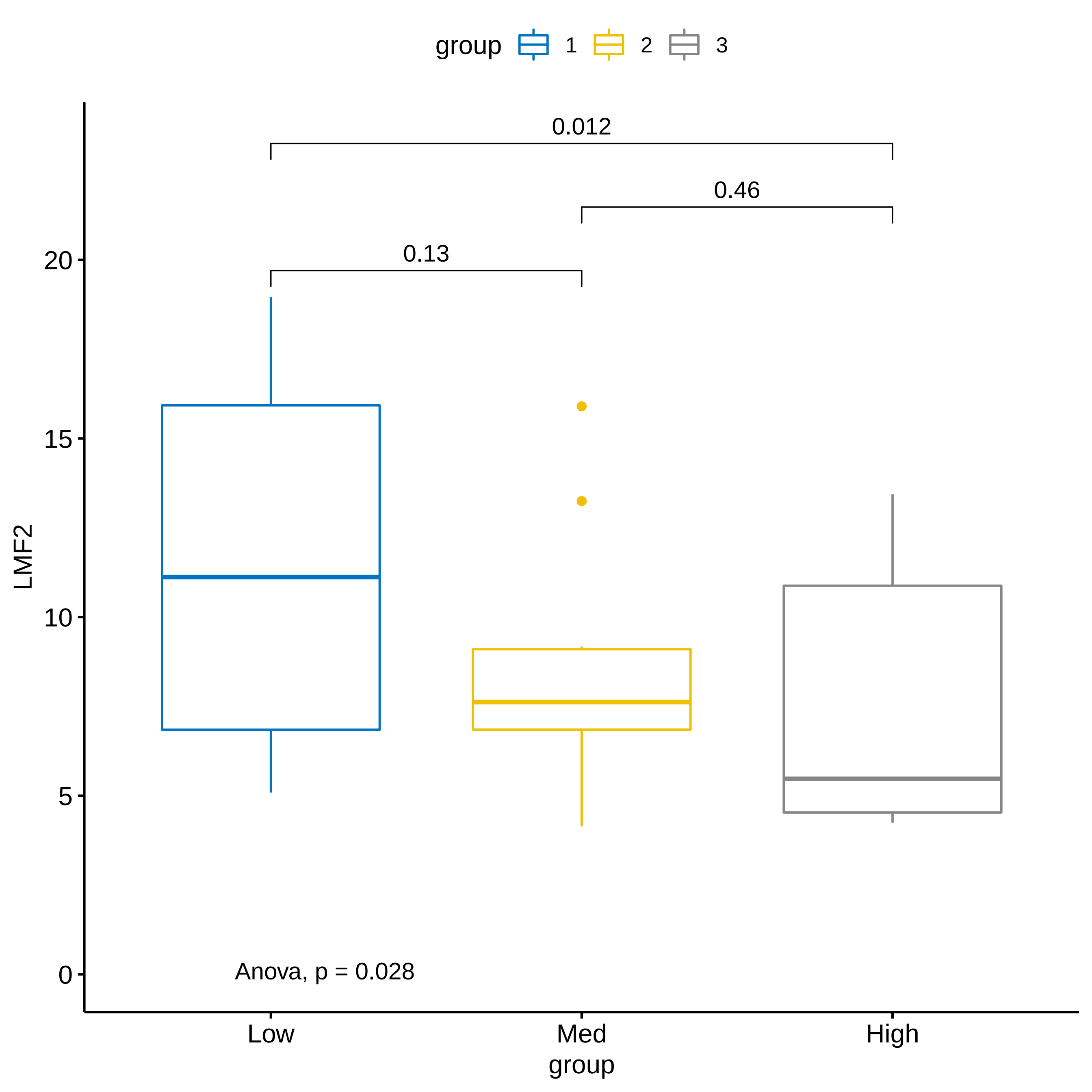
group
1
2
3
0.012
0.46
0.13
20
15
10
5
0
LMF2
Anova, p = 0.028
Low
Med
High
group

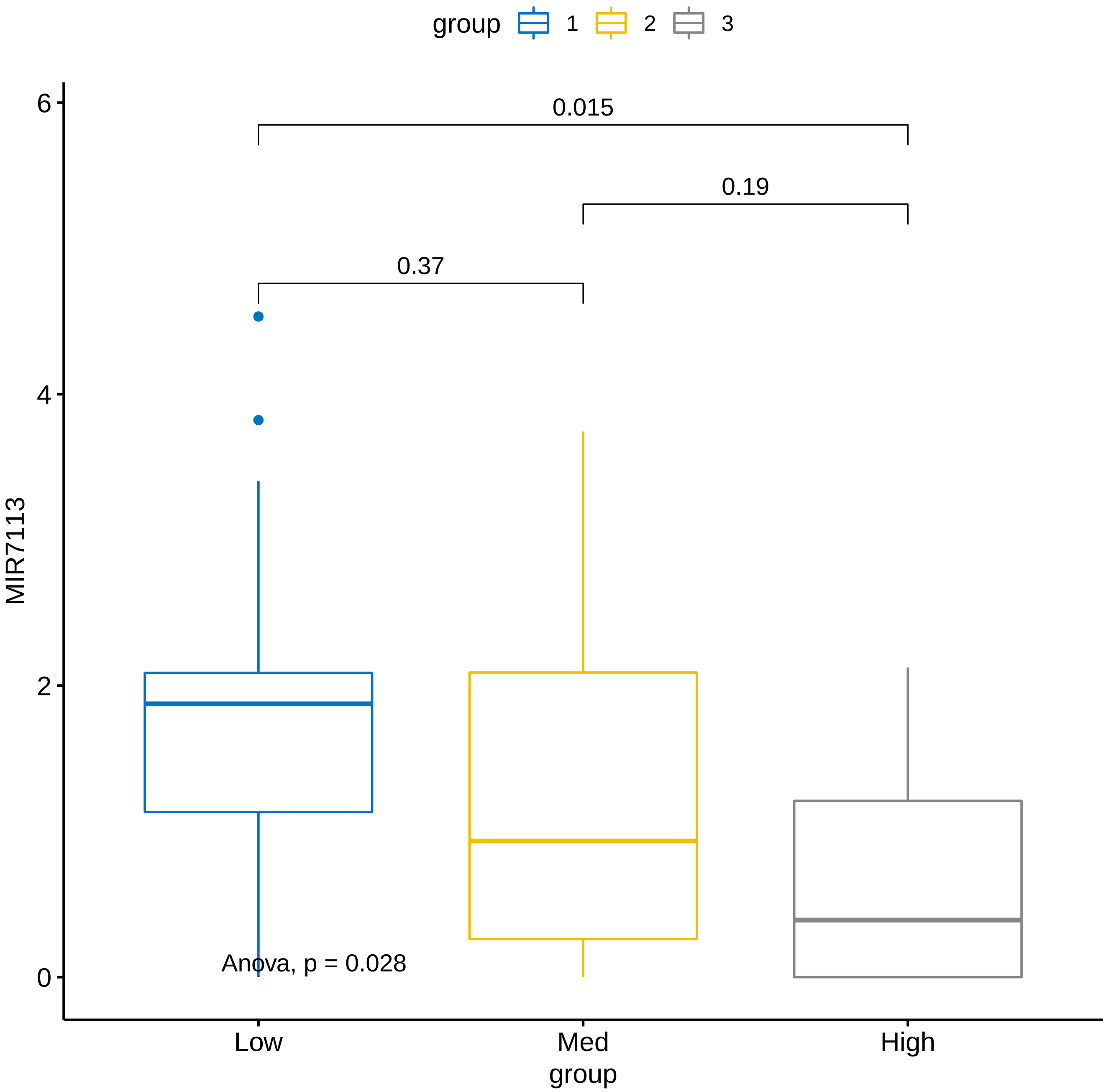

group
1
2
3
0.015
0.19
0.37
6
4
2
0
MIR7113
Anova, p = 0.028
Low
Med
High
group

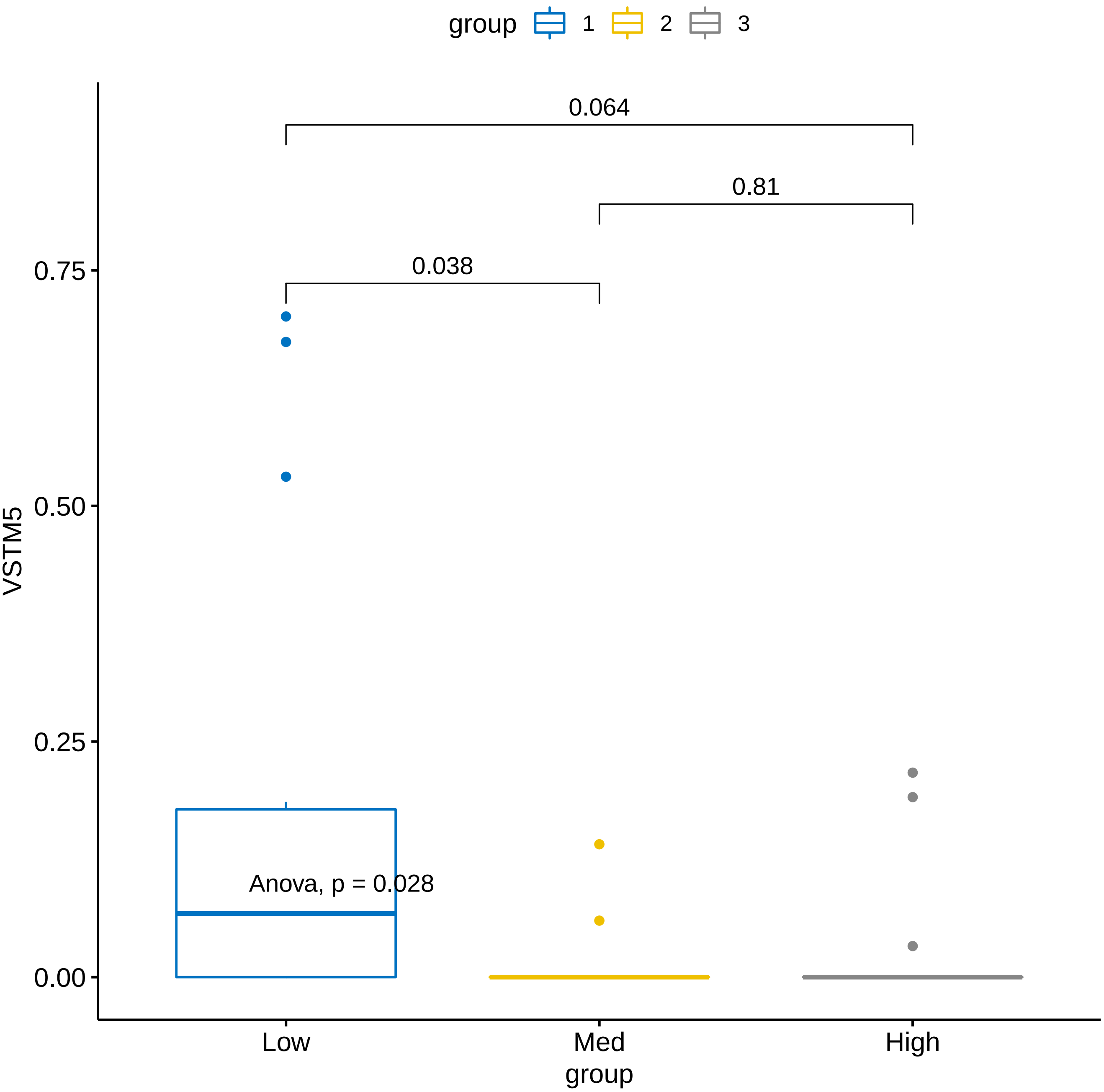
group 1 2 3
0.064
0.81
0.038
0.75
0.50
0.25
0.00
VSTM5
Anova, p = 0.028
Low
Med
High
group

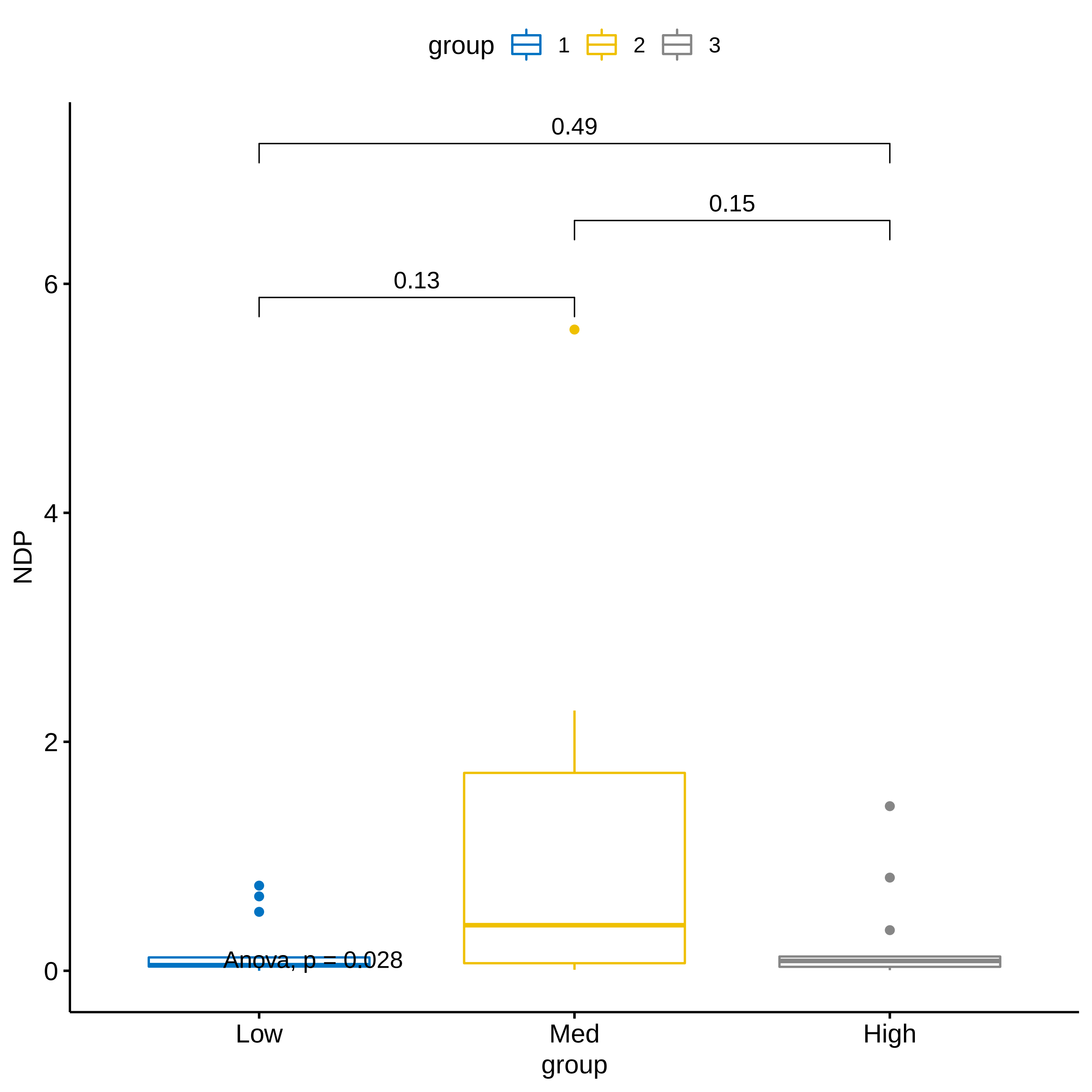
group
1
2
3
0.49
0.15
0.13
NDP
6
4
2
0
Anova, p = 0.028
Low
Med
High
group

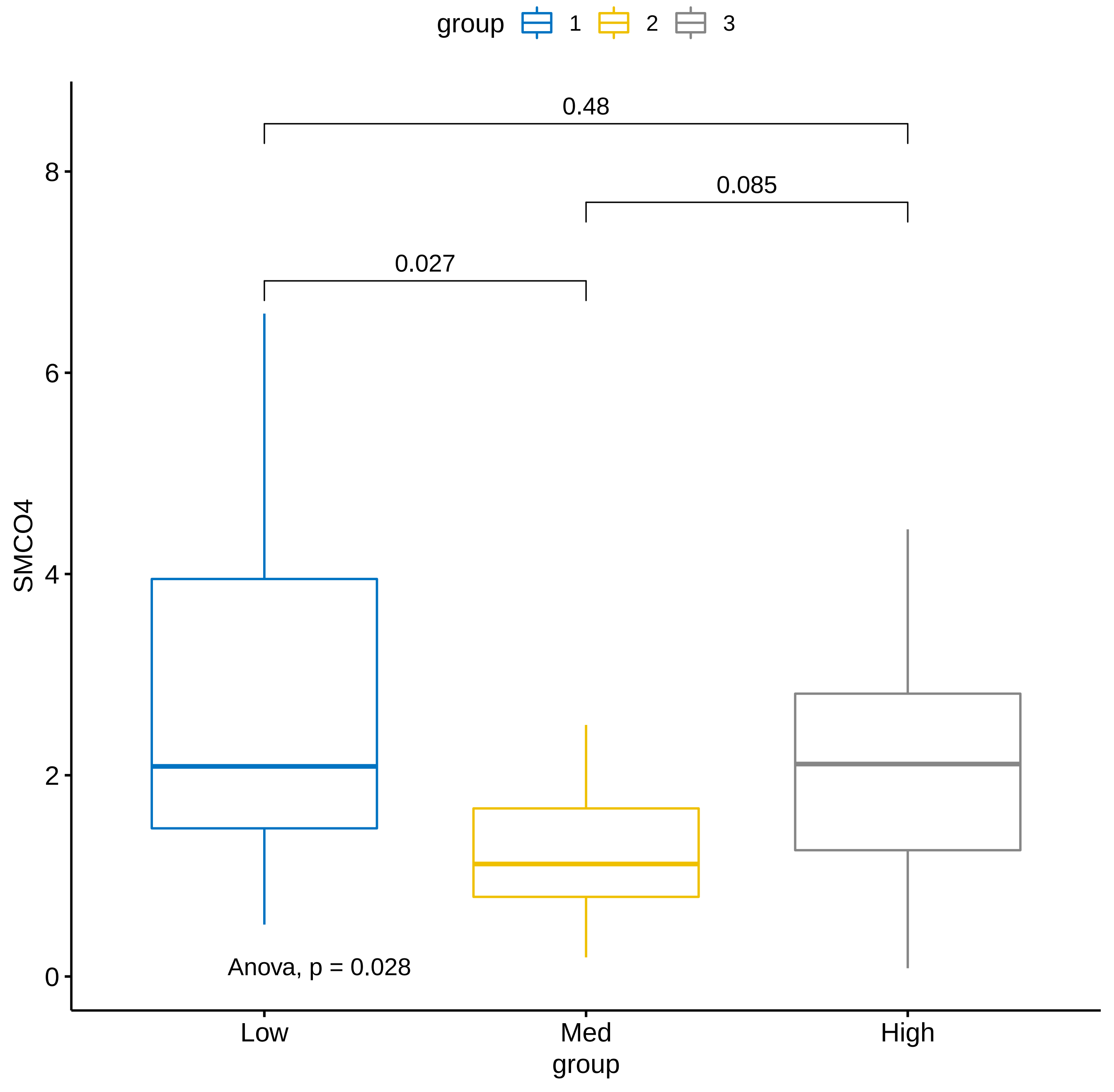

group
1
2
3
0.48
0.085
0.027
SMCO4
8
6
4
2
0
Anova, p = 0.028
Low
Med
High
group

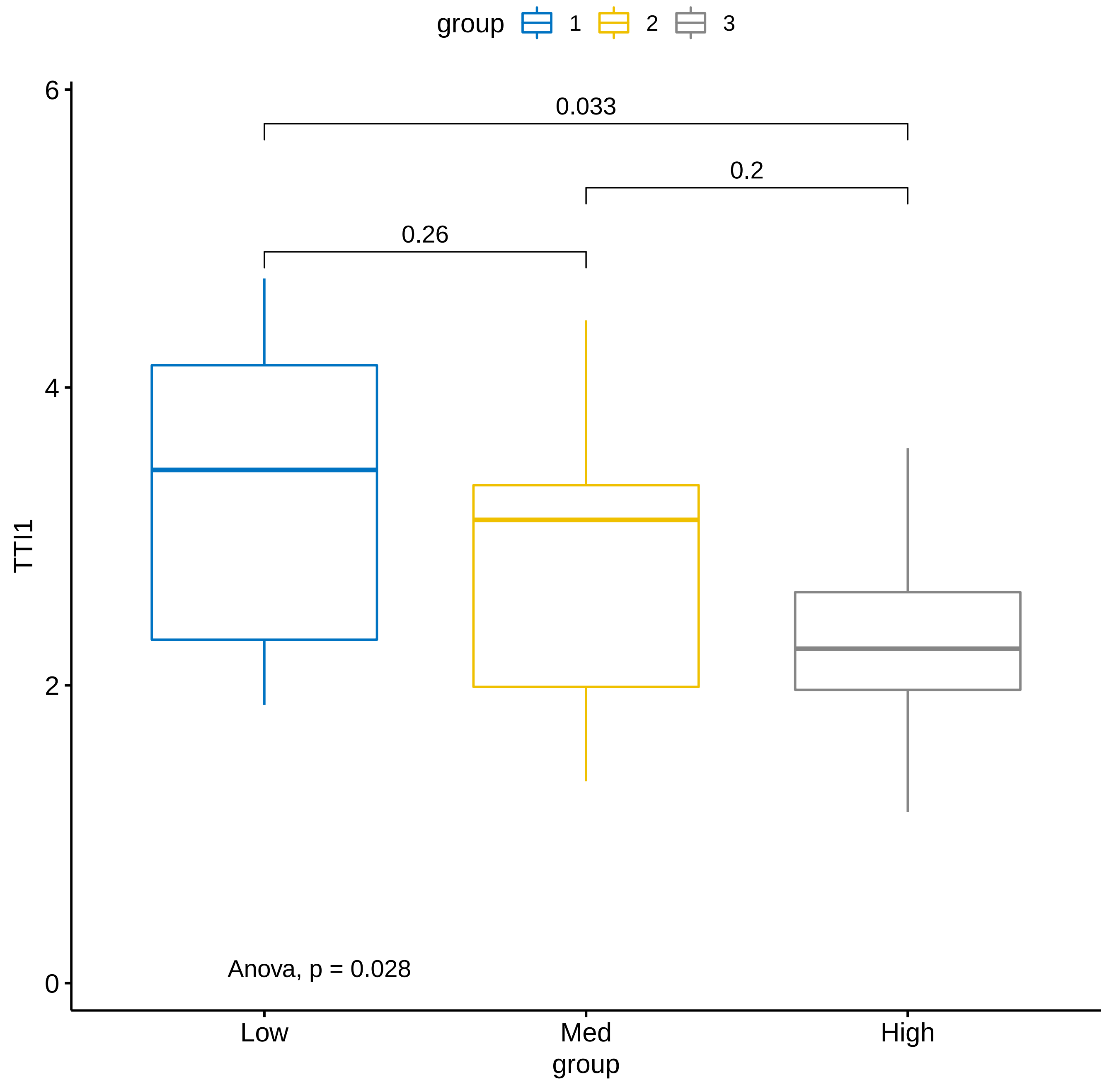
group
1
2
3
6
4
2
0
TTI1
0.033
0.2
0.26
Anova, p = 0.028
Low
Med
High
group

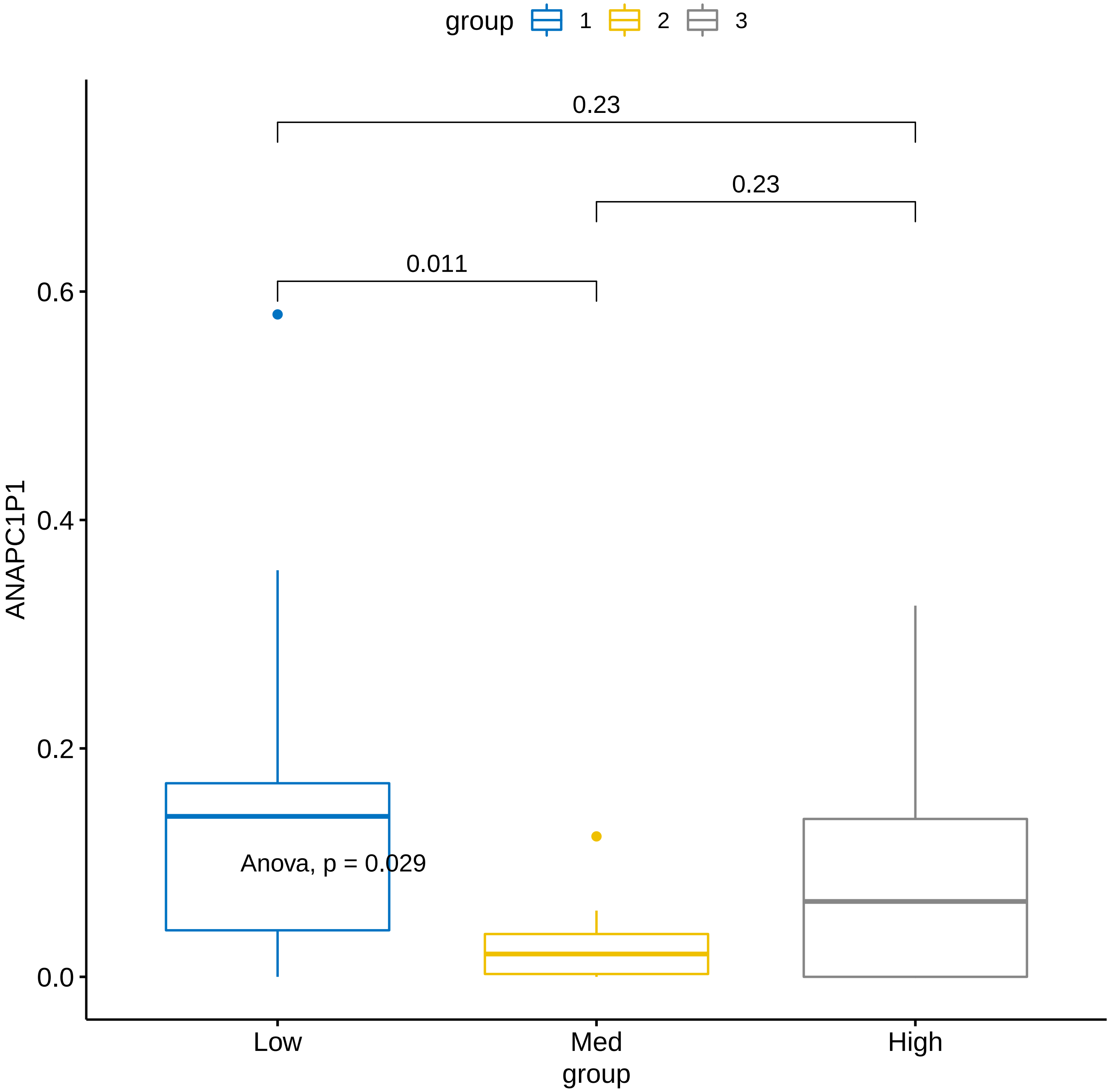
group
1
2
3
0.23
0.23
0.011
Anova, p = 0.029
ANAPC1P1
0.6
0.4
0.2
0.0
Low
Med
High
group

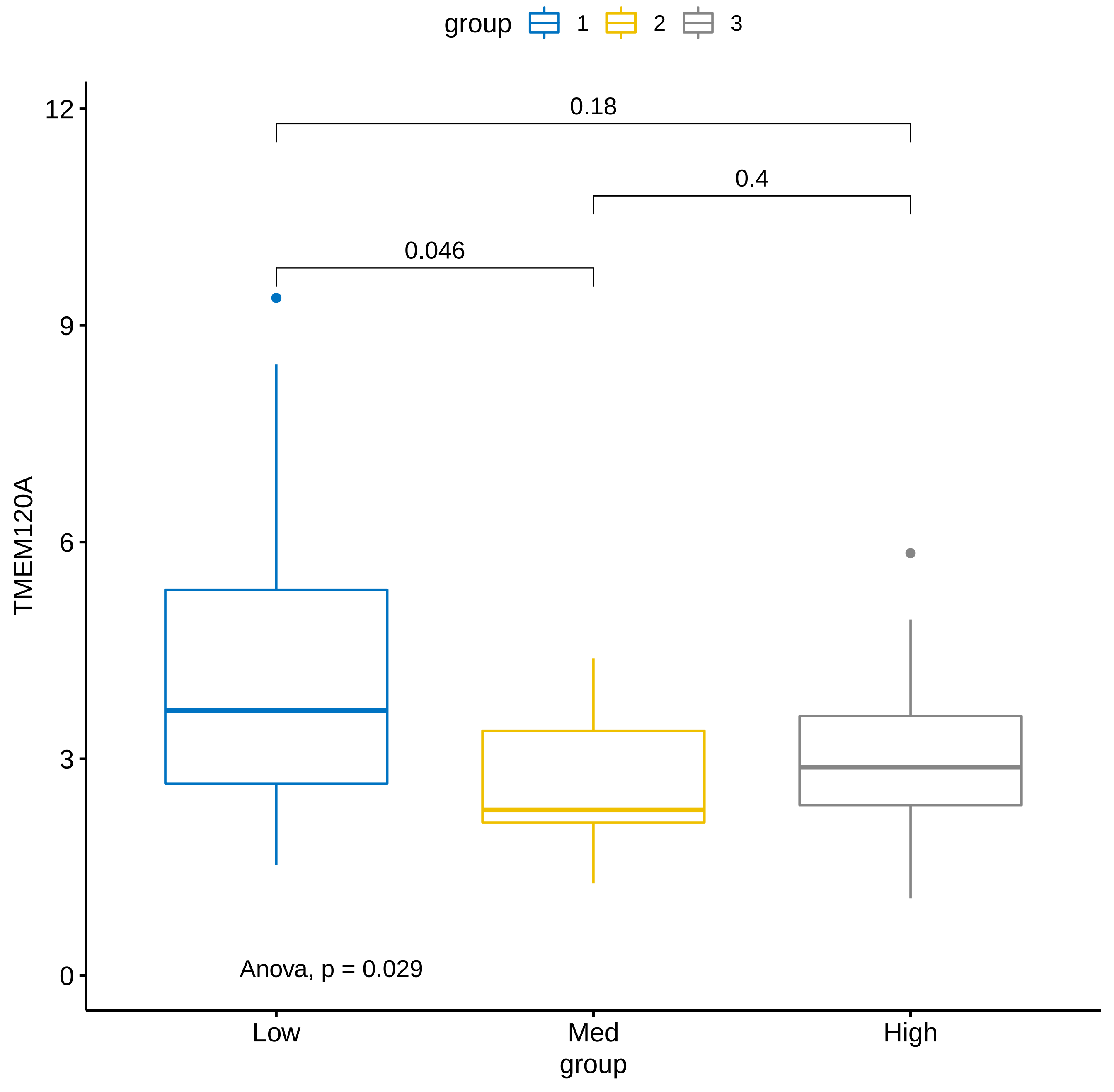

group 1 2 3
0.18
0.4
0.046
12
9
6
3
0
TMEM120A
Anova, p = 0.029
Low
Med
High
group

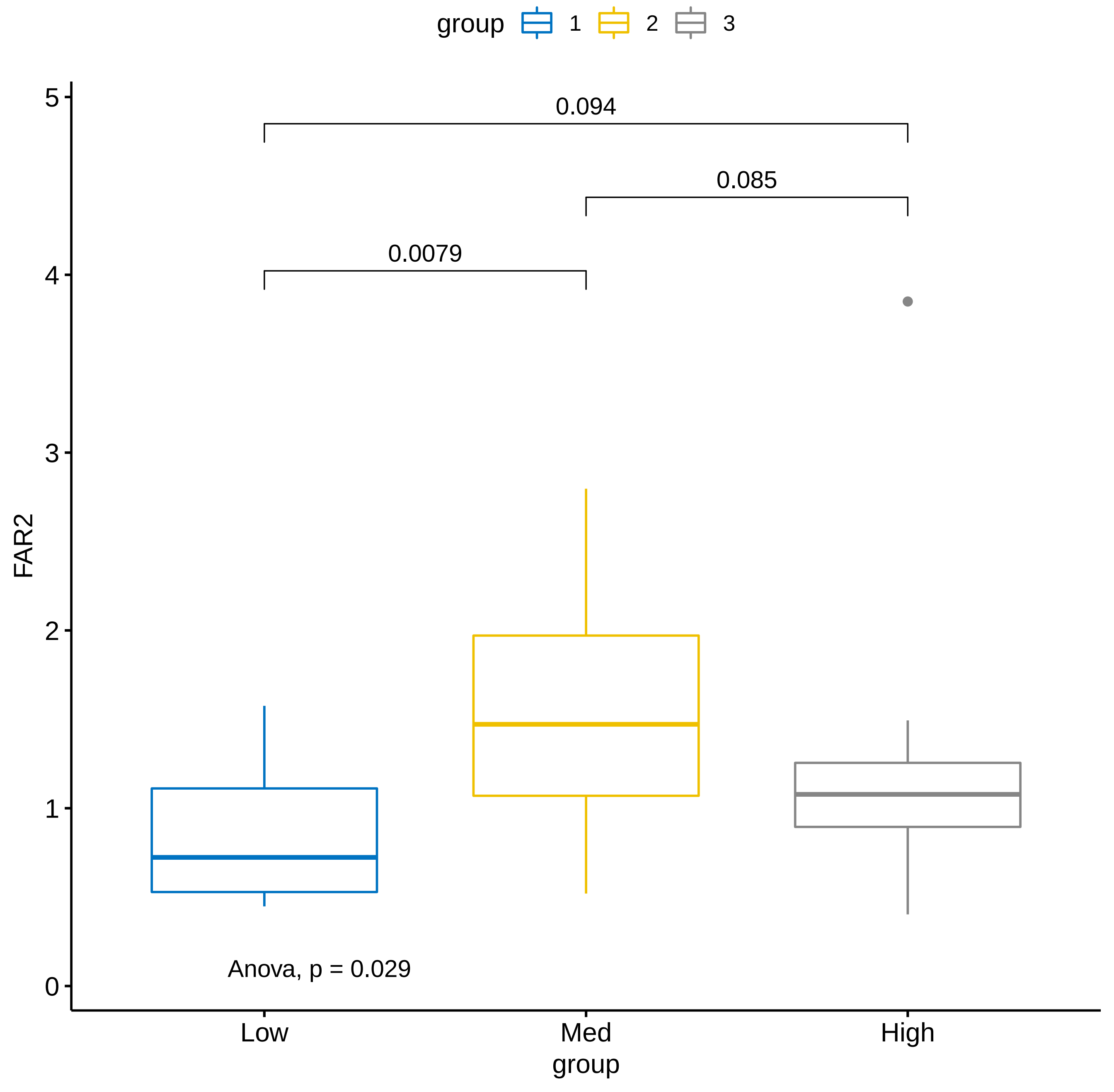
group
1
2
3
0.094
0.085
0.0079
5
4
3
2
1
0
FAR2
Anova, p = 0.029
Low
Med
High
group

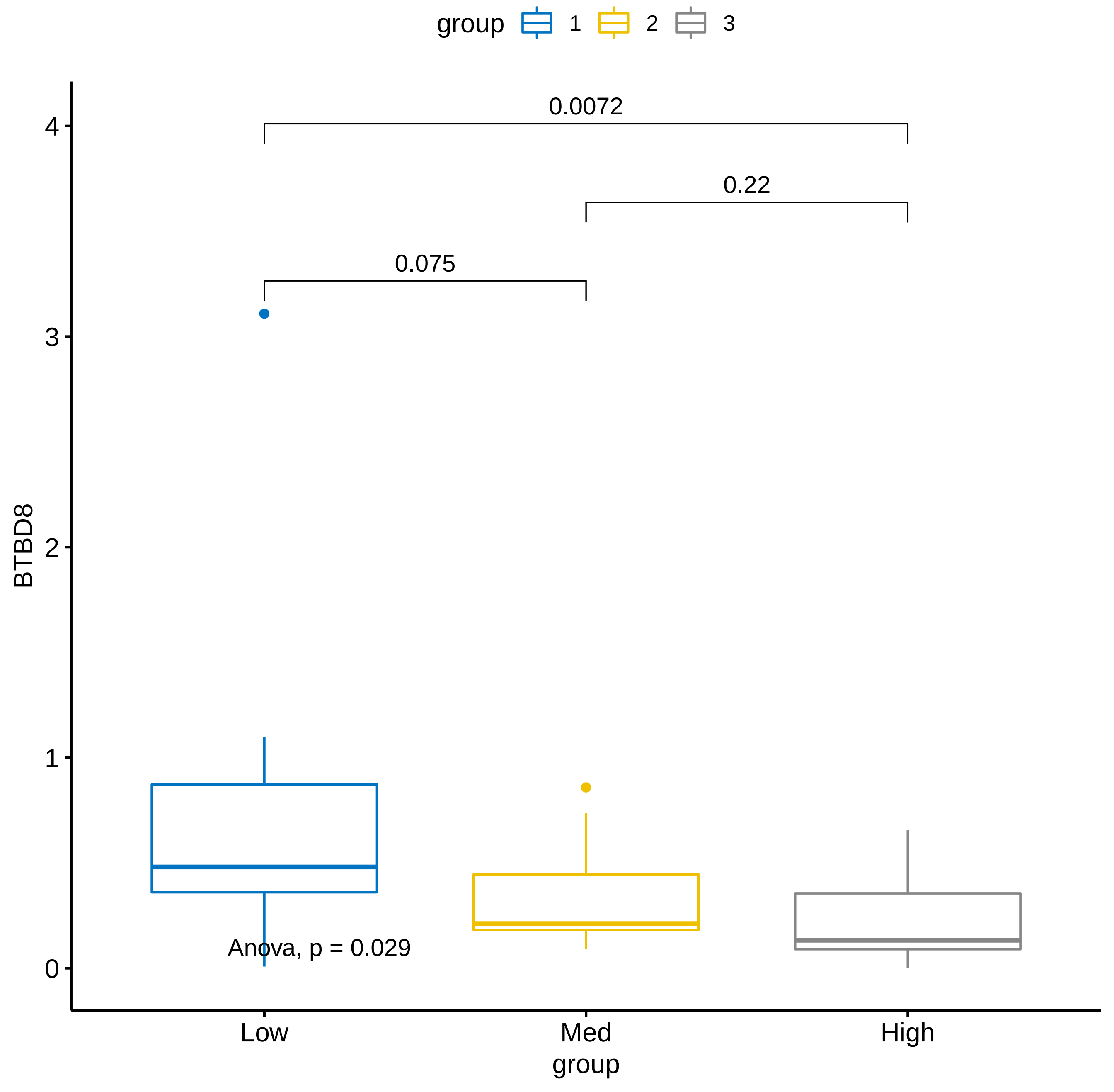
group
1
2
3
0.0072
0.22
0.075
BTBD8
4
3
2
1
0
Anova, p = 0.029
Low
Med
High
group

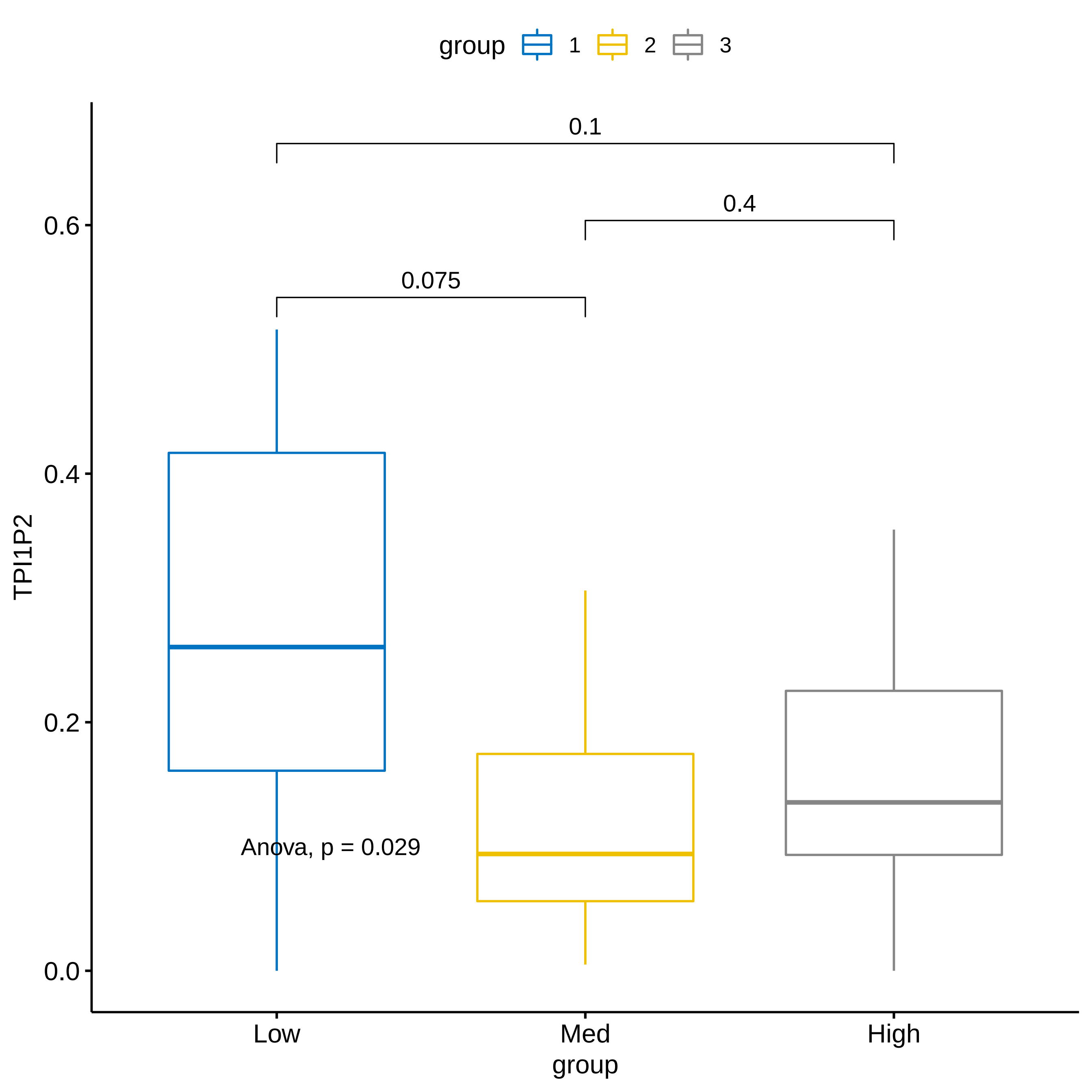
group
1
2
3
0.1
0.4
0.075
0.6
0.4
0.2
0.0
TPI1P2
Anova, p = 0.029
Low
Med
High
group

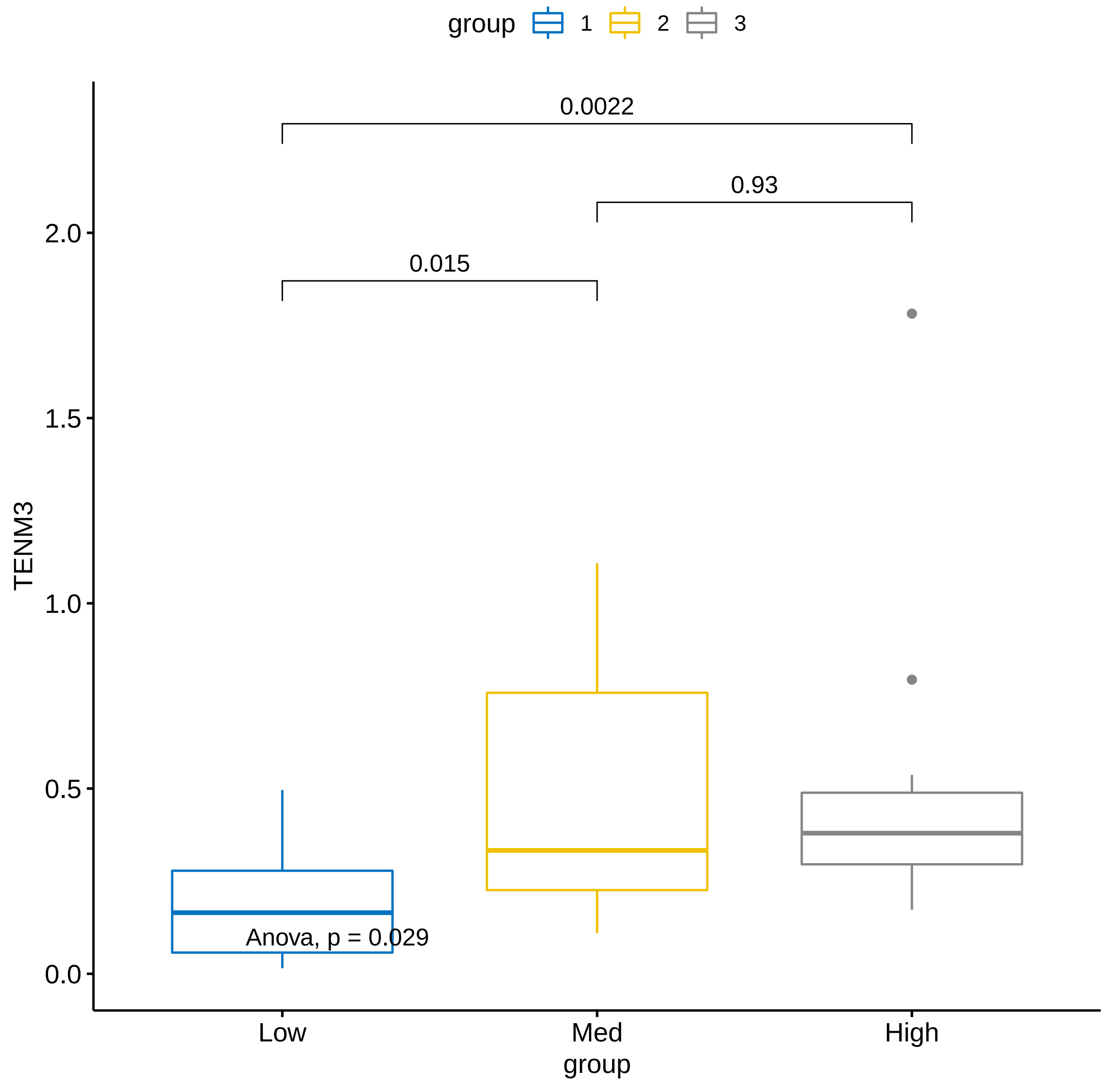

group
1
2
3
0.0022
0.93
0.015
2.0
1.5
1.0
0.5
0.0
TENM3
Anova, p = 0.029
Low
Med
High
group

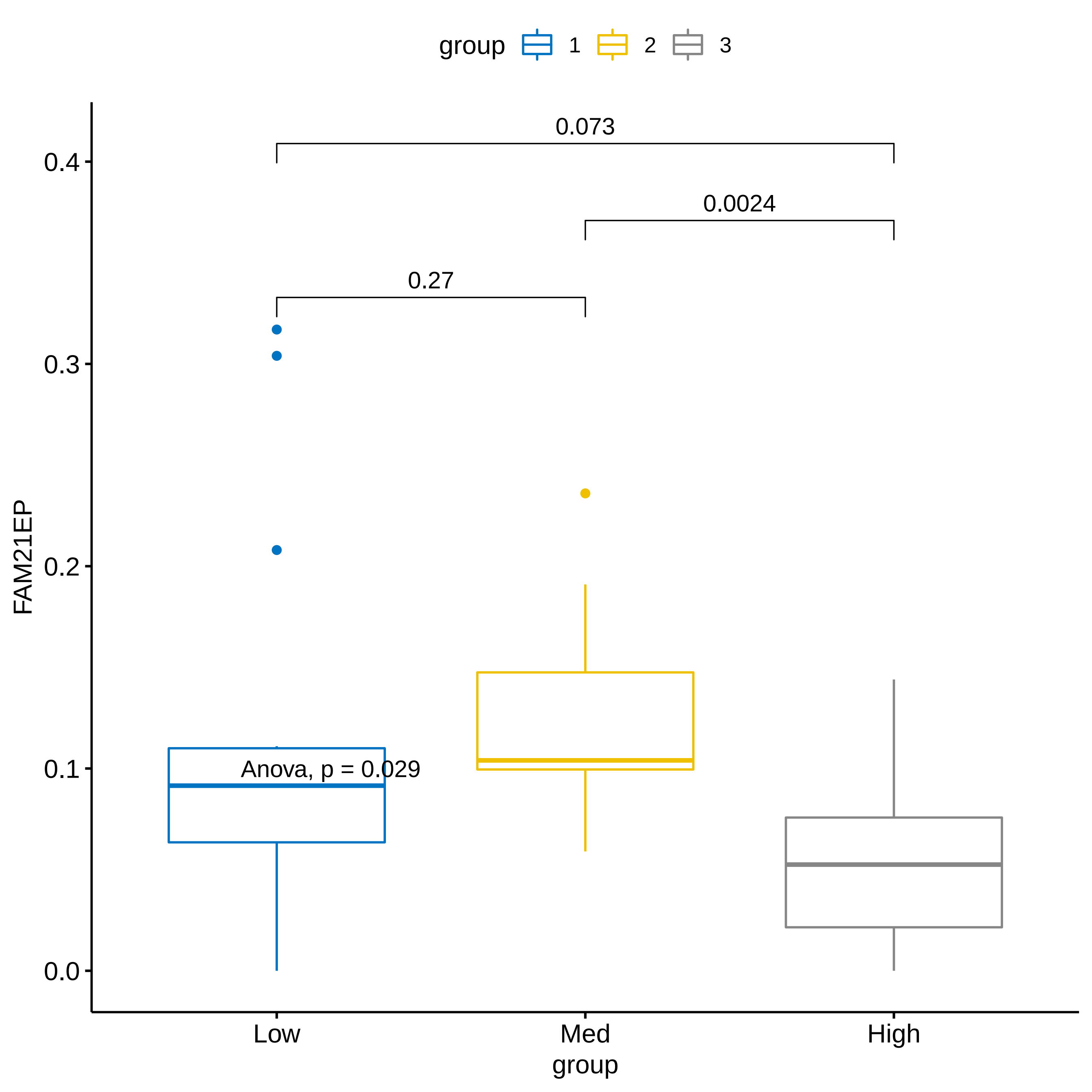
group
1
2
3
0.073
0.0024
0.27
Anova, p = 0.029
FAM21EP
0.0
0.1
0.2
0.3
0.4
Low
Med
High
group

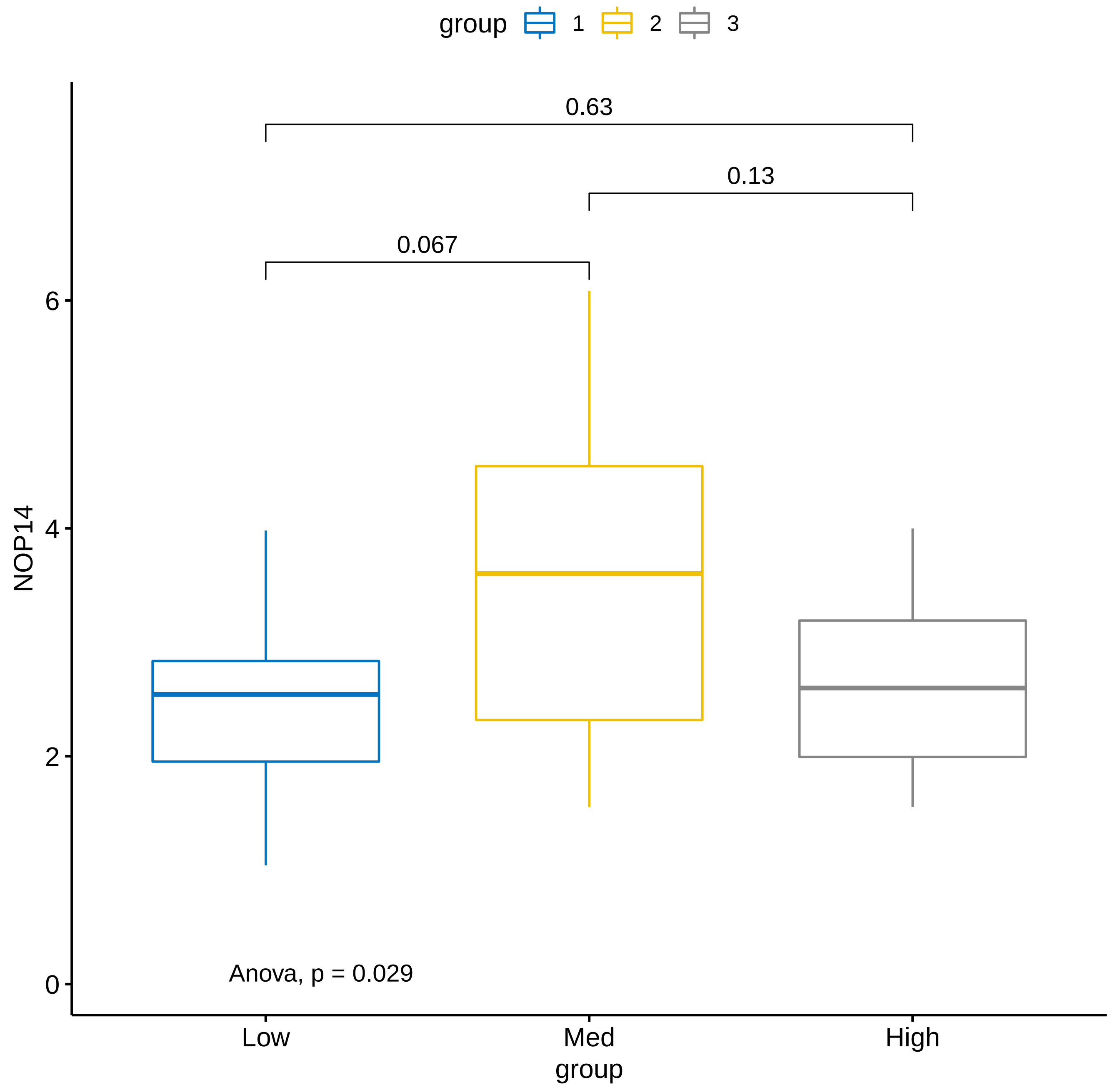

group 1 2 3
0.63
0.13
0.067
NOP14
6
4
2
0
Anova, p = 0.029
Low
Med
High
group

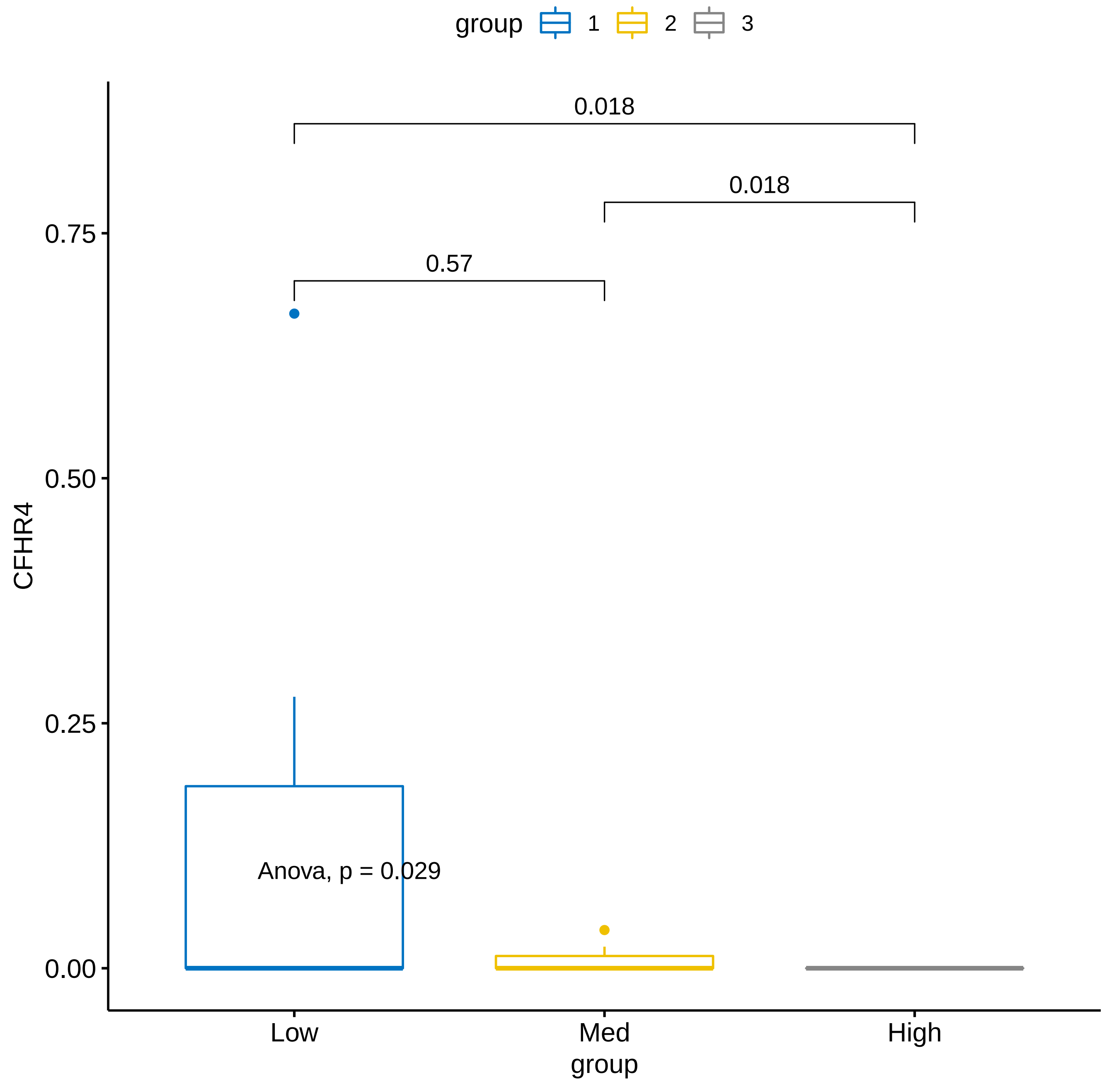
group
1
2
3
0.018
0.018
0.57
Anova, p = 0.029
0.75
0.50
0.25
0.00
CFHR4
Low
Med
High
group

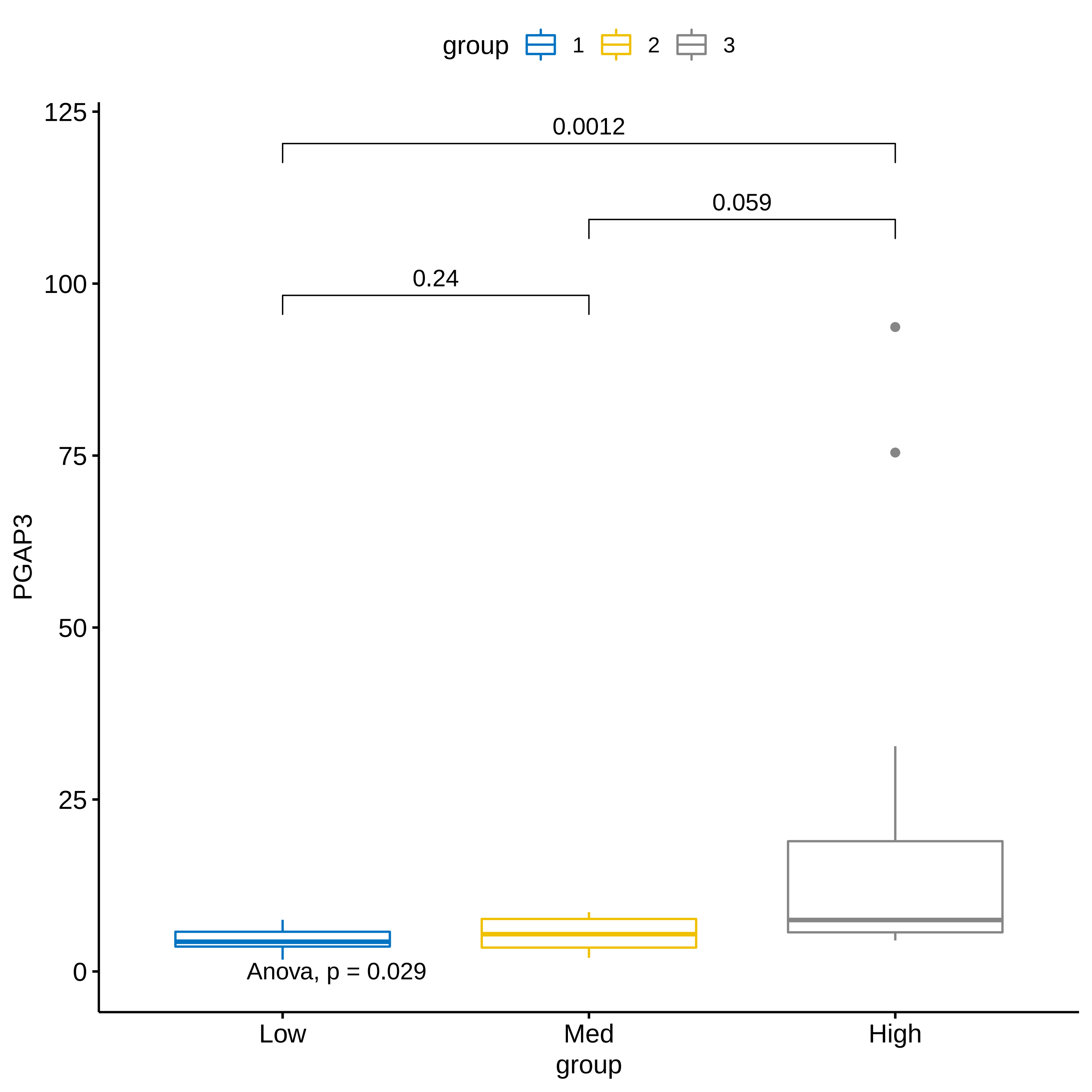
group
1
2
3
0.0012
0.059
0.24
125
100
75
50
25
0
PGAP3
Anova, p = 0.029
Low
Med
High
group

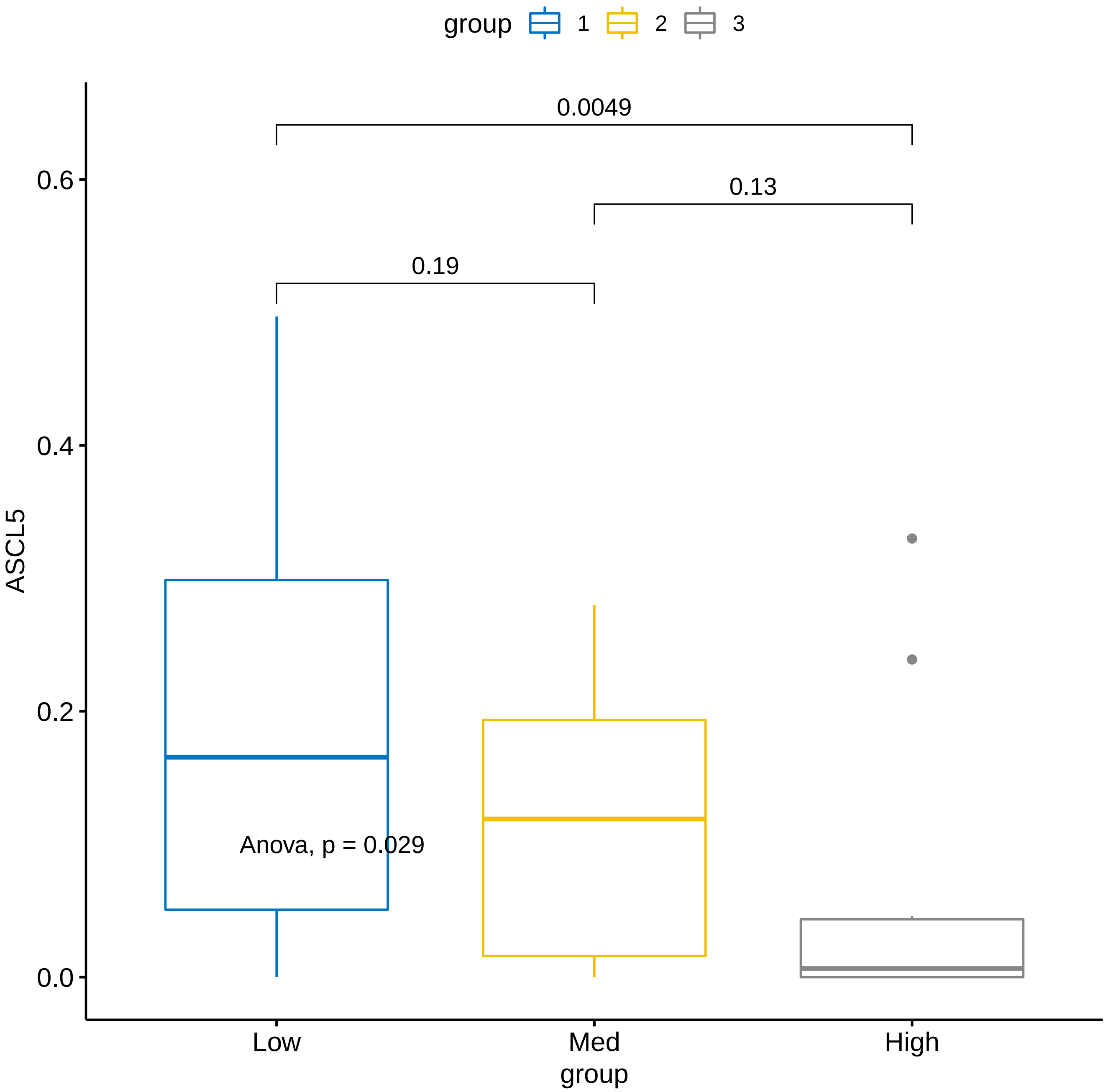
group 1 2 3
0.0049
0.13
0.19
Anova, p = 0.029
ASCL5
0.6
0.4
0.2
0.0
Low
Med
High
group

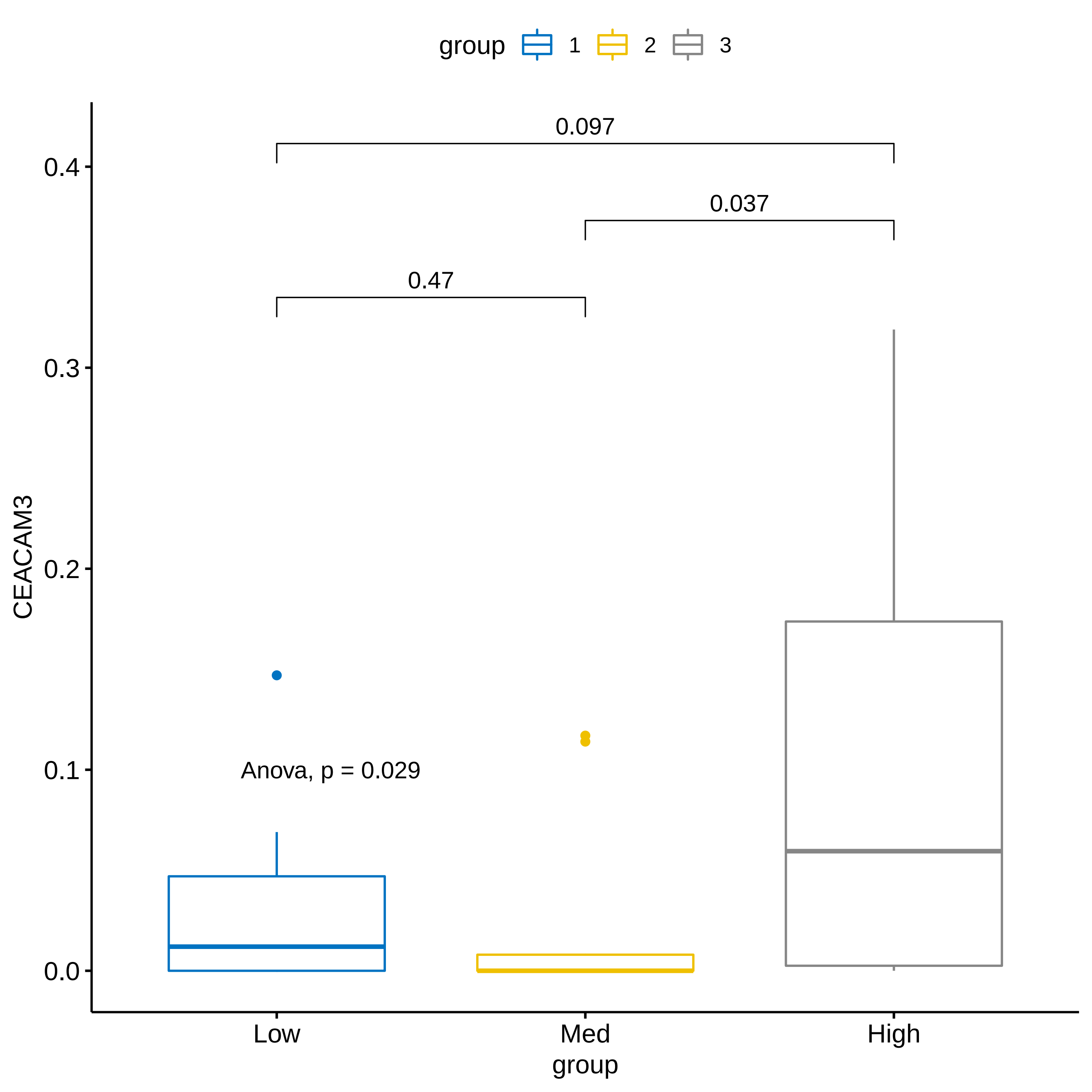

group
1
2
3
0.097
0.037
0.47
Anova, p = 0.029
0.4
0.3
0.2
0.1
0.0
CEACAM3
Low
Med
High
group

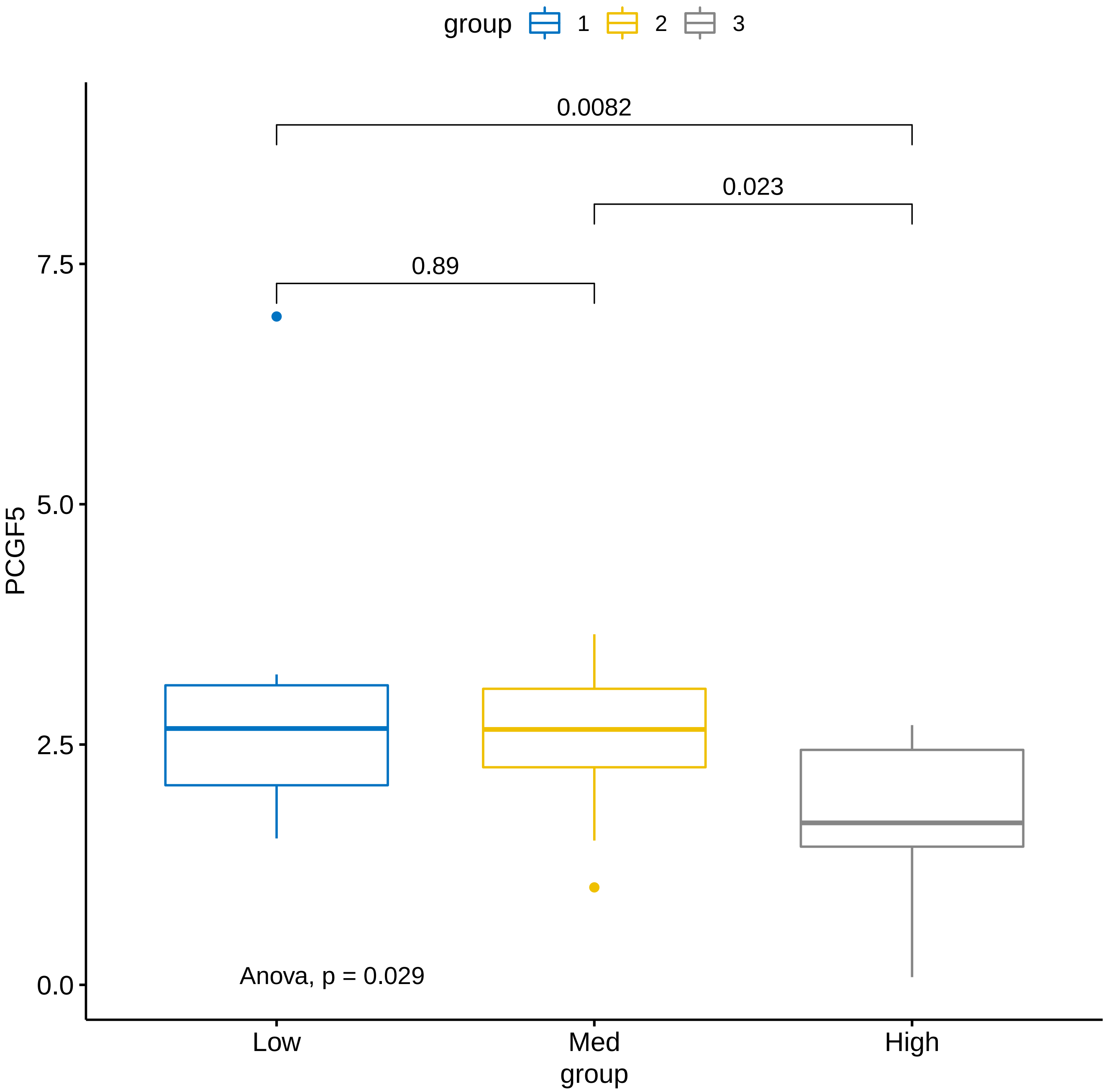
group
1
2
3
0.0082
0.023
0.89
7.5
5.0
2.5
0.0
PCGF5
Anova, p = 0.029
Low
Med
High
group

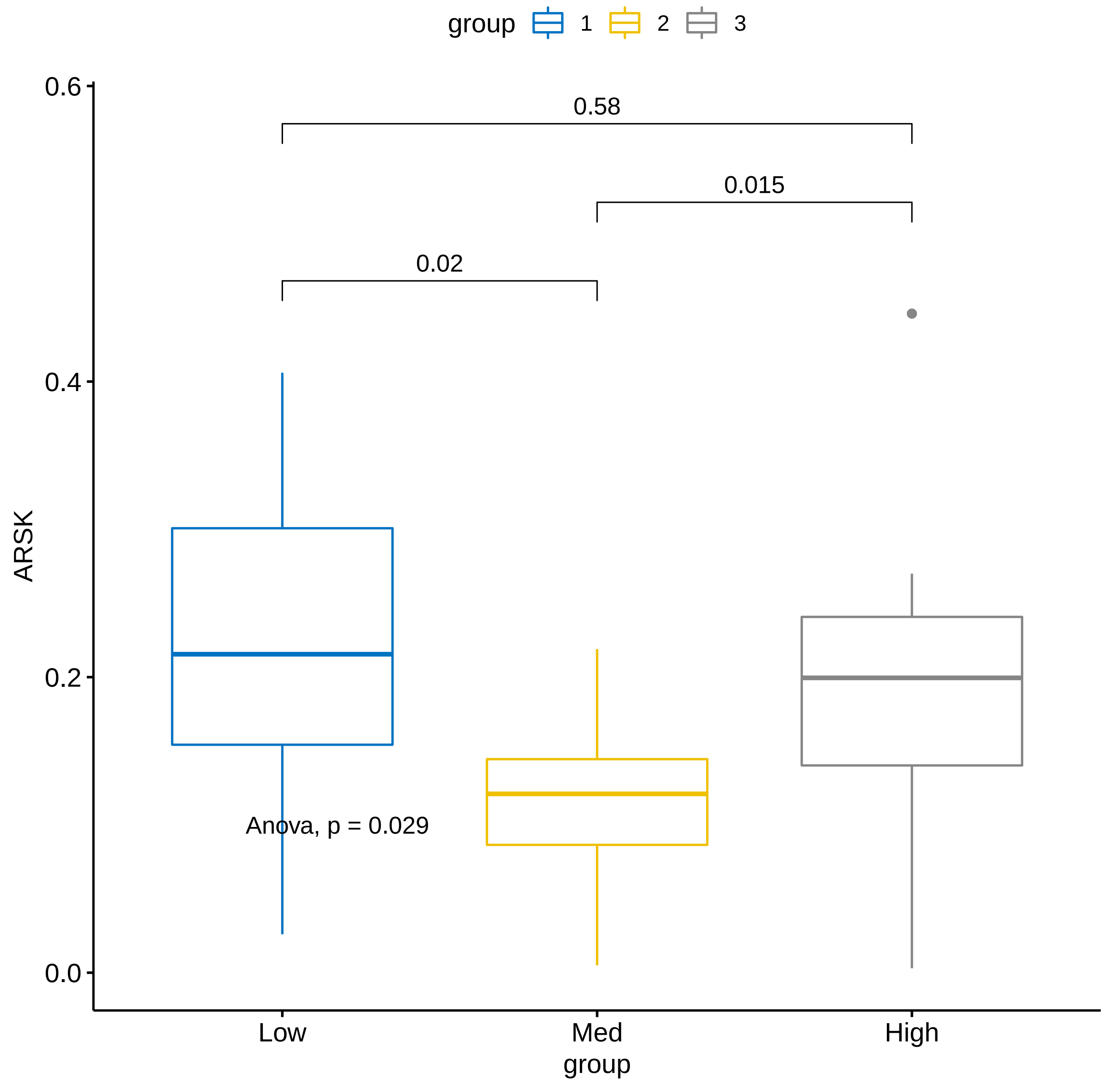
group 1 2 3
0.6
0.4
0.2
0.0
ARSK
0.58
0.015
0.02
Anova, p = 0.029
Low
Med
High
group

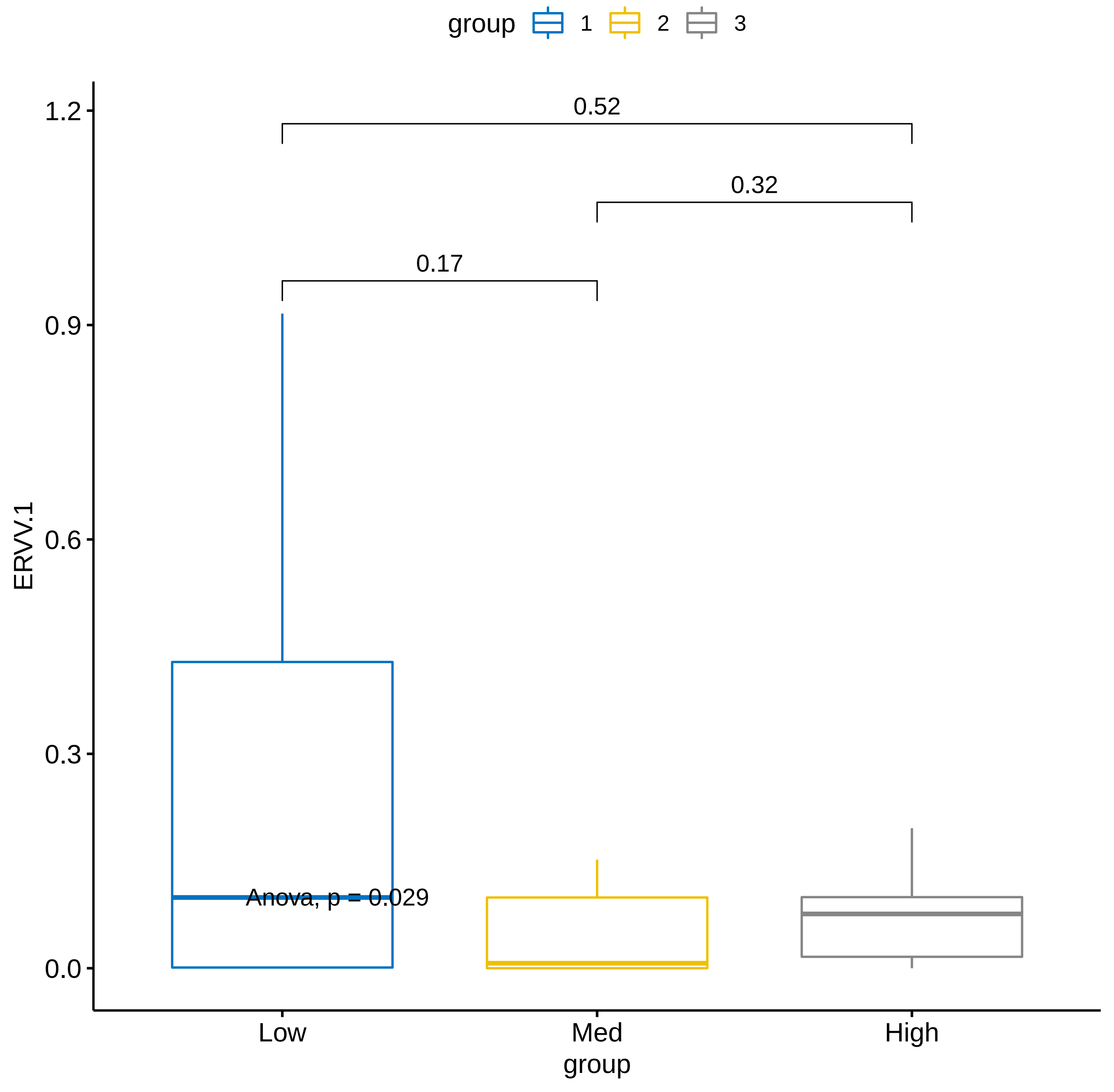

group 1 2 3
0.52
0.32
0.17
1.2
0.9
0.6
0.3
0.0
ERVV.1
Anova, p = 0.029
Low
Med
High
group

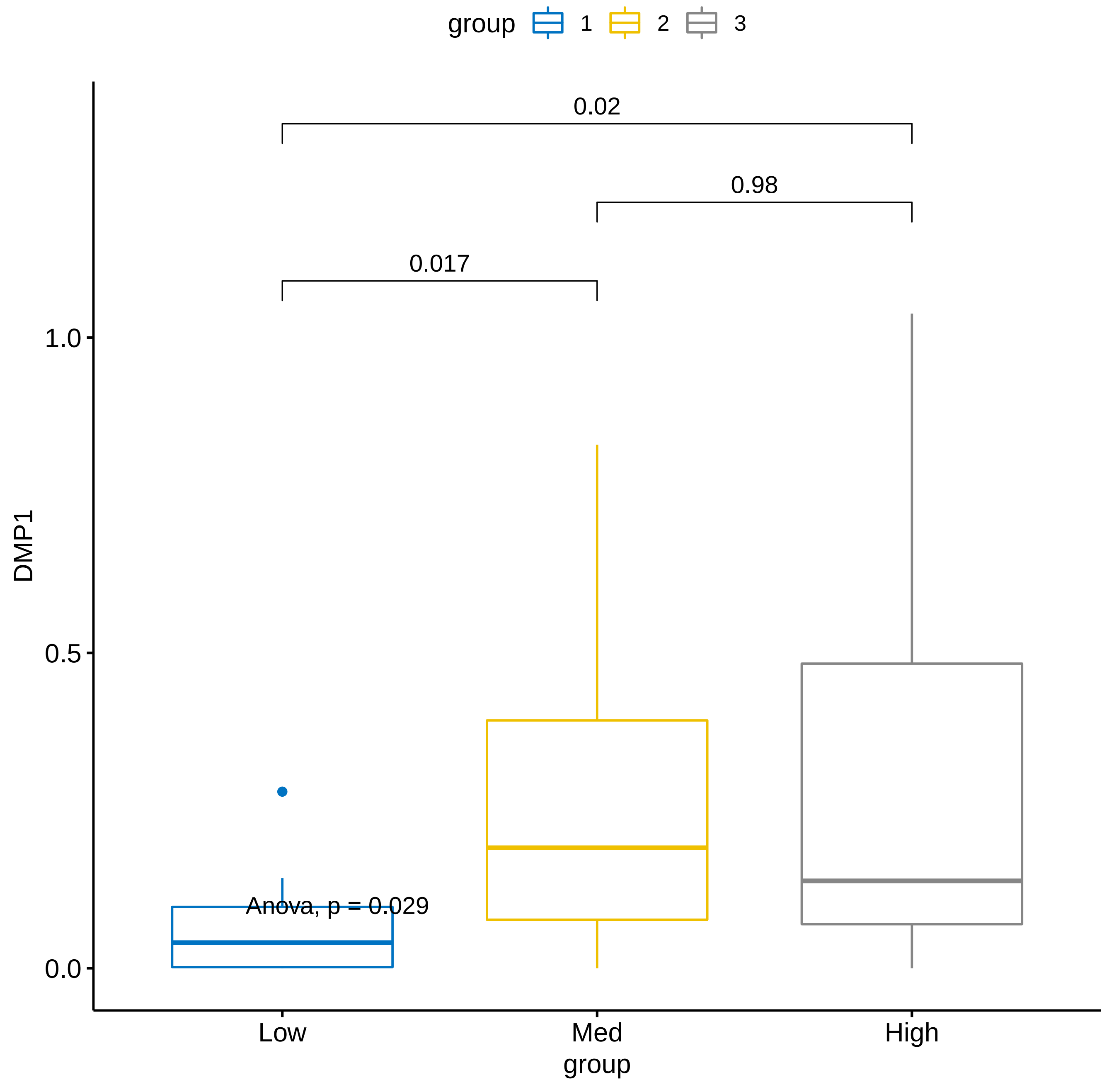
group
1
2
3
0.02
0.98
0.017
1.0
0.5
0.0
DMP1
Anova, p = 0.029
Low
Med
High
group

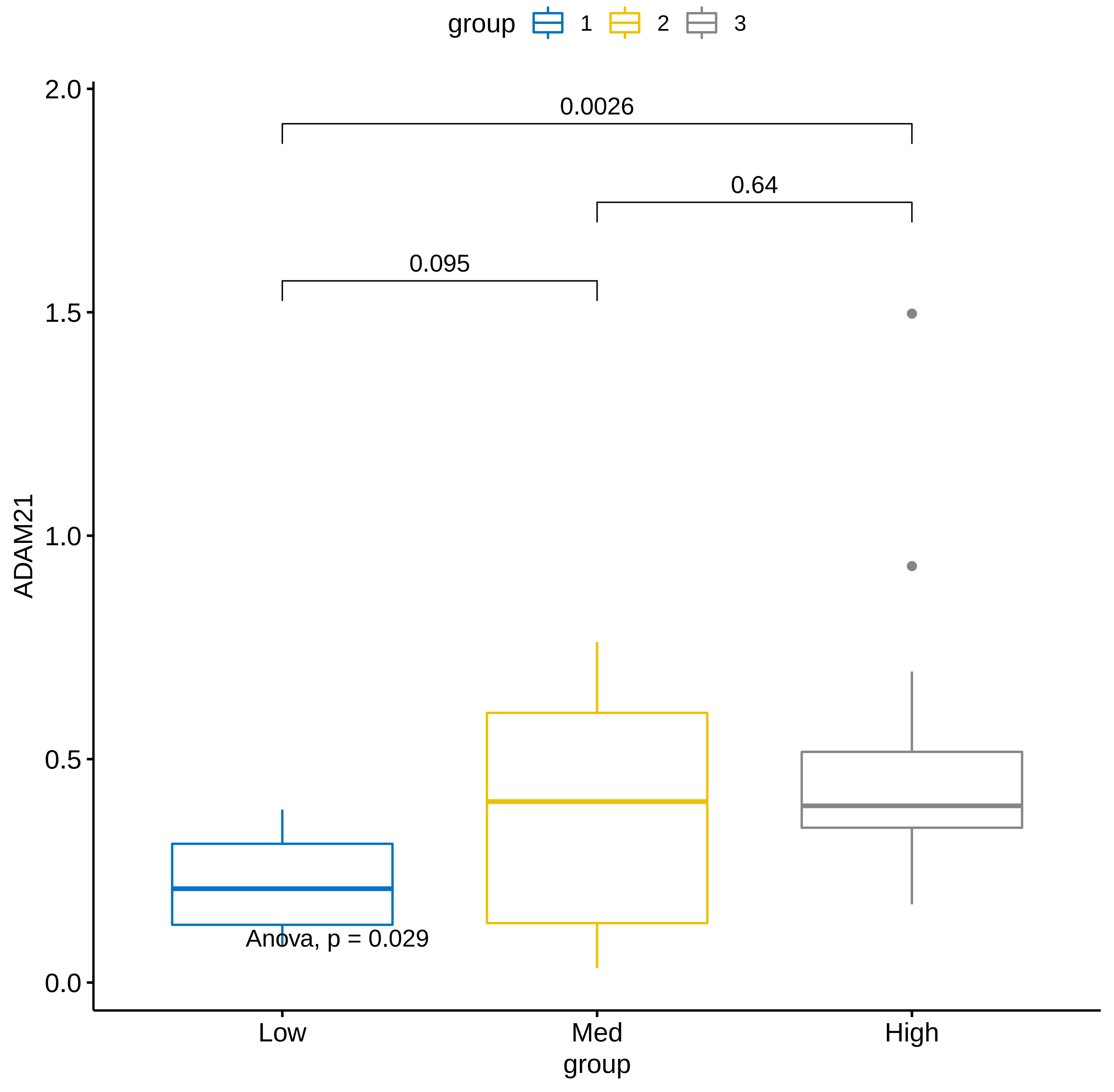
group
1
2
3
0.0026
0.64
0.095
ADAM21
2.0
1.5
1.0
0.5
0.0
Anova, p = 0.029
Low
Med
High
group

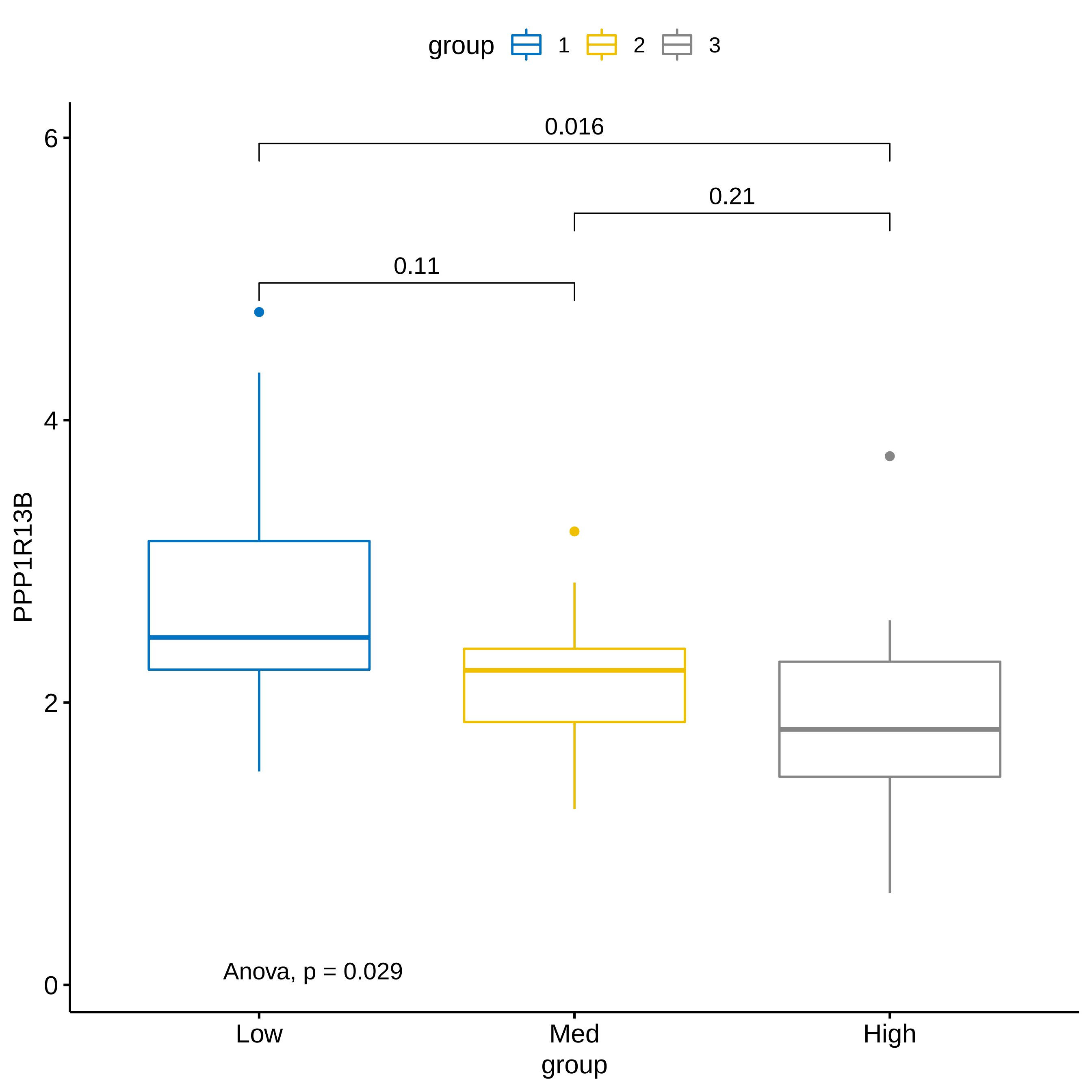
group
1
2
3
0.016
0.21
0.11
6
4
2
0
PPP1R13B
Anova, p = 0.029
Low
Med
High
group

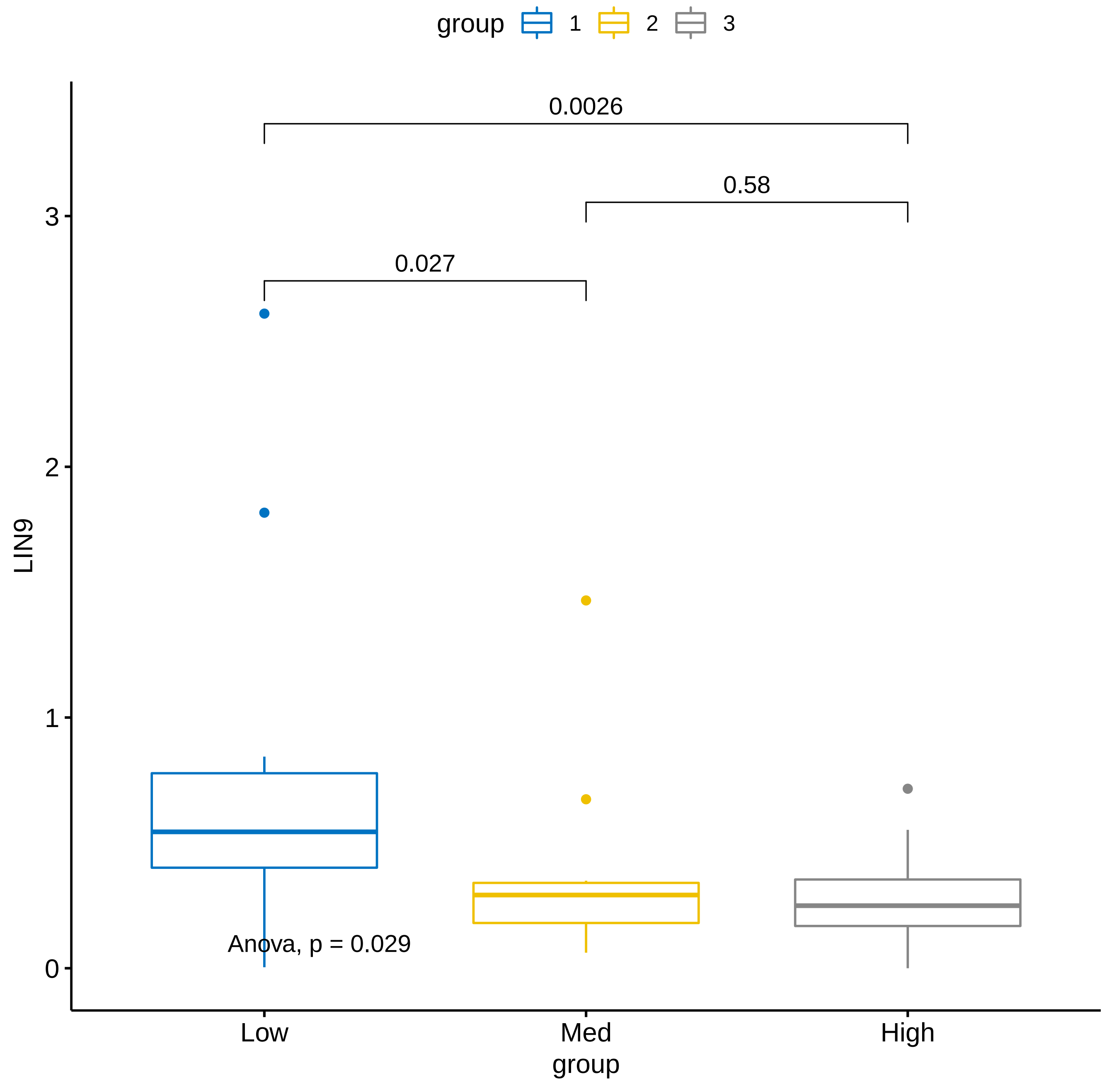
group
1
2
3
0.0026
0.58
0.027
3
2
1
0
LIN9
Anova, p = 0.029
Low
Med
High
group

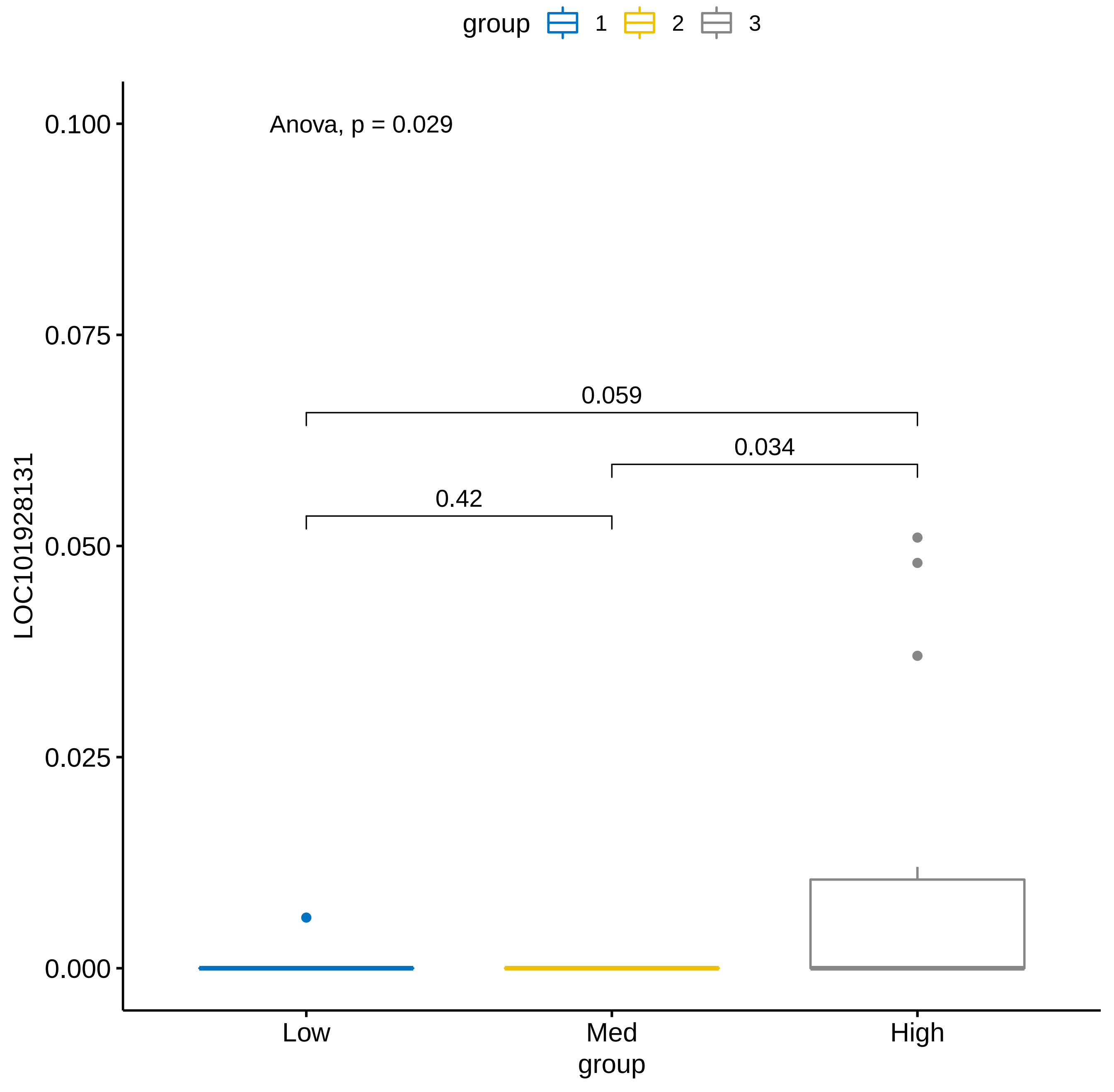
group
1
2
3
Anova, p = 0.029
0.059
0.034
0.42
0.100
0.075
0.050
0.025
0.000
LOC101928131
Low
Med
High
group

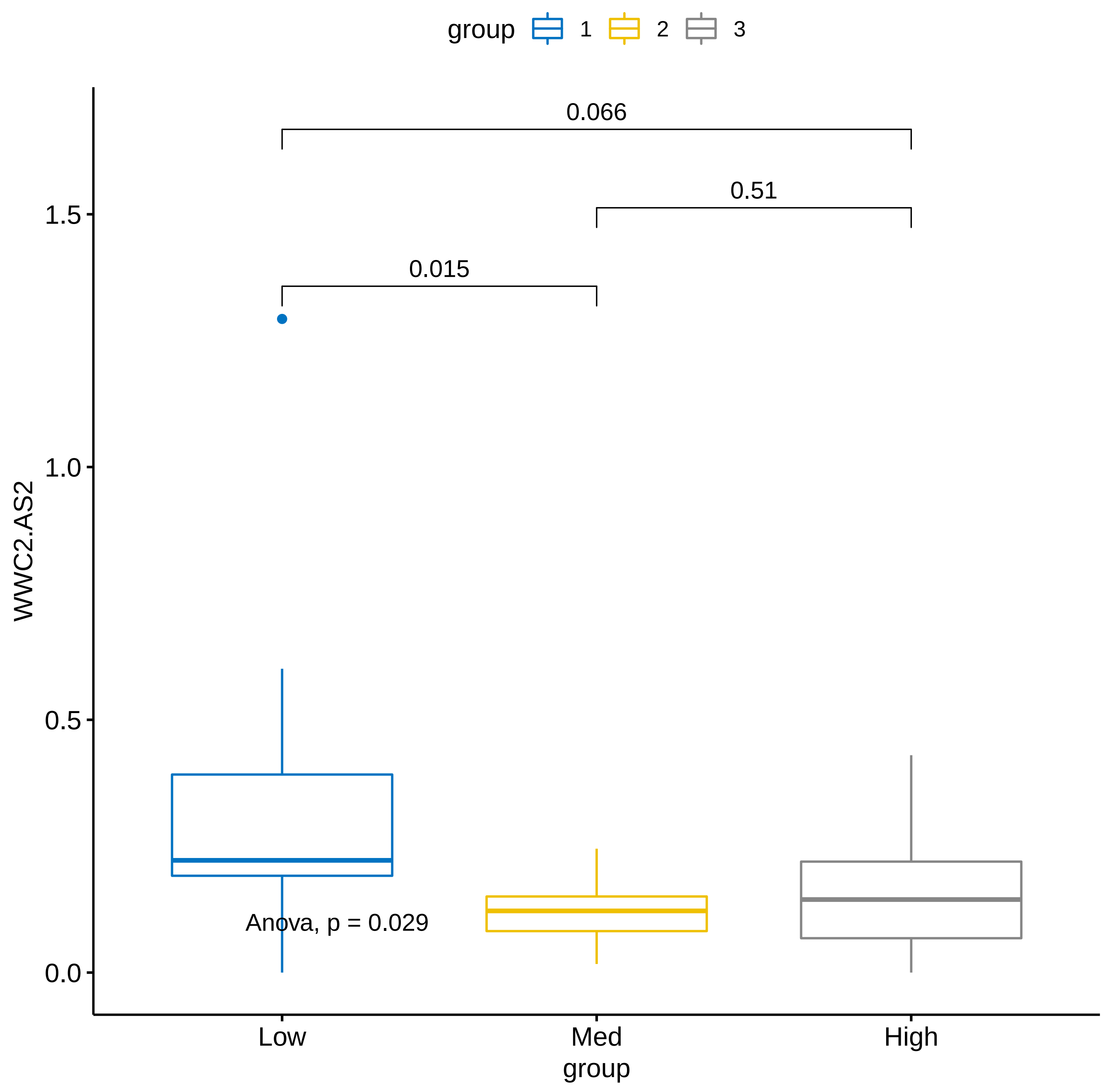
group
1
2
3
0.066
0.51
0.015
1.5
1.0
0.5
0.0
WWC2.AS2
Anova, p = 0.029
Low
Med
High
group

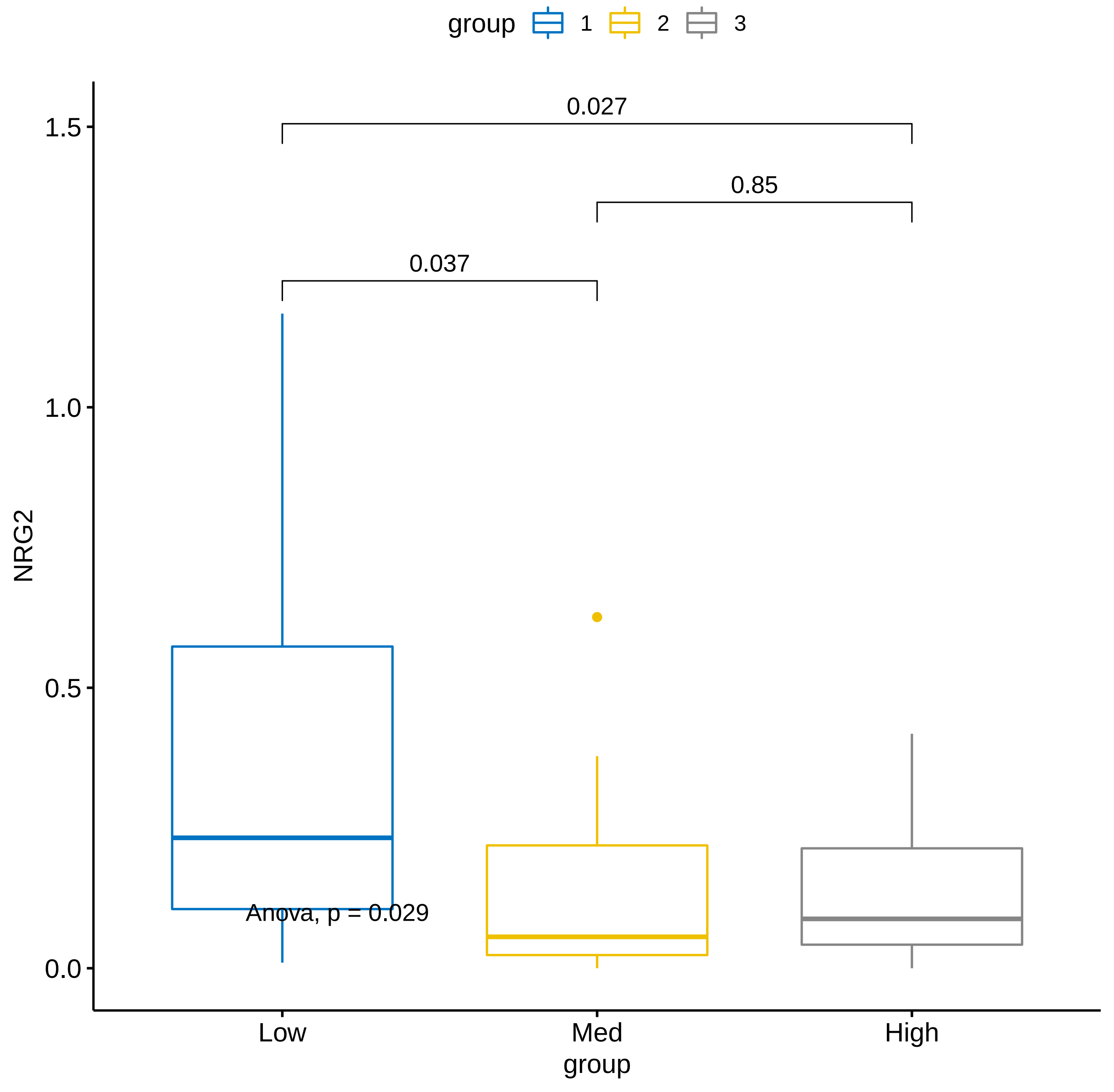

group 1 2 3
0.027
0.85
0.037
Anova, p = 0.029
1.5
1.0
0.5
0.0
NRG2
Low
Med
High
group

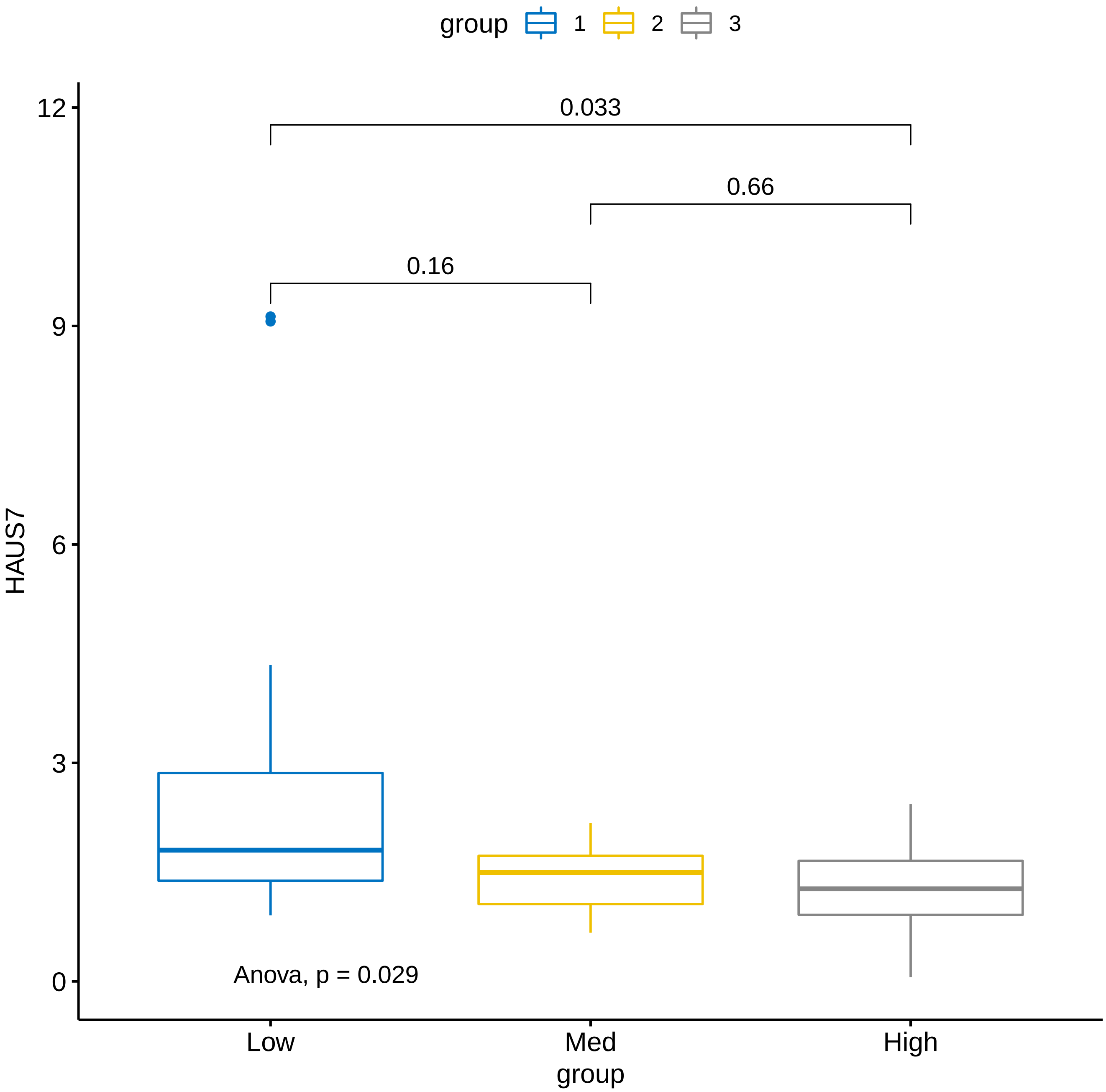
group
1
2
3
0.033
0.66
0.16
12
9
6
3
0
HAUS7
Anova, p = 0.029
Low
Med
High
group

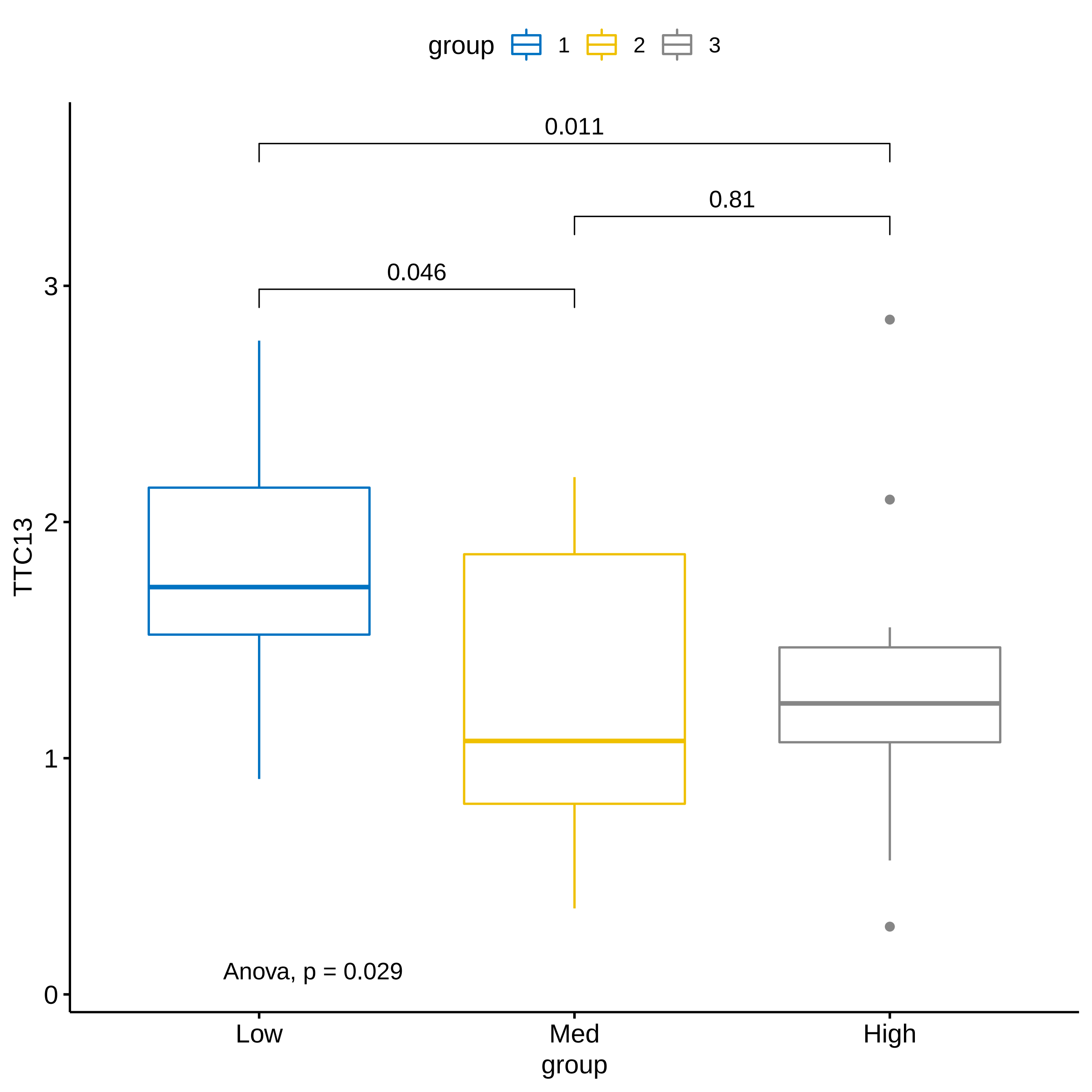
group
1
2
3
0.011
0.81
0.046
TTC13
3
2
1
0
Anova, p = 0.029
Low
Med
High
group

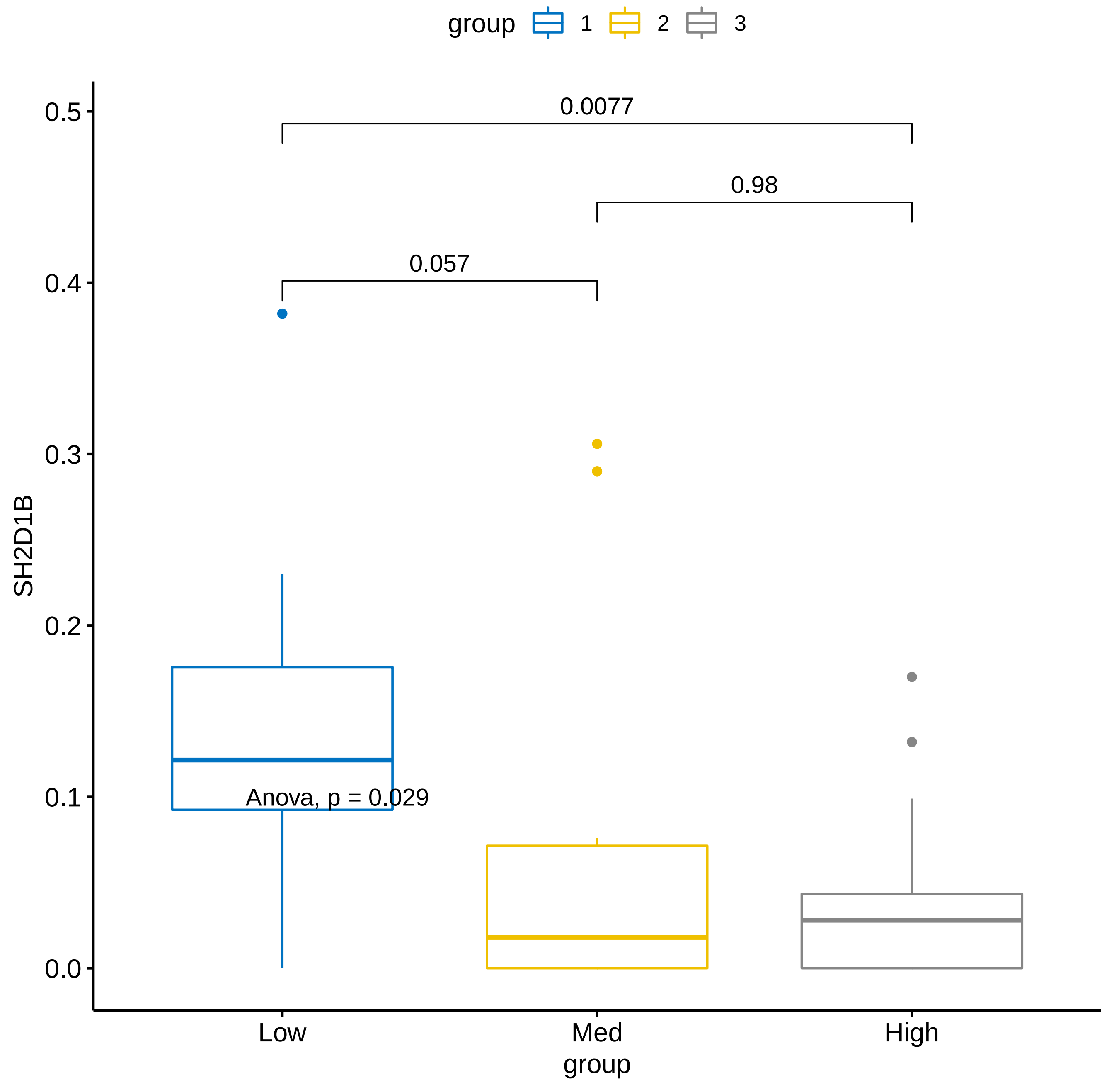
group
1
2
3
0.0077
0.98
0.057
0.5
0.4
0.3
0.2
0.1
0.0
SH2D1B
Anova, p = 0.029
Low
Med
High
group

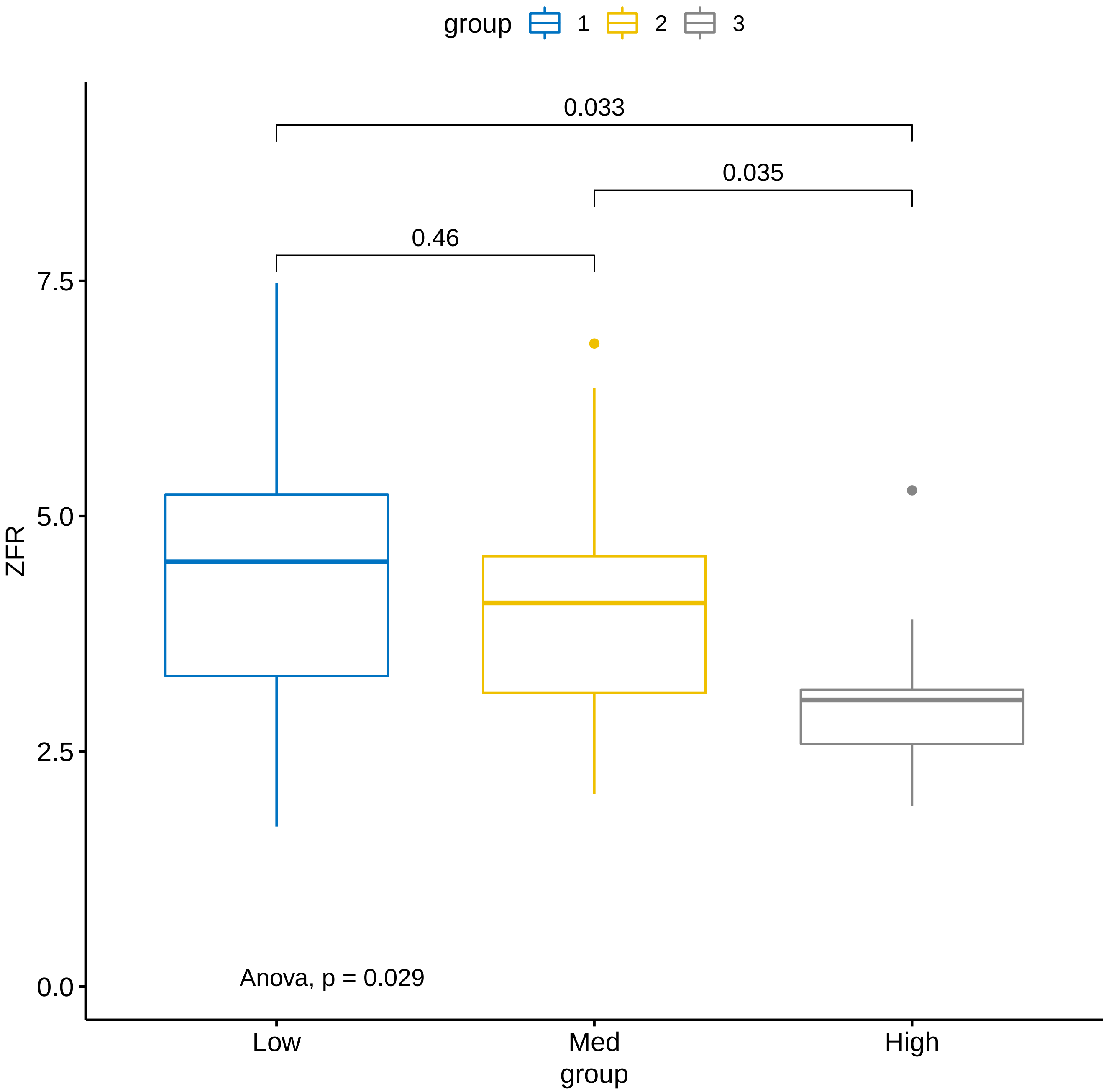
group
1
2
3
0.033
0.035
0.46
7.5
5.0
2.5
0.0
ZFR
Anova, p = 0.029
Low
Med
High
group

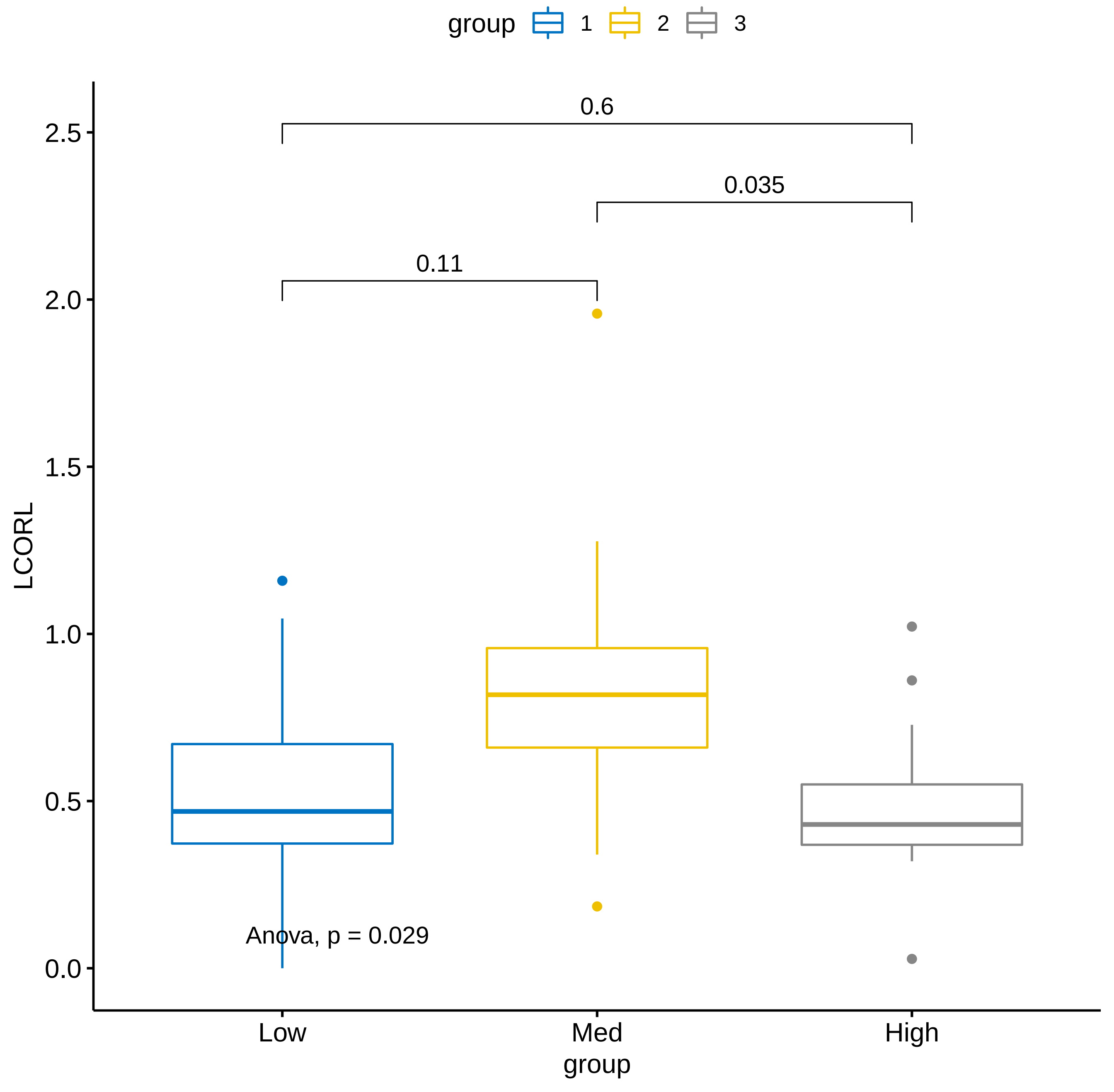

group
1
2
3
0.6
0.035
0.11
2.5
2.0
1.5
1.0
0.5
0.0
LCORL
Anova, p = 0.029
Low
Med
High
group

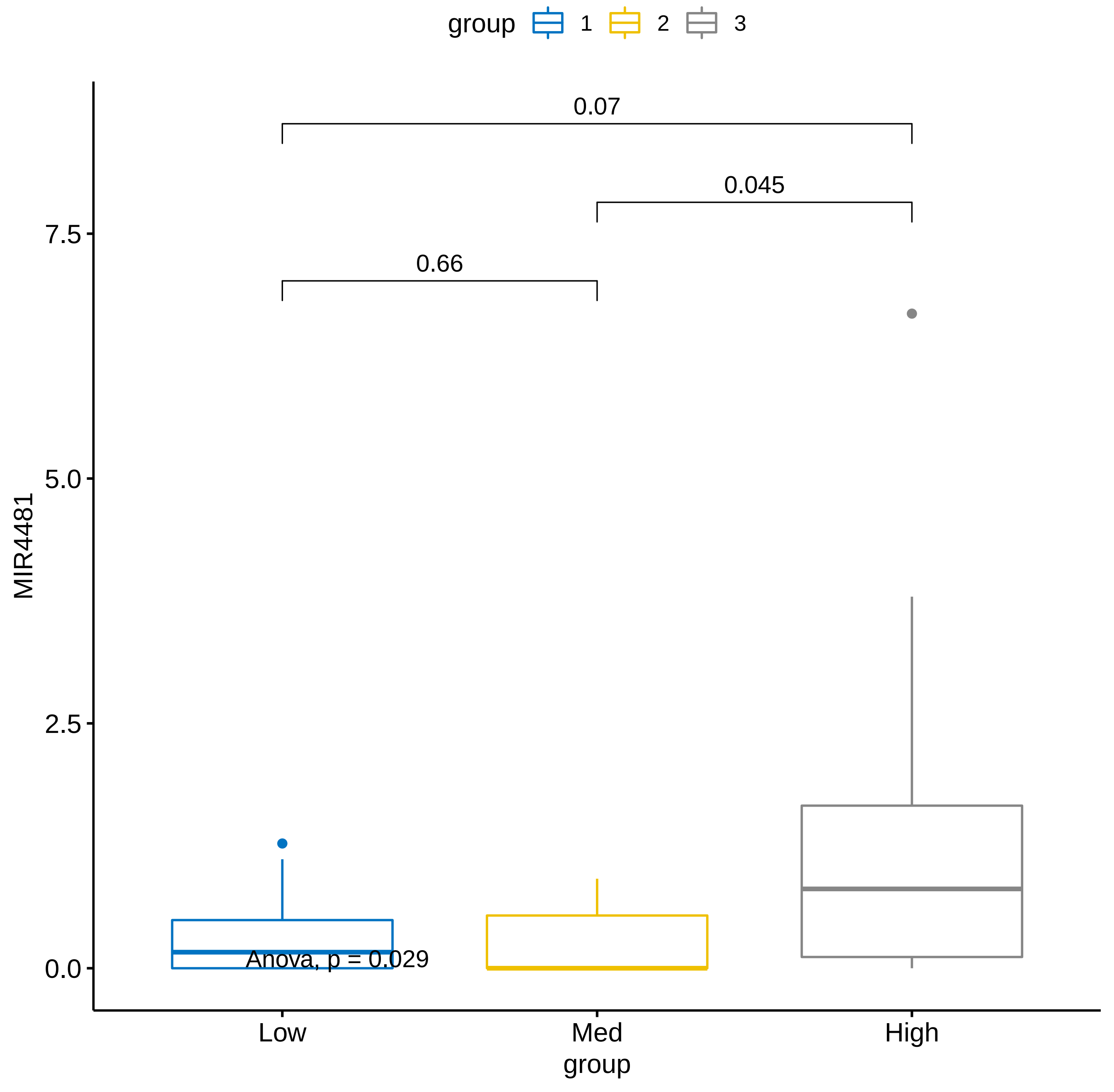

group 1 2 3
0.07
0.045
0.66
MIR4481
7.5
5.0
2.5
0.0
Anova, p = 0.029
Low
Med
High
group

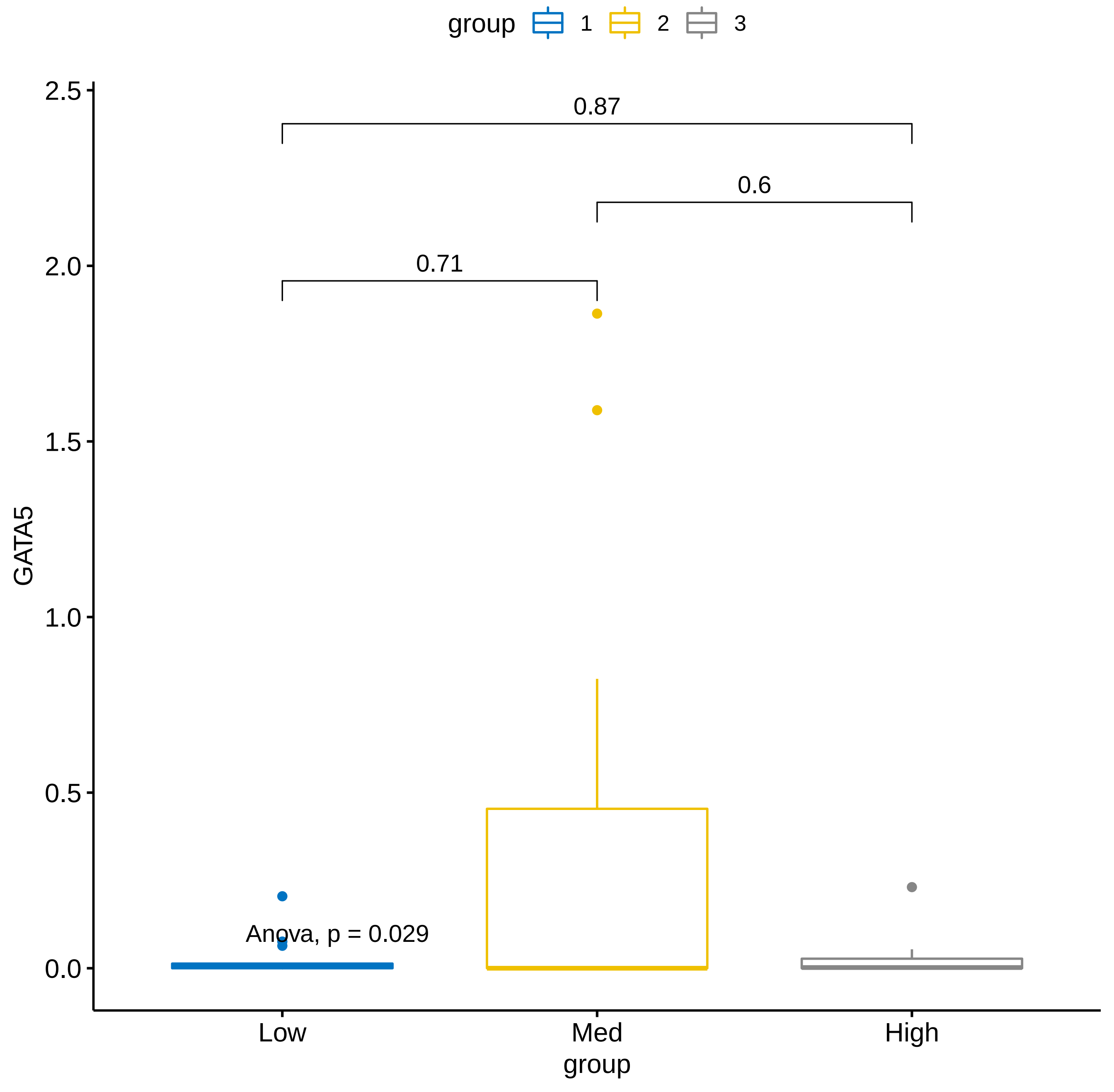
group
1
2
3
2.5
2.0
1.5
1.0
0.5
0.0
GATA5
0.87
0.6
0.71
Anova, p = 0.029
Low
Med
High
group

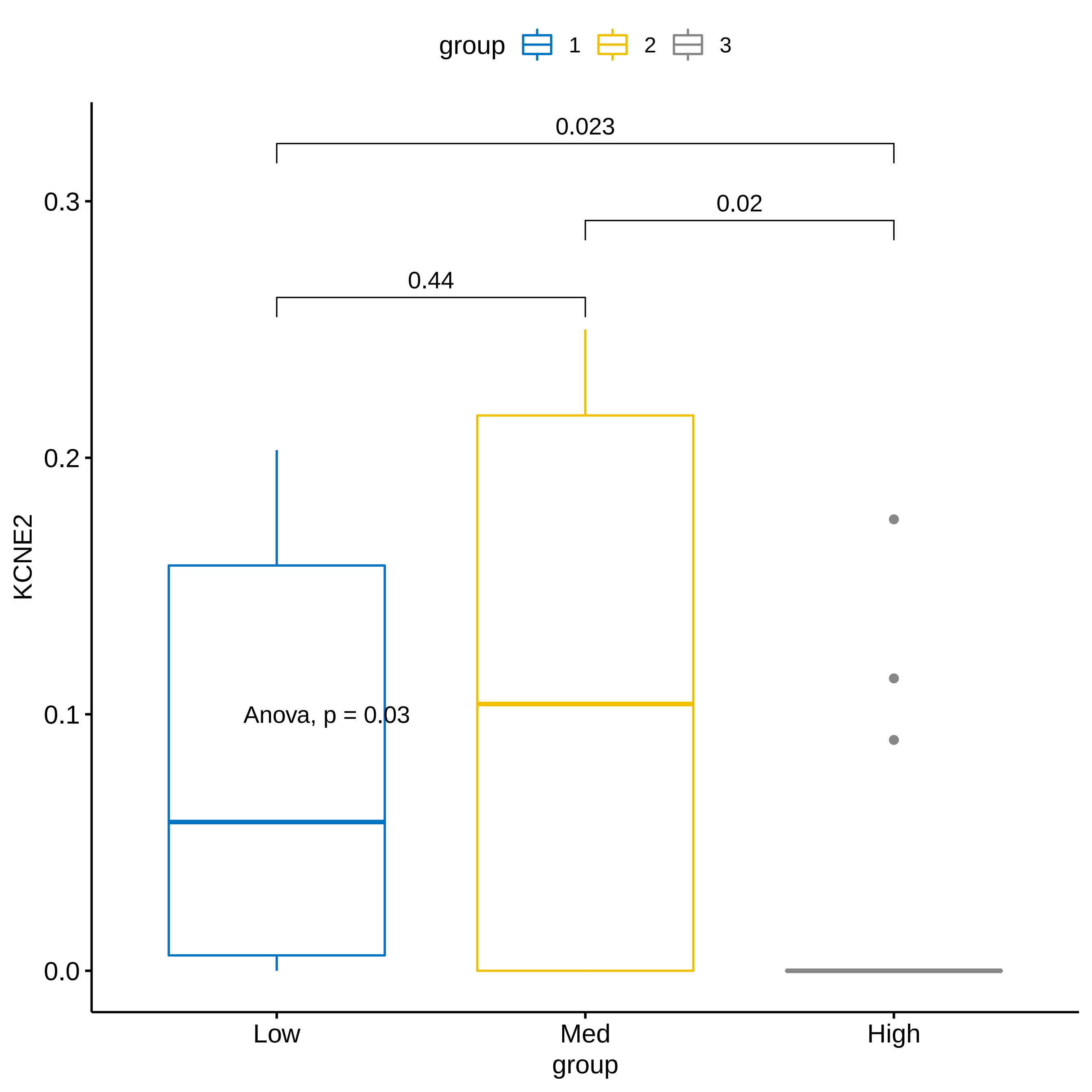
group 1 2 3
0.023
0.02
0.44
0.3
0.2
0.1
0.0
KCNE2
Anova, p = 0.03
Low
Med
High
group

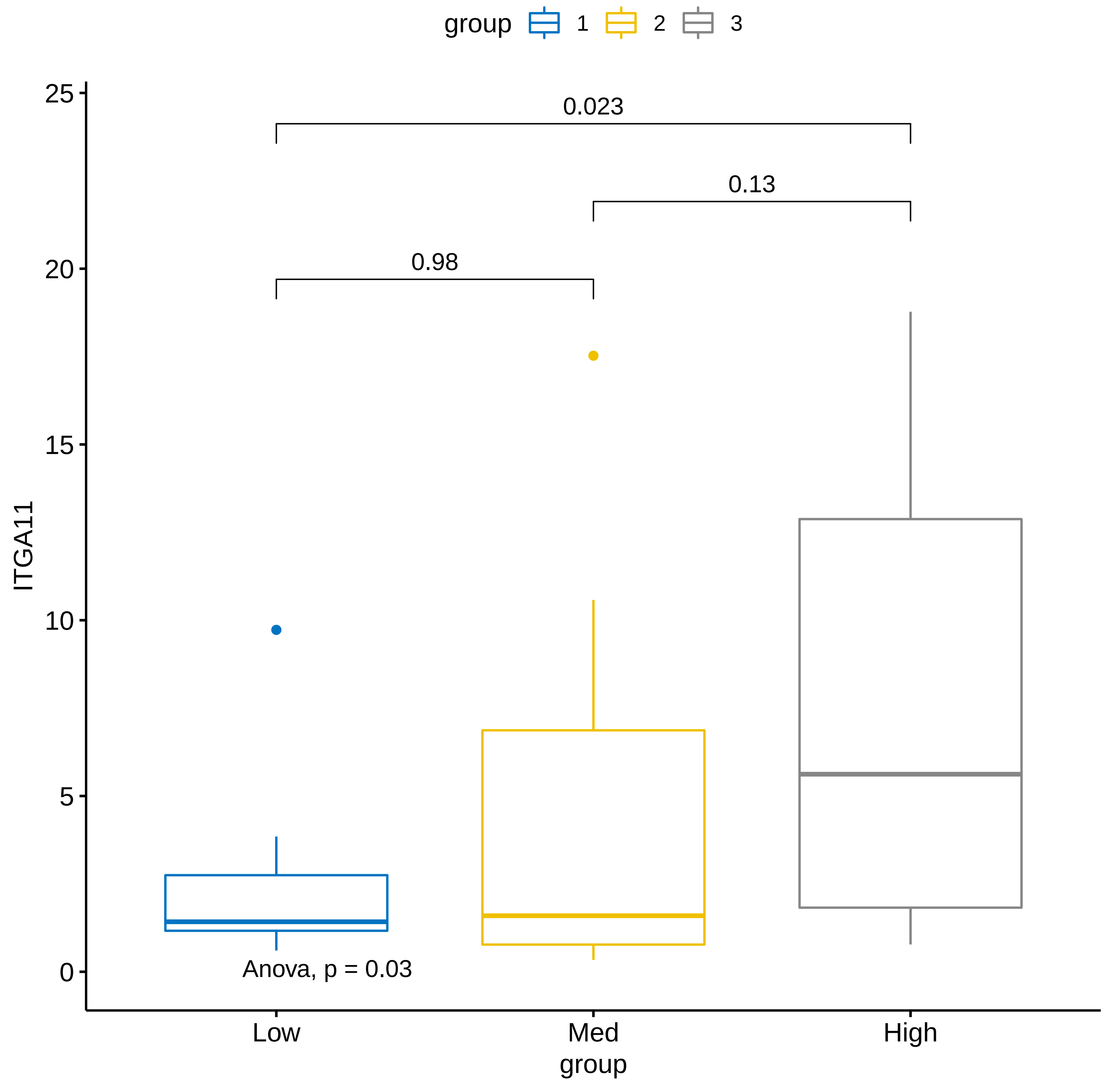
group
1
2
3
0.023
0.13
0.98
25
20
15
10
5
0
ITGA11
Anova, p = 0.03
Low
Med
High
group

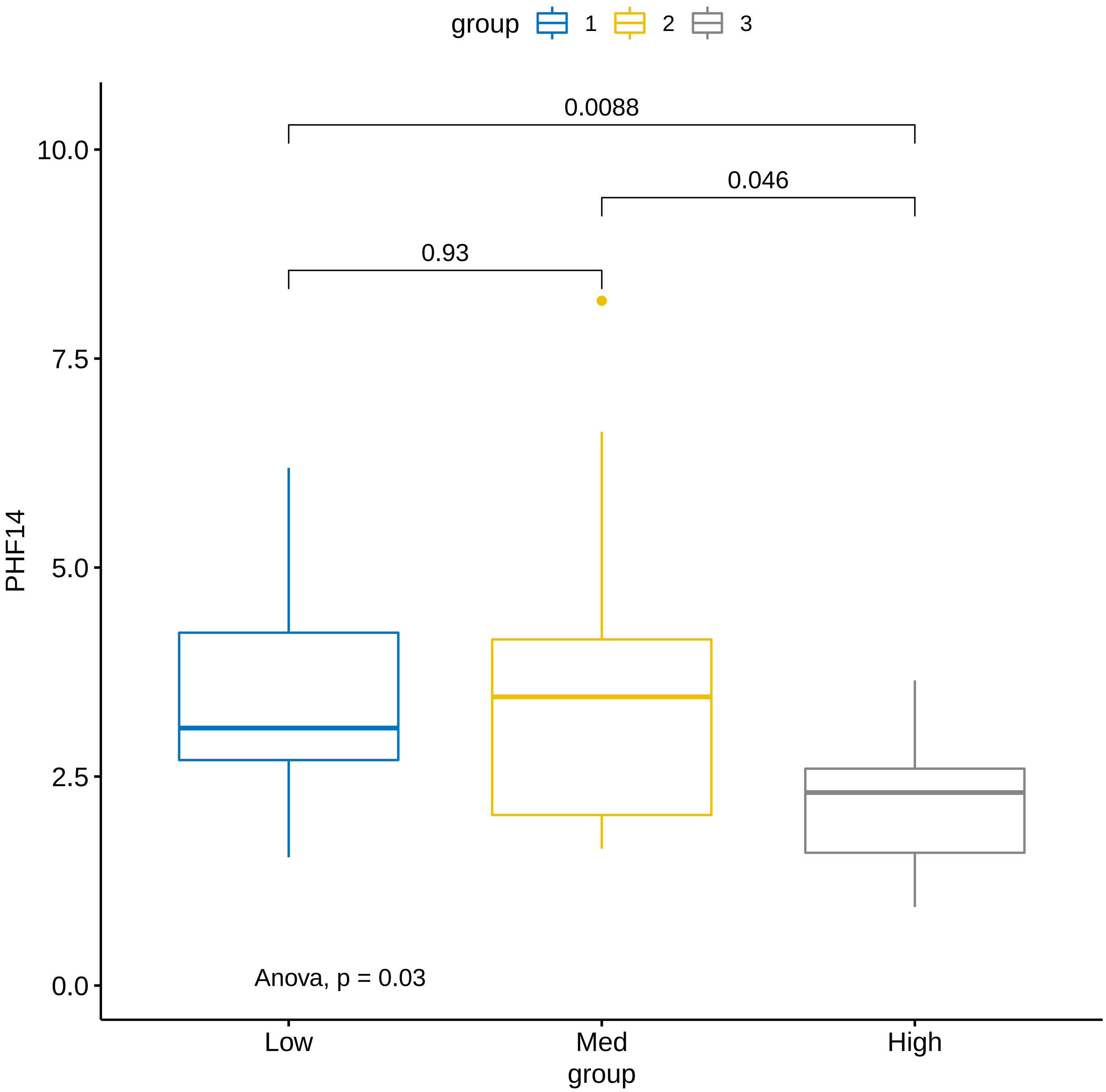

group 1 2 3
0.0088
0.046
0.93
10.0
7.5
5.0
2.5
0.0
PHF14
Anova, p = 0.03
Low
Med
High
group

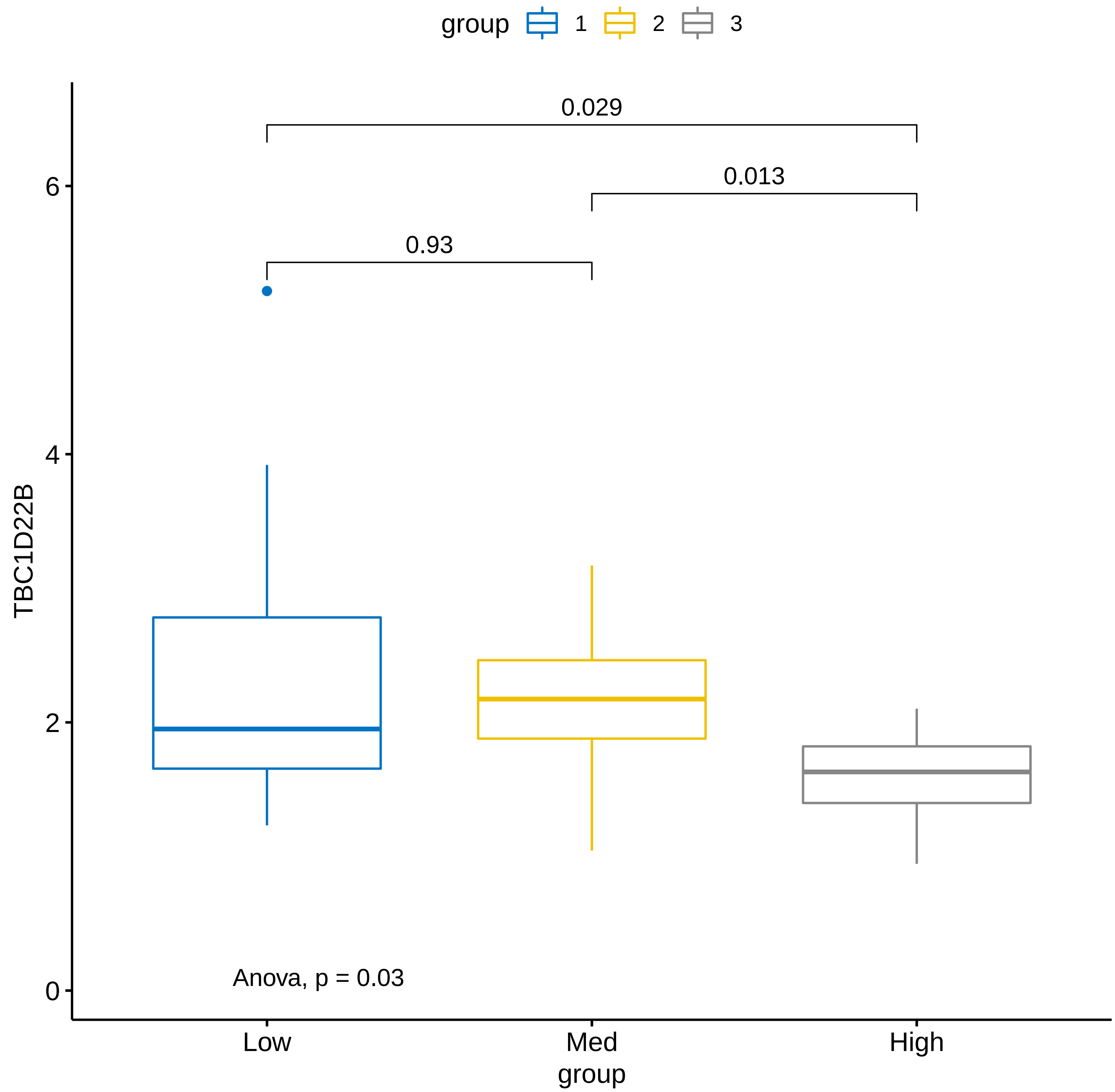
group
1
2
3
0.029
0.013
0.93
6
4
2
0
TBC1D22B
Anova, p = 0.03
Low
Med
High
group

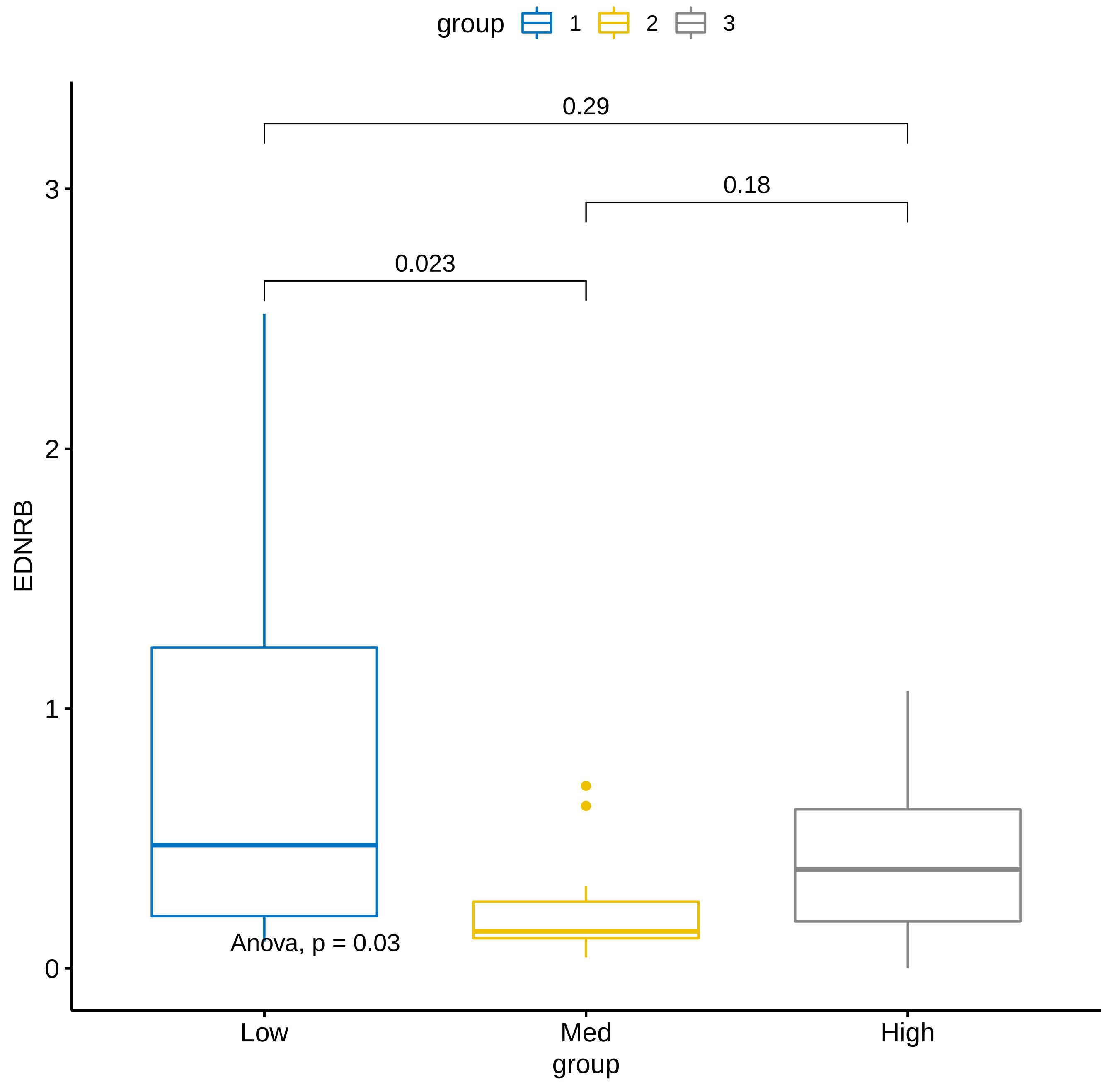
group
1
2
3
0.29
0.18
0.023
EDNRB
3
2
1
0
Anova, p = 0.03
Low
Med
High
group

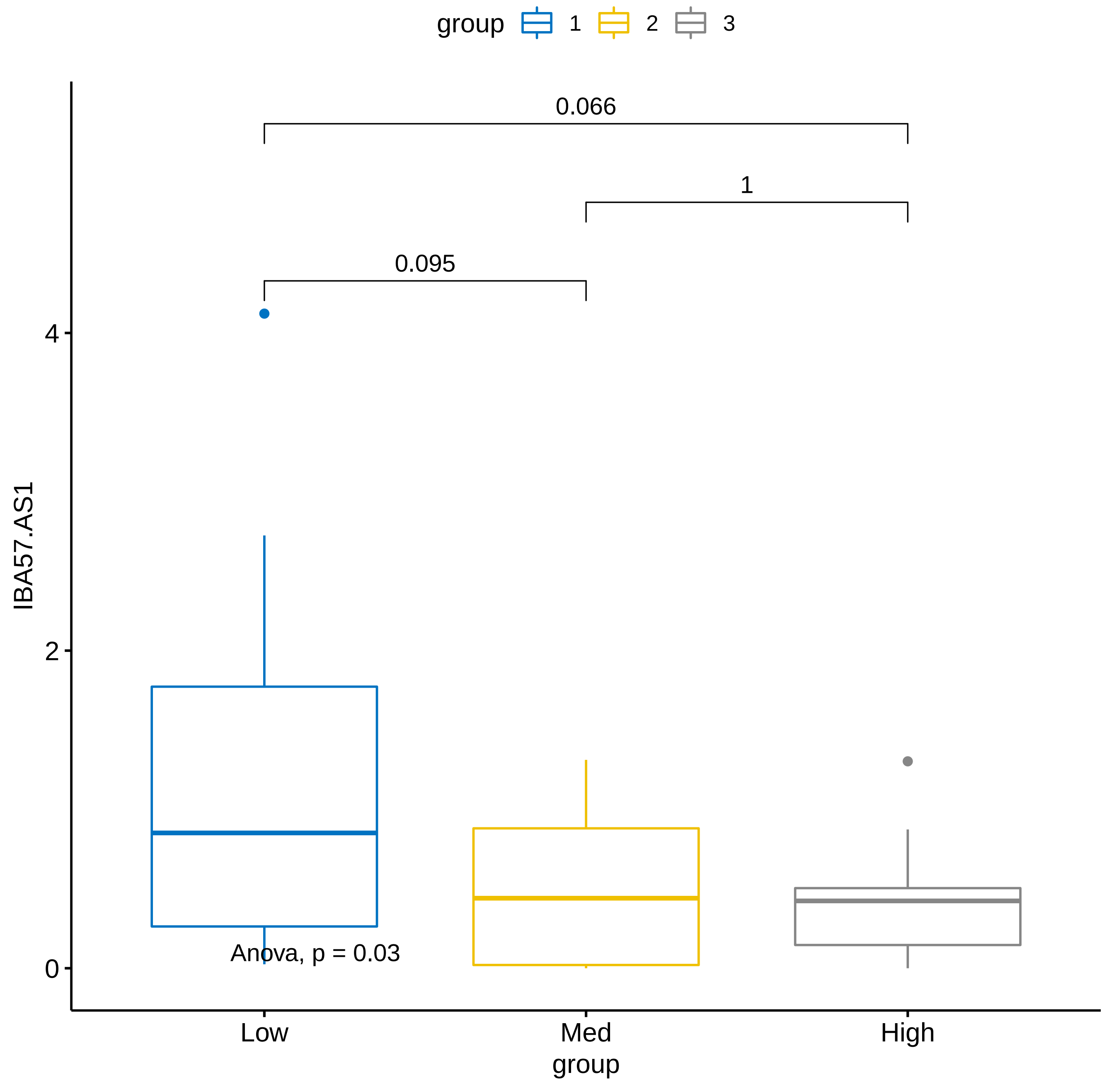
group
1
2
3
0.066
1
0.095
IBA57.AS1
4
2
0
Anova, p = 0.03
Low
Med
High
group

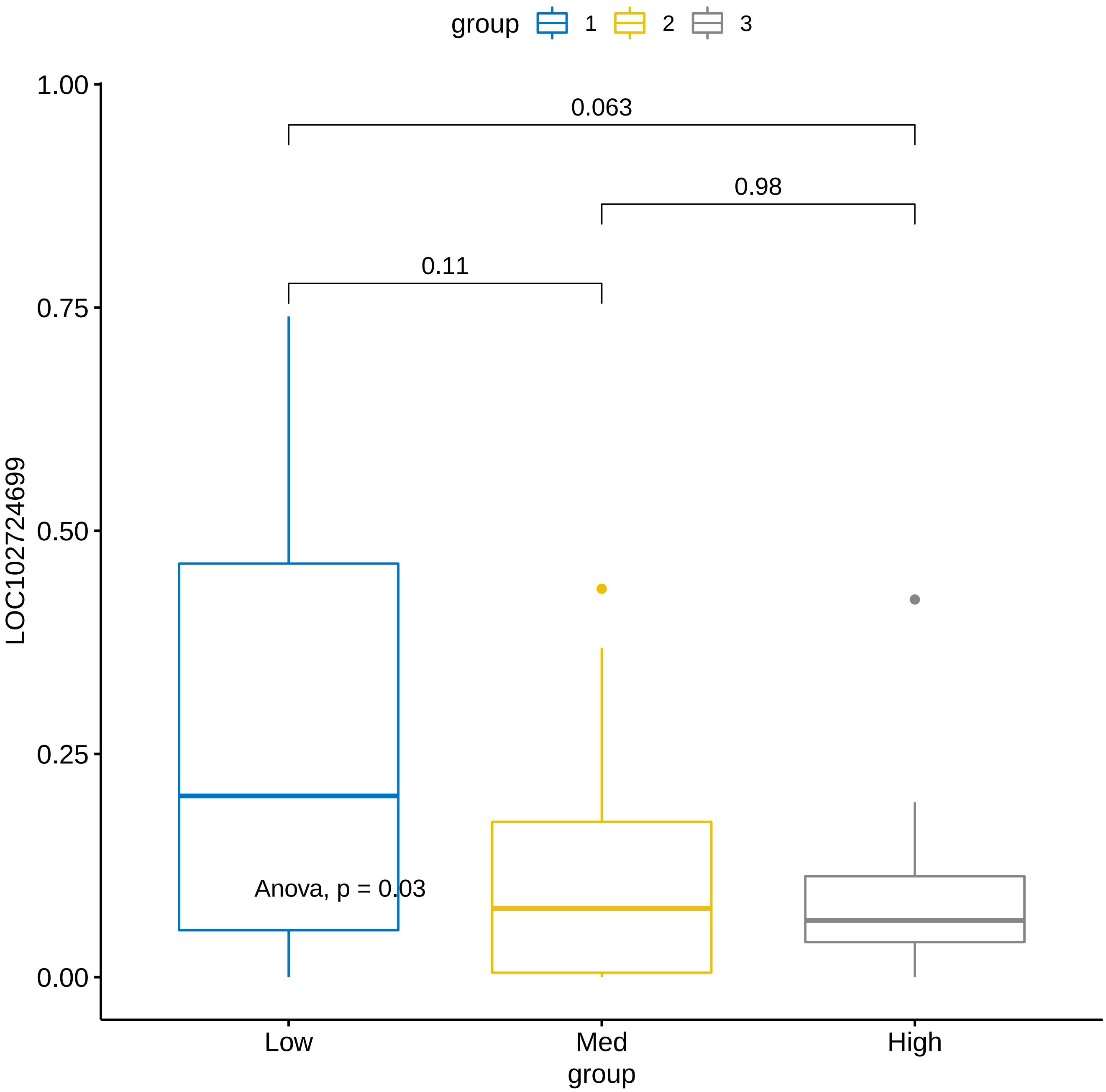
group
1
2
3
1.00
0.75
0.50
0.25
0.00
LOC102724699
0.063
0.98
0.11
Anova, p = 0.03
Low
Med
High
group

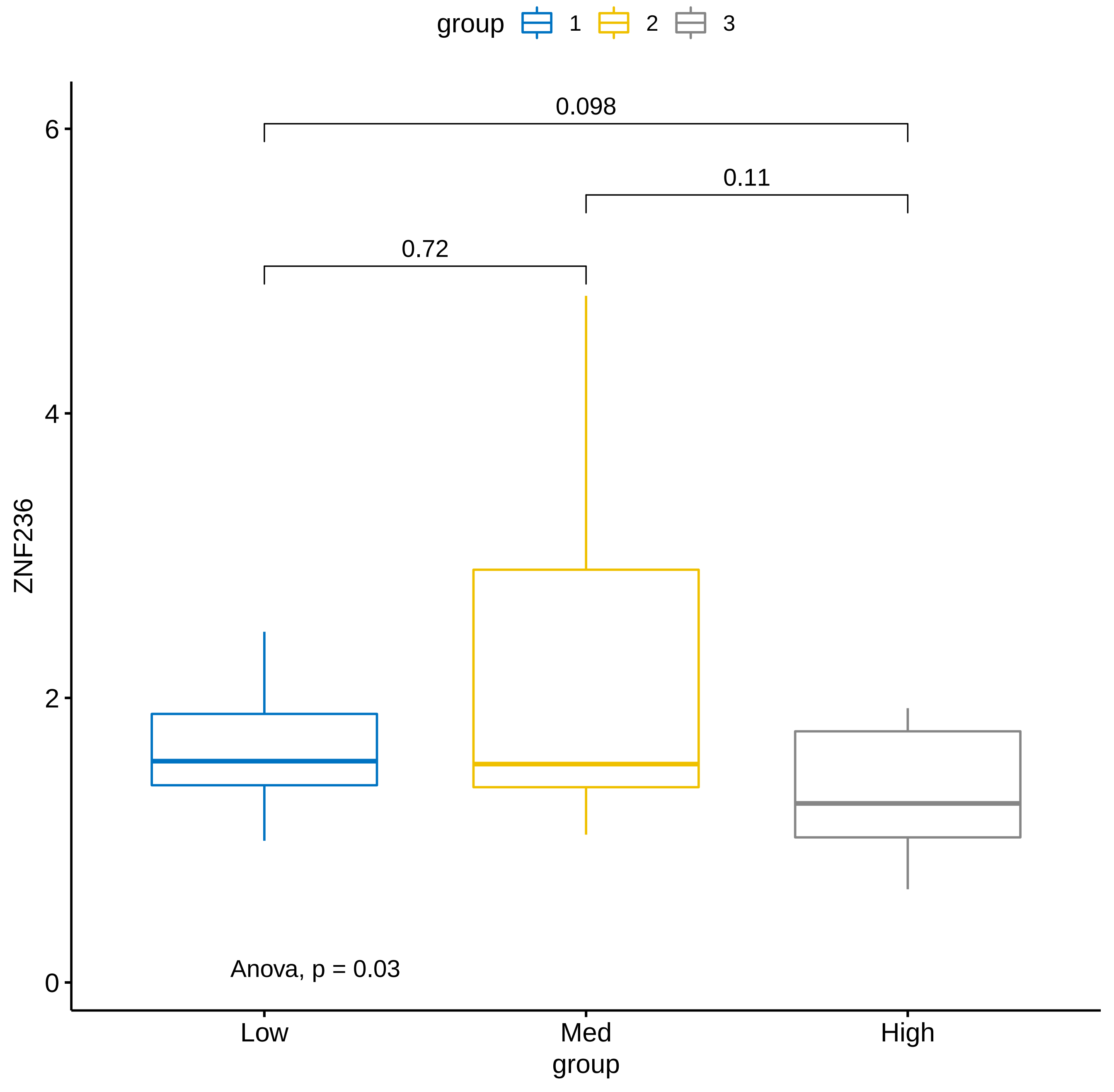
group
1
2
3
0.098
0.11
0.72
6
4
2
0
ZNF236
Anova, p = 0.03
Low
Med
High
group

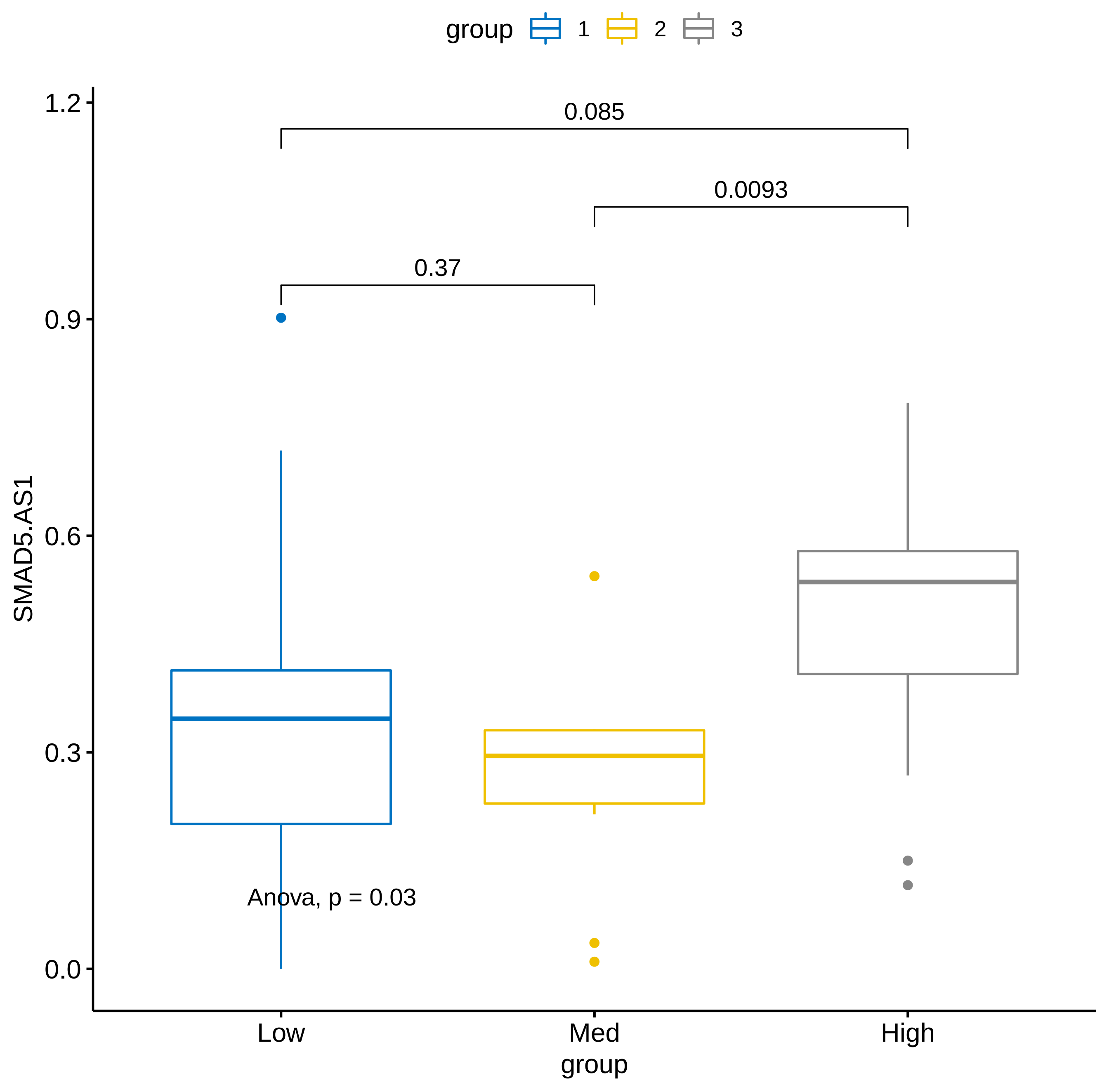

group
1
2
3
1.2
0.9
0.6
0.3
0.0
SMAD5.AS1
0.085
0.0093
0.37
Anova, p = 0.03
Low
Med
High
group

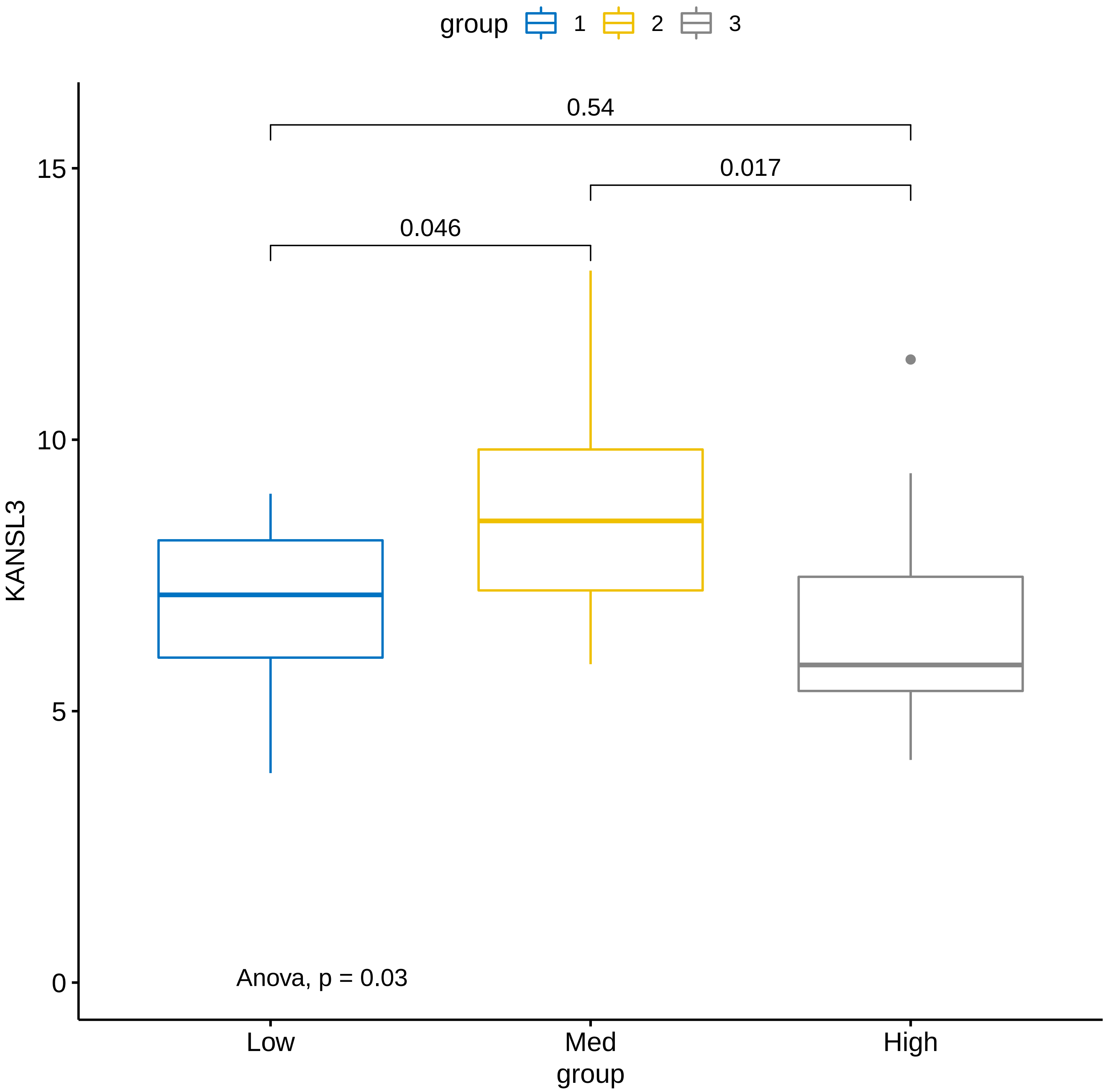

group
1
2
3
0.54
0.017
0.046
15
10
5
0
KANSL3
Anova, p = 0.03
Low
Med
High
group

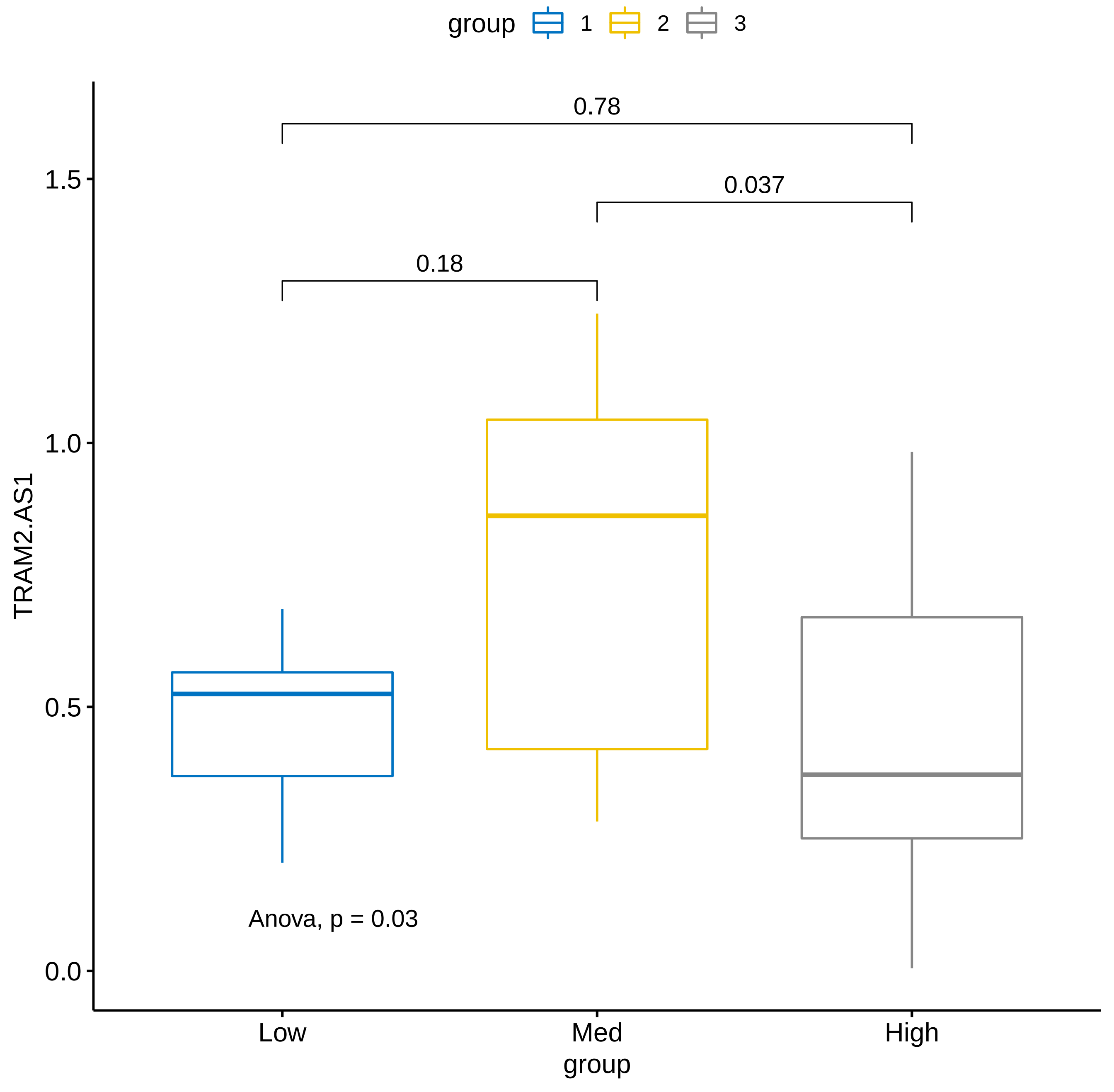
group
1
2
3
0.78
0.037
0.18
1.5
1.0
0.5
0.0
TRAM2.AS1
Anova, p = 0.03
Low
Med
High
group

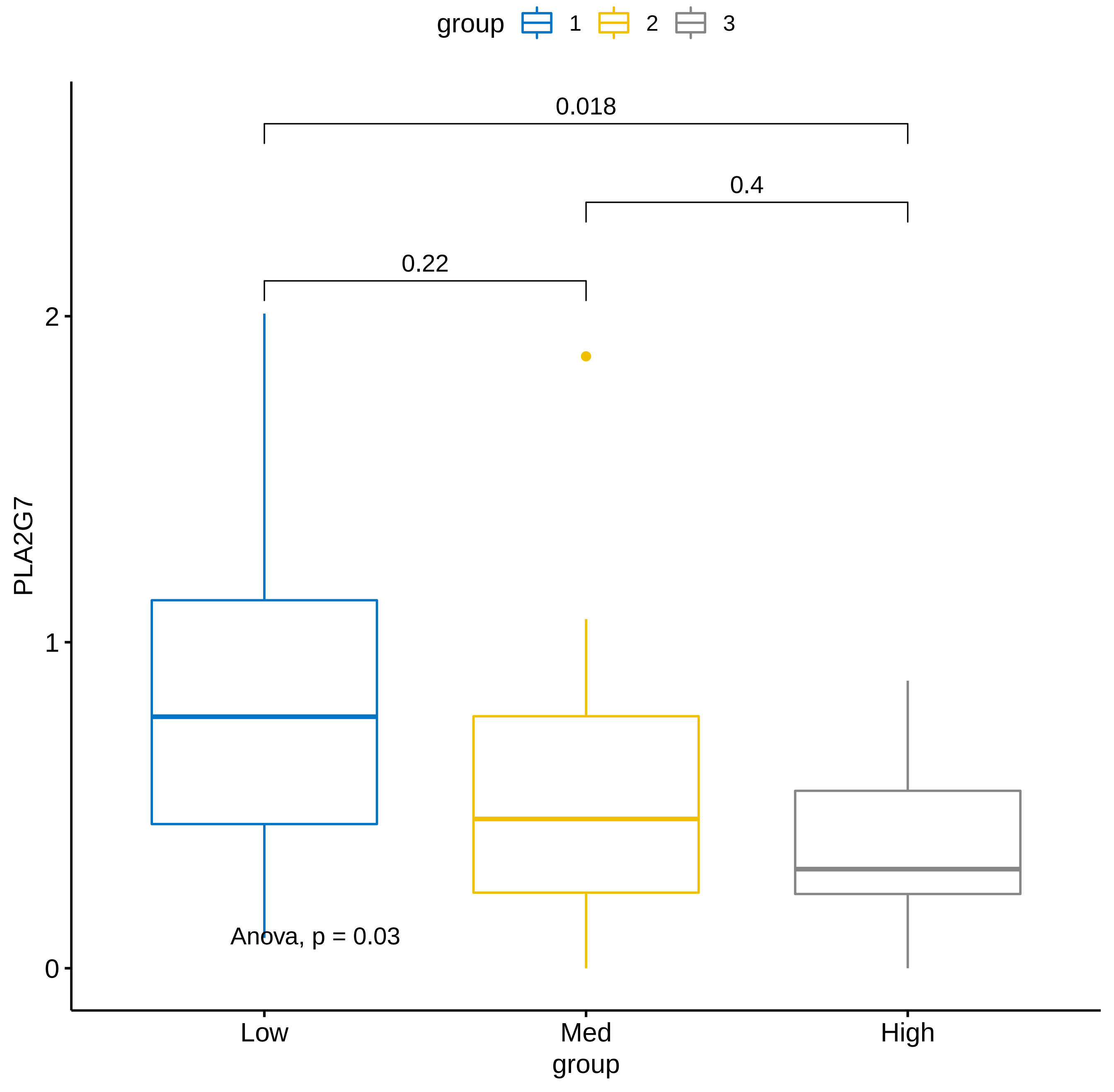
group
1
2
3
0.018
0.4
0.22
2
1
0
PLA2G7
Anova, p = 0.03
Low
Med
High
group

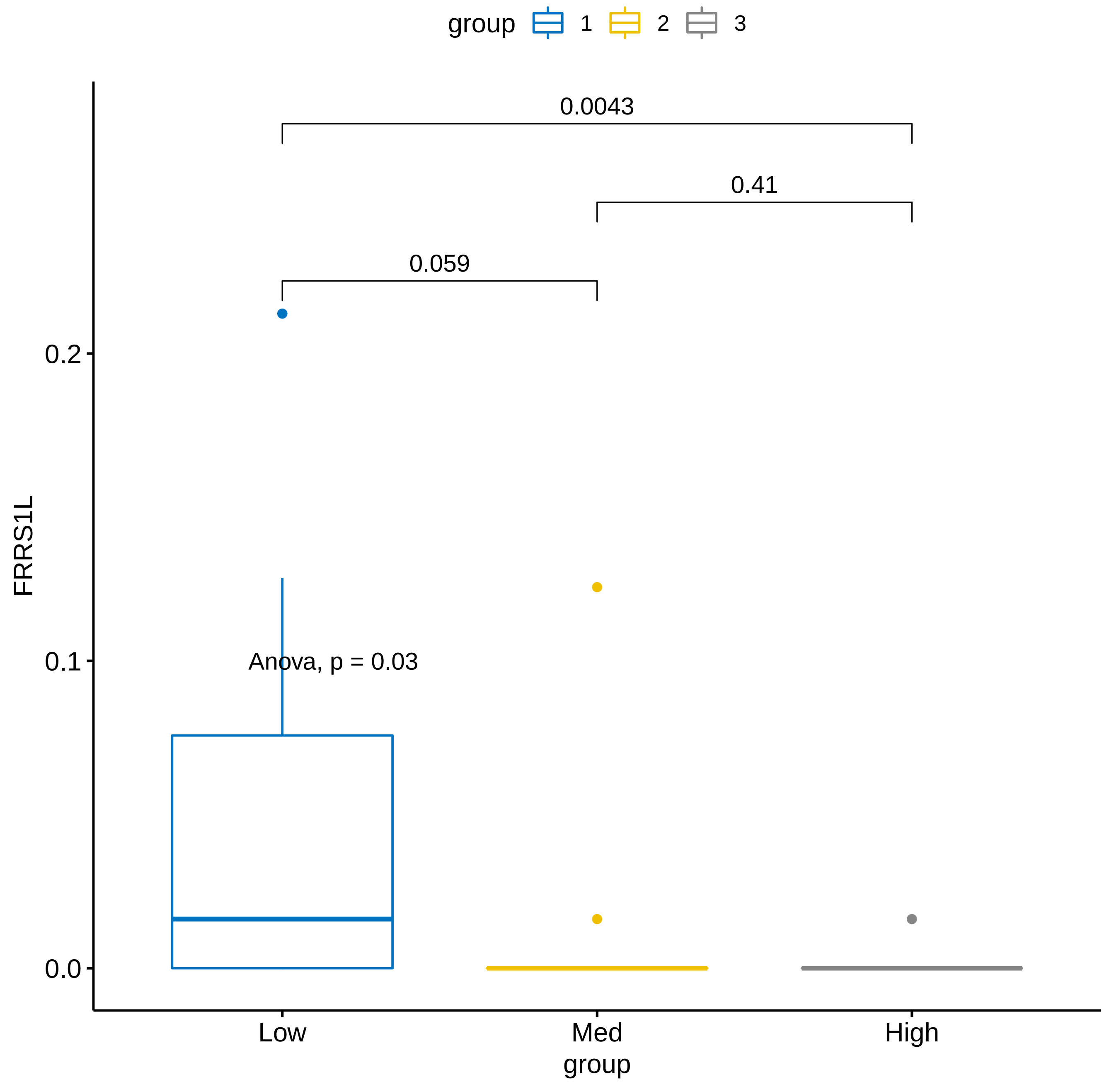
group
1
2
3
0.0043
0.41
0.059
0.2
0.1
0.0
FRRS1L
Anova, p = 0.03
Low
Med
High
group

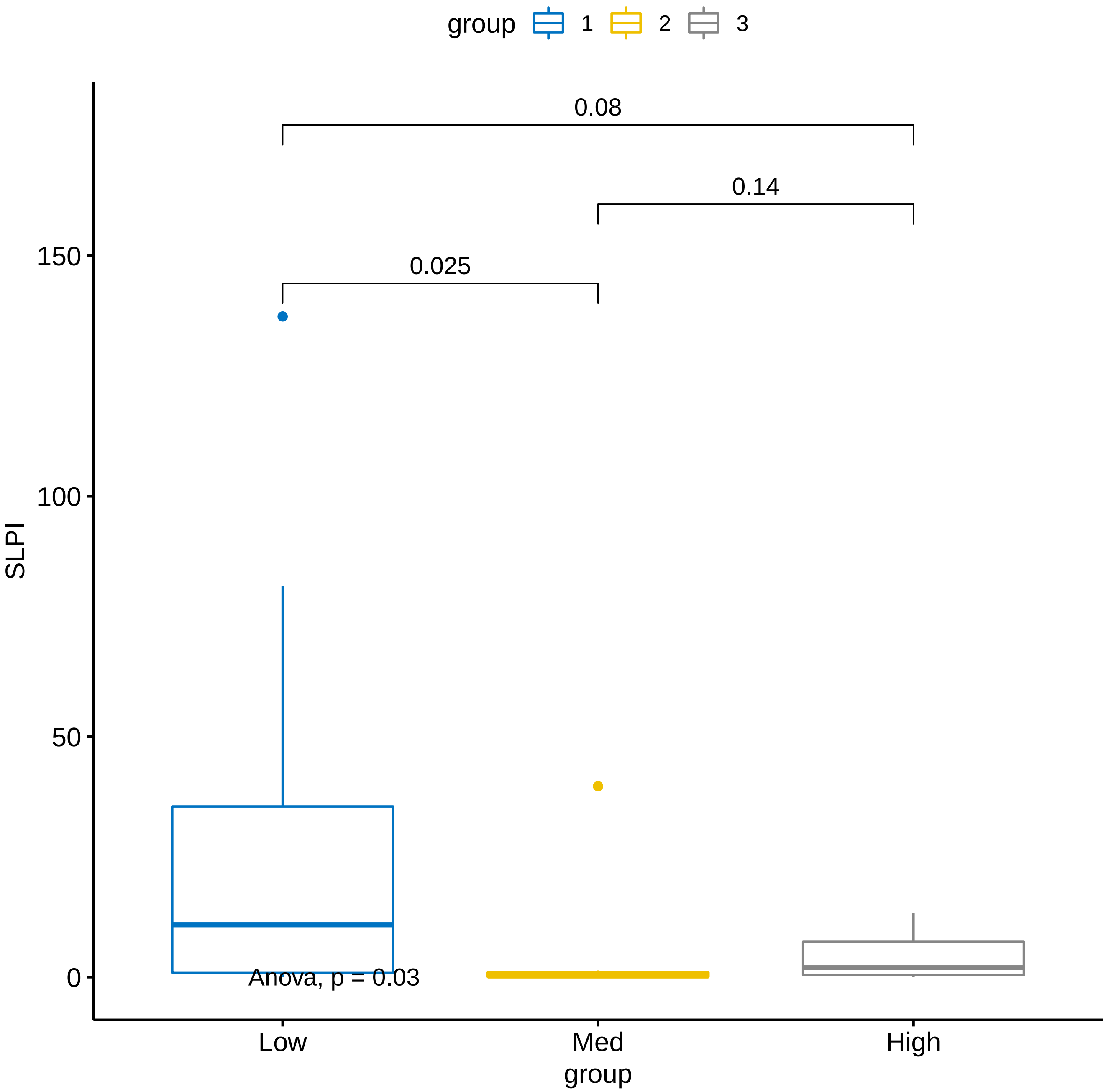
group
1
2
3
0.08
0.14
0.025
150
100
50
0
SLPI
Anova, p = 0.03
Low
Med
High
group

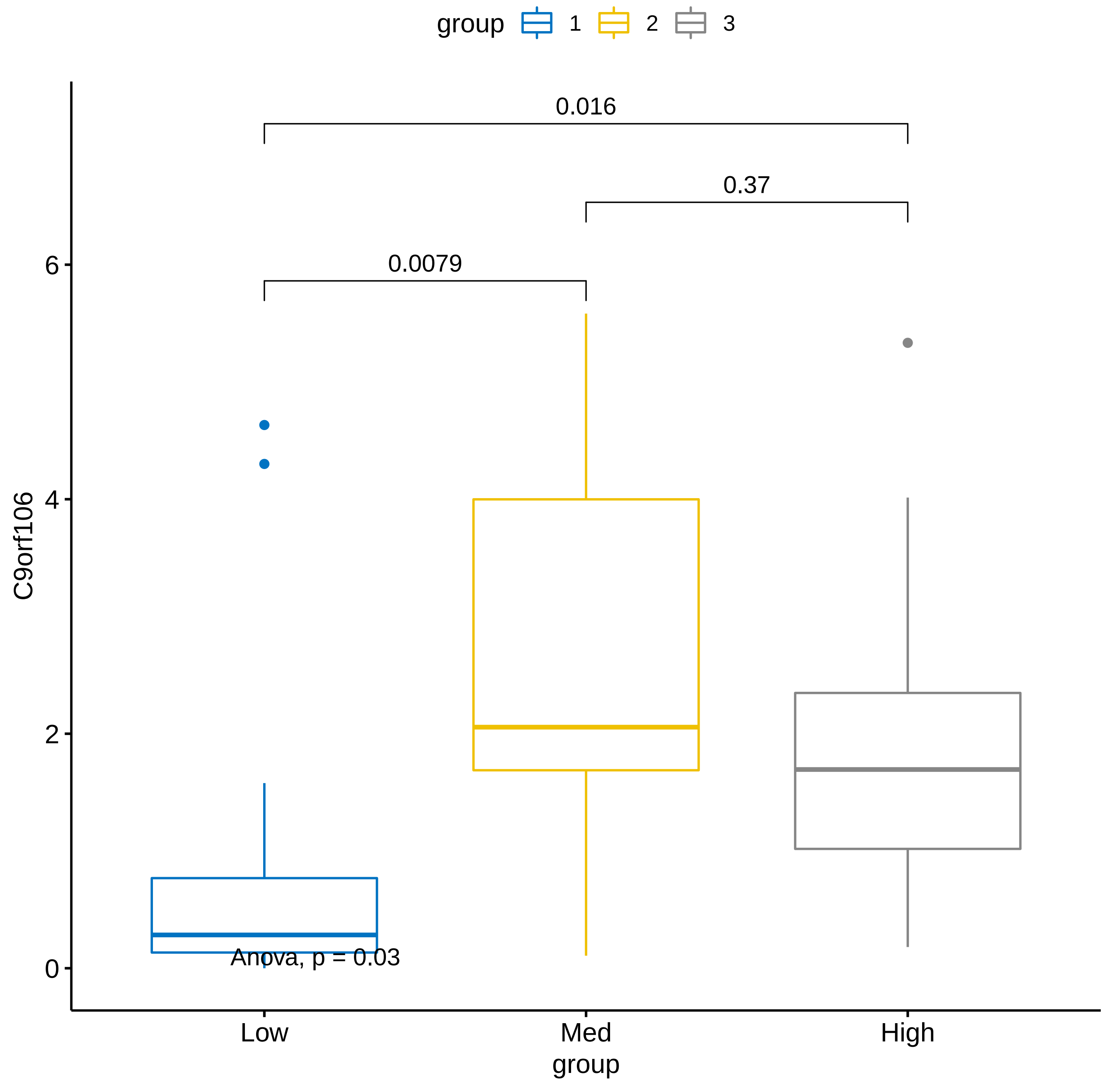

group 1 2 3
0.016
0.37
0.0079
C9orf106
6
4
2
0
Anova, p = 0.03
Low
Med
High
group

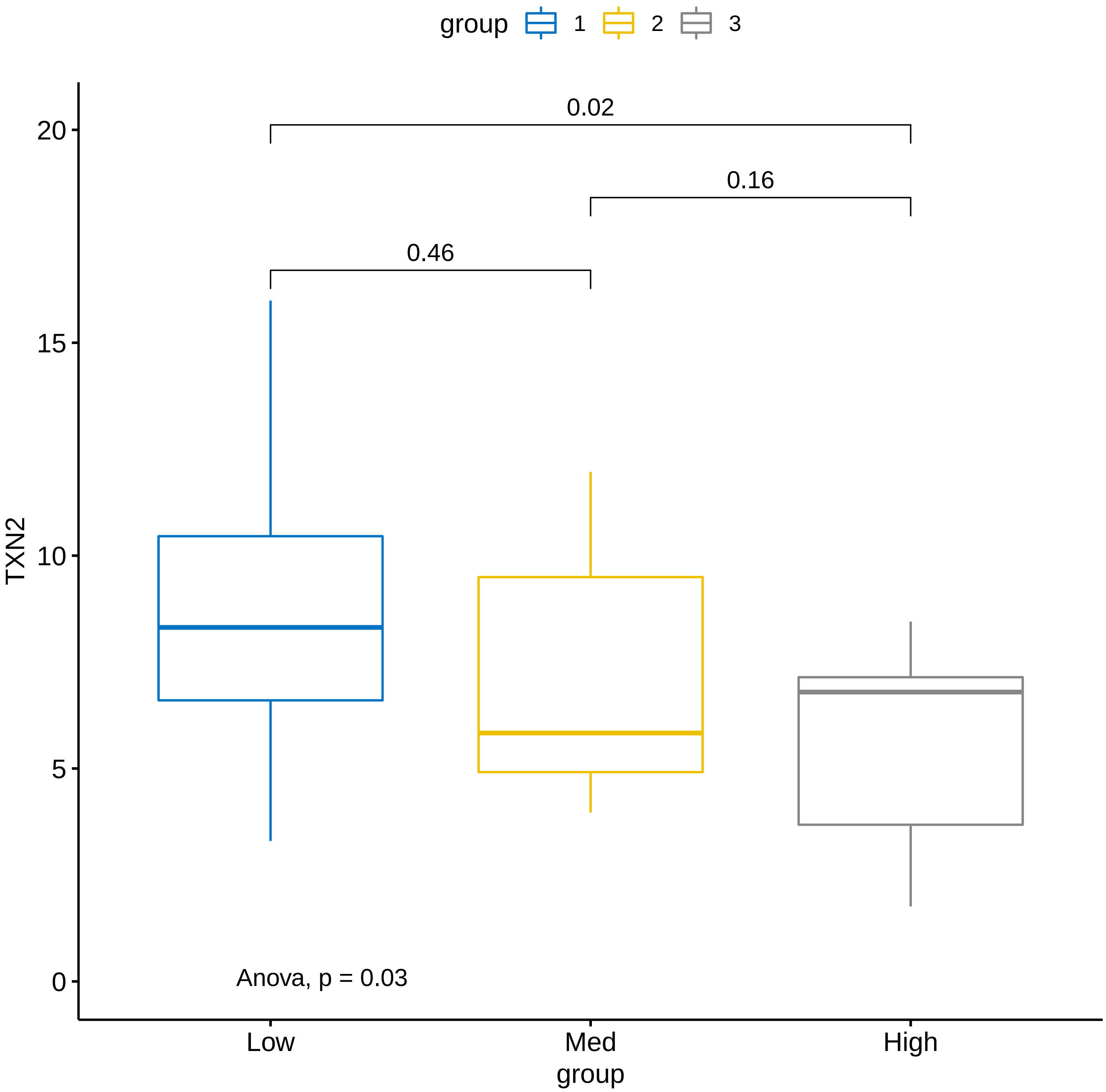
group 1 2 3
0.02
0.16
0.46
20
15
10
5
0
TXN2
Anova, p = 0.03
Low
Med
High
group

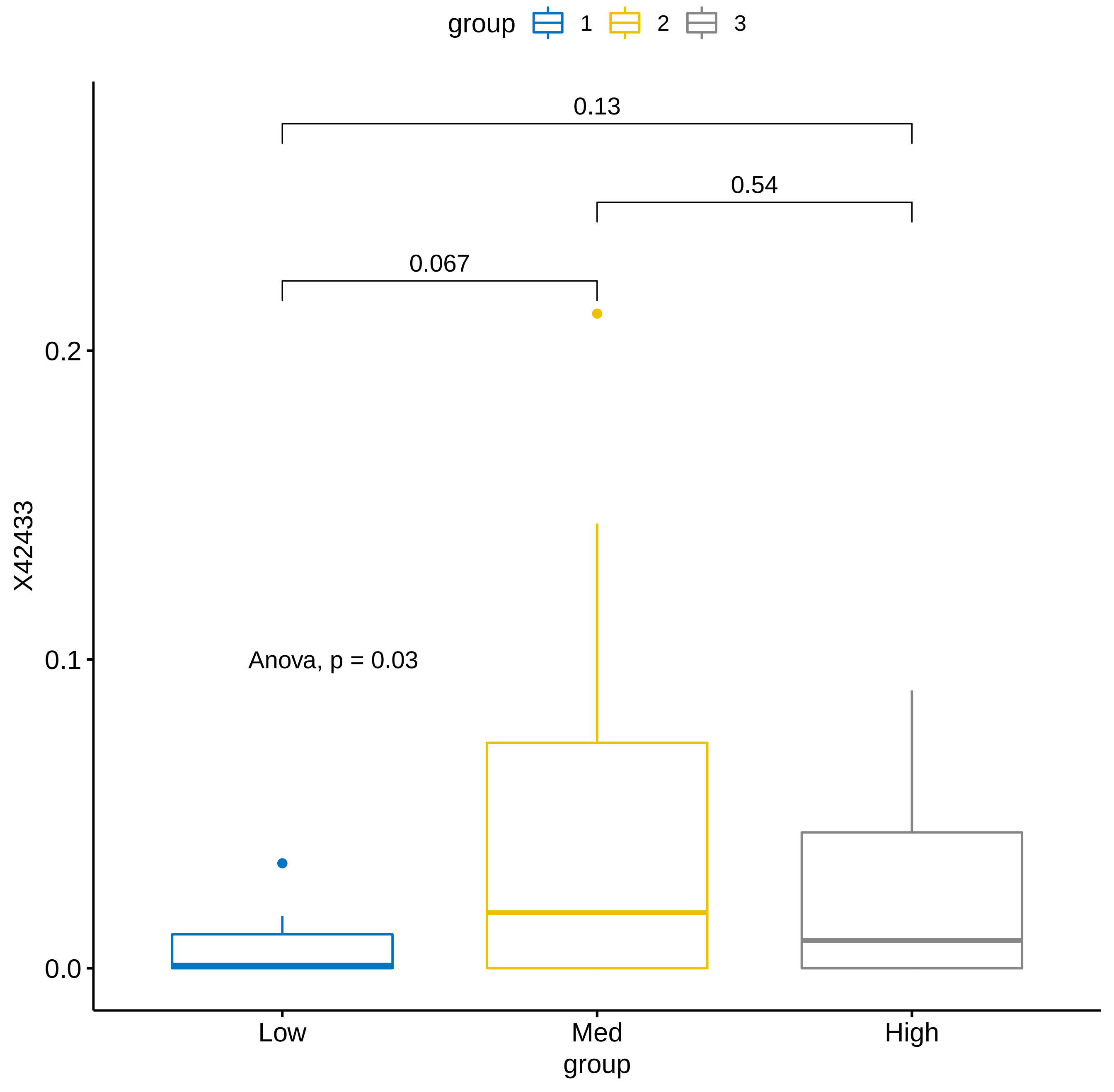
group
1
2
3
0.13
0.54
0.067
Anova, p = 0.03
X42433
0.2
0.1
0.0
Low
Med
High
group

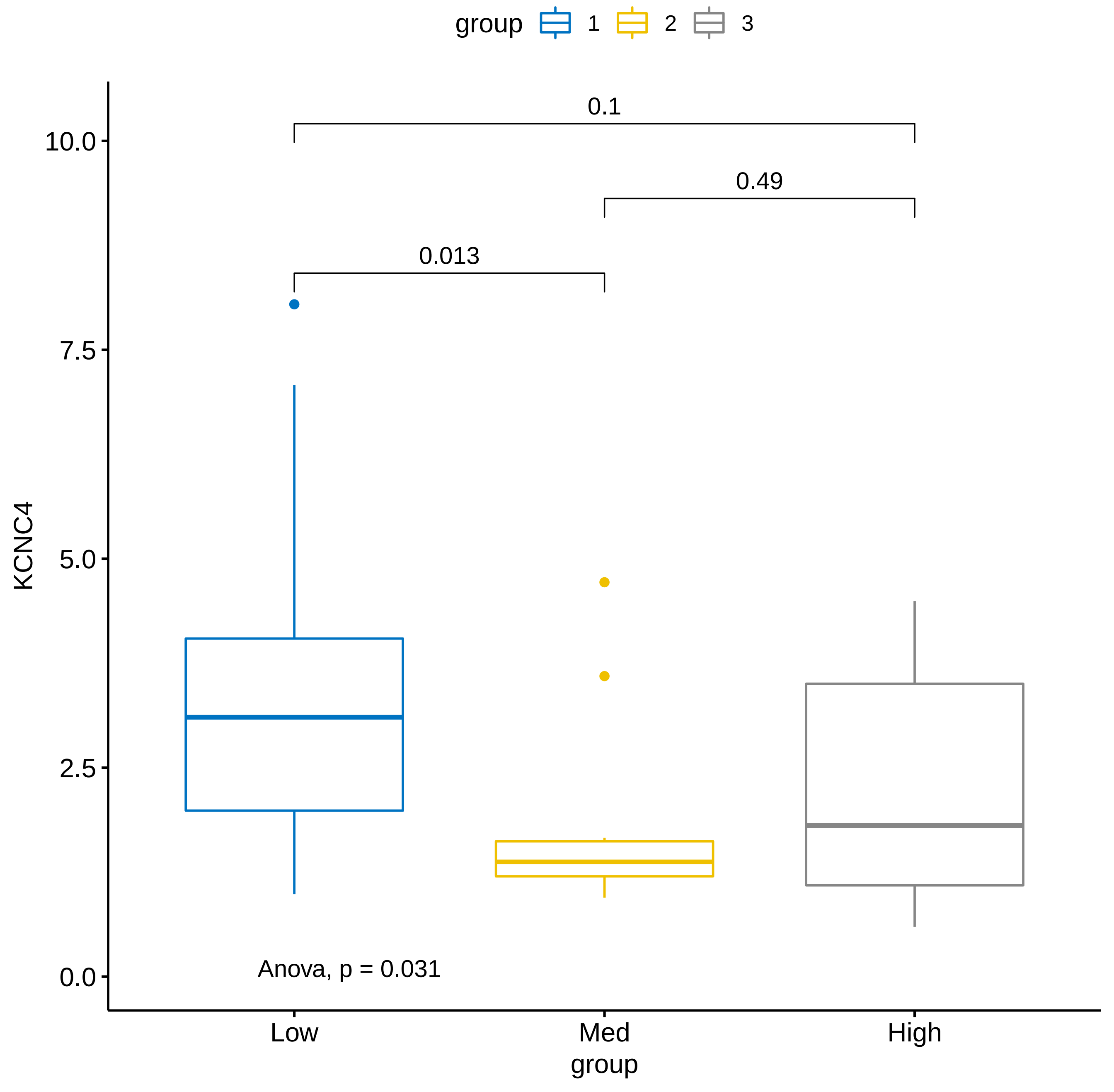

group 1 2 3
0.1
0.49
0.013
10.0
7.5
5.0
2.5
0.0
KCNC4
Anova, p = 0.031
Low
Med
High
group

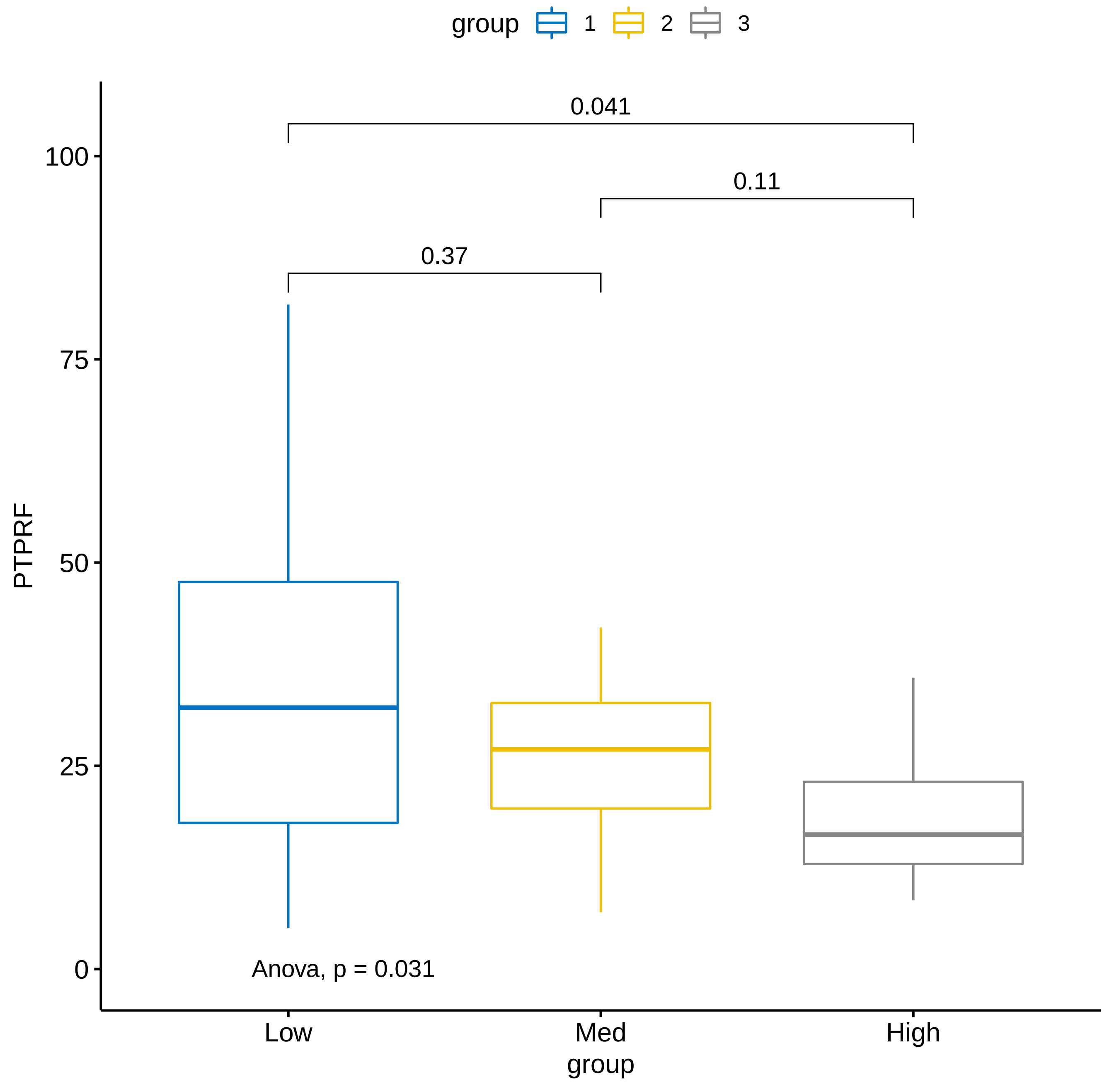

group 1 2 3
0.041
0.11
0.37
100
75
50
25
0
PTPRF
Anova, p = 0.031
Low
Med
High
group

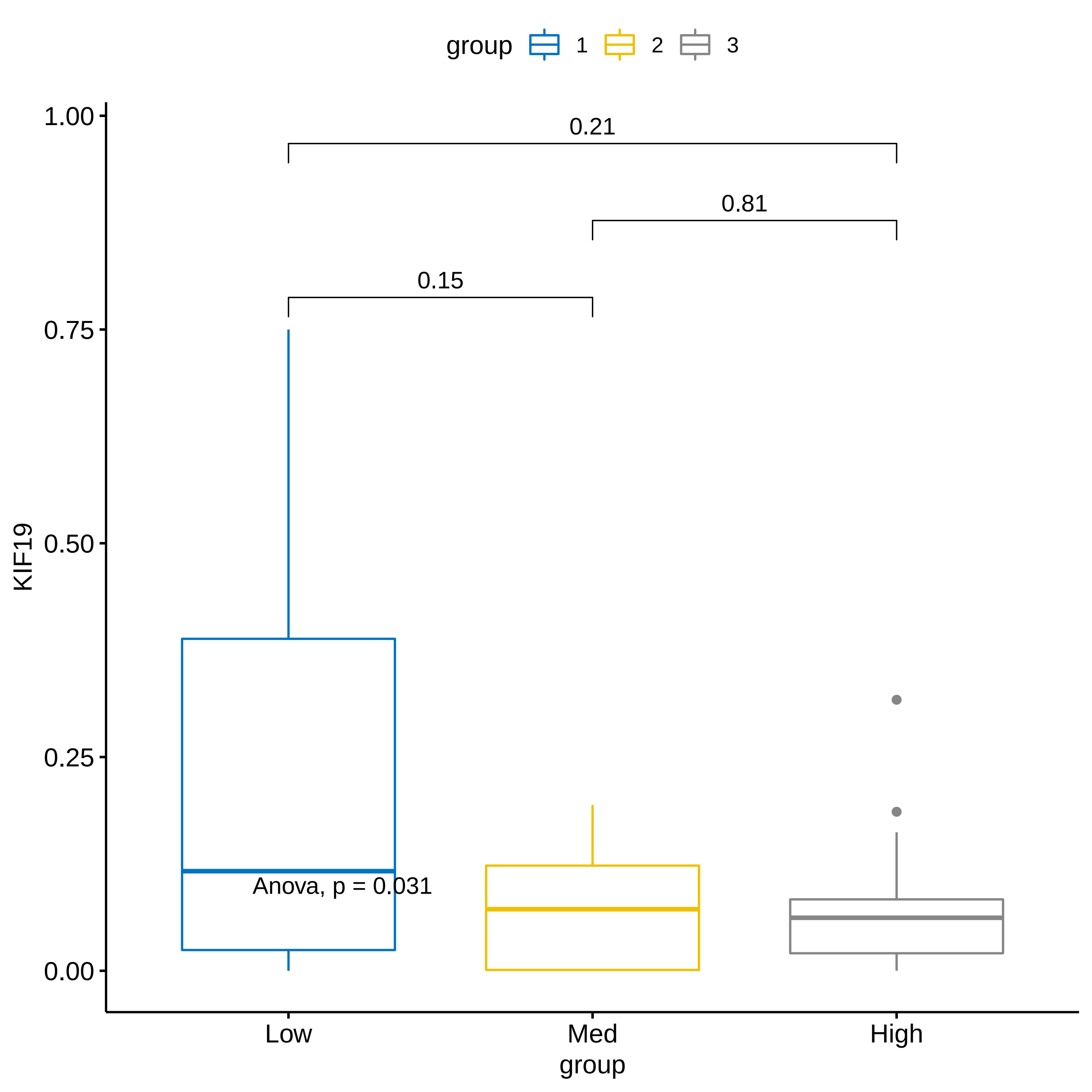

group
1
2
3
0.21
0.81
0.15
1.00
0.75
0.50
0.25
0.00
KIF19
Anova, p = 0.031
Low
Med
High
group

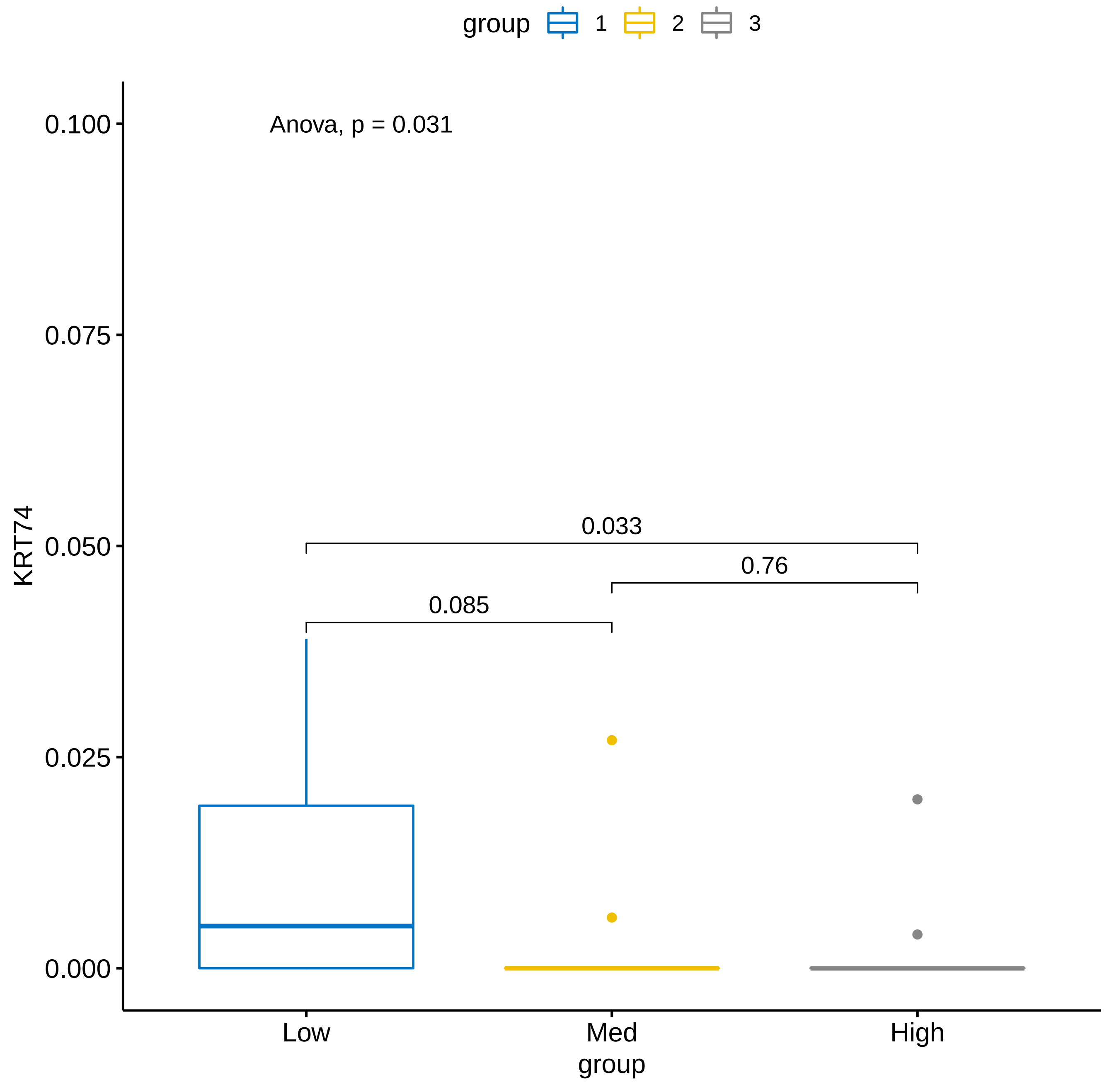
group 1 2 3
Anova, p = 0.031
0.100
0.075
0.050
0.025
0.000
KRT74
0.033
0.76
0.085
Low
Med
High
group

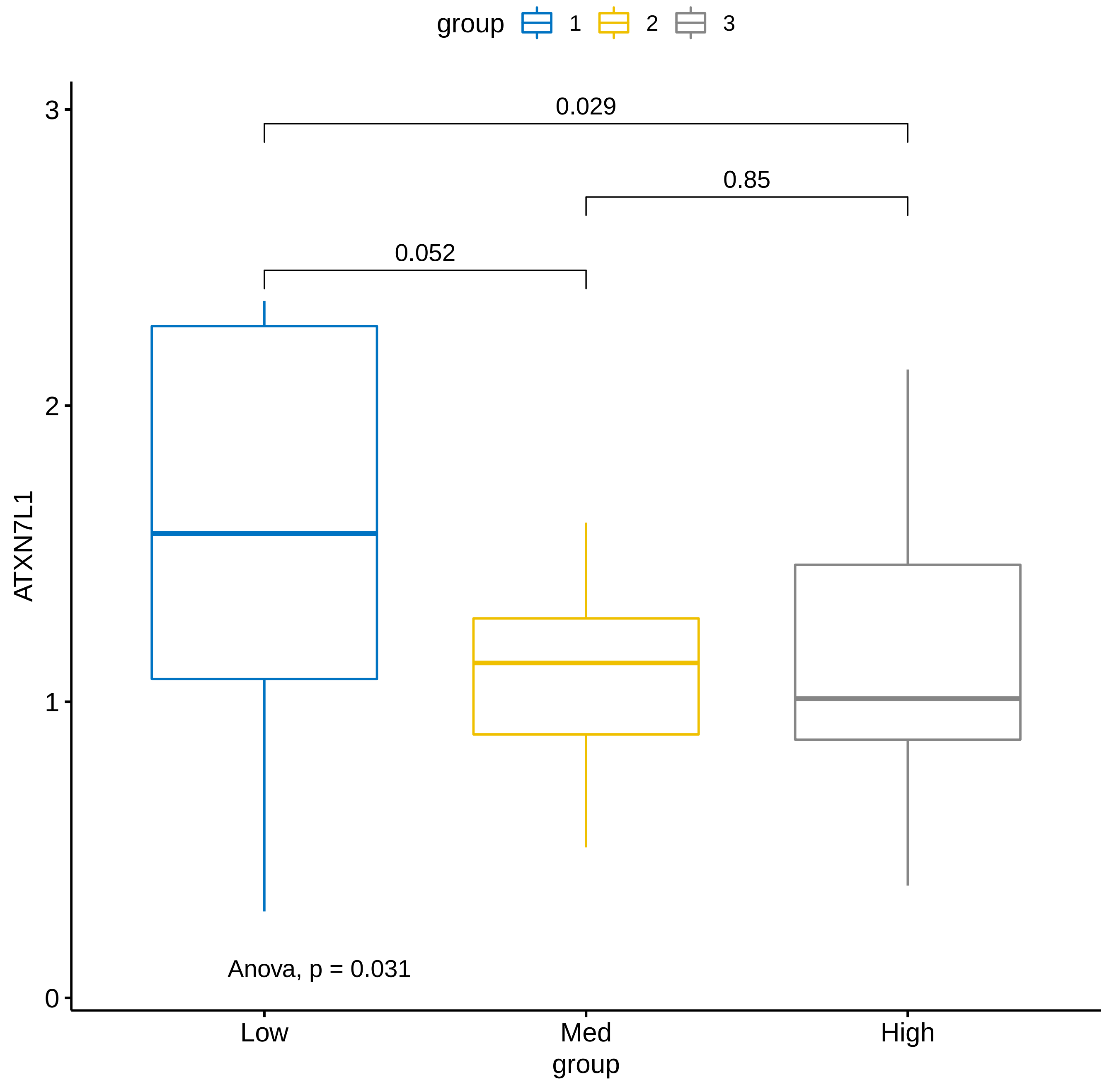
group 1 2 3
0.029
0.85
0.052
ATXN7L1
3
2
1
0
Anova, p = 0.031
Low
Med
High
group

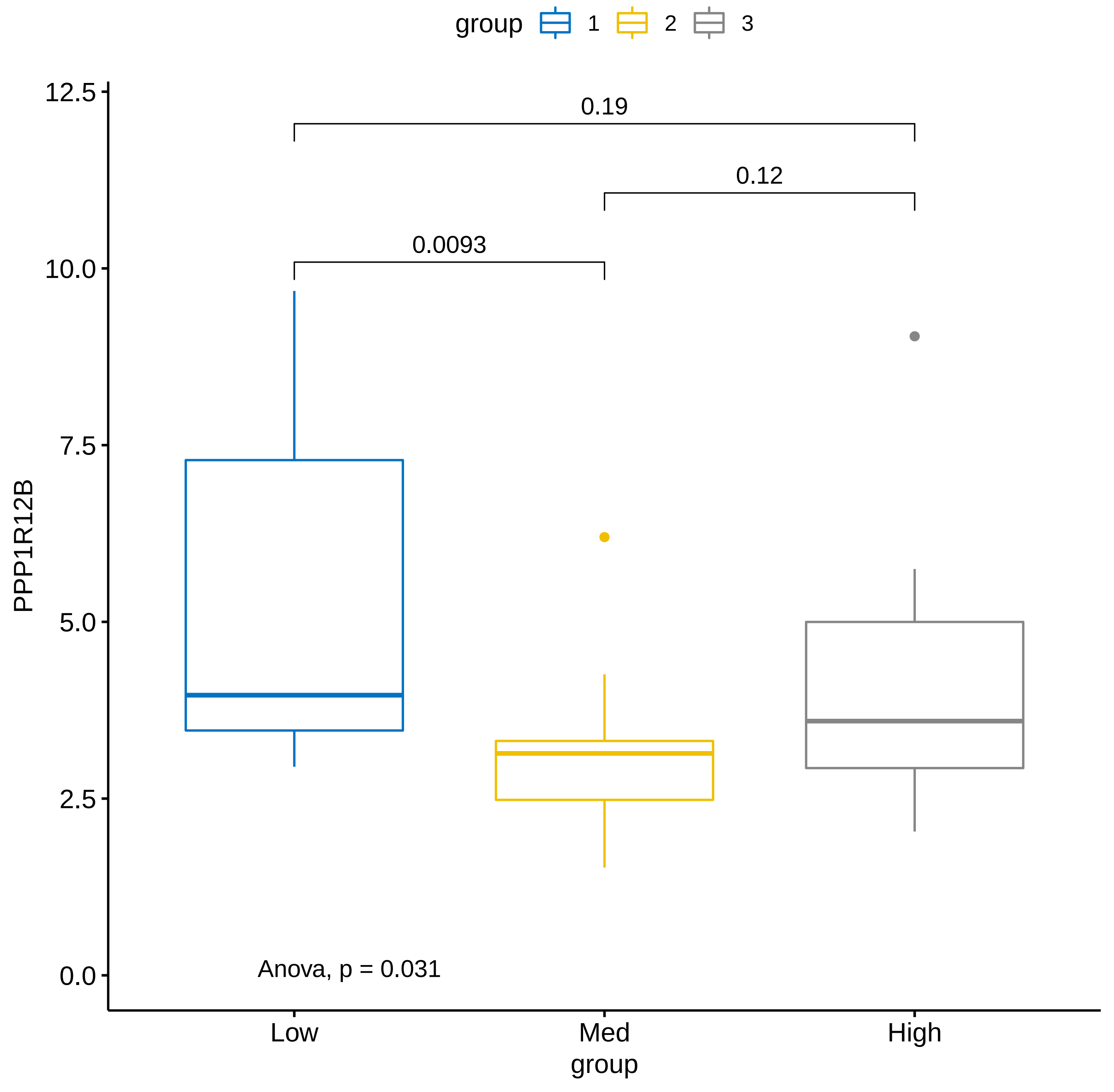

group
1
2
3
12.5
10.0
7.5
5.0
2.5
0.0
PPP1R12B
0.19
0.12
0.0093
Anova, p = 0.031
Low
Med
High
group

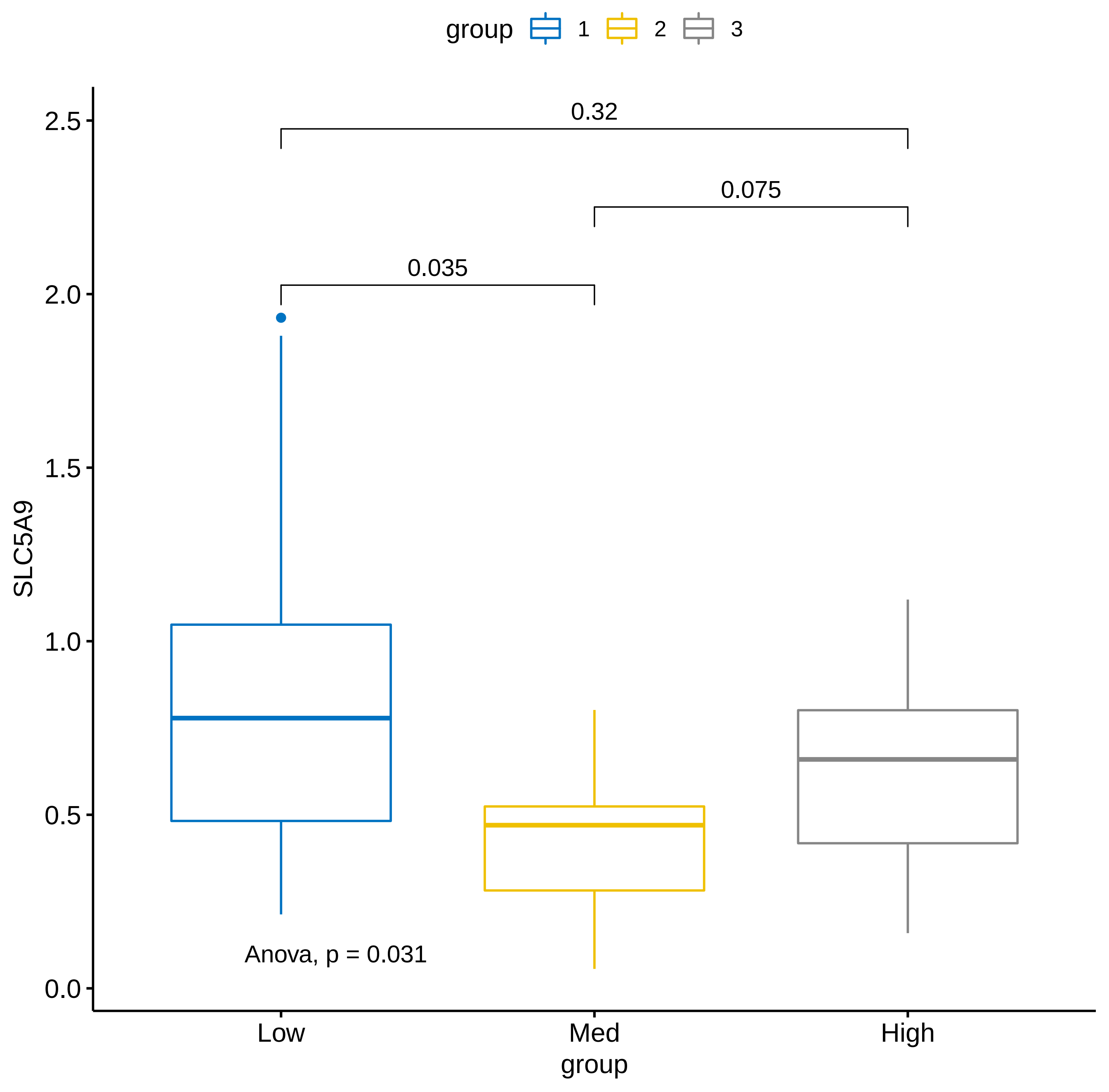
group
1
2
3
0.32
0.075
0.035
2.5
2.0
1.5
1.0
0.5
0.0
SLC5A9
Anova, p = 0.031
Low
Med
High
group

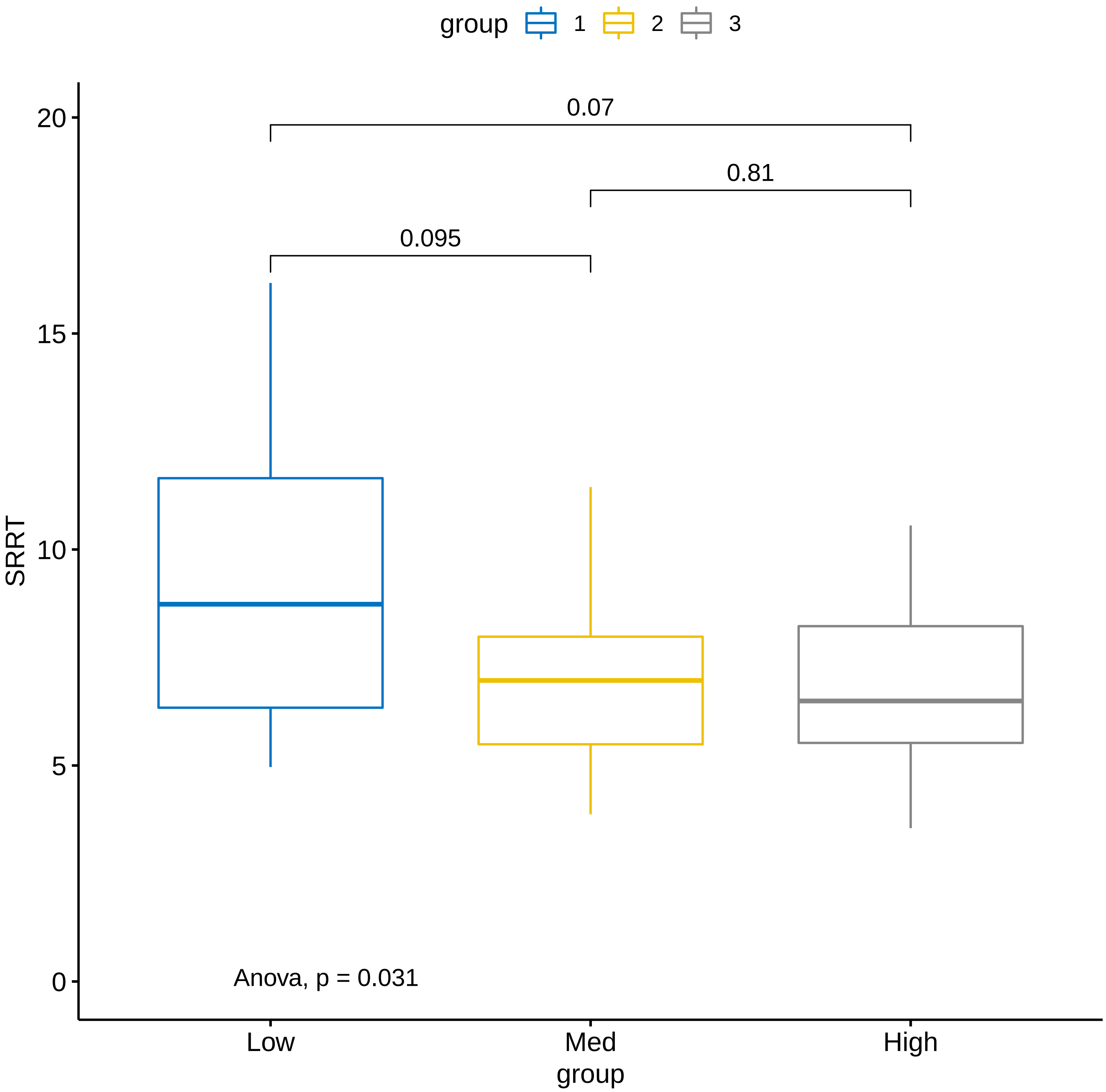

group 1 2 3
0.07
0.81
0.095
20
15
10
5
0
SRRT
Anova, p = 0.031
Low
Med
High
group

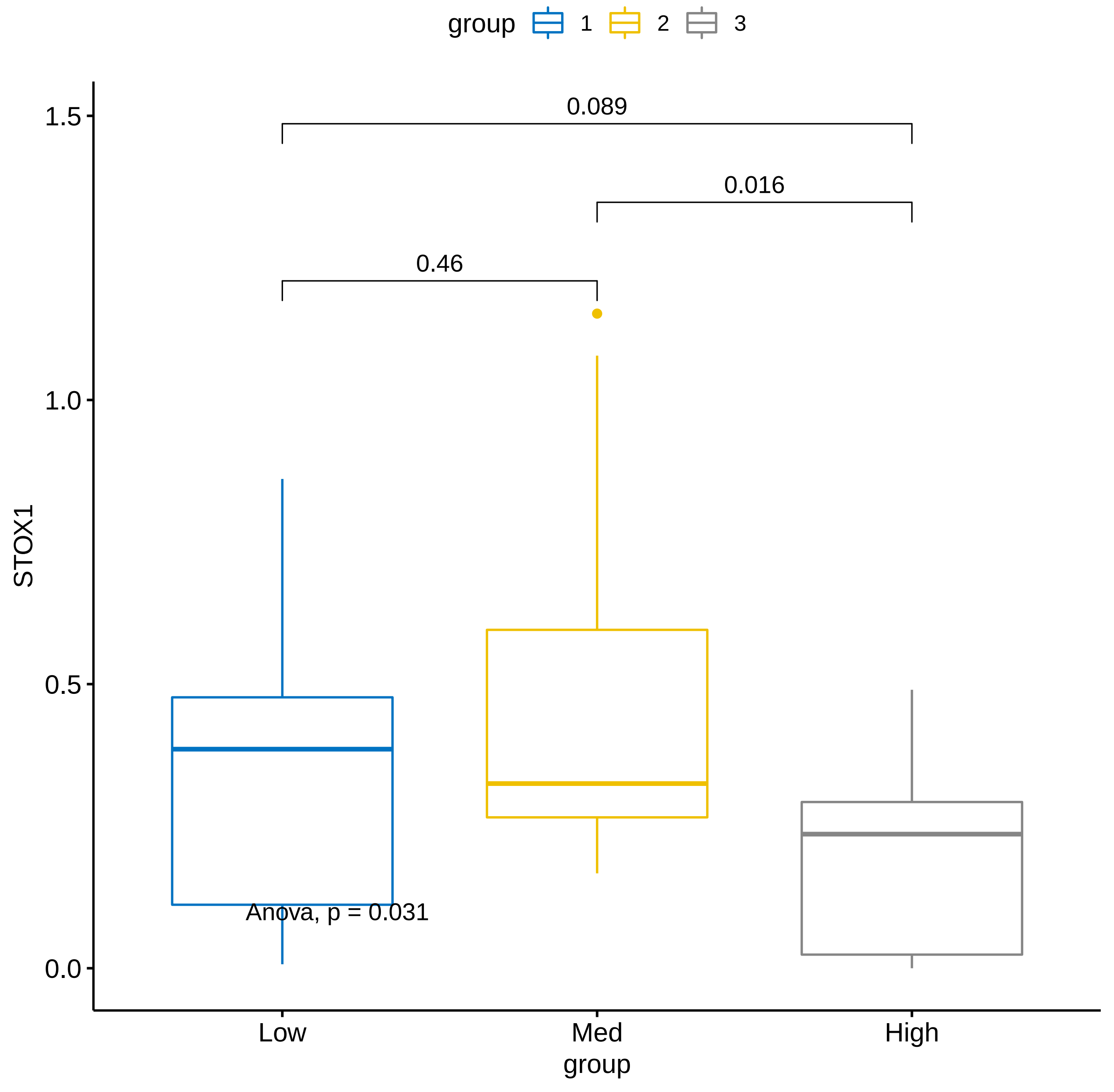
group
1
2
3
0.089
0.016
0.46
1.5
1.0
0.5
0.0
STOX1
Anova, p = 0.031
Low
Med
High
group

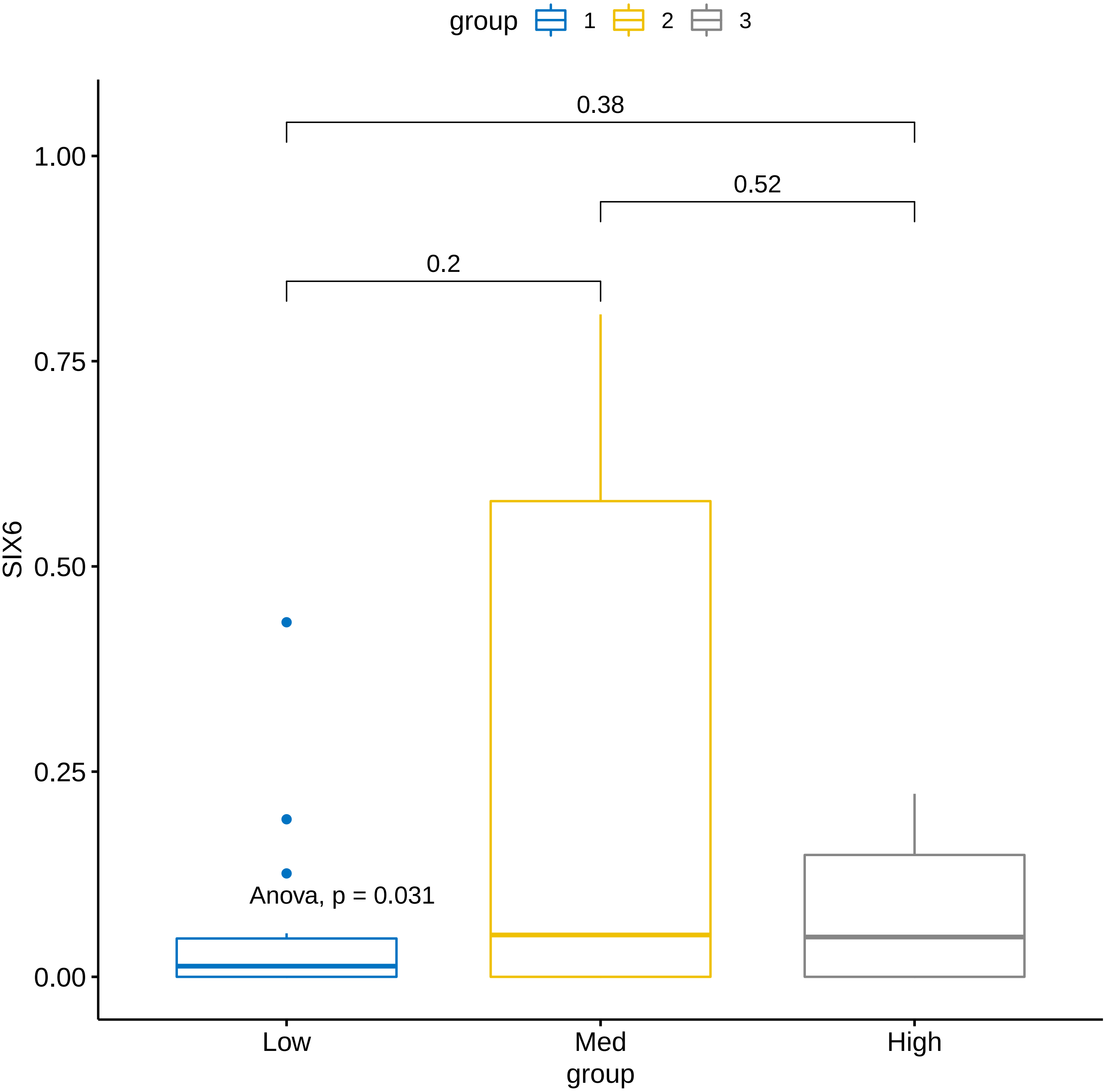
group
1
2
3
0.38
0.52
0.2
Anova, p = 0.031
SIX6
1.00
0.75
0.50
0.25
0.00
Low
Med
High
group

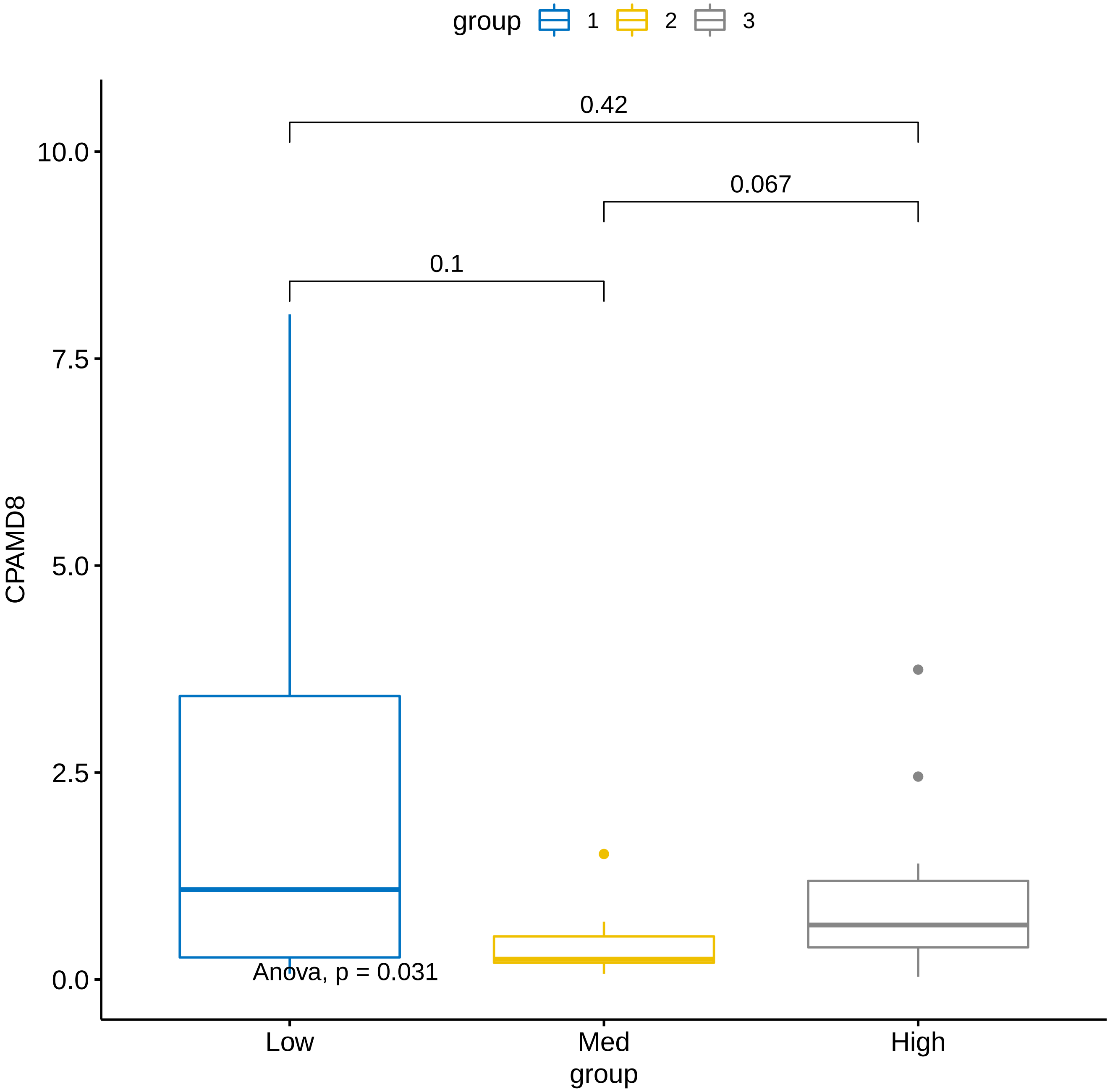
group
1
2
3
0.42
0.067
0.1
10.0
7.5
5.0
2.5
0.0
CPAMD8
Anova, p = 0.031
Low
Med
High
group

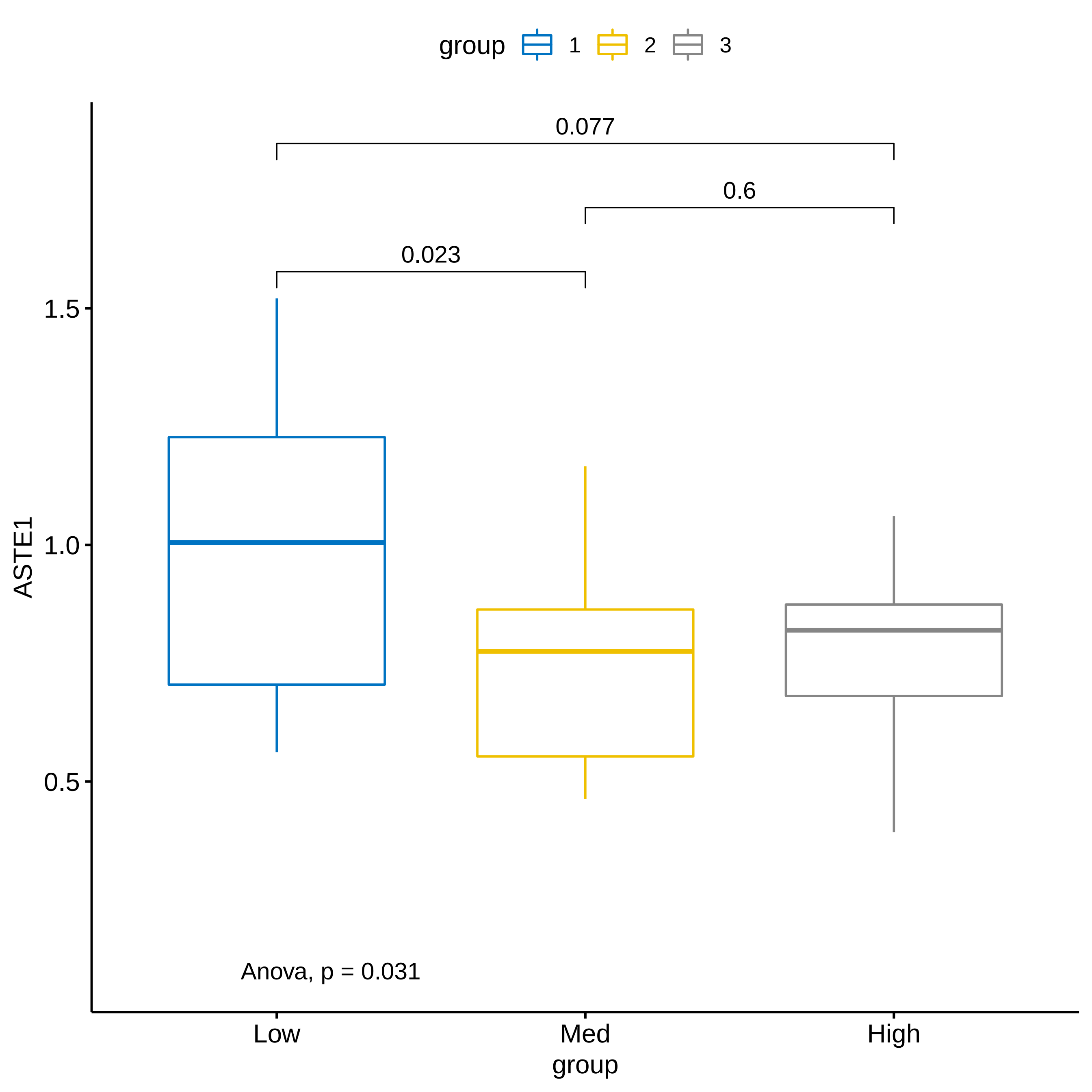
group
1
2
3
0.077
0.6
0.023
1.5
1.0
0.5
ASTE1
Anova, p = 0.031
Low
Med
High
group

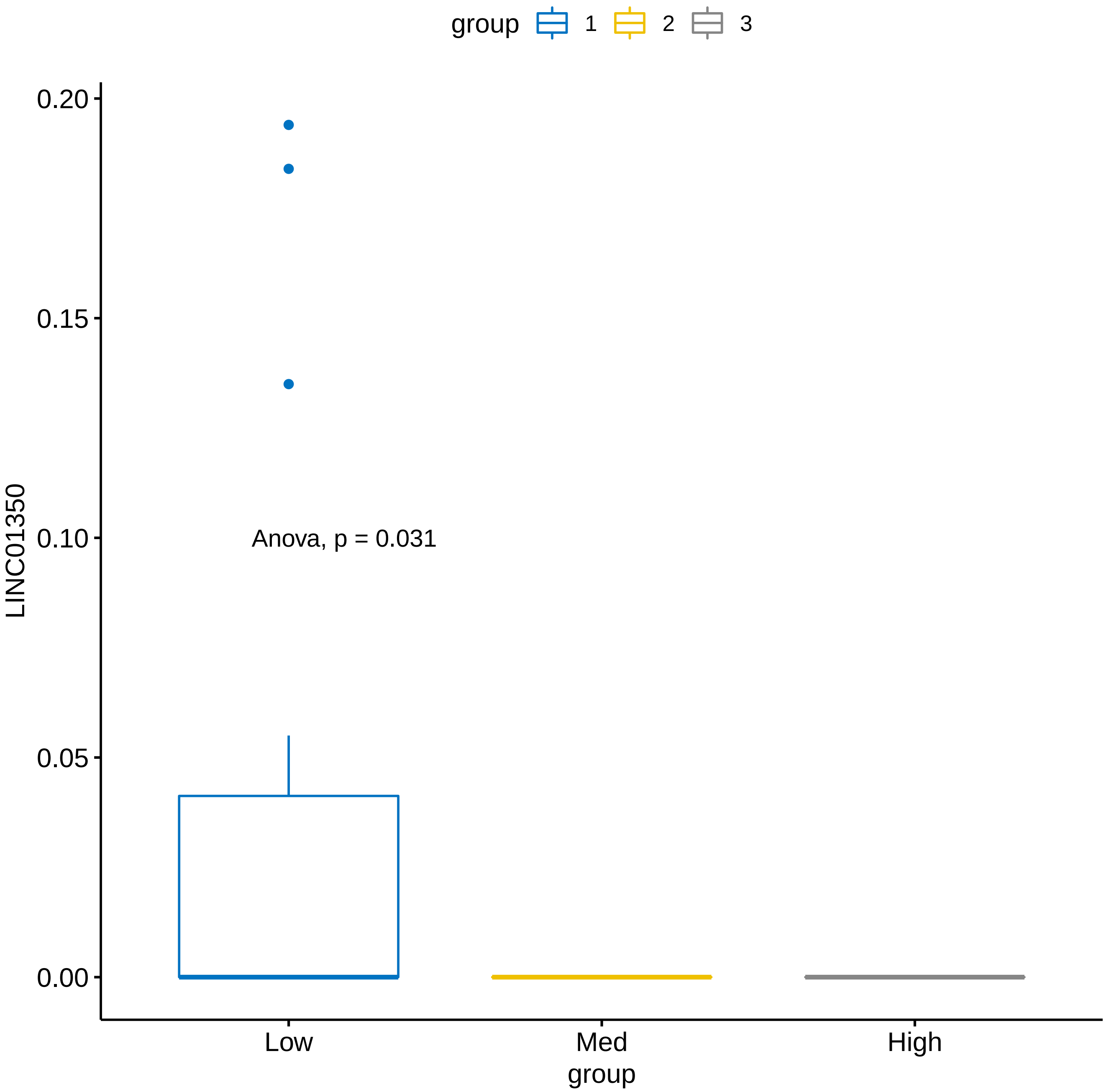

group
1
2
3
0.20
0.15
0.10
0.05
0.00
LINC01350
Anova, p = 0.031
Low
Med
High
group

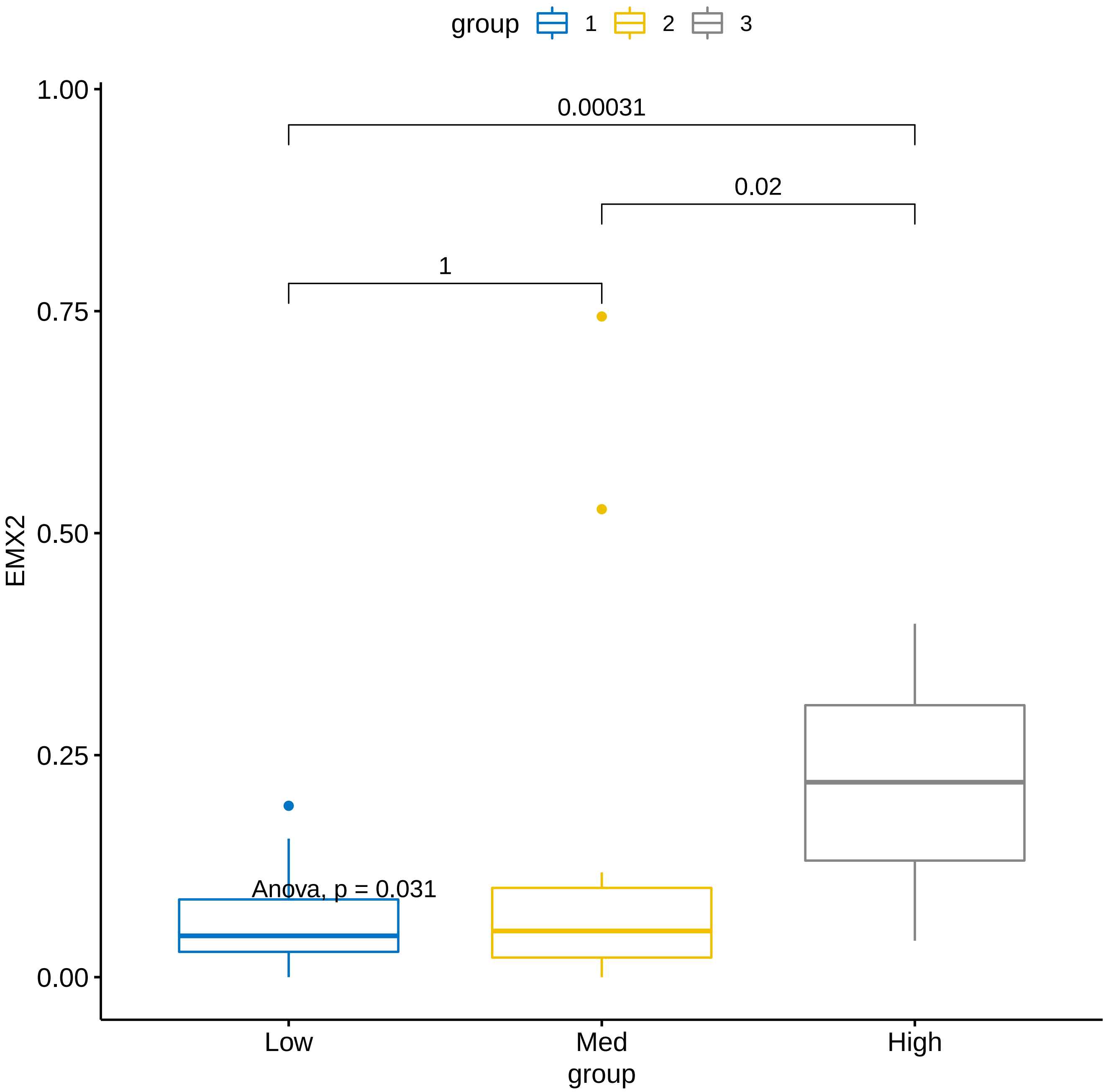
group 1 2 3
1.00
0.75
0.50
0.25
0.00
EMX2
0.00031
0.02
1
Anova, p = 0.031
Low
Med
High
group

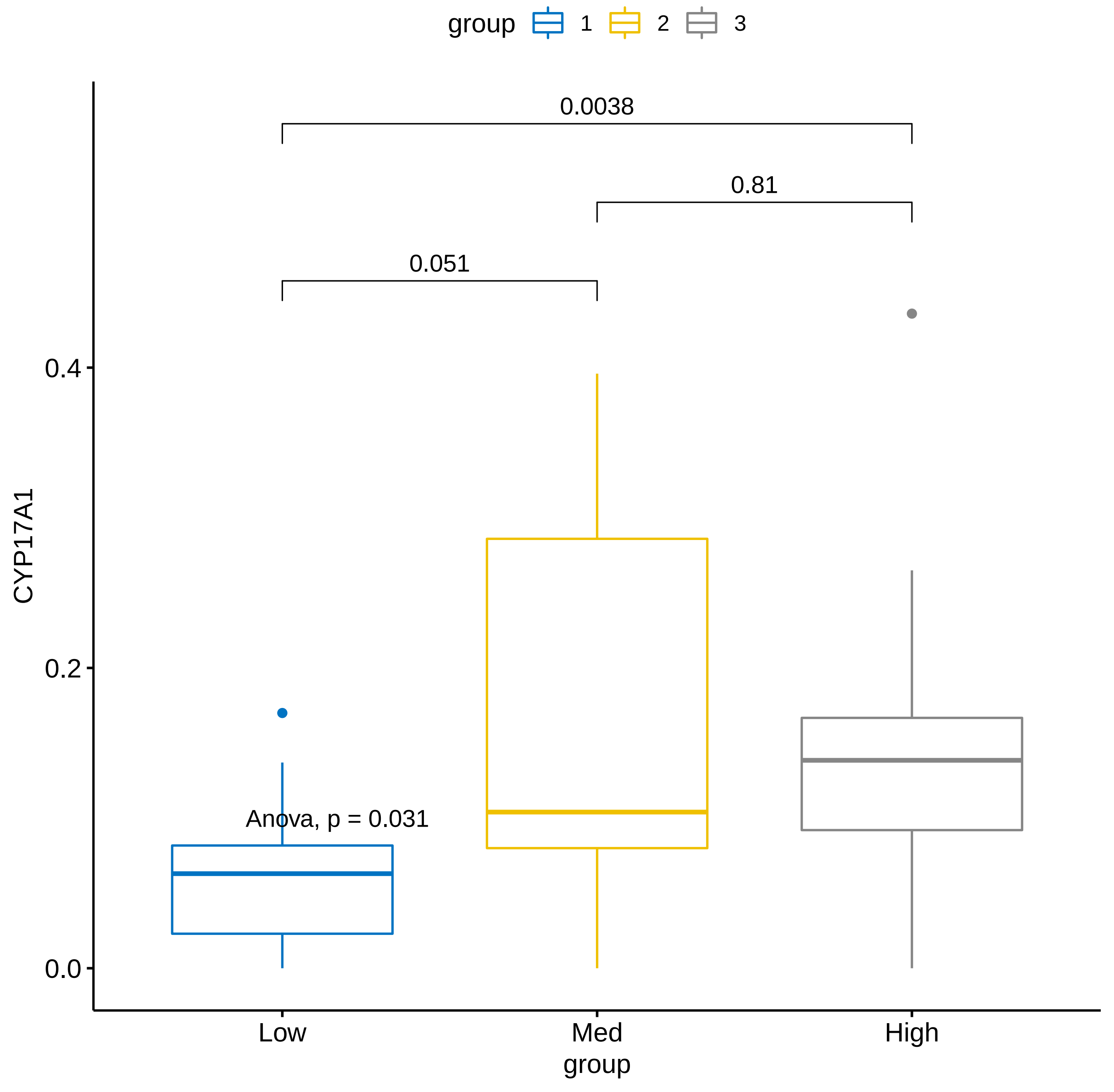
group
1
2
3
0.0038
0.81
0.051
Anova, p = 0.031
CYP17A1
0.4
0.2
0.0
Low
Med
High
group

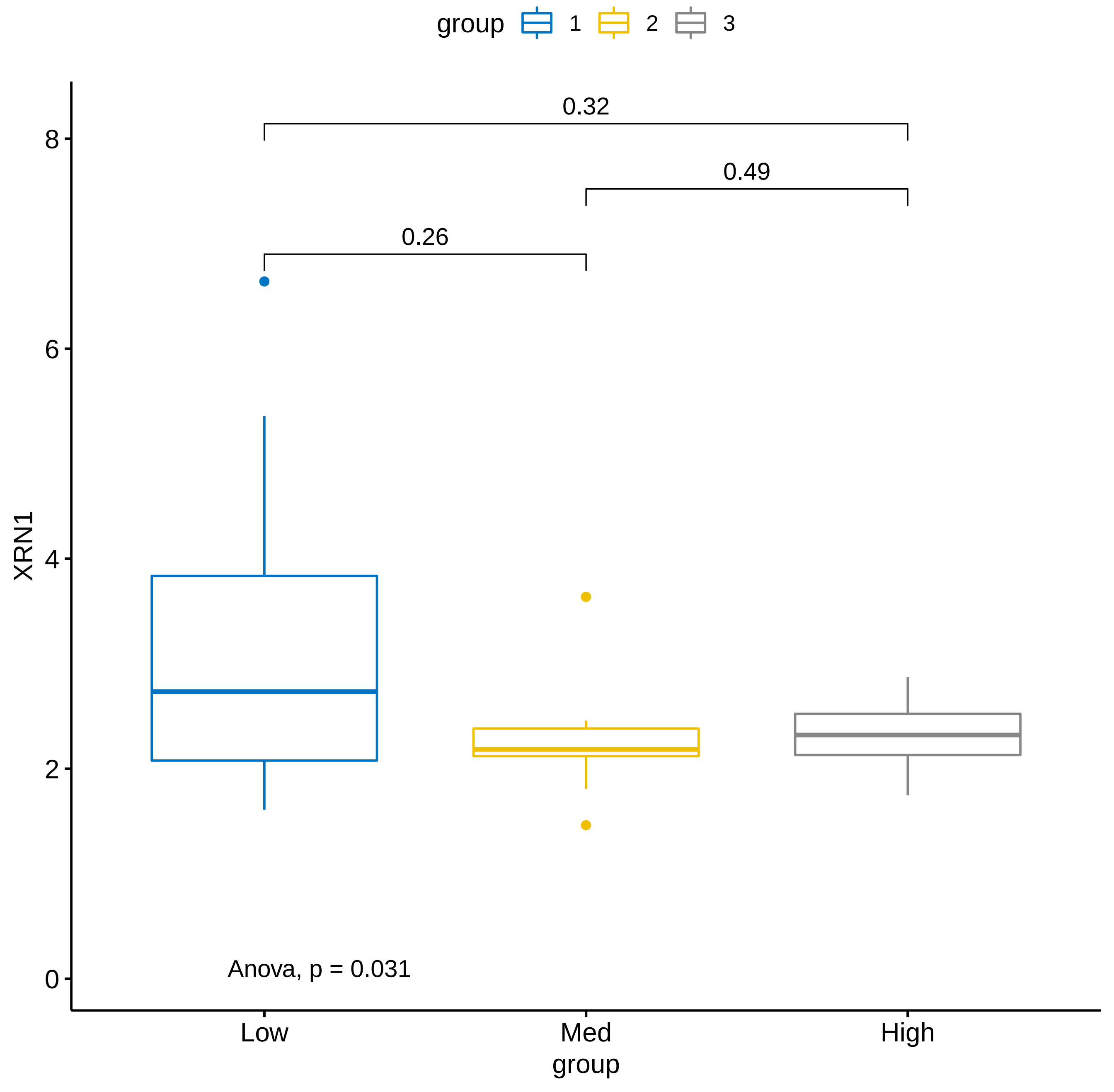
group 1 2 3
0.32
0.49
0.26
XRN1
8
6
4
2
0
Anova, p = 0.031
Low
Med
High
group

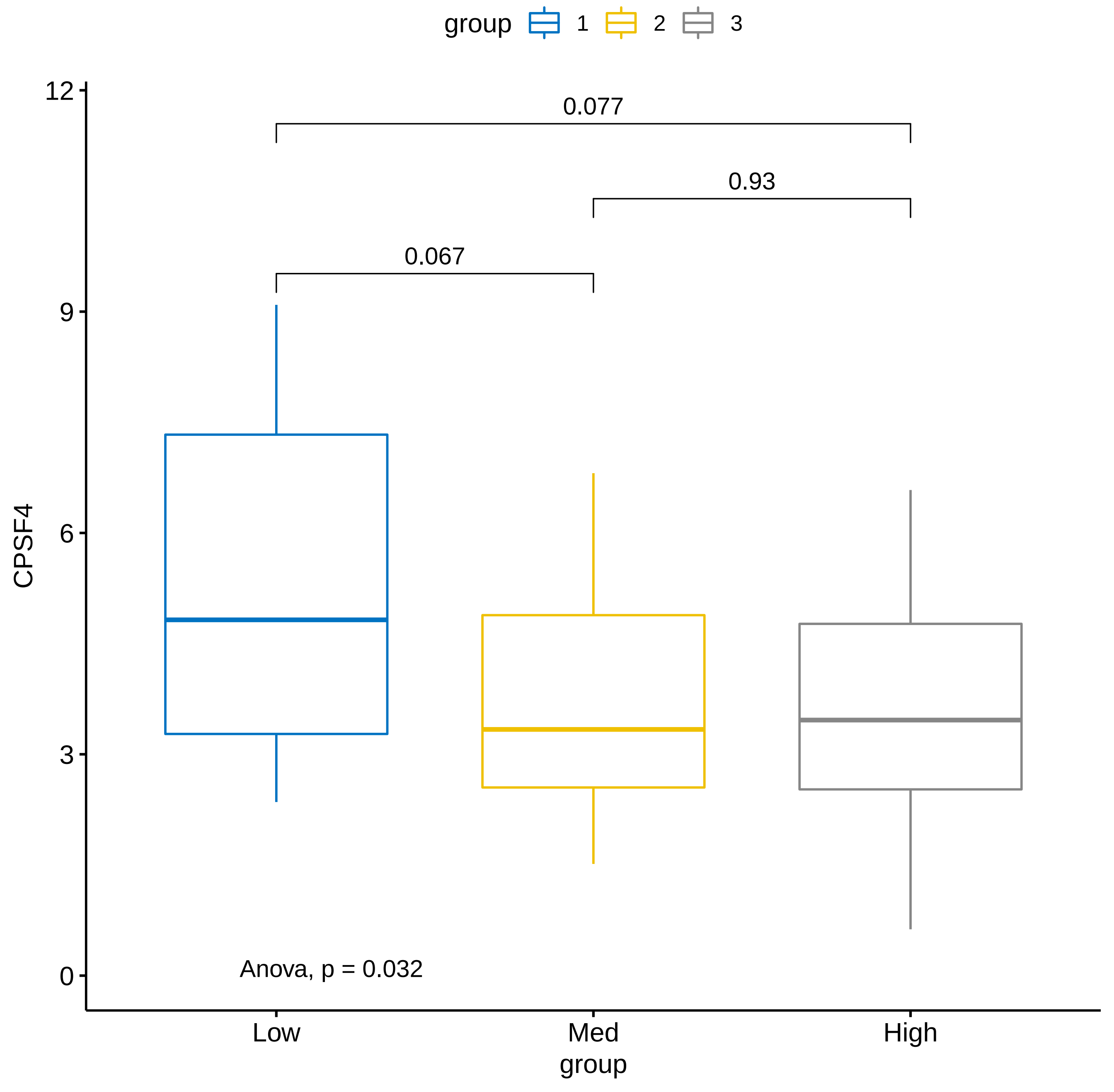

group
1
2
3
12
9
6
3
0
CPSF4
0.077
0.93
0.067
Anova, p = 0.032
Low
Med
High
group

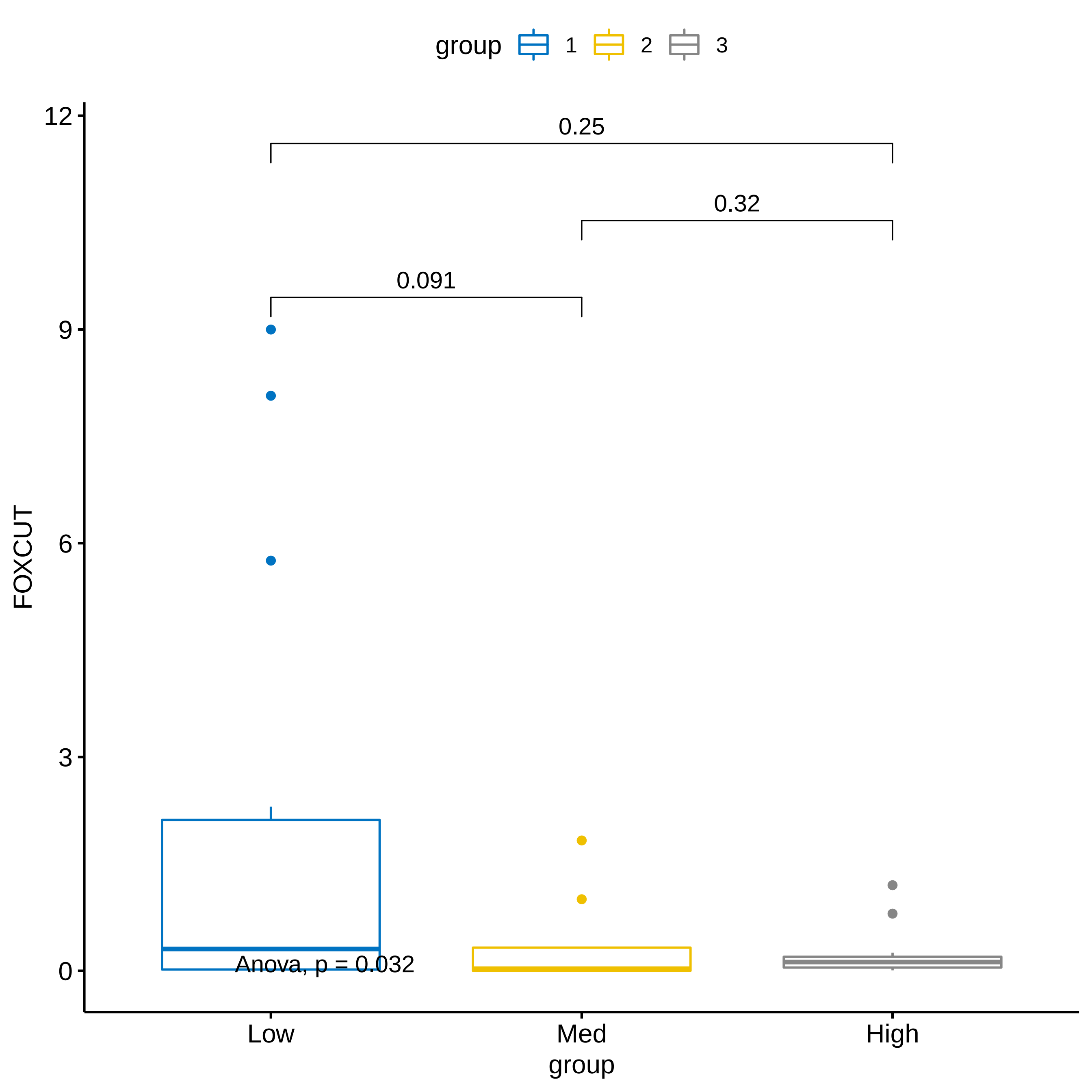
group 1 2 3
0.25
0.32
0.091
12
9
6
3
0
FOXCUT
Anova, p = 0.032
Low
Med
High
group

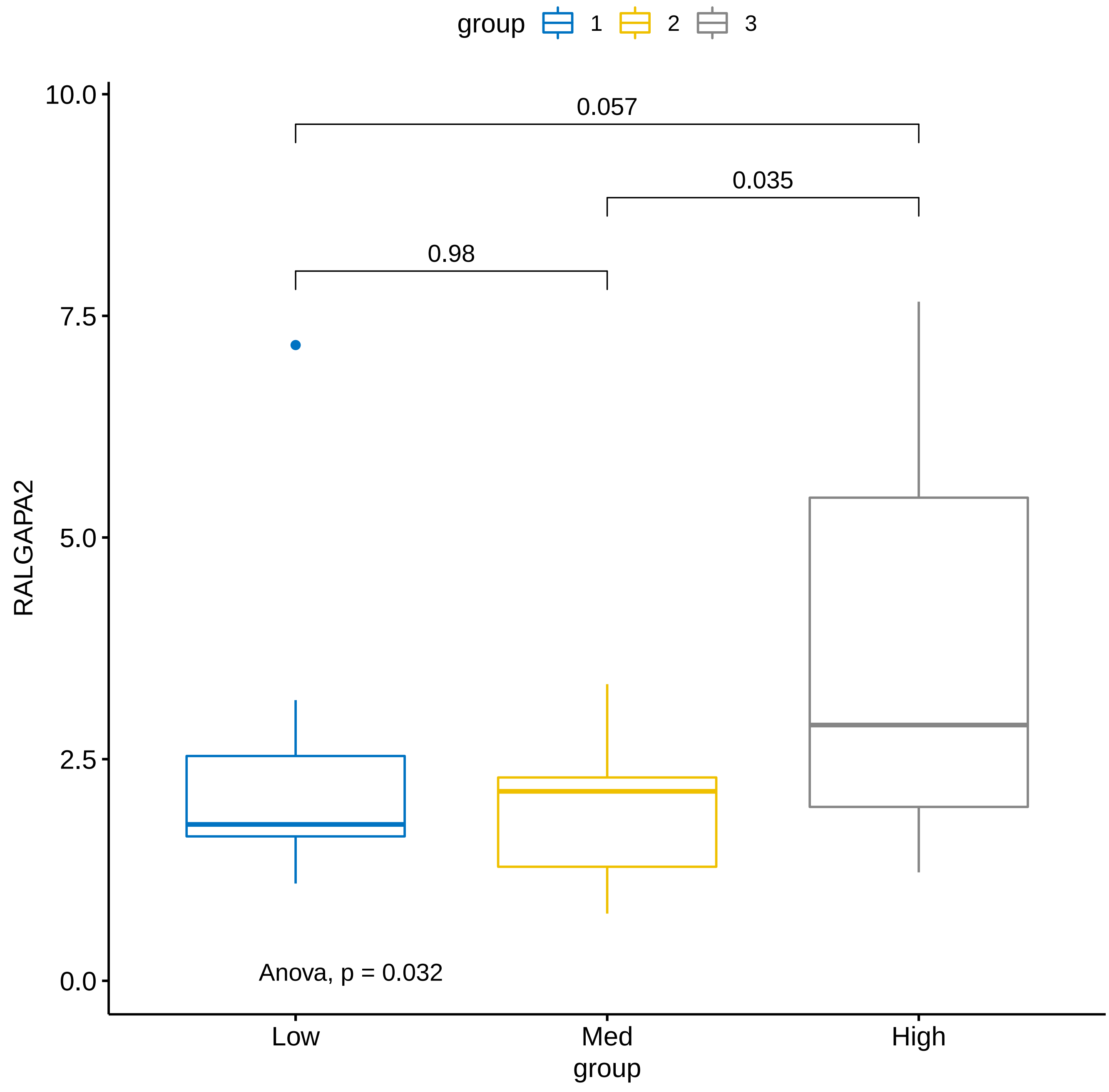

group
1
2
3
10.0
7.5
5.0
2.5
0.0
RALGAPA2
0.057
0.035
0.98
Anova, p = 0.032
Low
Med
High
group

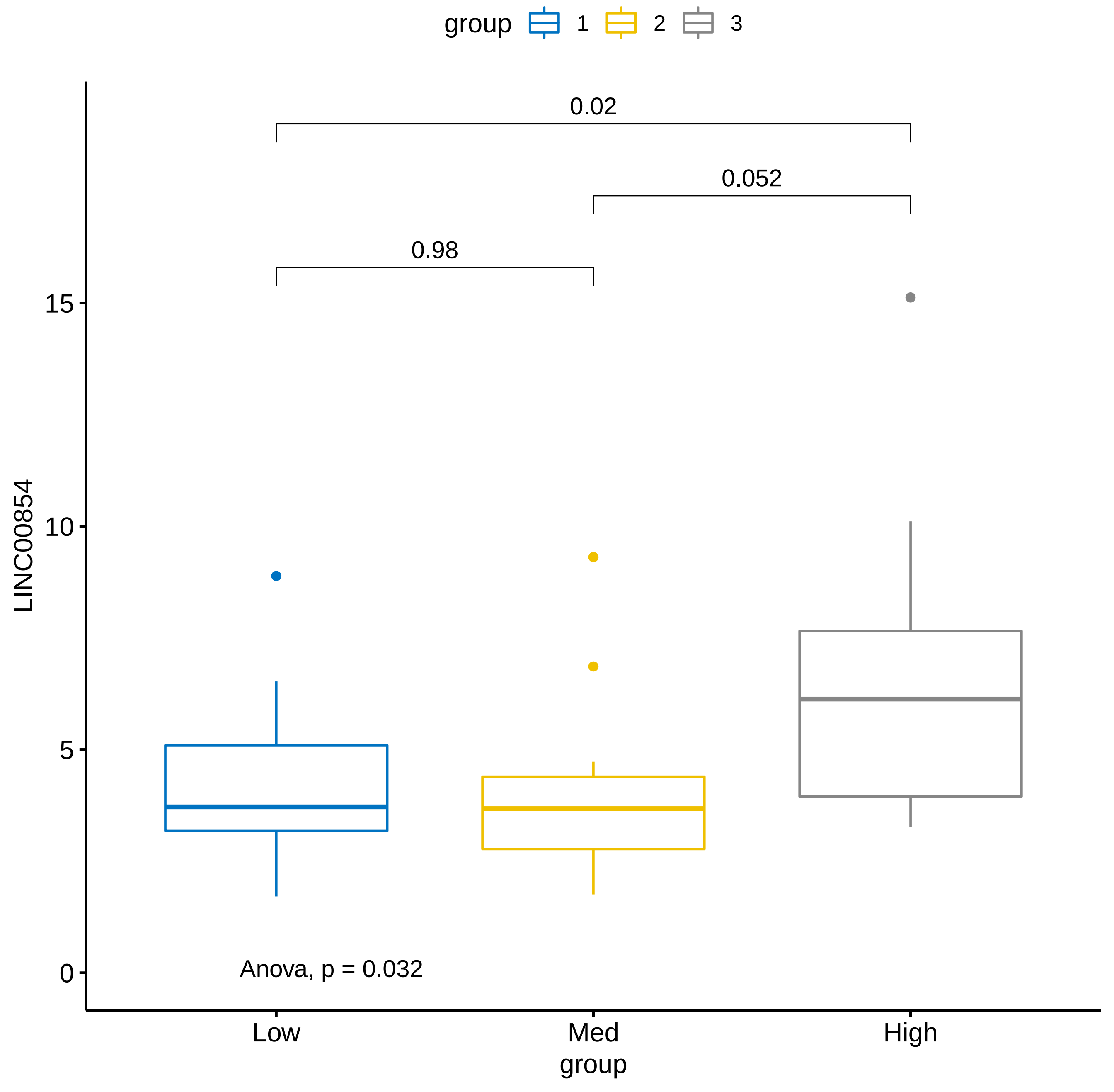

group
1
2
3
0.02
0.052
0.98
15
10
5
0
LINC00854
Anova, p = 0.032
Low
Med
High
group

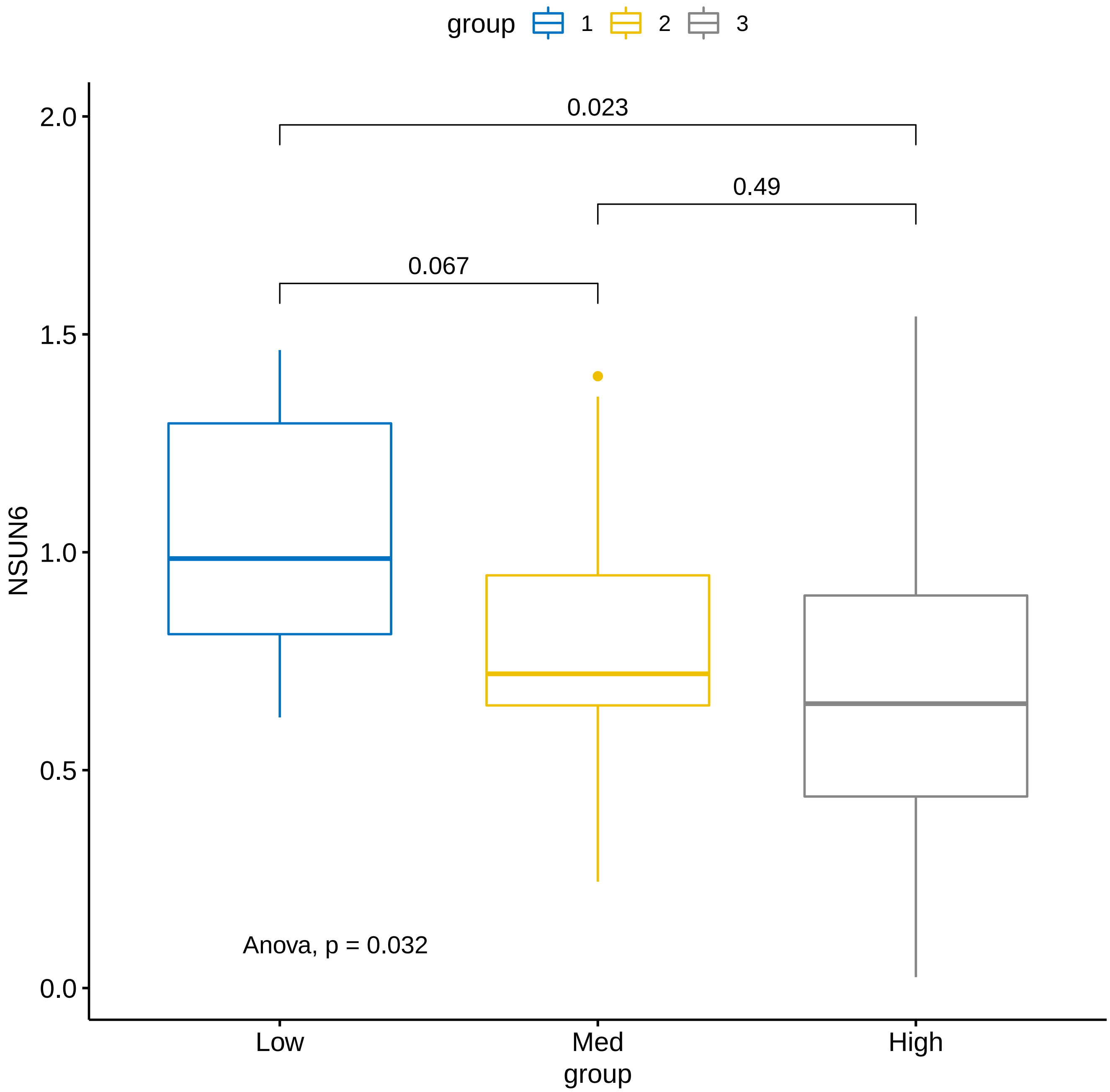
group
1
2
3
0.023
0.49
0.067
2.0
1.5
1.0
0.5
0.0
NSUN6
Anova, p = 0.032
Low
Med
High
group

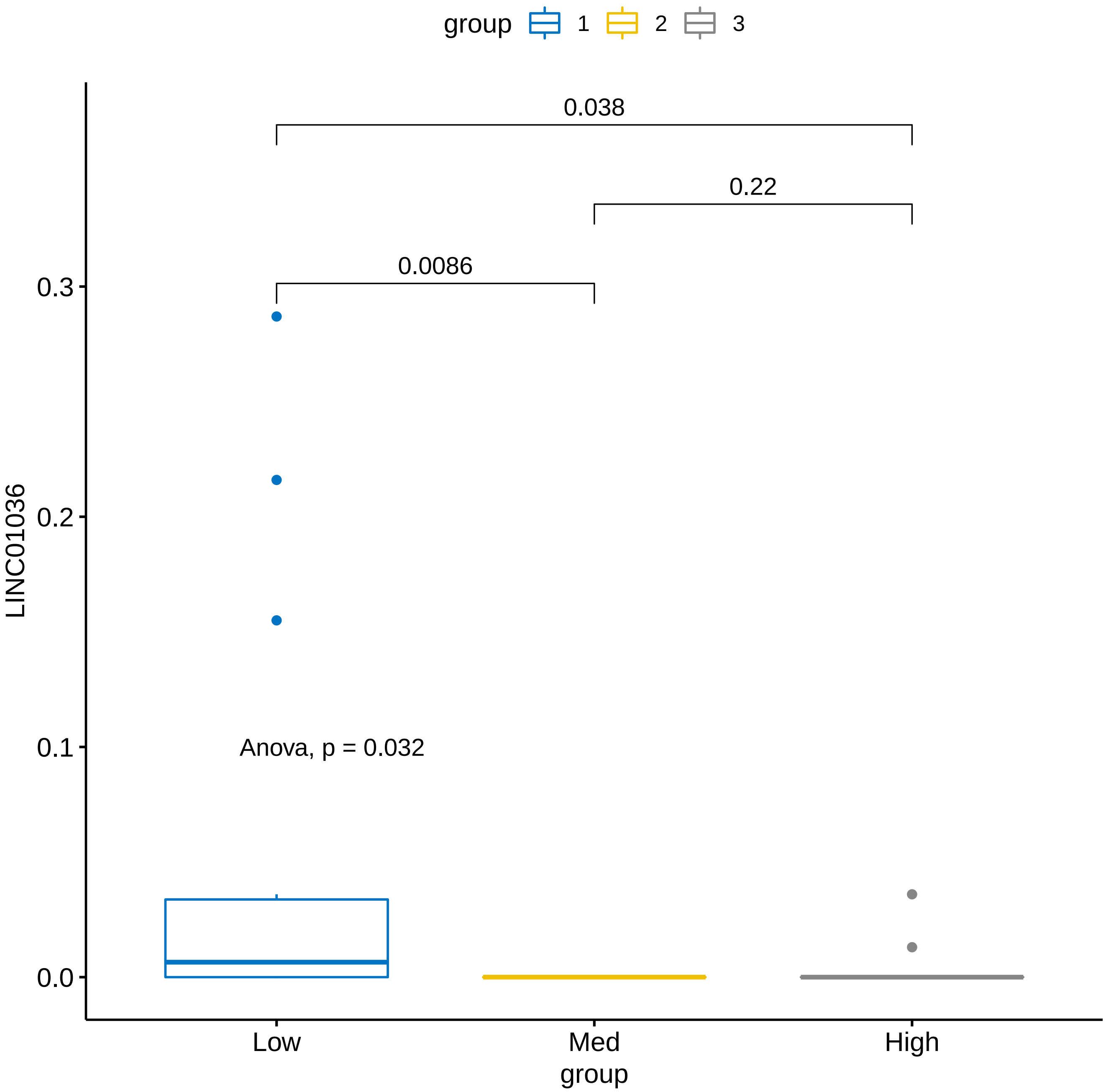

group 1 2 3
0.038
0.22
0.0086
Anova, p = 0.032
LINC01036
0.0
0.1
0.2
0.3
Low
Med
High
group

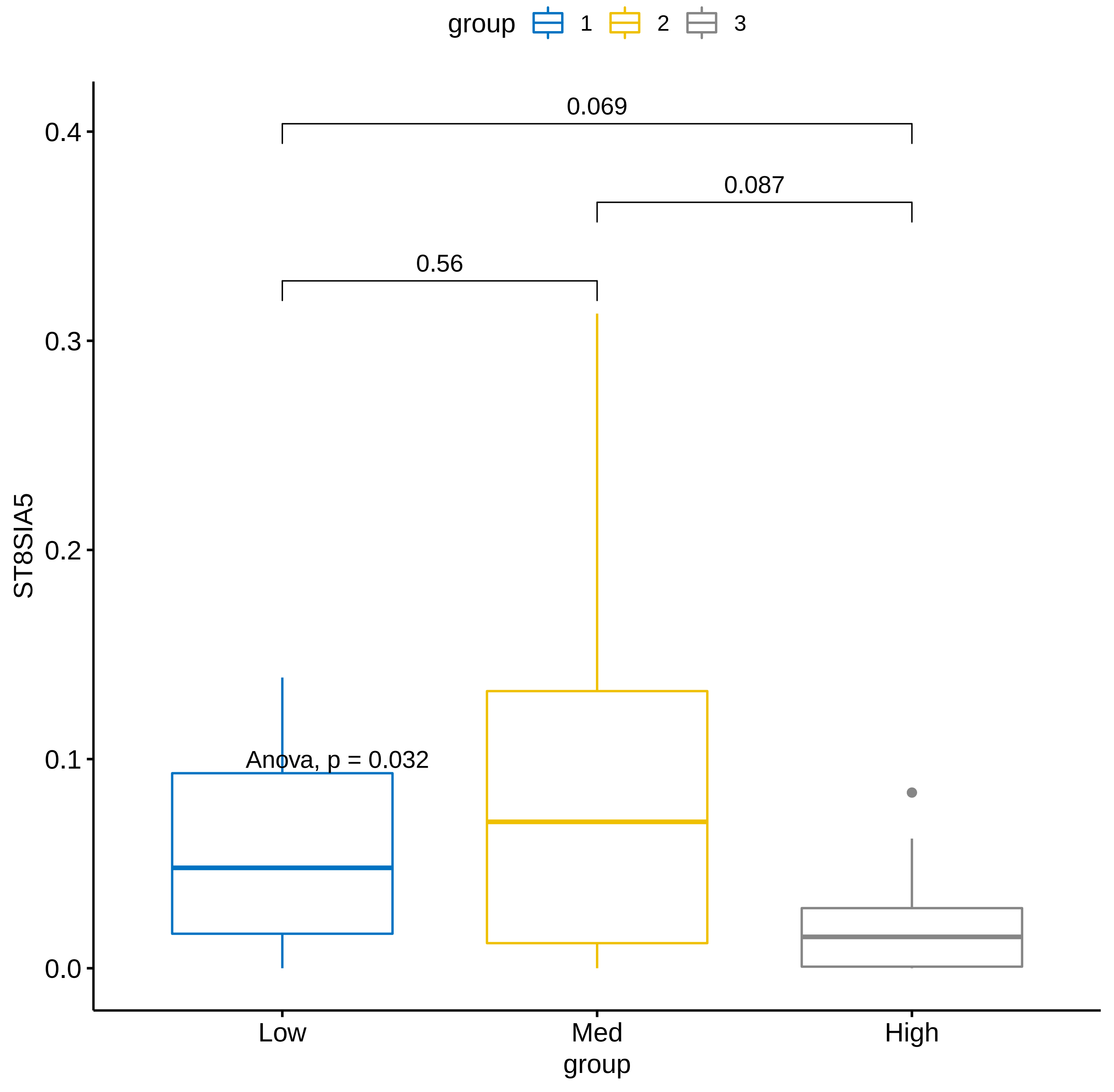
group
1
2
3
0.069
0.087
0.56
Anova, p = 0.032
ST8SIA5
0.4
0.3
0.2
0.1
0.0
Low
Med
High
group

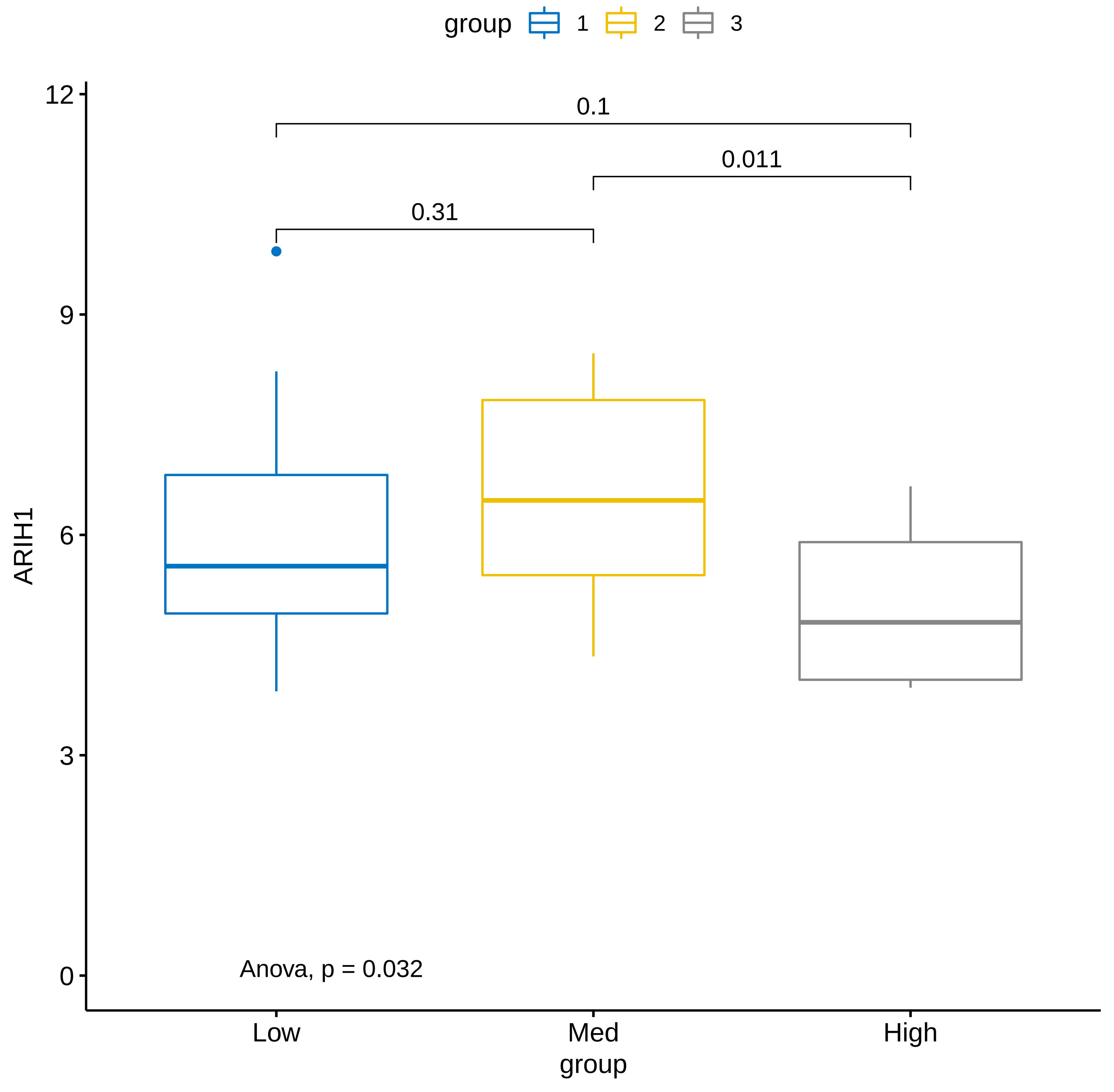

group 1 2 3
12
9
6
3
0
ARIH1
0.1
0.011
0.31
Anova, p = 0.032
Low
Med
High
group

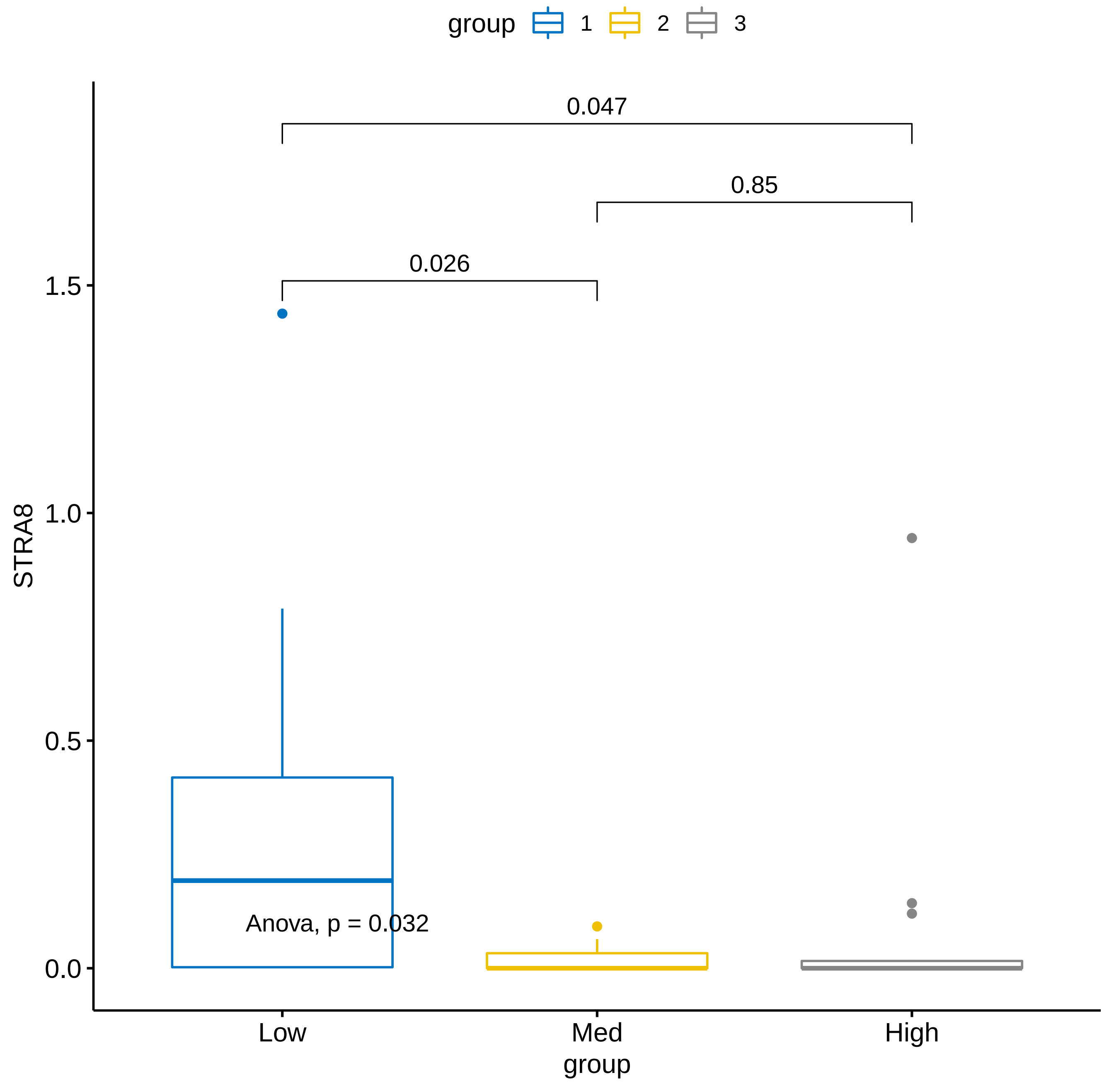
group
1
2
3
0.047
0.85
0.026
1.5
1.0
0.5
0.0
STRA8
Anova, p = 0.032
Low
Med
High
group

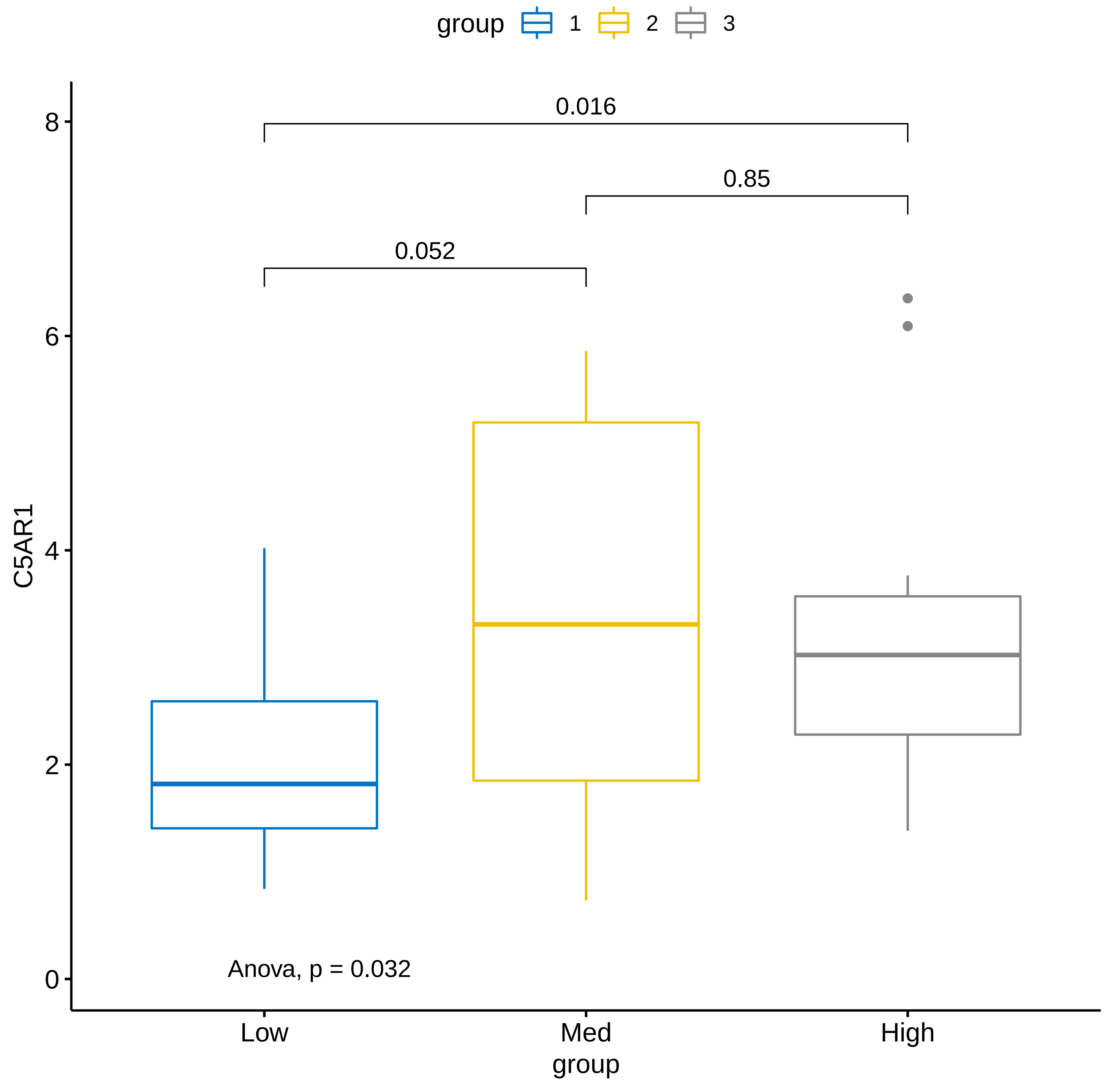

group
1
2
3
0.016
0.85
0.052
C5AR1
8
6
4
2
0
Anova, p = 0.032
Low
Med
High
group

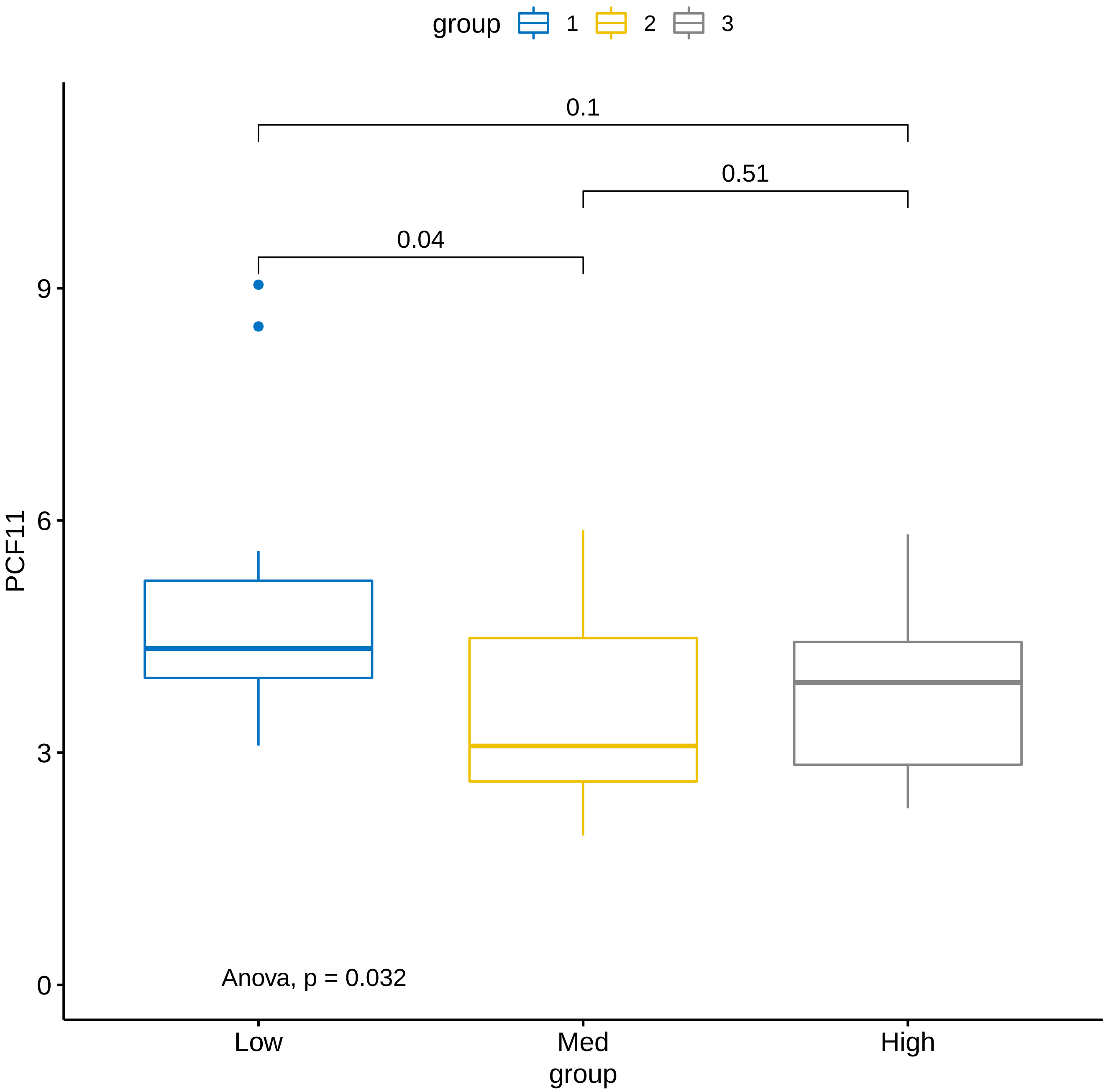
group
1
2
3
0.1
0.51
0.04
9
6
3
0
PCF11
Anova, p = 0.032
Low
Med
High
group

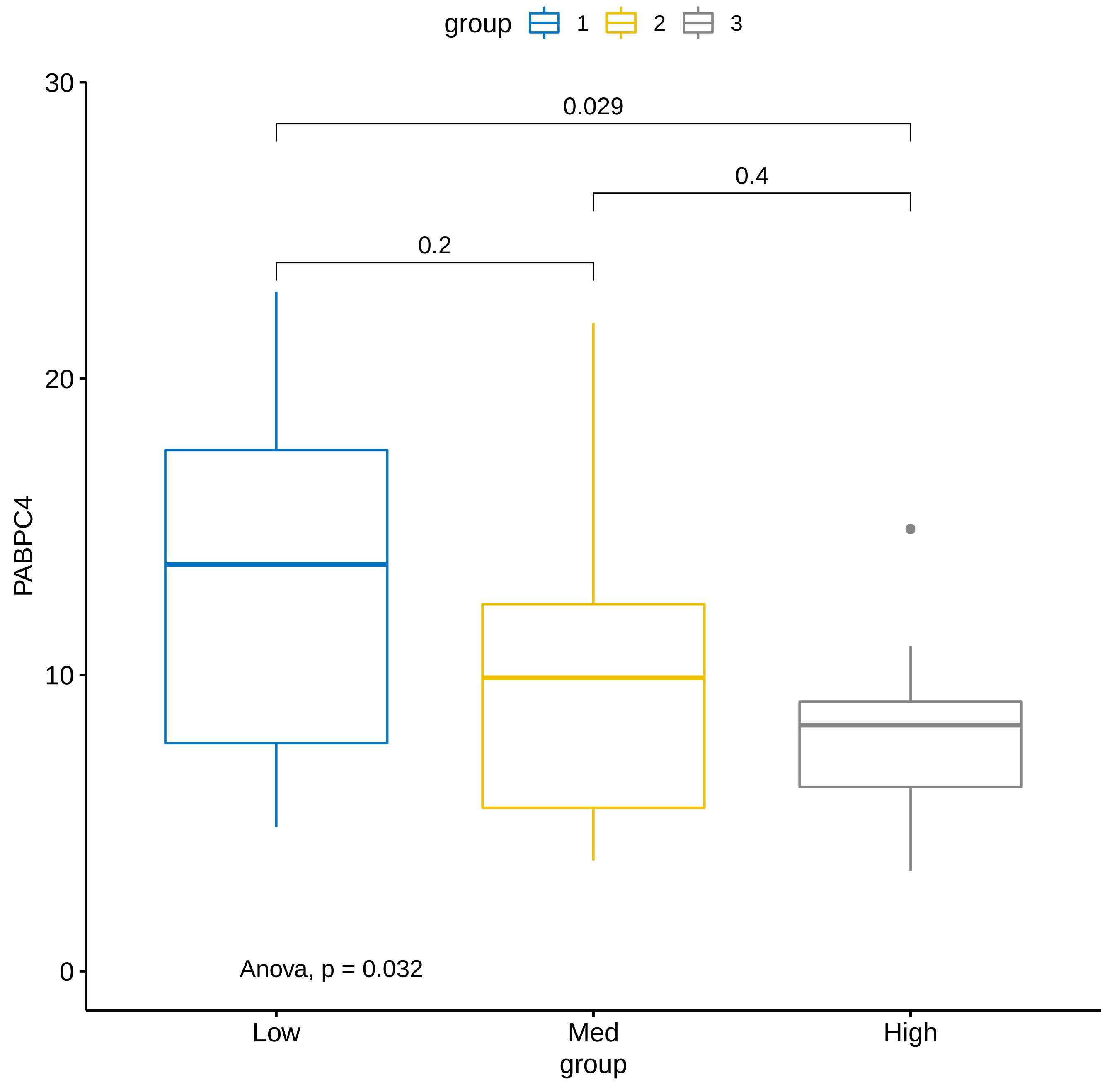
group
1
2
3
30
20
10
0
PABPC4
0.029
0.4
0.2
Anova, p = 0.032
Low
Med
High
group

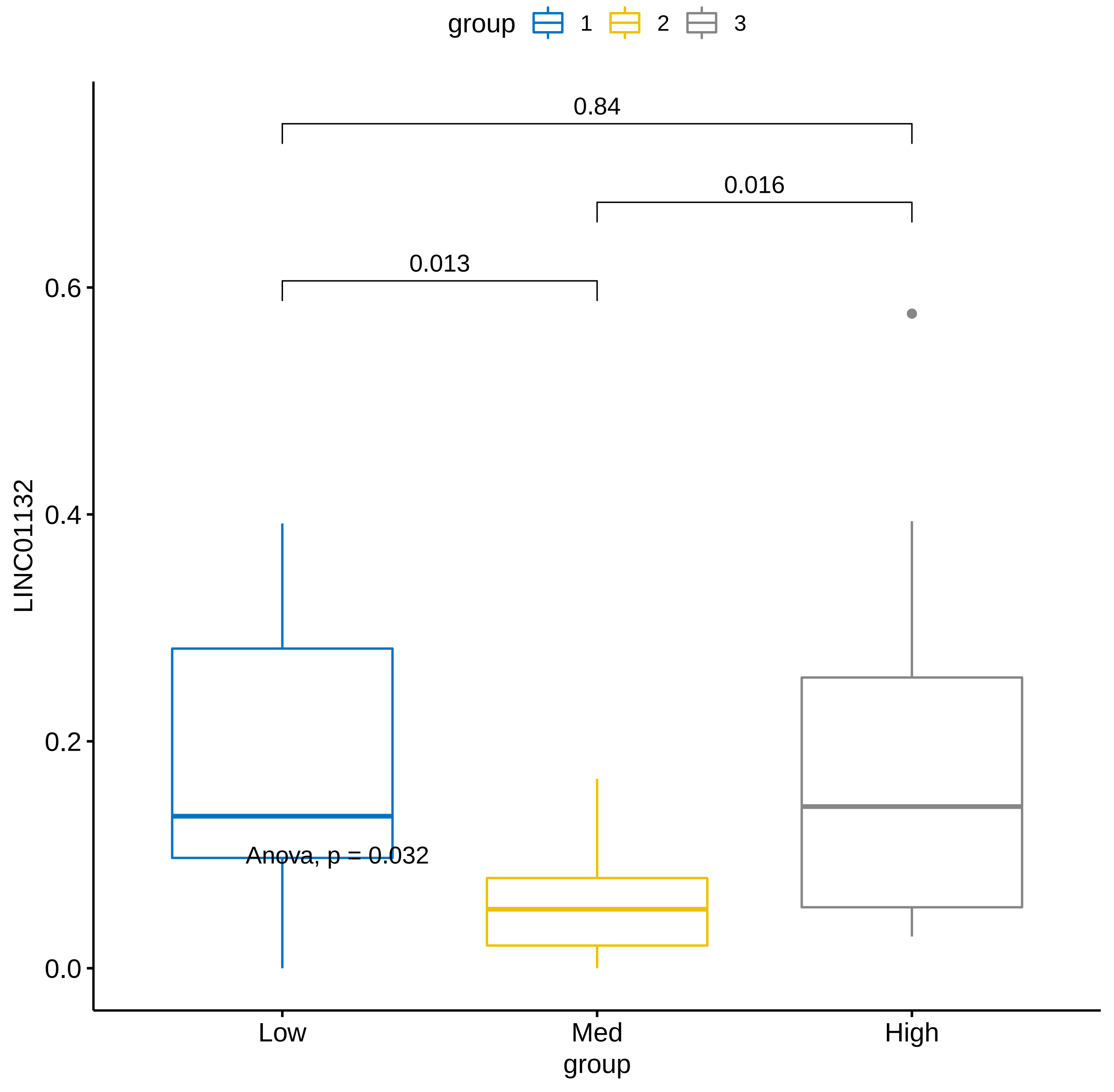
group
1
2
3
0.84
0.016
0.013
0.6
0.4
0.2
0.0
LINC01132
Anova, p = 0.032
Low
Med
High
group

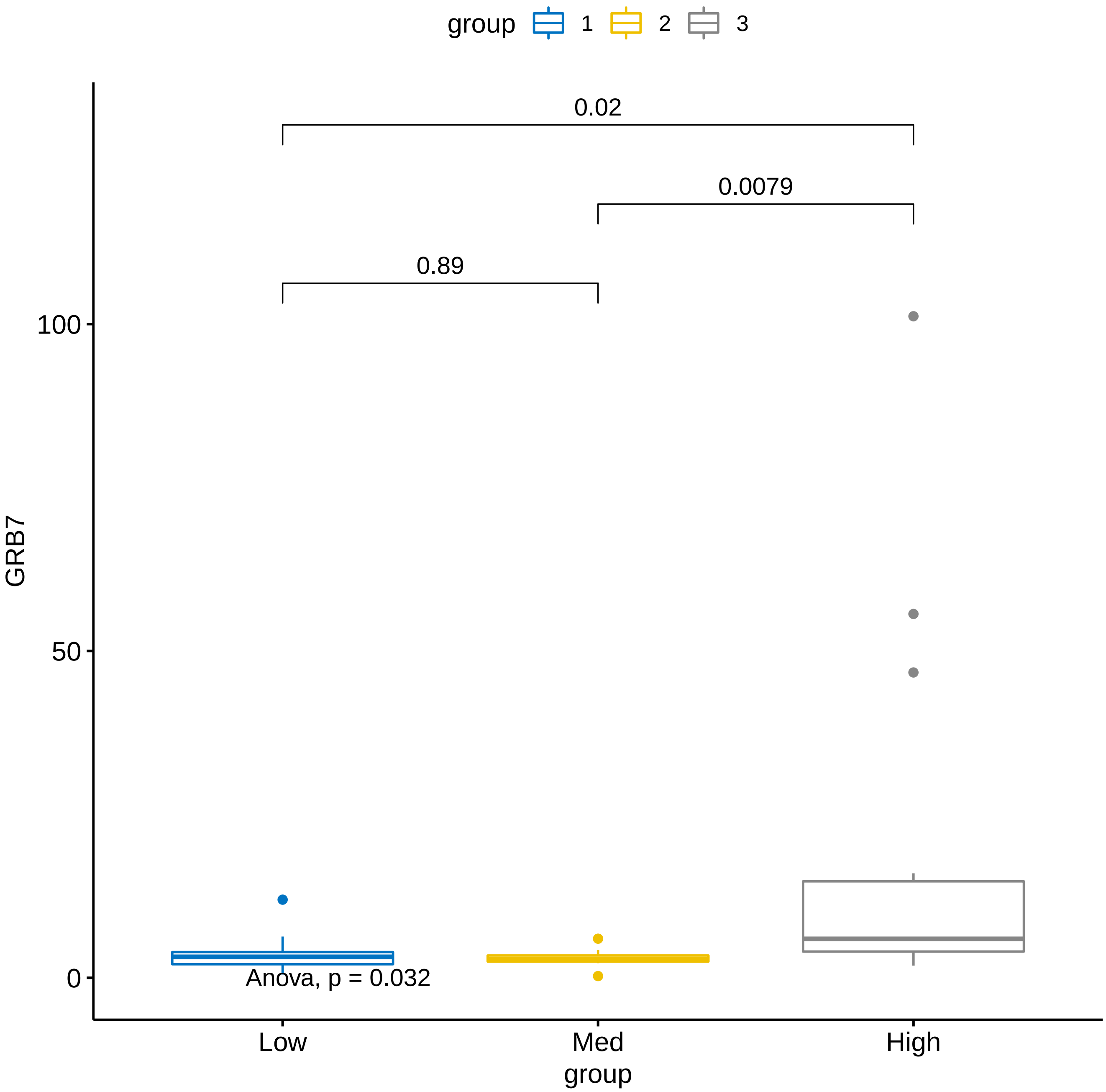
group 1 2 3
0.02
0.0079
0.89
100
50
0
GRB7
Anova, p = 0.032
Low
Med
High
group

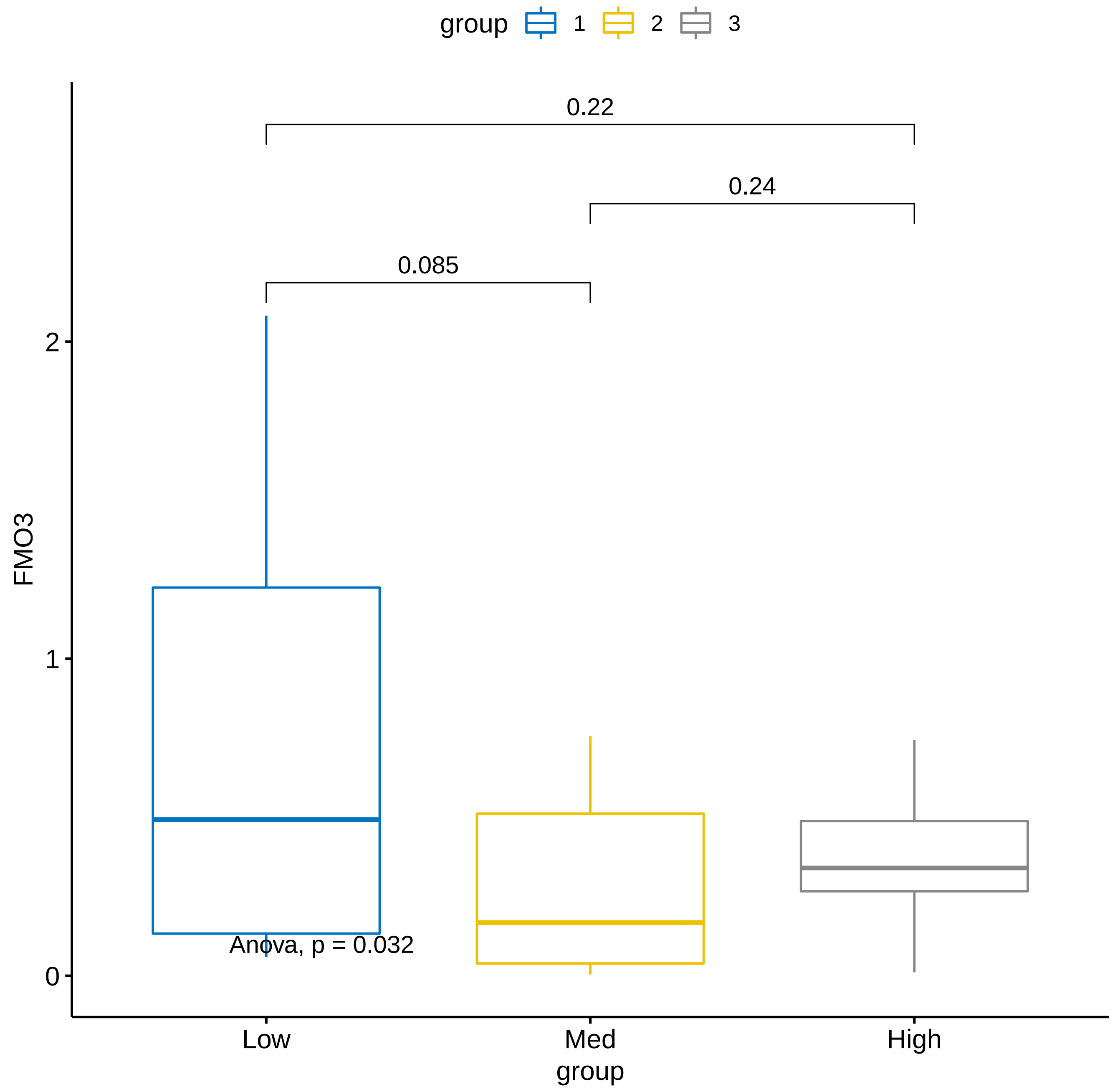

group 1 2 3
0.22
0.24
0.085
2
1
0
FMO3
Anova, p = 0.032
Low
Med
High
group

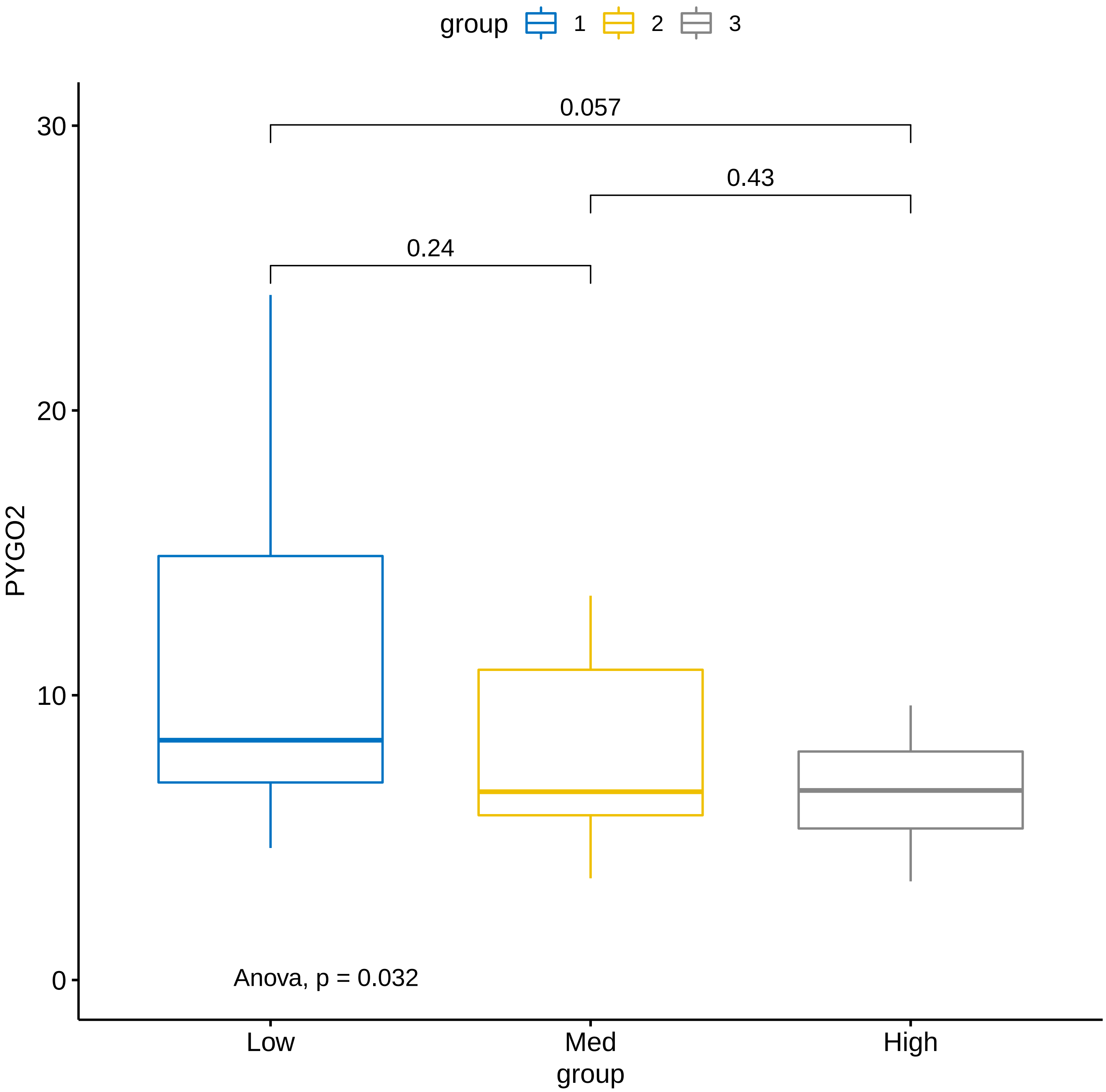
group
1
2
3
0.057
0.43
0.24
30
20
10
0
PYGO2
Anova, p = 0.032
Low
Med
High
group

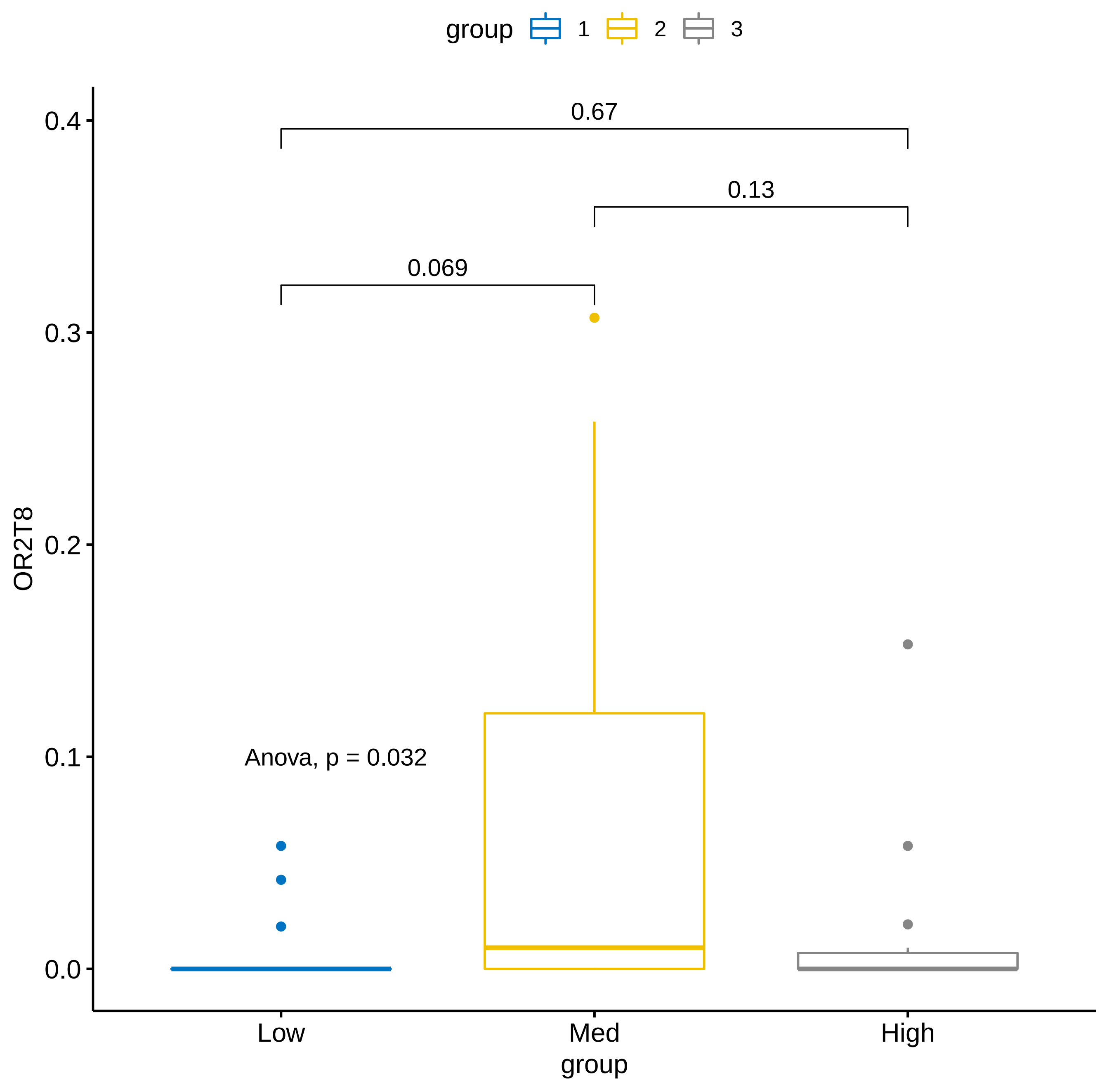
group
1
2
3
0.67
0.13
0.069
Anova, p = 0.032
OR2T8
0.4
0.3
0.2
0.1
0.0
Low
Med
High
group

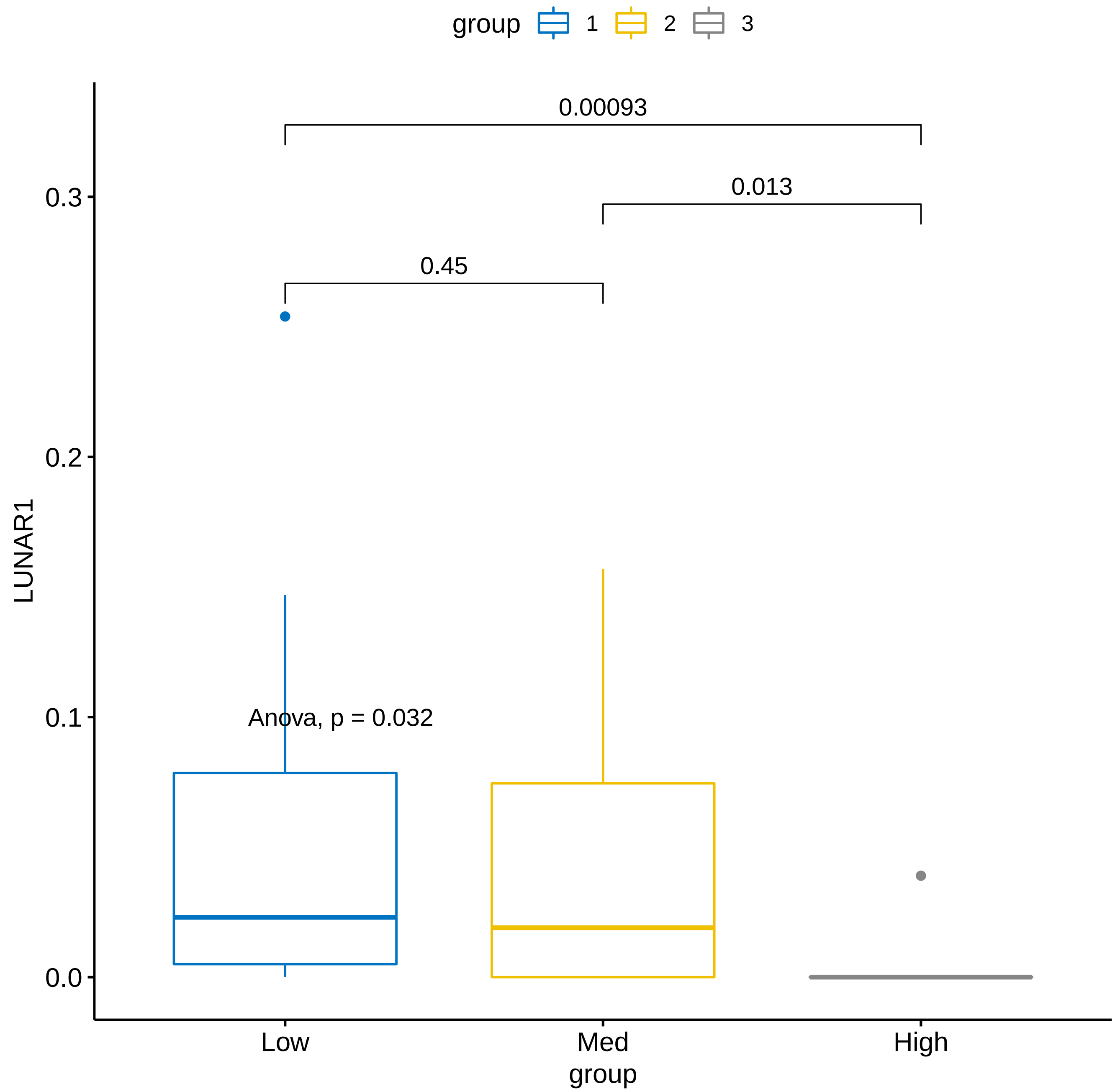
group
1
2
3
0.00093
0.013
0.45
Anova, p = 0.032
0.3
0.2
0.1
0.0
LUNAR1
Low
Med
High
group

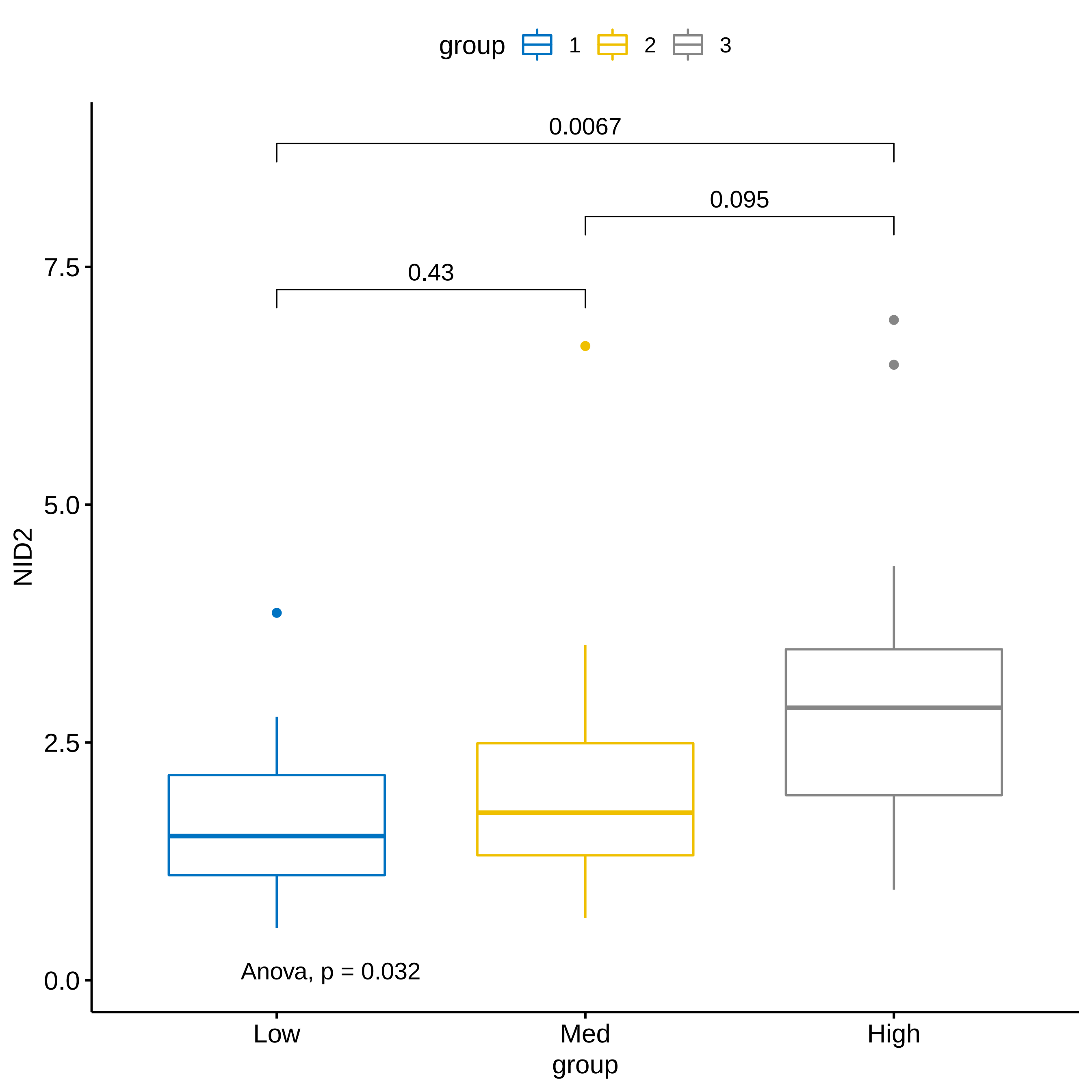
group
1
2
3
0.0067
0.095
0.43
7.5
5.0
2.5
0.0
NID2
Anova, p = 0.032
Low
Med
High
group

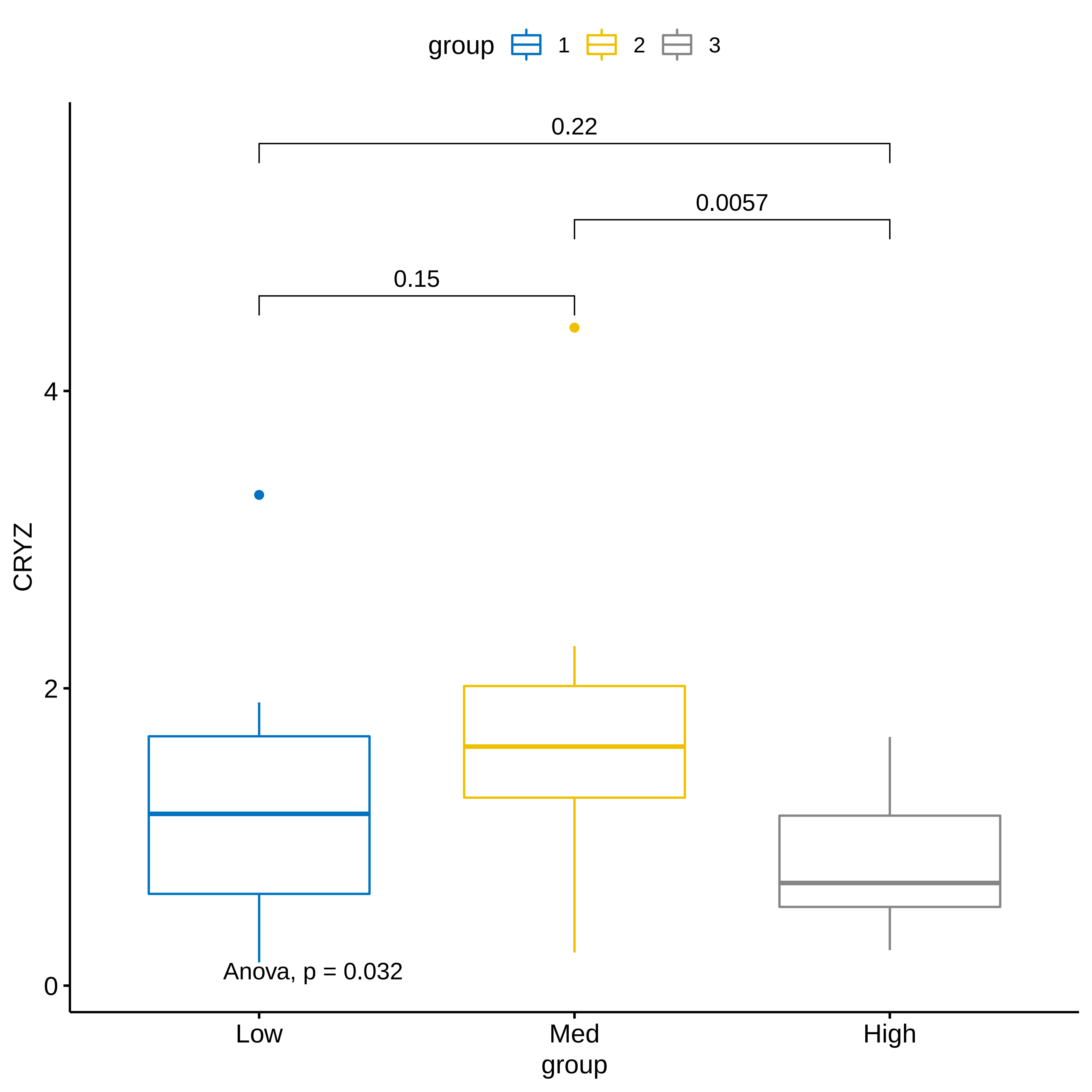

group 1 2 3
0.22
0.0057
0.15
CRYZ
4
2
0
Anova, p = 0.032
Low
Med
High
group

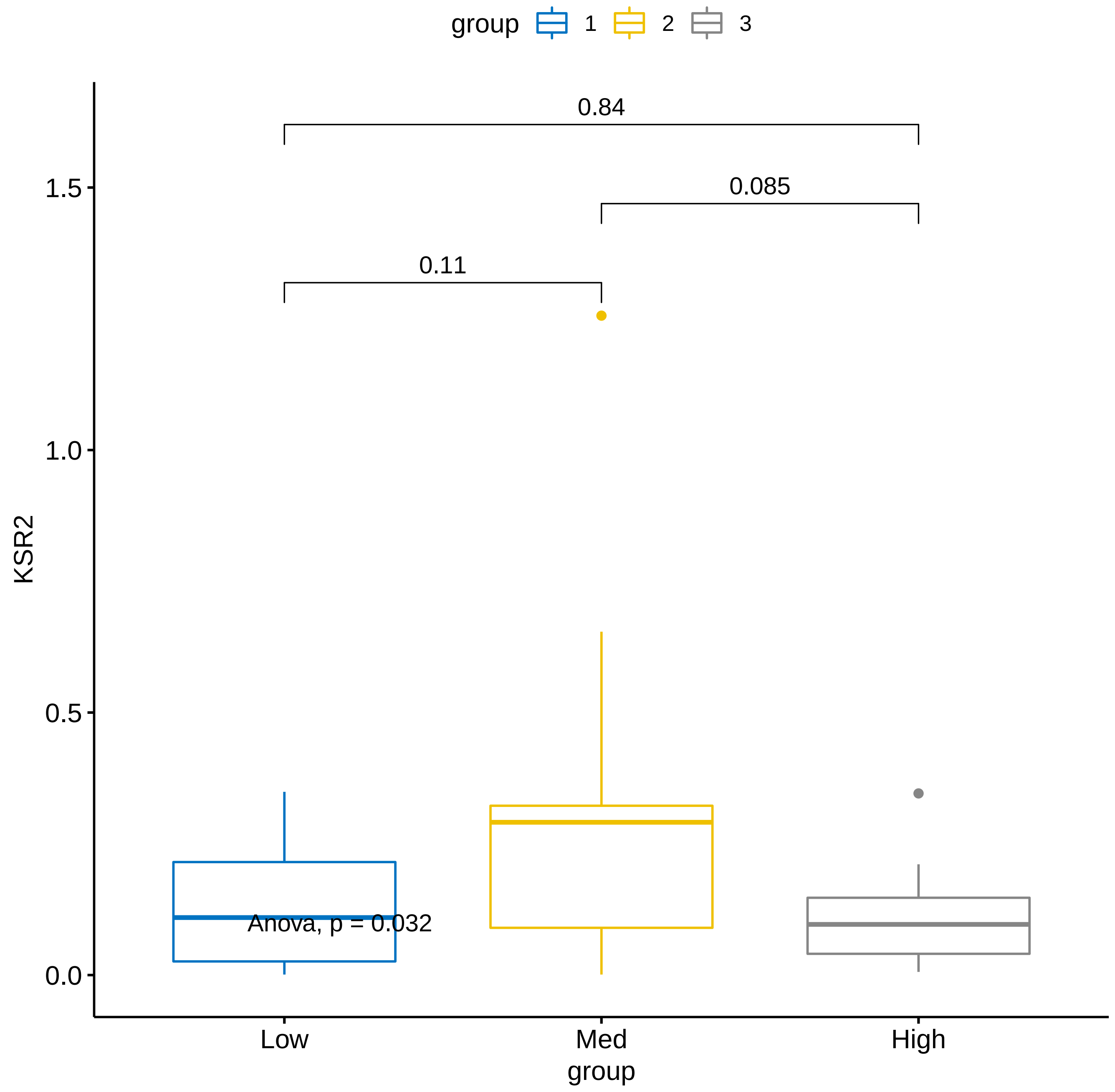

group 1 2 3
0.84
0.085
0.11
1.5
1.0
0.5
0.0
KSR2
Anova, p = 0.032
Low
Med
High
group

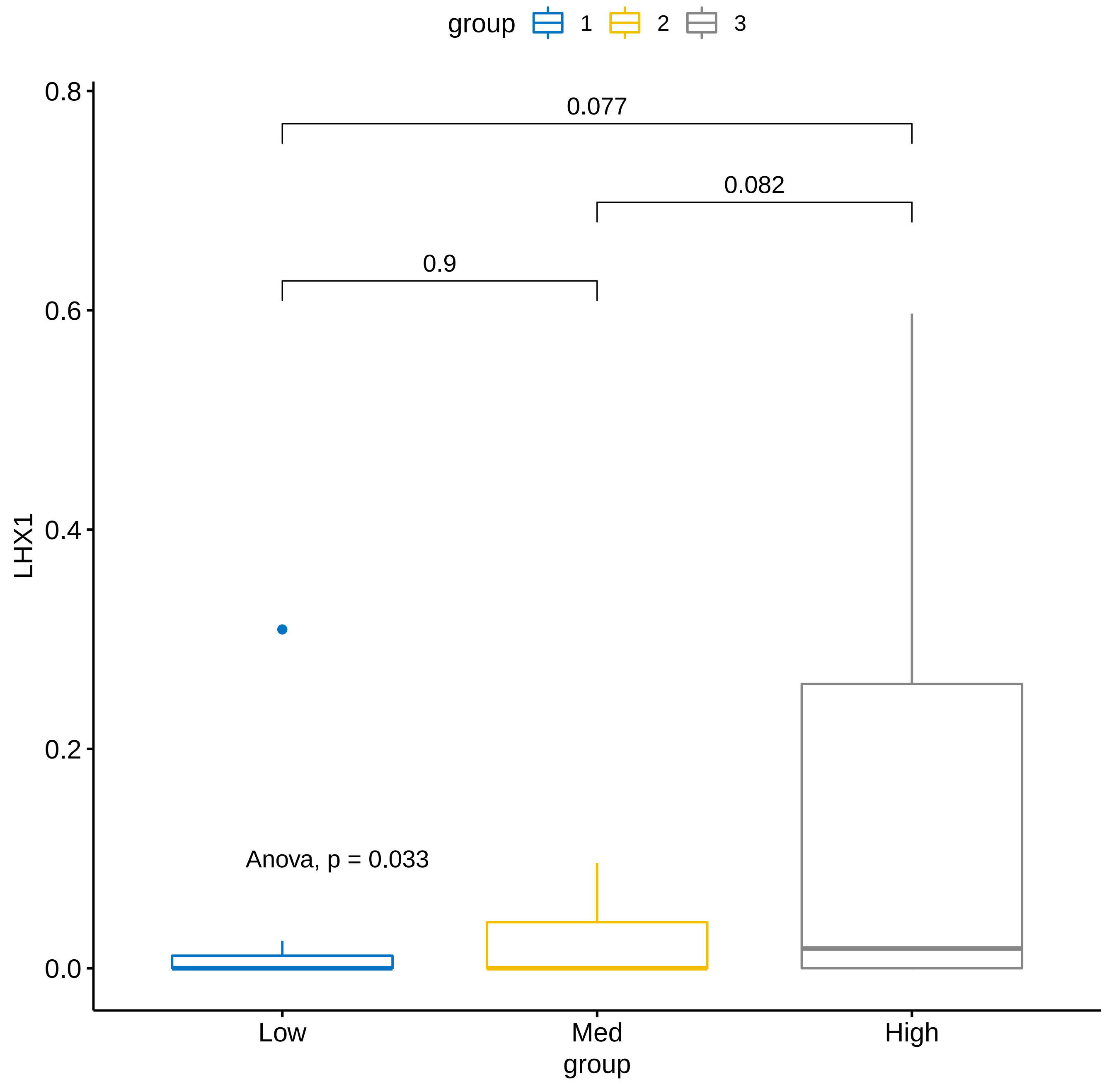

group
1
2
3
0.077
0.082
0.9
Anova, p = 0.033
LHX1
0.8
0.6
0.4
0.2
0.0
Low
Med
High
group

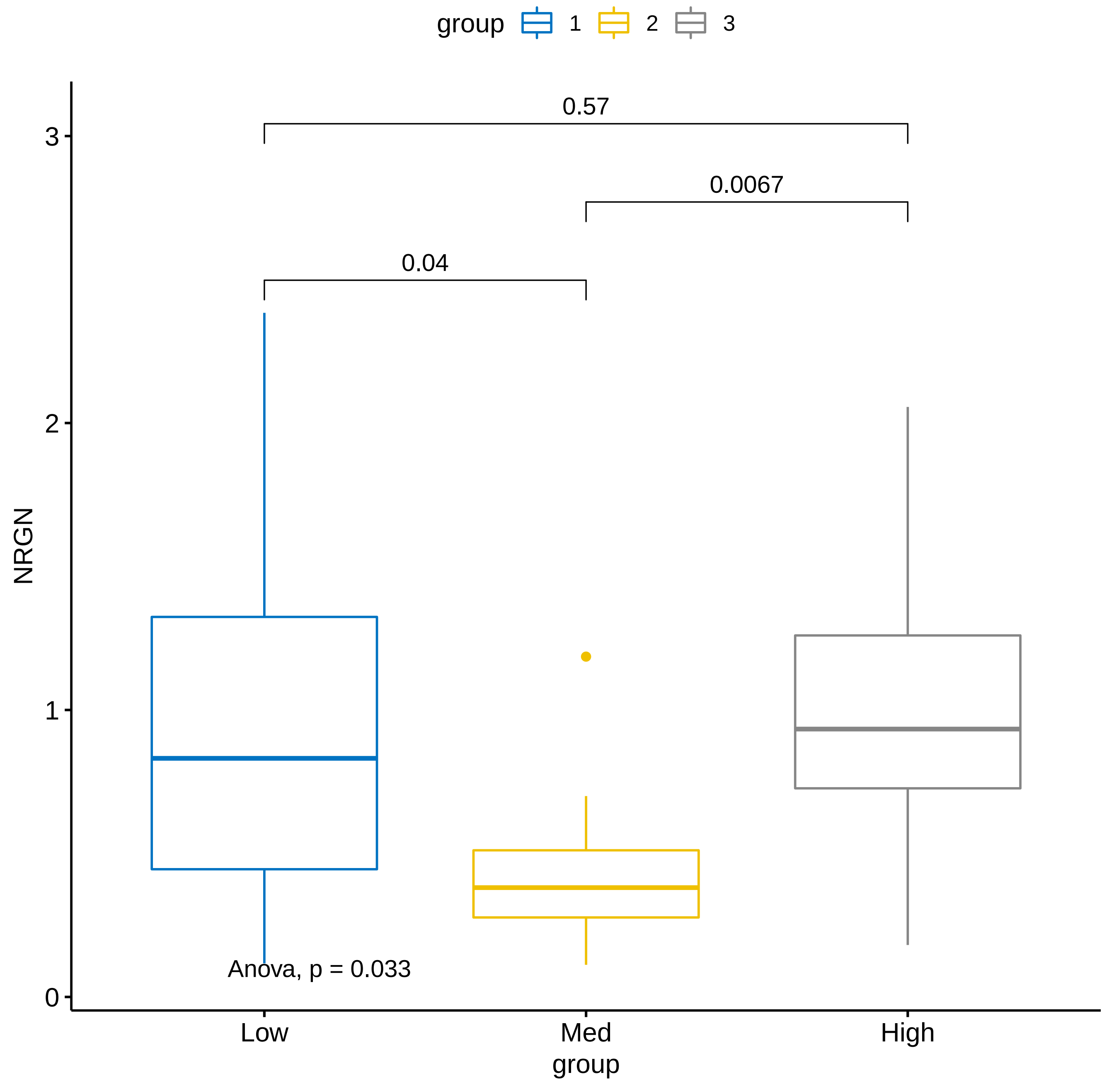

group 1 2 3
0.57
0.0067
0.04
NRGN
3
2
1
0
Anova, p = 0.033
Low
Med
High
group

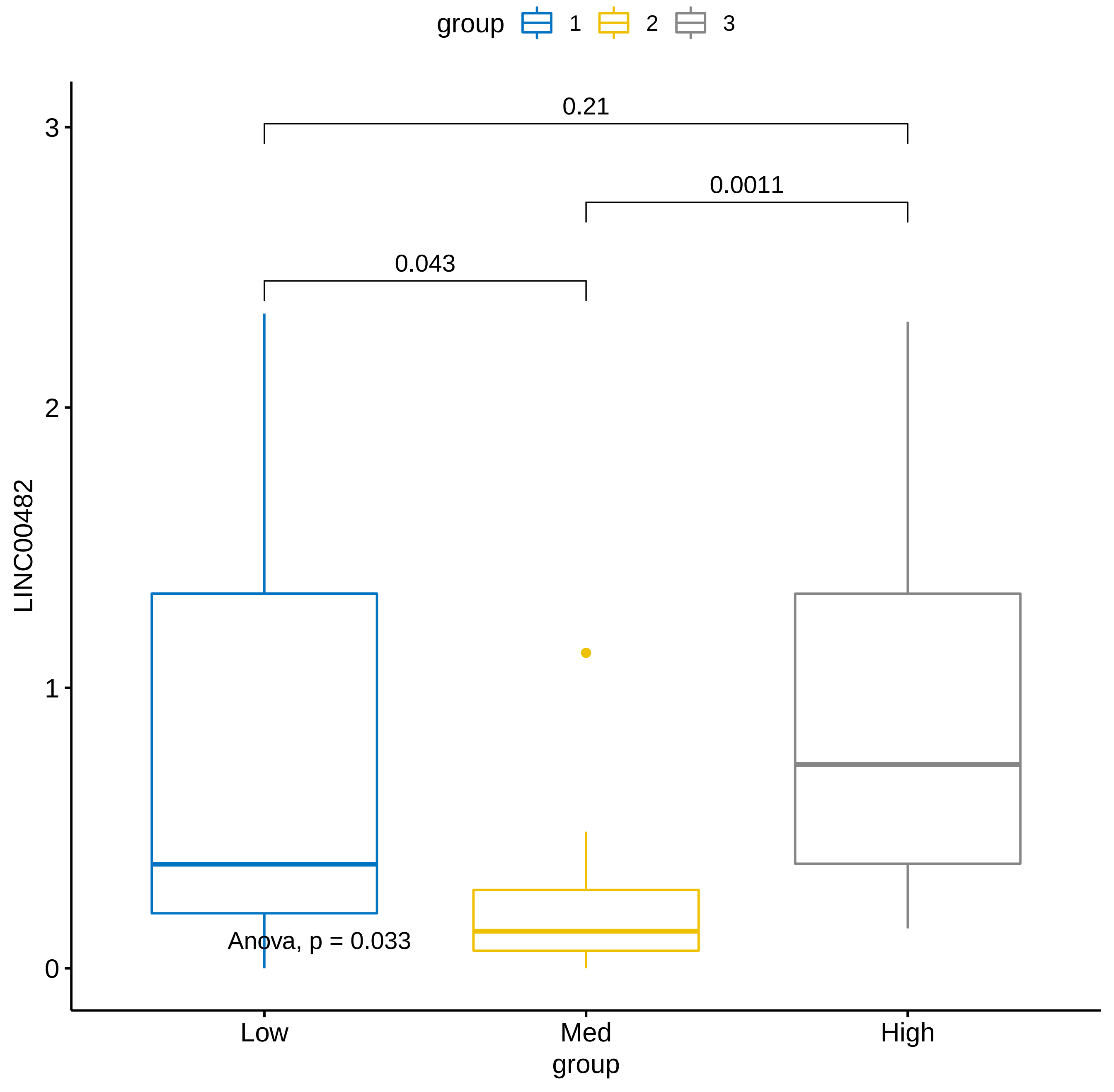
group 1 2 3
0.21
0.0011
0.043
Anova, p = 0.033
LINC00482
3
2
1
0
Low
Med
High
group

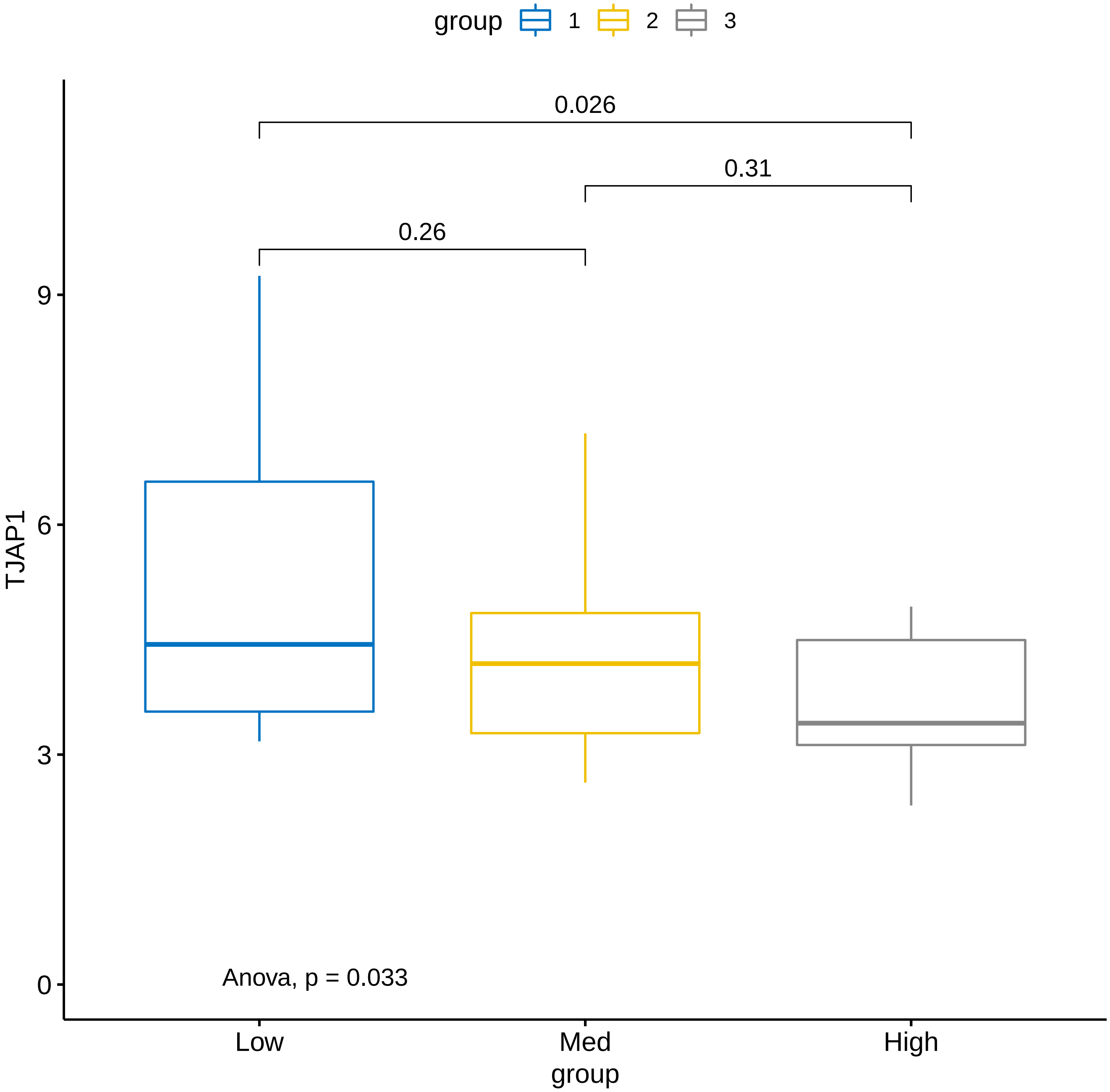
group
1
2
3
0.026
0.31
0.26
9
6
3
0
TJAP1
Anova, p = 0.033
Low
Med
High
group

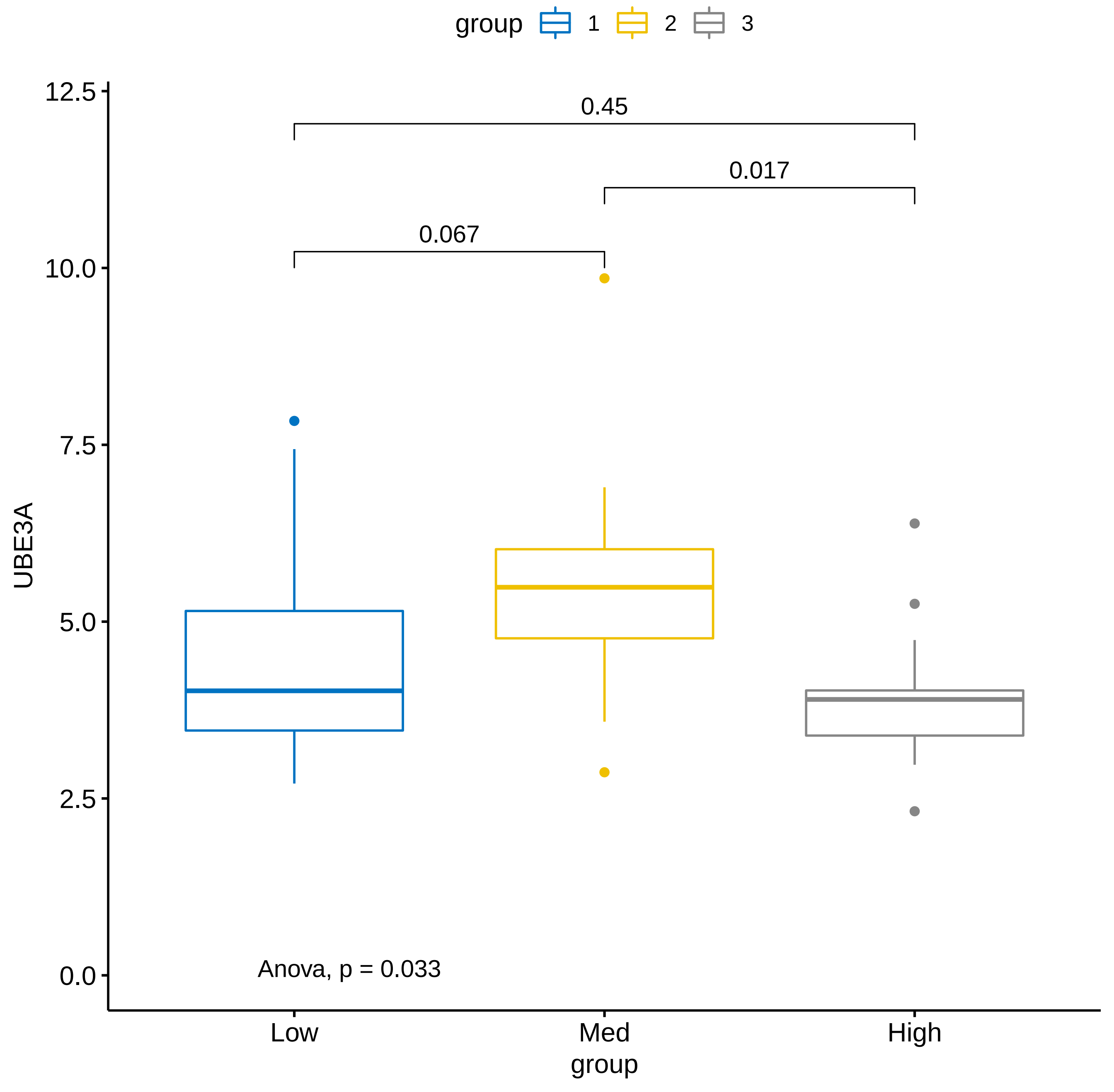
group
1
2
3
12.5
10.0
7.5
5.0
2.5
0.0
UBE3A
0.45
0.017
0.067
Anova, p = 0.033
Low
Med
High
group

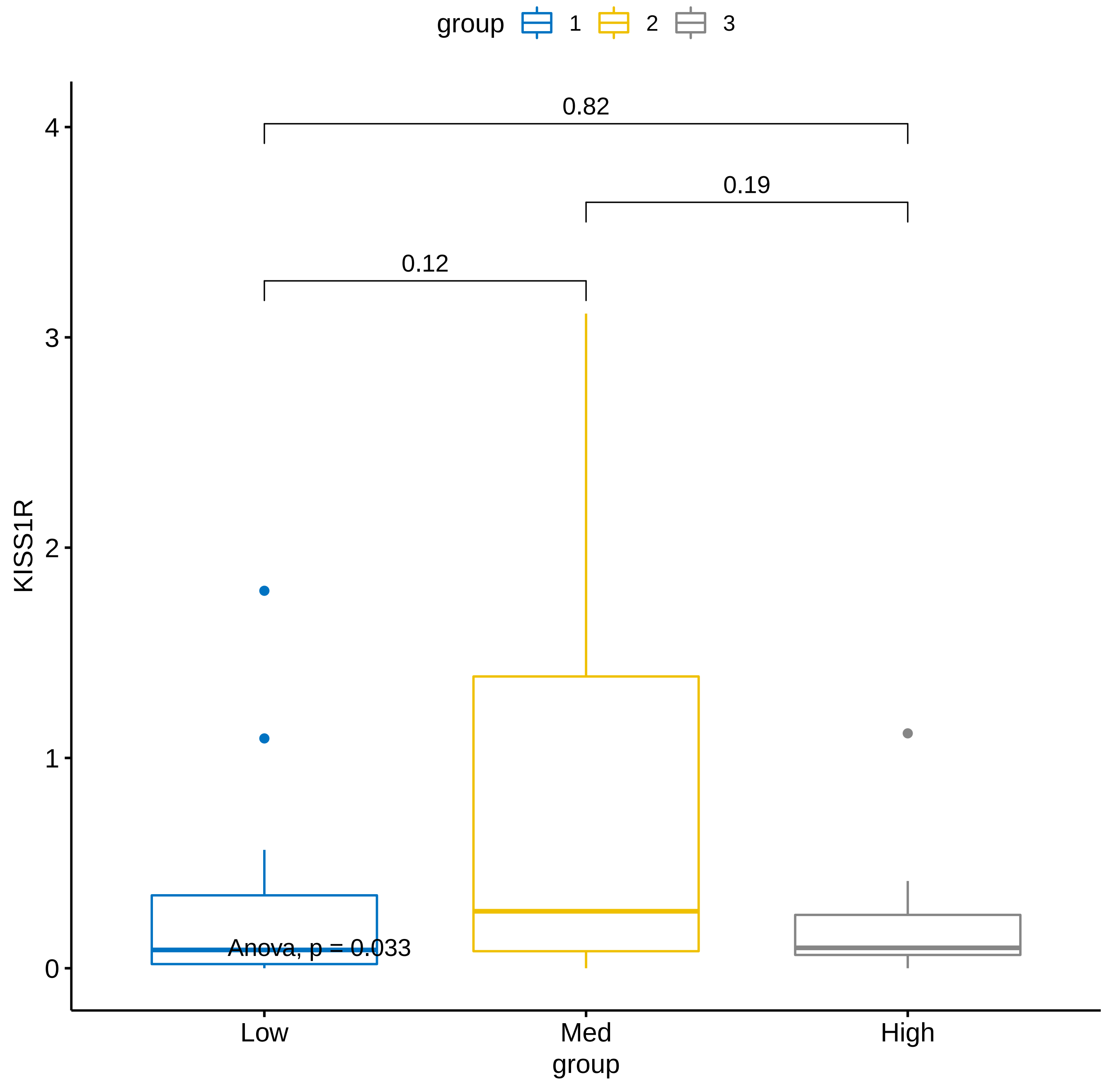

group 1 2 3
0.82
0.19
0.12
KISS1R
4
3
2
1
0
Anova, p = 0.033
Low
Med
High
group

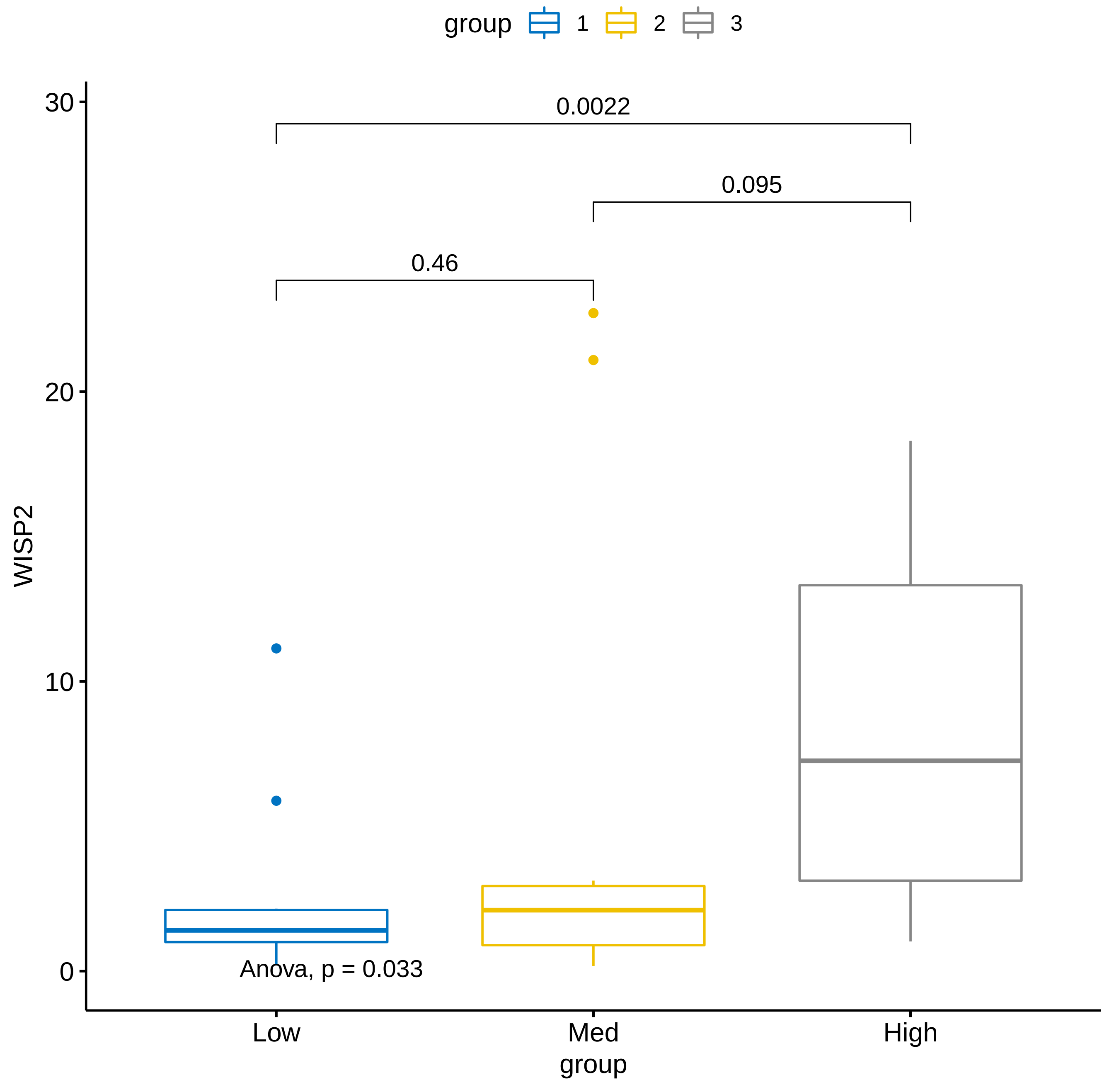
group
1
2
3
0.0022
0.095
0.46
30
20
10
0
WISP2
Anova, p = 0.033
Low
Med
High
group

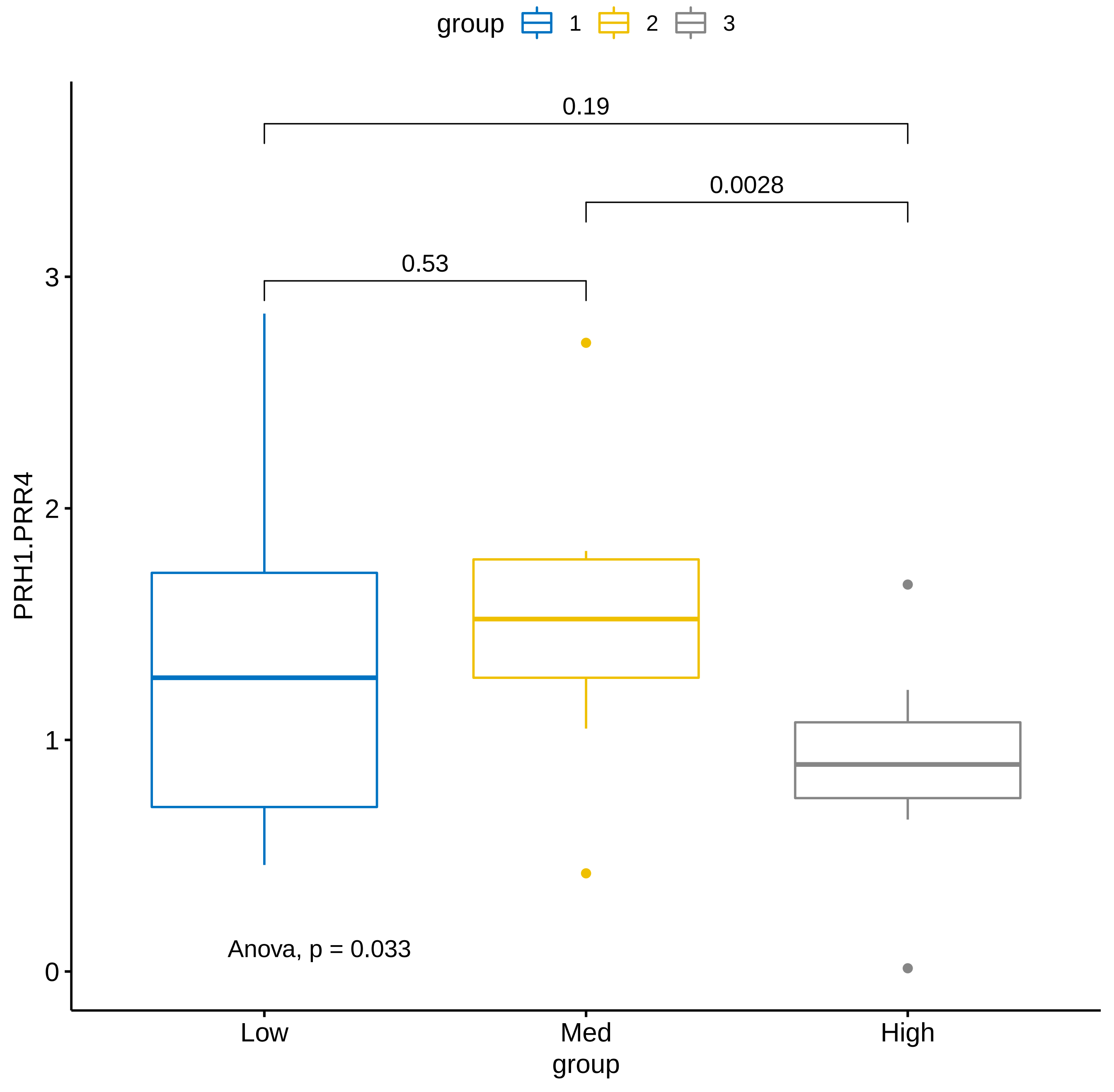

group 1 2 3
0.19
0.0028
0.53
PRH1.PRR4
3
2
1
0
Anova, p = 0.033
Low
Med
High
group

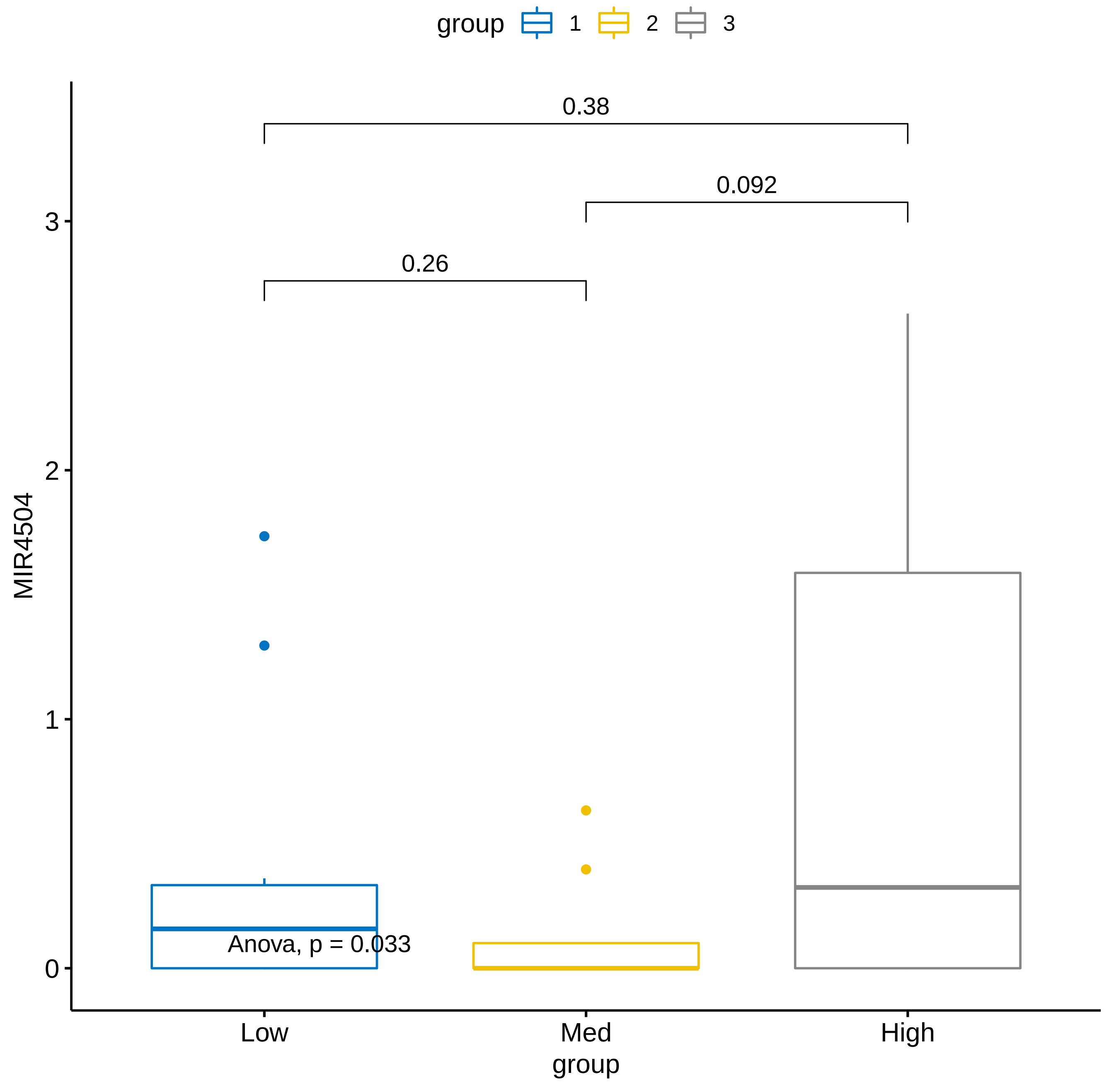
group
1
2
3
0.38
0.092
0.26
3
2
1
0
MIR4504
Anova, p = 0.033
Low
Med
High
group

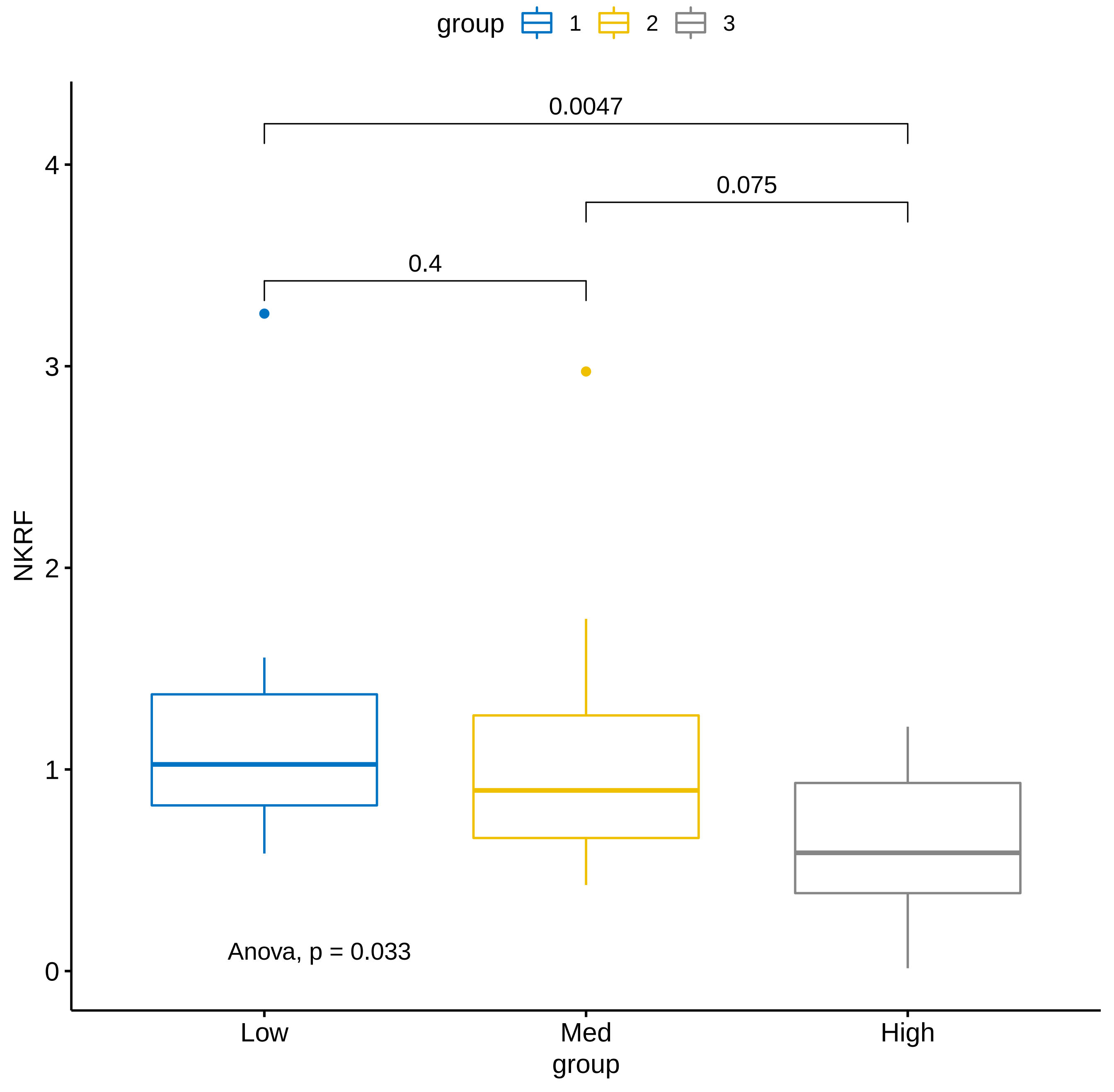
group 1 2 3
0.0047
0.075
0.4
4
3
2
1
0
NKRF
Anova, p = 0.033
Low
Med
High
group

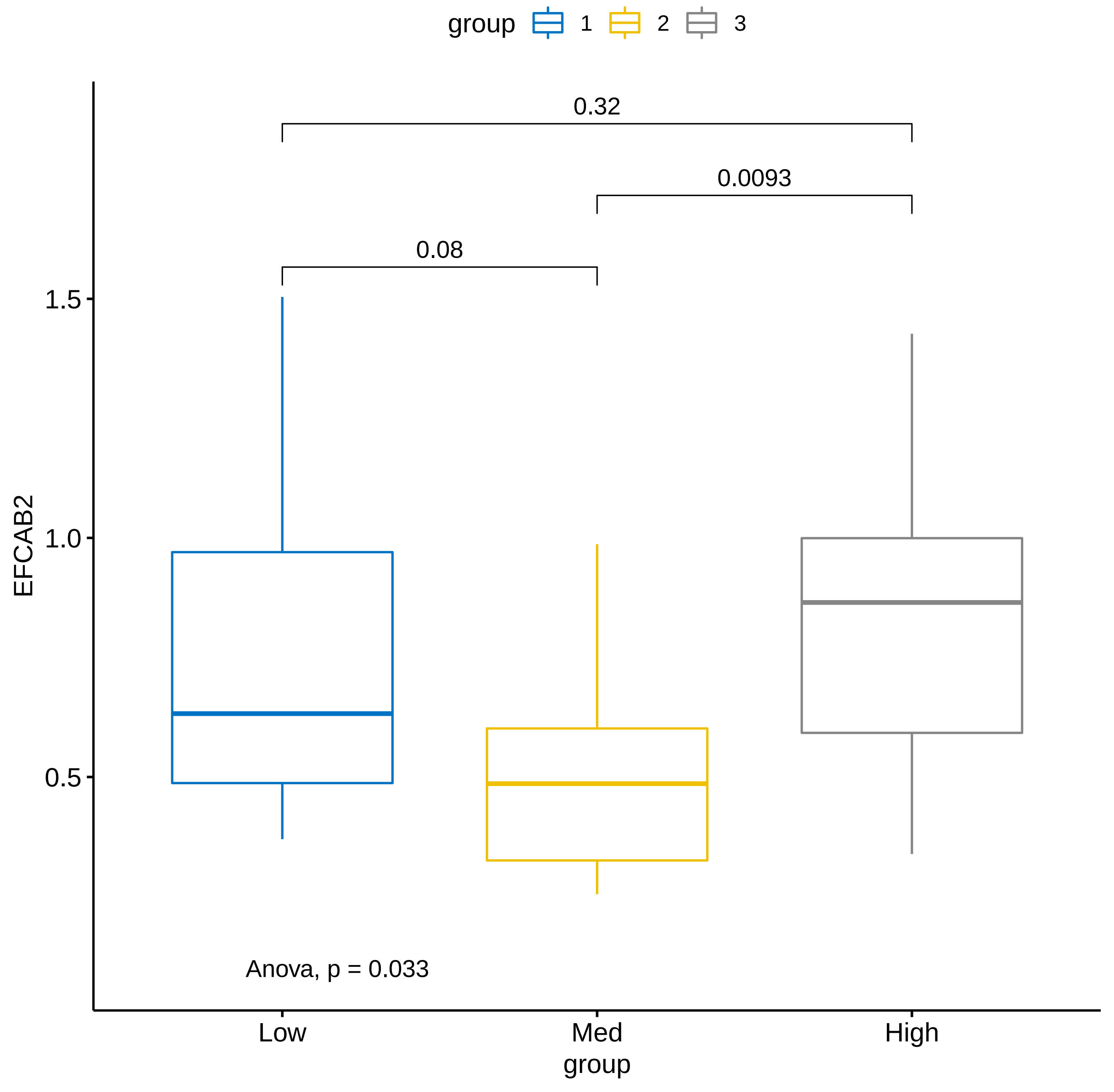
group 1 2 3
0.32
0.0093
0.08
1.5
1.0
0.5
EFCAB2
Anova, p = 0.033
Low
Med
High
group

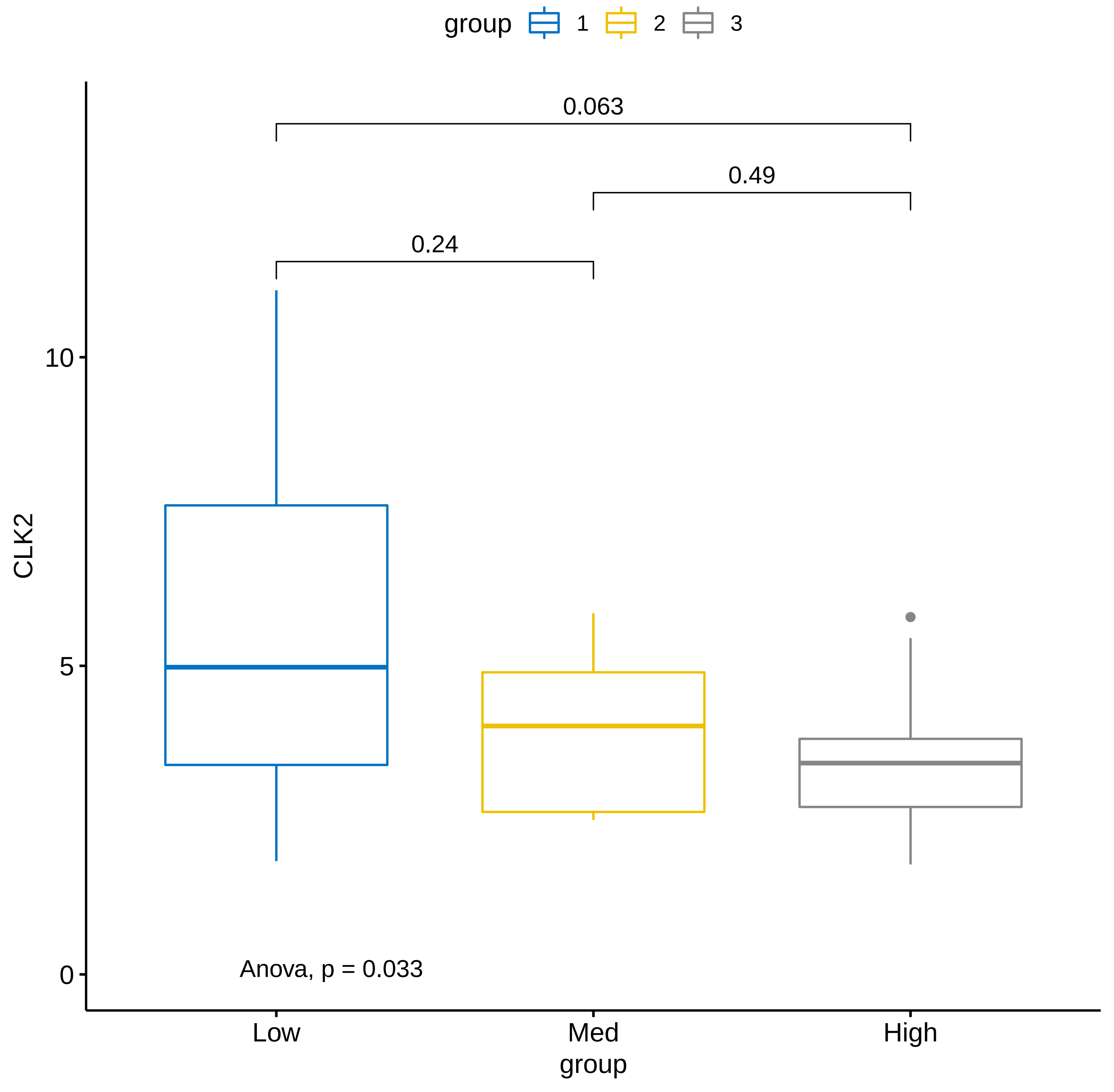

group
1
2
3
0.063
0.49
0.24
10
5
0
CLK2
Anova, p = 0.033
Low
Med
High
group

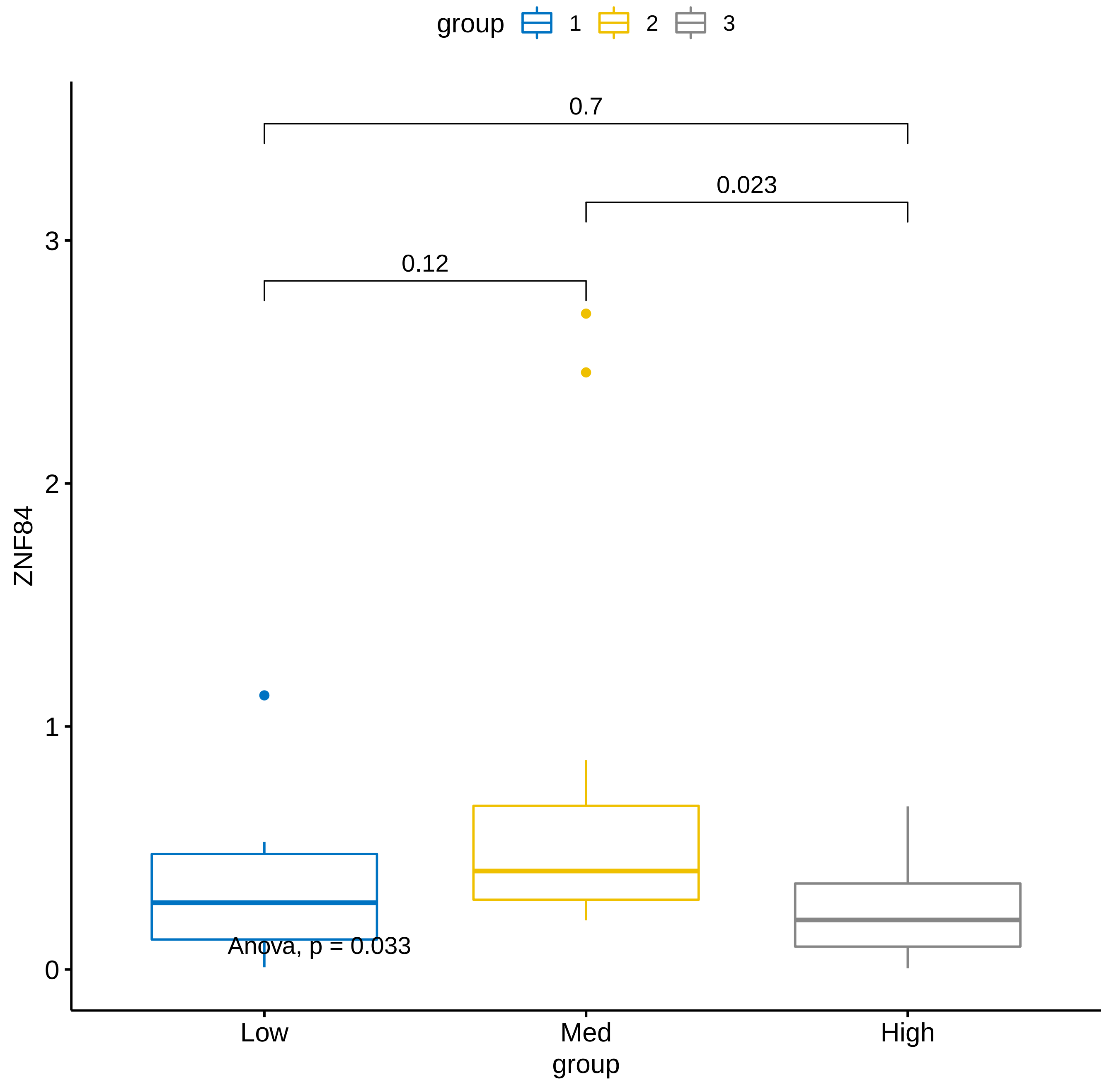
group 1 2 3
0.7
0.023
0.12
ZNF84
3
2
1
0
Anova, p = 0.033
Low
Med
High
group

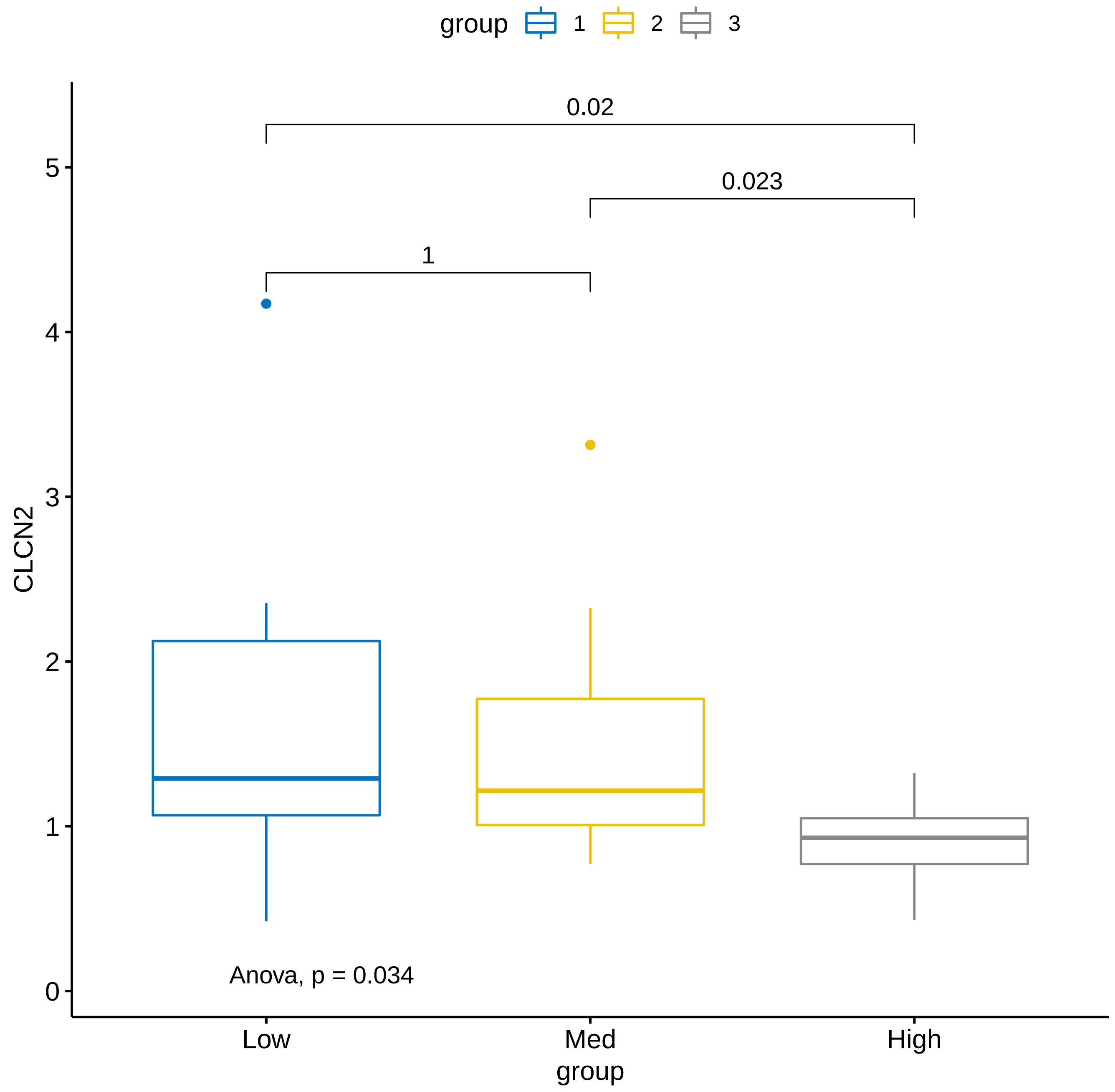

group
1
2
3
0.02
0.023
1
5
4
3
2
1
0
CLCN2
Anova, p = 0.034
Low
Med
High
group

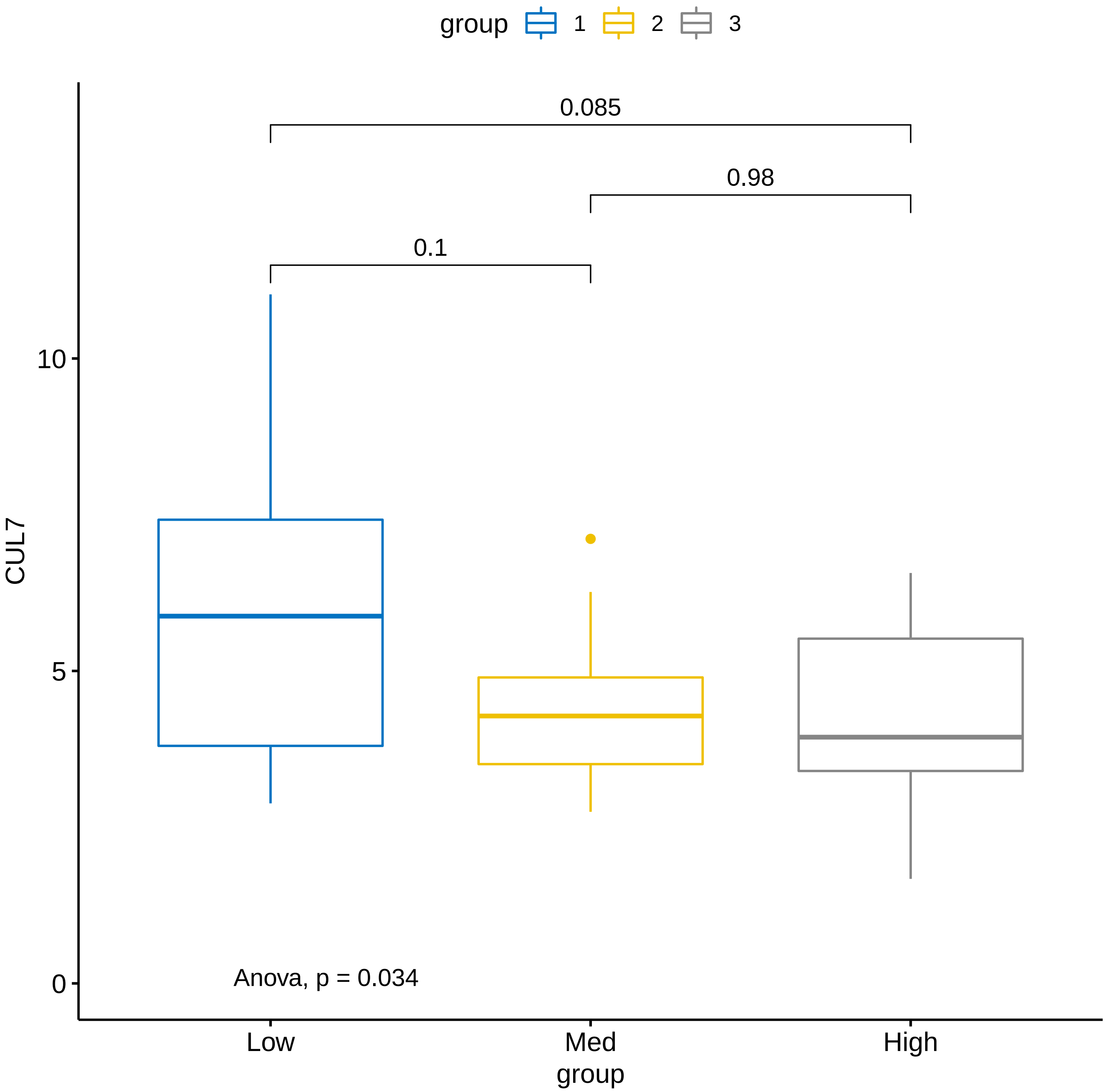
group
1
2
3
0.085
0.98
0.1
10
5
0
CUL7
Anova, p = 0.034
Low
Med
High
group

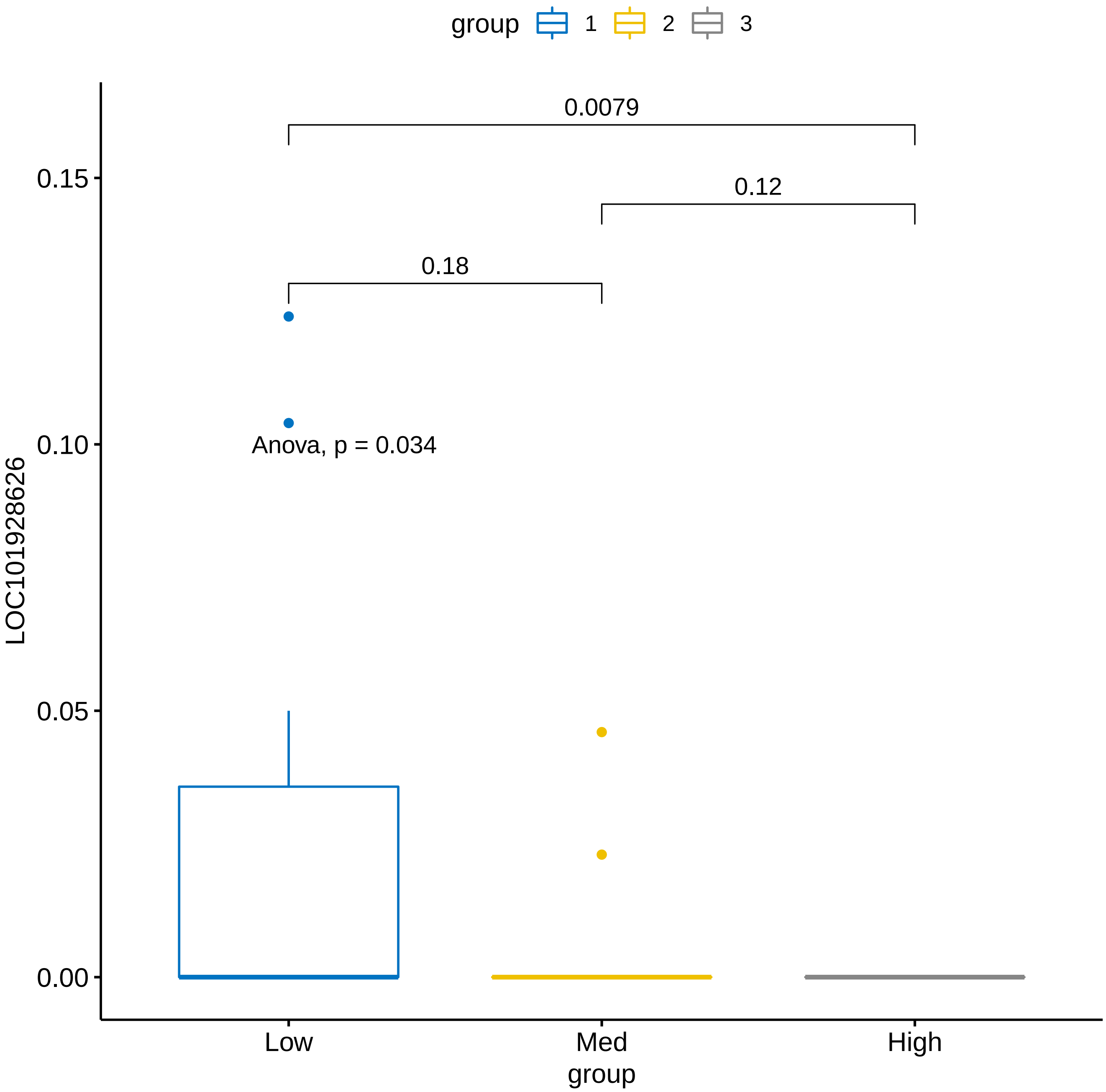

group
1
2
3
0.0079
0.12
0.18
Anova, p = 0.034
0.15
0.10
0.05
0.00
LOC101928626
Low
Med
High
group

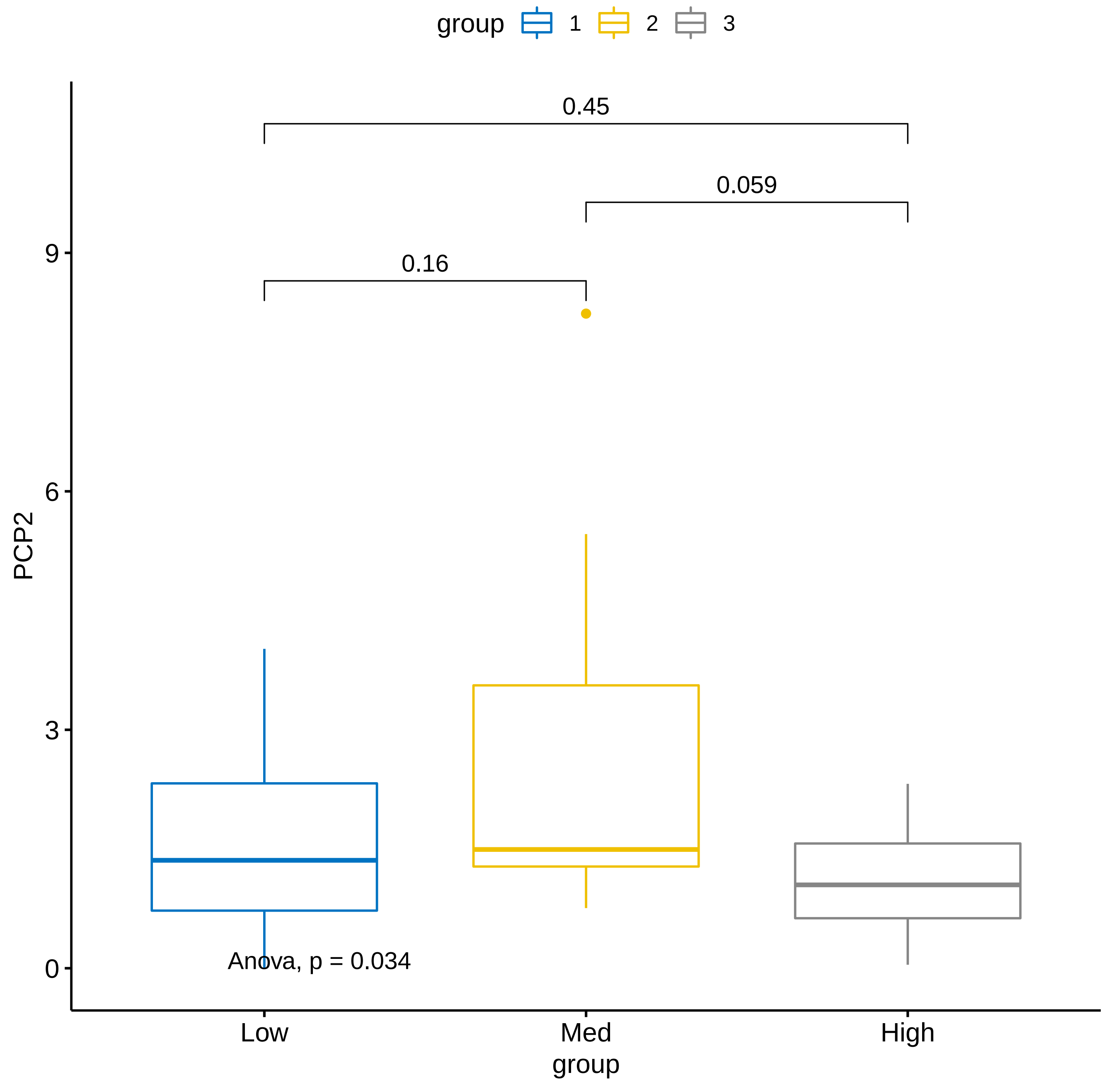

group 1 2 3
0.45
0.059
0.16
9
6
3
0
PCP2
Anova, p = 0.034
Low
Med
High
group

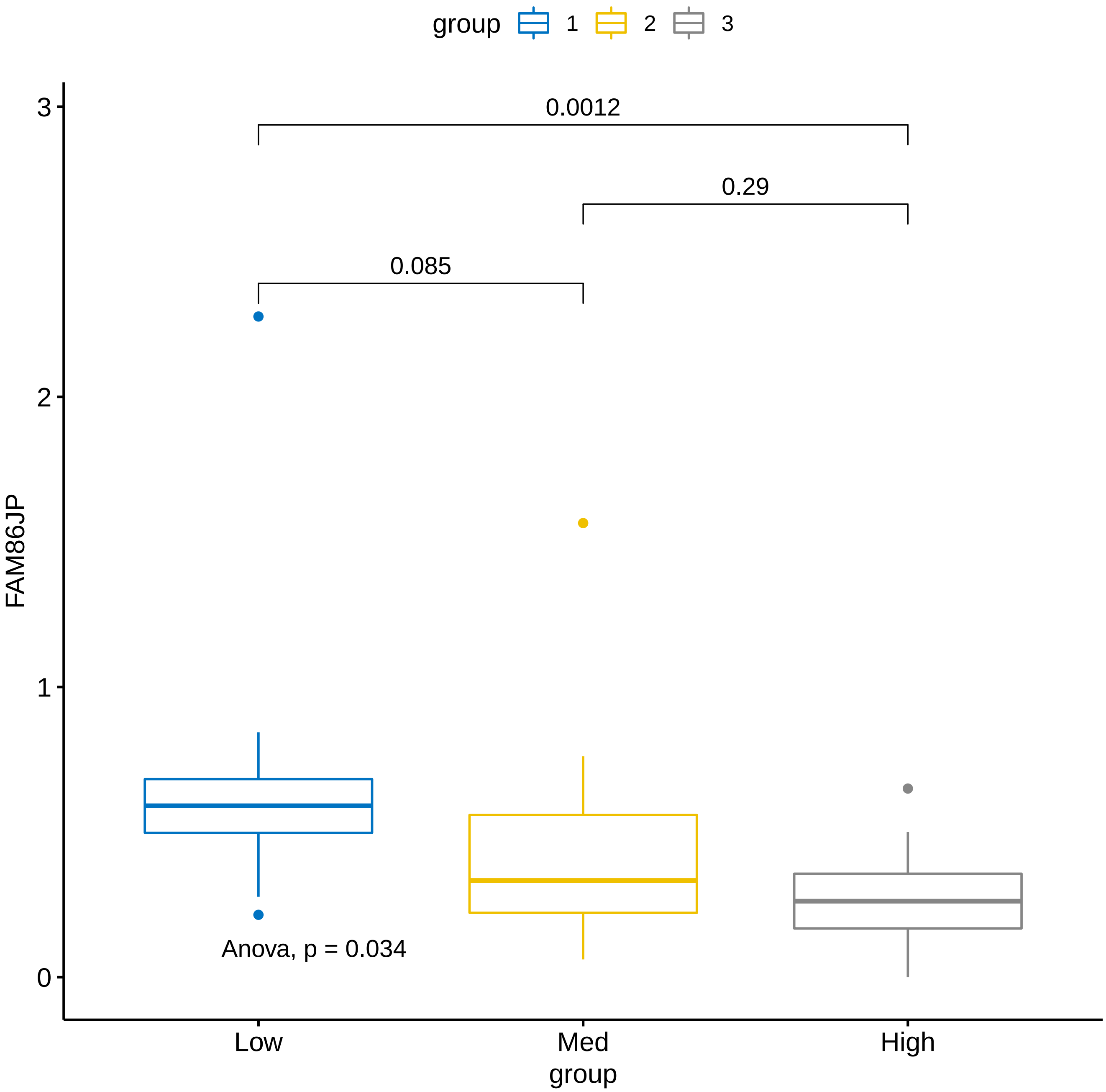

group 1 2 3
0.0012
0.29
0.085
FAM86JP
3
2
1
0
Anova, p = 0.034
Low
Med
High
group

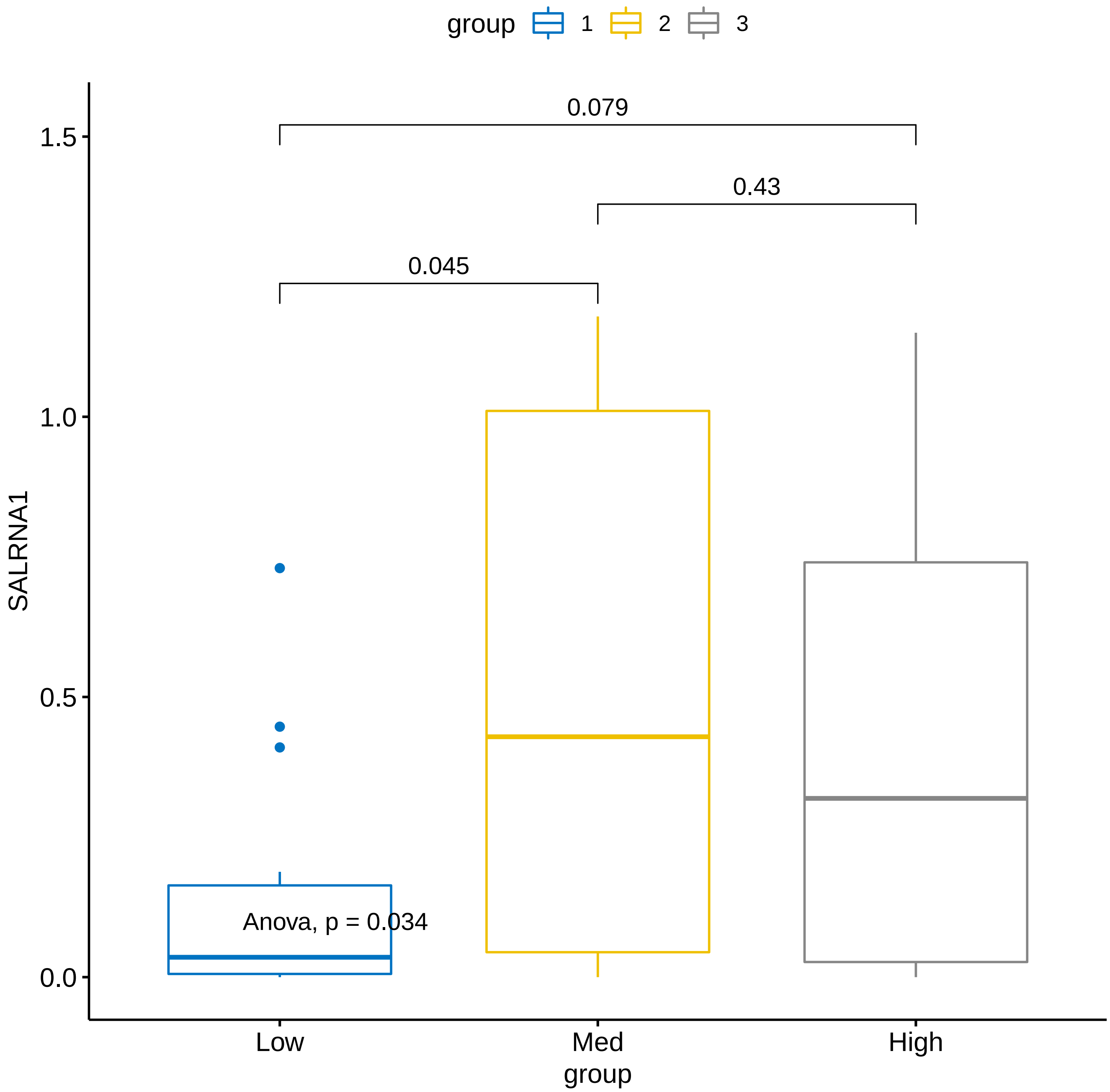
group 1 2 3
0.079
0.43
0.045
1.5
1.0
0.5
0.0
SALRNA1
Anova, p = 0.034
Low
Med
High
group

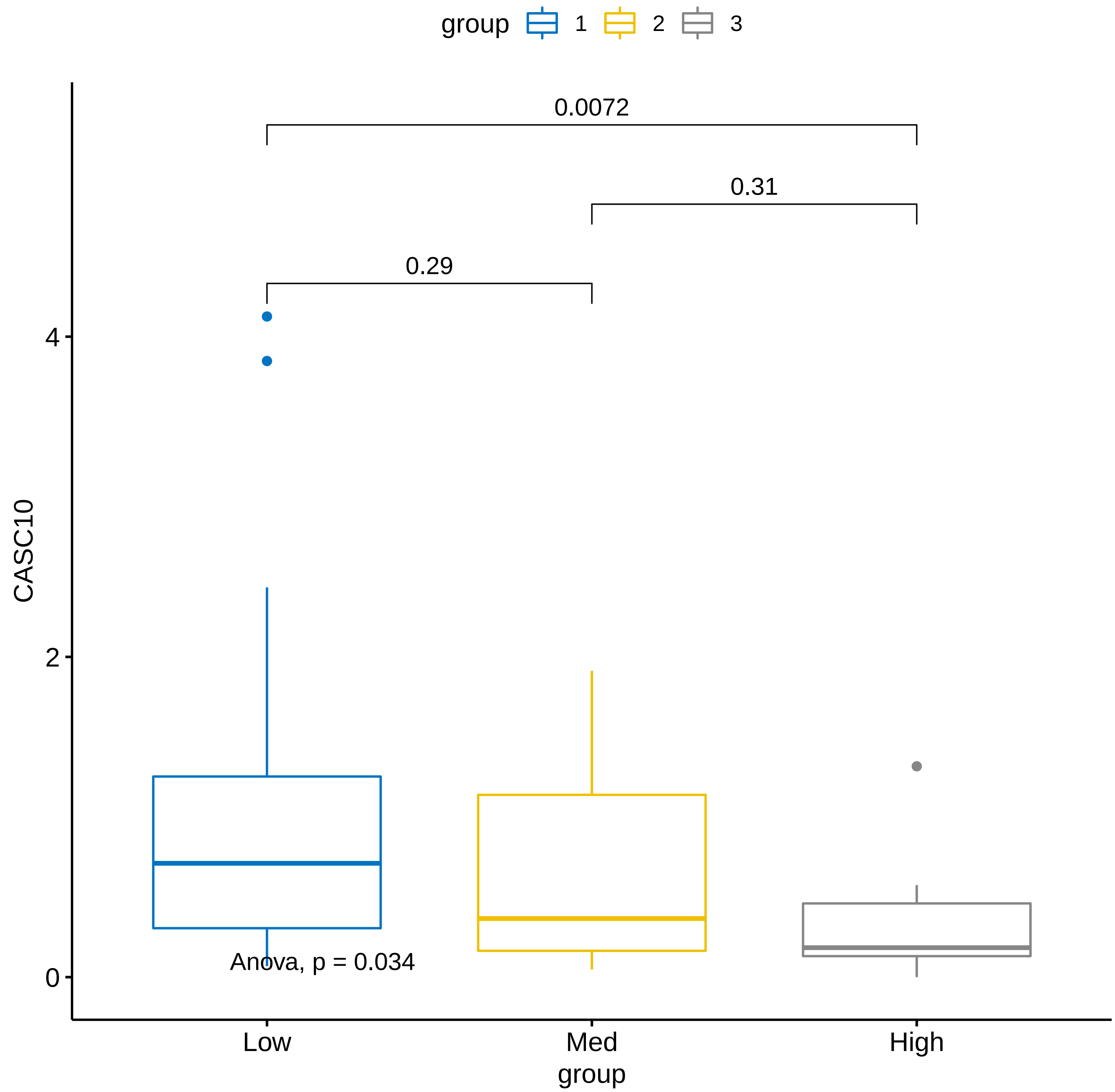
group
1
2
3
0.0072
0.31
0.29
4
2
0
CASC10
Anova, p = 0.034
Low
Med
High
group

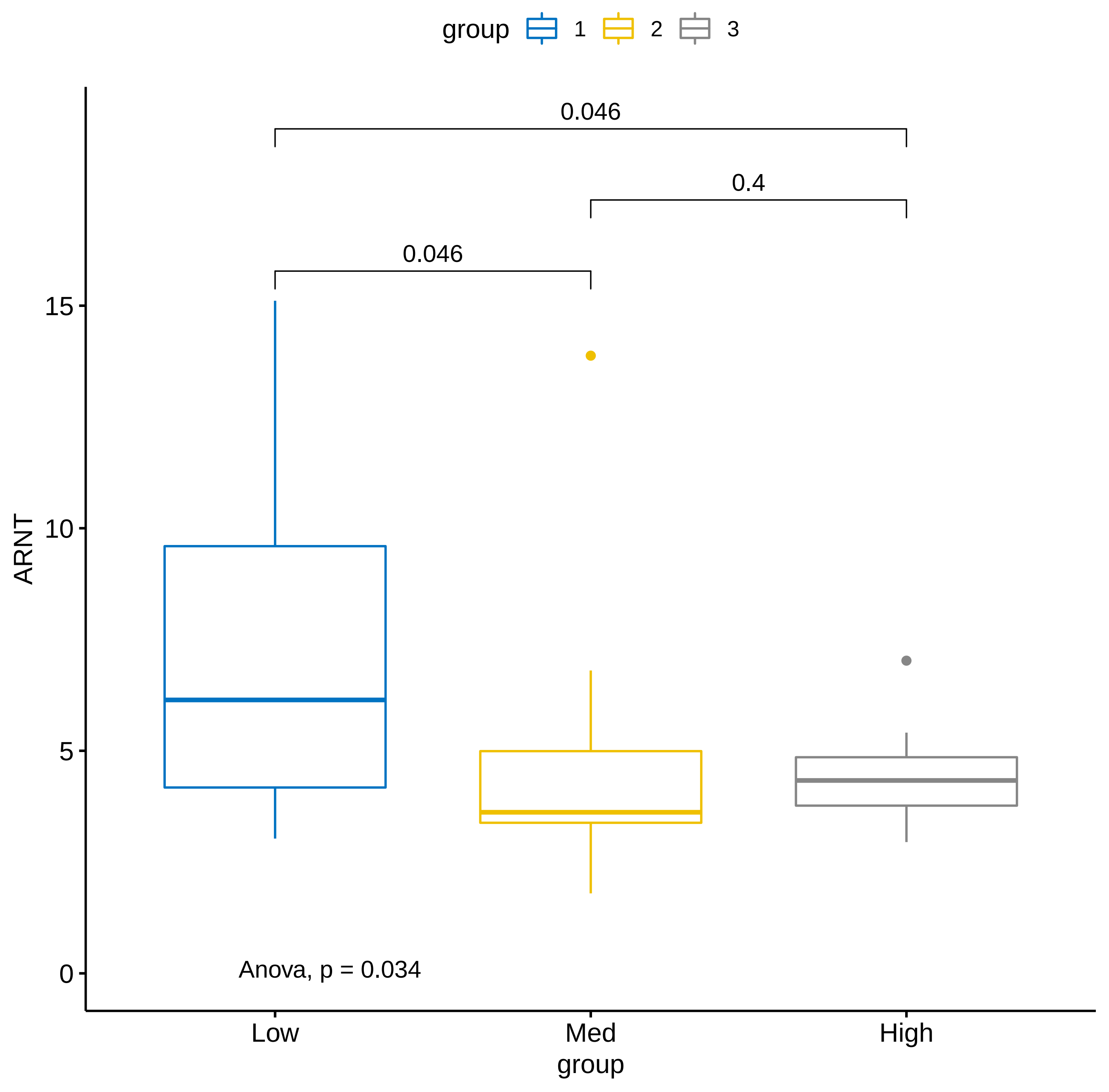
group 1 2 3
0.046
0.4
0.046
15
10
5
0
ARNT
Anova, p = 0.034
Low
Med
High
group

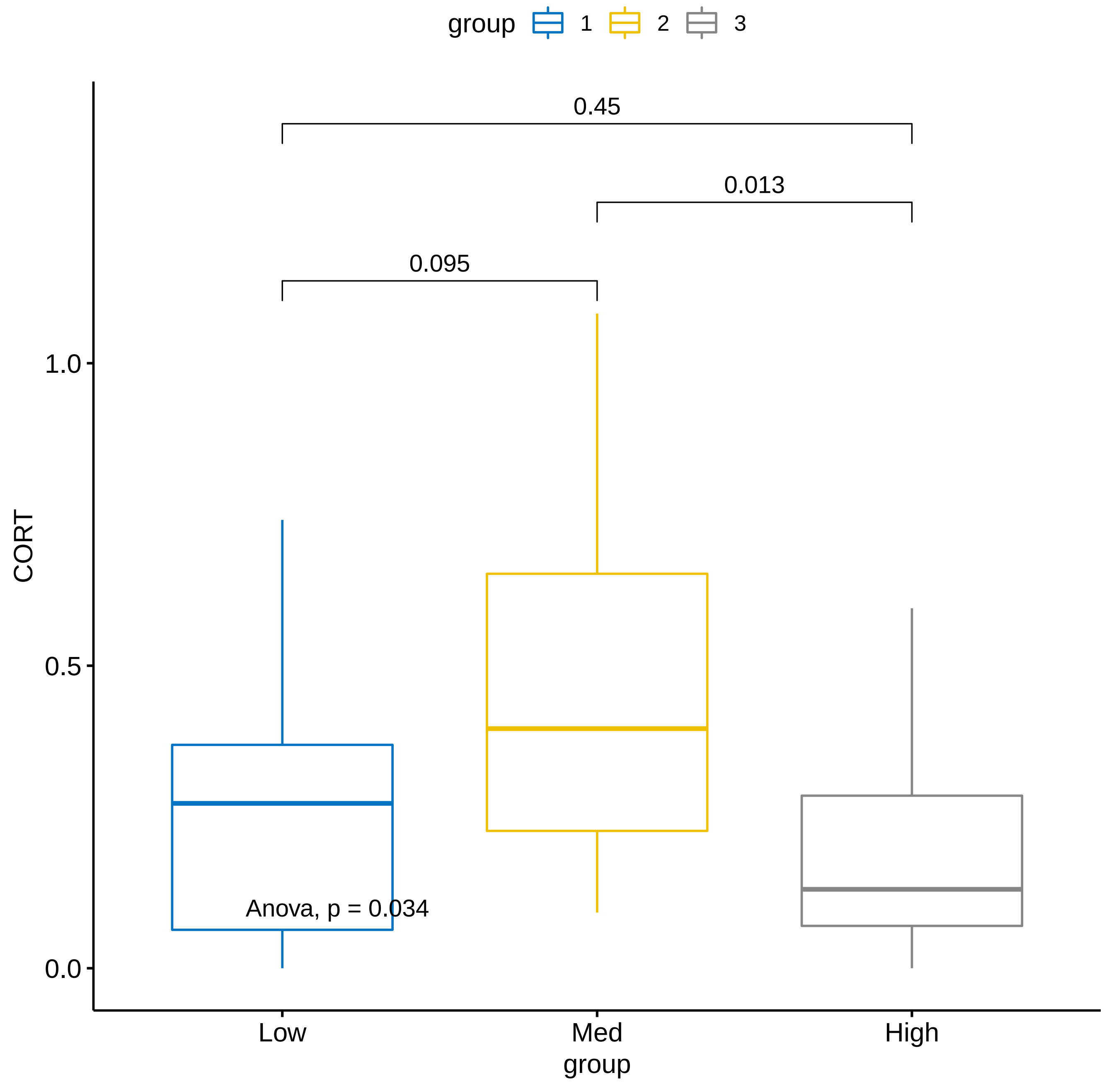

group 1 2 3
0.45
0.013
0.095
1.0
0.5
0.0
CORT
Anova, p = 0.034
Low
Med
High
group

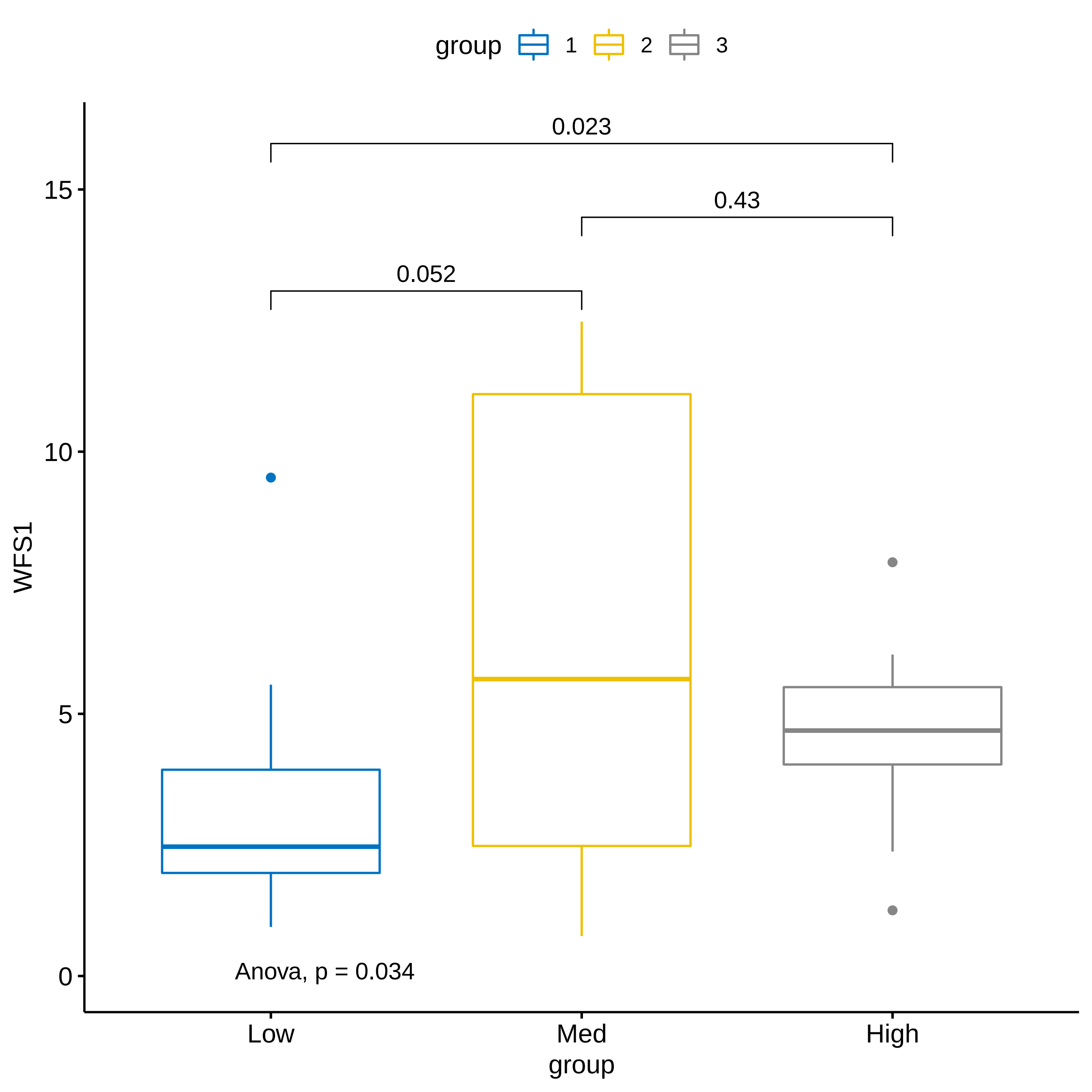
group
1
2
3
0.023
0.43
0.052
15
10
5
0
WFS1
Anova, p = 0.034
Low
Med
High
group

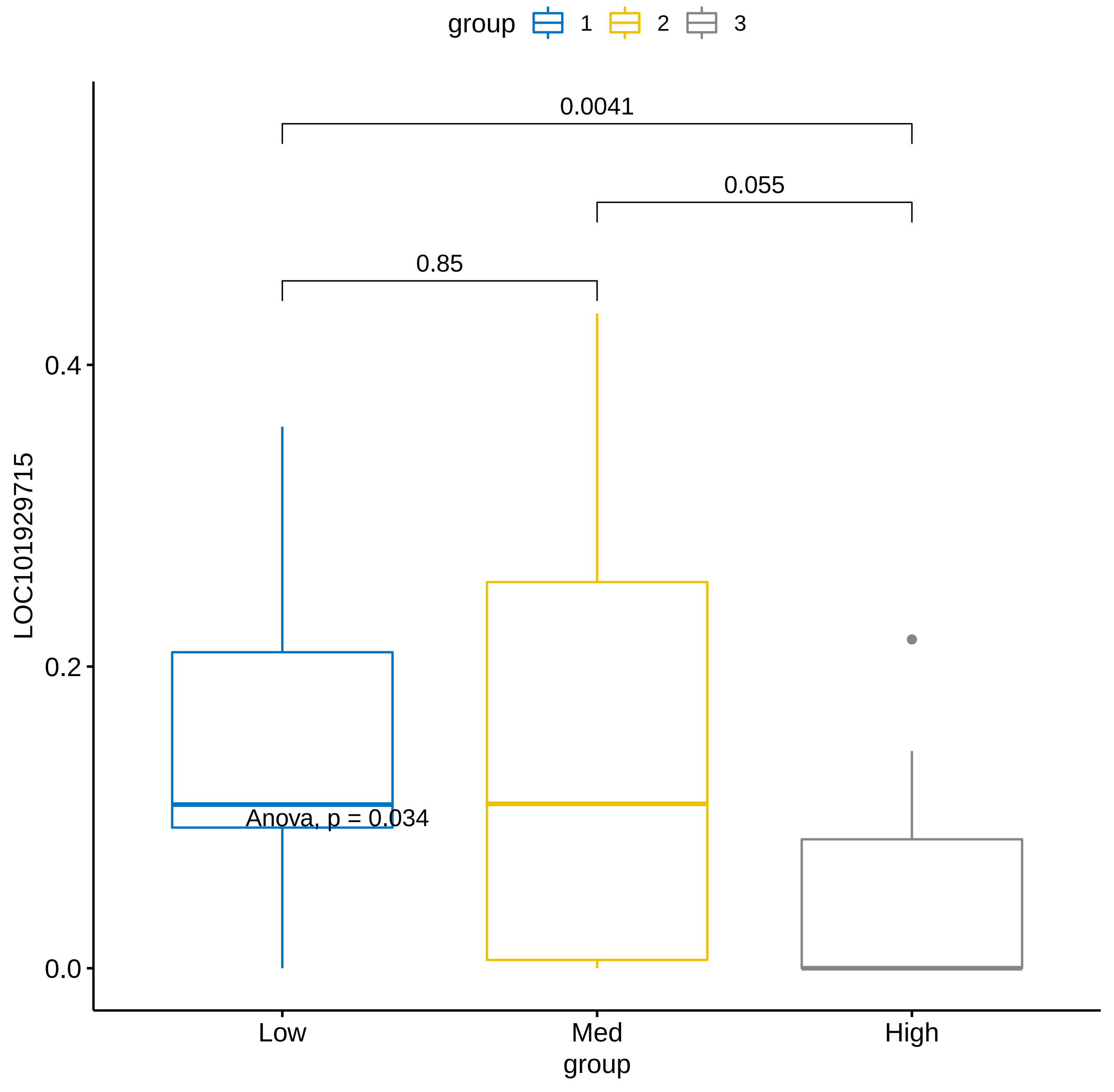
group 1 2 3
0.0041
0.055
0.85
0.4
0.2
0.0
LOC101929715
Anova, p = 0.034
Low
Med
High
group

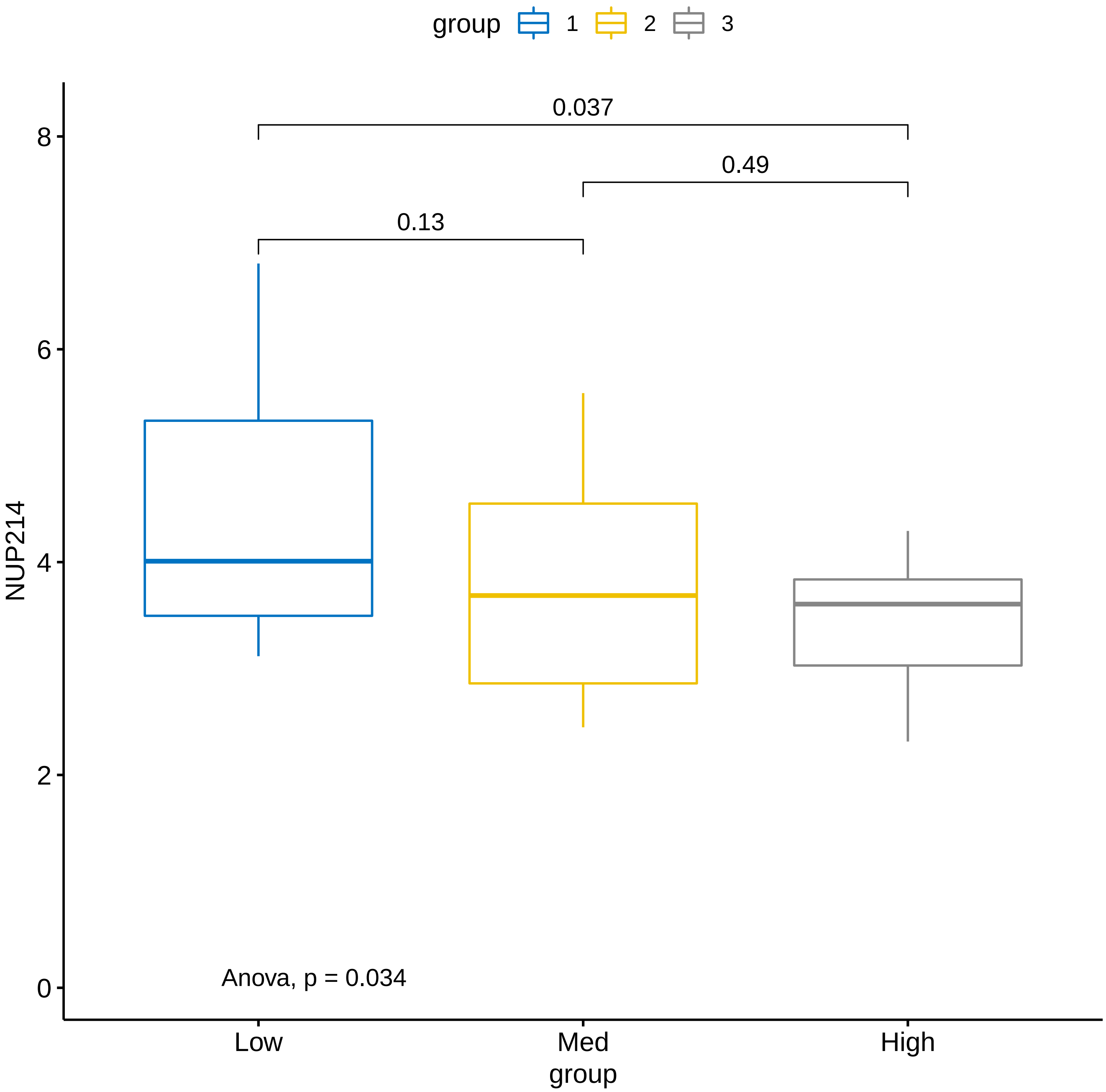
group
1
2
3
0.037
0.49
0.13
8
6
4
2
0
NUP214
Anova, p = 0.034
Low
Med
High
group

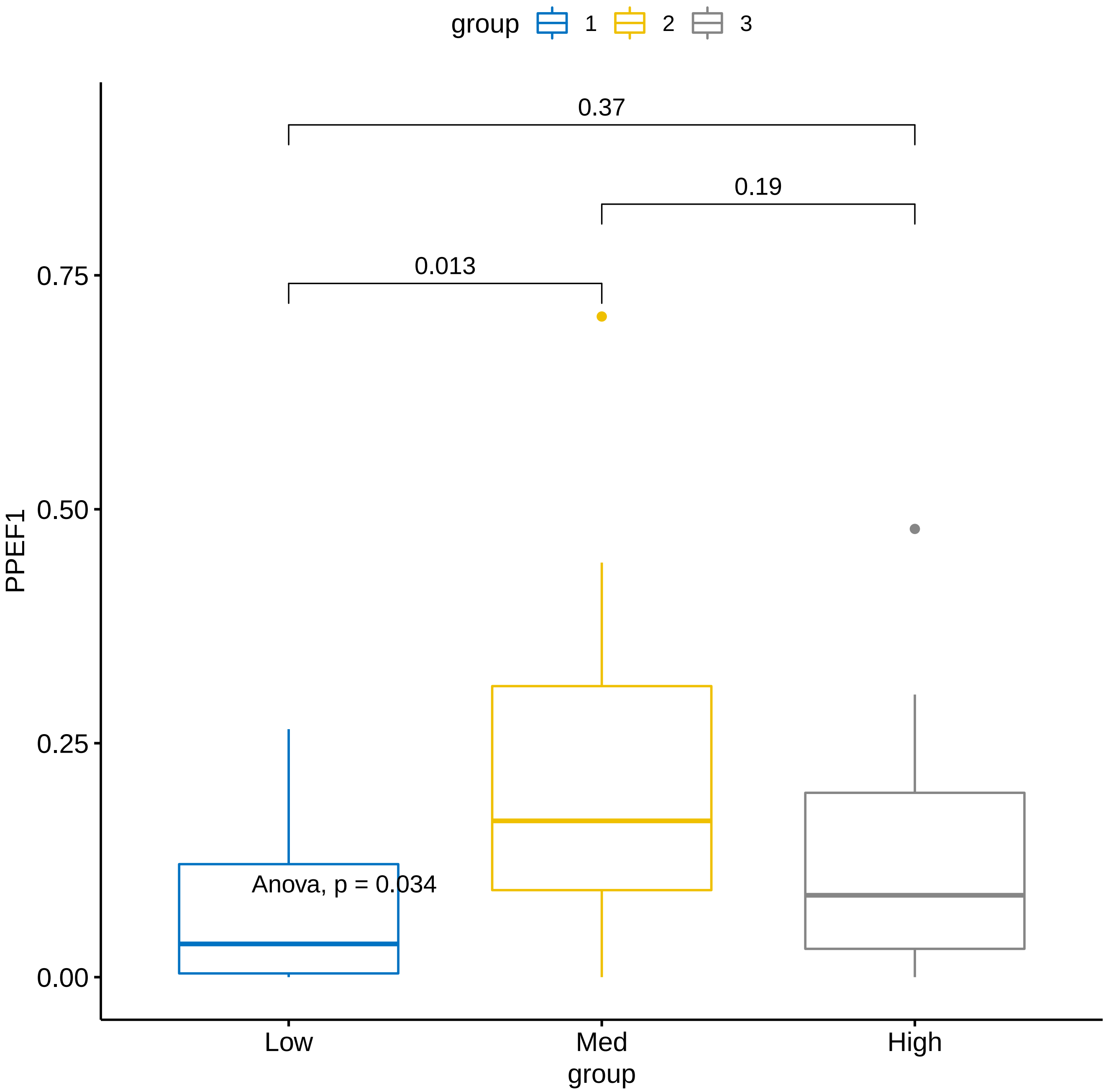

group 1 2 3
0.37
0.19
0.013
0.75
0.50
0.25
0.00
PPEF1
Anova, p = 0.034
Low
Med
High
group

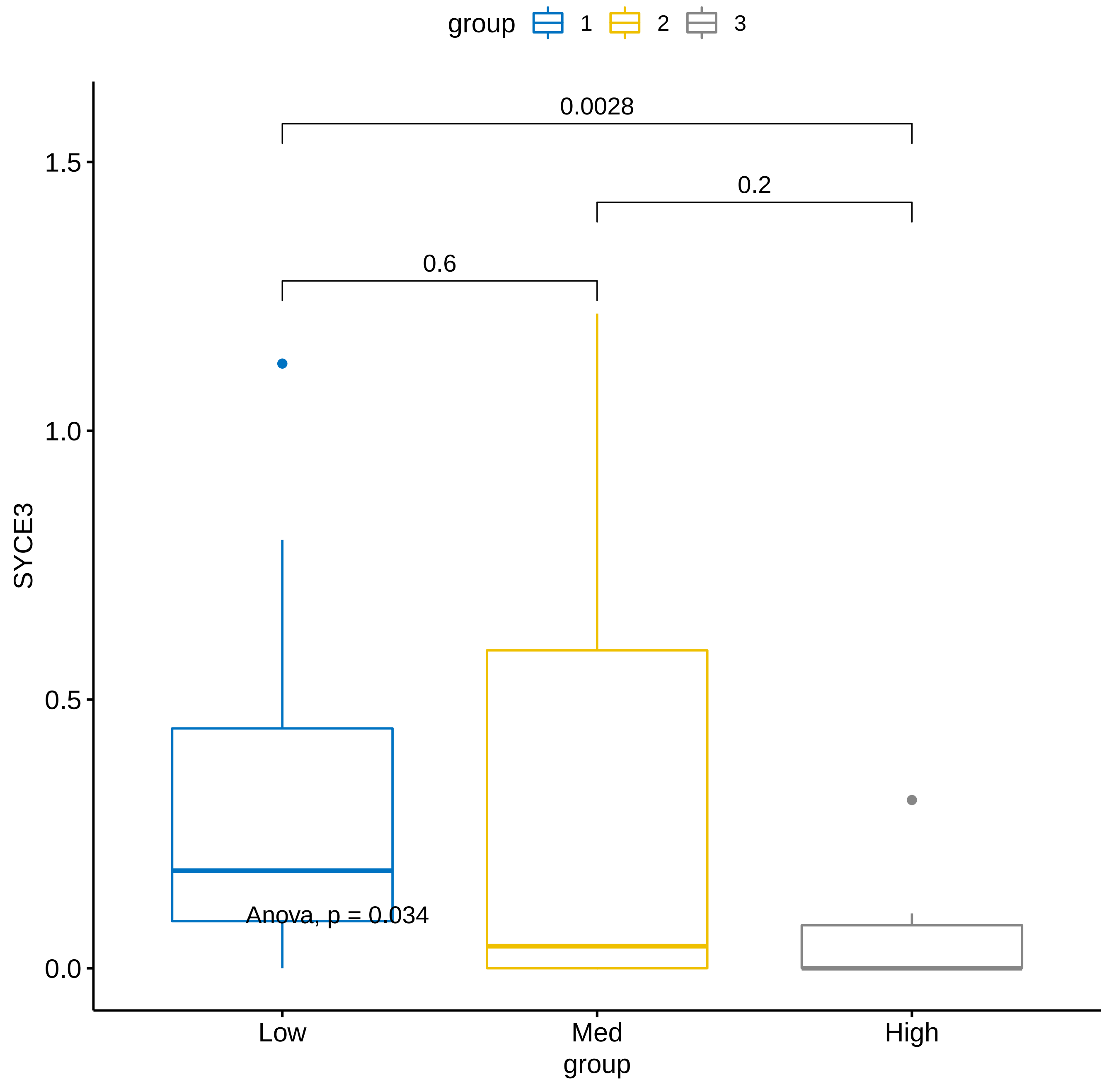
group 1 2 3
0.0028
0.2
0.6
1.5
1.0
0.5
0.0
SYCE3
Anova, p = 0.034
Low
Med
High
group

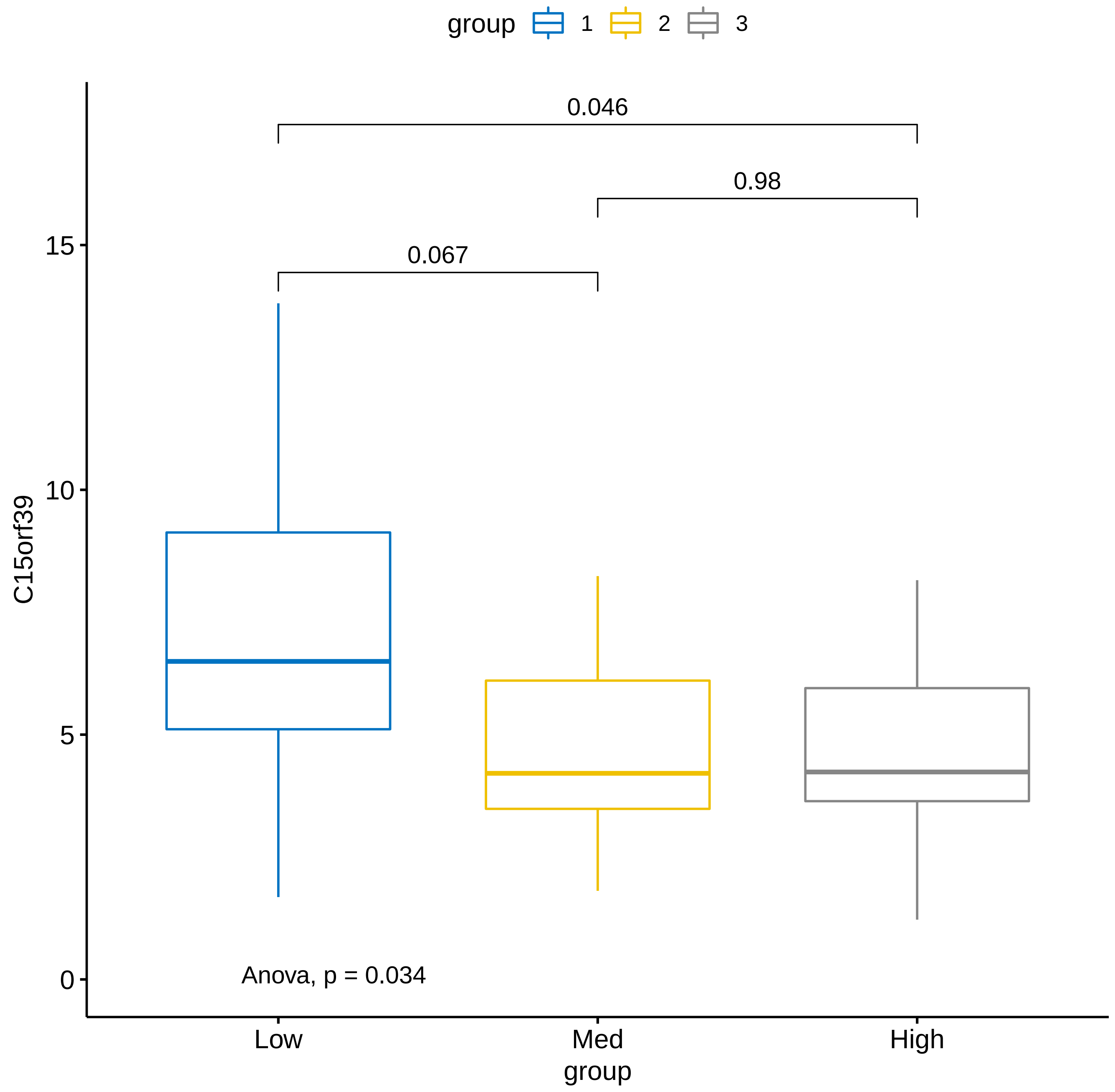

group 1 2 3
0.046
0.98
0.067
15
10
5
0
C15orf39
Anova, p = 0.034
Low
Med
High
group

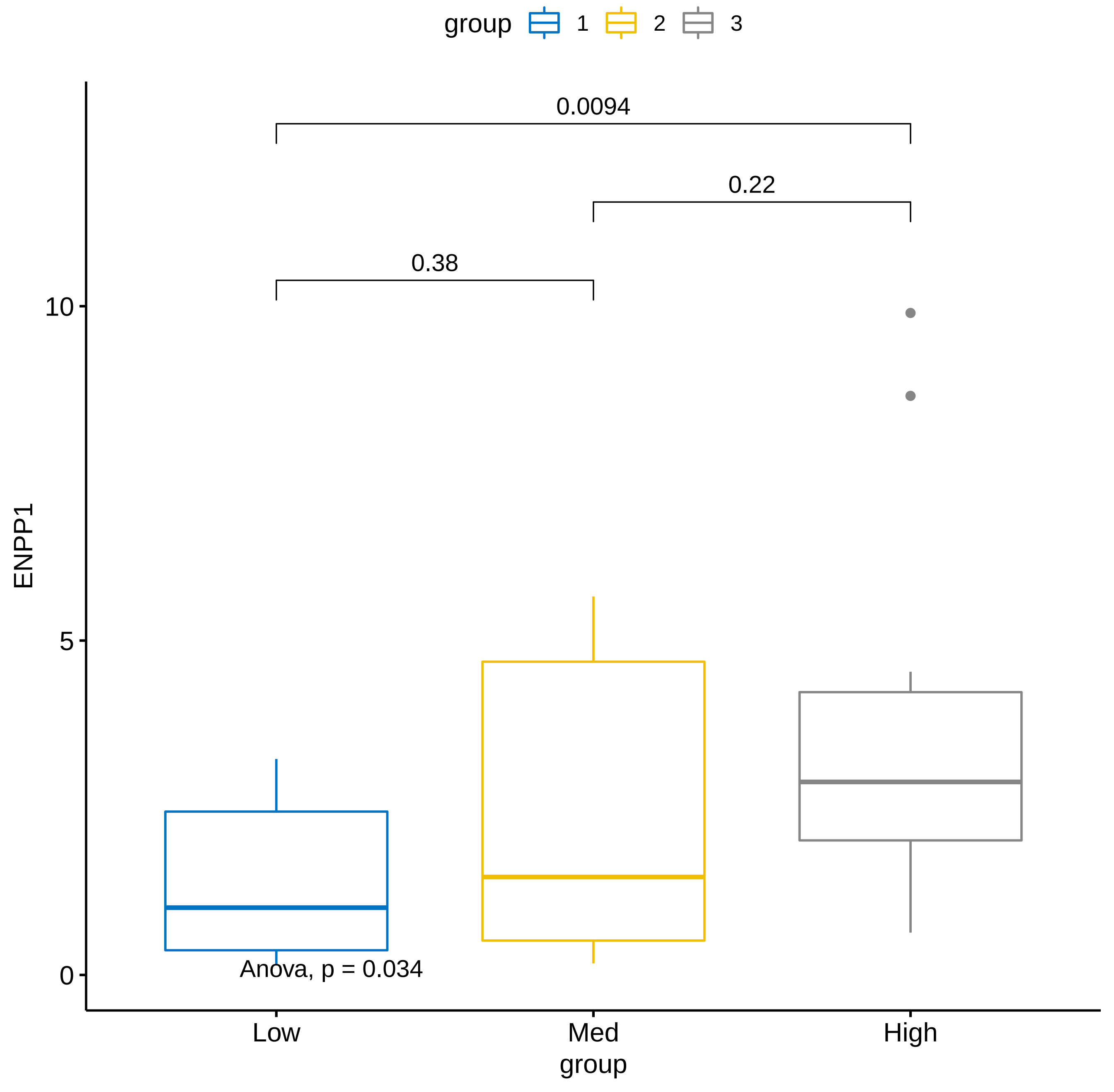
group
1
2
3
0.0094
0.22
0.38
10
5
0
ENPP1
Anova, p = 0.034
Low
Med
High
group

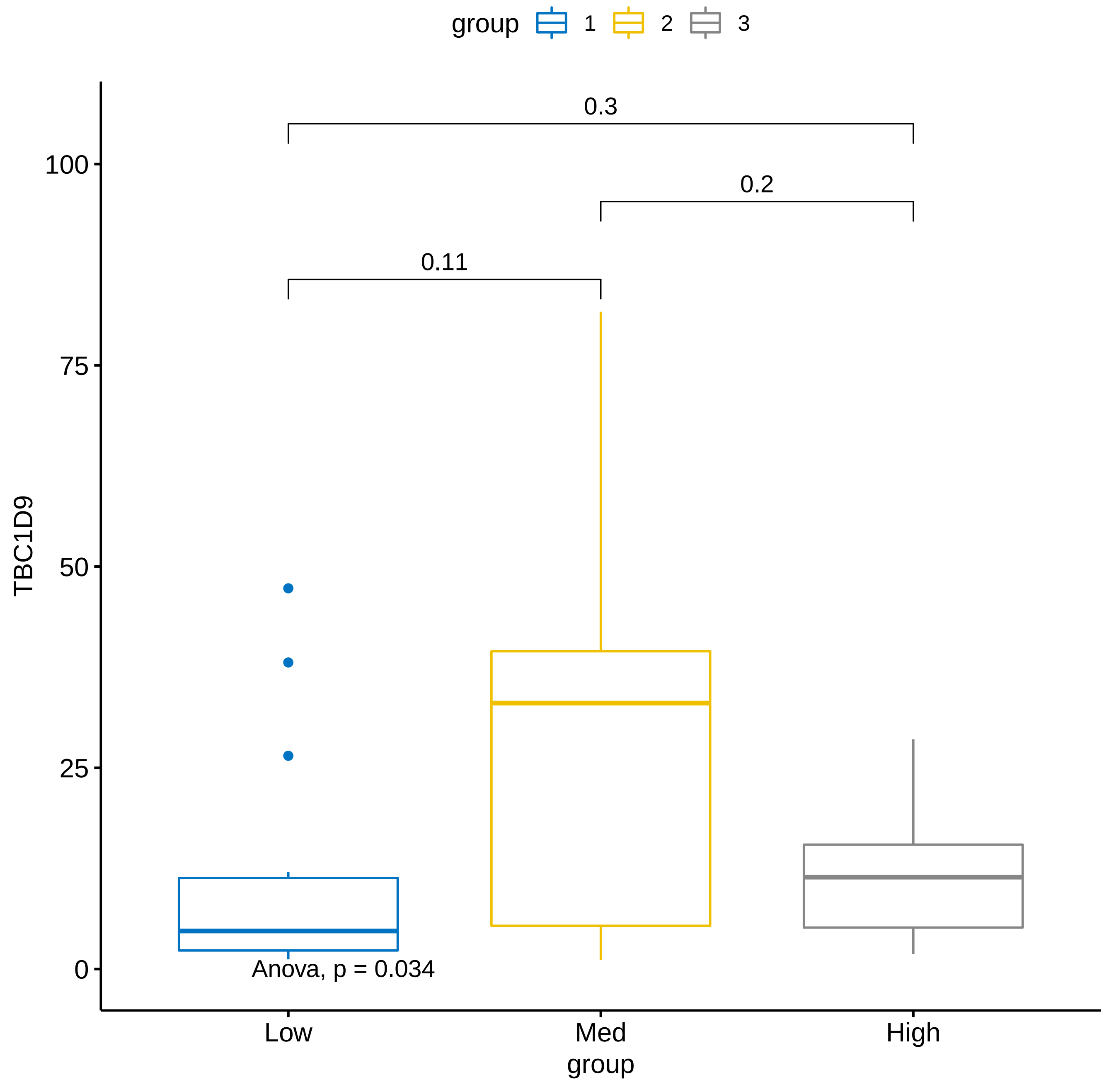
group 1 2 3
0.3
0.2
0.11
100
75
50
25
0
TBC1D9
Anova, p = 0.034
Low
Med
High
group

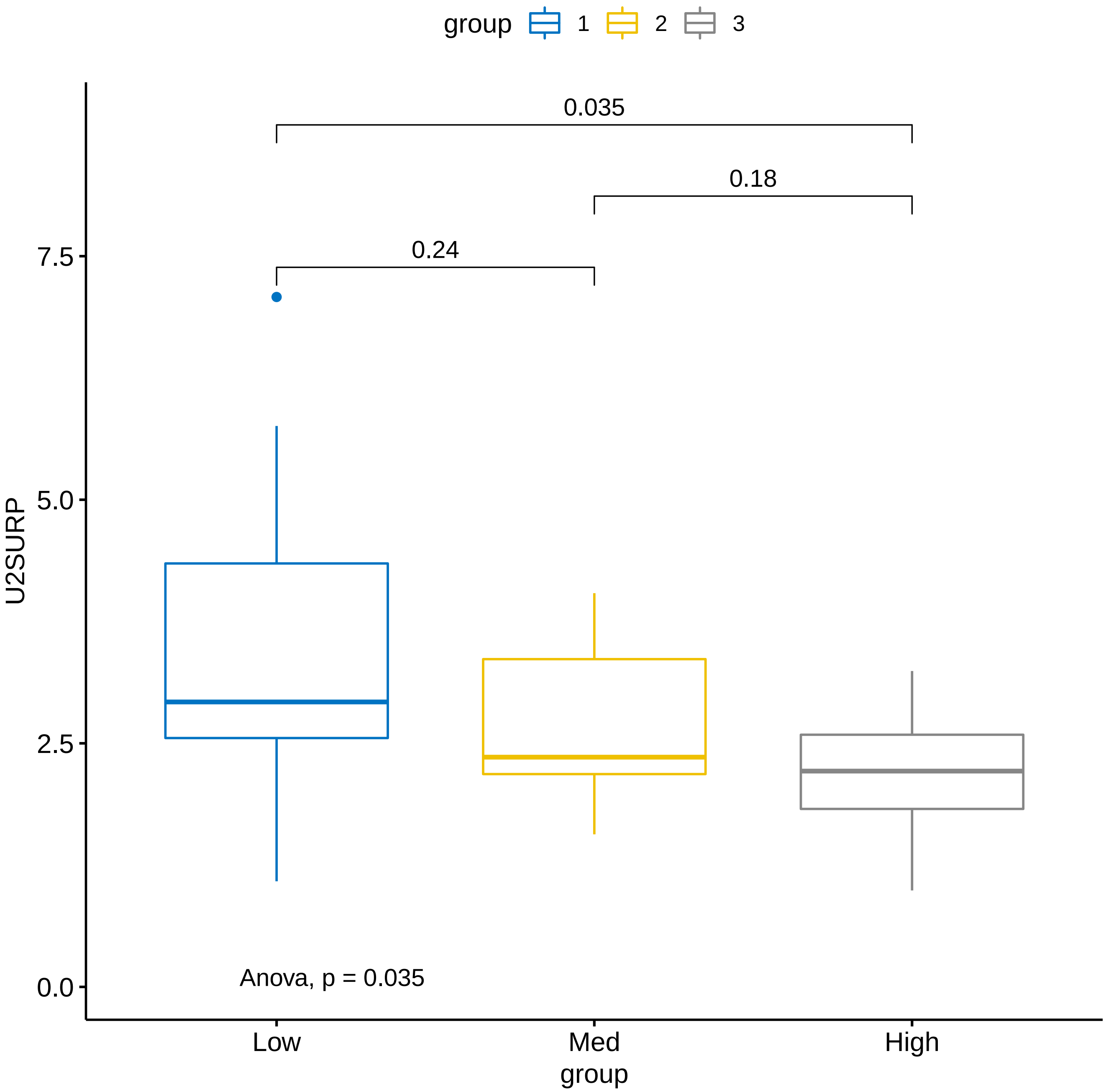
group
1
2
3
0.035
0.18
0.24
7.5
5.0
2.5
0.0
U2SURP
Anova, p = 0.035
Low
Med
High
group

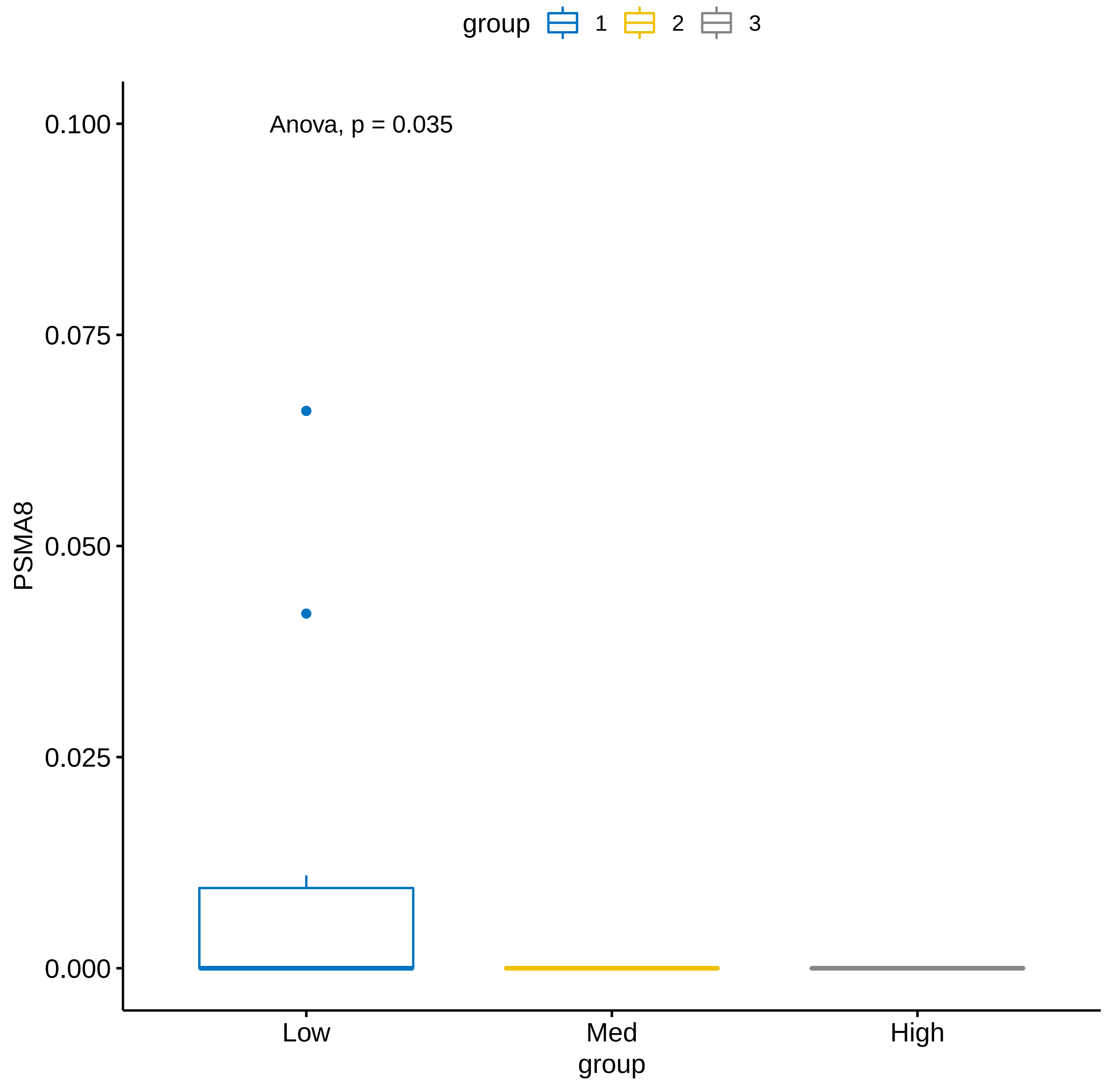
group
1
2
3
Anova, p = 0.035
0.100
0.075
0.050
0.025
0.000
PSMA8
Low
Med
High
group

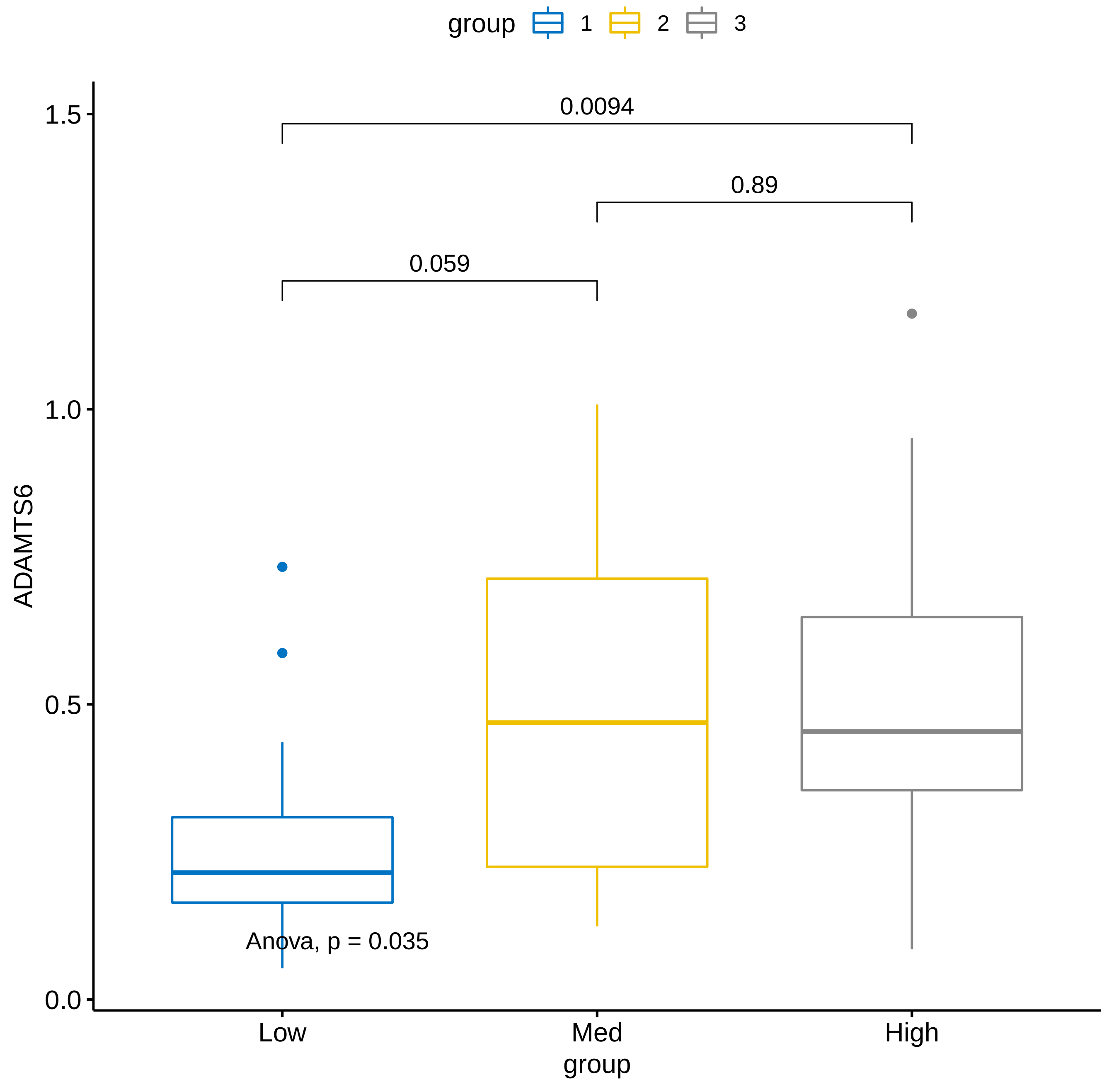
group
1
2
3
0.0094
0.89
0.059
ADAMTS6
1.5
1.0
0.5
0.0
Anova, p = 0.035
Low
Med
High
group

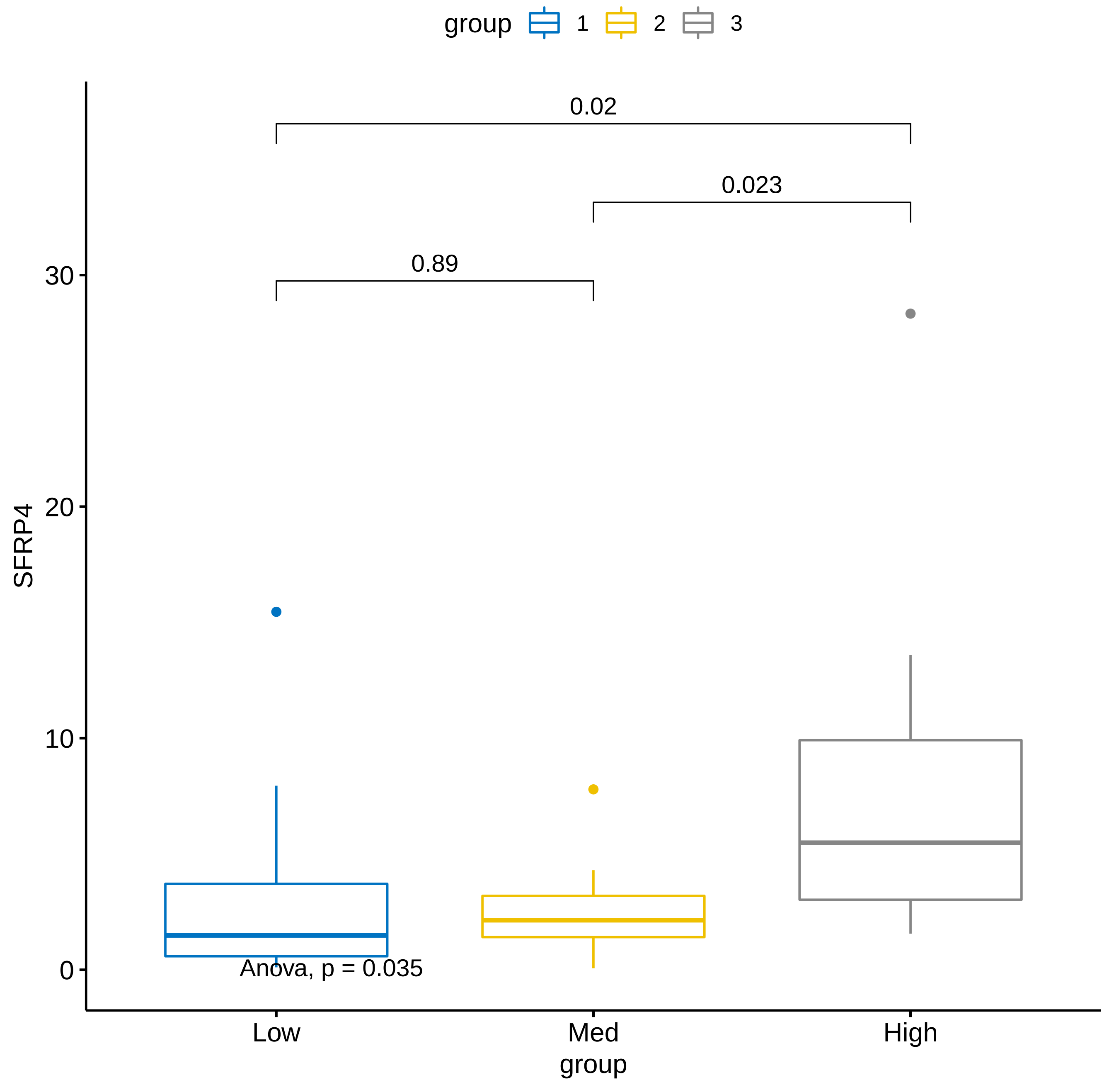
group
1
2
3
0.02
0.023
0.89
30
20
10
0
SFRP4
Anova, p = 0.035
Low
Med
High
group

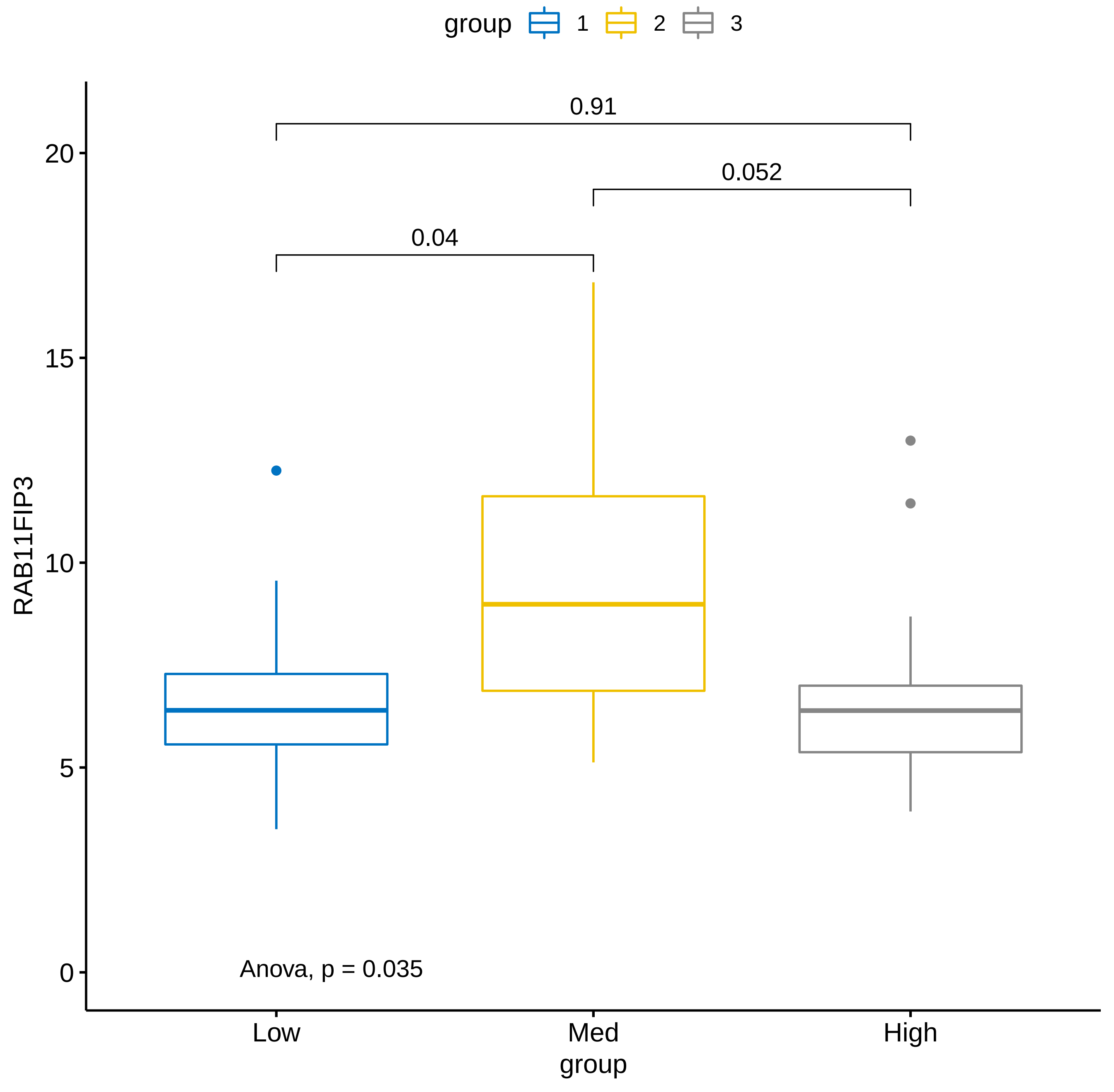
group
1
2
3
0.91
0.052
0.04
20
15
10
5
0
RAB11FIP3
Anova, p = 0.035
Low
Med
High
group

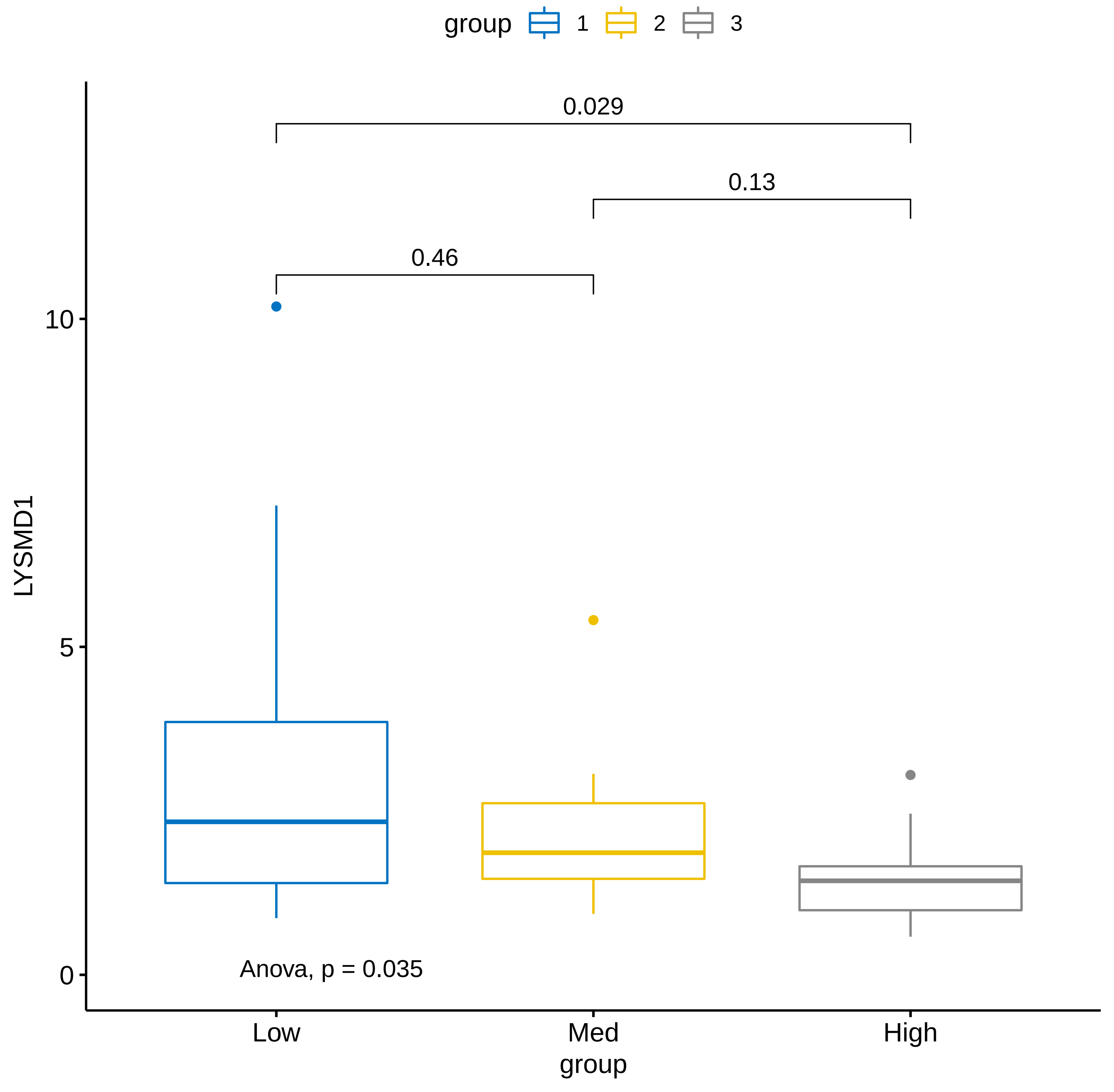
group
1
2
3
0.029
0.13
0.46
10
5
0
LYSMD1
Anova, p = 0.035
Low
Med
High
group

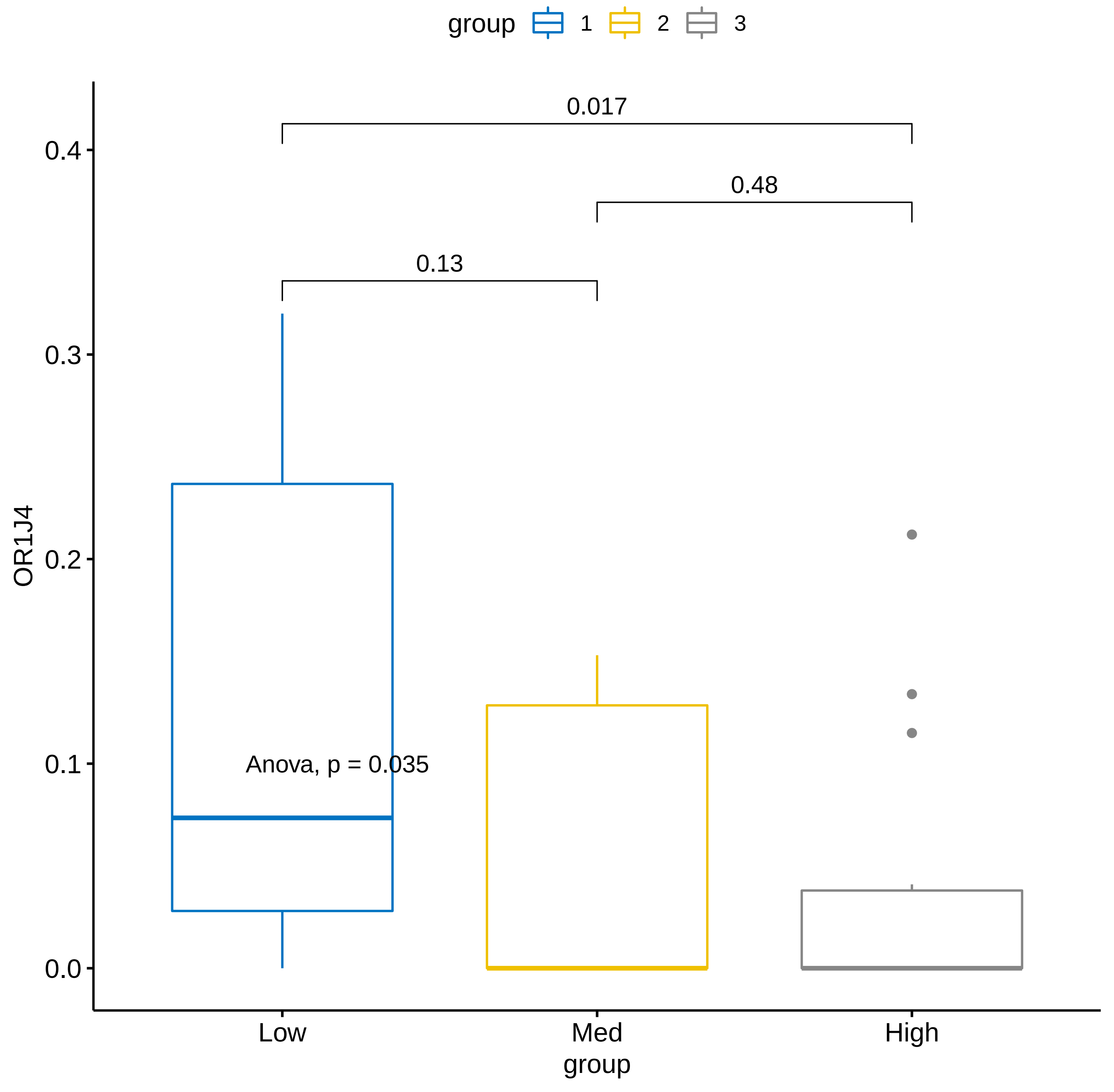

group
1
2
3
0.017
0.48
0.13
Anova, p = 0.035
OR1J4
0.0
0.1
0.2
0.3
0.4
Low
Med
High
group

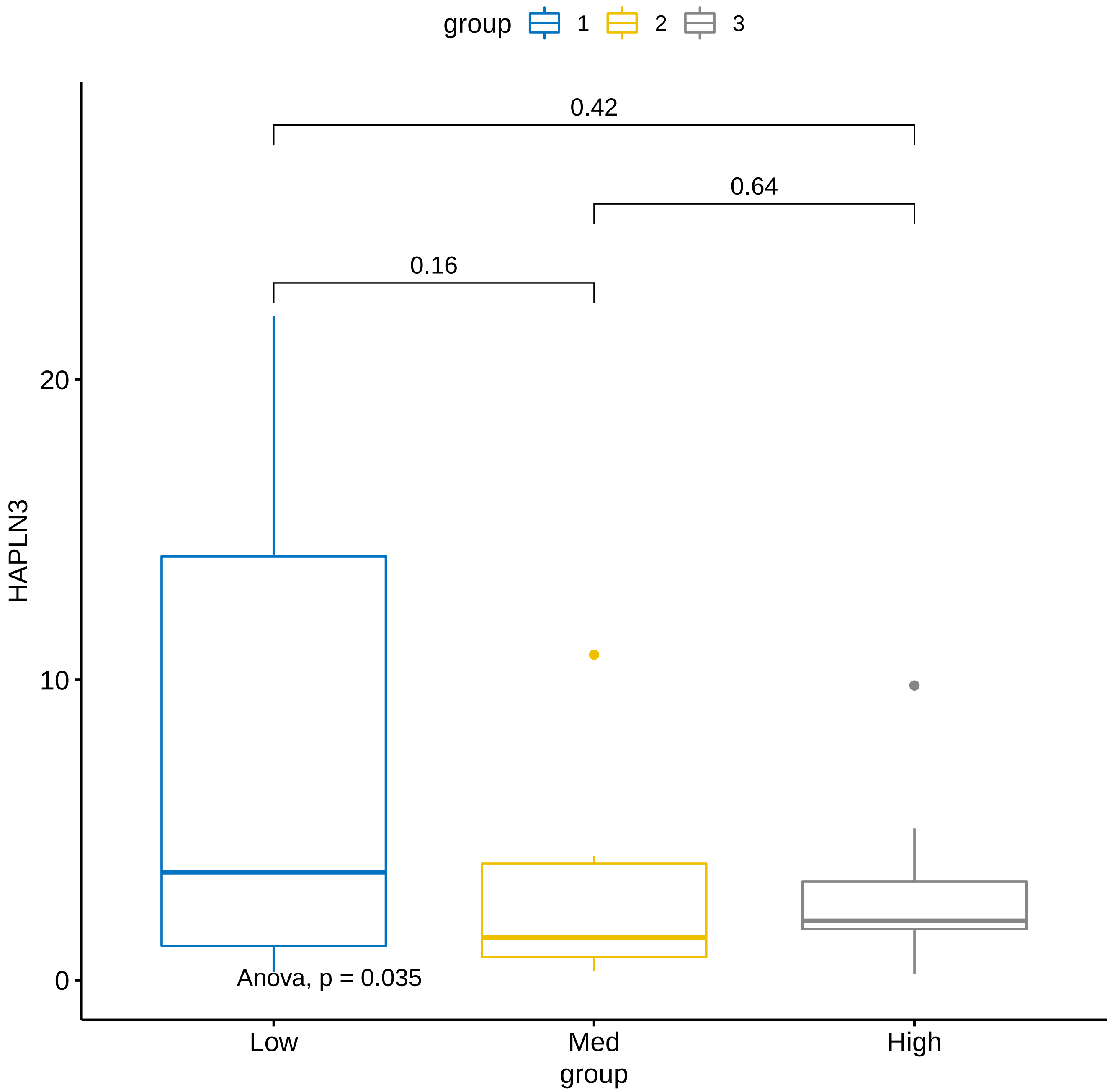
group
1
2
3
0.42
0.64
0.16
20
10
0
HAPLN3
Anova, p = 0.035
Low
Med
High
group

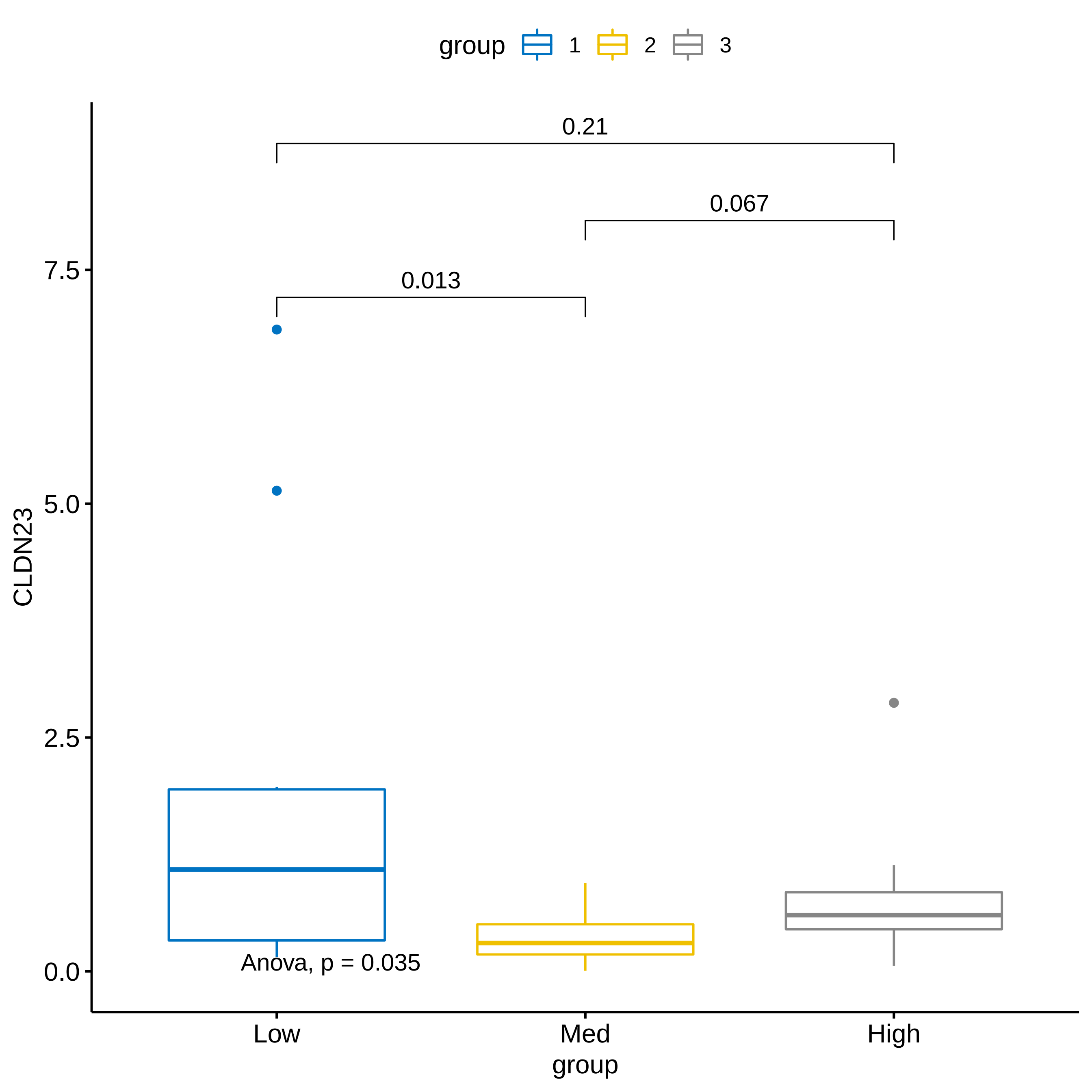

group
1
2
3
0.21
0.067
0.013
7.5
5.0
2.5
0.0
CLDN23
Anova, p = 0.035
Low
Med
High
group

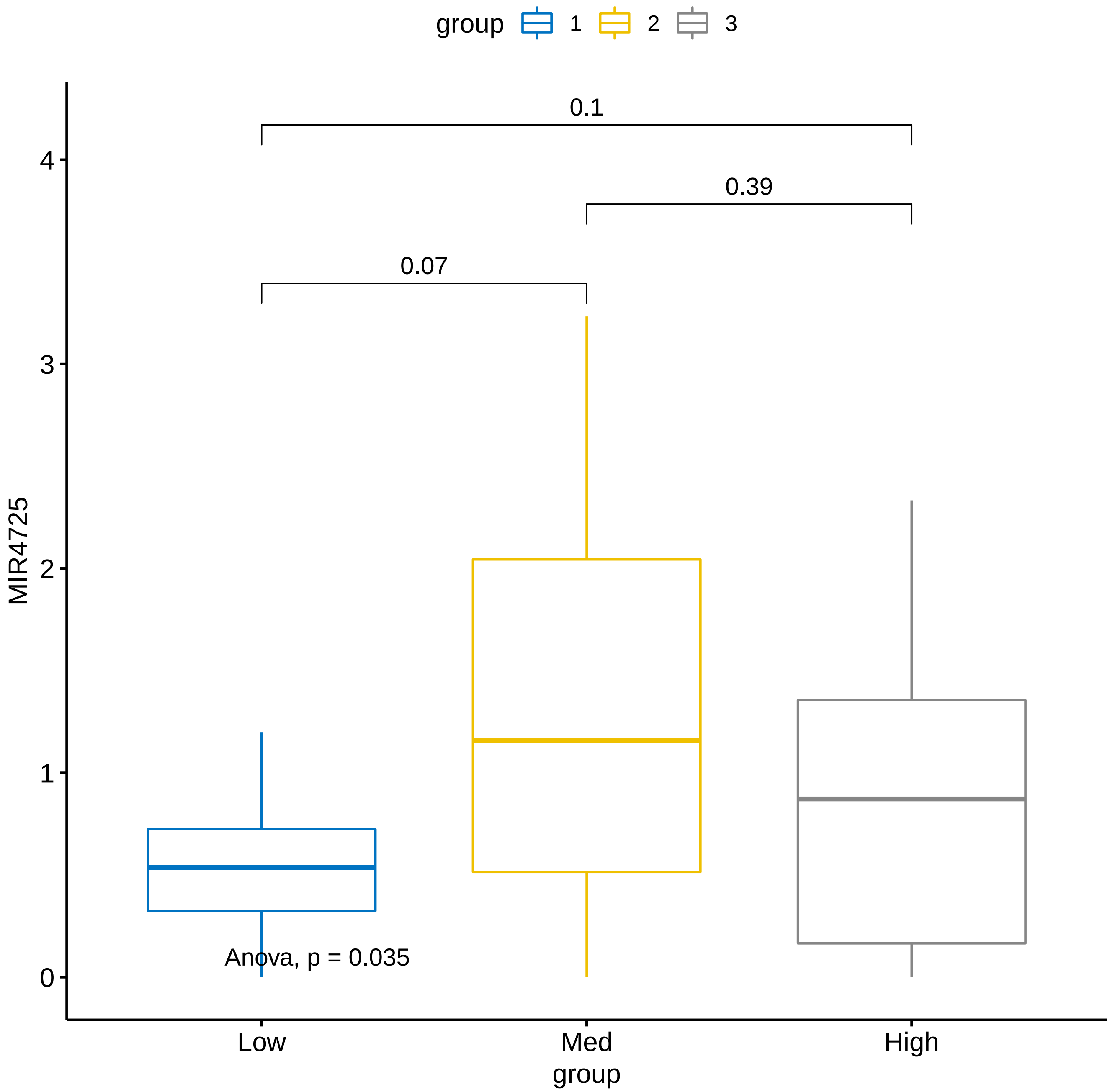
group
1
2
3
0.1
0.39
0.07
Anova, p = 0.035
MIR4725
4
3
2
1
0
Low
Med
High
group

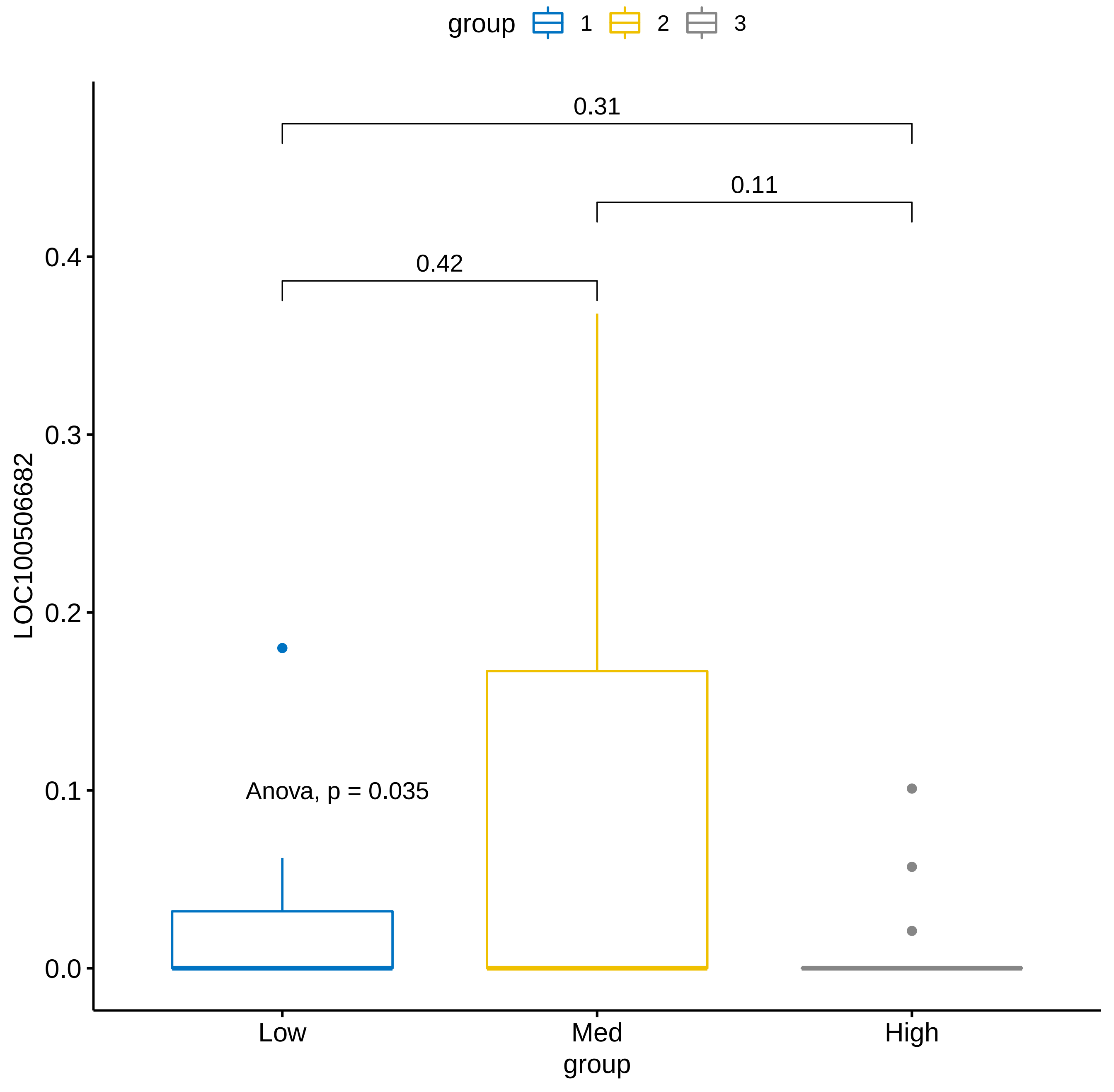

group 1 2 3
0.31
0.11
0.42
Anova, p = 0.035
LOC100506682
0.0
0.1
0.2
0.3
0.4
Low
Med
High
group

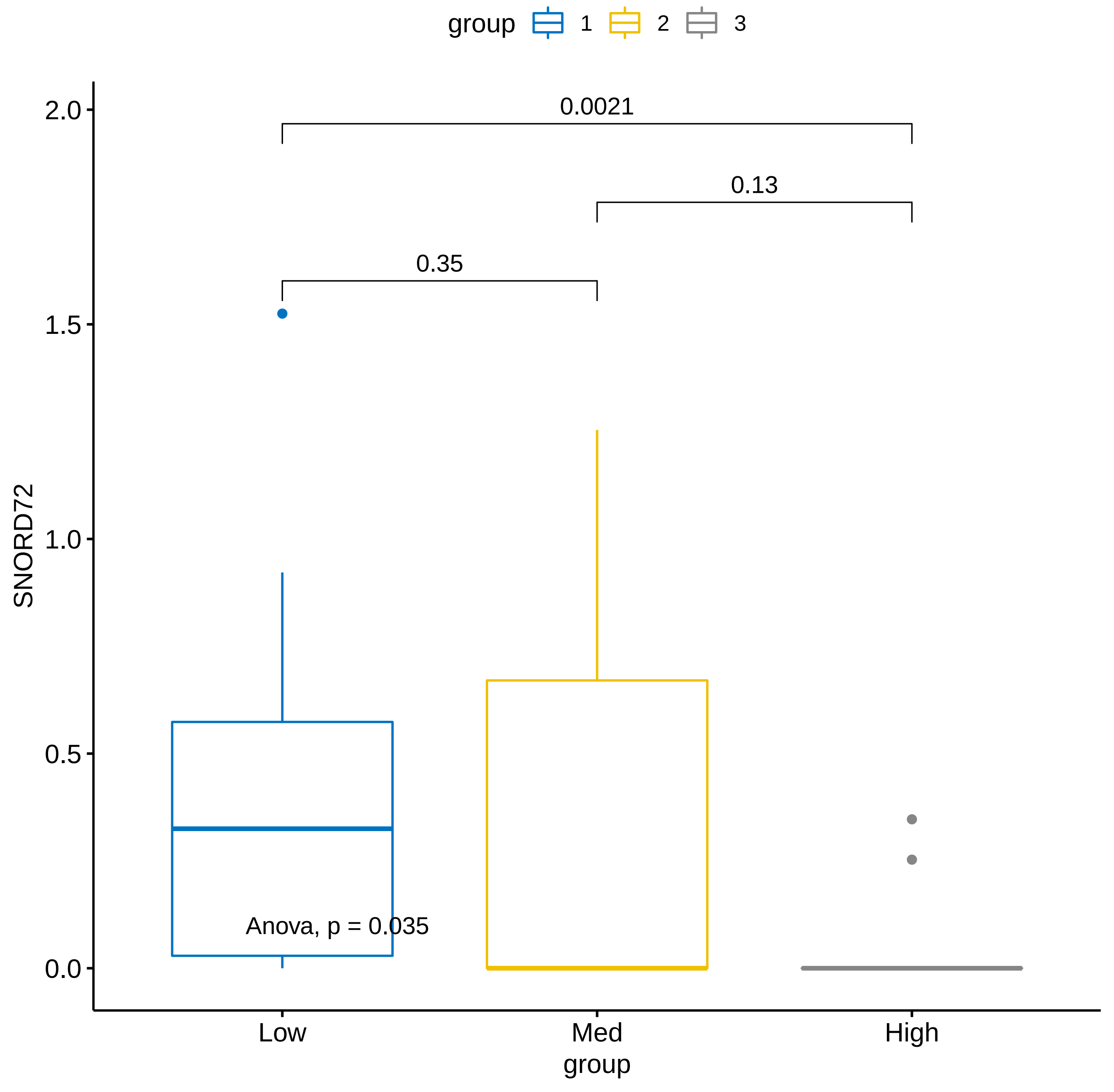

group 1 2 3
0.0021
0.13
0.35
SNORD72
2.0
1.5
1.0
0.5
0.0
Anova, p = 0.035
Low
Med
High
group

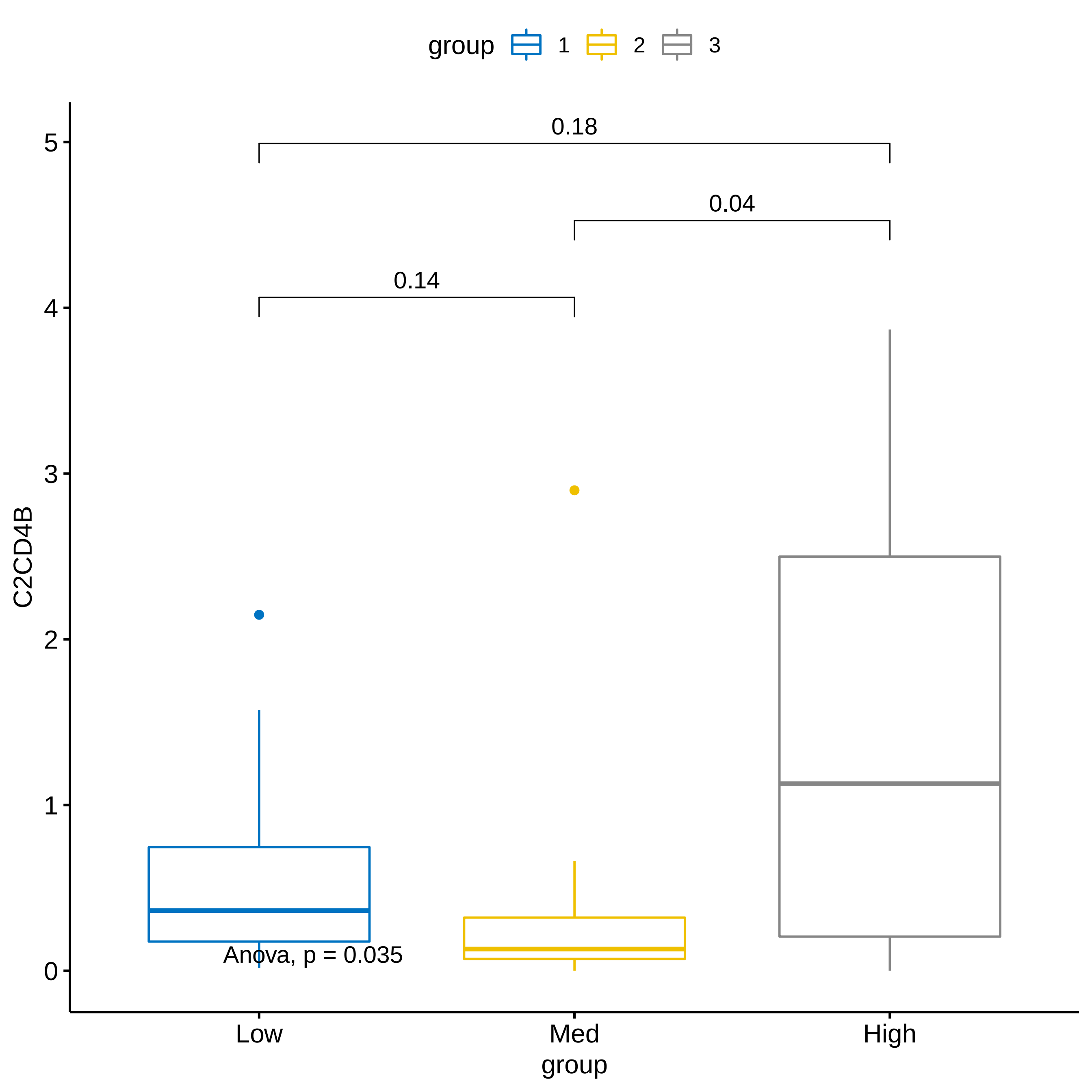

group 1 2 3
0.18
0.04
0.14
C2CD4B
5
4
3
2
1
0
Anova, p = 0.035
Low
Med
High
group

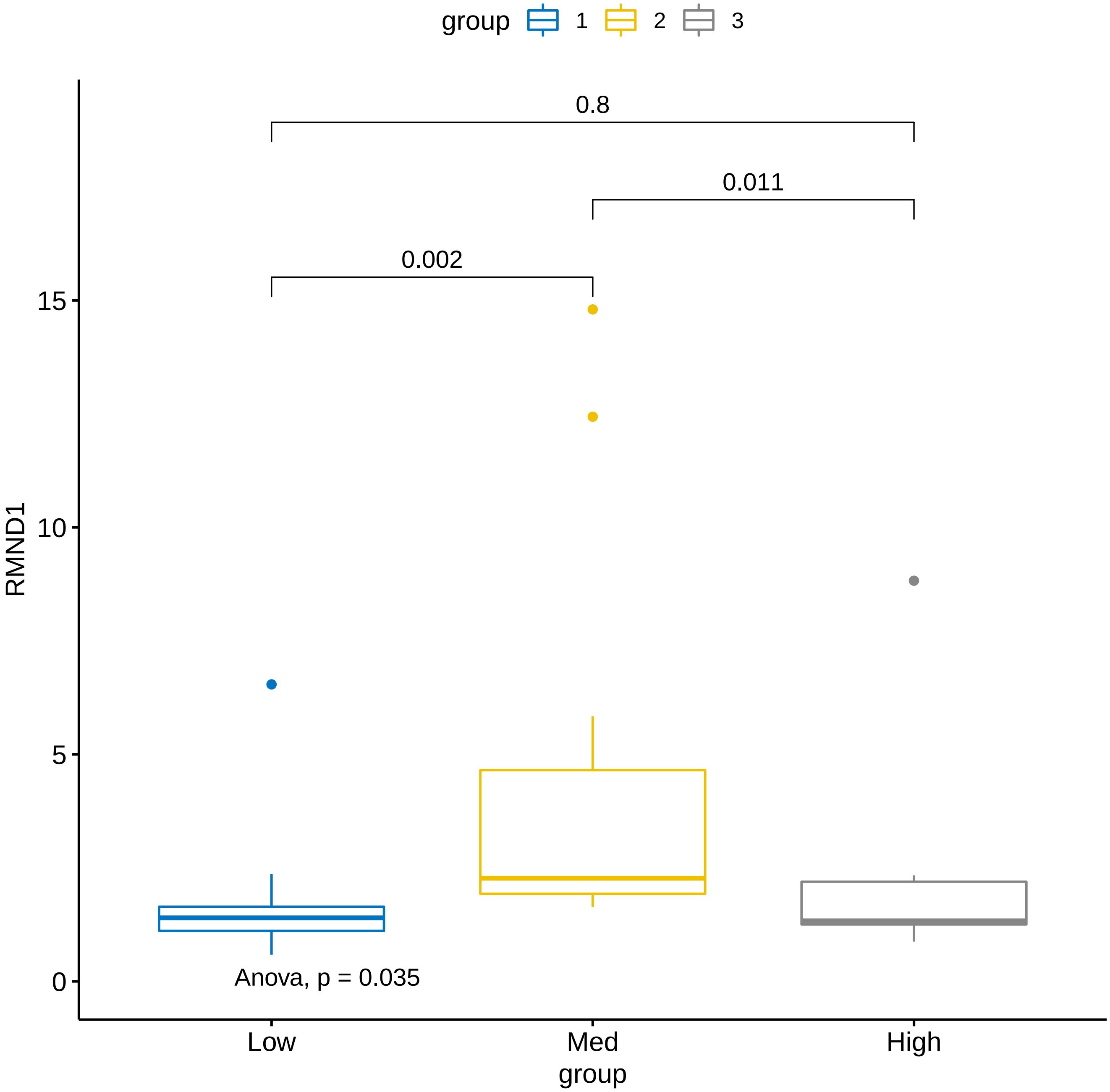

group
1
2
3
0.8
0.011
0.002
15
10
5
0
RMND1
Anova, p = 0.035
Low
Med
High
group

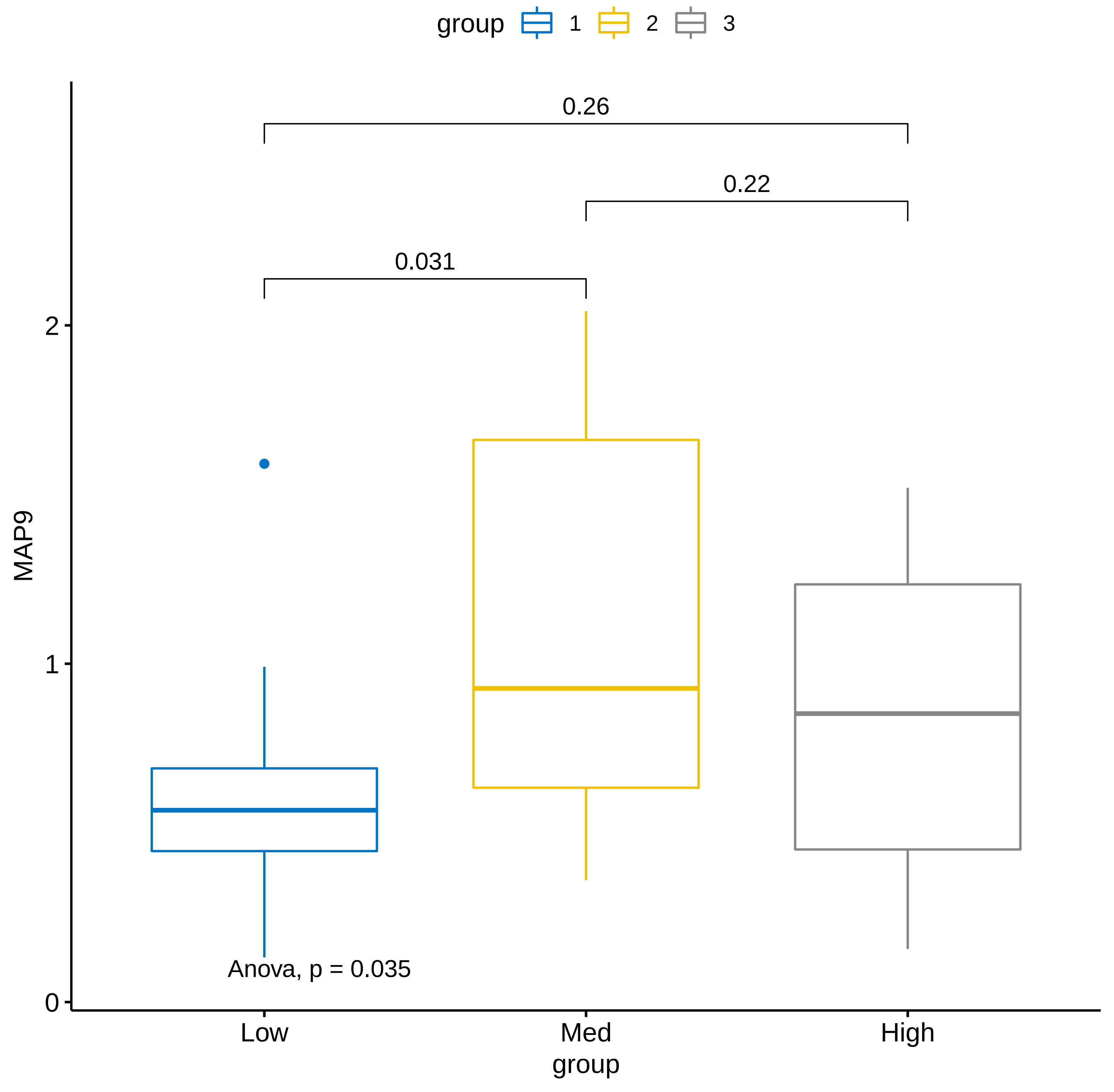

group 1 2 3
0.26
0.22
0.031
MAP9
2
1
0
Anova, p = 0.035
Low
Med
High
group

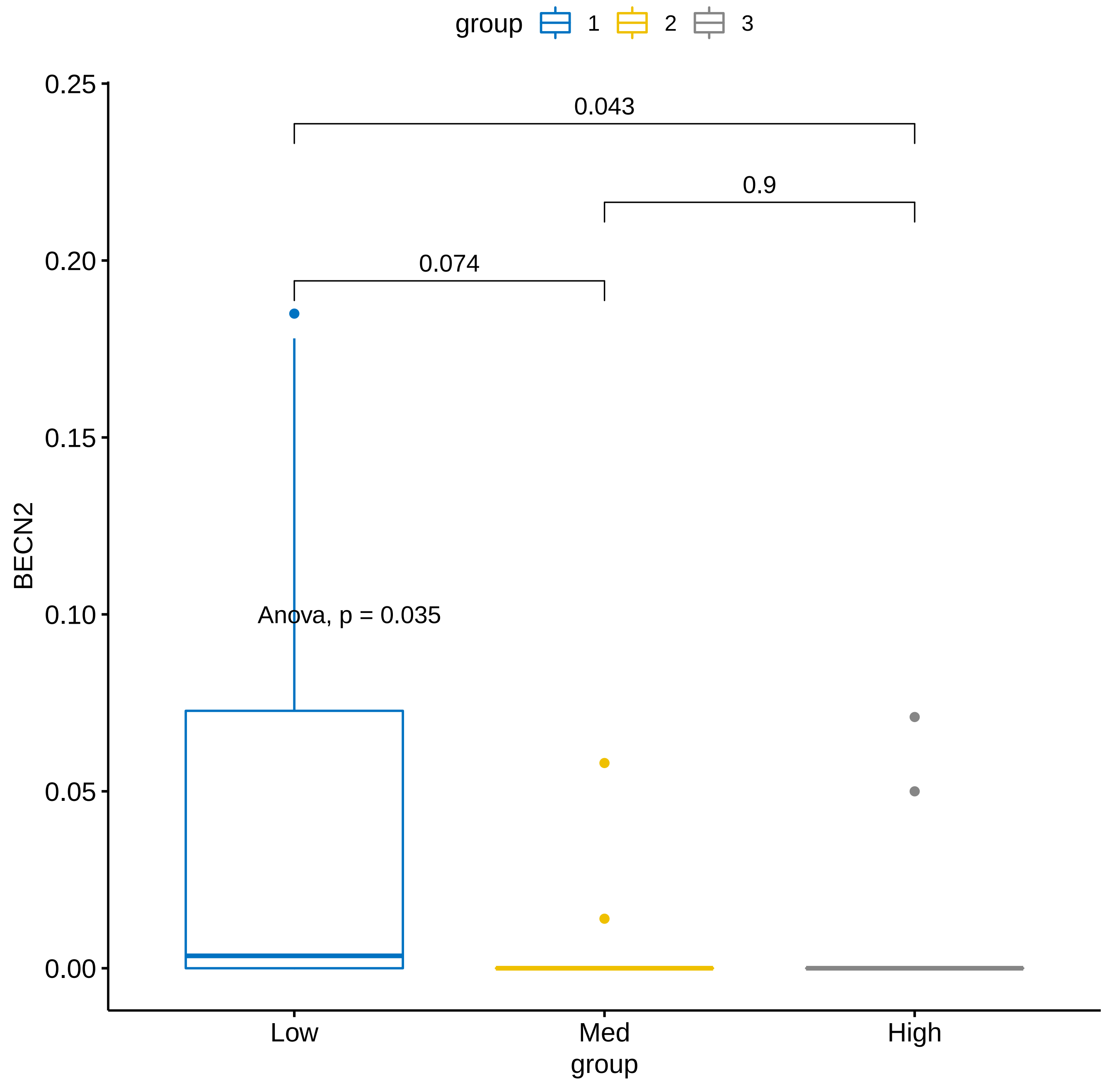
group
1
2
3
0.25
0.20
0.15
0.10
0.05
0.00
BECN2
0.043
0.9
0.074
Anova, p = 0.035
Low
Med
High
group

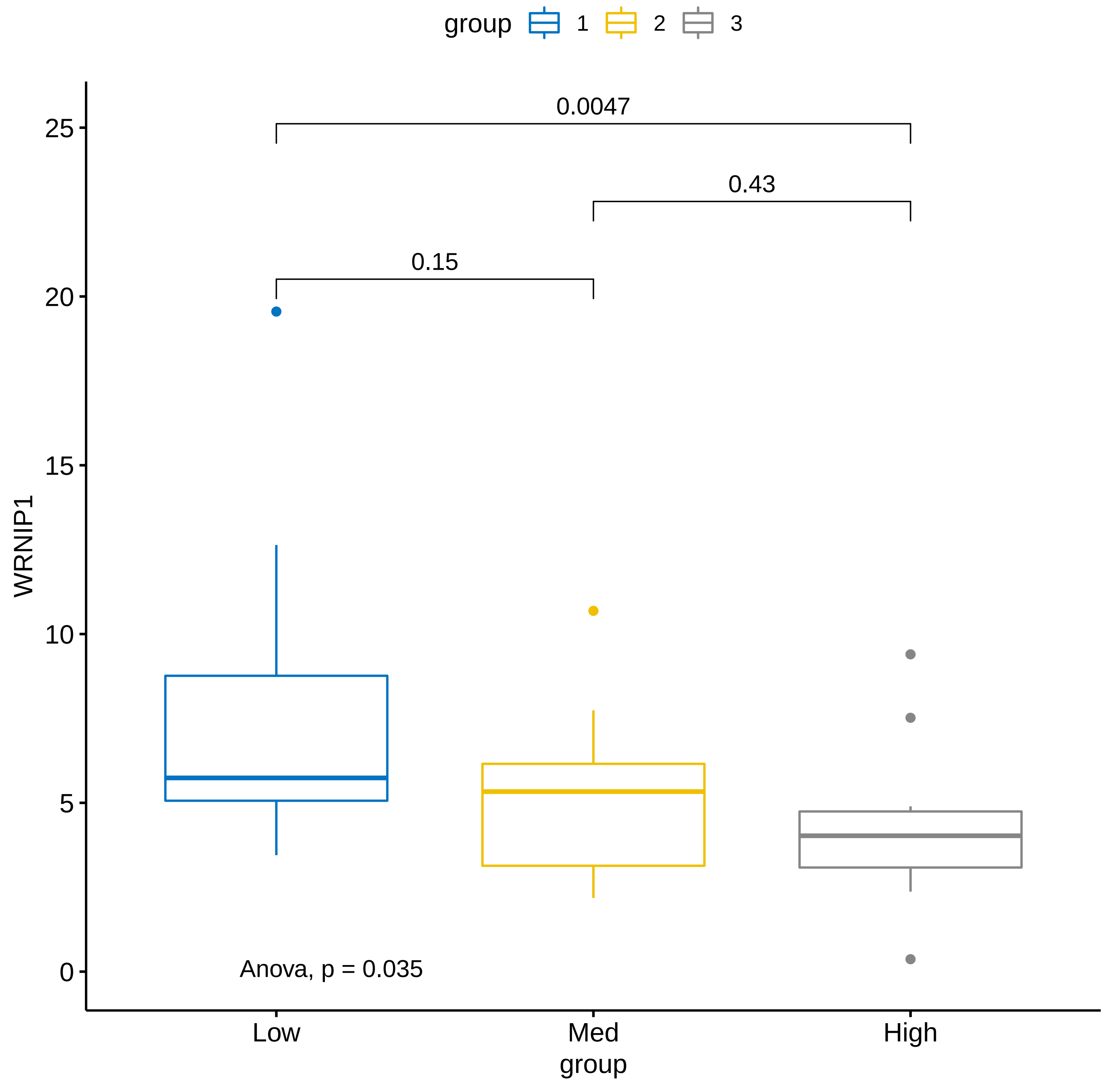
group
1
2
3
0.0047
0.43
0.15
25
20
15
10
5
0
WRNIP1
Anova, p = 0.035
Low
Med
High
group

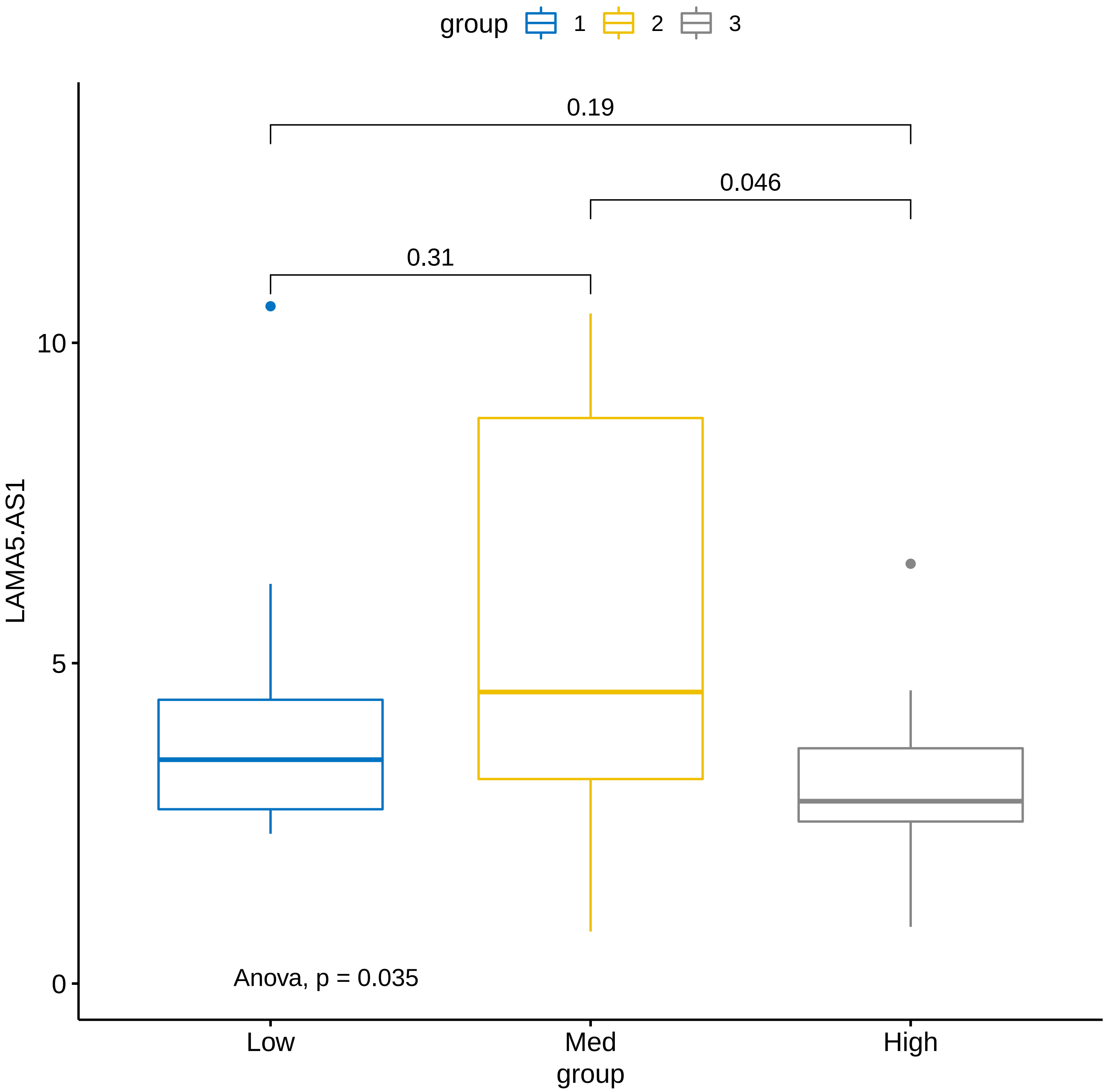
group
1
2
3
0.19
0.046
0.31
10
5
0
LAMA5.AS1
Anova, p = 0.035
Low
Med
High
group

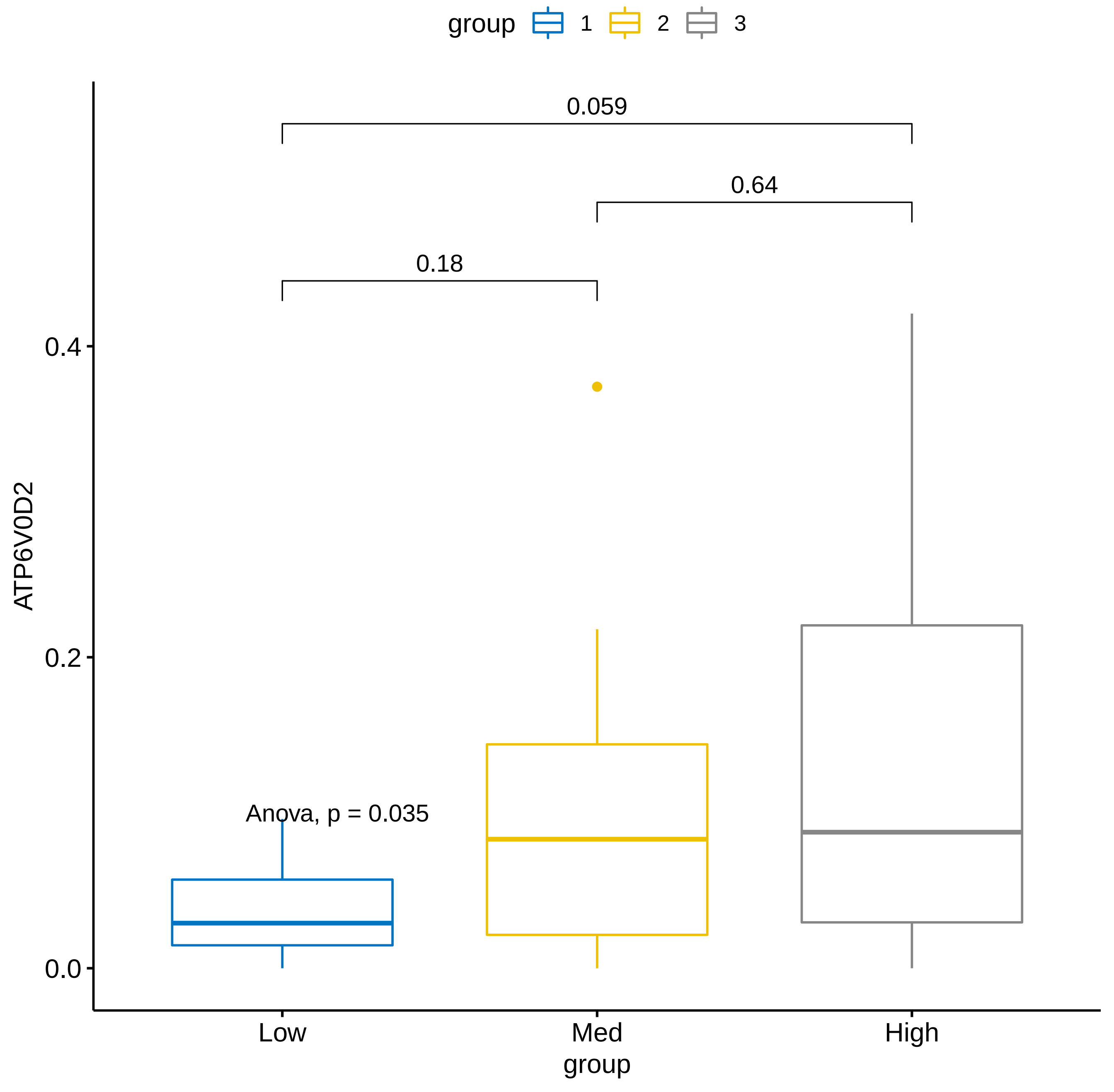
group
1
2
3
0.059
0.64
0.18
0.4
0.2
0.0
ATP6V0D2
Anova, p = 0.035
Low
Med
High
group

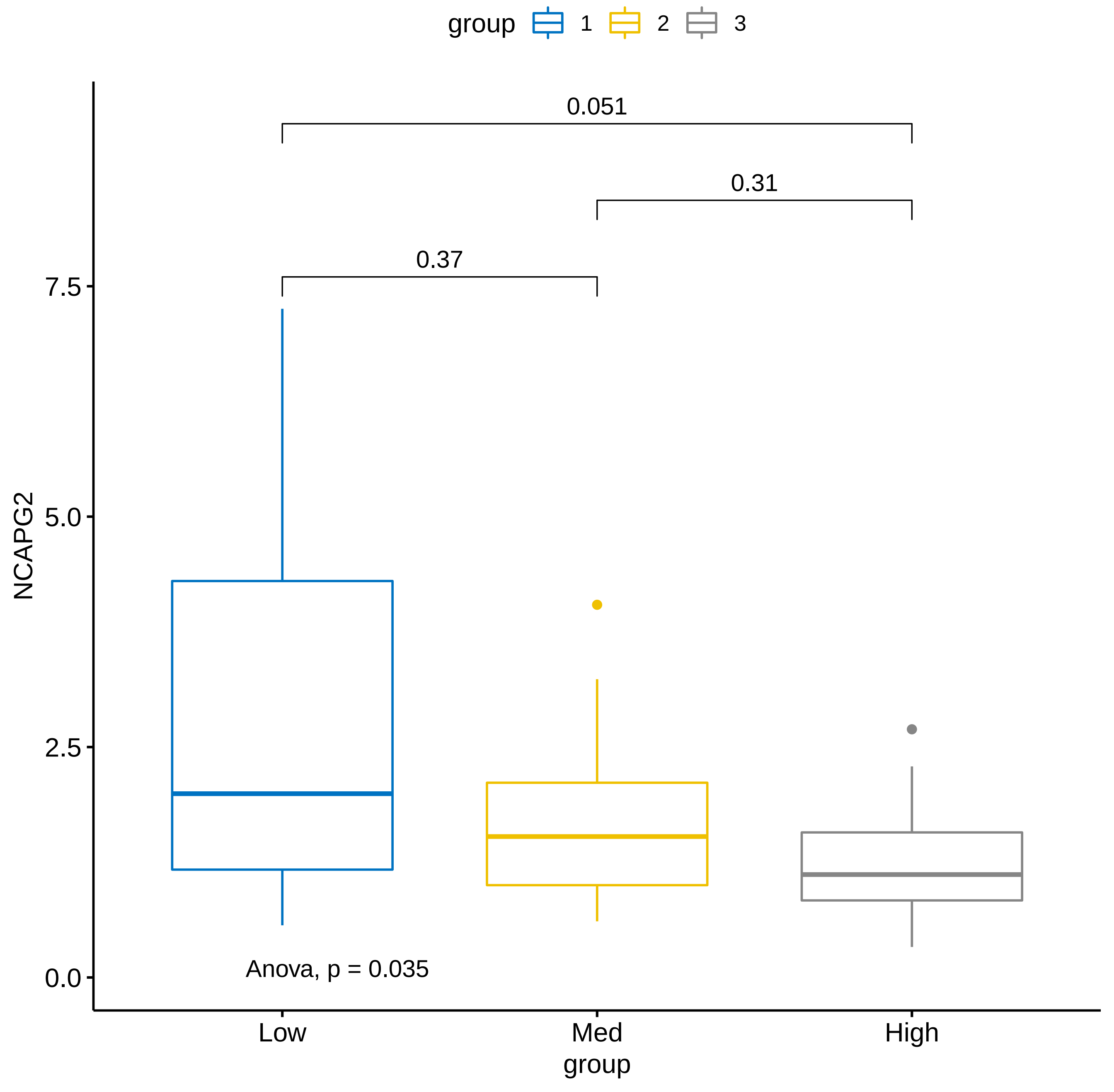
group
1
2
3
0.051
0.31
0.37
7.5
5.0
2.5
0.0
NCAPG2
Anova, p = 0.035
Low
Med
High
group

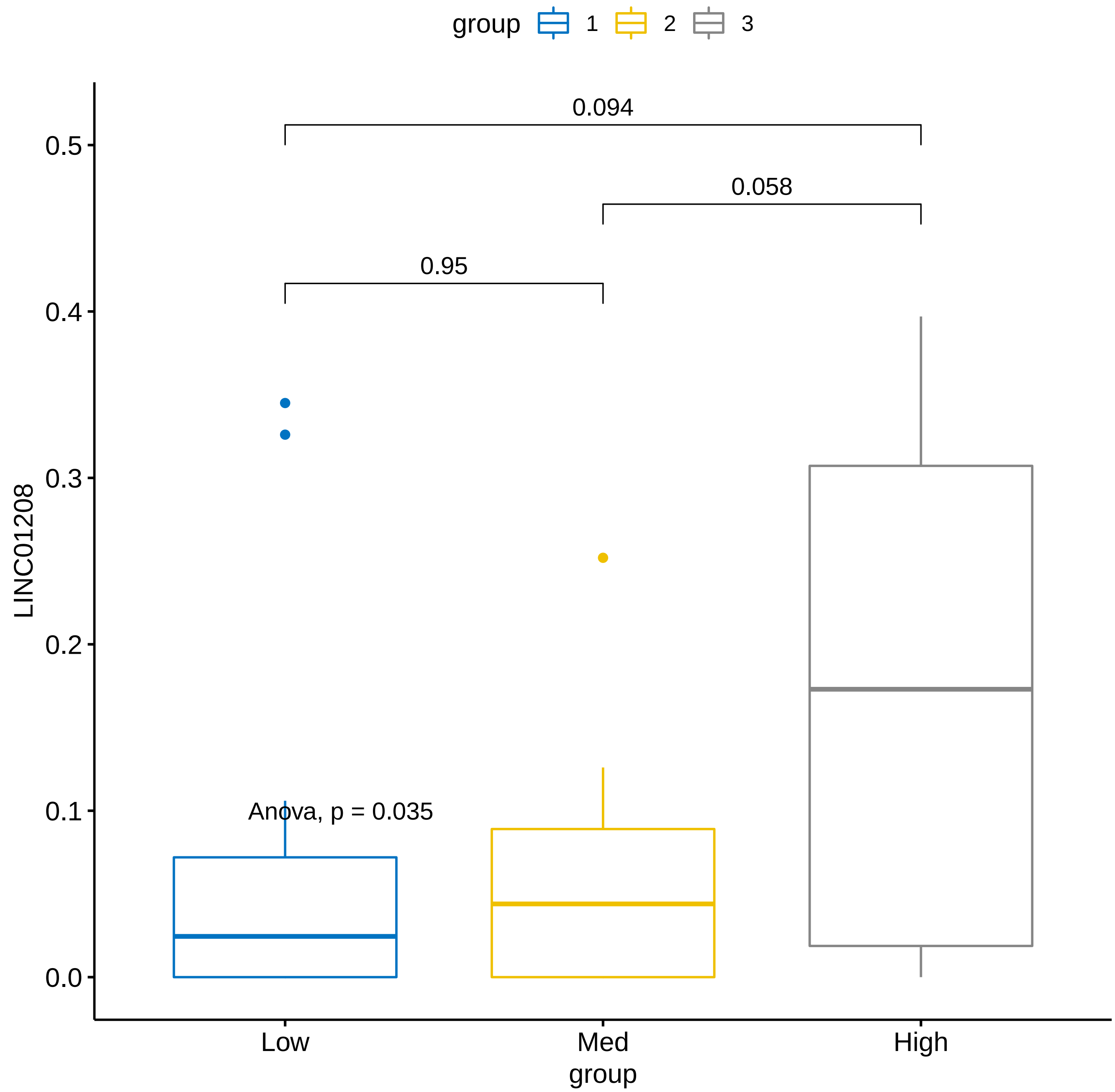

group 1 2 3
0.094
0.058
0.95
Anova, p = 0.035
LINC01208
0.0
0.1
0.2
0.3
0.4
0.5
Low
Med
High
group

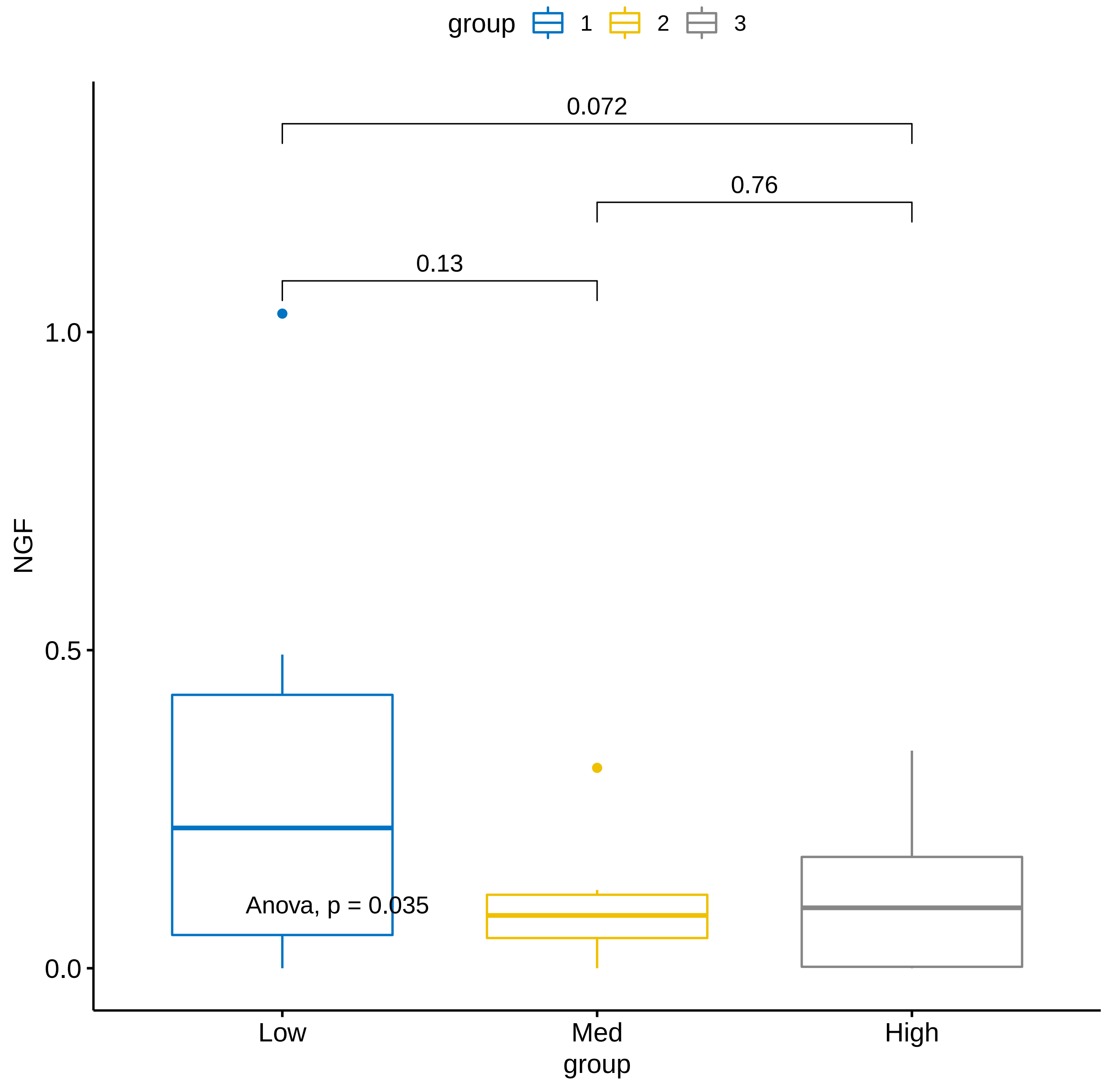

group
1
2
3
0.072
0.76
0.13
1.0
0.5
0.0
NGF
Anova, p = 0.035
Low
Med
High
group

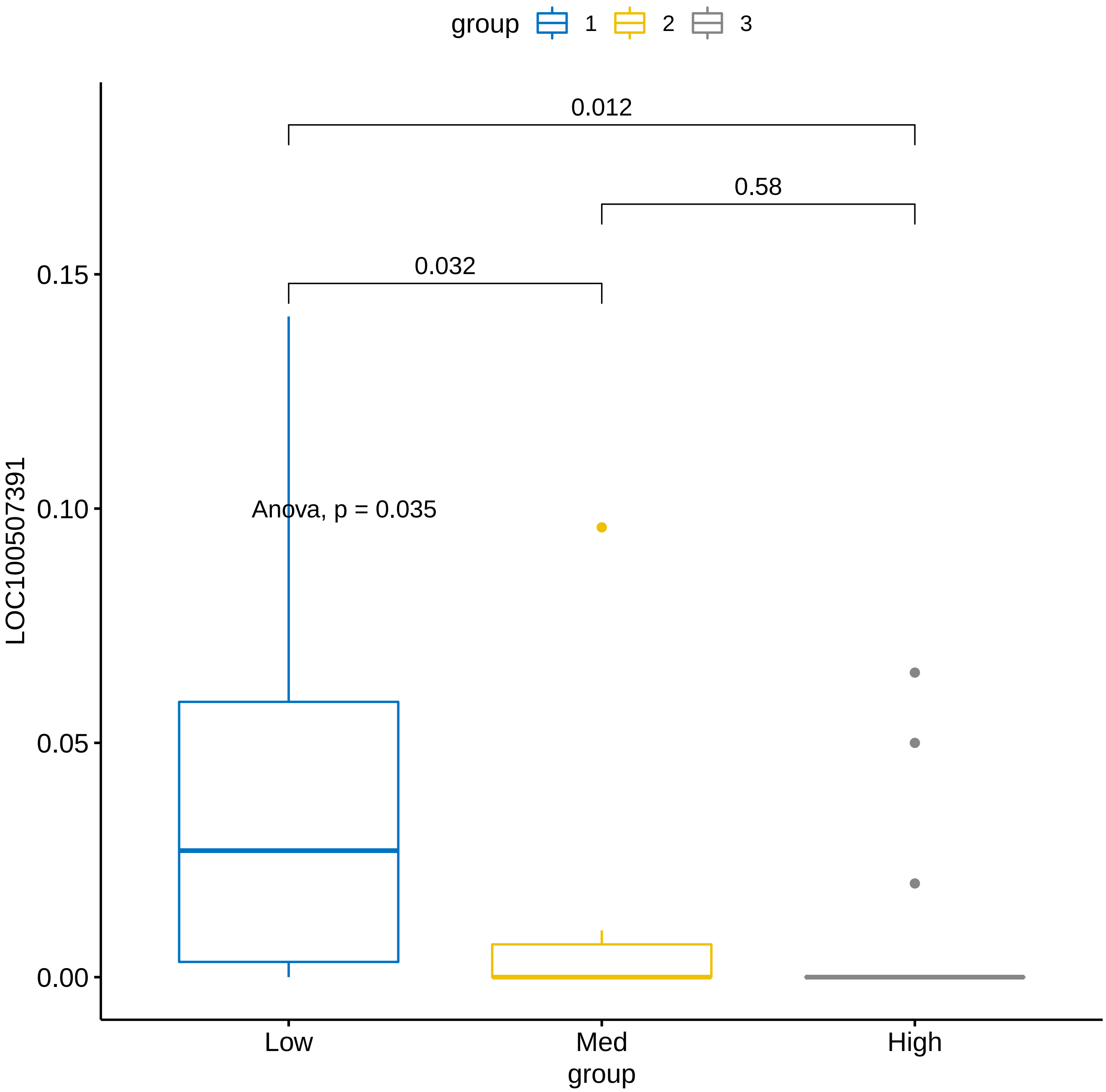
group
1
2
3
0.012
0.58
0.032
Anova, p = 0.035
0.15
0.10
0.05
0.00
LOC100507391
Low
Med
High
group

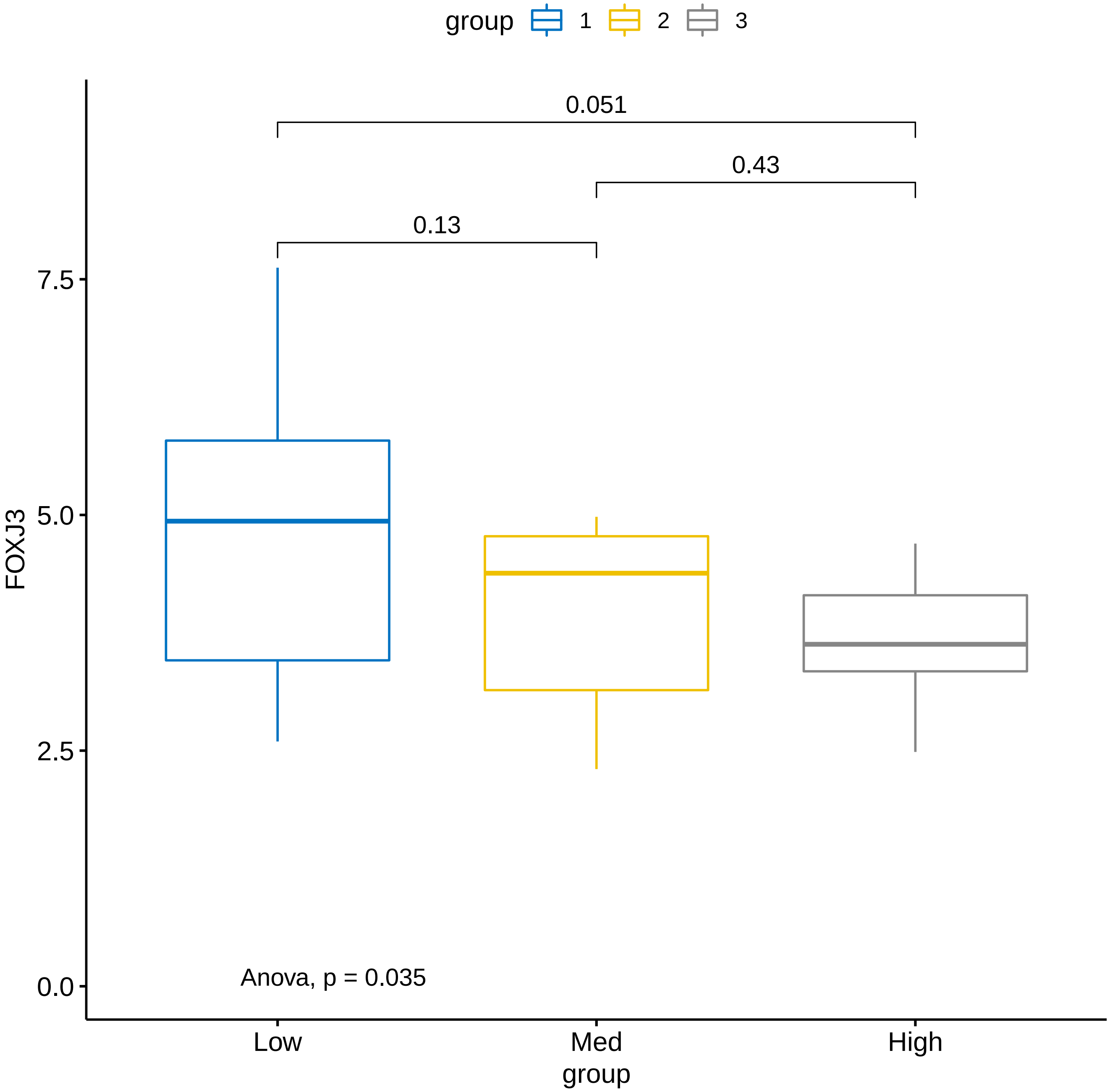
group
1
2
3
0.051
0.43
0.13
7.5
5.0
2.5
0.0
FOXJ3
Anova, p = 0.035
Low
Med
High
group

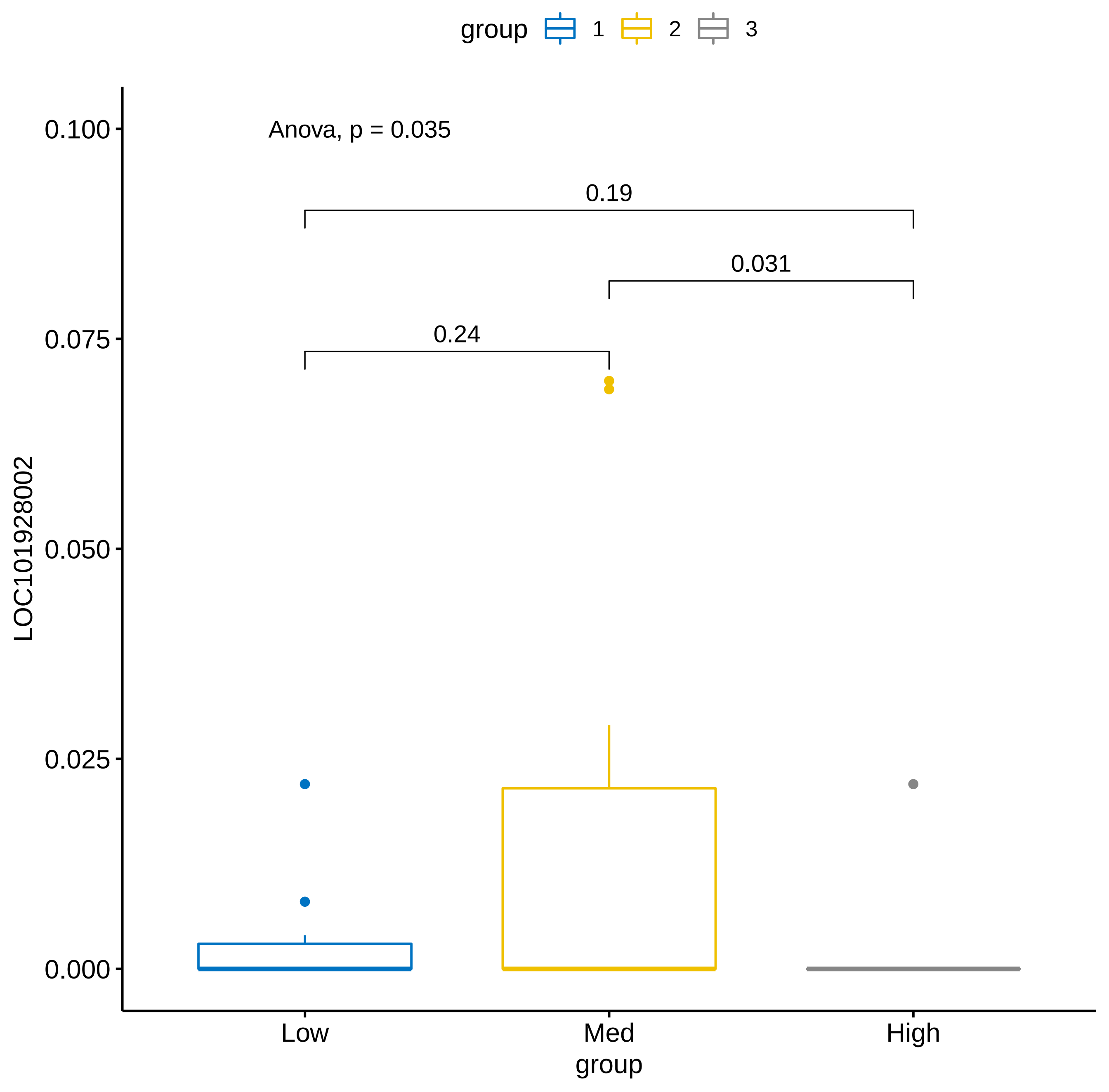

group 1 2 3
Anova, p = 0.035
0.19
0.031
0.24
0.100
0.075
0.050
0.025
0.000
LOC101928002
Low
Med
High
group

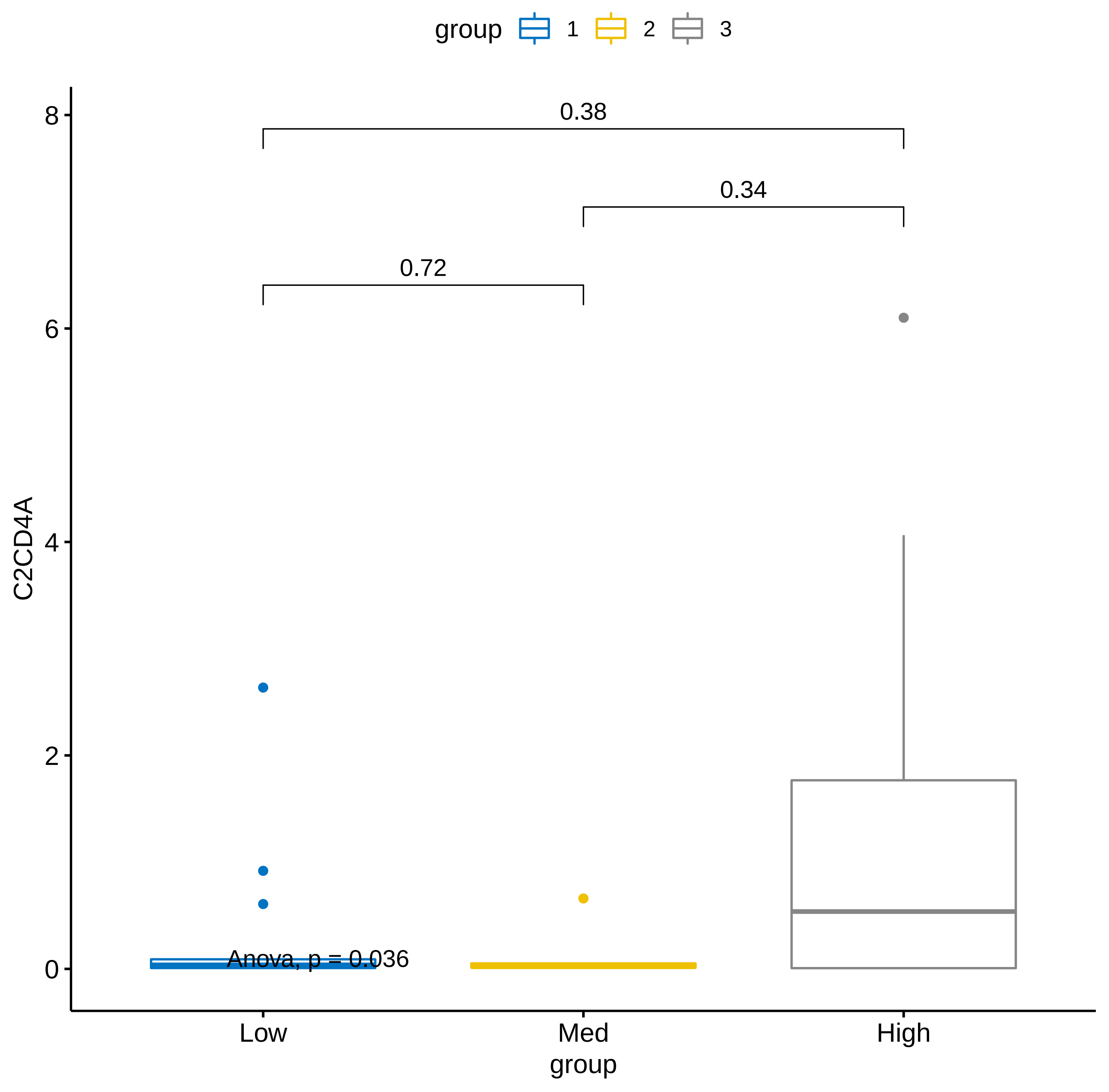
group
1
2
3
0.38
0.34
0.72
8
6
4
2
0
C2CD4A
Anova, p = 0.036
Low
Med
High
group

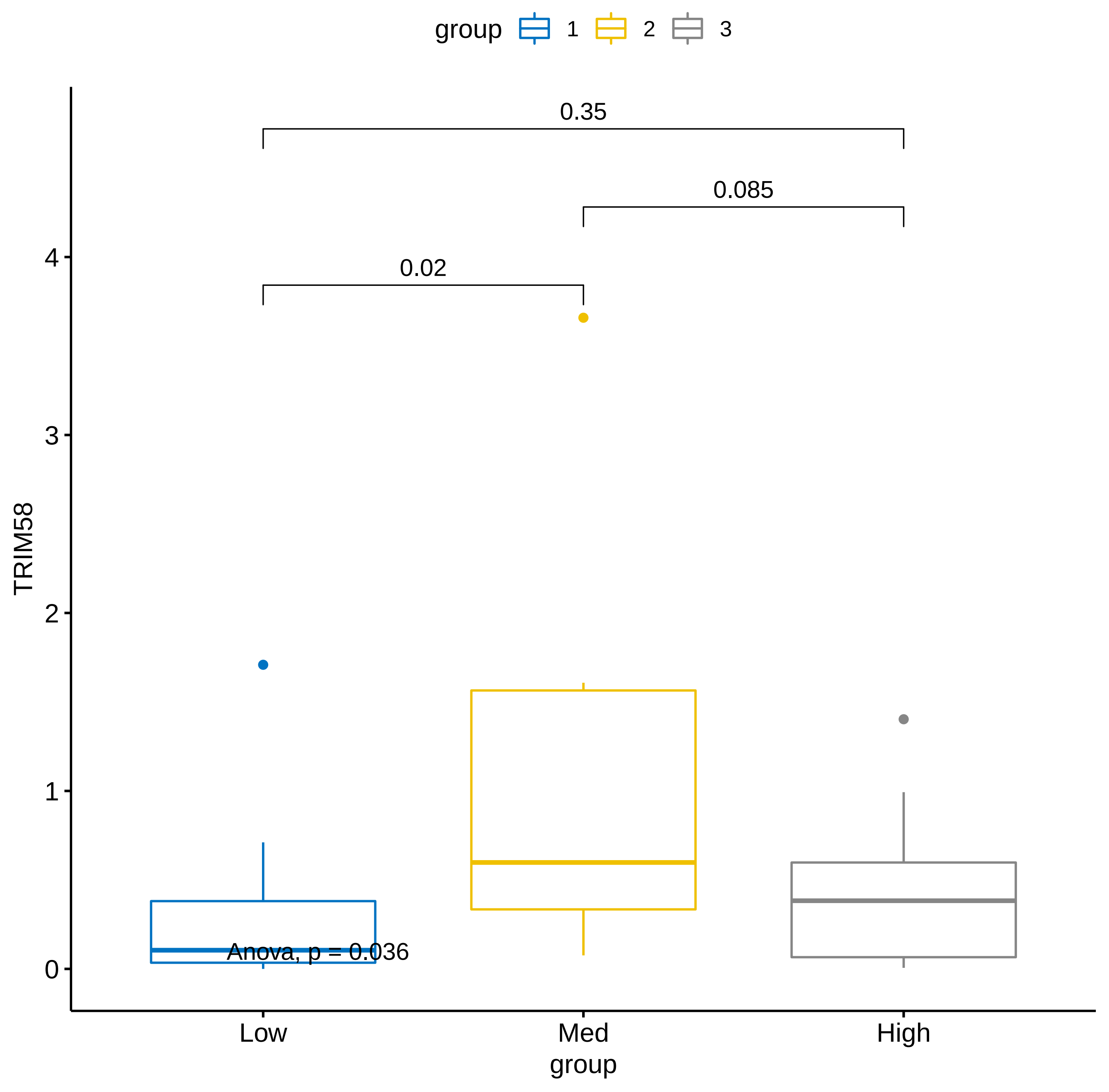
group 1 2 3
0.35
0.085
0.02
TRIM58
4
3
2
1
0
Anova, p = 0.036
Low
Med
High
group

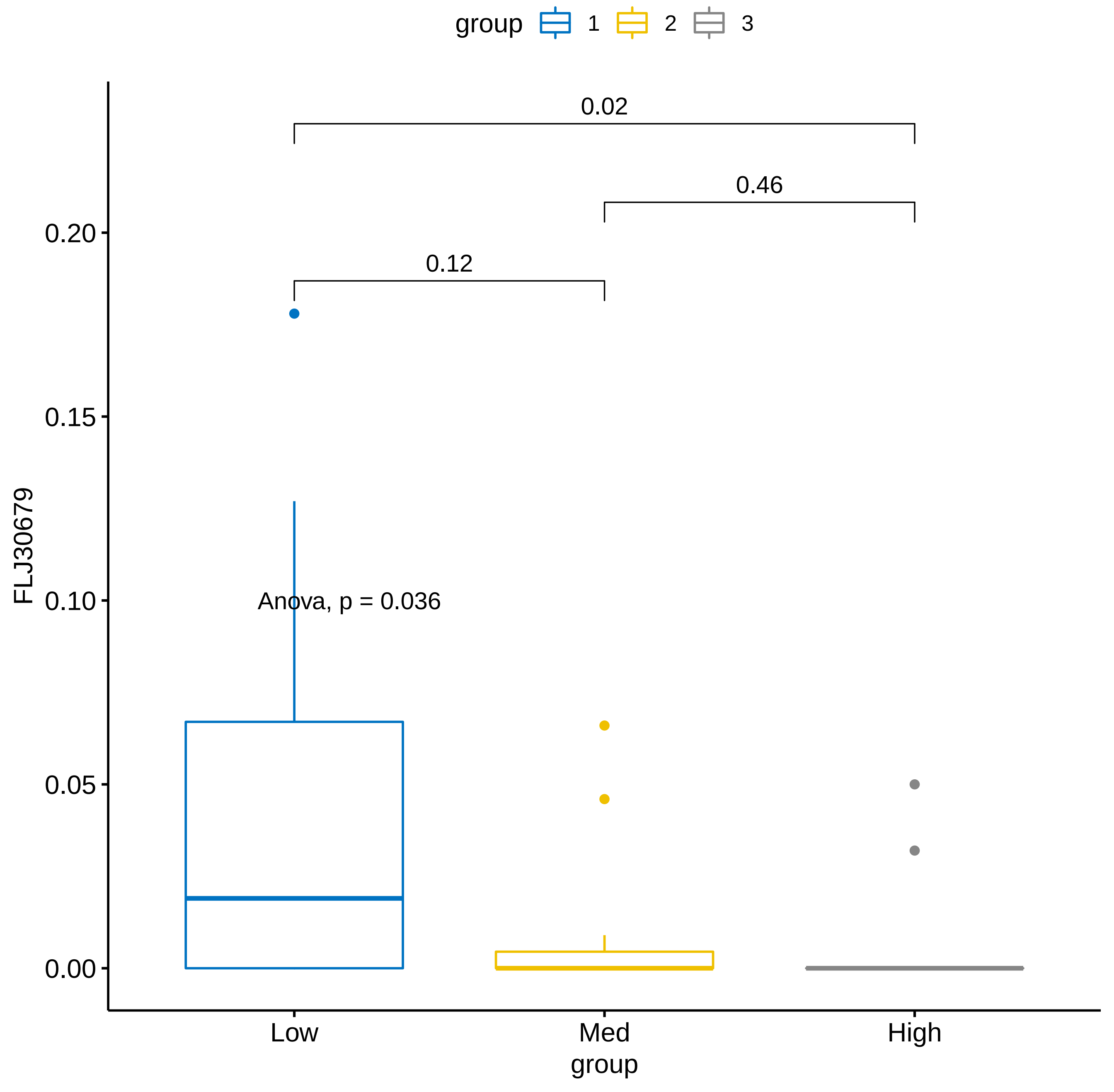

group
1
2
3
0.02
0.46
0.12
Anova, p = 0.036
FLJ30679
0.20
0.15
0.10
0.05
0.00
Low
Med
High
group

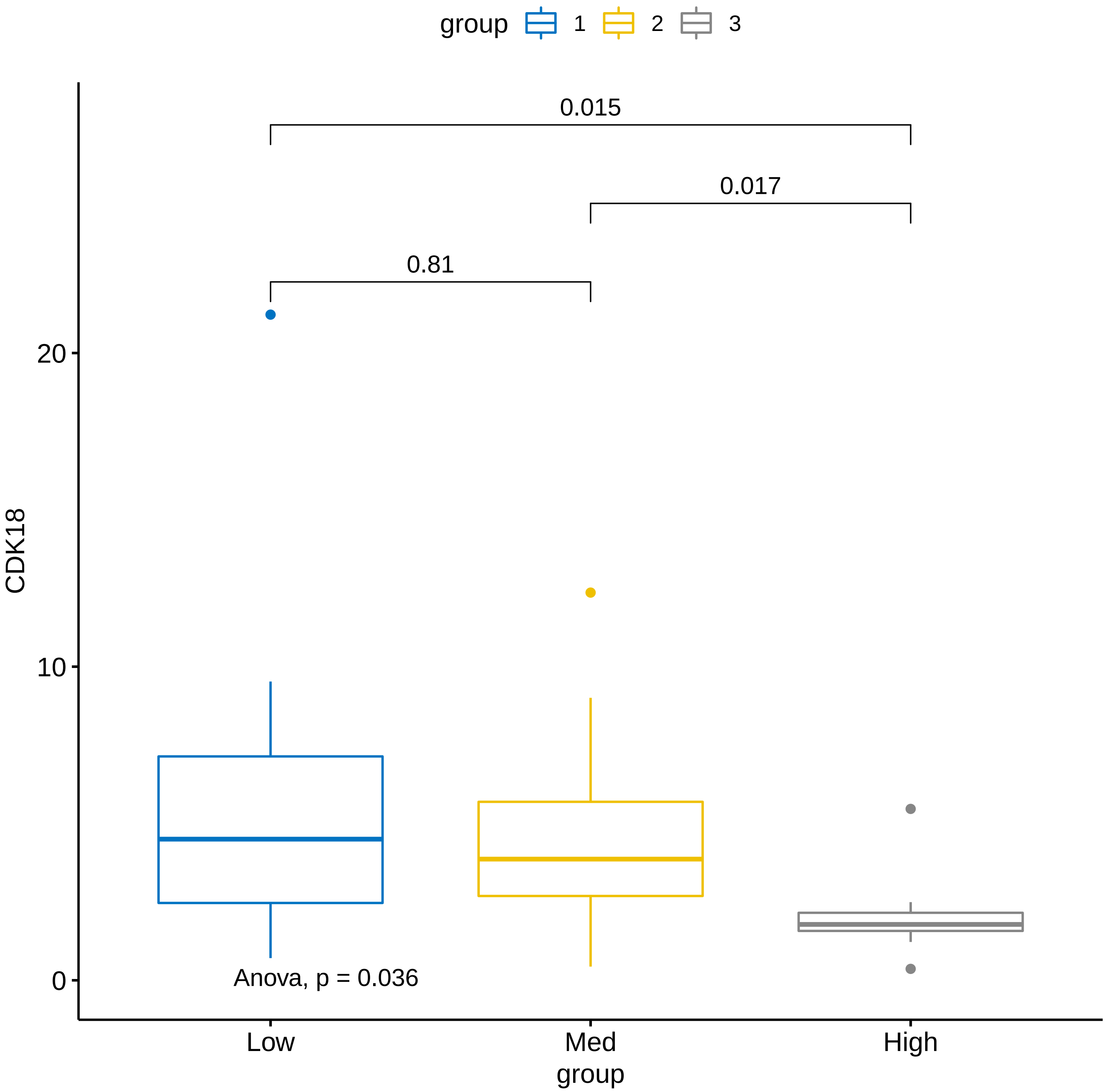

group
1
2
3
0.015
0.017
0.81
CDK18
20
10
0
Anova, p = 0.036
Low
Med
High
group

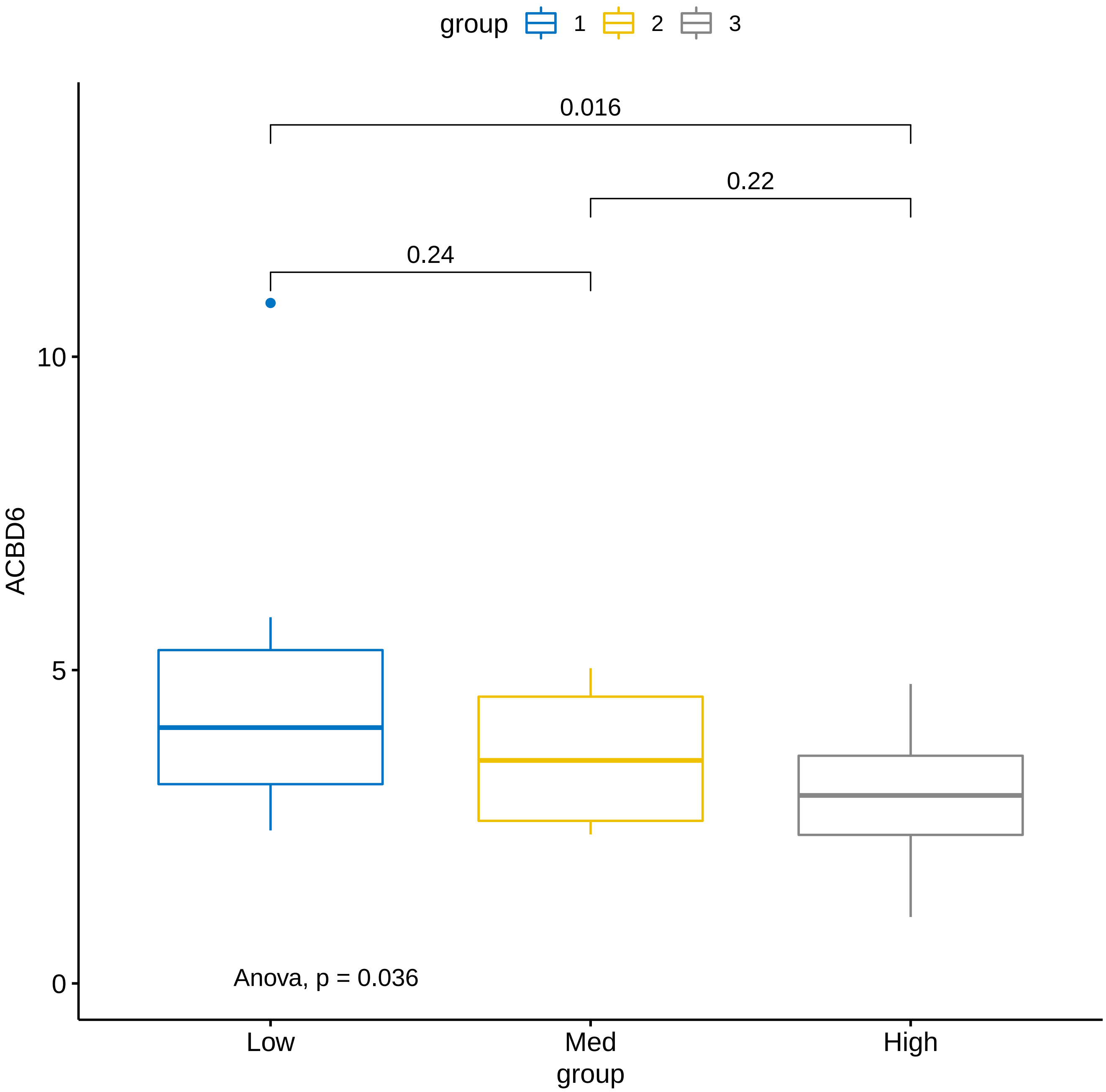
group 1 2 3
0.016
0.22
0.24
ACBD6
10
5
0
Anova, p = 0.036
Low
Med
High
group

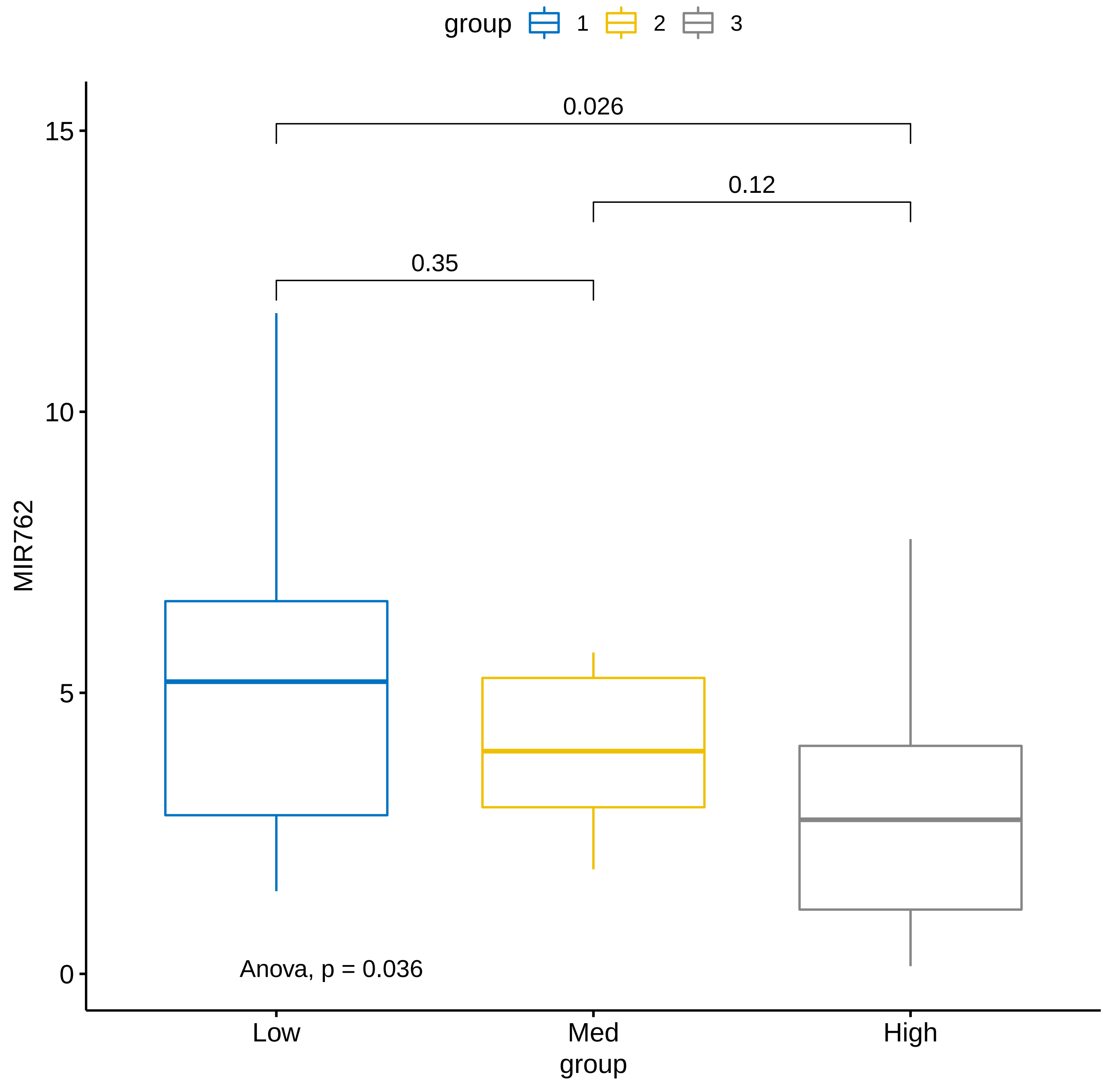
group
1
2
3
0.026
0.12
0.35
15
10
5
0
MIR762
Anova, p = 0.036
Low
Med
High
group

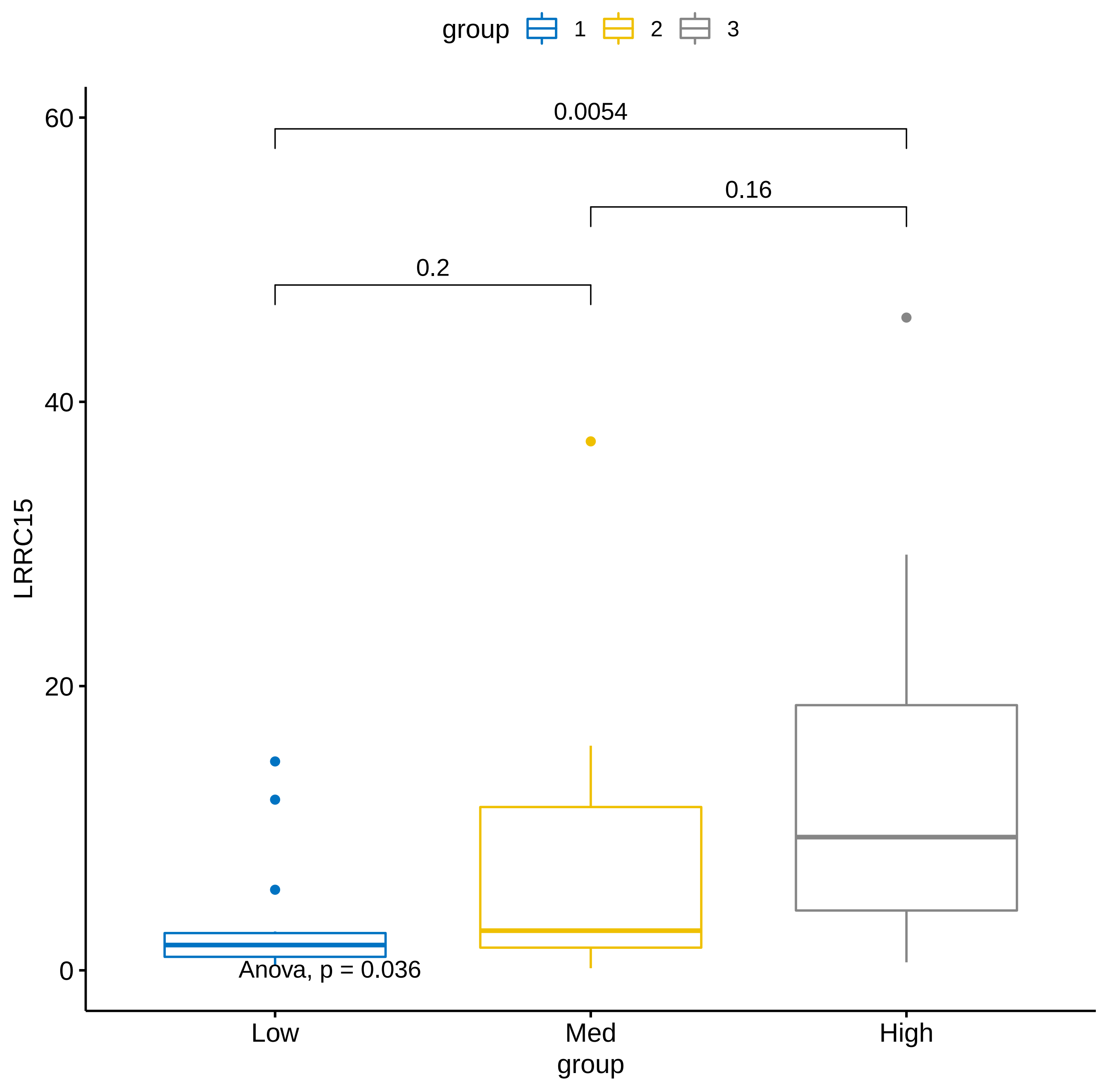
group
1
2
3
0.0054
0.16
0.2
60
40
20
0
LRRC15
Anova, p = 0.036
Low
Med
High
group

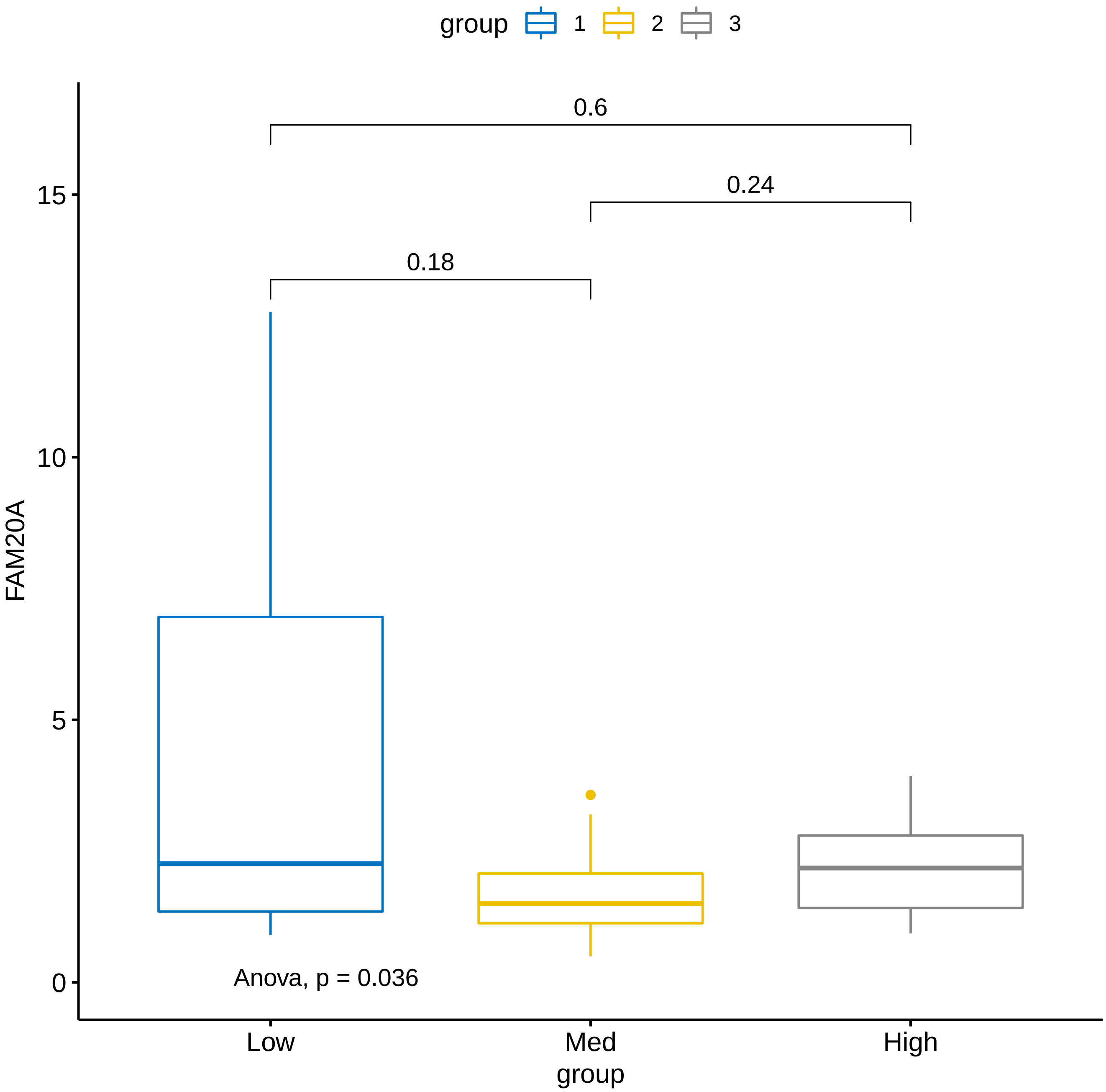
group
1
2
3
0.6
0.24
0.18
15
10
5
0
FAM20A
Anova, p = 0.036
Low
Med
High
group

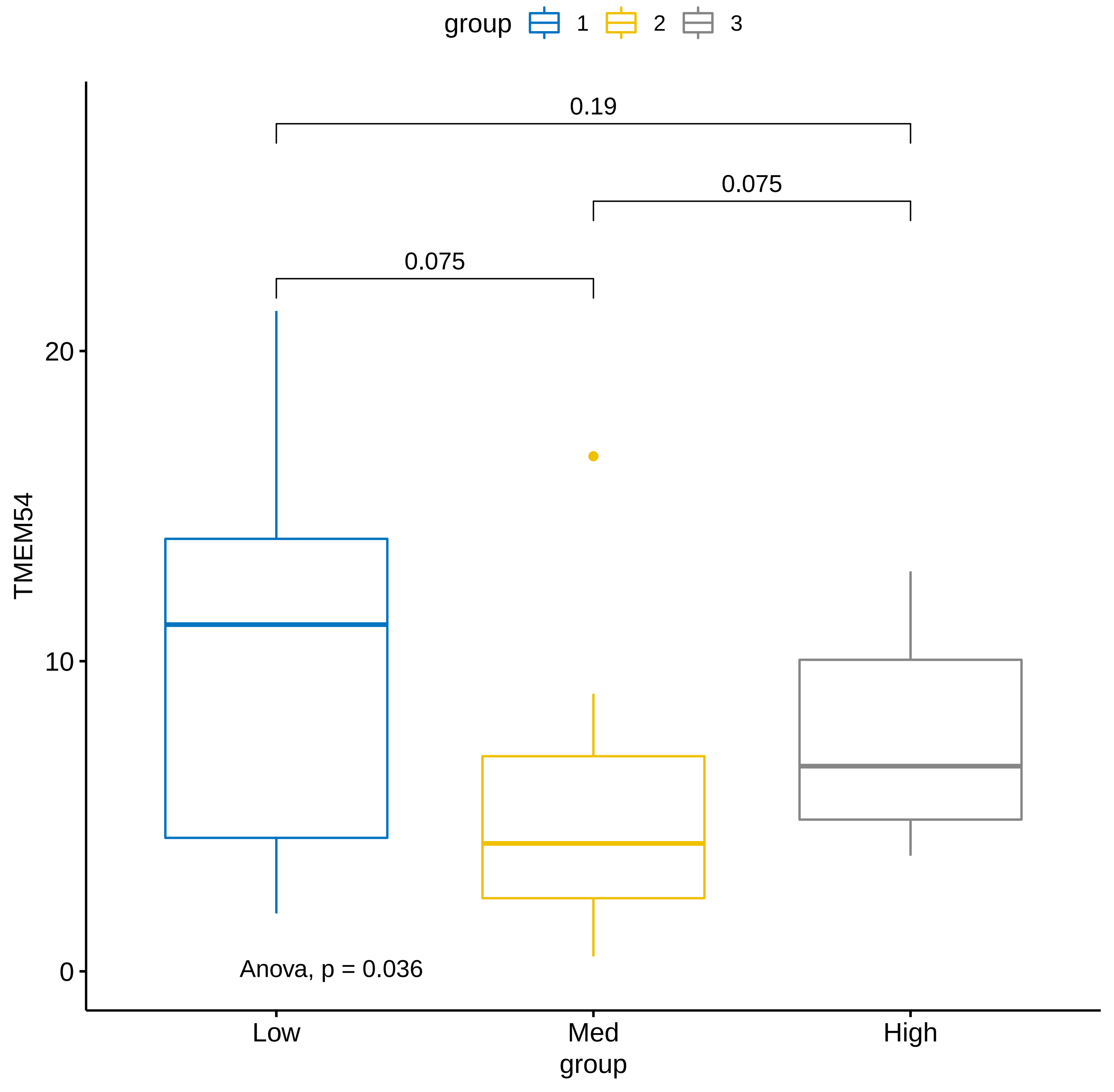
group
1
2
3
0.19
0.075
0.075
20
10
0
TMEM54
Anova, p = 0.036
Low
Med
High
group

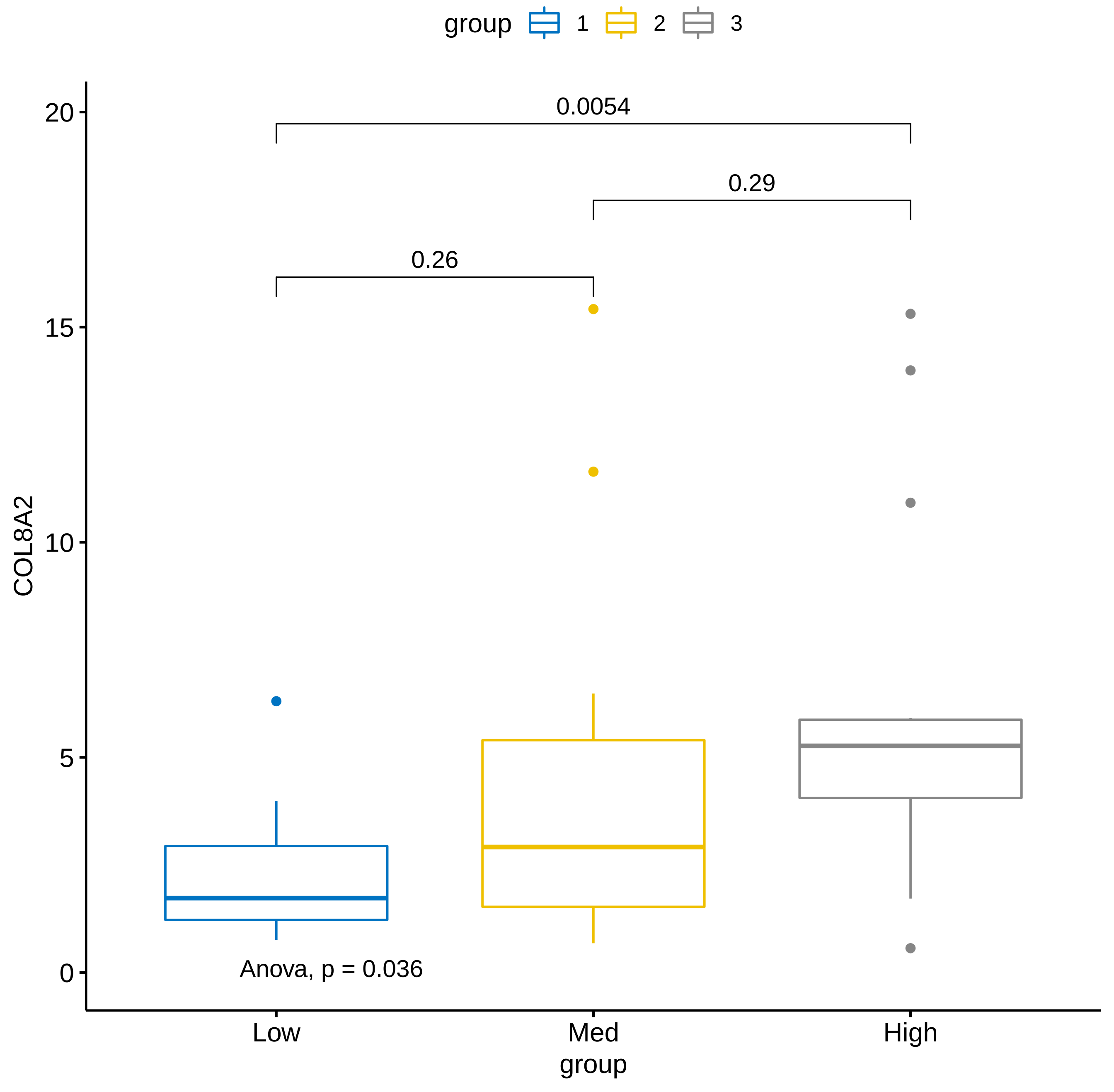

group
1
2
3
0.0054
0.29
0.26
Anova, p = 0.036
COL8A2
20
15
10
5
0
Low
Med
High
group

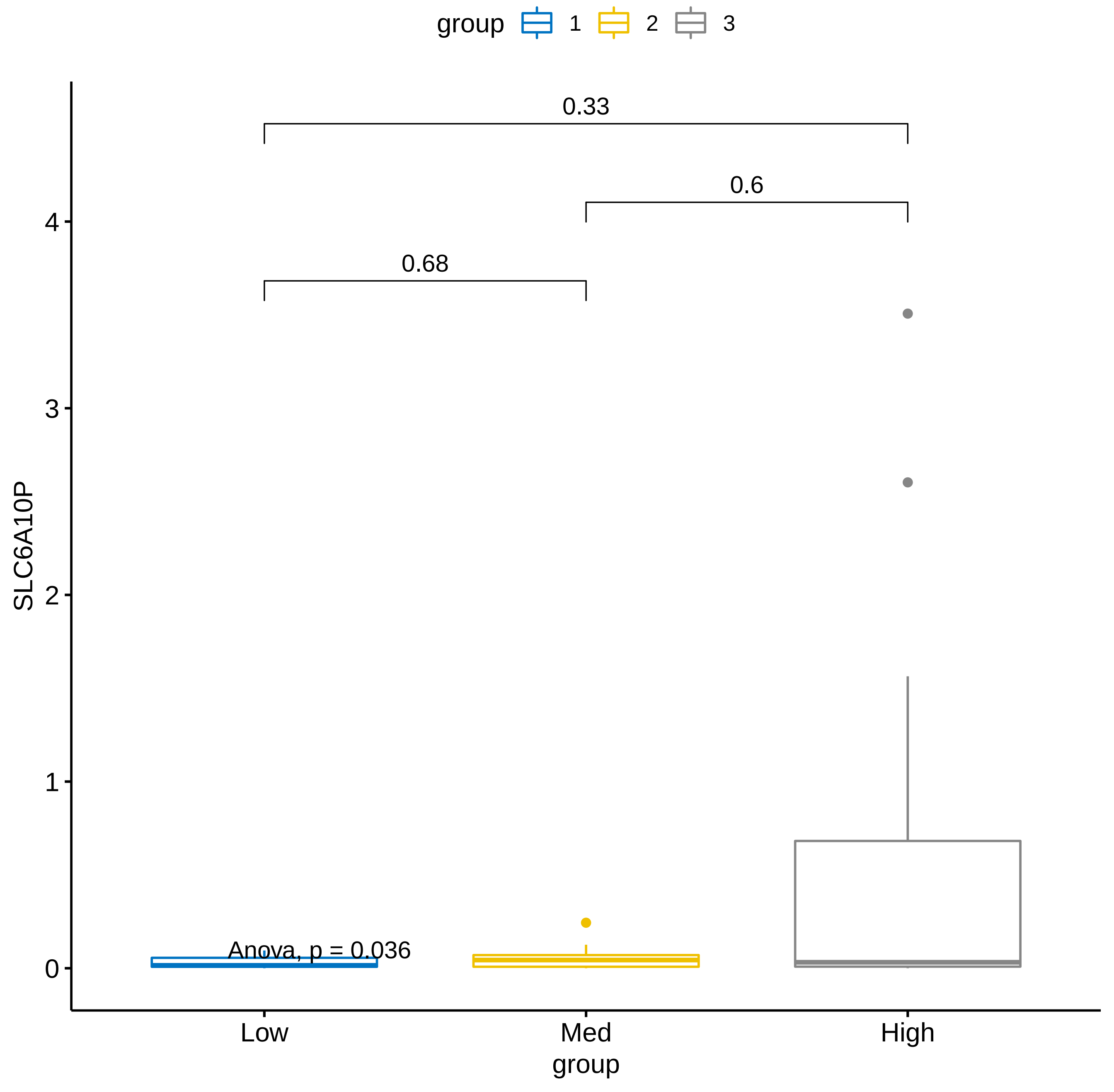

group 1 2 3
0.33
0.6
0.68
4
3
2
1
0
SLC6A10P
Anova, p = 0.036
Low
Med
High
group

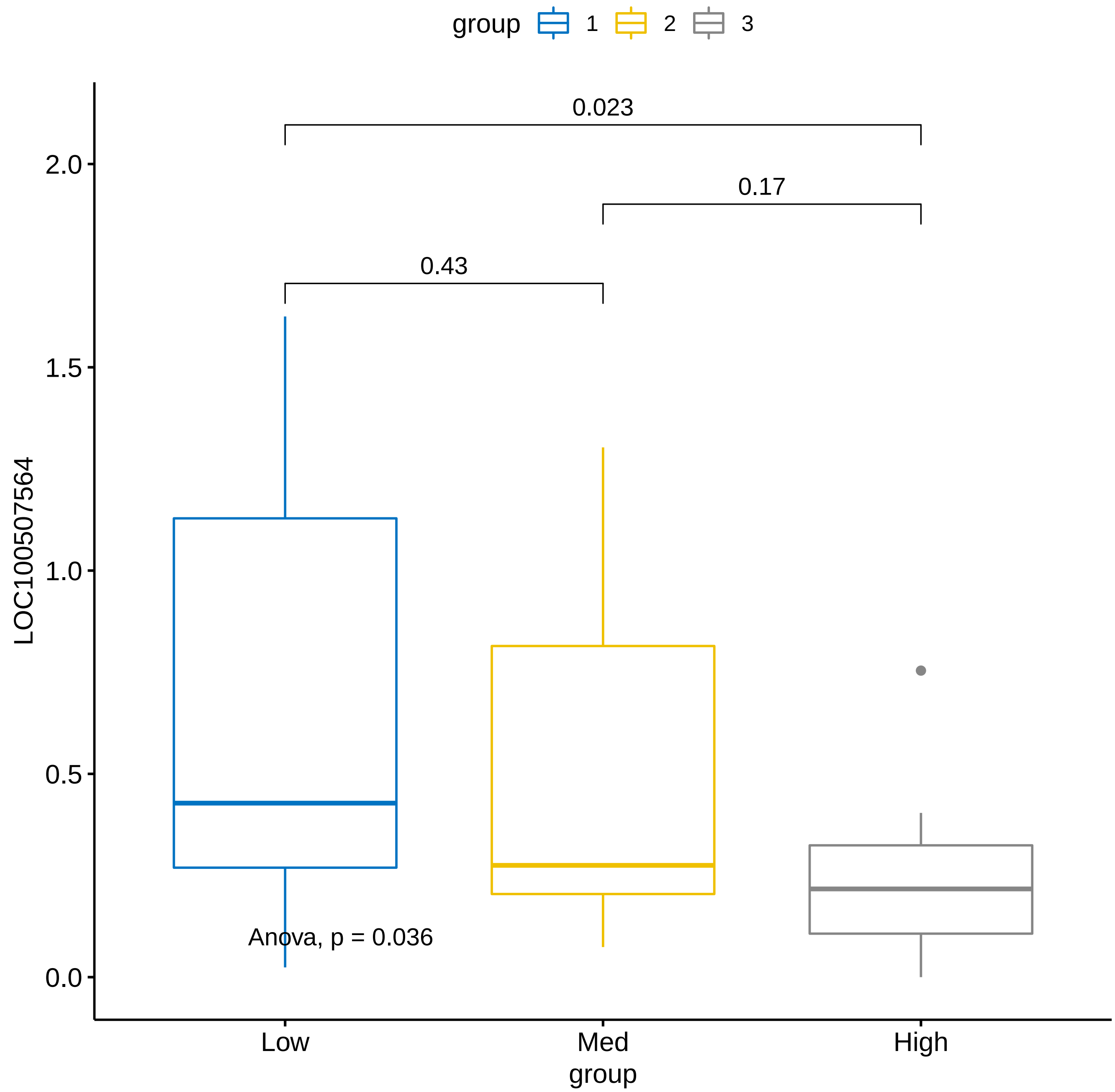

group
1
2
3
0.023
0.17
0.43
2.0
1.5
1.0
0.5
0.0
LOC100507564
Anova, p = 0.036
Low
Med
High
group

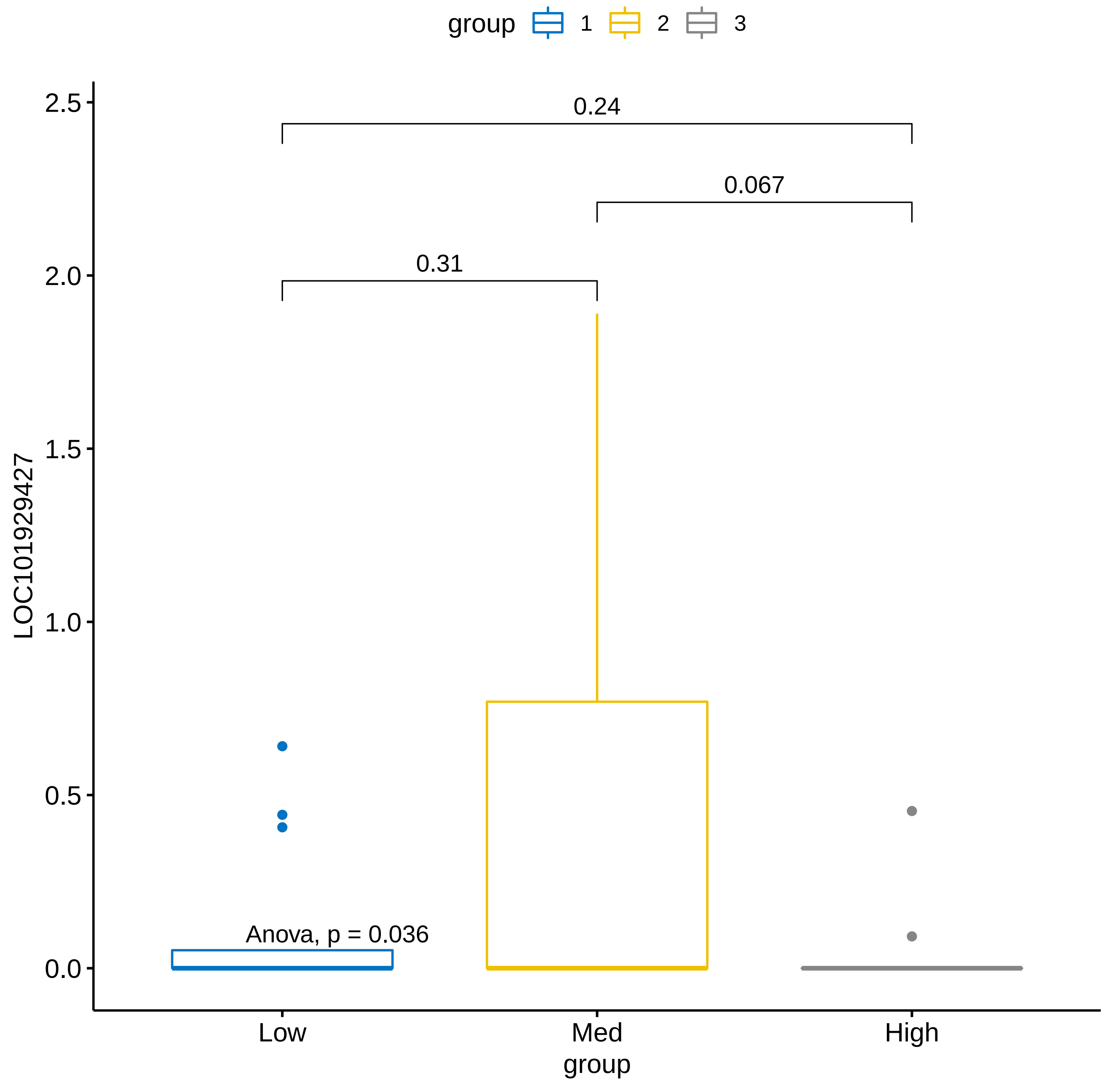

group 1 2 3
2.5
2.0
1.5
1.0
0.5
0.0
LOC101929427
0.24
0.067
0.31
Anova, p = 0.036
Low
Med
High
group

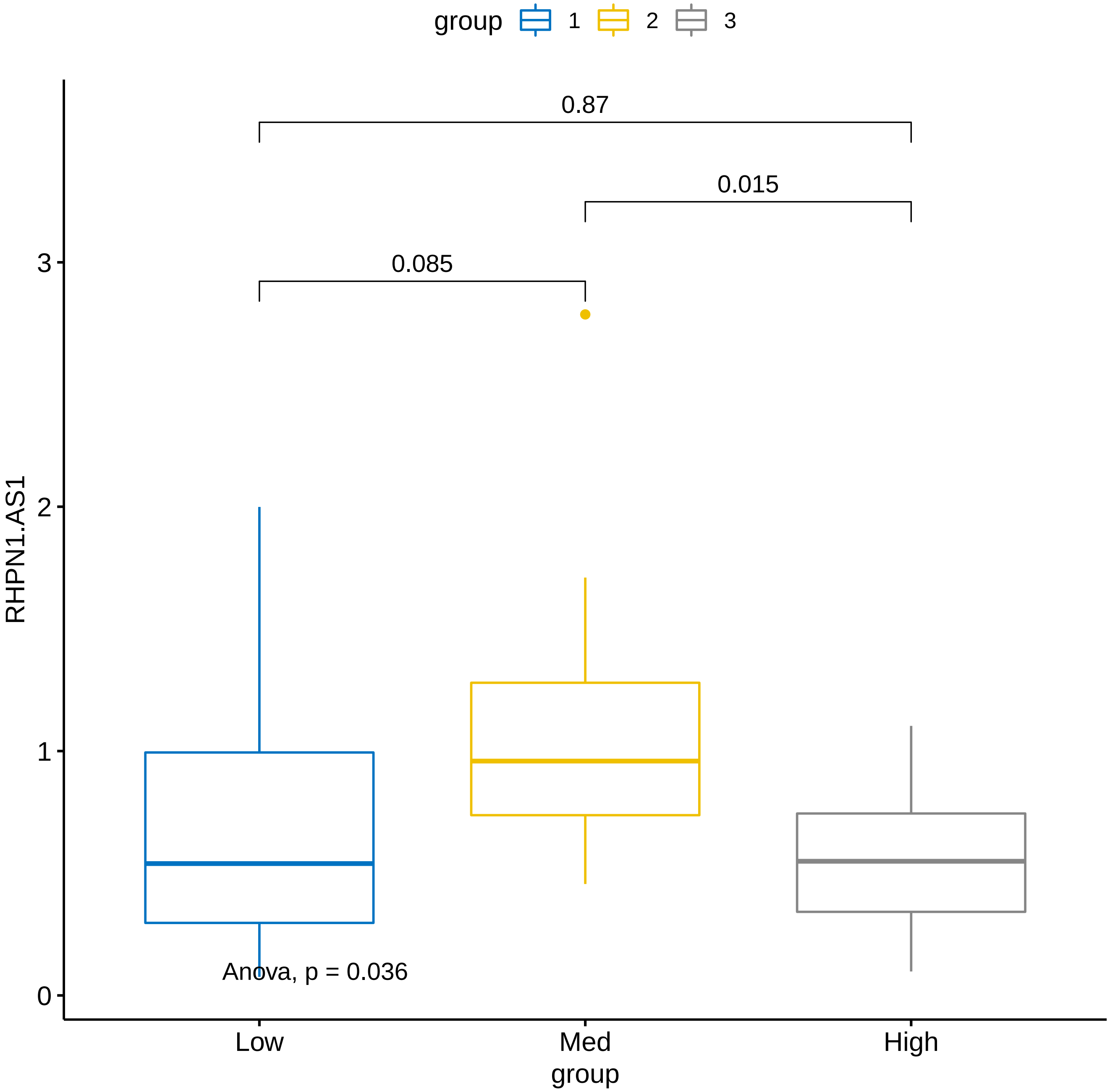

group
1
2
3
0.87
0.015
0.085
RHPN1.AS1
3
2
1
0
Anova, p = 0.036
Low
Med
High
group

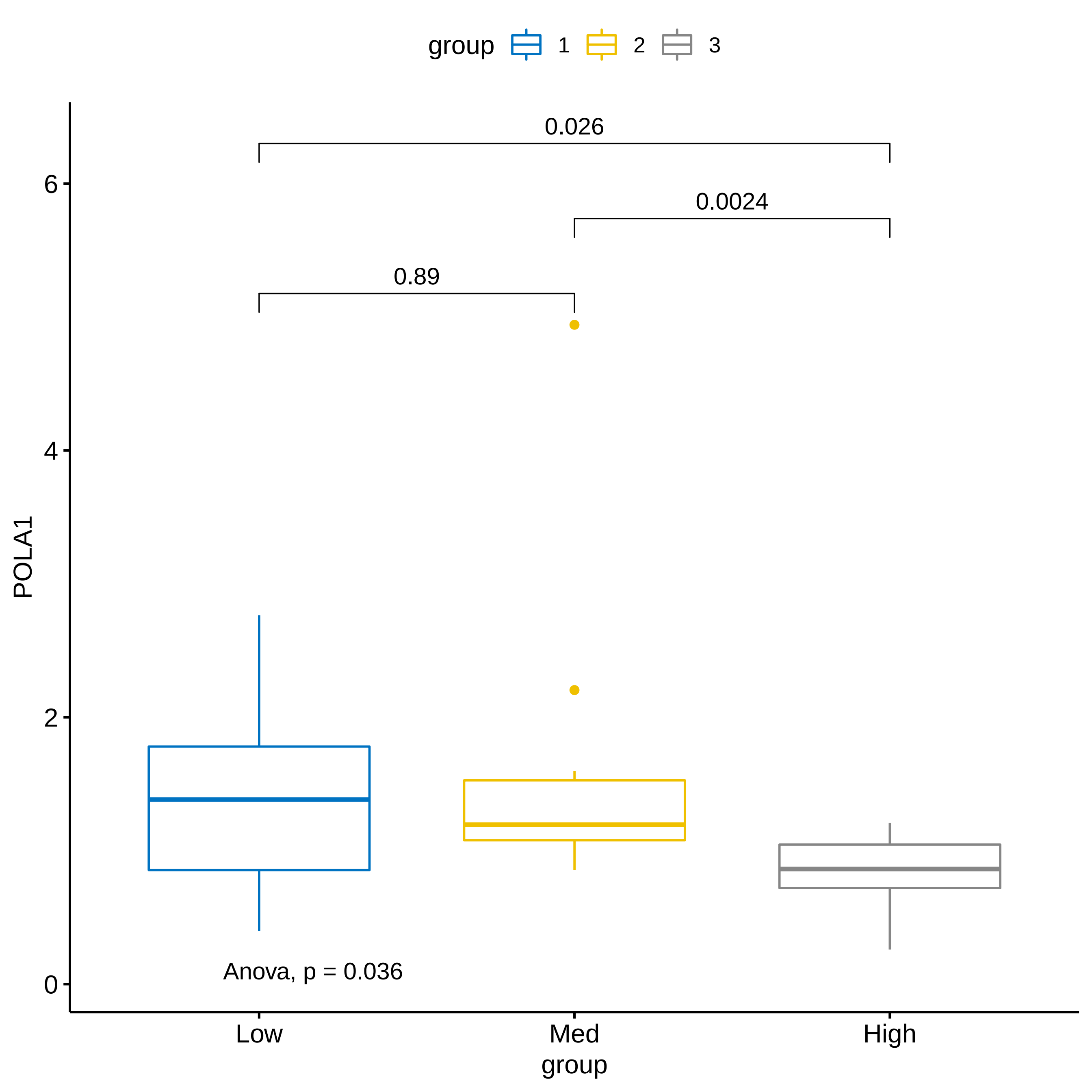

group 1 2 3
0.026
0.0024
0.89
6
4
2
0
POLA1
Anova, p = 0.036
Low
Med
High
group

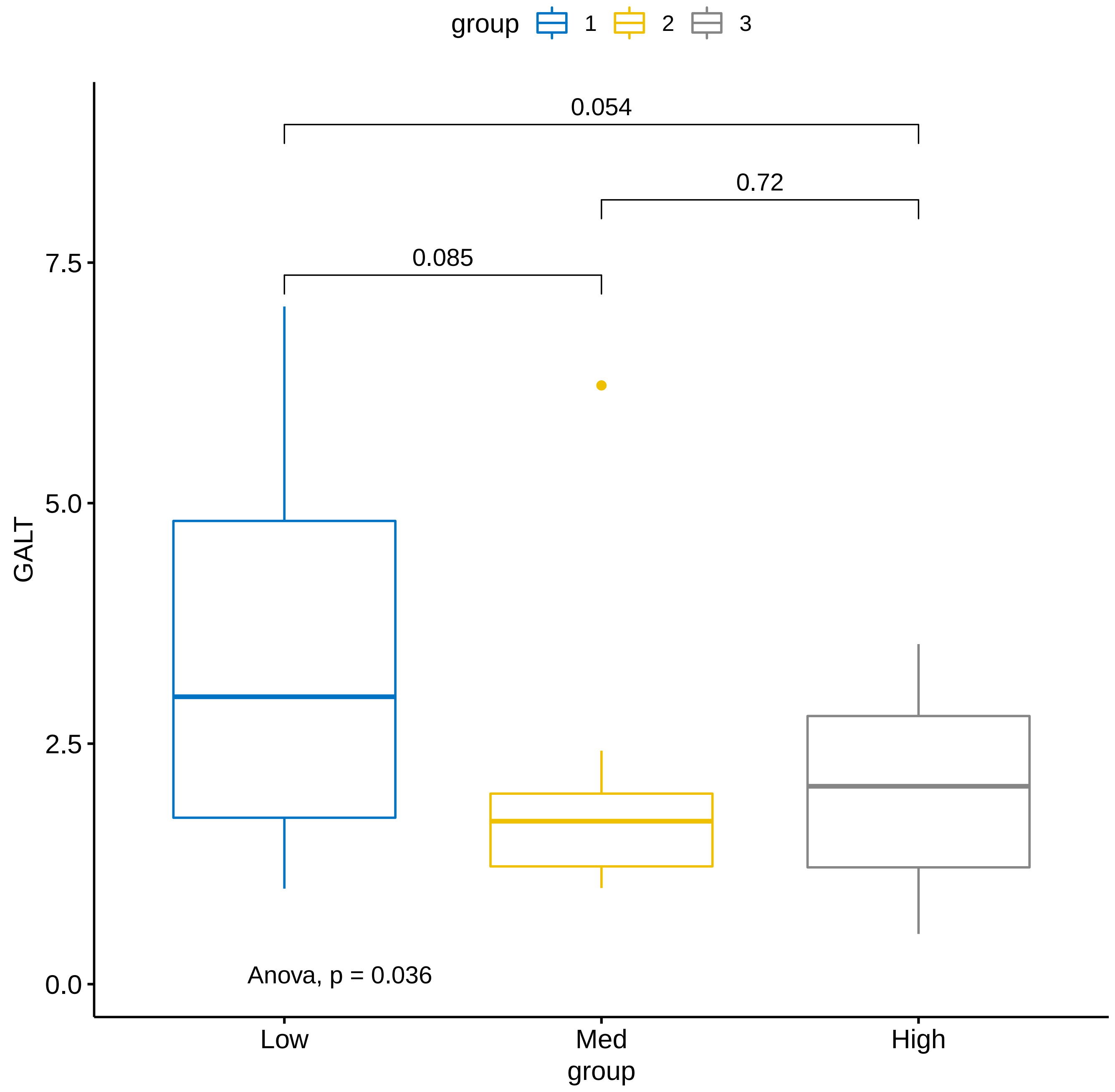
group 1 2 3
0.054
0.72
0.085
7.5
5.0
2.5
0.0
GALT
Anova, p = 0.036
Low
Med
High
group

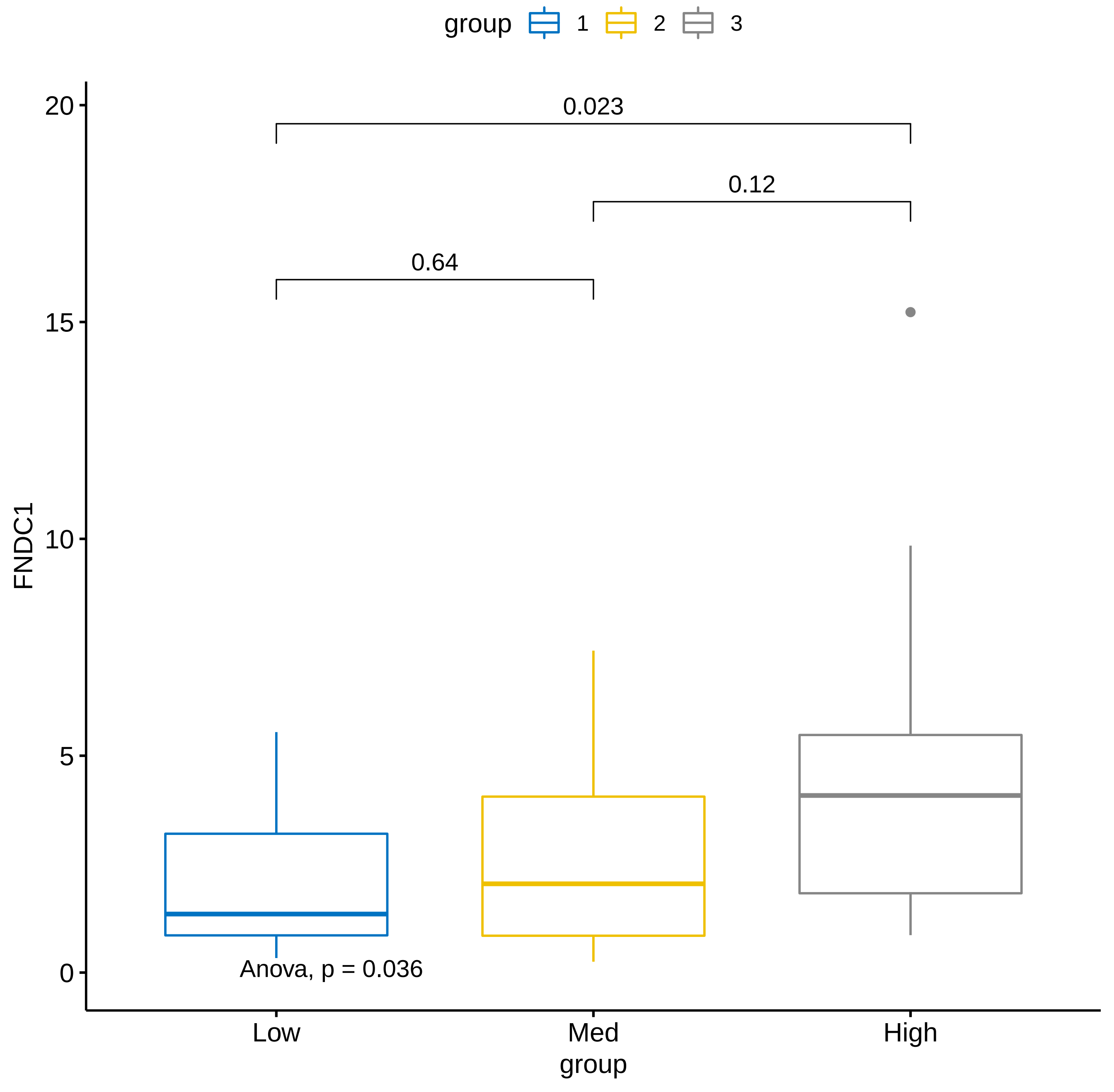
group
1
2
3
0.023
0.12
0.64
20
15
10
5
0
FNDC1
Anova, p = 0.036
Low
Med
High
group

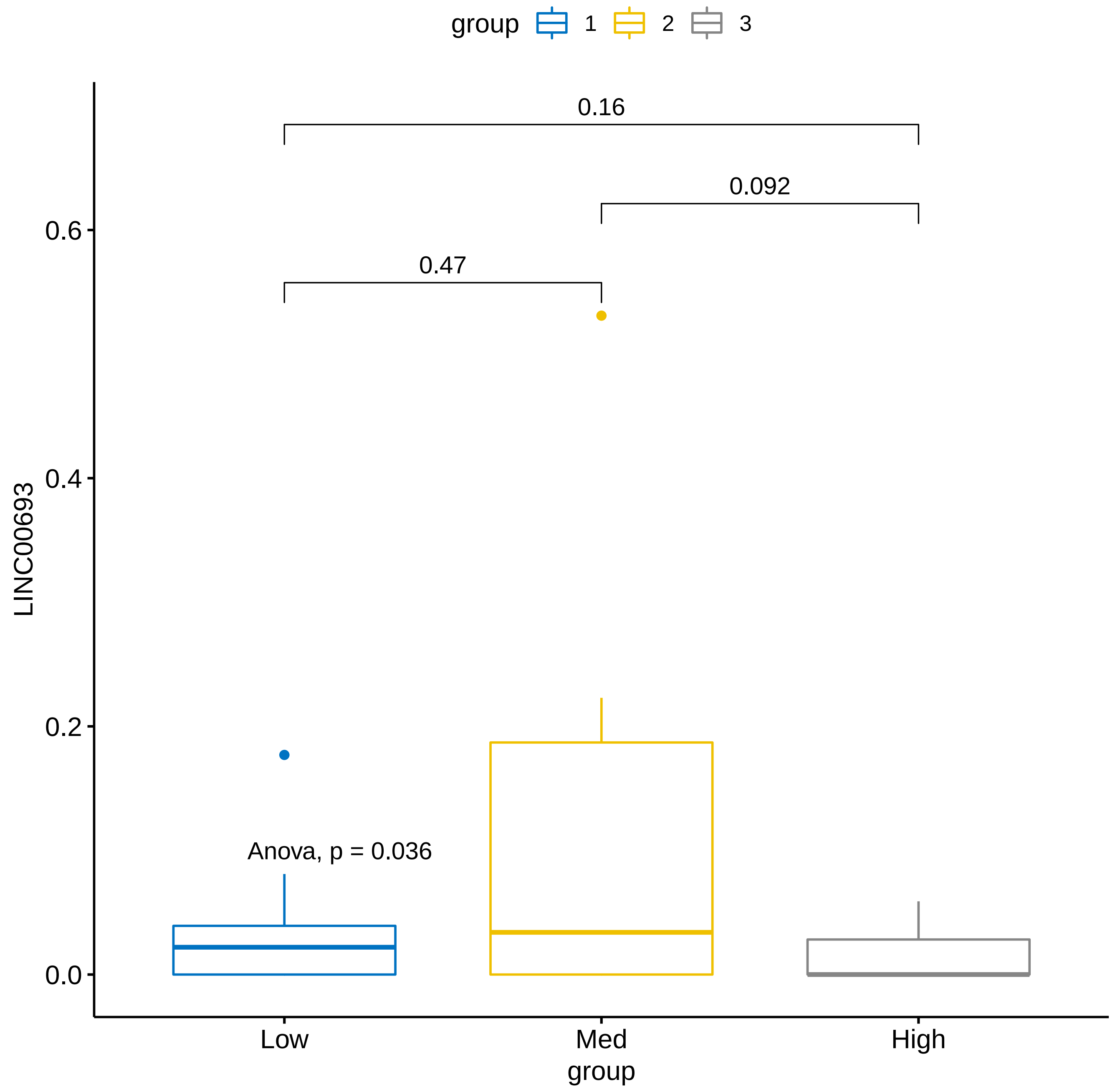
group 1 2 3
0.16
0.092
0.47
Anova, p = 0.036
0.6
0.4
0.2
0.0
LINC00693
Low
Med
High
group

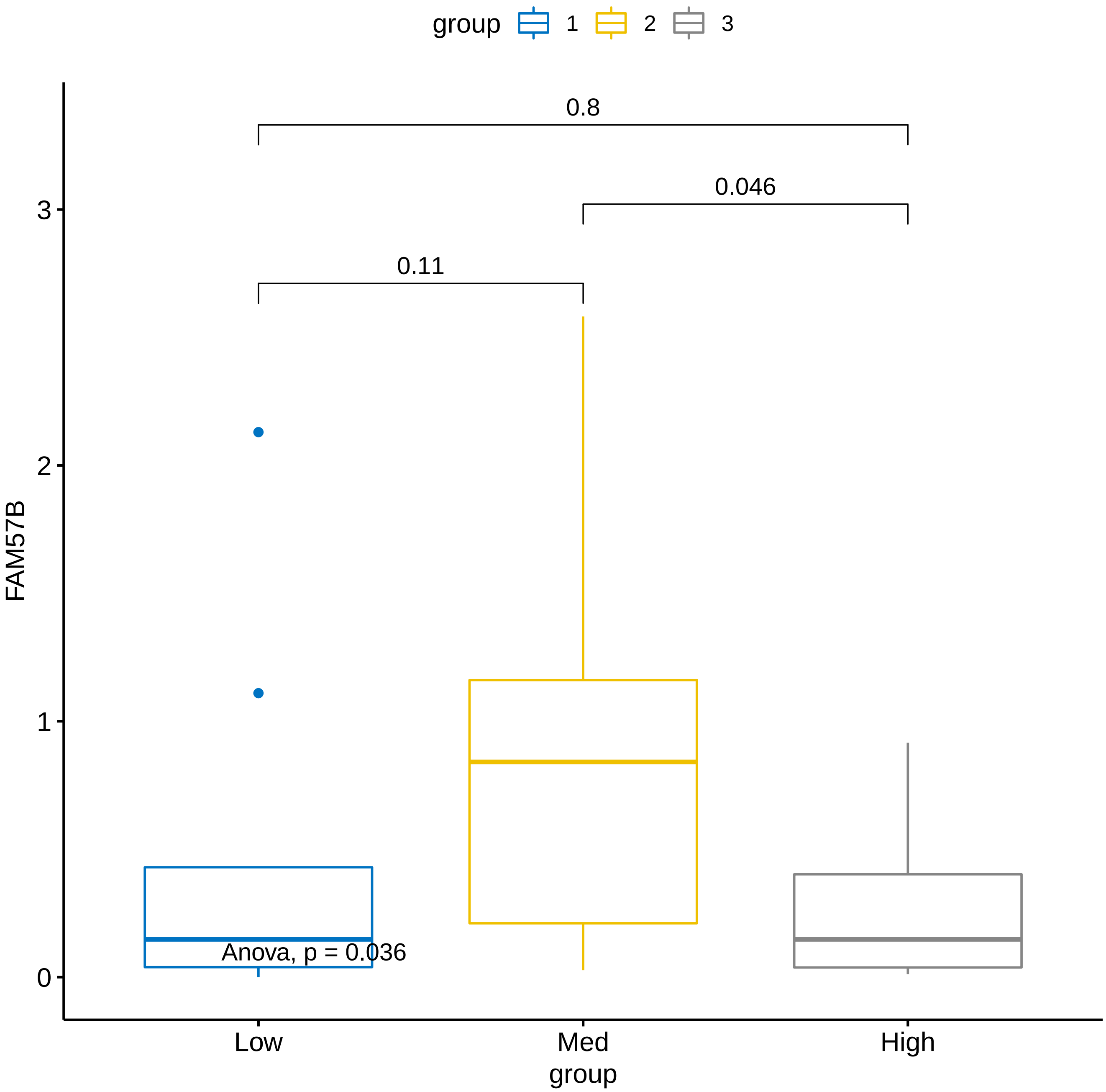
group
1
2
3
0.8
0.046
0.11
Anova, p = 0.036
FAM57B
3
2
1
0
Low
Med
High
group

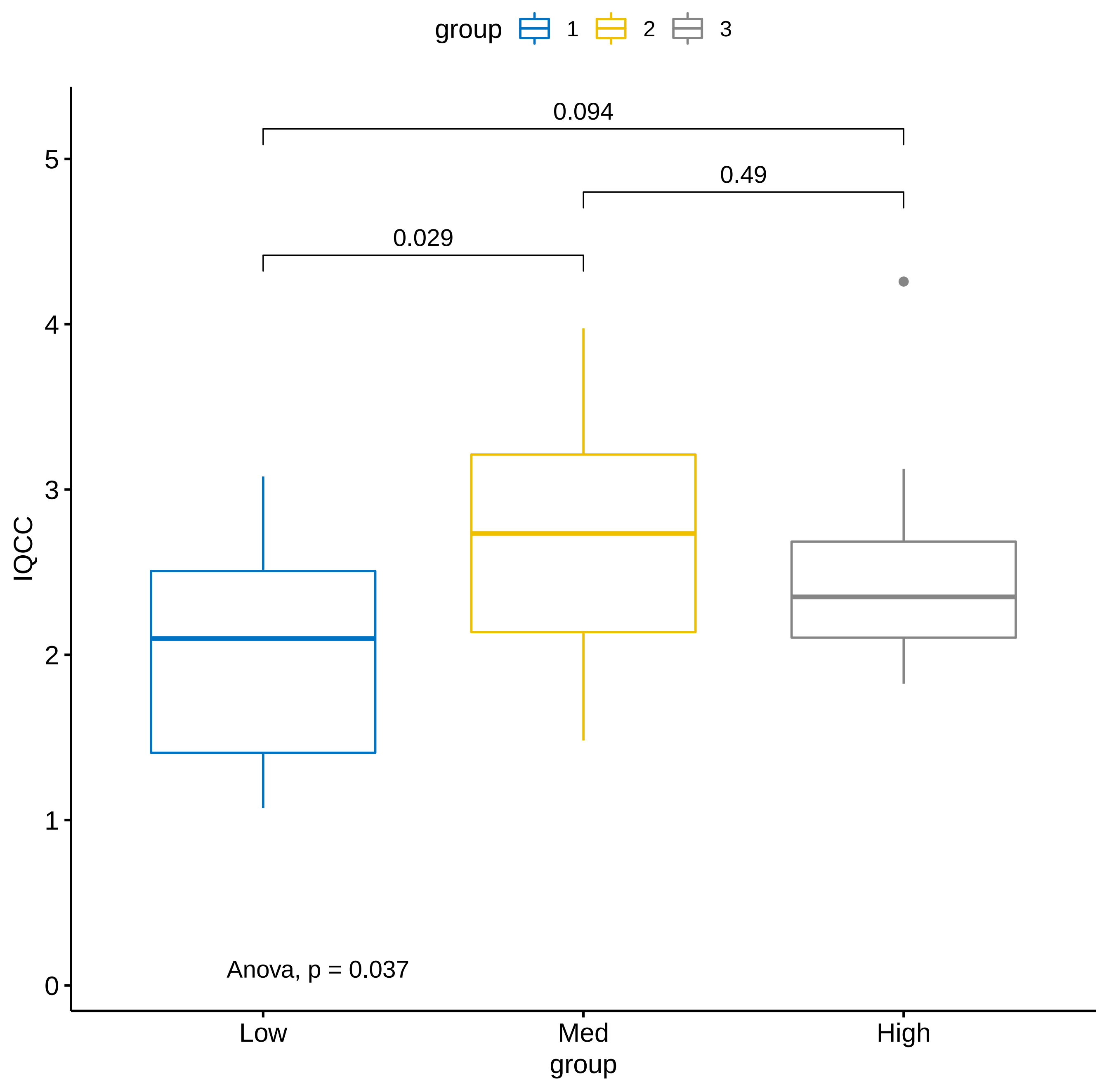

group 1 2 3
0.094
0.49
0.029
5
4
3
2
1
0
IQCC
Anova, p = 0.037
Low
Med
High
group

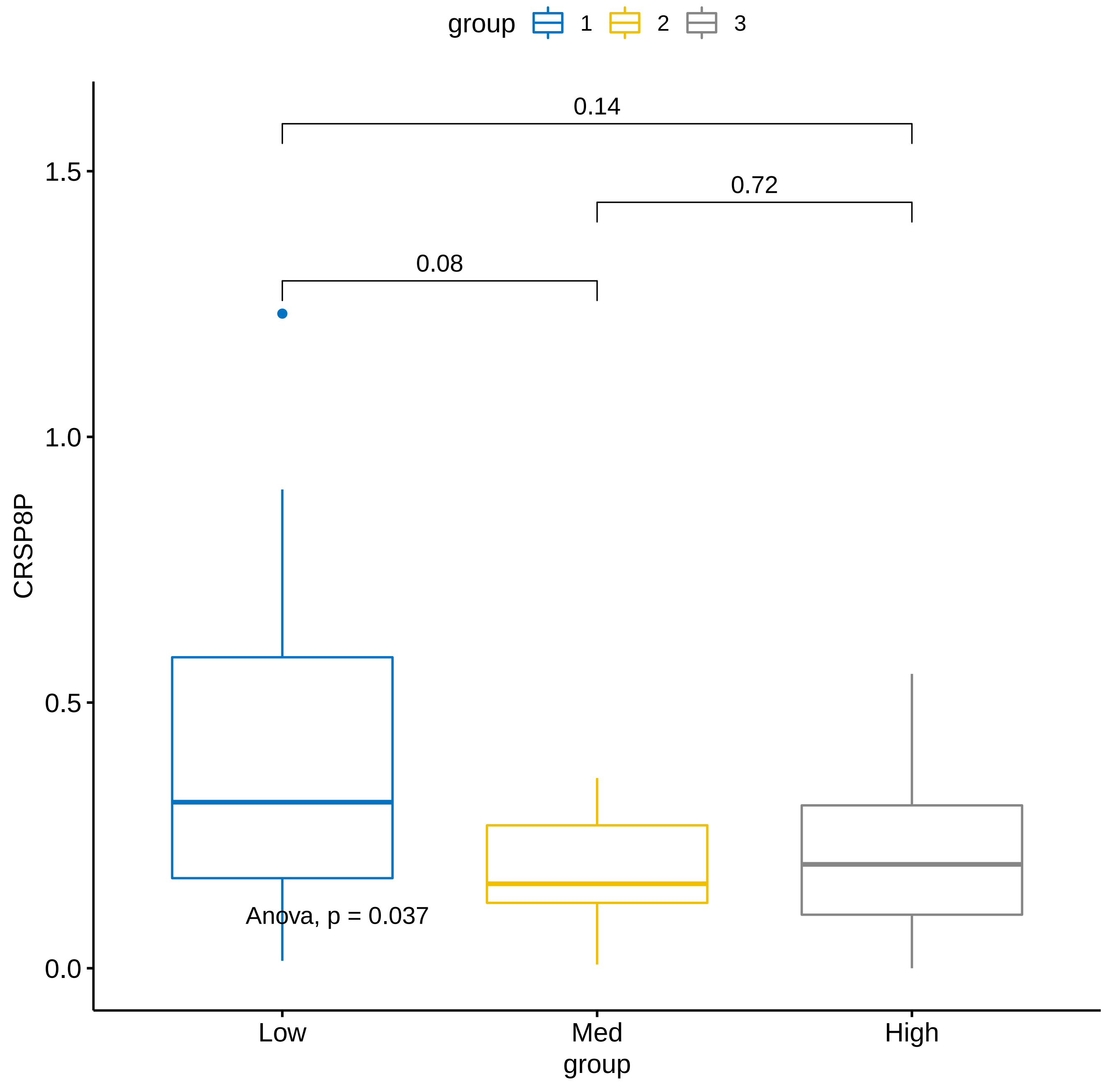
group
1
2
3
0.14
0.72
0.08
1.5
1.0
0.5
0.0
CRSP8P
Anova, p = 0.037
Low
Med
High
group

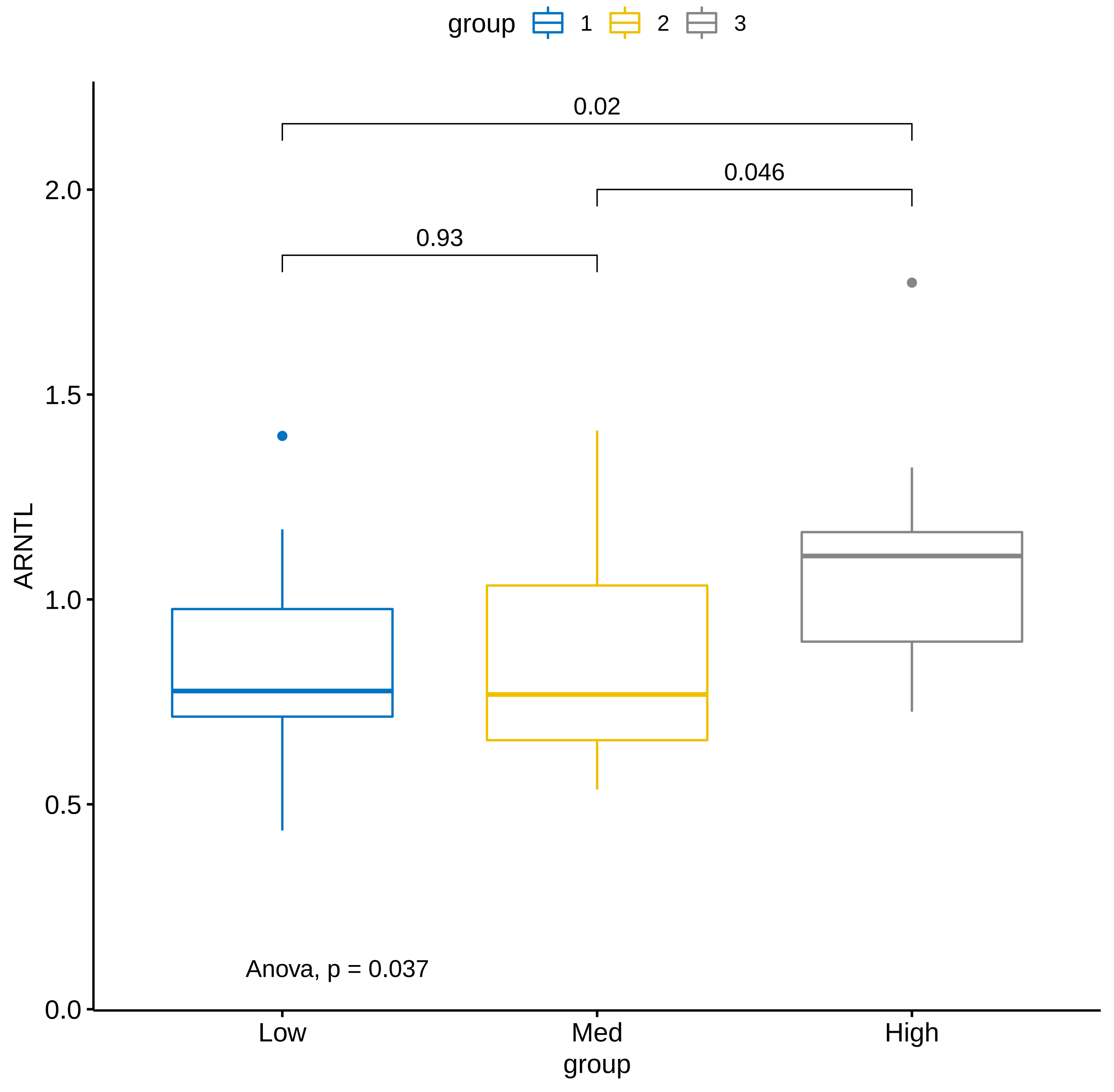

group
1
2
3
0.02
0.046
0.93
2.0
1.5
1.0
0.5
0.0
ARNTL
Anova, p = 0.037
Low
Med
High
group

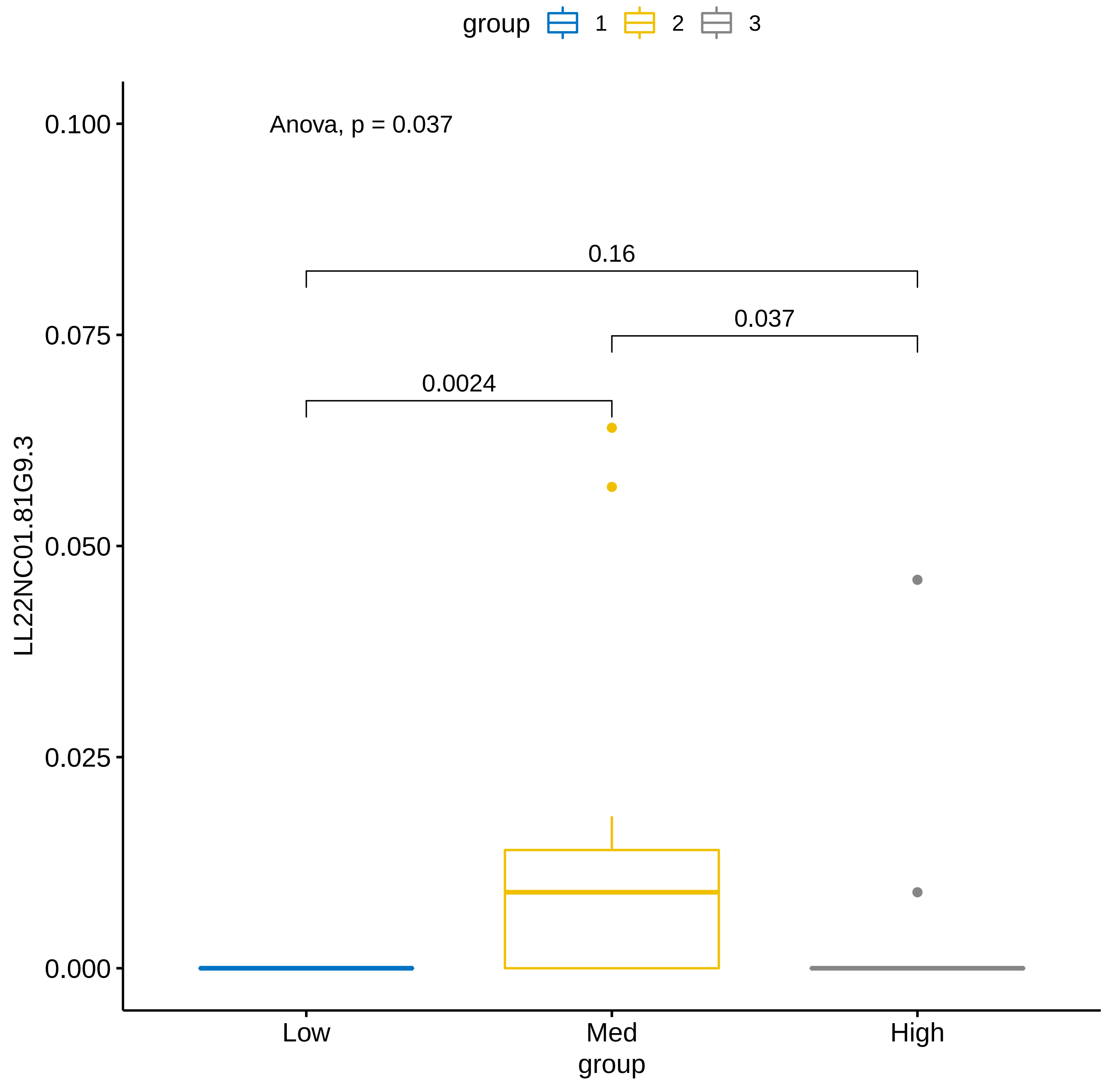
group
1
2
3
Anova, p = 0.037
0.16
0.037
0.0024
0.100
0.075
0.050
0.025
0.000
LL22NC01.81G9.3
Low
Med
High
group

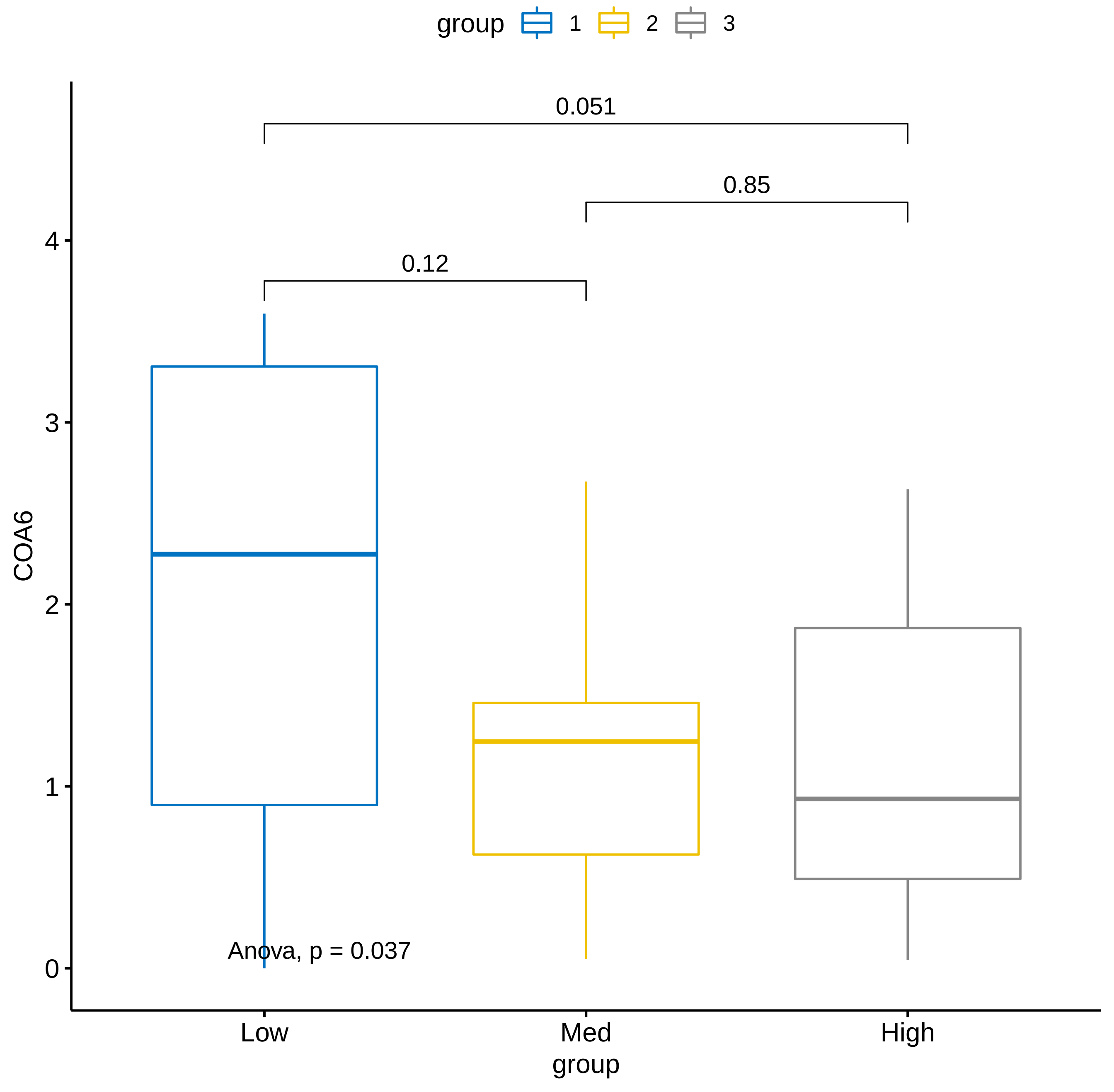
group
1
2
3
0.051
0.85
0.12
COA6
4
3
2
1
0
Anova, p = 0.037
Low
Med
High
group

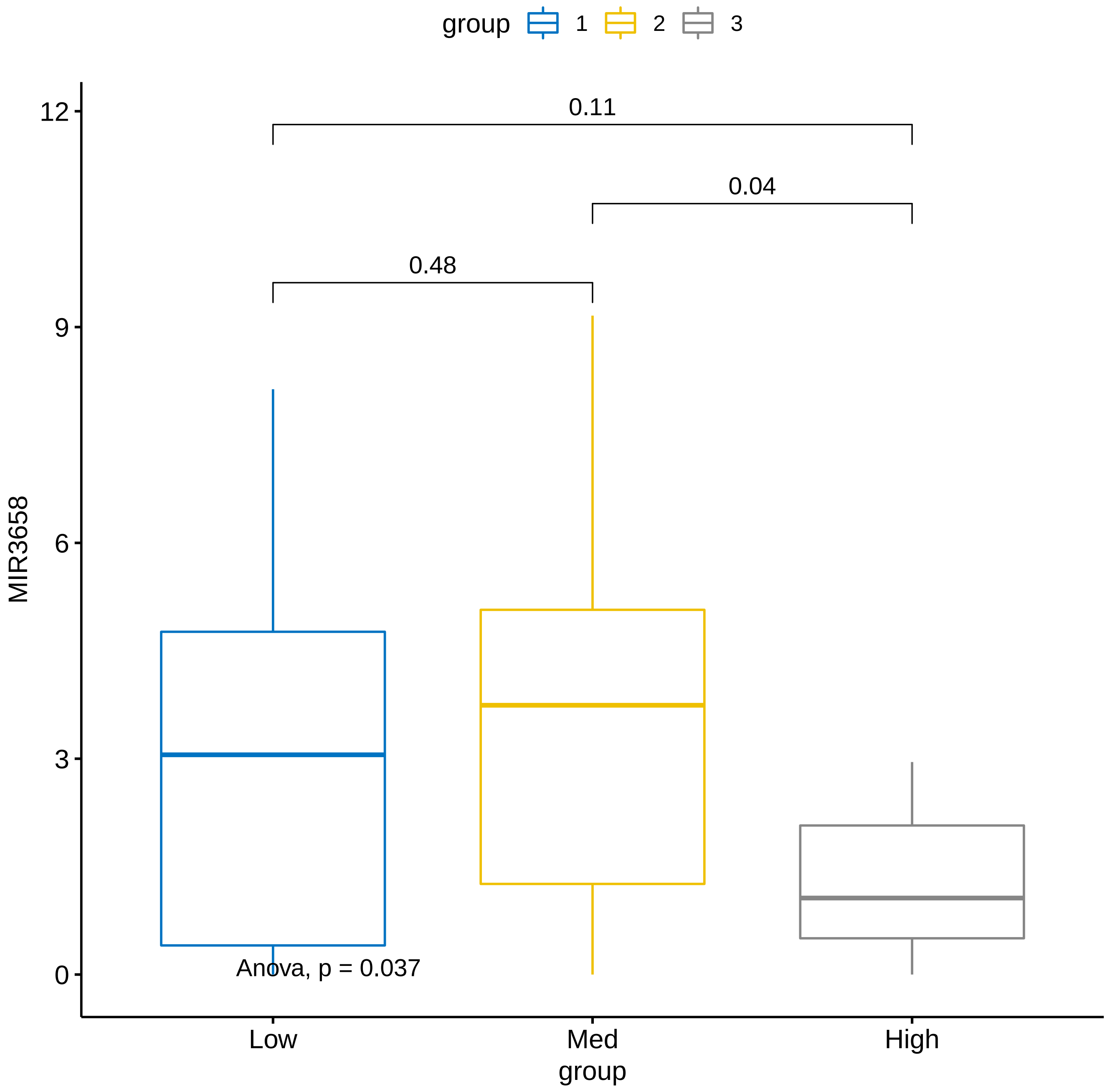
group 1 2 3
0.11
0.04
0.48
Anova, p = 0.037
MIR3658
12
9
6
3
0
Low
Med
High
group

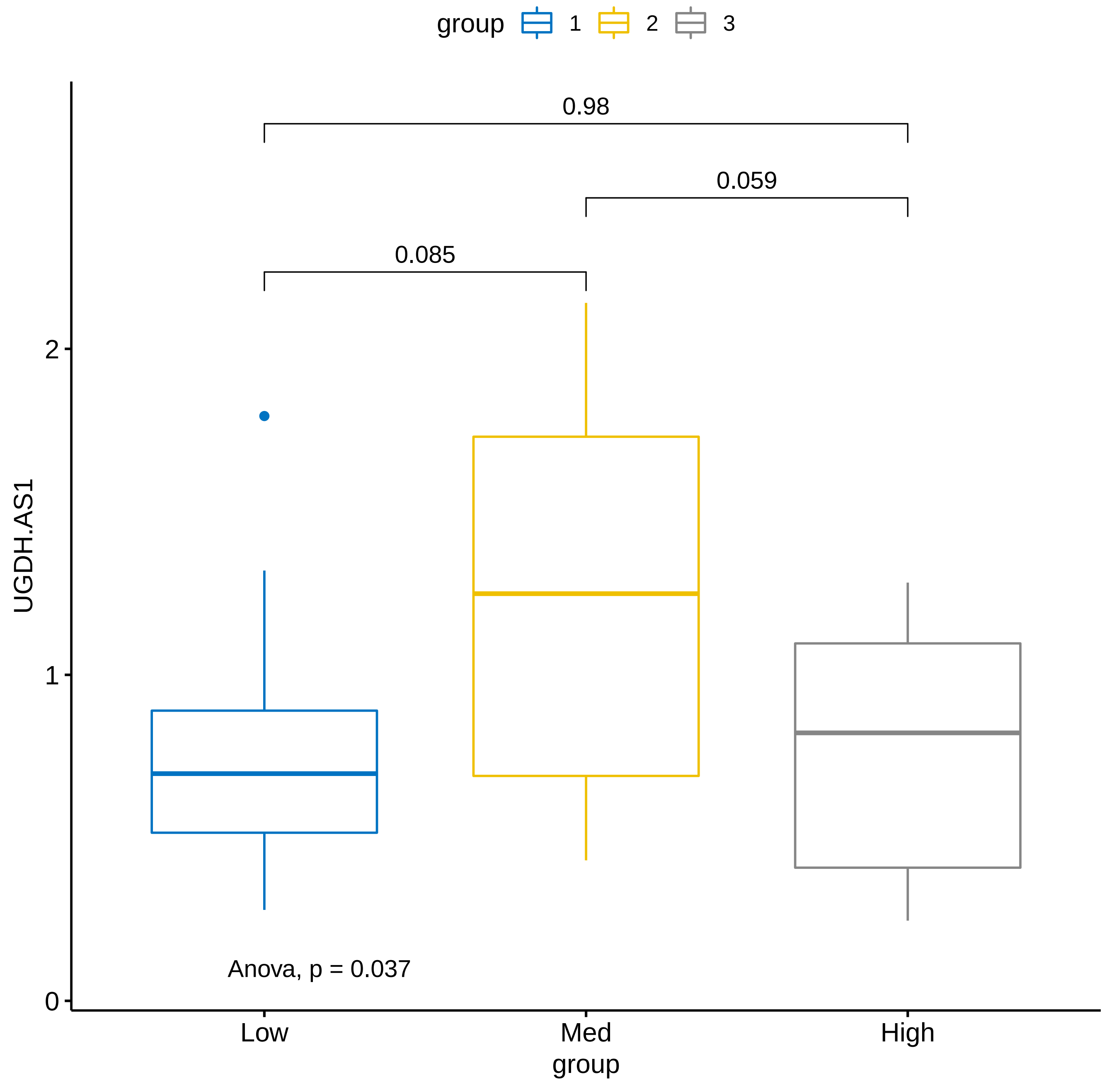

group
1
2
3
0.98
0.059
0.085
UGDH.AS1
2
1
0
Anova, p = 0.037
Low
Med
High
group

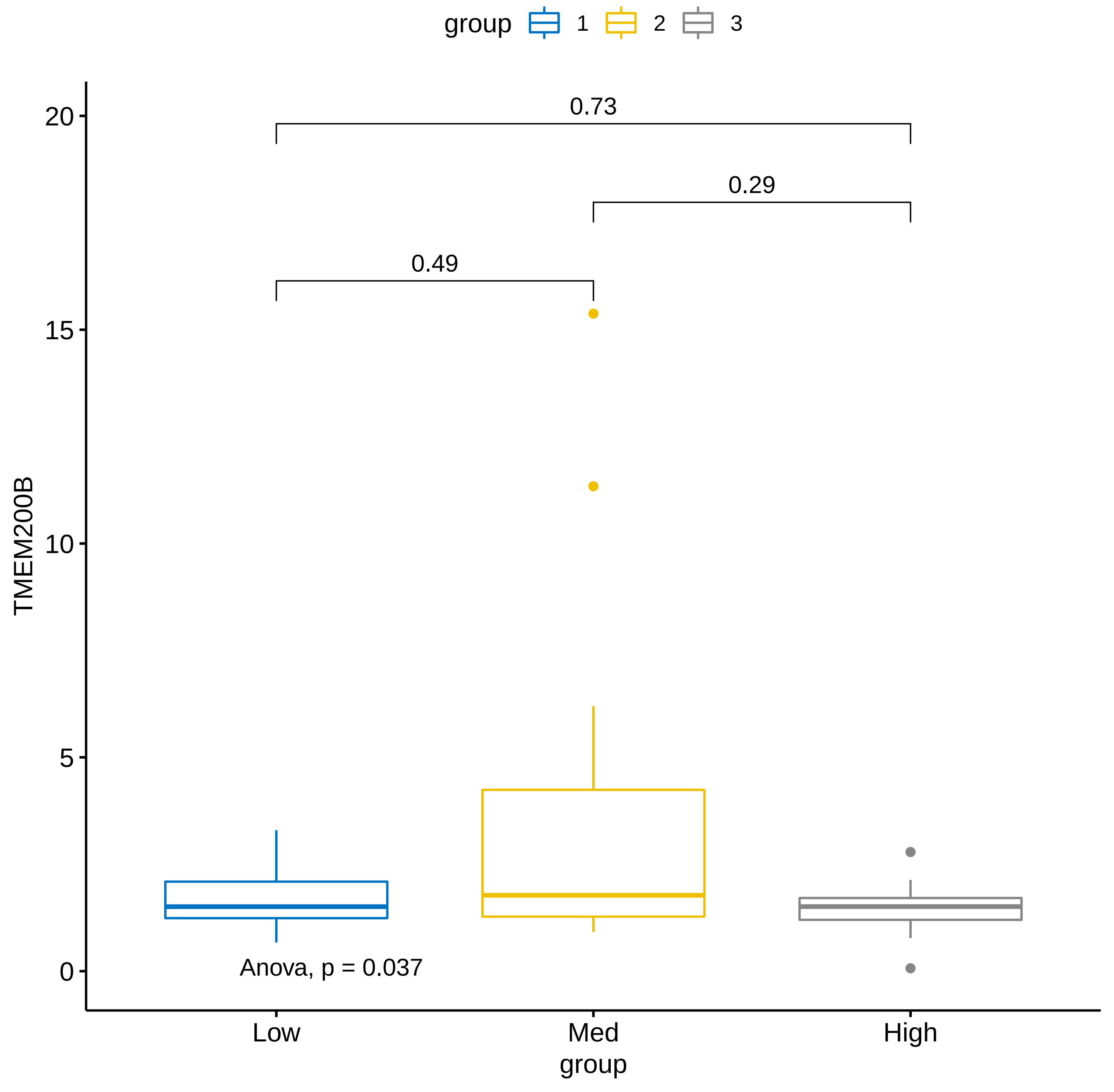

group
1
2
3
0.73
0.29
0.49
20
15
10
5
0
TMEM200B
Anova, p = 0.037
Low
Med
High
group

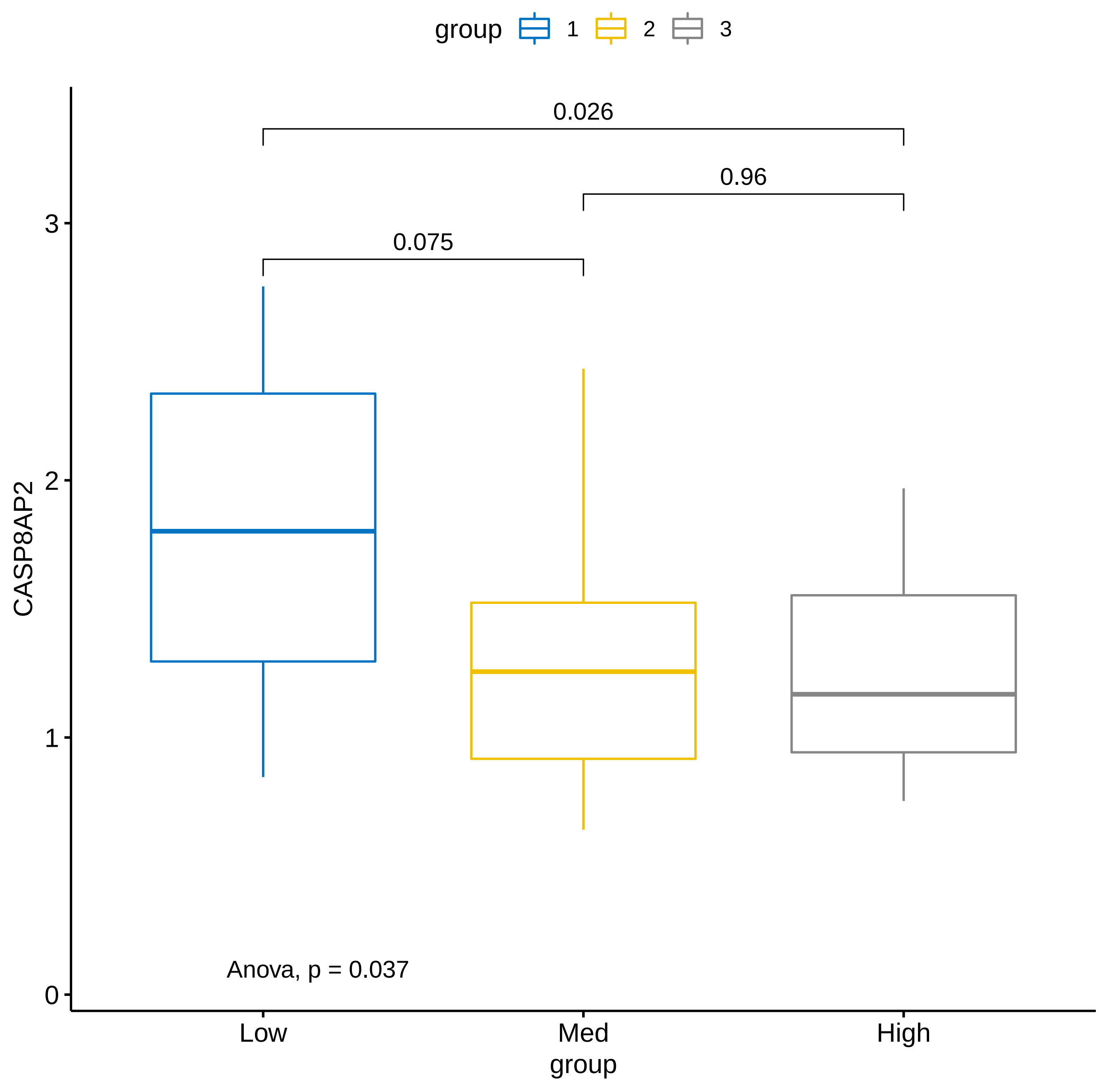
group
1
2
3
0.026
0.96
0.075
CASP8AP2
3
2
1
0
Anova, p = 0.037
Low
Med
High
group

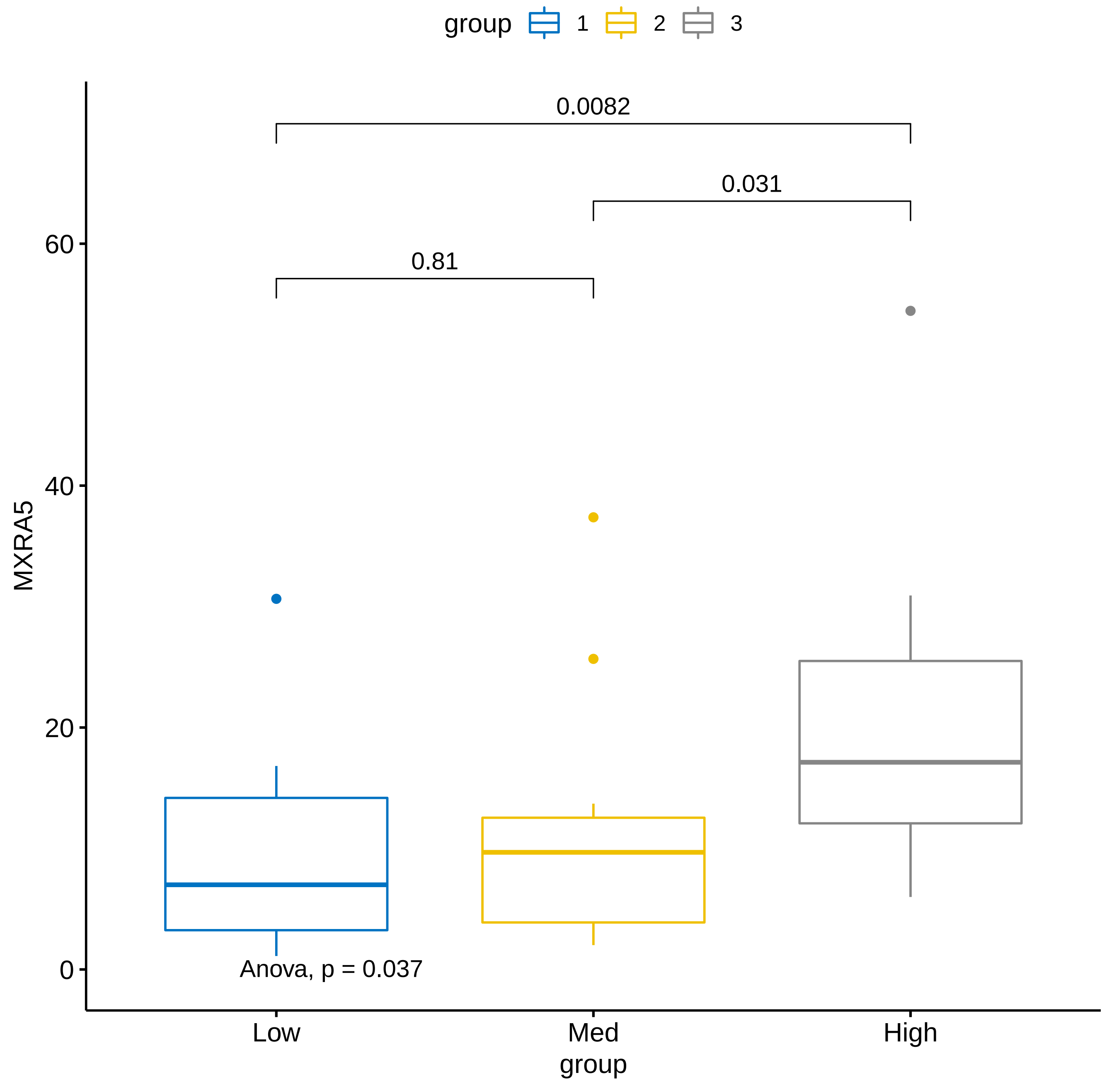
group
1
2
3
0.0082
0.031
0.81
60
40
20
0
MXRA5
Anova, p = 0.037
Low
Med
High
group

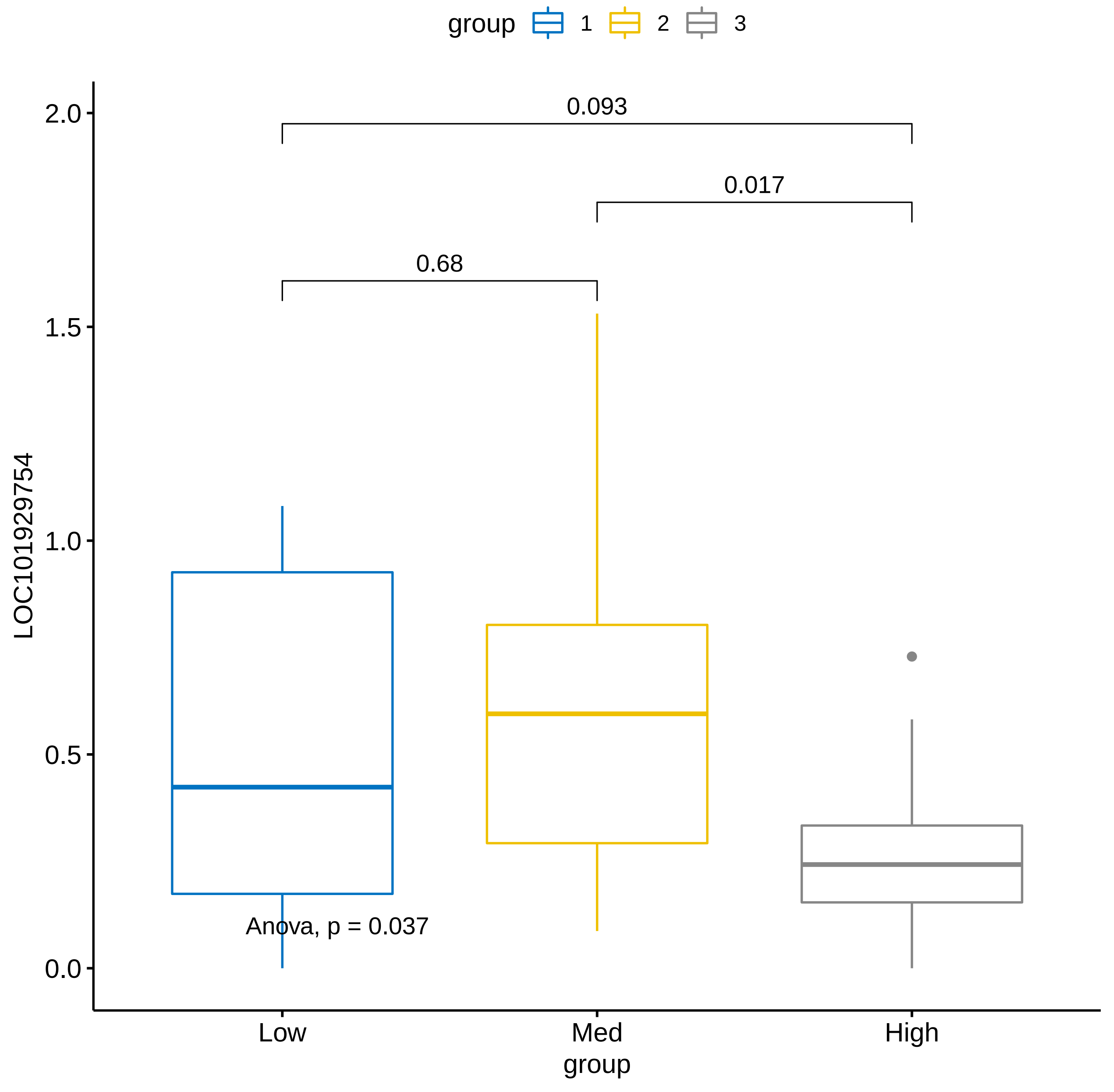
group
1
2
3
0.093
0.017
0.68
Anova, p = 0.037
2.0
1.5
1.0
0.5
0.0
LOC101929754
Low
Med
High
group

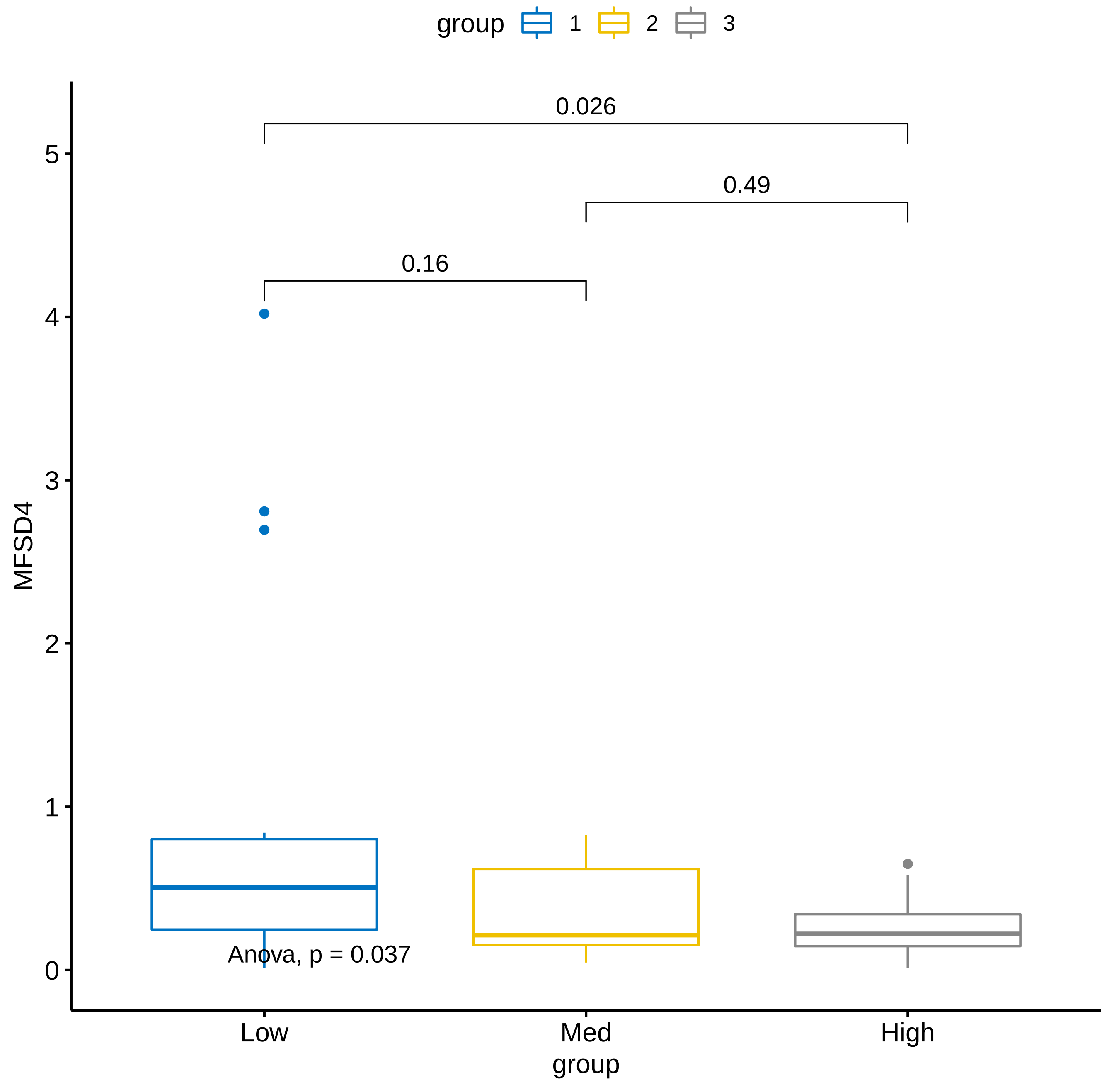

group 1 2 3
0.026
0.49
0.16
MFSD4
5
4
3
2
1
0
Anova, p = 0.037
Low
Med
High
group

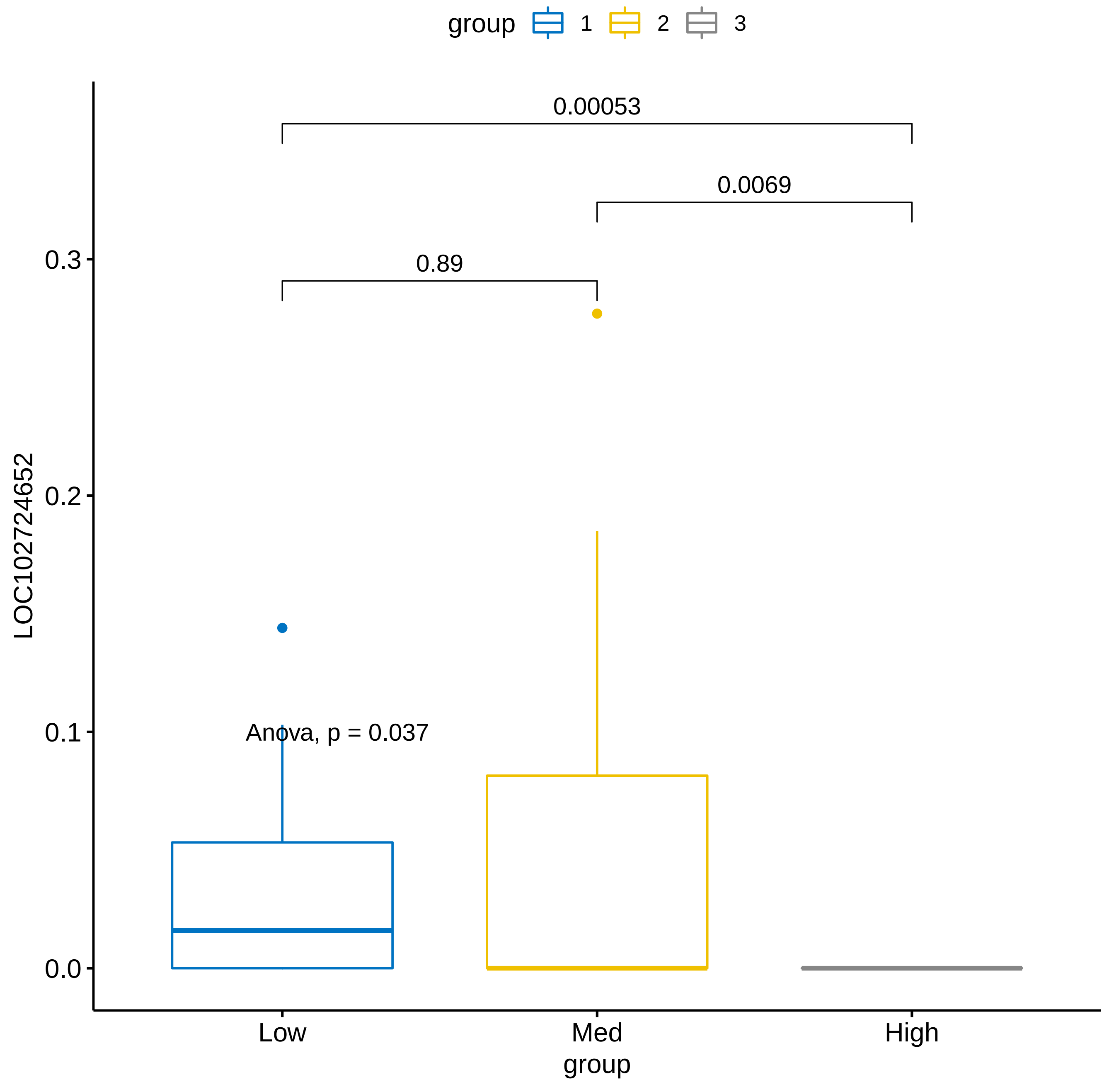
group
1
2
3
0.00053
0.0069
0.89
Anova, p = 0.037
LOC102724652
0.3
0.2
0.1
0.0
Low
Med
High
group

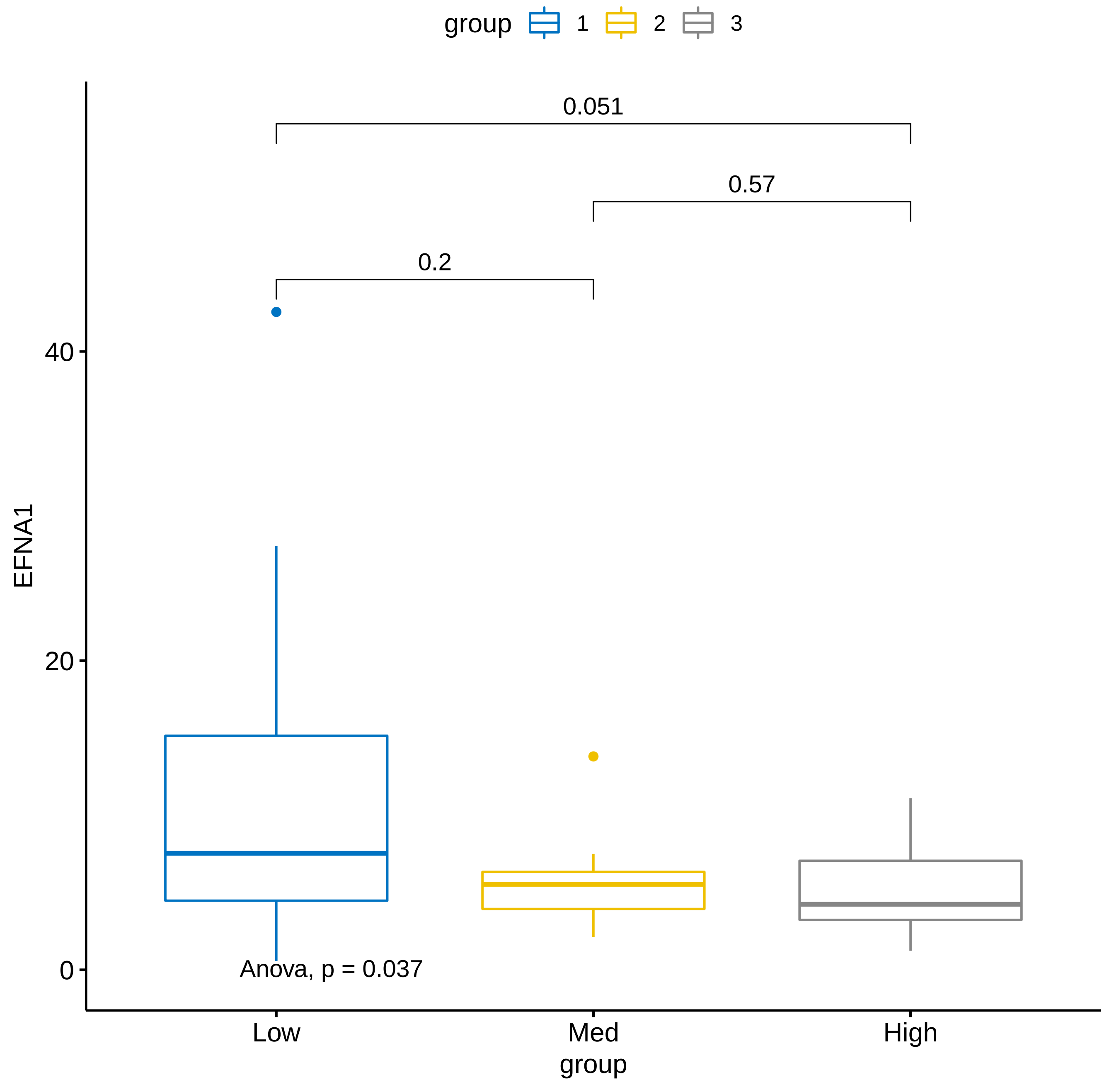

group 1 2 3
0.051
0.57
0.2
EFNA1
40
20
0
Anova, p = 0.037
Low
Med
High
group

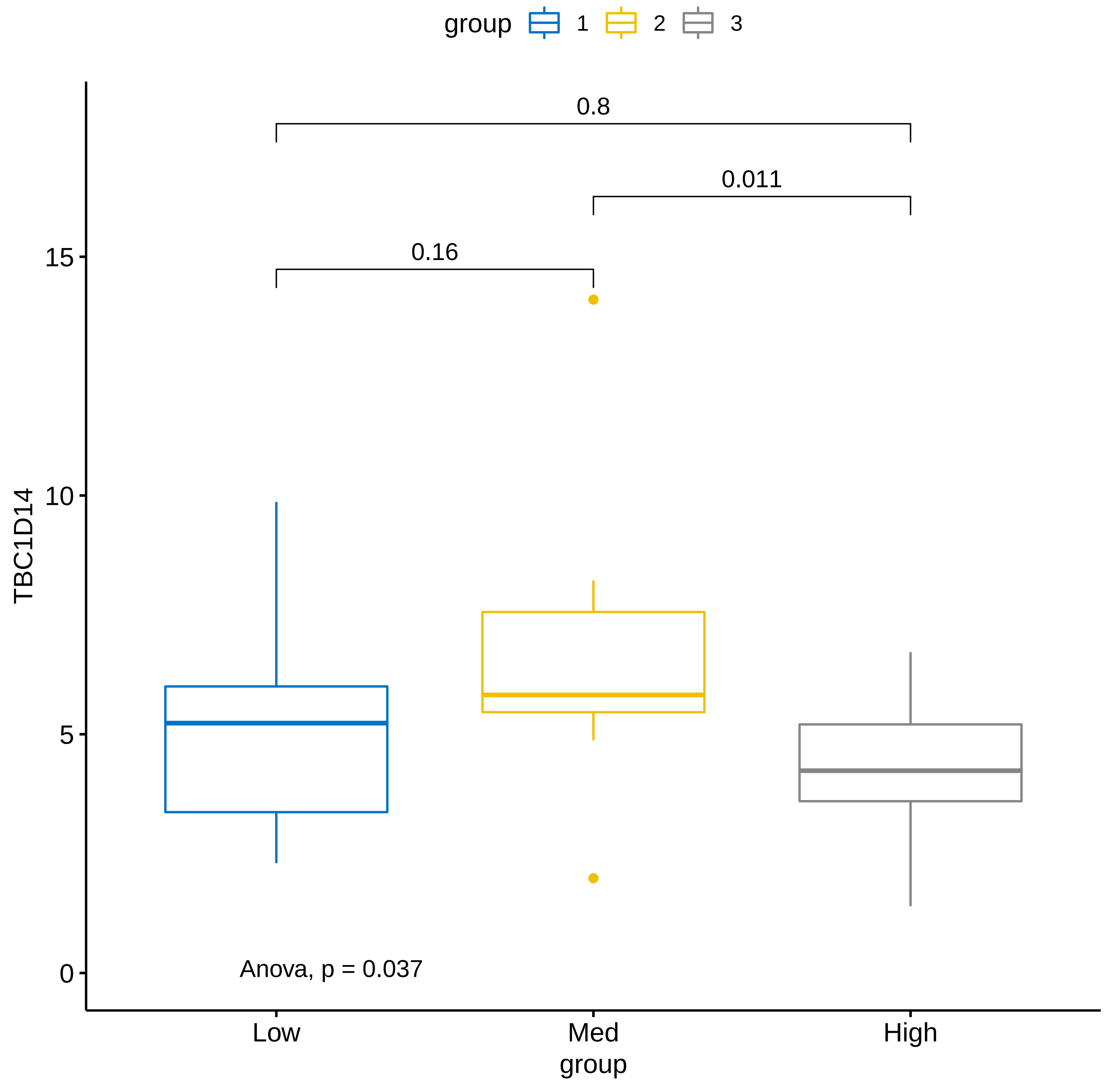
group 1 2 3
0.8
0.011
0.16
15
10
5
0
TBC1D14
Anova, p = 0.037
Low
Med
High
group

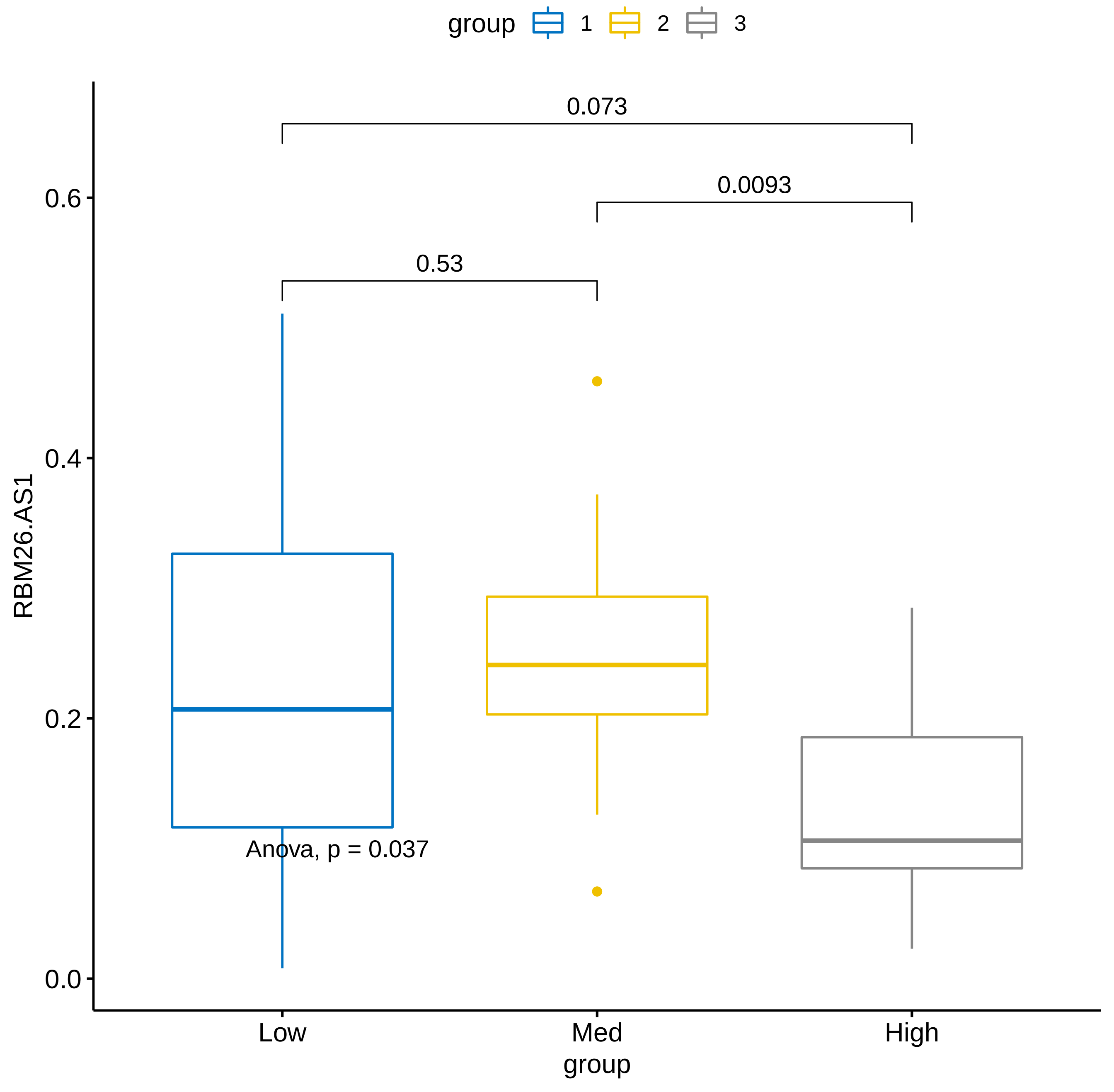
group
1
2
3
0.073
0.0093
0.53
0.6
0.4
0.2
0.0
RBM26.AS1
Anova, p = 0.037
Low
Med
High
group

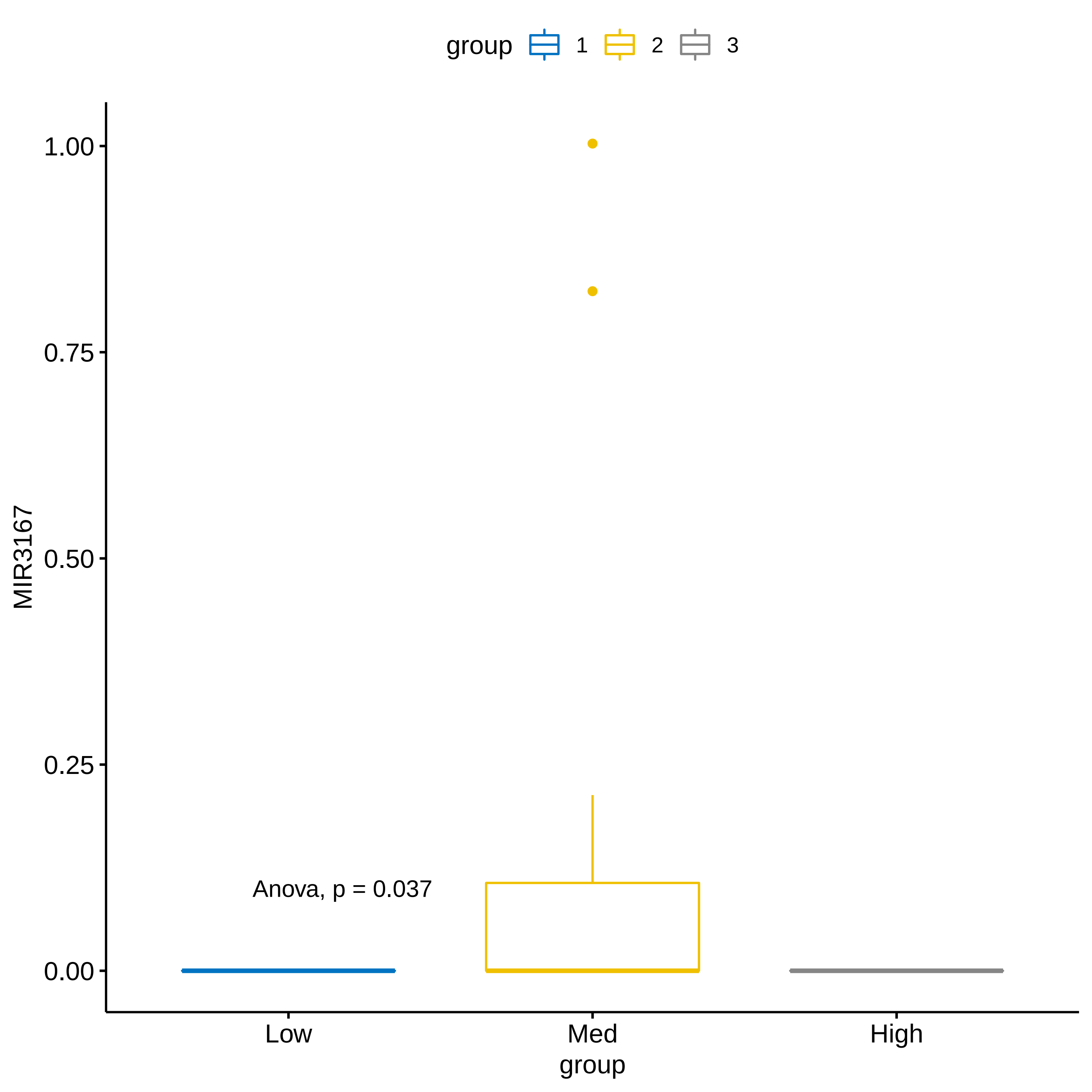
group
1
2
3
1.00
0.75
0.50
0.25
0.00
MIR3167
Anova, p = 0.037
Low
Med
High
group

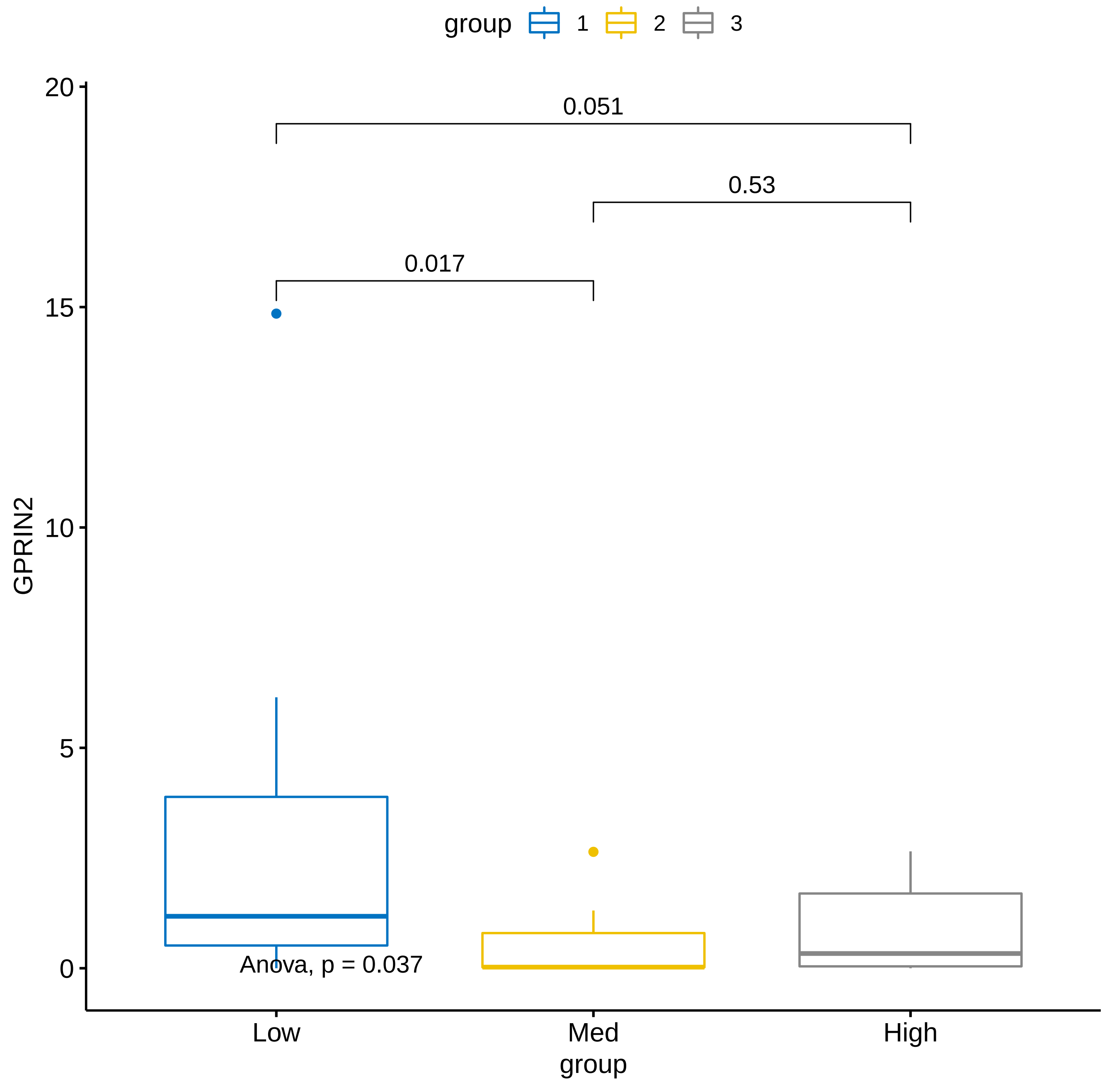

group
1
2
3
20
15
10
5
0
GPRIN2
0.051
0.53
0.017
Anova, p = 0.037
Low
Med
High
group

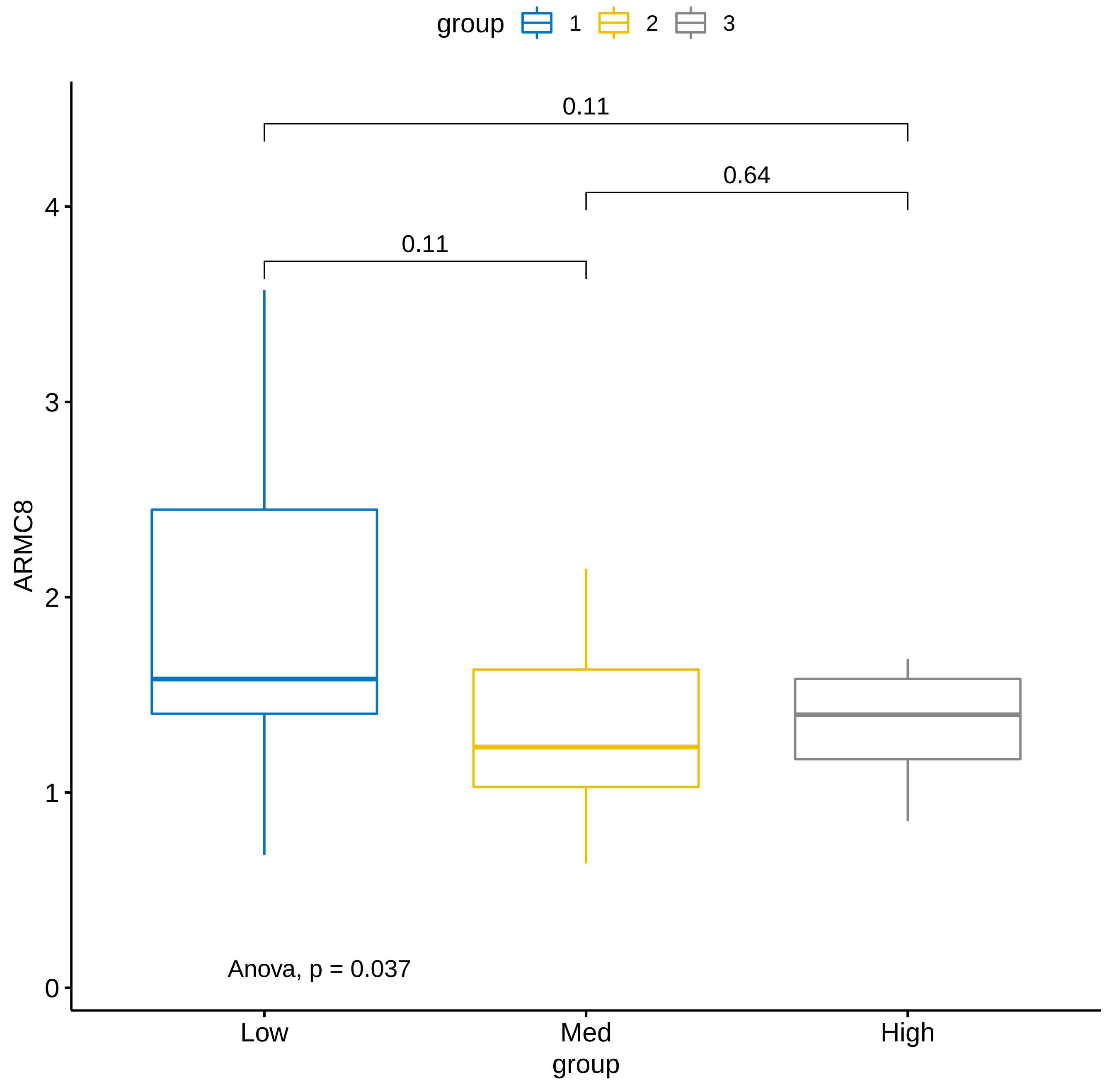
group
1
2
3
0.11
0.64
0.11
4
3
2
1
0
ARMC8
Anova, p = 0.037
Low
Med
High
group

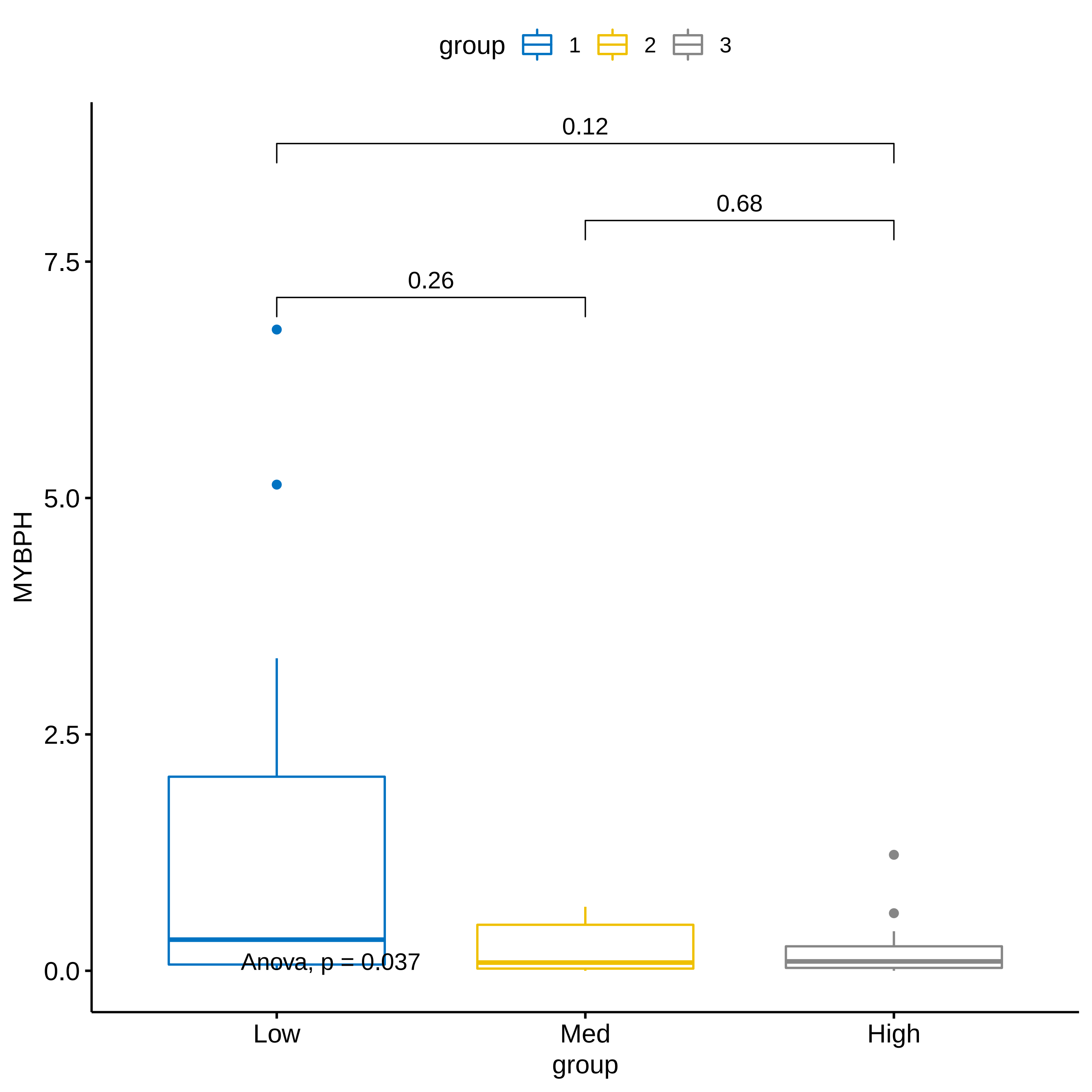

group 1 2 3
0.12
0.68
0.26
MYBPH
7.5
5.0
2.5
0.0
Anova, p = 0.037
Low
Med
High
group

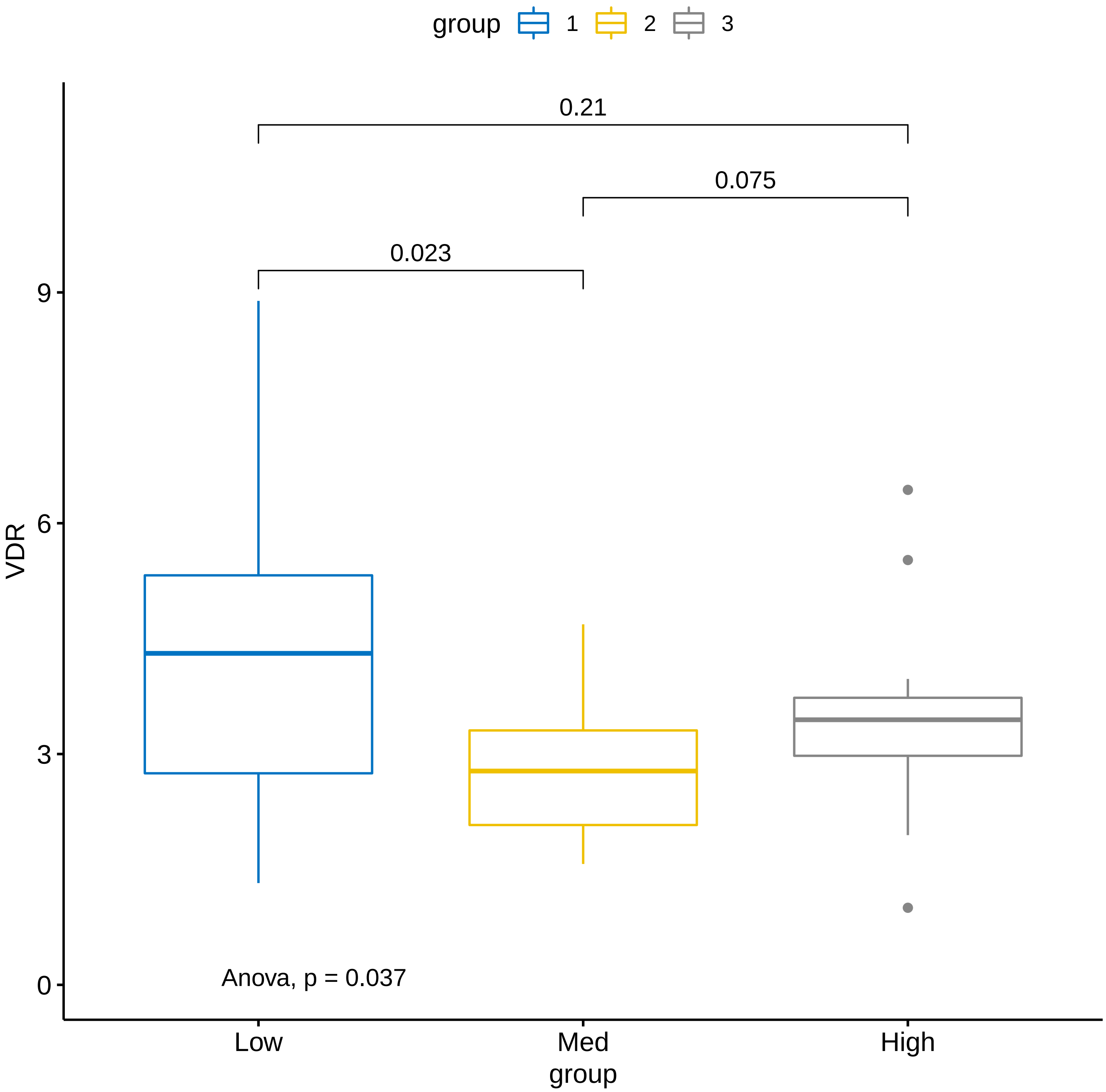
group
1
2
3
0.21
0.075
0.023
9
6
3
0
VDR
Anova, p = 0.037
Low
Med
High
group

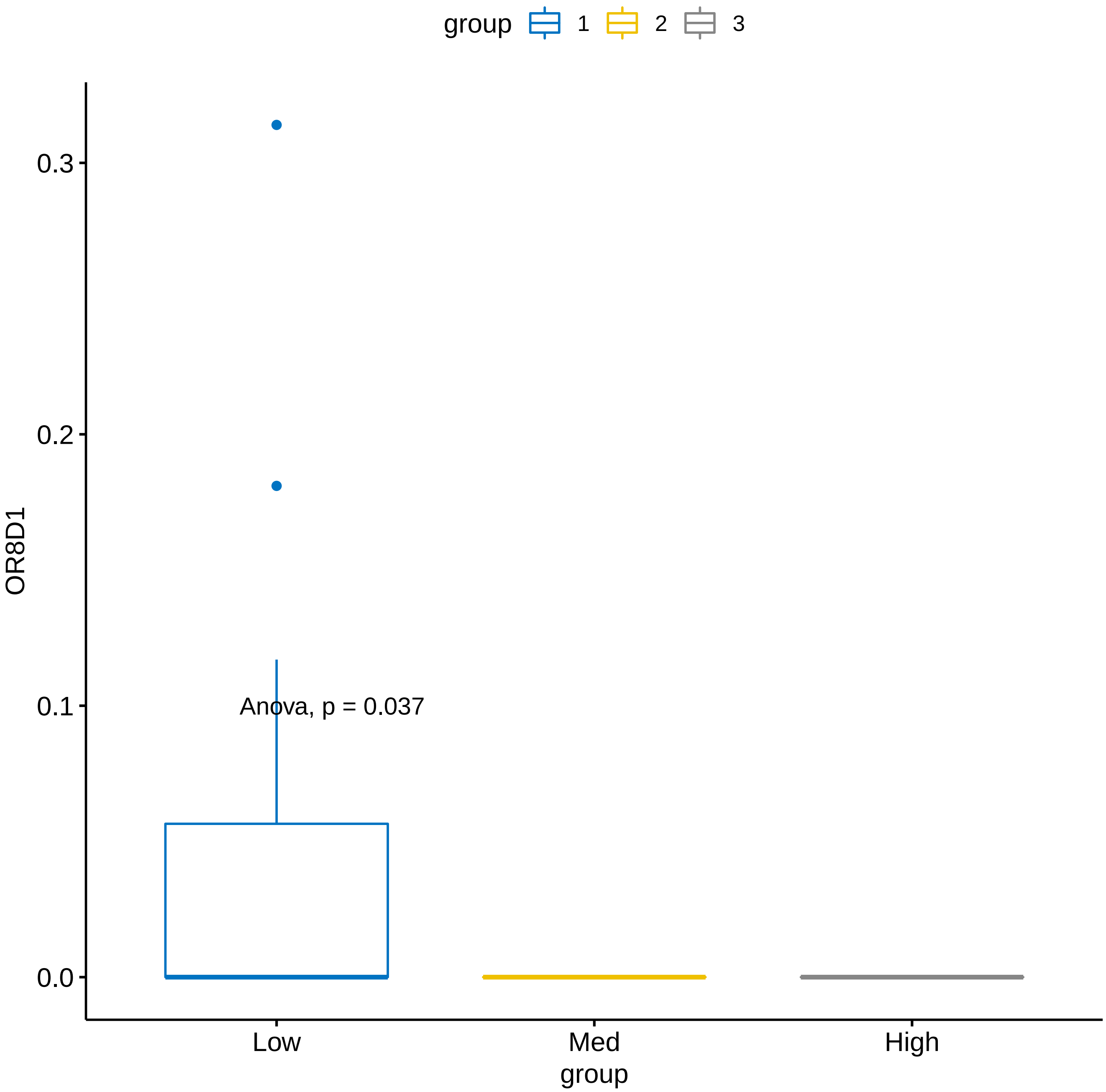
group
1
2
3
0.3
0.2
0.1
0.0
OR8D1
Anova, p = 0.037
Low
Med
High
group

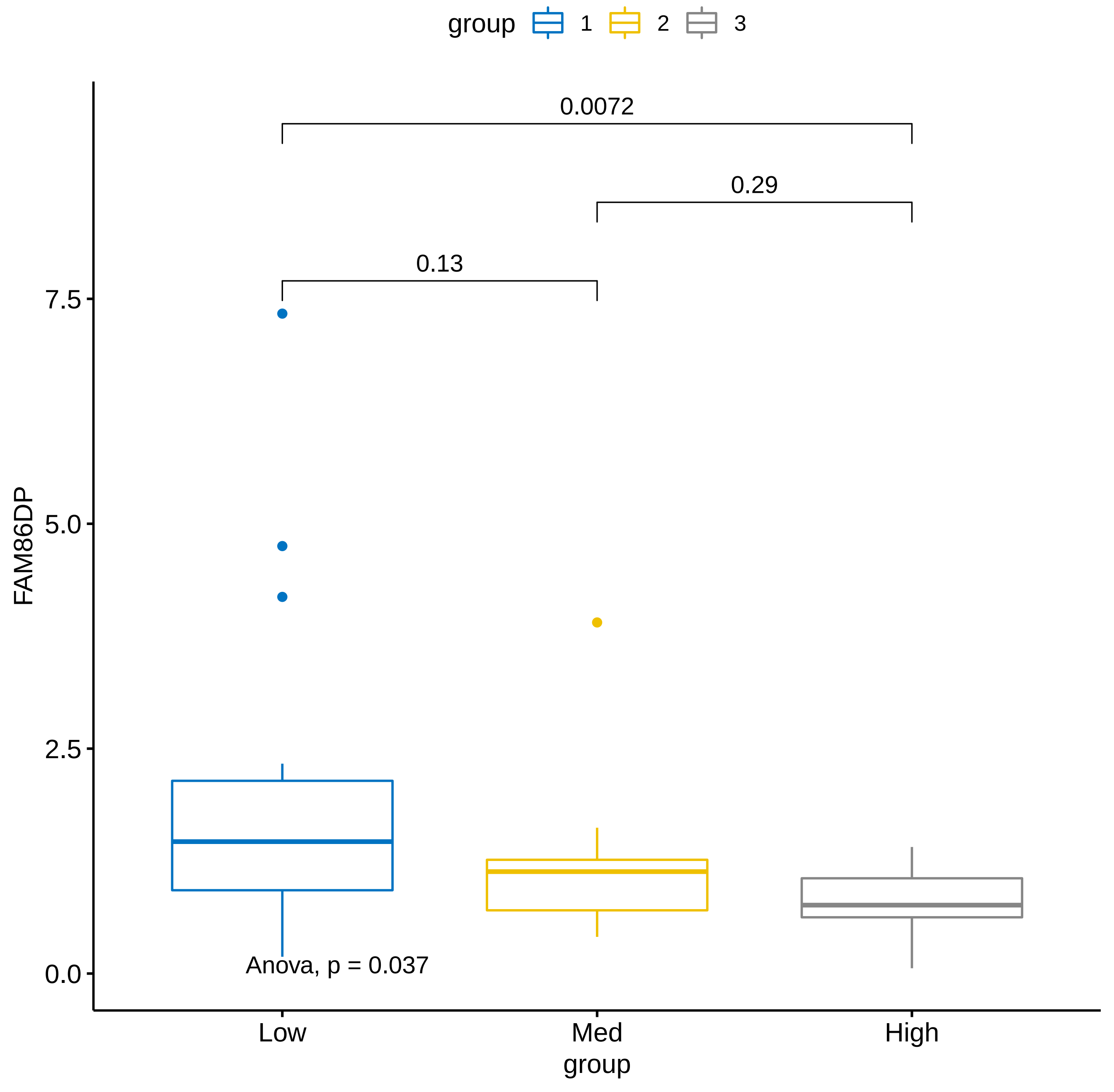

group 1 2 3
0.0072
0.29
0.13
7.5
5.0
2.5
0.0
FAM86DP
Anova, p = 0.037
Low
Med
High
group

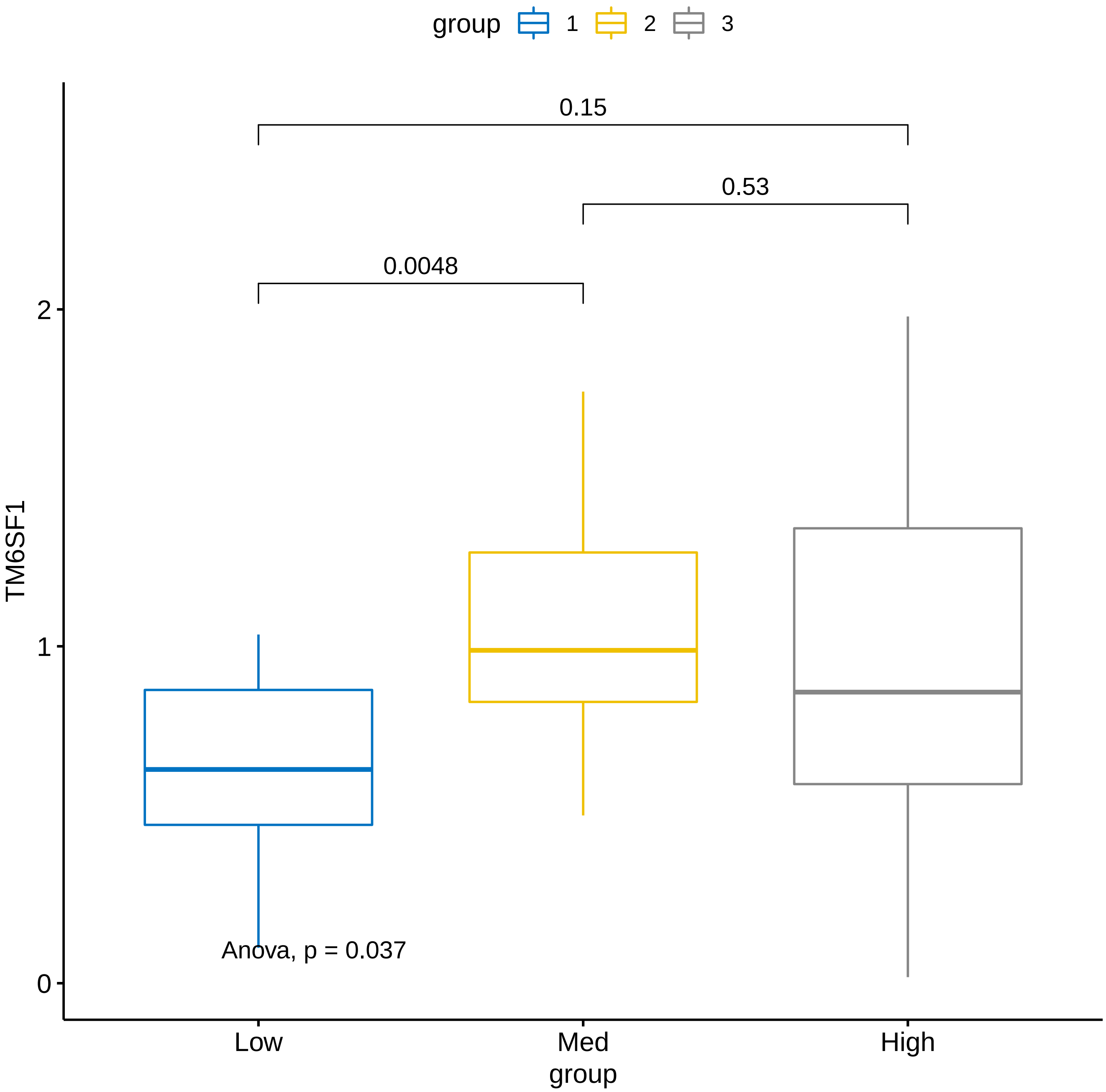
group 1 2 3
0.15
0.53
0.0048
TM6SF1
2
1
0
Anova, p = 0.037
Low
Med
High
group

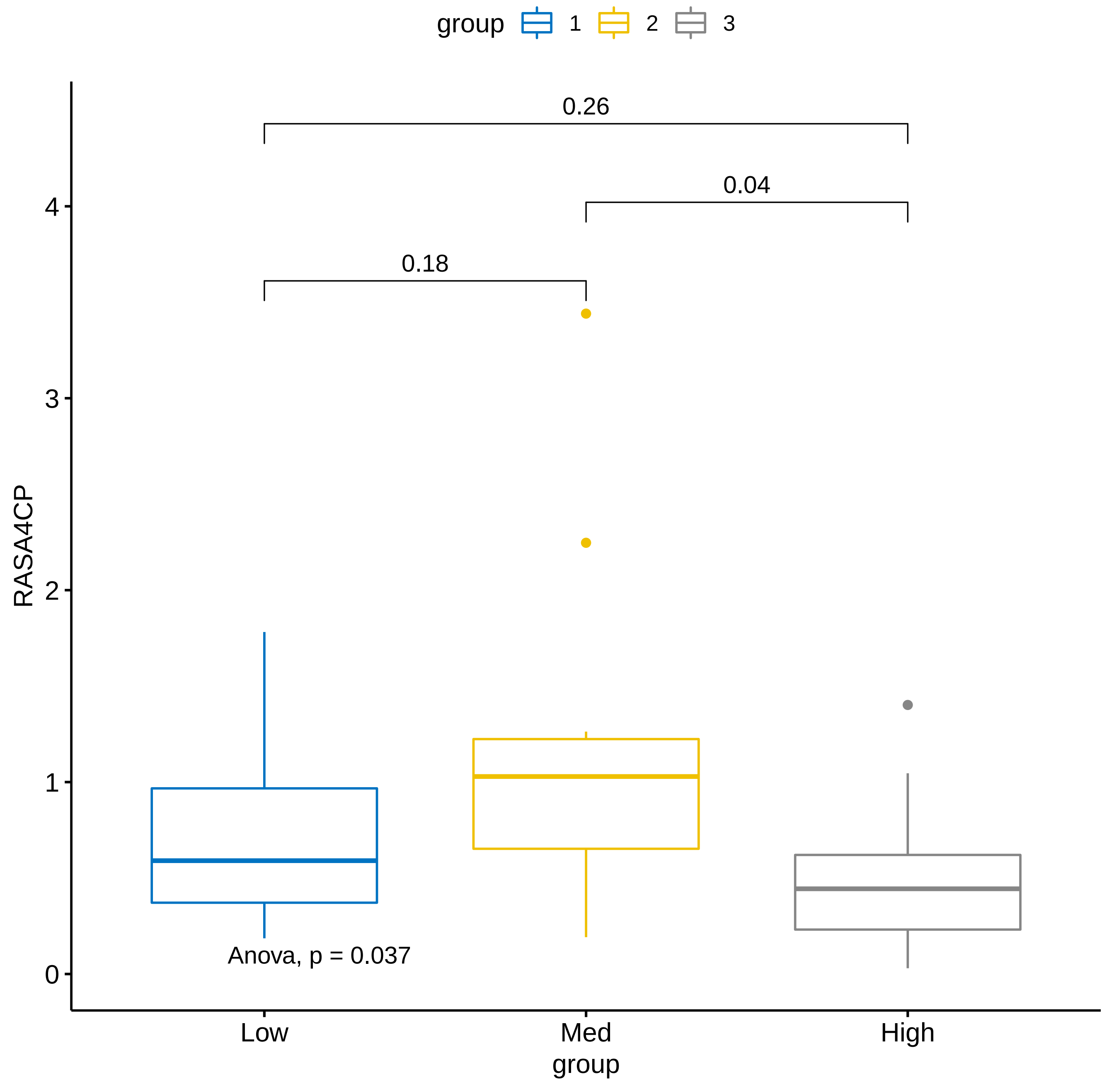
group
1
2
3
0.26
0.04
0.18
RASA4CP
4
3
2
1
0
Anova, p = 0.037
Low
Med
High
group

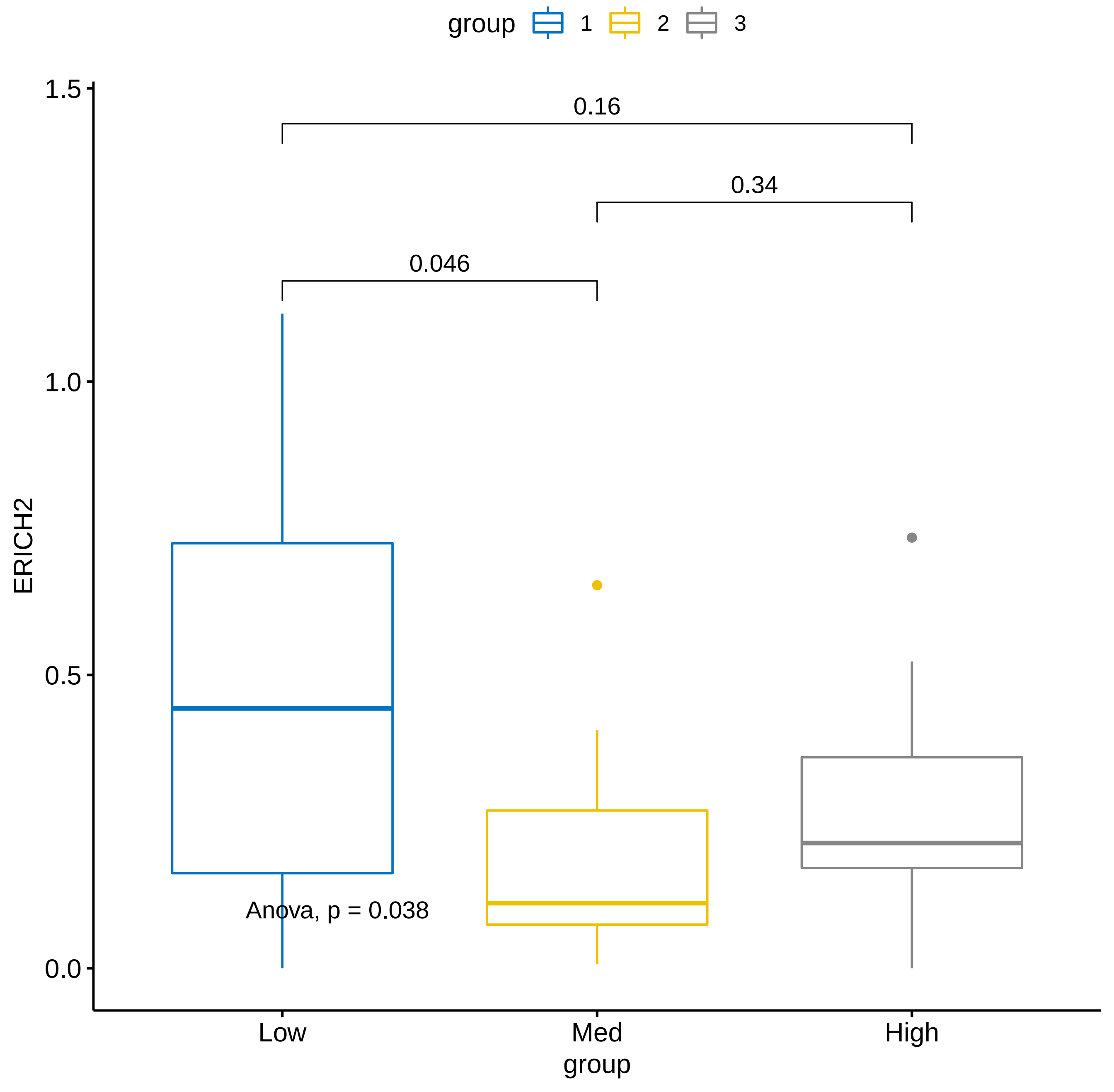
group
1
2
3
0.16
0.34
0.046
1.5
1.0
0.5
0.0
ERICH2
Anova, p = 0.038
Low
Med
High
group

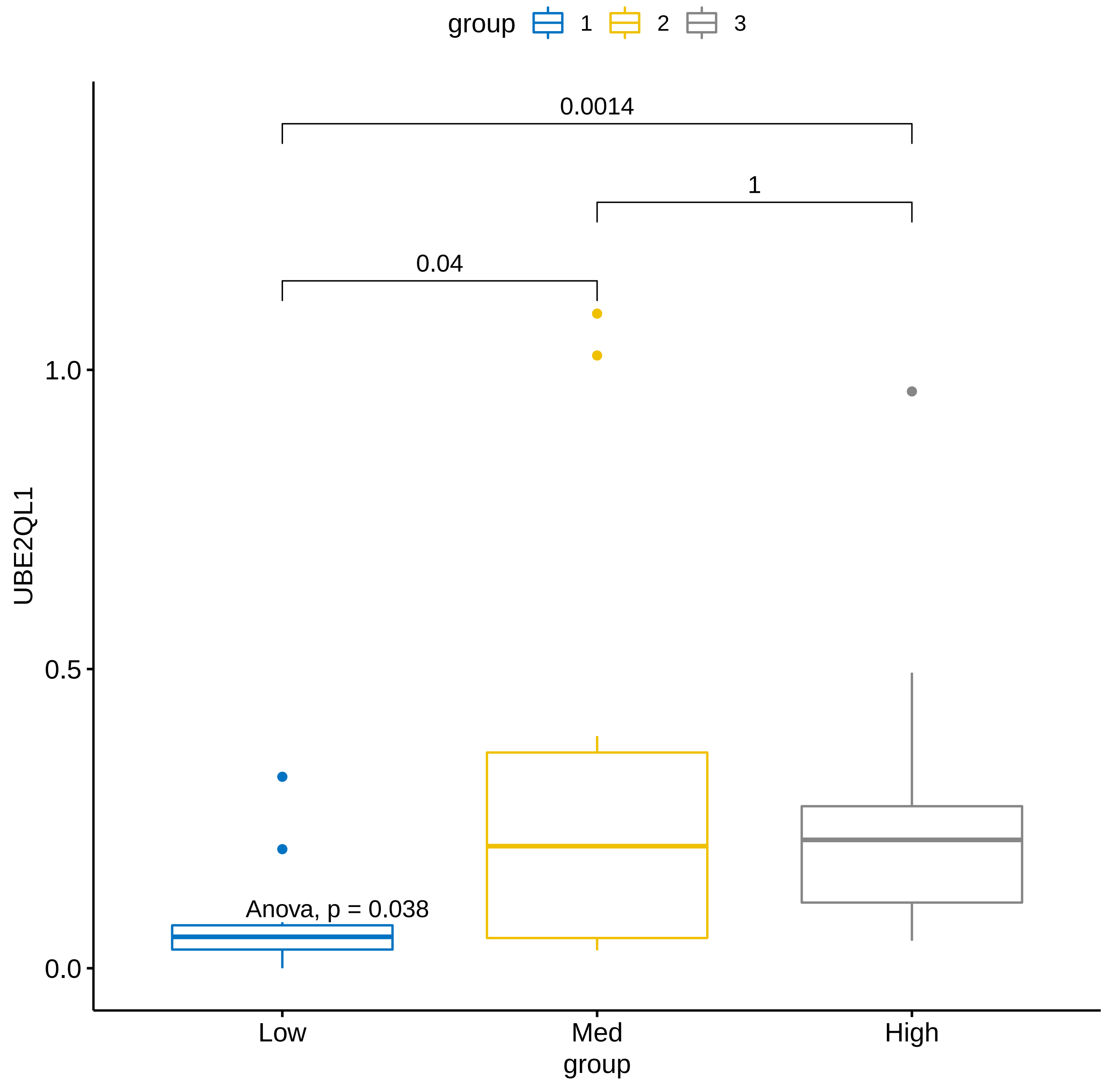

group 1 2 3
0.0014
1
0.04
Anova, p = 0.038
UBE2QL1
1.0
0.5
0.0
Low
Med
High
group

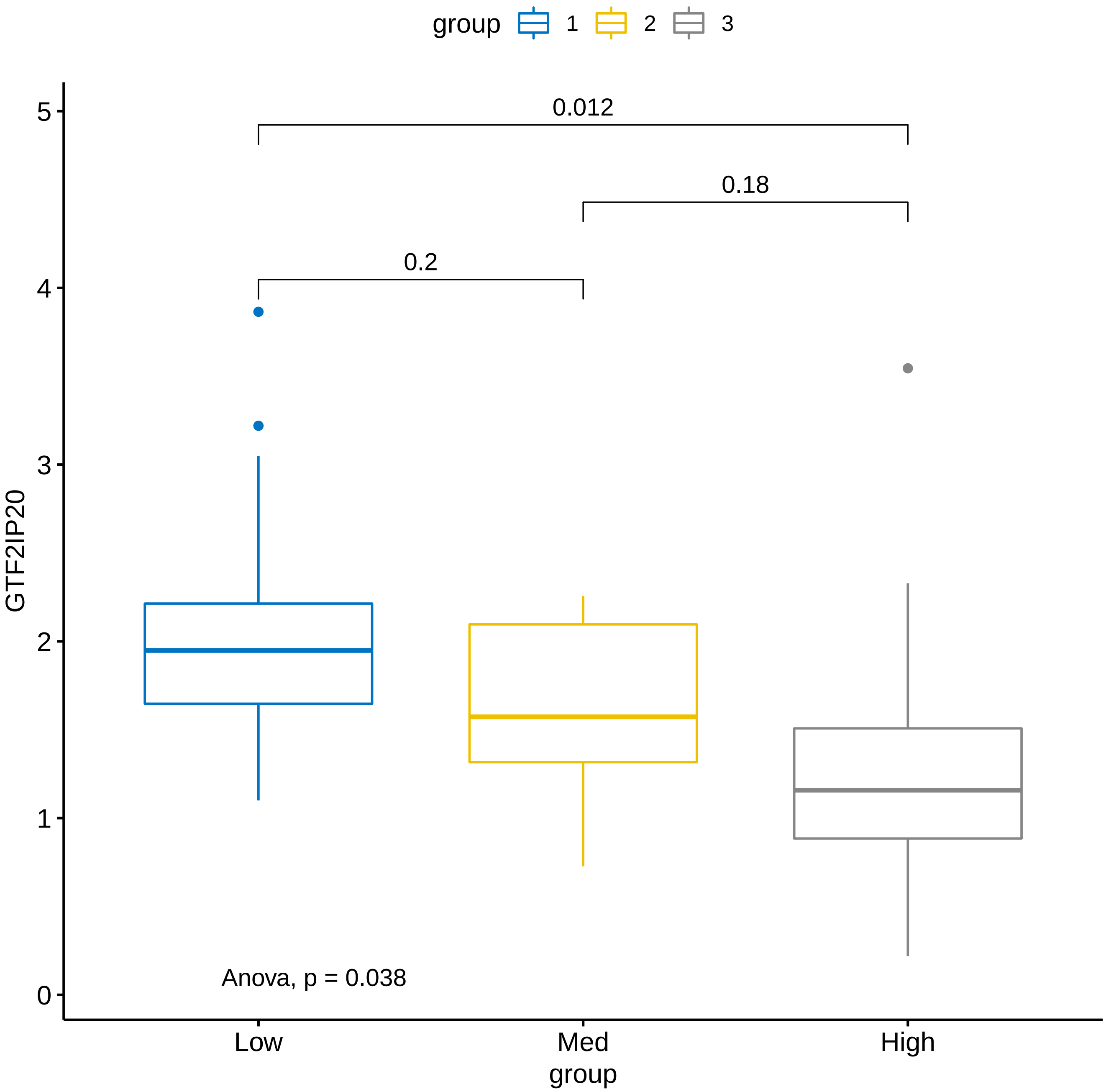

group 1 2 3
0.012
0.18
0.2
GTF2IP20
5
4
3
2
1
0
Anova, p = 0.038
Low
Med
High
group

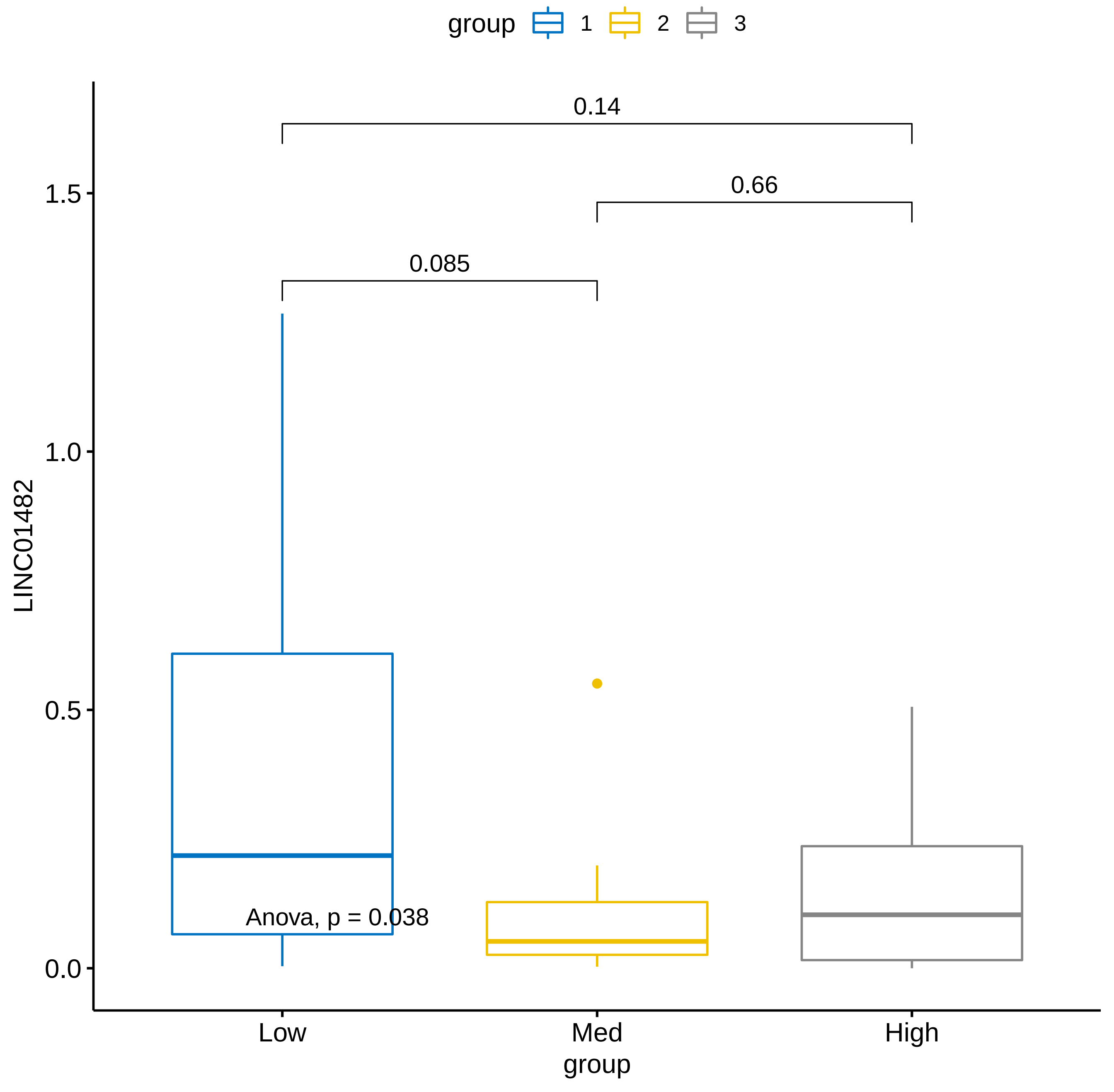

group
1
2
3
0.14
0.66
0.085
1.5
1.0
0.5
0.0
LINC01482
Anova, p = 0.038
Low
Med
High
group

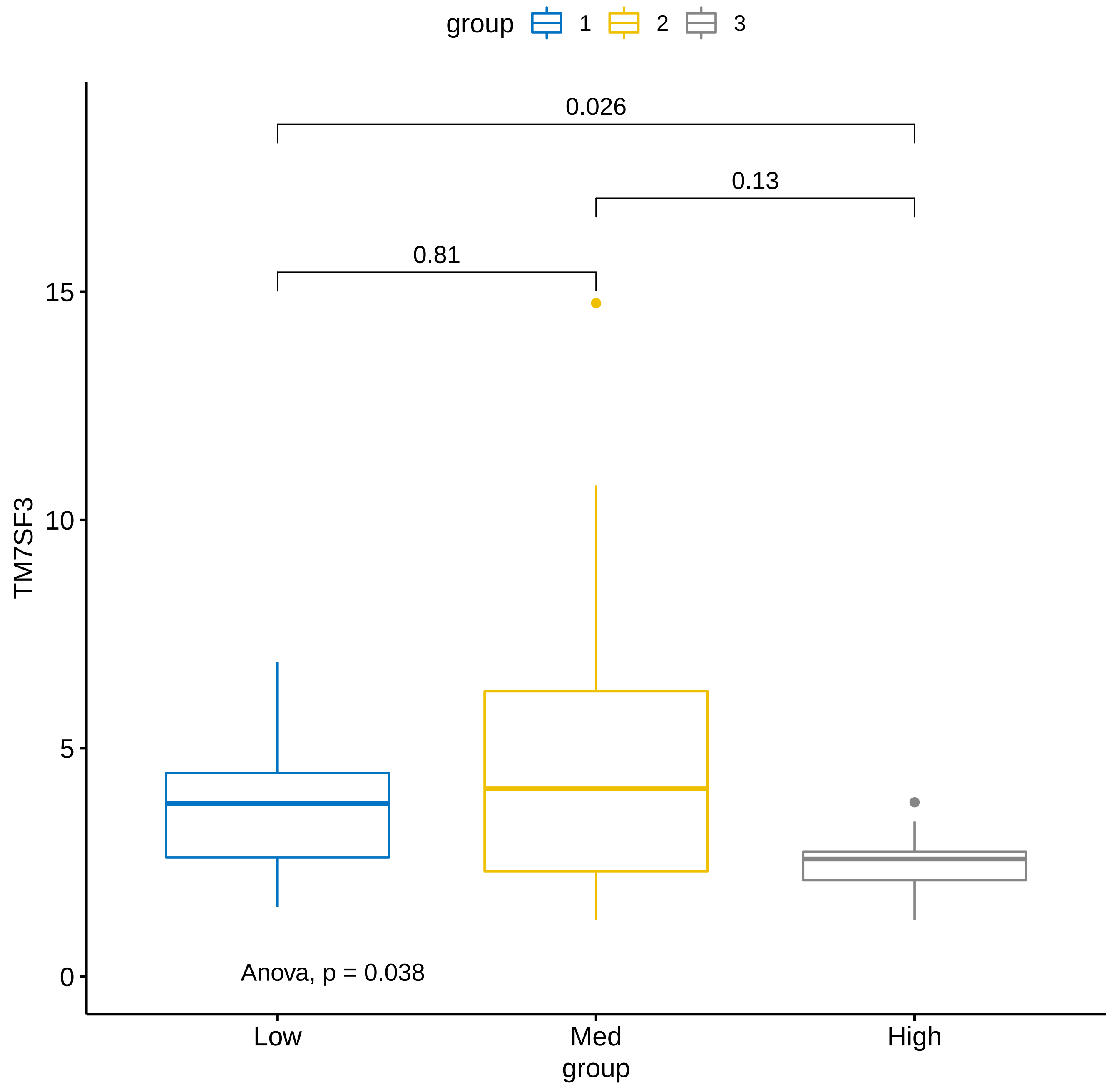
group
1
2
3
0.026
0.13
0.81
15
10
5
0
TM7SF3
Anova, p = 0.038
Low
Med
High
group

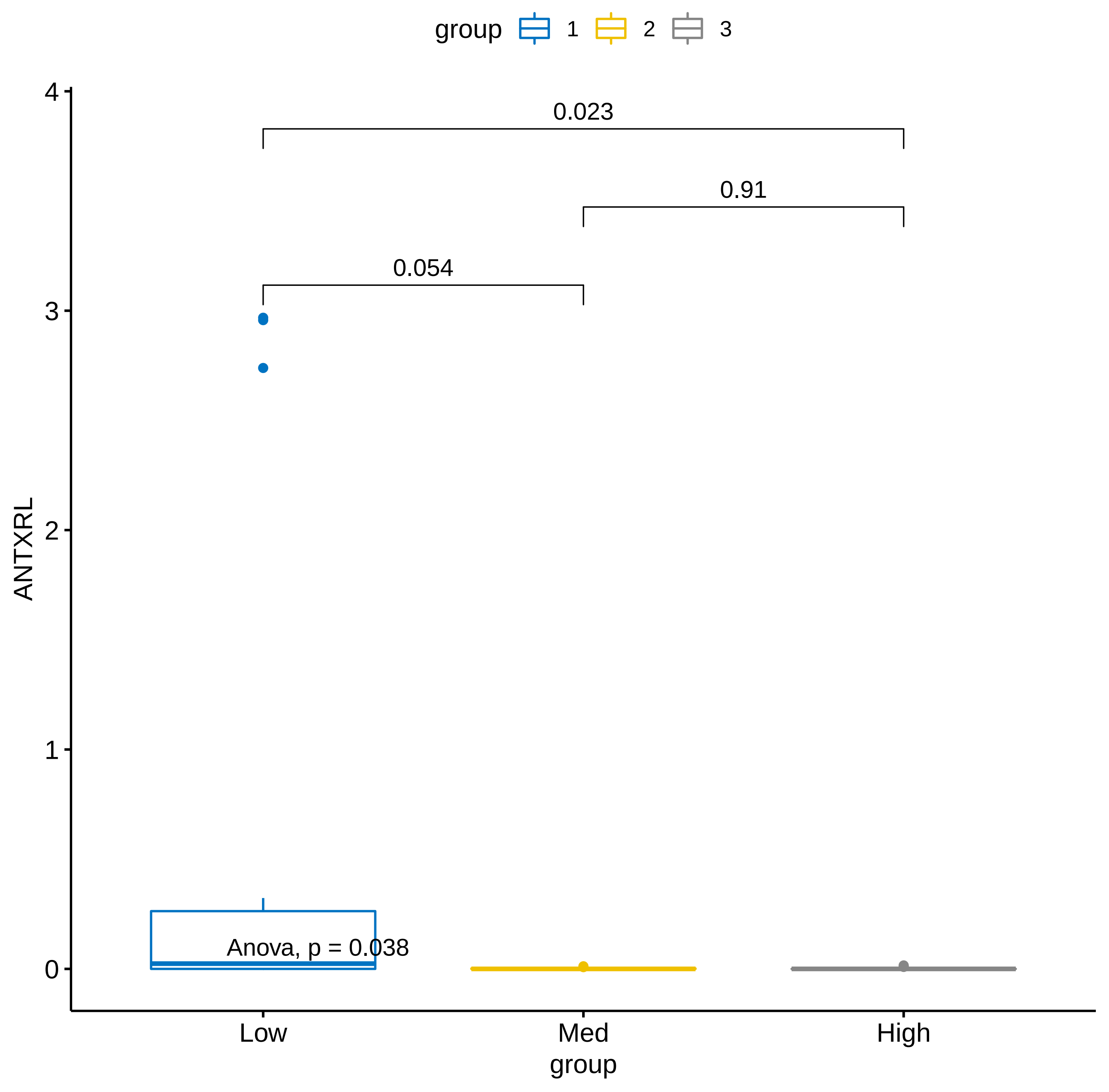
group
1
2
3
0.023
0.91
0.054
Anova, p = 0.038
ANTXRL
4
3
2
1
0
Low
Med
High
group

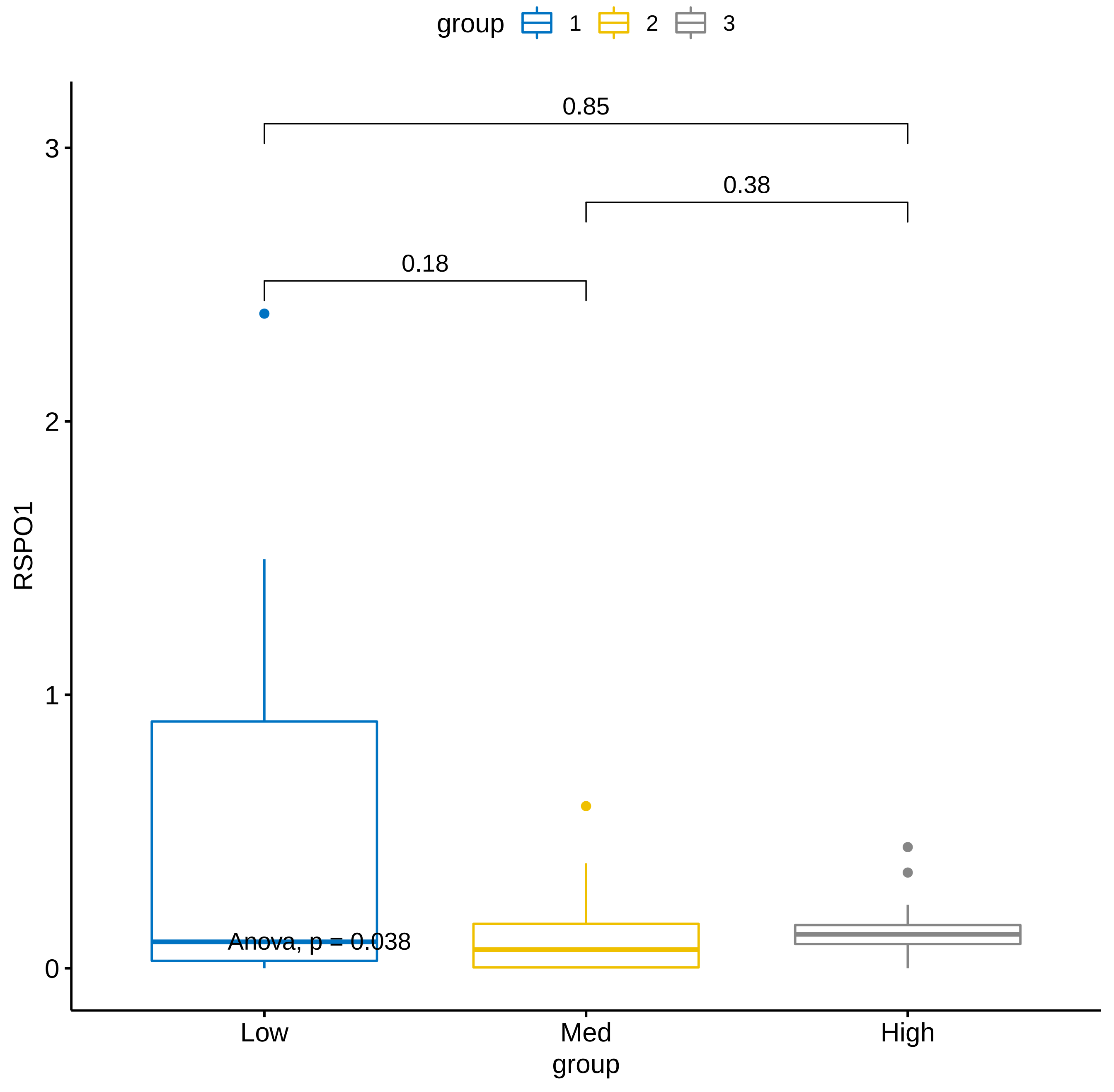
group 1 2 3
0.85
0.38
0.18
Anova, p = 0.038
RSPO1
3
2
1
0
Low
Med
High
group

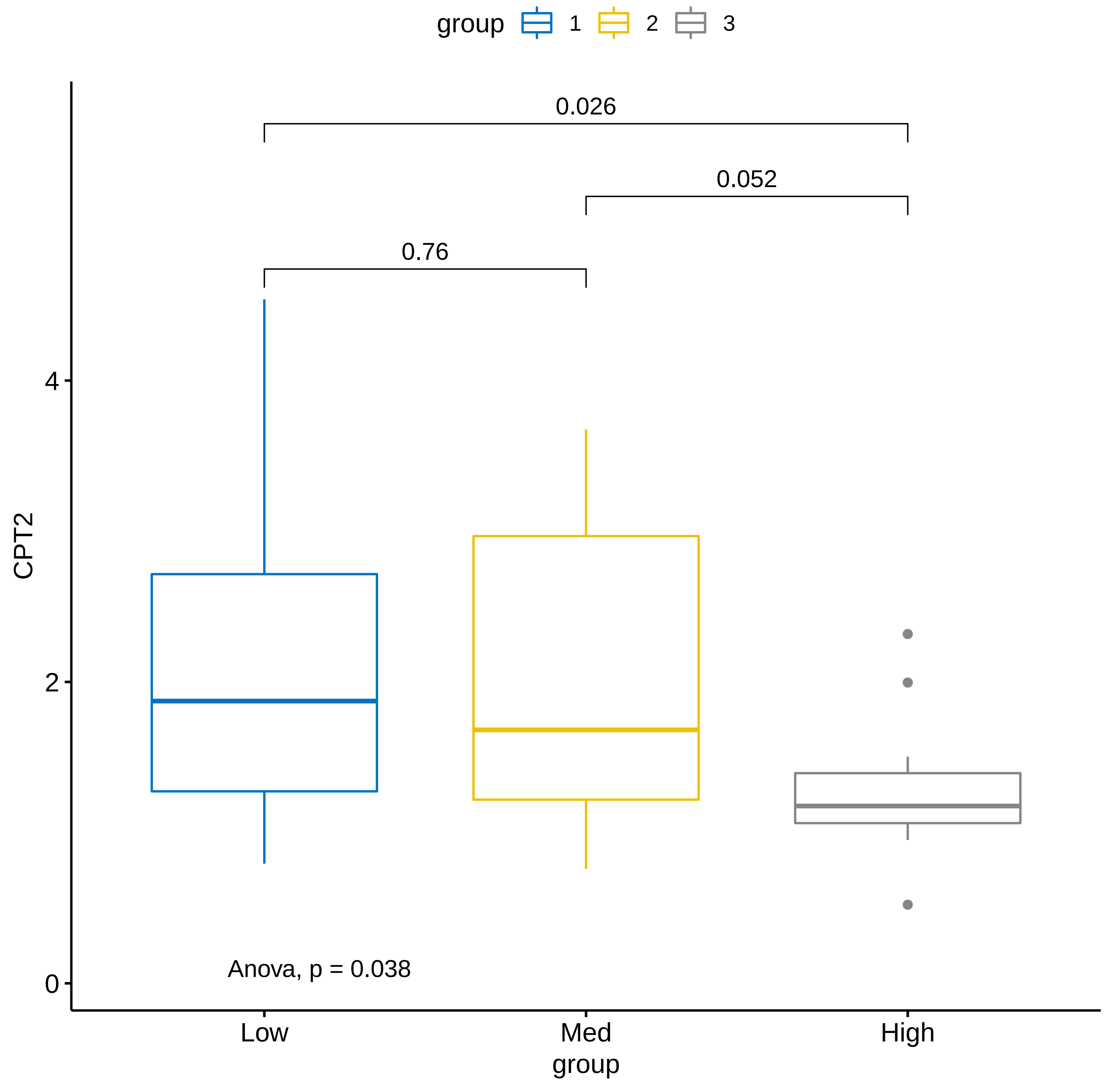

group 1 2 3
0.026
0.052
0.76
CPT2
4
2
0
Anova, p = 0.038
Low
Med
High
group

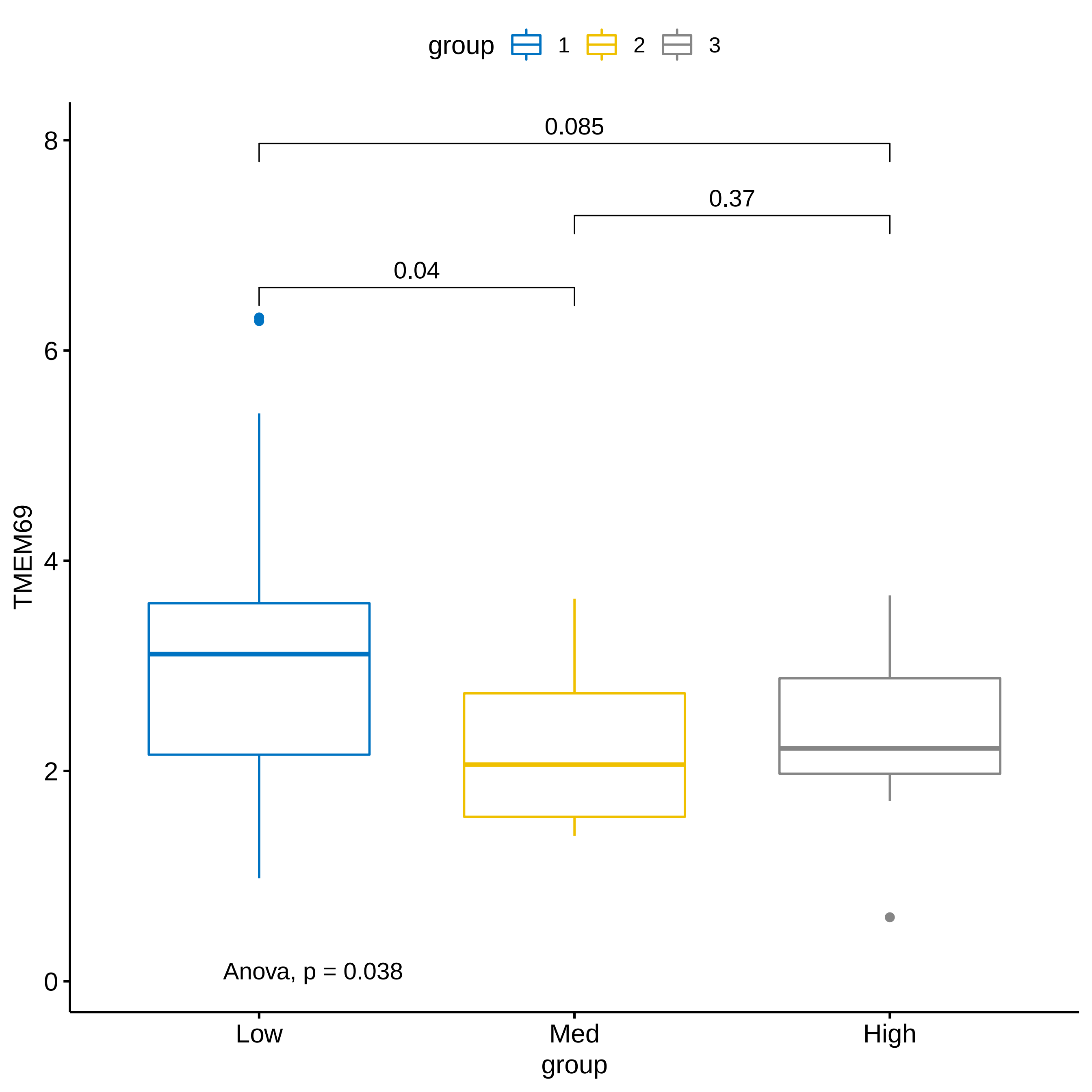

group
1
2
3
0.085
0.37
0.04
8
6
4
2
0
TMEM69
Anova, p = 0.038
Low
Med
High
group

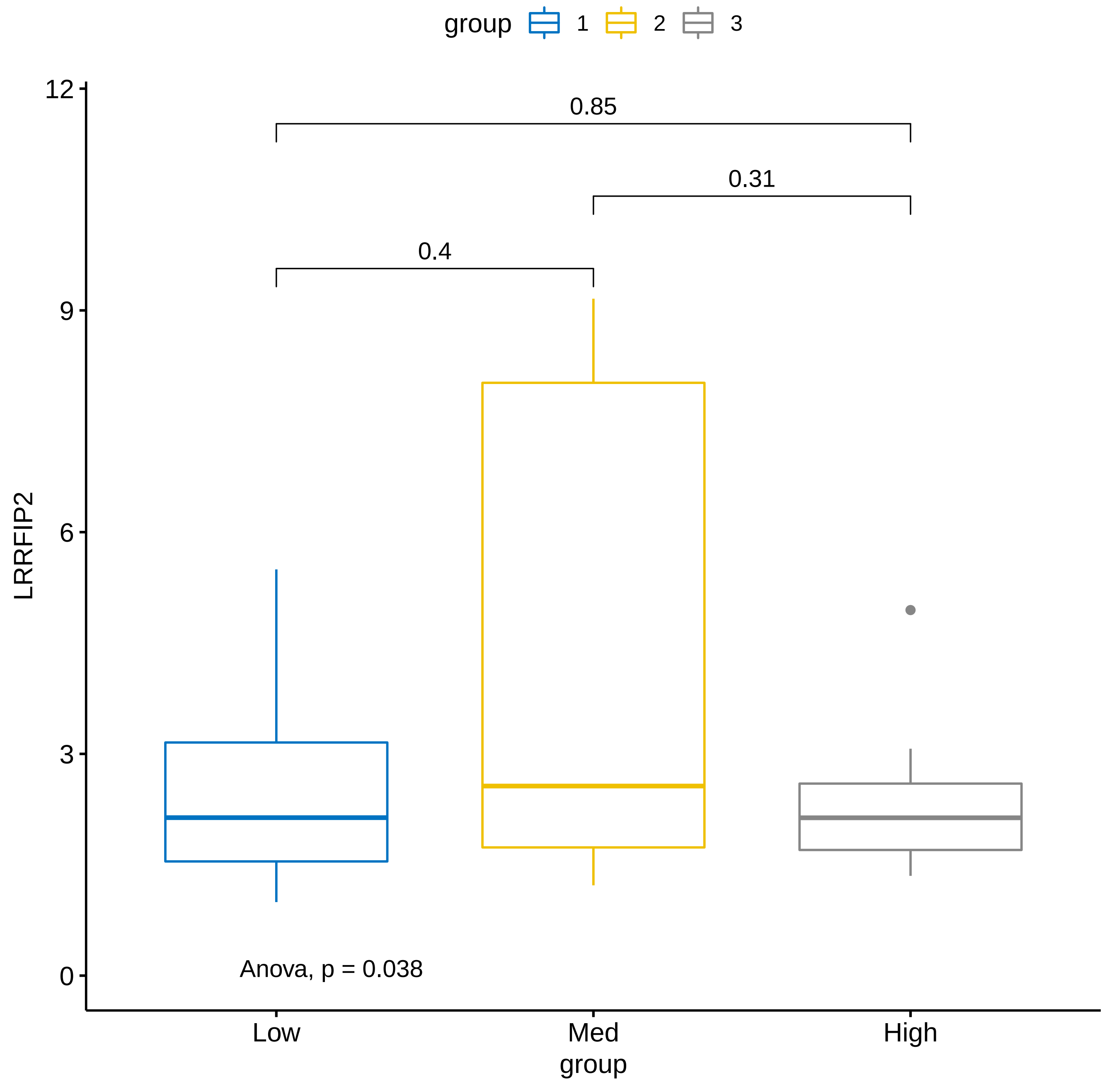

group
1
2
3
LRRFIP2
0
3
6
9
12
0.85
0.31
0.4
Anova, p = 0.038
Low
Med
High
group

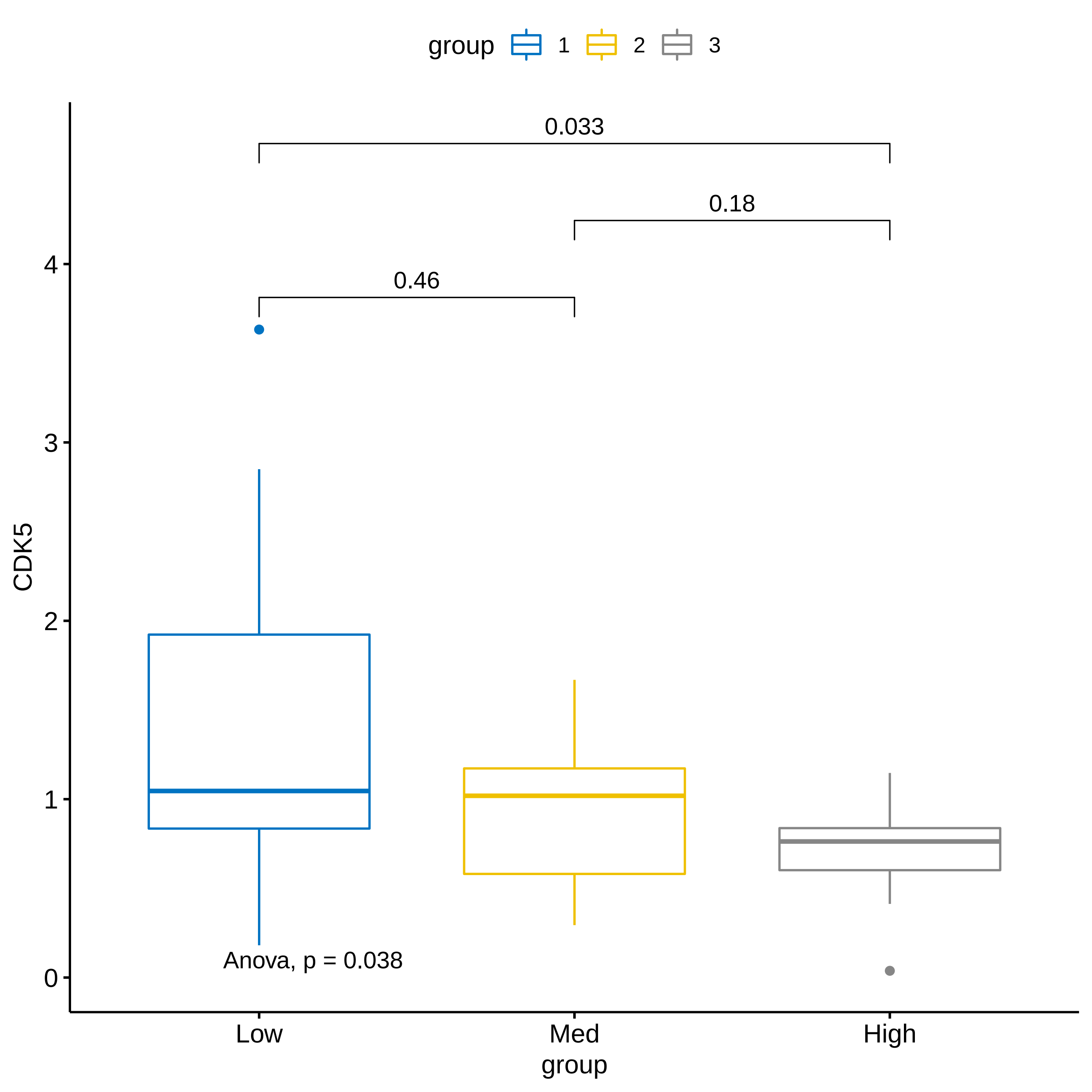

group 1 2 3
0.033
0.18
0.46
4
3
2
1
0
CDK5
Anova, p = 0.038
Low
Med
High
group

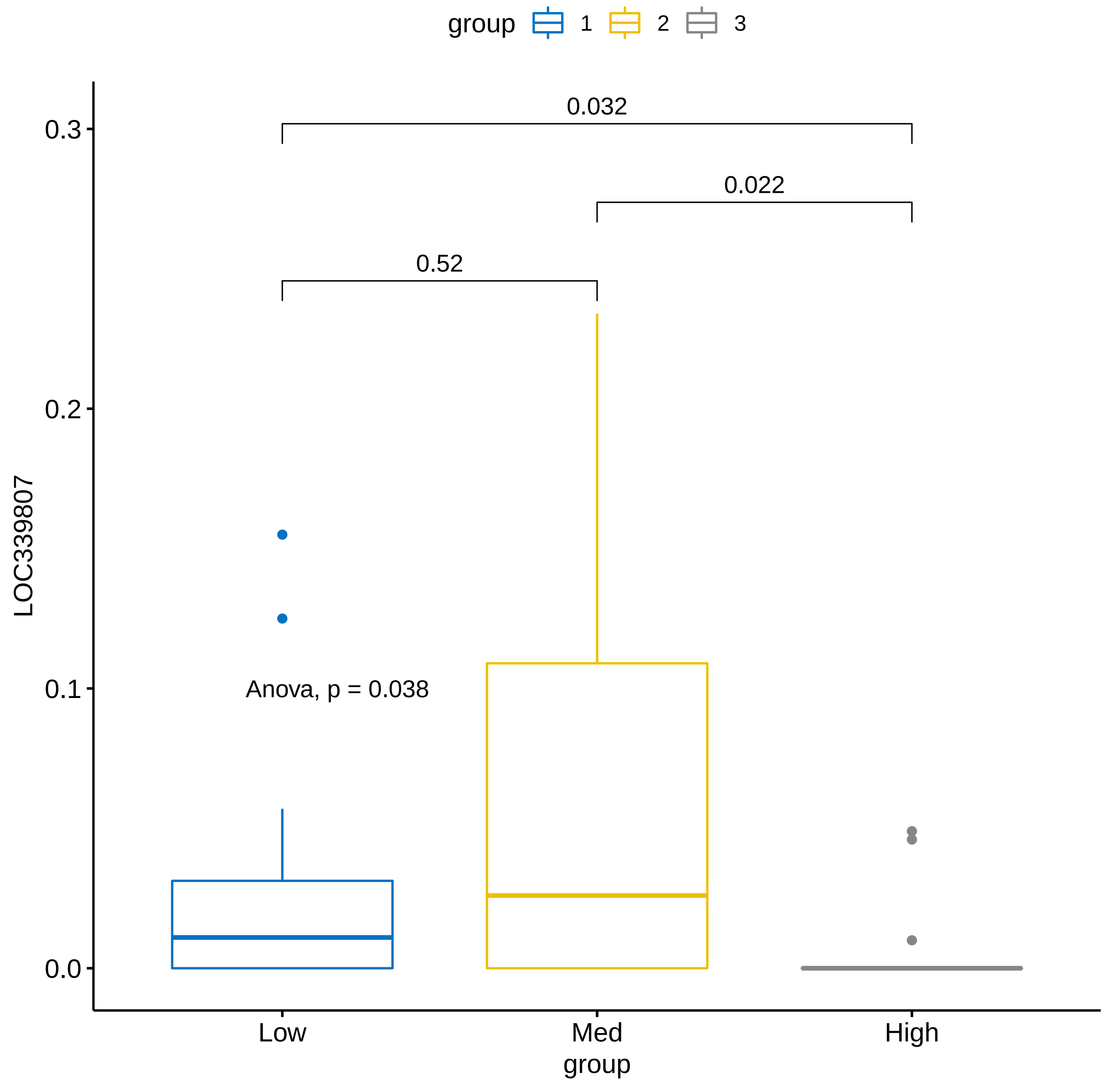
group 1 2 3
0.032
0.022
0.52
Anova, p = 0.038
0.3
0.2
0.1
0.0
LOC339807
Low
Med
High
group

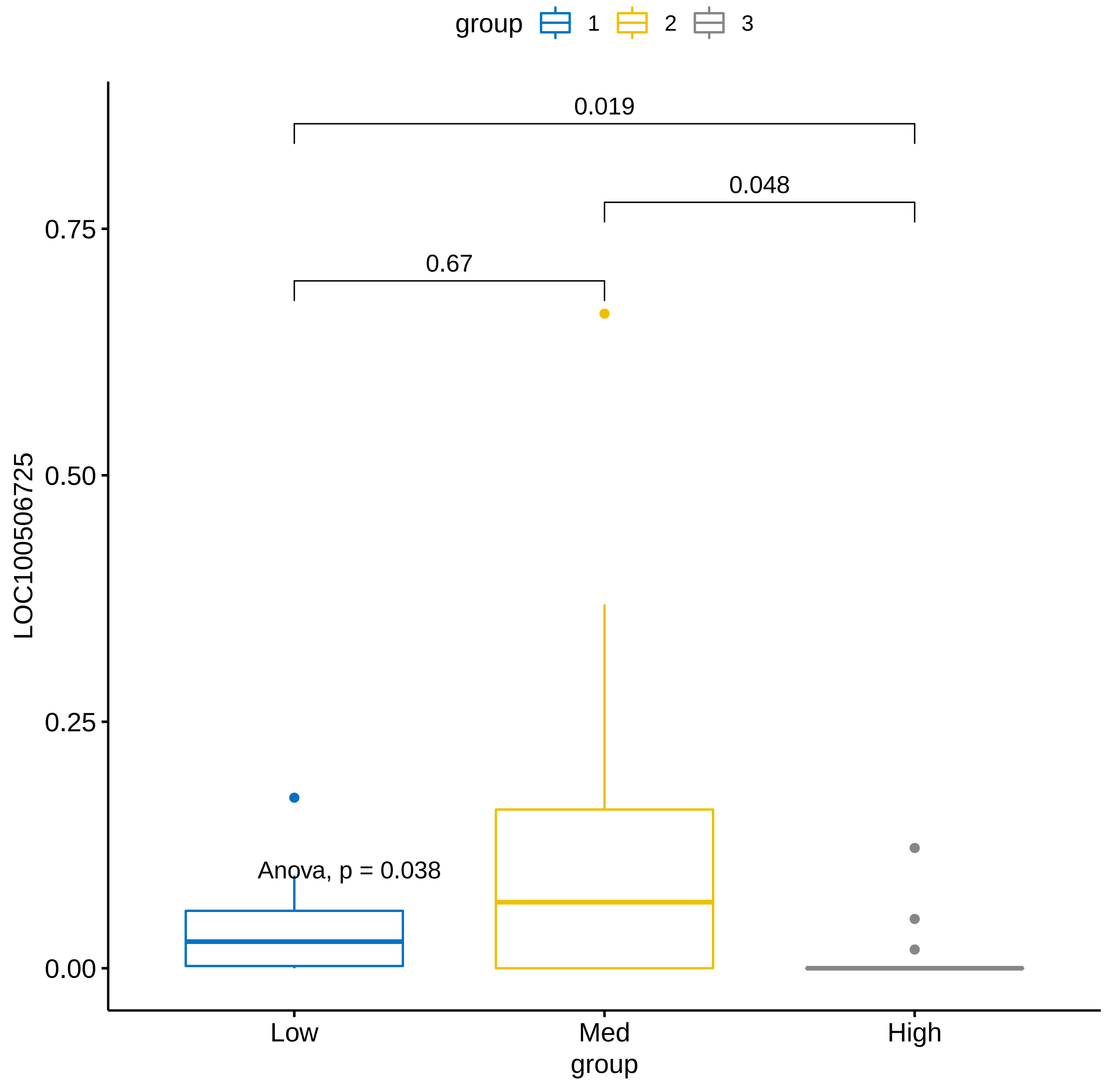

group
1
2
3
0.019
0.048
0.67
Anova, p = 0.038
LOC100506725
0.75
0.50
0.25
0.00
Low
Med
High
group

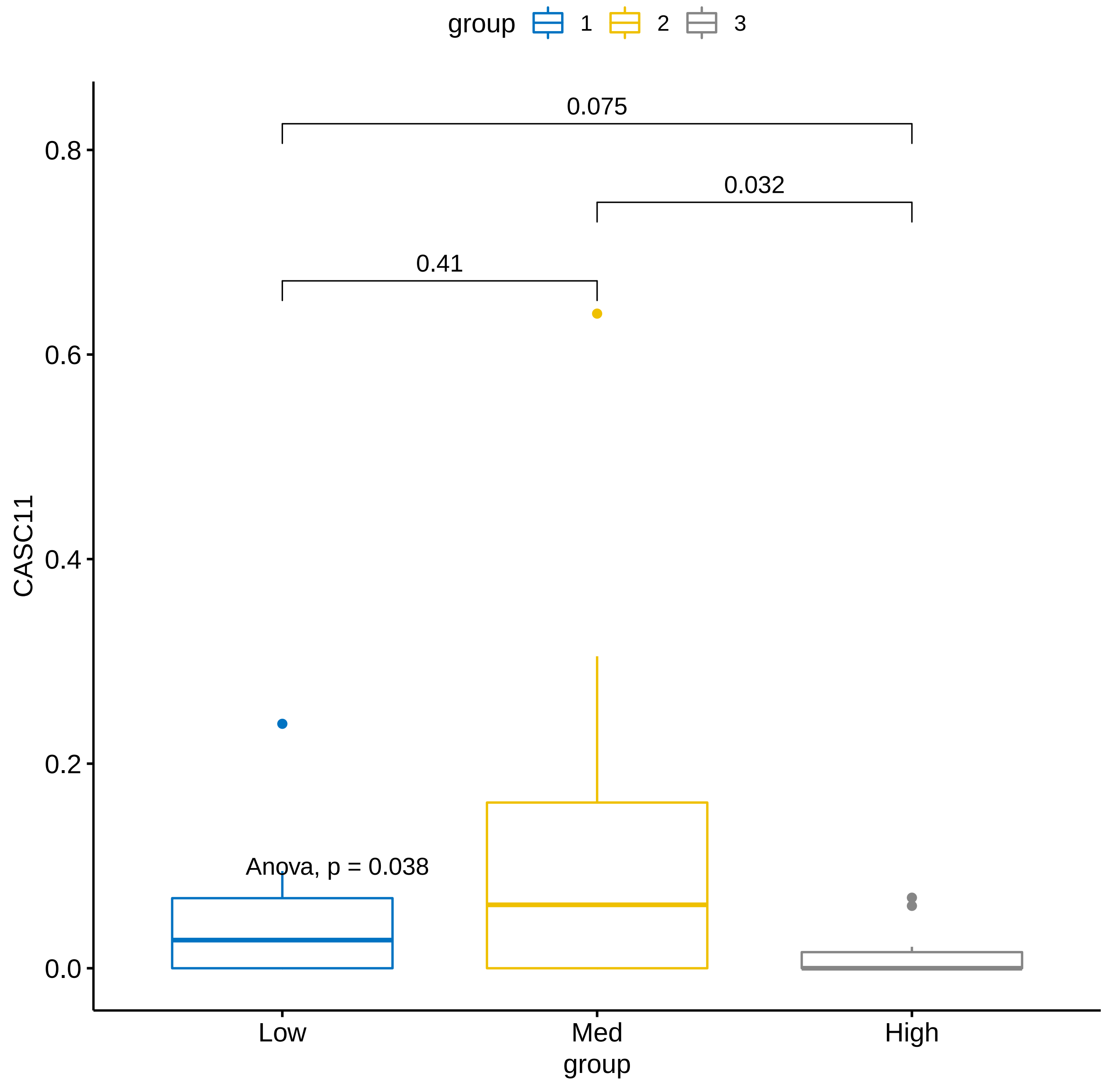
group
1
2
3
0.075
0.032
0.41
Anova, p = 0.038
0.8
0.6
0.4
0.2
0.0
CASC11
Low
Med
High
group

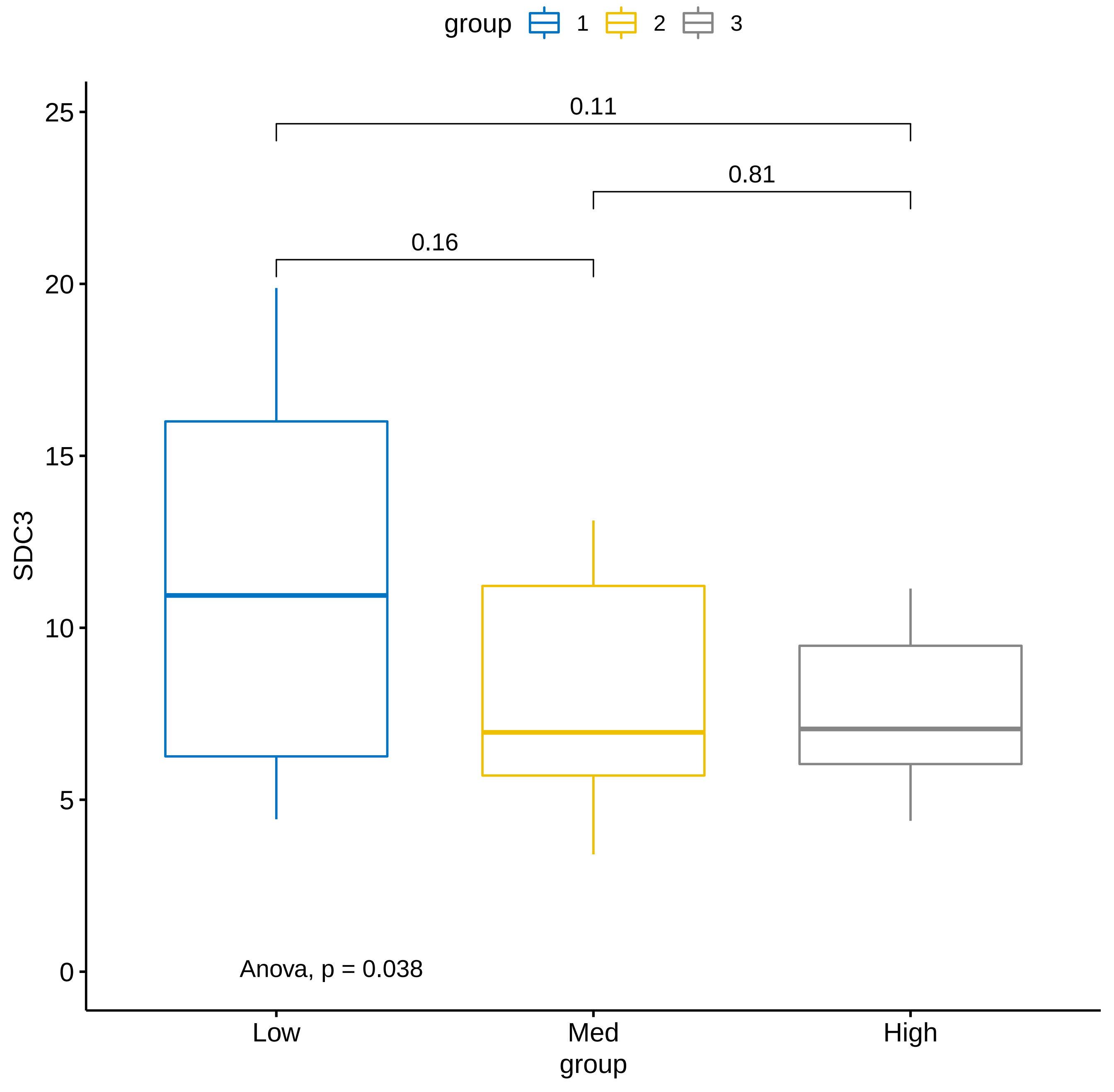

group 1 2 3
0.11
0.81
0.16
25
20
15
10
5
0
SDC3
Anova, p = 0.038
Low
Med
High
group

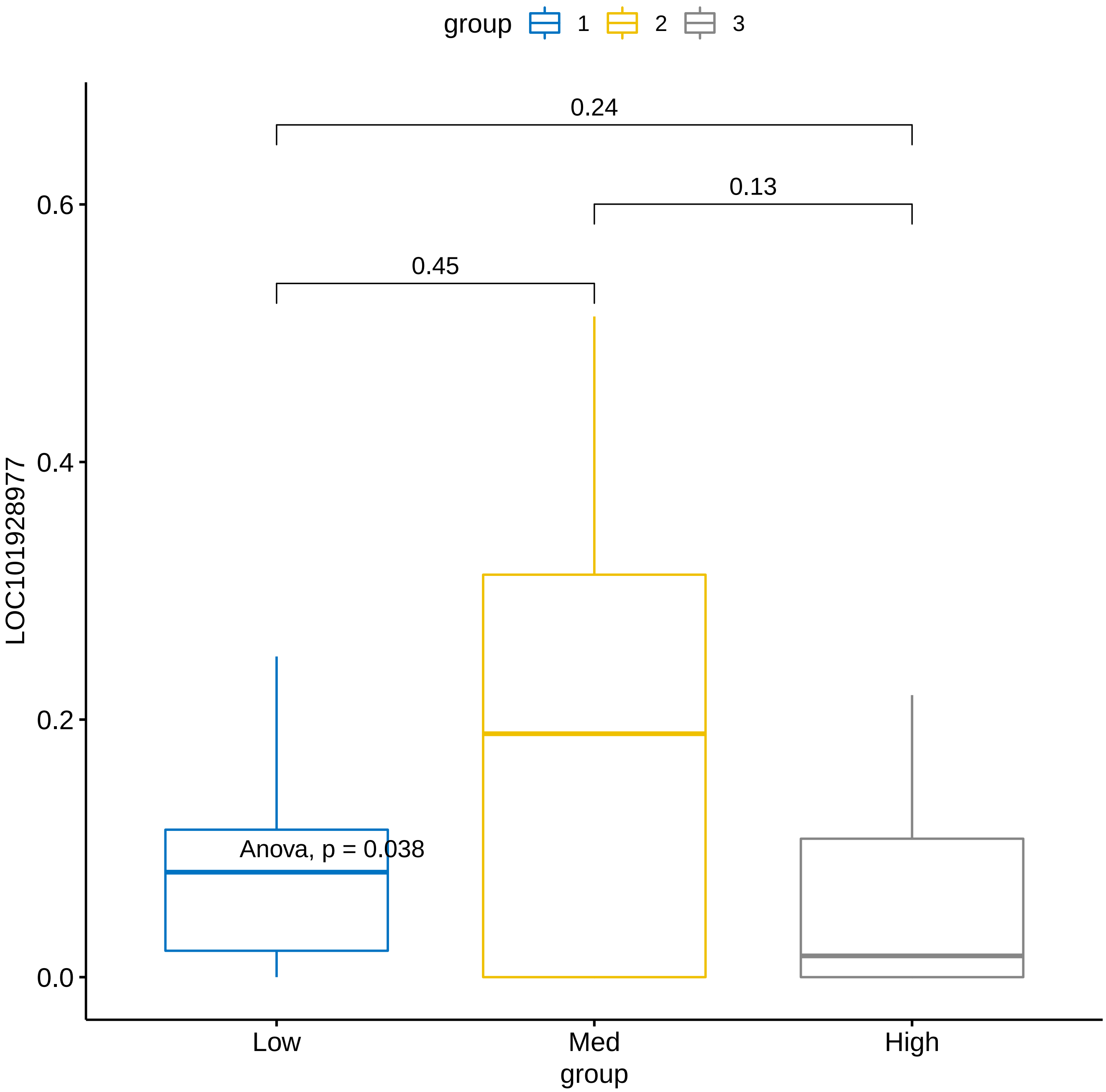
group 1 2 3
0.24
0.13
0.45
Anova, p = 0.038
0.6
0.4
0.2
0.0
LOC101928977
Low
Med
High
group

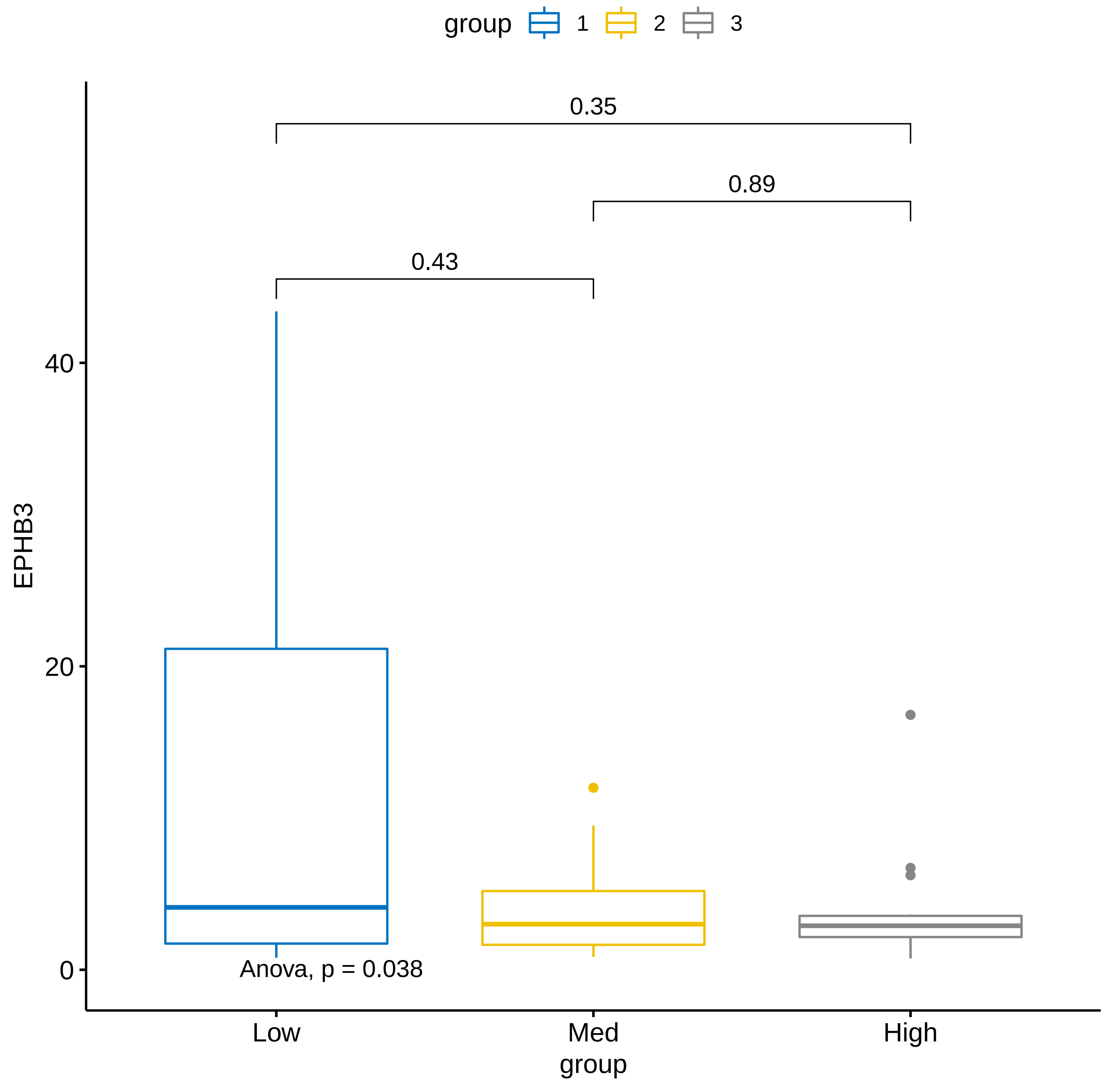
group
1
2
3
0.35
0.89
0.43
40
20
0
EPHB3
Anova, p = 0.038
Low
Med
High
group

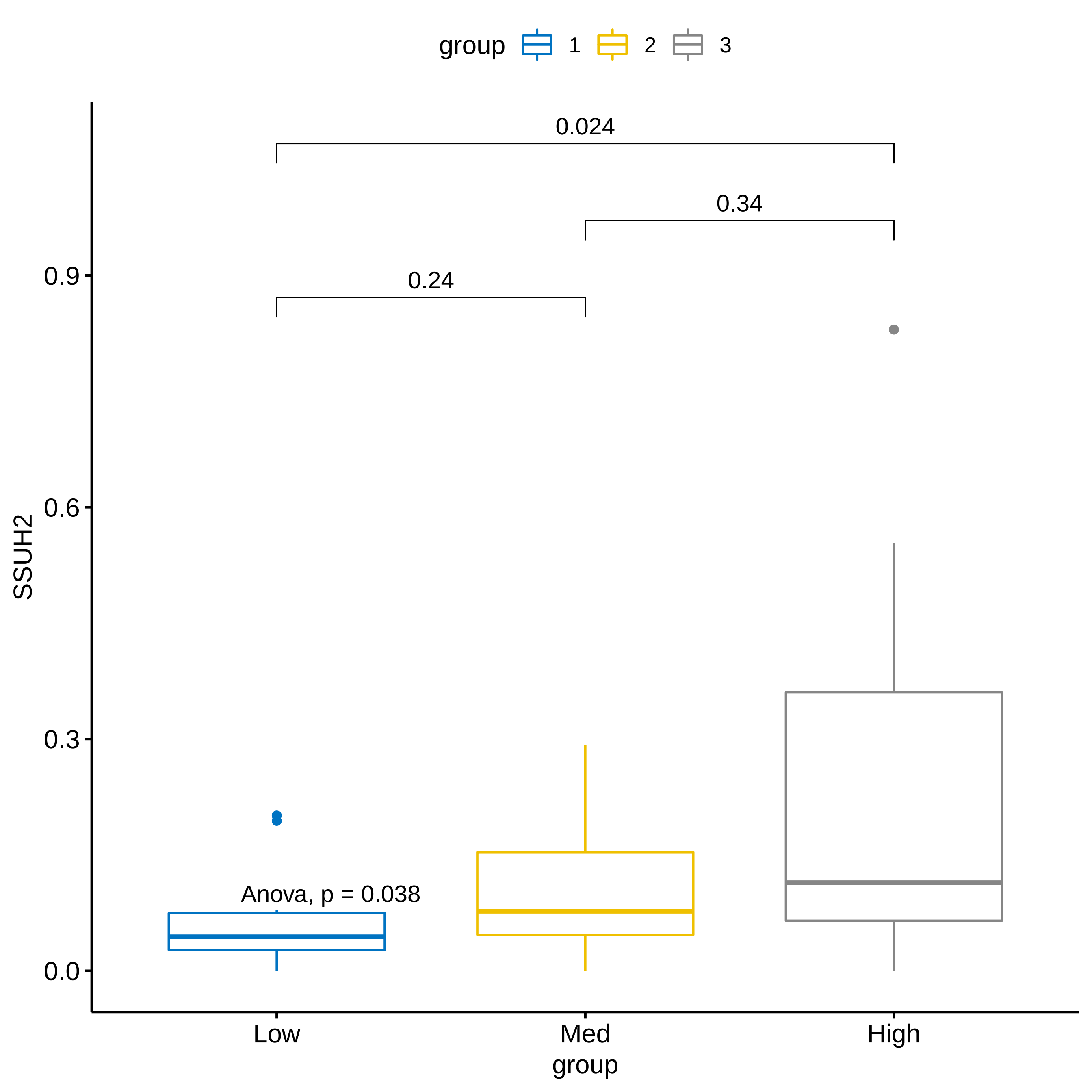
group 1 2 3
0.024
0.34
0.24
0.9
0.6
0.3
0.0
SSUH2
Anova, p = 0.038
Low
Med
High
group

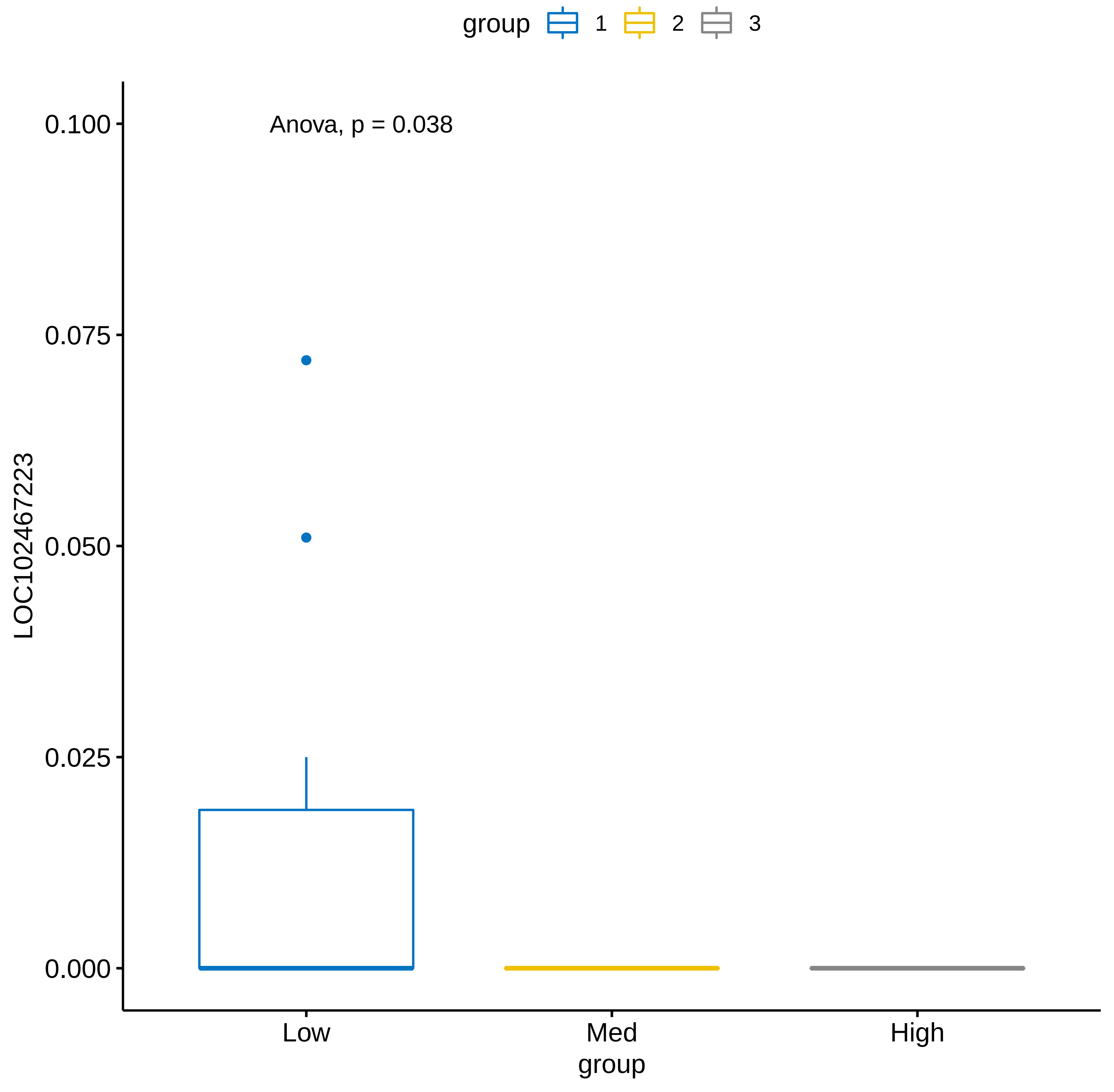
group 1 2 3
Anova, p = 0.038
0.100
0.075
0.050
0.025
0.000
LOC102467223
Low
Med
High
group

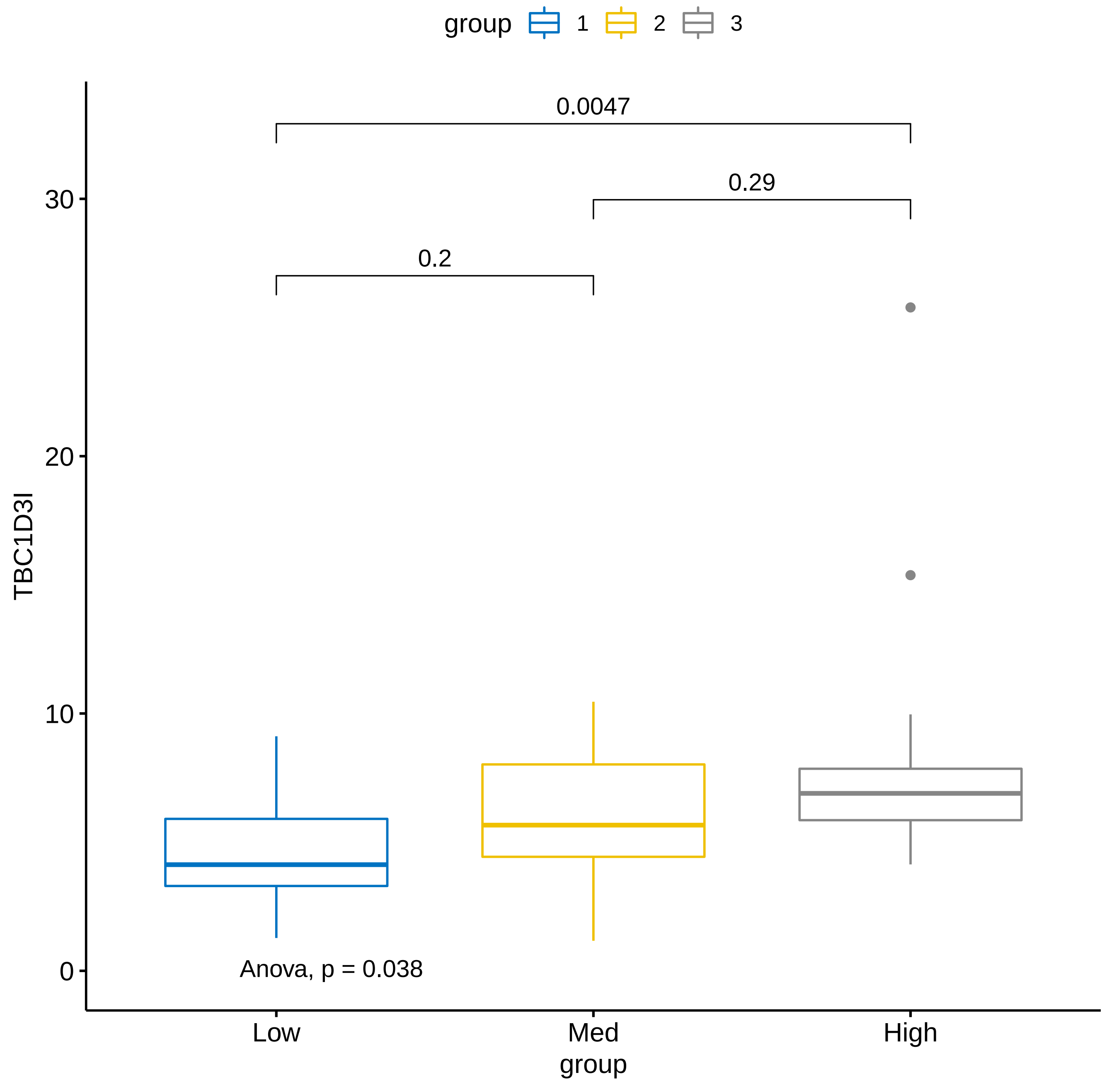
group
1
2
3
0.0047
0.29
0.2
30
20
10
0
TBC1D3I
Anova, p = 0.038
Low
Med
High
group

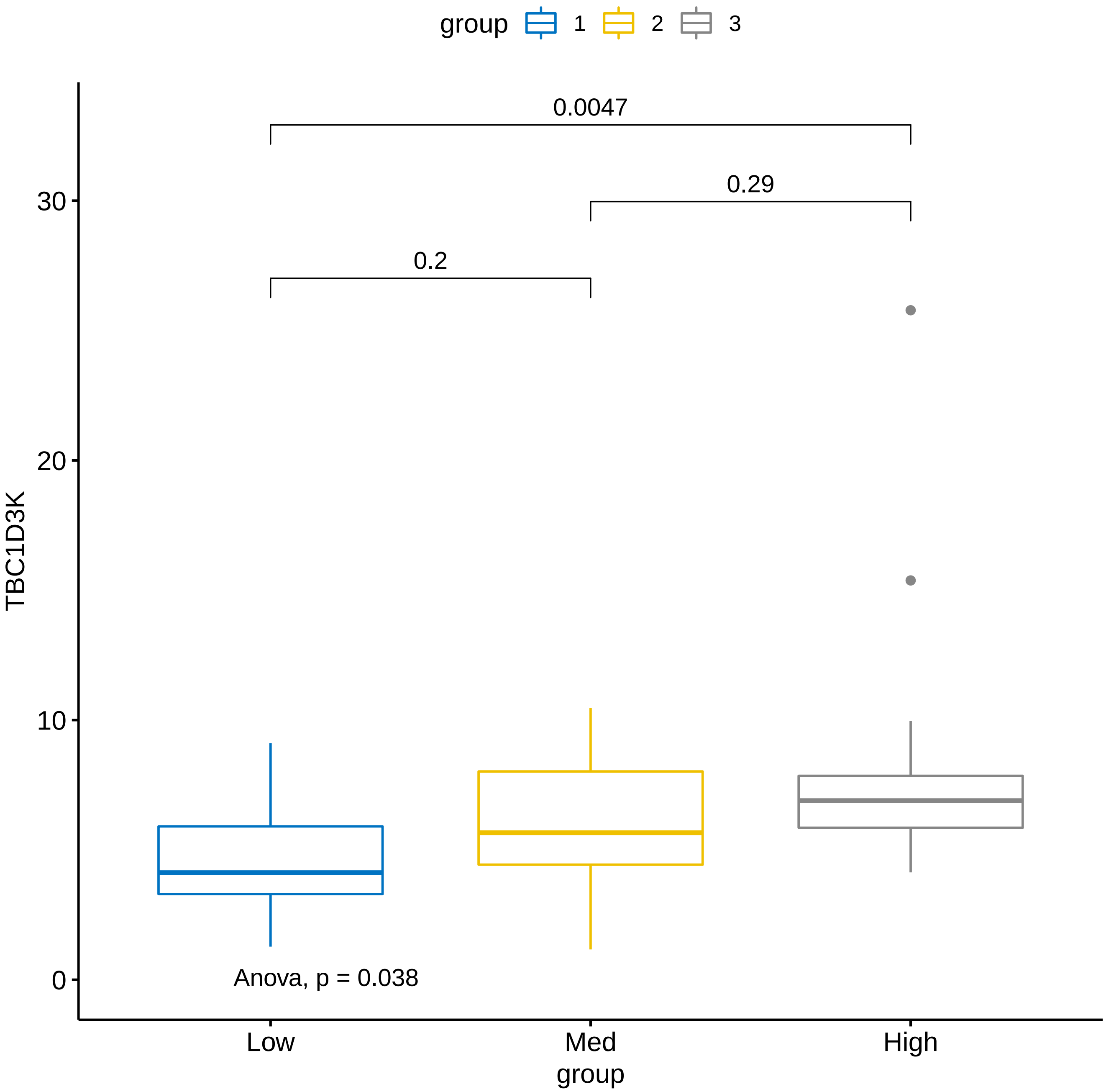
group 1 2 3
0.0047
0.29
0.2
30
20
10
0
TBC1D3K
Anova, p = 0.038
Low
Med
High
group

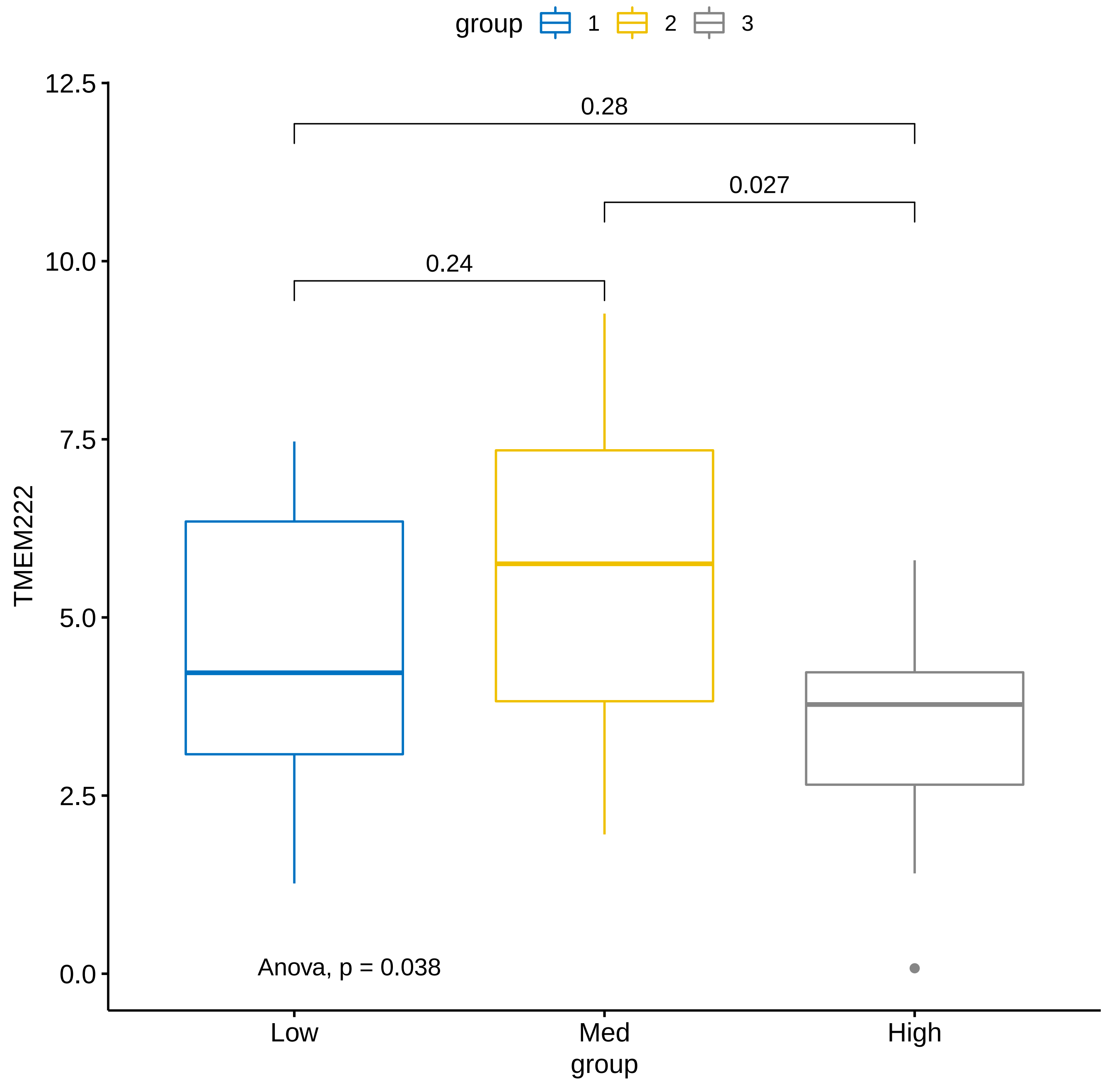
group
1
2
3
12.5
10.0
7.5
5.0
2.5
0.0
TMEM222
0.28
0.027
0.24
Anova, p = 0.038
Low
Med
High
group

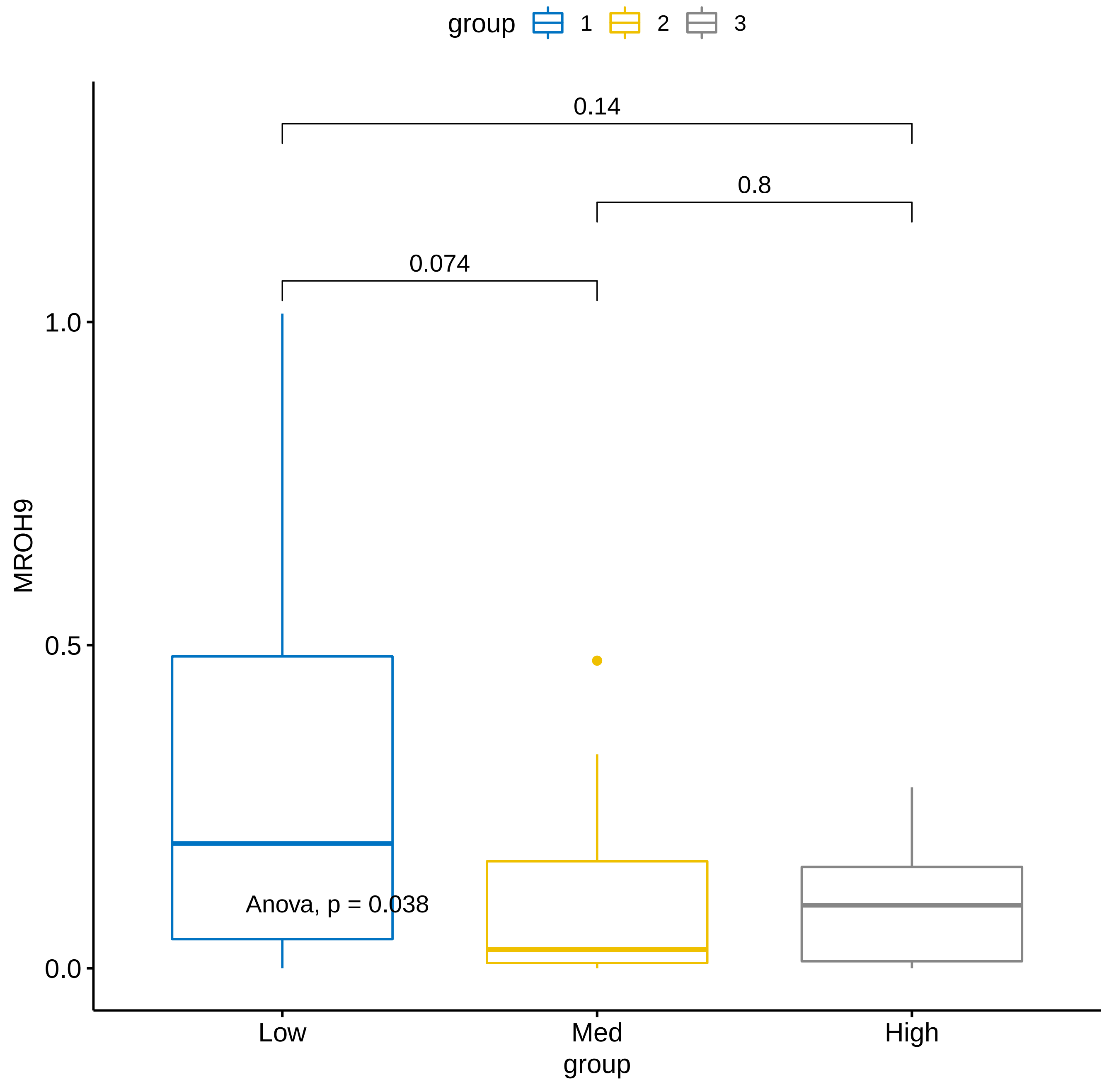
group
1
2
3
0.14
0.8
0.074
1.0
0.5
0.0
MROH9
Anova, p = 0.038
Low
Med
High
group

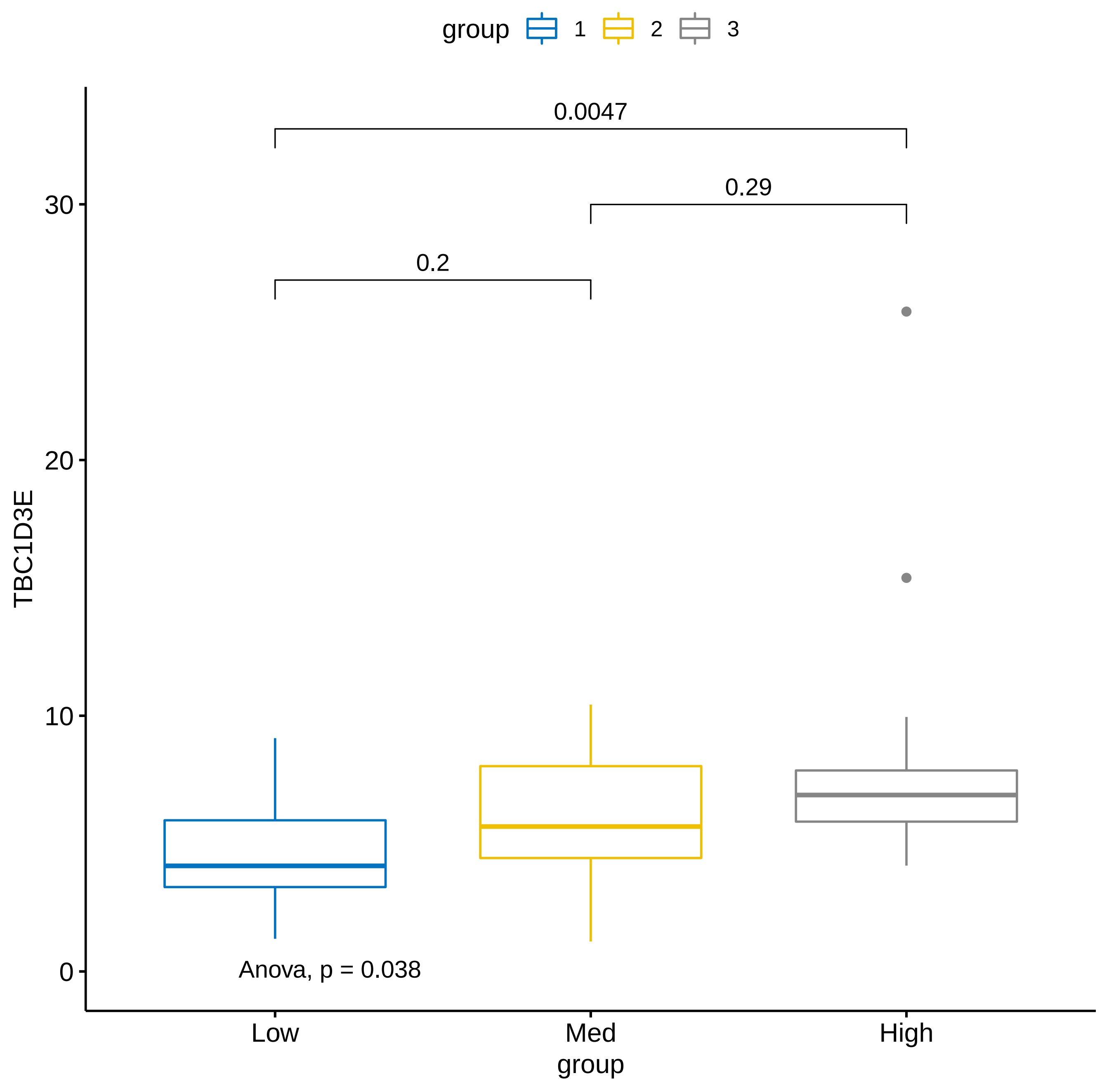

group
1
2
3
0.0047
0.29
0.2
30
20
10
0
TBC1D3E
Anova, p = 0.038
Low
Med
High
group

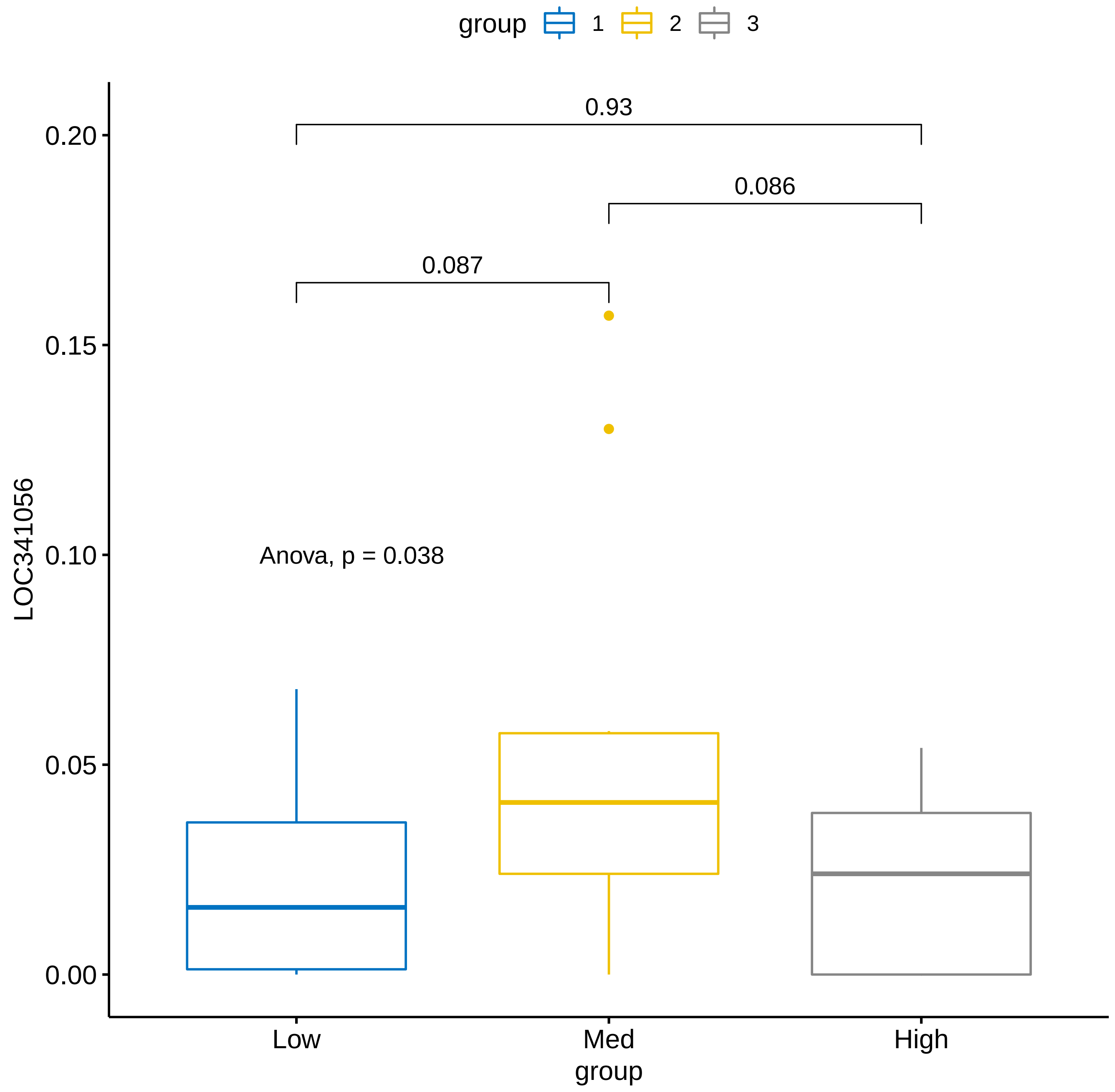
group 1 2 3
0.93
0.086
0.087
Anova, p = 0.038
LOC341056
0.20
0.15
0.10
0.05
0.00
Low
Med
High
group

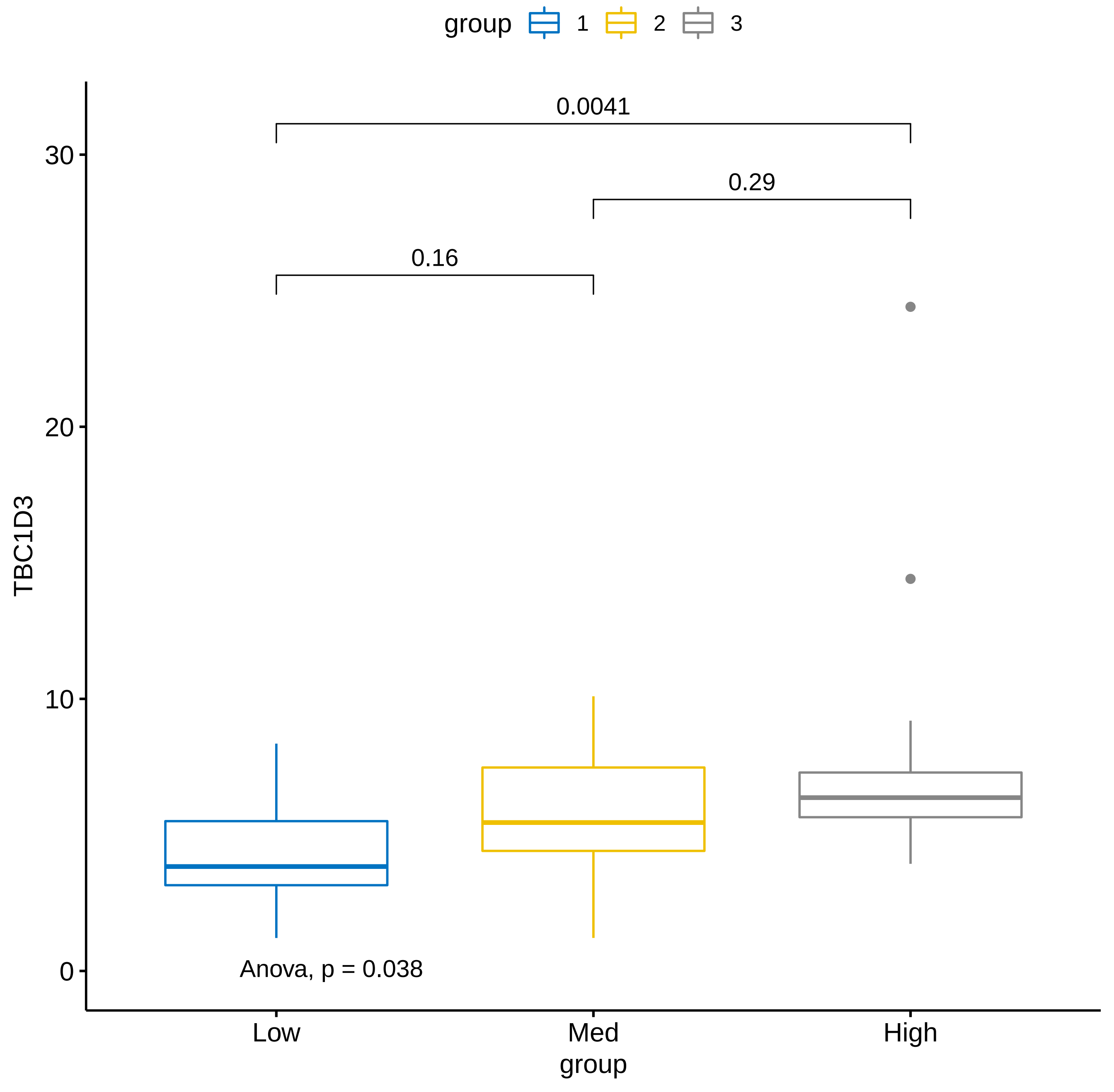
group
1
2
3
0.0041
0.29
0.16
30
20
10
0
TBC1D3
Anova, p = 0.038
Low
Med
High
group

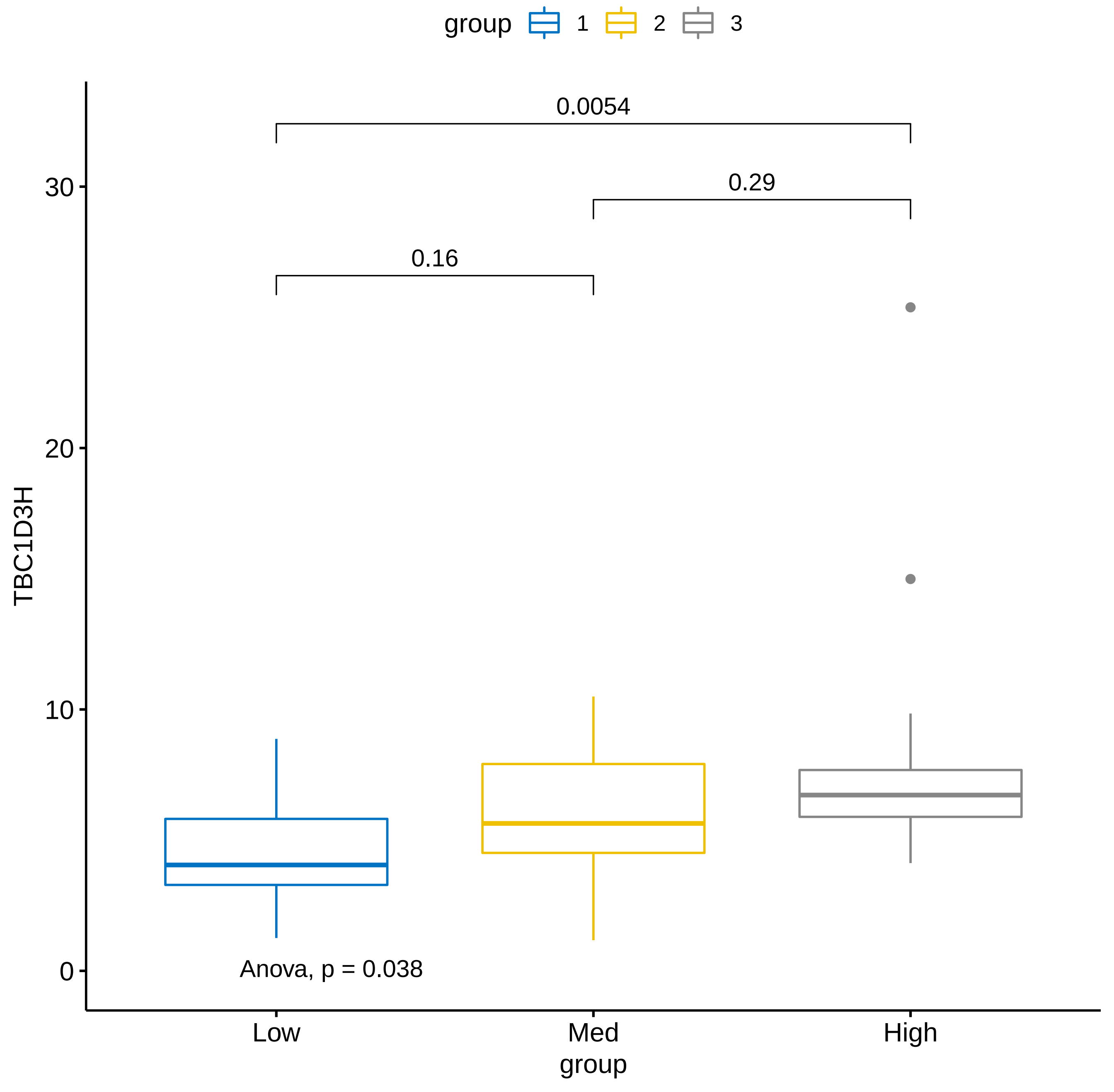
group 1 2 3
0.0054
0.29
0.16
30
20
10
0
TBC1D3H
Anova, p = 0.038
Low
Med
High
group

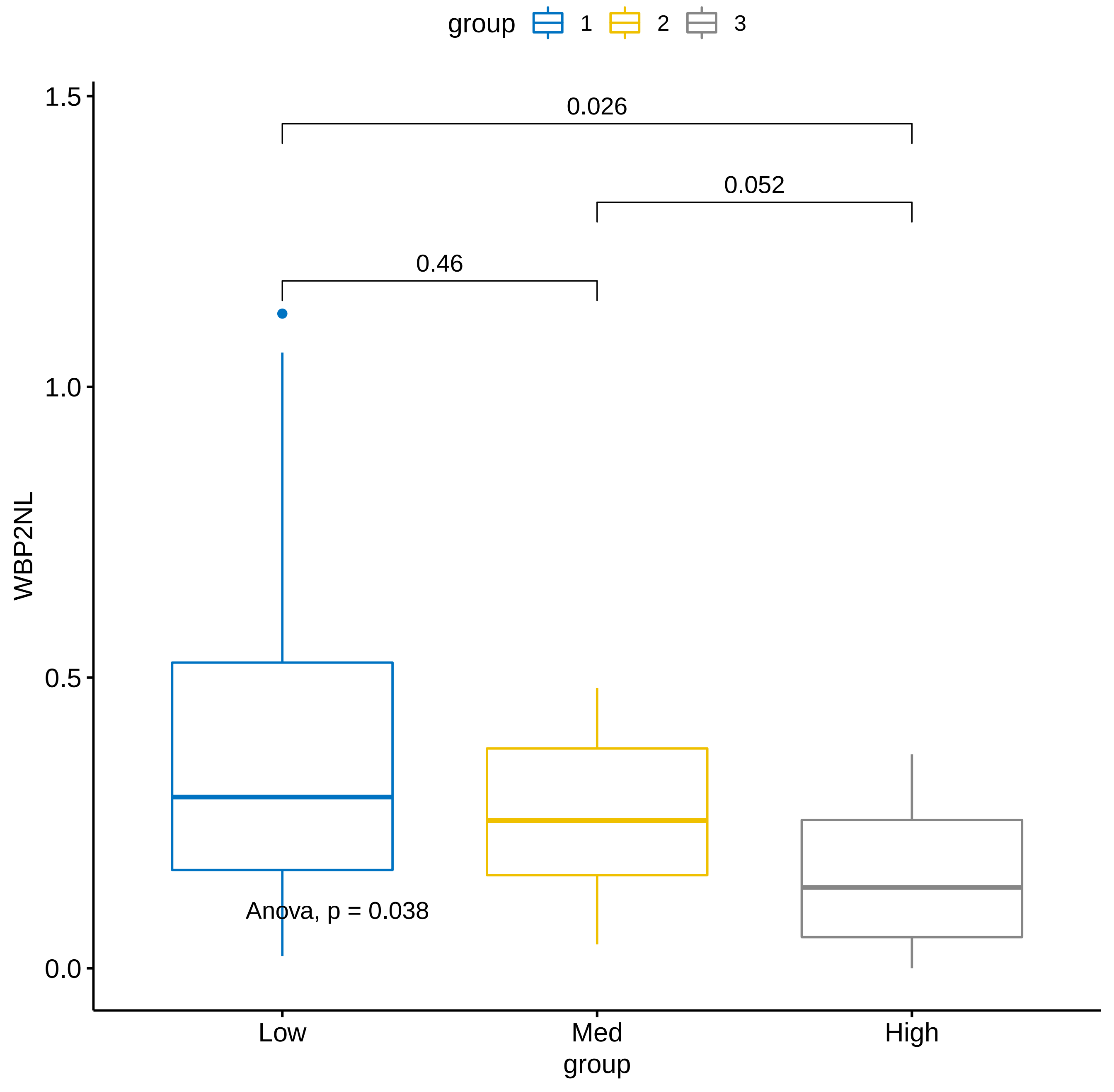

group
1
2
3
0.026
0.052
0.46
1.5
1.0
0.5
0.0
WBP2NL
Anova, p = 0.038
Low
Med
High
group

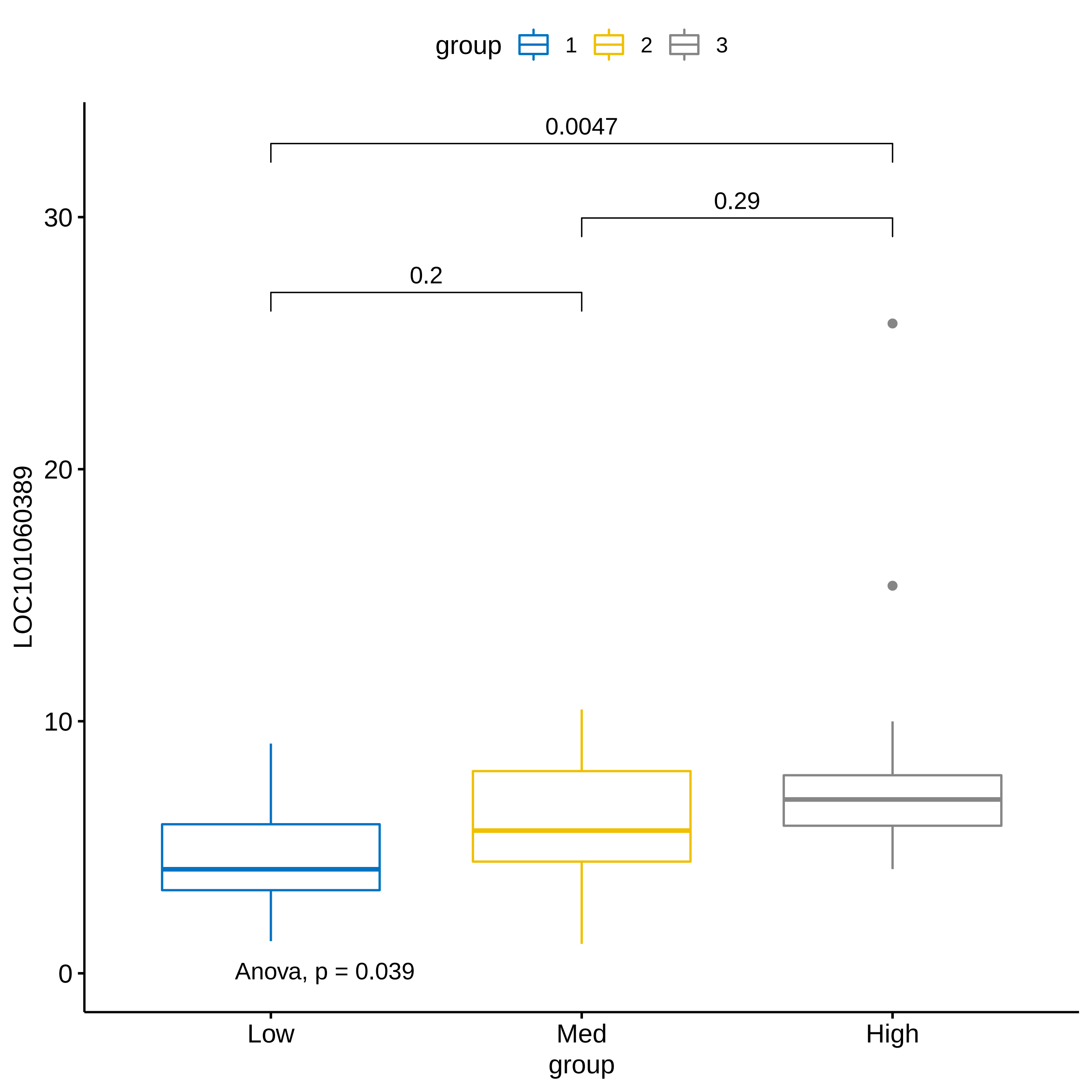
group
1
2
3
0.0047
0.29
0.2
30
20
10
0
LOC101060389
Anova, p = 0.039
Low
Med
High
group

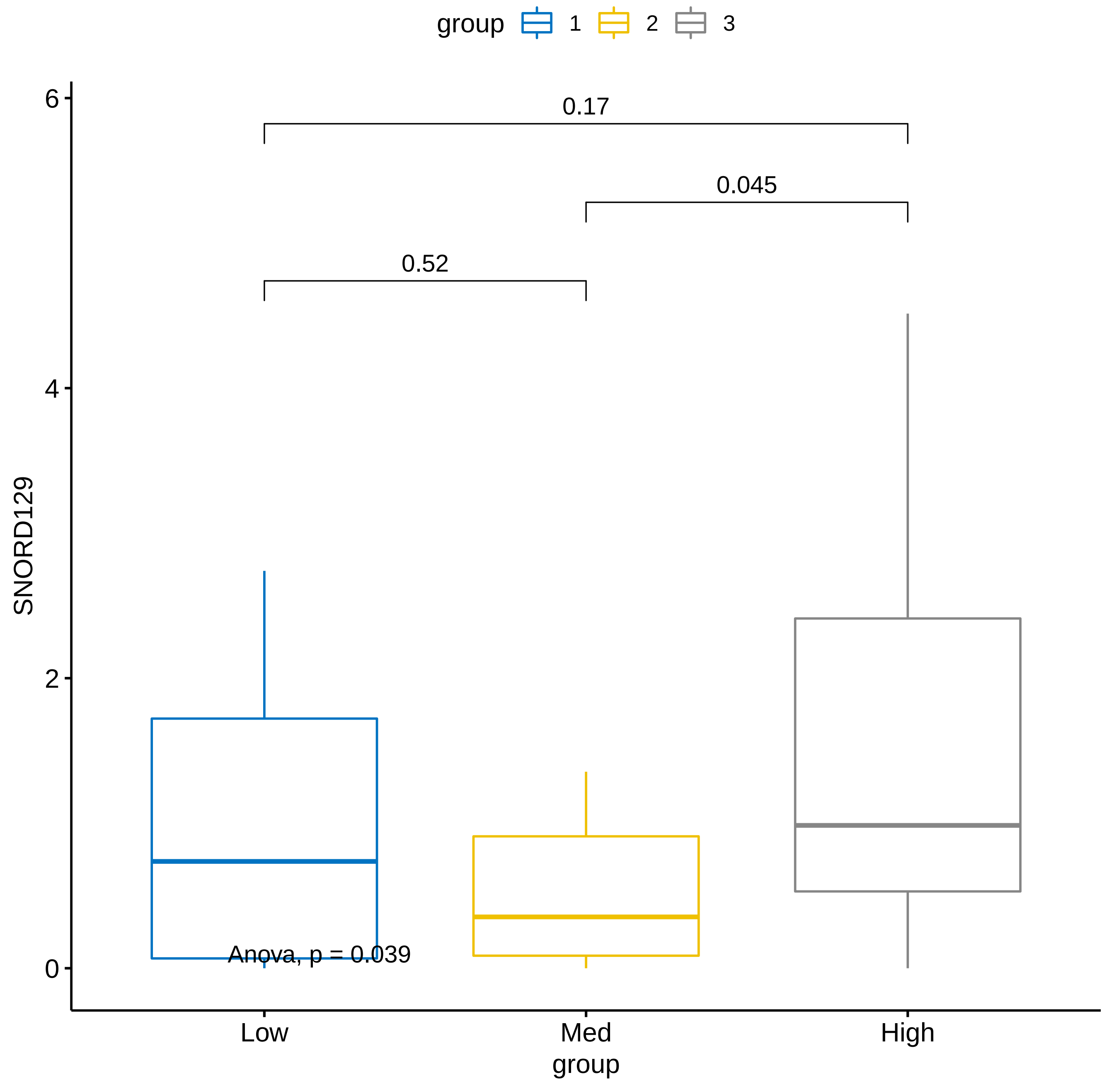
group
1
2
3
0.17
0.045
0.52
Anova, p = 0.039
6
4
2
0
SNORD129
Low
Med
High
group

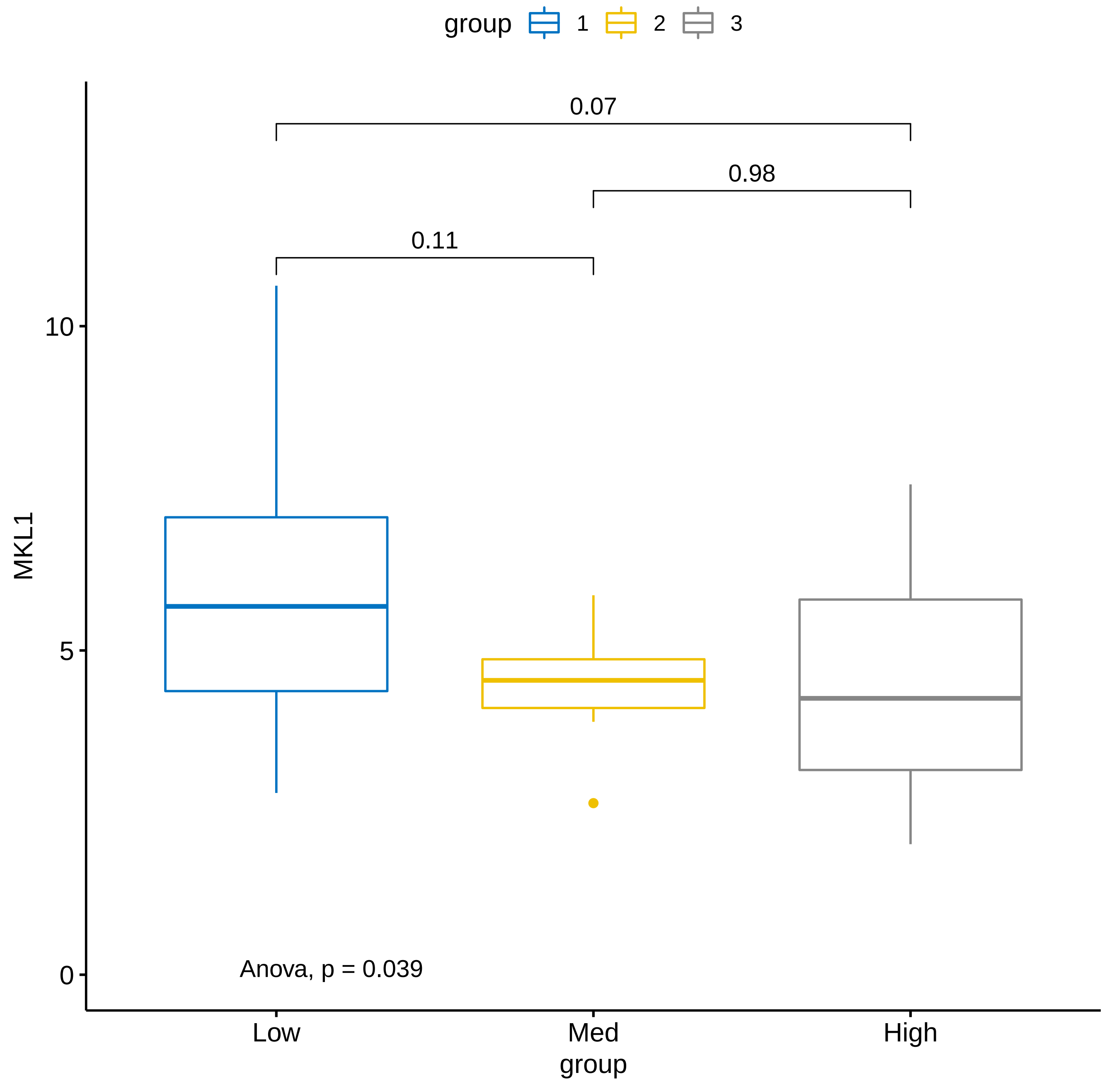

group
1
2
3
0.07
0.98
0.11
10
5
0
MKL1
Anova, p = 0.039
Low
Med
High
group

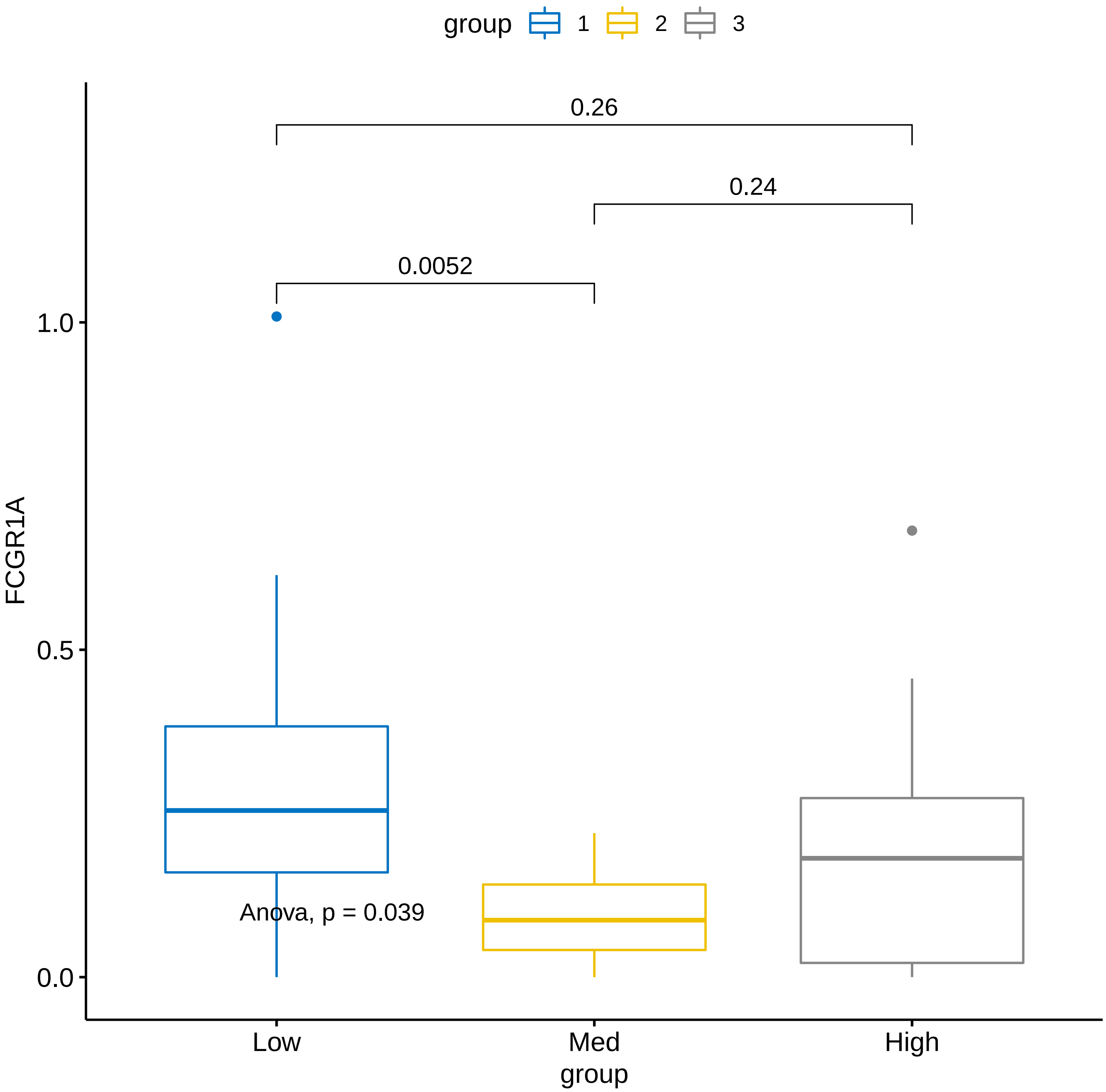
group
1
2
3
0.26
0.24
0.0052
FCGR1A
1.0
0.5
0.0
Anova, p = 0.039
Low
Med
High
group

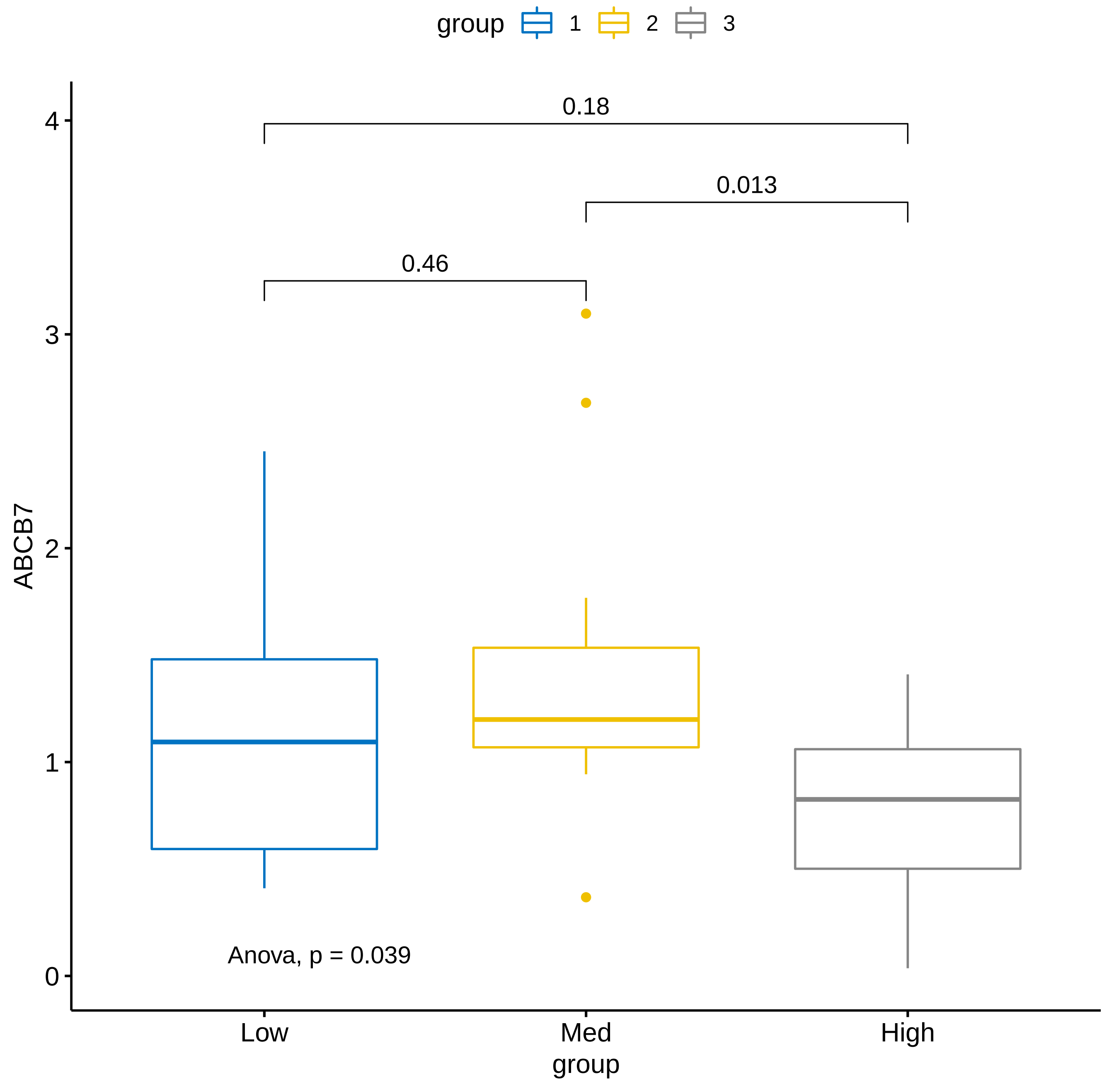
group
1
2
3
0.18
0.013
0.46
ABCB7
4
3
2
1
0
Anova, p = 0.039
Low
Med
High
group

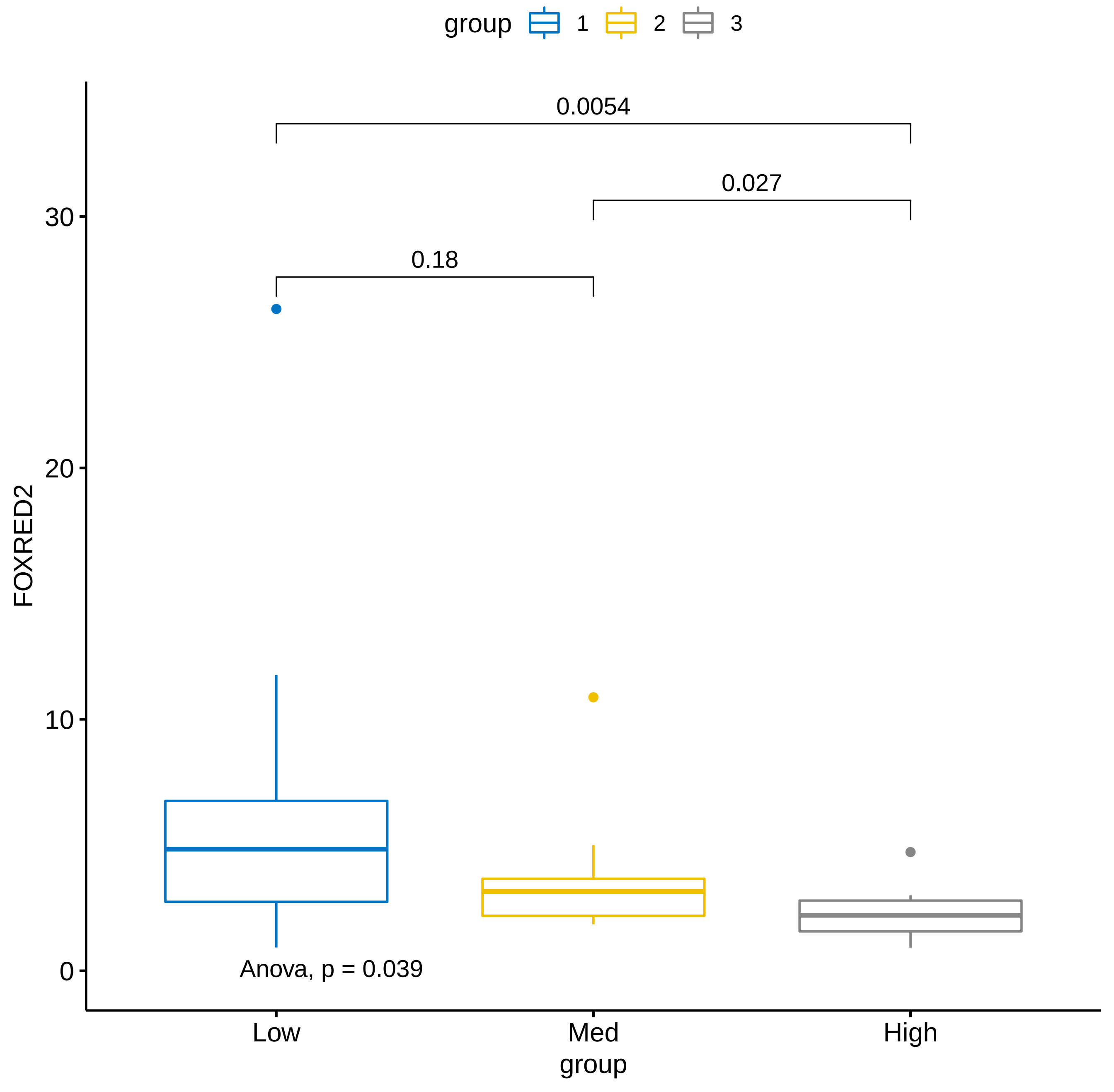
group
1
2
3
0.0054
0.027
0.18
30
20
10
0
FOXRED2
Anova, p = 0.039
Low
Med
High
group

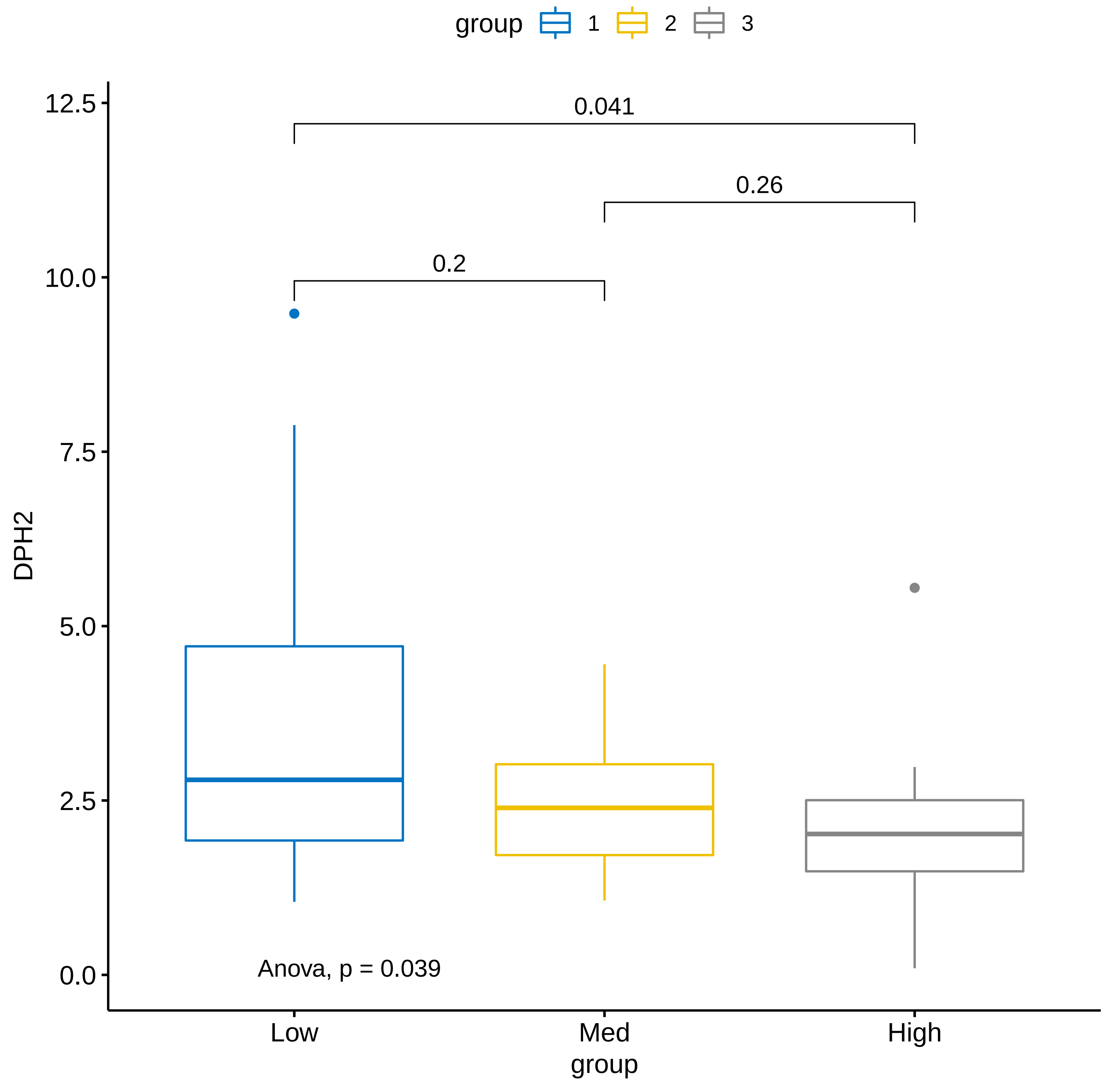
group
1
2
3
0.041
0.26
0.2
12.5
10.0
7.5
5.0
2.5
0.0
DPH2
Anova, p = 0.039
Low
Med
High
group

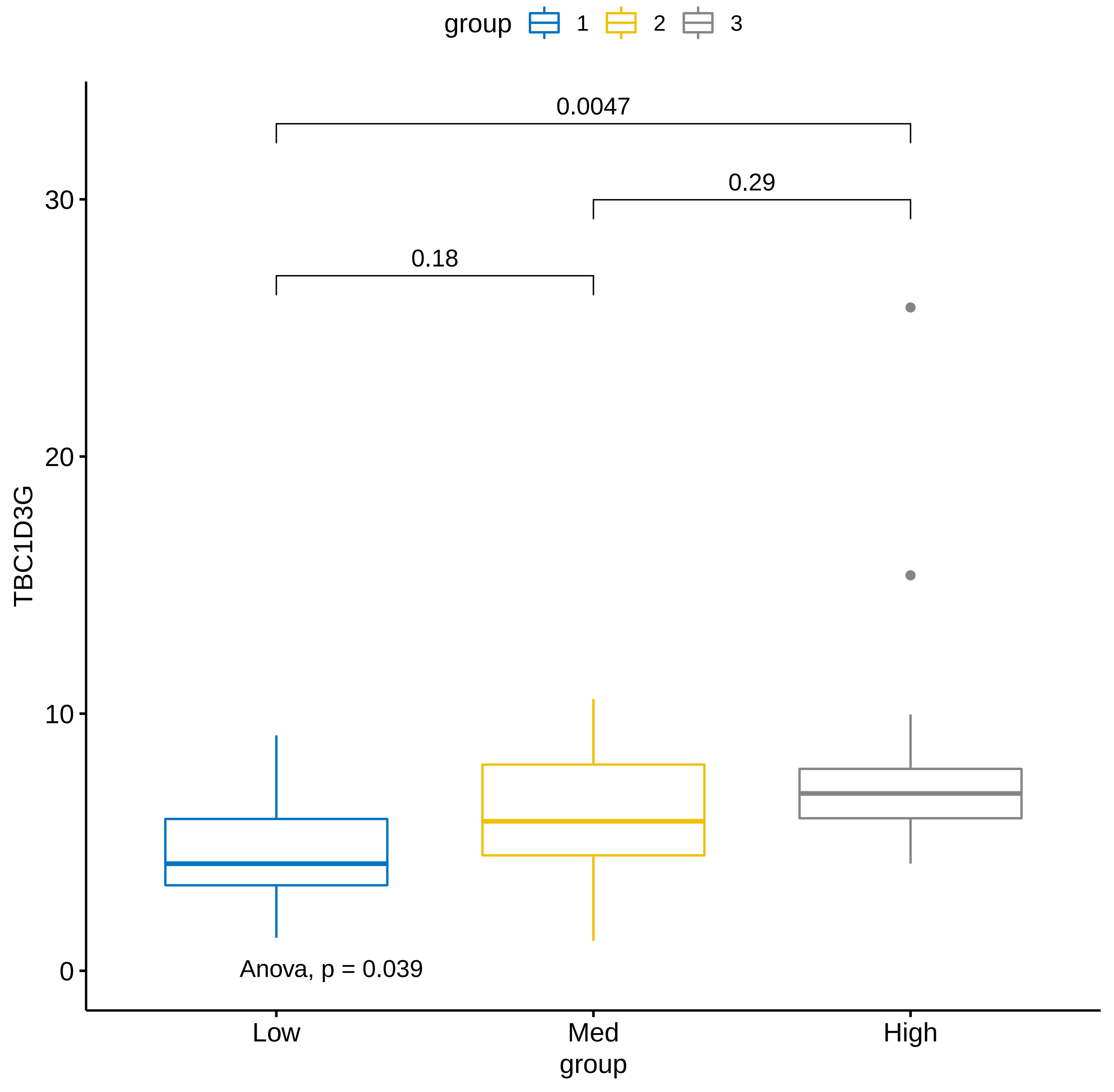
group 1 2 3
0.0047
0.29
0.18
30
20
10
0
TBC1D3G
Anova, p = 0.039
Low
Med
High
group

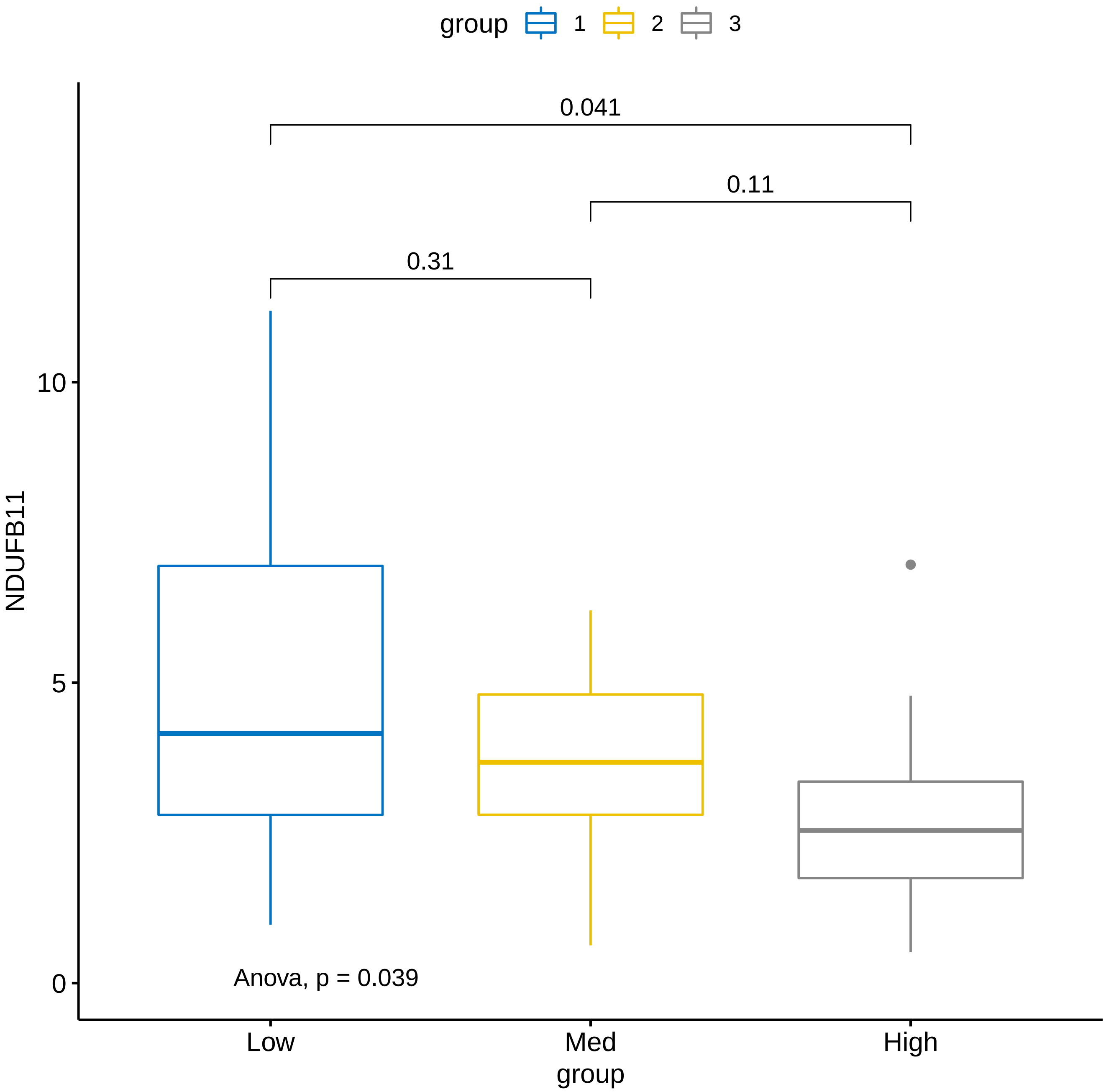

group 1 2 3
0.041
0.11
0.31
NDUFB11
10
5
0
Anova, p = 0.039
Low
Med
High
group

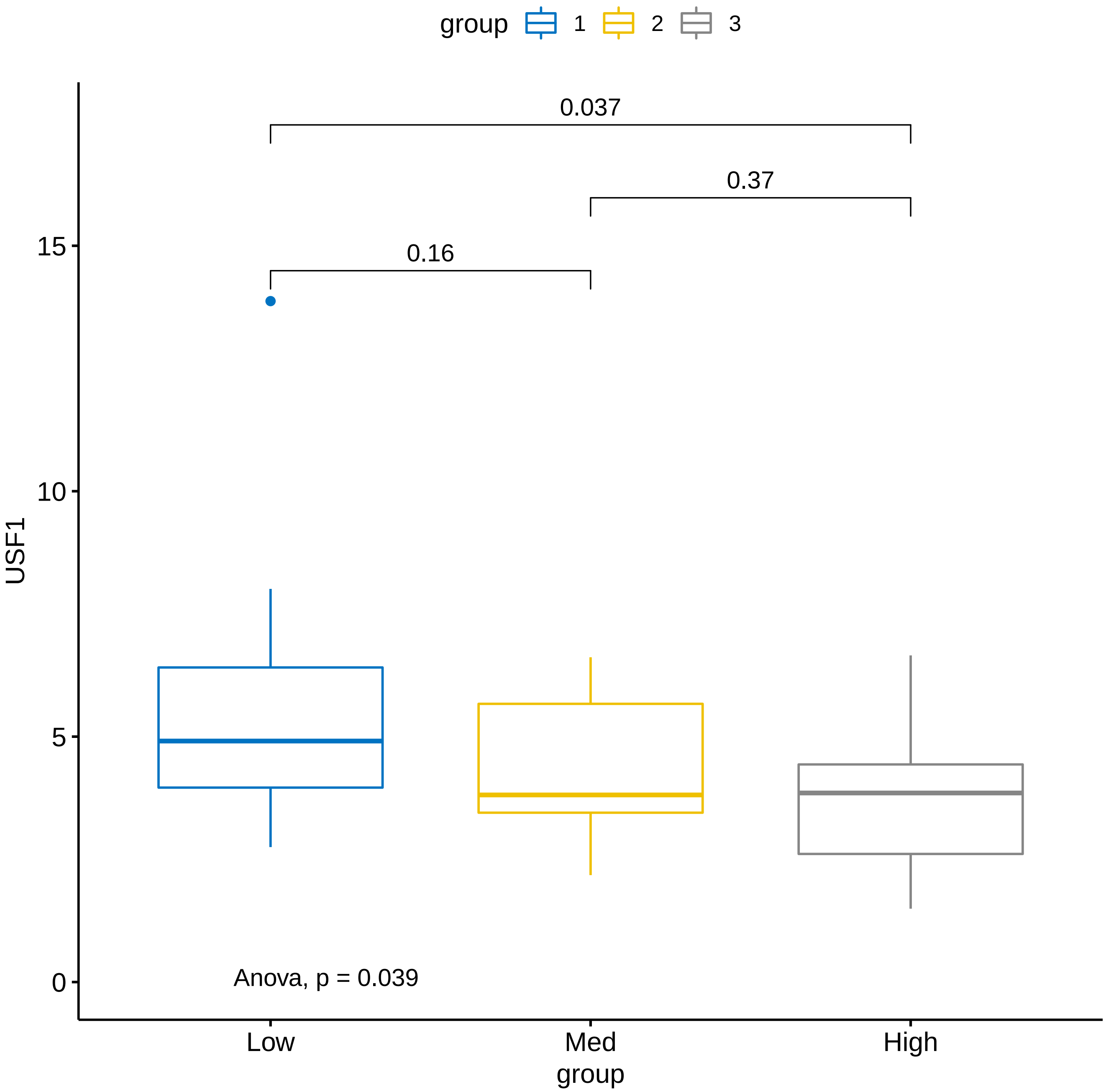
group
1
2
3
0.037
0.37
0.16
15
10
5
0
USF1
Anova, p = 0.039
Low
Med
High
group

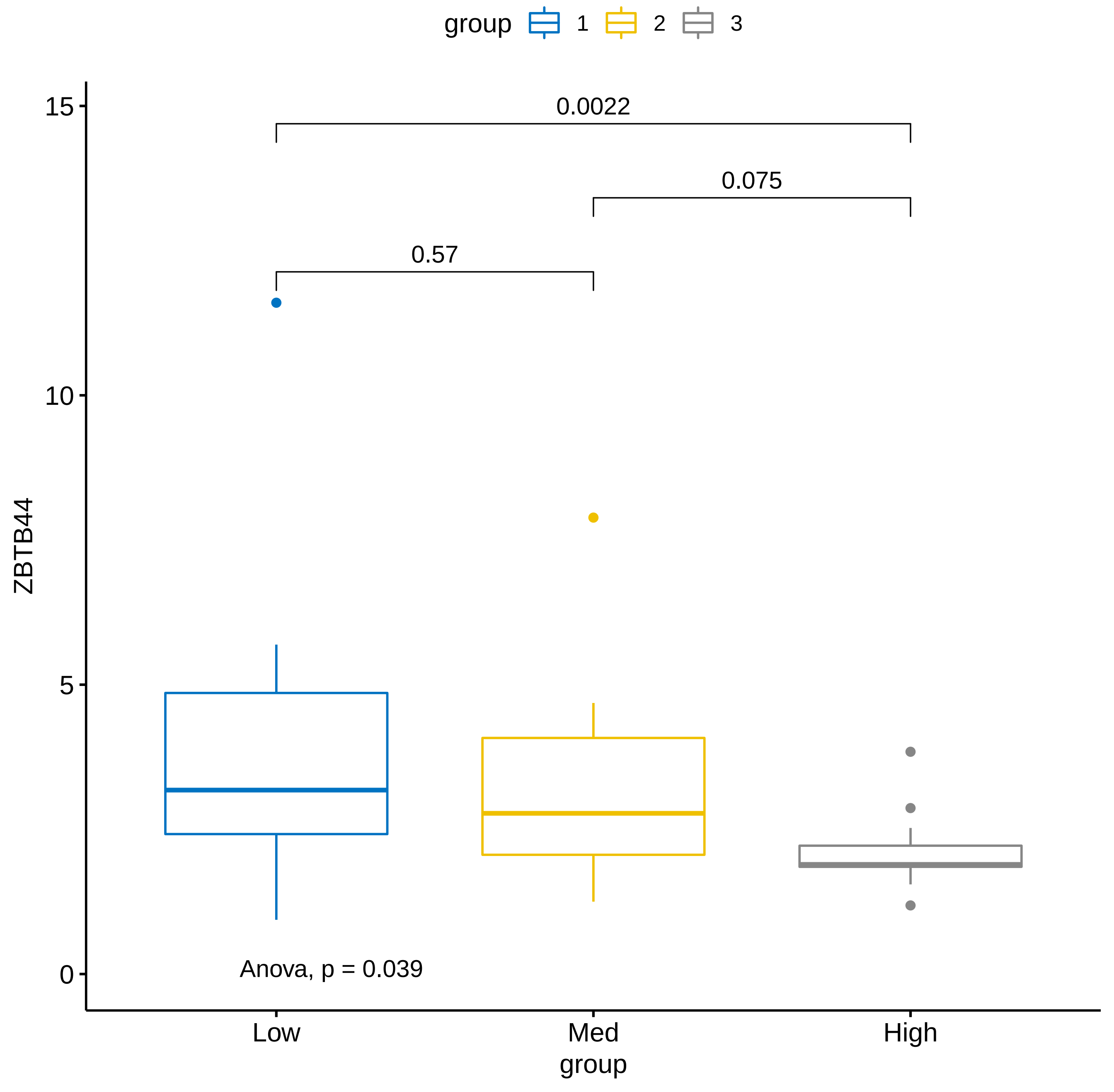

group 1 2 3
0.0022
0.075
0.57
15
10
5
0
ZBTB44
Anova, p = 0.039
Low
Med
High
group

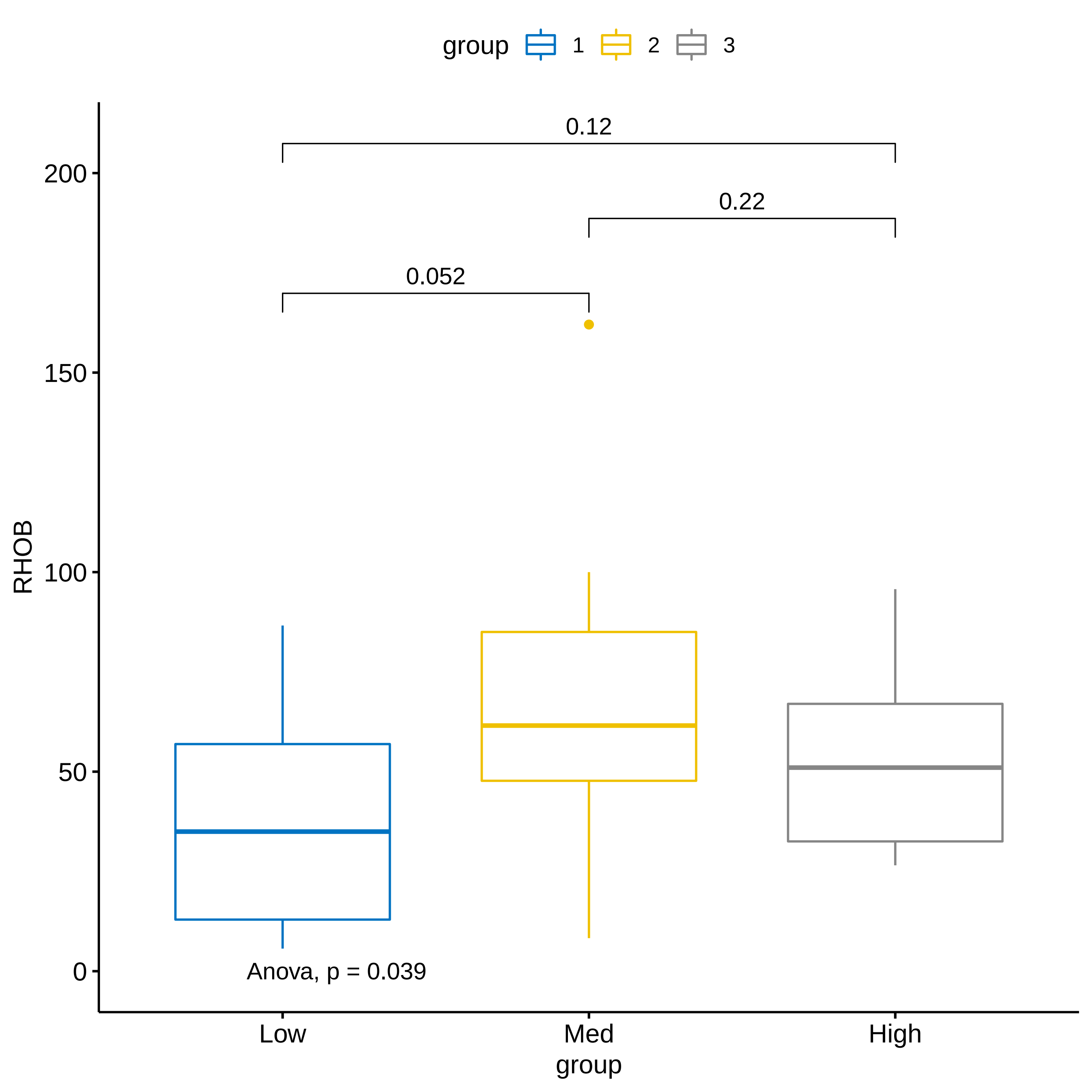
group
1
2
3
0.12
0.22
0.052
200
150
100
50
0
RHOB
Anova, p = 0.039
Low
Med
High
group

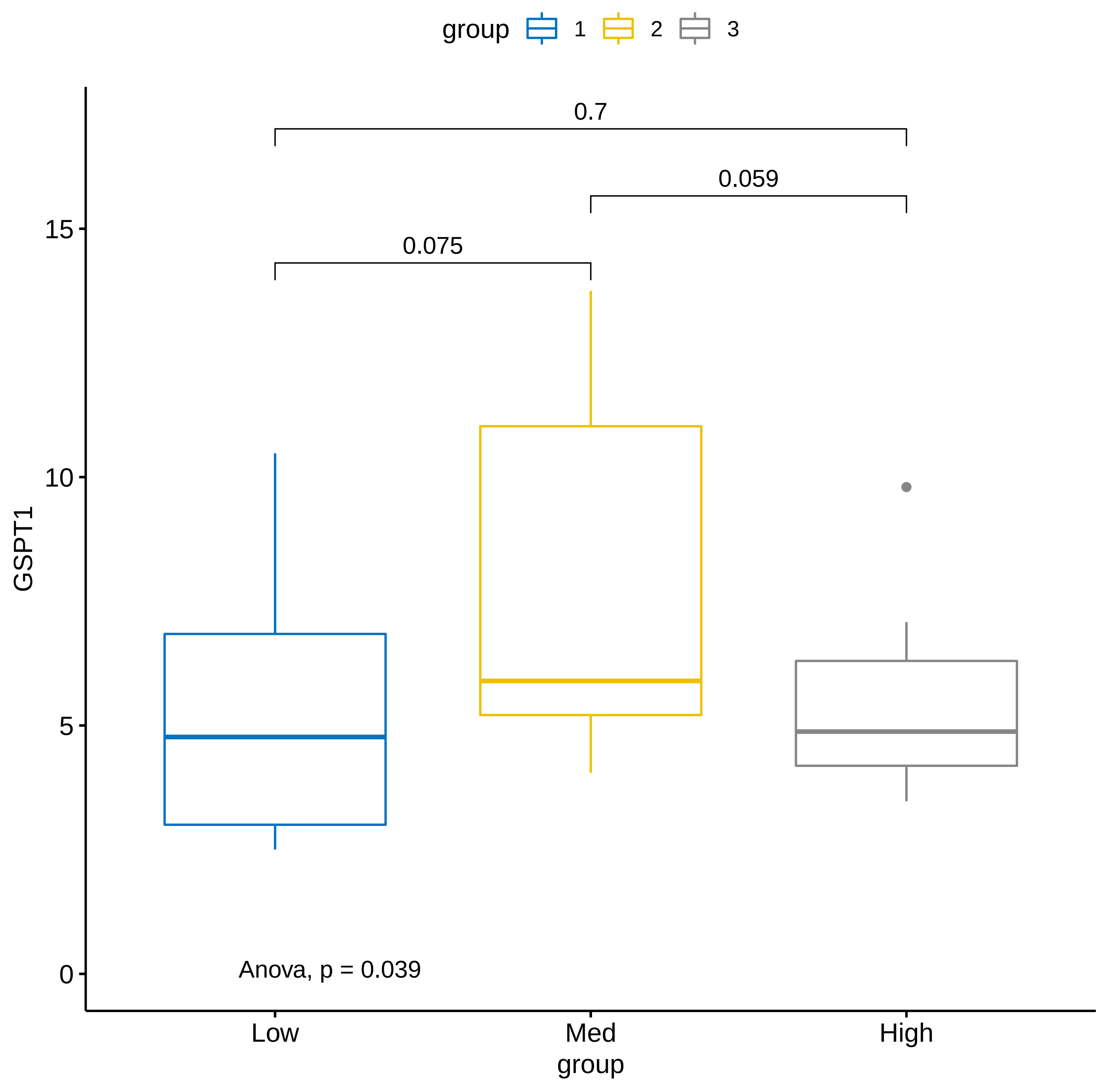

group 1 2 3
0.7
0.059
0.075
15
10
5
0
GSPT1
Anova, p = 0.039
Low
Med
High
group

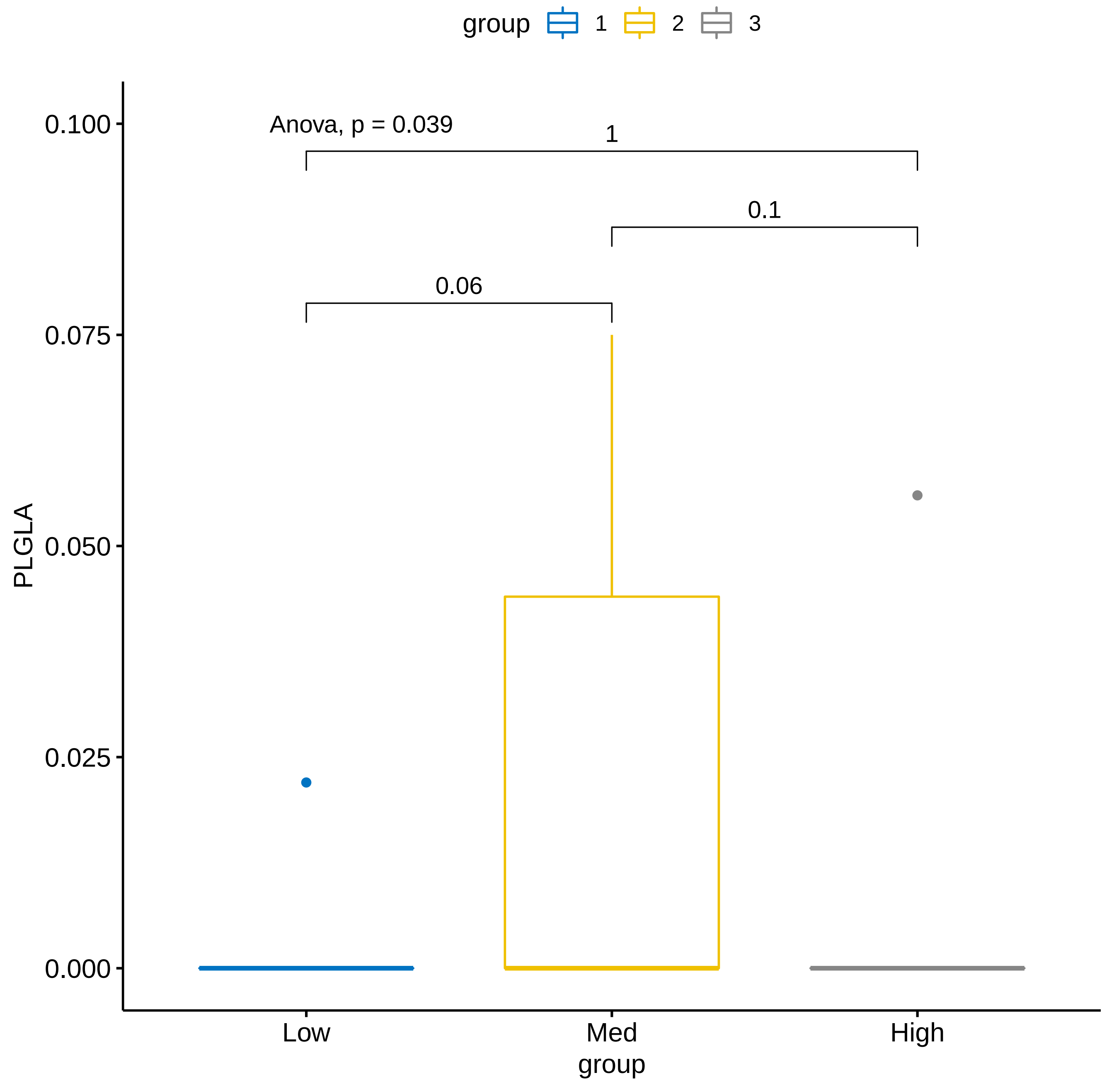

group 1 2 3
Anova, p = 0.039
1
0.1
0.06
PLGLA
0.100
0.075
0.050
0.025
0.000
Low
Med
High
group

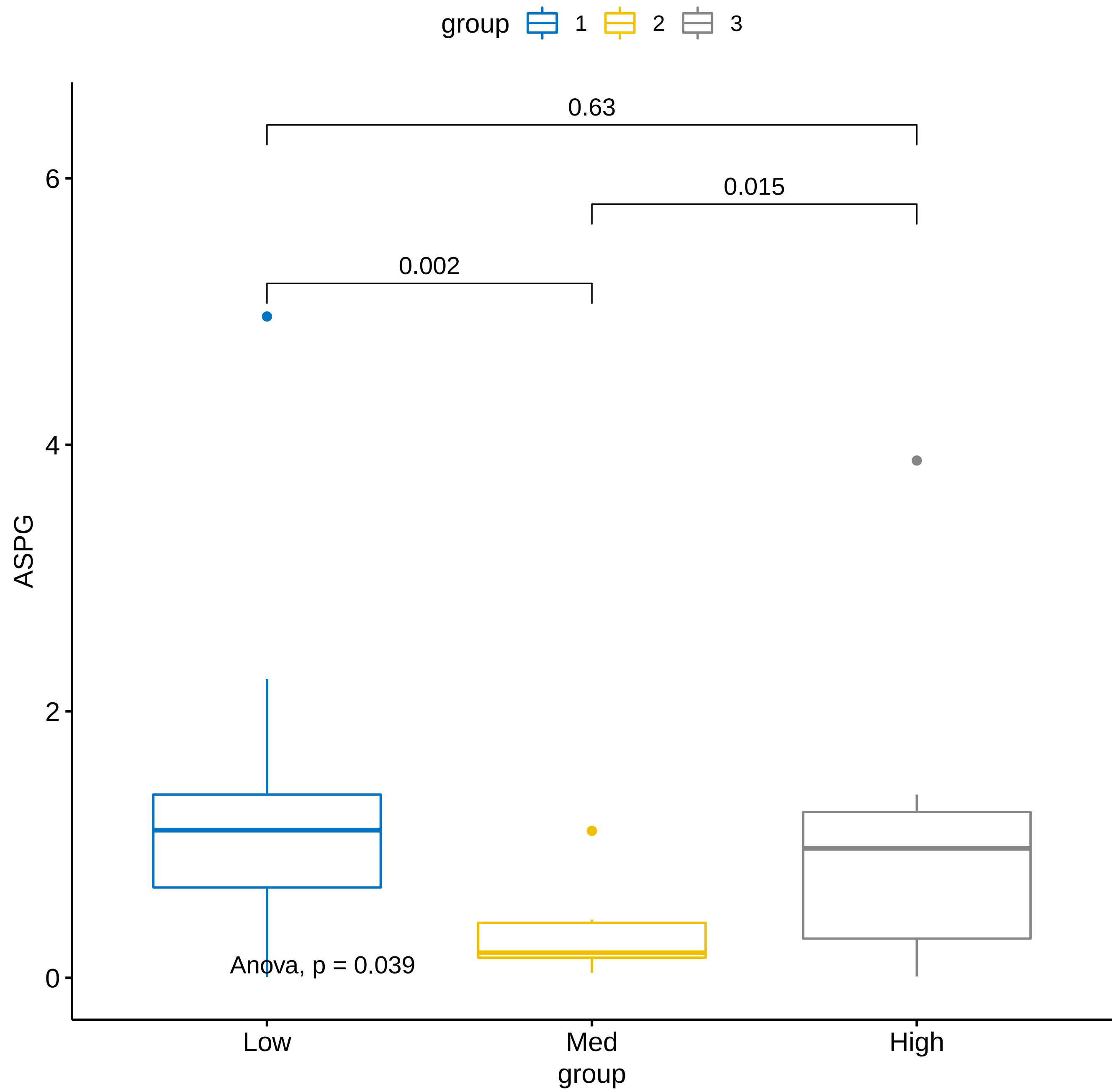

group
1
2
3
0.63
0.015
0.002
6
4
2
0
ASPG
Anova, p = 0.039
Low
Med
High
group

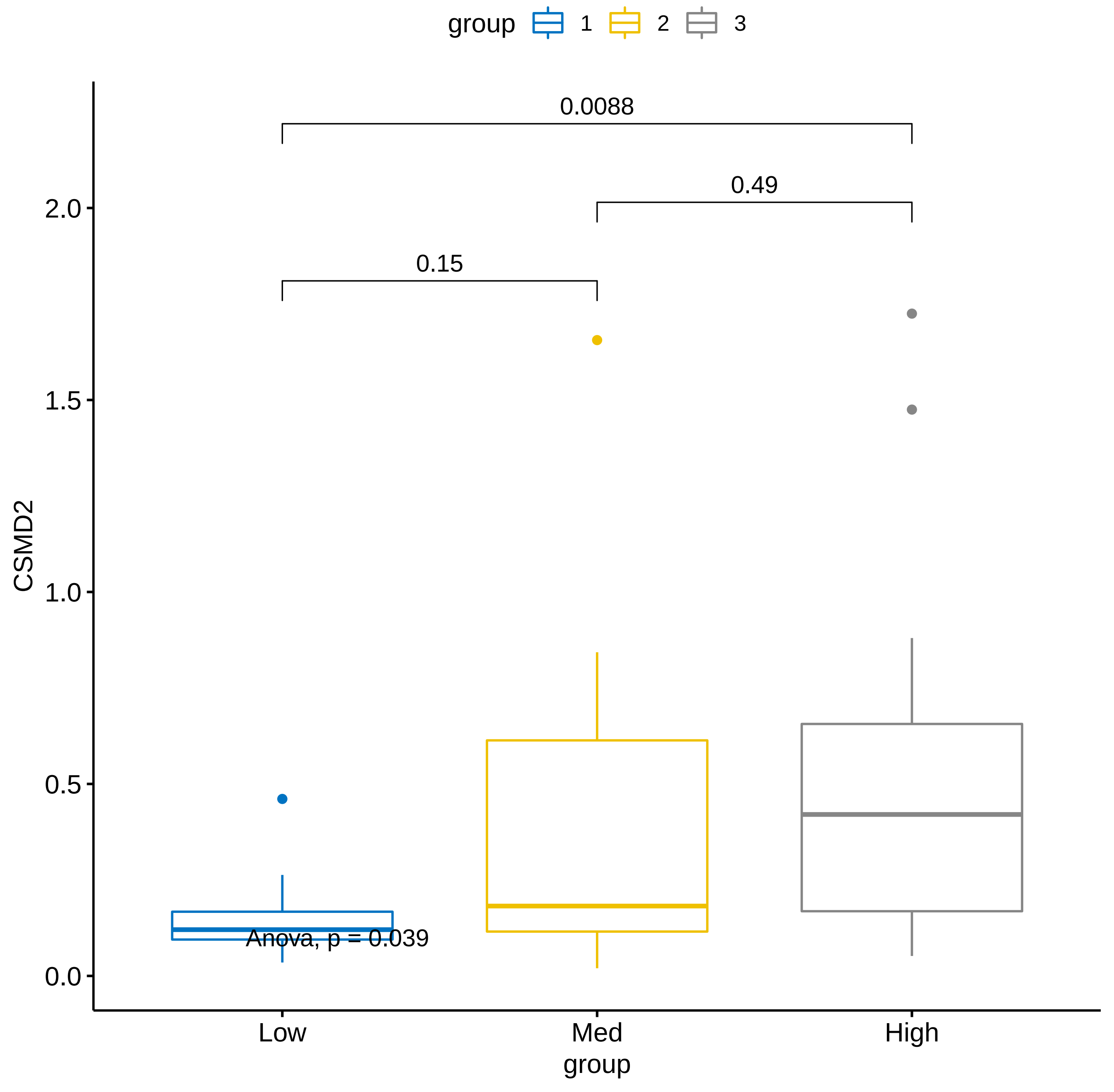

group 1 2 3
0.0088
0.49
0.15
CSMD2
2.0
1.5
1.0
0.5
0.0
Anova, p = 0.039
Low
Med
High
group

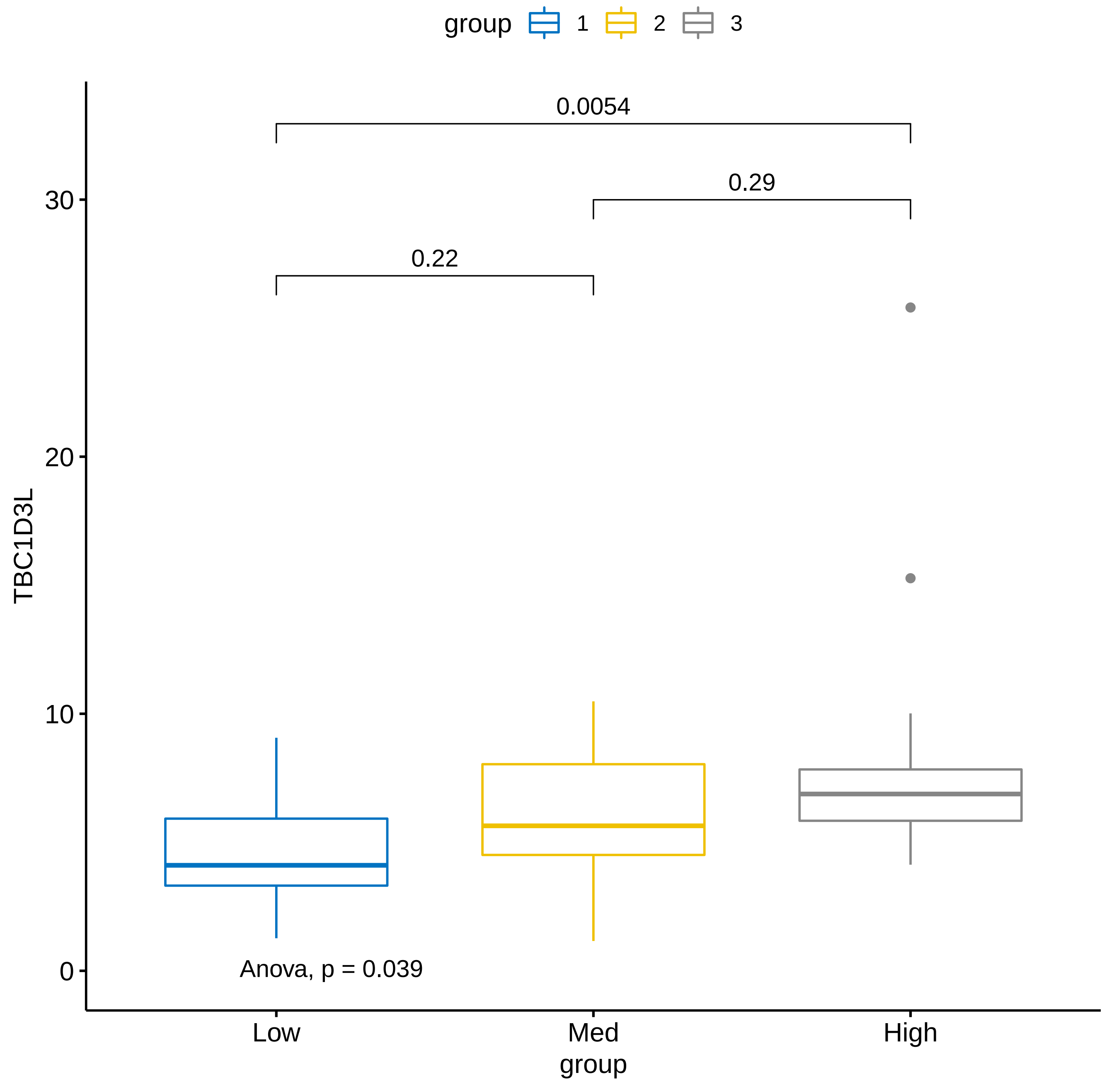

group
1
2
3
0.0054
0.29
0.22
30
20
10
0
TBC1D3L
Anova, p = 0.039
Low
Med
High
group

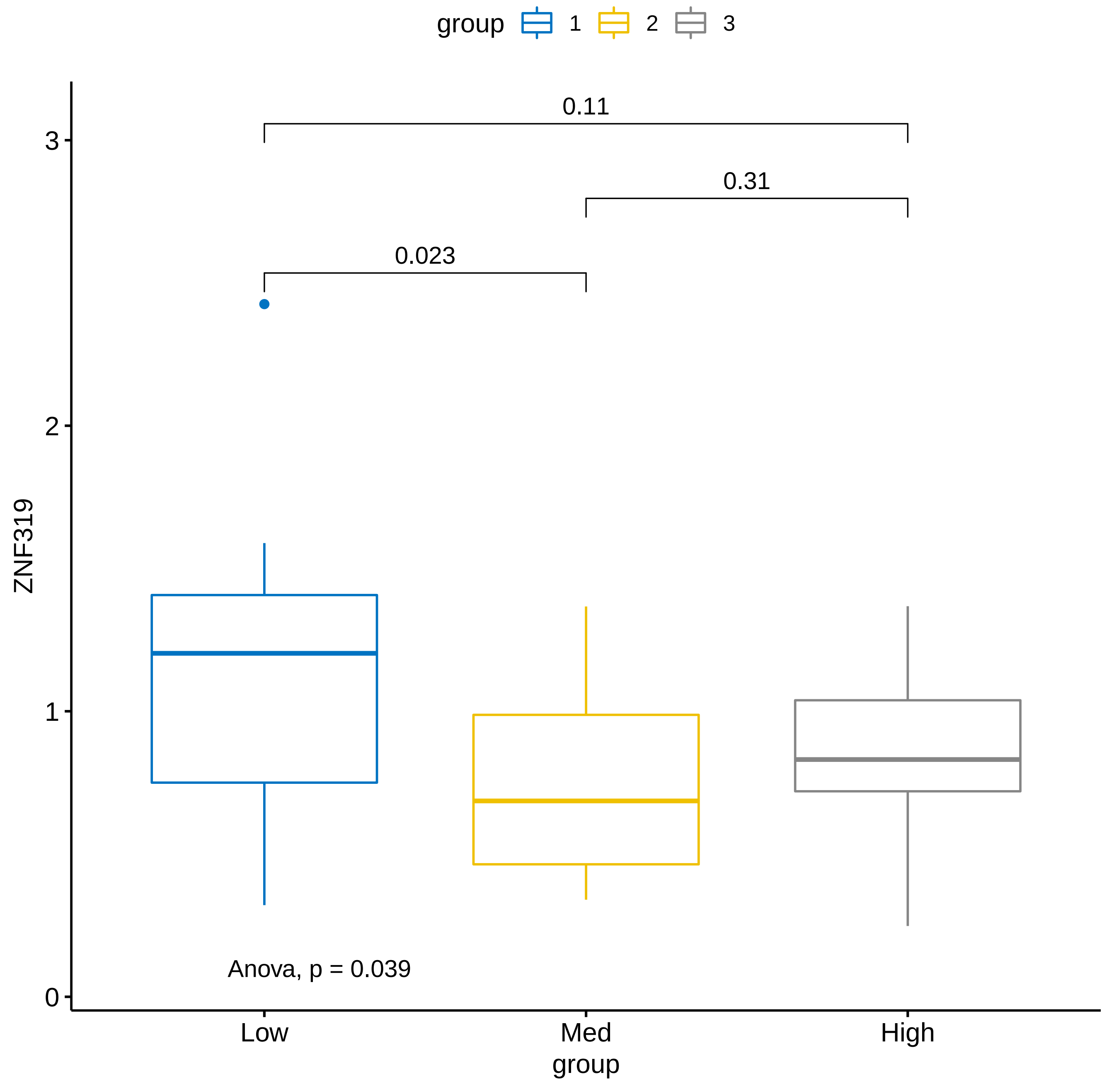
group 1 2 3
0.11
0.31
0.023
ZNF319
3
2
1
0
Anova, p = 0.039
Low
Med
High
group

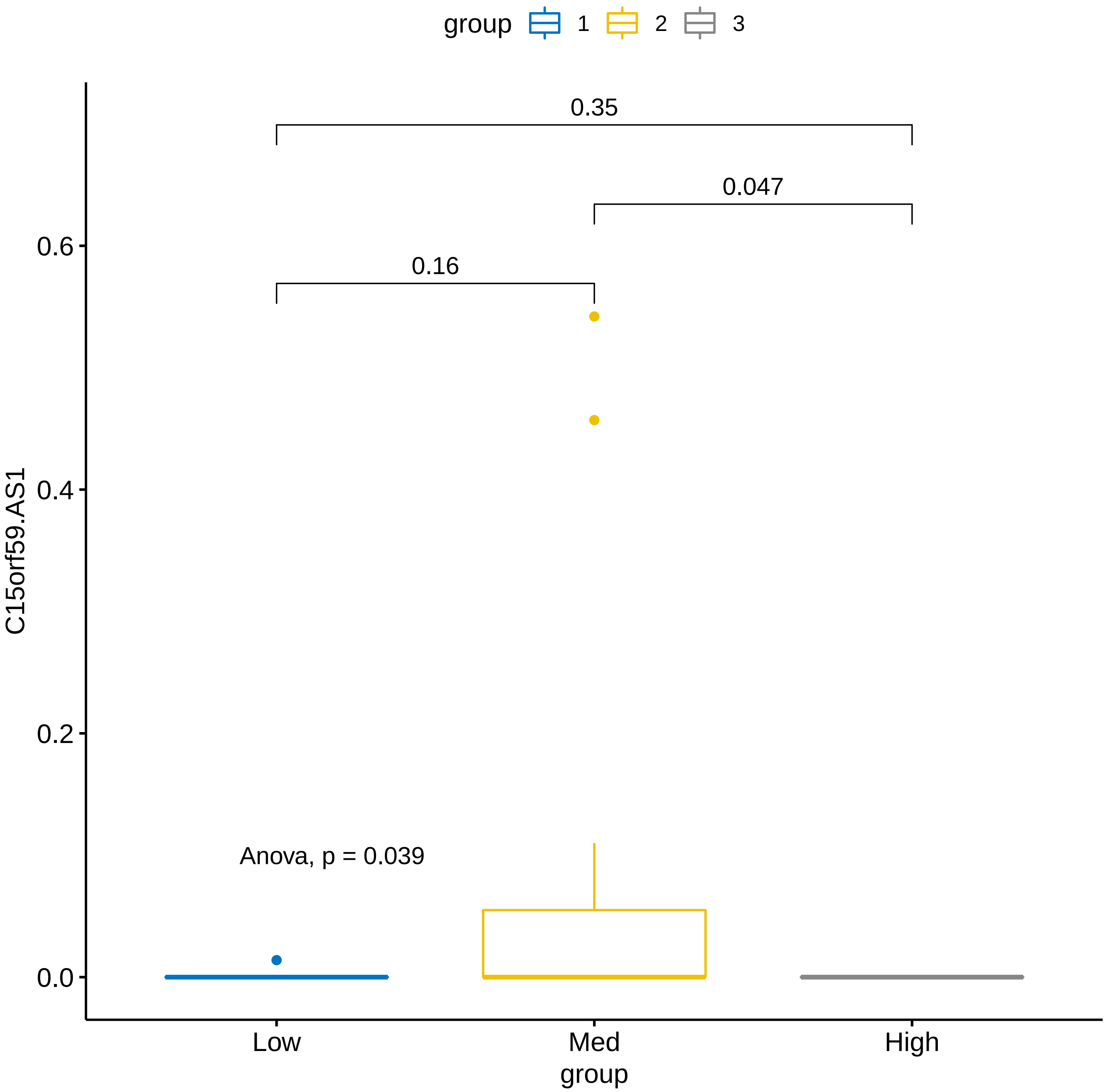
group 1 2 3
0.35
0.047
0.16
Anova, p = 0.039
C15orf59.AS1
0.6
0.4
0.2
0.0
Low
Med
High
group

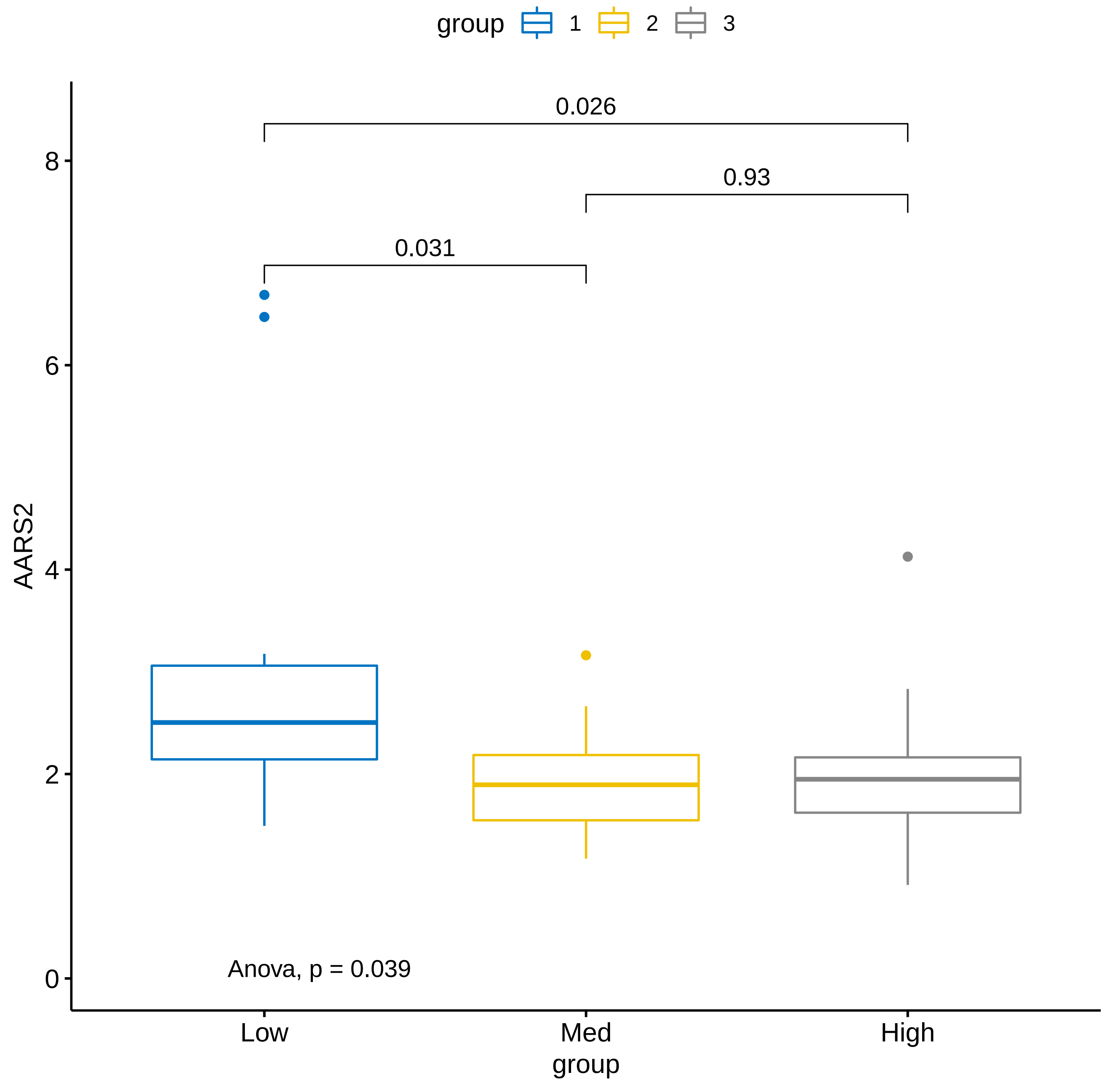
group
1
2
3
0.026
0.93
0.031
8
6
4
2
0
AARS2
Anova, p = 0.039
Low
Med
High
group

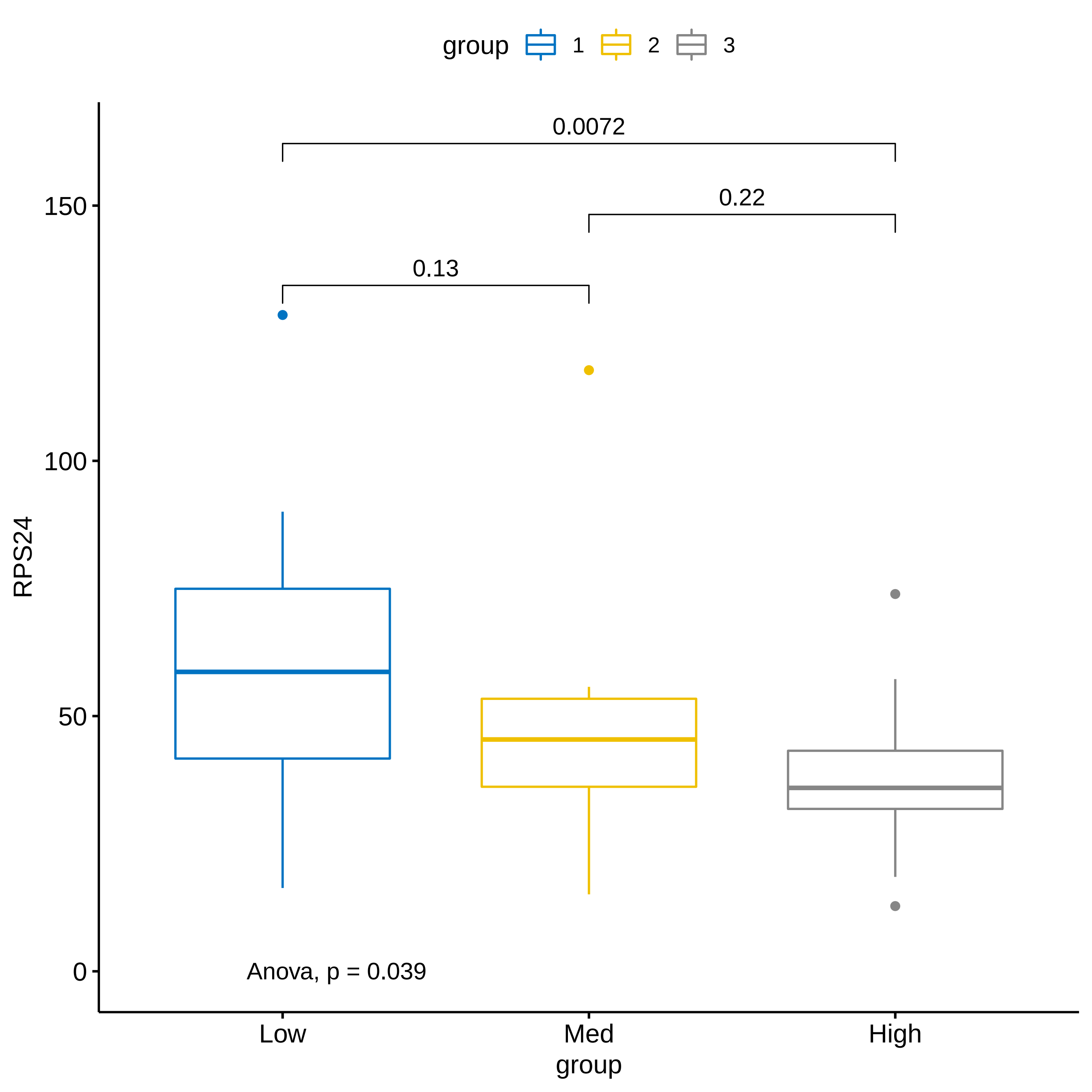
group 1 2 3
0.0072
0.22
0.13
150
100
50
0
RPS24
Anova, p = 0.039
Low
Med
High
group

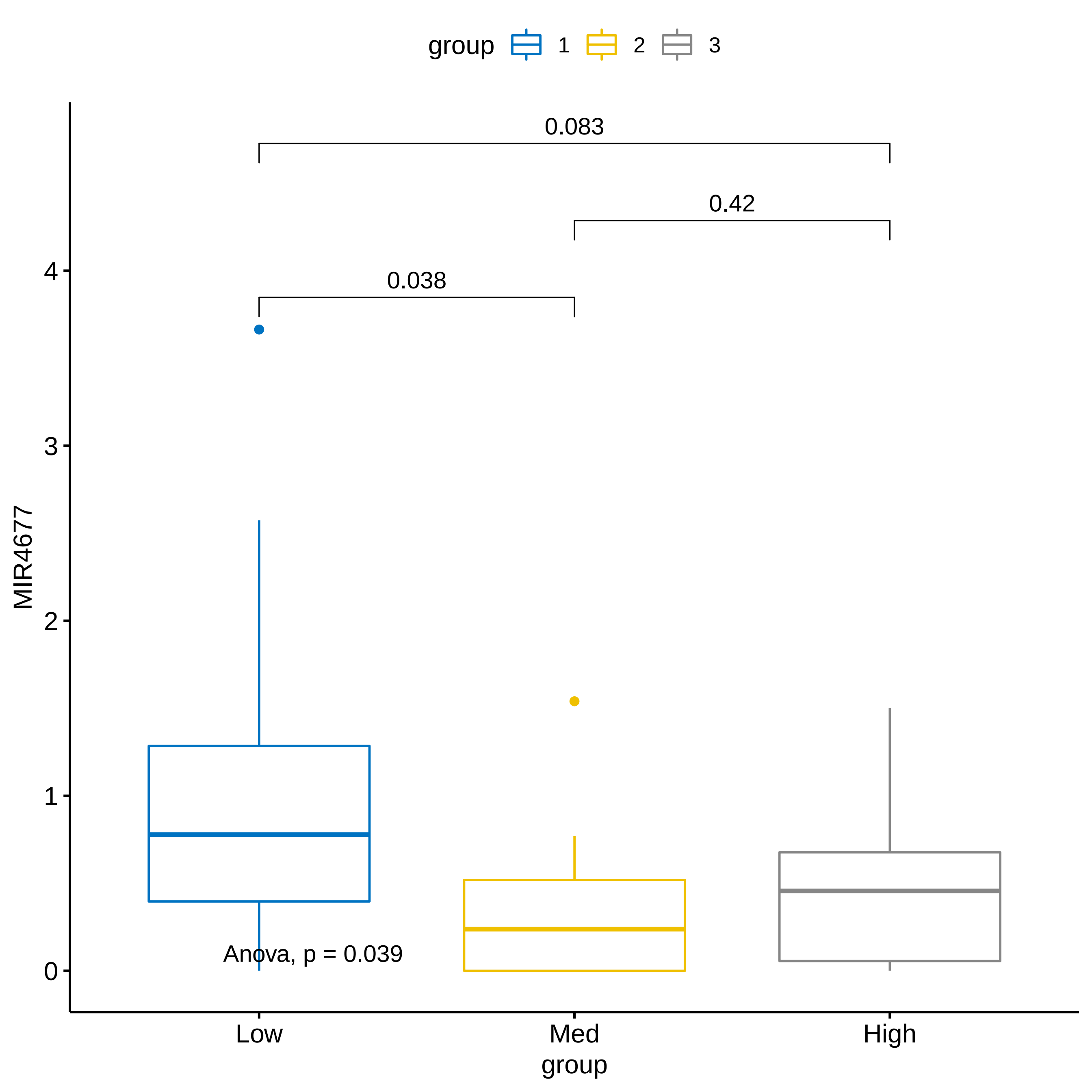
group 1 2 3
0.083
0.42
0.038
MIR4677
4
3
2
1
0
Anova, p = 0.039
Low
Med
High
group

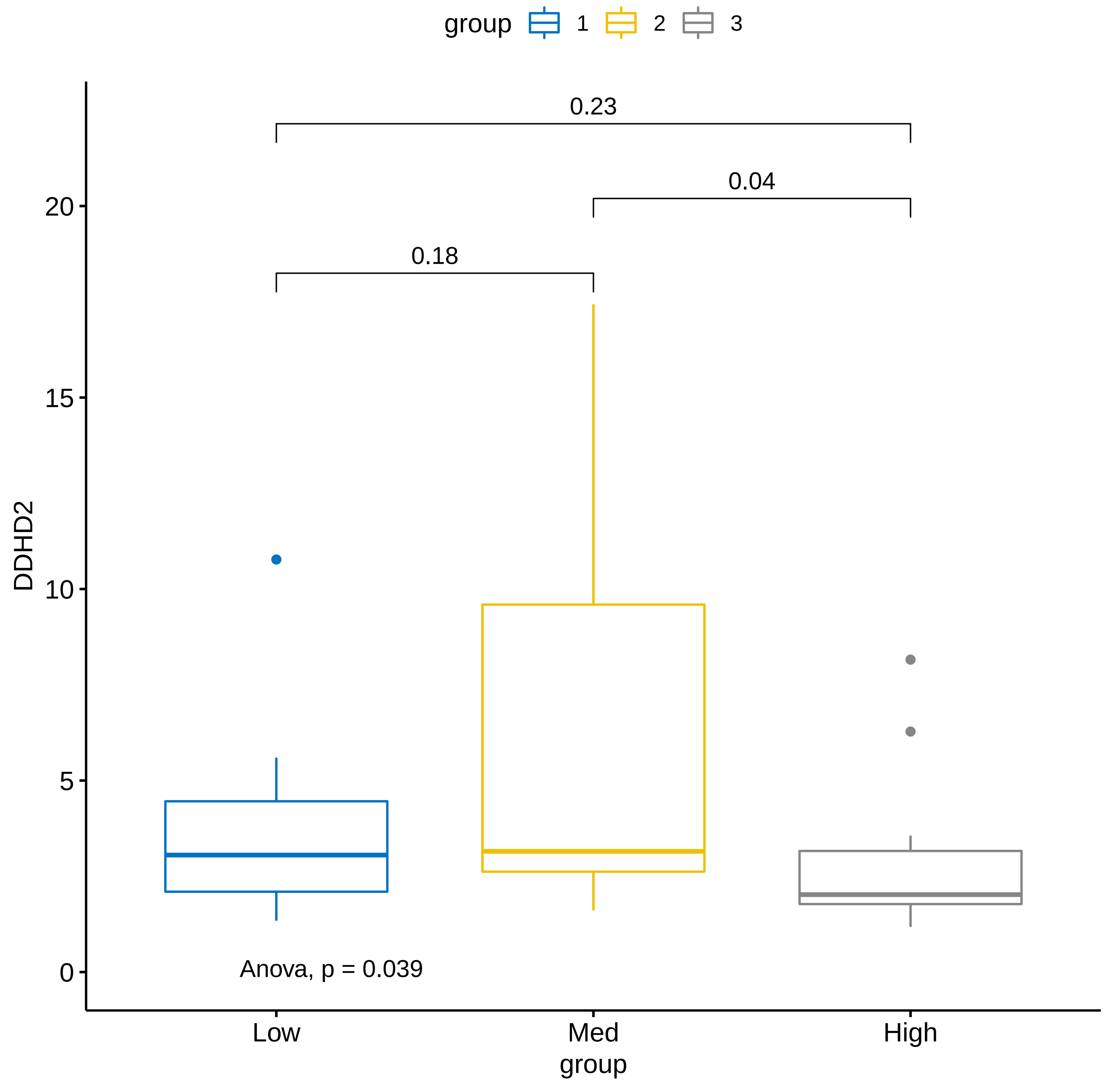
group 1 2 3
0.23
0.04
0.18
DDHD2
20
15
10
5
0
Anova, p = 0.039
Low
Med
High
group

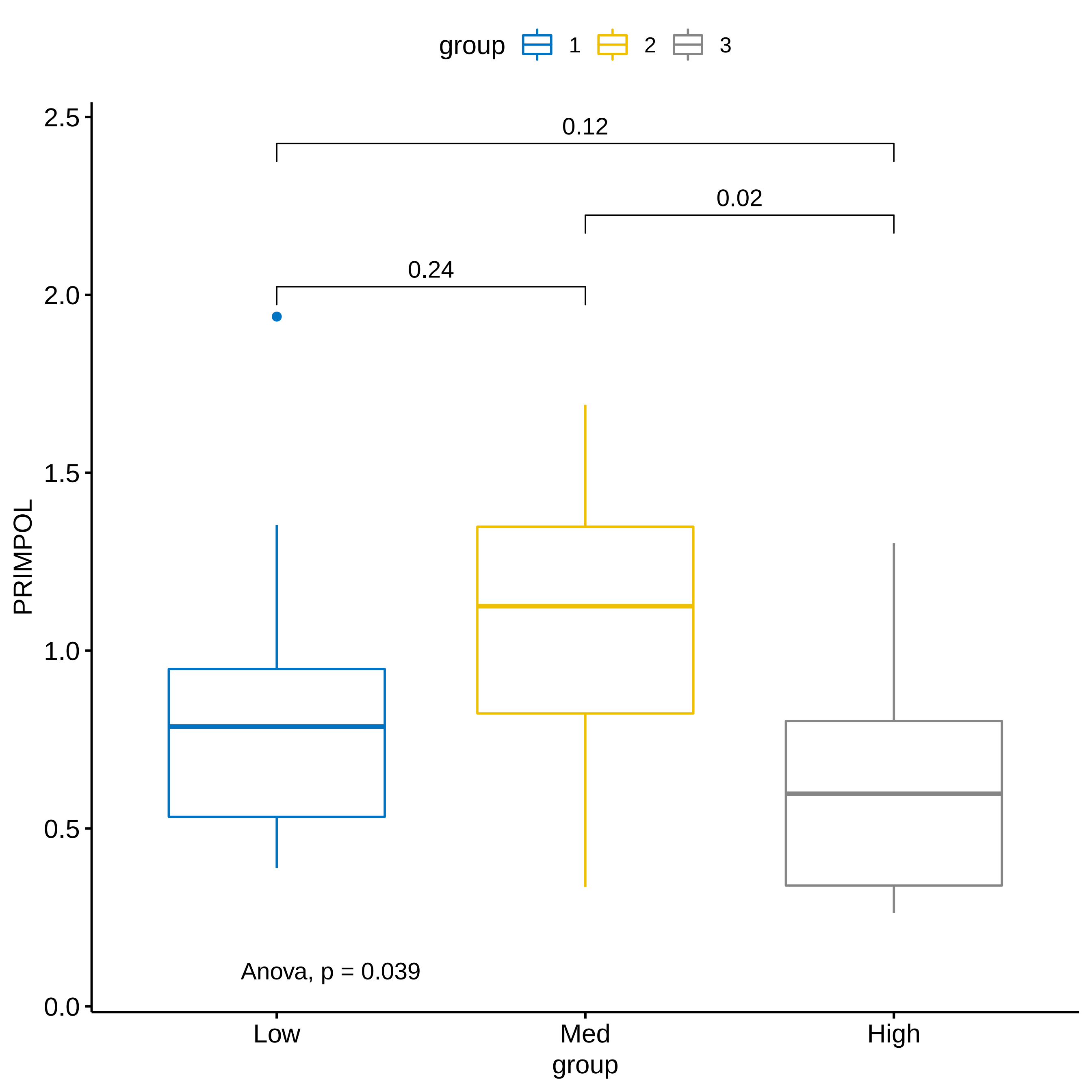
group
1
2
3
2.5
2.0
1.5
1.0
0.5
0.0
PRIMPOL
0.12
0.02
0.24
Anova, p = 0.039
Low
Med
High
group

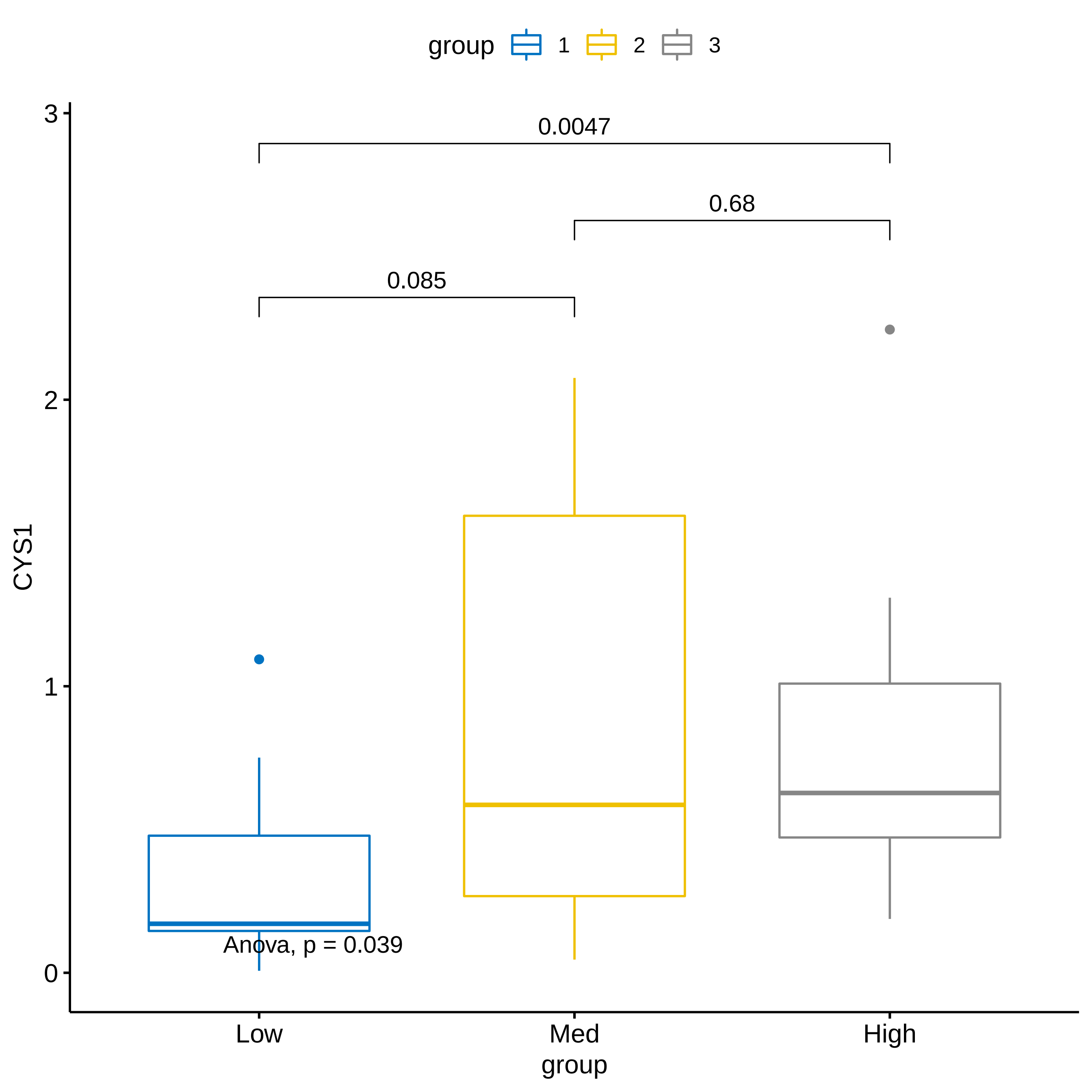
group
1
2
3
3
2
1
0
CYS1
0.0047
0.68
0.085
Anova, p = 0.039
Low
Med
High
group

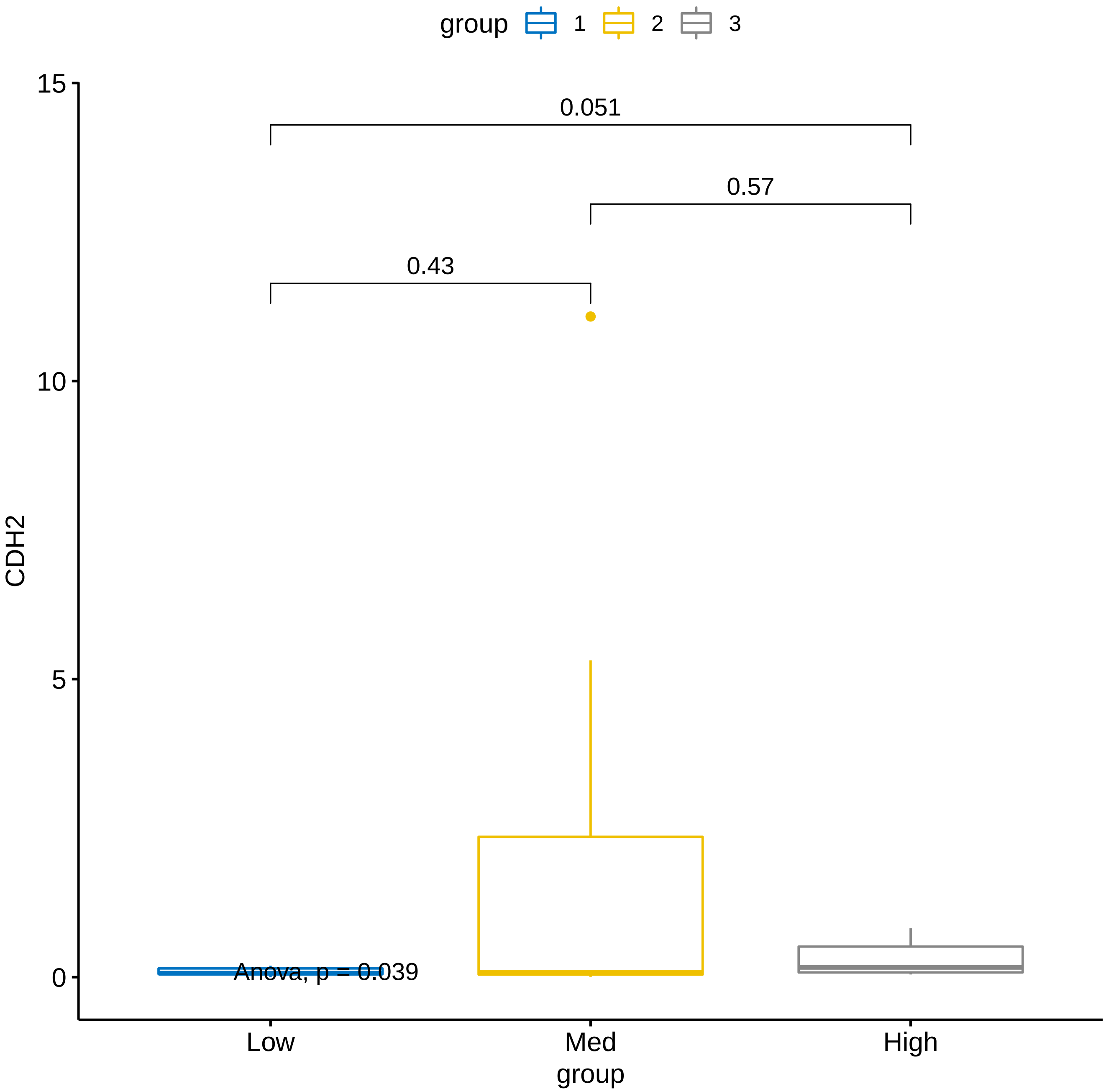
group
1
2
3
15
10
5
0
CDH2
0.051
0.57
0.43
Anova, p = 0.039
Low
Med
High
group

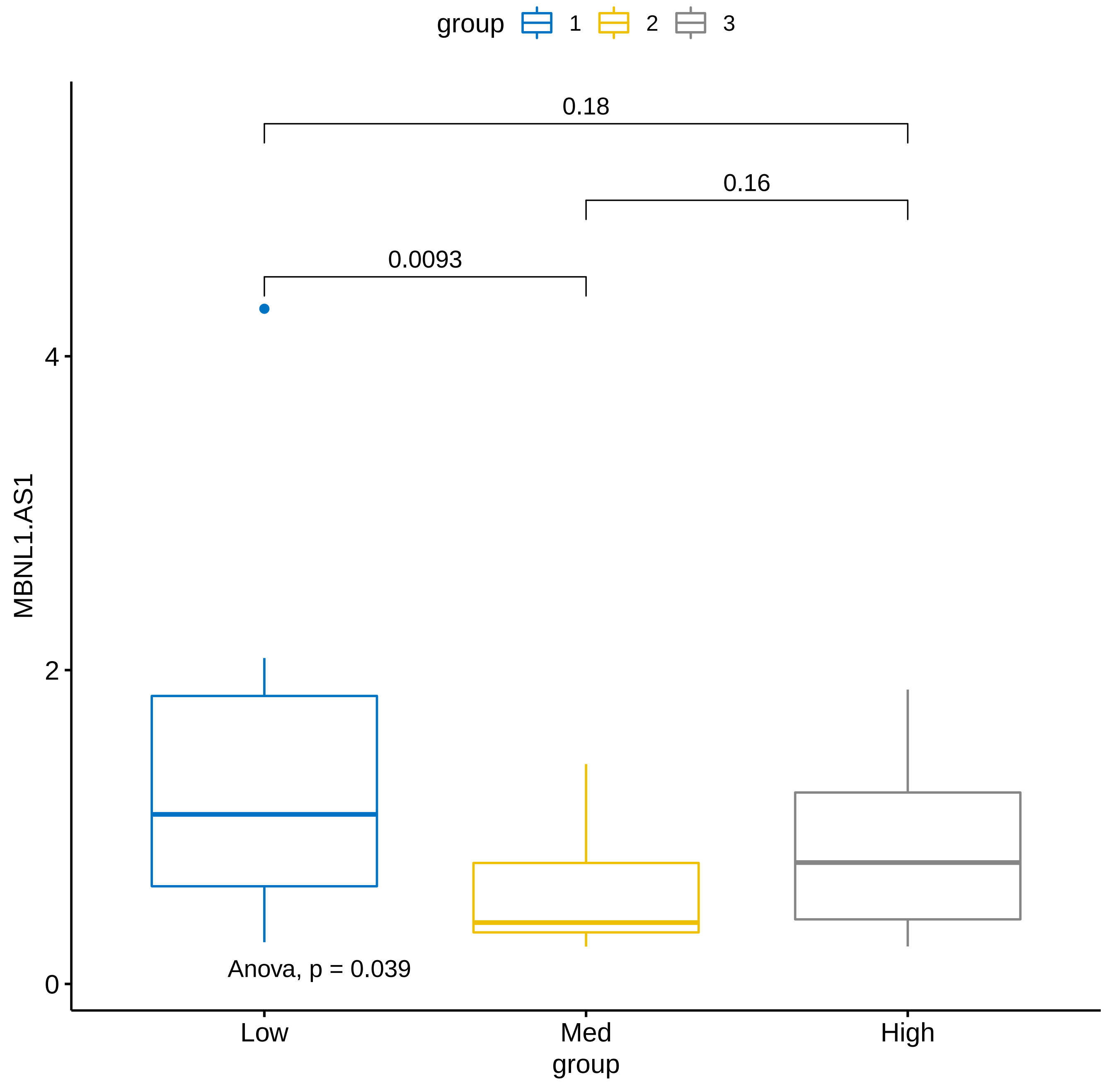
group 1 2 3
0.18
0.16
0.0093
4
2
0
MBNL1.AS1
Anova, p = 0.039
Low
Med
High
group

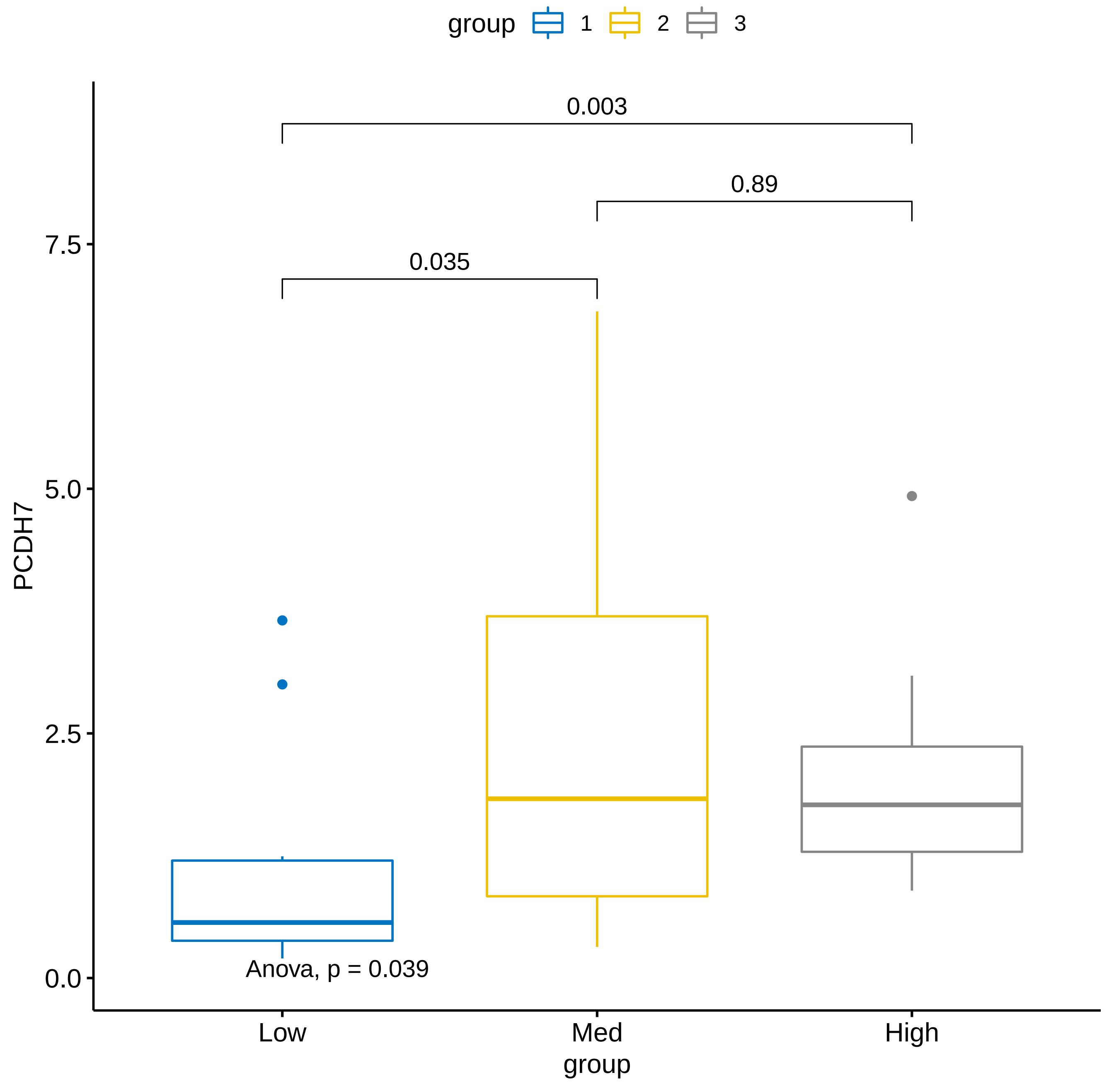

group 1 2 3
0.003
0.89
0.035
7.5
5.0
2.5
0.0
PCDH7
Anova, p = 0.039
Low
Med
High
group

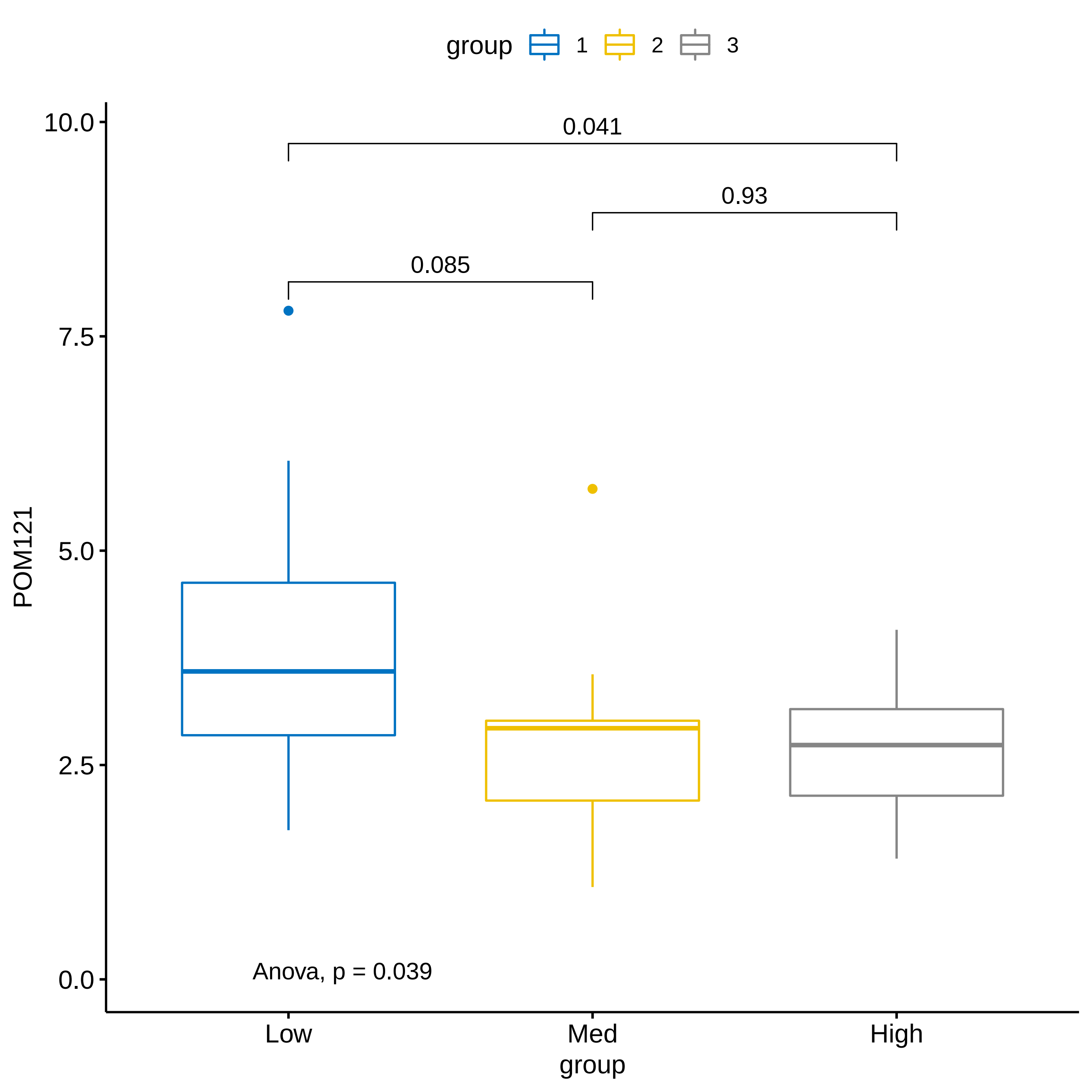

group
1
2
3
10.0
7.5
5.0
2.5
0.0
POM121
0.041
0.93
0.085
Anova, p = 0.039
Low
Med
High
group

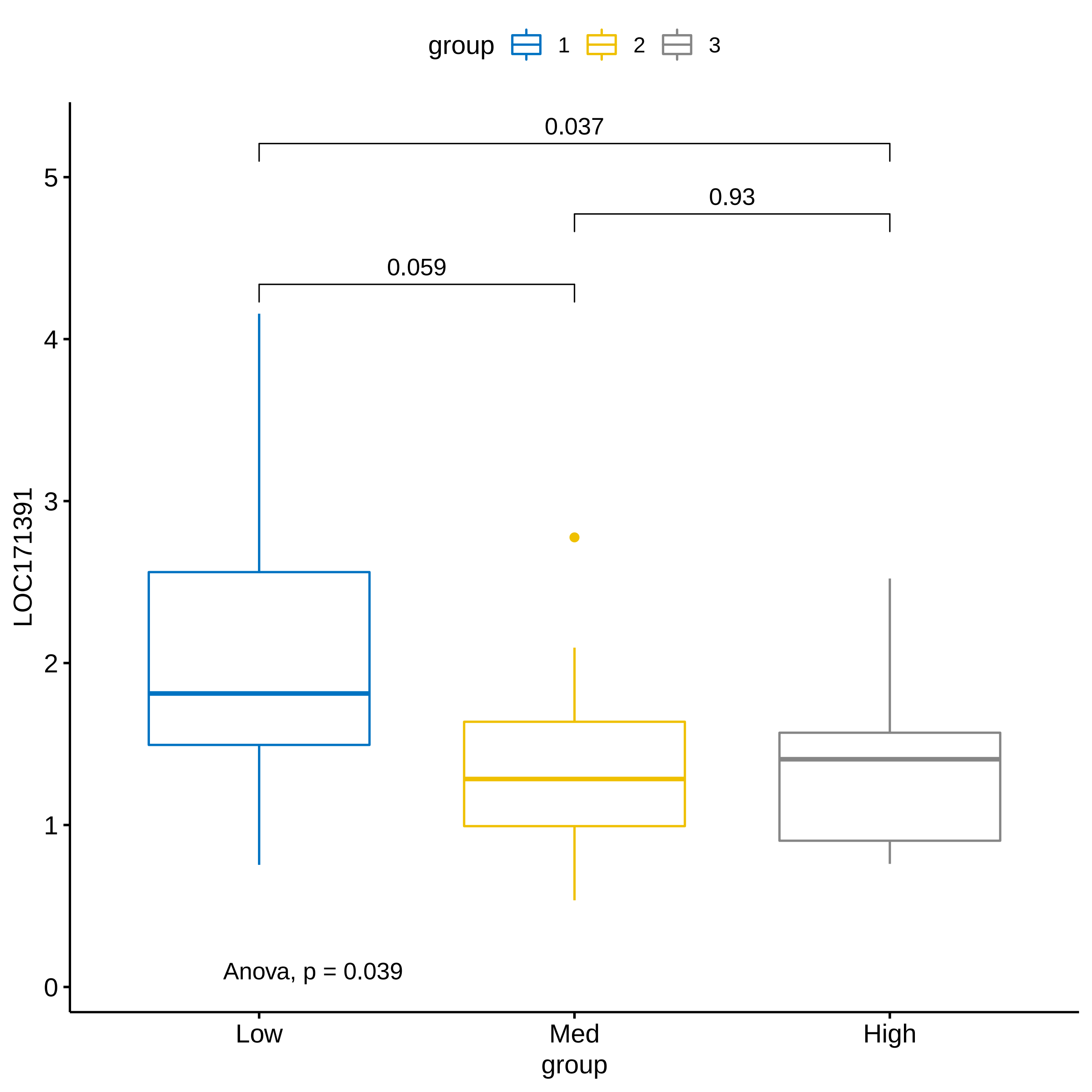
group 1 2 3
0.037
0.93
0.059
5
4
3
2
1
0
LOC171391
Anova, p = 0.039
Low
Med
High
group

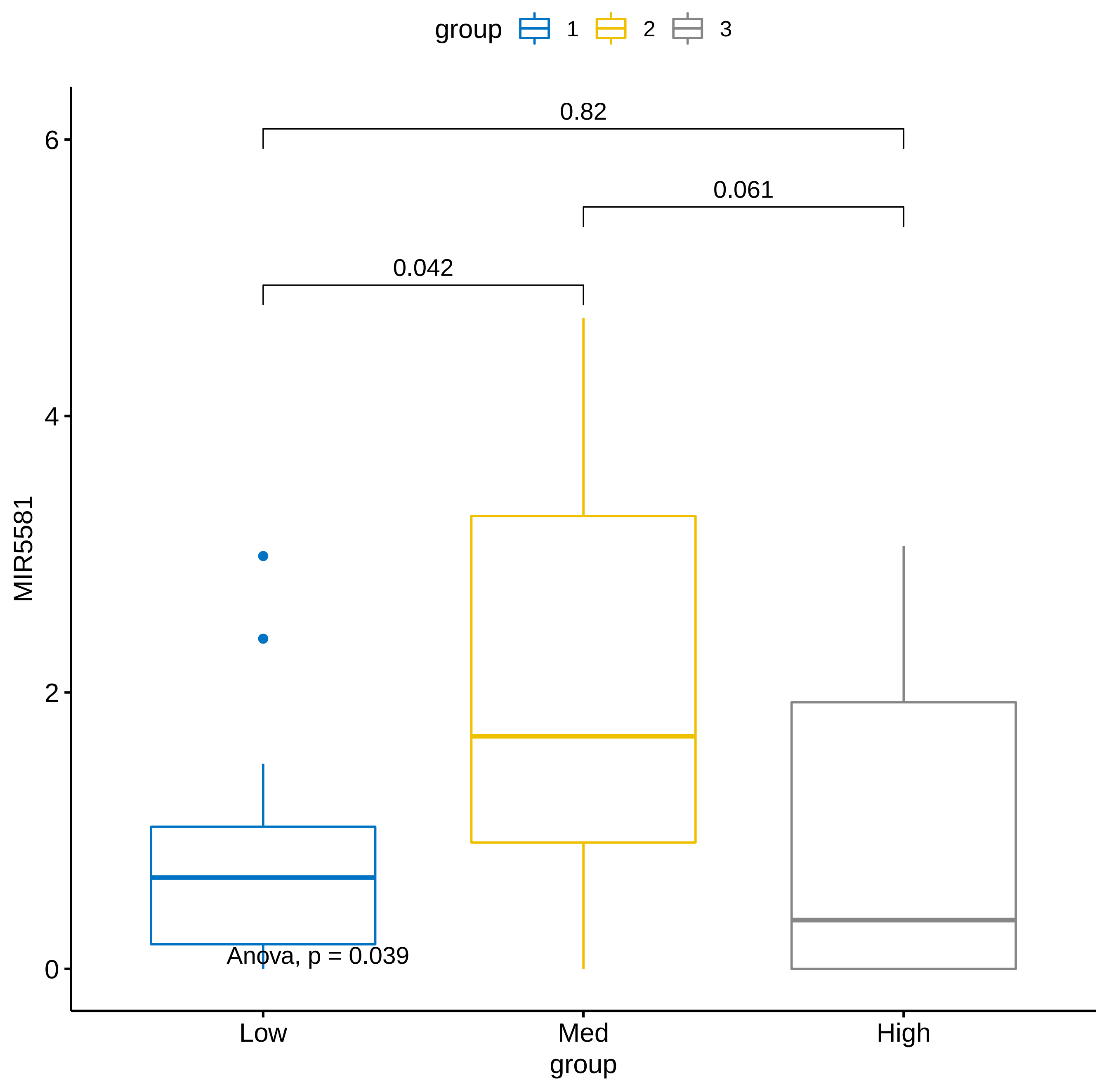
group
1
2
3
0.82
0.061
0.042
6
4
2
0
MIR5581
Anova, p = 0.039
Low
Med
High
group

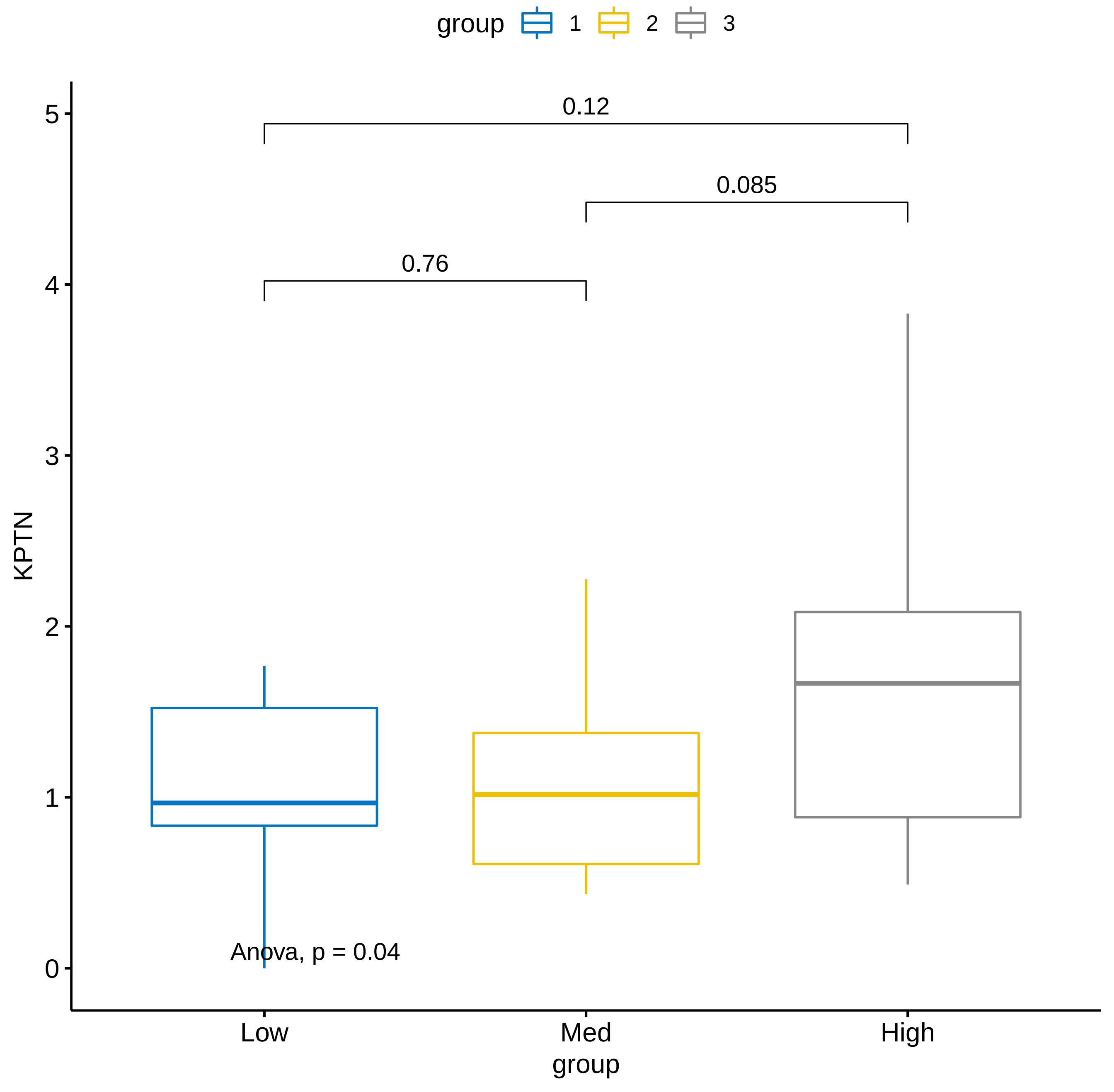
group
1
2
3
0.12
0.085
0.76
Anova, p = 0.04
KPTN
0
1
2
3
4
5
Low
Med
High
group

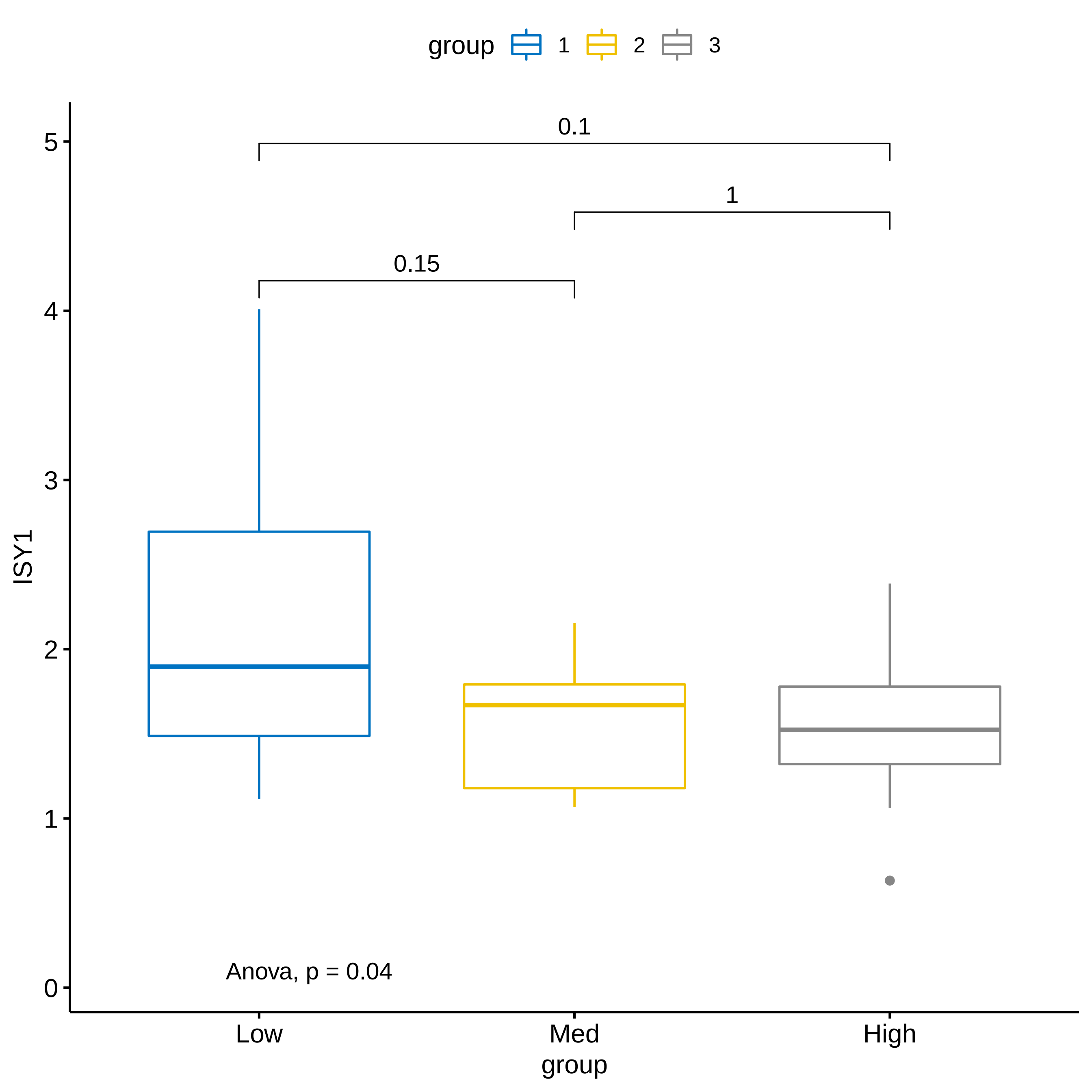
group
1
2
3
0.1
1
0.15
ISY1
5
4
3
2
1
0
Anova, p = 0.04
Low
Med
High
group

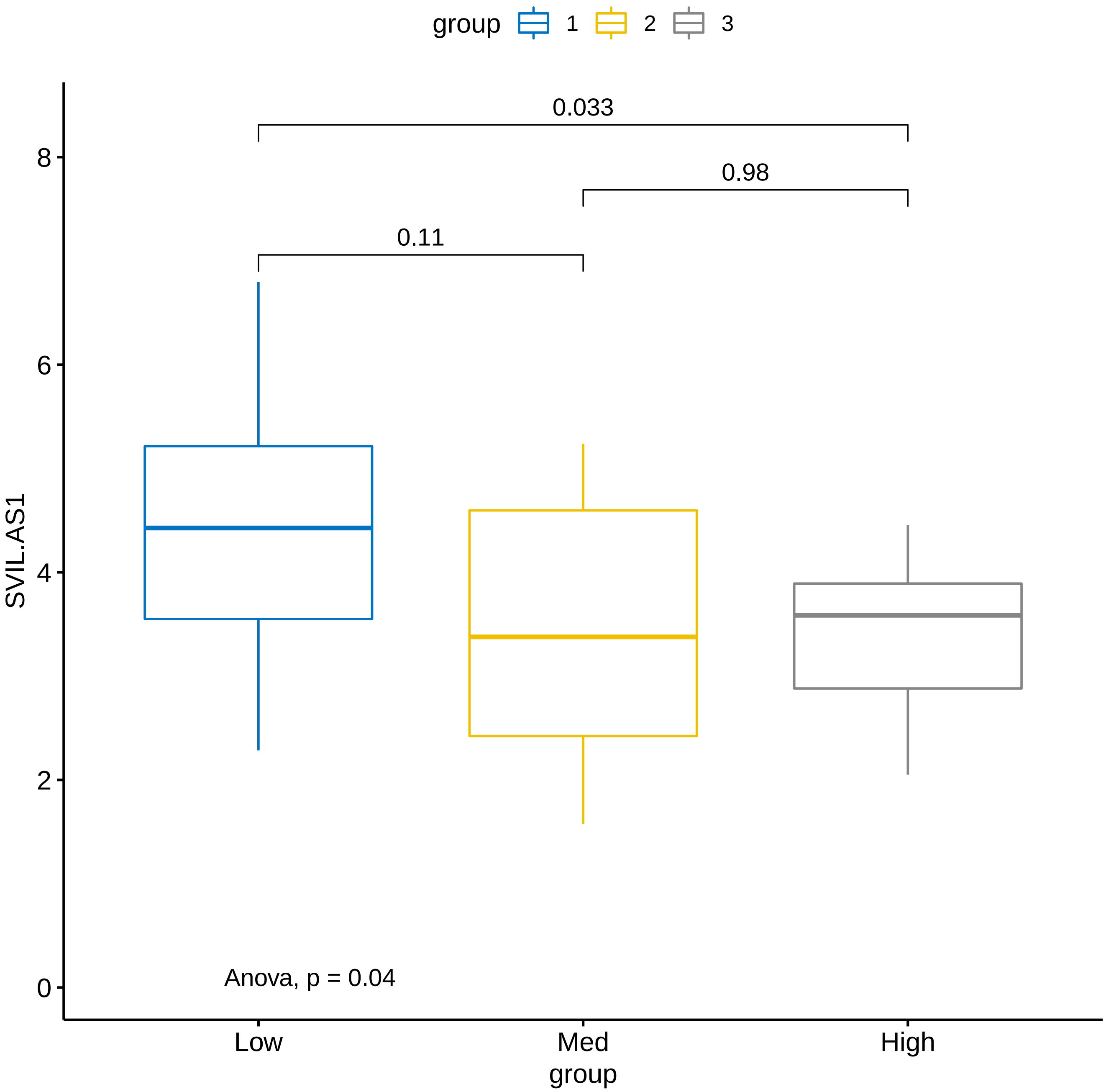

group 1 2 3
0.033
0.98
0.11
8
6
4
2
0
SVIL.AS1
Anova, p = 0.04
Low
Med
High
group

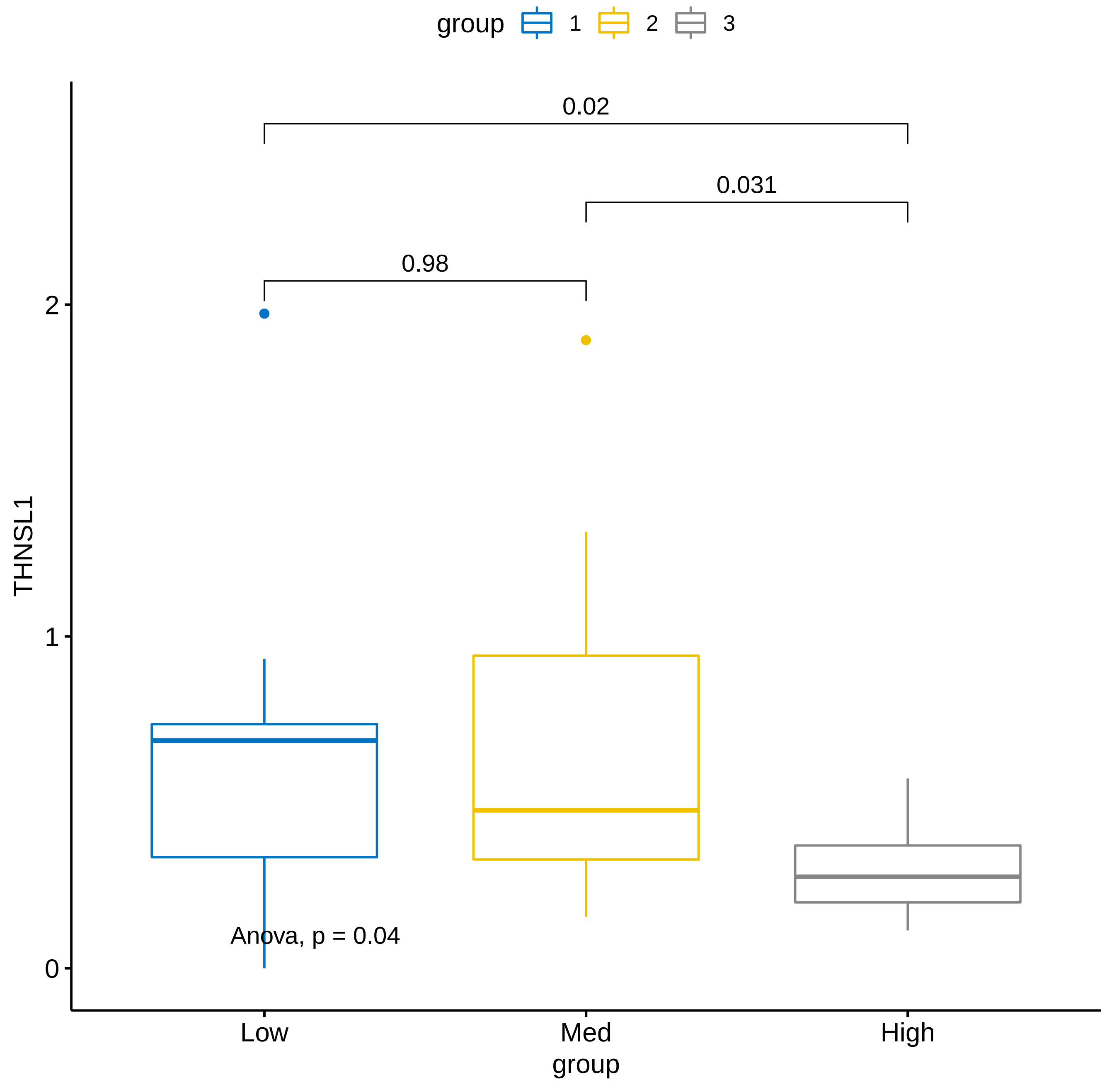

group
1
2
3
0.02
0.031
0.98
2
1
0
THNSL1
Anova, p = 0.04
Low
Med
High
group

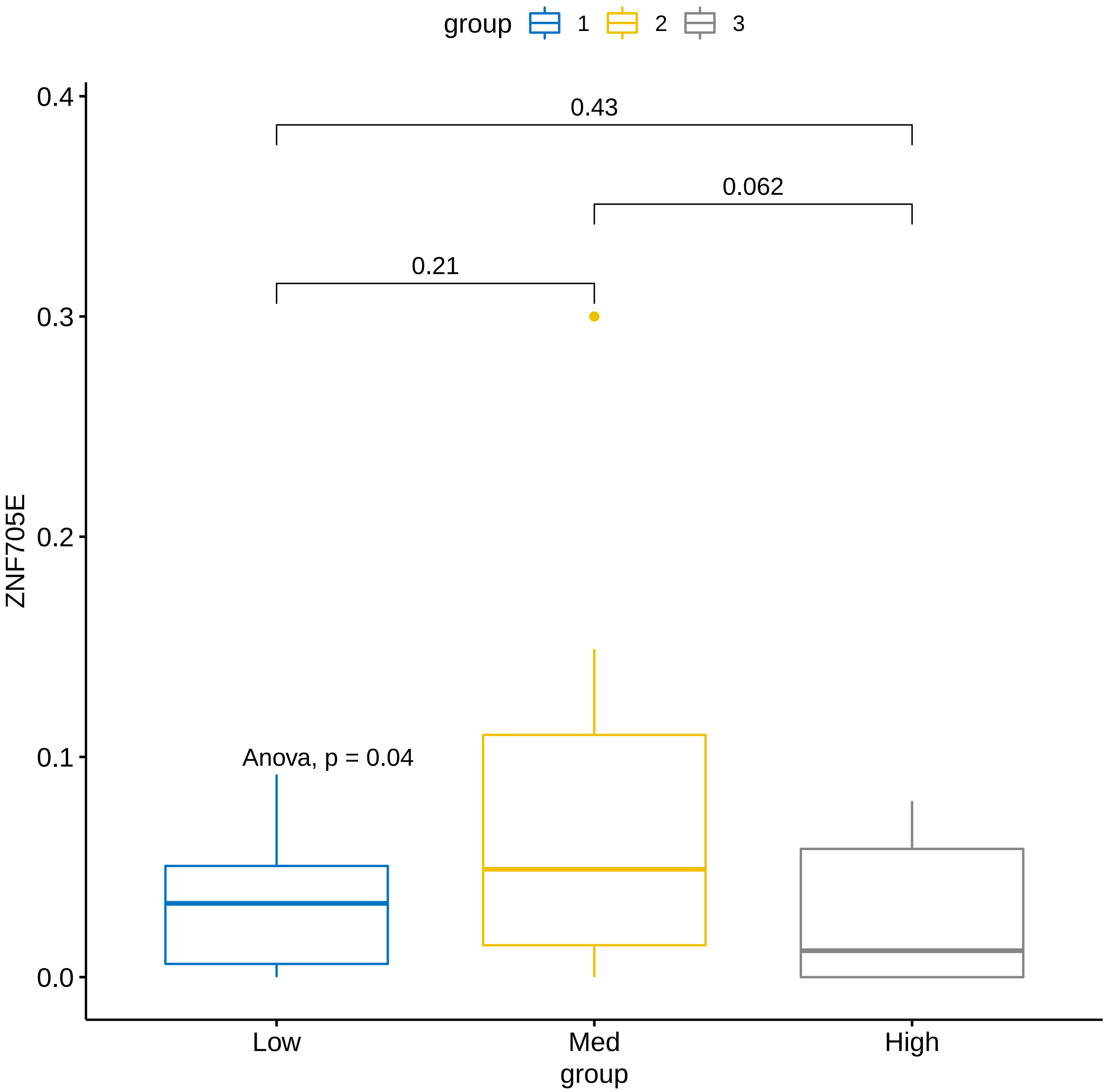
group
1
2
3
0.43
0.062
0.21
Anova, p = 0.04
0.4
0.3
0.2
0.1
0.0
ZNF705E
Low
Med
High
group

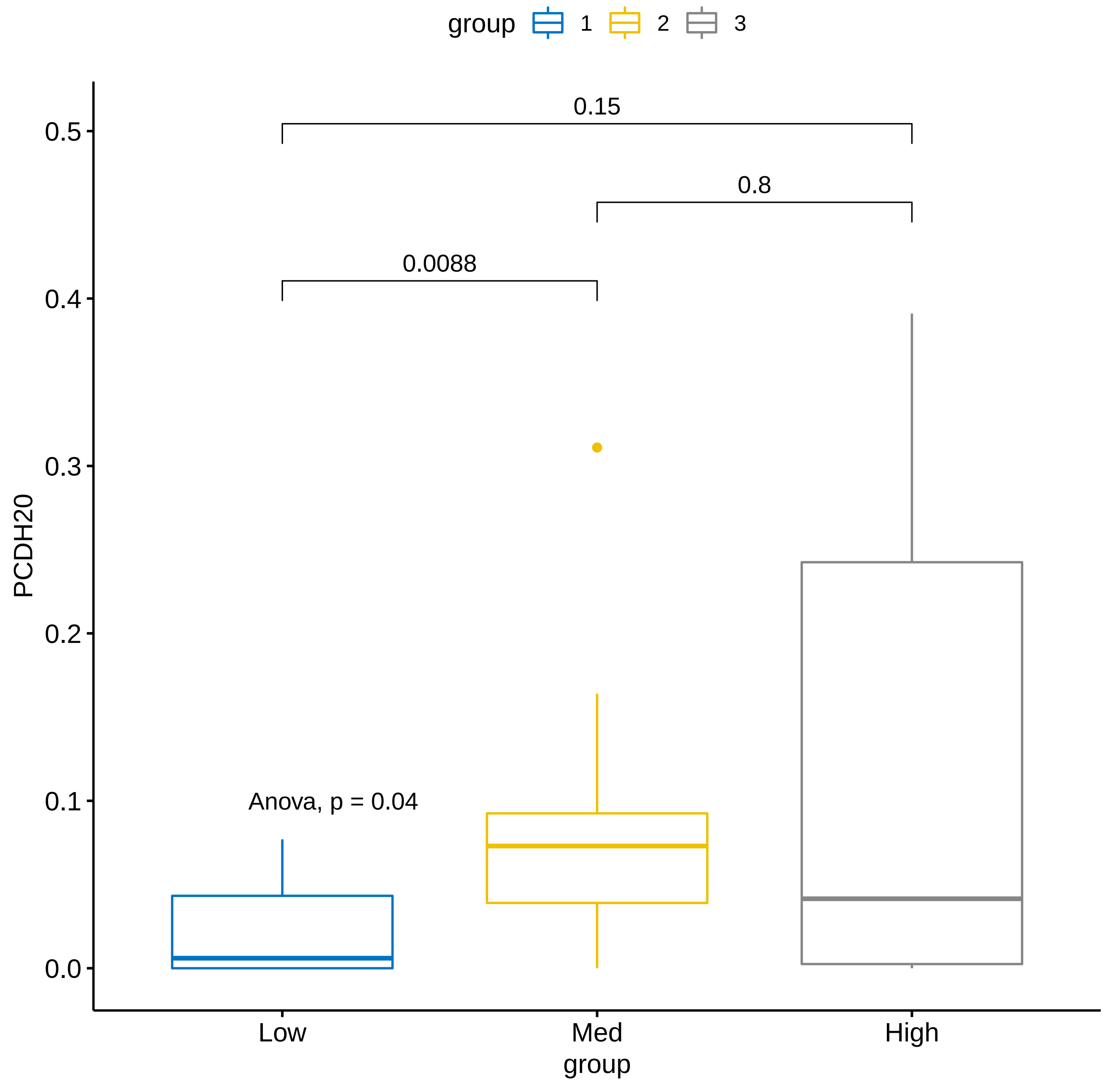
group
1
2
3
0.15
0.8
0.0088
Anova, p = 0.04
0.5
0.4
0.3
0.2
0.1
0.0
PCDH20
Low
Med
High
group

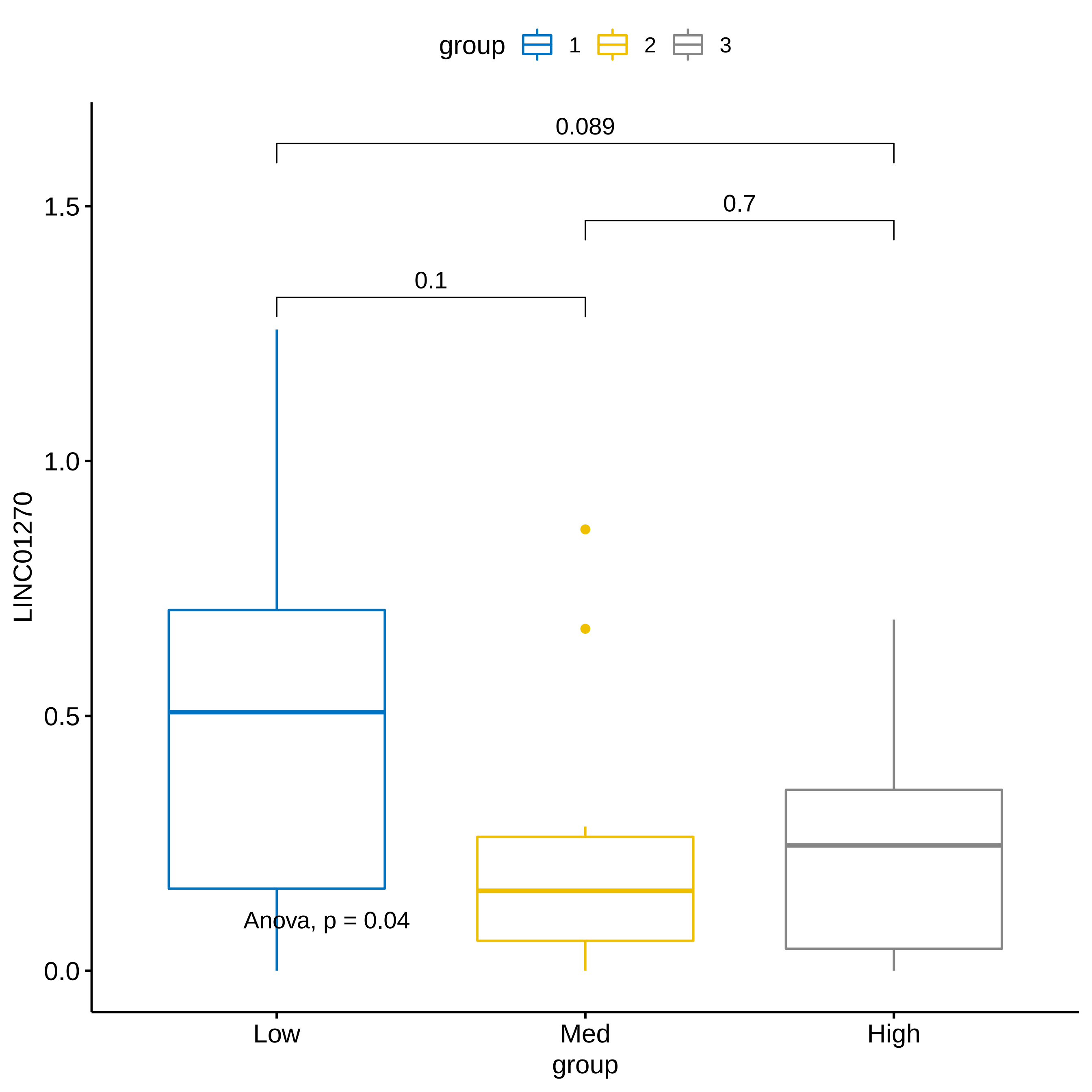
group
1
2
3
0.089
0.7
0.1
1.5
1.0
0.5
0.0
LINC01270
Anova, p = 0.04
Low
Med
High
group

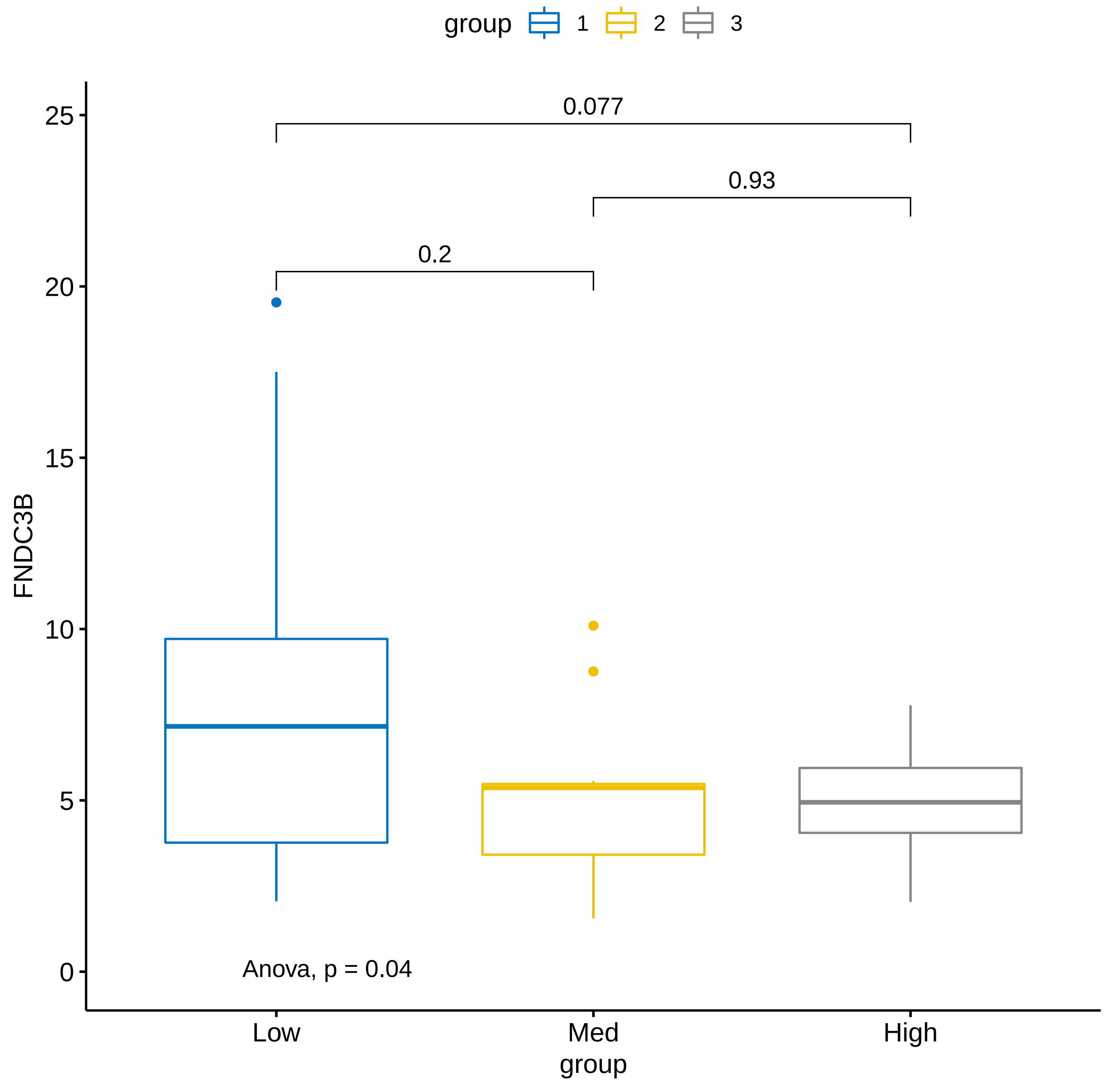
group
1
2
3
0.077
0.93
0.2
25
20
15
10
5
0
FNDC3B
Anova, p = 0.04
Low
Med
High
group

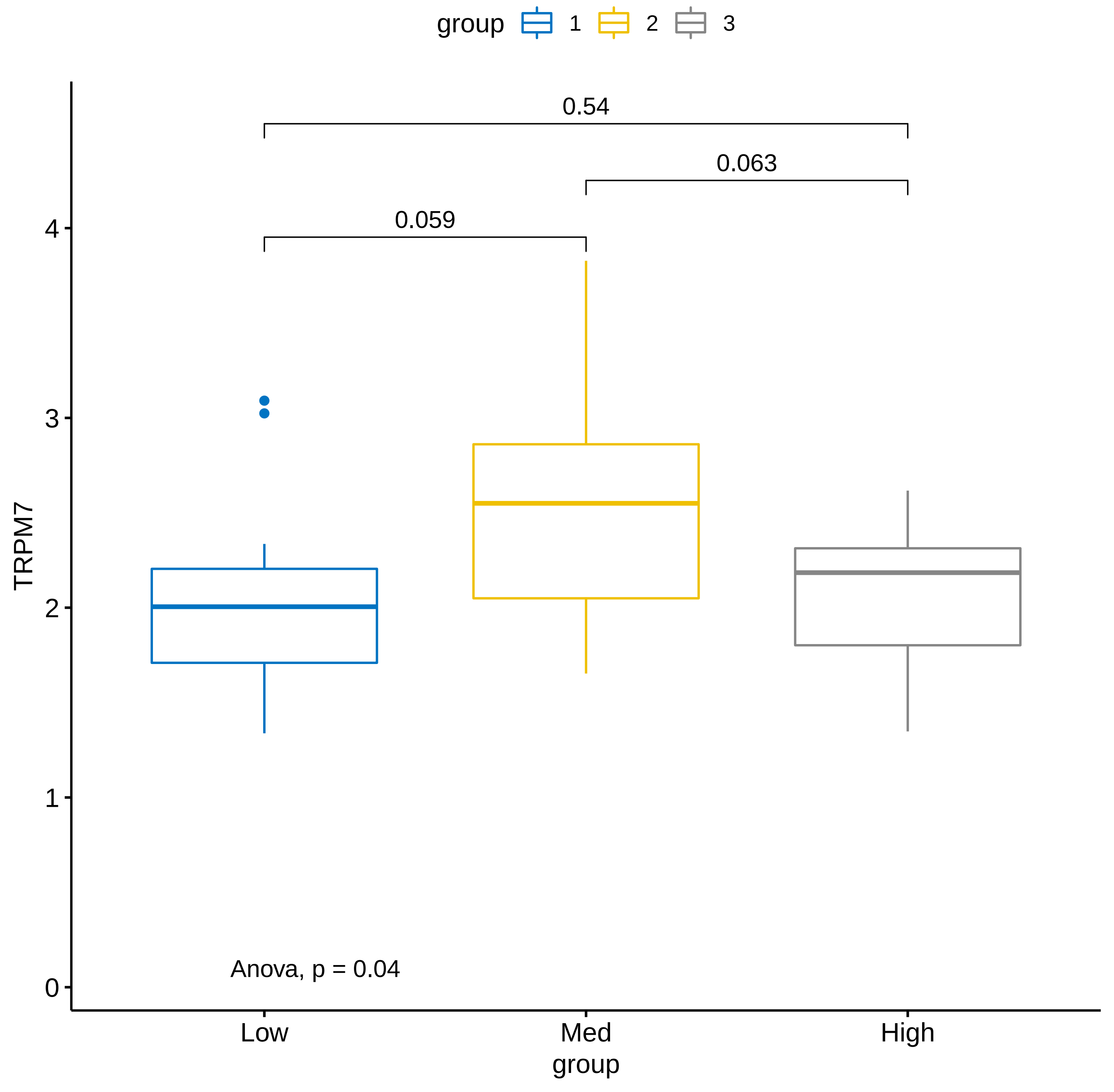
group 1 2 3
0.54
0.063
0.059
TRPM7
4
3
2
1
0
Anova, p = 0.04
Low
Med
High
group

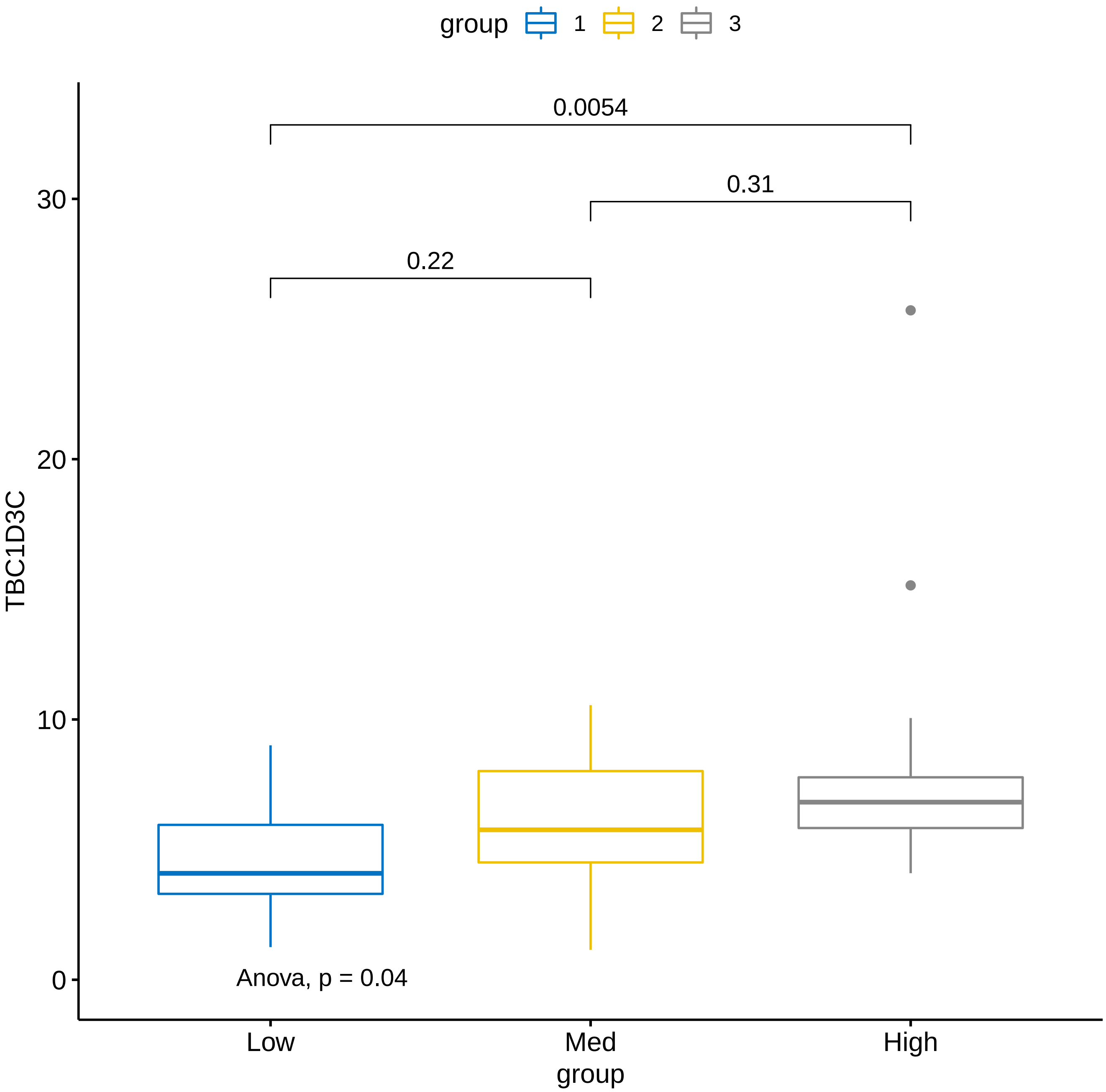
group
1
2
3
0.0054
0.31
0.22
30
20
10
0
TBC1D3C
Anova, p = 0.04
Low
Med
High
group

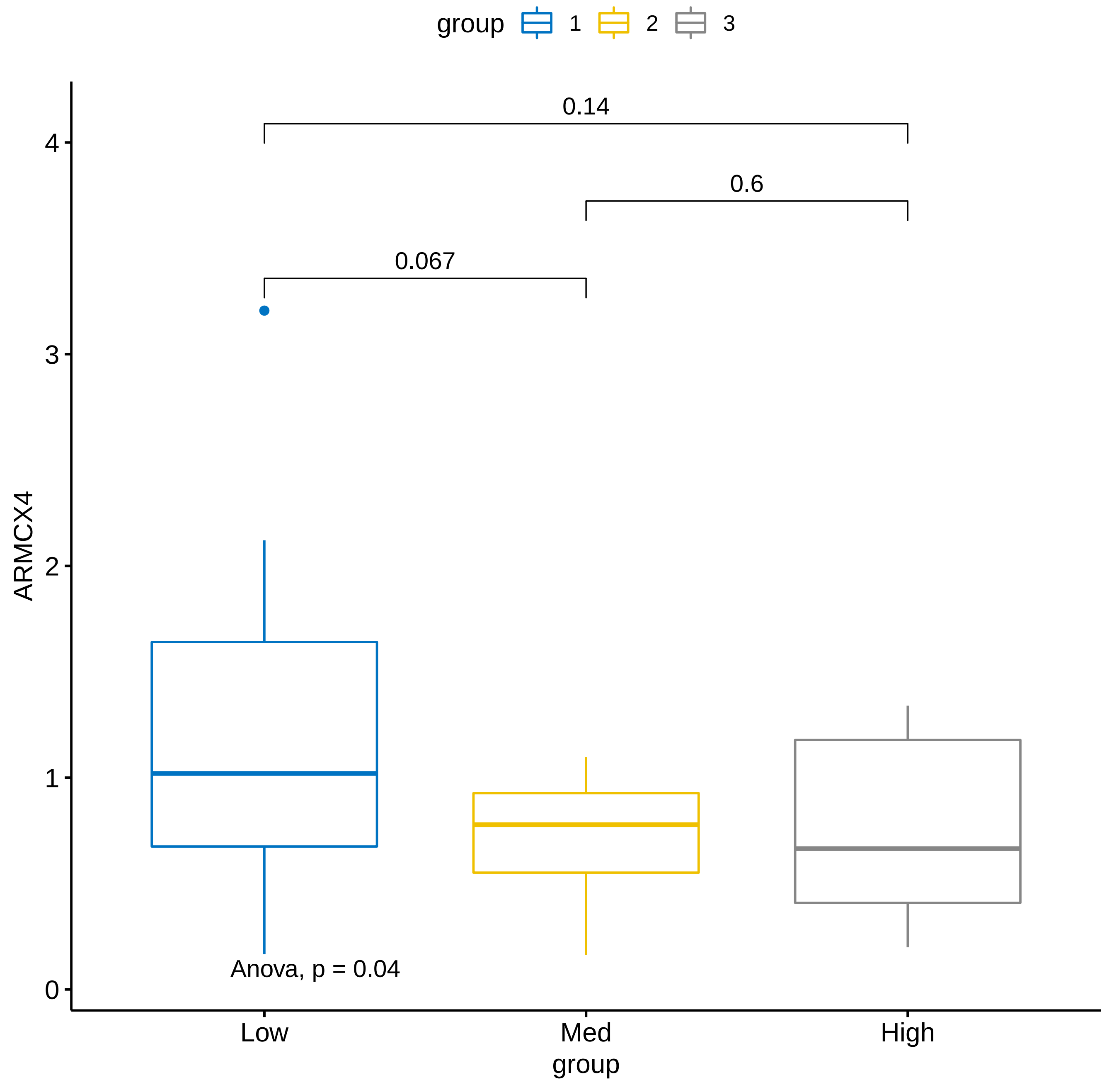

group 1 2 3
0.14
0.6
0.067
ARMCX4
4
3
2
1
0
Anova, p = 0.04
Low
Med
High
group

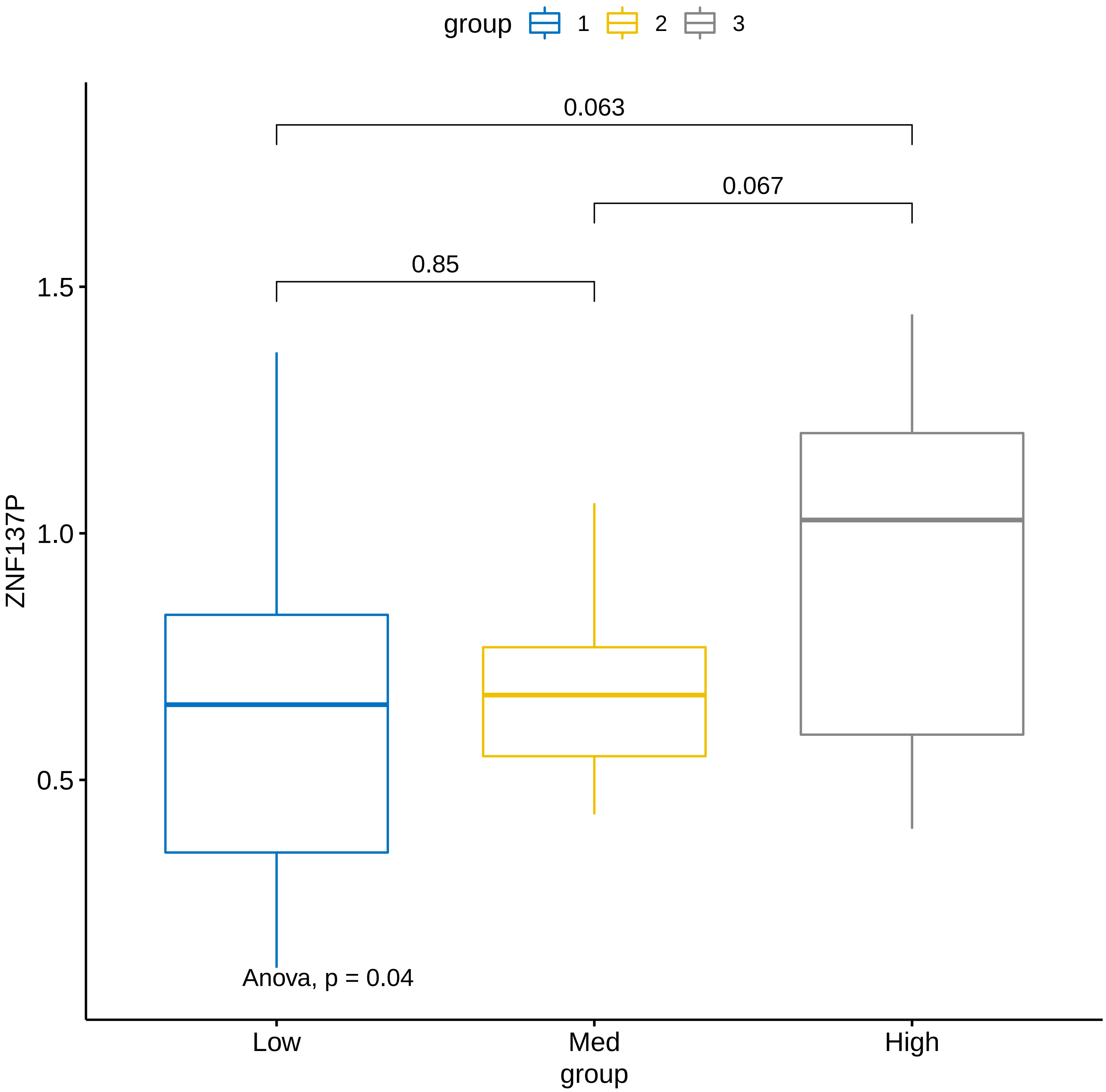
group
1
2
3
0.063
0.067
0.85
1.5
1.0
0.5
ZNF137P
Anova, p = 0.04
Low
Med
High
group

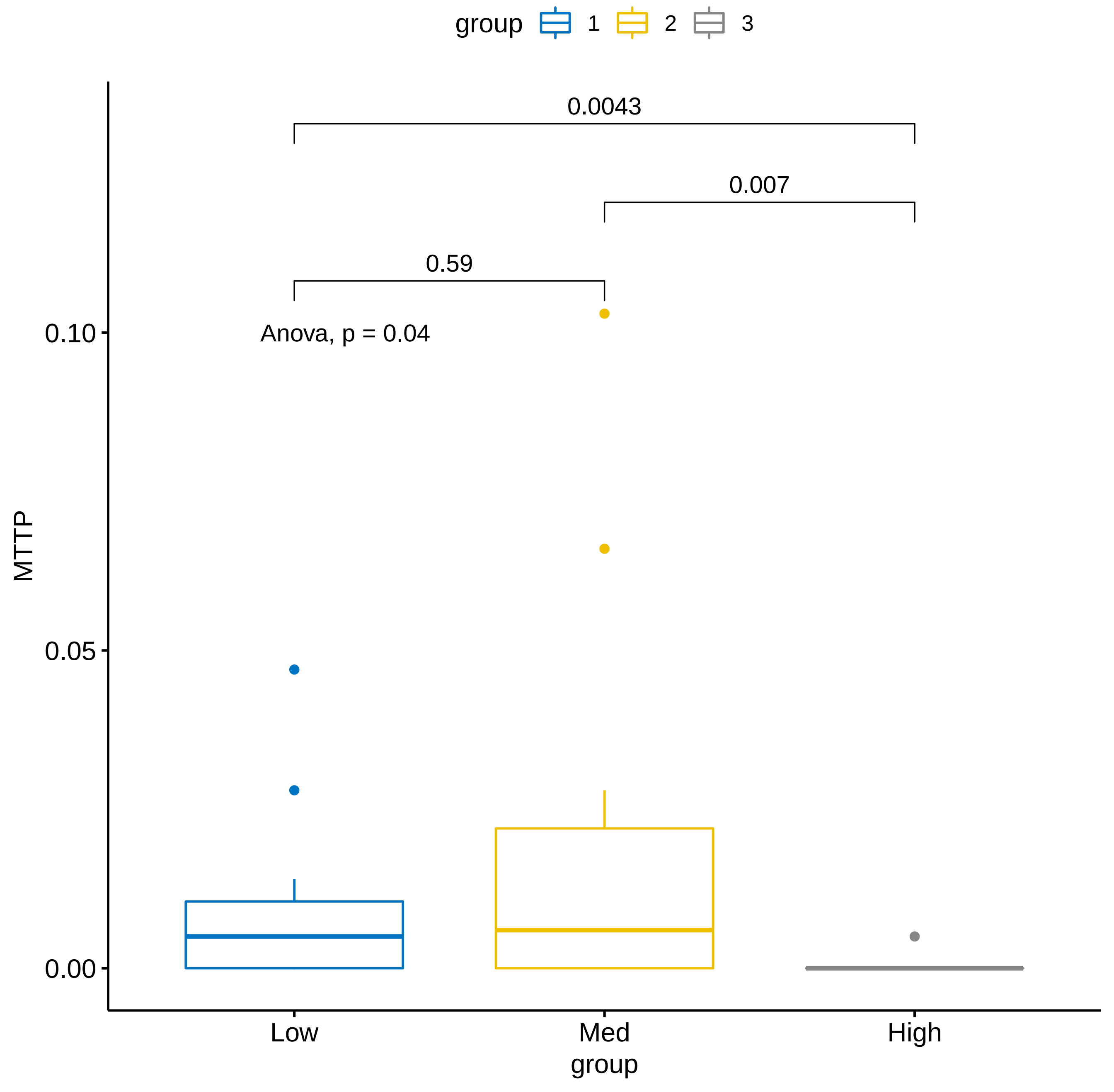
group
1
2
3
0.0043
0.007
0.59
Anova, p = 0.04
0.10
0.05
0.00
MTTP
Low
Med
High
group

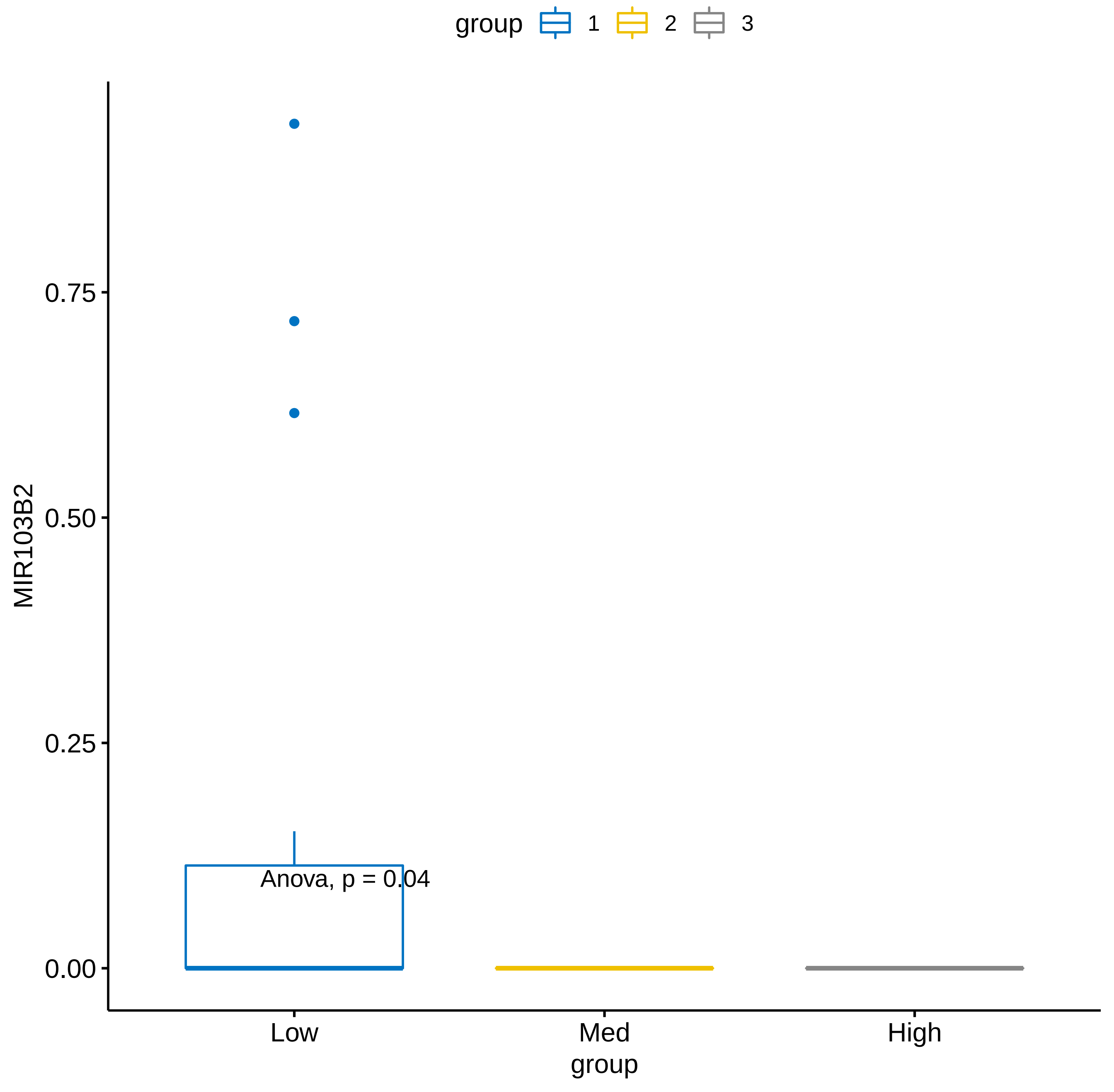
group
1
2
3
MIR103B2
0.75
0.50
0.25
0.00
Anova, p = 0.04
Low
Med
High
group

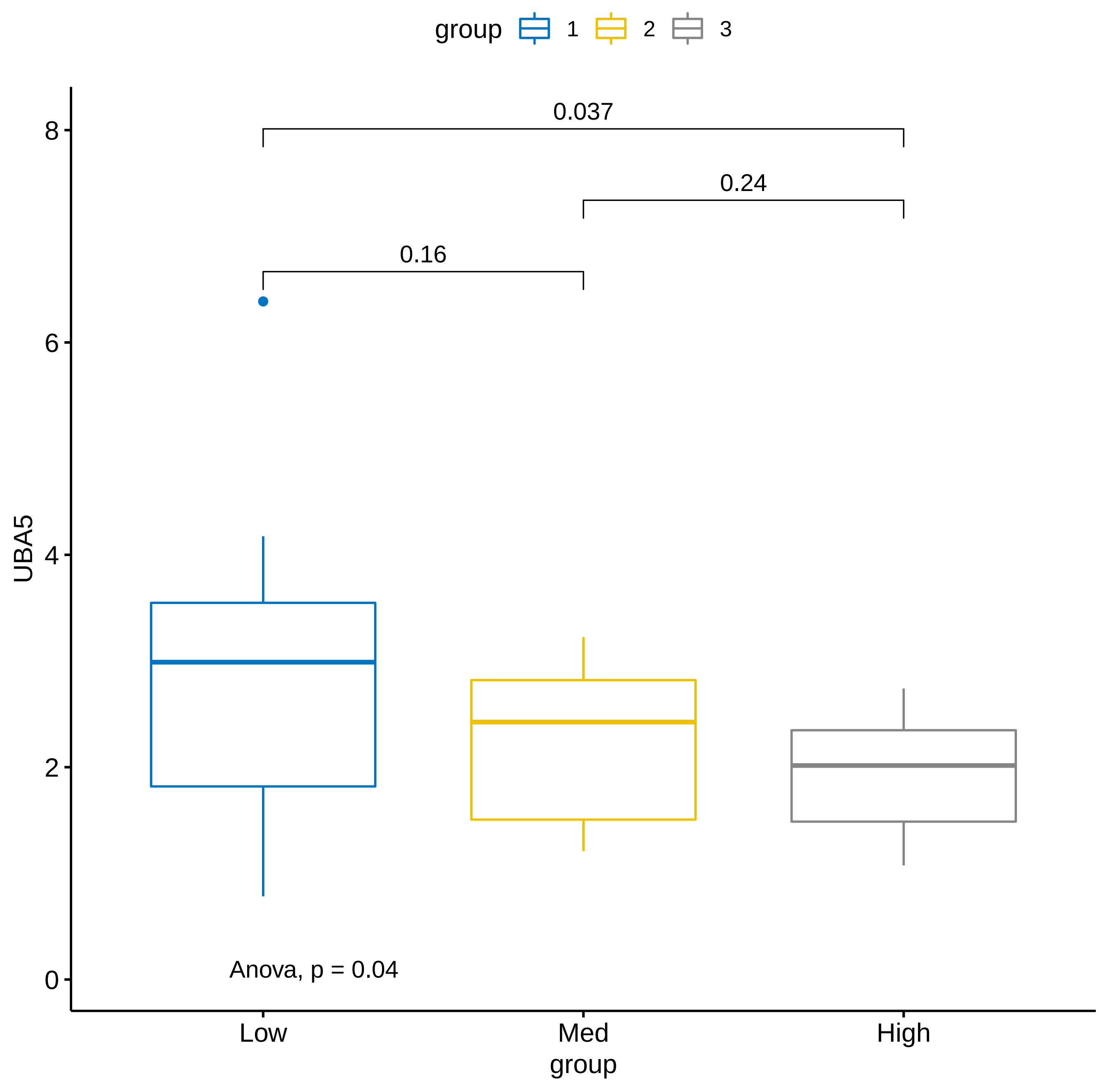
group
1
2
3
0.037
0.24
0.16
8
6
4
2
0
UBA5
Anova, p = 0.04
Low
Med
High
group

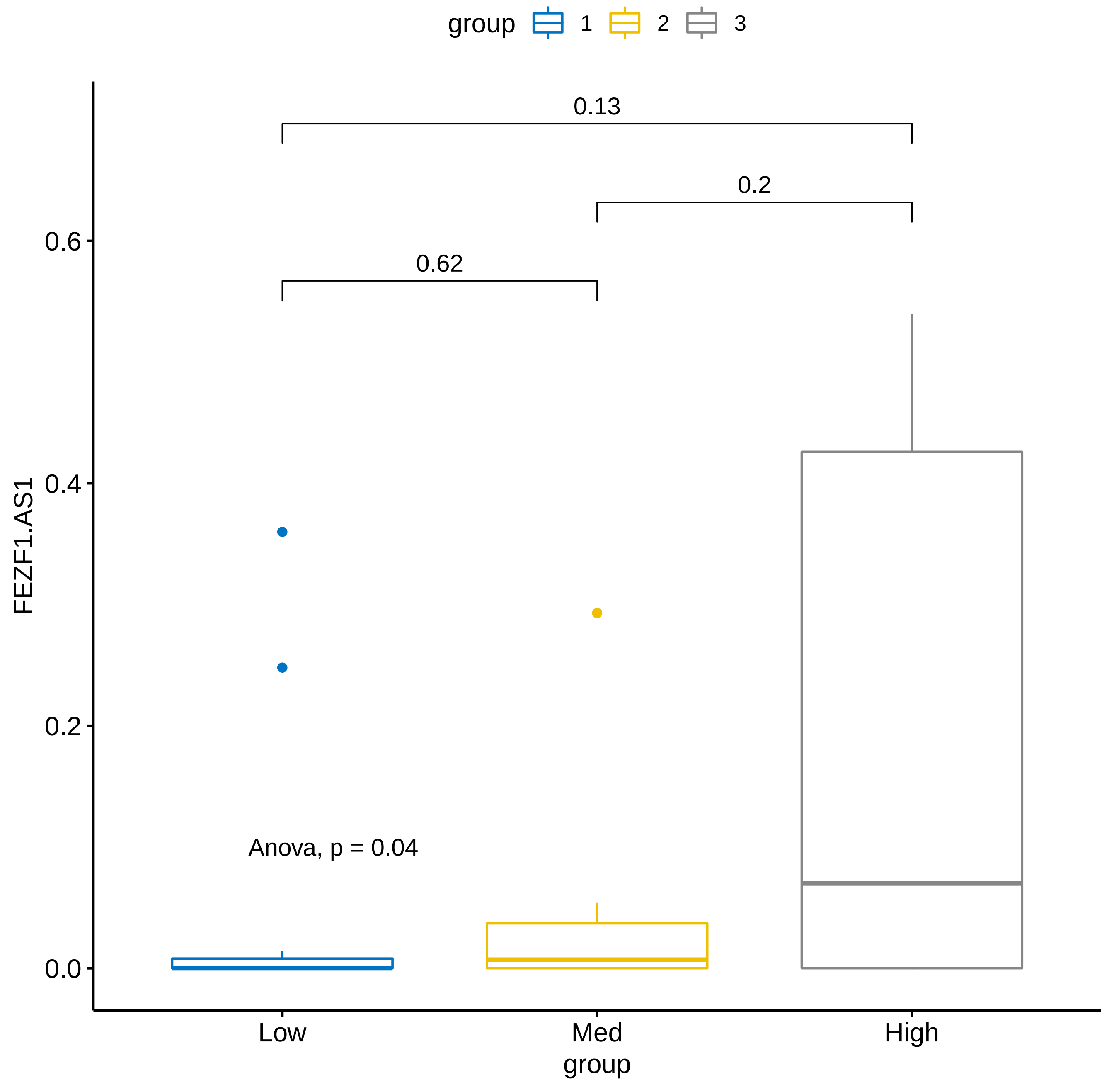

group 1 2 3
0.13
0.2
0.62
0.6
0.4
0.2
0.0
FEZF1.AS1
Anova, p = 0.04
Low
Med
High
group

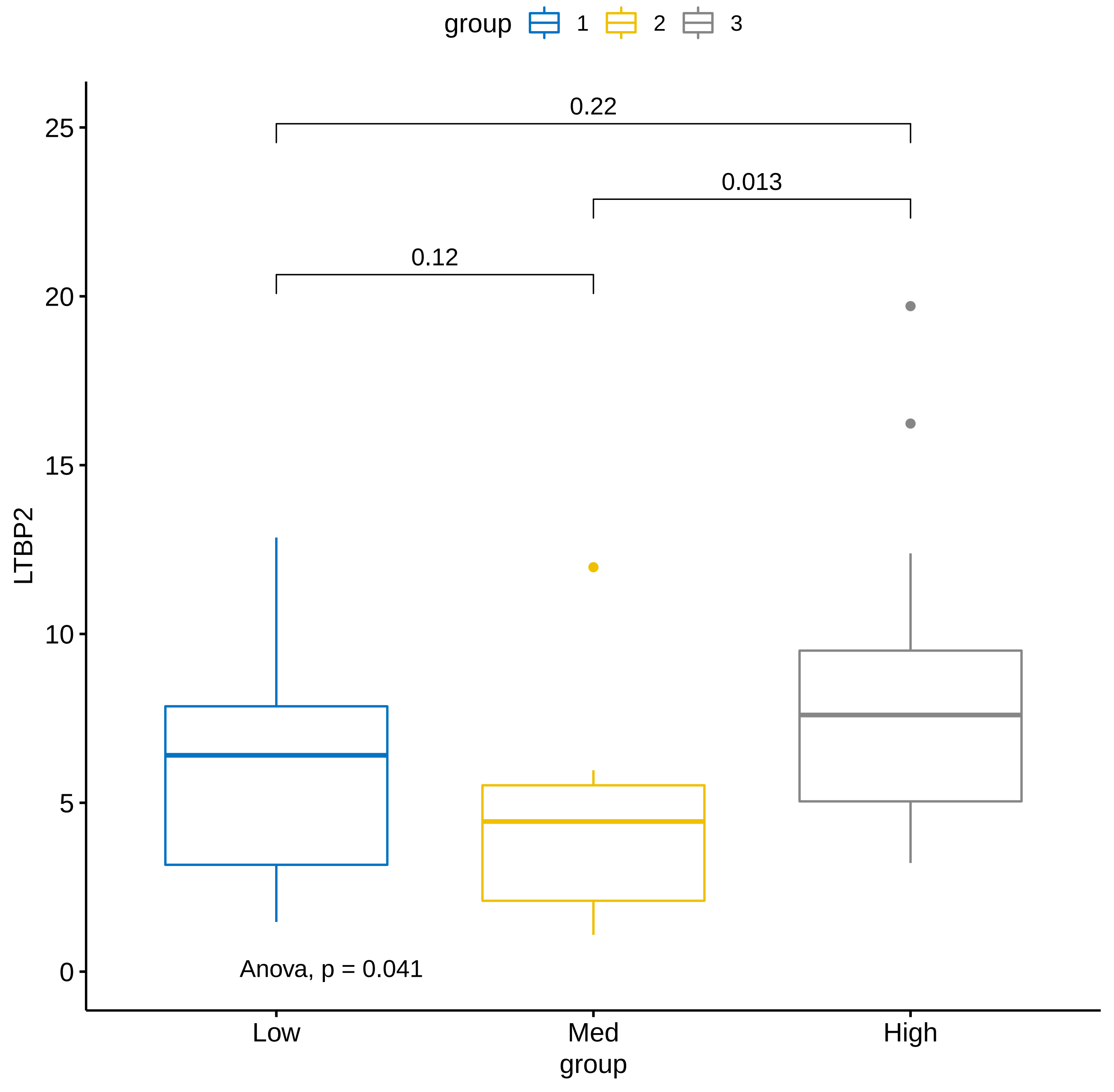
group
1
2
3
0.22
0.013
0.12
25
20
15
10
5
0
LTBP2
Anova, p = 0.041
Low
Med
High
group

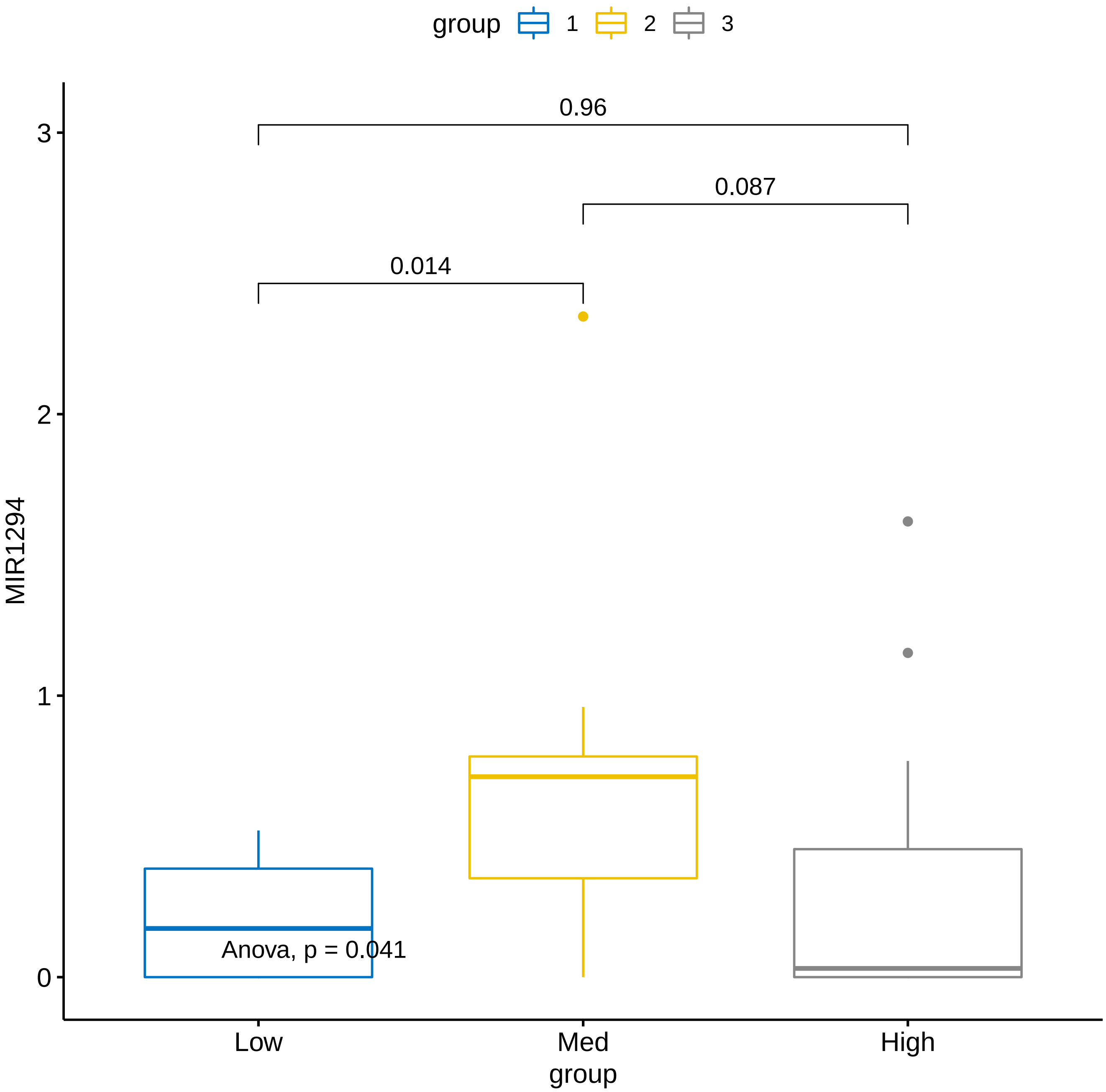

group
1
2
3
0.96
0.087
0.014
MIR1294
3
2
1
0
Anova, p = 0.041
Low
Med
High
group

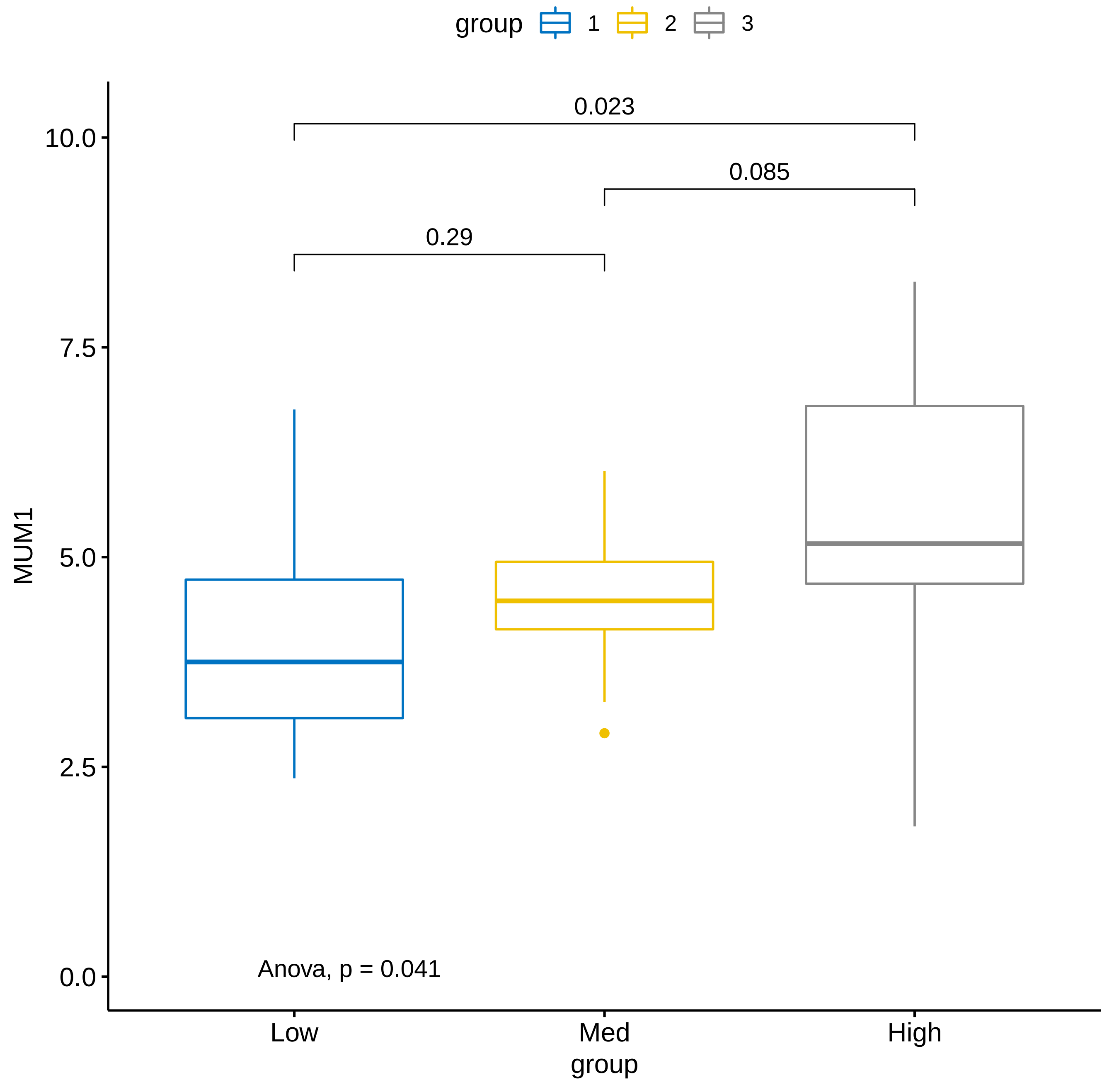

group
1
2
3
0.023
0.085
0.29
10.0
7.5
5.0
2.5
0.0
MUM1
Anova, p = 0.041
Low
Med
High
group

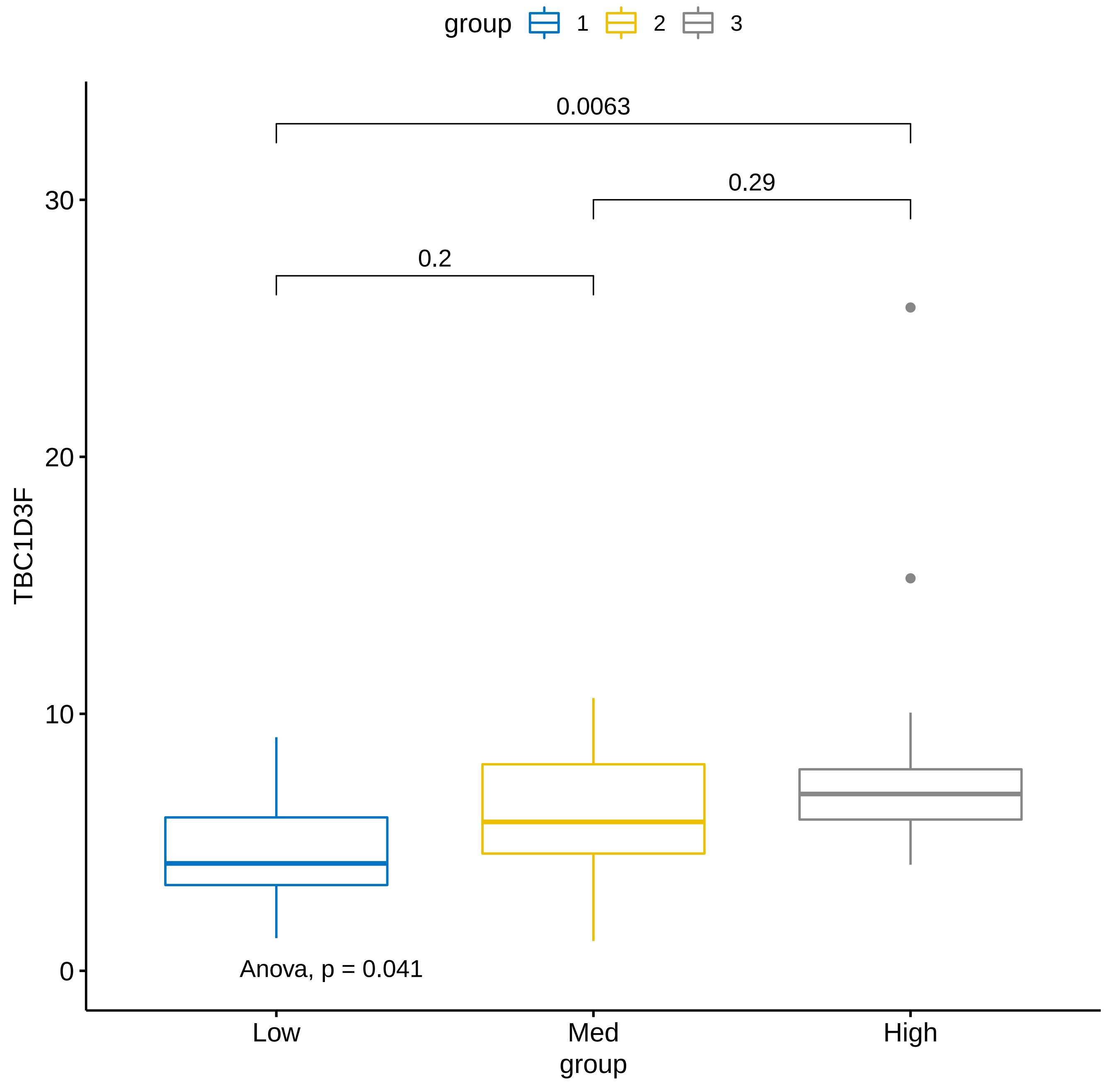
group 1 2 3
0.0063
0.29
0.2
30
20
10
0
TBC1D3F
Anova, p = 0.041
Low
Med
High
group

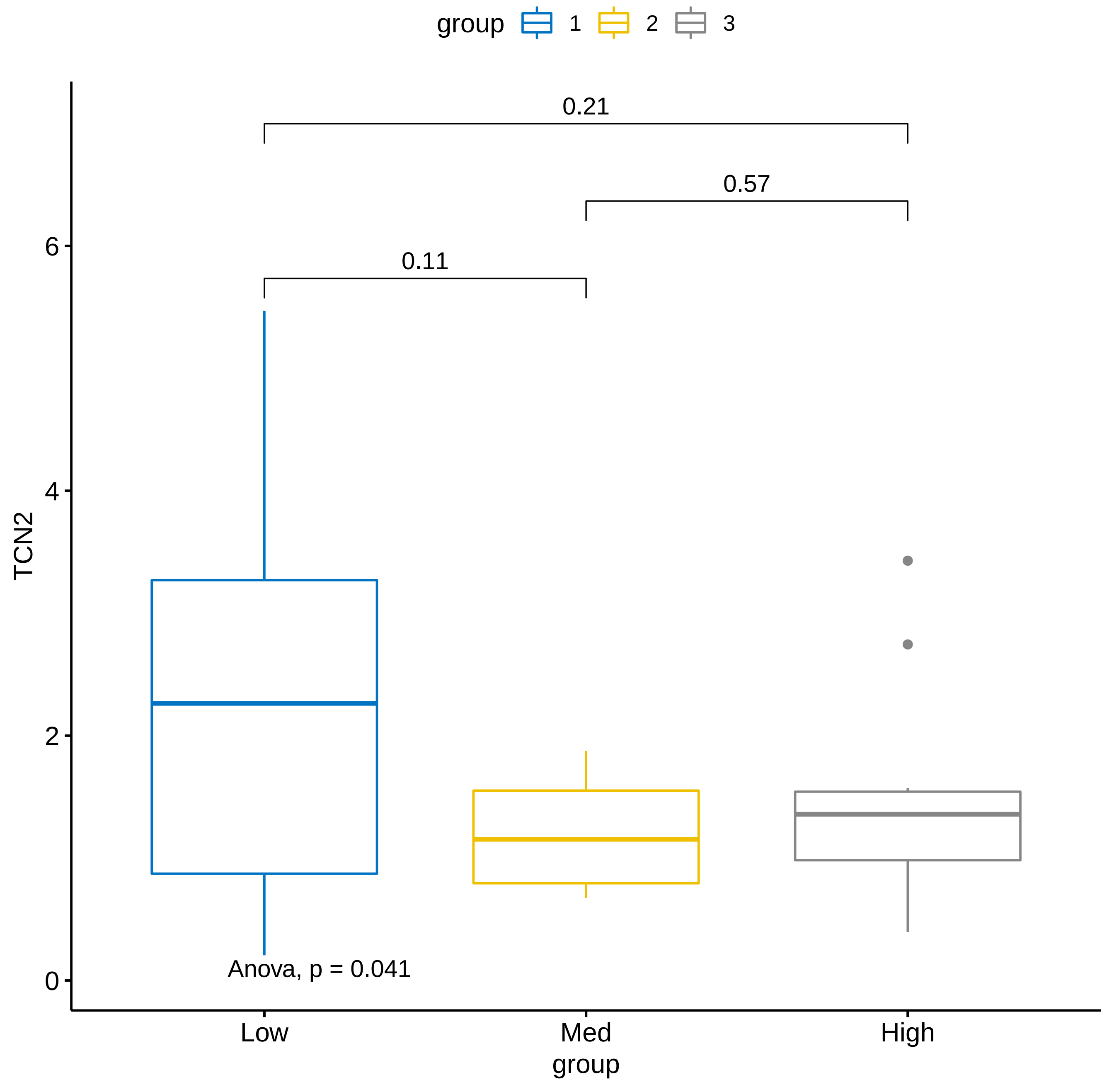

group 1 2 3
0.21
0.57
0.11
TCN2
6
4
2
0
Anova, p = 0.041
Low
Med
High
group

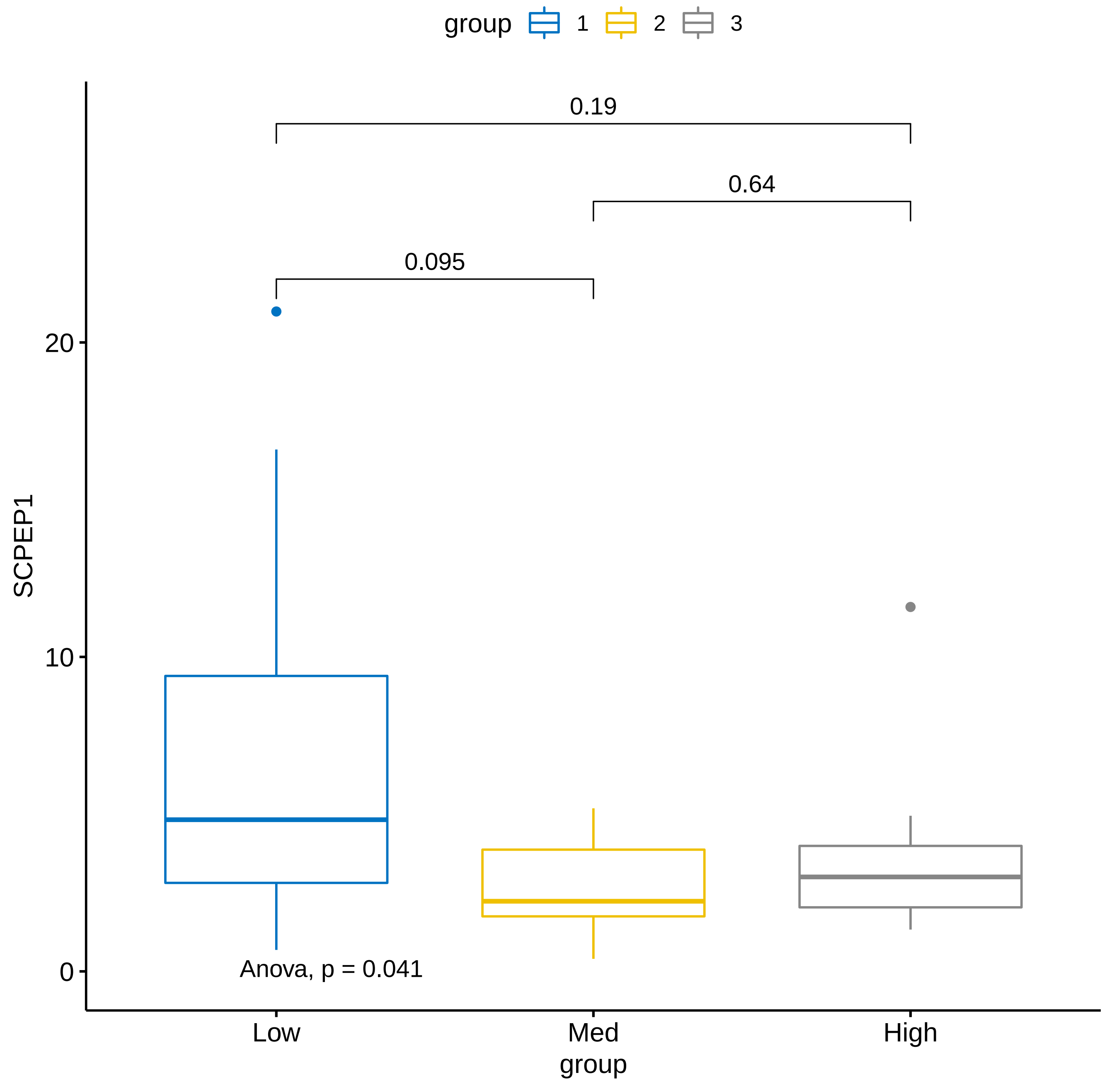
group 1 2 3
0.19
0.64
0.095
20
10
0
SCPEP1
Anova, p = 0.041
Low
Med
High
group

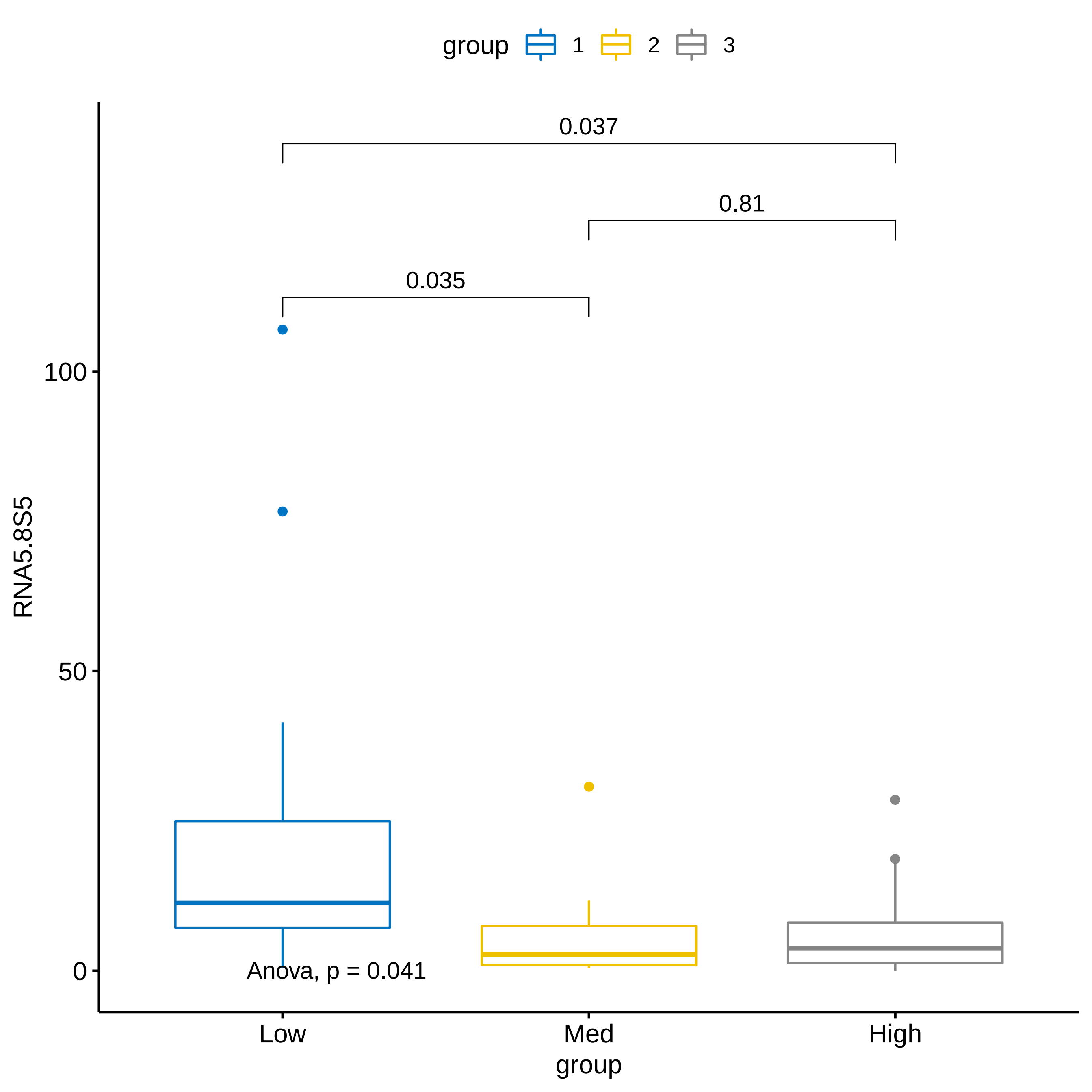
group
1
2
3
0.037
0.81
0.035
100
50
0
RNA5.8S5
Anova, p = 0.041
Low
Med
High
group

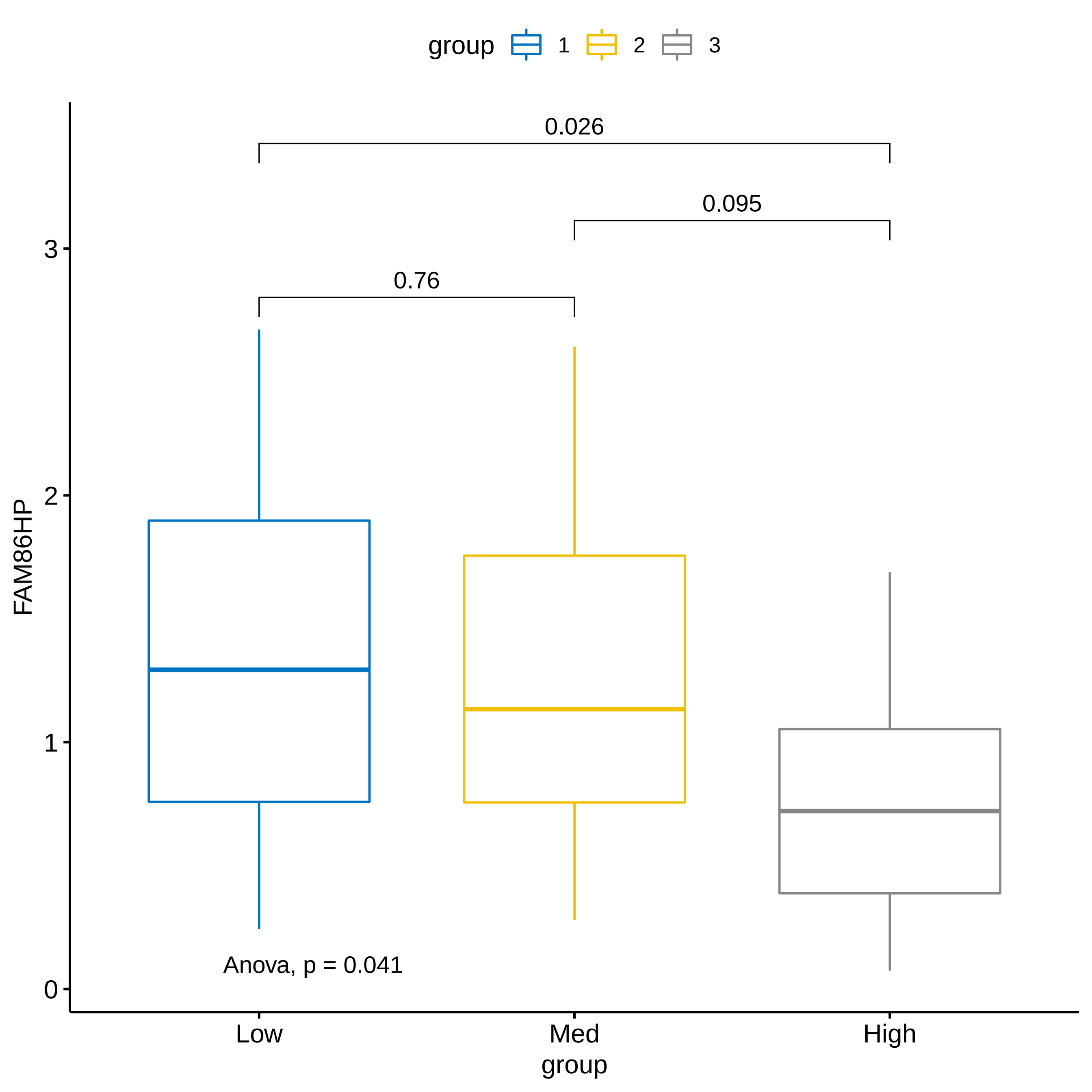
group 1 2 3
0.026
0.095
0.76
Anova, p = 0.041
FAM86HP
3
2
1
0
Low
Med
High
group

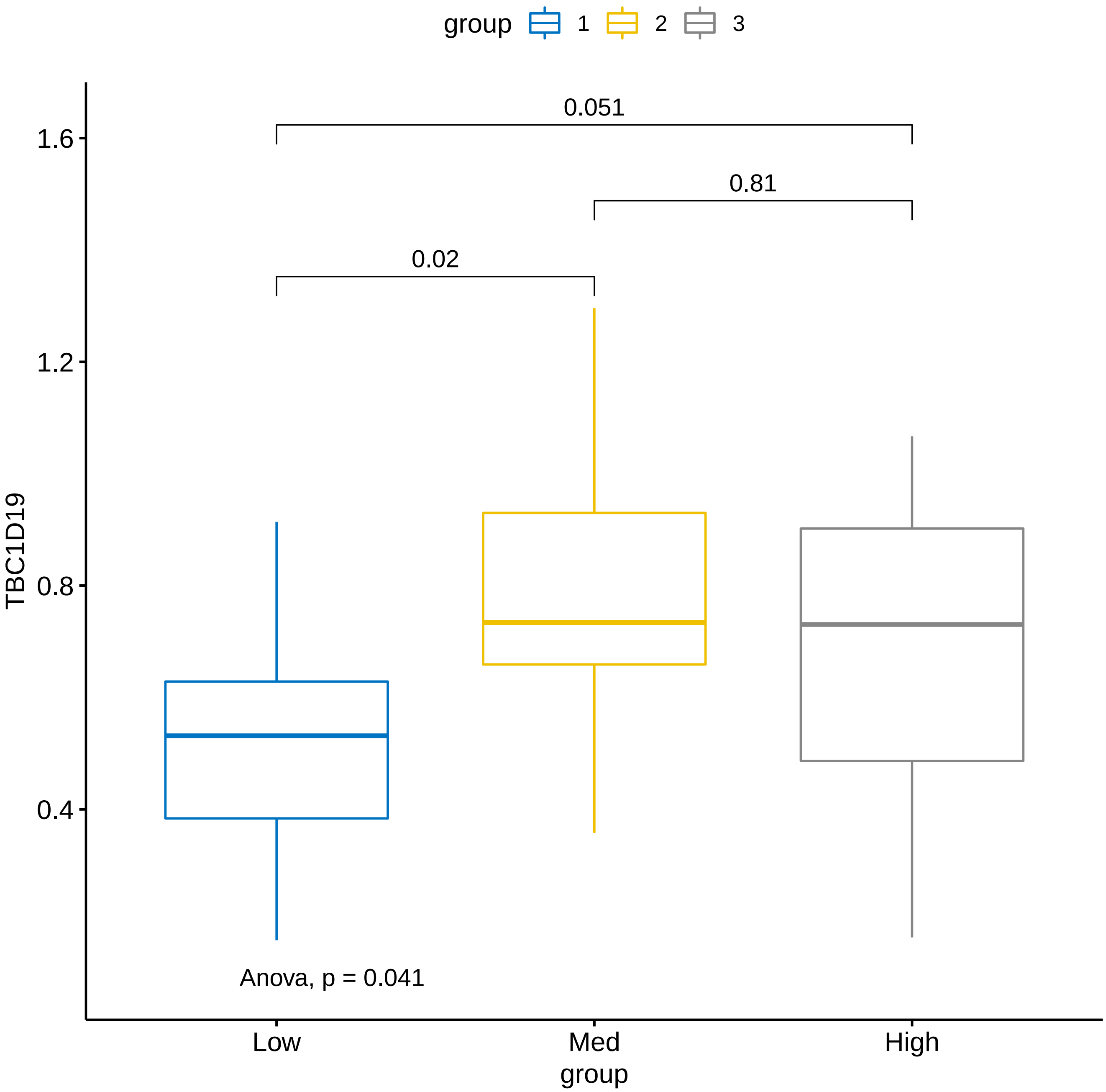

group 1 2 3
0.051
0.81
0.02
1.6
1.2
0.8
0.4
TBC1D19
Anova, p = 0.041
Low
Med
High
group

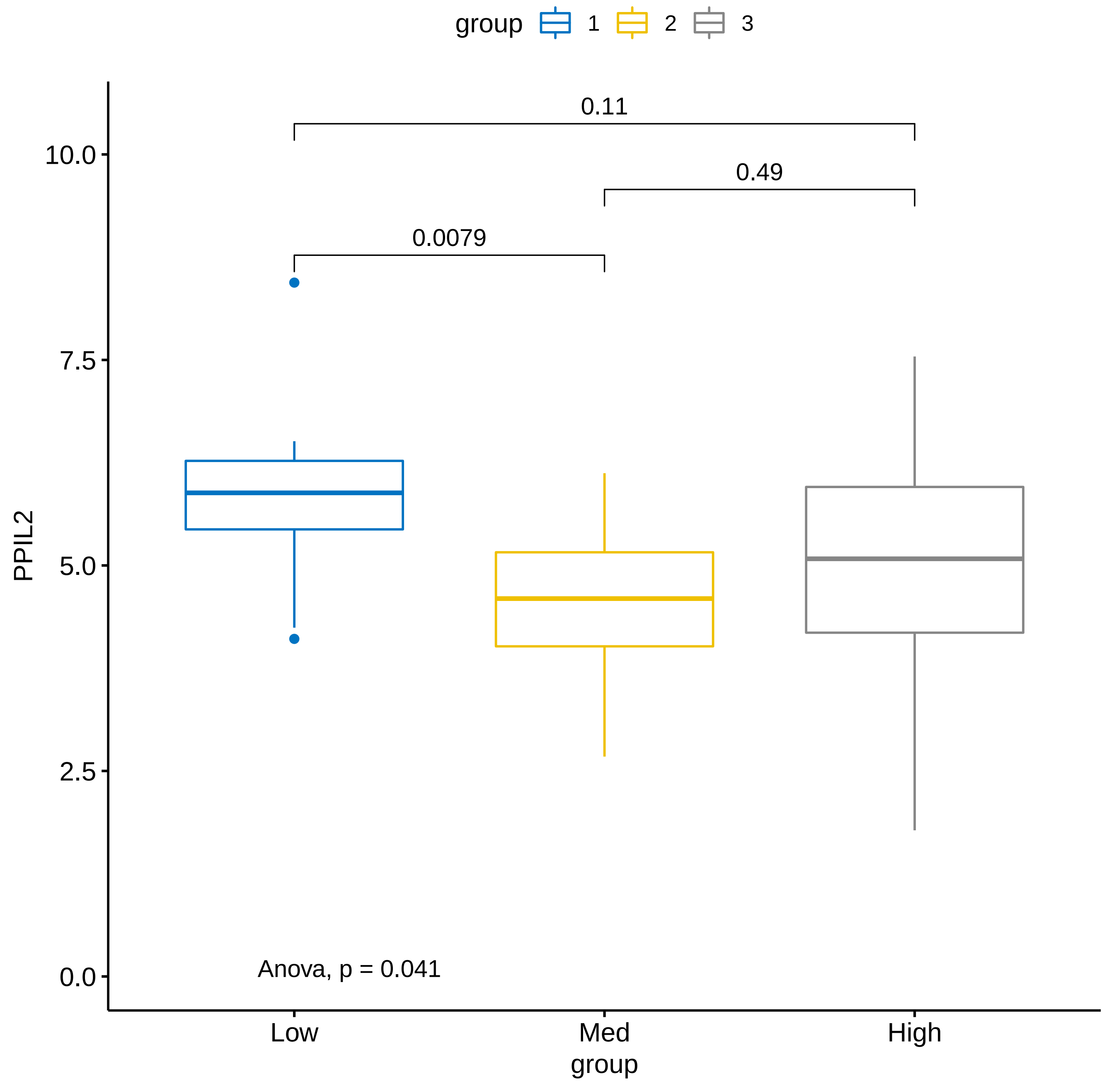

group
1
2
3
0.11
0.49
0.0079
10.0
7.5
5.0
2.5
0.0
PPIL2
Anova, p = 0.041
Low
Med
High
group

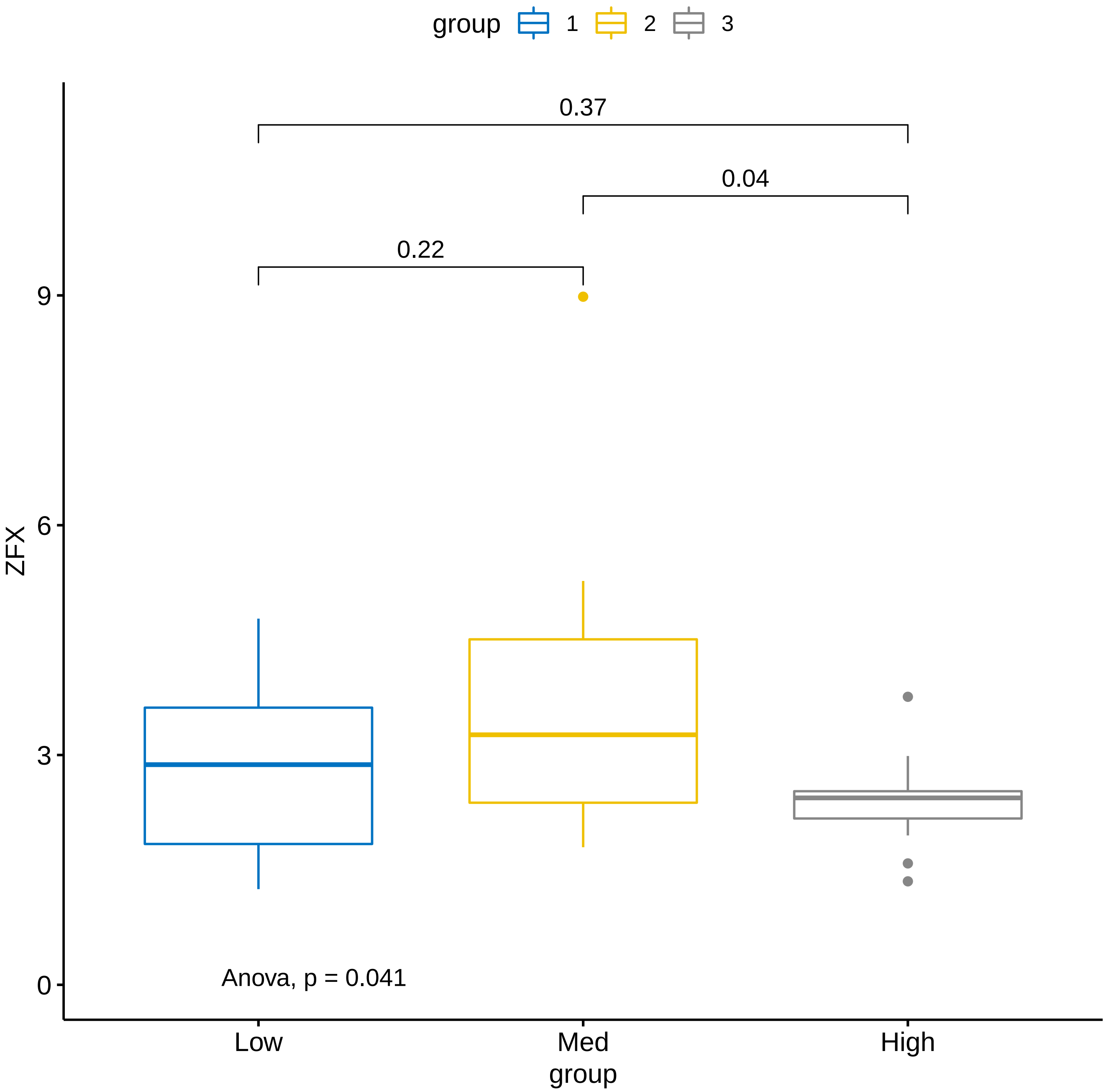
group
1
2
3
0.37
0.04
0.22
9
6
3
0
ZFX
Anova, p = 0.041
Low
Med
High
group

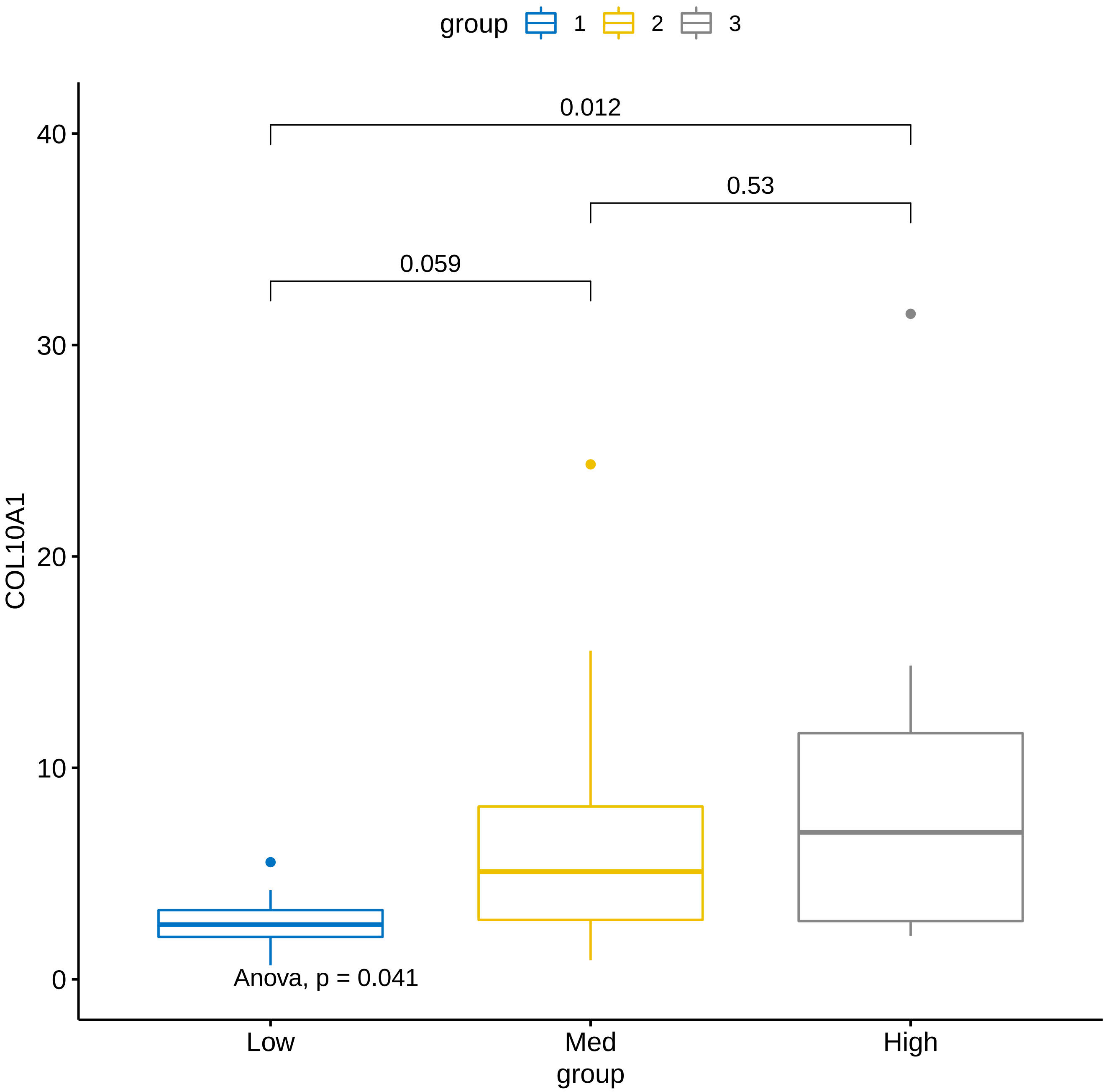

group 1 2 3
0.012
0.53
0.059
Anova, p = 0.041
COL10A1
40
30
20
10
0
Low
Med
High
group

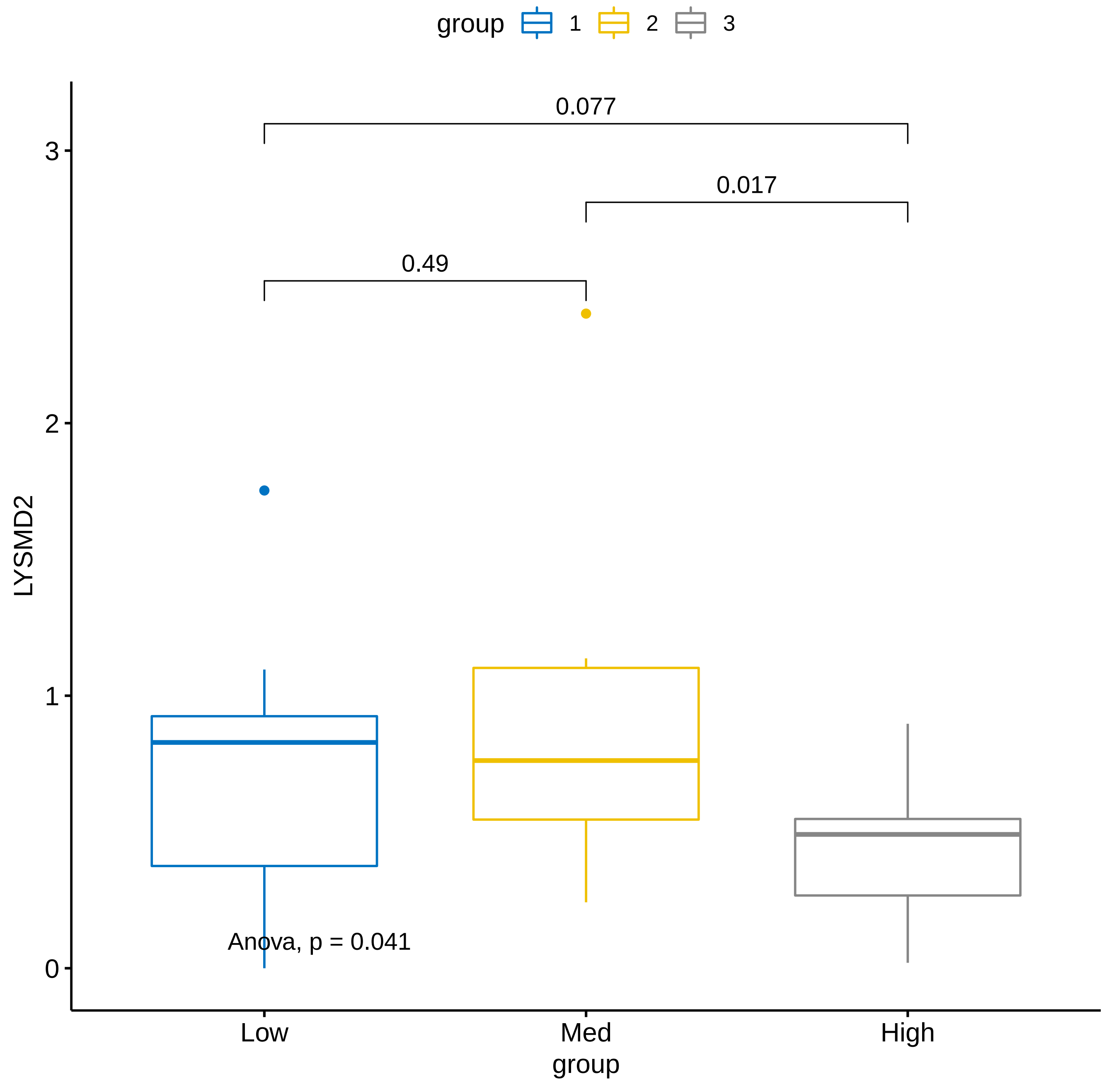
group
1
2
3
0.077
0.017
0.49
3
2
1
0
LYSMD2
Anova, p = 0.041
Low
Med
High
group

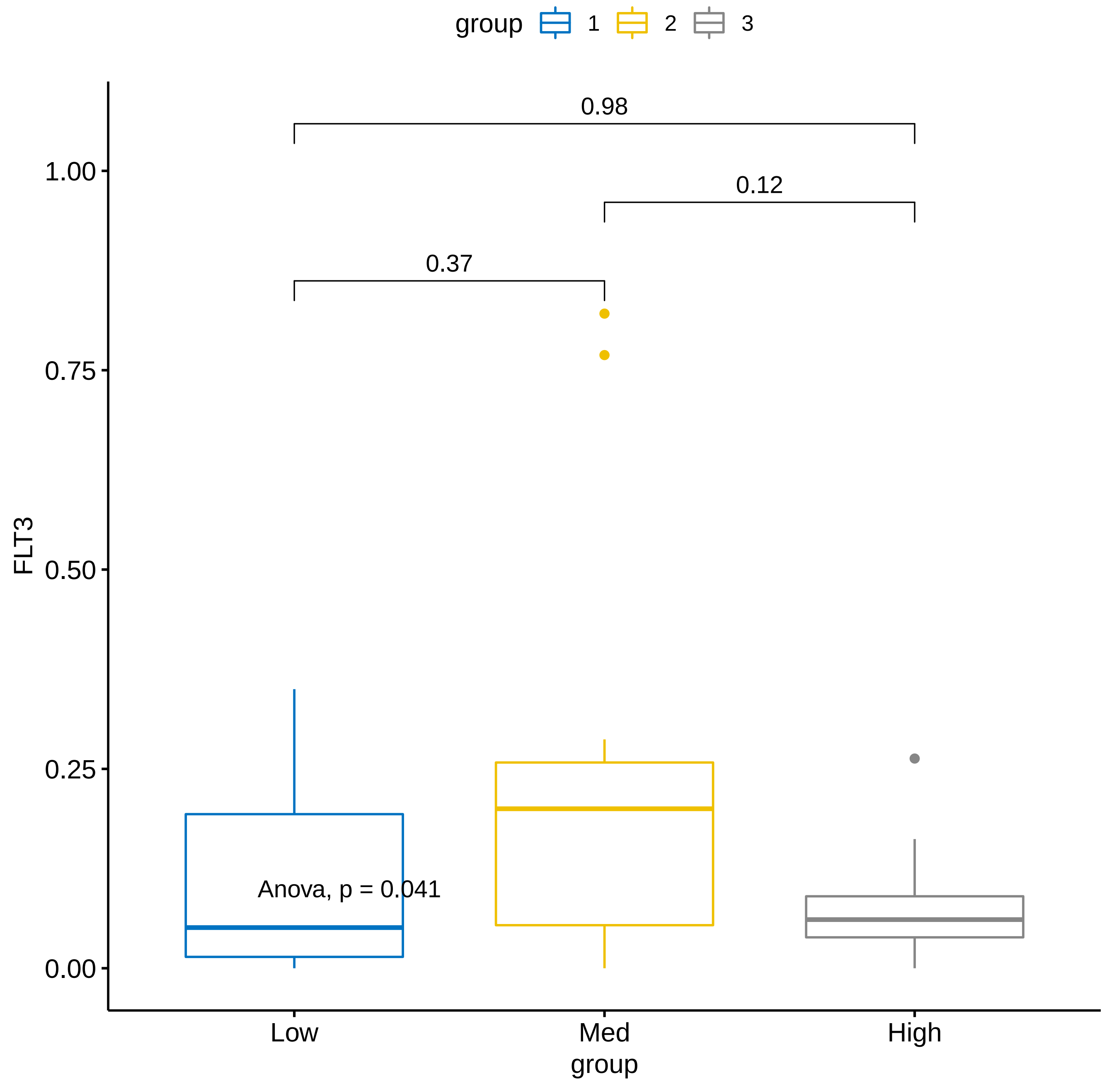
group 1 2 3
0.98
0.12
0.37
1.00
0.75
0.50
0.25
0.00
FLT3
Anova, p = 0.041
Low
Med
High
group

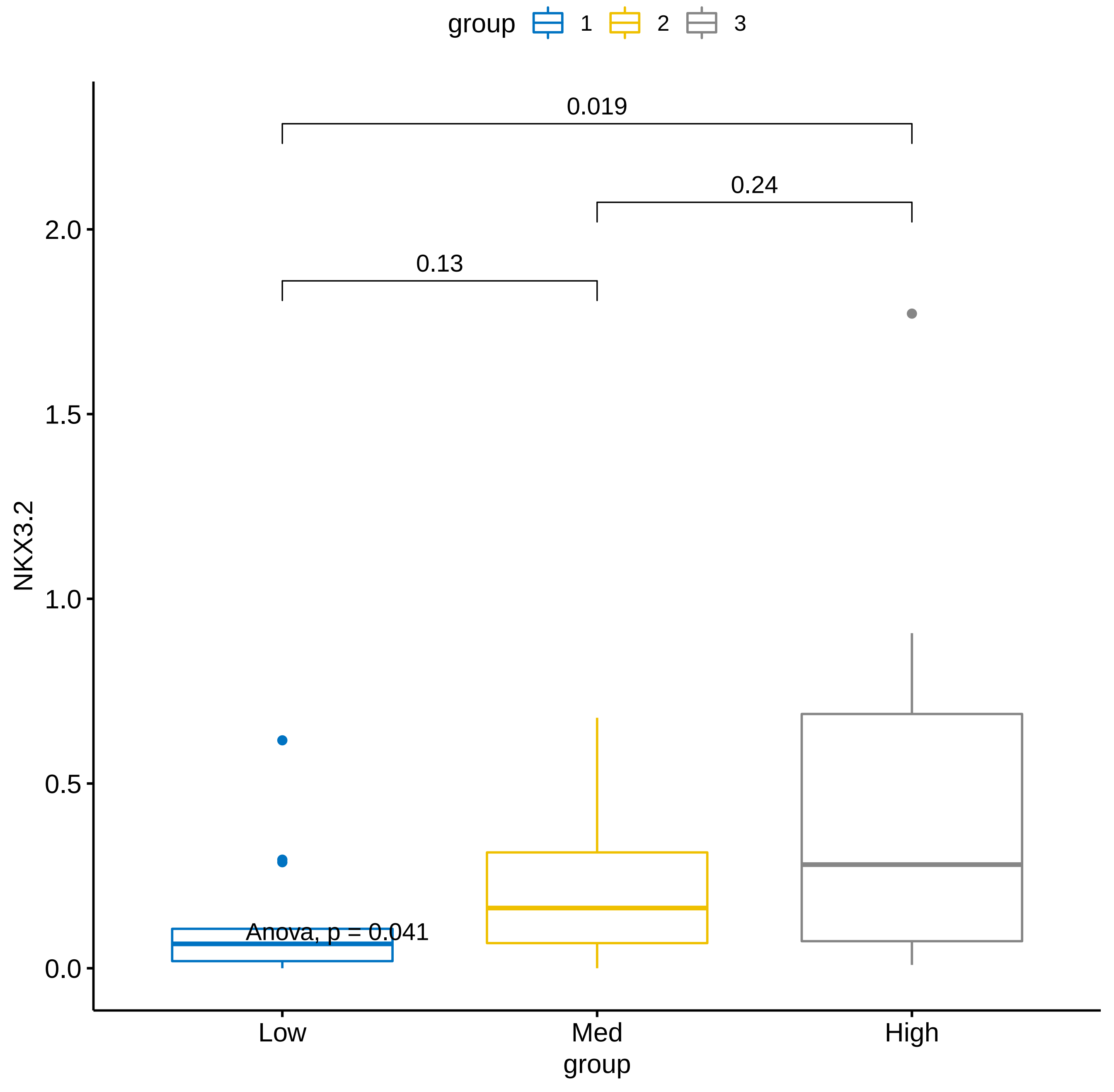
group
1
2
3
0.019
0.24
0.13
2.0
1.5
1.0
0.5
0.0
NKX3.2
Anova, p = 0.041
Low
Med
High
group

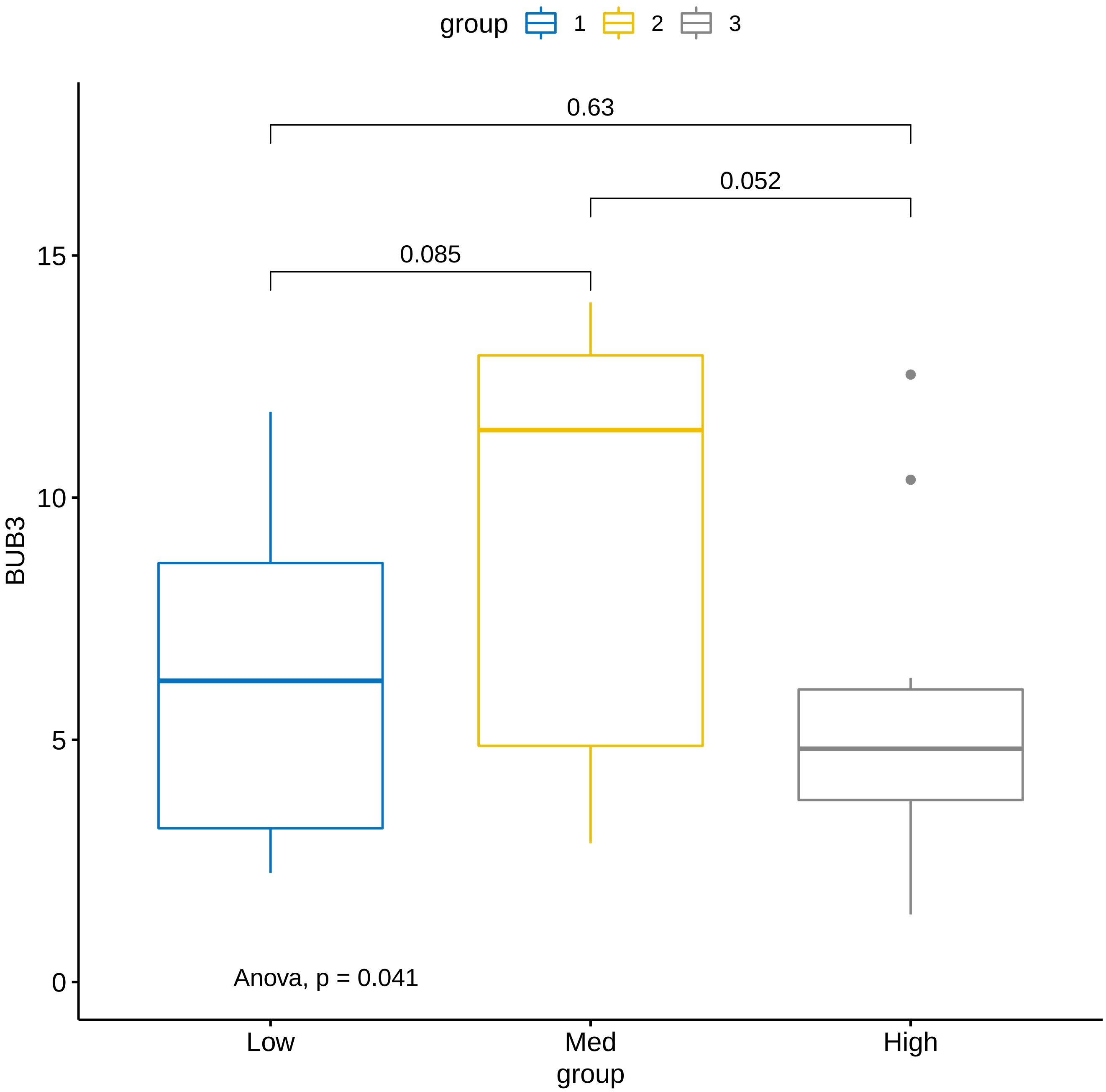

group 1 2 3
0.63
0.052
0.085
15
10
5
0
BUB3
Anova, p = 0.041
Low
Med
High
group

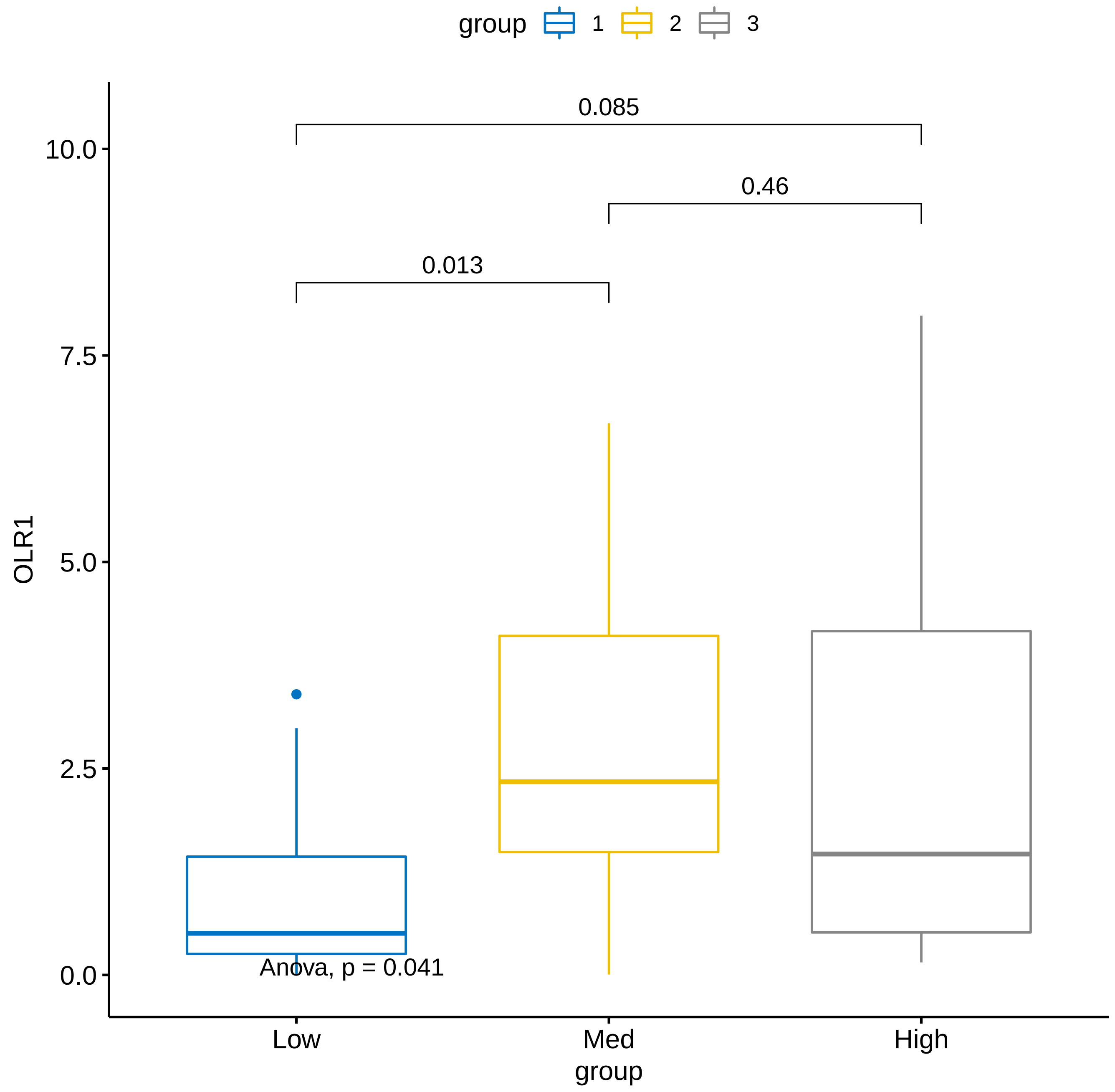
group
1
2
3
0.085
0.46
0.013
10.0
7.5
5.0
2.5
0.0
OLR1
Anova, p = 0.041
Low
Med
High
group

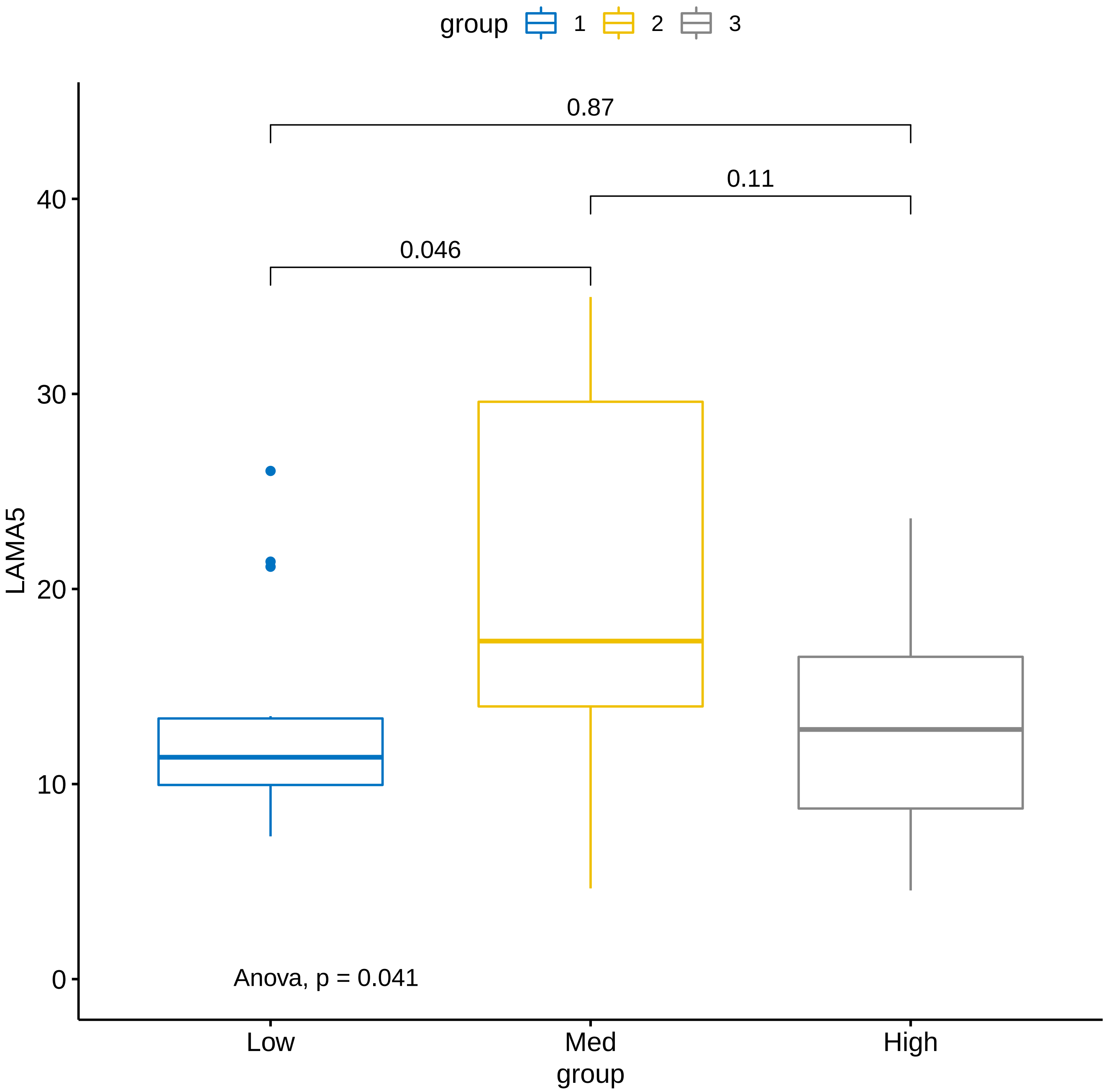

group
1
2
3
0.87
0.11
0.046
40
30
20
10
0
LAMA5
Anova, p = 0.041
Low
Med
High
group

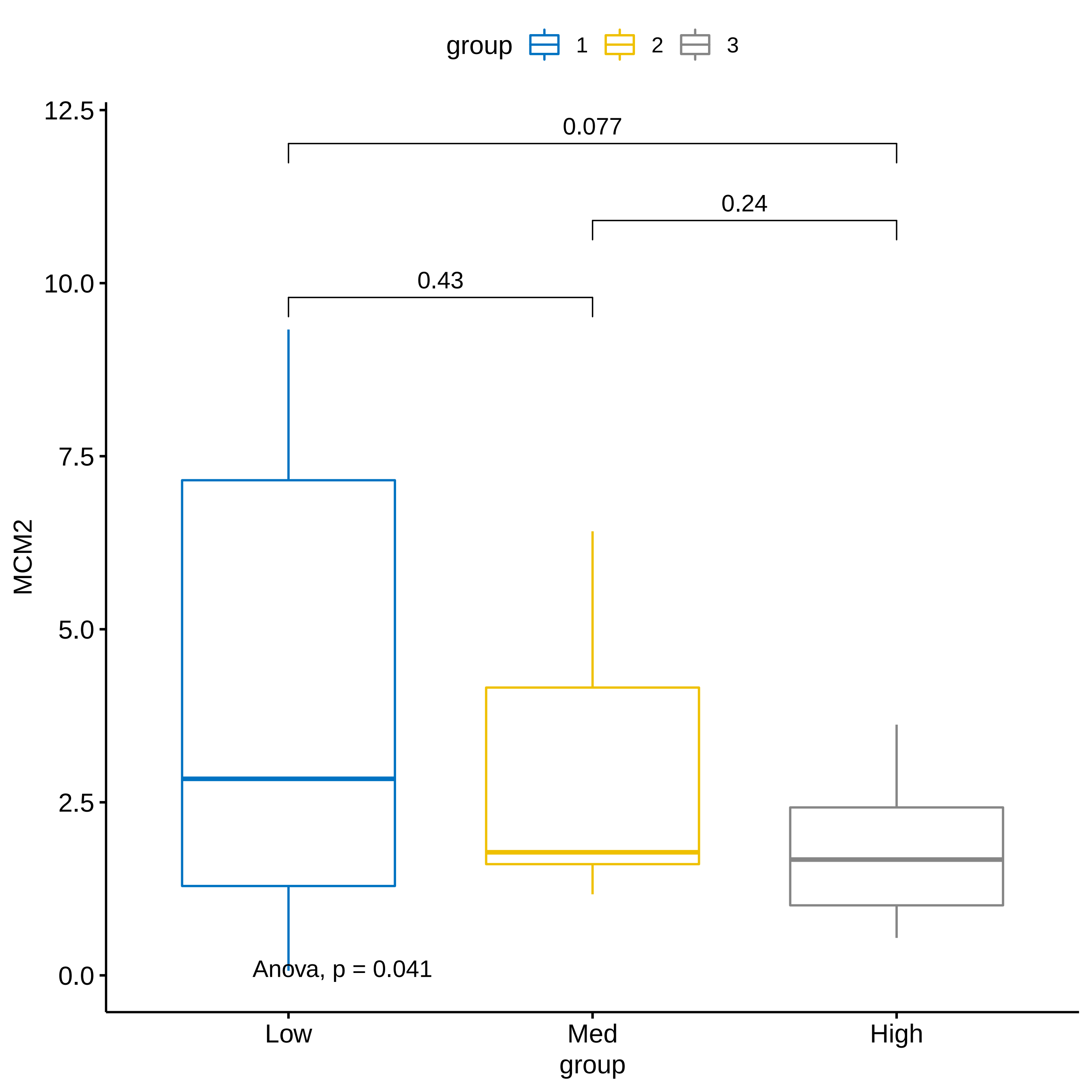
group
1
2
3
0.077
0.24
0.43
12.5
10.0
7.5
5.0
2.5
0.0
MCM2
Anova, p = 0.041
Low
Med
High
group

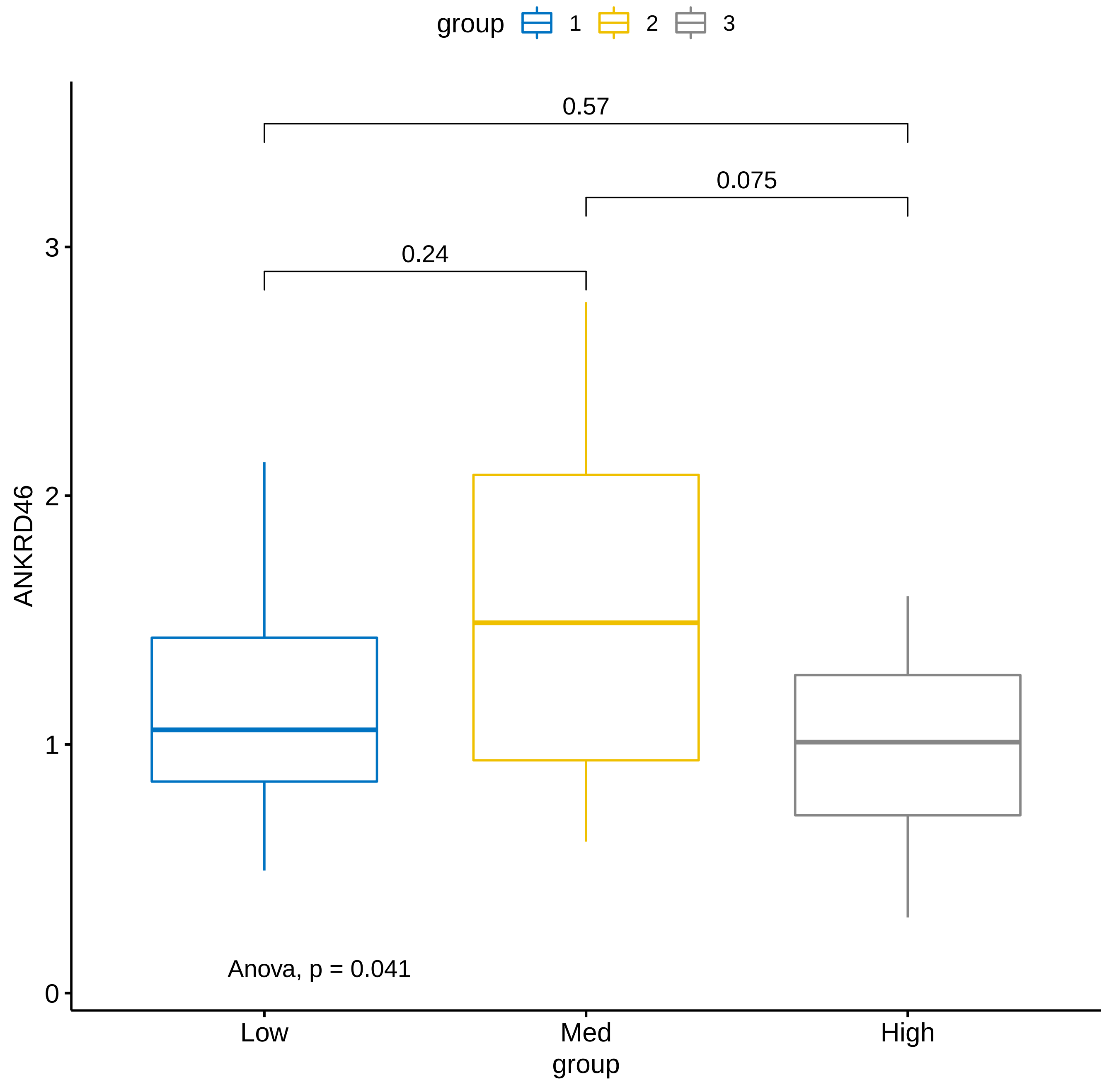

group 1 2 3
0.57
0.075
0.24
ANKRD46
3
2
1
0
Anova, p = 0.041
Low
Med
High
group

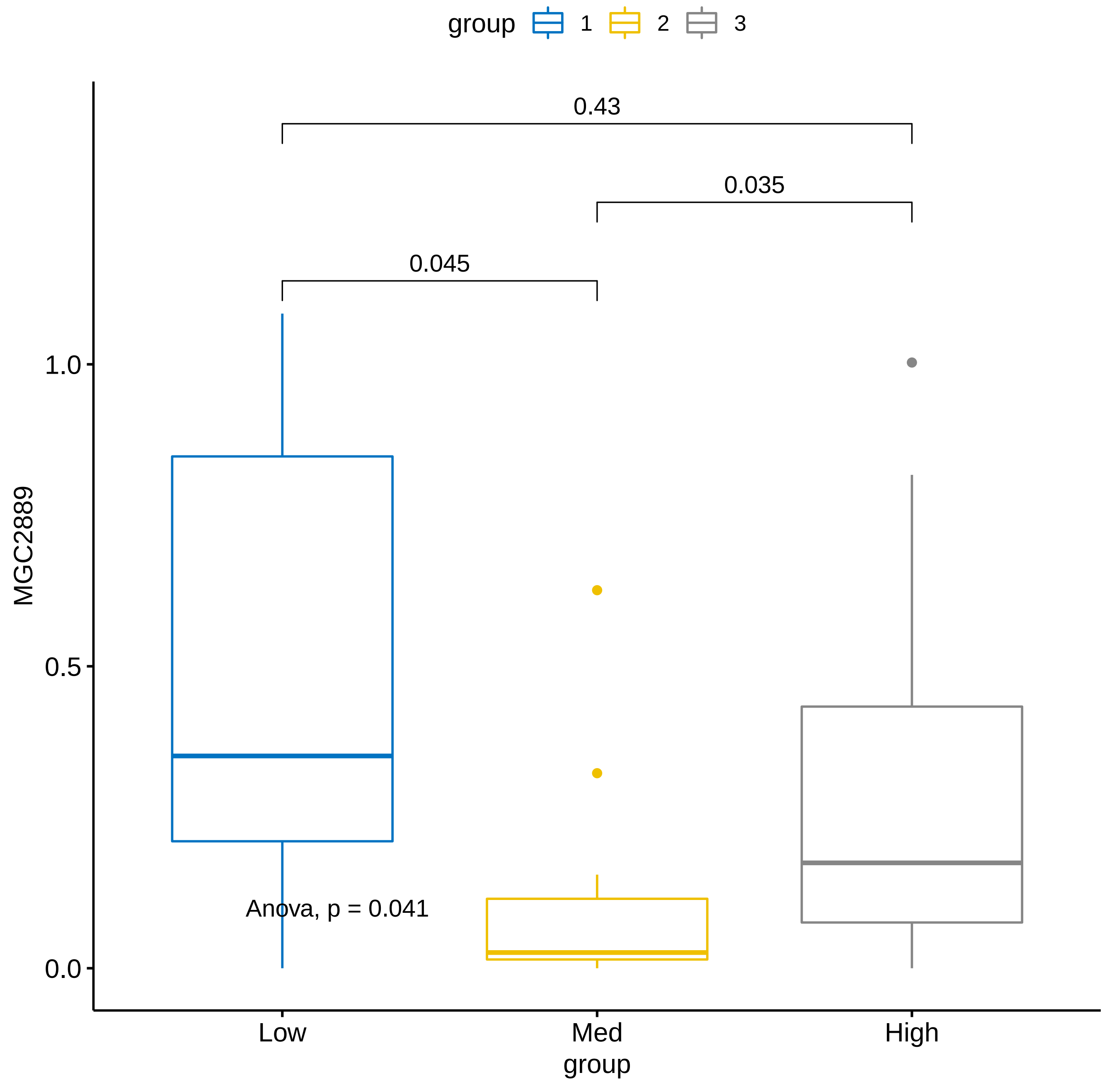
group 1 2 3
0.43
0.035
0.045
1.0
0.5
0.0
MGC2889
Anova, p = 0.041
Low
Med
High
group

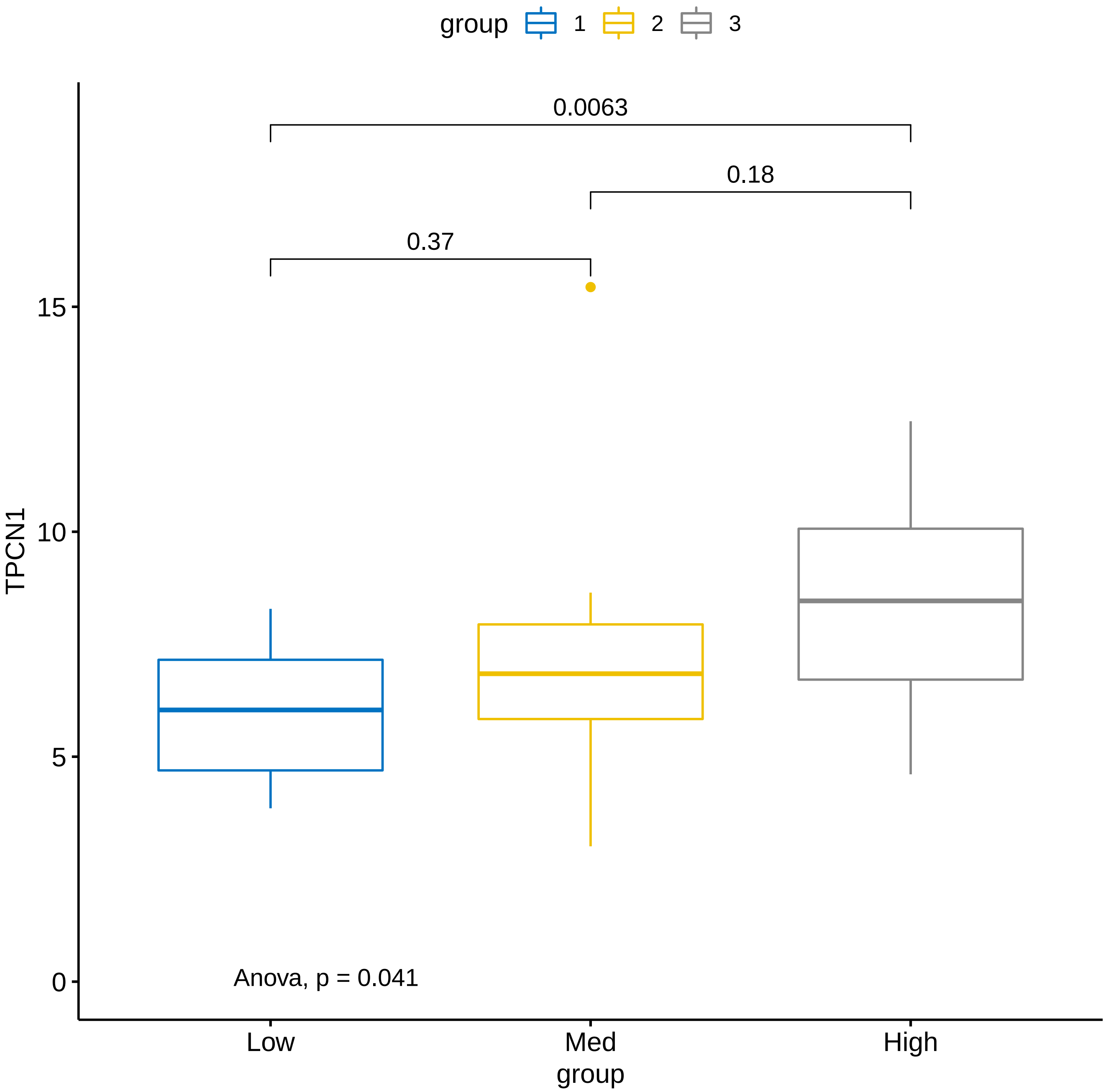
group
1
2
3
0.0063
0.18
0.37
15
10
5
0
TPCN1
Anova, p = 0.041
Low
Med
High
group

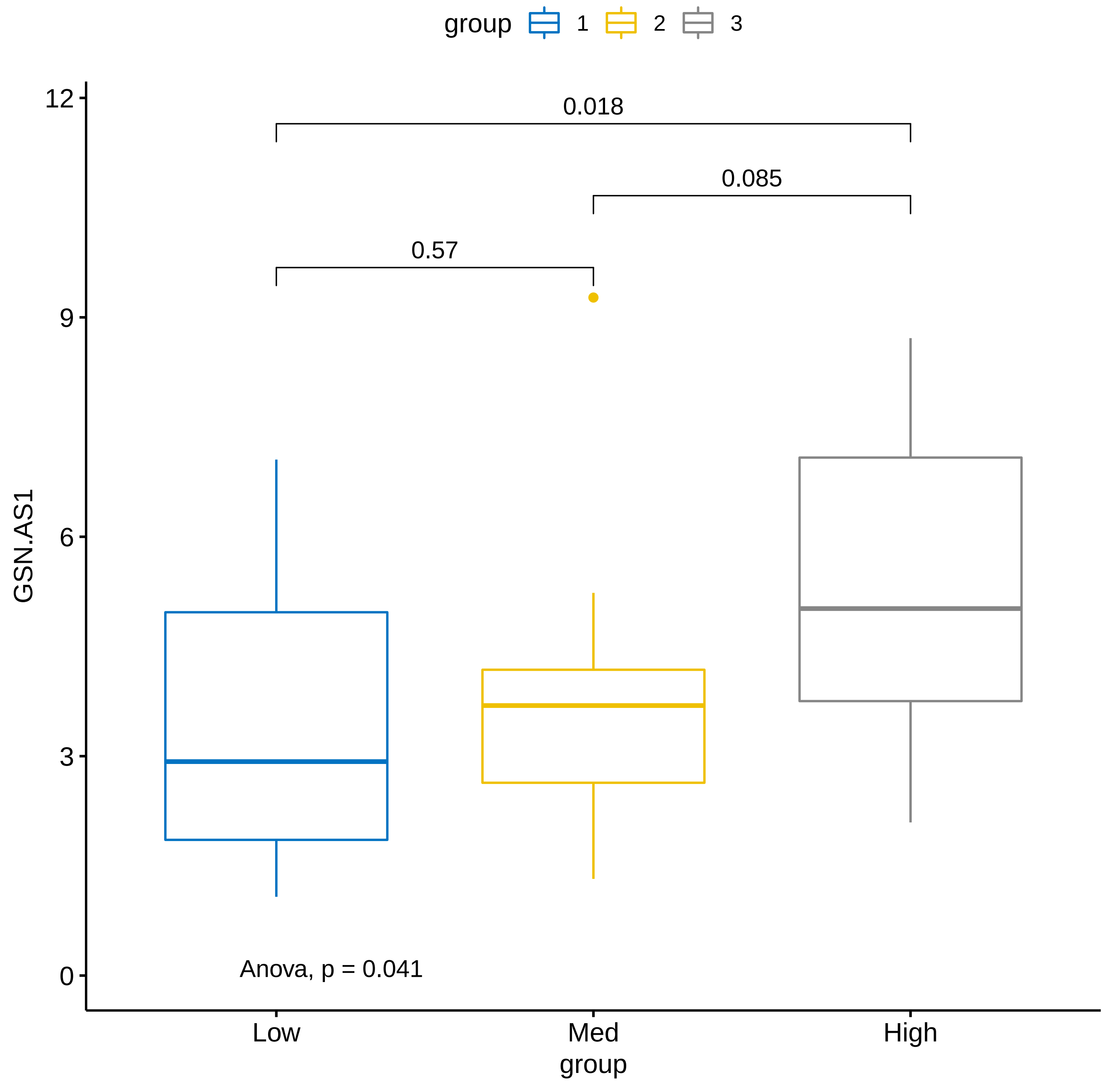
group
1
2
3
0.018
0.085
0.57
12
9
6
3
0
GSN.AS1
Anova, p = 0.041
Low
Med
High
group

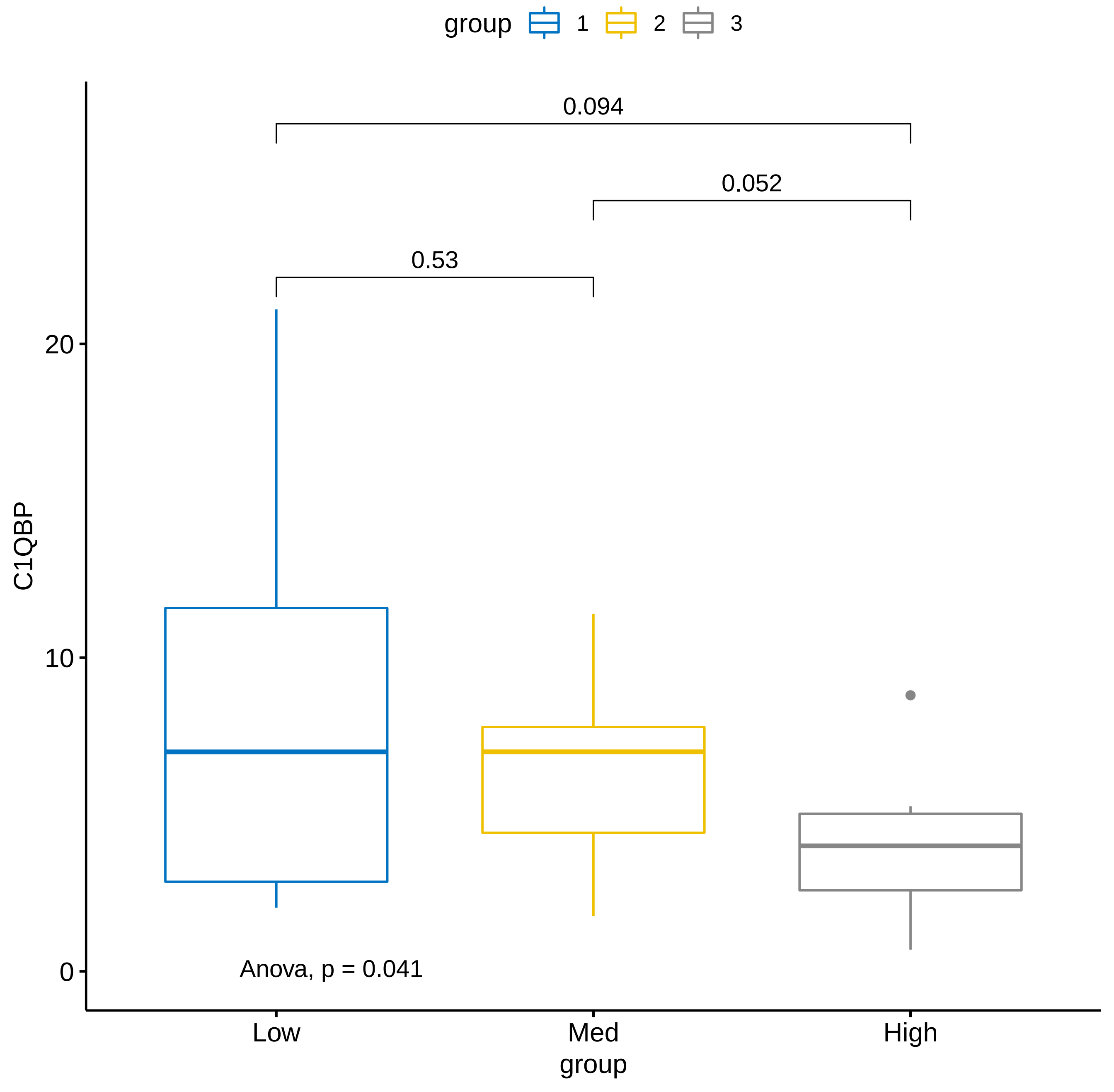
group
1
2
3
0.094
0.052
0.53
20
10
0
C1QBP
Anova, p = 0.041
Low
Med
High
group

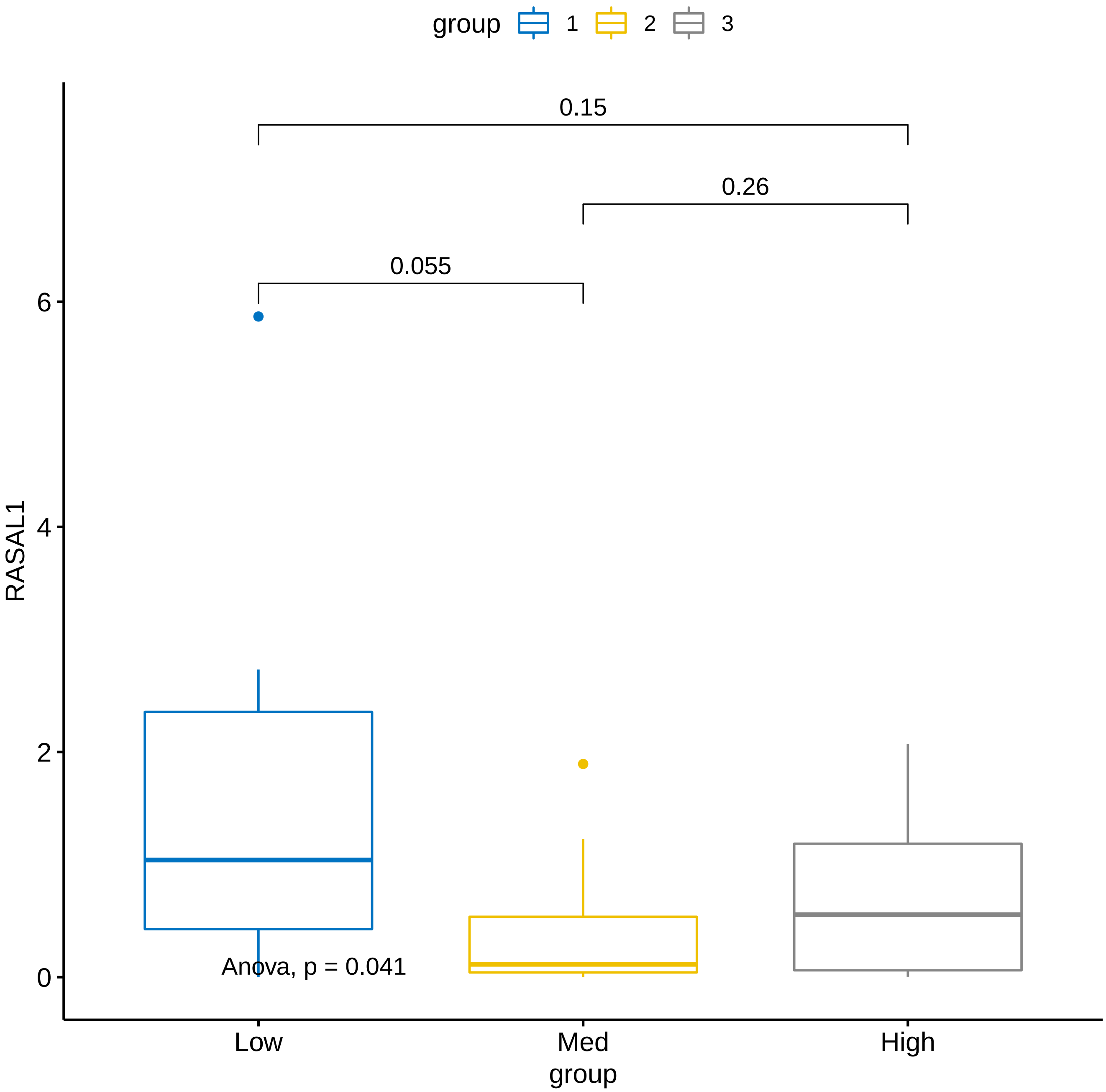
group 1 2 3
0.15
0.26
0.055
RASAL1
6
4
2
0
Anova, p = 0.041
Low
Med
High
group

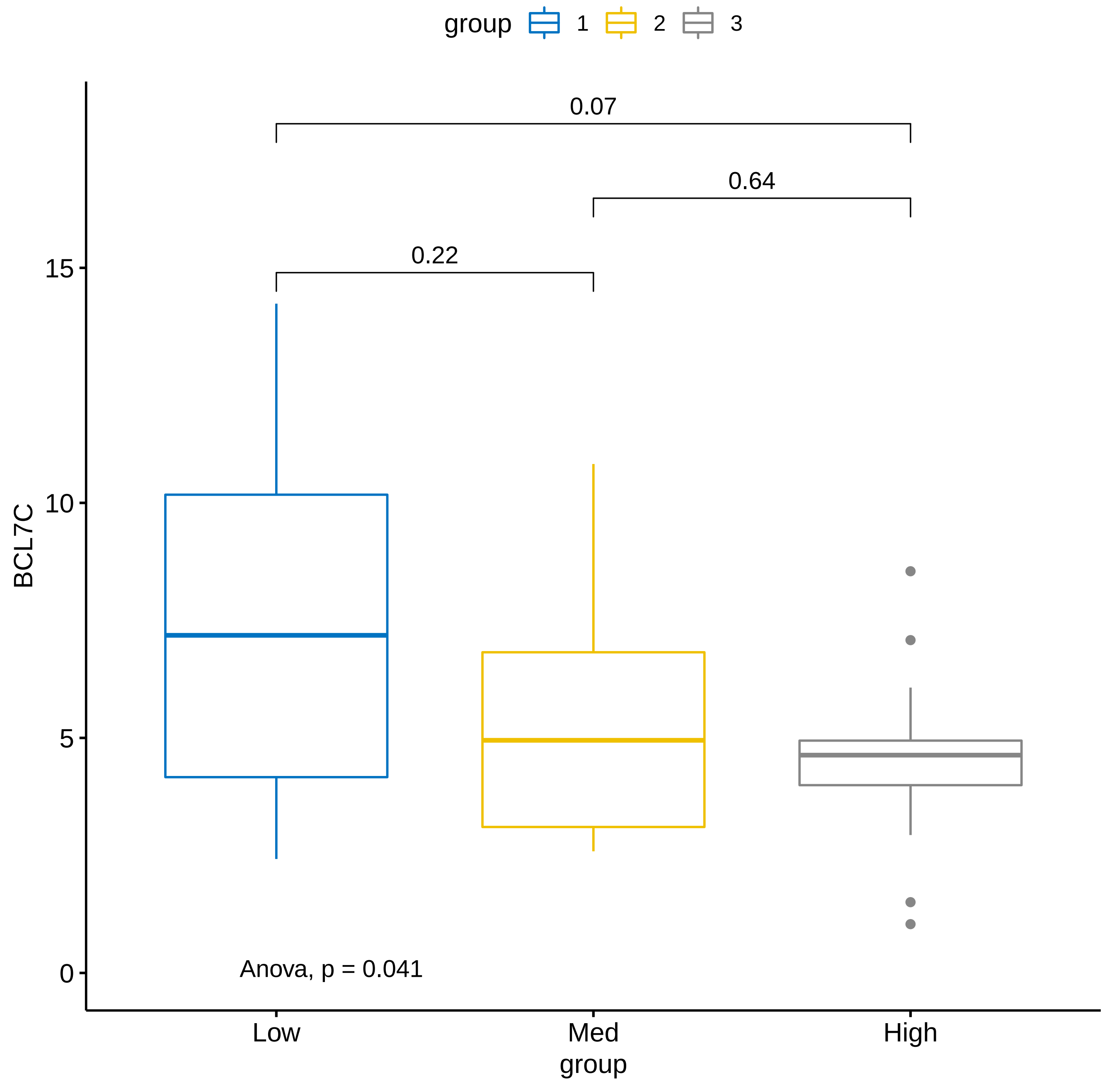
group
1
2
3
0.07
0.64
0.22
15
10
5
0
BCL7C
Anova, p = 0.041
Low
Med
High
group

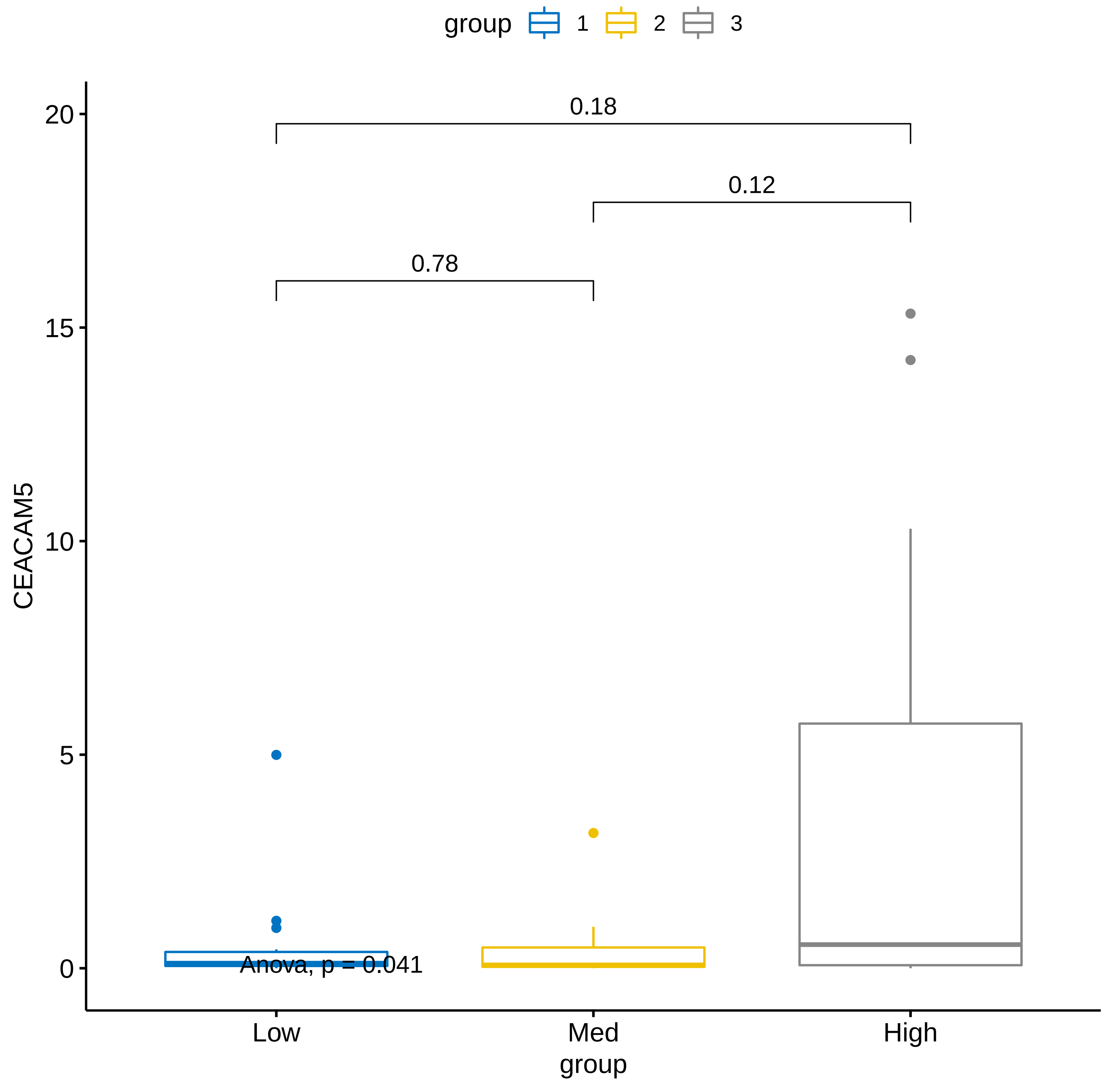

group
1
2
3
0.18
0.12
0.78
20
15
10
5
0
CEACAM5
Anova, p = 0.041
Low
Med
High
group

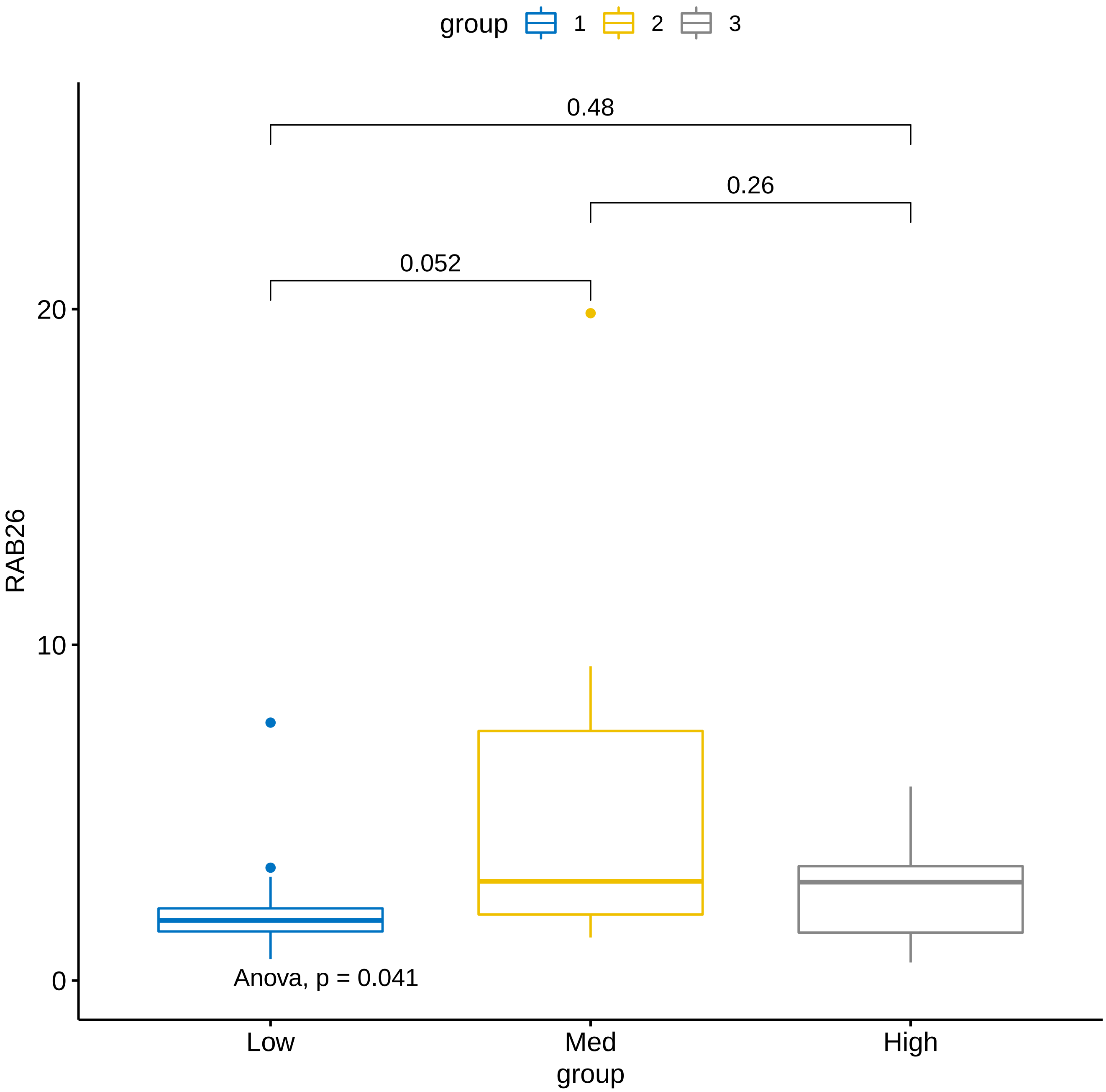
group
1
2
3
0.48
0.26
0.052
20
10
0
RAB26
Anova, p = 0.041
Low
Med
High
group

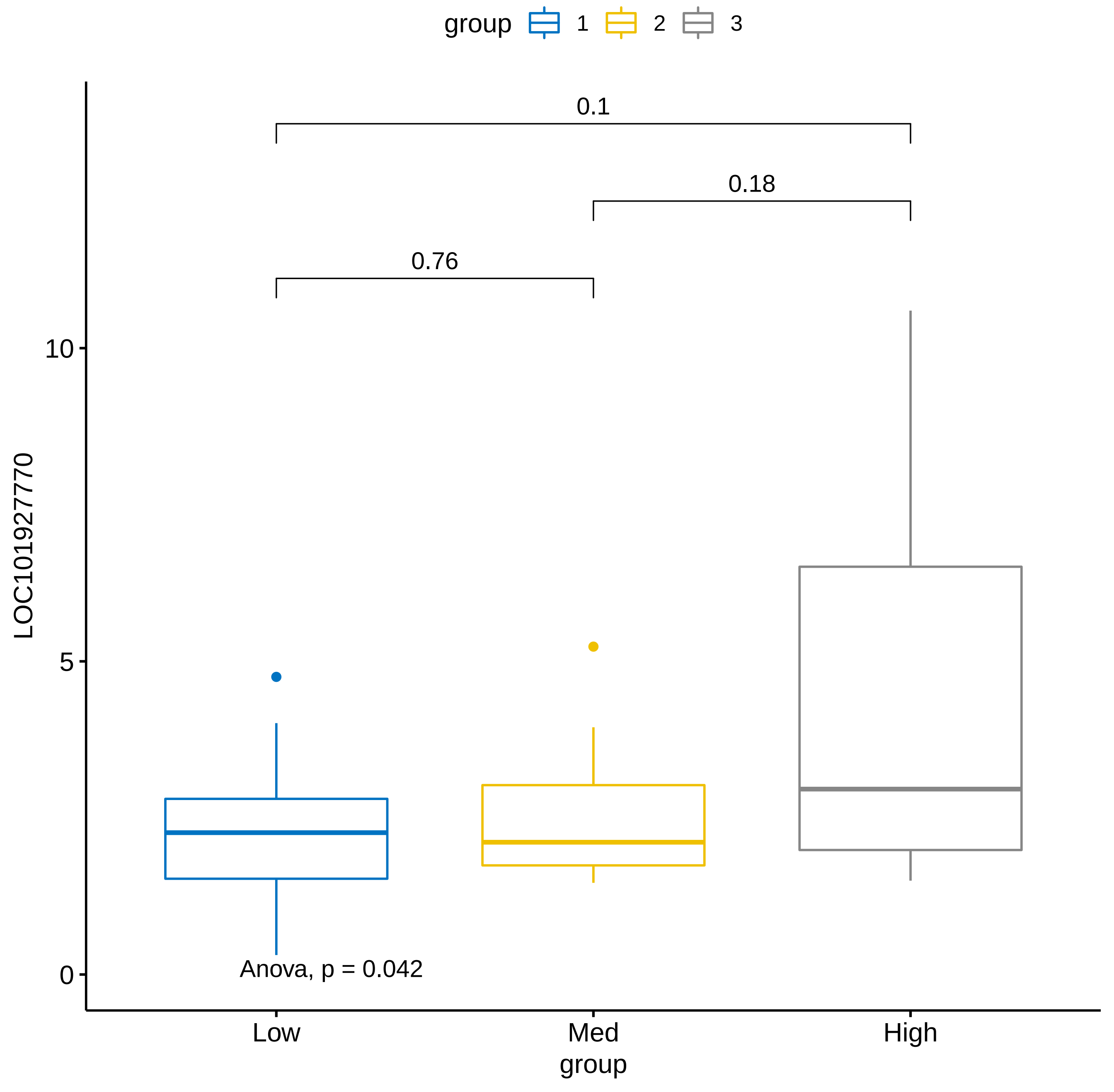

group 1 2 3
0.1
0.18
0.76
10
5
0
LOC101927770
Anova, p = 0.042
Low
Med
High
group

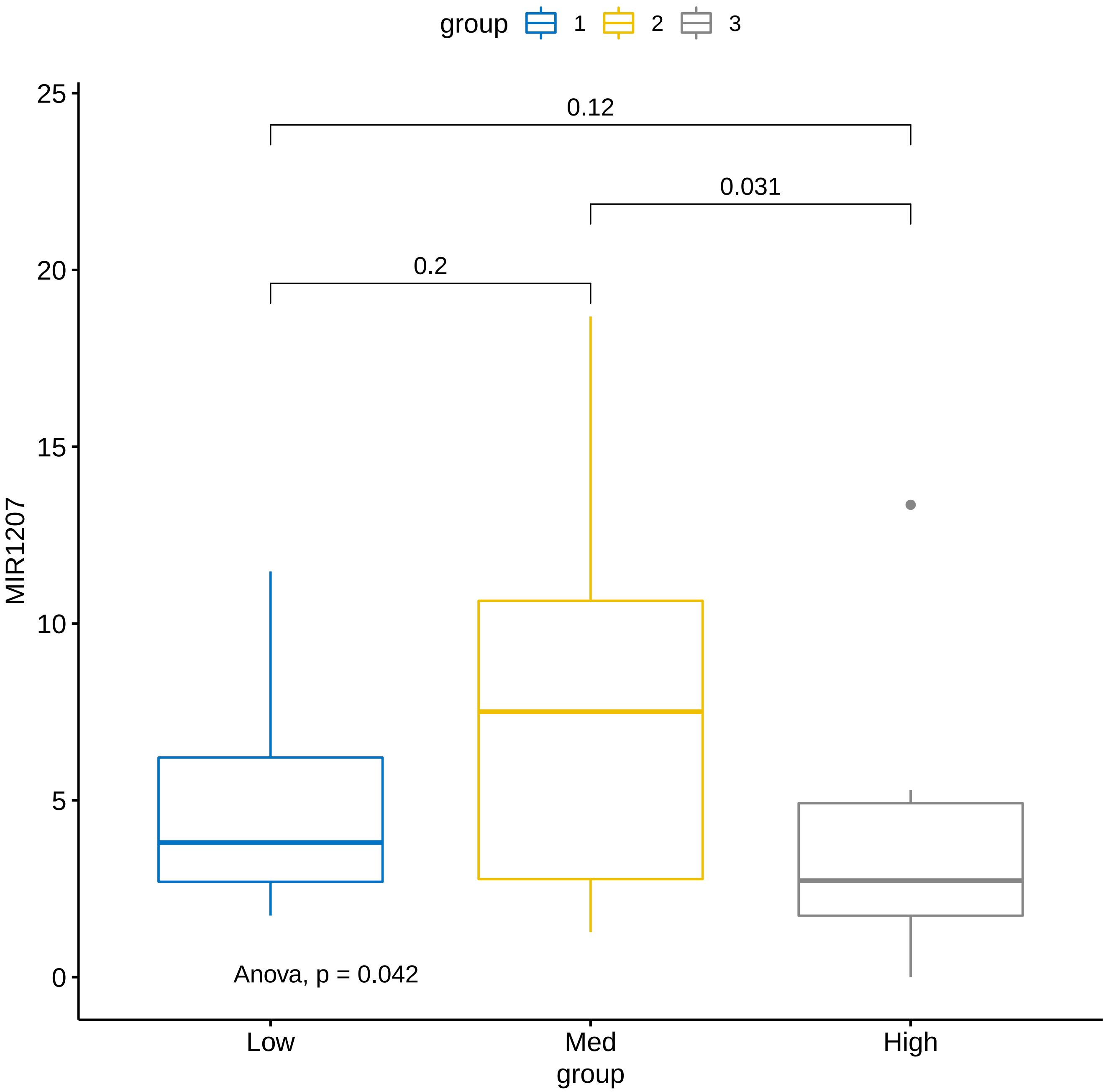
group 1 2 3
0.12
0.031
0.2
25
20
15
10
5
0
MIR1207
Anova, p = 0.042
Low
Med
High
group

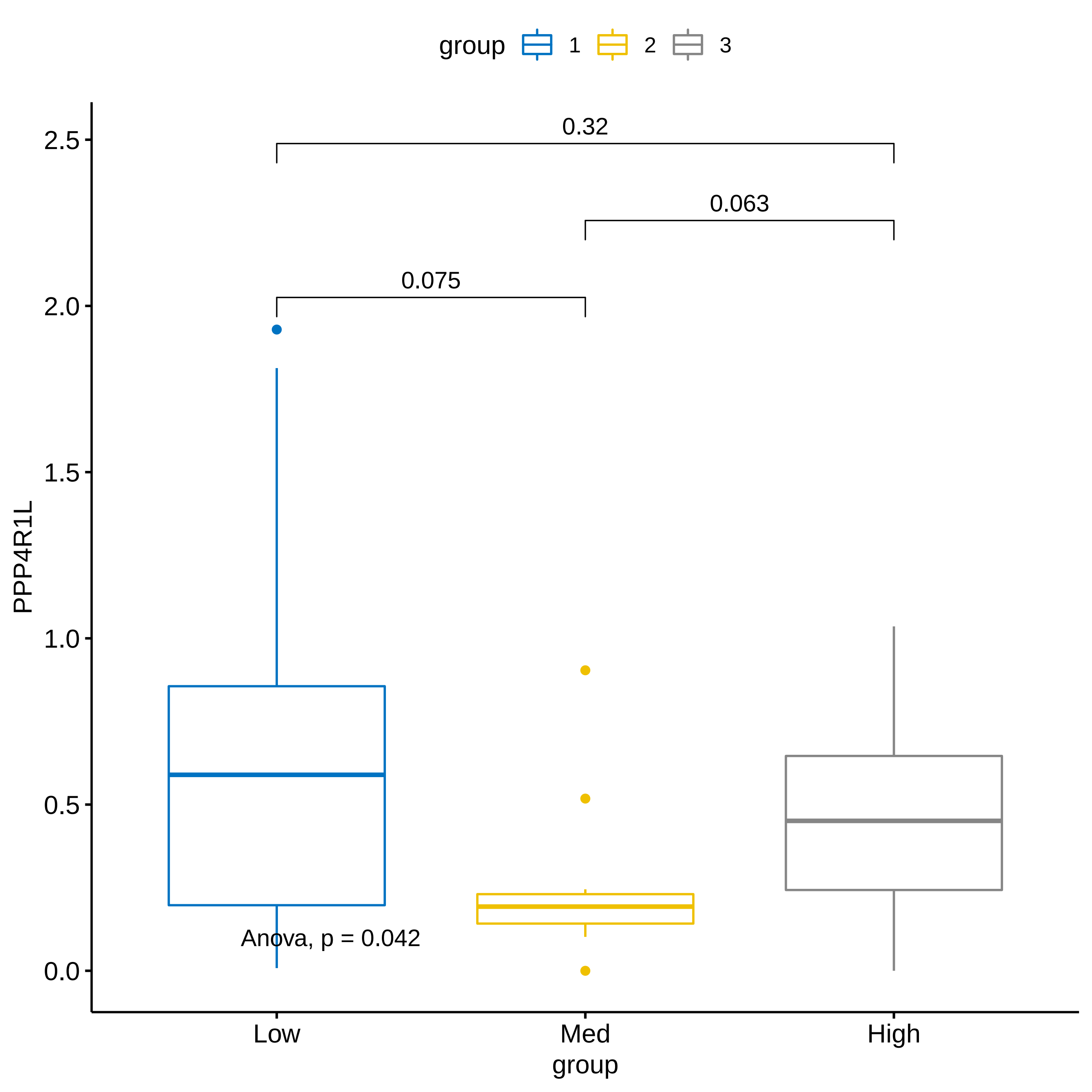

group
1
2
3
0.32
0.063
0.075
2.5
2.0
1.5
1.0
0.5
0.0
PPP4R1L
Anova, p = 0.042
Low
Med
High
group

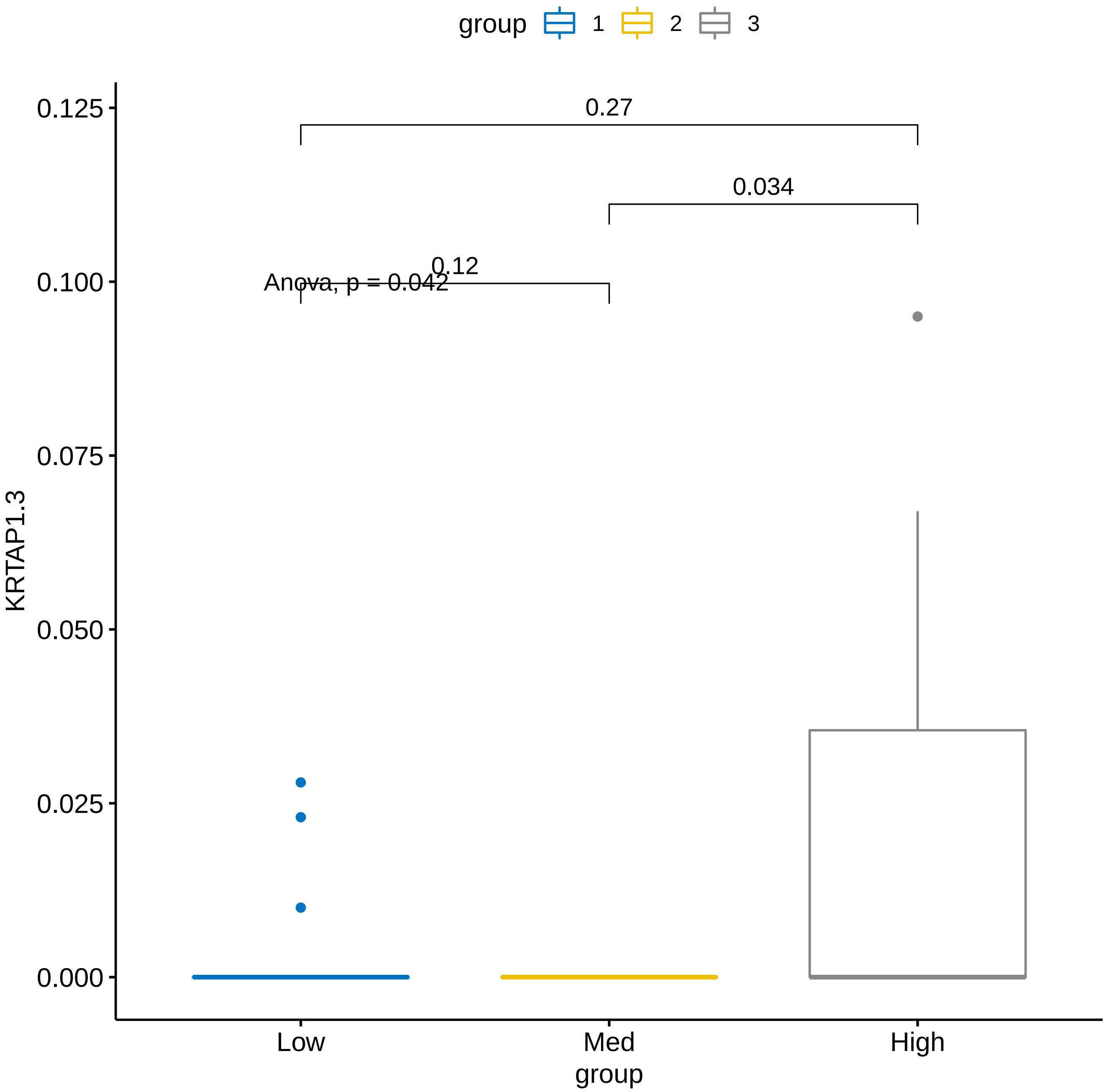
group
1
2
3
0.27
0.034
0.12
Anova, p = 0.042
0.125
0.100
0.075
0.050
0.025
0.000
KRTAP1.3
Low
Med
High
group

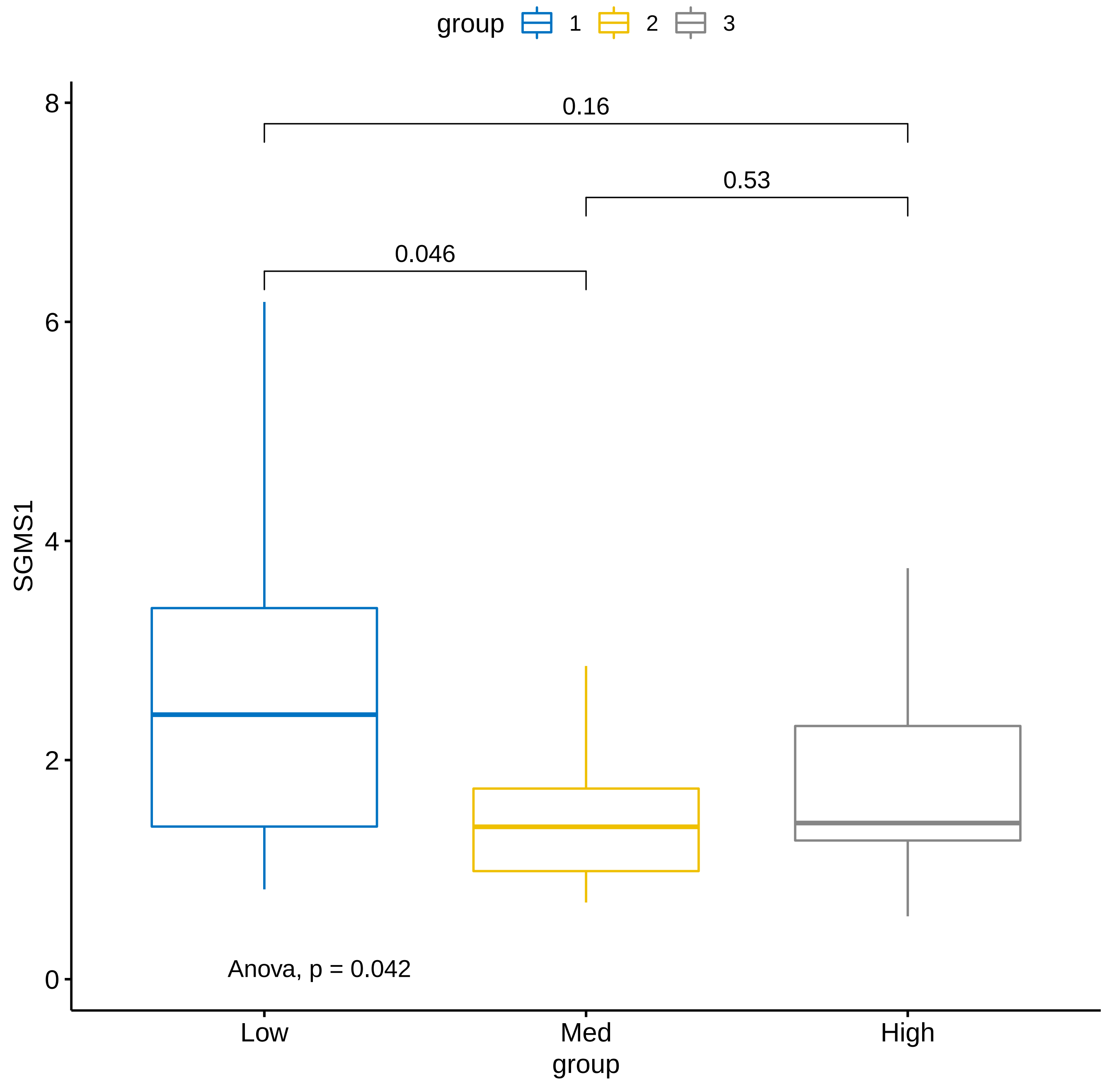

group
1
2
3
0.16
0.53
0.046
SGMS1
8
6
4
2
0
Anova, p = 0.042
Low
Med
High
group

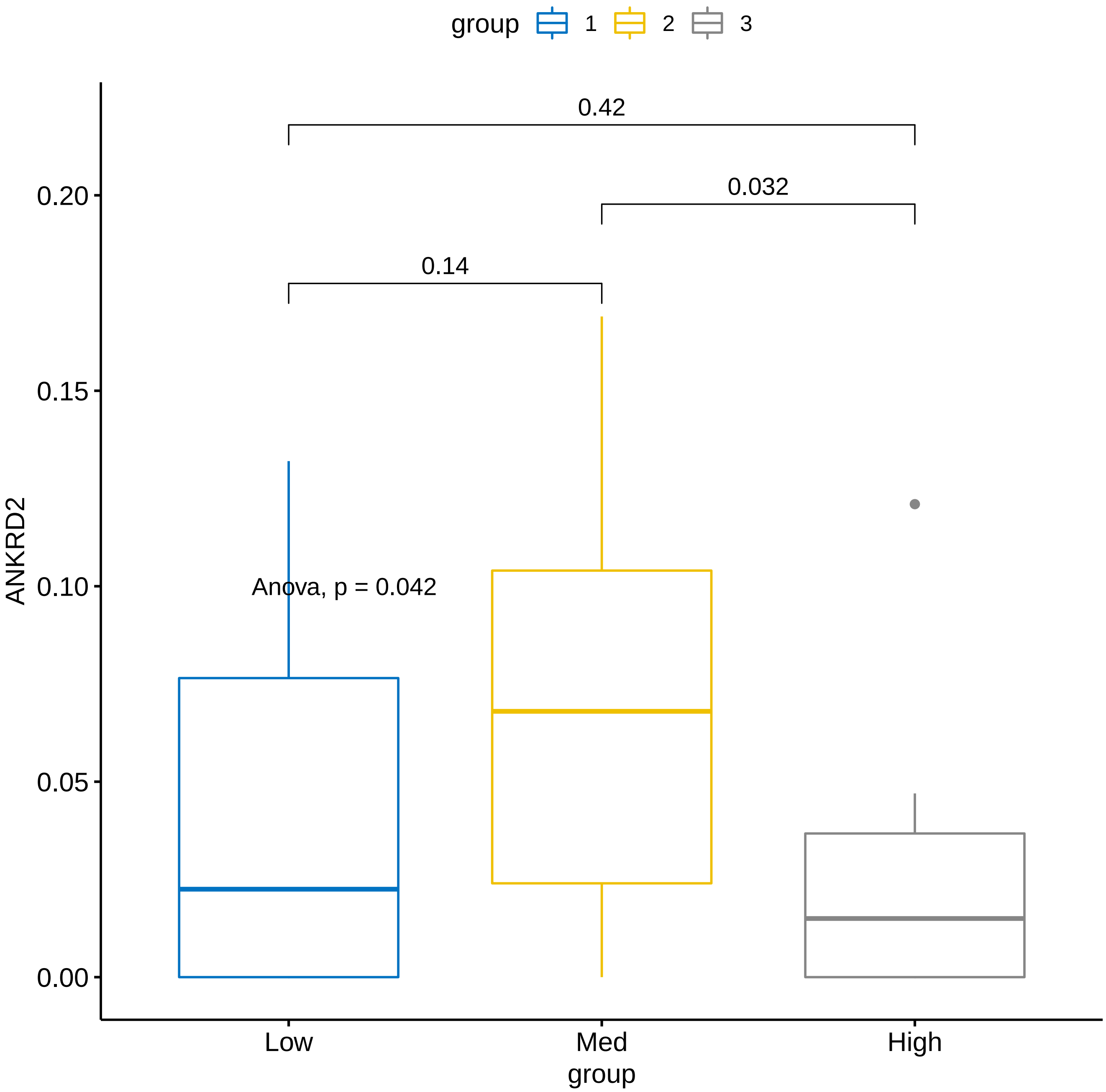
group
1
2
3
0.42
0.032
0.14
Anova, p = 0.042
ANKRD2
0.20
0.15
0.10
0.05
0.00
Low
Med
High
group

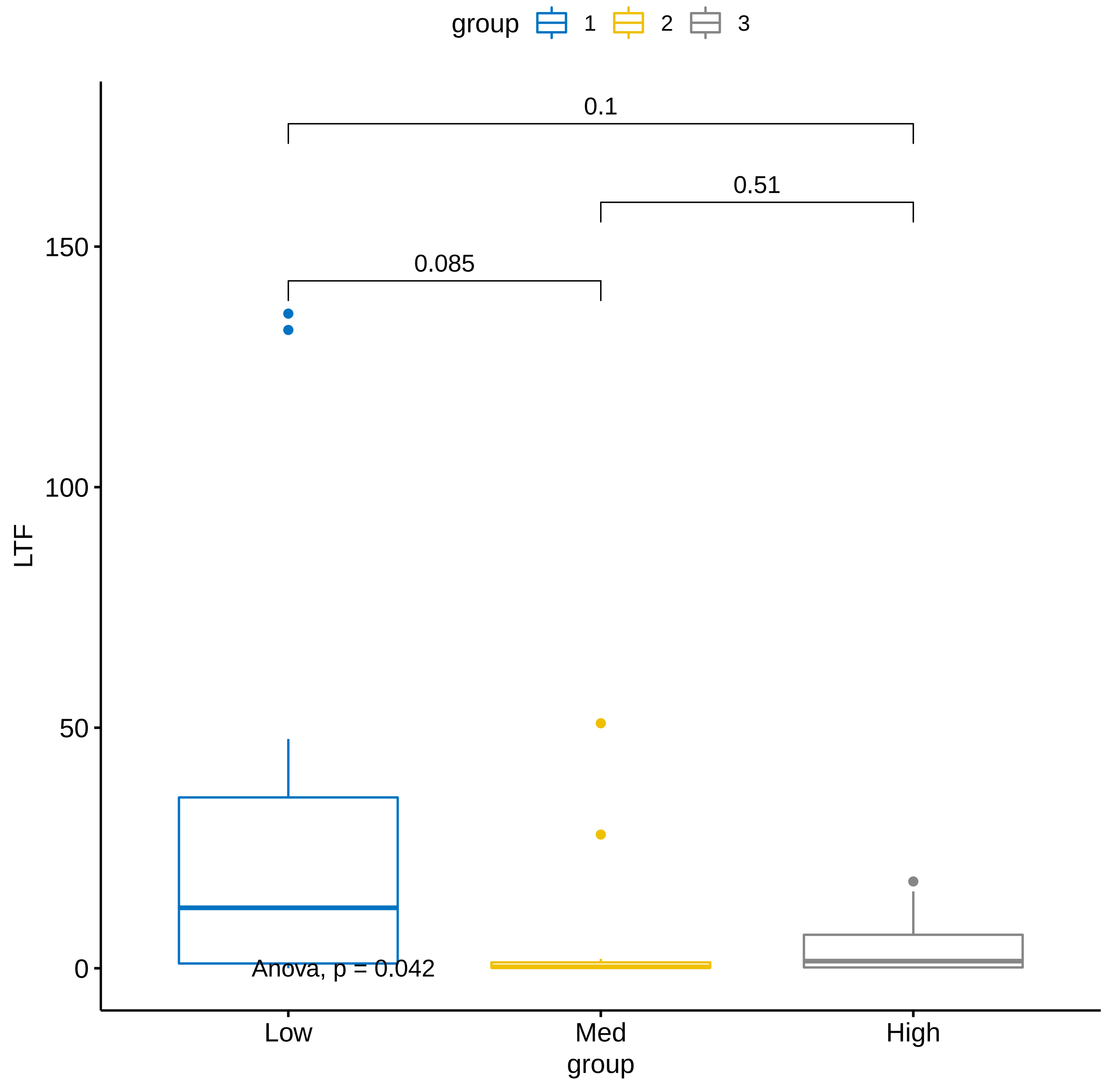

group
1
2
3
0.1
0.51
0.085
150
100
50
0
LTF
Anova, p = 0.042
Low
Med
High
group

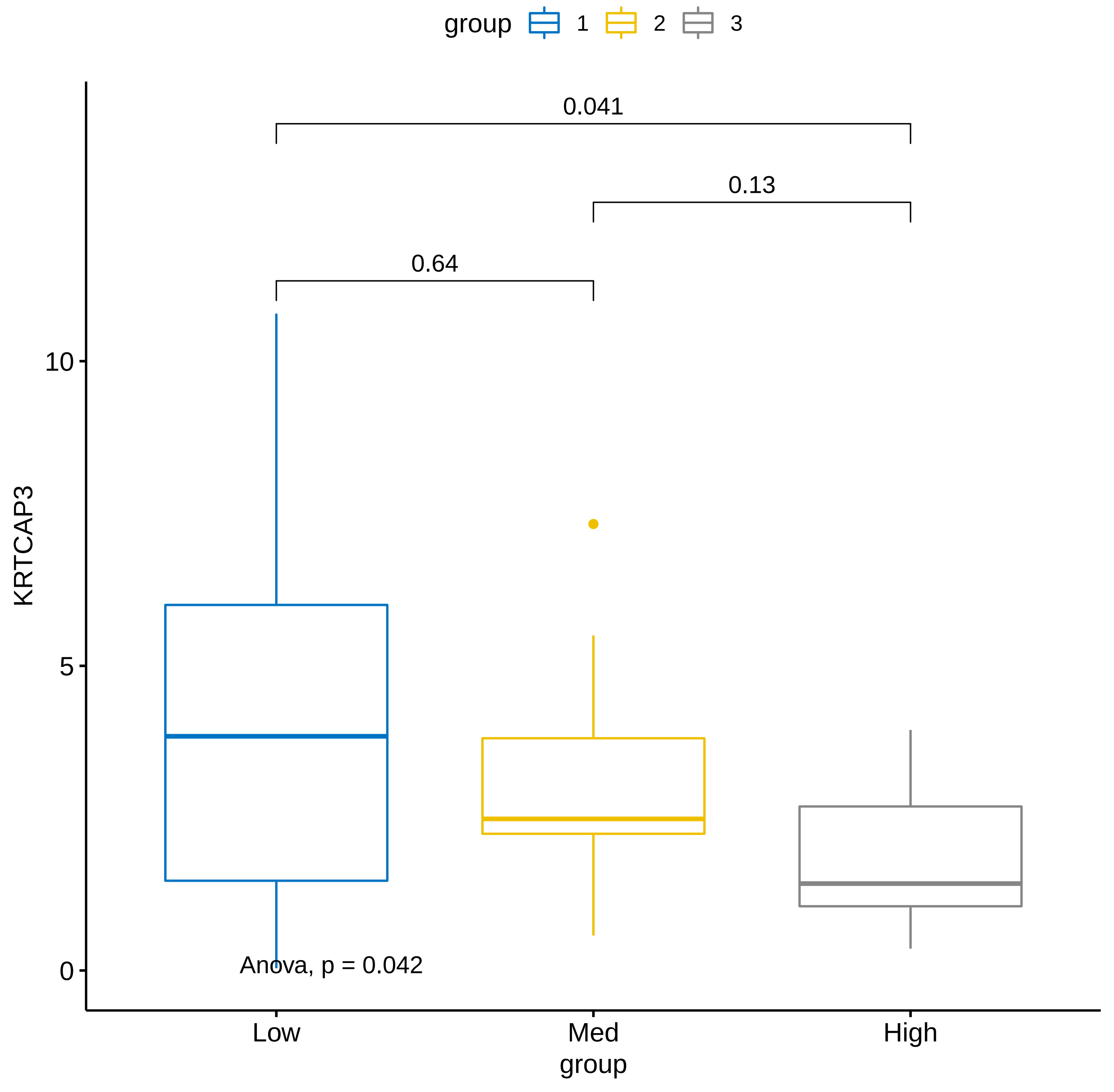
group
1
2
3
0.041
0.13
0.64
KRTCAP3
10
5
0
Anova, p = 0.042
Low
Med
High
group

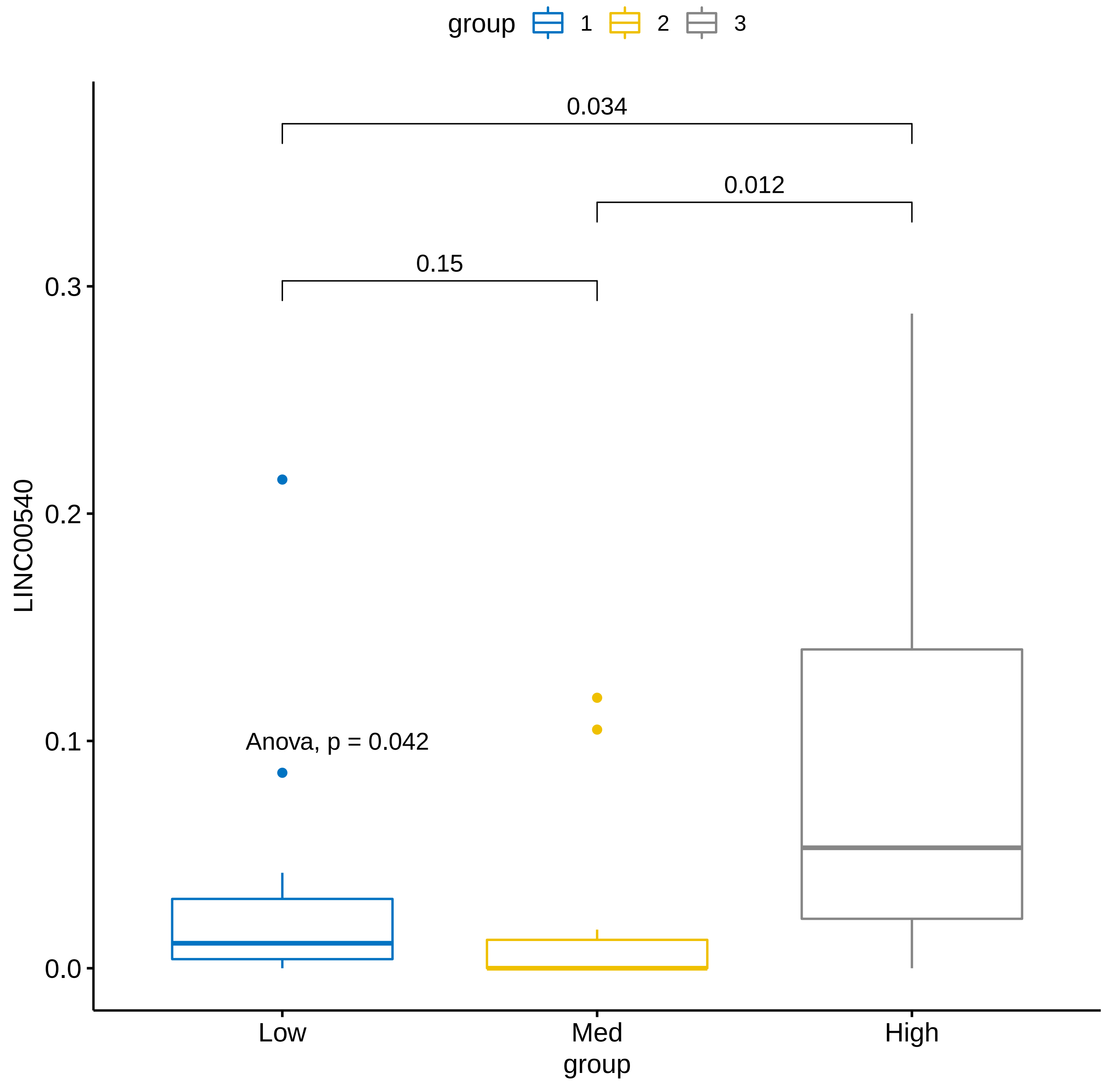

group 1 2 3
0.034
0.012
0.15
Anova, p = 0.042
0.3
0.2
0.1
0.0
LINC00540
Low
Med
High
group

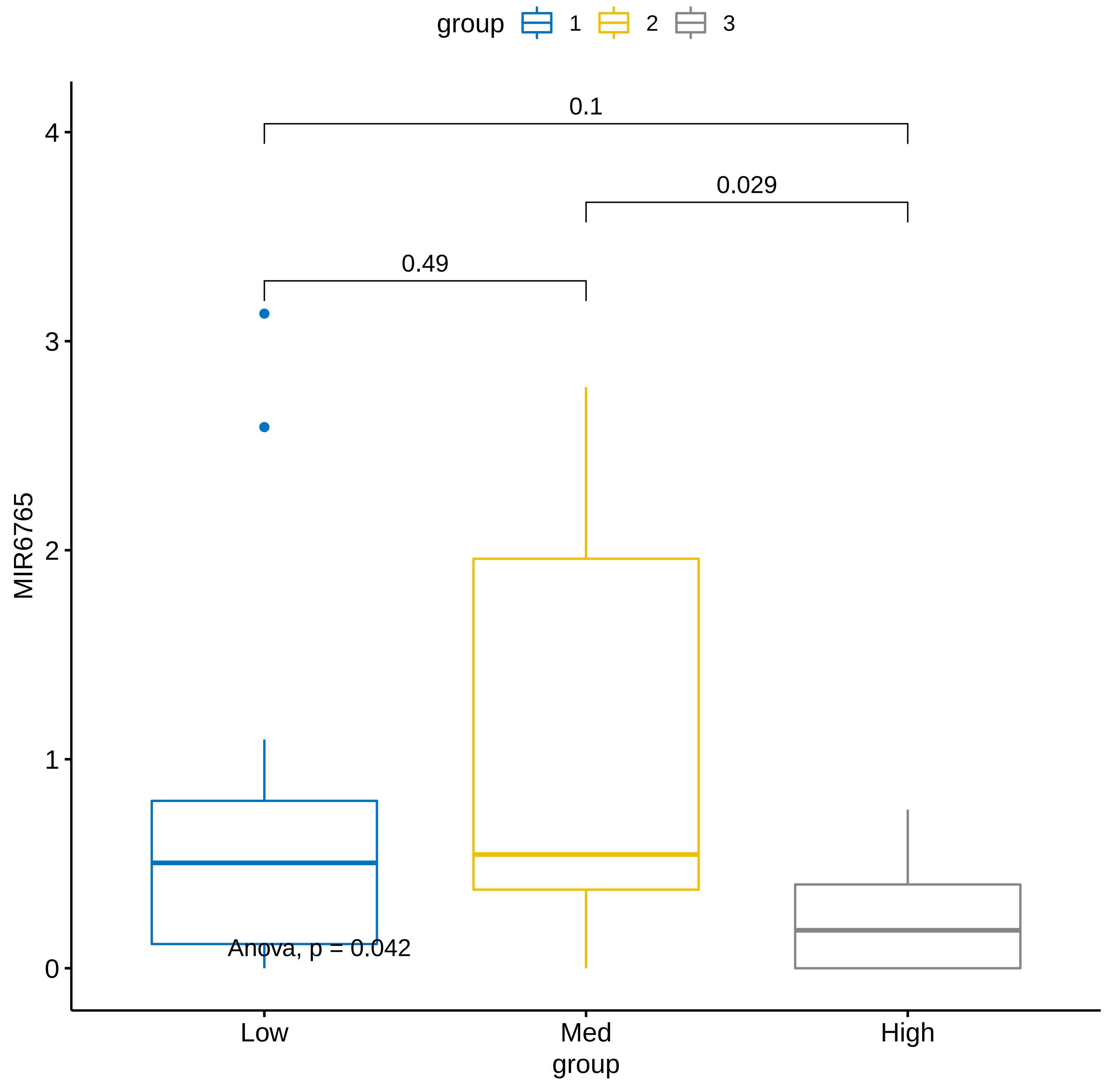

group
1
2
3
0.1
0.029
0.49
MIR6765
4
3
2
1
0
Anova, p = 0.042
Low
Med
High
group

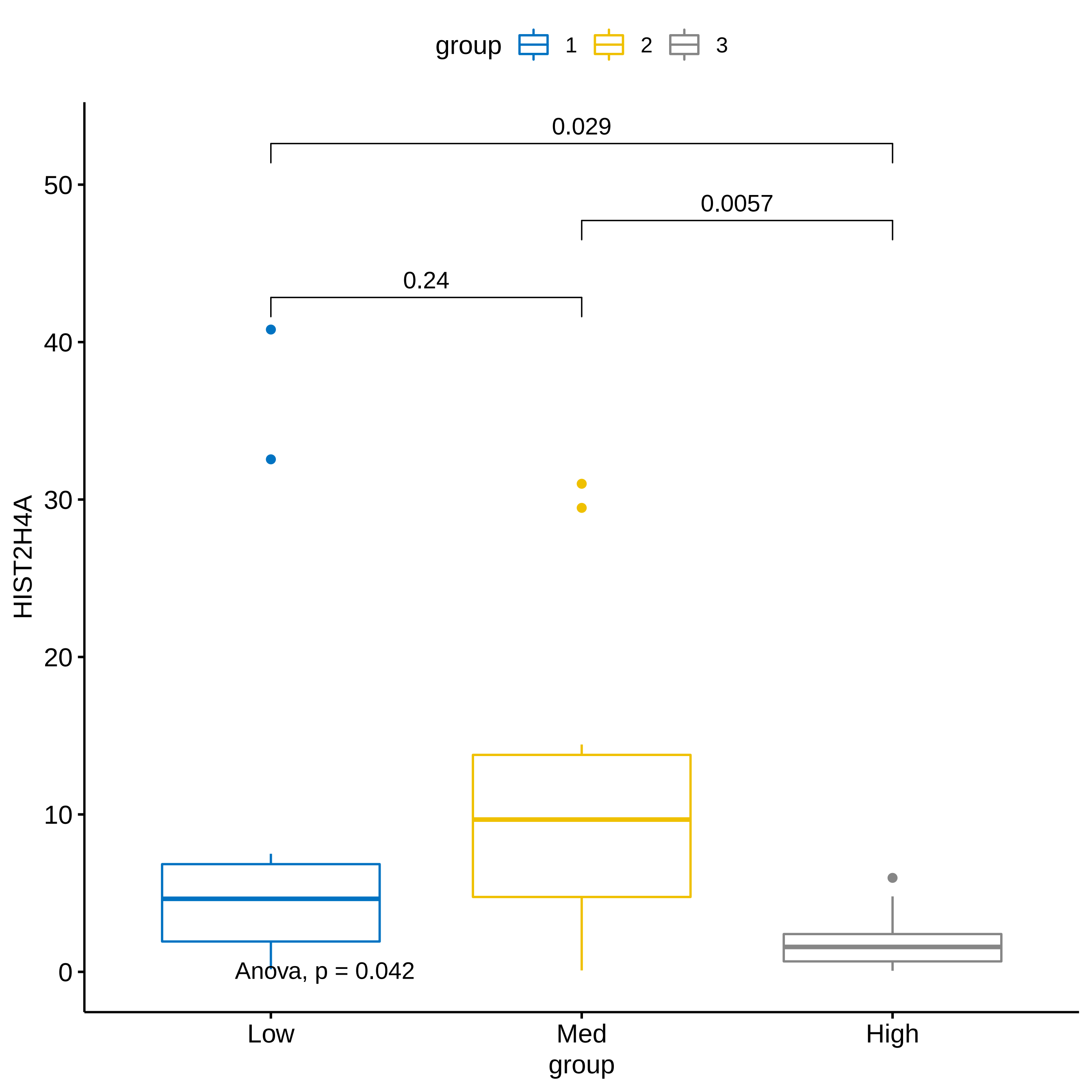

group 1 2 3
0.029
0.0057
0.24
50
40
30
20
10
0
HIST2H4A
Anova, p = 0.042
Low
Med
High
group

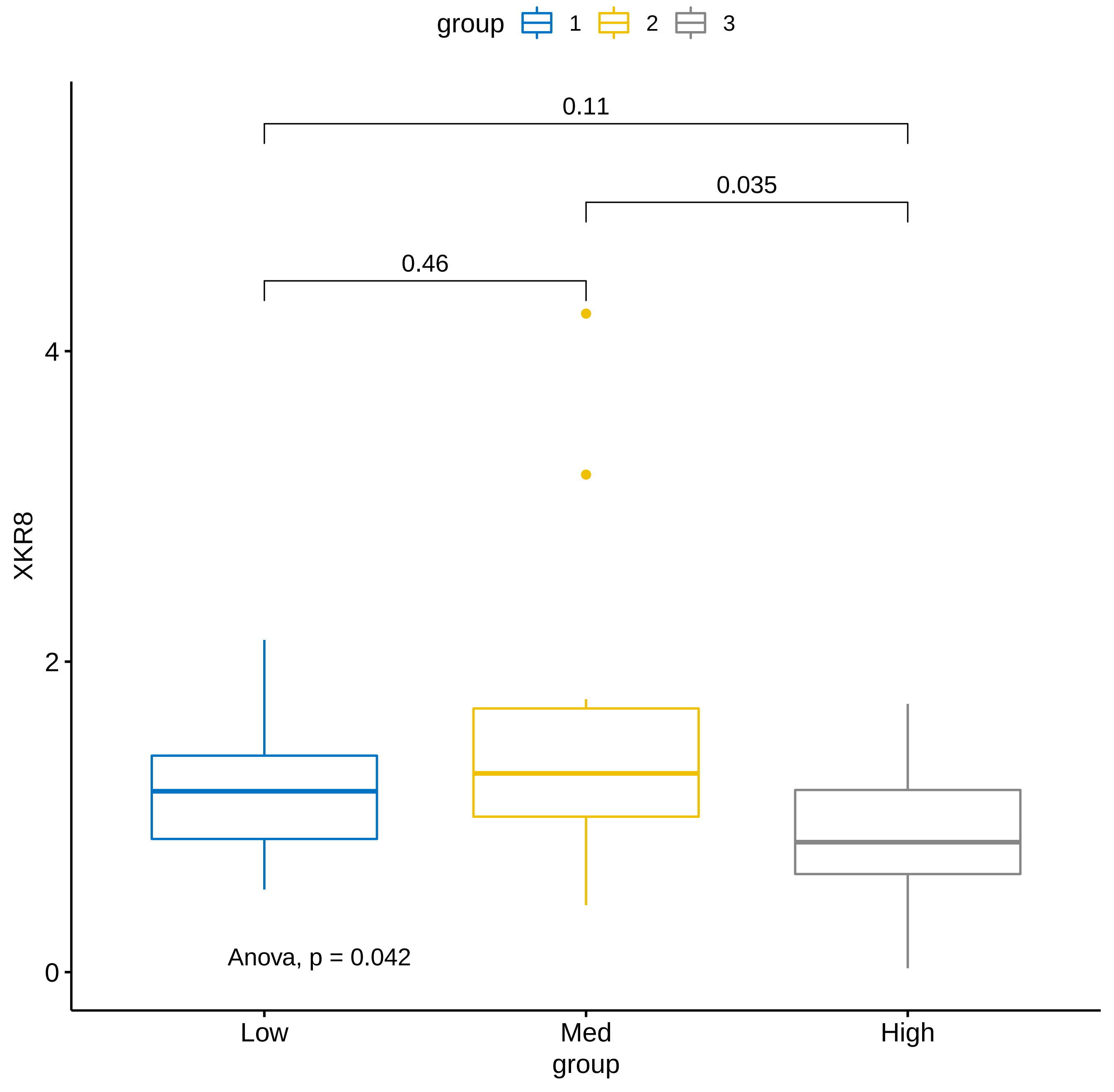

group 1 2 3
0.11
0.035
0.46
XKR8
4
2
0
Anova, p = 0.042
Low
Med
High
group

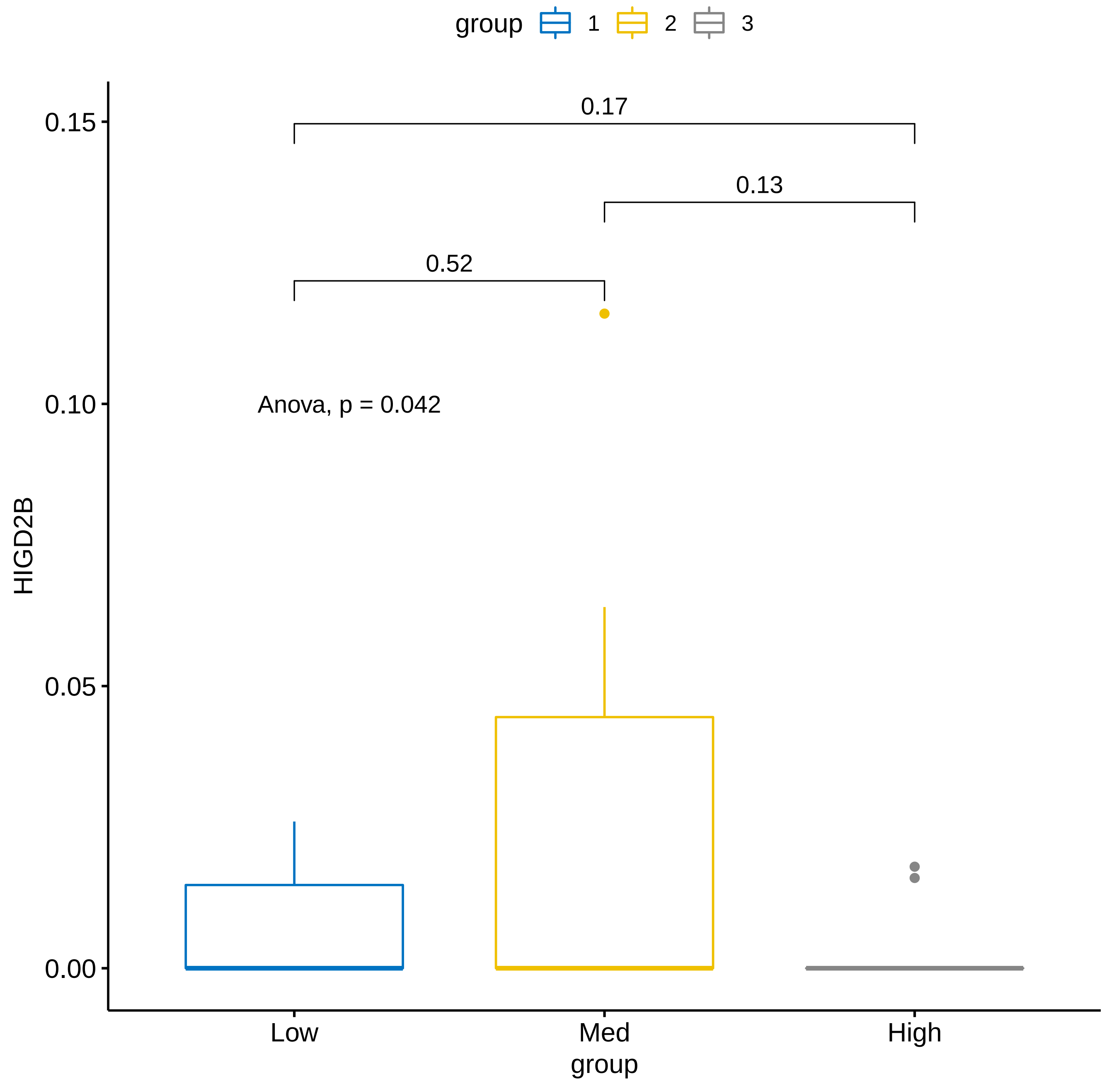
group
1
2
3
0.17
0.13
0.52
Anova, p = 0.042
0.15
0.10
0.05
0.00
HIGD2B
Low
Med
High
group

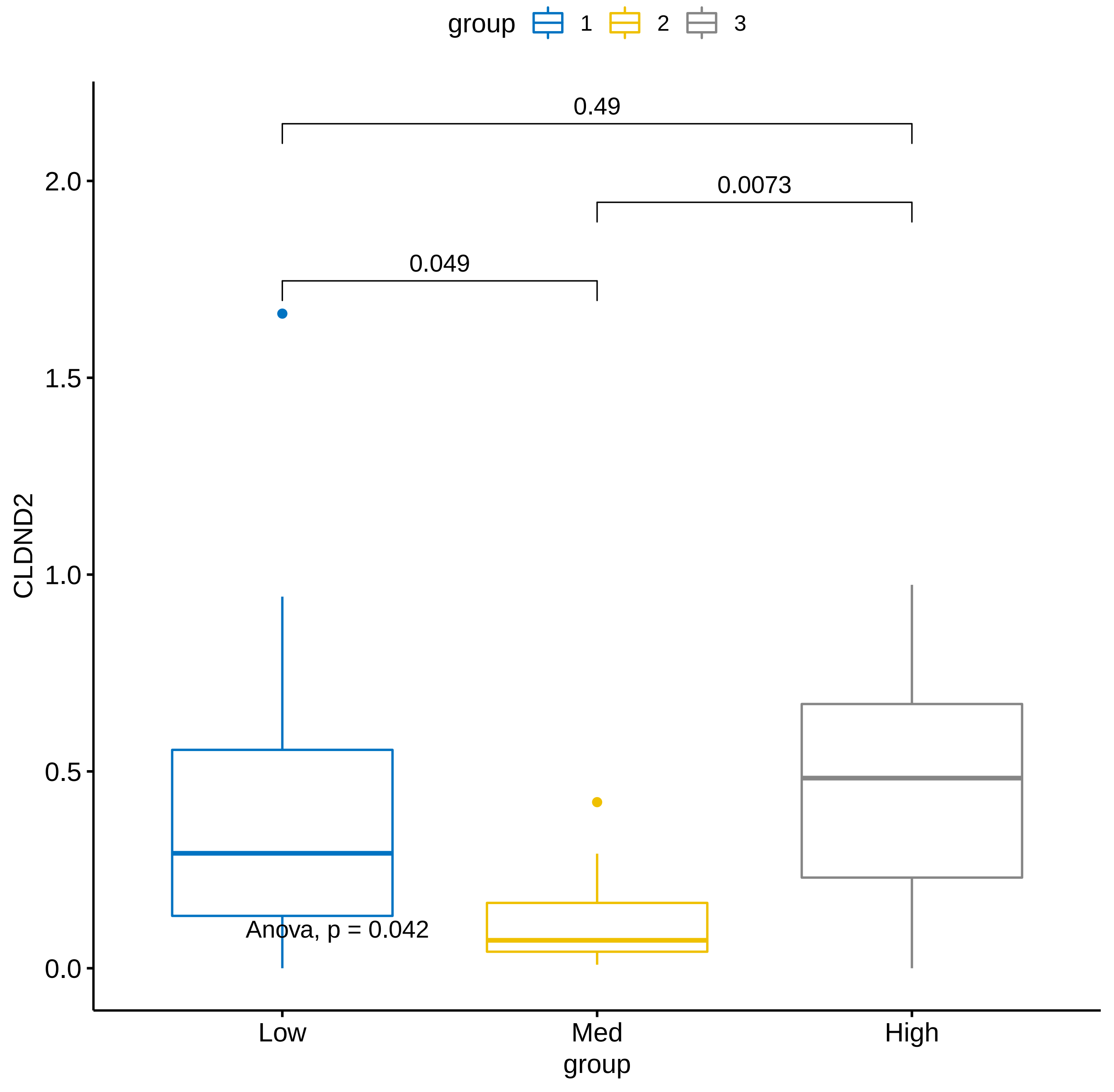

group
1
2
3
0.49
0.0073
0.049
2.0
1.5
1.0
0.5
0.0
CLDND2
Anova, p = 0.042
Low
Med
High
group

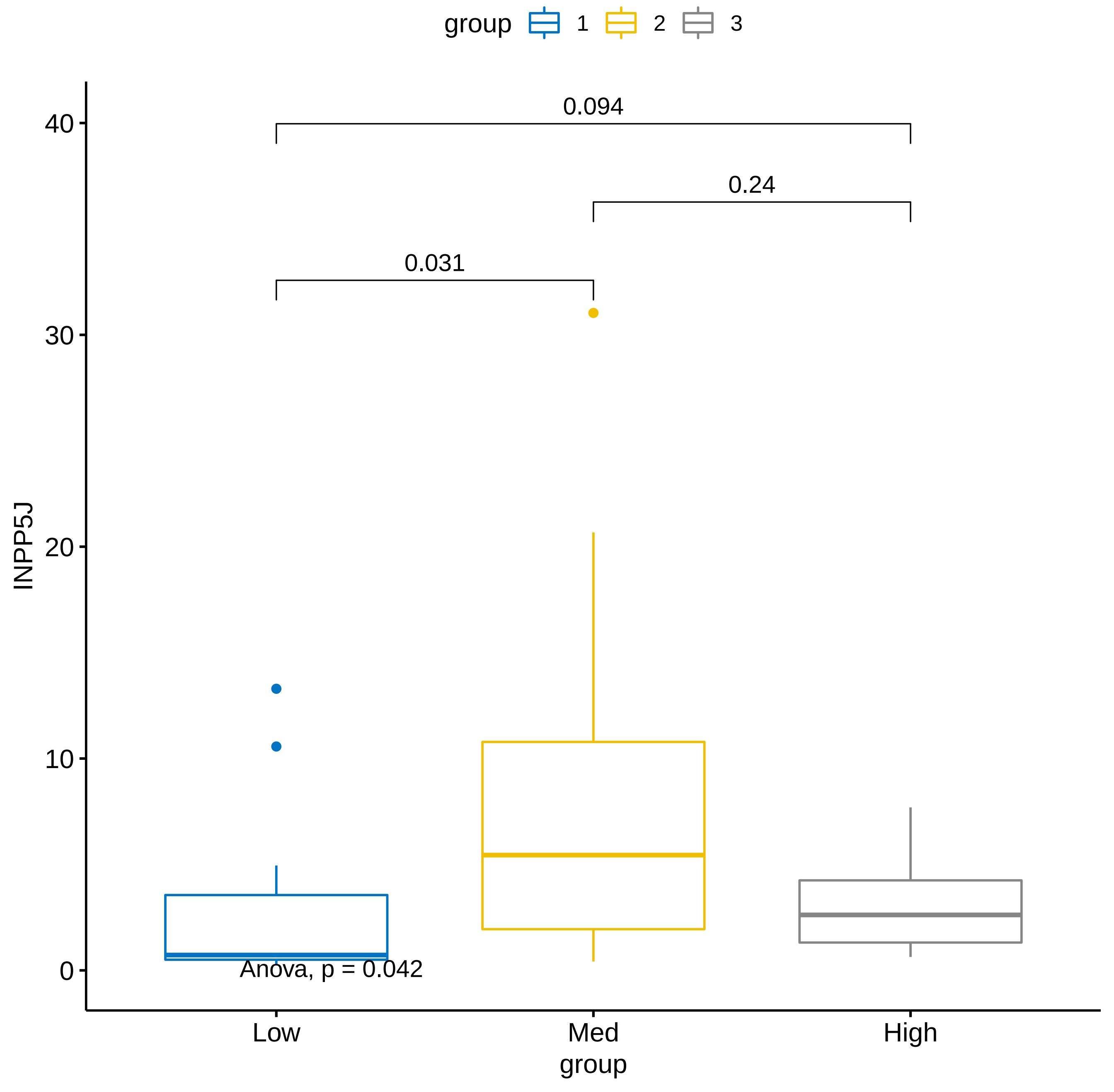

group
1
2
3
0.094
0.24
0.031
40
30
20
10
0
INPP5J
Anova, p = 0.042
Low
Med
High
group

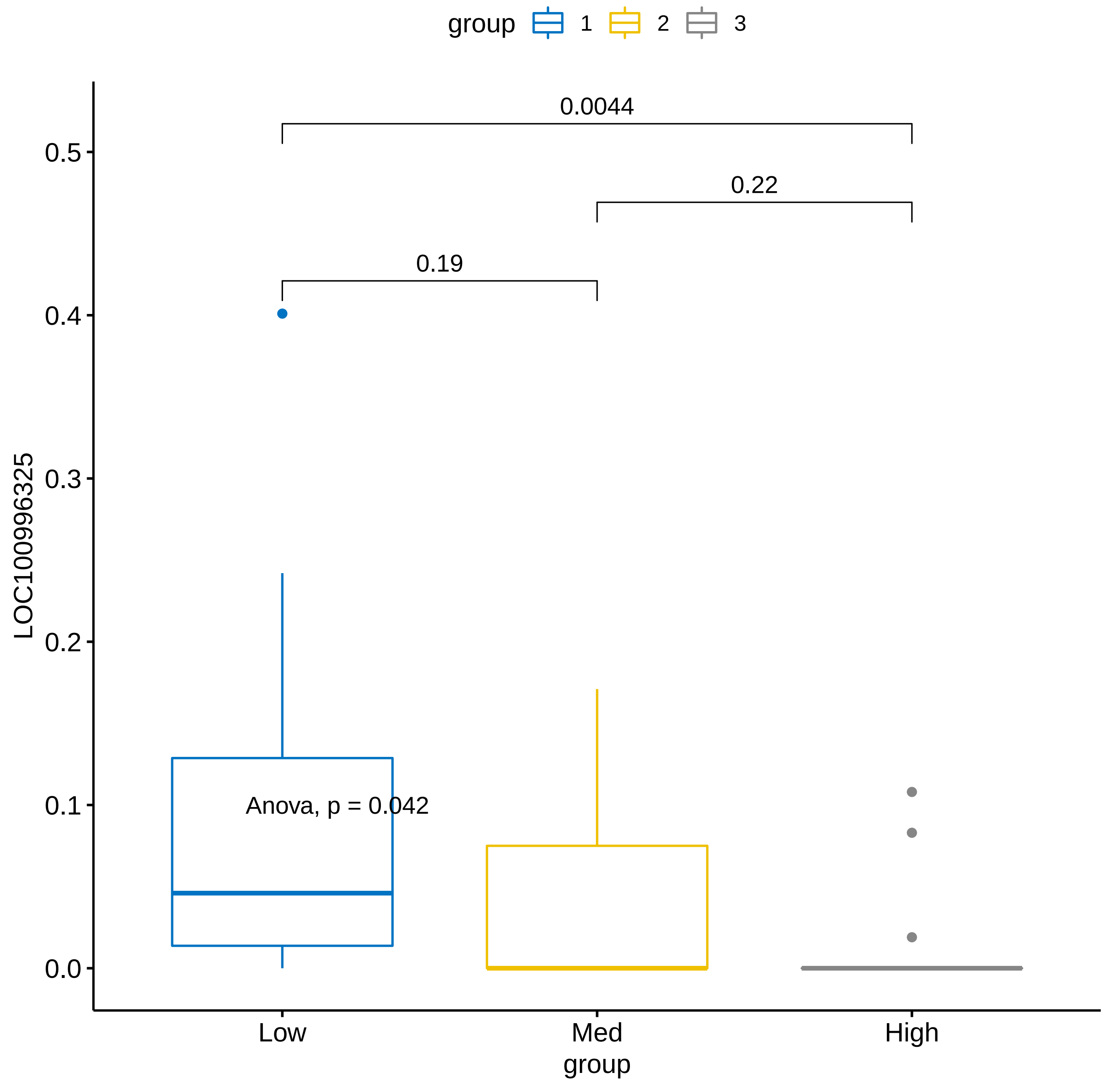
group
1
2
3
0.0044
0.22
0.19
Anova, p = 0.042
LOC100996325
0.0
0.1
0.2
0.3
0.4
0.5
Low
Med
High
group

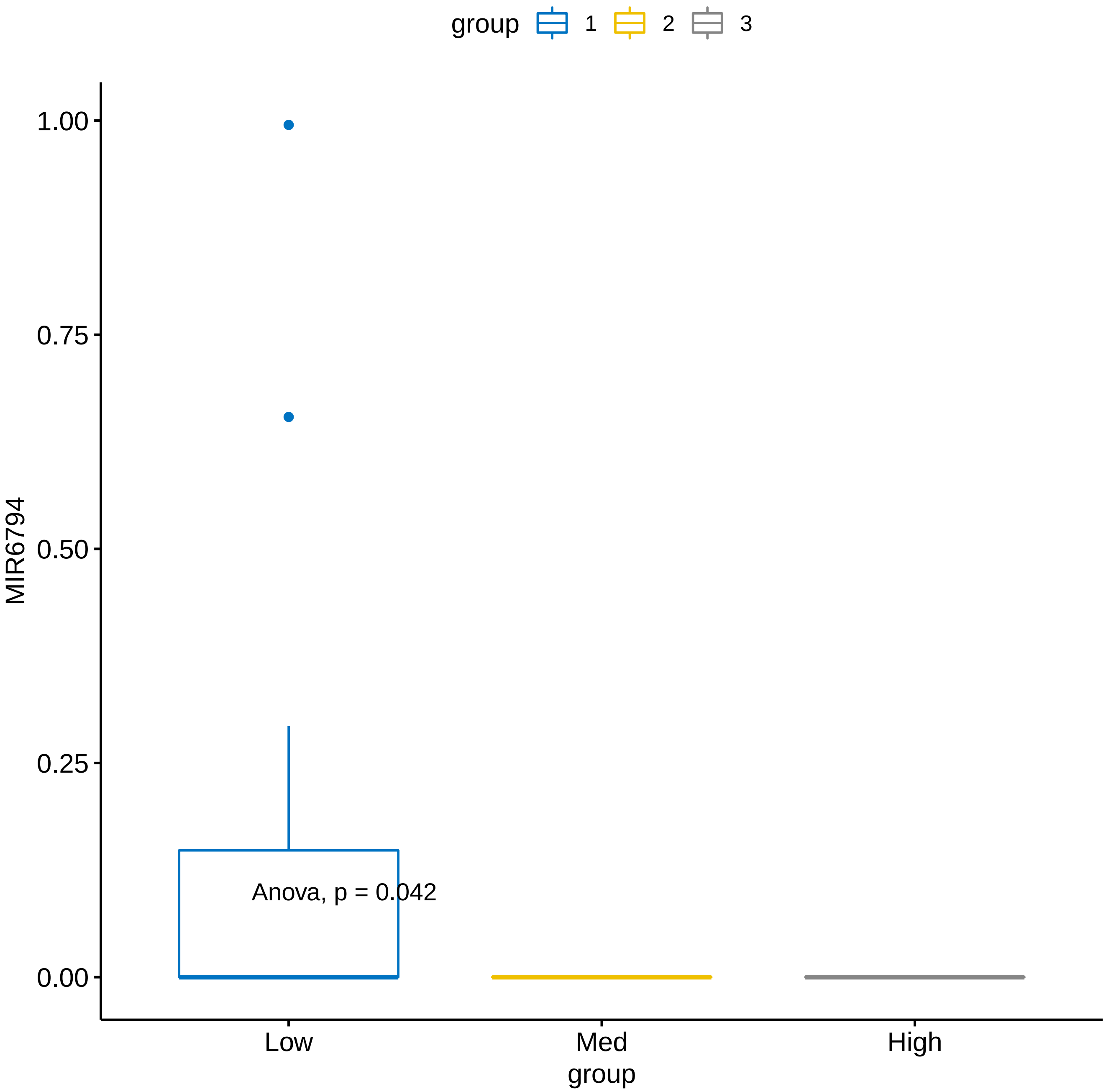

group
1
2
3
1.00
0.75
0.50
0.25
0.00
MIR6794
Anova, p = 0.042
Low
Med
High
group

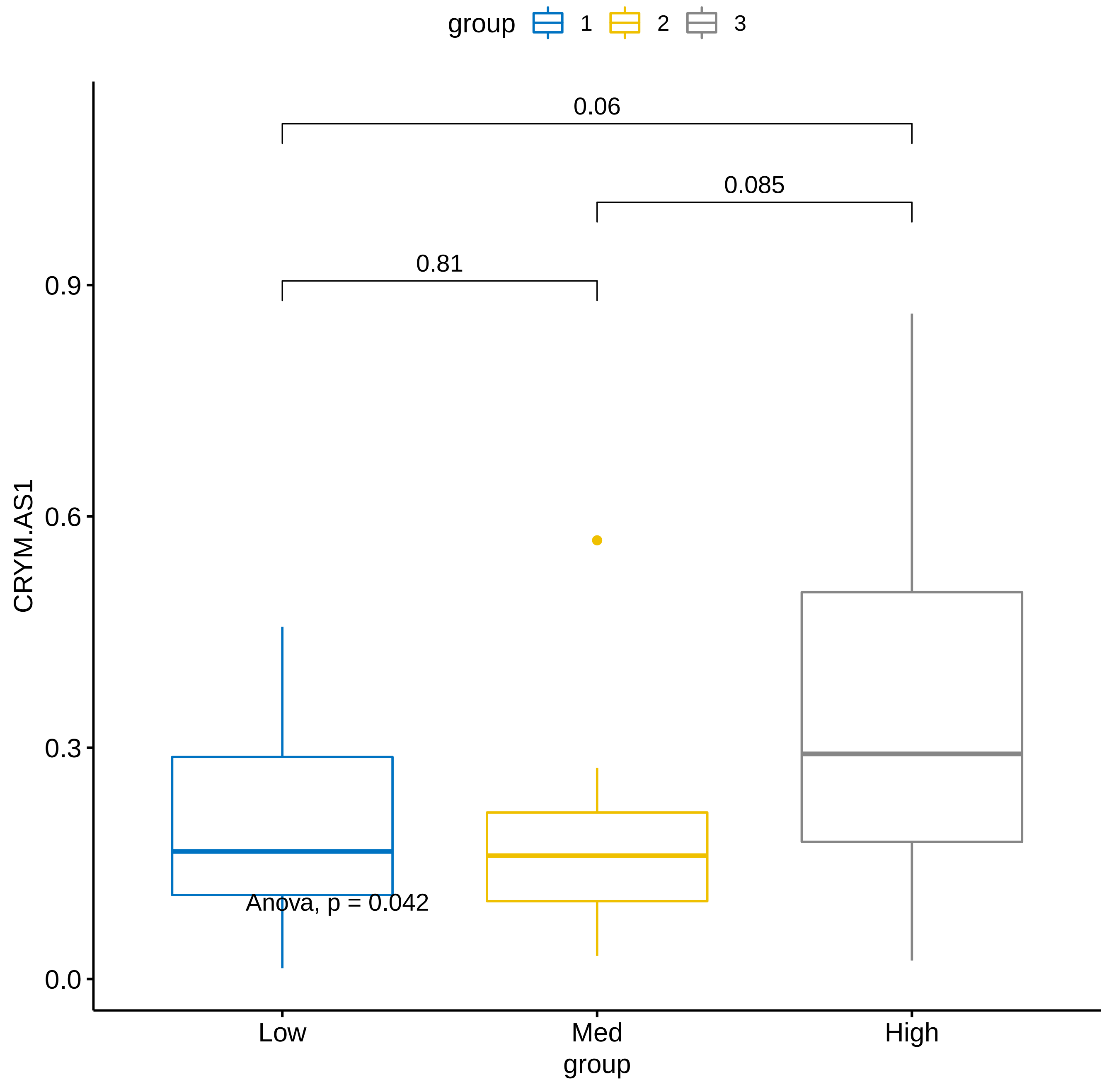
group
1
2
3
0.06
0.085
0.81
0.9
0.6
0.3
0.0
CRYM.AS1
Anova, p = 0.042
Low
Med
High
group

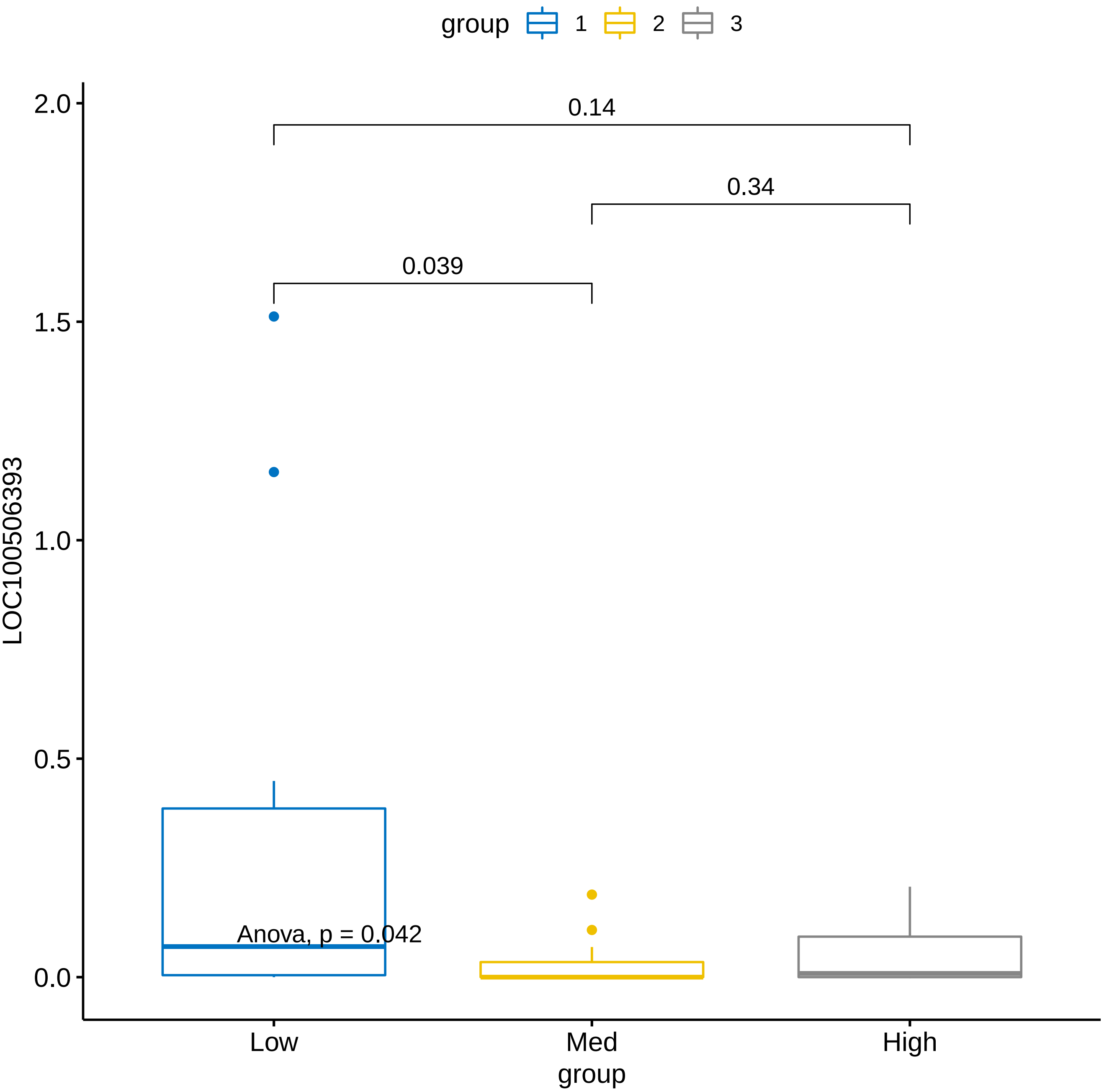
group
1
2
3
0.14
0.34
0.039
Anova, p = 0.042
LOC100506393
2.0
1.5
1.0
0.5
0.0
Low
Med
High
group

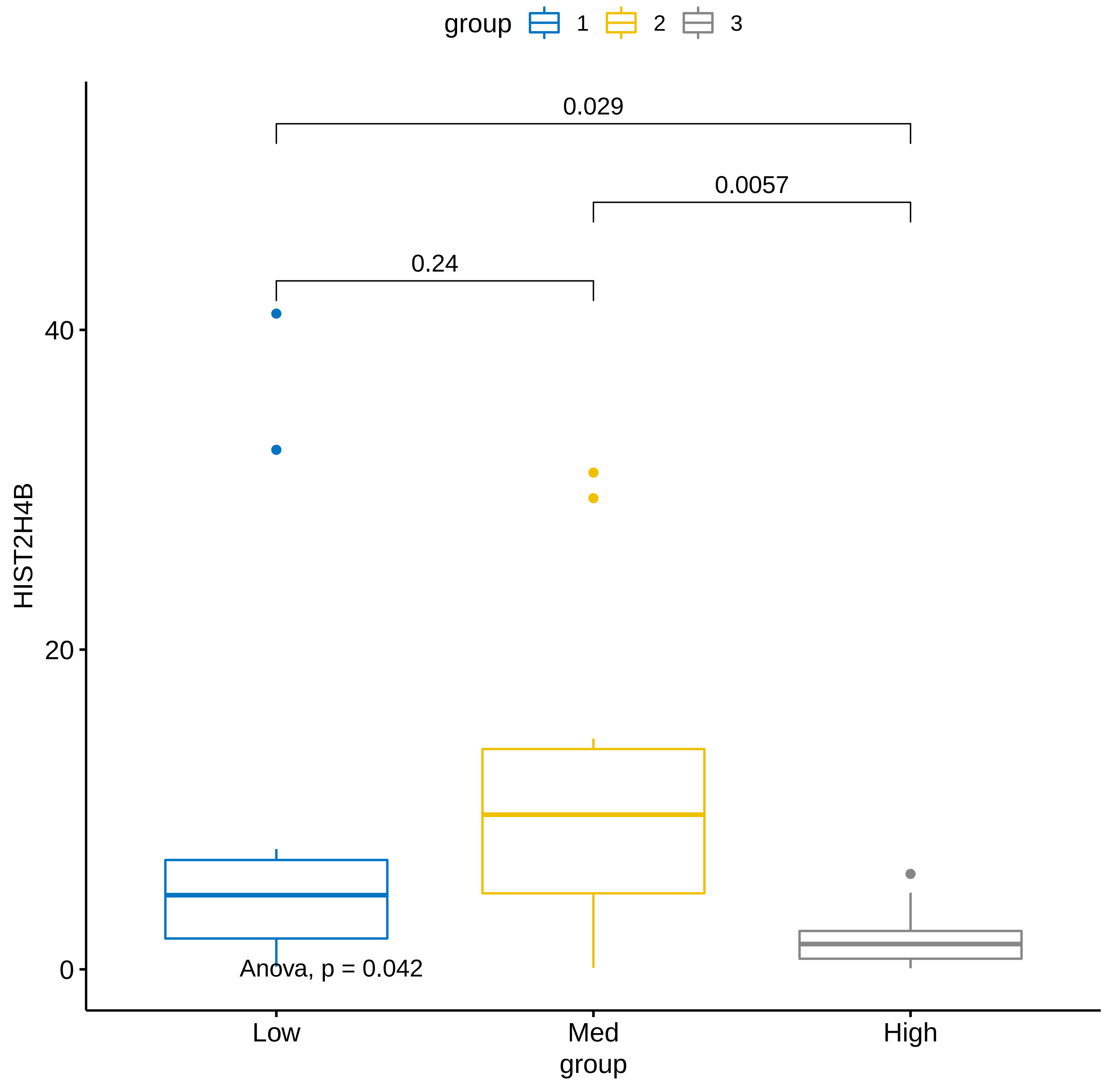

group 1 2 3
0.029
0.0057
0.24
40
20
0
HIST2H4B
Anova, p = 0.042
Low
Med
High
group

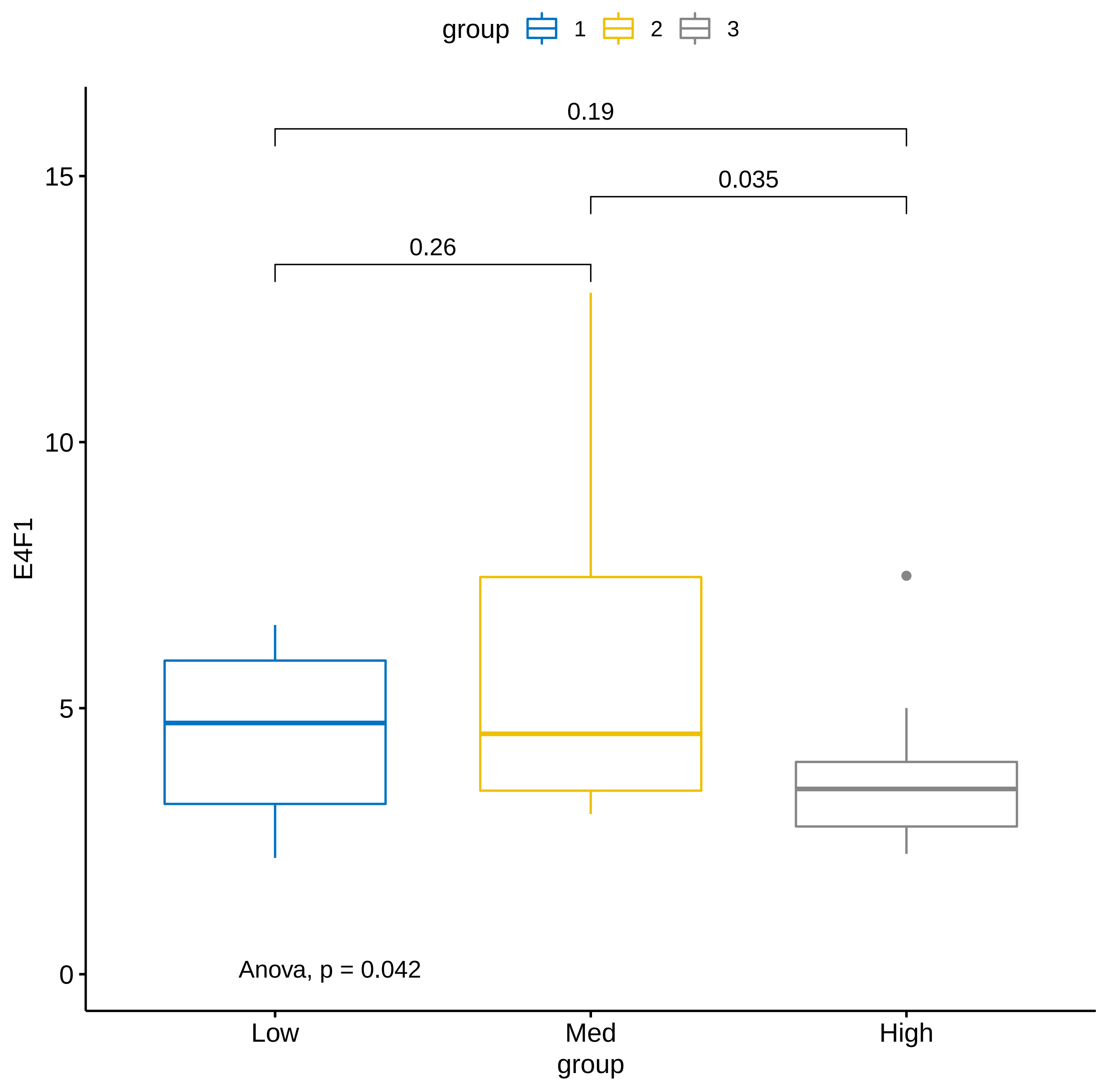
group 1 2 3
0.19
0.035
0.26
15
10
5
0
E4F1
Anova, p = 0.042
Low
Med
High
group

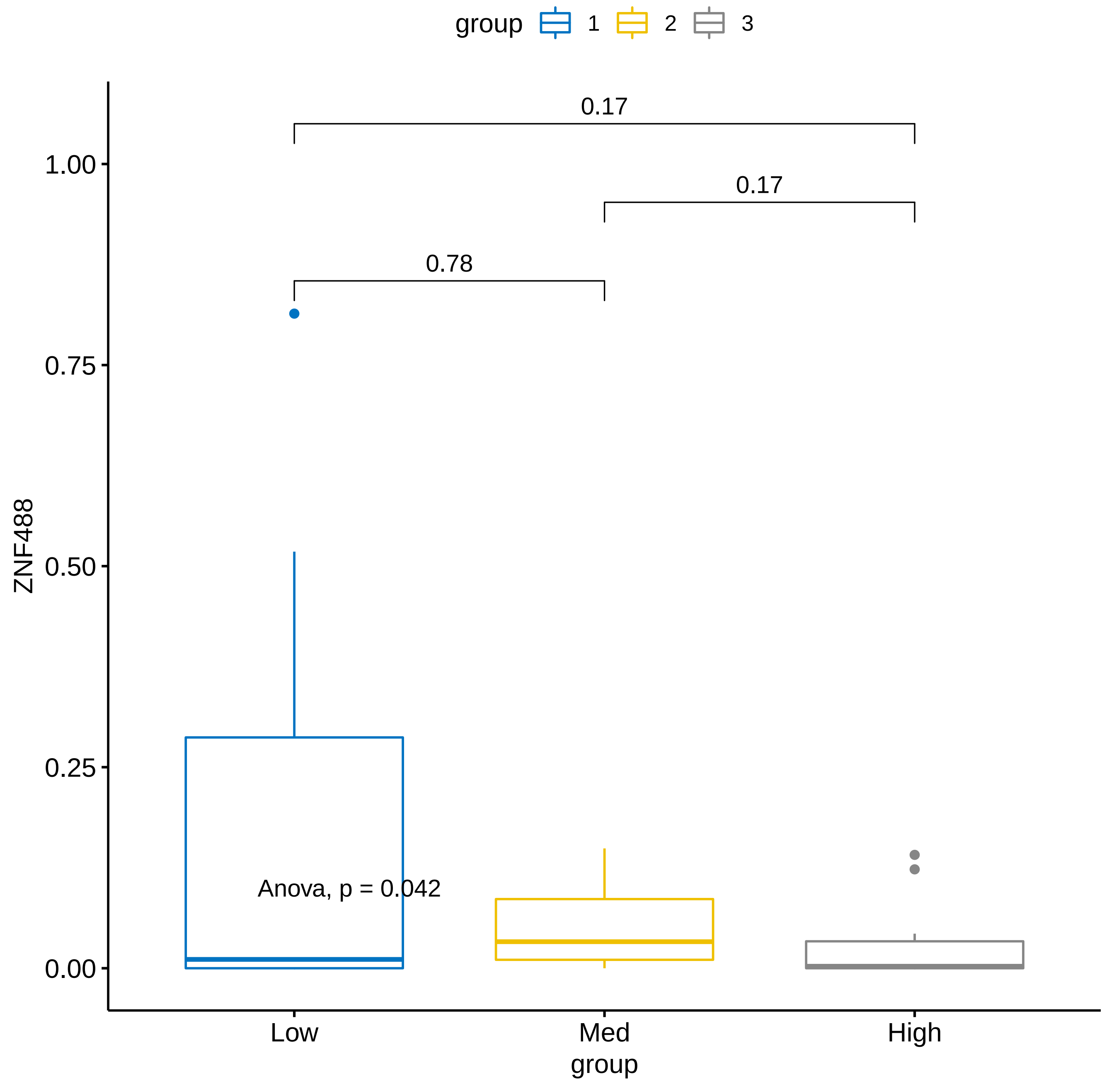
group
1
2
3
0.17
0.17
0.78
1.00
0.75
0.50
0.25
0.00
ZNF488
Anova, p = 0.042
Low
Med
High
group

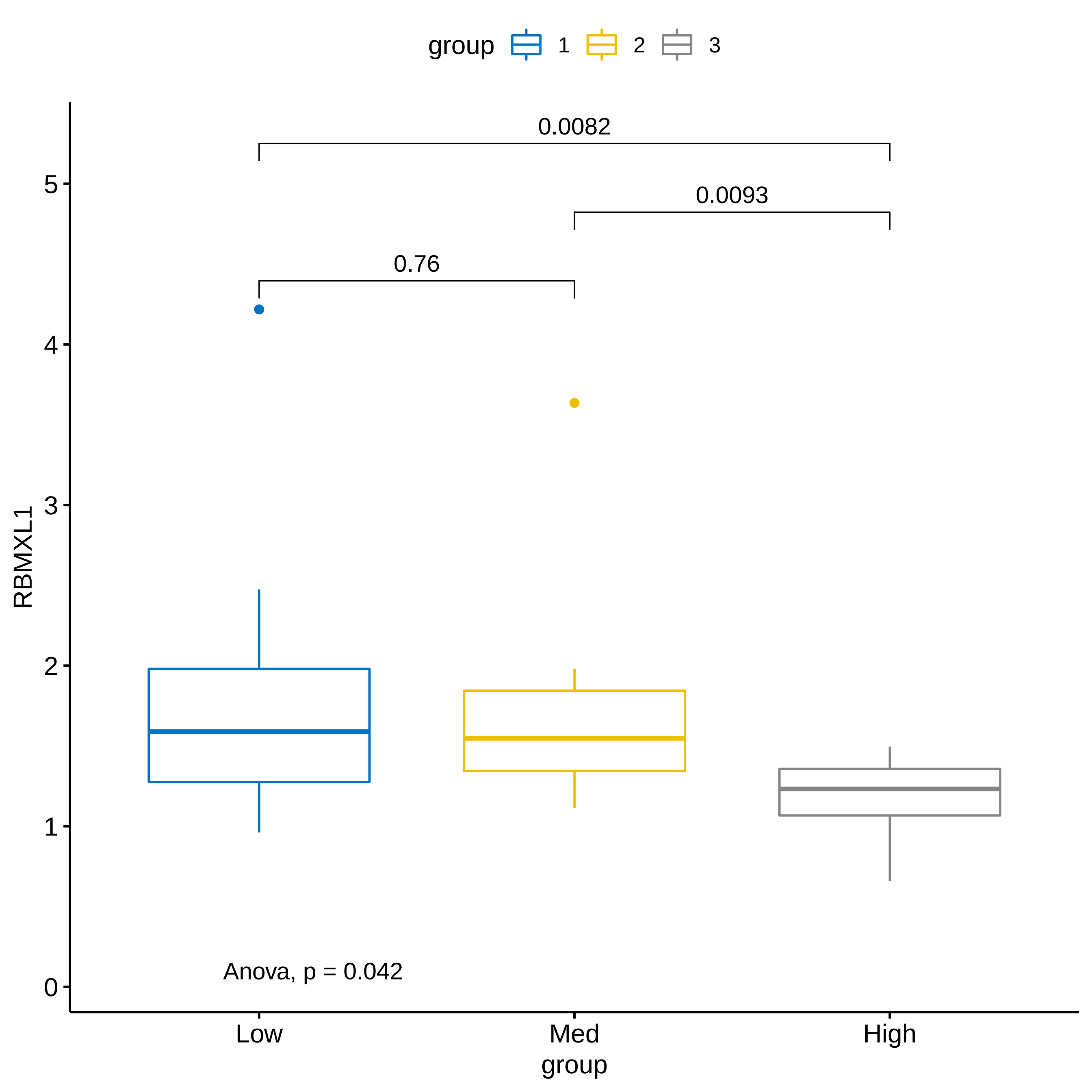

group 1 2 3
0.0082
0.0093
0.76
RBMXL1
5
4
3
2
1
0
Anova, p = 0.042
Low
Med
High
group

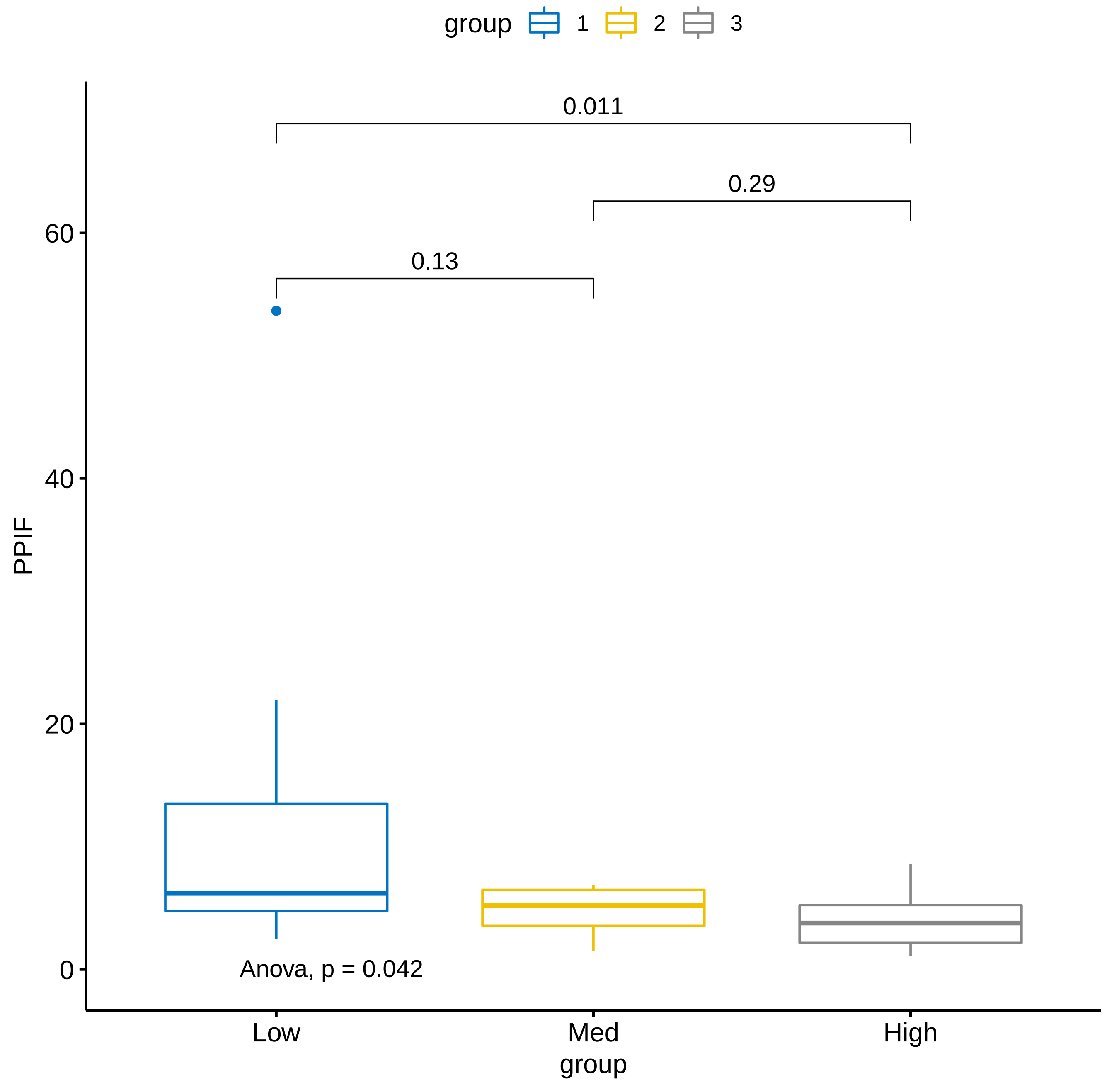
group
1
2
3
0.011
0.29
0.13
60
40
20
0
PPIF
Anova, p = 0.042
Low
Med
High
group

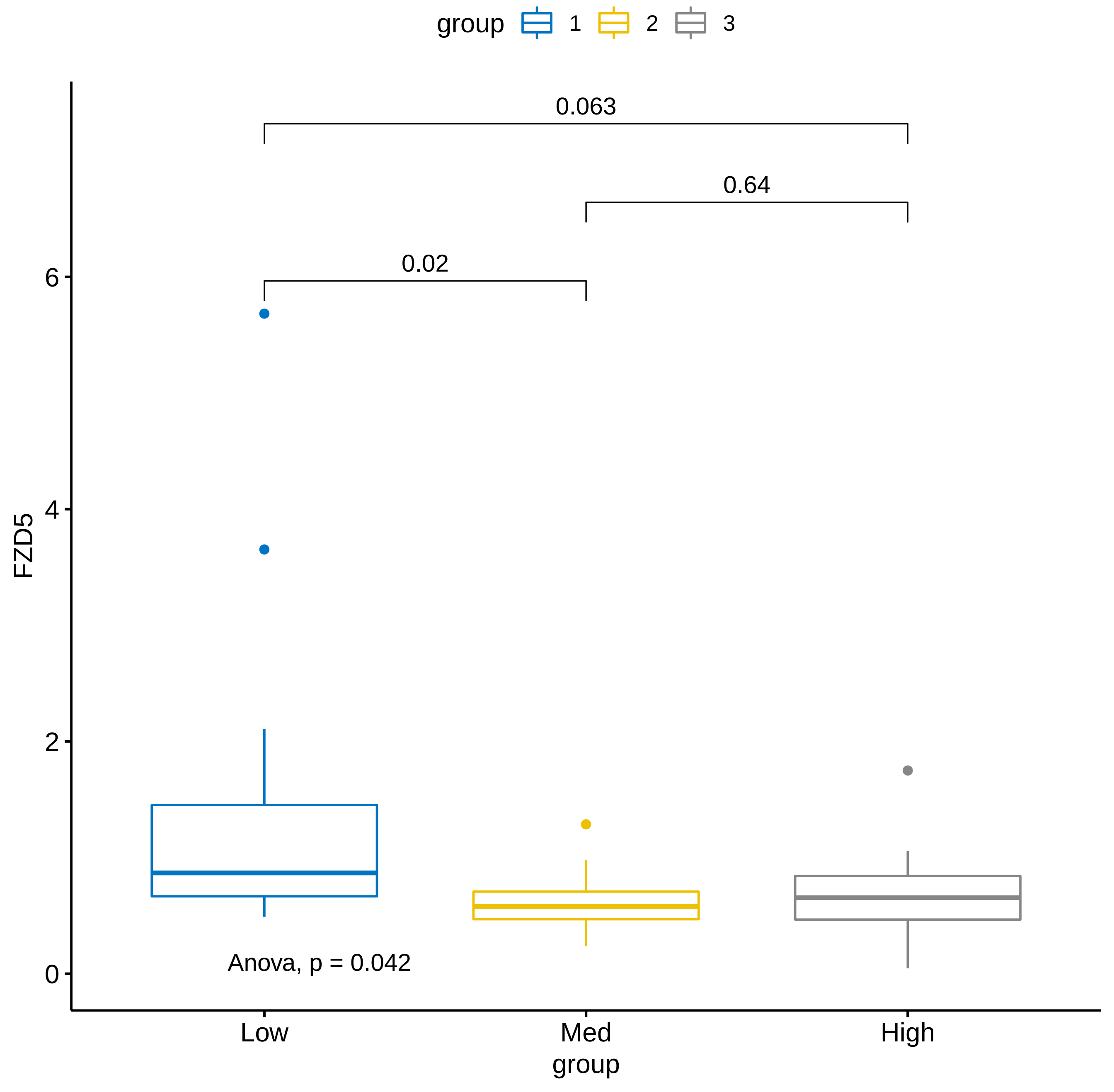

group 1 2 3
0.063
0.64
0.02
FZD5
6
4
2
0
Anova, p = 0.042
Low
Med
High
group

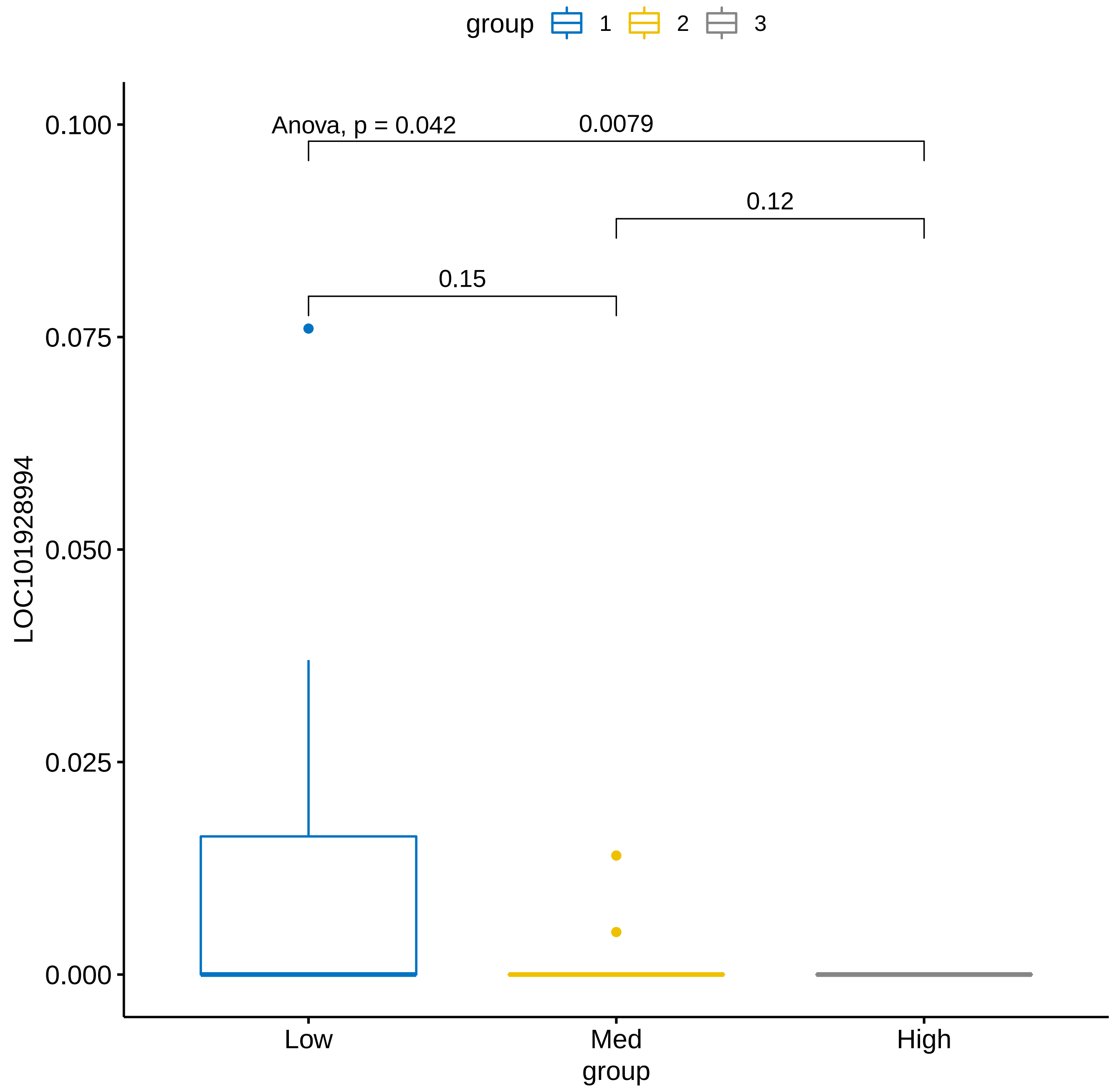

group 1 2 3
Anova, p = 0.042
0.0079
0.12
0.15
LOC101928994
0.100
0.075
0.050
0.025
0.000
Low
Med
High
group

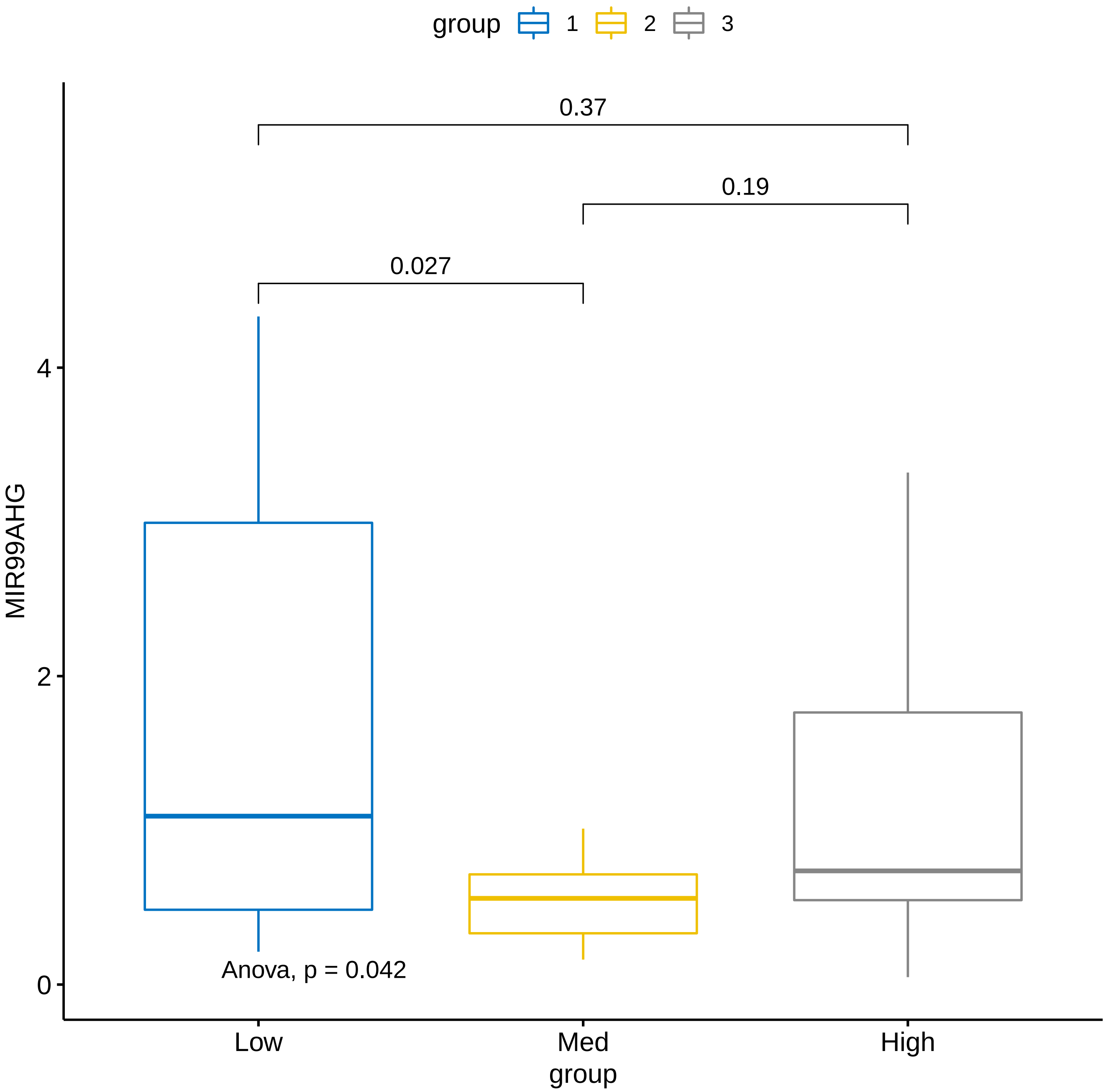
group
1
2
3
0.37
0.19
0.027
4
2
0
MIR99AHG
Anova, p = 0.042
Low
Med
High
group

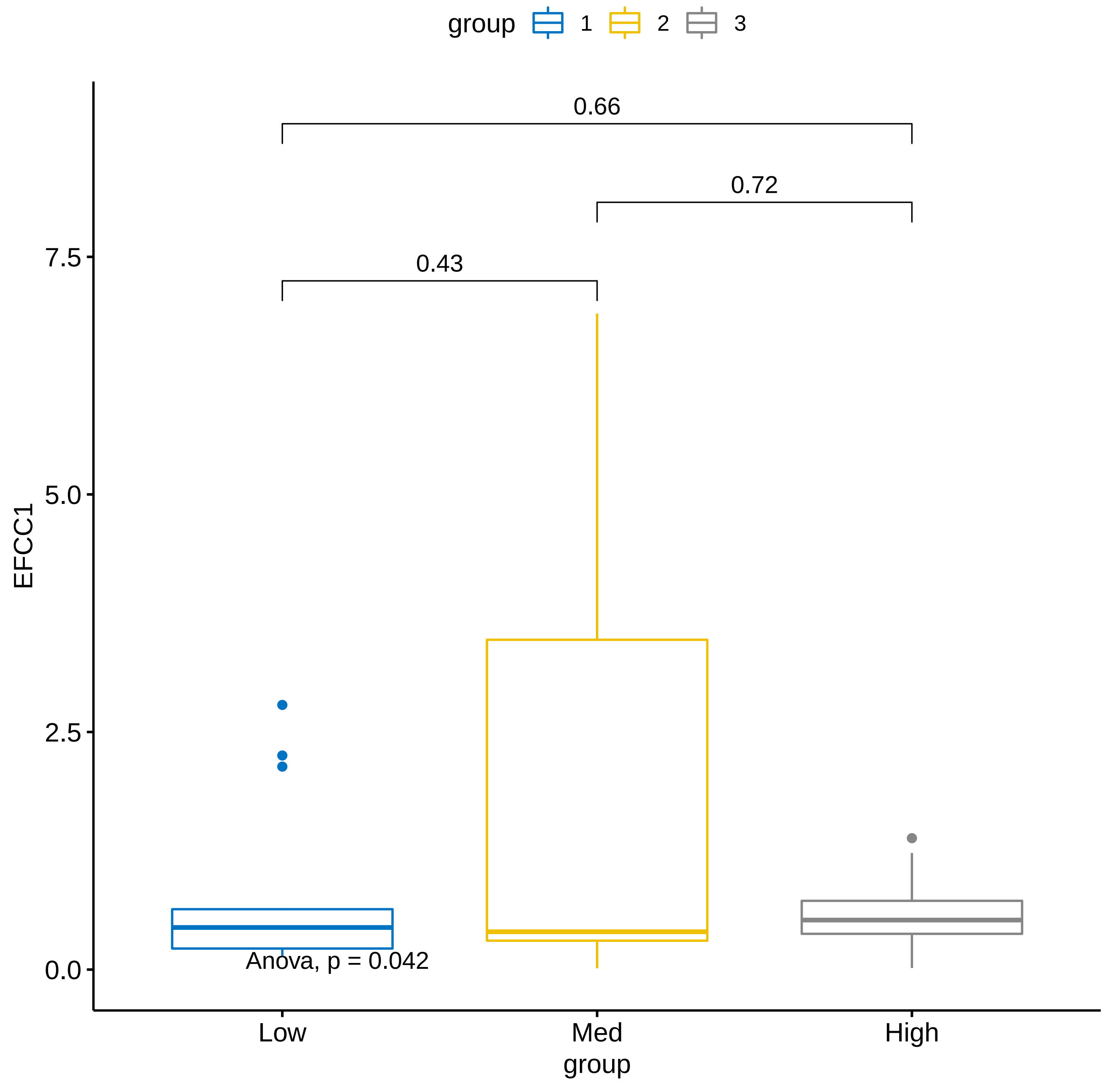

group 1 2 3
0.66
0.72
0.43
7.5
5.0
2.5
0.0
EFCC1
Anova, p = 0.042
Low
Med
High
group

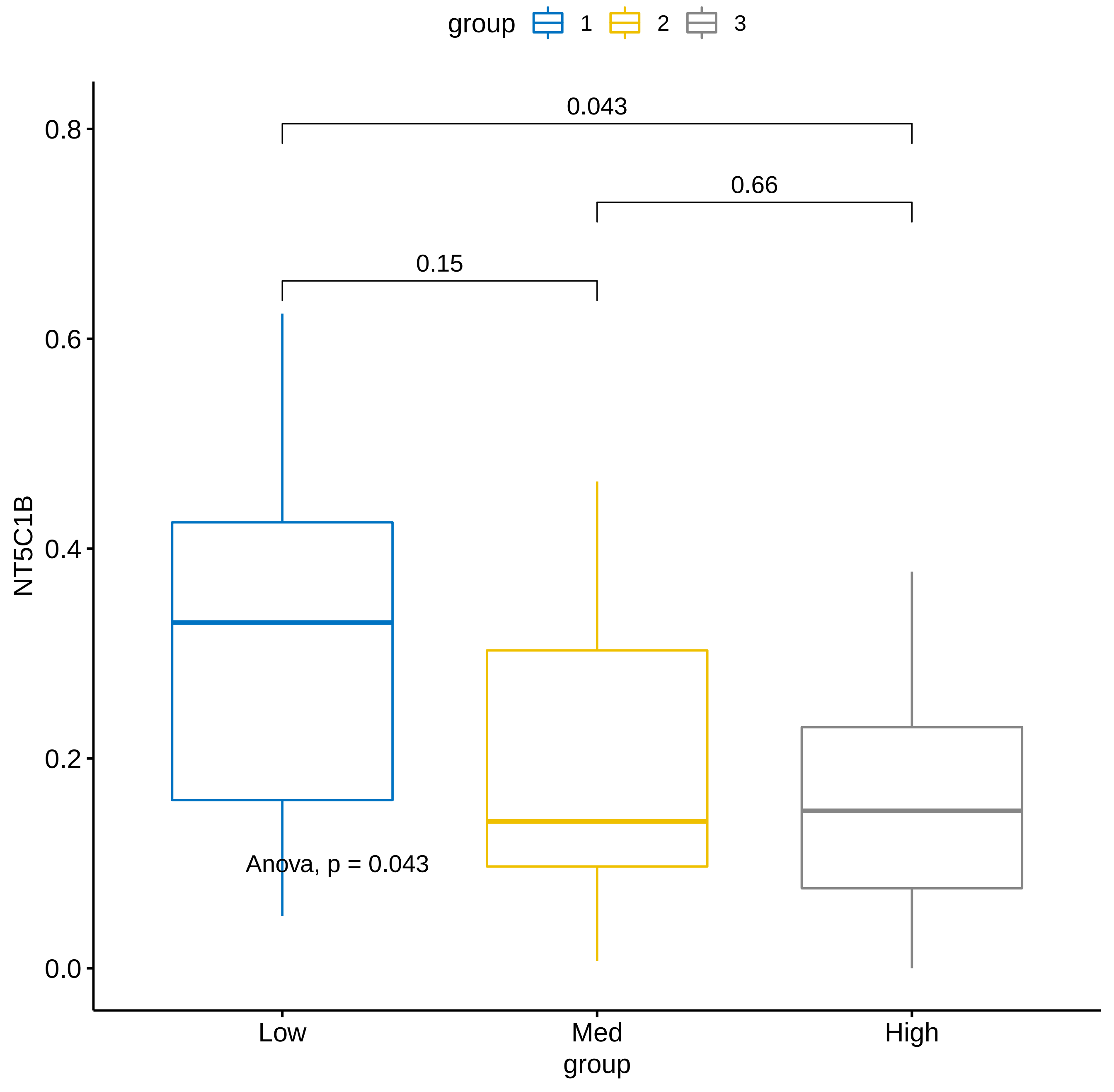

group 1 2 3
0.043
0.66
0.15
Anova, p = 0.043
0.8
0.6
0.4
0.2
0.0
NT5C1B
Low
Med
High
group

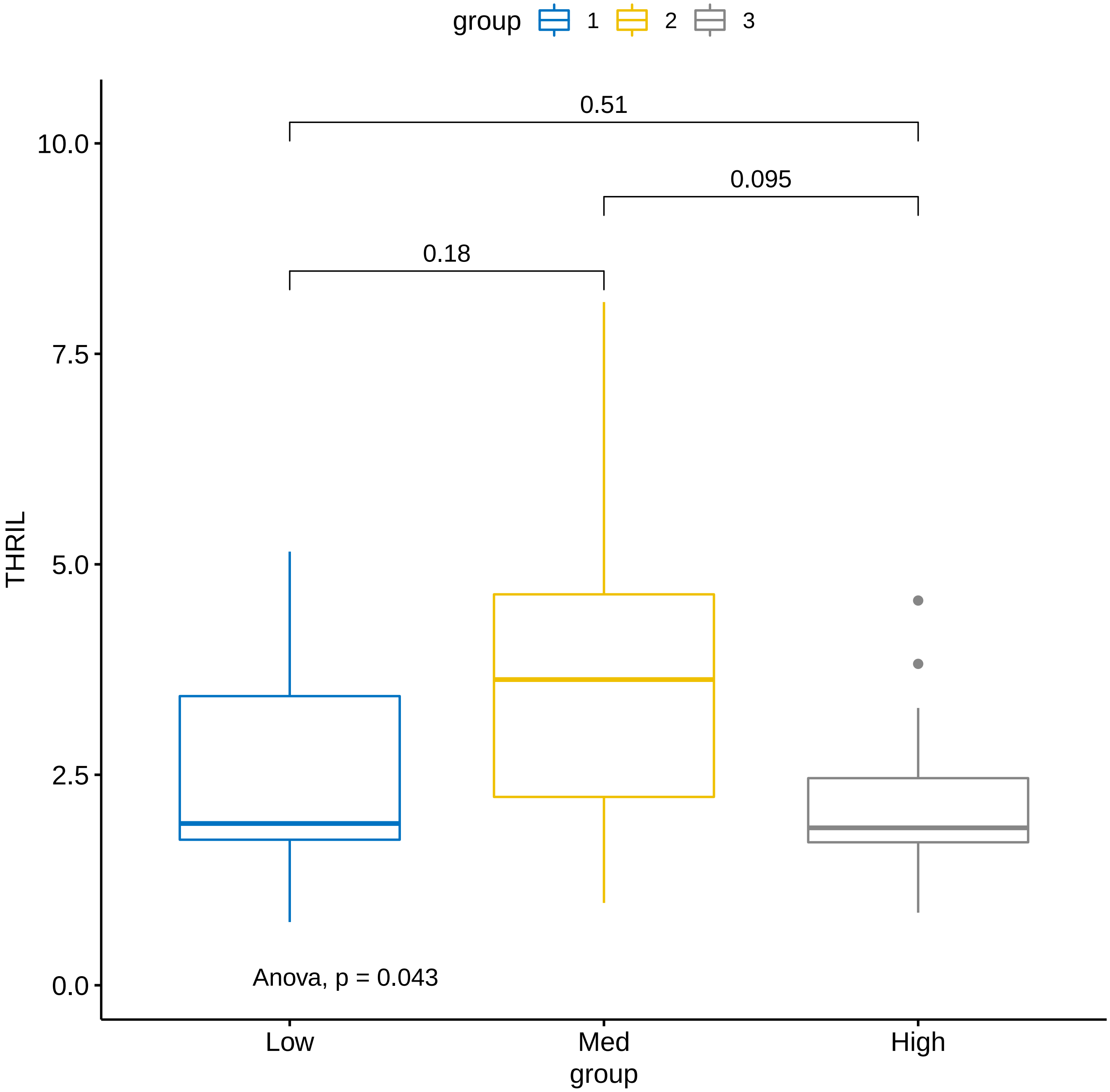

group 1 2 3
0.51
0.095
0.18
10.0
7.5
5.0
2.5
0.0
THRIL
Anova, p = 0.043
Low
Med
High
group

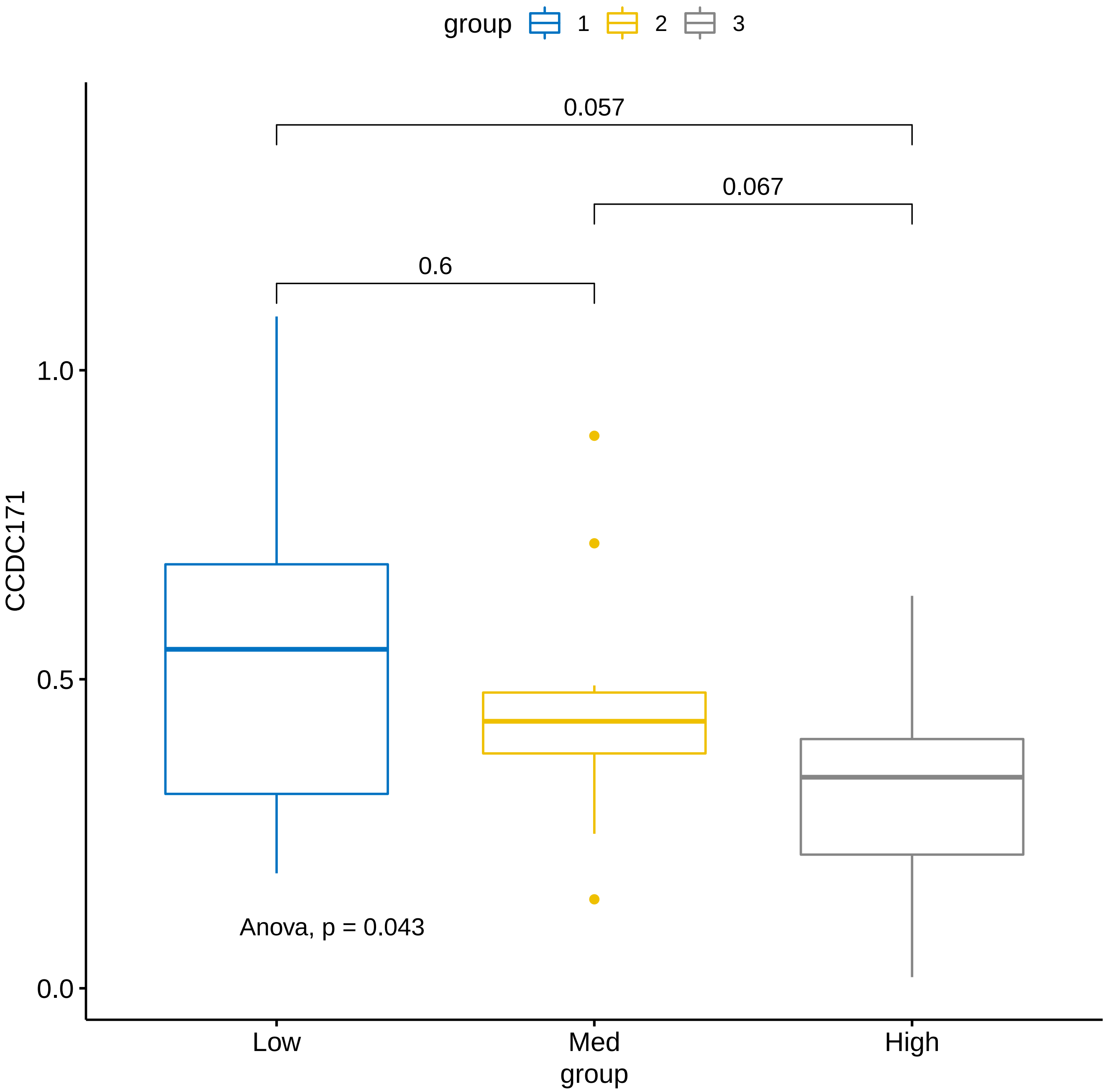
group
1
2
3
0.057
0.067
0.6
1.0
0.5
0.0
CCDC171
Anova, p = 0.043
Low
Med
High
group

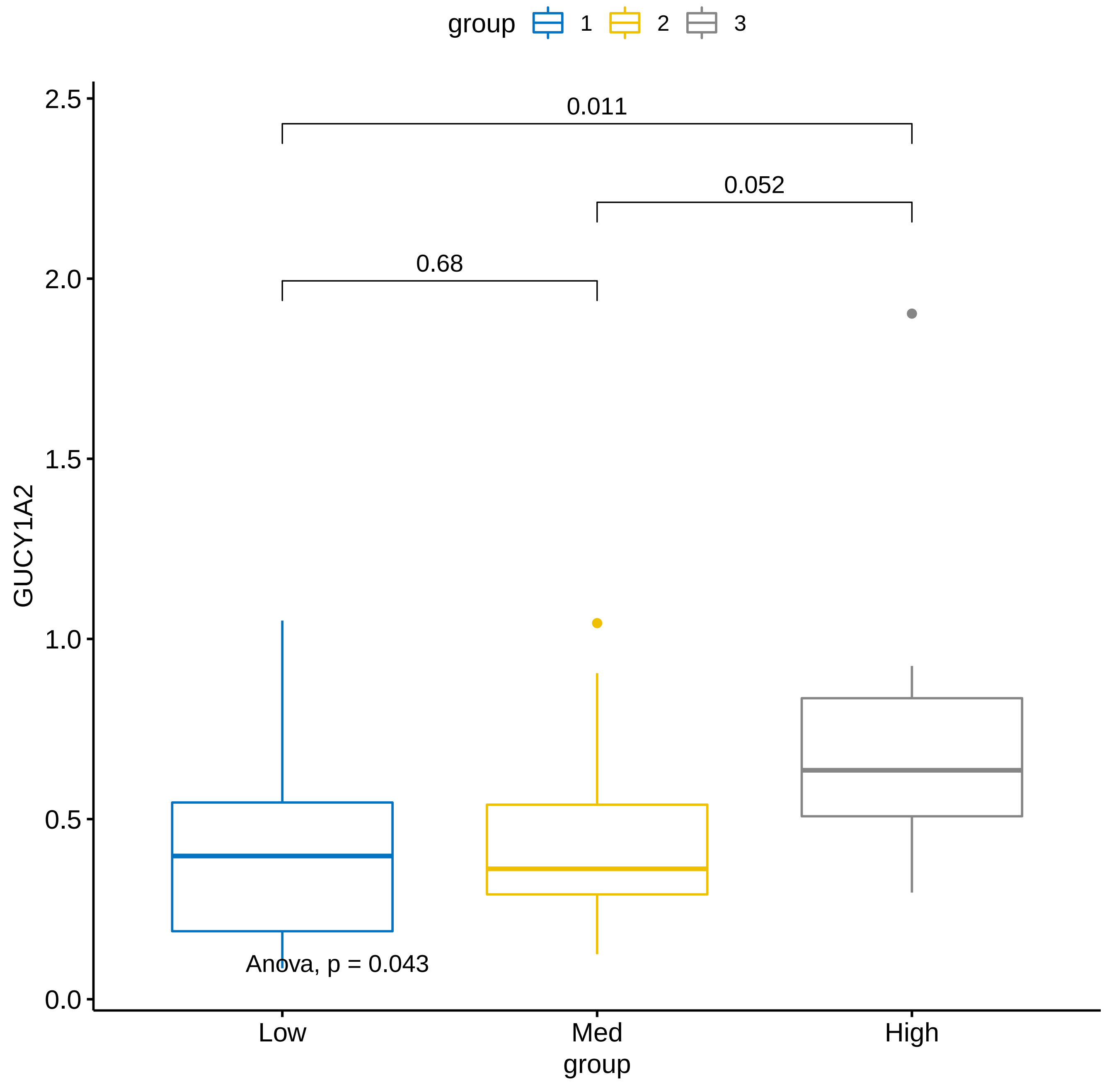
group 1 2 3
2.5
2.0
1.5
1.0
0.5
0.0
GUCY1A2
0.011
0.052
0.68
Anova, p = 0.043
Low
Med
High
group

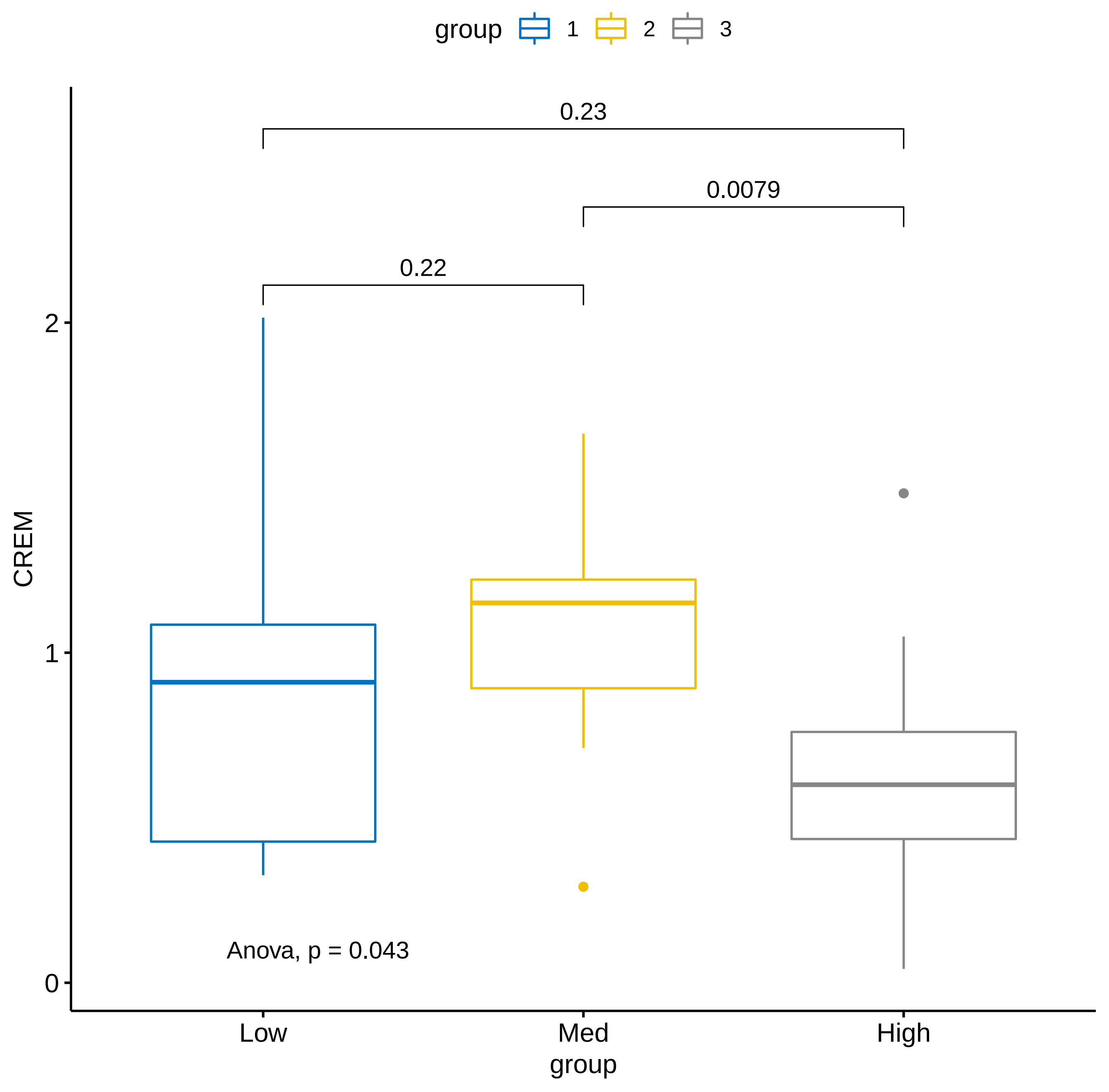
group
1
2
3
0.23
0.0079
0.22
CREM
2
1
0
Anova, p = 0.043
Low
Med
High
group

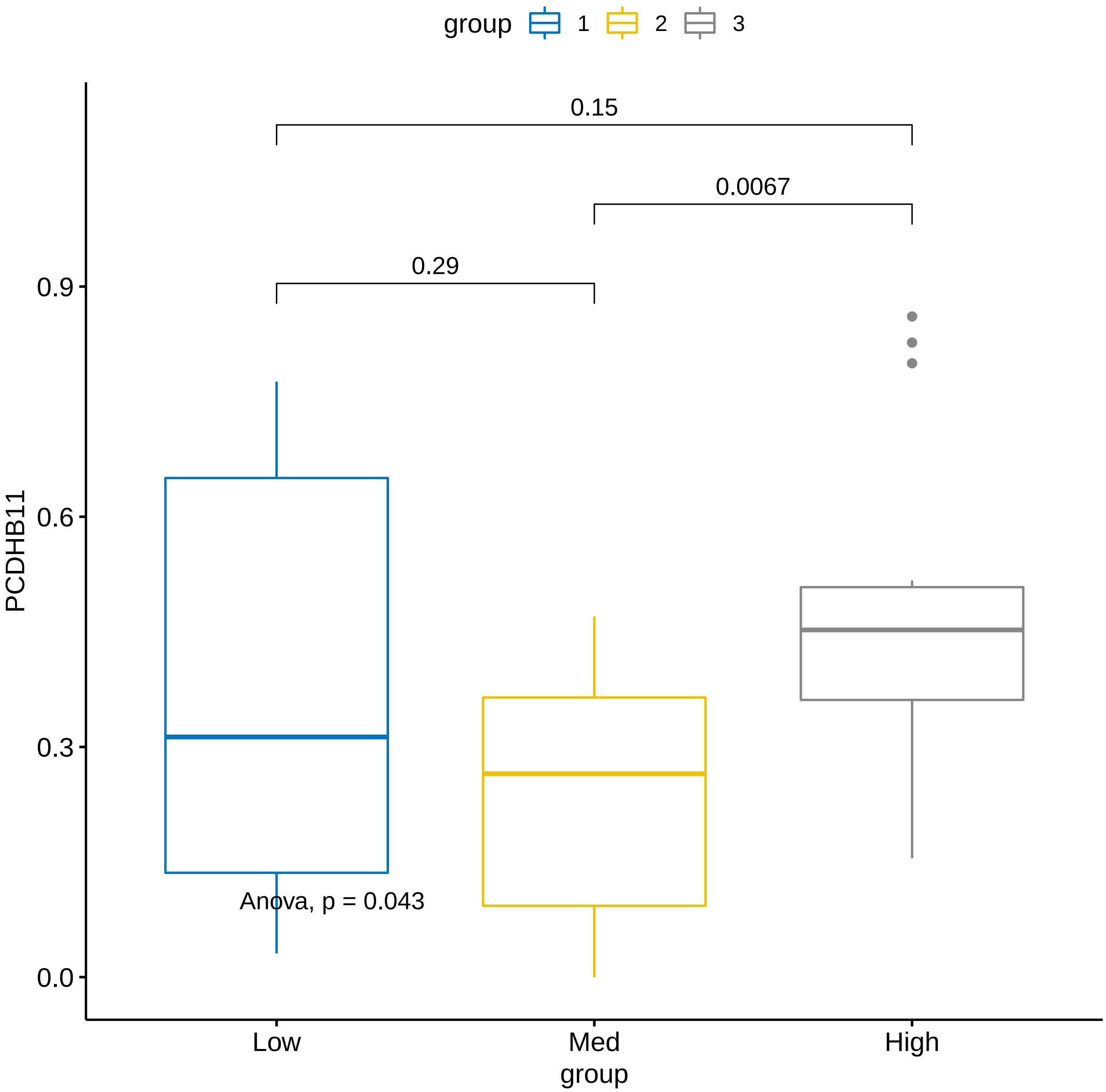

group
1
2
3
0.15
0.0067
0.29
0.9
0.6
0.3
0.0
PCDHB11
Anova, p = 0.043
Low
Med
High
group

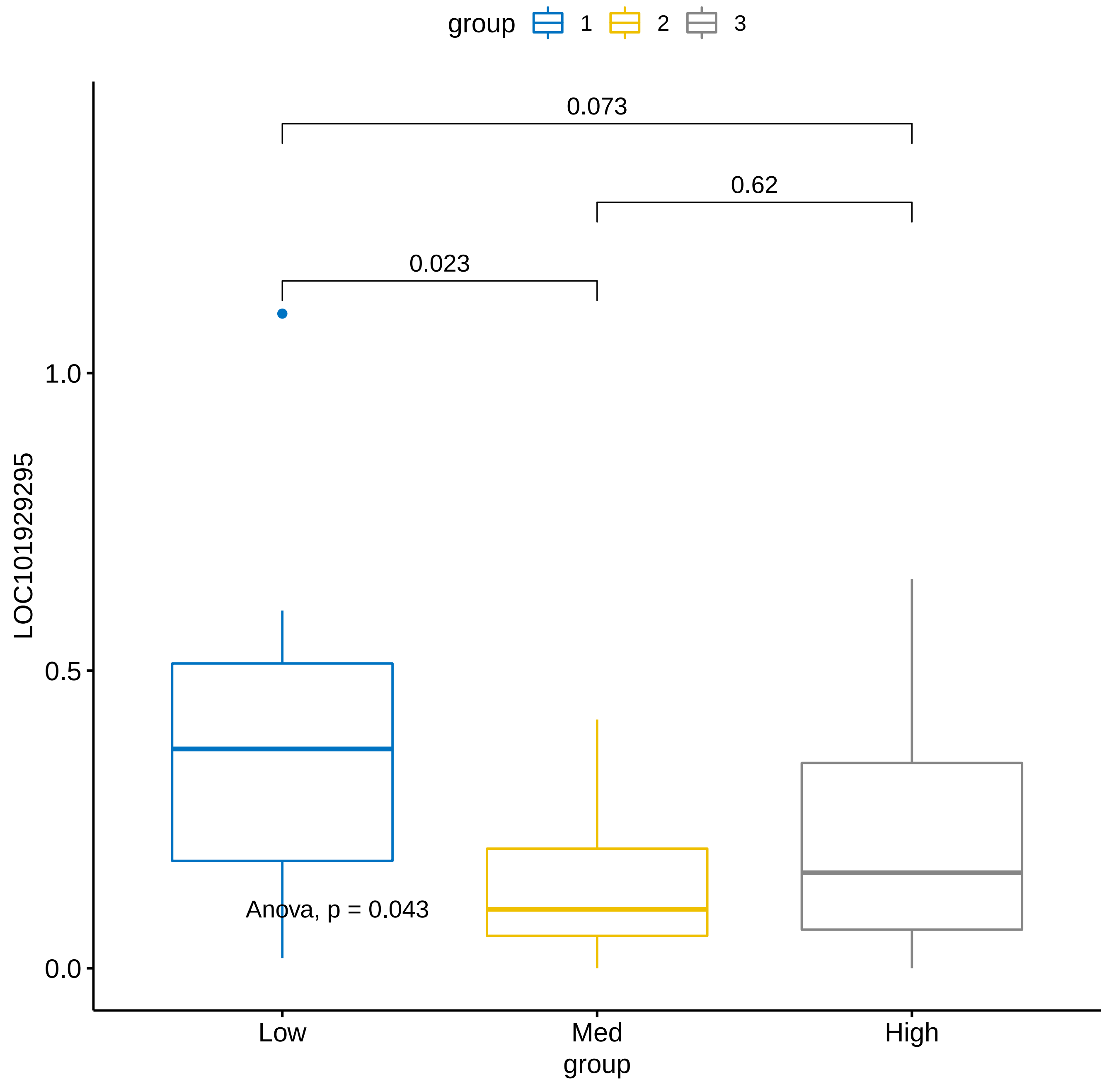

group 1 2 3
0.073
0.62
0.023
LOC101929295
1.0
0.5
0.0
Anova, p = 0.043
Low
Med
High
group

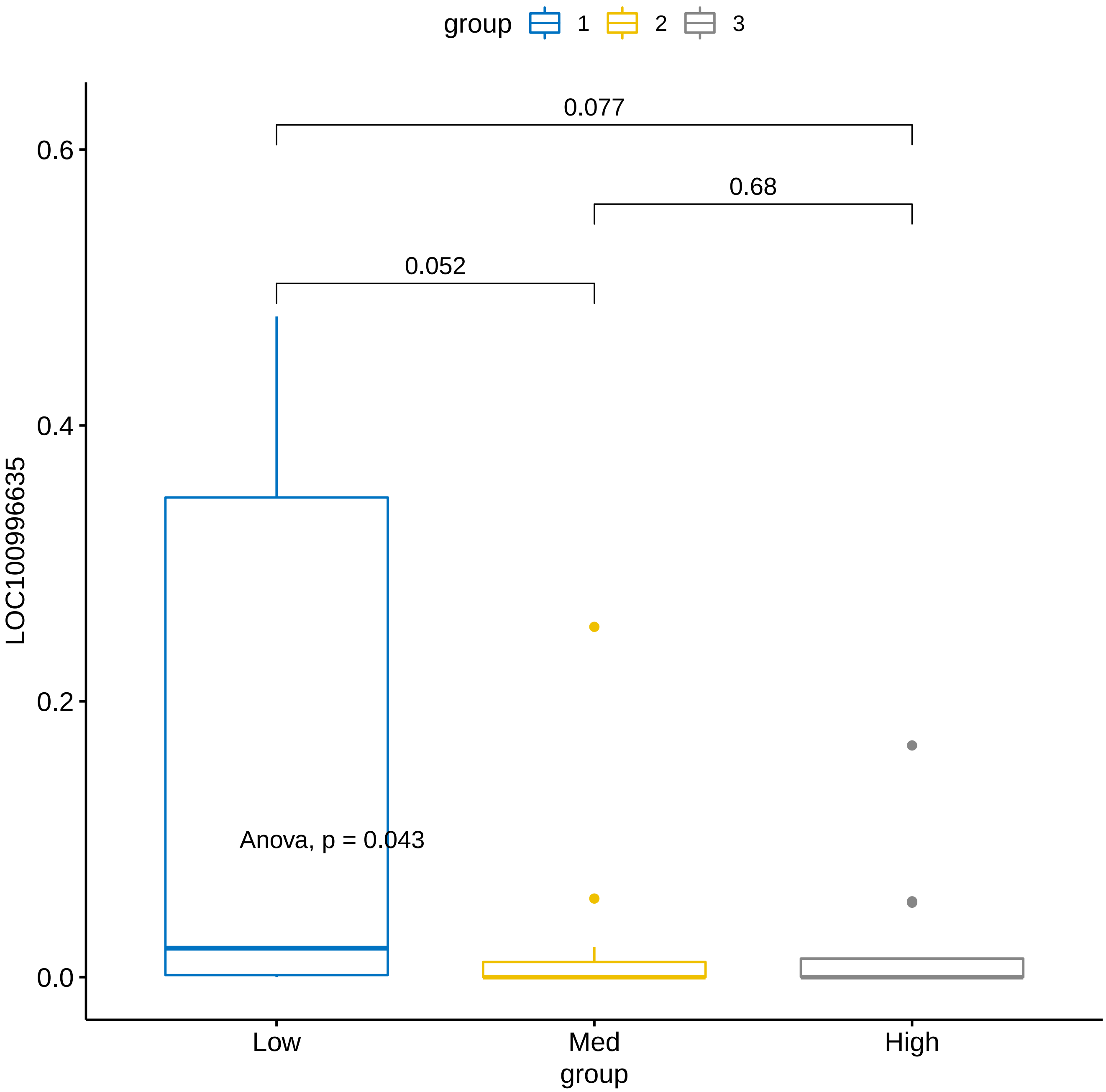
group 1 2 3
0.077
0.68
0.052
Anova, p = 0.043
LOC100996635
0.6
0.4
0.2
0.0
Low
Med
High
group

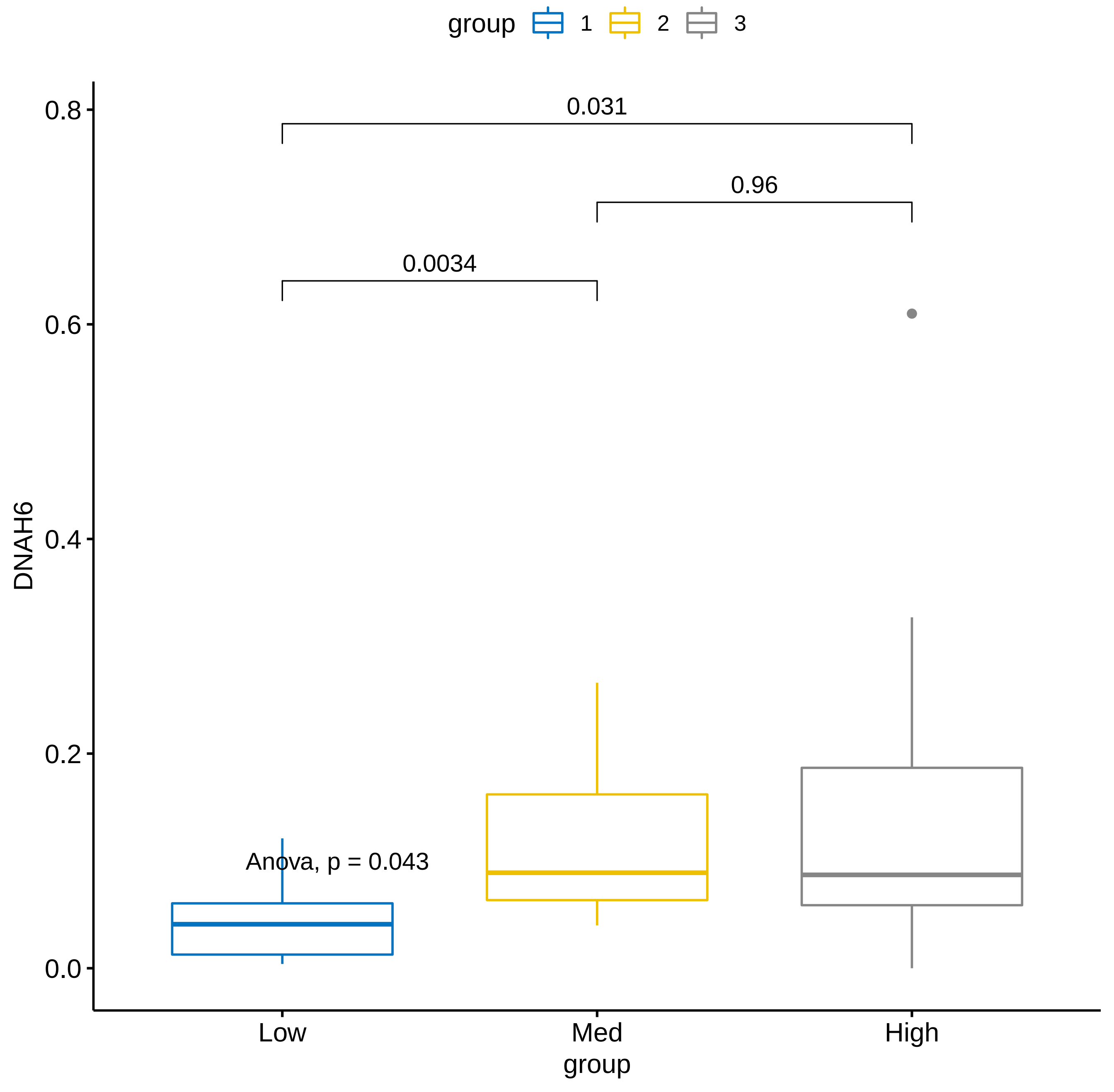

group
1
2
3
0.031
0.96
0.0034
Anova, p = 0.043
DNAH6
0.8
0.6
0.4
0.2
0.0
Low
Med
High
group

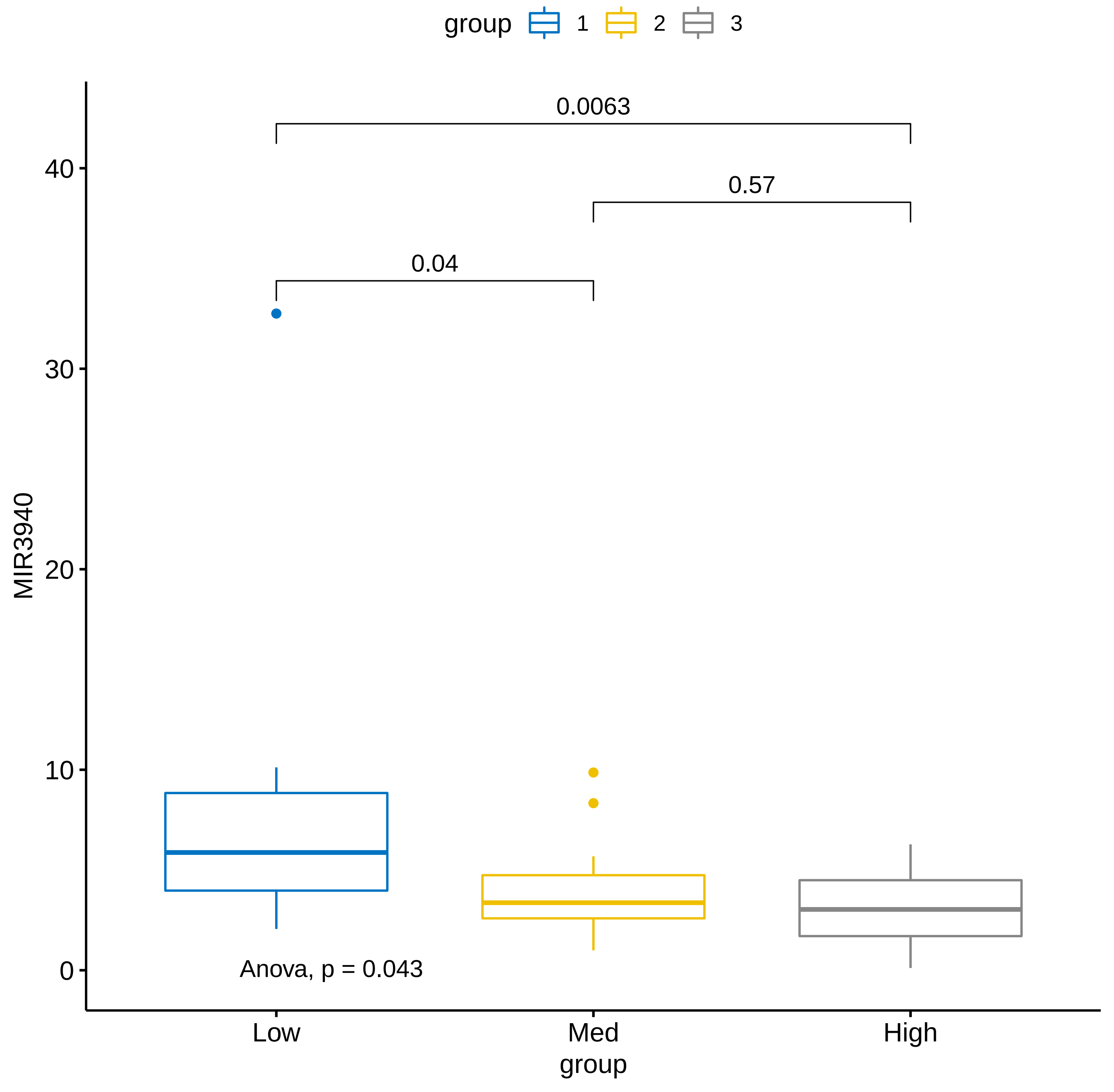
group
1
2
3
0.0063
0.57
0.04
40
30
20
10
0
MIR3940
Anova, p = 0.043
Low
Med
High
group

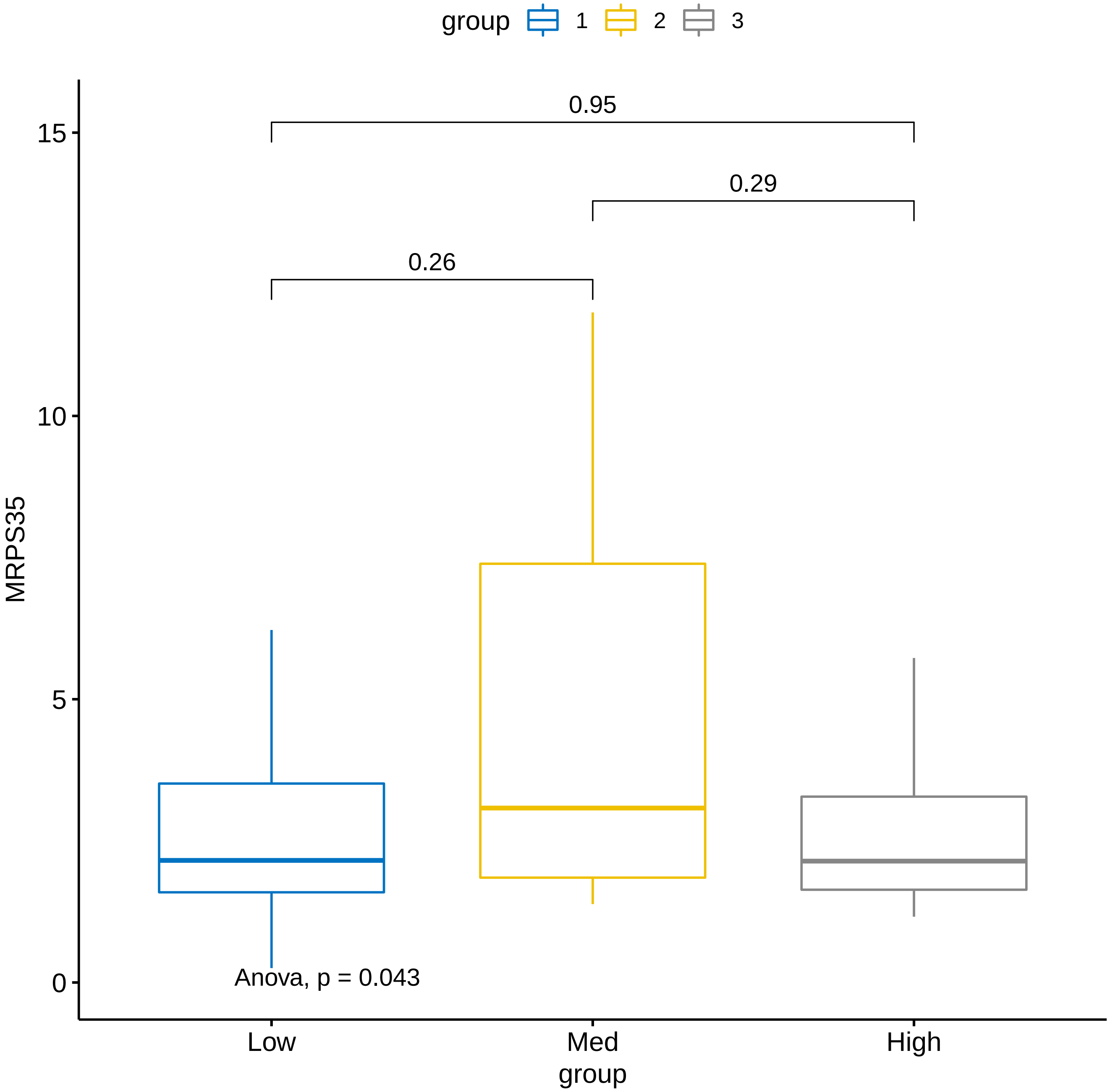
group
1
2
3
0.95
0.29
0.26
15
10
5
0
MRPS35
Anova, p = 0.043
Low
Med
High
group

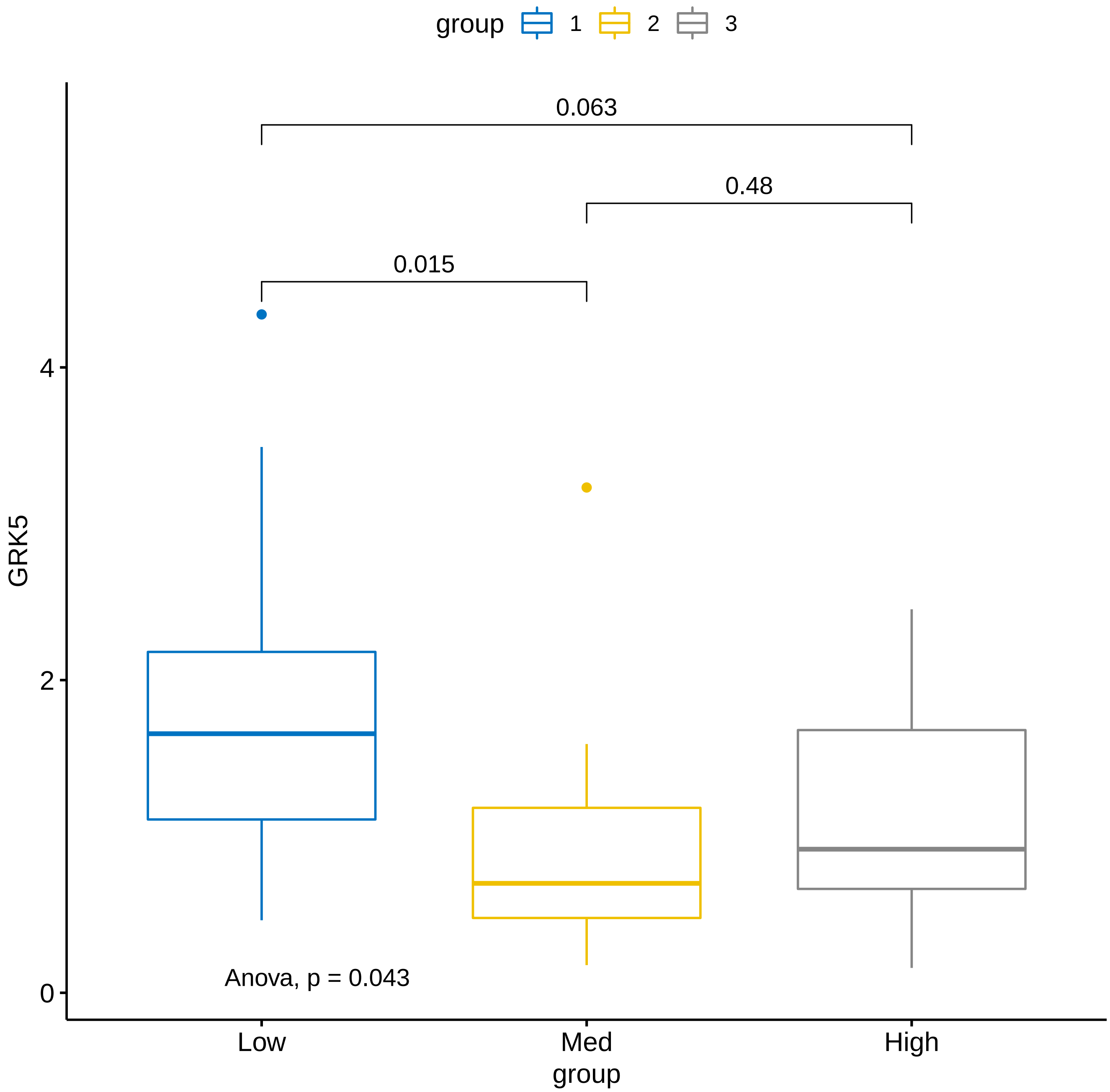

group
1
2
3
0.063
0.48
0.015
GRK5
4
2
0
Anova, p = 0.043
Low
Med
High
group

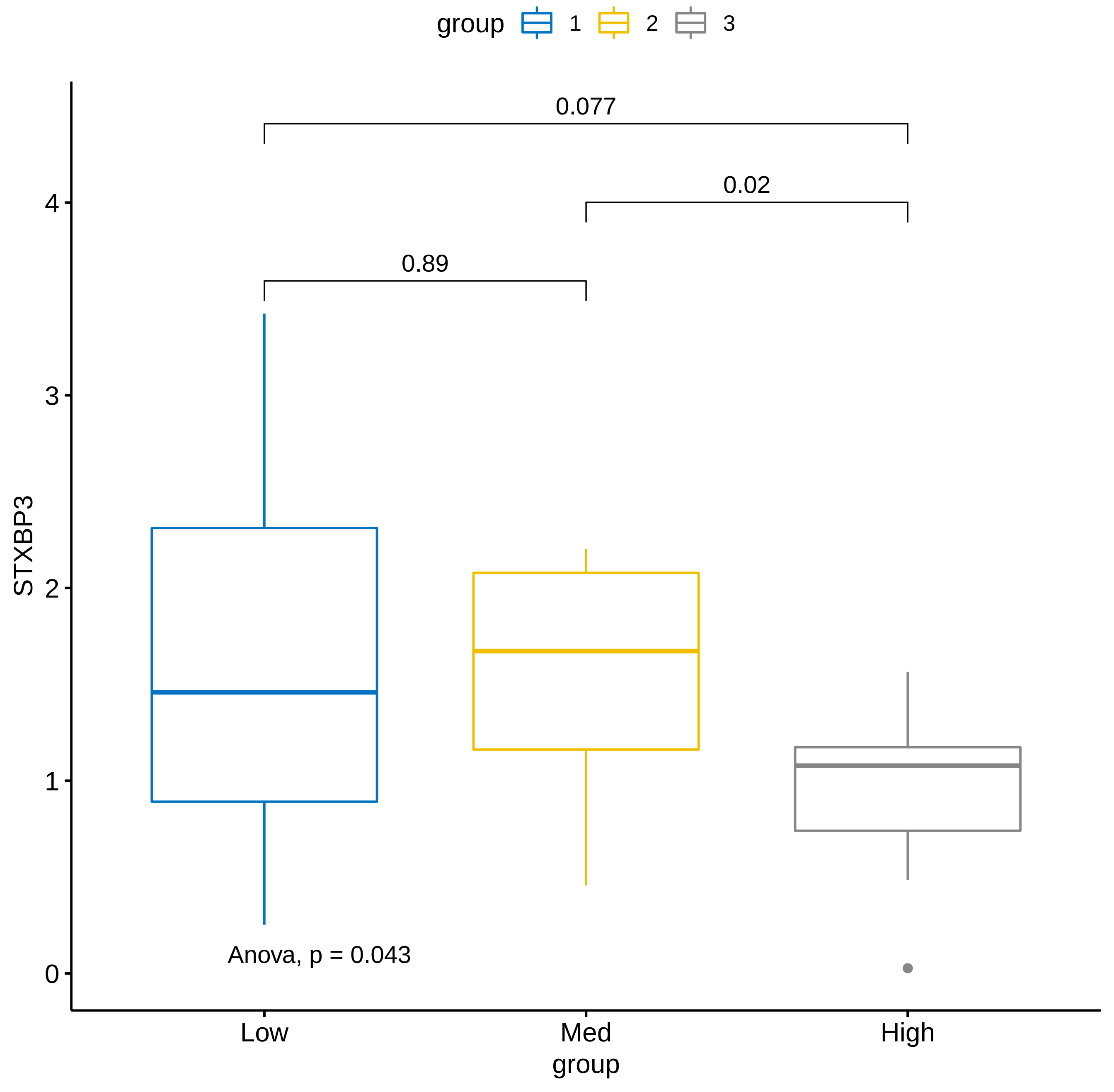
group
1
2
3
0.077
0.02
0.89
STXBP3
4
3
2
1
0
Anova, p = 0.043
Low
Med
High
group

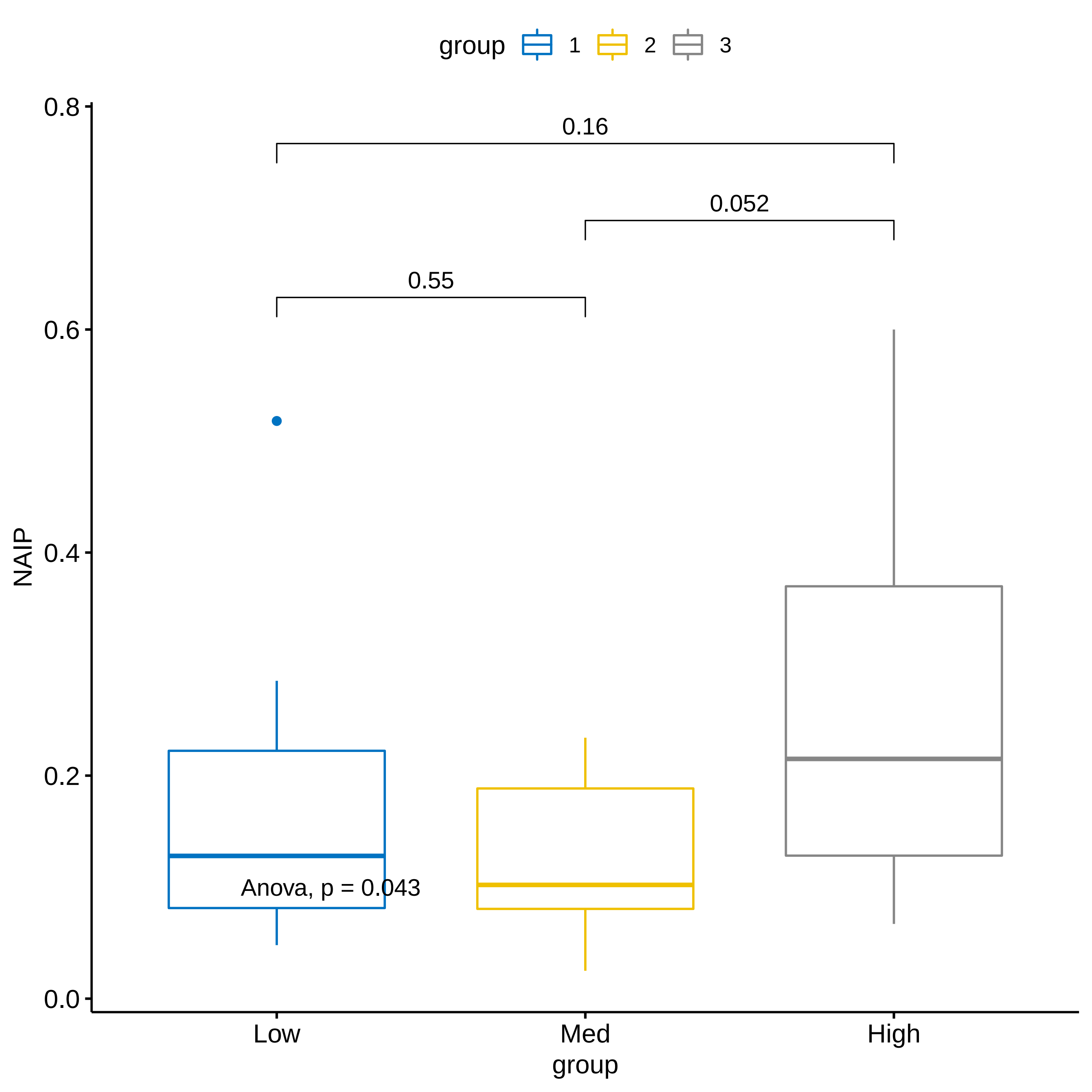

group 1 2 3
0.8
0.6
0.4
0.2
0.0
NAIP
0.16
0.052
0.55
Anova, p = 0.043
Low
Med
High
group

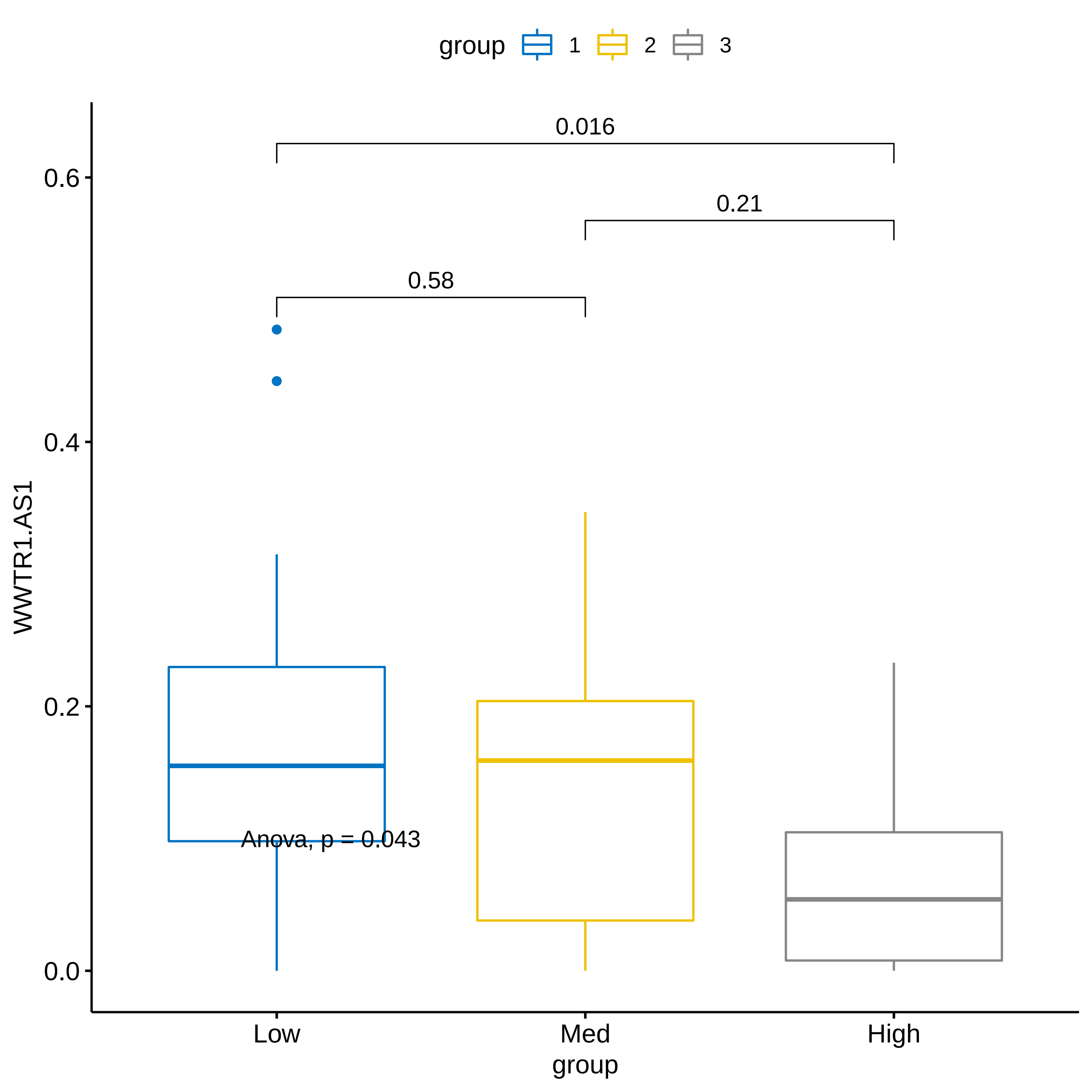
group
1
2
3
0.016
0.21
0.58
0.6
0.4
0.2
0.0
WWTR1.AS1
Anova, p = 0.043
Low
Med
High
group

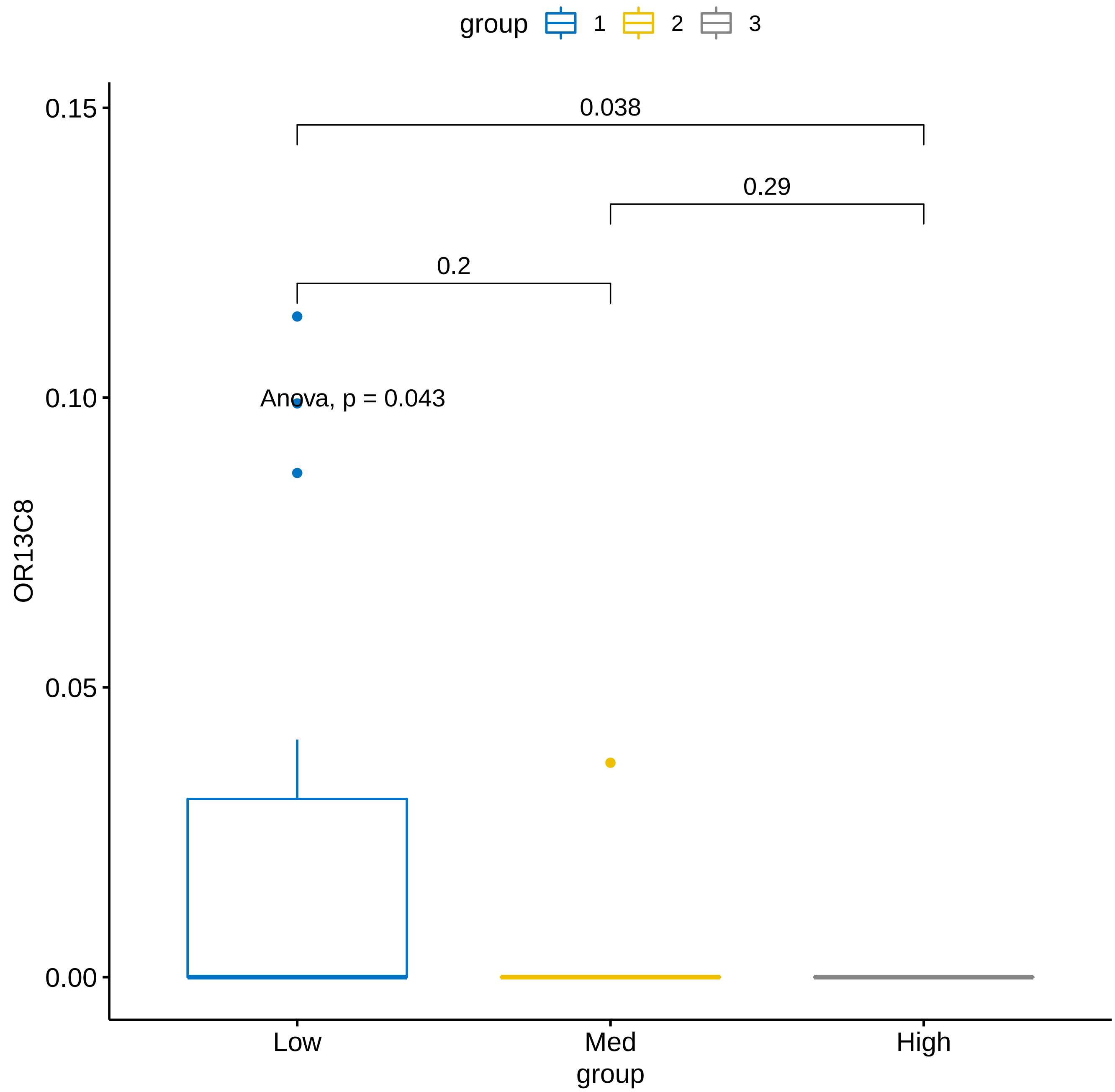

group 1 2 3
0.038
0.29
0.2
Anova, p = 0.043
0.15
0.10
0.05
0.00
OR13C8
Low
Med
High
group

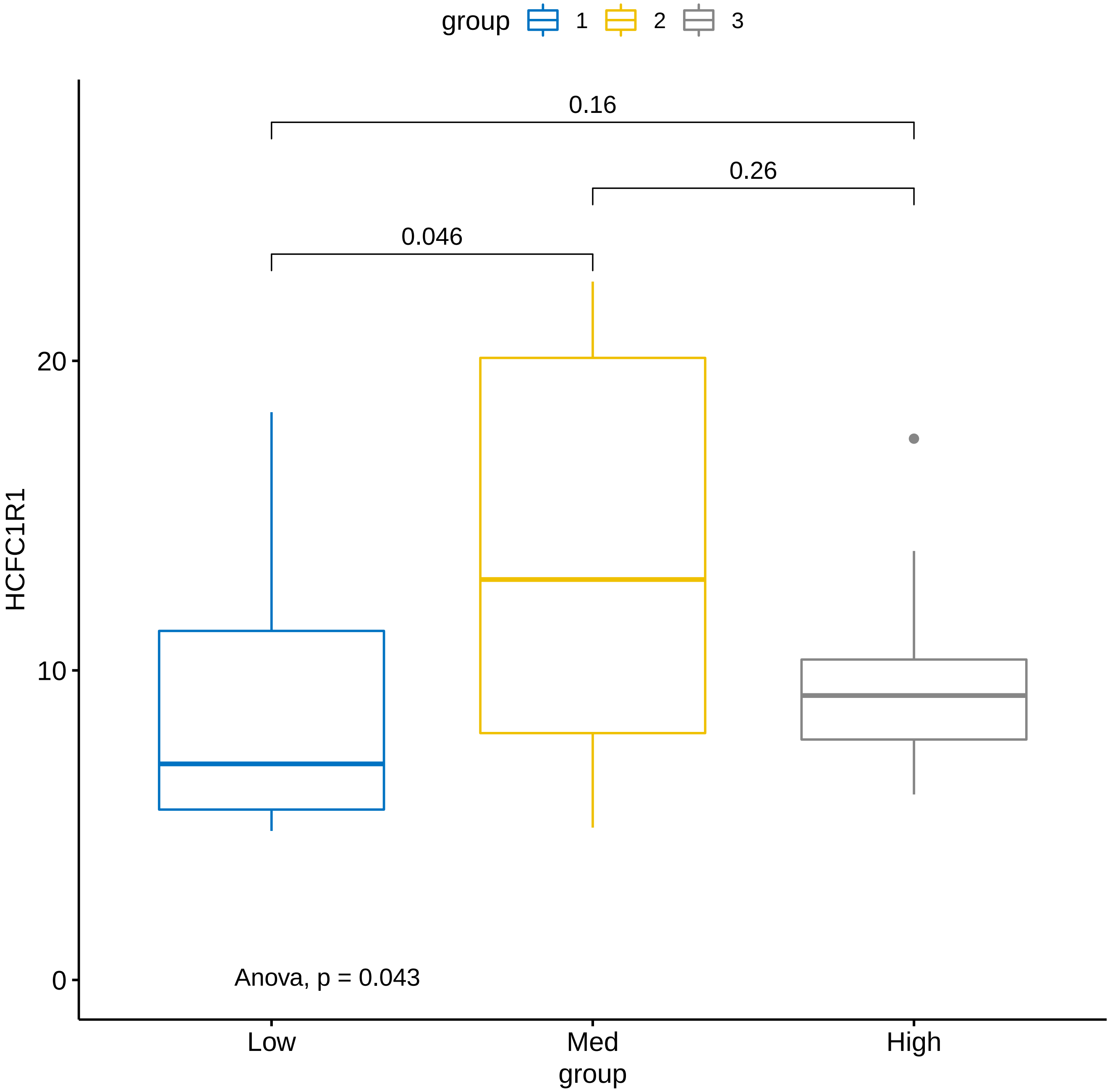
group
1
2
3
0.16
0.26
0.046
20
10
0
HCFC1R1
Anova, p = 0.043
Low
Med
High
group

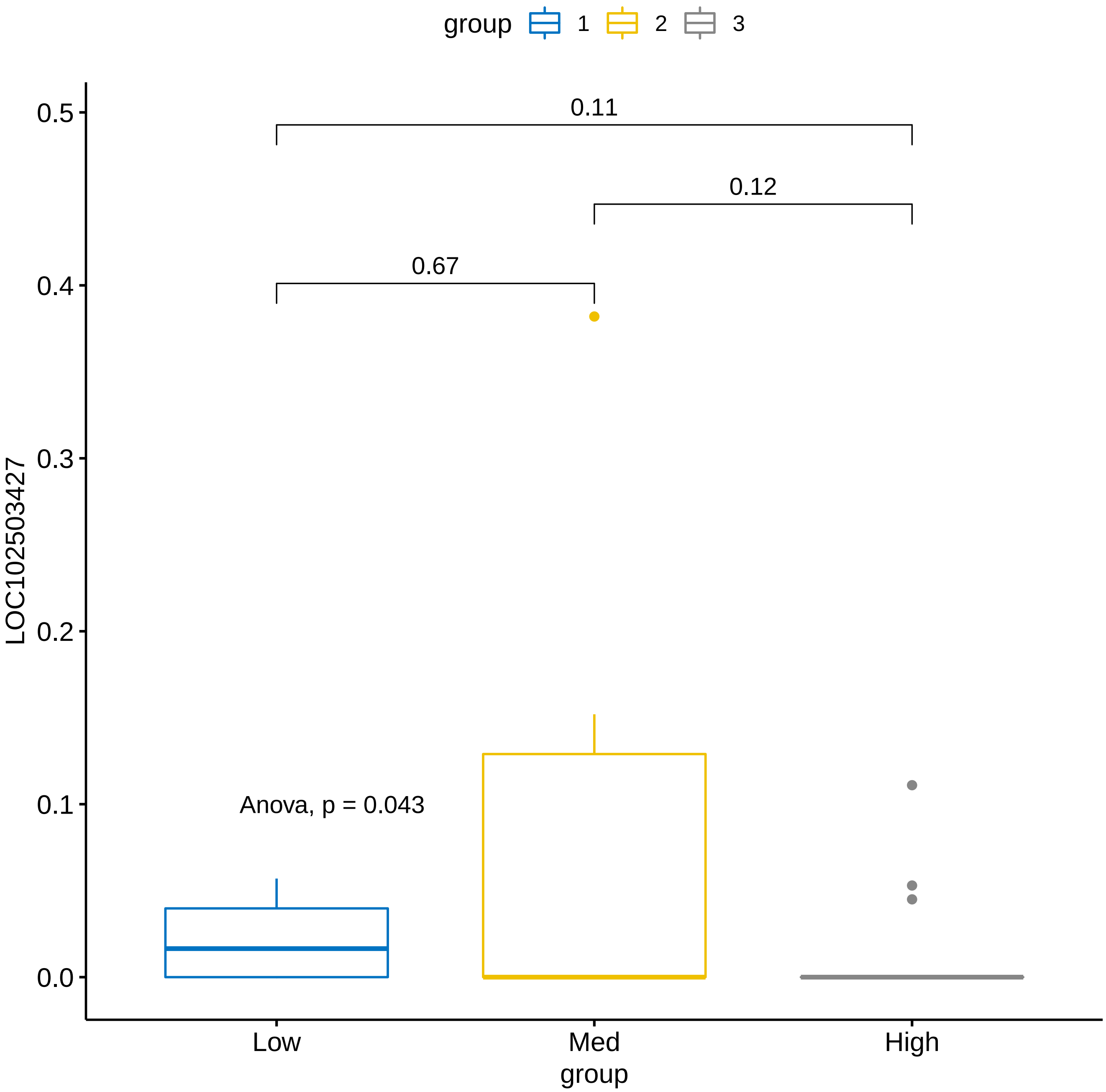
group 1 2 3
0.11
0.12
0.67
Anova, p = 0.043
LOC102503427
0.5
0.4
0.3
0.2
0.1
0.0
Low
Med
High
group

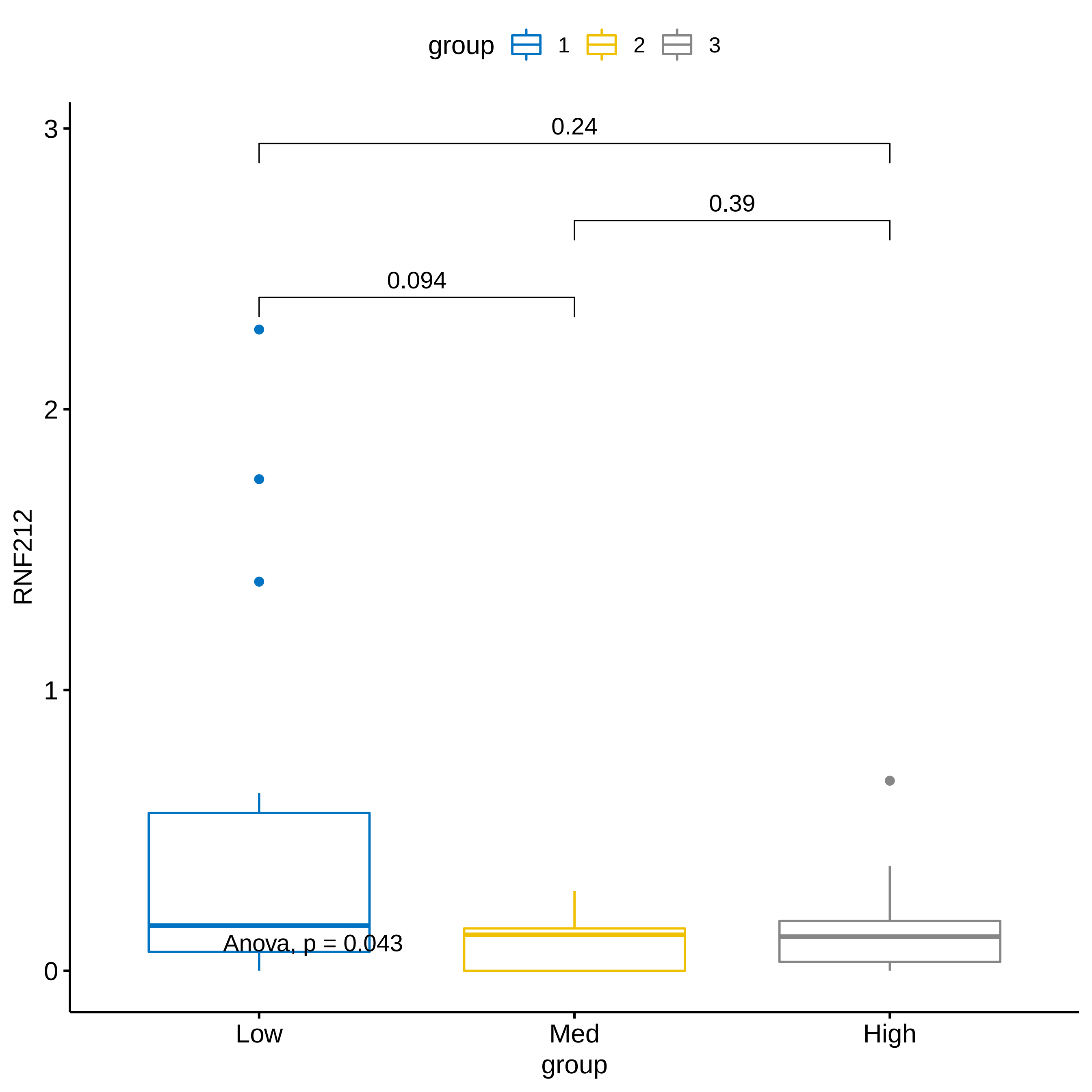
group
1
2
3
0.24
0.39
0.094
3
2
1
0
RNF212
Anova, p = 0.043
Low
Med
High
group

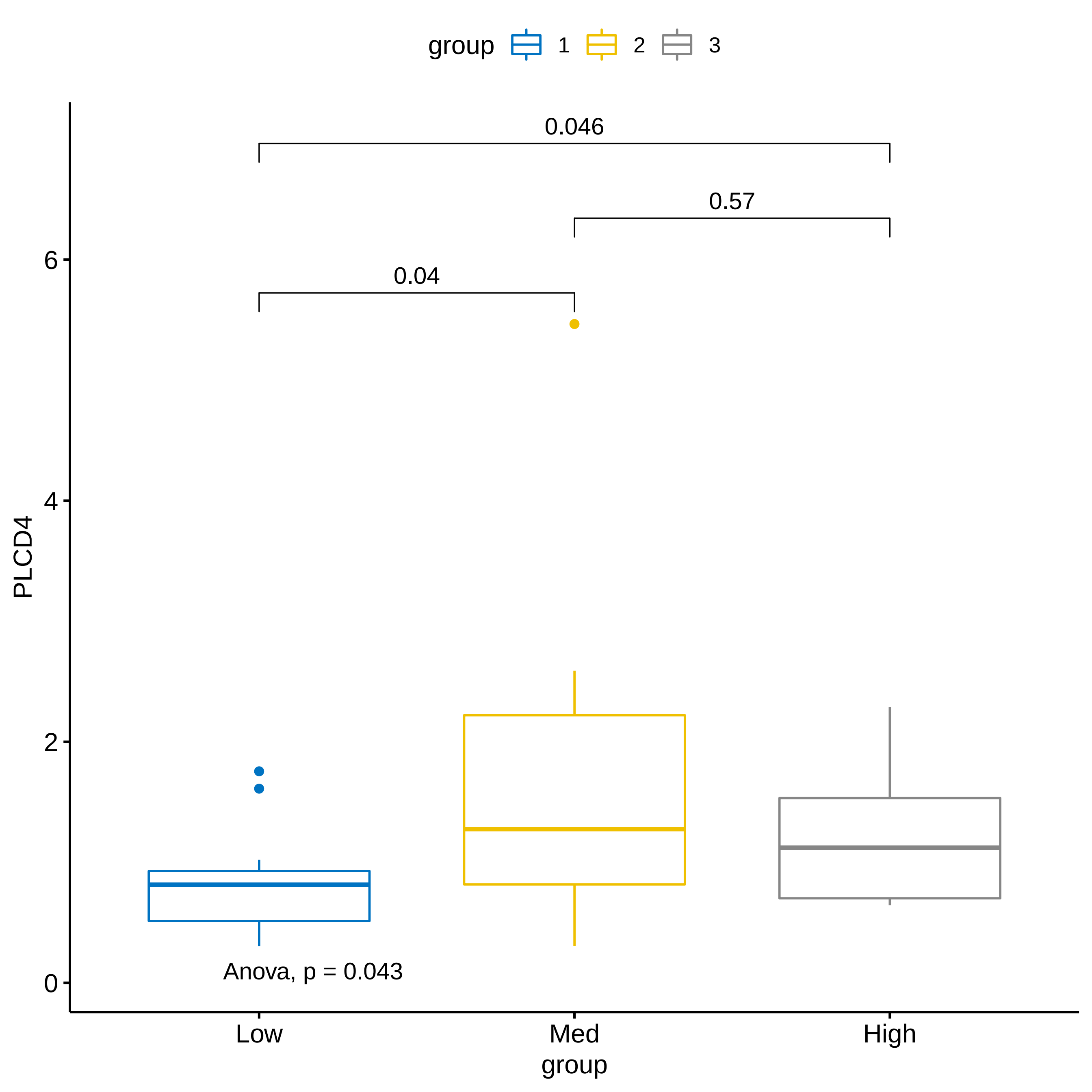
group
1
2
3
0.046
0.57
0.04
6
4
2
0
PLCD4
Anova, p = 0.043
Low
Med
High
group

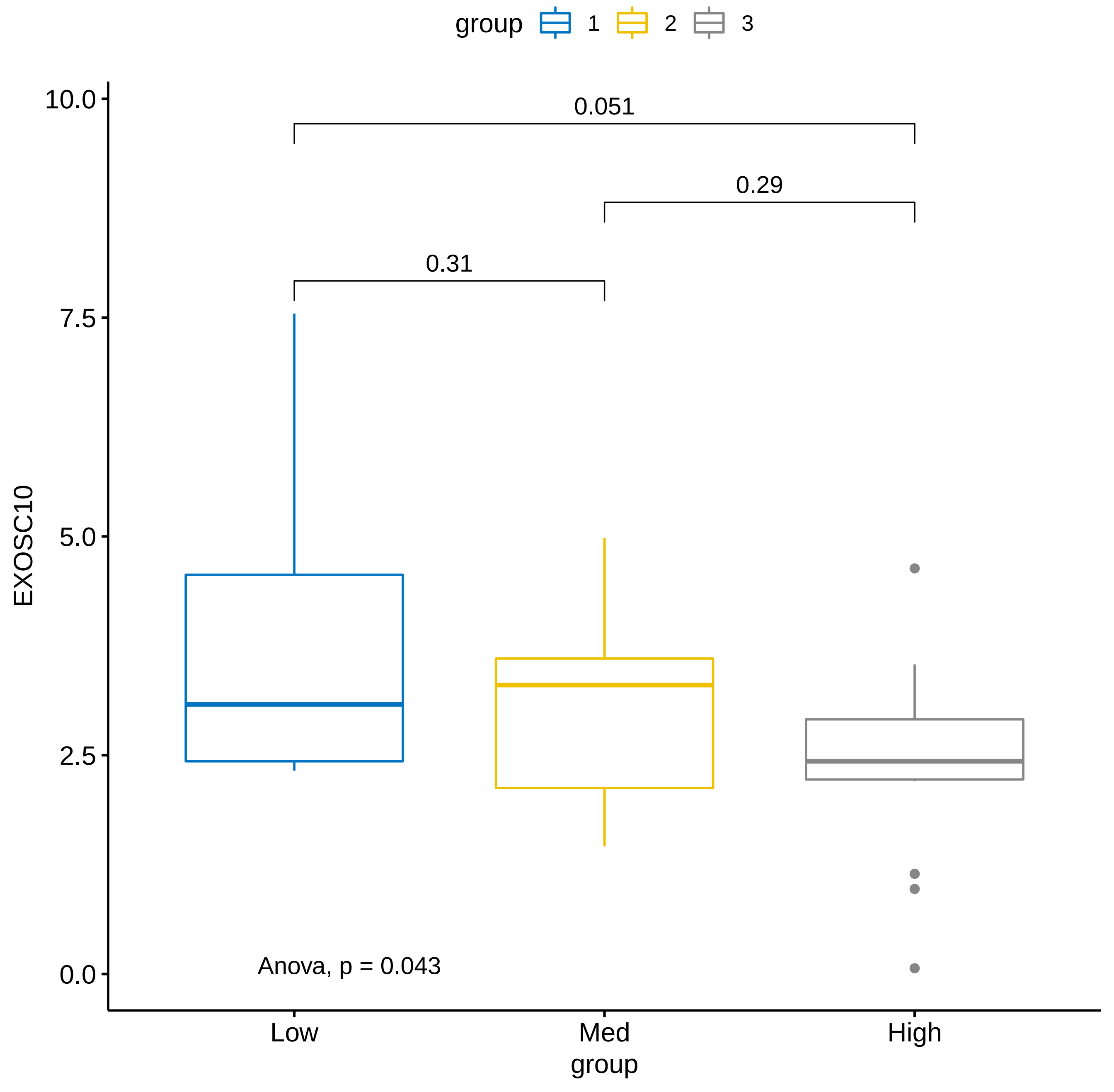
group
1
2
3
10.0
7.5
5.0
2.5
0.0
EXOSC10
0.051
0.29
0.31
Anova, p = 0.043
Low
Med
High
group

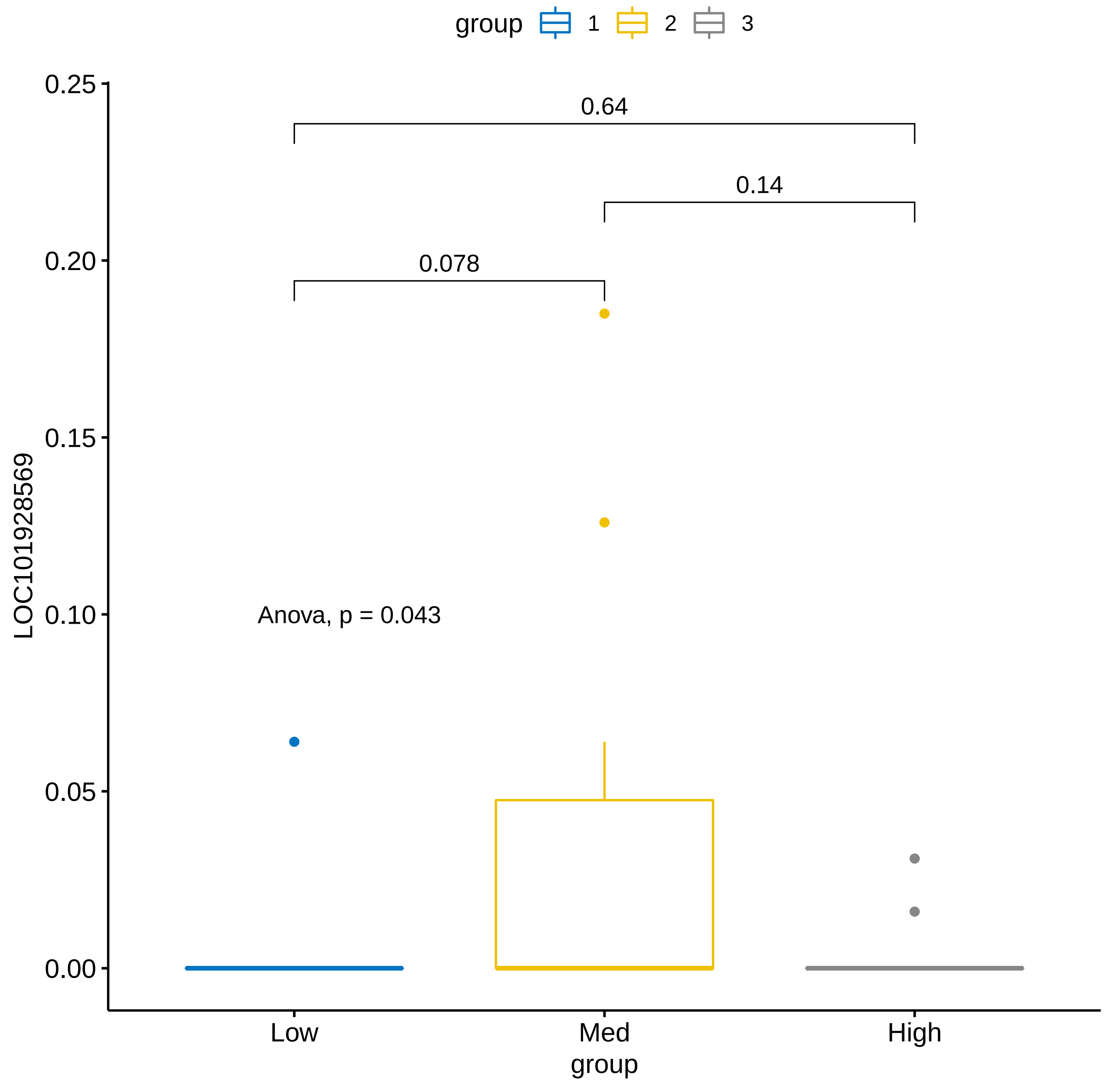
group 1 2 3
0.25
0.20
0.15
0.10
0.05
0.00
LOC101928569
0.64
0.14
0.078
Anova, p = 0.043
Low
Med
High
group

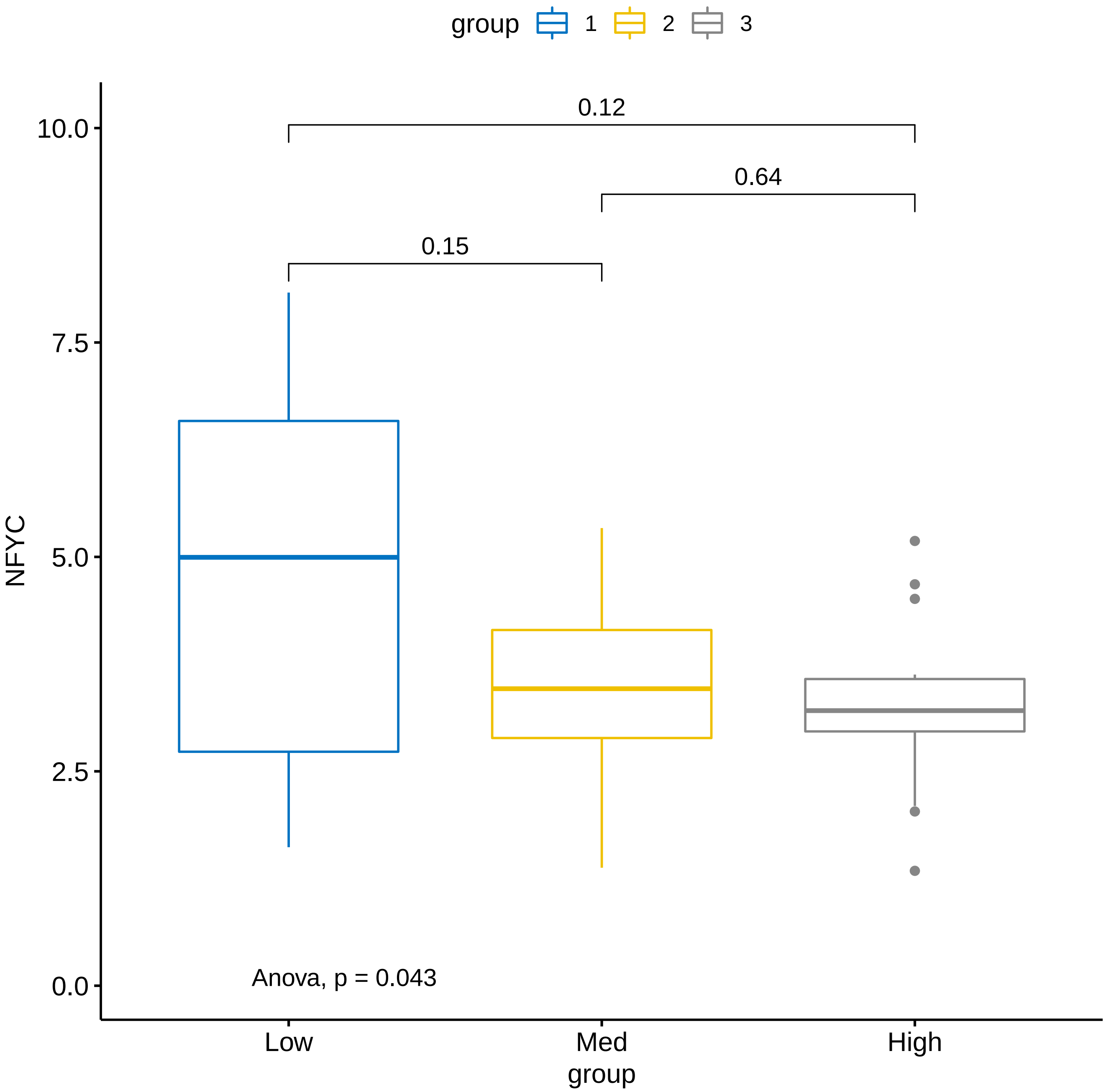

group 1 2 3
0.12
0.64
0.15
10.0
7.5
5.0
2.5
0.0
NFYC
Anova, p = 0.043
Low
Med
High
group

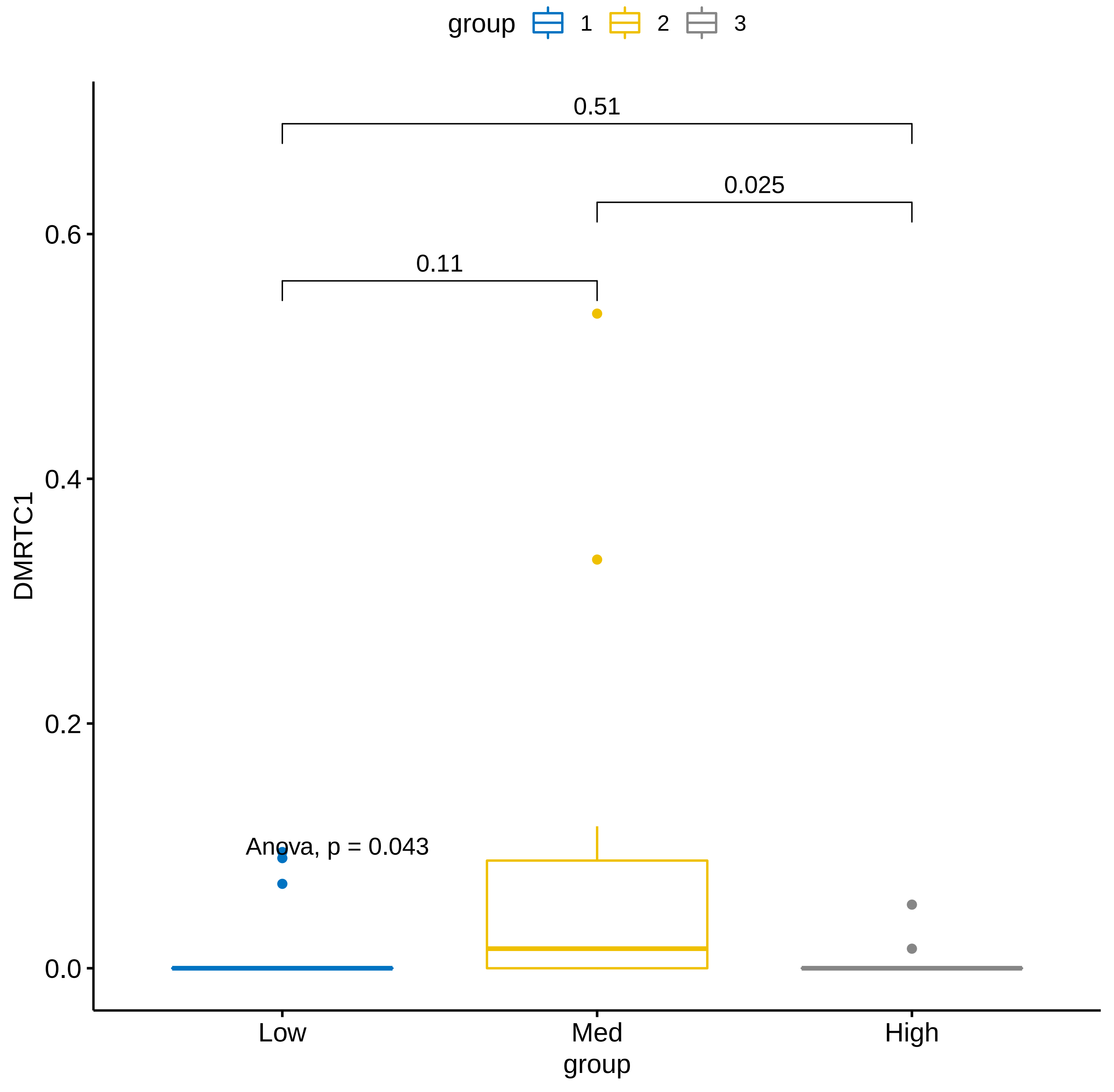

group 1 2 3
0.51
0.025
0.11
Anova, p = 0.043
0.6
0.4
0.2
0.0
DMRTC1
Low
Med
High
group

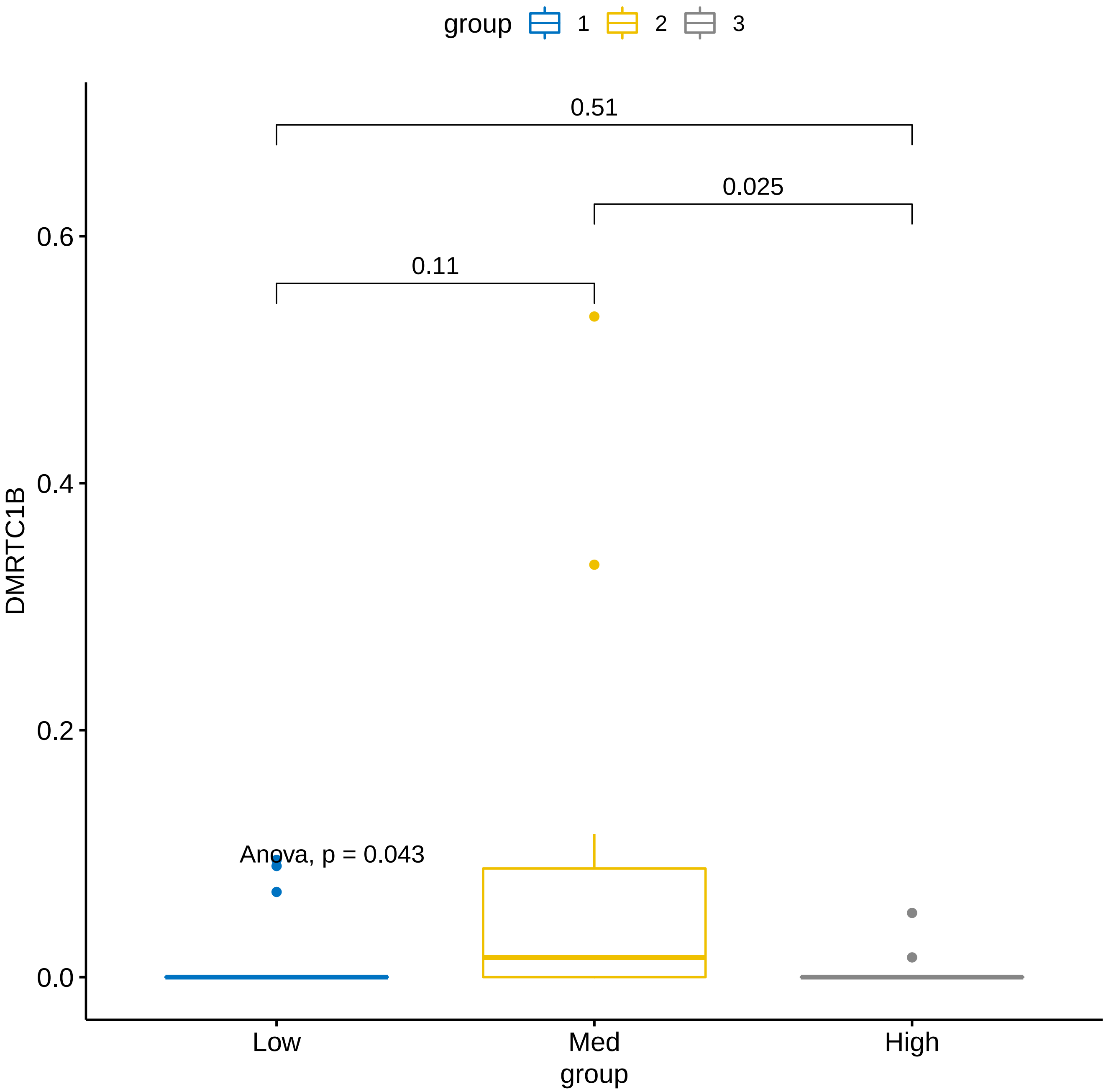
group
1
2
3
0.51
0.025
0.11
Anova, p = 0.043
0.6
0.4
0.2
0.0
DMRTC1B
Low
Med
High
group

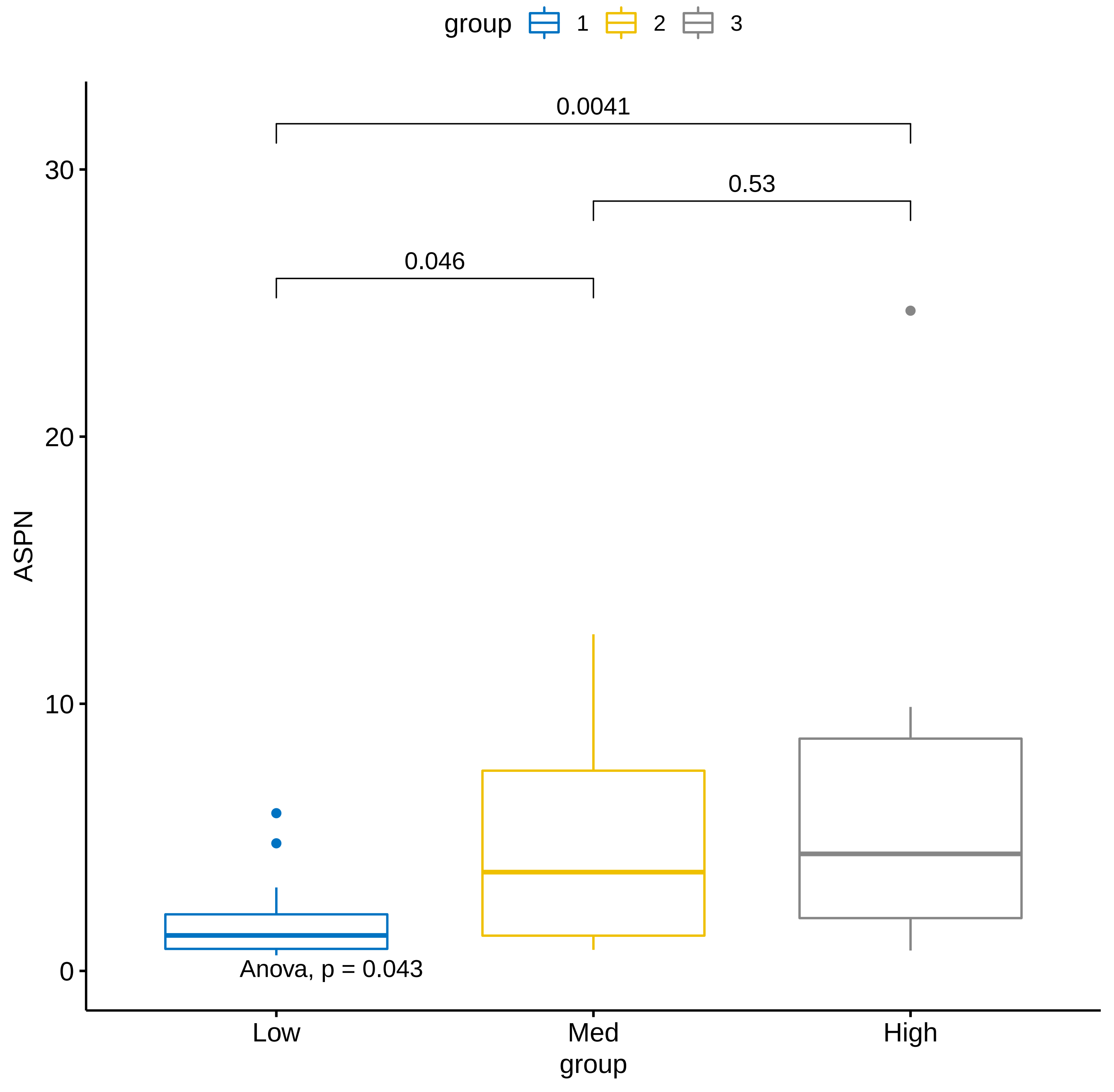
group
1
2
3
0.0041
0.53
0.046
30
20
10
0
ASPN
Anova, p = 0.043
Low
Med
High
group

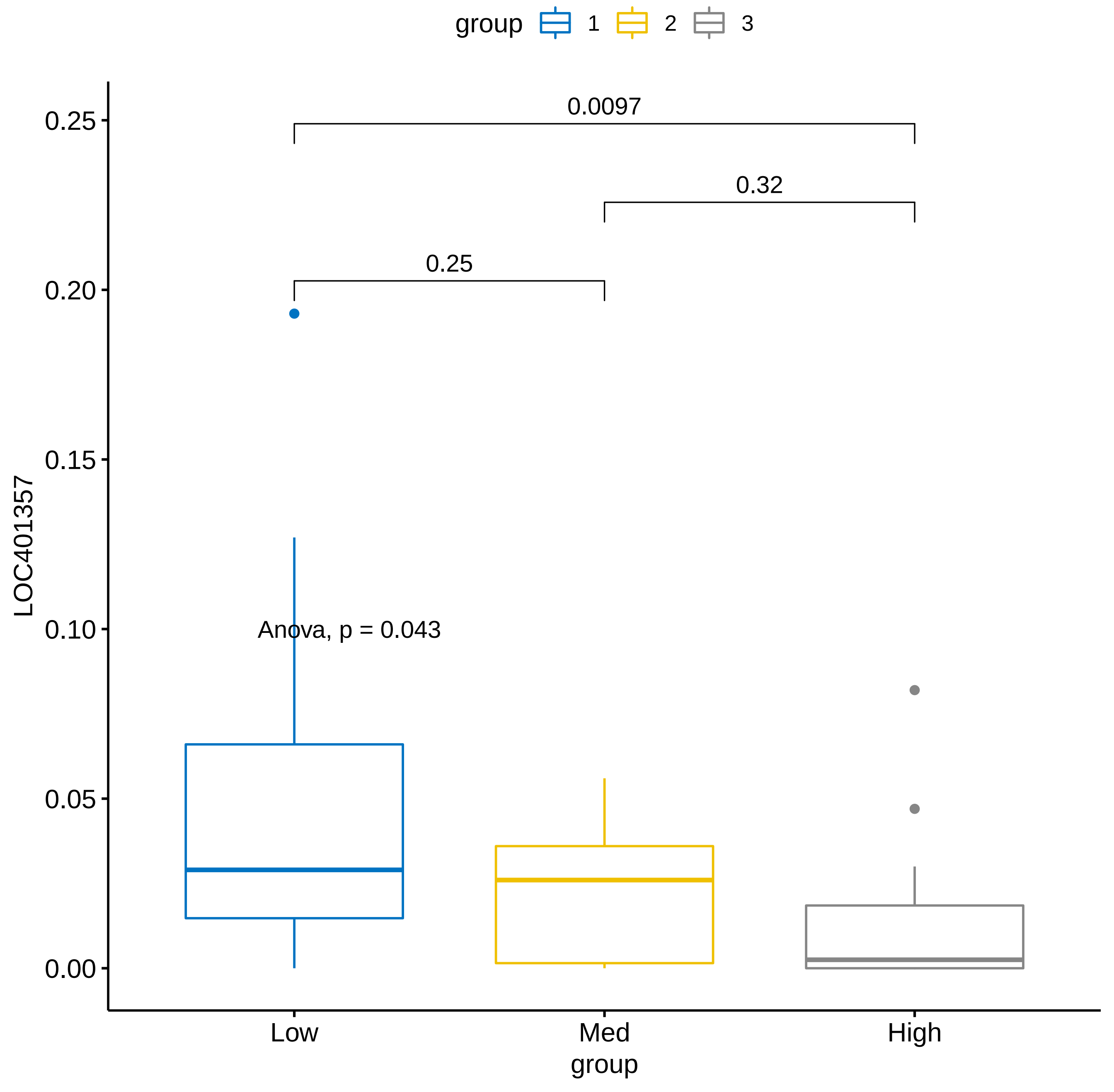
group
1
2
3
0.0097
0.32
0.25
Anova, p = 0.043
LOC401357
0.25
0.20
0.15
0.10
0.05
0.00
Low
Med
High
group

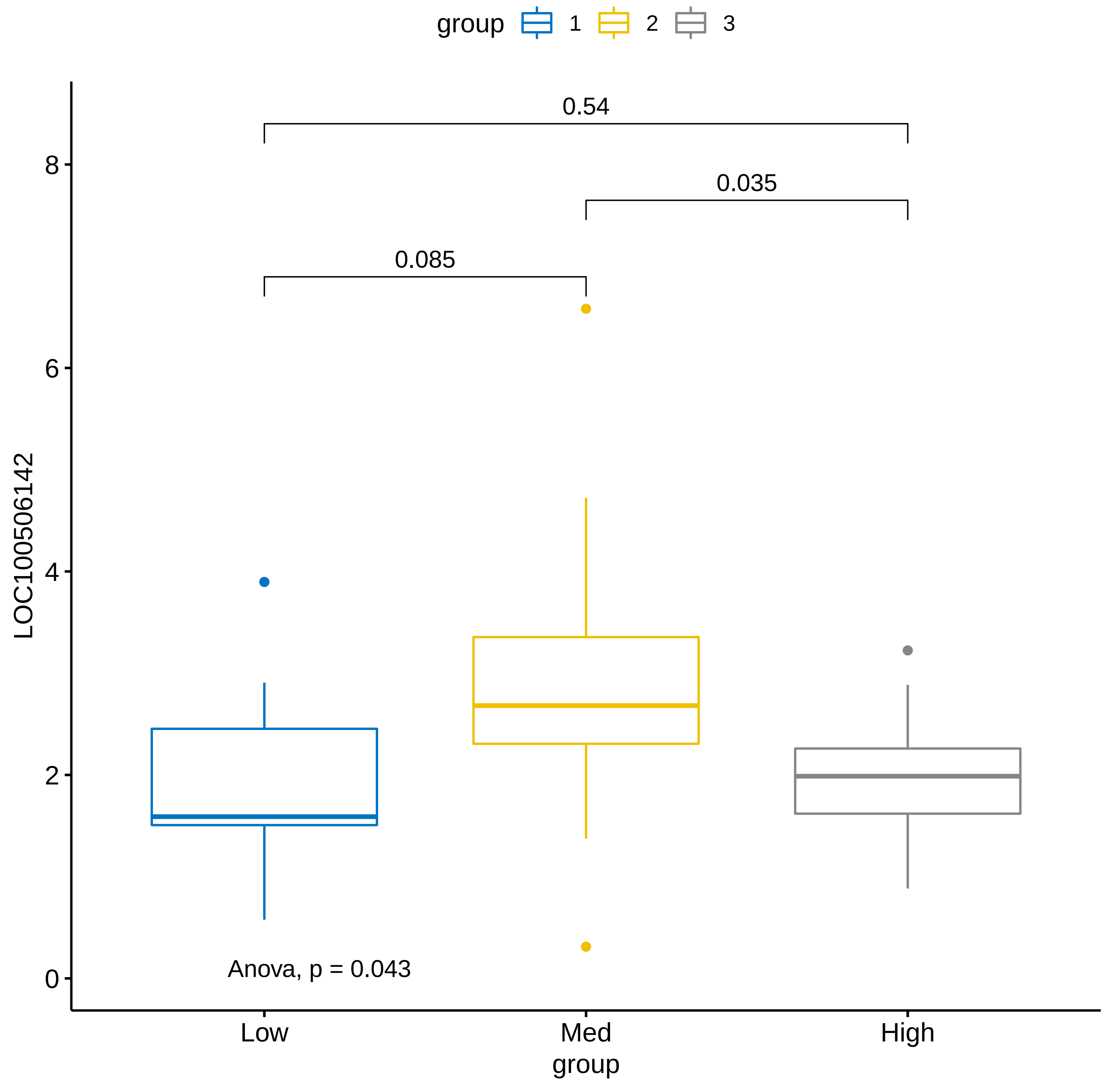
group 1 2 3
0.54
0.035
0.085
8
6
4
2
0
LOC100506142
Anova, p = 0.043
Low
Med
High
group

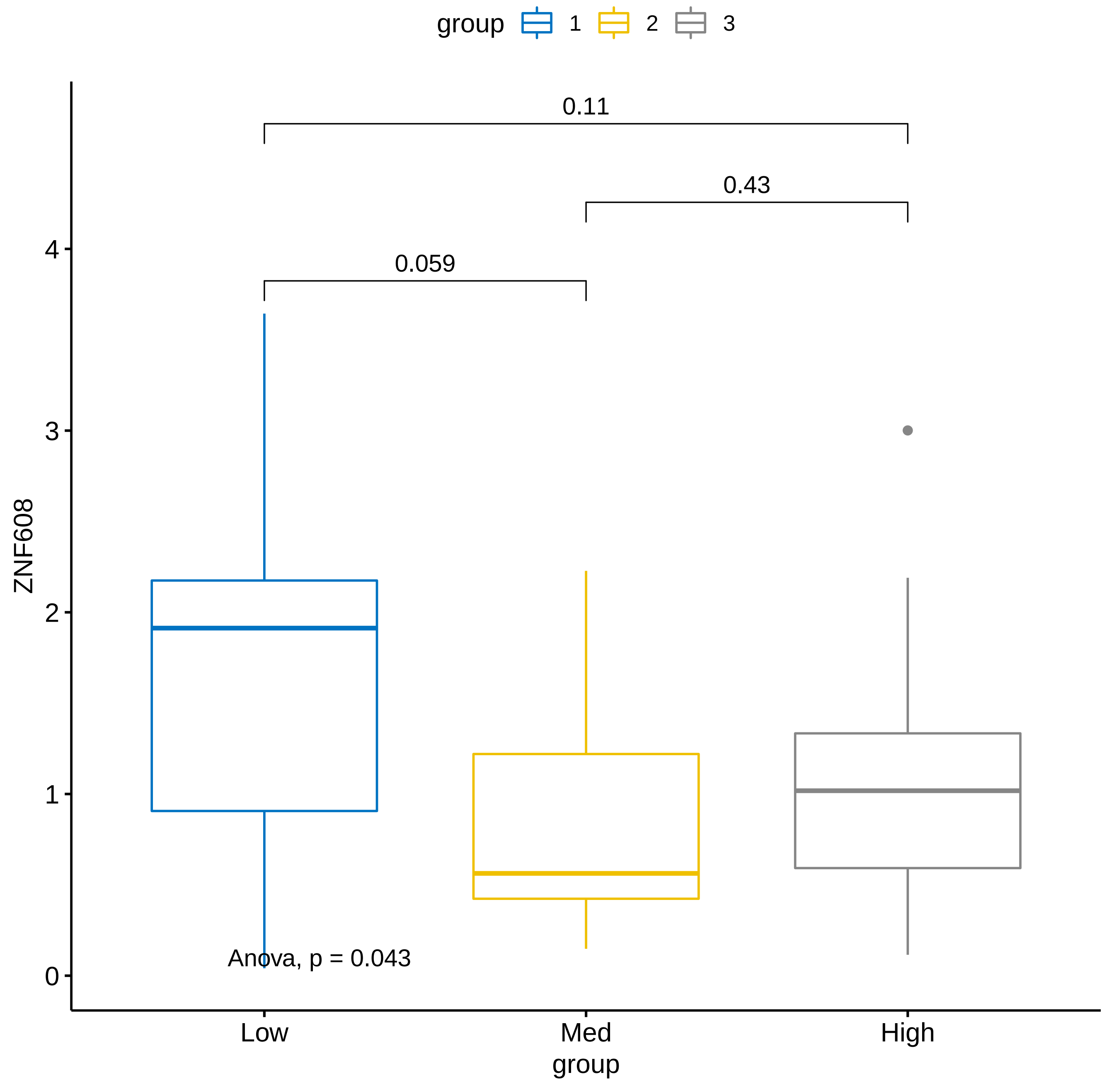

group 1 2 3
0.11
0.43
0.059
ZNF608
4
3
2
1
0
Anova, p = 0.043
Low
Med
High
group

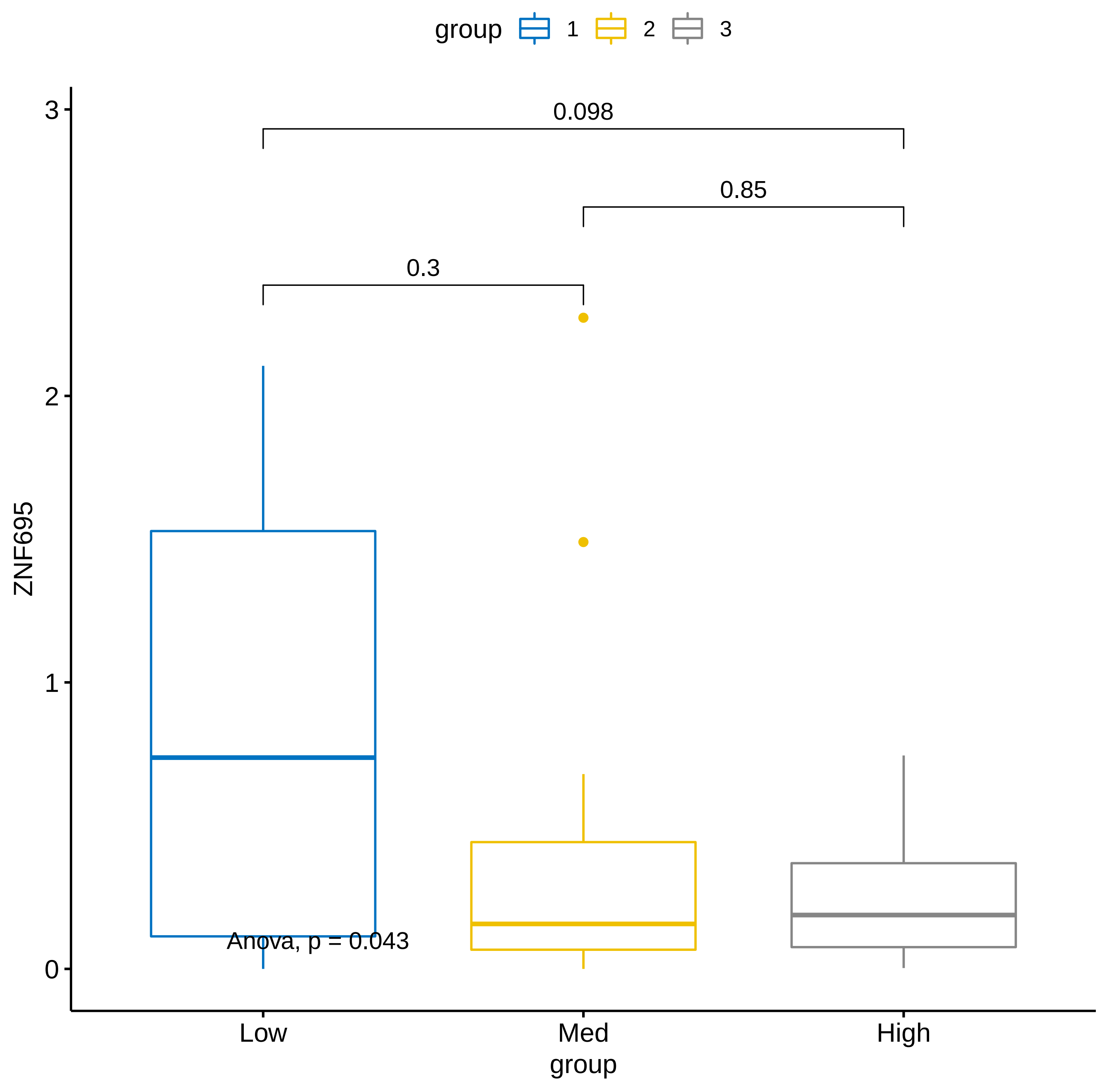
group
1
2
3
0.098
0.85
0.3
ZNF695
3
2
1
0
Anova, p = 0.043
Low
Med
High
group

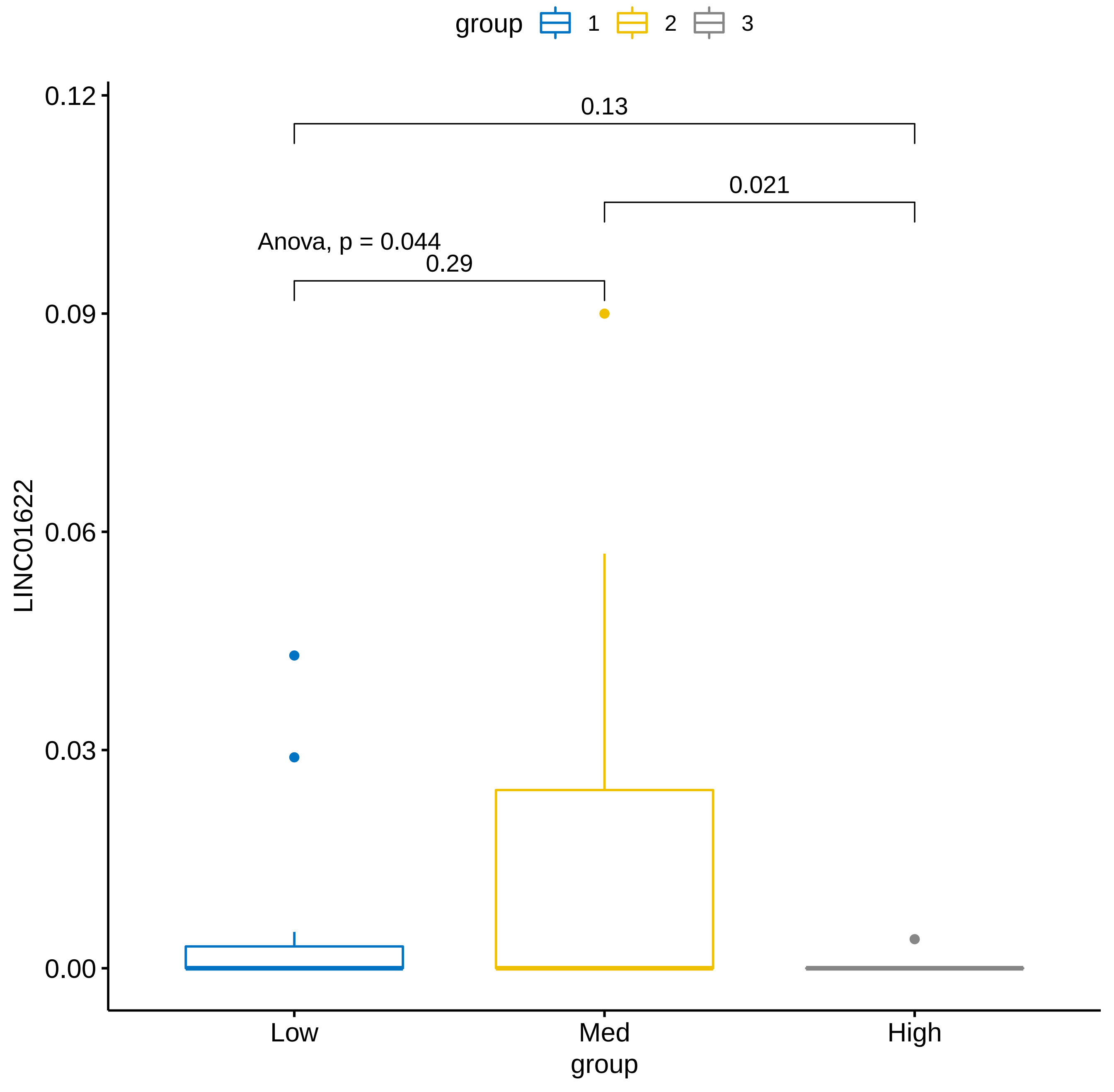

group
1
2
3
0.12
0.09
0.06
0.03
0.00
LINC01622
0.13
0.021
Anova, p = 0.044
0.29
Low
Med
High
group

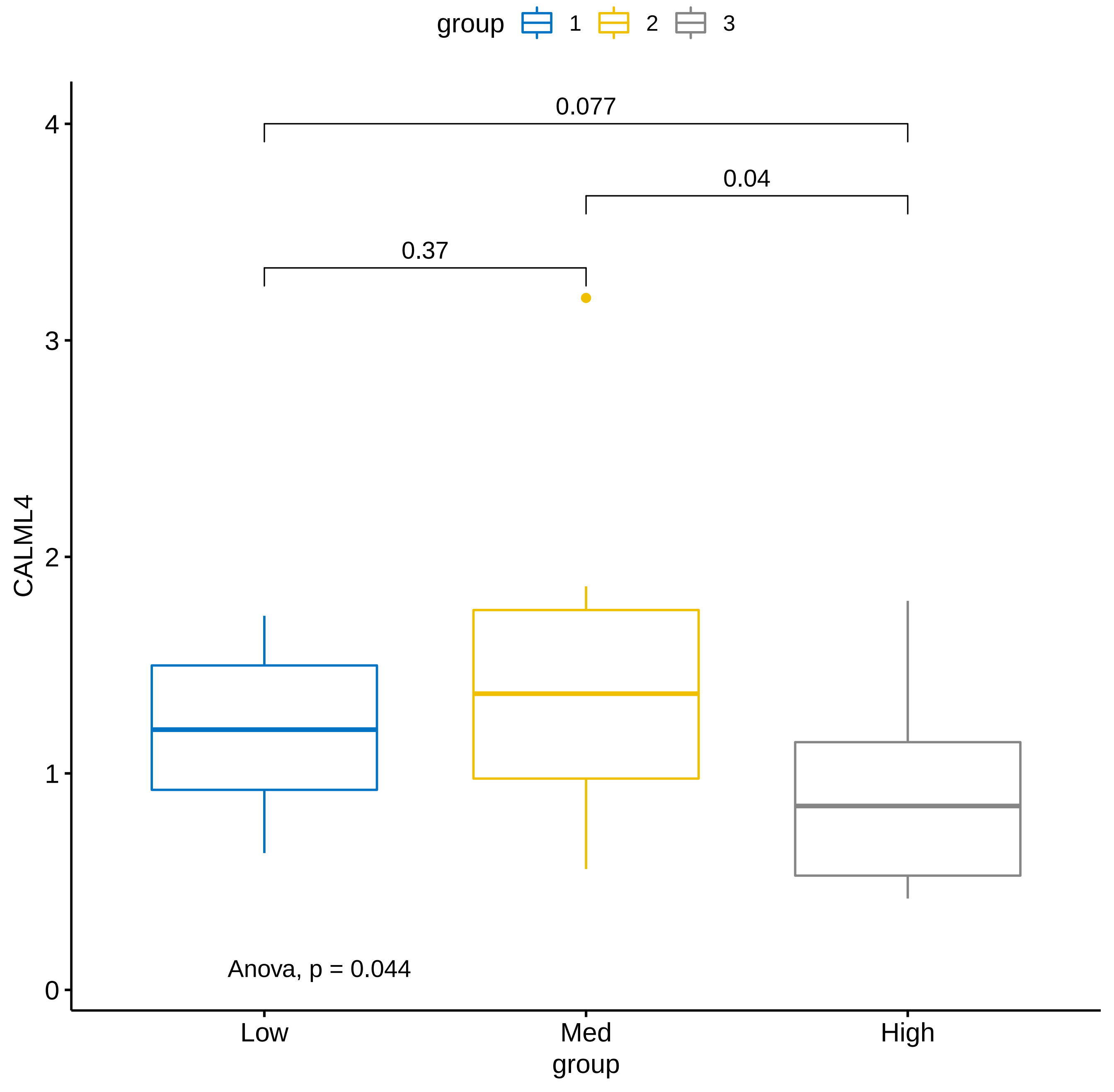

group 1 2 3
0.077
0.04
0.37
4
3
2
1
0
CALML4
Anova, p = 0.044
Low
Med
High
group

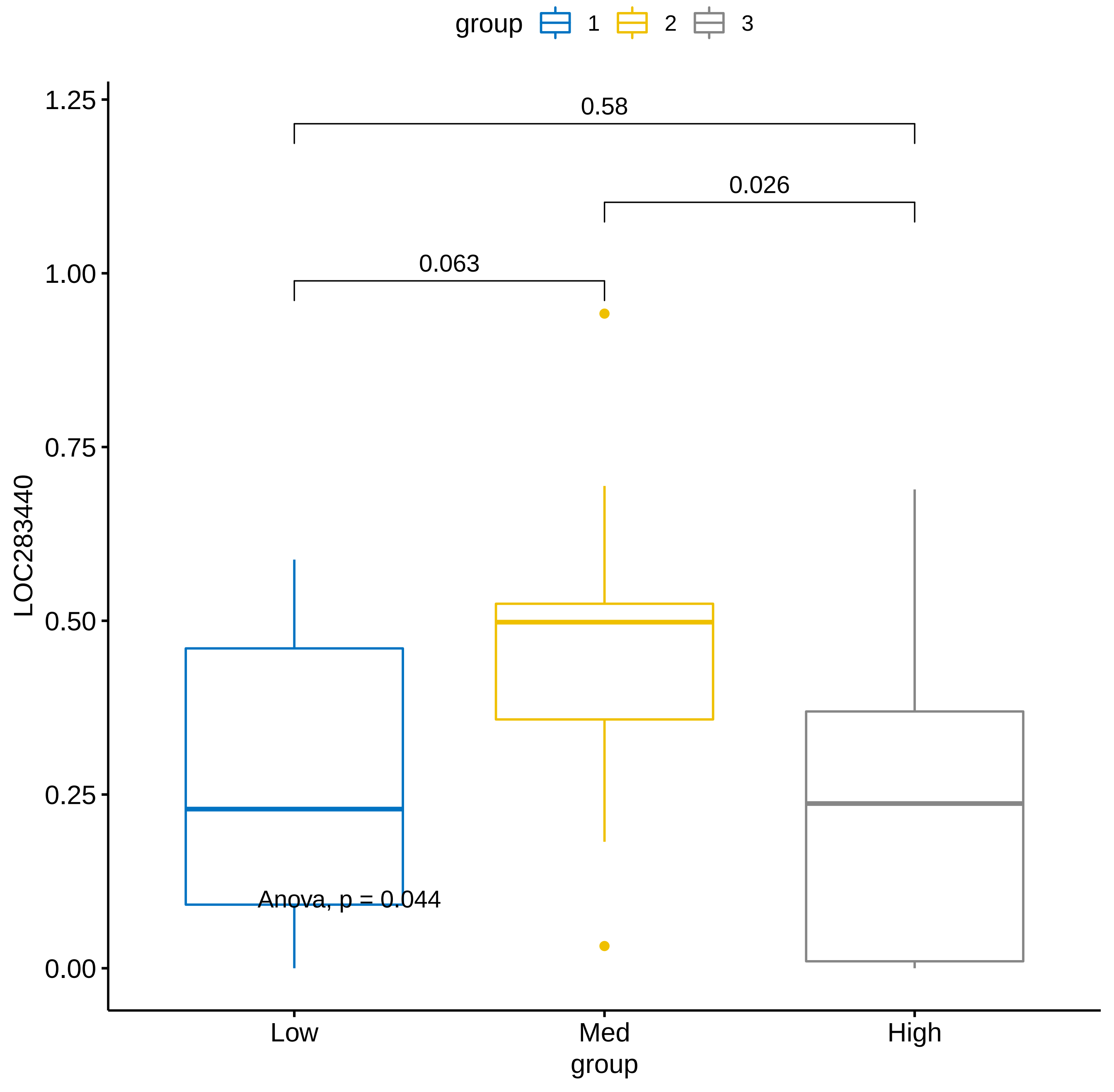
group 1 2 3
1.25
1.00
0.75
0.50
0.25
0.00
LOC283440
0.58
0.026
0.063
Anova, p = 0.044
Low
Med
High
group

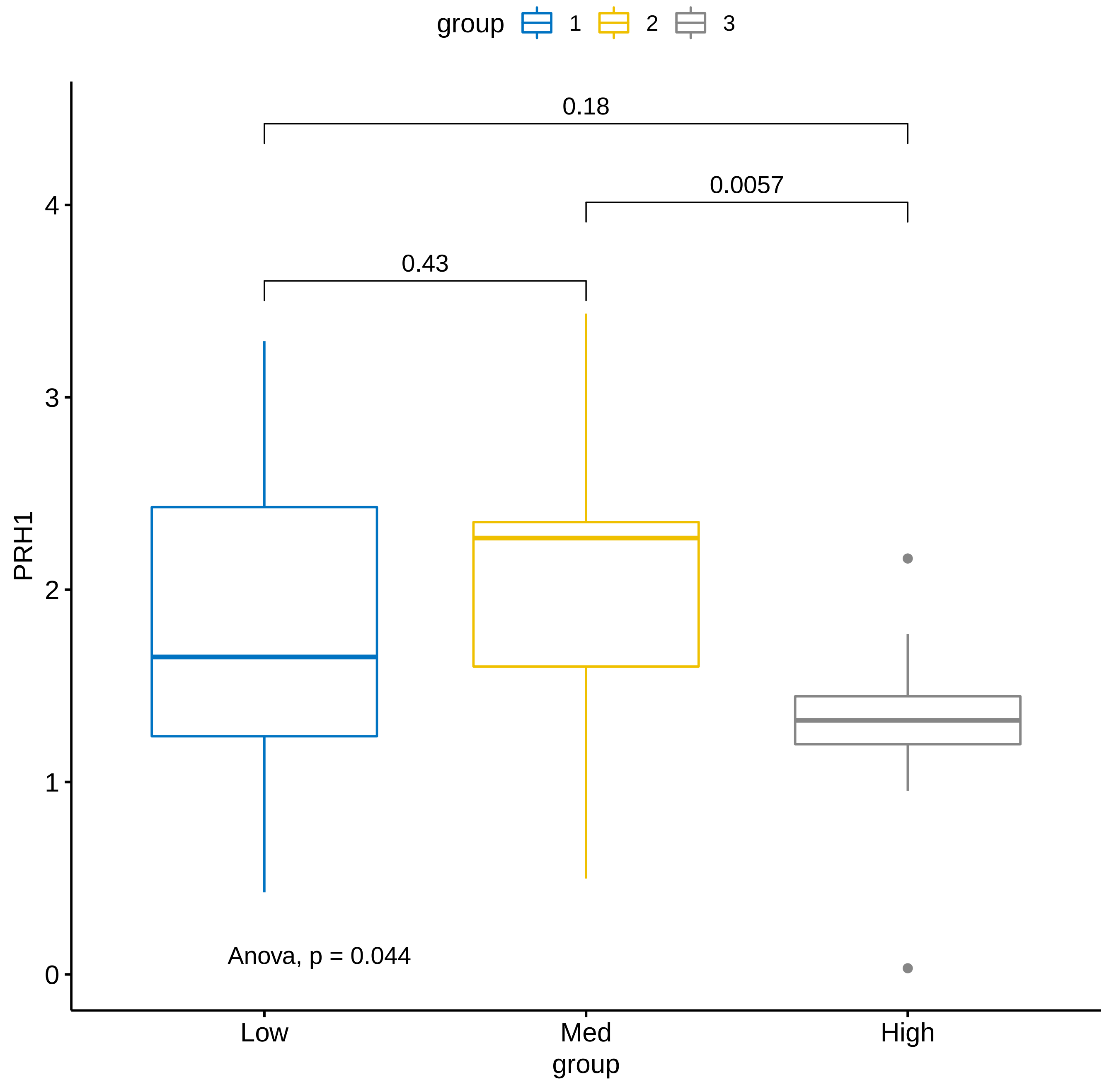

group
1
2
3
0.18
0.0057
0.43
Anova, p = 0.044
PRH1
0
1
2
3
4
Low
Med
High
group

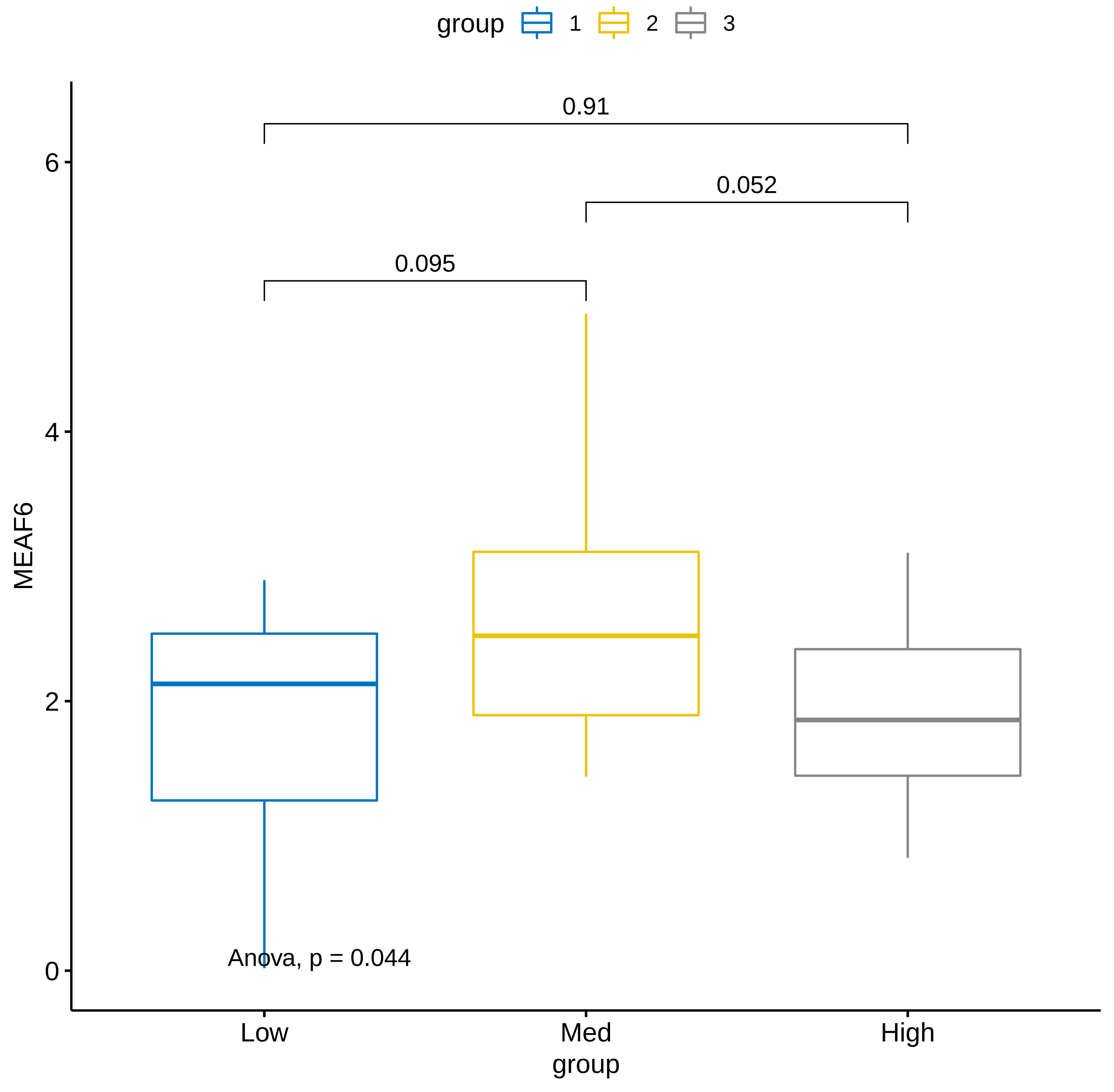
group
1
2
3
0.91
0.052
0.095
6
4
2
0
MEAF6
Anova, p = 0.044
Low
Med
High
group

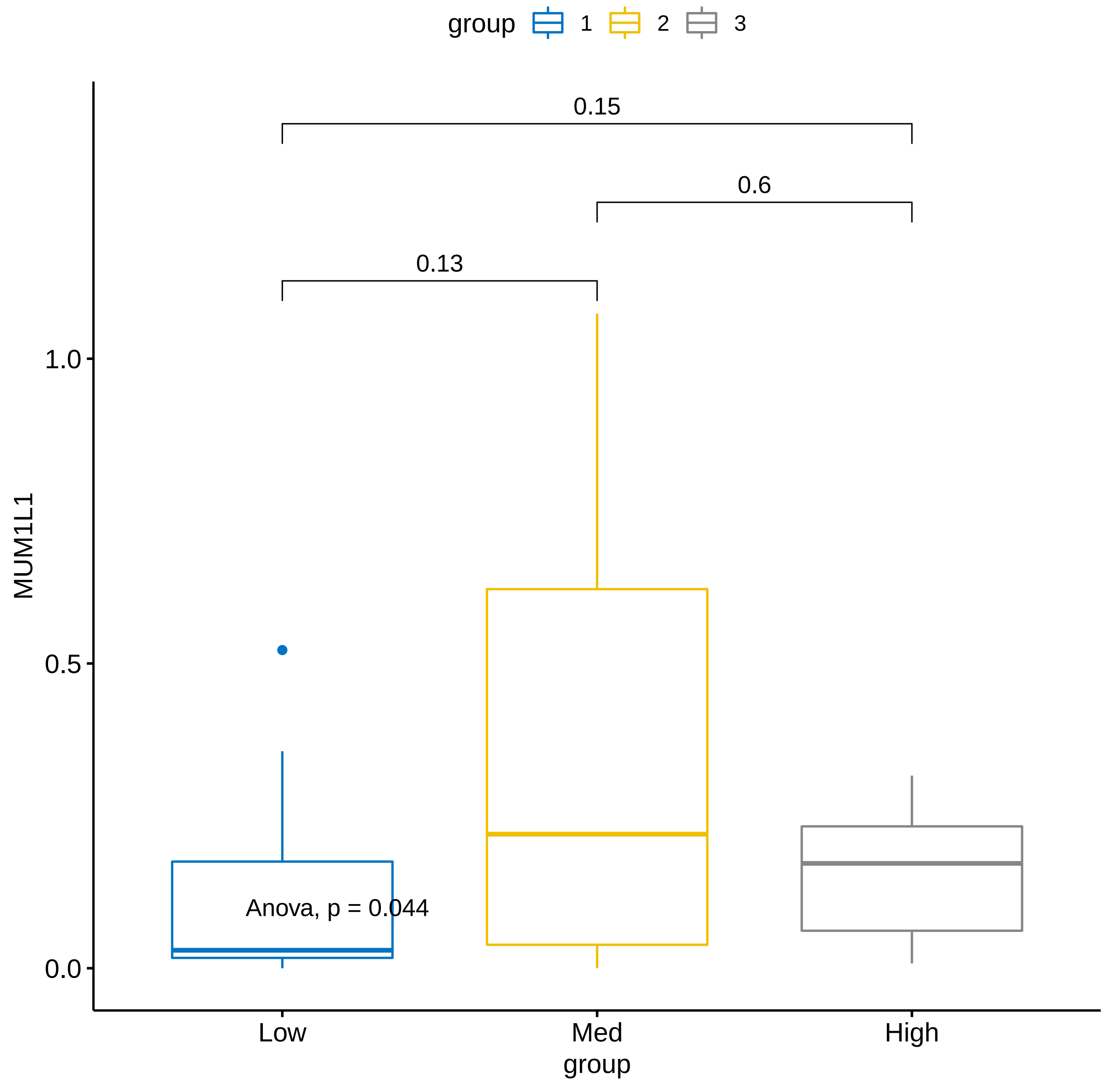
group 1 2 3
0.15
0.6
0.13
1.0
0.5
0.0
MUM1L1
Anova, p = 0.044
Low
Med
High
group

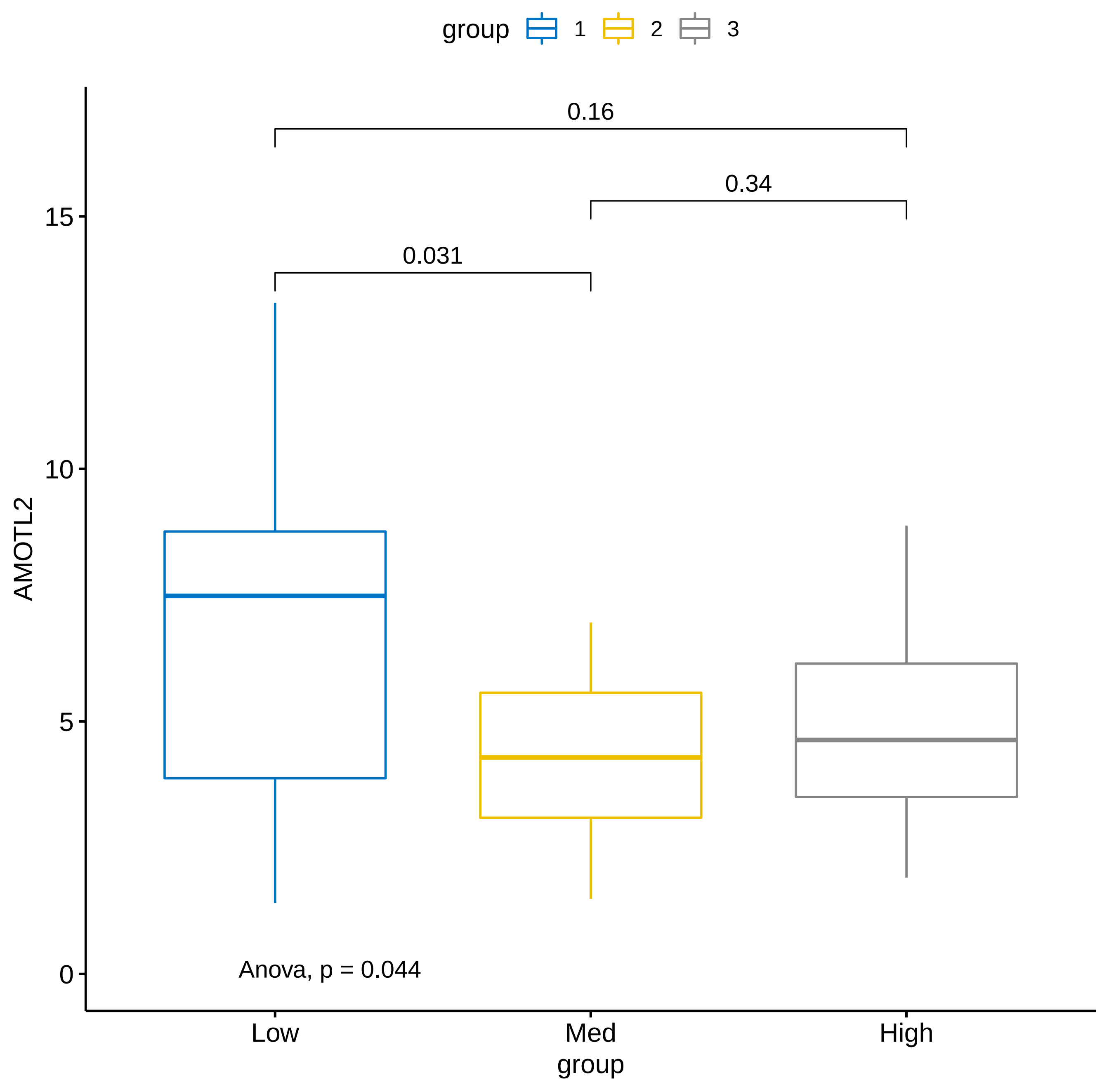

group 1 2 3
0.16
0.34
0.031
15
10
5
0
AMOTL2
Anova, p = 0.044
Low
Med
High
group

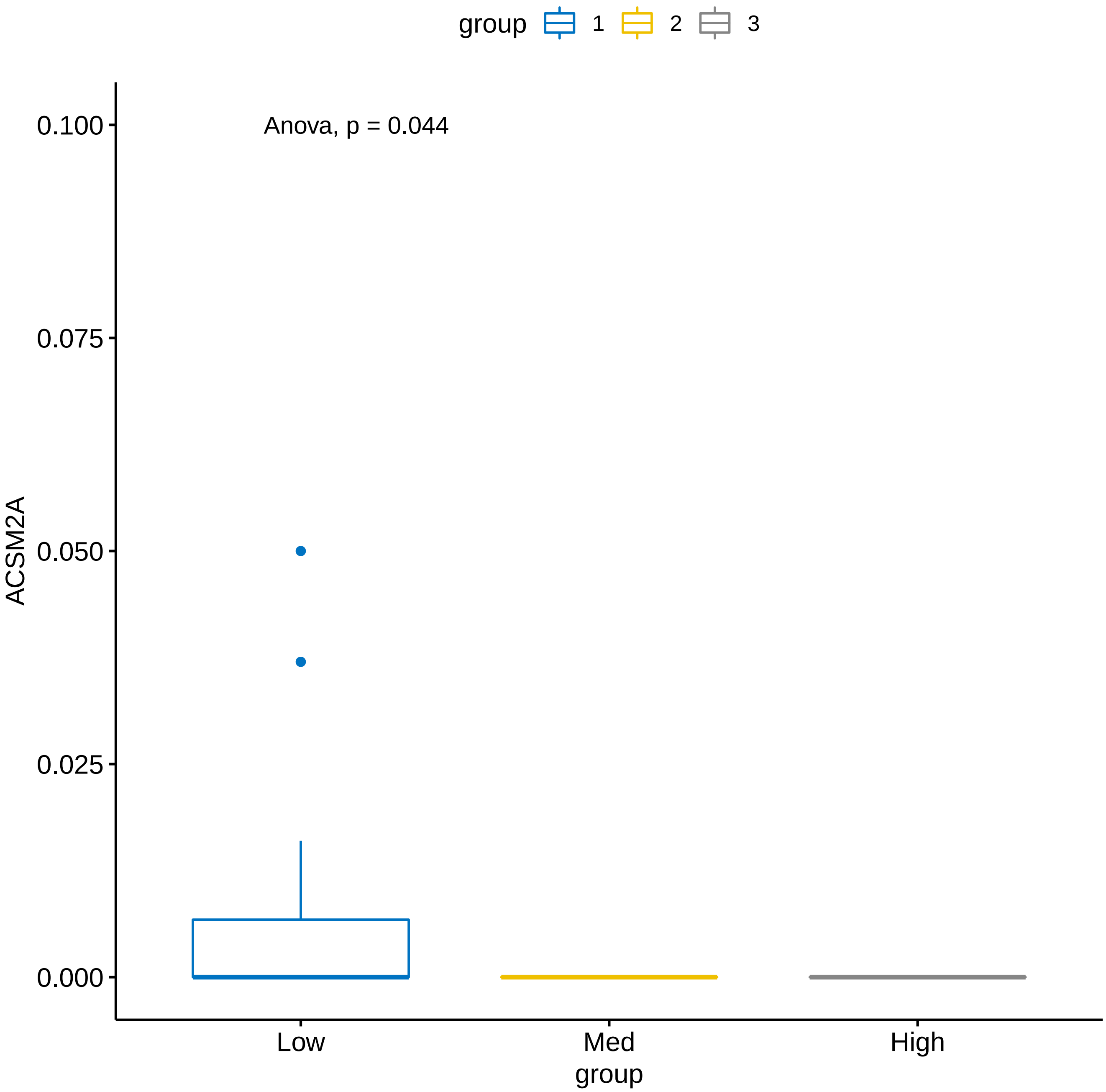
group
1
2
3
Anova, p = 0.044
ACSM2A
0.100
0.075
0.050
0.025
0.000
Low
Med
High
group

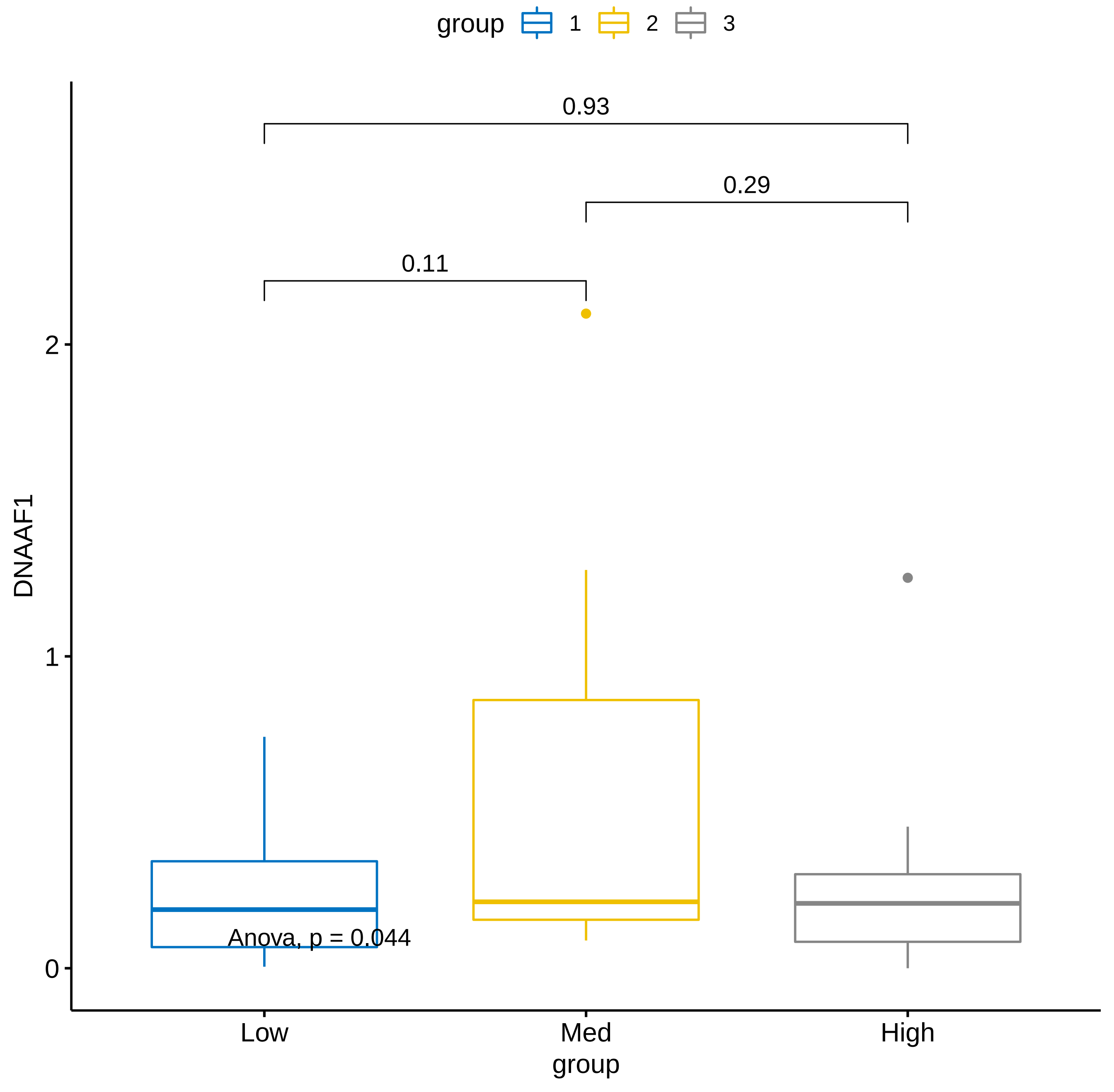

group
1
2
3
0.93
0.29
0.11
2
1
0
DNAAF1
Anova, p = 0.044
Low
Med
High
group

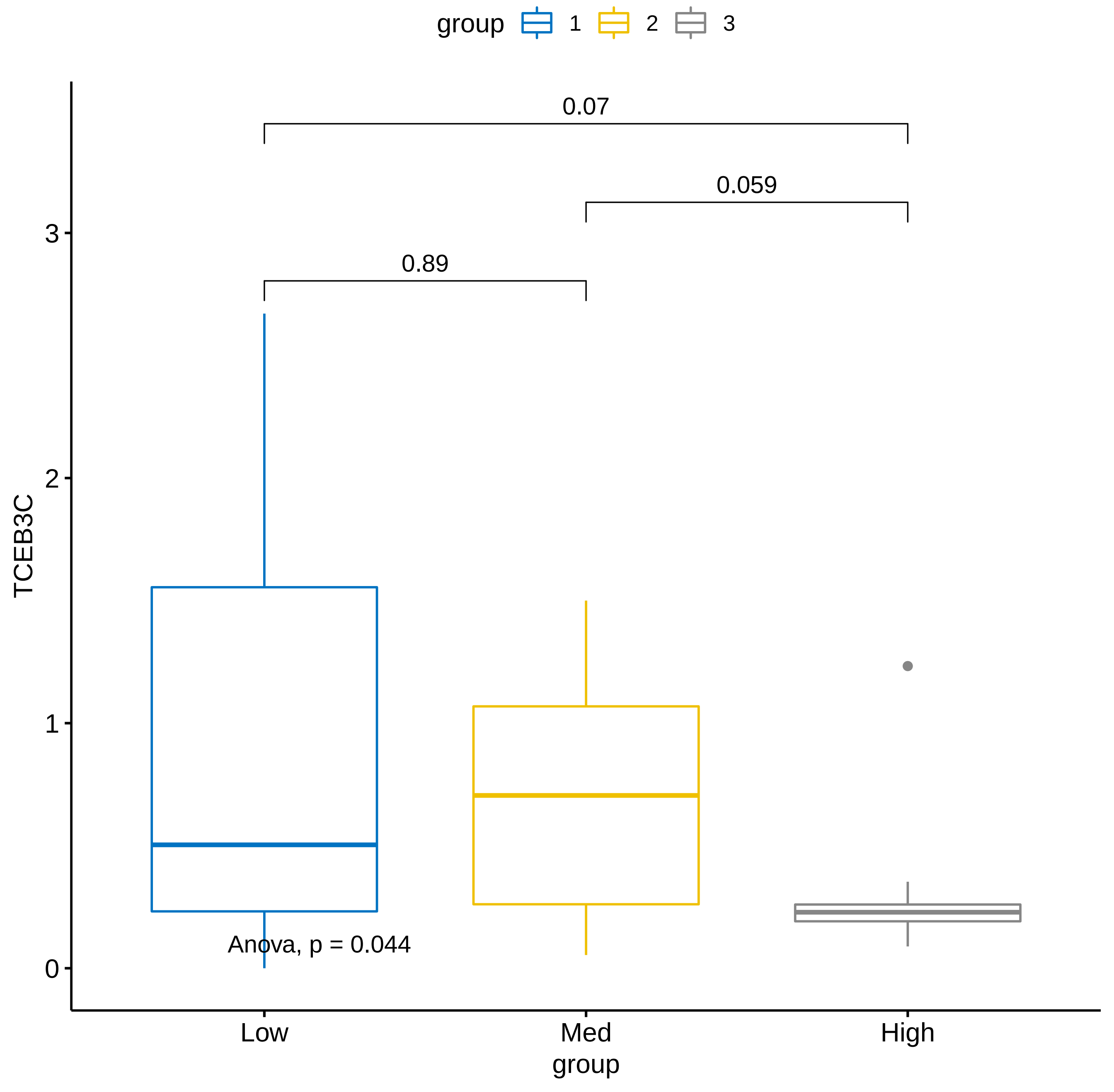

group 1 2 3
0.07
0.059
0.89
TCEB3C
3
2
1
0
Anova, p = 0.044
Low
Med
High
group

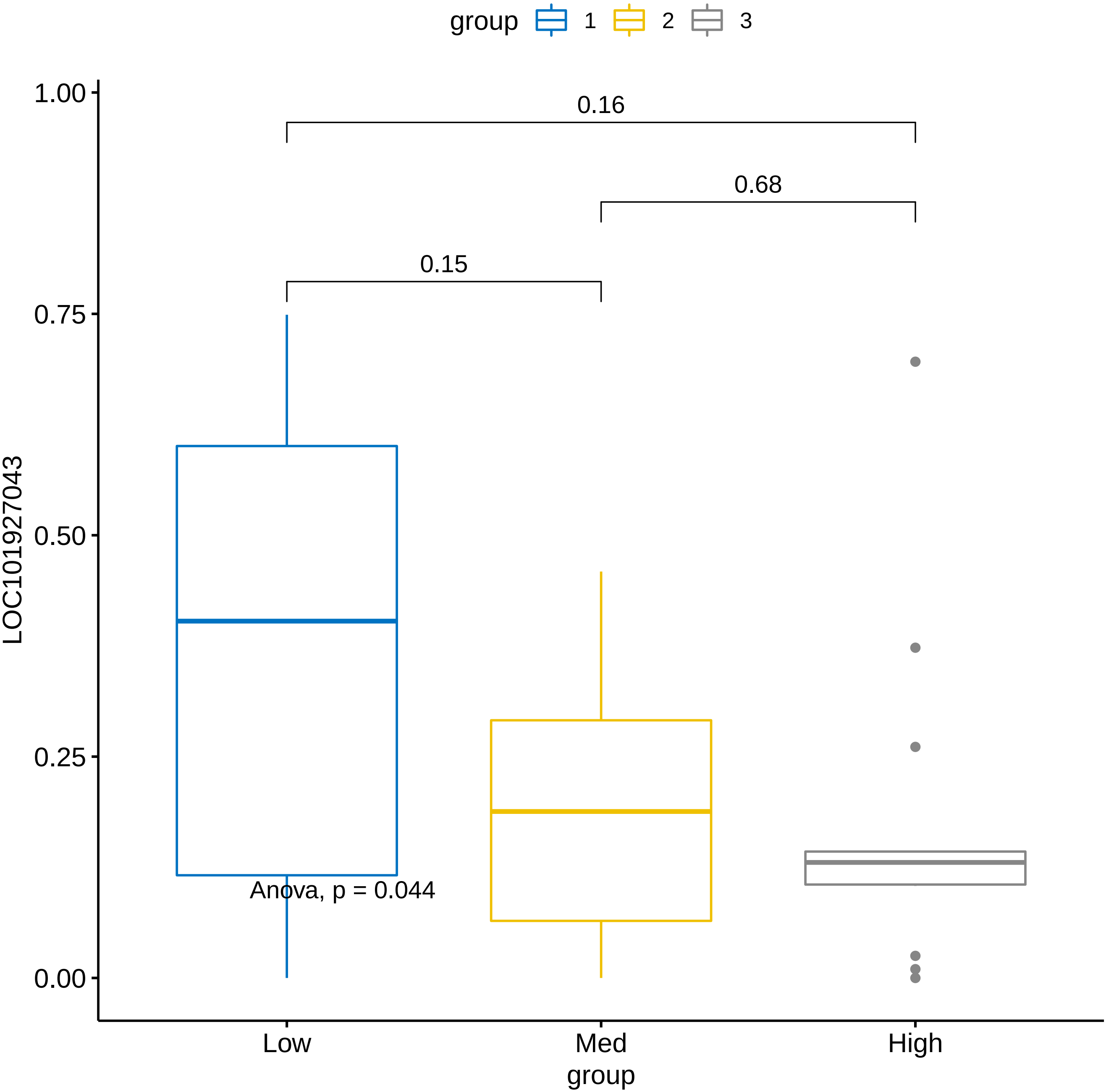
group
1
2
3
1.00
0.75
0.50
0.25
0.00
LOC101927043
0.16
0.68
0.15
Anova, p = 0.044
Low
Med
High
group

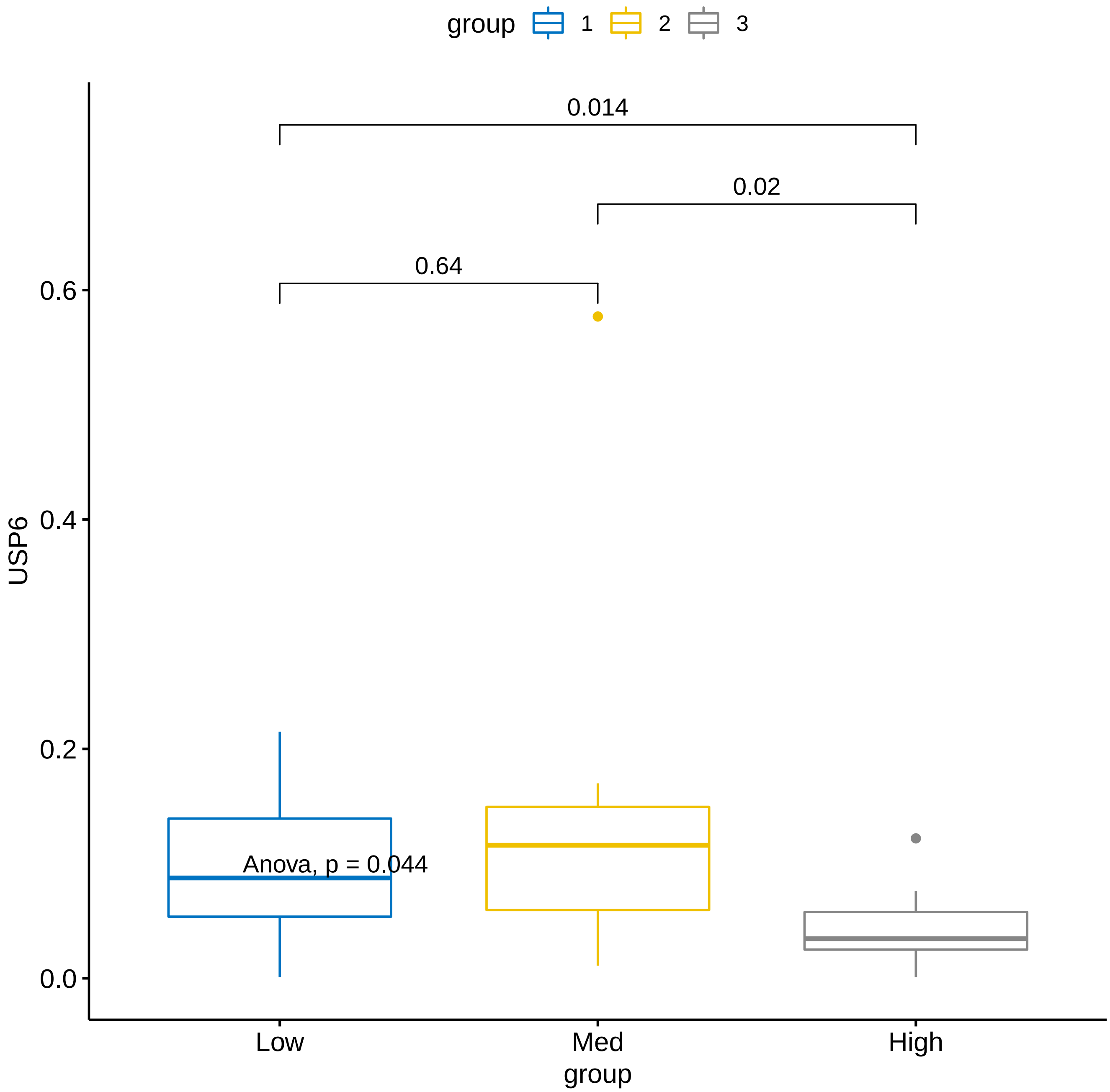

group 1 2 3
0.014
0.02
0.64
Anova, p = 0.044
USP6
0.6
0.4
0.2
0.0
Low
Med
High
group

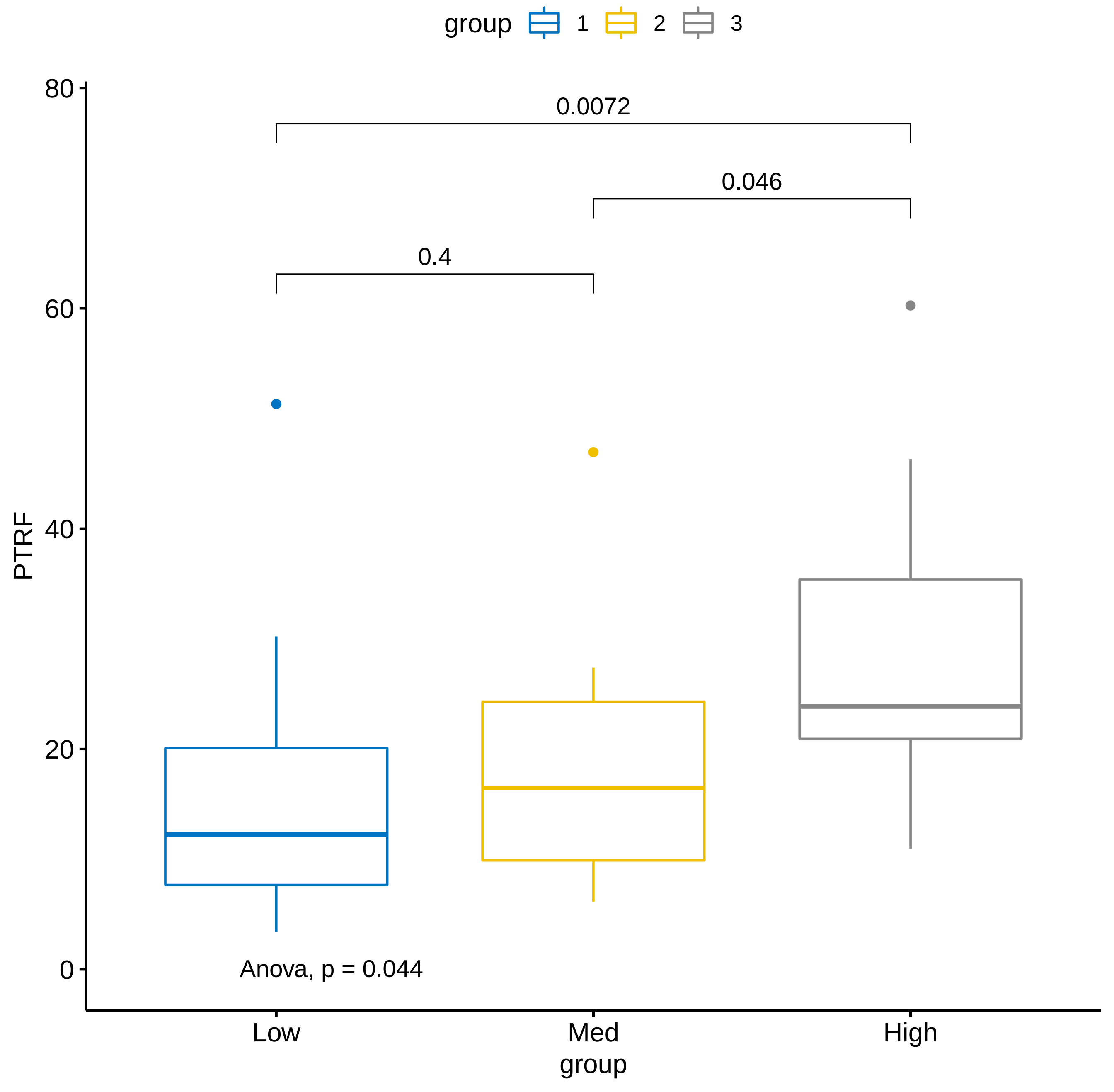
group
1
2
3
0.0072
0.046
0.4
80
60
40
20
0
PTRF
Anova, p = 0.044
Low
Med
High
group

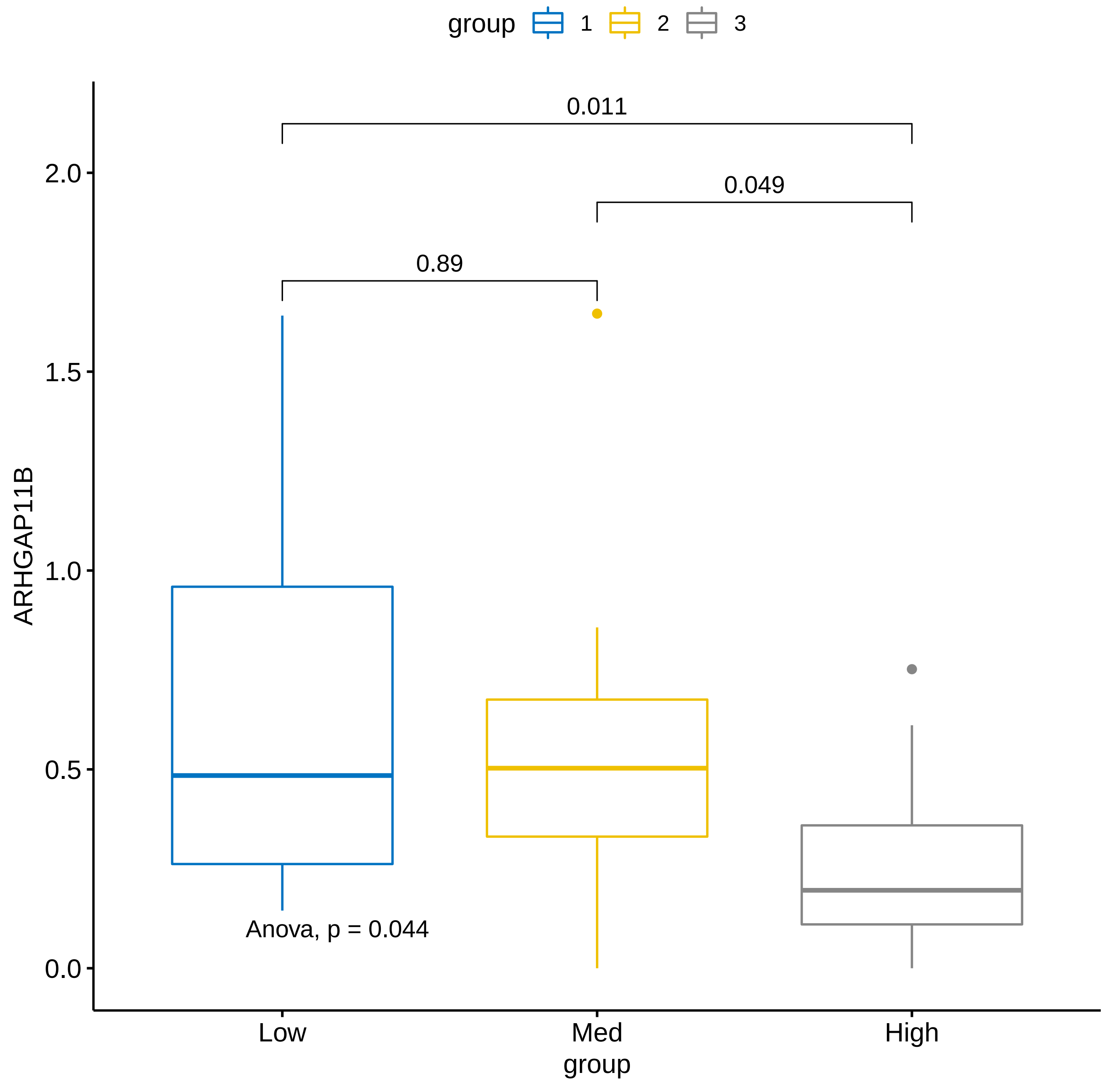

group 1 2 3
0.011
0.049
0.89
2.0
1.5
1.0
0.5
0.0
ARHGAP11B
Anova, p = 0.044
Low
Med
High
group

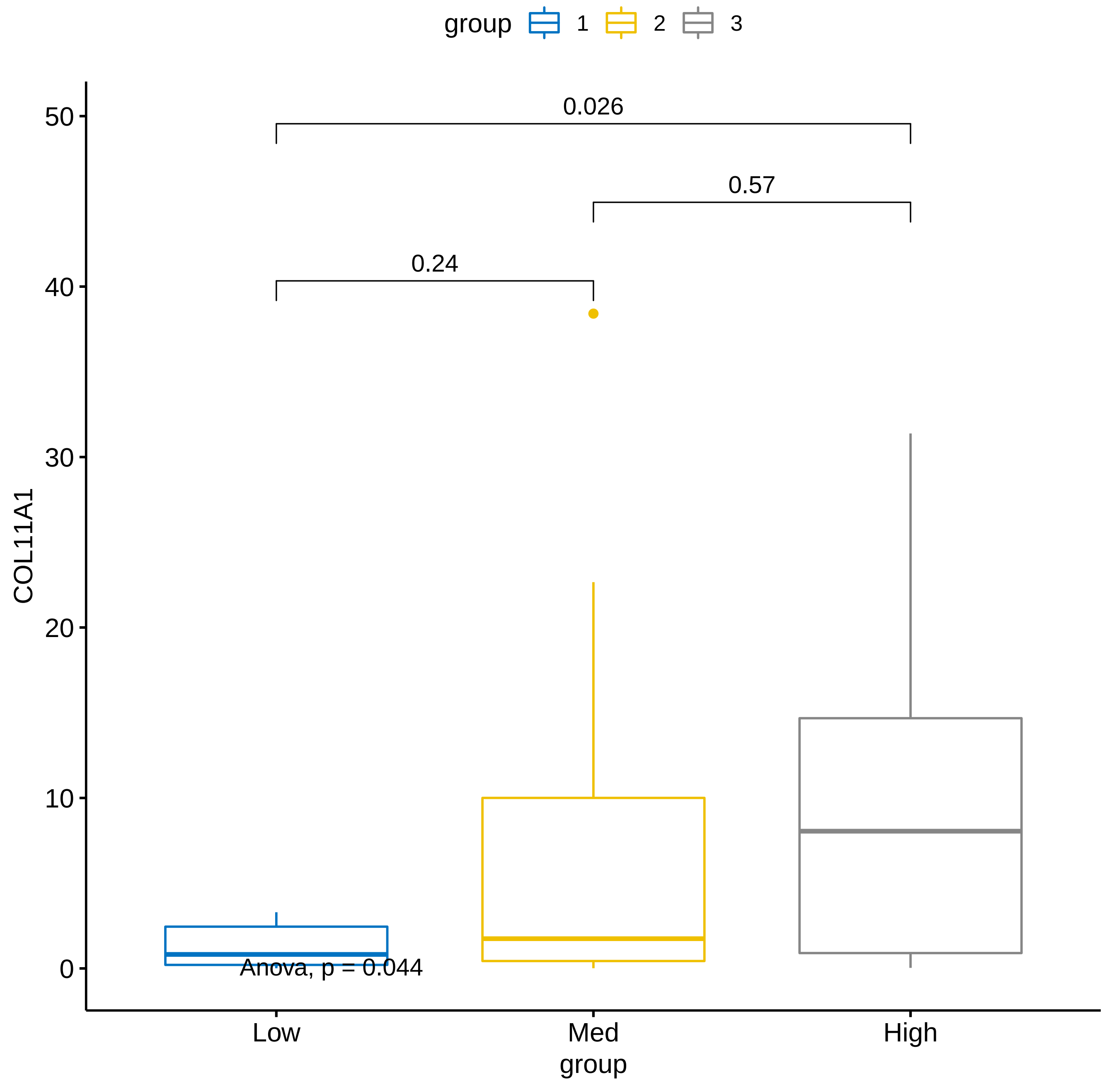
group
1
2
3
0.026
0.57
0.24
50
40
30
20
10
0
COL11A1
Anova, p = 0.044
Low
Med
High
group

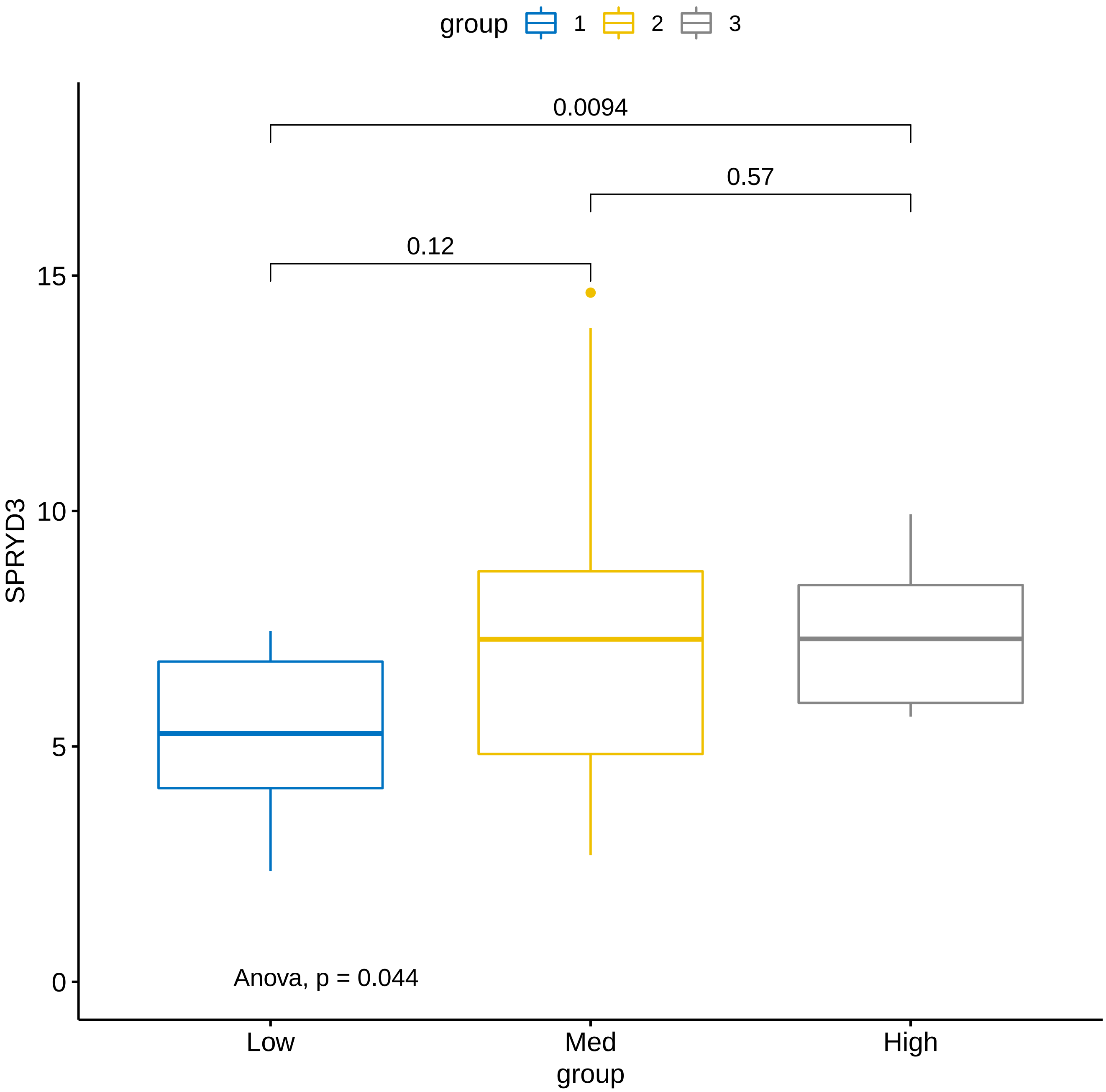

group
1
2
3
0.0094
0.57
0.12
15
10
5
0
SPRYD3
Anova, p = 0.044
Low
Med
High
group

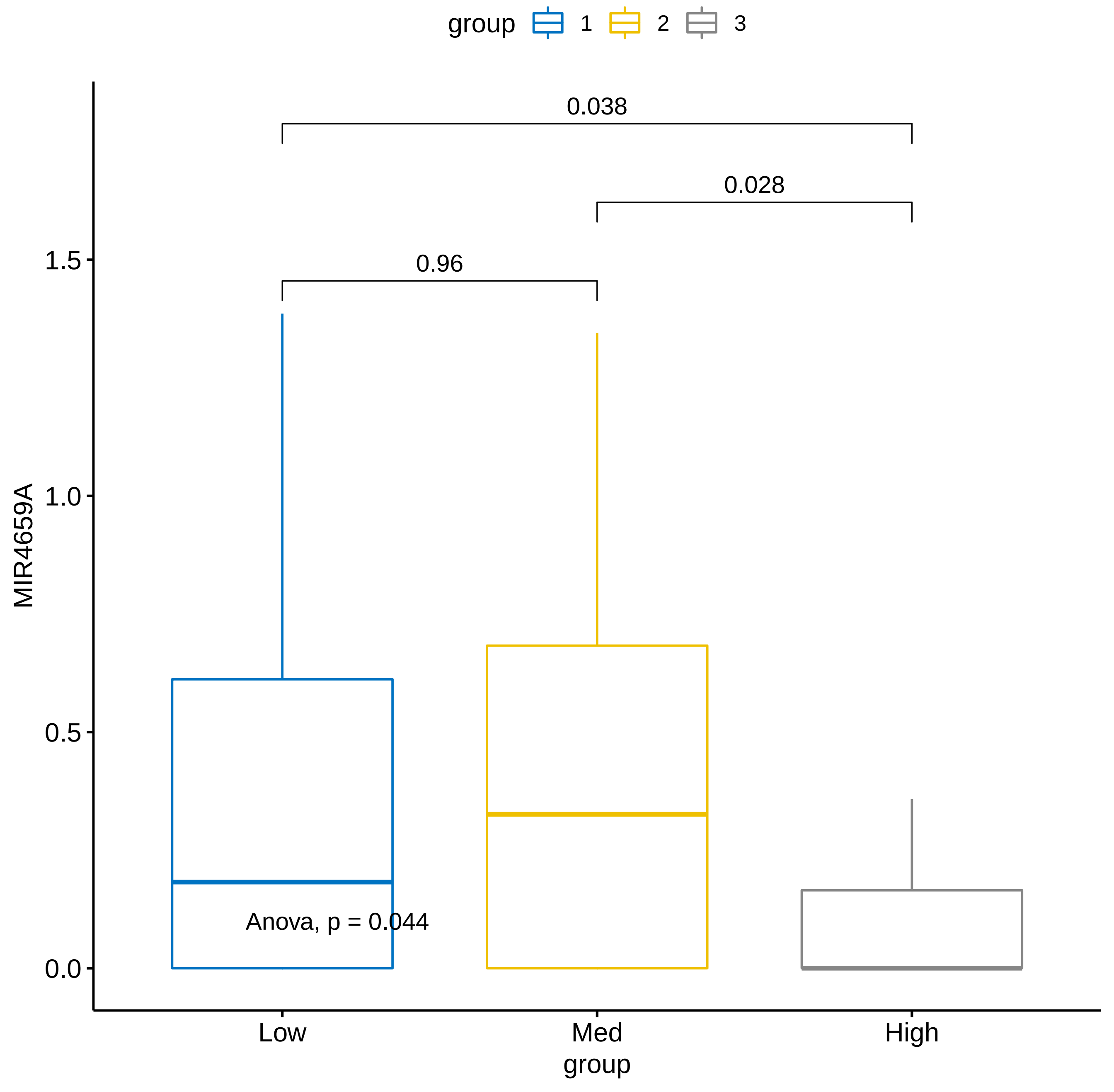
group 1 2 3
0.038
0.028
0.96
1.5
1.0
0.5
0.0
MIR4659A
Anova, p = 0.044
Low
Med
High
group

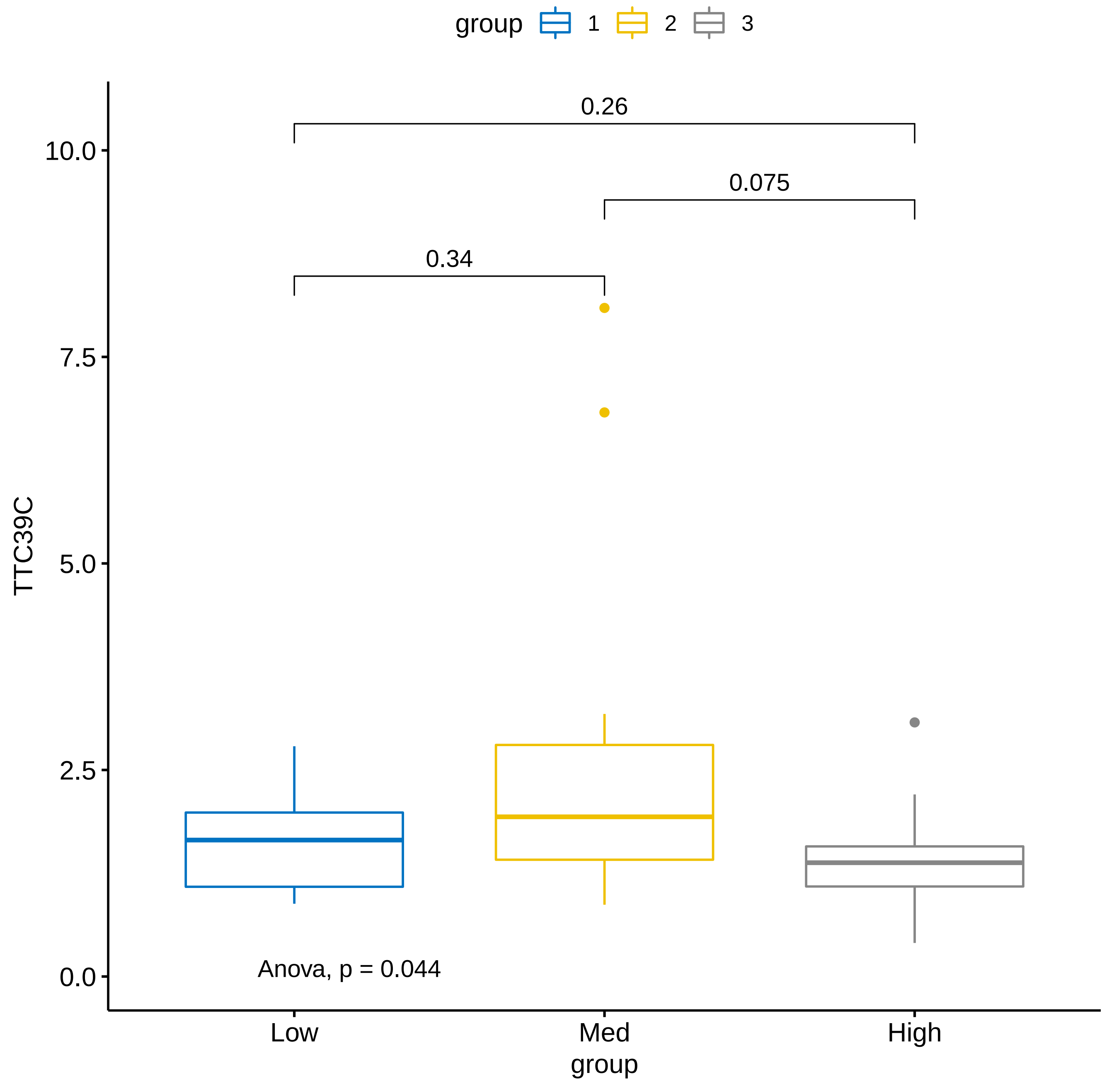
group
1
2
3
0.26
0.075
0.34
10.0
7.5
5.0
2.5
0.0
TTC39C
Anova, p = 0.044
Low
Med
High
group

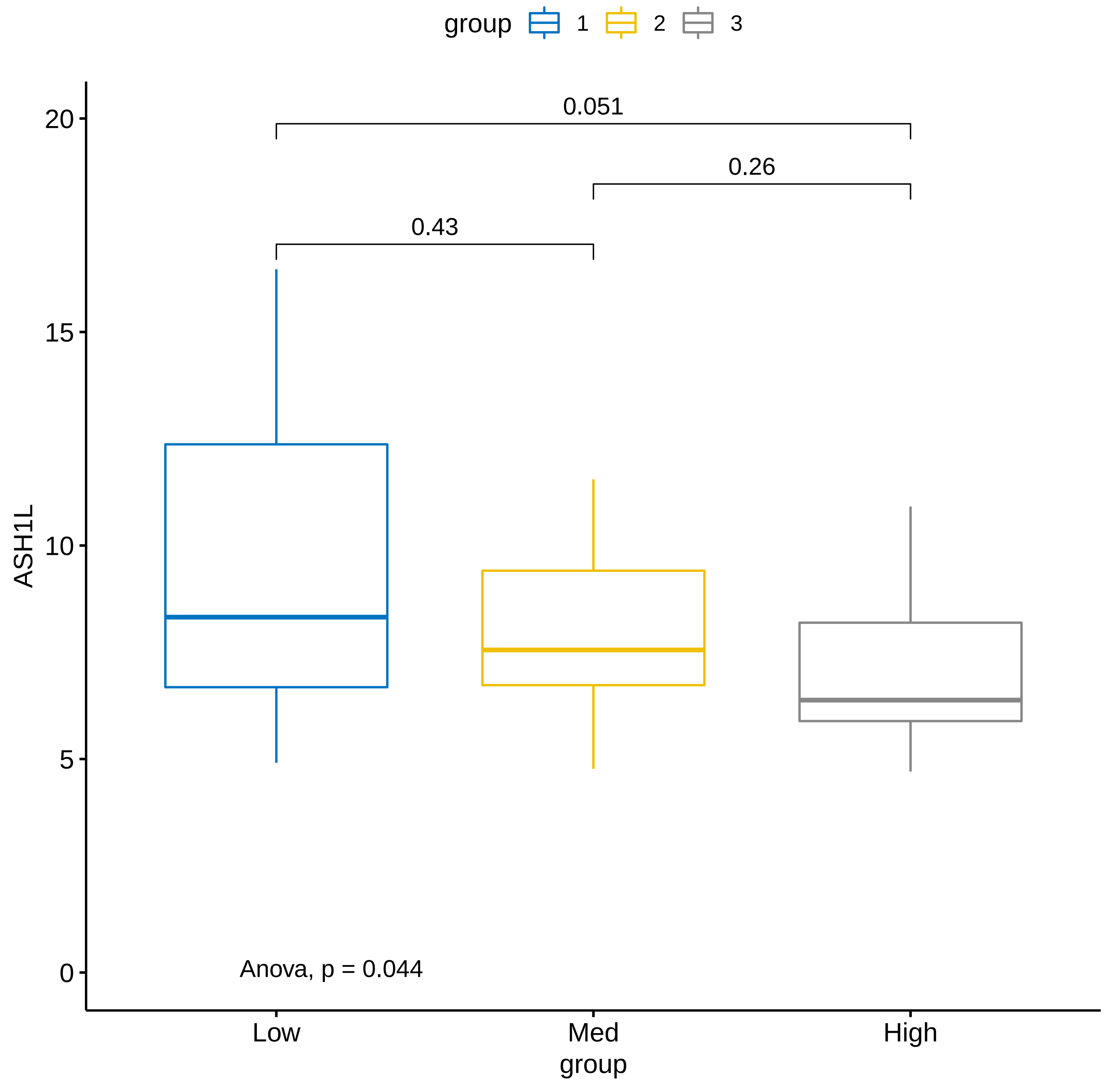
group
1
2
3
0.051
0.26
0.43
20
15
10
5
0
ASH1L
Anova, p = 0.044
Low
Med
High
group

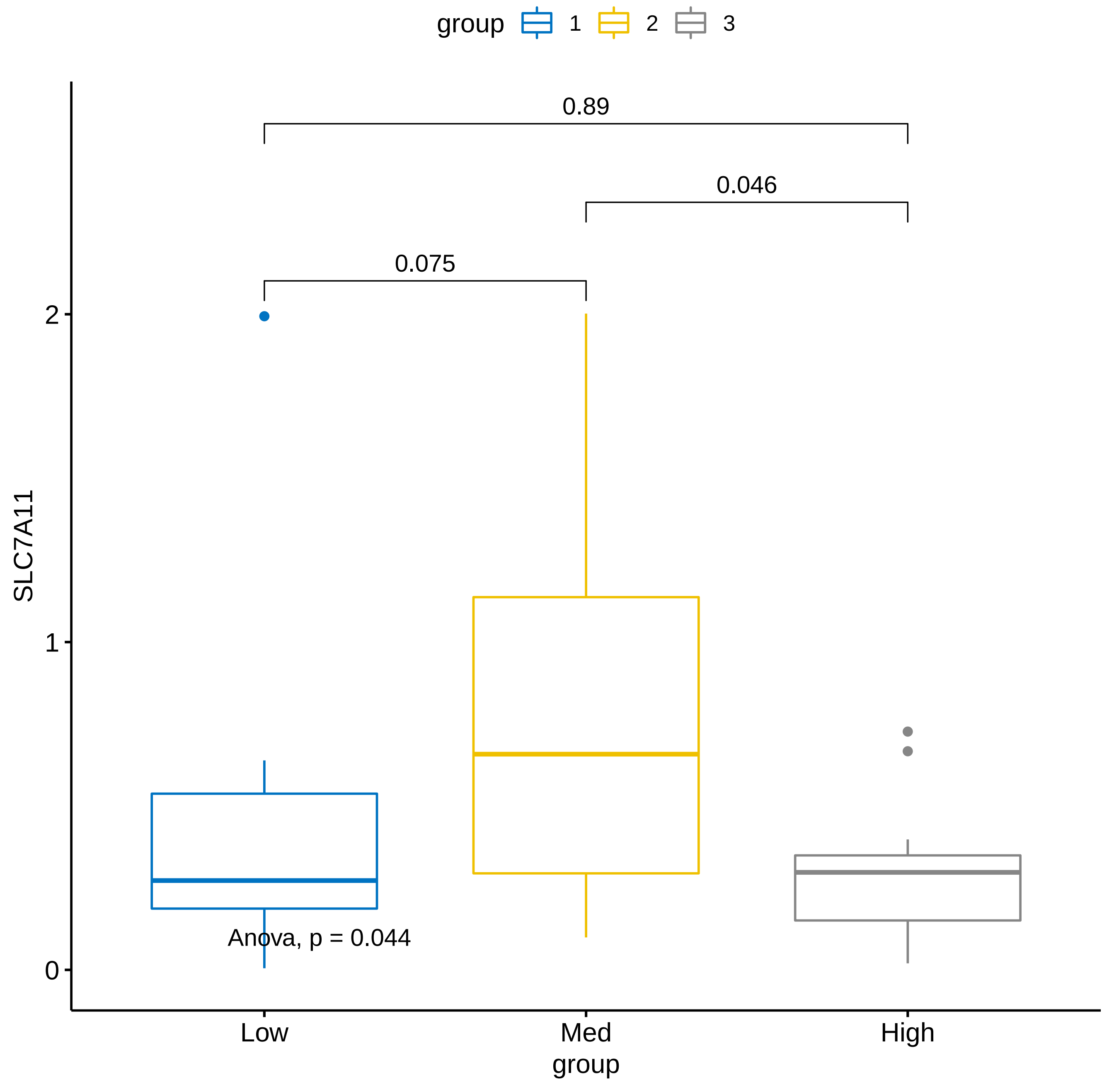

group 1 2 3
0.89
0.046
0.075
SLC7A11
2
1
0
Anova, p = 0.044
Low
Med
High
group

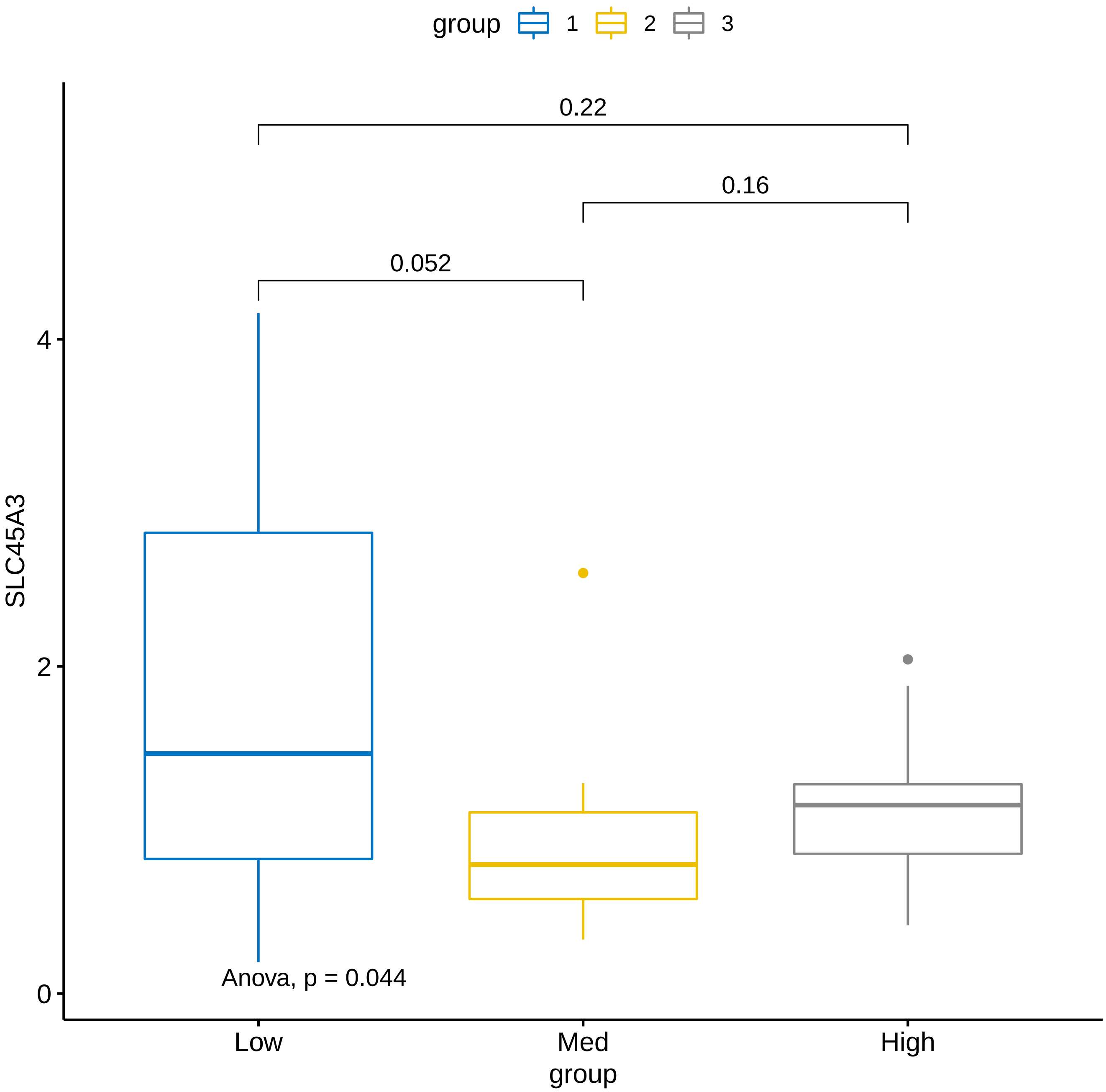
group
1
2
3
0.22
0.16
0.052
4
2
0
SLC45A3
Anova, p = 0.044
Low
Med
High
group

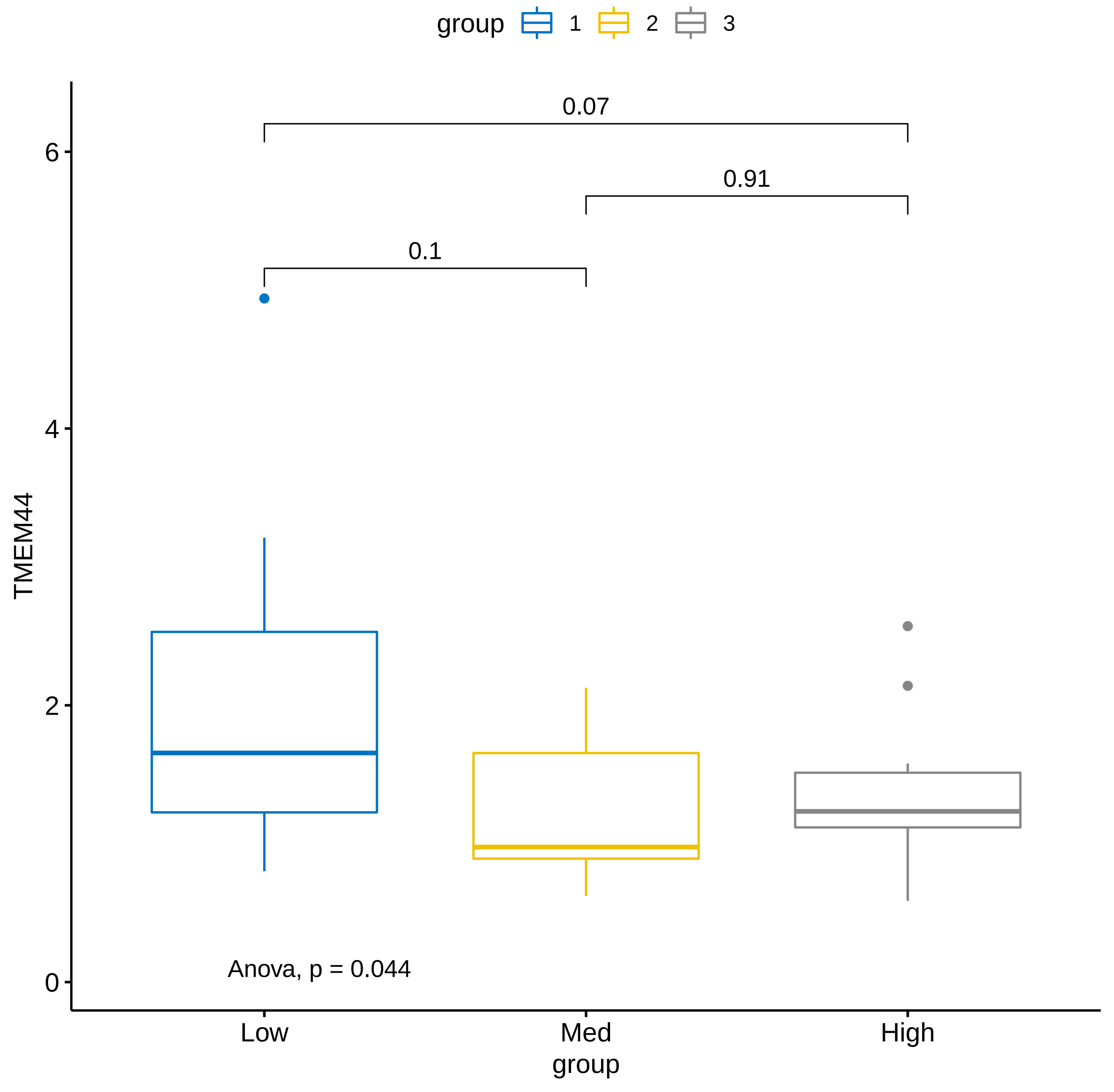
group
1
2
3
0.07
0.91
0.1
6
4
2
0
TMEM44
Anova, p = 0.044
Low
Med
High
group

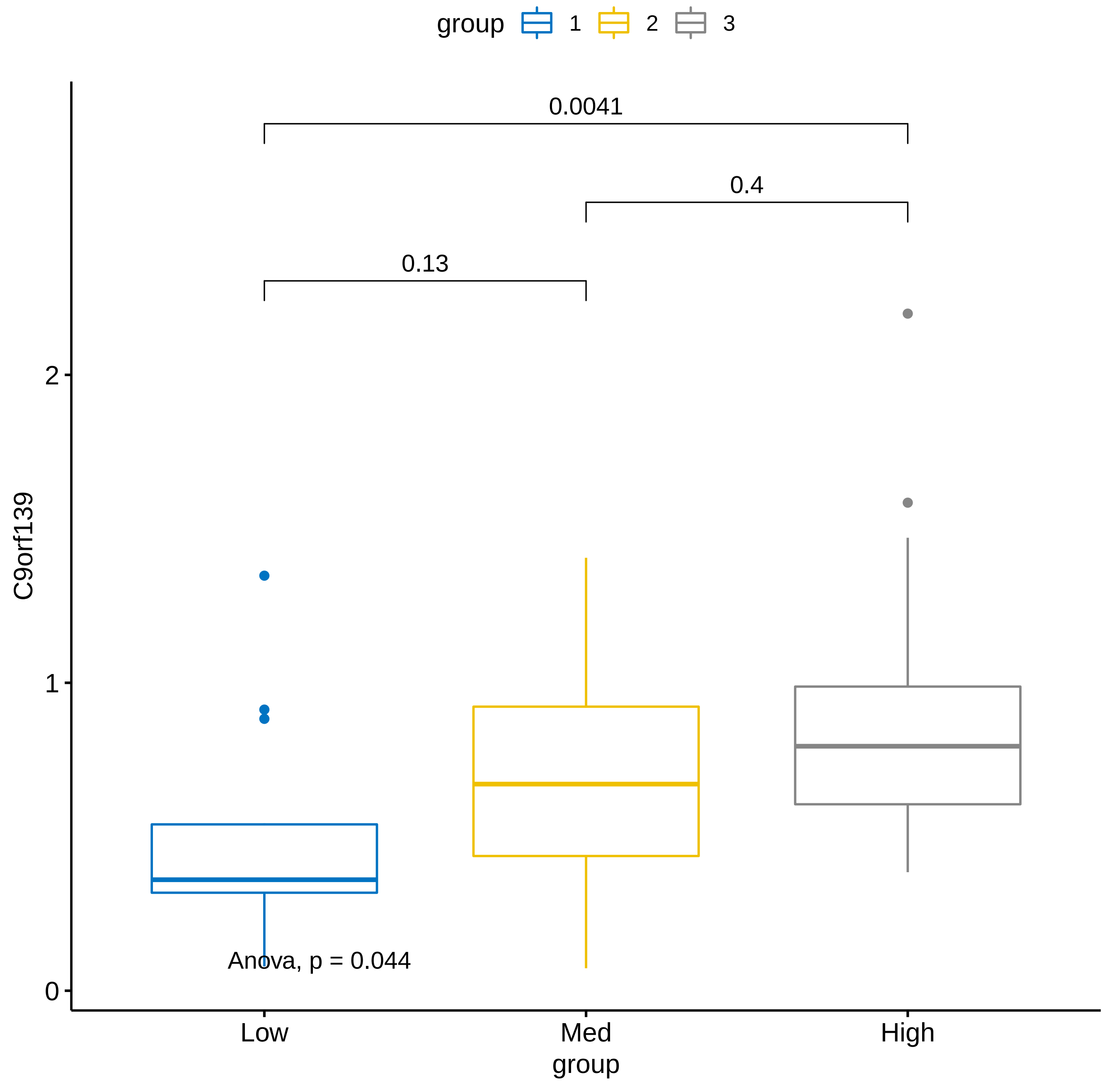

group
1
2
3
0.0041
0.4
0.13
2
1
0
C9orf139
Anova, p = 0.044
Low
Med
High
group

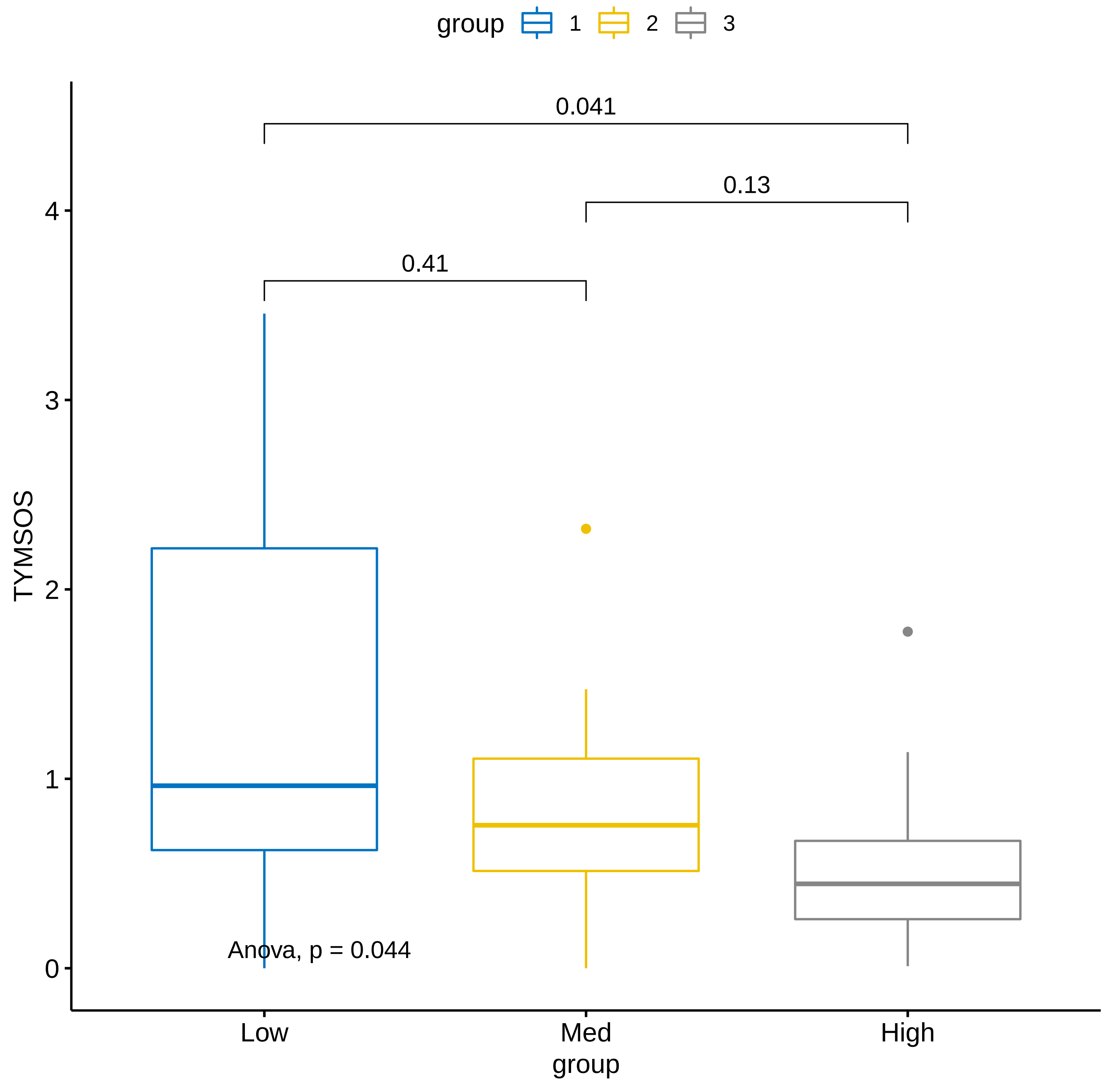
group
1
2
3
0.041
0.13
0.41
TYMSOS
4
3
2
1
0
Anova, p = 0.044
Low
Med
High
group

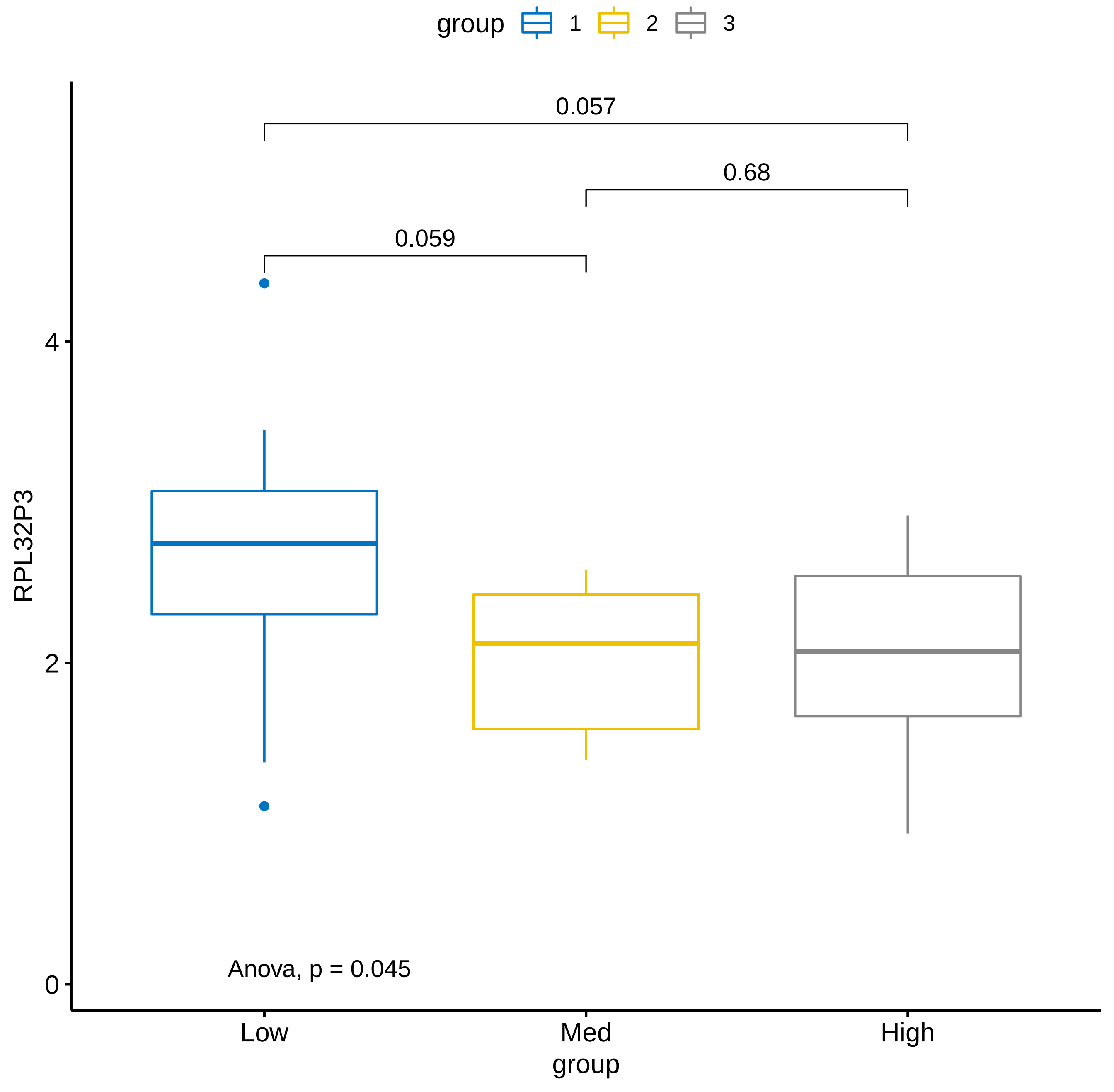
group 1 2 3
0.057
0.68
0.059
4
2
0
RPL32P3
Anova, p = 0.045
Low
Med
High
group

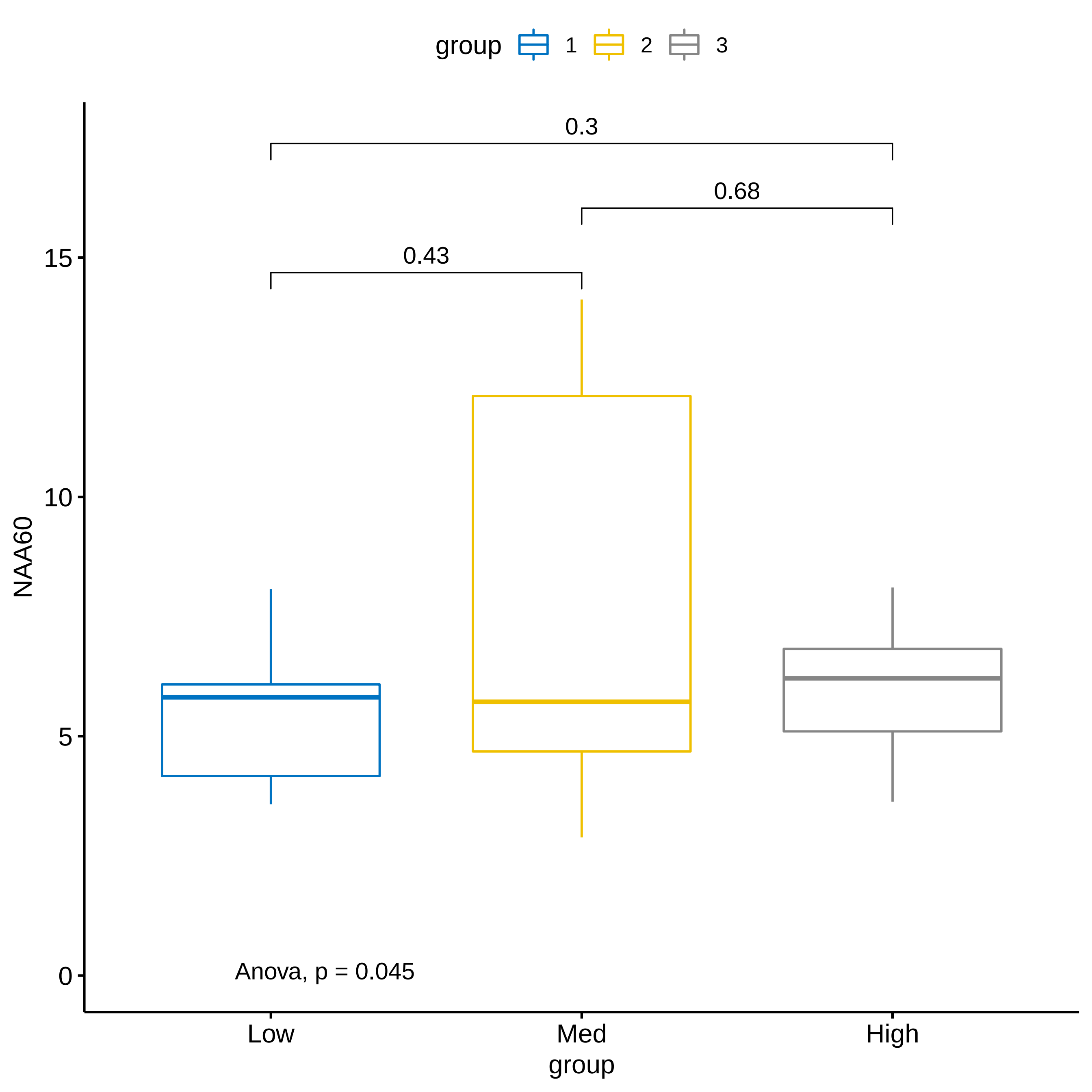
group 1 2 3
0.3
0.68
0.43
15
10
5
0
NAA60
Anova, p = 0.045
Low
Med
High
group

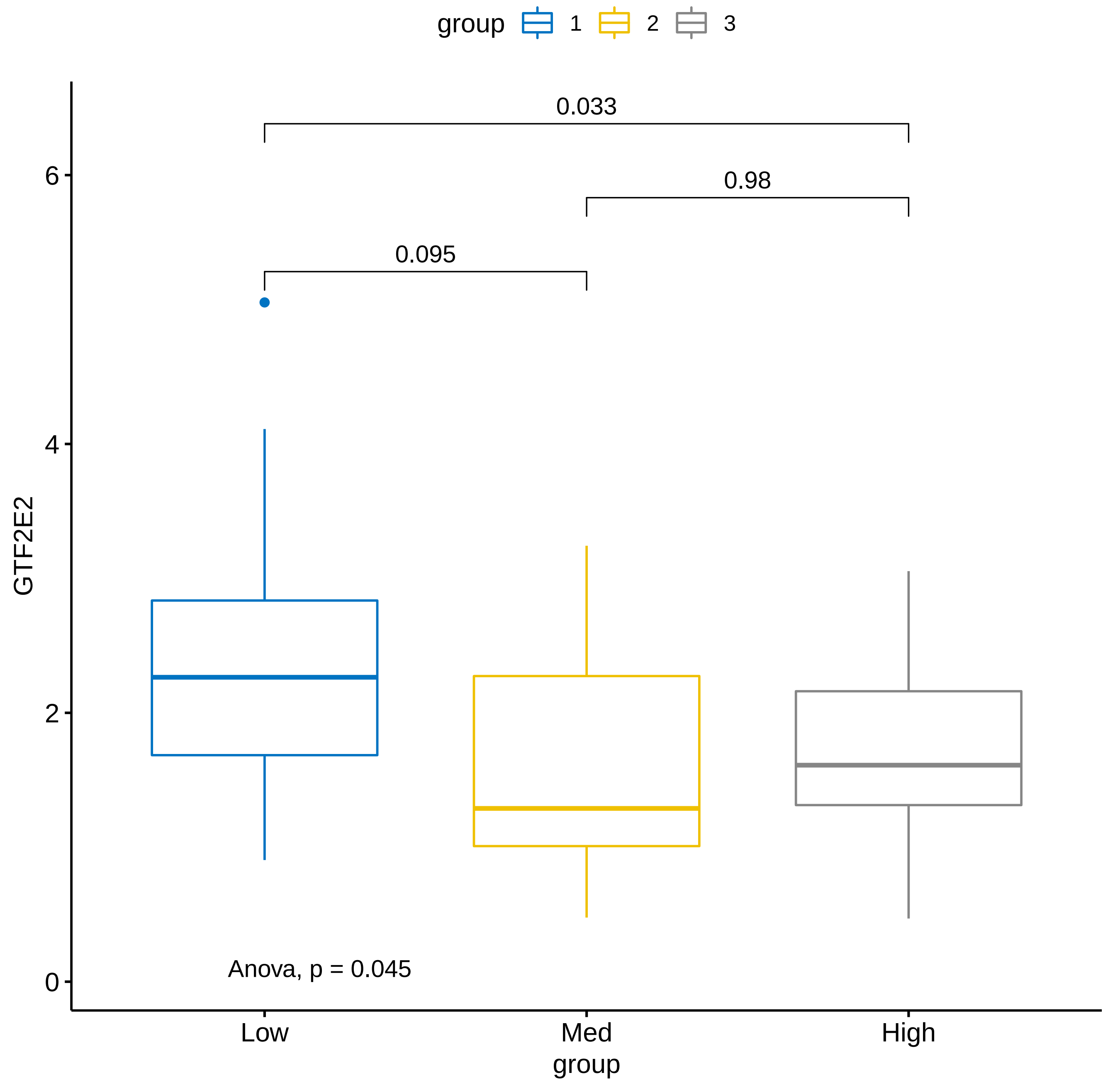
group
1
2
3
0.033
0.98
0.095
6
4
2
0
GTF2E2
Anova, p = 0.045
Low
Med
High
group

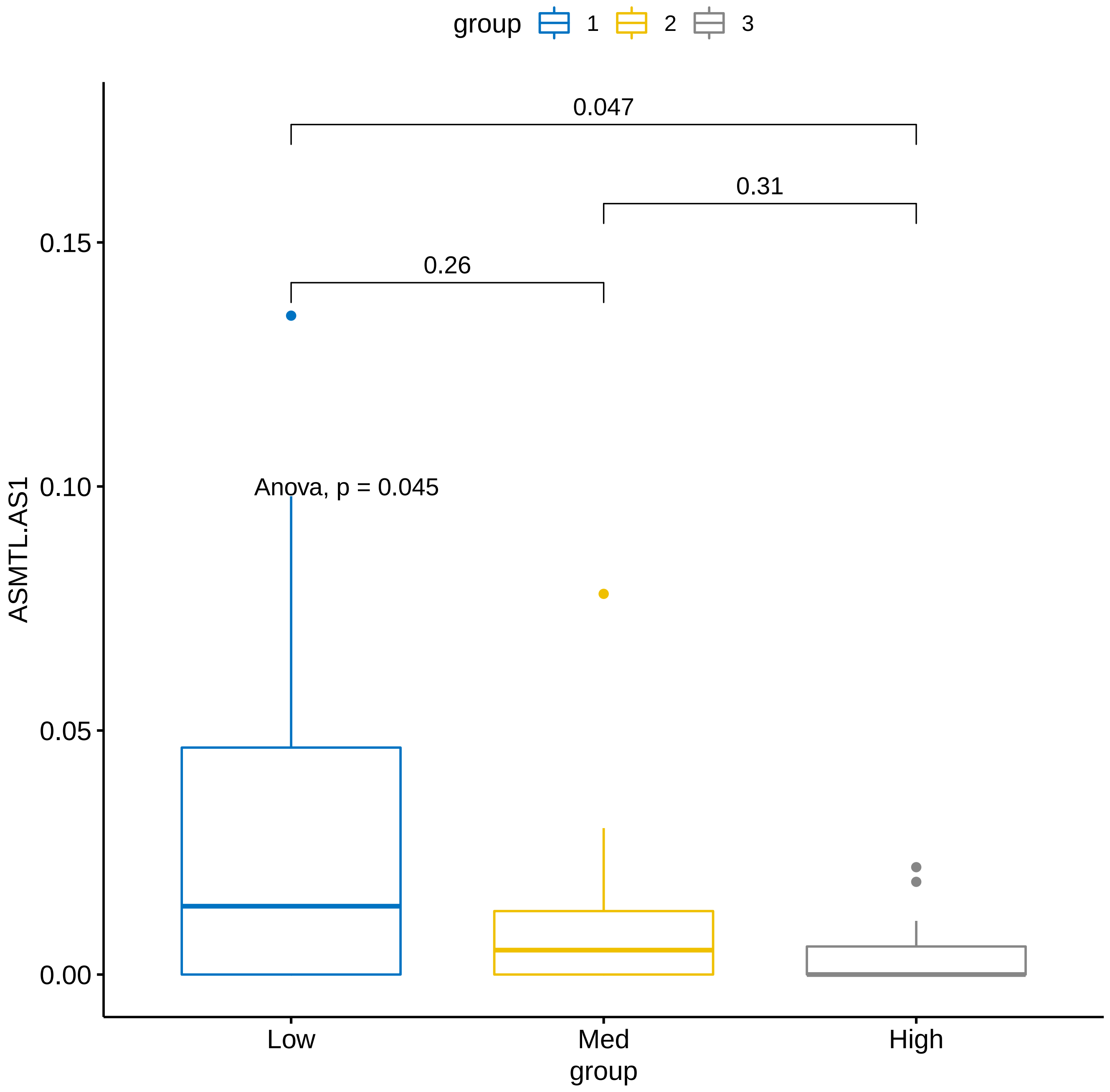
group 1 2 3
0.047
0.31
0.26
Anova, p = 0.045
0.15
0.10
0.05
0.00
ASMTL.AS1
Low
Med
High
group

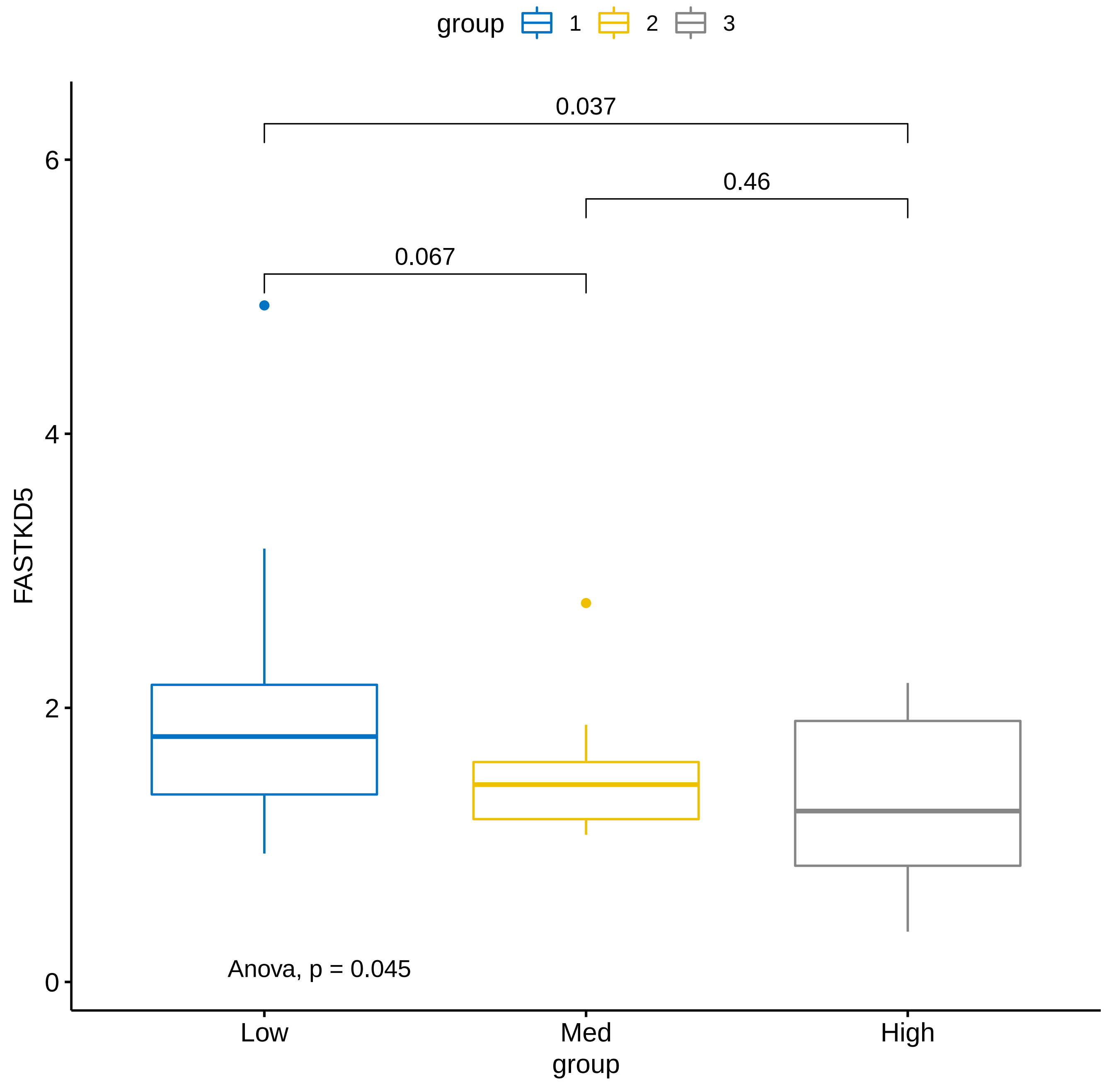
group
1
2
3
0.037
0.46
0.067
6
4
2
0
FASTKD5
Anova, p = 0.045
Low
Med
High
group

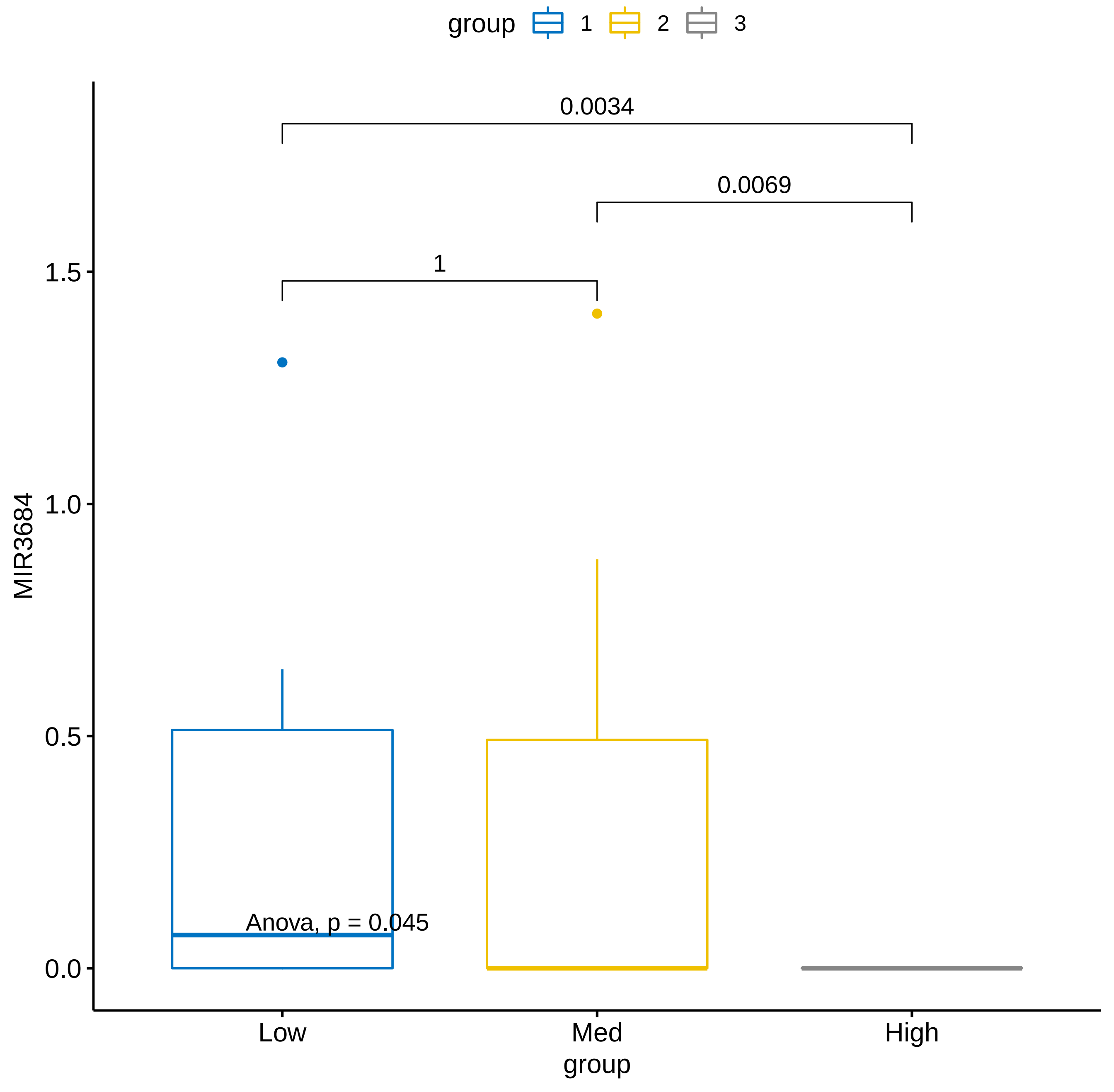
group
1
2
3
0.0034
0.0069
1
1.5
1.0
0.5
0.0
MIR3684
Anova, p = 0.045
Low
Med
High
group

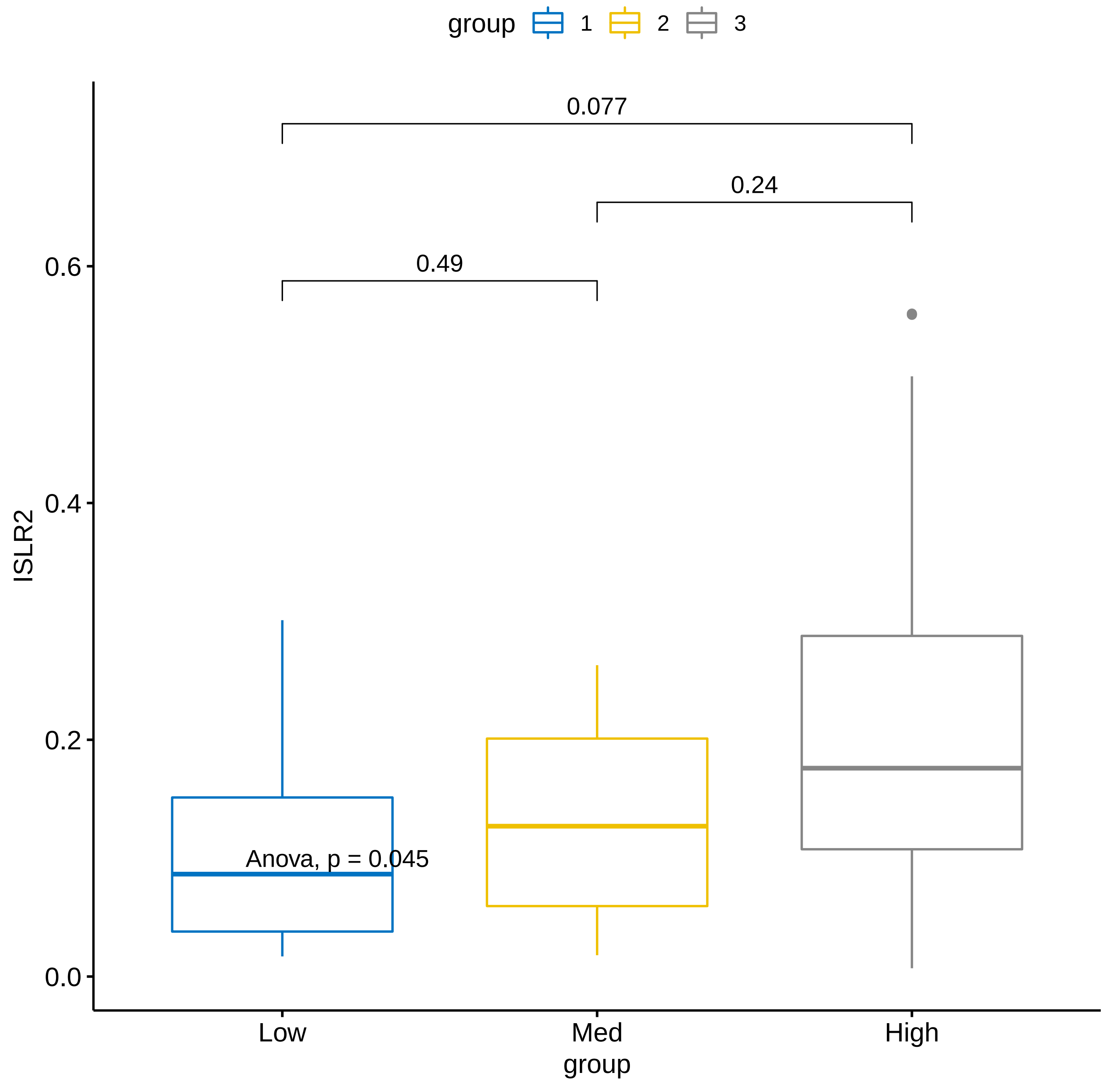
group
1
2
3
0.077
0.24
0.49
0.6
0.4
0.2
0.0
ISLR2
Anova, p = 0.045
Low
Med
High
group

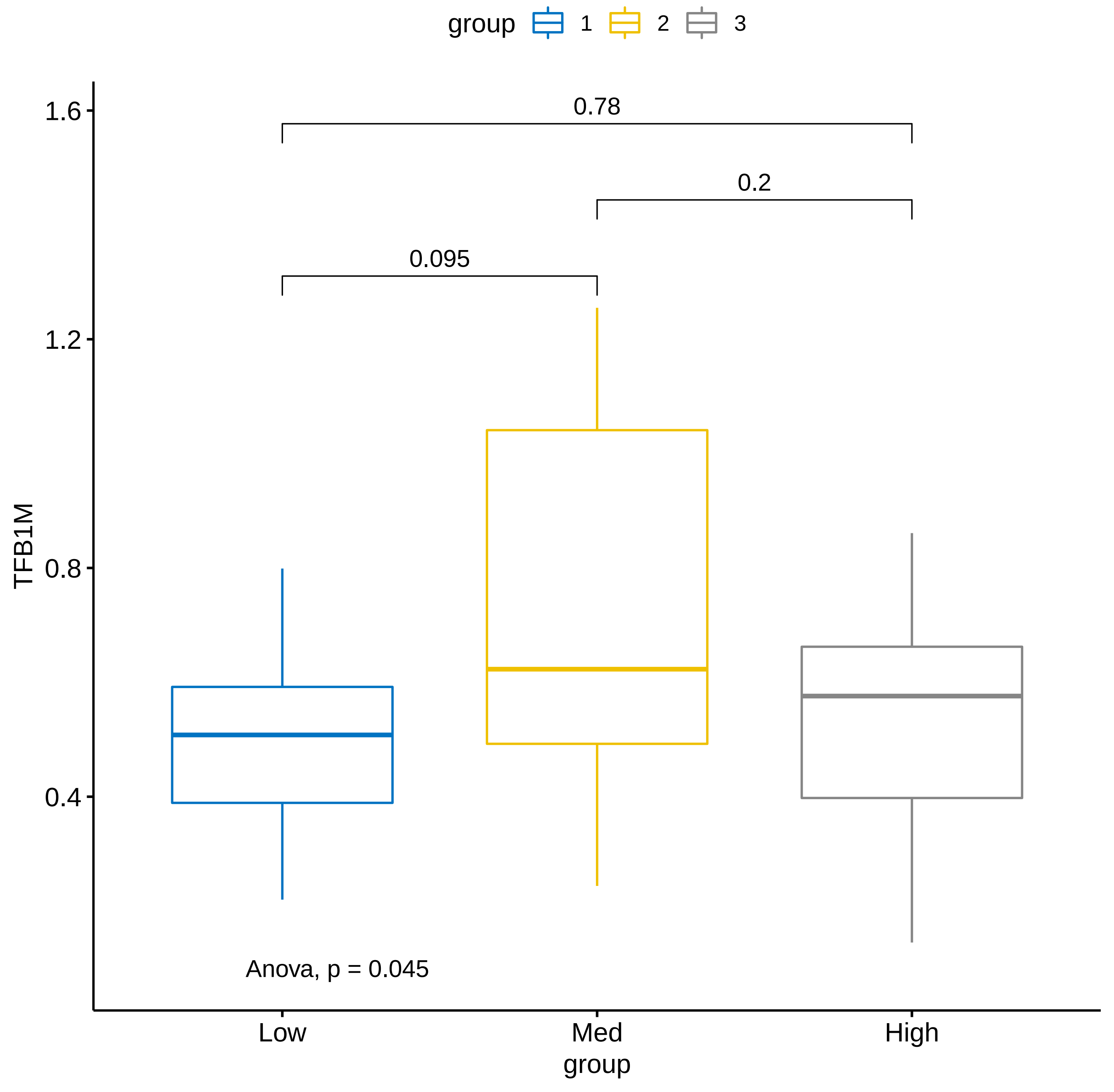

group
1
2
3
1.6
1.2
0.8
0.4
TFB1M
0.78
0.2
0.095
Anova, p = 0.045
Low
Med
High
group

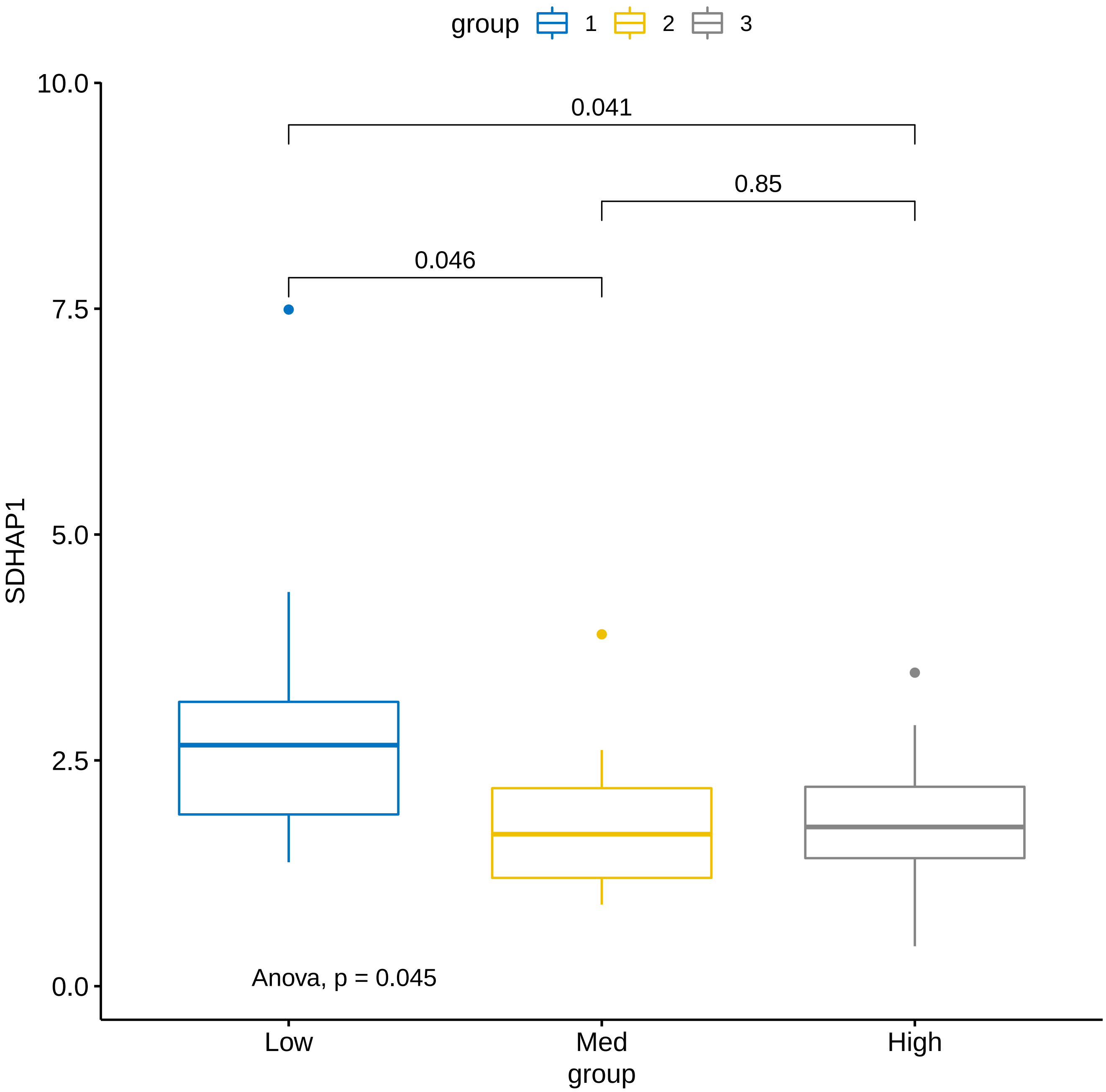
group
1
2
3
10.0
7.5
5.0
2.5
0.0
SDHAP1
0.041
0.85
0.046
Anova, p = 0.045
Low
Med
High
group

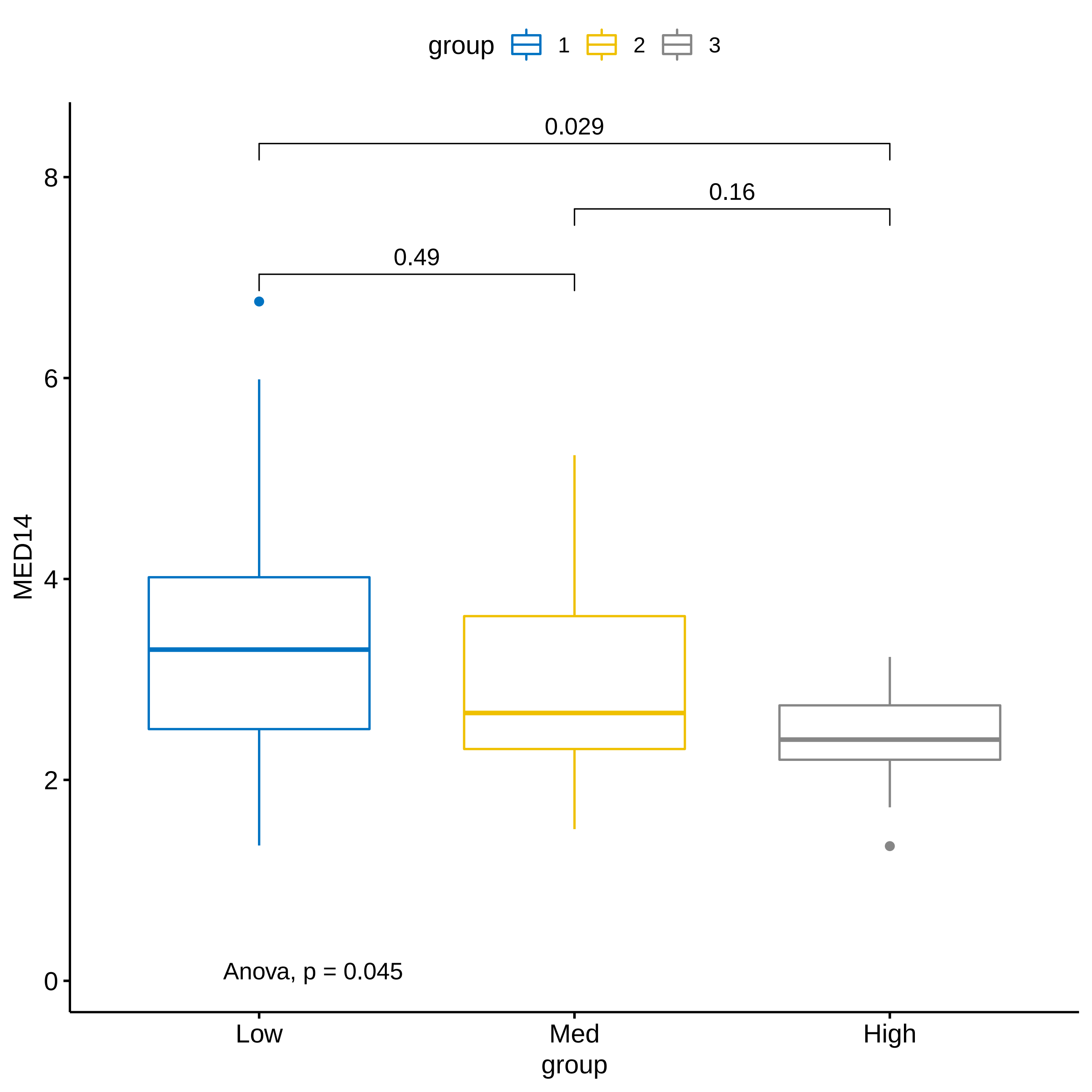
group 1 2 3
0.029
0.16
0.49
MED14
8
6
4
2
0
Anova, p = 0.045
Low
Med
High
group

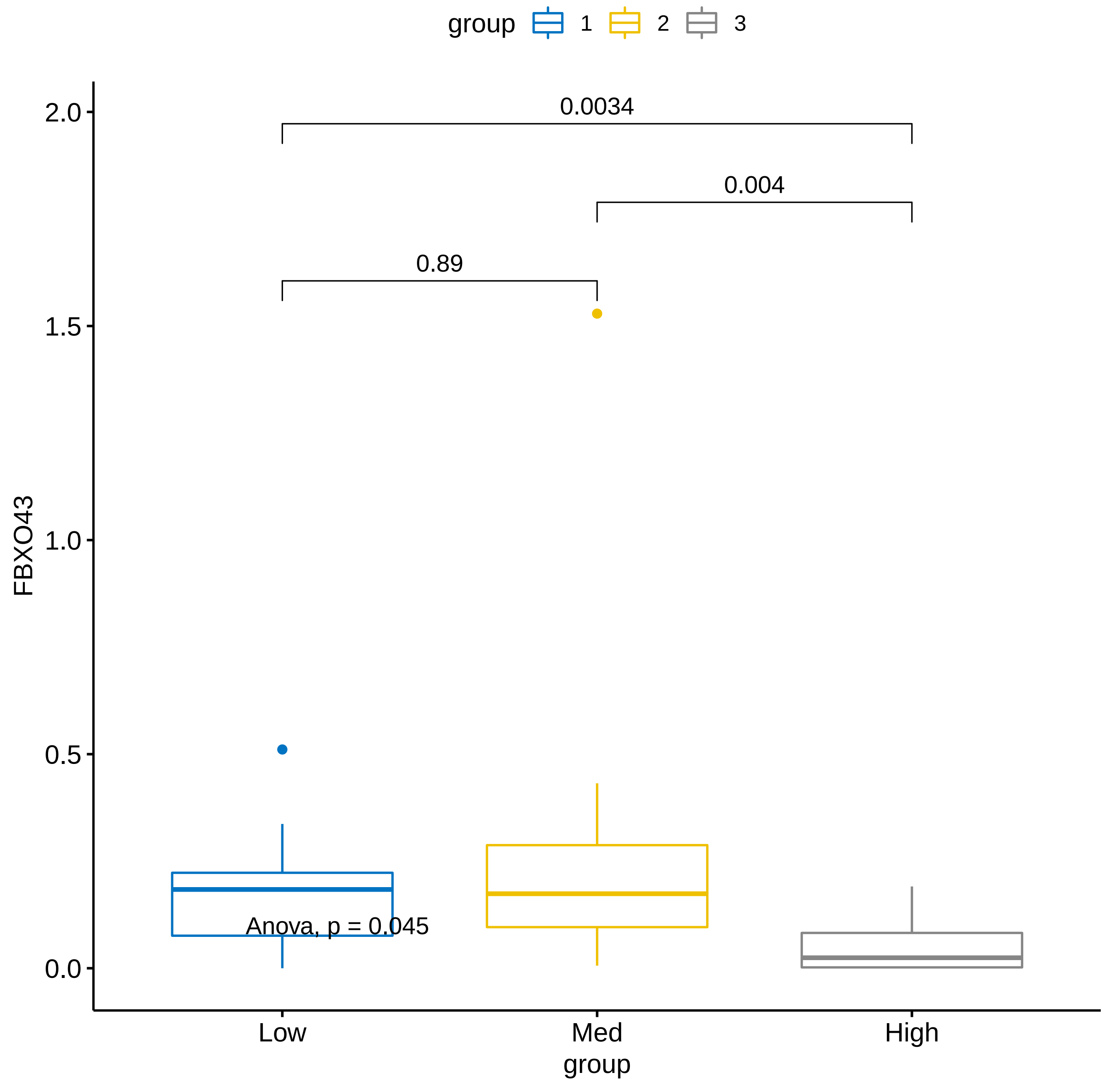

group 1 2 3
0.0034
0.004
0.89
Anova, p = 0.045
FBXO43
2.0
1.5
1.0
0.5
0.0
Low
Med
High
group

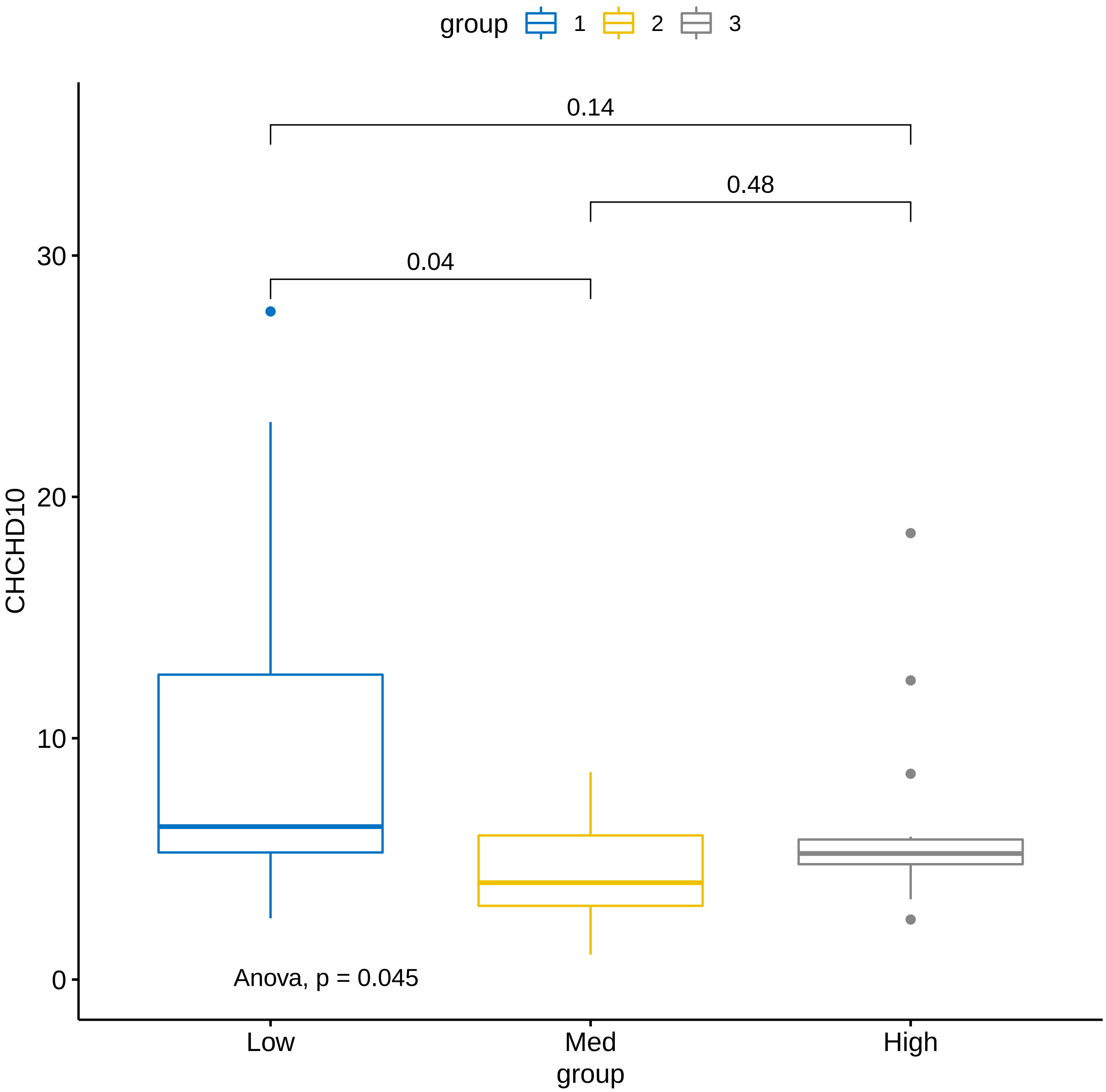
group
1
2
3
0.14
0.48
0.04
30
20
10
0
CHCHD10
Anova, p = 0.045
Low
Med
High
group

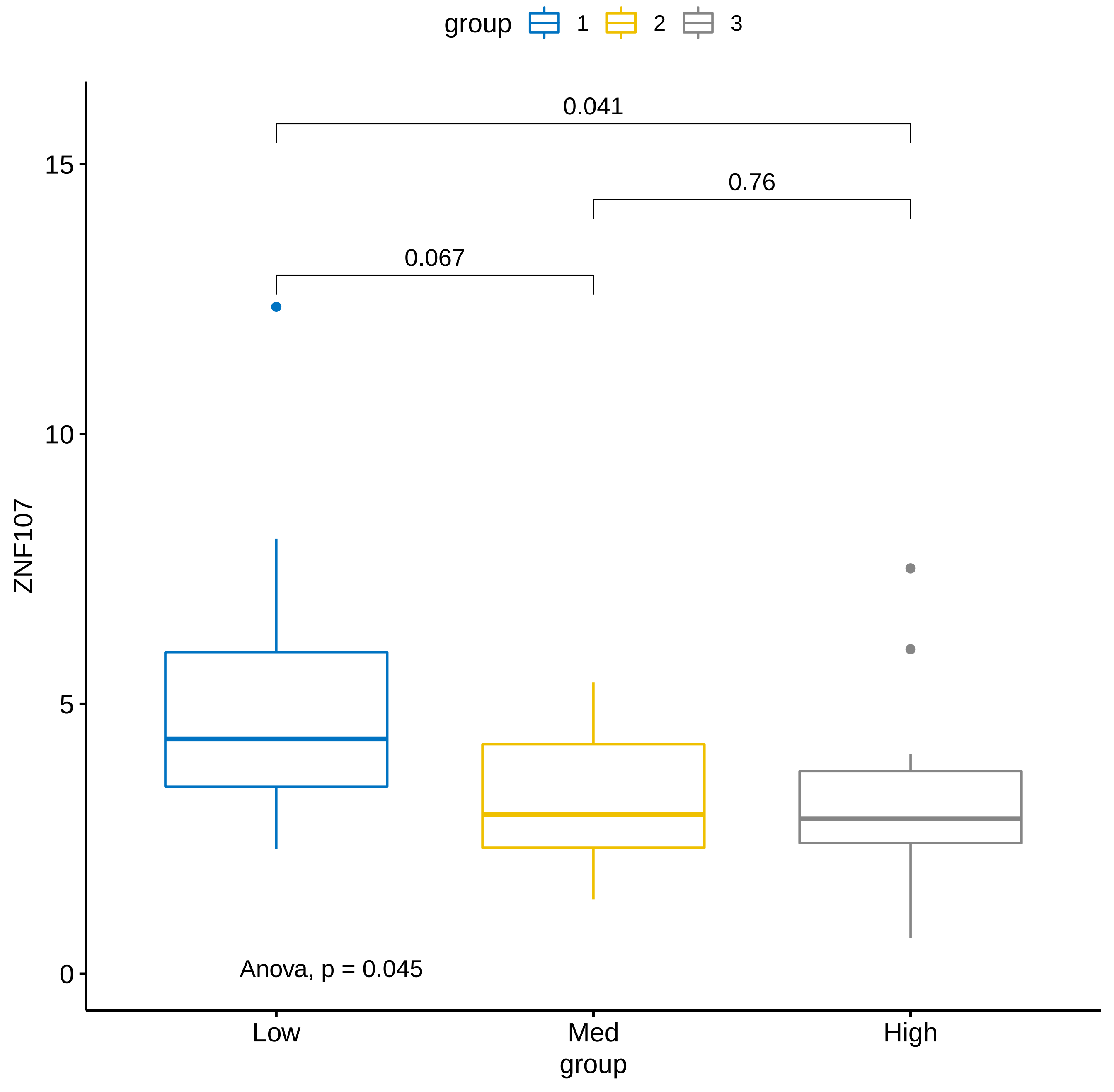

group 1 2 3
0.041
0.76
0.067
15
10
5
0
ZNF107
Anova, p = 0.045
Low
Med
High
group

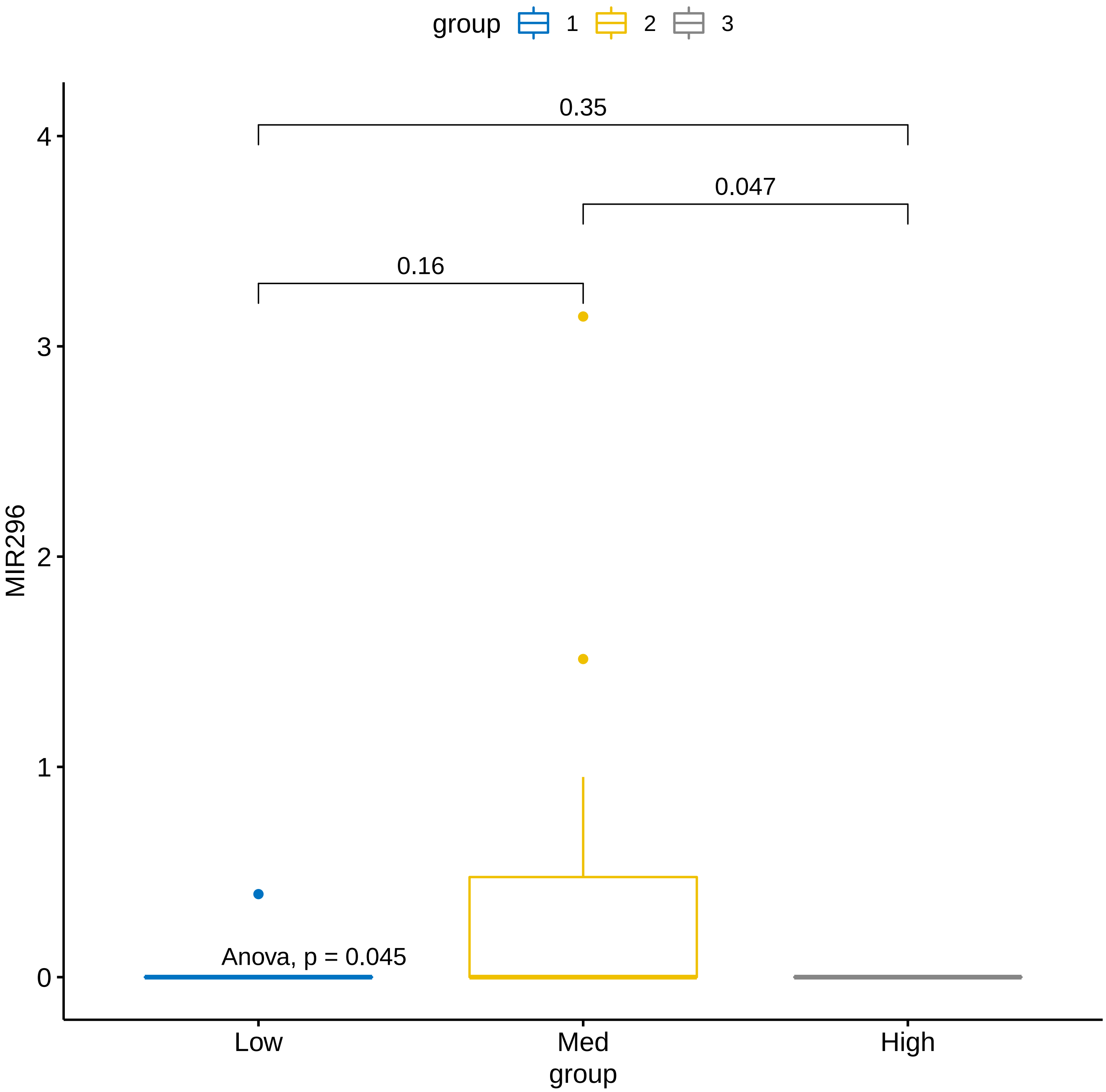
group
1
2
3
0.35
0.047
0.16
Anova, p = 0.045
4
3
2
1
0
MIR296
Low
Med
High
group

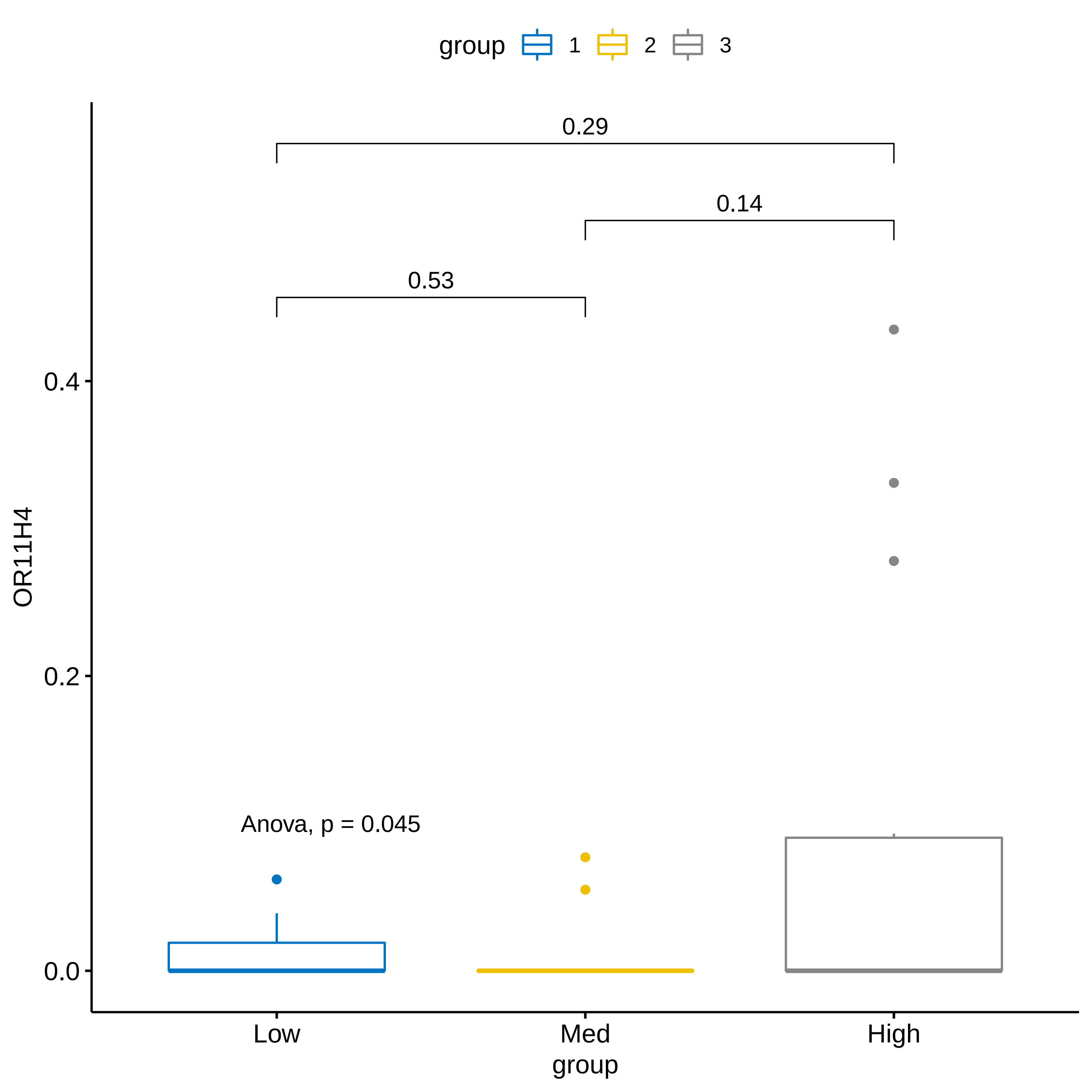
group
1
2
3
0.29
0.14
0.53
Anova, p = 0.045
OR11H4
0.4
0.2
0.0
Low
Med
High
group

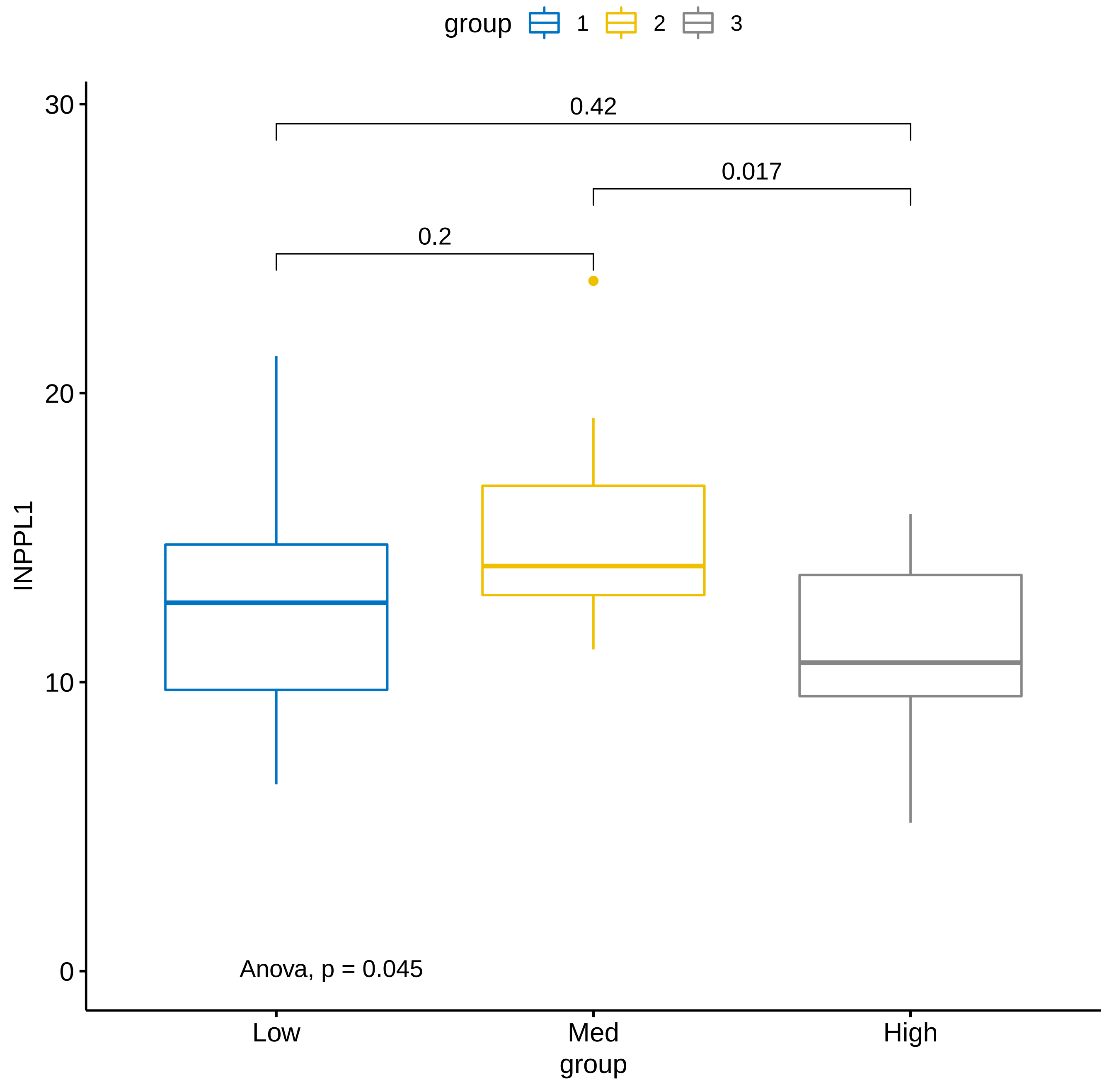
group
1
2
3
0.42
0.017
0.2
30
20
10
0
INPPL1
Anova, p = 0.045
Low
Med
High
group

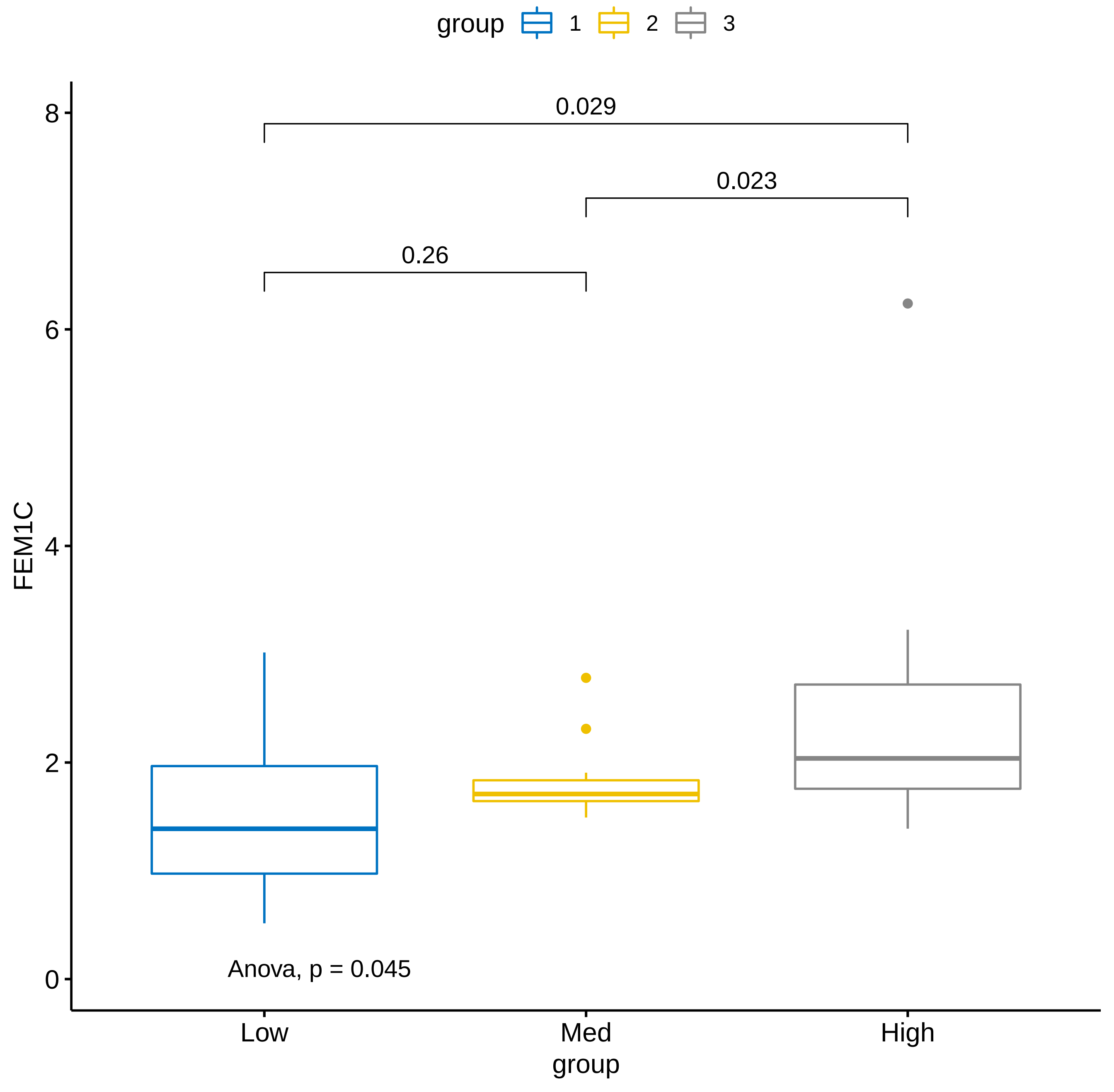
group
1
2
3
0.029
0.023
0.26
FEM1C
8
6
4
2
0
Anova, p = 0.045
Low
Med
High
group

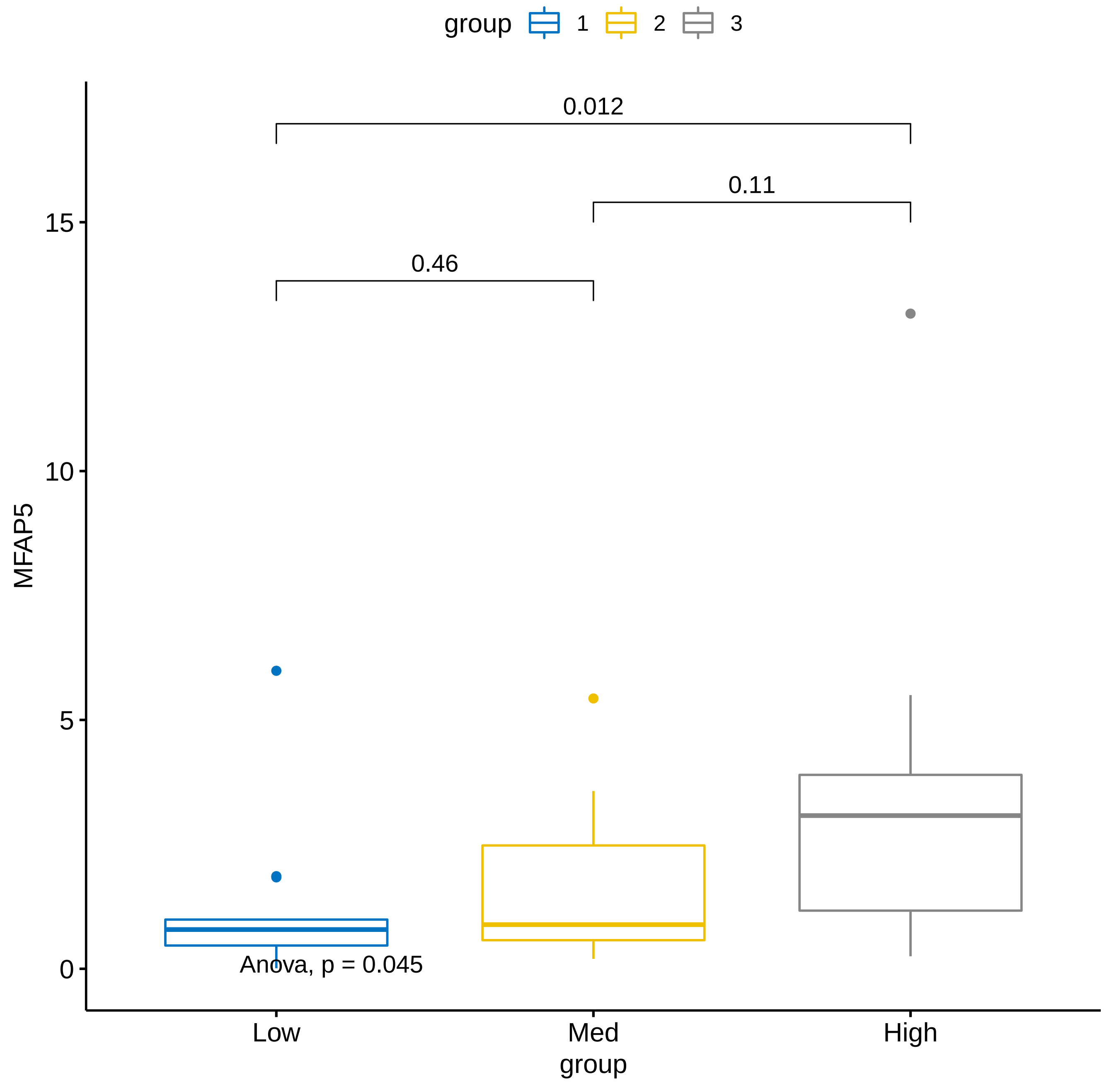
group
1
2
3
0.012
0.11
0.46
15
10
5
0
MFAP5
Anova, p = 0.045
Low
Med
High
group

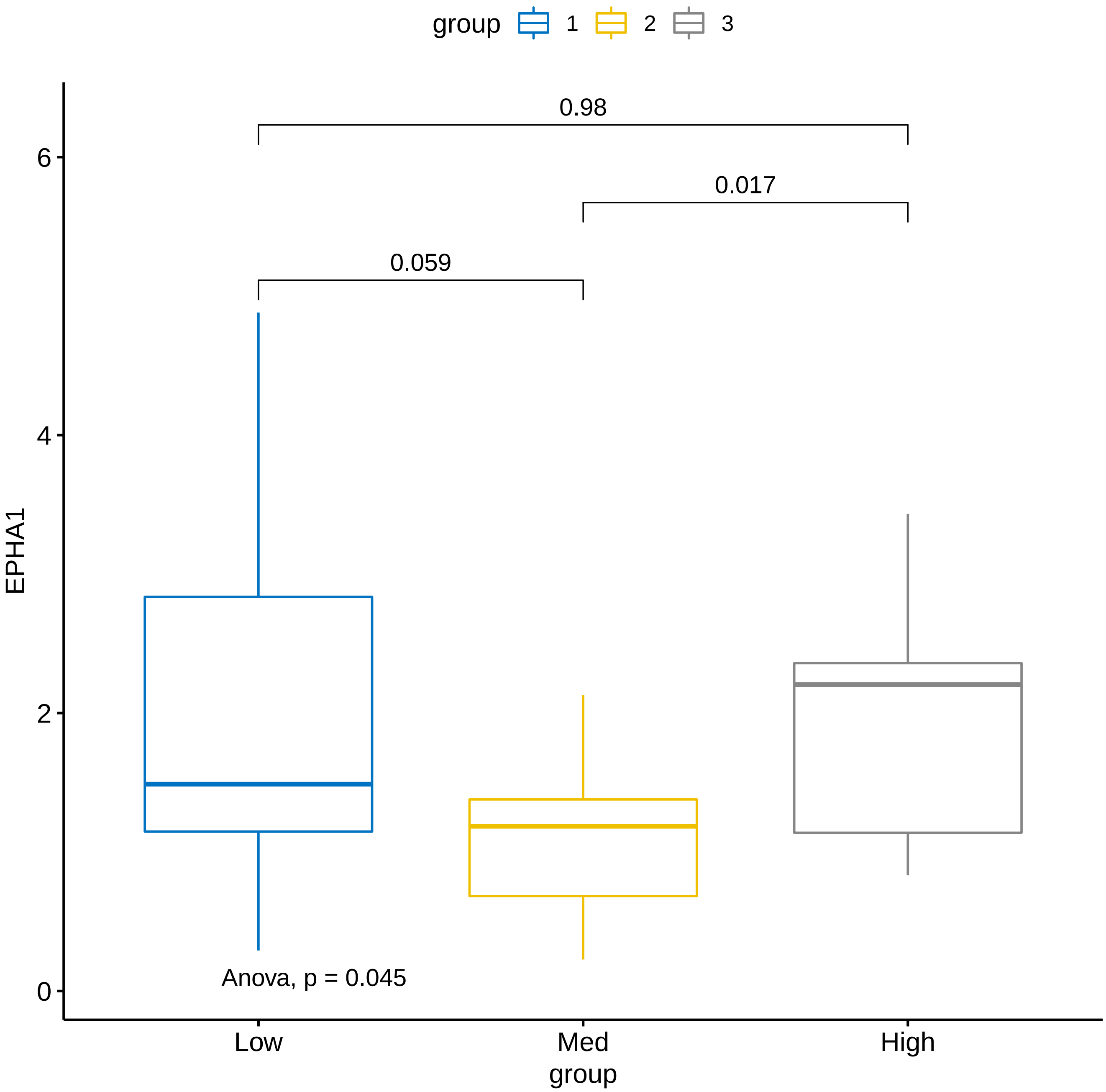
group
1
2
3
0.98
0.017
0.059
EPHA1
6
4
2
0
Anova, p = 0.045
Low
Med
High
group

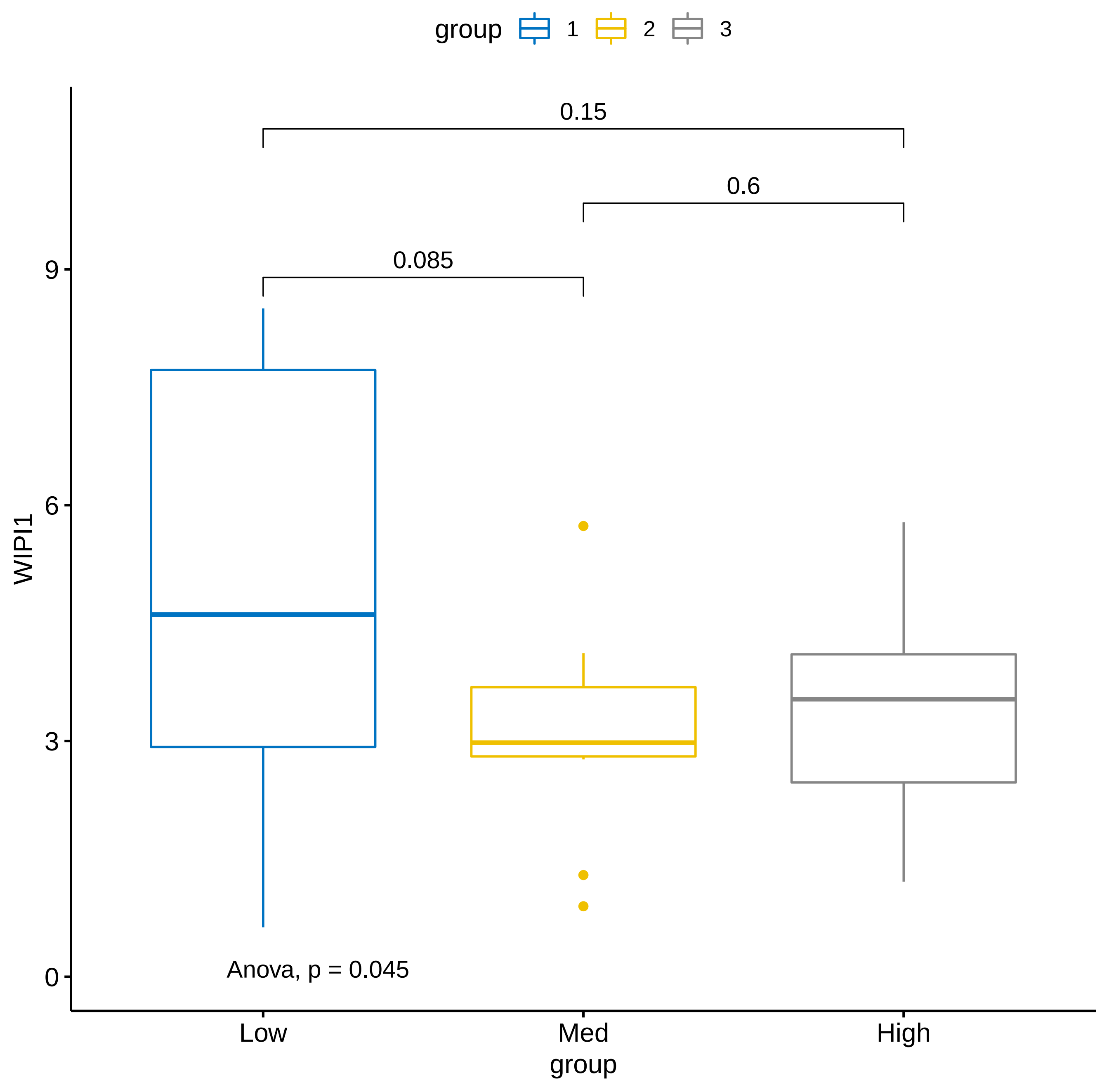

group
1
2
3
0.15
0.6
0.085
9
6
3
0
WIPI1
Anova, p = 0.045
Low
Med
High
group

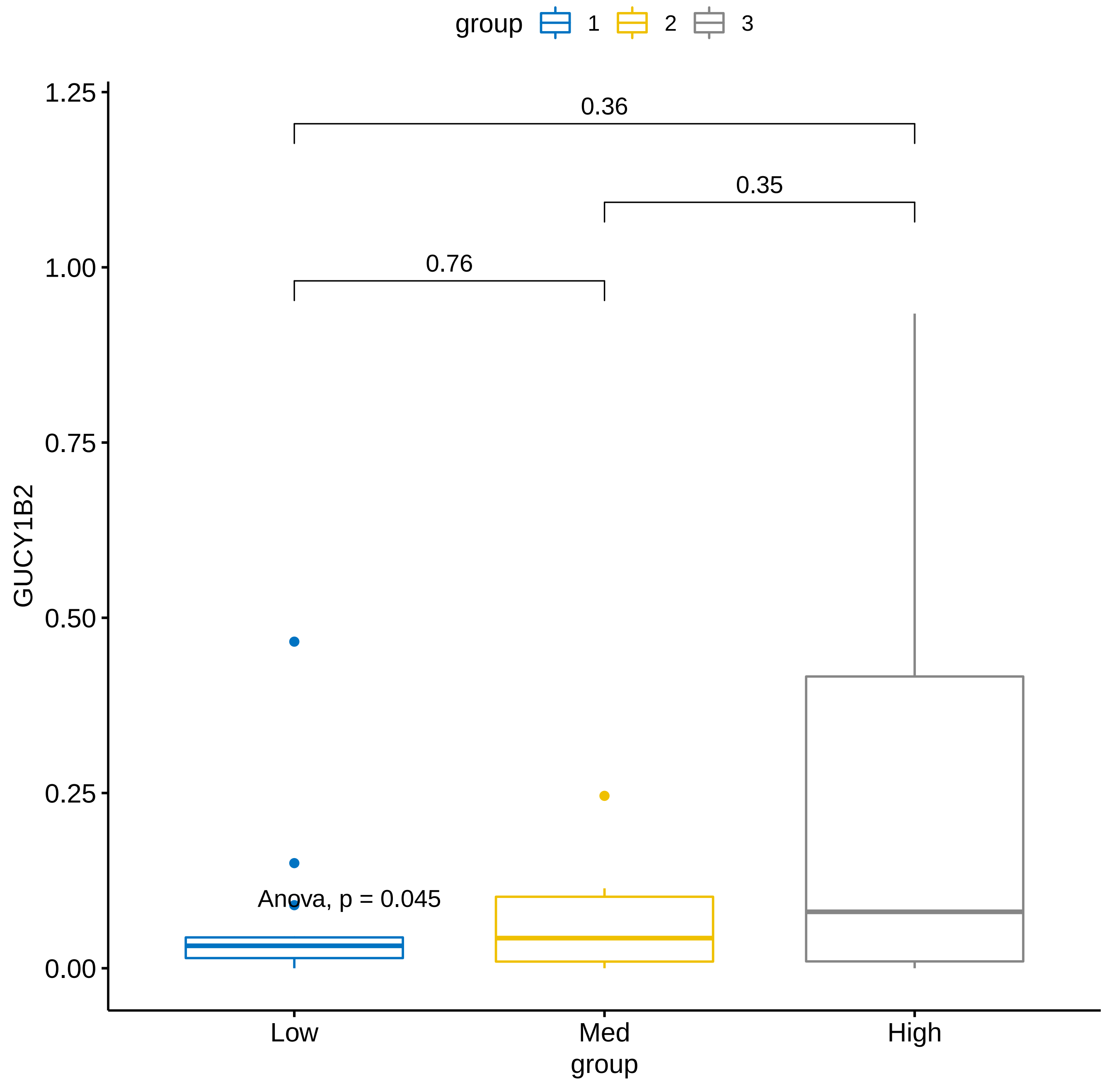

group 1 2 3
0.36
0.35
0.76
1.25
1.00
0.75
0.50
0.25
0.00
GUCY1B2
Anova, p = 0.045
Low
Med
High
group

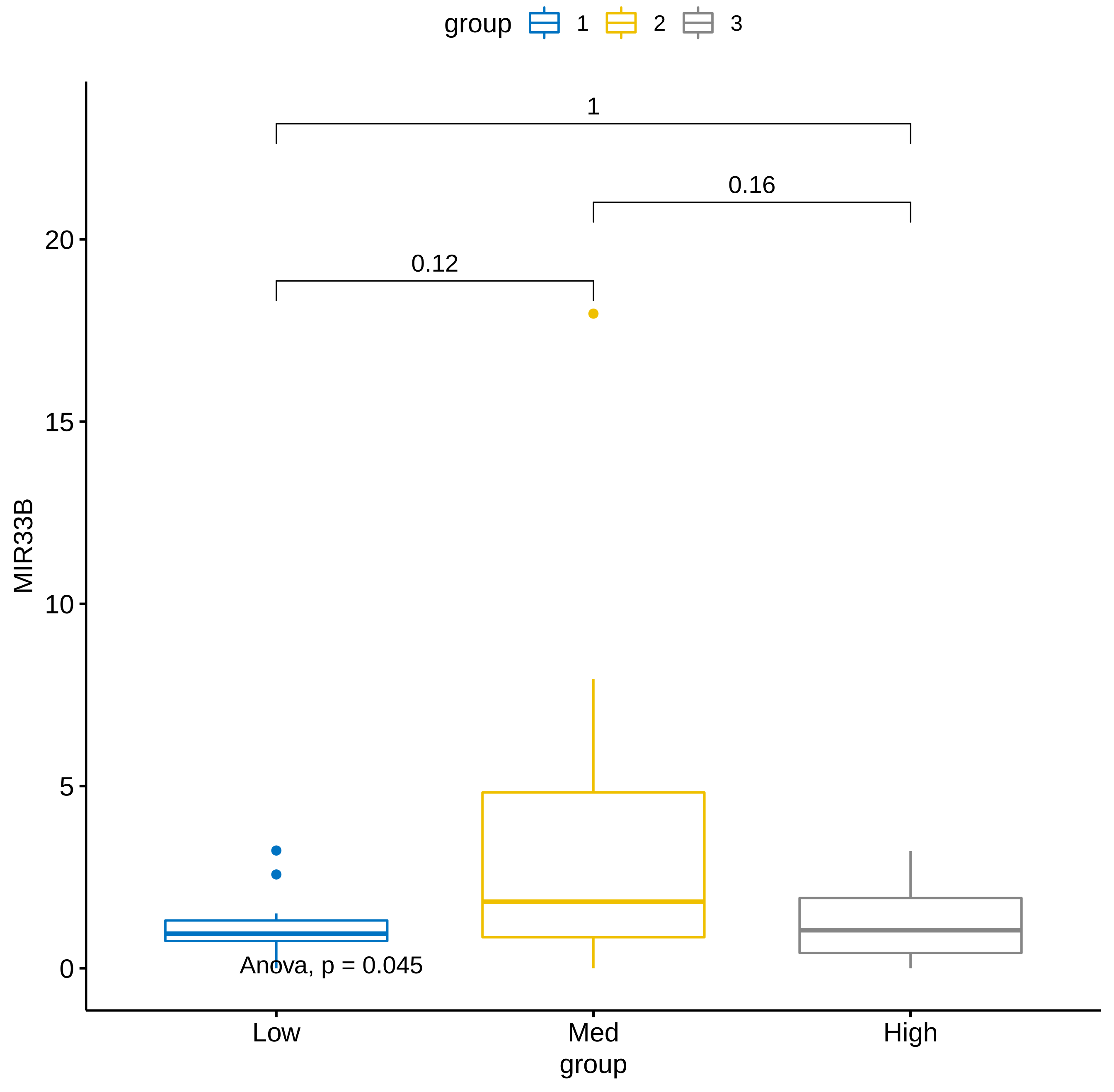

group 1 2 3
1
0.16
0.12
20
15
10
5
0
MIR33B
Anova, p = 0.045
Low
Med
High
group

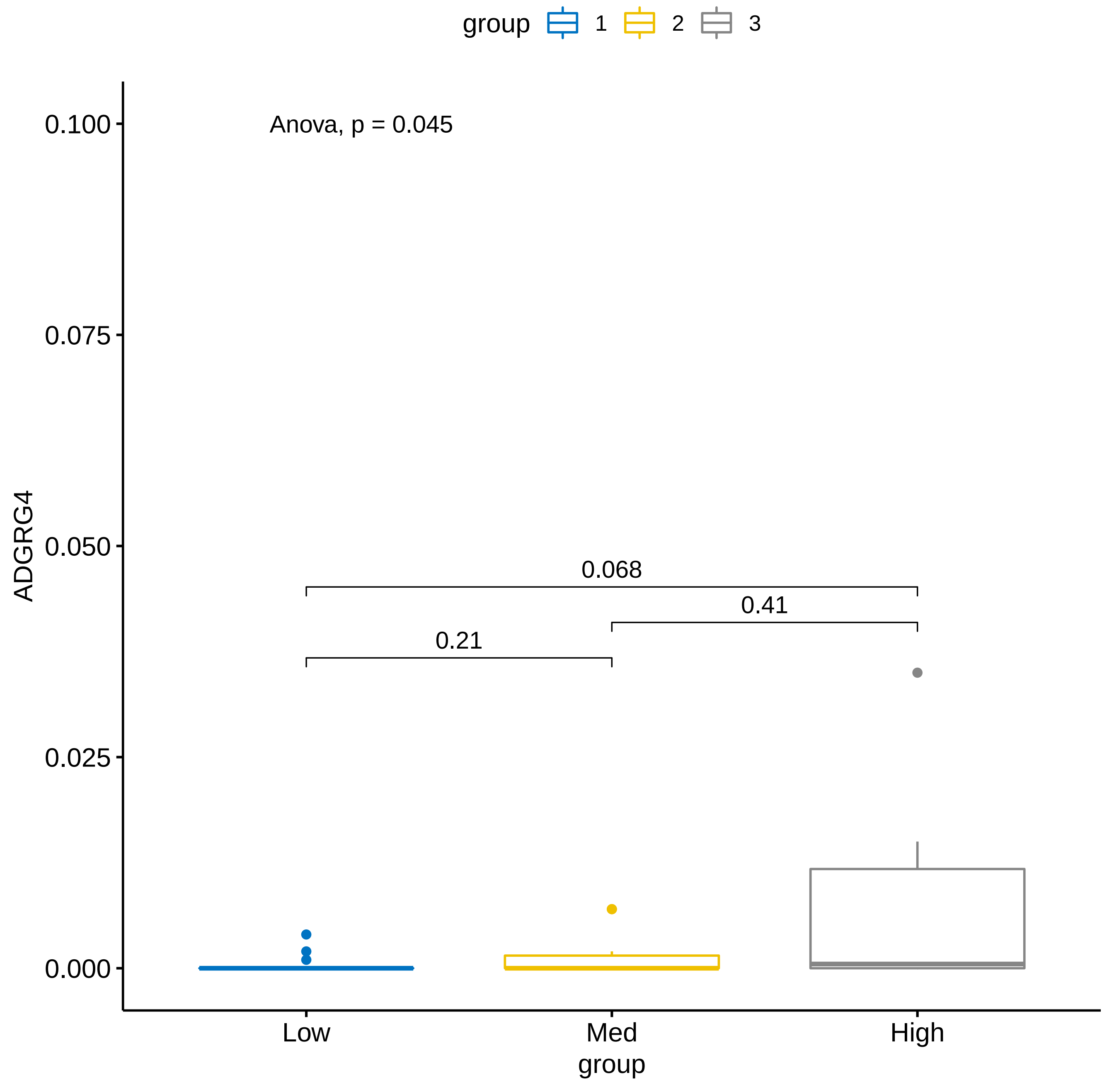
group
1
2
3
Anova, p = 0.045
0.100
0.075
0.050
0.025
0.000
ADGRG4
0.068
0.41
0.21
Low
Med
High
group

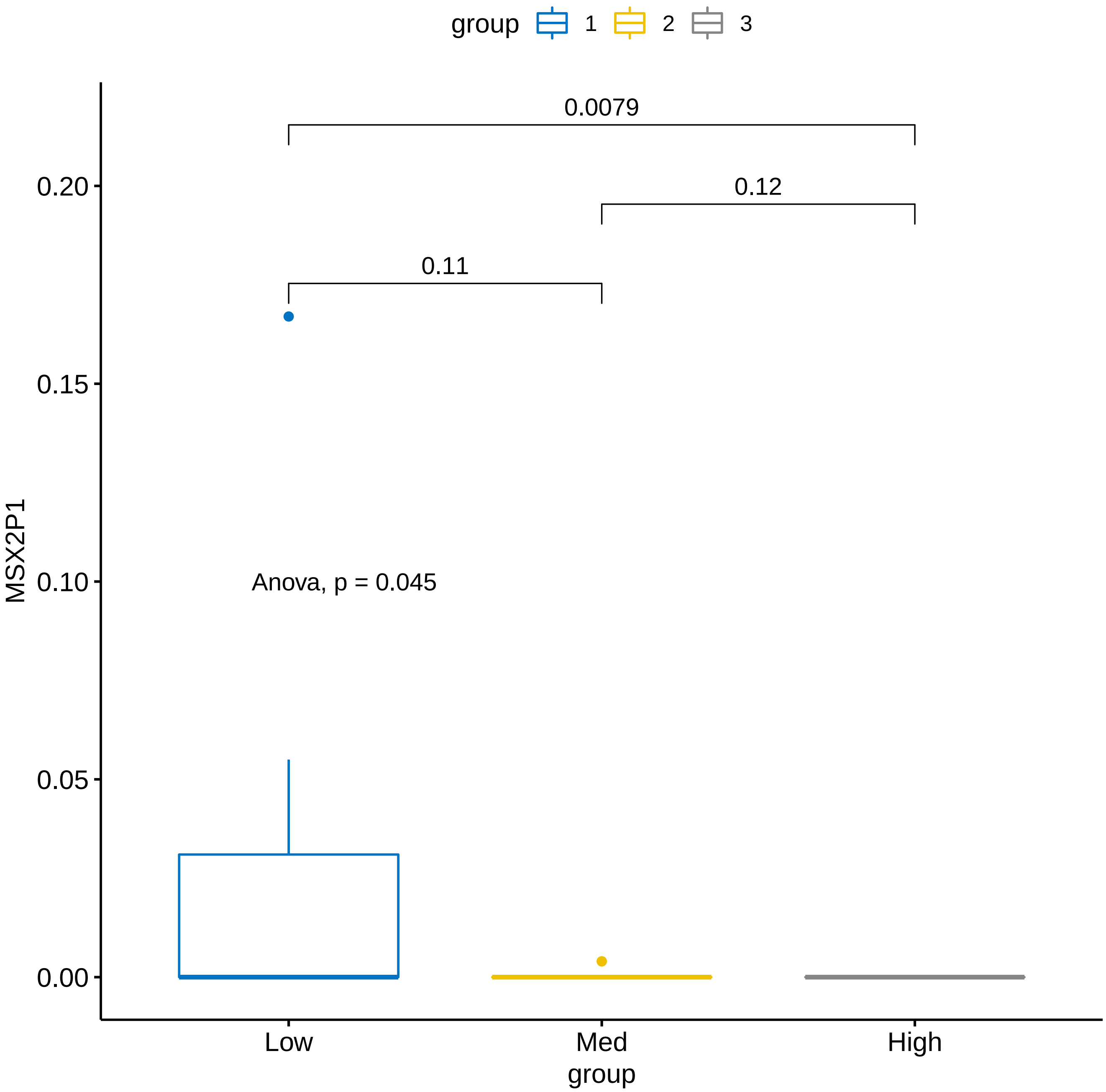

group
1
2
3
0.0079
0.12
0.11
Anova, p = 0.045
MSX2P1
0.20
0.15
0.10
0.05
0.00
Low
Med
High
group

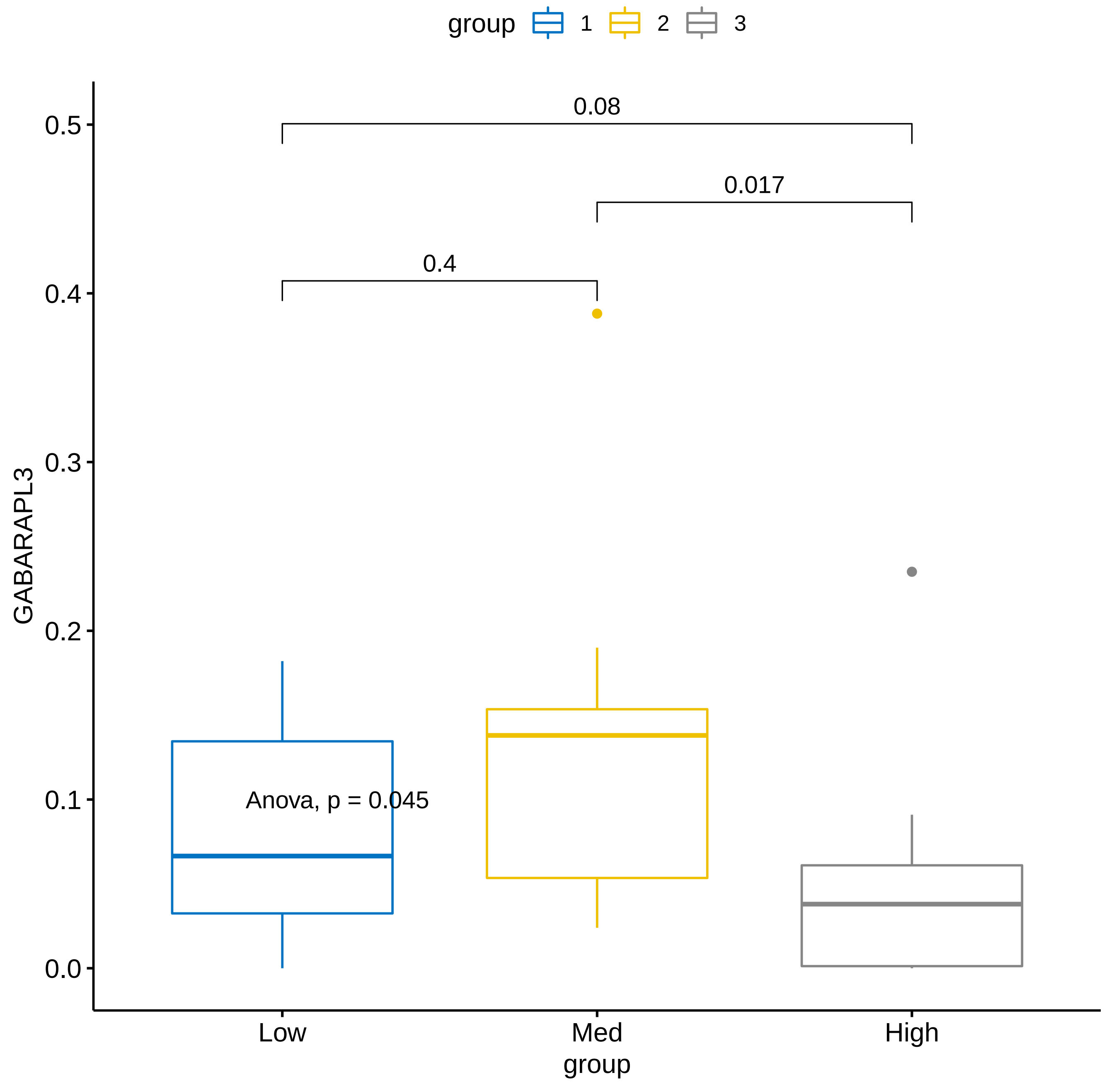
group
1
2
3
0.08
0.017
0.4
Anova, p = 0.045
GABARAPL3
0.5
0.4
0.3
0.2
0.1
0.0
Low
Med
High
group

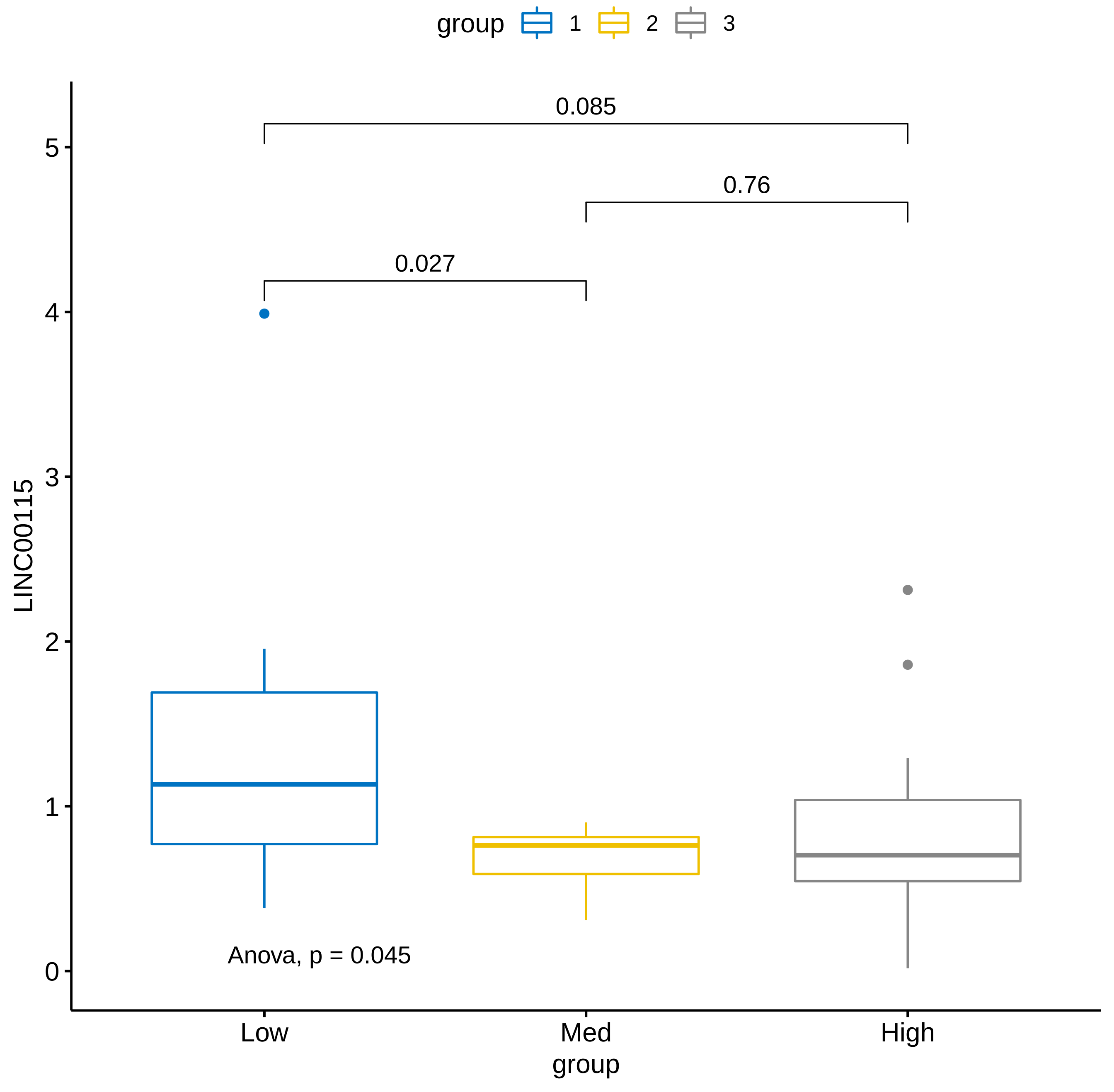
group
1
2
3
0.085
0.76
0.027
LINC00115
5
4
3
2
1
0
Anova, p = 0.045
Low
Med
High
group

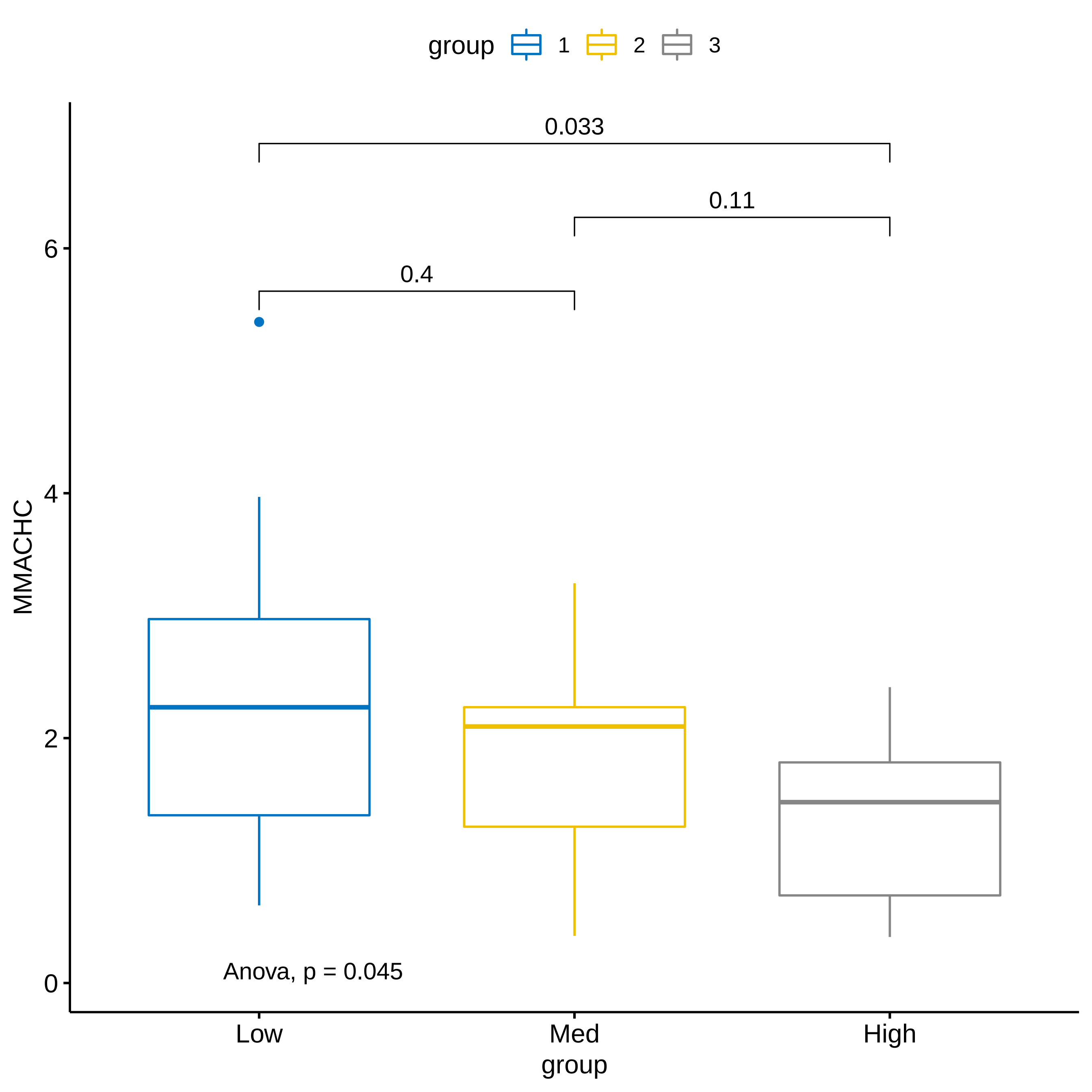

group
1
2
3
0.033
0.11
0.4
MMACHC
6
4
2
0
Anova, p = 0.045
Low
Med
High
group

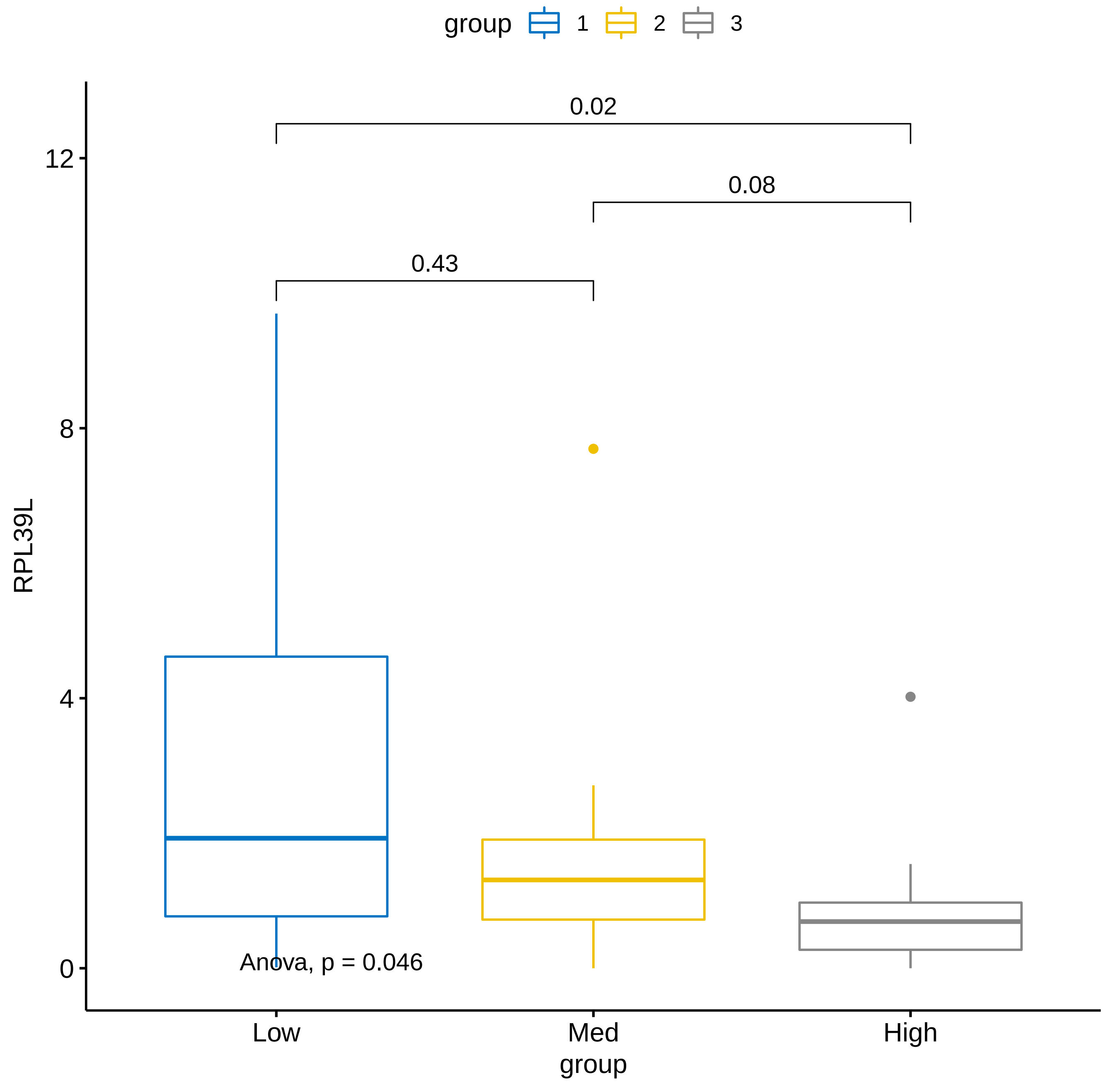

group 1 2 3
0.02
0.08
0.43
12
8
4
0
RPL39L
Anova, p = 0.046
Low
Med
High
group

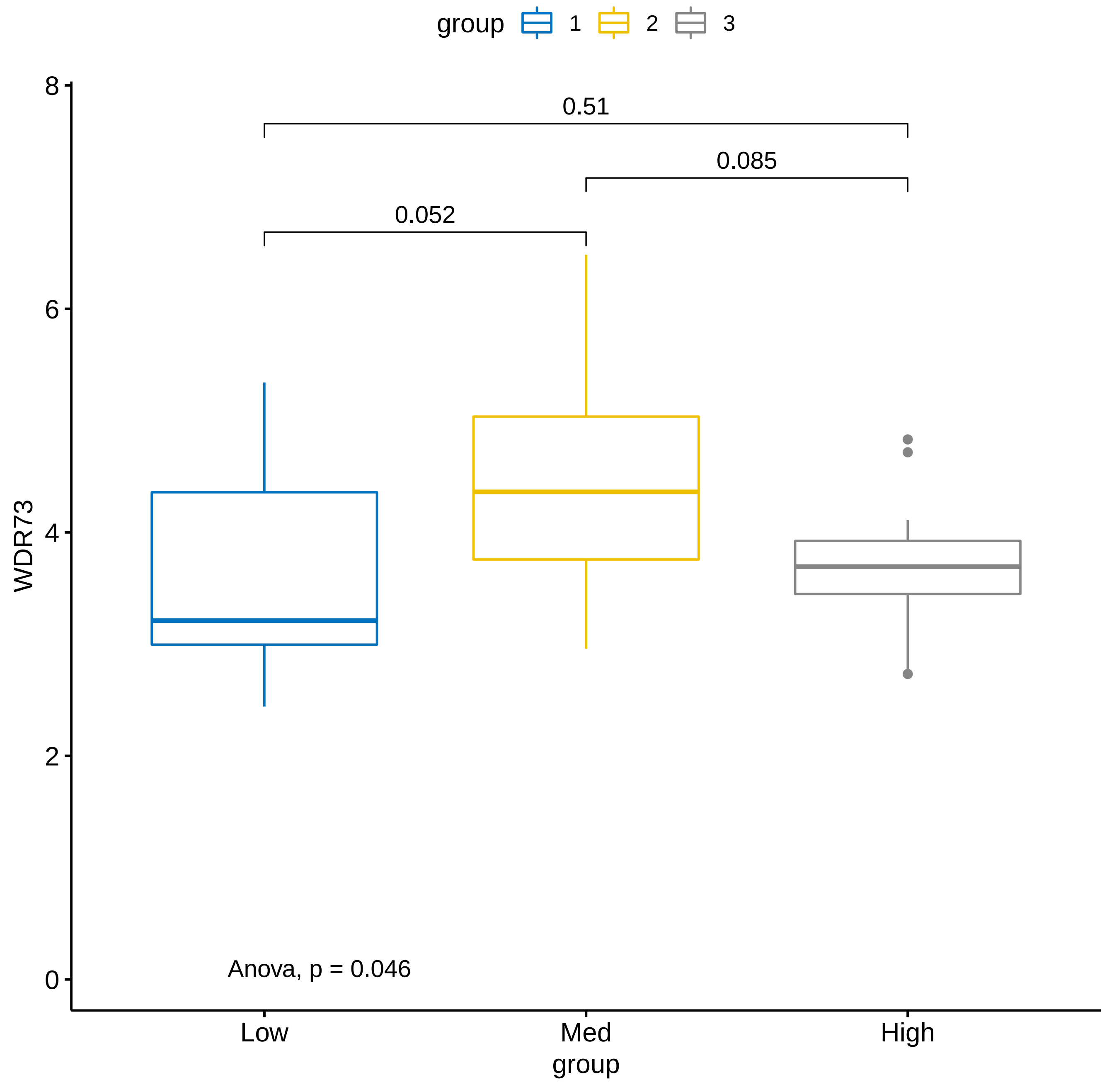
group
1
2
3
8
6
4
2
0
WDR73
0.51
0.085
0.052
Anova, p = 0.046
Low
Med
High
group

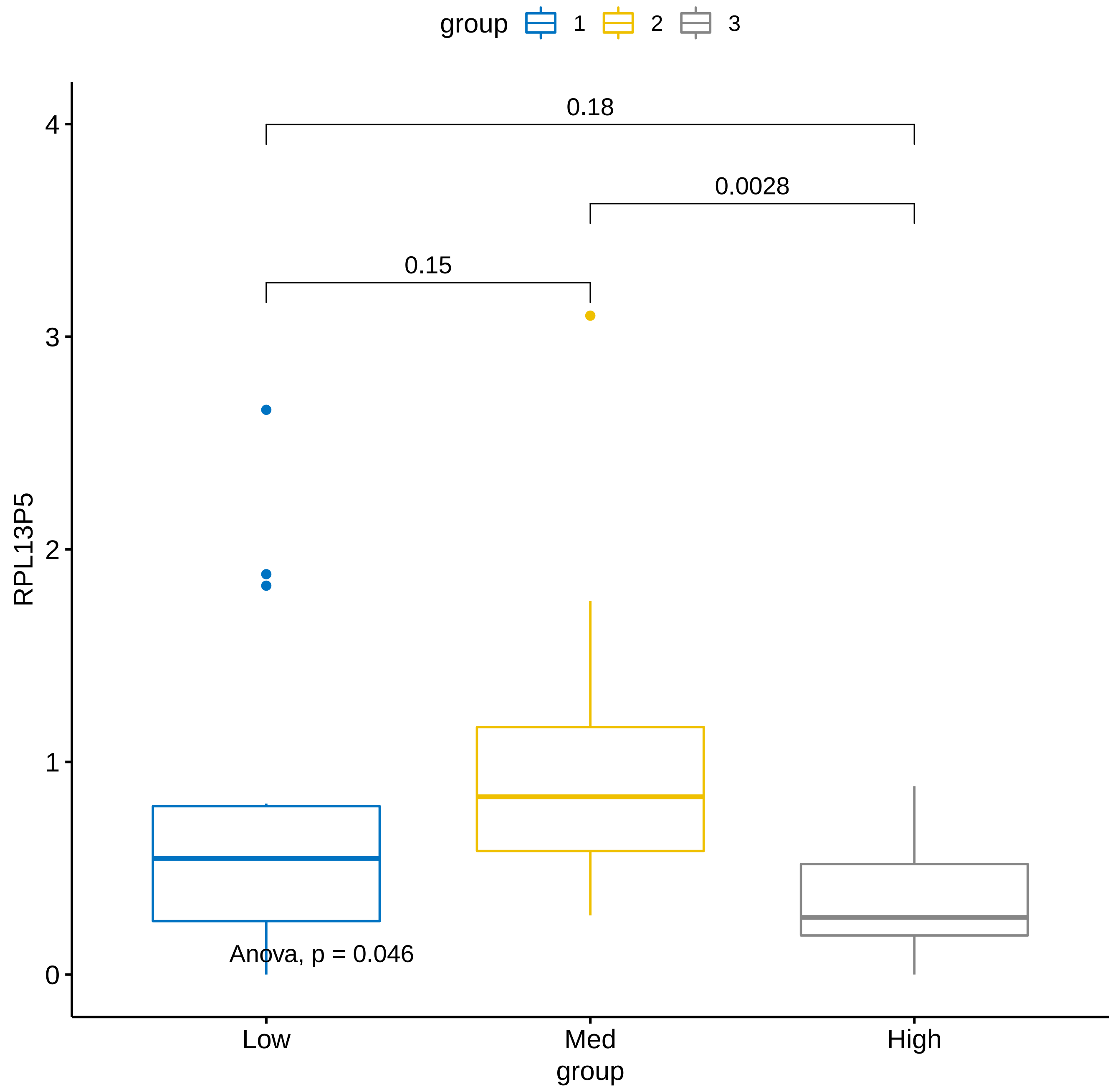
group
1
2
3
0.18
0.0028
0.15
RPL13P5
4
3
2
1
0
Anova, p = 0.046
Low
Med
High
group

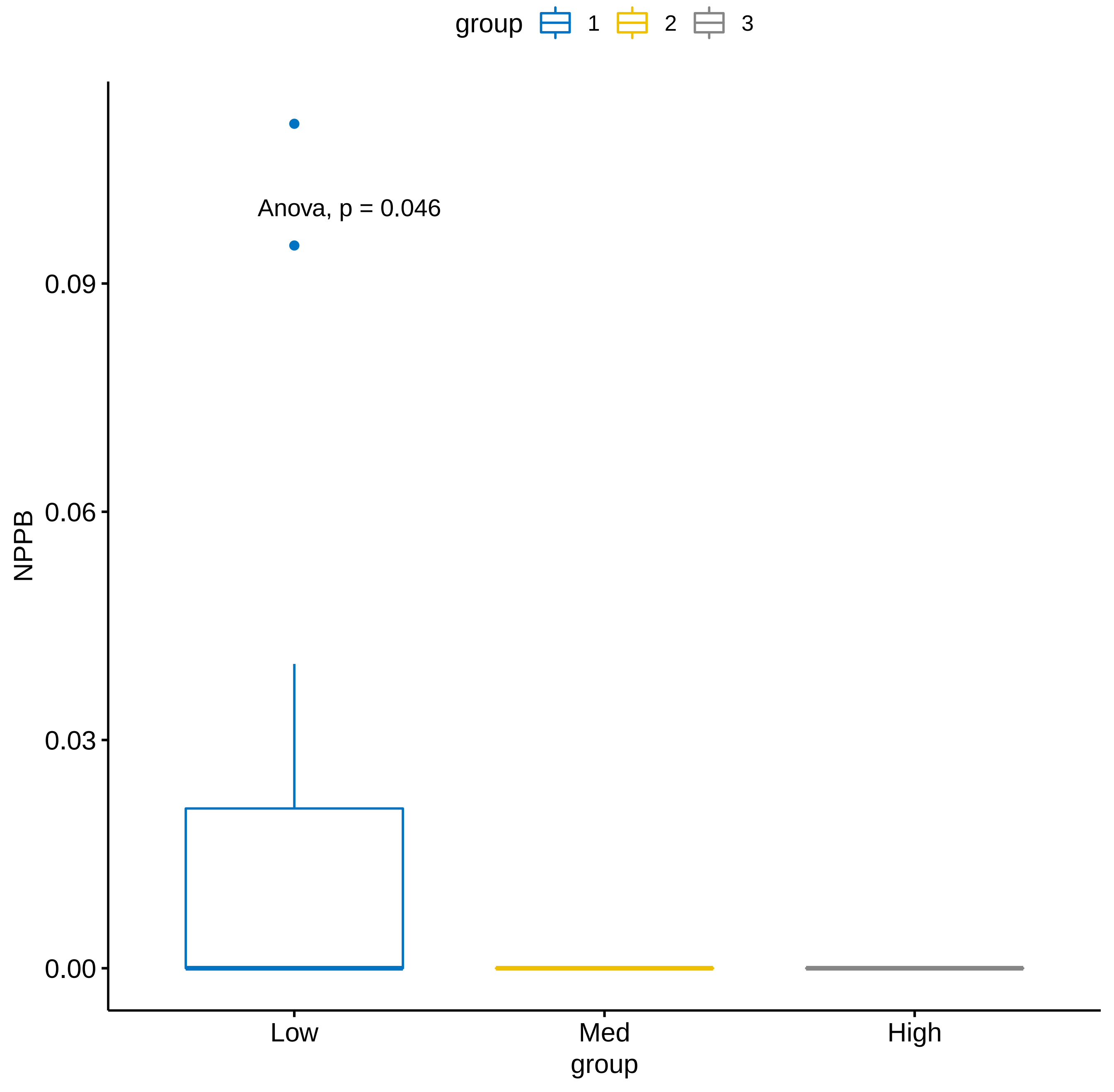

group
1
2
3
Anova, p = 0.046
NPPB
0.09
0.06
0.03
0.00
Low
Med
High
group

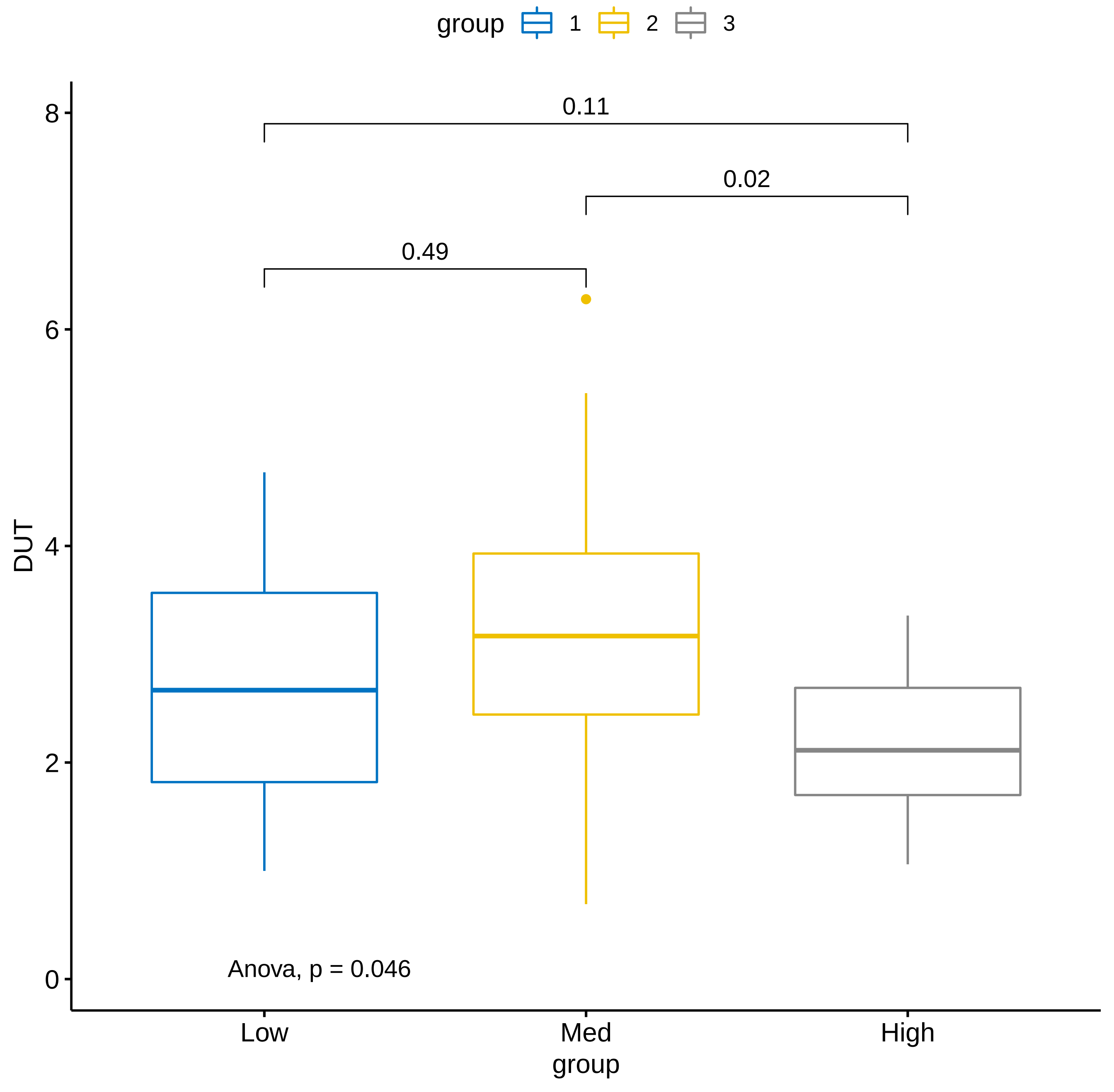

group 1 2 3
0.11
0.02
0.49
8
6
4
2
0
DUT
Anova, p = 0.046
Low
Med
High
group

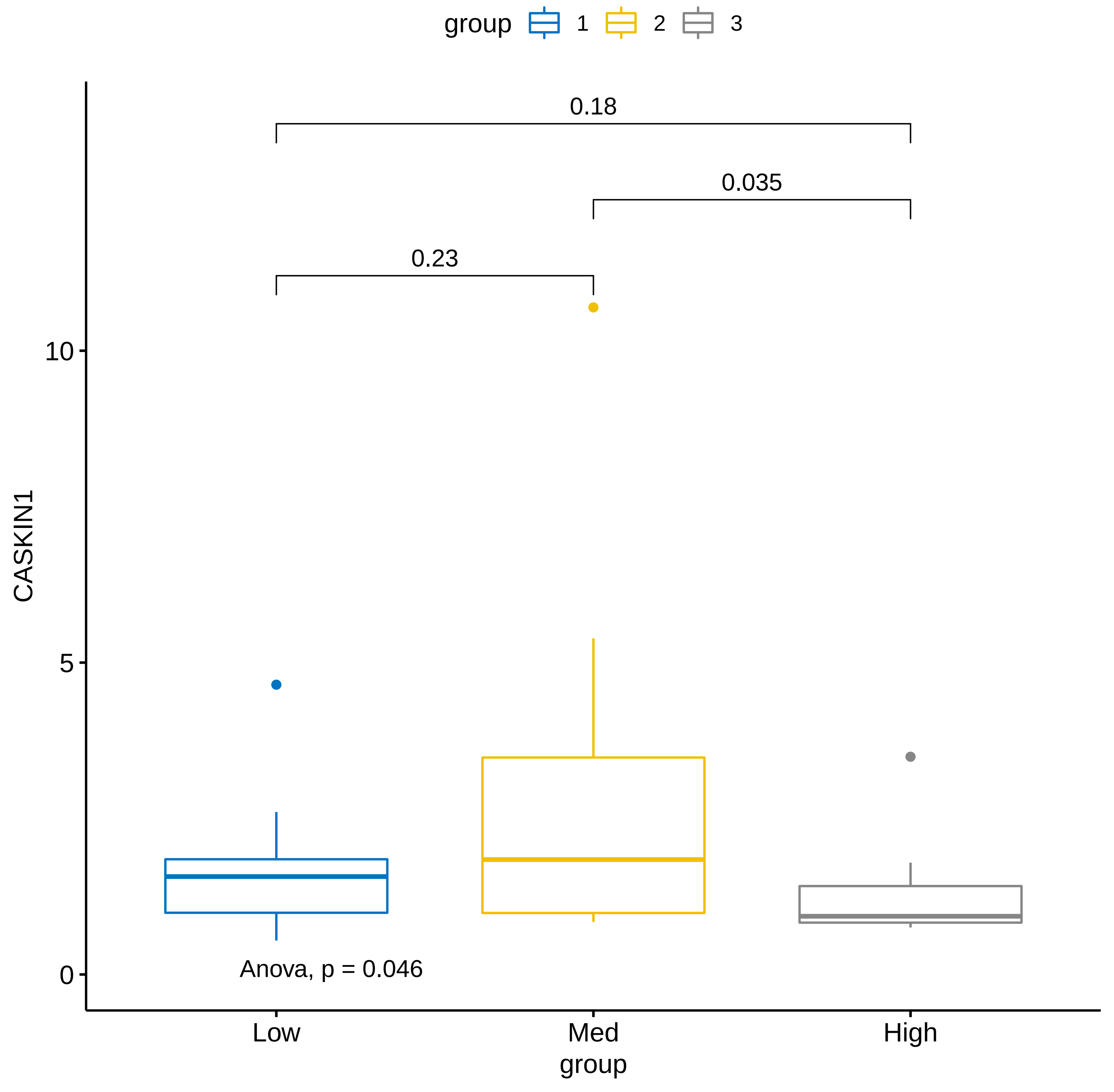

group 1 2 3
0.18
0.035
0.23
10
5
0
CASKIN1
Anova, p = 0.046
Low
Med
High
group

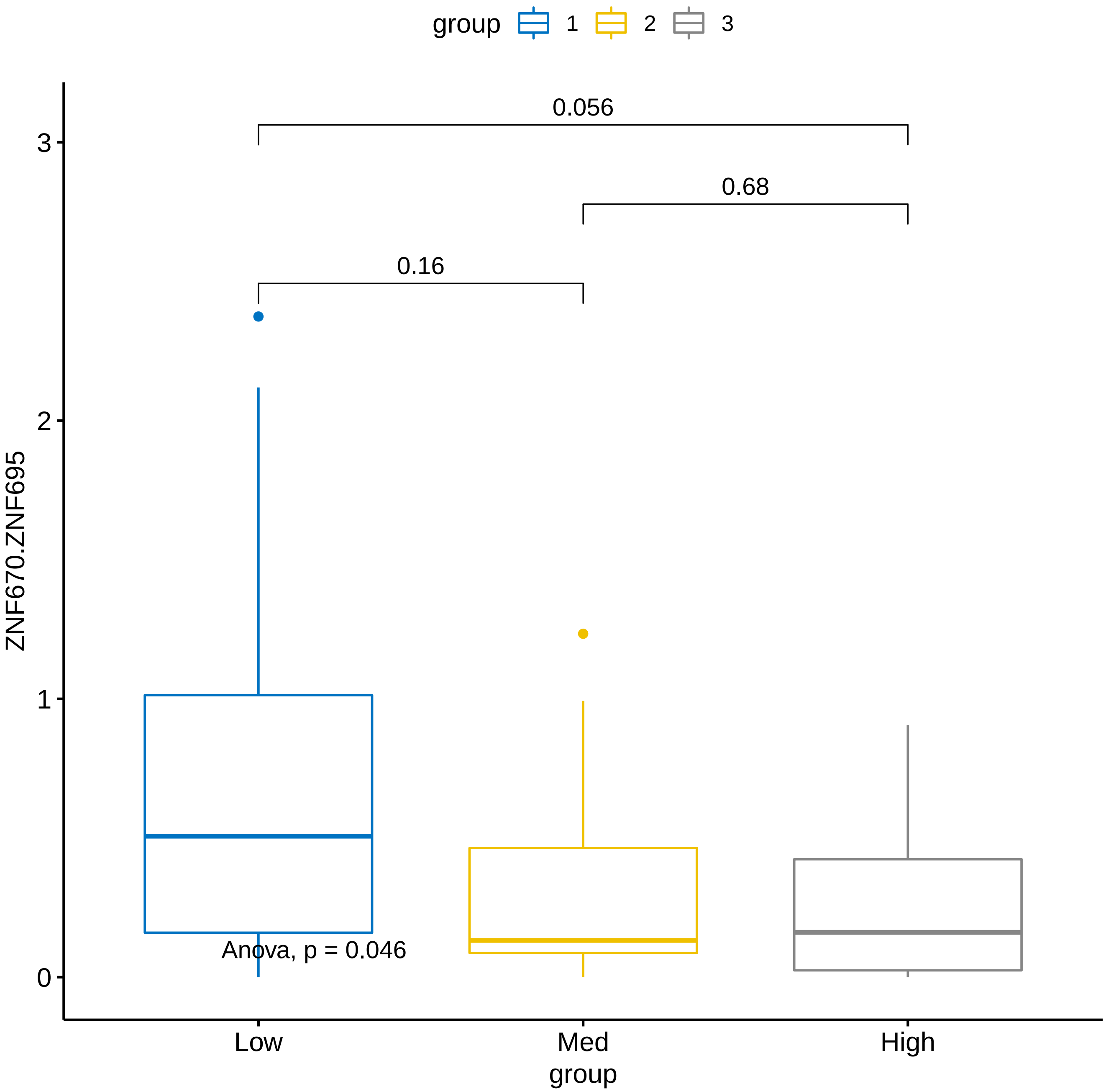

group 1 2 3
0.056
0.68
0.16
3
2
1
0
ZNF670.ZNF695
Anova, p = 0.046
Low
Med
High
group

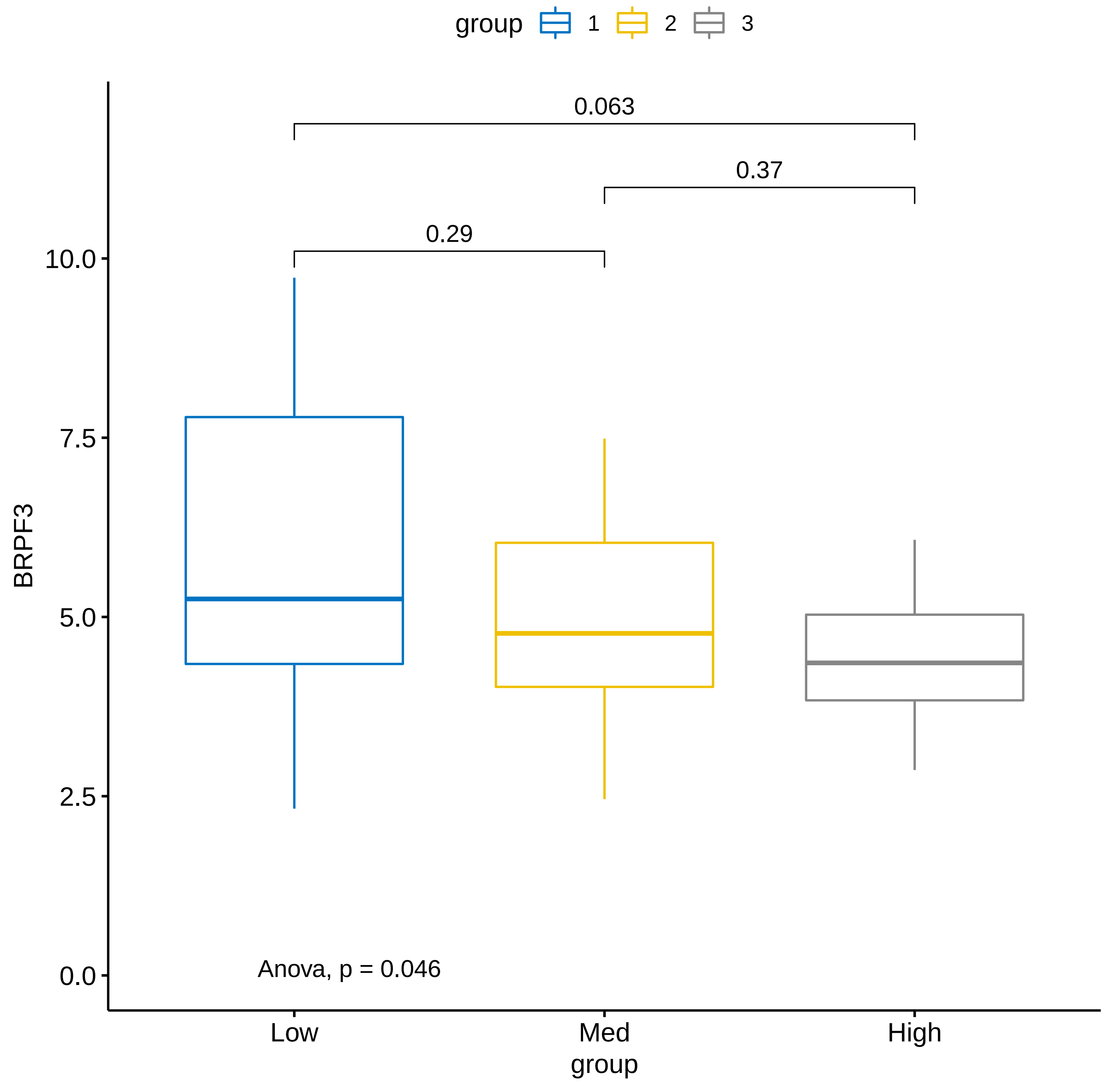

group 1 2 3
0.063
0.37
0.29
10.0
7.5
5.0
2.5
0.0
BRPF3
Anova, p = 0.046
Low
Med
High
group

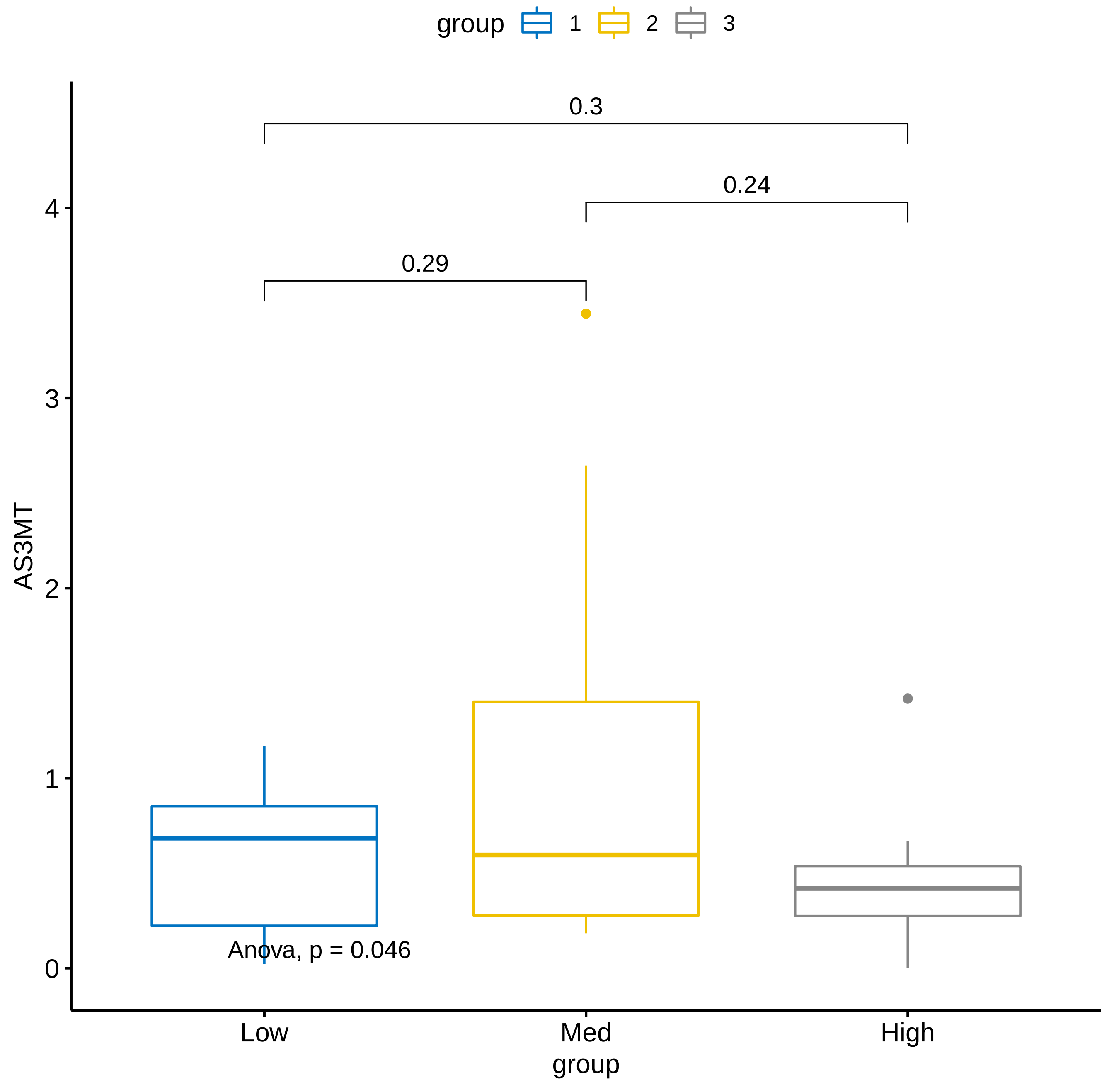

group
1
2
3
0.3
0.24
0.29
AS3MT
4
3
2
1
0
Anova, p = 0.046
Low
Med
High
group

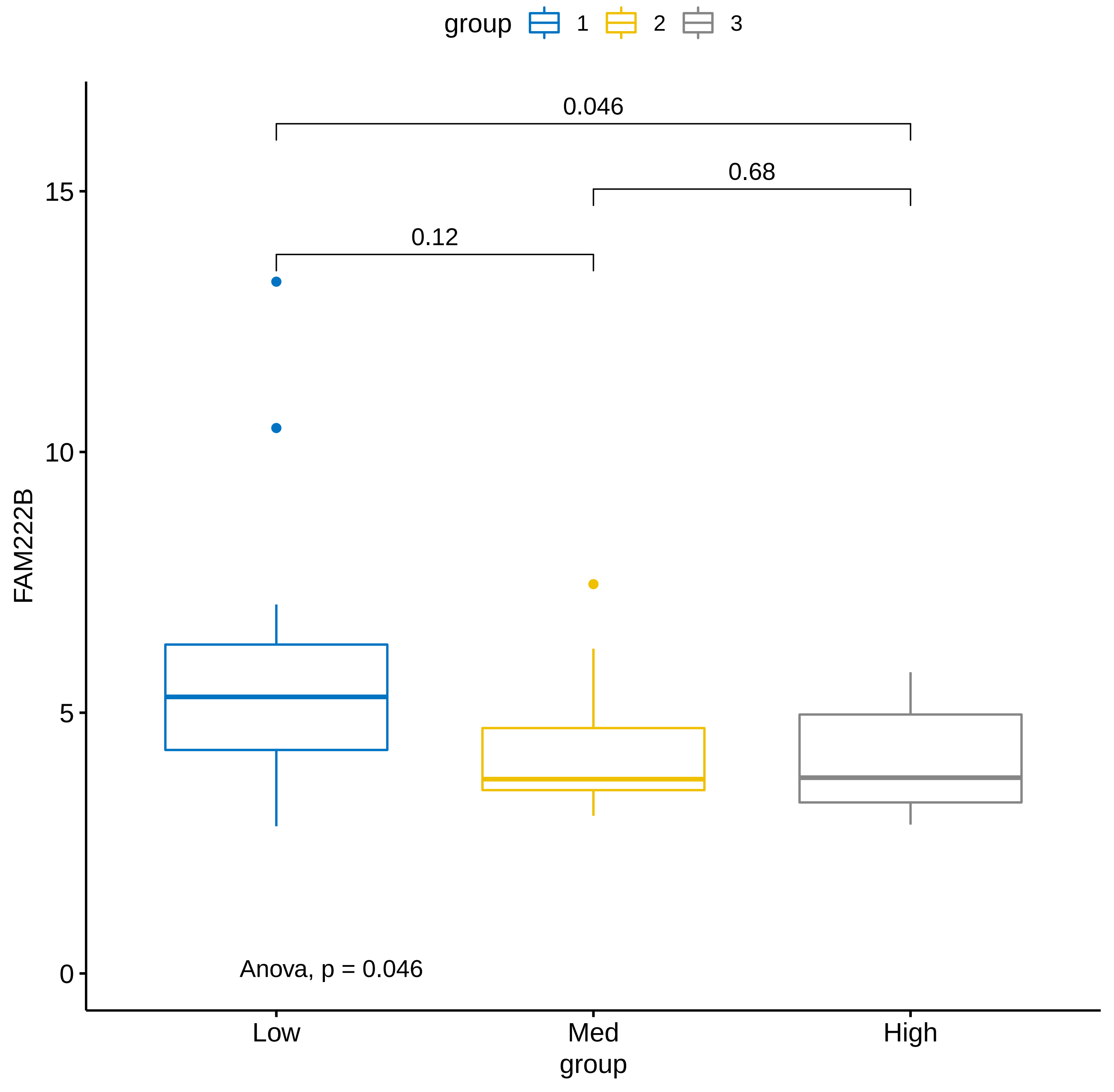

group 1 2 3
0.046
0.68
0.12
15
10
5
0
FAM222B
Anova, p = 0.046
Low
Med
High
group

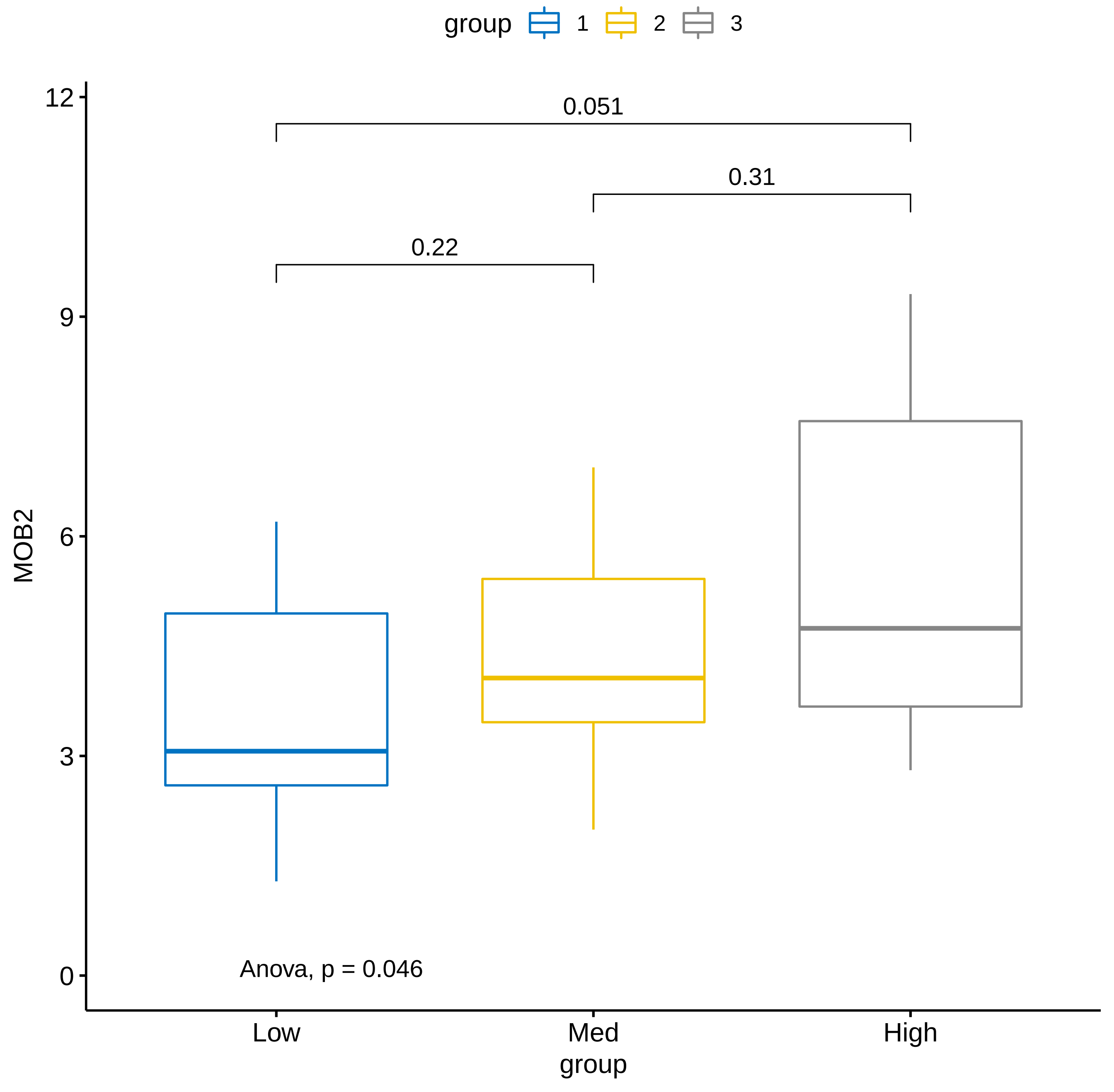
group
1
2
3
0.051
0.31
0.22
12
9
6
3
0
MOB2
Anova, p = 0.046
Low
Med
High
group

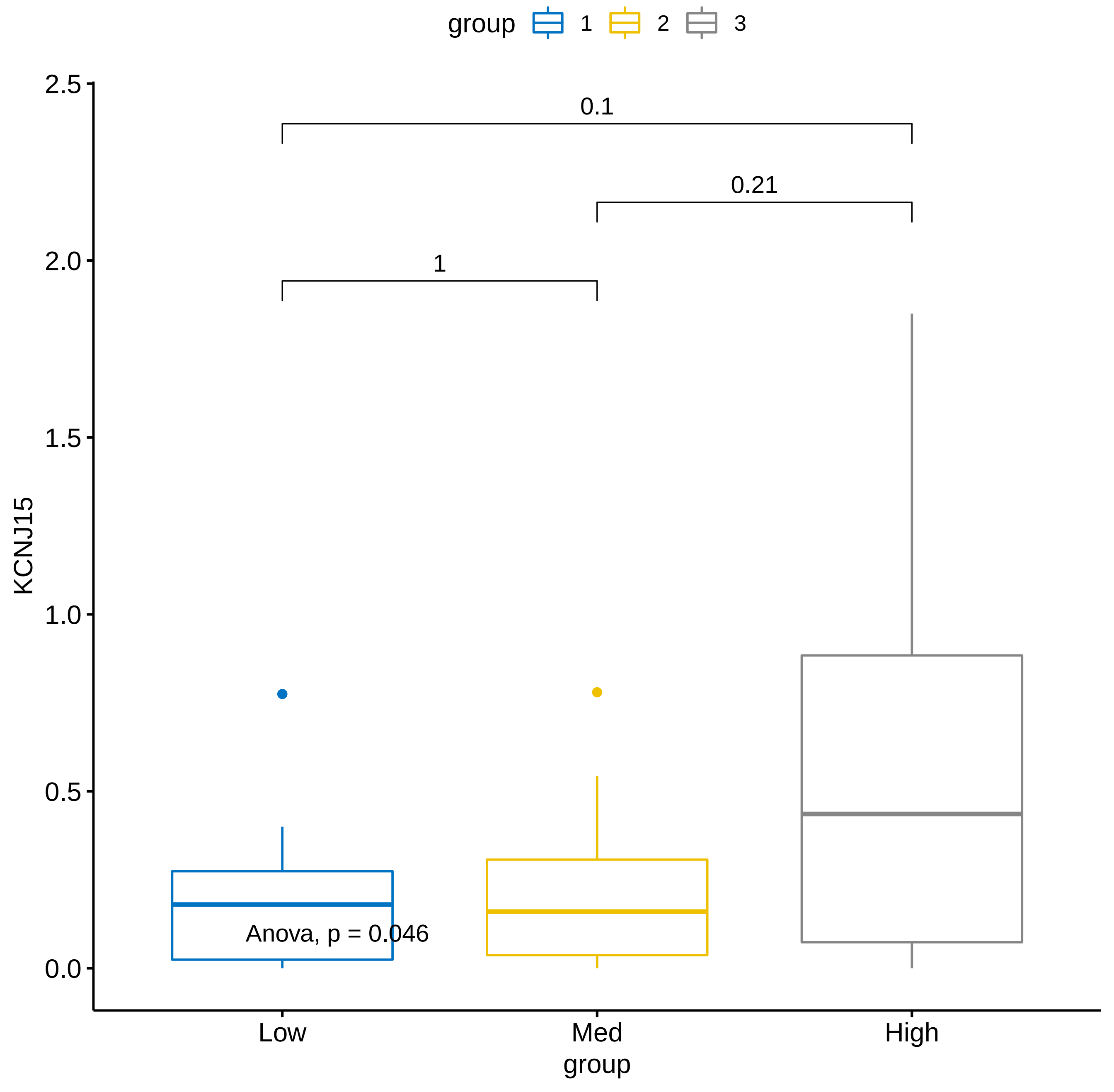
group 1 2 3
2.5
2.0
1.5
1.0
0.5
0.0
KCNJ15
0.1
0.21
1
Anova, p = 0.046
Low
Med
High
group

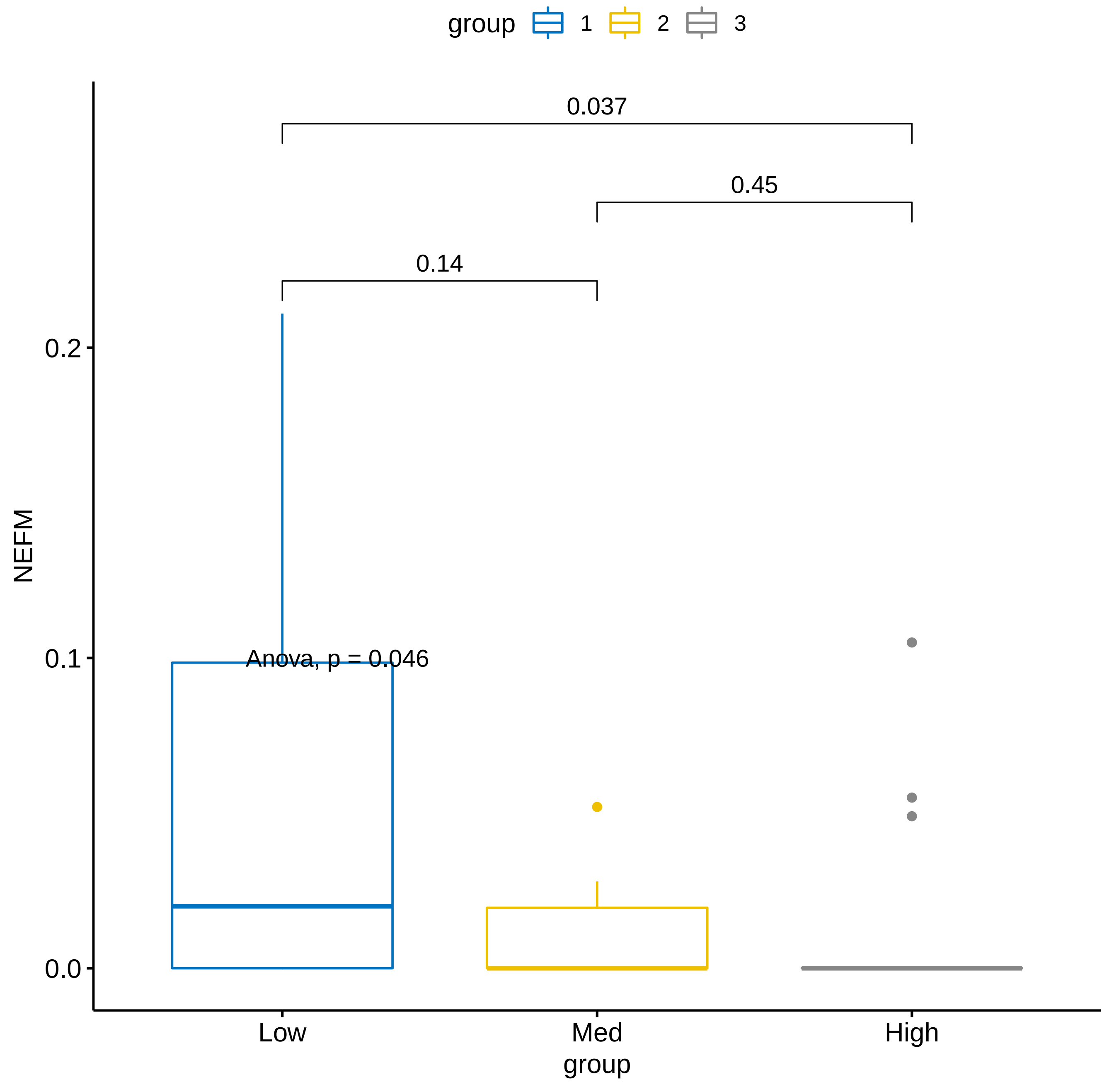

group 1 2 3
0.037
0.45
0.14
Anova, p = 0.046
NEFM
0.2
0.1
0.0
Low
Med
High
group

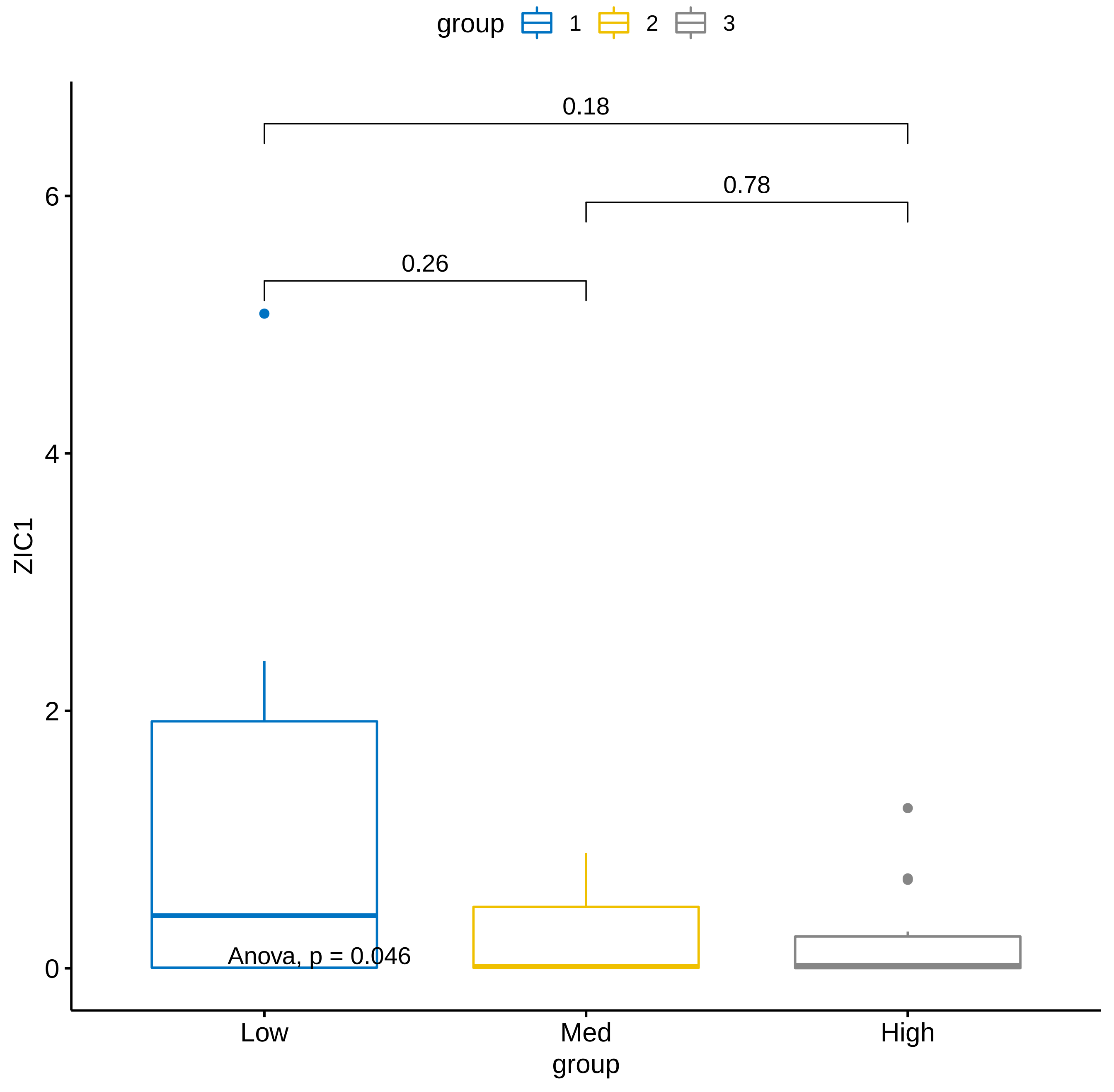
group
1
2
3
0.18
0.78
0.26
6
4
2
0
ZIC1
Anova, p = 0.046
Low
Med
High
group

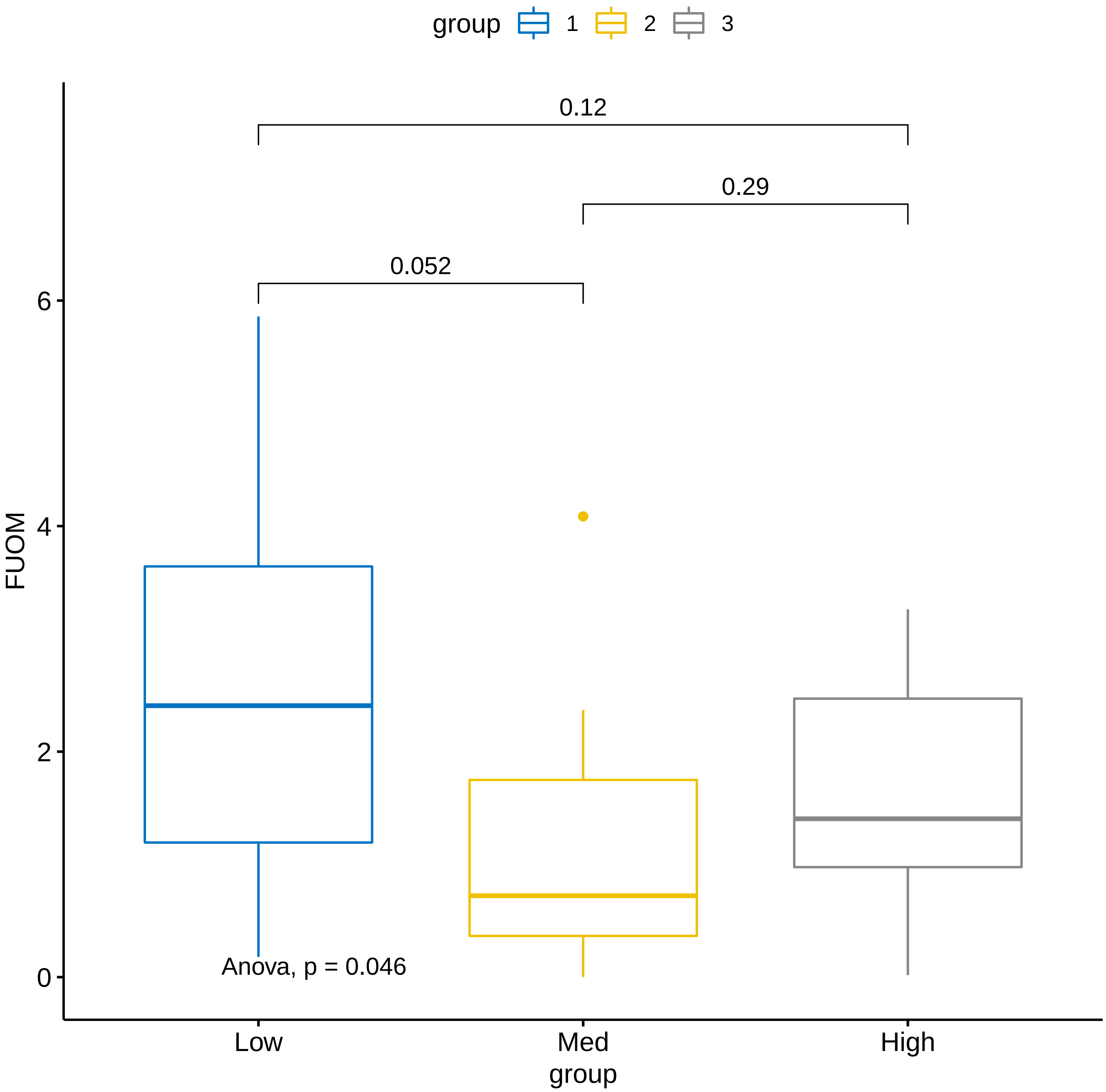
group
1
2
3
0.12
0.29
0.052
6
4
2
0
FUOM
Anova, p = 0.046
Low
Med
High
group

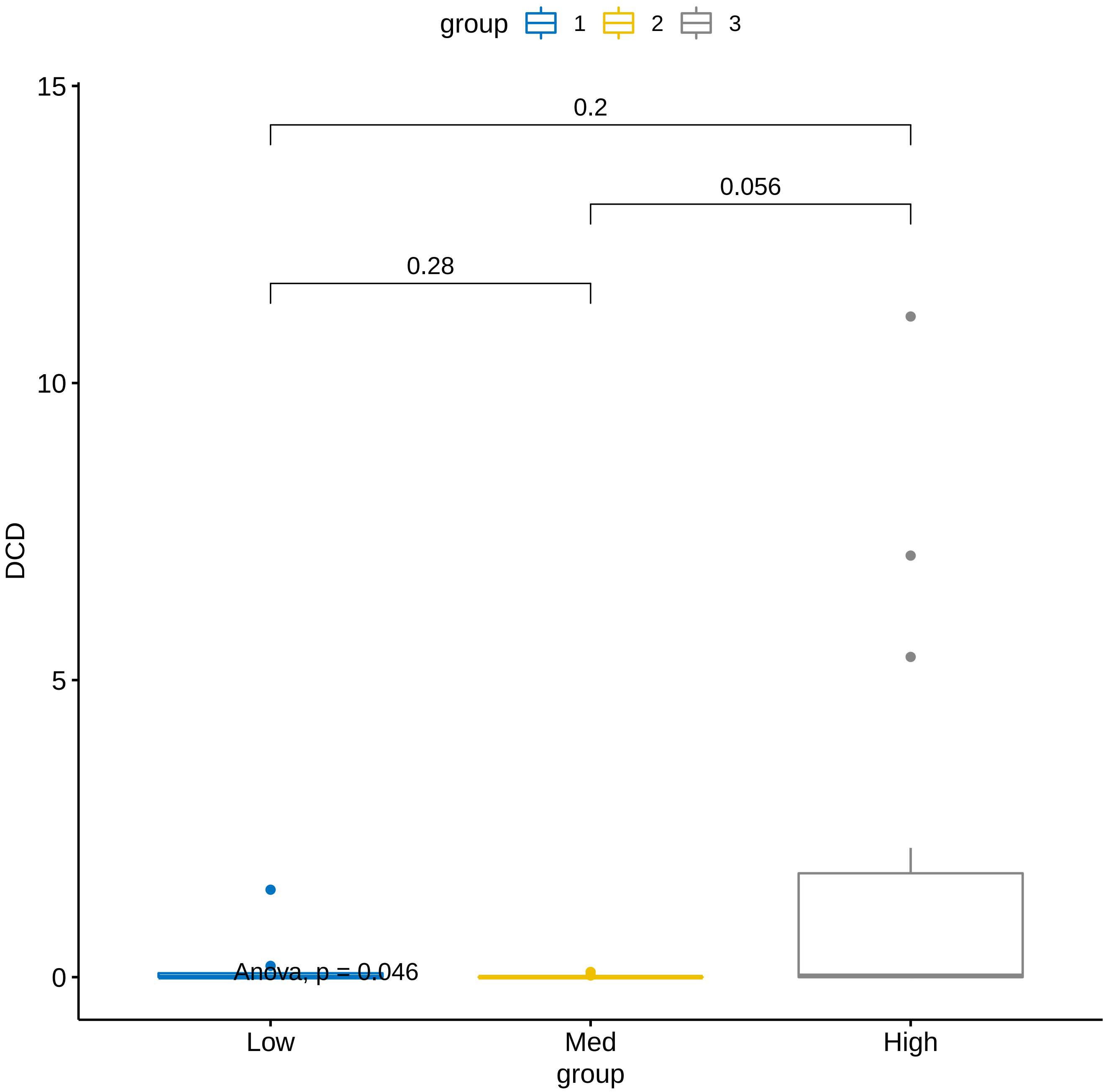

group
1
2
3
15
10
5
0
DCD
0.2
0.056
0.28
Anova, p = 0.046
Low
Med
High
group

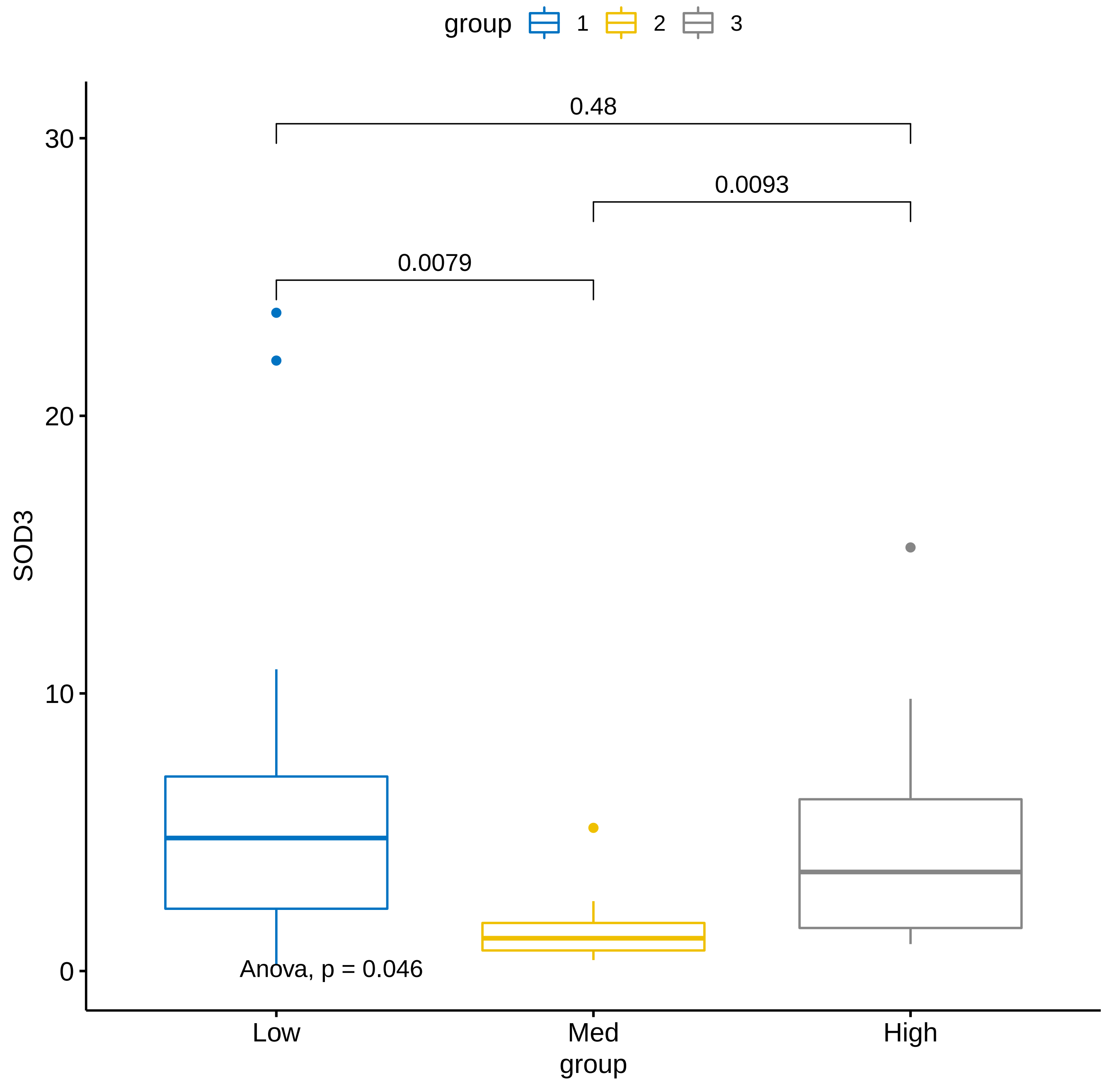

group
1
2
3
0.48
0.0093
0.0079
30
20
10
0
SOD3
Anova, p = 0.046
Low
Med
High
group

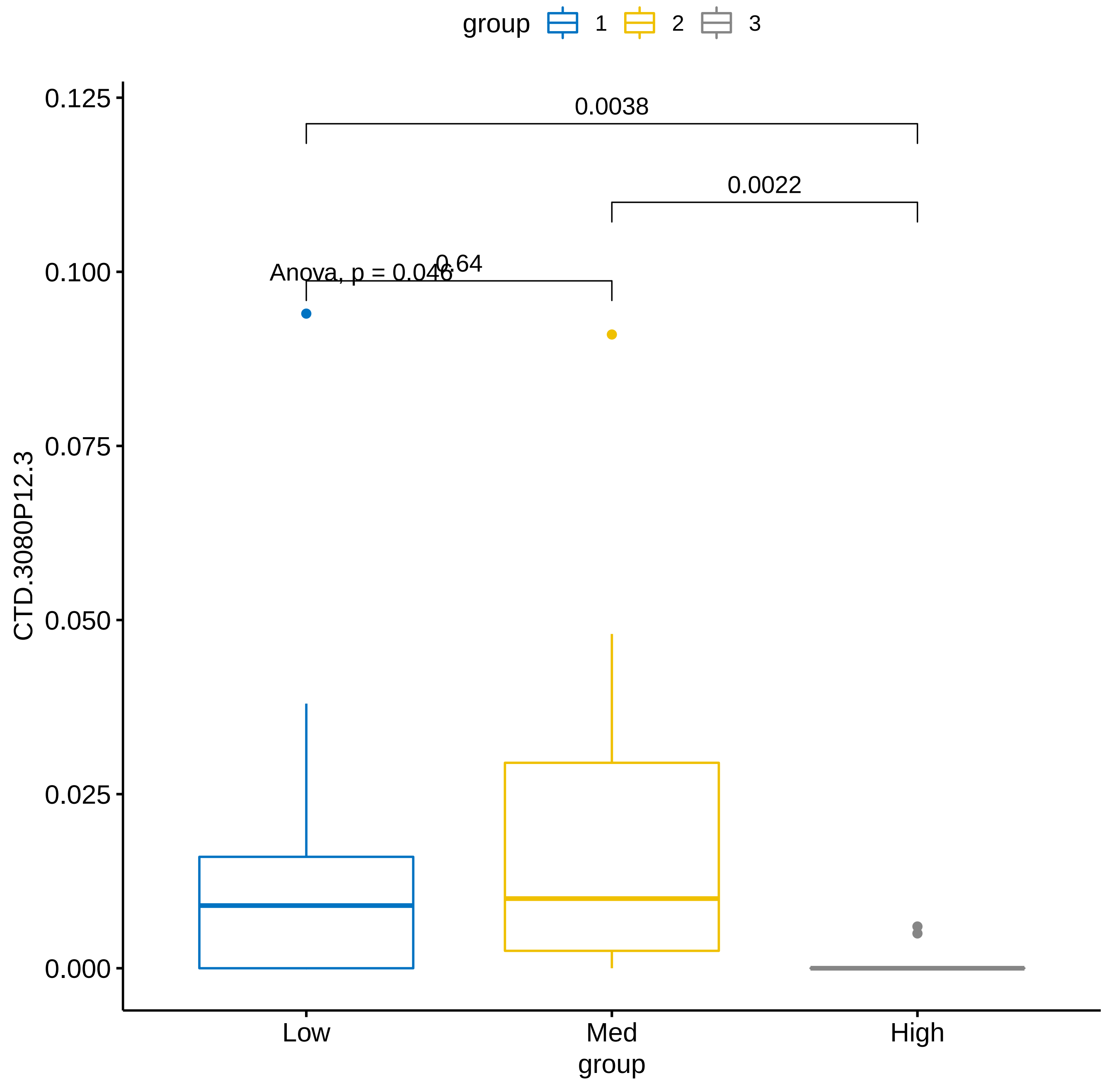

group
1
2
3
0.125
0.100
0.075
0.050
0.025
0.000
CTD.3080P12.3
0.0038
0.0022
Anova, p = 0.046
0.64
Low
Med
High
group

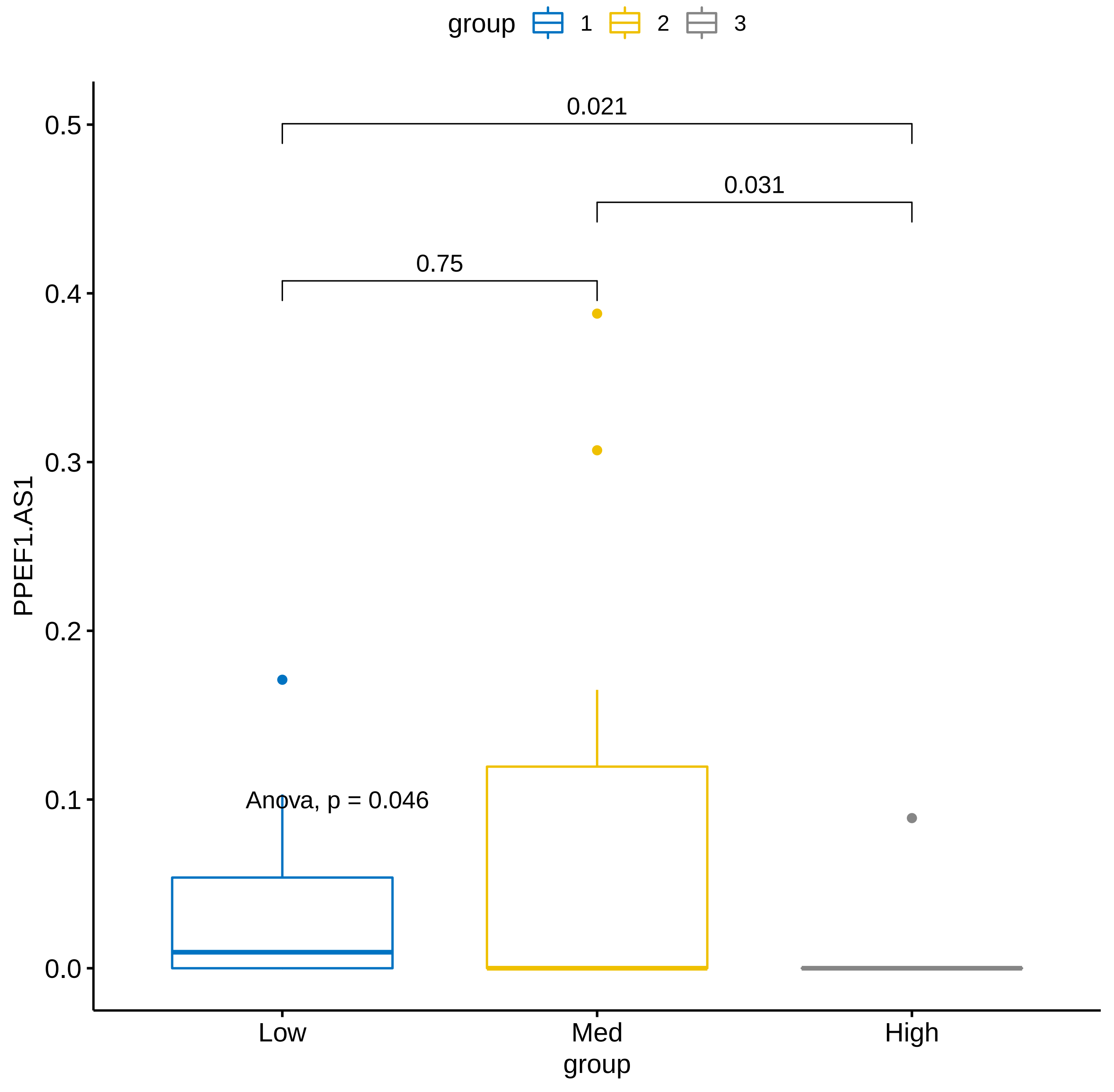
group
1
2
3
0.021
0.031
0.75
0.5
0.4
0.3
0.2
0.1
0.0
PPEF1.AS1
Anova, p = 0.046
Low
Med
High
group

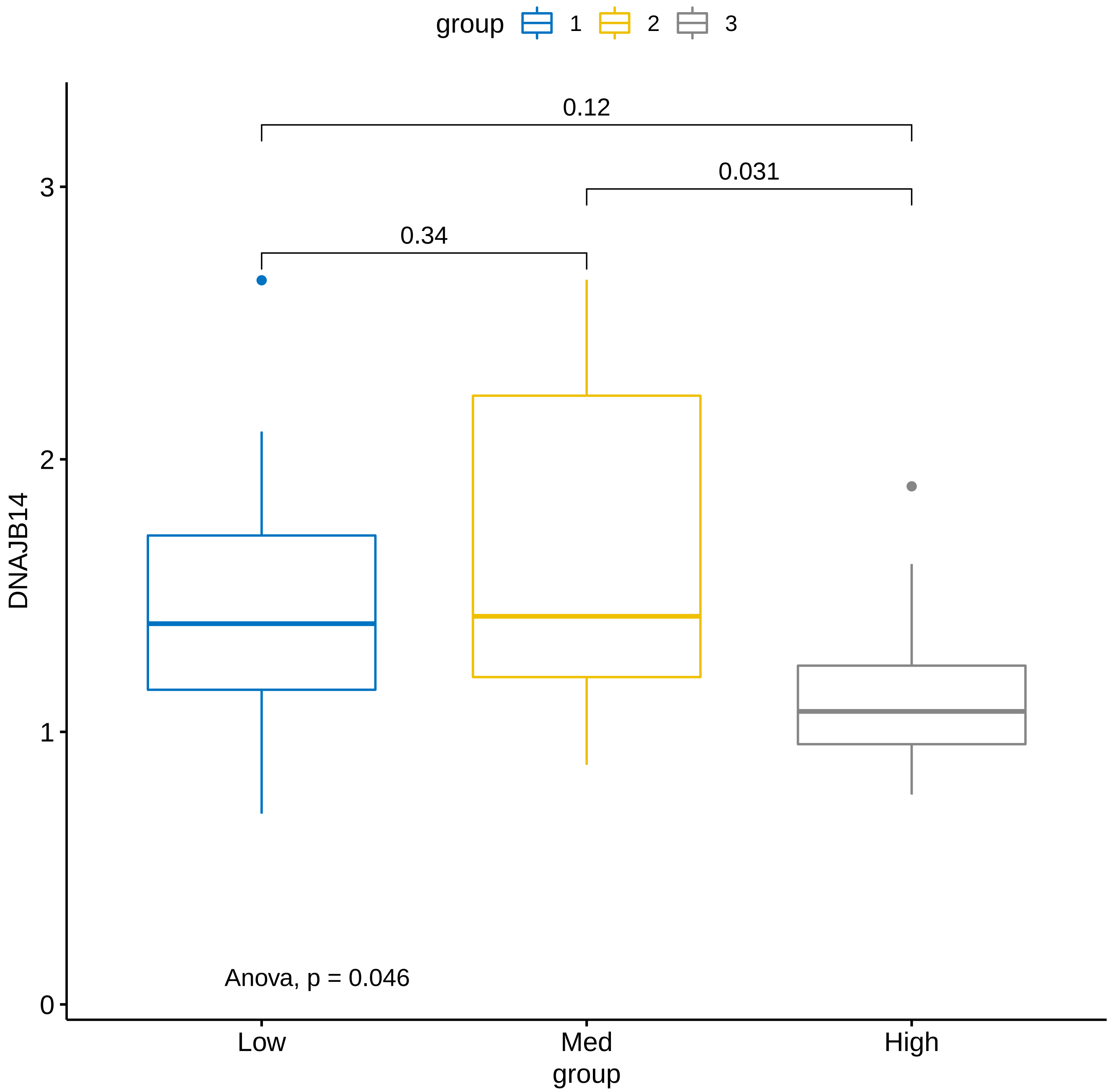
group
1
2
3
0.12
0.031
0.34
3
2
1
0
DNAJB14
Anova, p = 0.046
Low
Med
High
group

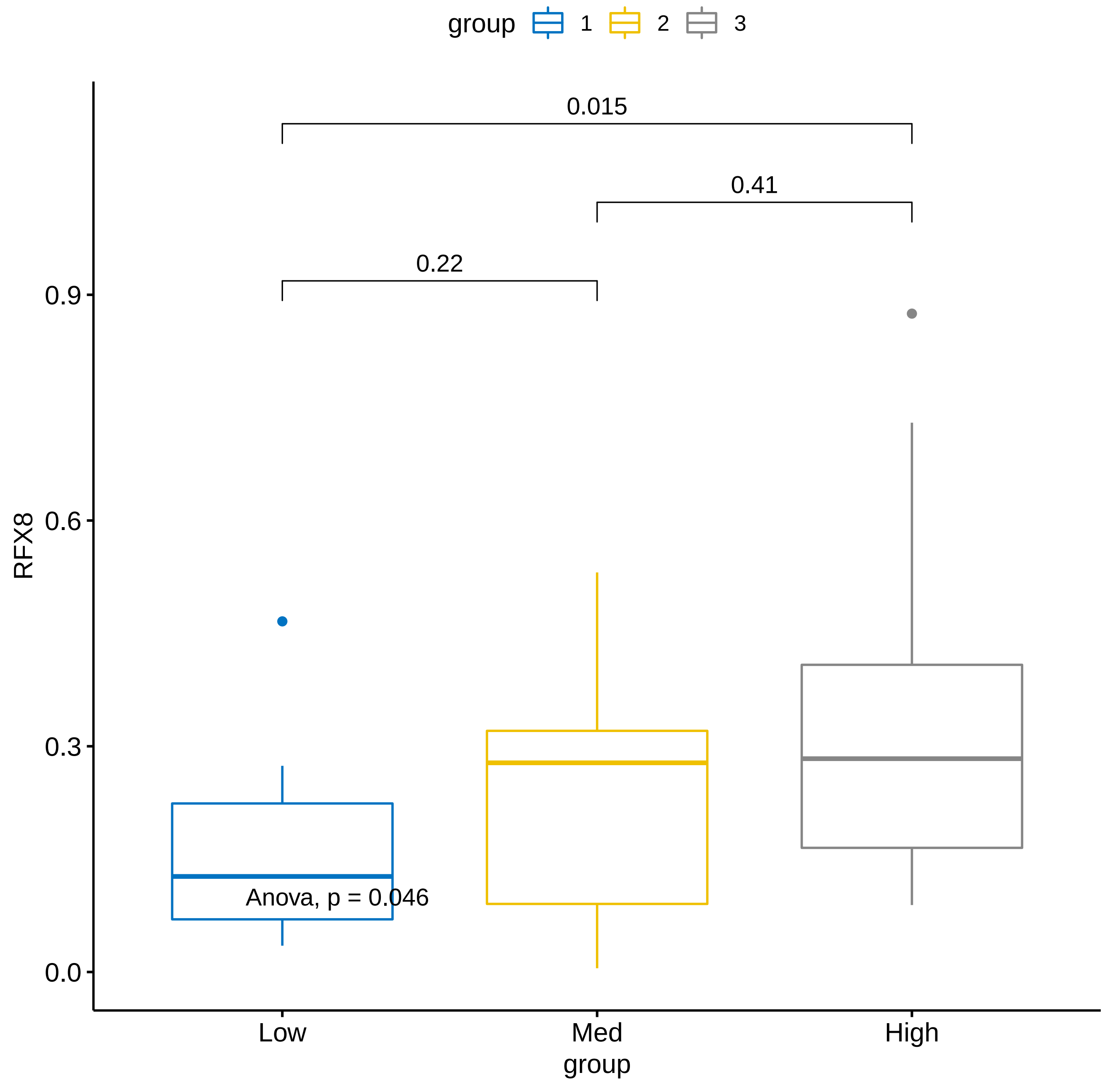
group
1
2
3
0.015
0.41
0.22
RFX8
0.9
0.6
0.3
0.0
Anova, p = 0.046
Low
Med
High
group

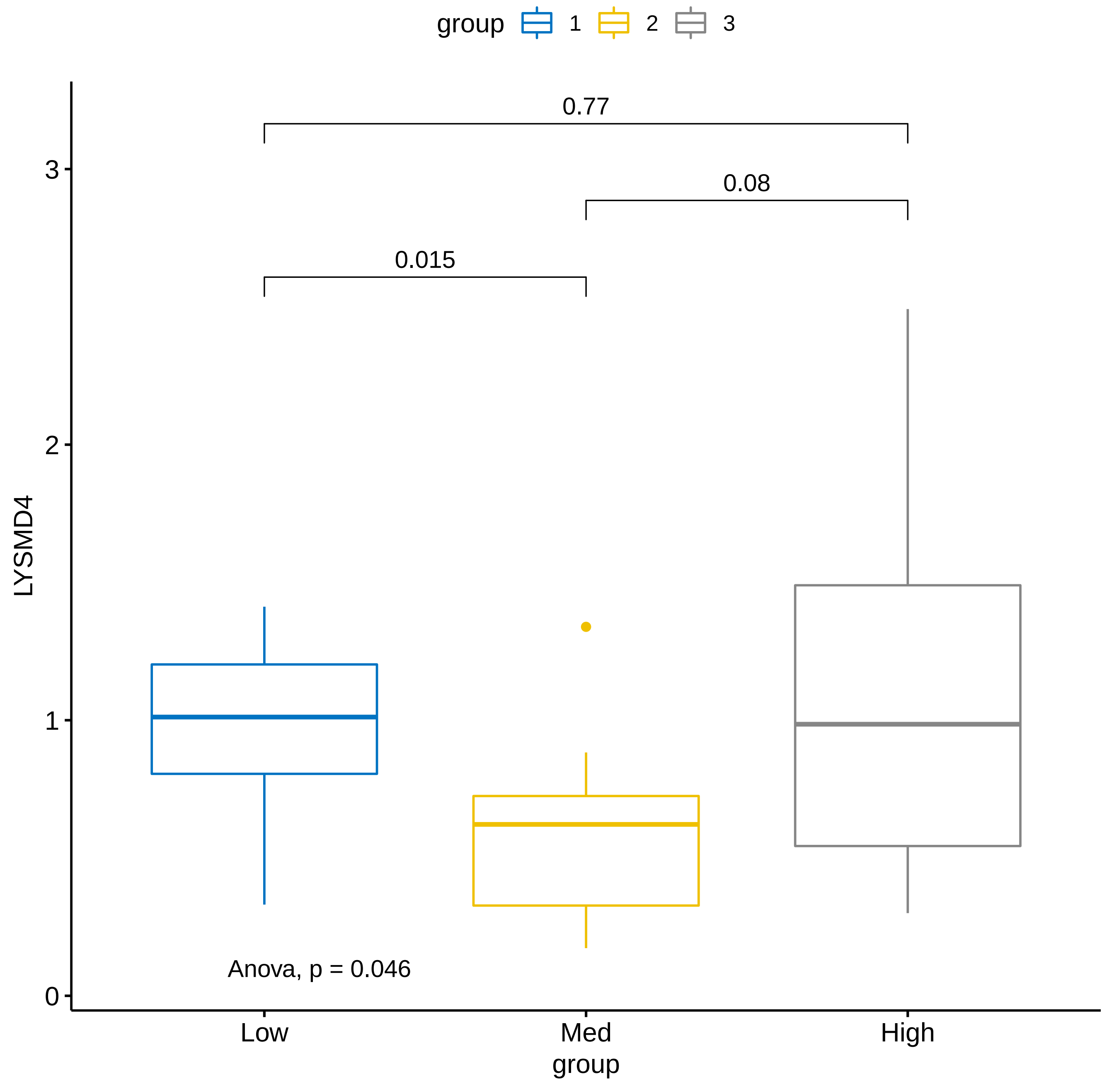
group
1
2
3
0.77
0.08
0.015
3
2
1
0
LYSMD4
Anova, p = 0.046
Low
Med
High
group

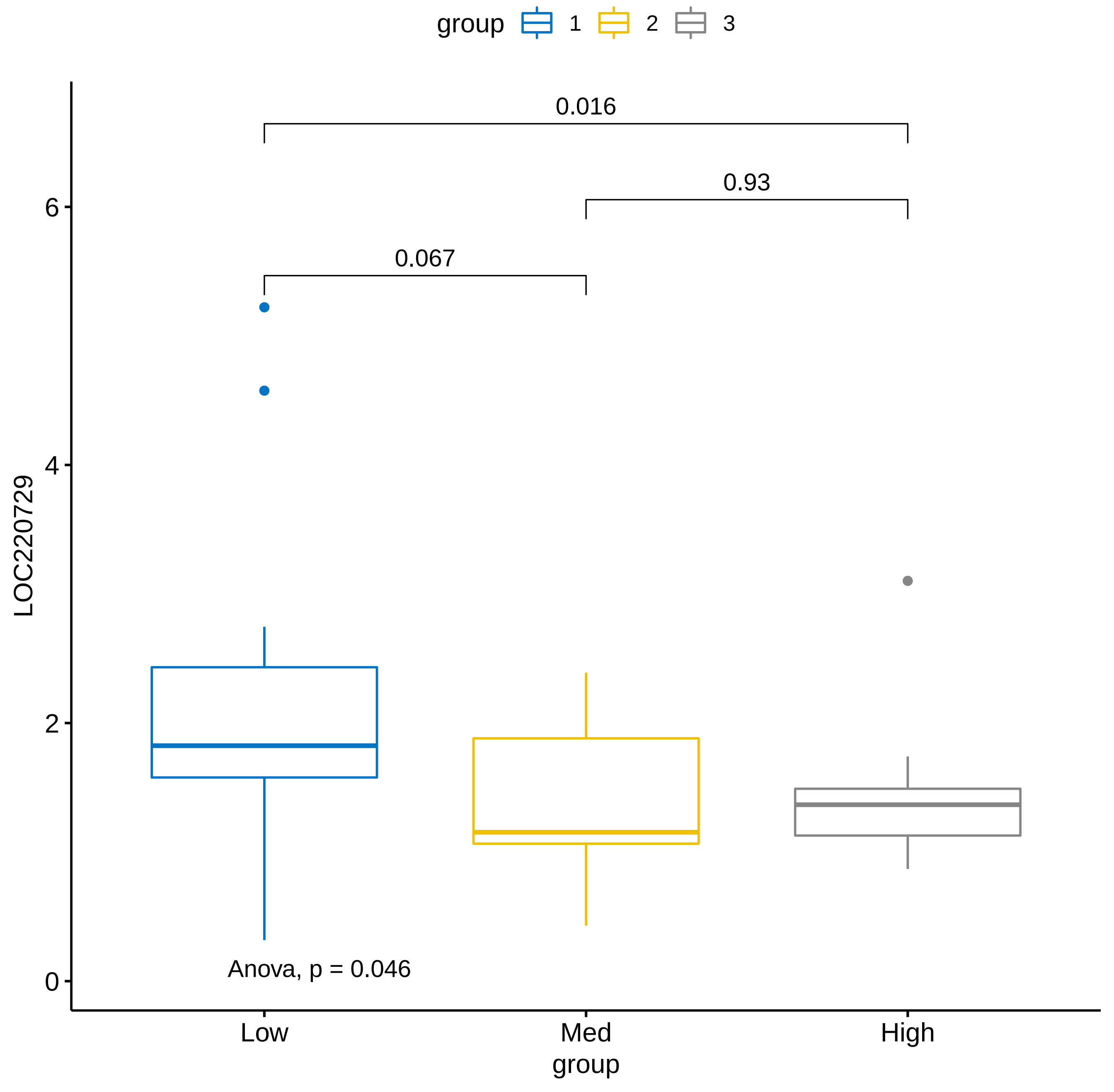
group
1
2
3
0.016
0.93
0.067
6
4
2
0
LOC220729
Anova, p = 0.046
Low
Med
High
group

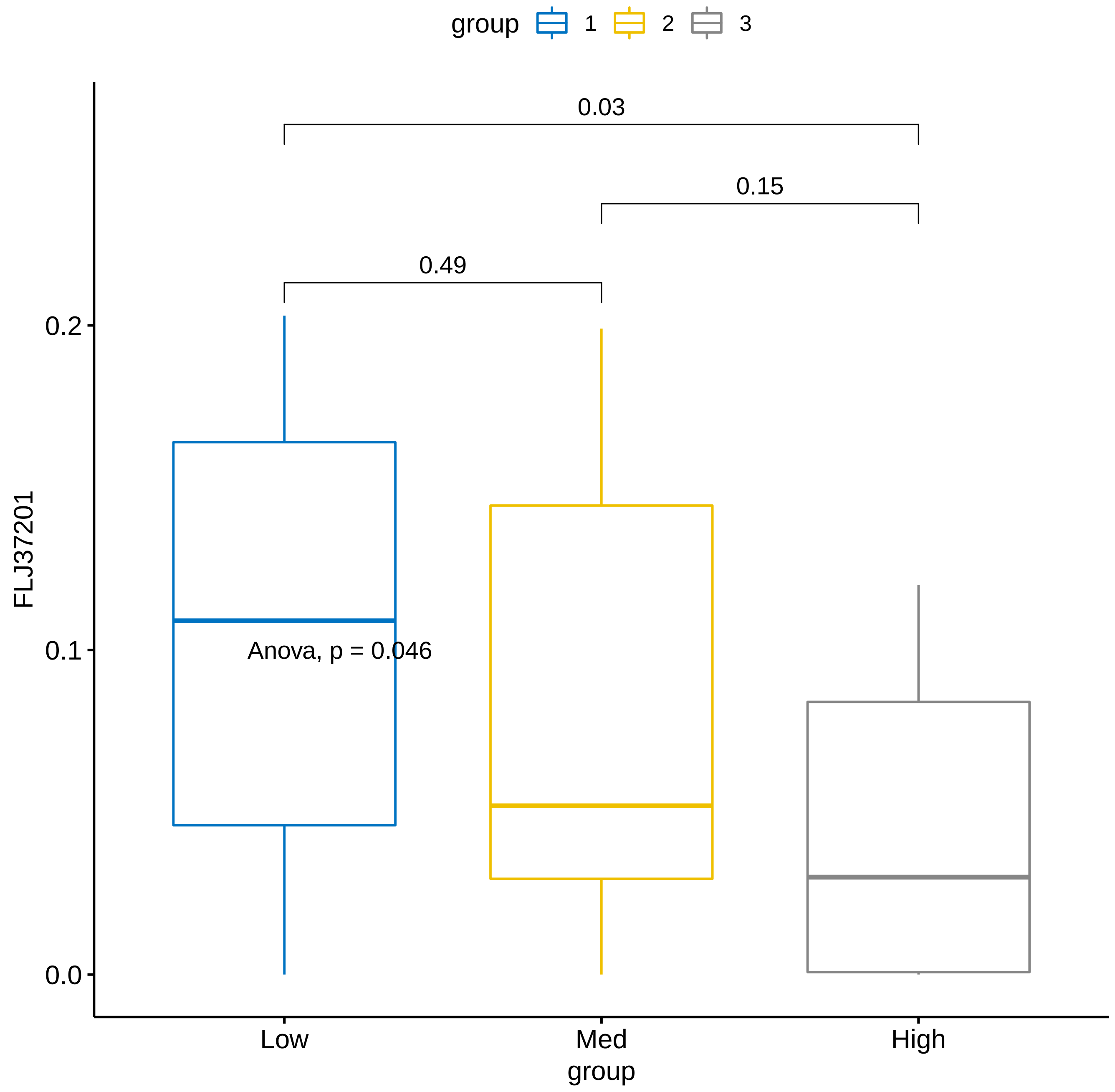
group
1
2
3
0.03
0.15
0.49
Anova, p = 0.046
0.2
0.1
0.0
FLJ37201
Low
Med
High
group

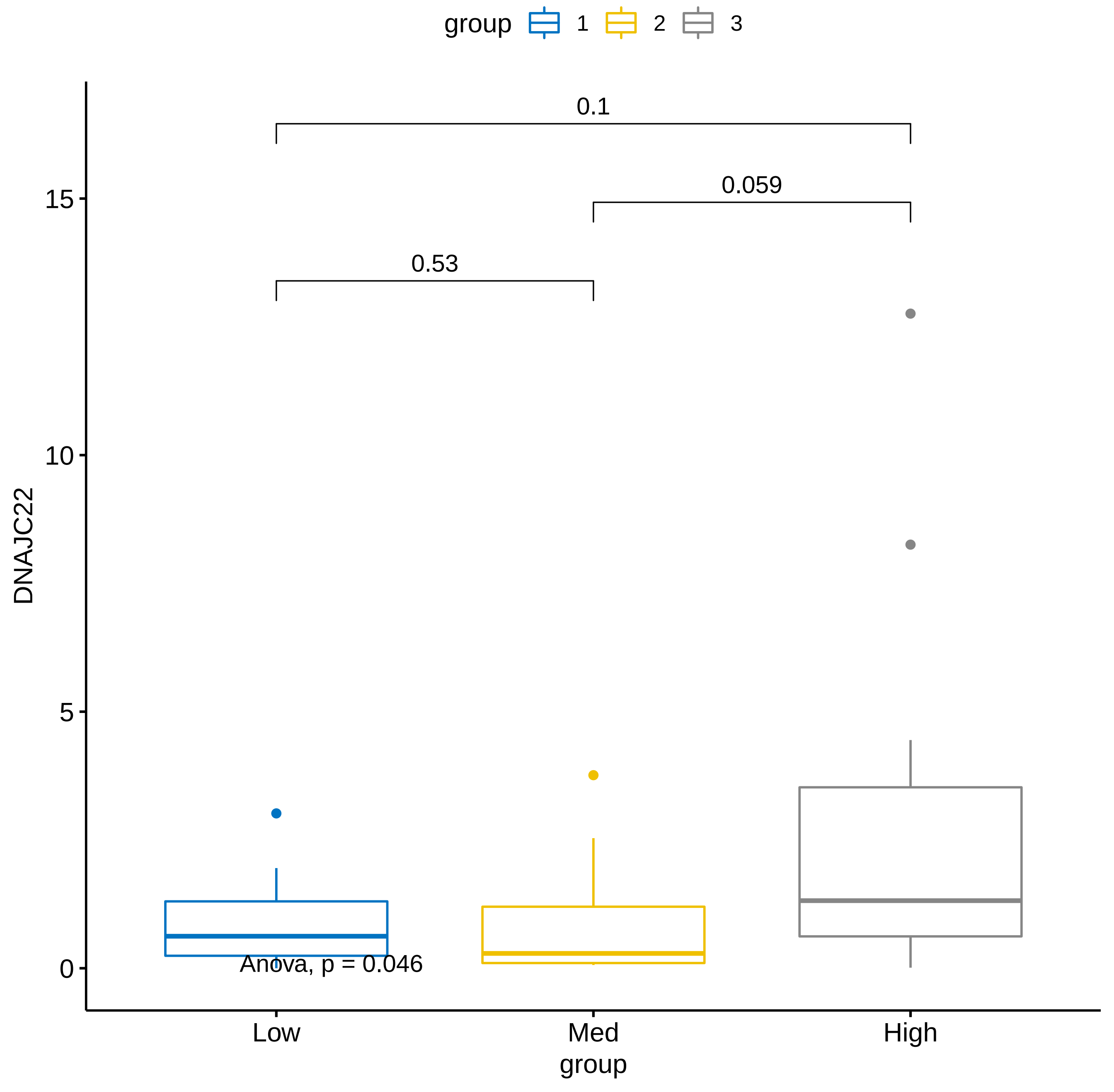
group 1 2 3
0.1
0.059
0.53
15
10
5
0
DNAJC22
Anova, p = 0.046
Low
Med
High
group

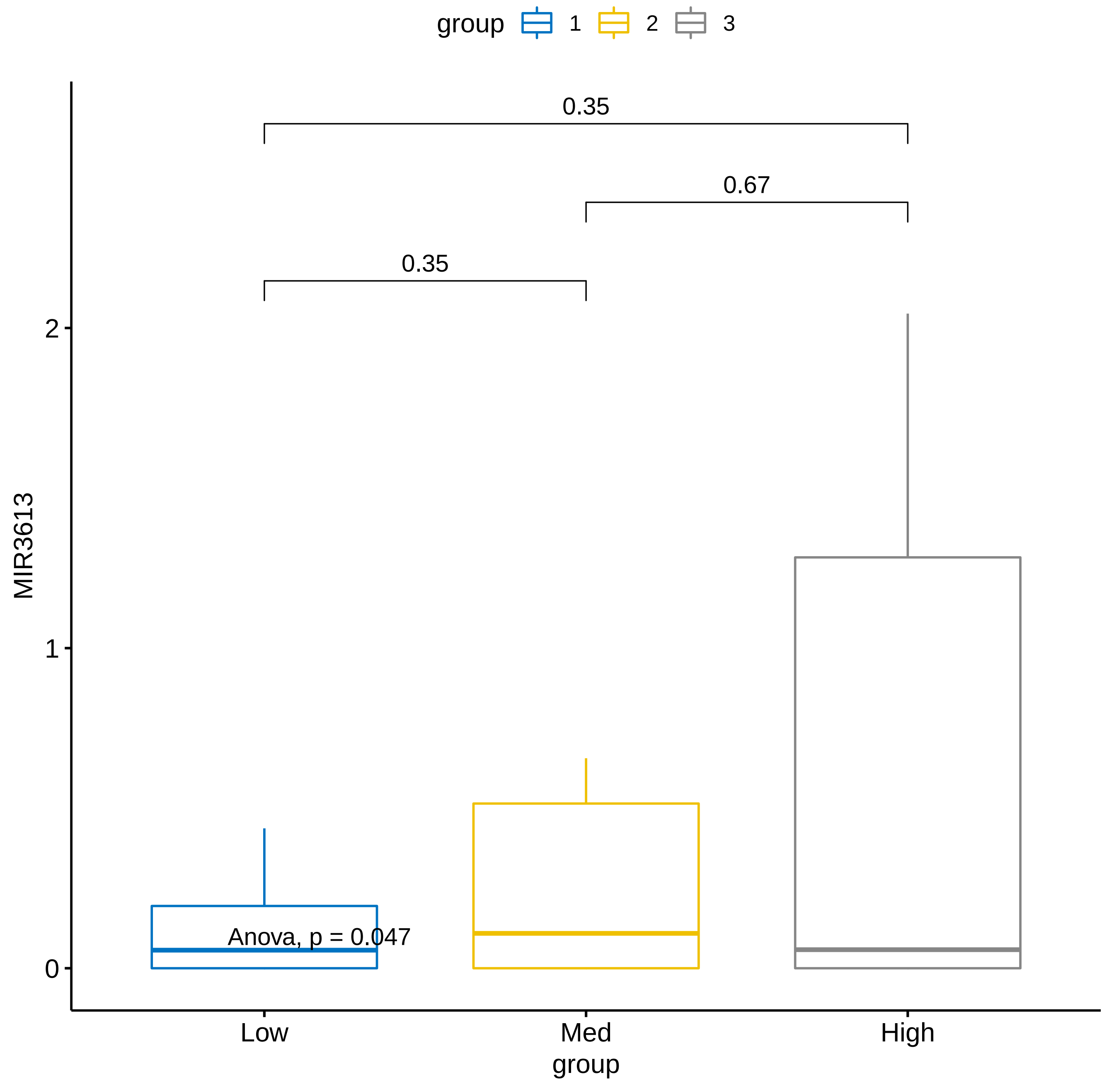
group 1 2 3
0.35
0.67
0.35
MIR3613
2
1
0
Anova, p = 0.047
Low
Med
High
group

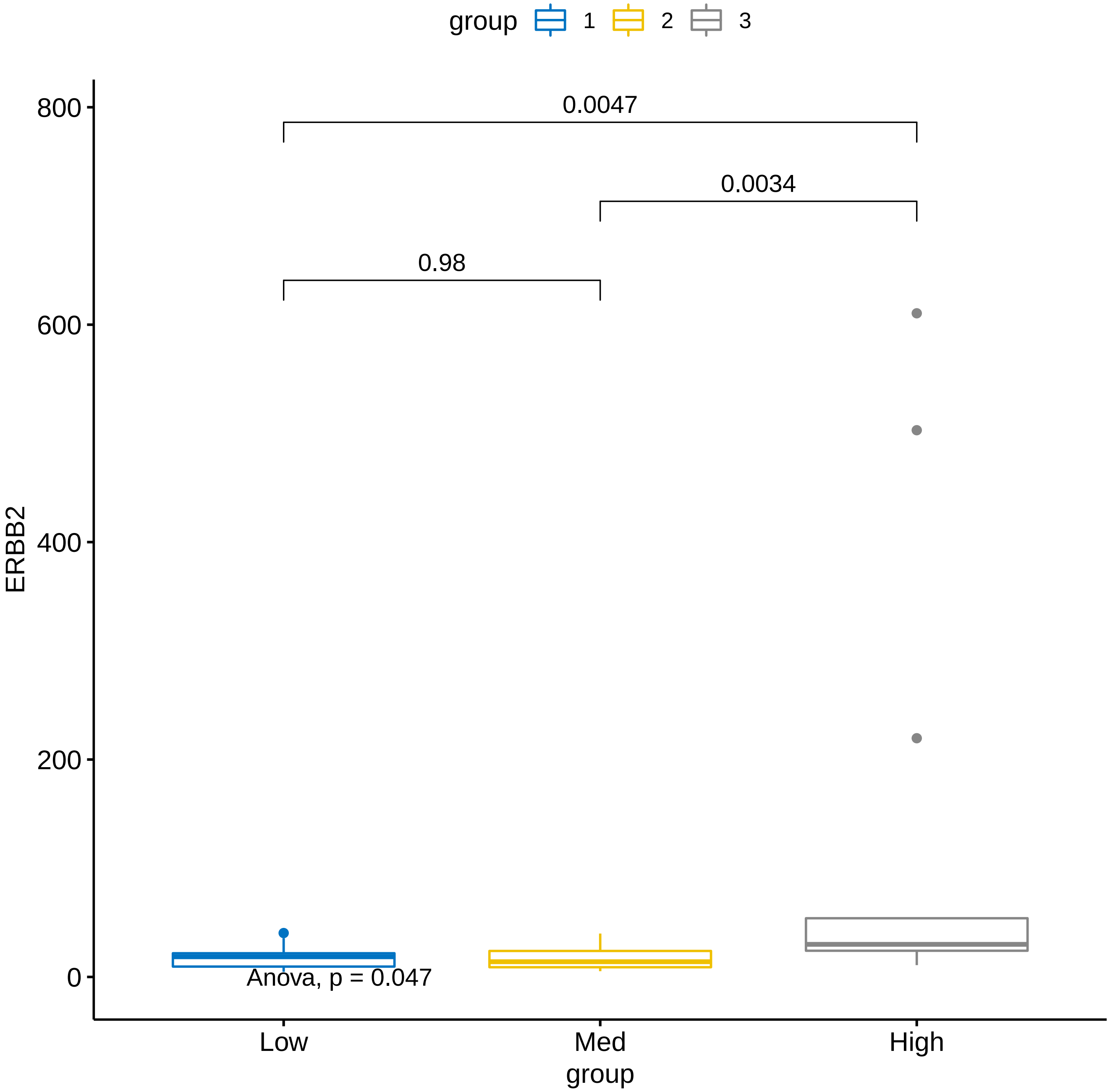

group 1 2 3
0.0047
0.0034
0.98
800
600
400
200
0
ERBB2
Anova, p = 0.047
Low
Med
High
group

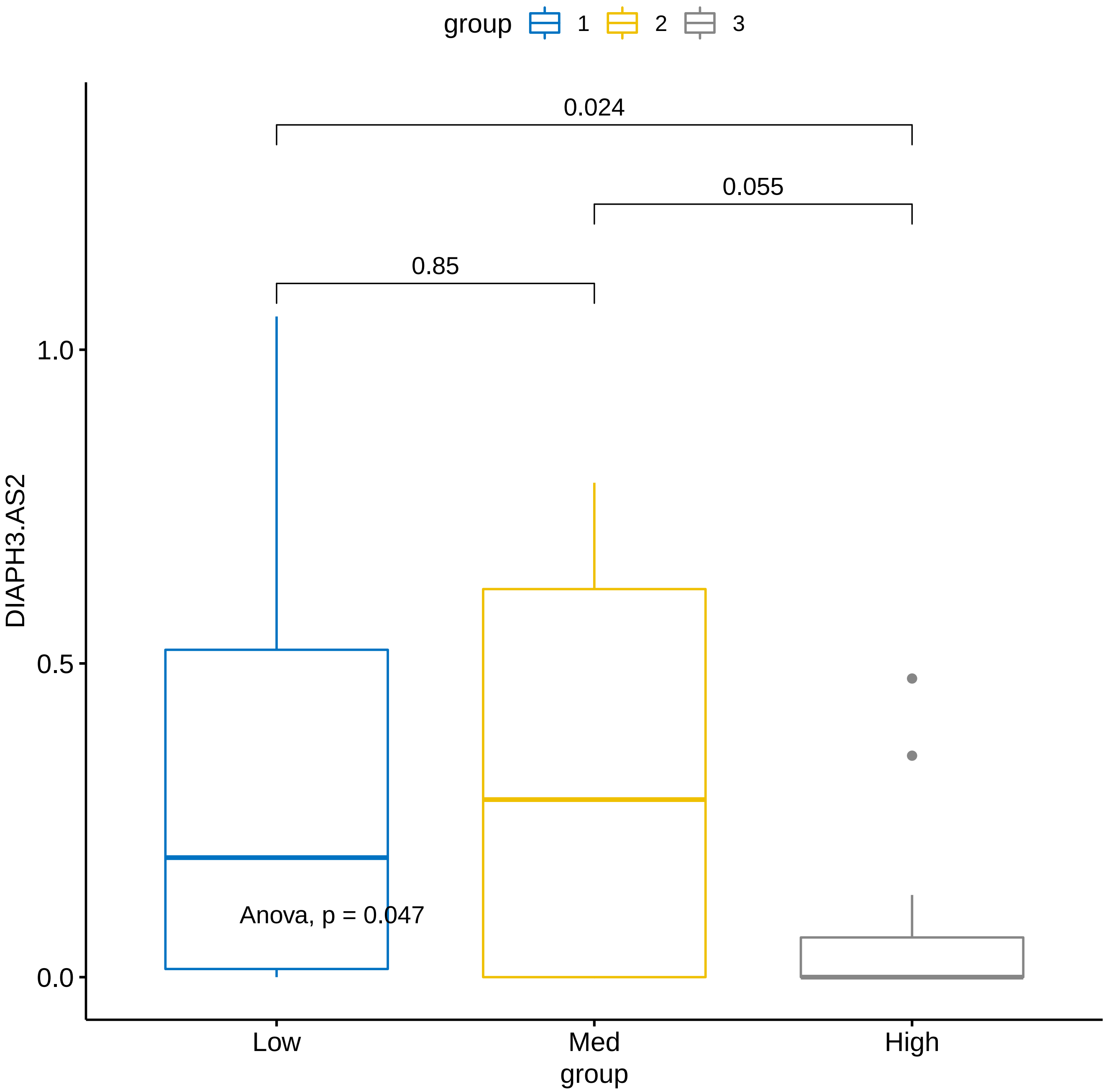
group
1
2
3
0.024
0.055
0.85
1.0
0.5
0.0
DIAPH3.AS2
Anova, p = 0.047
Low
Med
High
group

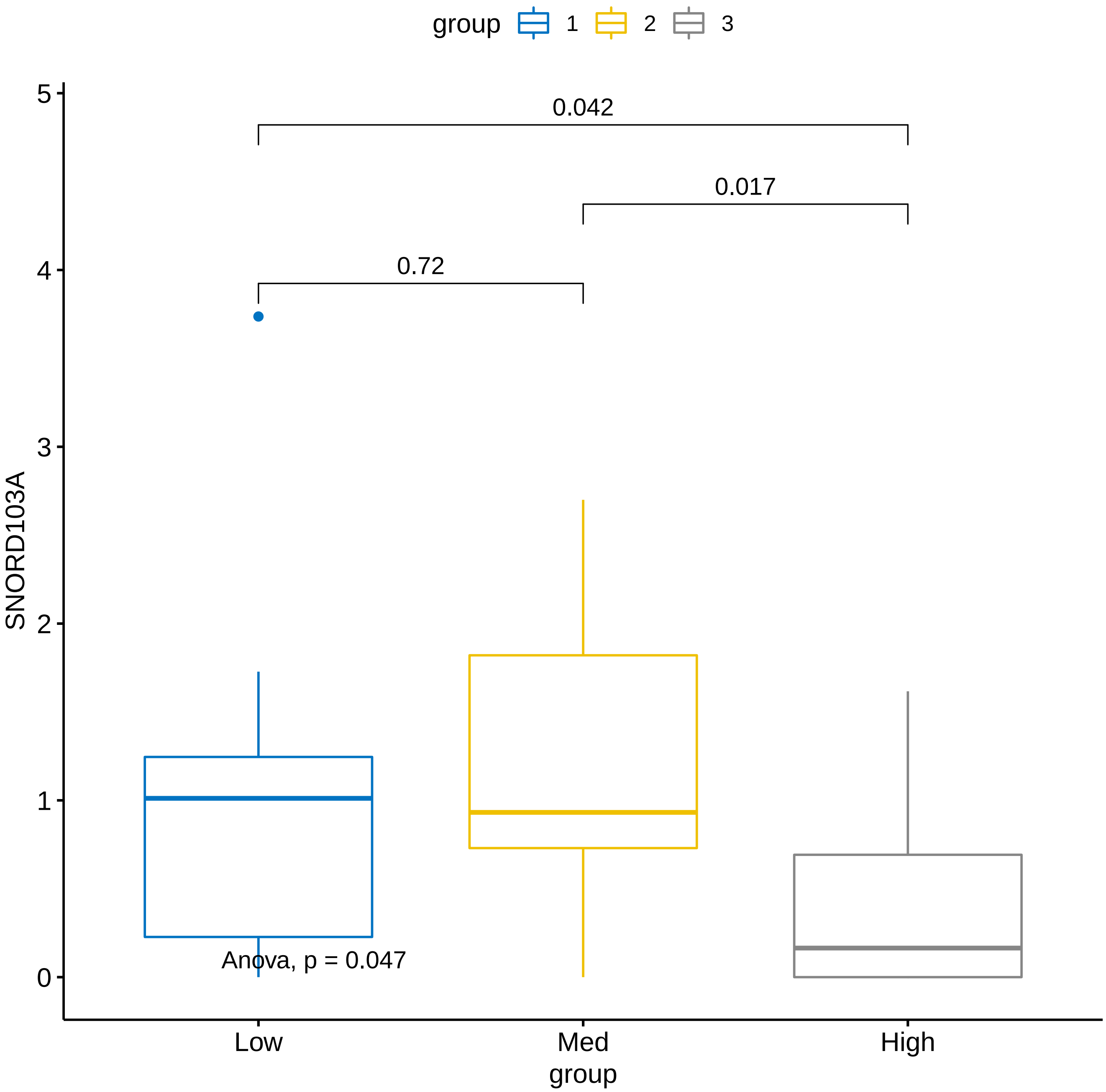
group 1 2 3
0.042
0.017
0.72
SNORD103A
0
1
2
3
4
5
Anova, p = 0.047
Low
Med
High
group

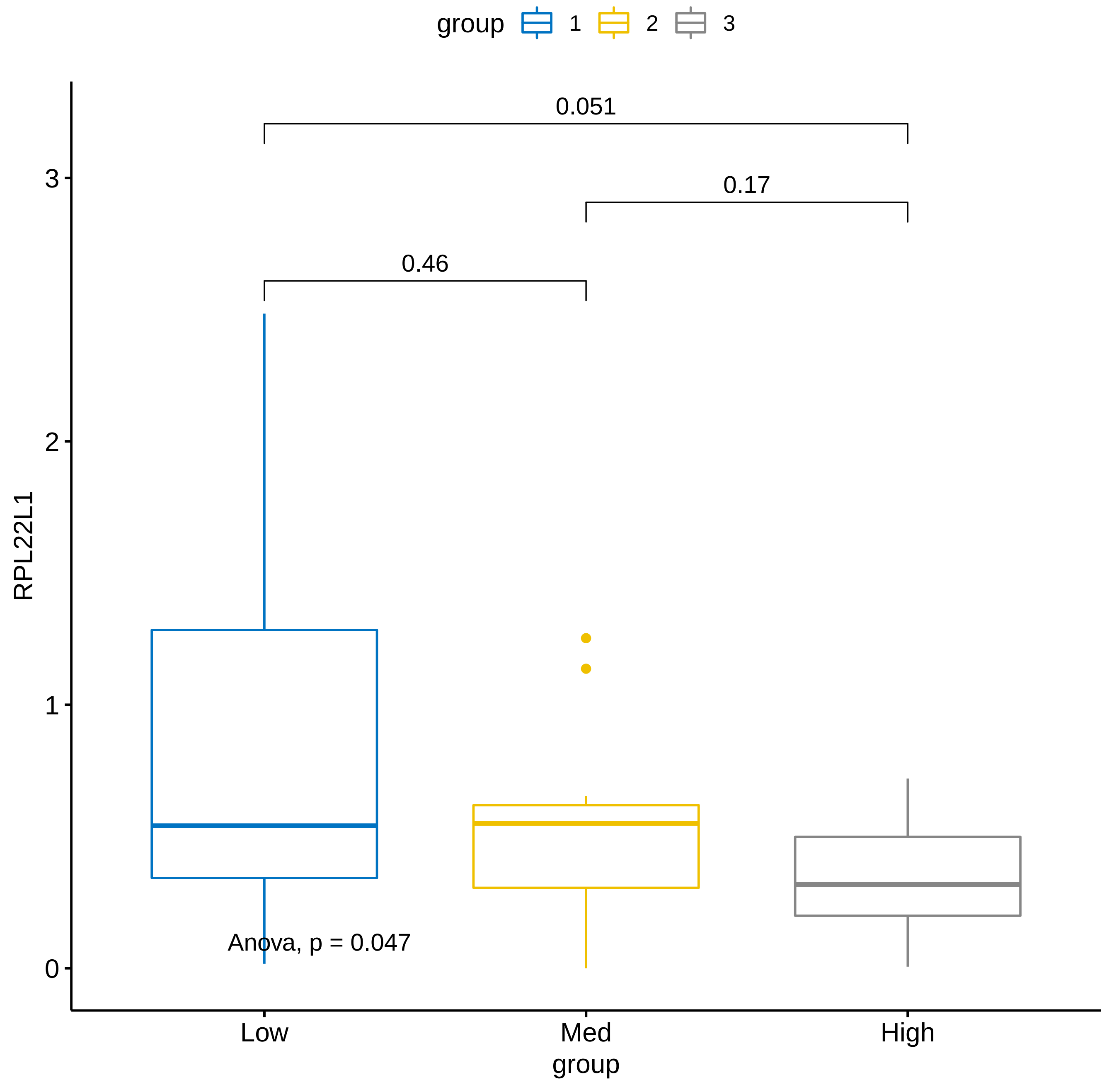
group 1 2 3
0.051
0.17
0.46
3
2
1
0
RPL22L1
Anova, p = 0.047
Low
Med
High
group

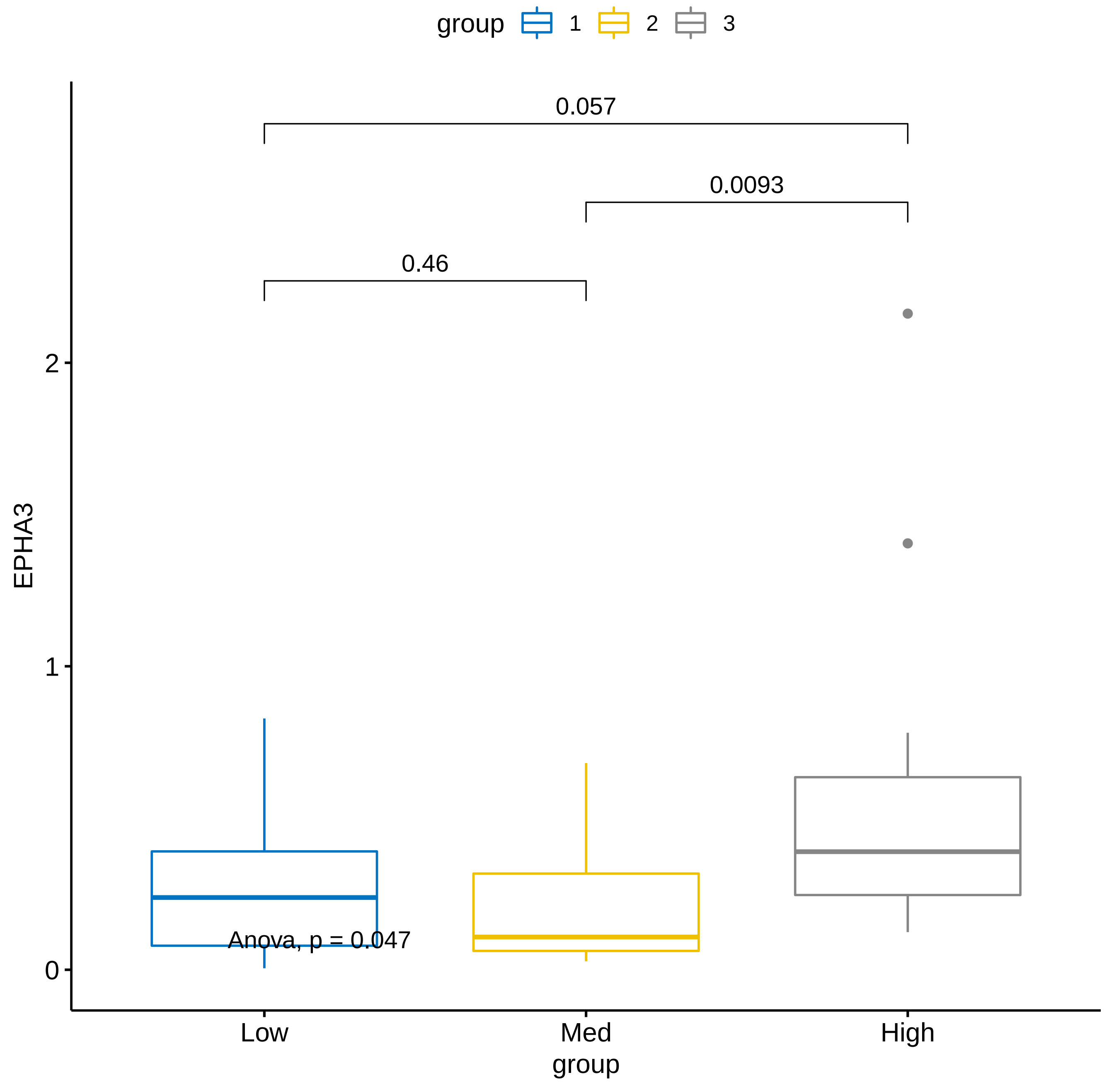
group
1
2
3
0.057
0.0093
0.46
EPHA3
2
1
0
Anova, p = 0.047
Low
Med
High
group

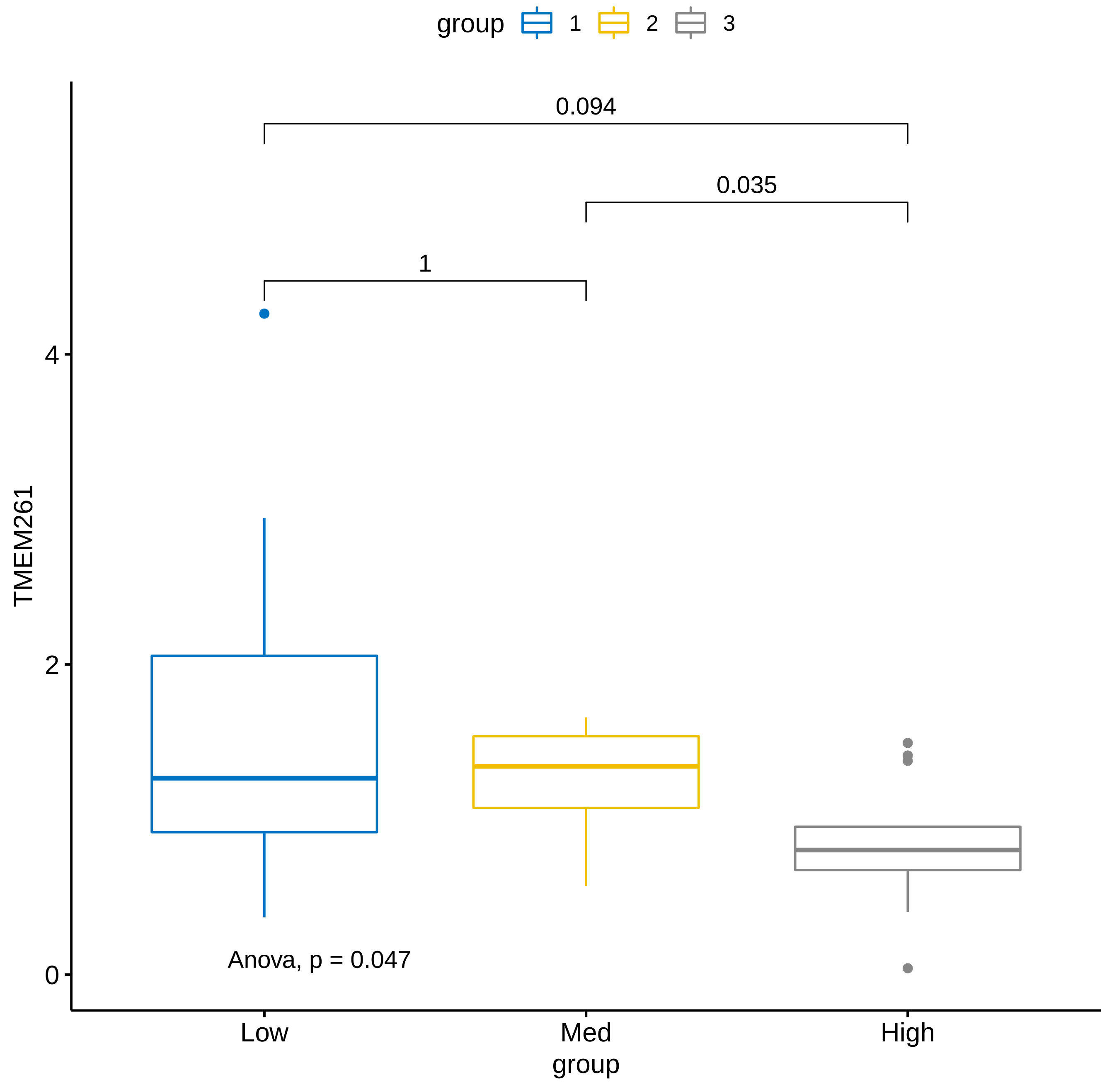
group
1
2
3
0.094
0.035
1
4
2
0
TMEM261
Anova, p = 0.047
Low
Med
High
group

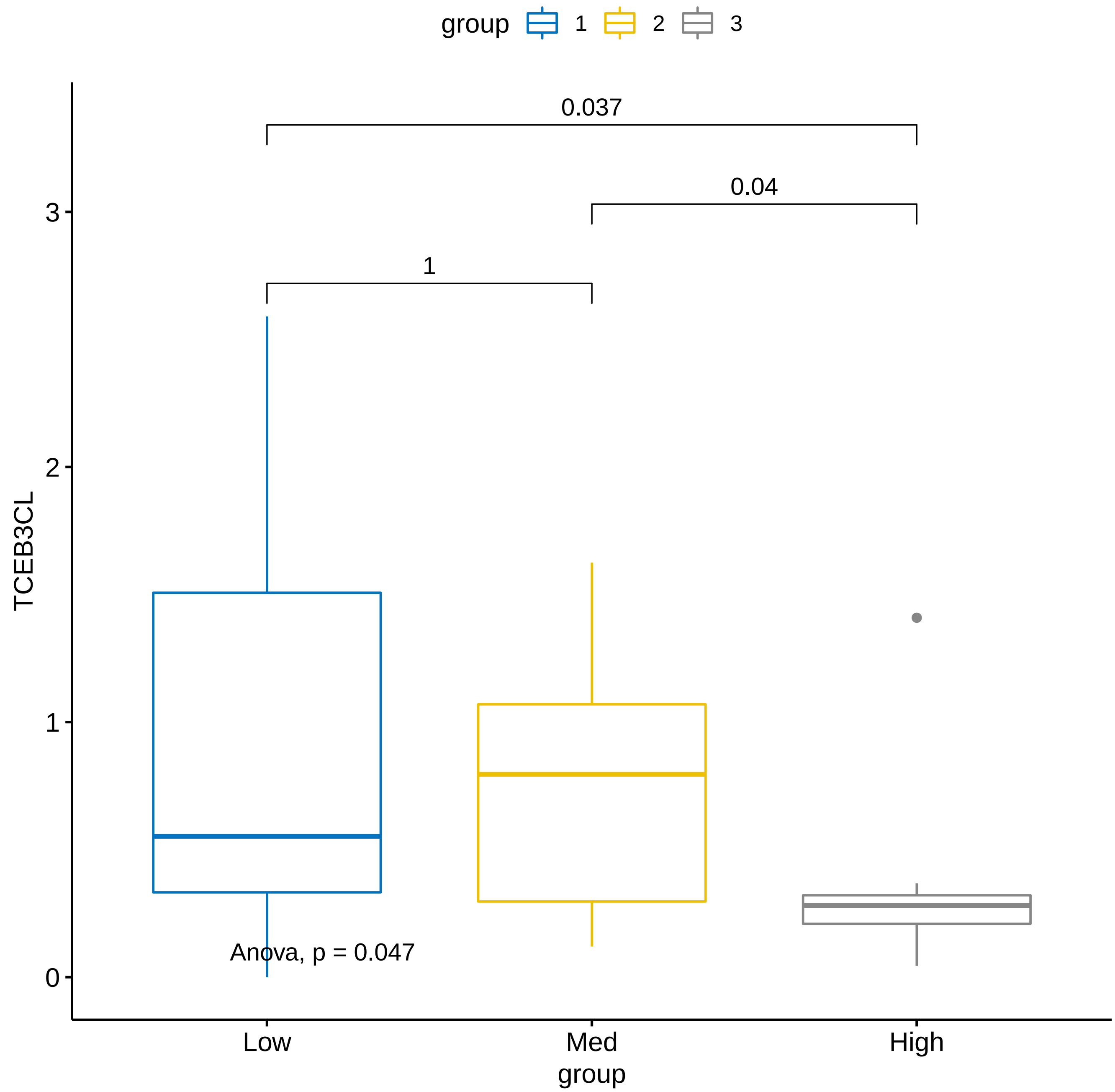
group
1
2
3
0.037
0.04
1
3
2
1
0
TCEB3CL
Anova, p = 0.047
Low
Med
High
group

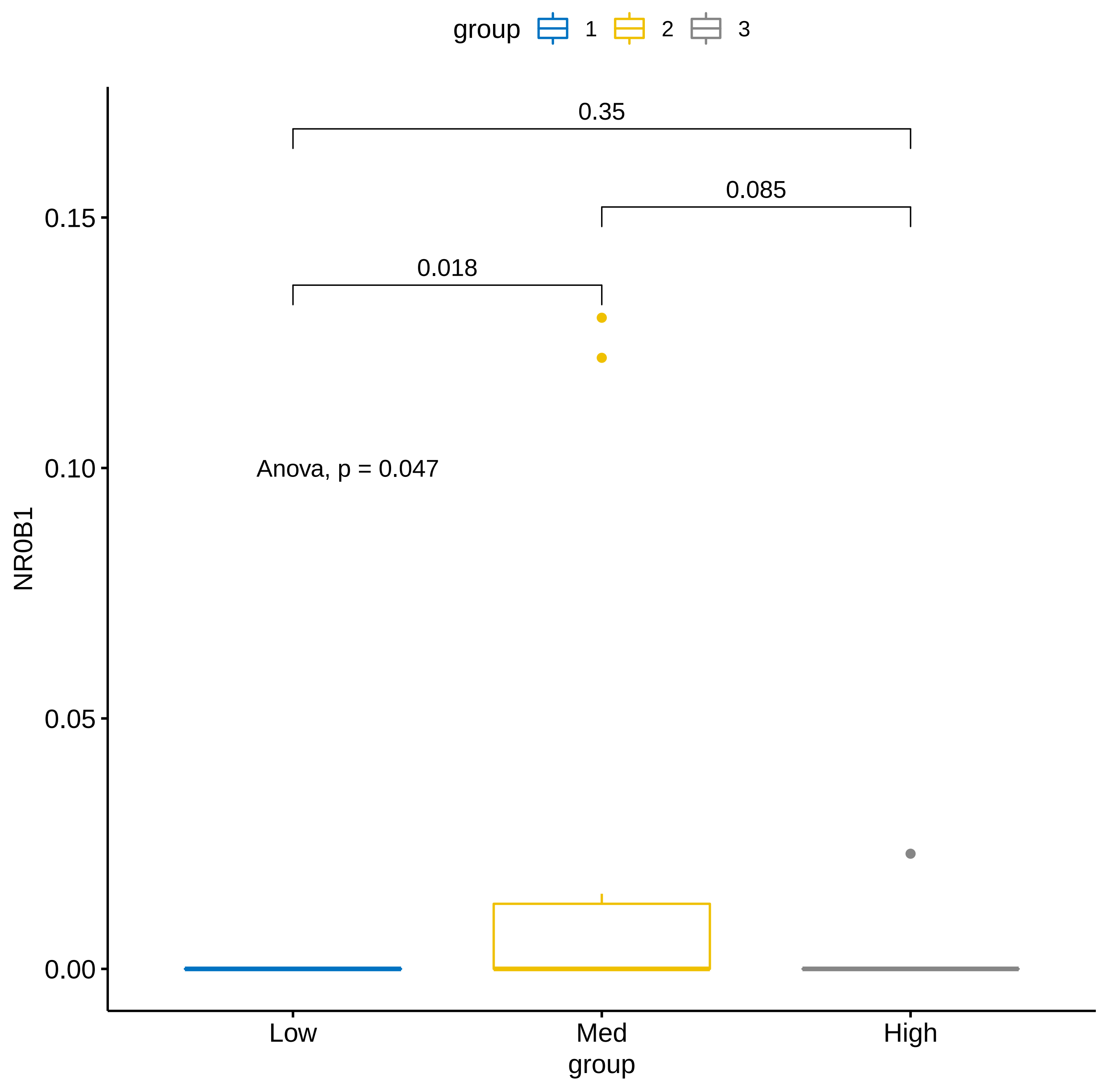

group
1
2
3
0.35
0.085
0.018
Anova, p = 0.047
0.15
0.10
0.05
0.00
NR0B1
Low
Med
High
group

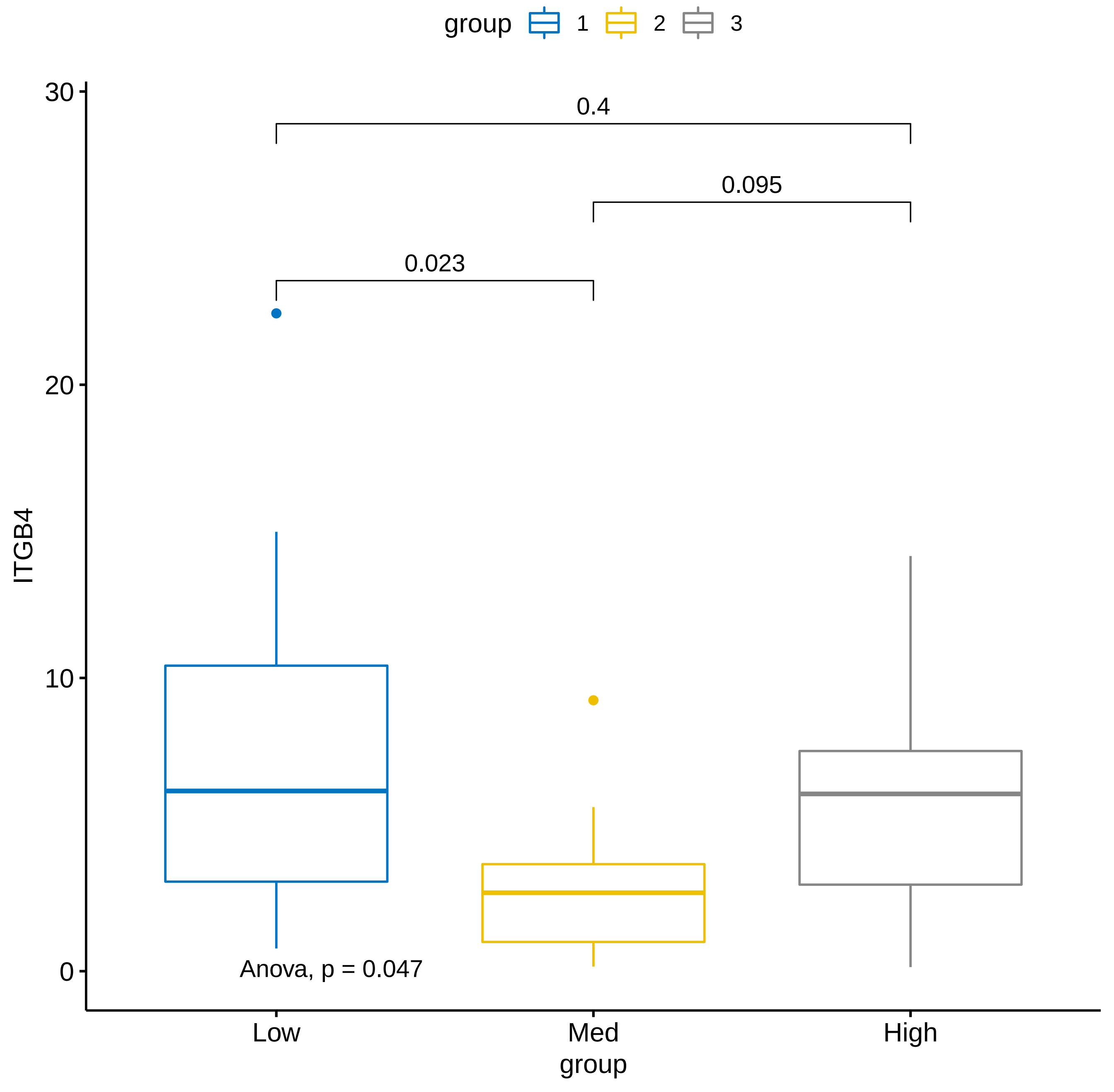
group
1
2
3
30
20
10
0
ITGB4
0.4
0.095
0.023
Anova, p = 0.047
Low
Med
High
group

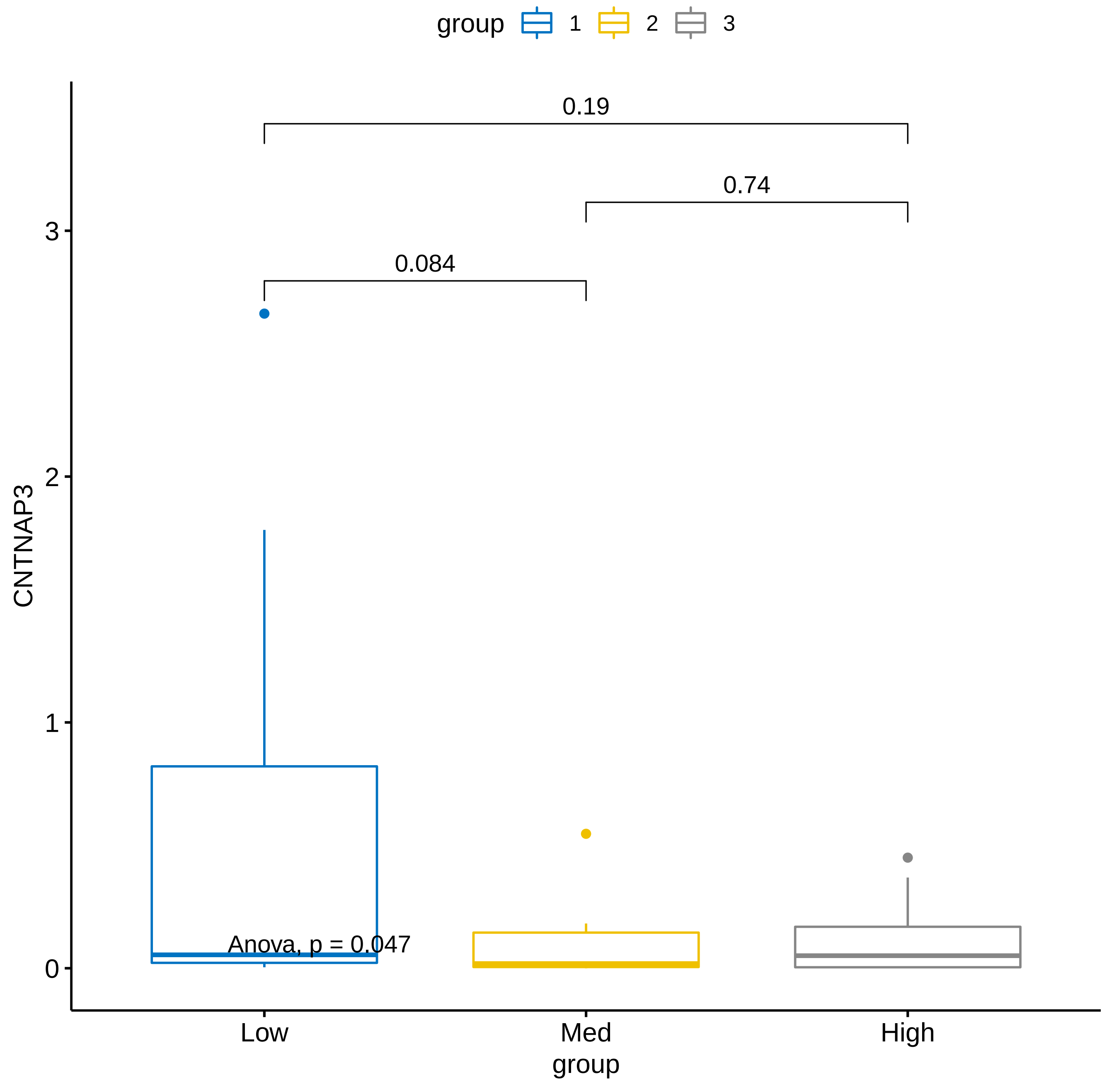
group
1
2
3
0.19
0.74
0.084
3
2
1
0
CNTNAP3
Anova, p = 0.047
Low
Med
High
group

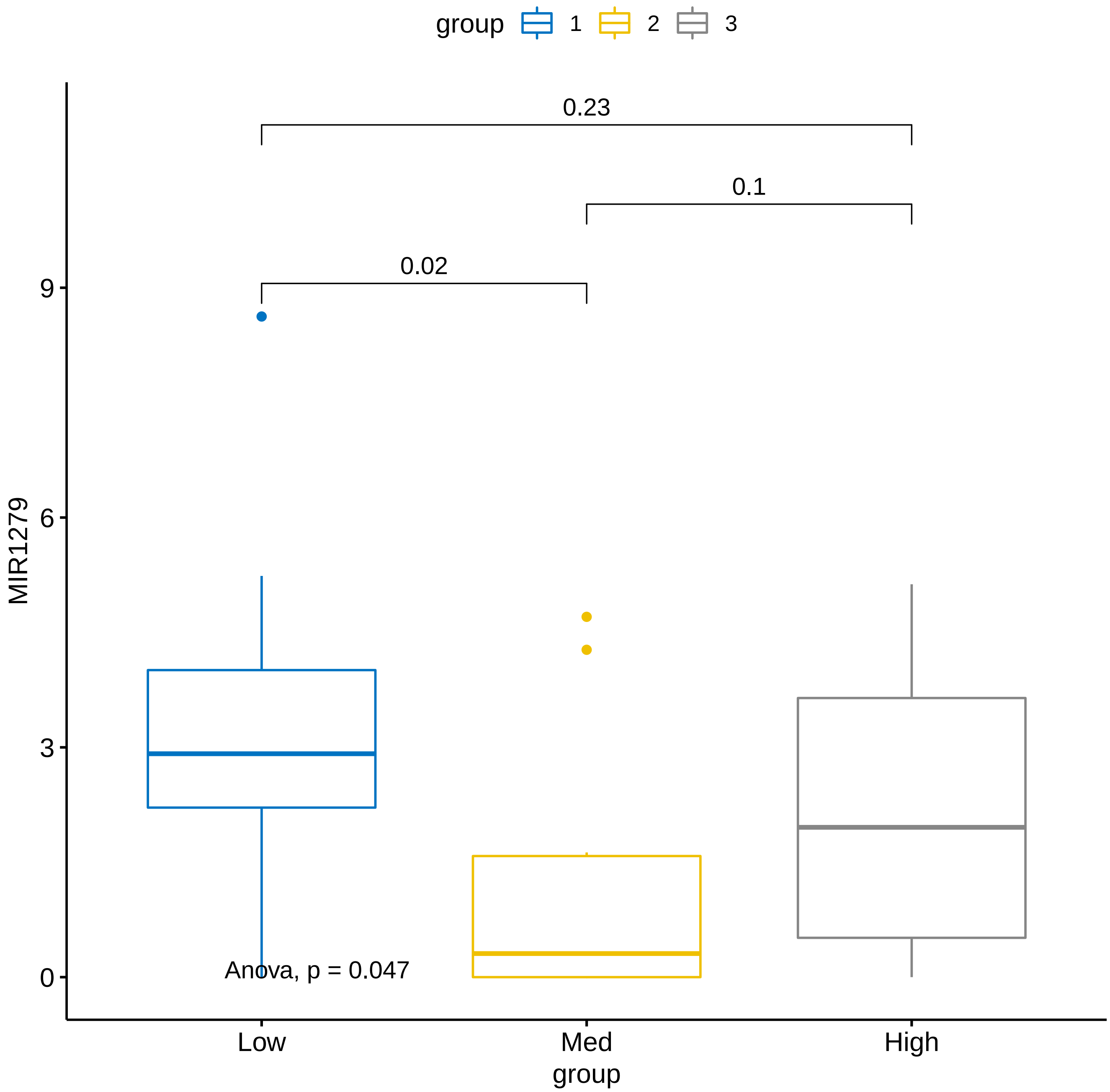
group
1
2
3
0.23
0.1
0.02
9
6
3
0
MIR1279
Anova, p = 0.047
Low
Med
High
group

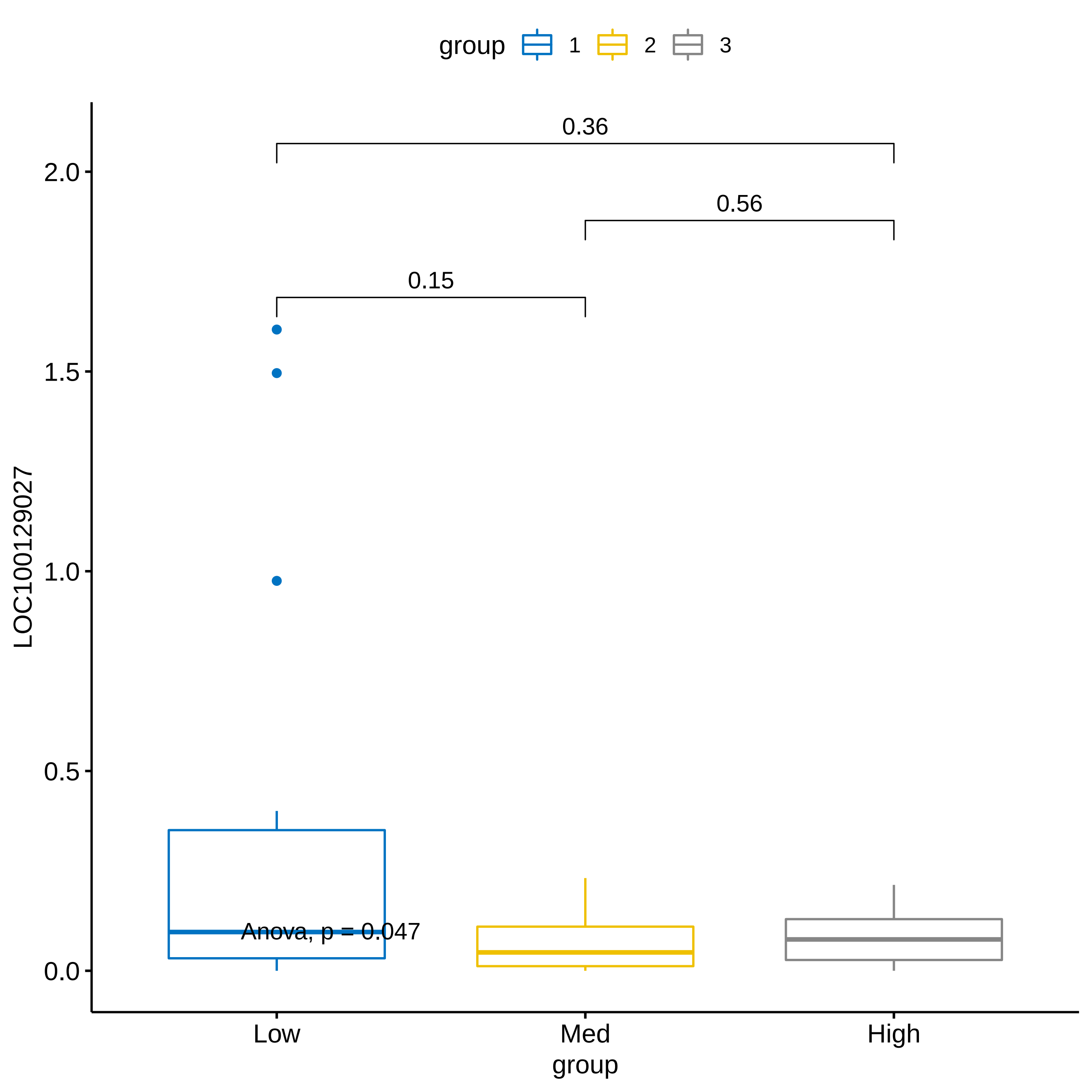

group
1
2
3
0.36
0.56
0.15
LOC100129027
2.0
1.5
1.0
0.5
0.0
Anova, p = 0.047
Low
Med
High
group

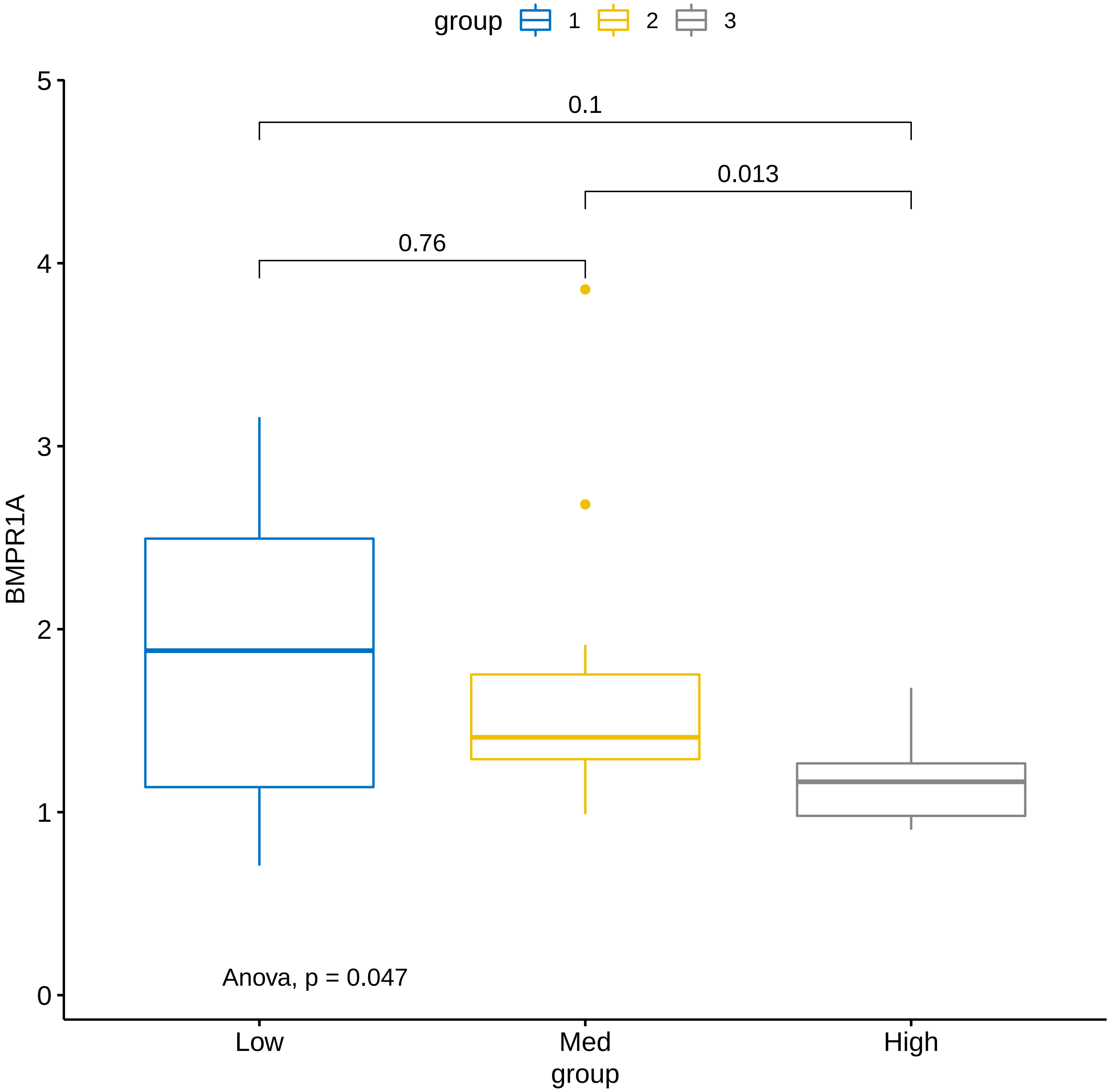
group
1
2
3
5
4
3
2
1
0
BMPR1A
0.1
0.013
0.76
Anova, p = 0.047
Low
Med
High
group

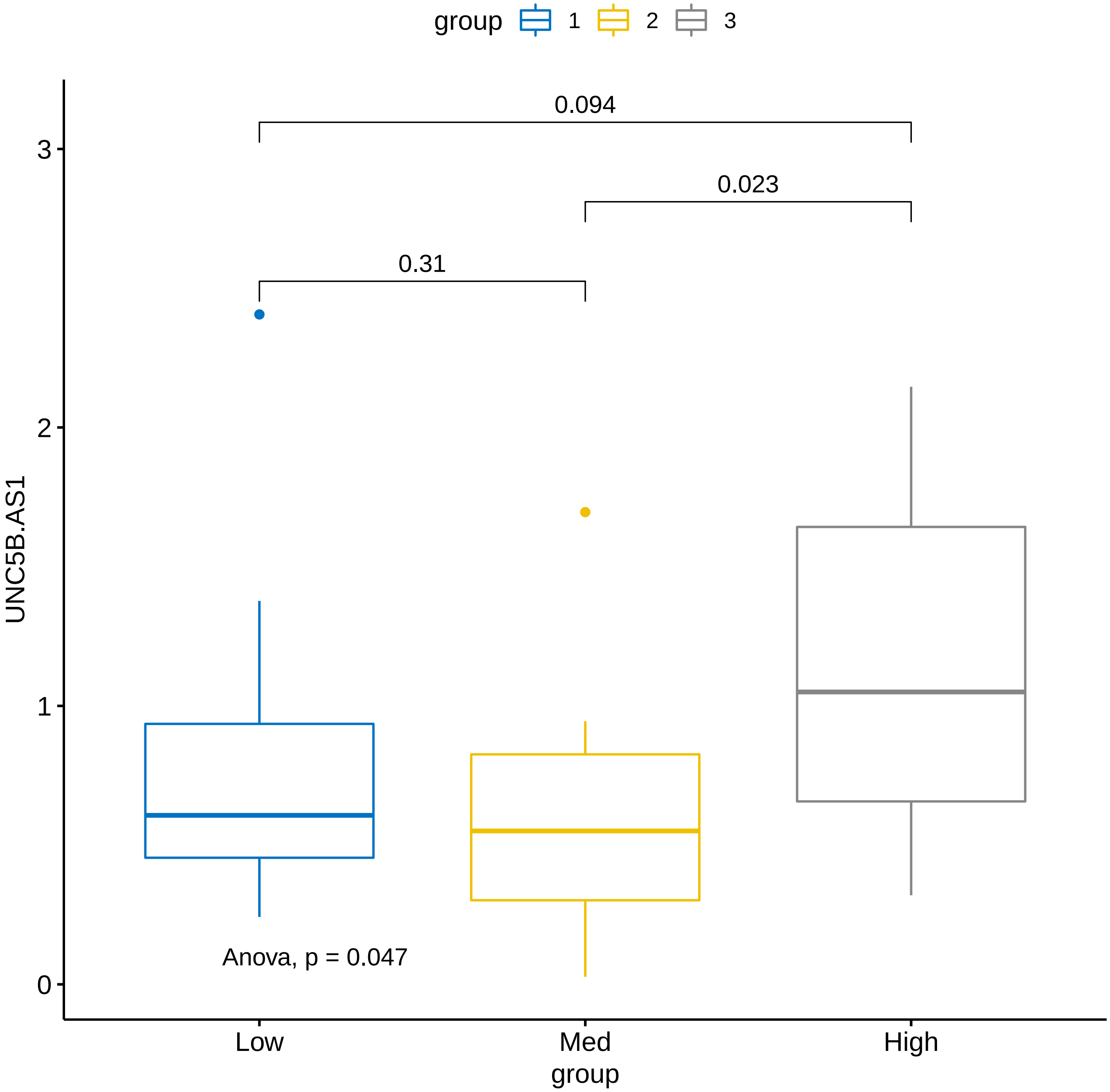
group
1
2
3
0.094
0.023
0.31
UNC5B.AS1
3
2
1
0
Anova, p = 0.047
Low
Med
High
group

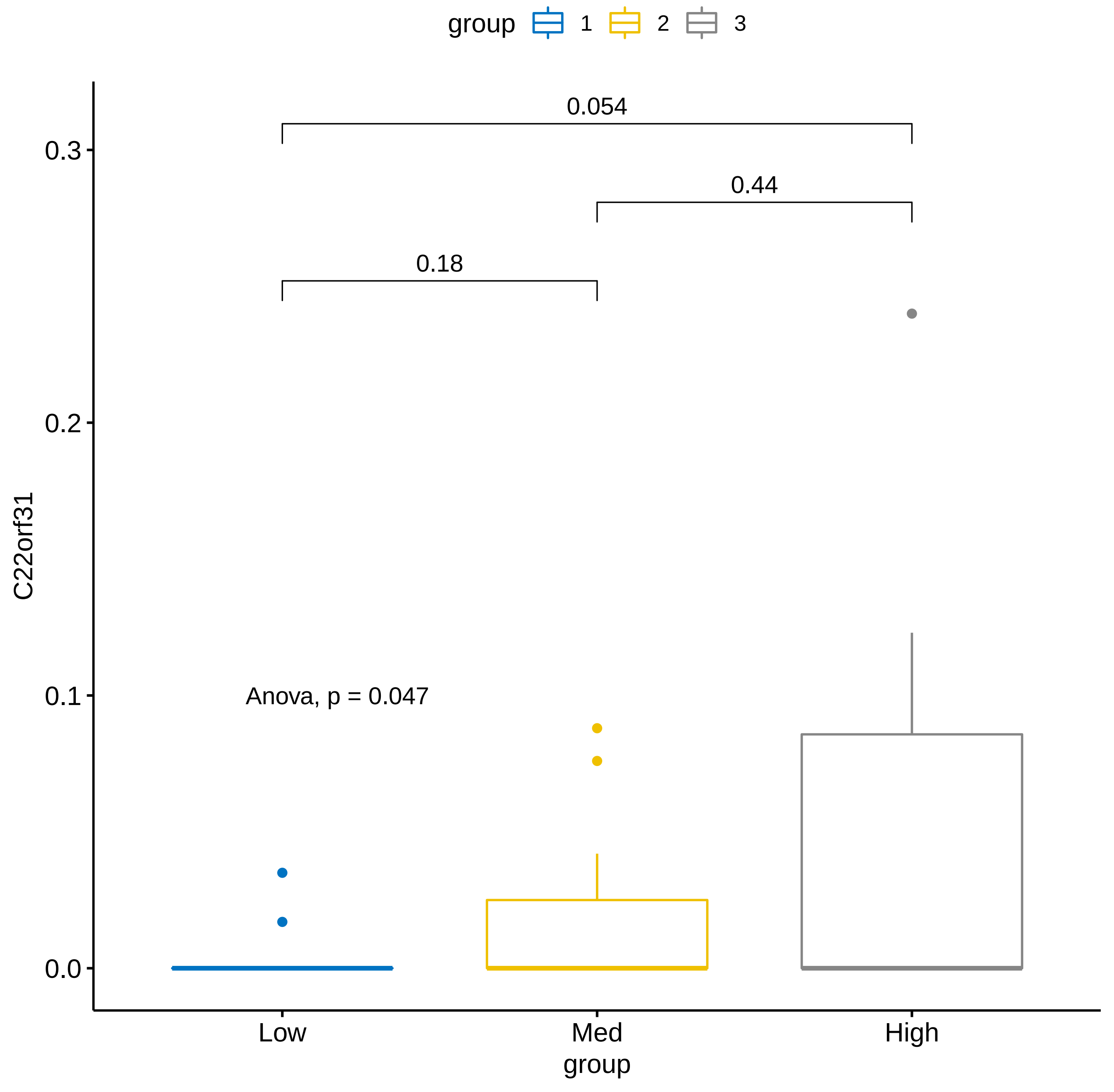
group 1 2 3
0.054
0.44
0.18
Anova, p = 0.047
0.3
0.2
0.1
0.0
C22orf31
Low
Med
High
group

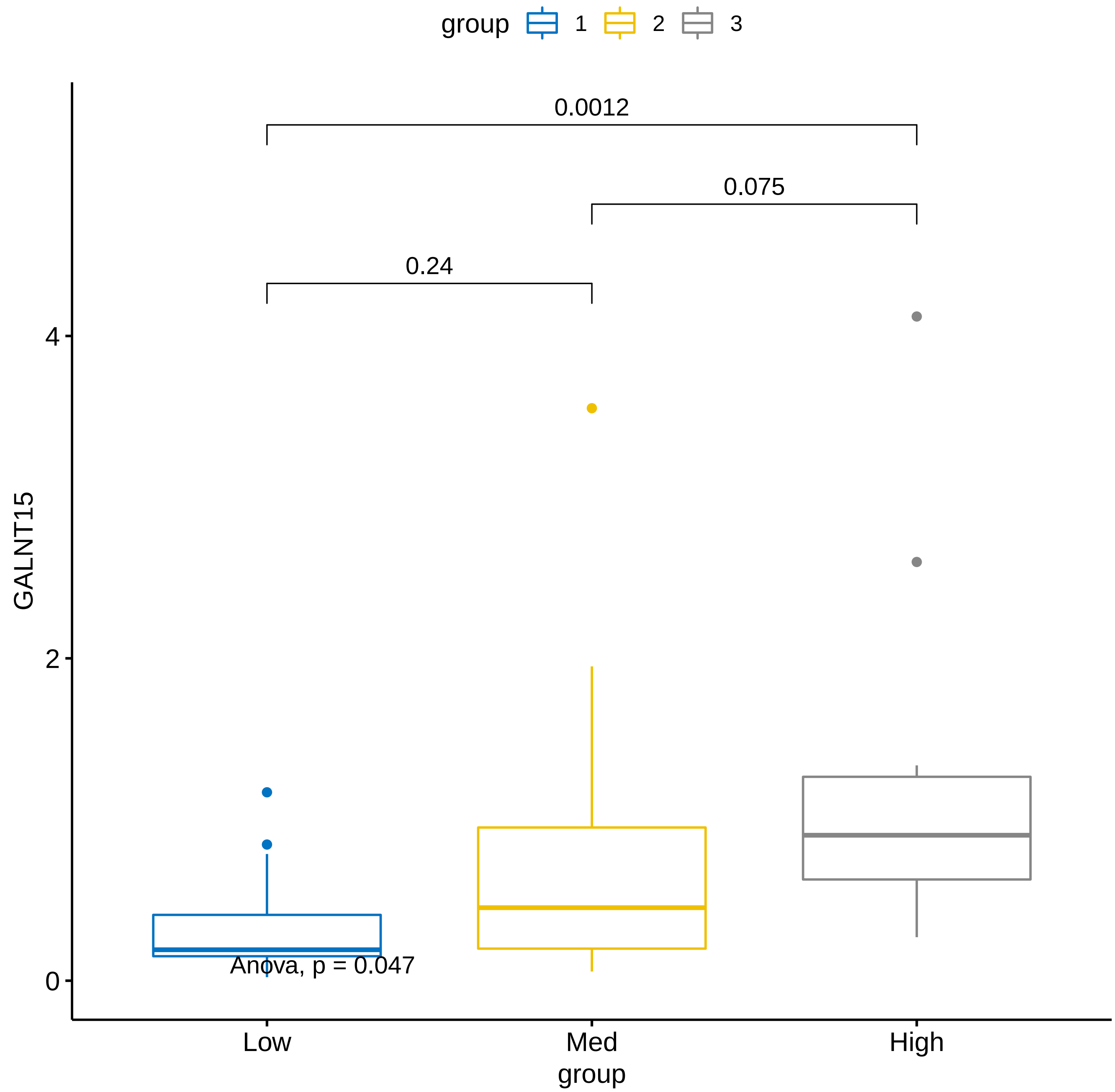
group
1
2
3
0.0012
0.075
0.24
4
2
0
GALNT15
Anova, p = 0.047
Low
Med
High
group

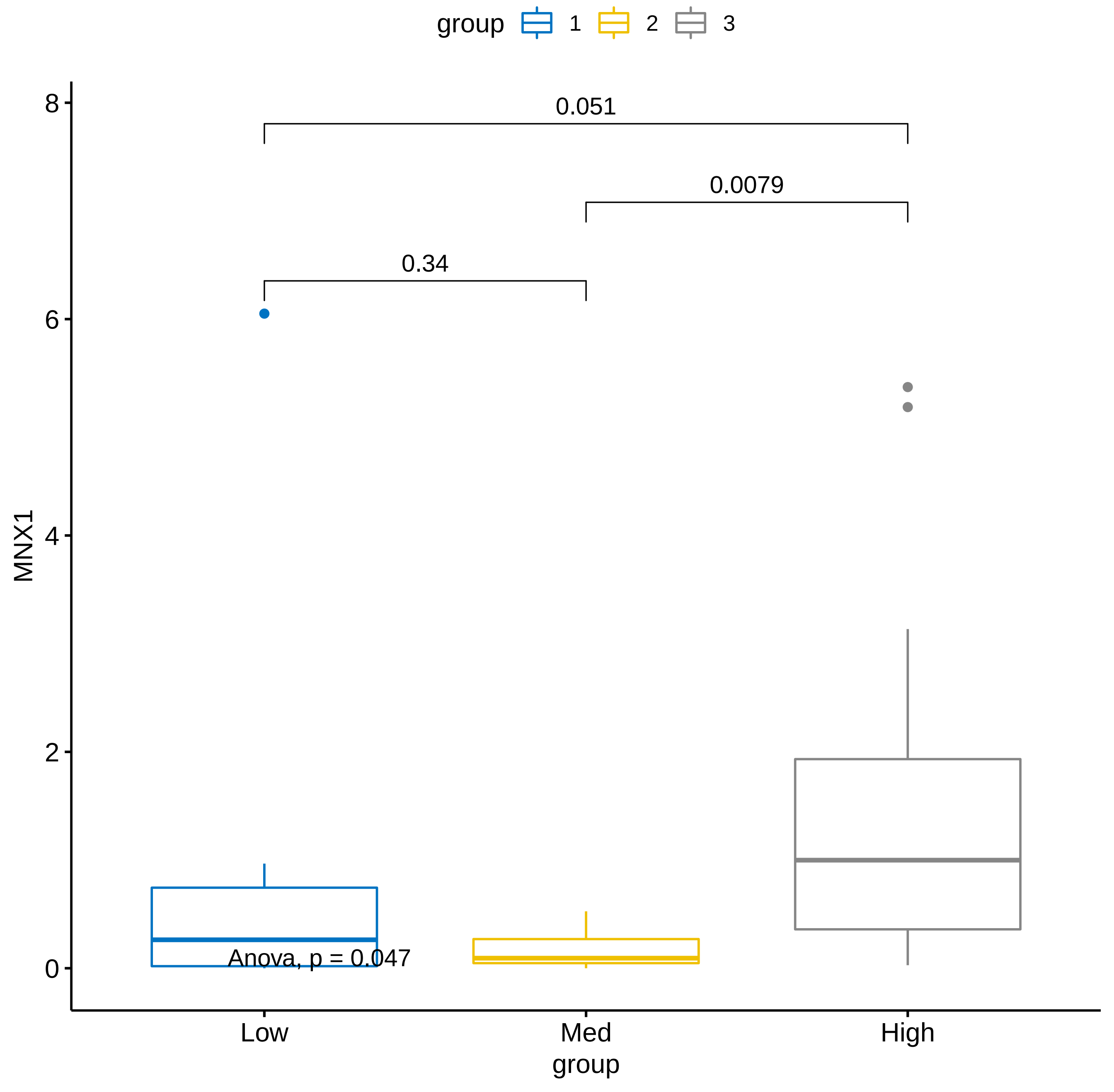
group
1
2
3
8
6
4
2
0
MNX1
0.051
0.0079
0.34
Anova, p = 0.047
Low
Med
High
group

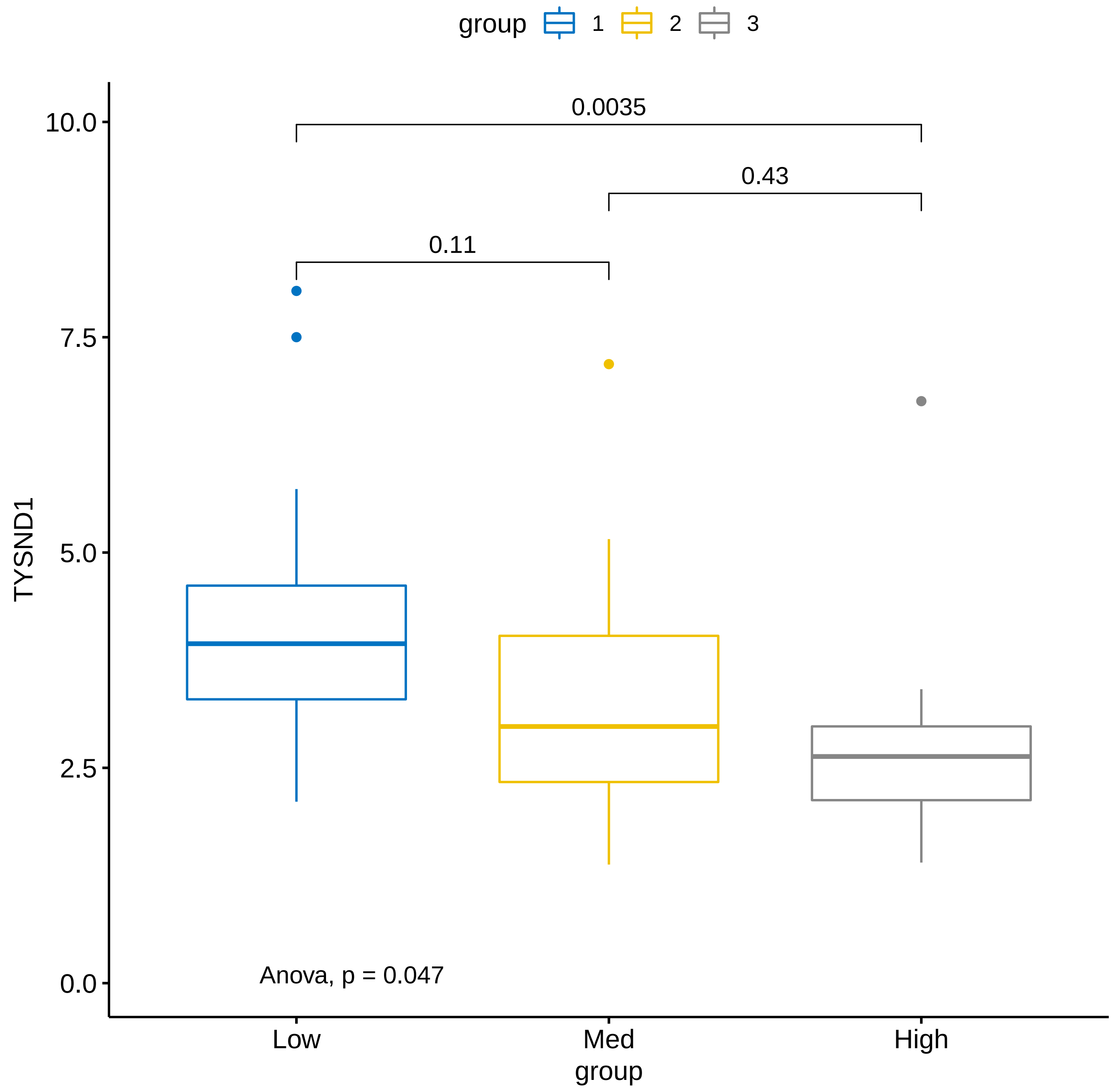
group
1
2
3
0.0035
0.43
0.11
10.0
7.5
5.0
2.5
0.0
TYSND1
Anova, p = 0.047
Low
Med
High
group

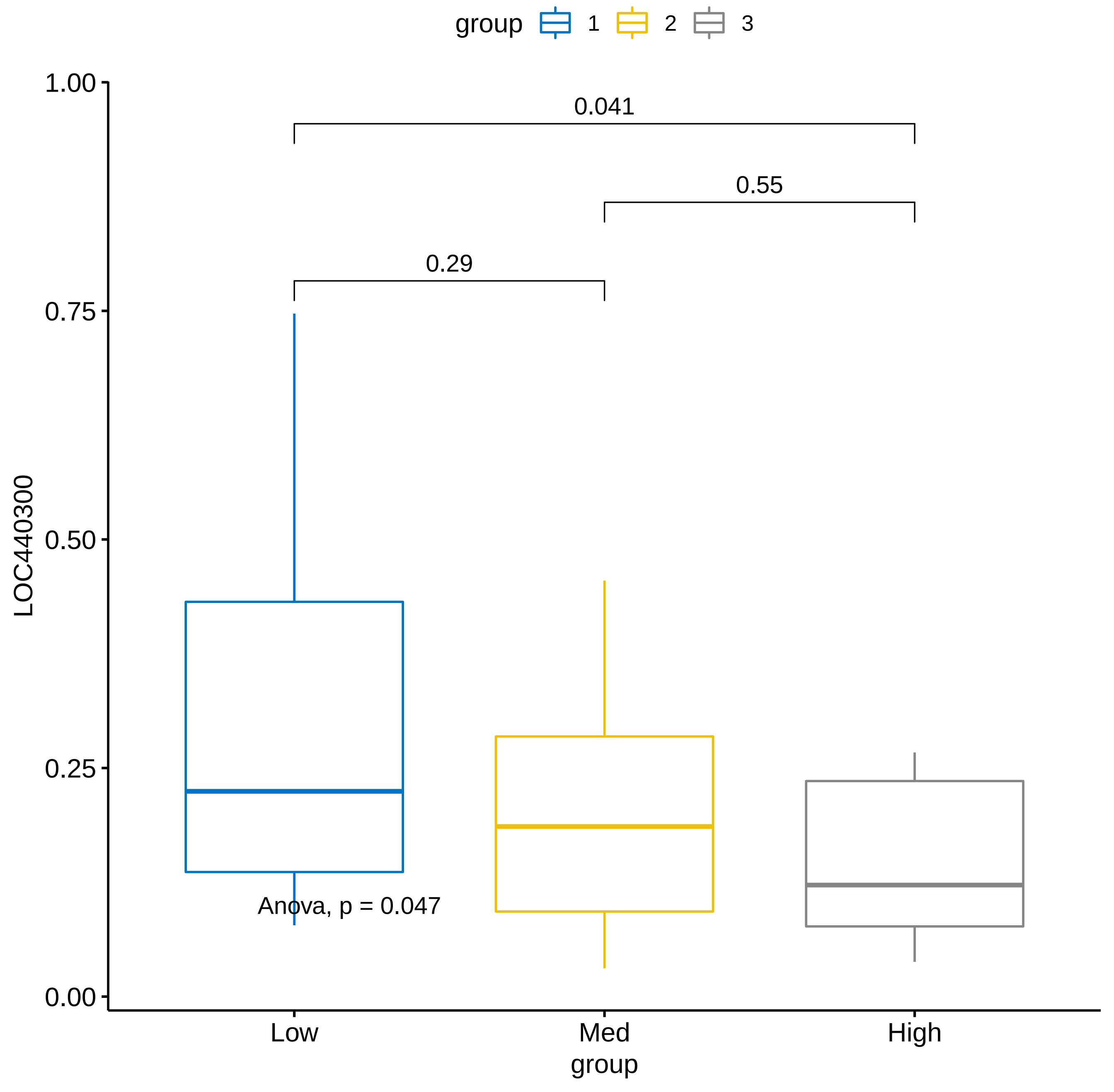
group
1
2
3
1.00
0.75
0.50
0.25
0.00
LOC440300
0.041
0.55
0.29
Anova, p = 0.047
Low
Med
High
group

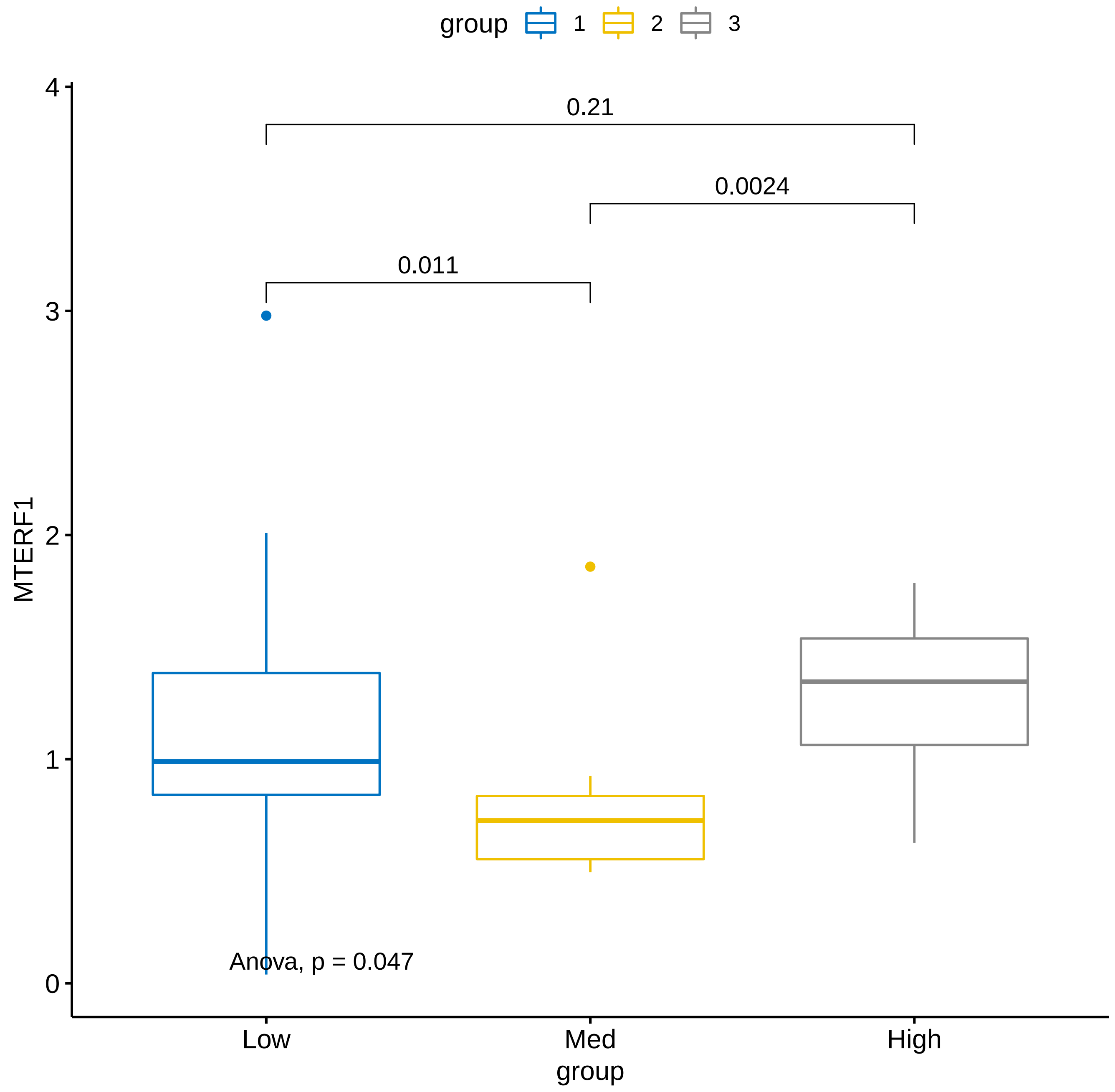
group
1
2
3
0.21
0.0024
0.011
Anova, p = 0.047
MTERF1
4
3
2
1
0
Low
Med
High
group

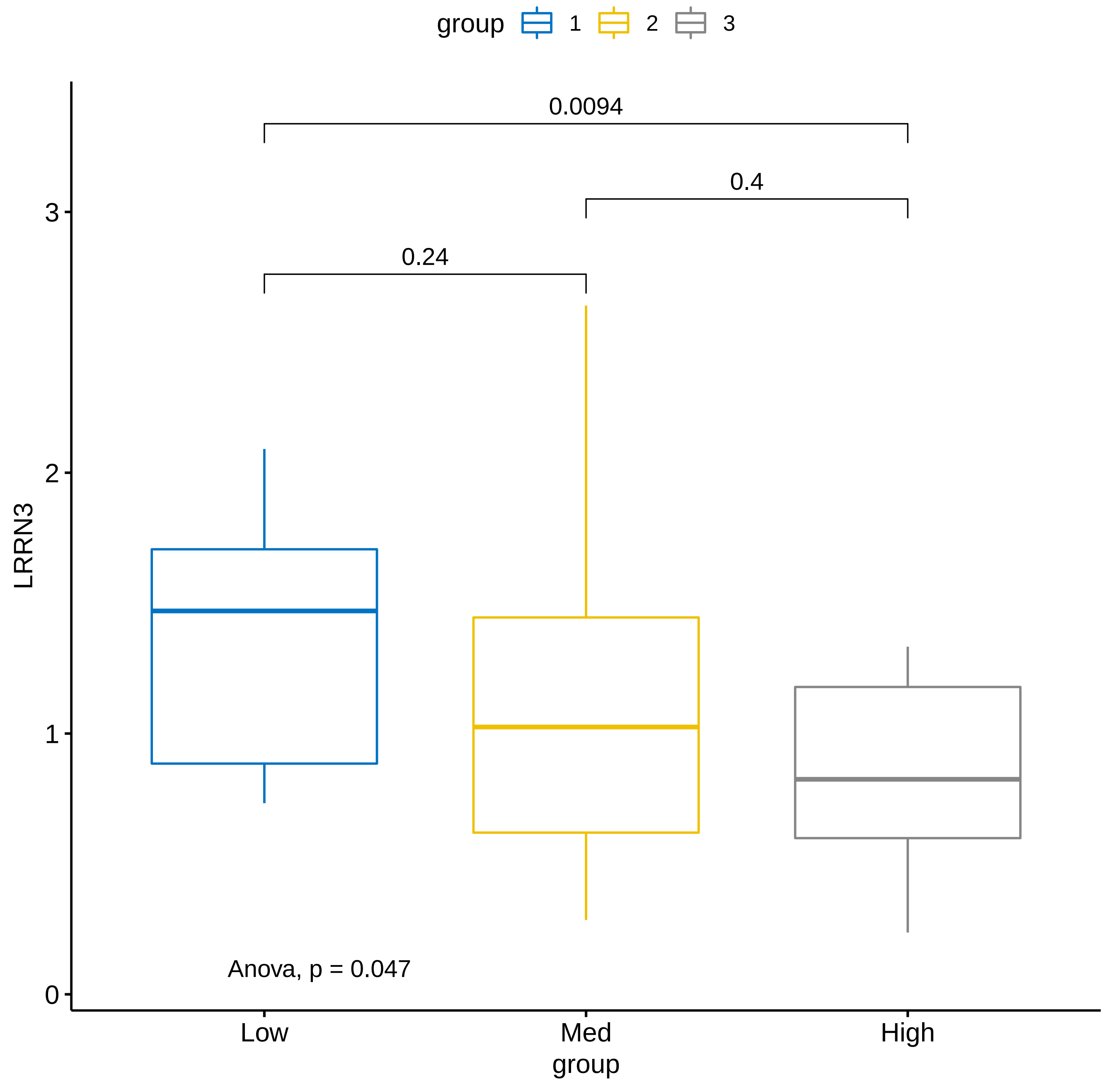

group 1 2 3
0.0094
0.4
0.24
3
2
1
0
LRRN3
Anova, p = 0.047
Low
Med
High
group

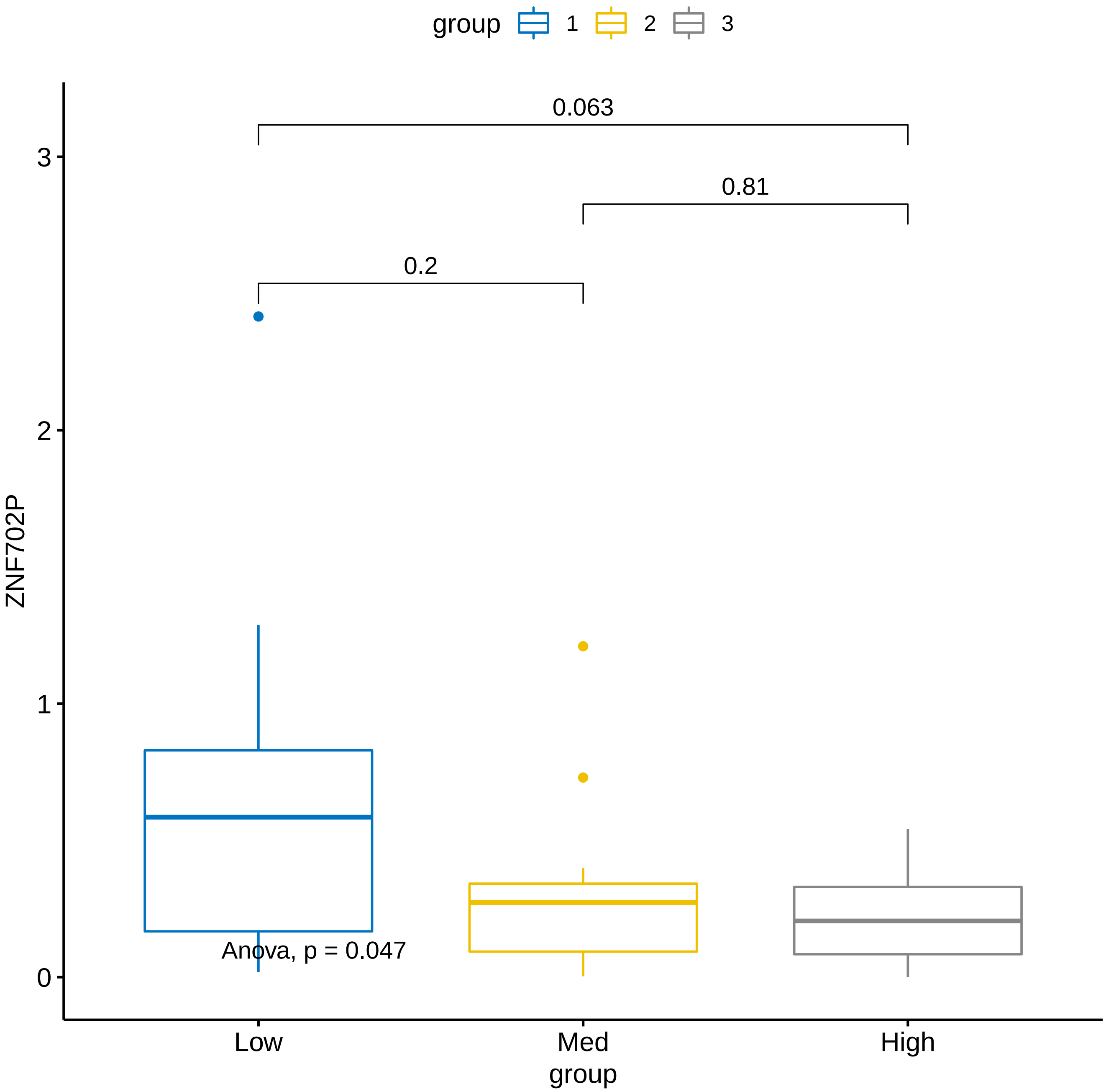
group
1
2
3
0.063
0.81
0.2
3
2
1
0
ZNF702P
Anova, p = 0.047
Low
Med
High
group

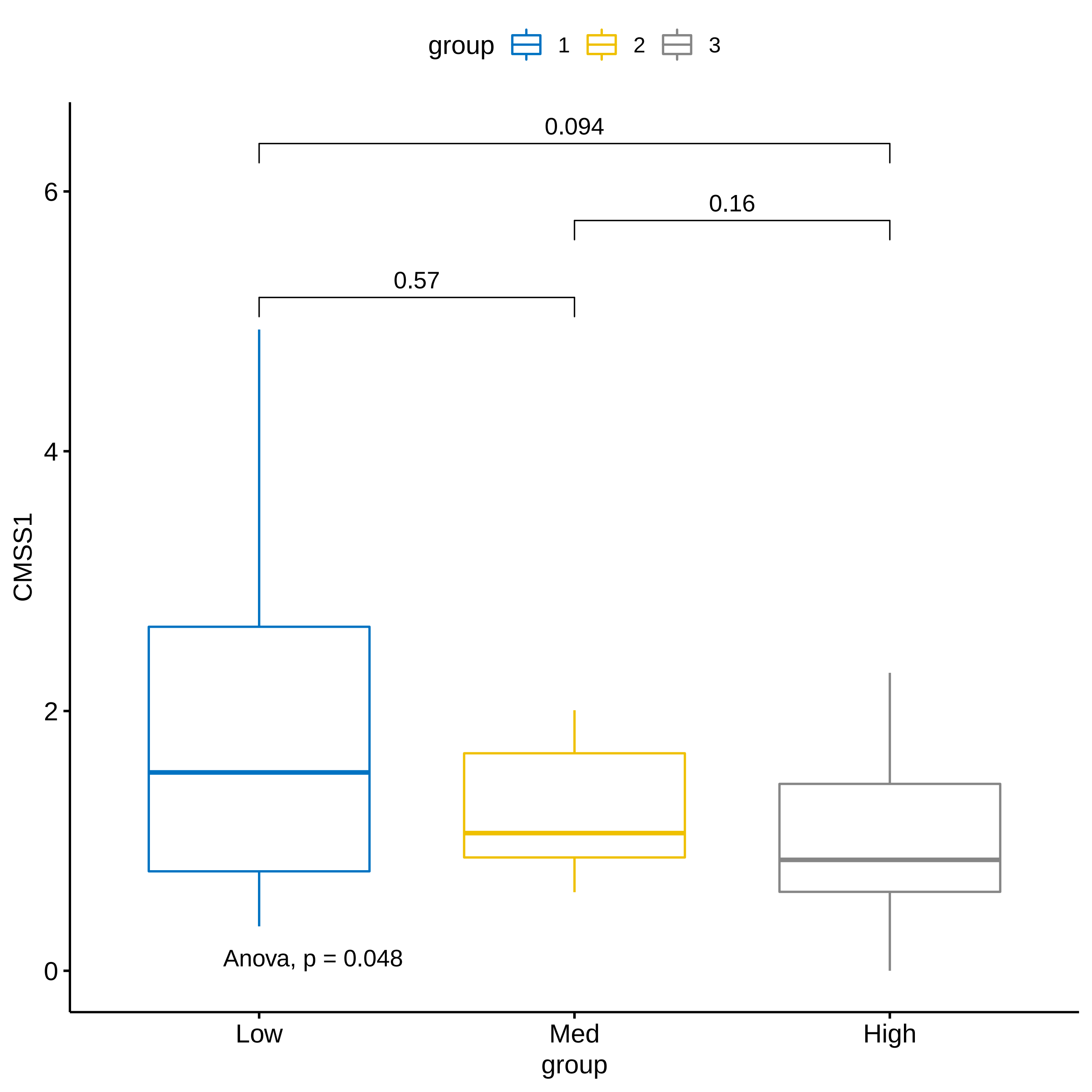
group
1
2
3
0.094
0.16
0.57
6
4
2
0
CMSS1
Anova, p = 0.048
Low
Med
High
group

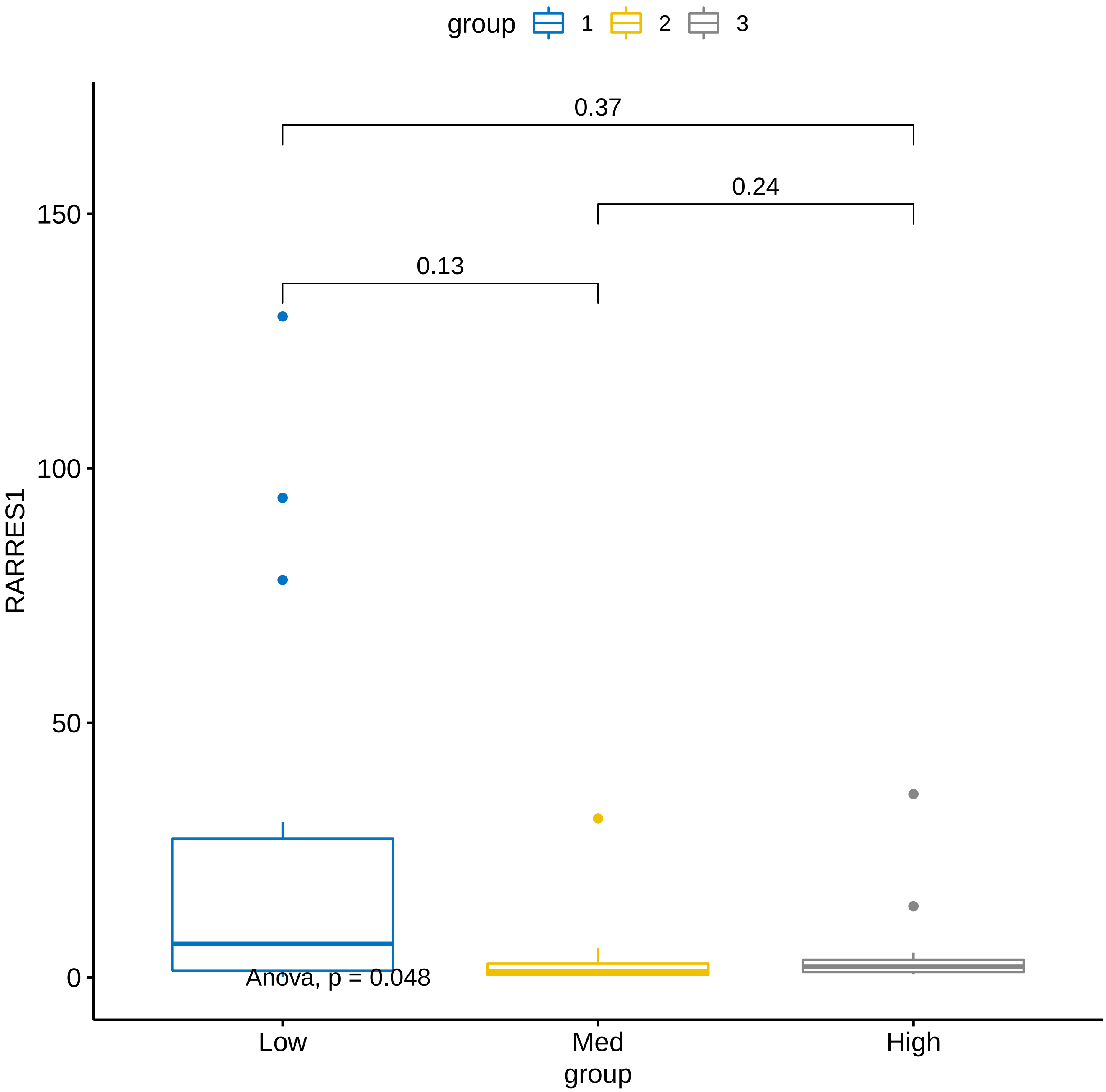

group
1
2
3
0.37
0.24
0.13
150
100
50
0
RARRES1
Anova, p = 0.048
Low
Med
High
group

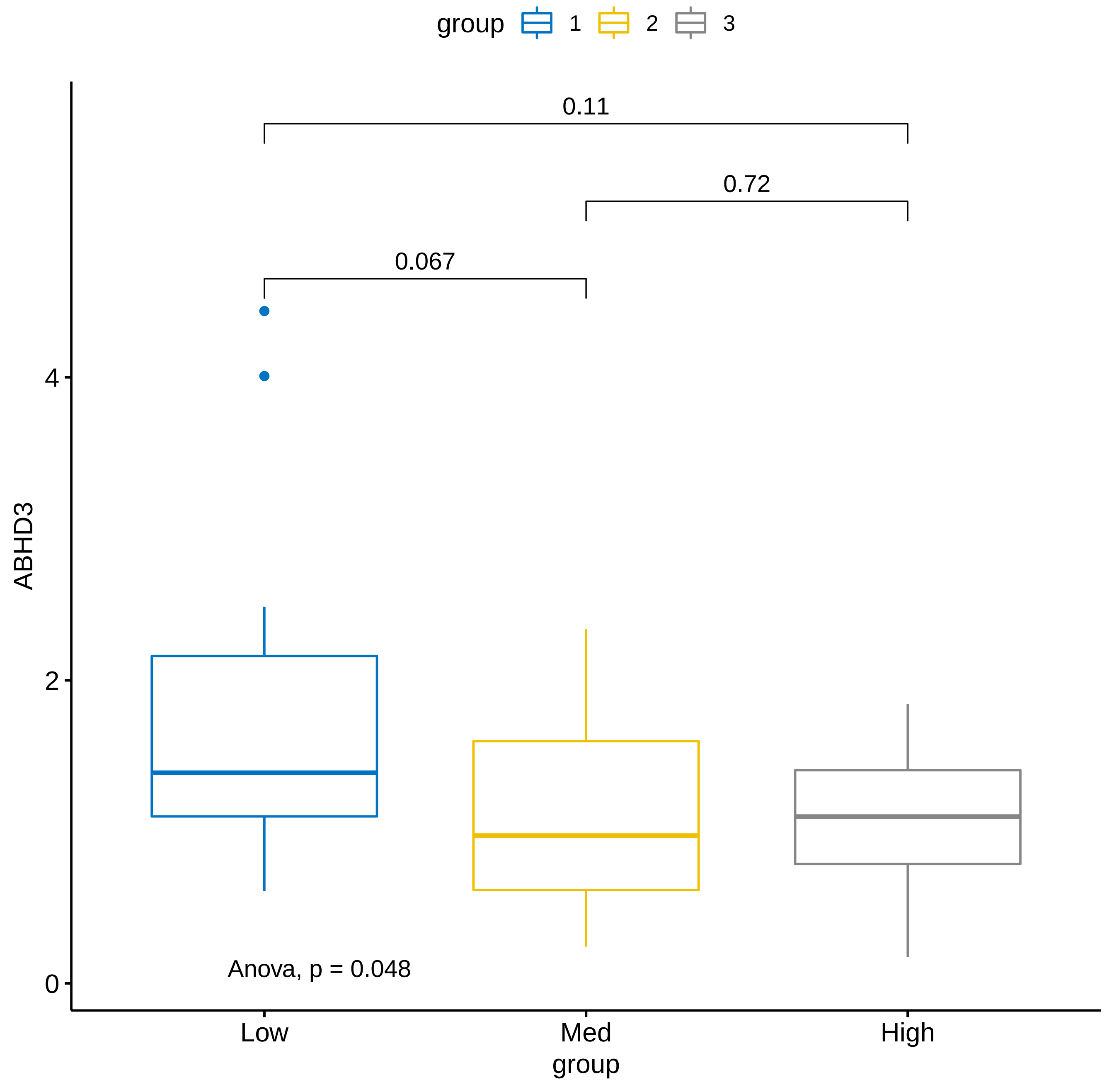
group 1 2 3
0.11
0.72
0.067
ABHD3
4
2
0
Anova, p = 0.048
Low
Med
High
group

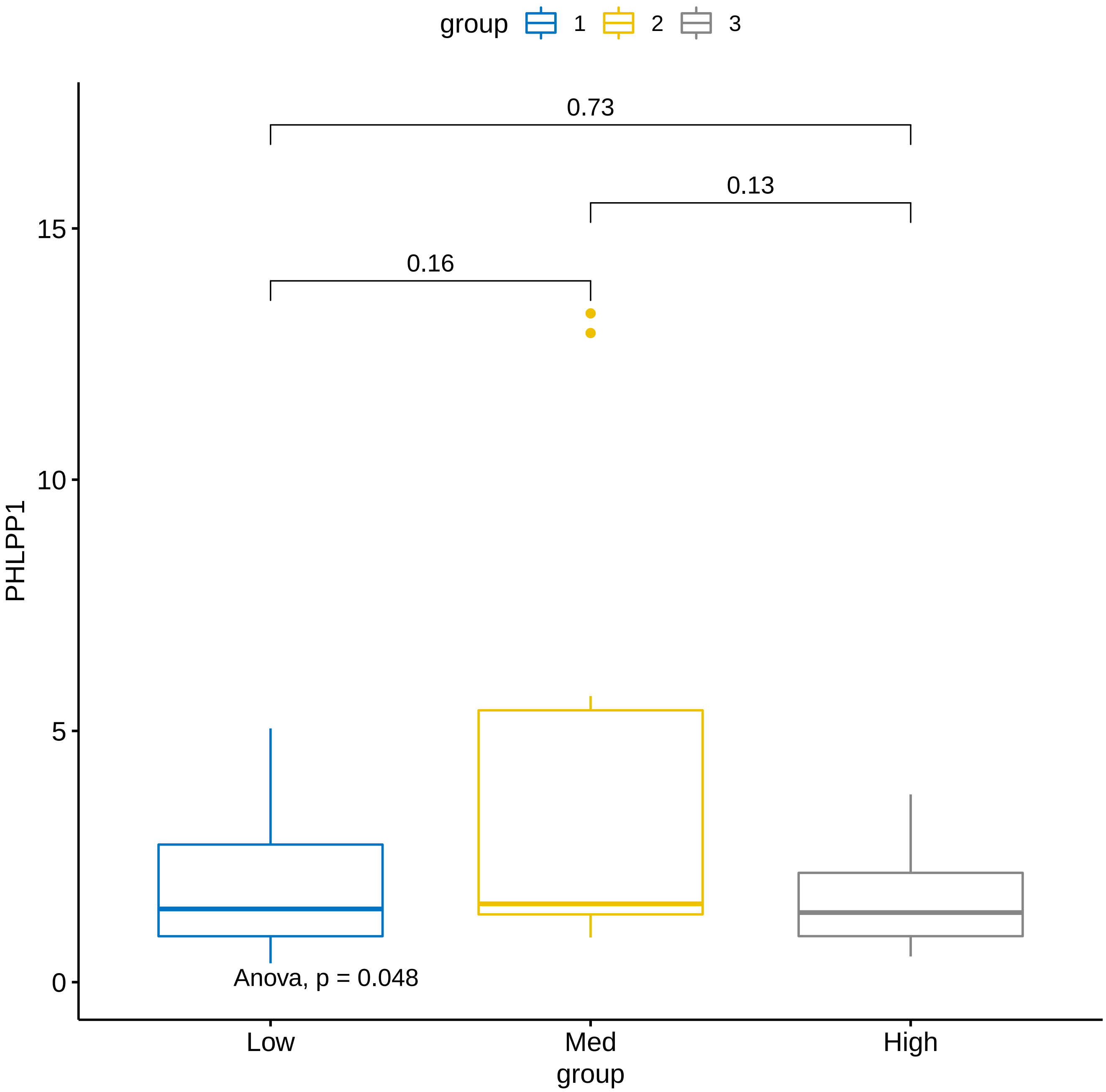
group
1
2
3
0.73
0.13
0.16
15
10
5
0
PHLPP1
Anova, p = 0.048
Low
Med
High
group

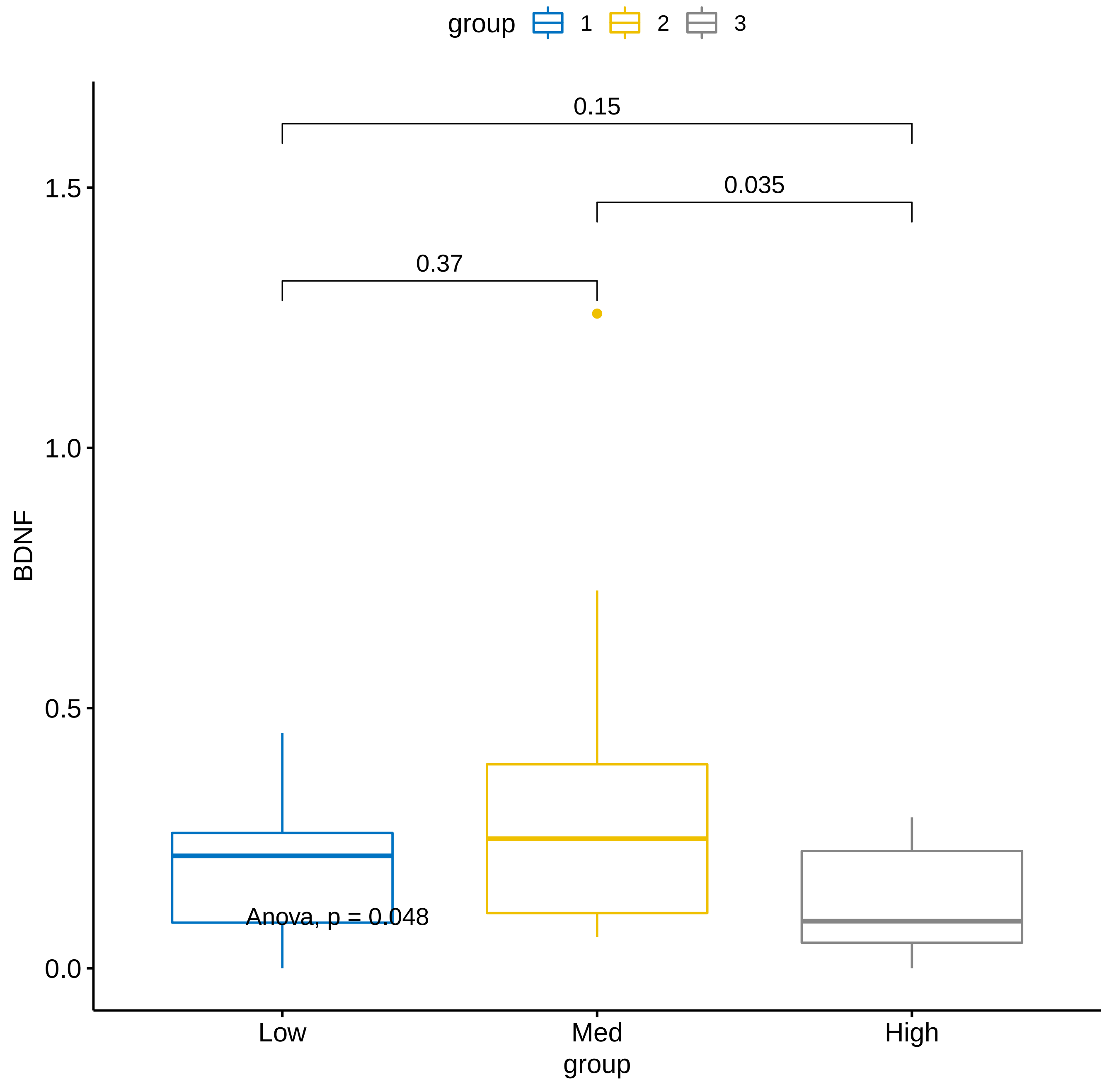
group
1
2
3
0.15
0.035
0.37
1.5
1.0
0.5
0.0
BDNF
Anova, p = 0.048
Low
Med
High
group

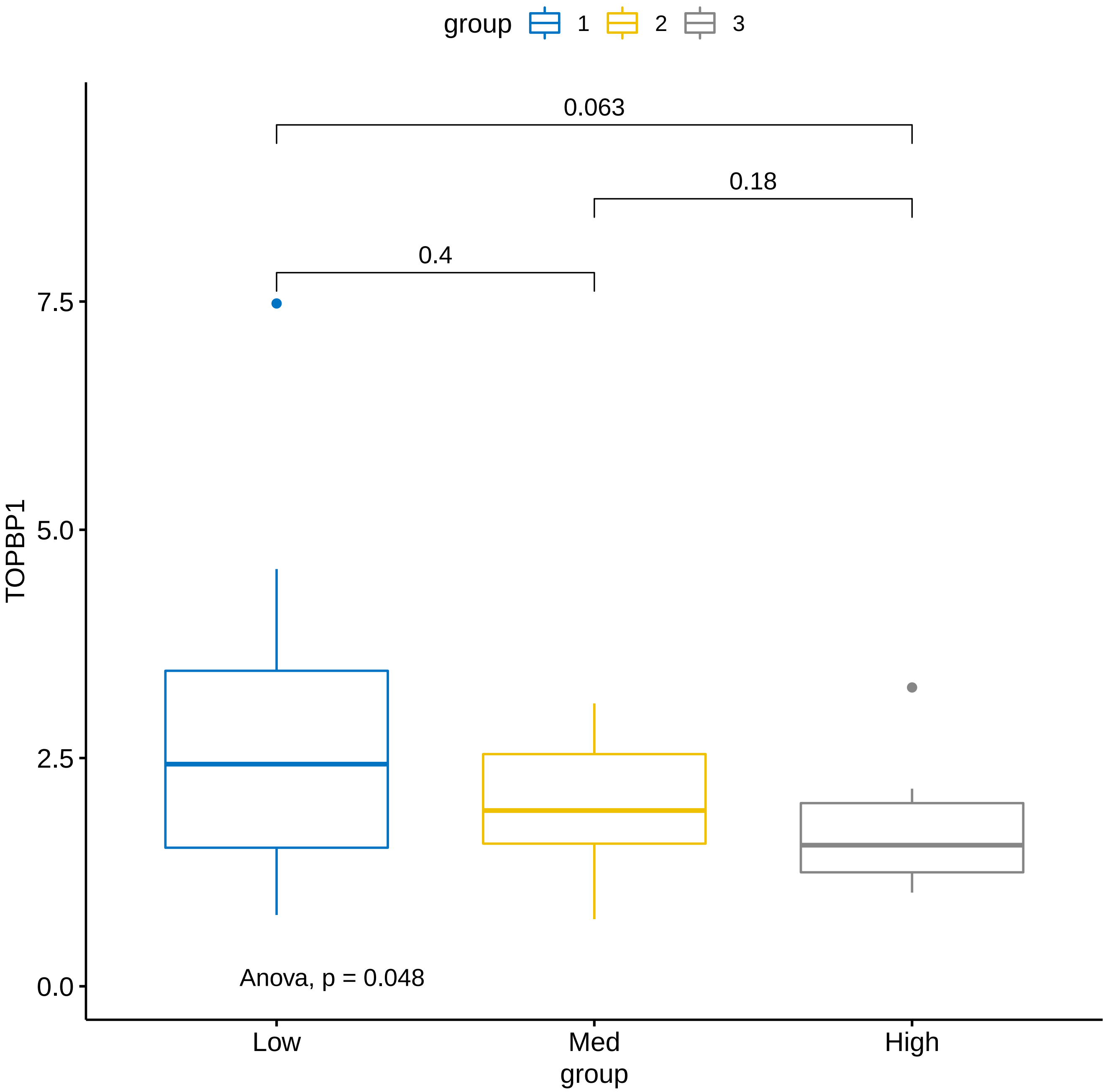
group
1
2
3
0.063
0.18
0.4
7.5
5.0
2.5
0.0
TOPBP1
Anova, p = 0.048
Low
Med
High
group

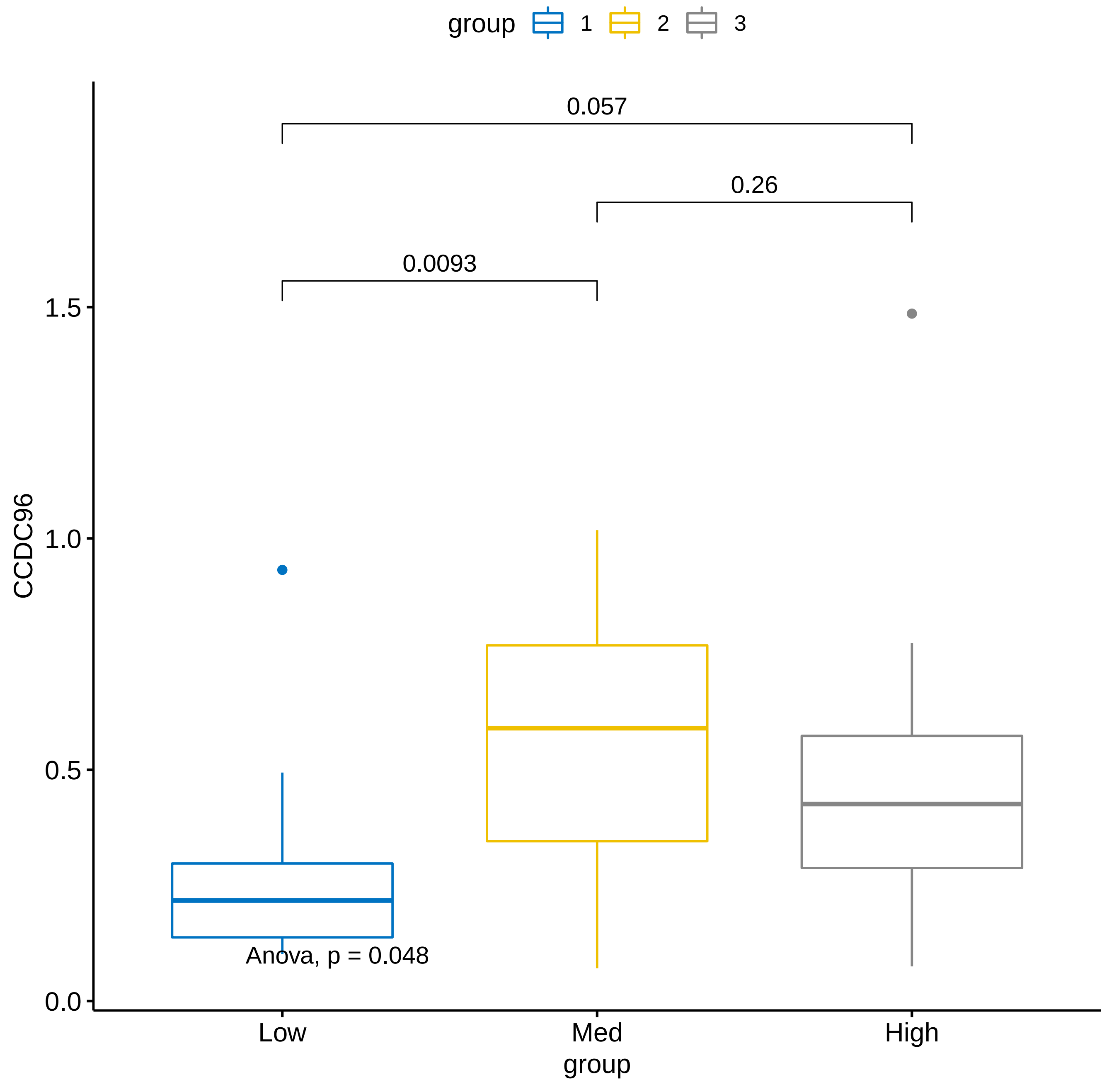
group
1
2
3
0.057
0.26
0.0093
CCDC96
1.5
1.0
0.5
0.0
Anova, p = 0.048
Low
Med
High
group

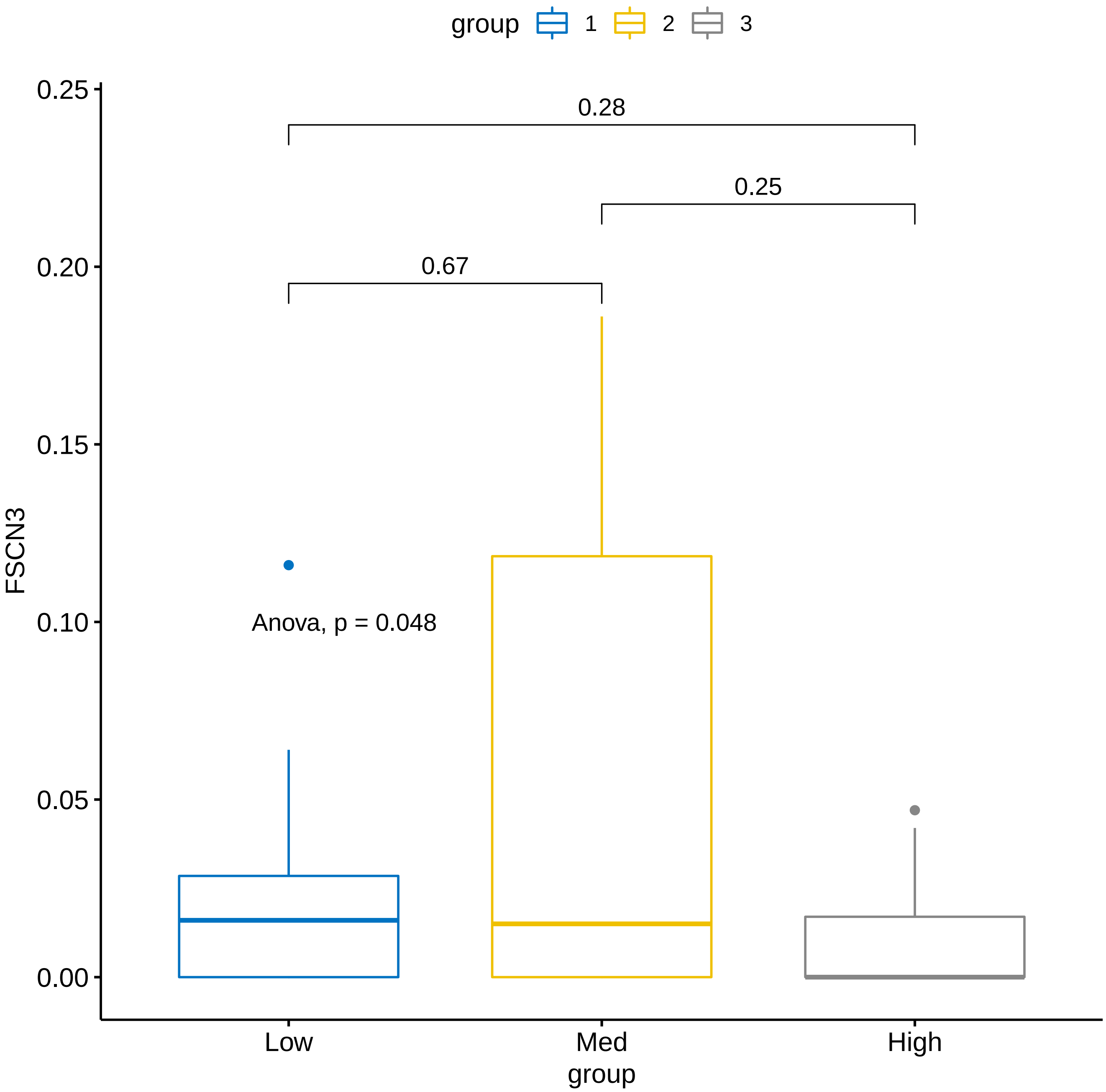
group
1
2
3
0.25
0.20
0.15
0.10
0.05
0.00
FSCN3
0.28
0.25
0.67
Anova, p = 0.048
Low
Med
High
group

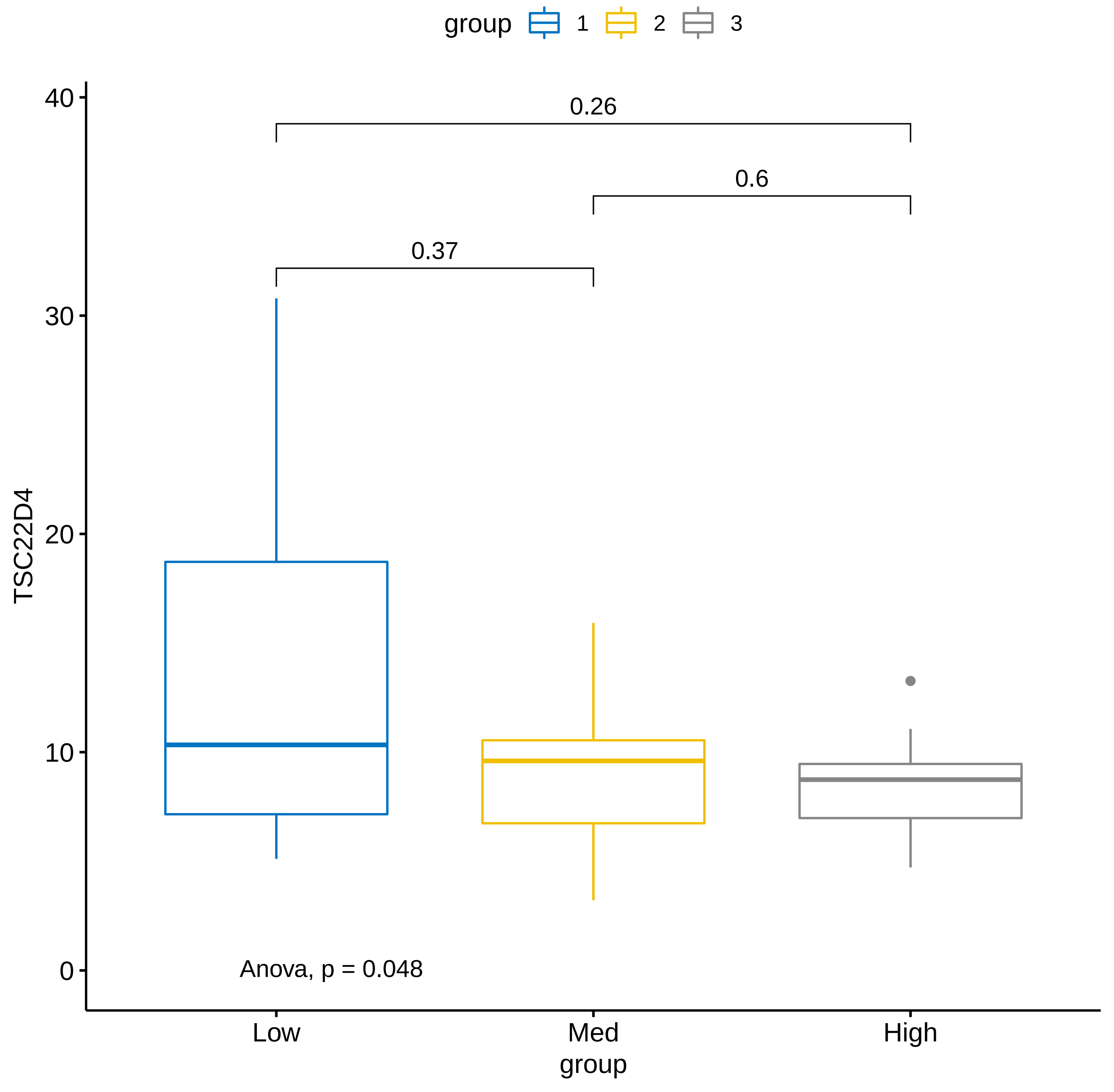
group
1
2
3
0.26
0.6
0.37
40
30
20
10
0
TSC22D4
Anova, p = 0.048
Low
Med
High
group

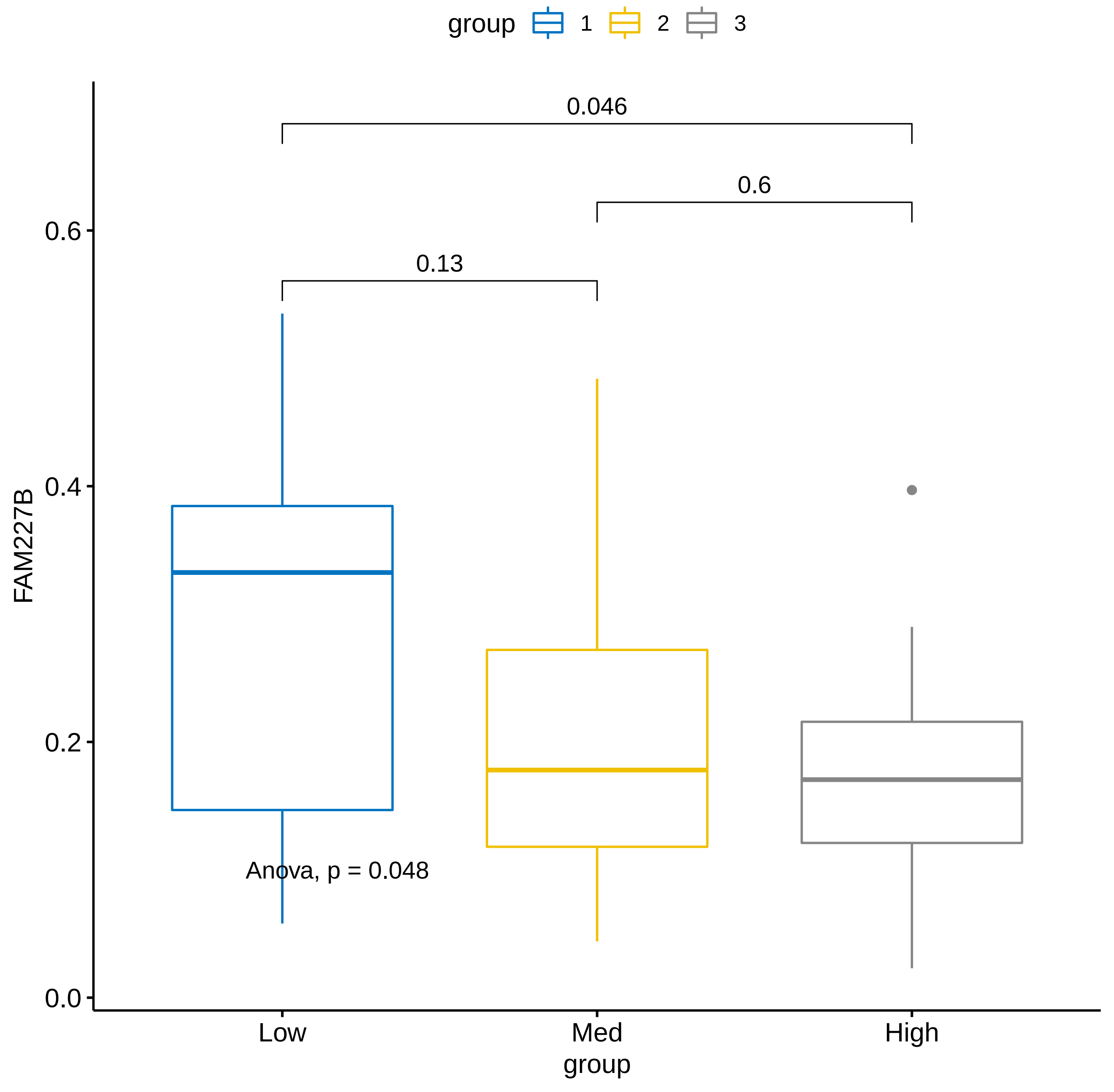

group
1
2
3
0.046
0.6
0.13
Anova, p = 0.048
FAM227B
0.0
0.2
0.4
0.6
Low
Med
High
group

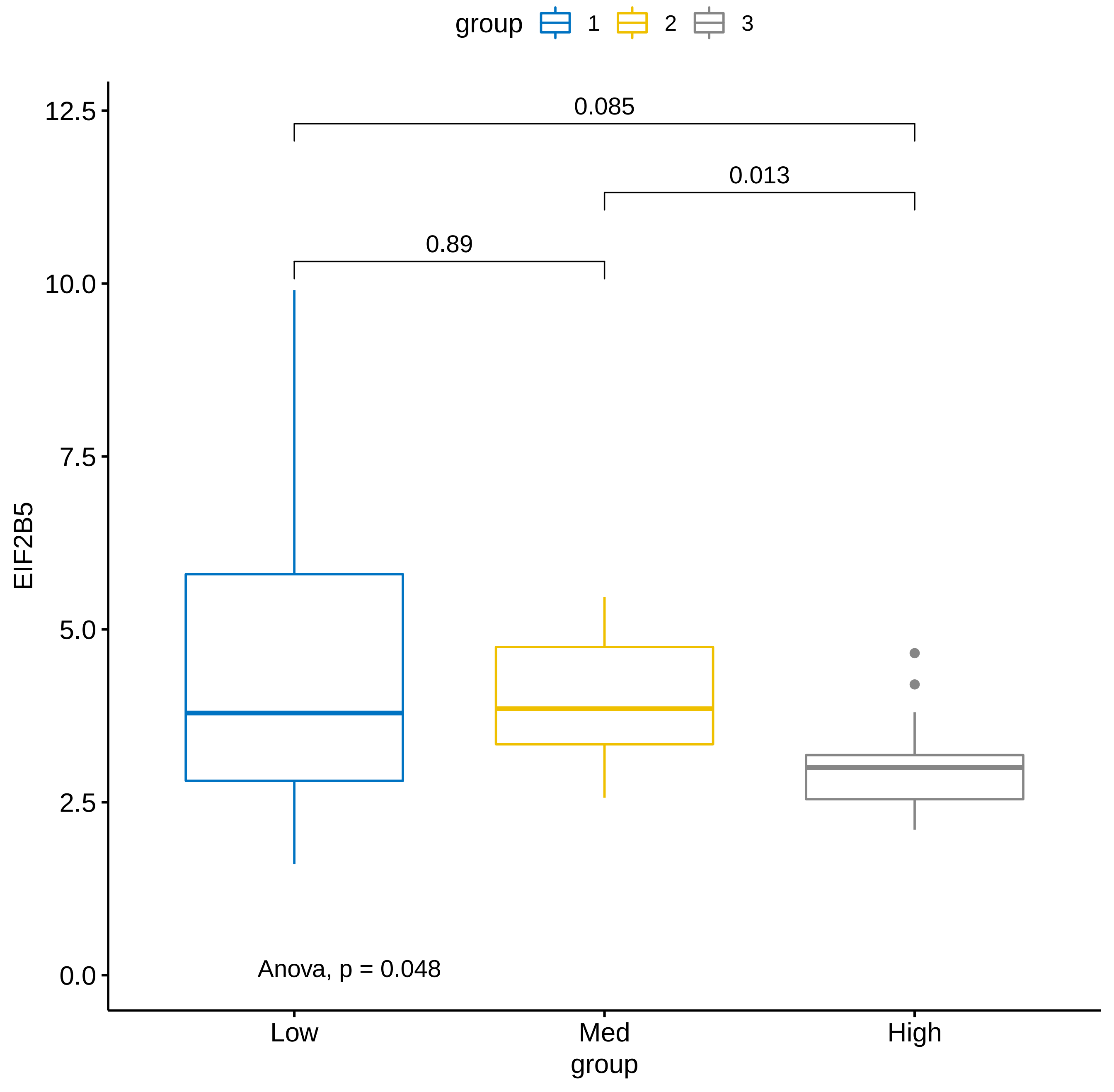
group
1
2
3
0.085
0.013
0.89
12.5
10.0
7.5
5.0
2.5
0.0
EIF2B5
Anova, p = 0.048
Low
Med
High
group

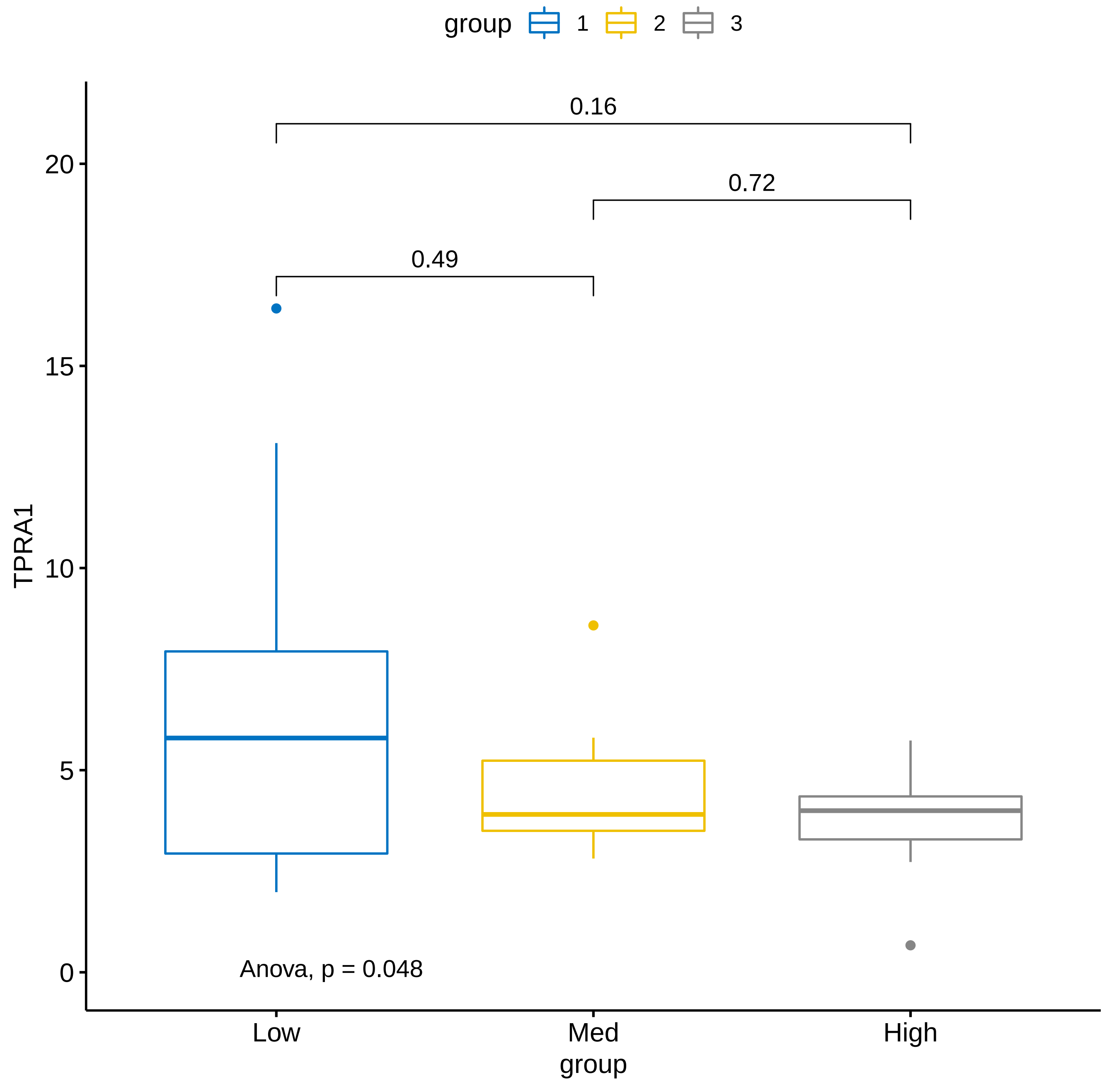
group
1
2
3
0.16
0.72
0.49
20
15
10
5
0
TPRA1
Anova, p = 0.048
Low
Med
High
group

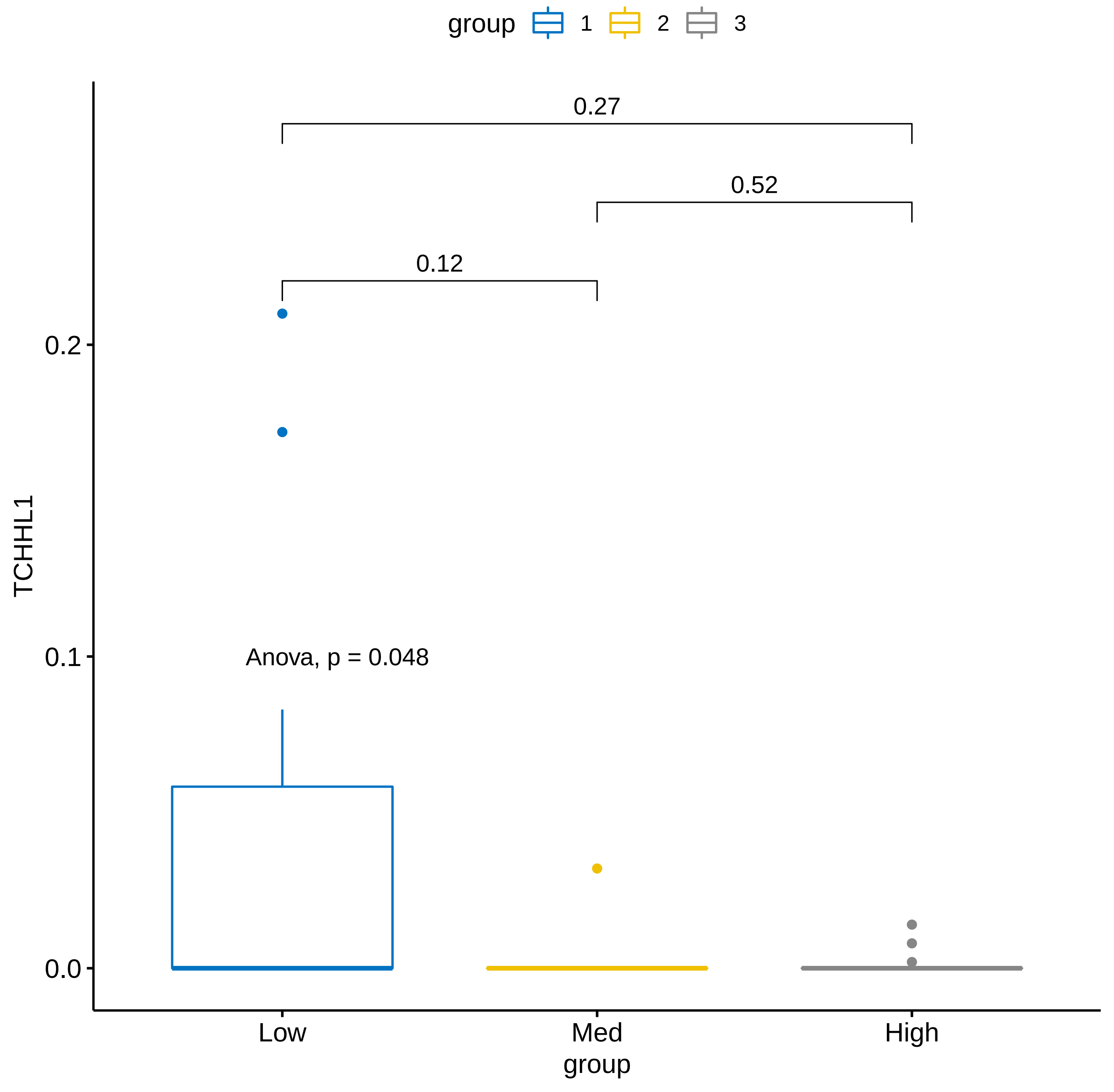
group 1 2 3
0.27
0.52
0.12
0.2
0.1
0.0
TCHHL1
Anova, p = 0.048
Low
Med
High
group

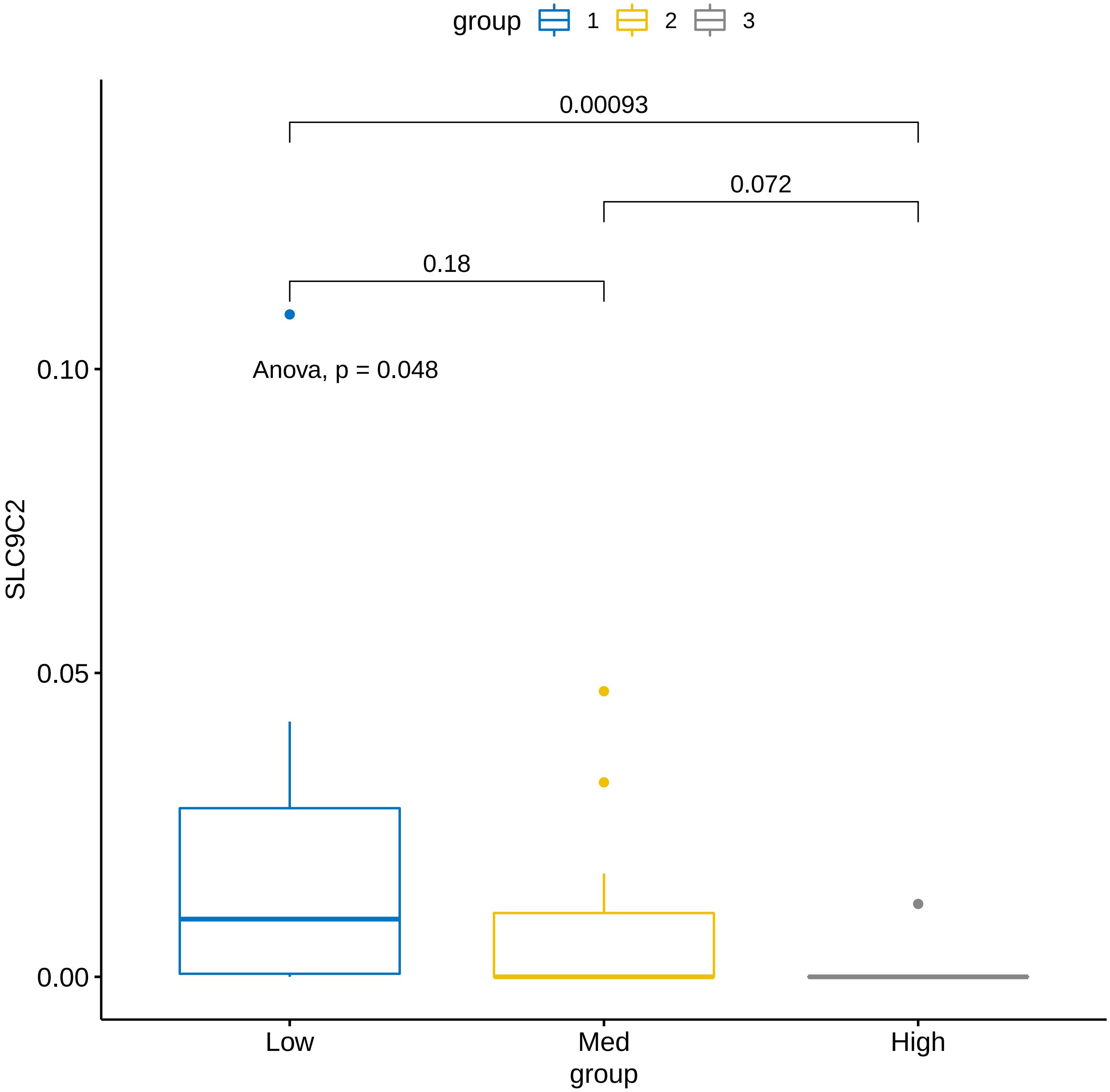

group
1
2
3
0.00093
0.072
0.18
Anova, p = 0.048
0.10
0.05
0.00
SLC9C2
Low
Med
High
group

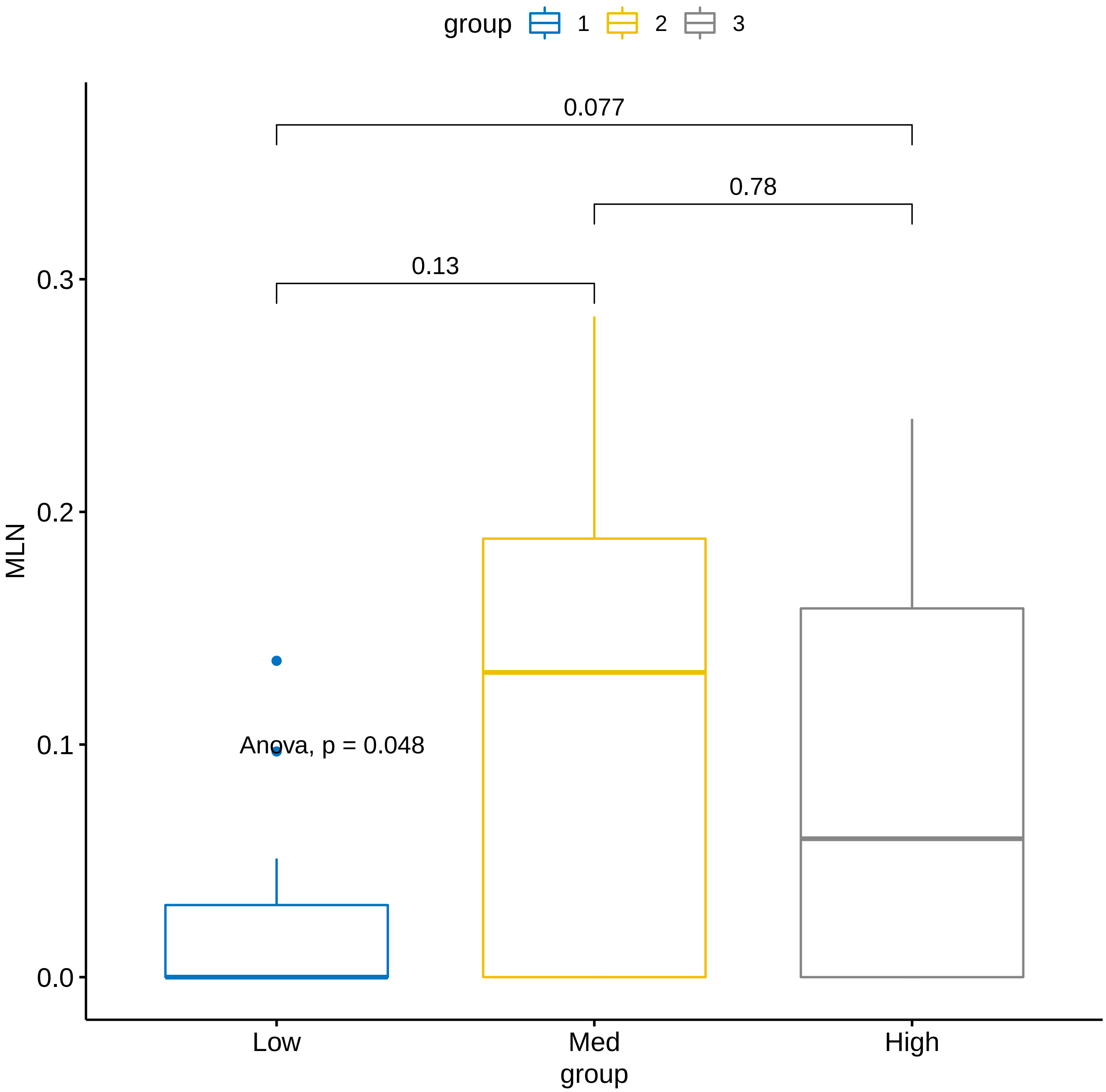
group
1
2
3
0.077
0.78
0.13
Anova, p = 0.048
MLN
0.3
0.2
0.1
0.0
Low
Med
High
group

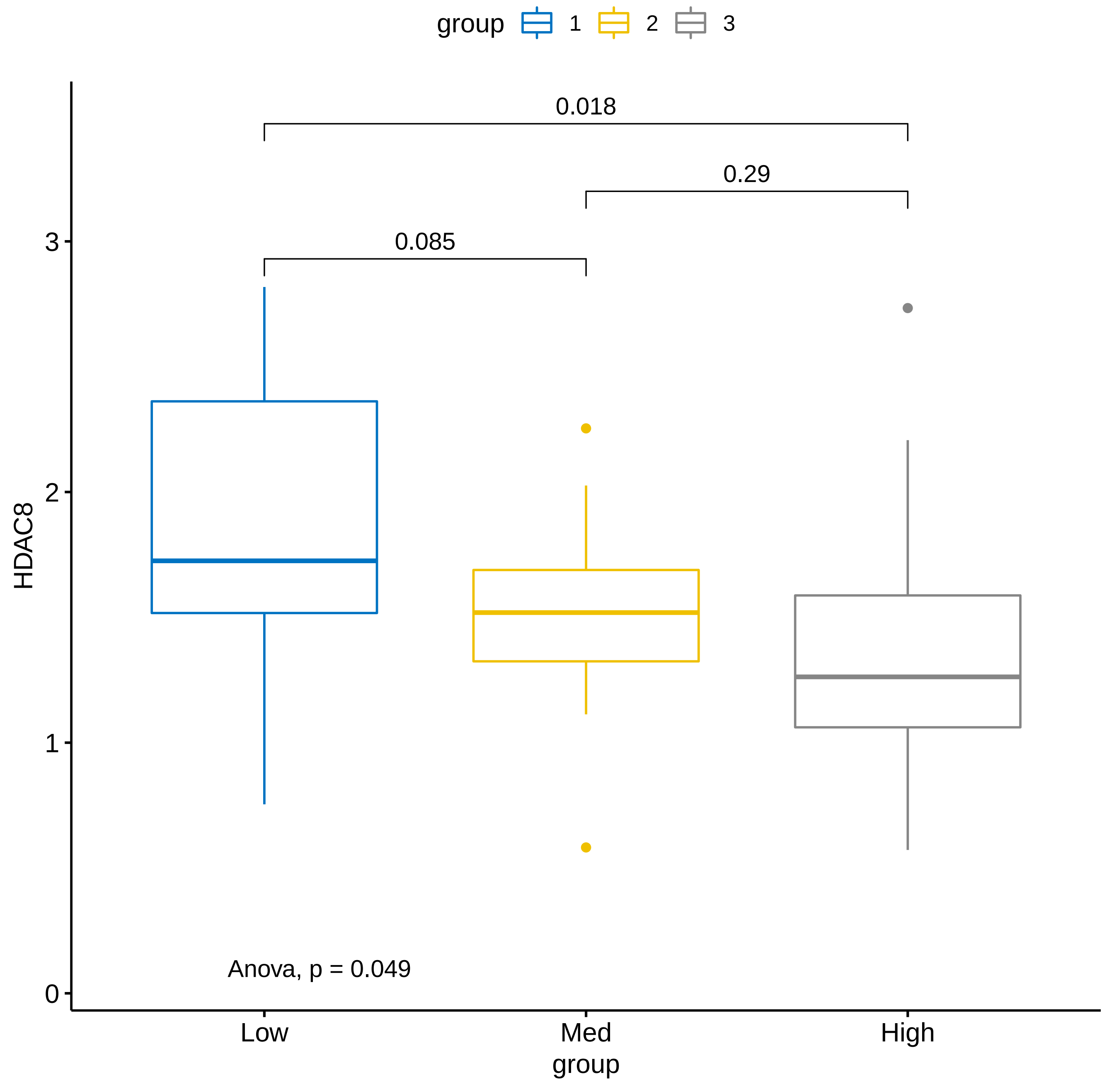

group
1
2
3
0.018
0.29
0.085
3
2
1
0
HDAC8
Anova, p = 0.049
Low
Med
High
group

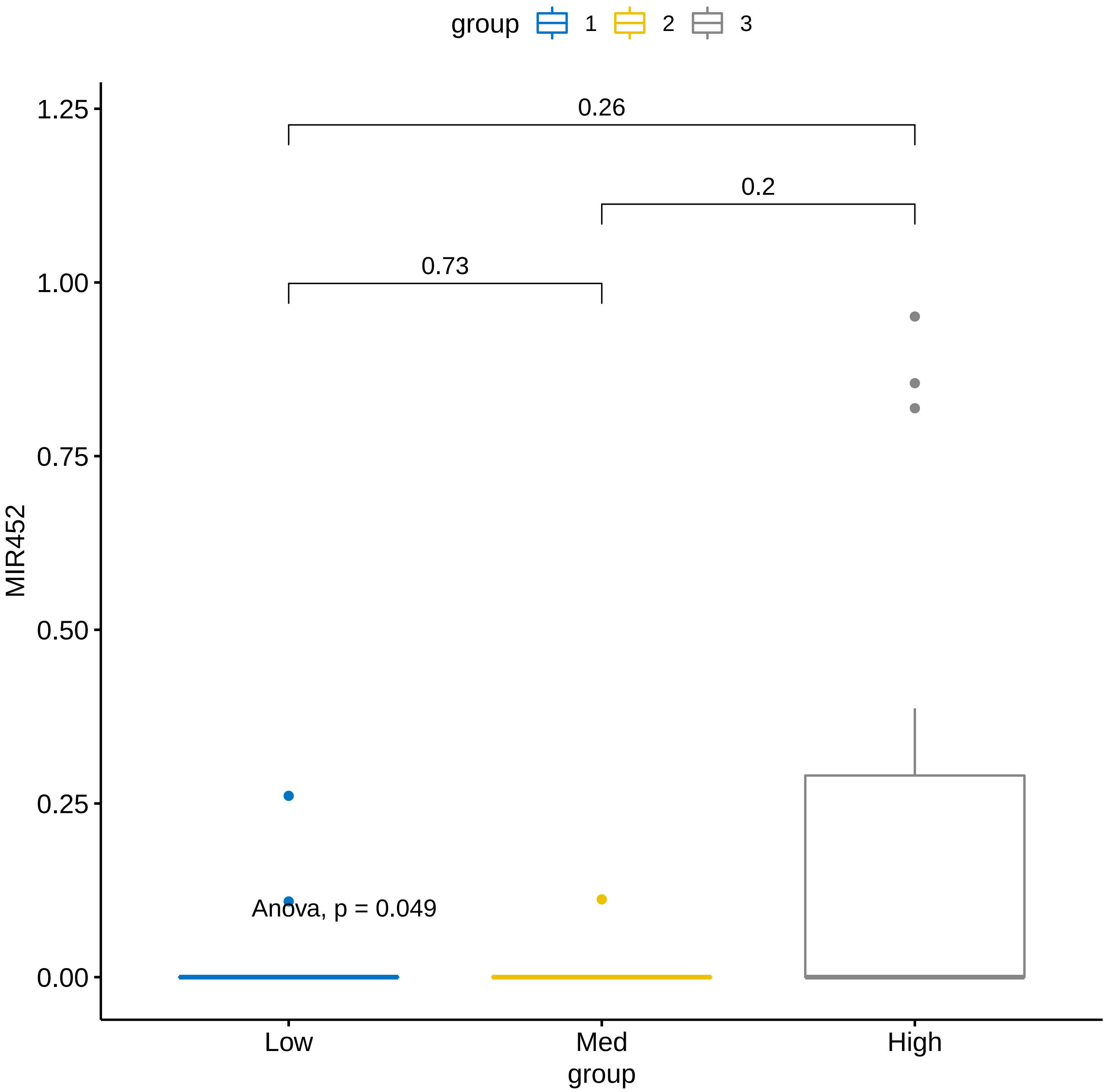

group
1
2
3
0.26
0.2
0.73
1.25
1.00
0.75
0.50
0.25
0.00
MIR452
Anova, p = 0.049
Low
Med
High
group

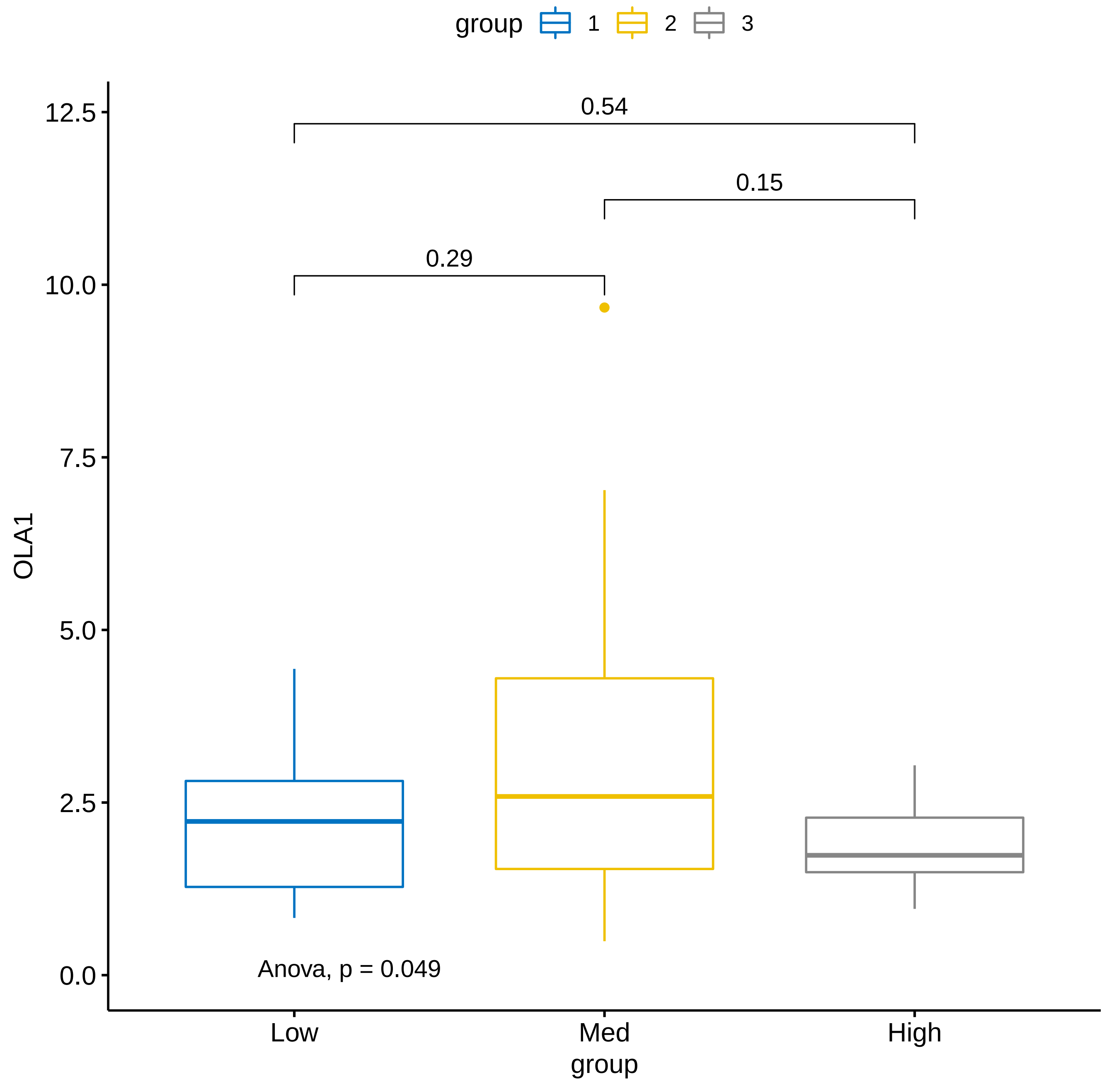
group 1 2 3
0.54
0.15
0.29
12.5
10.0
7.5
5.0
2.5
0.0
OLA1
Anova, p = 0.049
Low
Med
High
group

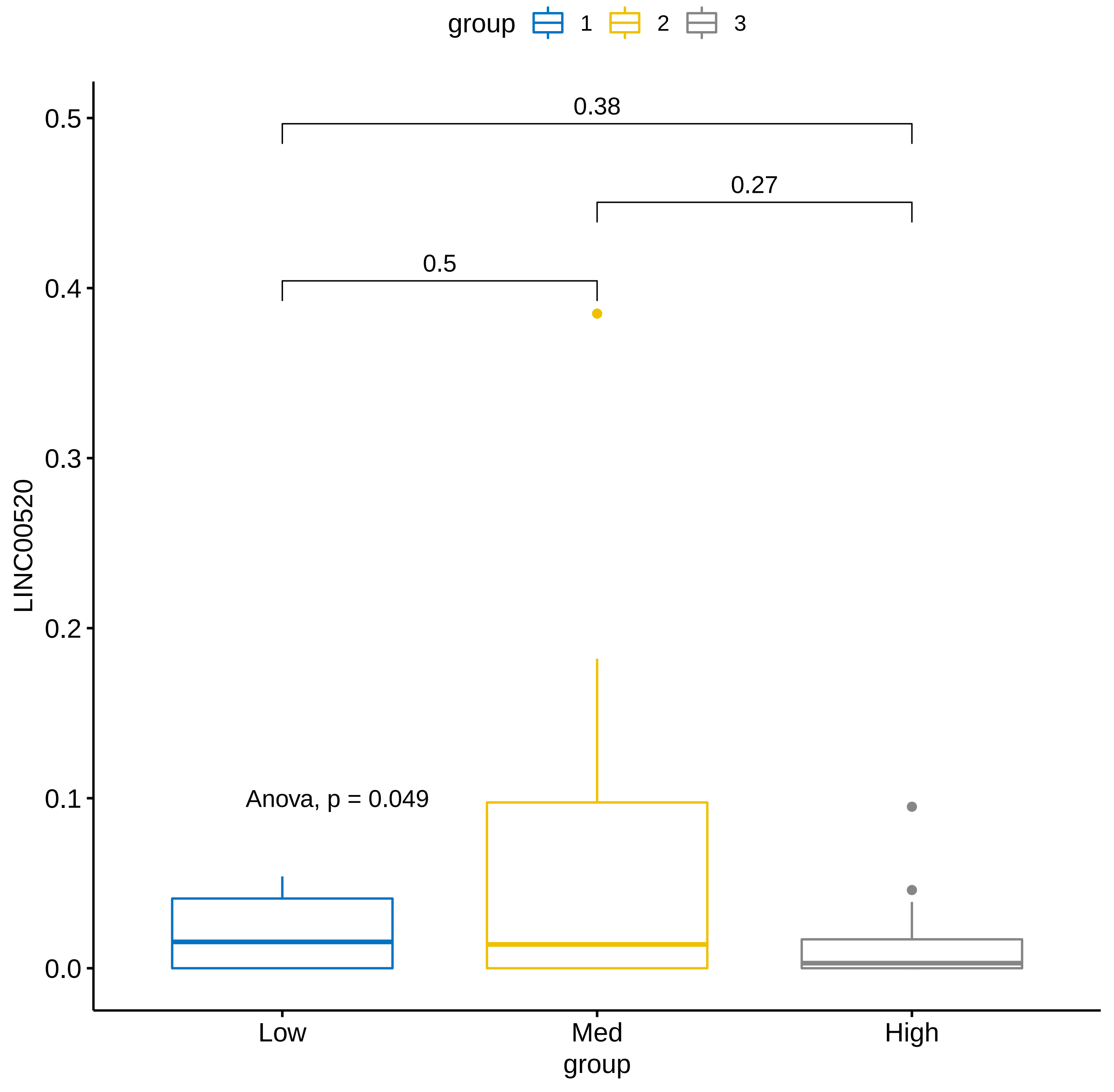

group
1
2
3
0.38
0.27
0.5
Anova, p = 0.049
0.5
0.4
0.3
0.2
0.1
0.0
LINC00520
Low
Med
High
group

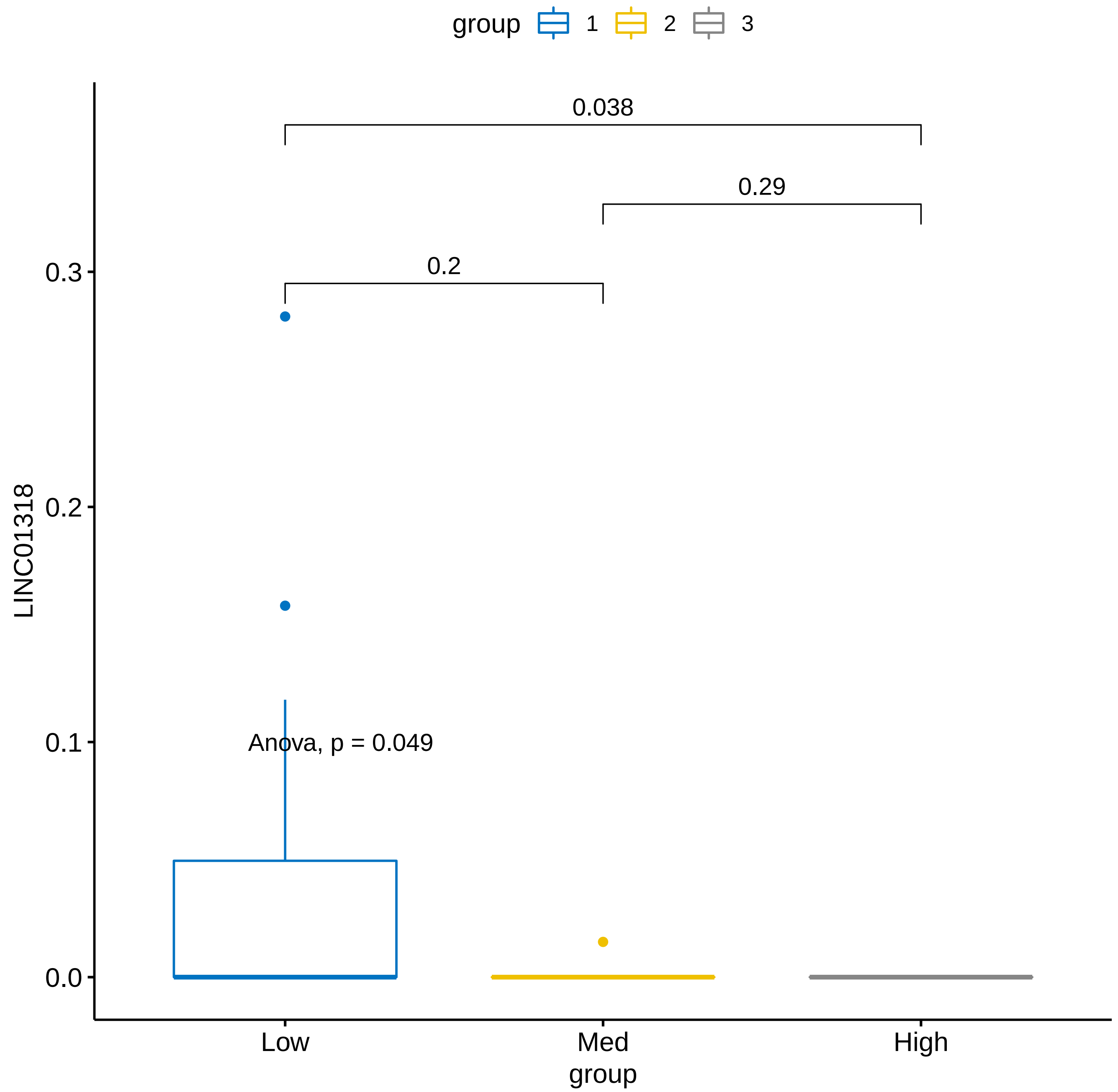
group
1
2
3
0.038
0.29
0.2
0.3
0.2
0.1
0.0
LINC01318
Anova, p = 0.049
Low
Med
High
group

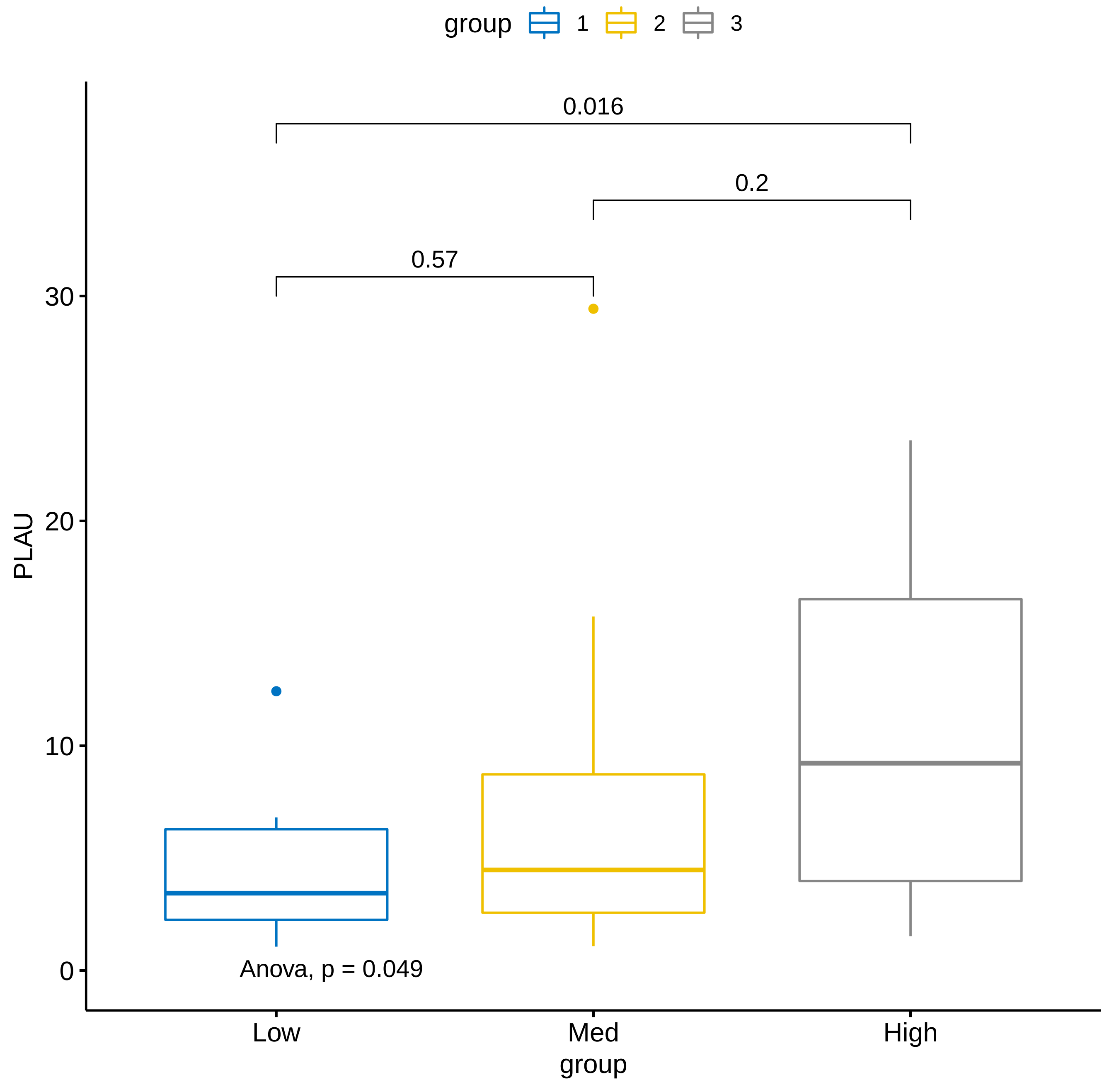

group
1
2
3
0.016
0.2
0.57
30
20
10
0
PLAU
Anova, p = 0.049
Low
Med
High
group

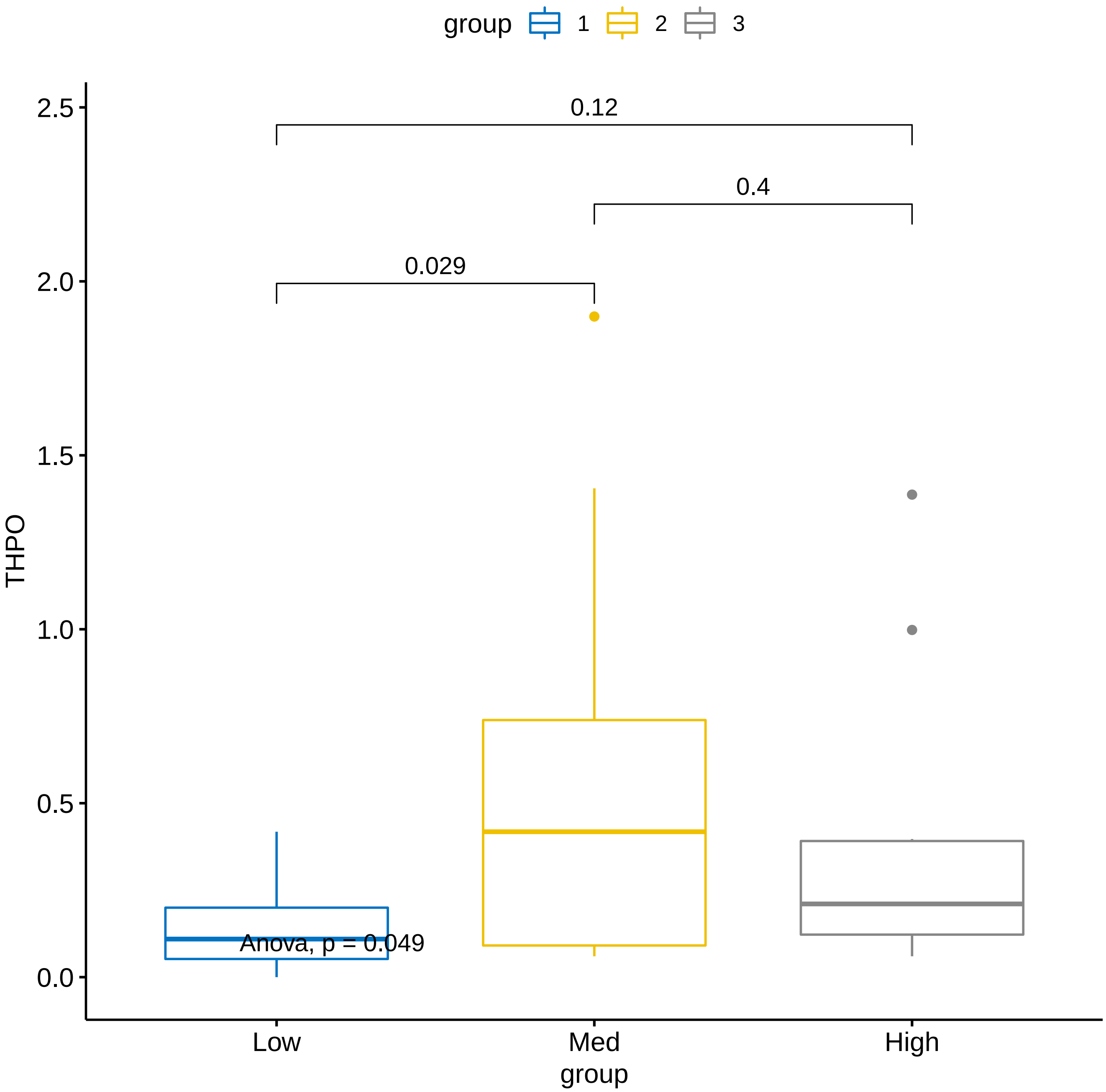
group
1
2
3
0.12
0.4
0.029
2.5
2.0
1.5
1.0
0.5
0.0
THPO
Anova, p = 0.049
Low
Med
High
group

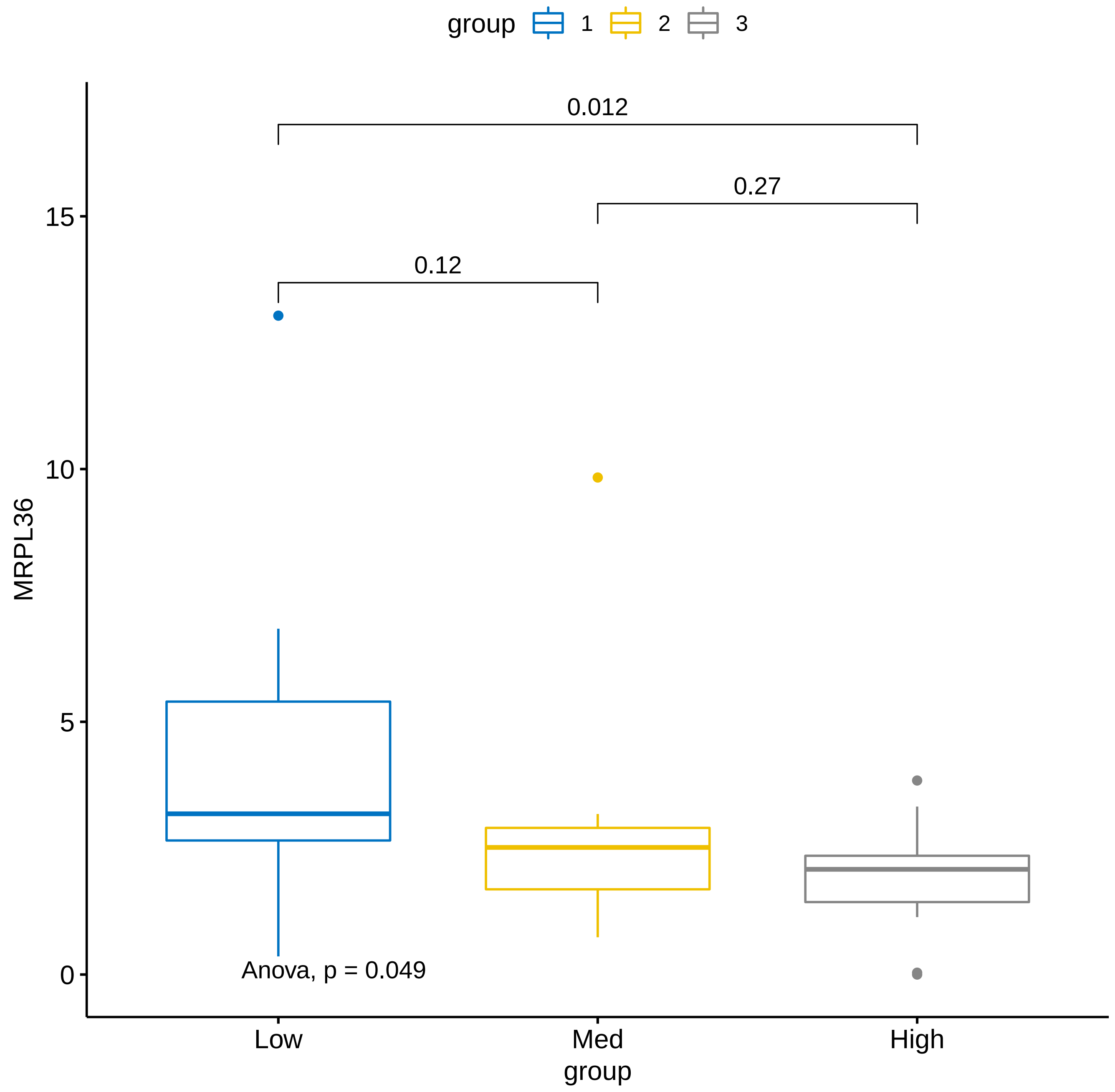

group
1
2
3
0.012
0.27
0.12
15
10
5
0
MRPL36
Anova, p = 0.049
Low
Med
High
group

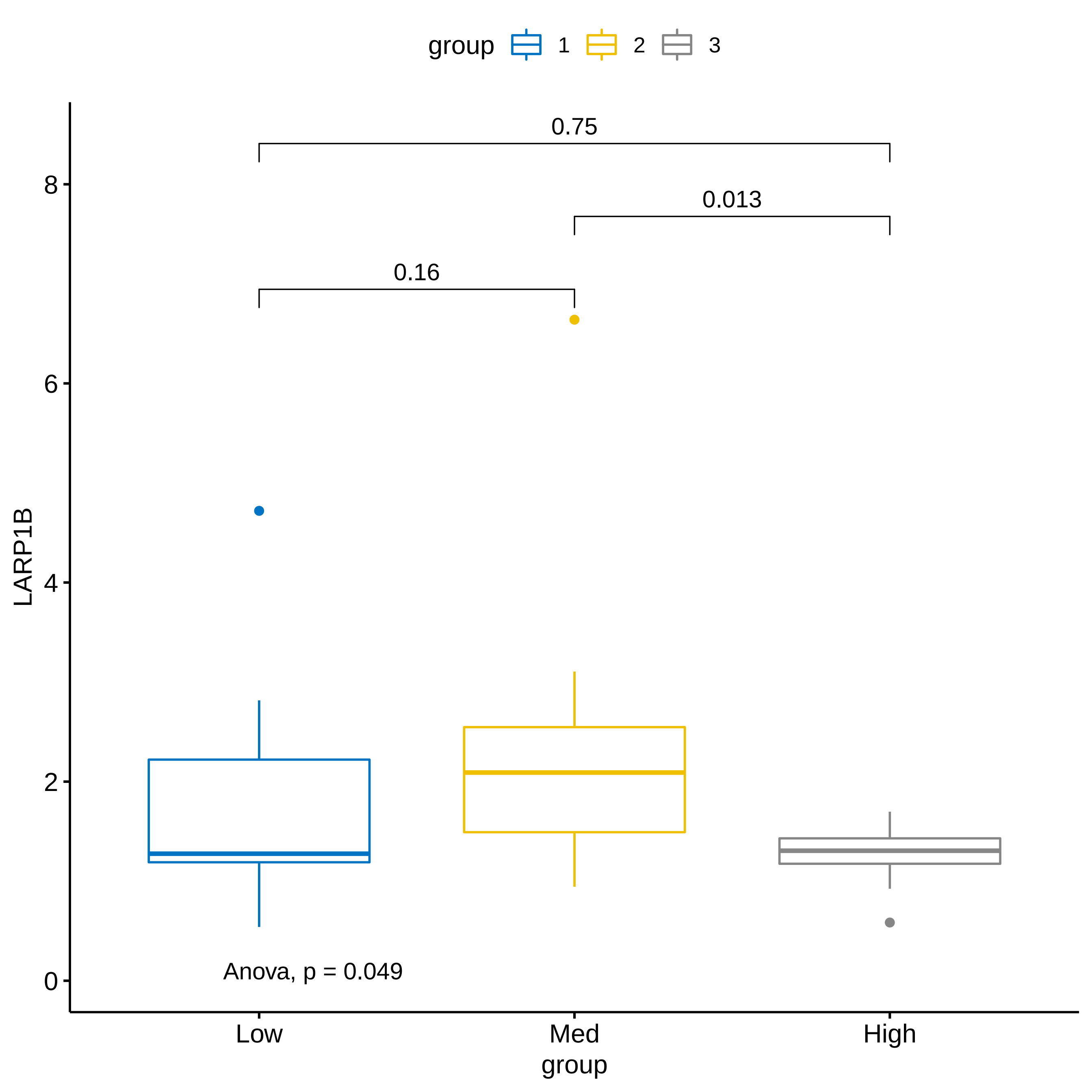

group 1 2 3
0.75
0.013
0.16
8
6
4
2
0
LARP1B
Anova, p = 0.049
Low
Med
High
group

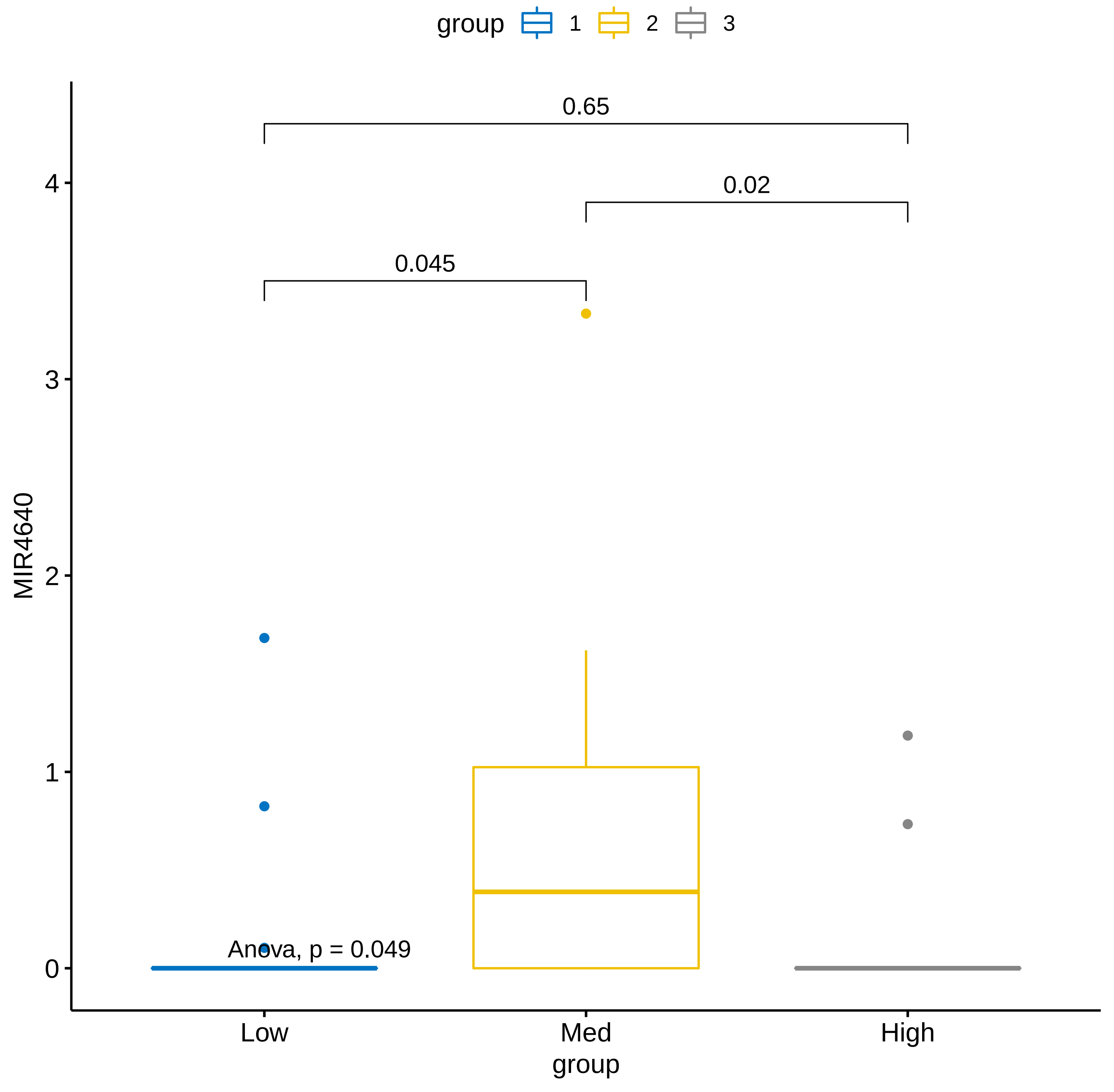
group
1
2
3
0.65
0.02
0.045
4
3
2
1
0
MIR4640
Anova, p = 0.049
Low
Med
High
group

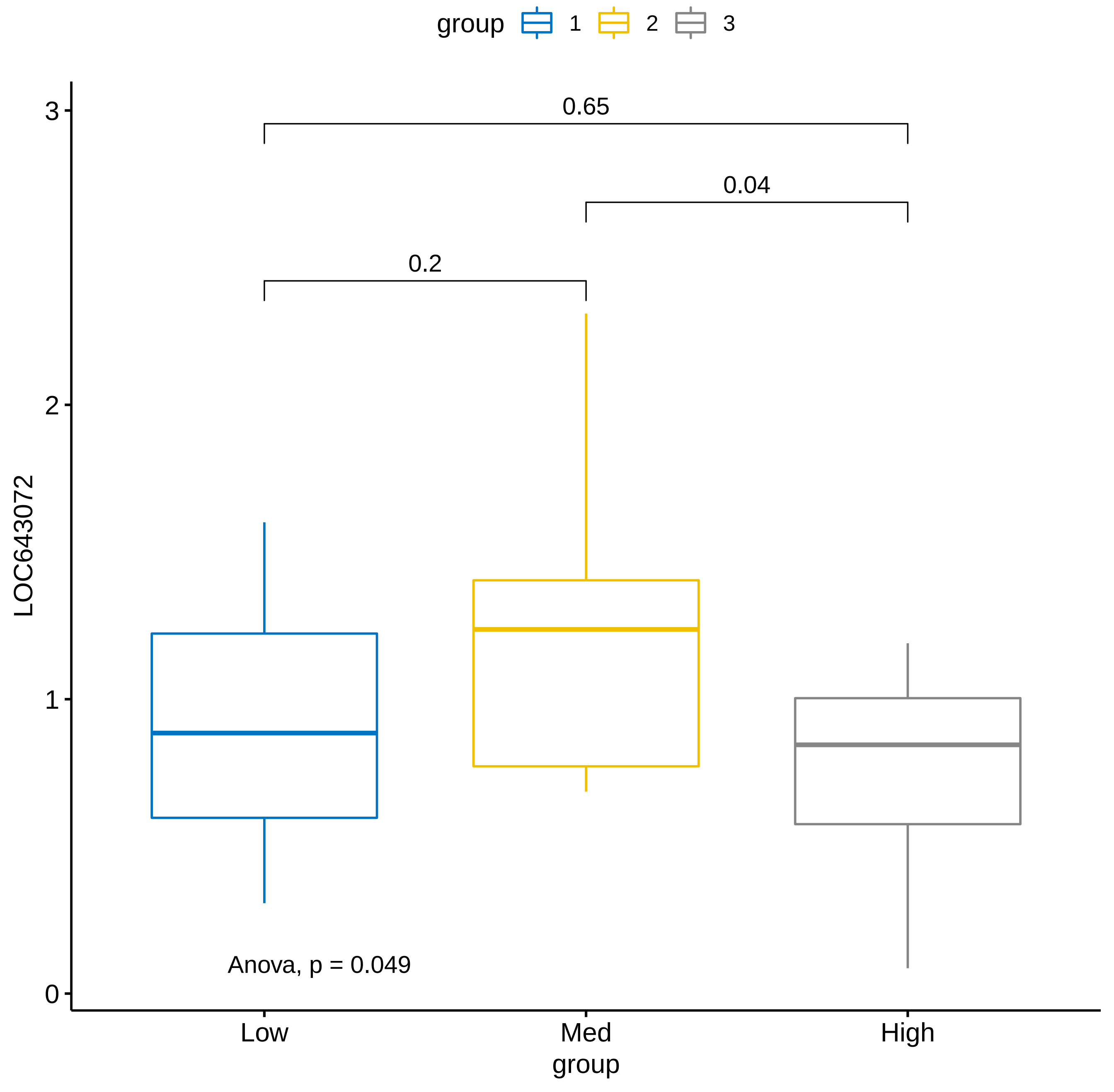
group
1
2
3
0.65
0.04
0.2
LOC643072
3
2
1
0
Anova, p = 0.049
Low
Med
High
group

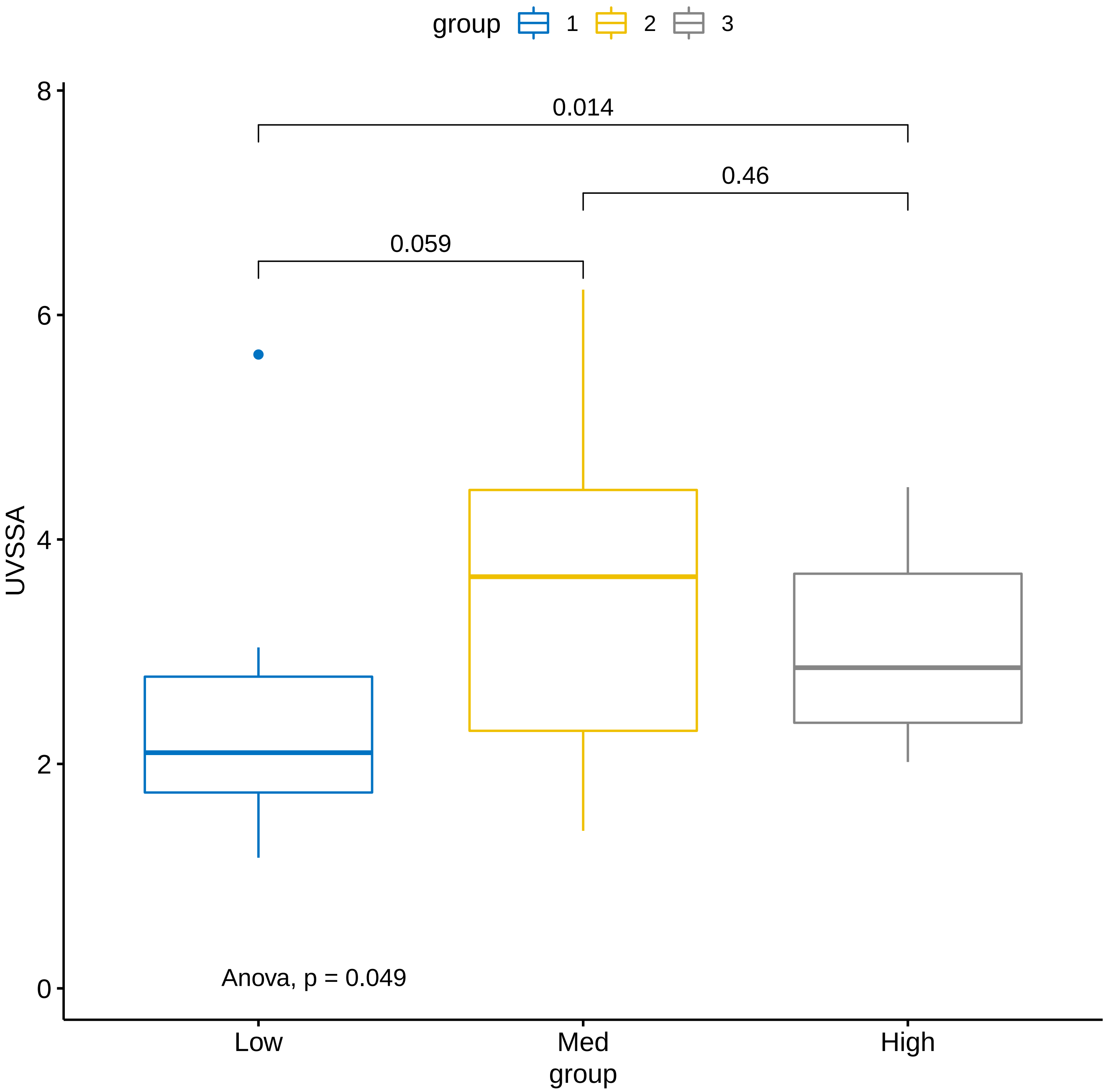
group
1
2
3
8
6
4
2
0
UVSSA
0.014
0.46
0.059
Anova, p = 0.049
Low
Med
High
group

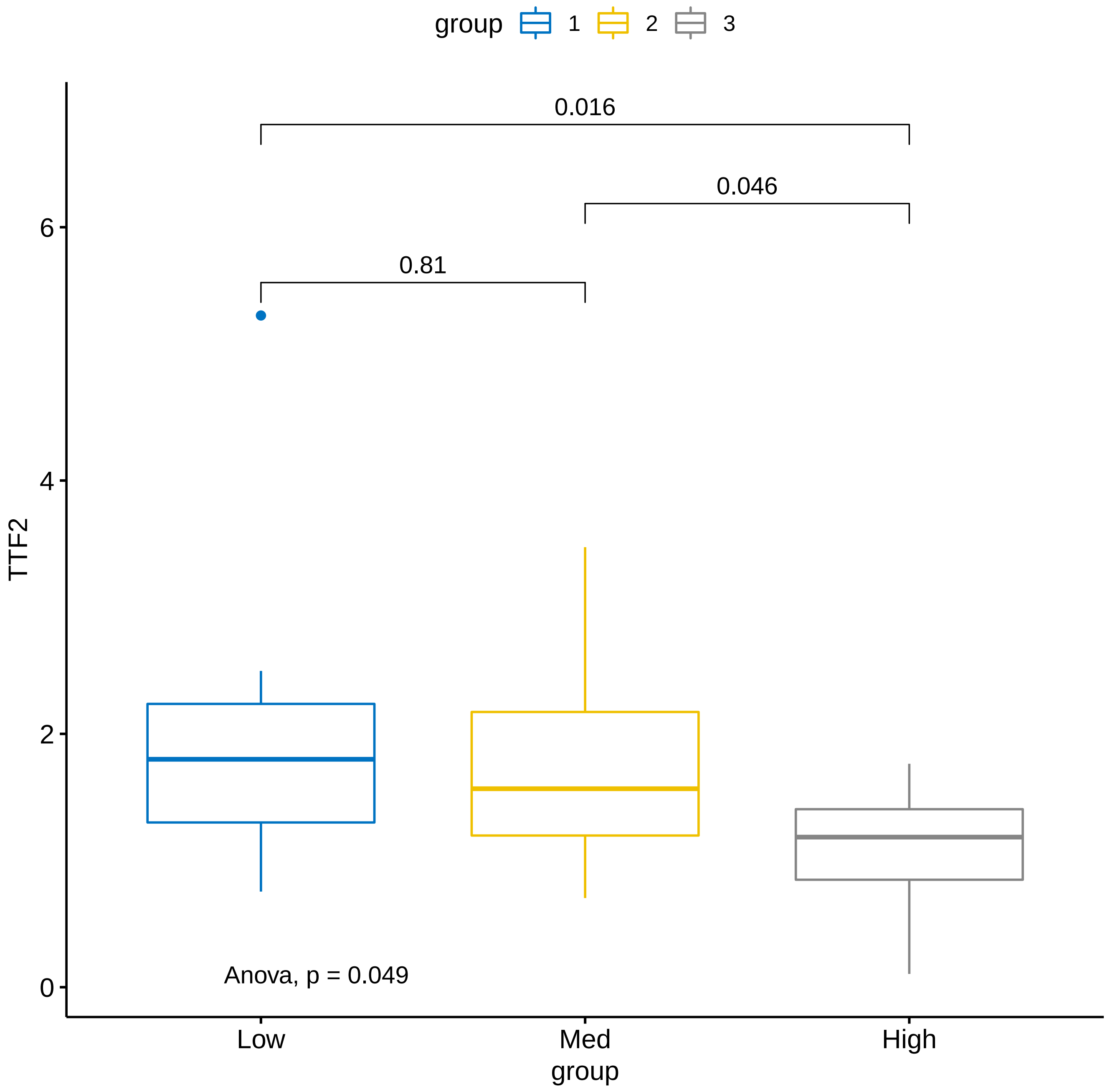

group
1
2
3
0.016
0.046
0.81
6
4
2
0
TTF2
Anova, p = 0.049
Low
Med
High
group

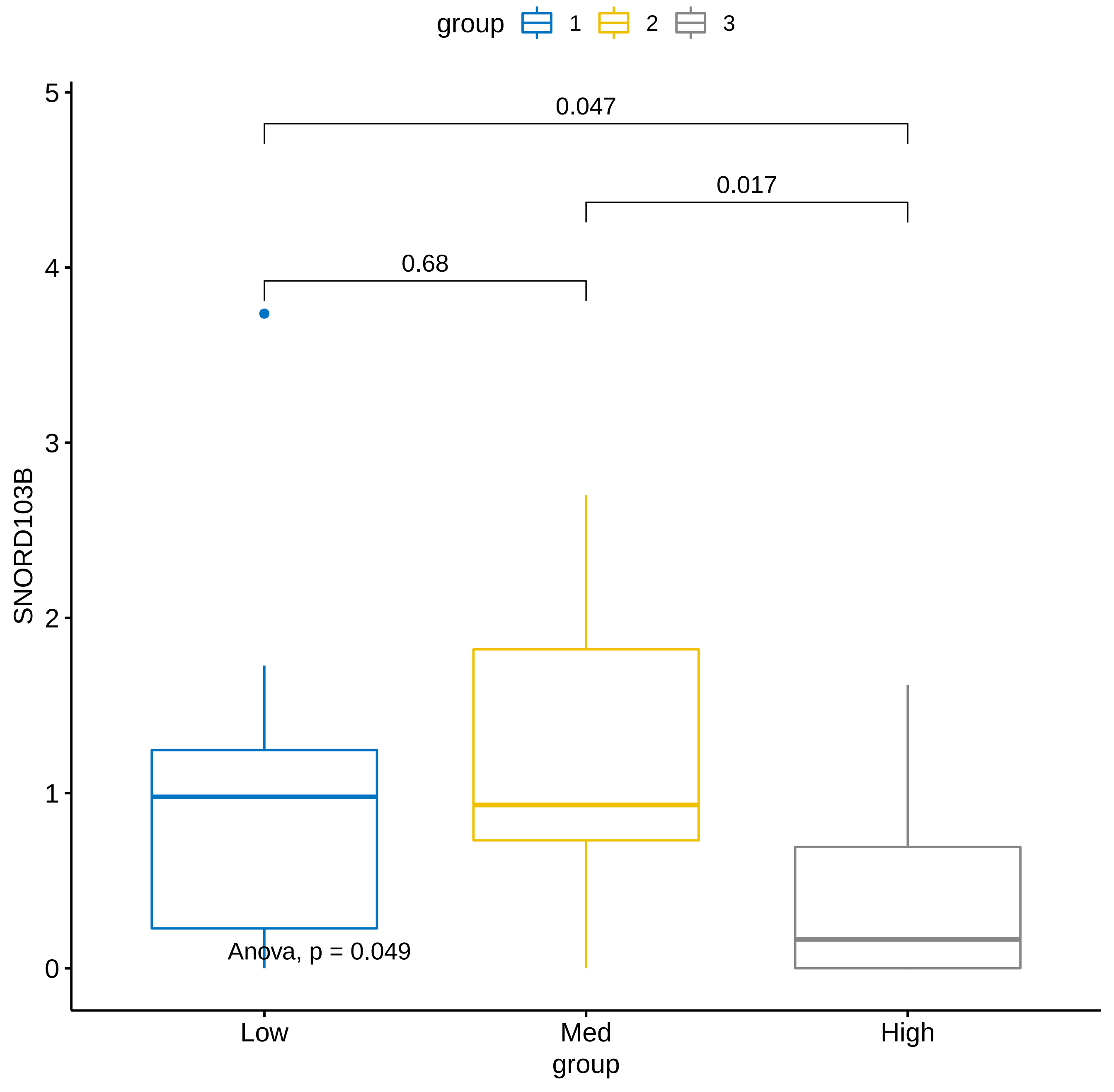
group
1
2
3
0.047
0.017
0.68
SNORD103B
5
4
3
2
1
0
Anova, p = 0.049
Low
Med
High
group

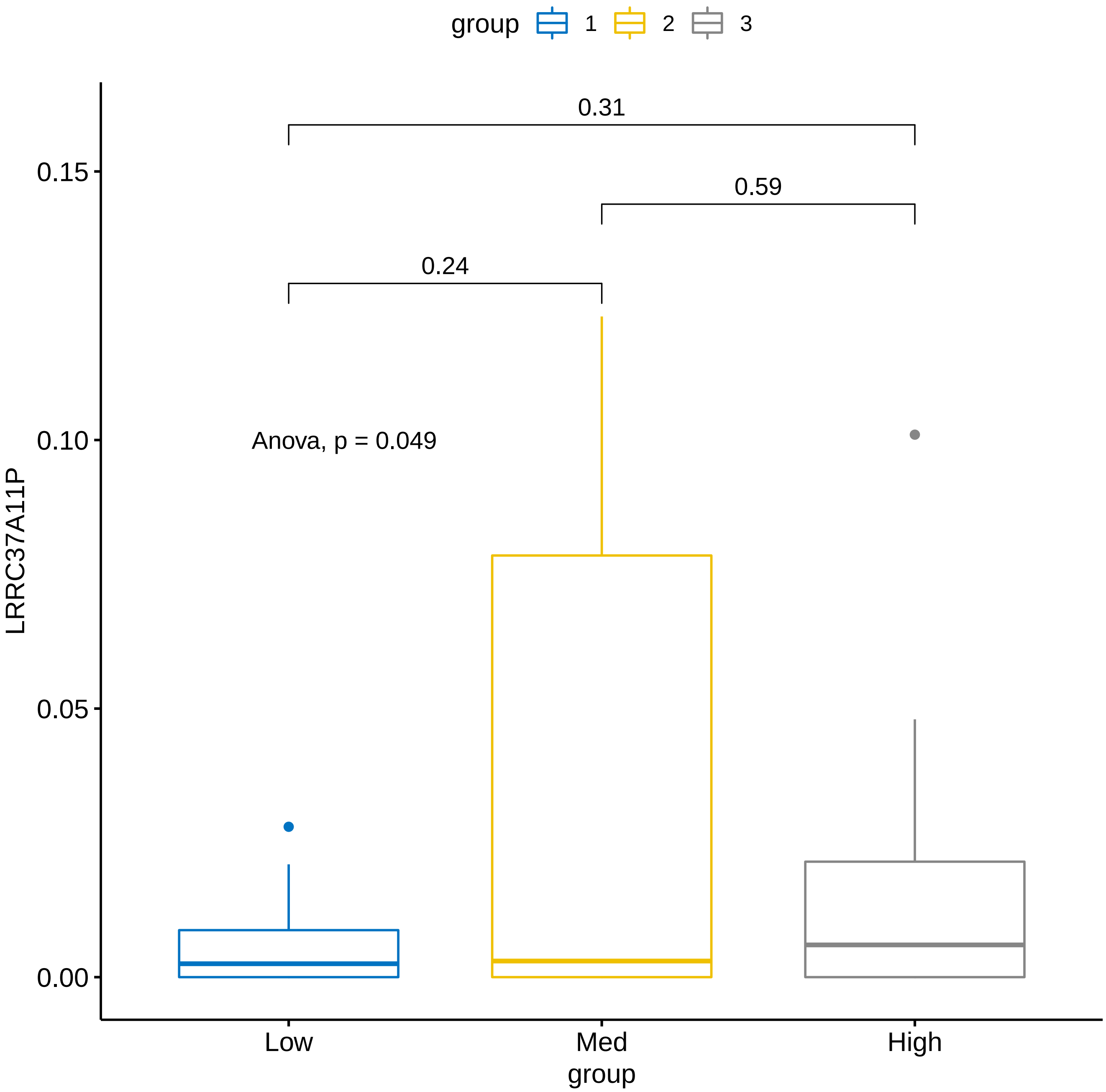
group 1 2 3
0.31
0.59
0.24
Anova, p = 0.049
0.15
0.10
0.05
0.00
LRRC37A11P
Low
Med
High
group

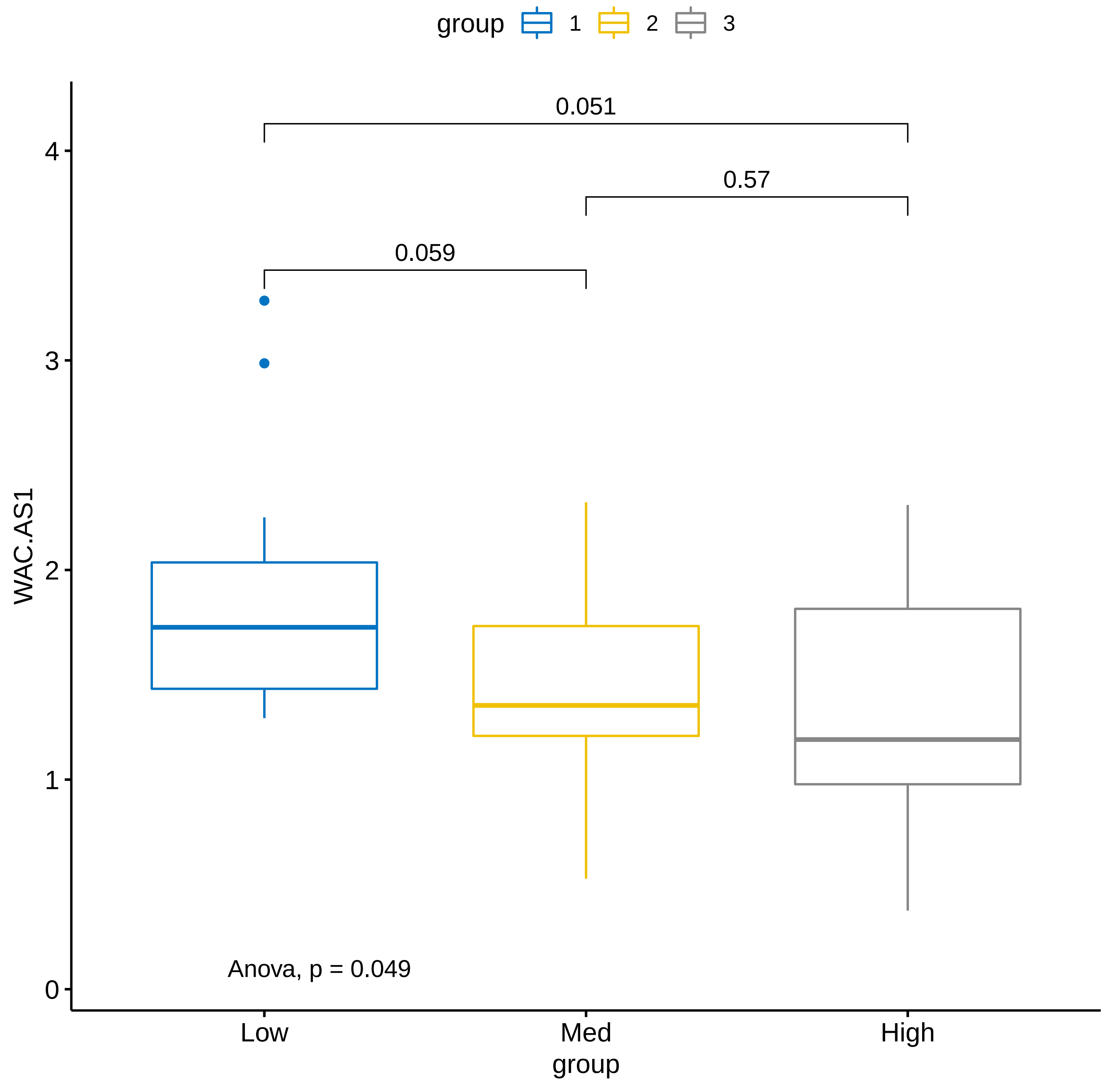
group 1 2 3
0.051
0.57
0.059
WAC.AS1
4
3
2
1
0
Anova, p = 0.049
Low
Med
High
group

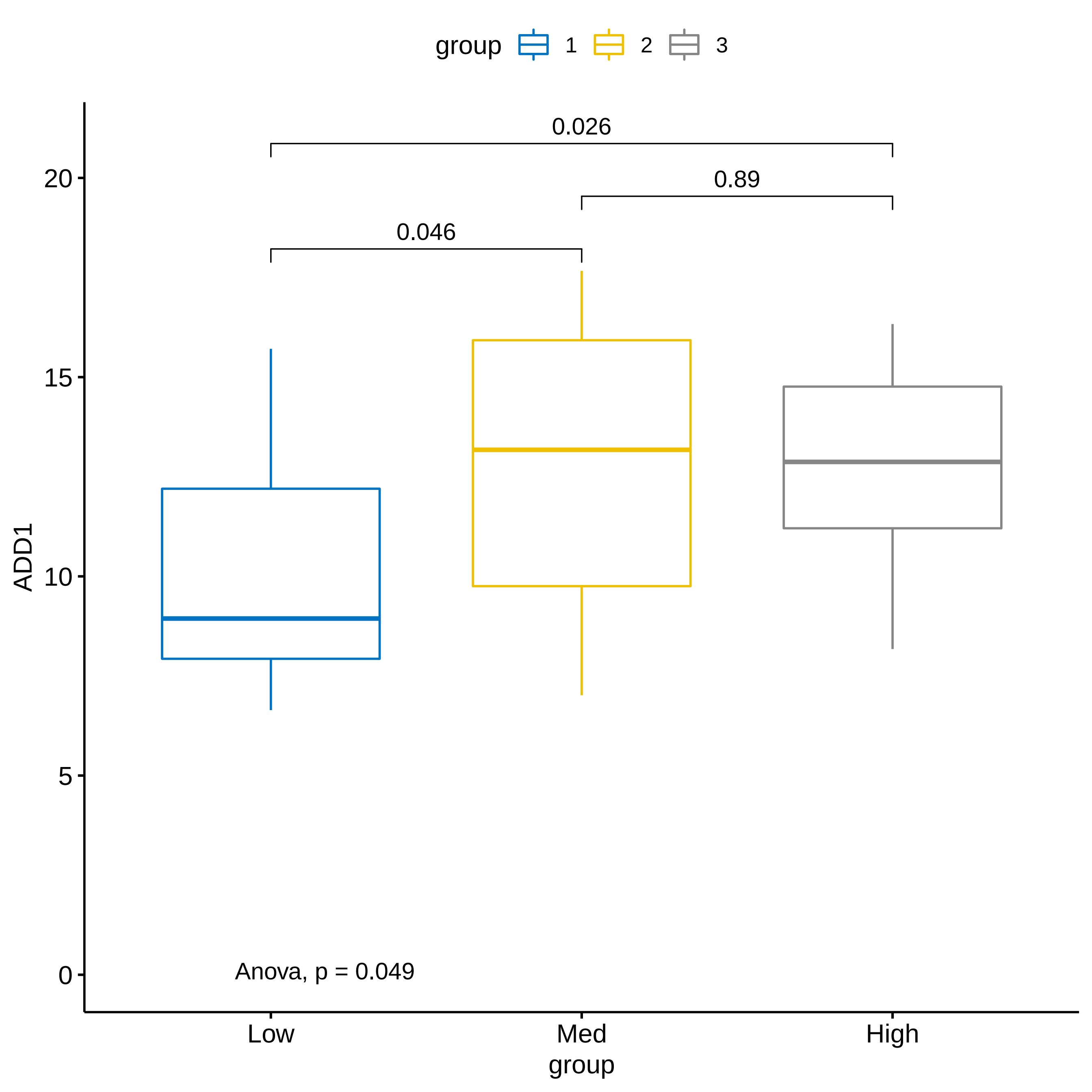

group 1 2 3
0.026
0.89
0.046
20
15
10
5
0
ADD1
Anova, p = 0.049
Low
Med
High
group

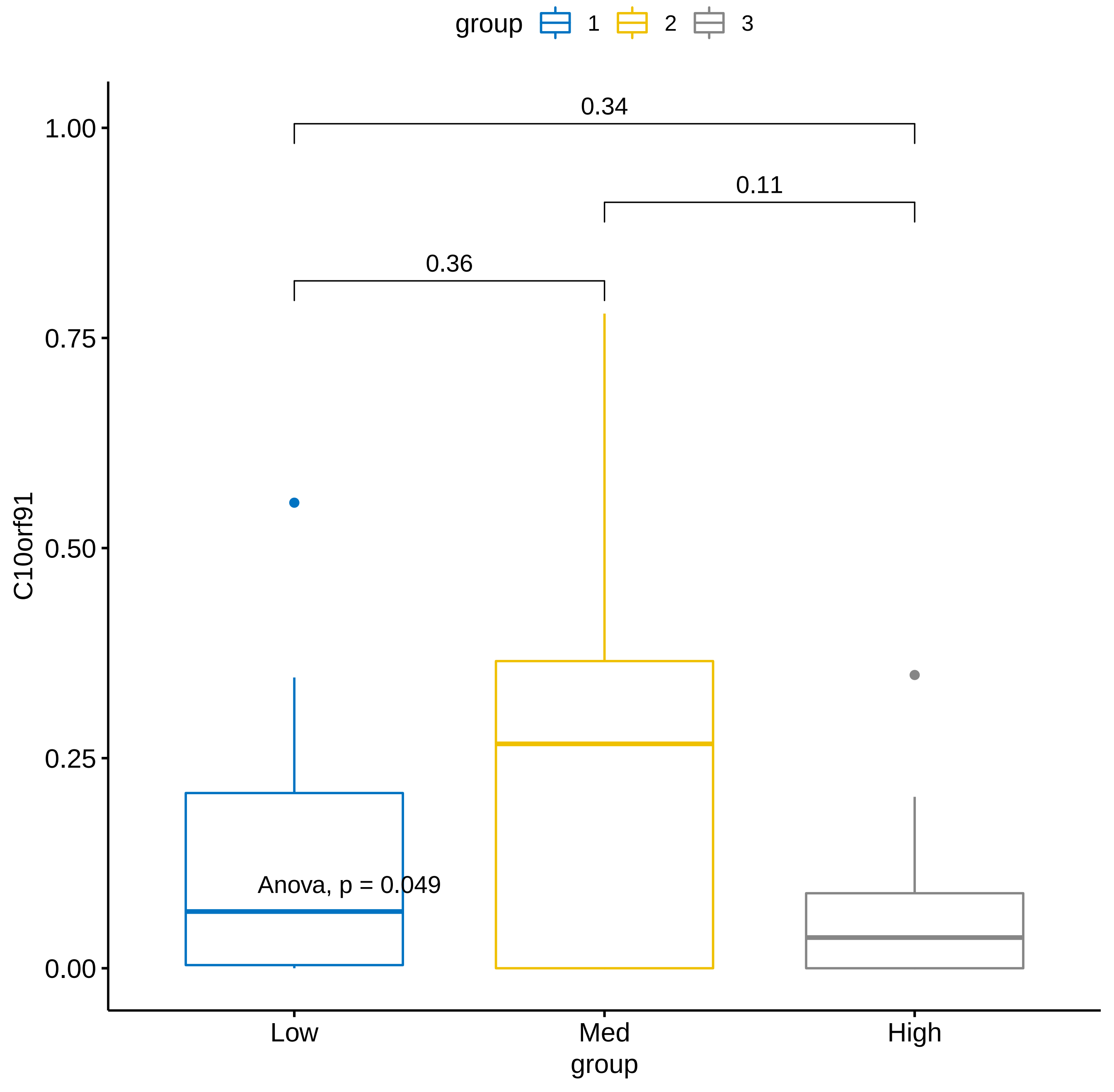
group
1
2
3
0.34
0.11
0.36
1.00
0.75
0.50
0.25
0.00
C10orf91
Anova, p = 0.049
Low
Med
High
group

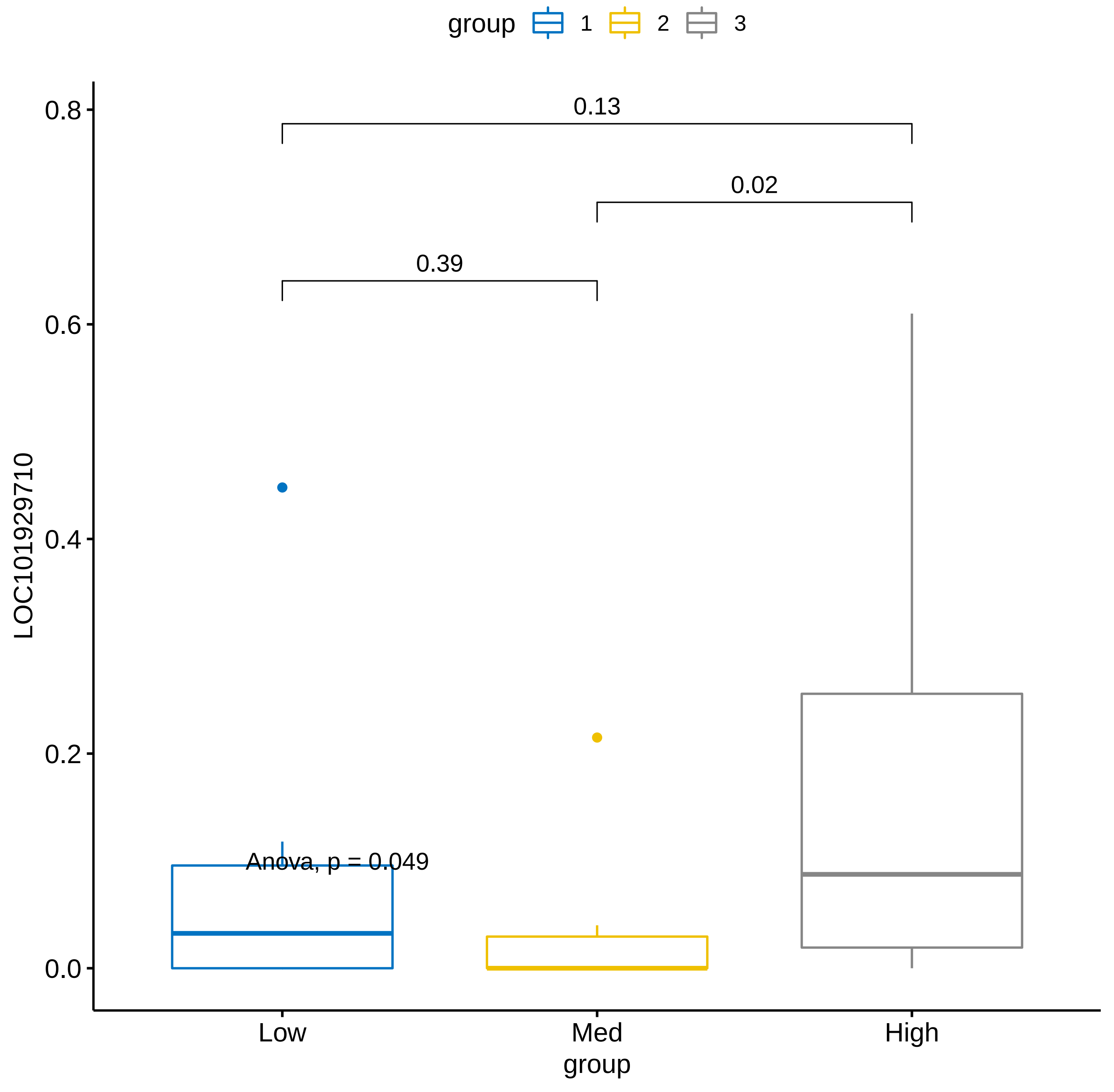

group
1
2
3
0.13
0.02
0.39
Anova, p = 0.049
0.8
0.6
0.4
0.2
0.0
LOC101929710
Low
Med
High
group

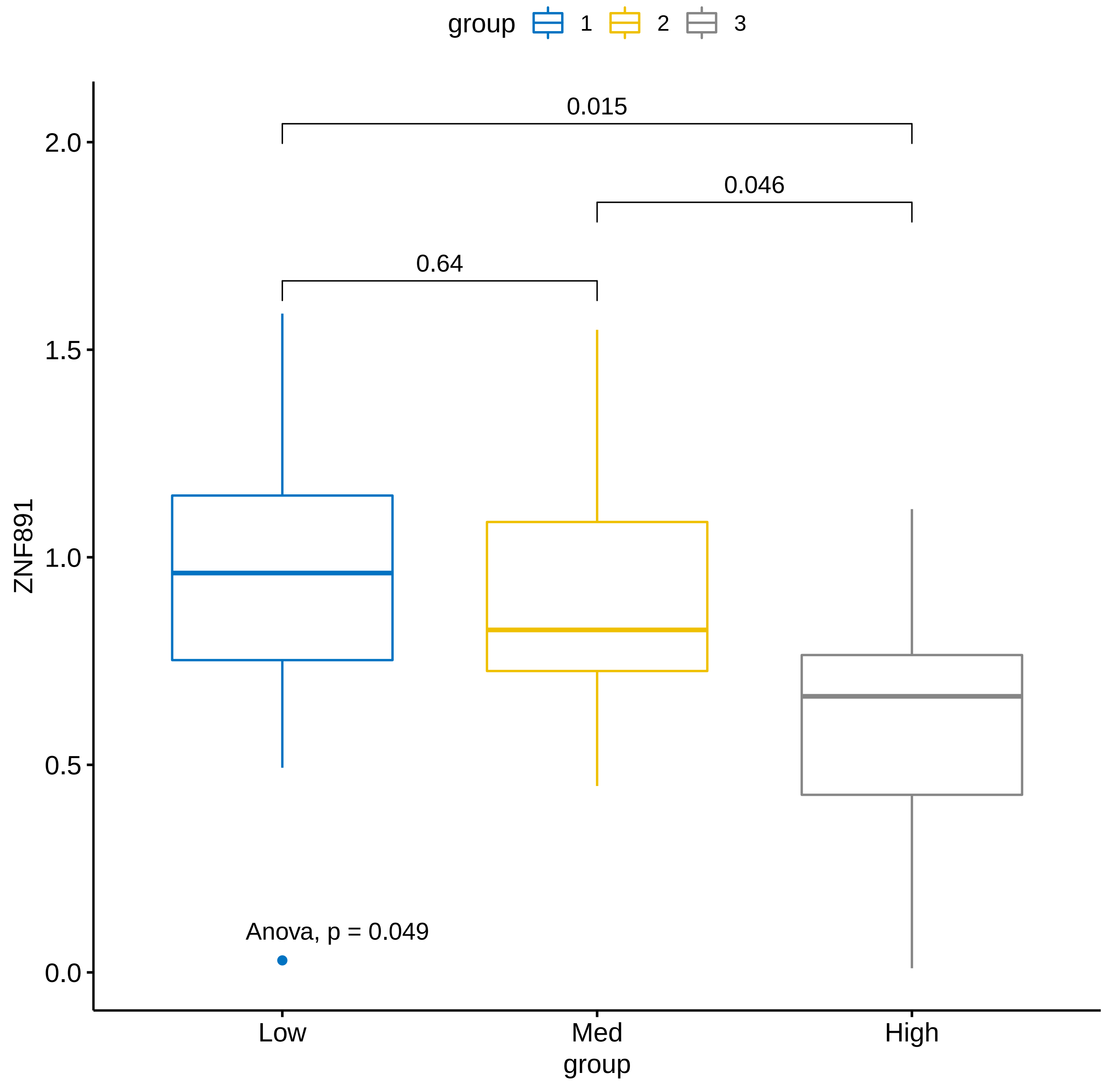

group 1 2 3
0.015
0.046
0.64
2.0
1.5
1.0
0.5
0.0
ZNF891
Anova, p = 0.049
Low
Med
High
group

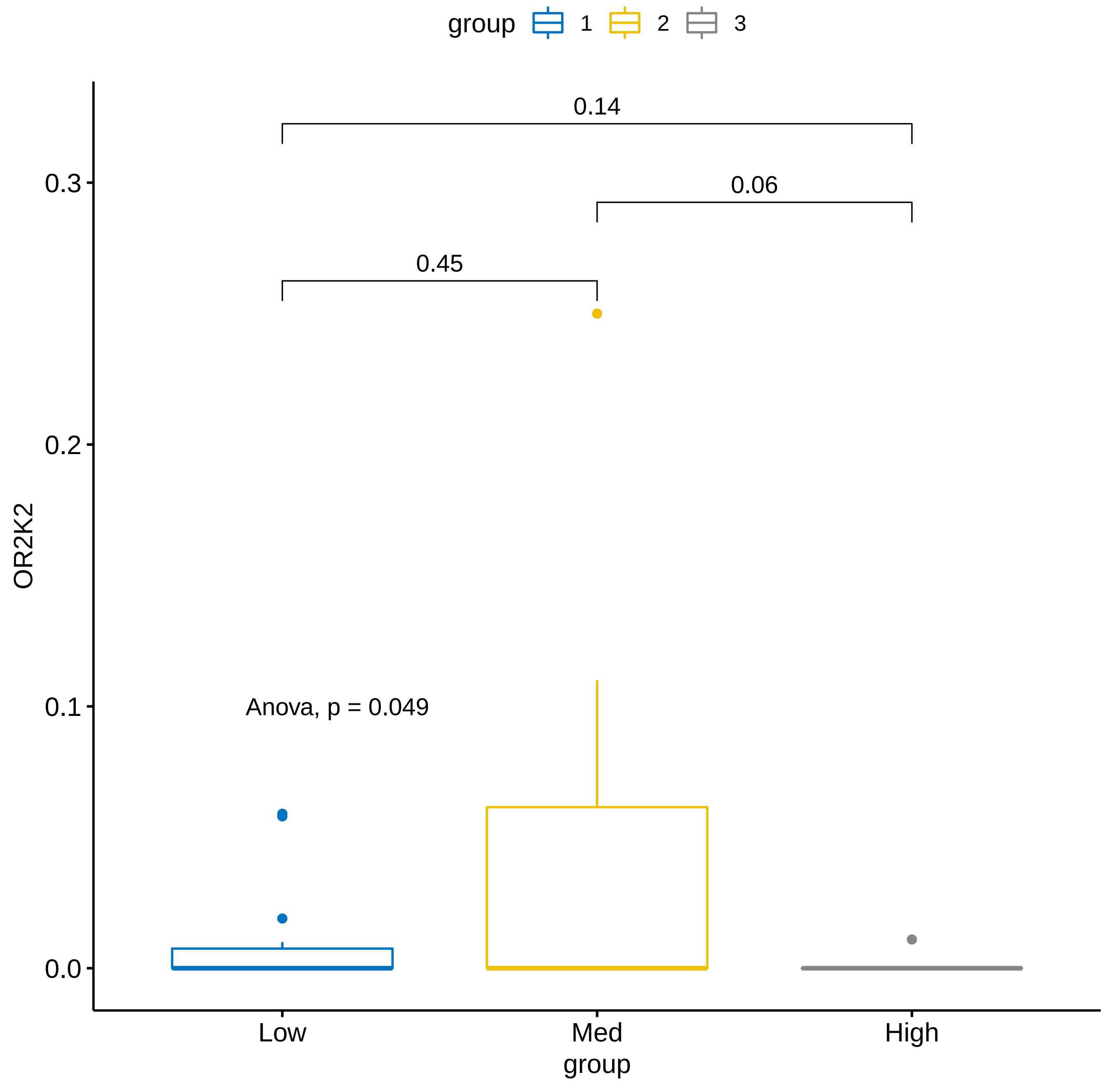
group
1
2
3
0.14
0.06
0.45
0.3
0.2
0.1
0.0
OR2K2
Anova, p = 0.049
Low
Med
High
group

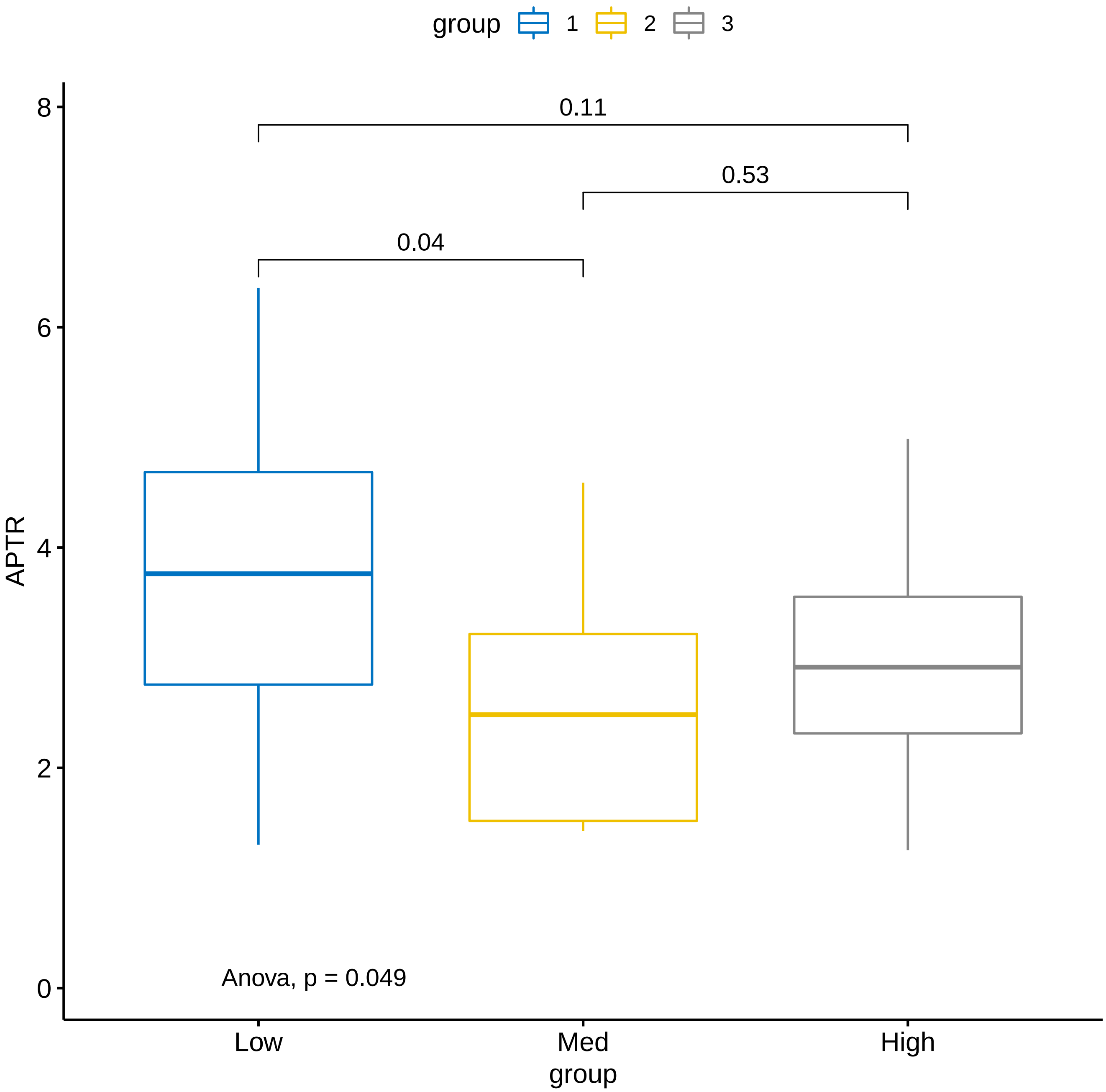
group 1 2 3
0.11
0.53
0.04
8
6
4
2
0
APTR
Anova, p = 0.049
Low
Med
High
group

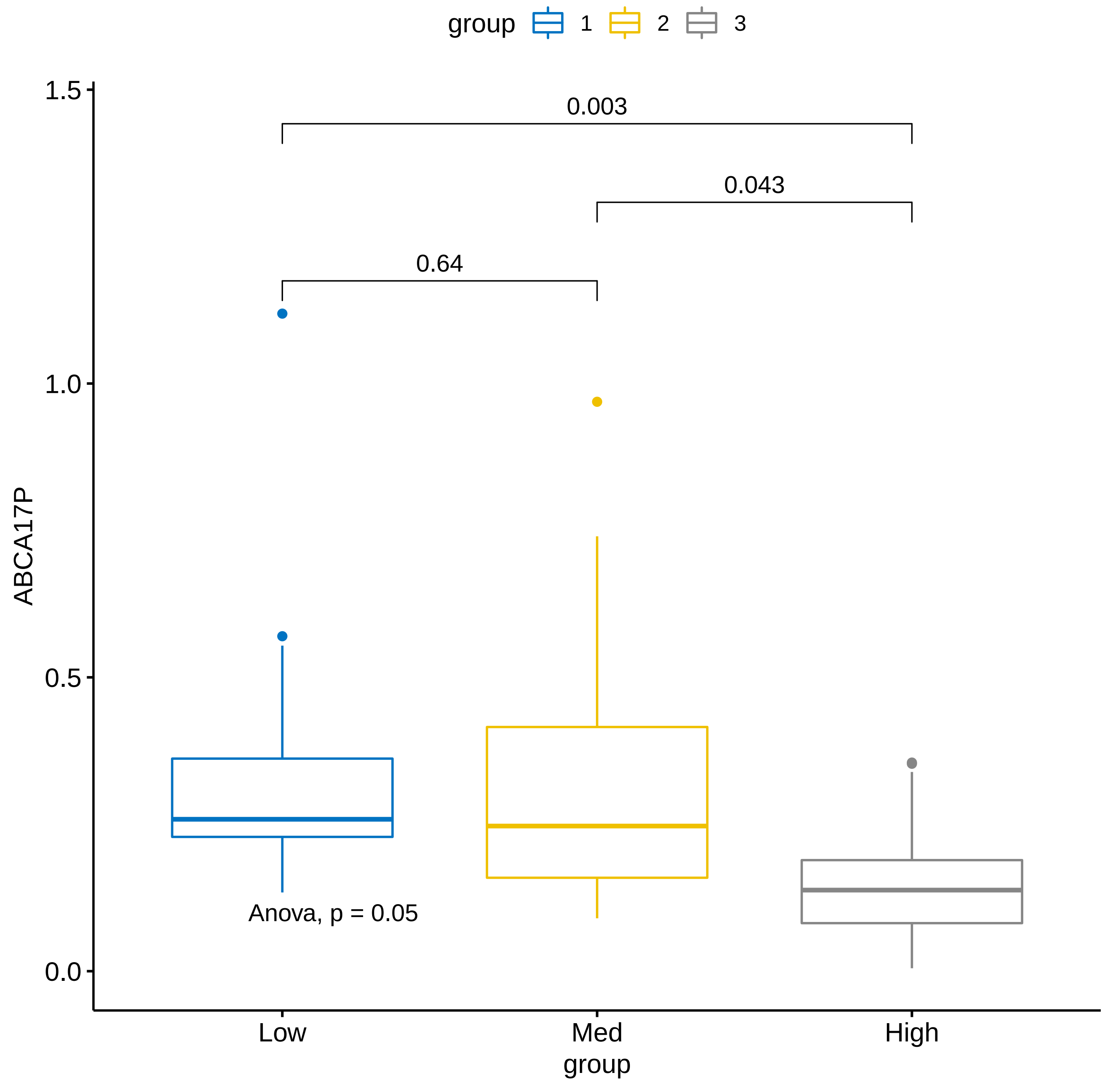
group
1
2
3
1.5
1.0
0.5
0.0
ABCA17P
0.003
0.043
0.64
Anova, p = 0.05
Low
Med
High
group

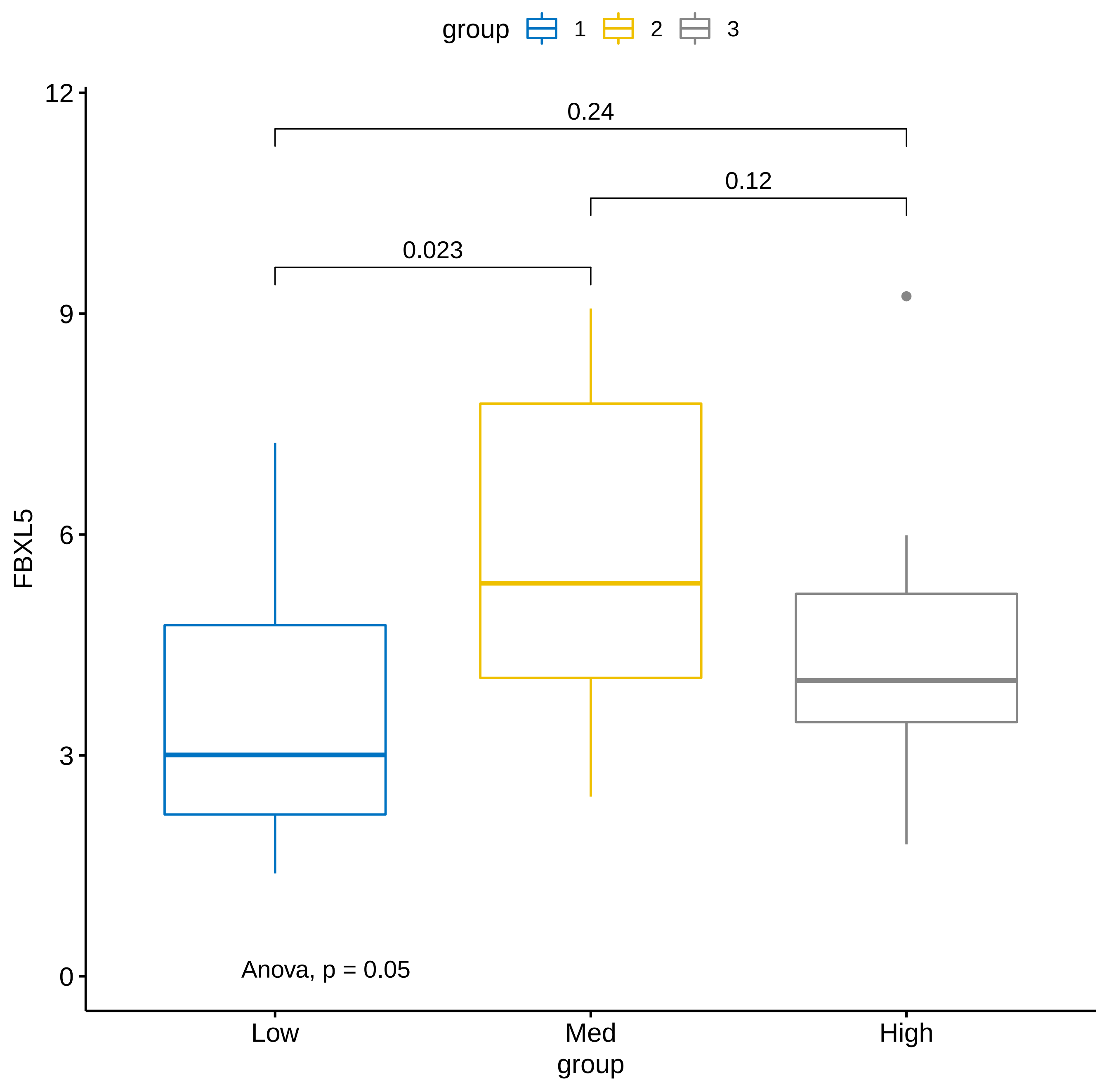
group
1
2
3
12
9
6
3
0
FBXL5
0.24
0.12
0.023
Anova, p = 0.05
Low
Med
High
group

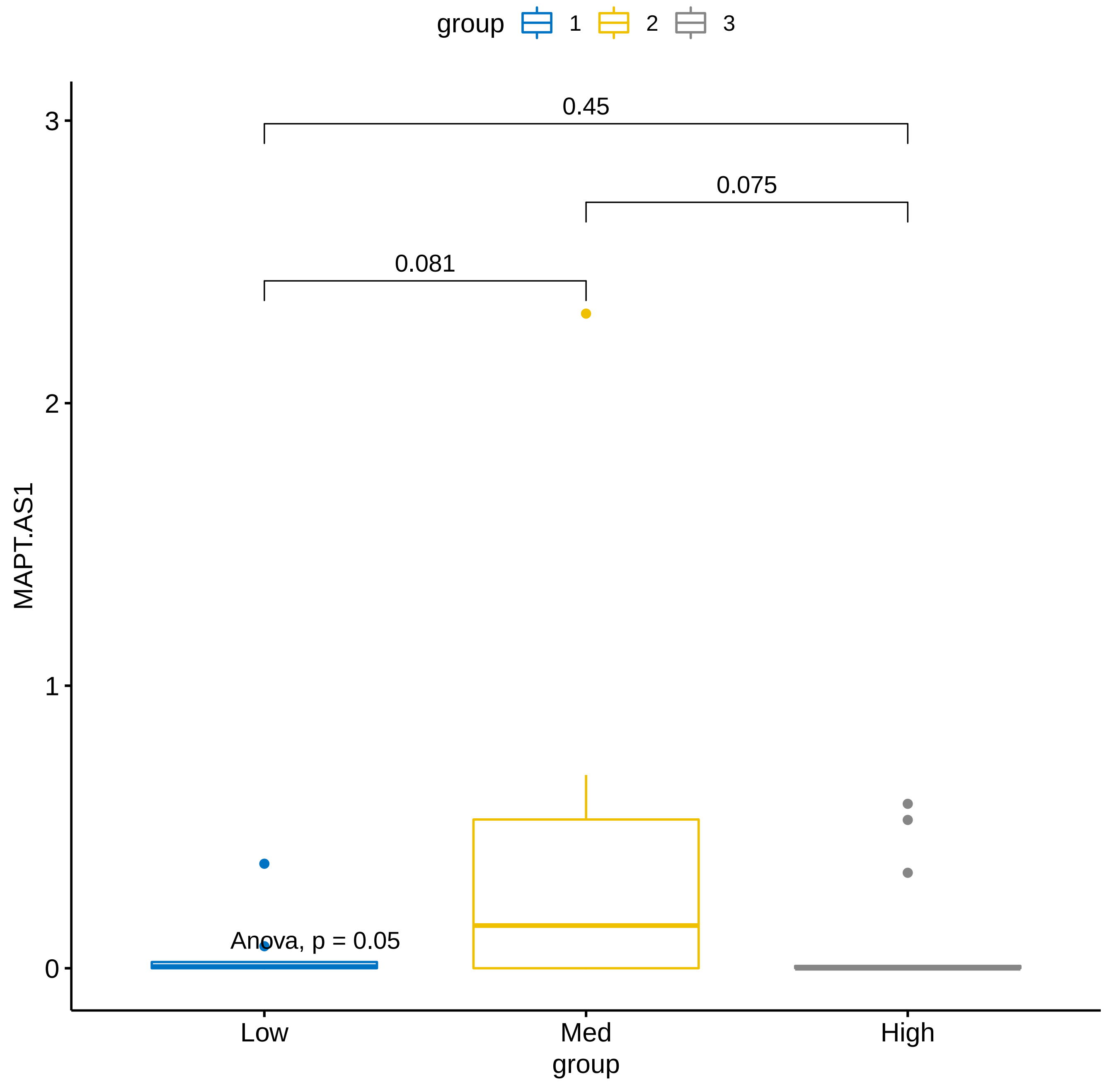

group 1 2 3
0.45
0.075
0.081
Anova, p = 0.05
3
2
1
0
MAPT.AS1
Low
Med
High
group

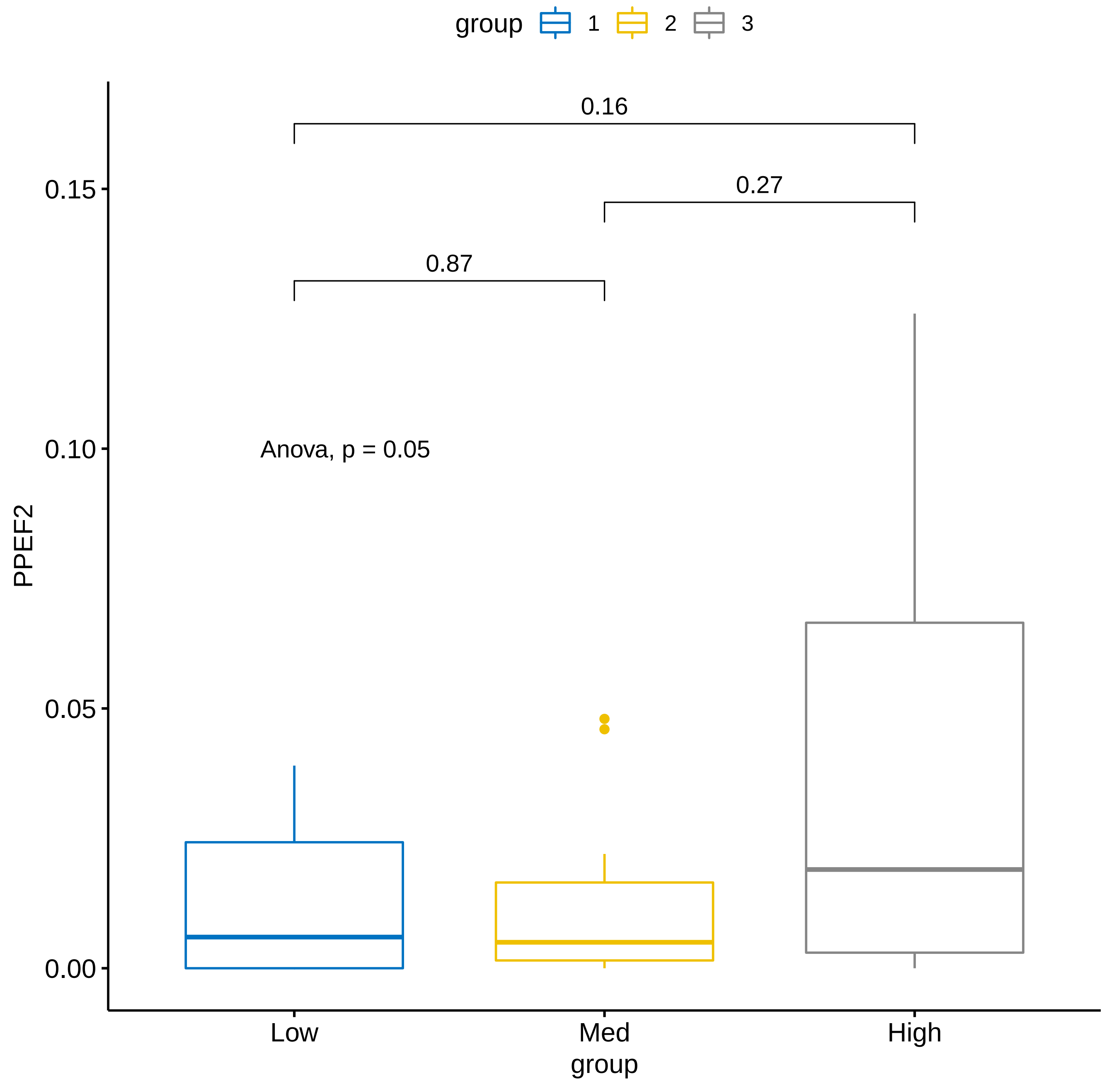

group
1
2
3
0.16
0.27
0.87
Anova, p = 0.05
0.15
0.10
0.05
0.00
PPEF2
Low
Med
High
group

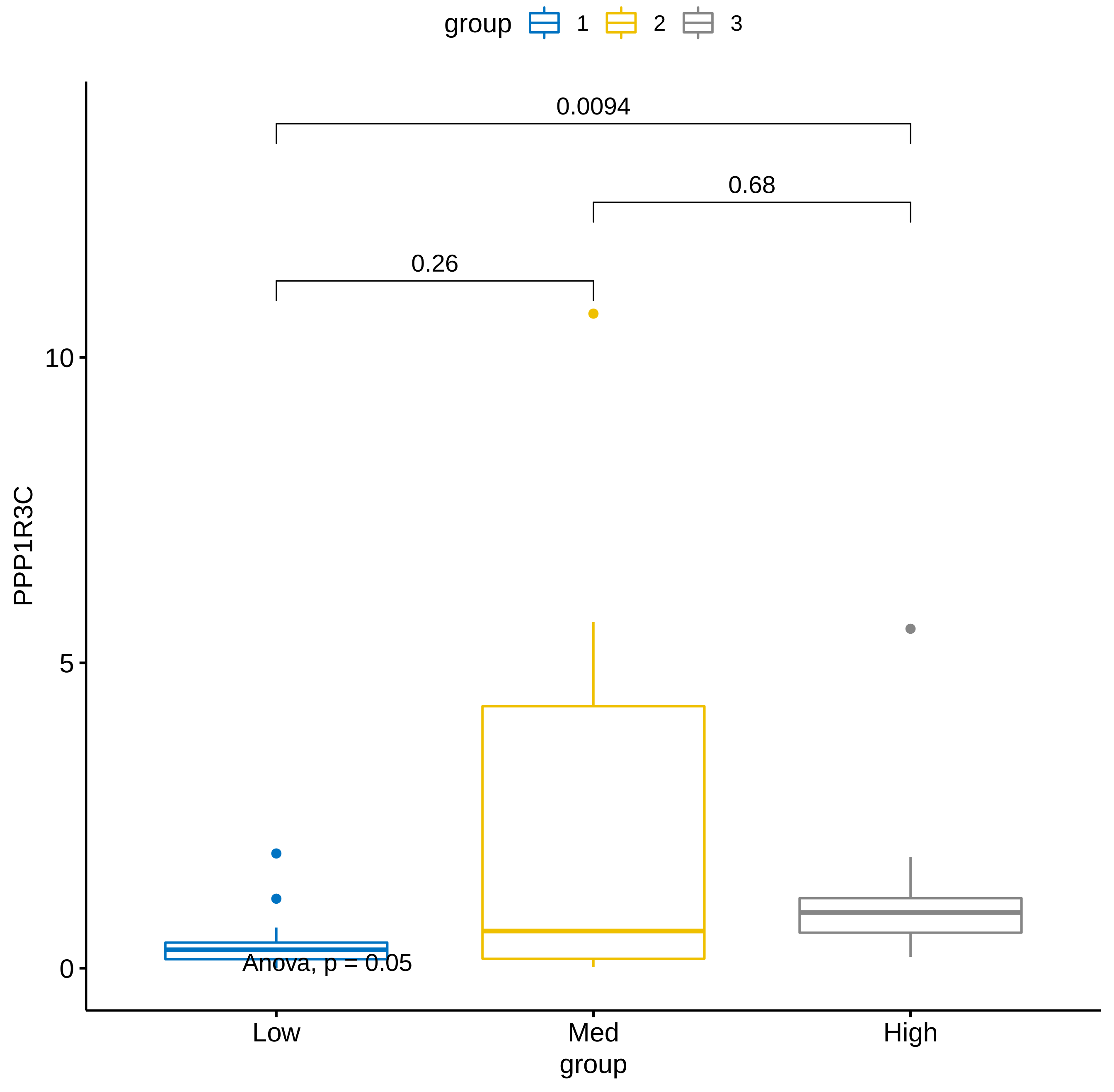
group 1 2 3
0.0094
0.68
0.26
PPP1R3C
10
5
0
Anova, p = 0.05
Low
Med
High
group

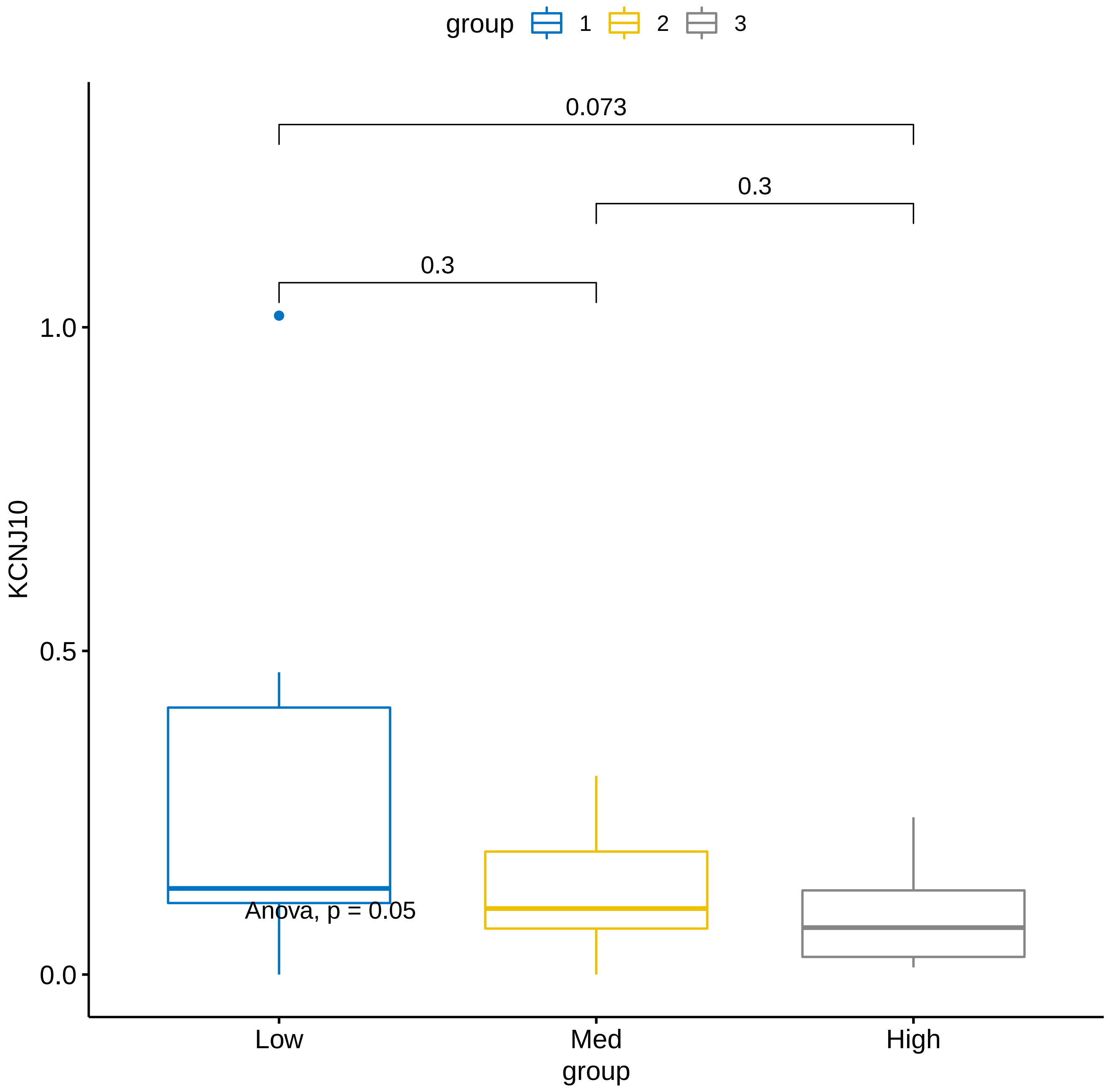
group
1
2
3
0.073
0.3
0.3
1.0
0.5
0.0
KCNJ10
Anova, p = 0.05
Low
Med
High
group
