# Supplementary material for: Epigenetic re-wiring of breast cancer by pharmacological targeting of C-terminal binding protein
Source: Cell Death Dis. 2019 Sep 18;10(10):689. doi: 10.1038/s41419-019-1892-7 (PMC6751206; doi:10.1038/s41419-019-1892-7)
Supplement: Supplementary file 4 — Supplementary Table 3. [file 41419_2019_1892_MOESM4_ESM.pdf]

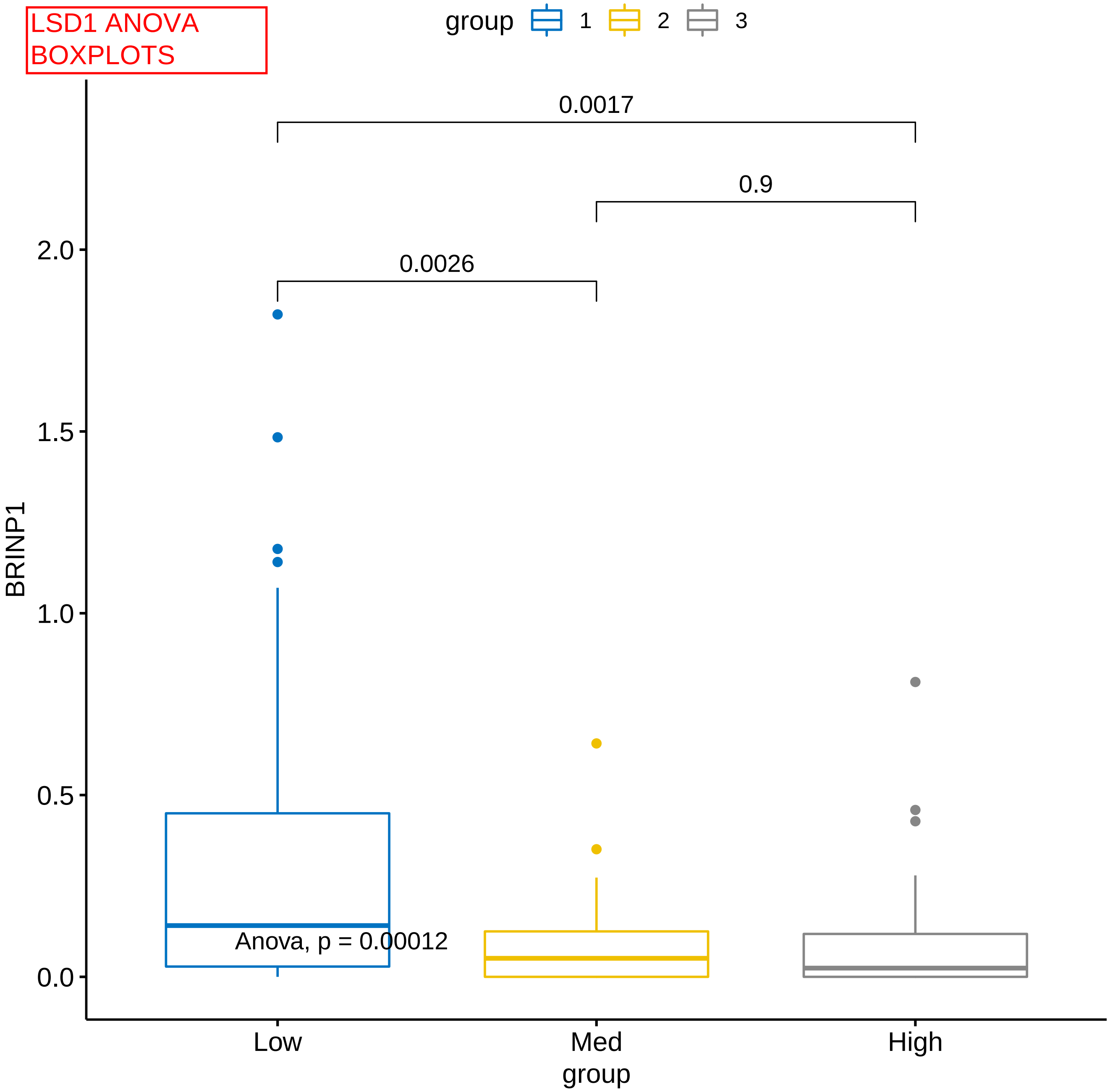
LSD1 ANOVA
BOXPLOTS
group 1 2 3
0.0017
0.9
0.0026
2.0
1.5
1.0
0.5
0.0
BRINP1
Anova, p = 0.00012
Low
Med
High
group

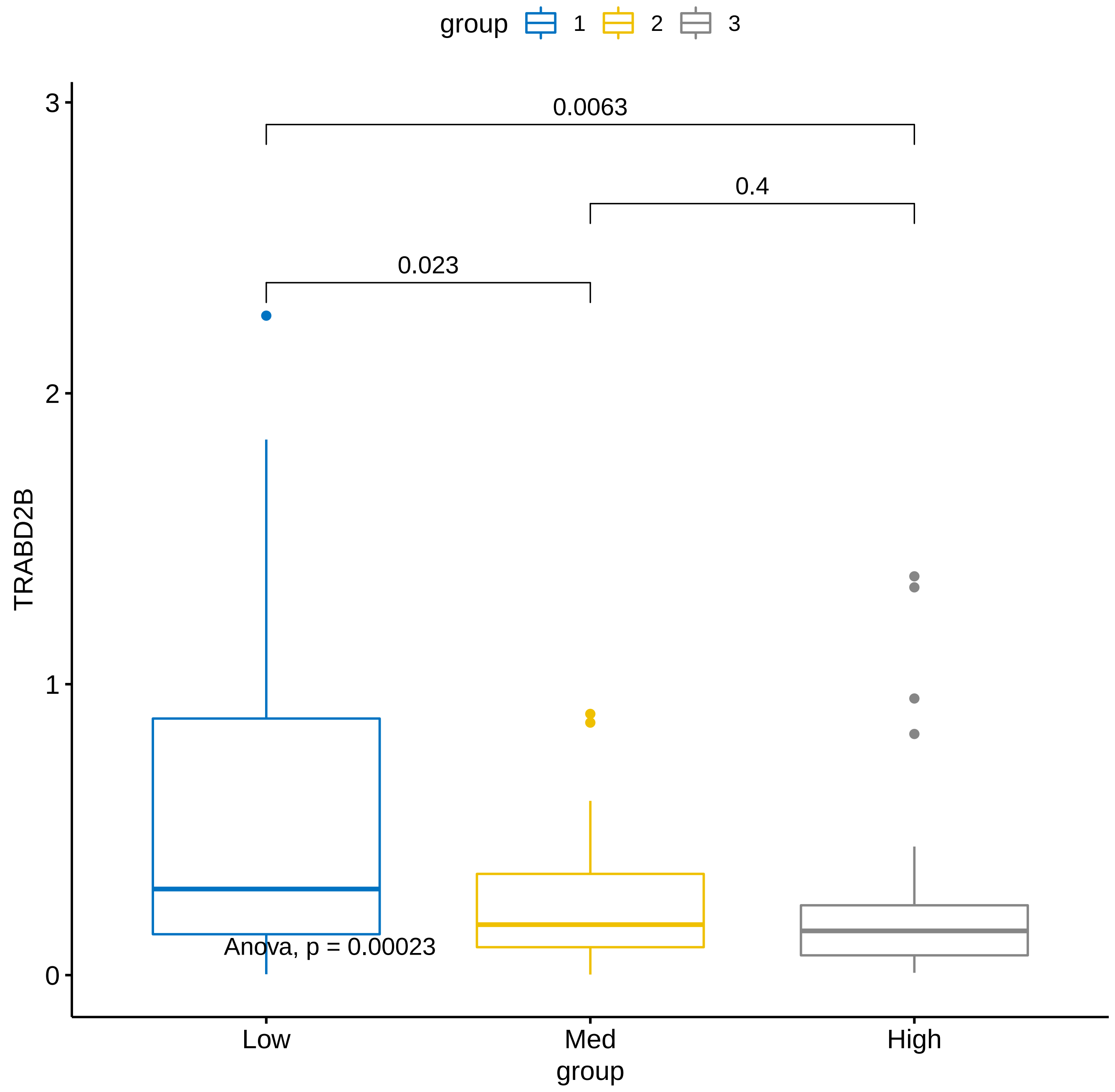
group
1
2
3
TRABD2B
0.0063
0.4
0.023
Anova, p = 0.00023
3
2
1
0
Low
Med
High
group

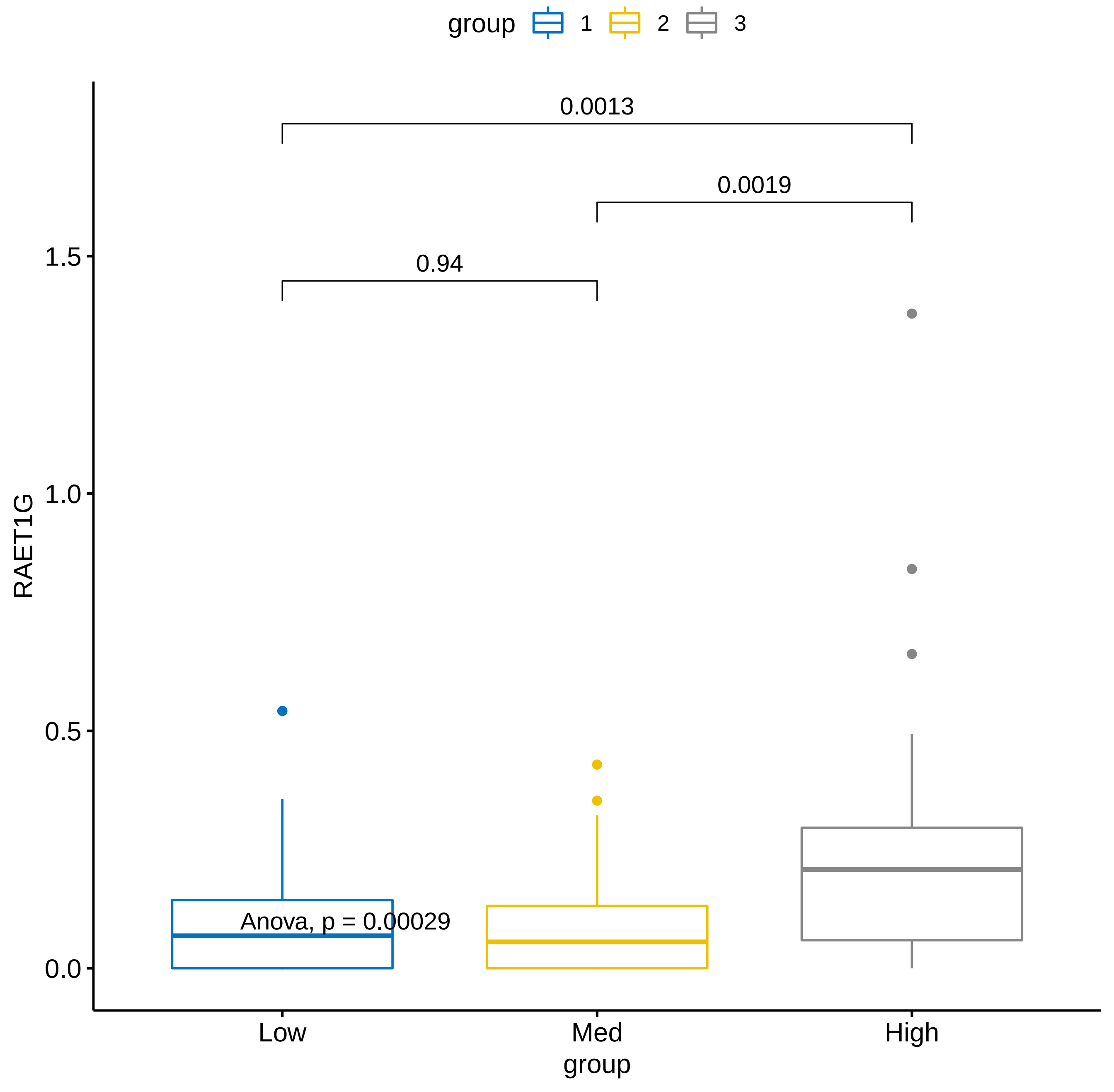

group
1
2
3
0.0013
0.0019
0.94
1.5
1.0
0.5
0.0
RAET1G
Anova, p = 0.00029
Low
Med
High
group

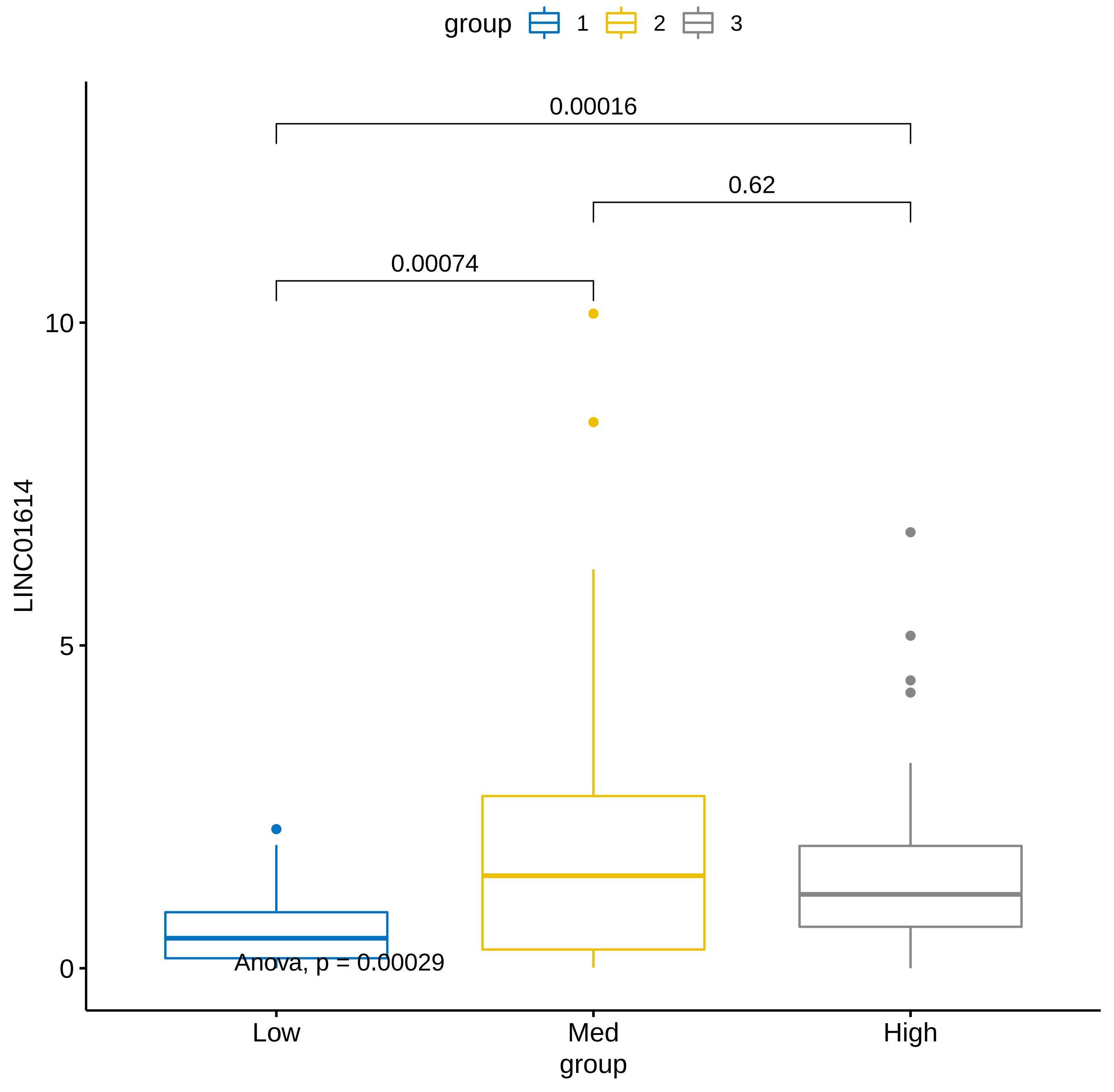
group
1
2
3
0.00016
0.62
0.00074
LINC01614
10
5
0
Anova, p = 0.00029
Low
Med
High
group

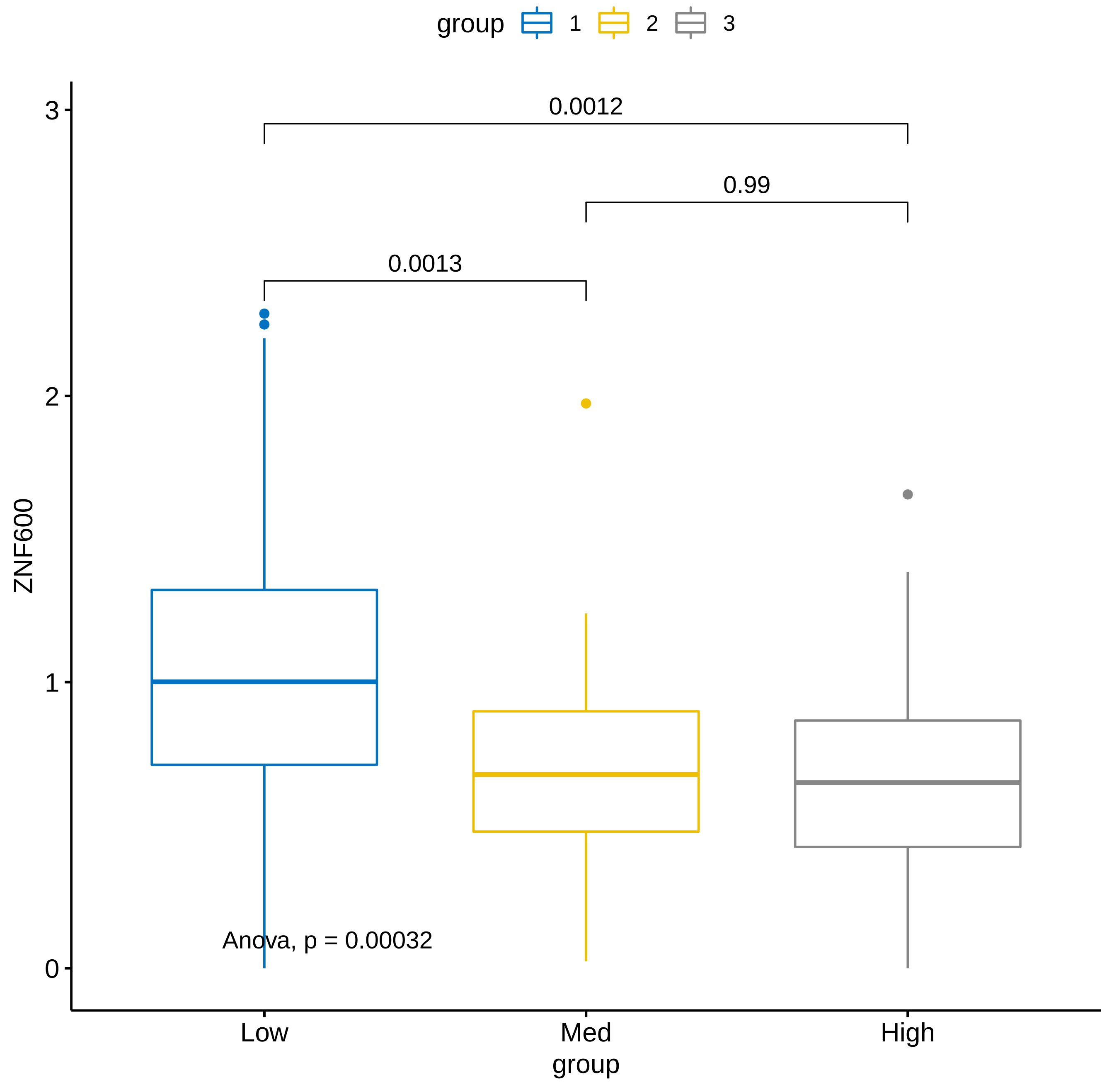

group
1
2
3
0.0012
0.99
0.0013
Anova, p = 0.00032
ZNF600
3
2
1
0
Low
Med
High
group

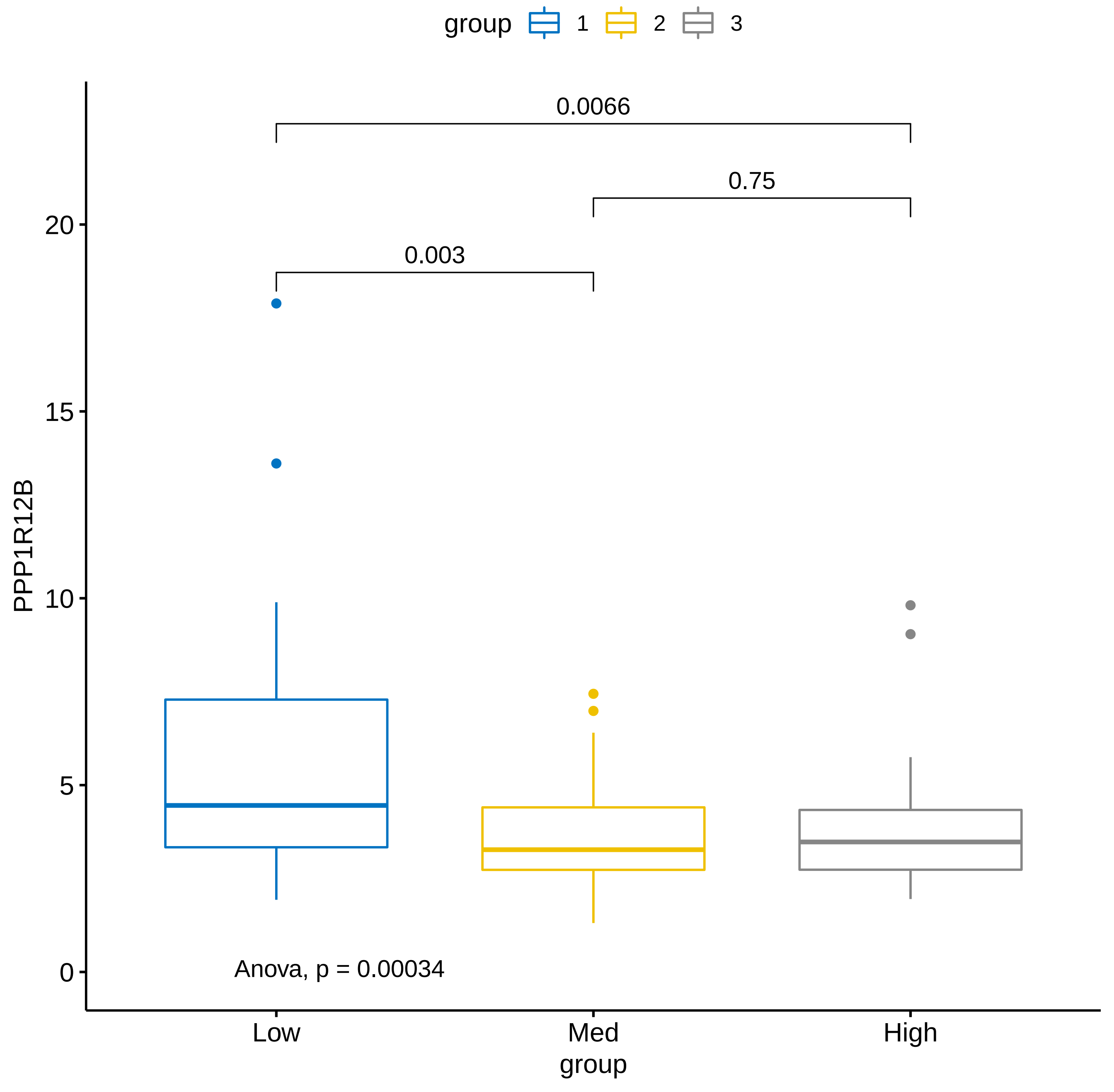

group
1
2
3
0.0066
0.75
0.003
20
15
10
5
0
PPP1R12B
Anova, p = 0.00034
Low
Med
High
group

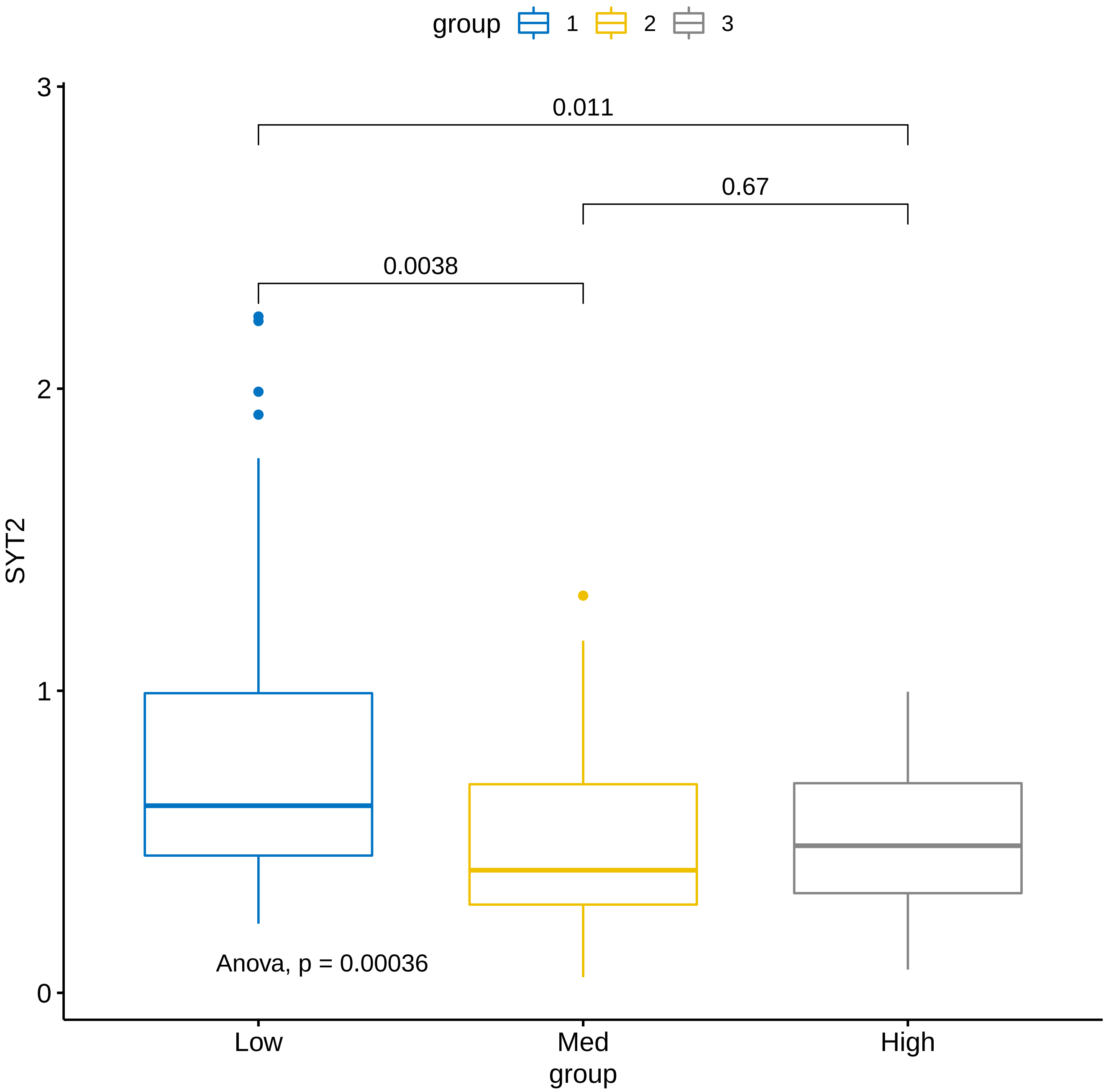
group
1
2
3
0.011
0.67
0.0038
SYT2
3
2
1
0
Anova, p = 0.00036
Low
Med
High
group

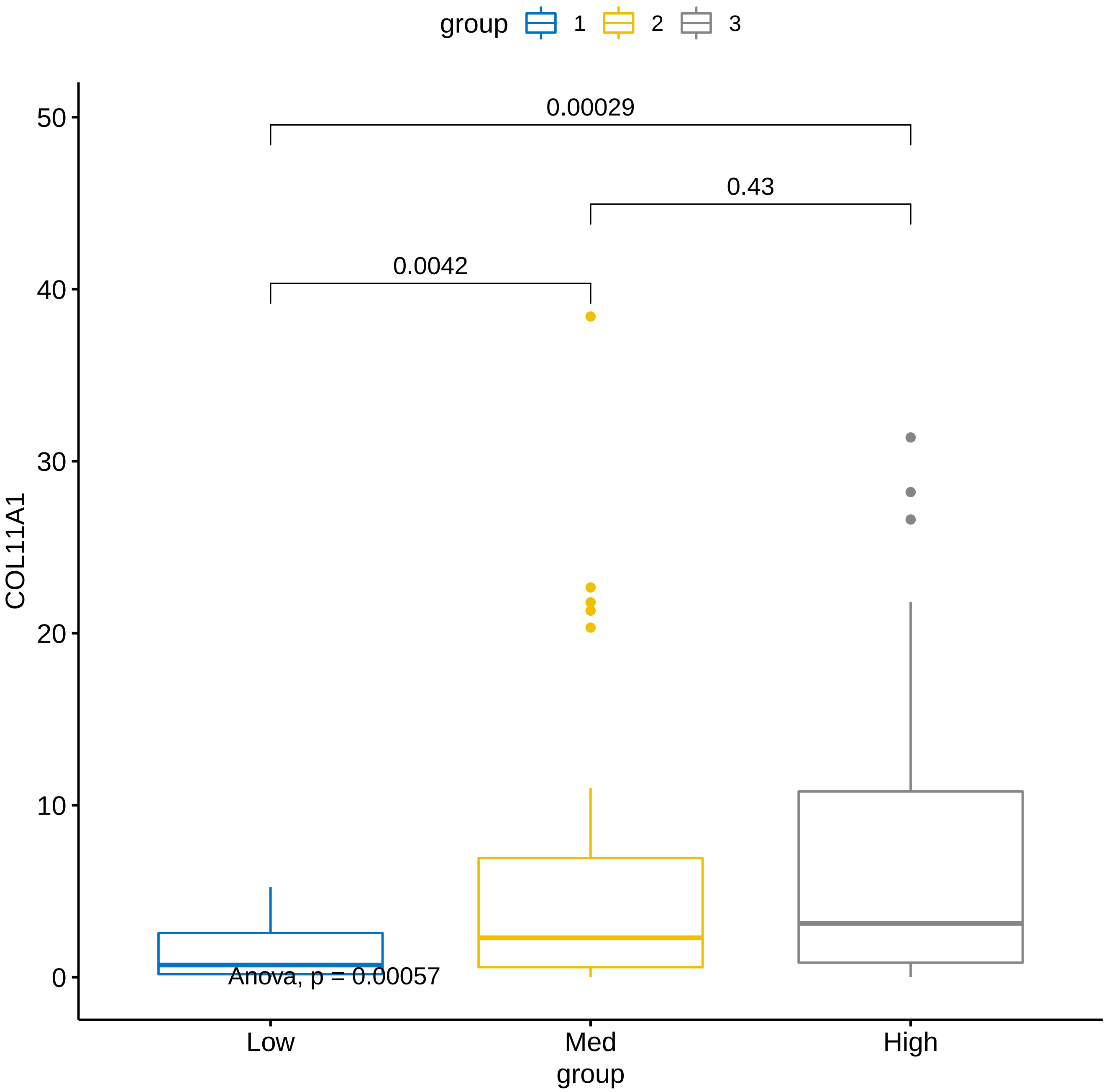

group
1
2
3
0.00029
0.43
0.0042
50
40
30
20
10
0
COL11A1
Anova, p = 0.00057
Low
Med
High
group

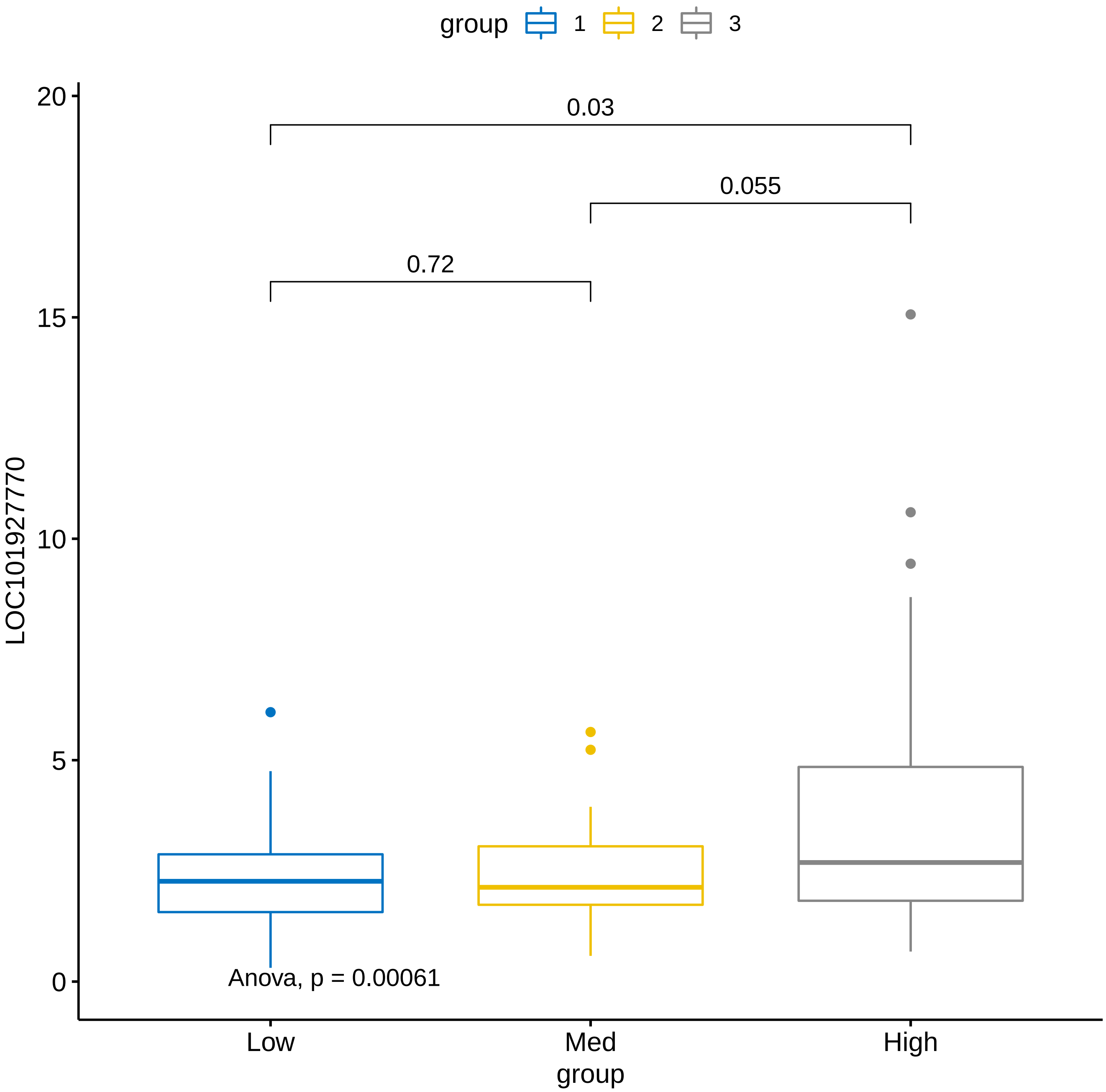
group
1
2
3
0.03
0.055
0.72
LOC101927770
20
15
10
5
0
Anova, p = 0.00061
Low
Med
High
group

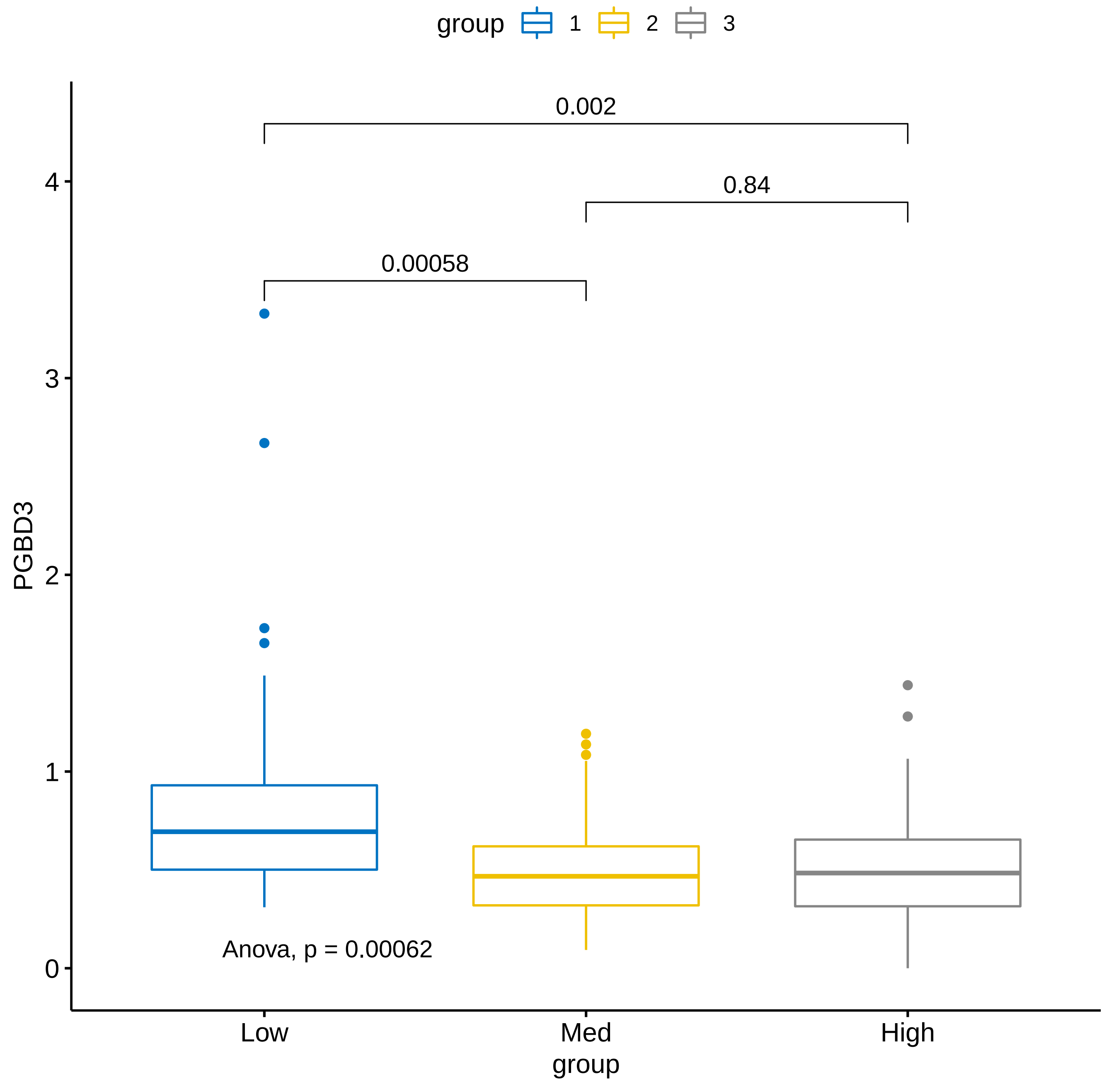
group
1
2
3
0.002
0.84
0.00058
Anova, p = 0.00062
PGBD3
4
3
2
1
0
Low
Med
High
group

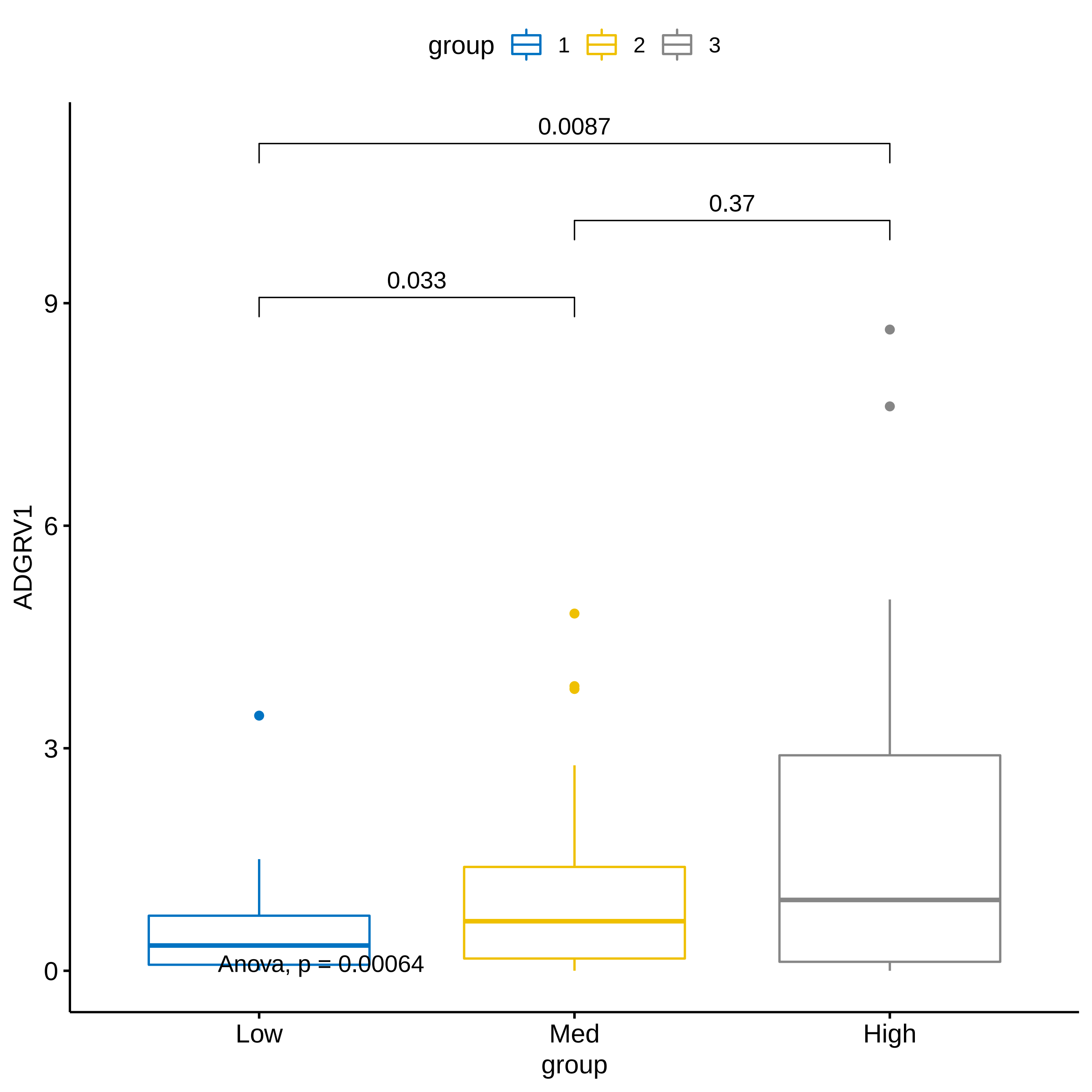
group 1 2 3
0.0087
0.37
0.033
9
6
3
0
ADGRV1
Anova, p = 0.00064
Low
Med
High
group

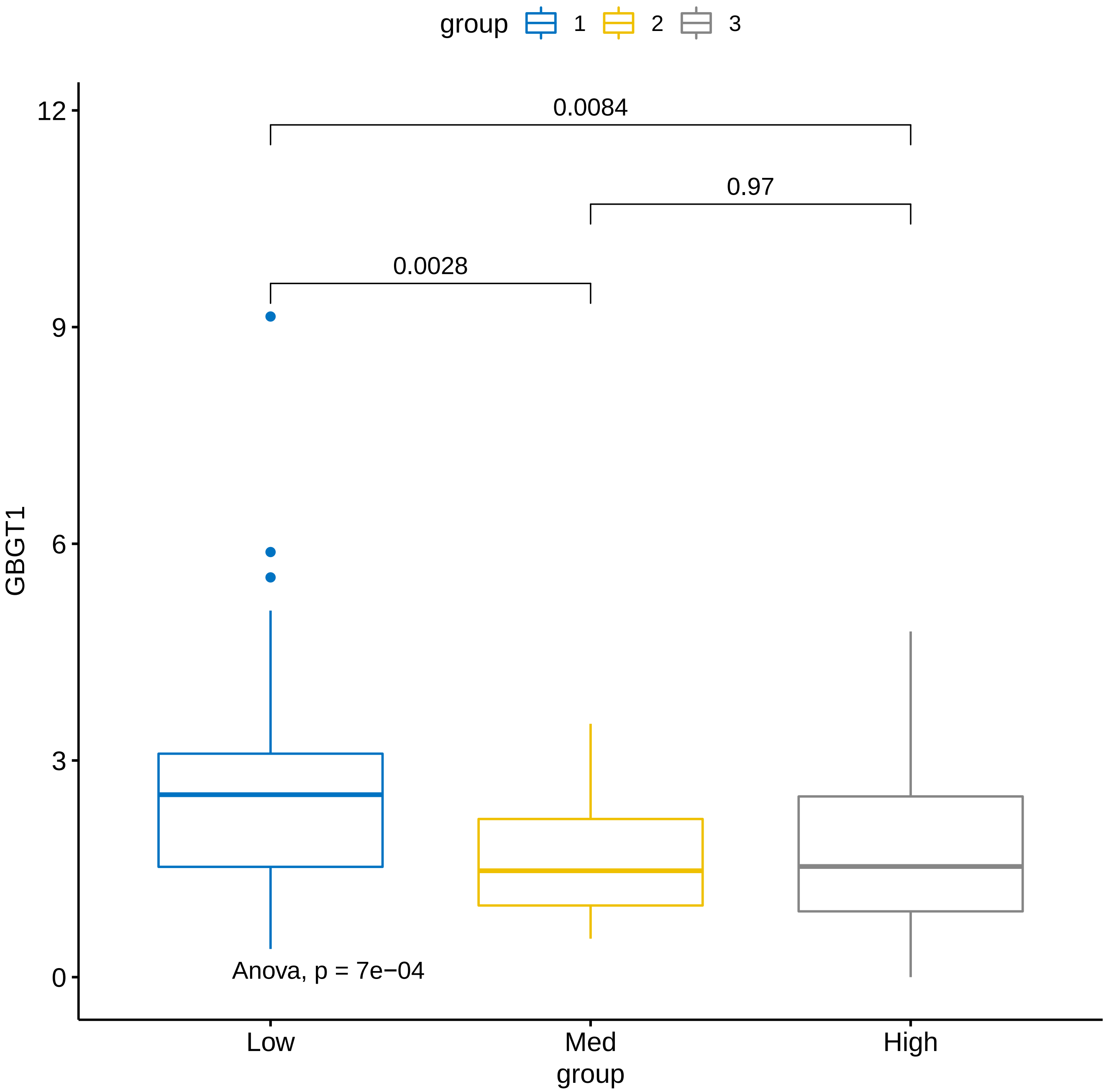
group
1
2
3
0.0084
0.97
0.0028
12
9
6
3
0
GBGT1
Anova, p = 7e−04
Low
Med
High
group

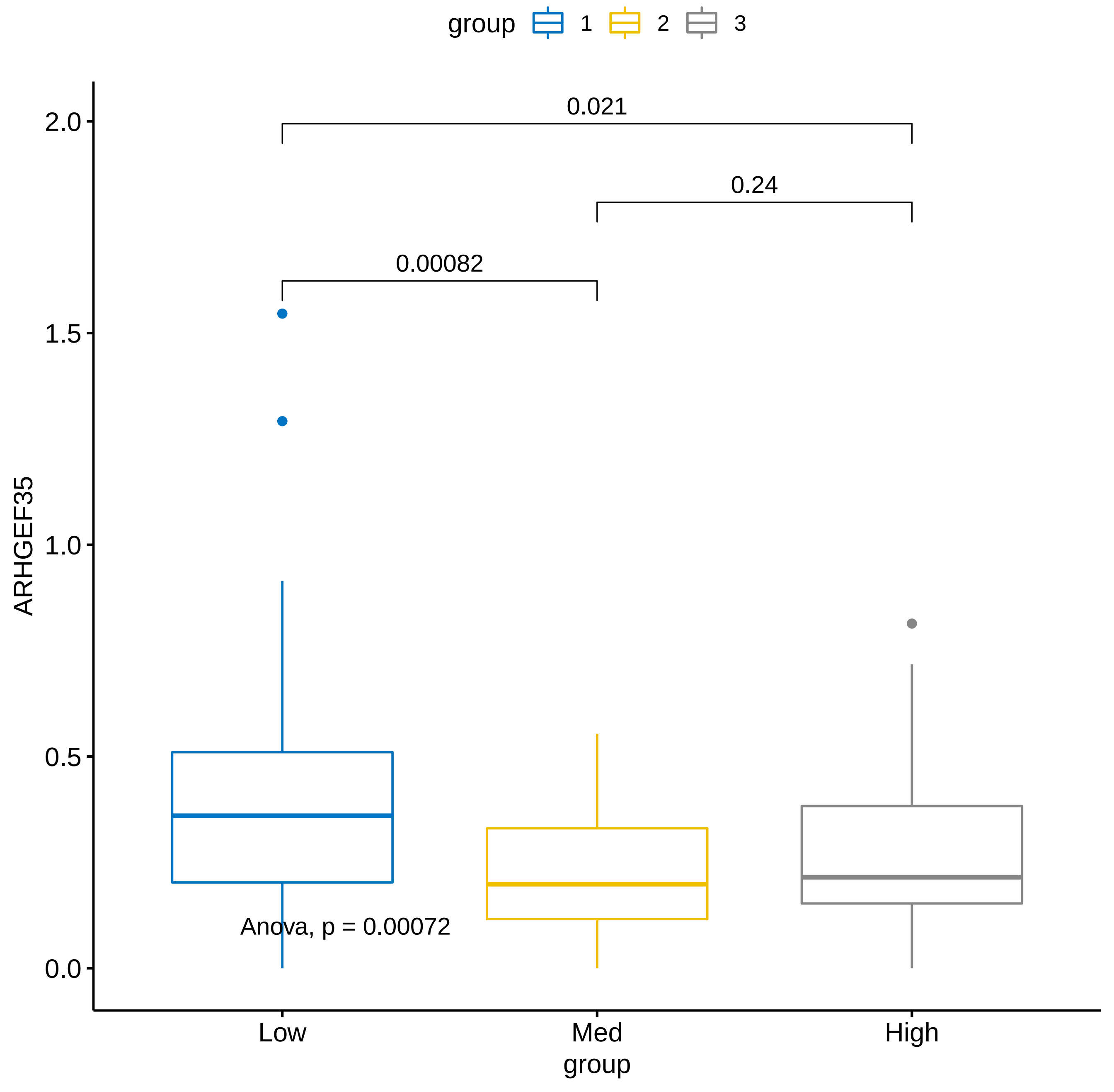

group 1 2 3
0.021
0.24
0.00082
2.0
1.5
1.0
0.5
0.0
ARHGEF35
Anova, p = 0.00072
Low
Med
High
group

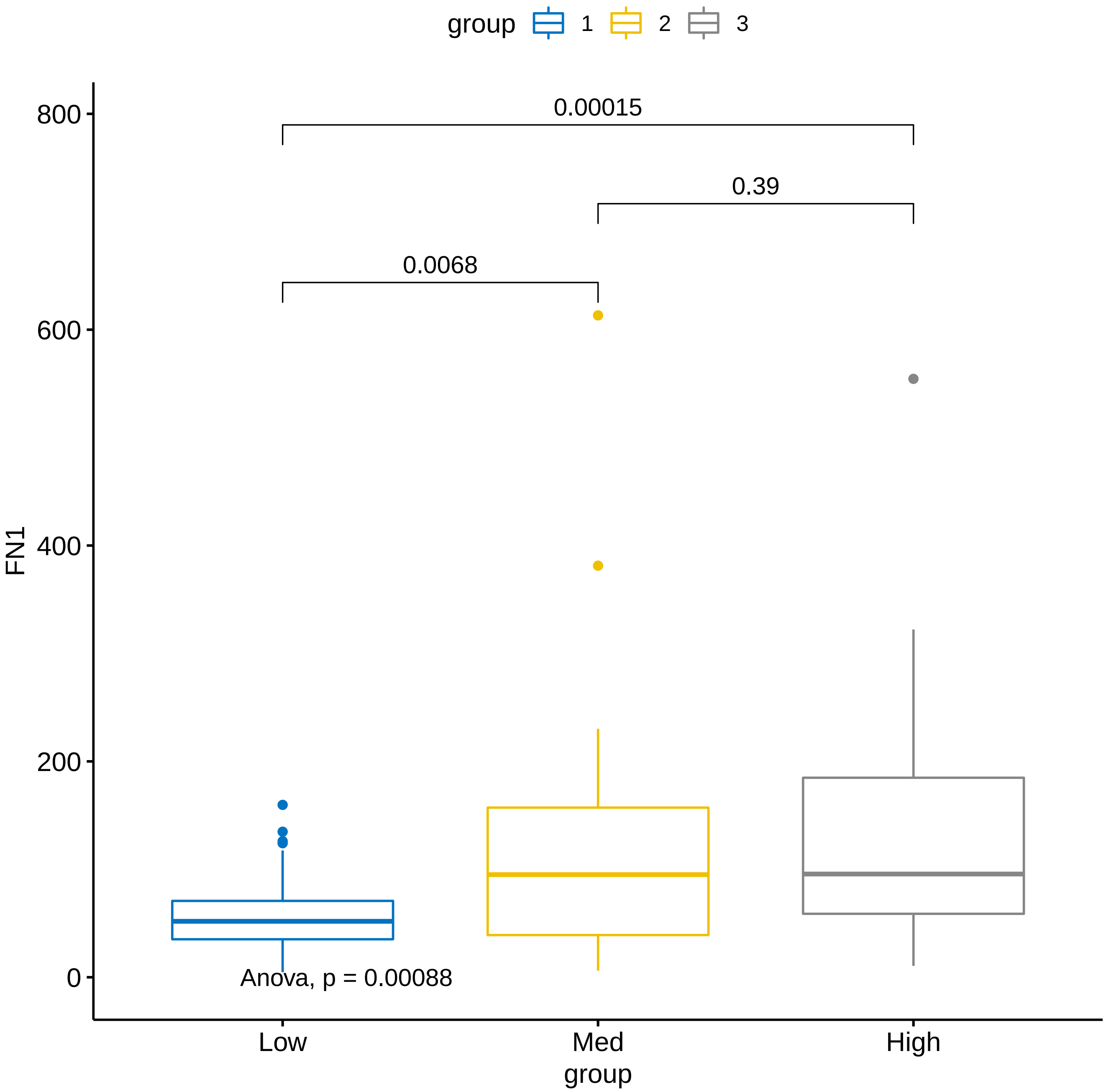

group
1
2
3
0.00015
0.39
0.0068
800
600
400
200
0
FN1
Anova, p = 0.00088
Low
Med
High
group

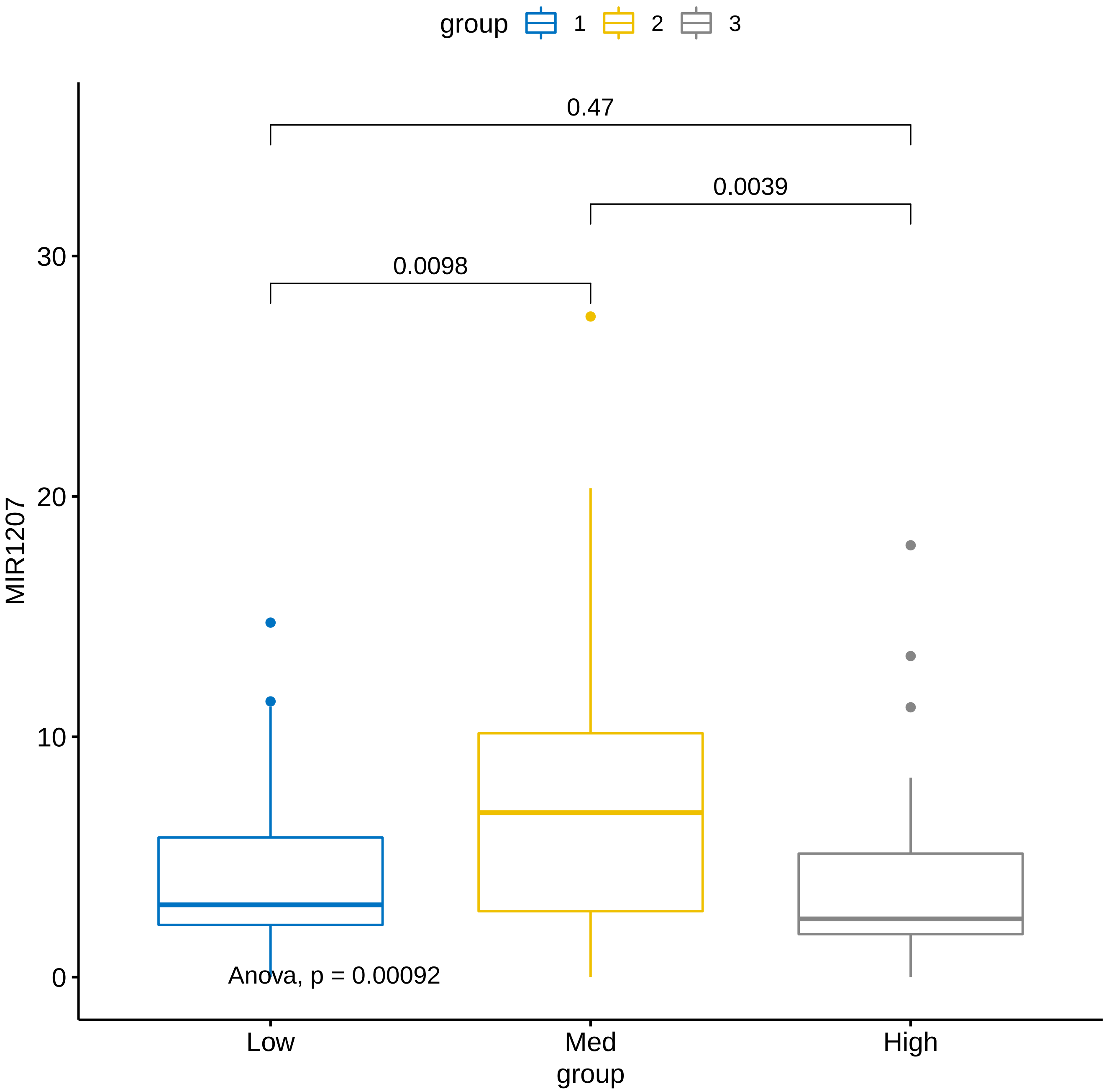
group
1
2
3
0.47
0.0039
0.0098
30
20
10
0
MIR1207
Anova, p = 0.00092
Low
Med
High
group

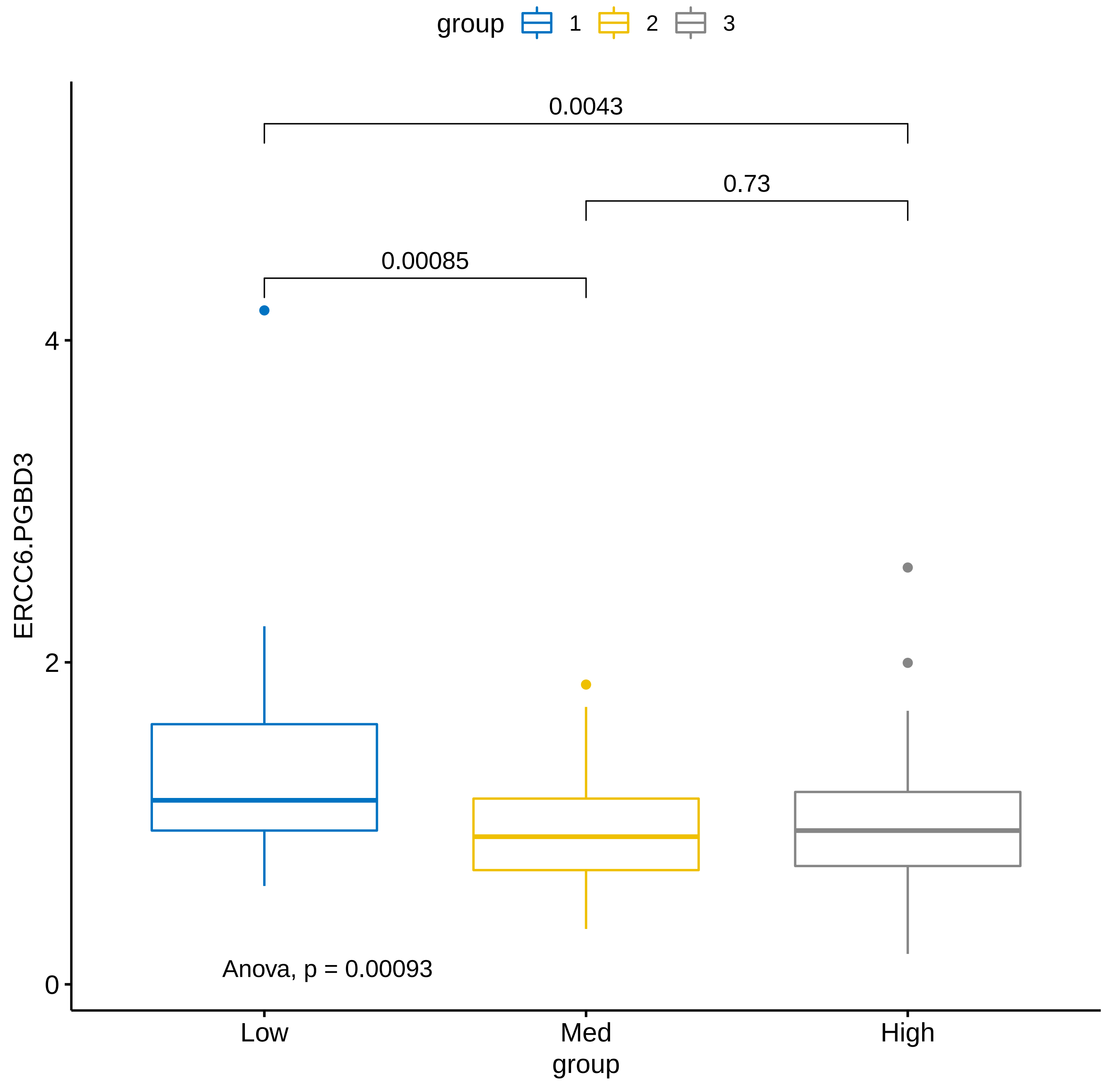
group
1
2
3
0.0043
0.73
0.00085
ERCC6.PGBD3
4
2
0
Anova, p = 0.00093
Low
Med
High
group

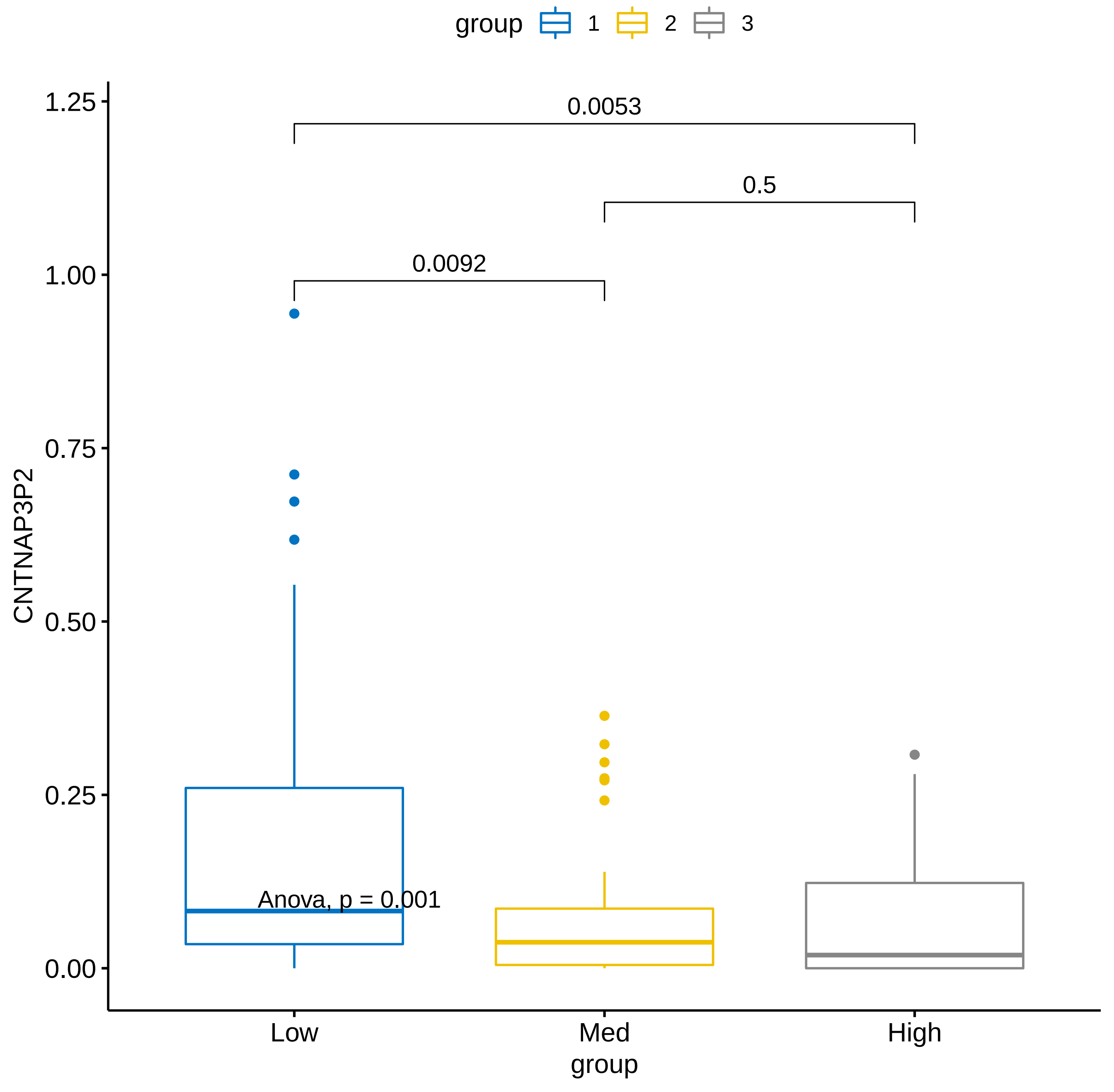
group
1
2
3
0.0053
0.5
0.0092
1.25
1.00
0.75
0.50
0.25
0.00
CNTNAP3P2
Anova, p = 0.001
Low
Med
High
group

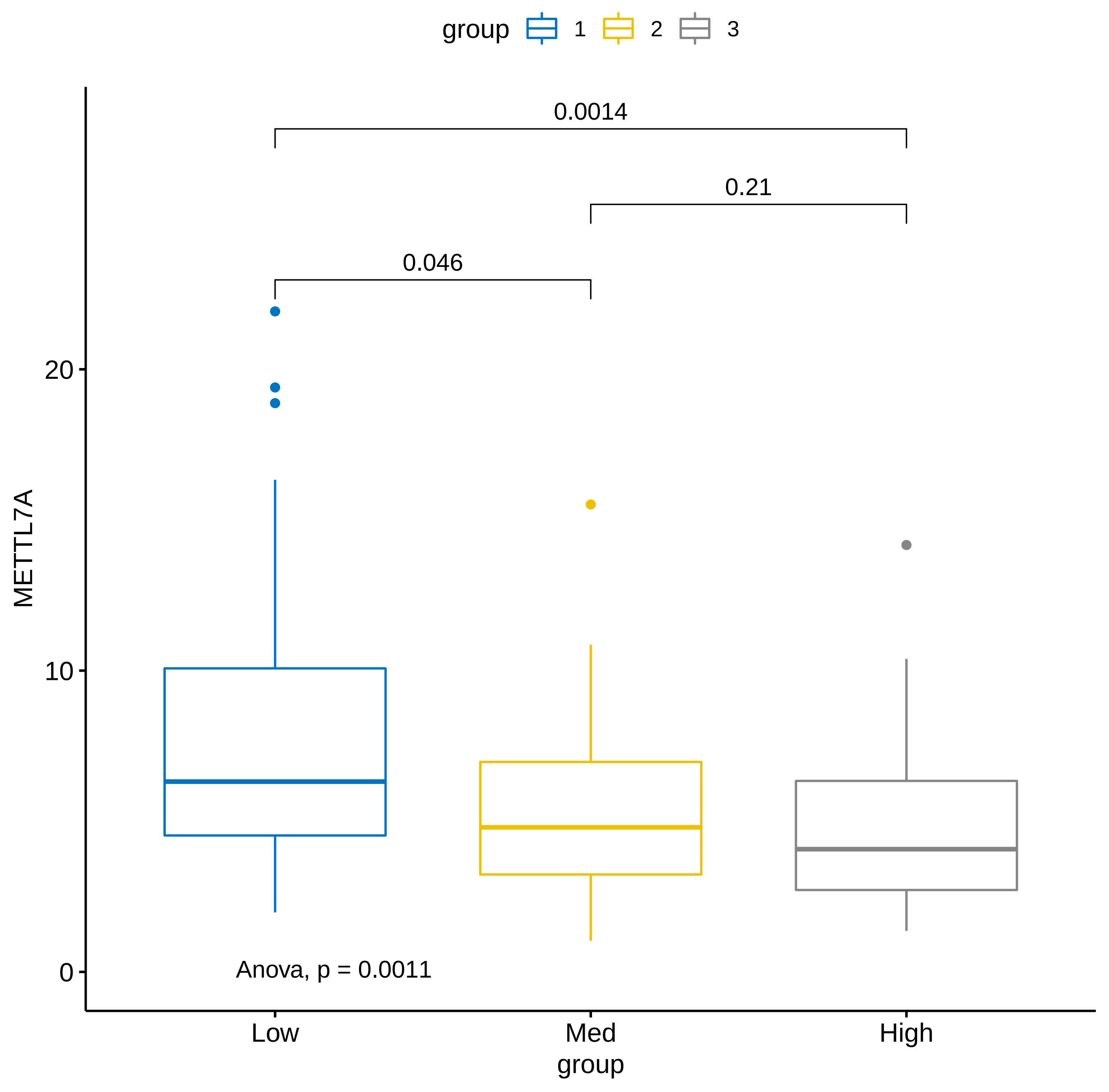
group
1
2
3
0.0014
0.21
0.046
20
10
0
METTL7A
Anova, p = 0.0011
Low
Med
High
group

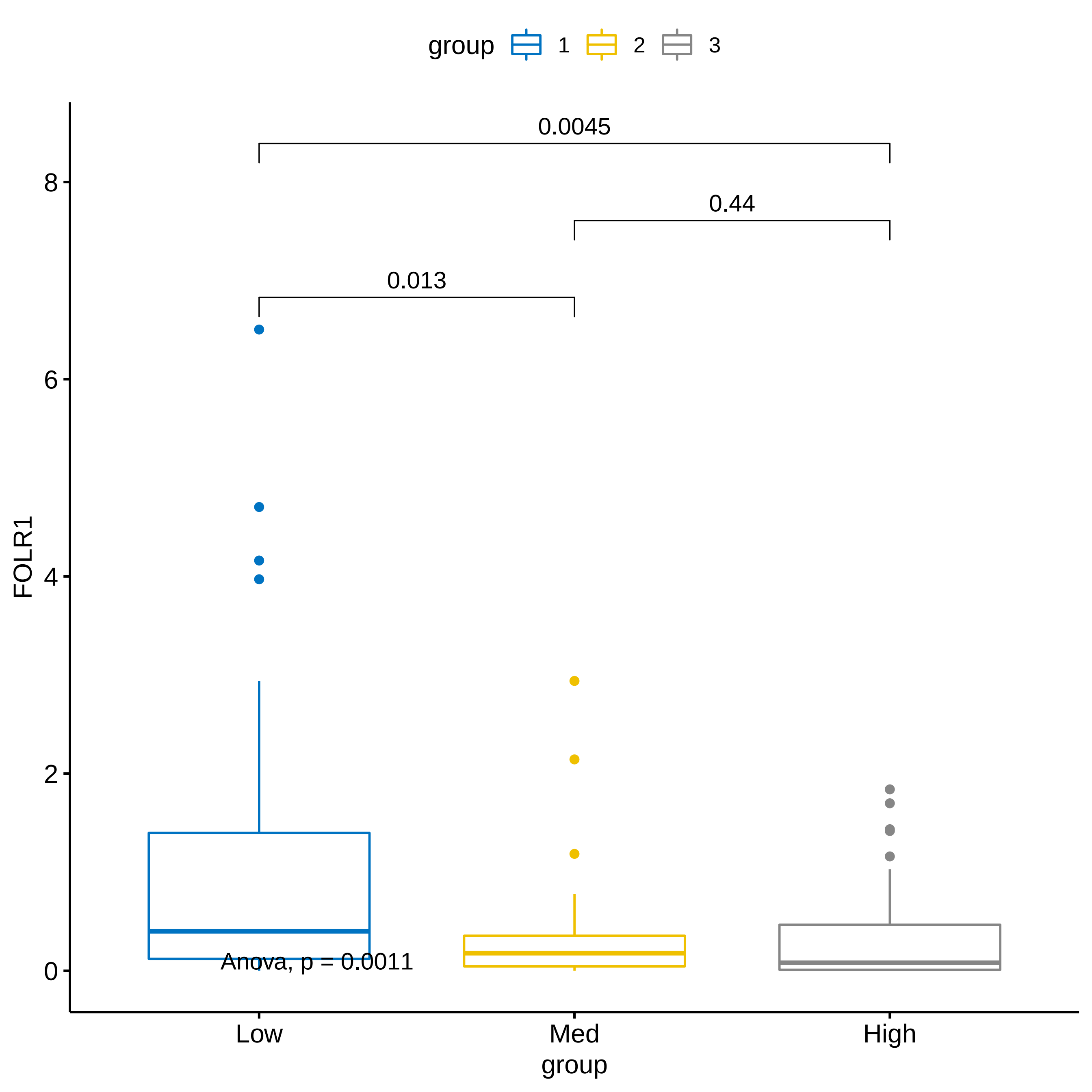
group
1
2
3
0.0045
0.44
0.013
8
6
4
2
0
FOLR1
Anova, p = 0.0011
Low
Med
High
group

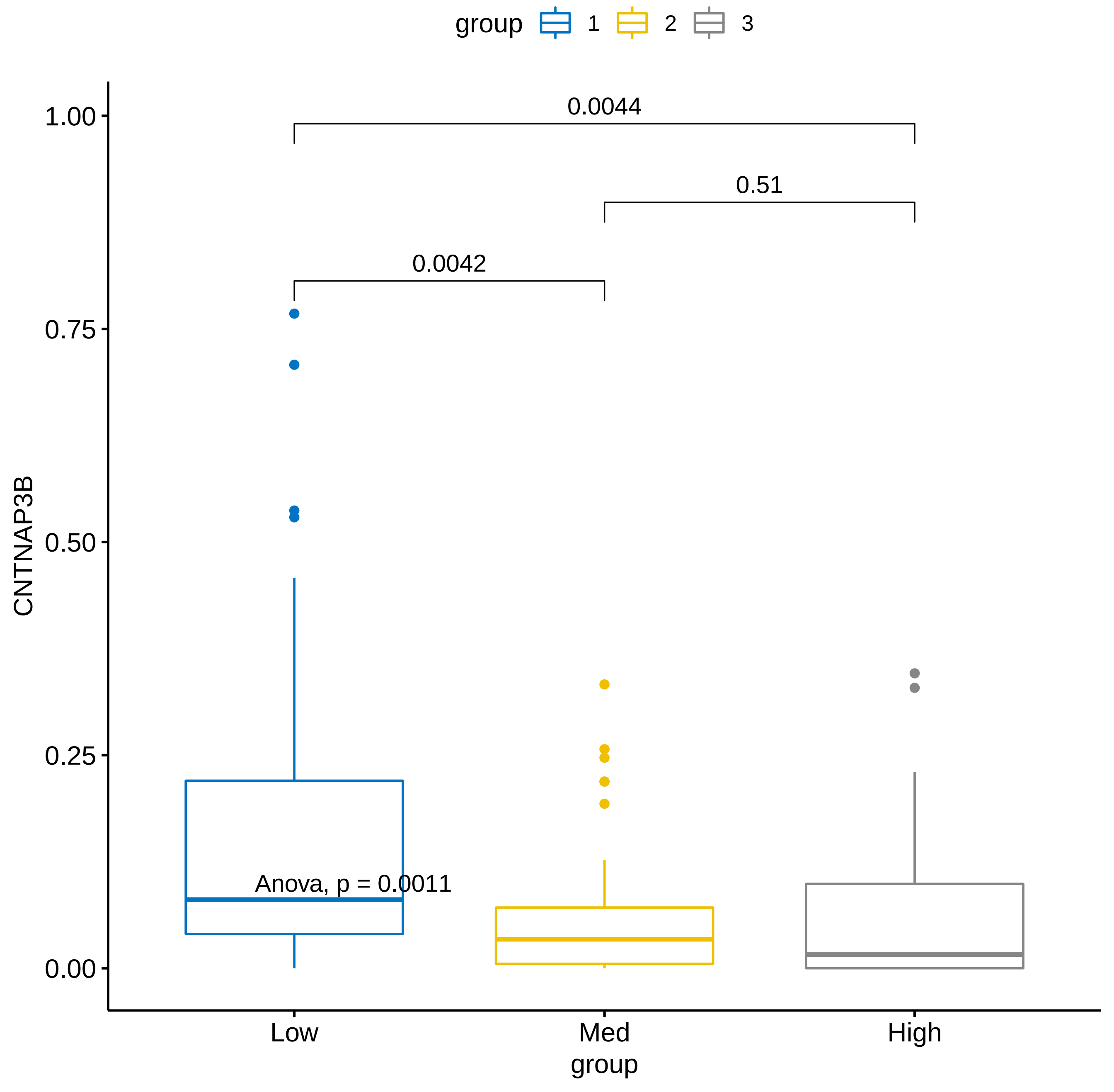
group
1
2
3
0.0044
0.51
0.0042
1.00
0.75
0.50
0.25
0.00
CNTNAP3B
Anova, p = 0.0011
Low
Med
High
group

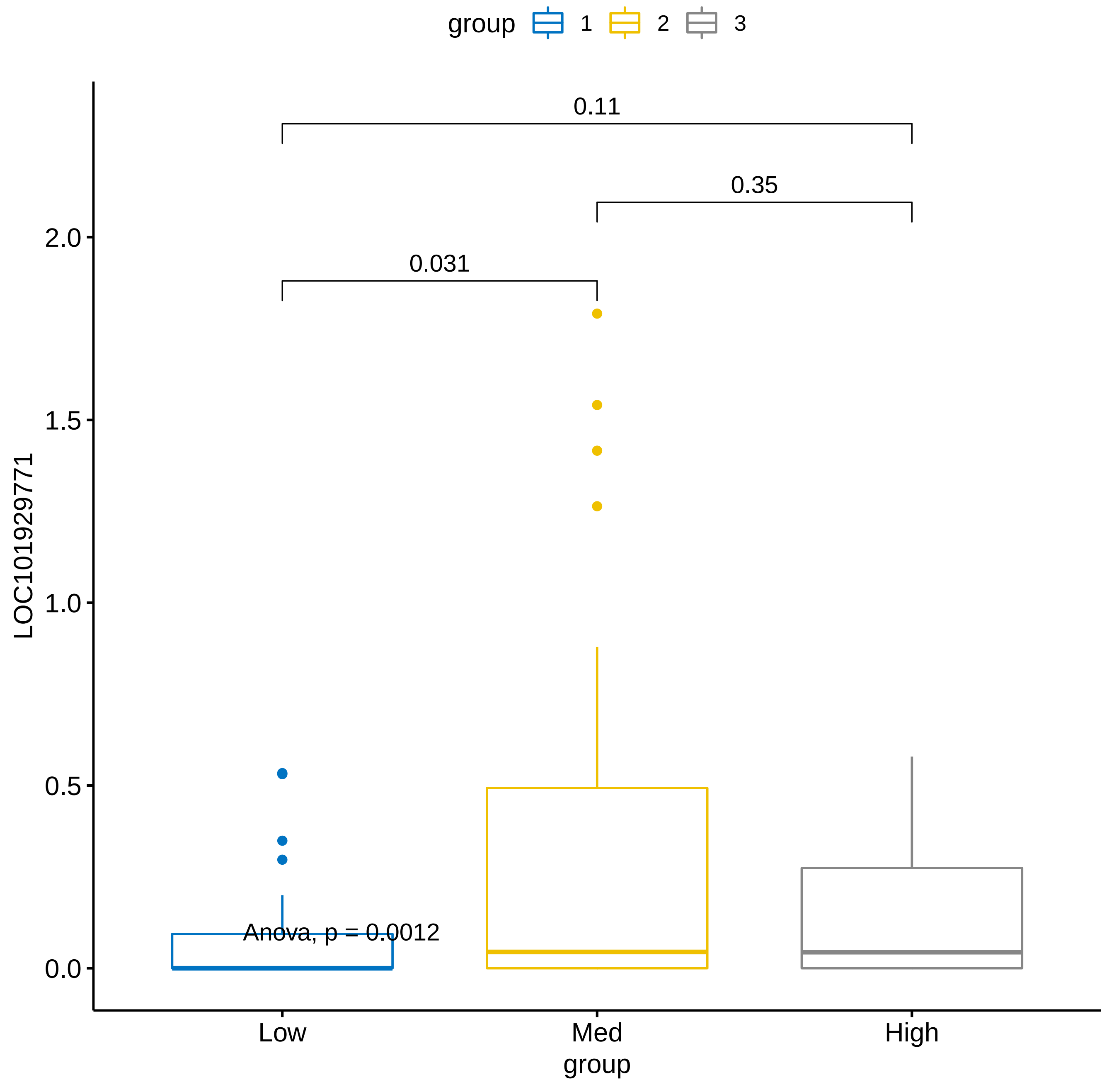

group 1 2 3
0.11
0.35
0.031
2.0
1.5
1.0
0.5
0.0
LOC101929771
Anova, p = 0.0012
Low
Med
High
group

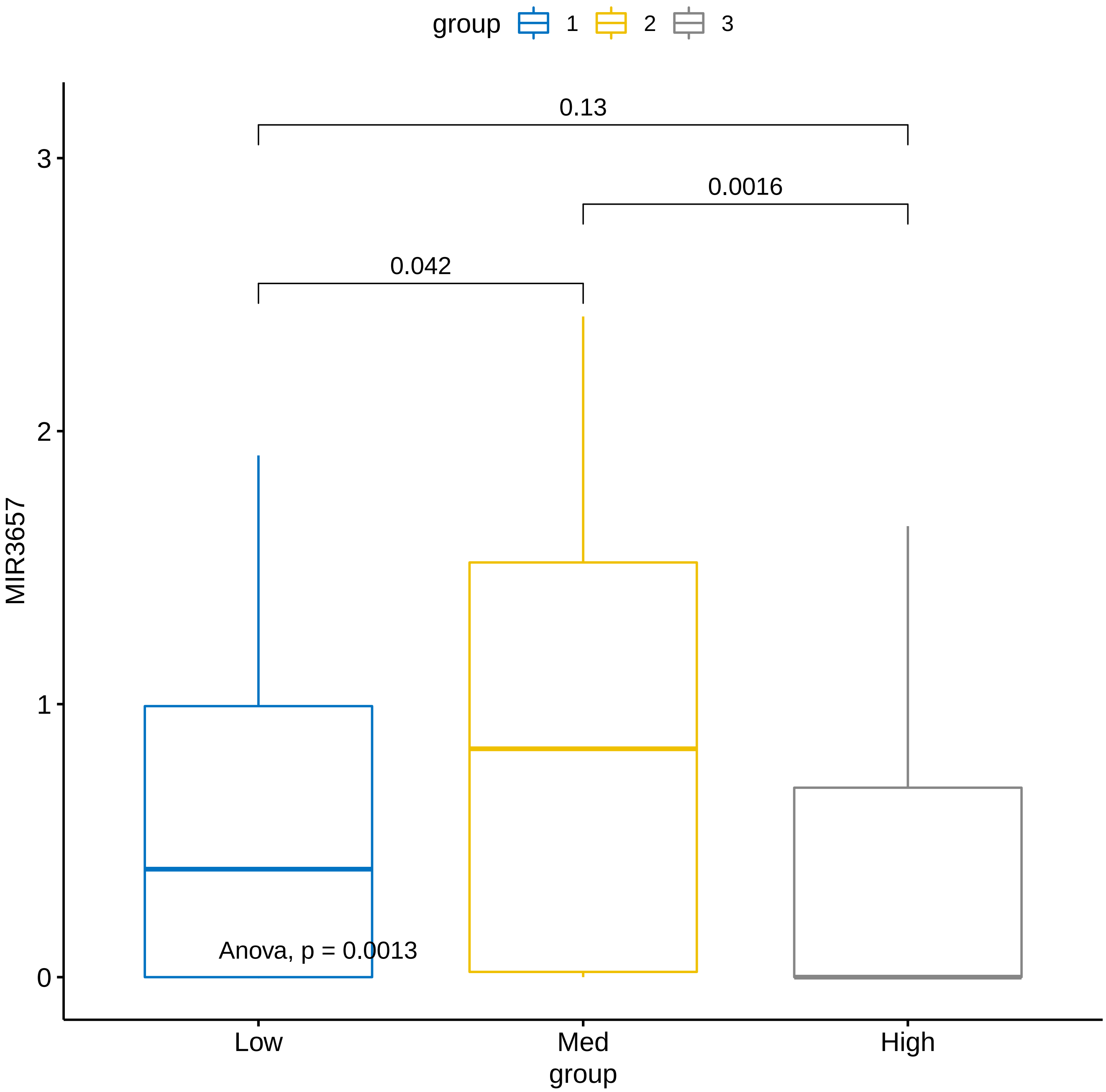
group
1
2
3
0.13
0.0016
0.042
MIR3657
3
2
1
0
Anova, p = 0.0013
Low
Med
High
group

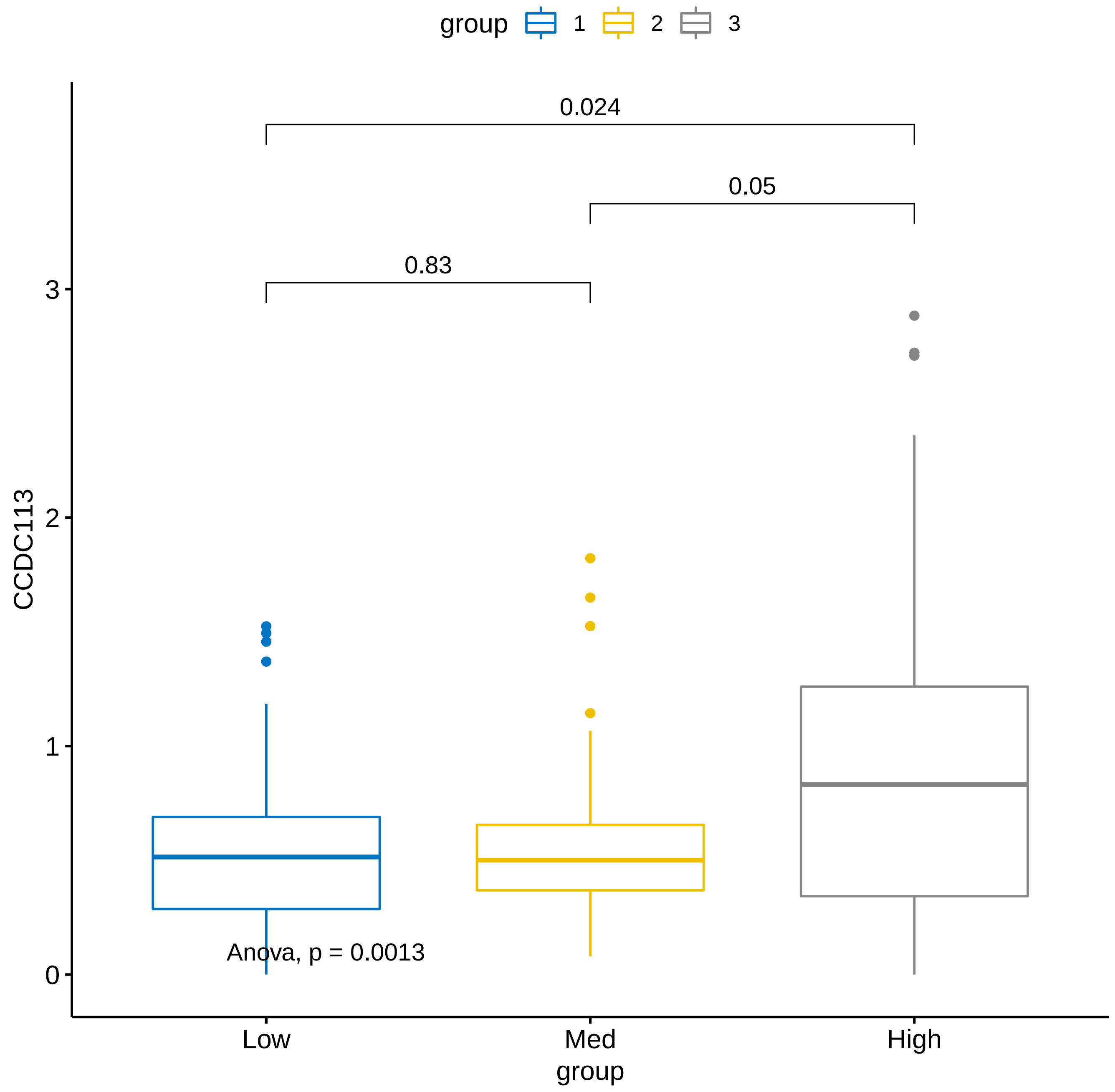

group 1 2 3
0.024
0.05
0.83
CCDC113
3
2
1
0
Anova, p = 0.0013
Low
Med
High
group

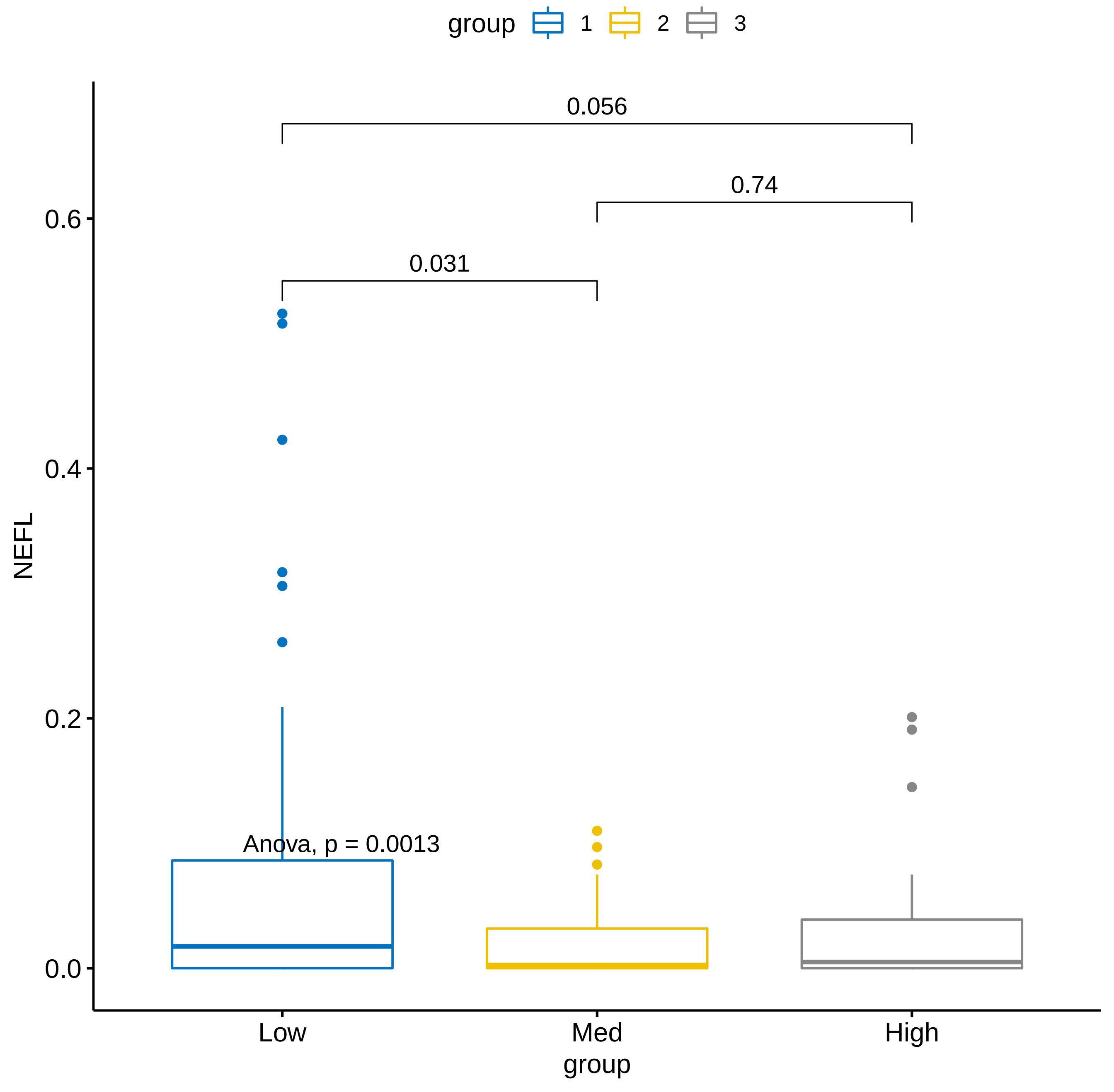
group 1 2 3
0.056
0.74
0.031
0.6
0.4
0.2
0.0
NEFL
Anova, p = 0.0013
Low
Med
High
group

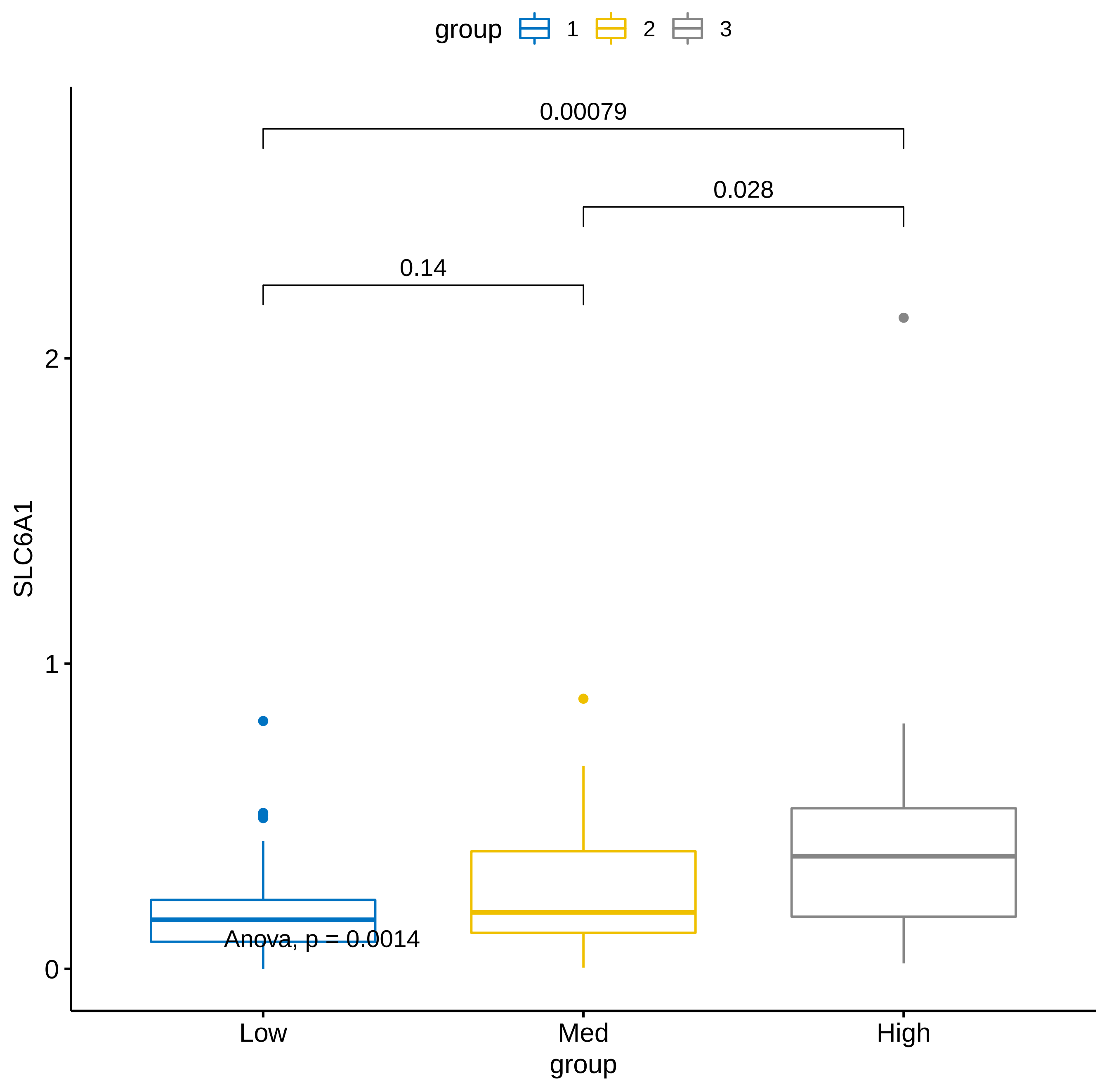

group 1 2 3
0.00079
0.028
0.14
SLC6A1
2
1
0
Anova, p = 0.0014
Low
Med
High
group

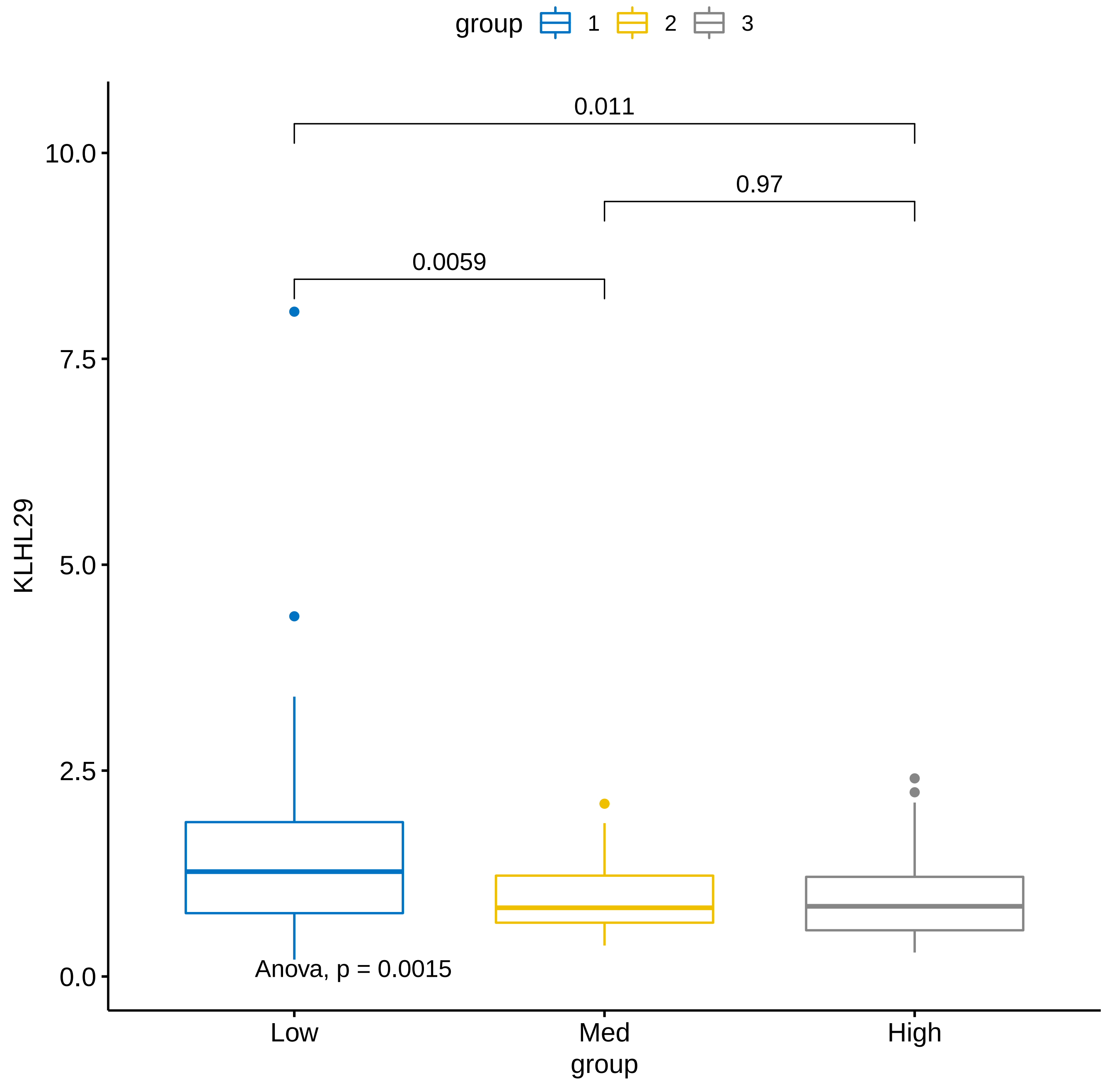

group
1
2
3
0.011
0.97
0.0059
10.0
7.5
5.0
2.5
0.0
KLHL29
Anova, p = 0.0015
Low
Med
High
group

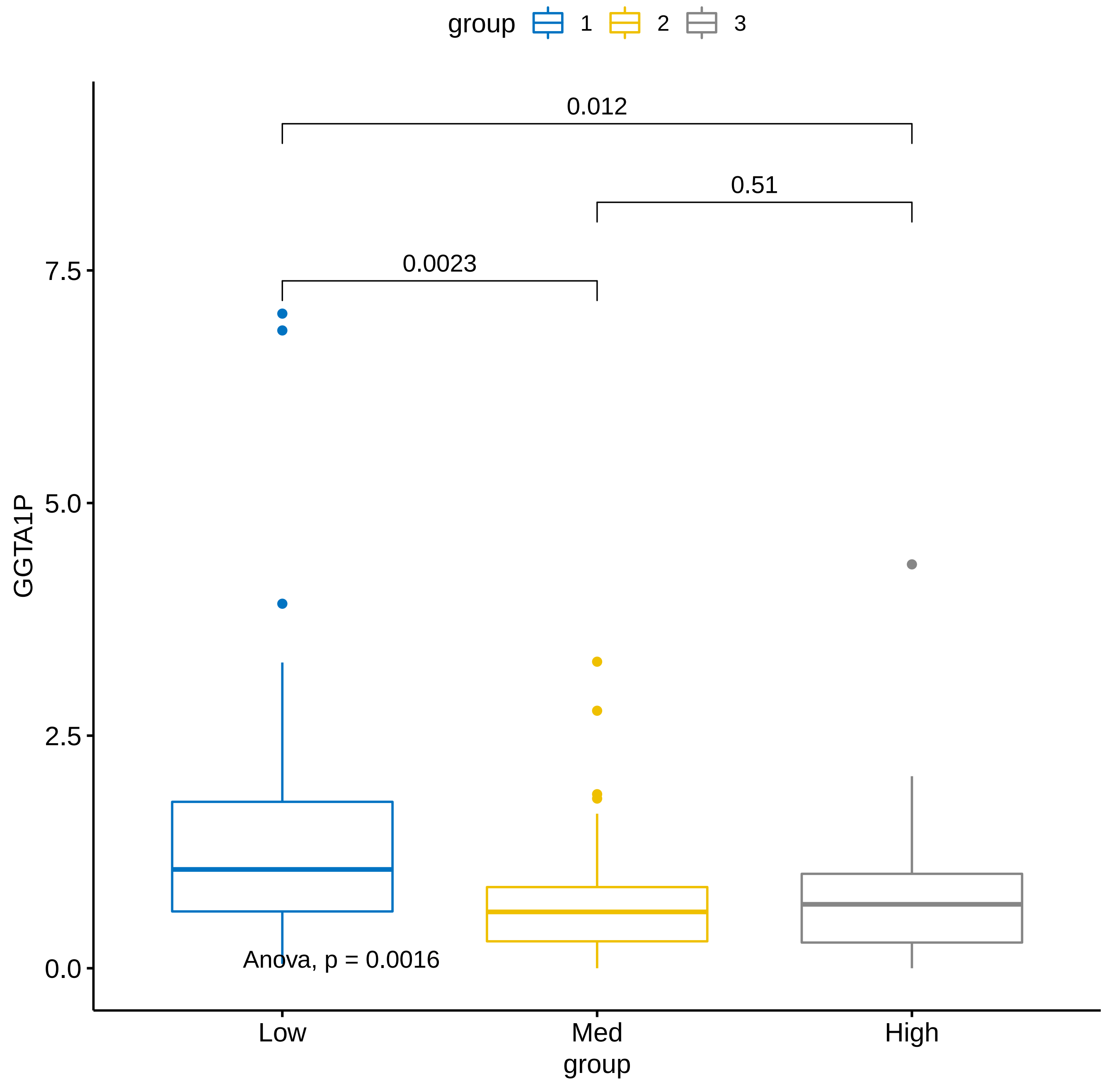

group 1 2 3
0.012
0.51
0.0023
7.5
5.0
2.5
0.0
GGTA1P
Anova, p = 0.0016
Low
Med
High
group

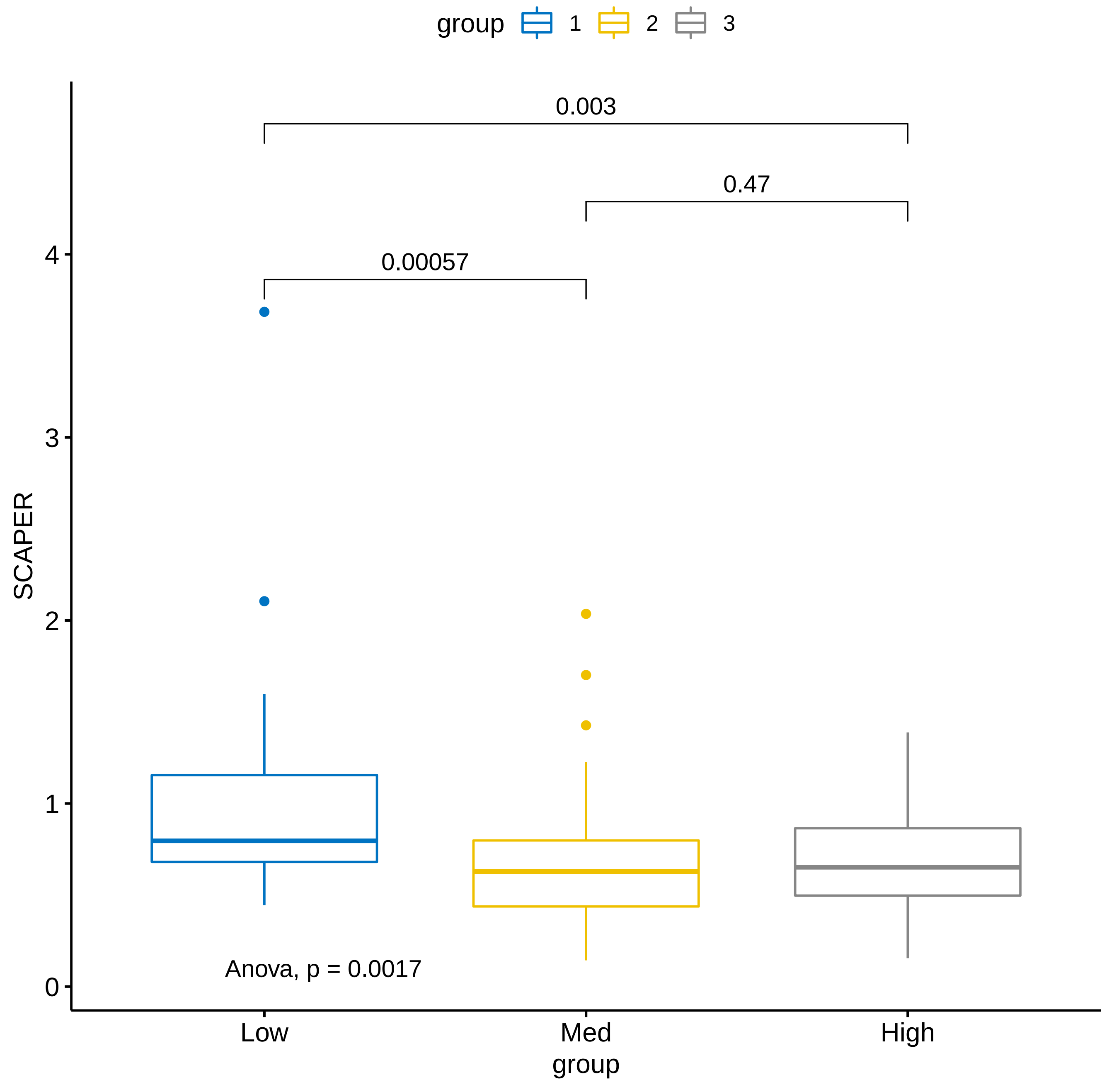
group
1
2
3
0.003
0.47
0.00057
4
3
2
1
0
SCAPER
Anova, p = 0.0017
Low
Med
High
group

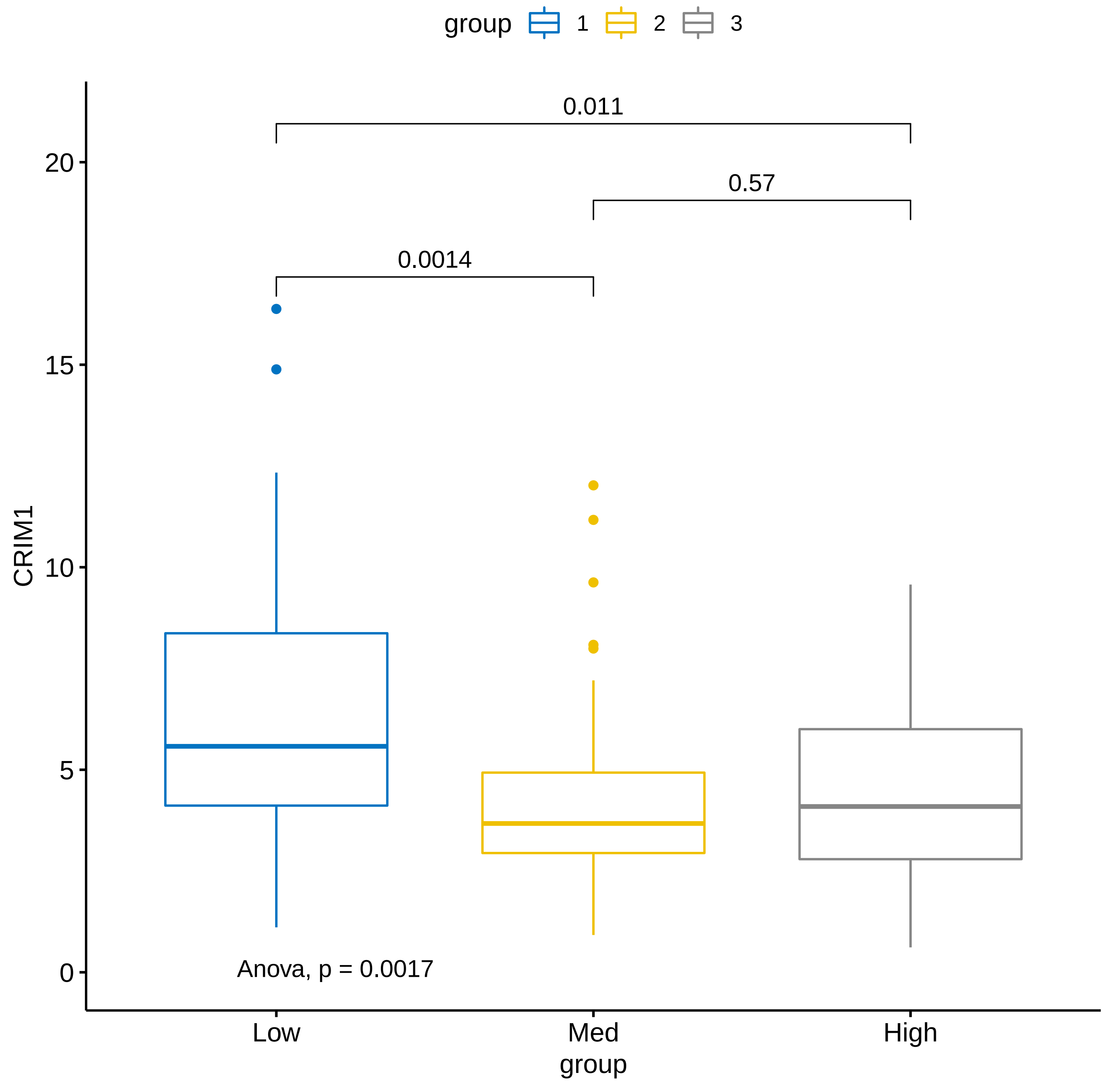
group 1 2 3
0.011
0.57
0.0014
20
15
10
5
0
CRIM1
Anova, p = 0.0017
Low
Med
High
group

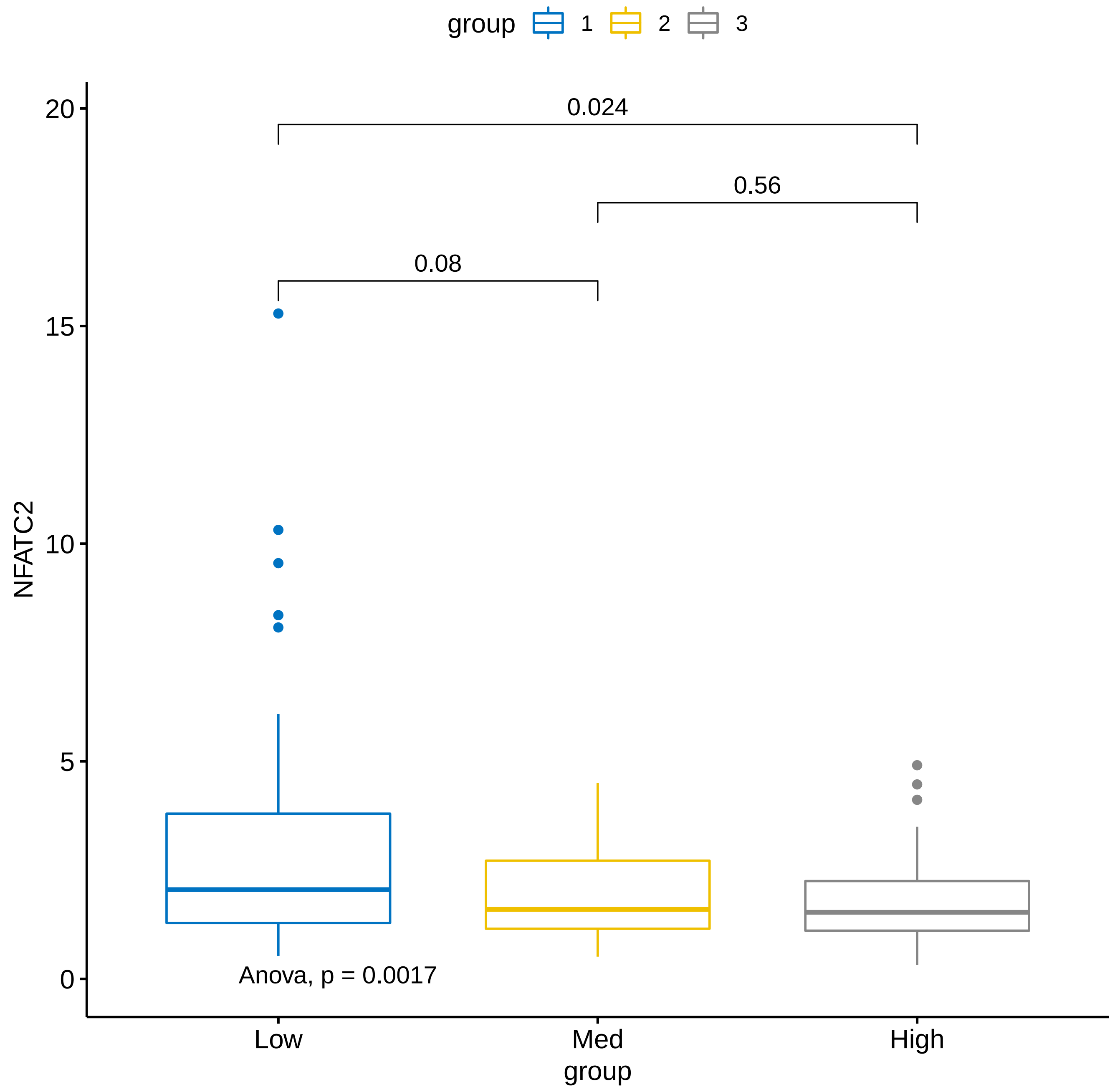
group
1
2
3
20
15
10
5
0
NFATC2
0.024
0.56
0.08
Anova, p = 0.0017
Low
Med
High
group

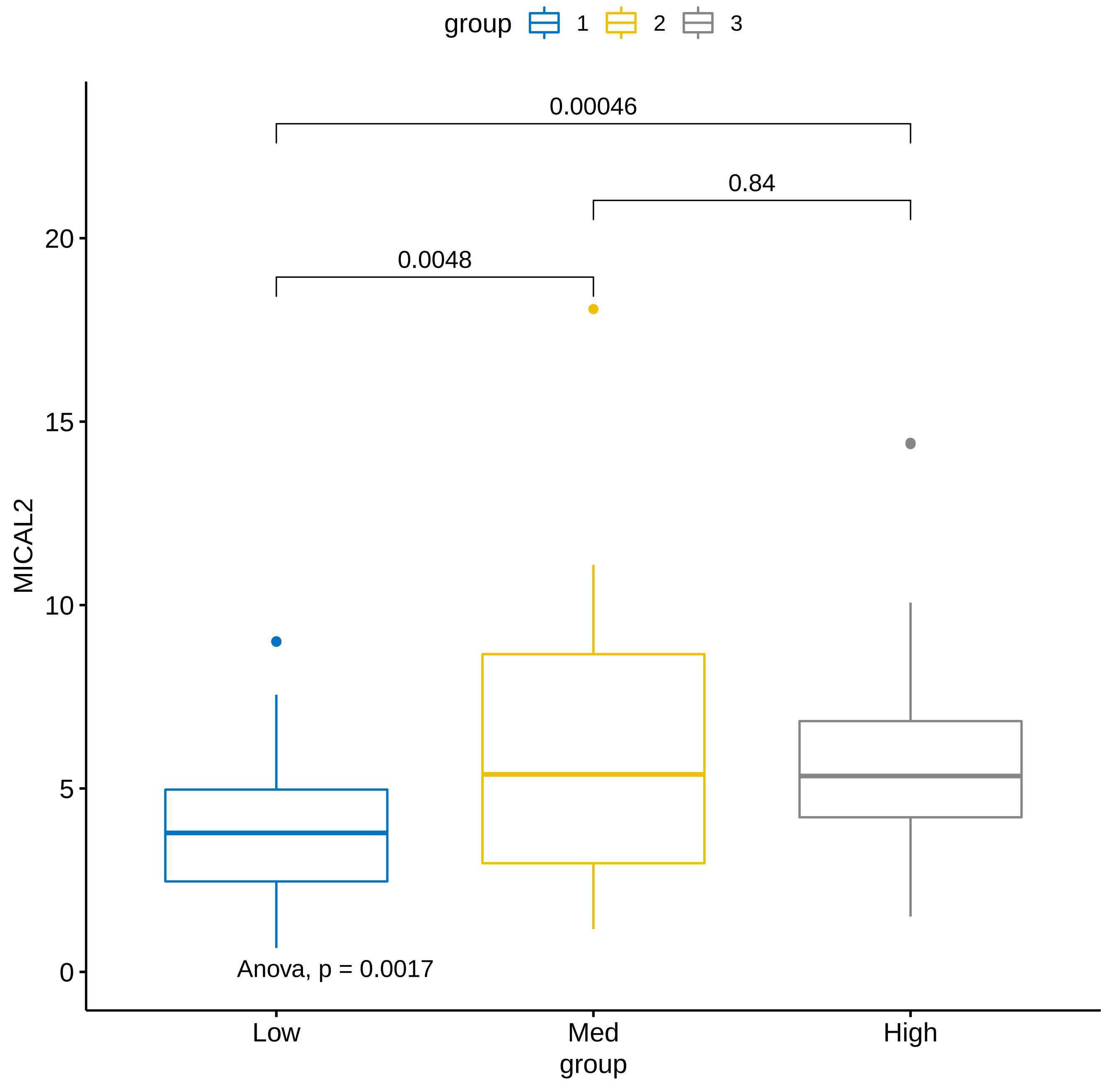
group
1
2
3
0.00046
0.84
0.0048
20
15
10
5
0
MICAL2
Anova, p = 0.0017
Low
Med
High
group

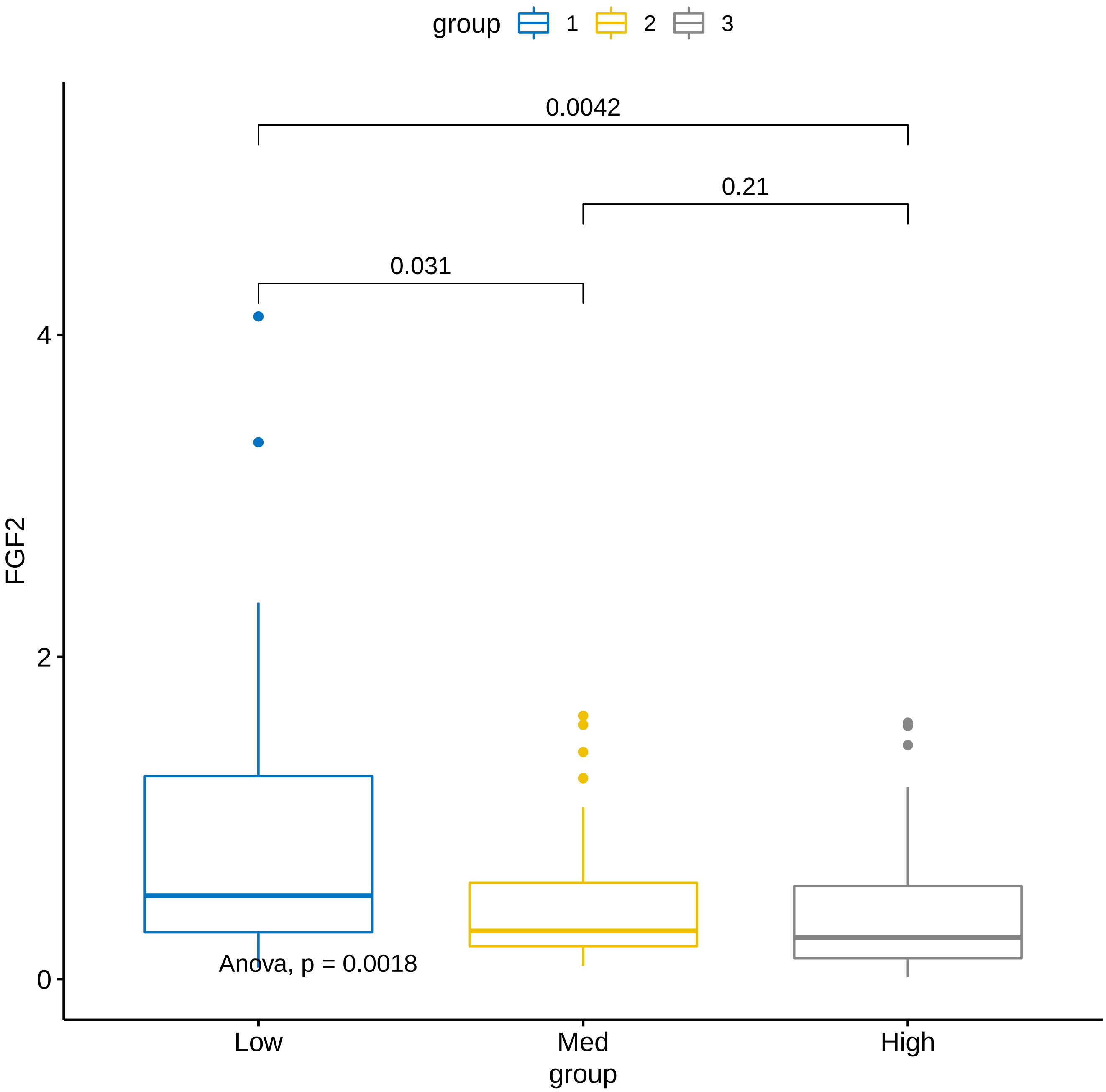

group 1 2 3
0.0042
0.21
0.031
FGF2
4
2
0
Anova, p = 0.0018
Low
Med
High
group

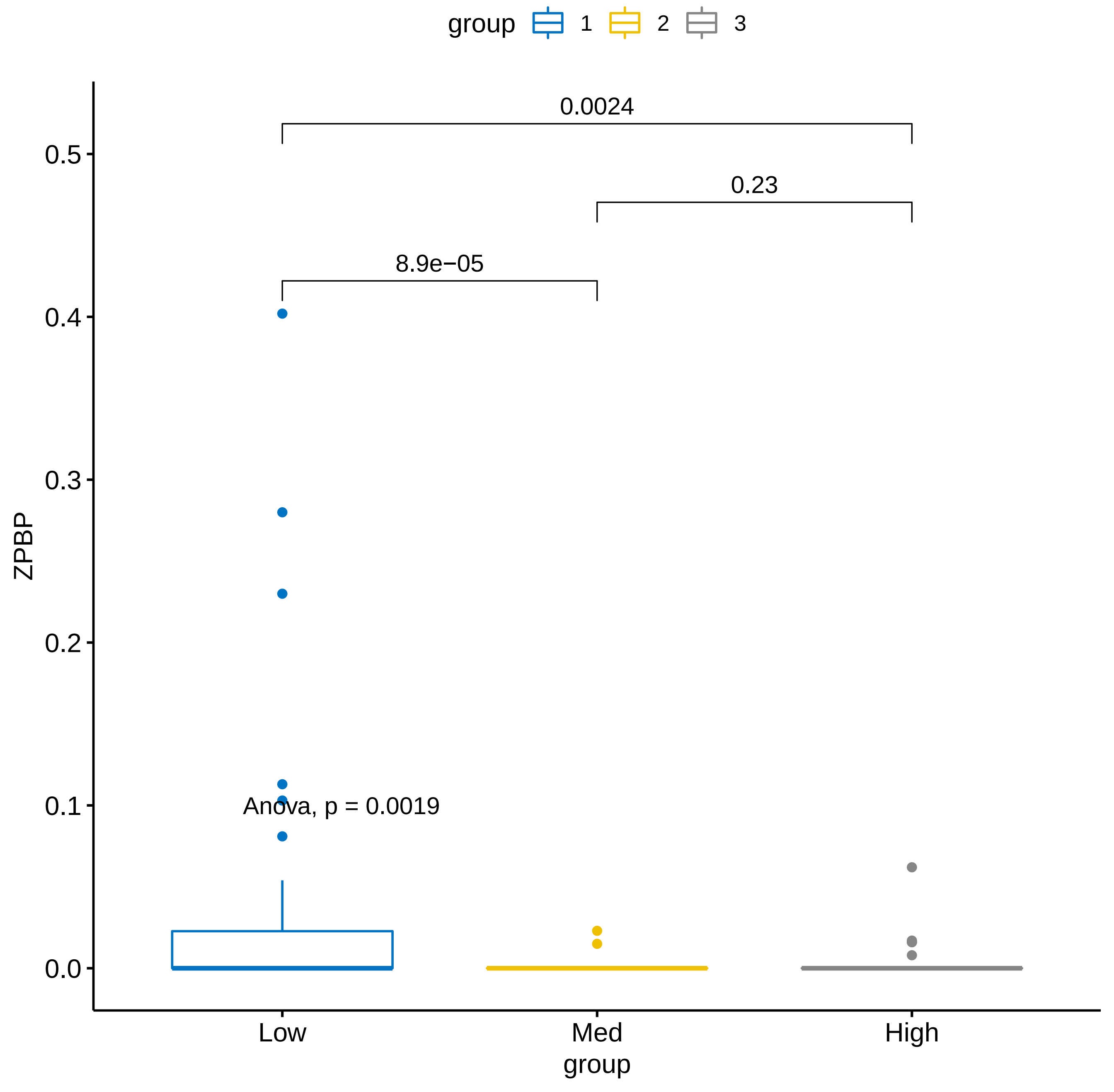

group 1 2 3
0.0024
0.23
8.9e−05
Anova, p = 0.0019
ZPBP
0.5
0.4
0.3
0.2
0.1
0.0
Low
Med
High
group

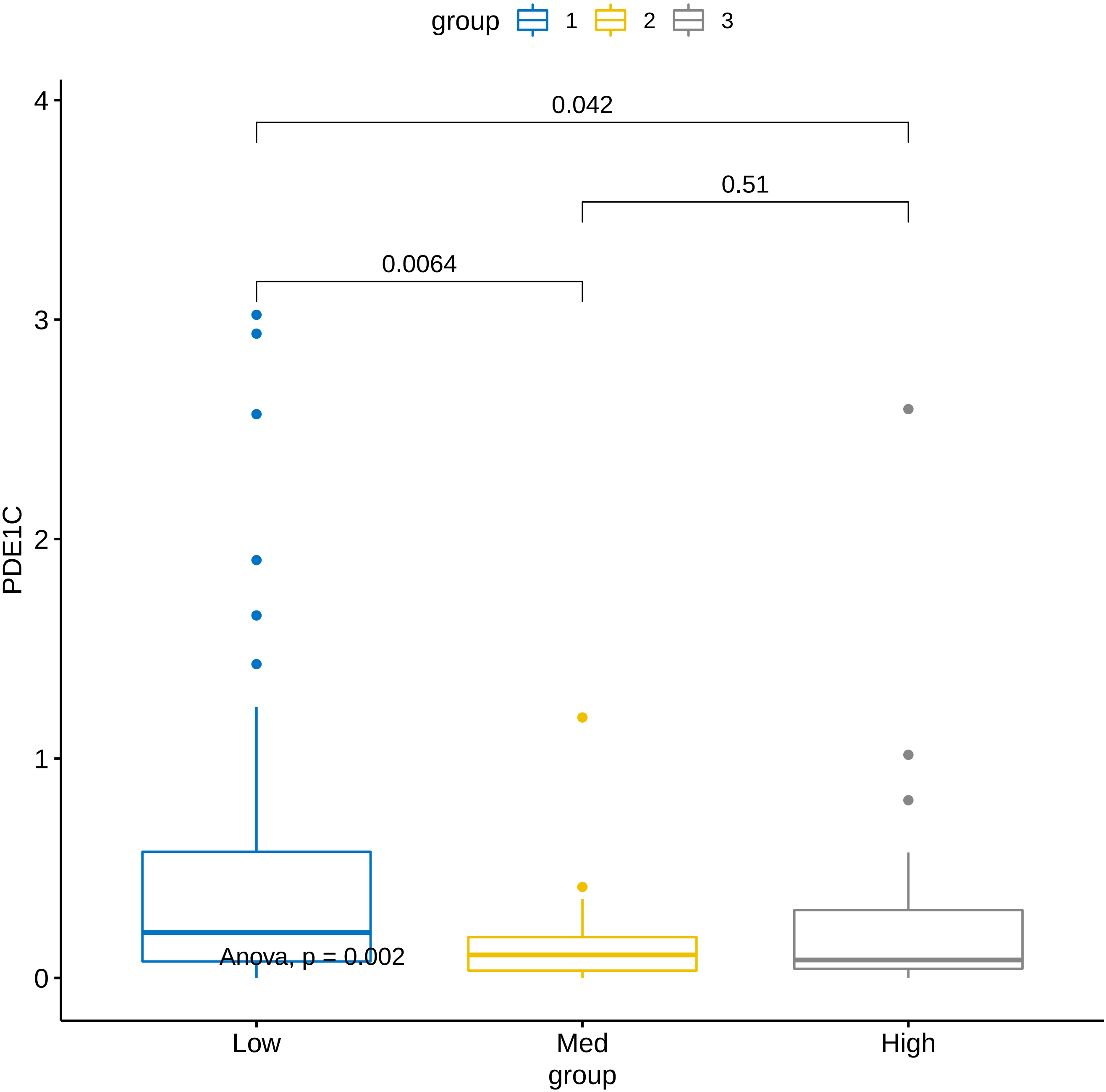

group 1 2 3
0.042
0.51
0.0064
PDE1C
4
3
2
1
0
Anova, p = 0.002
Low
Med
High
group

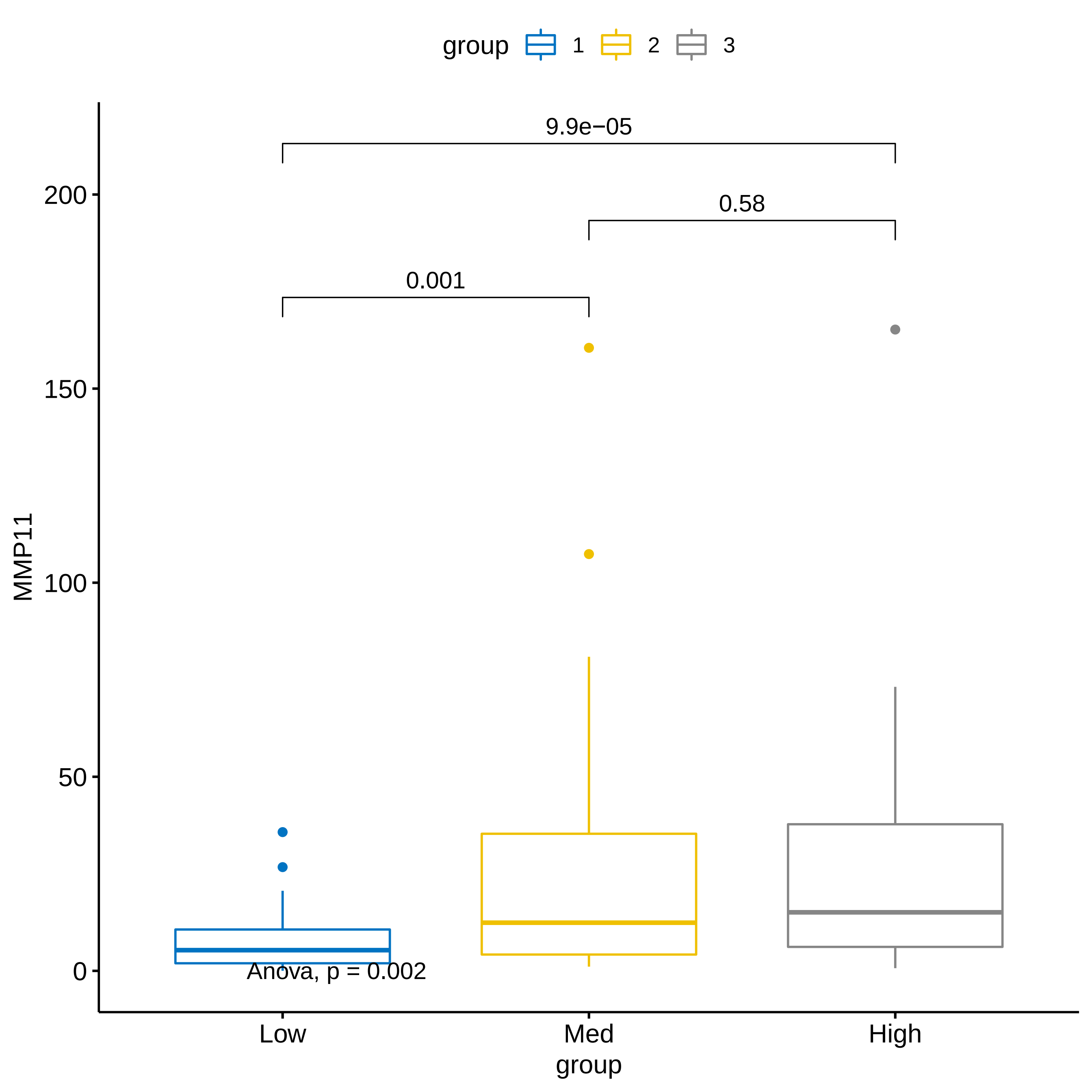
group
1
2
3
9.9e−05
0.58
0.001
200
150
100
50
0
MMP11
Anova, p = 0.002
Low
Med
High
group

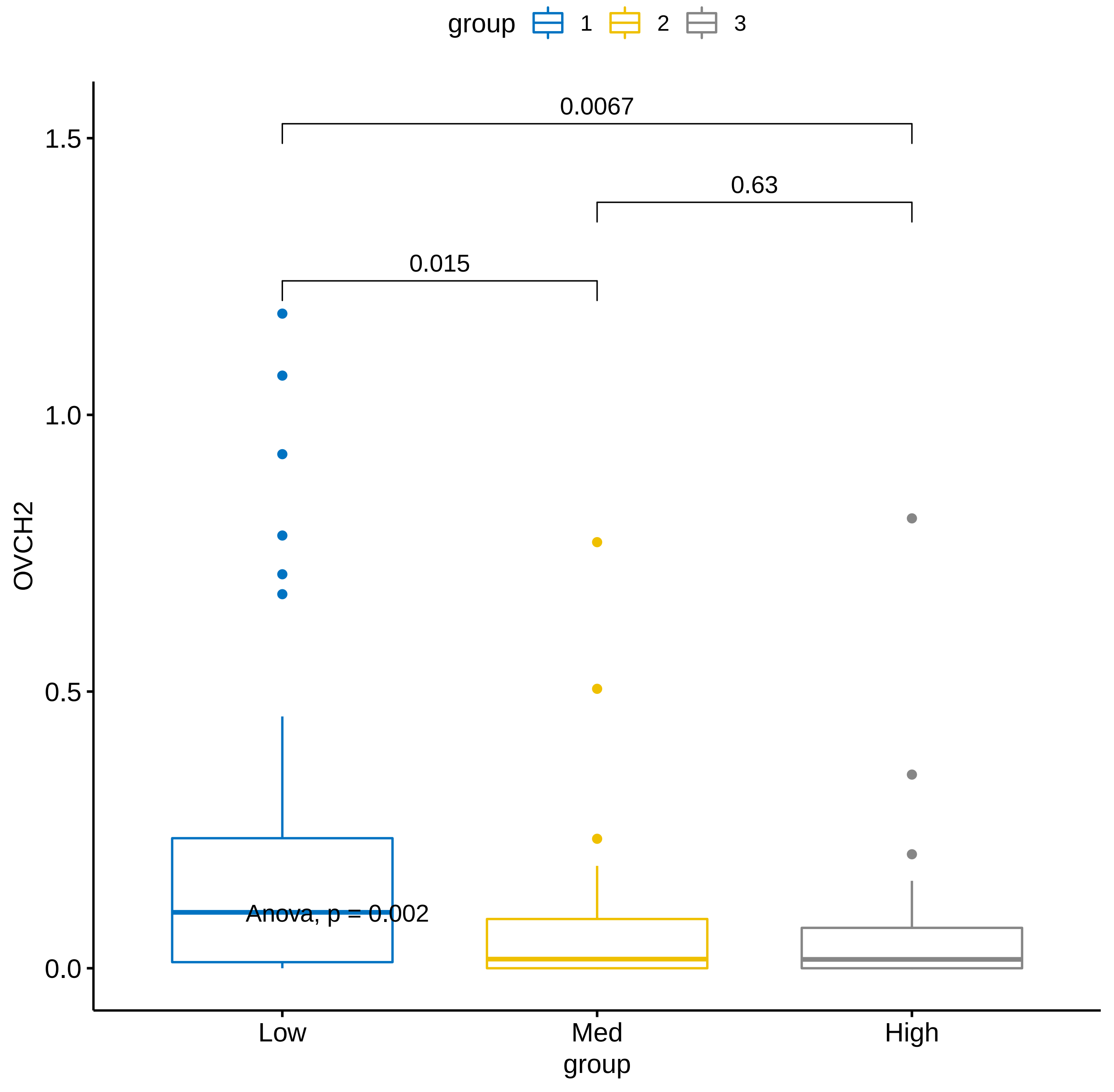
group
1
2
3
0.0067
0.63
0.015
1.5
1.0
0.5
0.0
OVCH2
Anova, p = 0.002
Low
Med
High
group

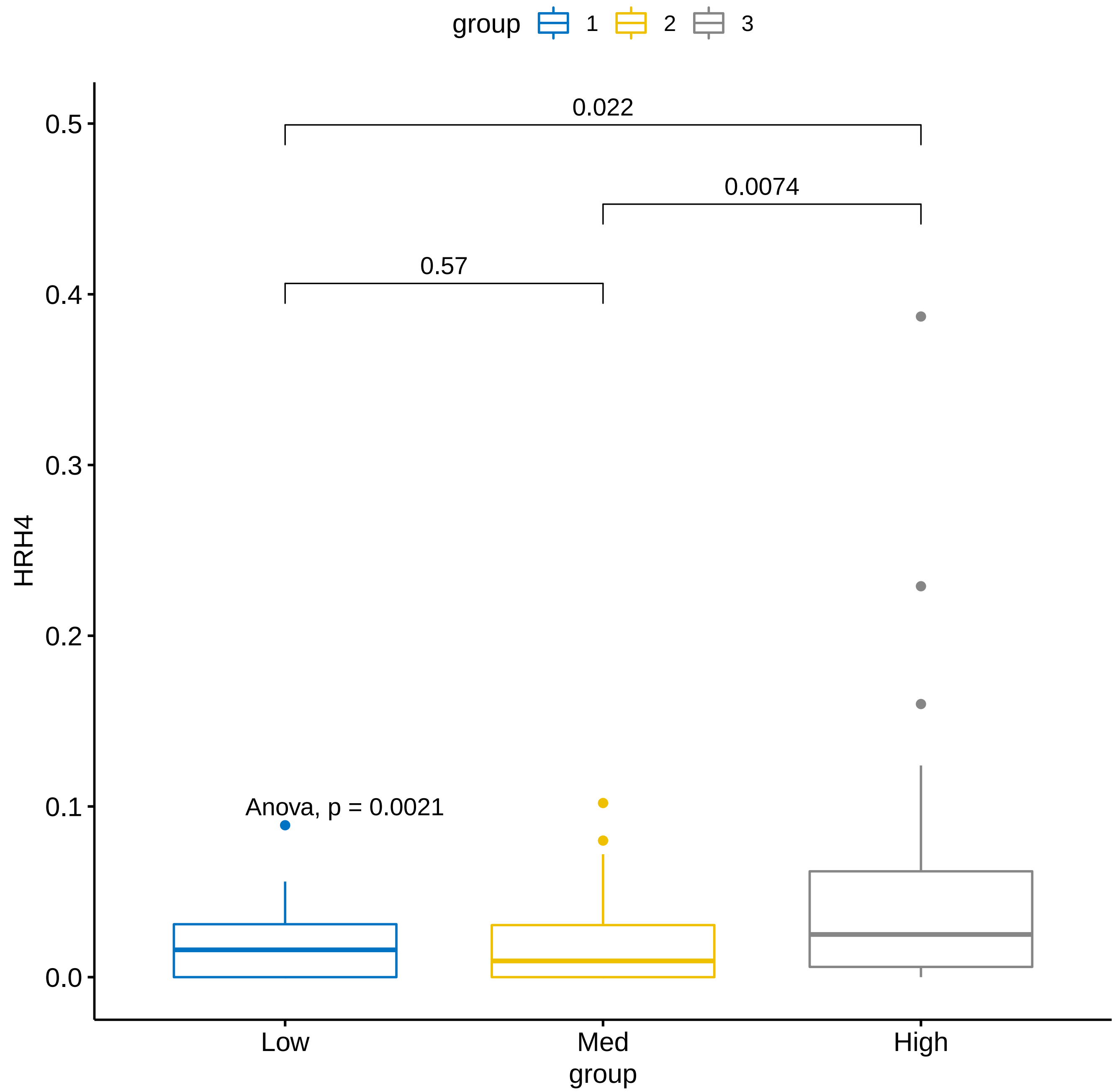
group
1
2
3
0.022
0.0074
0.57
0.5
0.4
0.3
0.2
0.1
0.0
HRH4
Anova, p = 0.0021
Low
Med
High
group

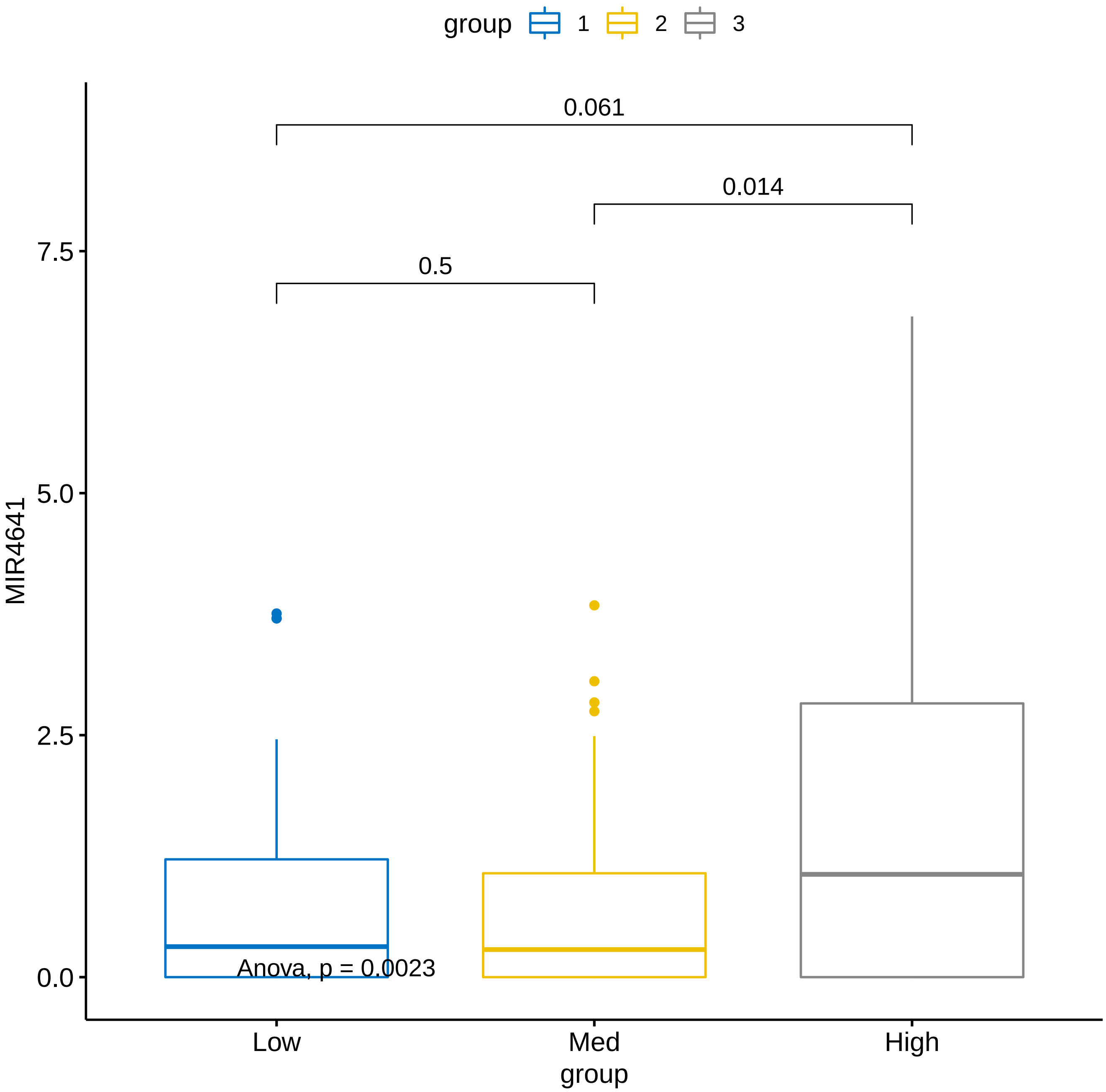
group
1
2
3
0.061
0.014
0.5
7.5
5.0
2.5
0.0
MIR4641
Anova, p = 0.0023
Low
Med
High
group

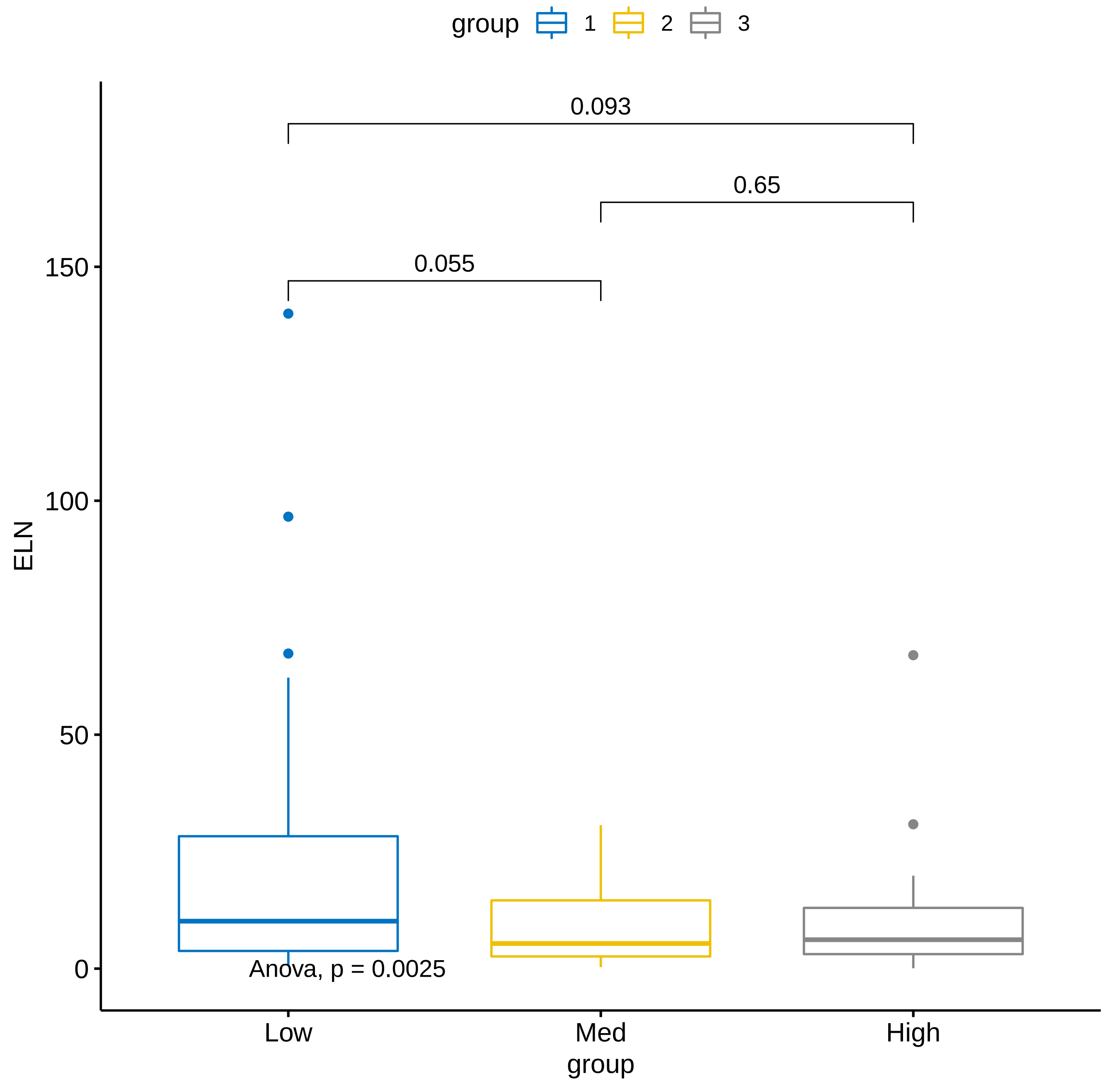

group 1 2 3
0.093
0.65
0.055
150
100
50
0
ELN
Anova, p = 0.0025
Low
Med
High
group

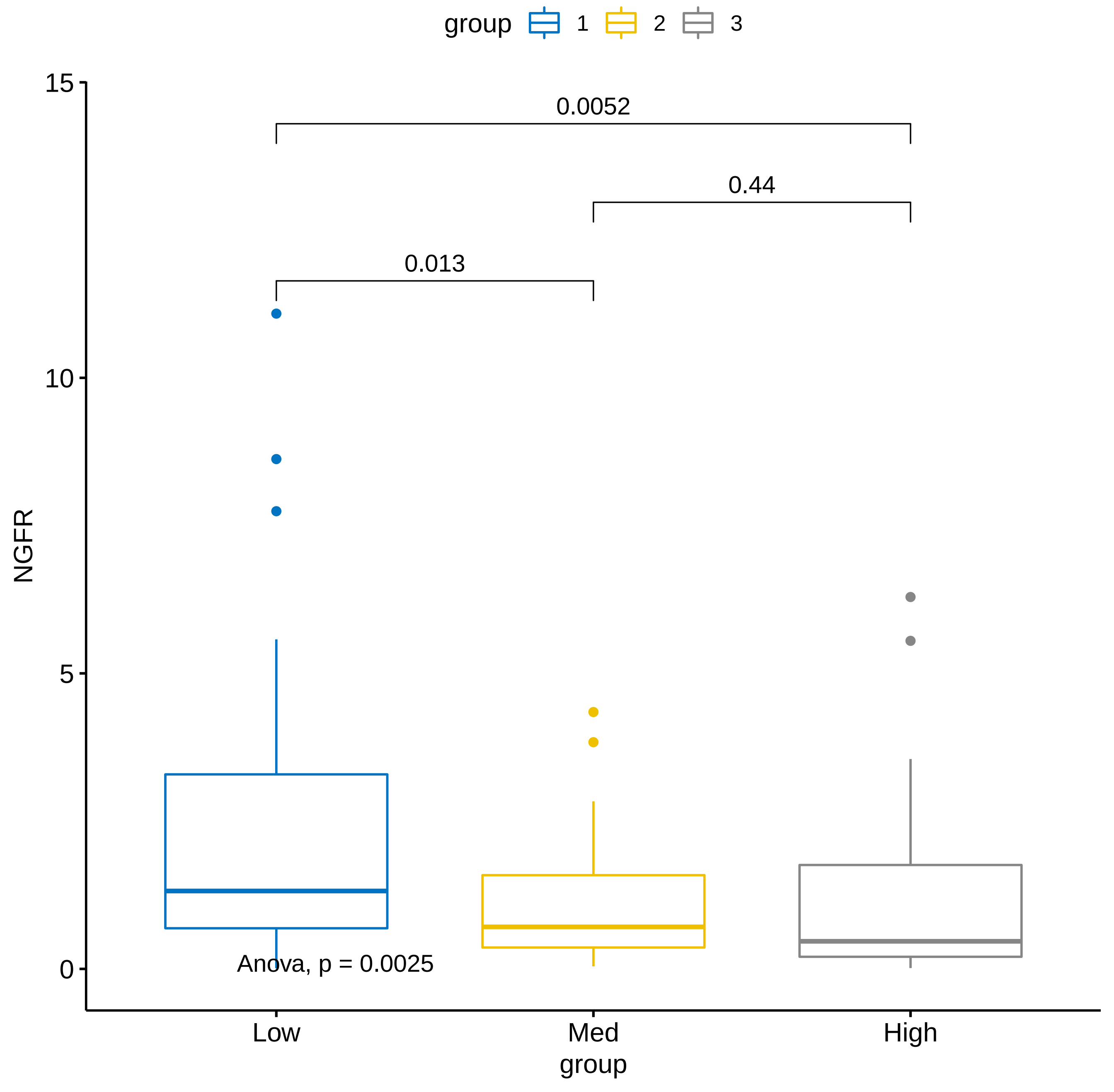
group 1 2 3
0.0052
0.44
0.013
NGFR
15
10
5
0
Anova, p = 0.0025
Low
Med
High
group

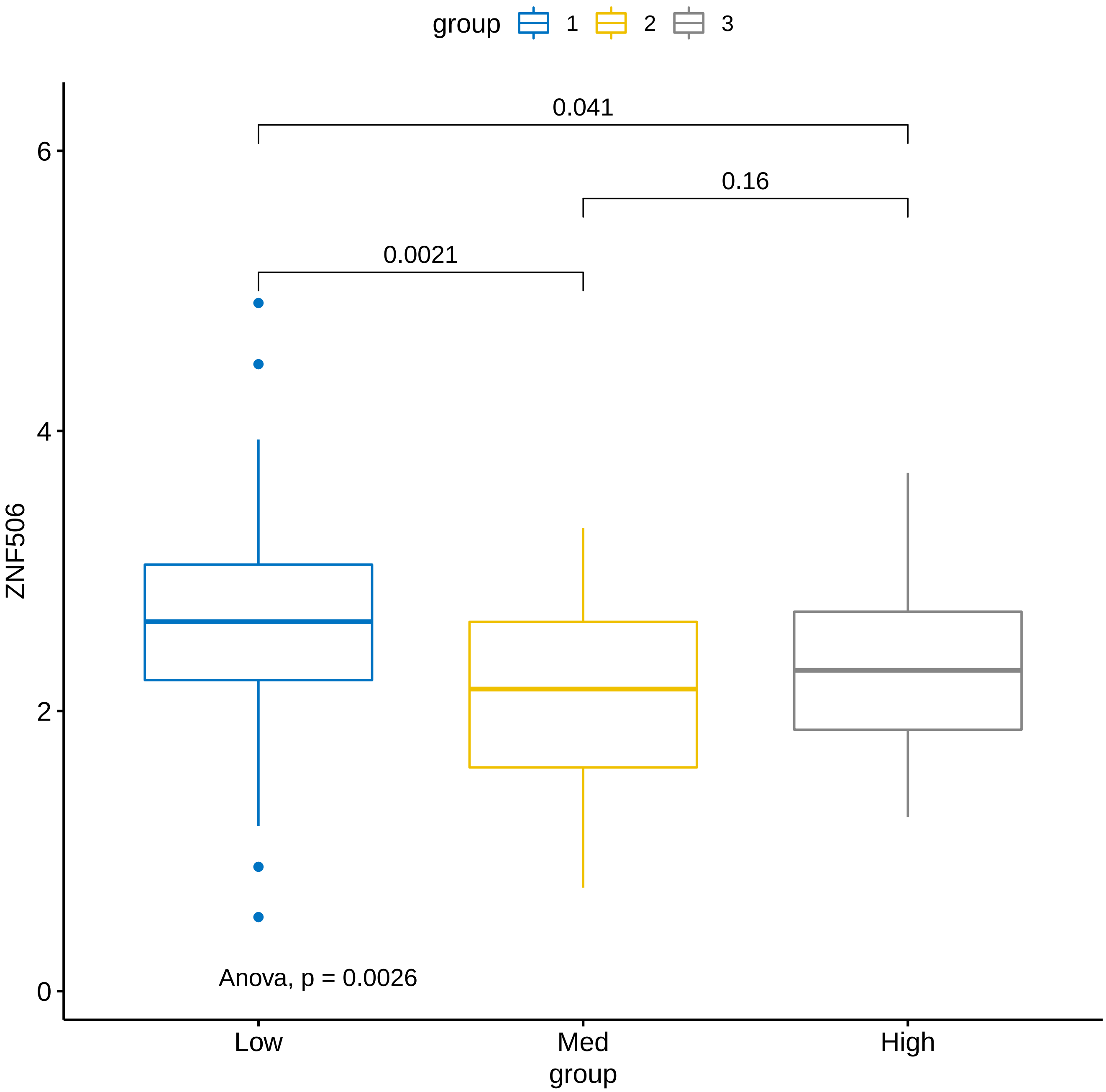
group
1
2
3
0.041
0.16
0.0021
6
4
2
0
ZNF506
Anova, p = 0.0026
Low
Med
High
group

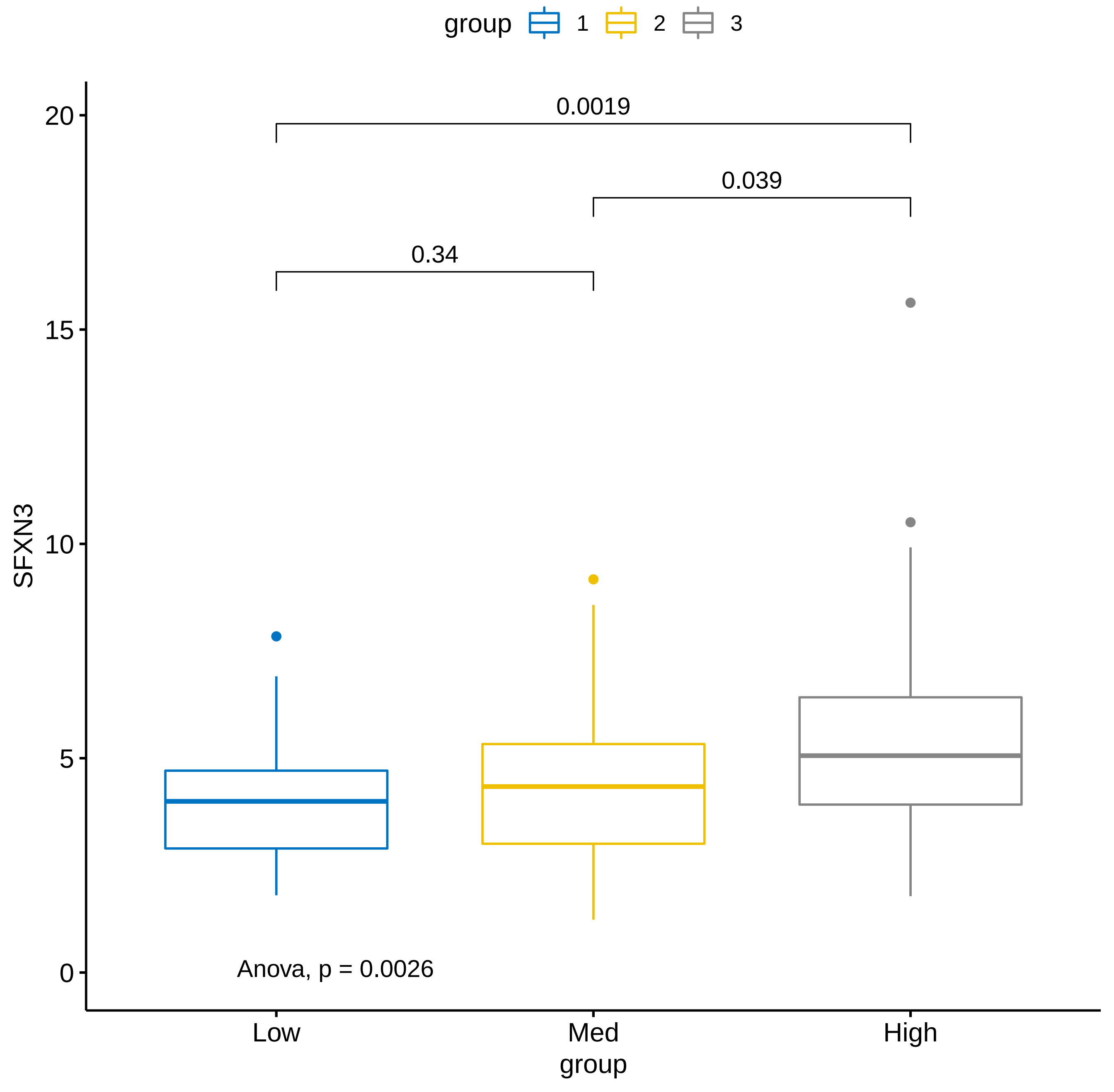

group
1
2
3
0.0019
0.039
0.34
20
15
10
5
0
SFXN3
Anova, p = 0.0026
Low
Med
High
group

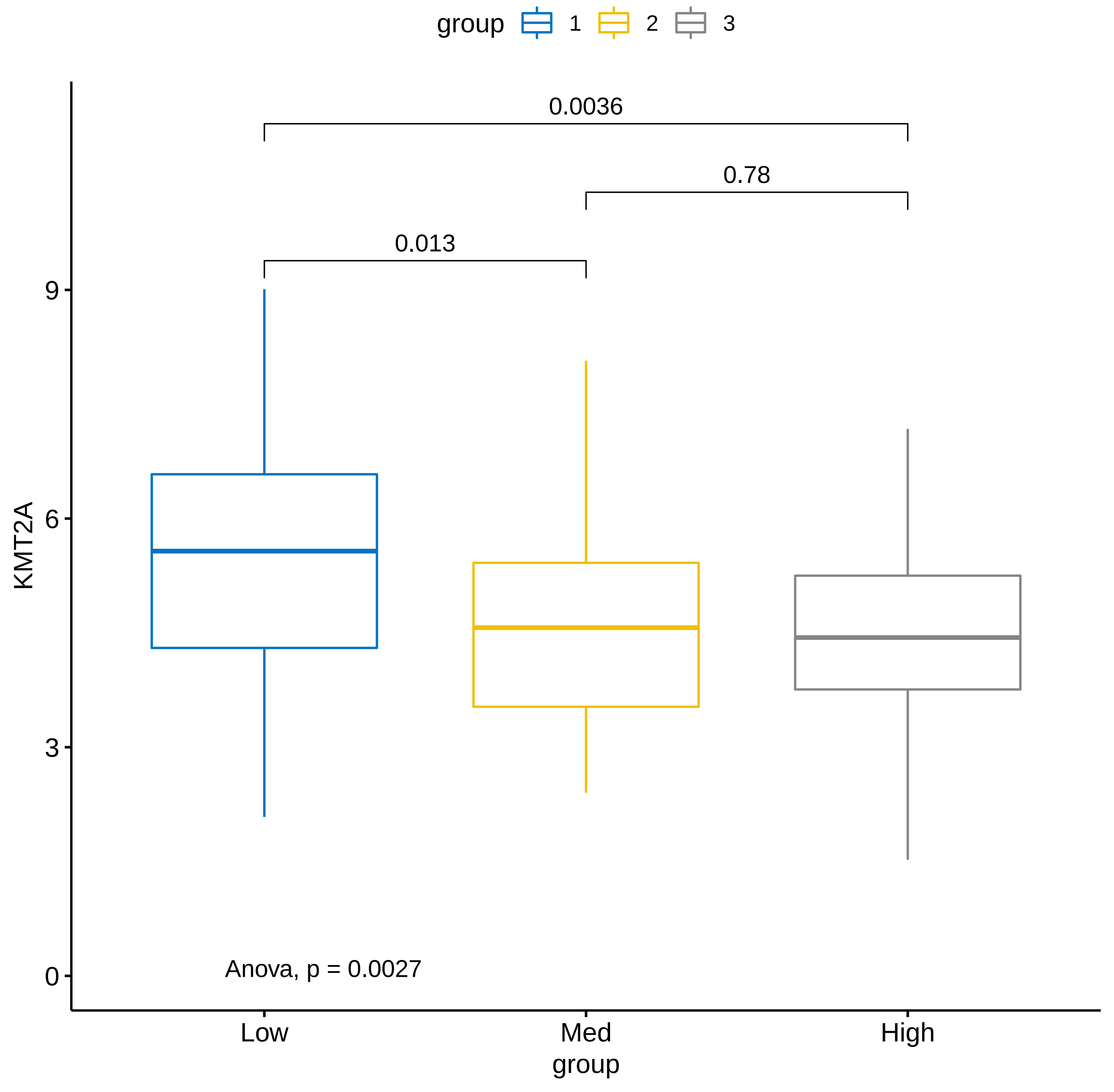
group
1
2
3
0.0036
0.78
0.013
9
6
3
0
KMT2A
Anova, p = 0.0027
Low
Med
High
group

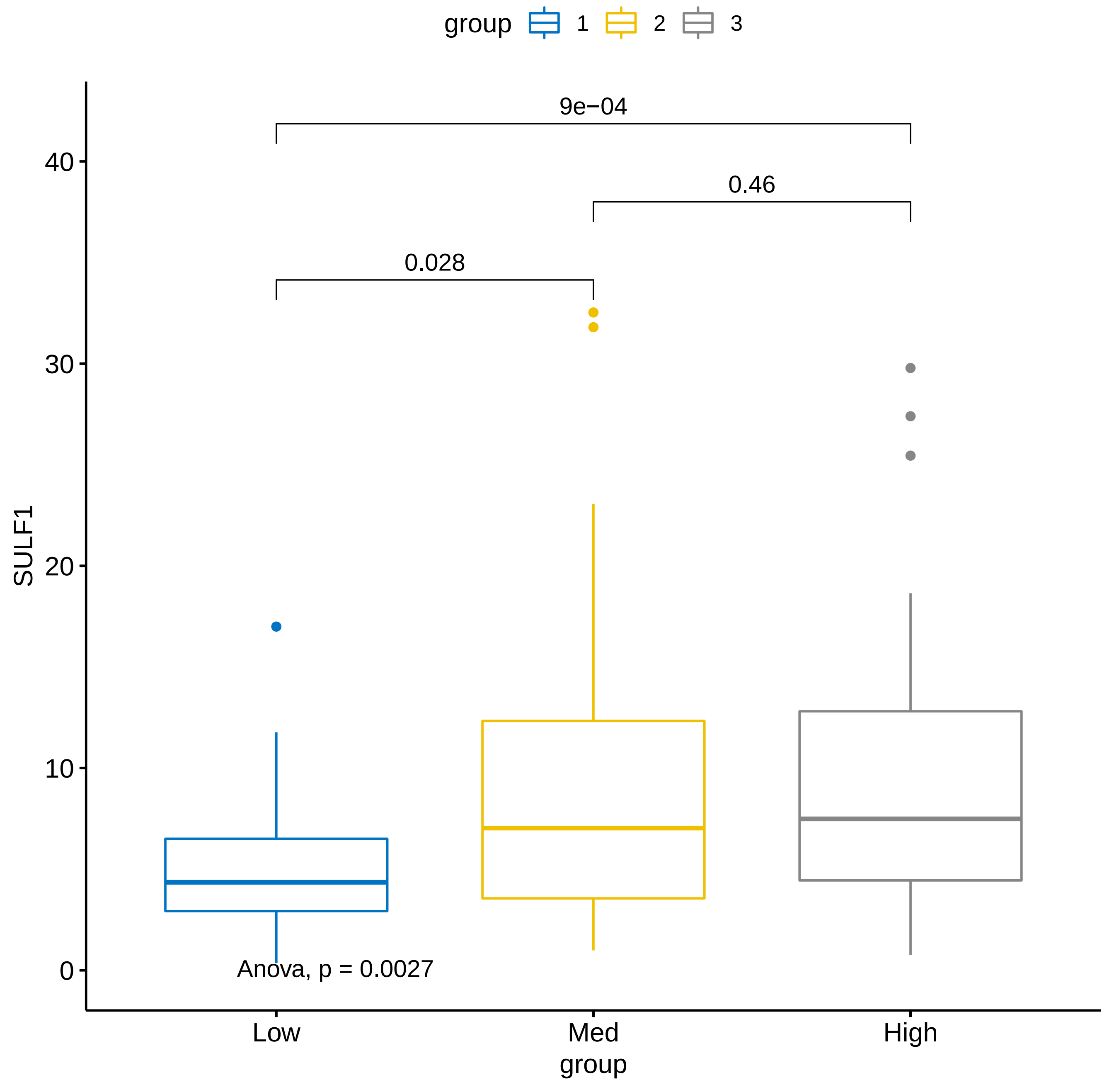

group 1 2 3
9e−04
0.46
0.028
40
30
20
10
0
SULF1
Anova, p = 0.0027
Low
Med
High
group

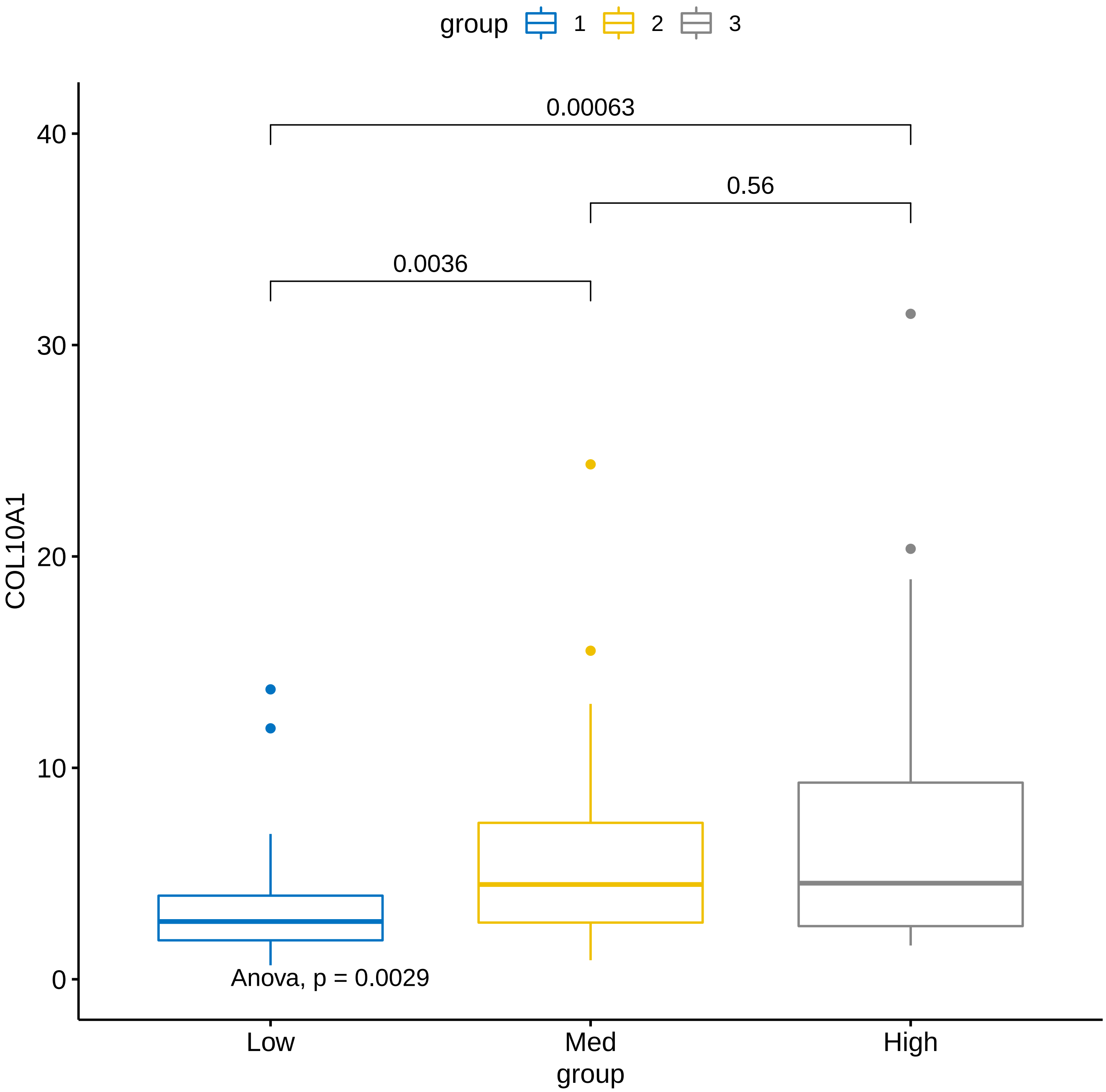
group
1
2
3
0.00063
0.56
0.0036
40
30
20
10
0
COL10A1
Anova, p = 0.0029
Low
Med
High
group

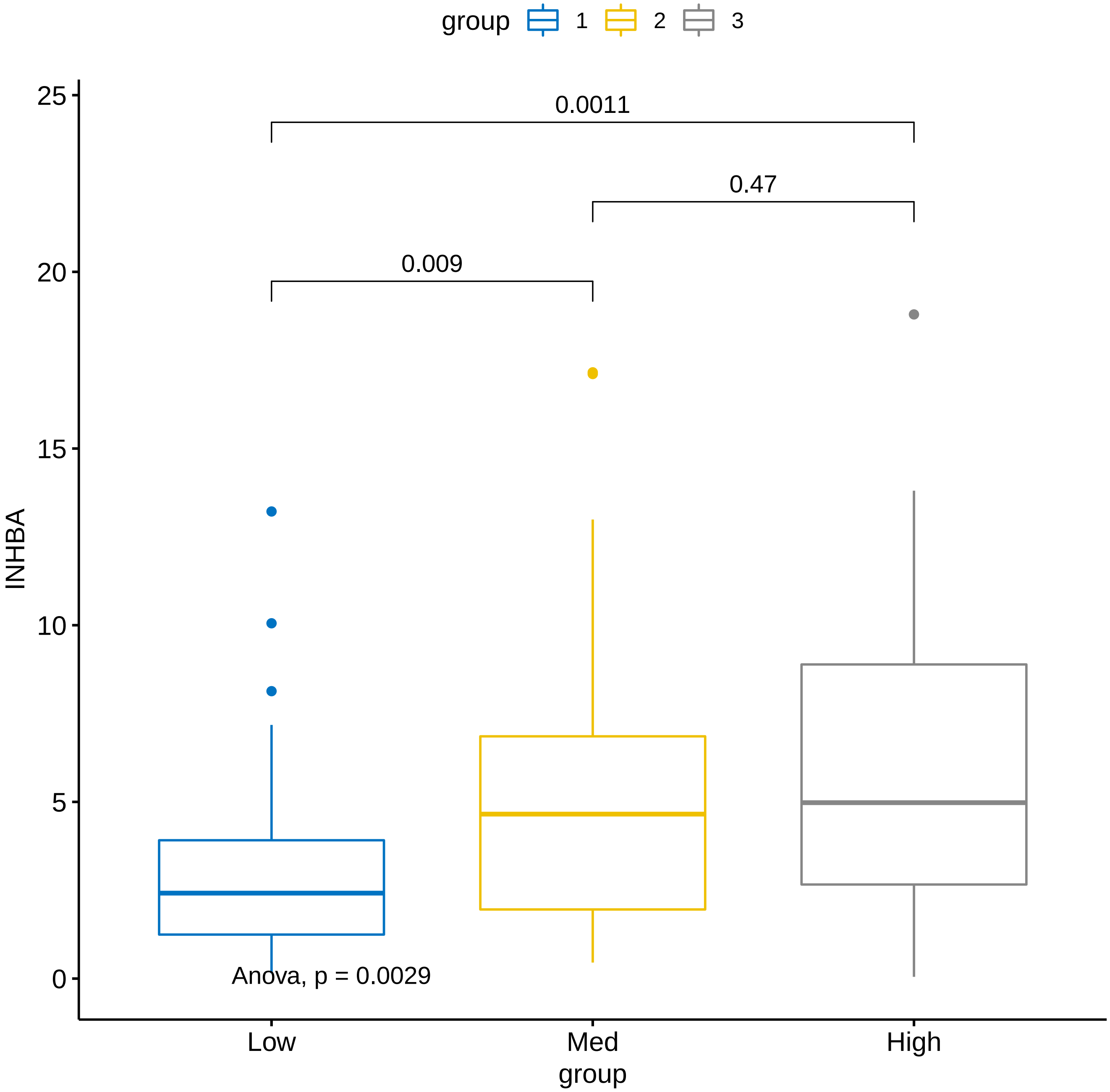
group 1 2 3
0.0011
0.47
0.009
25
20
15
10
5
0
INHBA
Anova, p = 0.0029
Low
Med
High
group

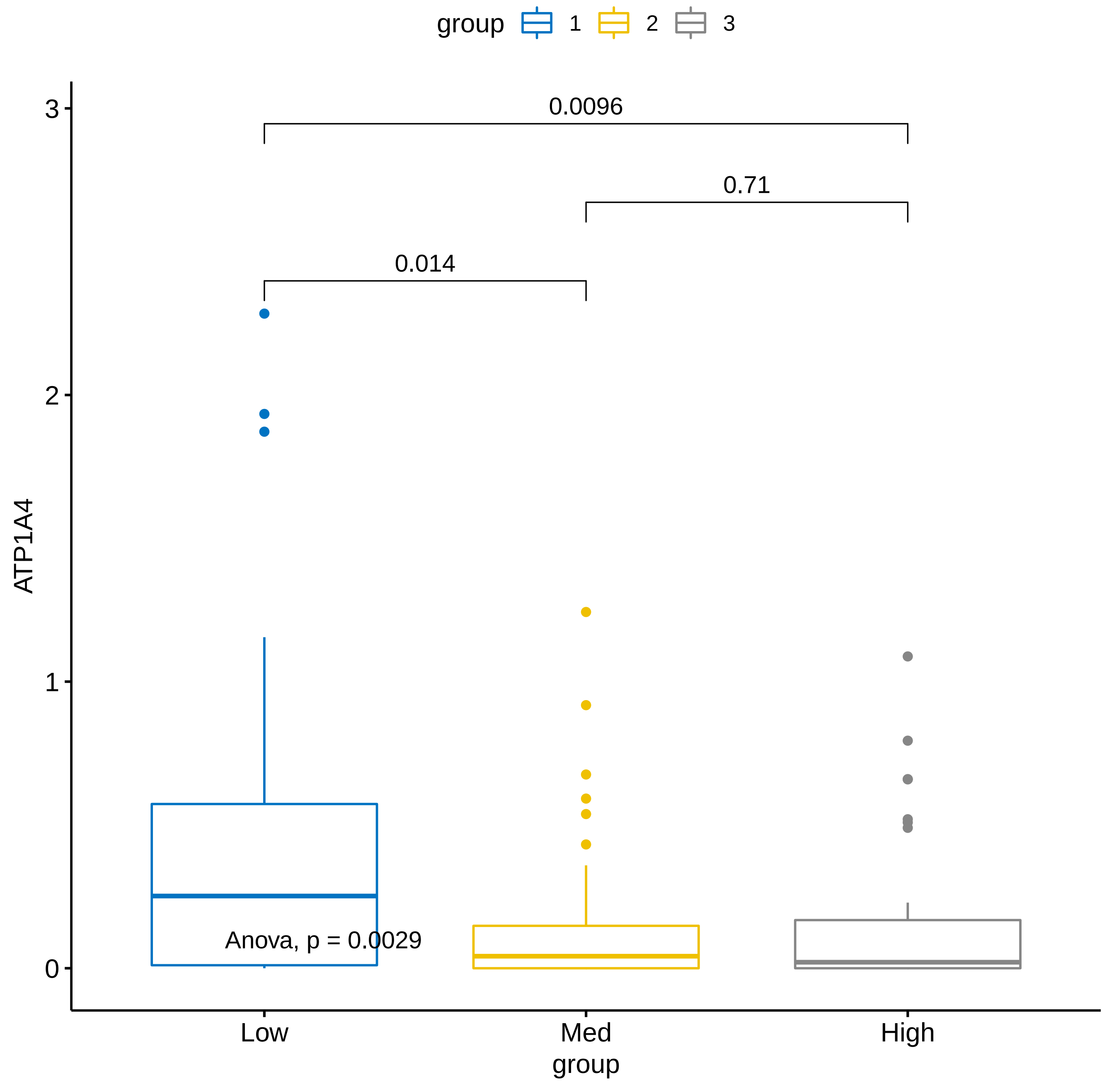

group 1 2 3
0.0096
0.71
0.014
ATP1A4
3
2
1
0
Anova, p = 0.0029
Low
Med
High
group

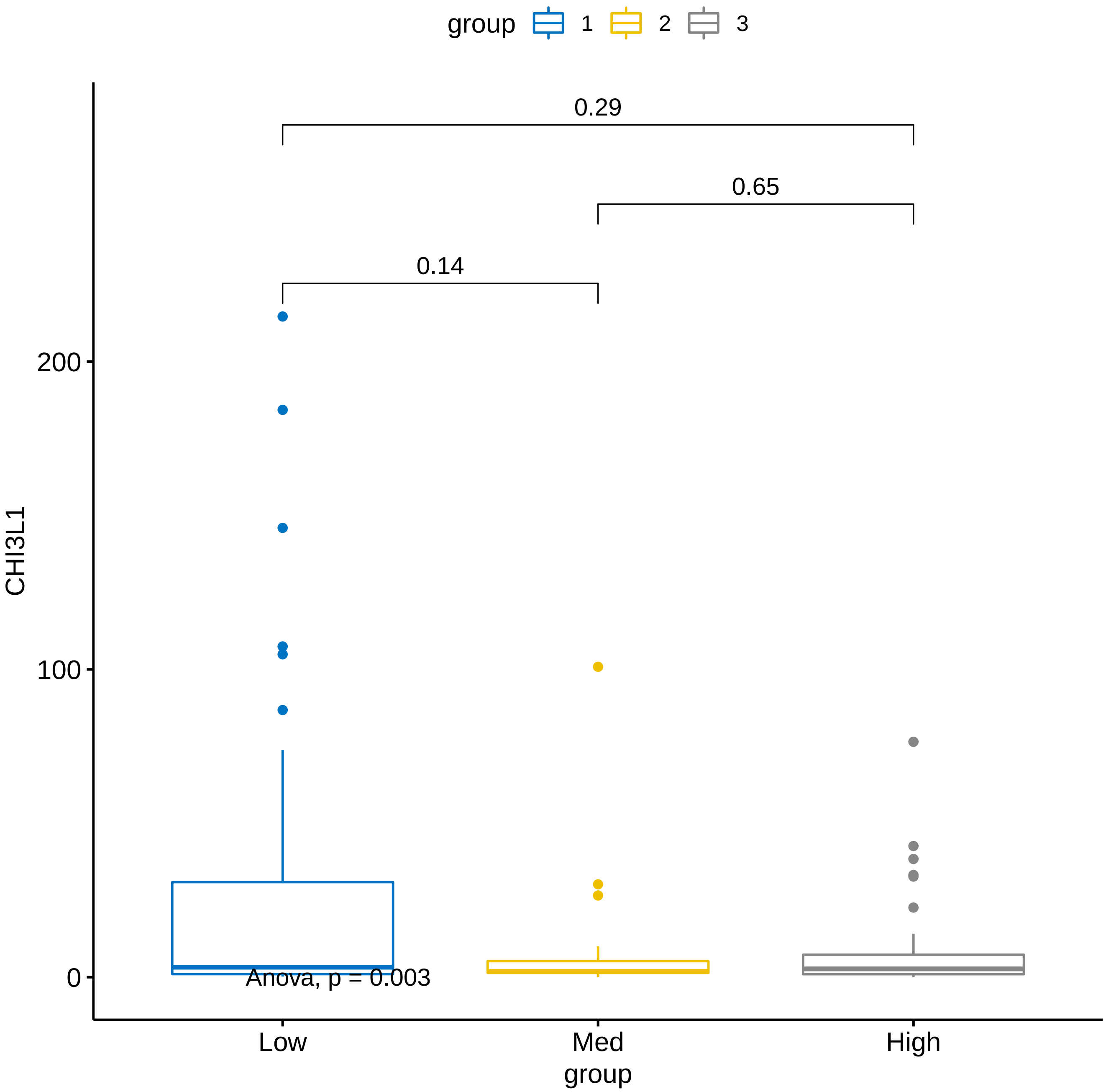
group
1
2
3
0.29
0.65
0.14
CHI3L1
200
100
0
Anova, p = 0.003
Low
Med
High
group

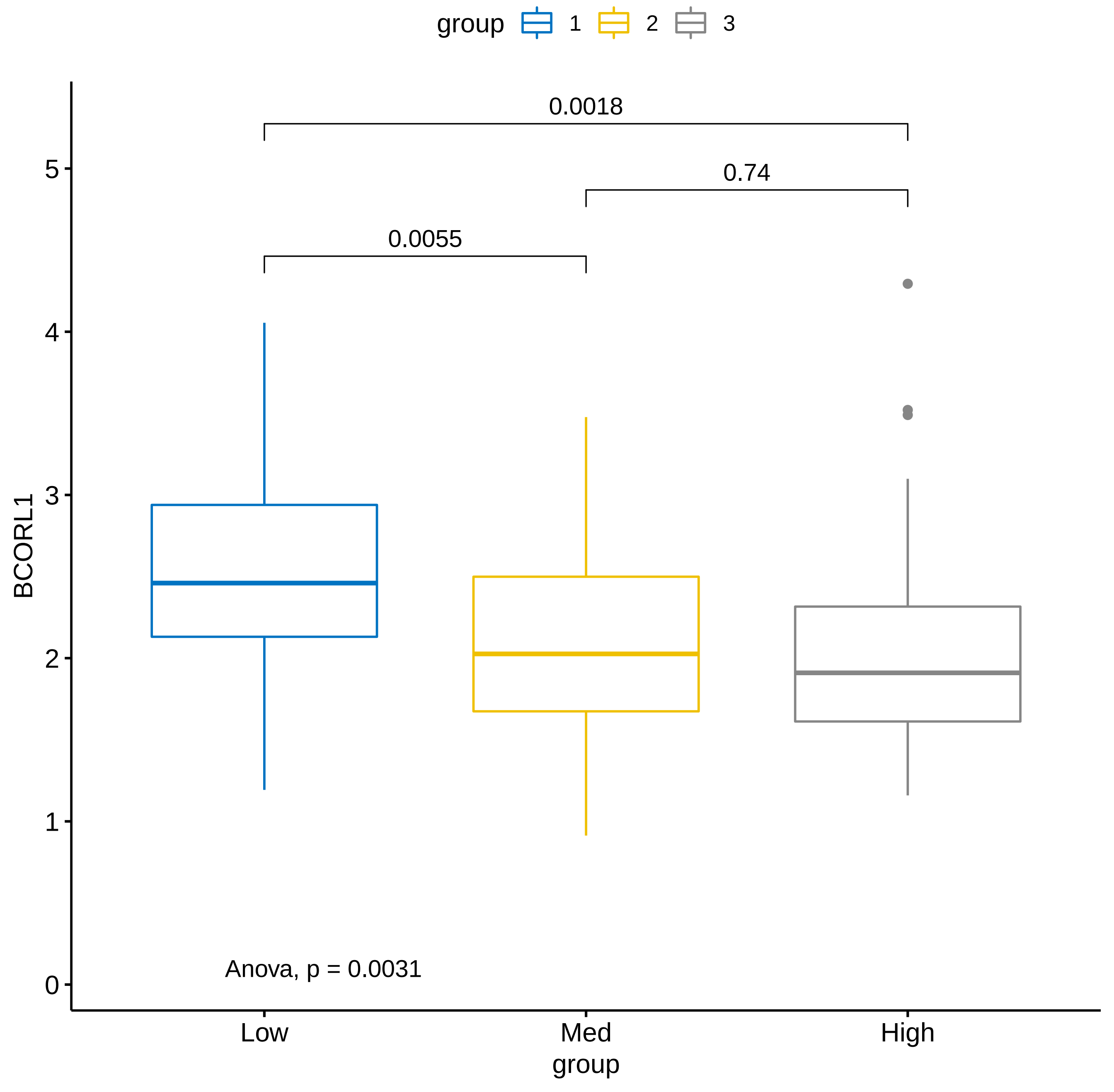
group
1
2
3
0.0018
0.74
0.0055
5
4
3
2
1
0
BCORL1
Anova, p = 0.0031
Low
Med
High
group

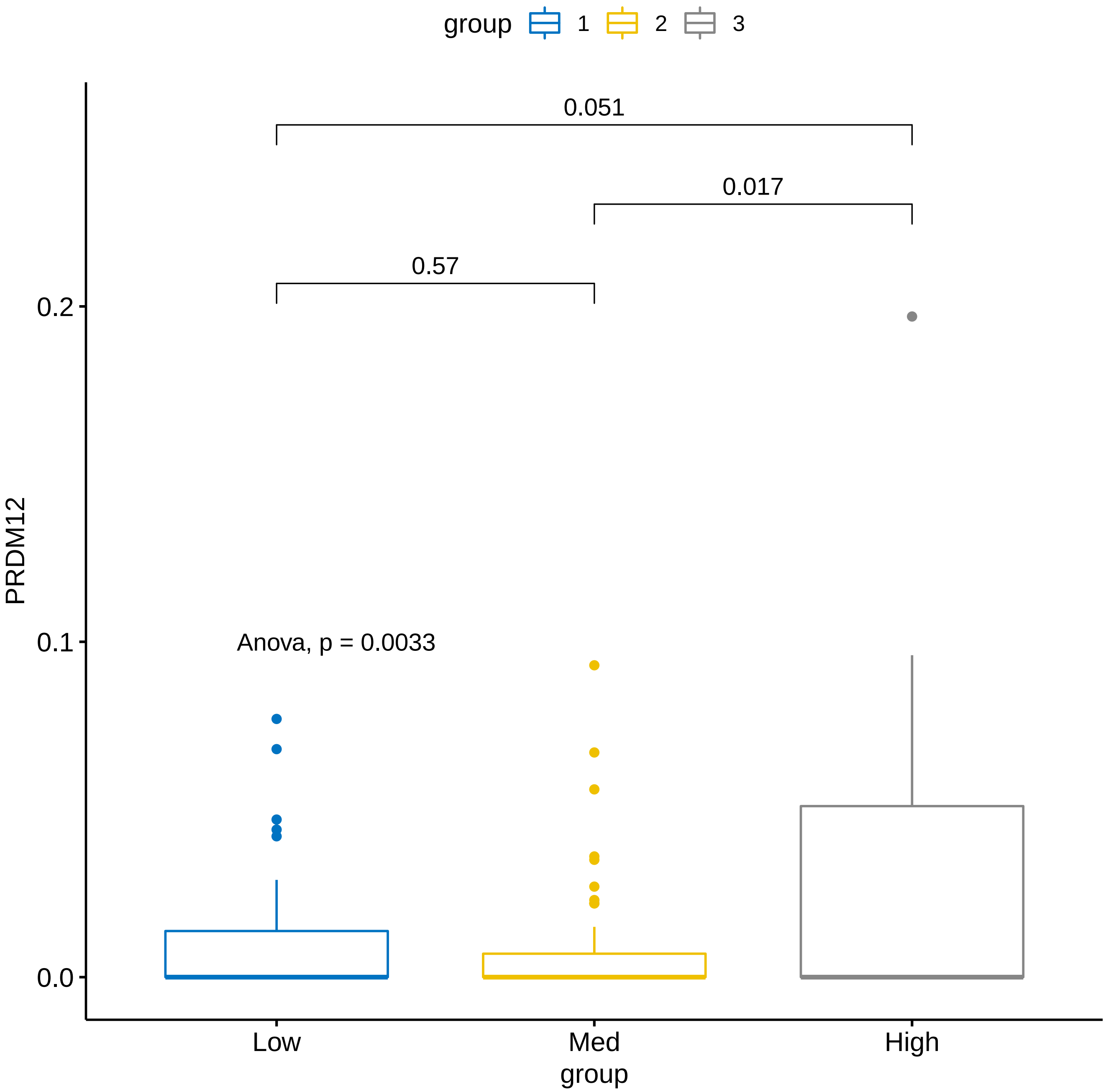
group 1 2 3
0.051
0.017
0.57
Anova, p = 0.0033
0.2
0.1
0.0
PRDM12
Low
Med
High
group

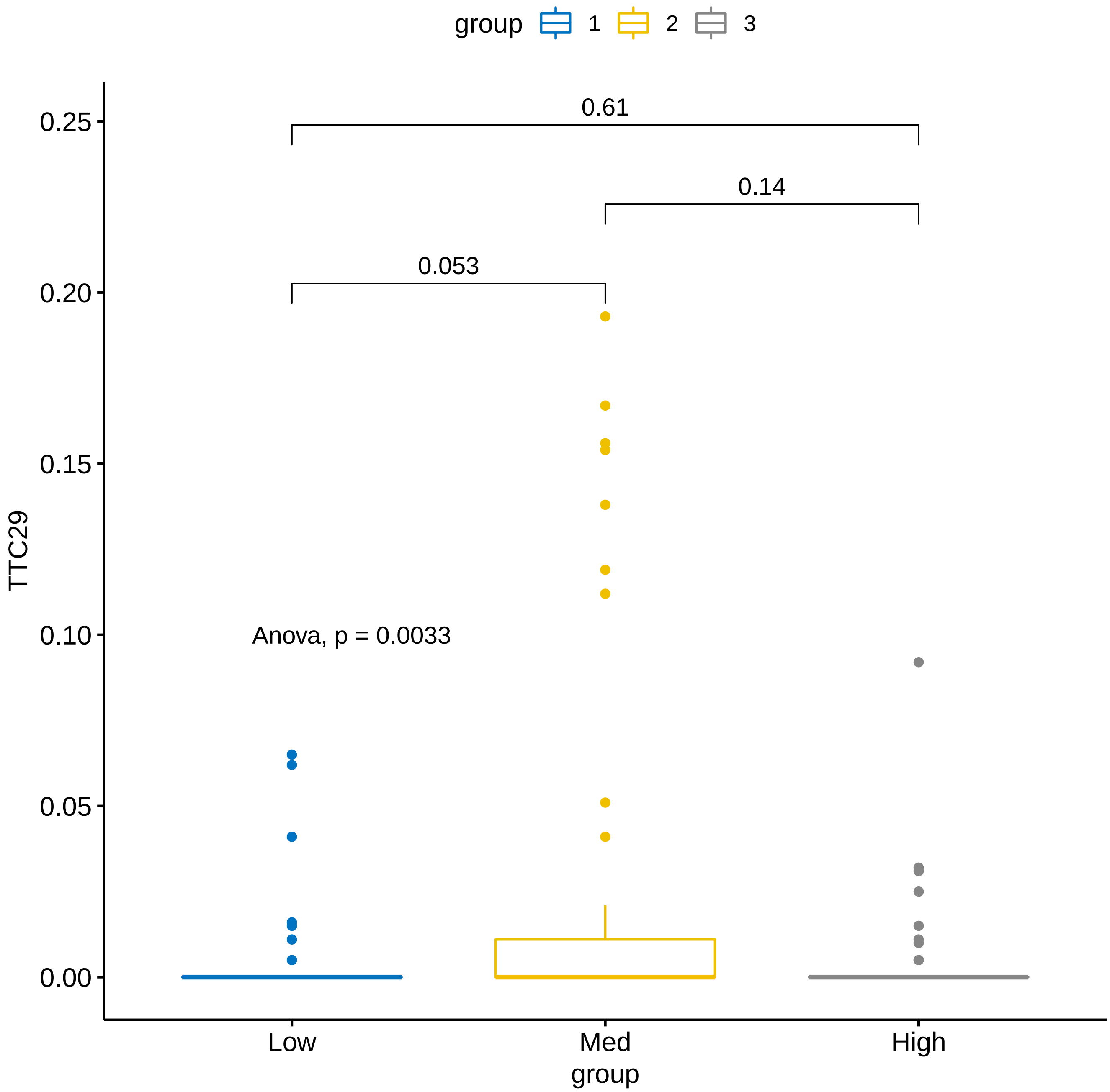
group
1
2
3
0.61
0.14
0.053
0.25
0.20
0.15
0.10
0.05
0.00
TTC29
Anova, p = 0.0033
Low
Med
High
group

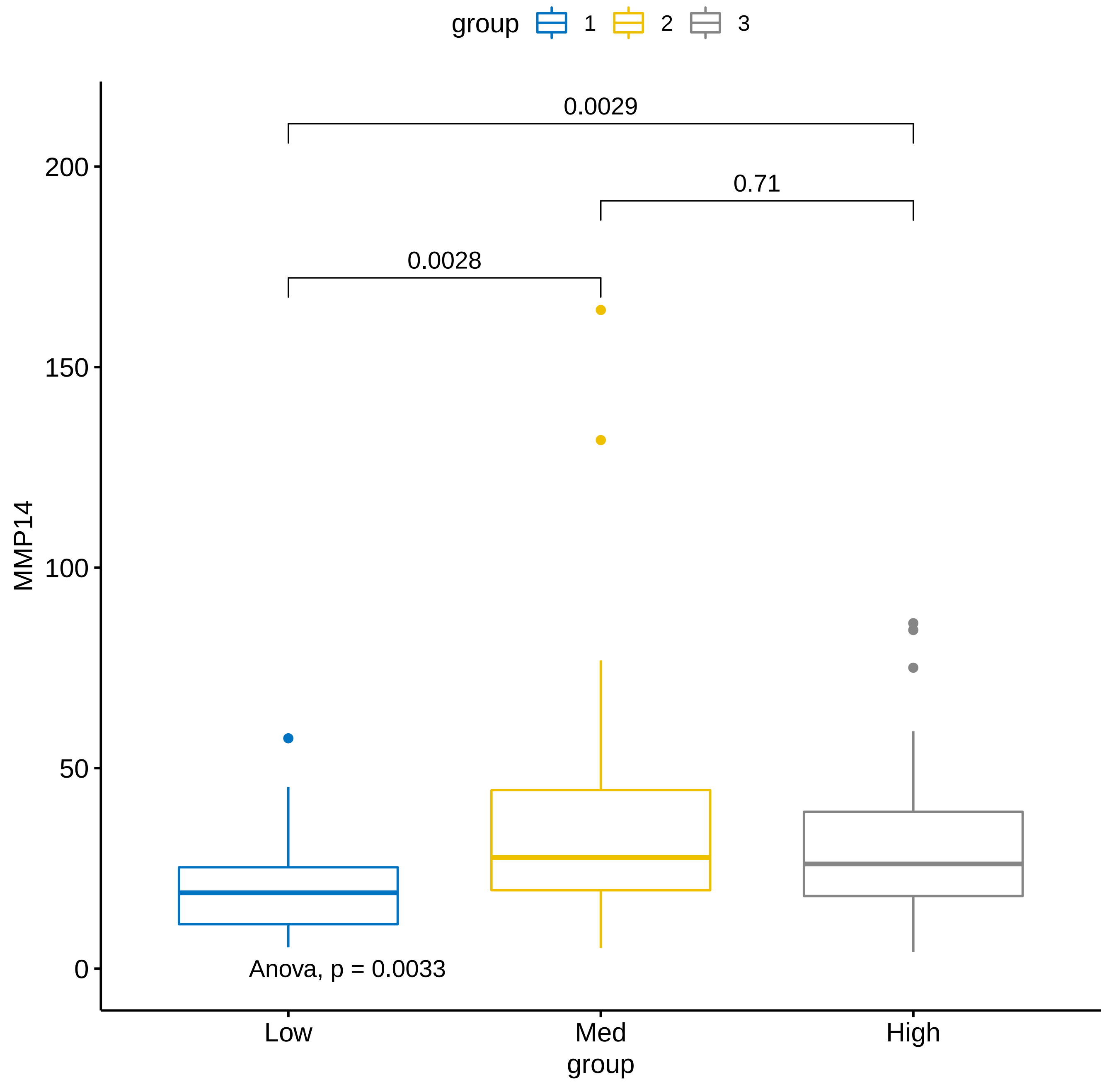
group
1
2
3
0.0029
0.71
0.0028
200
150
100
50
0
MMP14
Anova, p = 0.0033
Low
Med
High
group

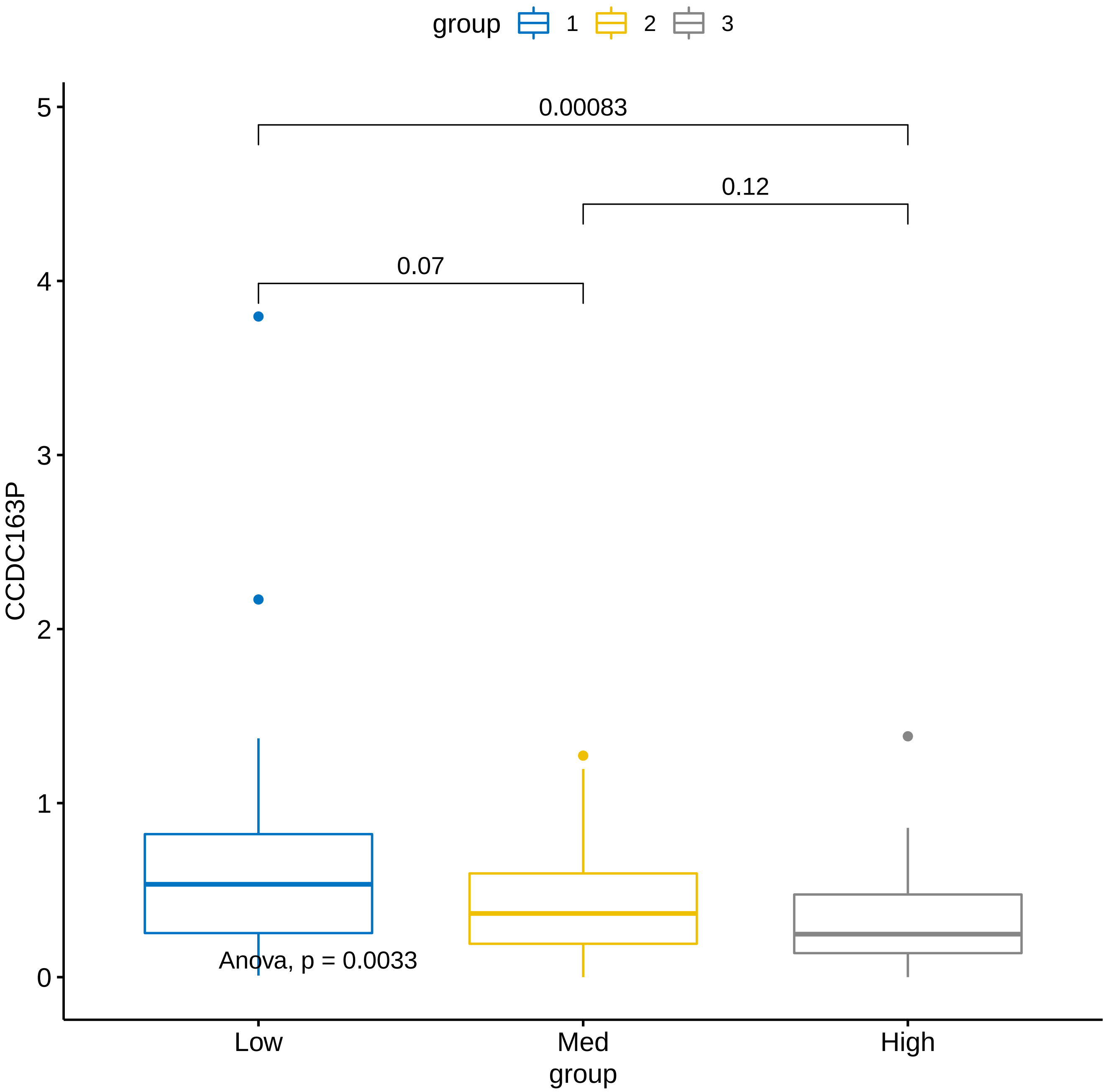

group
1
2
3
0.00083
0.12
0.07
Anova, p = 0.0033
CCDC163P
5
4
3
2
1
0
Low
Med
High
group

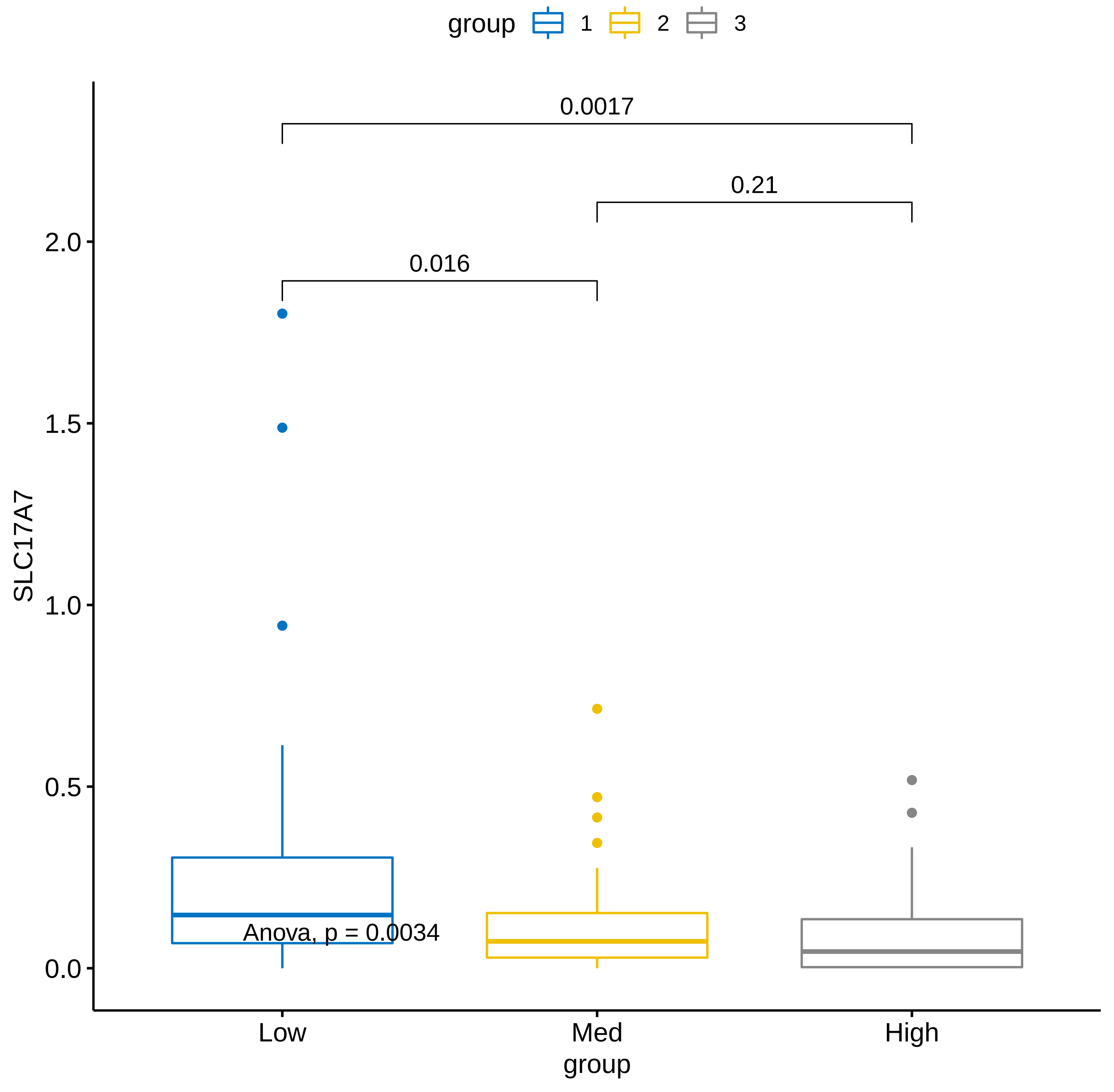

group
1
2
3
0.0017
0.21
0.016
SLC17A7
2.0
1.5
1.0
0.5
0.0
Anova, p = 0.0034
Low
Med
High
group

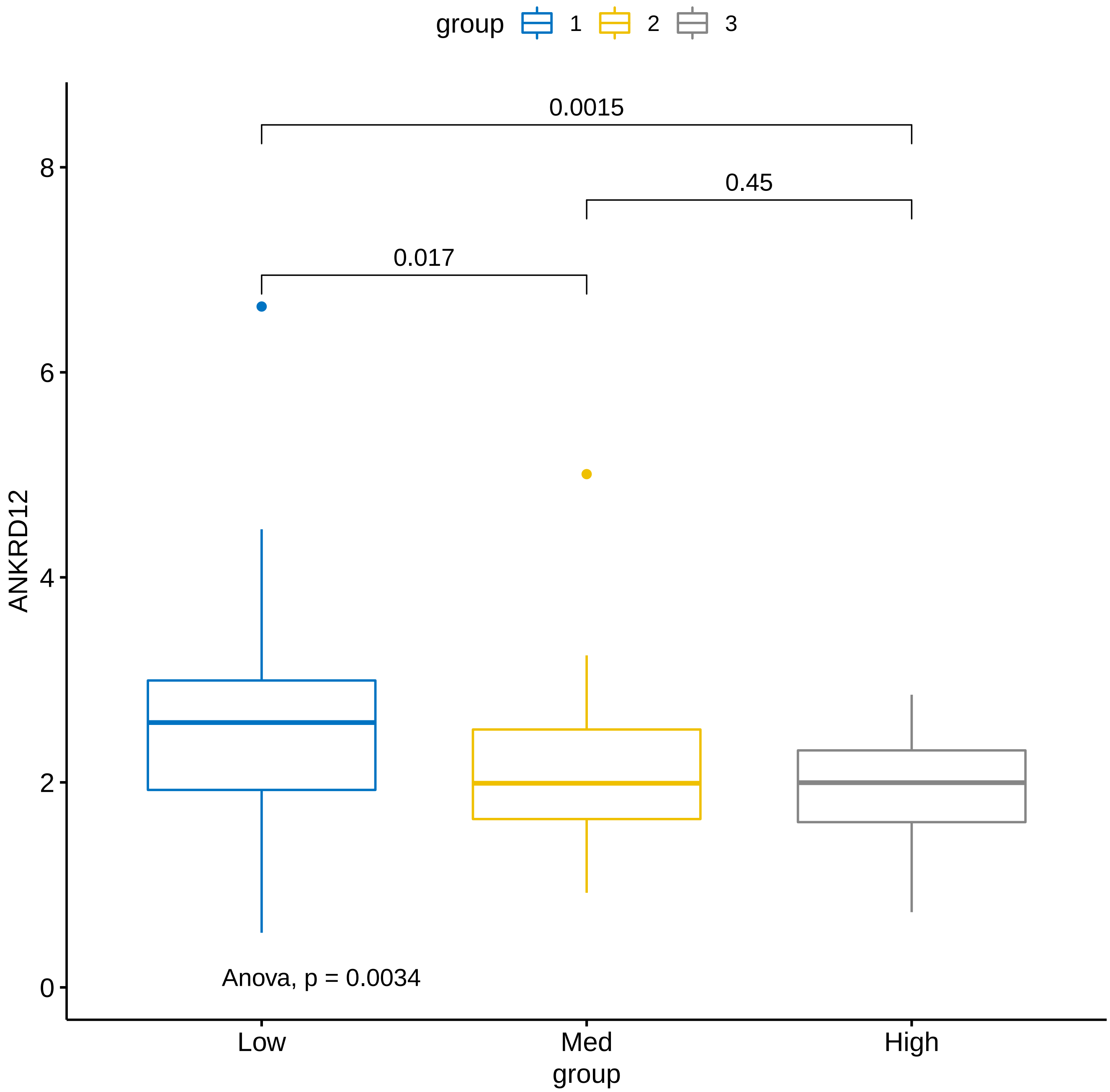
group
1
2
3
0.0015
0.45
0.017
8
6
4
2
0
ANKRD12
Anova, p = 0.0034
Low
Med
High
group

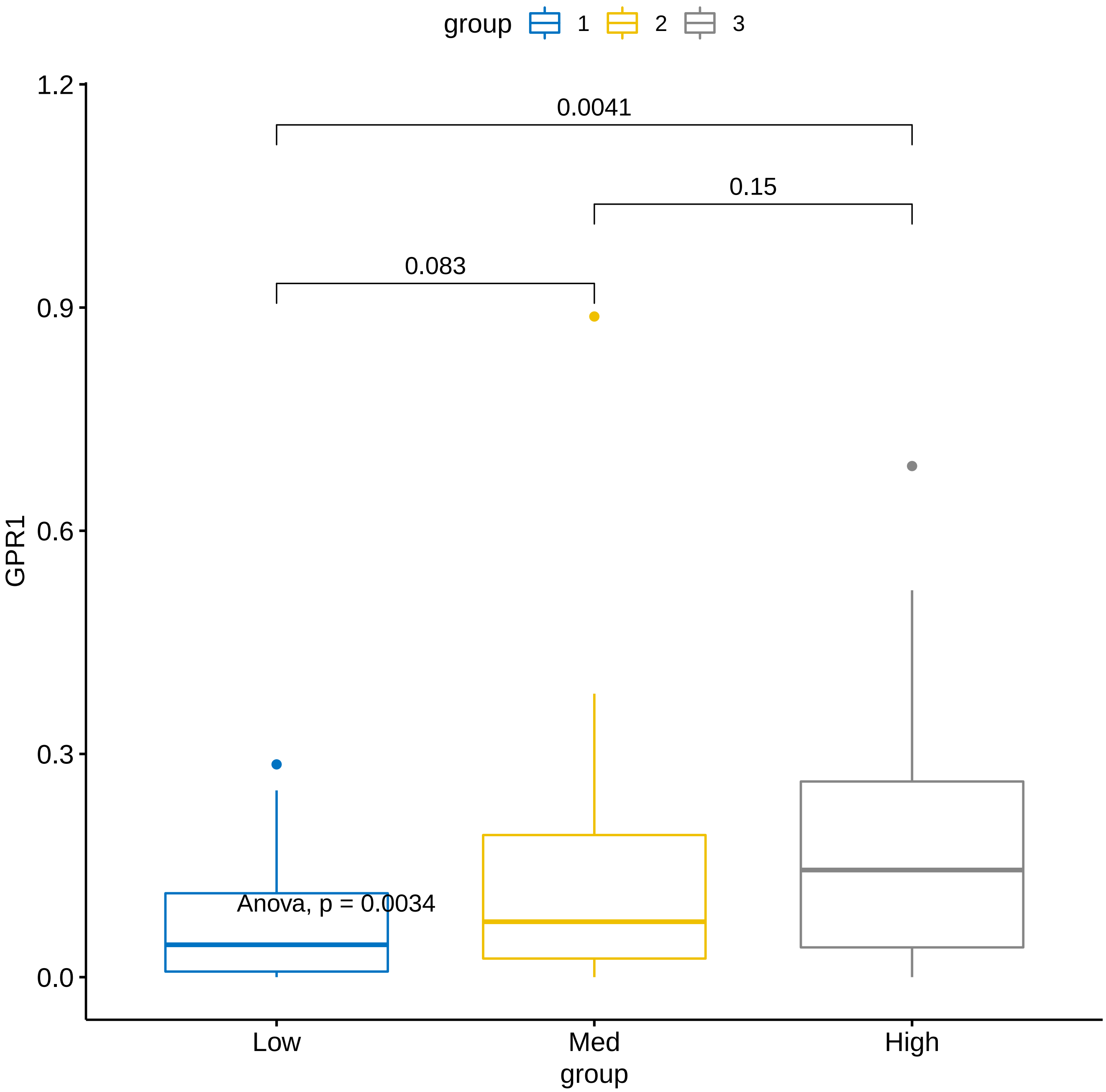

group 1 2 3
0.0041
0.15
0.083
Anova, p = 0.0034
1.2
0.9
0.6
0.3
0.0
GPR1
Low
Med
High
group

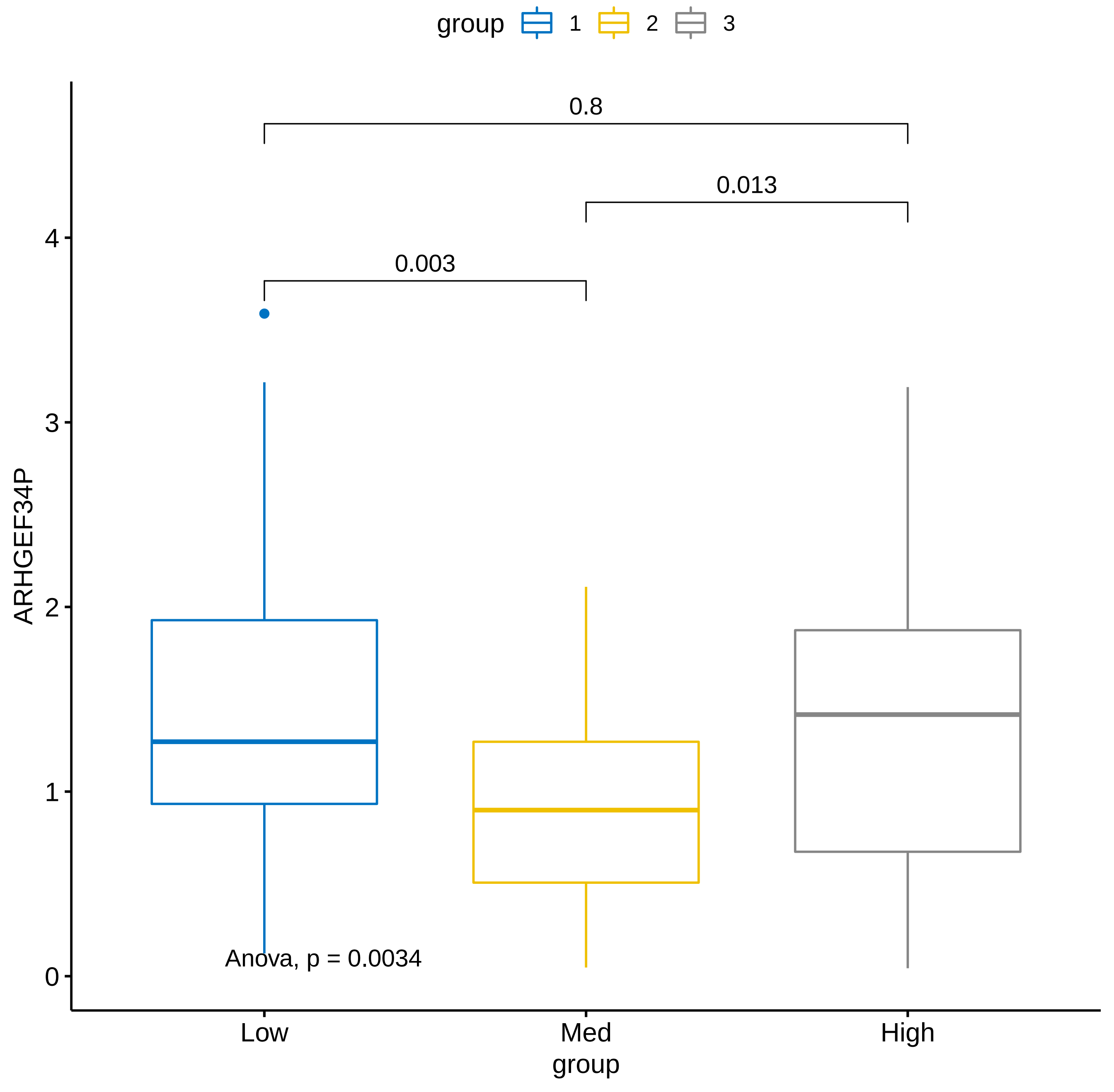
group
1
2
3
0.8
0.013
0.003
4
3
2
1
0
ARHGEF34P
Anova, p = 0.0034
Low
Med
High
group

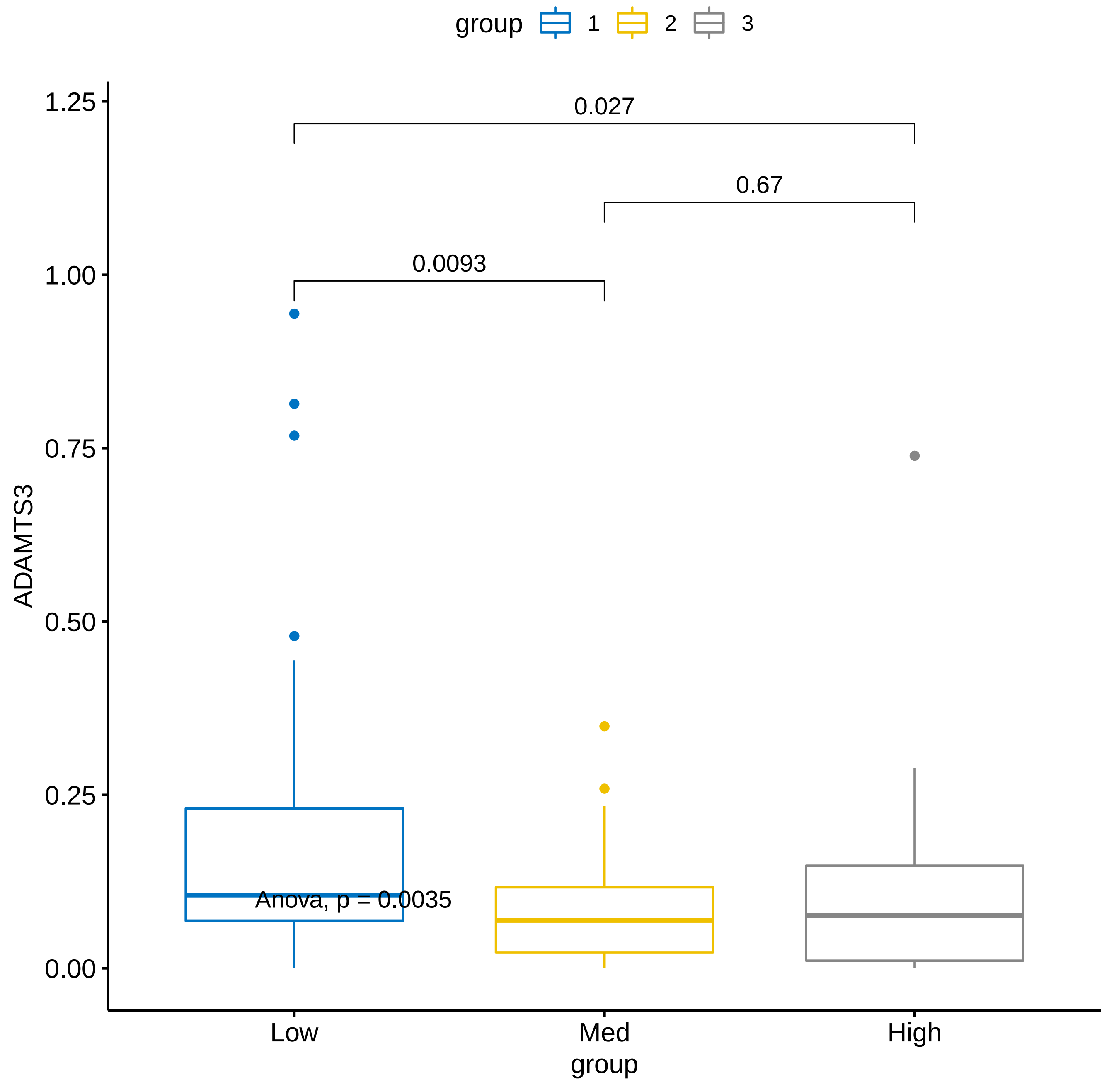
group 1 2 3
0.027
0.67
0.0093
1.25
1.00
0.75
0.50
0.25
0.00
ADAMTS3
Anova, p = 0.0035
Low
Med
High
group

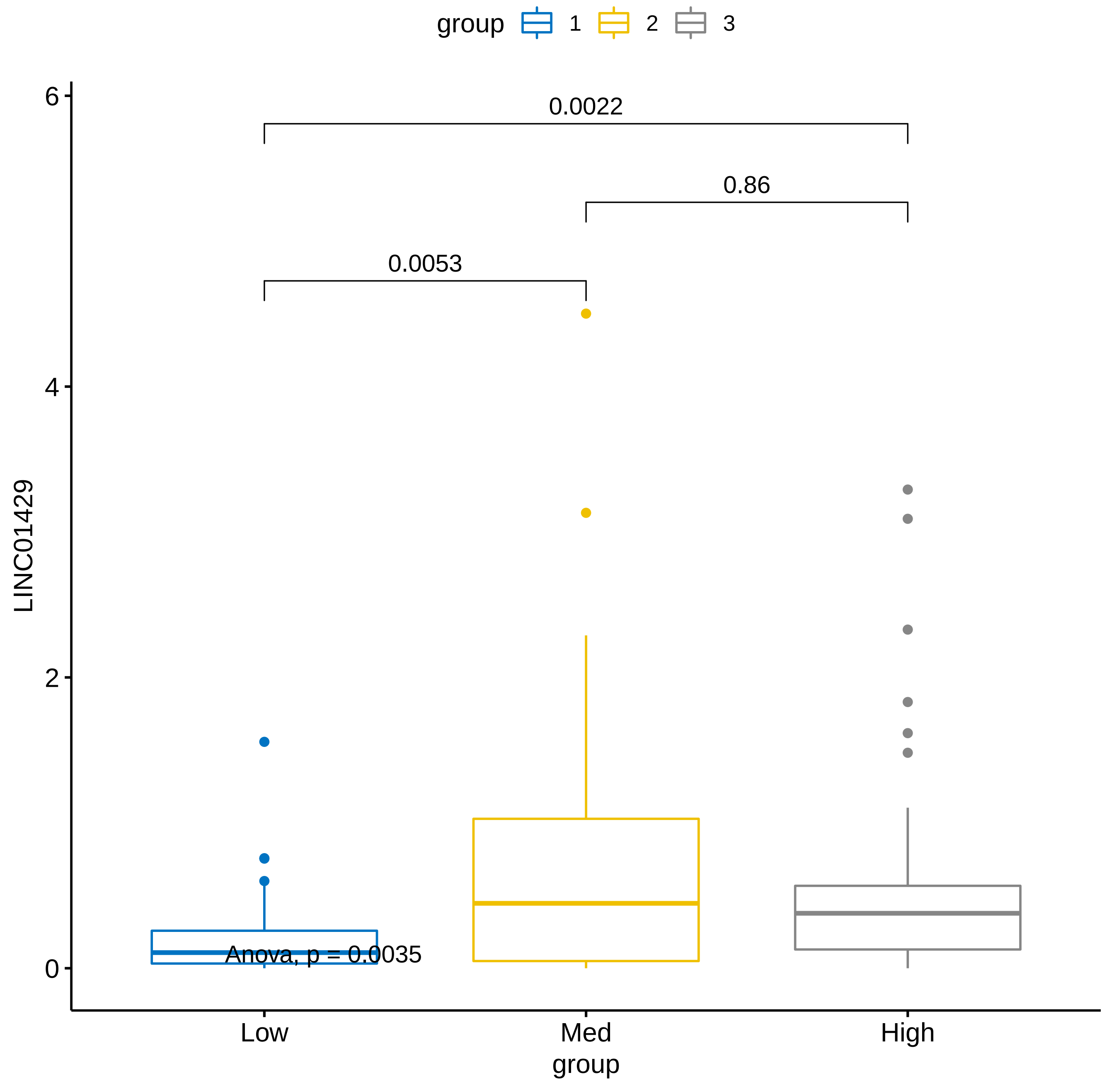
group
1
2
3
0.0022
0.86
0.0053
LINC01429
6
4
2
0
Anova, p = 0.0035
Low
Med
High
group

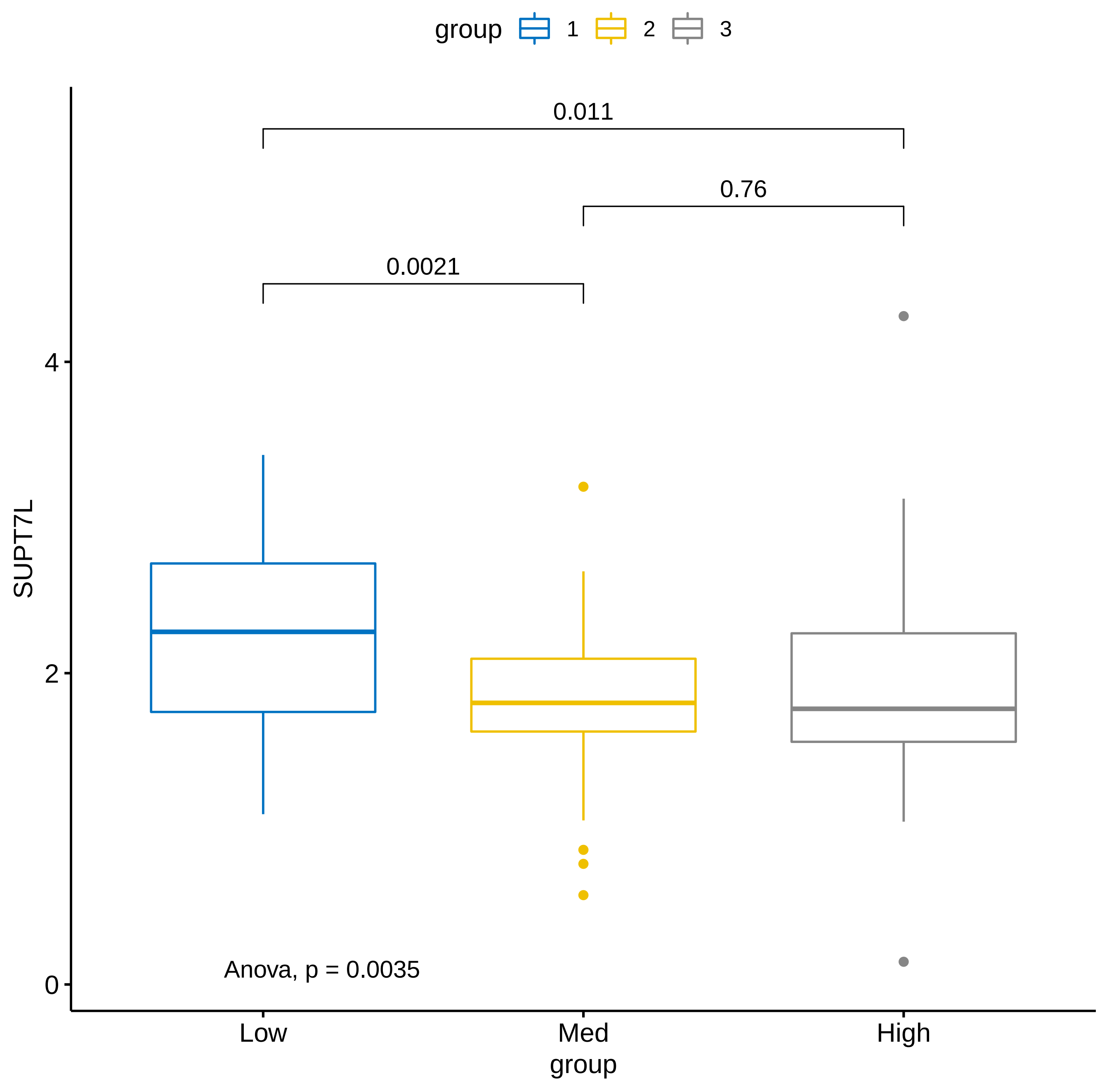

group
1
2
3
0.011
0.76
0.0021
SUPT7L
4
2
0
Anova, p = 0.0035
Low
Med
High
group

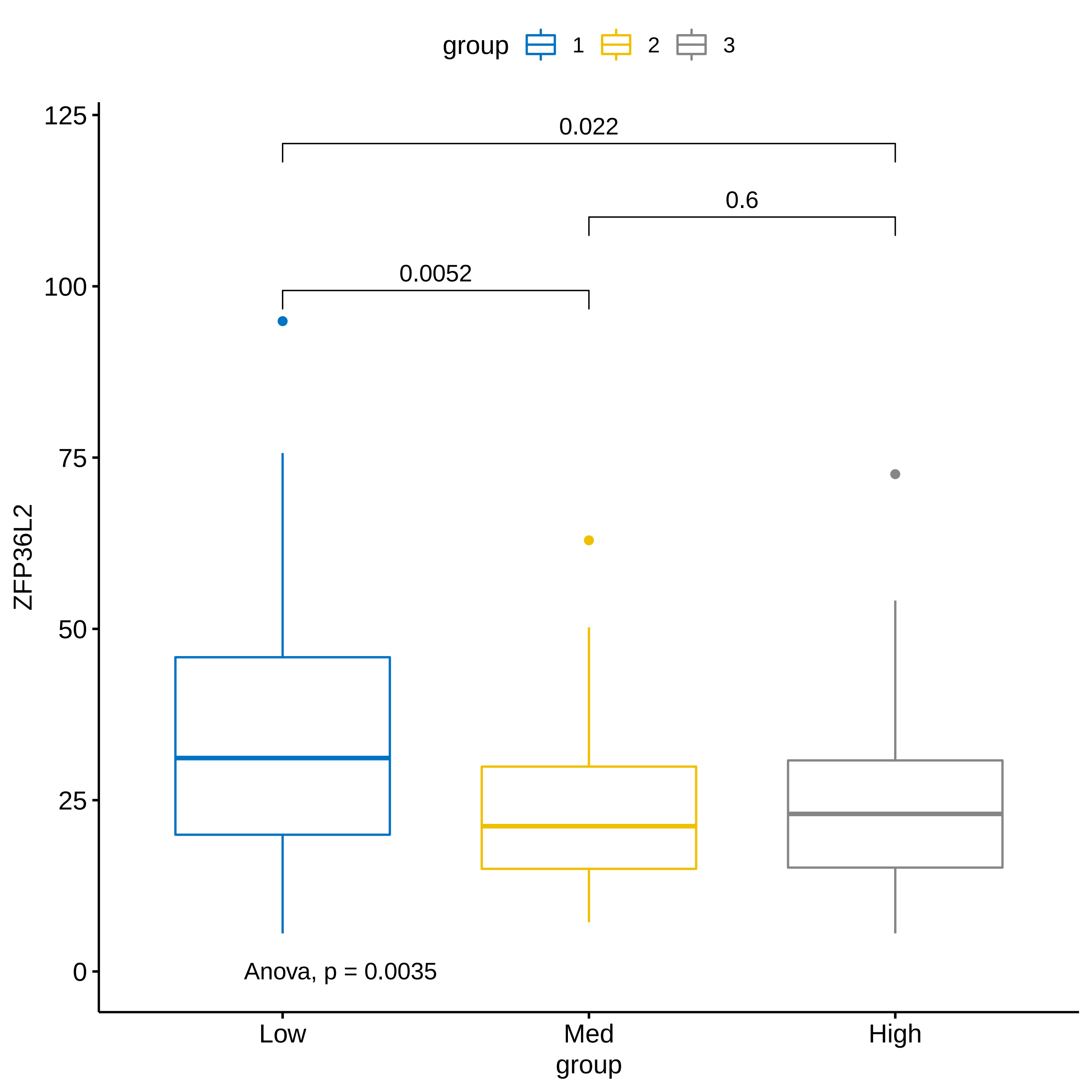

group 1 2 3
125
100
75
50
25
0
ZFP36L2
0.022
0.6
0.0052
Anova, p = 0.0035
Low
Med
High
group

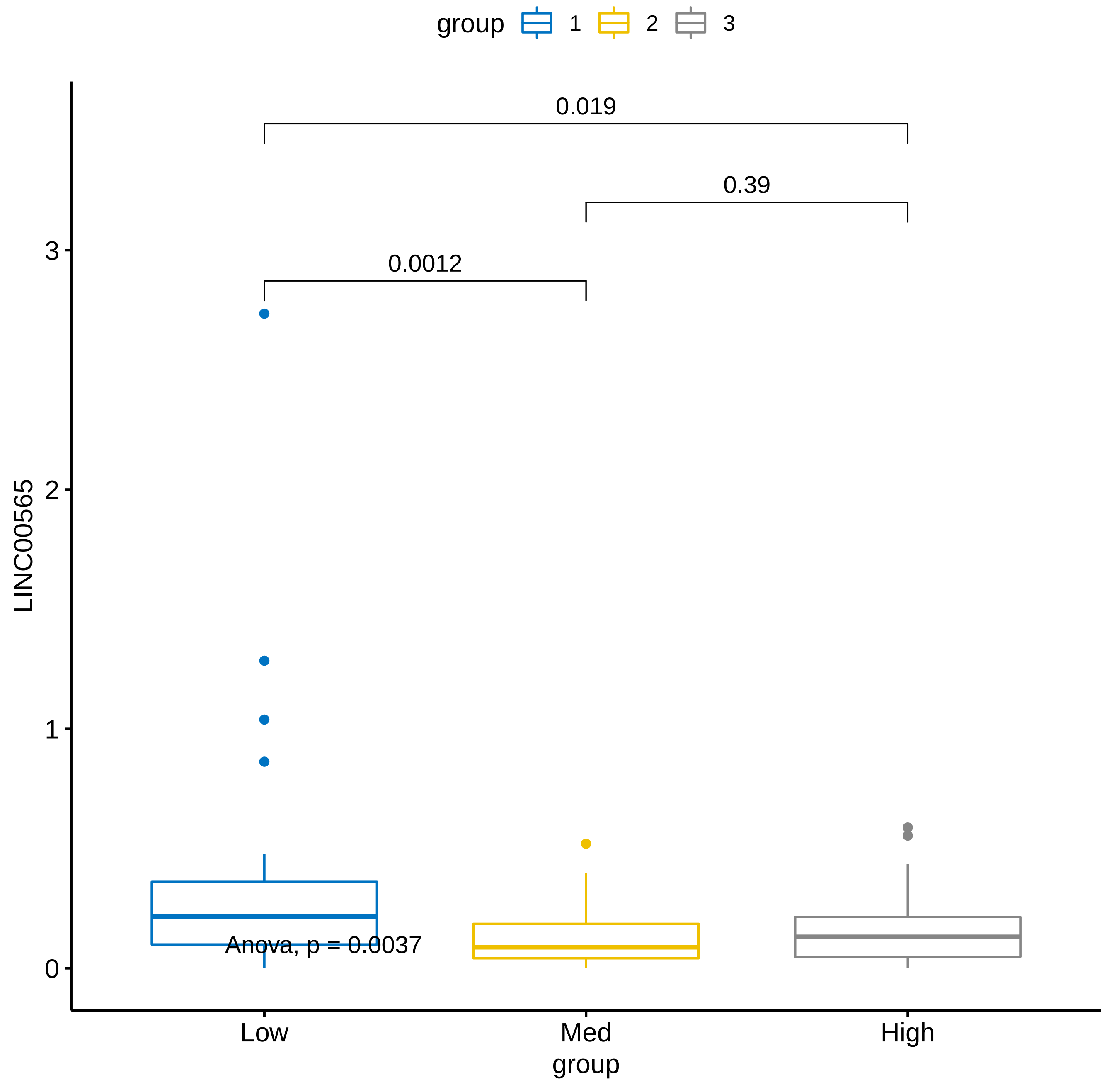

group 1 2 3
0.019
0.39
0.0012
Anova, p = 0.0037
LINC00565
0
1
2
3
Low
Med
High
group

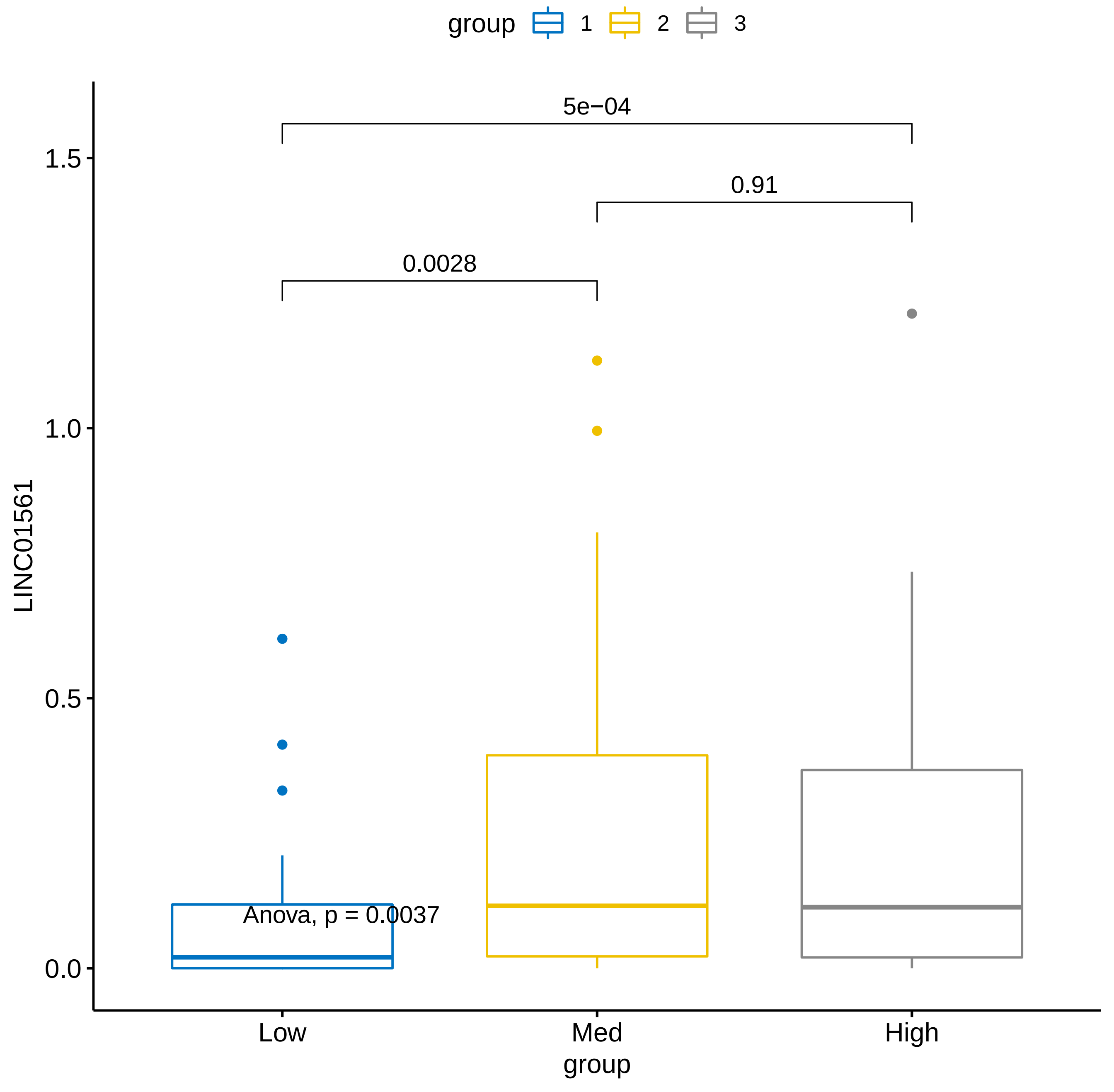
group
1
2
3
5e−04
0.91
0.0028
1.5
1.0
0.5
0.0
LINC01561
Anova, p = 0.0037
Low
Med
High
group

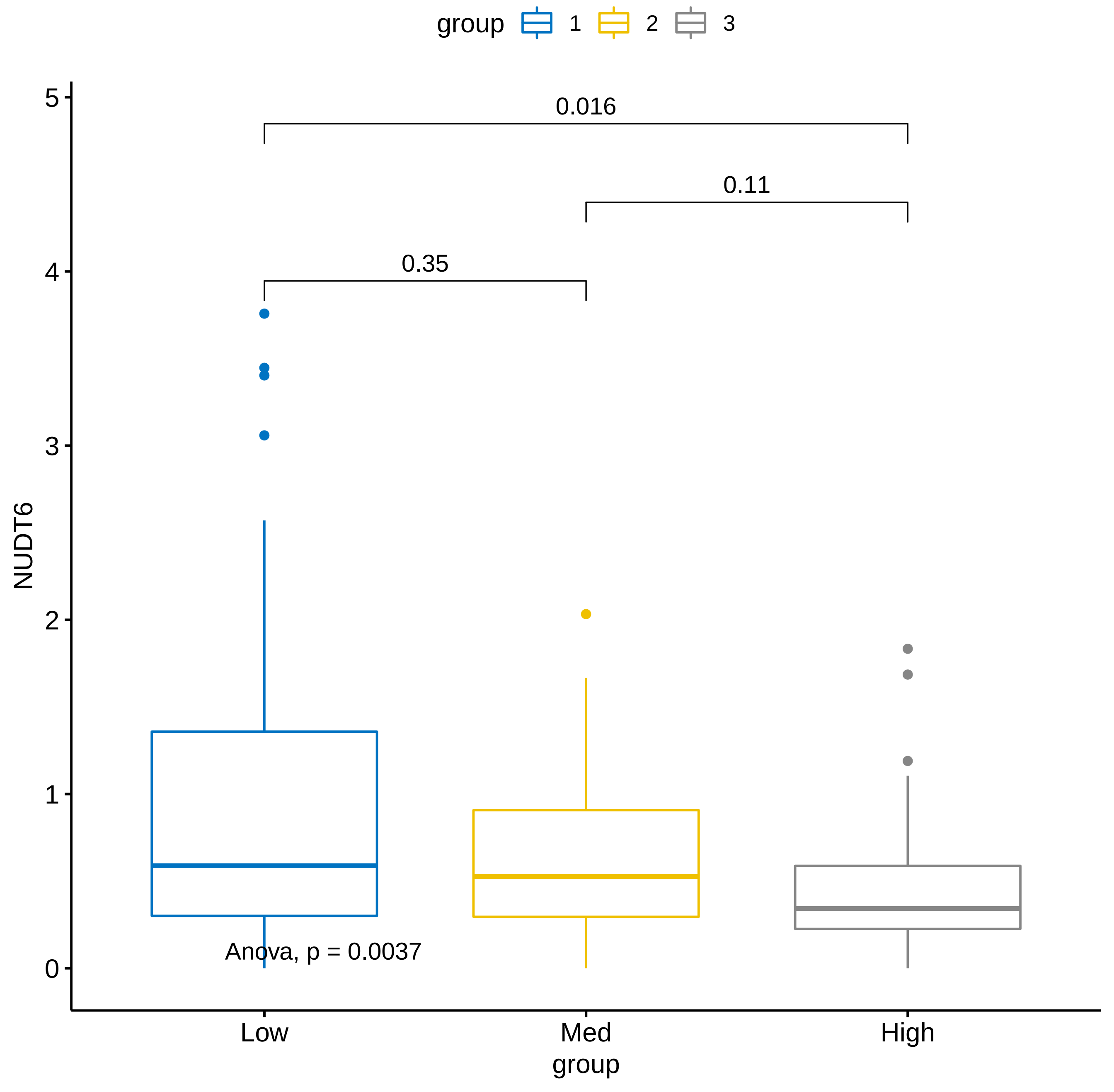

group
1
2
3
0.016
0.11
0.35
Anova, p = 0.0037
NUDT6
0
1
2
3
4
5
Low
Med
High
group

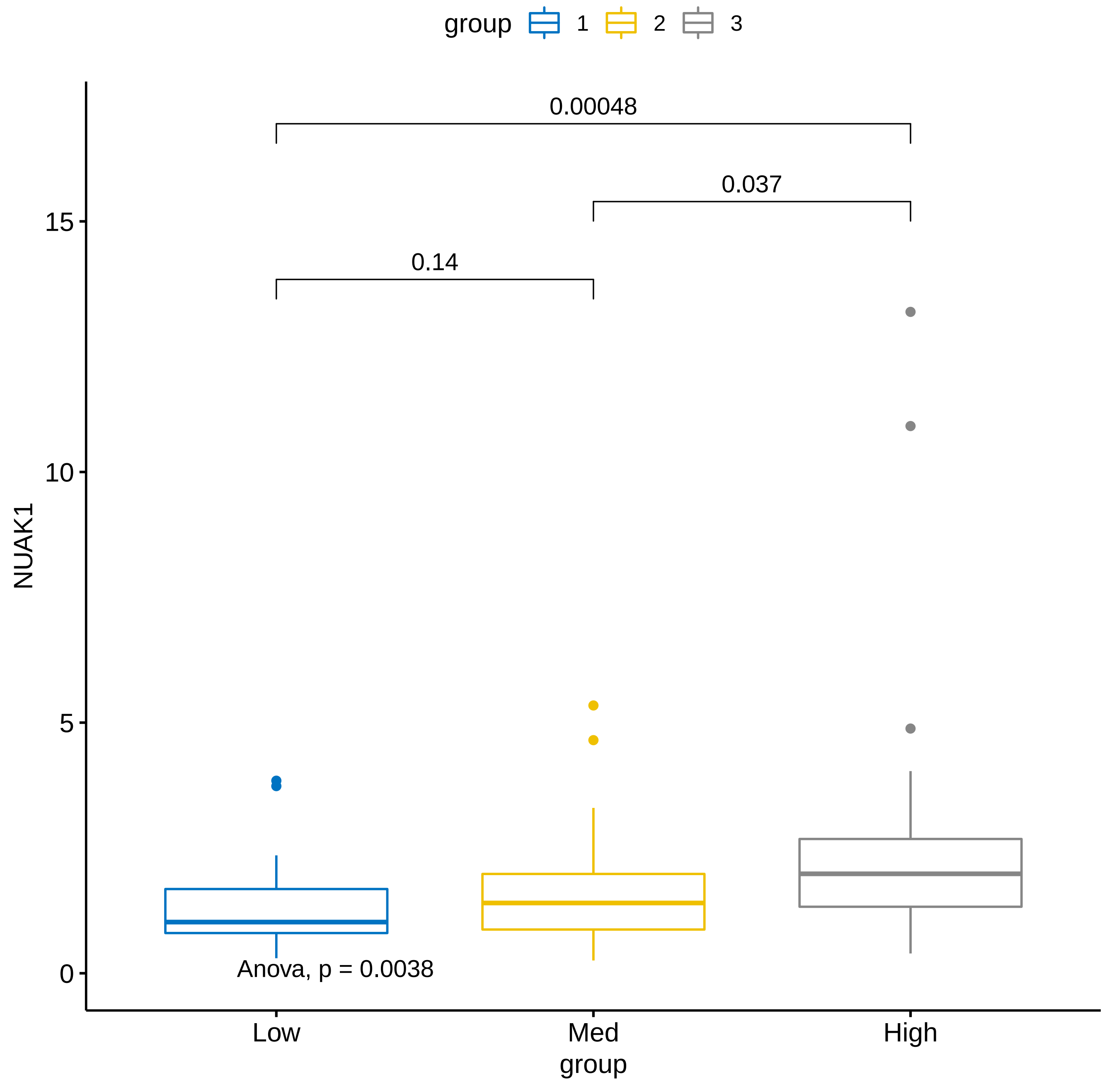
group
1
2
3
0.00048
0.037
0.14
15
10
5
0
NUAK1
Anova, p = 0.0038
Low
Med
High
group

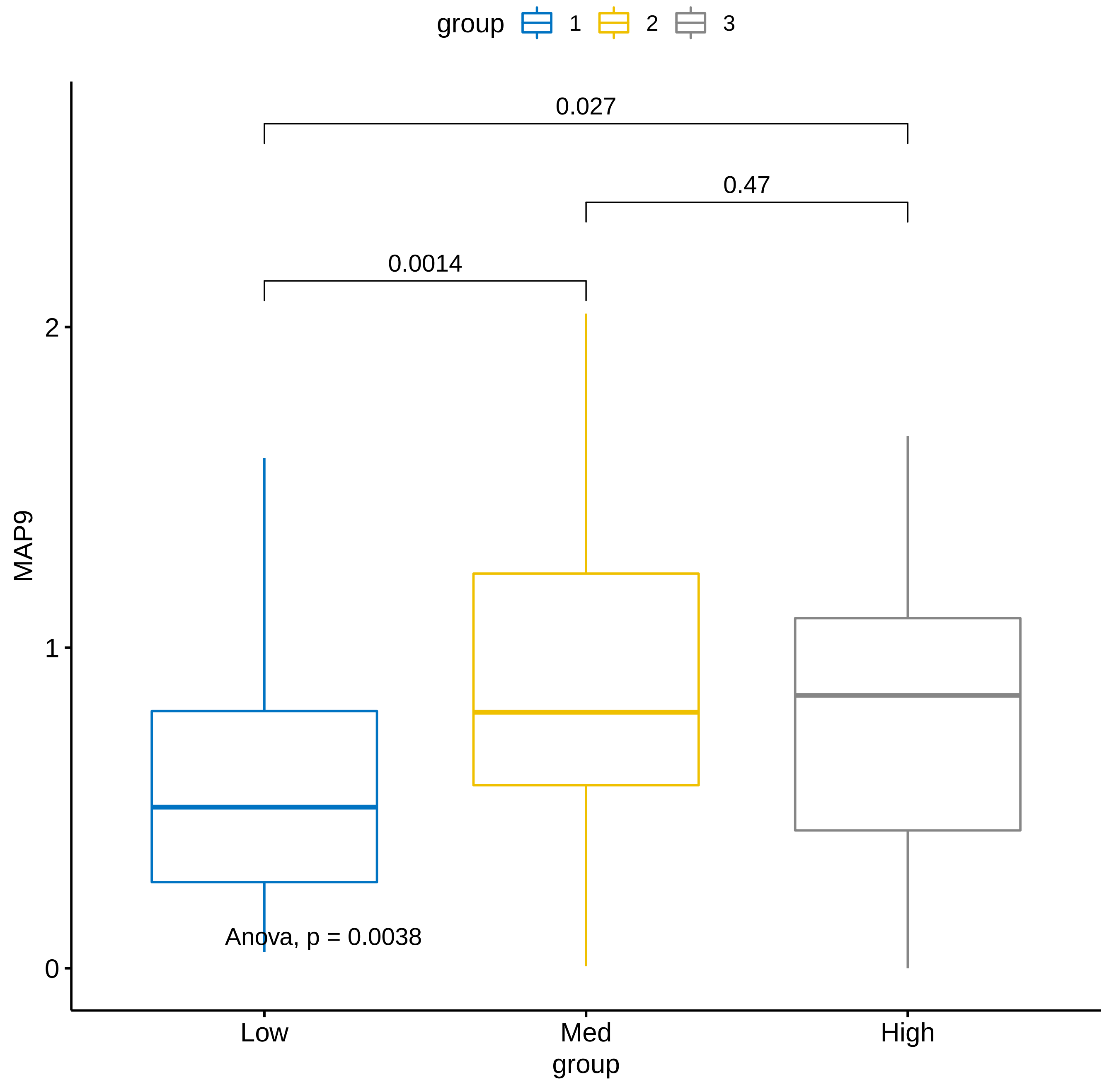
group
1
2
3
0.027
0.47
0.0014
2
1
0
MAP9
Anova, p = 0.0038
Low
Med
High
group

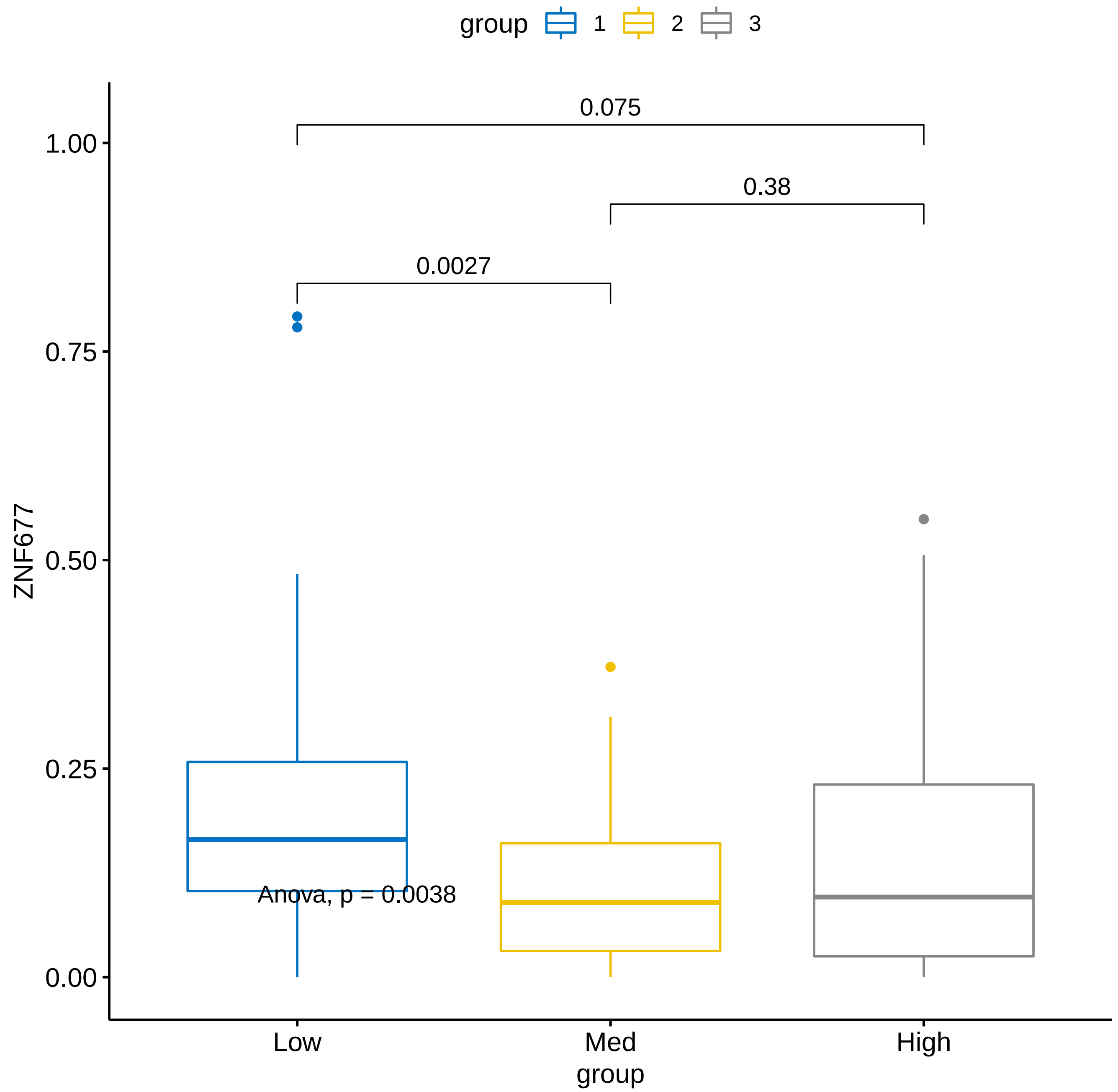
group
1
2
3
0.075
0.38
0.0027
1.00
0.75
0.50
0.25
0.00
ZNF677
Anova, p = 0.0038
Low
Med
High
group

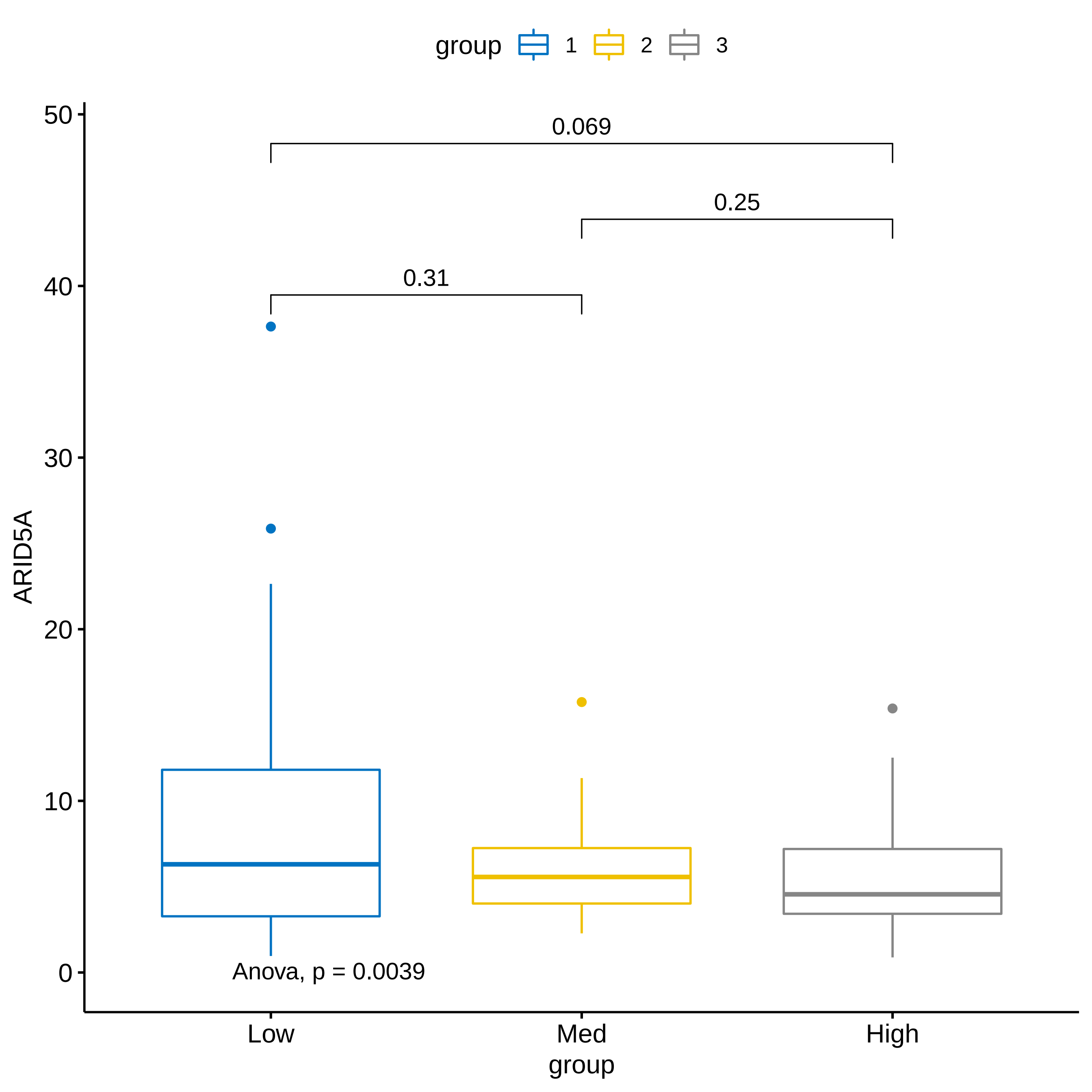
group 1 2 3
0.069
0.25
0.31
50
40
30
20
10
0
ARID5A
Anova, p = 0.0039
Low
Med
High
group

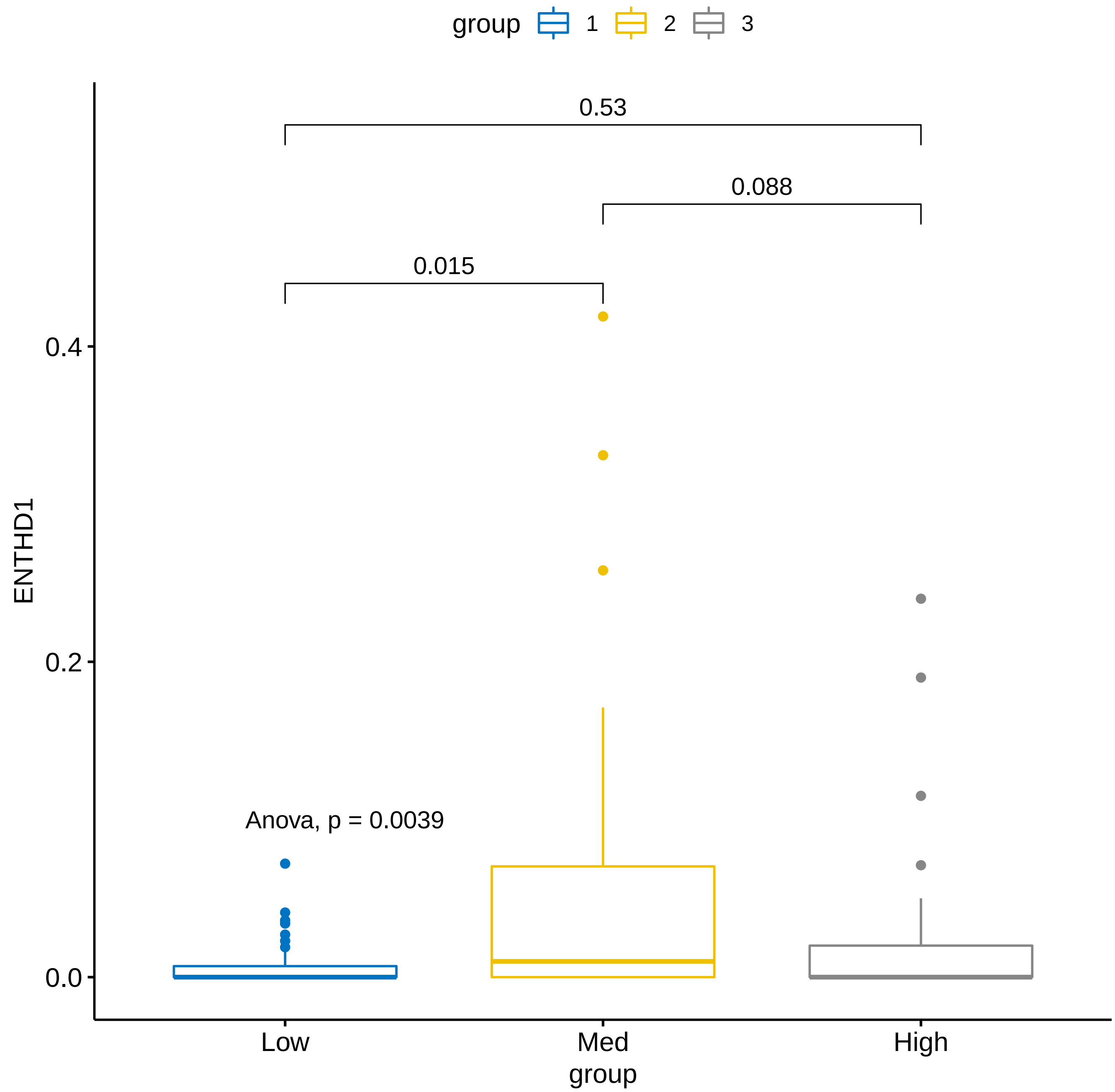

group
1
2
3
0.53
0.088
0.015
0.4
0.2
0.0
ENTHD1
Anova, p = 0.0039
Low
Med
High
group

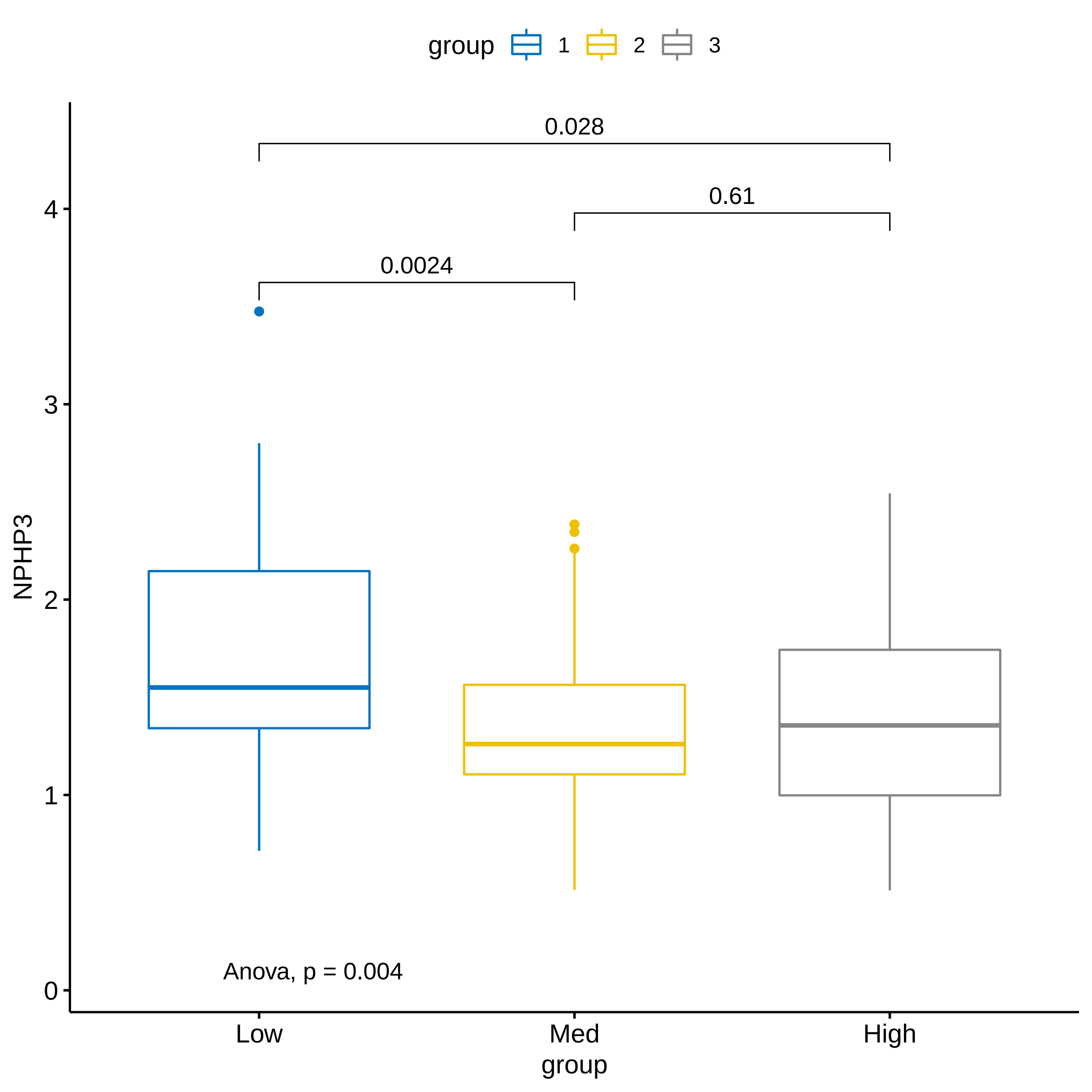
group
1
2
3
0.028
0.61
0.0024
4
3
2
1
0
NPHP3
Anova, p = 0.004
Low
Med
High
group

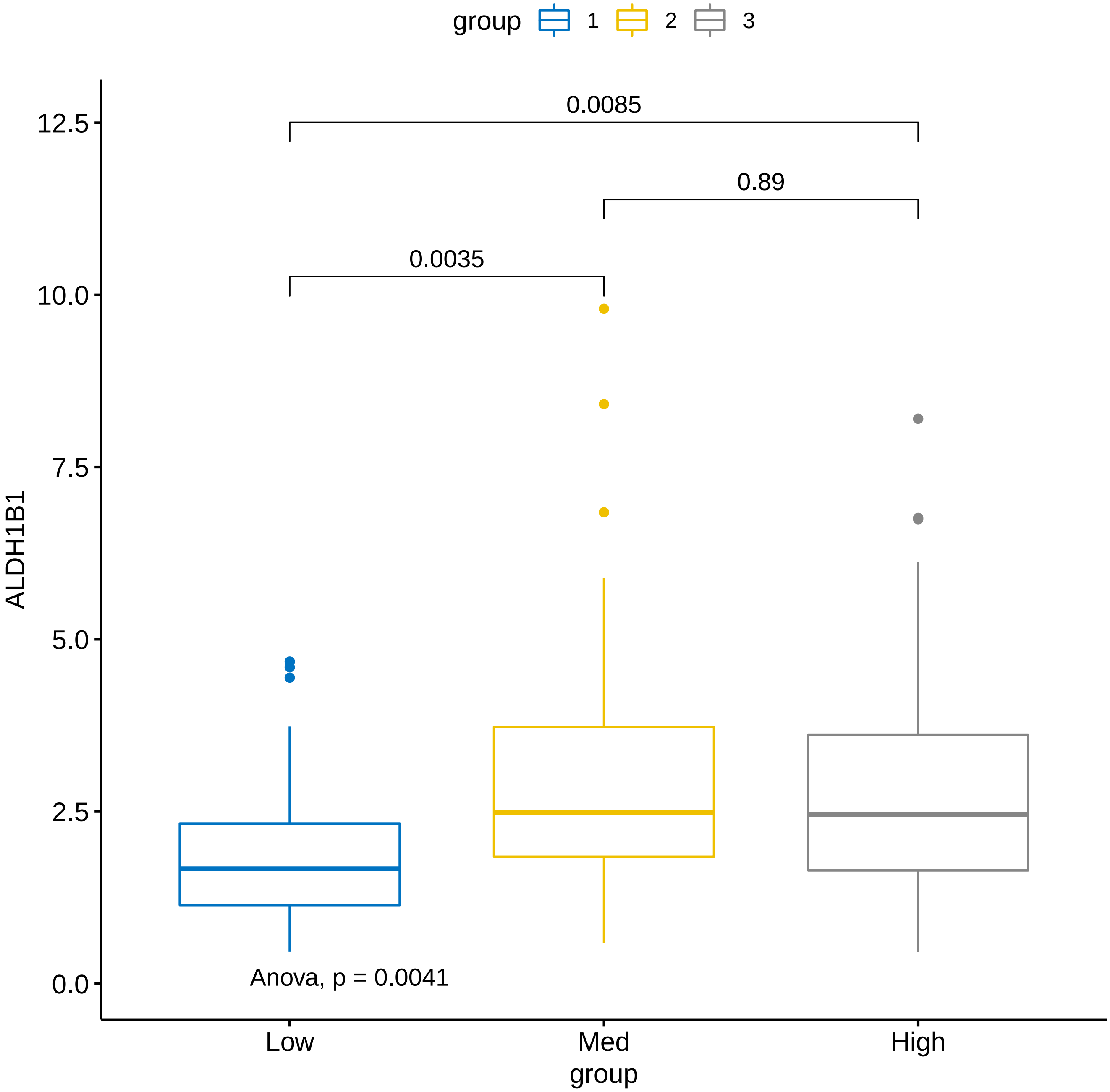
group
1
2
3
0.0085
0.89
0.0035
12.5
10.0
7.5
5.0
2.5
0.0
ALDH1B1
Anova, p = 0.0041
Low
Med
High
group

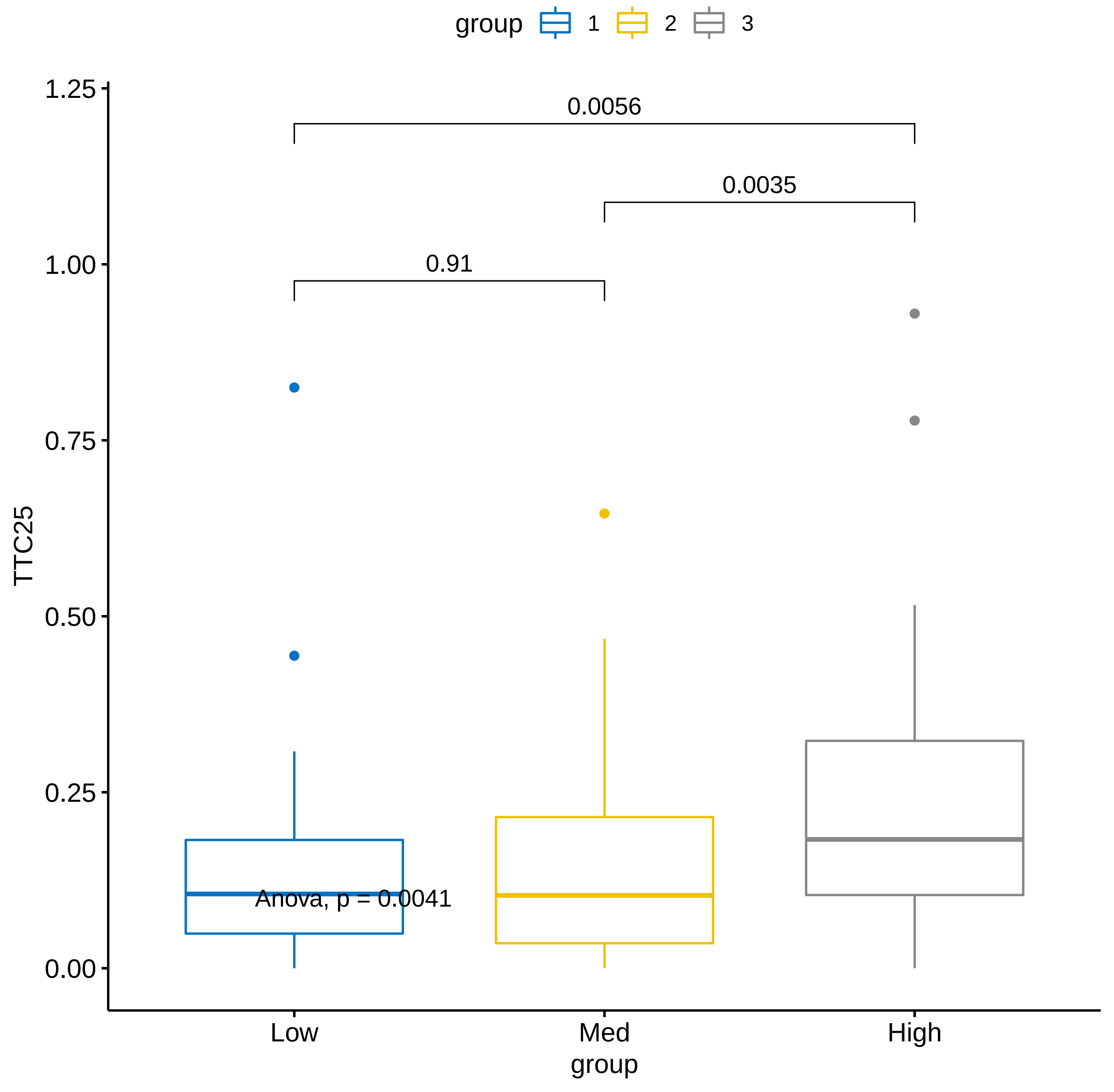

group 1 2 3
1.25
1.00
0.75
0.50
0.25
0.00
TTC25
0.0056
0.0035
0.91
Anova, p = 0.0041
Low
Med
High
group

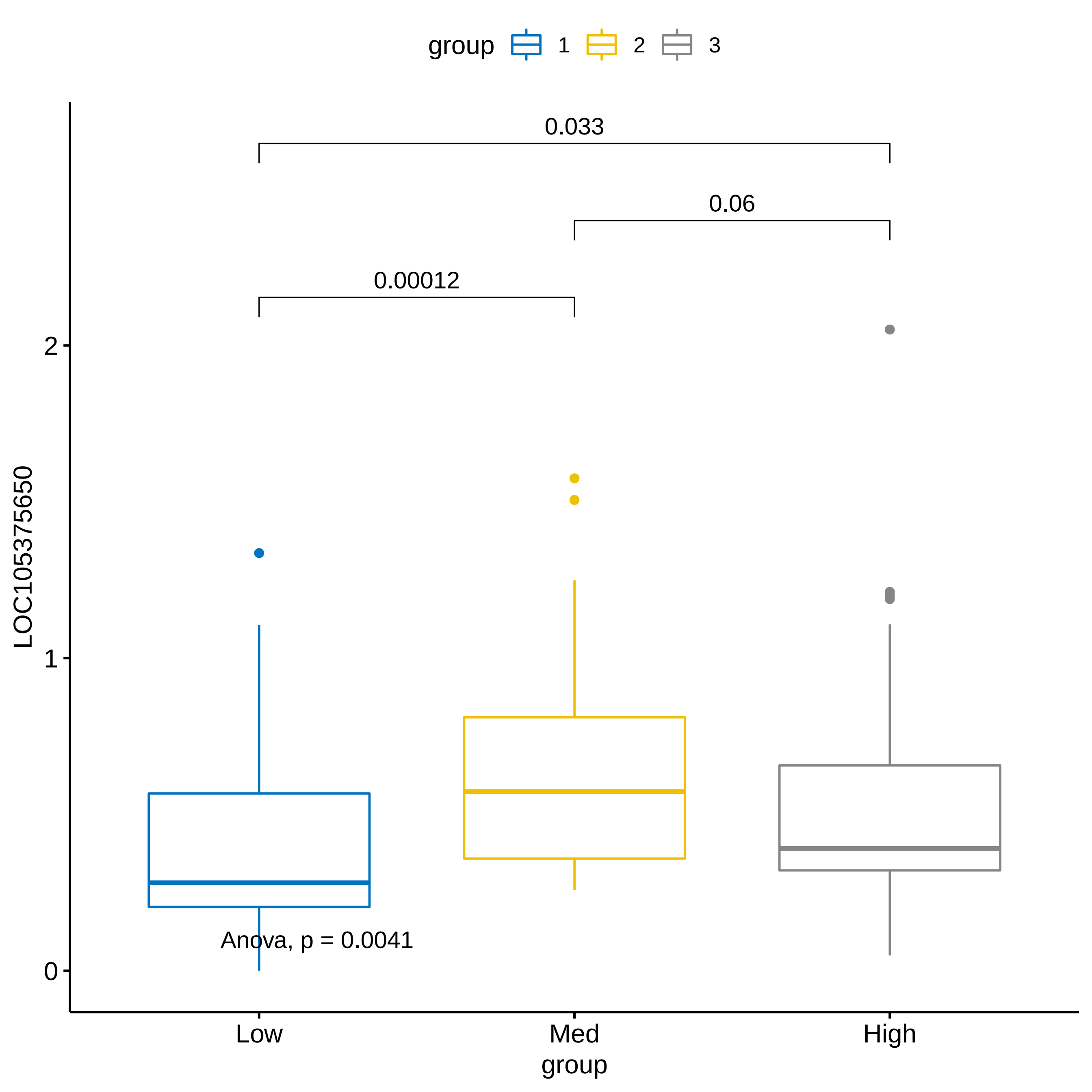

group
1
2
3
0.033
0.06
0.00012
2
1
0
LOC105375650
Anova, p = 0.0041
Low
Med
High
group

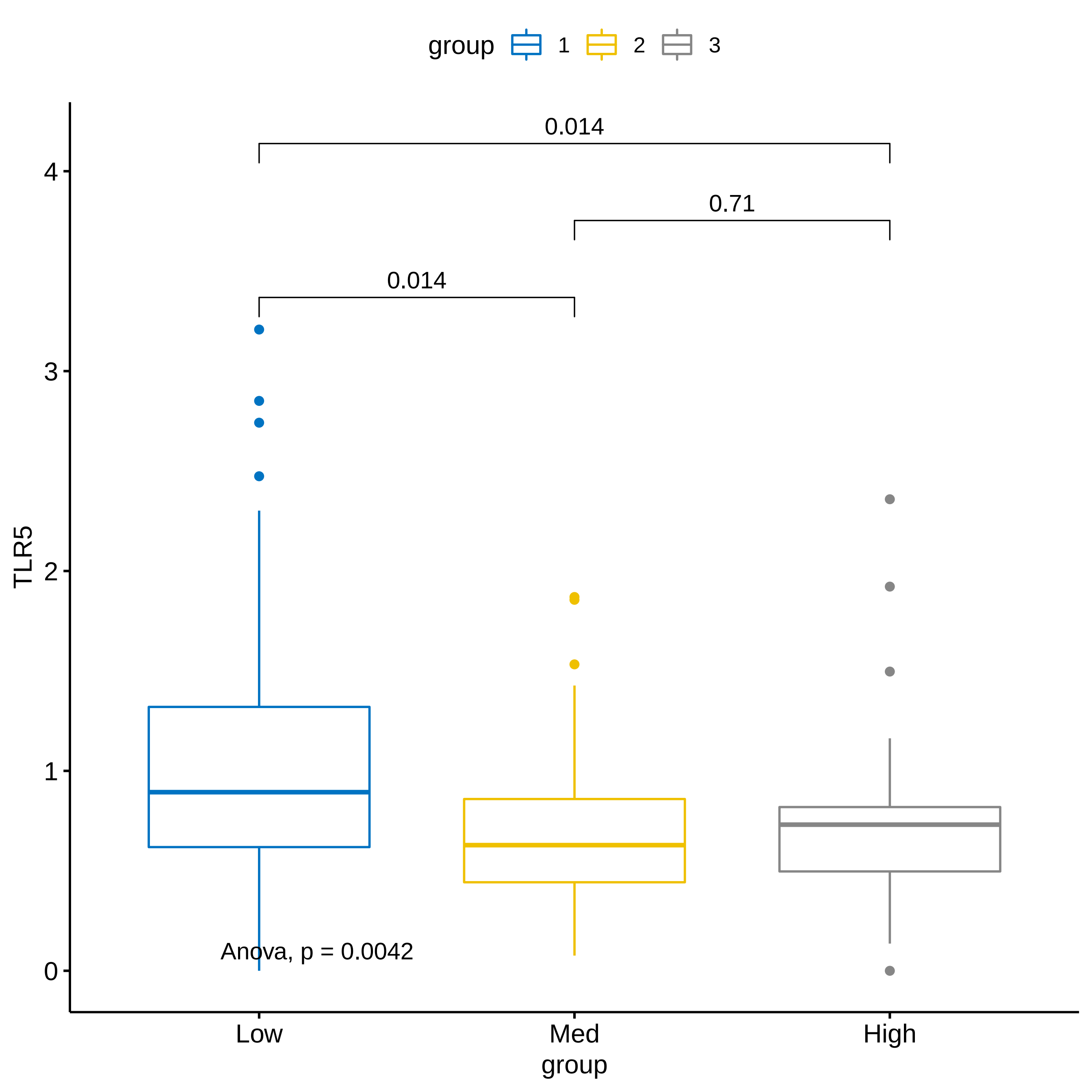
group
1
2
3
0.014
0.71
0.014
Anova, p = 0.0042
TLR5
0
1
2
3
4
Low
Med
High
group

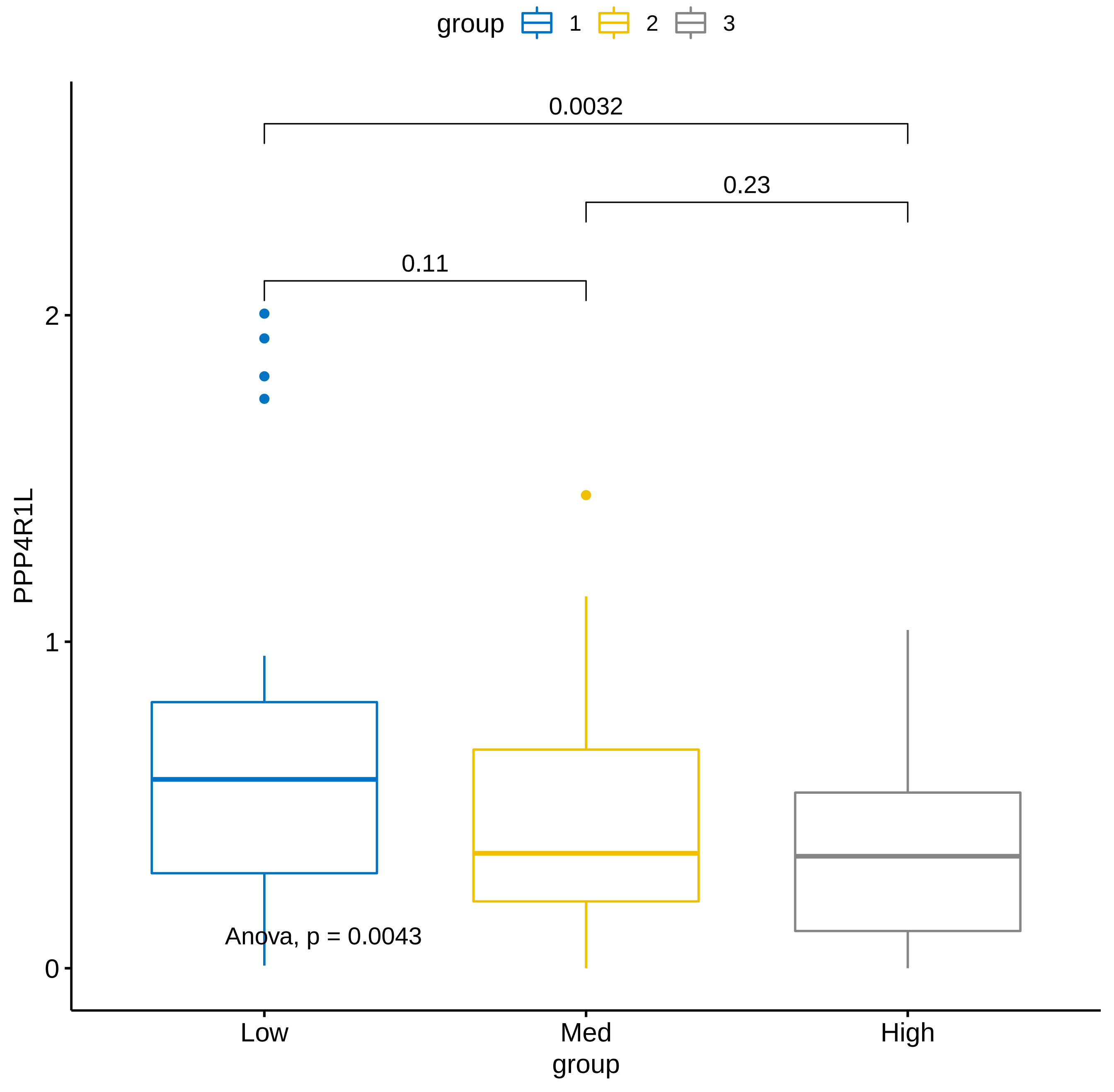
group
1
2
3
0.0032
0.23
0.11
2
1
0
PPP4R1L
Anova, p = 0.0043
Low
Med
High
group

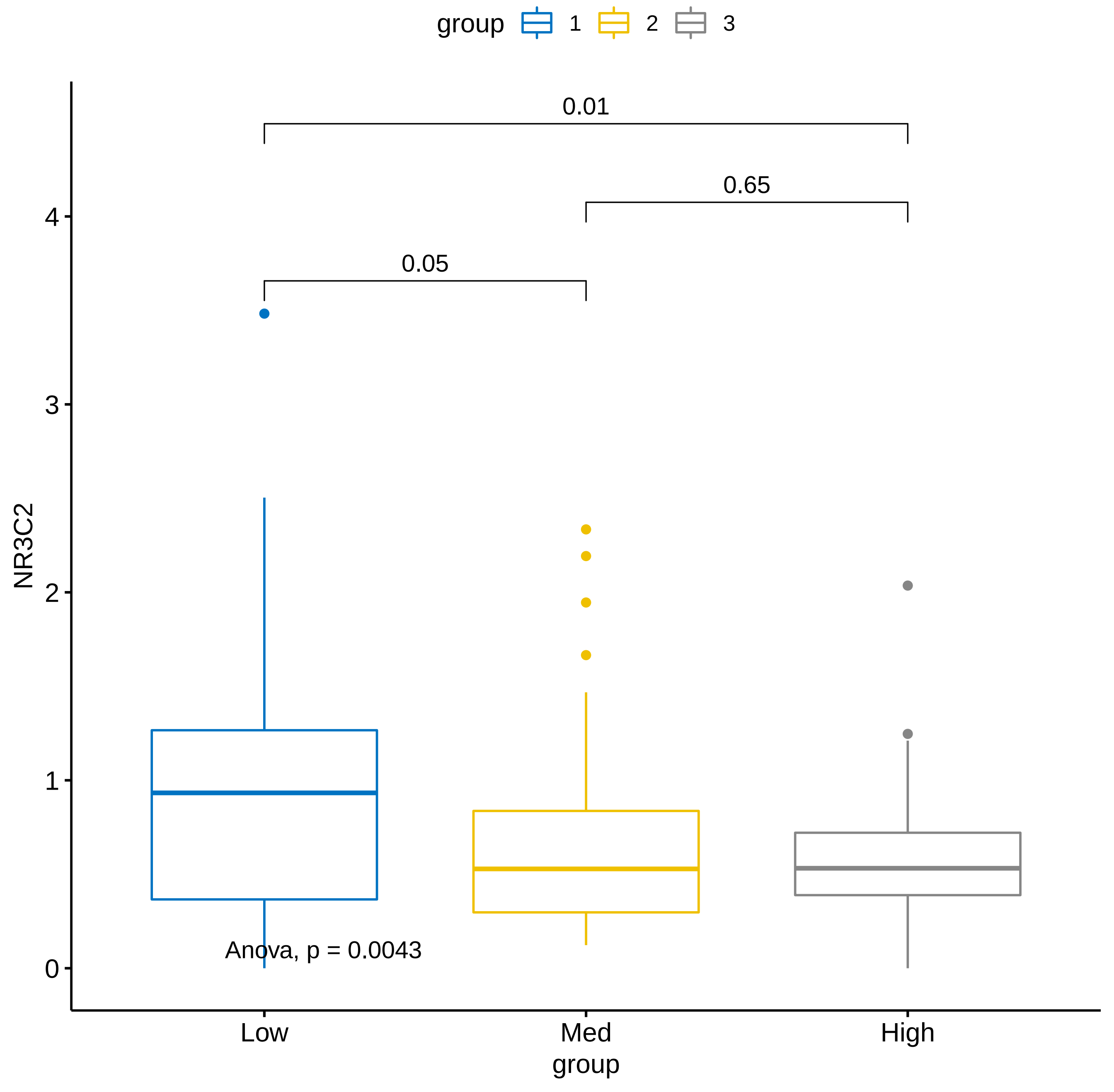
group
1
2
3
0.01
0.65
0.05
4
3
2
1
0
NR3C2
Anova, p = 0.0043
Low
Med
High
group

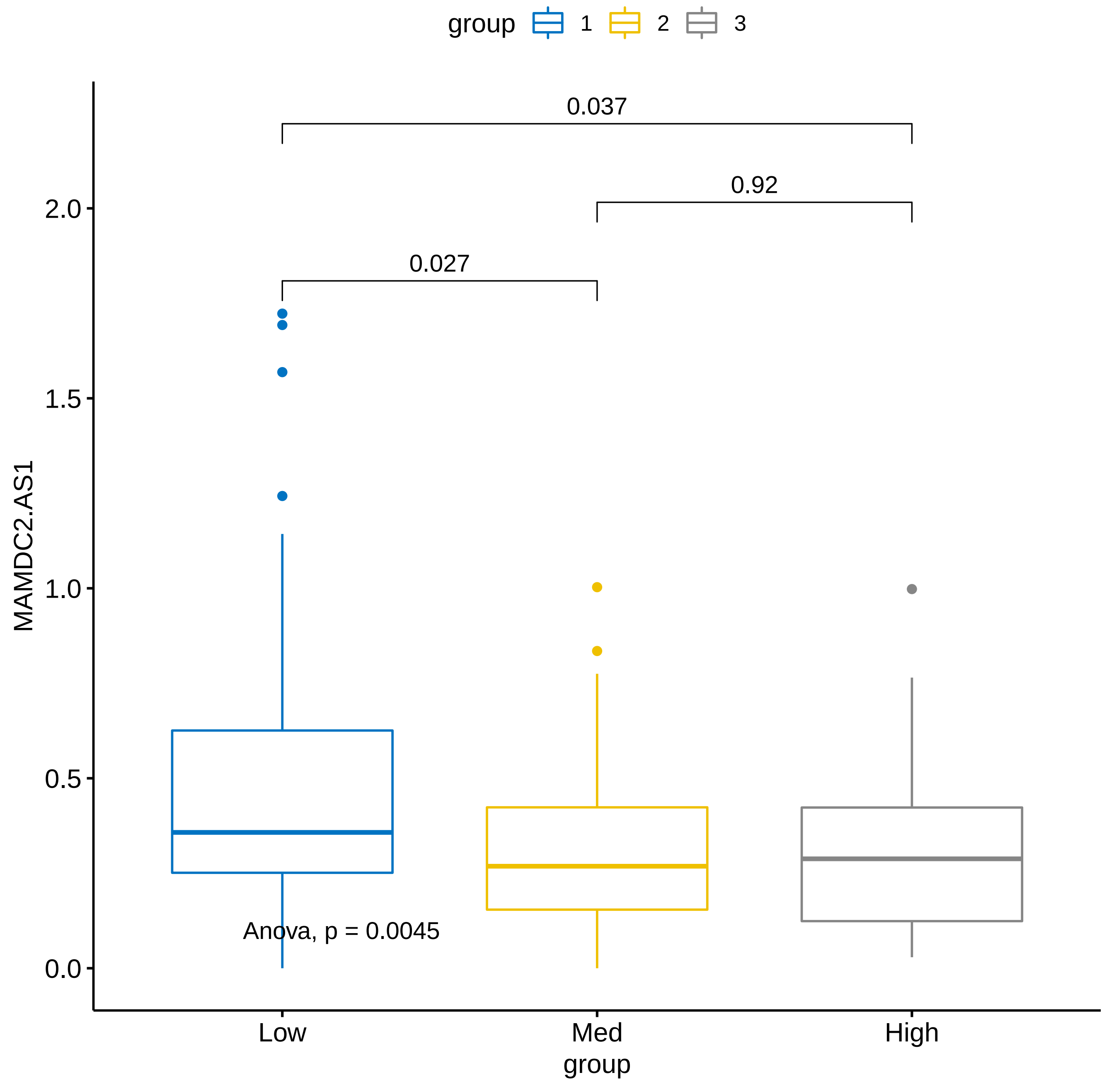
group
1
2
3
0.037
0.92
0.027
MAMDC2.AS1
2.0
1.5
1.0
0.5
0.0
Anova, p = 0.0045
Low
Med
High
group

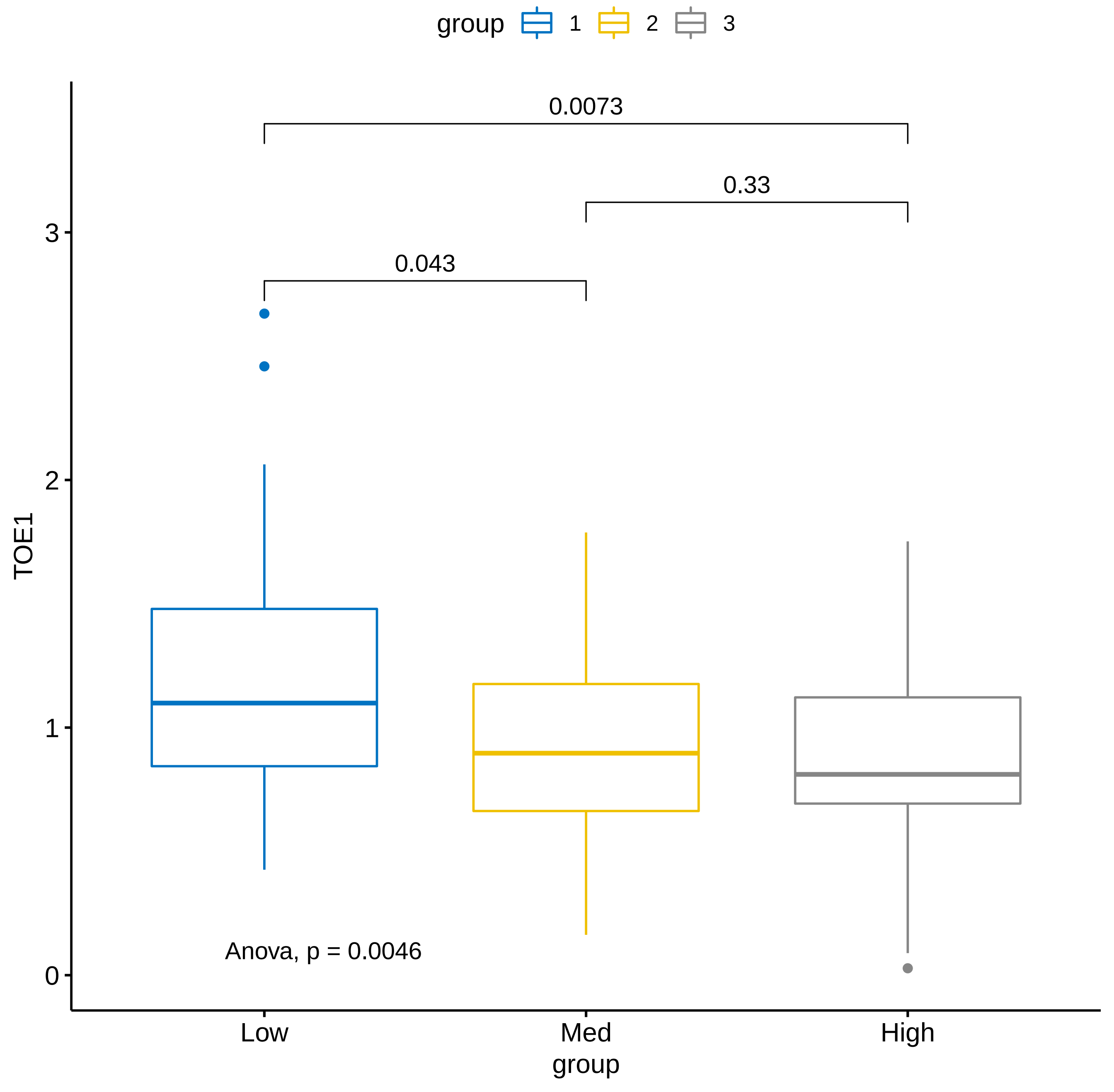
group
1
2
3
0.0073
0.33
0.043
TOE1
3
2
1
0
Anova, p = 0.0046
Low
Med
High
group

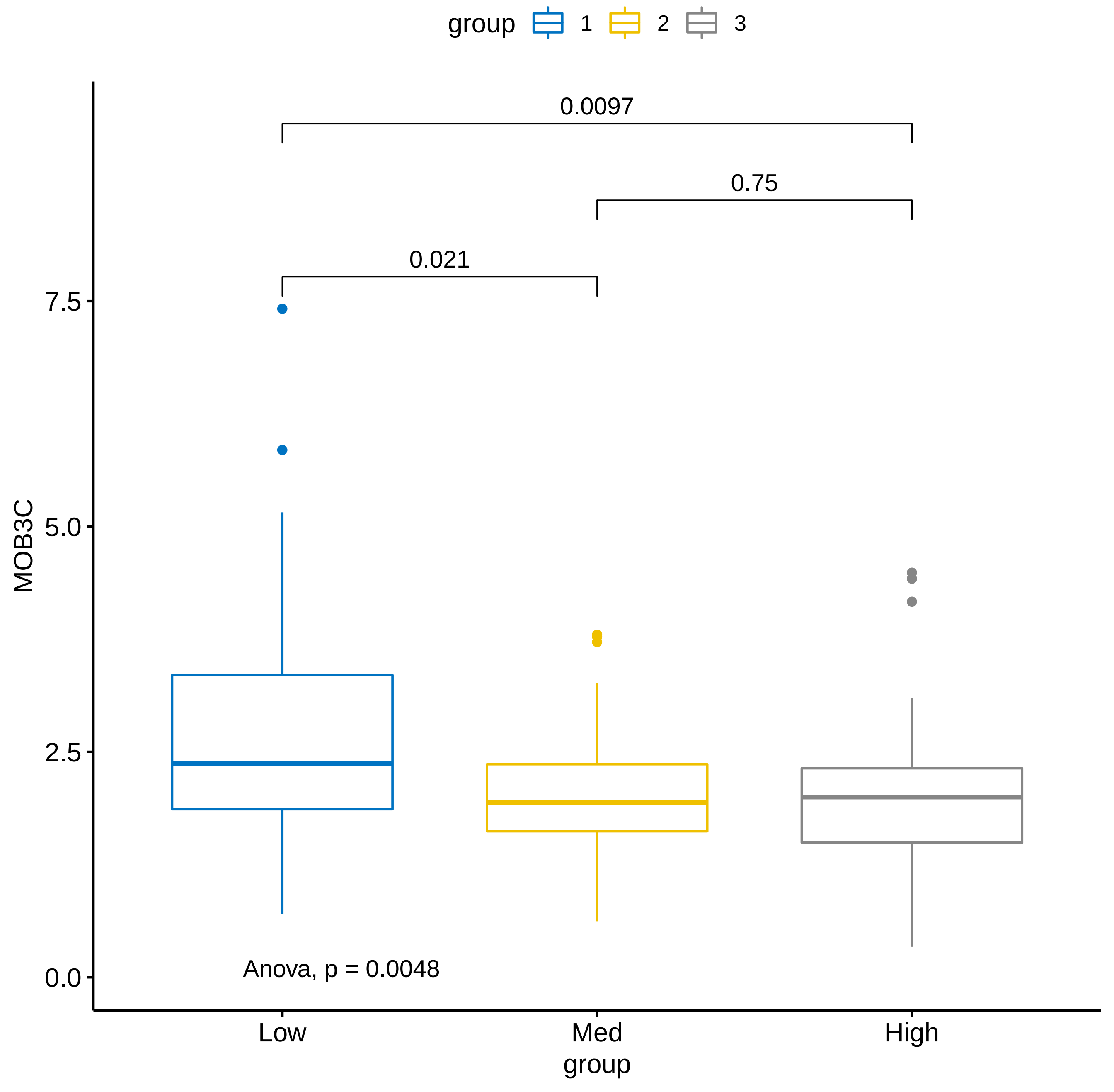

group
1
2
3
0.0097
0.75
0.021
7.5
5.0
2.5
0.0
MOB3C
Anova, p = 0.0048
Low
Med
High
group

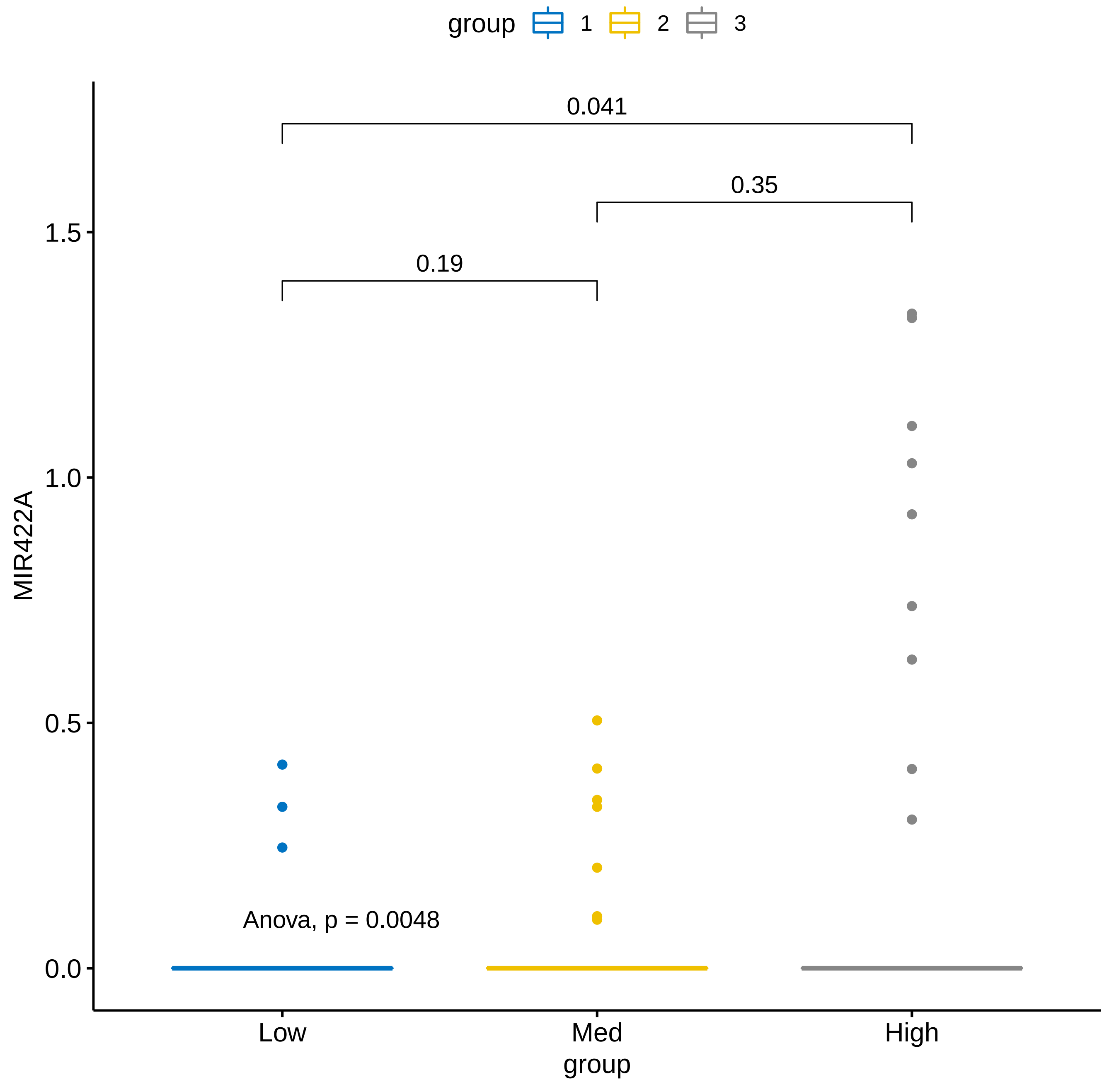

group 1 2 3
0.041
0.35
0.19
1.5
1.0
0.5
0.0
MIR422A
Anova, p = 0.0048
Low
Med
High
group

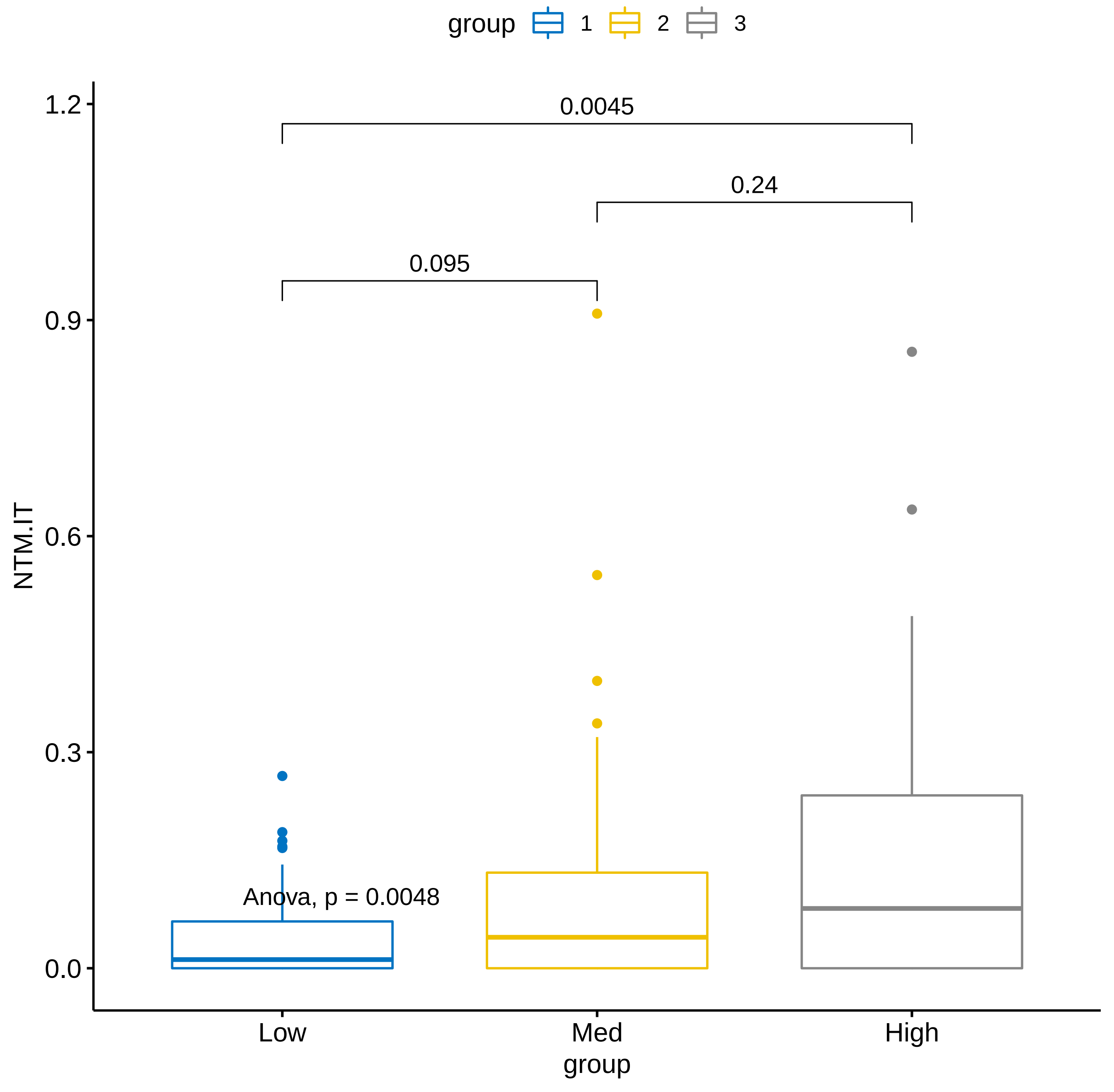

group
1
2
3
1.2
0.9
0.6
0.3
0.0
NTM.IT
0.0045
0.24
0.095
Anova, p = 0.0048
Low
Med
High
group

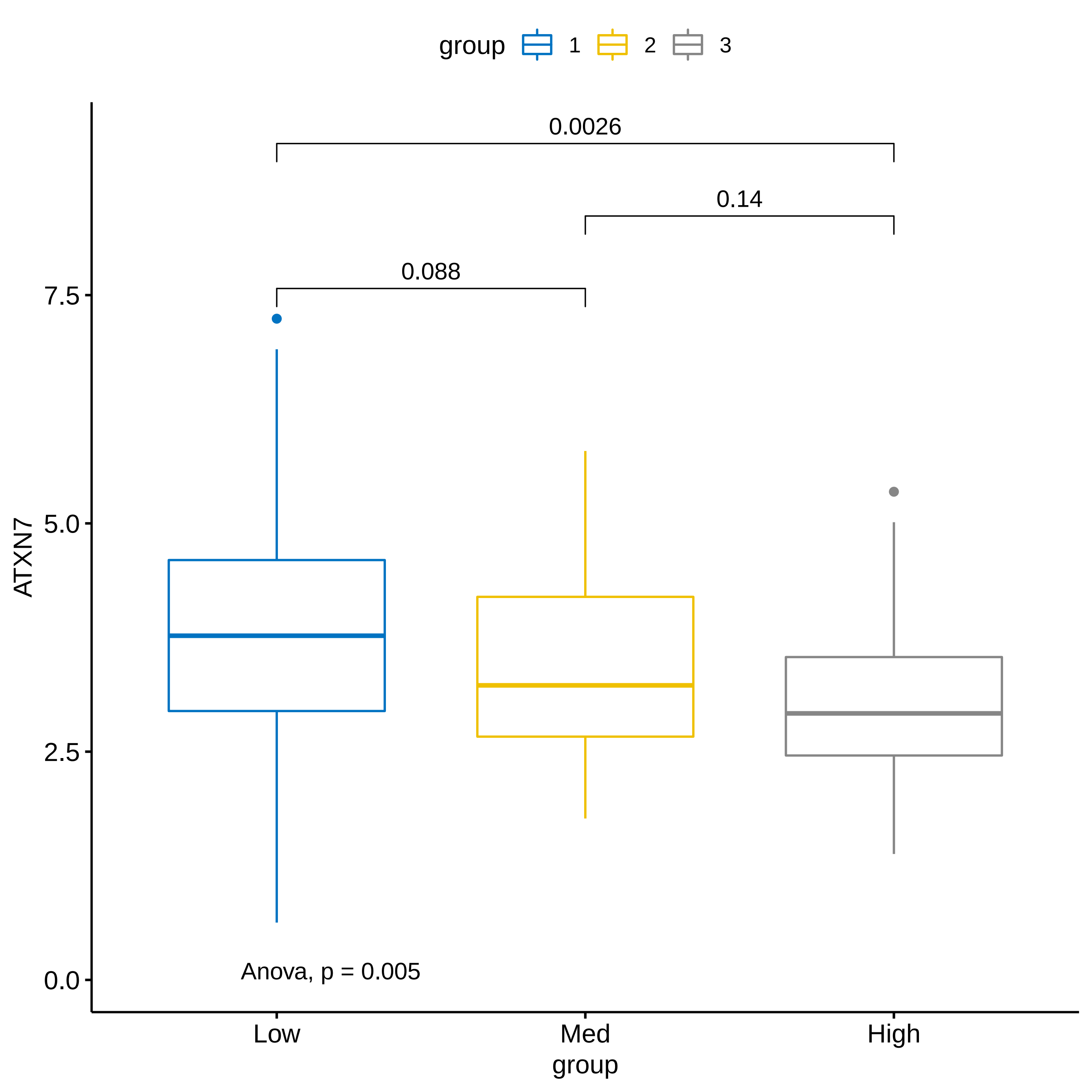
group
1
2
3
0.0026
0.14
0.088
7.5
5.0
2.5
0.0
ATXN7
Anova, p = 0.005
Low
Med
High
group

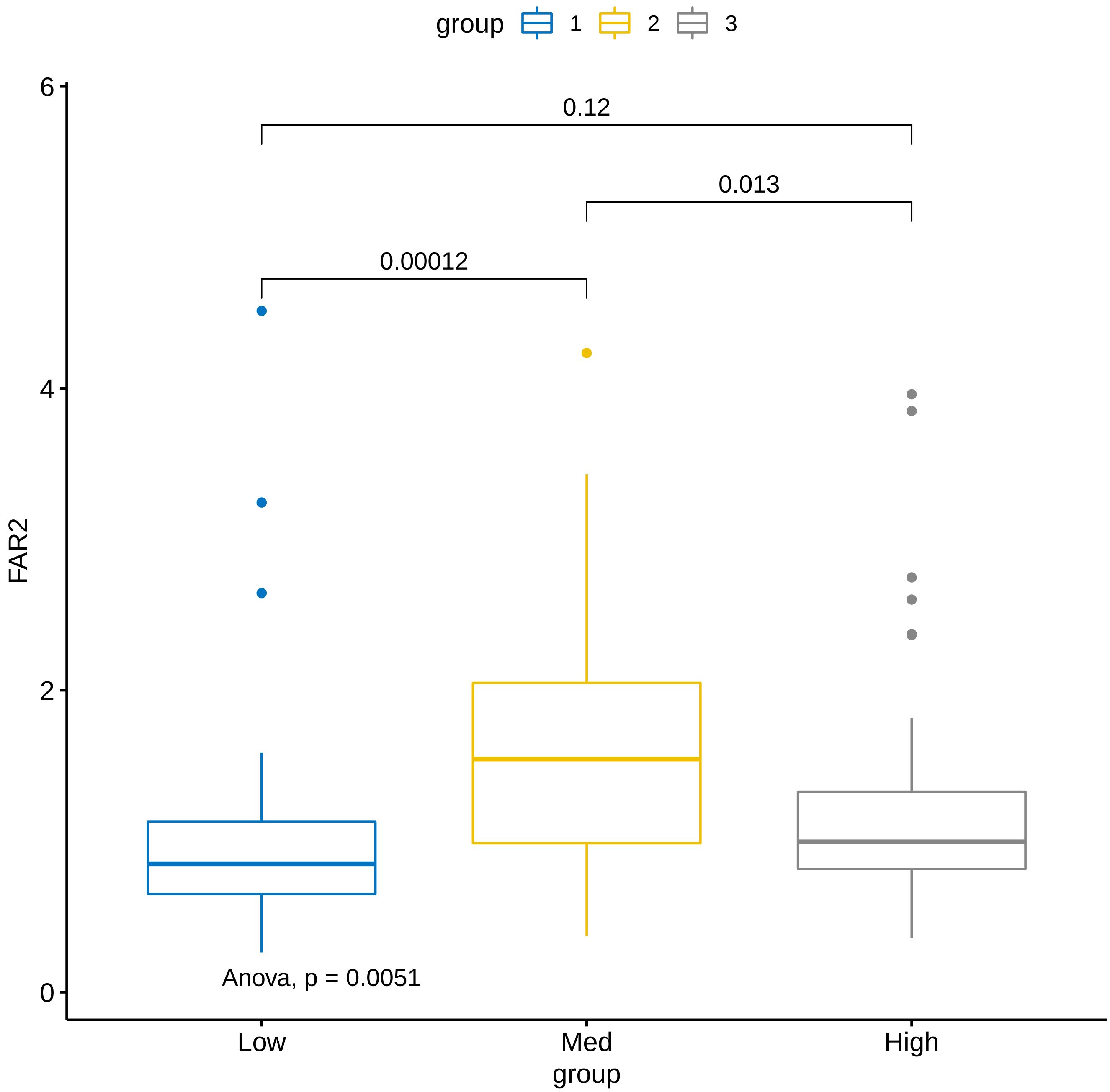

group 1 2 3
0.12
0.013
0.00012
FAR2
6
4
2
0
Anova, p = 0.0051
Low
Med
High
group

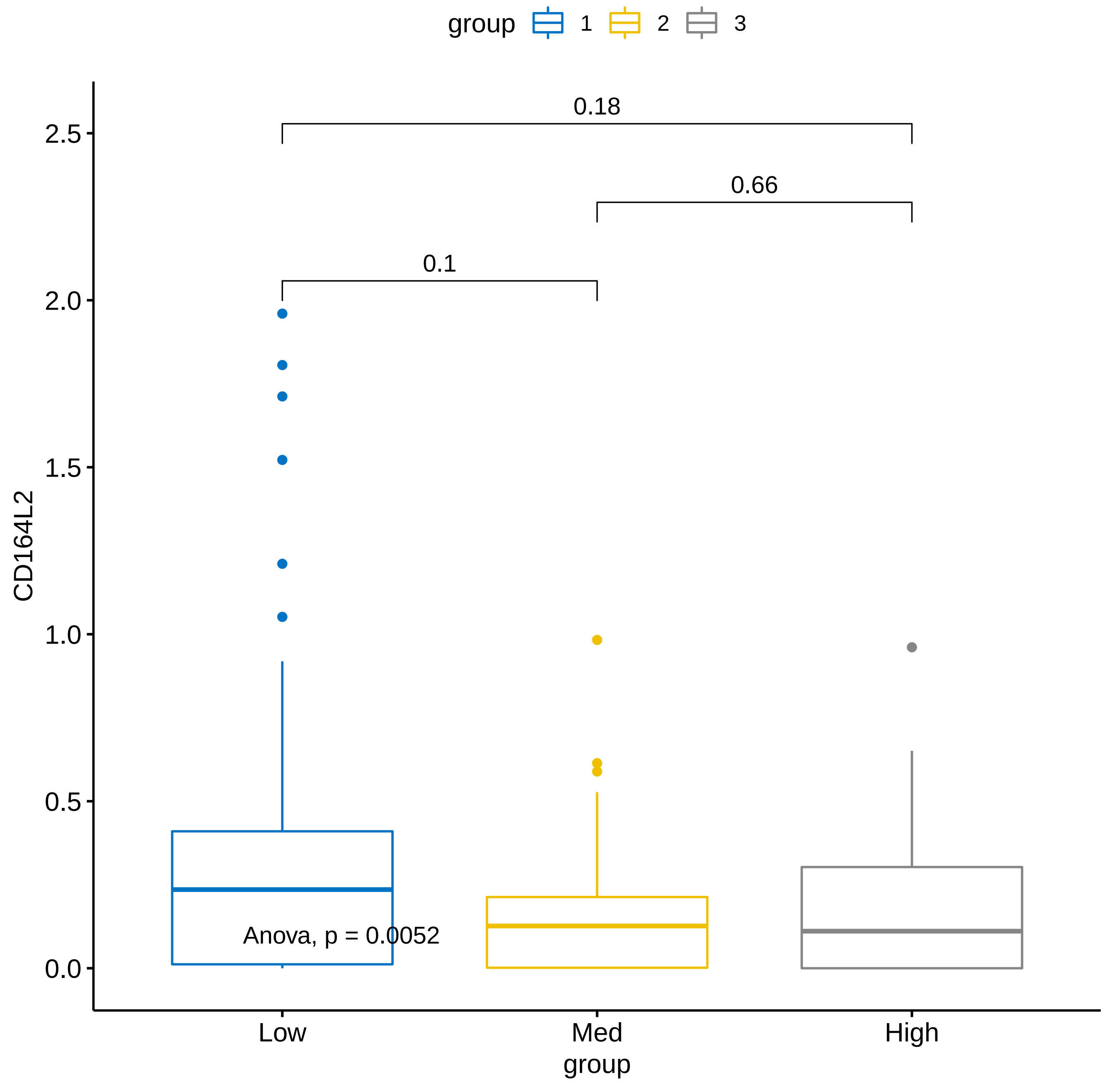

group 1 2 3
0.18
0.66
0.1
2.5
2.0
1.5
1.0
0.5
0.0
CD164L2
Anova, p = 0.0052
Low
Med
High
group

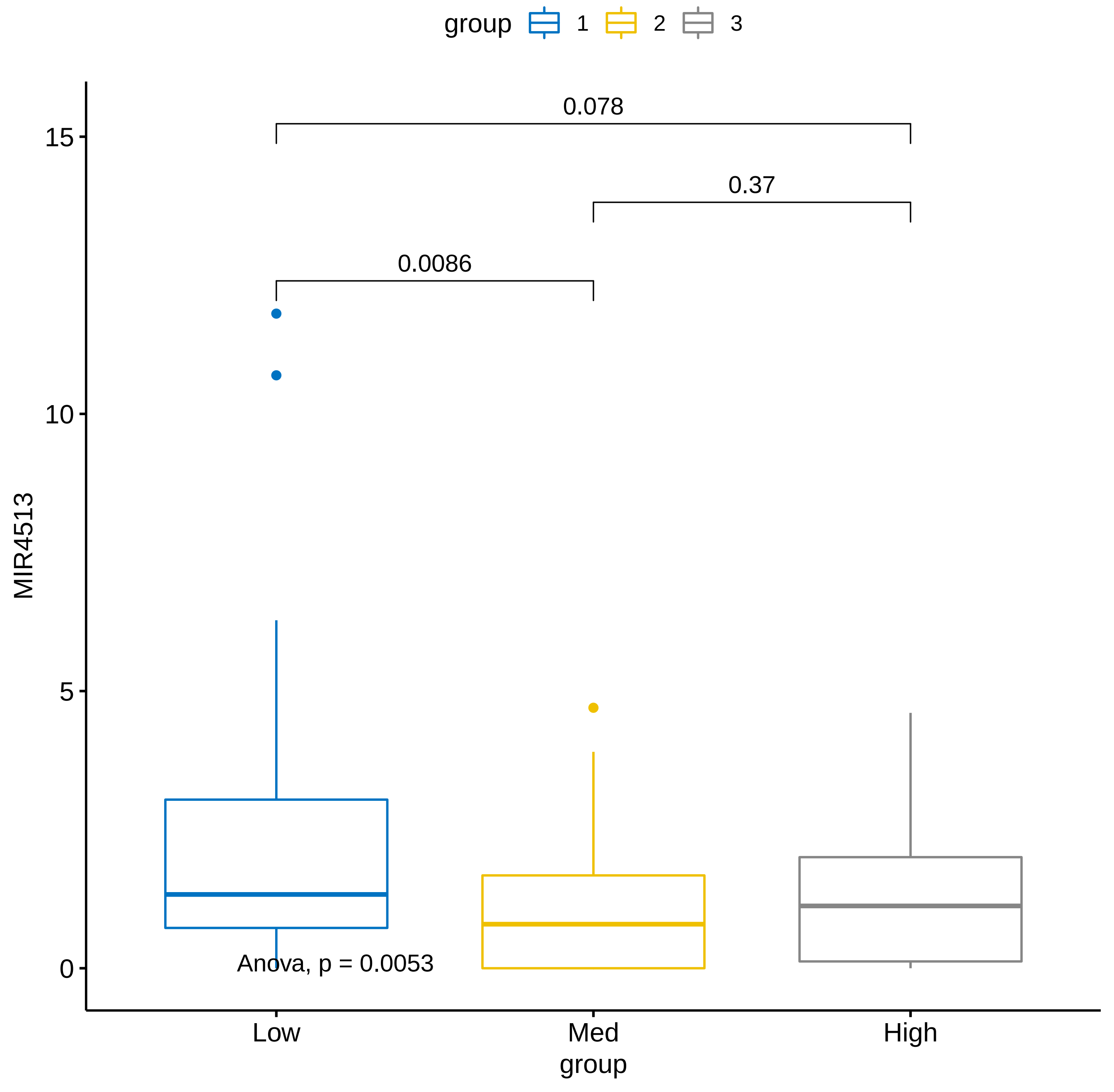

group
1
2
3
0.078
0.37
0.0086
15
10
5
0
MIR4513
Anova, p = 0.0053
Low
Med
High
group

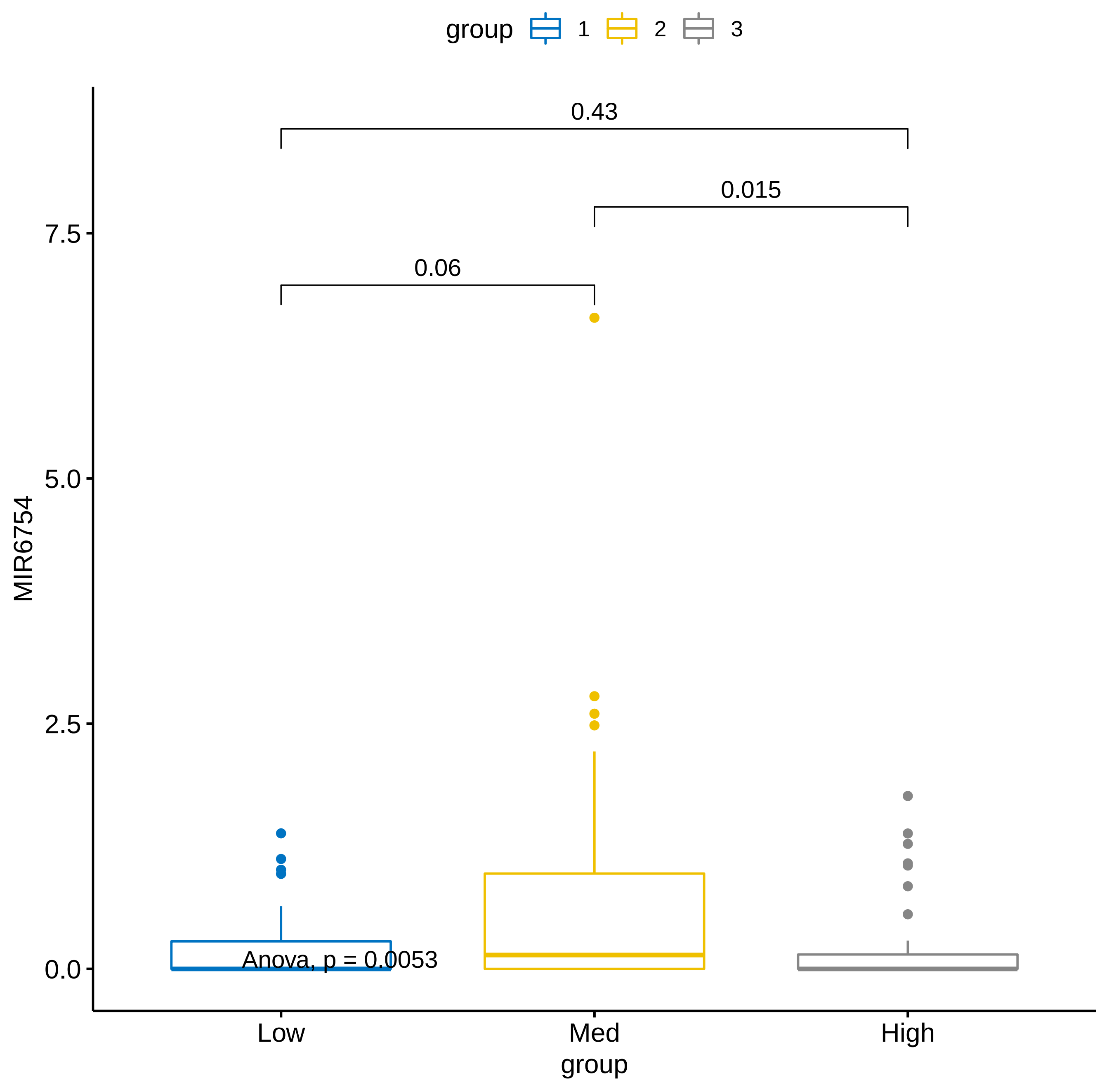
group 1 2 3
0.43
0.015
0.06
7.5
5.0
2.5
0.0
MIR6754
Anova, p = 0.0053
Low
Med
High
group

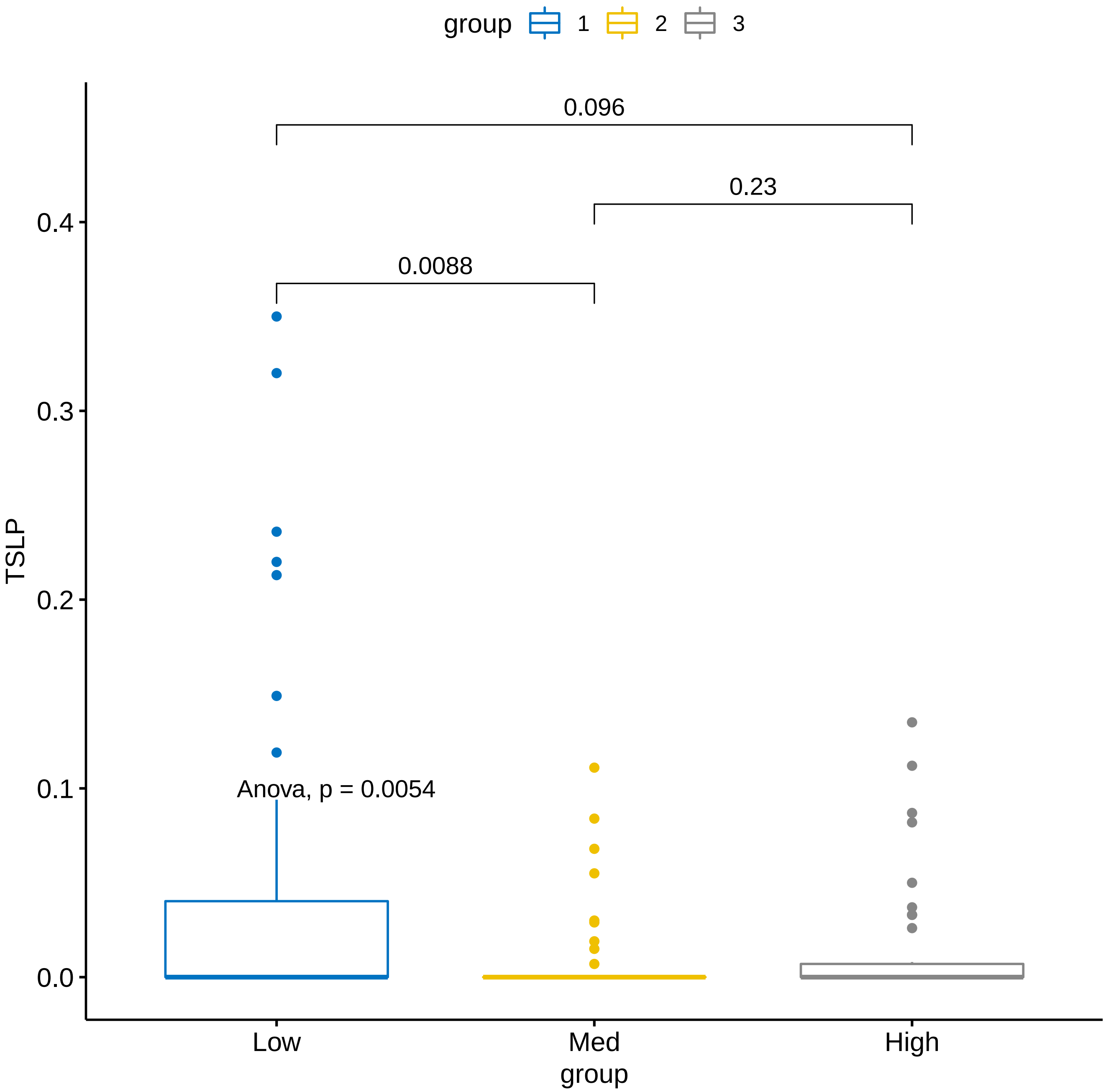
group
1
2
3
0.096
0.23
0.0088
Anova, p = 0.0054
TSLP
0.4
0.3
0.2
0.1
0.0
Low
Med
High
group

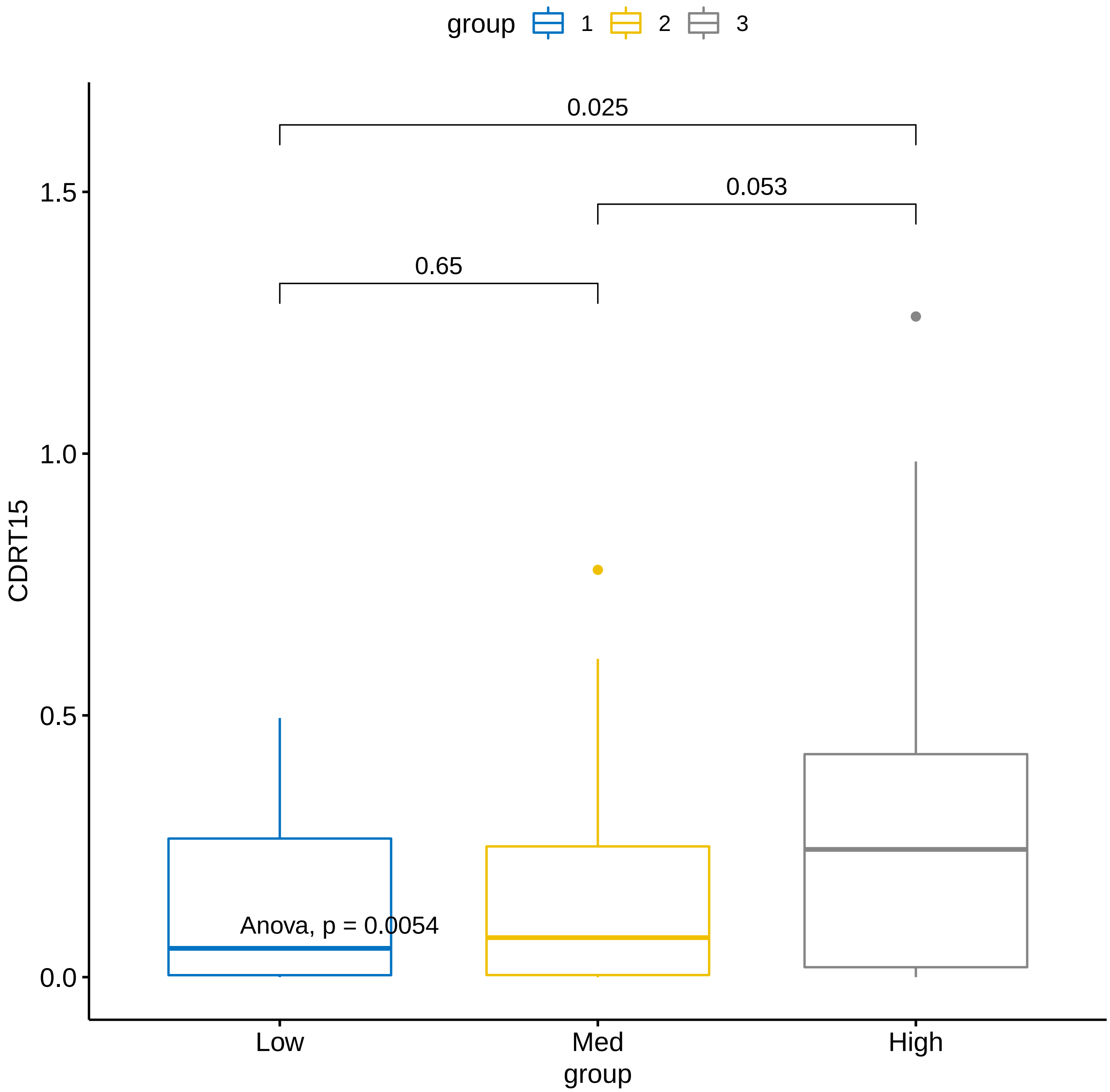

group
1
2
3
0.025
0.053
0.65
Anova, p = 0.0054
CDRT15
1.5
1.0
0.5
0.0
Low
Med
High
group

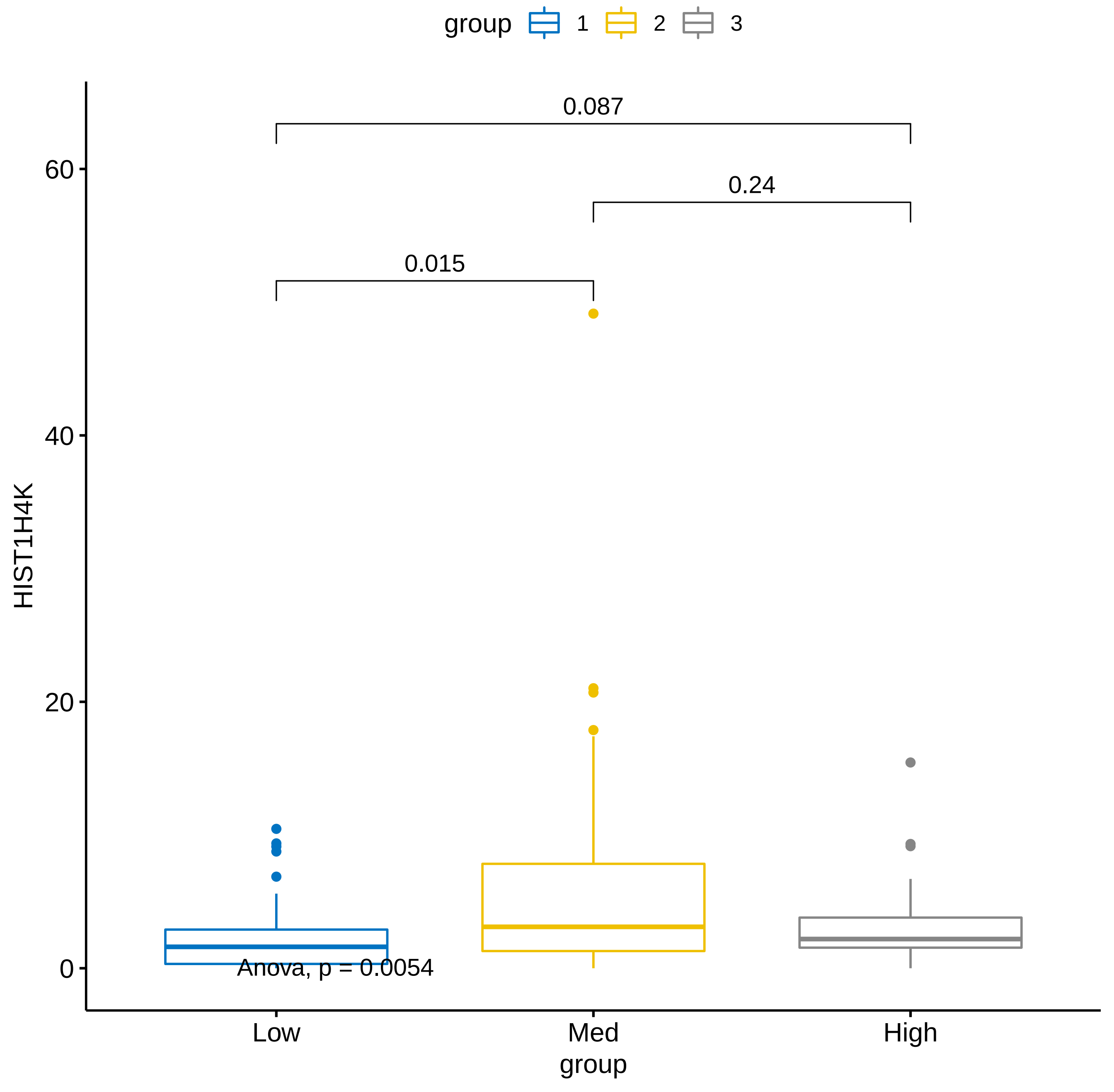
group
1
2
3
0.087
0.24
0.015
60
40
20
0
HIST1H4K
Anova, p = 0.0054
Low
Med
High
group

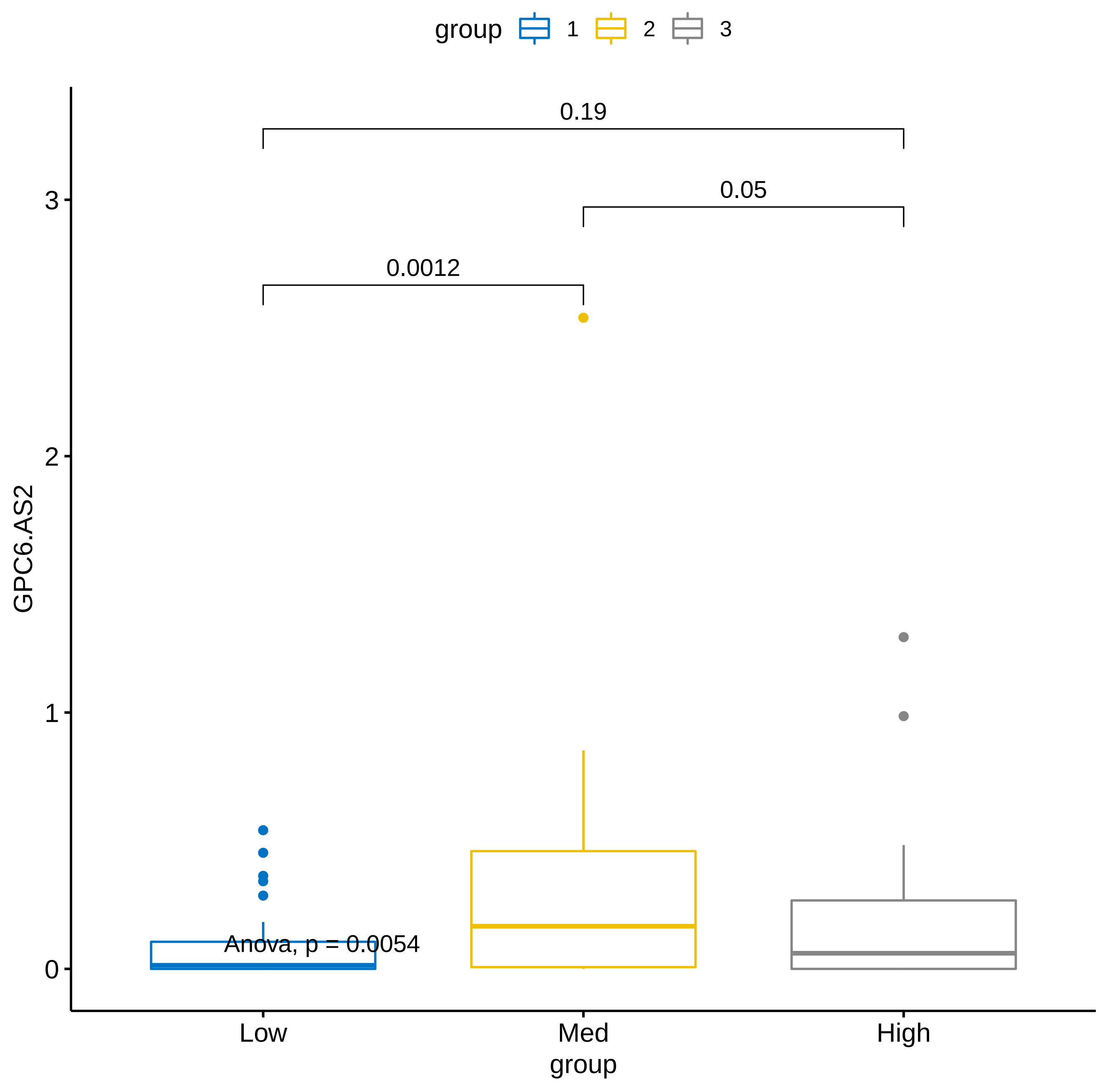
group 1 2 3
0.19
0.05
0.0012
Anova, p = 0.0054
GPC6.AS2
3
2
1
0
Low
Med
High
group

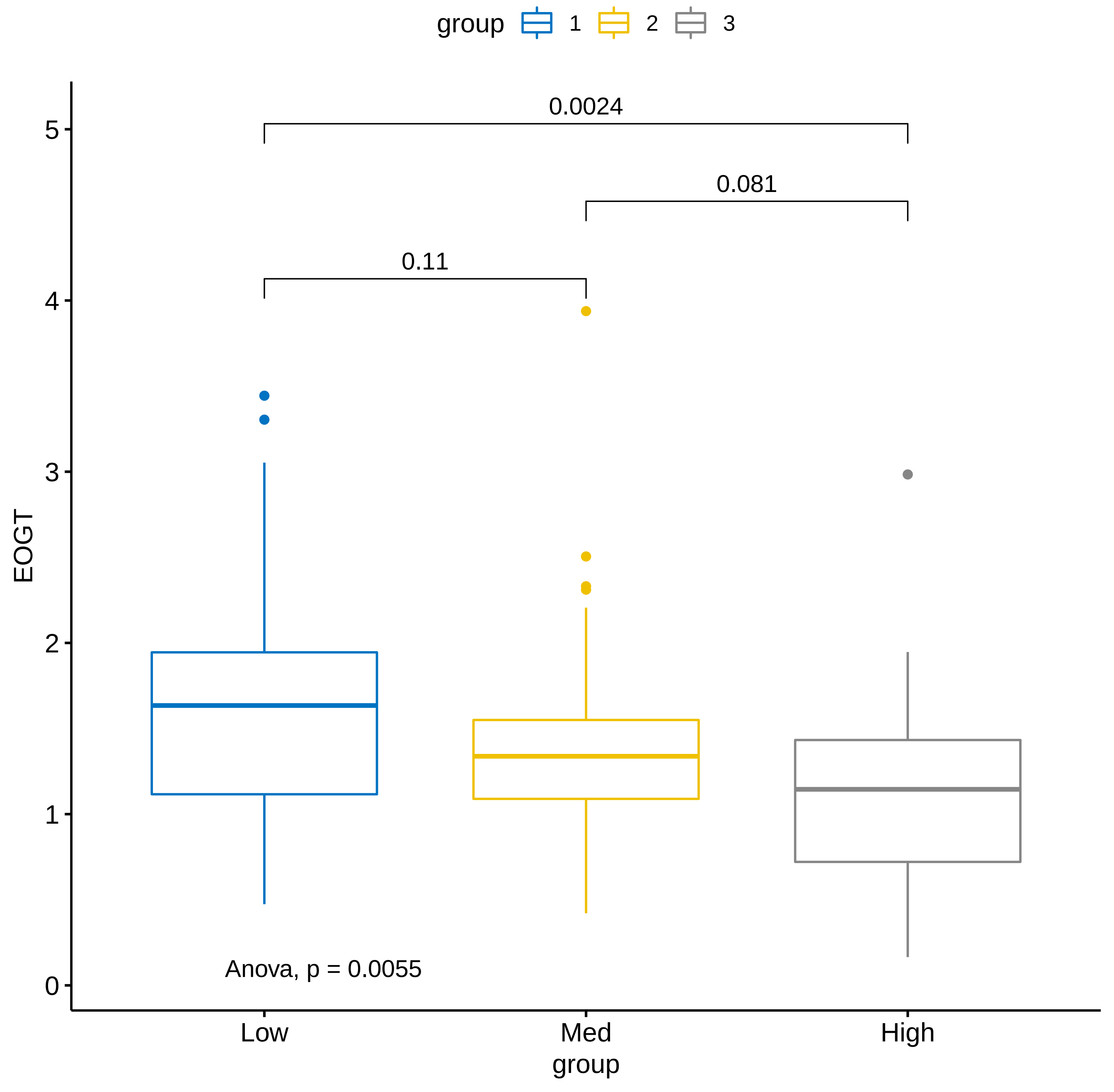

group
1
2
3
0.0024
0.081
0.11
5
4
3
2
1
0
EOGT
Anova, p = 0.0055
Low
Med
High
group

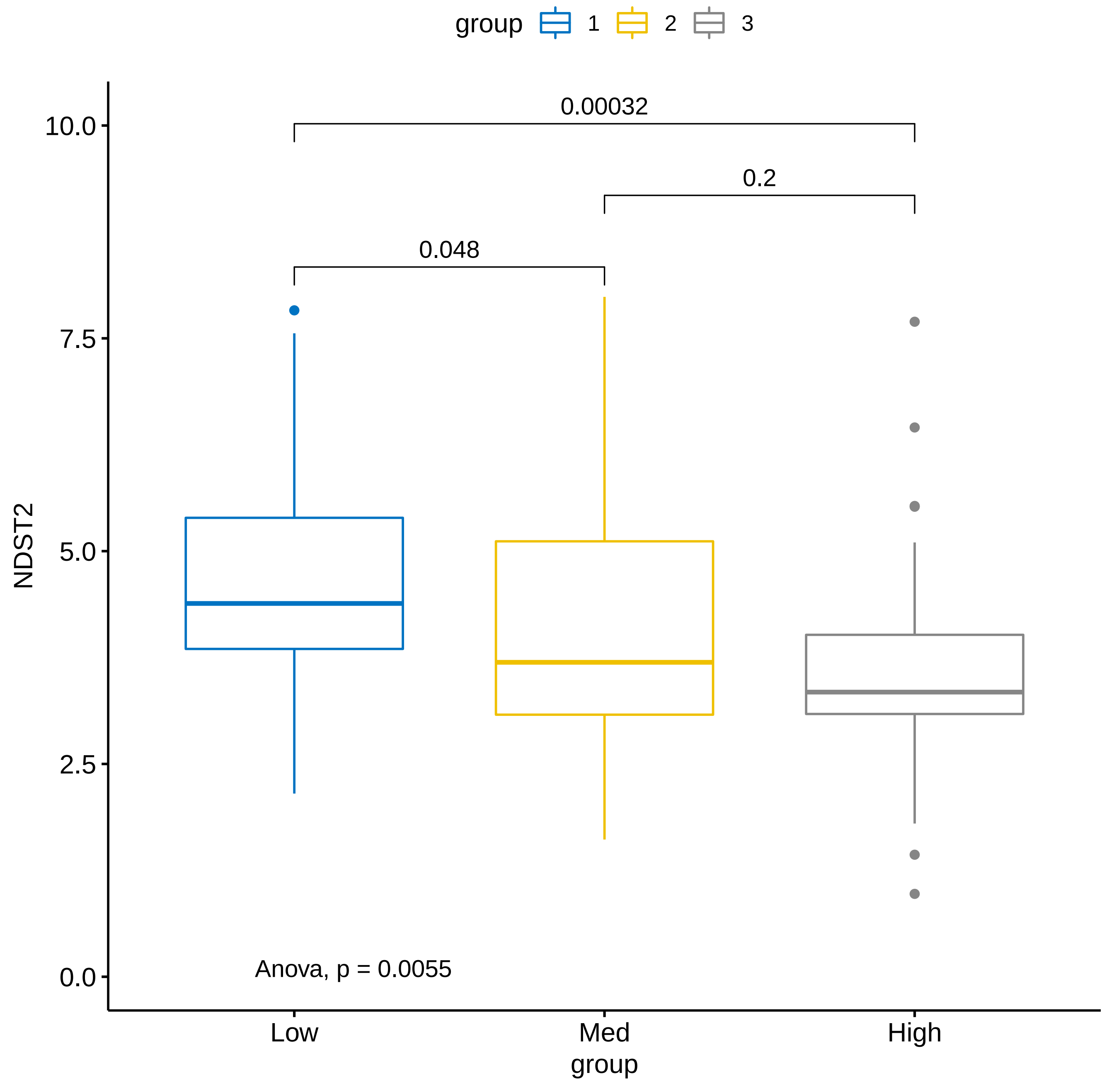
group
1
2
3
0.00032
0.2
0.048
10.0
7.5
5.0
2.5
0.0
NDST2
Anova, p = 0.0055
Low
Med
High
group

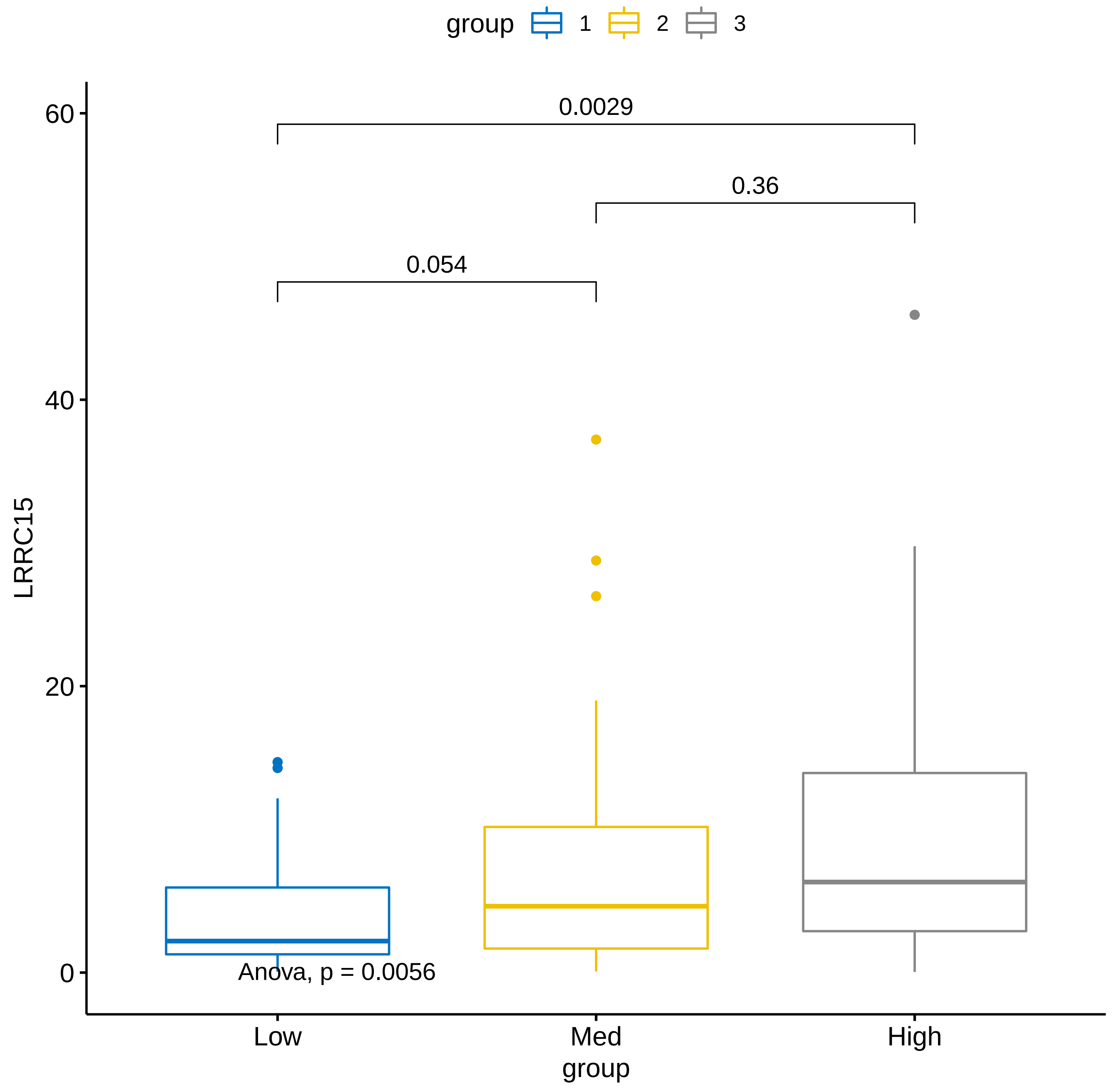

group 1 2 3
0.0029
0.36
0.054
60
40
20
0
LRRC15
Anova, p = 0.0056
Low
Med
High
group

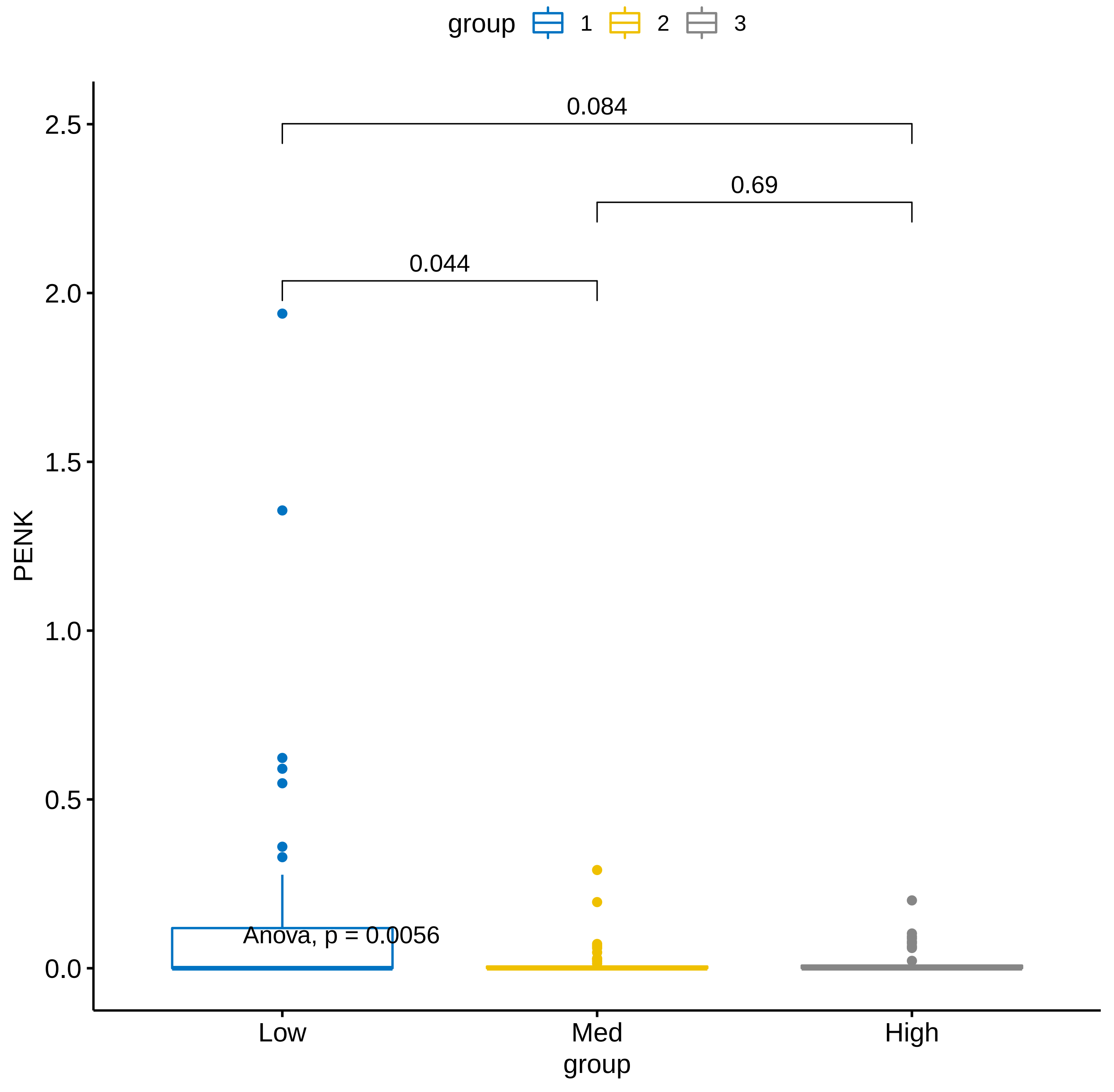
group
1
2
3
0.084
0.69
0.044
2.5
2.0
1.5
1.0
0.5
0.0
PENK
Anova, p = 0.0056
Low
Med
High
group

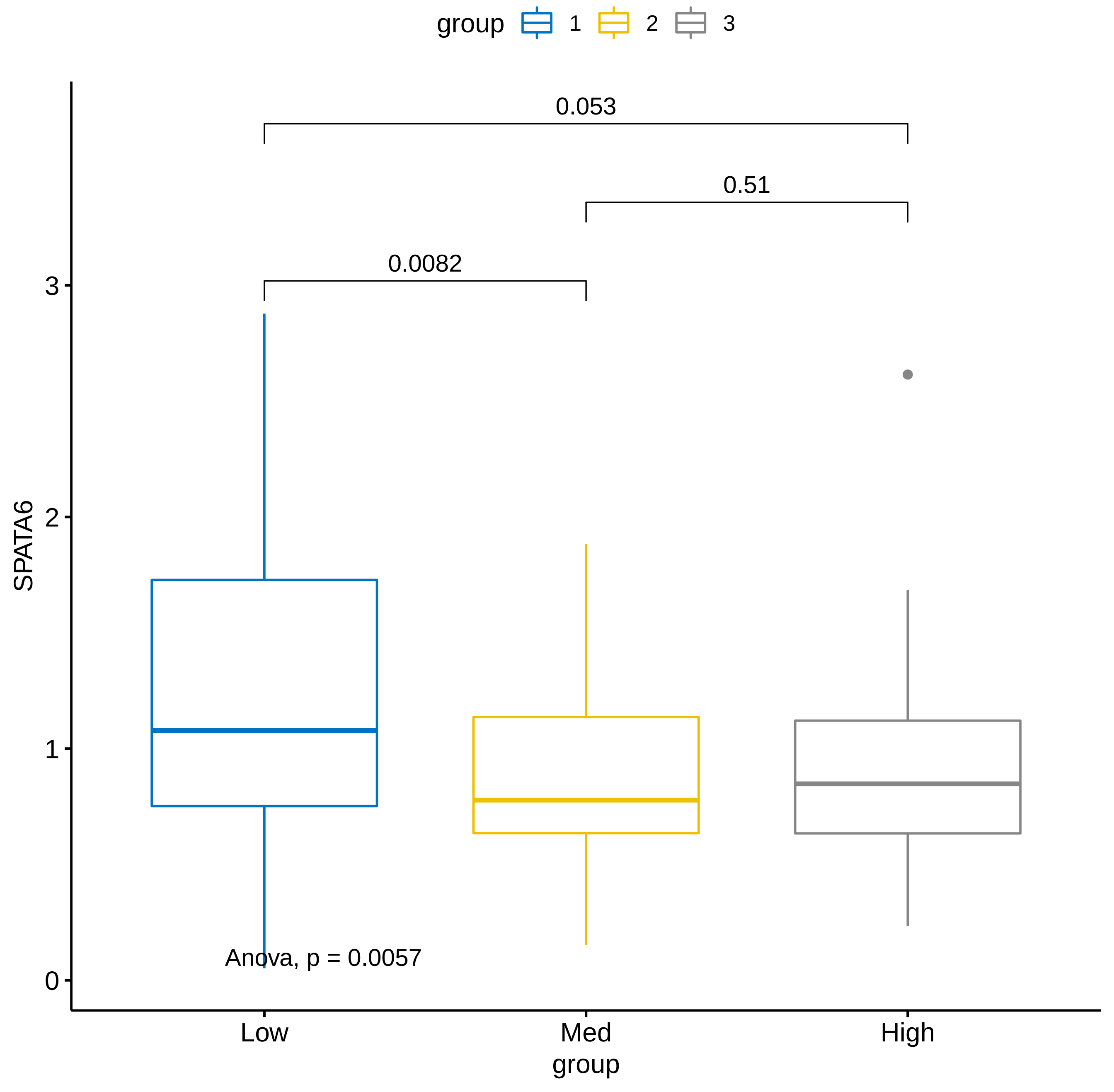

group 1 2 3
0.053
0.51
0.0082
3
2
1
0
SPATA6
Anova, p = 0.0057
Low
Med
High
group

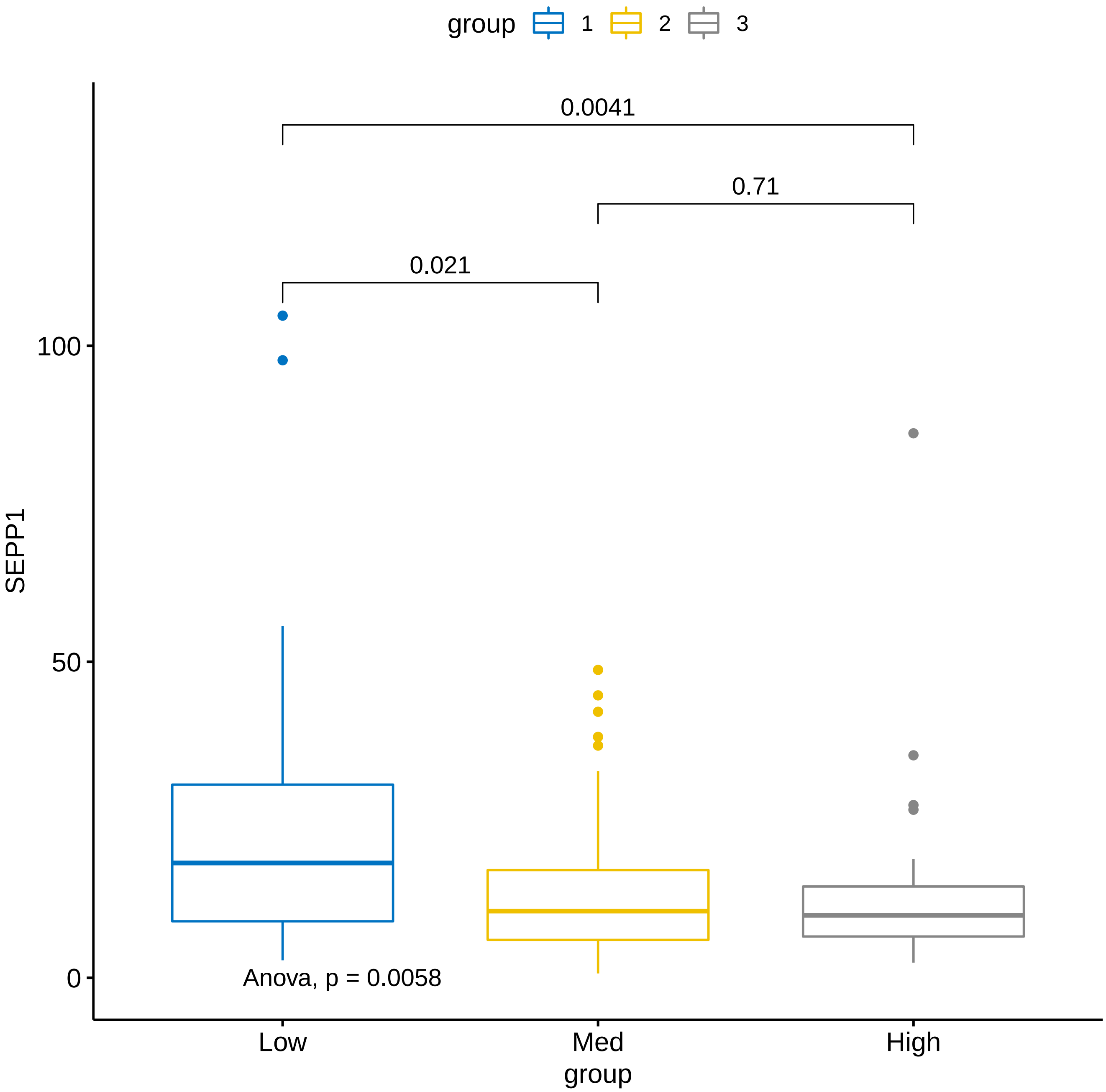
group
1
2
3
0.0041
0.71
0.021
100
50
0
SEPP1
Anova, p = 0.0058
Low
Med
High
group

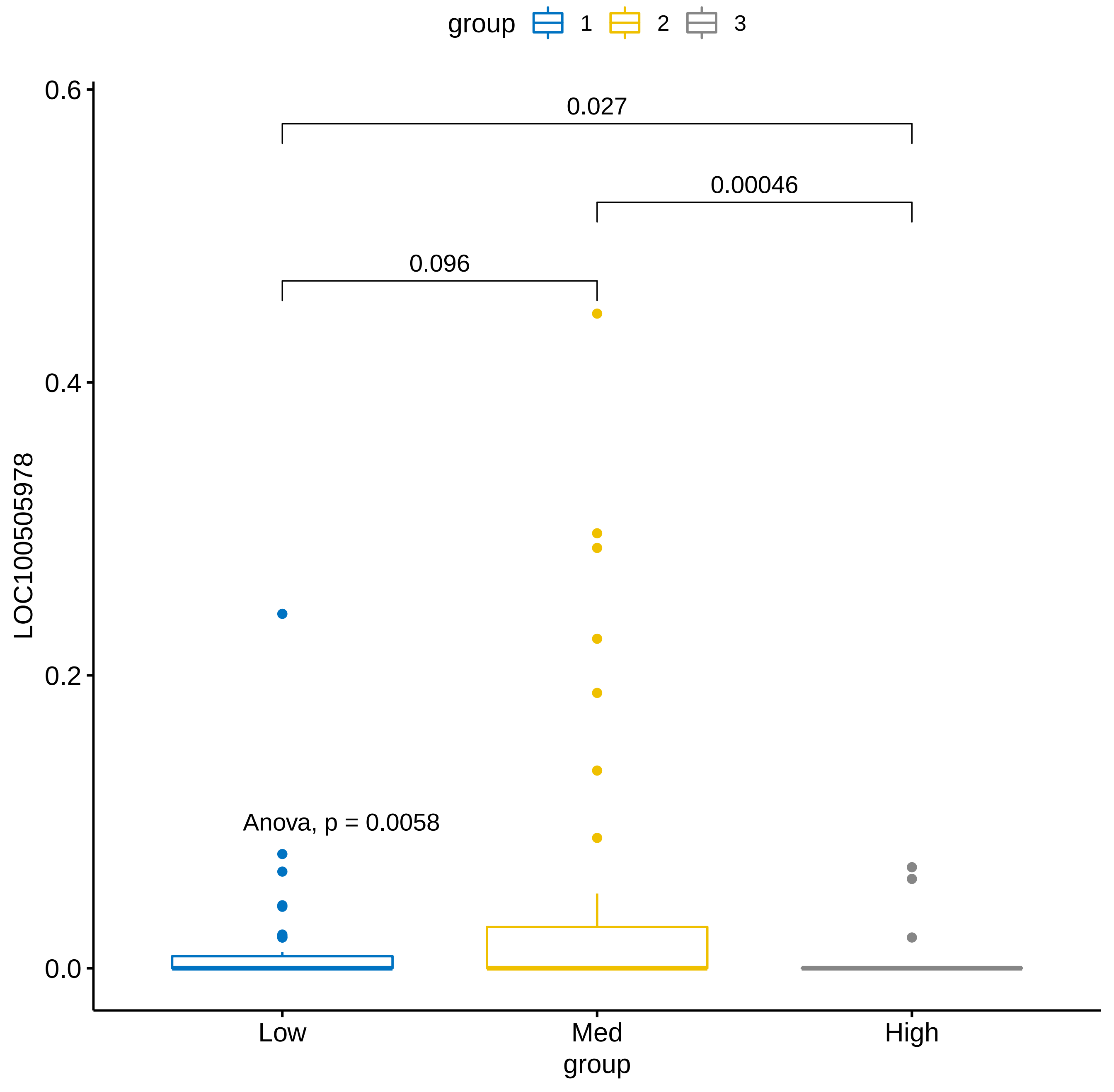

group
1
2
3
0.6
0.4
0.2
0.0
LOC100505978
0.027
0.00046
0.096
Anova, p = 0.0058
Low
Med
High
group

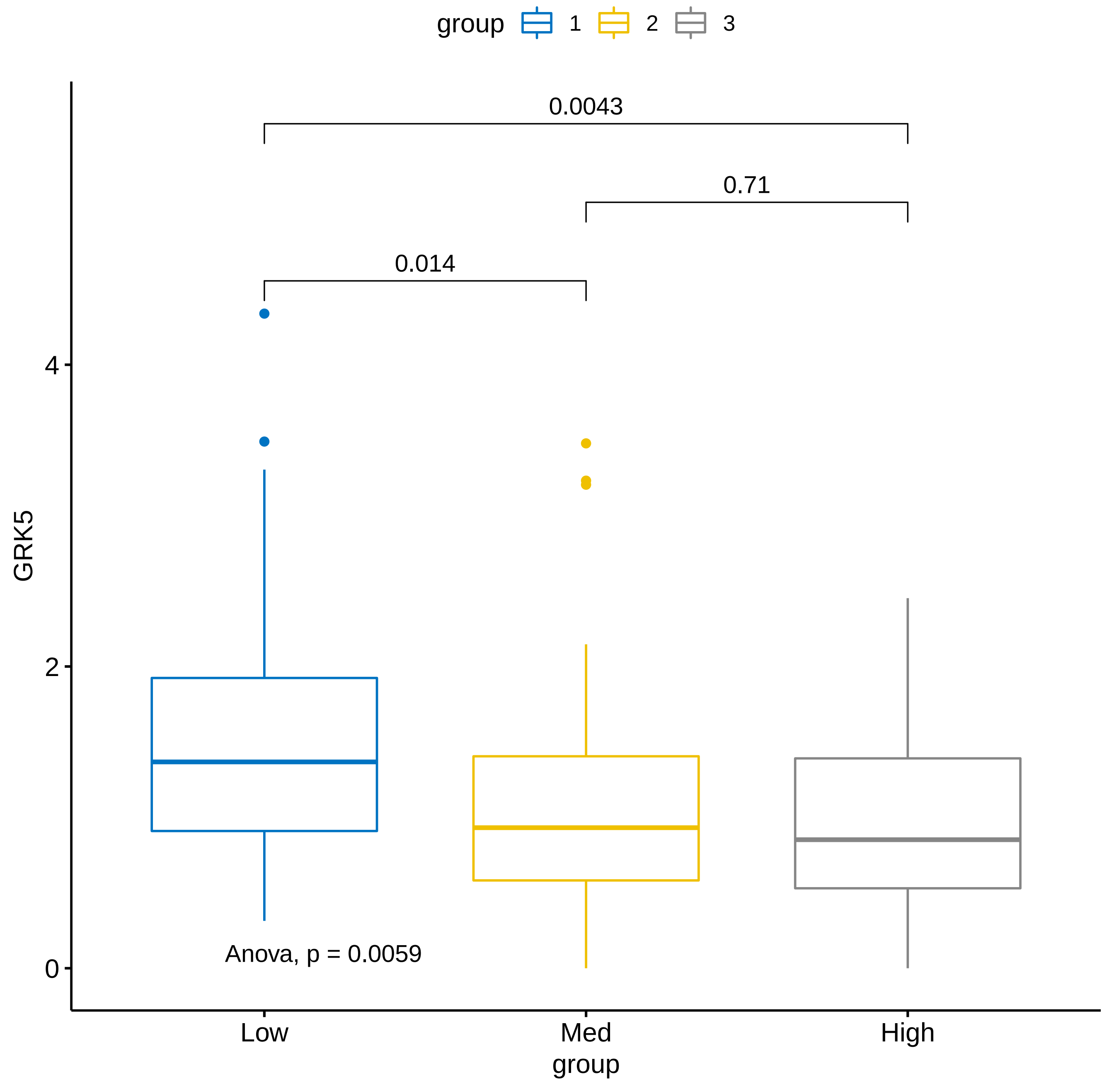

group
1
2
3
0.0043
0.71
0.014
4
2
0
GRK5
Anova, p = 0.0059
Low
Med
High
group

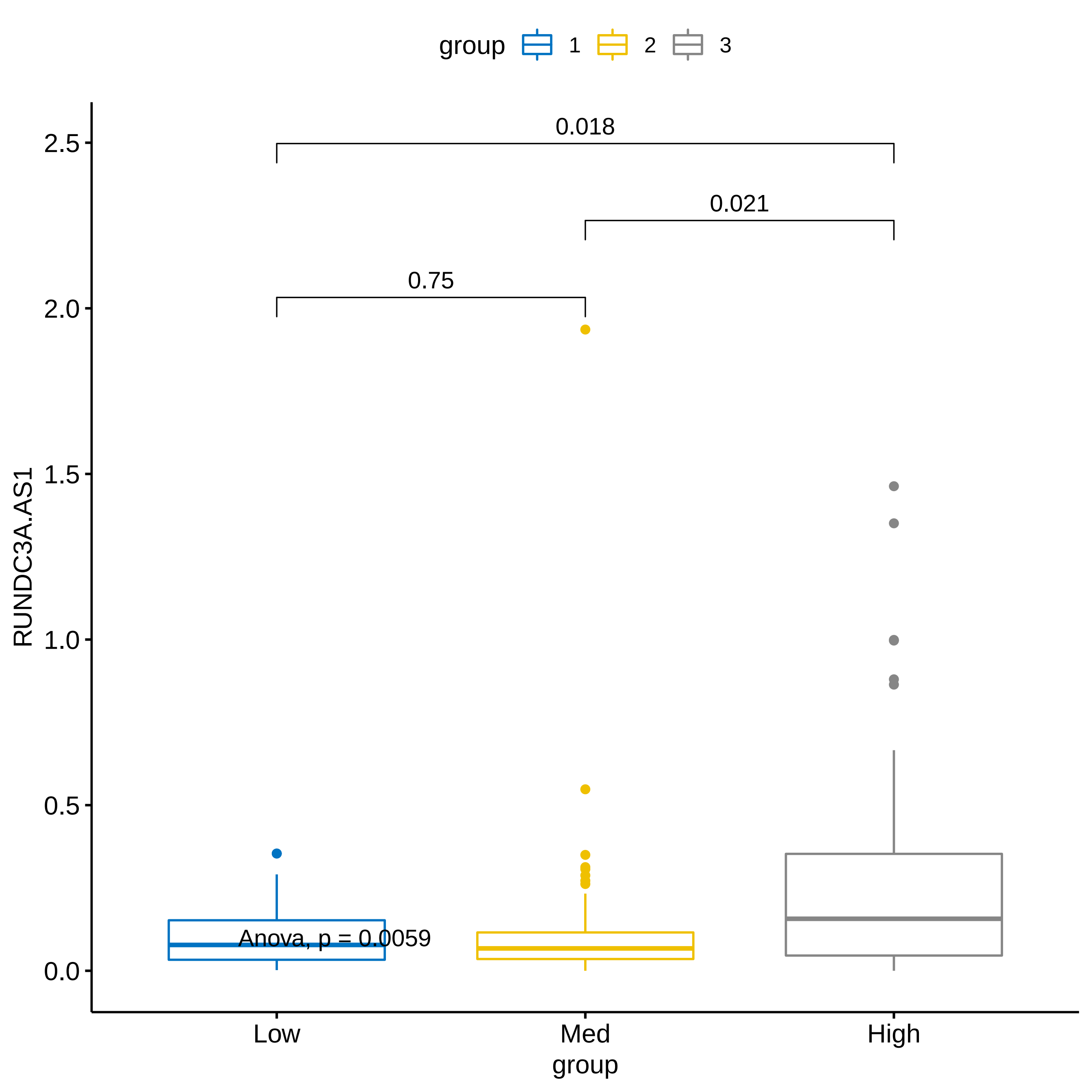
group
1
2
3
0.018
0.021
0.75
2.5
2.0
1.5
1.0
0.5
0.0
RUNDC3A.AS1
Anova, p = 0.0059
Low
Med
High
group

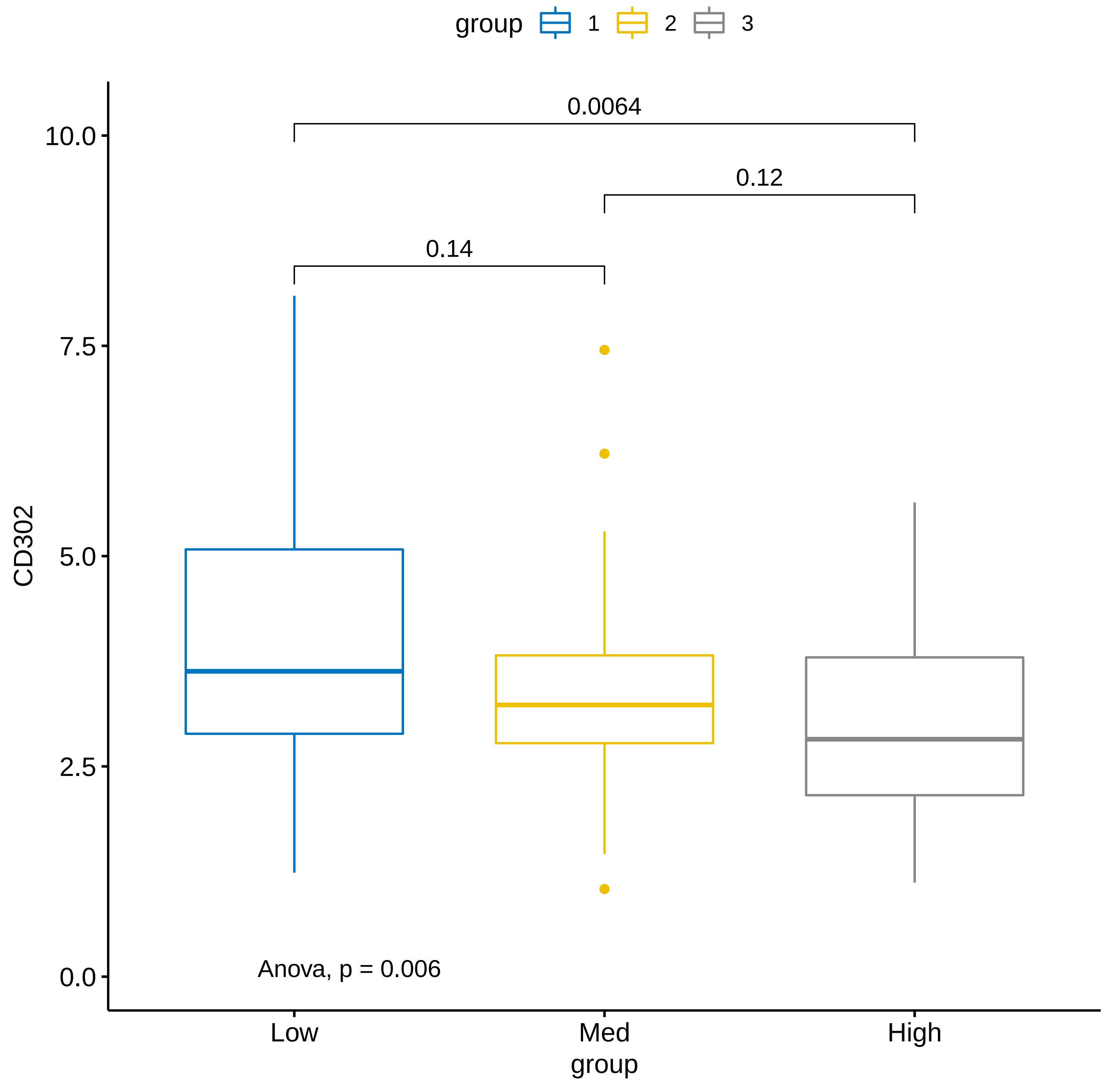

group 1 2 3
0.0064
0.12
0.14
10.0
7.5
5.0
2.5
0.0
CD302
Anova, p = 0.006
Low
Med
High
group

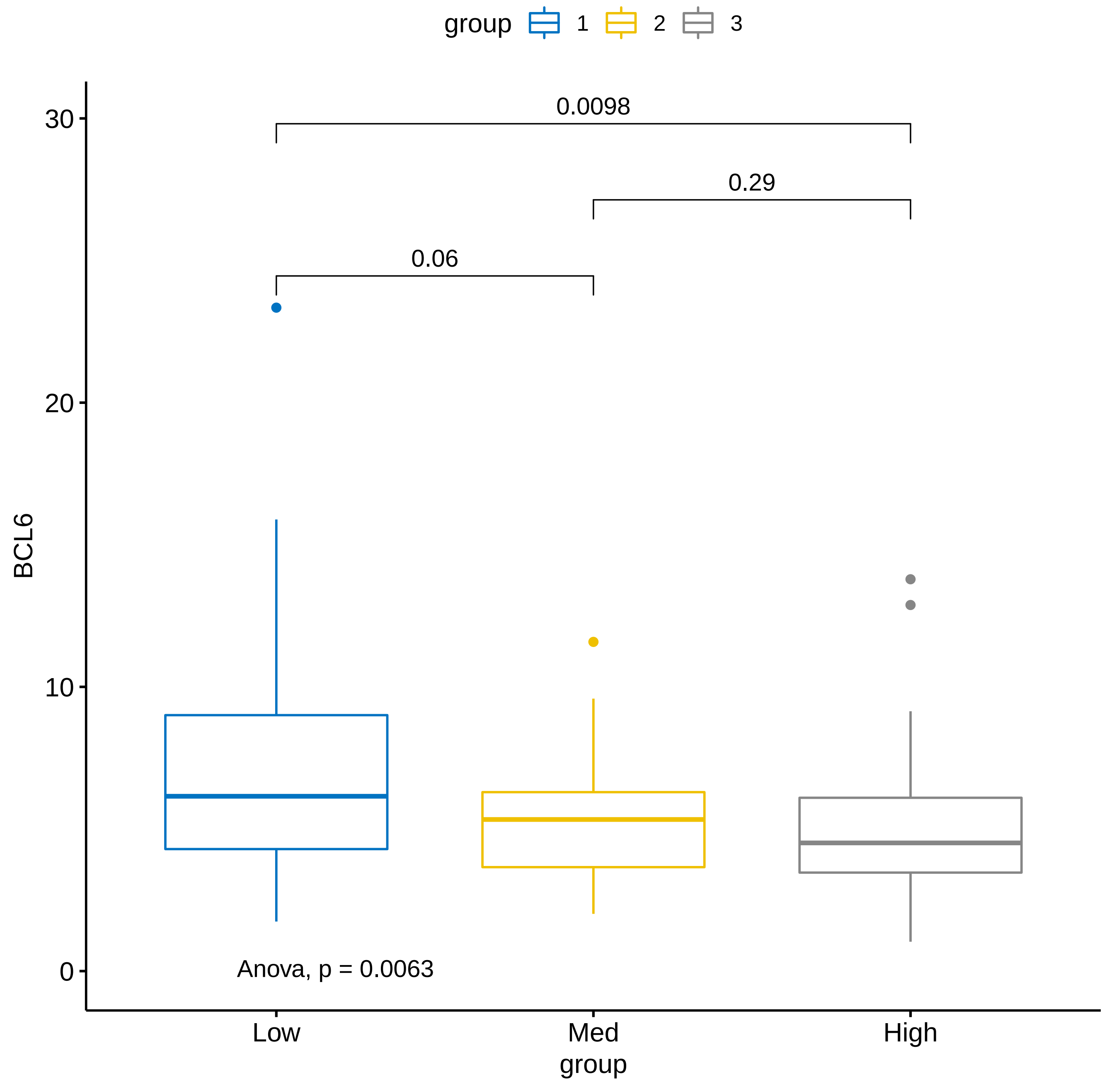
group 1 2 3
0.0098
0.29
0.06
30
20
10
0
BCL6
Anova, p = 0.0063
Low
Med
High
group

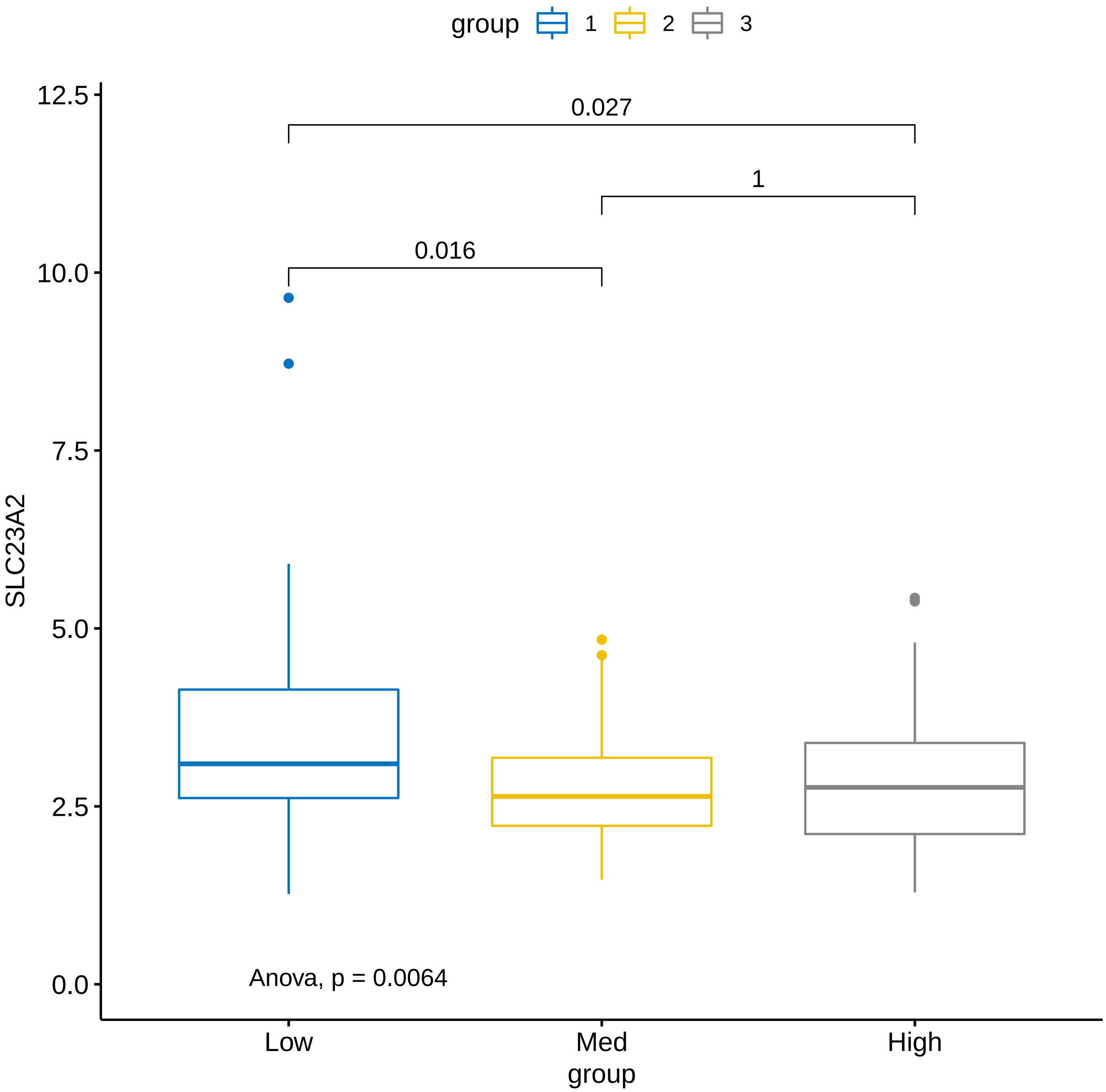
group 1 2 3
12.5
10.0
7.5
5.0
2.5
0.0
SLC23A2
0.027
1
0.016
Anova, p = 0.0064
Low
Med
High
group

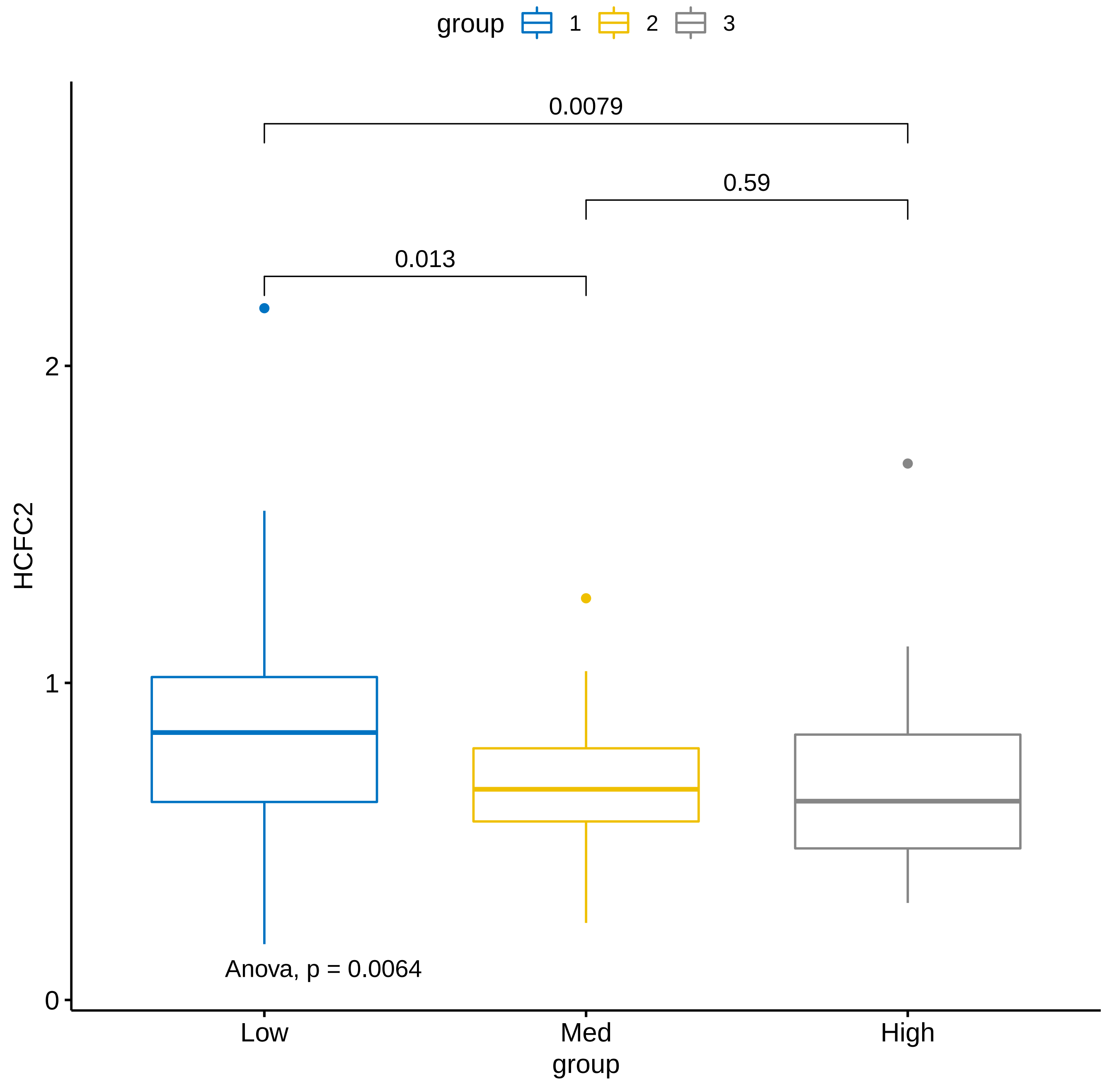
group
1
2
3
0.0079
0.59
0.013
HCFC2
2
1
0
Anova, p = 0.0064
Low
Med
High
group

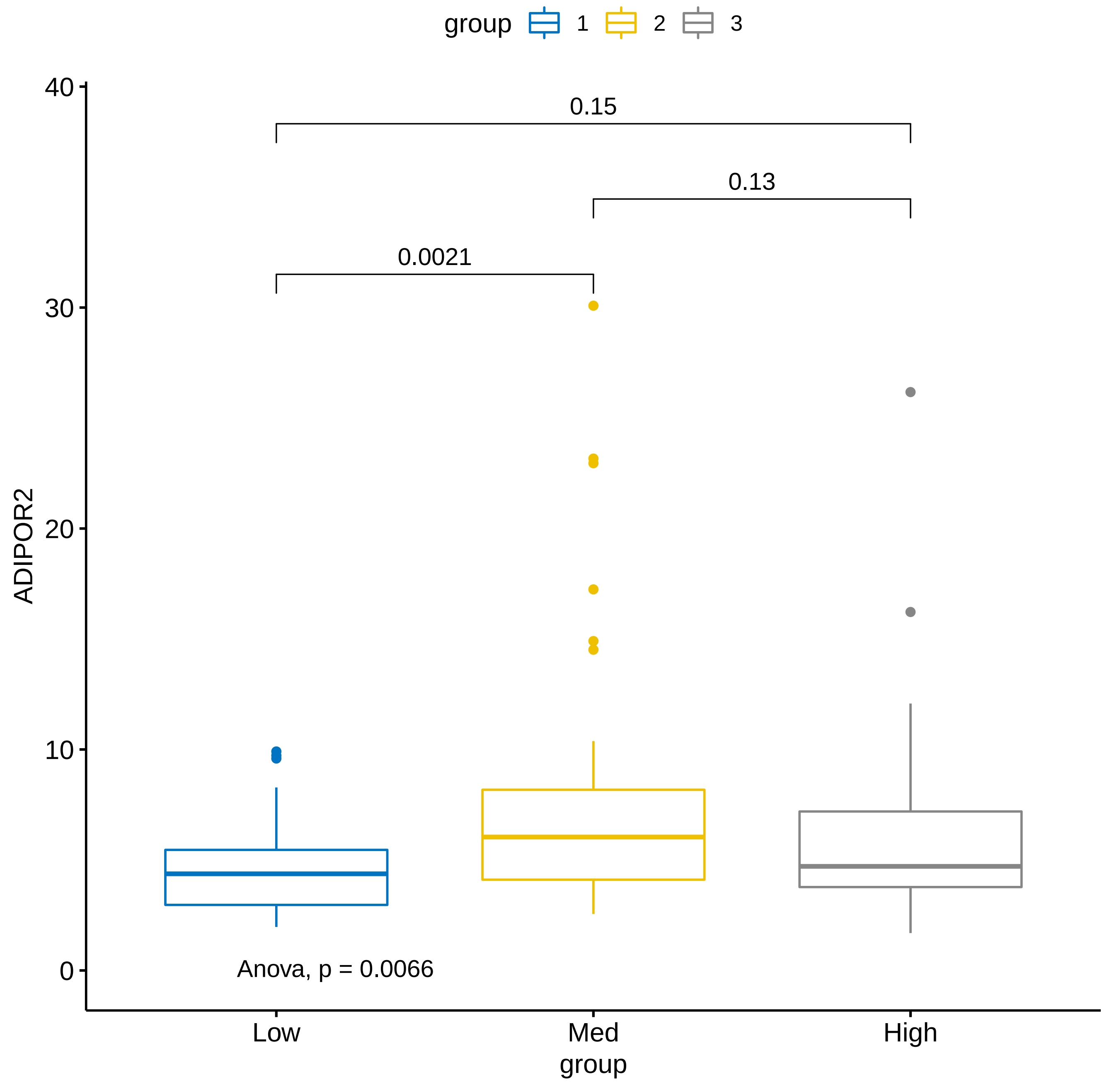

group
1
2
3
0.15
0.13
0.0021
40
30
20
10
0
ADIPOR2
Anova, p = 0.0066
Low
Med
High
group

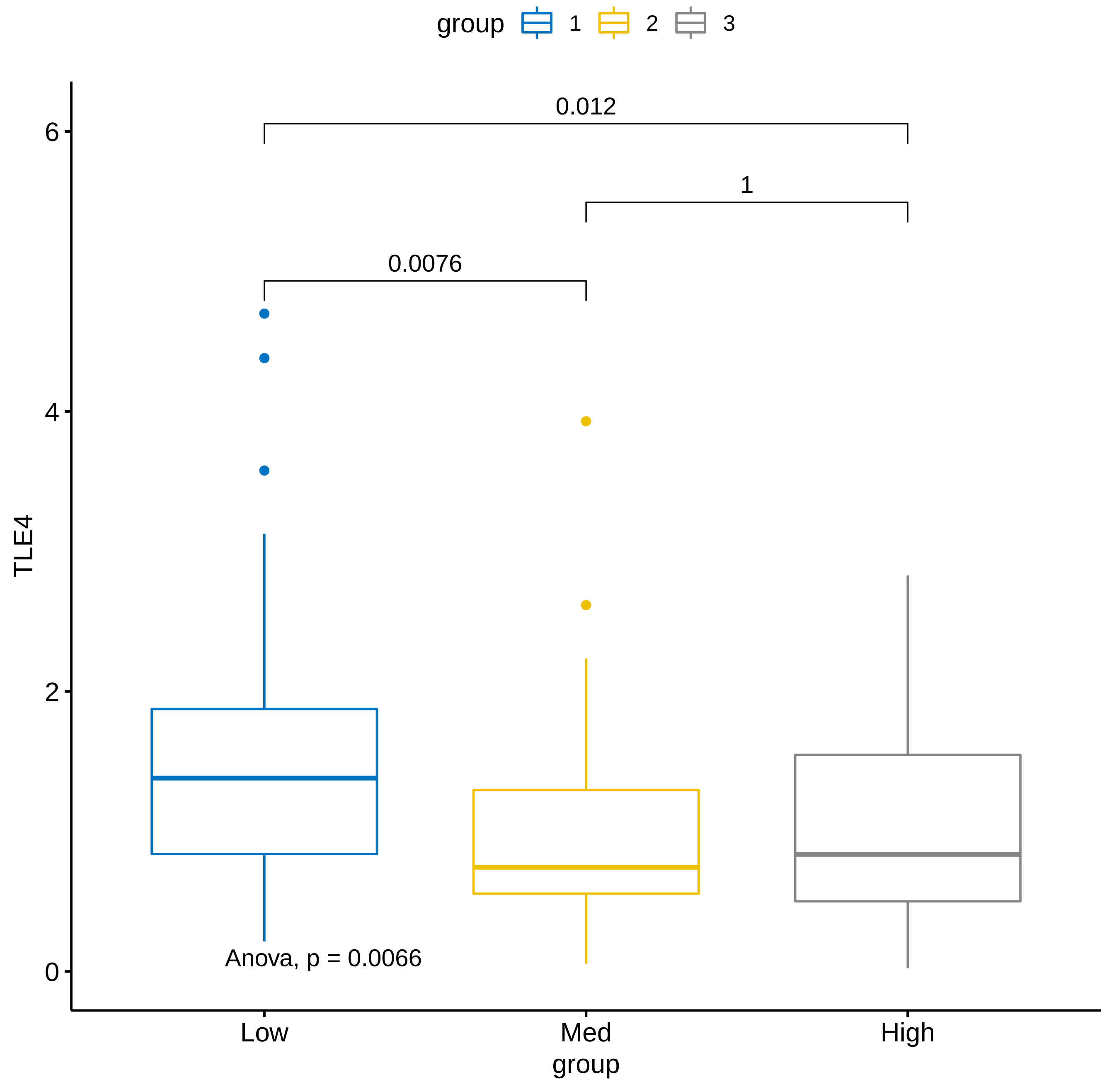
group
1
2
3
0.012
1
0.0076
6
4
2
0
TLE4
Anova, p = 0.0066
Low
Med
High
group

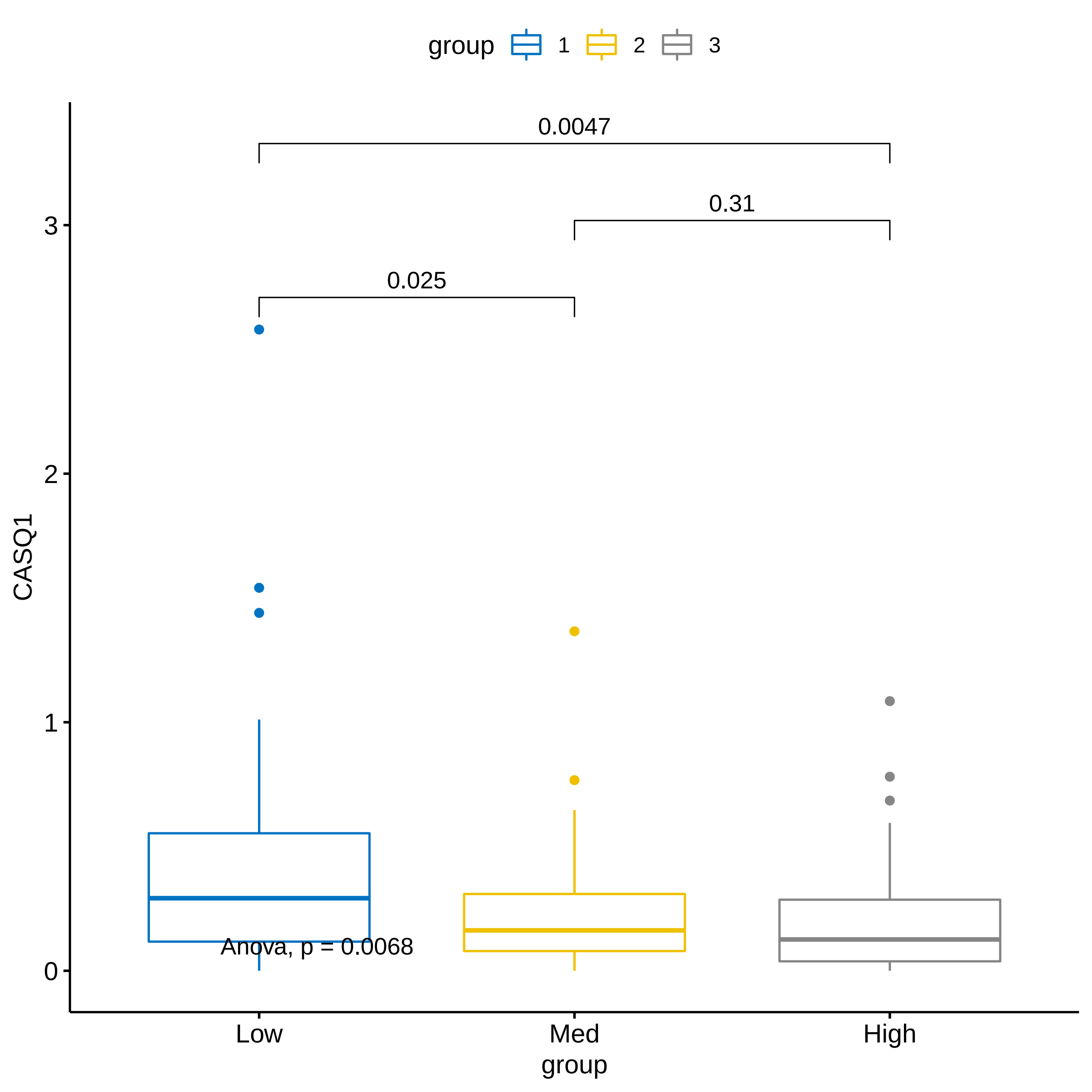
group 1 2 3
0.0047
0.31
0.025
3
2
1
0
CASQ1
Anova, p = 0.0068
Low
Med
High
group

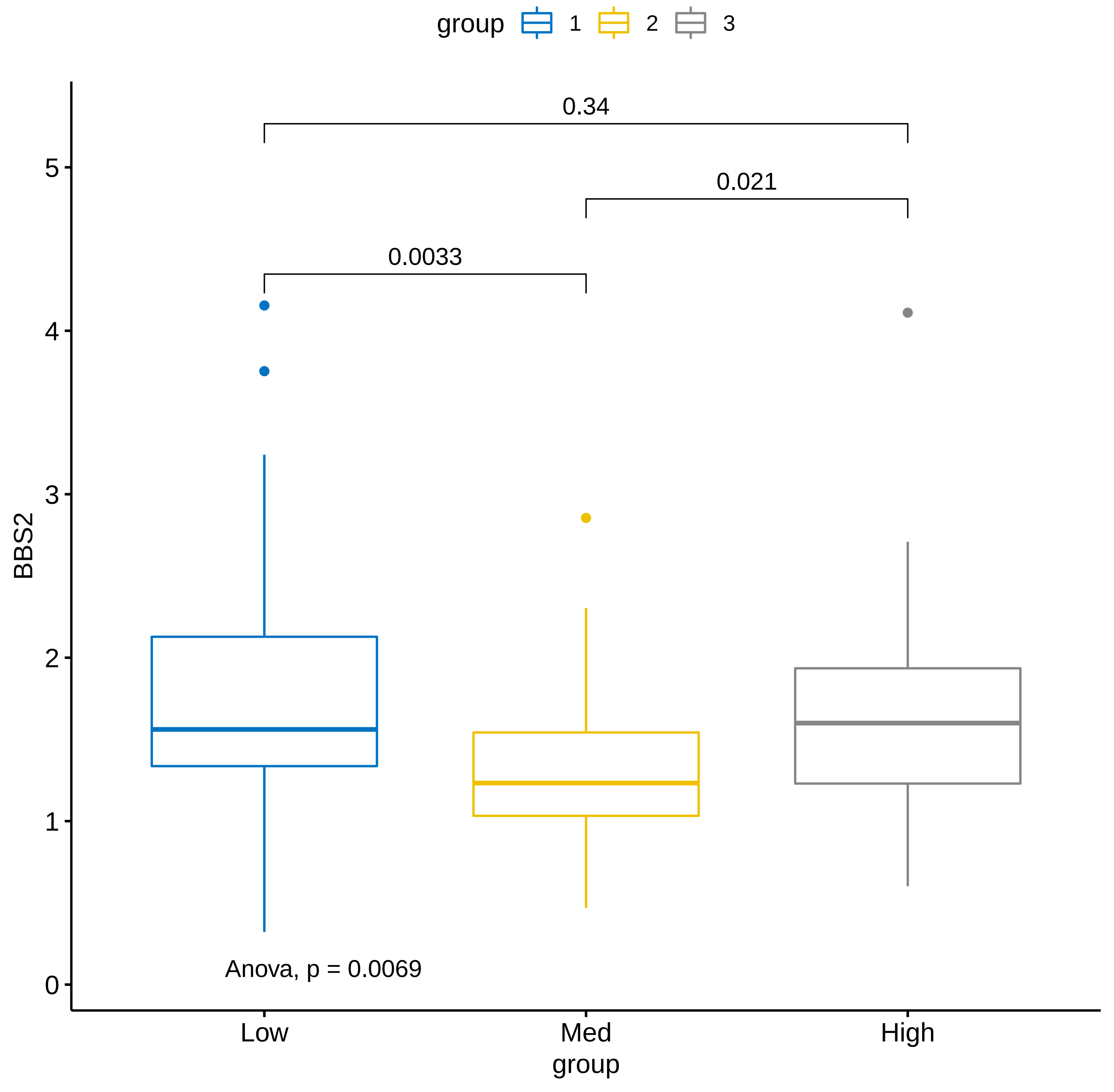
group
1
2
3
0.34
0.021
0.0033
5
4
3
2
1
0
BBS2
Anova, p = 0.0069
Low
Med
High
group

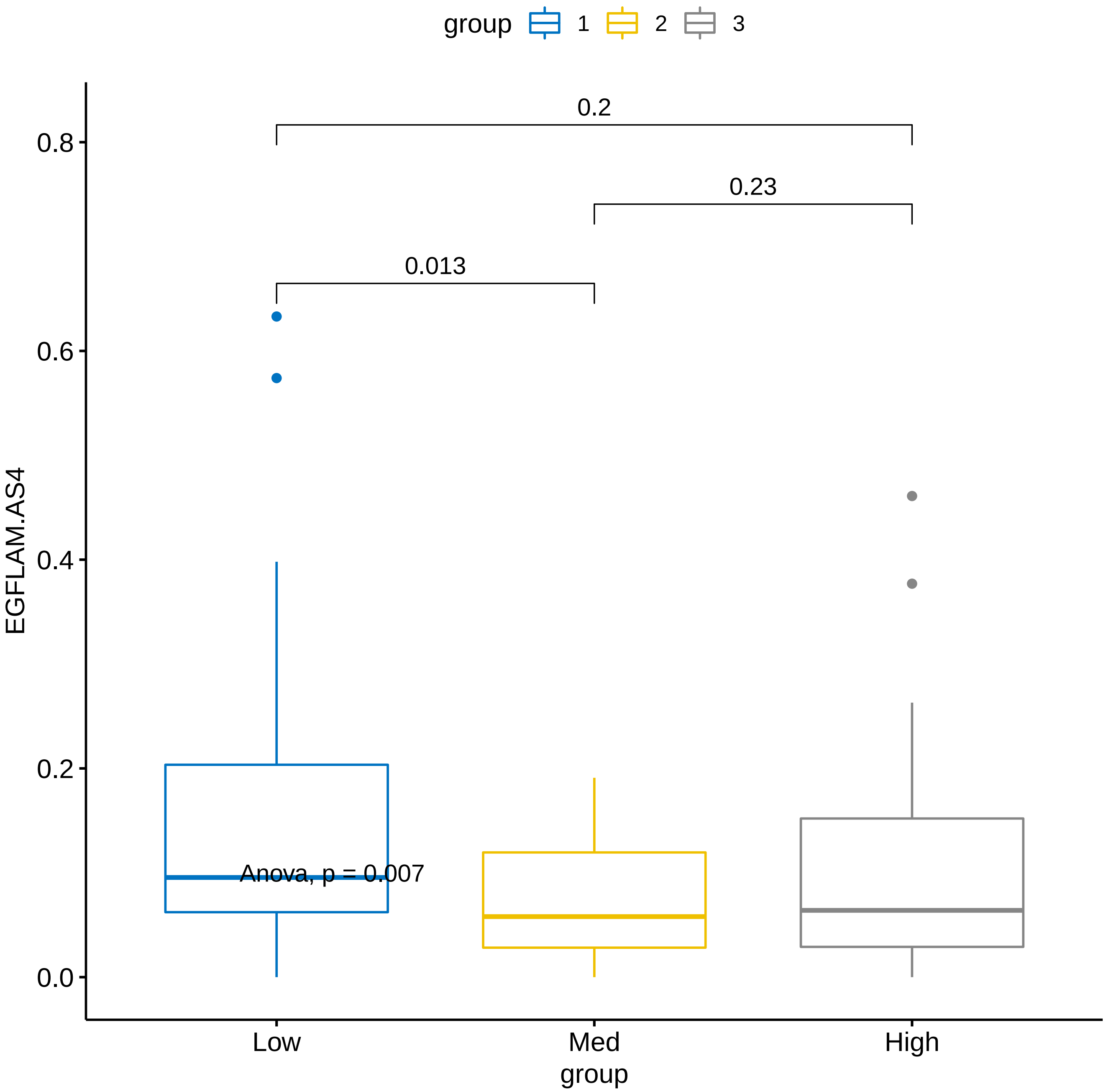

group
1
2
3
0.2
0.23
0.013
0.8
0.6
0.4
0.2
0.0
EGFLAM.AS4
Anova, p = 0.007
Low
Med
High
group

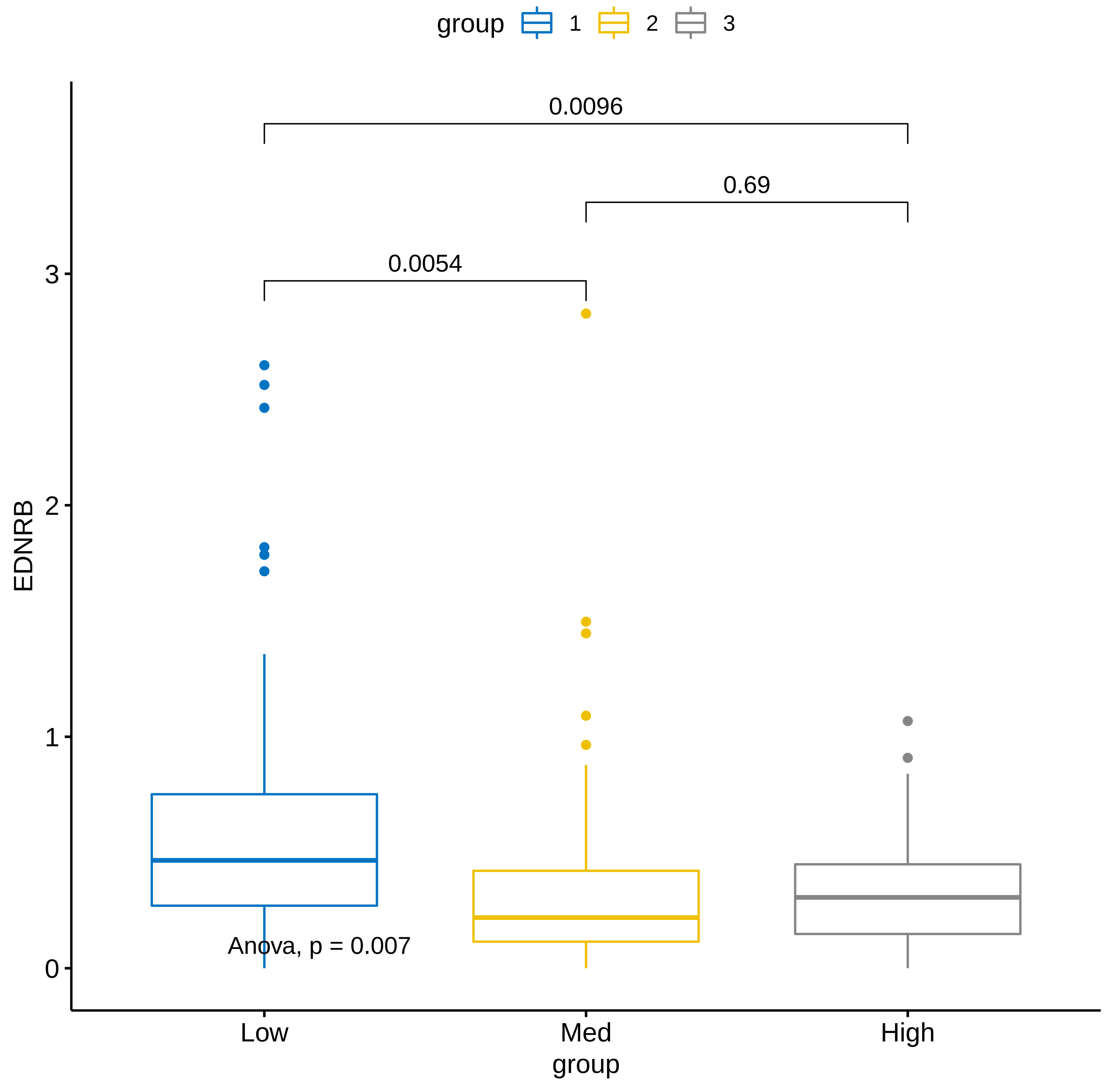
group 1 2 3
0.0096
0.69
0.0054
3
2
1
0
EDNRB
Anova, p = 0.007
Low
Med
High
group

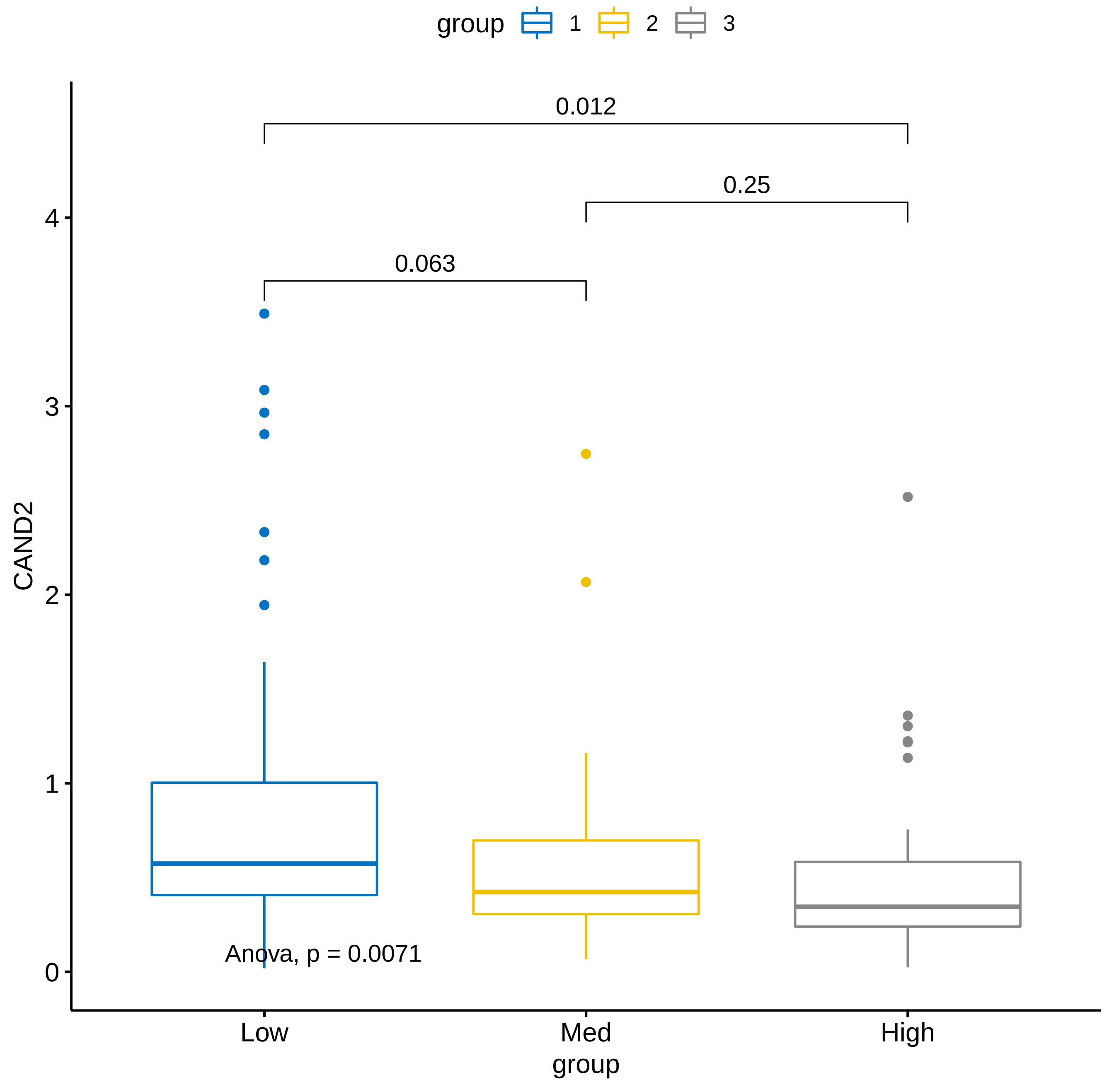

group 1 2 3
0.012
0.25
0.063
4
3
2
1
0
CAND2
Anova, p = 0.0071
Low
Med
High
group

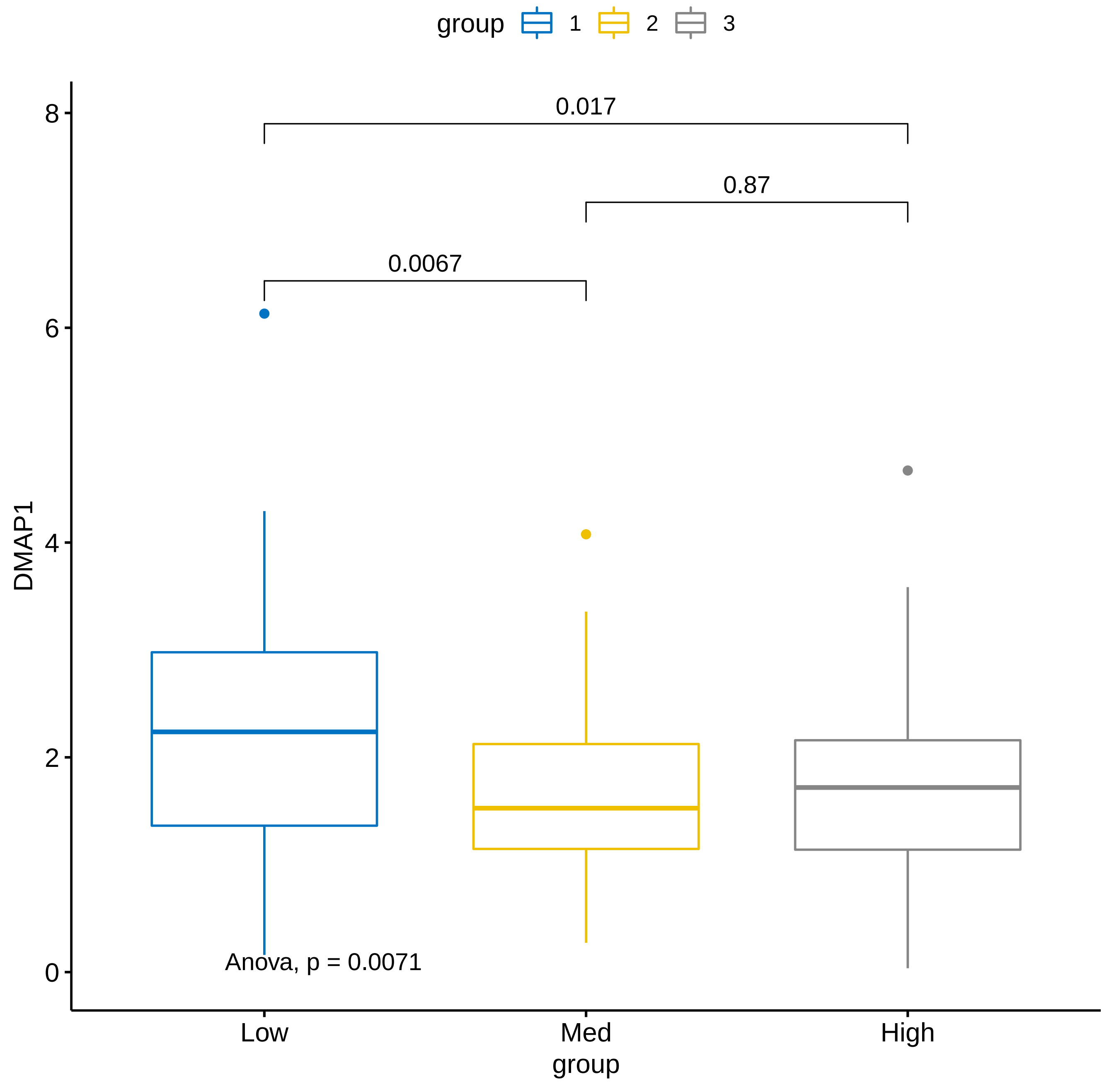
group 1 2 3
0.017
0.87
0.0067
DMAP1
8
6
4
2
0
Anova, p = 0.0071
Low
Med
High
group

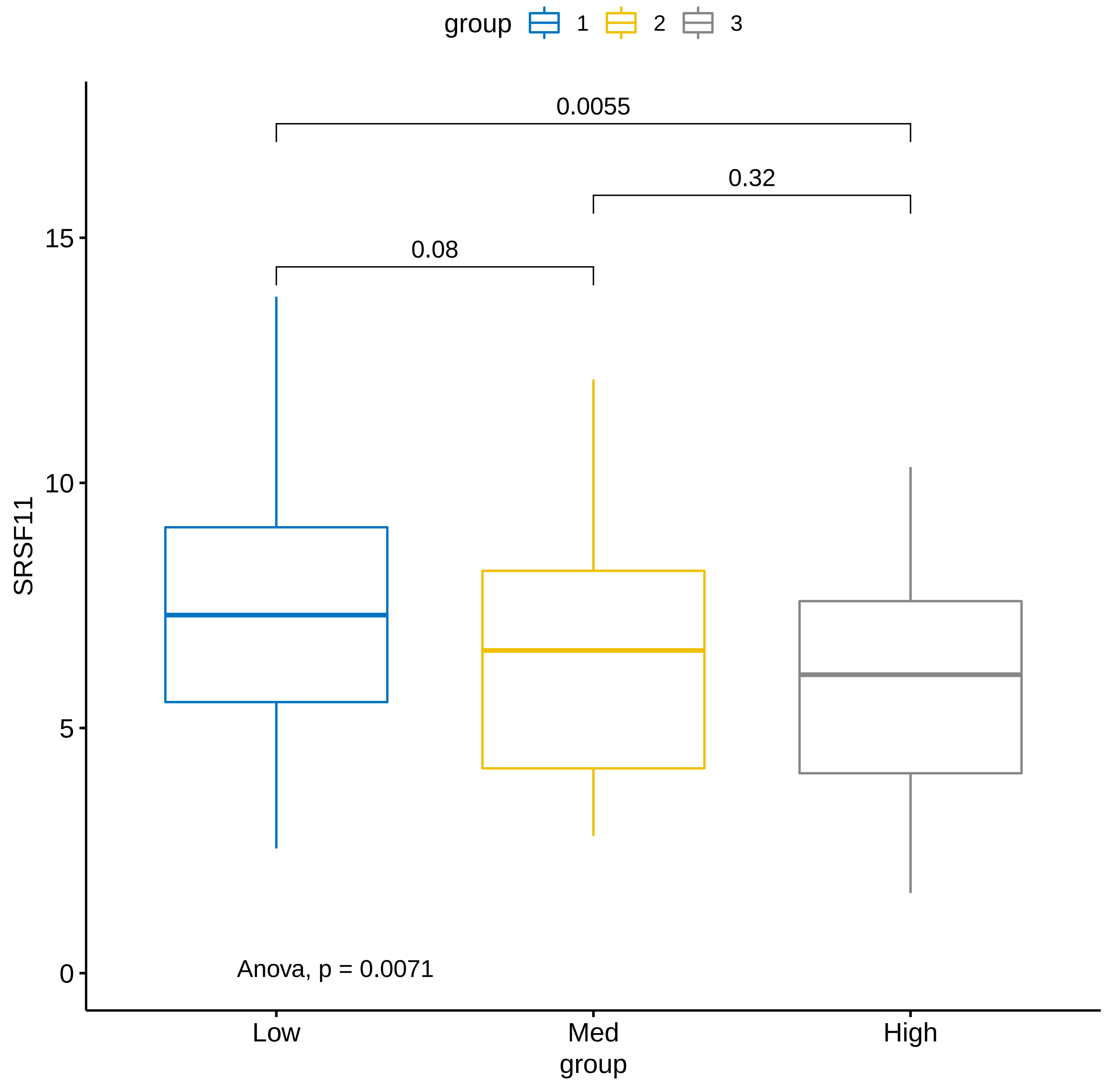
group 1 2 3
0.0055
0.32
0.08
15
10
5
0
SRSF11
Anova, p = 0.0071
Low
Med
High
group

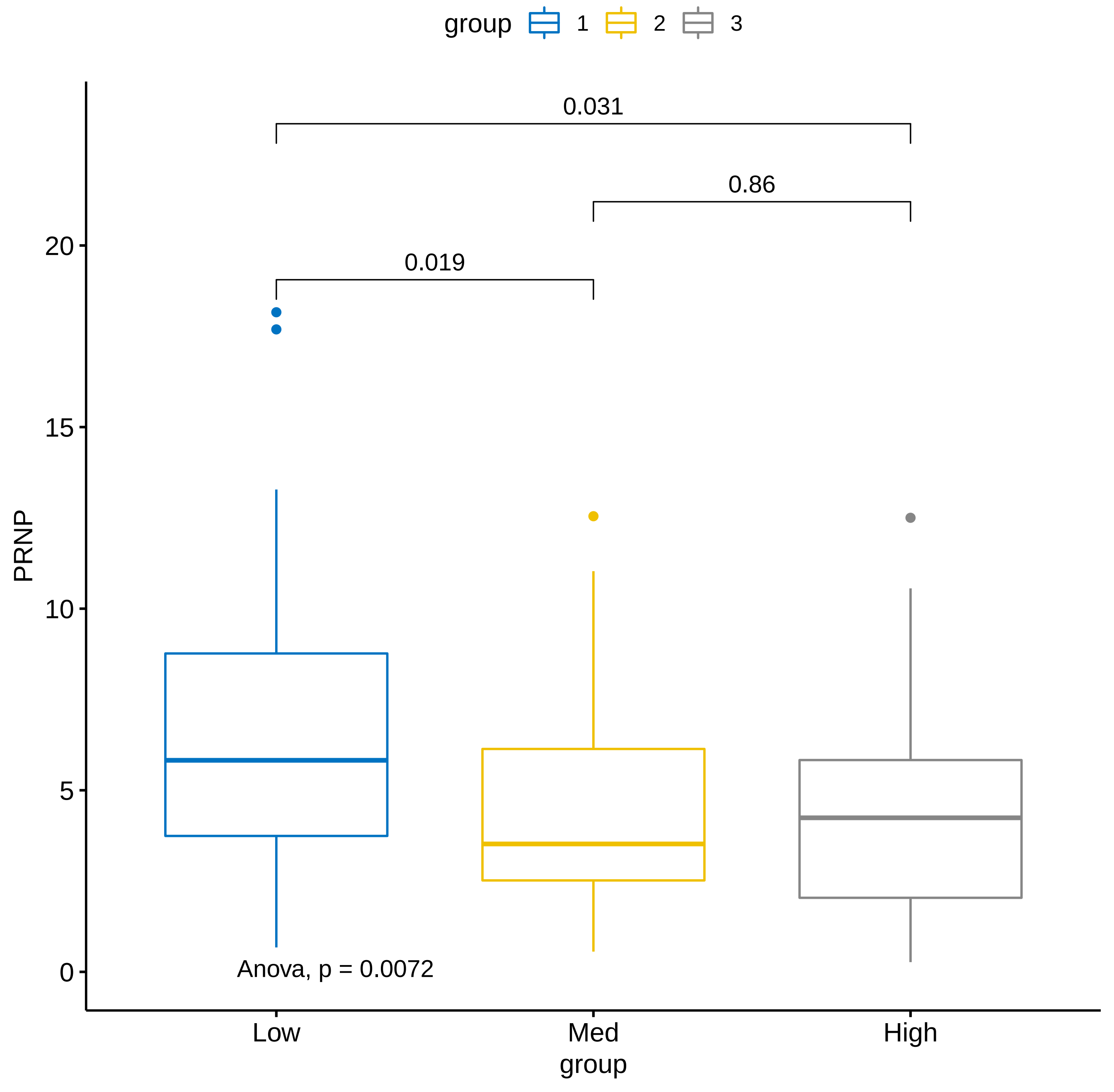

group
1
2
3
0.031
0.86
0.019
20
15
10
5
0
PRNP
Anova, p = 0.0072
Low
Med
High
group

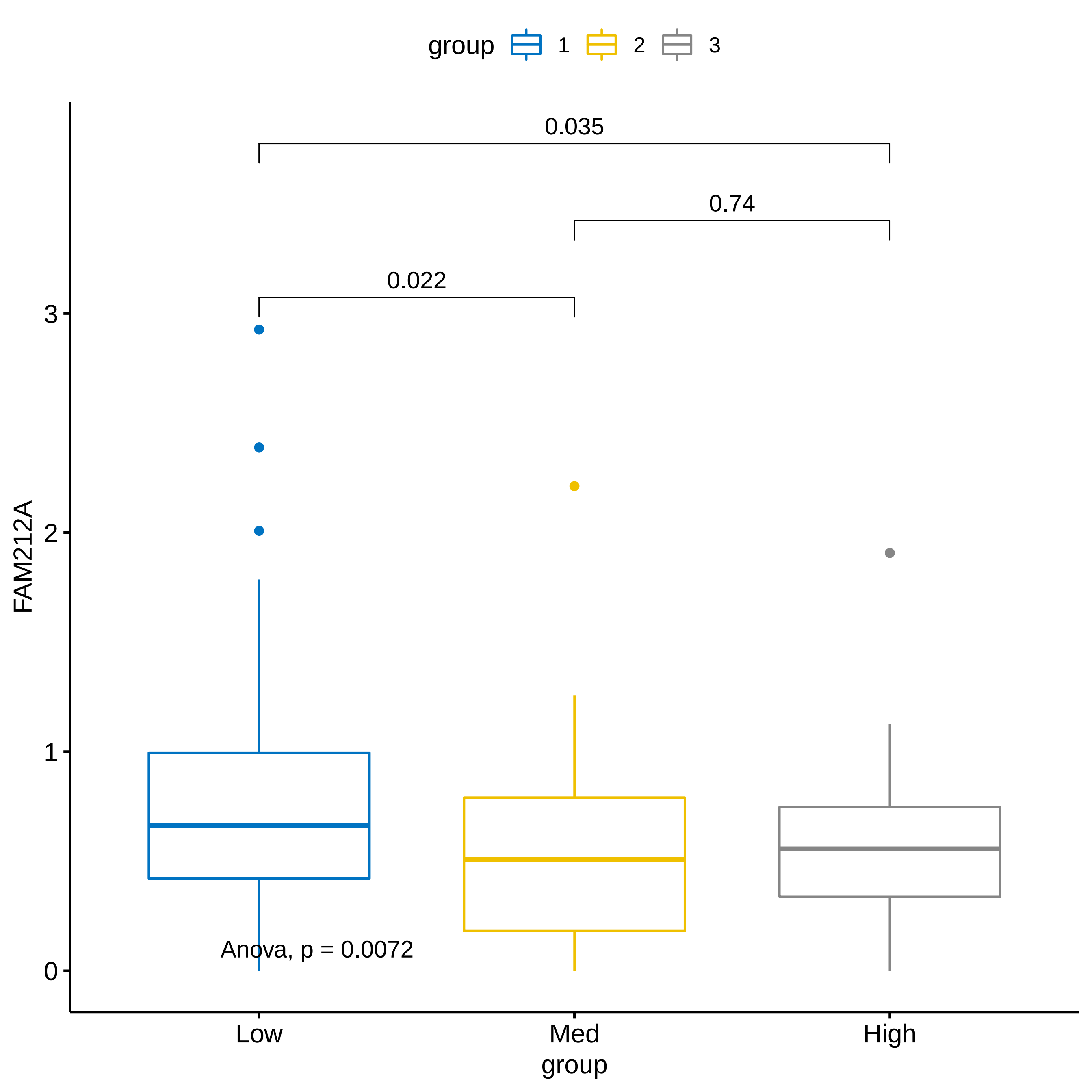
group
1
2
3
0.035
0.74
0.022
3
2
1
0
FAM212A
Anova, p = 0.0072
Low
Med
High
group

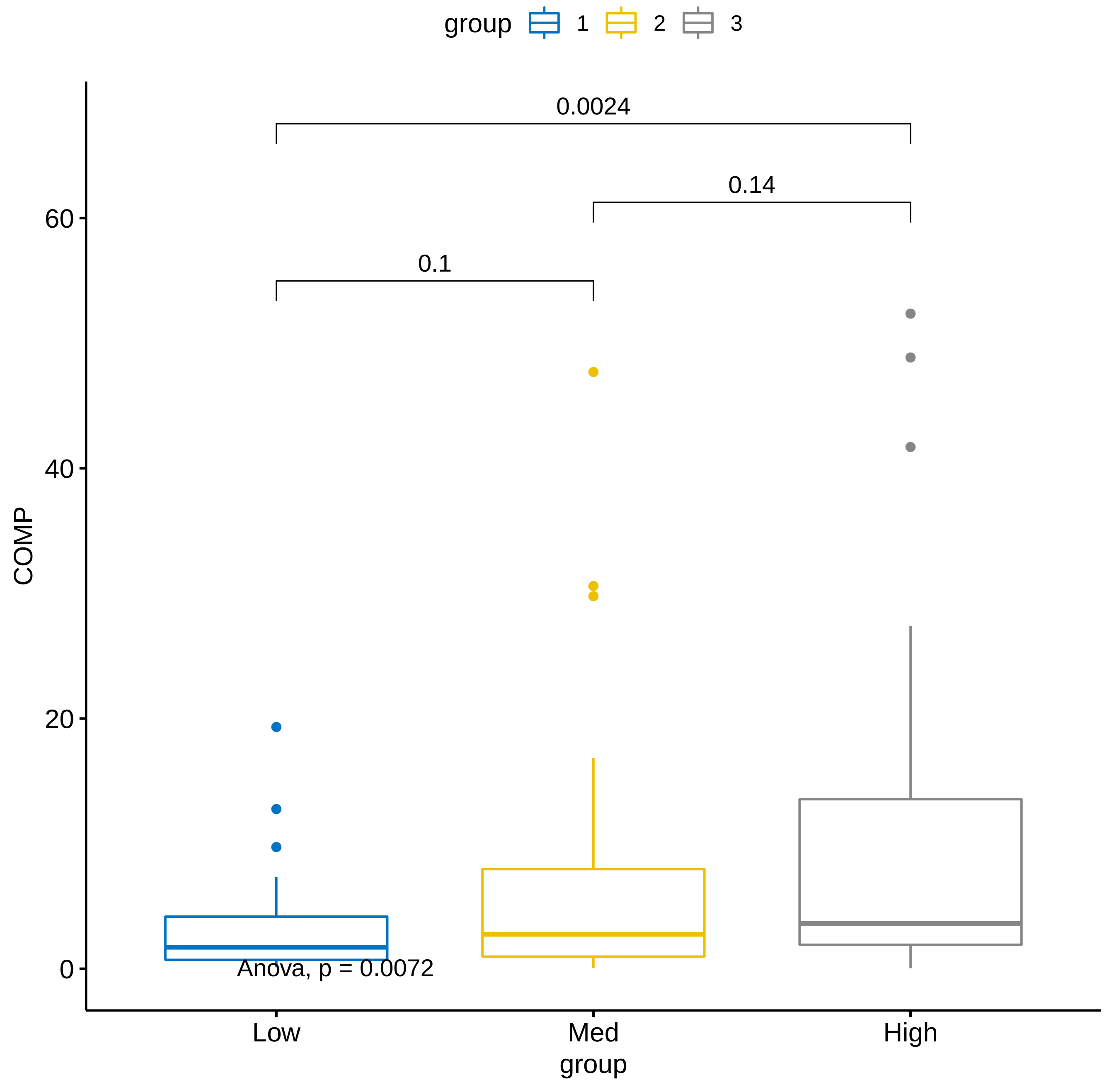
group
1
2
3
0.0024
0.14
0.1
60
40
20
0
COMP
Anova, p = 0.0072
Low
Med
High
group

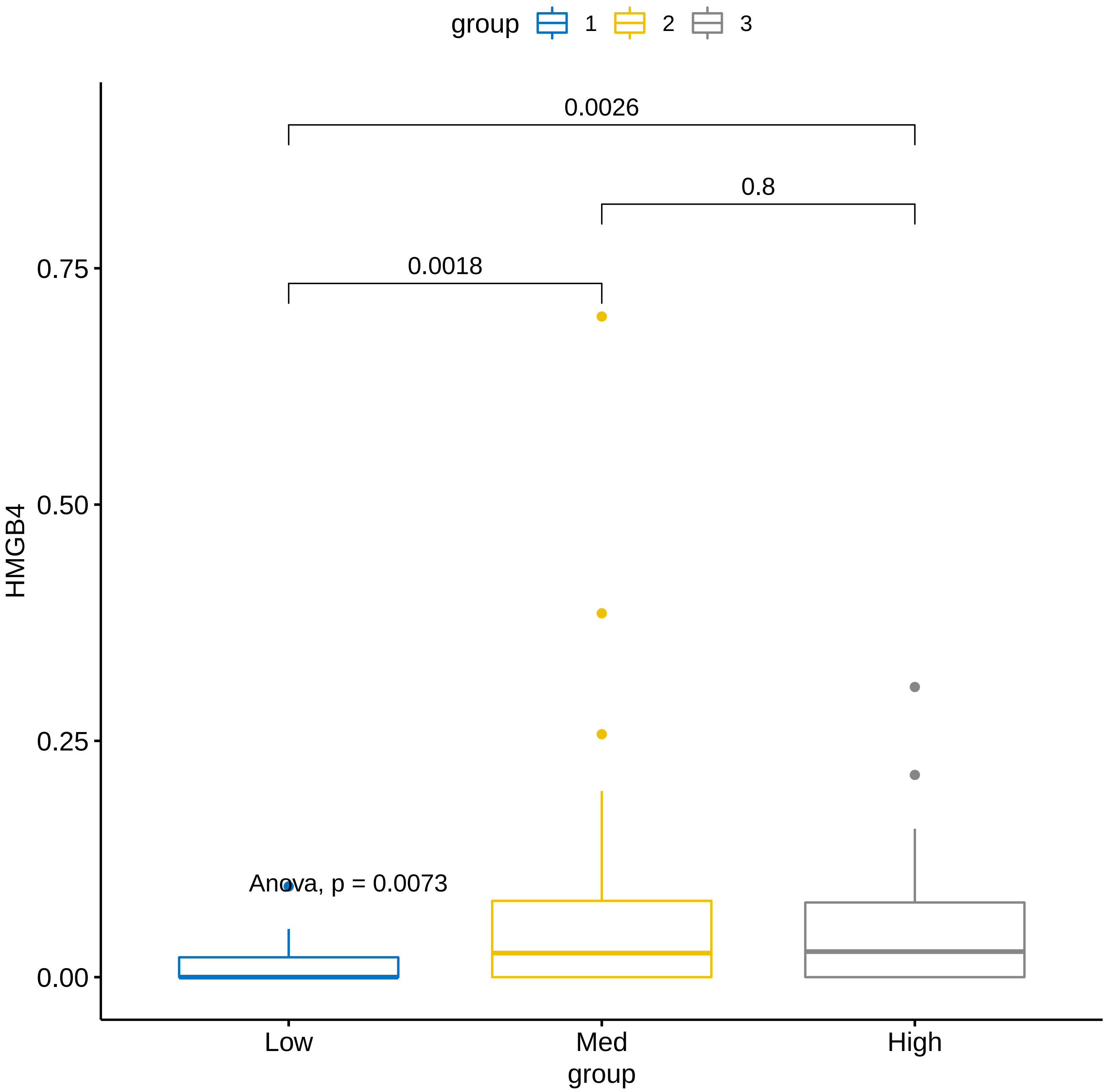
group
1
2
3
0.0026
0.8
0.0018
0.75
0.50
0.25
0.00
HMGB4
Anova, p = 0.0073
Low
Med
High
group

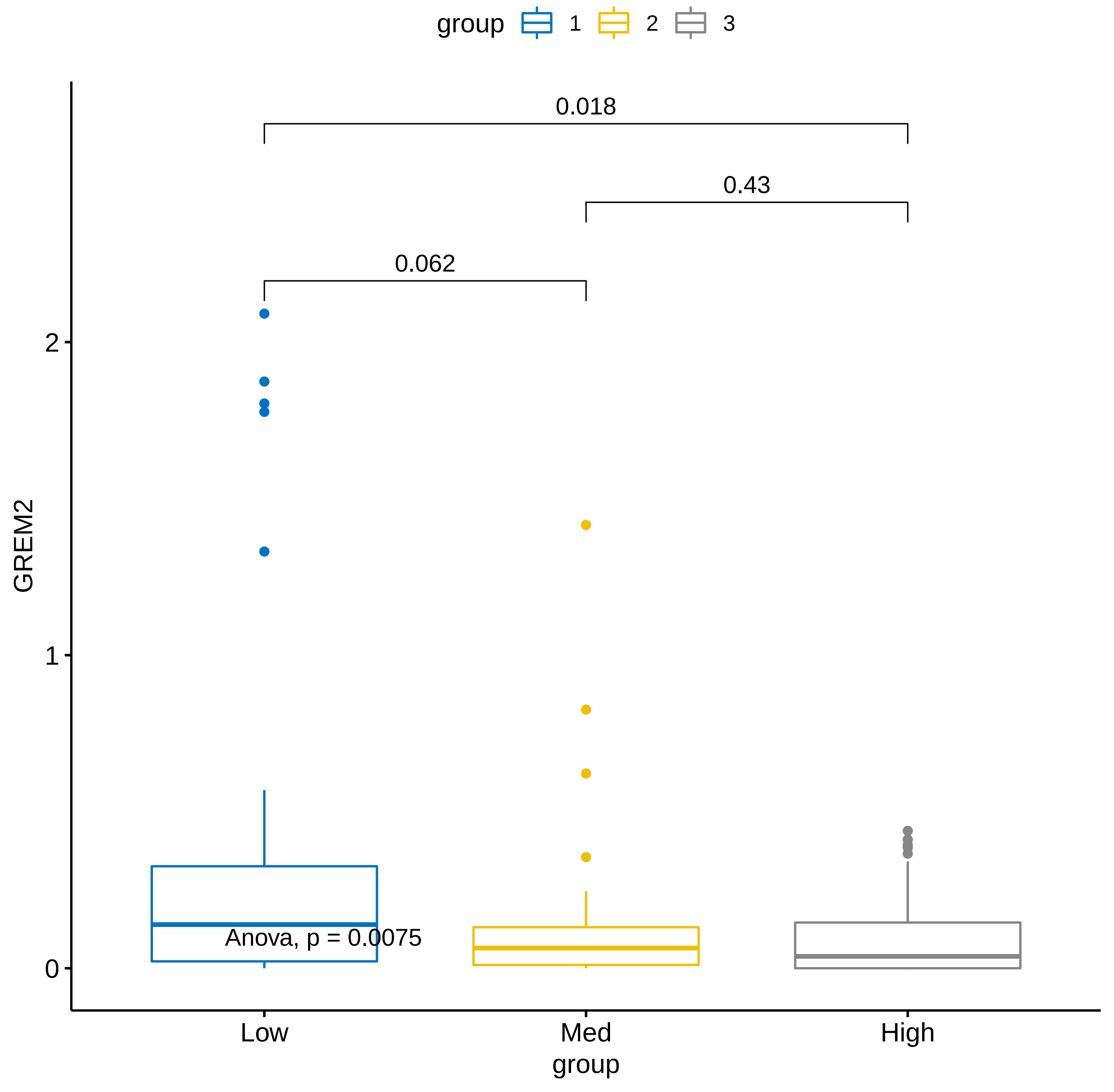

group 1 2 3
0.018
0.43
0.062
2
1
0
GREM2
Anova, p = 0.0075
Low
Med
High
group

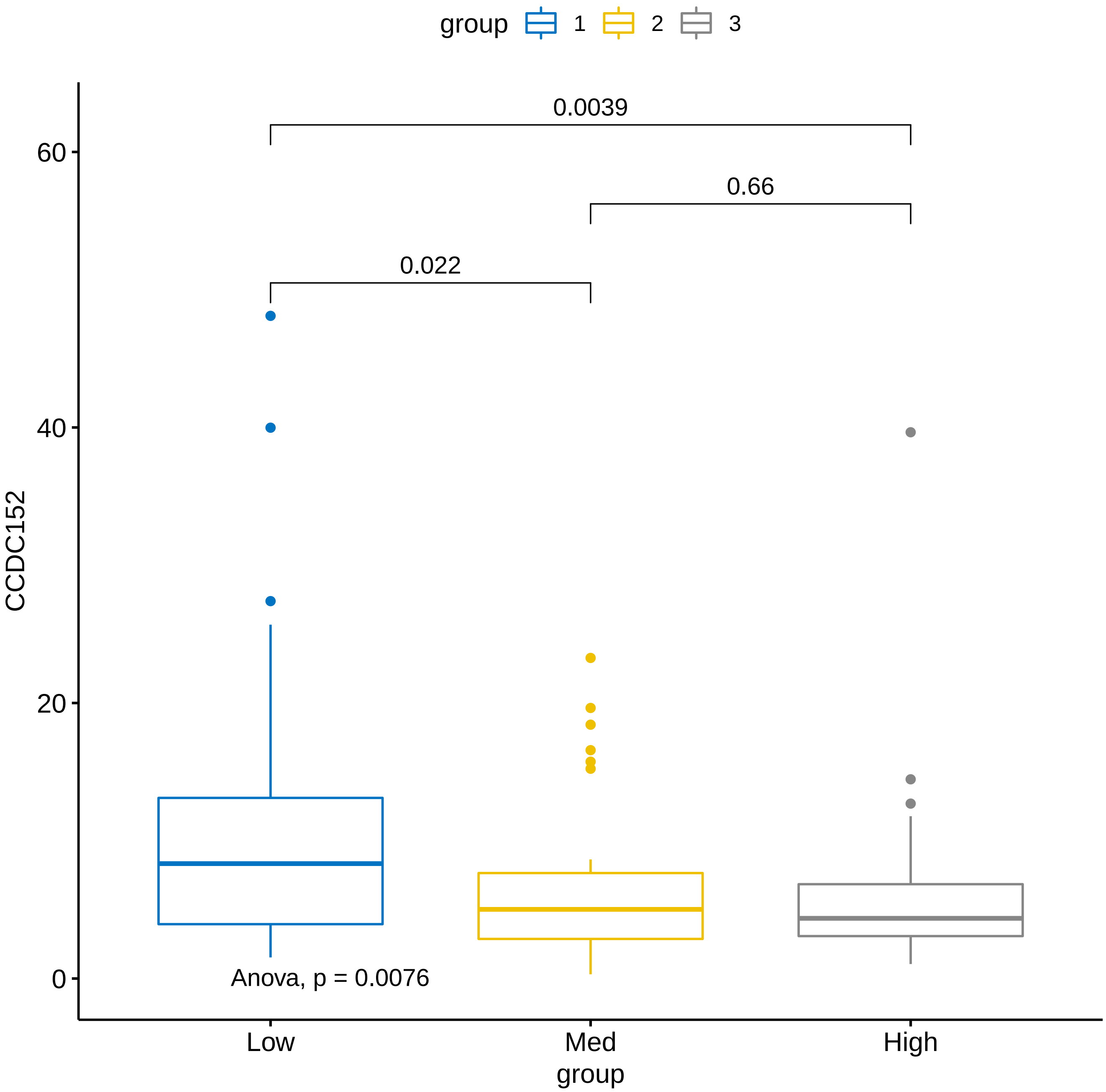
group
1
2
3
0.0039
0.66
0.022
60
40
20
0
CCDC152
Anova, p = 0.0076
Low
Med
High
group

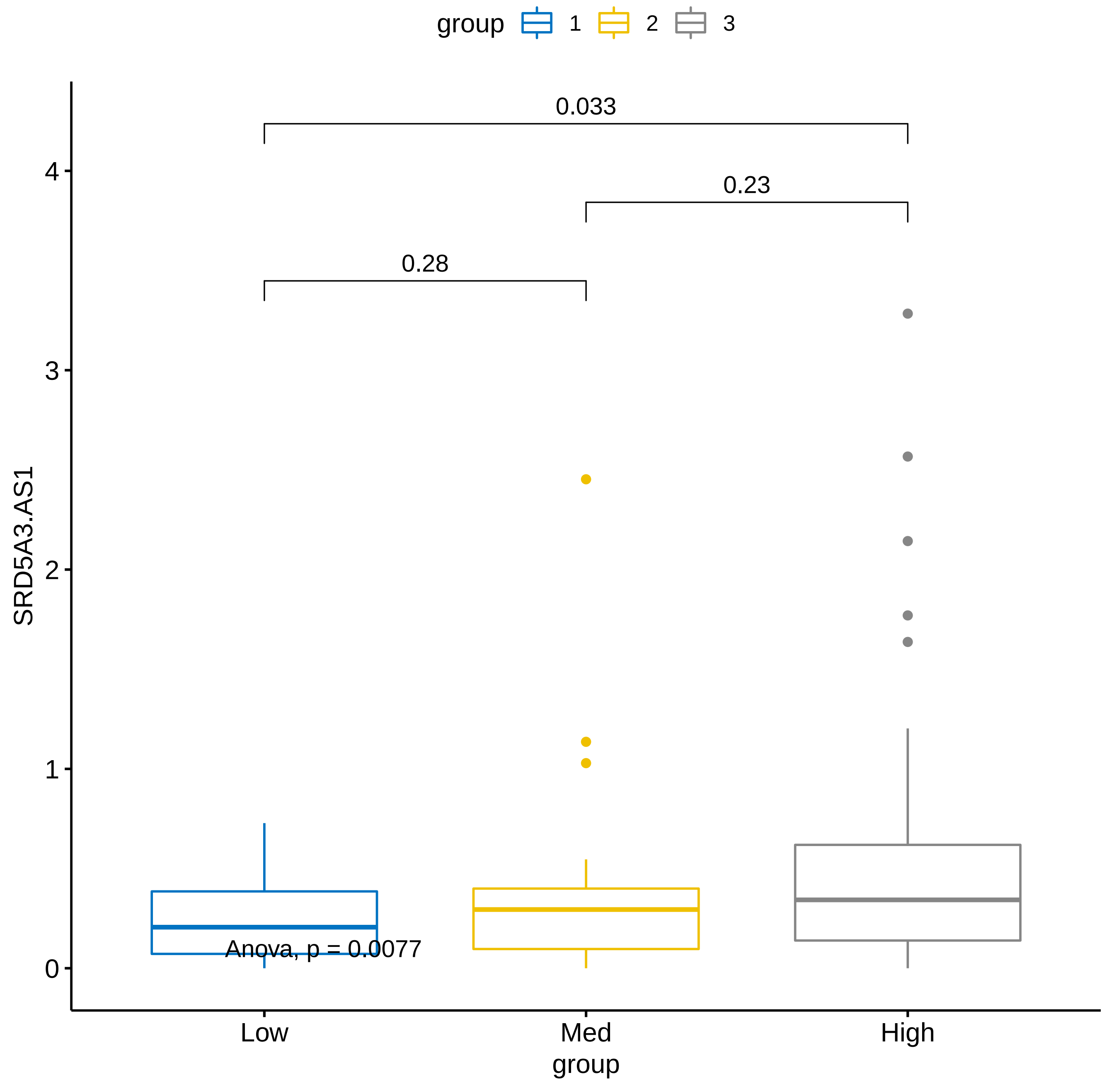
group 1 2 3
0.033
0.23
0.28
SRD5A3.AS1
4
3
2
1
0
Anova, p = 0.0077
Low
Med
High
group

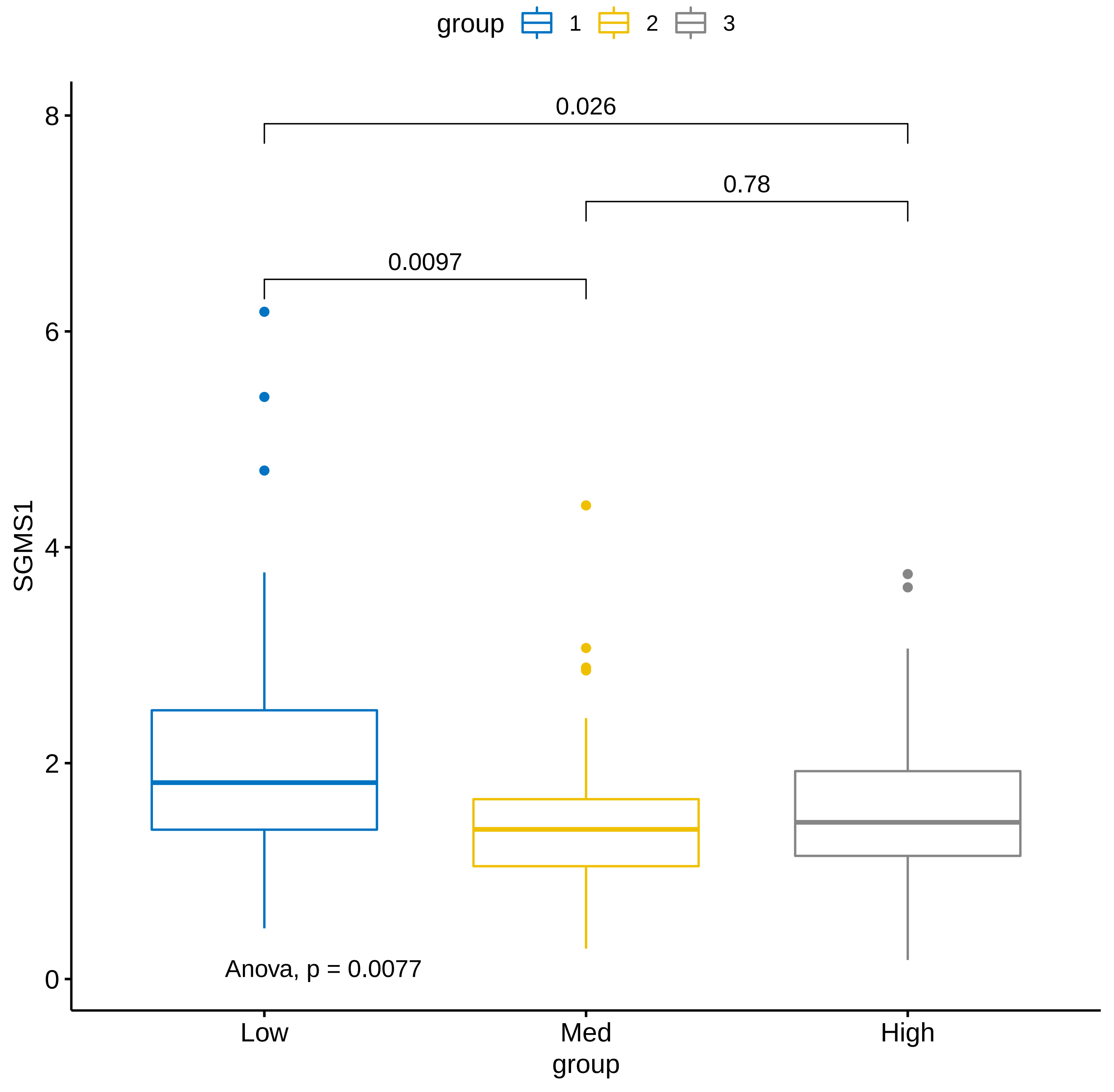

group 1 2 3
0.026
0.78
0.0097
SGMS1
8
6
4
2
0
Anova, p = 0.0077
Low
Med
High
group

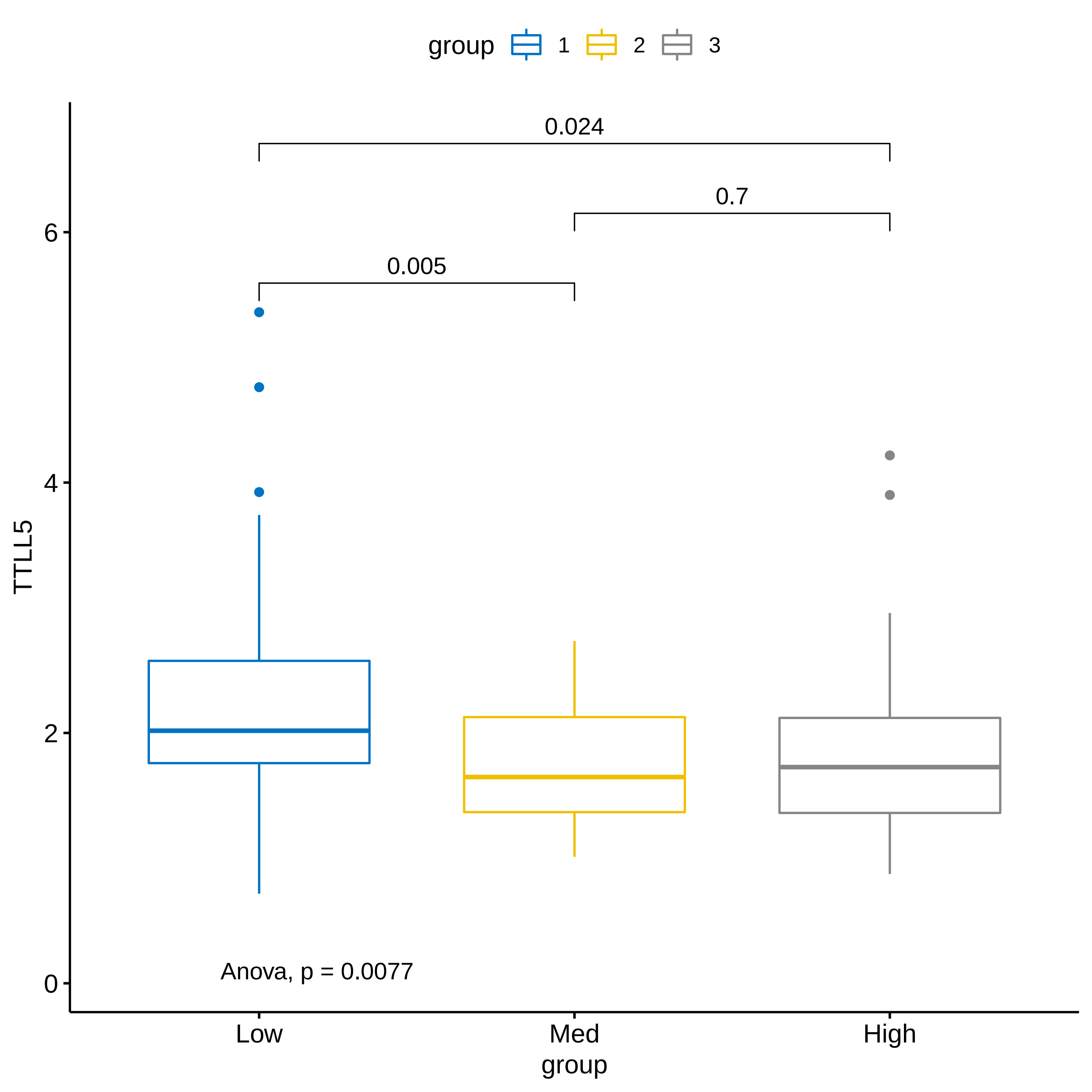
group 1 2 3
0.024
0.7
0.005
6
4
2
0
TTLL5
Anova, p = 0.0077
Low
Med
High
group

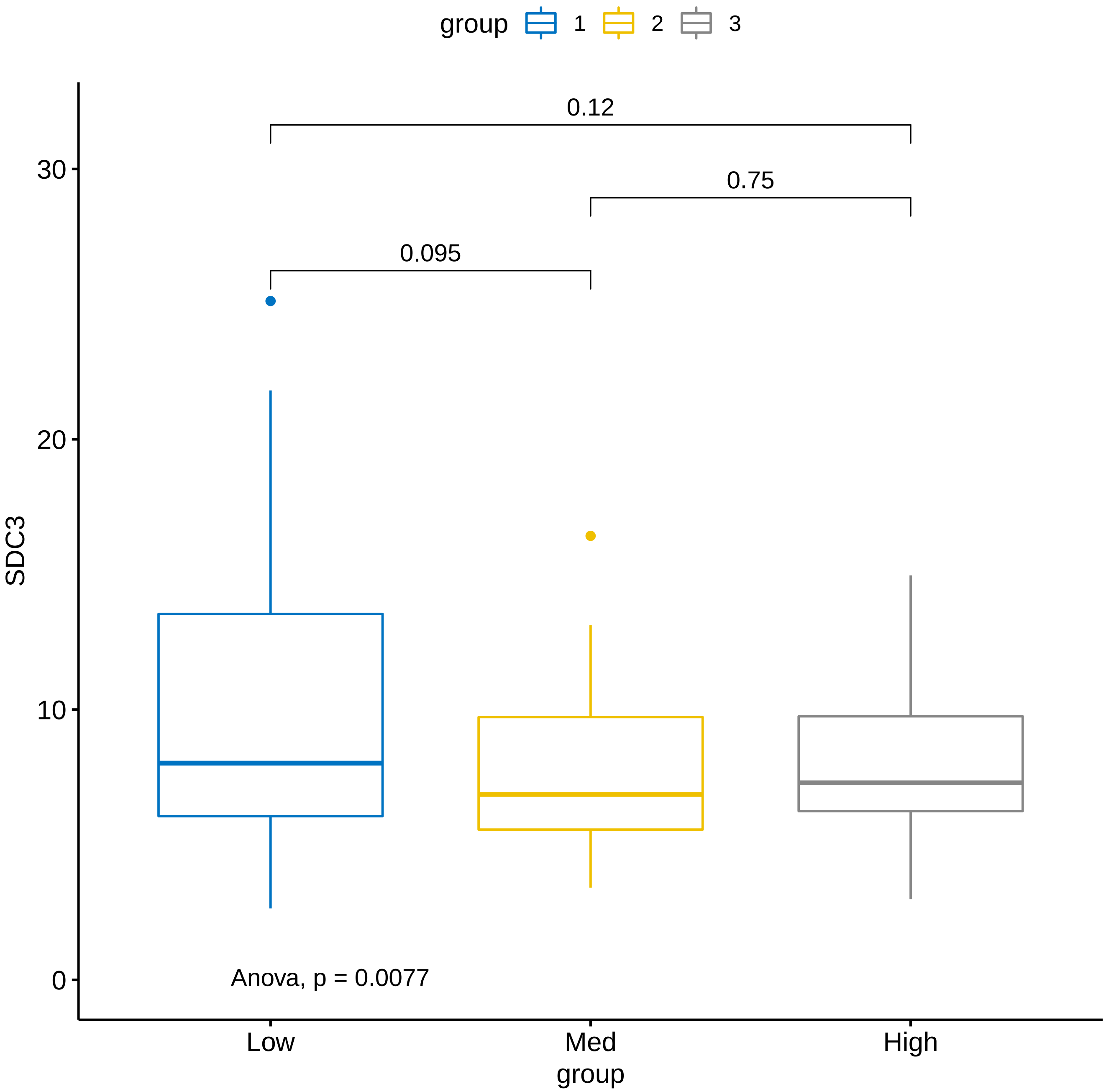

group
1
2
3
0.12
0.75
0.095
30
20
10
0
SDC3
Anova, p = 0.0077
Low
Med
High
group

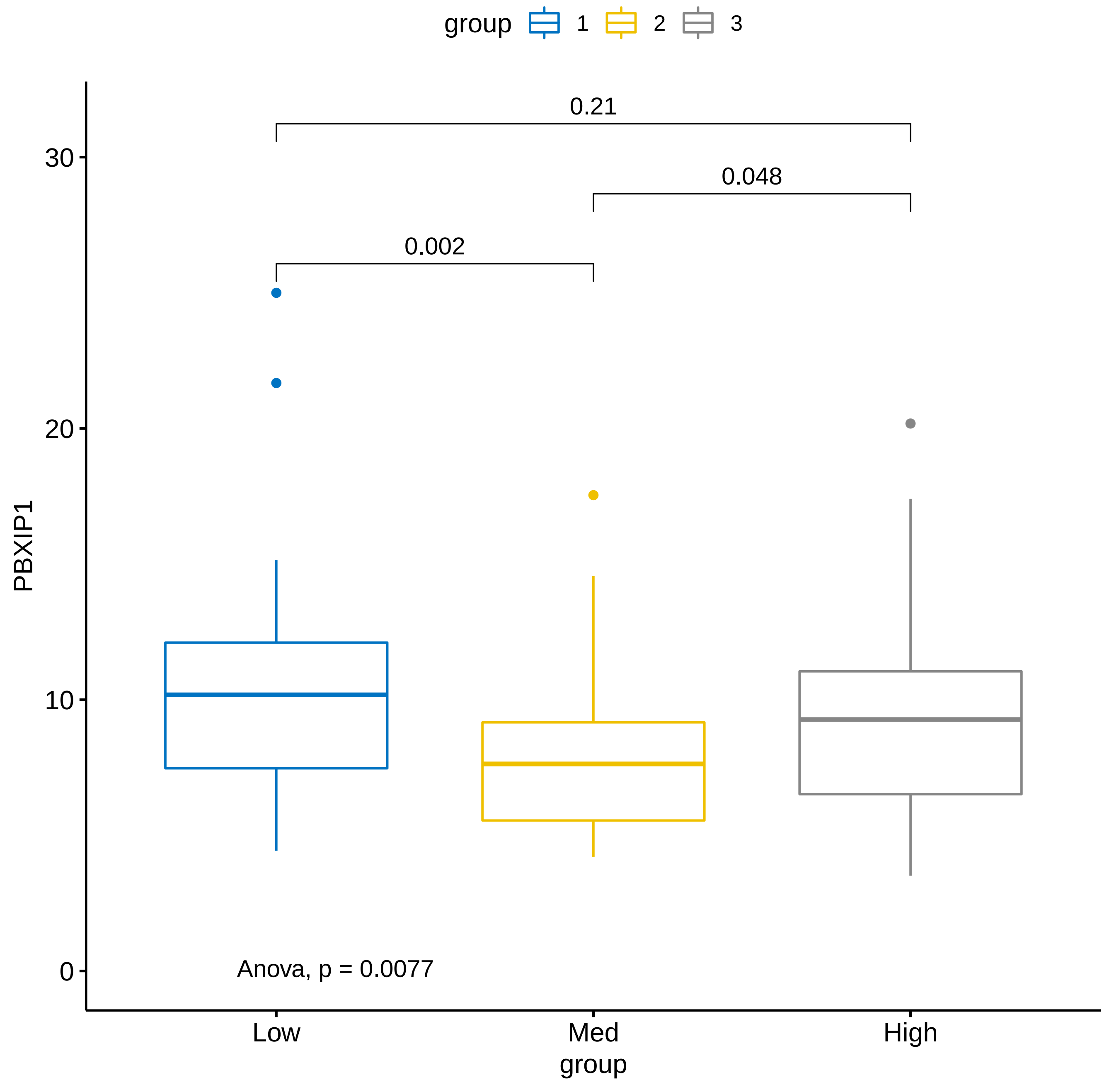
group
1
2
3
0.21
0.048
0.002
30
20
10
0
PBXIP1
Anova, p = 0.0077
Low
Med
High
group

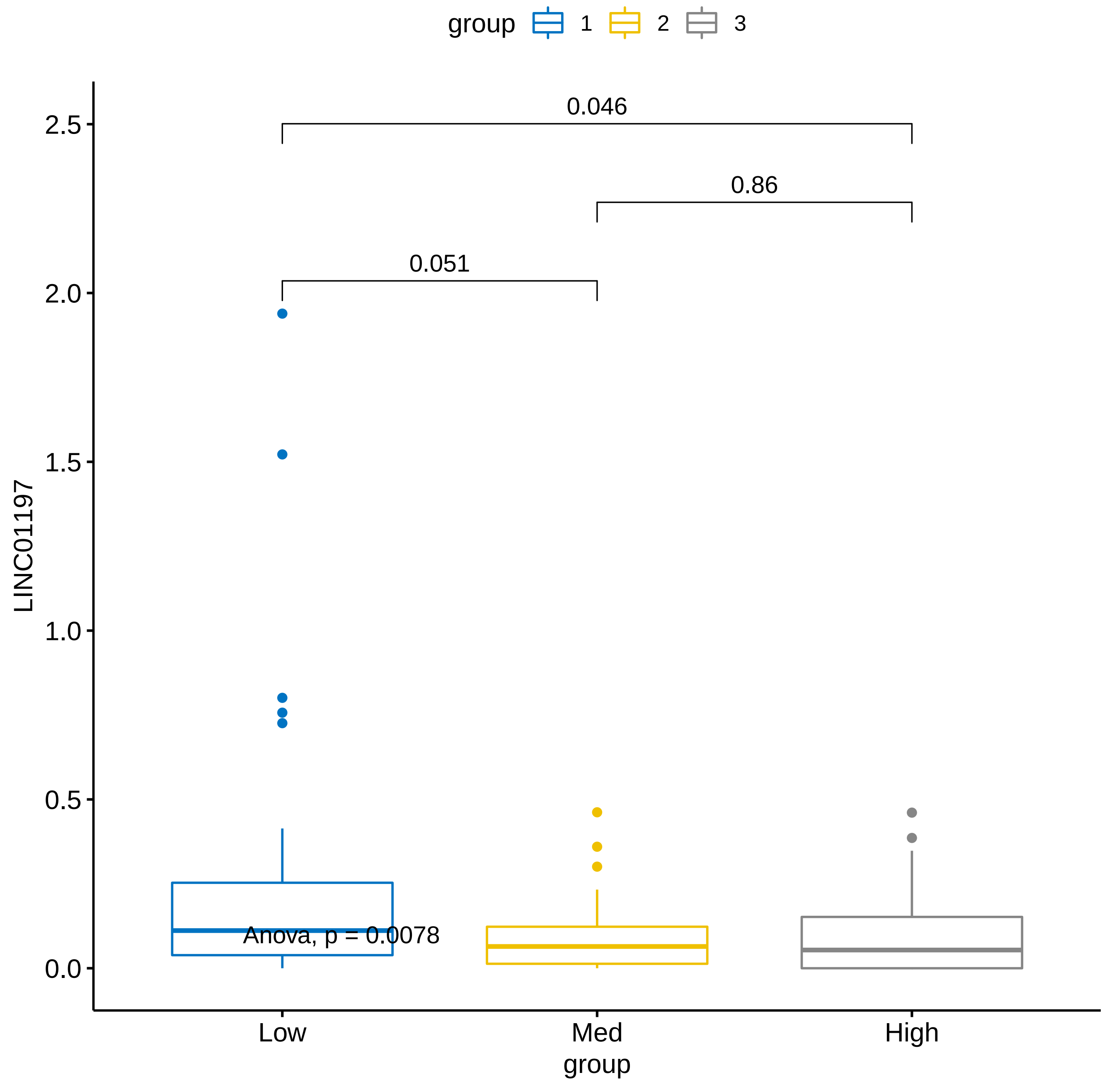

group
1
2
3
0.046
0.86
0.051
2.5
2.0
1.5
1.0
0.5
0.0
LINC01197
Anova, p = 0.0078
Low
Med
High
group

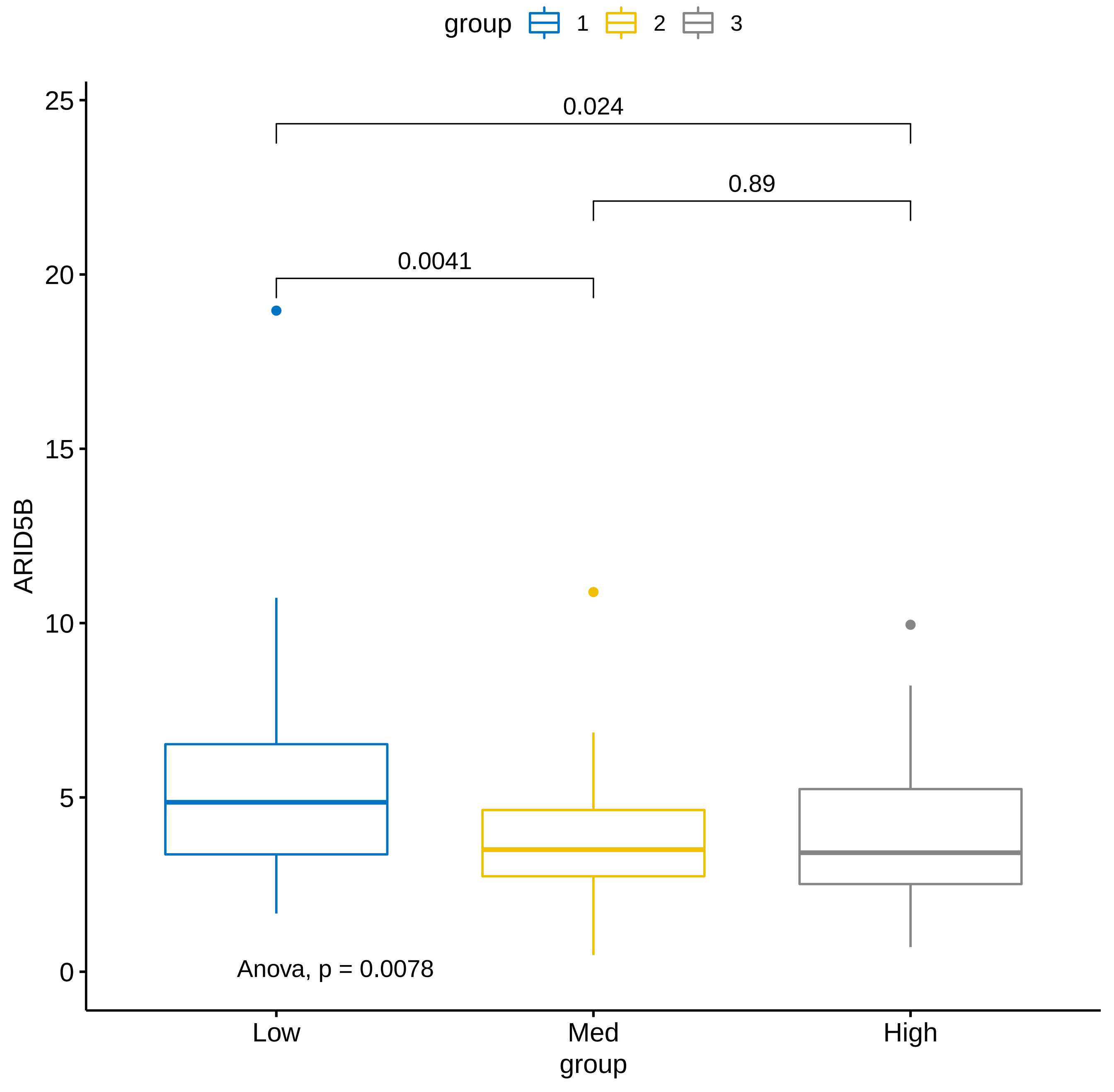
group
1
2
3
0.024
0.89
0.0041
ARID5B
25
20
15
10
5
0
Anova, p = 0.0078
Low
Med
High
group

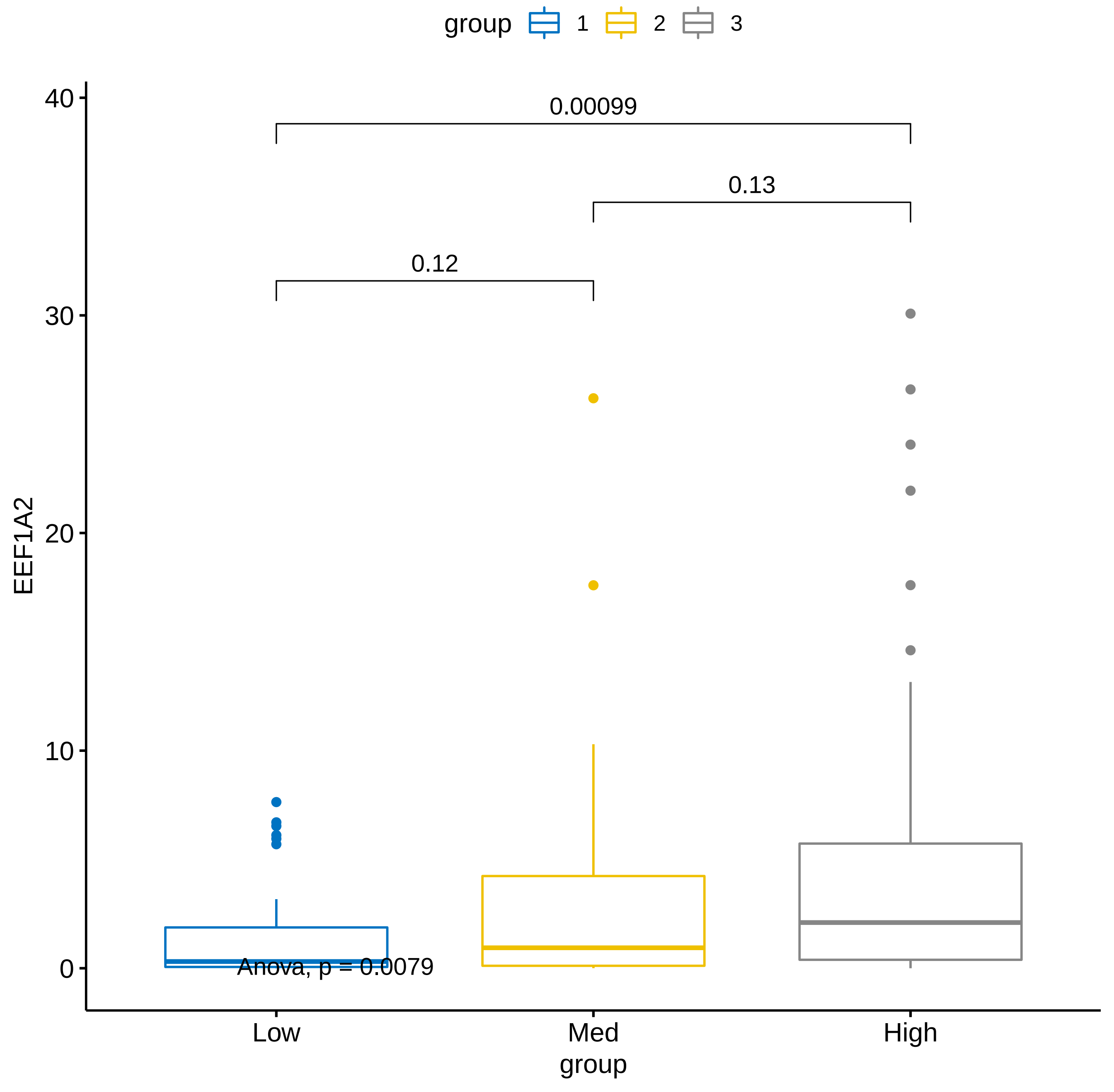

group 1 2 3
0.00099
0.13
0.12
Anova, p = 0.0079
EEF1A2
40
30
20
10
0
Low
Med
High
group

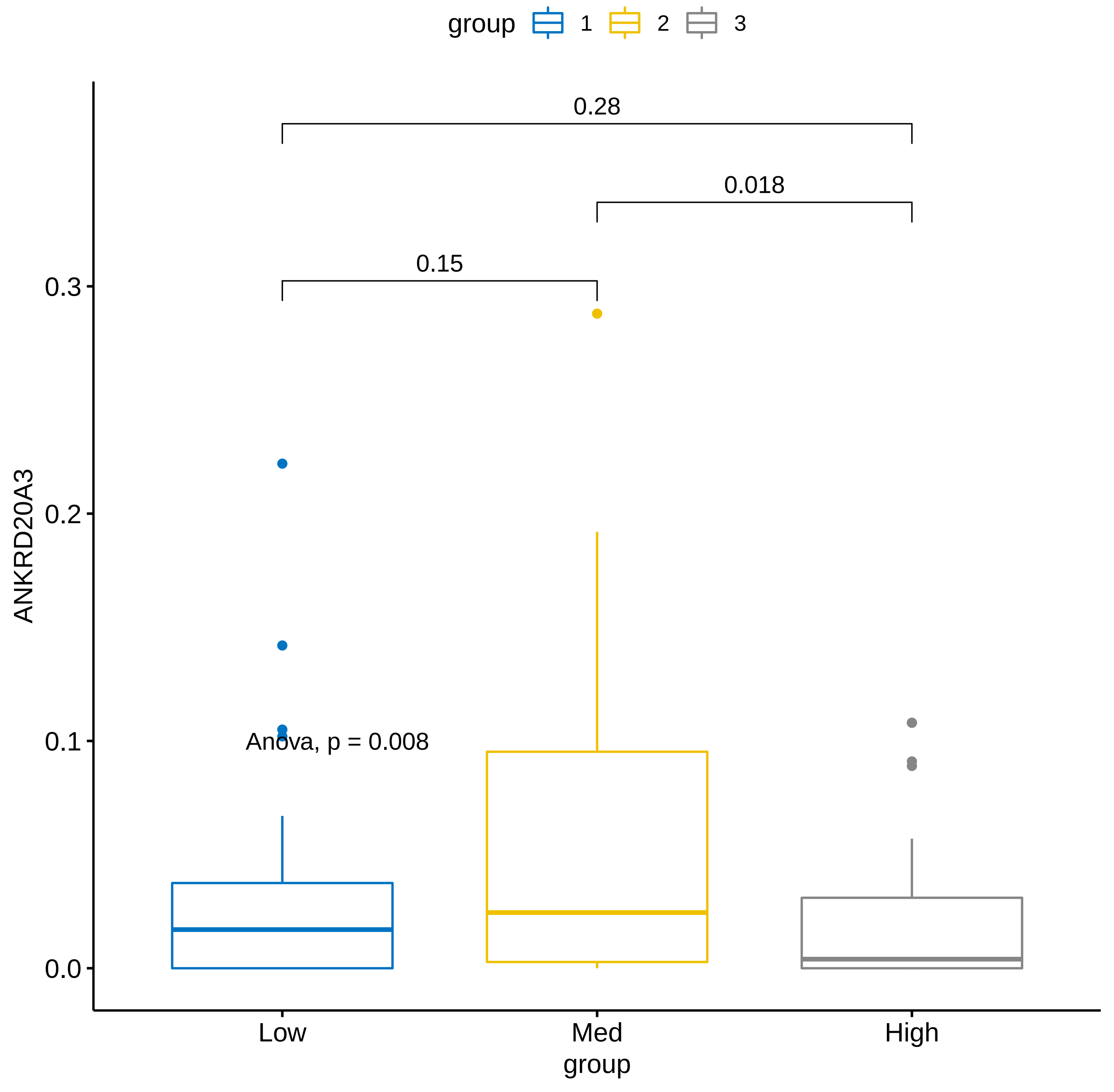
group
1
2
3
0.28
0.018
0.15
Anova, p = 0.008
ANKRD20A3
0.3
0.2
0.1
0.0
Low
Med
High
group

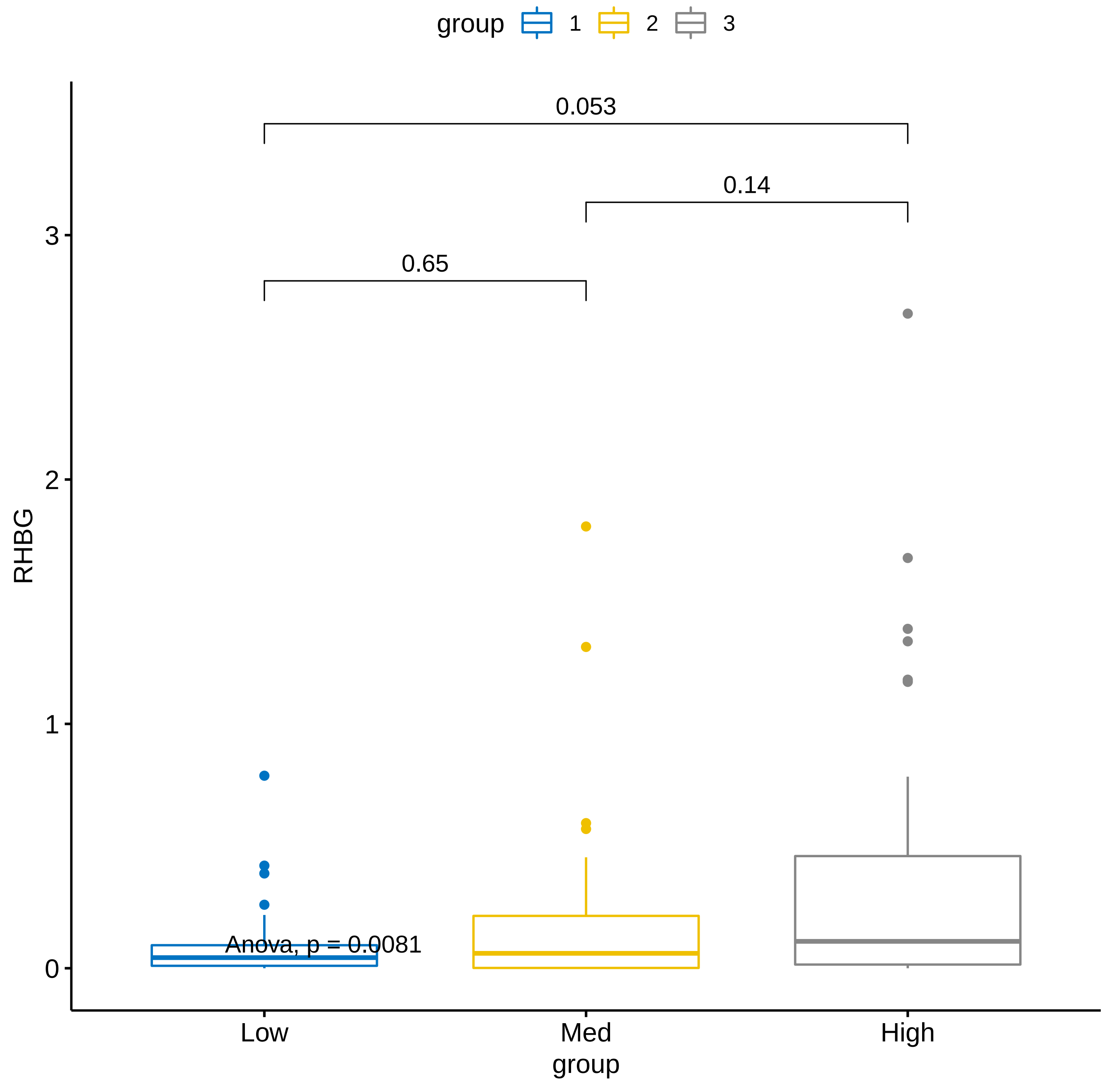

group 1 2 3
0.053
0.14
0.65
3
2
1
0
RHBG
Anova, p = 0.0081
Low
Med
High
group

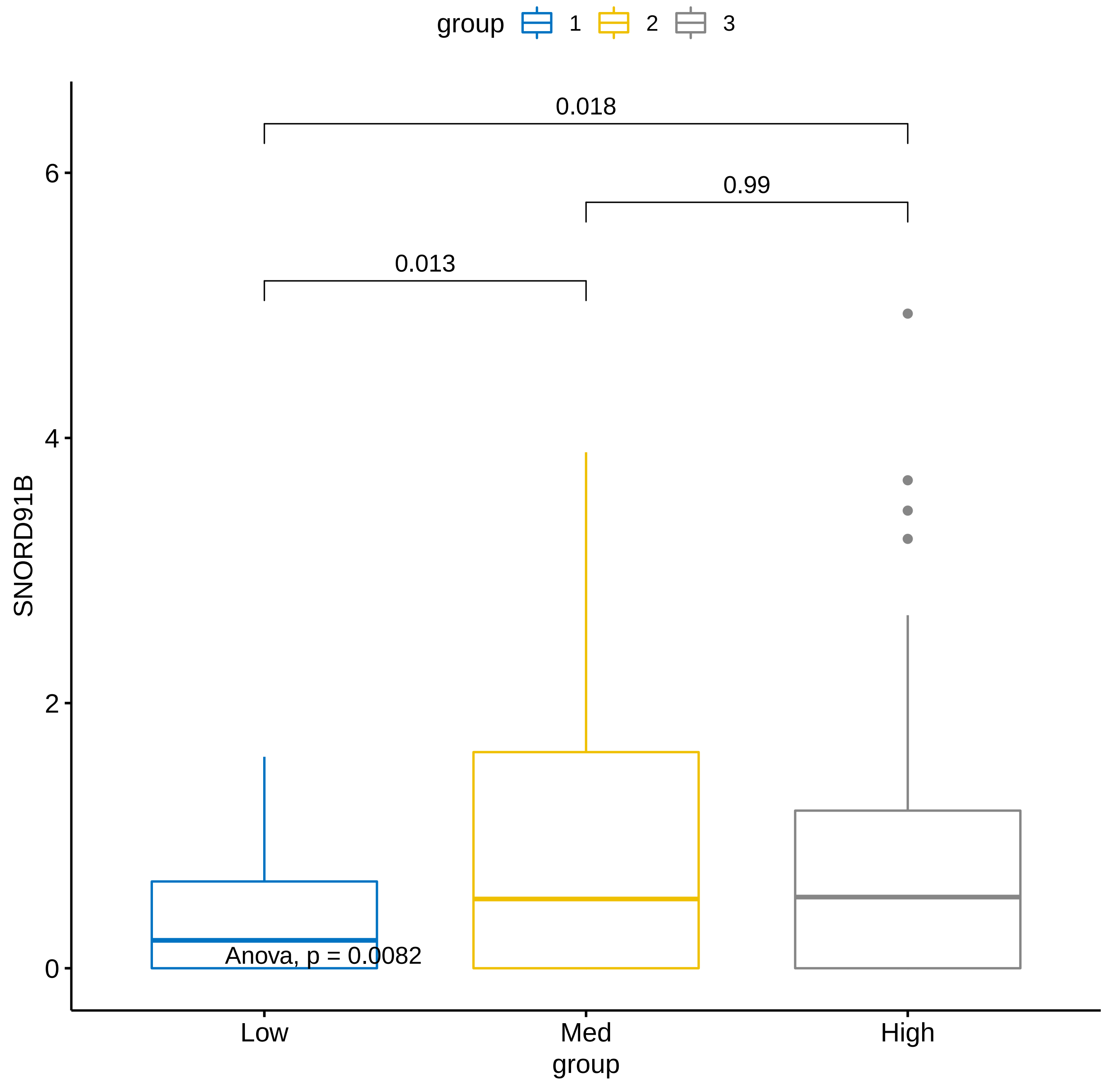

group 1 2 3
0.018
0.99
0.013
SNORD91B
6
4
2
0
Anova, p = 0.0082
Low
Med
High
group

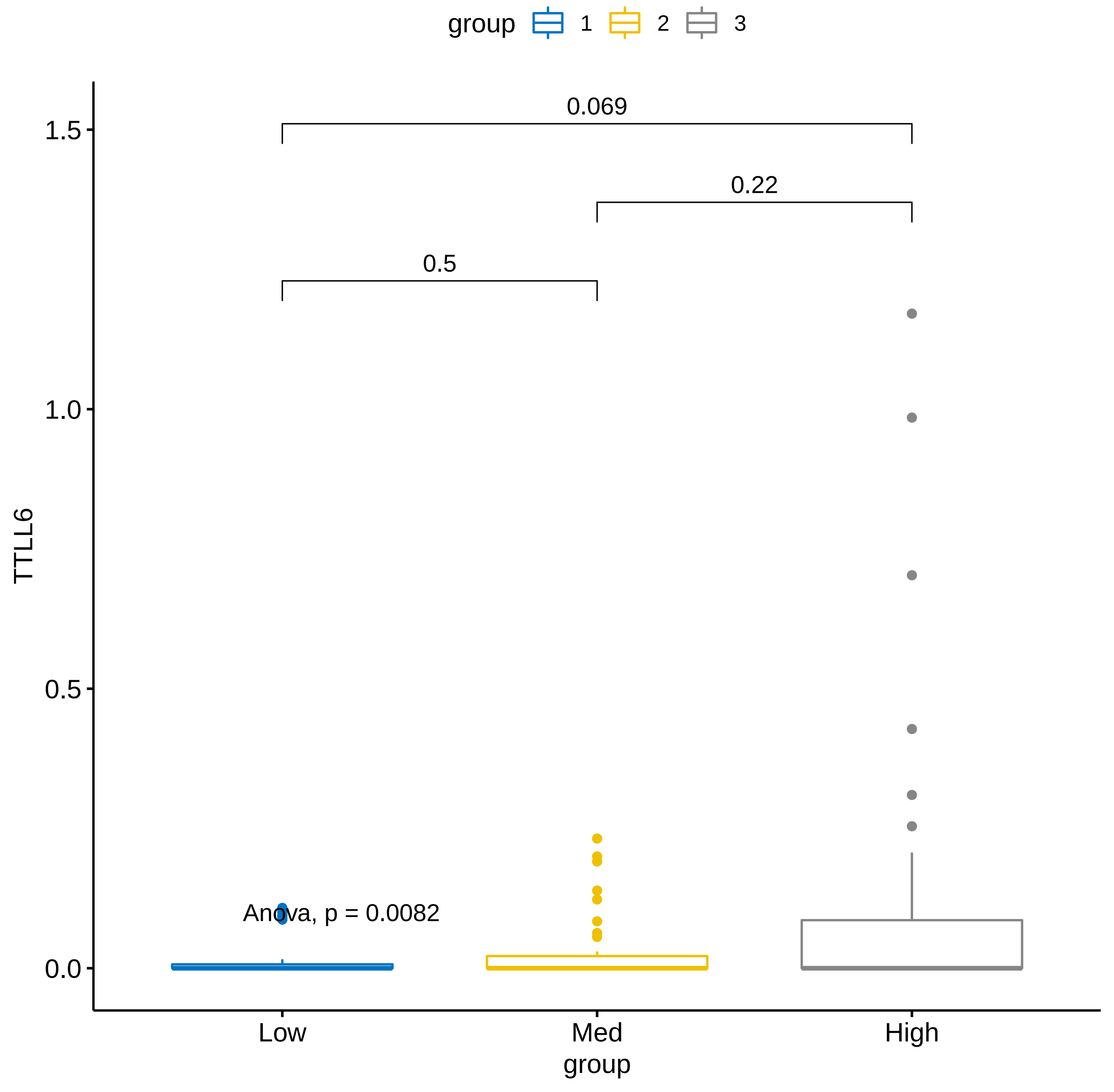
group
1
2
3
0.069
0.22
0.5
Anova, p = 0.0082
1.5
1.0
0.5
0.0
TTLL6
Low
Med
High
group

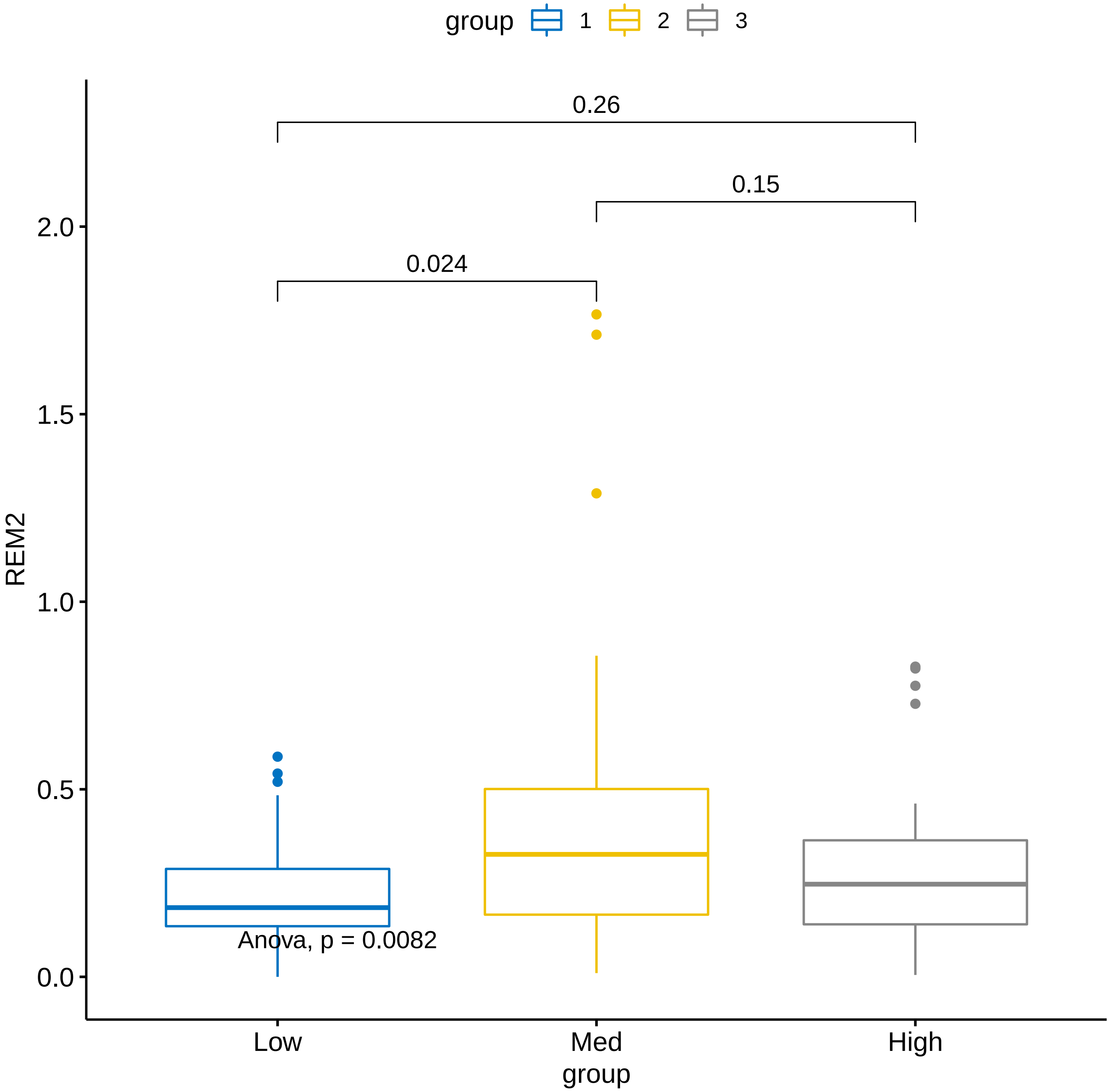

group 1 2 3
0.26
0.15
0.024
Anova, p = 0.0082
REM2
2.0
1.5
1.0
0.5
0.0
Low
Med
High
group

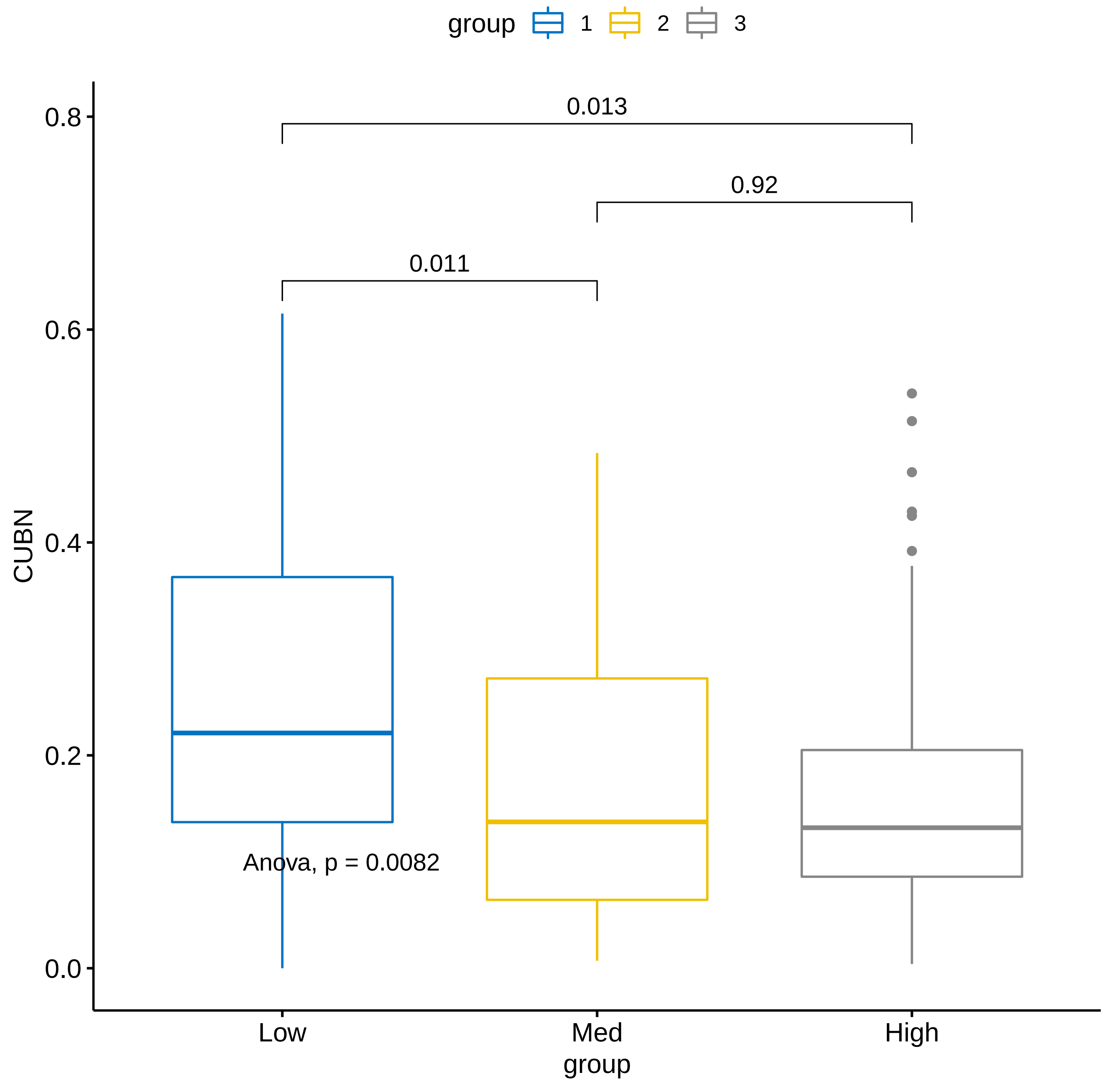
group
1
2
3
0.013
0.92
0.011
Anova, p = 0.0082
CUBN
0.0
0.2
0.4
0.6
0.8
Low
Med
High
group

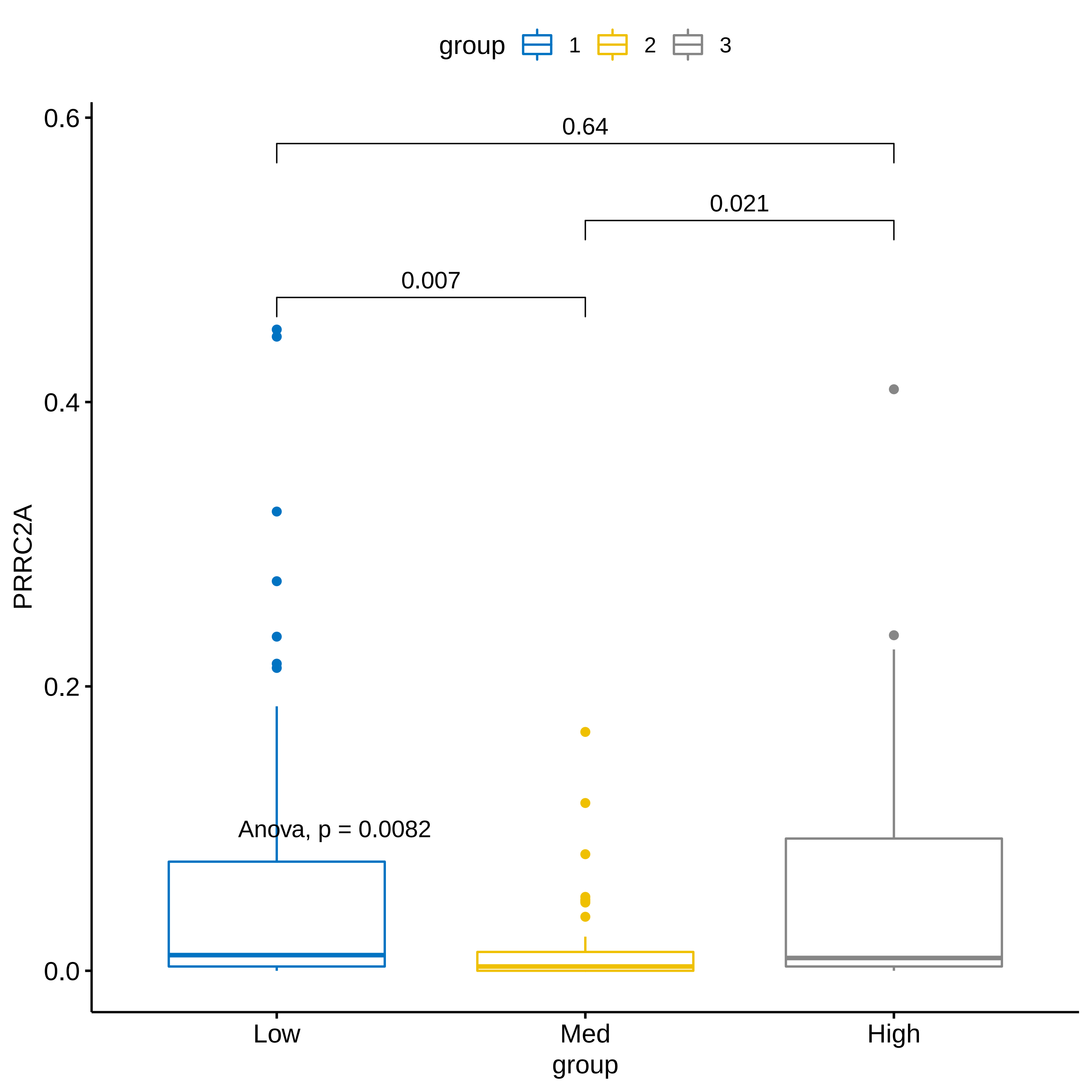

group
1
2
3
0.64
0.021
0.007
Anova, p = 0.0082
0.6
0.4
0.2
0.0
PRRC2A
Low
Med
High
group

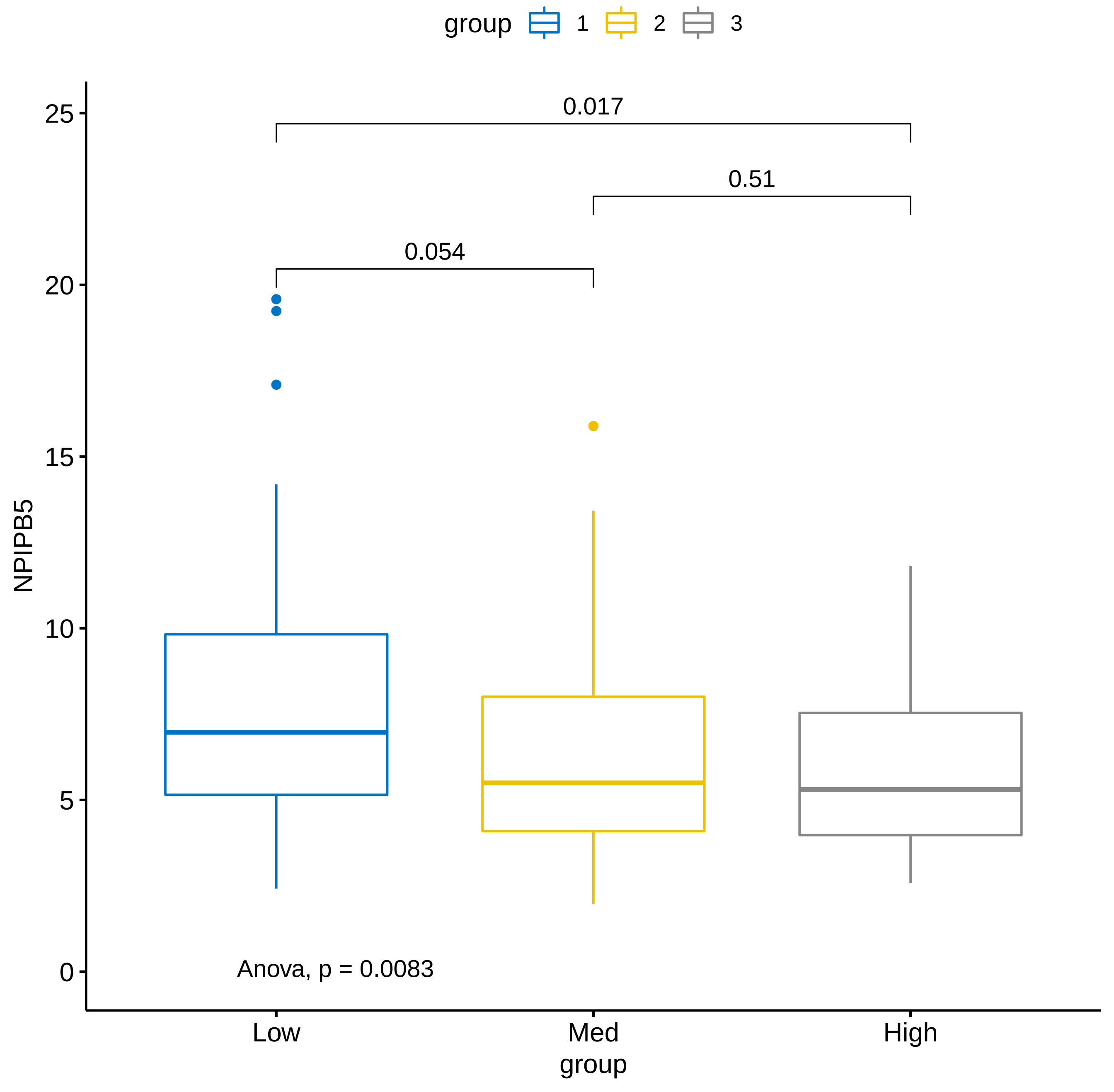
group
1
2
3
0.017
0.51
0.054
25
20
15
10
5
0
NPIPB5
Anova, p = 0.0083
Low
Med
High
group

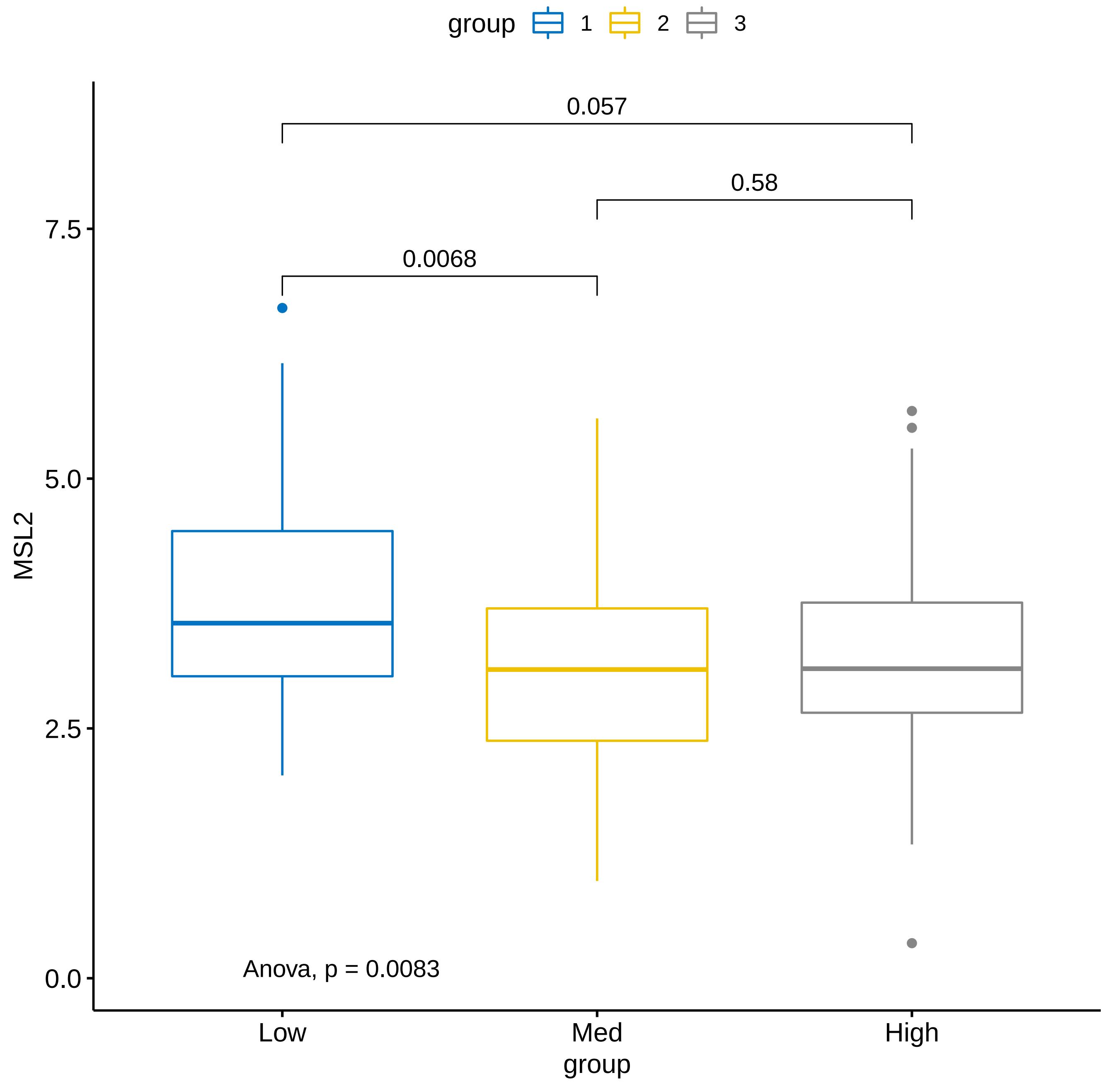

group 1 2 3
0.057
0.58
0.0068
7.5
5.0
2.5
0.0
MSL2
Anova, p = 0.0083
Low
Med
High
group

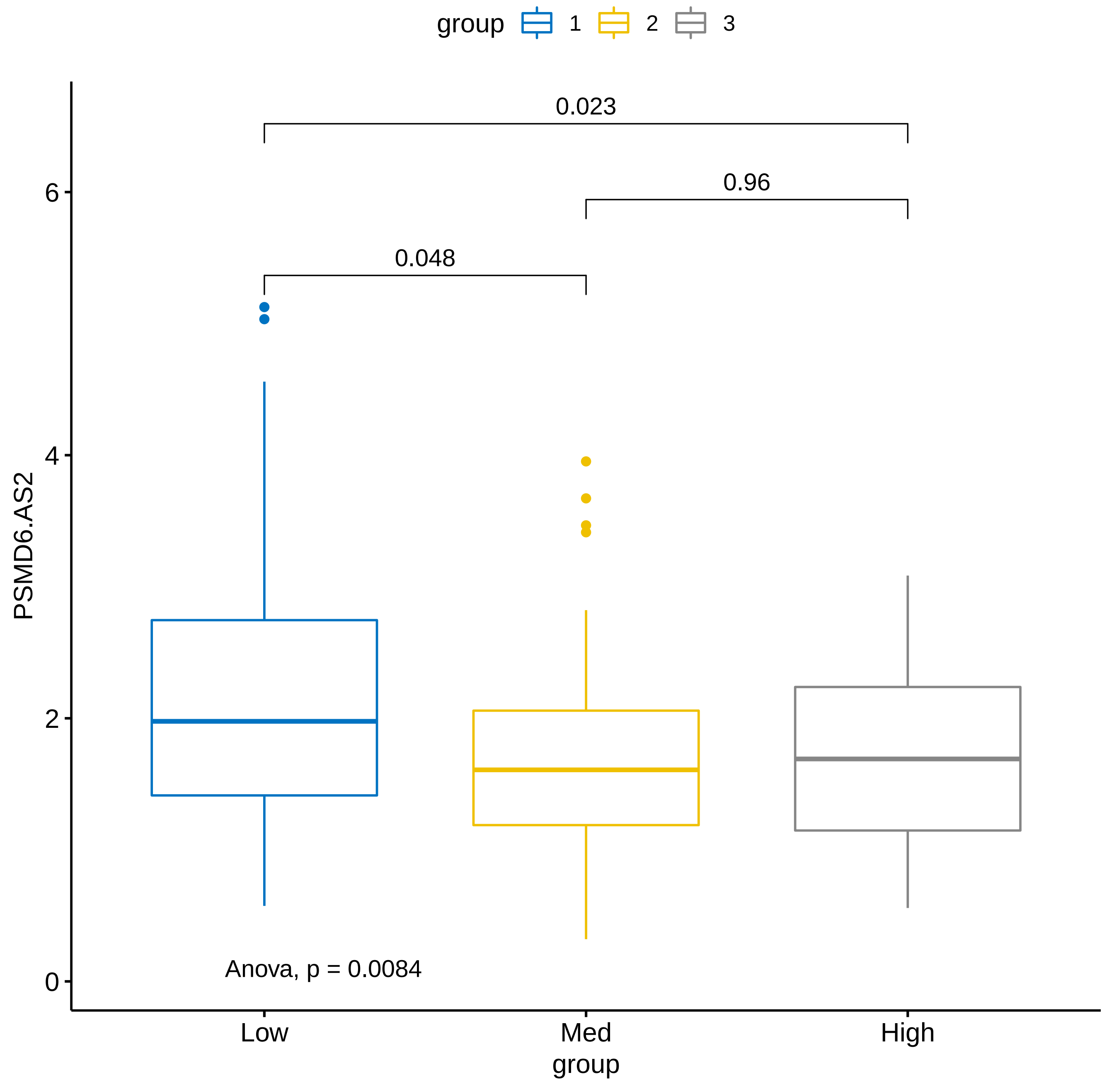

group 1 2 3
0.023
0.96
0.048
PSMD6.AS2
6
4
2
0
Anova, p = 0.0084
Low
Med
High
group

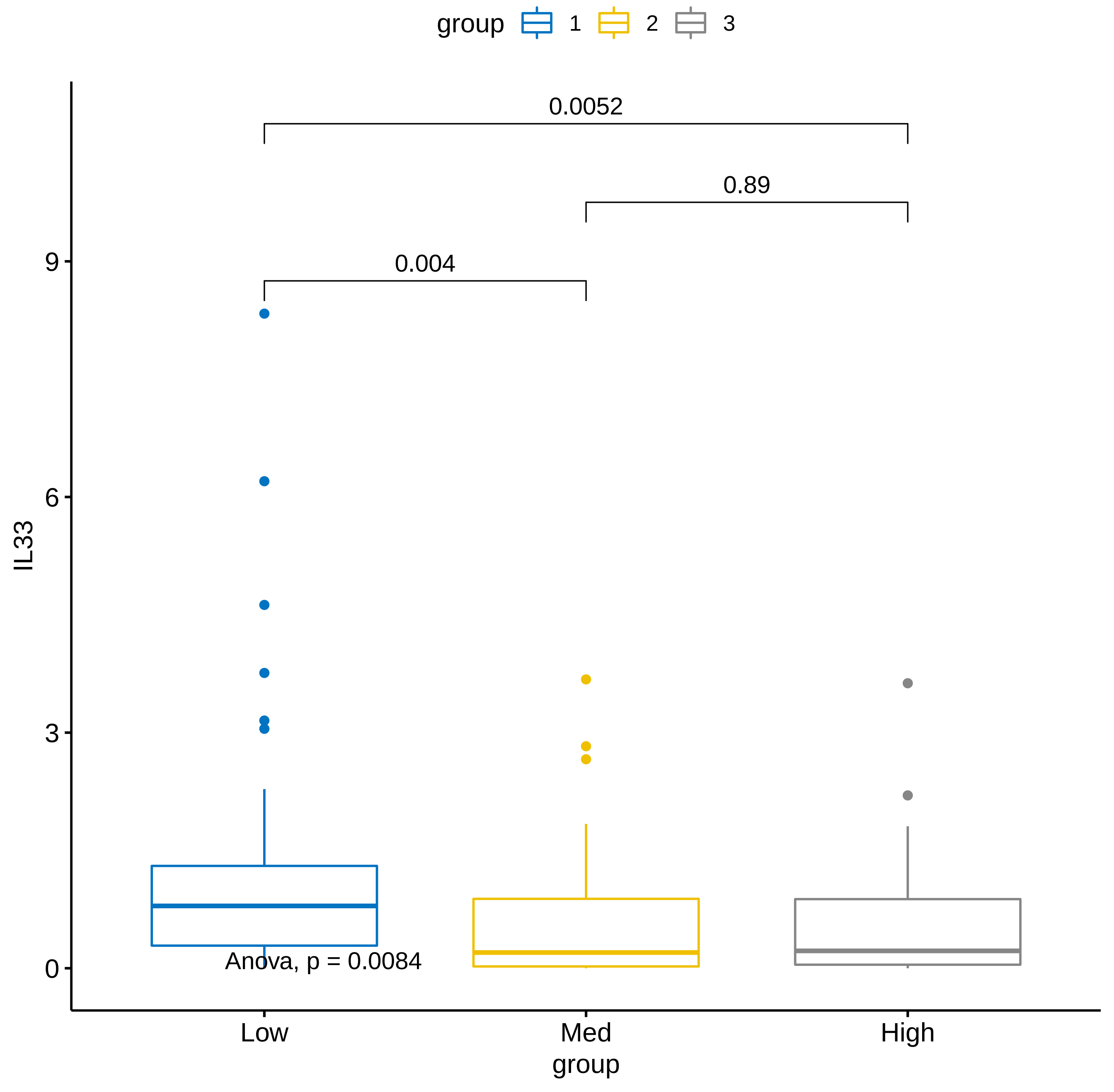
group 1 2 3
0.0052
0.89
0.004
Anova, p = 0.0084
IL33
9
6
3
0
Low
Med
High
group

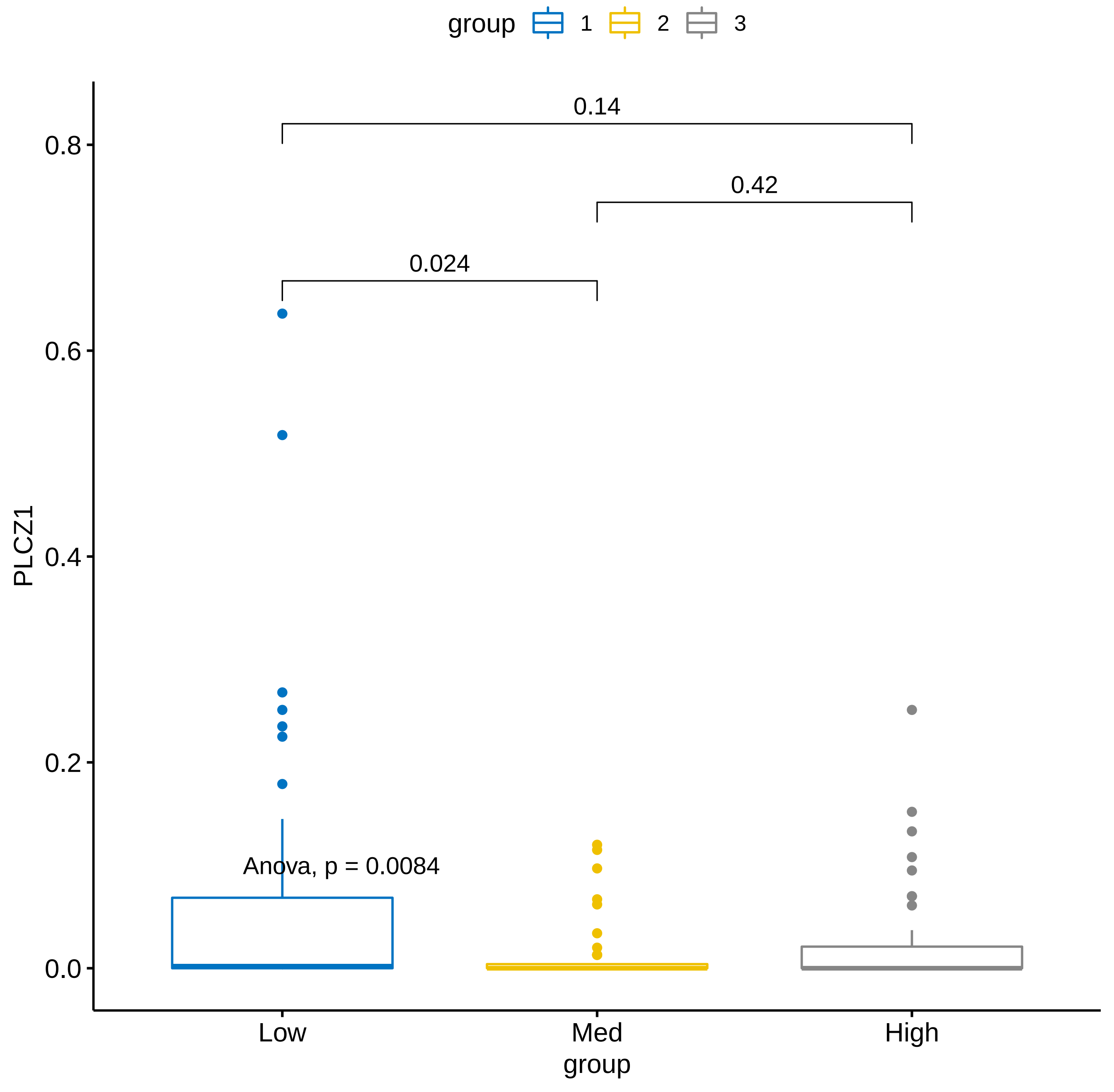

group
1
2
3
0.14
0.42
0.024
0.8
0.6
0.4
0.2
0.0
PLCZ1
Anova, p = 0.0084
Low
Med
High
group

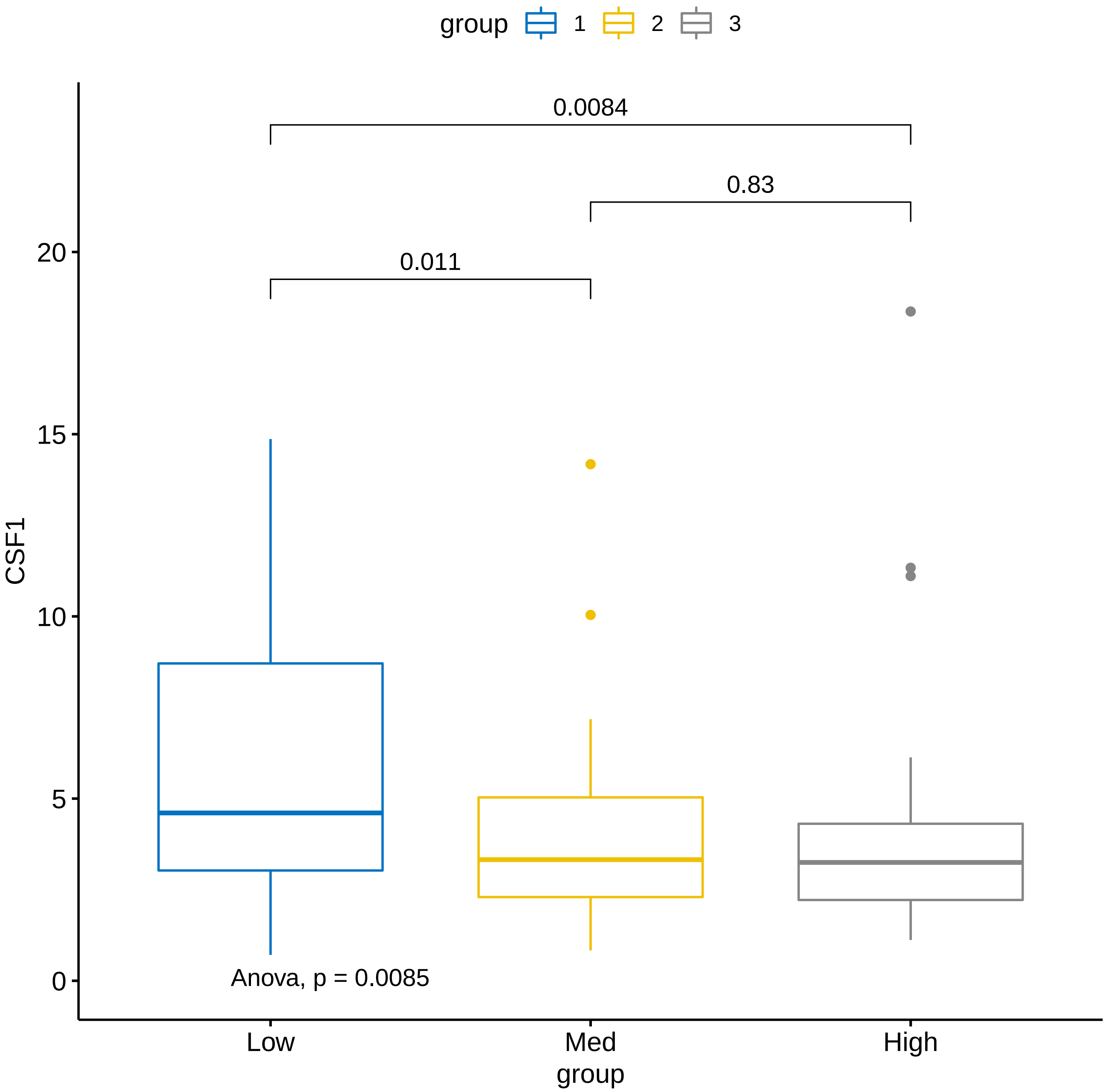
group 1 2 3
0.0084
0.83
0.011
20
15
10
5
0
CSF1
Anova, p = 0.0085
Low
Med
High
group

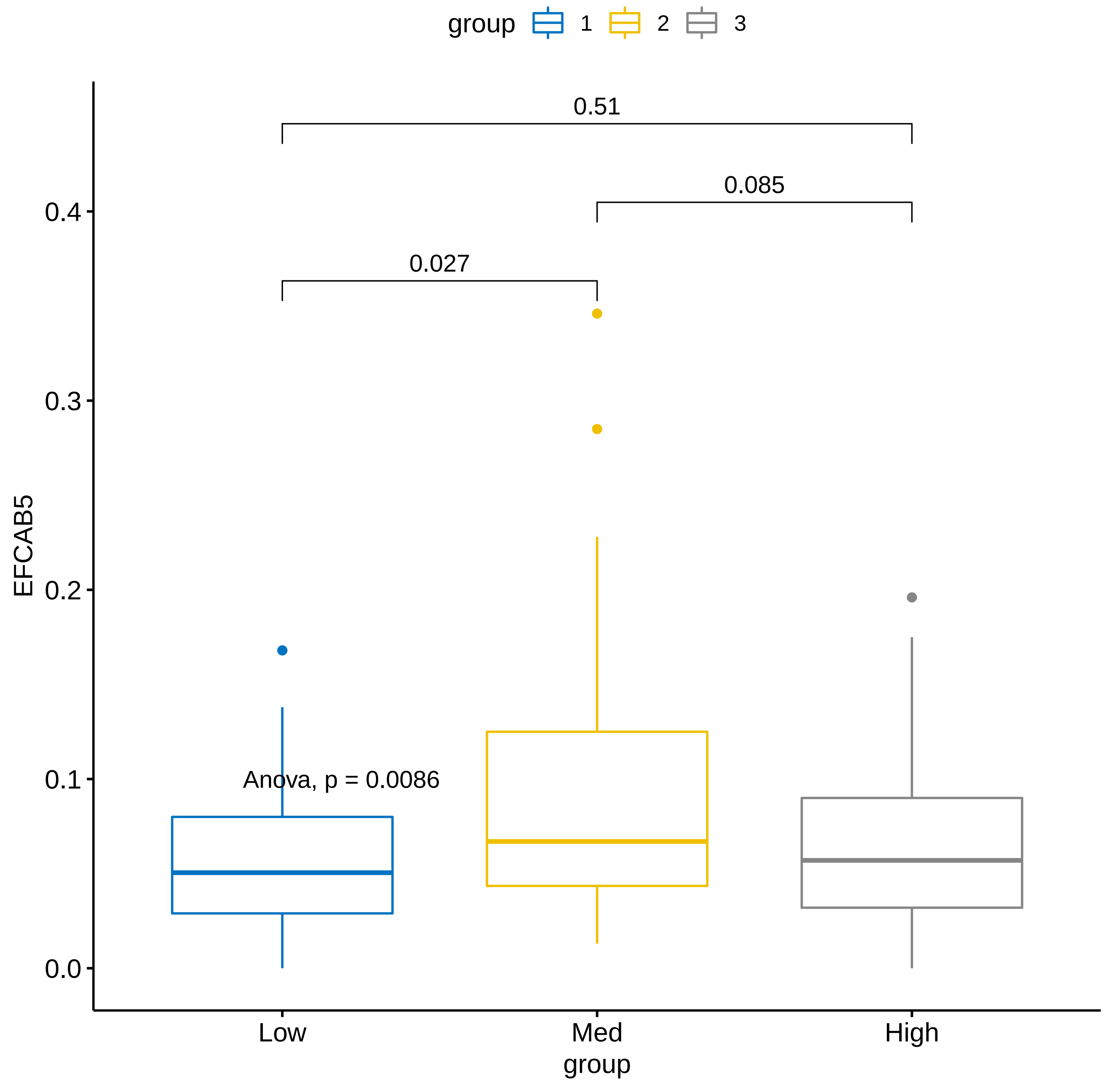
group
1
2
3
0.51
0.085
0.027
0.4
0.3
0.2
0.1
0.0
EFCAB5
Anova, p = 0.0086
Low
Med
High
group

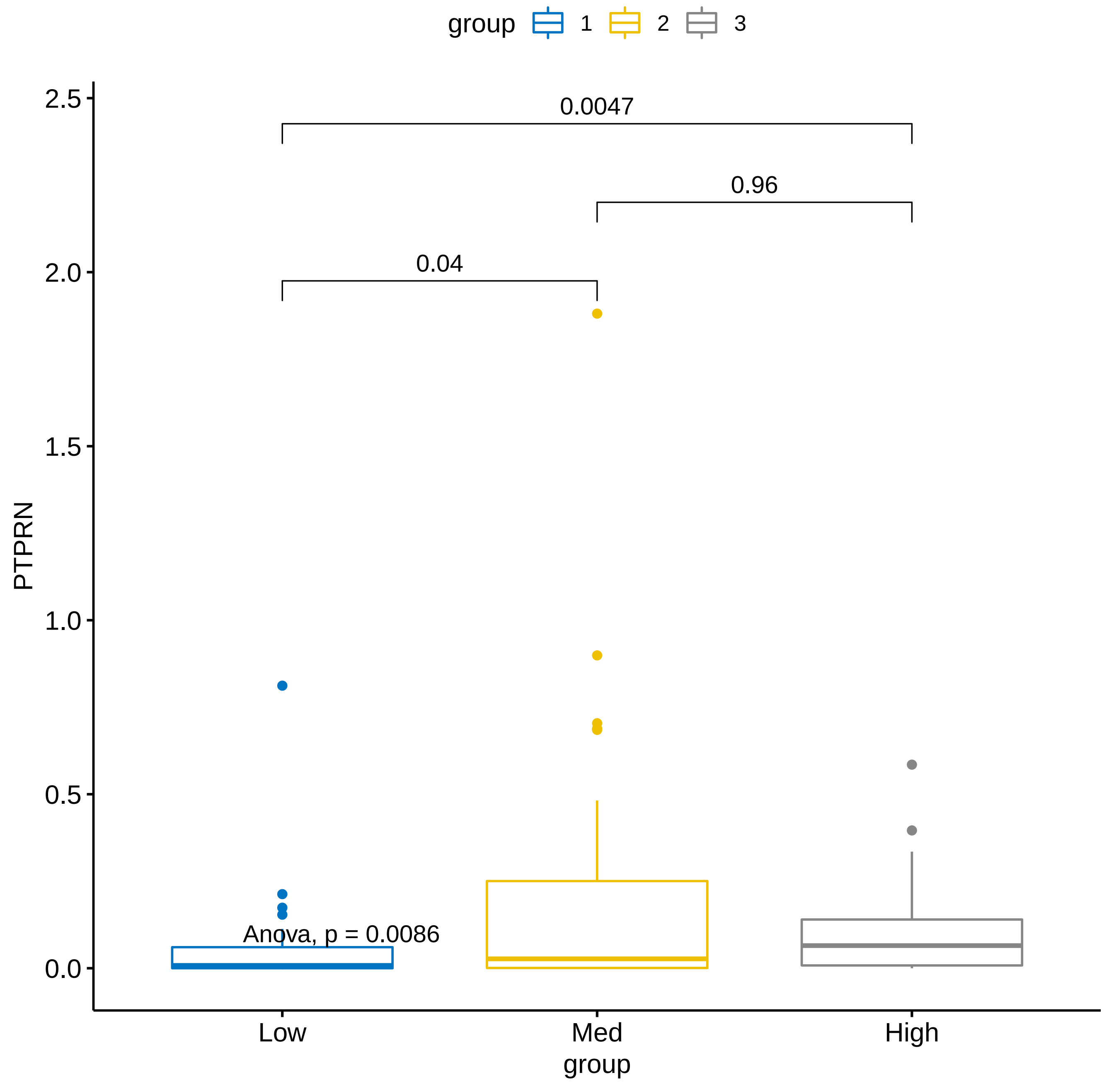
group
1
2
3
0.0047
0.96
0.04
Anova, p = 0.0086
2.5
2.0
1.5
1.0
0.5
0.0
PTPRN
Low
Med
High
group

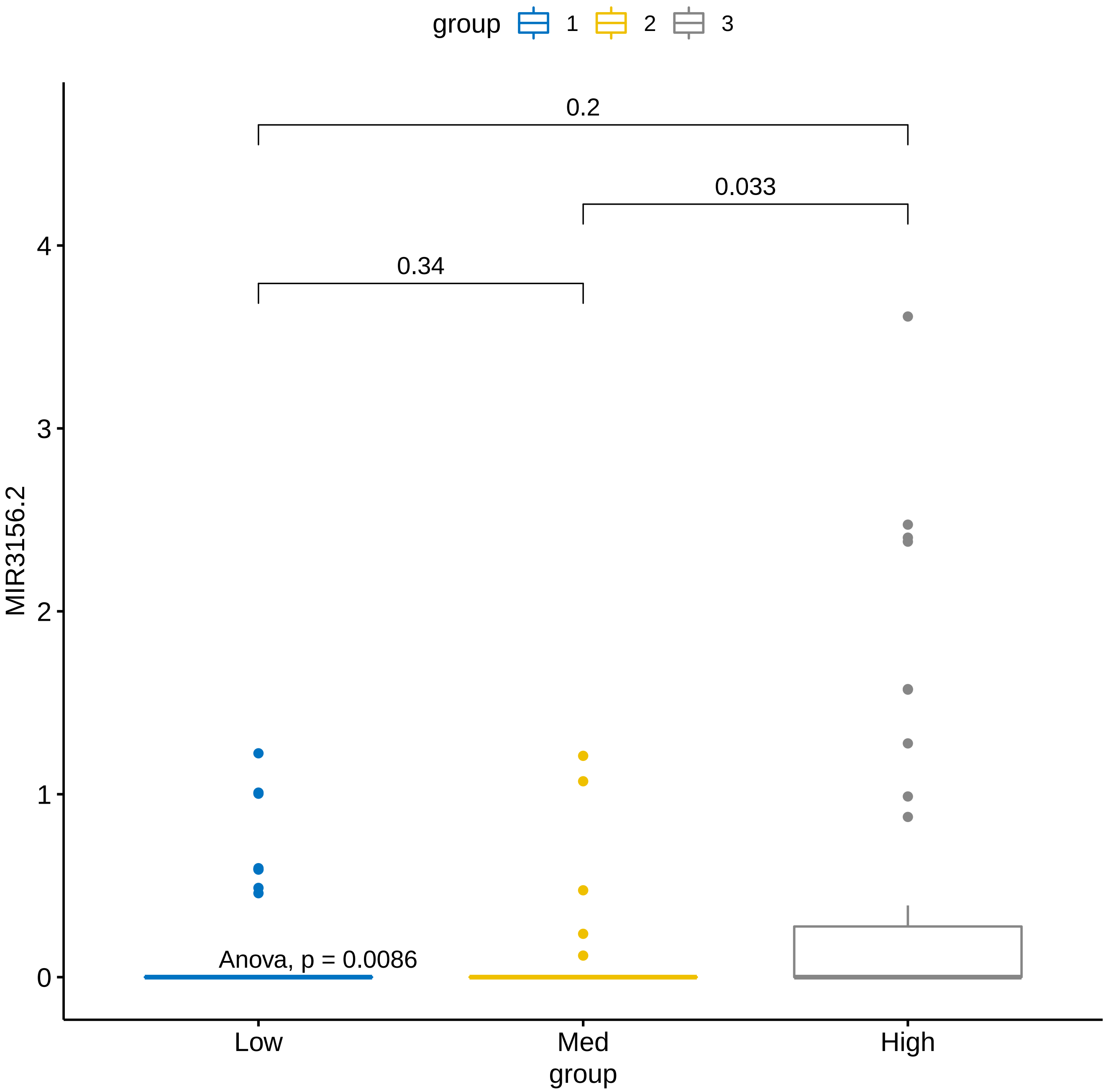
group 1 2 3
0.2
0.033
0.34
Anova, p = 0.0086
MIR3156.2
0
1
2
3
4
Low
Med
High
group

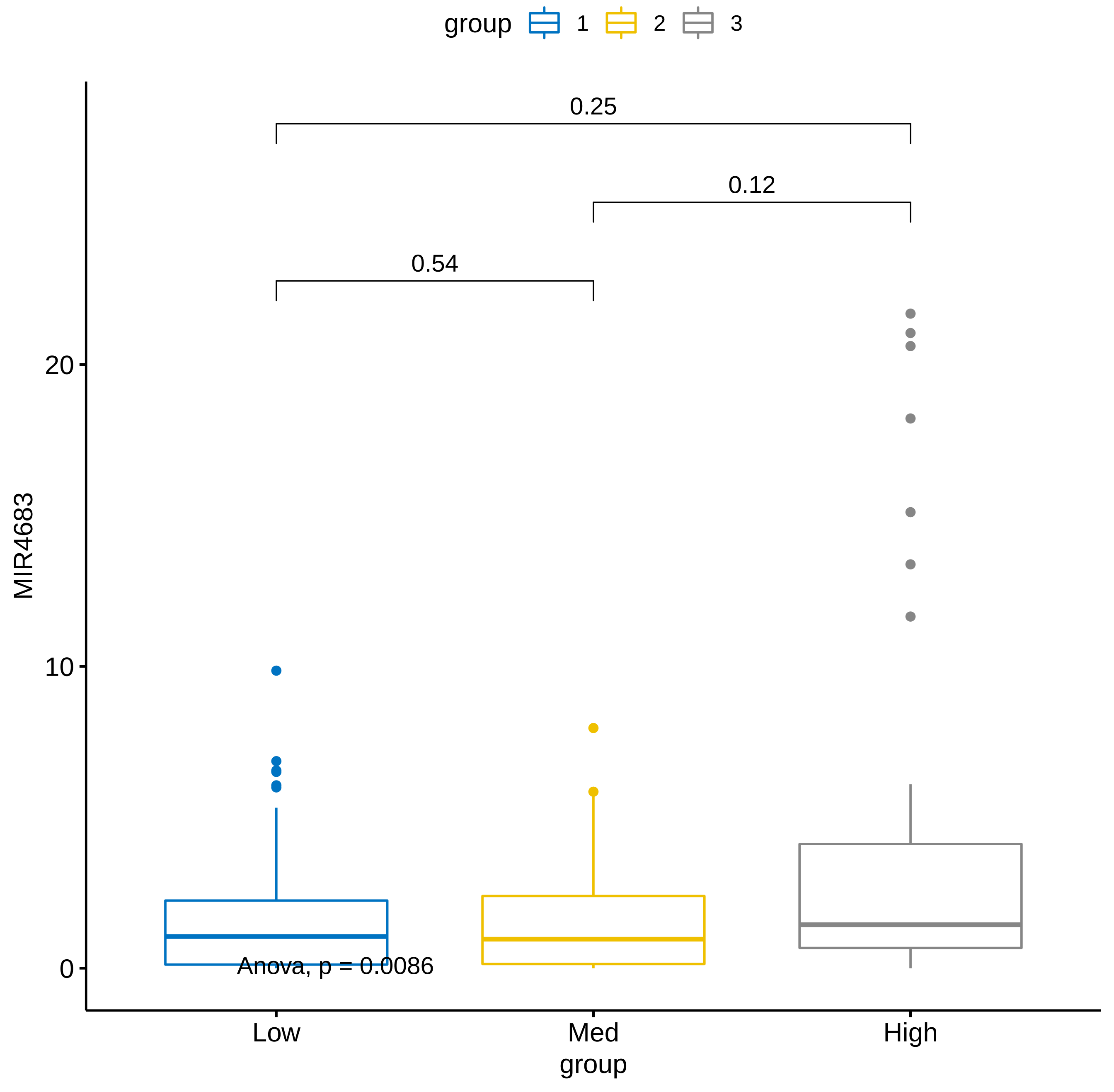
group
1
2
3
0.25
0.12
0.54
MIR4683
20
10
0
Anova, p = 0.0086
Low
Med
High
group

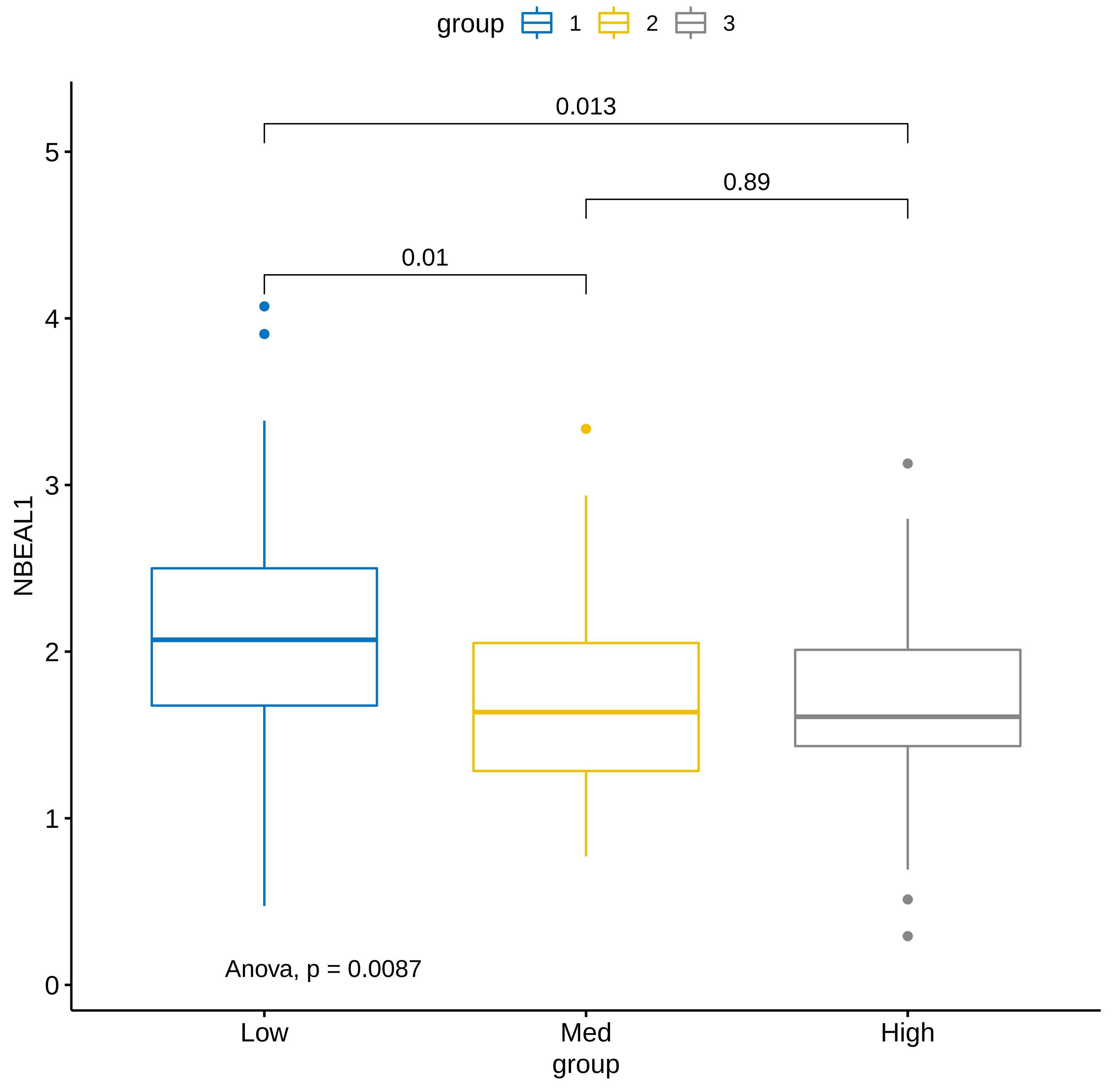
group
1
2
3
0.013
0.89
0.01
5
4
3
2
1
0
NBEAL1
Anova, p = 0.0087
Low
Med
High
group

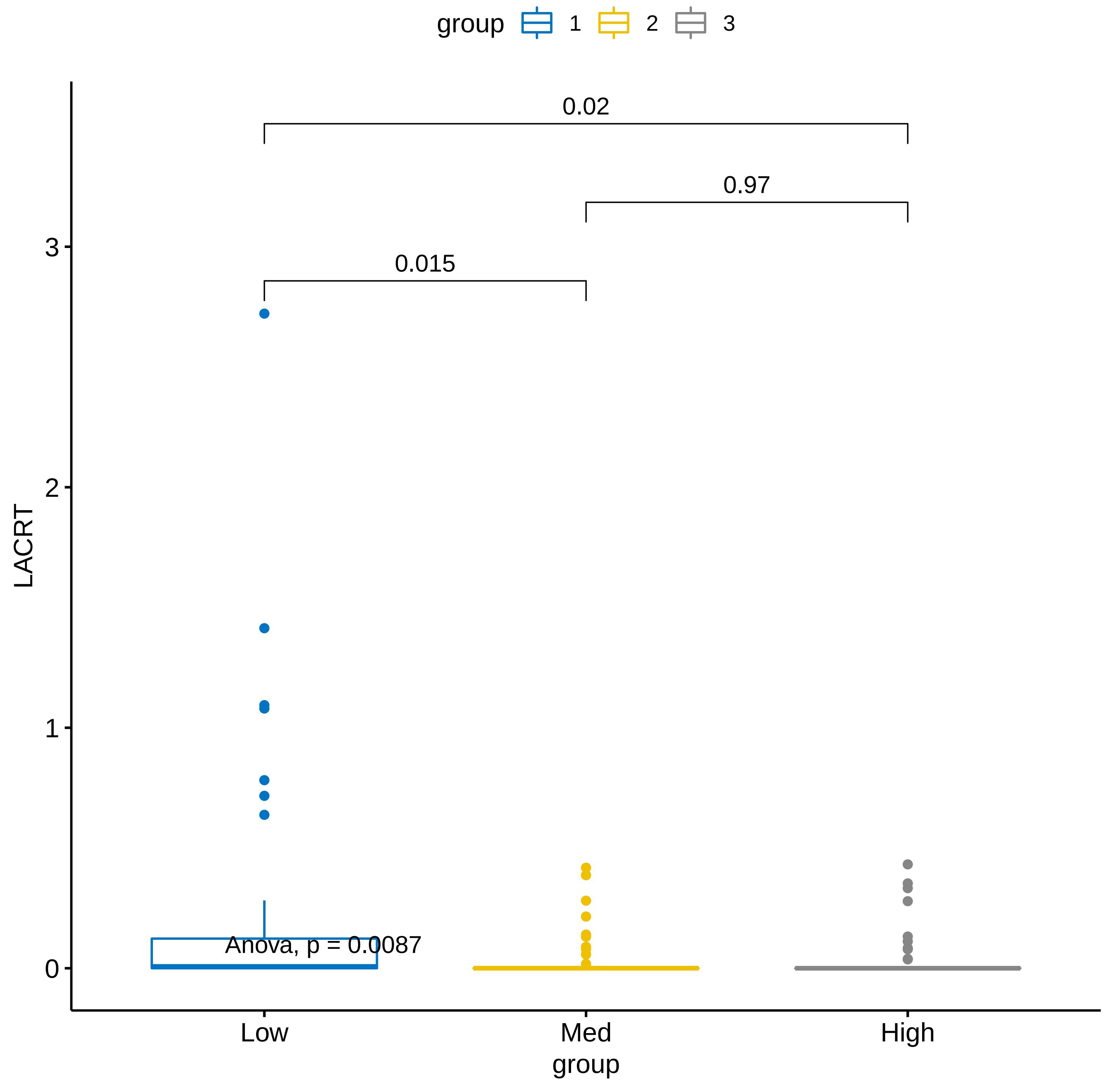

group 1 2 3
0.02
0.97
0.015
Anova, p = 0.0087
LACRT
0
1
2
3
Low
Med
High
group

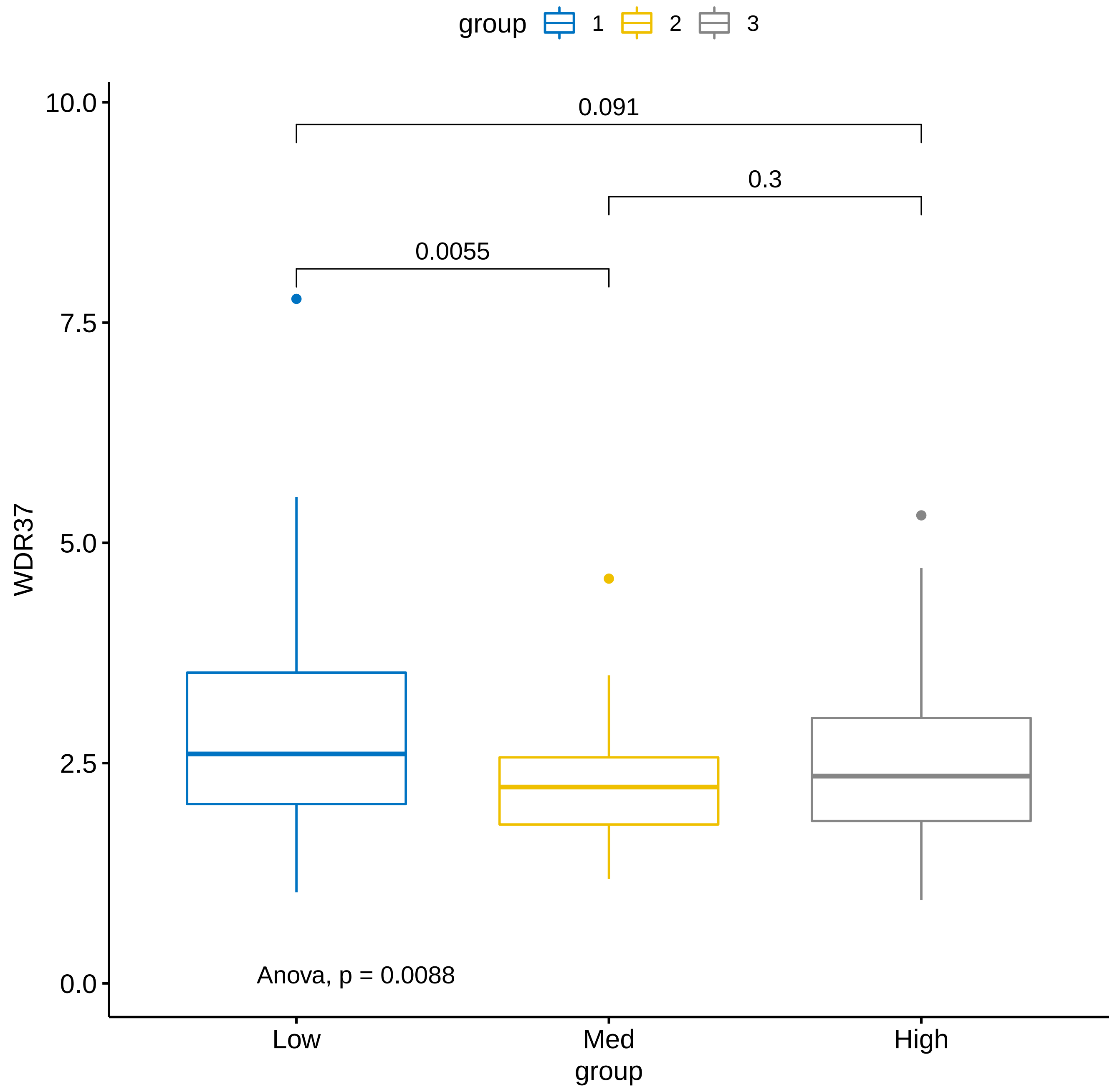
group 1 2 3
0.091
0.3
0.0055
10.0
7.5
5.0
2.5
0.0
WDR37
Anova, p = 0.0088
Low
Med
High
group

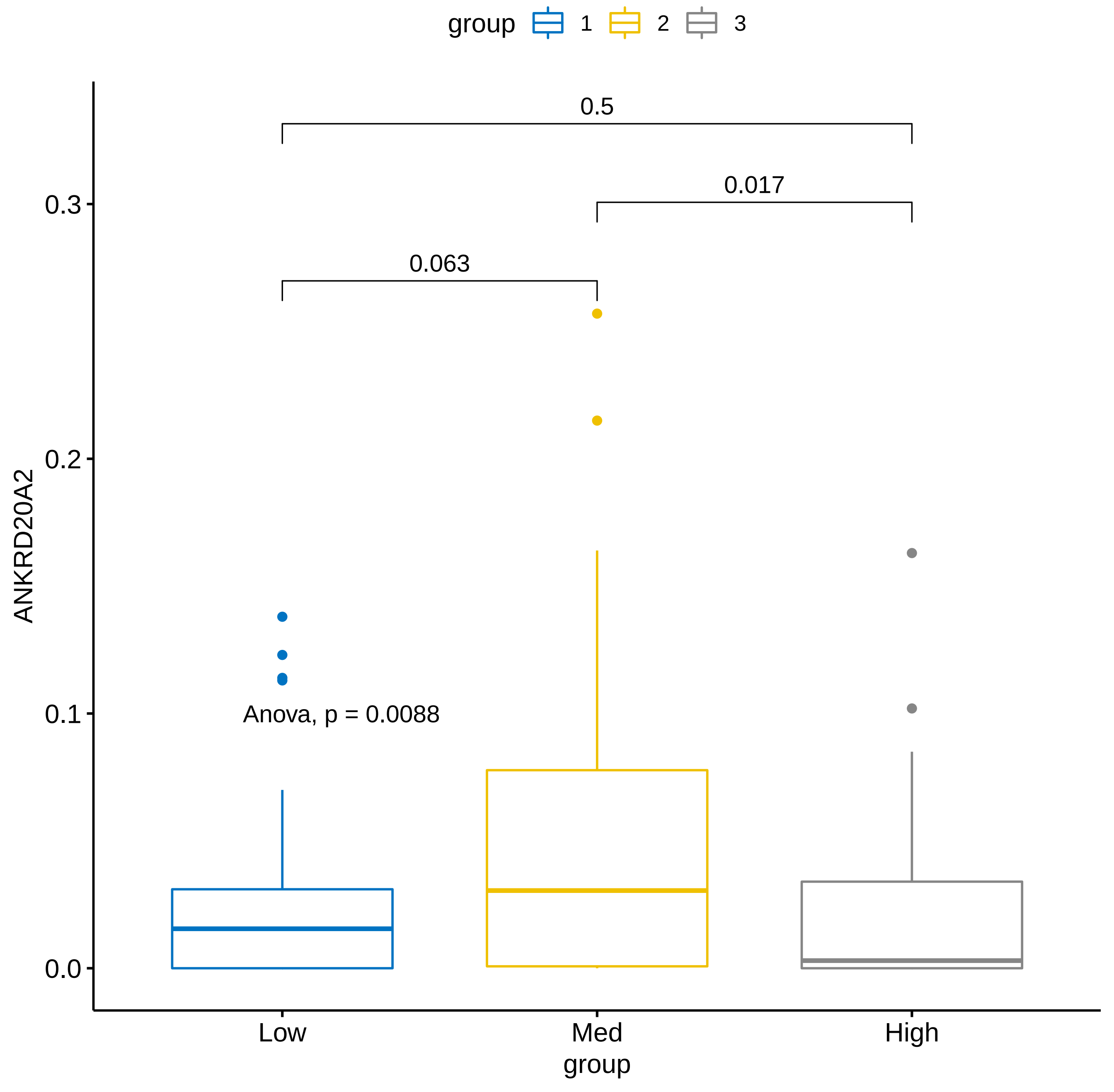
group
1
2
3
0.5
0.017
0.063
Anova, p = 0.0088
0.3
0.2
0.1
0.0
ANKRD20A2
Low
Med
High
group

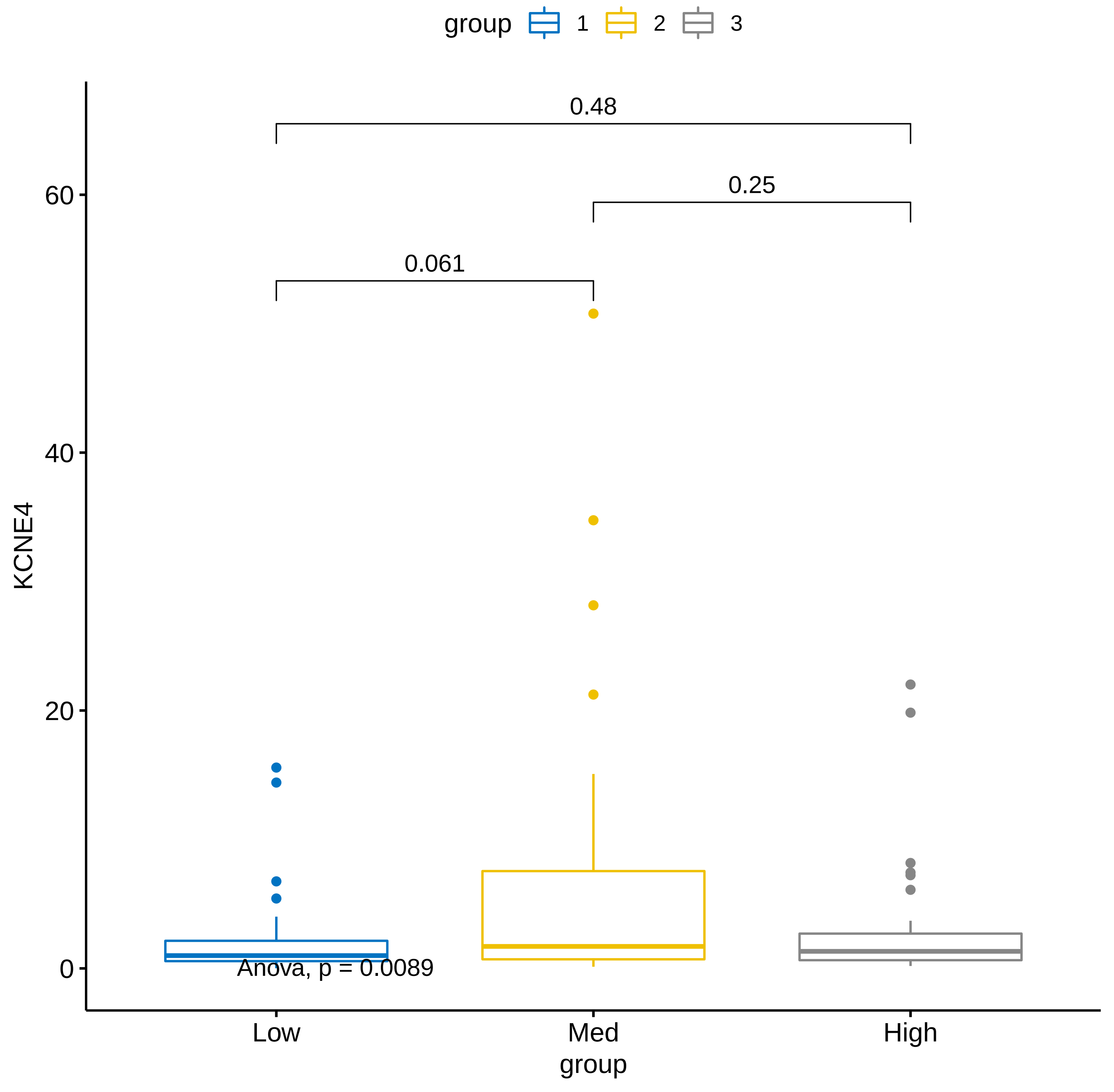
group
1
2
3
0.48
0.25
0.061
60
40
20
0
KCNE4
Anova, p = 0.0089
Low
Med
High
group

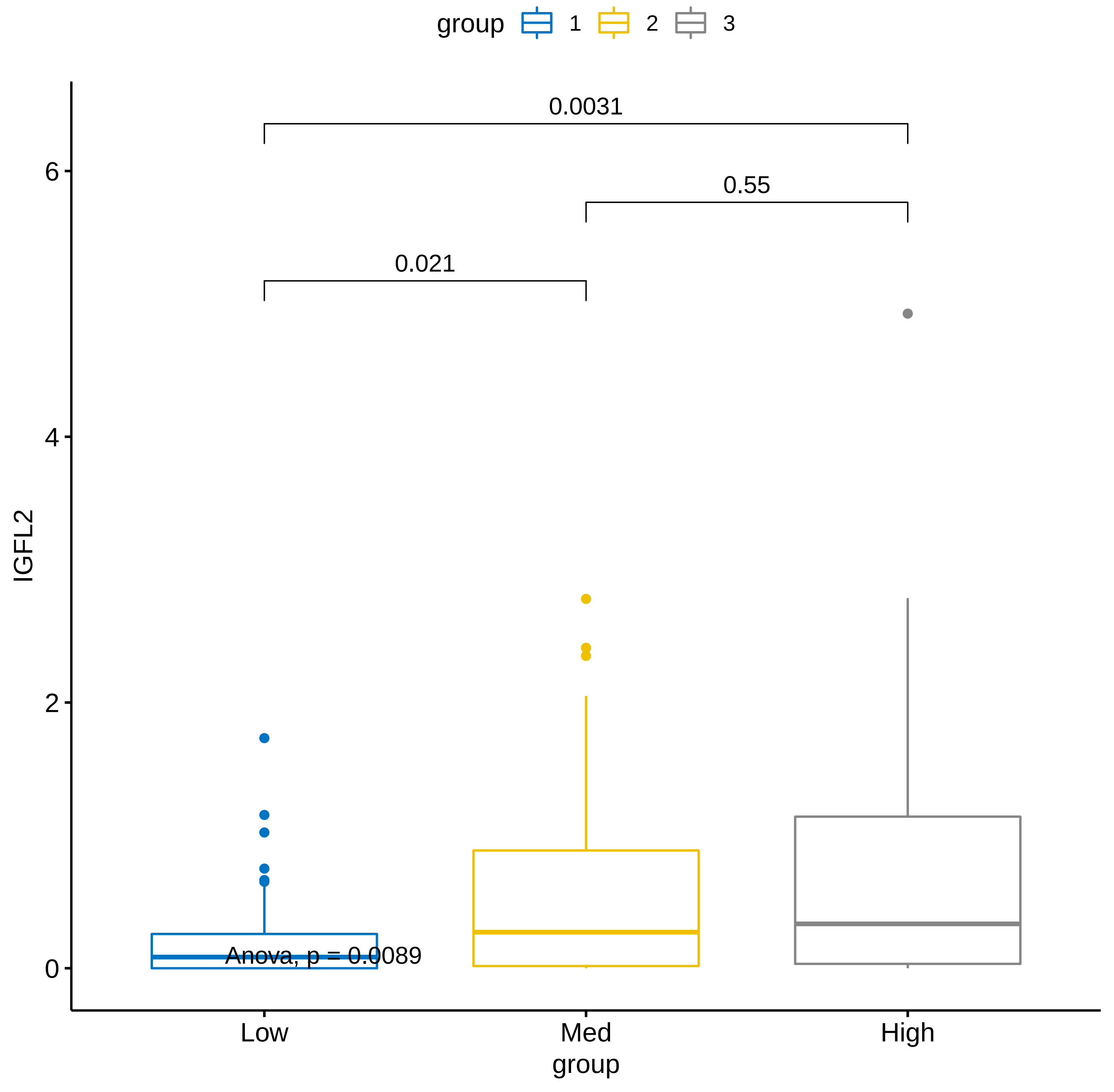
group 1 2 3
0.0031
0.55
0.021
6
4
2
0
IGFL2
Anova, p = 0.0089
Low
Med
High
group

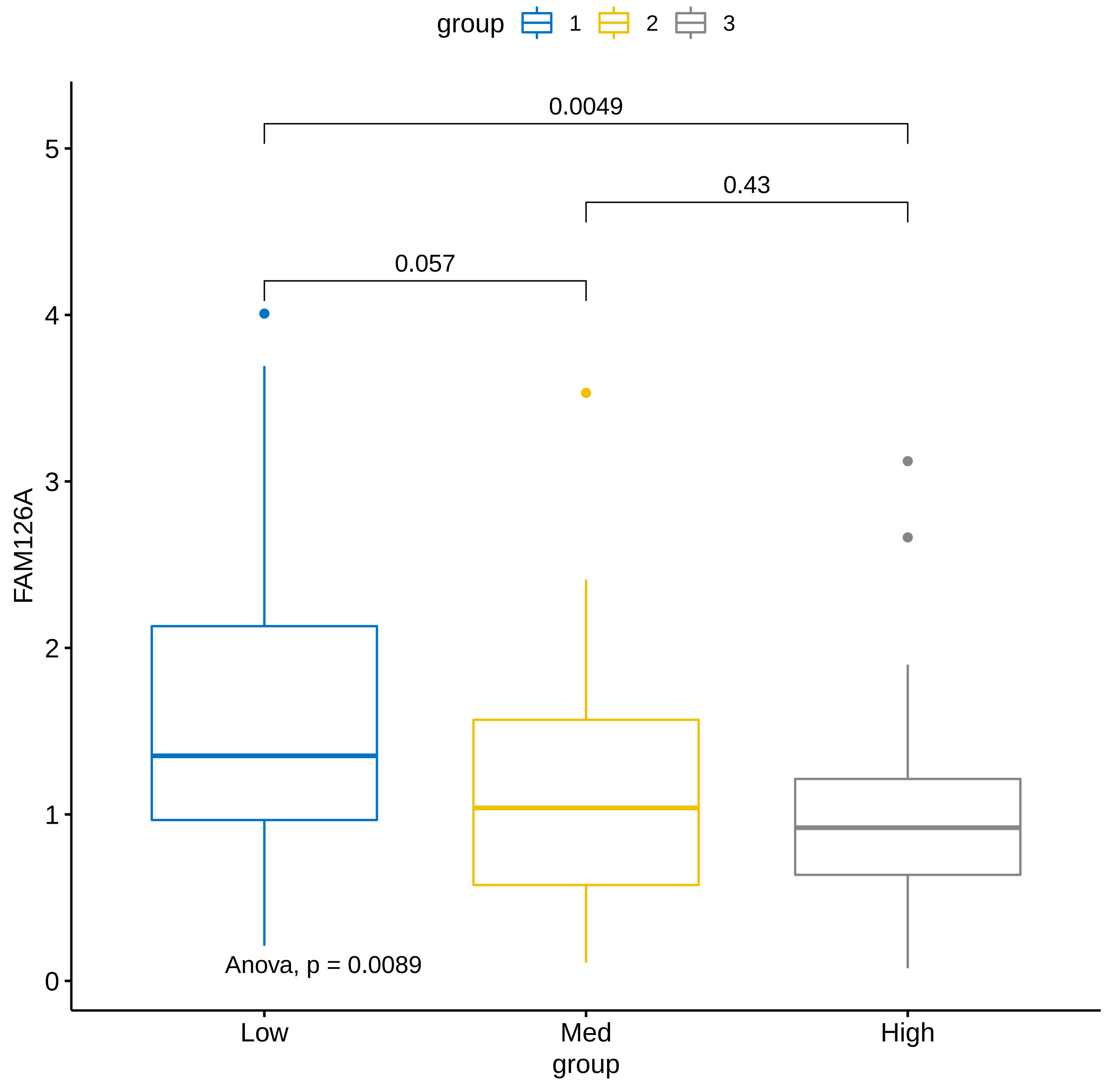

group 1 2 3
0.0049
0.43
0.057
FAM126A
5
4
3
2
1
0
Anova, p = 0.0089
Low
Med
High
group

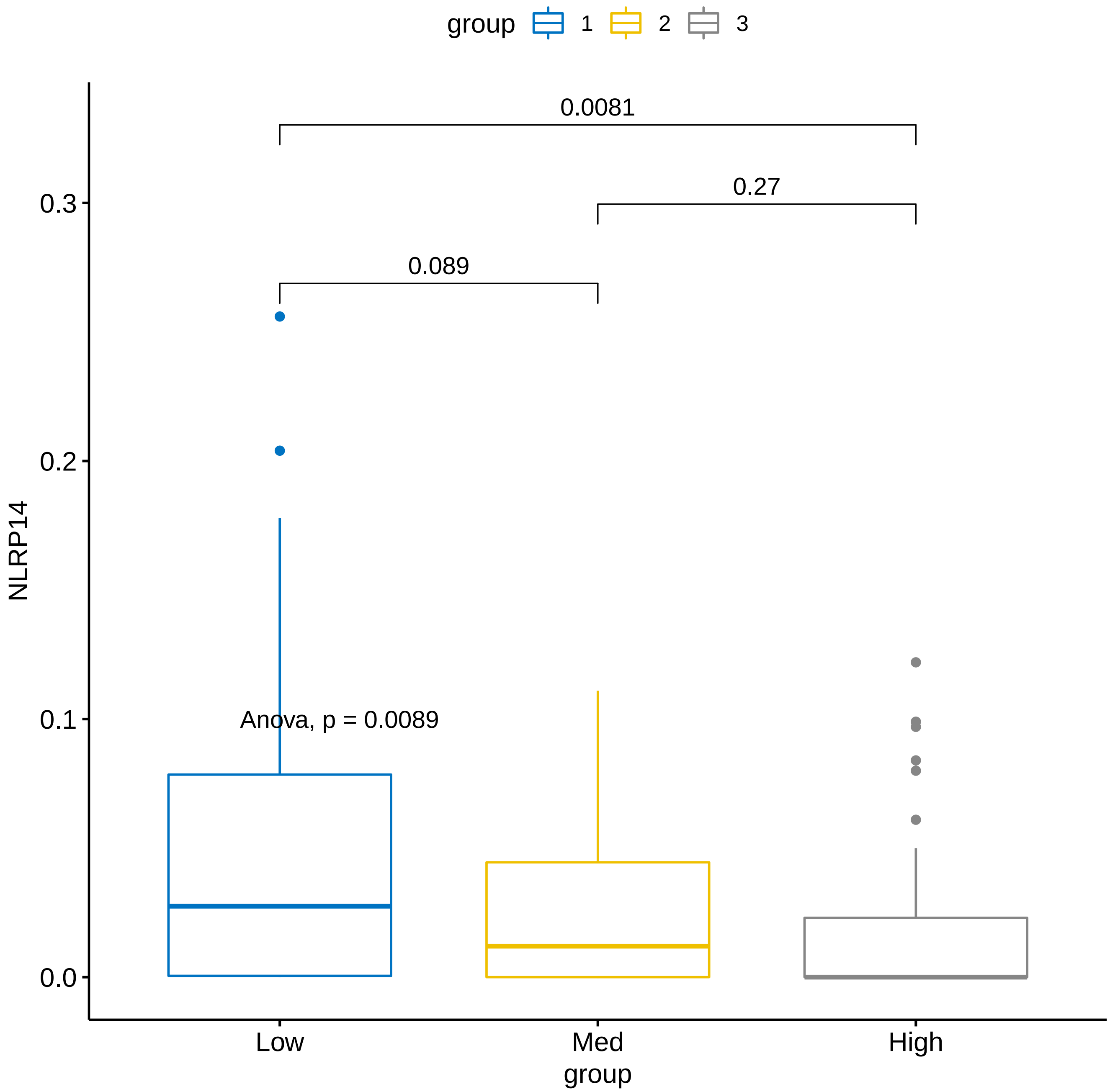
group
1
2
3
0.0081
0.27
0.089
Anova, p = 0.0089
NLRP14
0.3
0.2
0.1
0.0
Low
Med
High
group

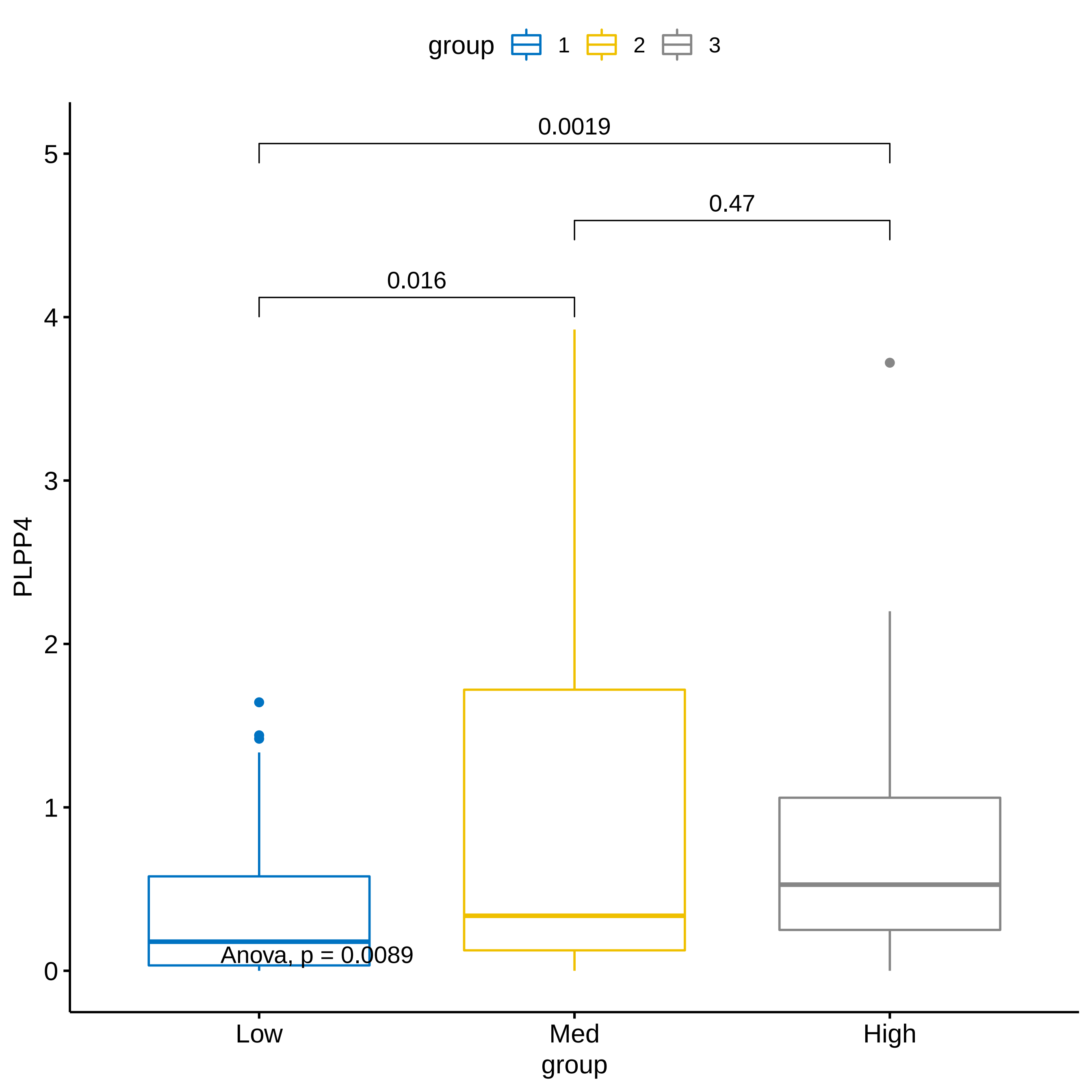
group
1
2
3
0.0019
0.47
0.016
Anova, p = 0.0089
PLPP4
5
4
3
2
1
0
Low
Med
High
group

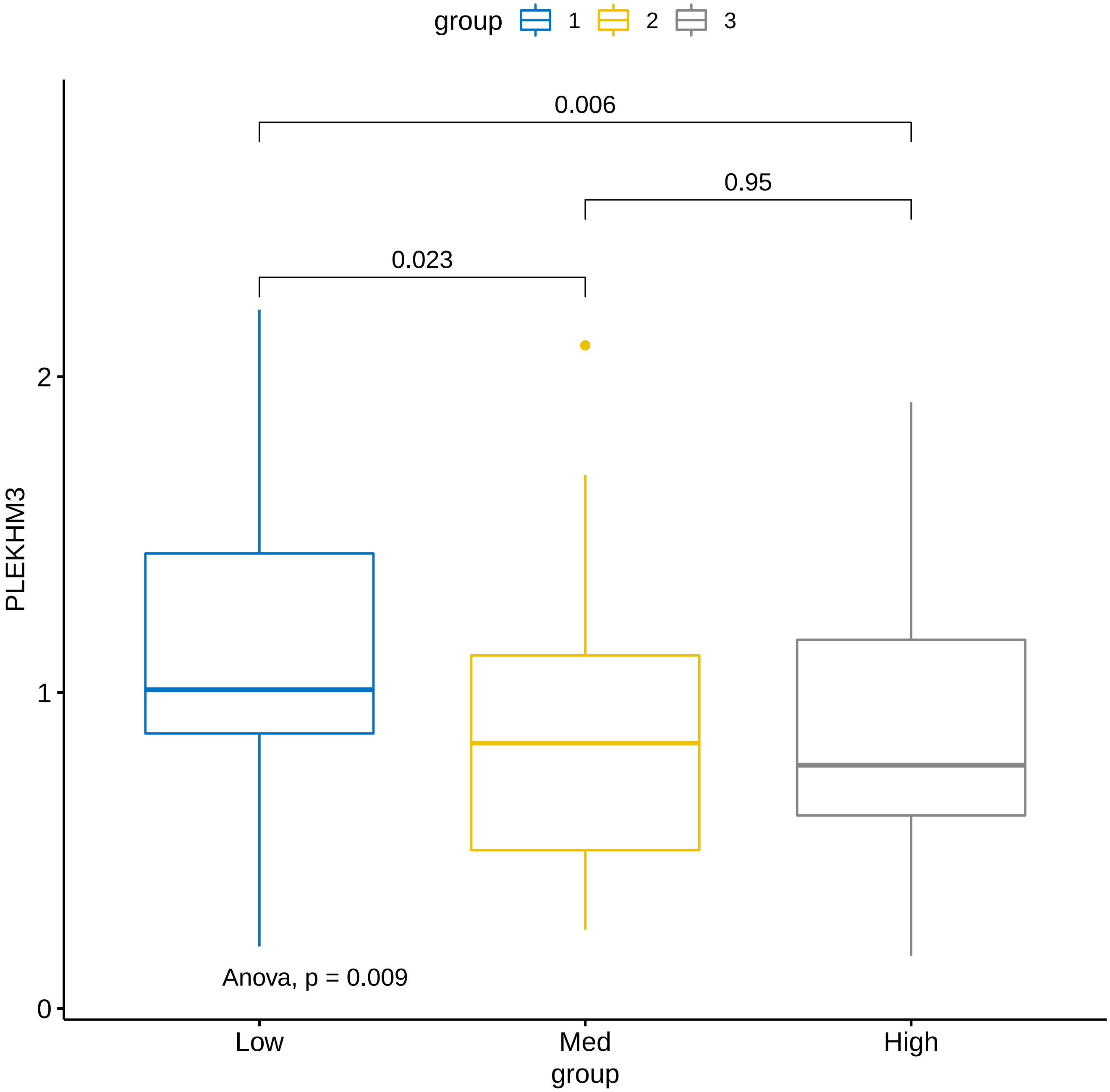
group
1
2
3
0.006
0.95
0.023
PLEKHM3
2
1
0
Anova, p = 0.009
Low
Med
High
group

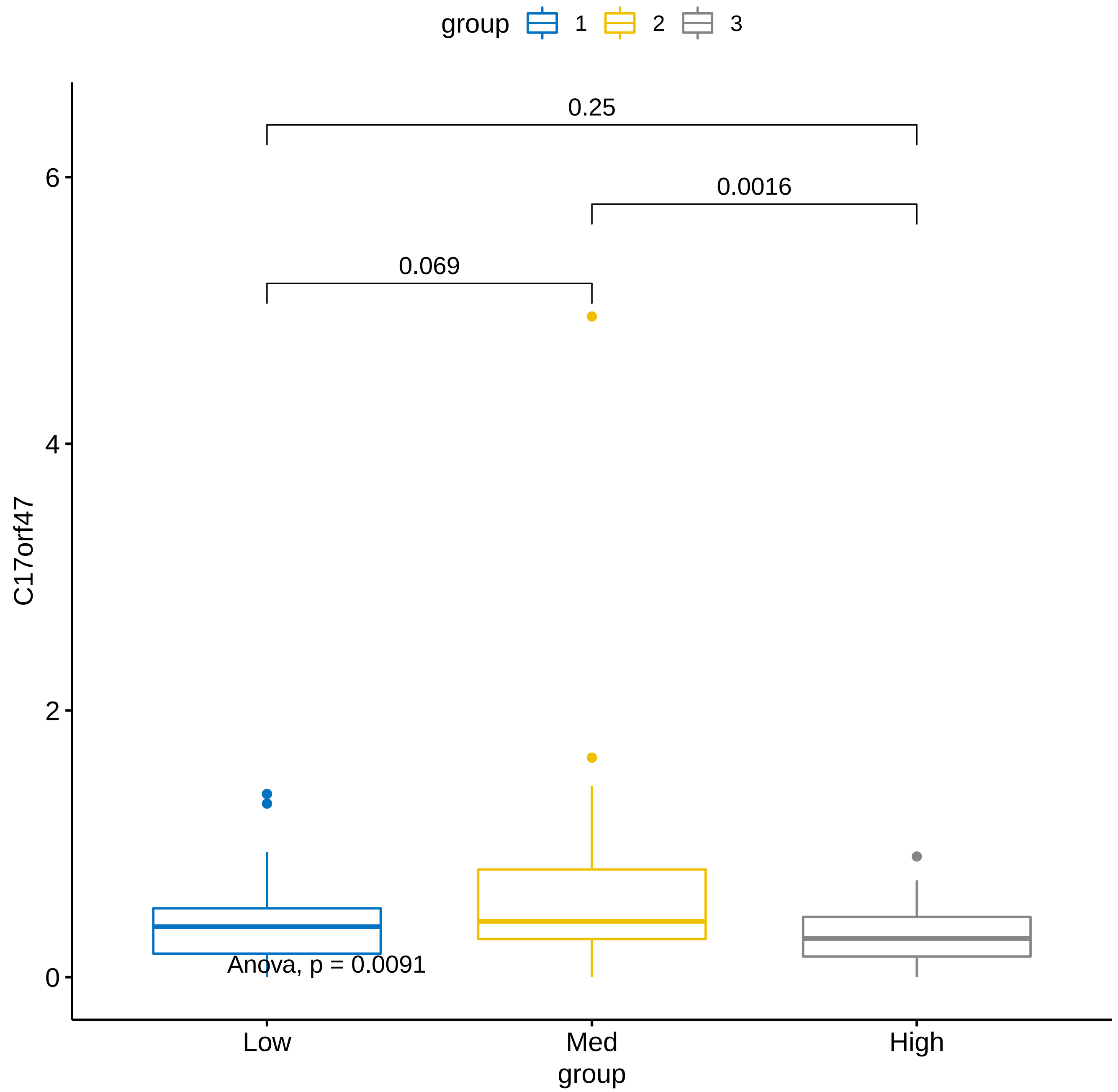
group 1 2 3
0.25
0.0016
0.069
6
4
2
0
C17orf47
Anova, p = 0.0091
Low
Med
High
group

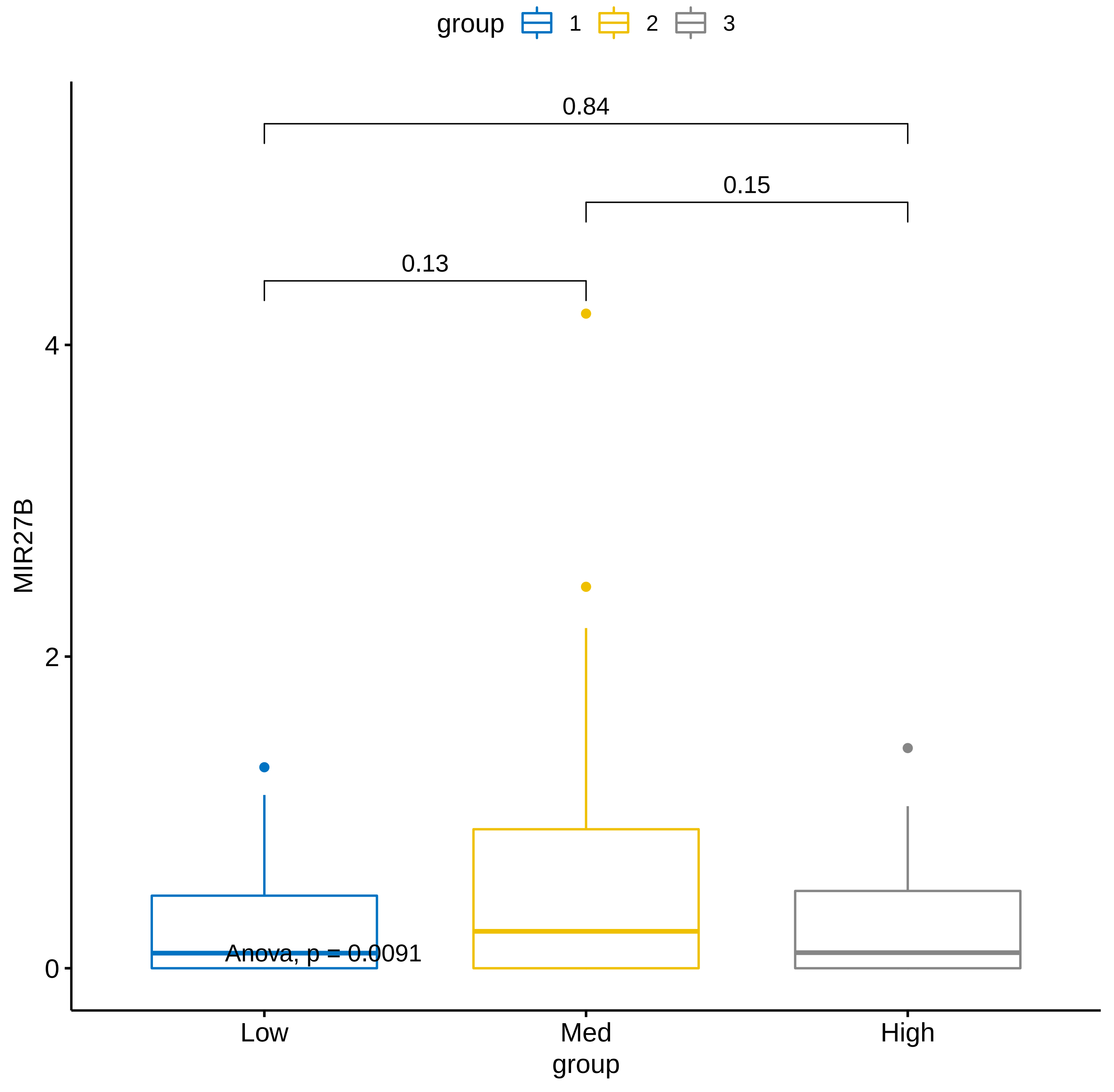

group
1
2
3
0.84
0.15
0.13
4
2
0
MIR27B
Anova, p = 0.0091
Low
Med
High
group

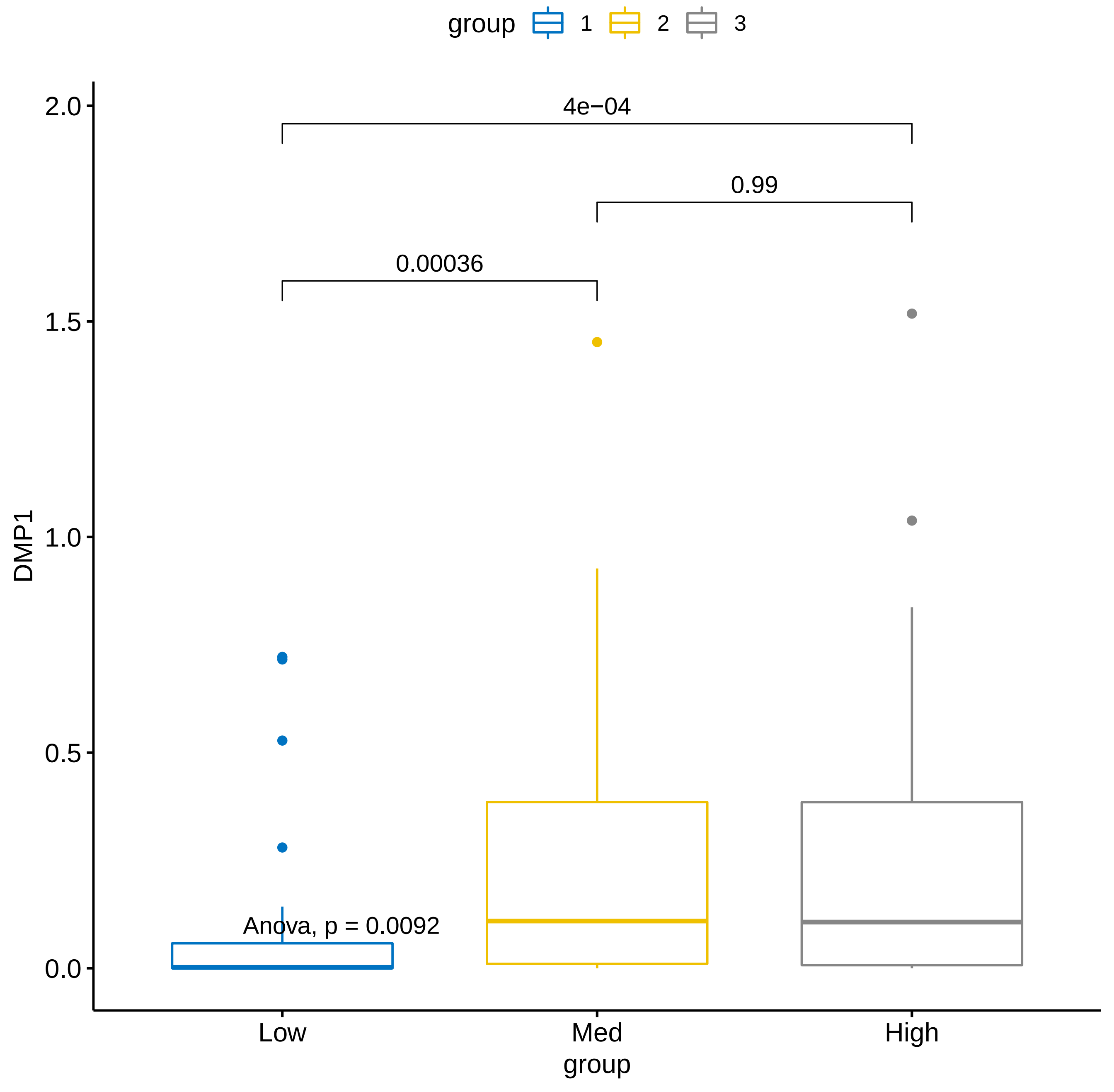

group
1
2
3
2.0
1.5
1.0
0.5
0.0
DMP1
4e−04
0.99
0.00036
Anova, p = 0.0092
Low
Med
High
group

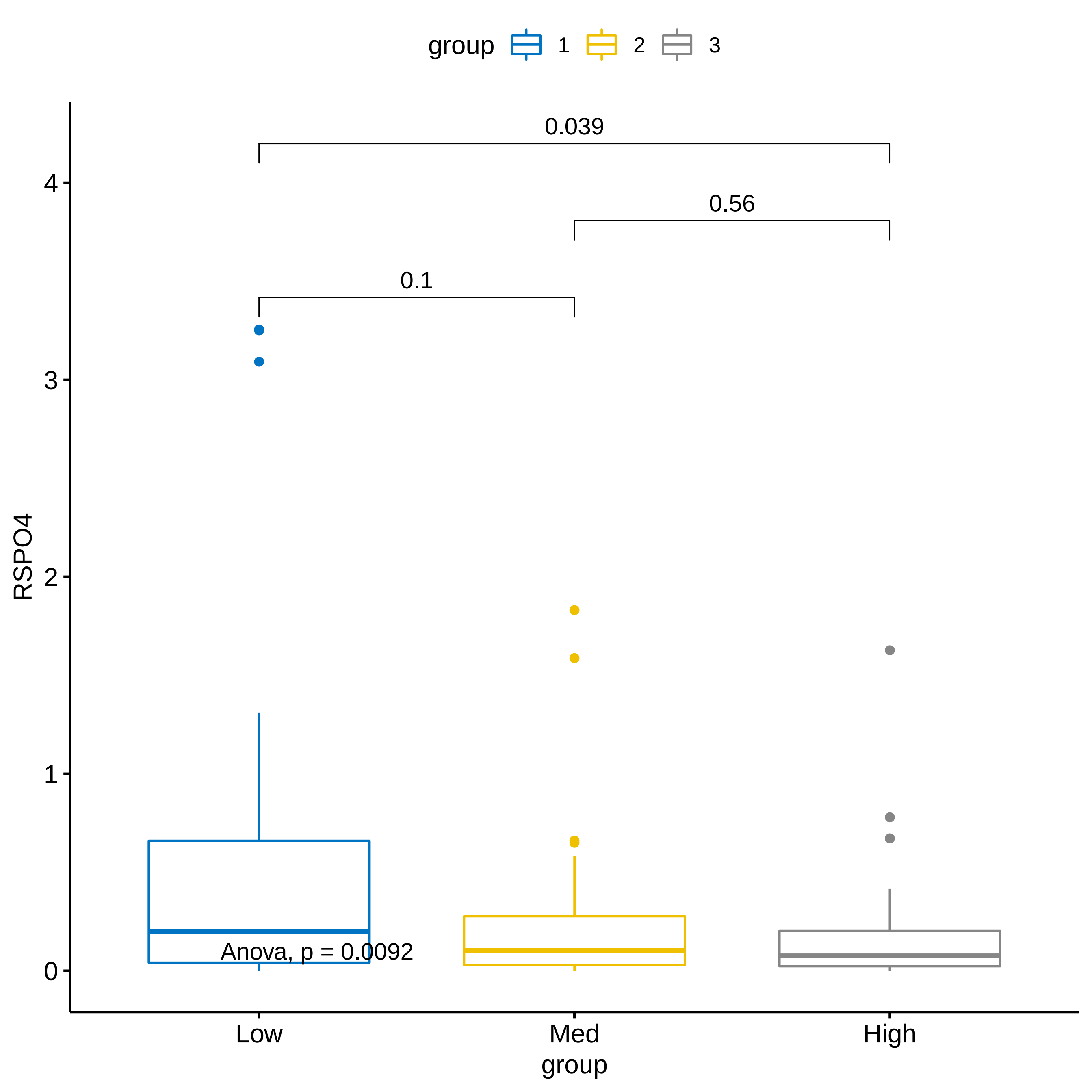

group 1 2 3
0.039
0.56
0.1
RSPO4
4
3
2
1
0
Anova, p = 0.0092
Low
Med
High
group

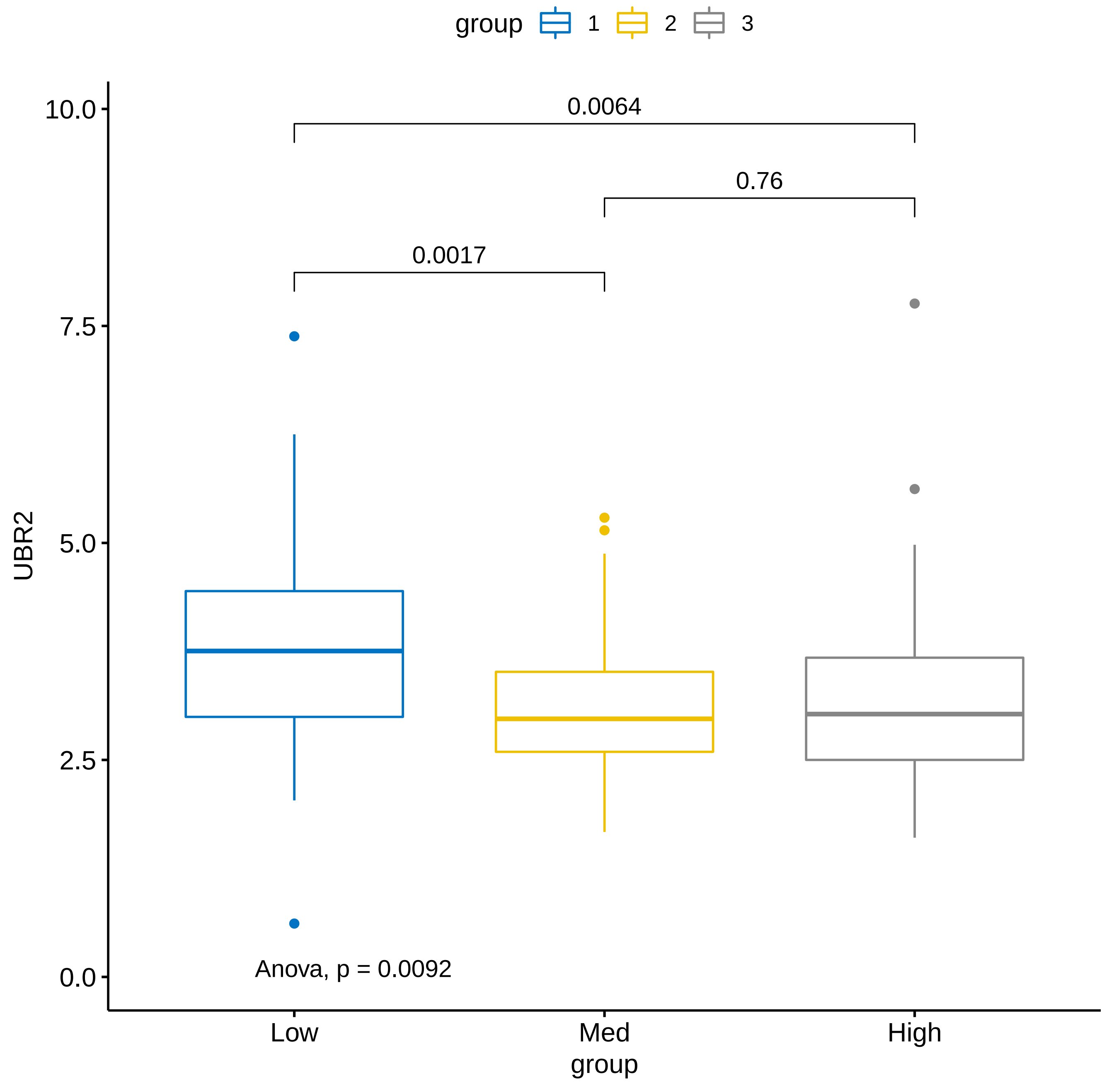
group 1 2 3
0.0064
0.76
0.0017
10.0
7.5
5.0
2.5
0.0
UBR2
Anova, p = 0.0092
Low
Med
High
group

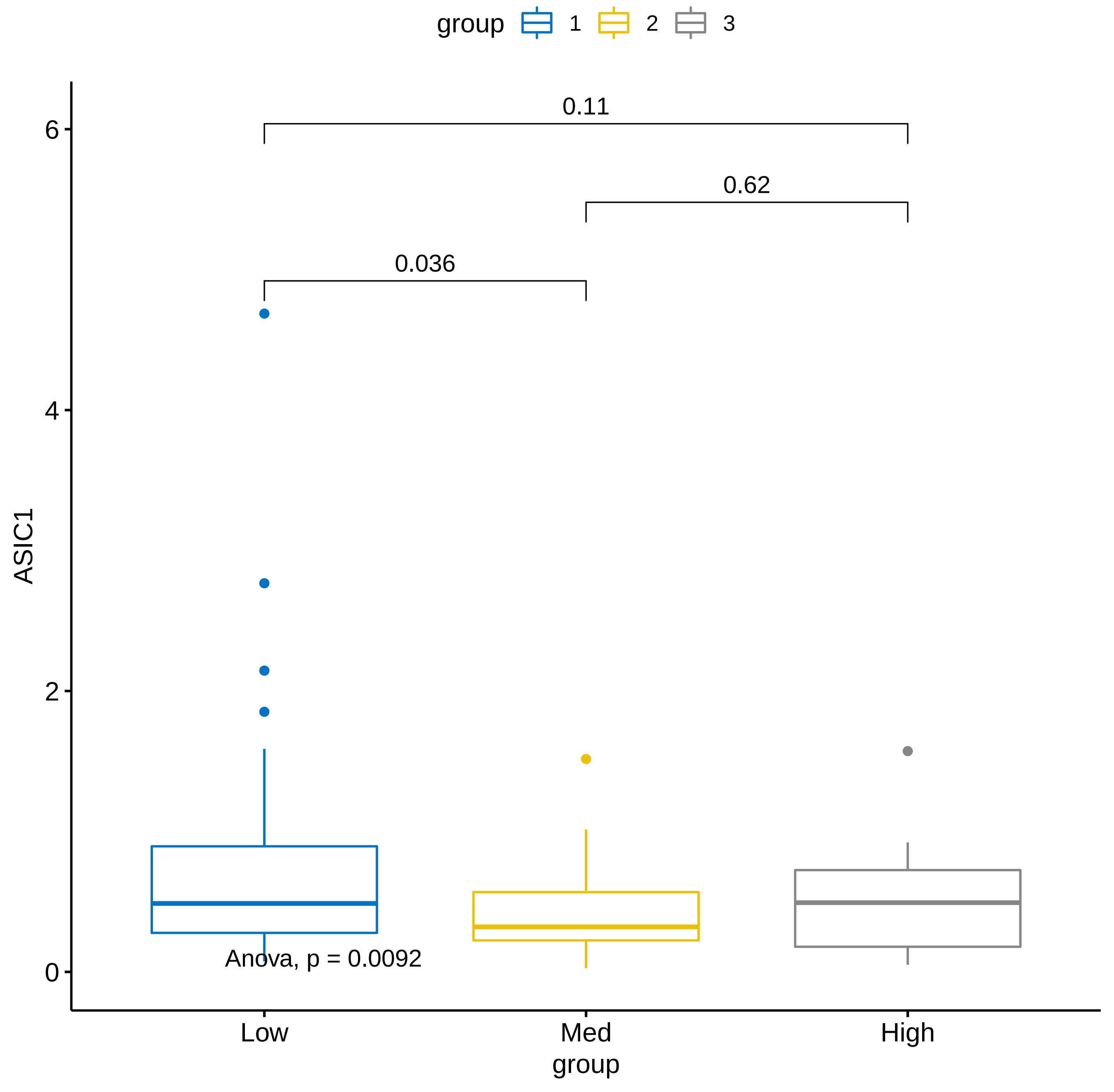

group 1 2 3
0.11
0.62
0.036
ASIC1
6
4
2
0
Anova, p = 0.0092
Low
Med
High
group

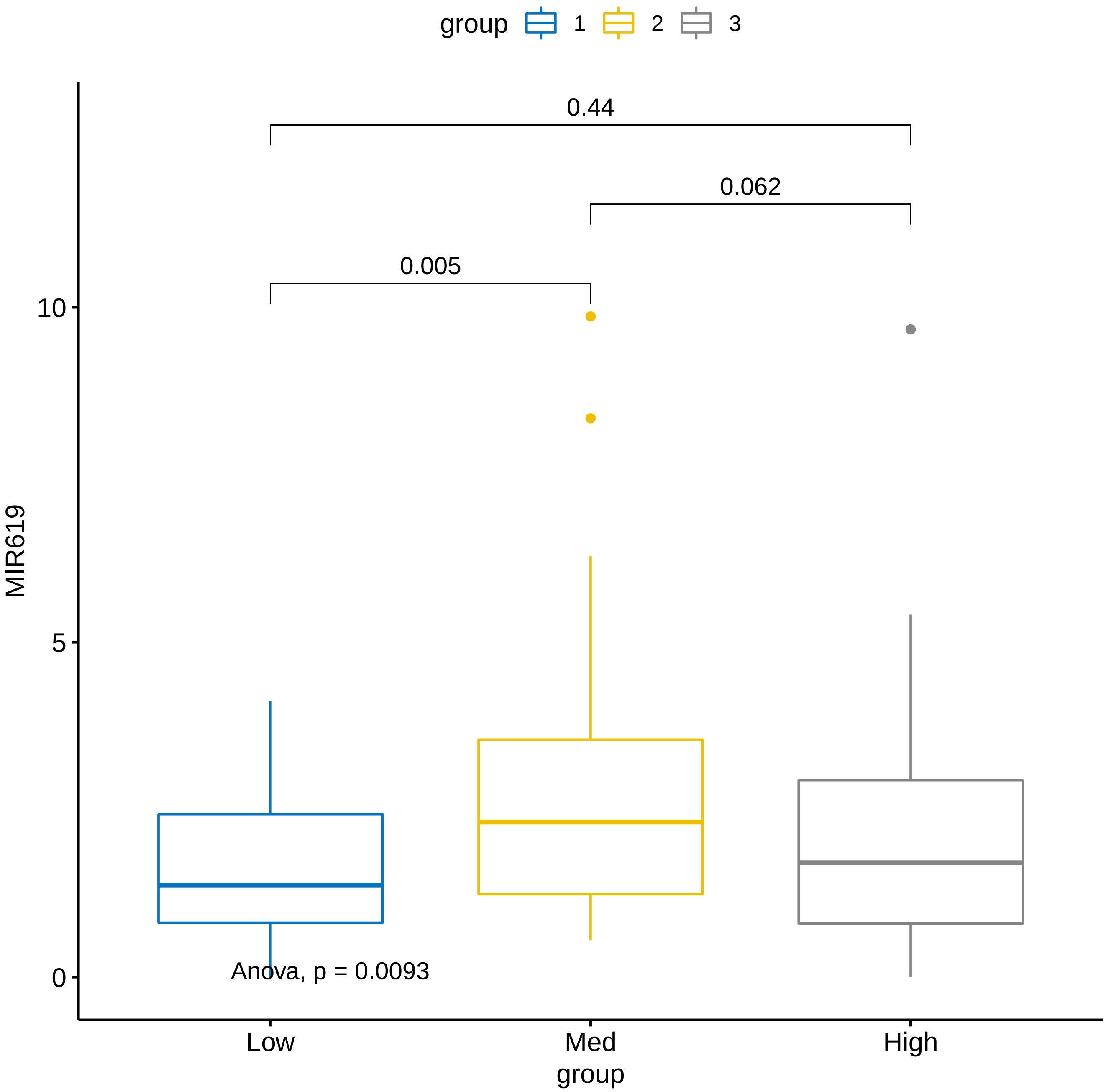

group
1
2
3
0.44
0.062
0.005
10
5
0
MIR619
Anova, p = 0.0093
Low
Med
High
group

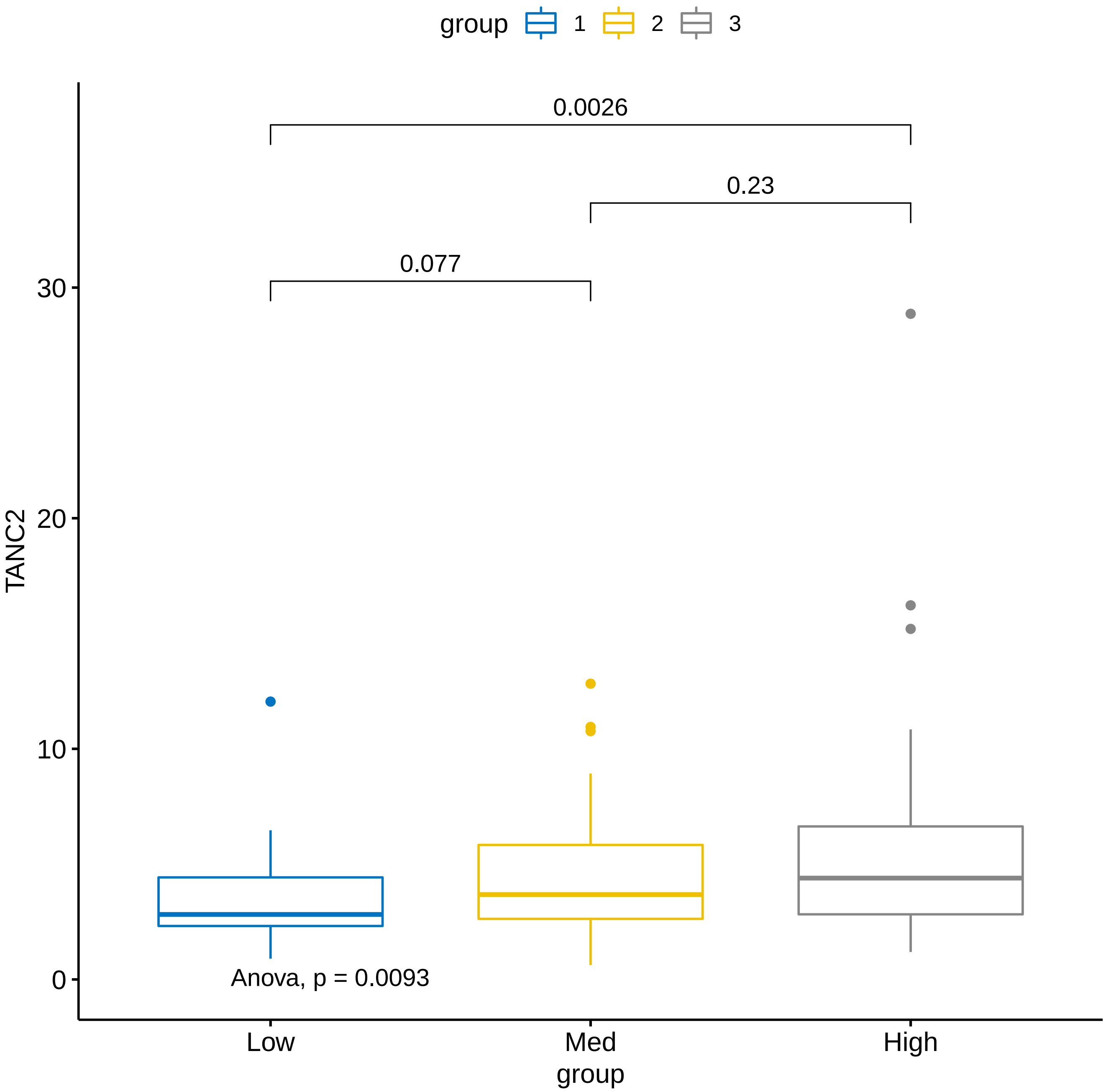
group 1 2 3
0.0026
0.23
0.077
30
20
10
0
TANC2
Anova, p = 0.0093
Low
Med
High
group

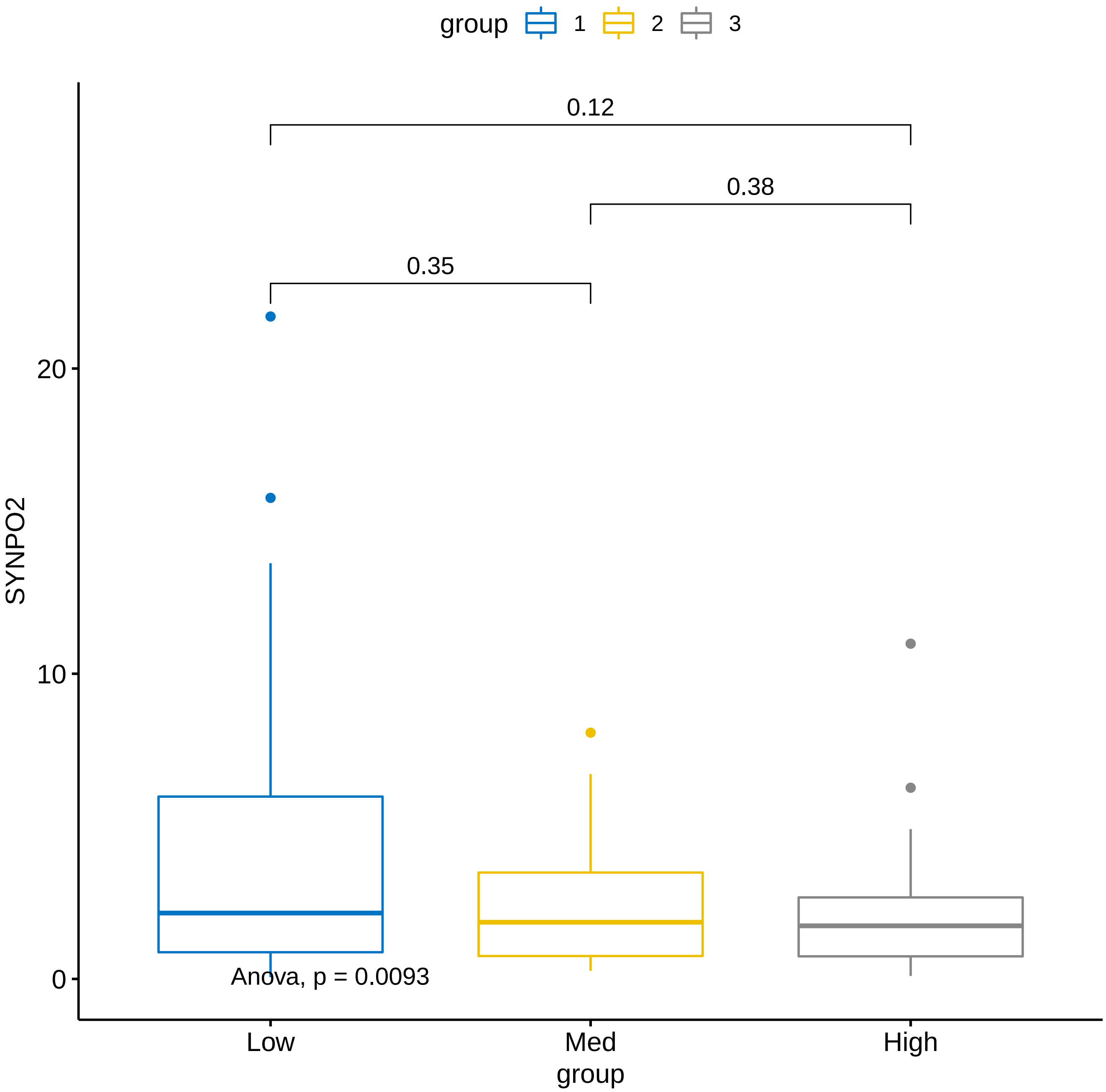
group 1 2 3
0.12
0.38
0.35
SYNPO2
20
10
0
Anova, p = 0.0093
Low
Med
High
group

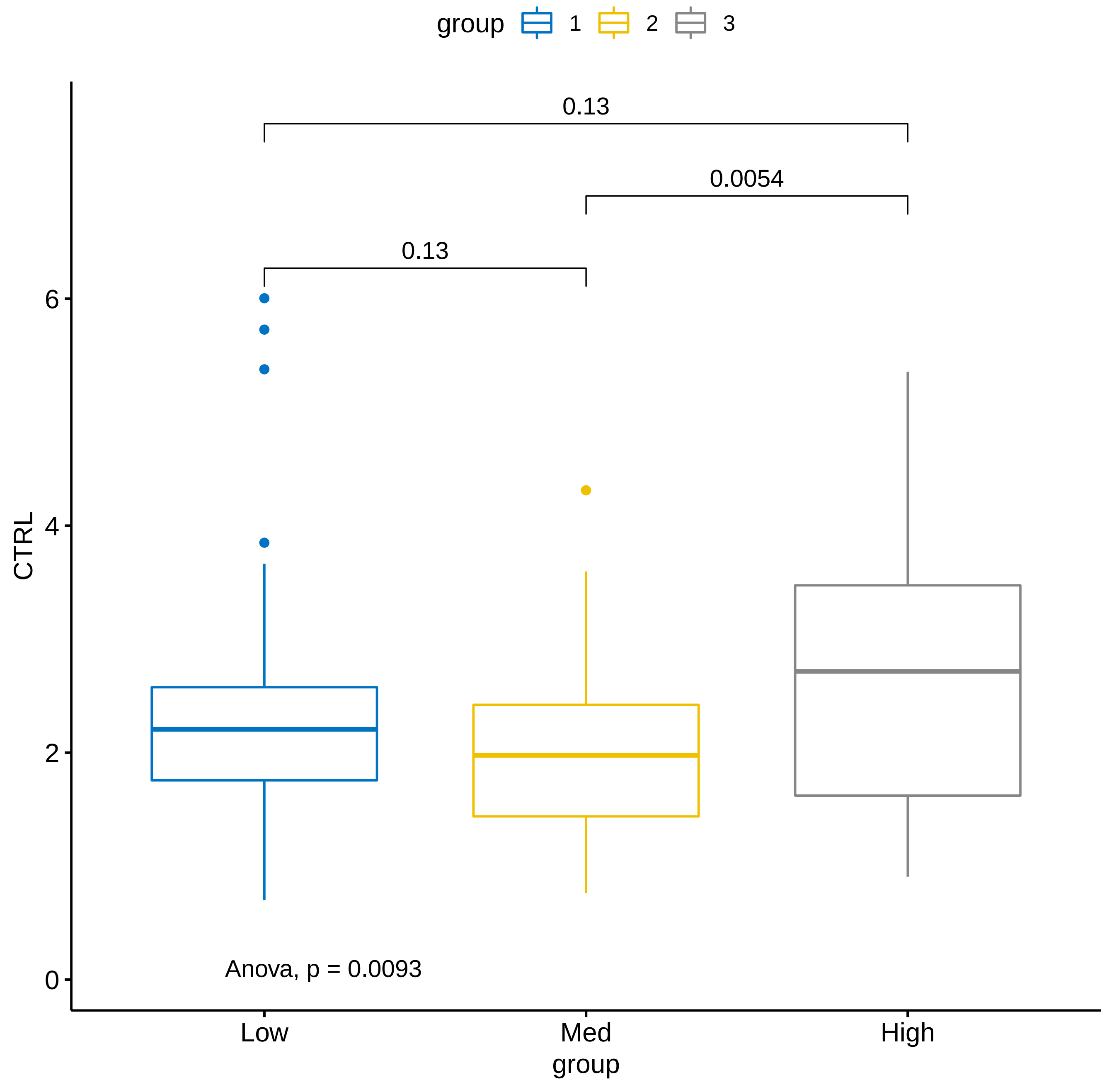

group 1 2 3
0.13
0.0054
0.13
CTRL
6
4
2
0
Anova, p = 0.0093
Low
Med
High
group

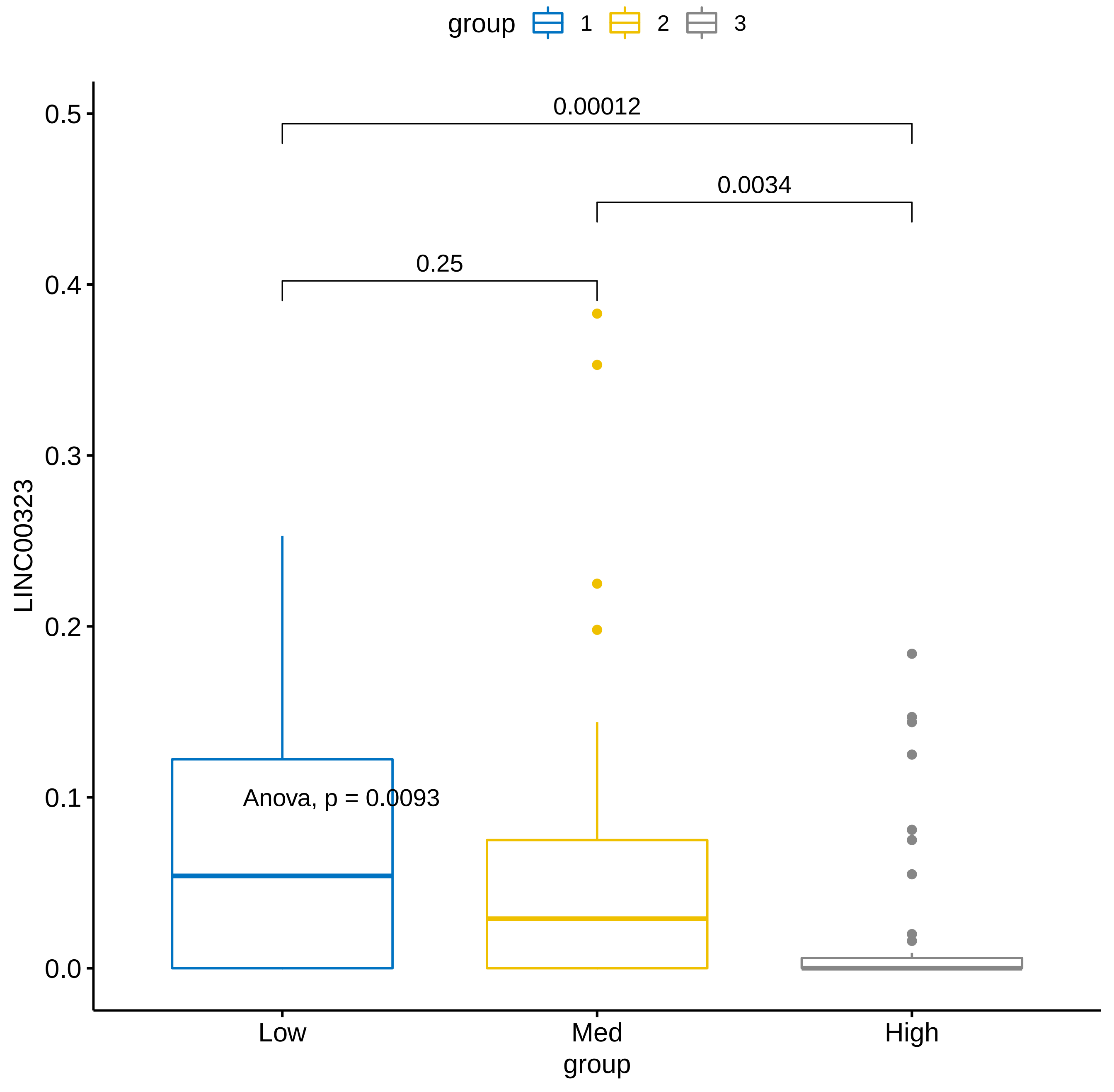

group 1 2 3
0.00012
0.0034
0.25
Anova, p = 0.0093
LINC00323
0.0
0.1
0.2
0.3
0.4
0.5
Low
Med
High
group

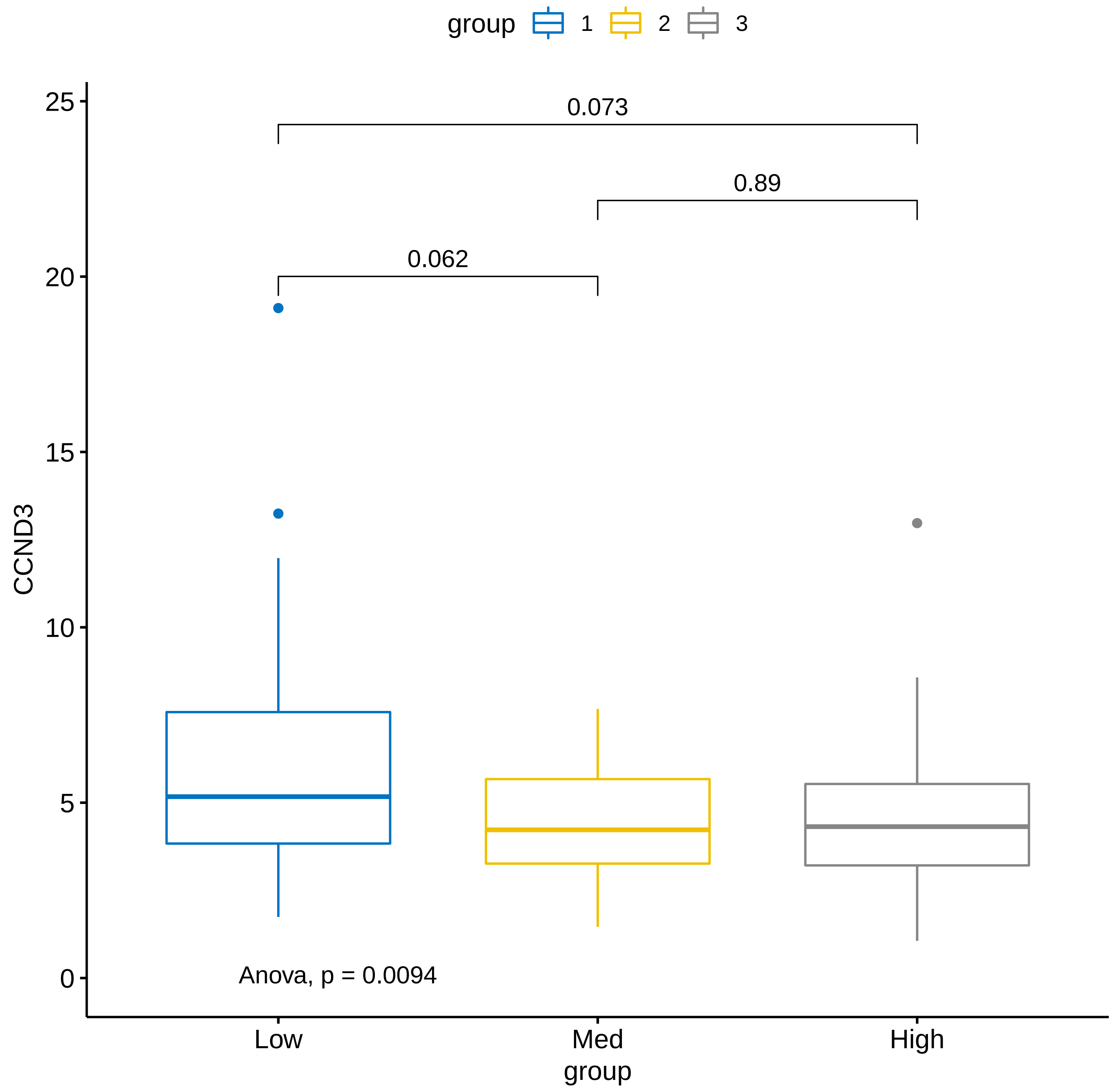

group 1 2 3
0.073
0.89
0.062
25
20
15
10
5
0
CCND3
Anova, p = 0.0094
Low
Med
High
group

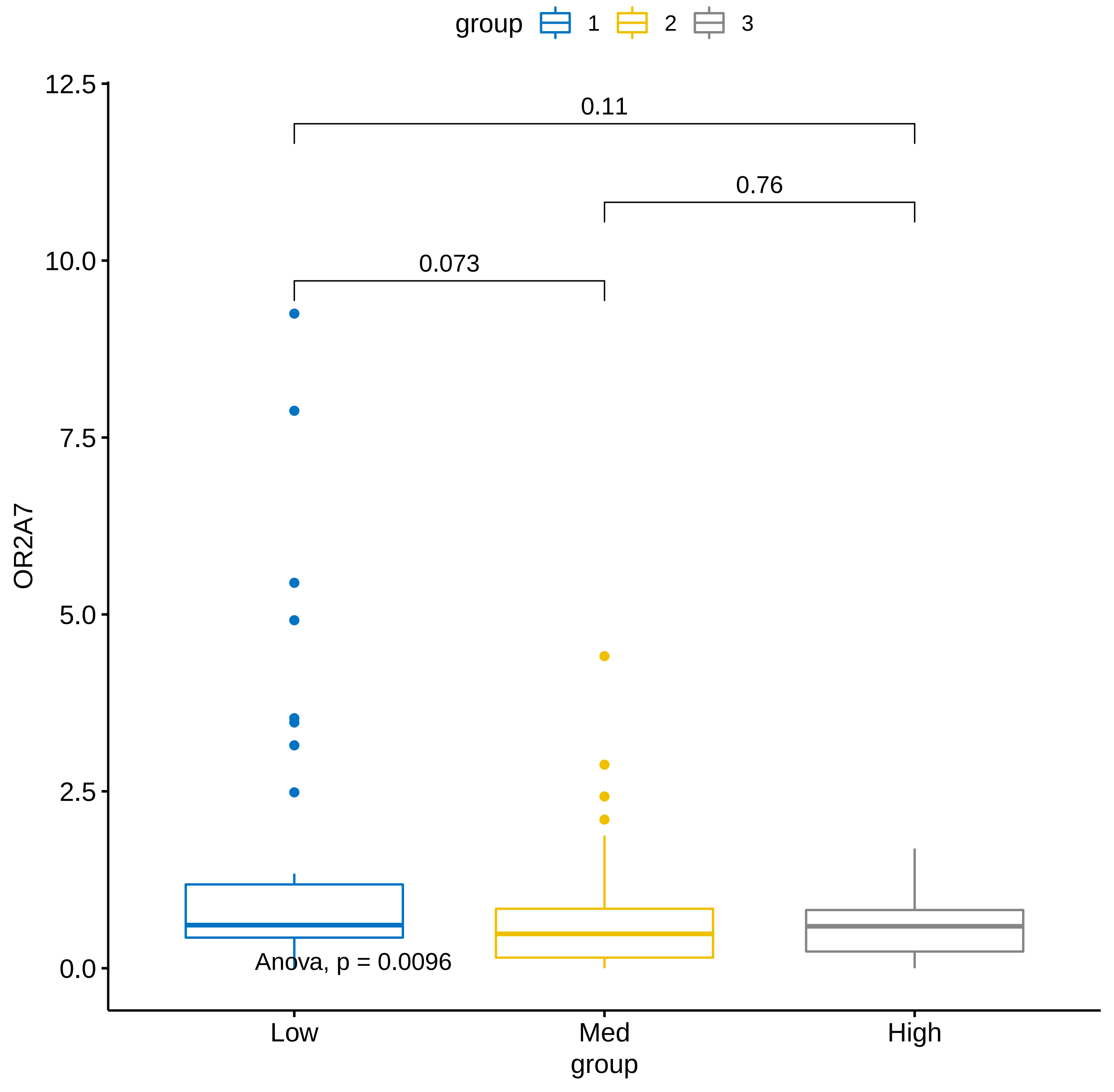

group
1
2
3
12.5
10.0
7.5
5.0
2.5
0.0
OR2A7
0.11
0.76
0.073
Anova, p = 0.0096
Low
Med
High
group

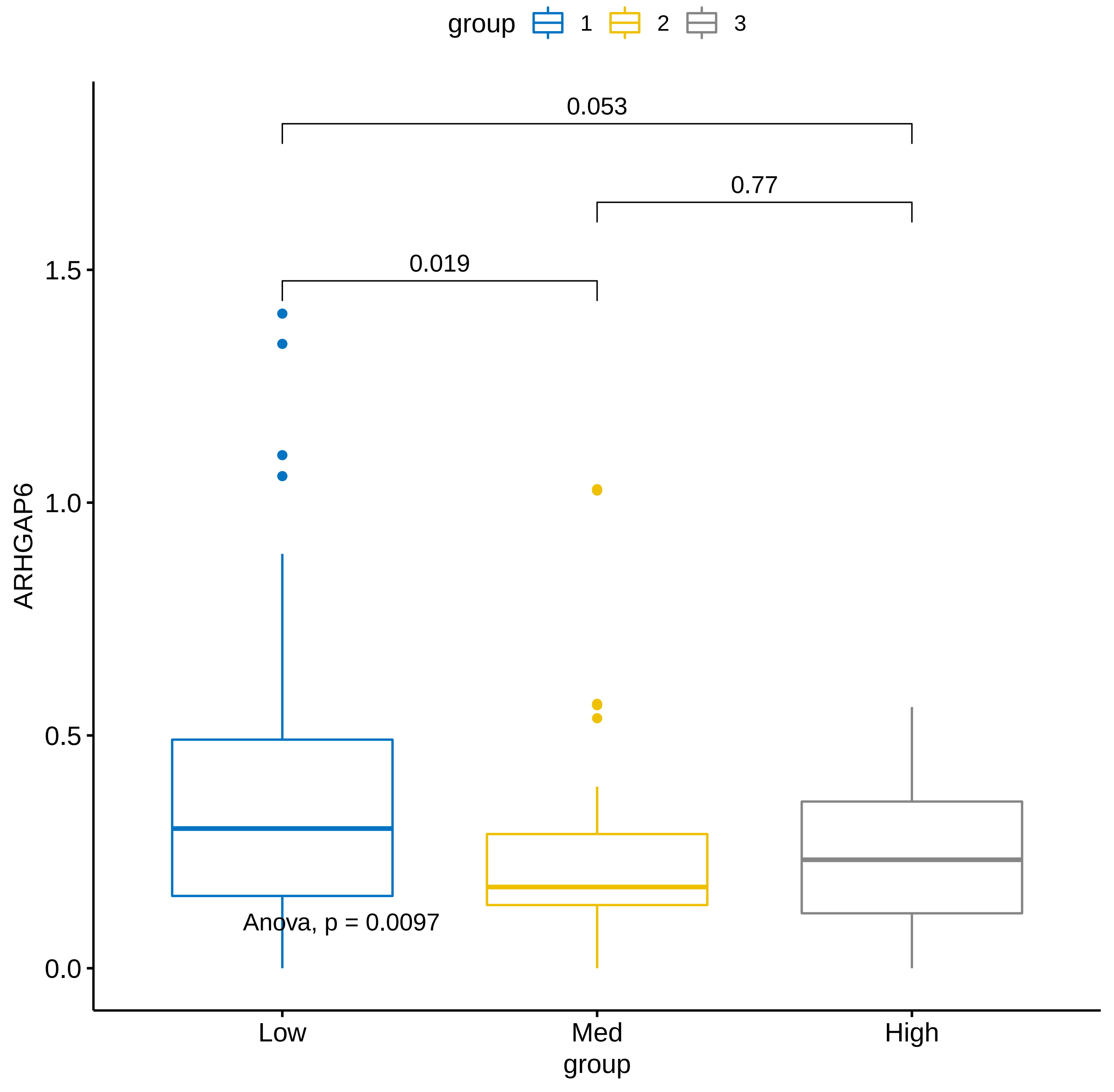
group 1 2 3
0.053
0.77
0.019
1.5
1.0
0.5
0.0
ARHGAP6
Anova, p = 0.0097
Low
Med
High
group

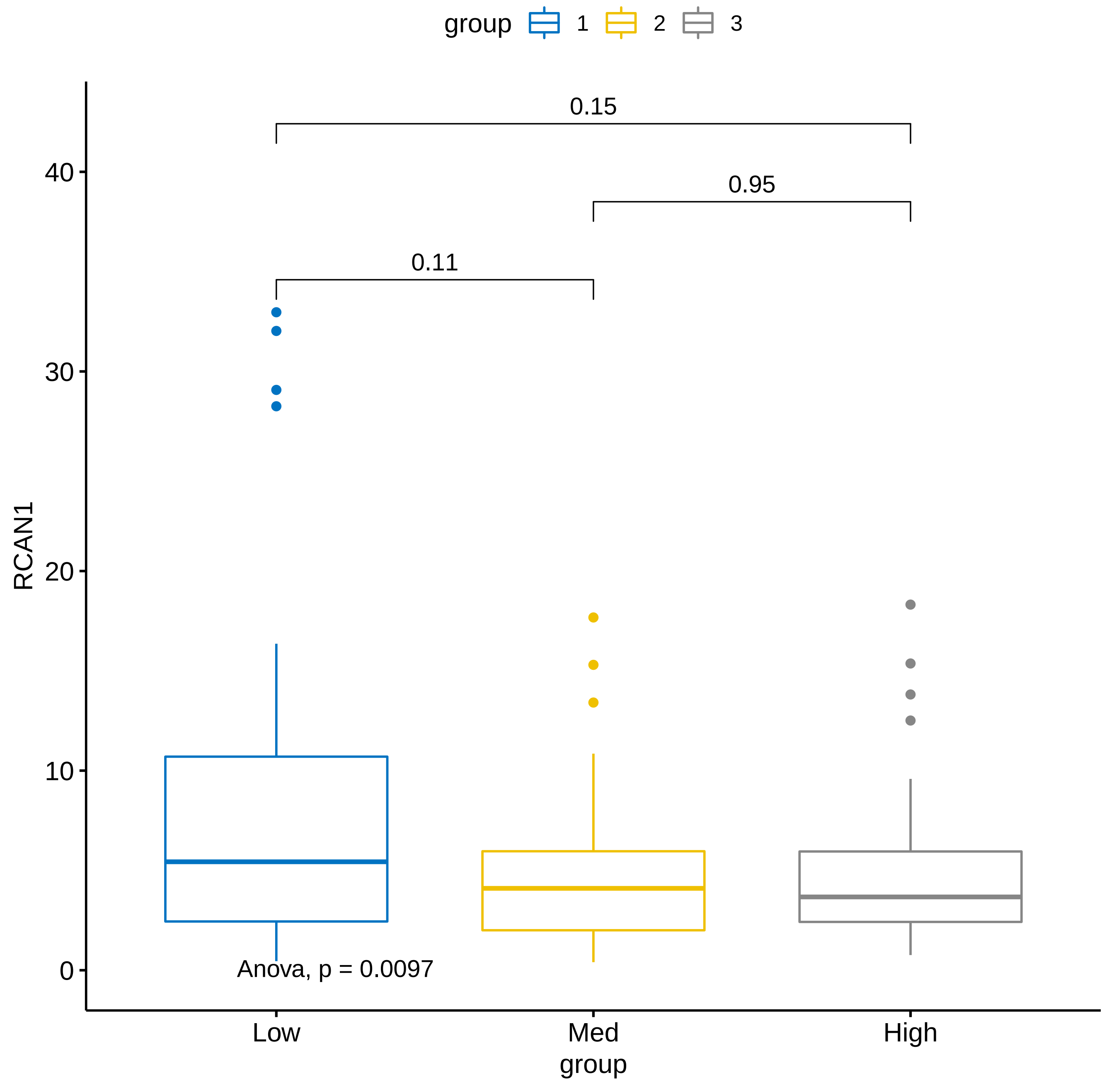

group 1 2 3
0.15
0.95
0.11
40
30
20
10
0
RCAN1
Anova, p = 0.0097
Low
Med
High
group

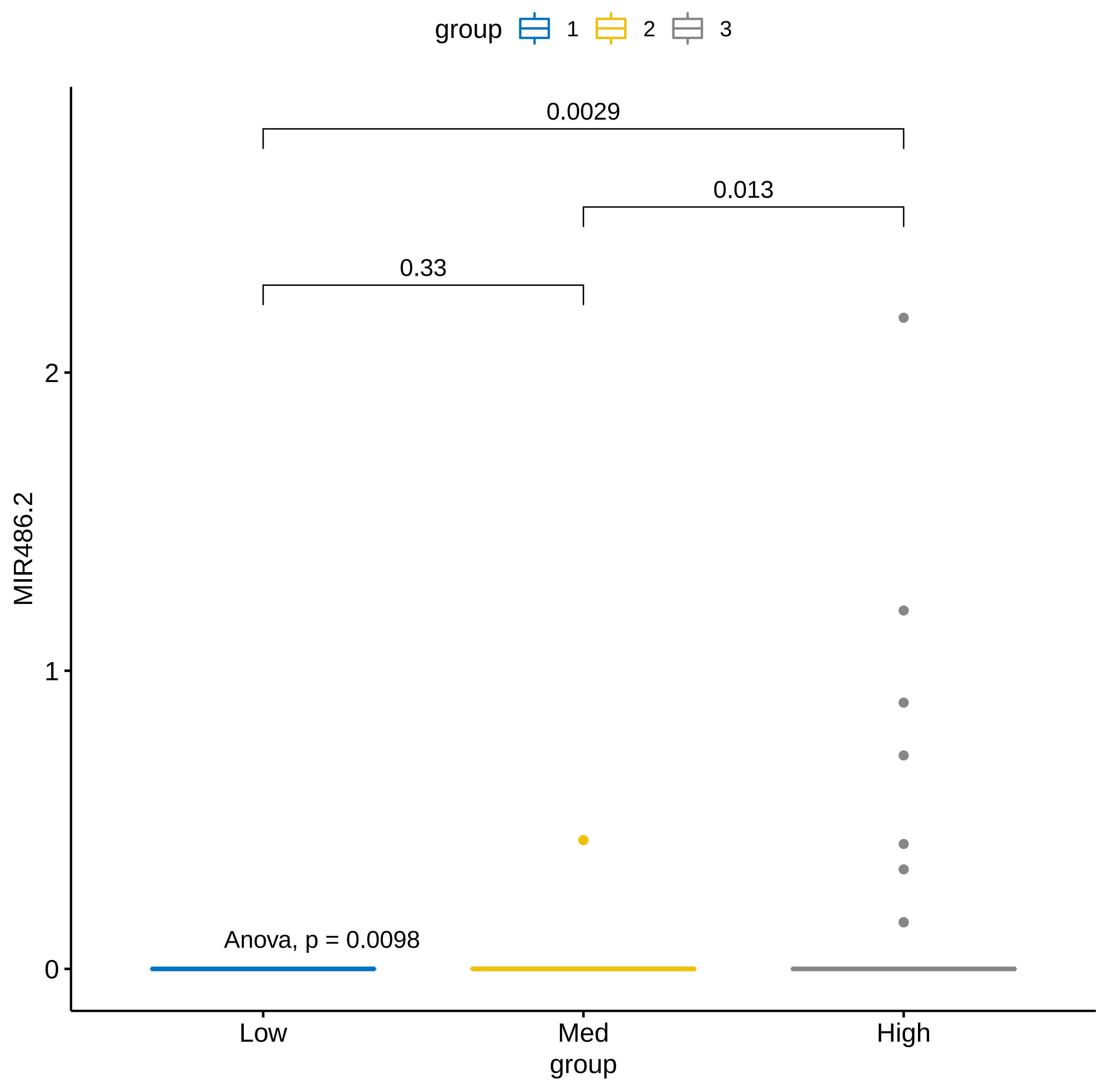

group
1
2
3
0.0029
0.013
0.33
Anova, p = 0.0098
MIR486.2
2
1
0
Low
Med
High
group

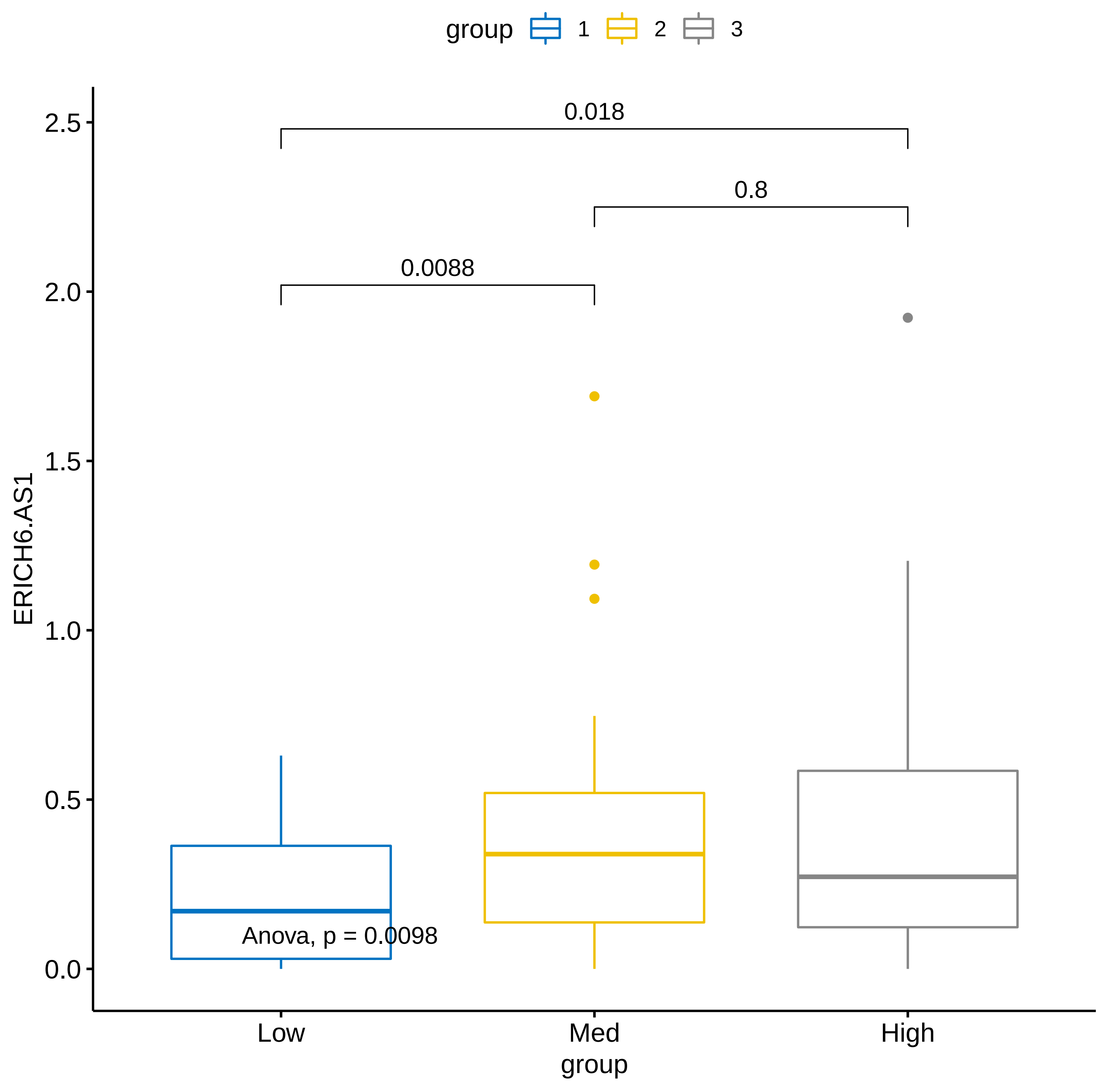
group
1
2
3
0.018
0.8
0.0088
2.5
2.0
1.5
1.0
0.5
0.0
ERICH6.AS1
Anova, p = 0.0098
Low
Med
High
group

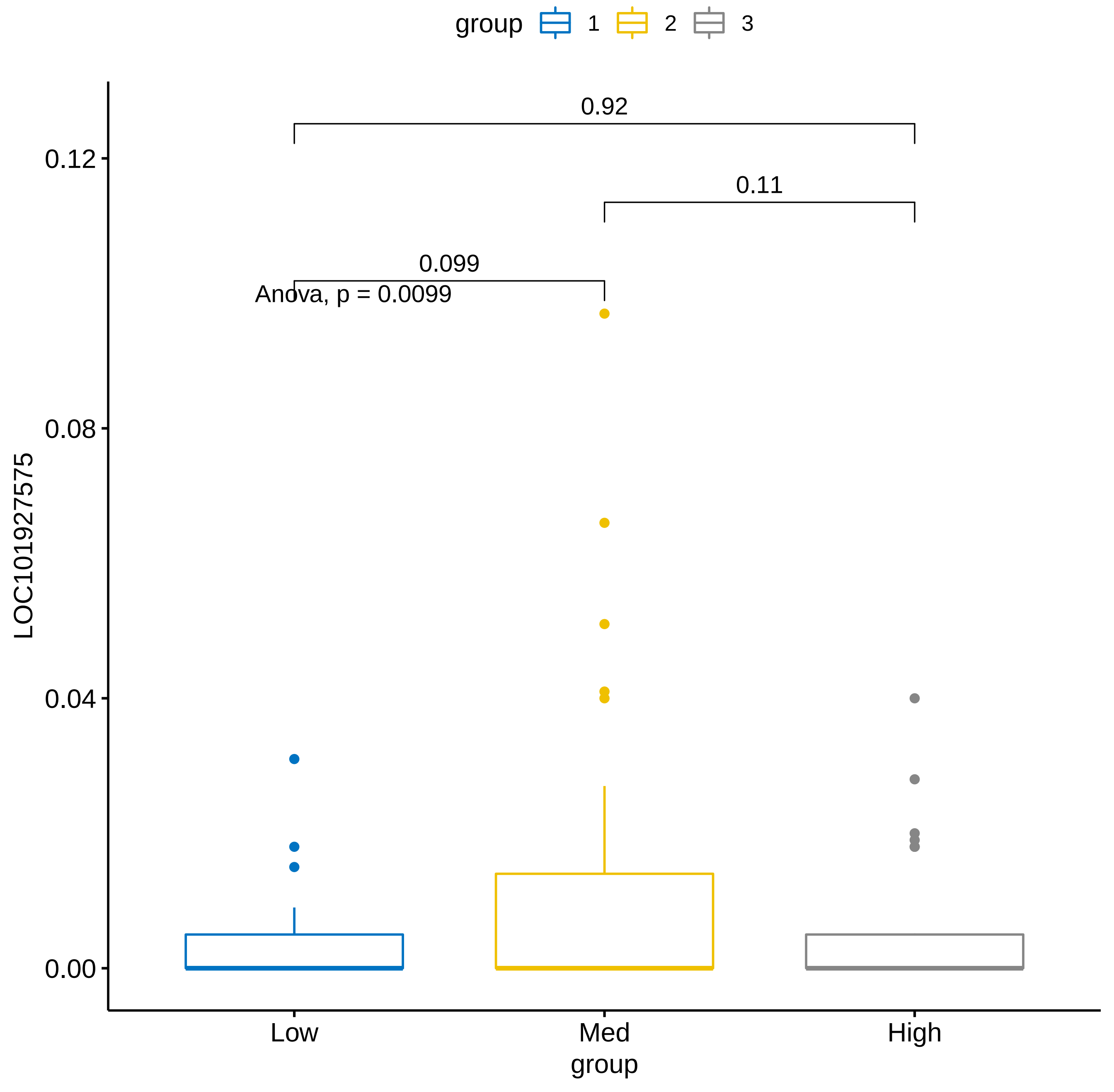

group 1 2 3
0.92
0.11
0.099
Anova, p = 0.0099
0.12
0.08
0.04
0.00
LOC101927575
Low
Med
High
group

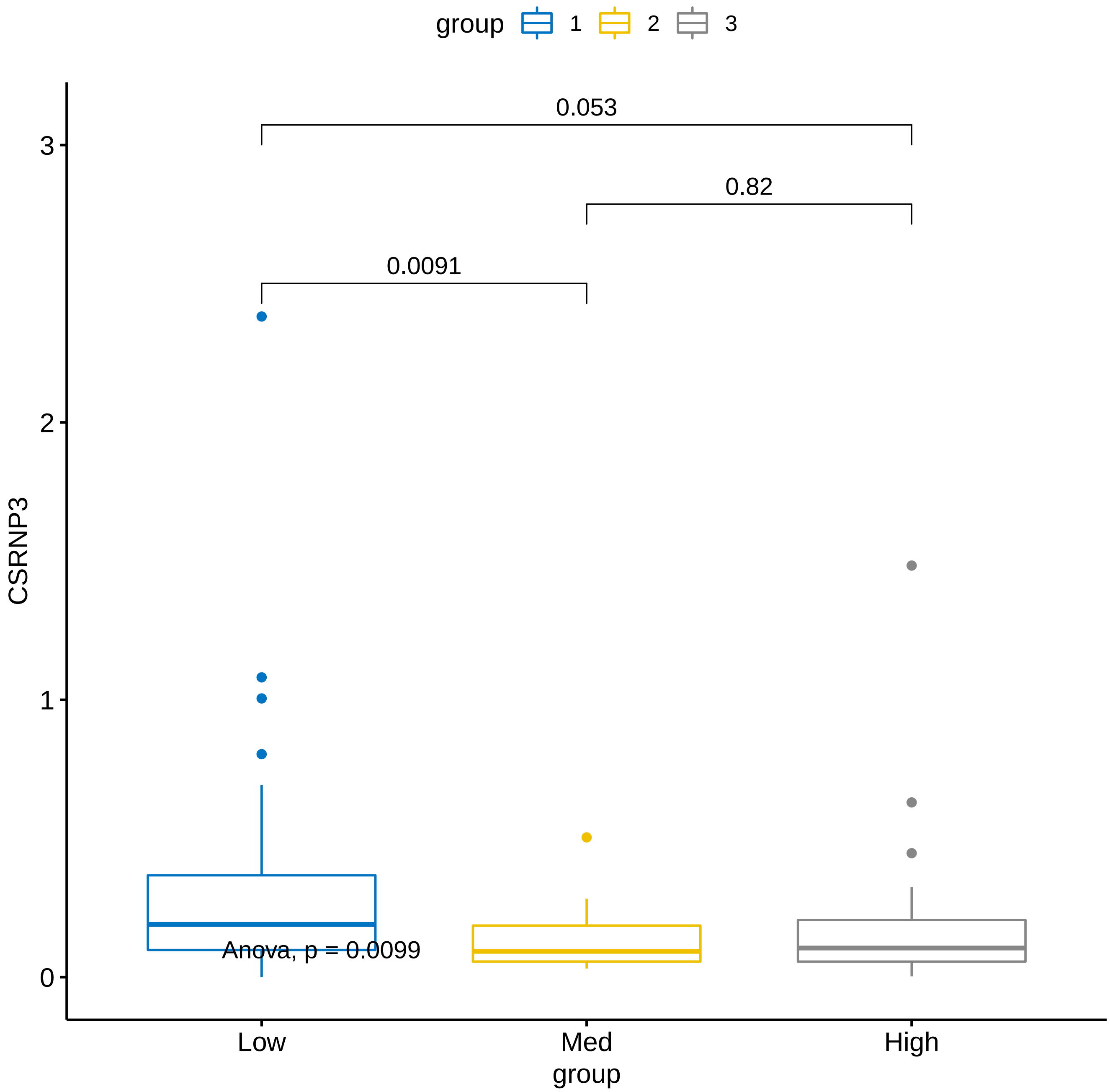
group
1
2
3
0.053
0.82
0.0091
3
2
1
0
CSRNP3
Anova, p = 0.0099
Low
Med
High
group

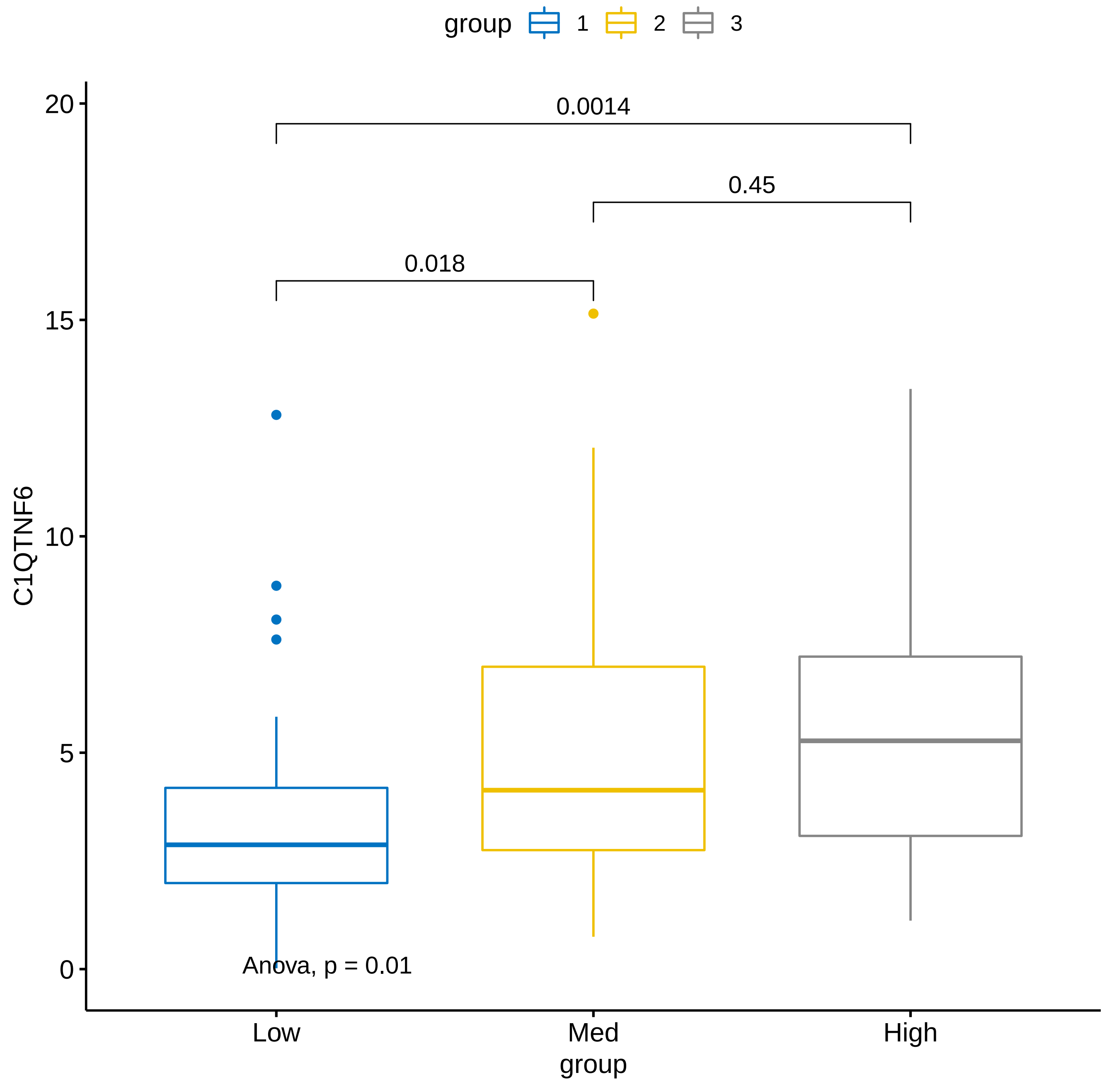
group
1
2
3
0.0014
0.45
0.018
20
15
10
5
0
C1QTNF6
Anova, p = 0.01
Low
Med
High
group

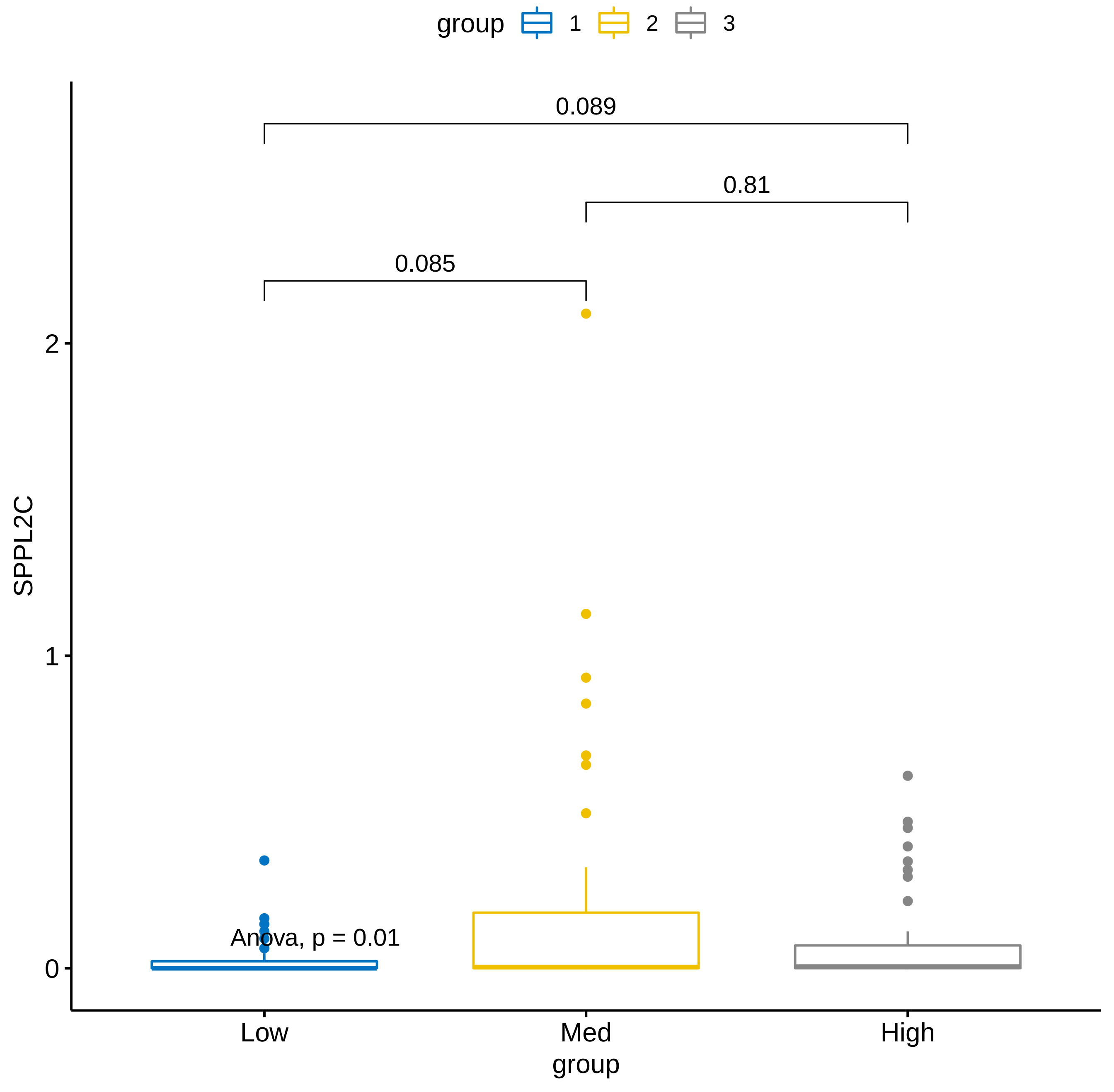

group 1 2 3
0.089
0.81
0.085
Anova, p = 0.01
SPPL2C
2
1
0
Low
Med
High
group

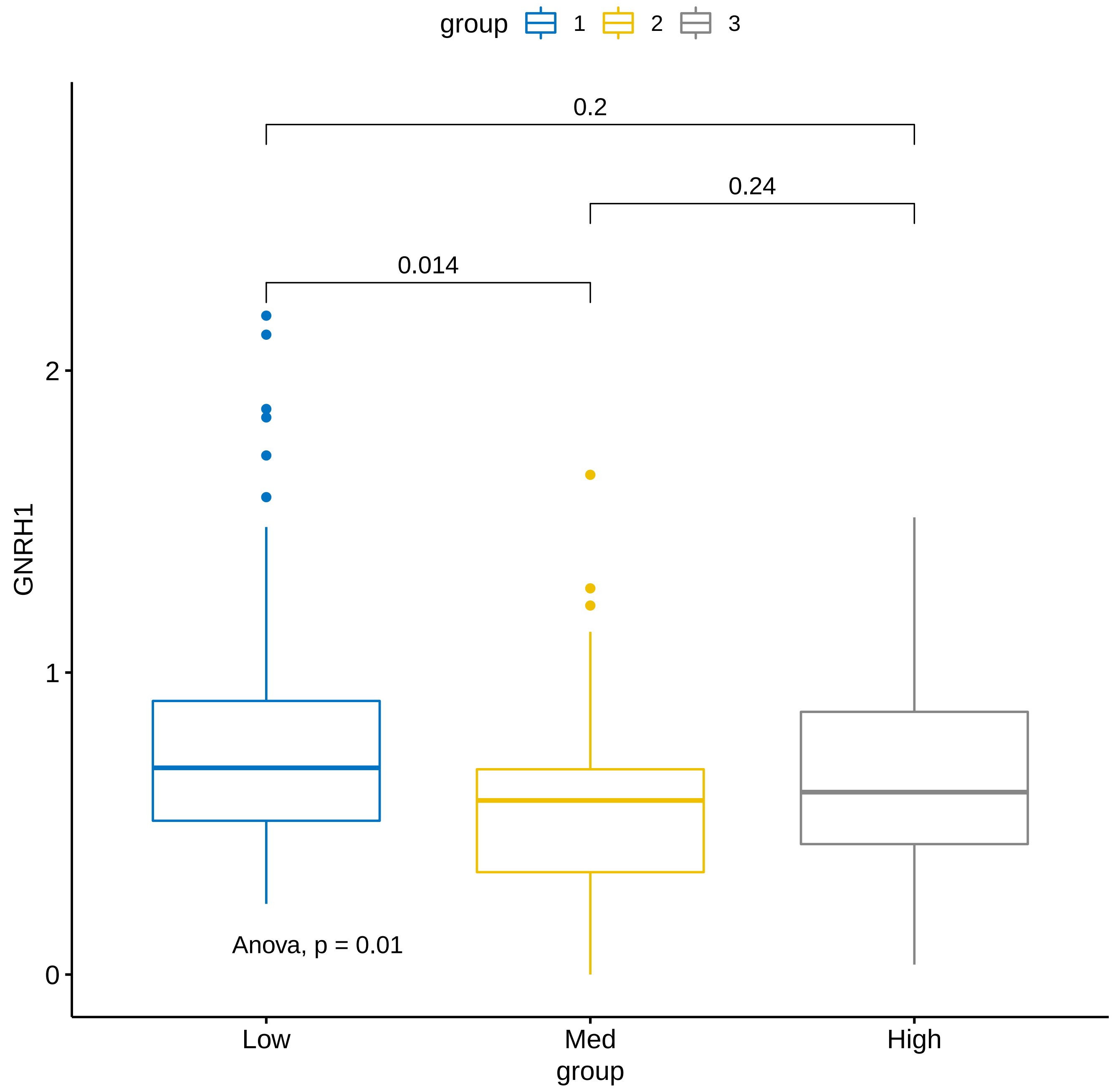

group
1
2
3
0.2
0.24
0.014
2
1
0
GNRH1
Anova, p = 0.01
Low
Med
High
group

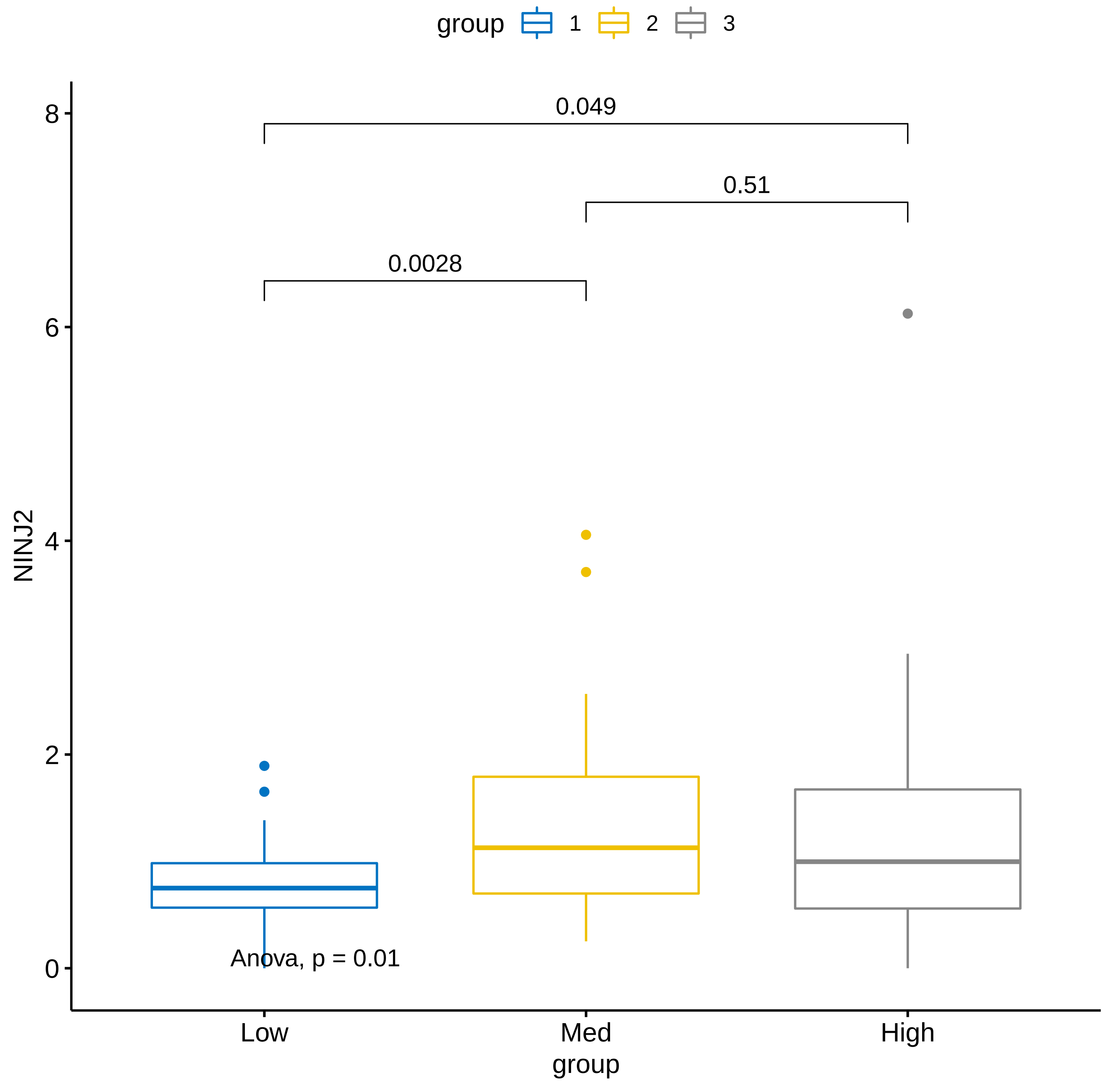

group 1 2 3
0.049
0.51
0.0028
NINJ2
8
6
4
2
0
Anova, p = 0.01
Low
Med
High
group

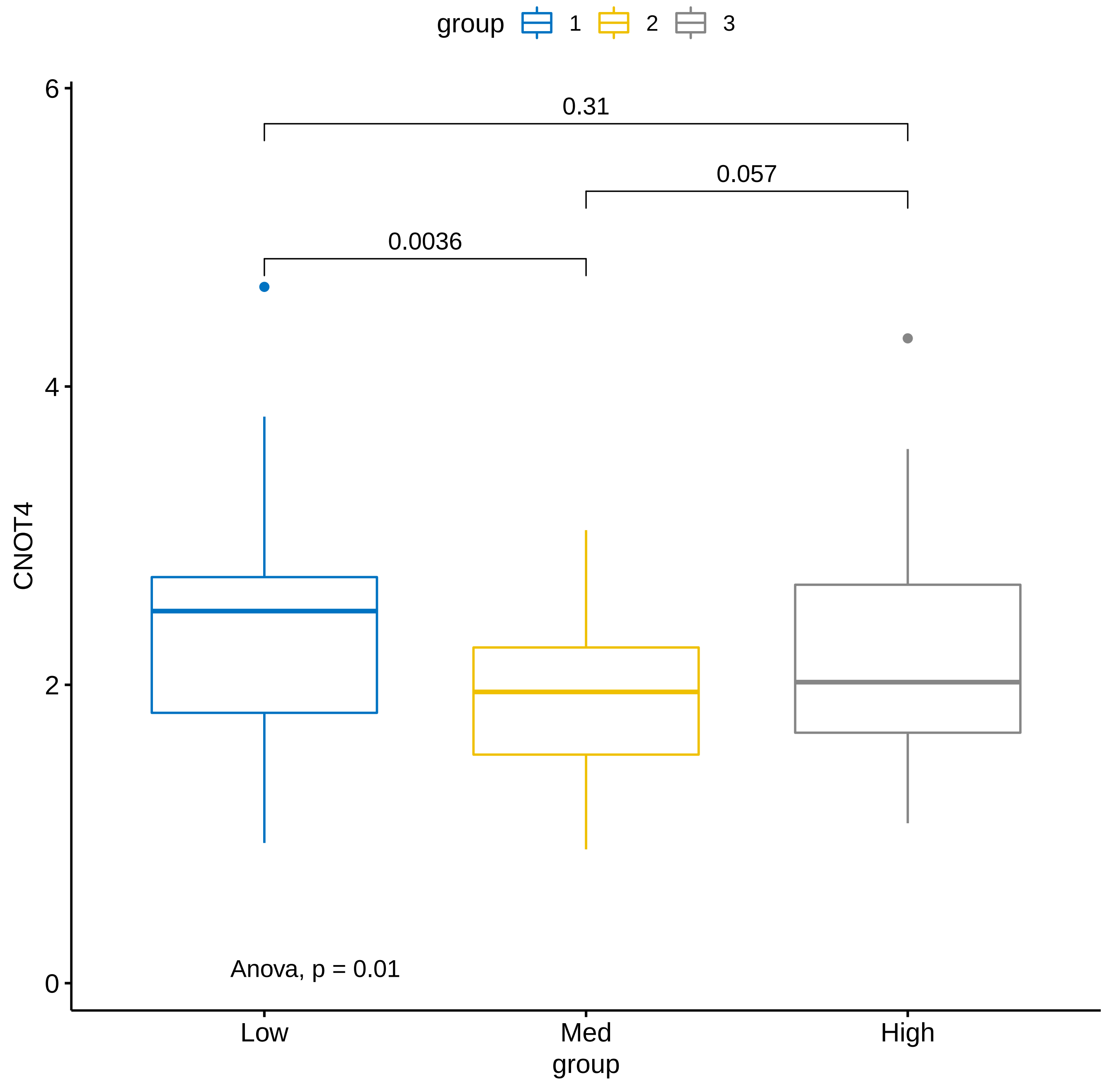
group 1 2 3
0.31
0.057
0.0036
6
4
2
0
CNOT4
Anova, p = 0.01
Low
Med
High
group

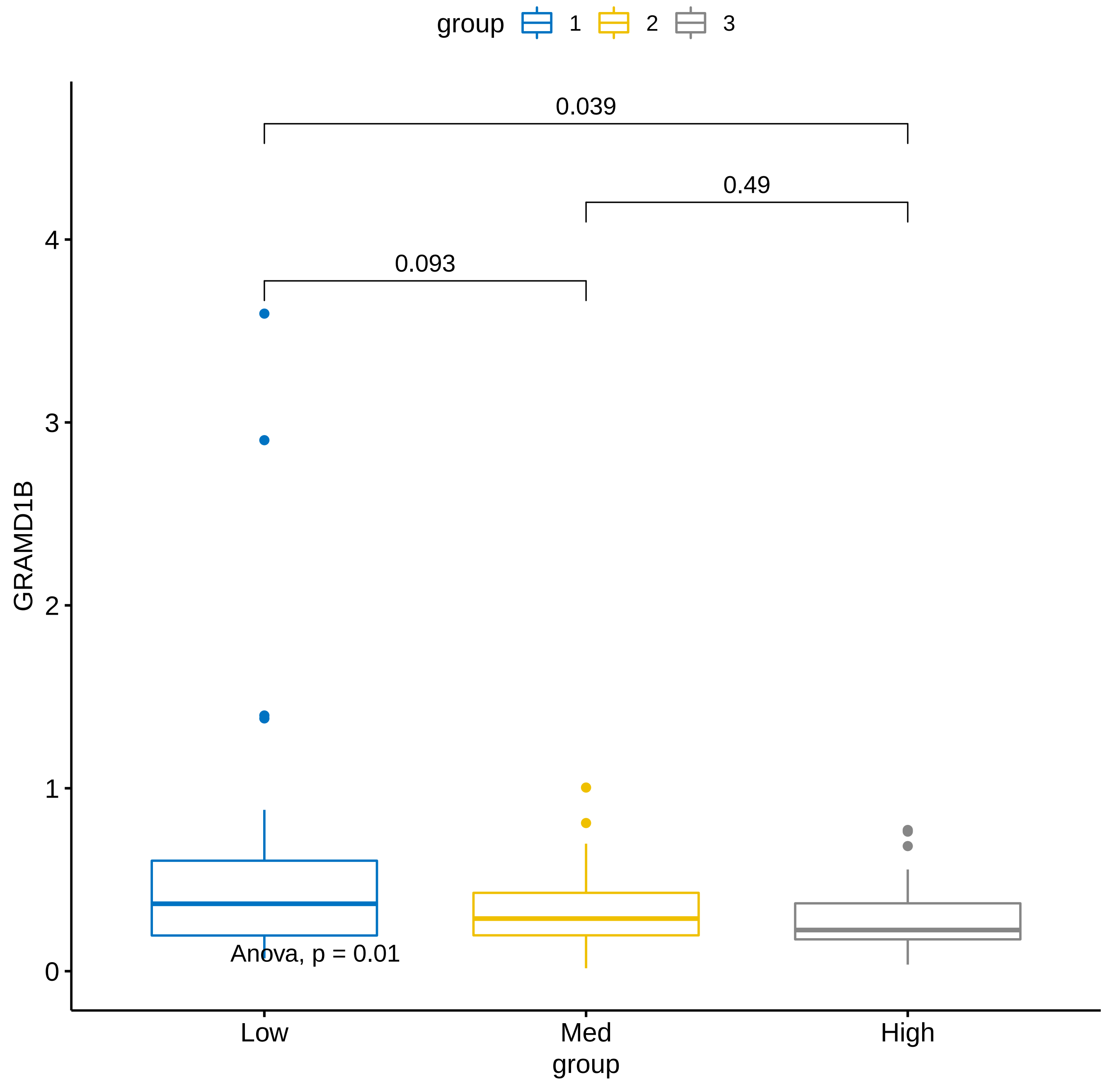
group
1
2
3
0.039
0.49
0.093
4
3
2
1
0
GRAMD1B
Anova, p = 0.01
Low
Med
High
group

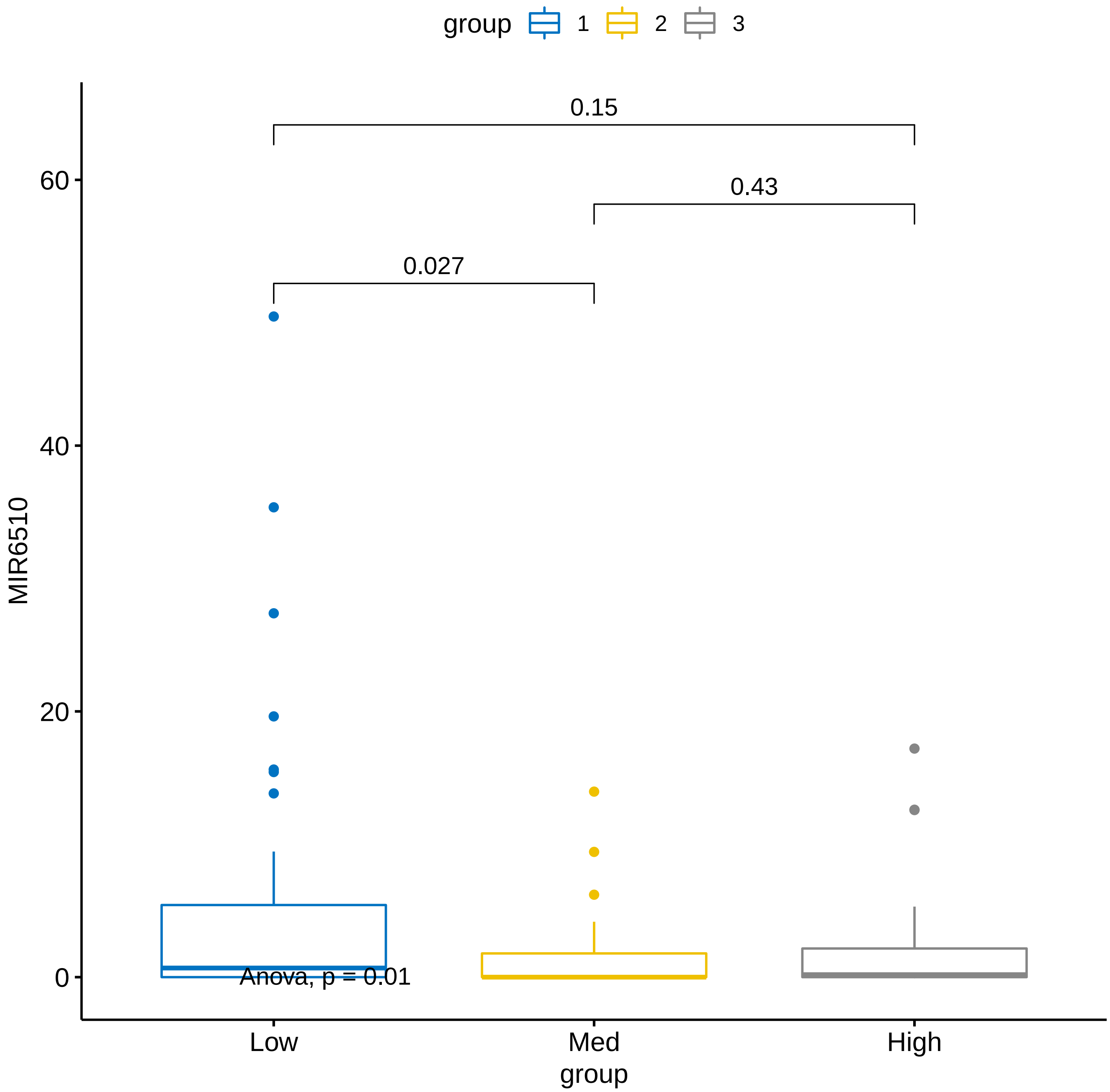

group
1
2
3
0.15
0.43
0.027
60
40
20
0
MIR6510
Anova, p = 0.01
Low
Med
High
group

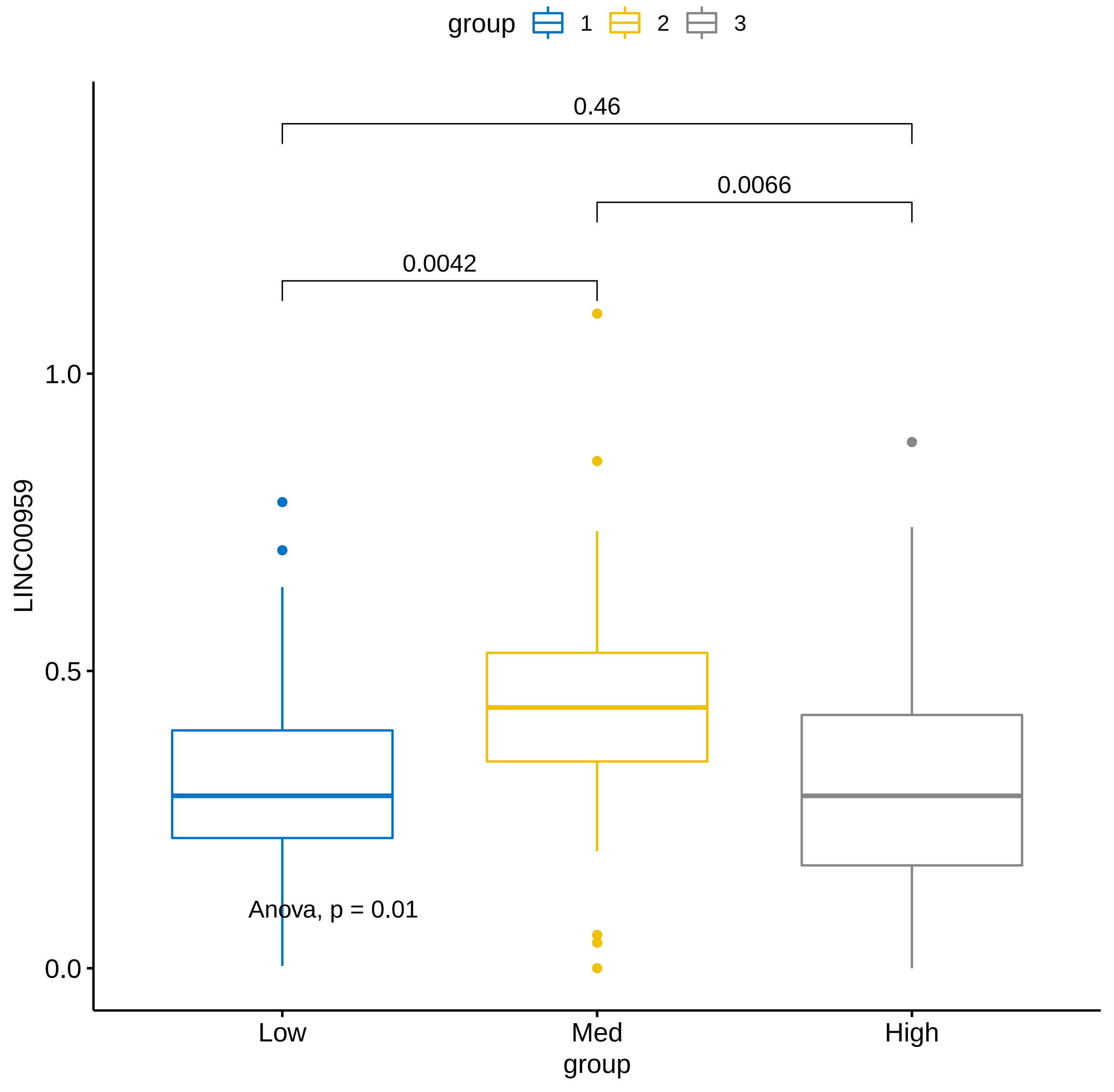
group 1 2 3
0.46
0.0066
0.0042
1.0
0.5
0.0
LINC00959
Anova, p = 0.01
Low
Med
High
group

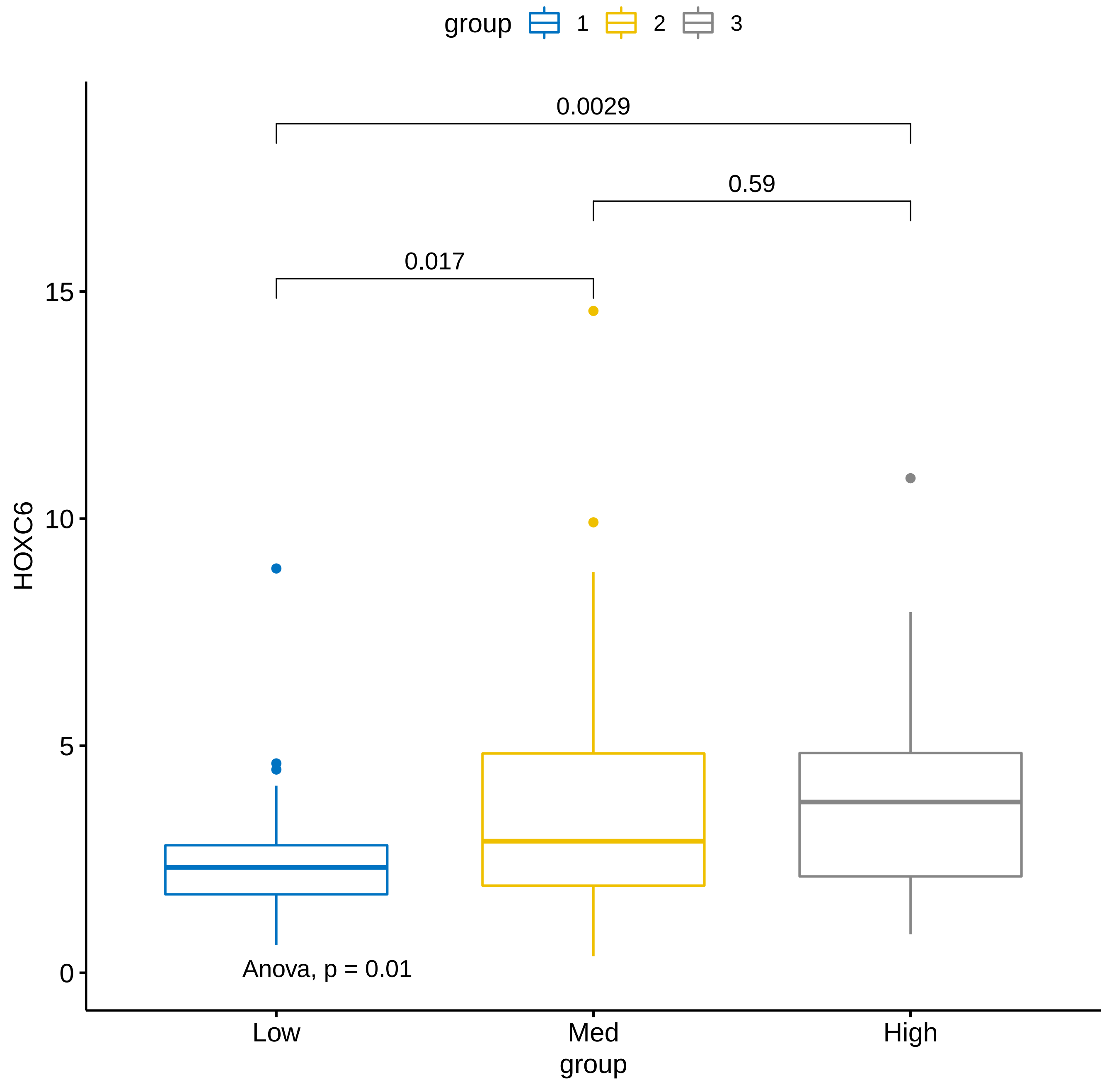

group
1
2
3
0.0029
0.59
0.017
15
10
5
0
HOXC6
Anova, p = 0.01
Low
Med
High
group

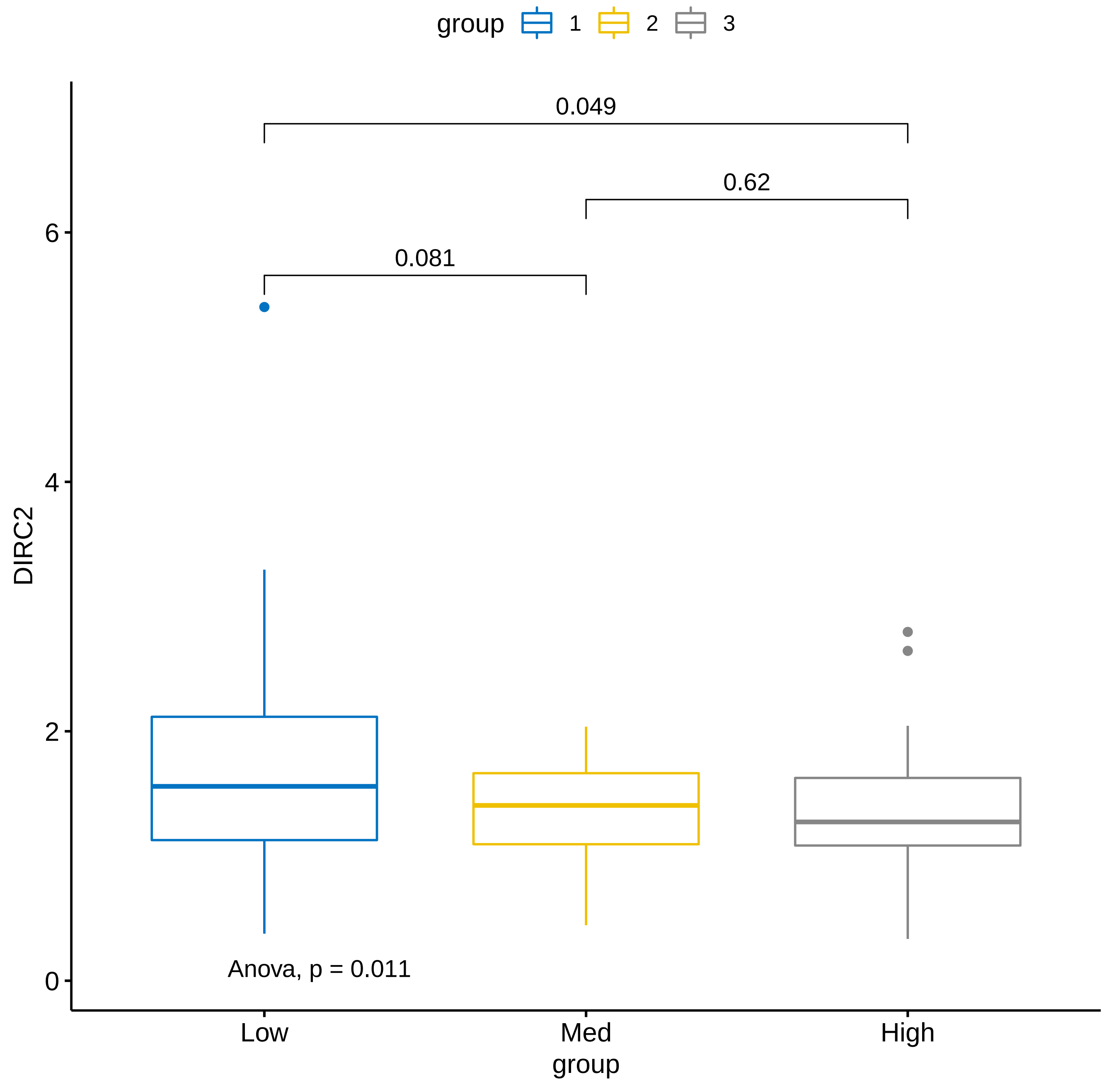

group 1 2 3
0.049
0.62
0.081
6
4
2
0
DIRC2
Anova, p = 0.011
Low
Med
High
group

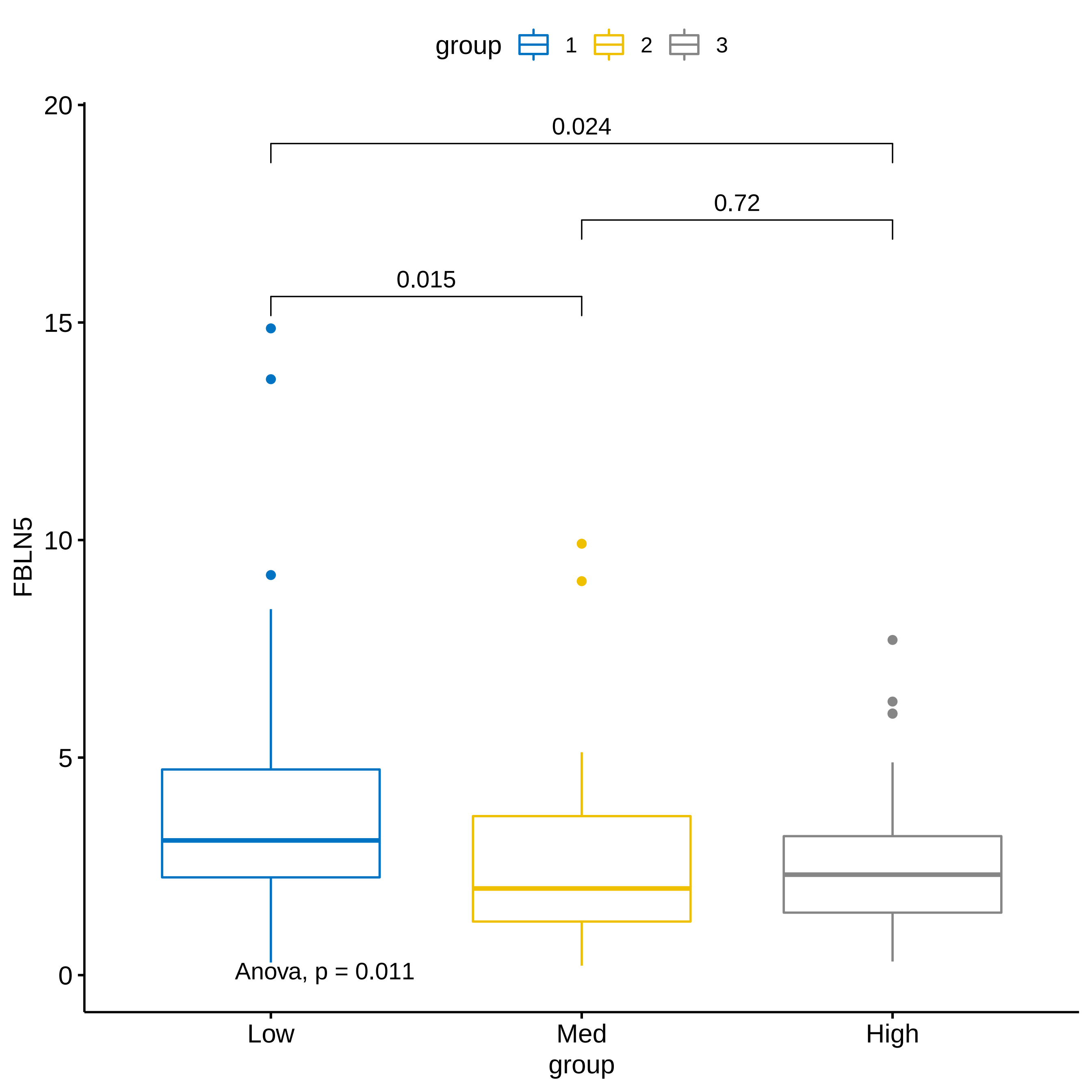

group
1
2
3
20
15
10
5
0
FBLN5
0.024
0.72
0.015
Anova, p = 0.011
Low
Med
High
group

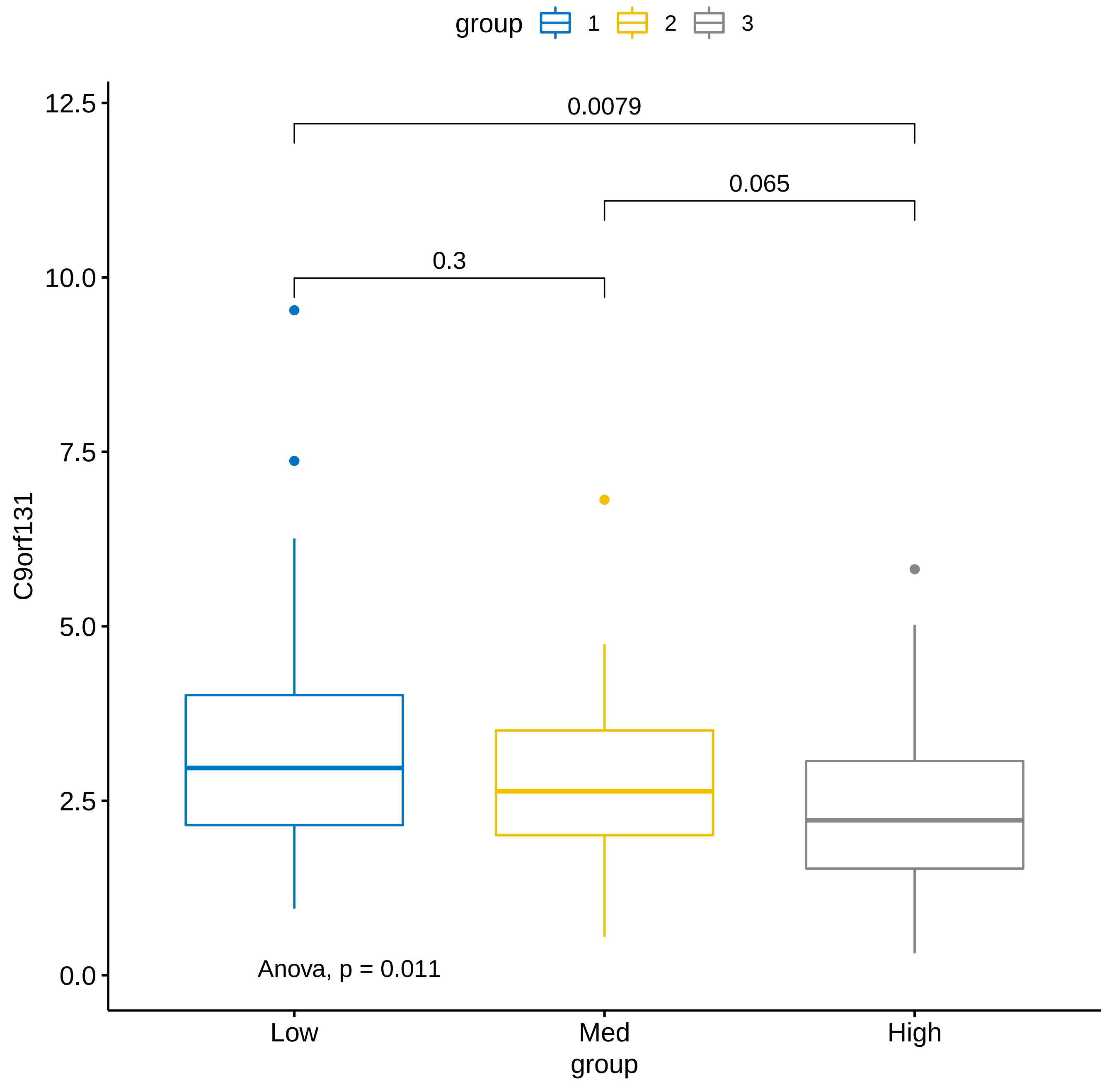
group
1
2
3
12.5
10.0
7.5
5.0
2.5
0.0
C9orf131
0.0079
0.065
0.3
Anova, p = 0.011
Low
Med
High
group

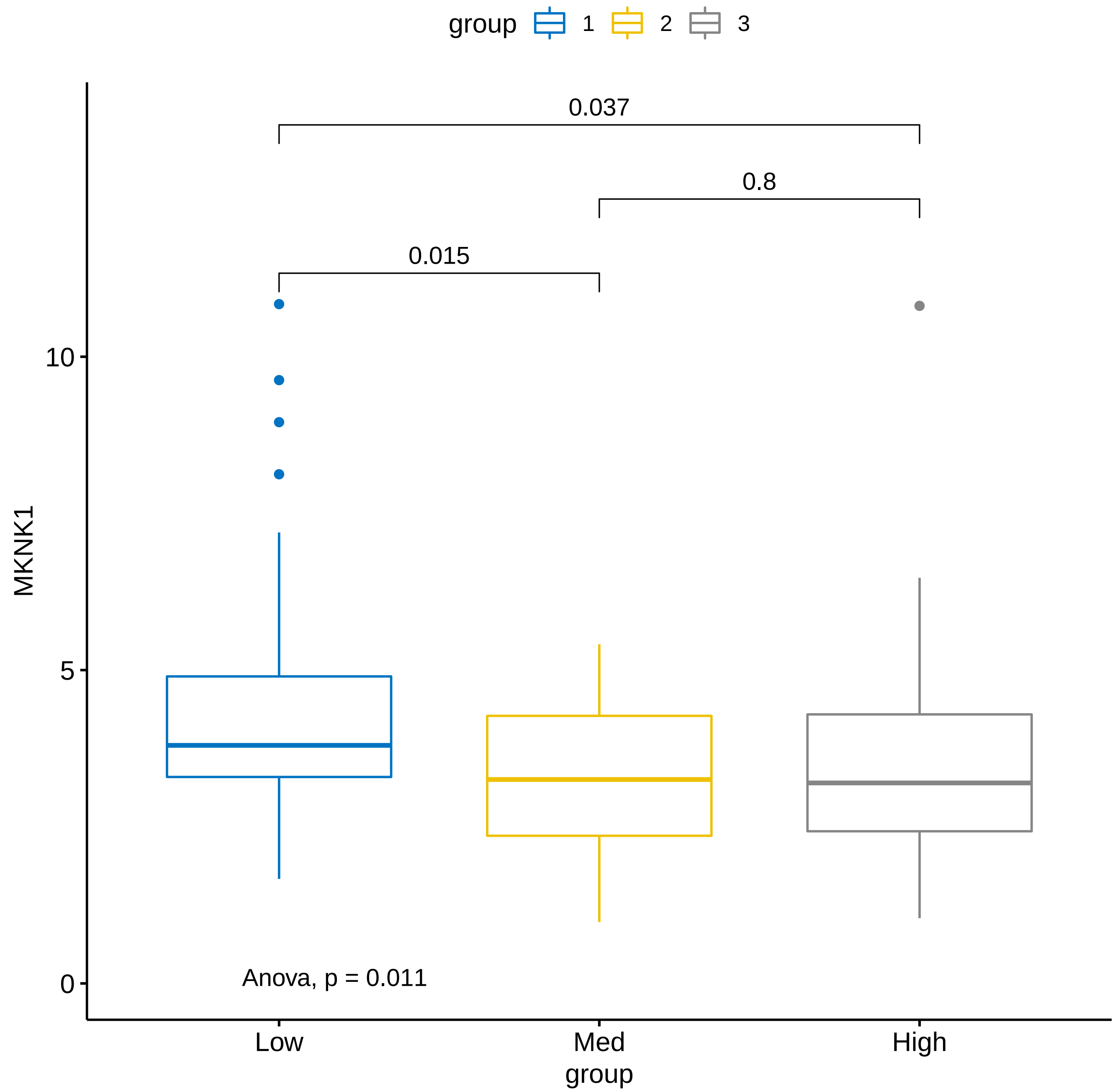

group
1
2
3
0.037
0.8
0.015
10
5
0
MKNK1
Anova, p = 0.011
Low
Med
High
group

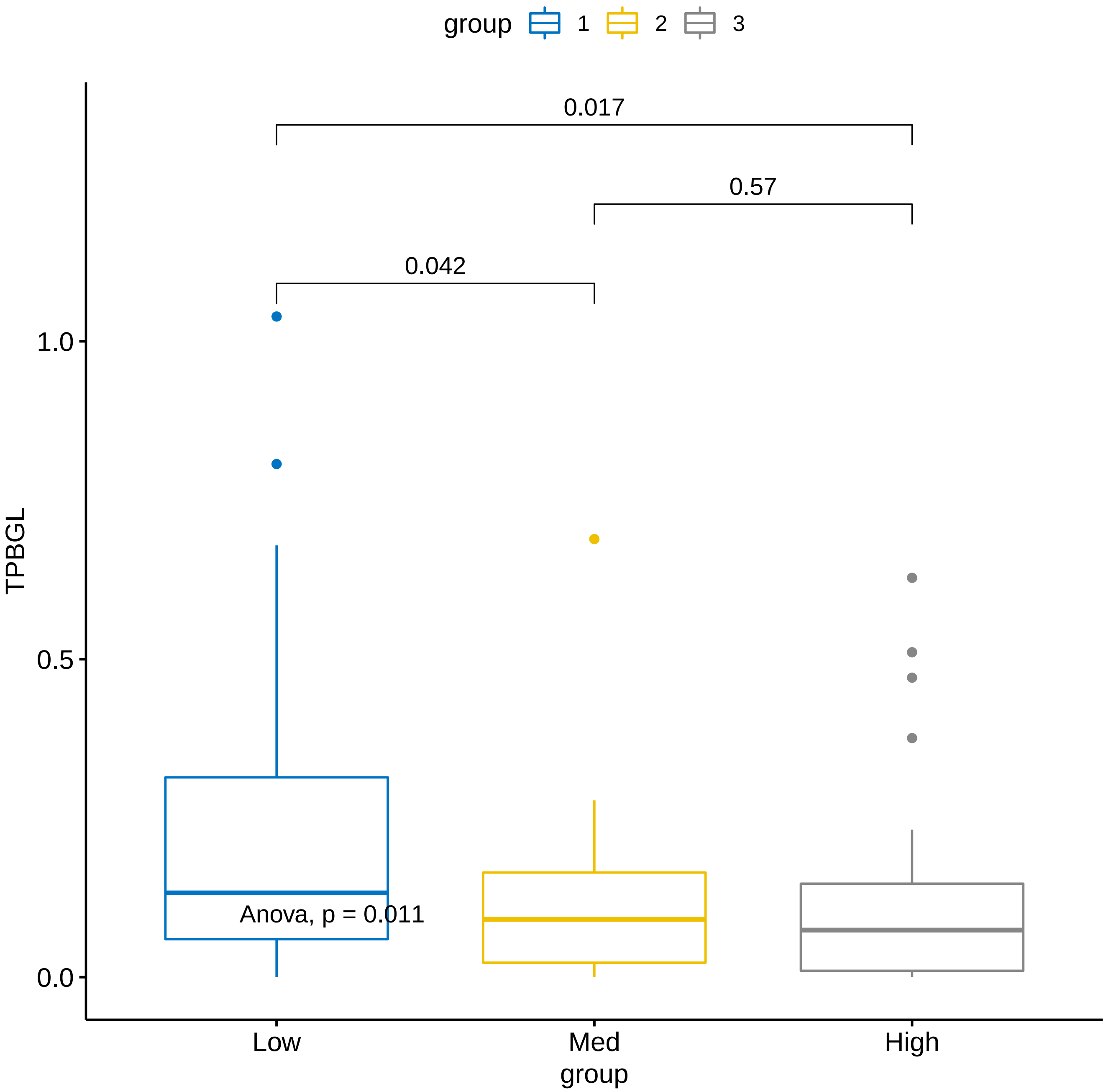

group
1
2
3
0.017
0.57
0.042
TPBGL
1.0
0.5
0.0
Anova, p = 0.011
Low
Med
High
group

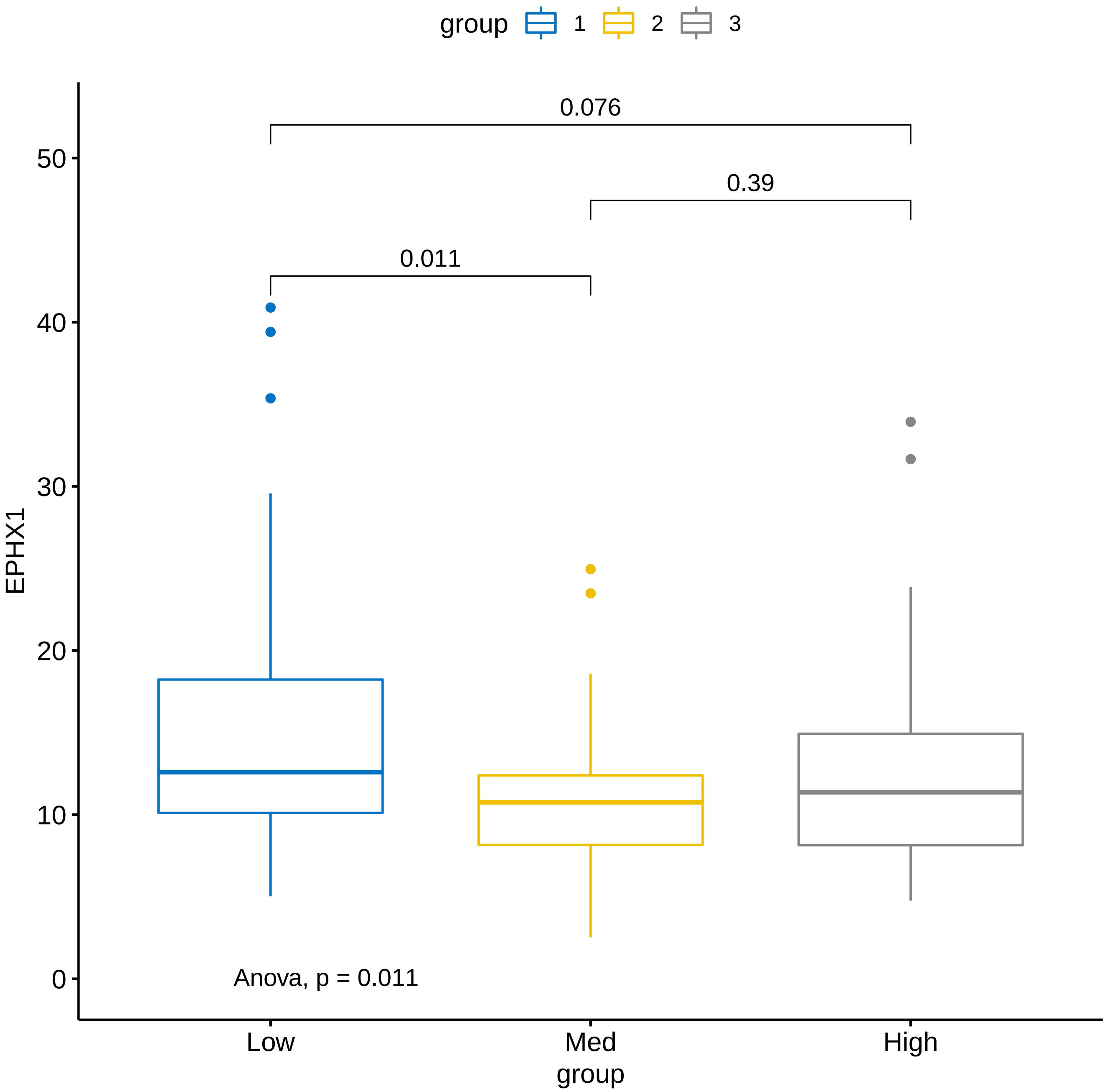
group
1
2
3
0.076
0.39
0.011
50
40
30
20
10
0
EPHX1
Anova, p = 0.011
Low
Med
High
group

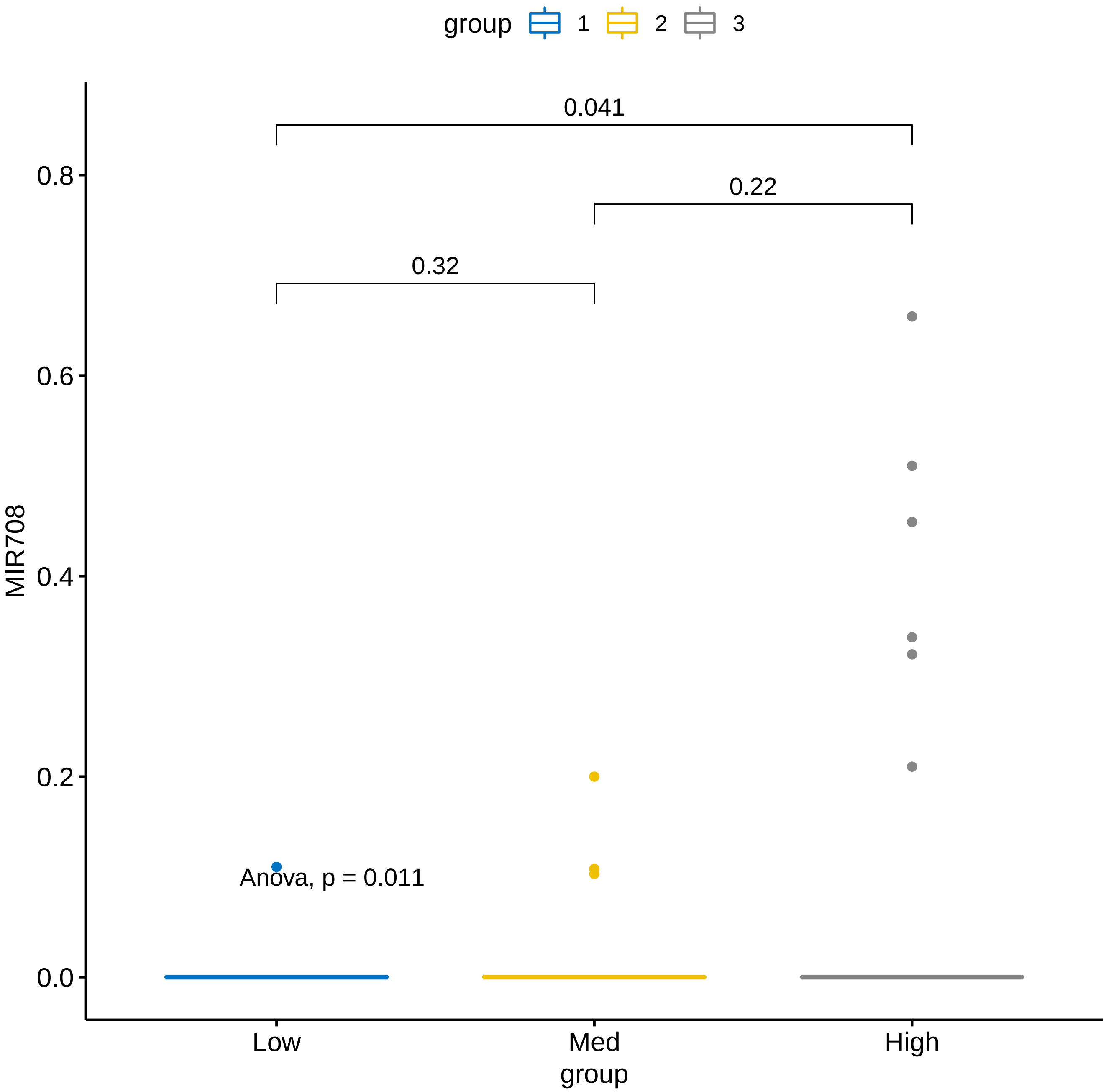
group 1 2 3
0.041
0.22
0.32
Anova, p = 0.011
MIR708
0.8
0.6
0.4
0.2
0.0
Low
Med
High
group

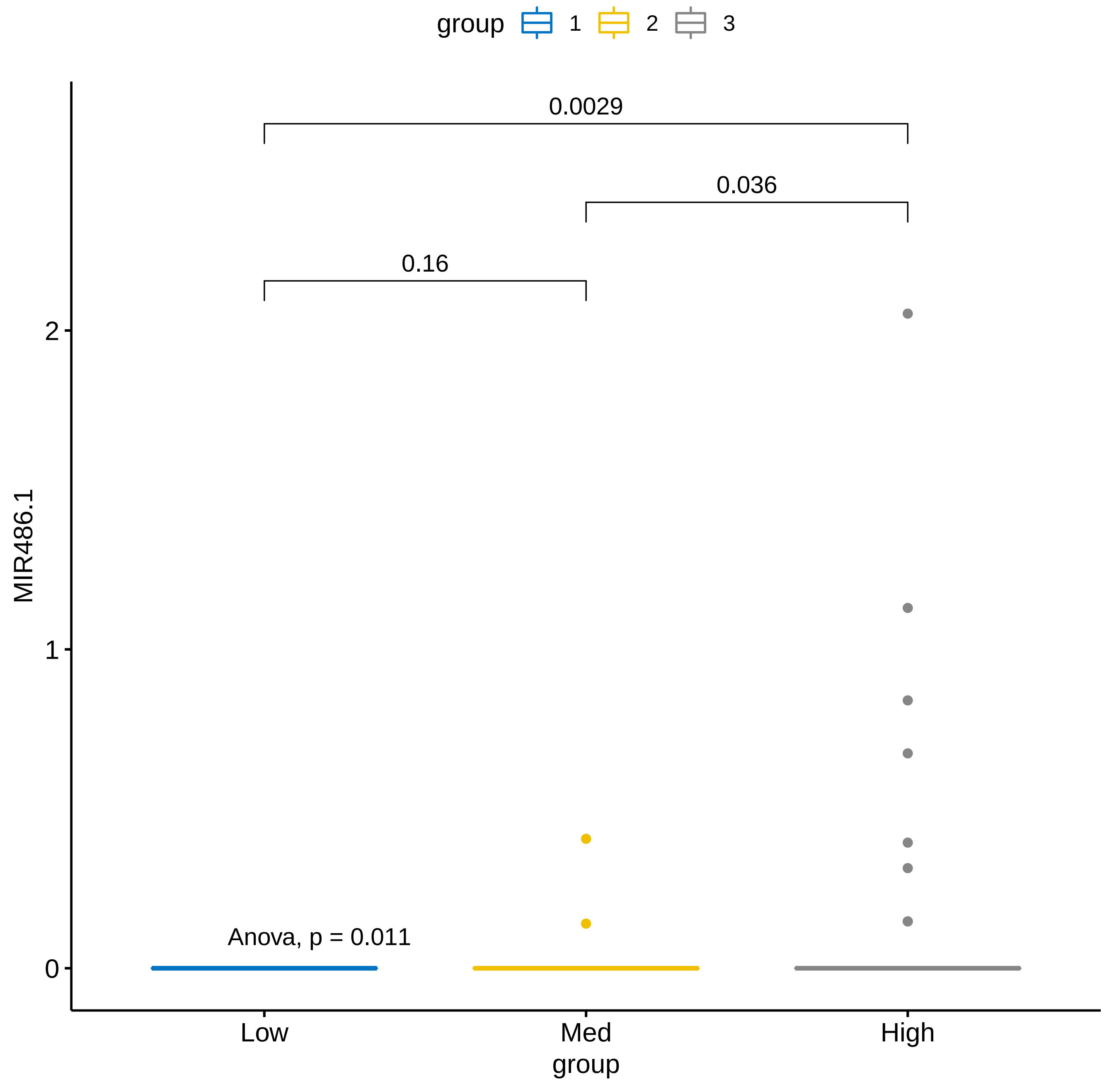
group
1
2
3
0.0029
0.036
0.16
Anova, p = 0.011
MIR486.1
2
1
0
Low
Med
High
group

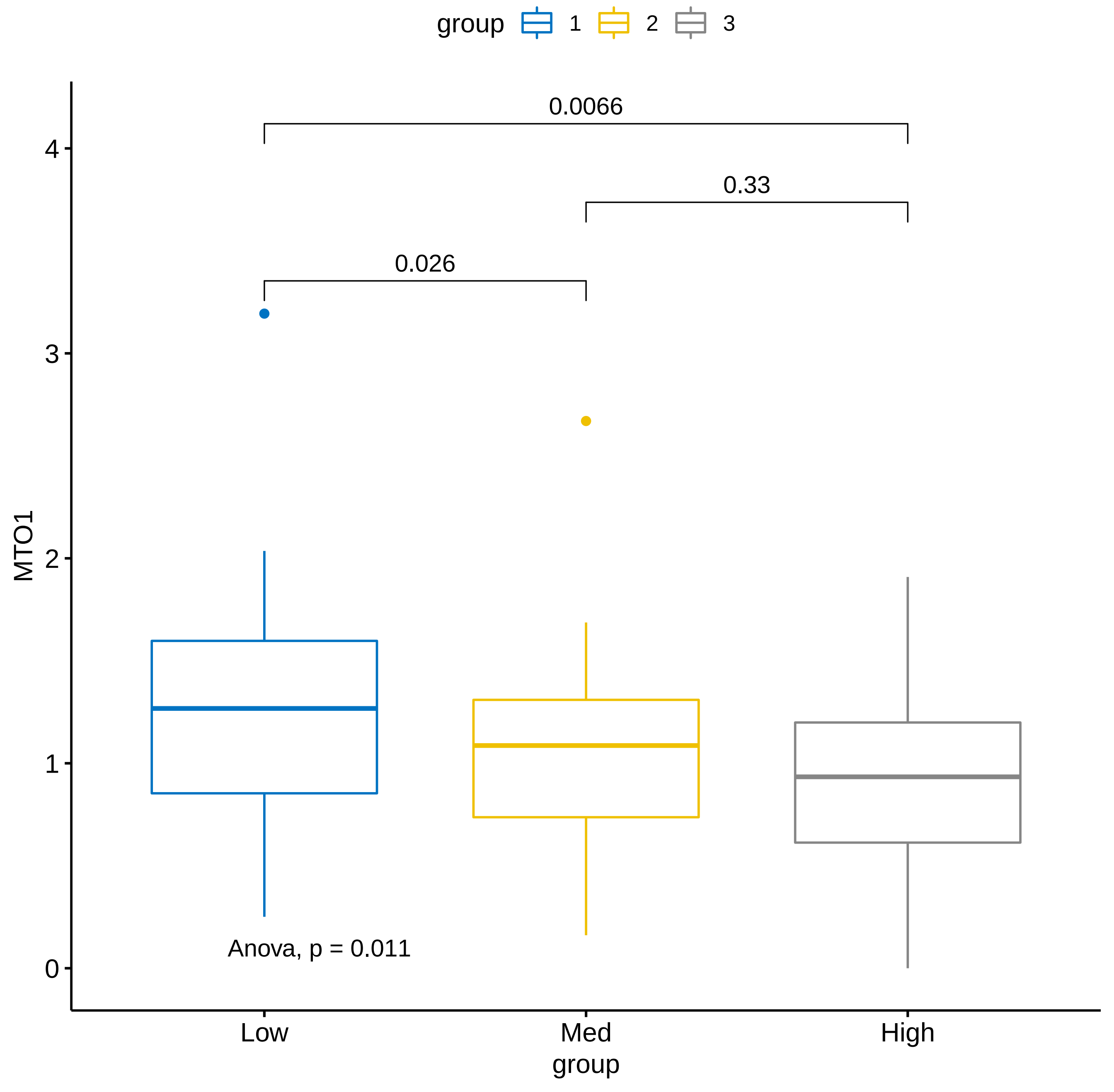
group
1
2
3
0.0066
0.33
0.026
MTO1
4
3
2
1
0
Anova, p = 0.011
Low
Med
High
group

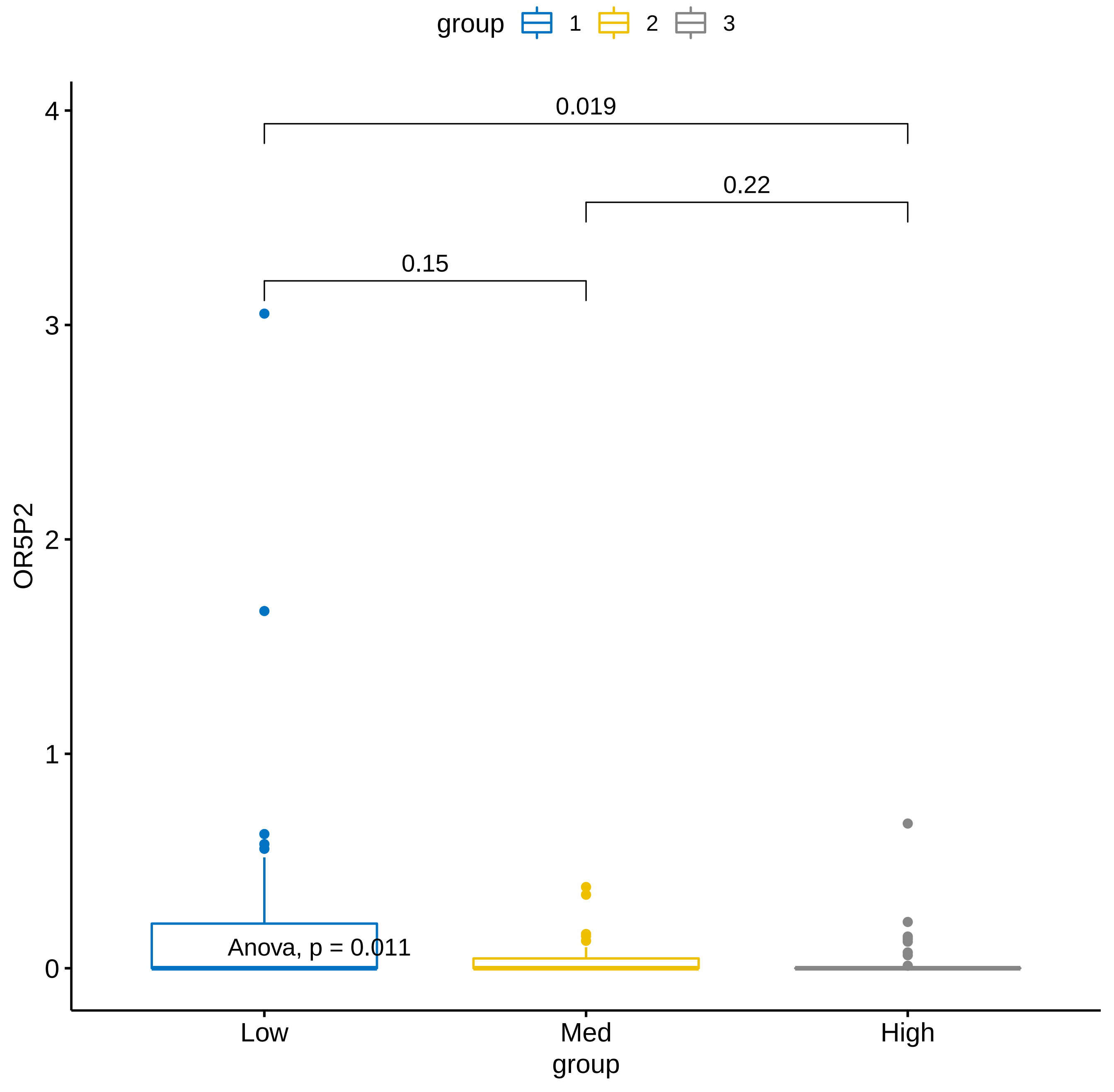
group
1
2
3
0.019
0.22
0.15
Anova, p = 0.011
OR5P2
4
3
2
1
0
Low
Med
High
group

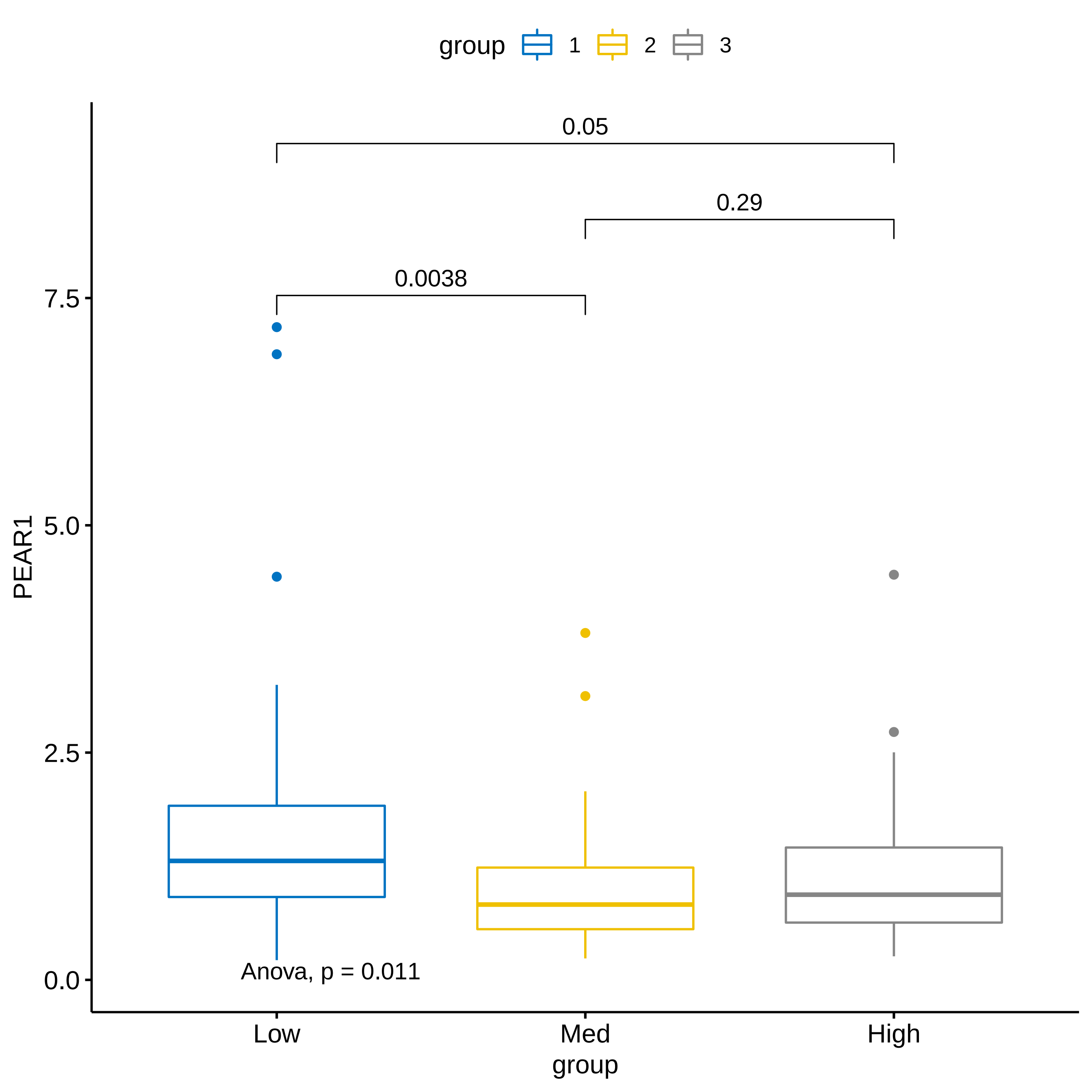
group
1
2
3
0.05
0.29
0.0038
7.5
5.0
2.5
0.0
PEAR1
Anova, p = 0.011
Low
Med
High
group

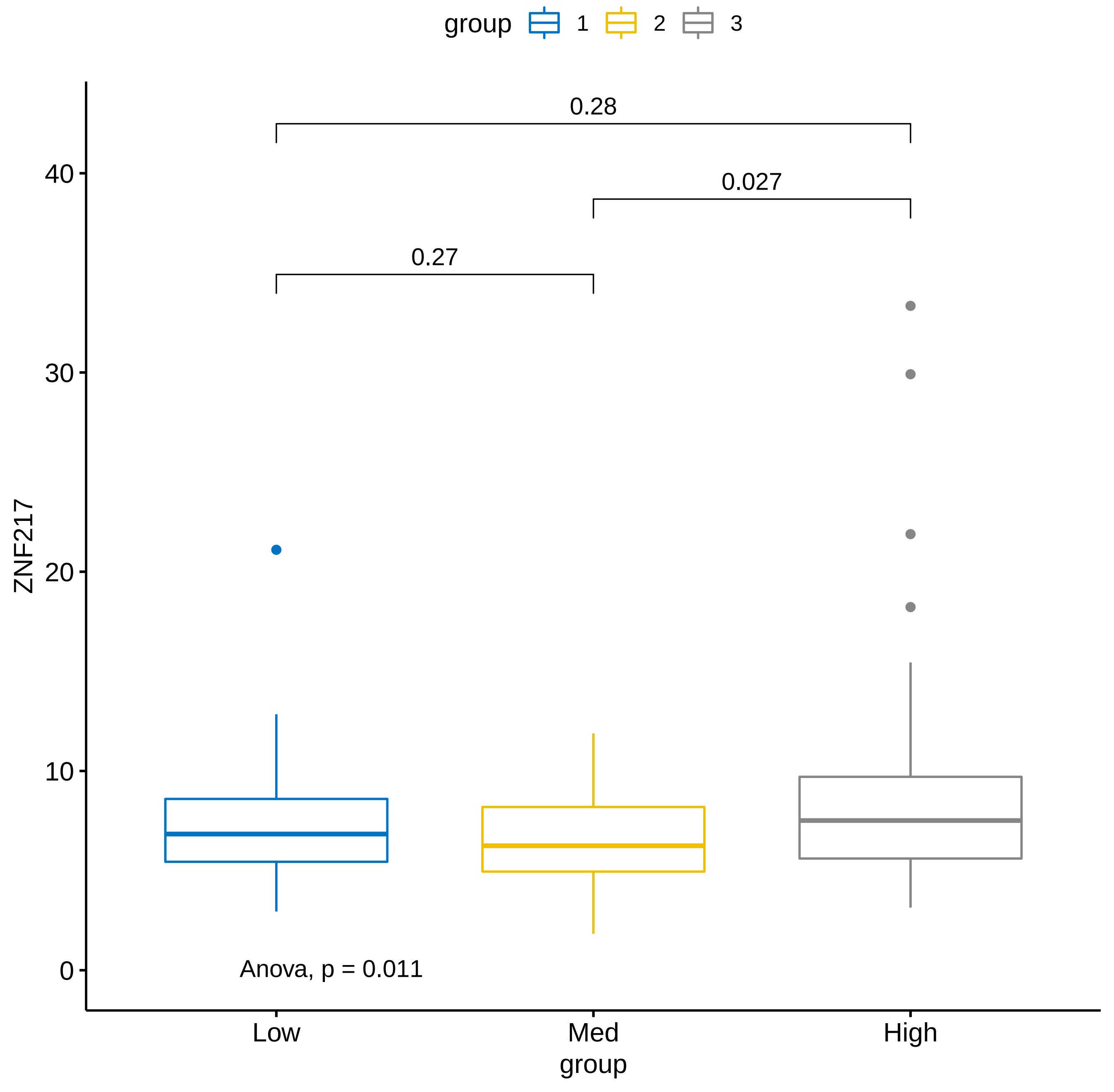
group
1
2
3
0.28
0.027
0.27
40
30
20
10
0
ZNF217
Anova, p = 0.011
Low
Med
High
group

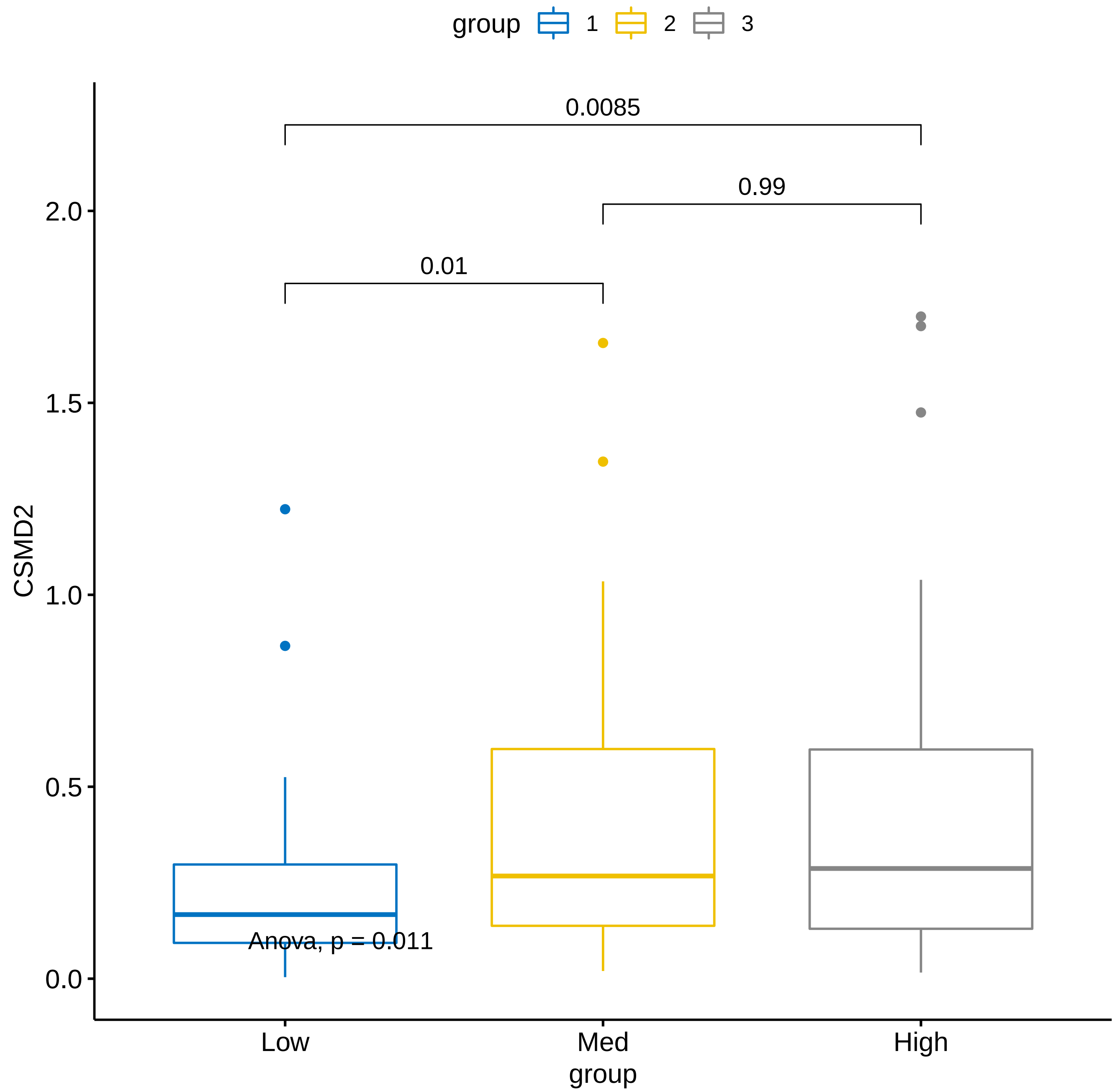
group 1 2 3
0.0085
0.99
0.01
2.0
1.5
1.0
0.5
0.0
CSMD2
Anova, p = 0.011
Low
Med
High
group

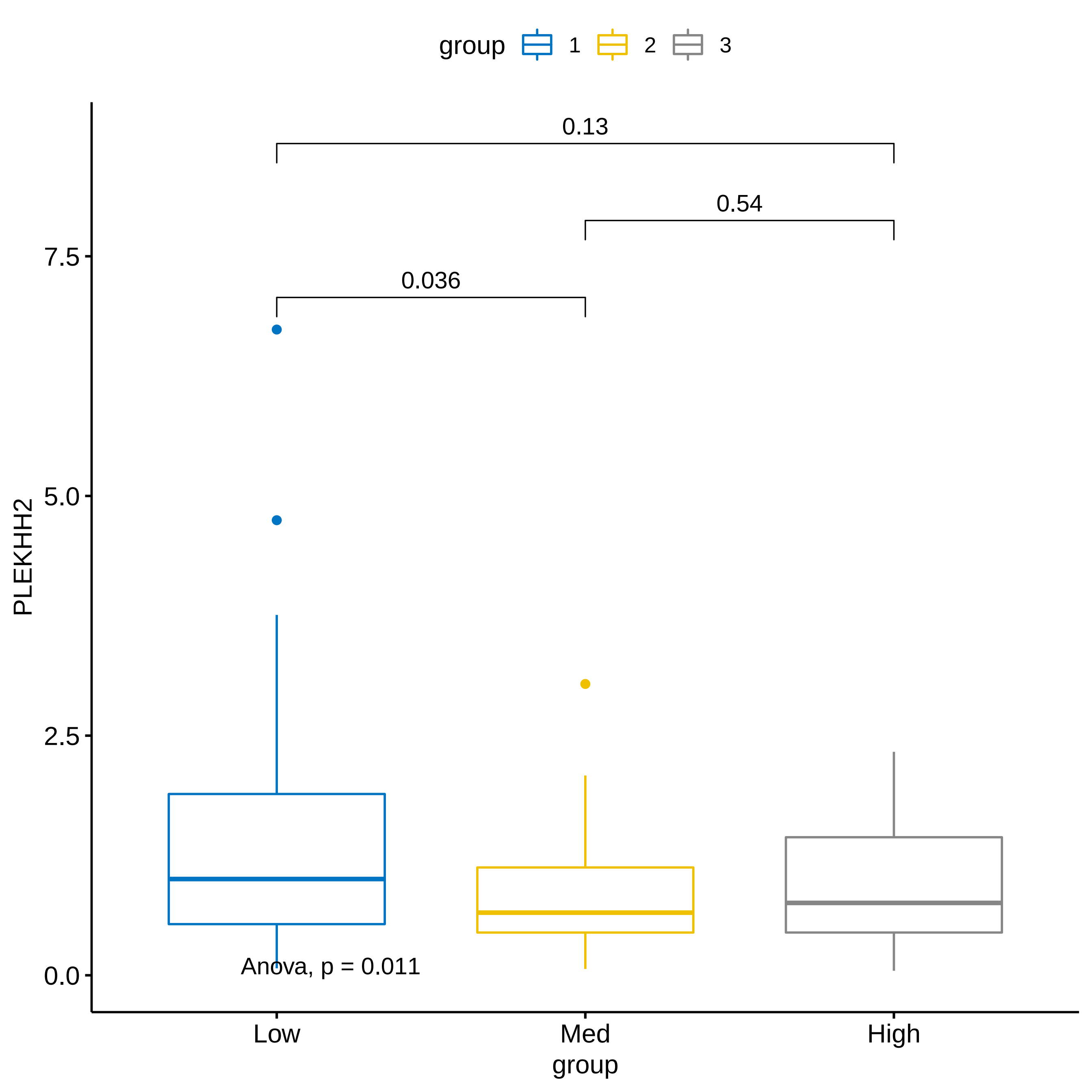

group 1 2 3
0.13
0.54
0.036
7.5
5.0
2.5
0.0
PLEKHH2
Anova, p = 0.011
Low
Med
High
group

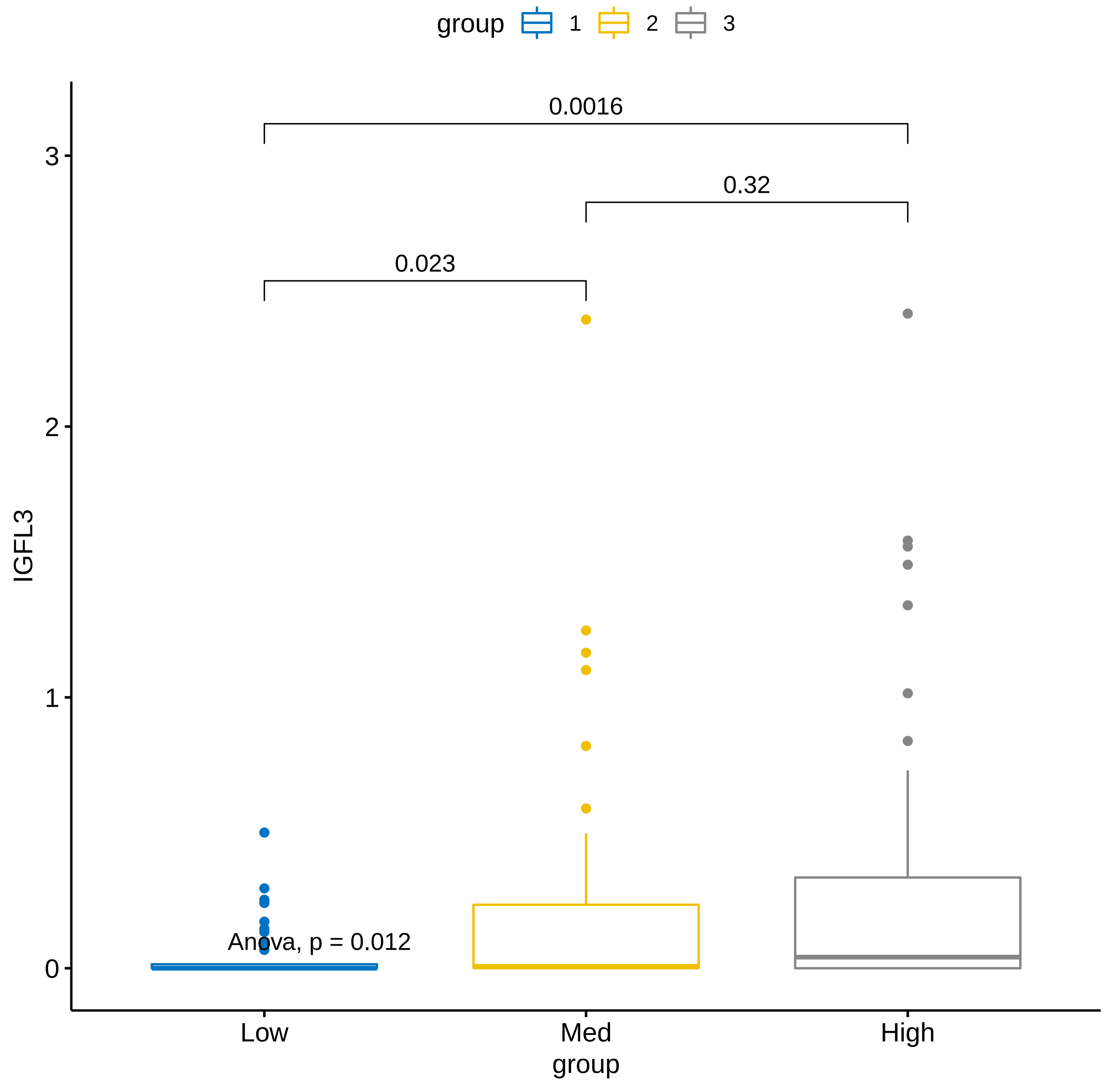
group
1
2
3
0.0016
0.32
0.023
Anova, p = 0.012
IGFL3
3
2
1
0
Low
Med
High
group

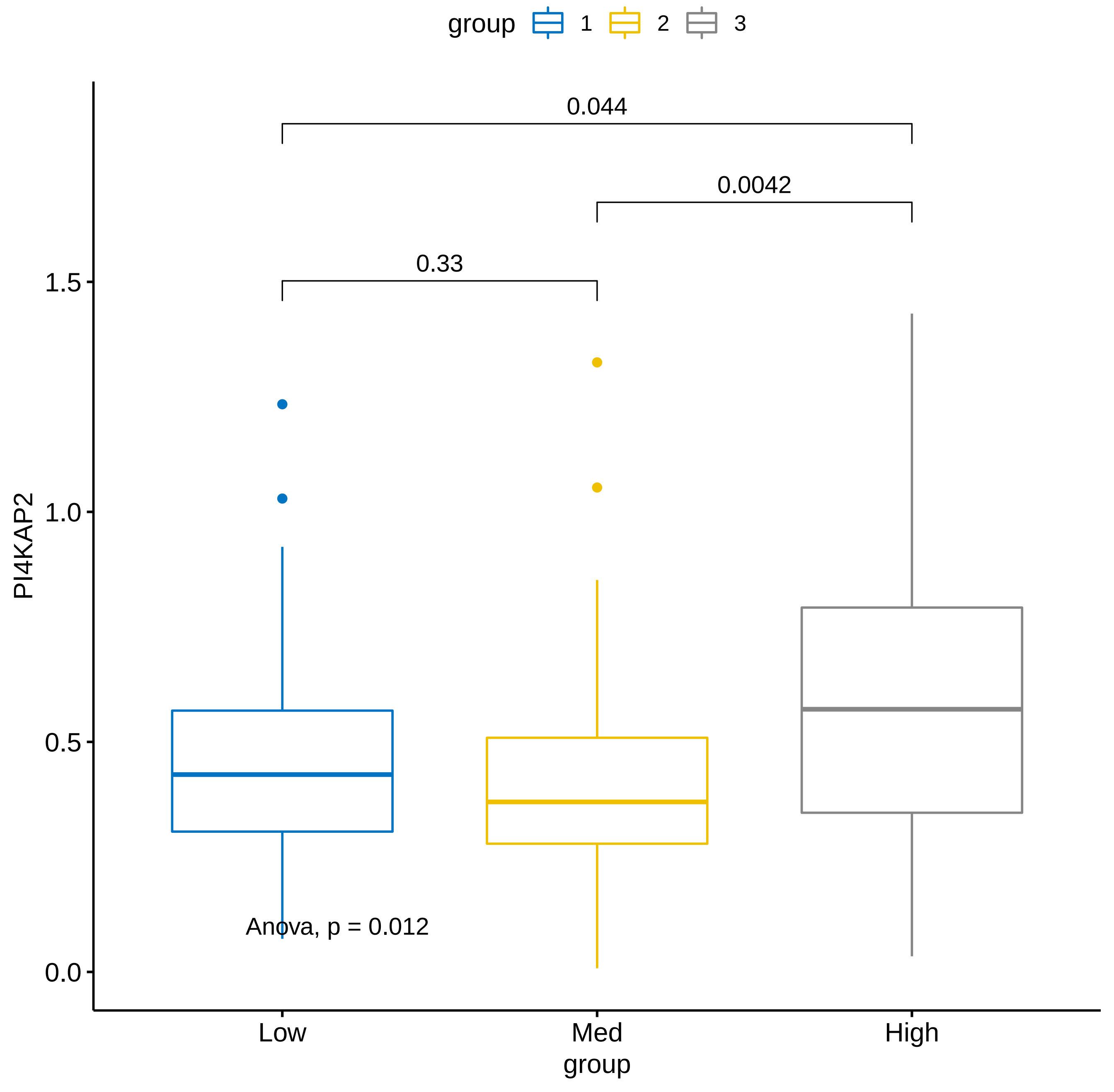

group 1 2 3
0.044
0.0042
0.33
1.5
1.0
0.5
0.0
PI4KAP2
Anova, p = 0.012
Low
Med
High
group

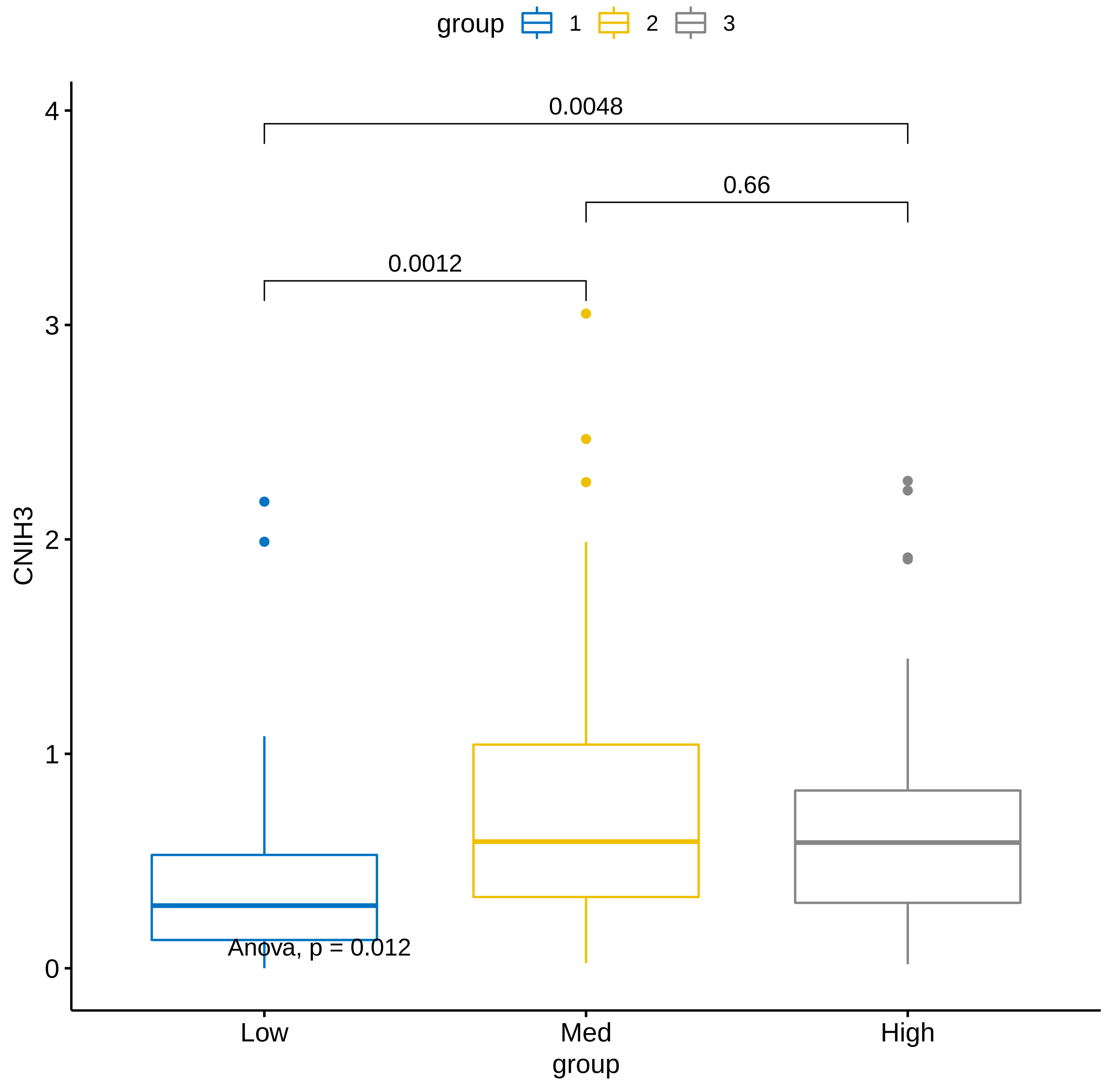

group 1 2 3
0.0048
0.66
0.0012
CNIH3
4
3
2
1
0
Anova, p = 0.012
Low
Med
High
group

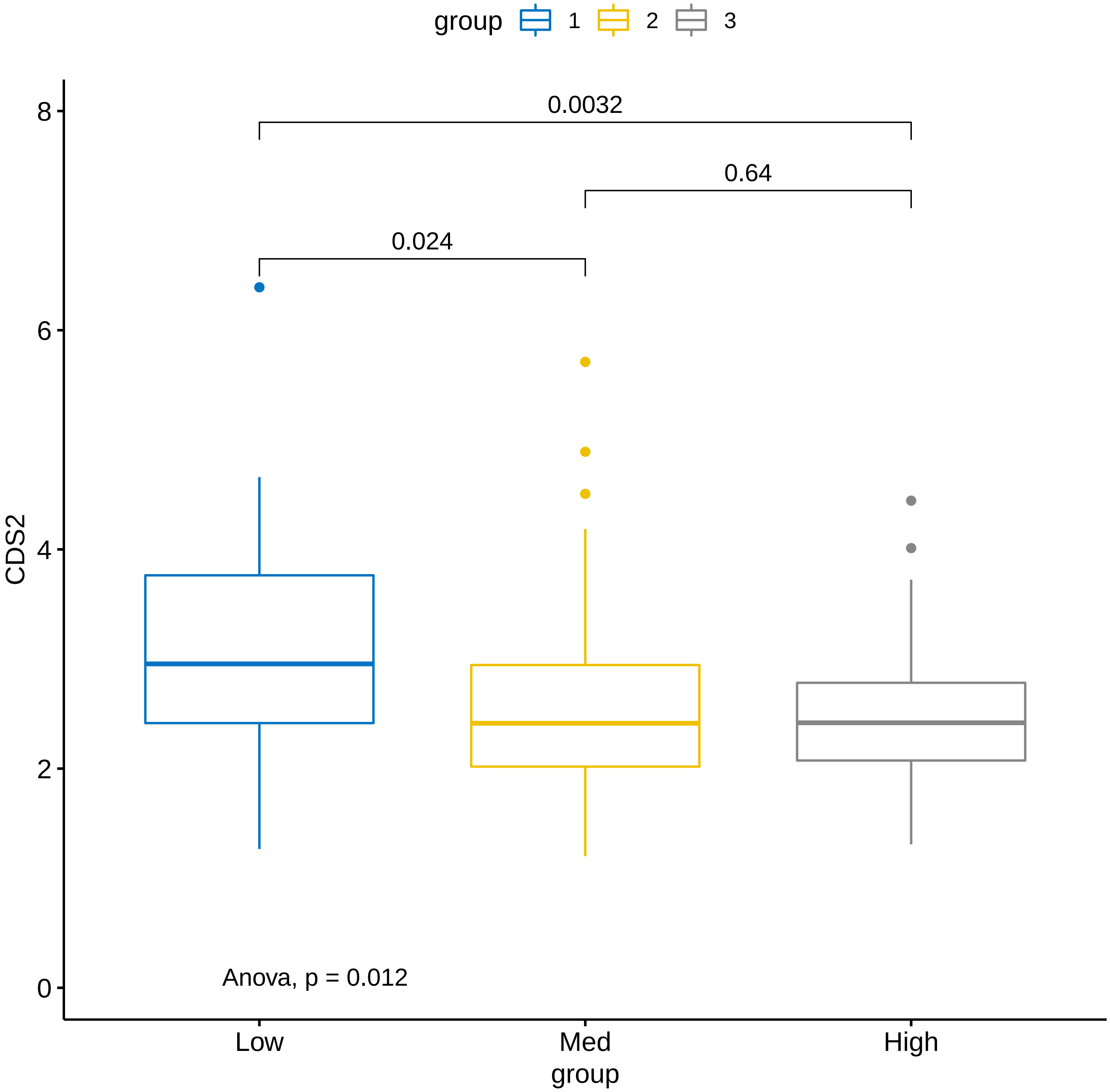

group
1
2
3
0.0032
0.64
0.024
8
6
4
2
0
CDS2
Anova, p = 0.012
Low
Med
High
group

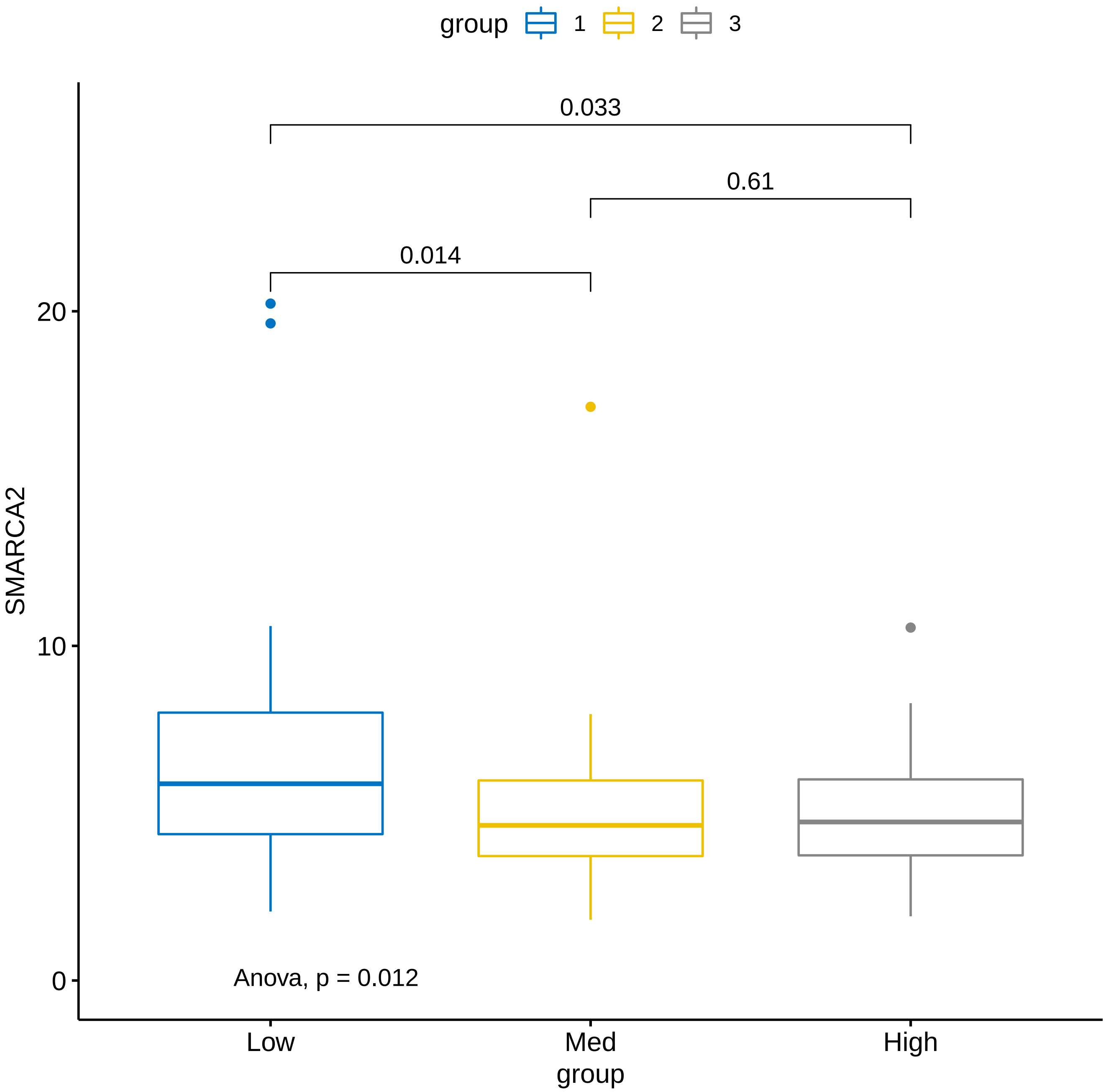

group 1 2 3
0.033
0.61
0.014
20
10
0
SMARCA2
Anova, p = 0.012
Low
Med
High
group

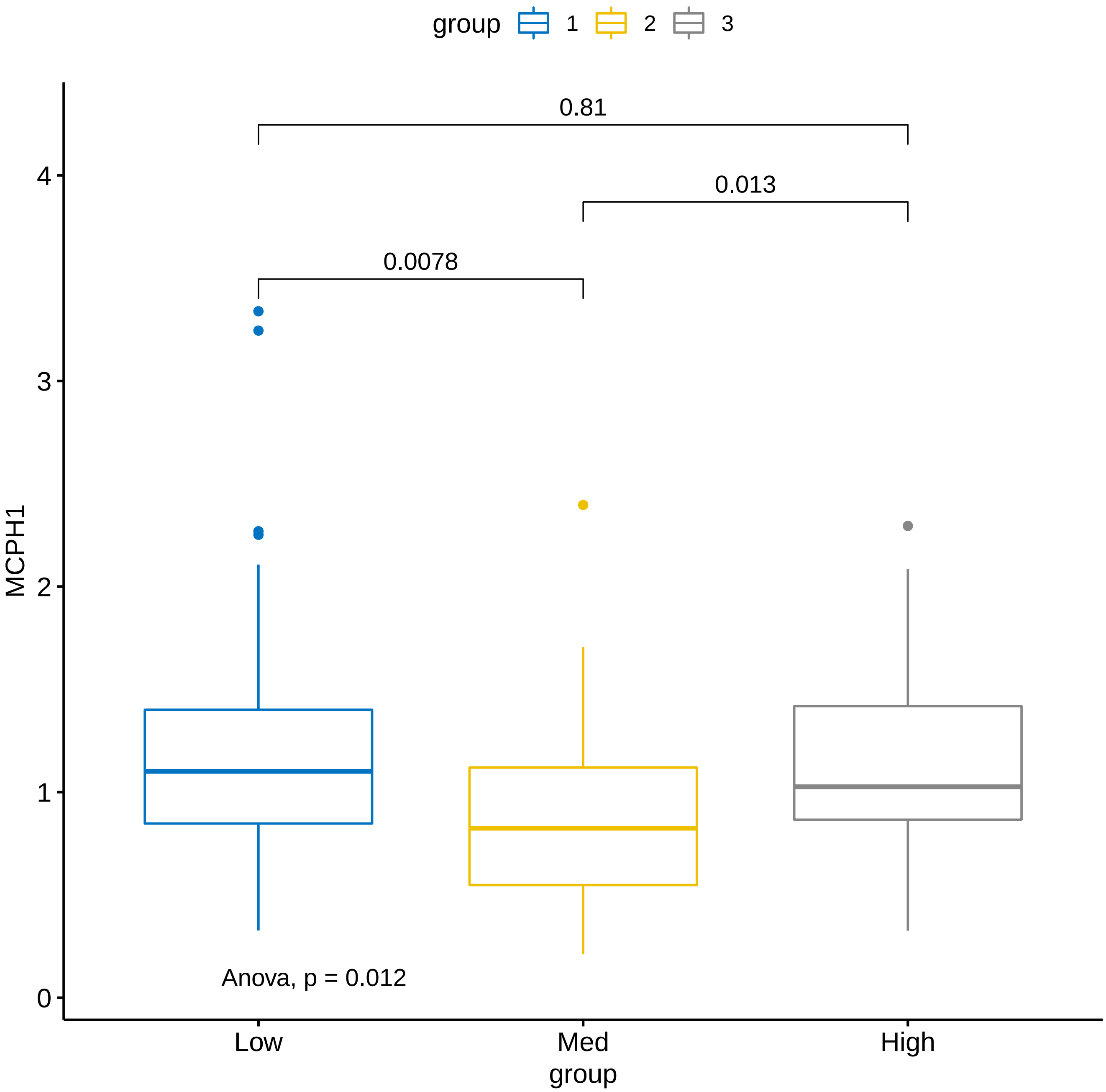

group 1 2 3
0.81
0.013
0.0078
MCPH1
4
3
2
1
0
Anova, p = 0.012
Low
Med
High
group

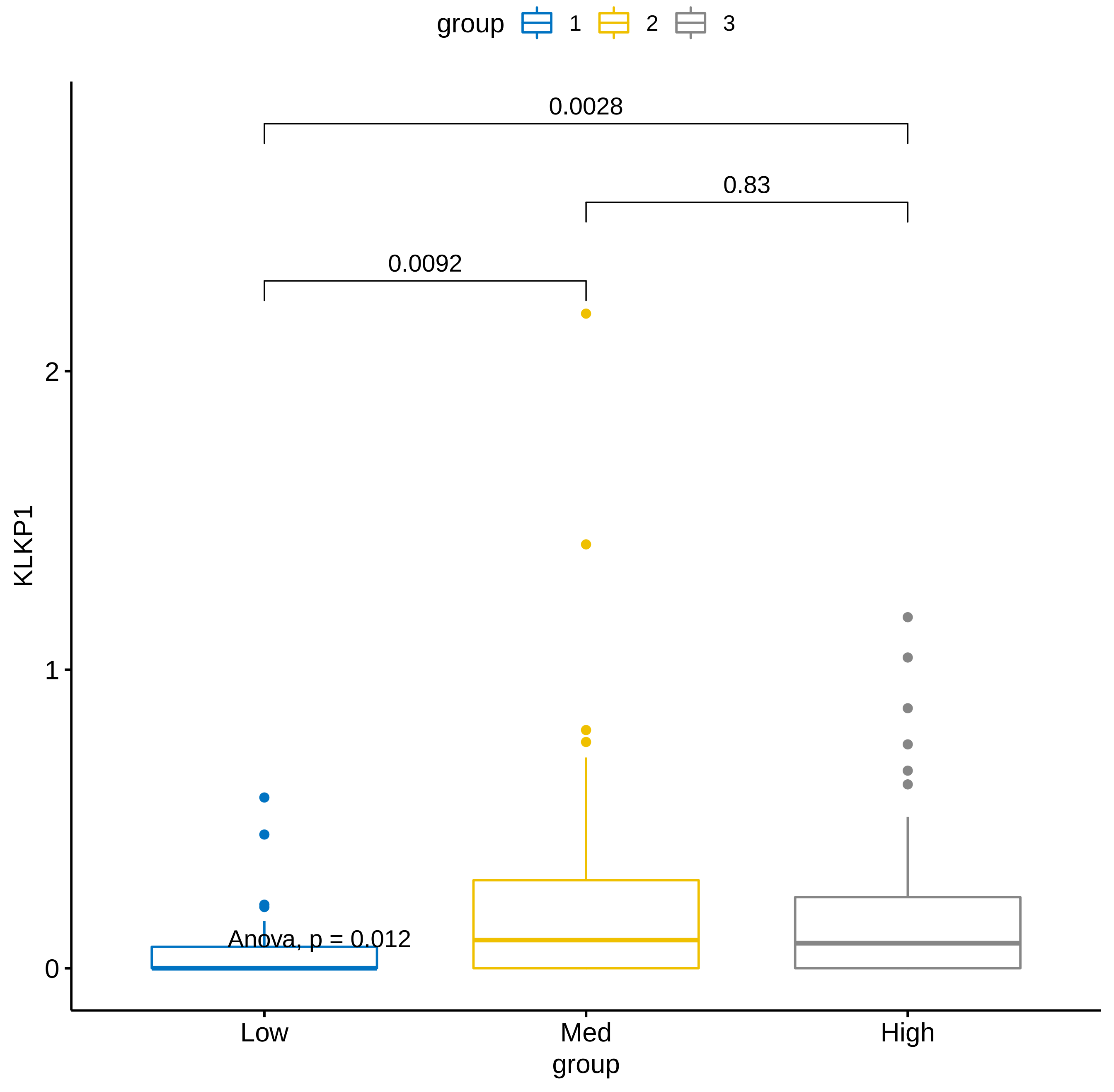

group
1
2
3
0.0028
0.83
0.0092
KLKP1
2
1
0
Anova, p = 0.012
Low
Med
High
group

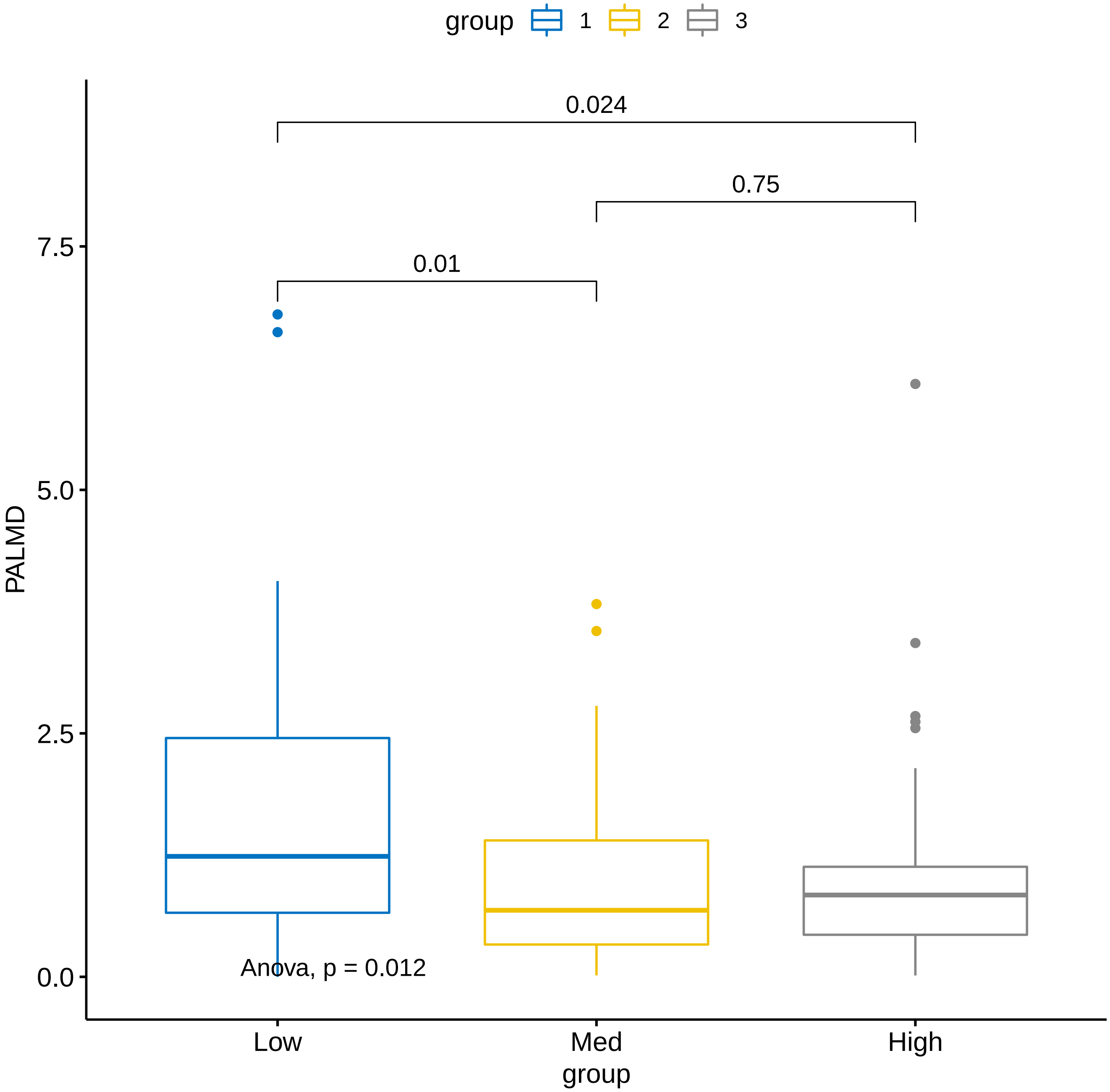
group
1
2
3
0.024
0.75
0.01
7.5
5.0
2.5
0.0
PALMD
Anova, p = 0.012
Low
Med
High
group

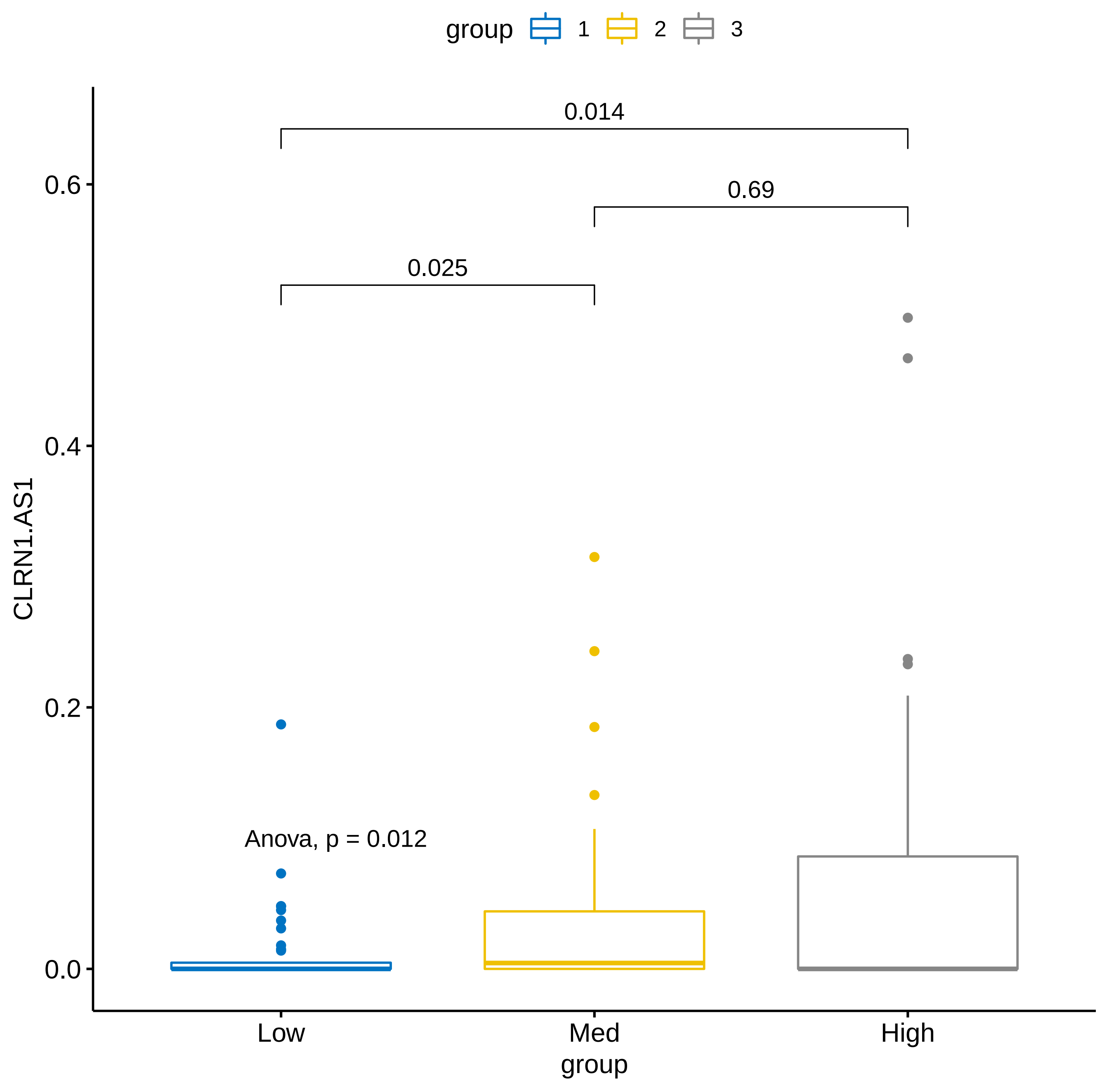

group 1 2 3
0.014
0.69
0.025
Anova, p = 0.012
0.6
0.4
0.2
0.0
CLRN1.AS1
Low
Med
High
group

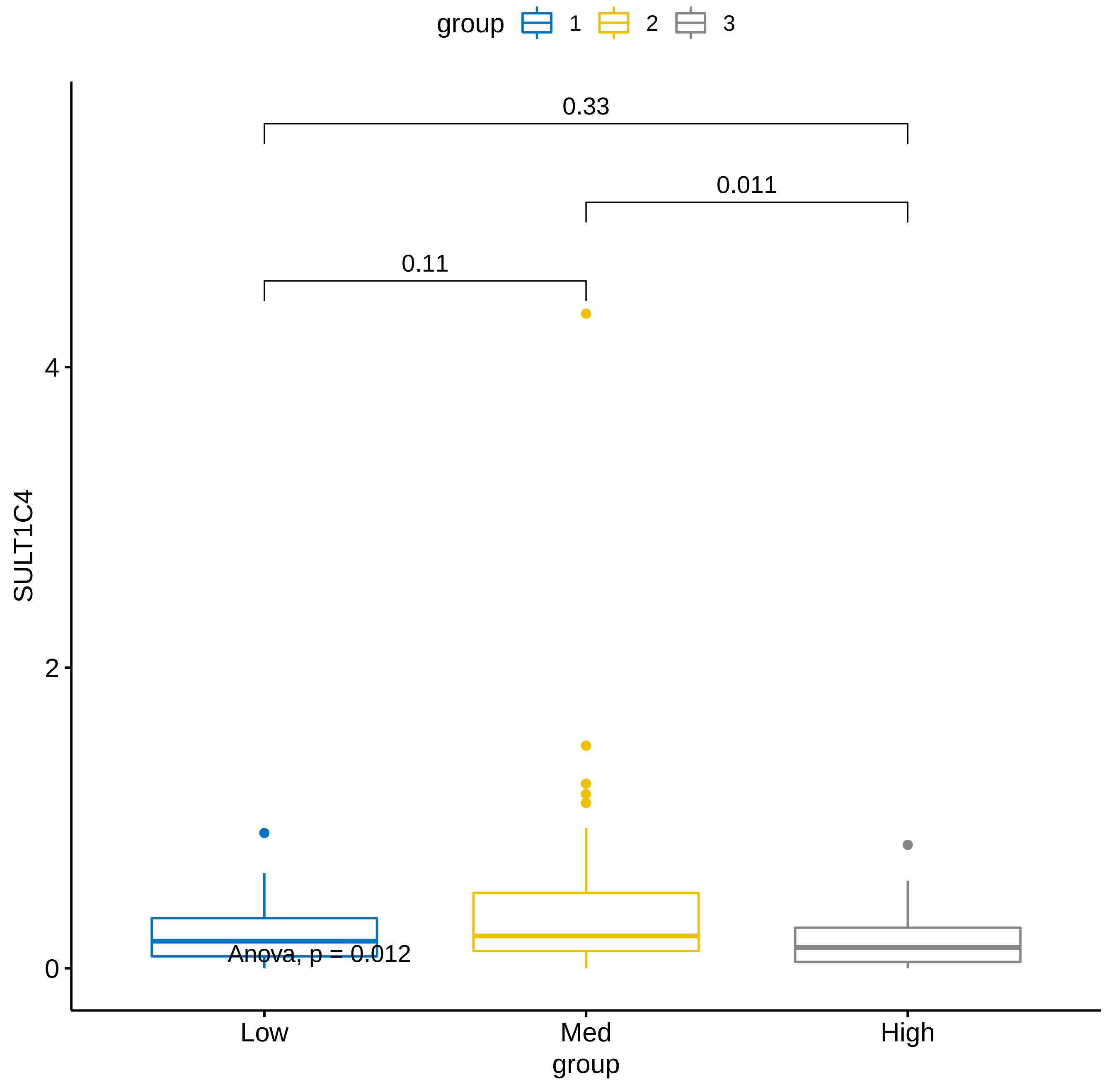

group 1 2 3
0.33
0.011
0.11
4
2
0
SULT1C4
Anova, p = 0.012
Low
Med
High
group

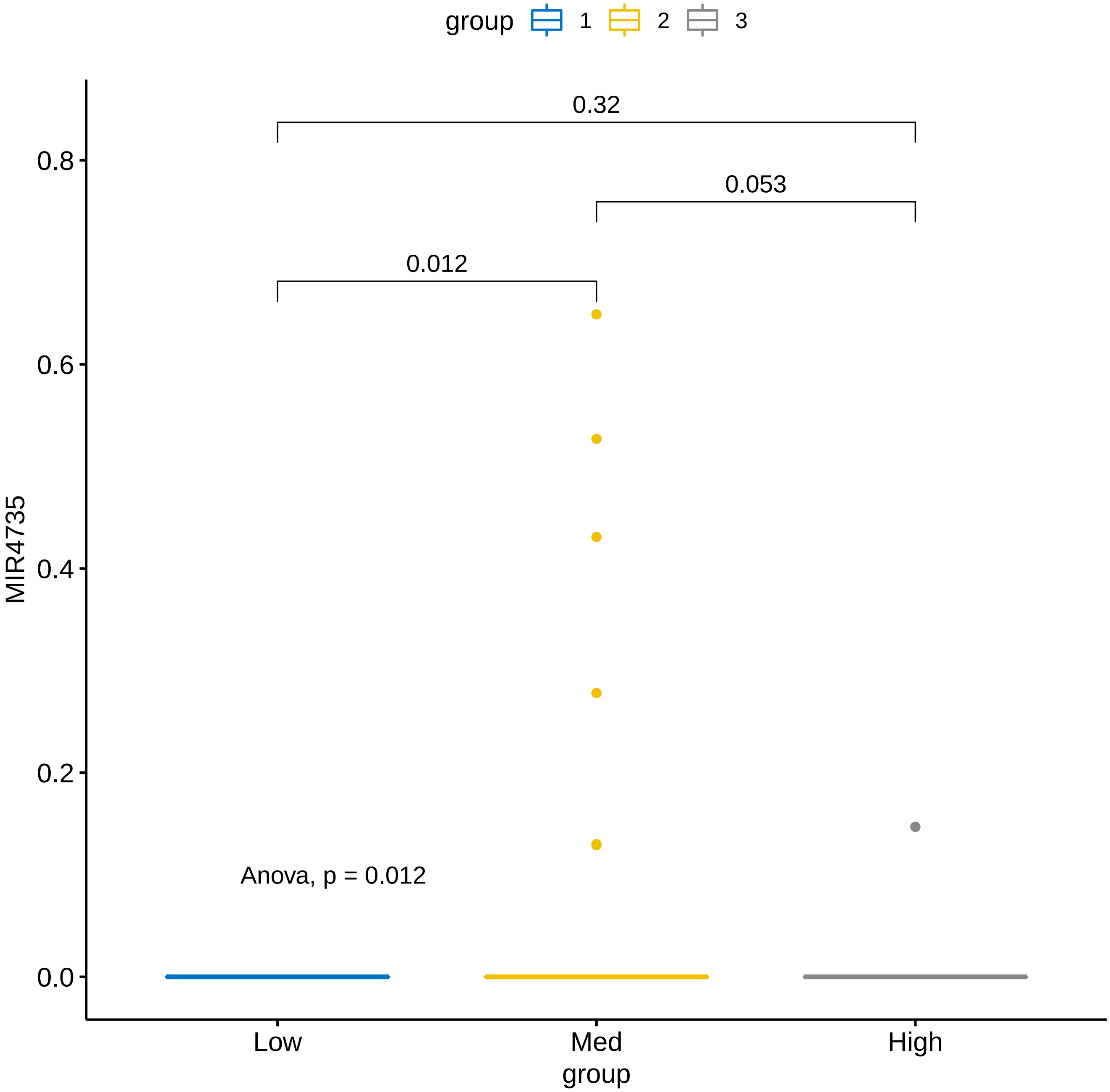
group
1
2
3
0.32
0.053
0.012
Anova, p = 0.012
MIR4735
0.8
0.6
0.4
0.2
0.0
Low
Med
High
group

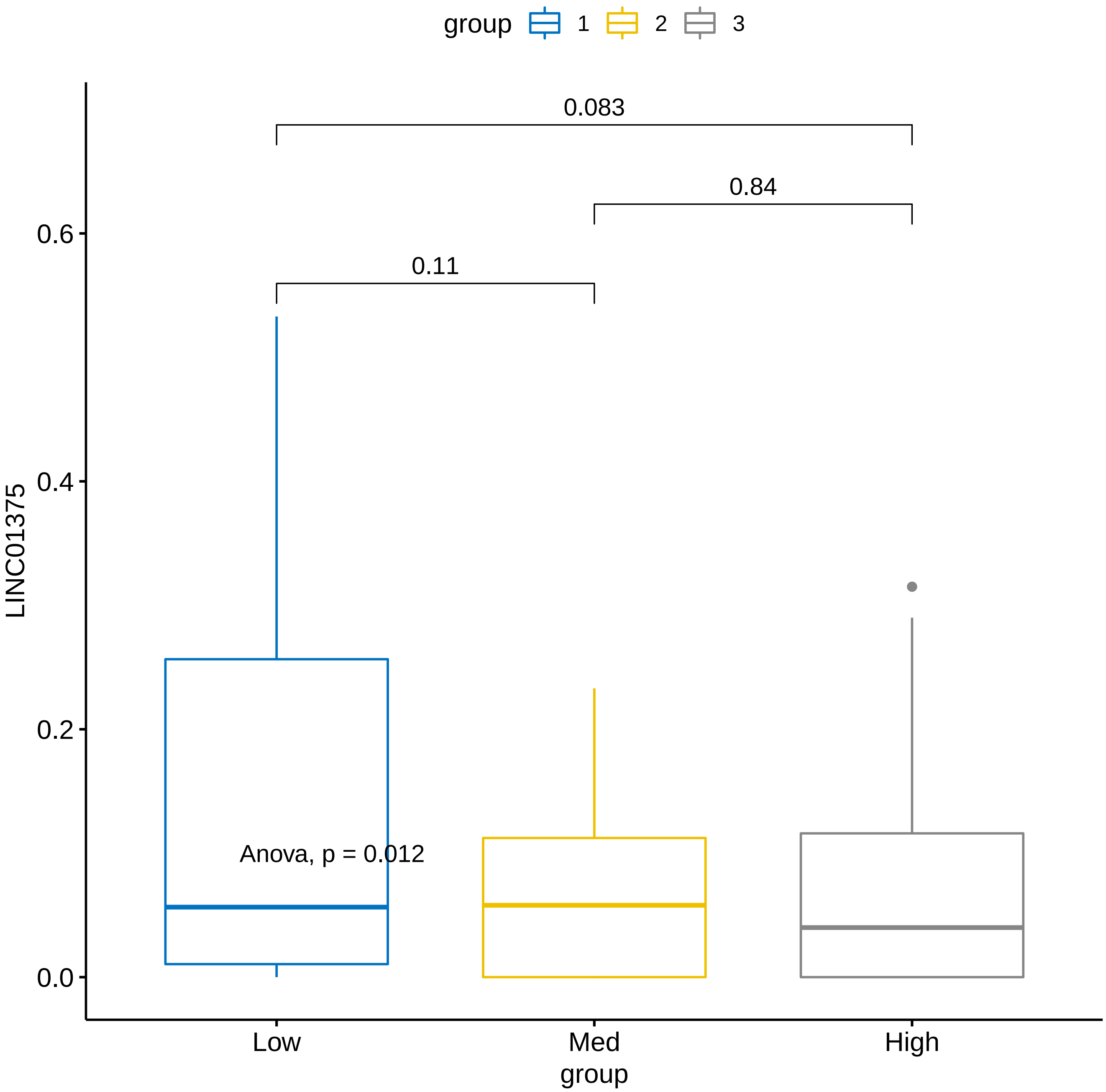
group
1
2
3
0.083
0.84
0.11
Anova, p = 0.012
LINC01375
0.6
0.4
0.2
0.0
Low
Med
High
group

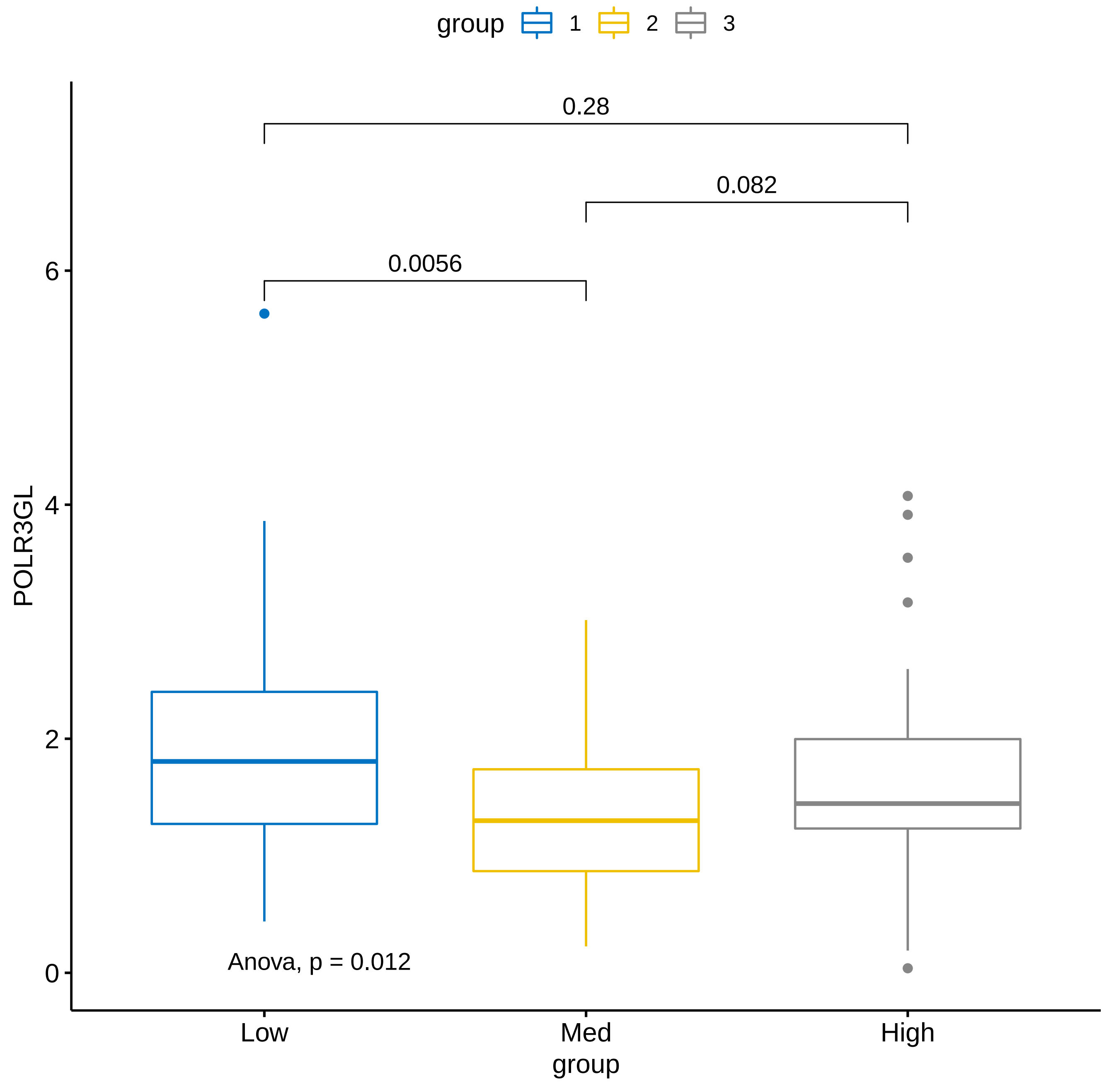

group 1 2 3
0.28
0.082
0.0056
POLR3GL
6
4
2
0
Anova, p = 0.012
Low
Med
High
group

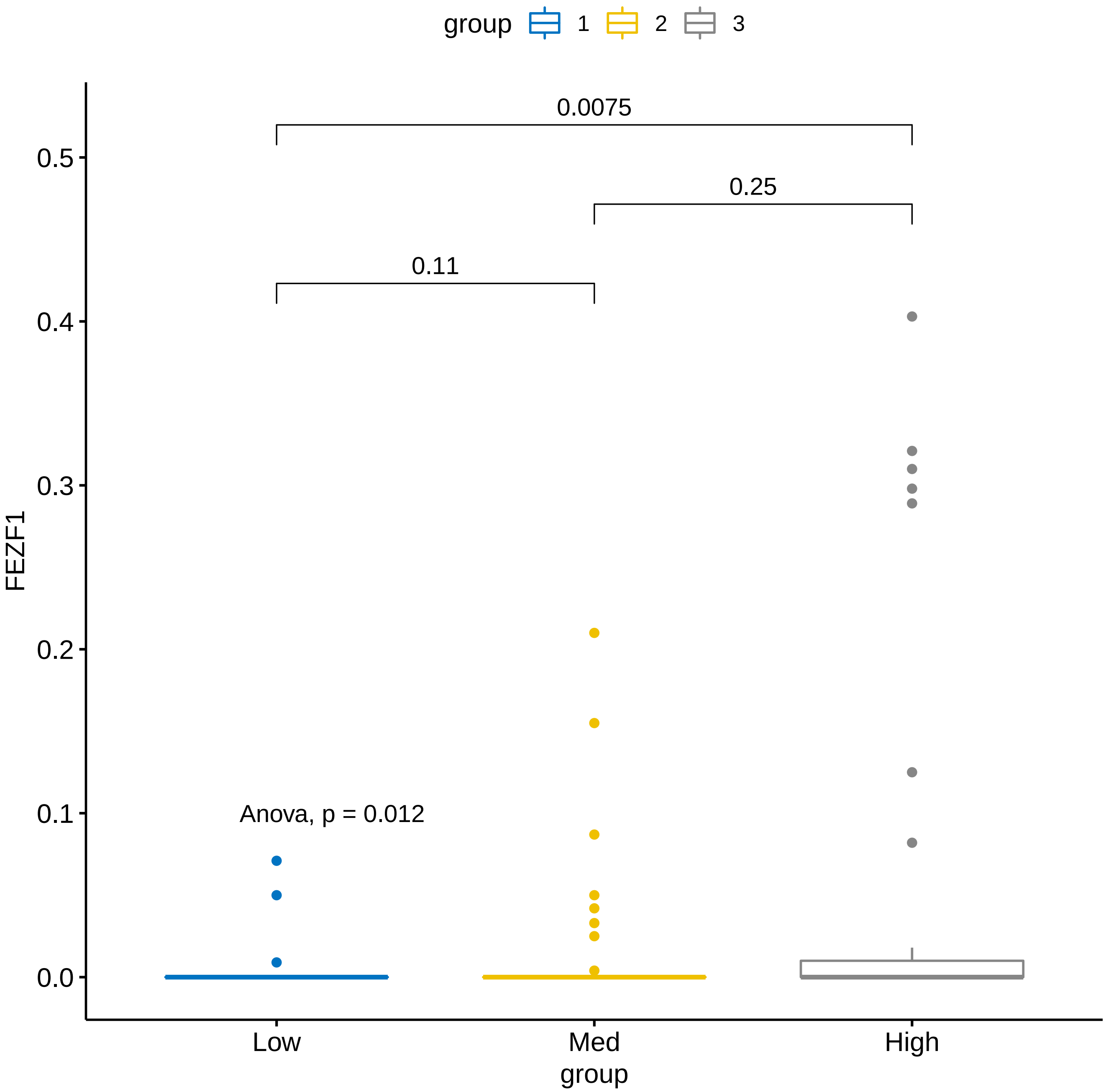
group
1
2
3
0.0075
0.25
0.11
Anova, p = 0.012
0.5
0.4
0.3
0.2
0.1
0.0
FEZF1
Low
Med
High
group

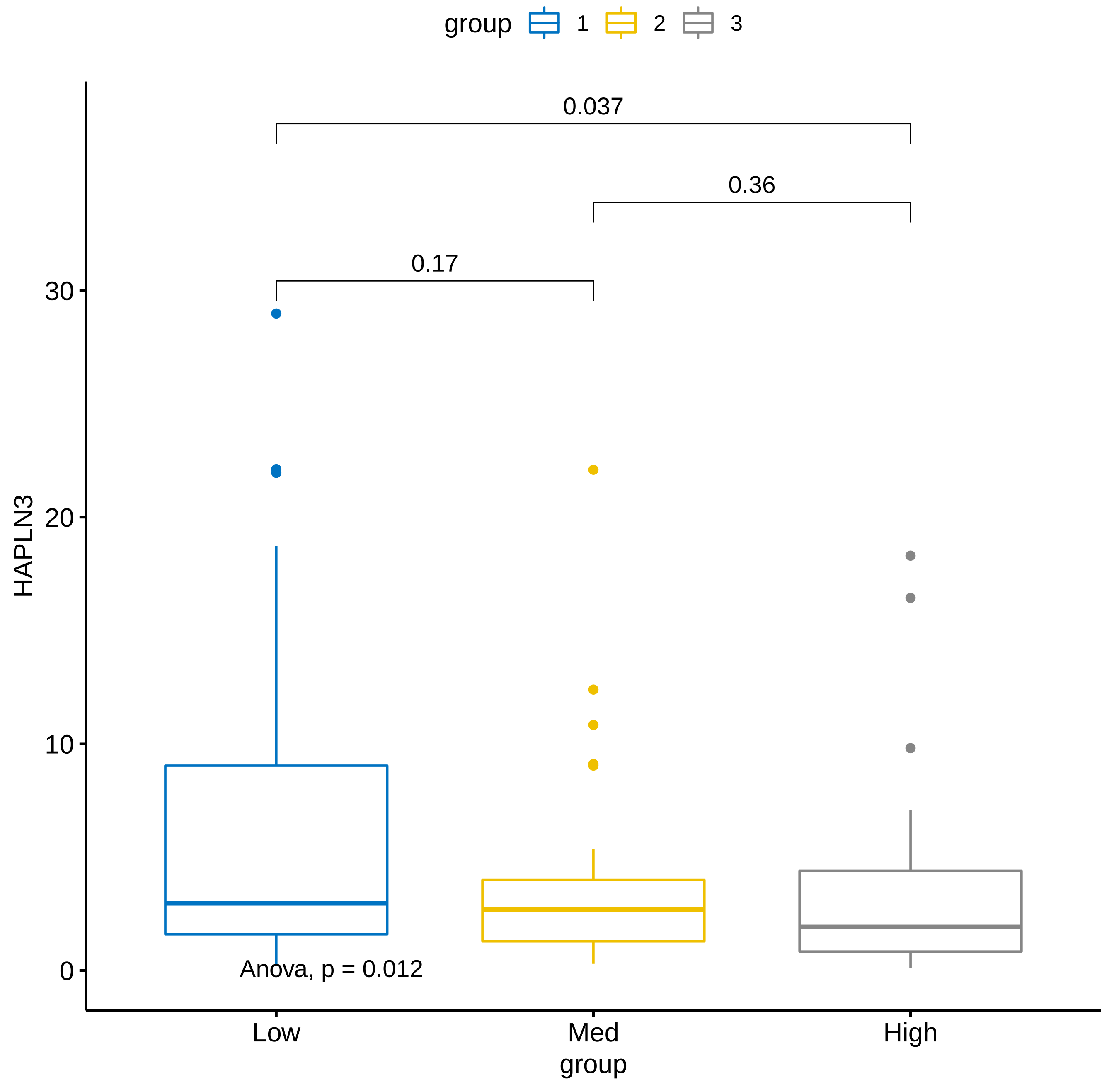
group 1 2 3
0.037
0.36
0.17
30
20
10
0
HAPLN3
Anova, p = 0.012
Low
Med
High
group

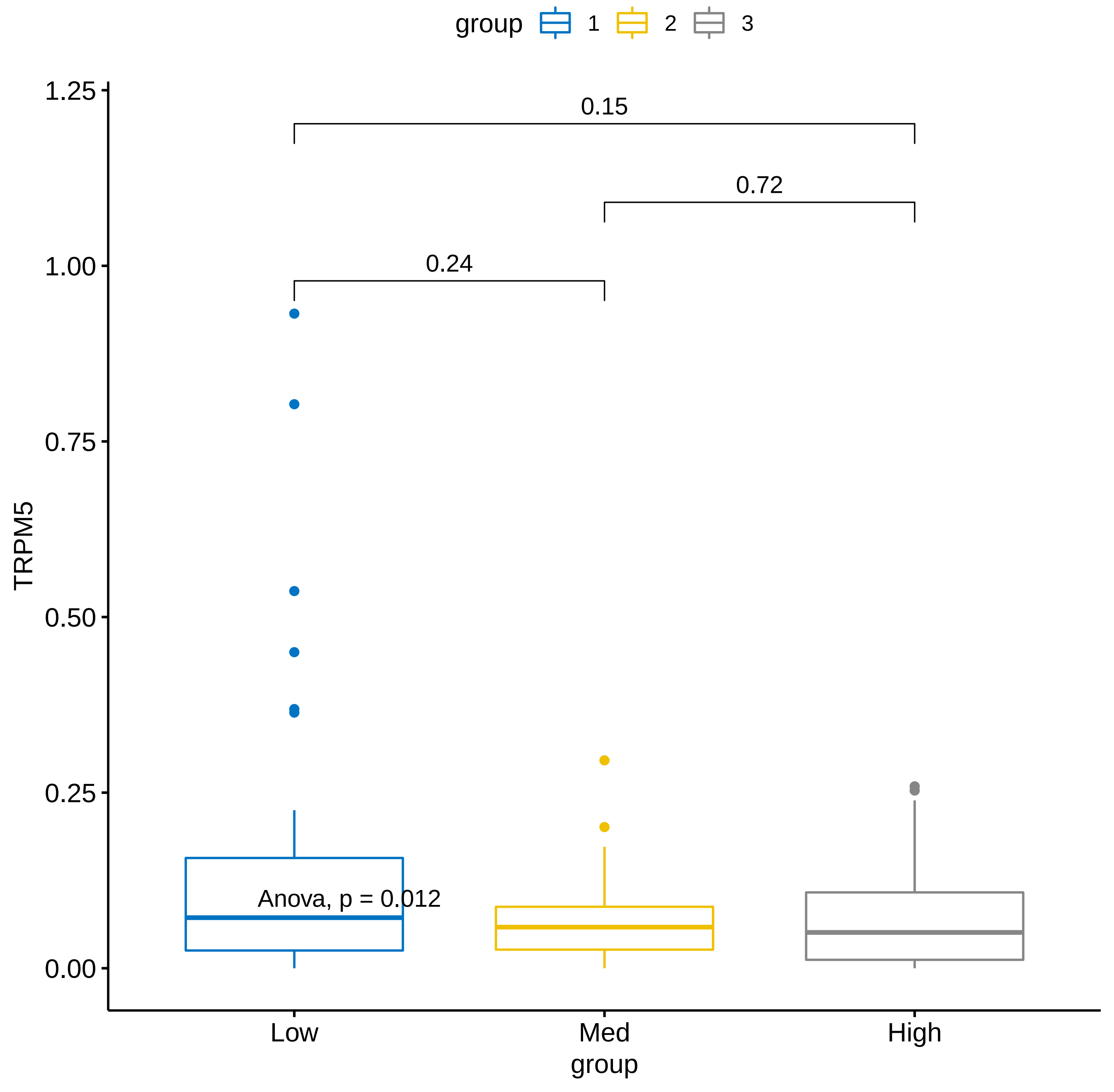
group 1 2 3
1.25
1.00
0.75
0.50
0.25
0.00
TRPM5
0.15
0.72
0.24
Anova, p = 0.012
Low
Med
High
group

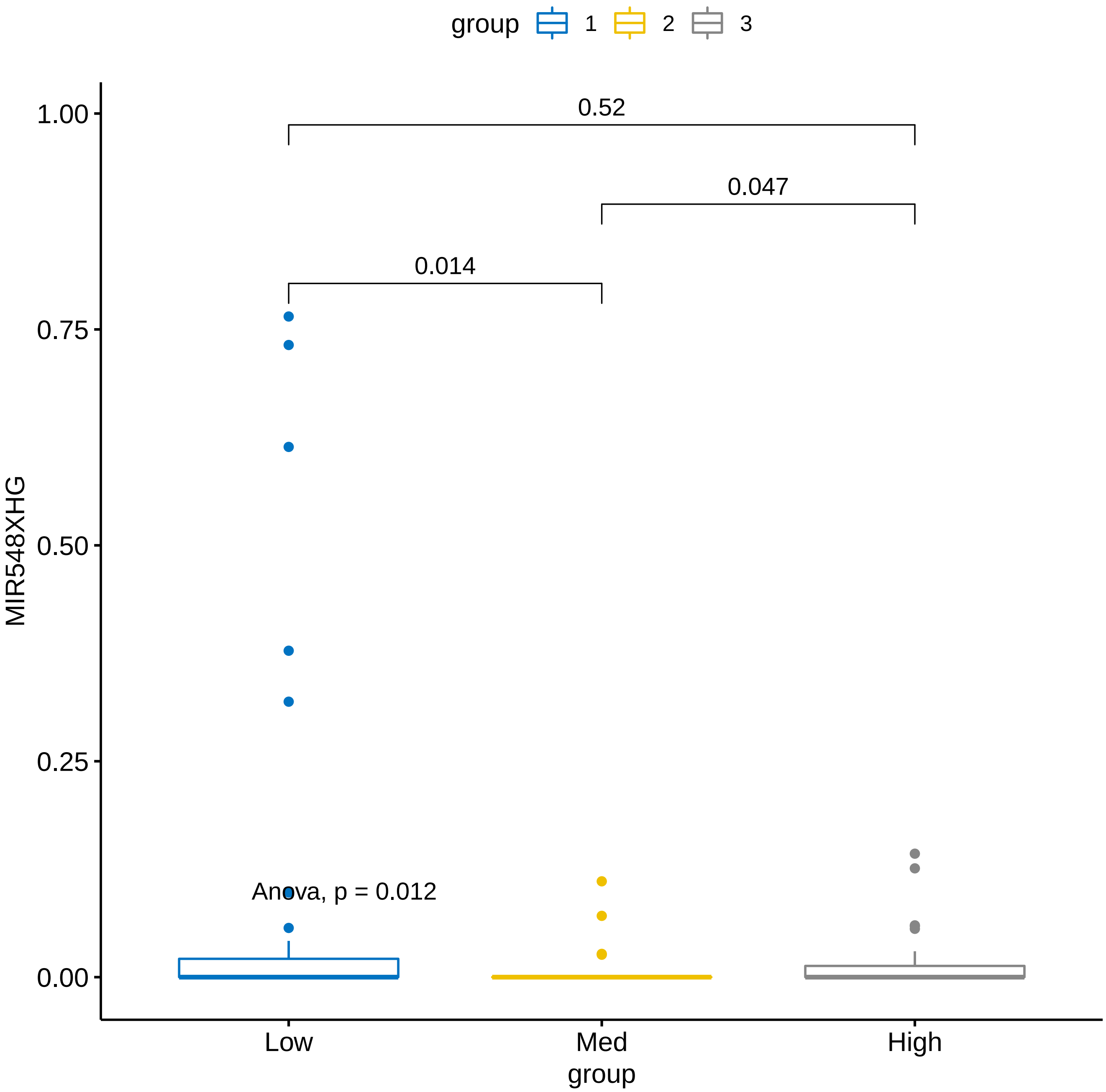

group
1
2
3
0.52
0.047
0.014
1.00
0.75
0.50
0.25
0.00
MIR548XHG
Anova, p = 0.012
Low
Med
High
group

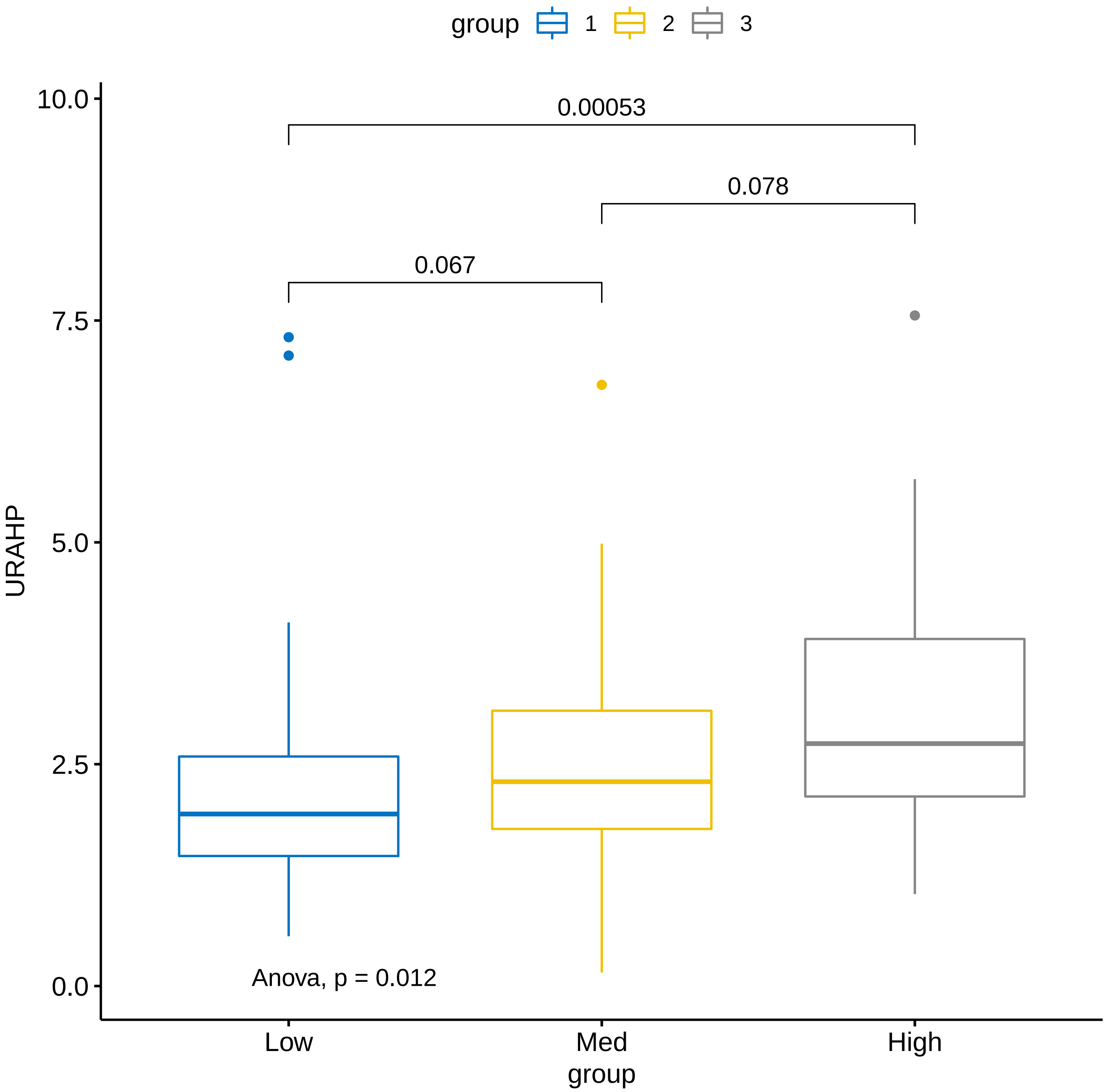

group
1
2
3
10.0
7.5
5.0
2.5
0.0
URAHP
0.00053
0.078
0.067
Anova, p = 0.012
Low
Med
High
group

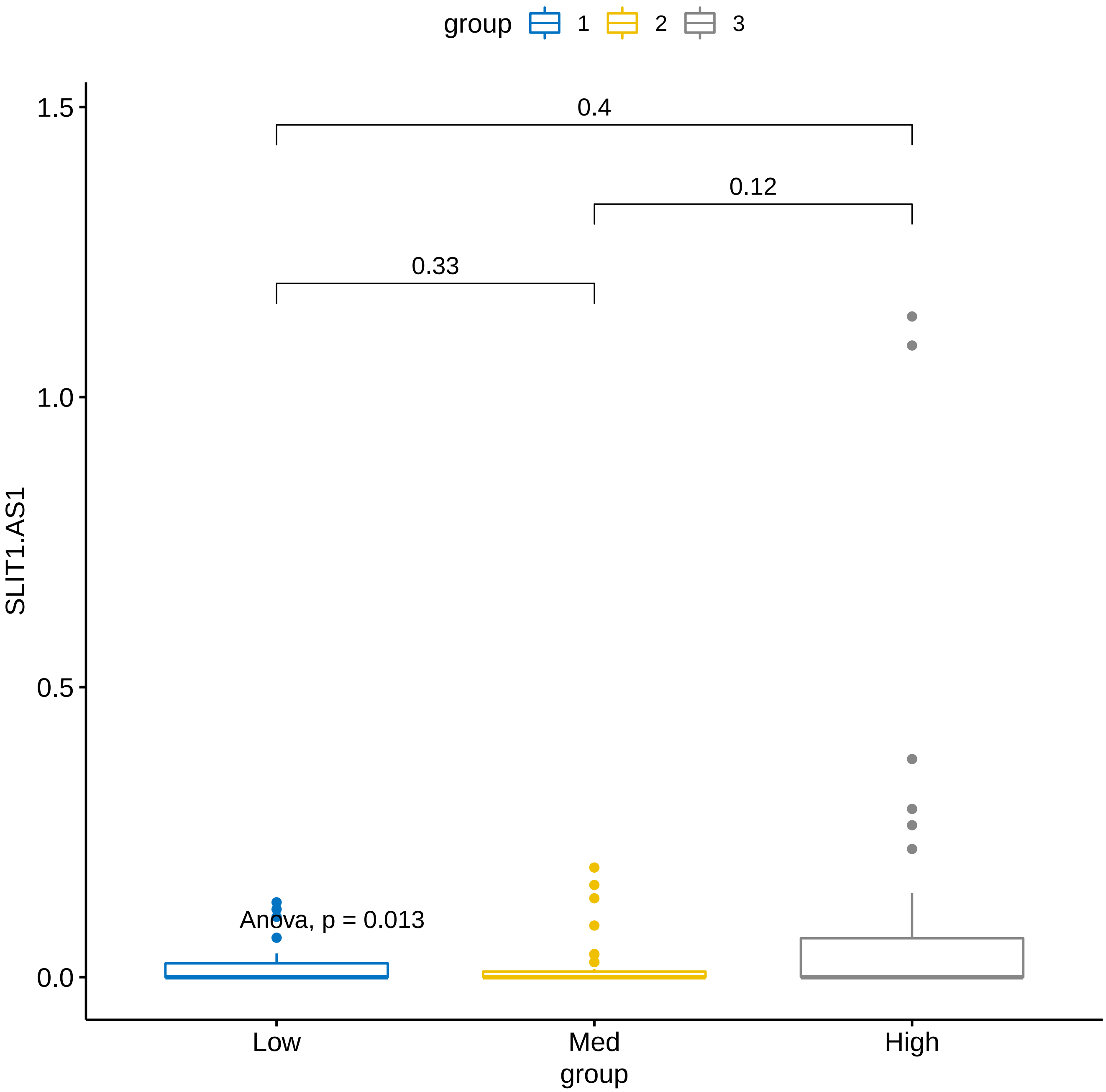
group
1
2
3
0.4
0.12
0.33
Anova, p = 0.013
1.5
1.0
0.5
0.0
SLIT1.AS1
Low
Med
High
group

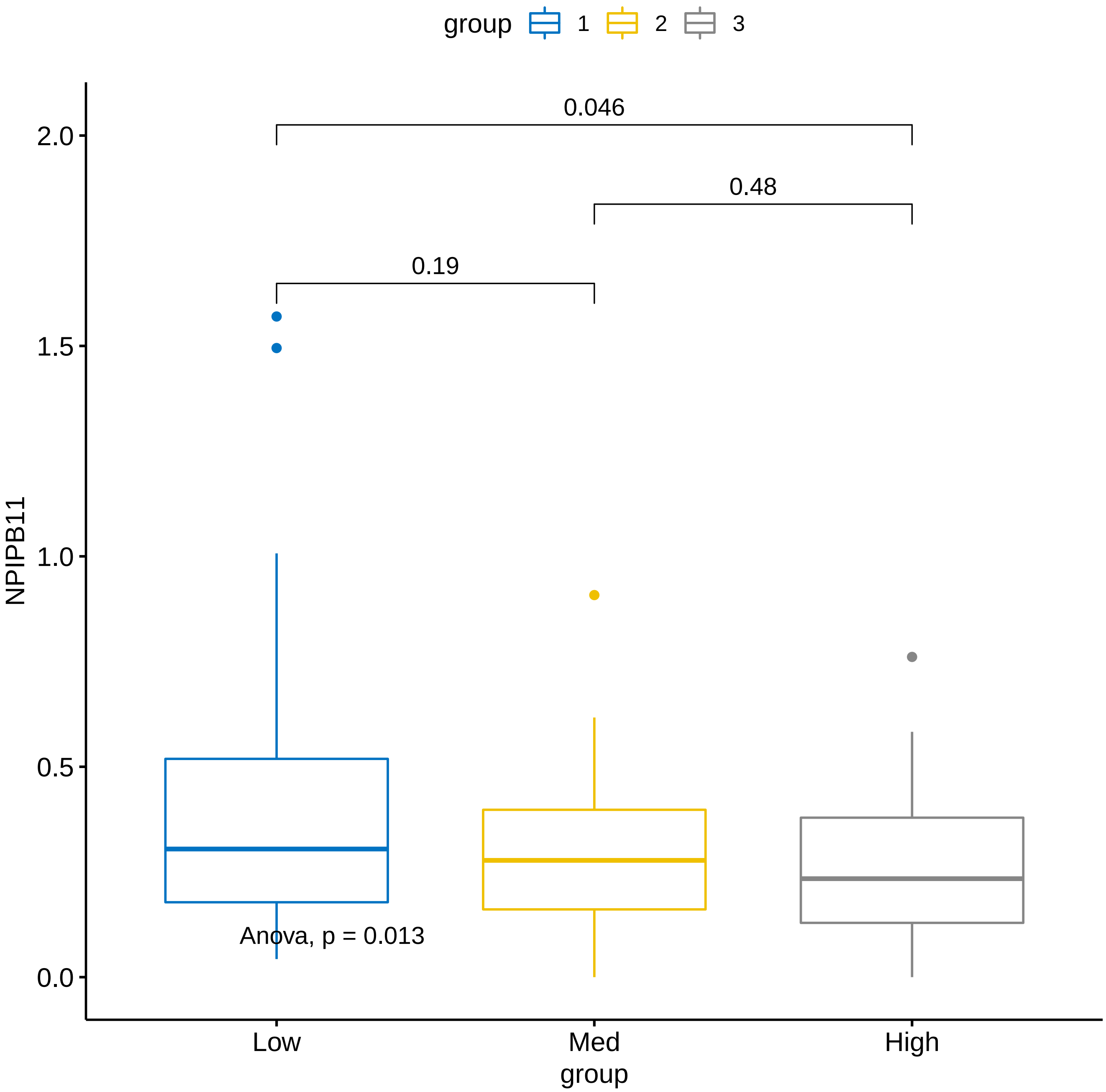
group
1
2
3
0.046
0.48
0.19
NPIPB11
2.0
1.5
1.0
0.5
0.0
Anova, p = 0.013
Low
Med
High
group

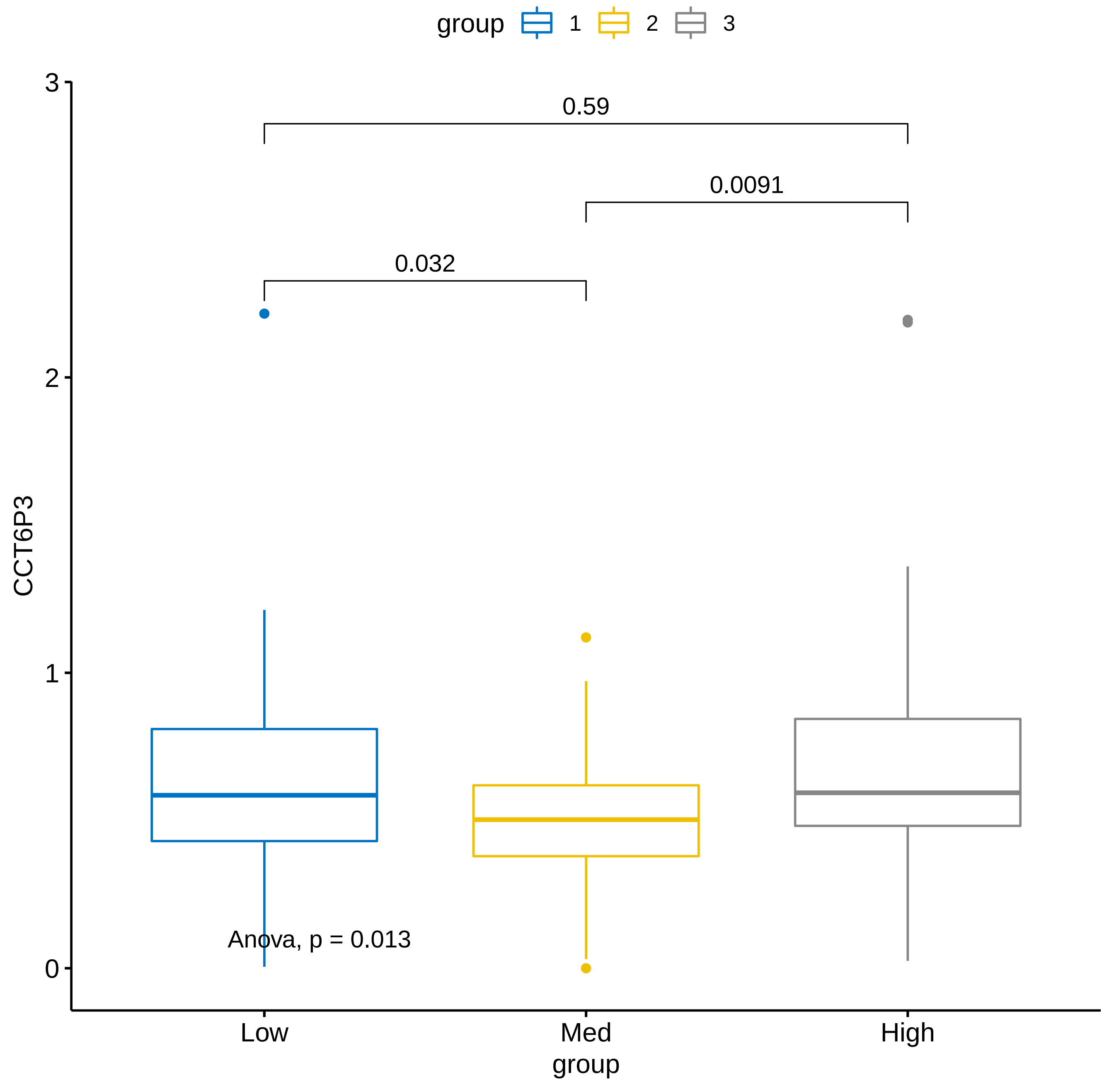

group 1 2 3
0.59
0.0091
0.032
Anova, p = 0.013
CCT6P3
0
1
2
3
Low
Med
High
group

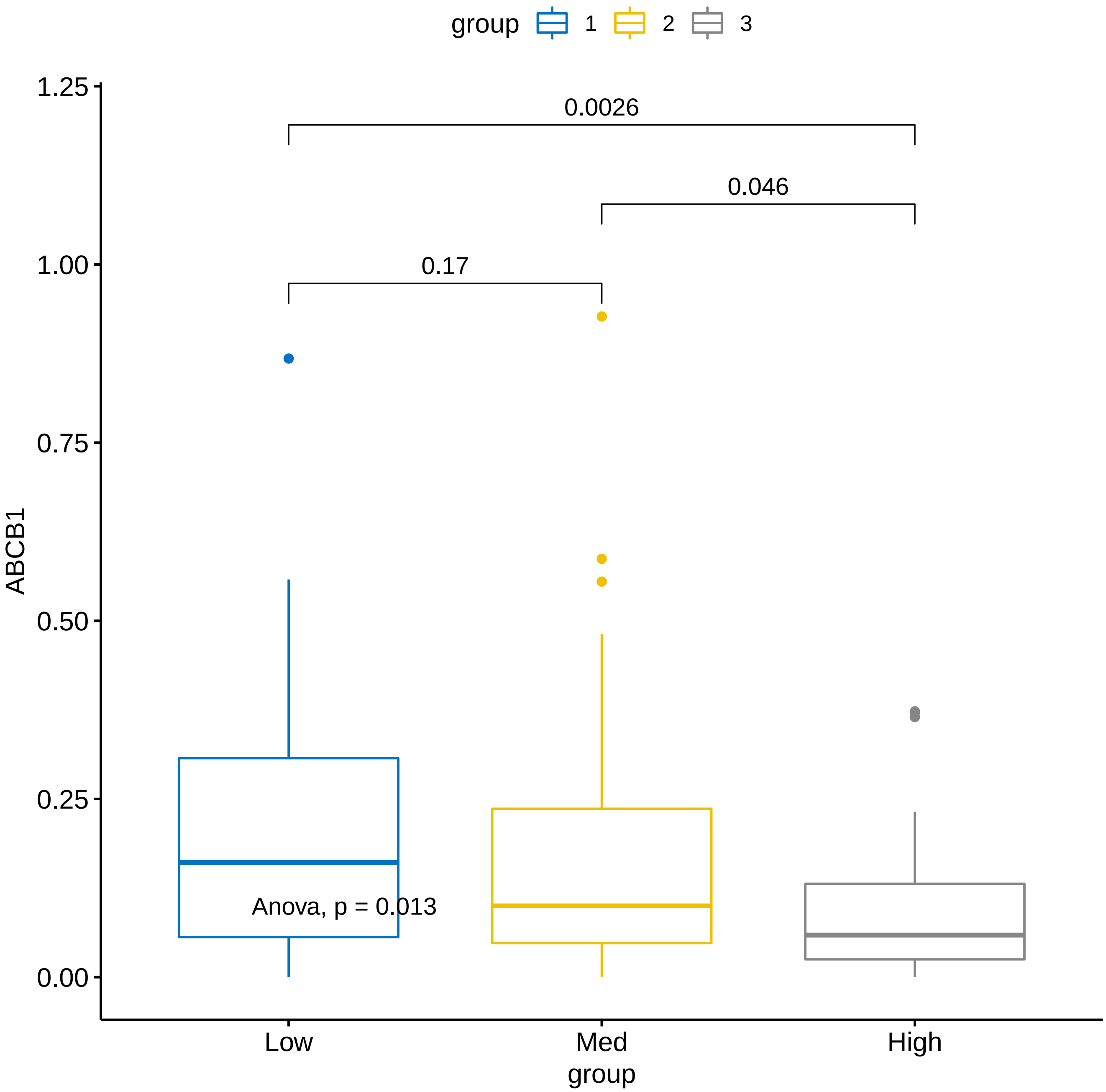

group
1
2
3
1.25
1.00
0.75
0.50
0.25
0.00
ABCB1
0.0026
0.046
0.17
Anova, p = 0.013
Low
Med
High
group

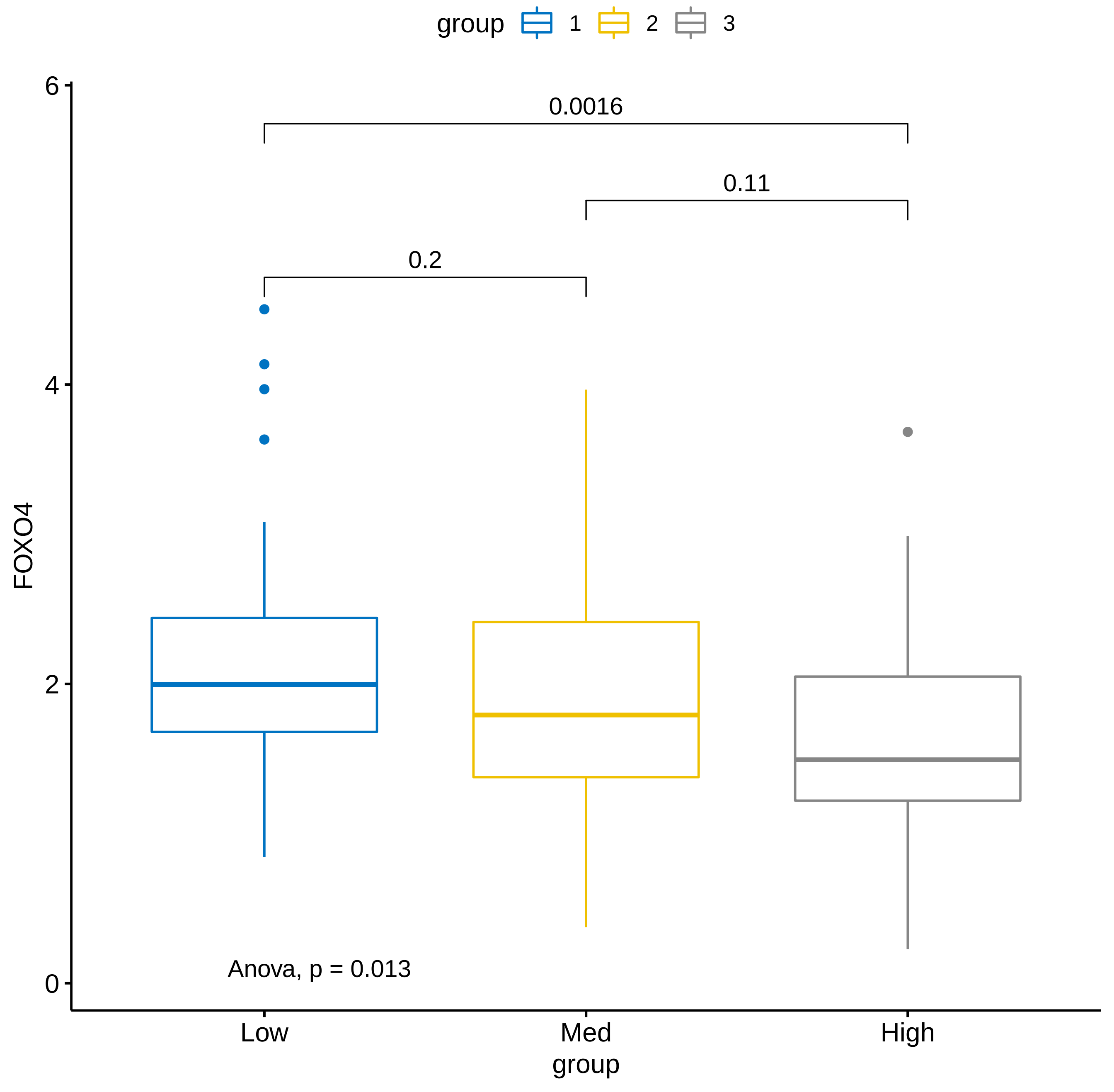

group 1 2 3
0.0016
0.11
0.2
6
4
2
0
FOXO4
Anova, p = 0.013
Low
Med
High
group

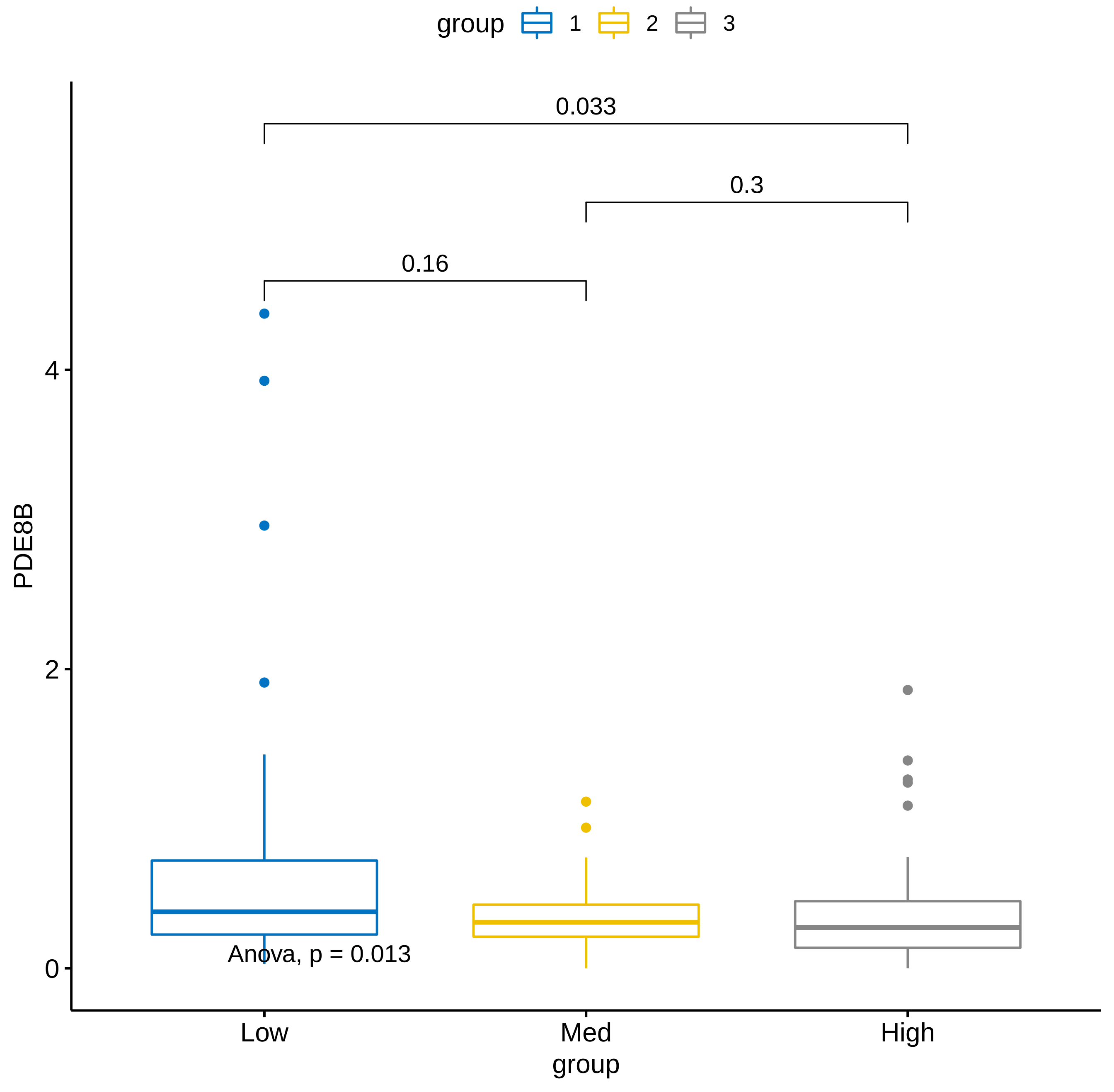

group 1 2 3
0.033
0.3
0.16
PDE8B
4
2
0
Anova, p = 0.013
Low
Med
High
group

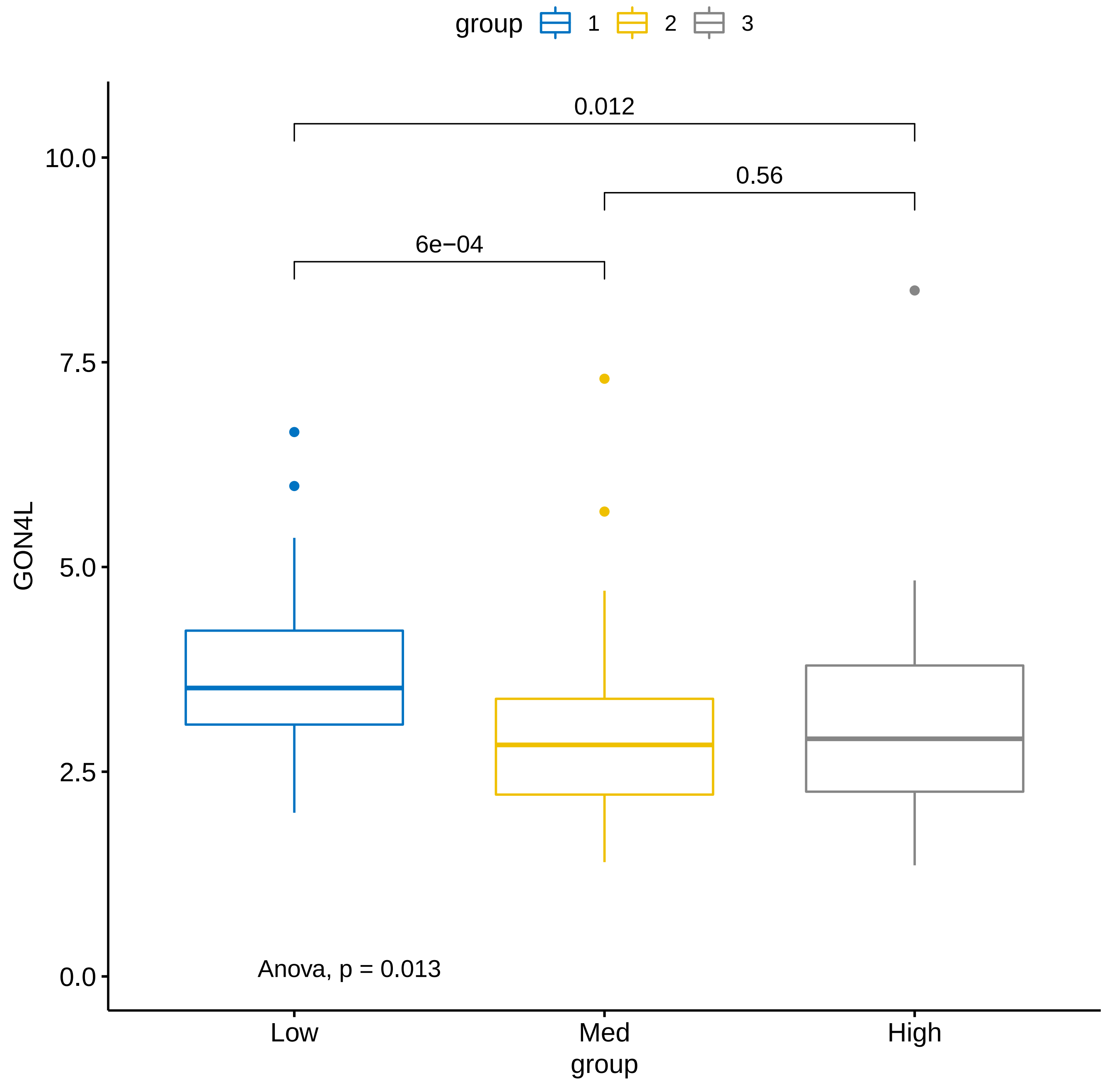

group 1 2 3
0.012
0.56
6e−04
10.0
7.5
5.0
2.5
0.0
GON4L
Anova, p = 0.013
Low
Med
High
group

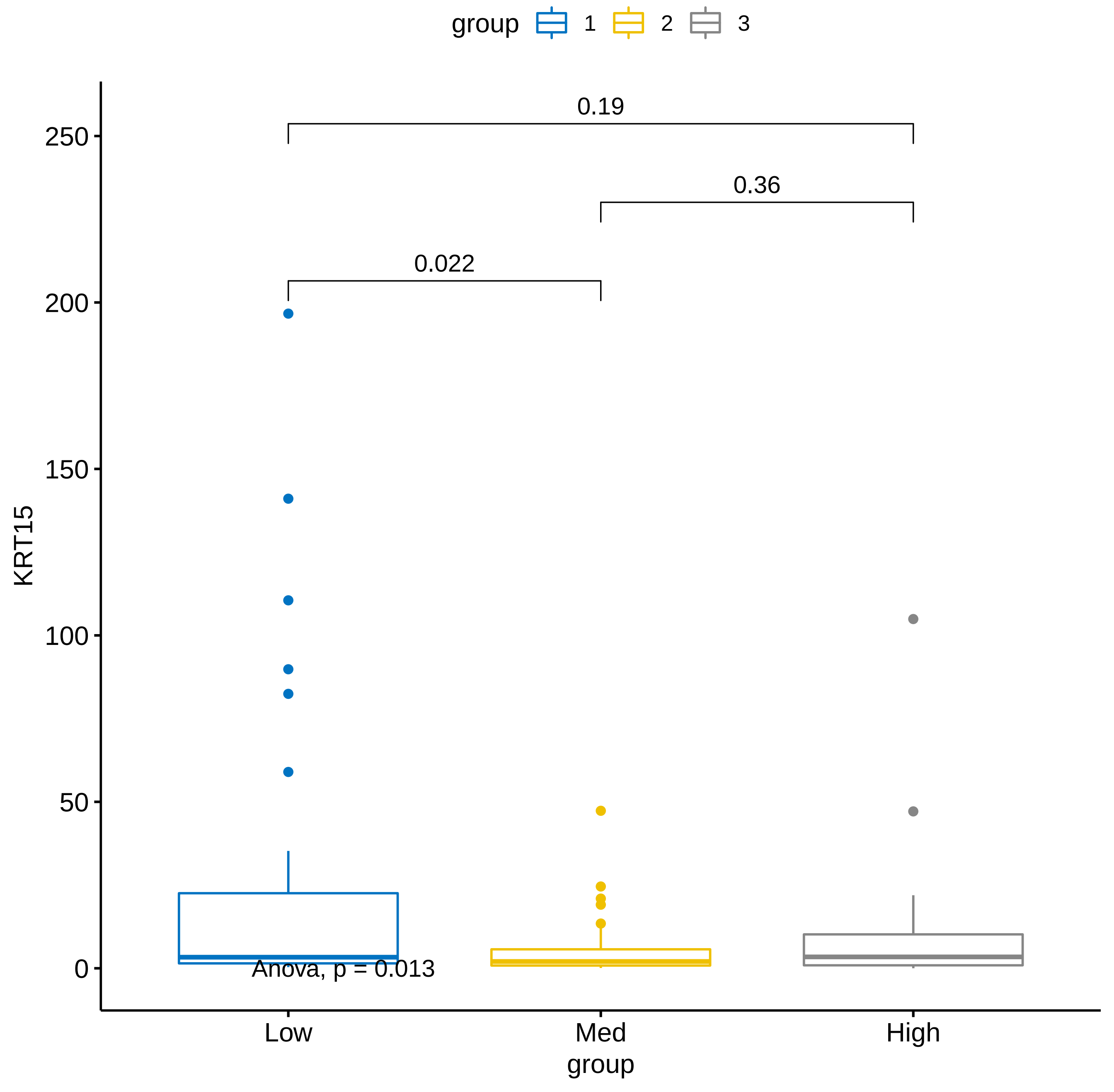
group 1 2 3
0.19
0.36
0.022
250
200
150
100
50
0
KRT15
Anova, p = 0.013
Low
Med
High
group

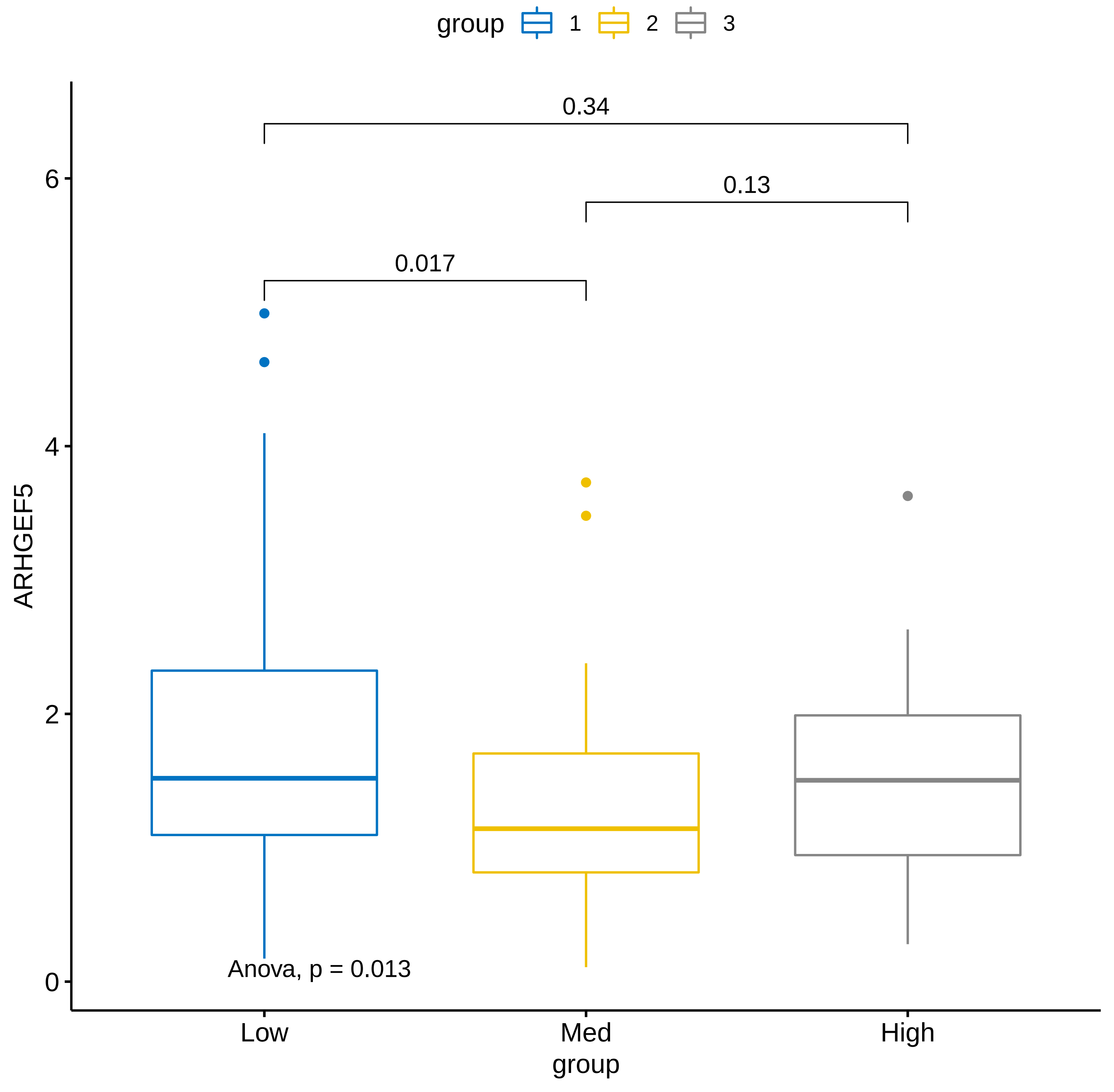

group 1 2 3
0.34
0.13
0.017
ARHGEF5
6
4
2
0
Anova, p = 0.013
Low
Med
High
group

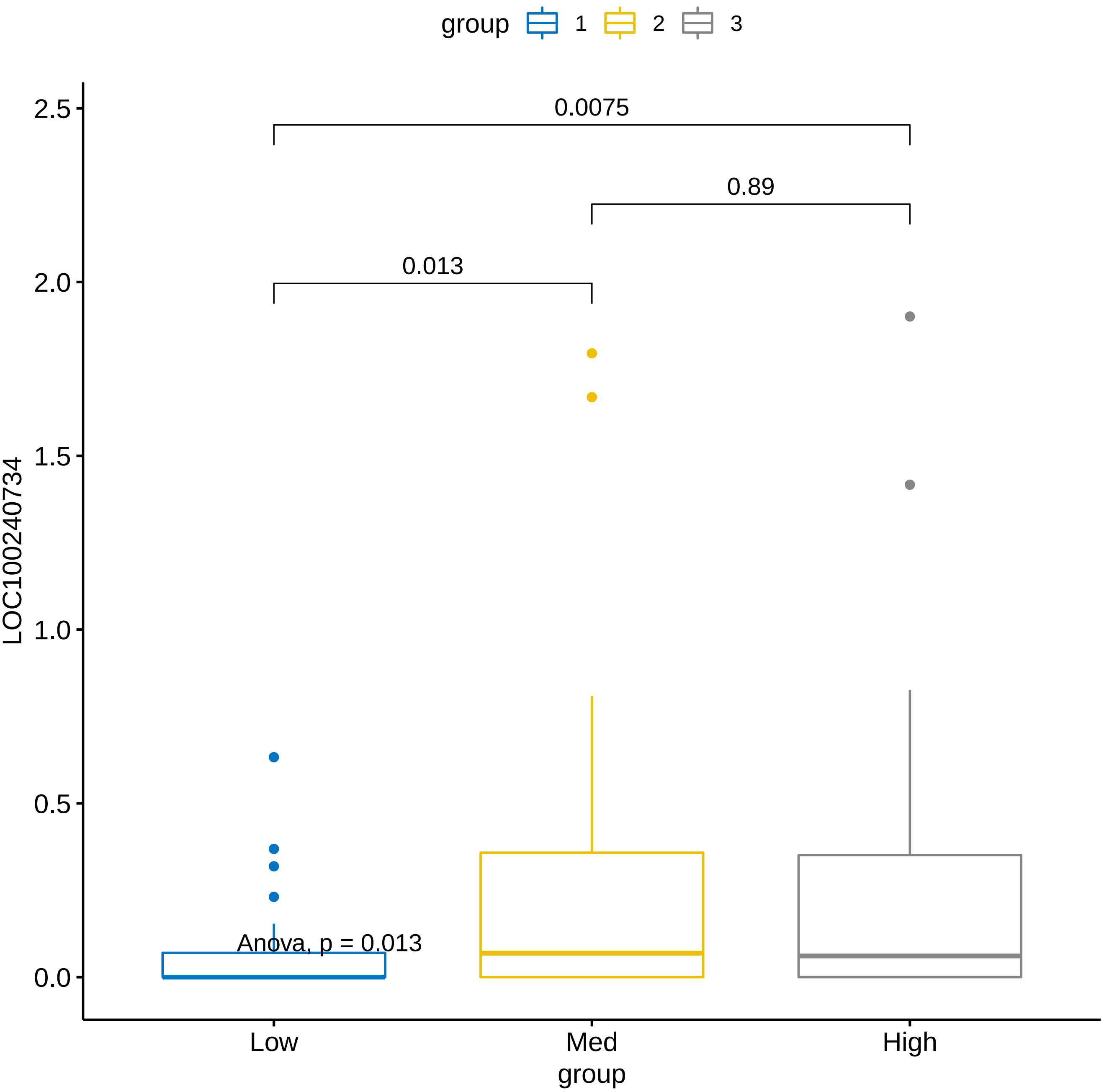
group
1
2
3
0.0075
0.89
0.013
Anova, p = 0.013
LOC100240734
2.5
2.0
1.5
1.0
0.5
0.0
Low
Med
High
group

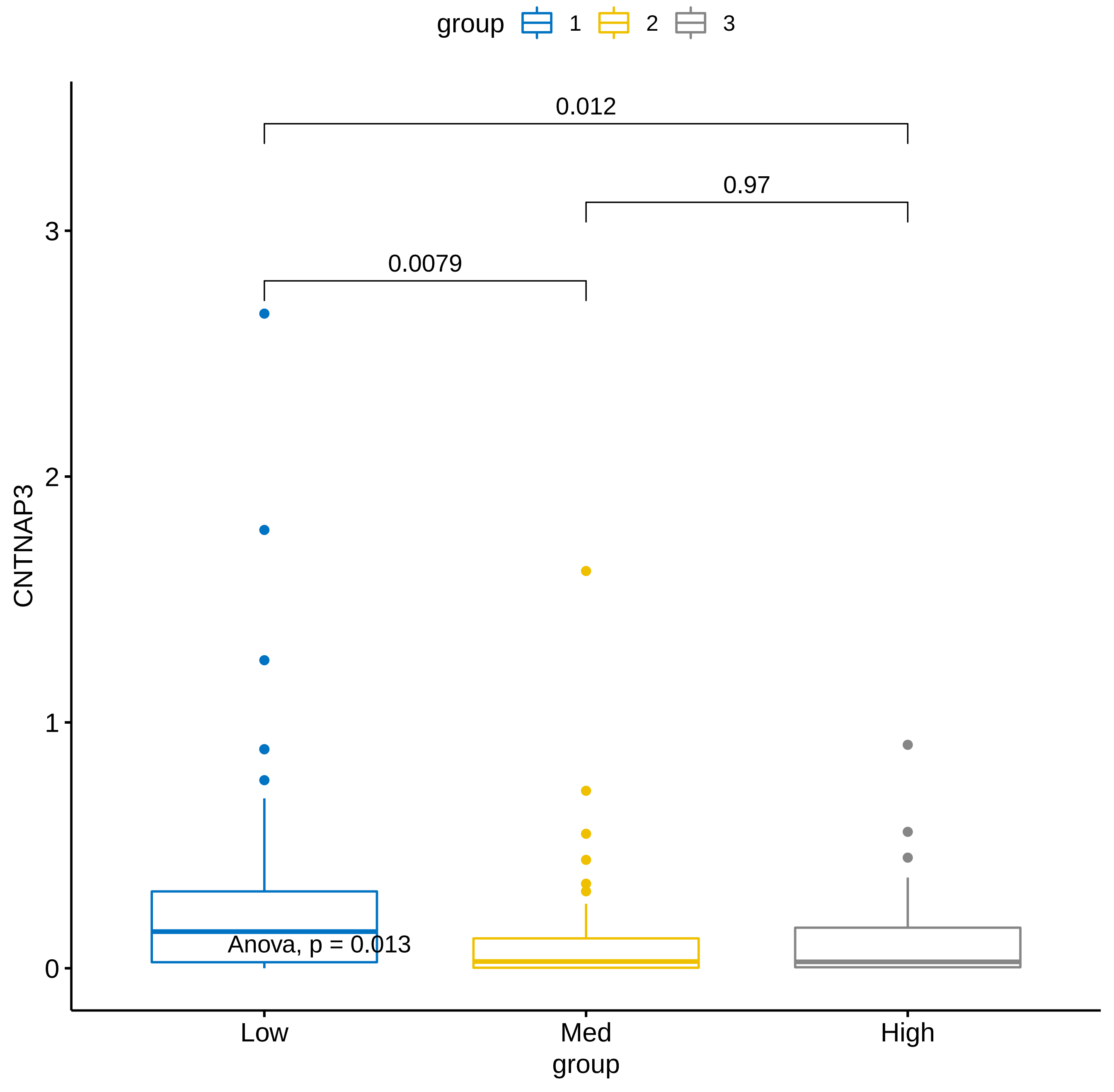

group 1 2 3
0.012
0.97
0.0079
CNTNAP3
3
2
1
0
Anova, p = 0.013
Low
Med
High
group

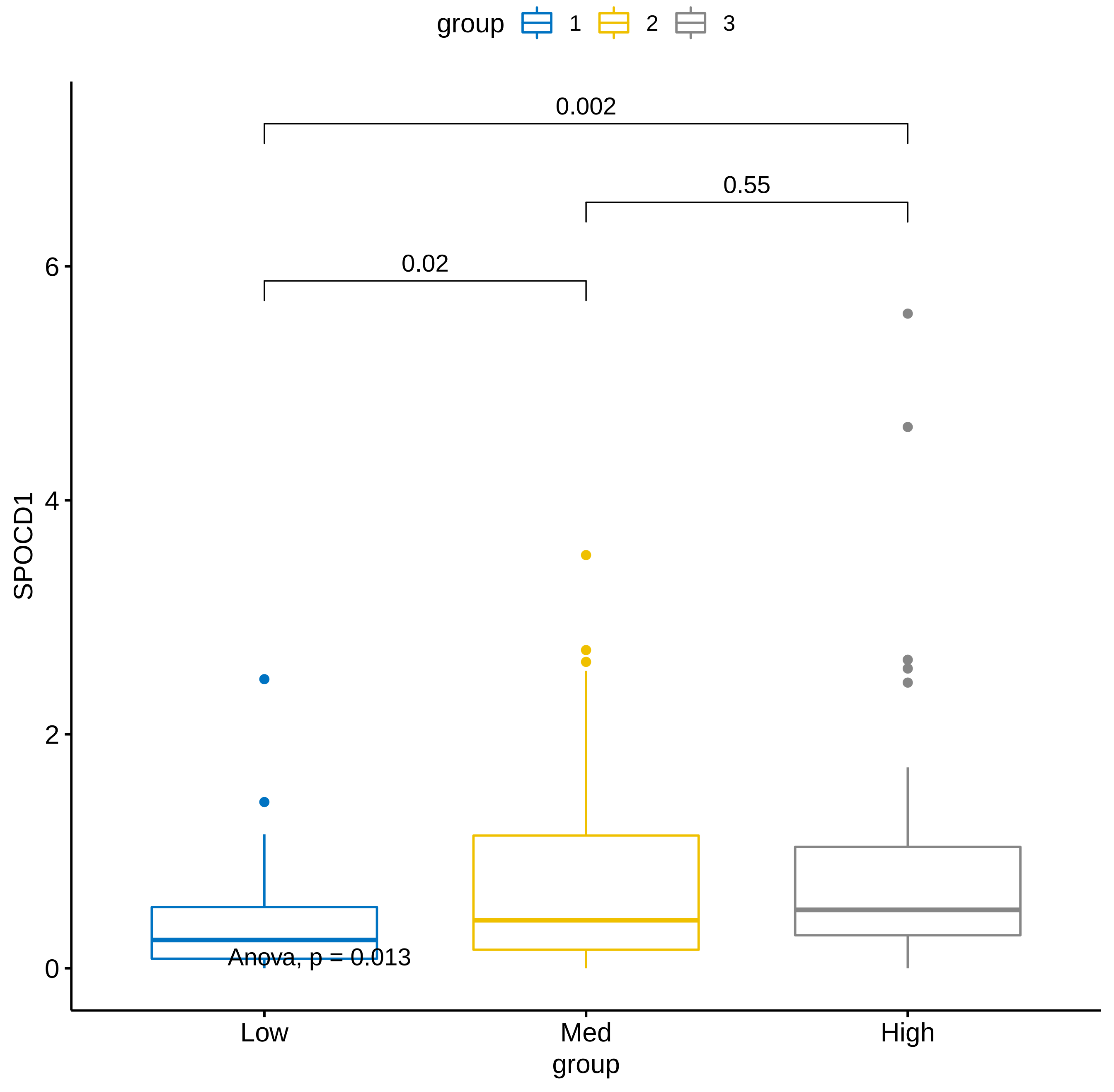
group 1 2 3
0.002
0.55
0.02
6
4
2
0
SPOCD1
Anova, p = 0.013
Low
Med
High
group

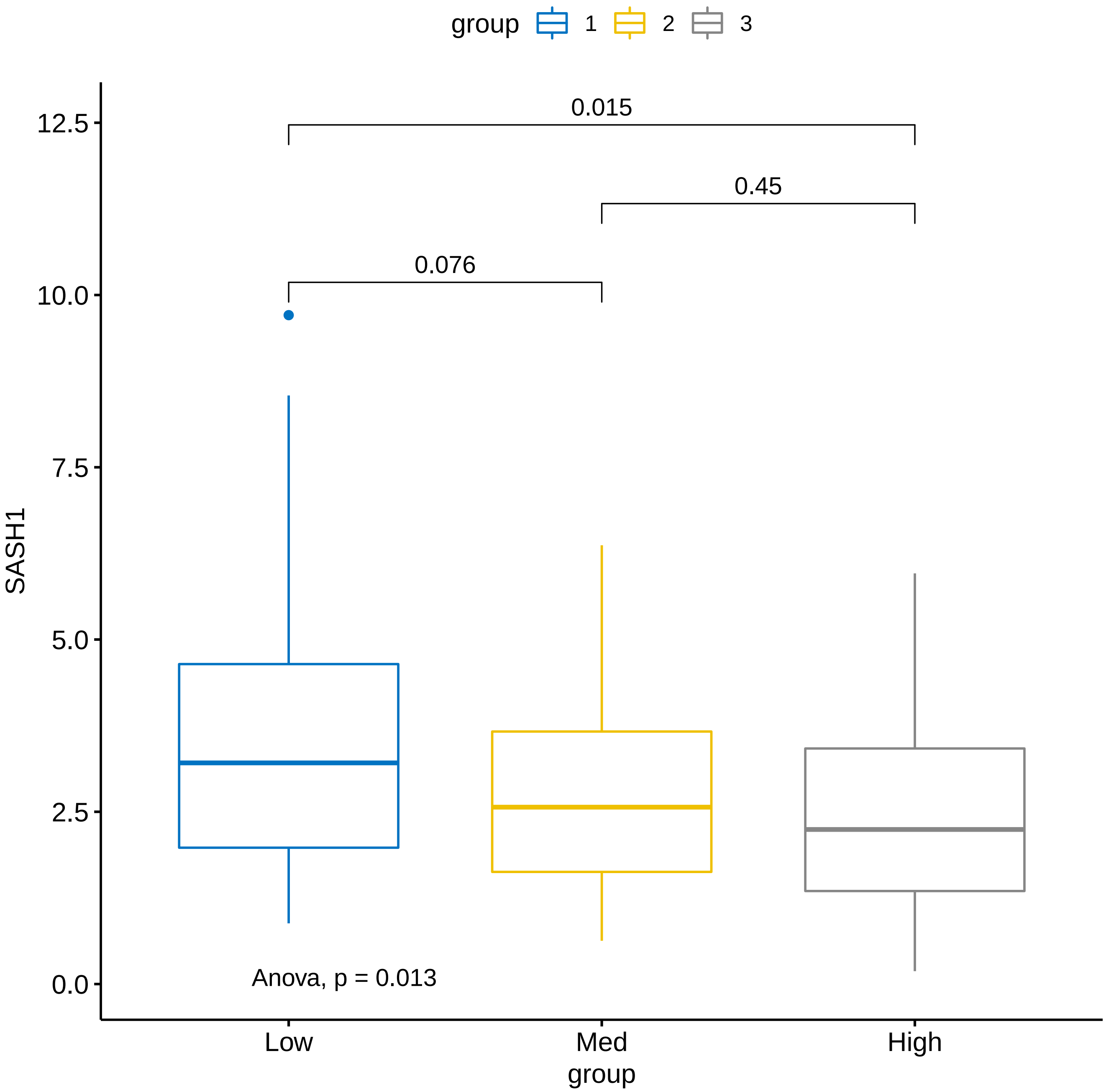
group
1
2
3
0.015
0.45
0.076
12.5
10.0
7.5
5.0
2.5
0.0
SASH1
Anova, p = 0.013
Low
Med
High
group

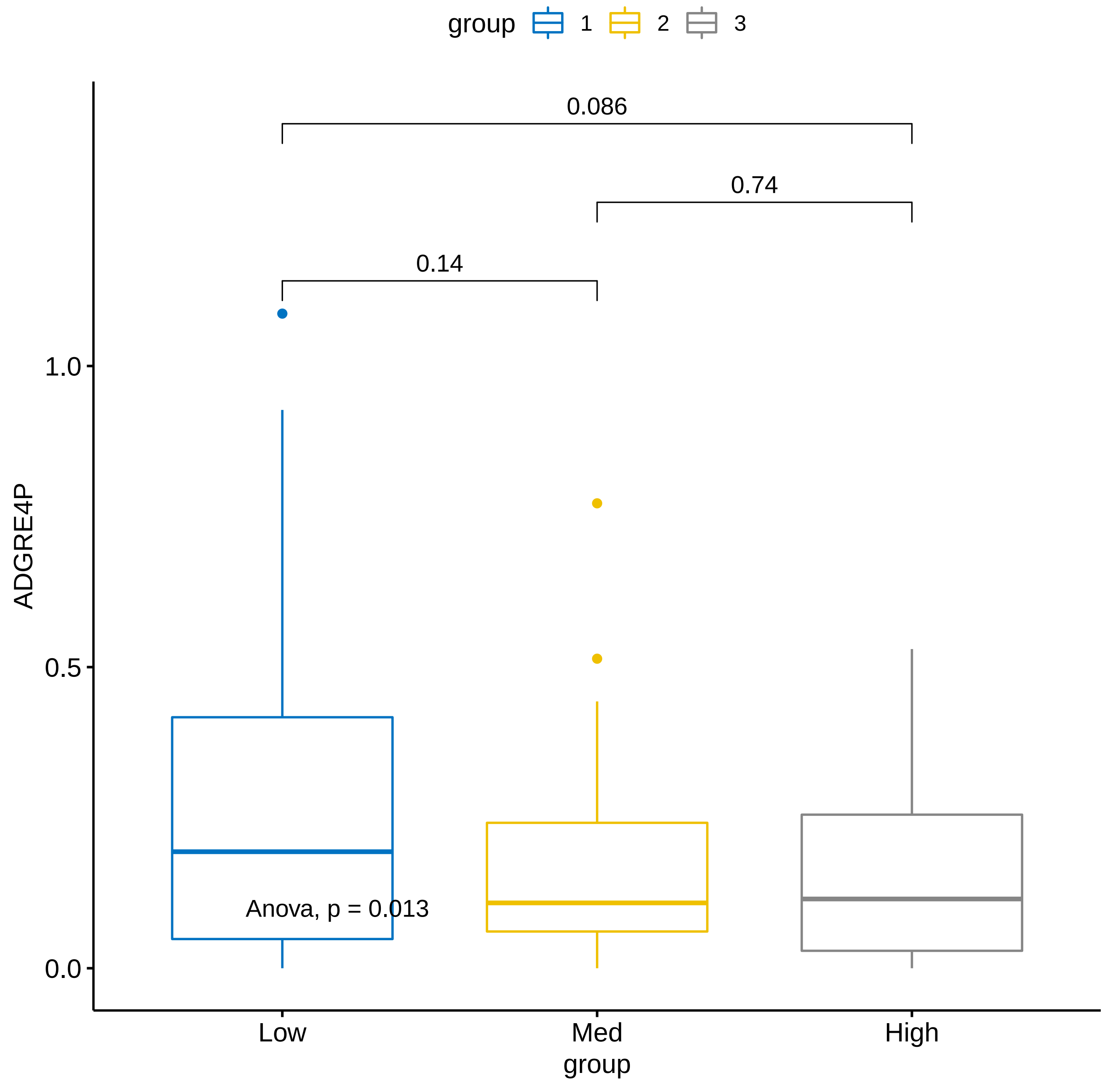

group 1 2 3
0.086
0.74
0.14
1.0
0.5
0.0
ADGRE4P
Anova, p = 0.013
Low
Med
High
group

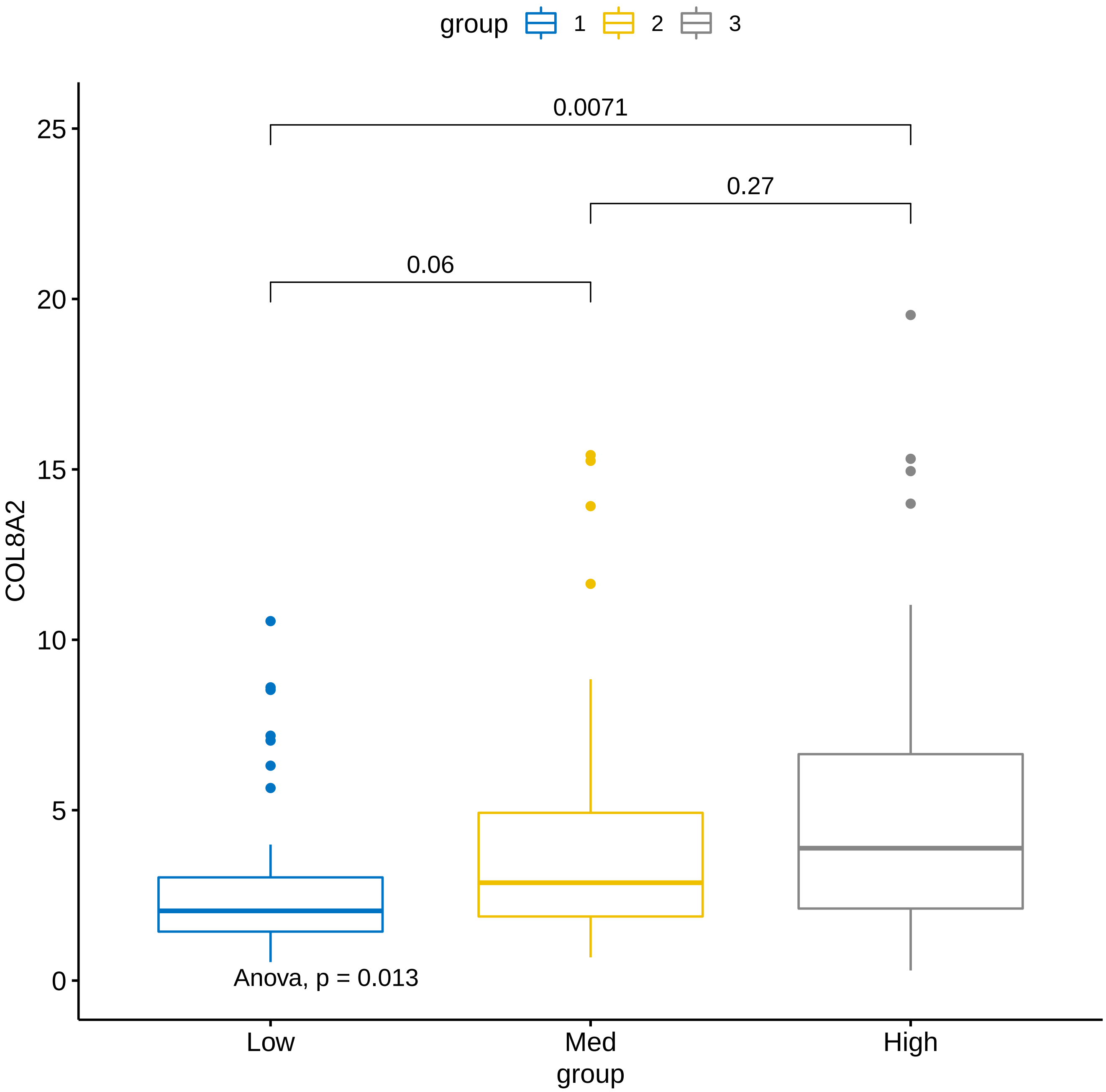

group 1 2 3
0.0071
0.27
0.06
25
20
15
10
5
0
COL8A2
Anova, p = 0.013
Low
Med
High
group

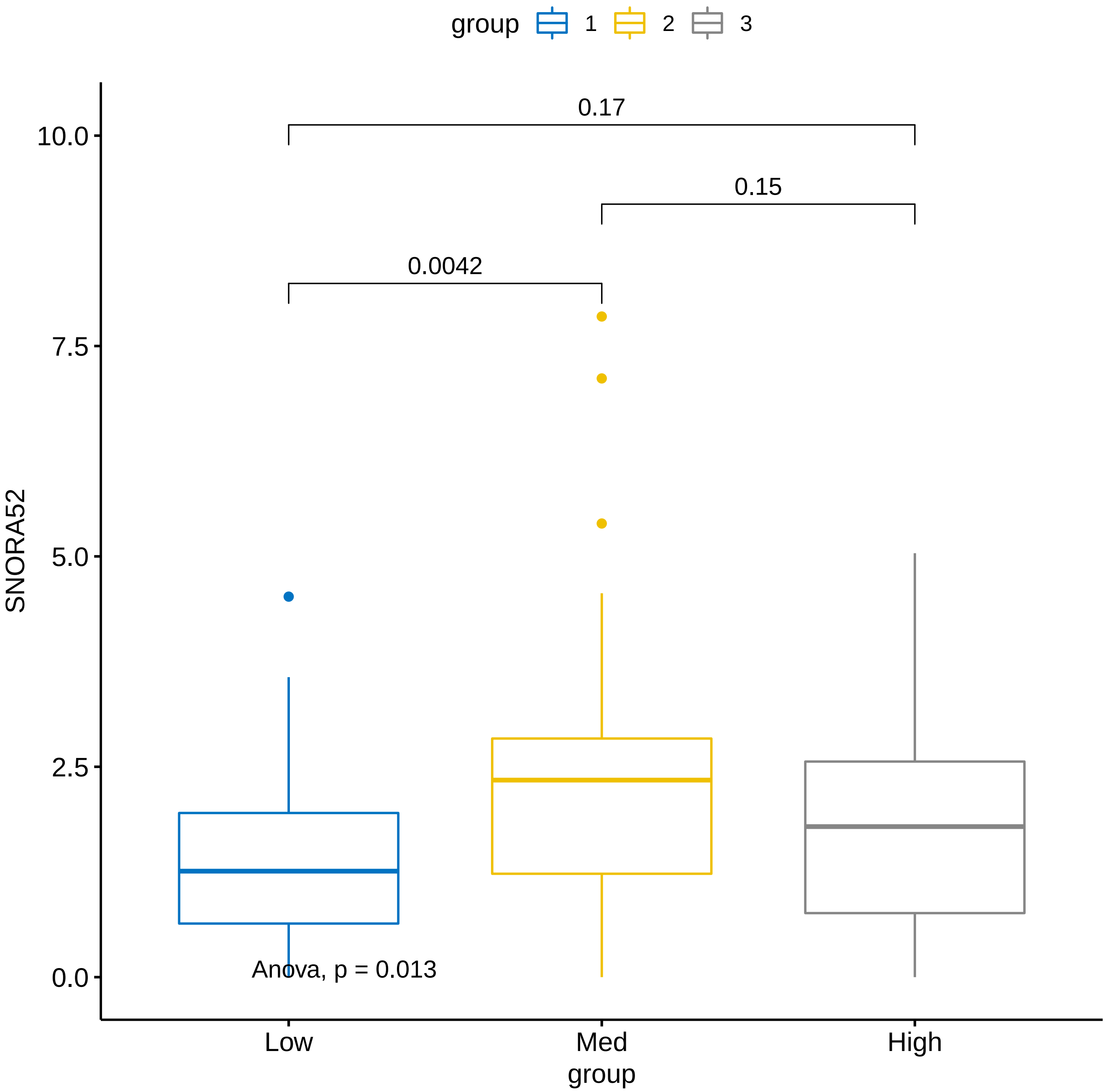

group
1
2
3
0.17
0.15
0.0042
10.0
7.5
5.0
2.5
0.0
SNORA52
Anova, p = 0.013
Low
Med
High
group

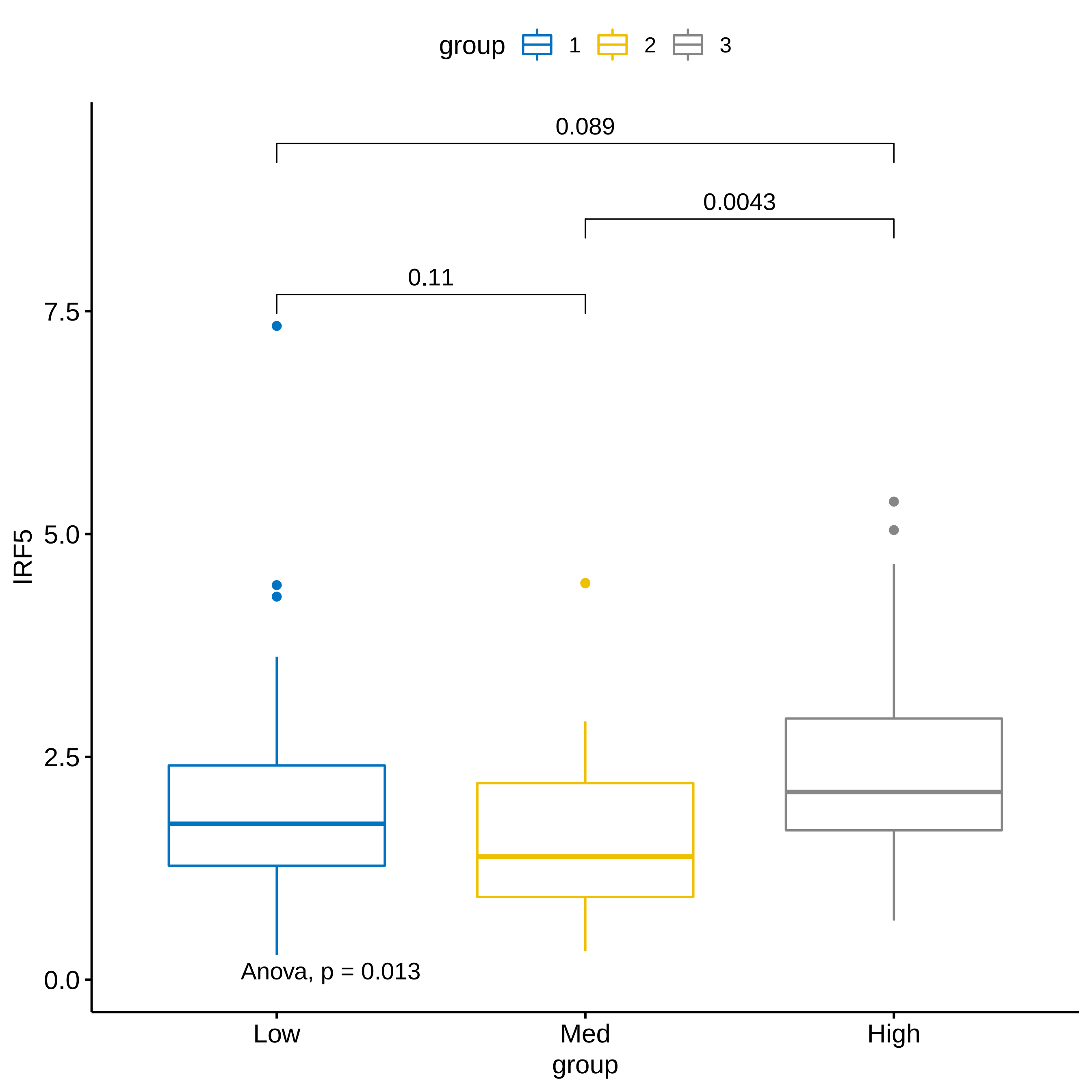

group 1 2 3
0.089
0.0043
0.11
7.5
5.0
2.5
0.0
IRF5
Anova, p = 0.013
Low
Med
High
group

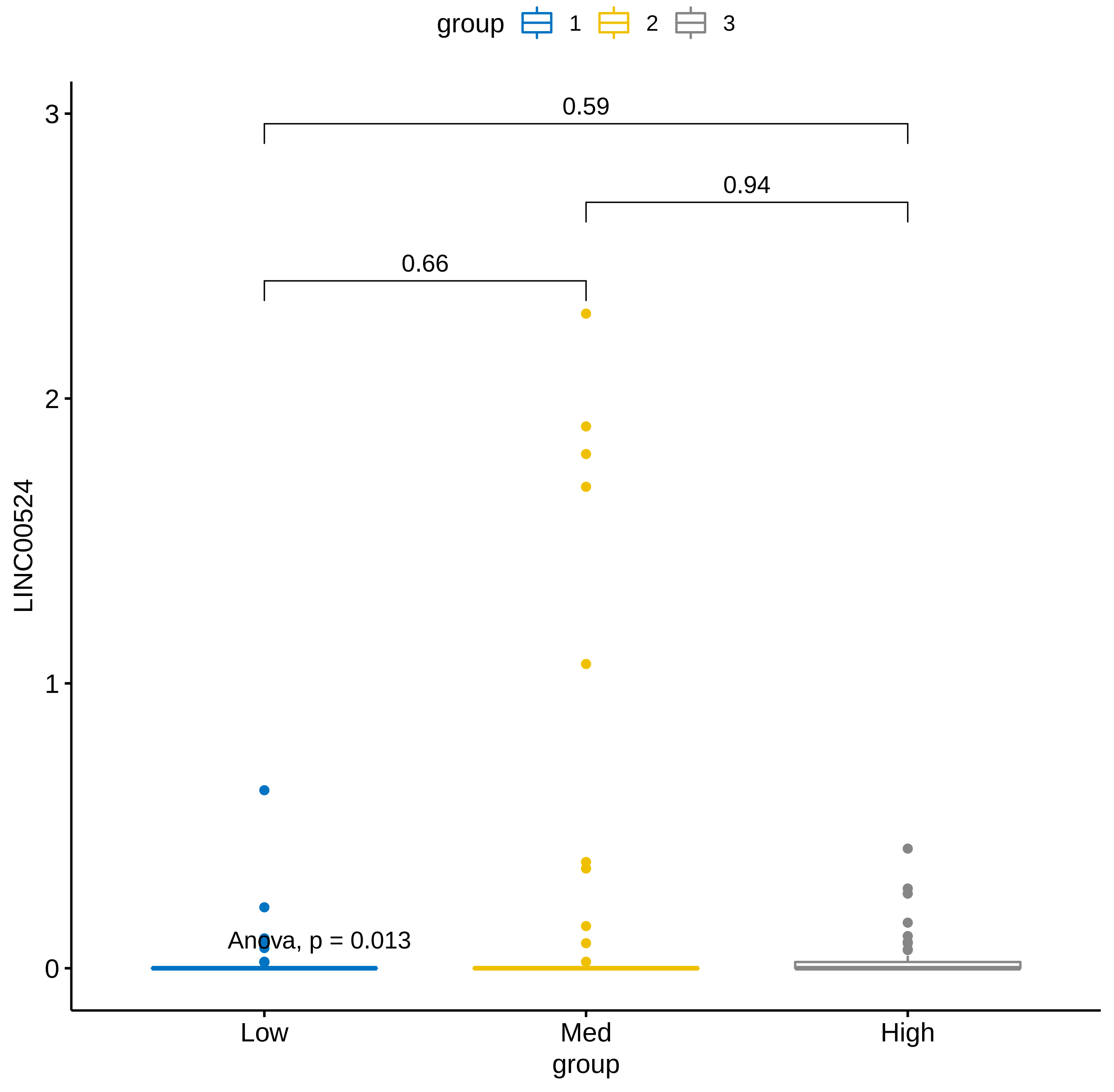

group 1 2 3
0.59
0.94
0.66
Anova, p = 0.013
LINC00524
3
2
1
0
Low
Med
High
group

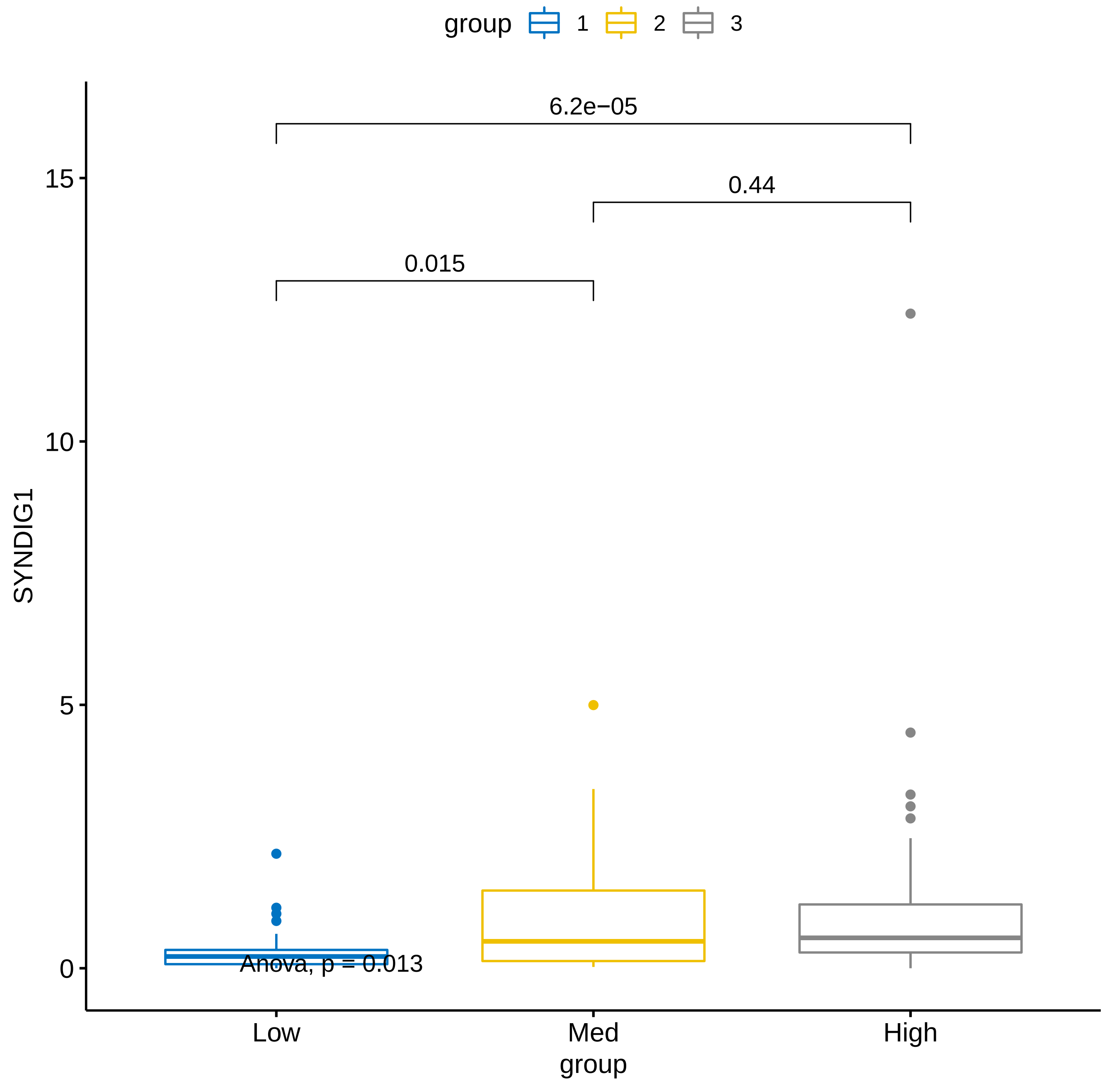
group
1
2
3
6.2e−05
0.44
0.015
15
10
5
0
SYNDIG1
Anova, p = 0.013
Low
Med
High
group

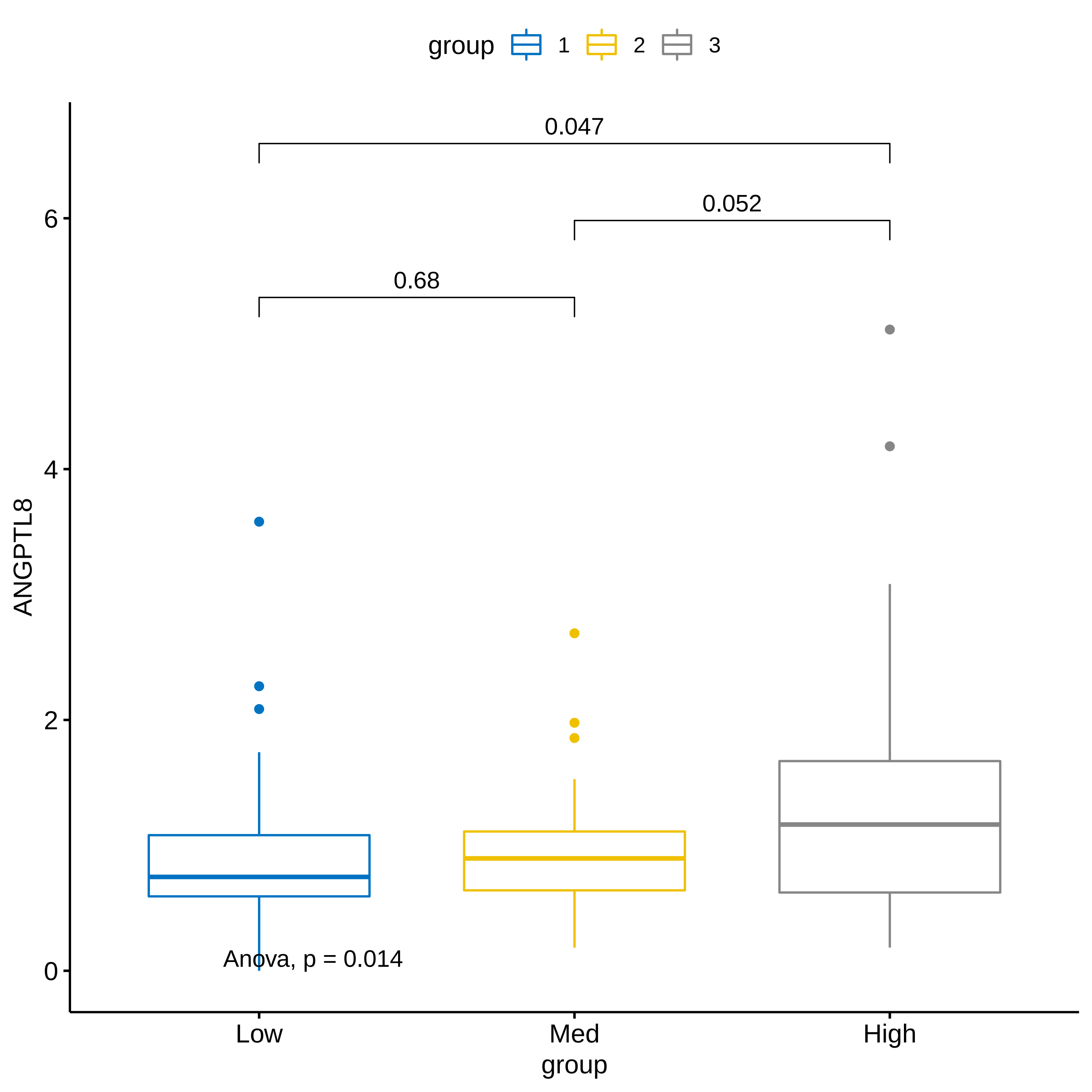
group
1
2
3
0.047
0.052
0.68
6
4
2
0
ANGPTL8
Anova, p = 0.014
Low
Med
High
group

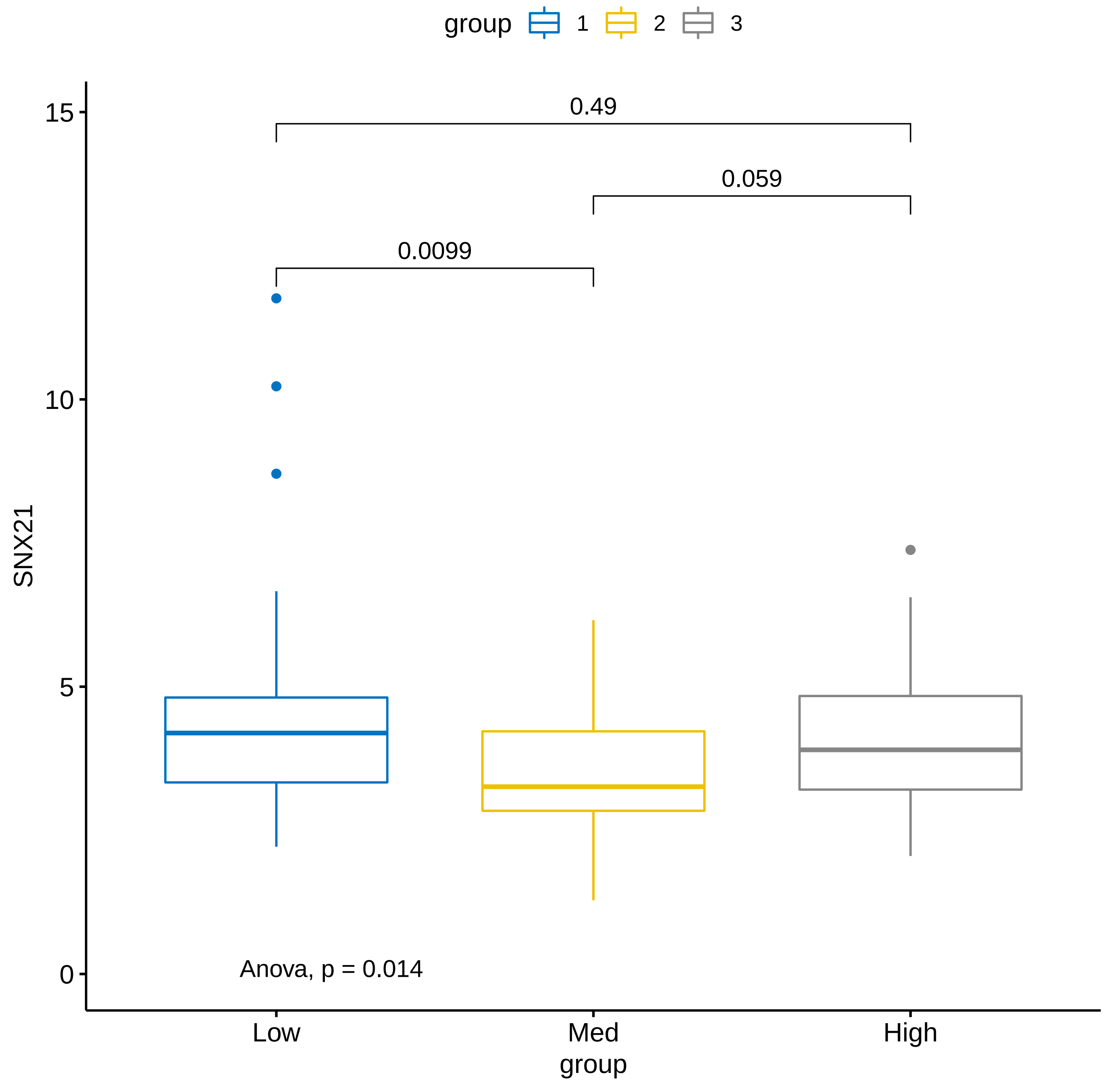
group
1
2
3
0.49
0.059
0.0099
15
10
5
0
SNX21
Anova, p = 0.014
Low
Med
High
group

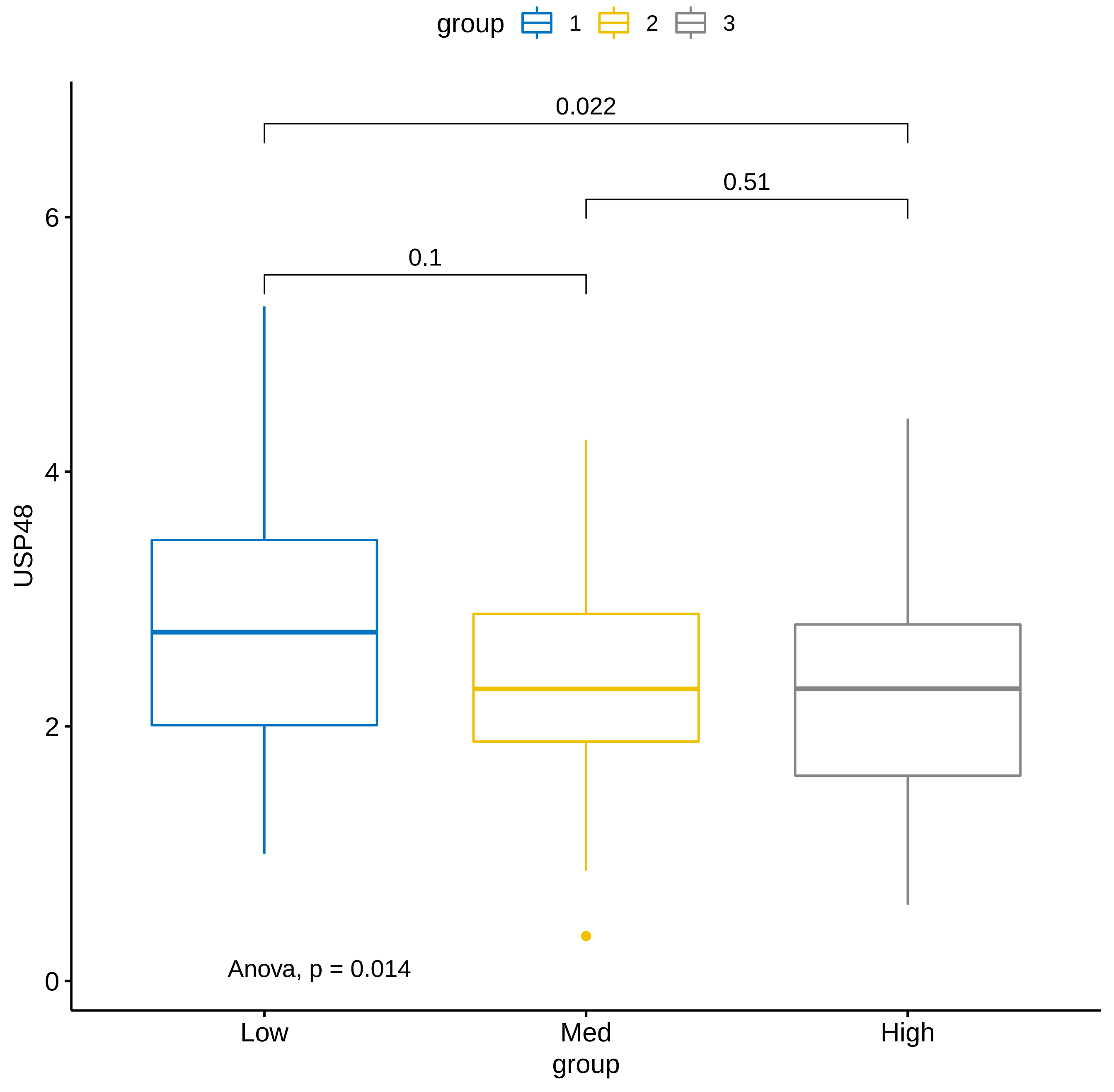
group
1
2
3
0.022
0.51
0.1
USP48
6
4
2
0
Anova, p = 0.014
Low
Med
High
group

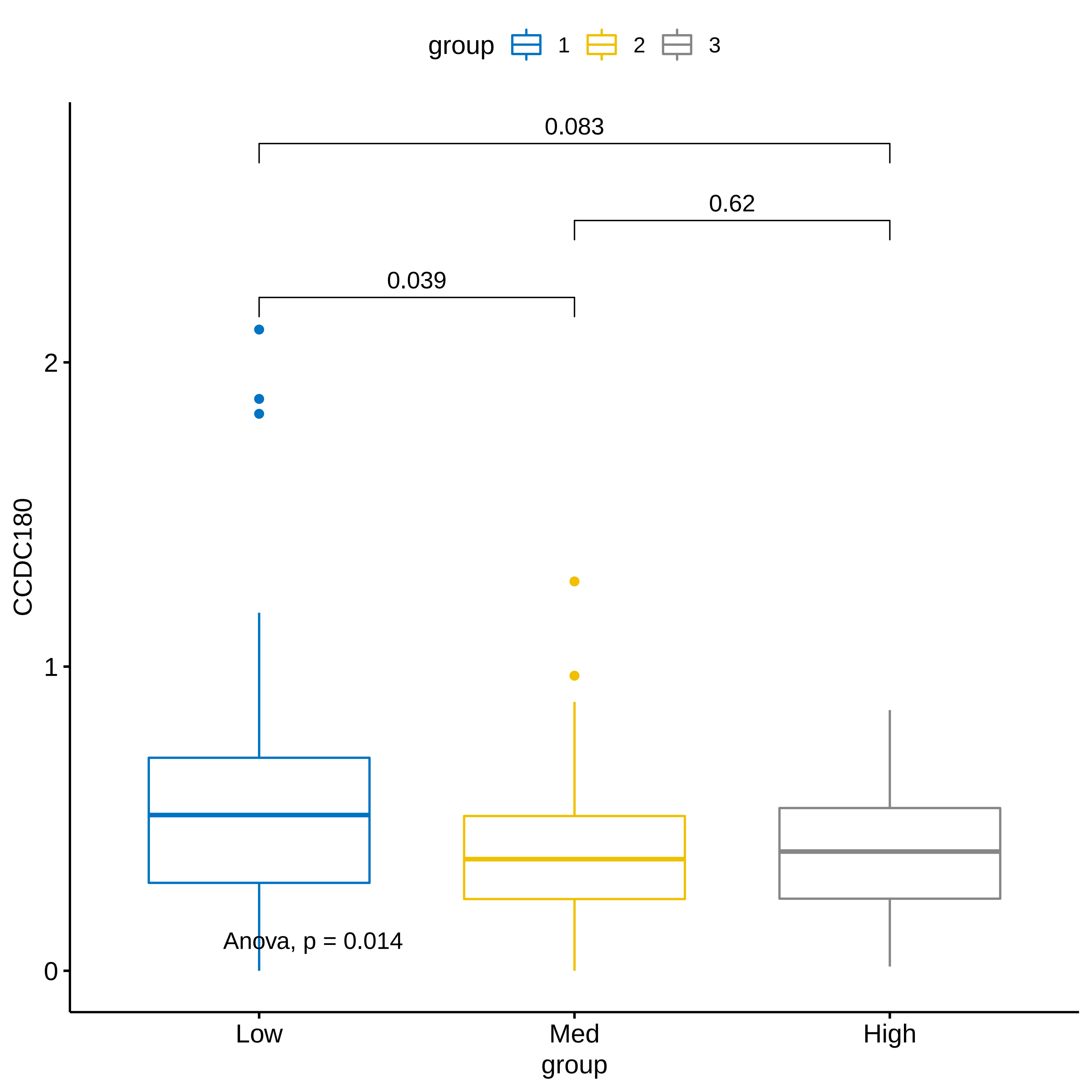
group
1
2
3
0.083
0.62
0.039
Anova, p = 0.014
CCDC180
0
1
2
Low
Med
High
group

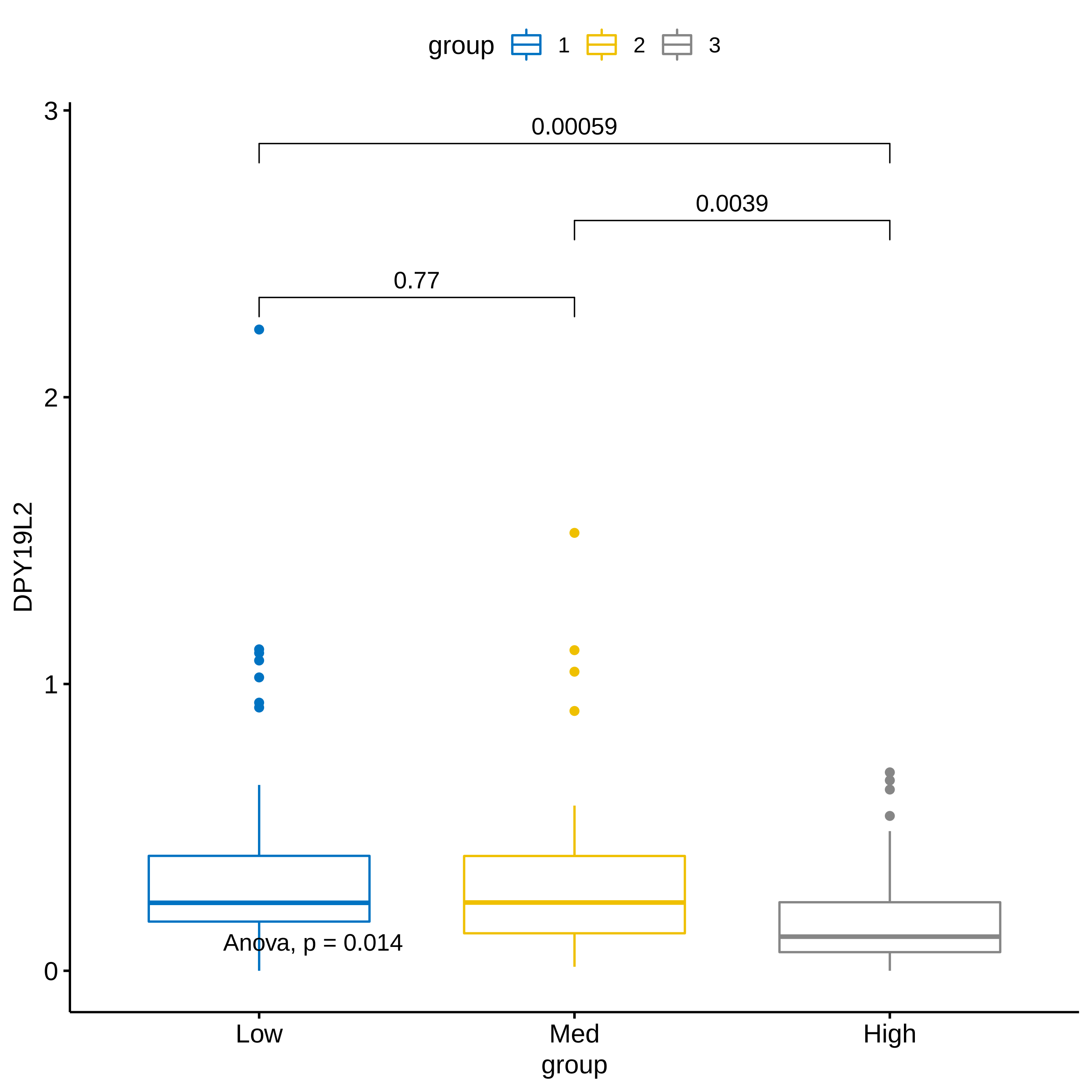
group
1
2
3
0.00059
0.0039
0.77
DPY19L2
3
2
1
0
Anova, p = 0.014
Low
Med
High
group

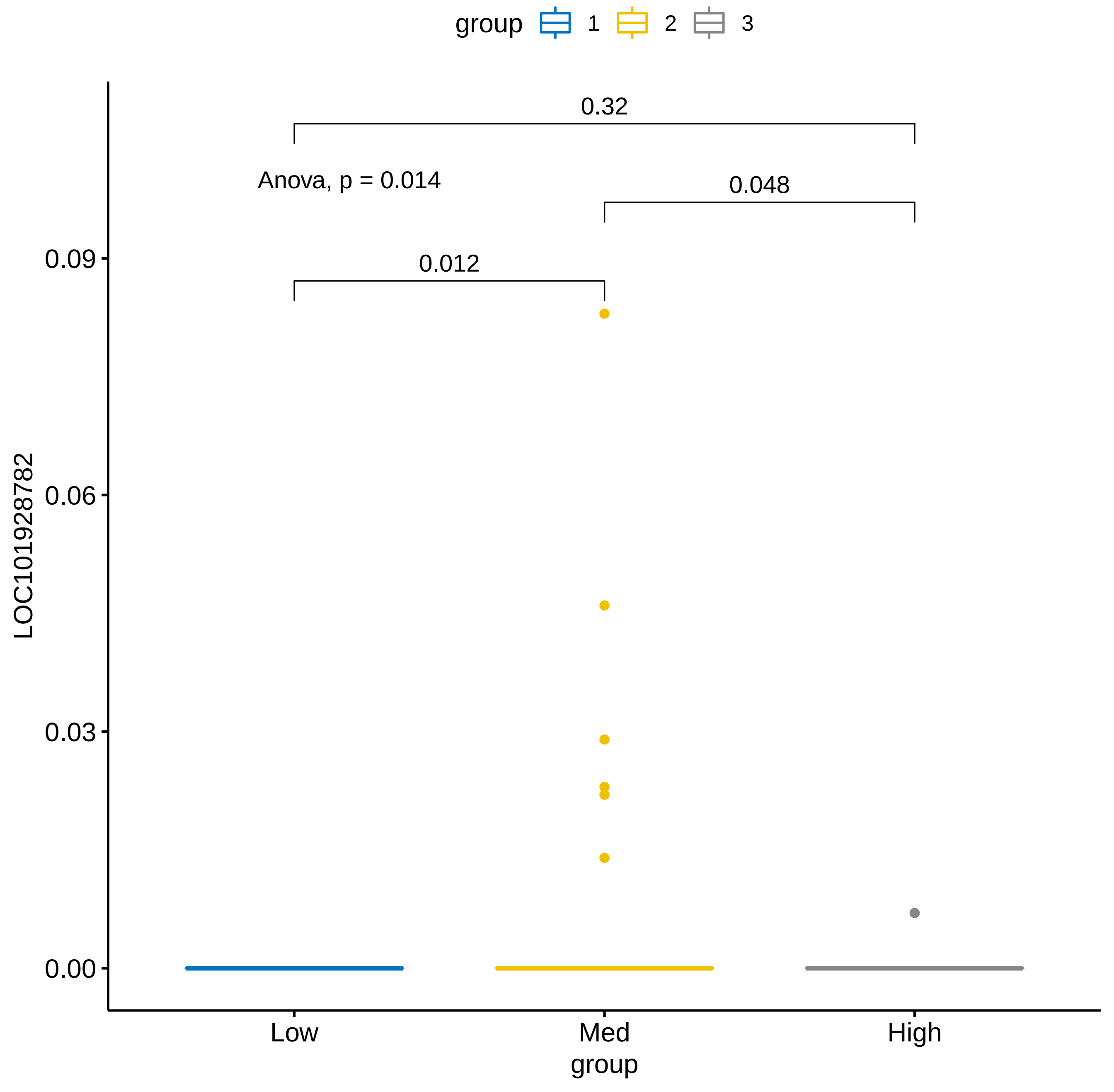

group 1 2 3
0.32
Anova, p = 0.014
0.048
0.012
LOC101928782
0.09
0.06
0.03
0.00
Low
Med
High
group

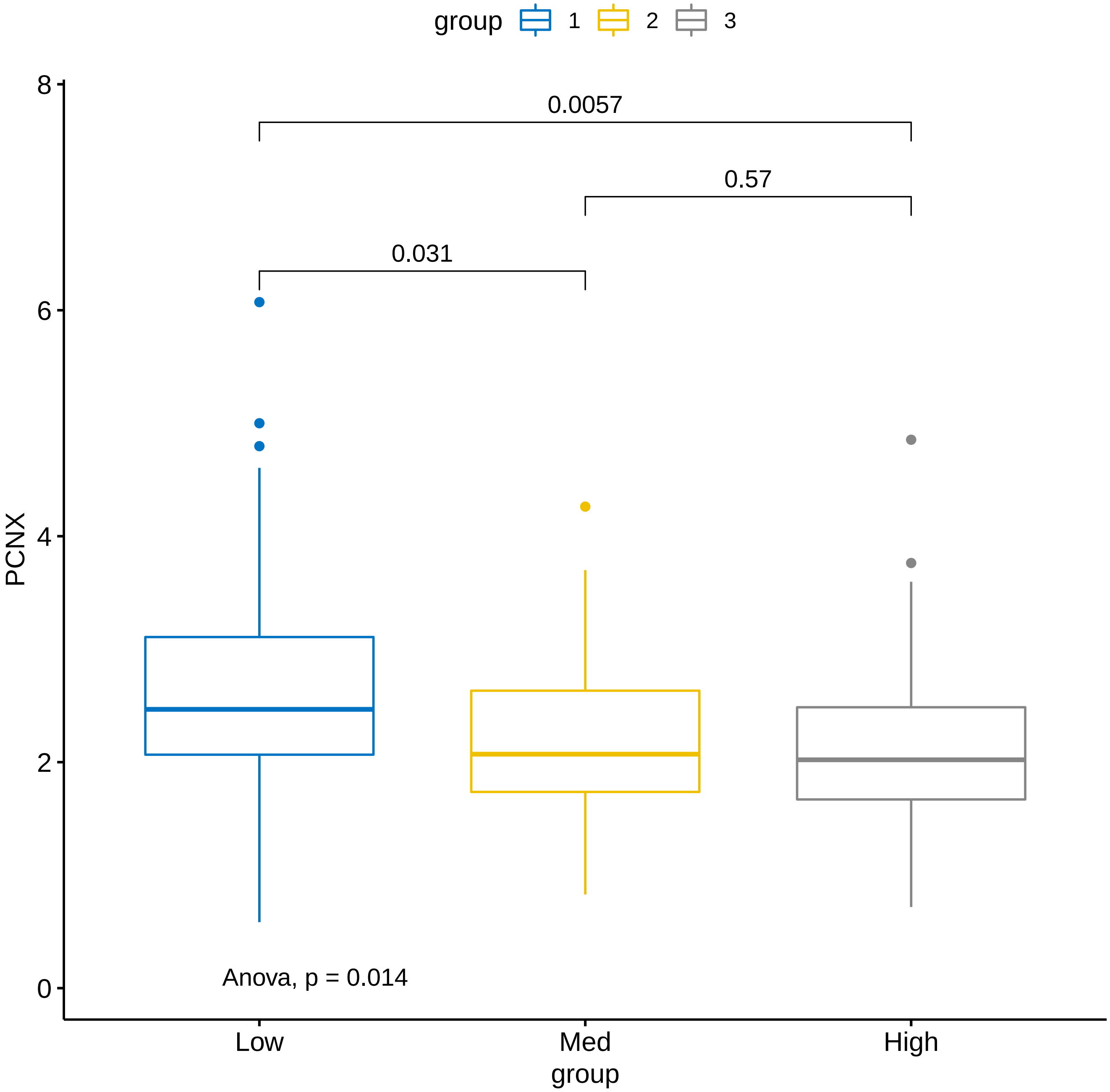

group 1 2 3
8
6
4
2
0
PCNX
0.0057
0.57
0.031
Anova, p = 0.014
Low
Med
High
group

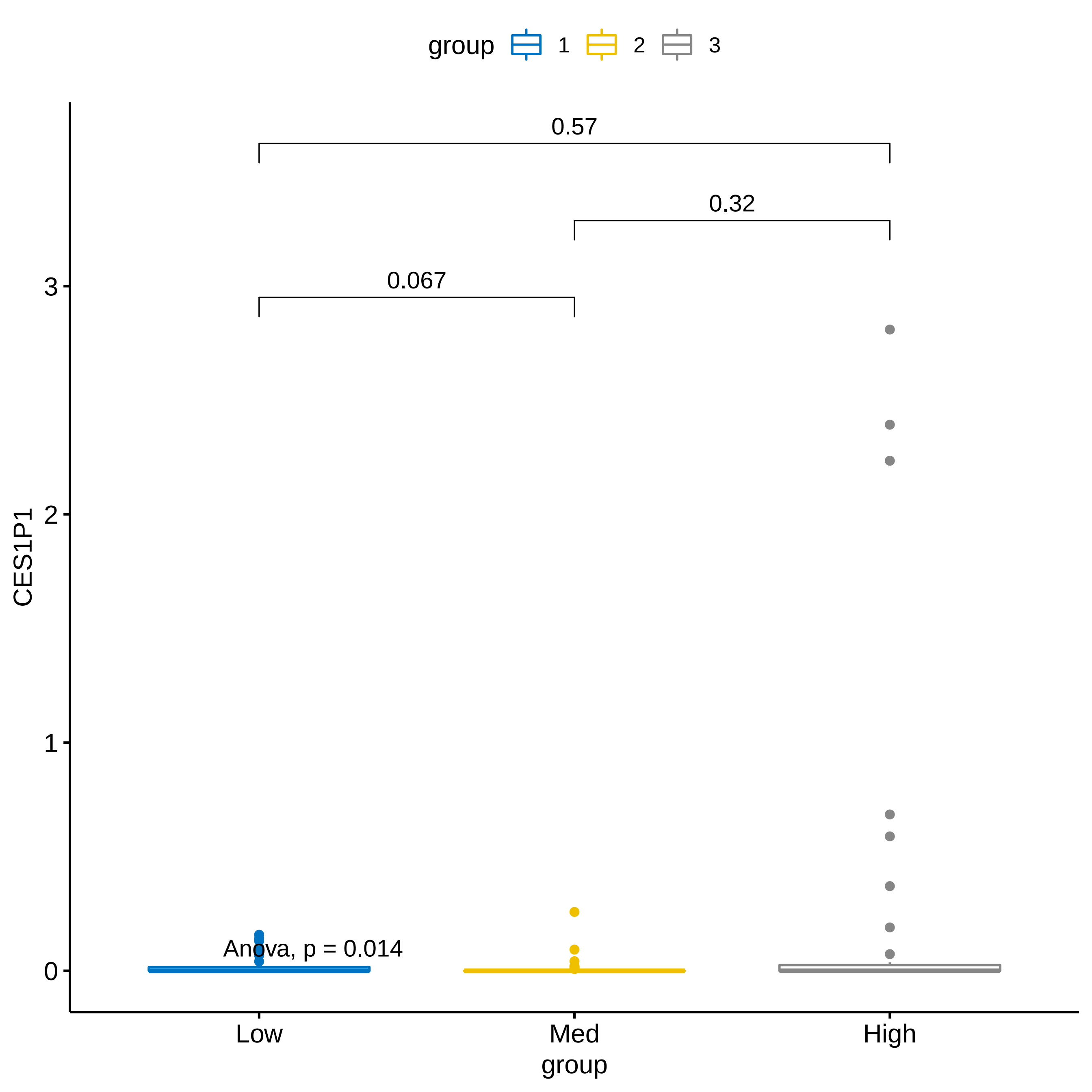

group
1
2
3
0.57
0.32
0.067
Anova, p = 0.014
CES1P1
3
2
1
0
Low
Med
High
group

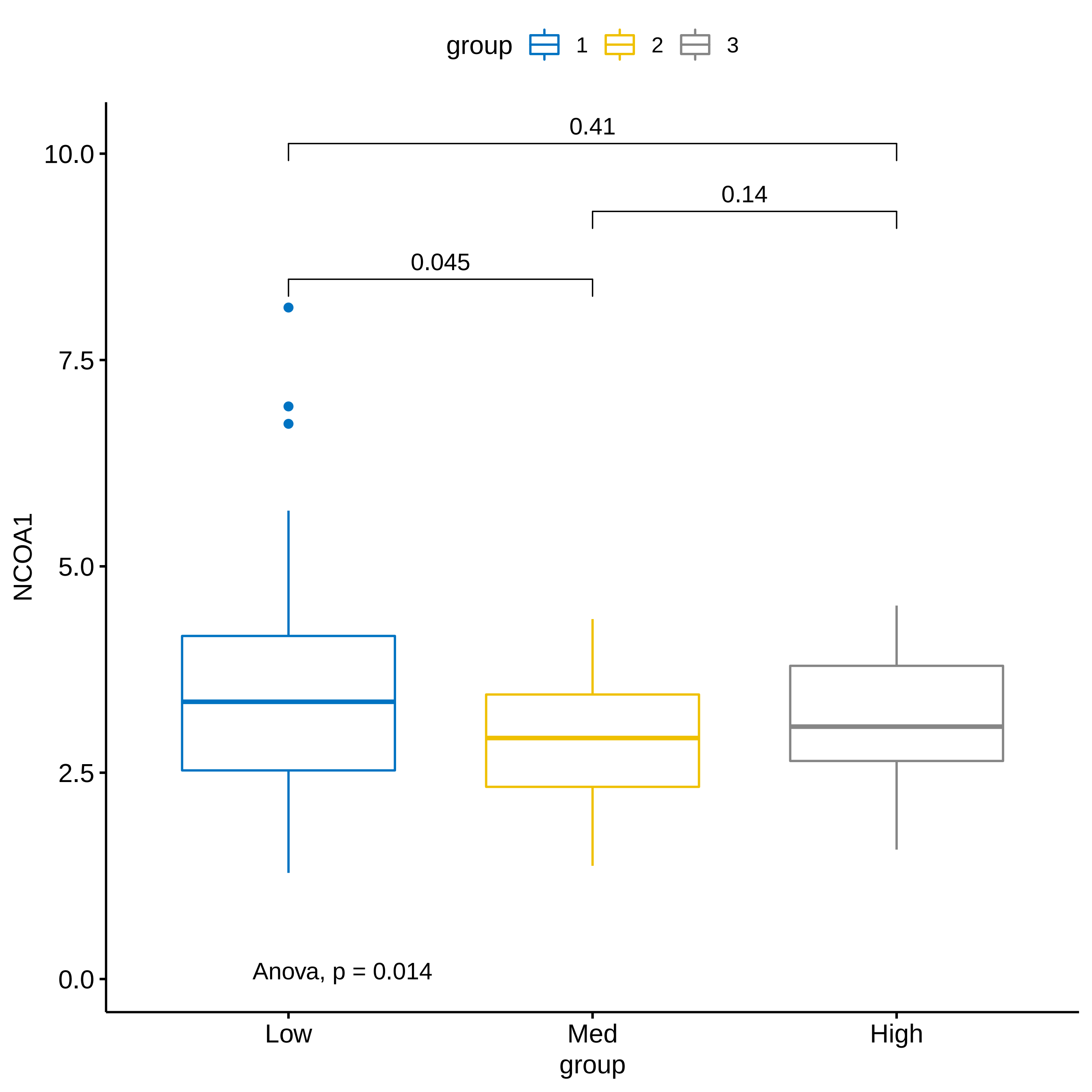
group
1
2
3
0.41
0.14
0.045
10.0
7.5
5.0
2.5
0.0
NCOA1
Anova, p = 0.014
Low
Med
High
group

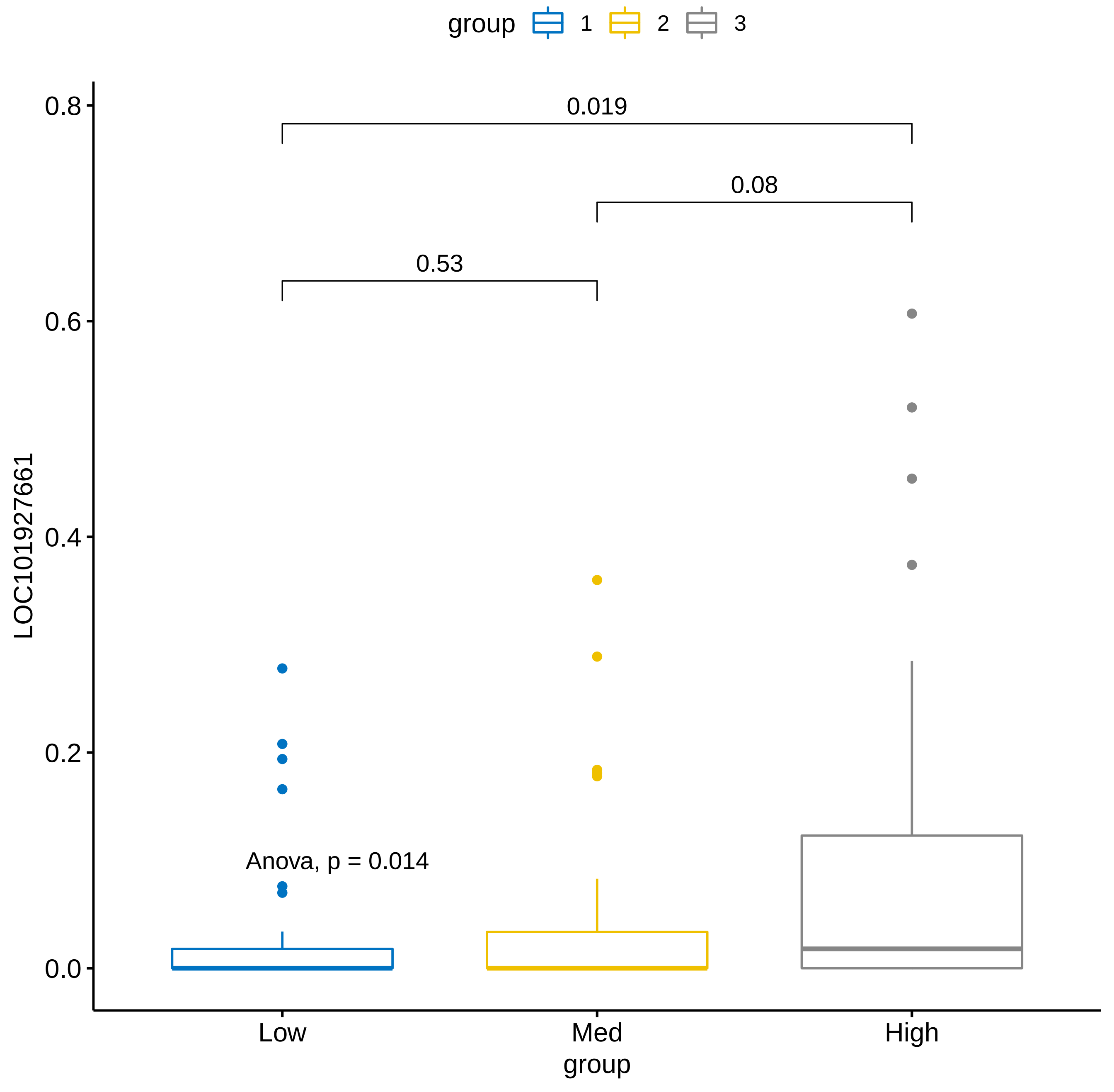

group
1
2
3
0.8
0.6
0.4
0.2
0.0
LOC101927661
0.019
0.08
0.53
Anova, p = 0.014
Low
Med
High
group

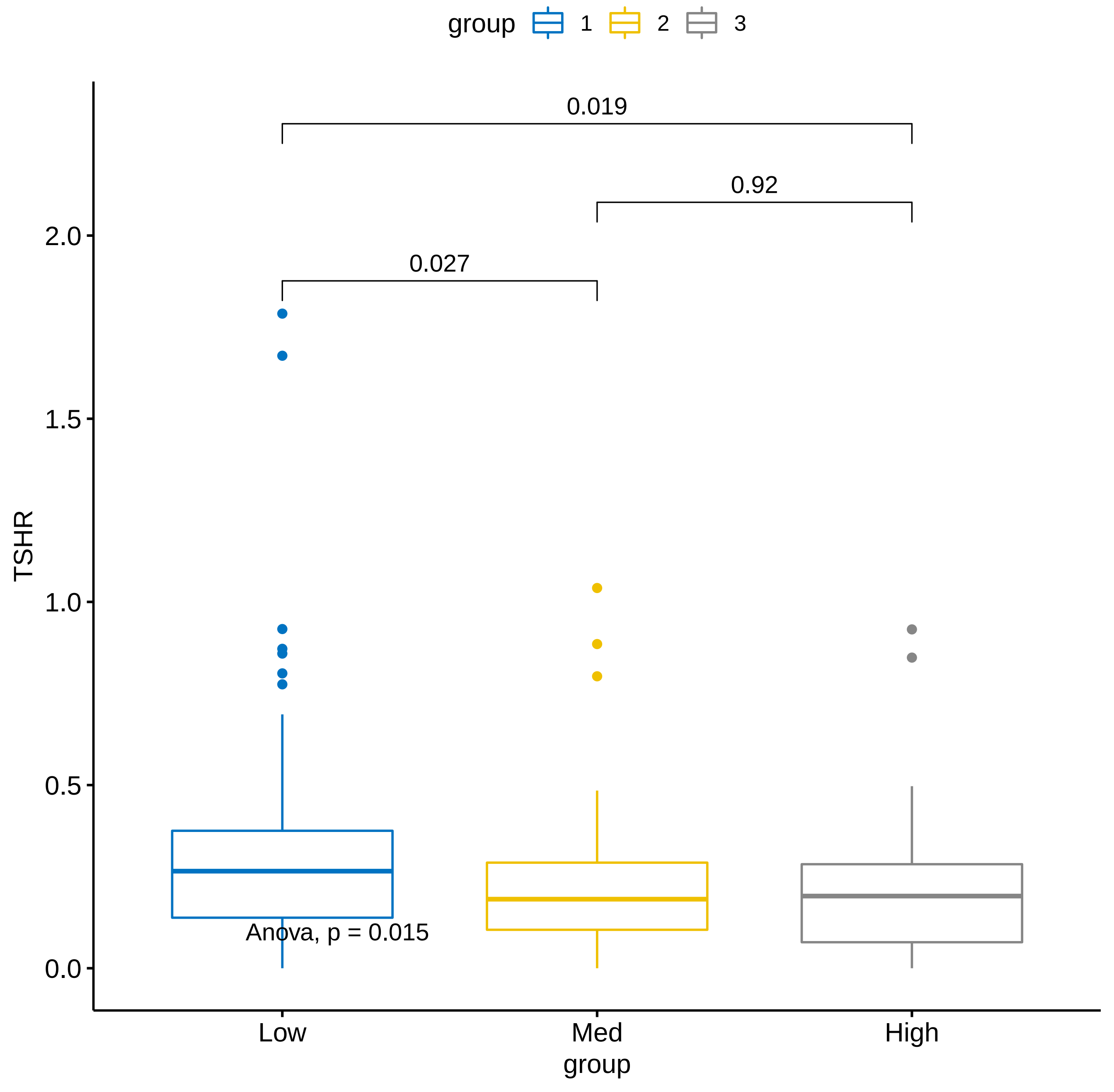
group
1
2
3
0.019
0.92
0.027
2.0
1.5
1.0
0.5
0.0
TSHR
Anova, p = 0.015
Low
Med
High
group

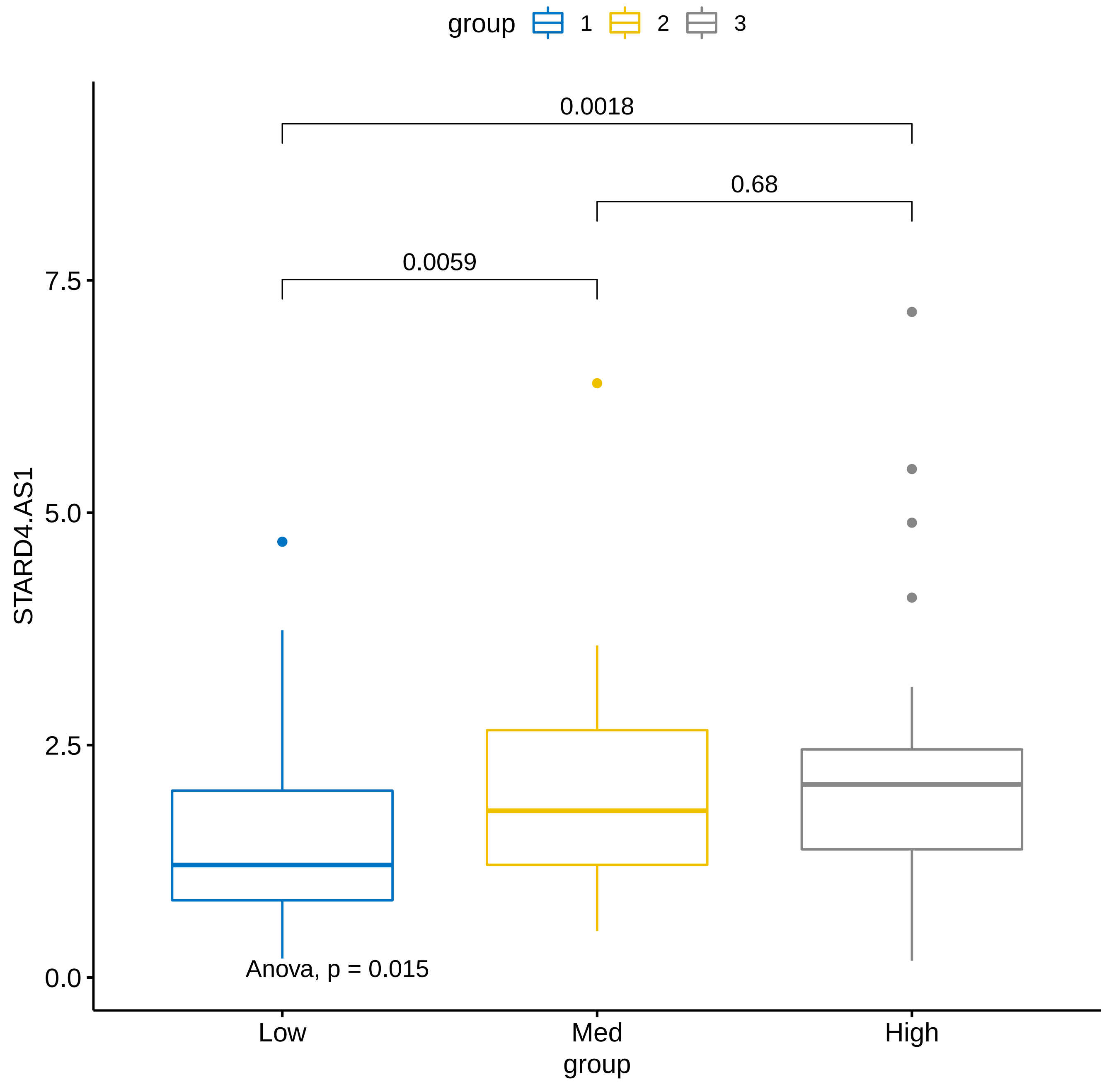
group
1
2
3
0.0018
0.68
0.0059
7.5
5.0
2.5
0.0
STARD4.AS1
Anova, p = 0.015
Low
Med
High
group

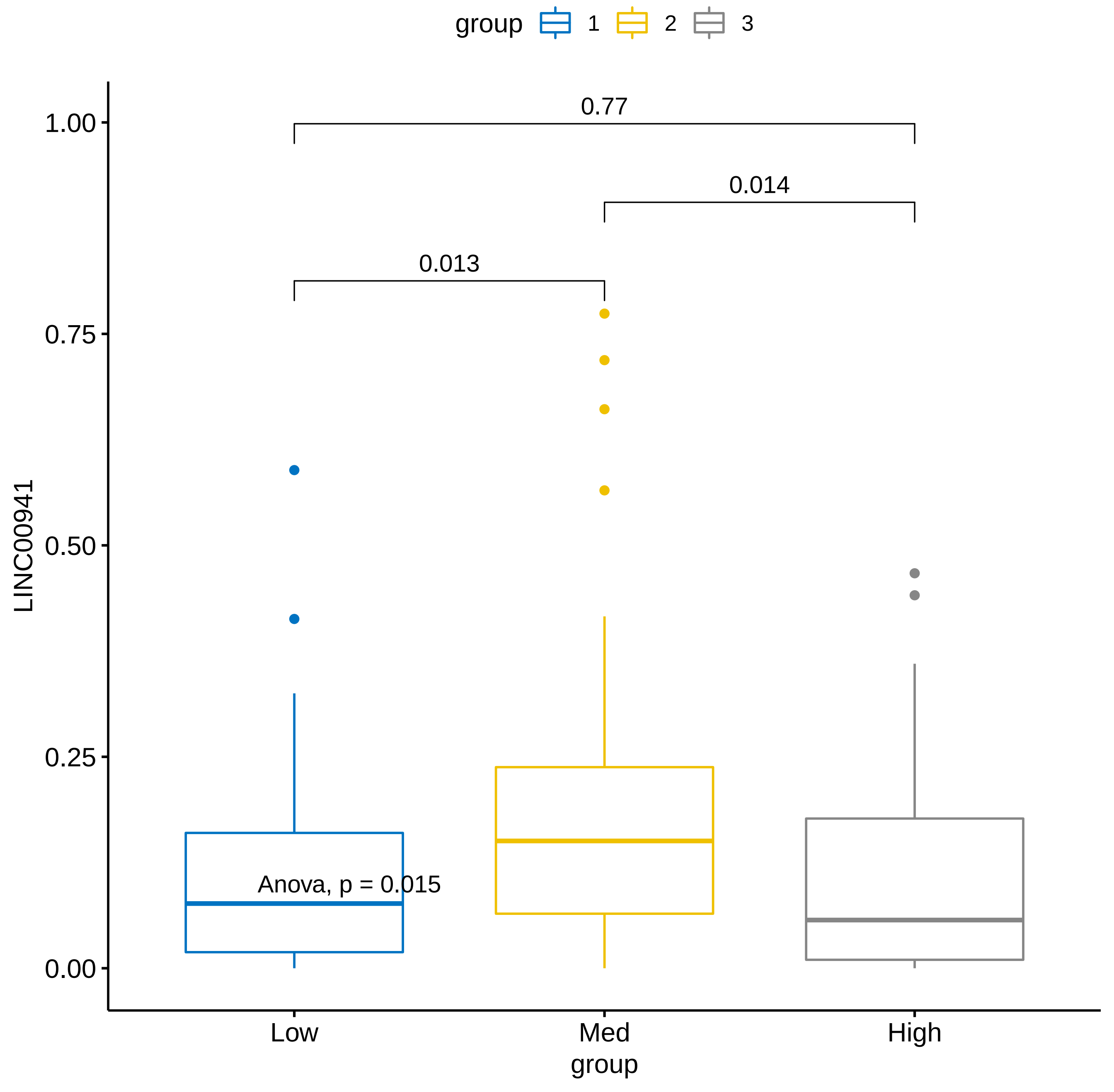
group
1
2
3
0.77
0.014
0.013
1.00
0.75
0.50
0.25
0.00
LINC00941
Anova, p = 0.015
Low
Med
High
group

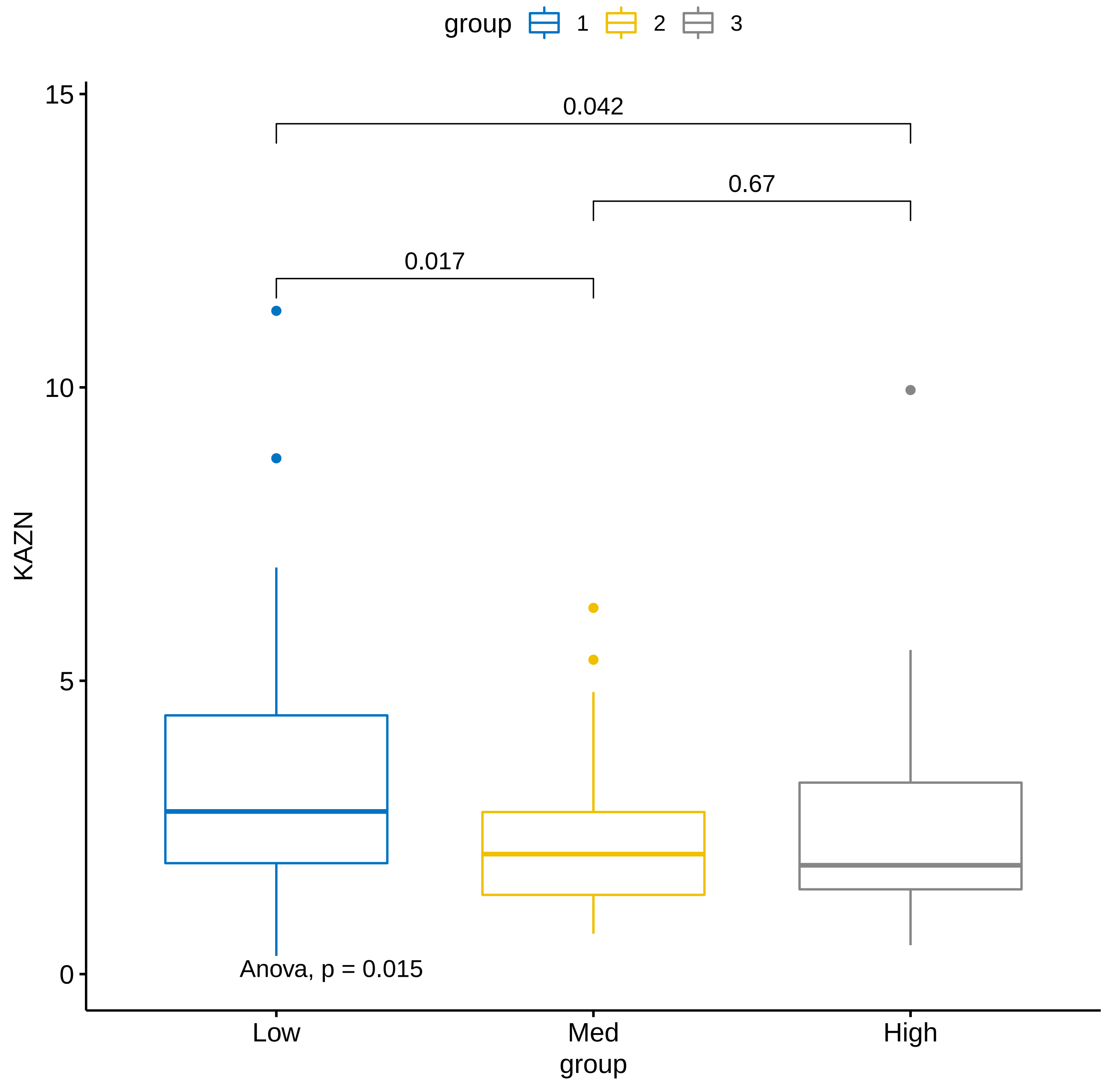
group
1
2
3
0.042
0.67
0.017
15
10
5
0
KAZN
Anova, p = 0.015
Low
Med
High
group

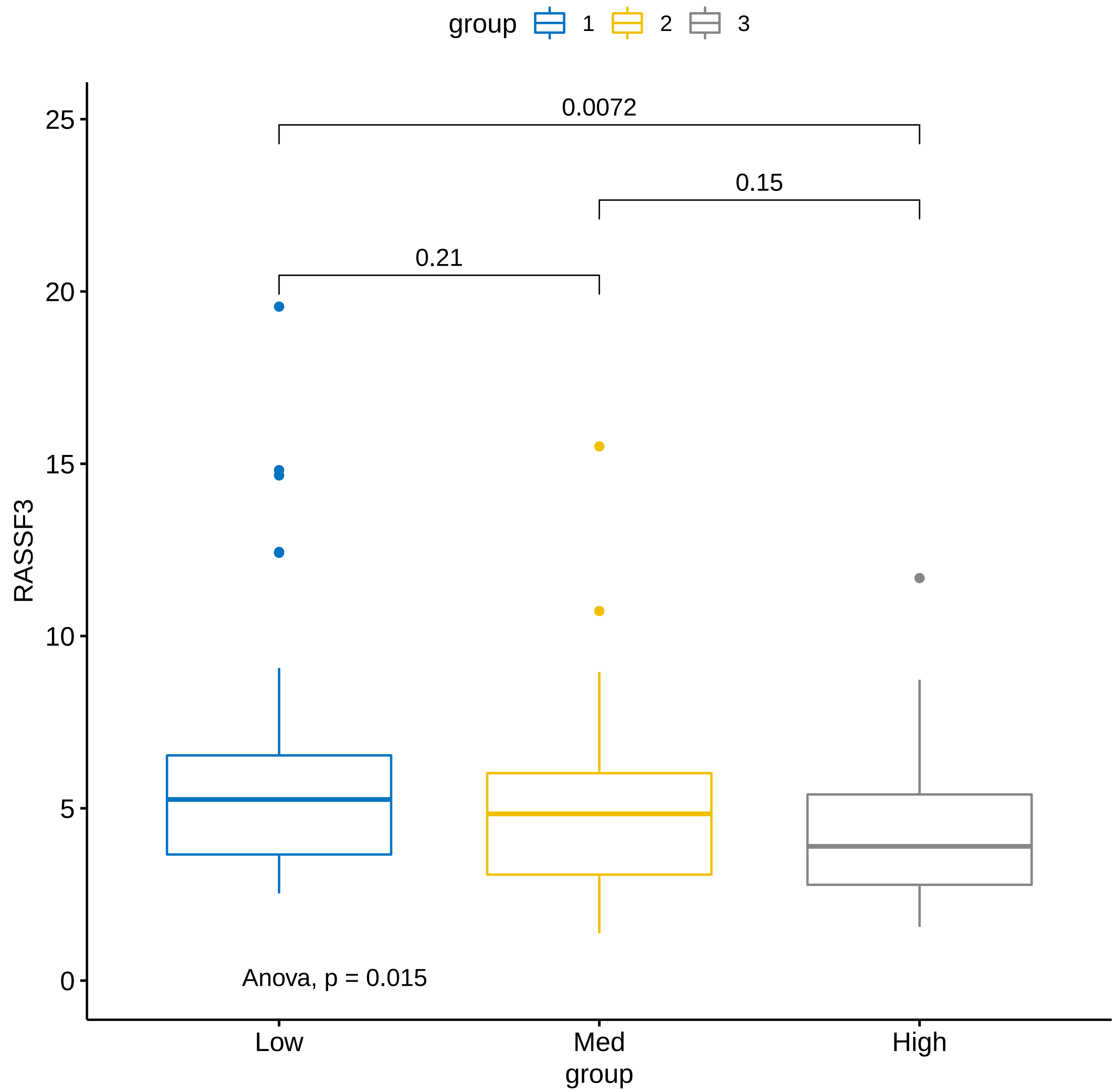

group 1 2 3
0.0072
0.15
0.21
RASSF3
25
20
15
10
5
0
Anova, p = 0.015
Low
Med
High
group

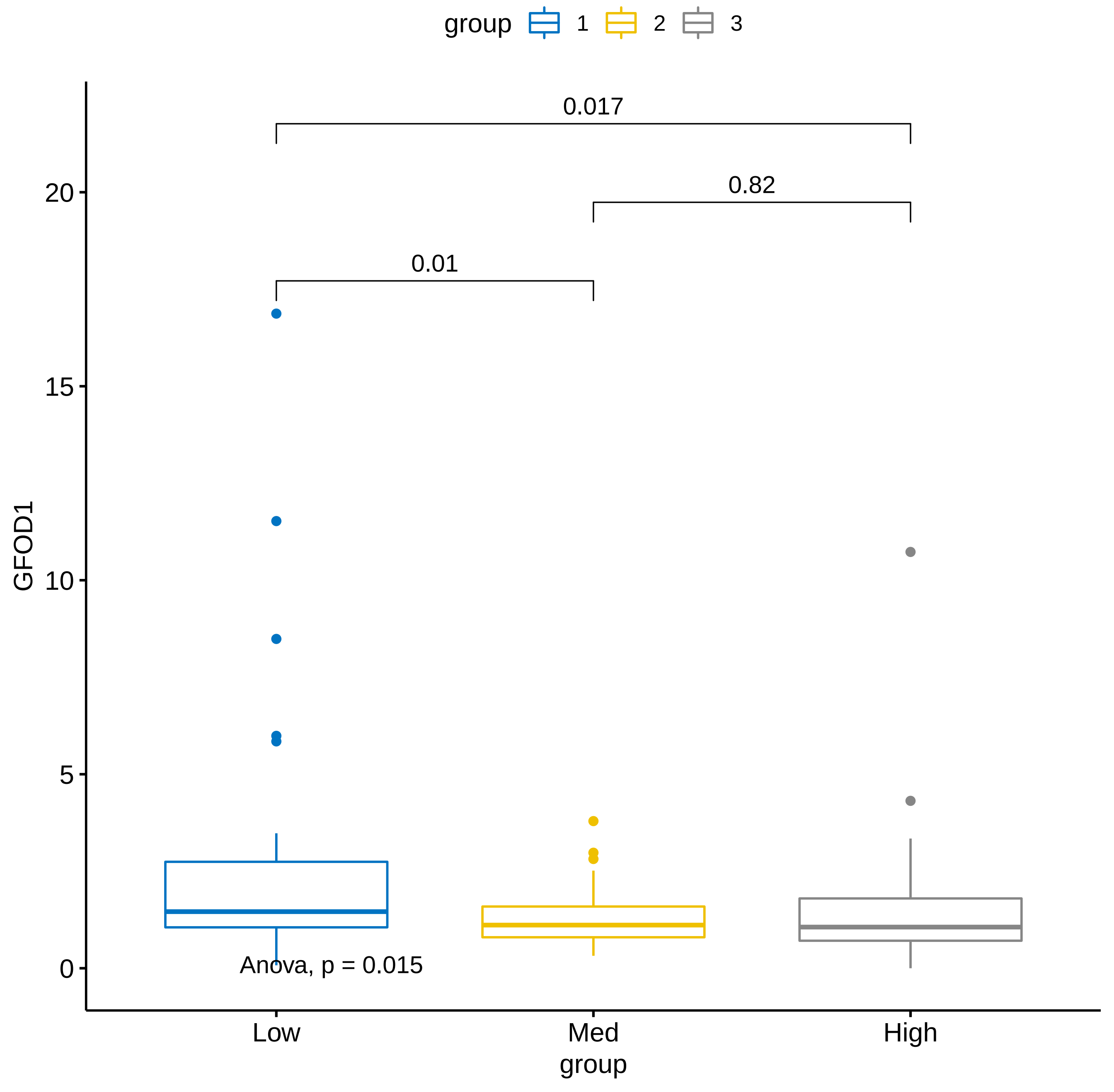

group
1
2
3
0.017
0.82
0.01
20
15
10
5
0
GFOD1
Anova, p = 0.015
Low
Med
High
group

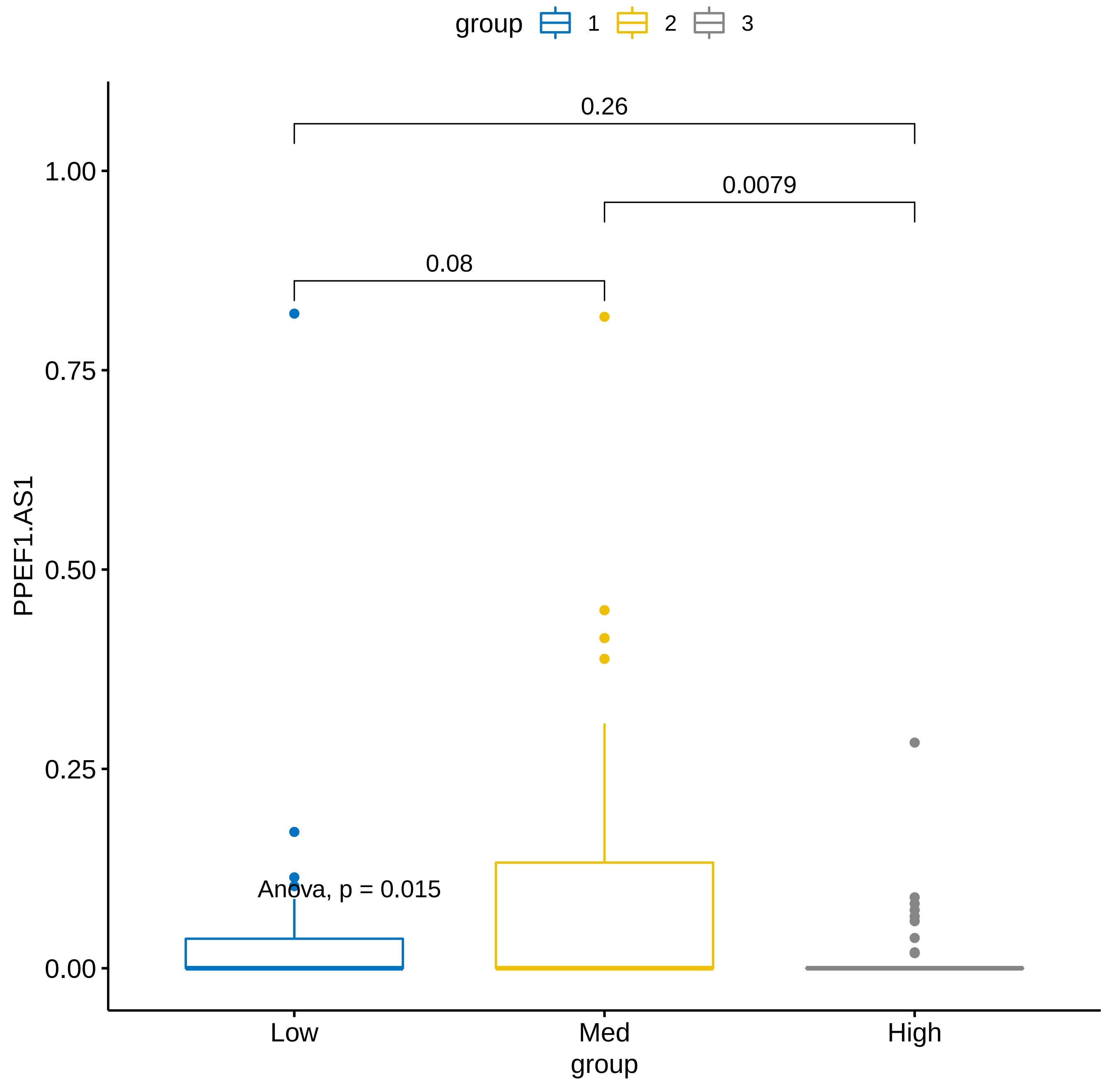

group 1 2 3
0.26
0.0079
0.08
1.00
0.75
0.50
0.25
0.00
PPEF1.AS1
Anova, p = 0.015
Low
Med
High
group

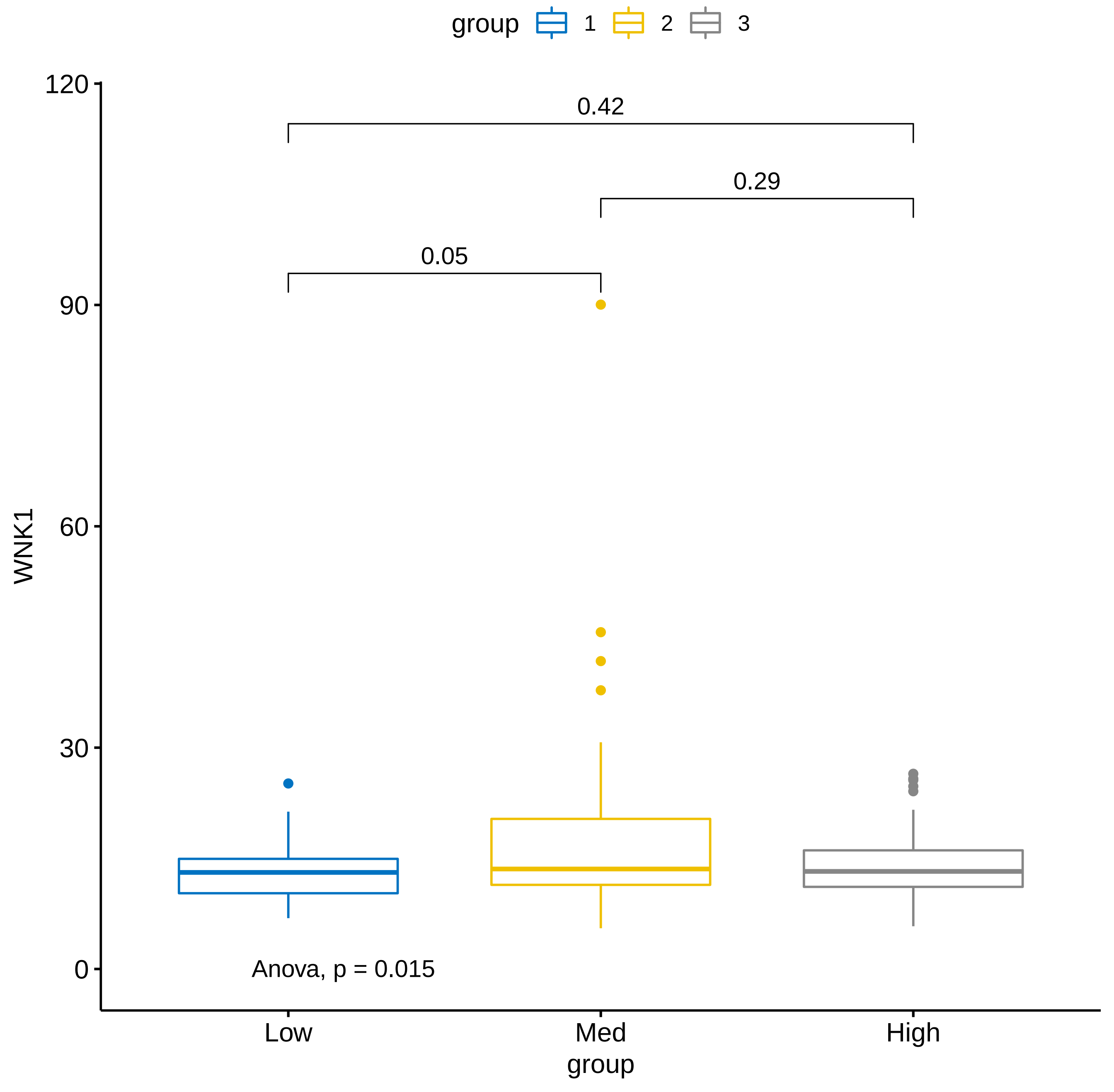
group 1 2 3
120
90
60
30
0
WNK1
0.42
0.29
0.05
Anova, p = 0.015
Low
Med
High
group

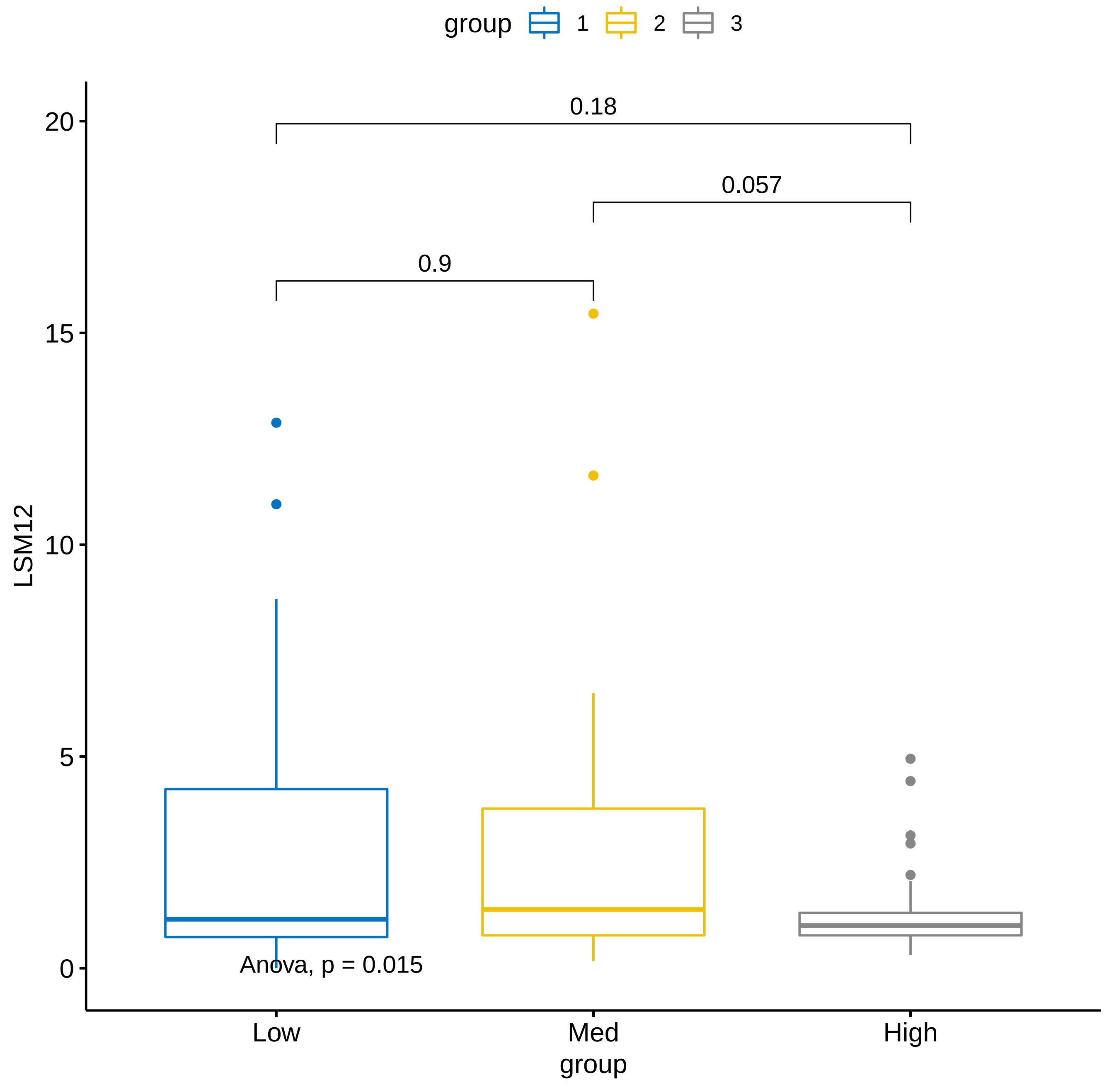
group
1
2
3
0.18
0.057
0.9
LSM12
20
15
10
5
0
Anova, p = 0.015
Low
Med
High
group

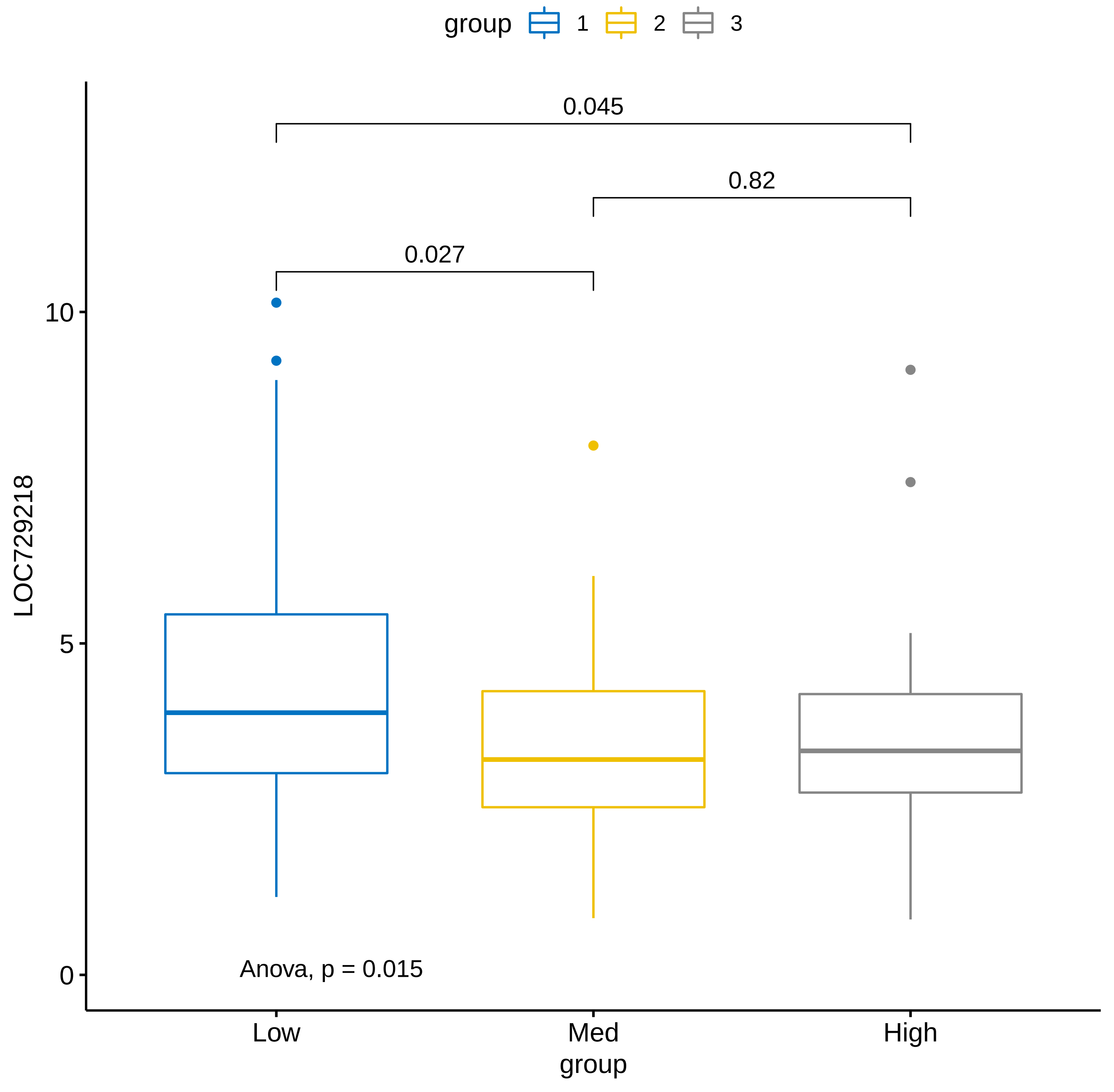

group
1
2
3
0.045
0.82
0.027
10
5
0
LOC729218
Anova, p = 0.015
Low
Med
High
group

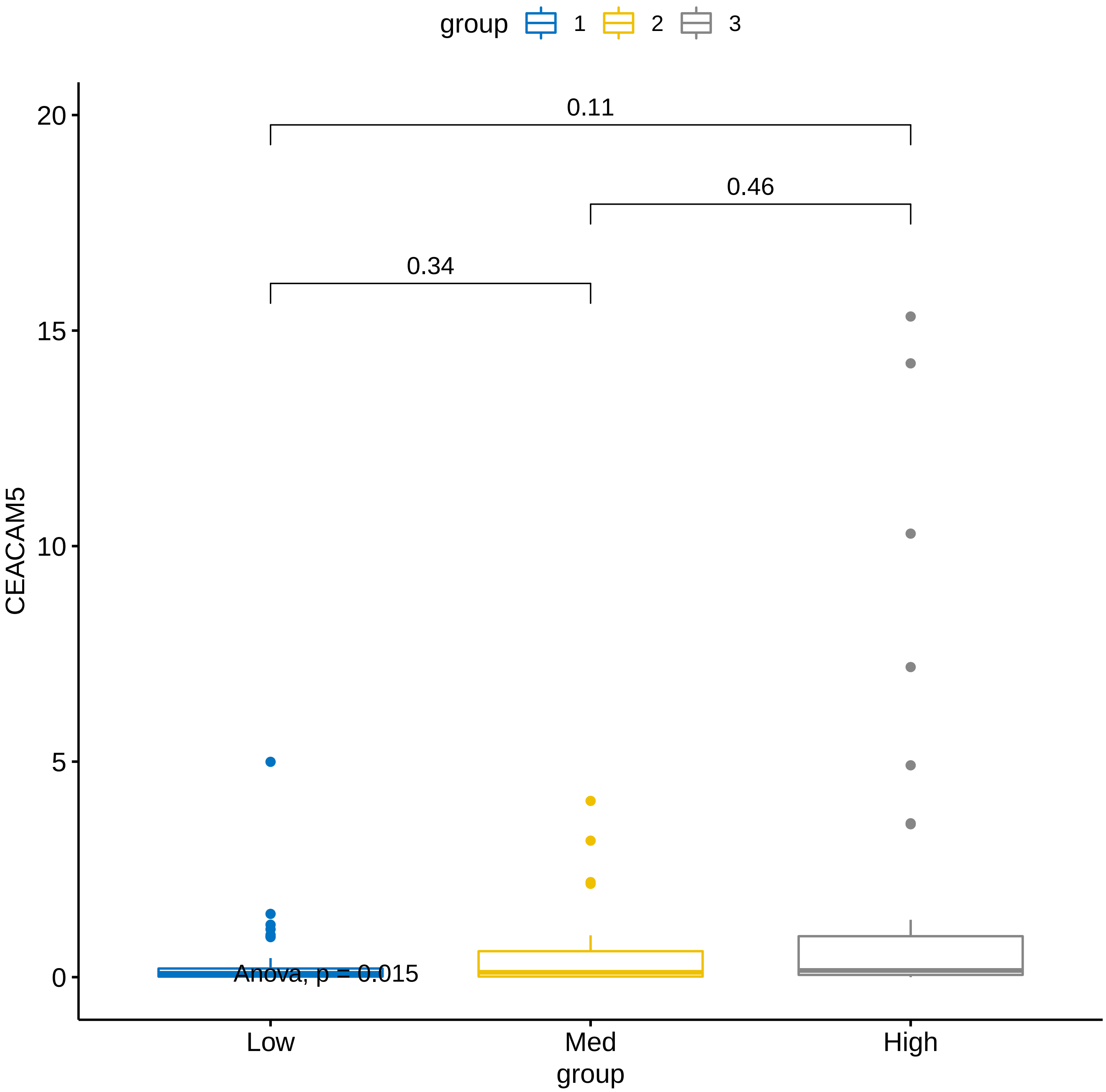
group
1
2
3
0.11
0.46
0.34
20
15
10
5
0
CEACAM5
Anova, p = 0.015
Low
Med
High
group

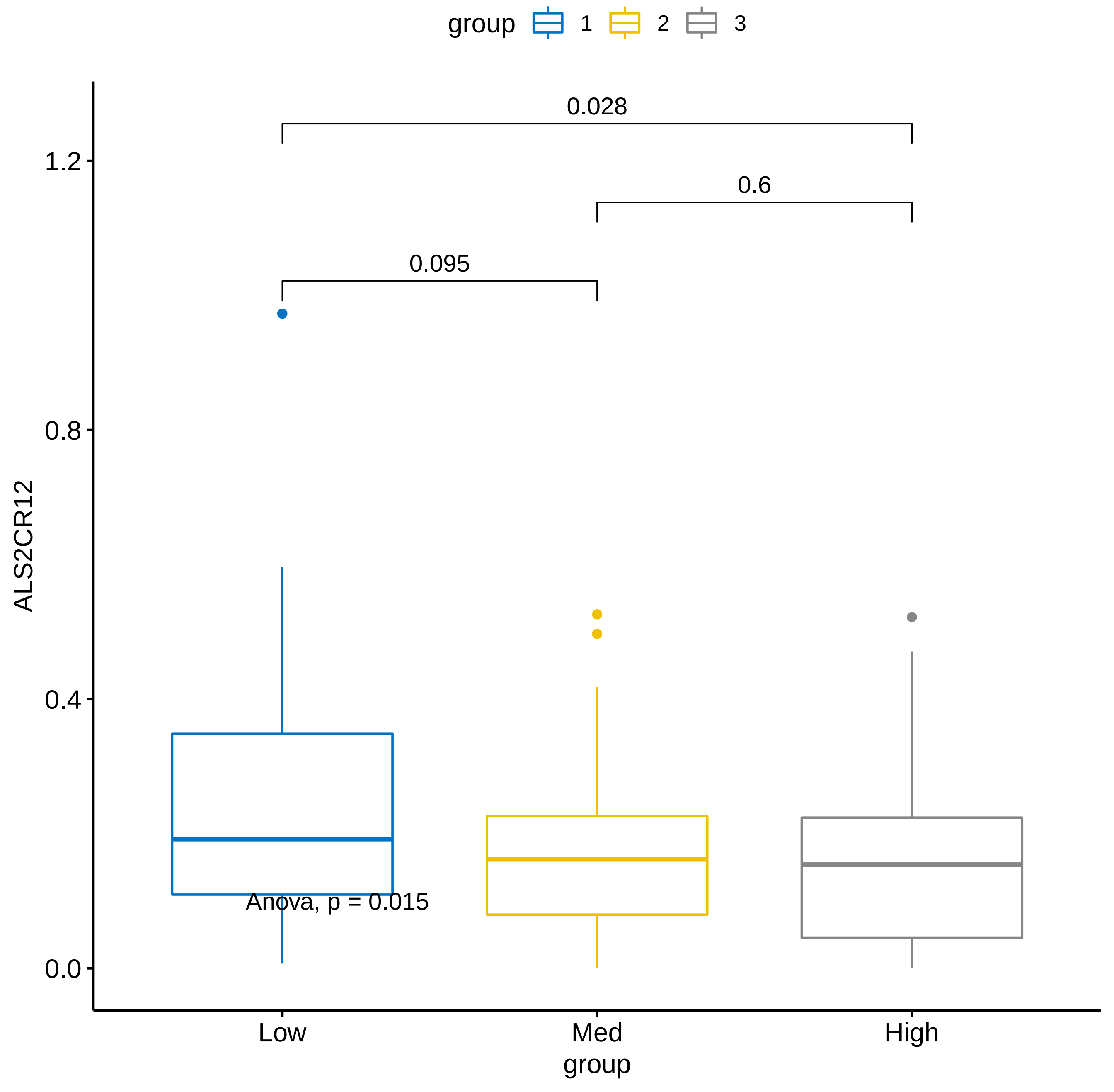

group
1
2
3
0.028
0.6
0.095
1.2
0.8
0.4
0.0
ALS2CR12
Anova, p = 0.015
Low
Med
High
group

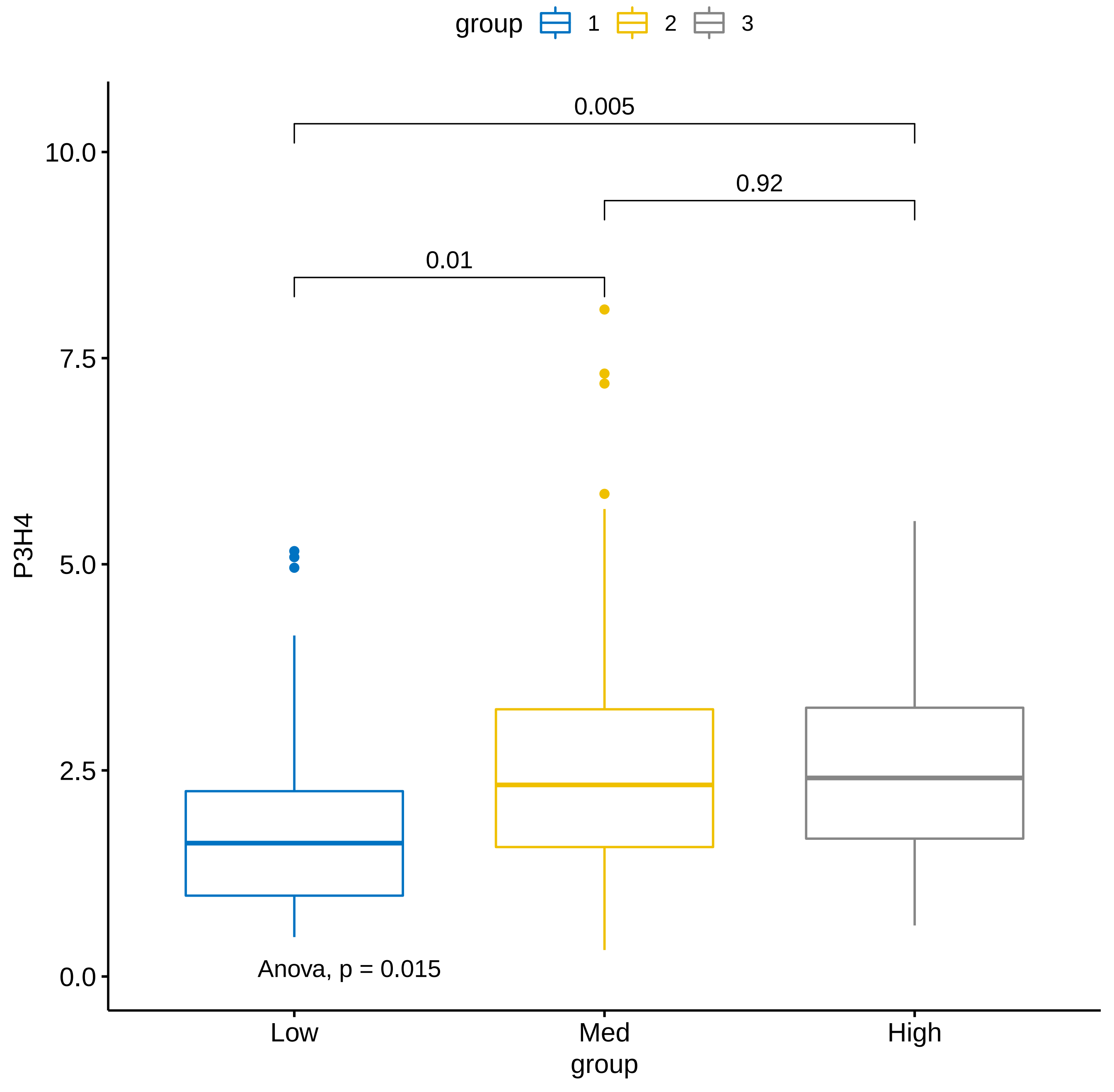

group 1 2 3
0.005
0.92
0.01
P3H4
10.0
7.5
5.0
2.5
0.0
Anova, p = 0.015
Low
Med
High
group

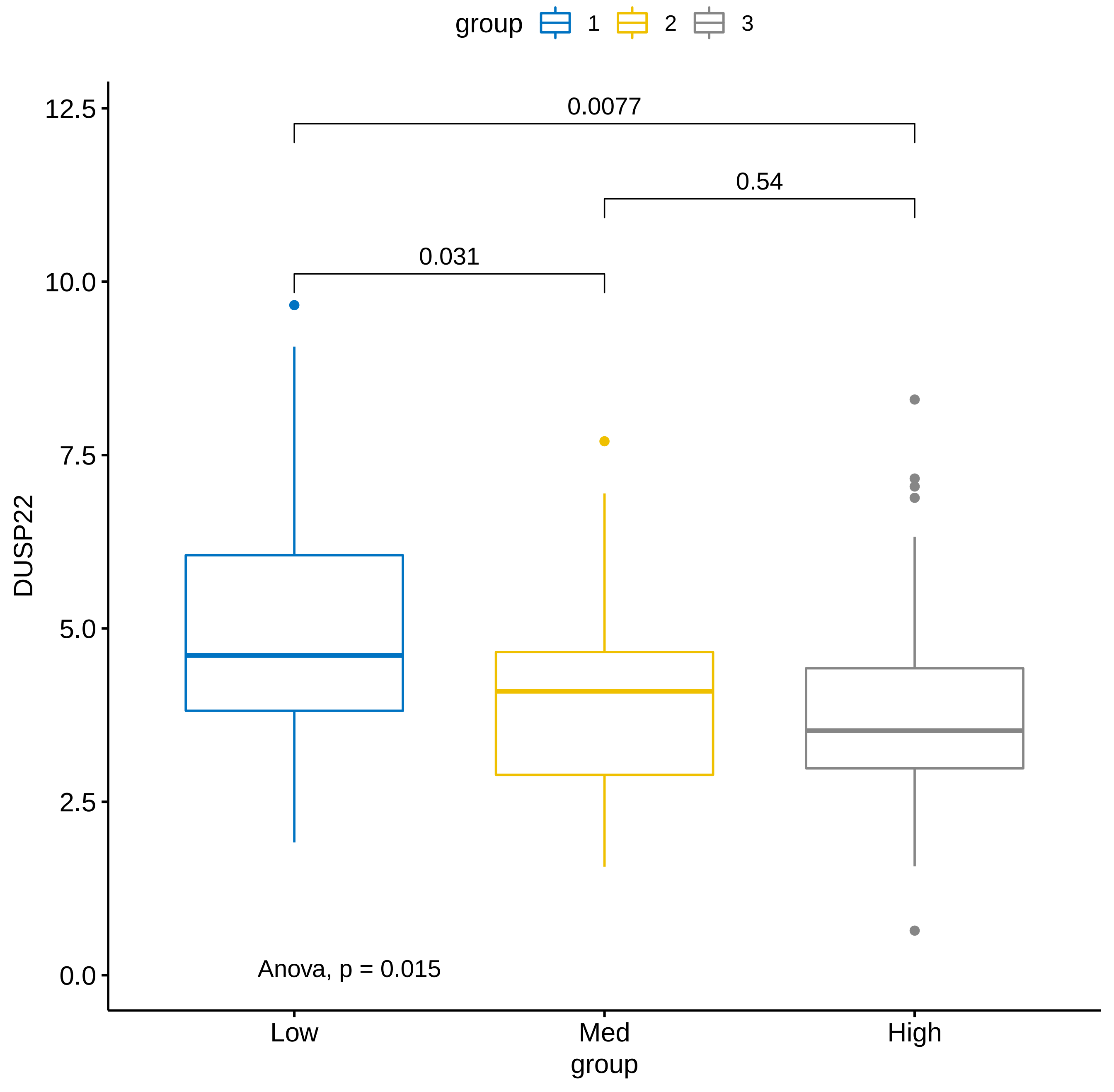

group 1 2 3
0.0077
0.54
0.031
Anova, p = 0.015
DUSP22
12.5
10.0
7.5
5.0
2.5
0.0
Low
Med
High
group

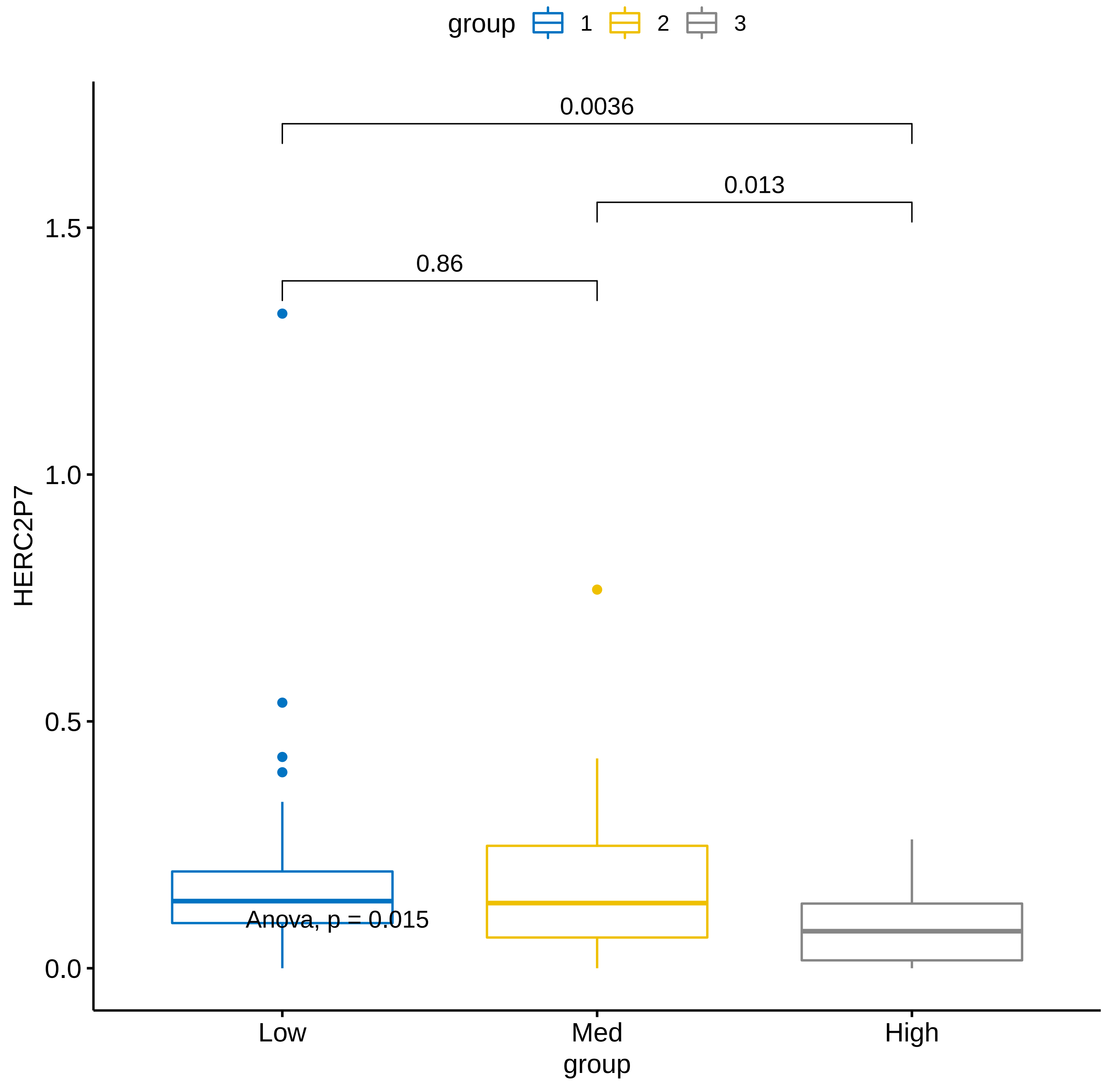
group
1
2
3
0.0036
0.013
0.86
1.5
1.0
0.5
0.0
HERC2P7
Anova, p = 0.015
Low
Med
High
group

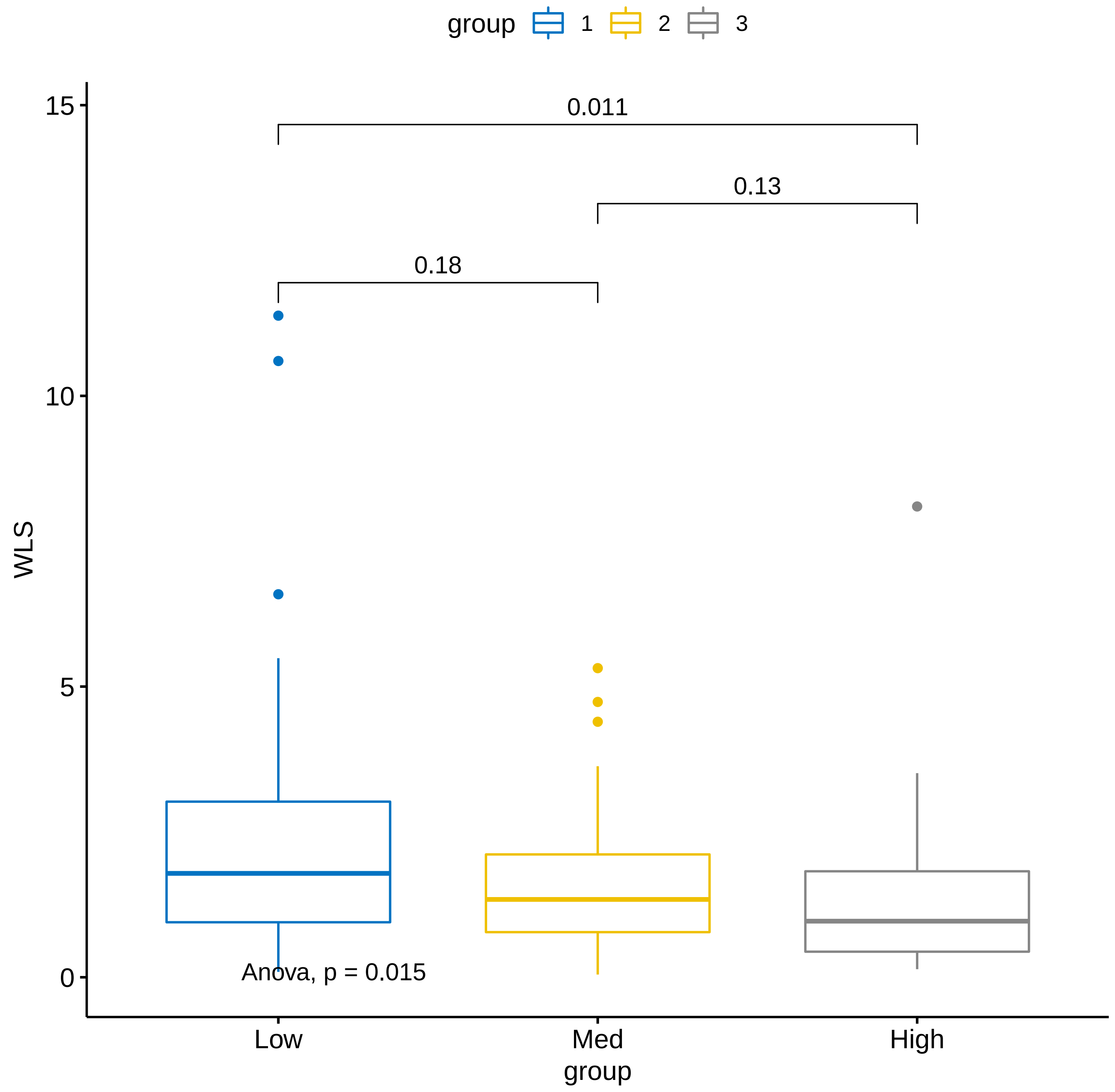

group
1
2
3
15
10
5
0
WLS
0.011
0.13
0.18
Anova, p = 0.015
Low
Med
High
group

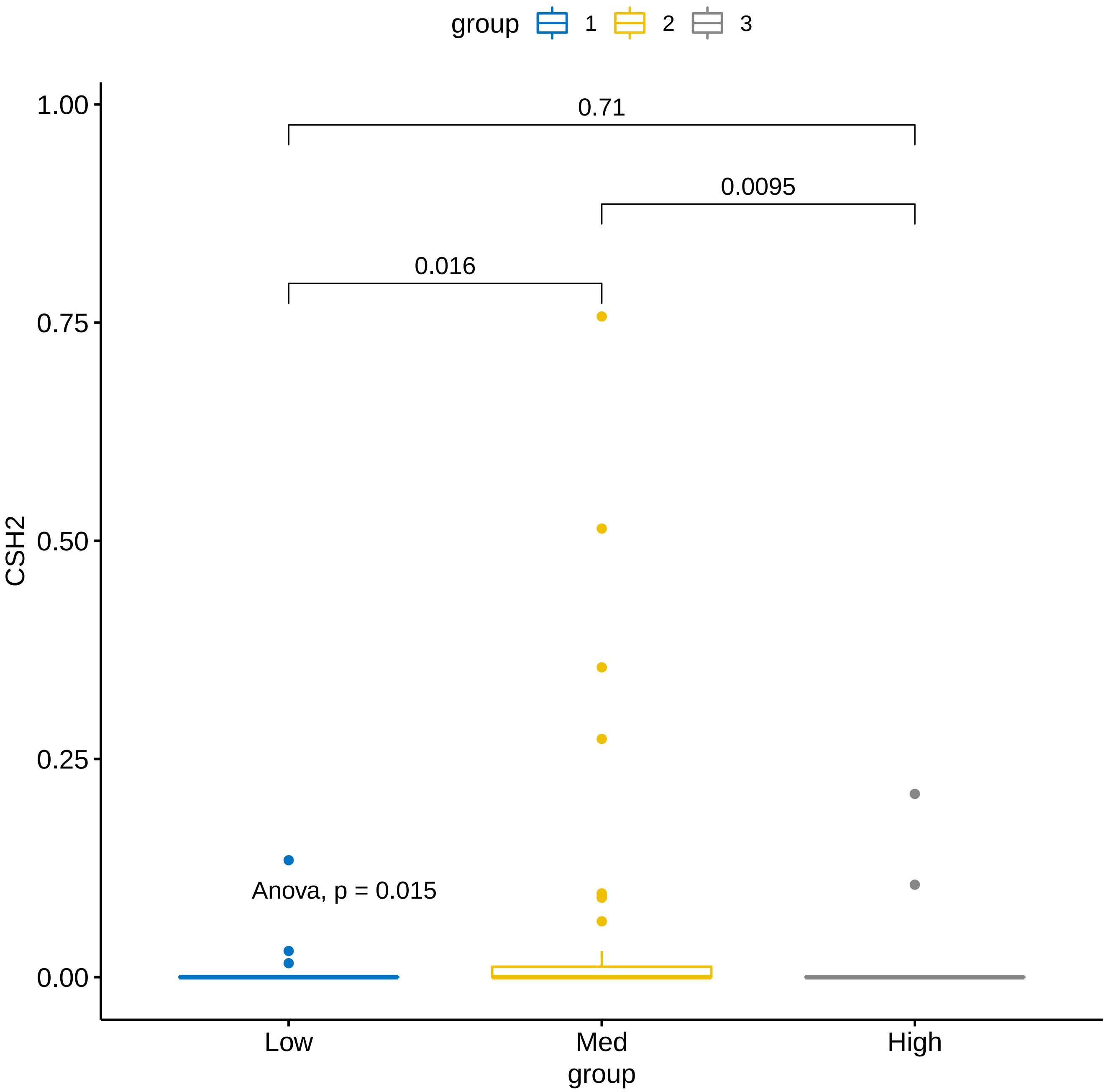

group
1
2
3
1.00
0.75
0.50
0.25
0.00
CSH2
0.71
0.0095
0.016
Anova, p = 0.015
Low
Med
High
group

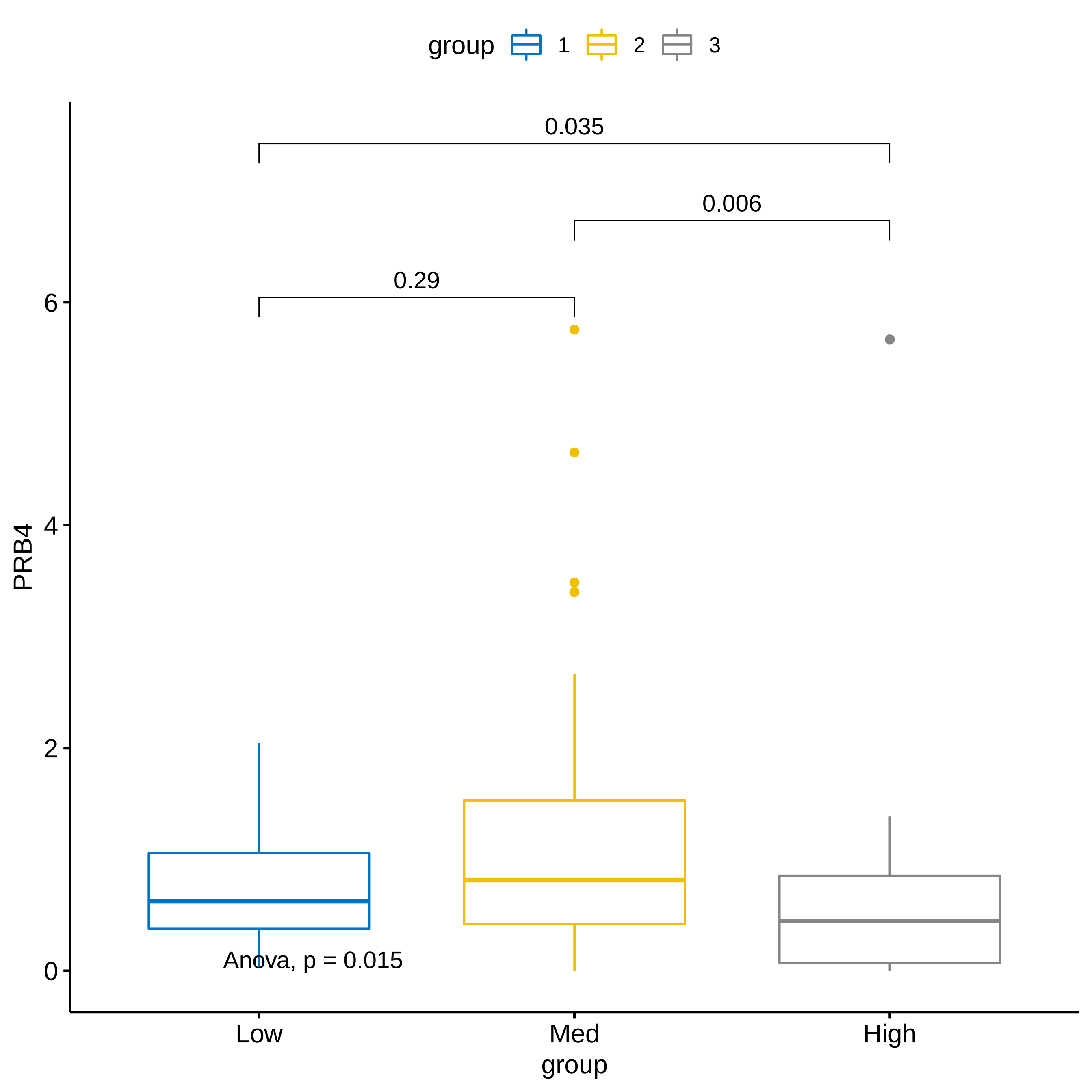
group
1
2
3
0.035
0.006
0.29
PRB4
6
4
2
0
Anova, p = 0.015
Low
Med
High
group

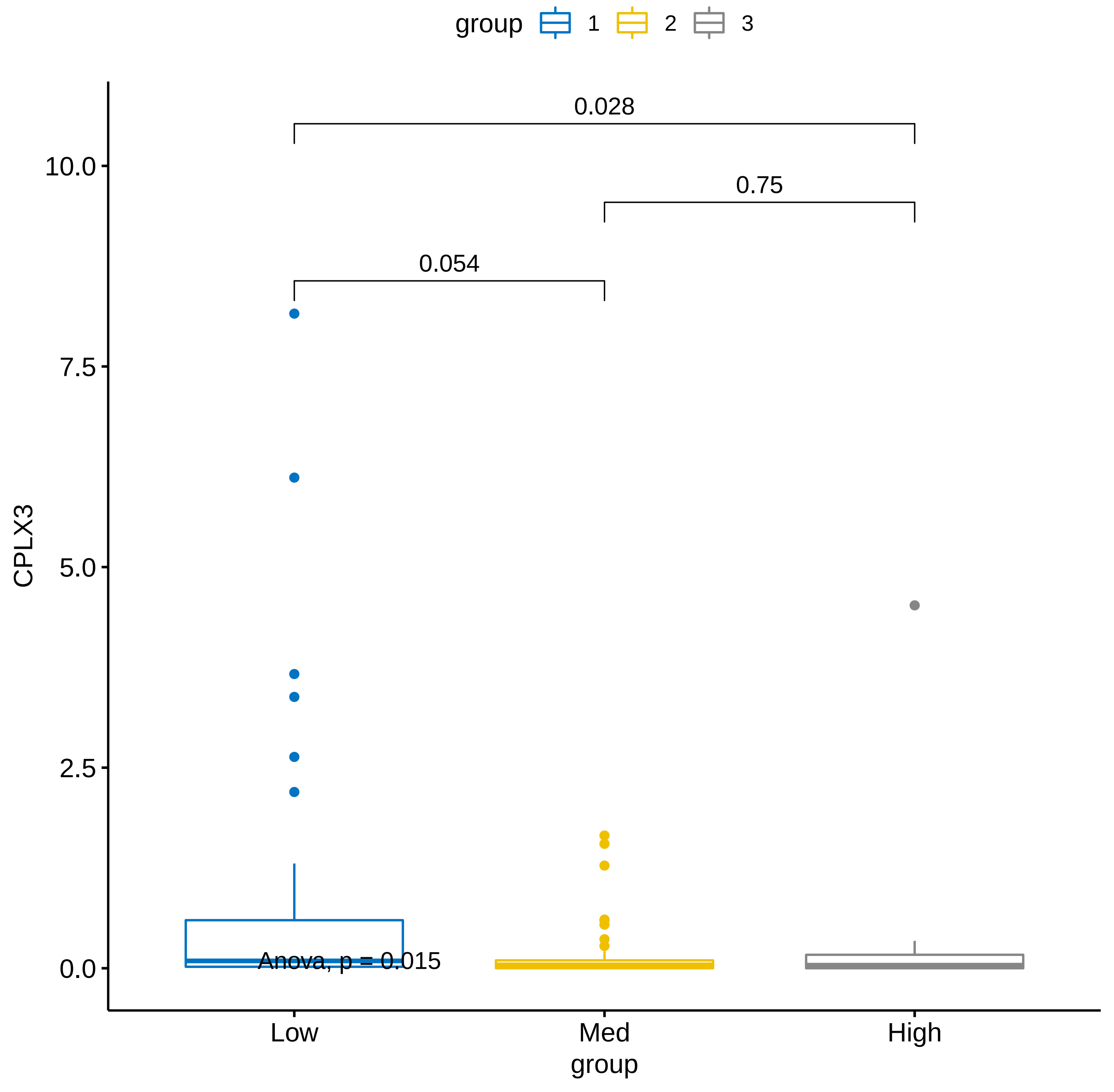

group 1 2 3
0.028
0.75
0.054
10.0
7.5
5.0
2.5
0.0
CPLX3
Anova, p = 0.015
Low
Med
High
group

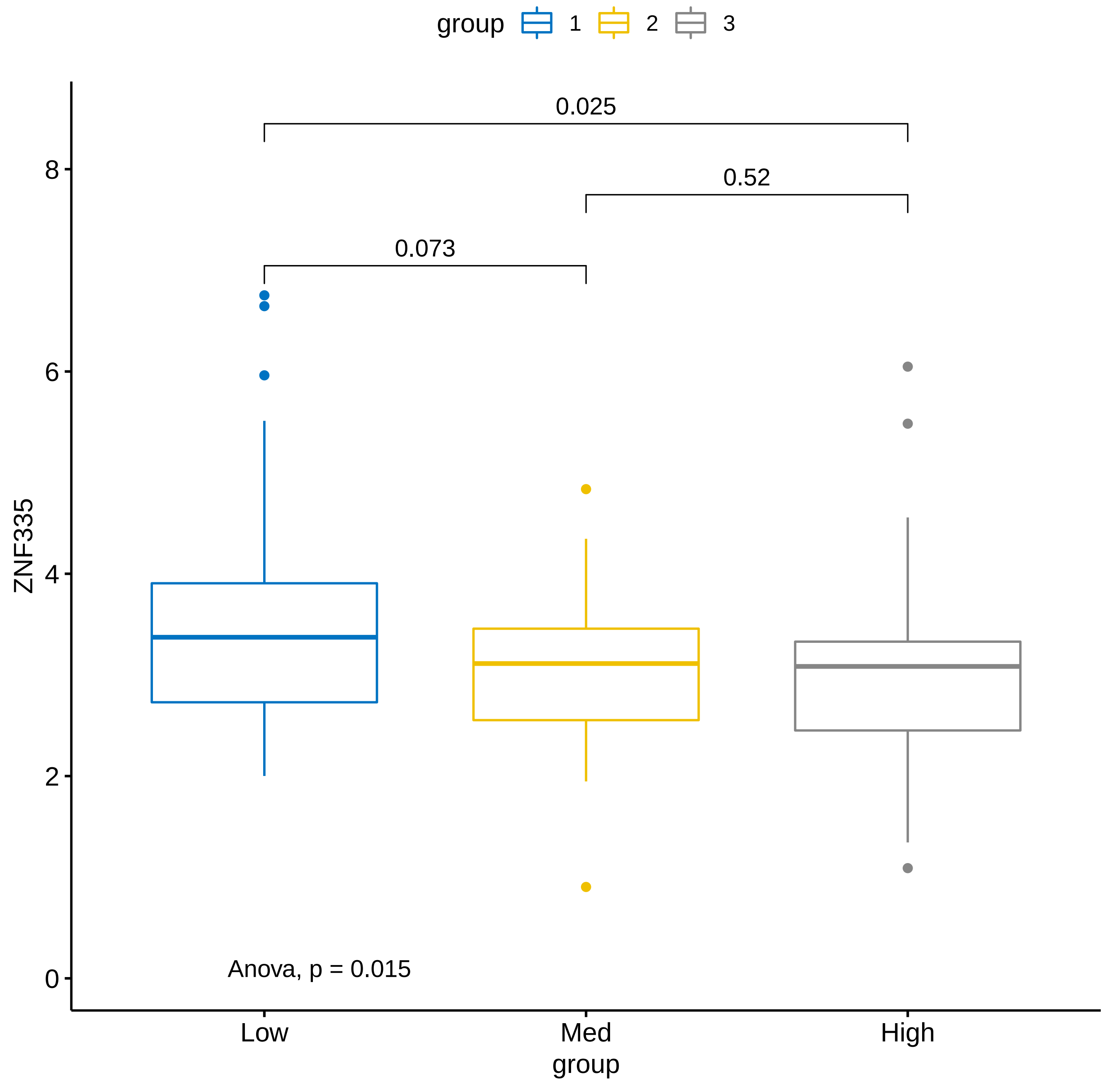
group
1
2
3
0.025
0.52
0.073
8
6
4
2
0
ZNF335
Anova, p = 0.015
Low
Med
High
group

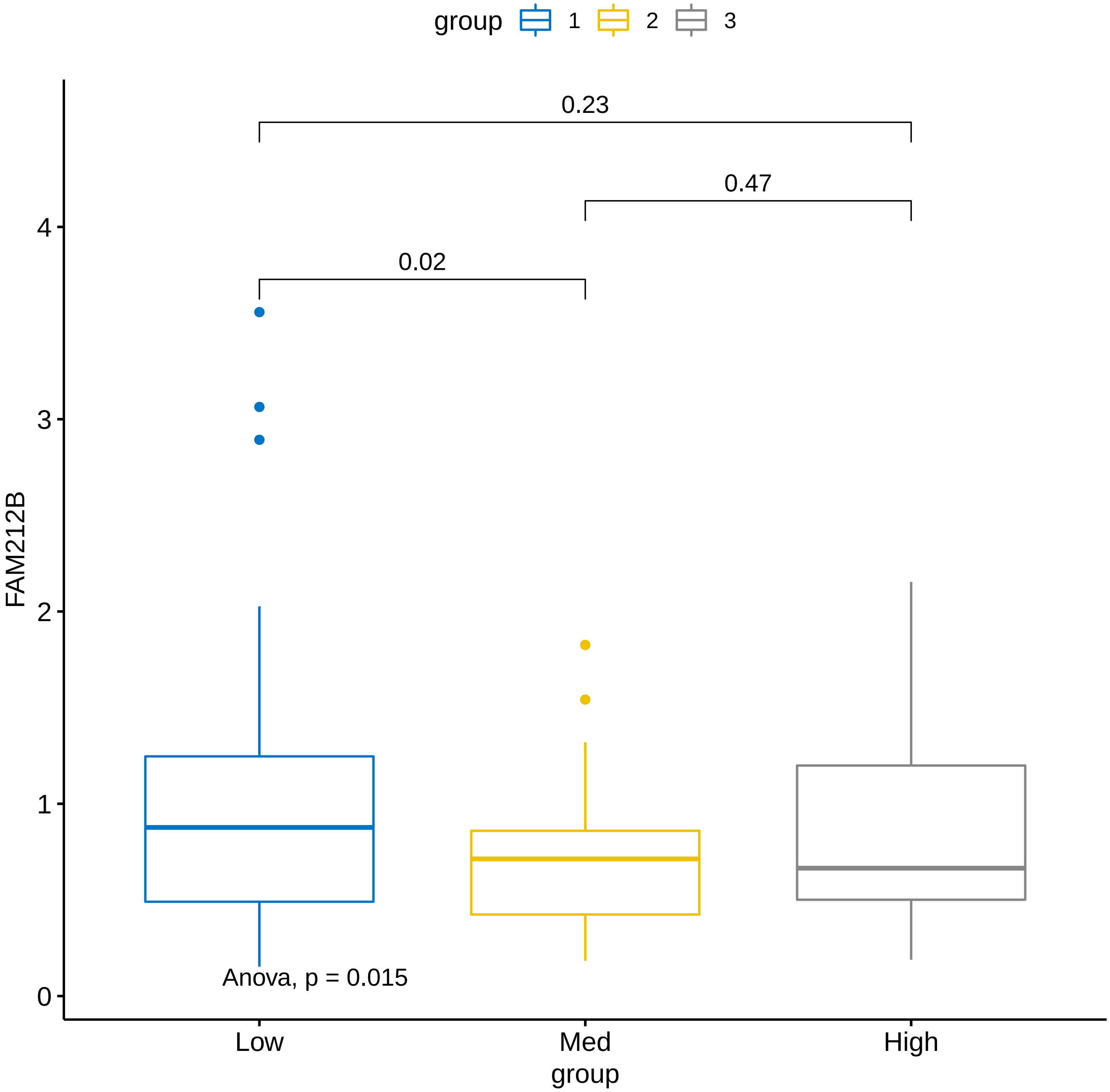
group
1
2
3
0.23
0.47
0.02
FAM212B
4
3
2
1
0
Anova, p = 0.015
Low
Med
High
group

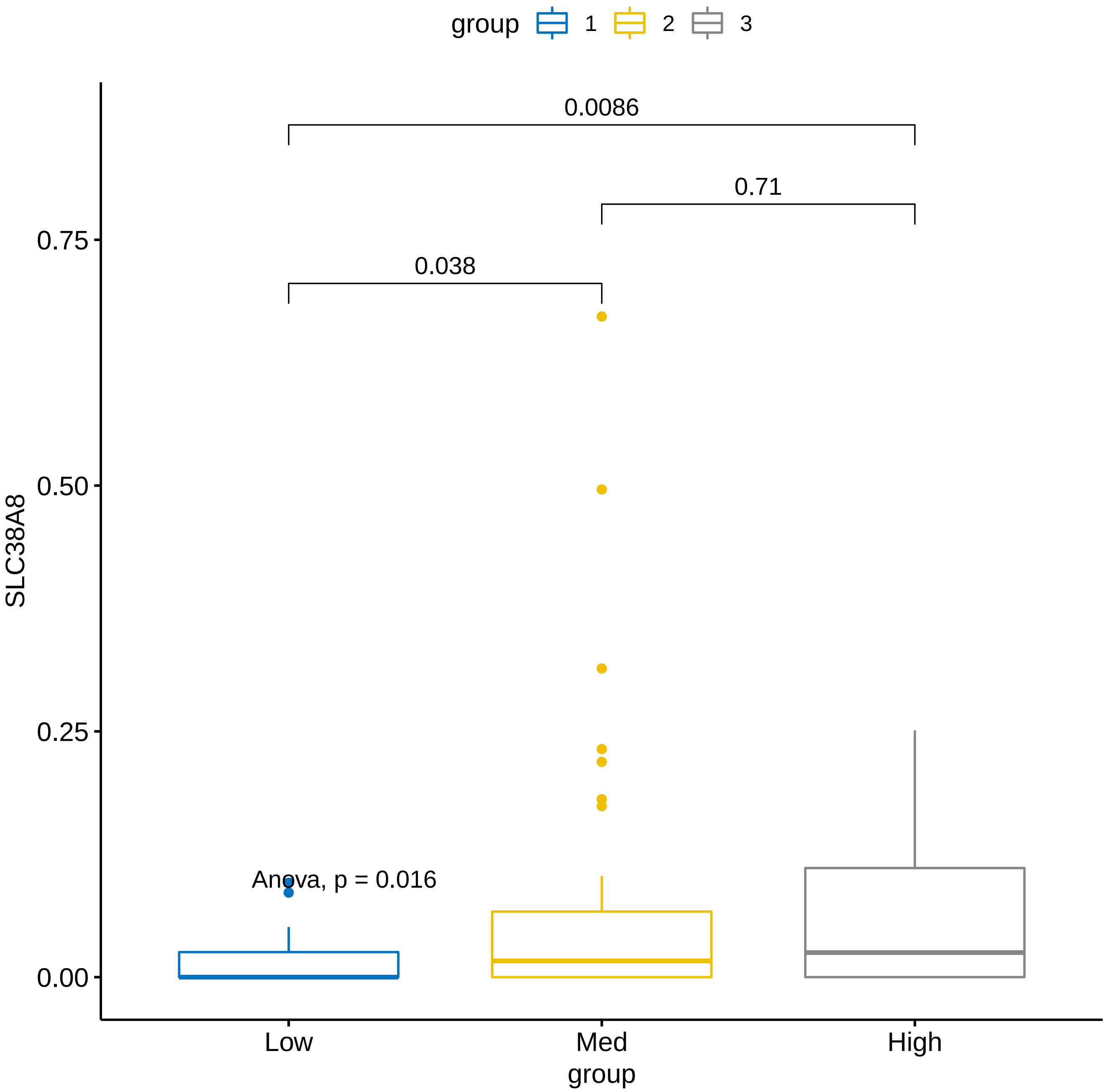
group
1
2
3
0.0086
0.71
0.038
0.75
0.50
0.25
0.00
SLC38A8
Anova, p = 0.016
Low
Med
High
group

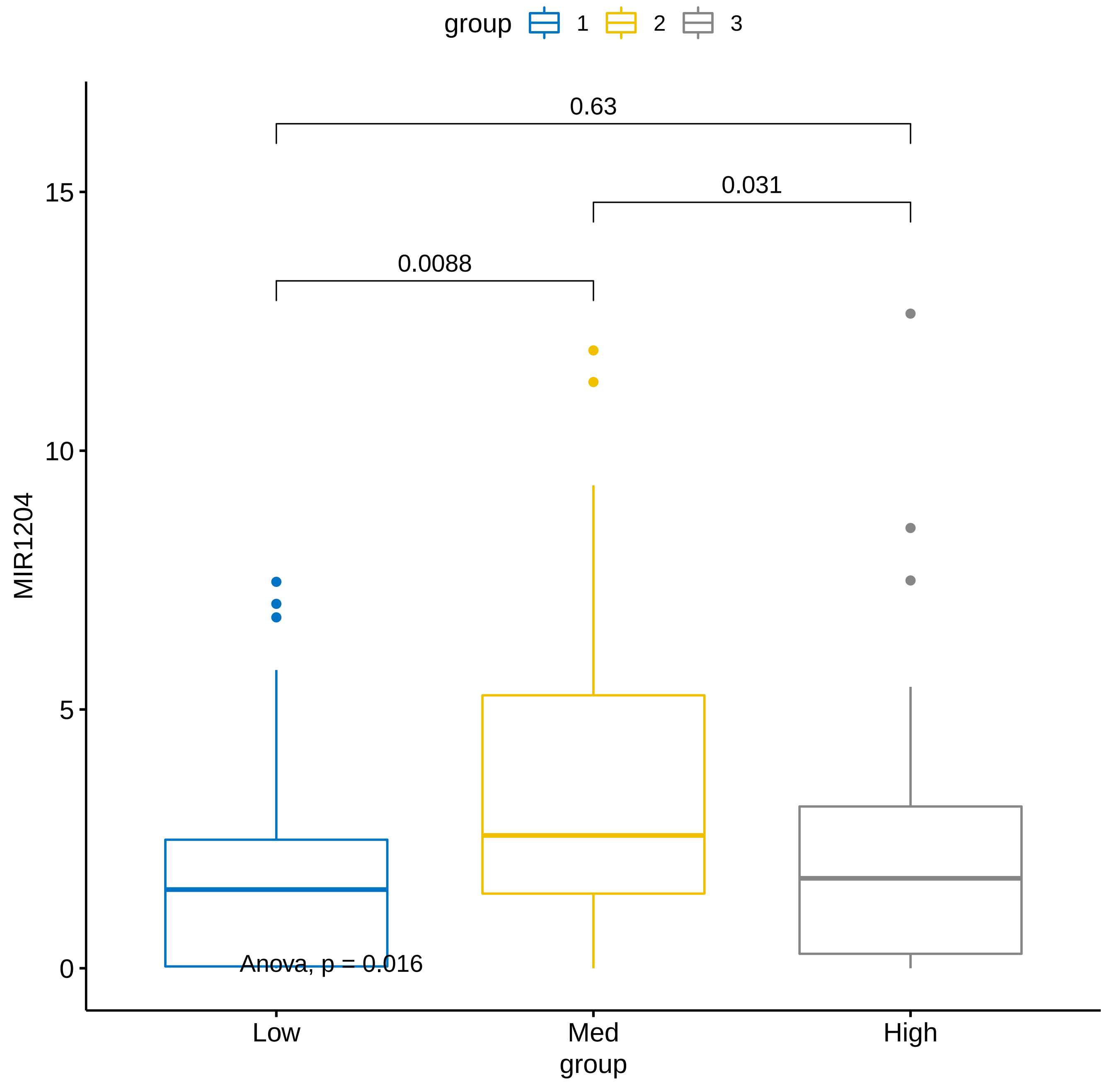
group
1
2
3
0.63
0.031
0.0088
15
10
5
0
MIR1204
Anova, p = 0.016
Low
Med
High
group

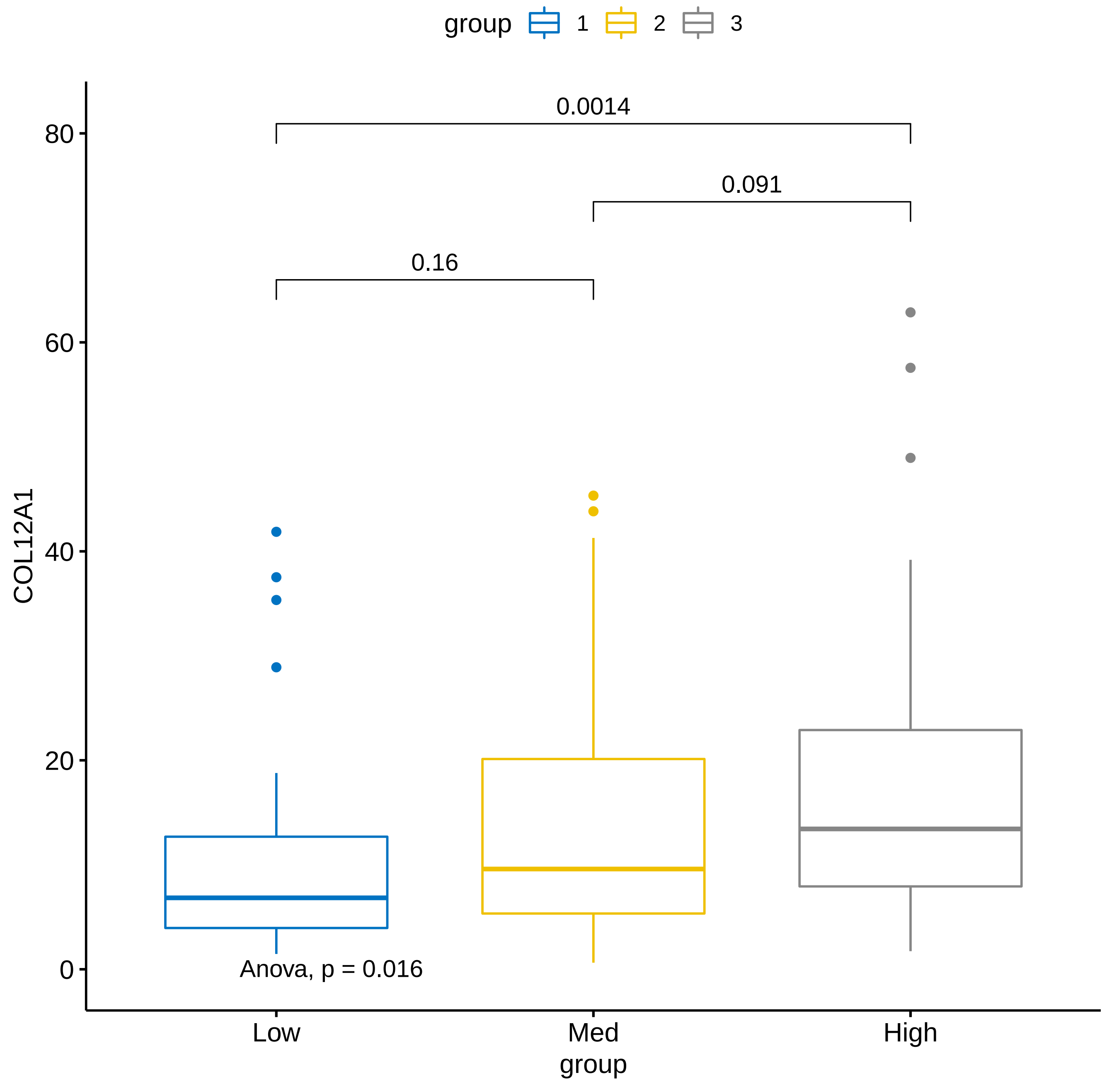
group
1
2
3
0.0014
0.091
0.16
80
60
40
20
0
COL12A1
Anova, p = 0.016
Low
Med
High
group

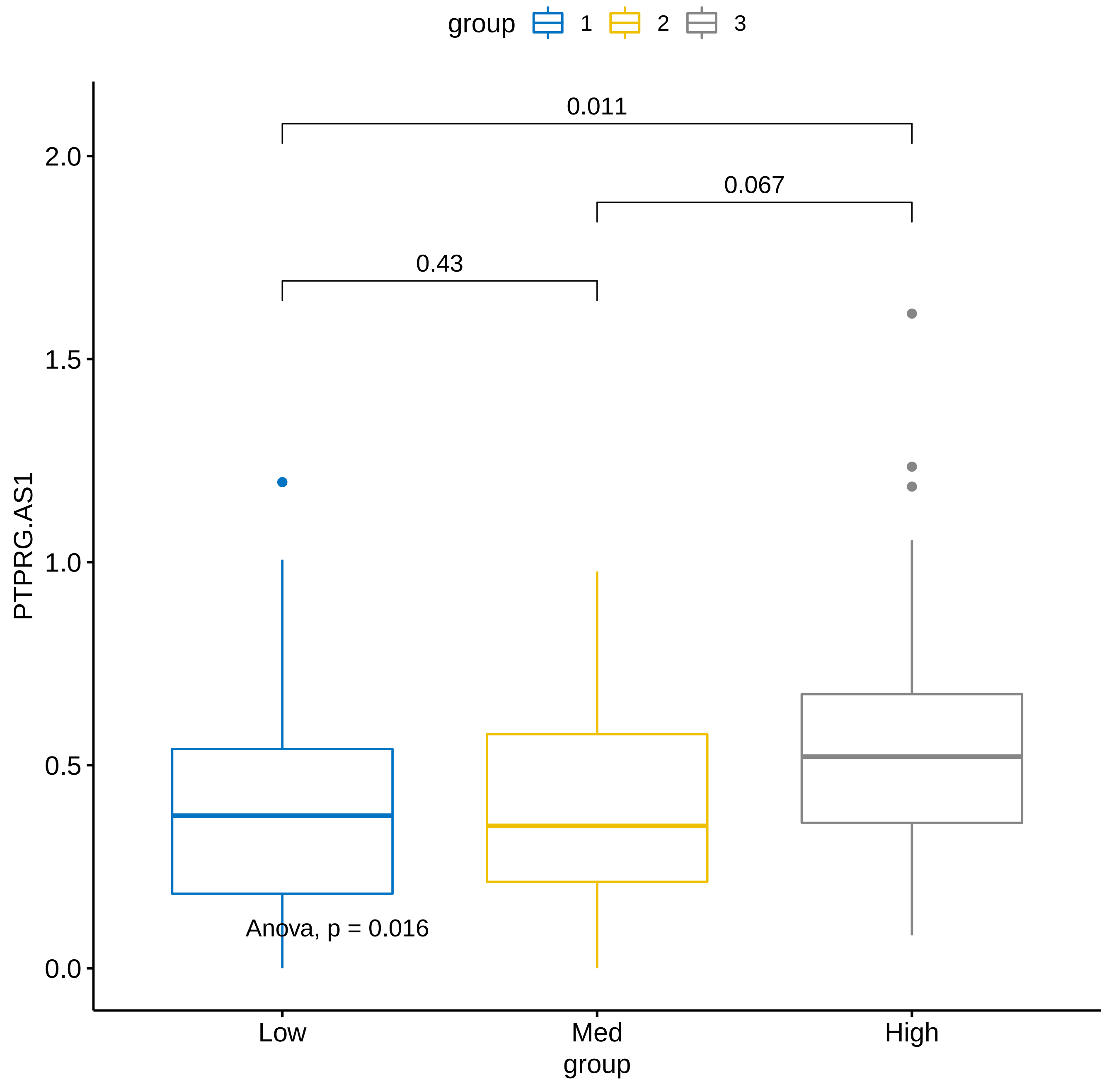

group 1 2 3
0.011
0.067
0.43
2.0
1.5
1.0
0.5
0.0
PTPRG.AS1
Anova, p = 0.016
Low
Med
High
group

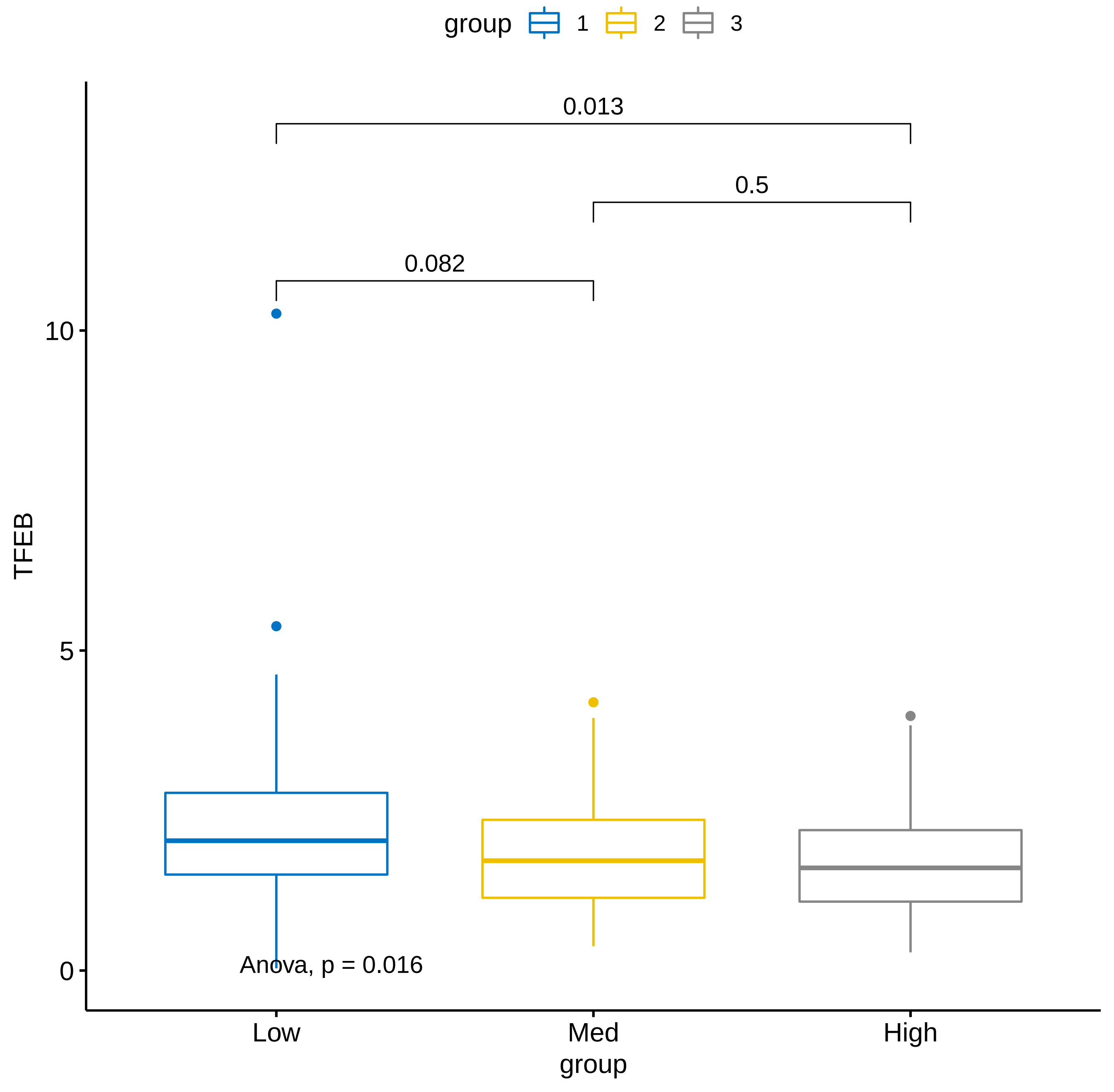

group
1
2
3
0.013
0.5
0.082
10
5
0
TFEB
Anova, p = 0.016
Low
Med
High
group

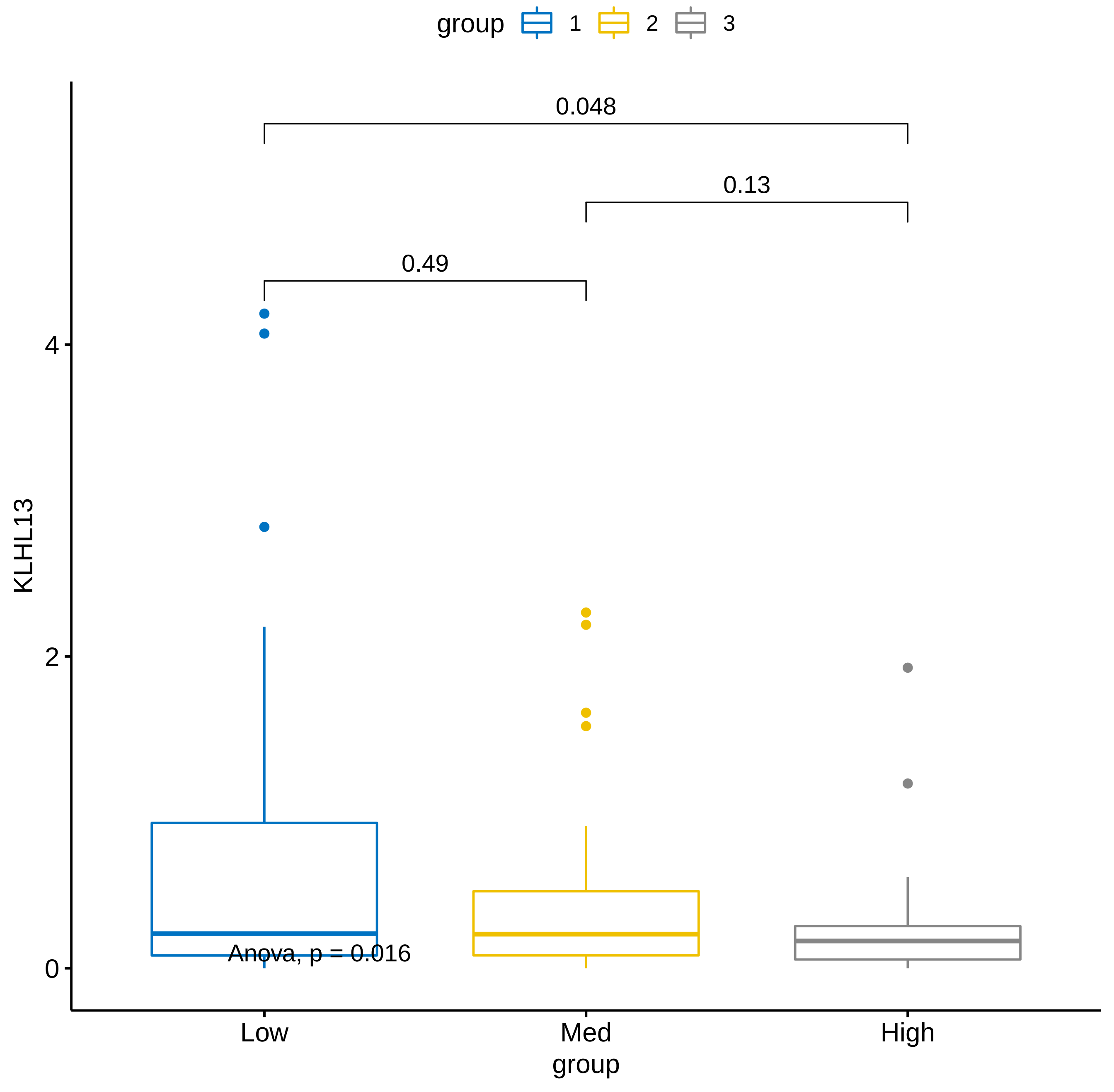
group
1
2
3
0.048
0.13
0.49
KLHL13
4
2
0
Anova, p = 0.016
Low
Med
High
group

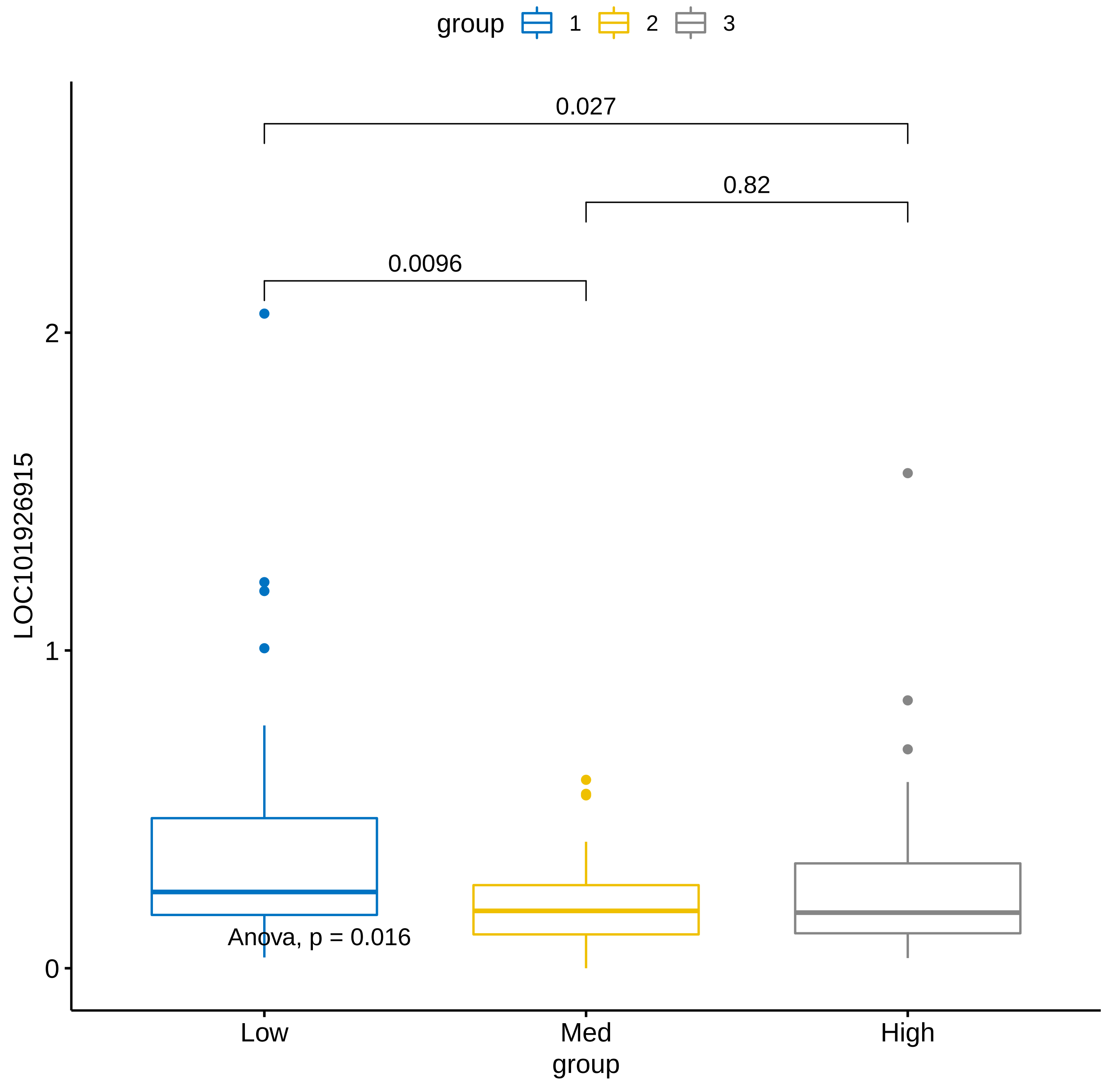

group
1
2
3
0.027
0.82
0.0096
2
1
0
LOC101926915
Anova, p = 0.016
Low
Med
High
group

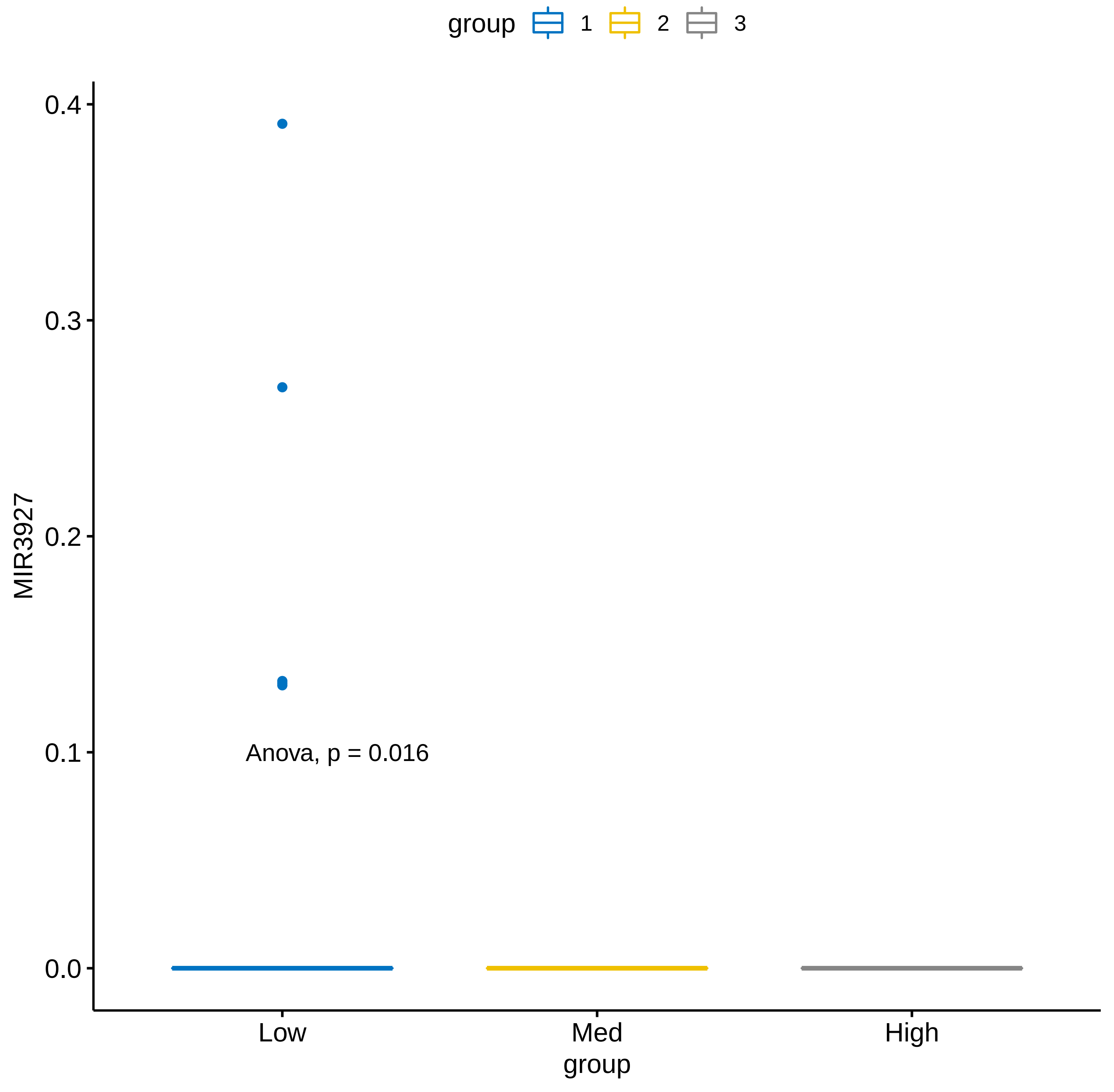

group
1
2
3
0.4
0.3
0.2
0.1
0.0
MIR3927
Anova, p = 0.016
Low
Med
High
group

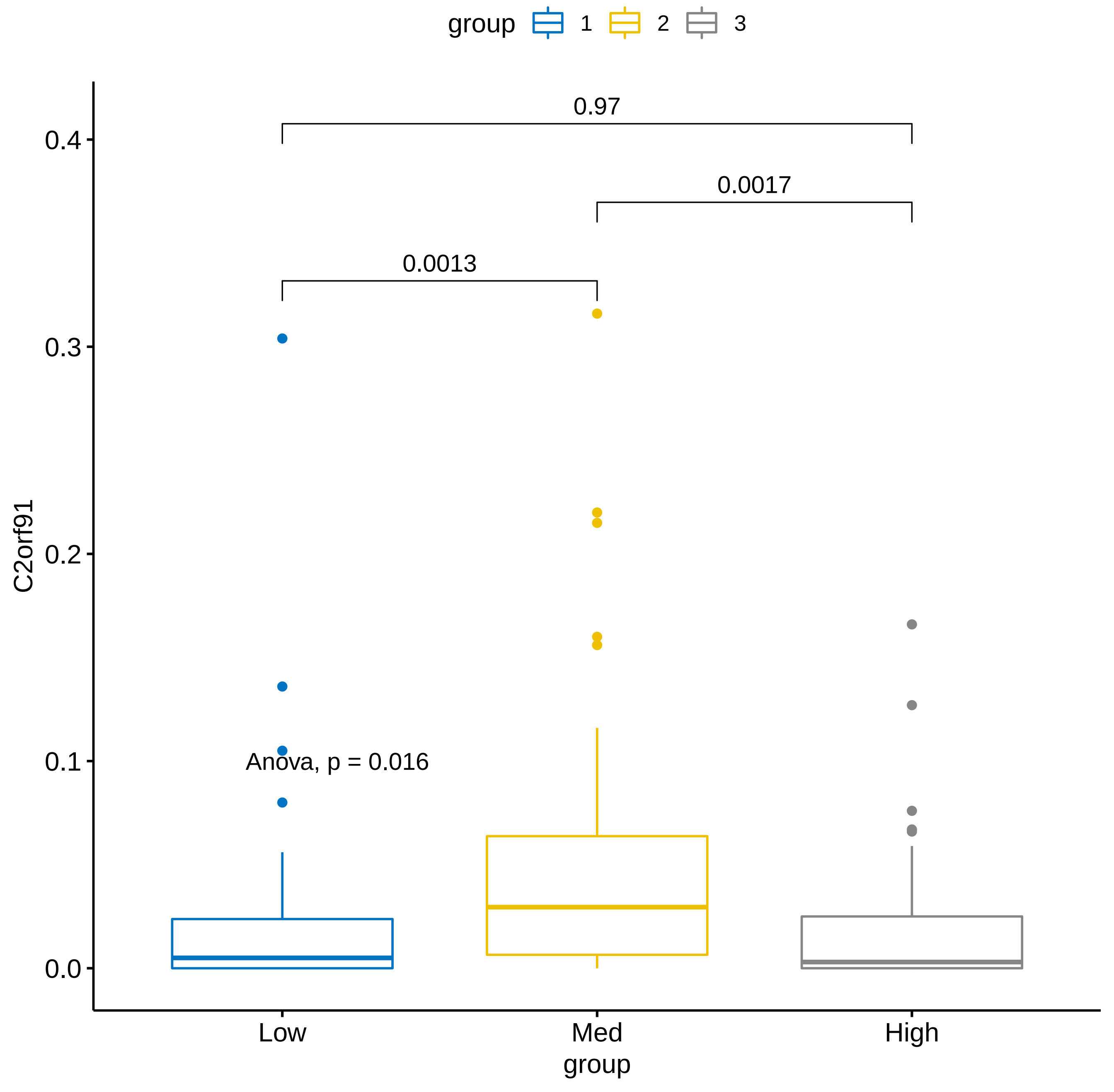
group 1 2 3
0.97
0.0017
0.0013
Anova, p = 0.016
C2orf91
0.4
0.3
0.2
0.1
0.0
Low
Med
High
group

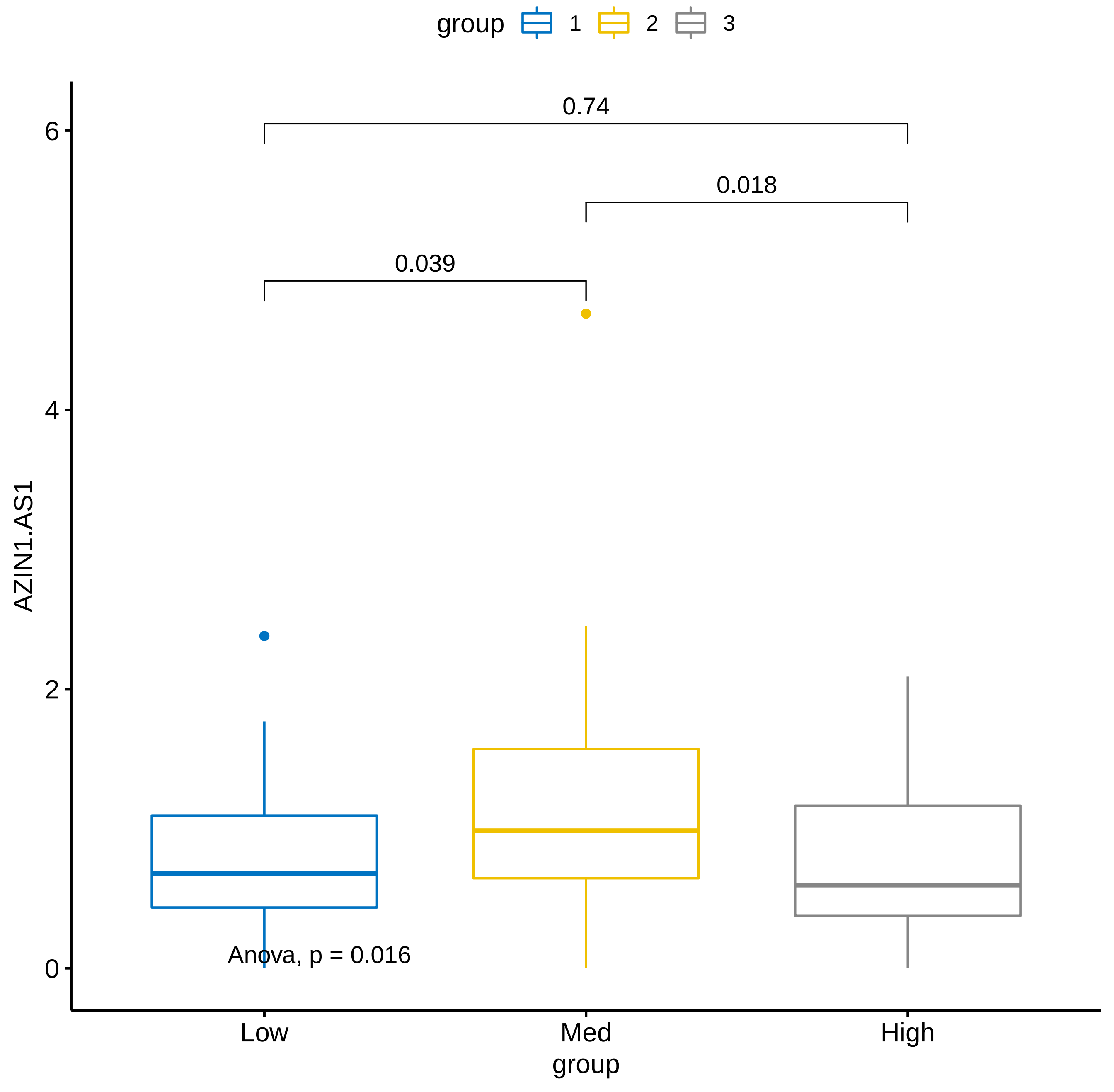
group
1
2
3
0.74
0.018
0.039
6
4
2
0
AZIN1.AS1
Anova, p = 0.016
Low
Med
High
group

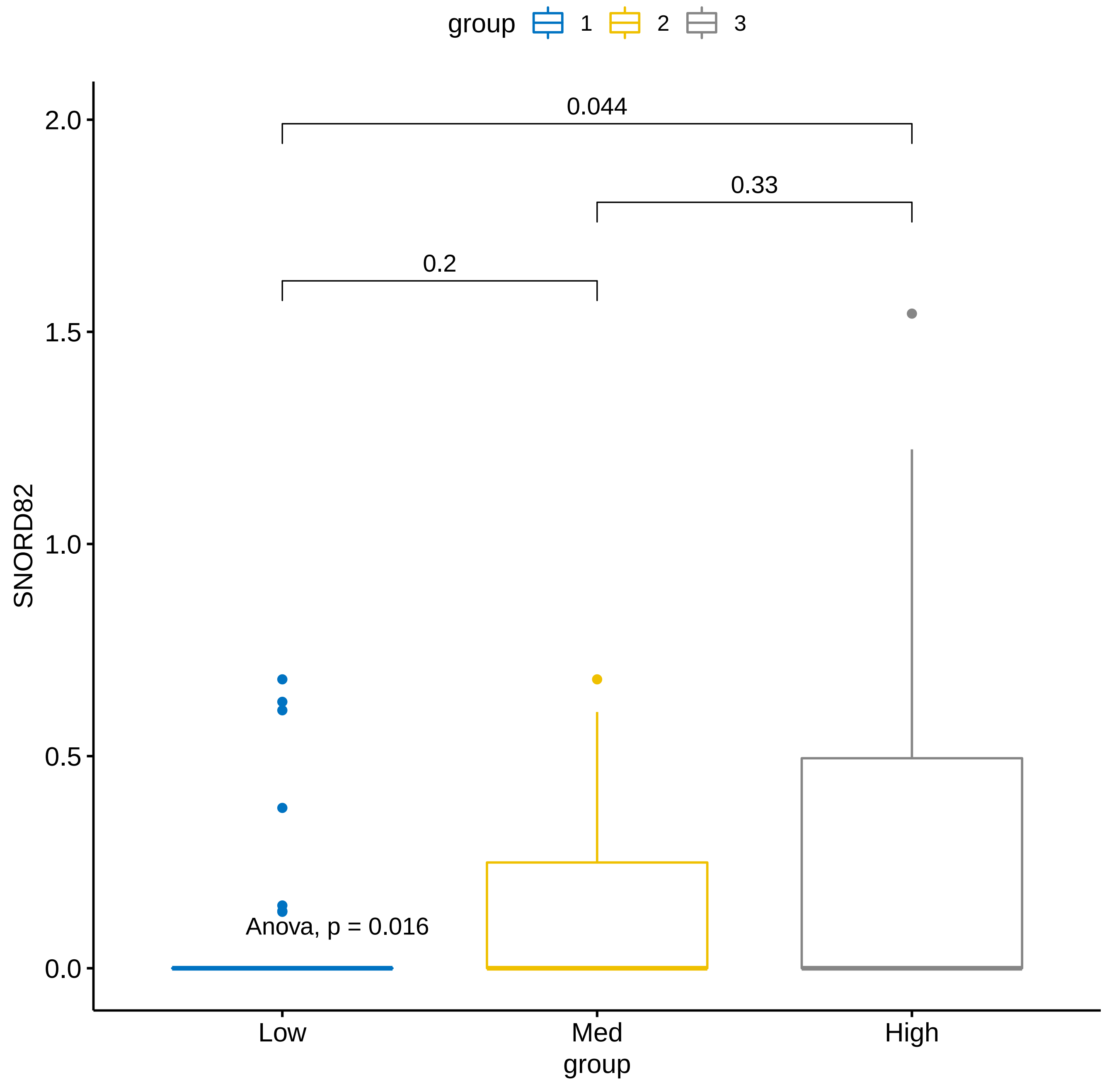

group
1
2
3
0.044
0.33
0.2
Anova, p = 0.016
SNORD82
2.0
1.5
1.0
0.5
0.0
Low
Med
High
group

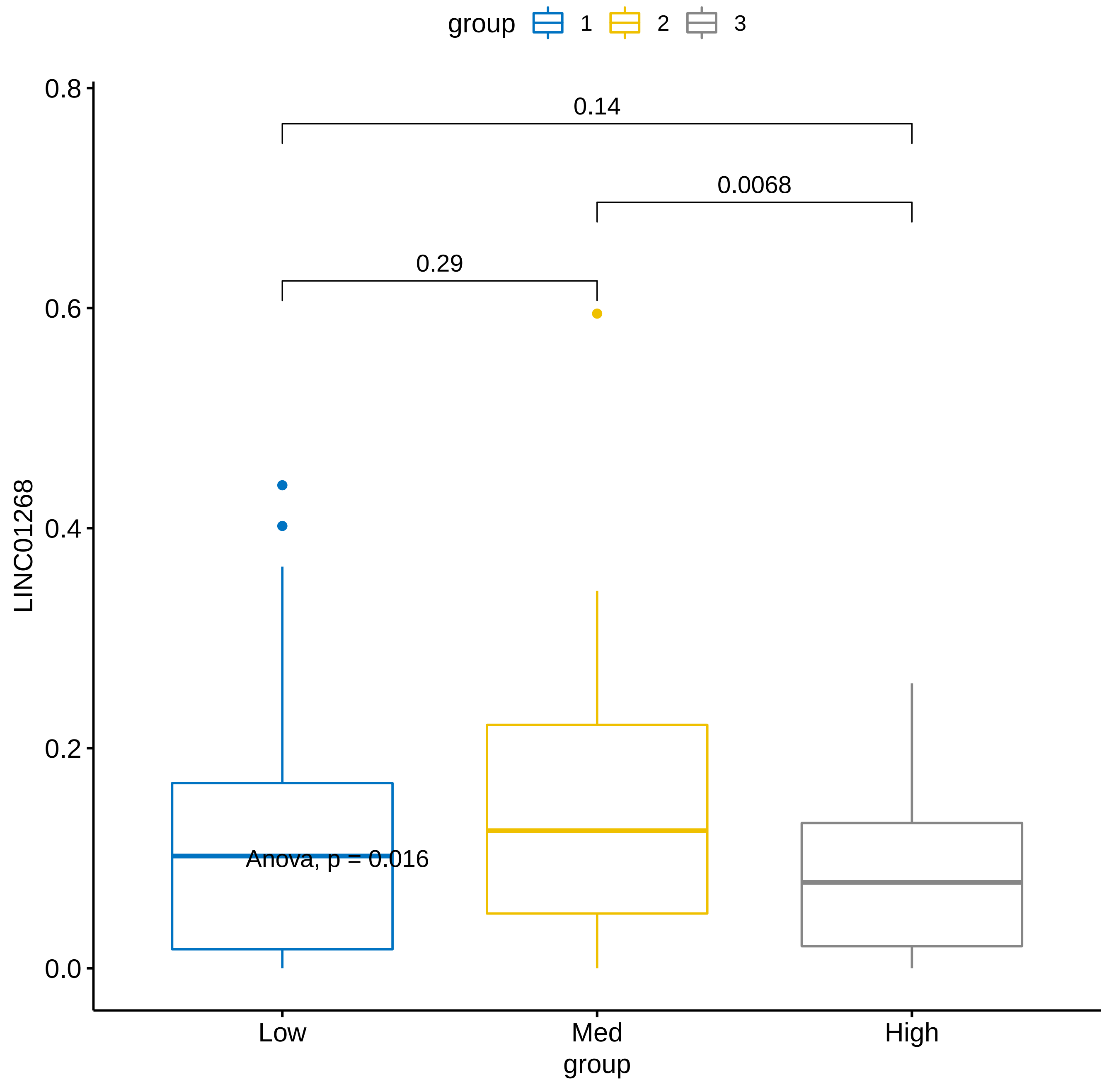

group 1 2 3
0.14
0.0068
0.29
Anova, p = 0.016
0.0
0.2
0.4
0.6
0.8
LINC01268
Low
Med
High
group

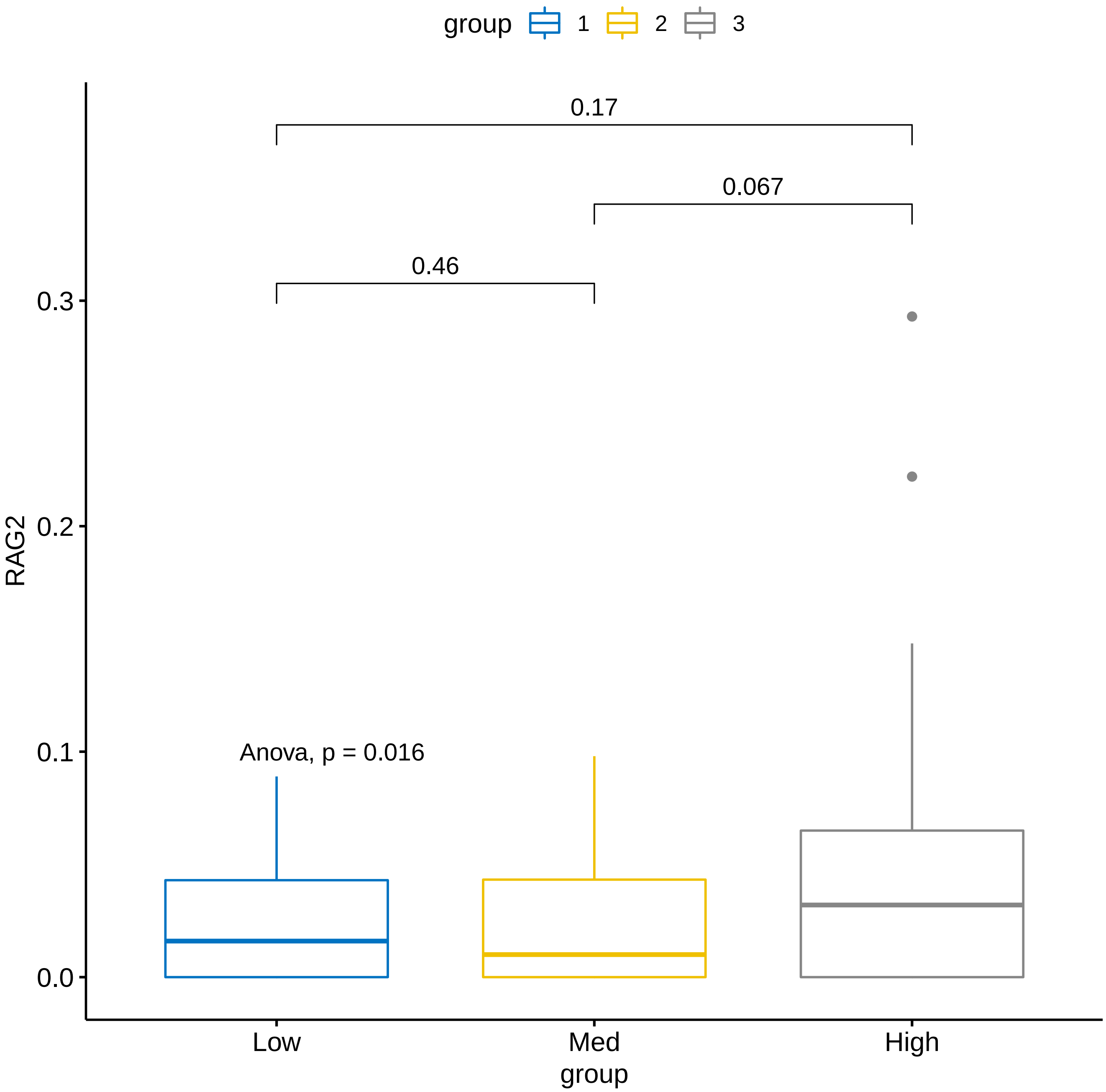
group 1 2 3
0.17
0.067
0.46
Anova, p = 0.016
RAG2
0.0
0.1
0.2
0.3
Low
Med
High
group

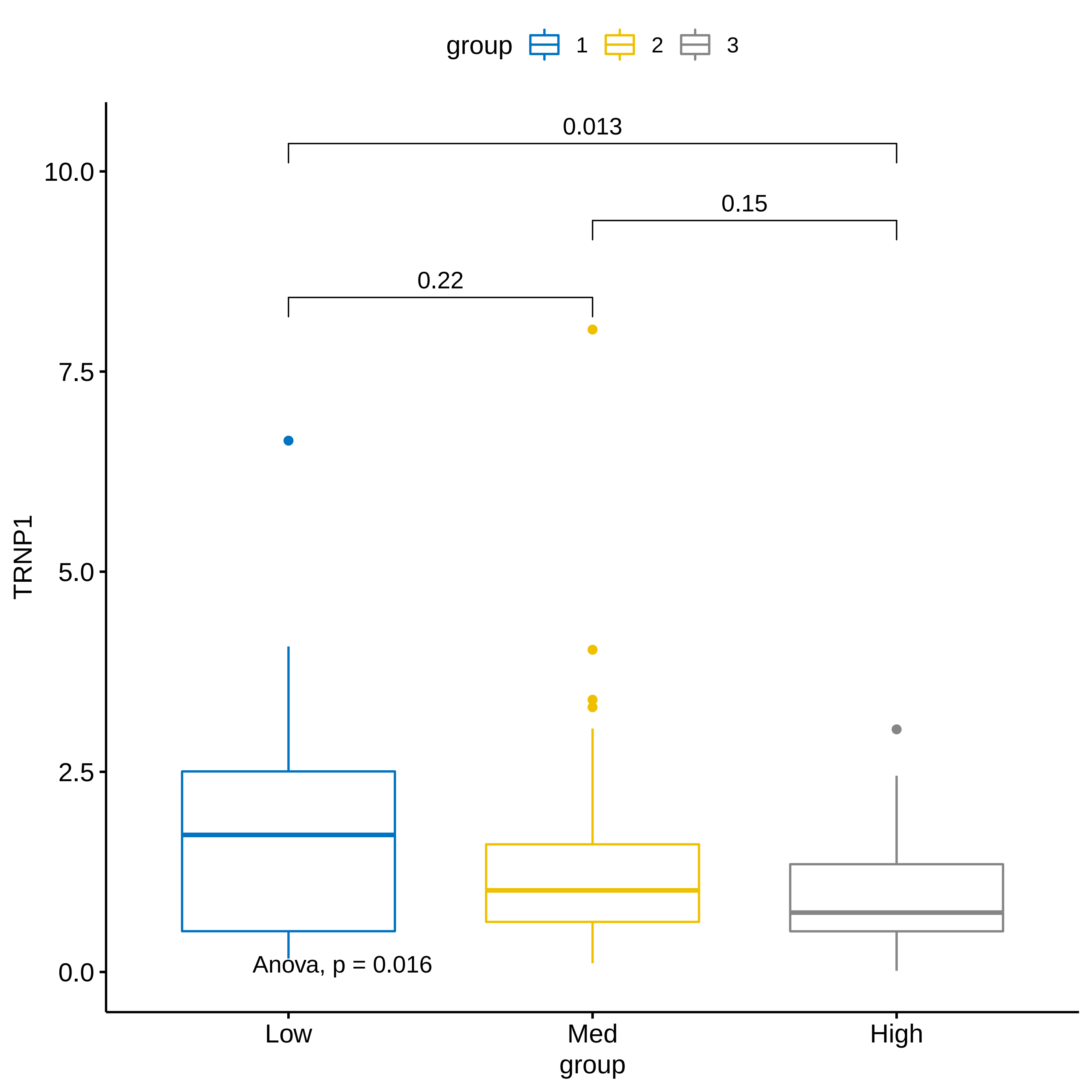

group 1 2 3
0.013
0.15
0.22
10.0
7.5
5.0
2.5
0.0
TRNP1
Anova, p = 0.016
Low
Med
High
group

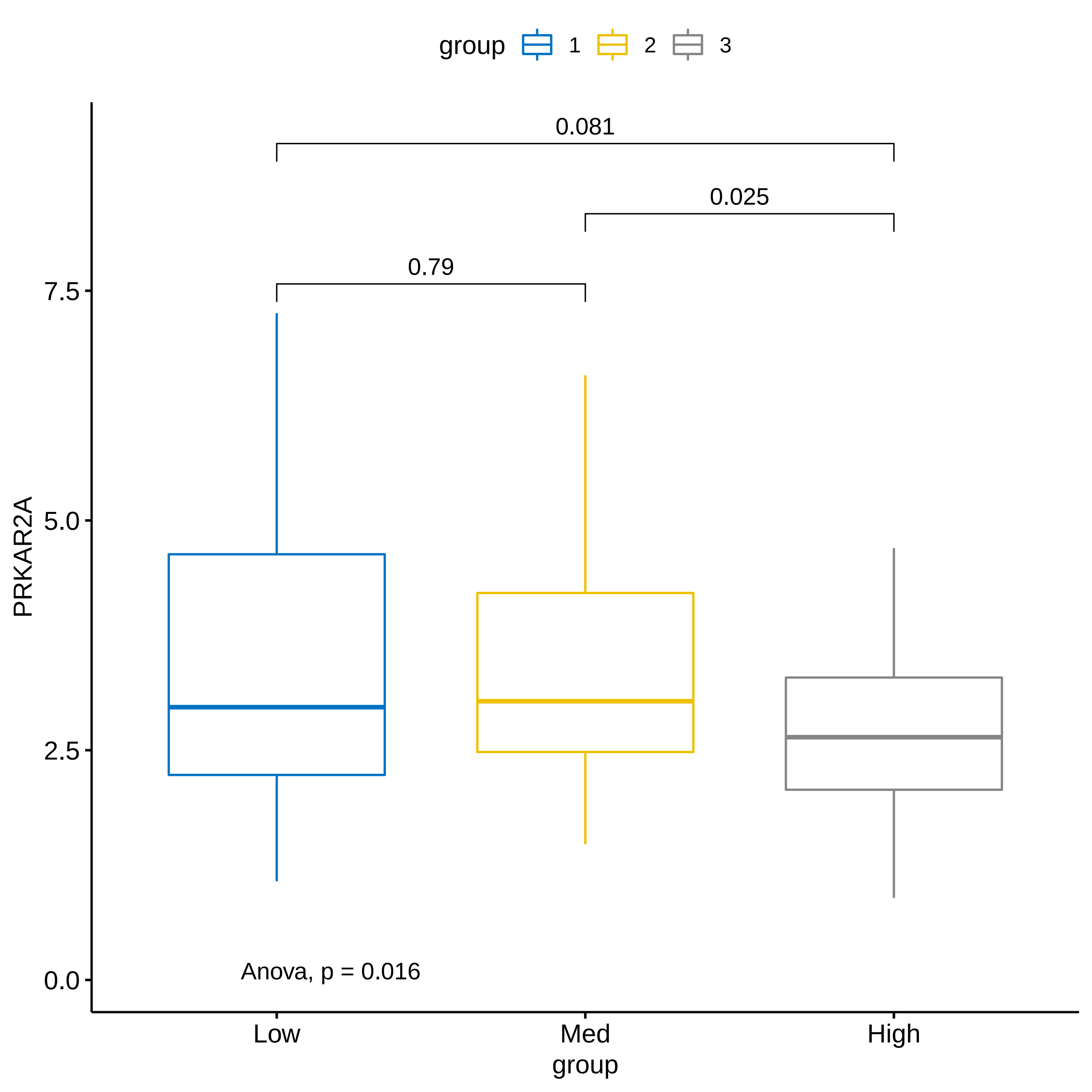
group
1
2
3
0.081
0.025
0.79
7.5
5.0
2.5
0.0
PRKAR2A
Anova, p = 0.016
Low
Med
High
group

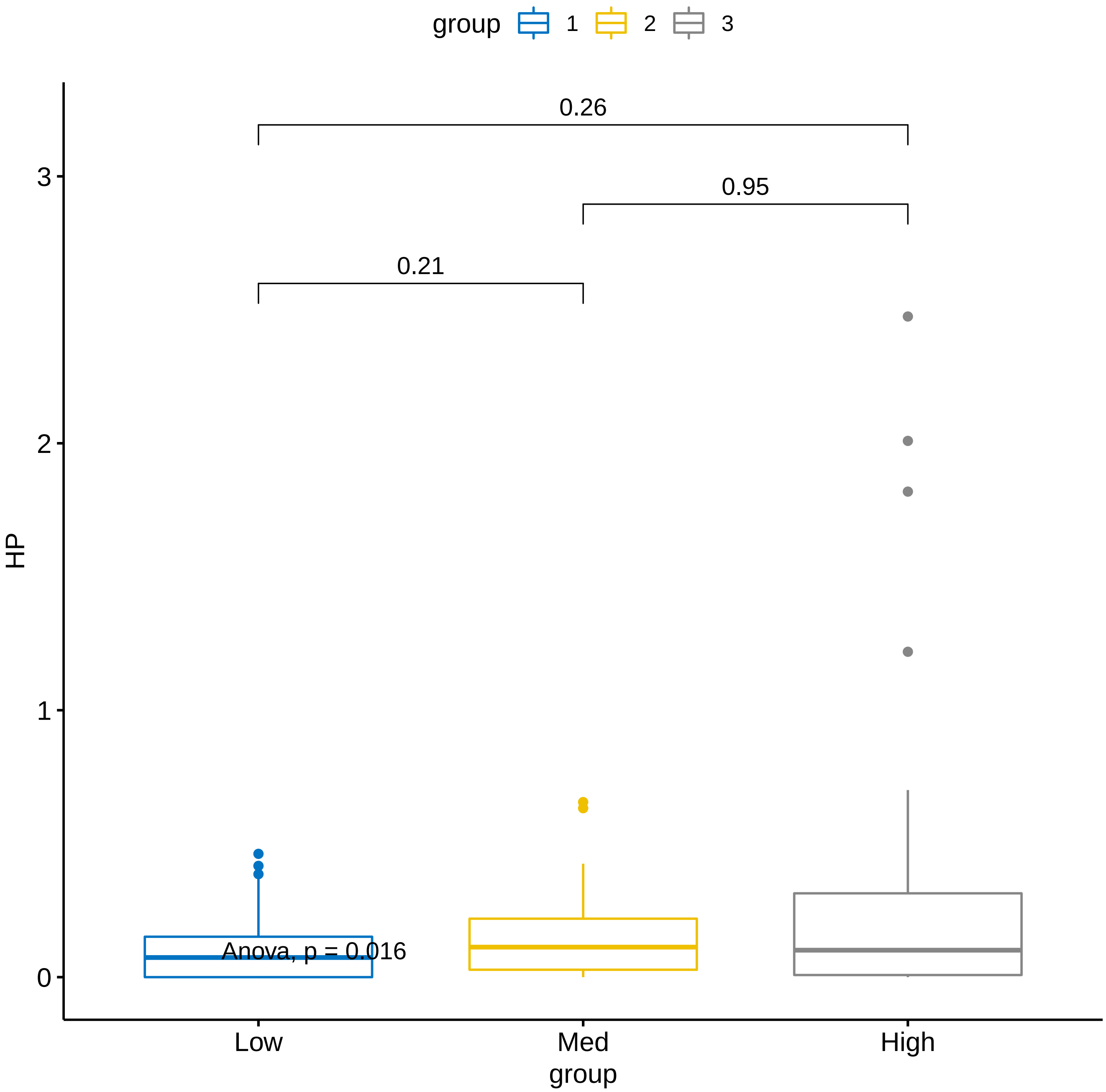

group 1 2 3
0.26
0.95
0.21
3
2
1
0
HP
Anova, p = 0.016
Low
Med
High
group

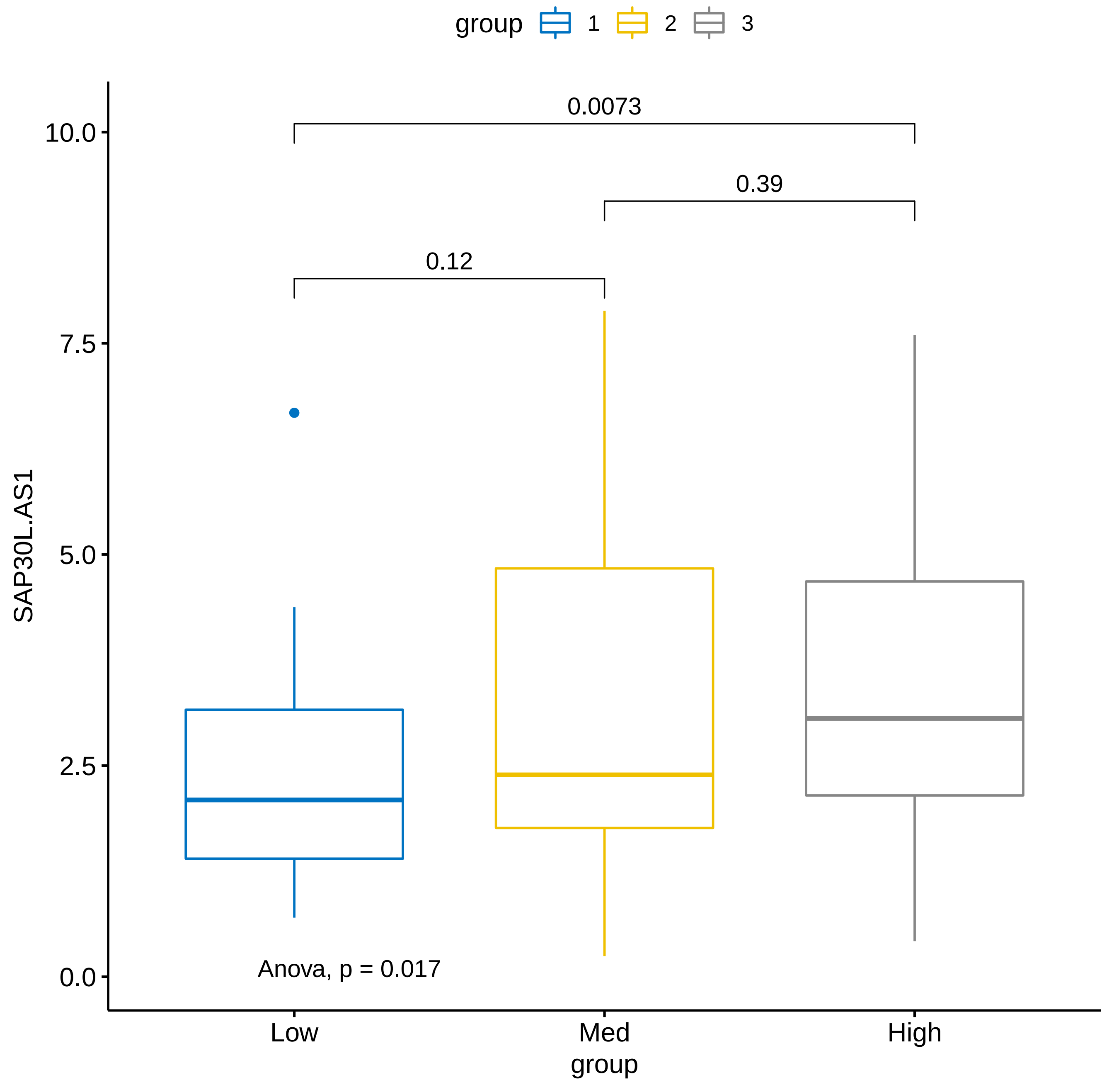
group
1
2
3
0.0073
0.39
0.12
10.0
7.5
5.0
2.5
0.0
SAP30L.AS1
Anova, p = 0.017
Low
Med
High
group

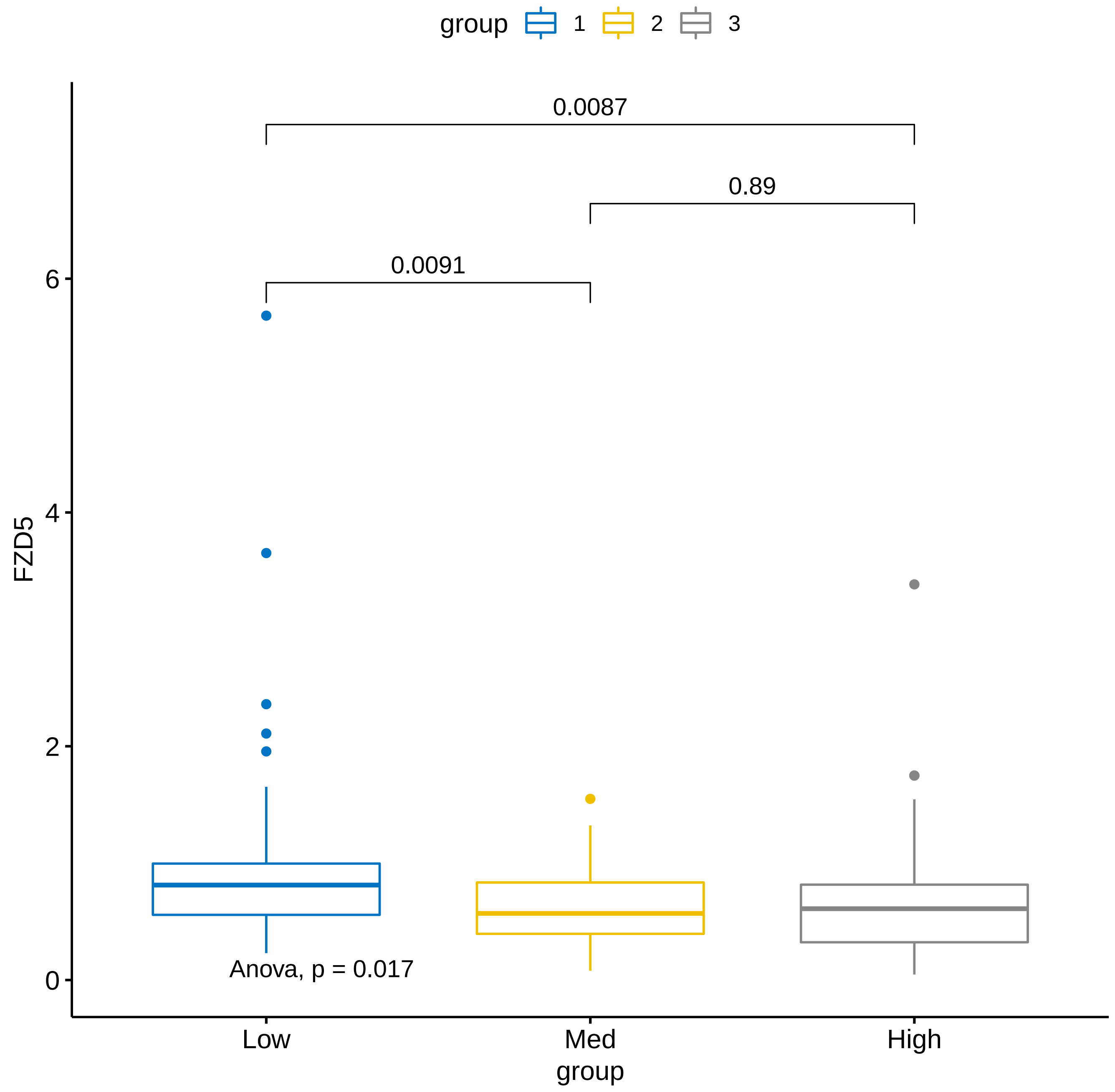
group
1
2
3
0.0087
0.89
0.0091
FZD5
6
4
2
0
Anova, p = 0.017
Low
Med
High
group

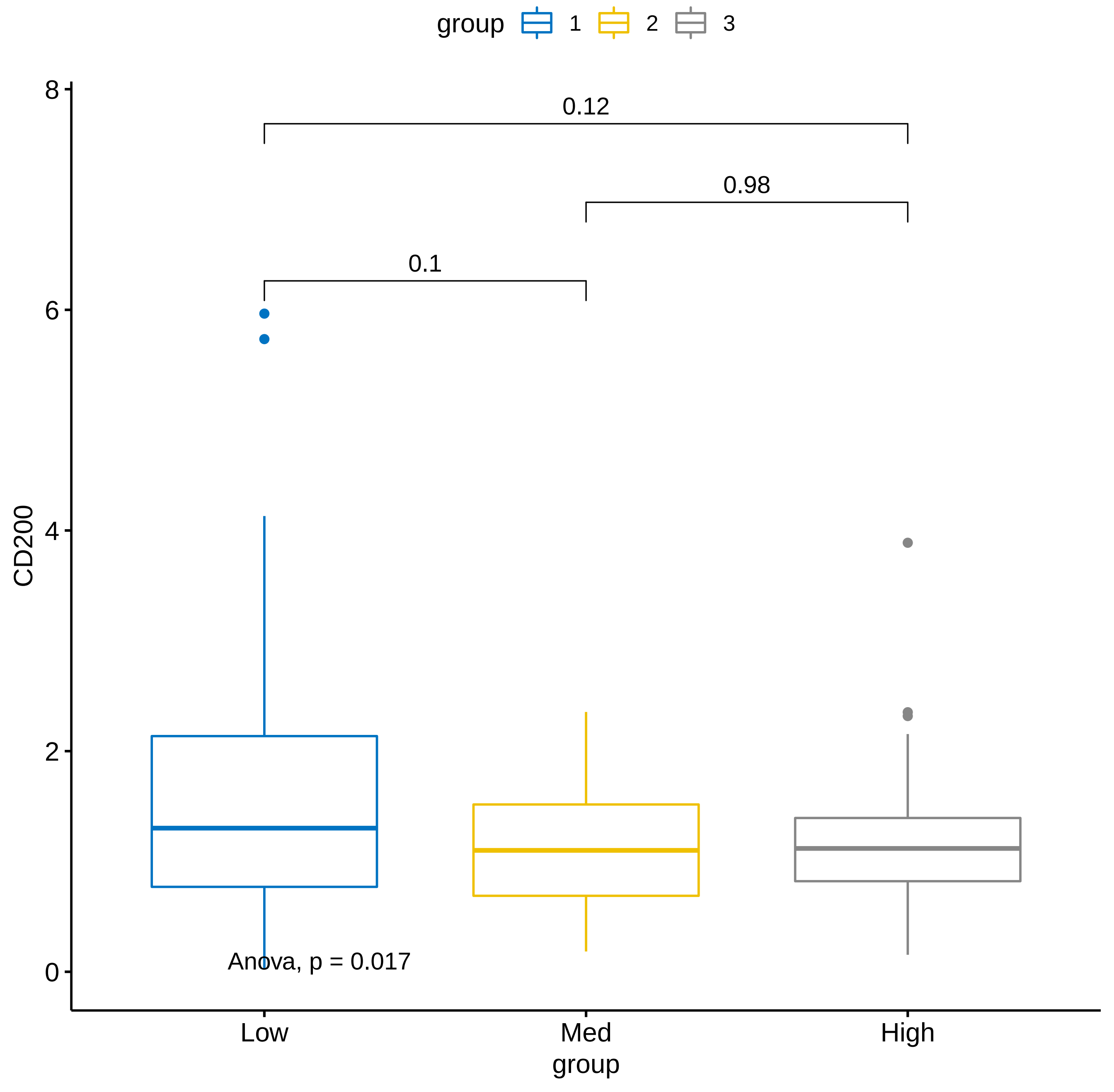

group
1
2
3
8
6
4
2
0
CD200
0.12
0.98
0.1
Anova, p = 0.017
Low
Med
High
group

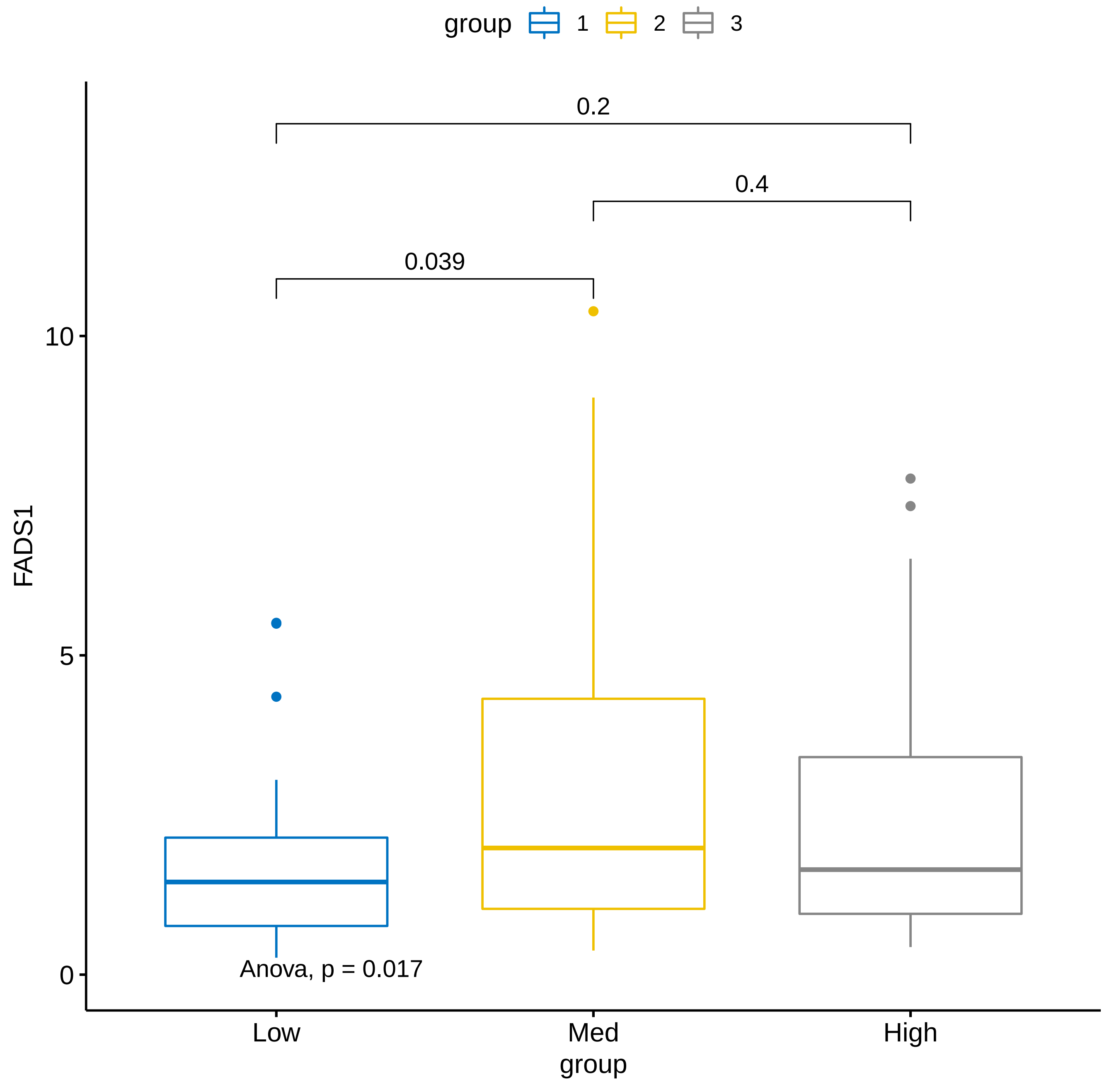

group
1
2
3
0.2
0.4
0.039
FADS1
10
5
0
Anova, p = 0.017
Low
Med
High
group

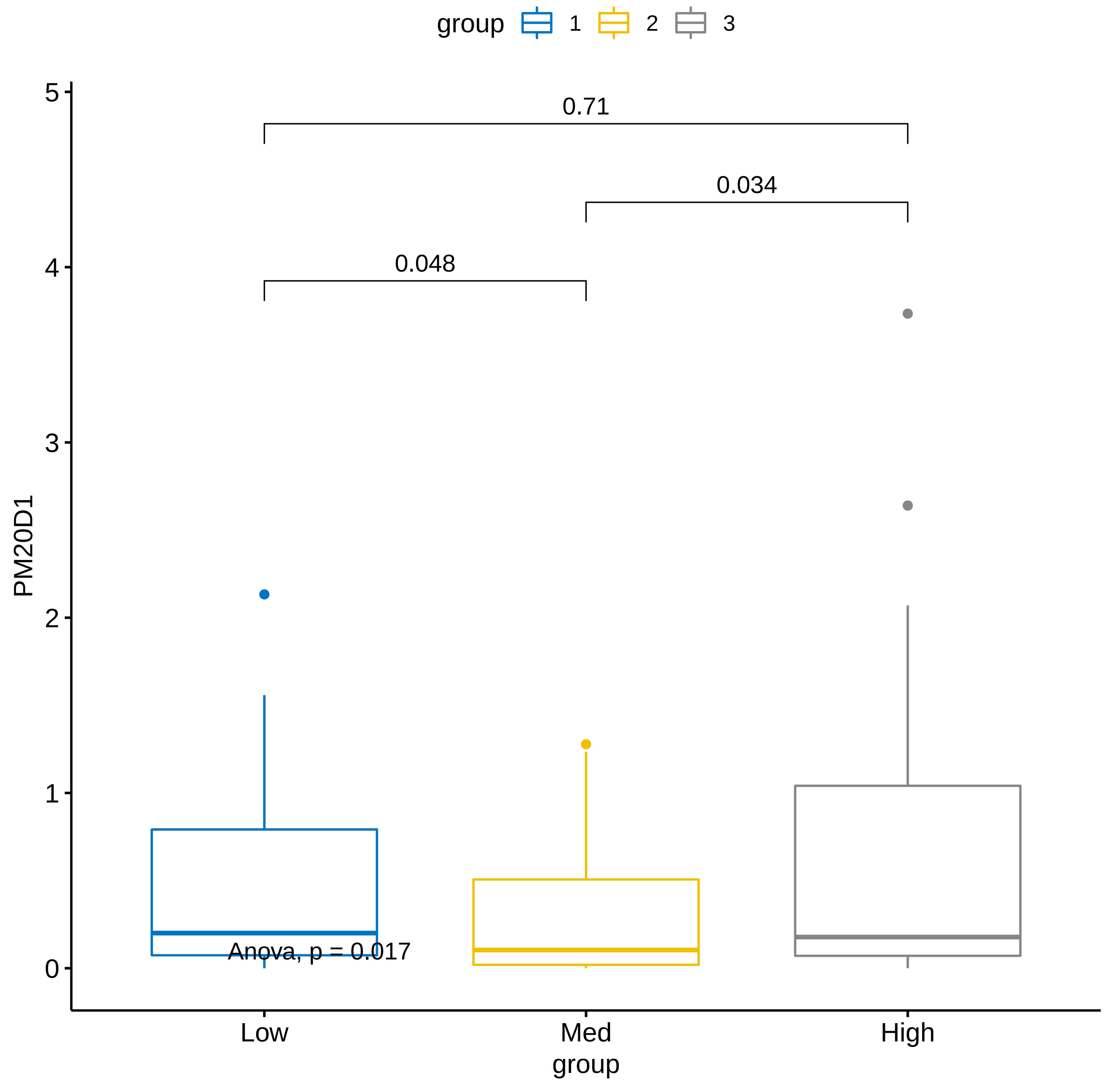

group
1
2
3
0.71
0.034
0.048
Anova, p = 0.017
PM20D1
0
1
2
3
4
5
Low
Med
High
group

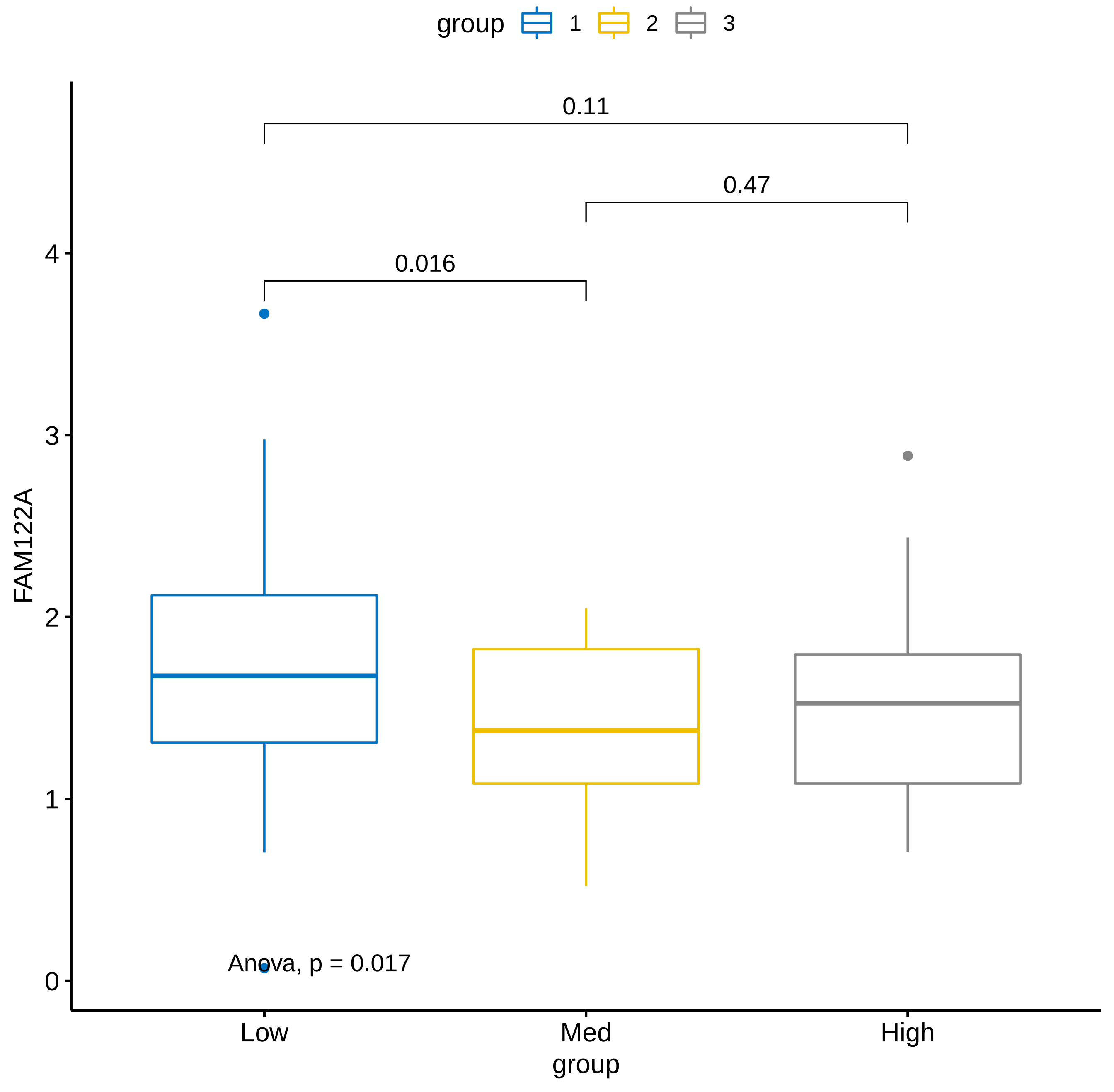

group 1 2 3
0.11
0.47
0.016
FAM122A
4
3
2
1
0
Anova, p = 0.017
Low
Med
High
group

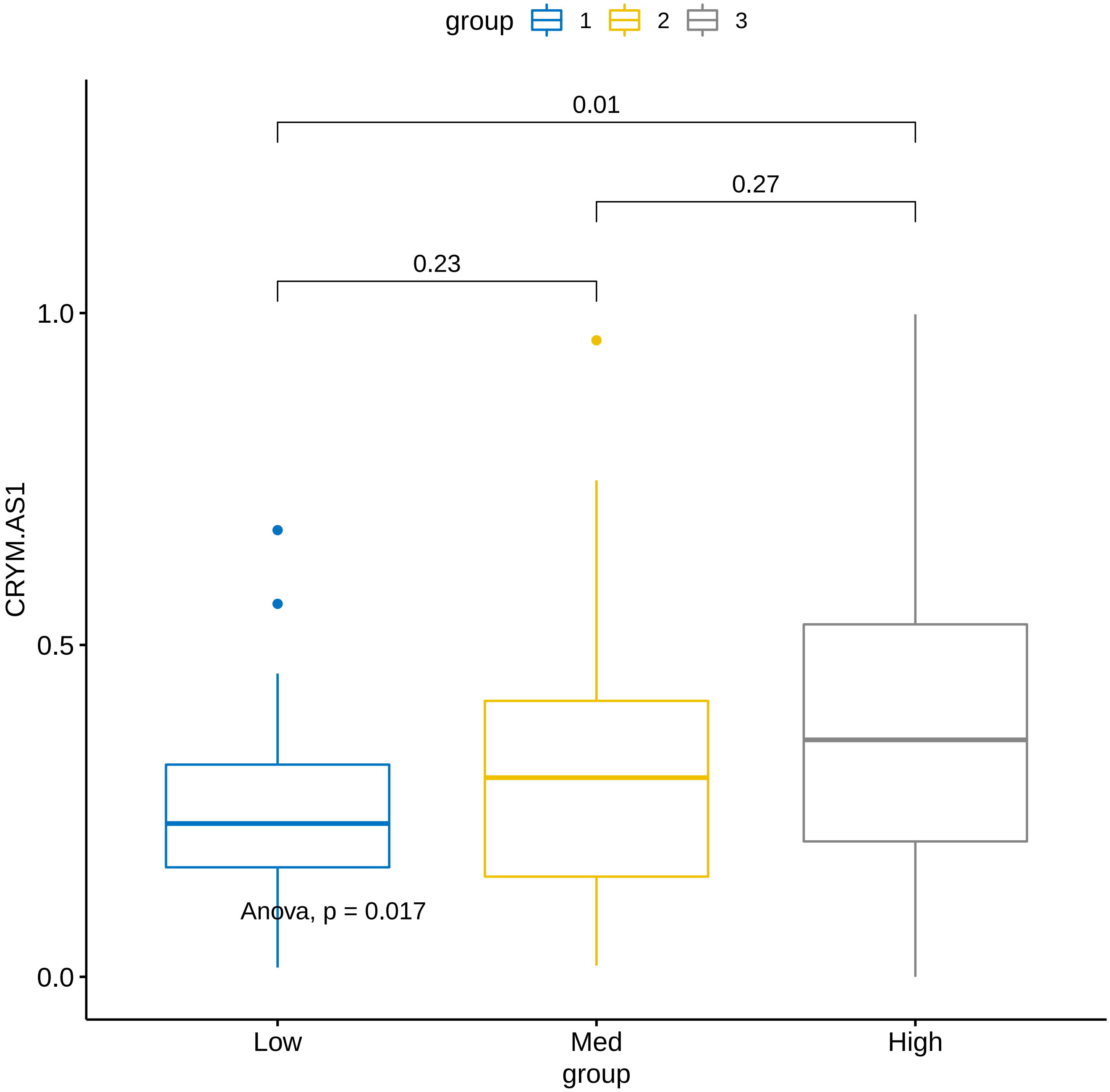
group
1
2
3
0.01
0.27
0.23
1.0
0.5
0.0
CRYM.AS1
Anova, p = 0.017
Low
Med
High
group

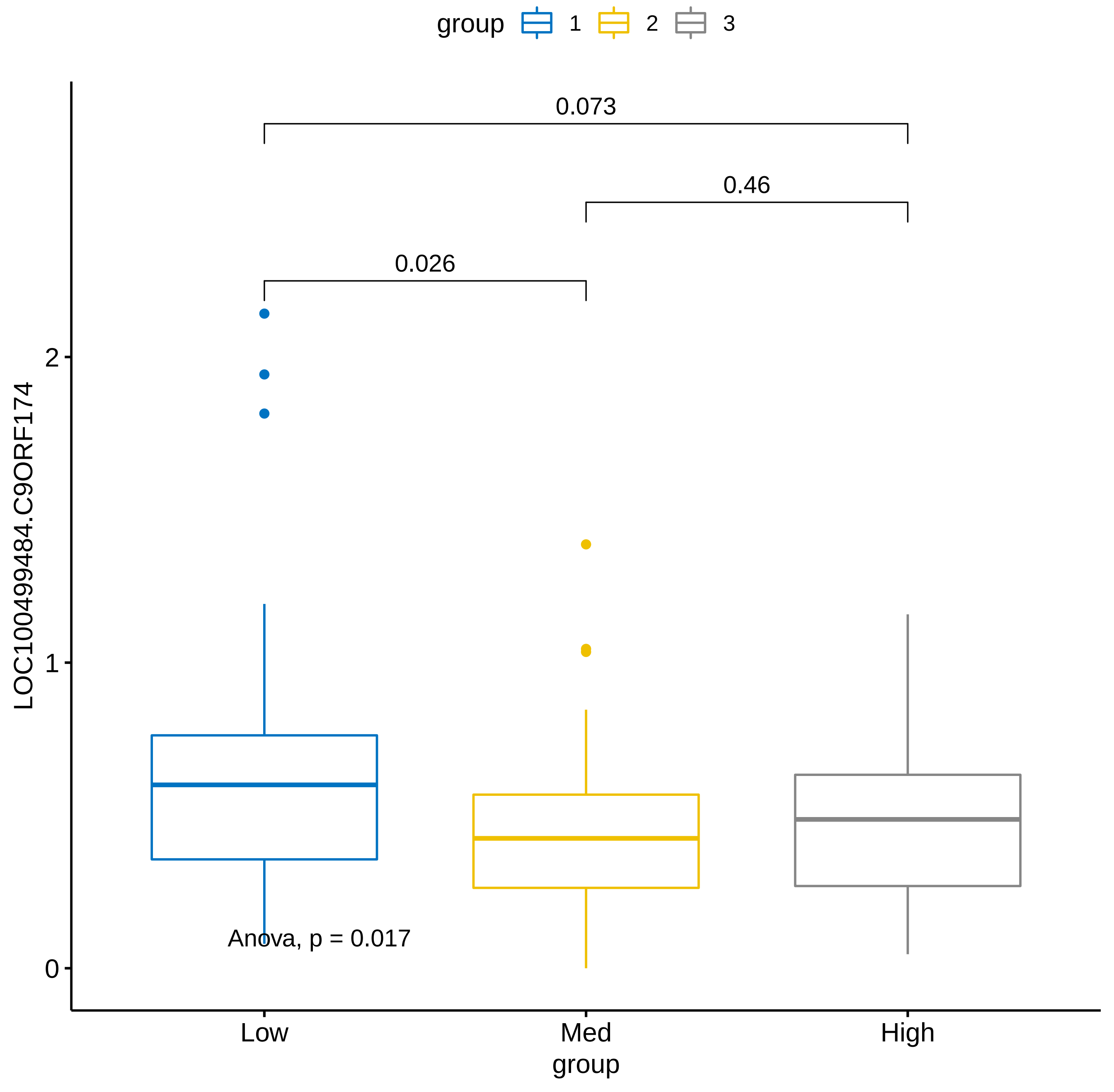

group
1
2
3
0.073
0.46
0.026
LOC100499484.C9ORF174
2
1
0
Anova, p = 0.017
Low
Med
High
group

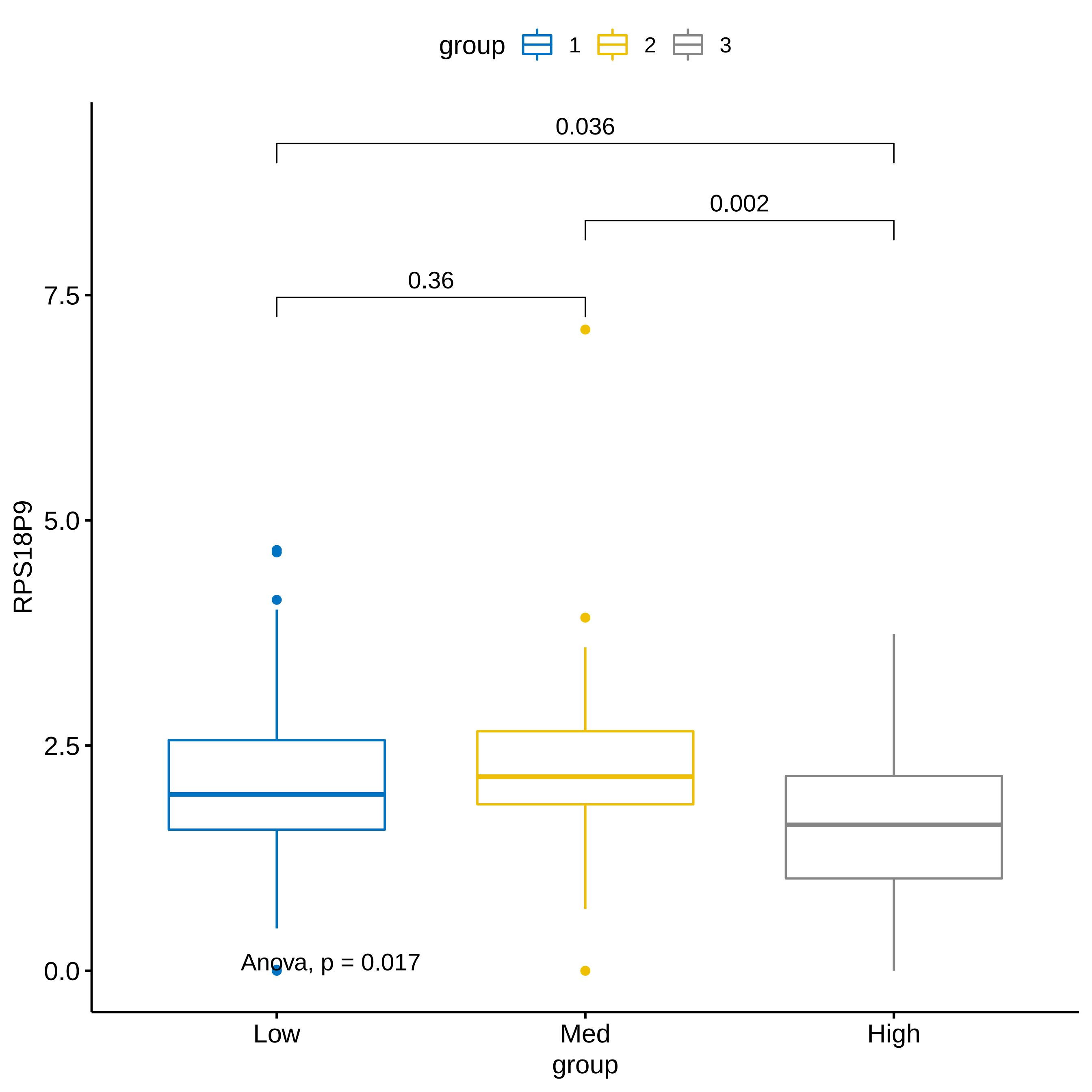

group
1
2
3
0.036
0.002
0.36
7.5
5.0
2.5
0.0
RPS18P9
Anova, p = 0.017
Low
Med
High
group

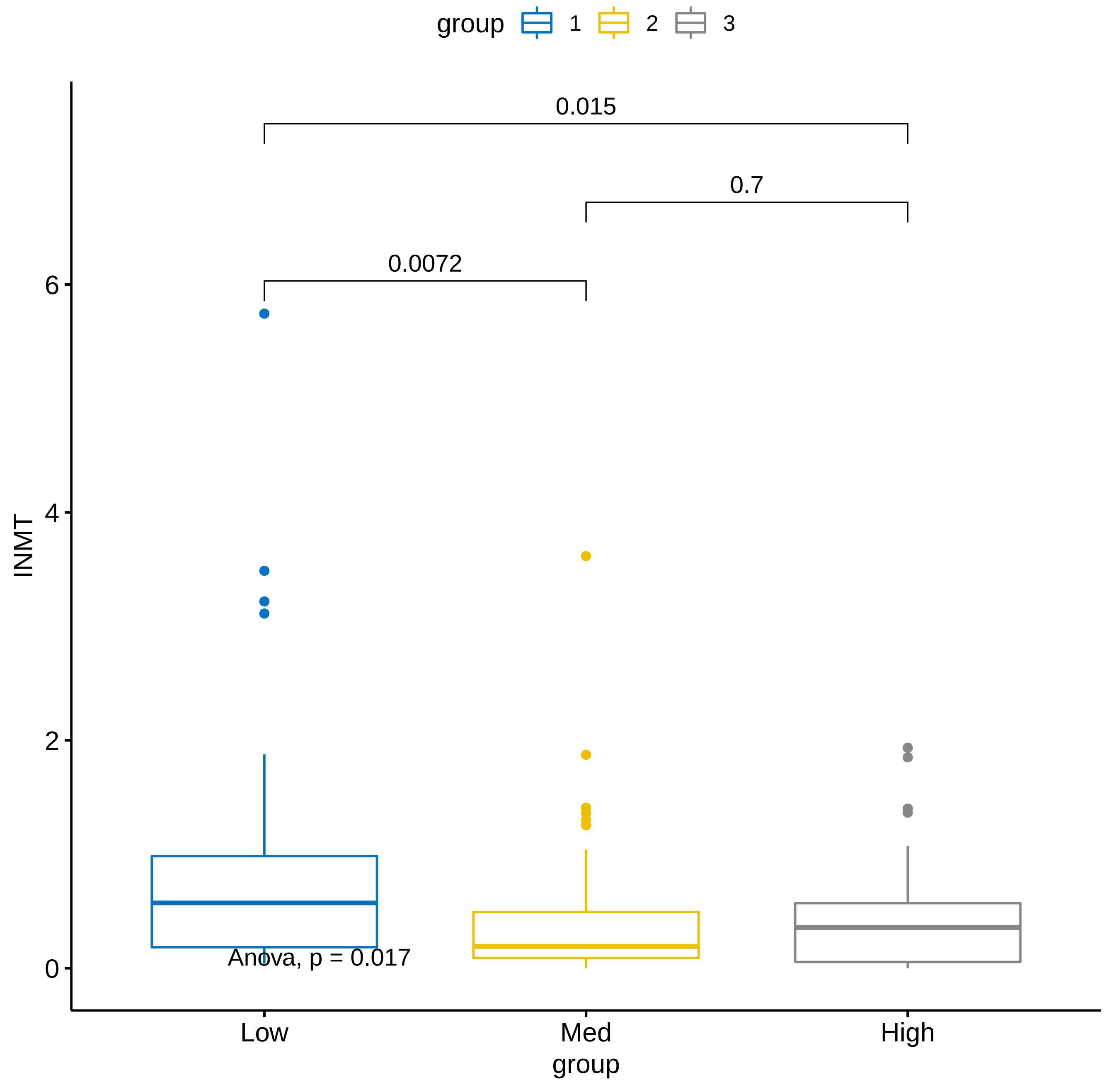
group
1
2
3
0.015
0.7
0.0072
6
4
2
0
INMT
Anova, p = 0.017
Low
Med
High
group

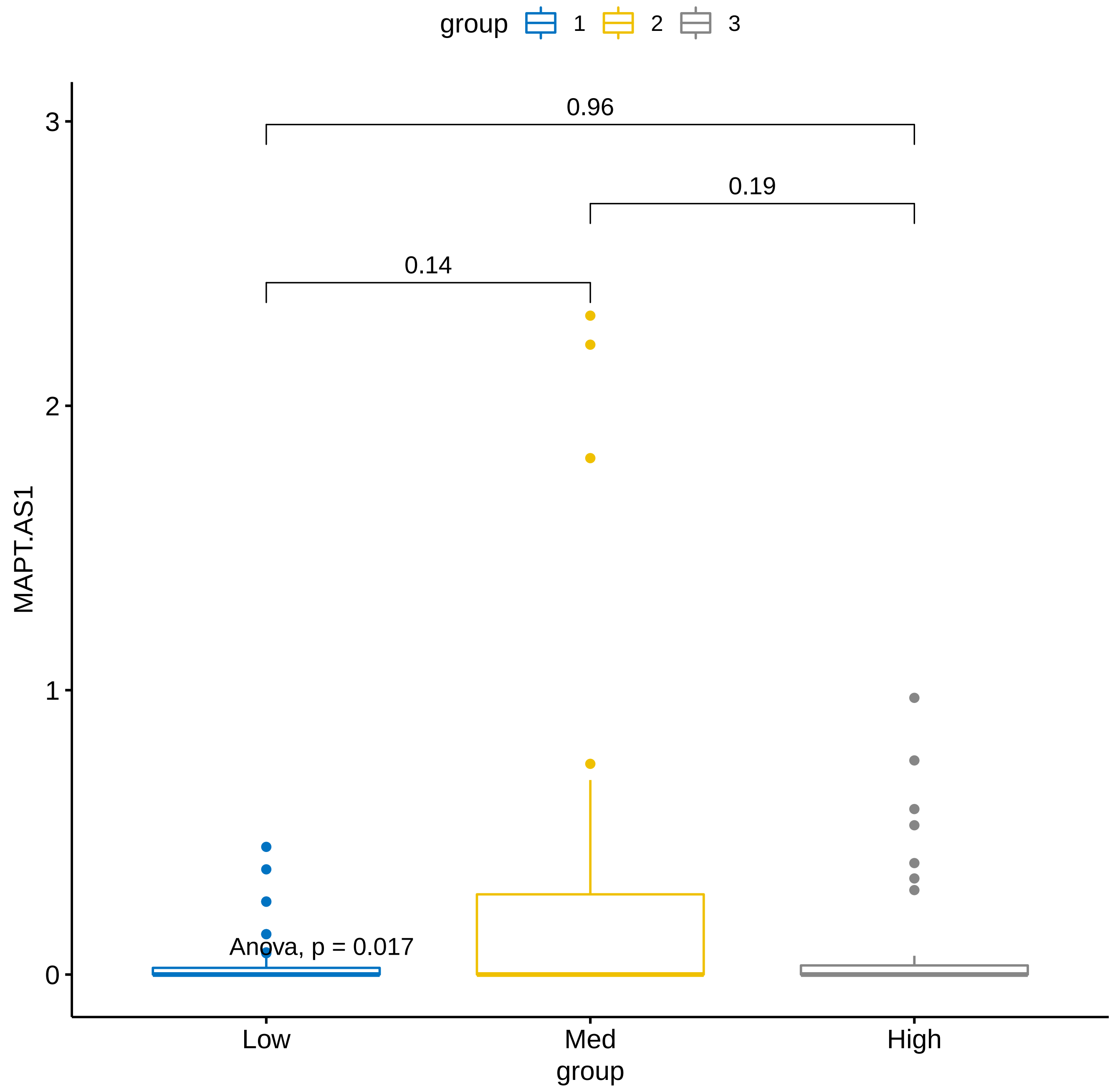

group
1
2
3
0.96
0.19
0.14
3
2
1
0
MAPT.AS1
Anova, p = 0.017
Low
Med
High
group

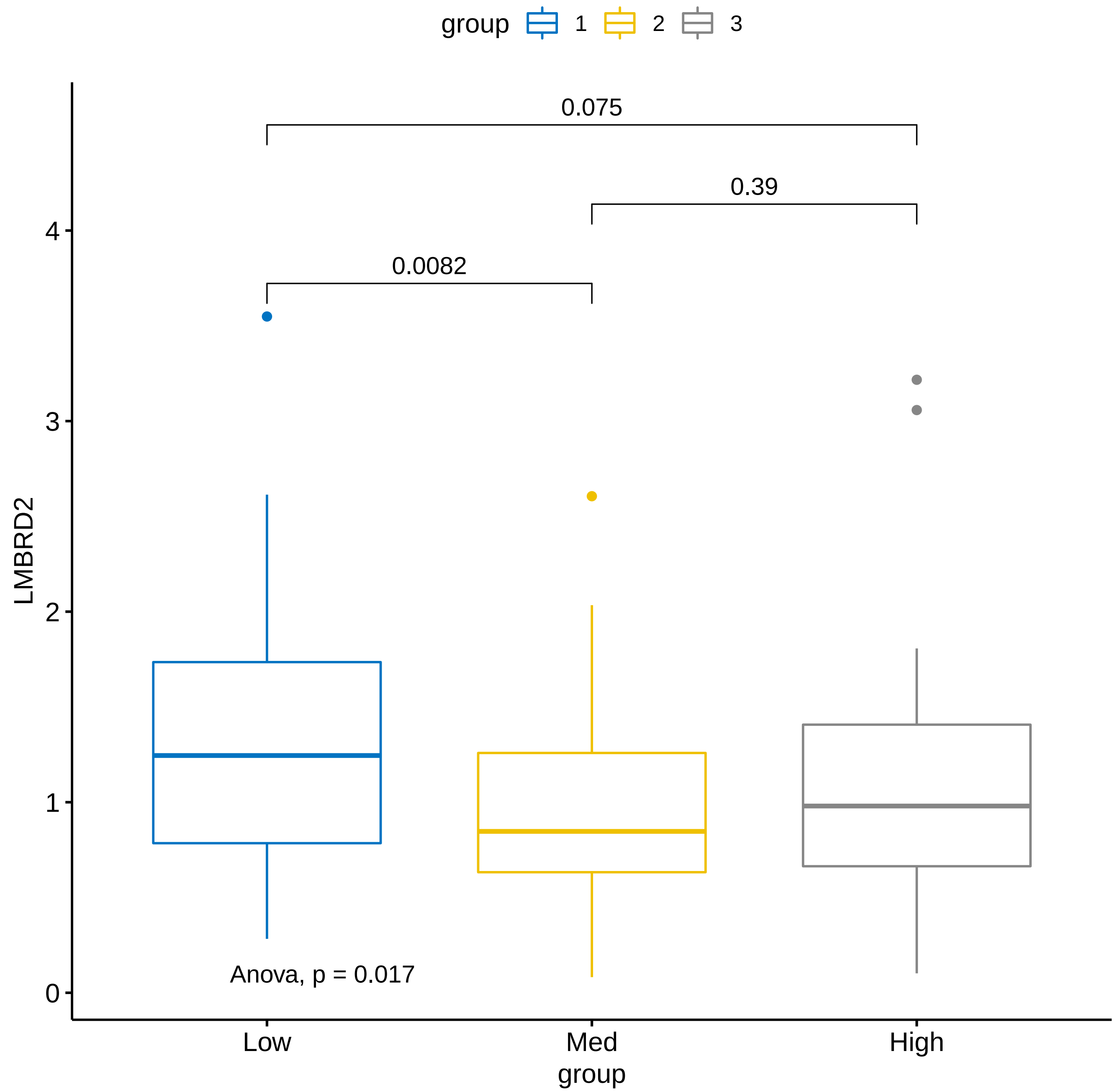

group 1 2 3
0.075
0.39
0.0082
LMBRD2
4
3
2
1
0
Anova, p = 0.017
Low
Med
High
group

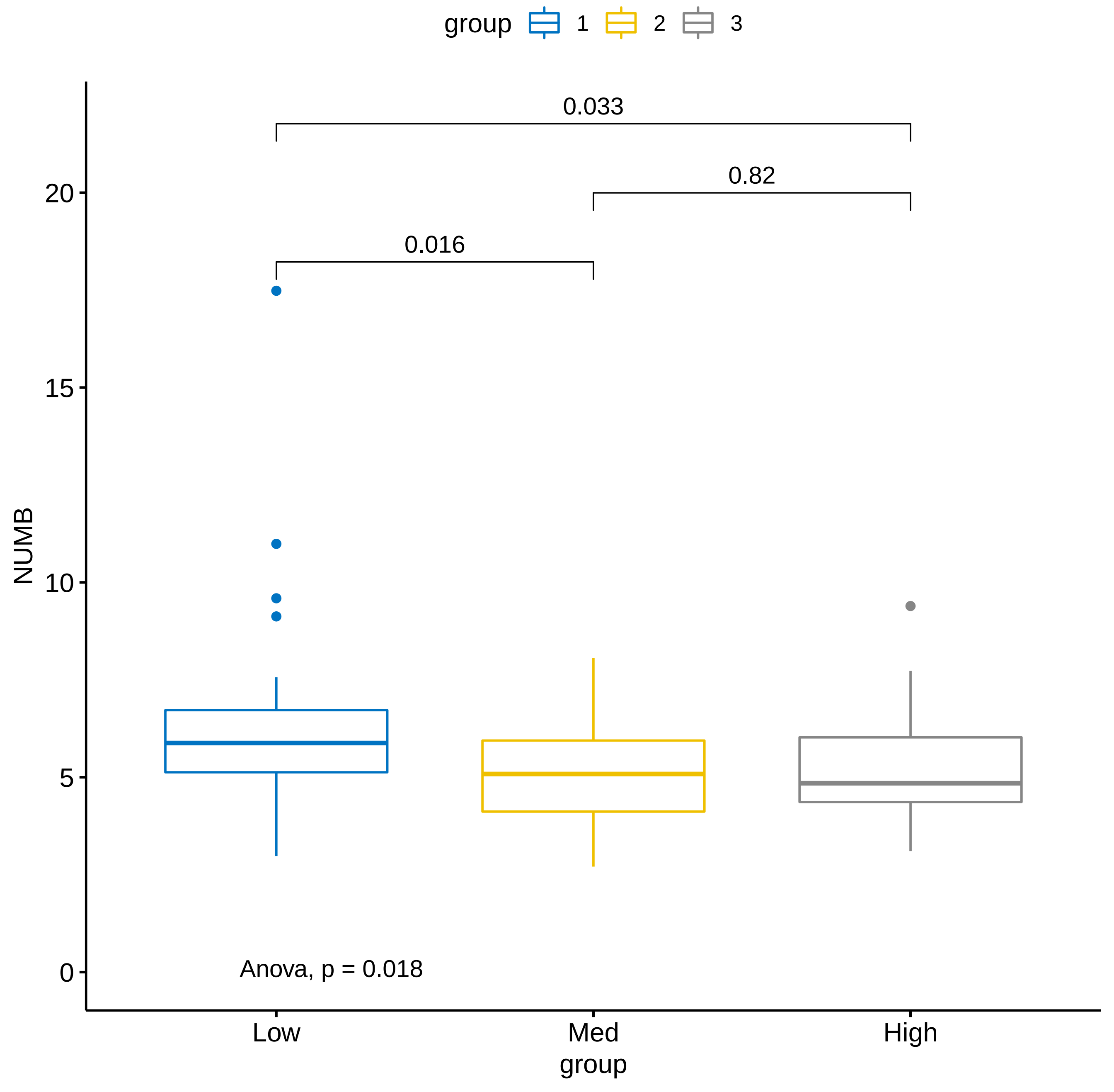
group 1 2 3
0.033
0.82
0.016
20
15
10
5
0
NUMB
Anova, p = 0.018
Low
Med
High
group

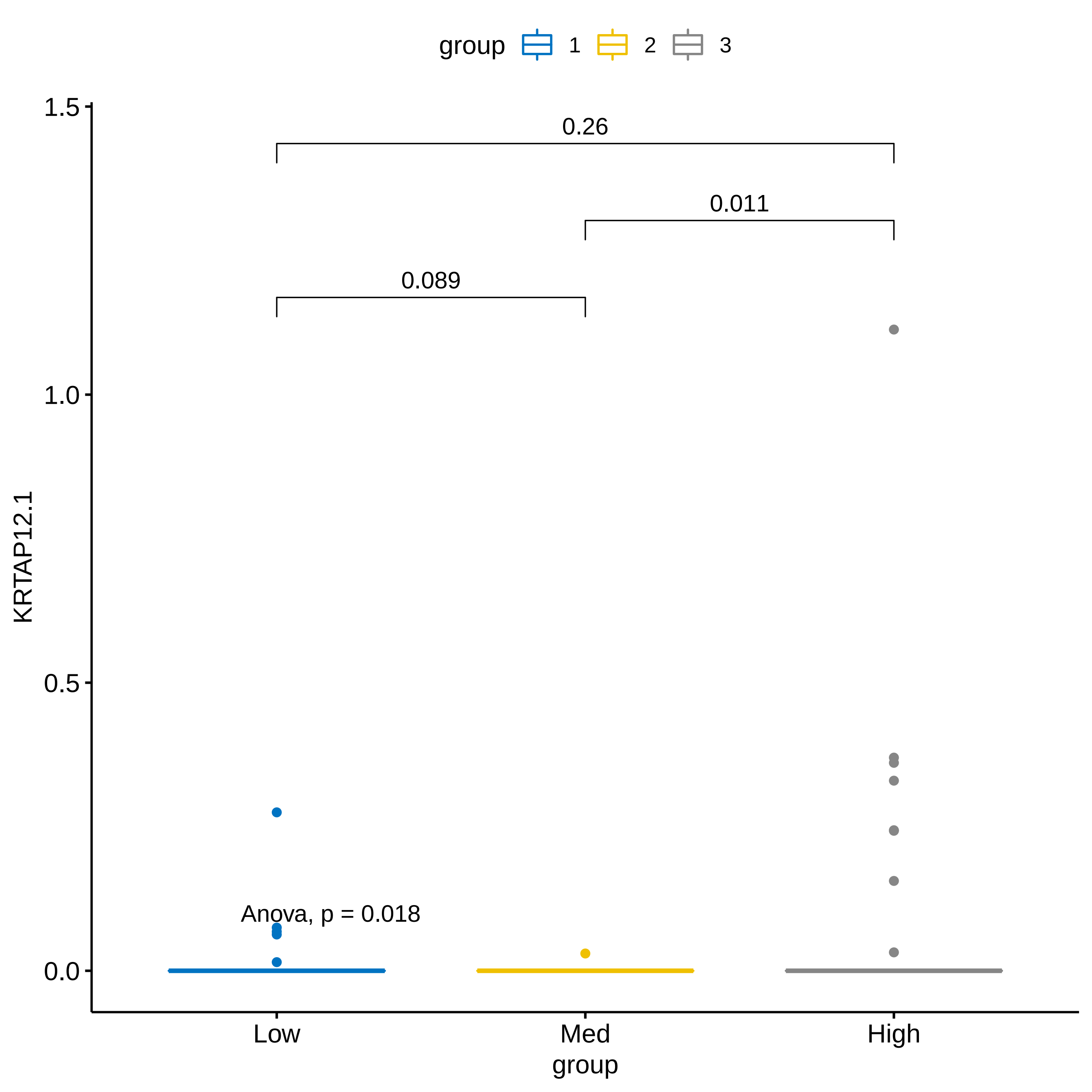
group
1
2
3
1.5
1.0
0.5
0.0
KRTAP12.1
0.26
0.011
0.089
Anova, p = 0.018
Low
Med
High
group

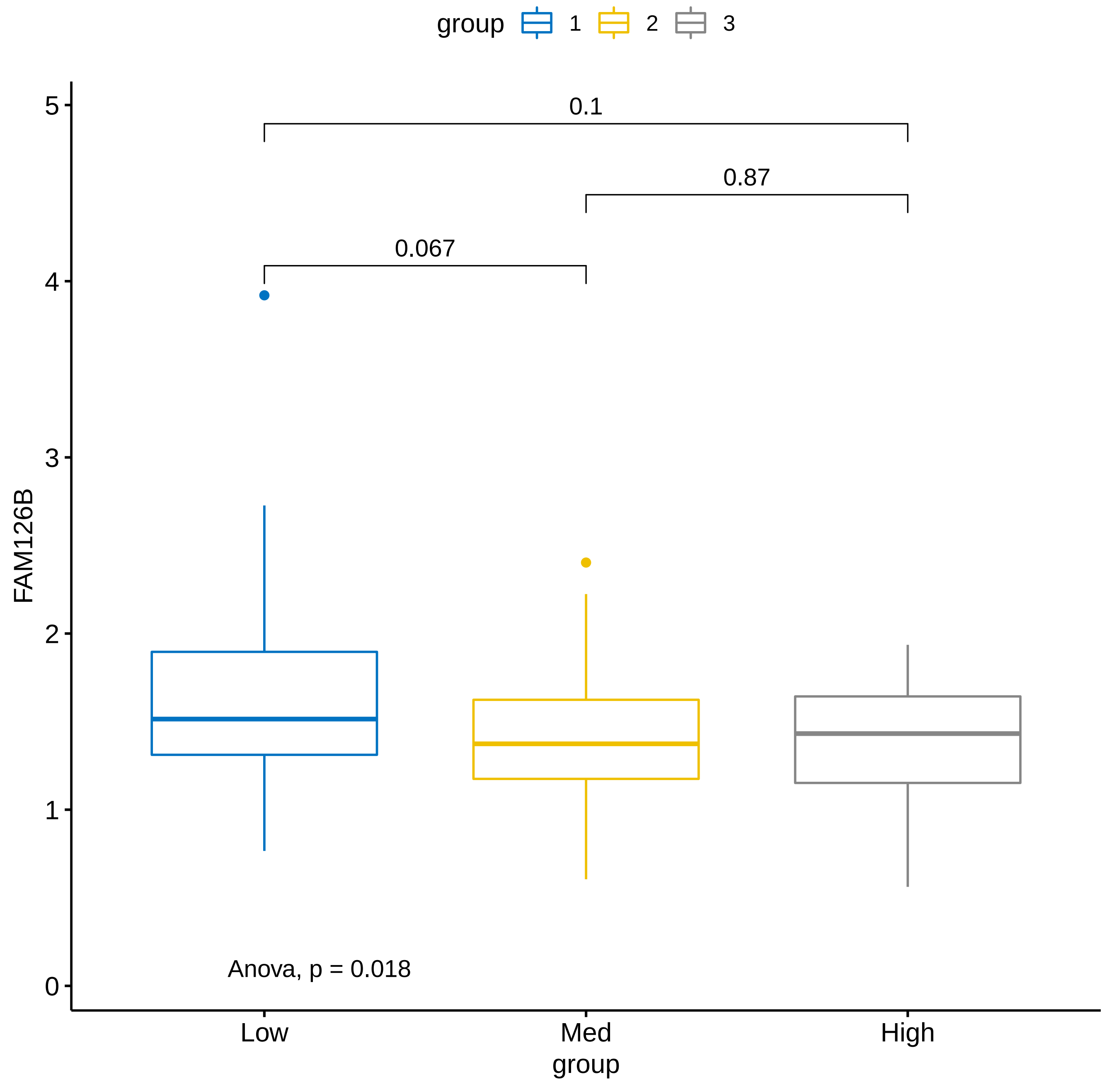

group 1 2 3
0.1
0.87
0.067
FAM126B
5
4
3
2
1
0
Anova, p = 0.018
Low
Med
High
group

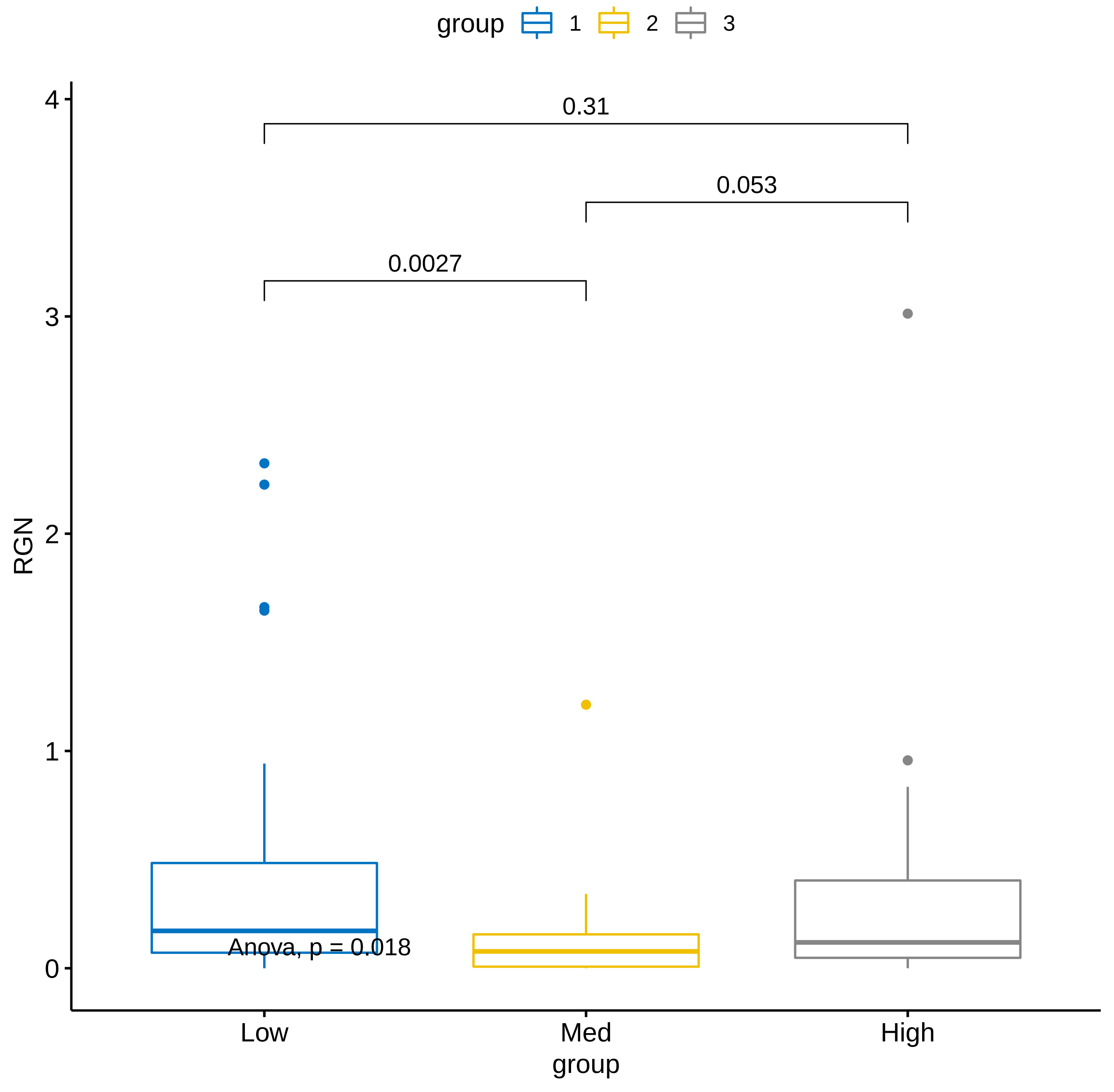
group 1 2 3
0.31
0.053
0.0027
Anova, p = 0.018
RGN
0
1
2
3
4
Low
Med
High
group

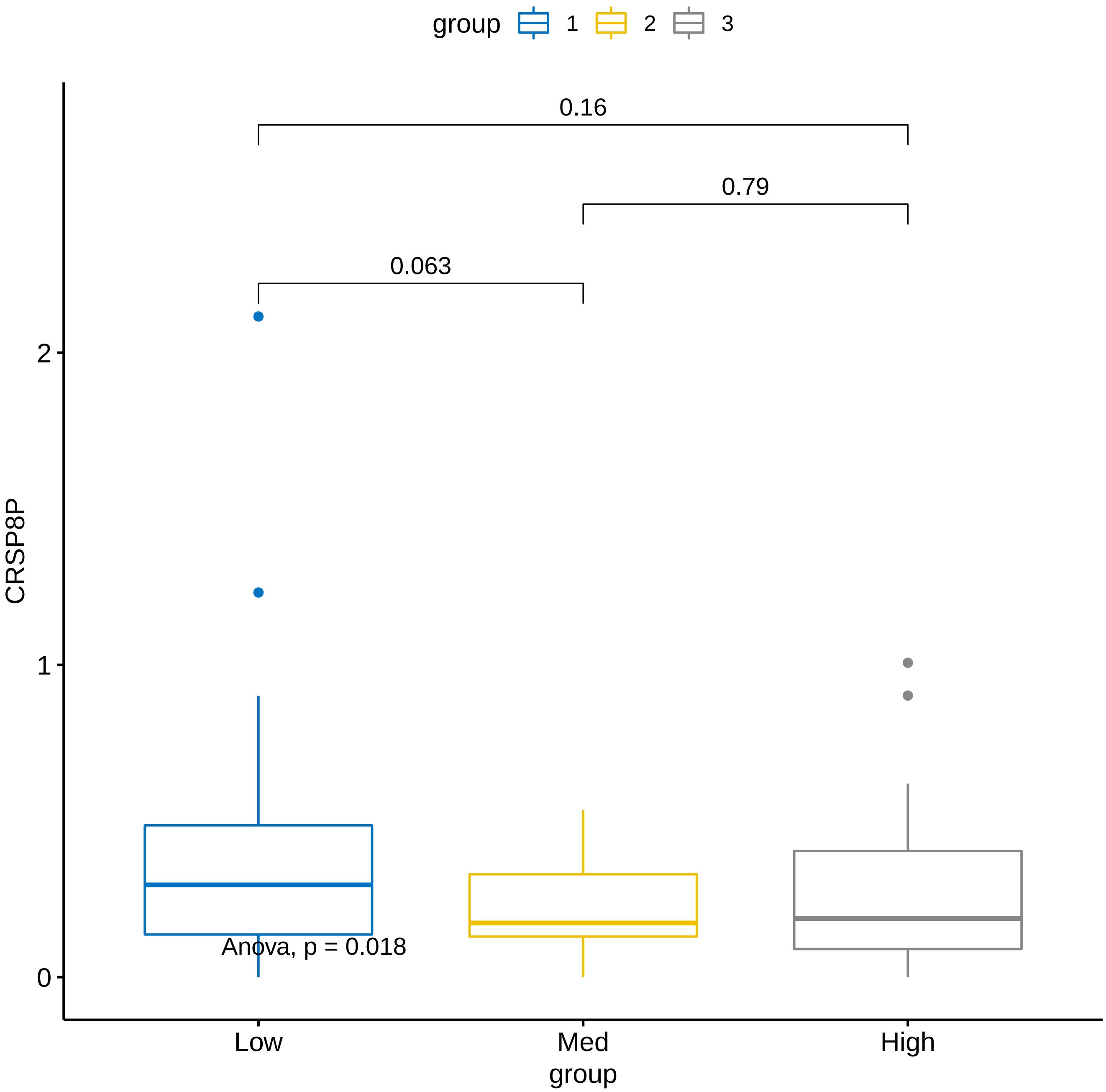
group
1
2
3
0.16
0.79
0.063
CRSP8P
2
1
0
Anova, p = 0.018
Low
Med
High
group

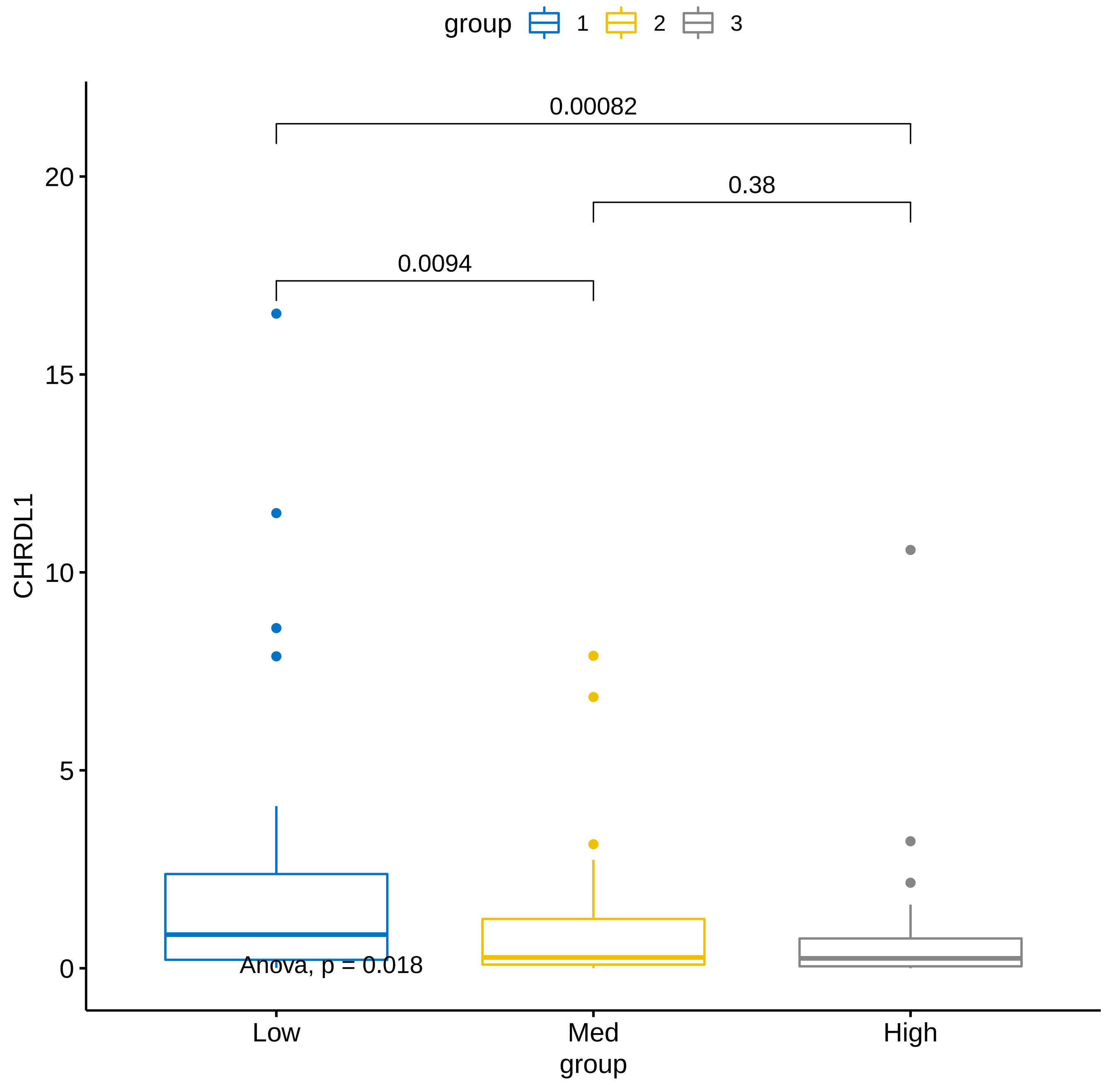

group 1 2 3
0.00082
0.38
0.0094
20
15
10
5
0
CHRDL1
Anova, p = 0.018
Low
Med
High
group

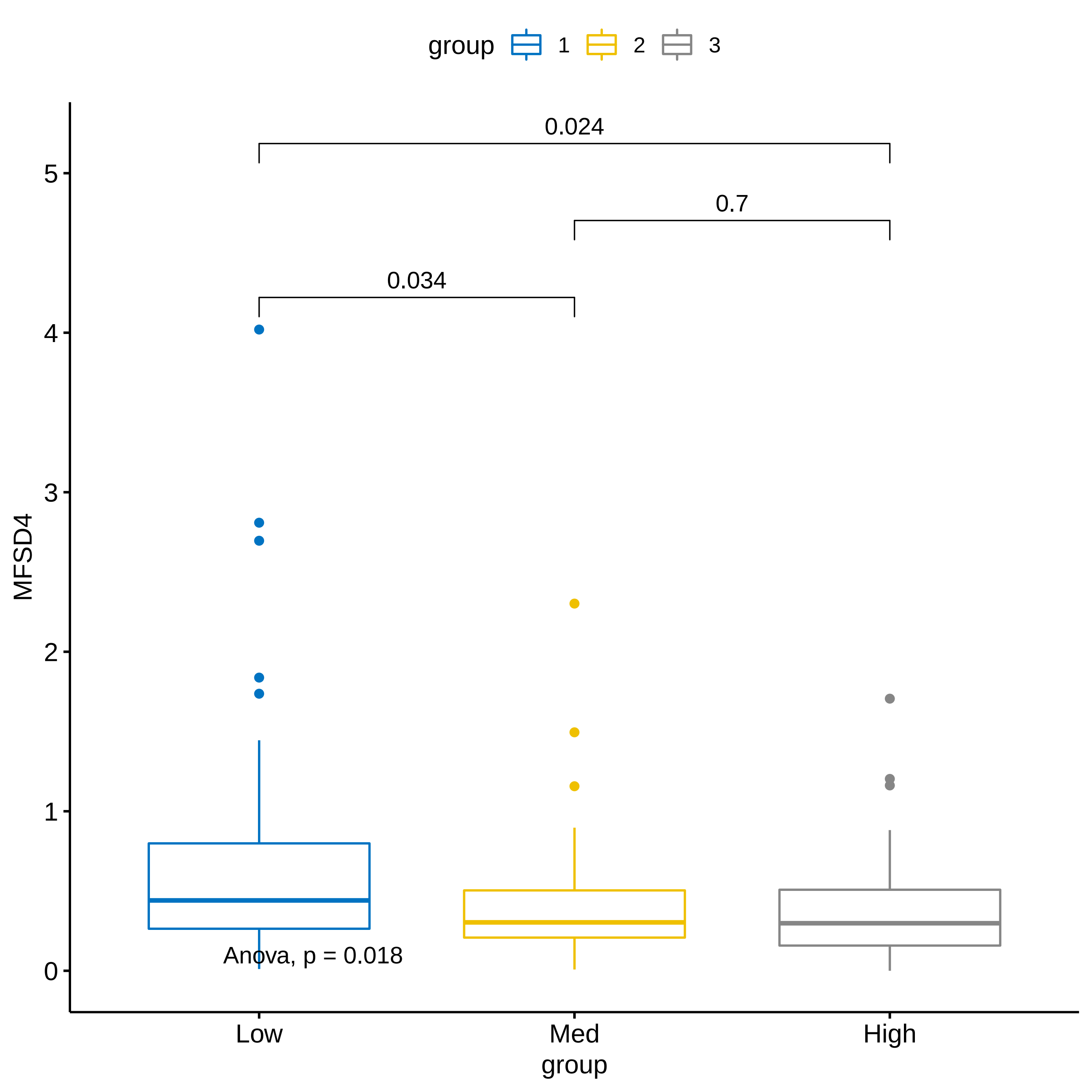
group 1 2 3
0.024
0.7
0.034
MFSD4
5
4
3
2
1
0
Anova, p = 0.018
Low
Med
High
group

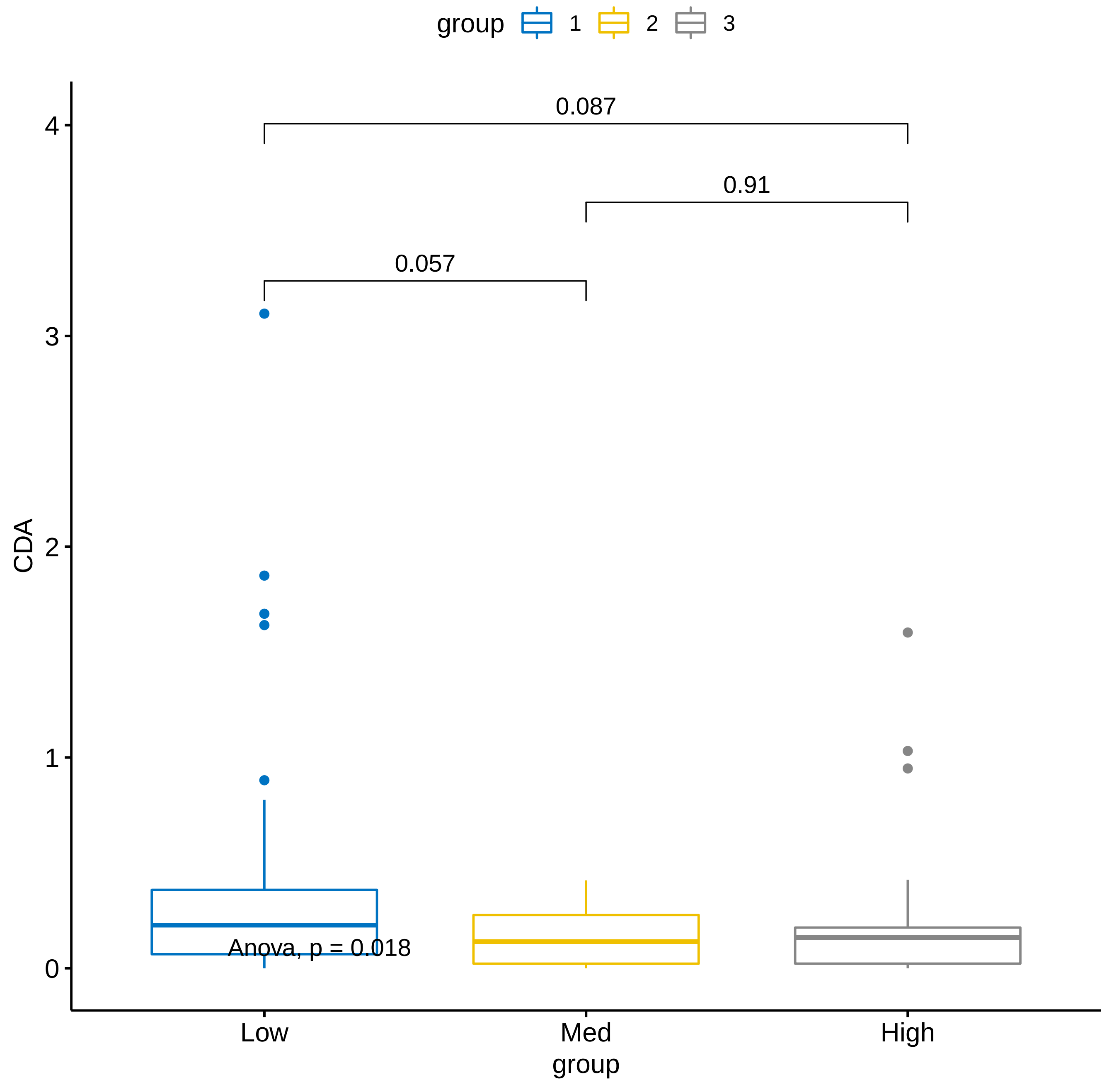

group 1 2 3
0.087
0.91
0.057
CDA
4
3
2
1
0
Anova, p = 0.018
Low
Med
High
group

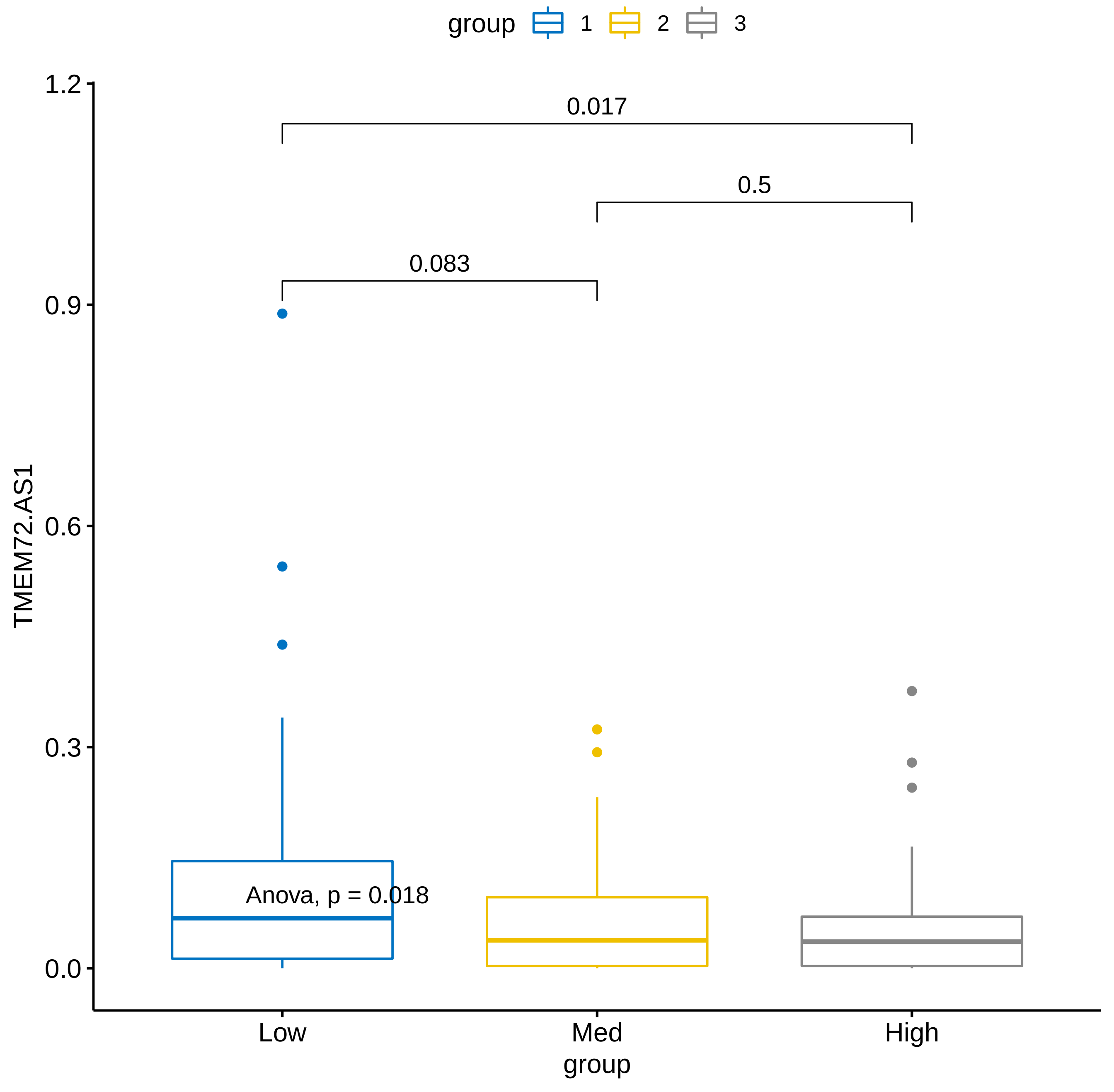
group
1
2
3
1.2
0.9
0.6
0.3
0.0
TMEM72.AS1
0.017
0.5
0.083
Anova, p = 0.018
Low
Med
High
group

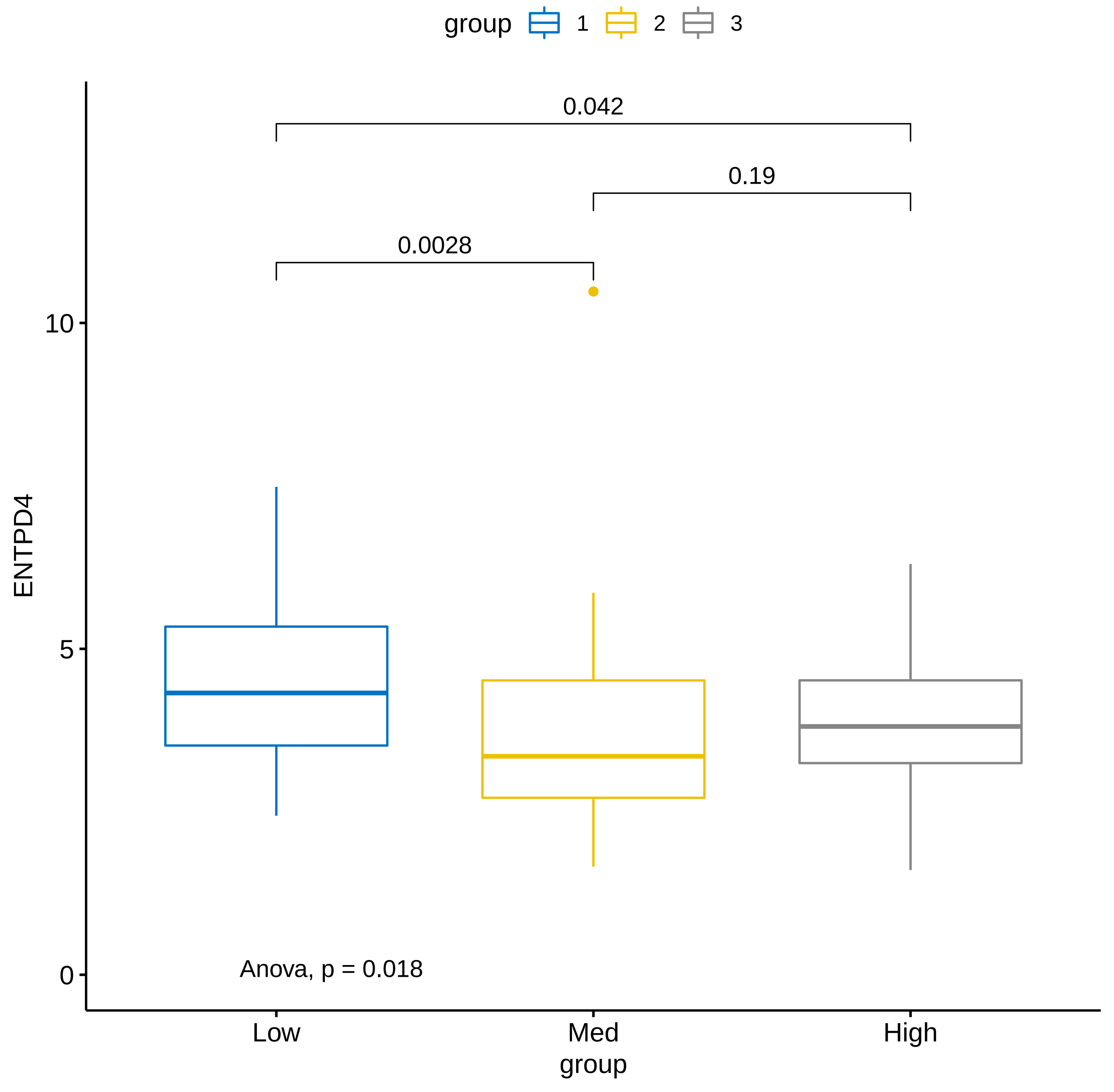
group
1
2
3
0.042
0.19
0.0028
10
5
0
ENTPD4
Anova, p = 0.018
Low
Med
High
group

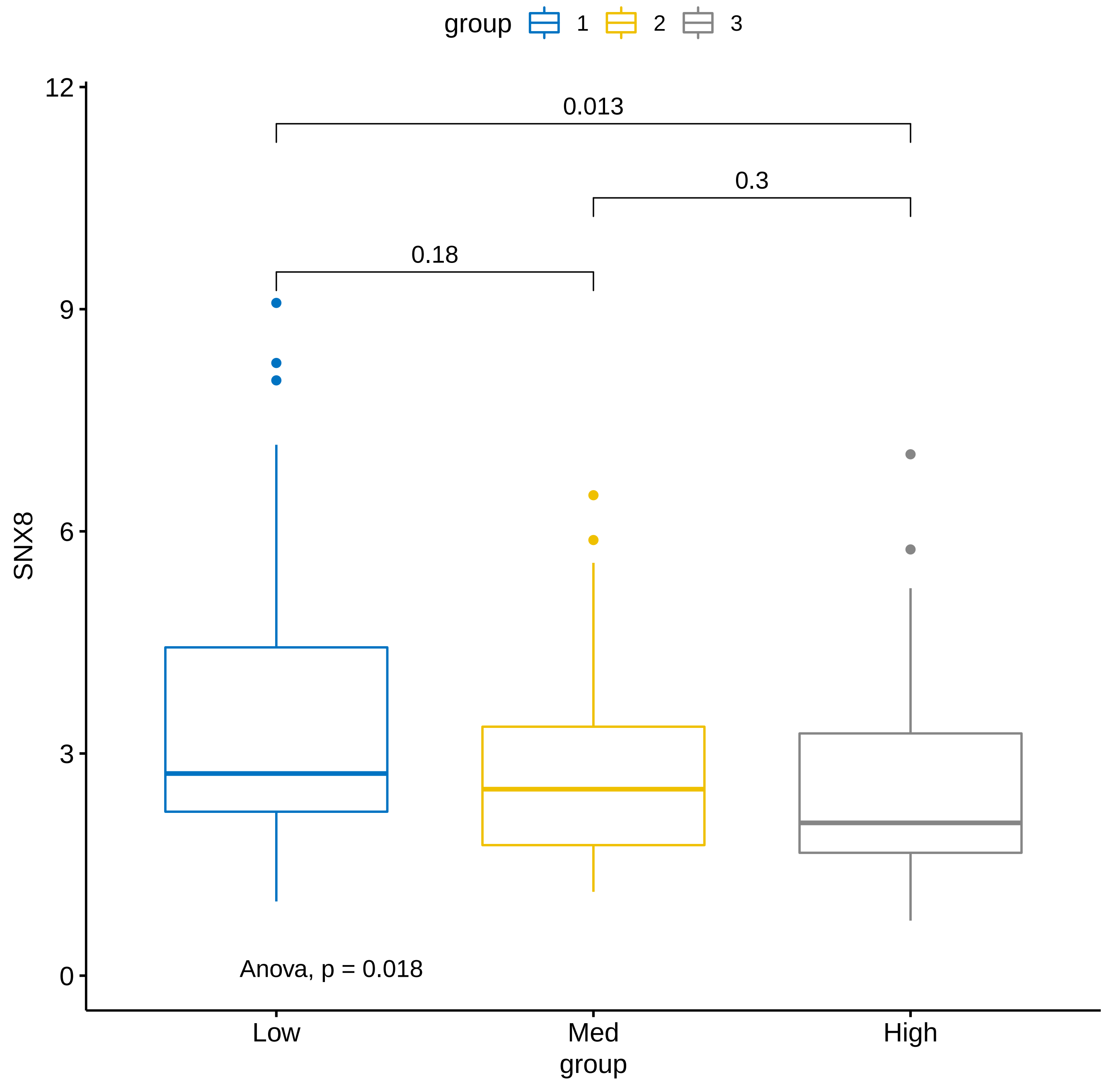
group
1
2
3
0.013
0.3
0.18
12
9
6
3
0
SNX8
Anova, p = 0.018
Low
Med
High
group

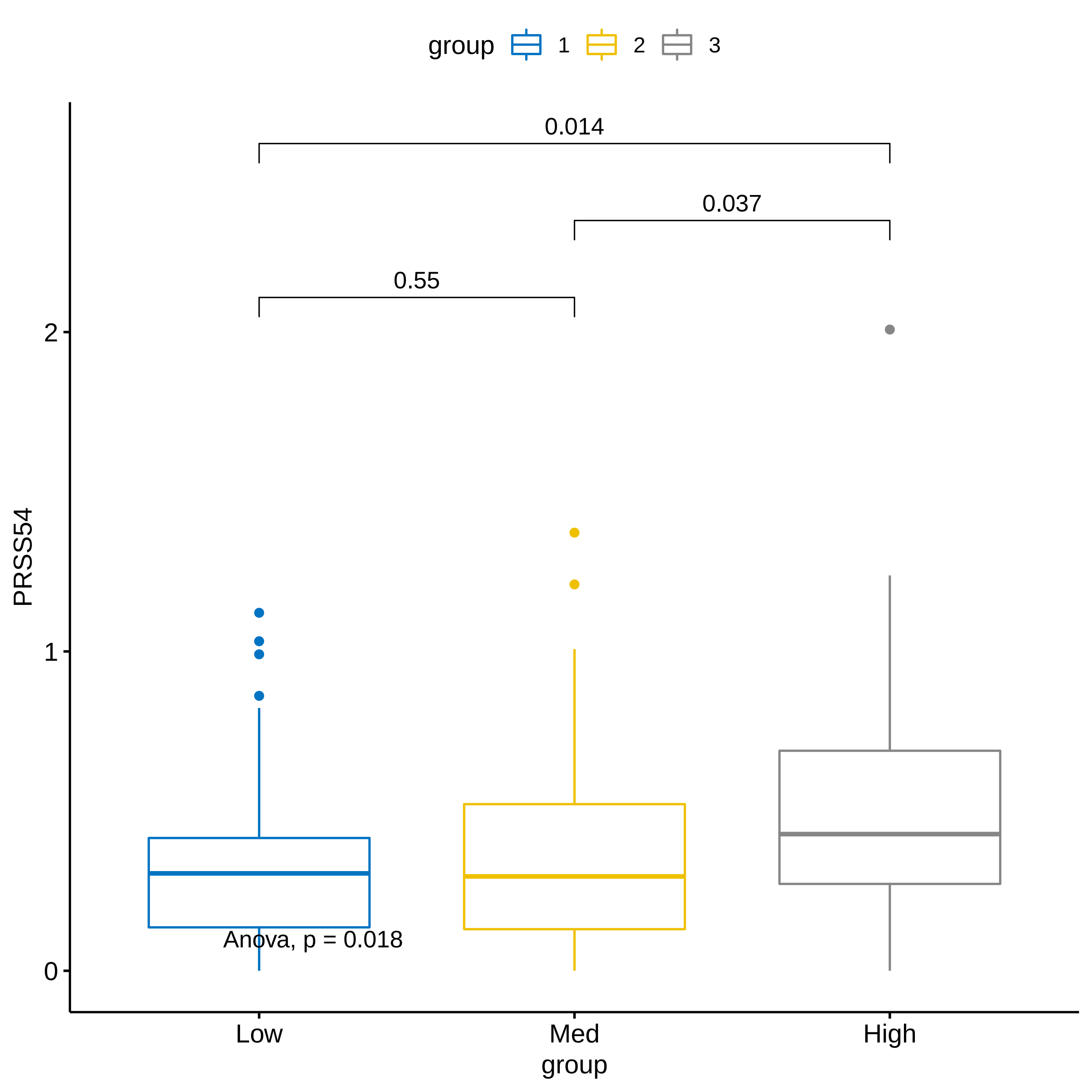
group
1
2
3
0.014
0.037
0.55
2
1
0
PRSS54
Anova, p = 0.018
Low
Med
High
group

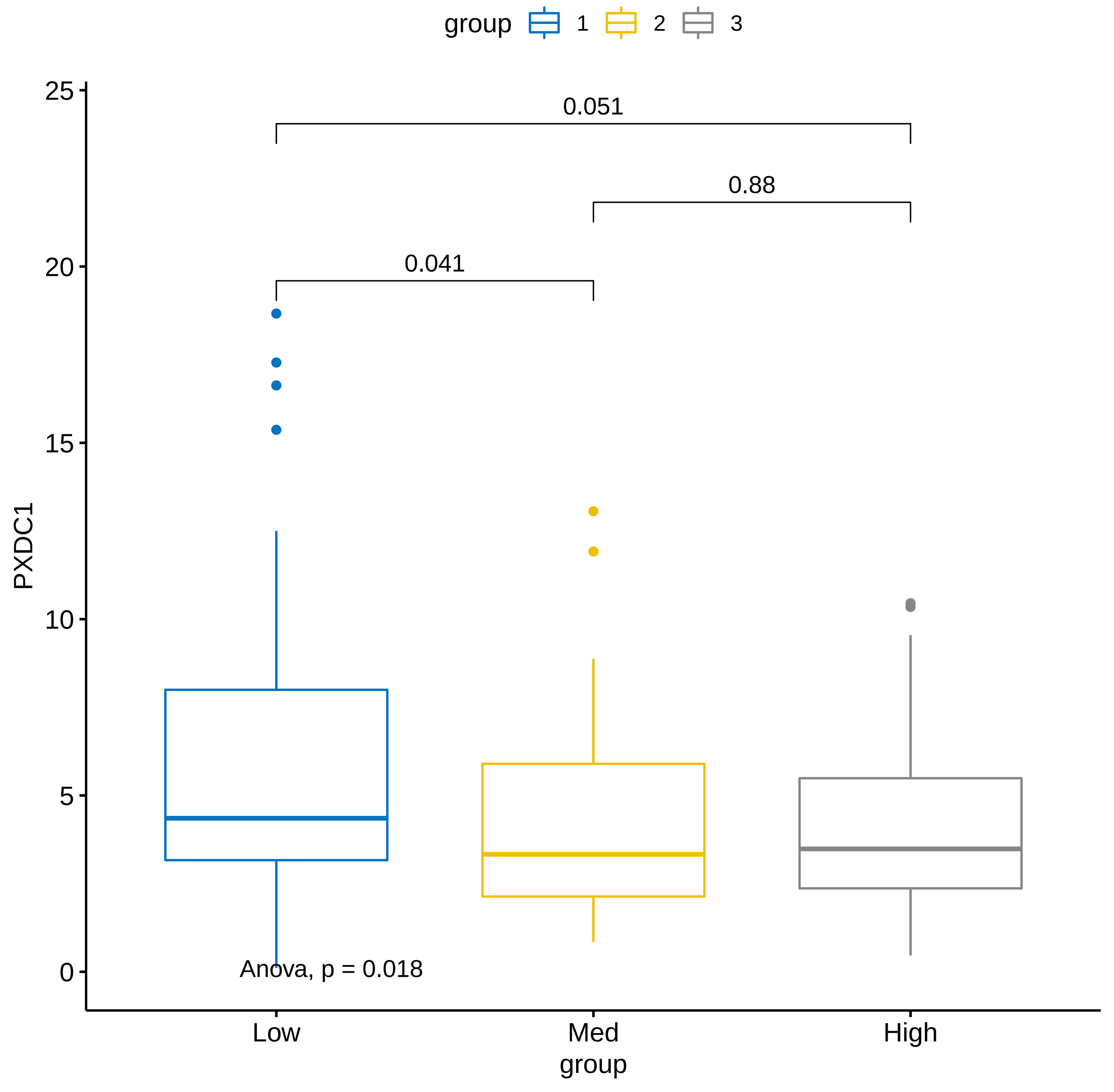

group
1
2
3
25
20
15
10
5
0
PXDC1
0.051
0.88
0.041
Anova, p = 0.018
Low
Med
High
group

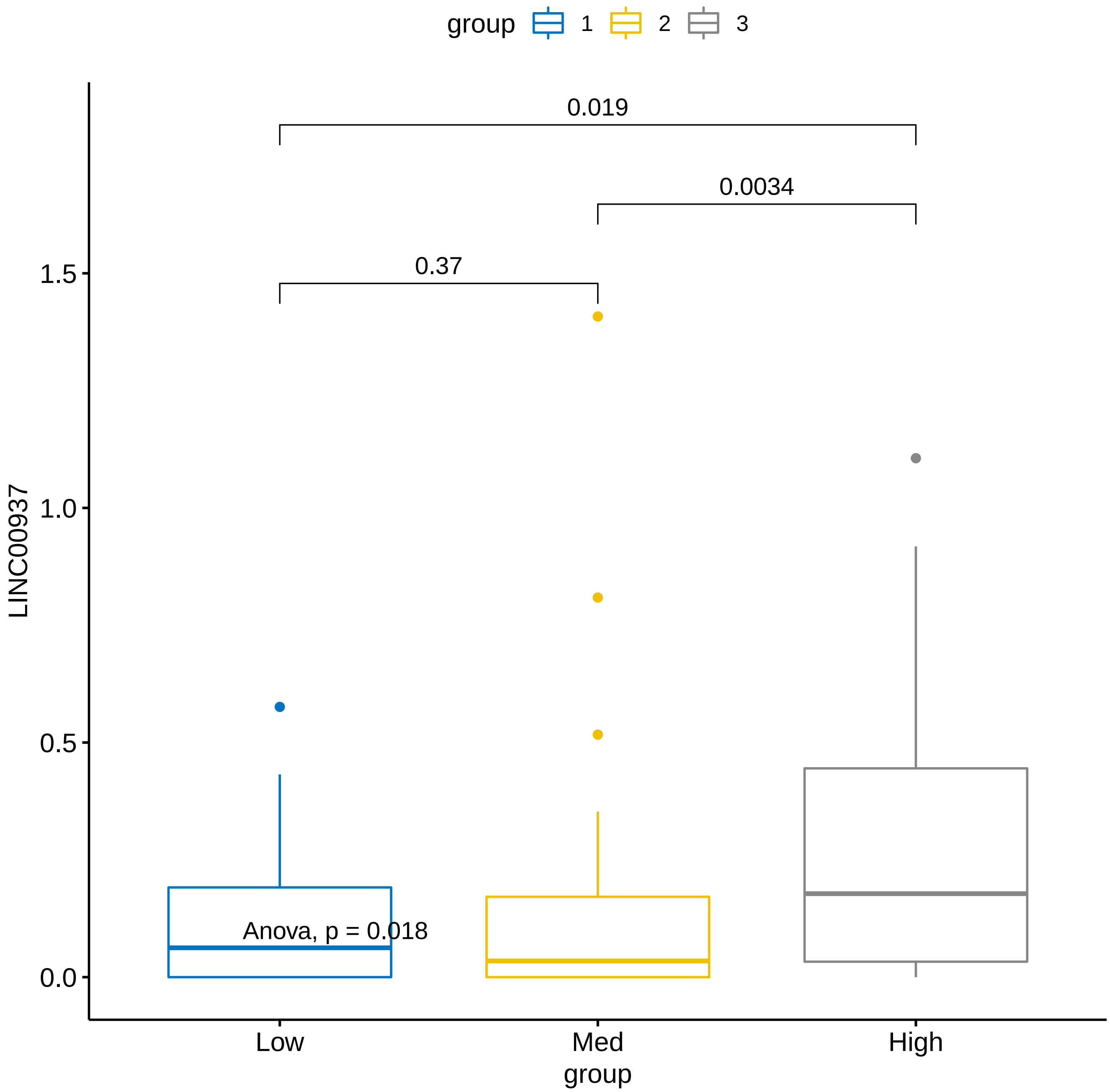

group
1
2
3
0.019
0.0034
0.37
1.5
1.0
0.5
0.0
LINC00937
Anova, p = 0.018
Low
Med
High
group

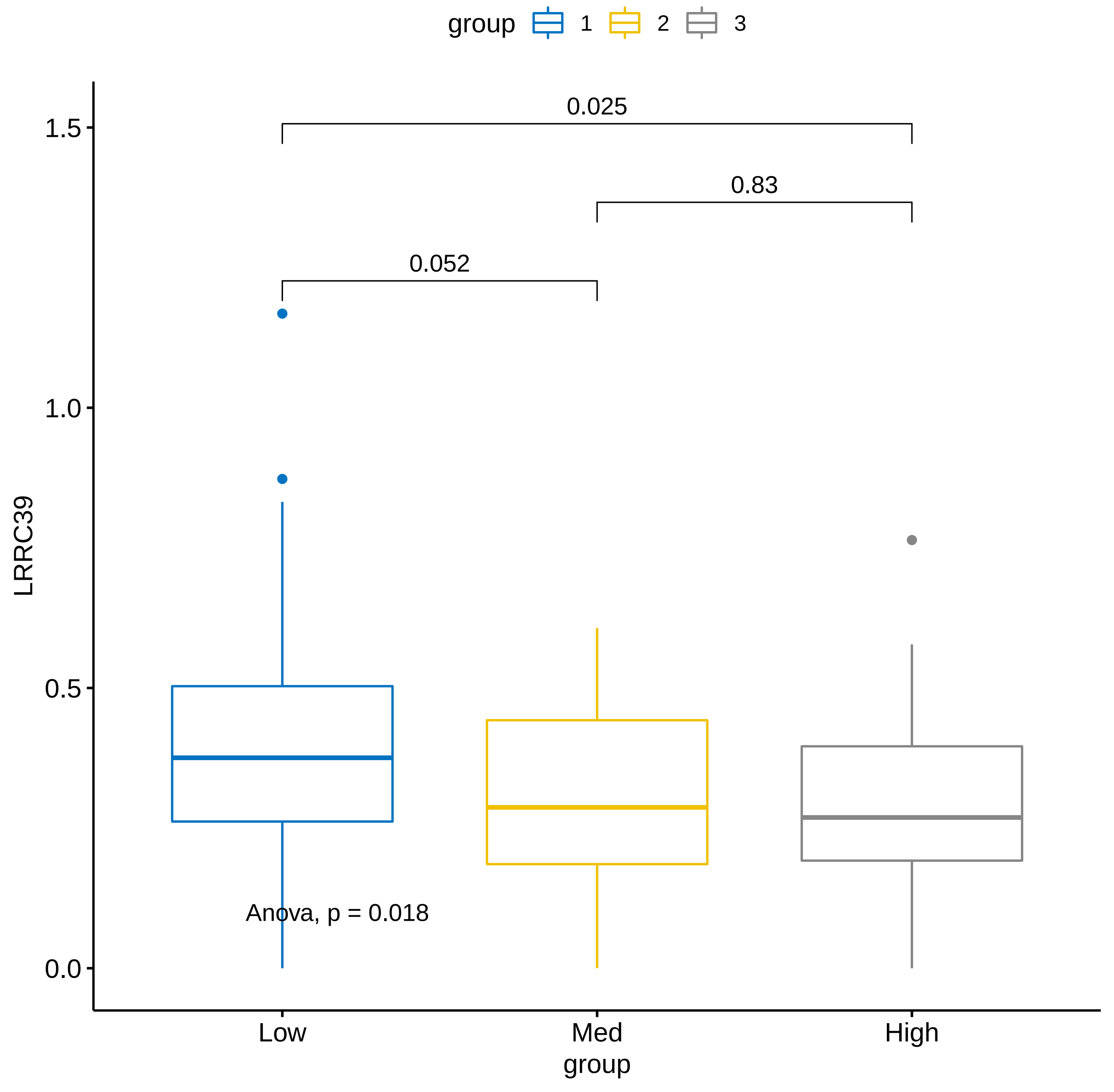

group 1 2 3
0.025
0.83
0.052
1.5
1.0
0.5
0.0
LRRC39
Anova, p = 0.018
Low
Med
High
group

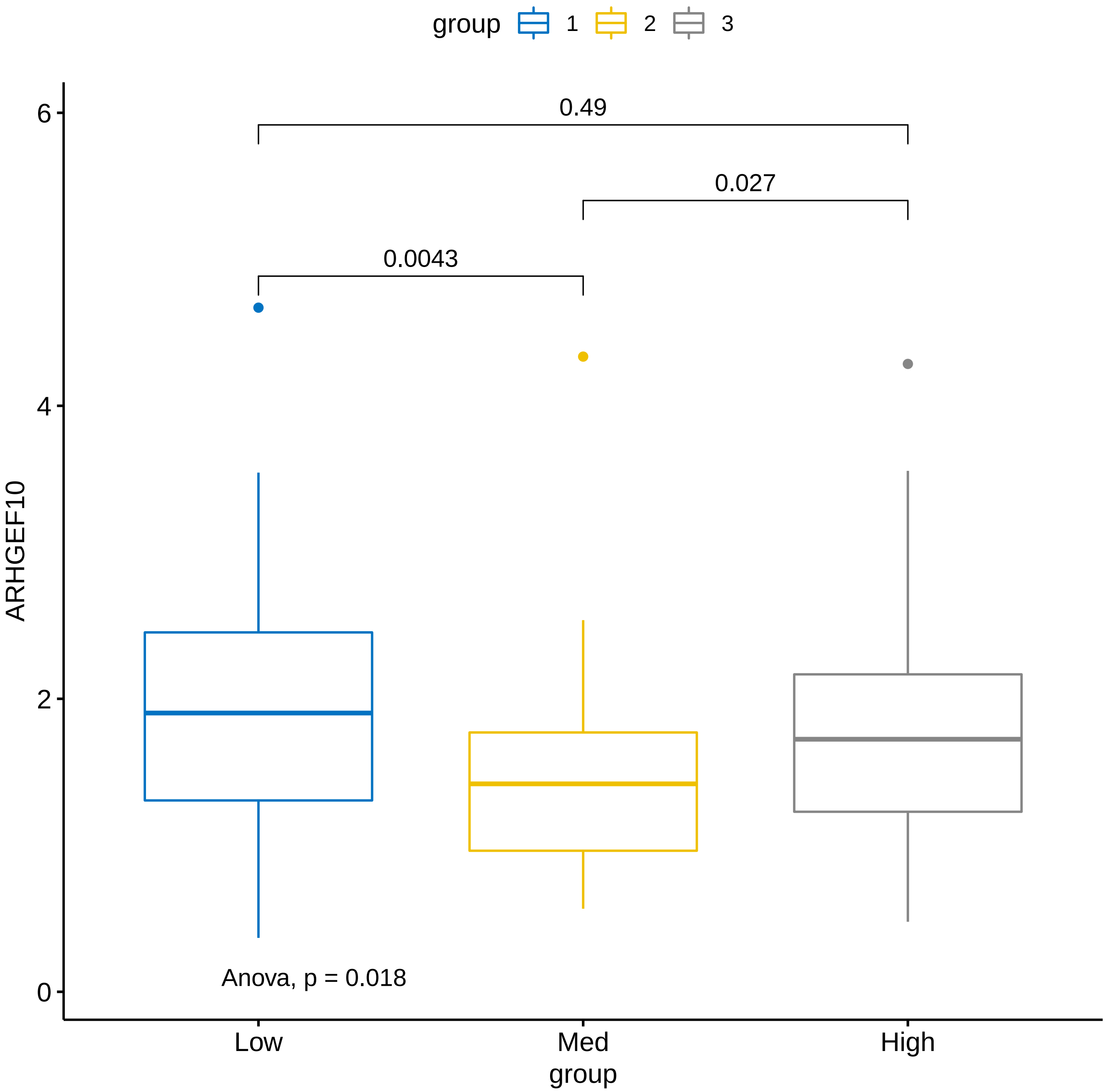

group
1
2
3
0.49
0.027
0.0043
6
4
2
0
ARHGEF10
Anova, p = 0.018
Low
Med
High
group

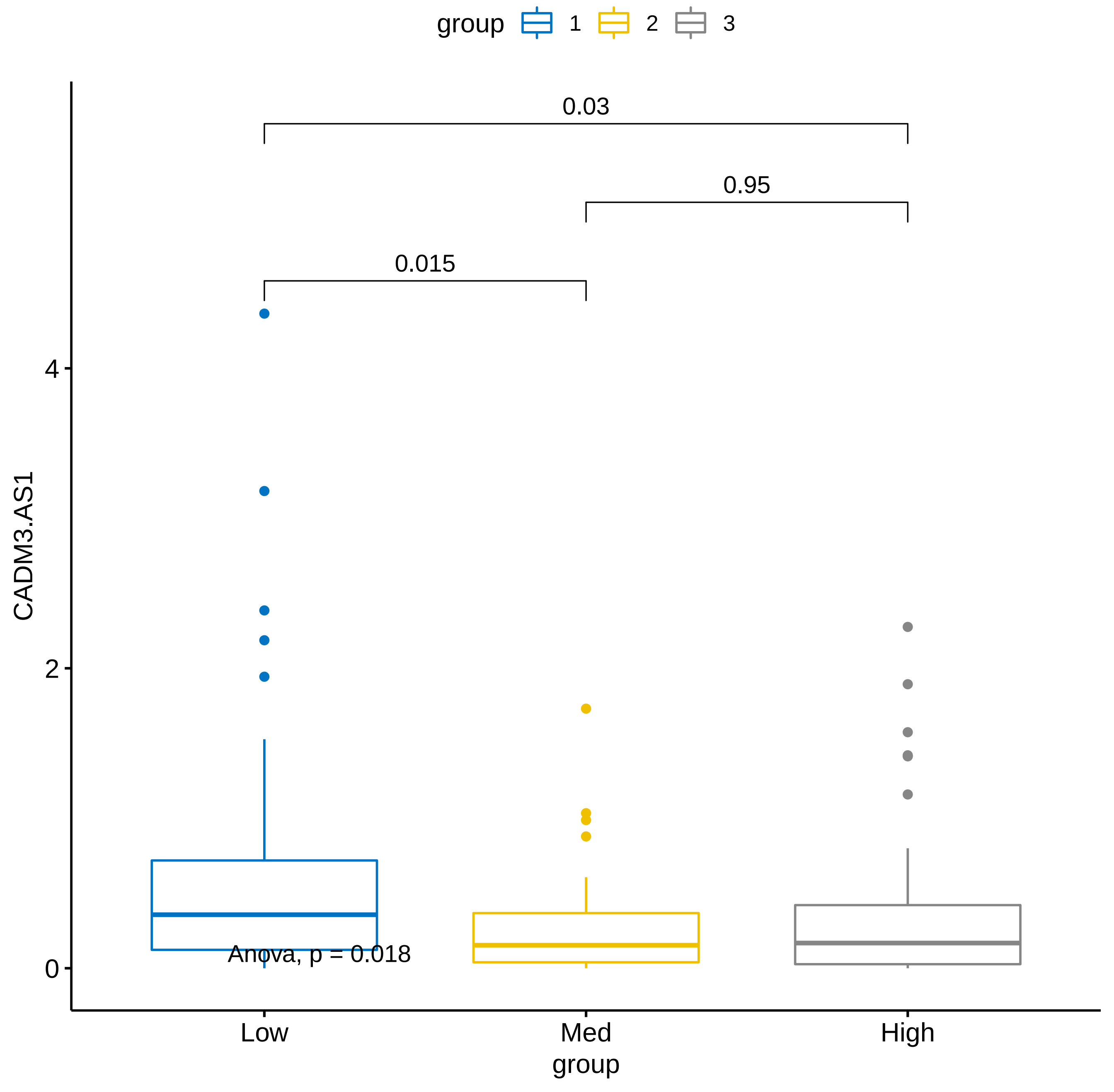
group
1
2
3
0.03
0.95
0.015
4
2
0
CADM3.AS1
Anova, p = 0.018
Low
Med
High
group

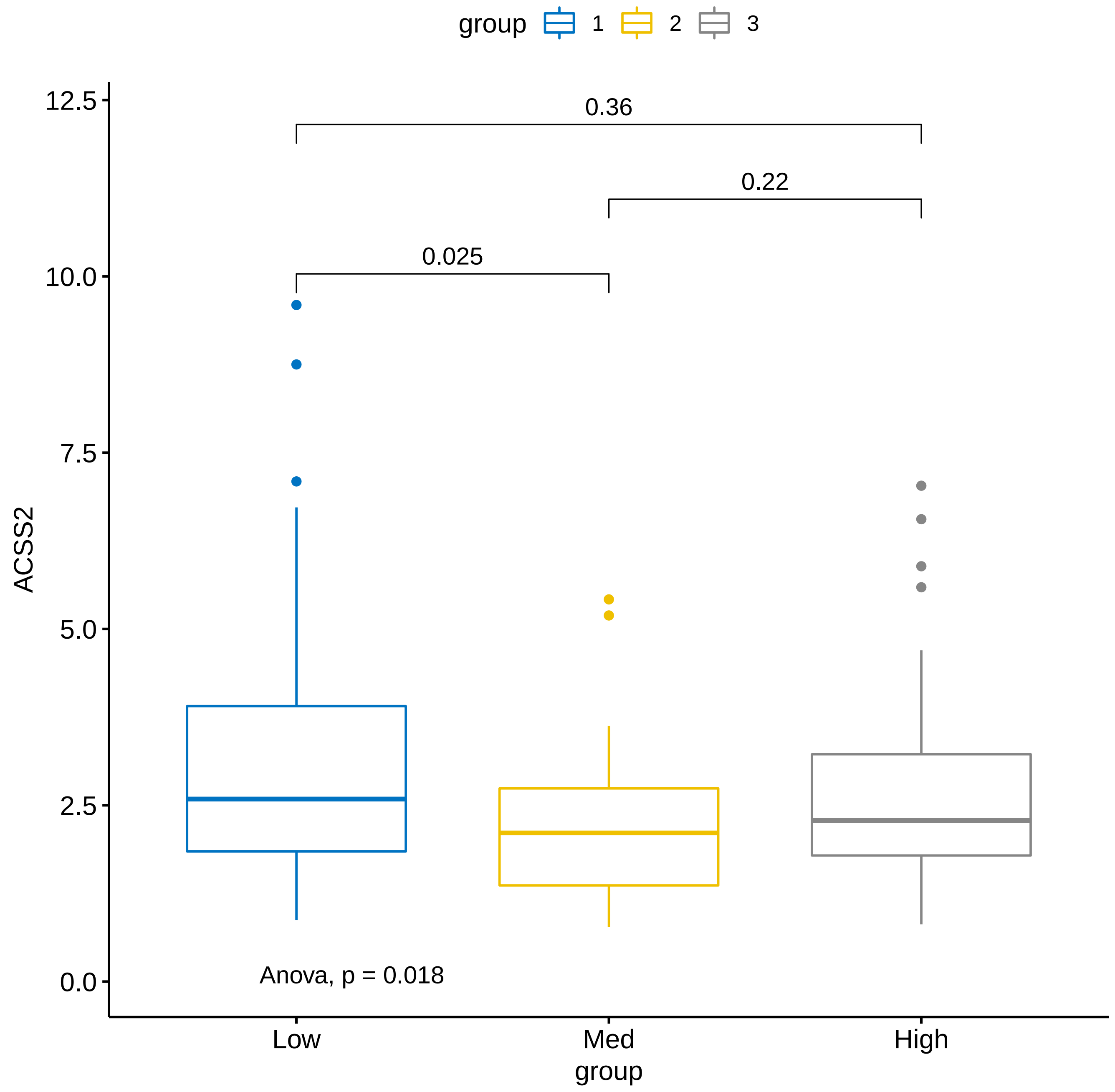

group
1
2
3
0.36
0.22
0.025
12.5
10.0
7.5
5.0
2.5
0.0
ACSS2
Anova, p = 0.018
Low
Med
High
group

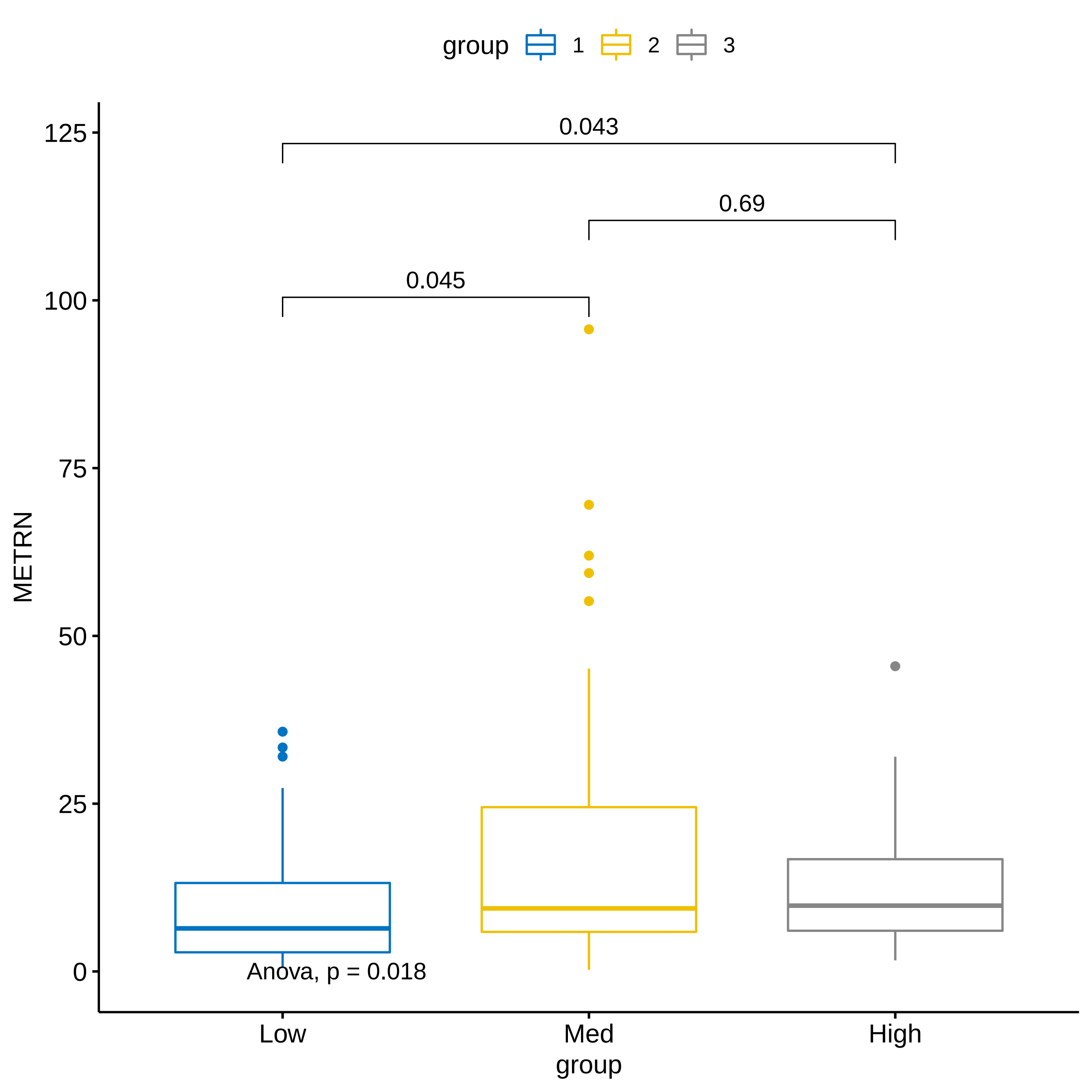

group 1 2 3
0.043
0.69
0.045
125
100
75
50
25
0
METRN
Anova, p = 0.018
Low
Med
High
group

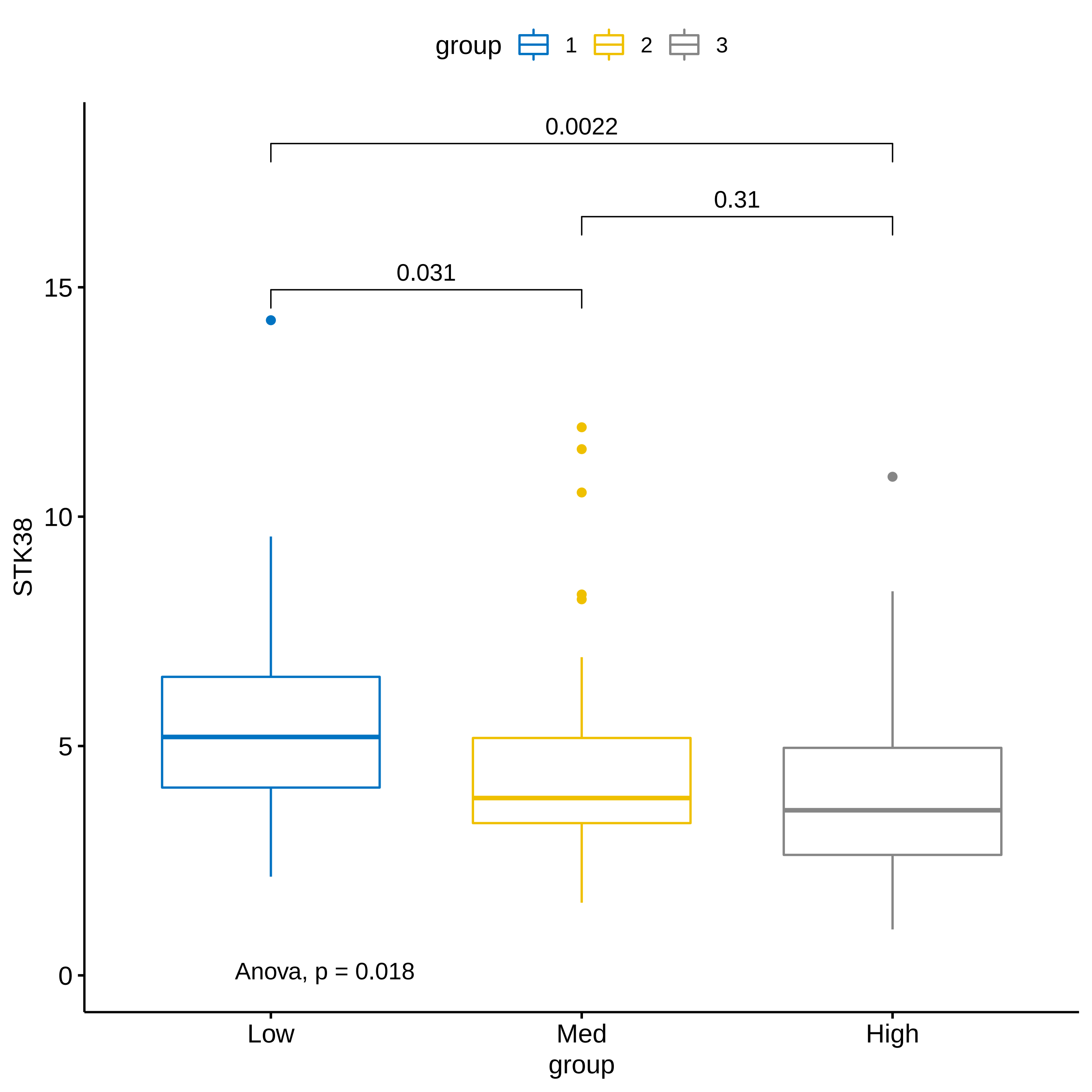

group
1
2
3
0.0022
0.31
0.031
15
10
5
0
STK38
Anova, p = 0.018
Low
Med
High
group

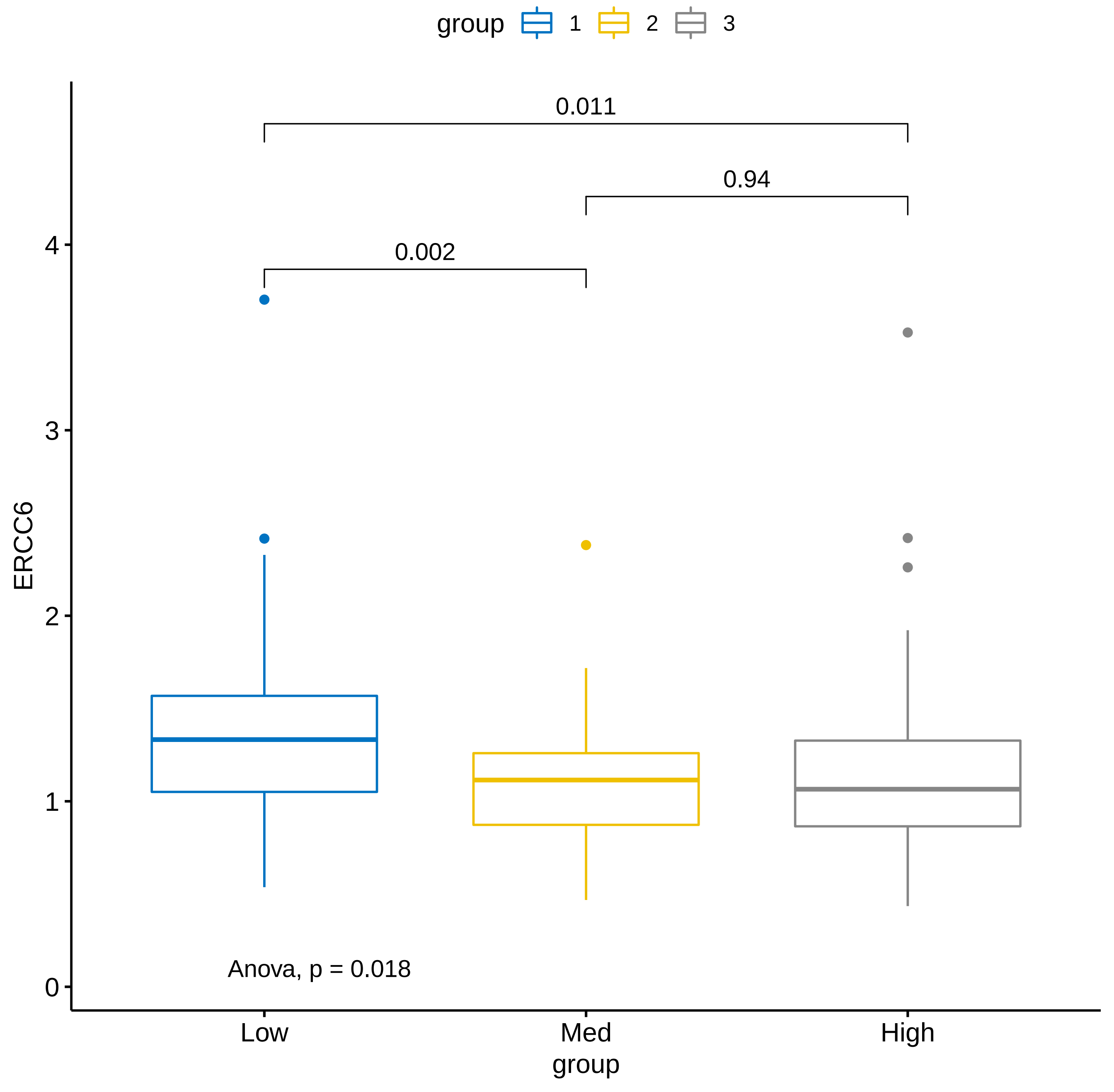

group 1 2 3
0.011
0.94
0.002
ERCC6
4
3
2
1
0
Anova, p = 0.018
Low
Med
High
group

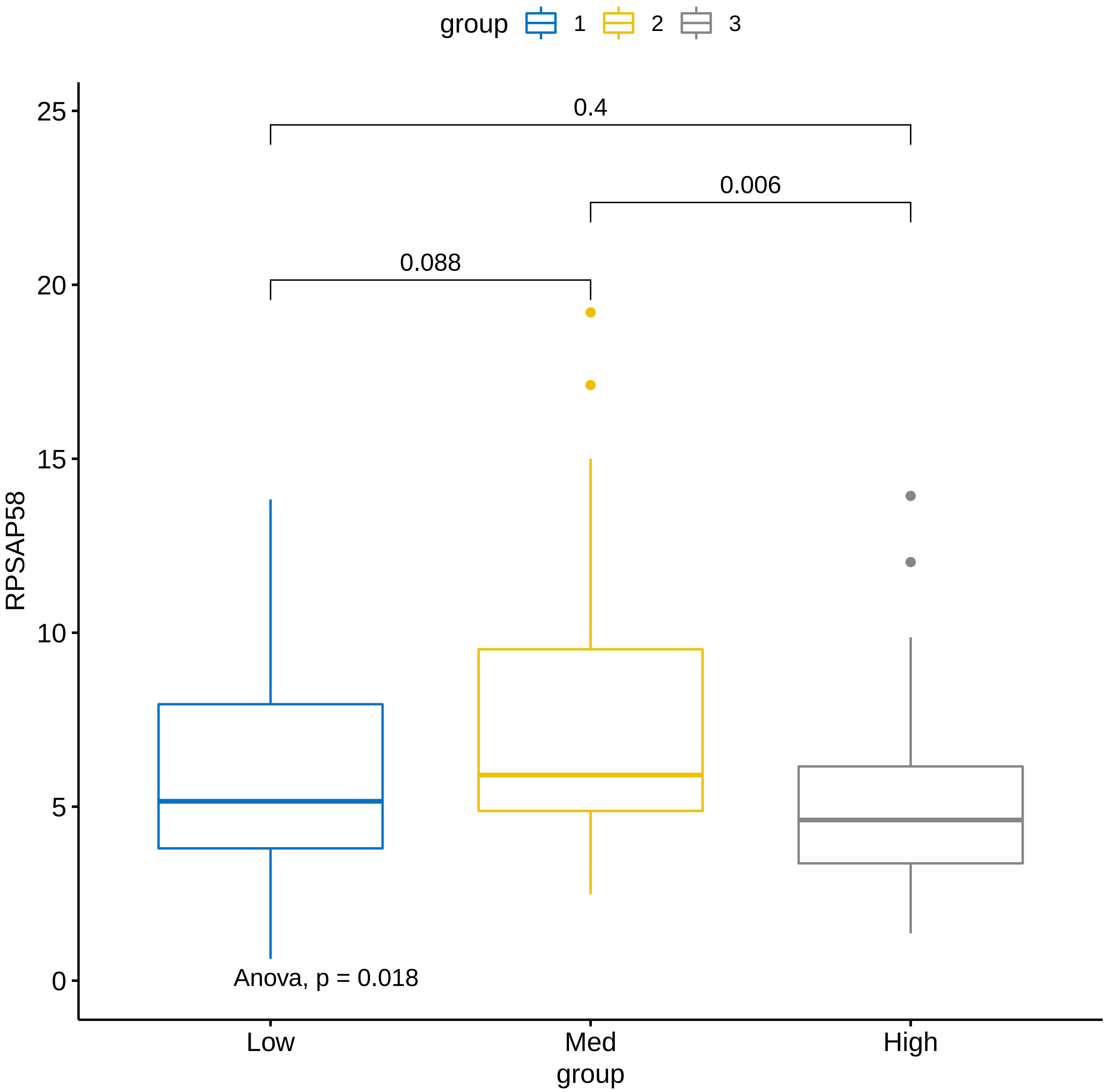
group
1
2
3
0.4
0.006
0.088
25
20
15
10
5
0
RPSAP58
Anova, p = 0.018
Low
Med
High
group

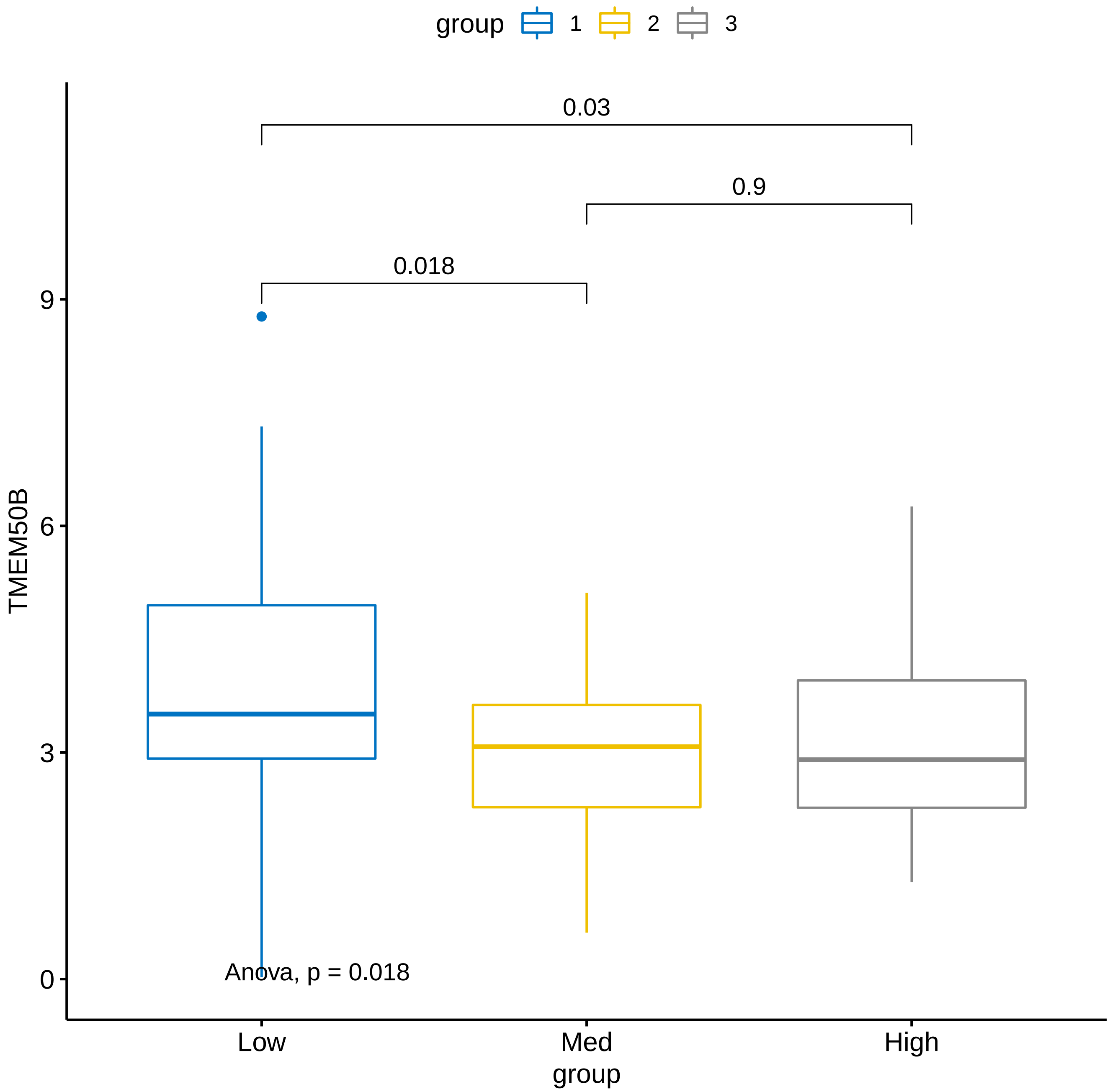

group 1 2 3
0.03
0.9
0.018
9
6
3
0
TMEM50B
Anova, p = 0.018
Low
Med
High
group

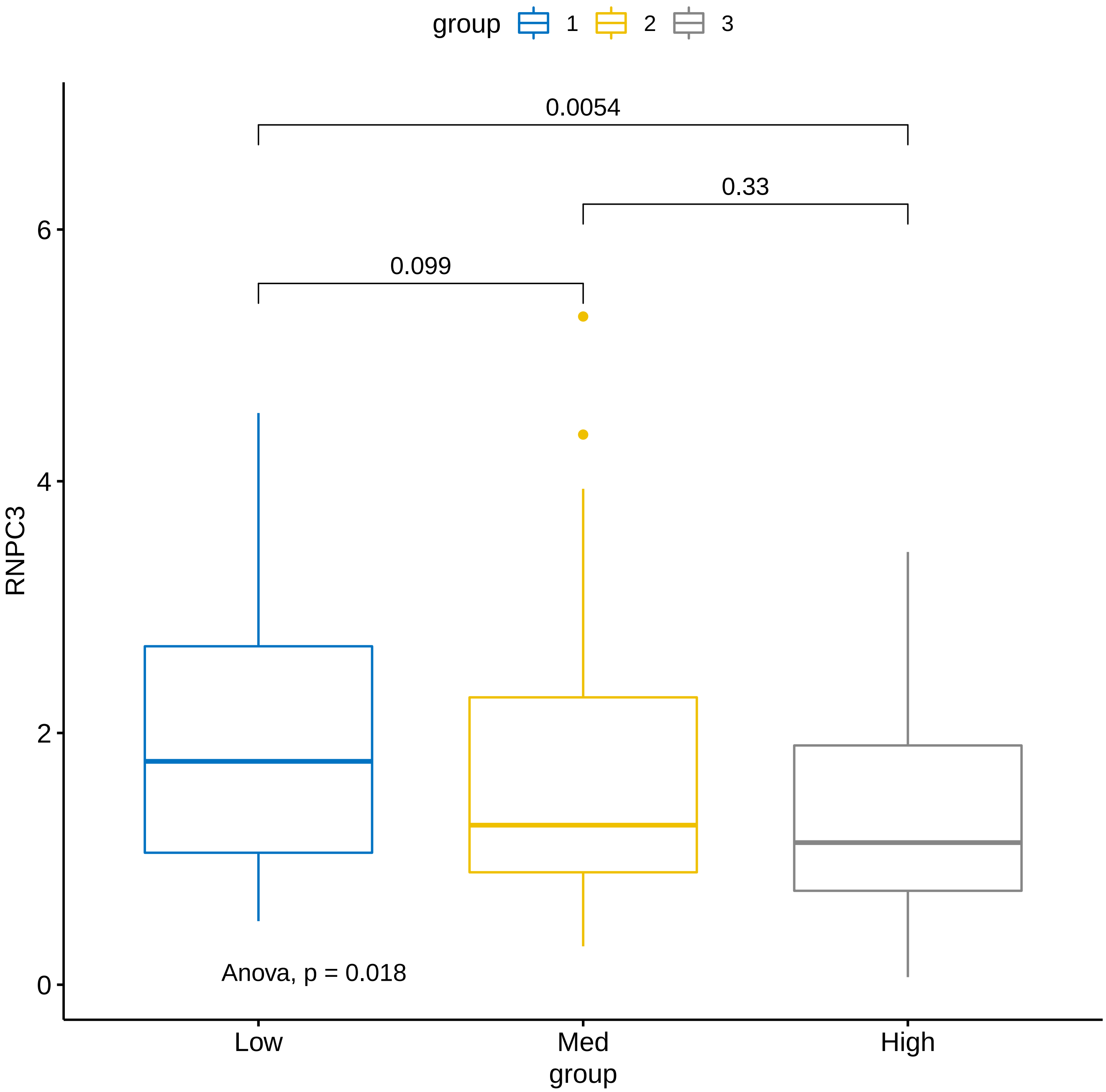
group
1
2
3
0.0054
0.33
0.099
6
4
2
0
RNPC3
Anova, p = 0.018
Low
Med
High
group

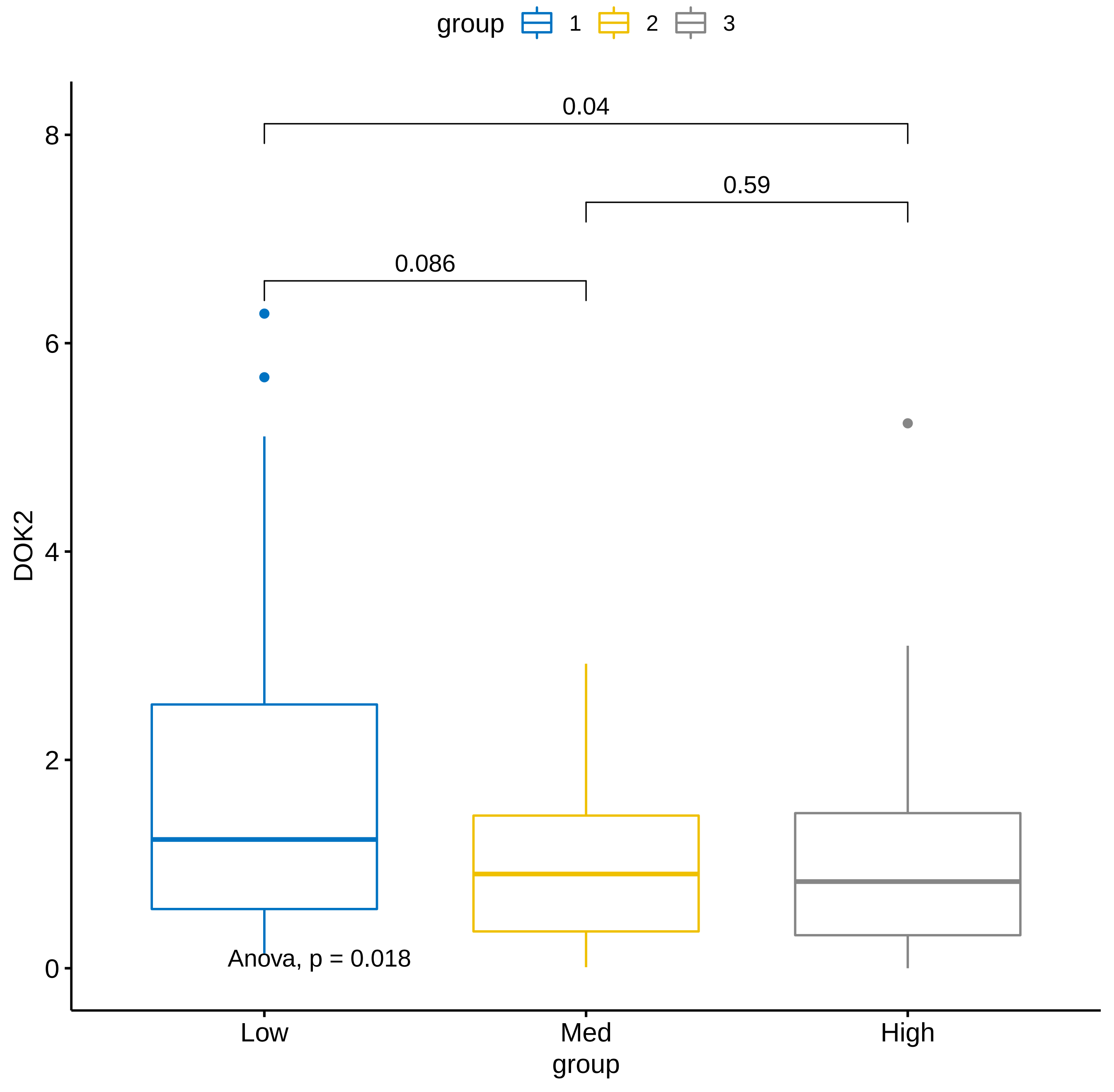

group
1
2
3
0.04
0.59
0.086
DOK2
8
6
4
2
0
Anova, p = 0.018
Low
Med
High
group

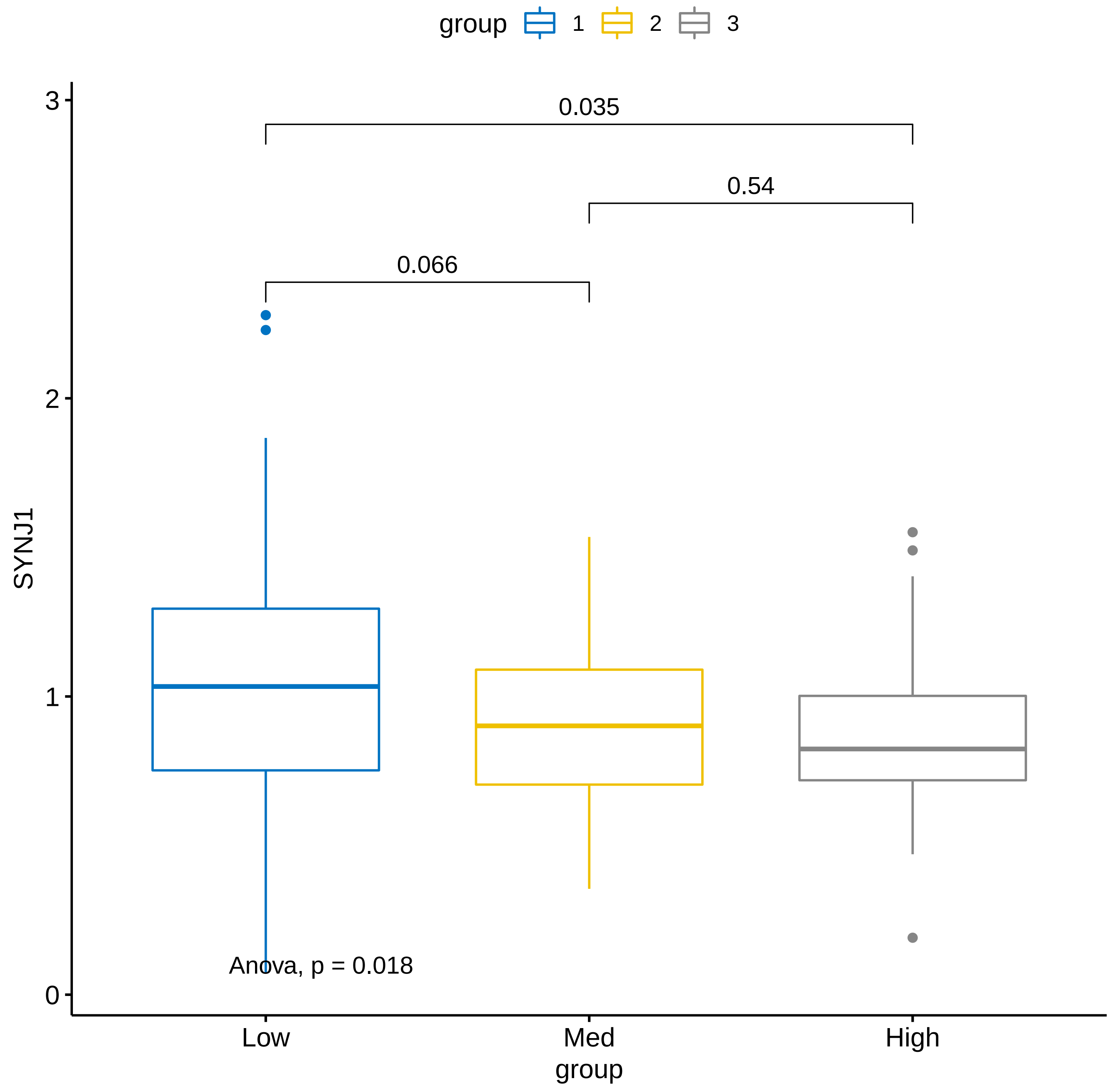

group
1
2
3
0.035
0.54
0.066
SYNJ1
3
2
1
0
Anova, p = 0.018
Low
Med
High
group

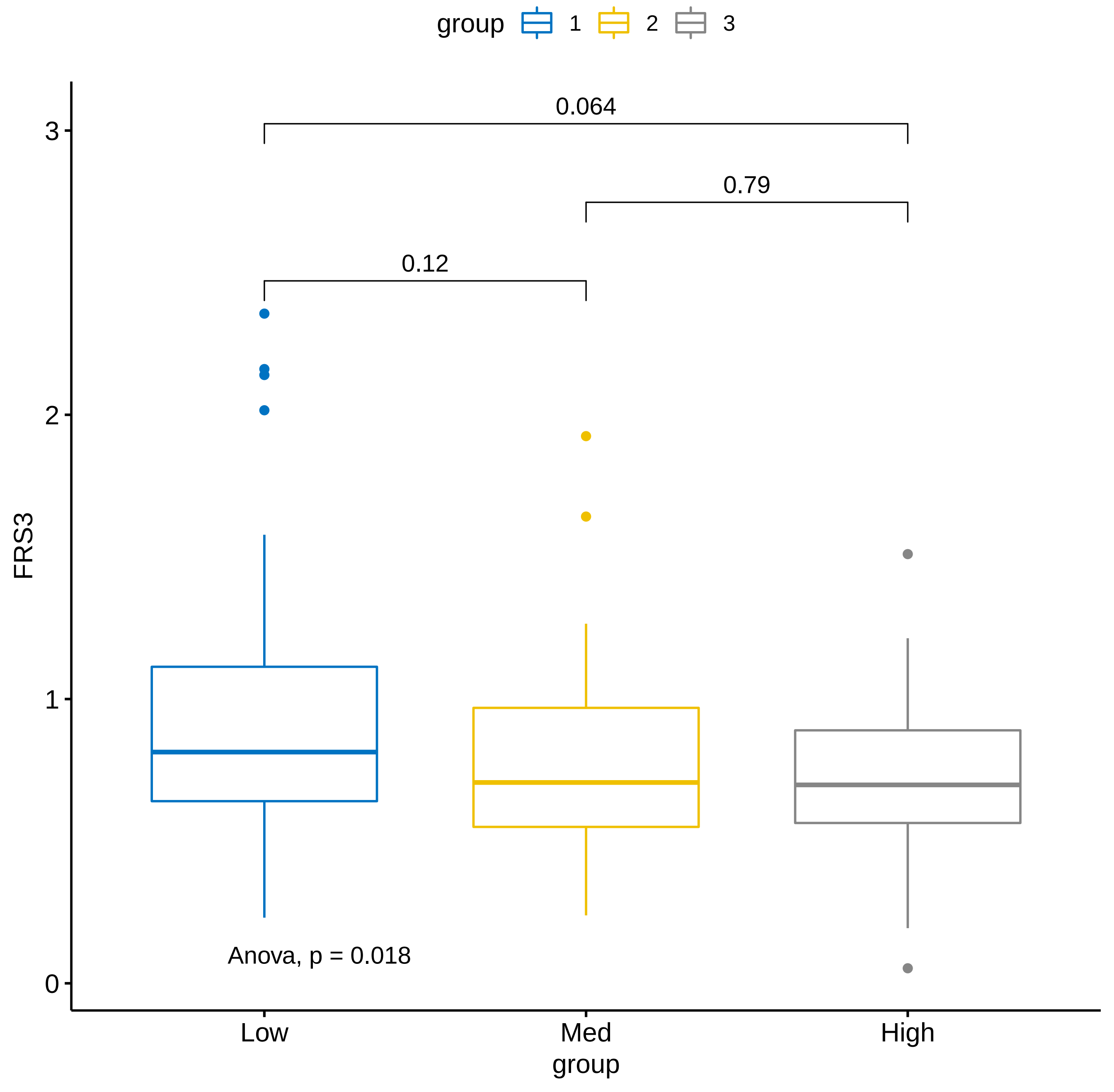

group 1 2 3
0.064
0.79
0.12
3
2
1
0
FRS3
Anova, p = 0.018
Low
Med
High
group

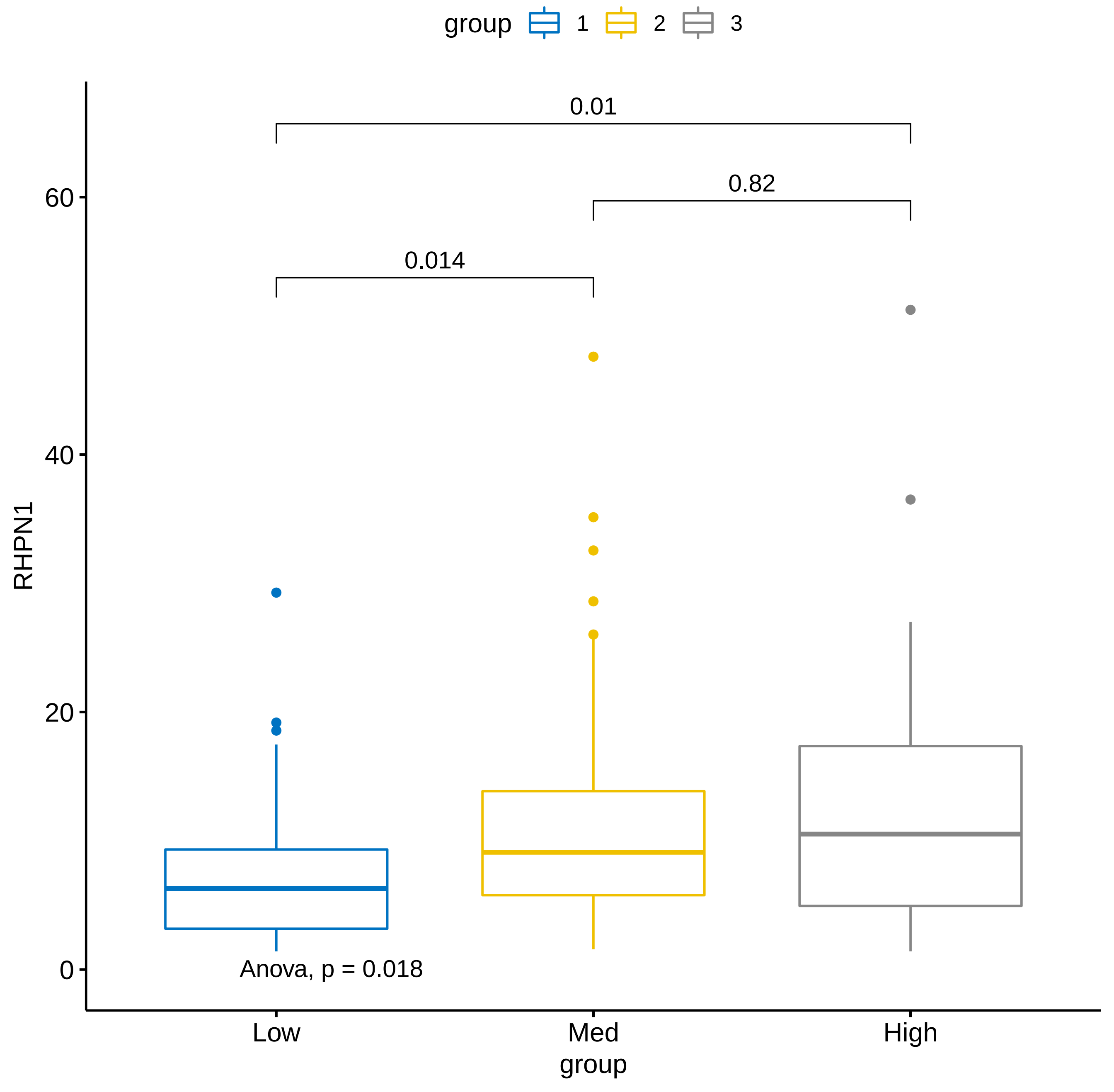

group
1
2
3
0.01
0.82
0.014
60
40
20
0
RHPN1
Anova, p = 0.018
Low
Med
High
group

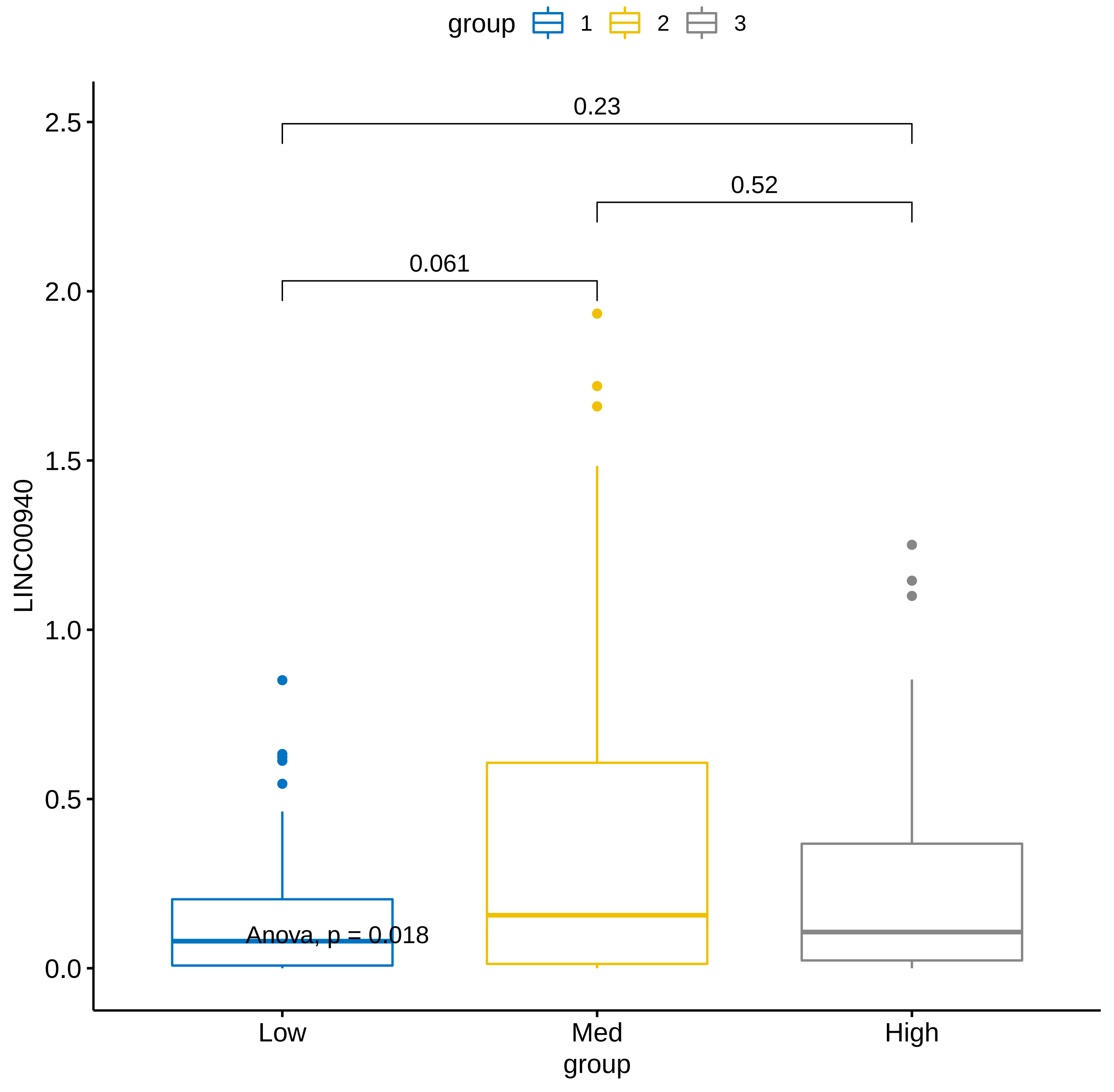
group 1 2 3
0.23
0.52
0.061
Anova, p = 0.018
LINC00940
2.5
2.0
1.5
1.0
0.5
0.0
Low
Med
High
group

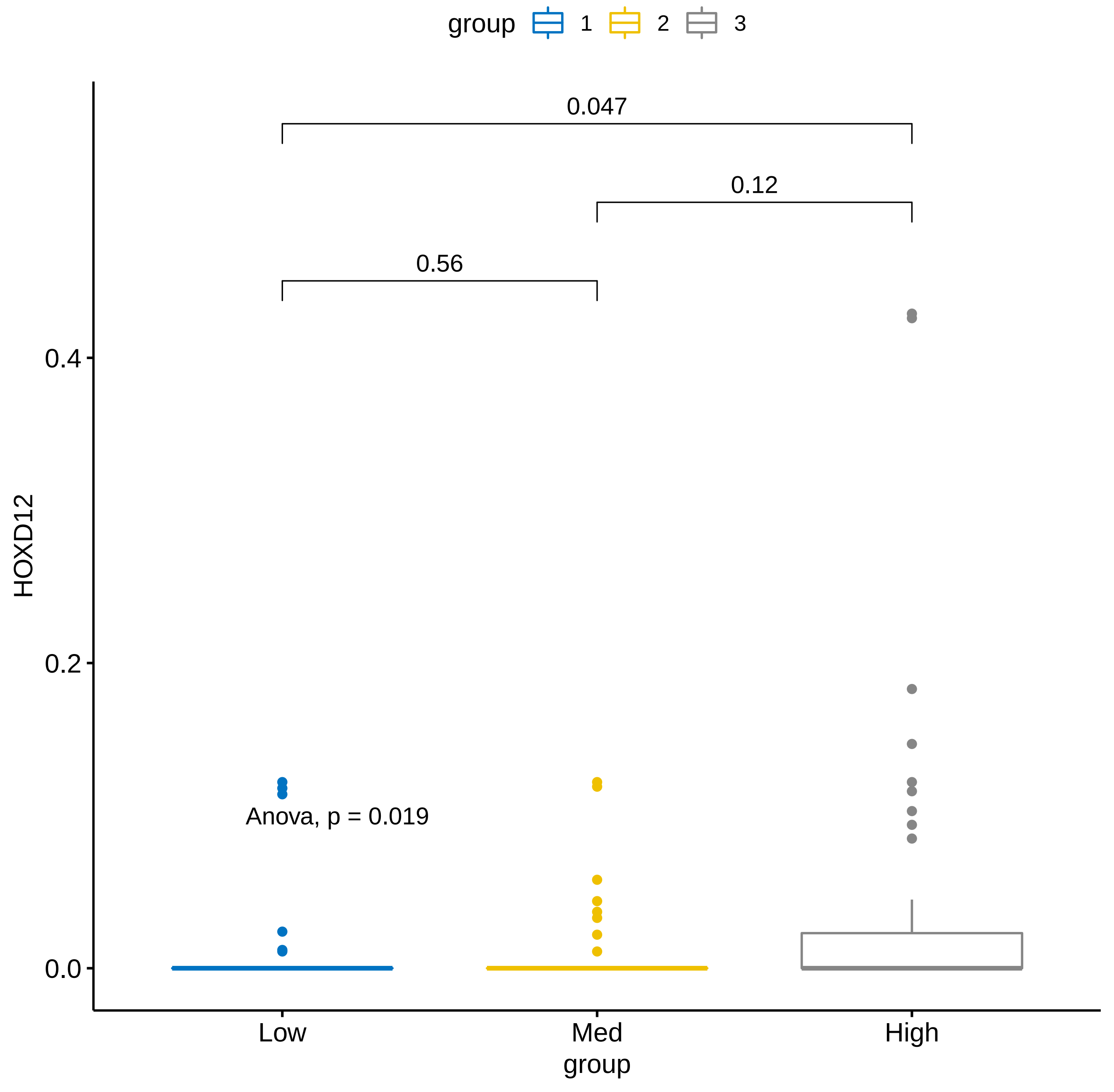
group 1 2 3
0.047
0.12
0.56
Anova, p = 0.019
HOXD12
0.4
0.2
0.0
Low
Med
High
group

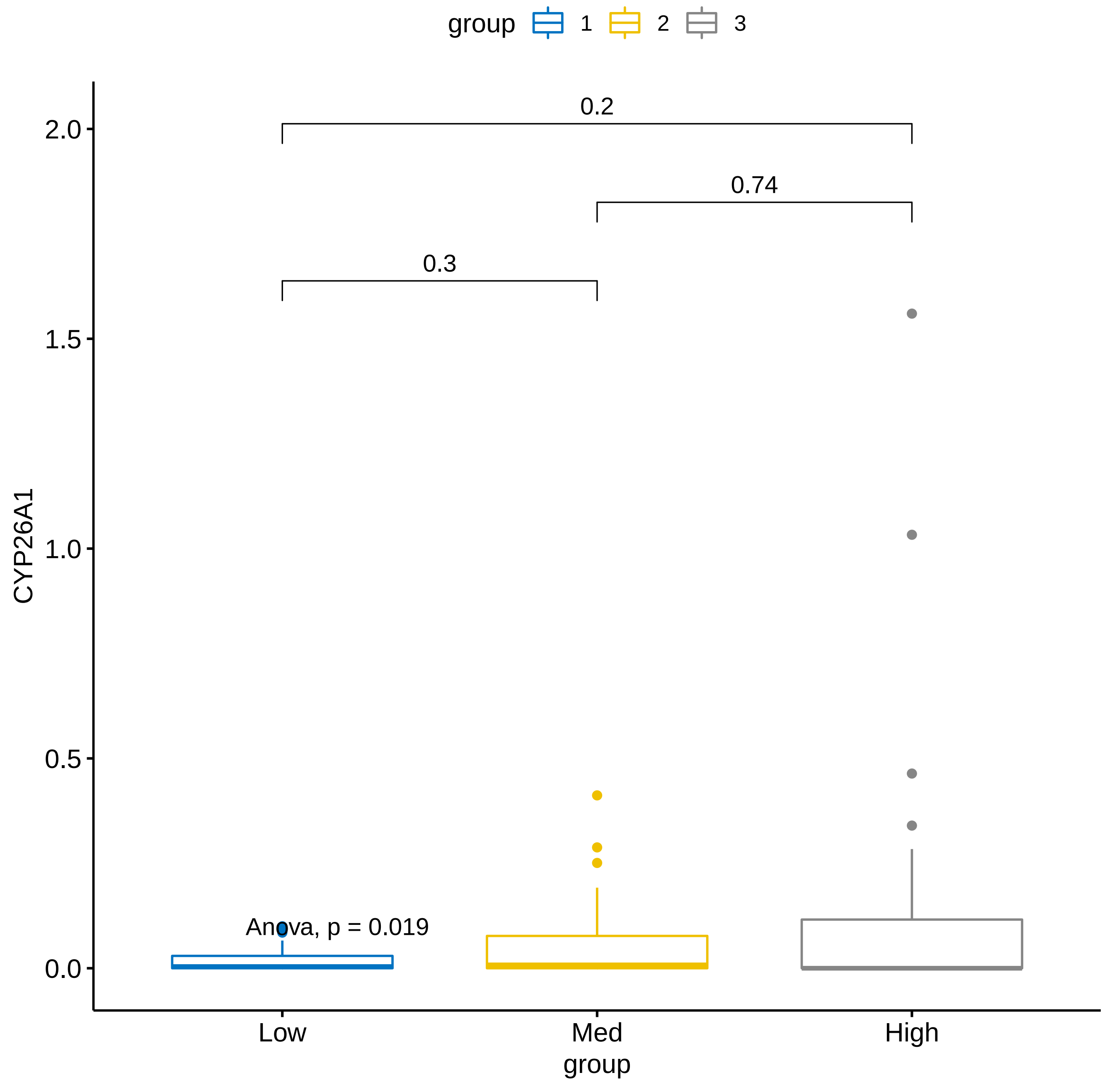
group
1
2
3
0.2
0.74
0.3
Anova, p = 0.019
CYP26A1
2.0
1.5
1.0
0.5
0.0
Low
Med
High
group

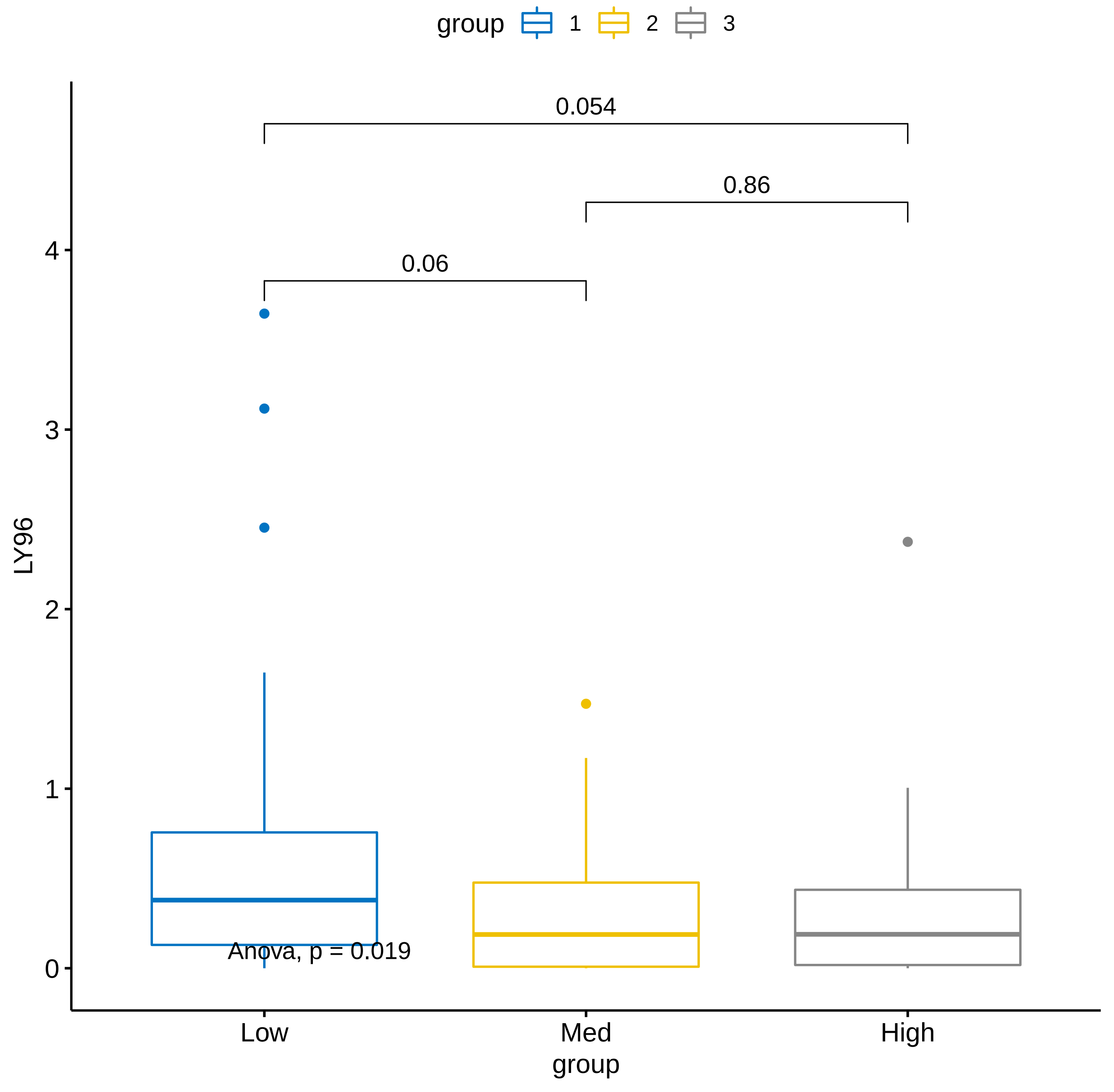
group 1 2 3
0.054
0.86
0.06
LY96
4
3
2
1
0
Anova, p = 0.019
Low
Med
High
group

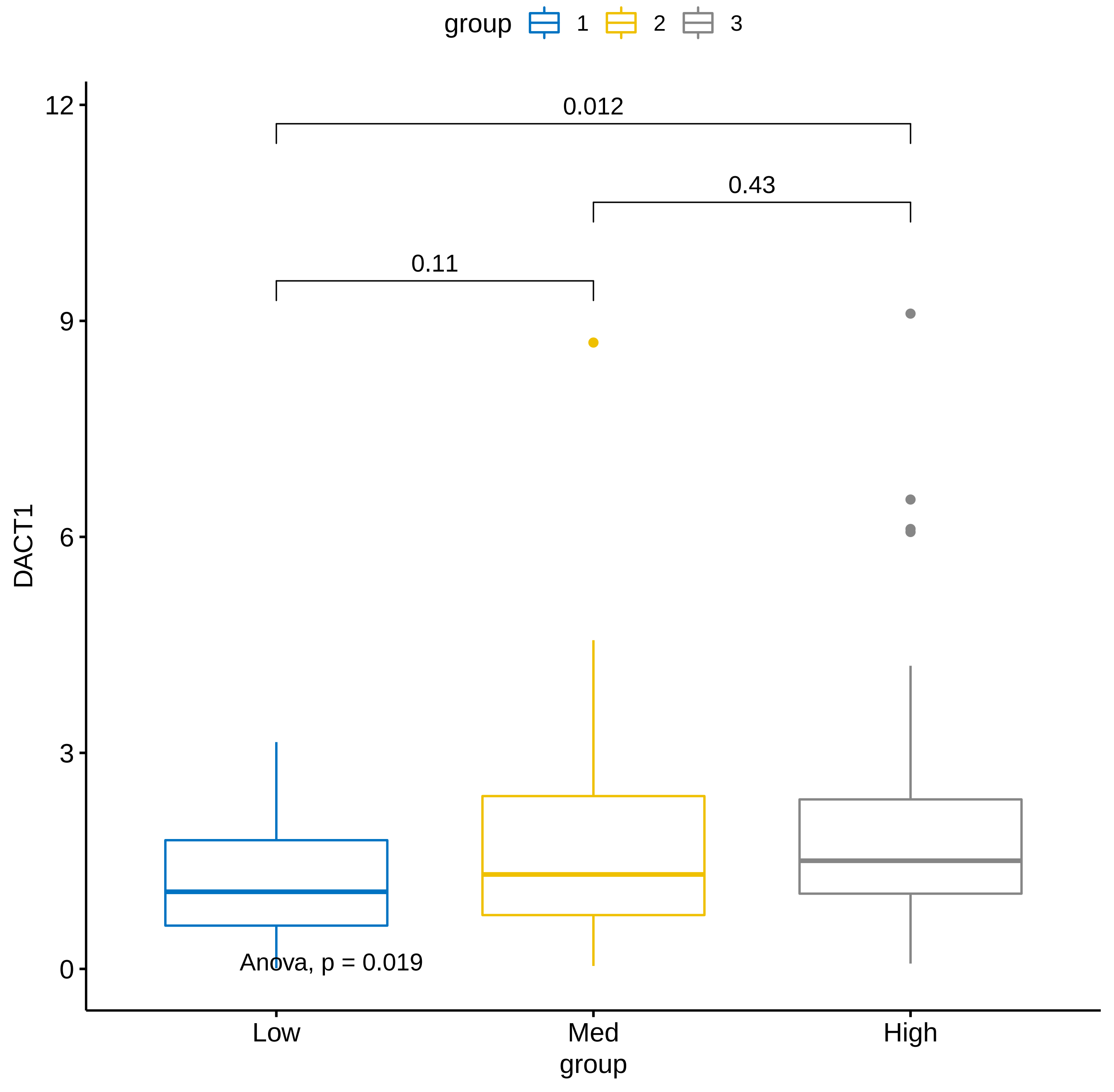

group 1 2 3
0.012
0.43
0.11
DACT1
12
9
6
3
0
Anova, p = 0.019
Low
Med
High
group

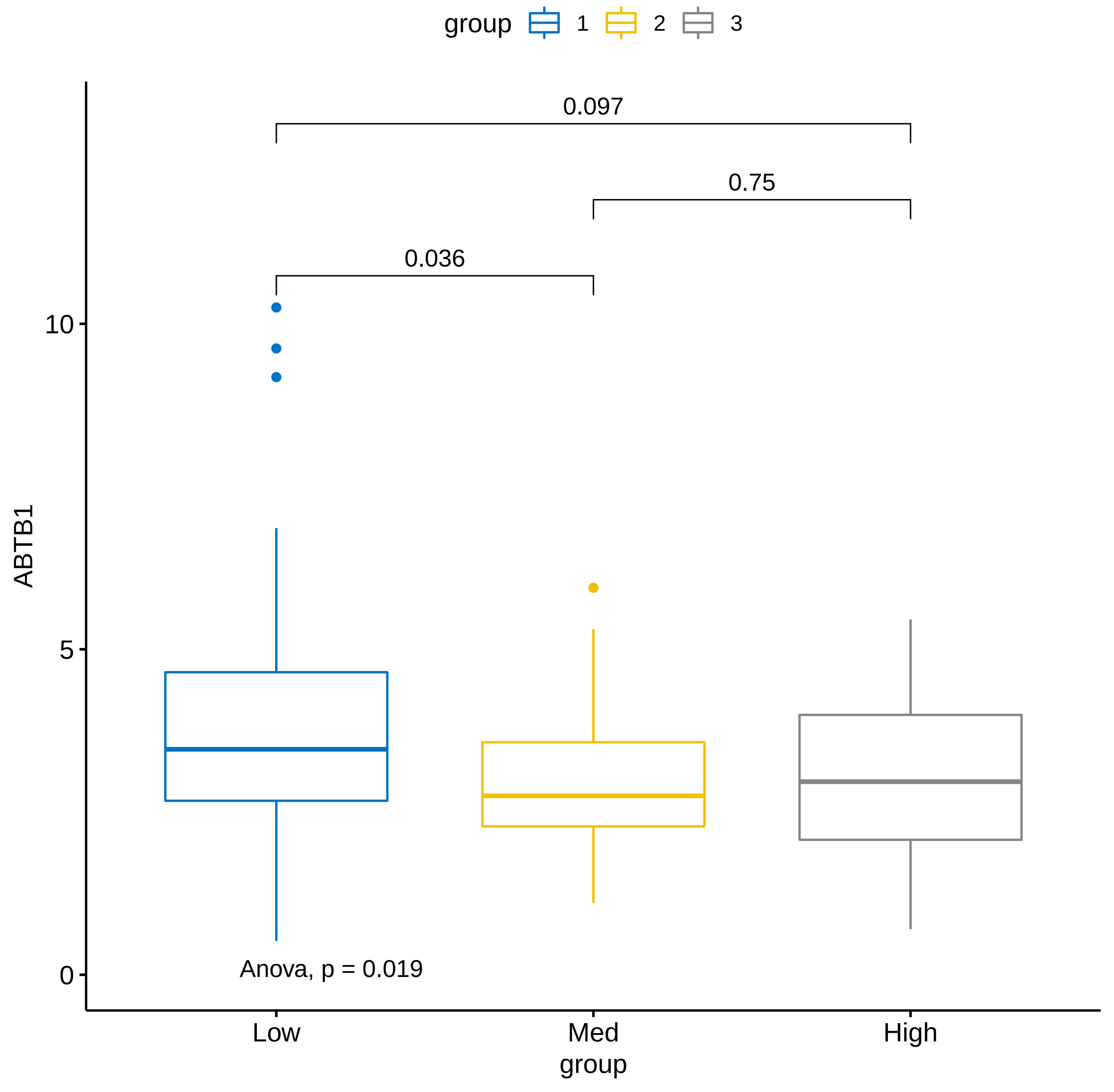
group 1 2 3
0.097
0.75
0.036
ABTB1
10
5
0
Anova, p = 0.019
Low
Med
High
group

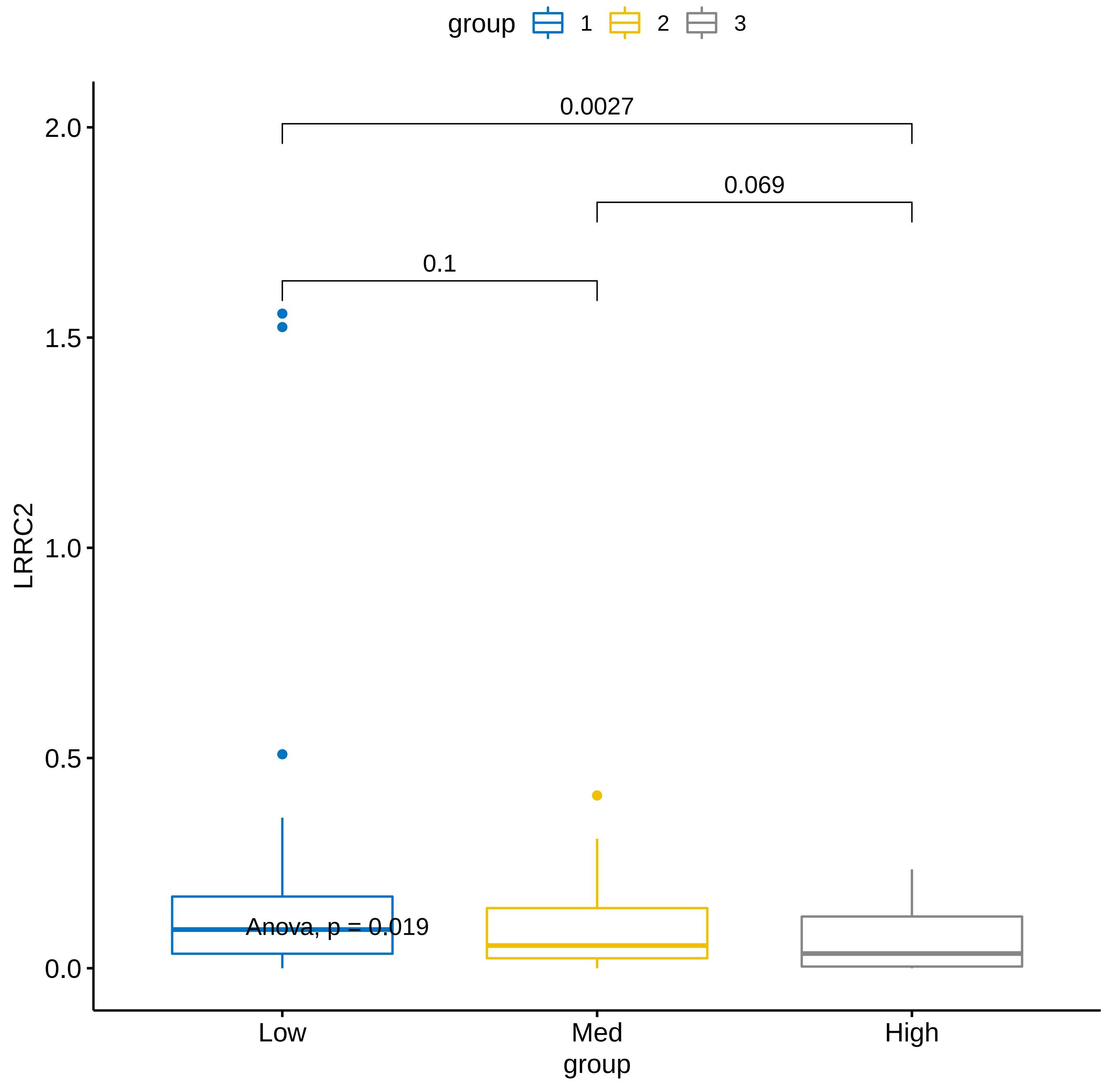
group
1
2
3
0.0027
0.069
0.1
2.0
1.5
1.0
0.5
0.0
LRRC2
Anova, p = 0.019
Low
Med
High
group

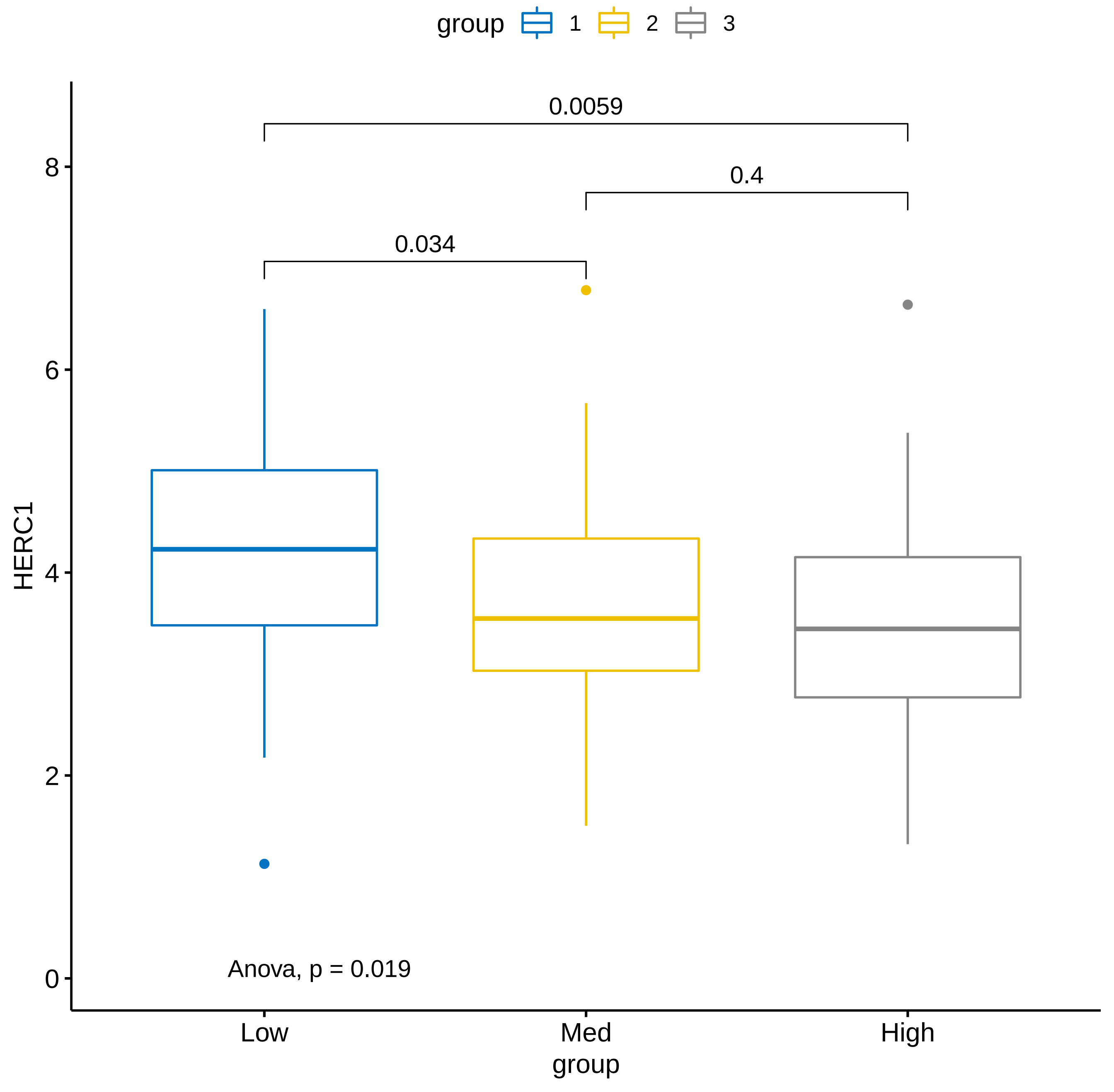

group 1 2 3
0.0059
0.4
0.034
HERC1
8
6
4
2
0
Anova, p = 0.019
Low
Med
High
group

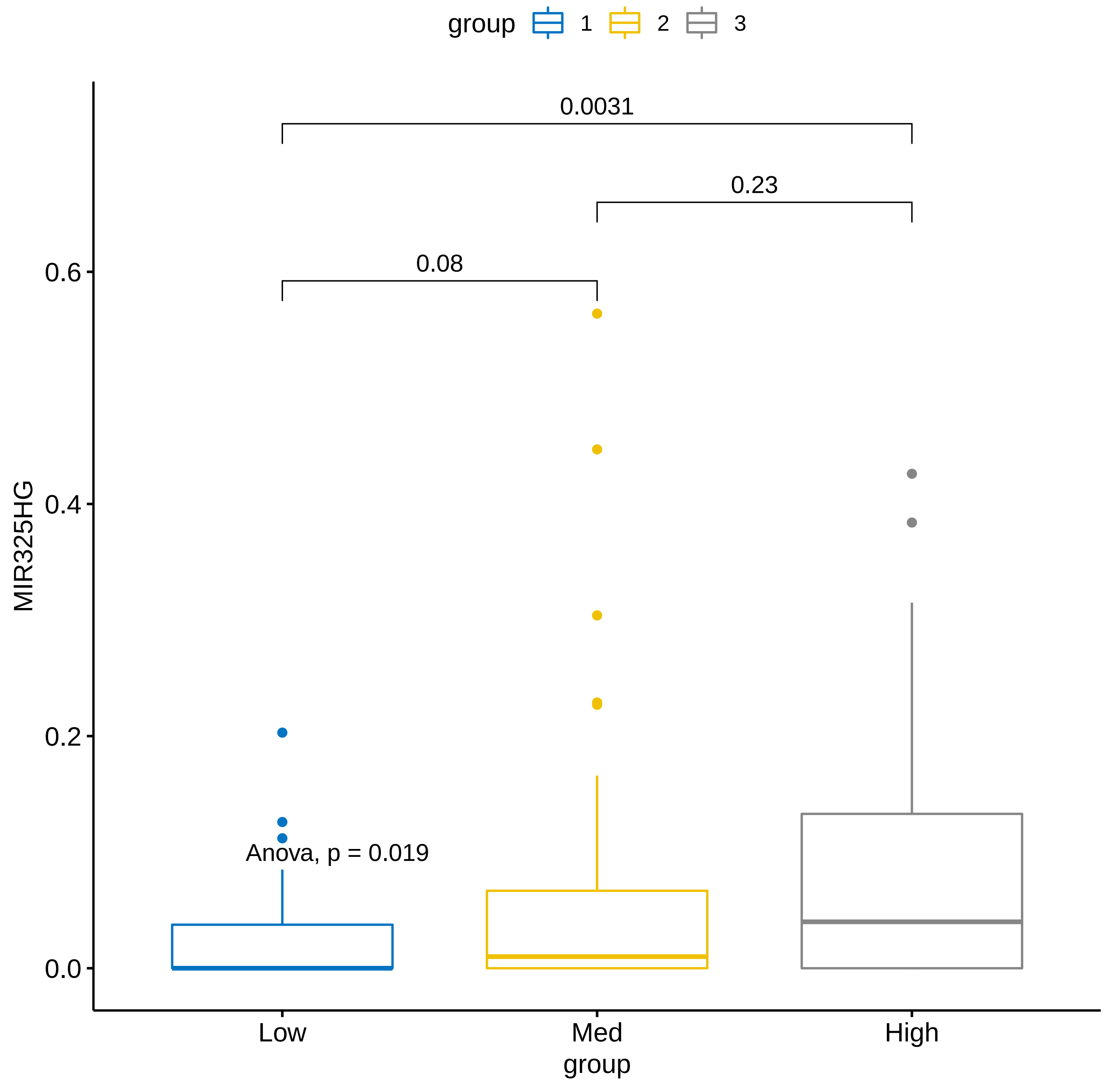
group 1 2 3
0.0031
0.23
0.08
Anova, p = 0.019
MIR325HG
0.6
0.4
0.2
0.0
Low
Med
High
group

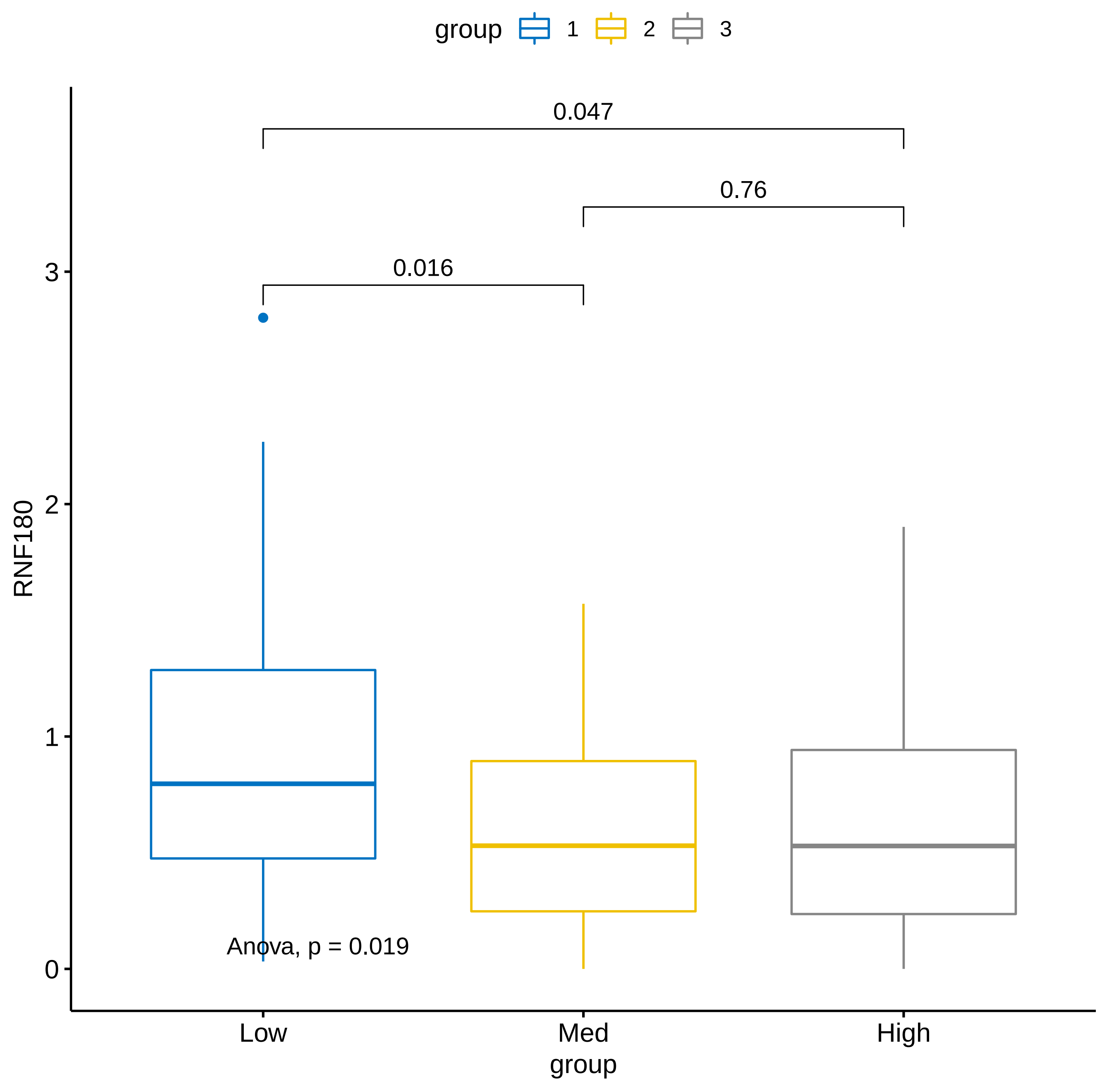

group 1 2 3
0.047
0.76
0.016
Anova, p = 0.019
RNF180
3
2
1
0
Low
Med
High
group

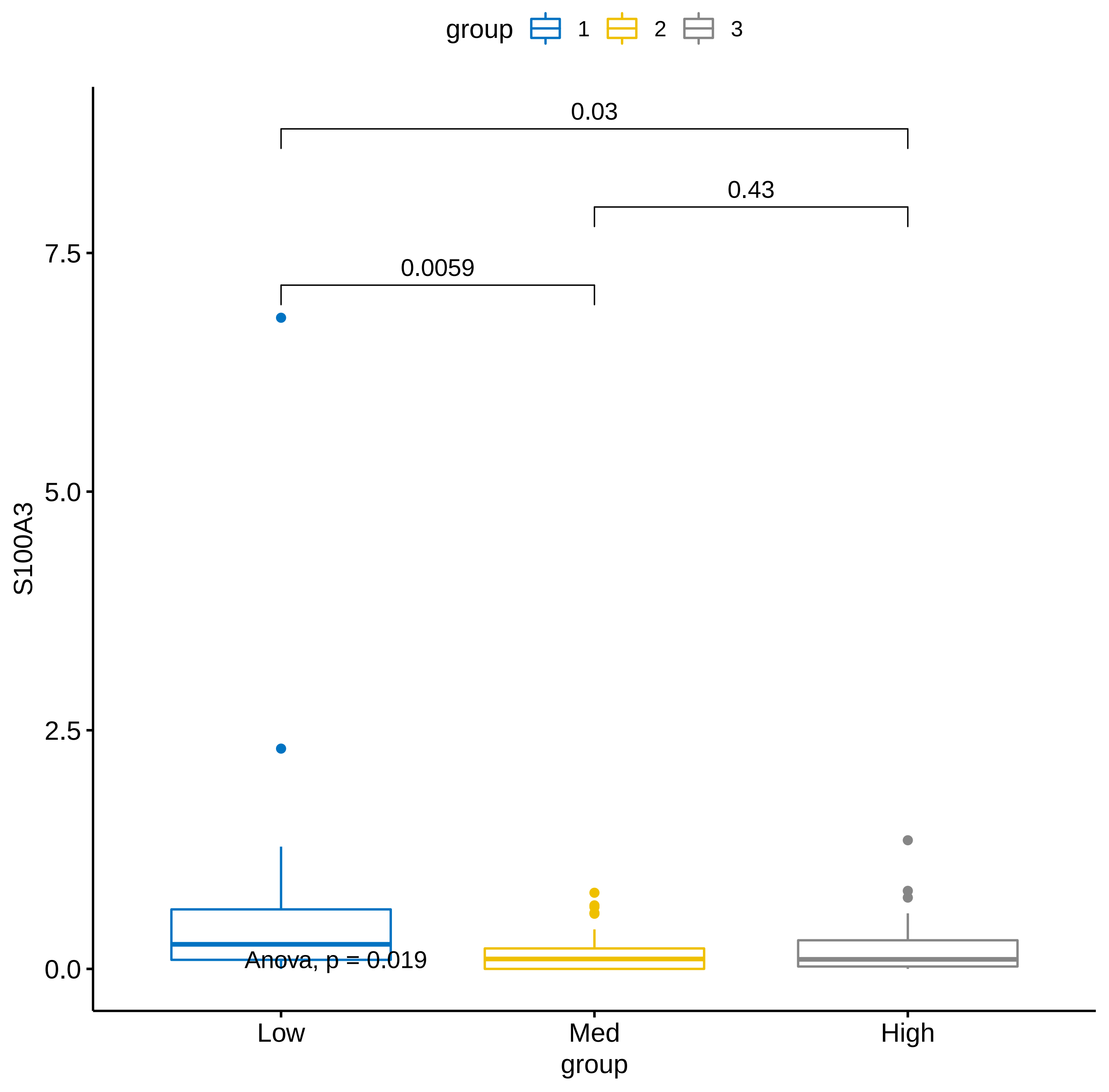

group 1 2 3
0.03
0.43
0.0059
S100A3
7.5
5.0
2.5
0.0
Anova, p = 0.019
Low
Med
High
group

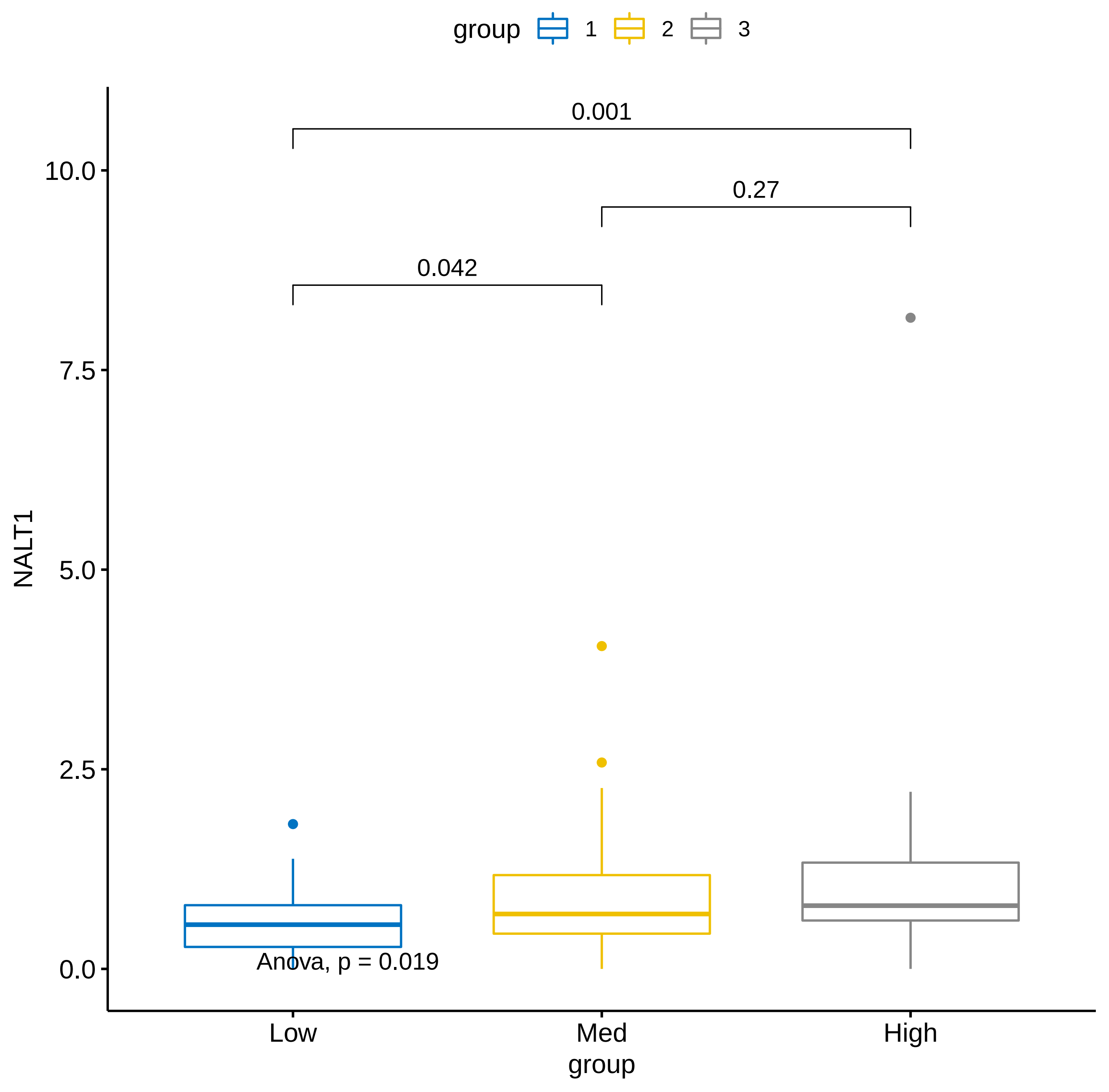

group
1
2
3
0.001
0.27
0.042
10.0
7.5
5.0
2.5
0.0
NALT1
Anova, p = 0.019
Low
Med
High
group

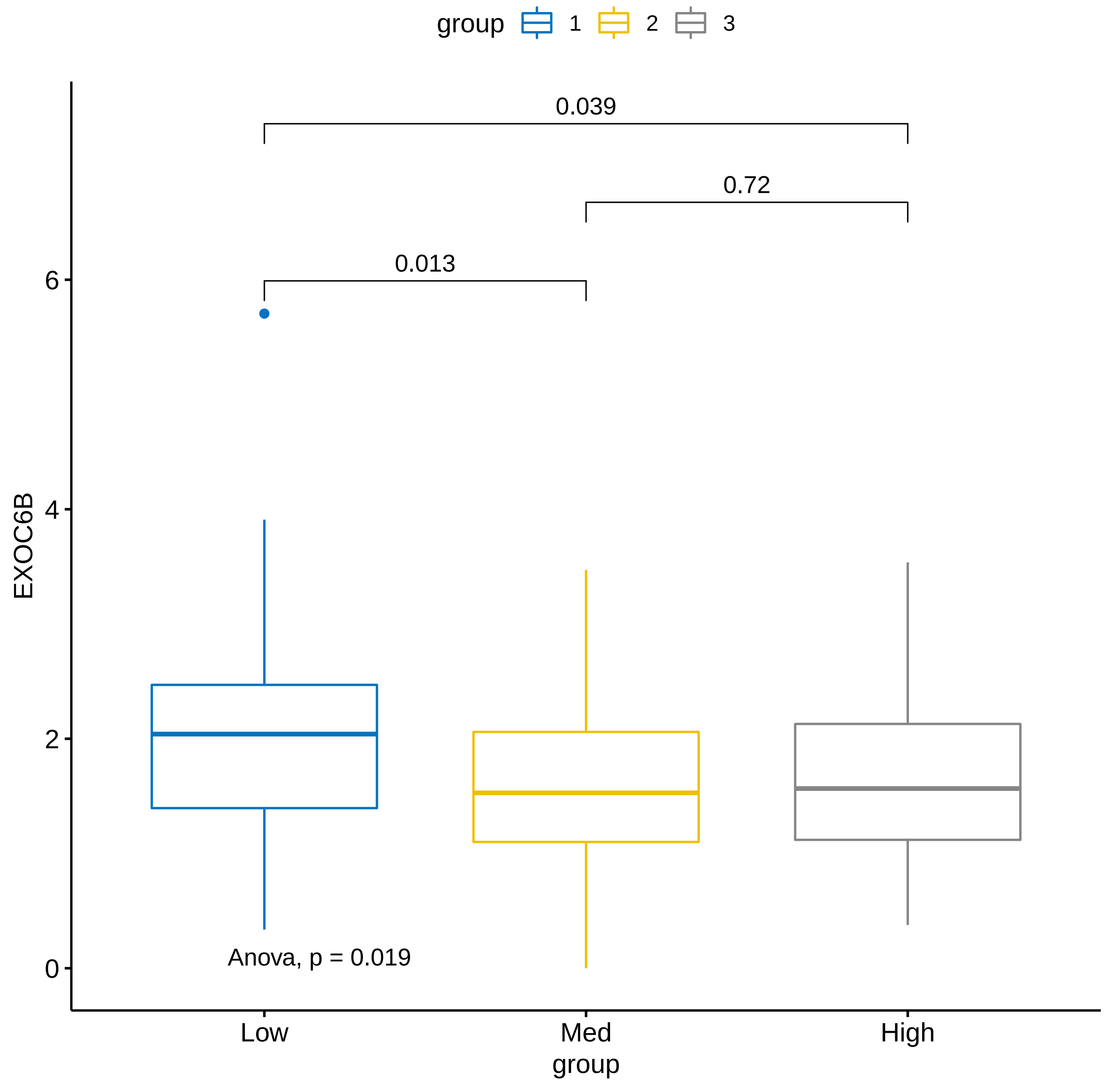
group 1 2 3
0.039
0.72
0.013
6
4
2
0
EXOC6B
Anova, p = 0.019
Low
Med
High
group

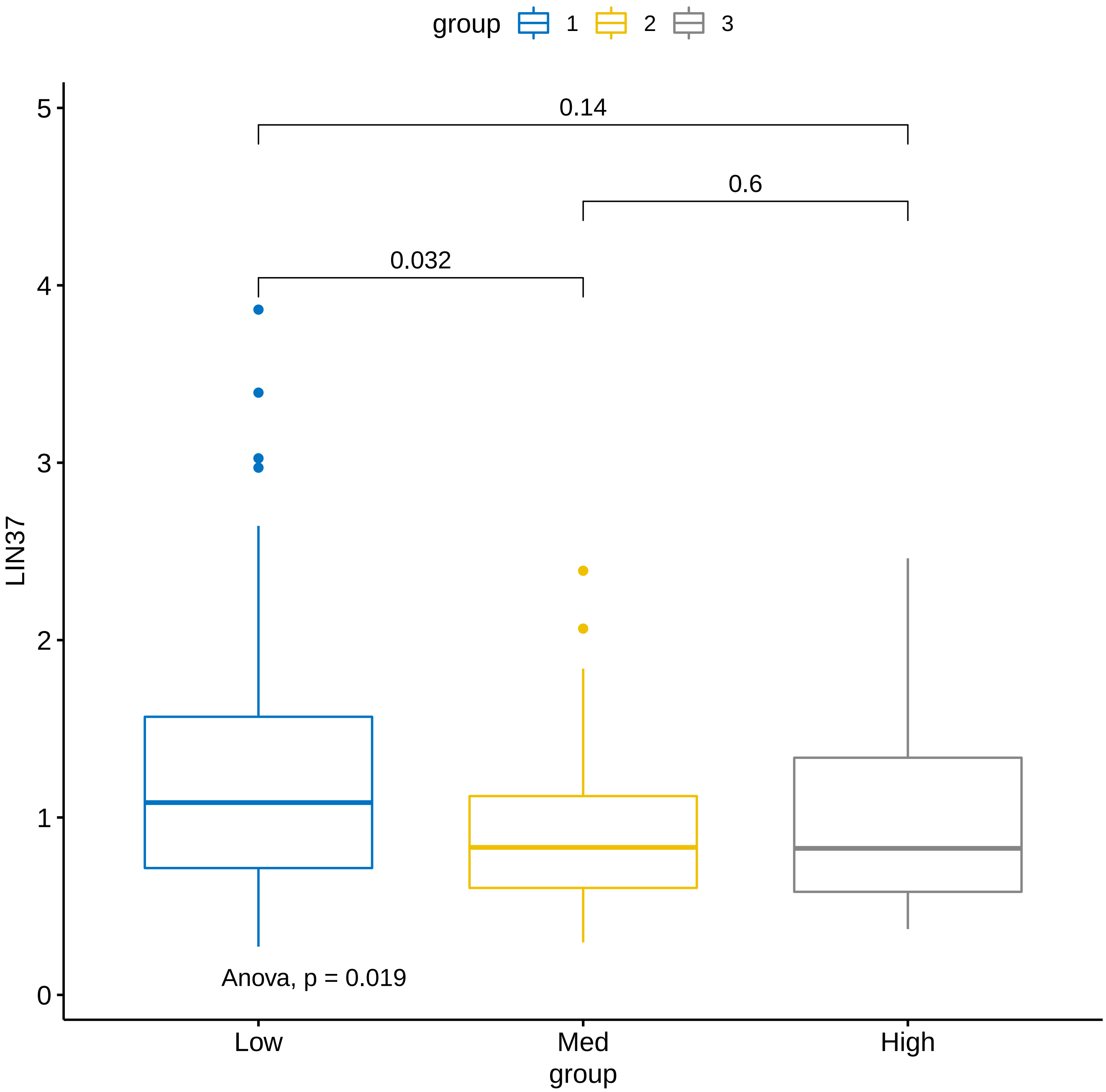
group
1
2
3
0.14
0.6
0.032
Anova, p = 0.019
LIN37
5
4
3
2
1
0
Low
Med
High
group

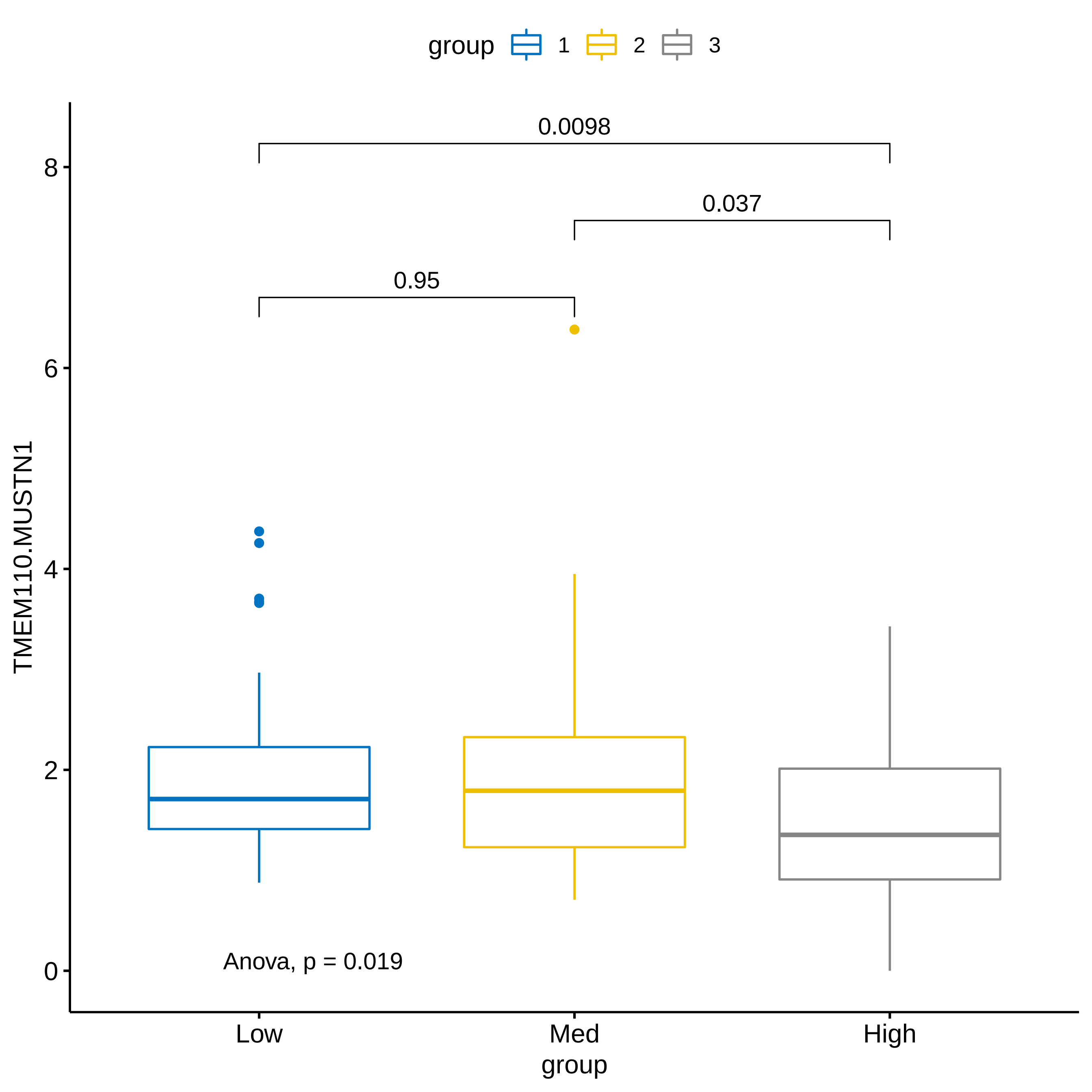

group 1 2 3
0.0098
0.037
0.95
Anova, p = 0.019
TMEM110.MUSTN1
0
2
4
6
8
Low
Med
High
group

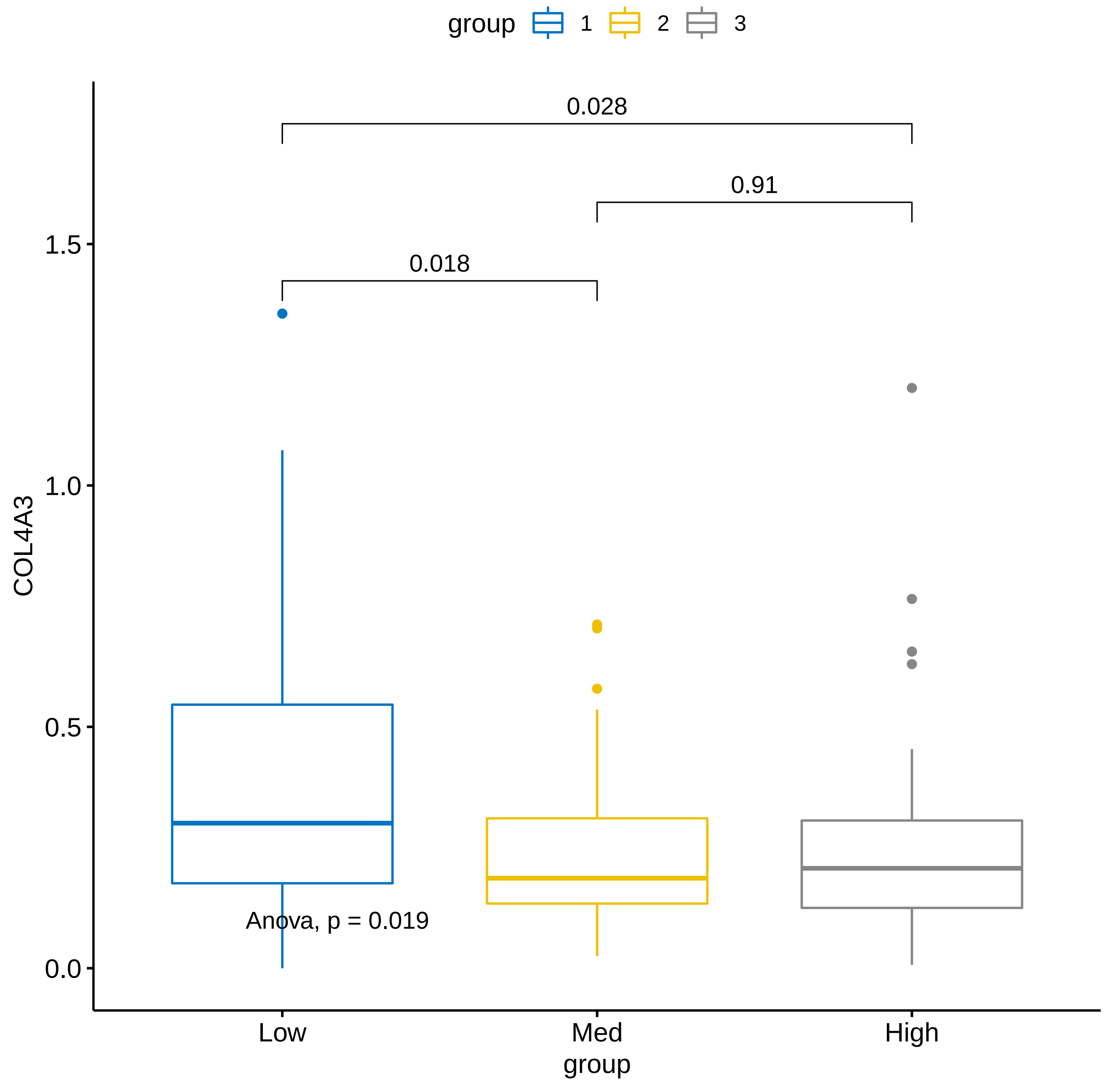
group
1
2
3
0.028
0.91
0.018
1.5
1.0
0.5
0.0
COL4A3
Anova, p = 0.019
Low
Med
High
group

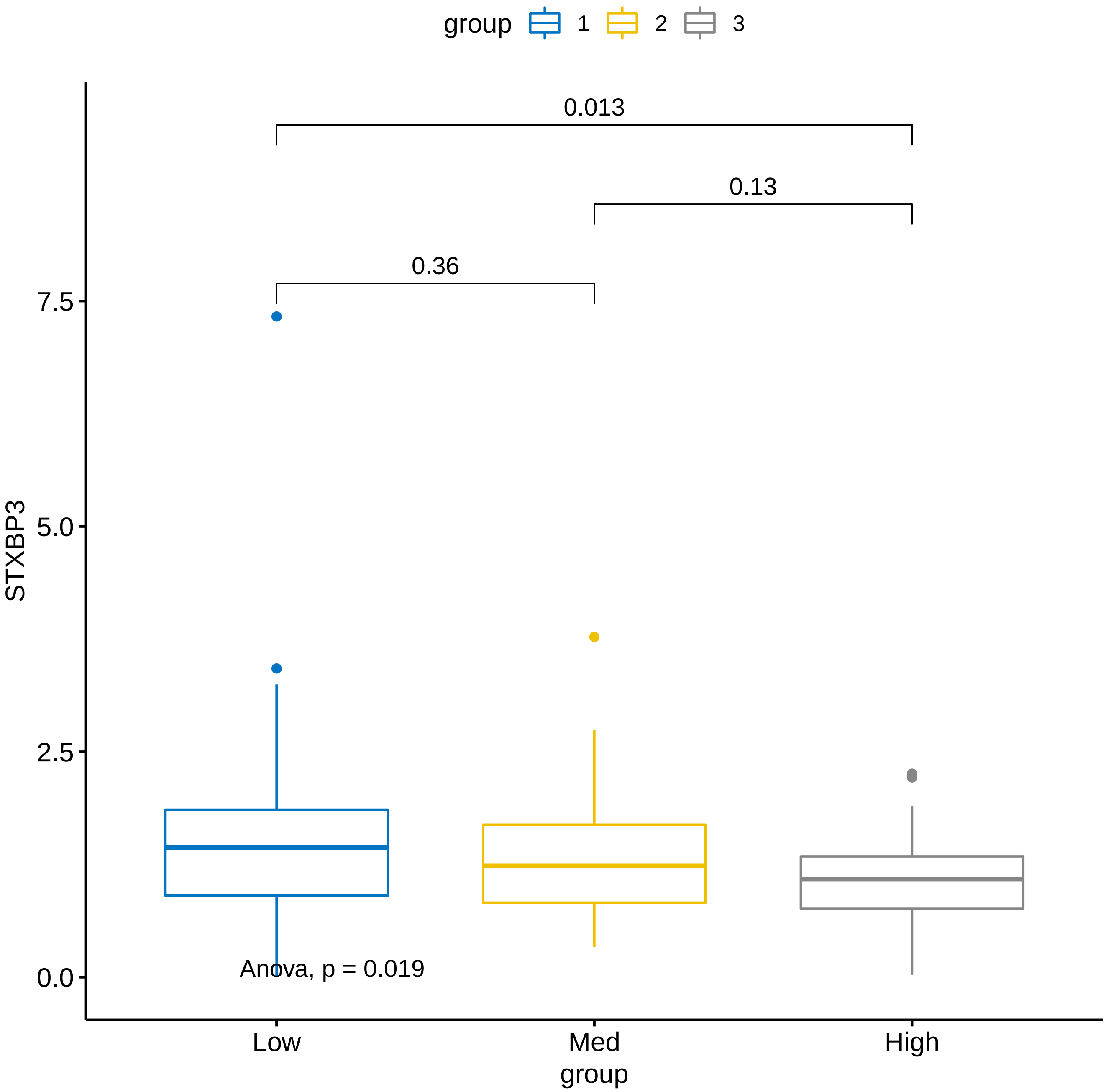

group
1
2
3
0.013
0.13
0.36
7.5
5.0
2.5
0.0
STXBP3
Anova, p = 0.019
Low
Med
High
group

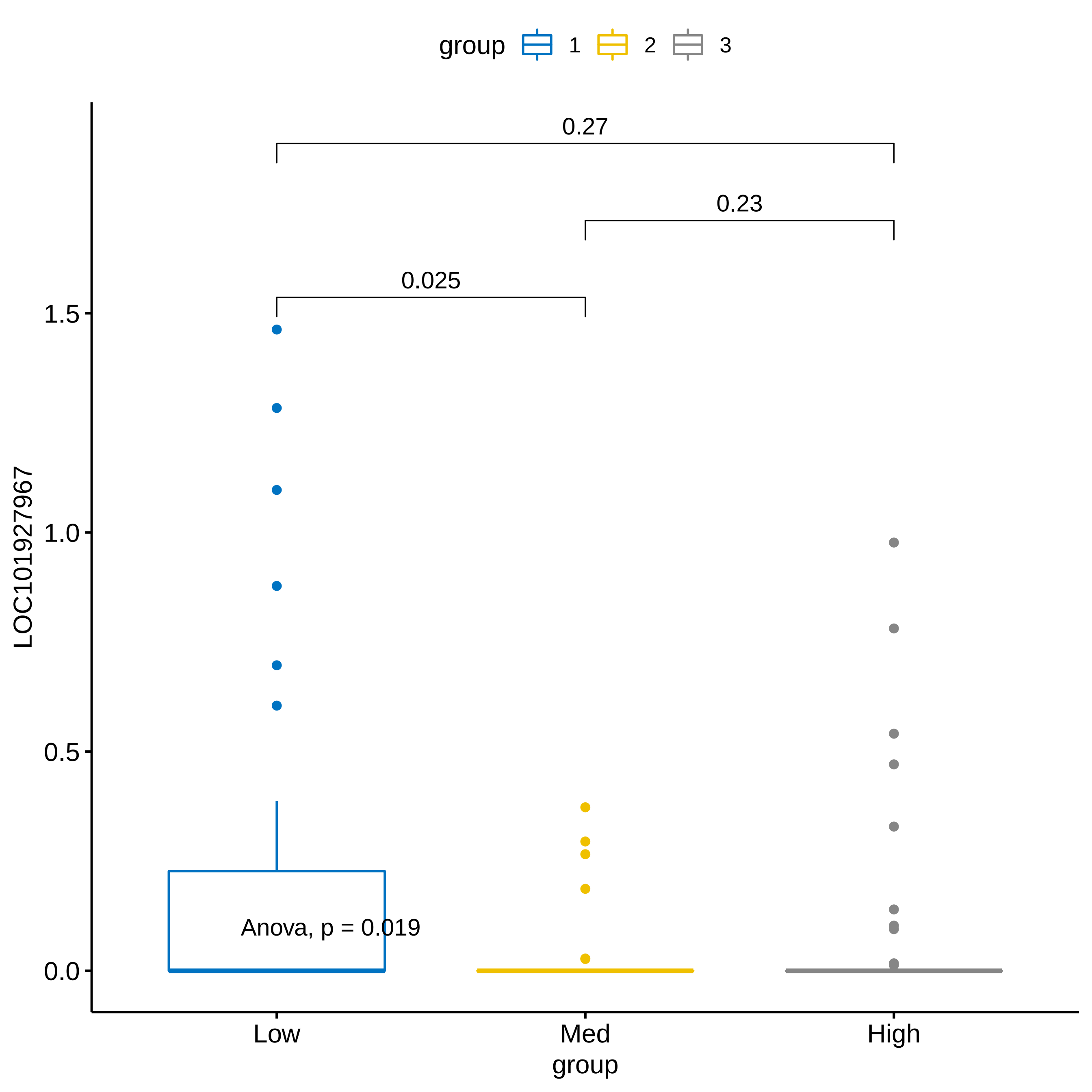

group 1 2 3
0.27
0.23
0.025
1.5
1.0
0.5
0.0
LOC101927967
Anova, p = 0.019
Low
Med
High
group

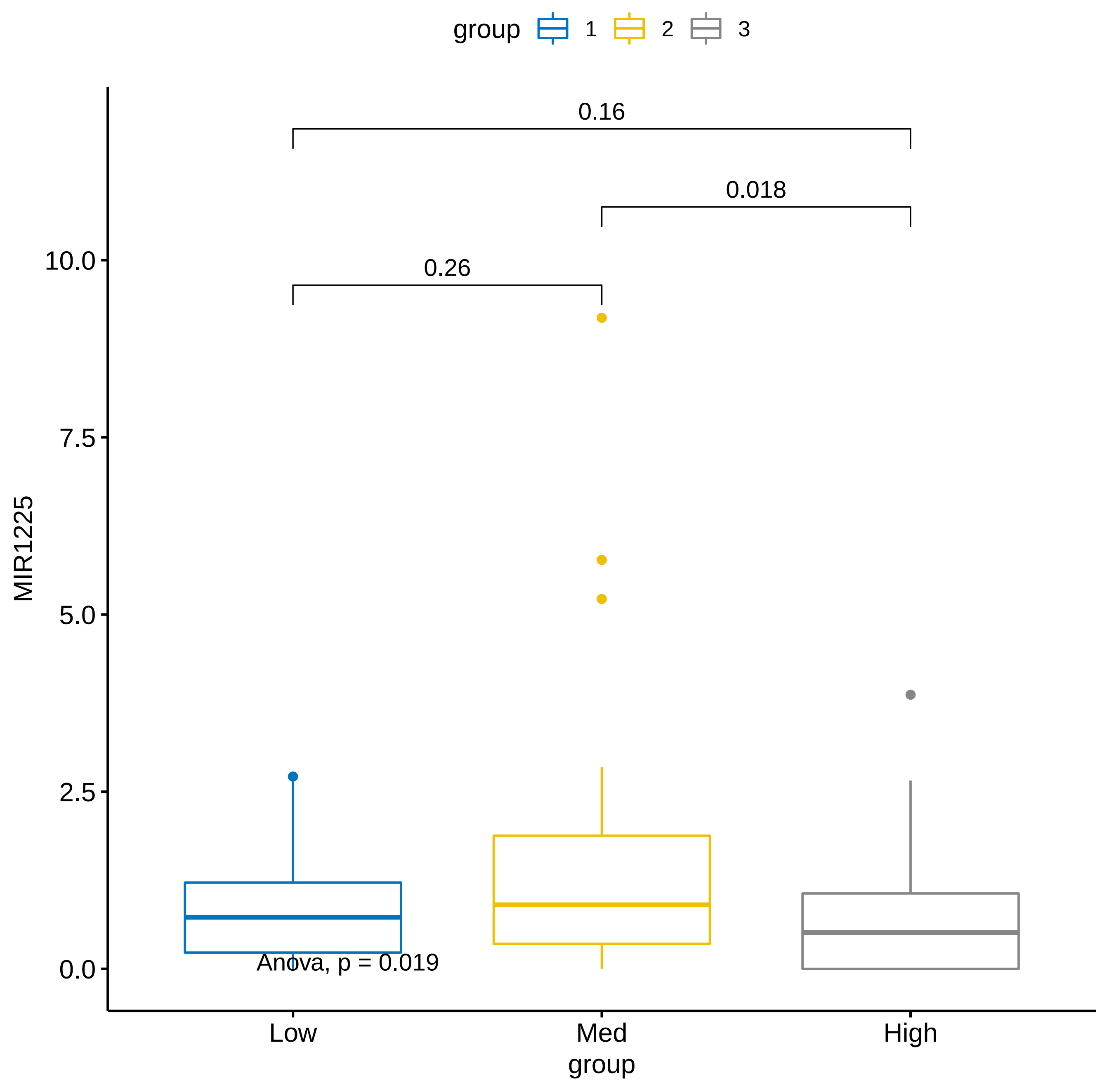

group
1
2
3
0.16
0.018
0.26
10.0
7.5
5.0
2.5
0.0
MIR1225
Anova, p = 0.019
Low
Med
High
group

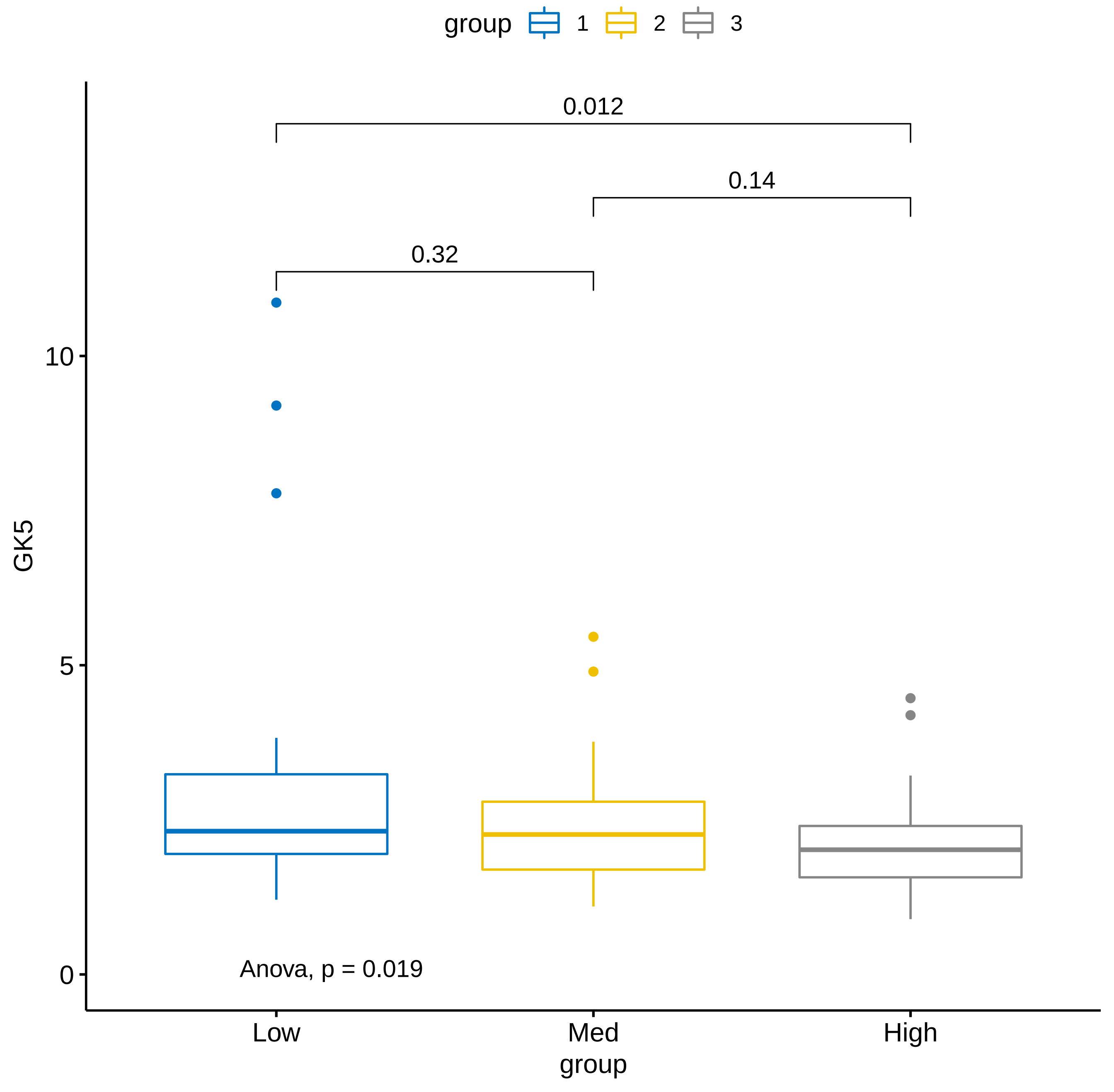
group
1
2
3
0.012
0.14
0.32
10
5
0
GK5
Anova, p = 0.019
Low
Med
High
group

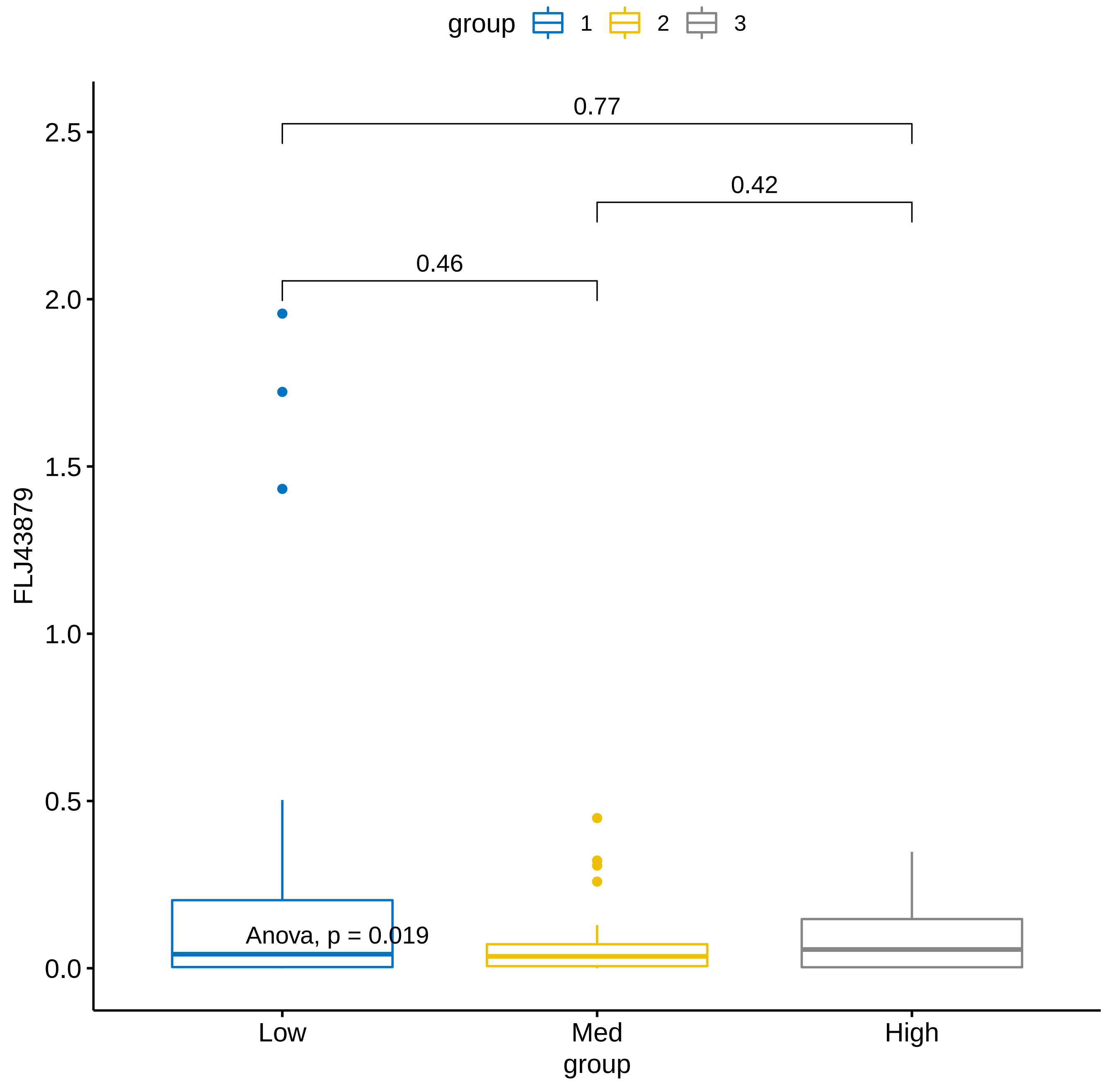
group
1
2
3
0.77
0.42
0.46
2.5
2.0
1.5
1.0
0.5
0.0
FLJ43879
Anova, p = 0.019
Low
Med
High
group

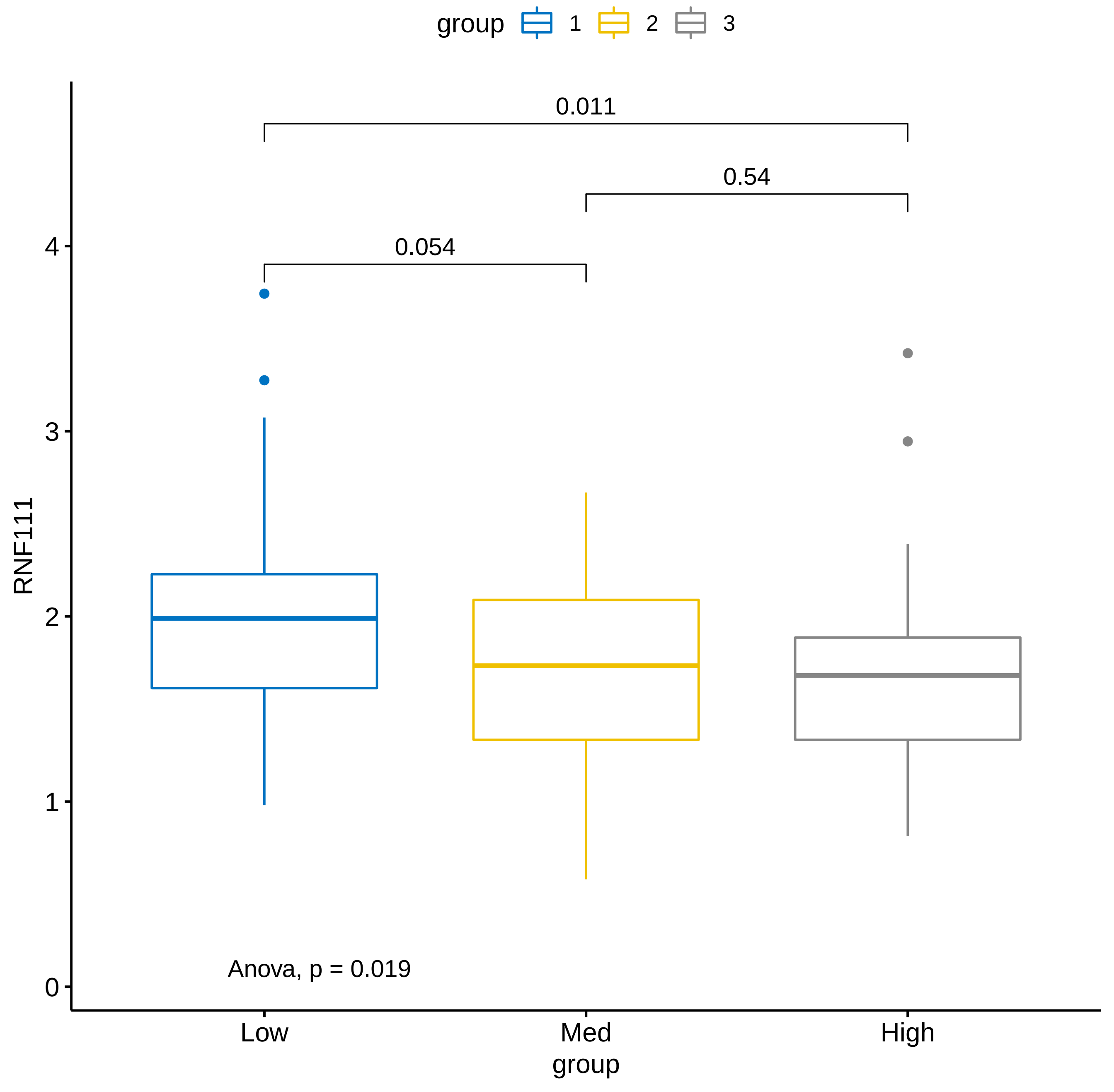
group
1
2
3
0.011
0.54
0.054
RNF111
4
3
2
1
0
Anova, p = 0.019
Low
Med
High
group

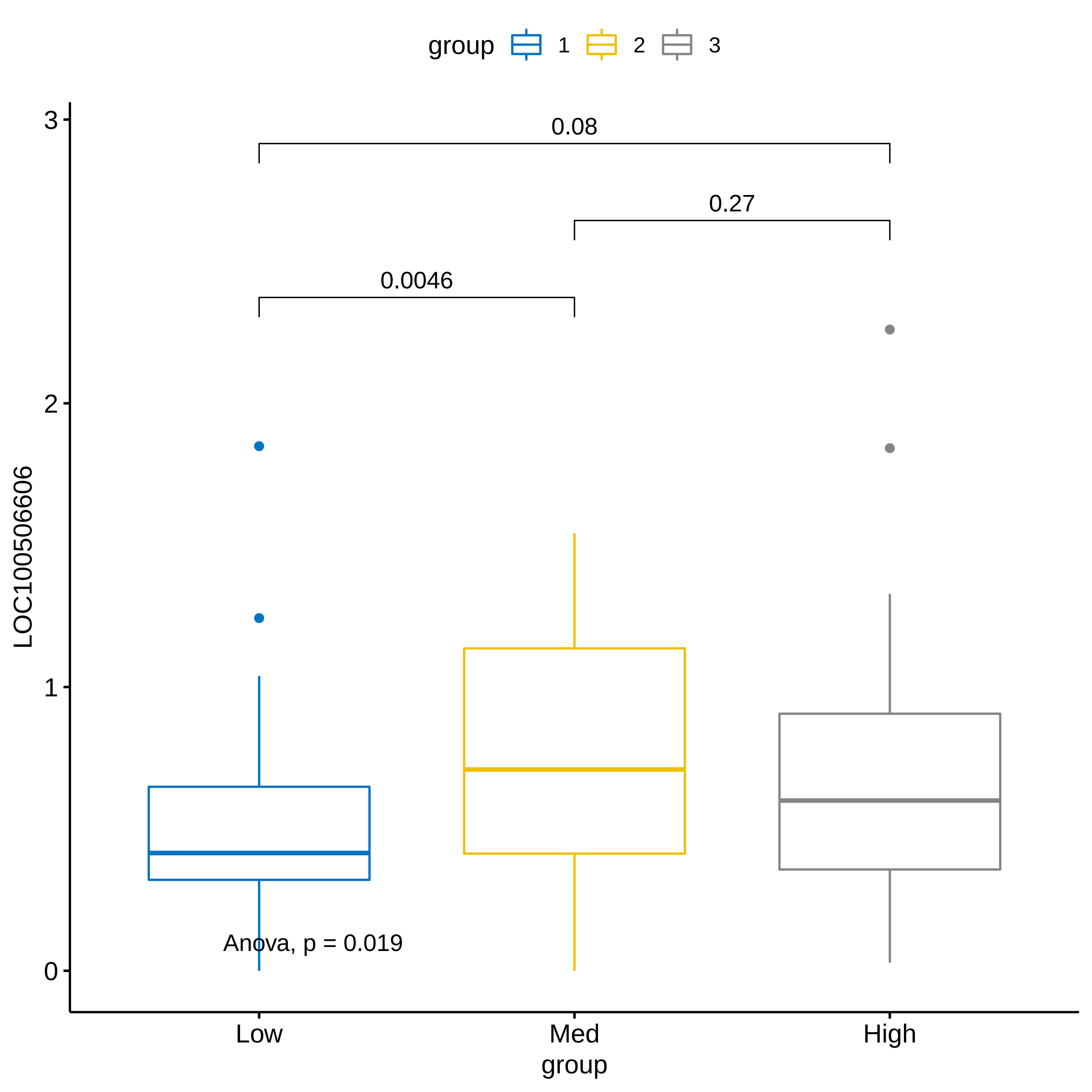

group 1 2 3
0.08
0.27
0.0046
Anova, p = 0.019
LOC100506606
3
2
1
0
Low
Med
High
group

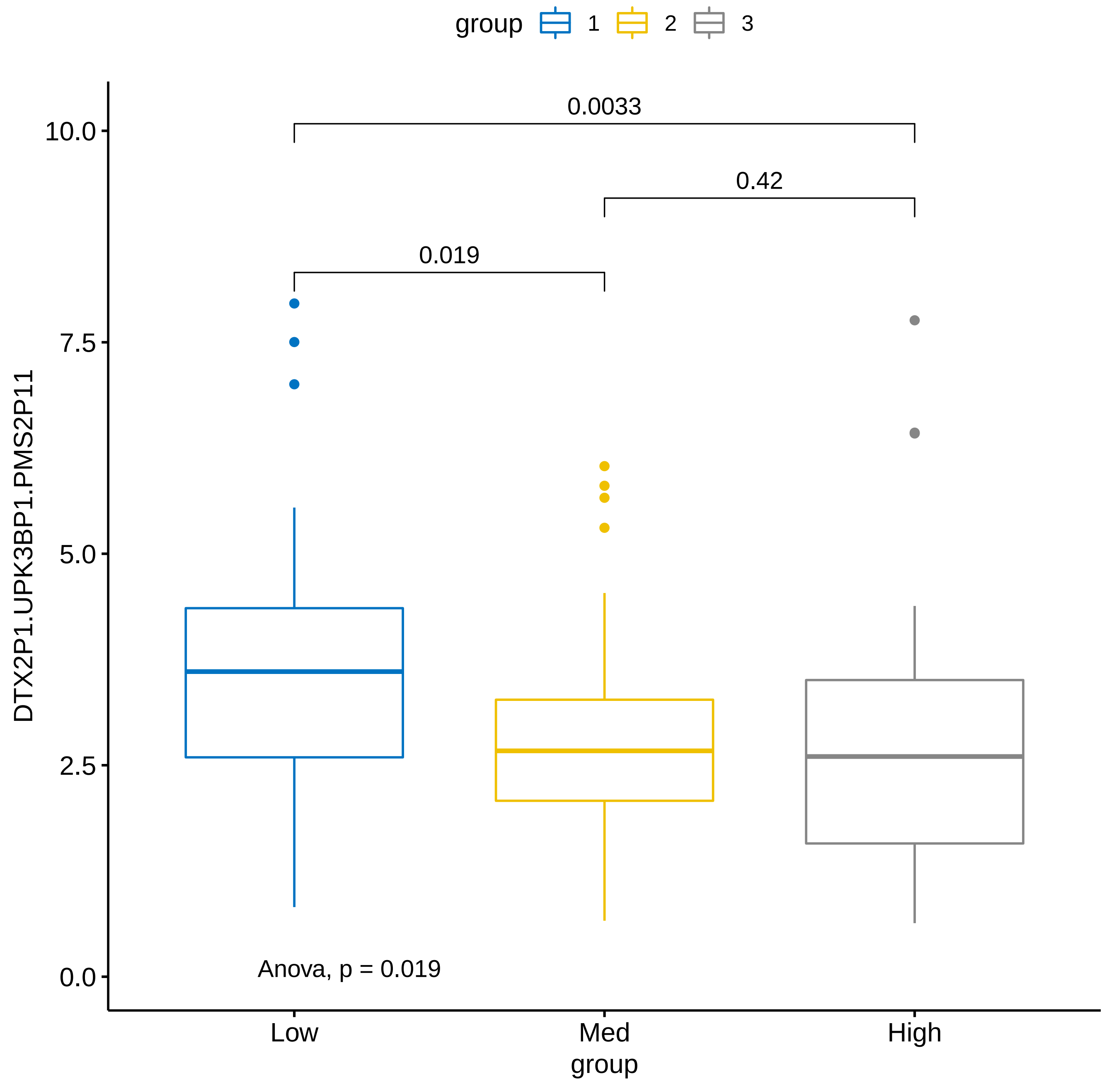

group 1 2 3
0.0033
0.42
0.019
10.0
7.5
5.0
2.5
0.0
DTX2P1.UPK3BP1.PMS2P11
Anova, p = 0.019
Low
Med
High
group

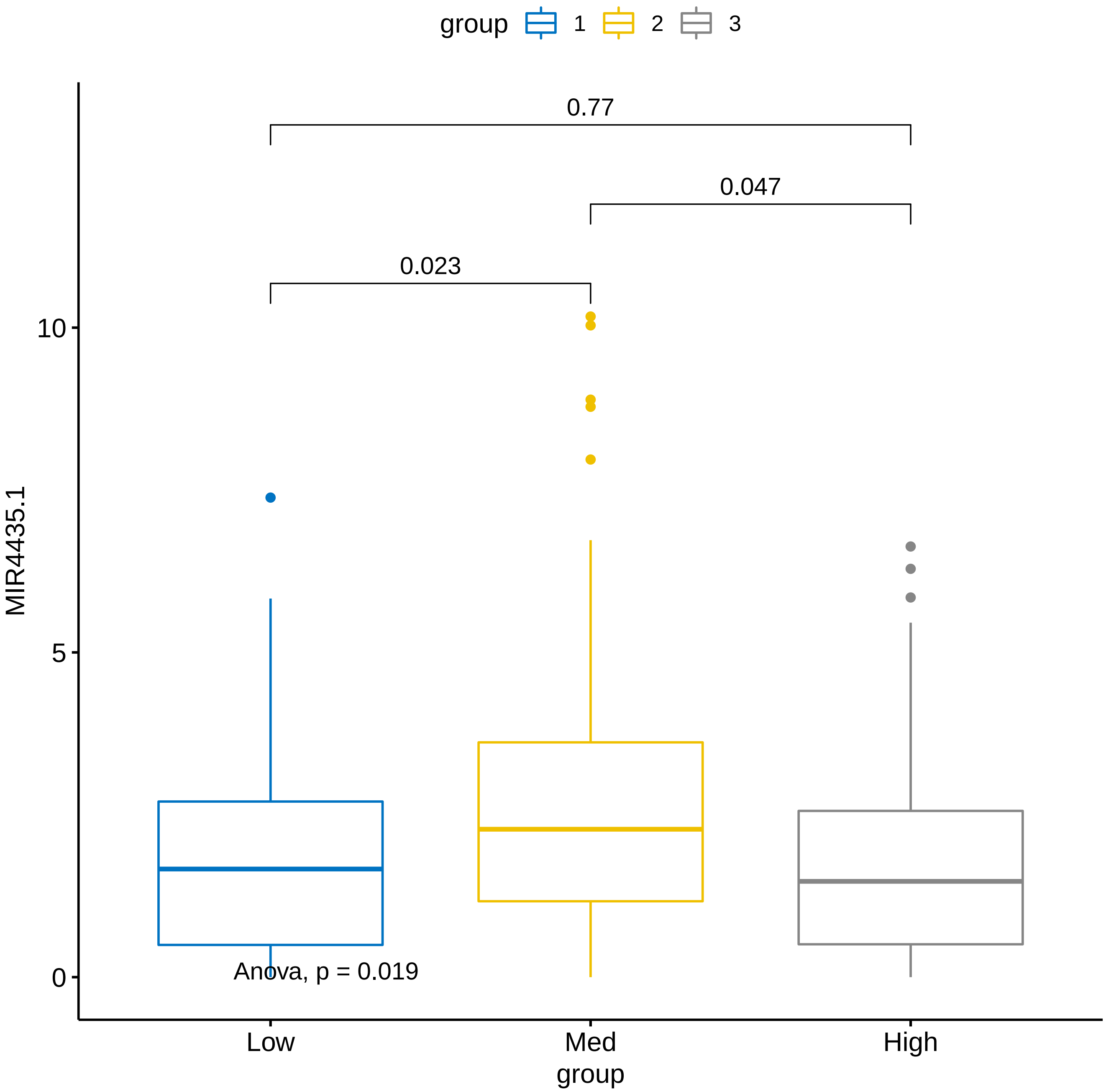

group 1 2 3
0.77
0.047
0.023
10
5
0
MIR4435.1
Anova, p = 0.019
Low
Med
High
group

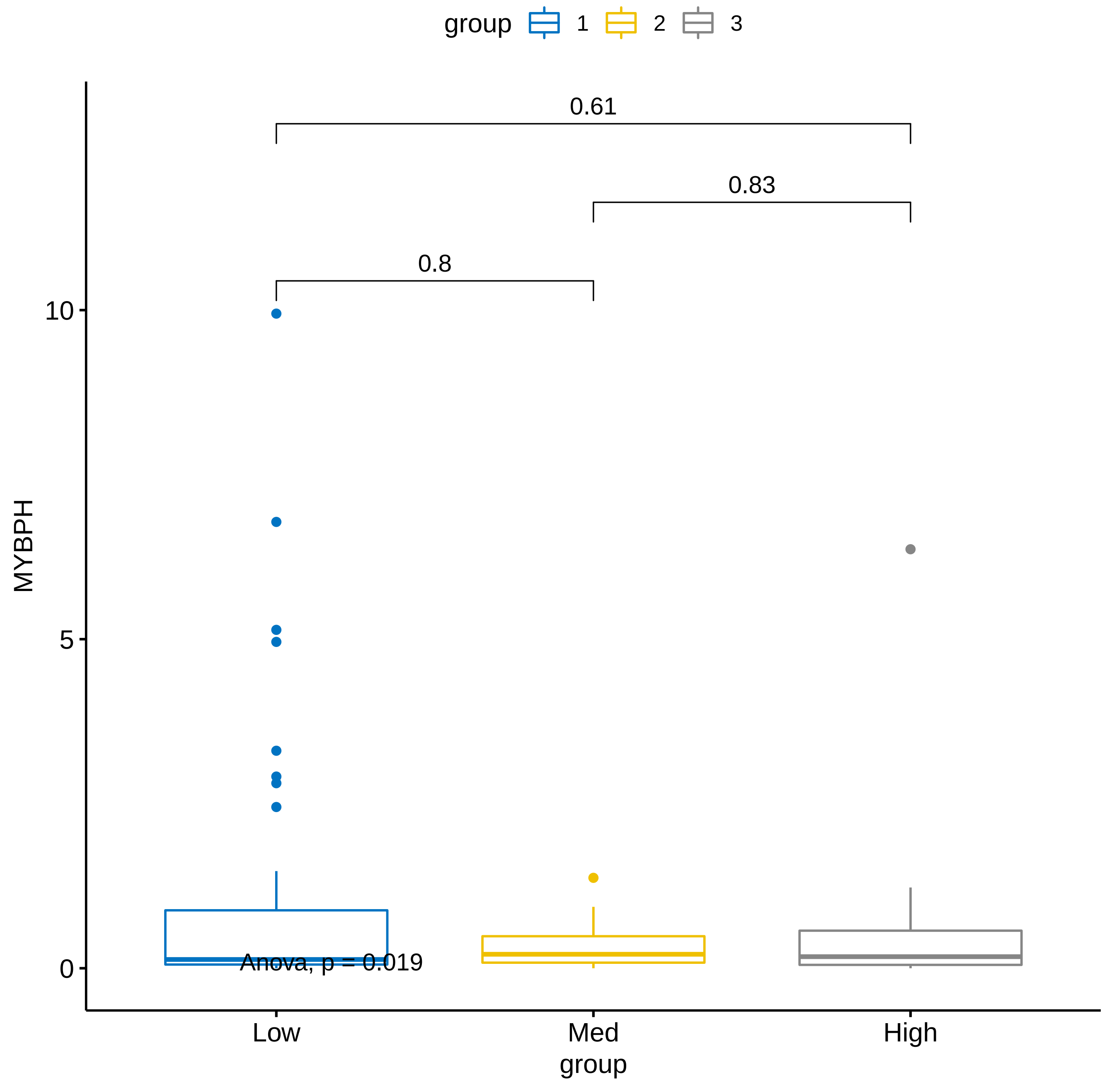

group 1 2 3
0.61
0.83
0.8
10
5
0
MYBPH
Anova, p = 0.019
Low
Med
High
group

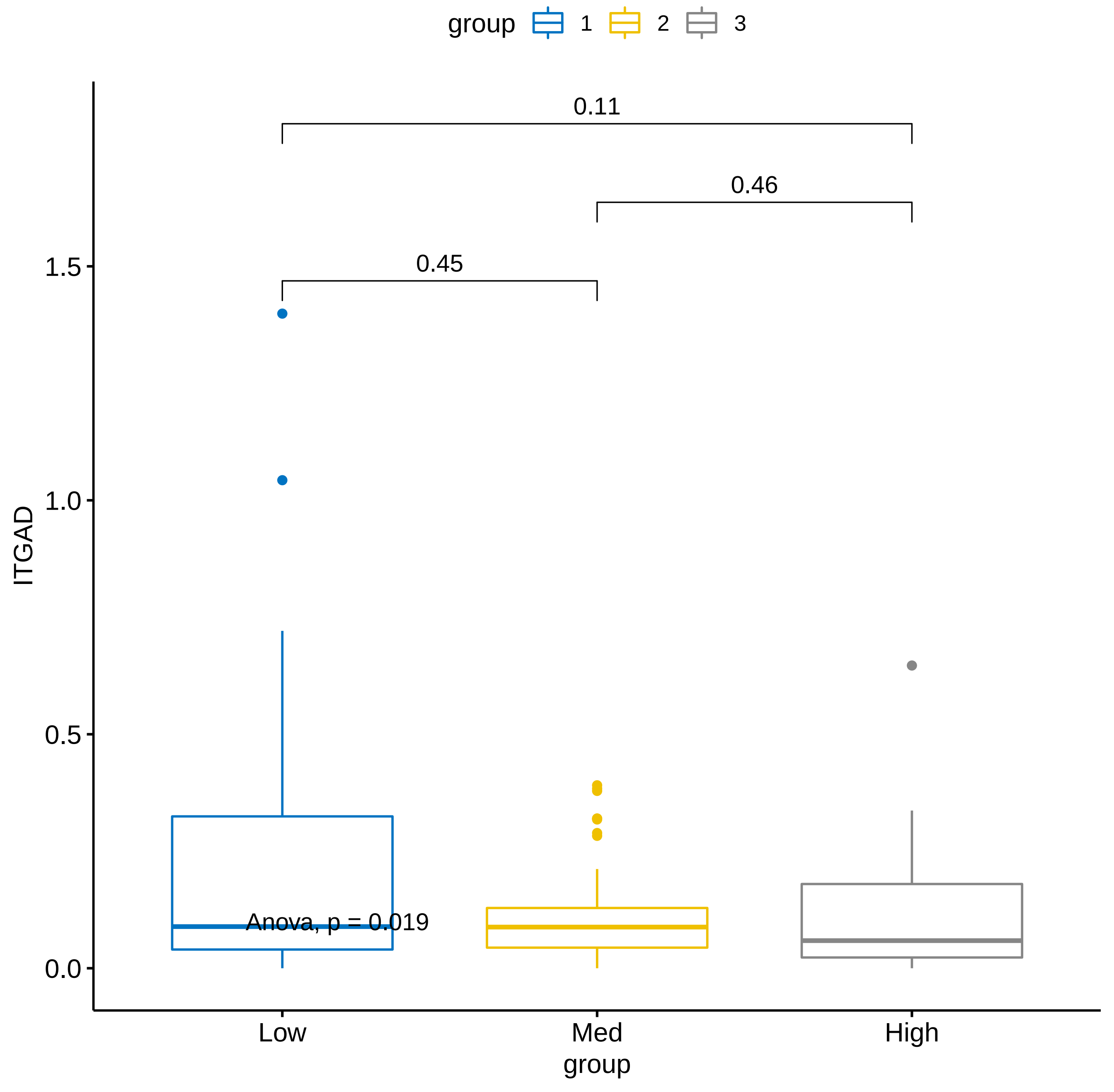

group 1 2 3
0.11
0.46
0.45
1.5
1.0
0.5
0.0
ITGAD
Anova, p = 0.019
Low
Med
High
group

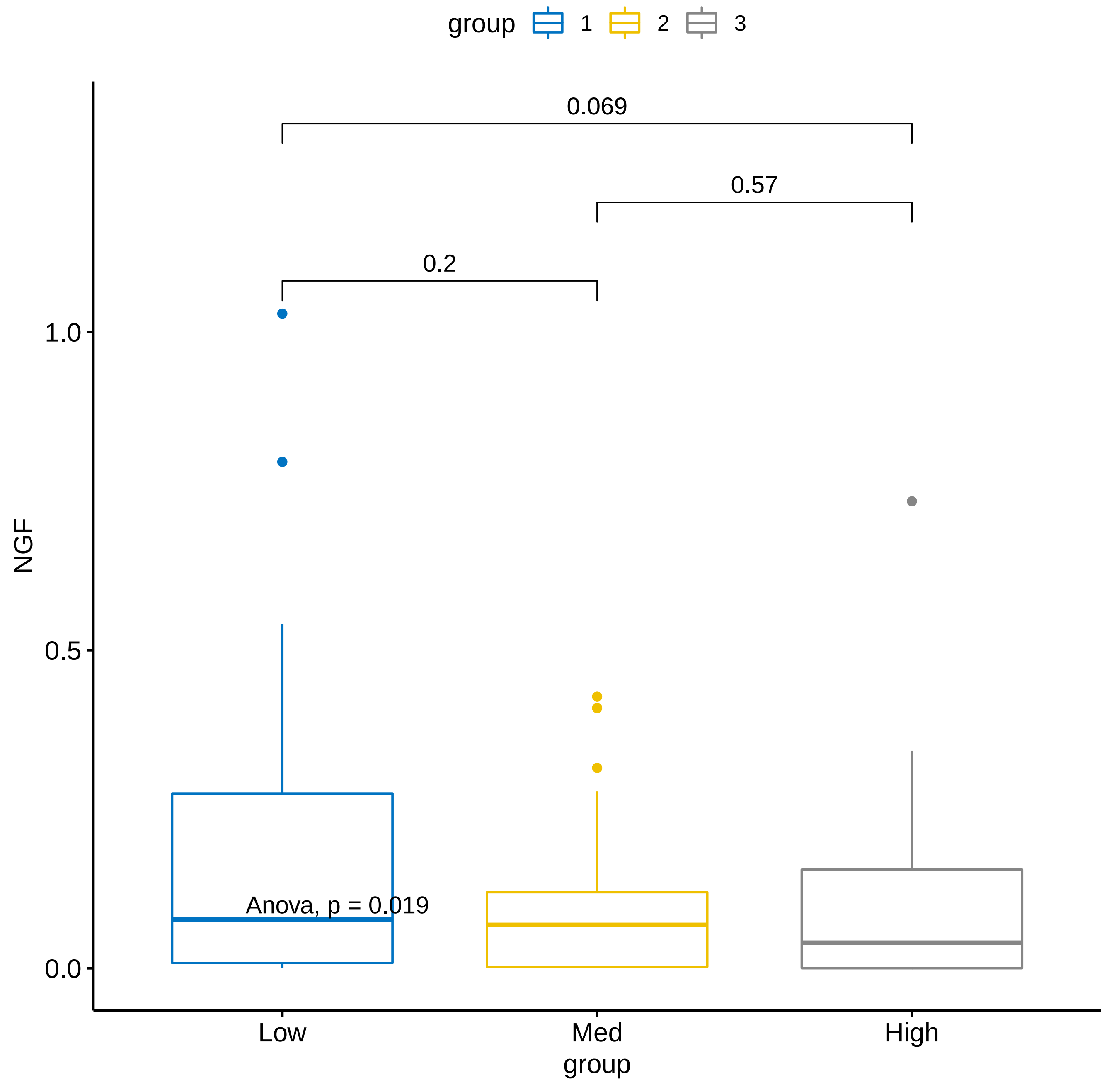
group 1 2 3
0.069
0.57
0.2
1.0
0.5
0.0
NGF
Anova, p = 0.019
Low
Med
High
group

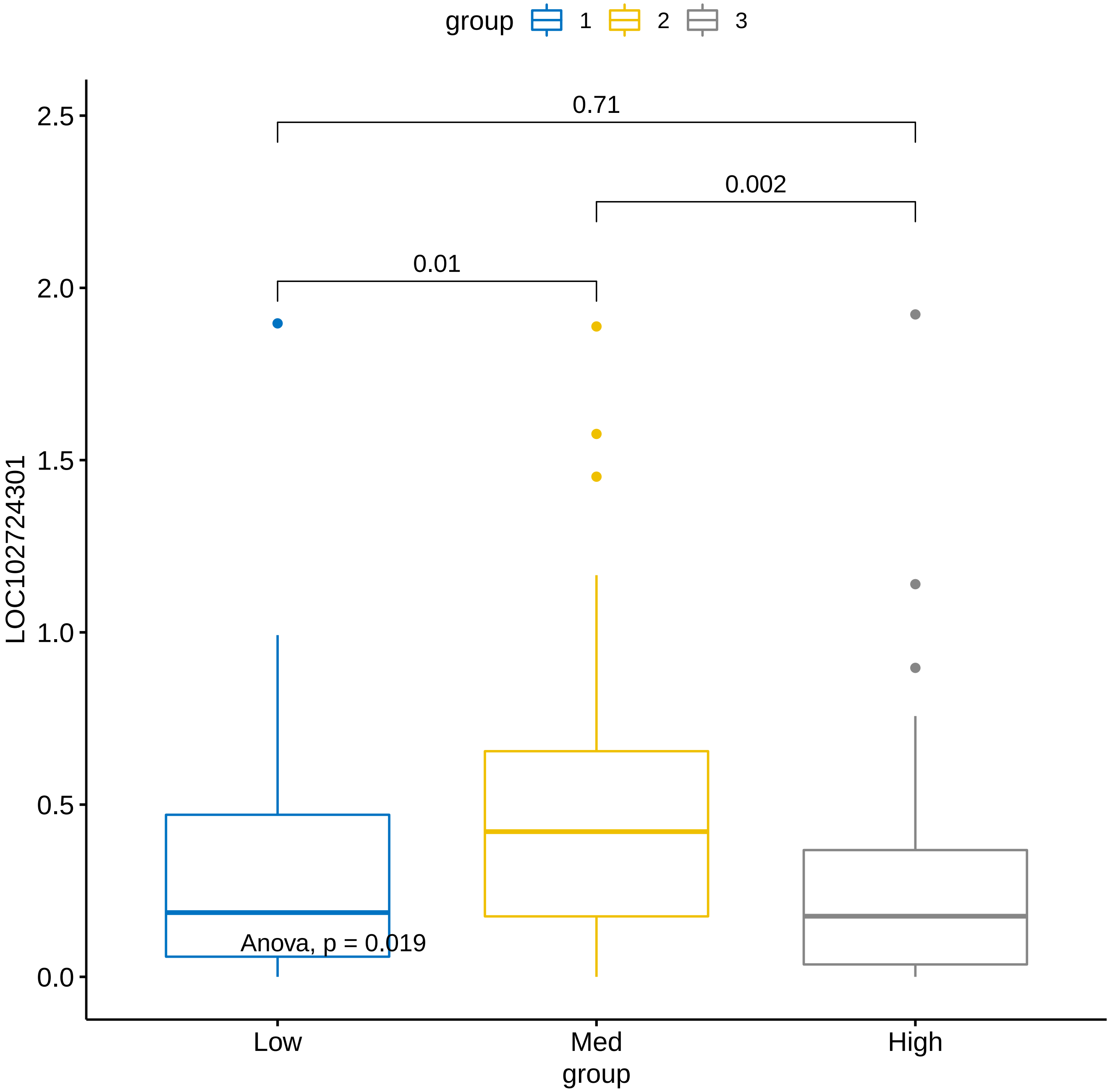
group
1
2
3
0.71
0.002
0.01
2.5
2.0
1.5
1.0
0.5
0.0
LOC102724301
Anova, p = 0.019
Low
Med
High
group

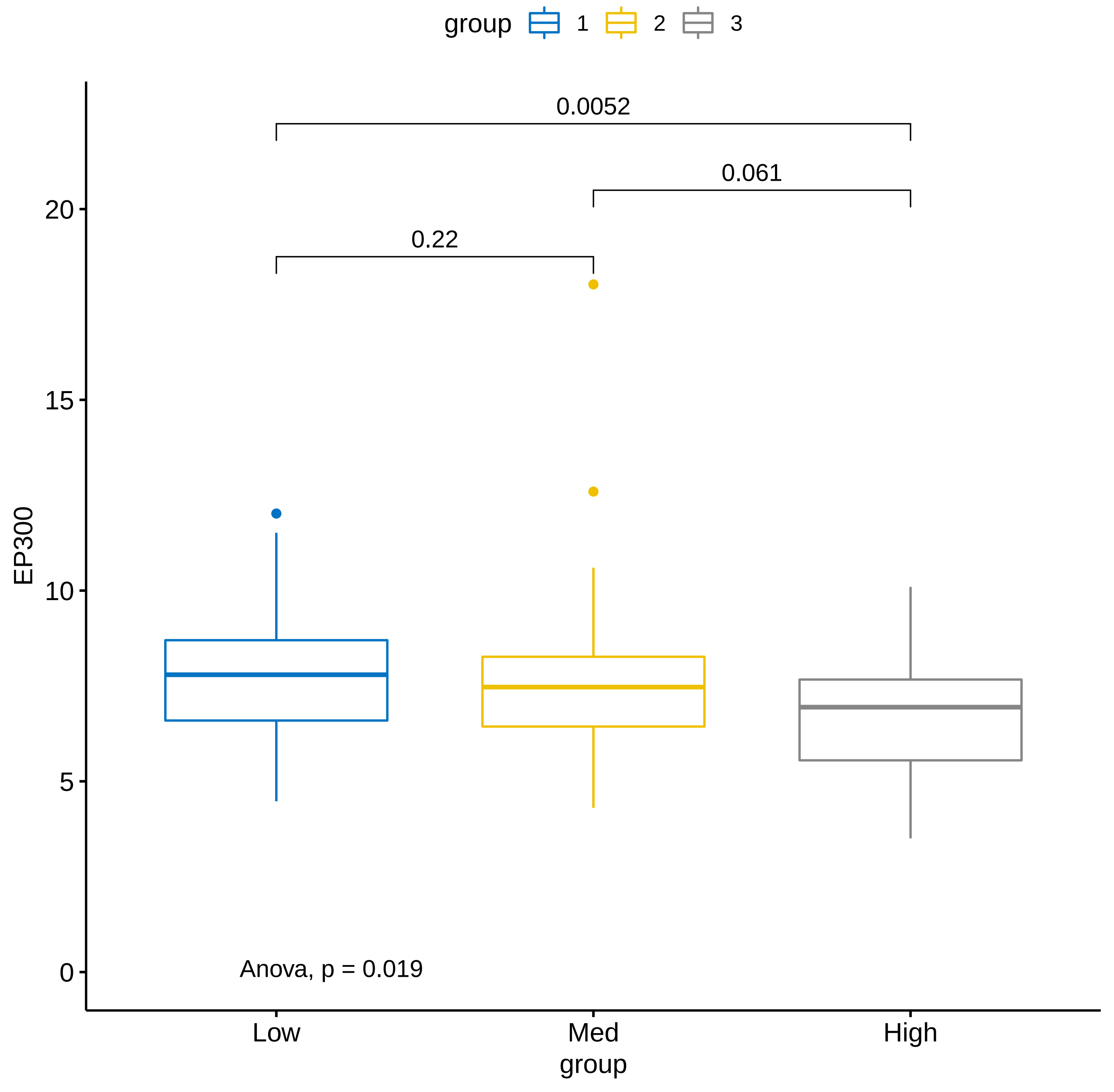

group
1
2
3
0.0052
0.061
0.22
20
15
10
5
0
EP300
Anova, p = 0.019
Low
Med
High
group

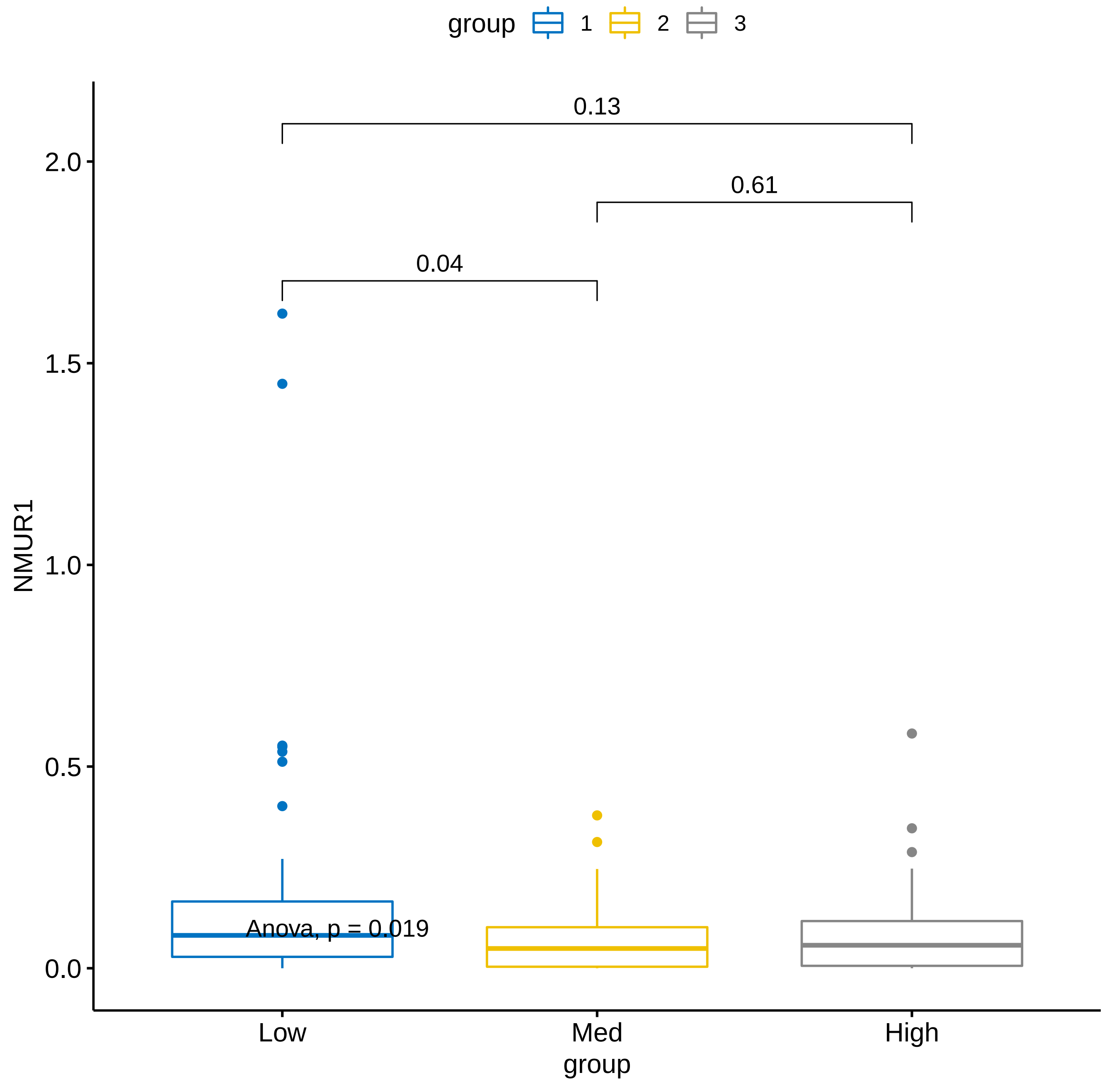
group
1
2
3
0.13
0.61
0.04
2.0
1.5
1.0
0.5
0.0
NMUR1
Anova, p = 0.019
Low
Med
High
group

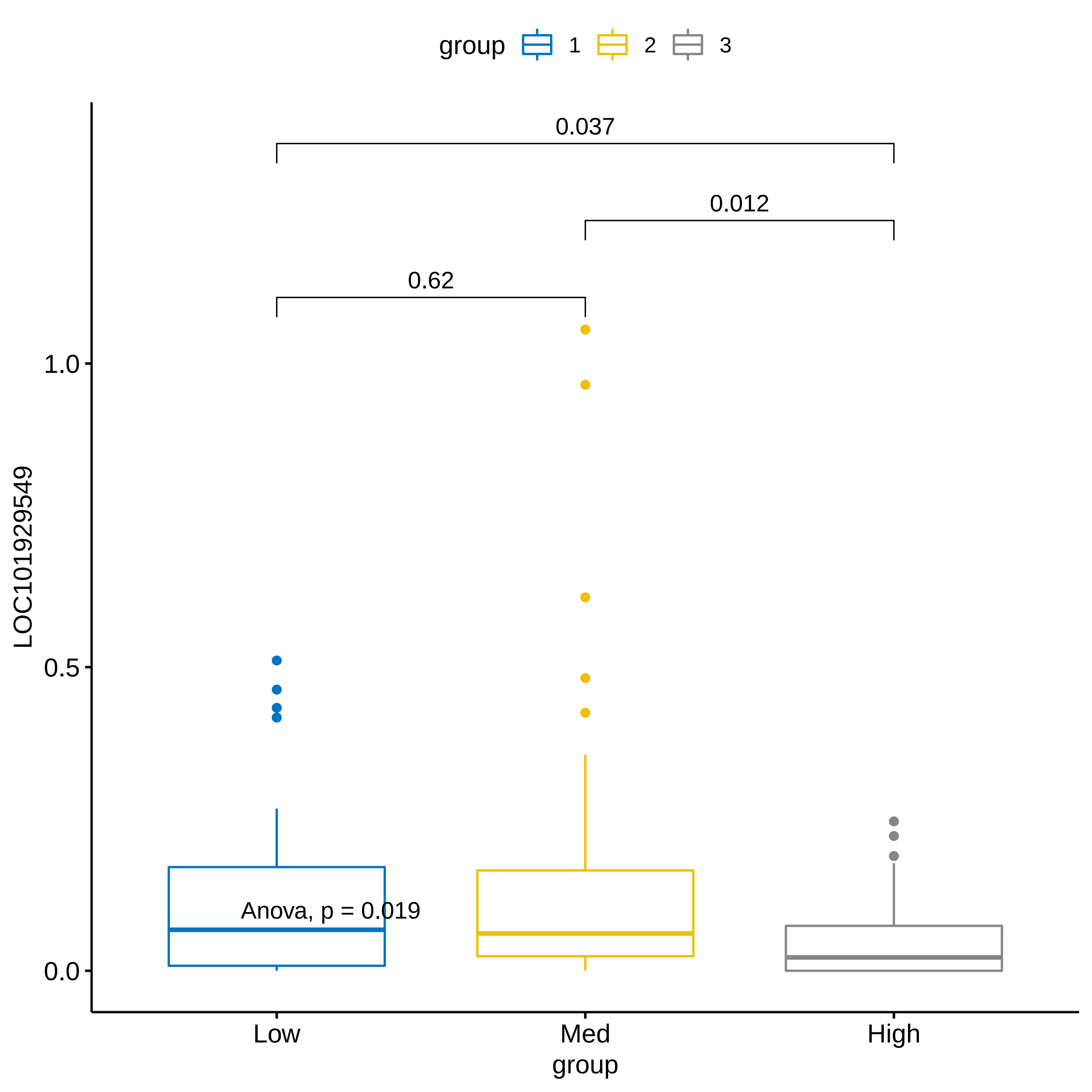

group
1
2
3
0.037
0.012
0.62
LOC101929549
1.0
0.5
0.0
Anova, p = 0.019
Low
Med
High
group

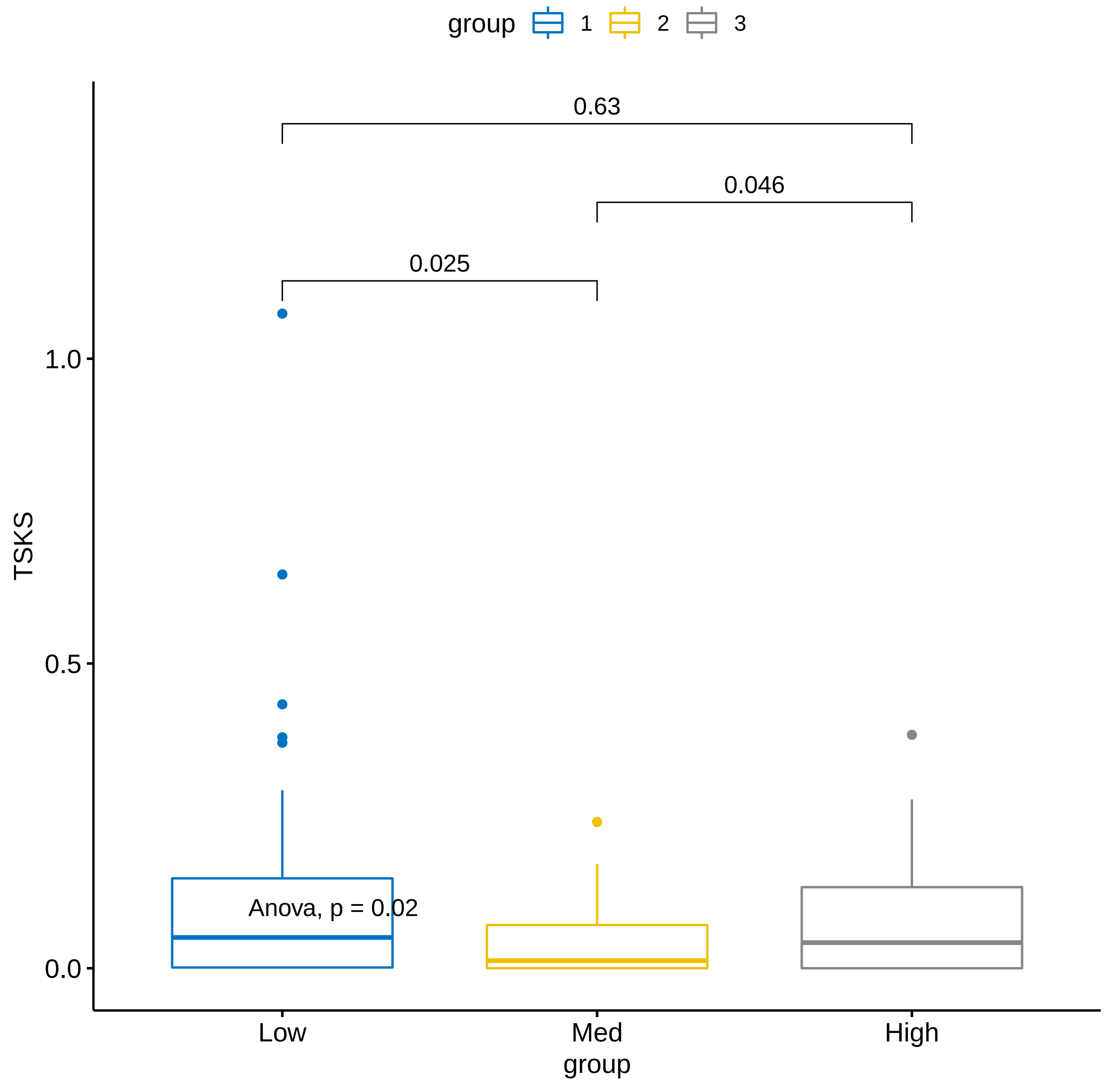

group
1
2
3
0.63
0.046
0.025
1.0
0.5
0.0
TSKS
Anova, p = 0.02
Low
Med
High
group

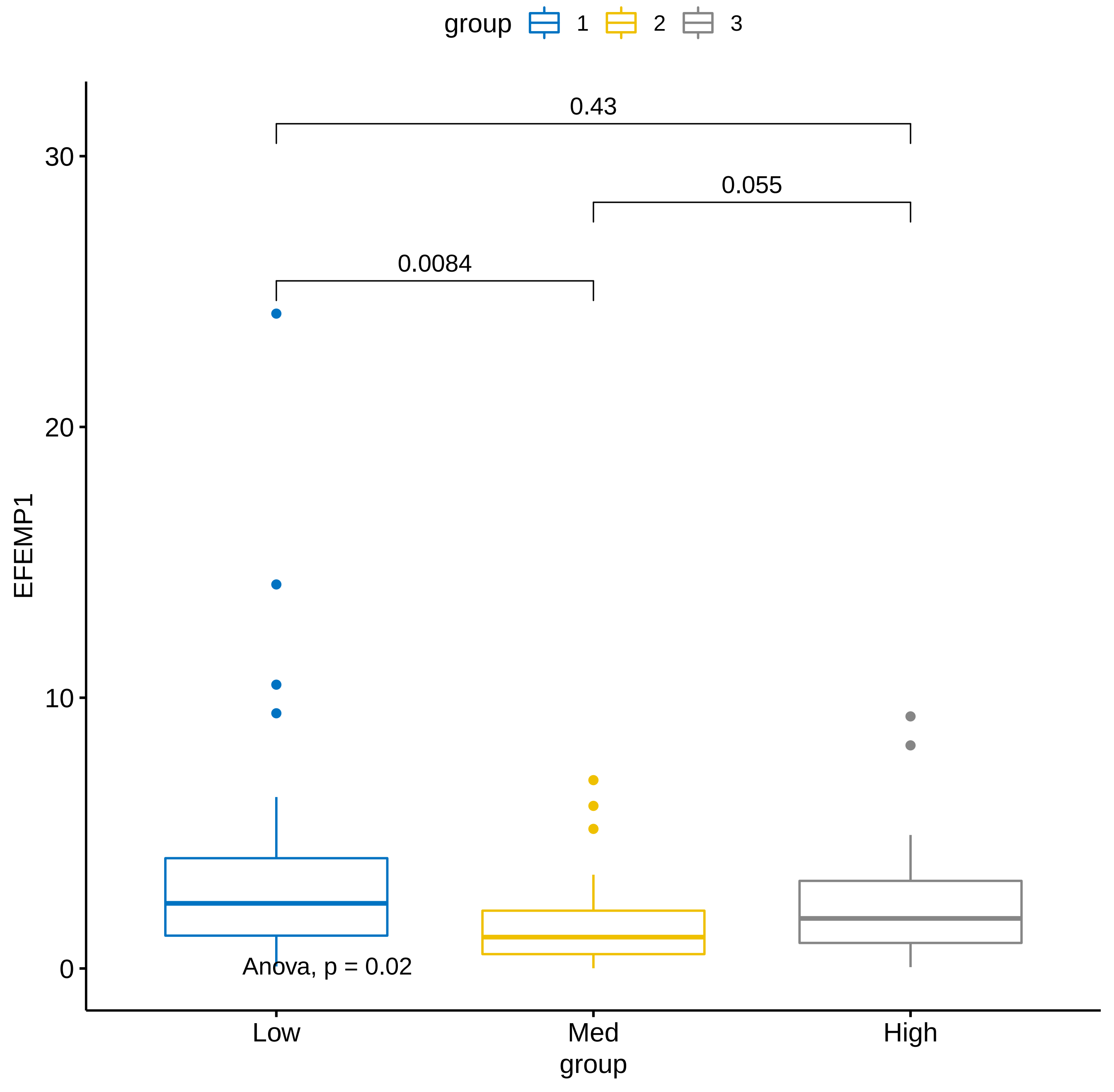
group 1 2 3
0.43
0.055
0.0084
30
20
10
0
EFEMP1
Anova, p = 0.02
Low
Med
High
group

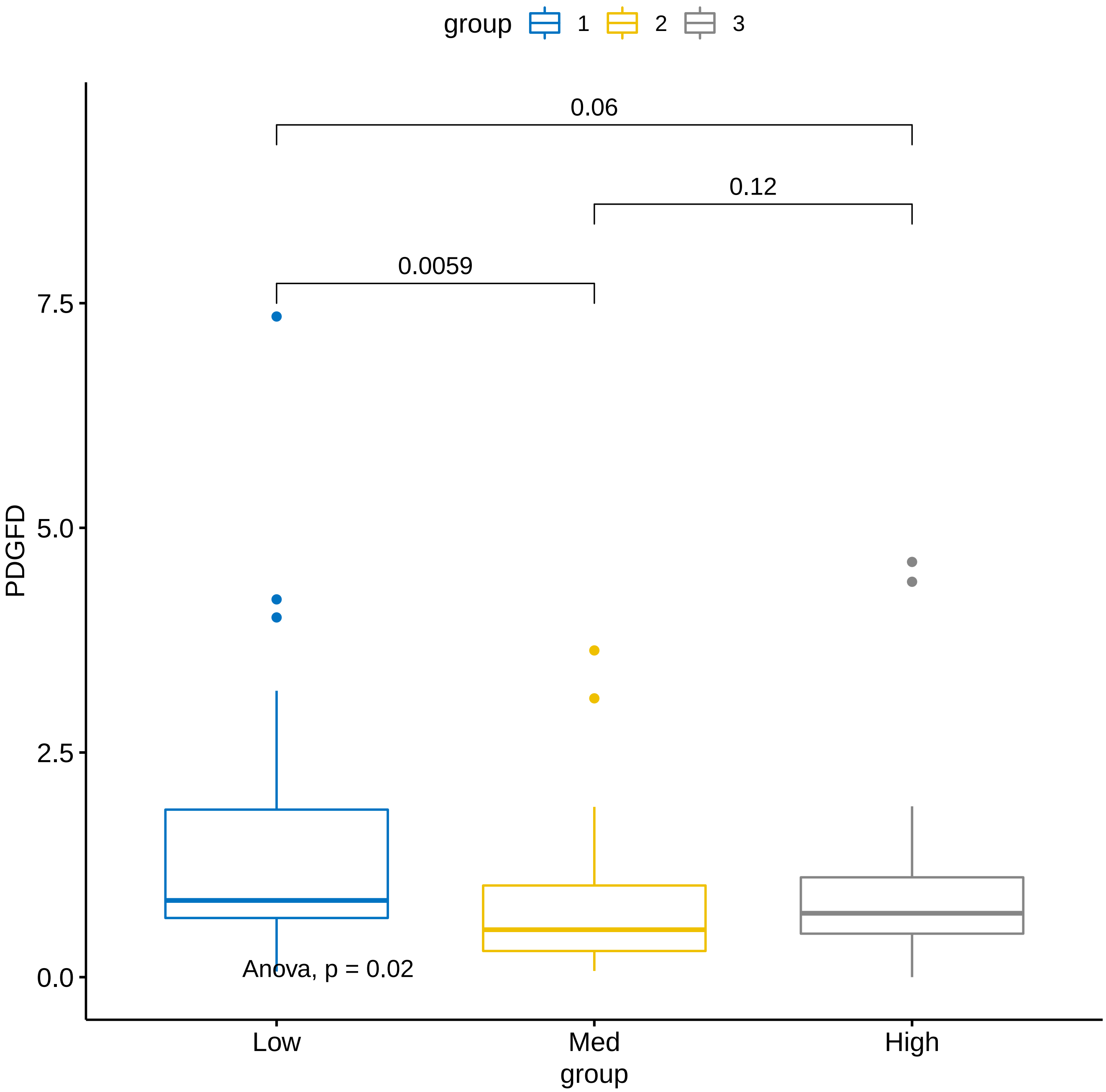
group
1
2
3
0.06
0.12
0.0059
7.5
5.0
2.5
0.0
PDGFD
Anova, p = 0.02
Low
Med
High
group

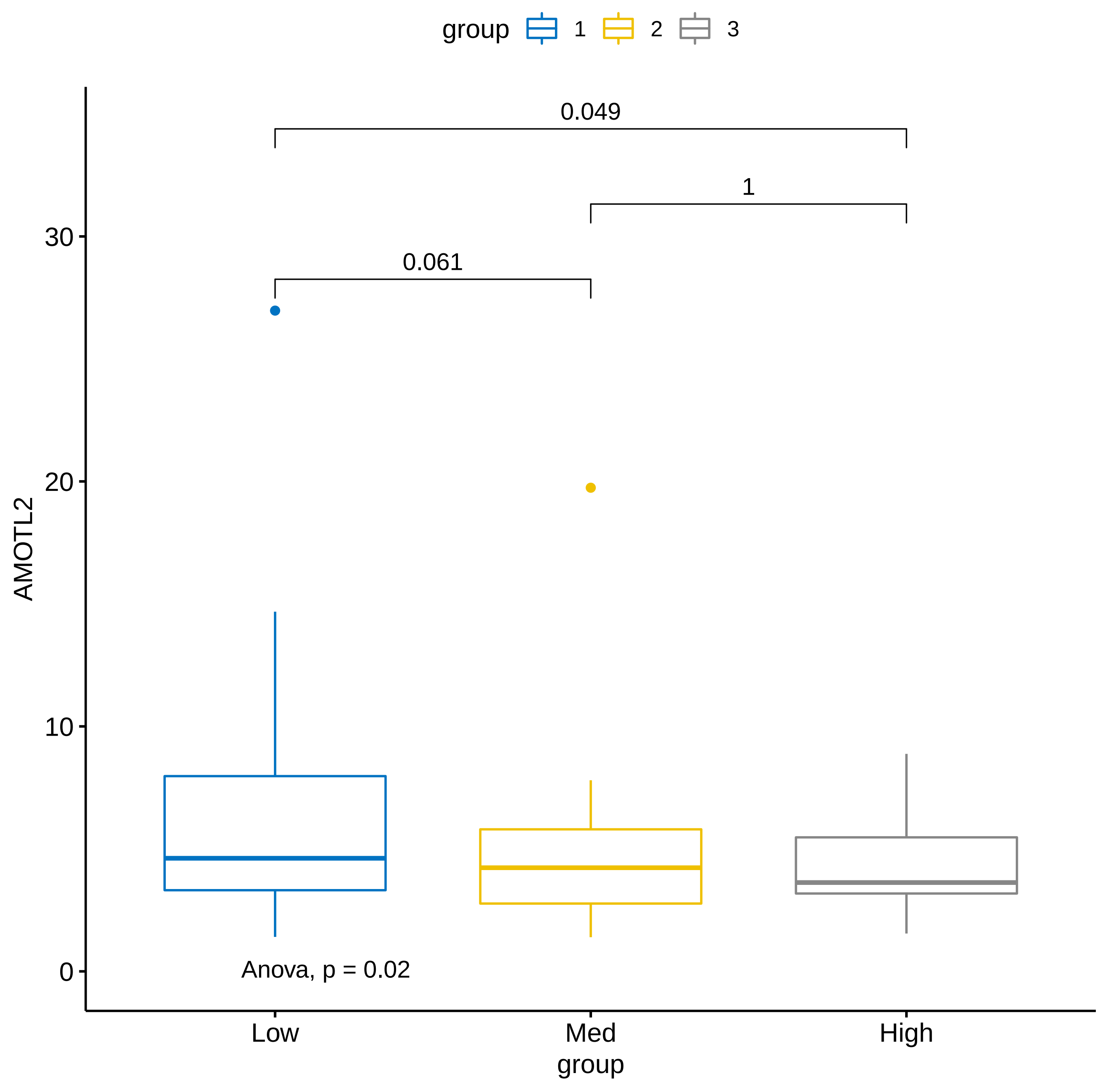
group
1
2
3
0.049
1
0.061
30
20
10
0
AMOTL2
Anova, p = 0.02
Low
Med
High
group

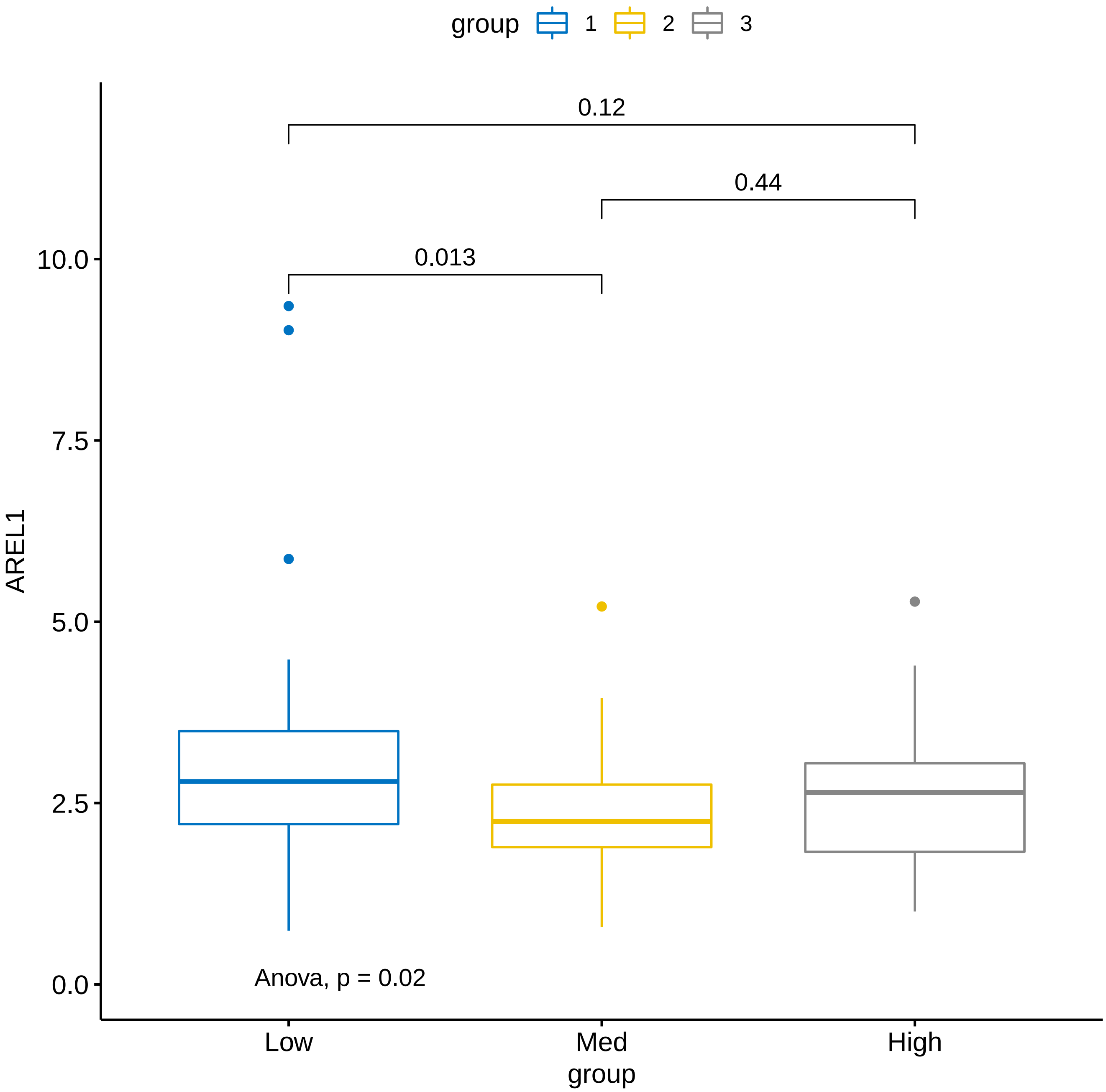
group
1
2
3
0.12
0.44
0.013
10.0
7.5
5.0
2.5
0.0
AREL1
Anova, p = 0.02
Low
Med
High
group

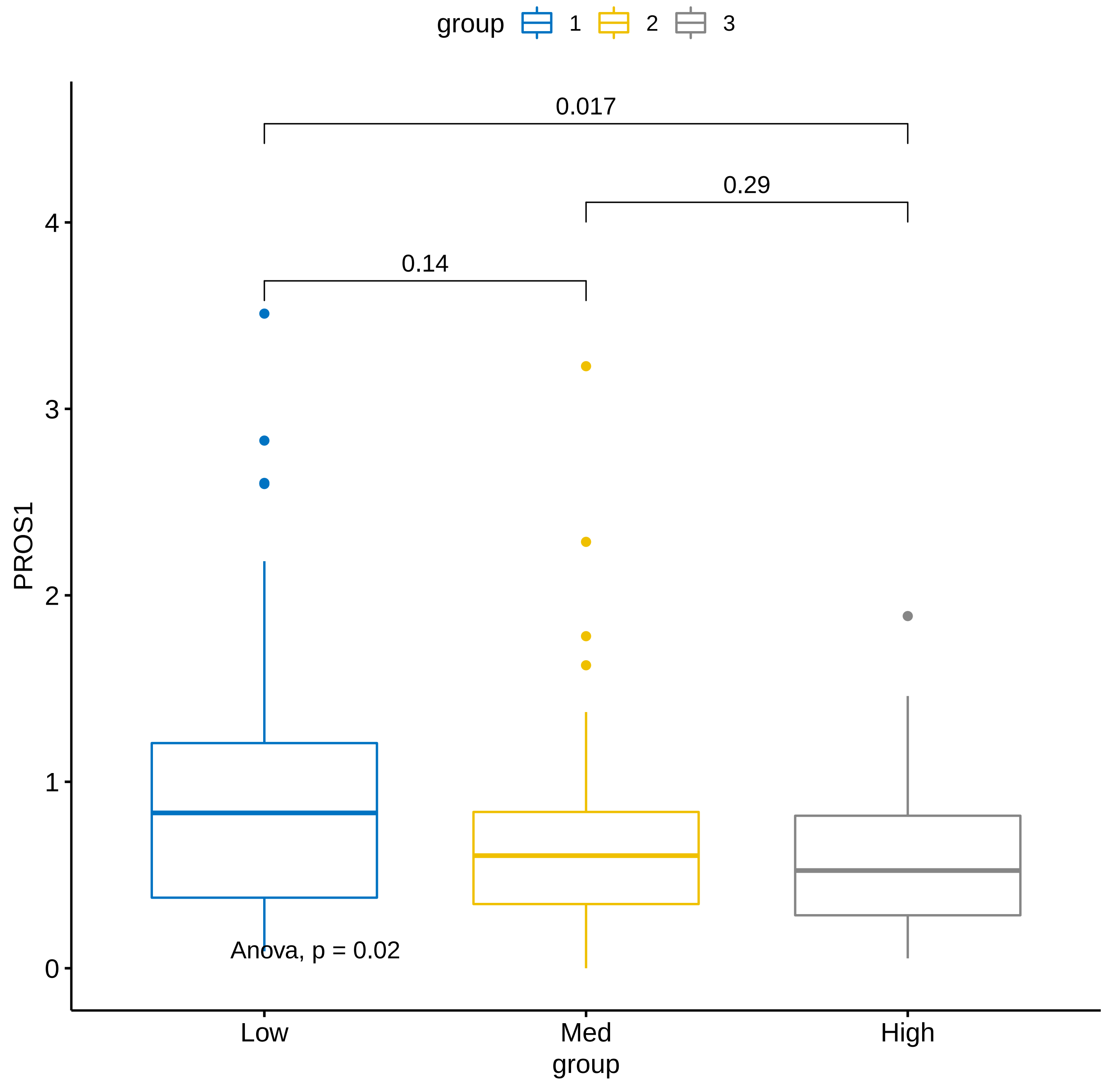
group
1
2
3
0.017
0.29
0.14
PROS1
4
3
2
1
0
Anova, p = 0.02
Low
Med
High
group

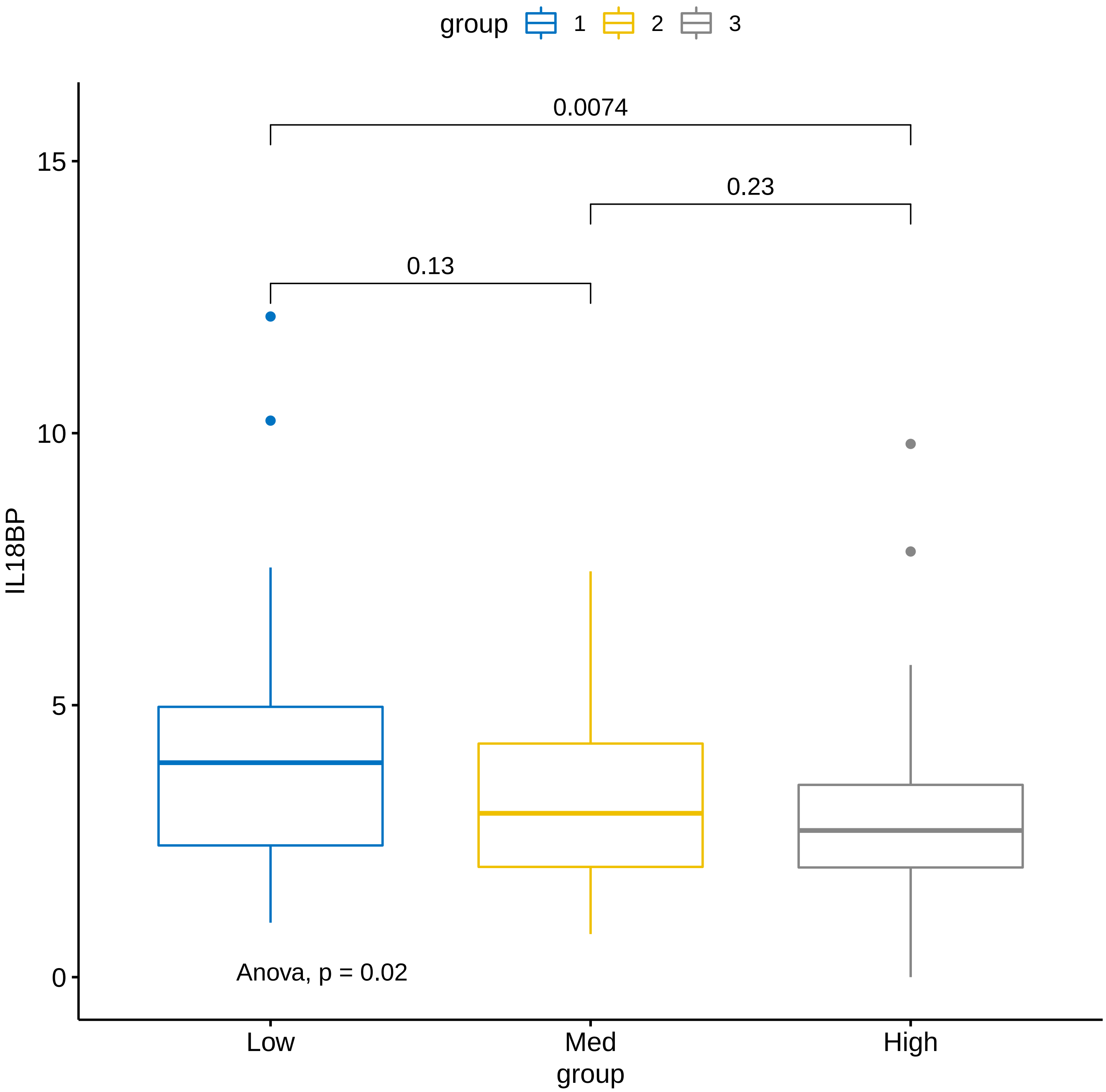
group
1
2
3
0.0074
0.23
0.13
15
10
5
0
IL18BP
Anova, p = 0.02
Low
Med
High
group

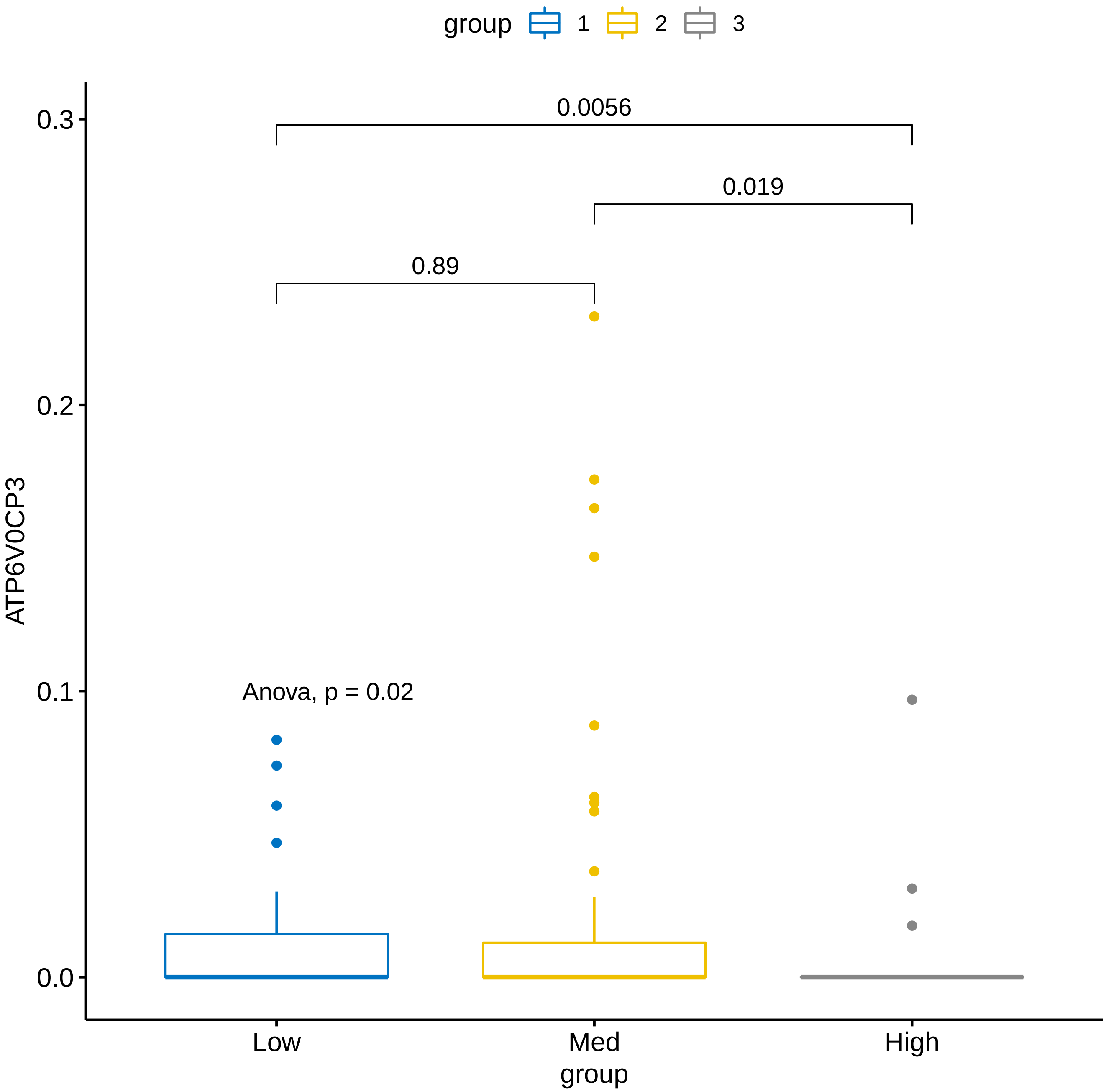
group
1
2
3
0.0056
0.019
0.89
Anova, p = 0.02
0.3
0.2
0.1
0.0
ATP6V0CP3
Low
Med
High
group

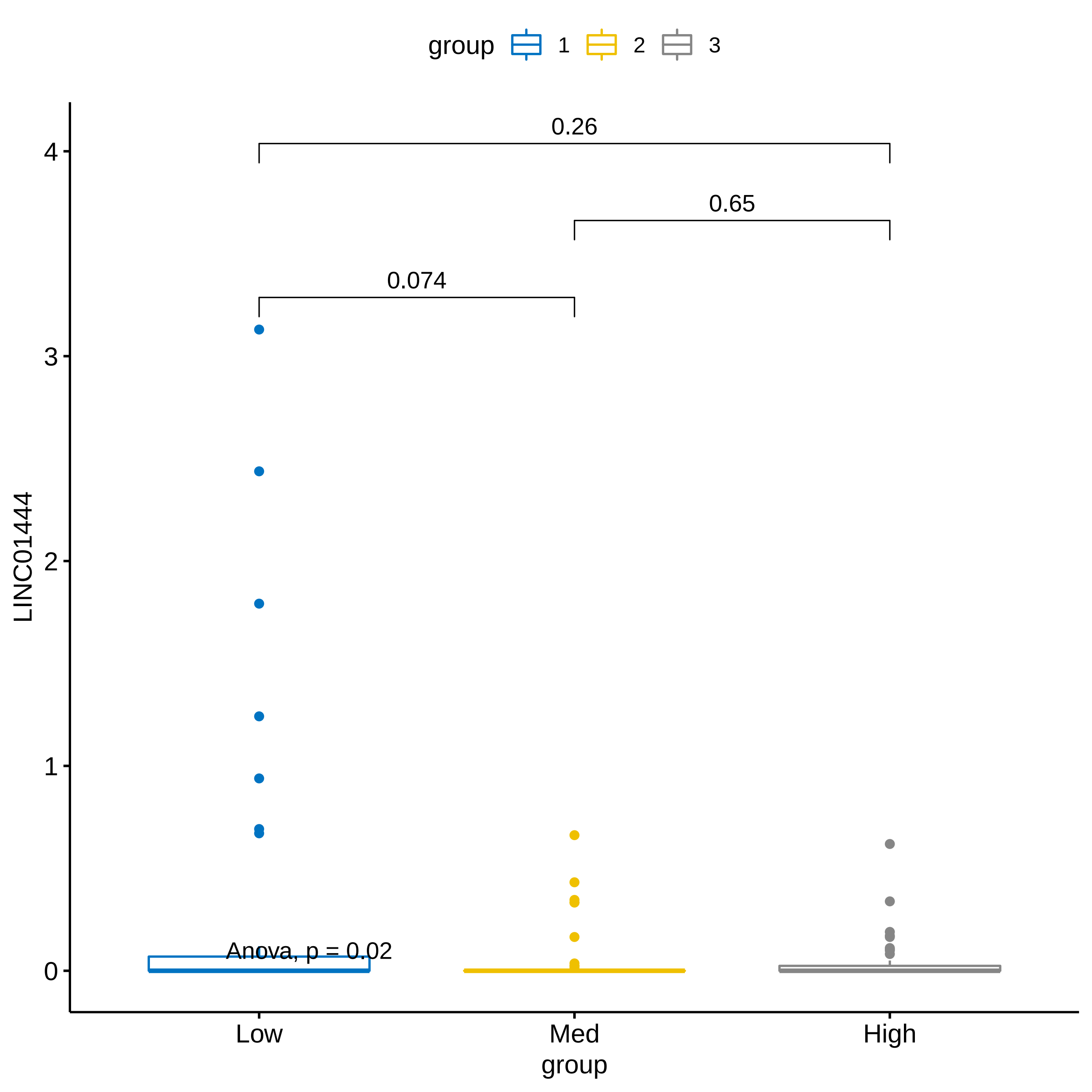

group
1
2
3
0.26
0.65
0.074
Anova, p = 0.02
LINC01444
4
3
2
1
0
Low
Med
High
group

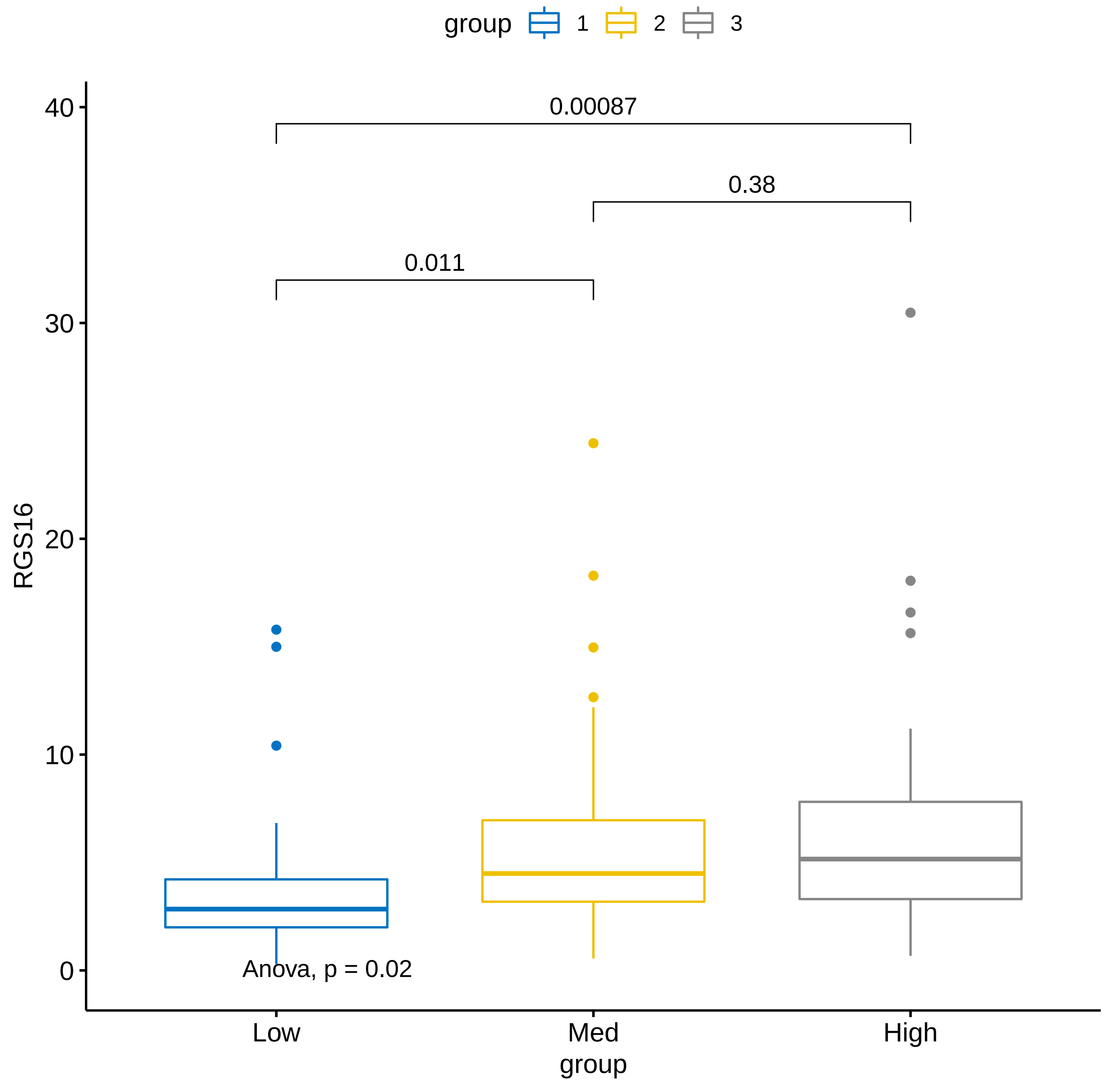
group
1
2
3
0.00087
0.38
0.011
40
30
20
10
0
RGS16
Anova, p = 0.02
Low
Med
High
group

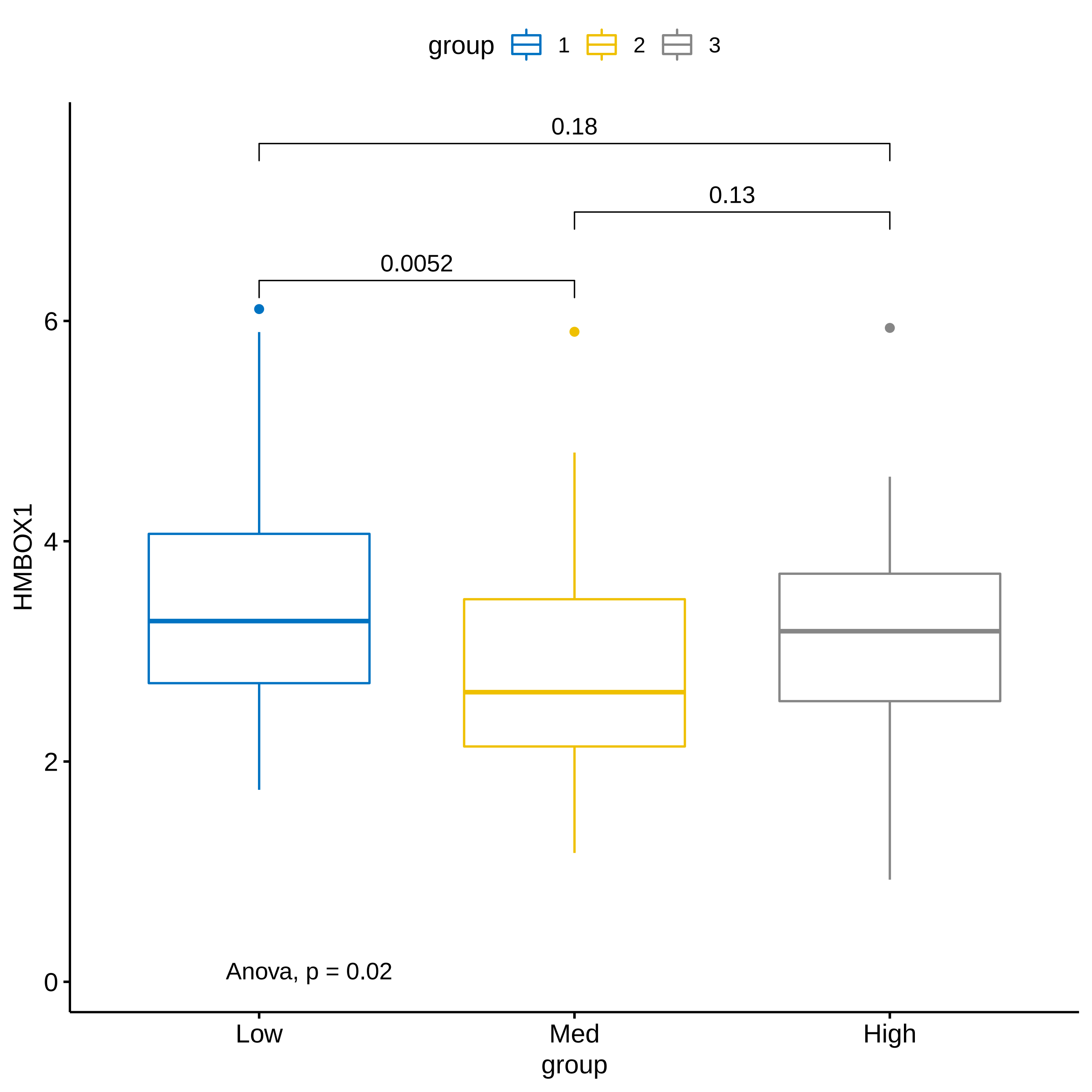

group
1
2
3
0.18
0.13
0.0052
HMBOX1
6
4
2
0
Anova, p = 0.02
Low
Med
High
group

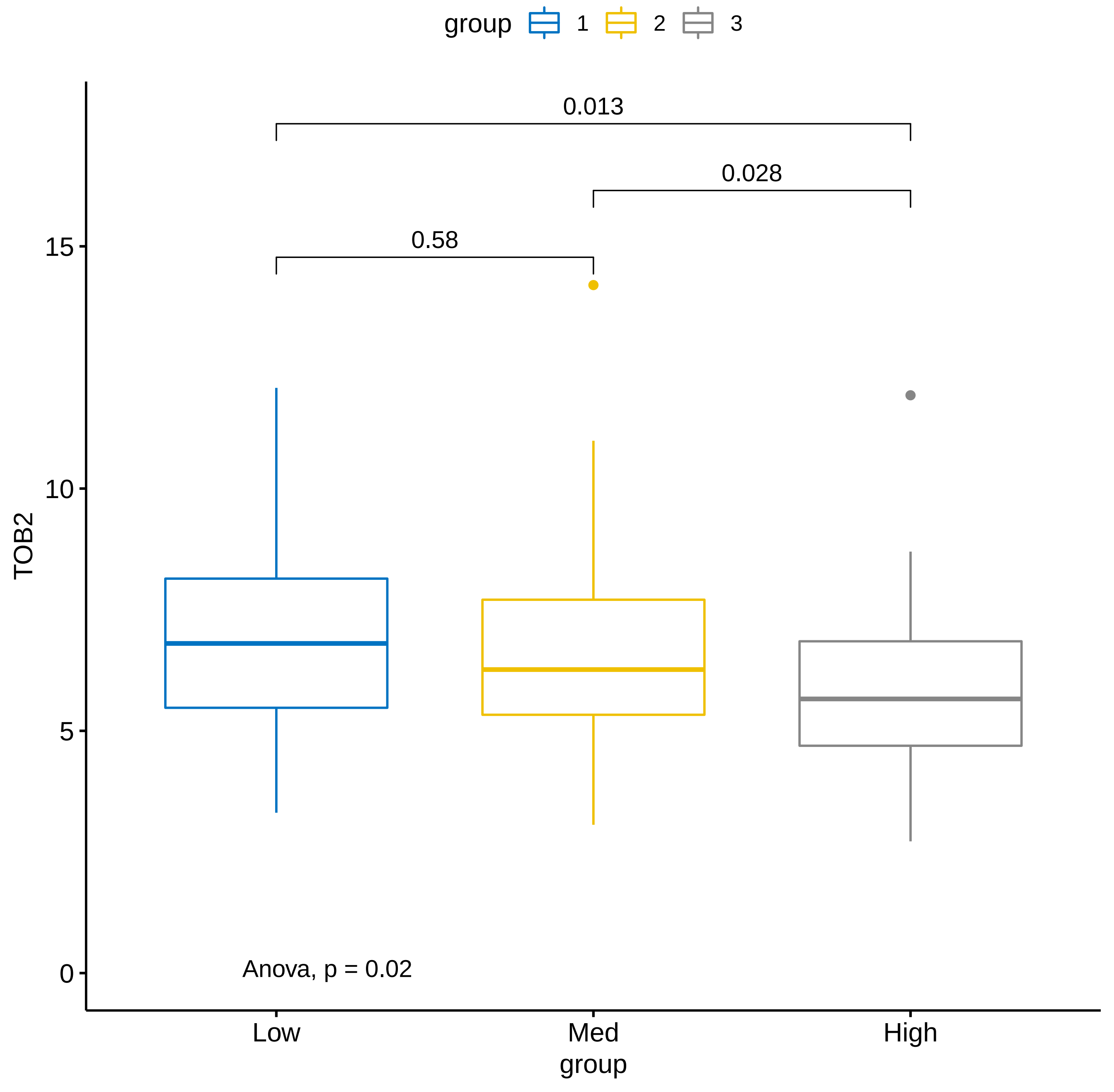
group
1
2
3
0.013
0.028
0.58
15
10
5
0
TOB2
Anova, p = 0.02
Low
Med
High
group

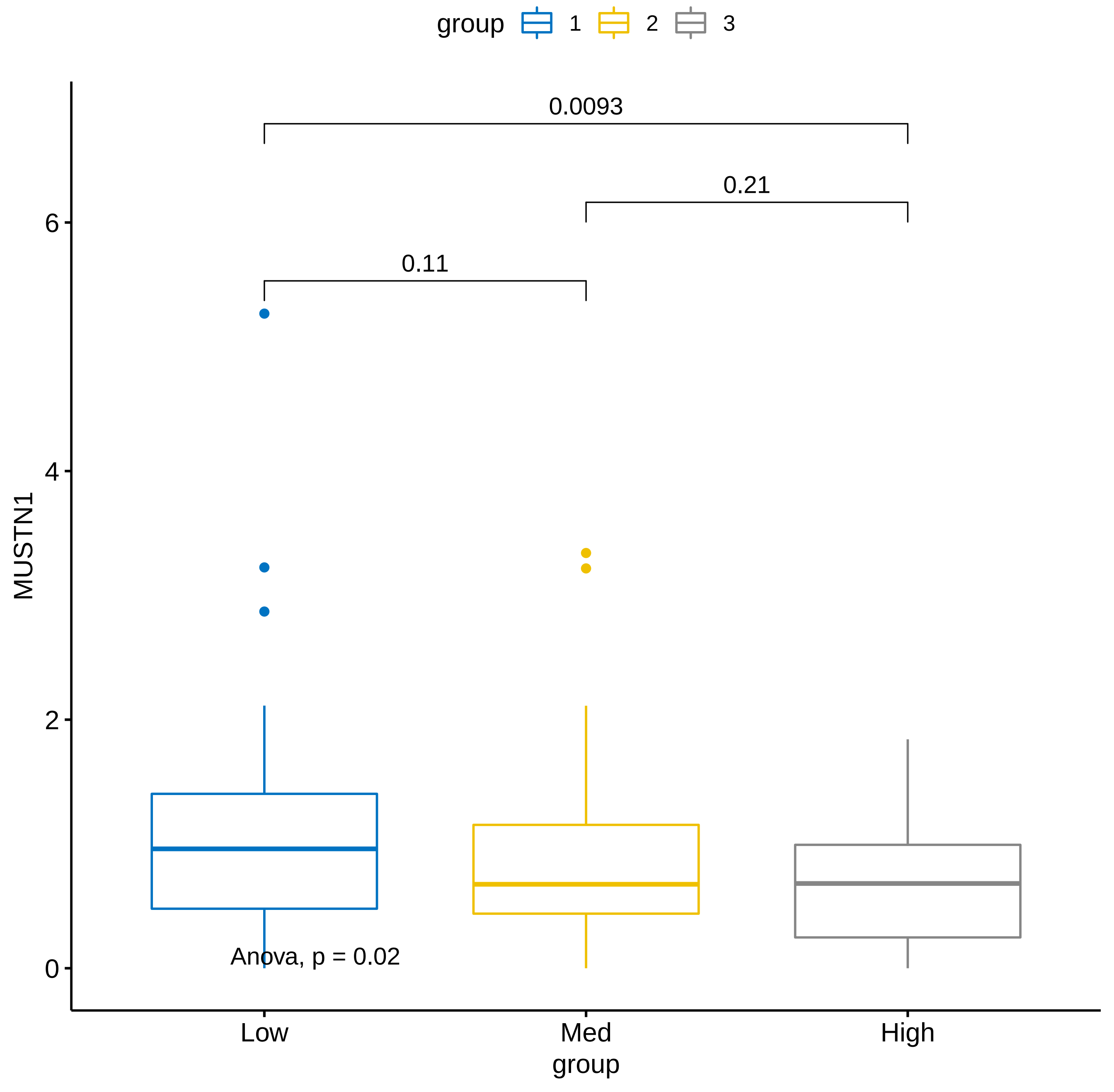
group 1 2 3
0.0093
0.21
0.11
6
4
2
0
MUSTN1
Anova, p = 0.02
Low
Med
High
group

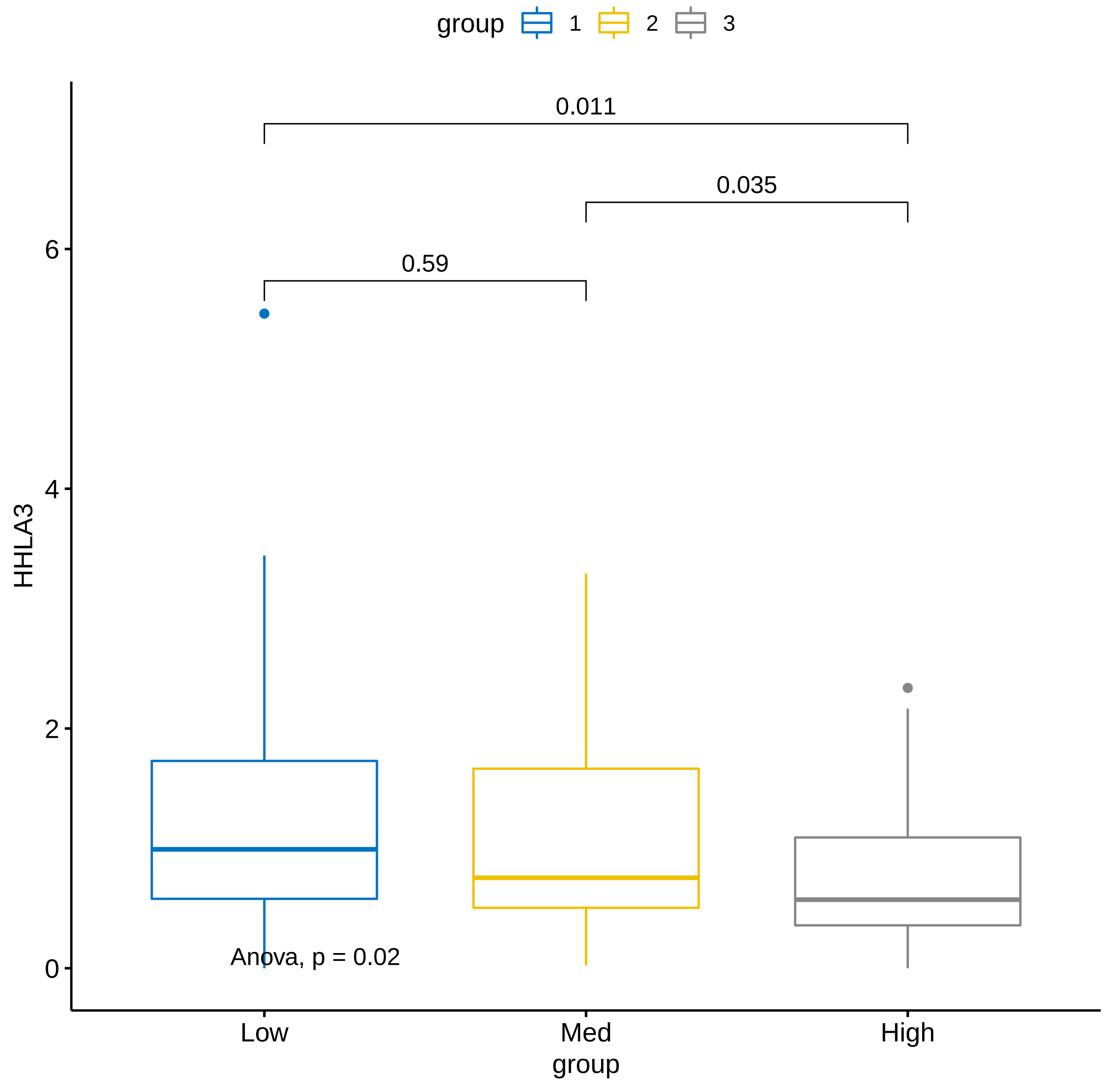

group 1 2 3
0.011
0.035
0.59
6
4
2
0
HHLA3
Anova, p = 0.02
Low
Med
High
group

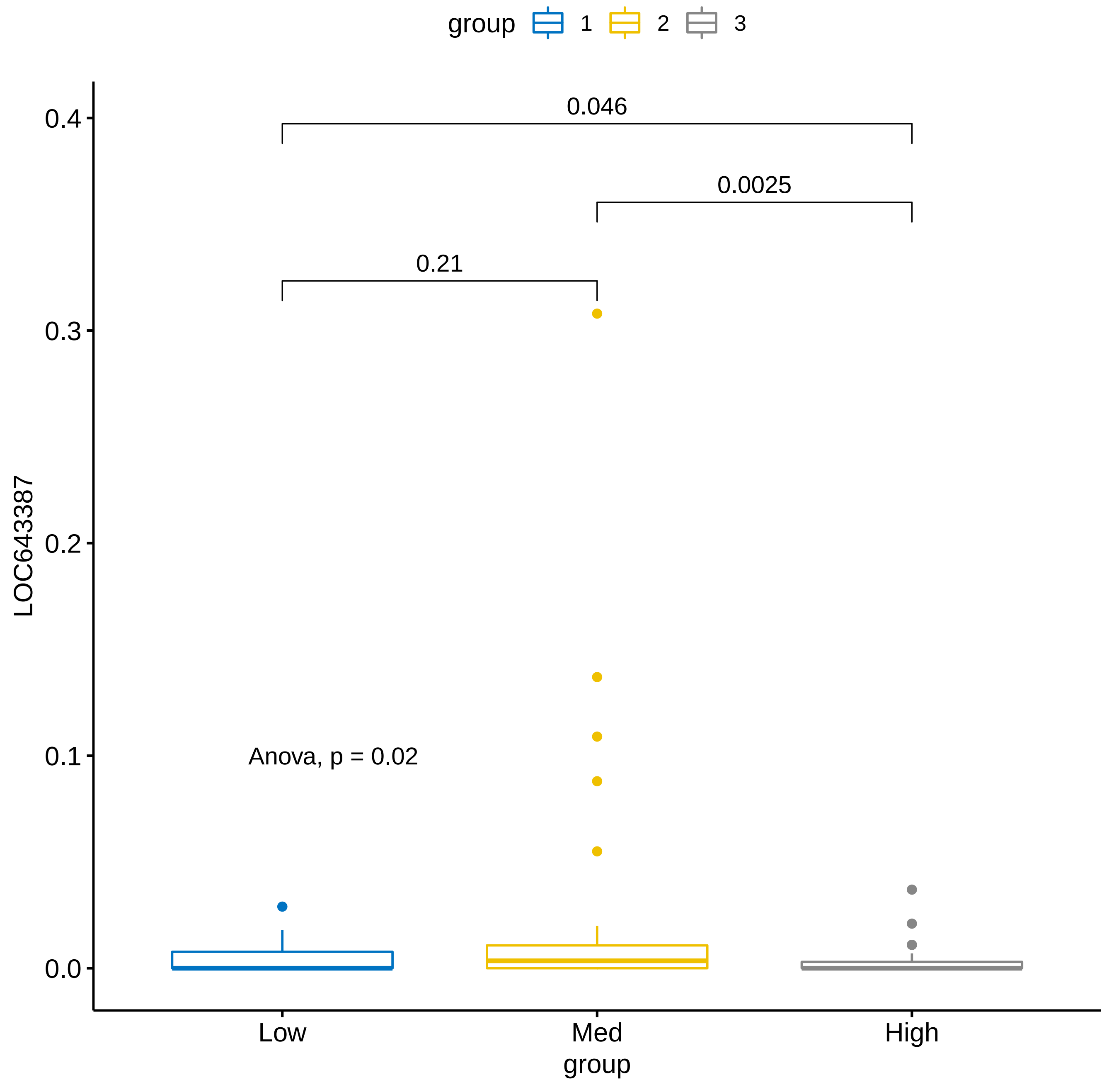

group
1
2
3
0.046
0.0025
0.21
Anova, p = 0.02
0.4
0.3
0.2
0.1
0.0
LOC643387
Low
Med
High
group

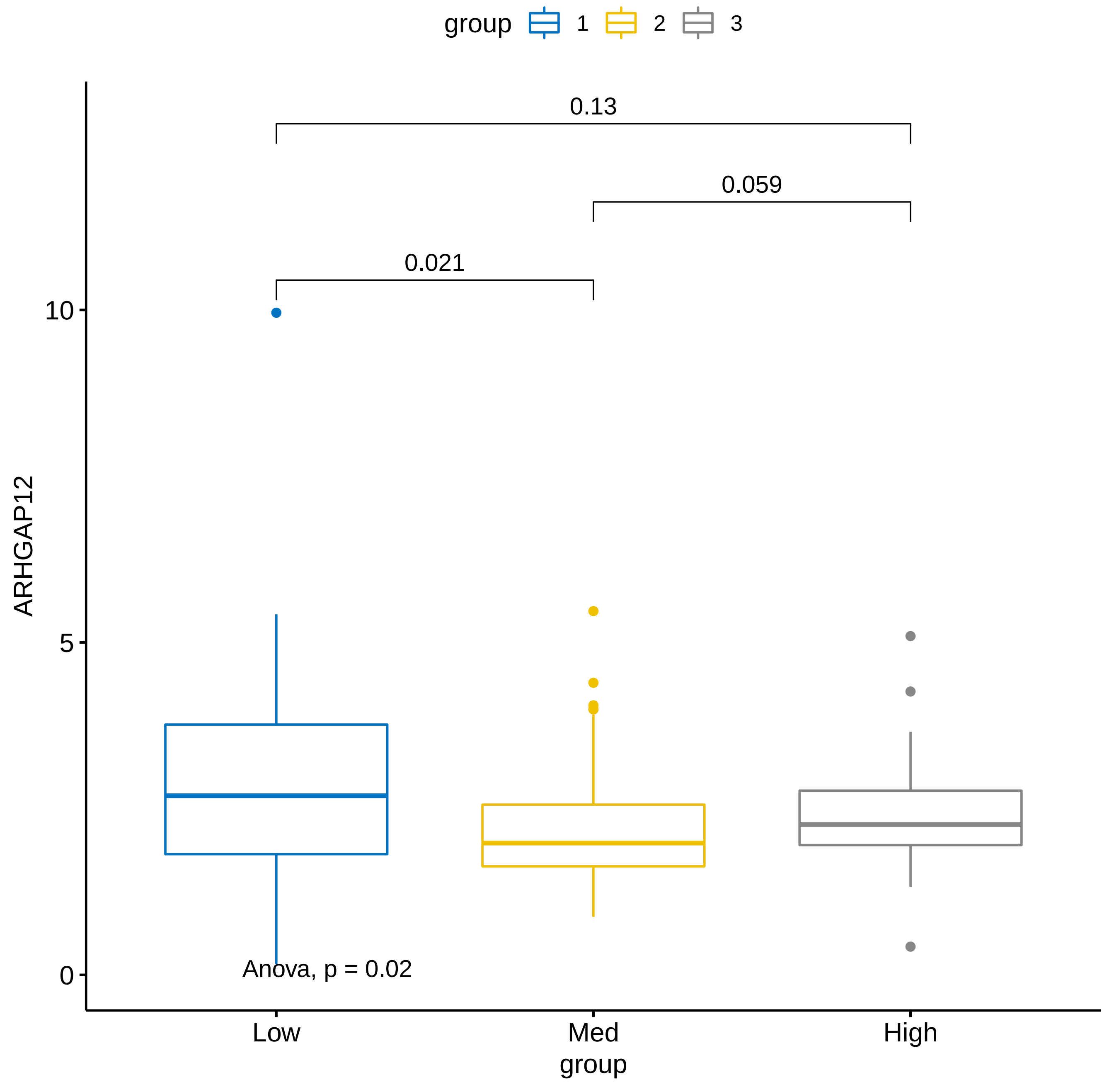

group 1 2 3
0.13
0.059
0.021
ARHGAP12
10
5
0
Anova, p = 0.02
Low
Med
High
group

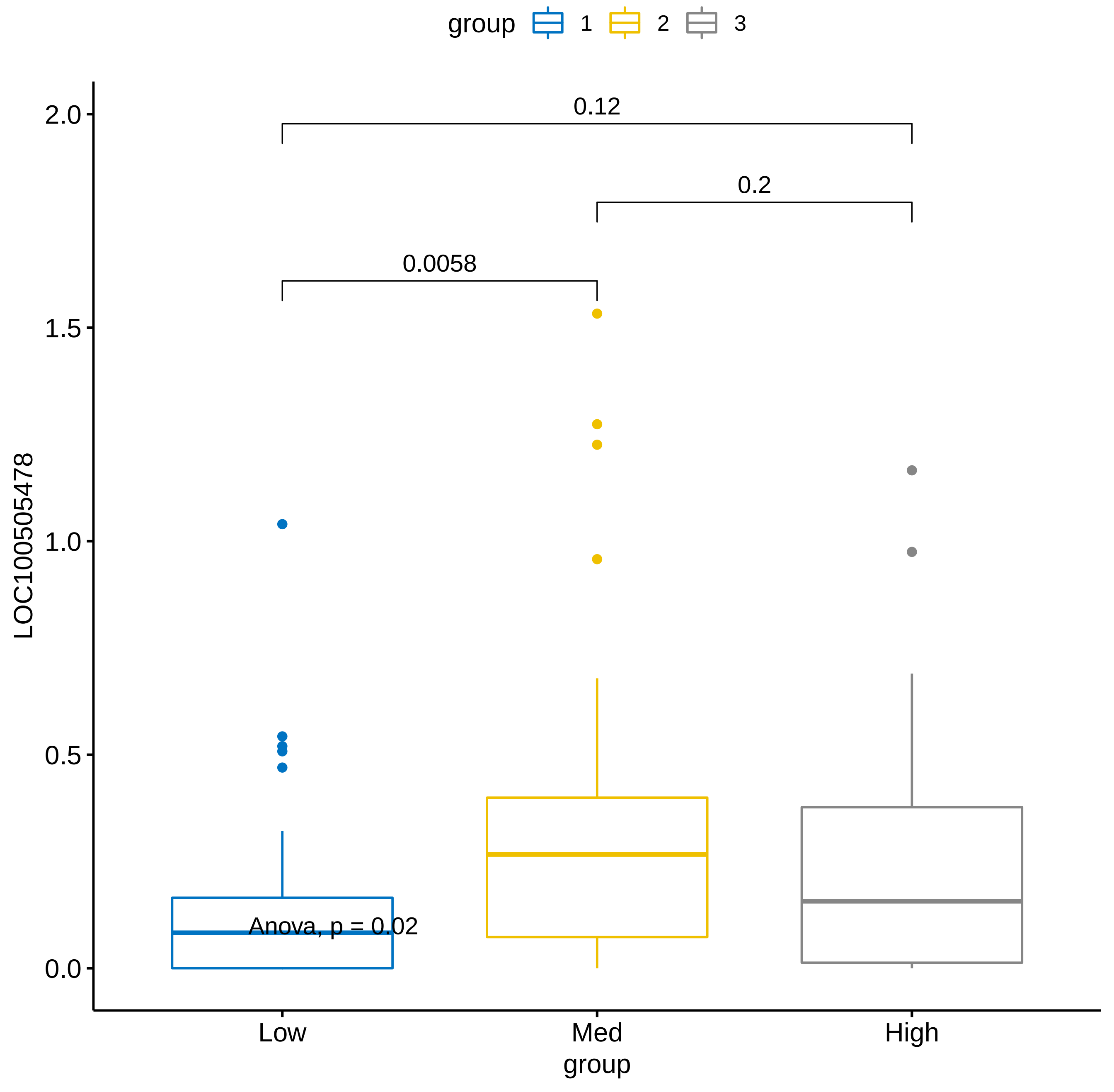

group
1
2
3
0.12
0.2
0.0058
2.0
1.5
1.0
0.5
0.0
LOC100505478
Anova, p = 0.02
Low
Med
High
group

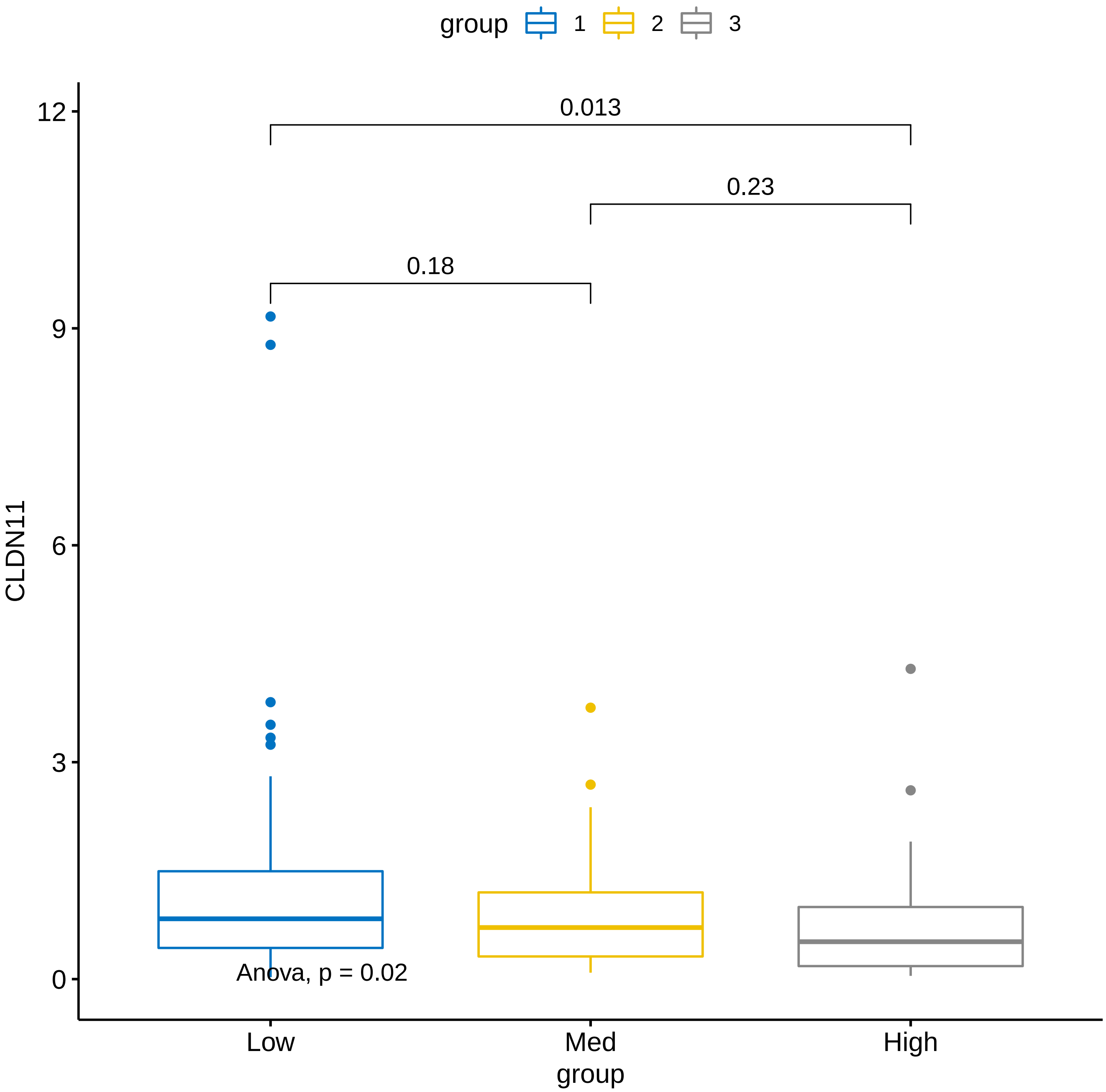
group
1
2
3
0.013
0.23
0.18
12
9
6
3
0
CLDN11
Anova, p = 0.02
Low
Med
High
group

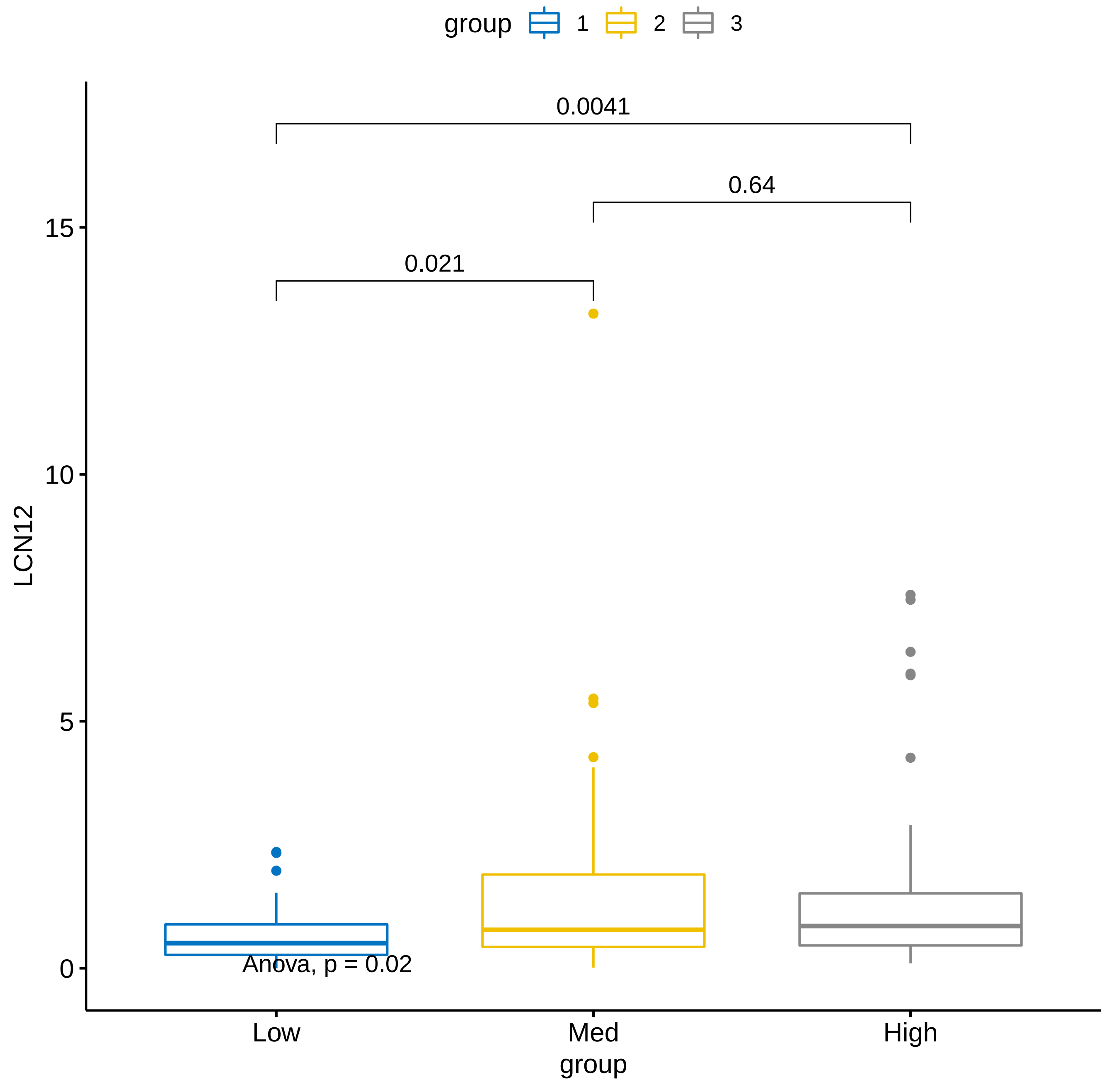
group
1
2
3
0.0041
0.64
0.021
15
10
5
0
LCN12
Anova, p = 0.02
Low
Med
High
group

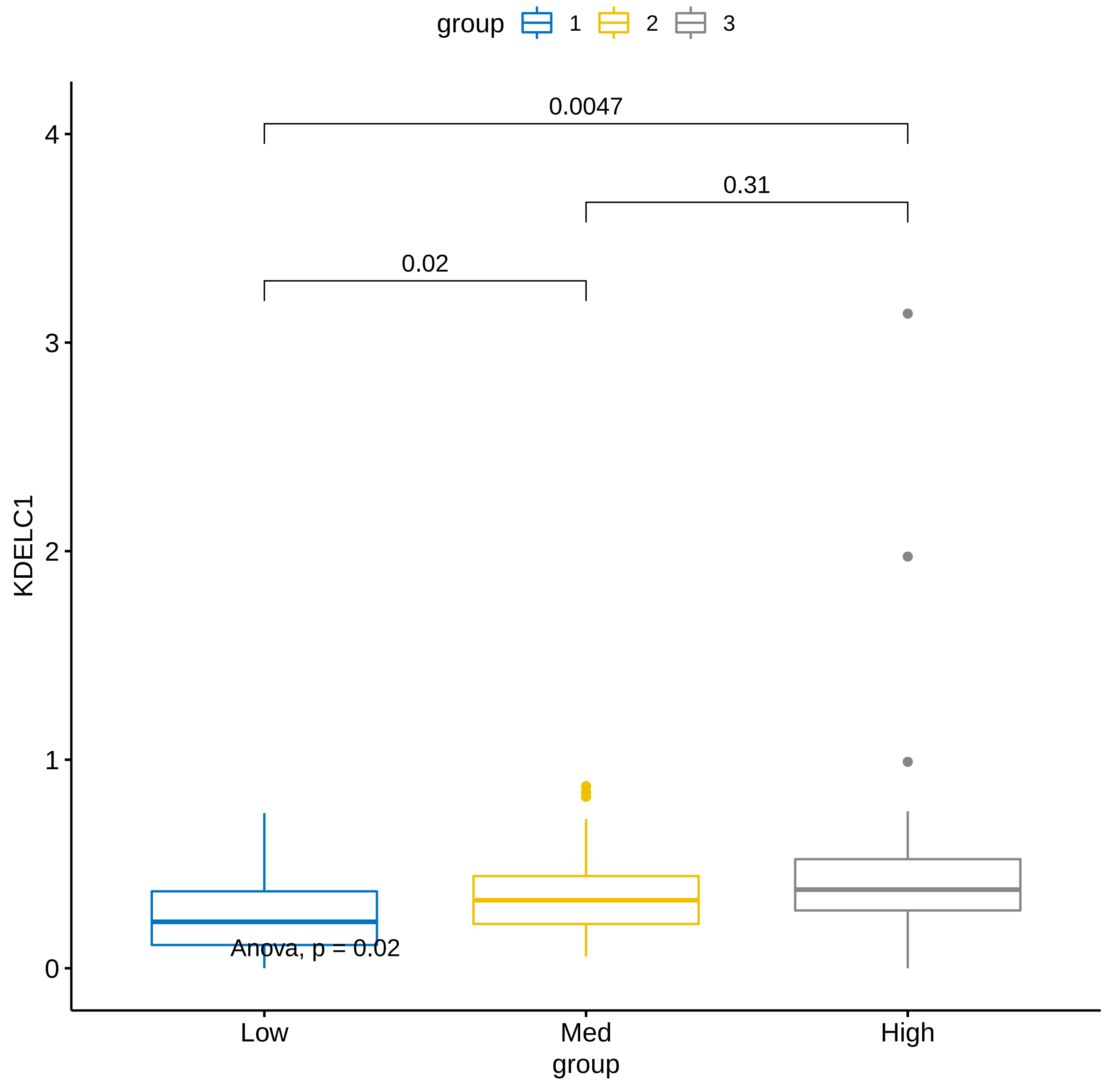
group
1
2
3
0.0047
0.31
0.02
Anova, p = 0.02
KDELC1
4
3
2
1
0
Low
Med
High
group

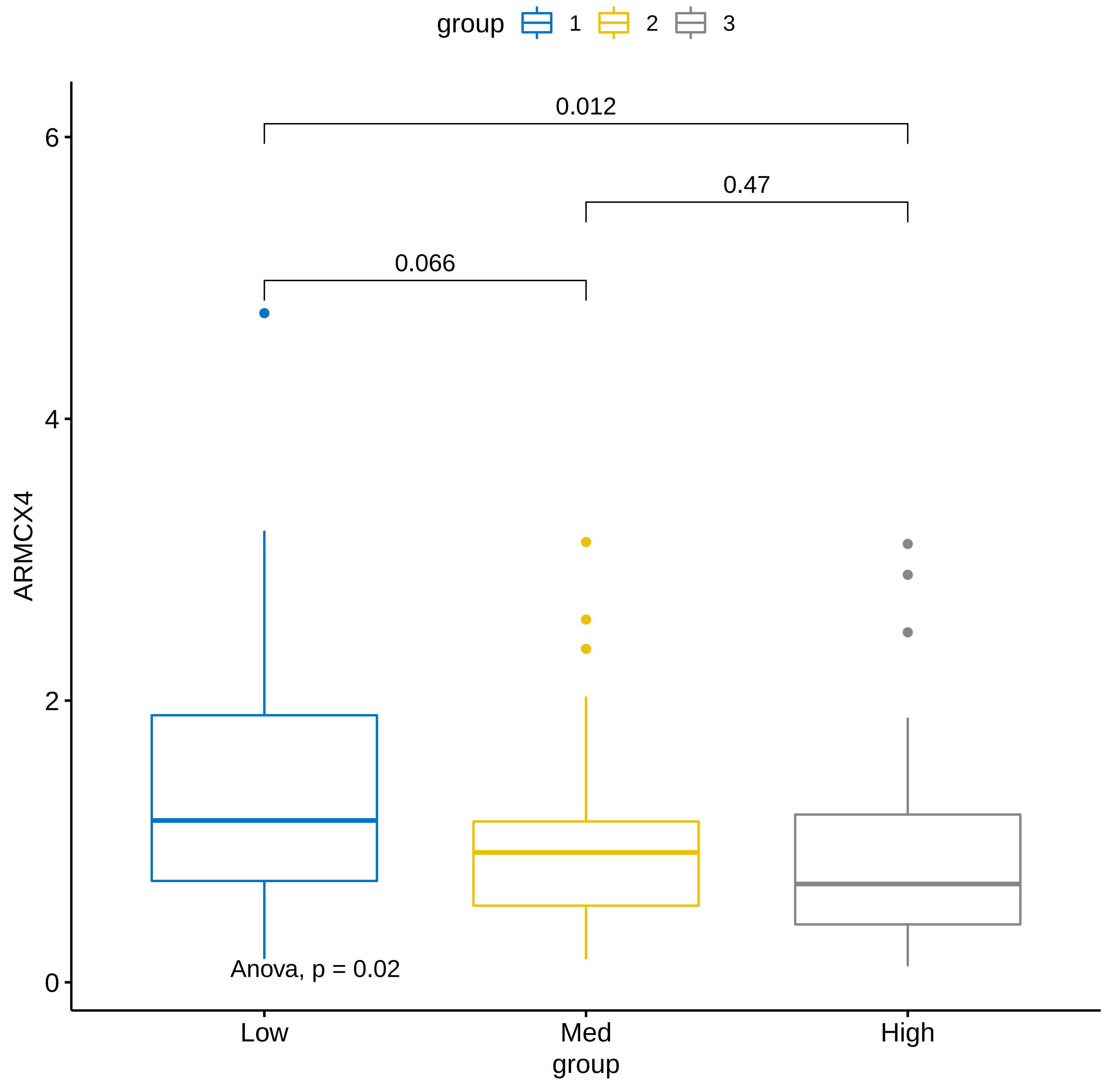

group
1
2
3
0.012
0.47
0.066
6
4
2
0
ARMCX4
Anova, p = 0.02
Low
Med
High
group

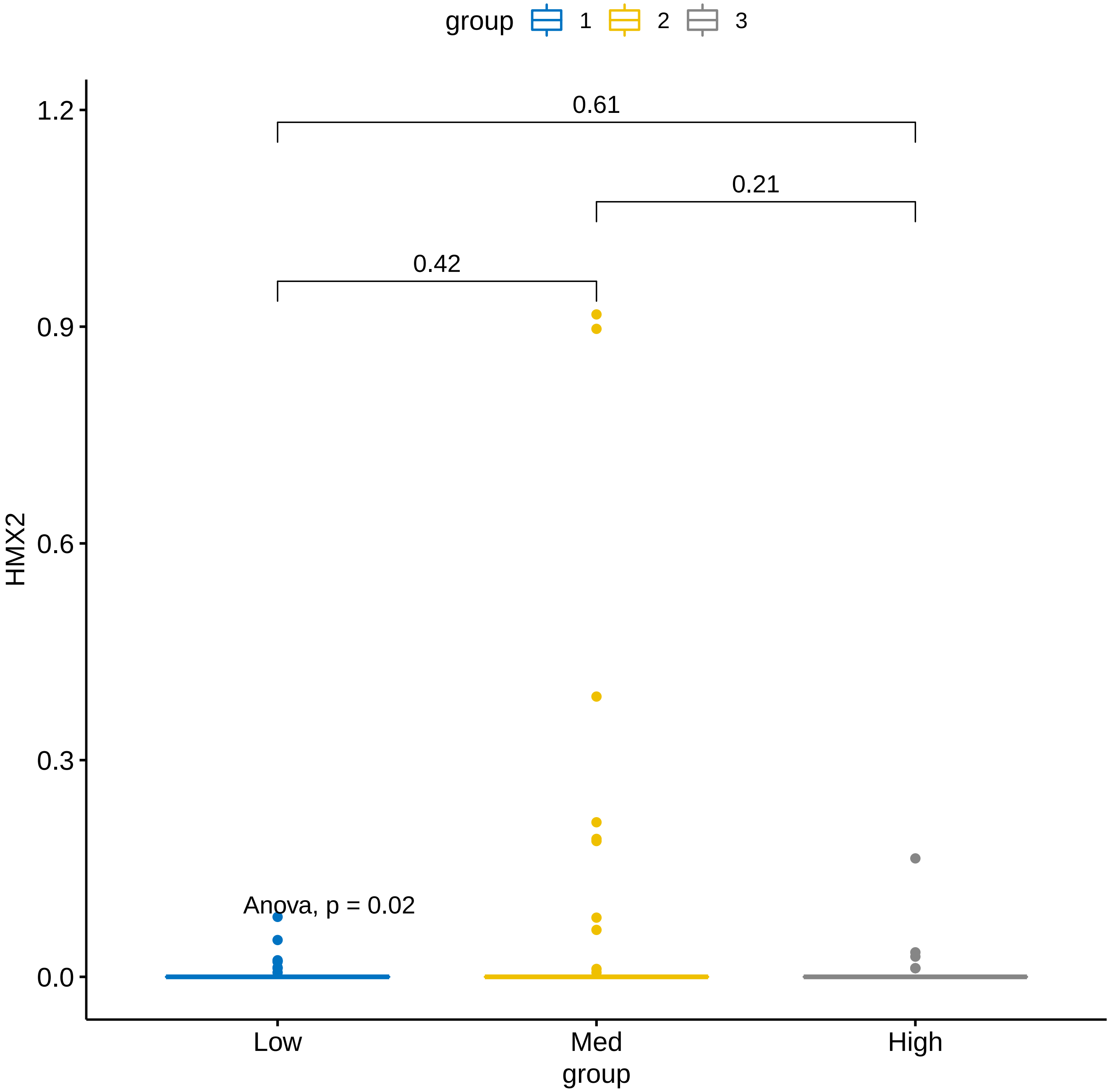

group 1 2 3
0.61
0.21
0.42
Anova, p = 0.02
1.2
0.9
0.6
0.3
0.0
HMX2
Low
Med
High
group

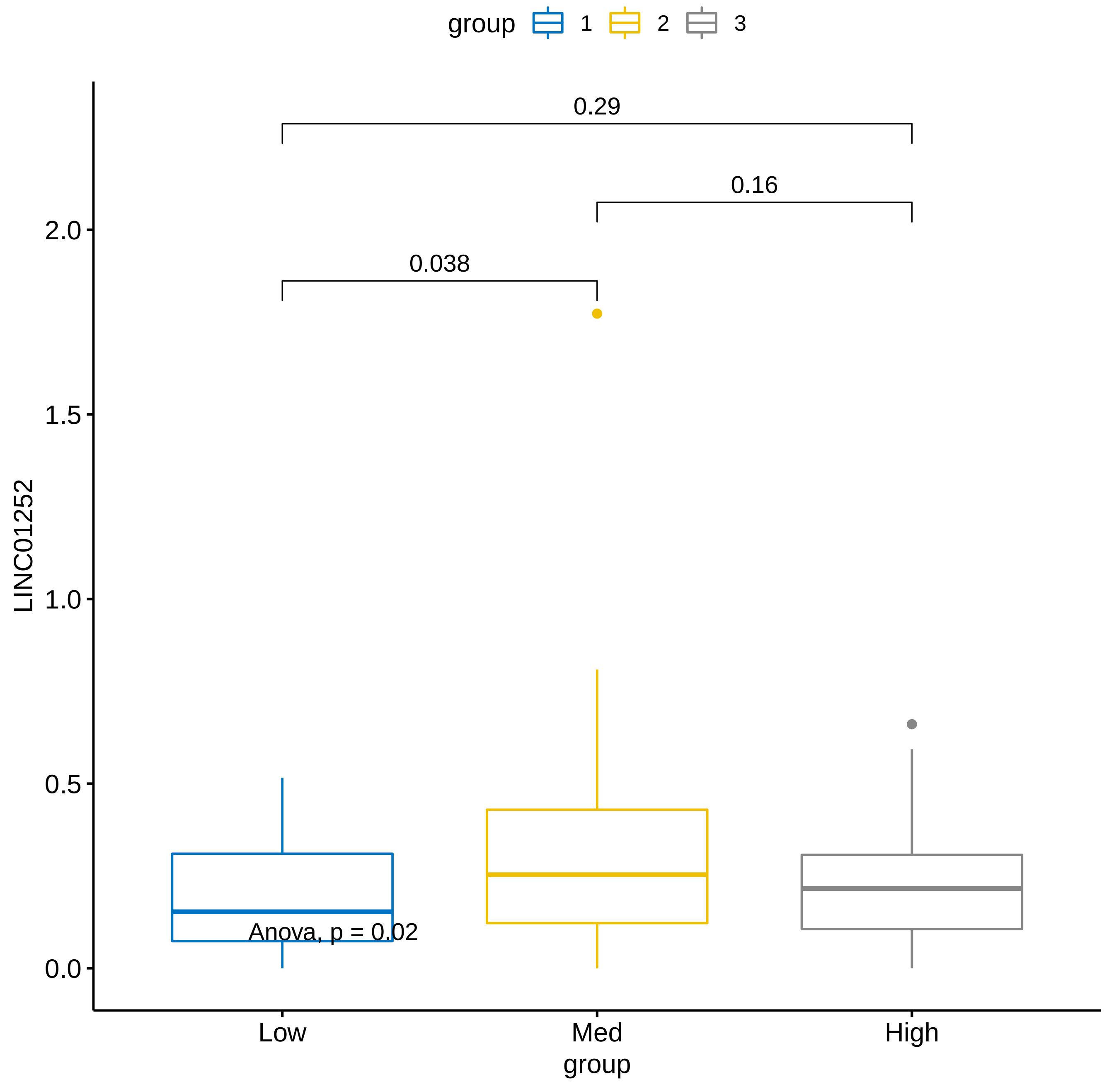
group
1
2
3
0.29
0.16
0.038
2.0
1.5
1.0
0.5
0.0
LINC01252
Anova, p = 0.02
Low
Med
High
group

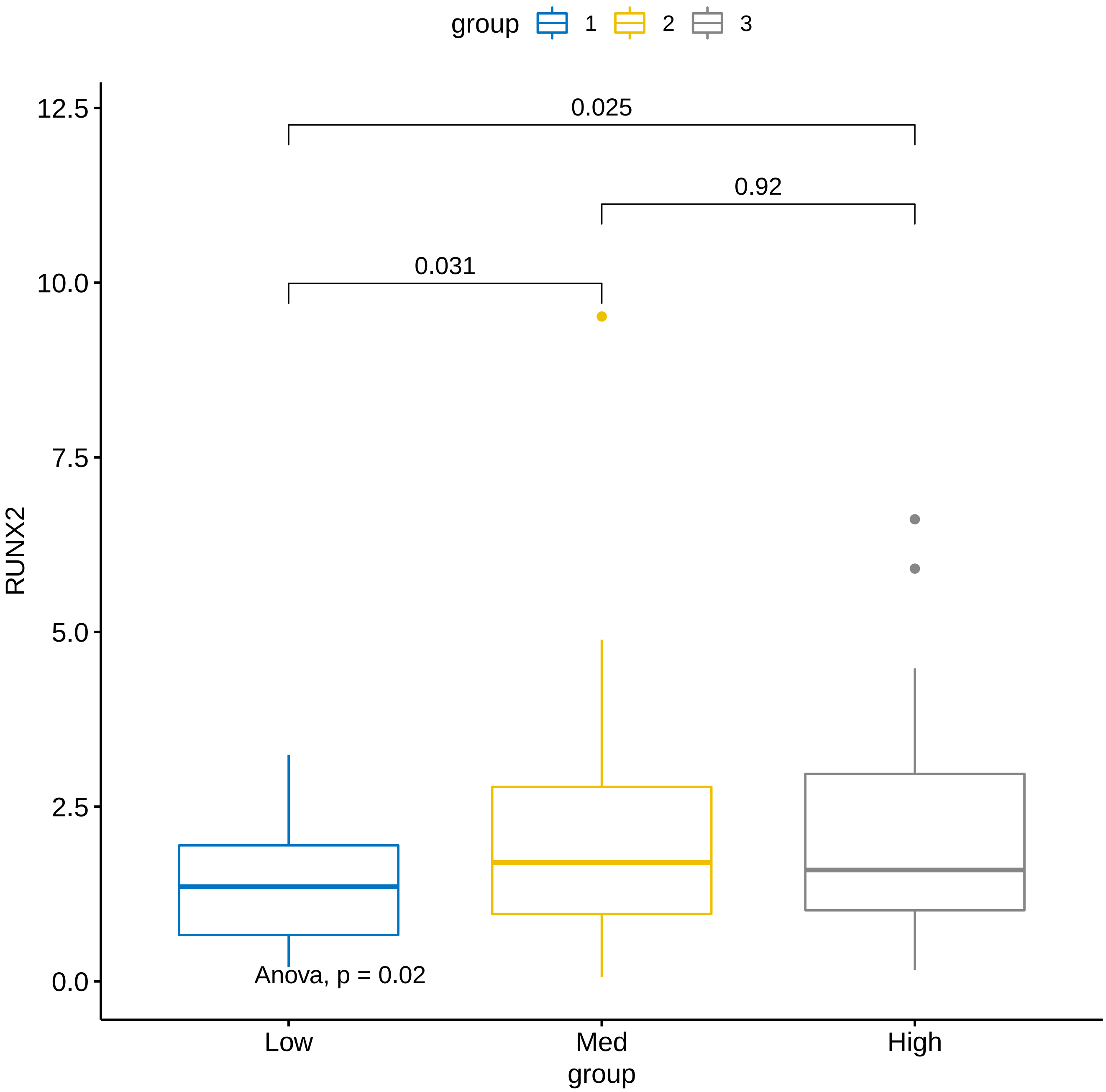

group 1 2 3
0.025
0.92
0.031
12.5
10.0
7.5
5.0
2.5
0.0
RUNX2
Anova, p = 0.02
Low
Med
High
group

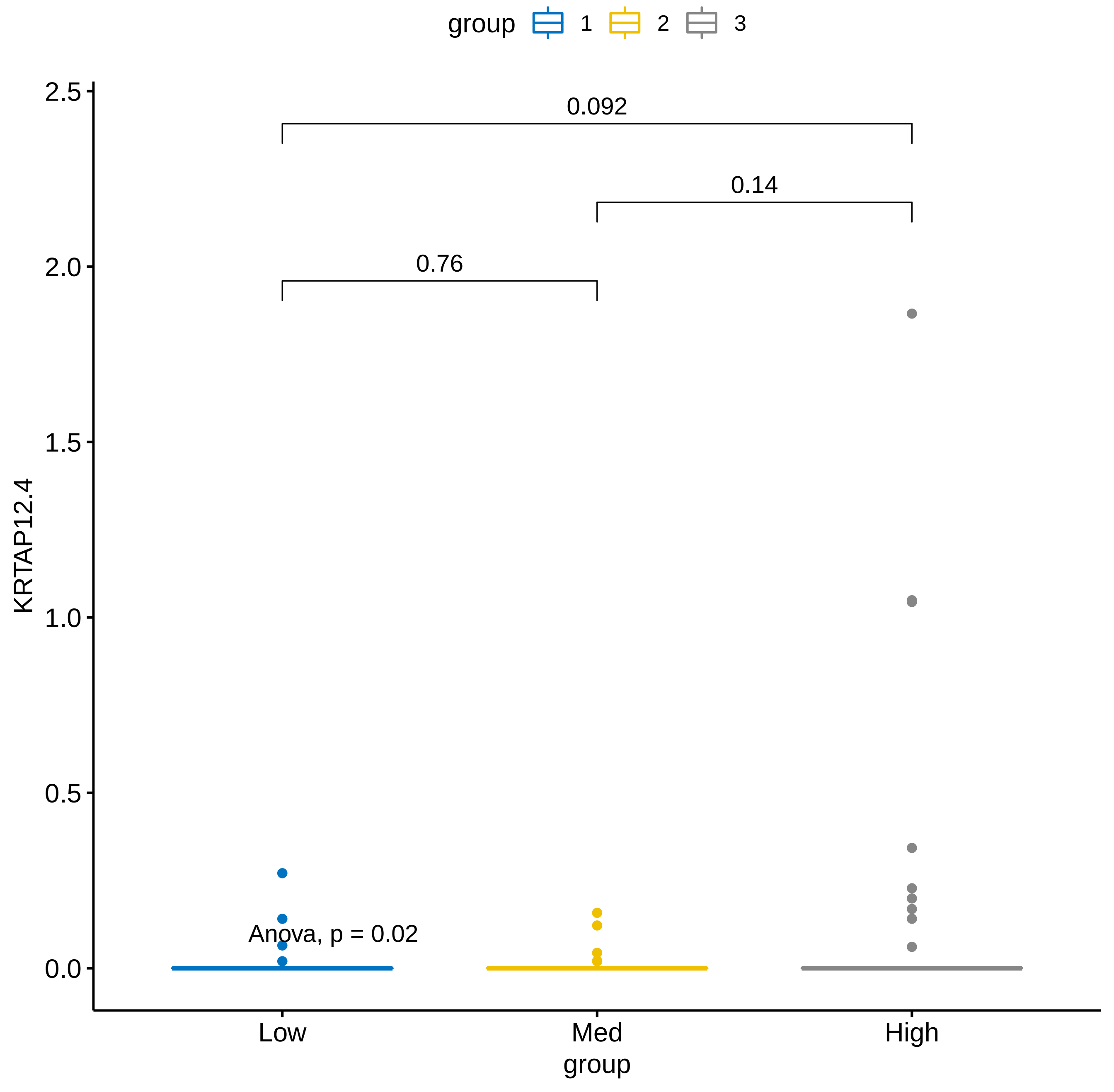

group 1 2 3
2.5
2.0
1.5
1.0
0.5
0.0
KRTAP12.4
0.092
0.14
0.76
Anova, p = 0.02
Low
Med
High
group

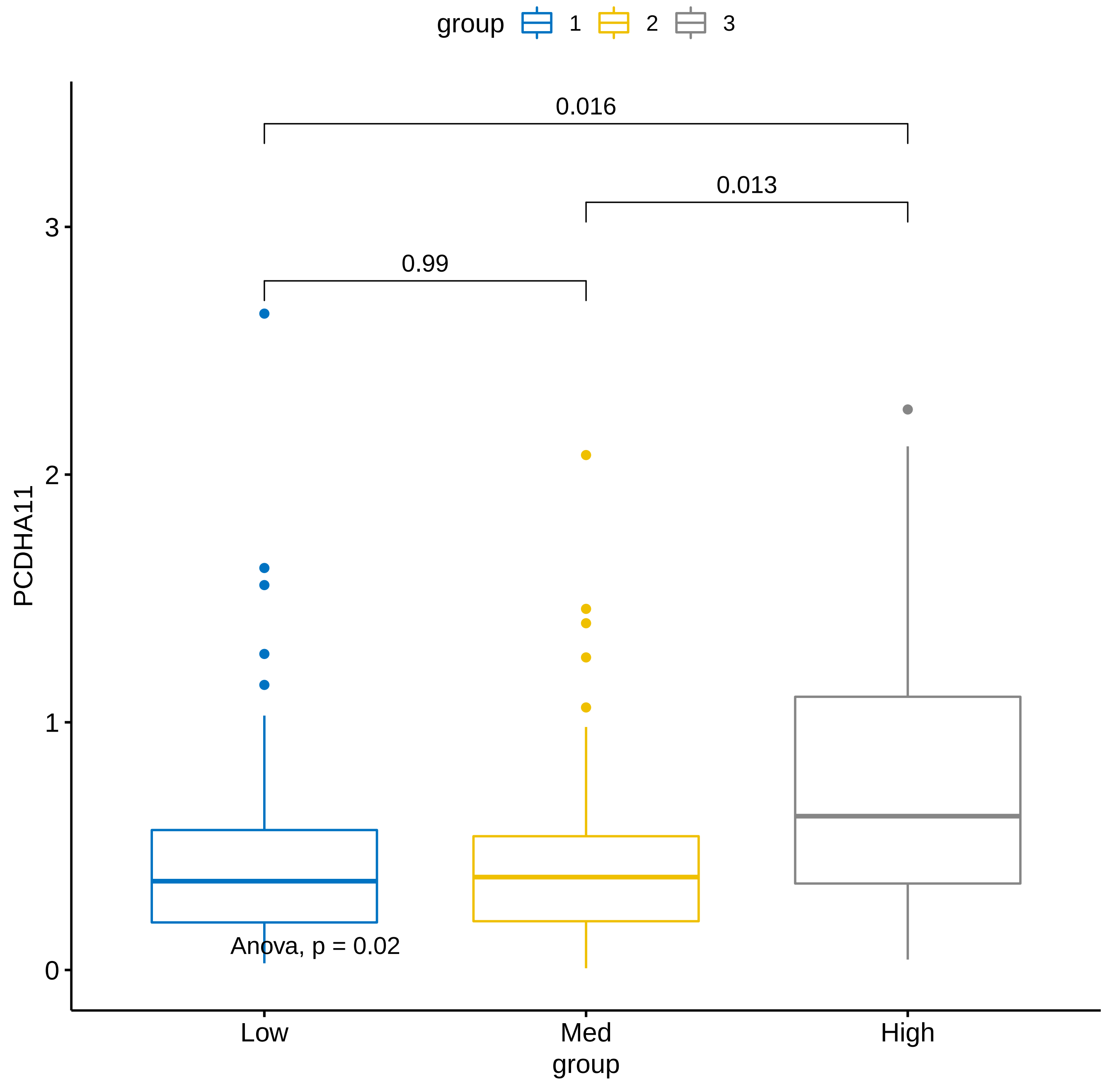
group 1 2 3
0.016
0.013
0.99
PCDHA11
3
2
1
0
Anova, p = 0.02
Low
Med
High
group

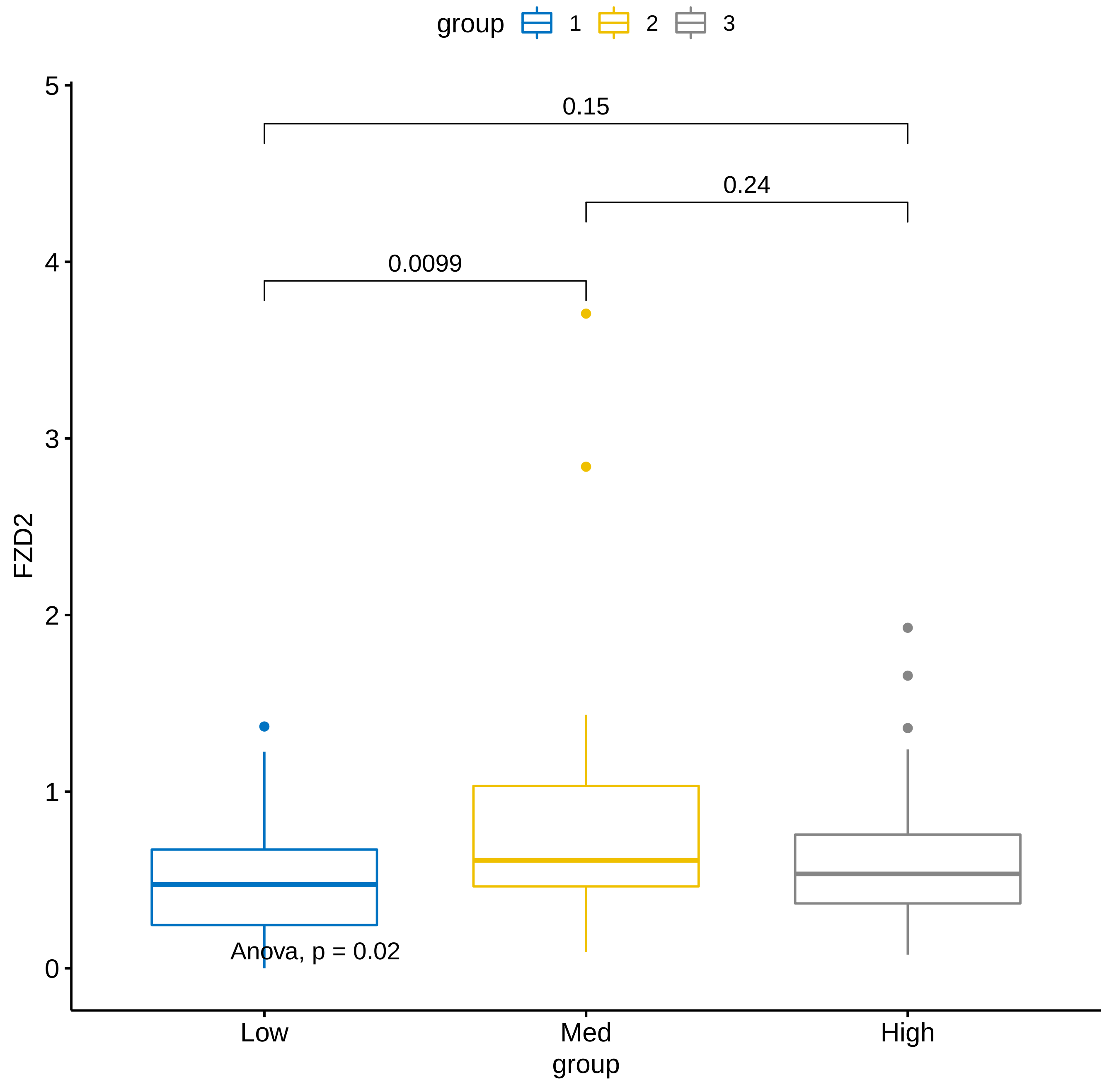

group 1 2 3
0.15
0.24
0.0099
FZD2
5
4
3
2
1
0
Anova, p = 0.02
Low
Med
High
group

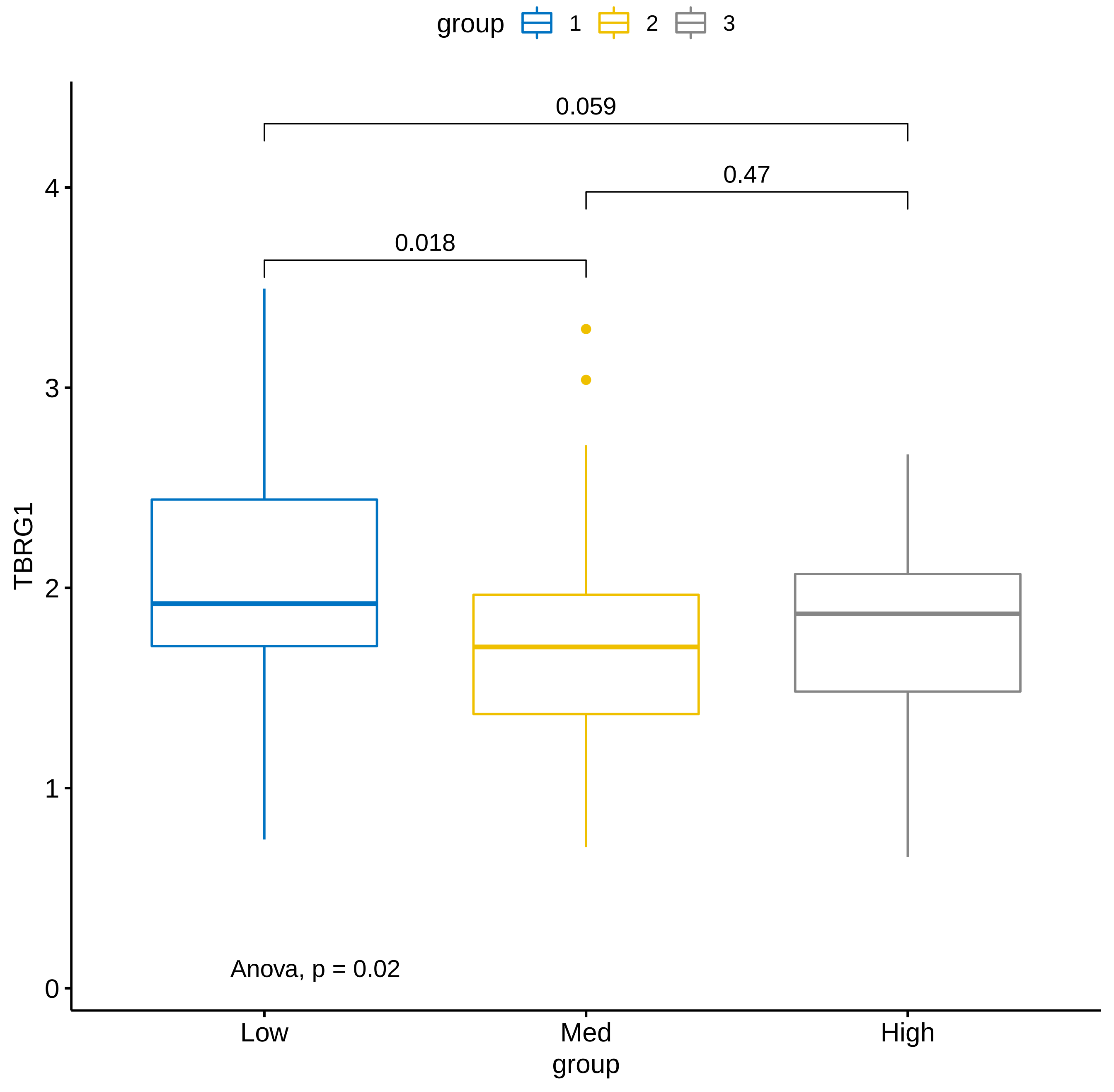

group 1 2 3
0.059
0.47
0.018
4
3
2
1
0
TBRG1
Anova, p = 0.02
Low
Med
High
group

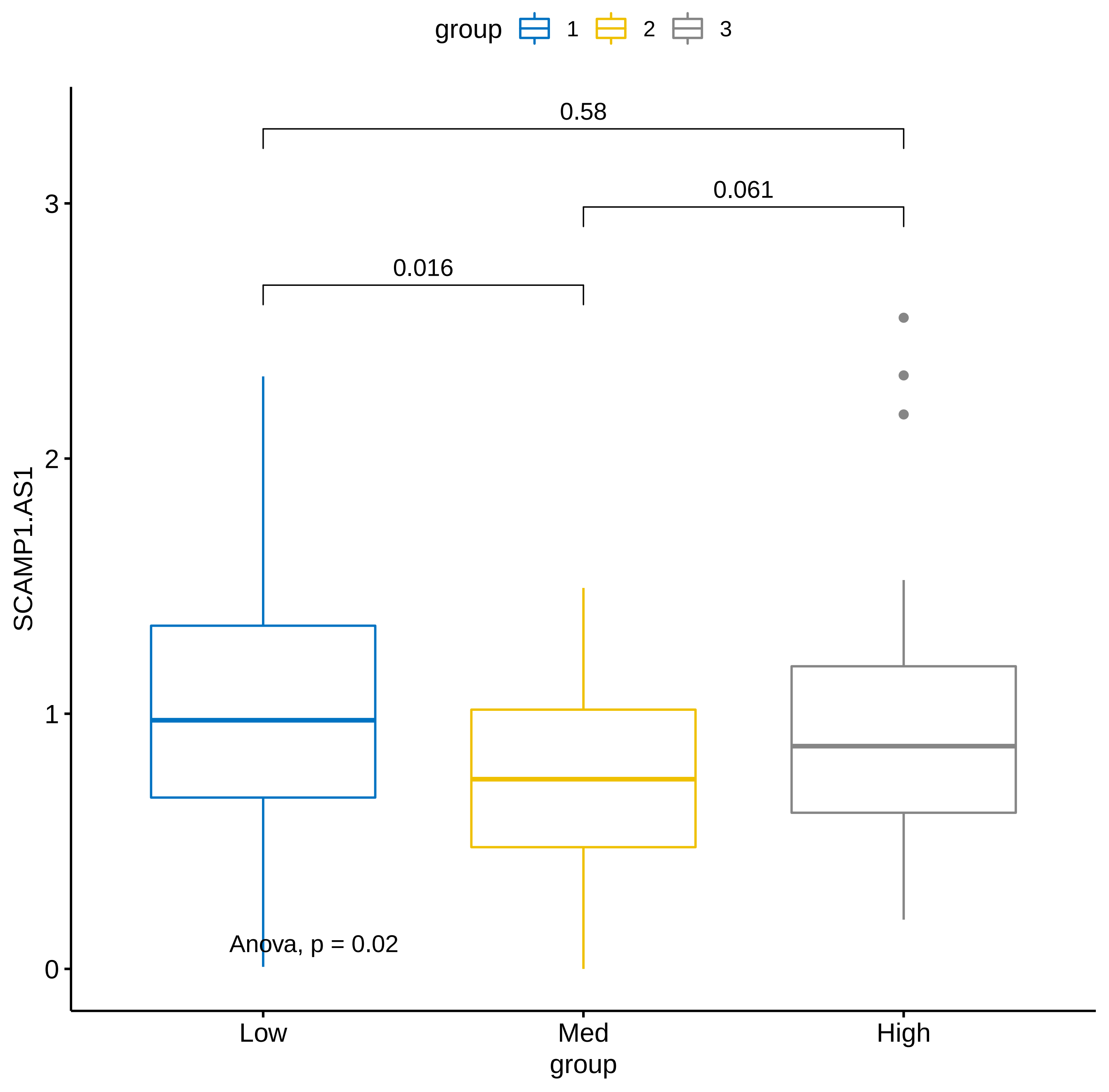
group 1 2 3
0.58
0.061
0.016
3
2
1
0
SCAMP1.AS1
Anova, p = 0.02
Low
Med
High
group

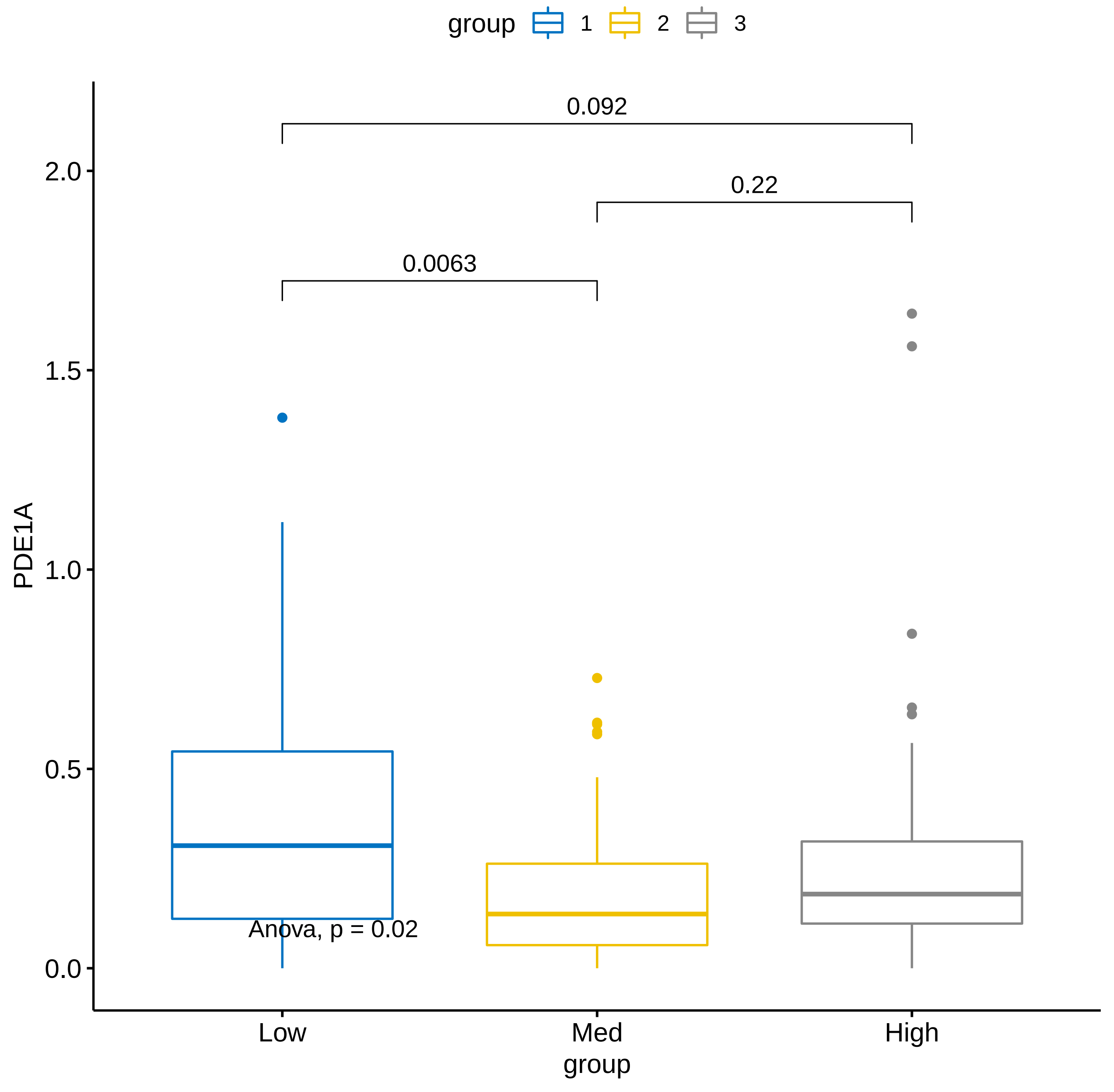
group
1
2
3
0.092
0.22
0.0063
2.0
1.5
1.0
0.5
0.0
PDE1A
Anova, p = 0.02
Low
Med
High
group

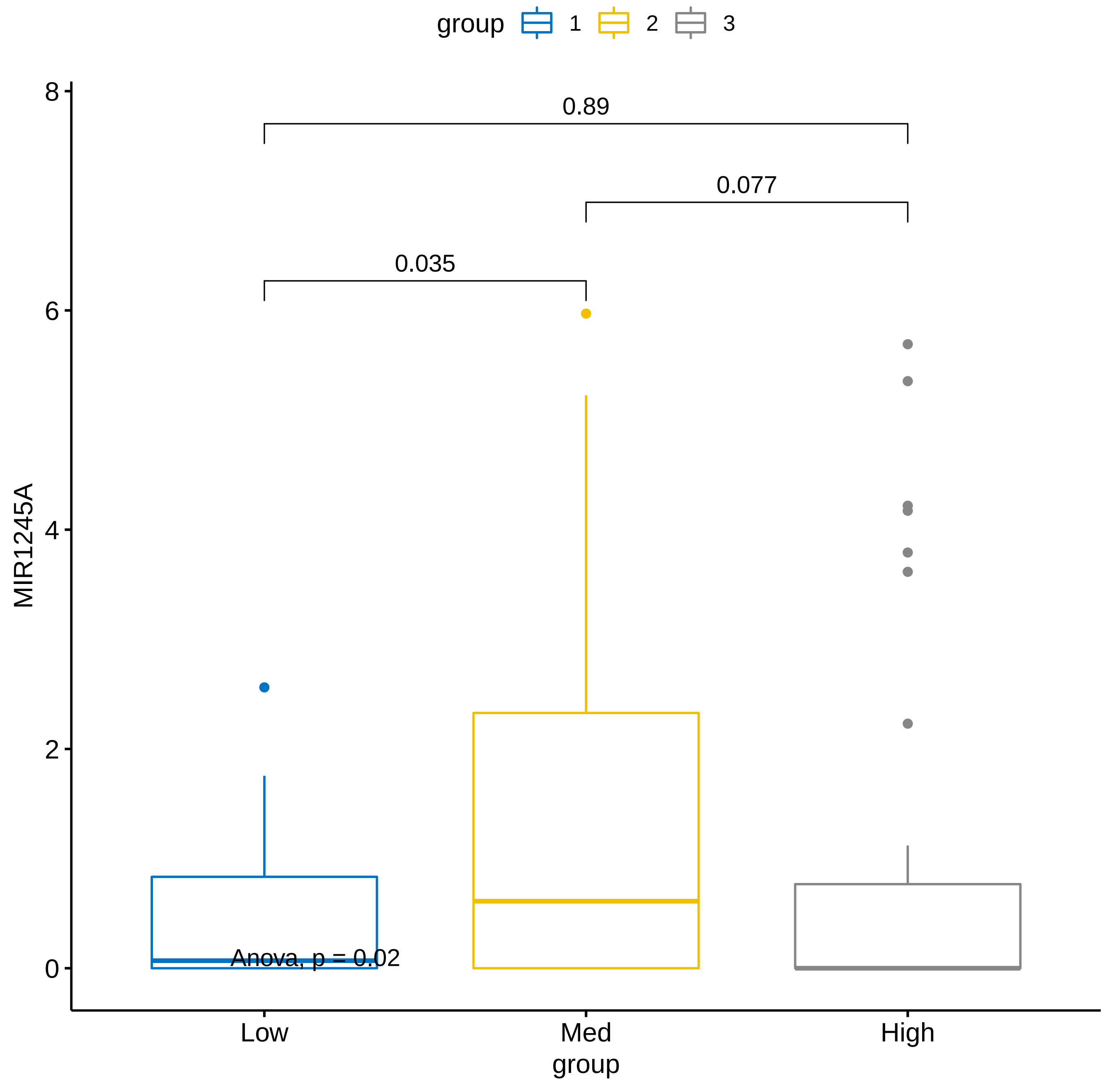

group 1 2 3
0.89
0.077
0.035
8
6
4
2
0
MIR1245A
Anova, p = 0.02
Low
Med
High
group

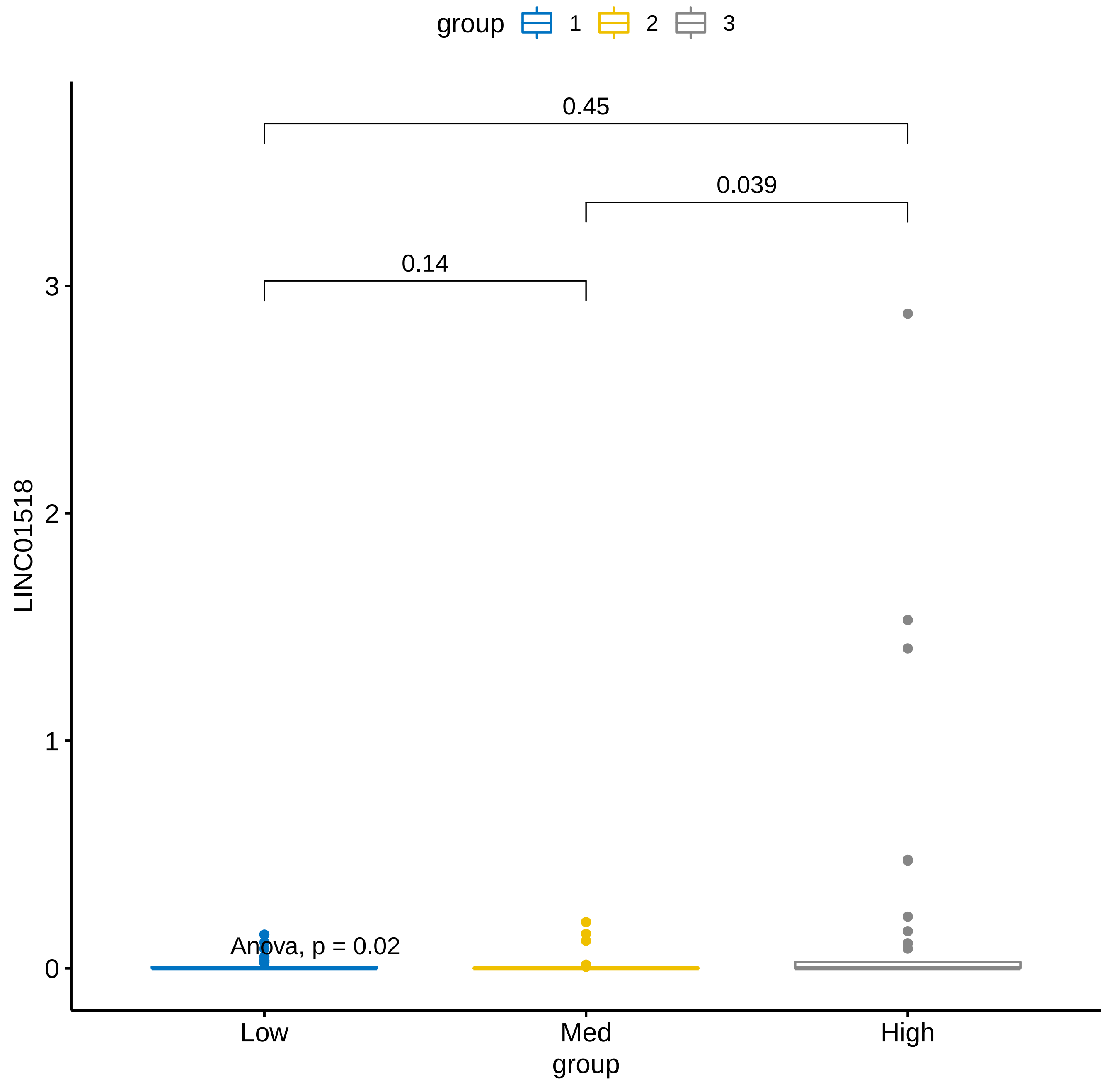

group
1
2
3
0.45
0.039
0.14
3
2
1
0
LINC01518
Anova, p = 0.02
Low
Med
High
group

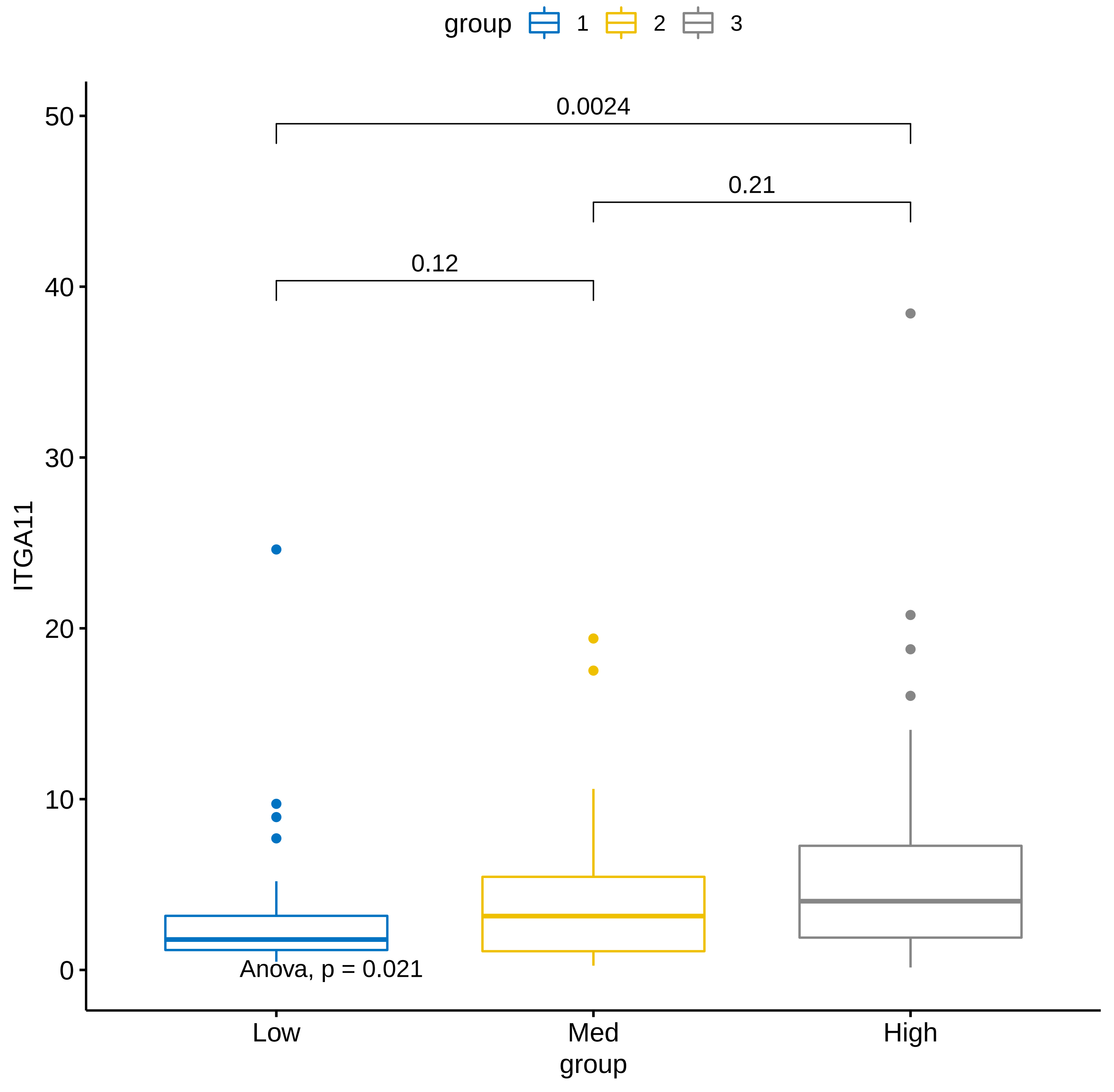
group
1
2
3
0.0024
0.21
0.12
50
40
30
20
10
0
ITGA11
Anova, p = 0.021
Low
Med
High
group

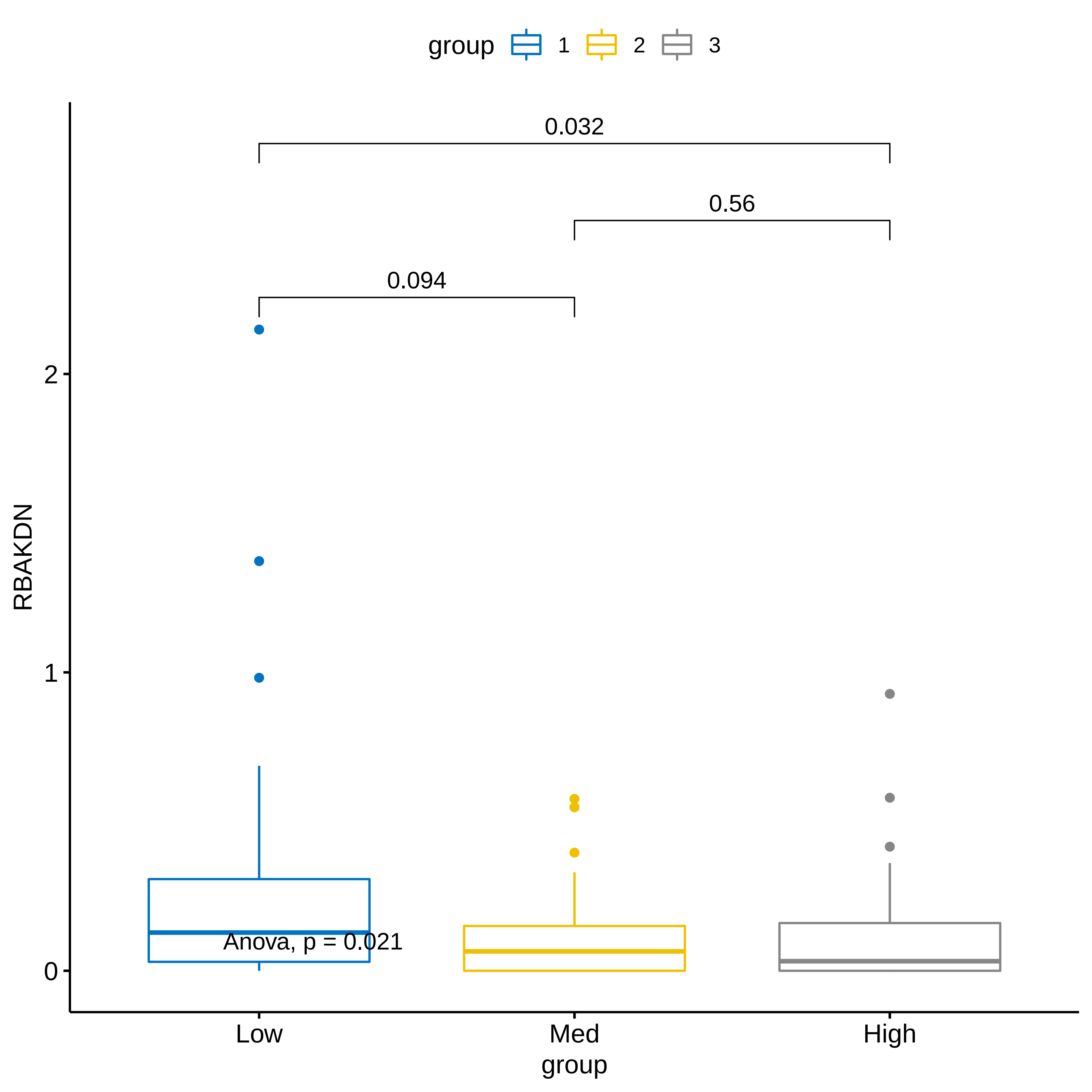

group
1
2
3
0.032
0.56
0.094
Anova, p = 0.021
RBAKDN
2
1
0
Low
Med
High
group

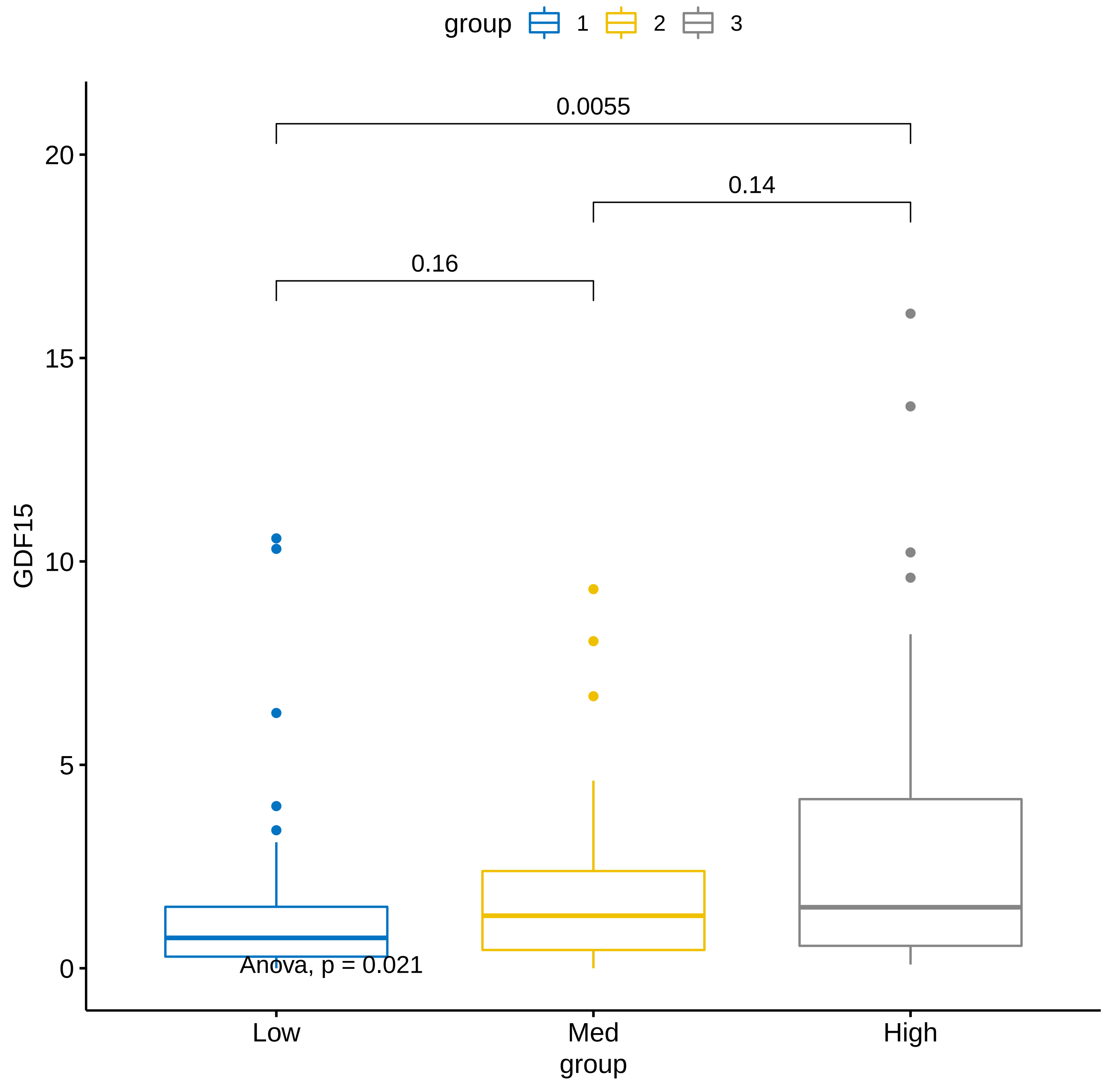

group
1
2
3
0.0055
0.14
0.16
GDF15
20
15
10
5
0
Anova, p = 0.021
Low
Med
High
group

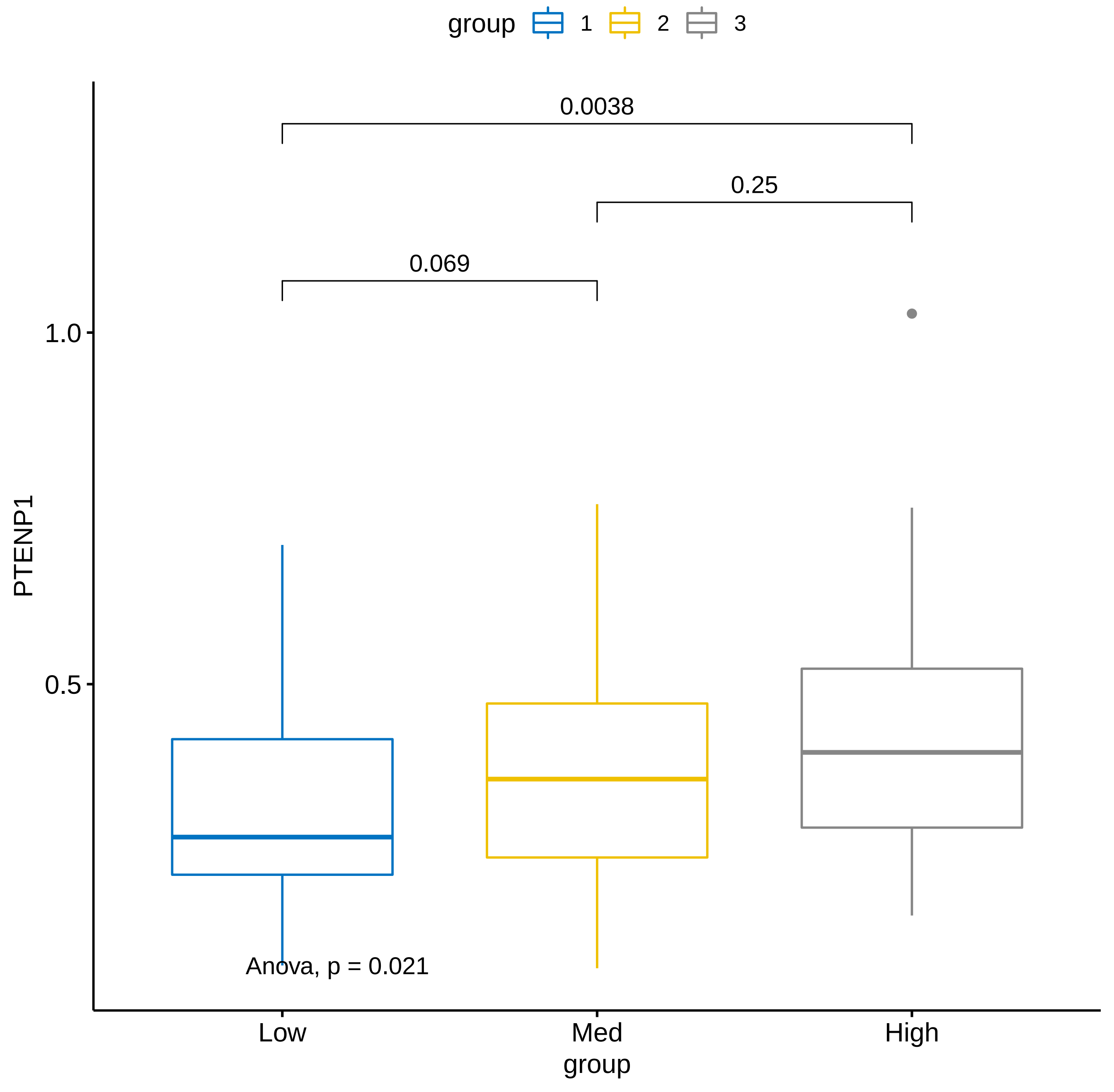
group
1
2
3
0.0038
0.25
0.069
1.0
0.5
PTENP1
Anova, p = 0.021
Low
Med
High
group

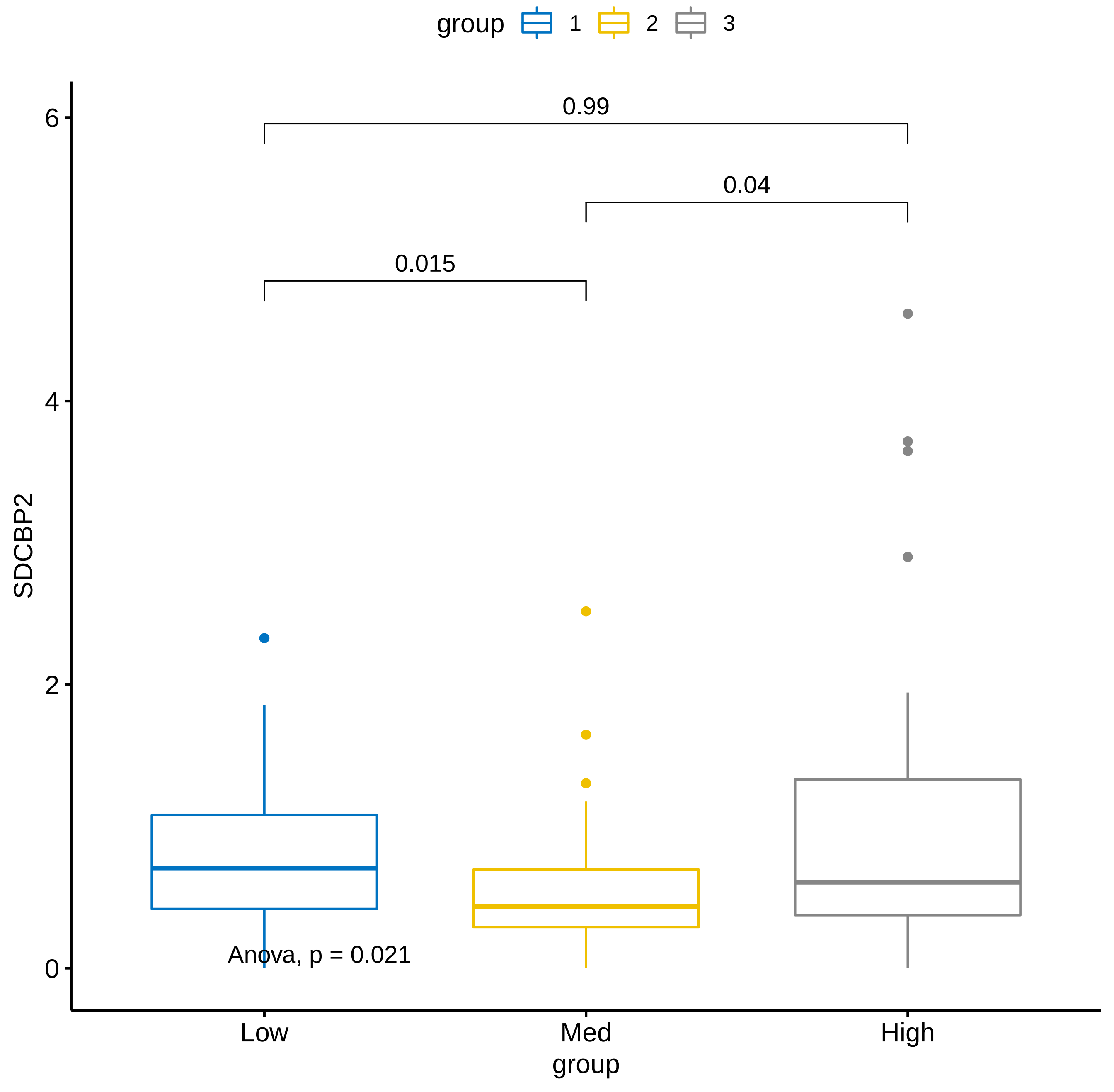
group 1 2 3
0.99
0.04
0.015
SDCBP2
6
4
2
0
Anova, p = 0.021
Low
Med
High
group

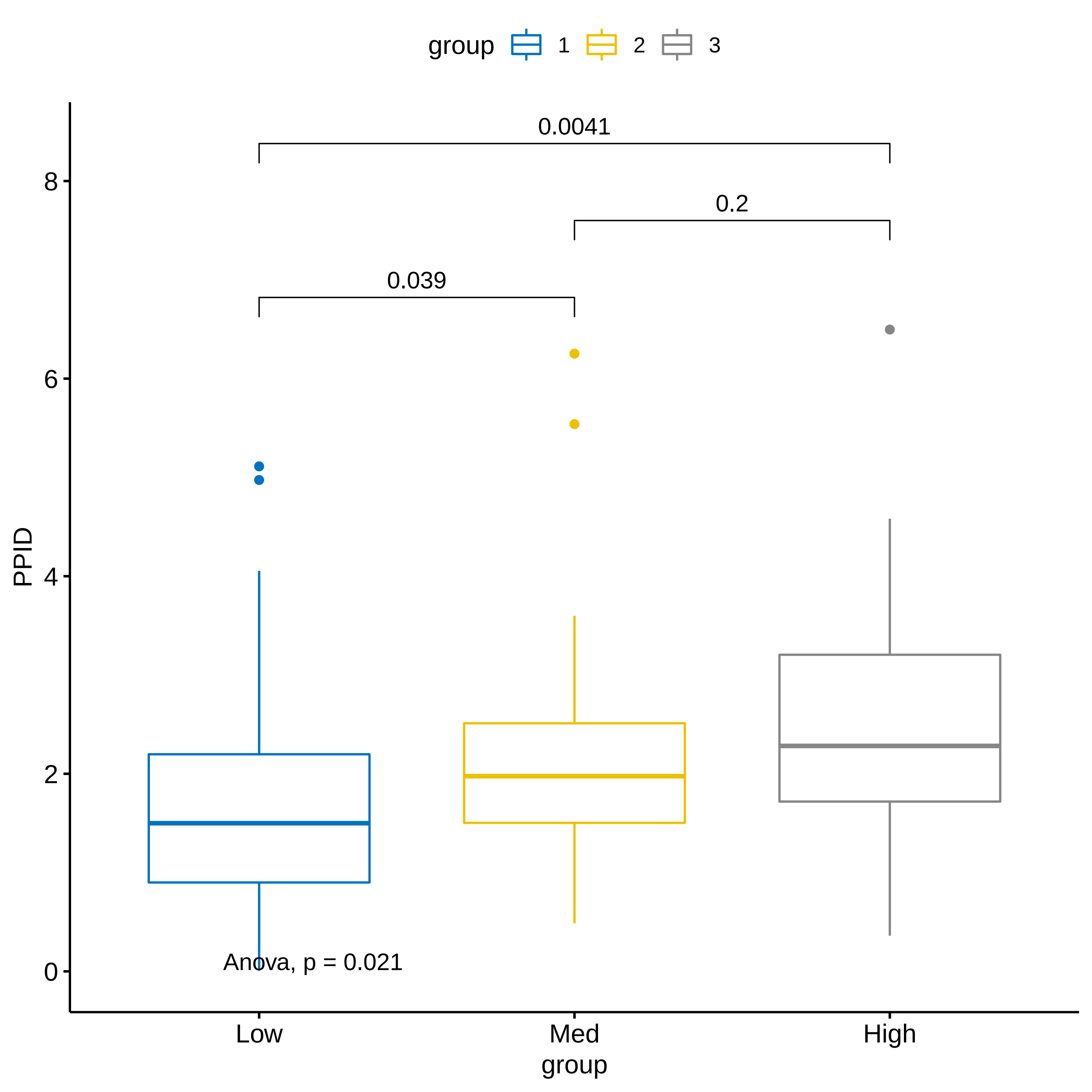
group 1 2 3
0.0041
0.2
0.039
PPID
8
6
4
2
0
Anova, p = 0.021
Low
Med
High
group

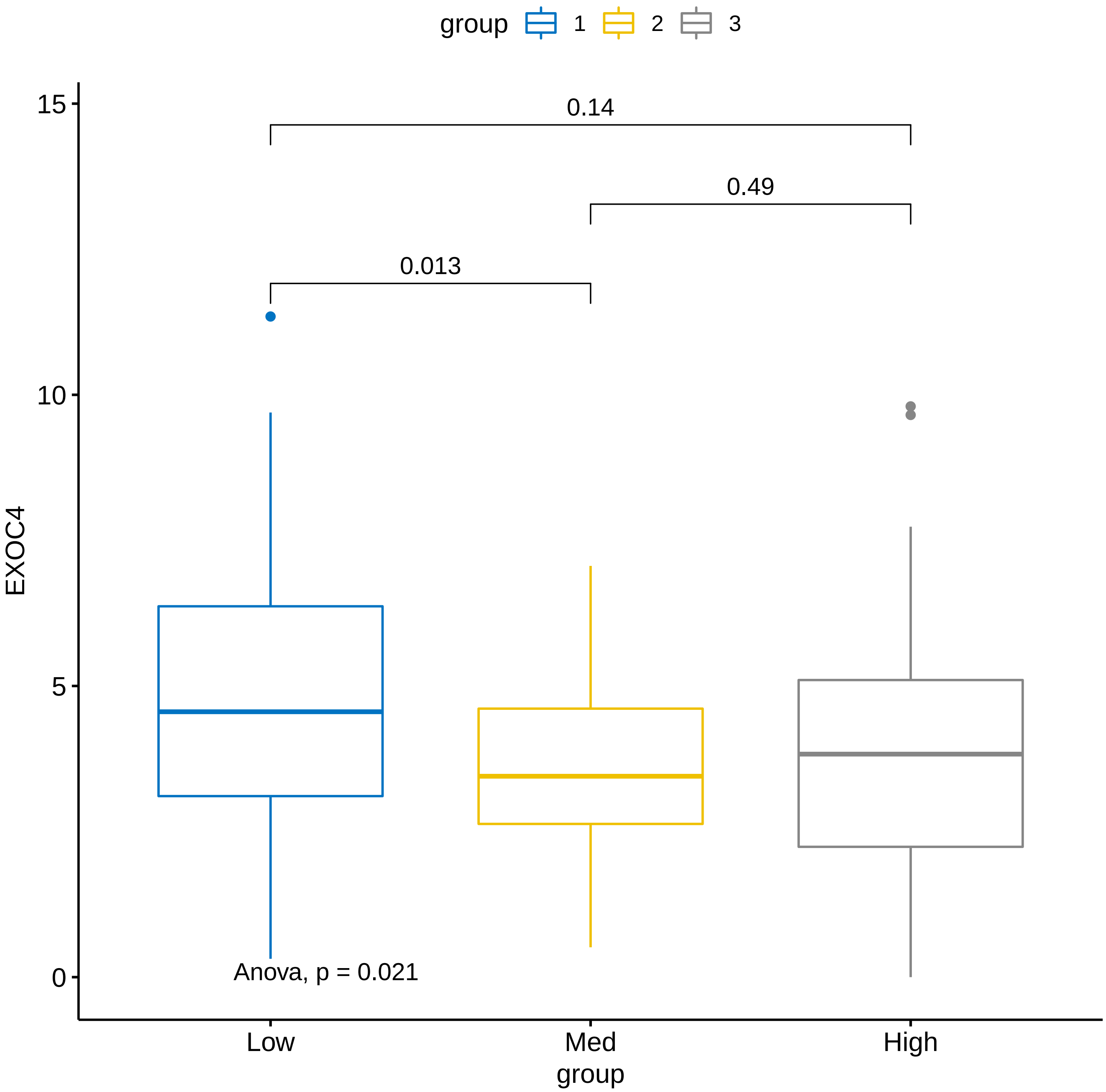

group 1 2 3
0.14
0.49
0.013
Anova, p = 0.021
EXOC4
15
10
5
0
Low
Med
High
group

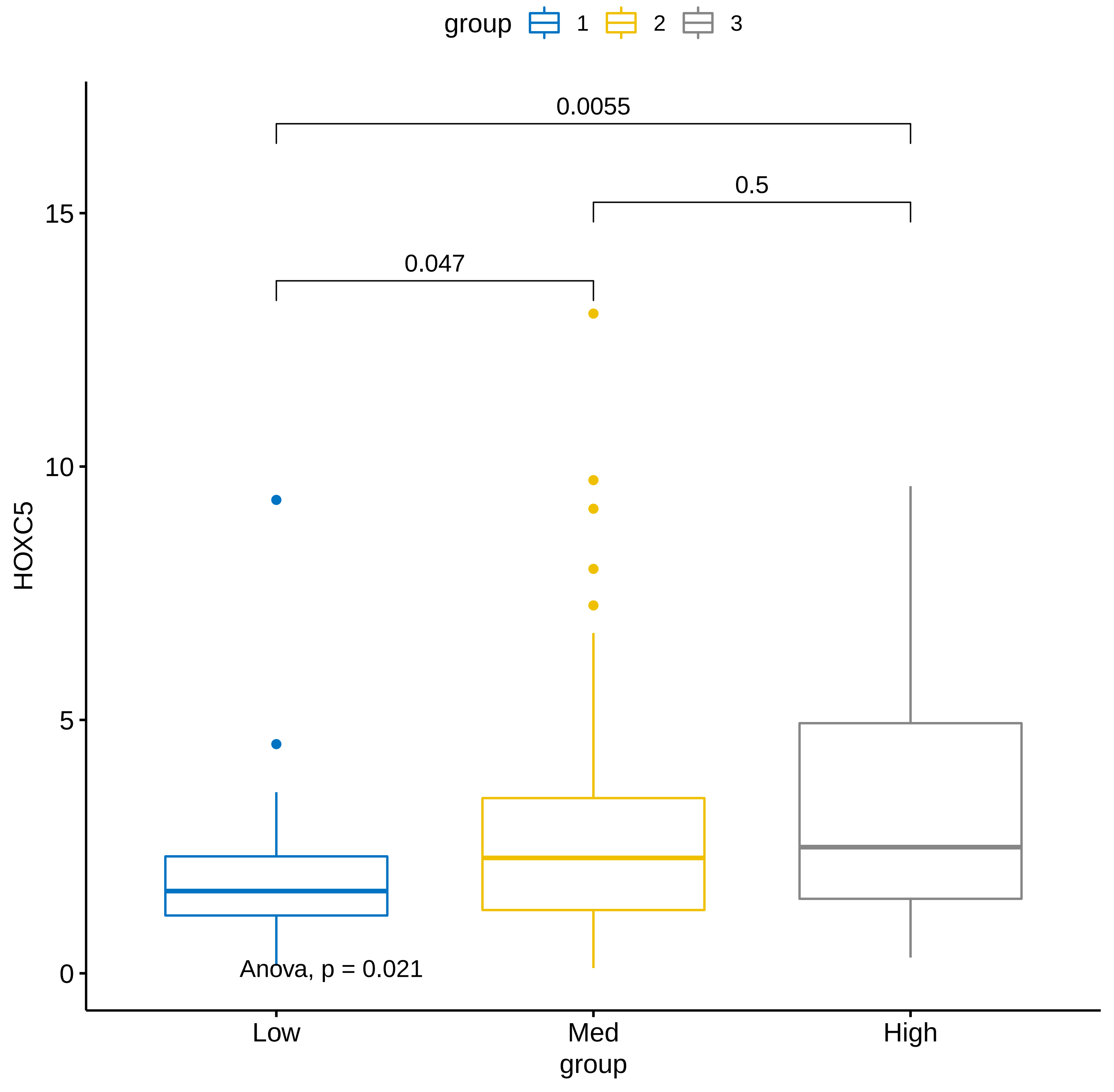

group
1
2
3
0.0055
0.5
0.047
HOXC5
15
10
5
0
Anova, p = 0.021
Low
Med
High
group

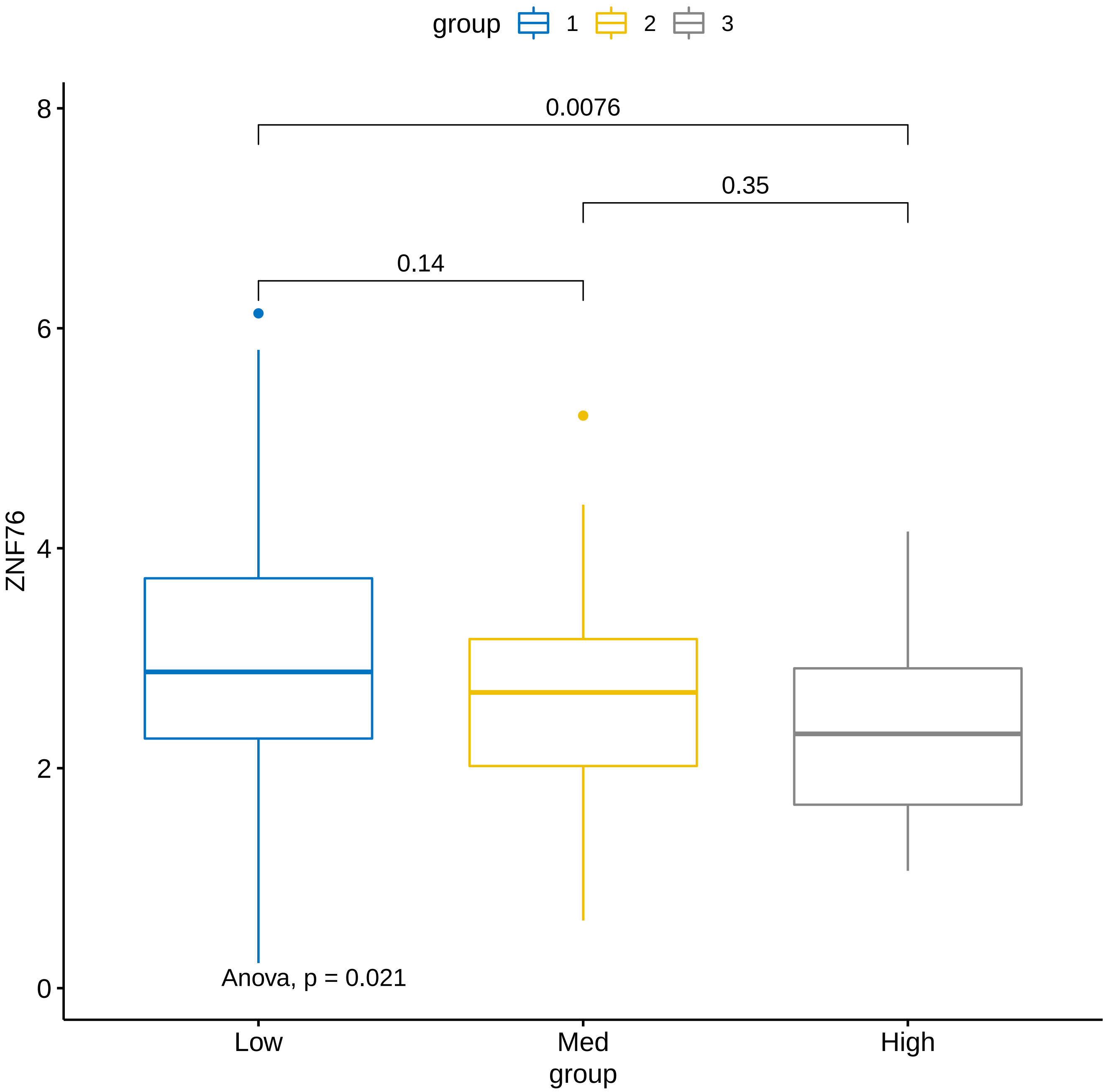
group
1
2
3
0.0076
0.35
0.14
8
6
4
2
0
ZNF76
Anova, p = 0.021
Low
Med
High
group

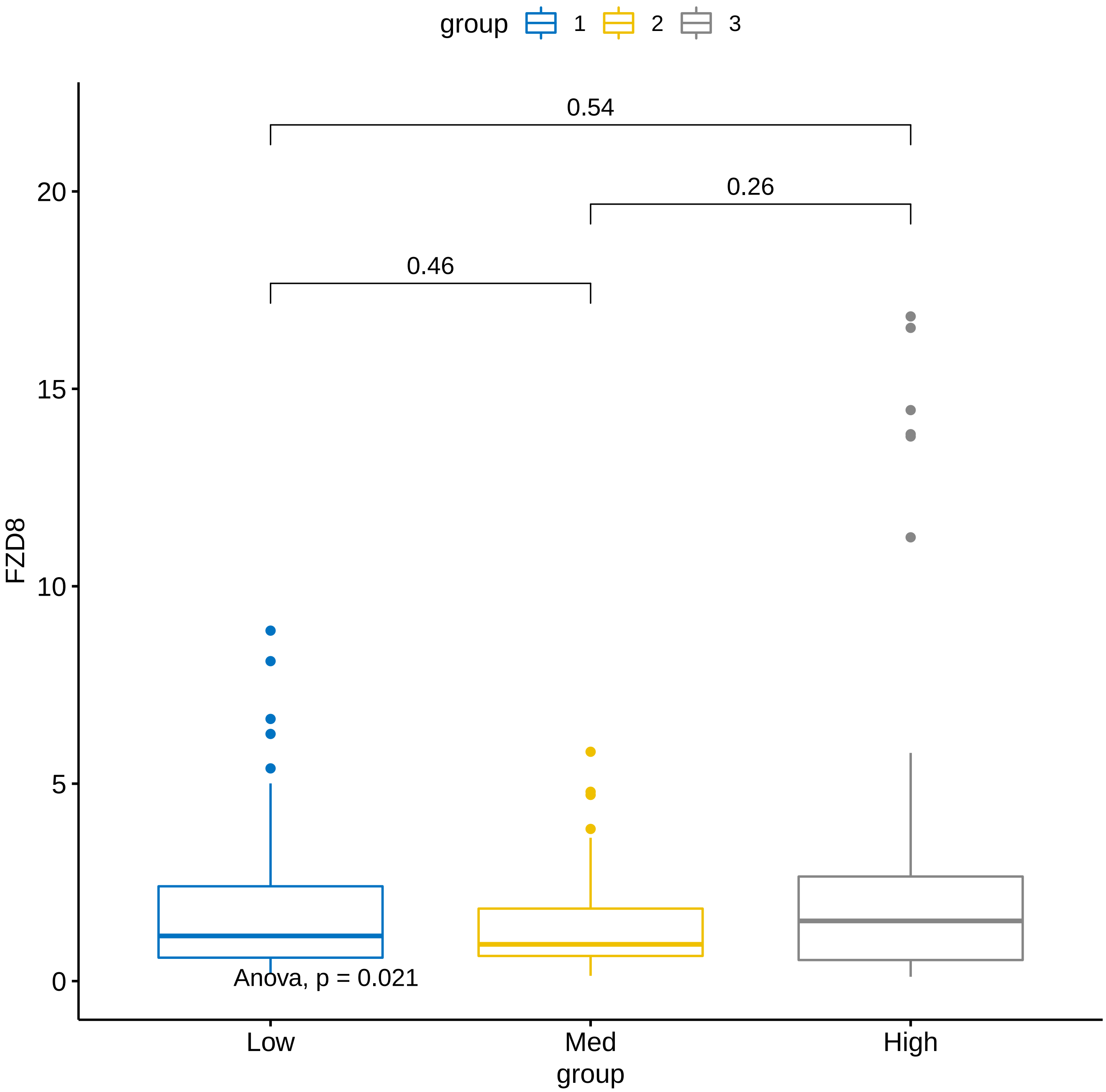
group
1
2
3
0.54
0.26
0.46
20
15
10
5
0
FZD8
Anova, p = 0.021
Low
Med
High
group

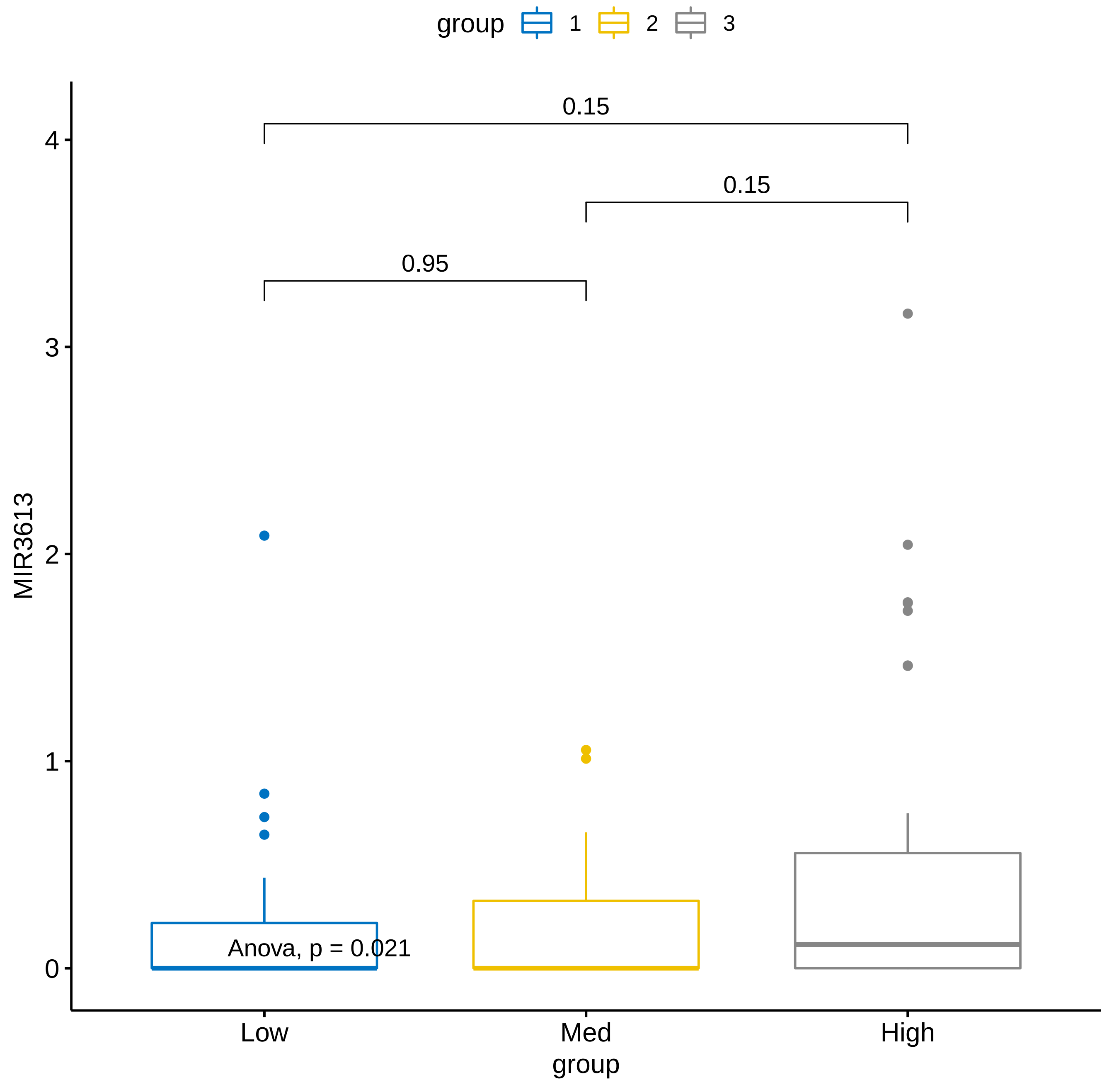

group 1 2 3
0.15
0.15
0.95
4
3
2
1
0
MIR3613
Anova, p = 0.021
Low
Med
High
group

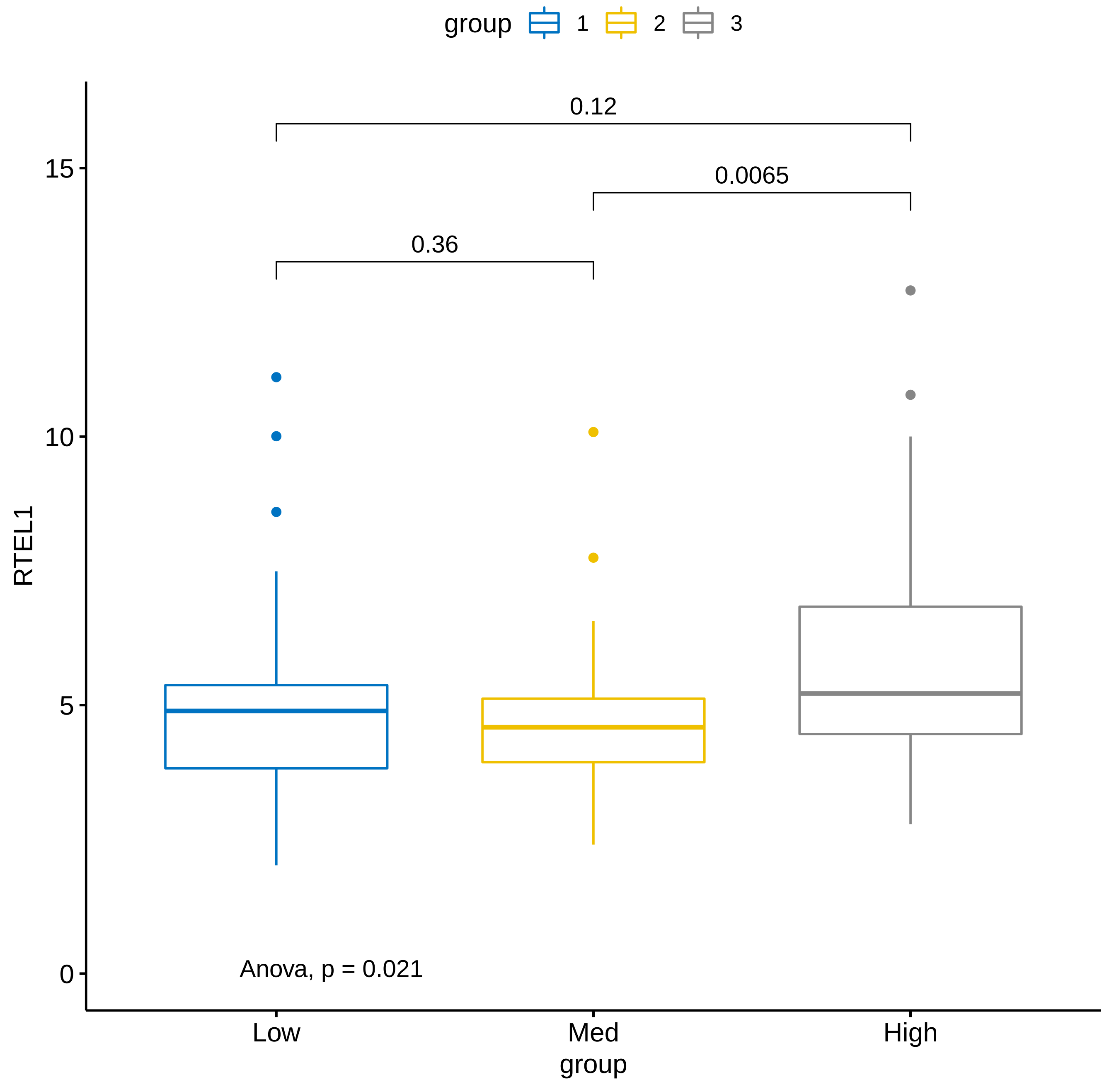

group 1 2 3
0.12
0.0065
0.36
15
10
5
0
RTEL1
Anova, p = 0.021
Low
Med
High
group

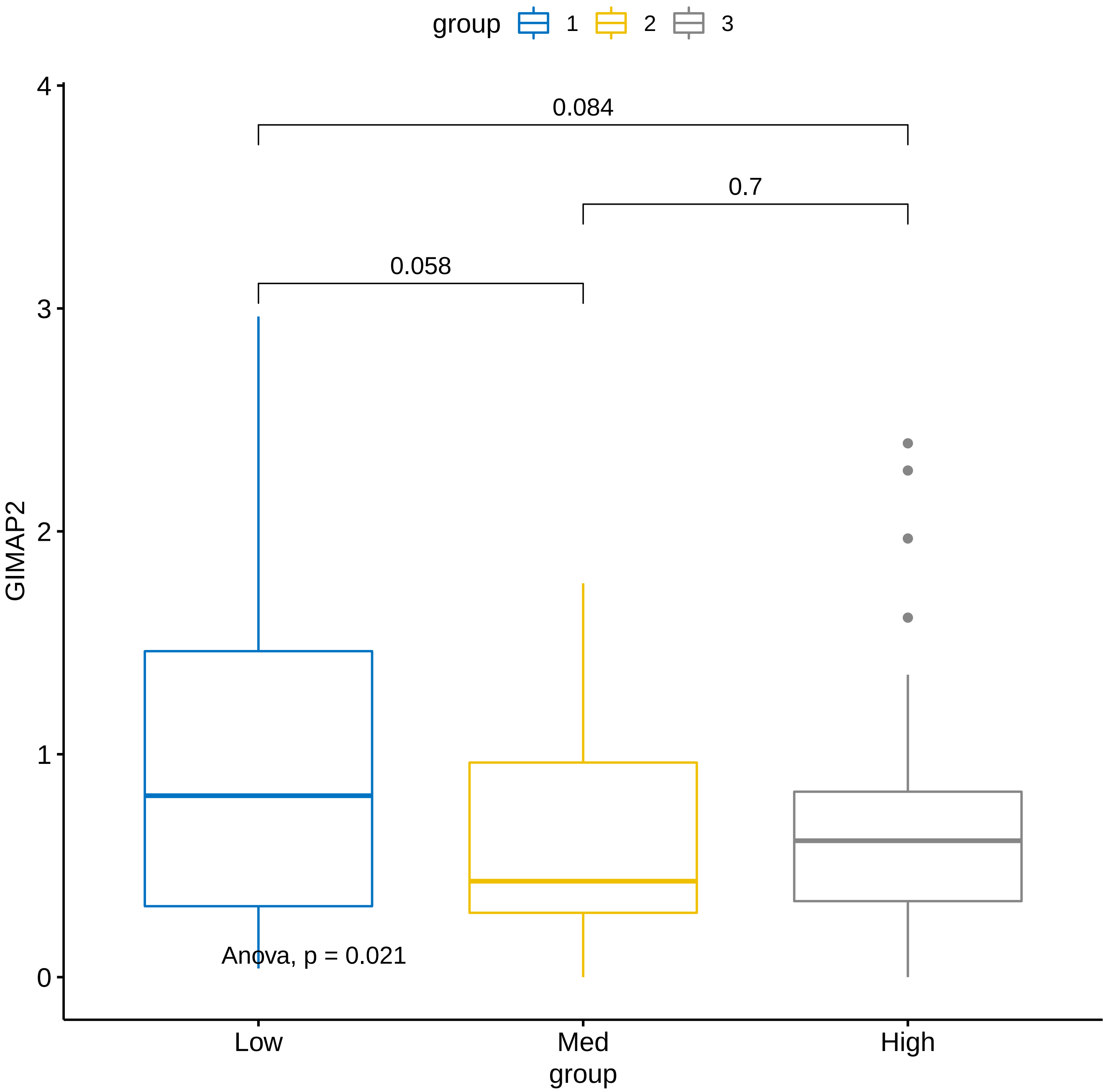
group
1
2
3
0.084
0.7
0.058
Anova, p = 0.021
GIMAP2
4
3
2
1
0
Low
Med
High
group

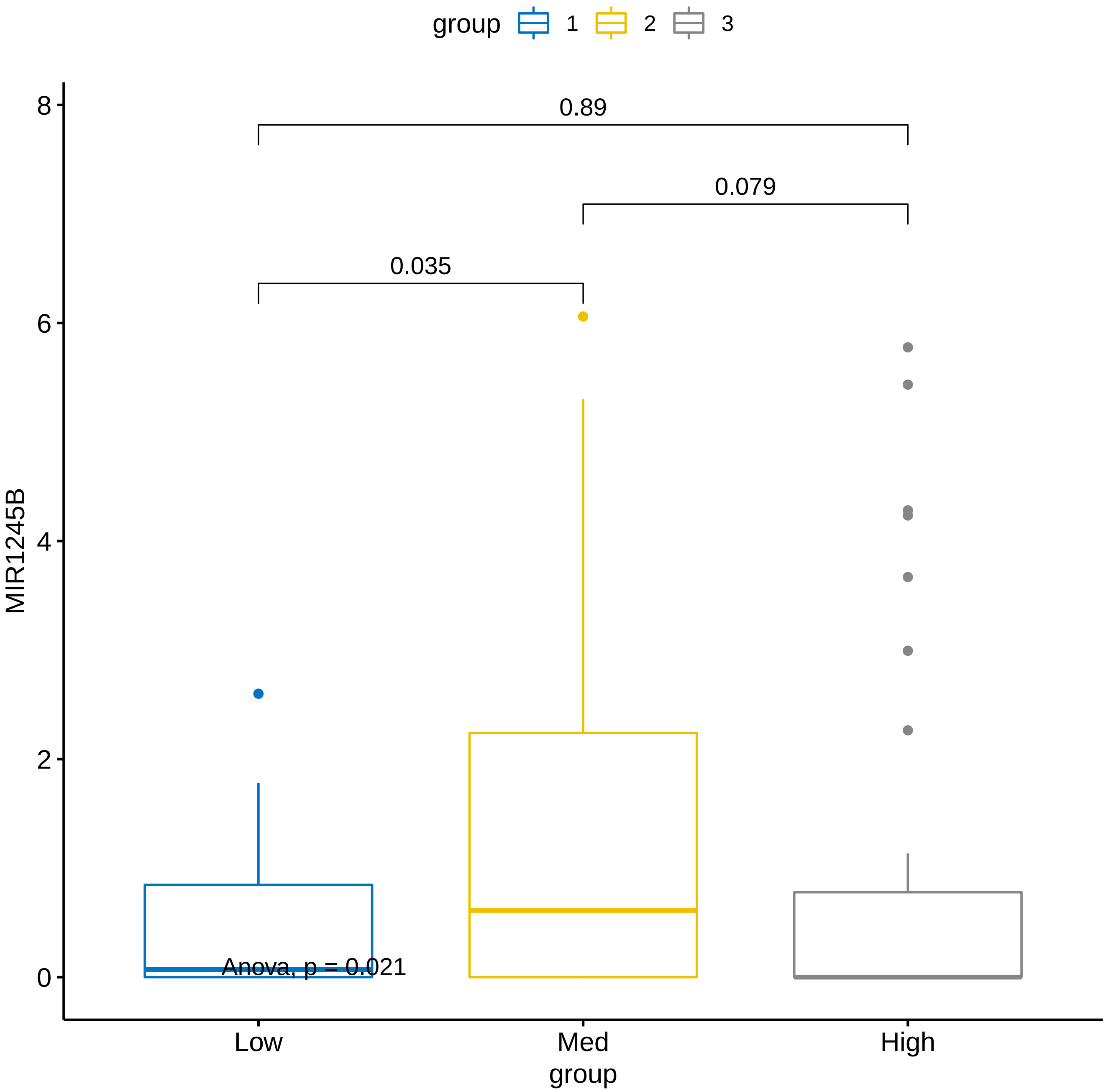

group
1
2
3
0.89
0.079
0.035
8
6
4
2
0
MIR1245B
Anova, p = 0.021
Low
Med
High
group

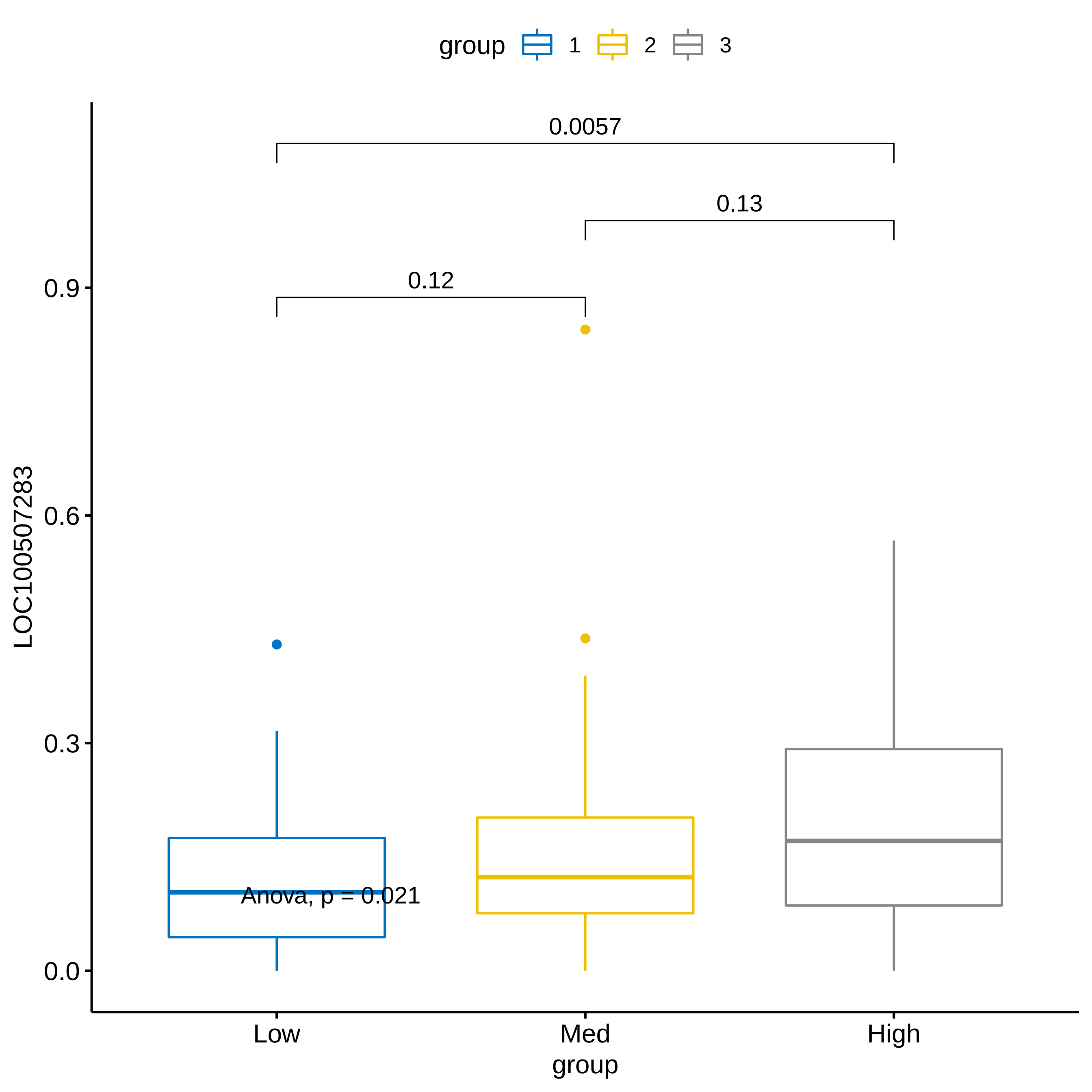

group
1
2
3
0.0057
0.13
0.12
0.9
0.6
0.3
0.0
LOC100507283
Anova, p = 0.021
Low
Med
High
group

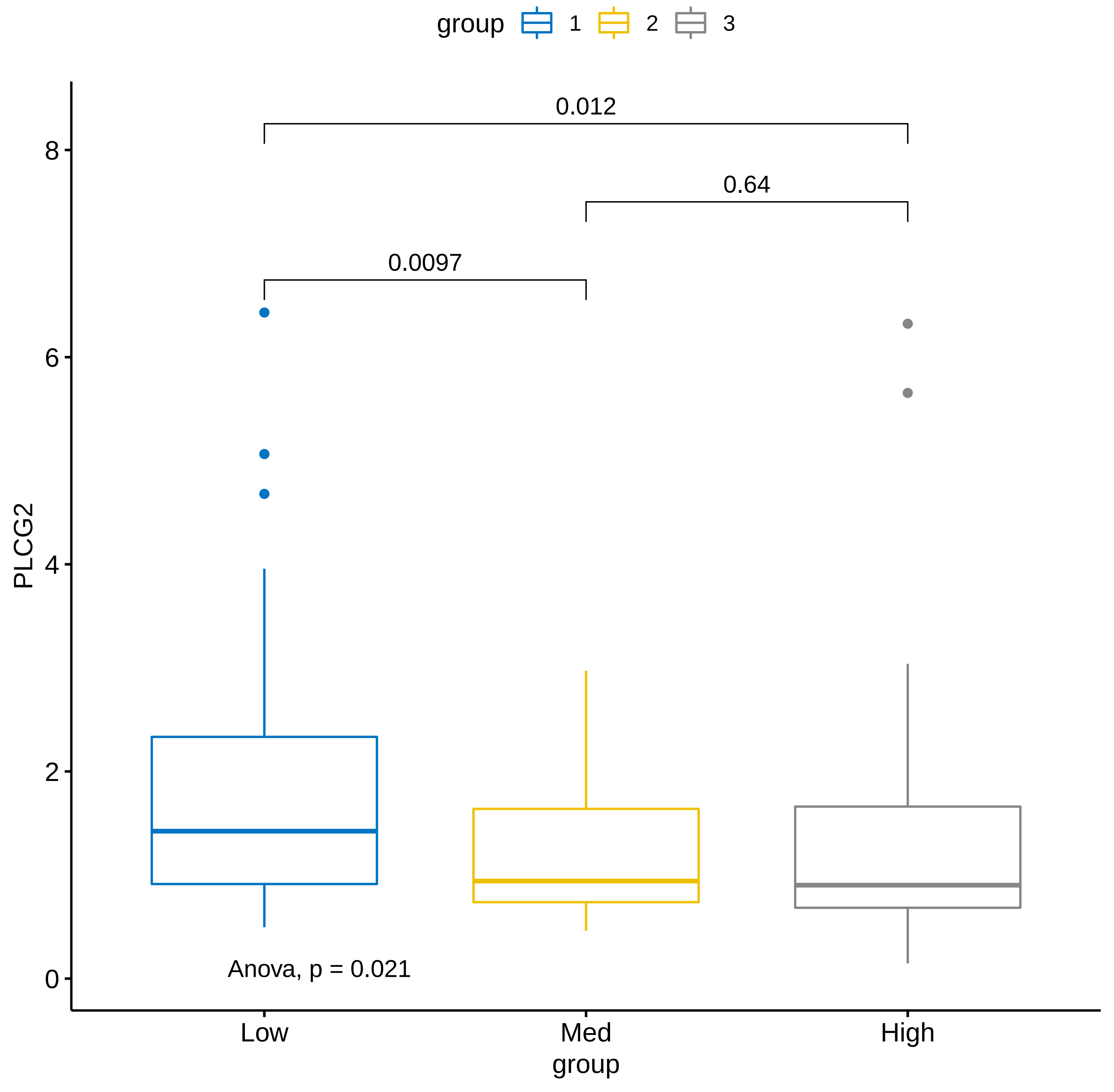
group
1
2
3
0.012
0.64
0.0097
PLCG2
8
6
4
2
0
Anova, p = 0.021
Low
Med
High
group

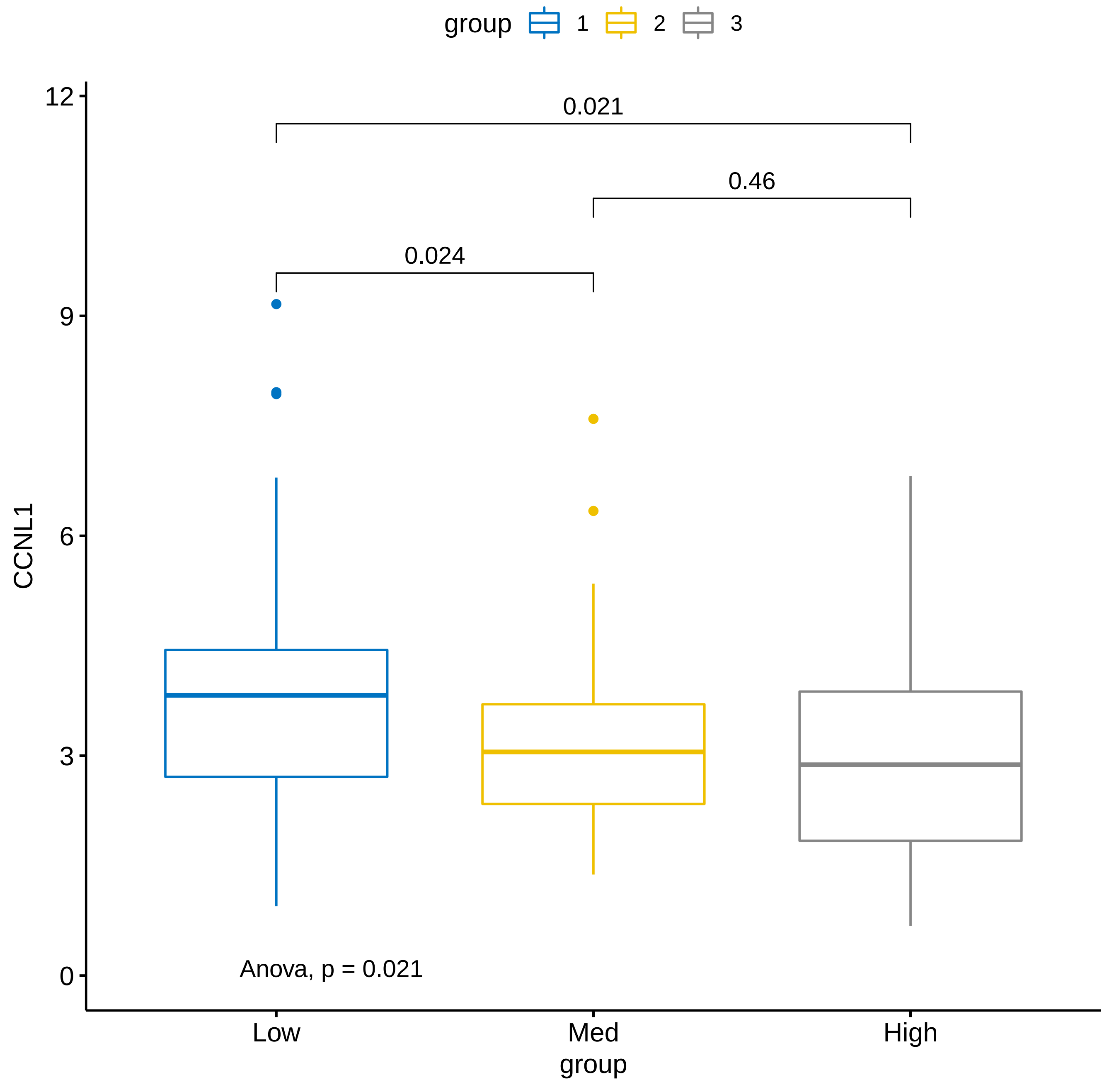
group 1 2 3
0.021
0.46
0.024
12
9
6
3
0
CCNL1
Anova, p = 0.021
Low
Med
High
group

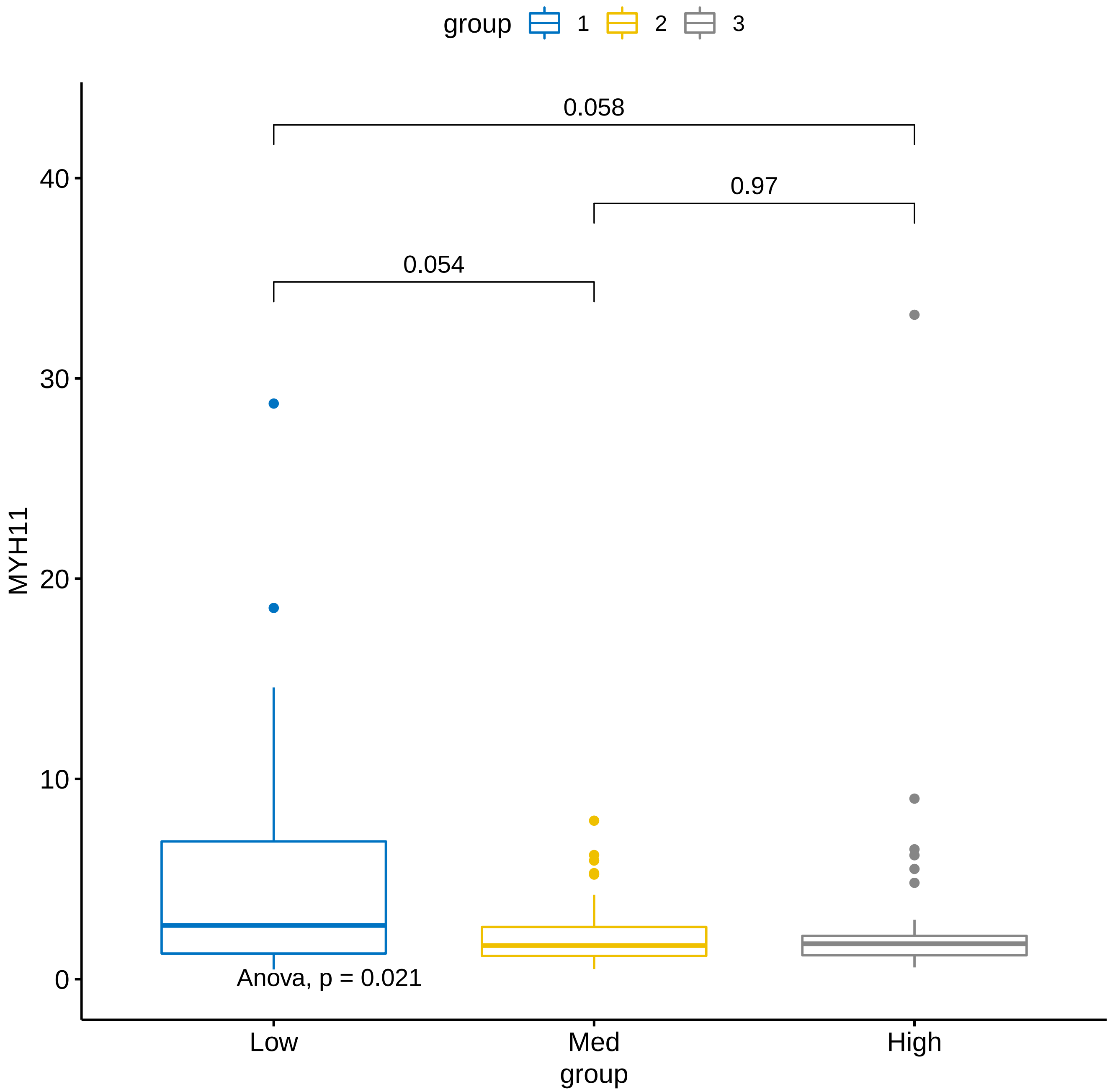

group
1
2
3
0.058
0.97
0.054
40
30
20
10
0
MYH11
Anova, p = 0.021
Low
Med
High
group

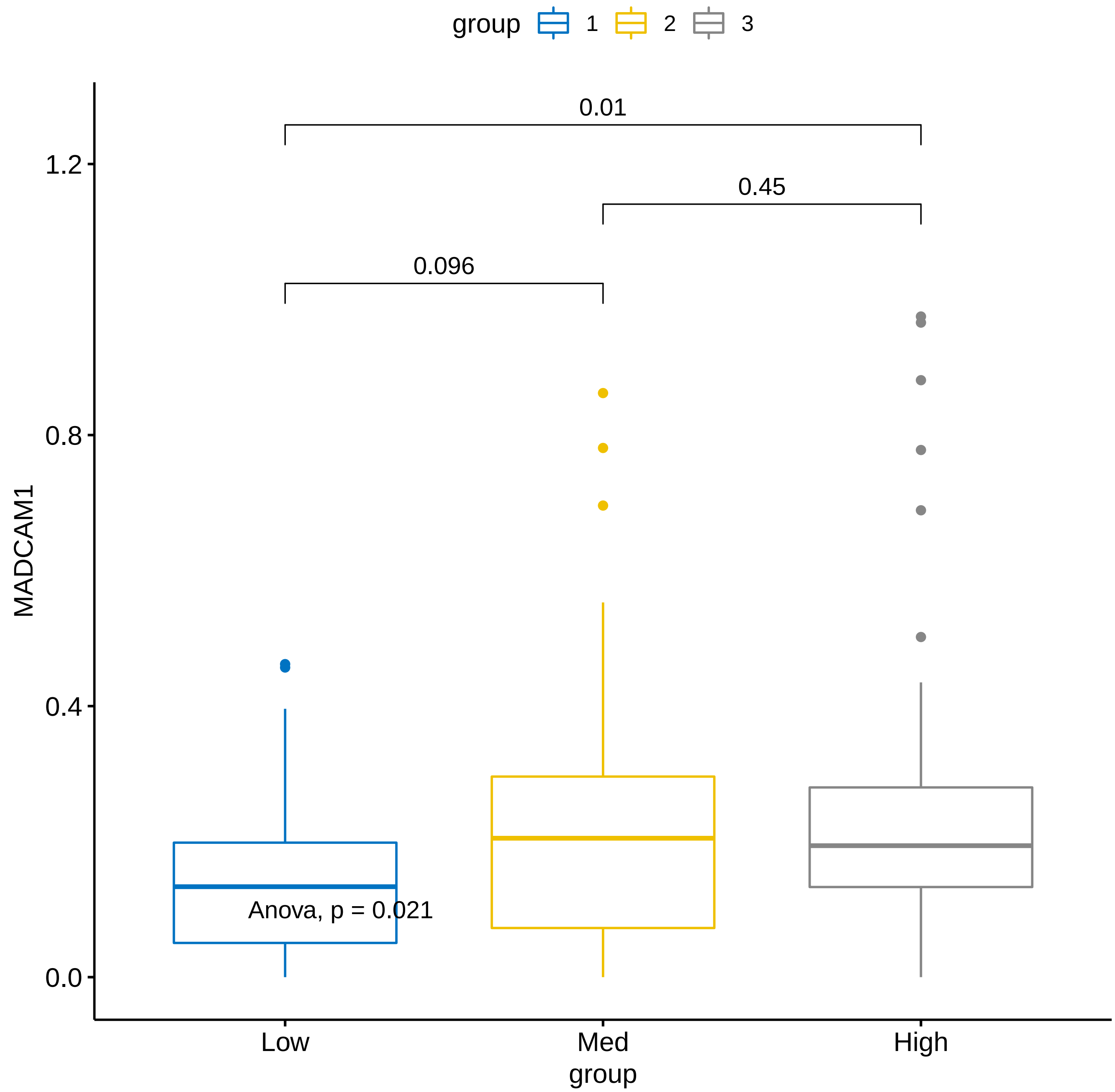
group
1
2
3
0.01
0.45
0.096
1.2
0.8
0.4
0.0
MADCAM1
Anova, p = 0.021
Low
Med
High
group

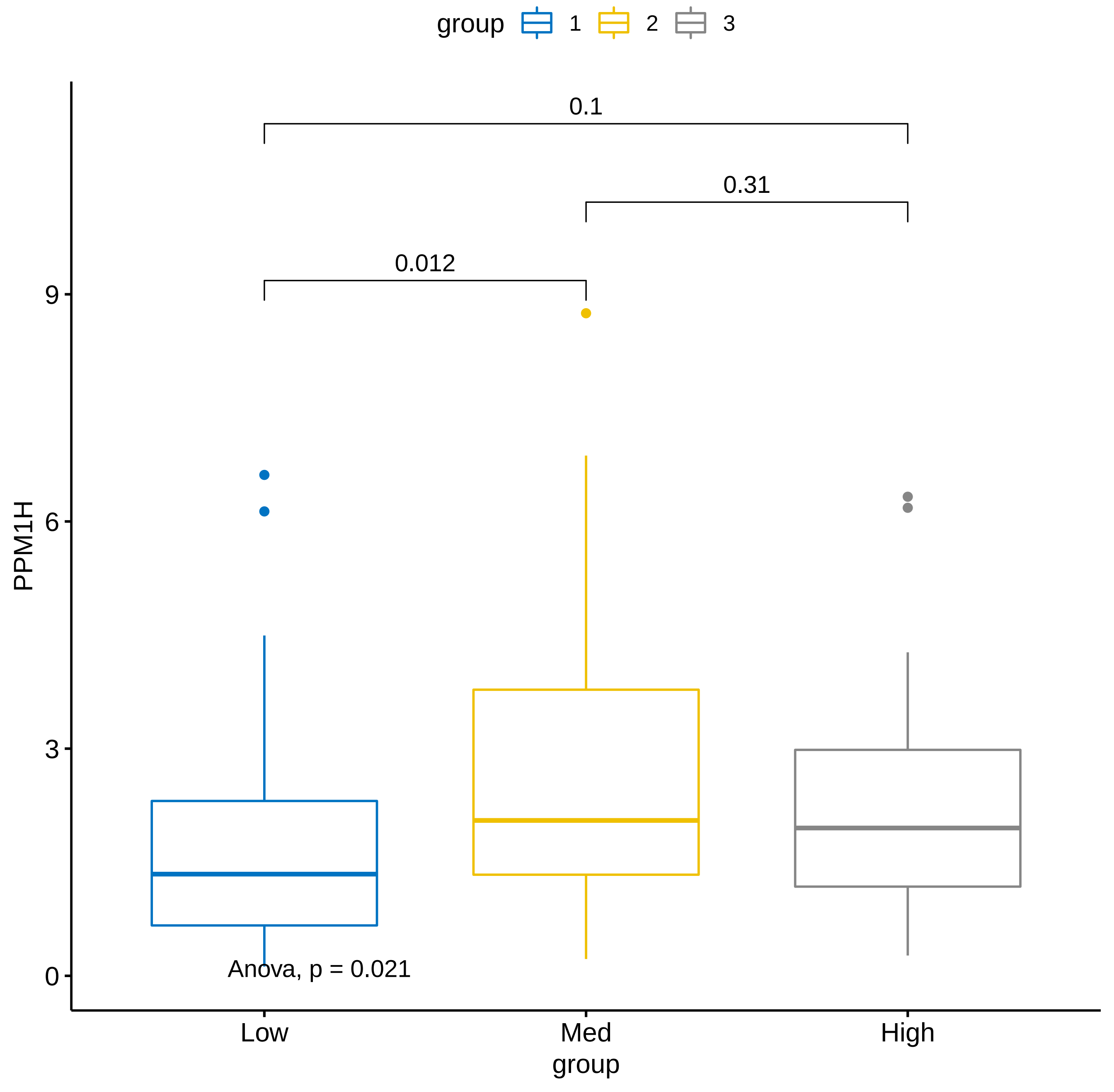

group
1
2
3
0.1
0.31
0.012
9
6
3
0
PPM1H
Anova, p = 0.021
Low
Med
High
group

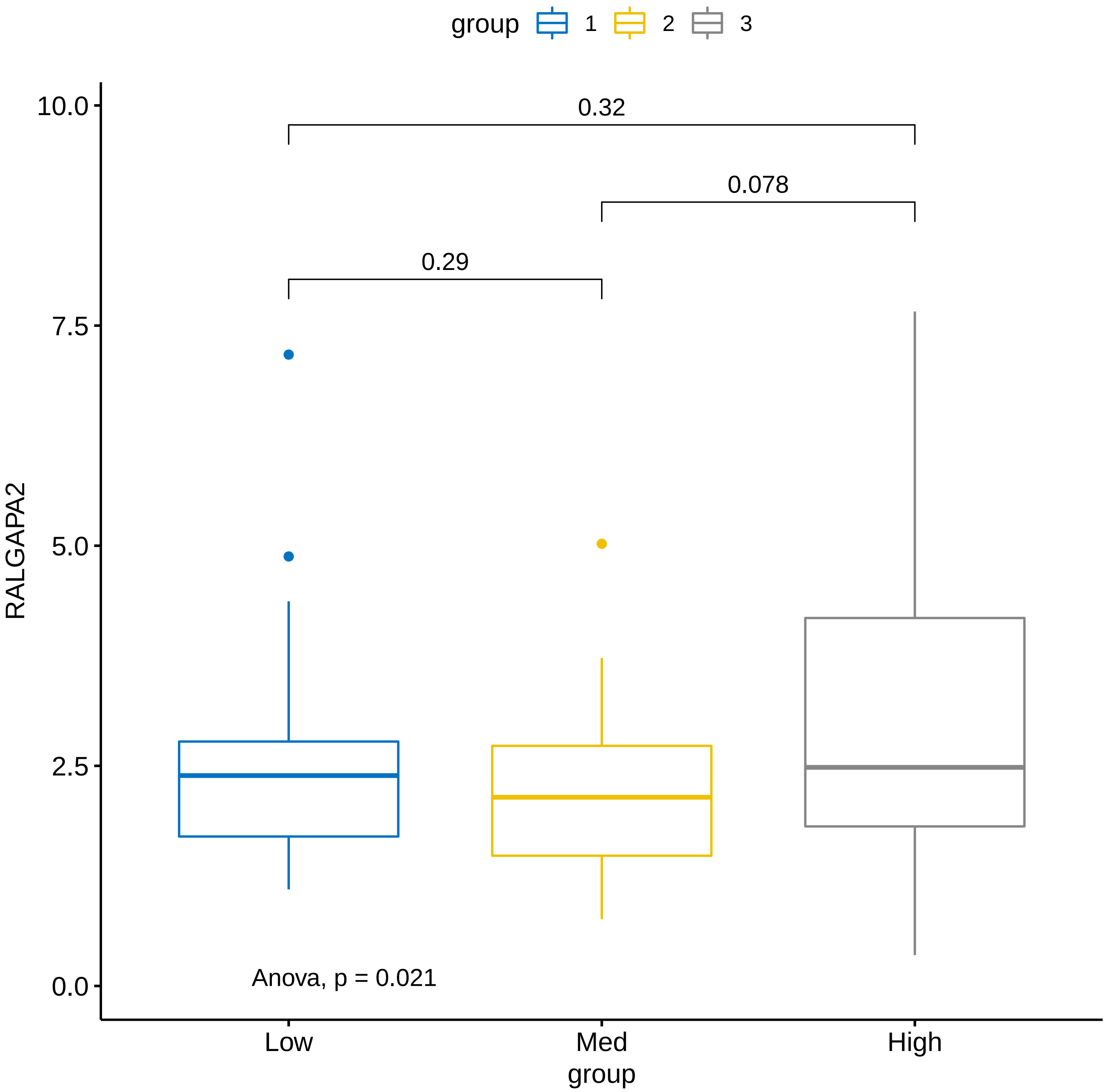

group
1
2
3
10.0
7.5
5.0
2.5
0.0
RALGAPA2
0.32
0.078
0.29
Anova, p = 0.021
Low
Med
High
group

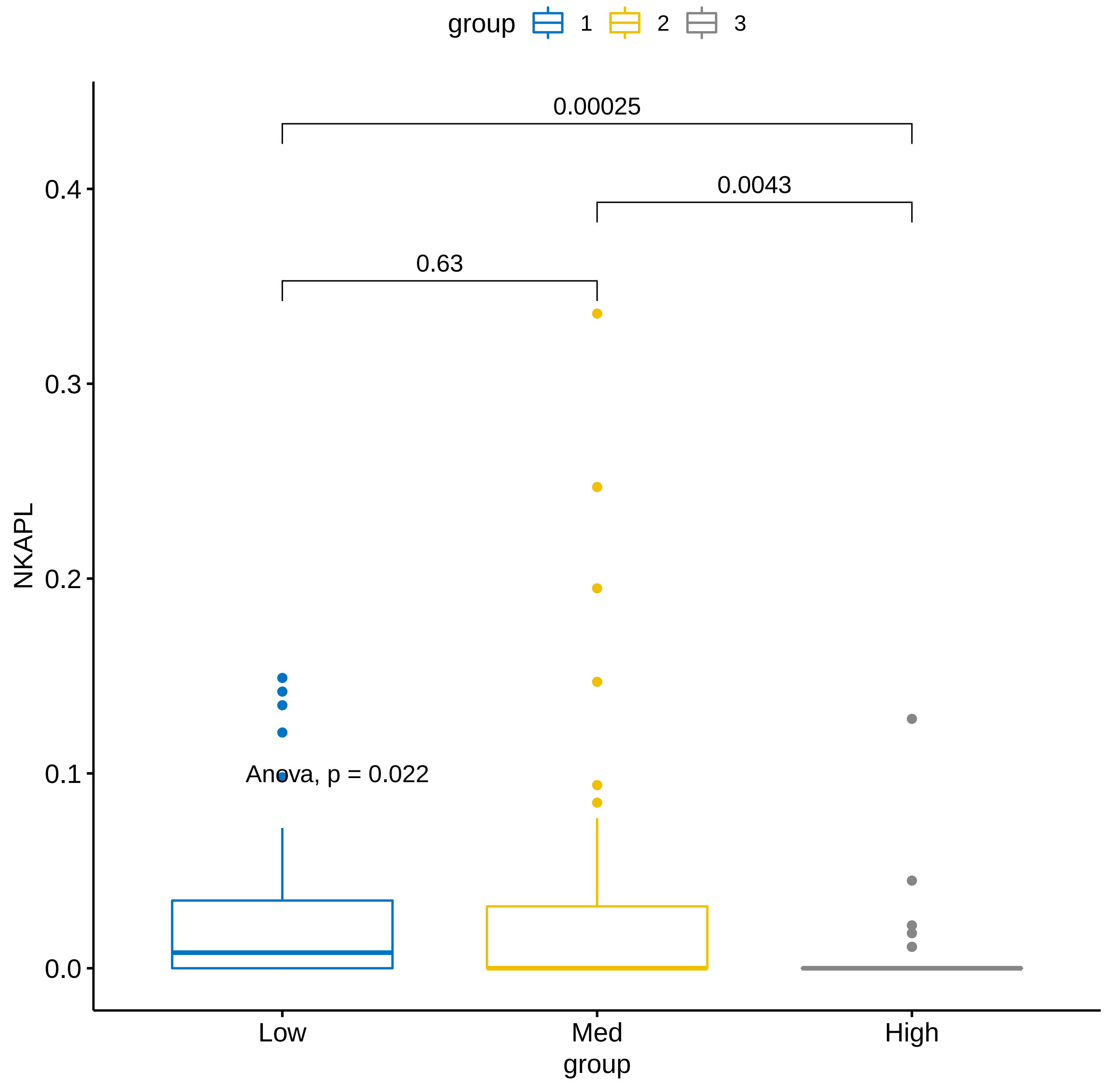

group
1
2
3
0.00025
0.0043
0.63
Anova, p = 0.022
NKAPL
0.4
0.3
0.2
0.1
0.0
Low
Med
High
group

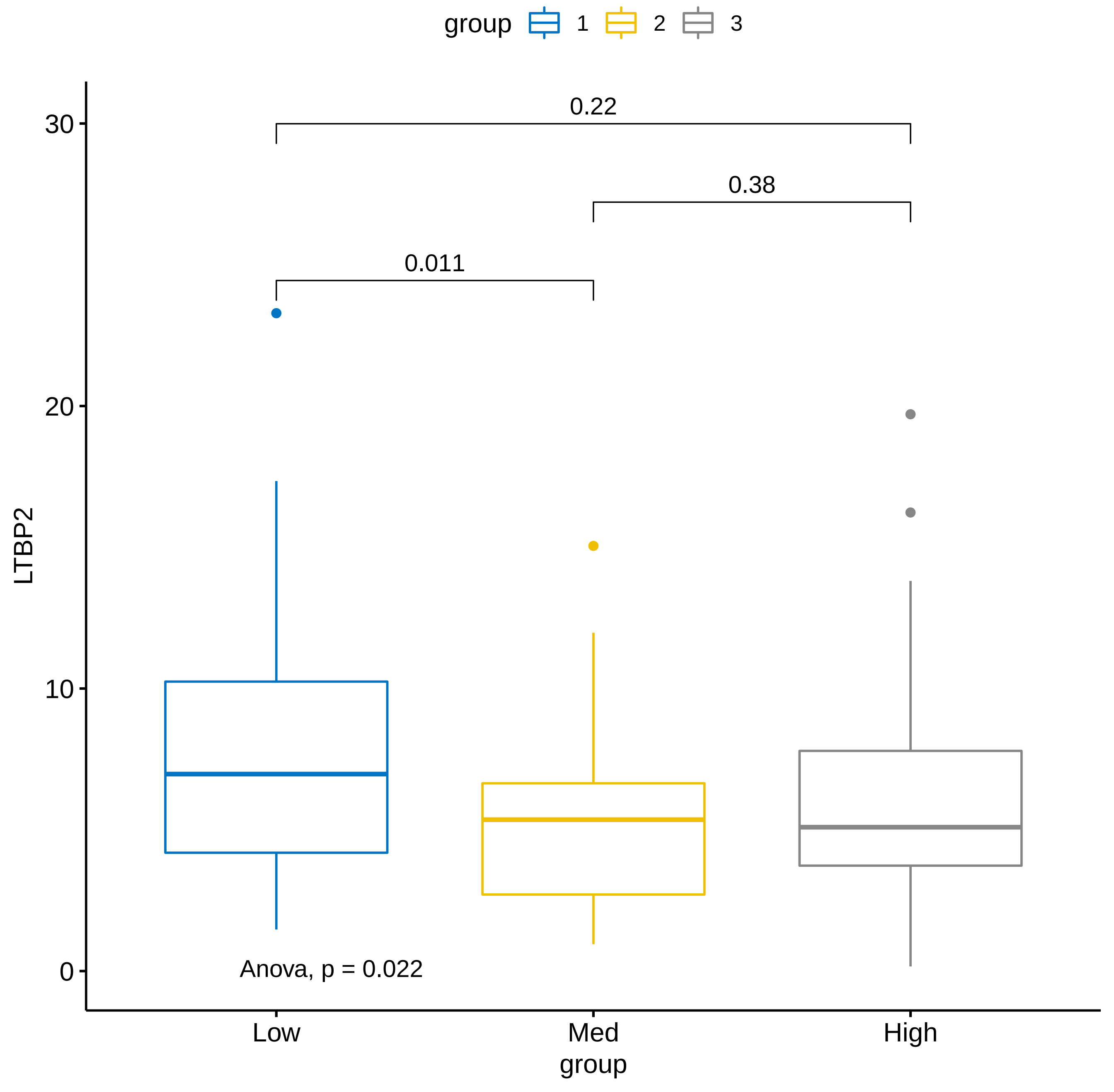
group
1
2
3
0.22
0.38
0.011
30
20
10
0
LTBP2
Anova, p = 0.022
Low
Med
High
group

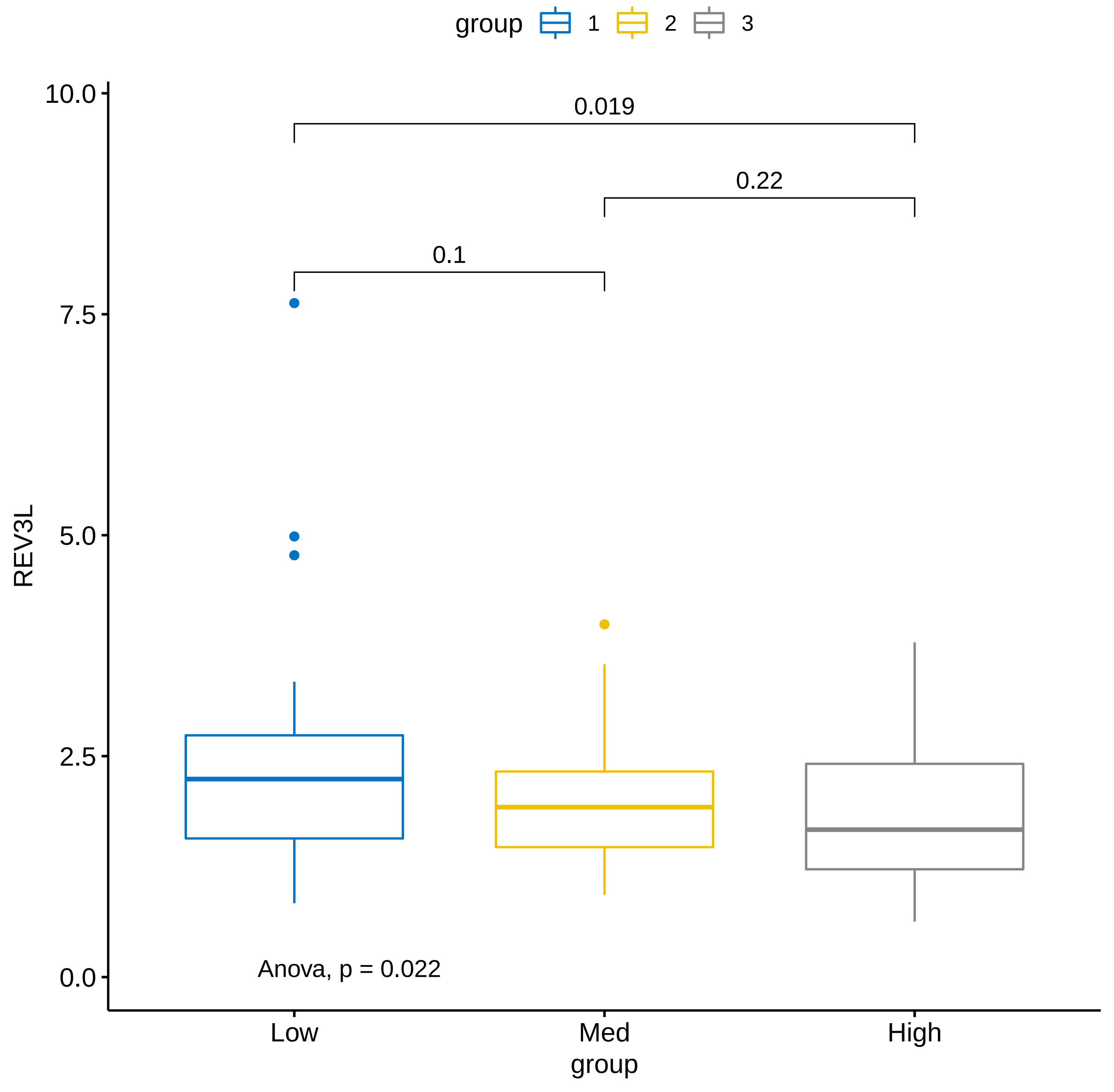
group
1
2
3
10.0
7.5
5.0
2.5
0.0
REV3L
0.019
0.22
0.1
Anova, p = 0.022
Low
Med
High
group

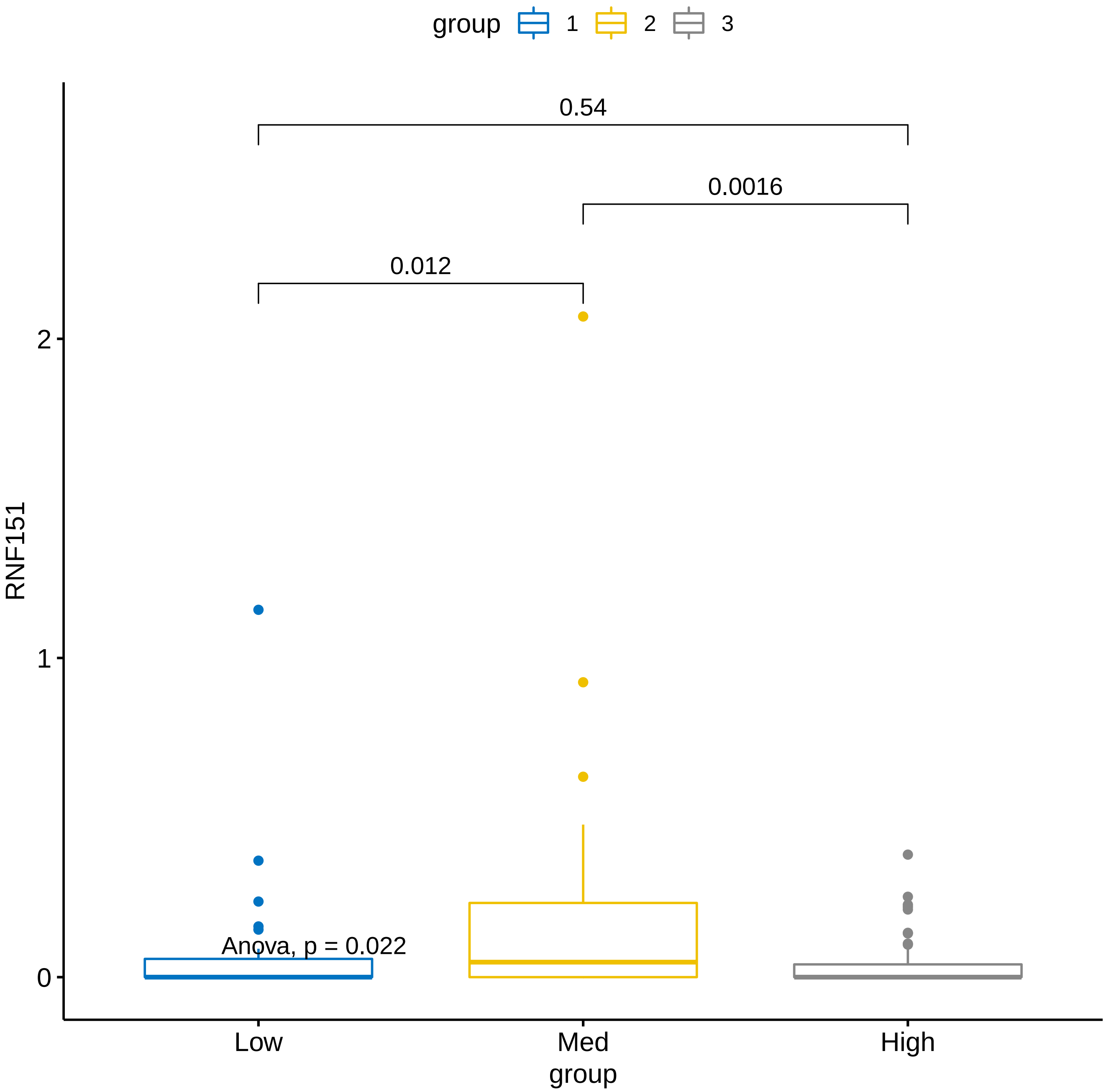
group
1
2
3
0.54
0.0016
0.012
RNF151
2
1
0
Anova, p = 0.022
Low
Med
High
group

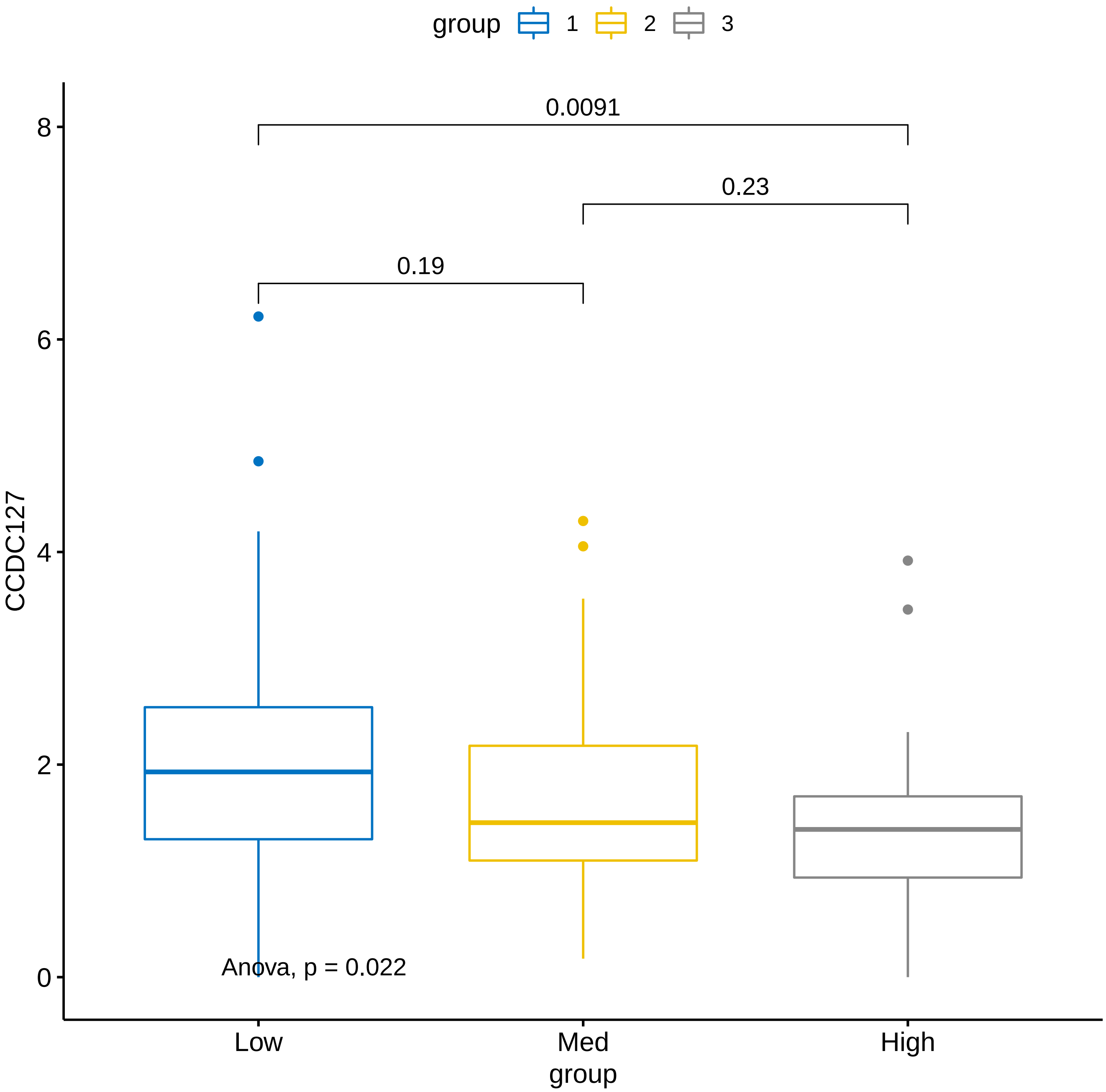
group
1
2
3
0.0091
0.23
0.19
8
6
4
2
0
CCDC127
Anova, p = 0.022
Low
Med
High
group

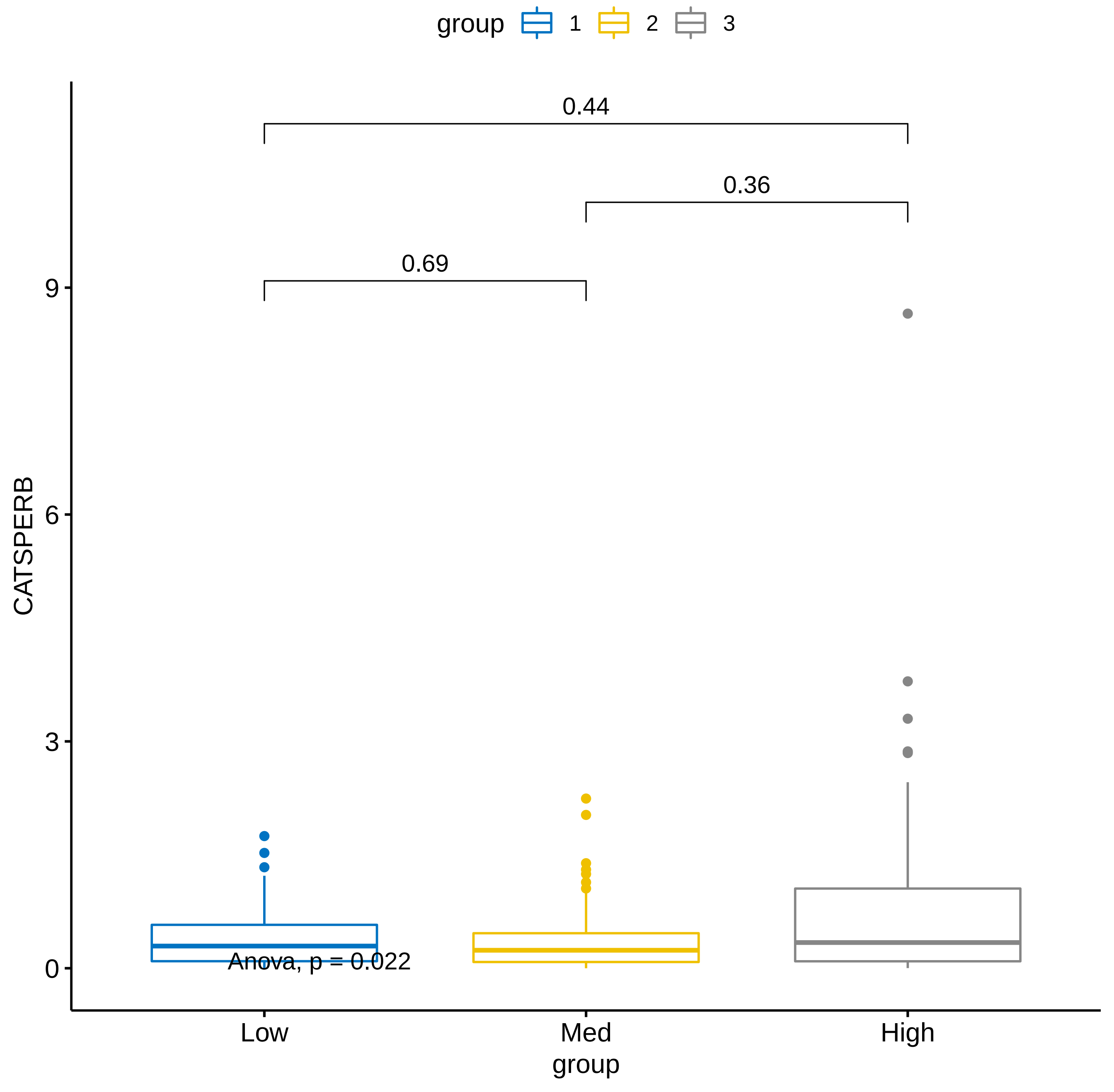

group 1 2 3
0.44
0.36
0.69
9
6
3
0
CATSPERB
Anova, p = 0.022
Low
Med
High
group

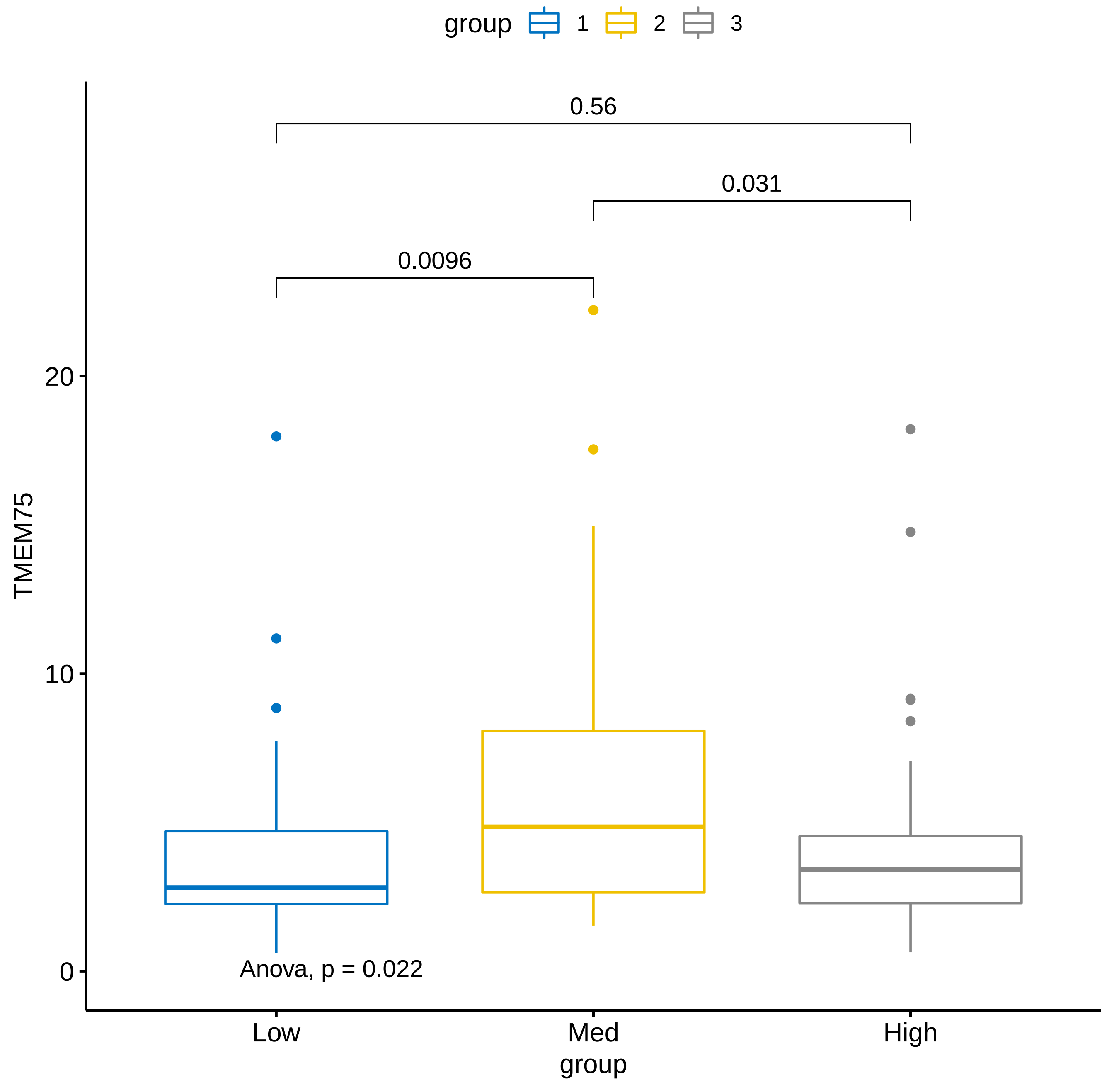

group 1 2 3
0.56
0.031
0.0096
20
10
0
TMEM75
Anova, p = 0.022
Low
Med
High
group

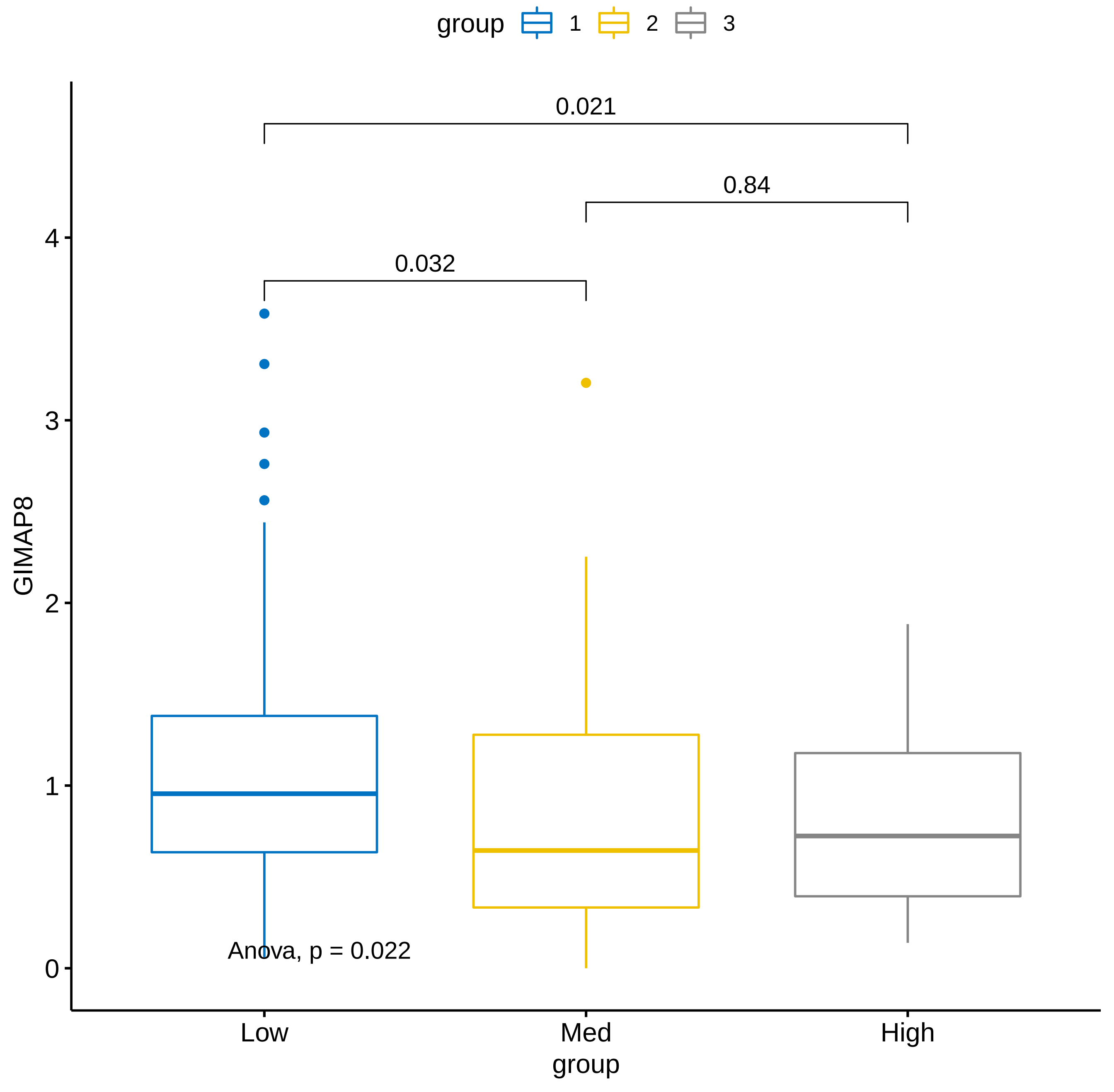

group
1
2
3
0.021
0.84
0.032
4
3
2
1
0
GIMAP8
Anova, p = 0.022
Low
Med
High
group

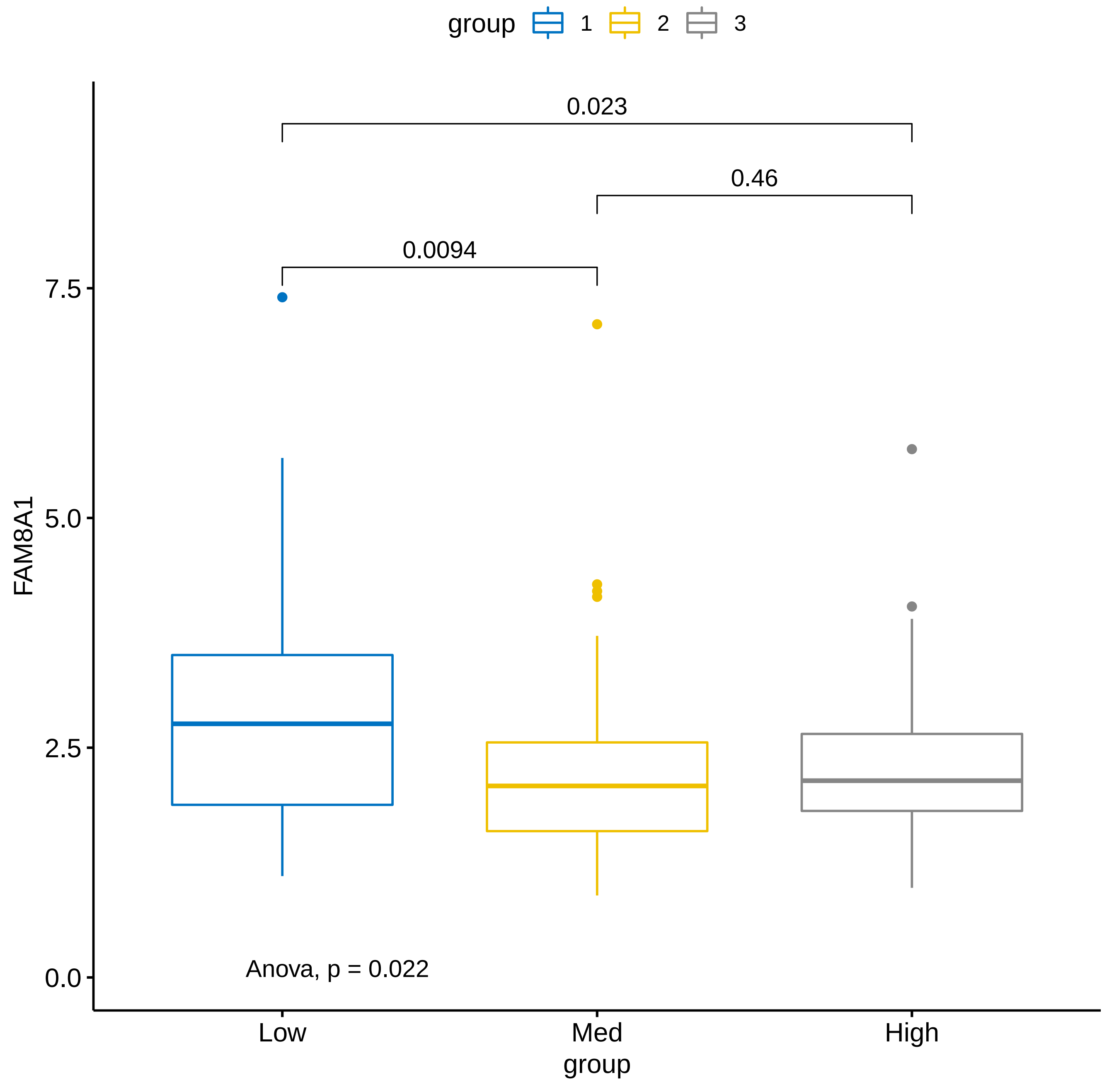

group
1
2
3
0.023
0.46
0.0094
7.5
5.0
2.5
0.0
FAM8A1
Anova, p = 0.022
Low
Med
High
group

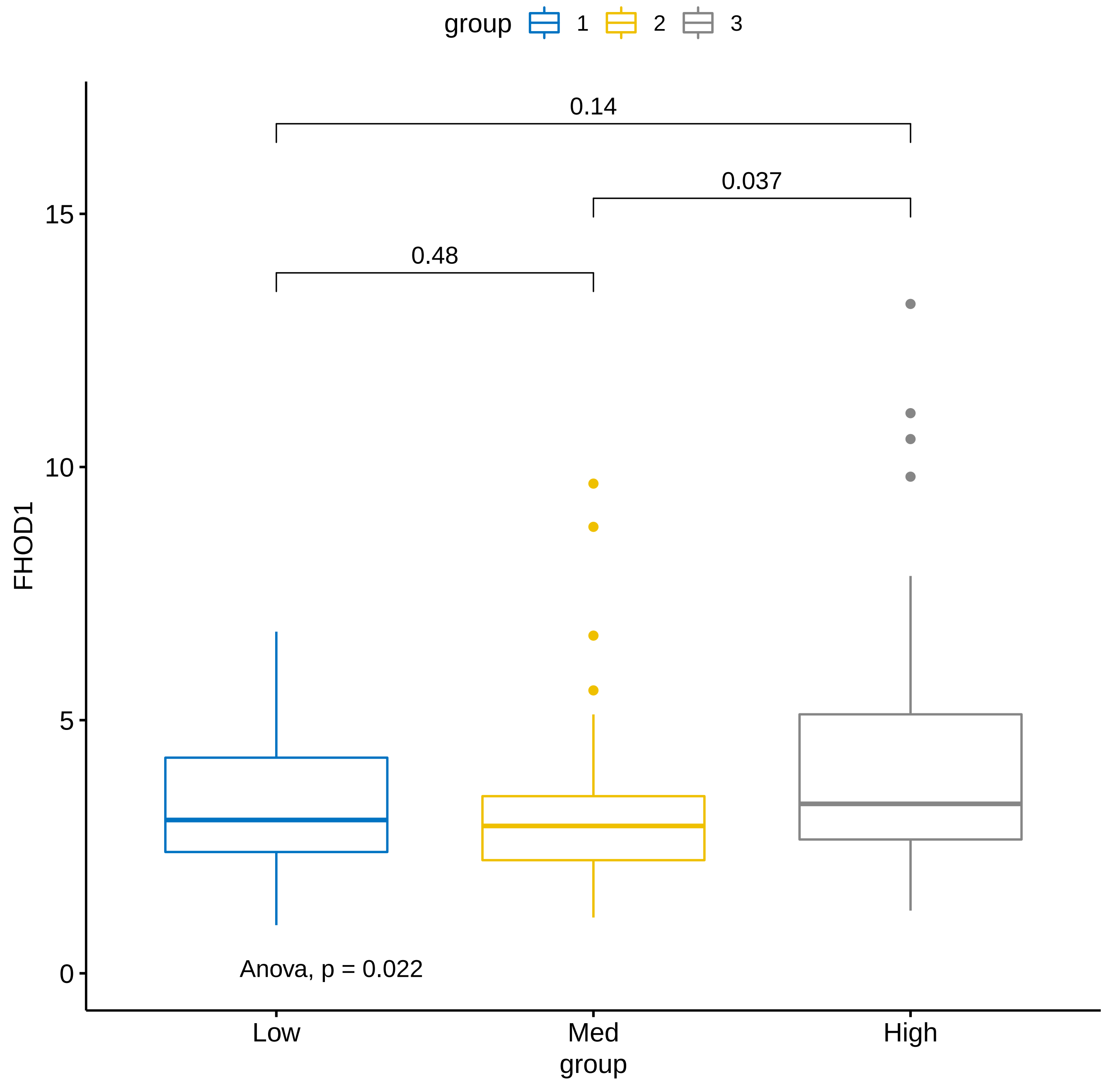

group
1
2
3
0.14
0.037
0.48
15
10
5
0
FHOD1
Anova, p = 0.022
Low
Med
High
group

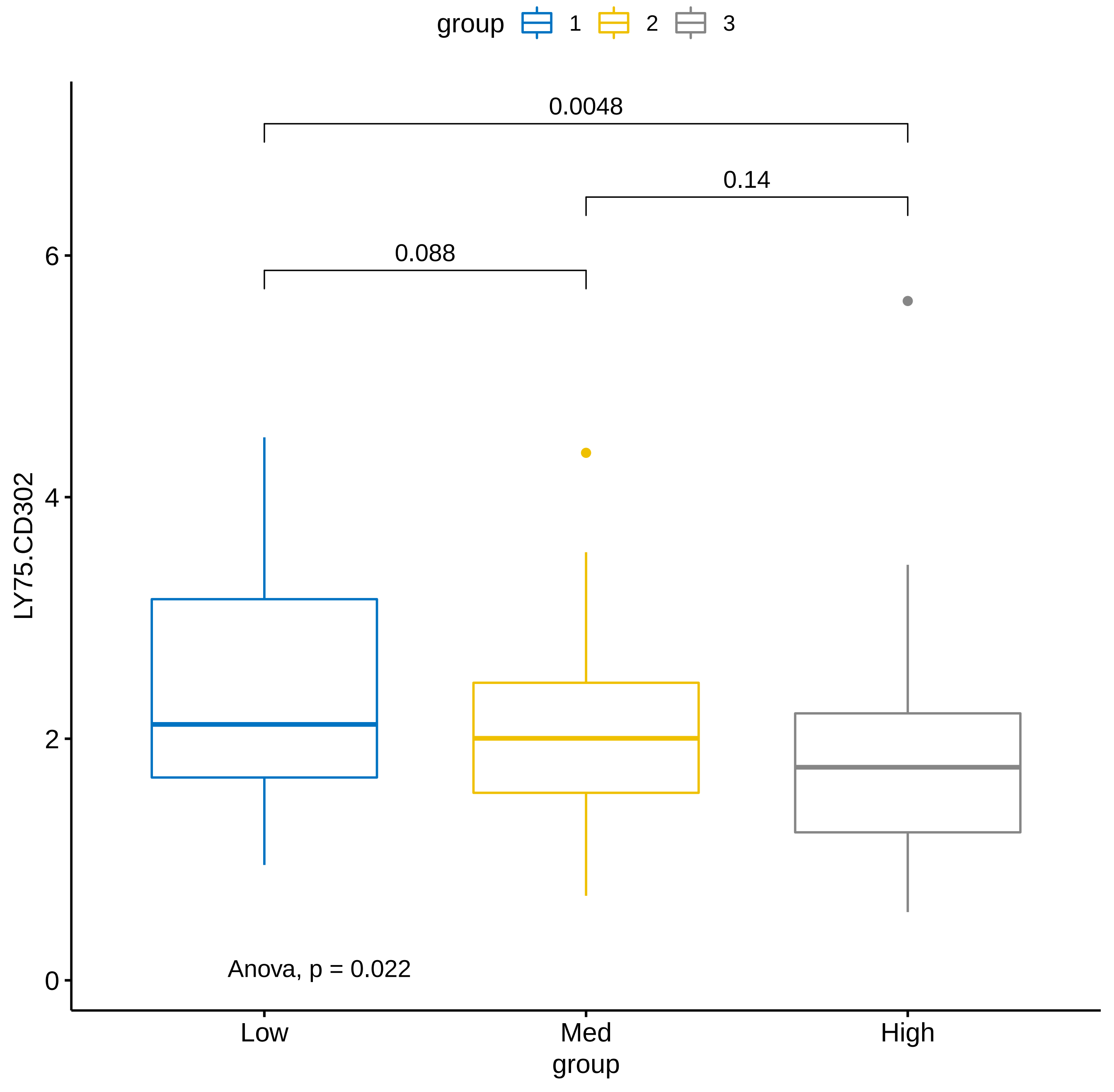
group
1
2
3
0.0048
0.14
0.088
LY75.CD302
6
4
2
0
Anova, p = 0.022
Low
Med
High
group

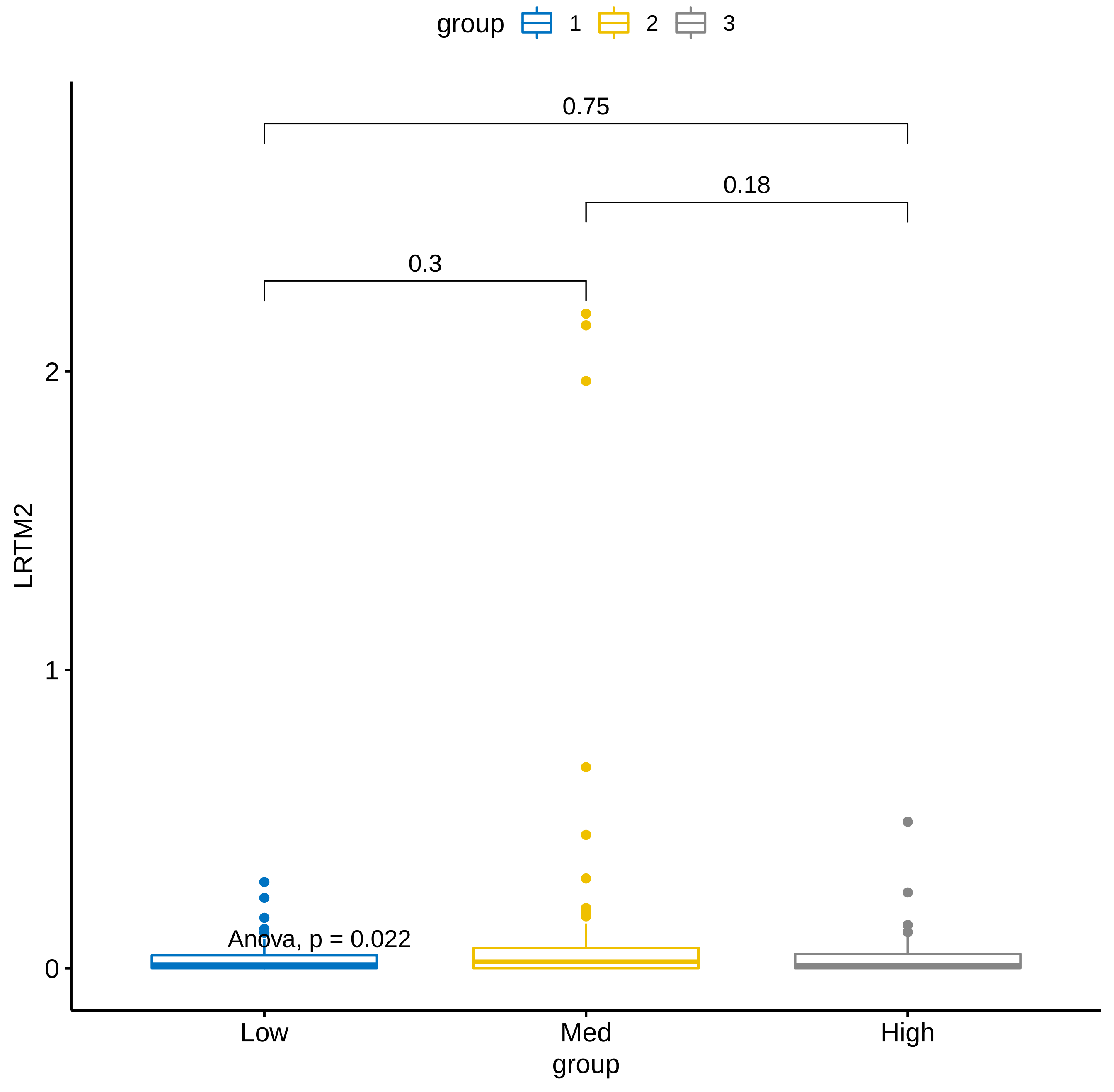

group
1
2
3
0.75
0.18
0.3
LRTM2
2
1
0
Anova, p = 0.022
Low
Med
High
group

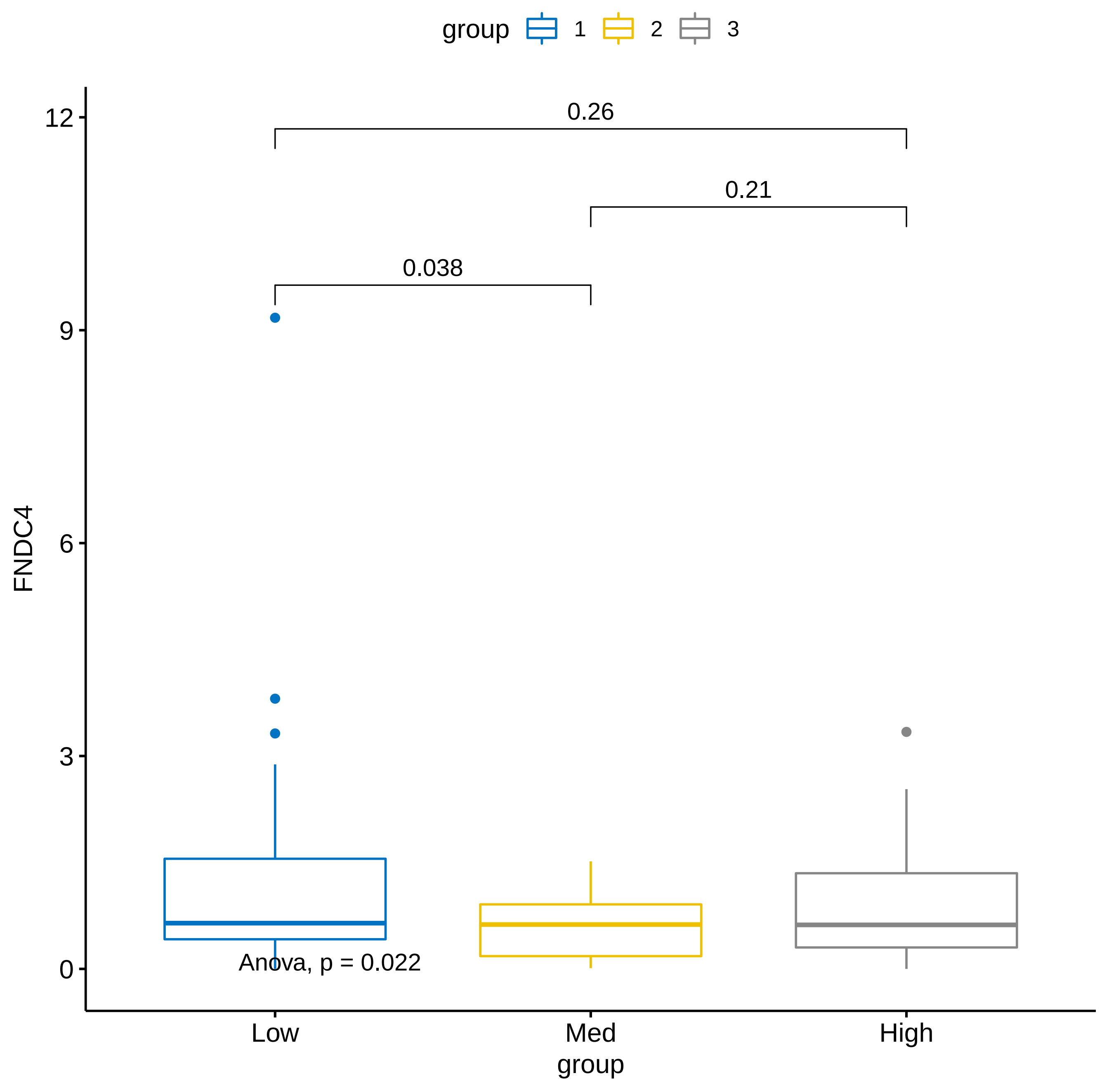

group
1
2
3
0.26
0.21
0.038
12
9
6
3
0
FNDC4
Anova, p = 0.022
Low
Med
High
group

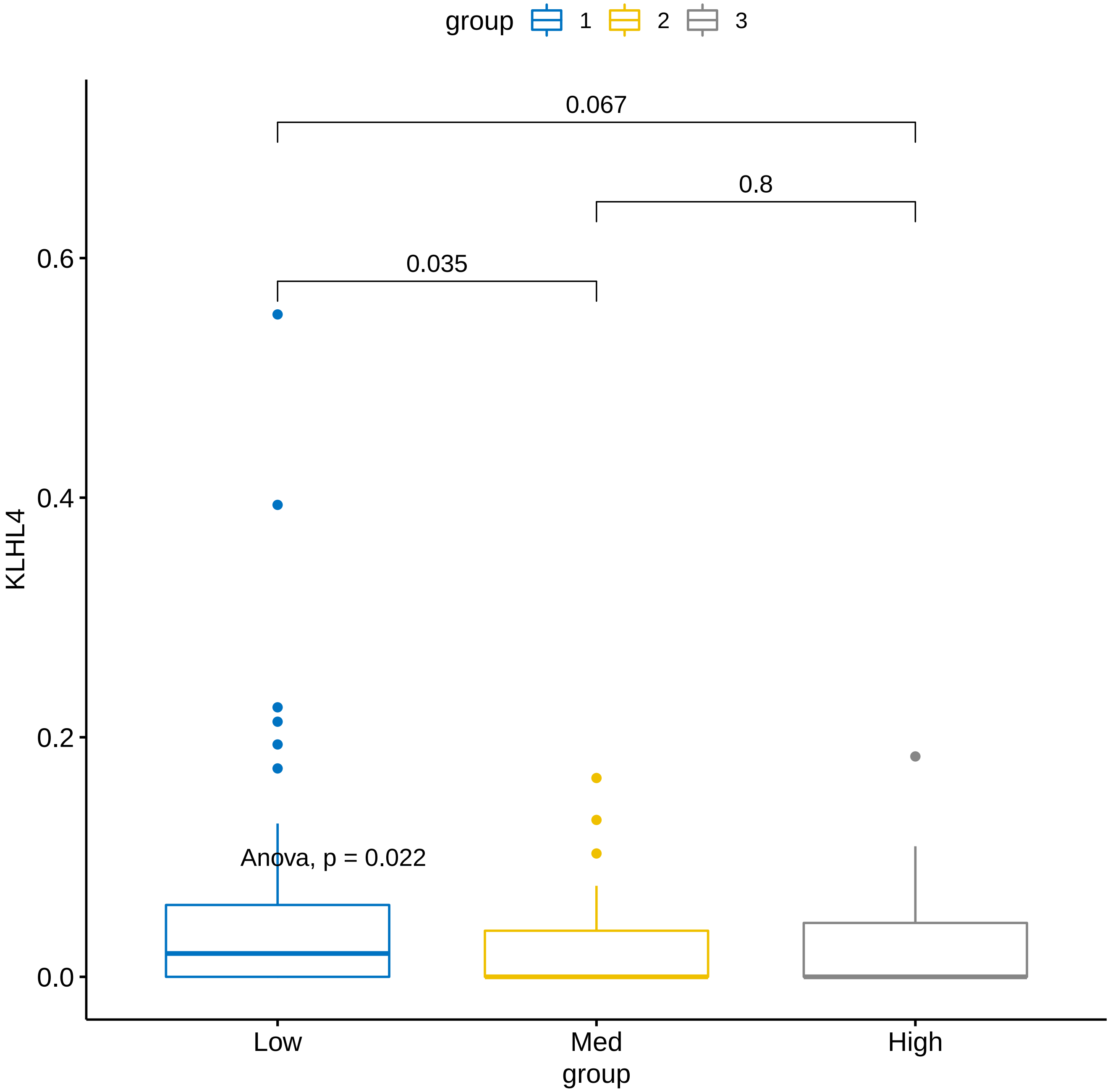

group 1 2 3
0.067
0.8
0.035
Anova, p = 0.022
KLHL4
0.6
0.4
0.2
0.0
Low
Med
High
group

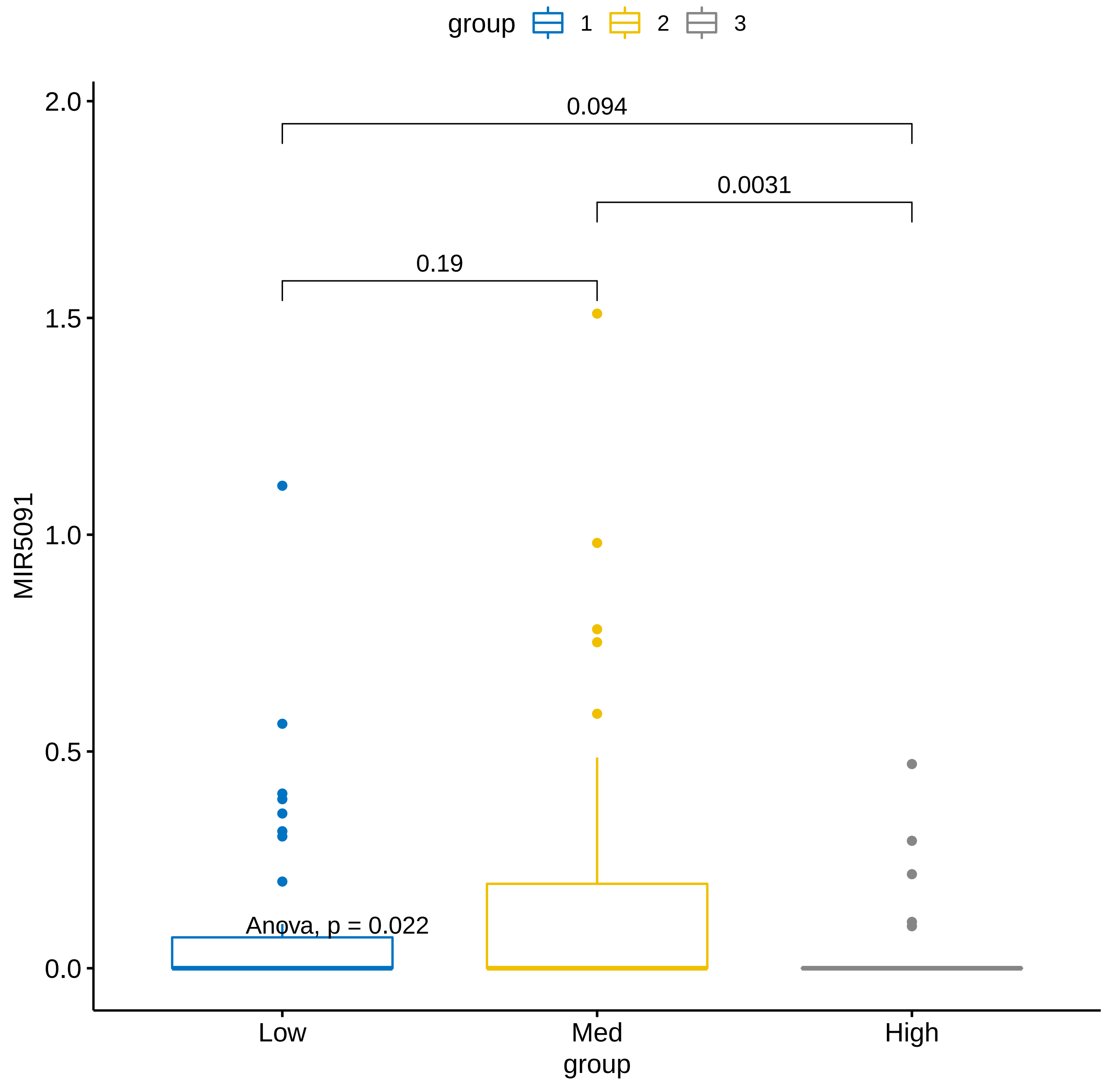

group 1 2 3
2.0
1.5
1.0
0.5
0.0
MIR5091
0.094
0.0031
0.19
Anova, p = 0.022
Low
Med
High
group

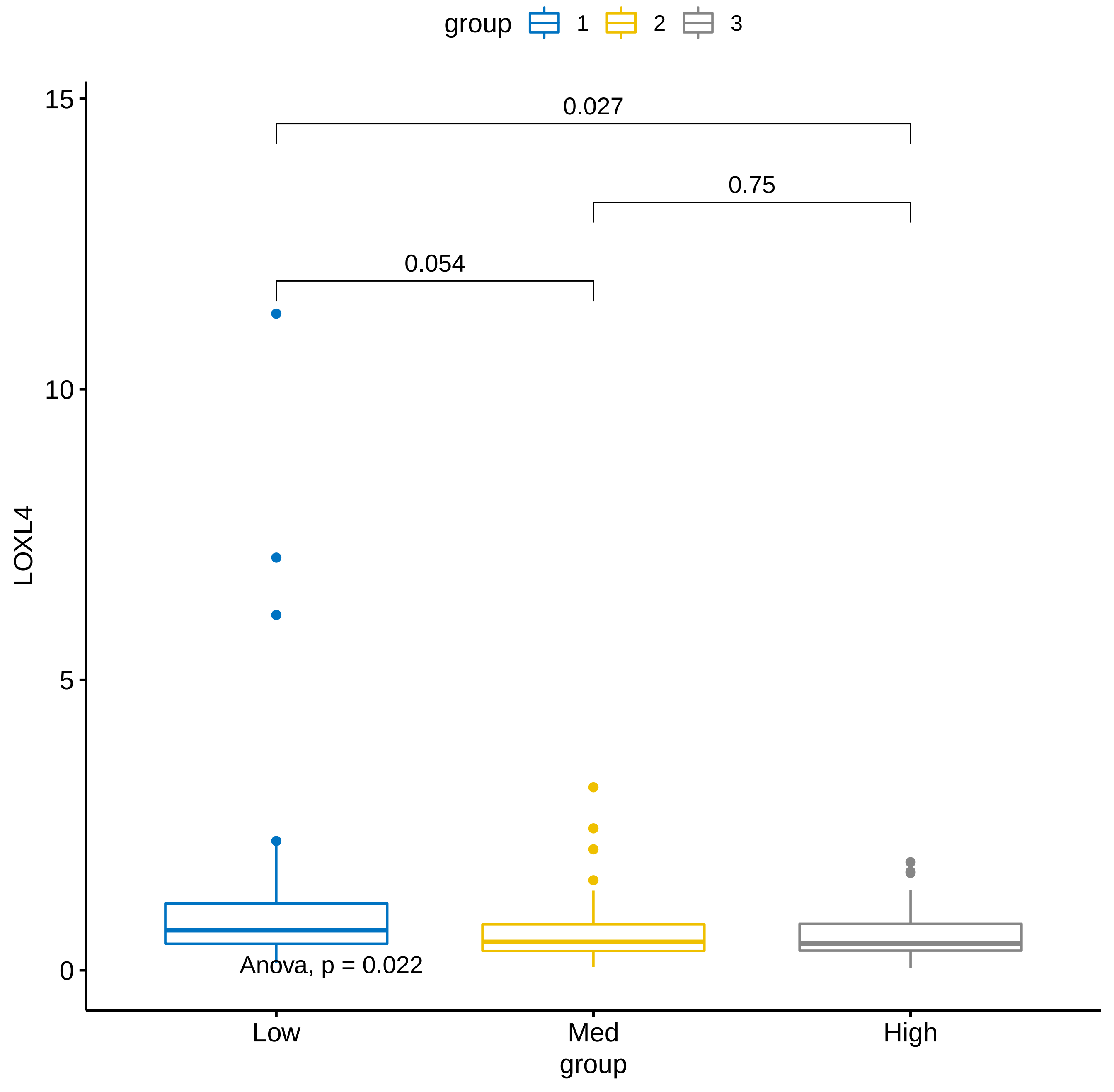

group
1
2
3
15
10
5
0
LOXL4
0.027
0.75
0.054
Anova, p = 0.022
Low
Med
High
group

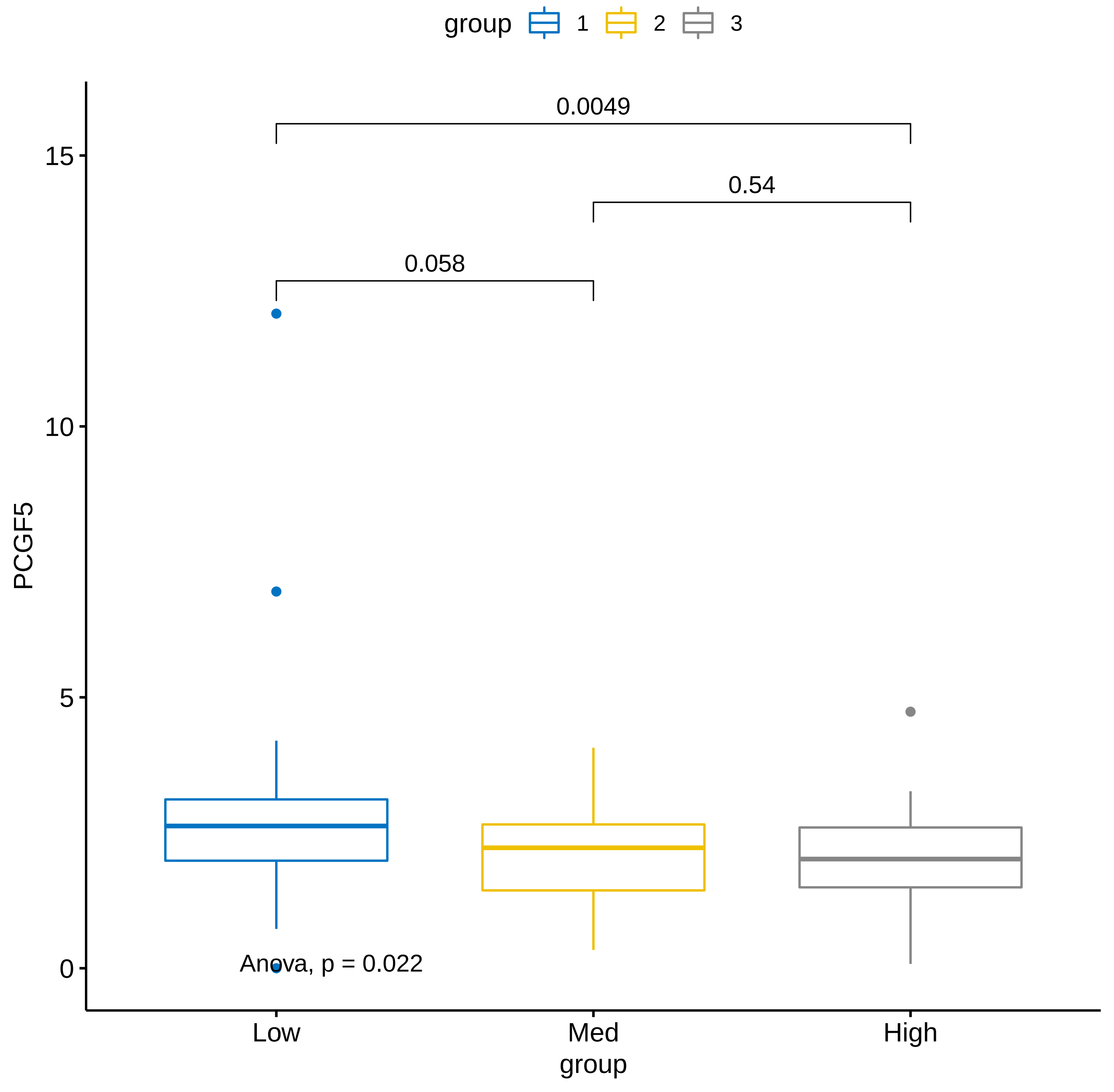
group
1
2
3
0.0049
0.54
0.058
15
10
5
0
PCGF5
Anova, p = 0.022
Low
Med
High
group

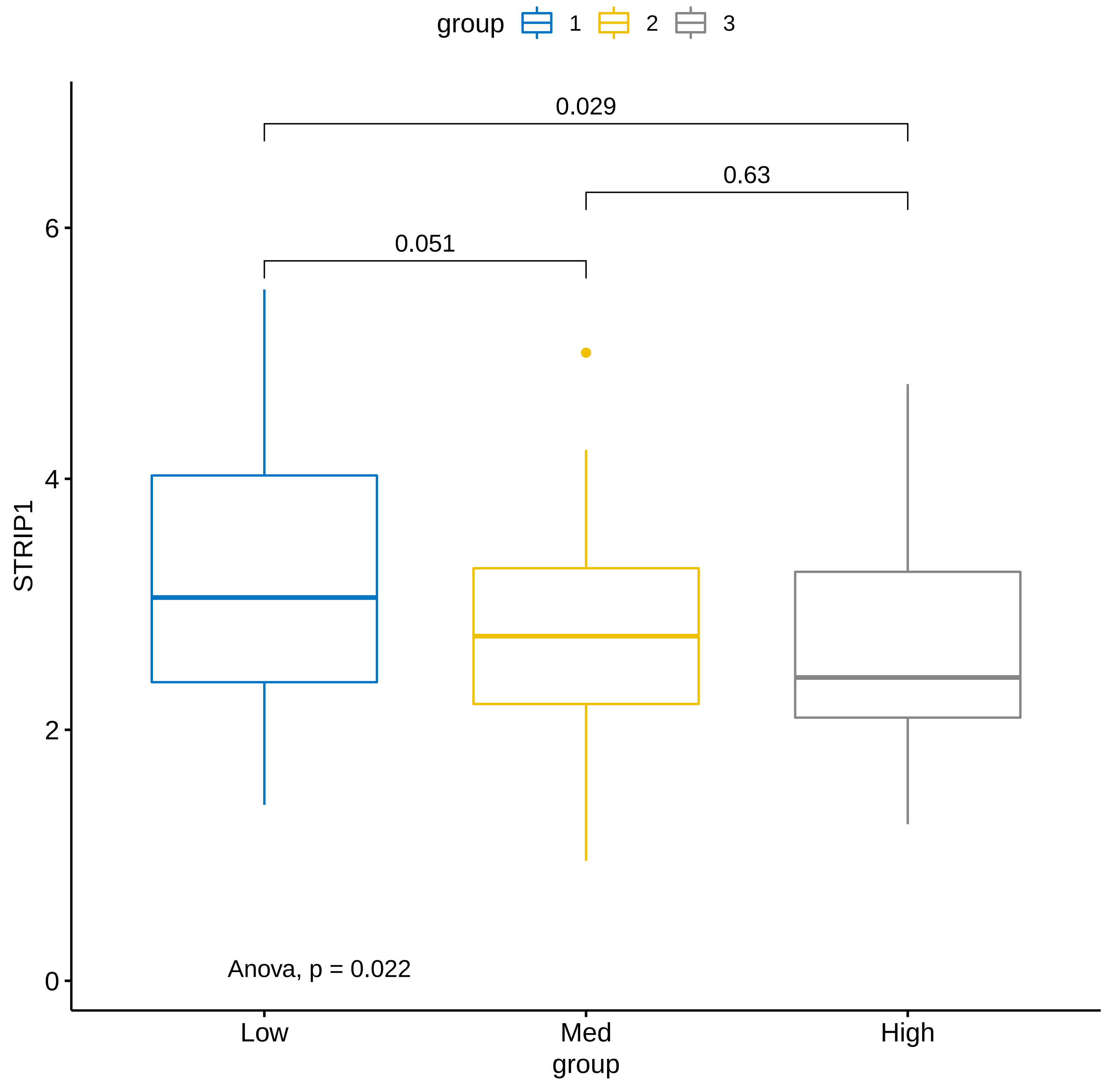
group
1
2
3
0.029
0.63
0.051
6
4
2
0
STRIP1
Anova, p = 0.022
Low
Med
High
group

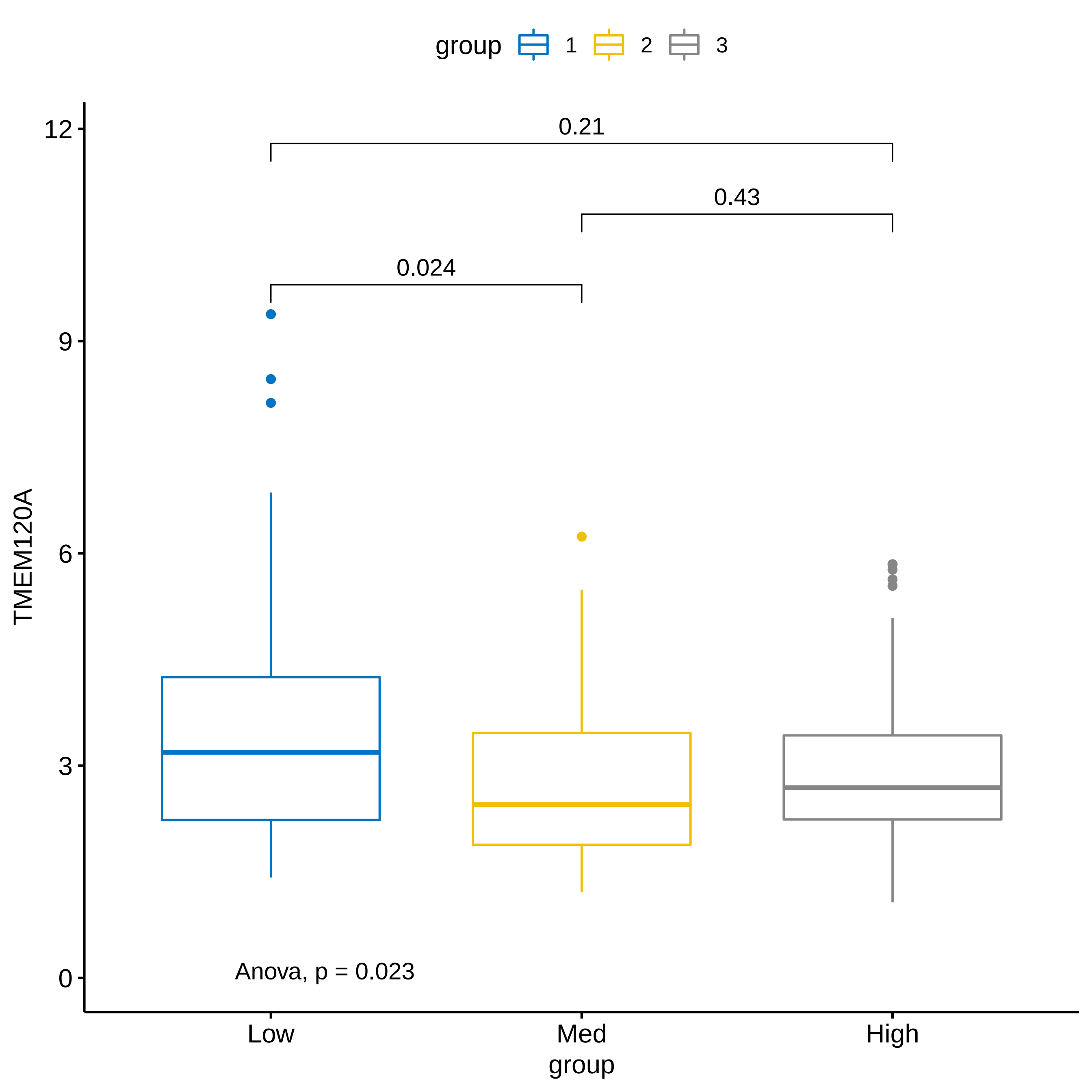

group 1 2 3
0.21
0.43
0.024
12
9
6
3
0
TMEM120A
Anova, p = 0.023
Low
Med
High
group

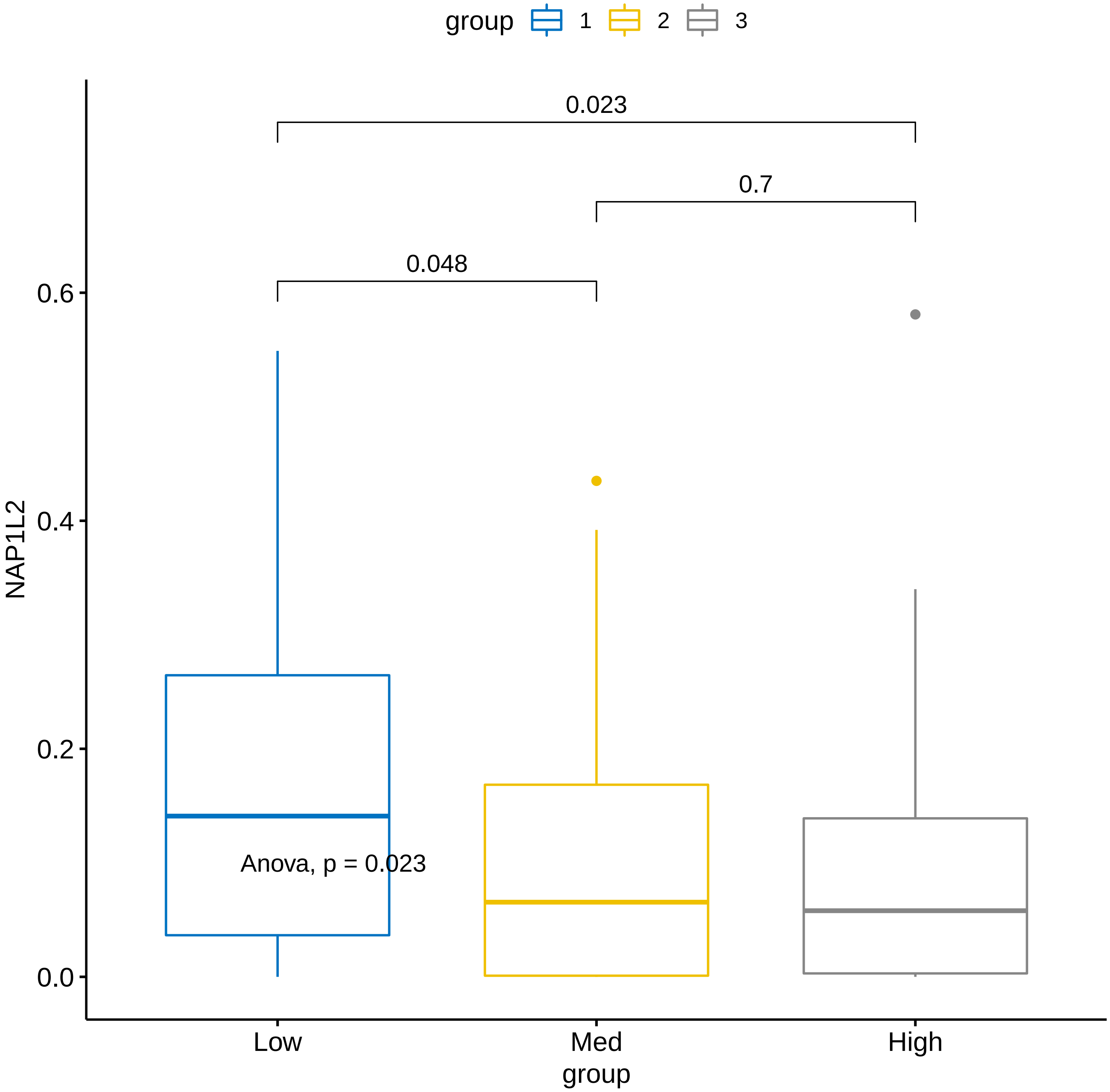
group
1
2
3
0.023
0.7
0.048
Anova, p = 0.023
NAP1L2
0.6
0.4
0.2
0.0
Low
Med
High
group

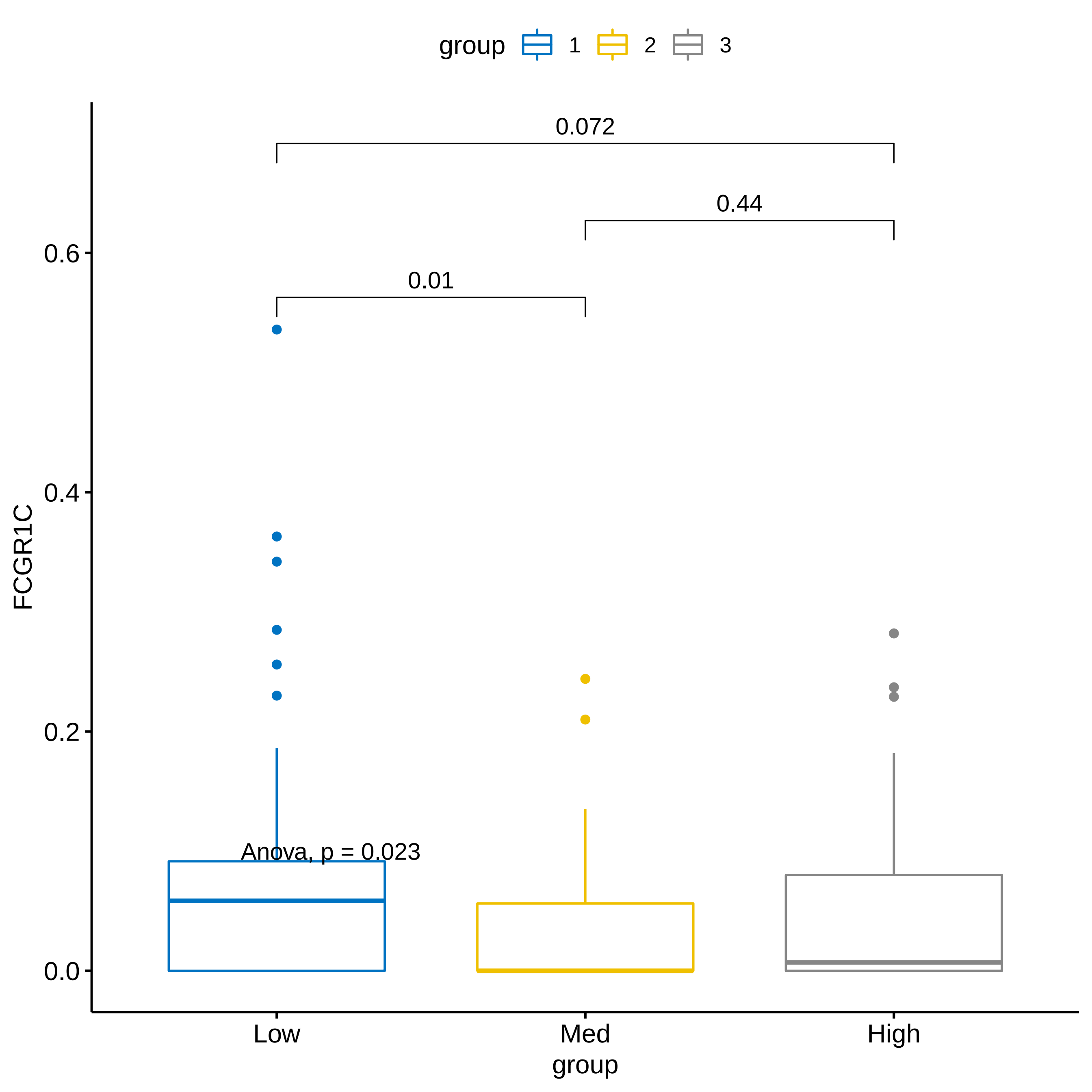
group
1
2
3
0.072
0.44
0.01
0.6
0.4
0.2
0.0
FCGR1C
Anova, p = 0.023
Low
Med
High
group

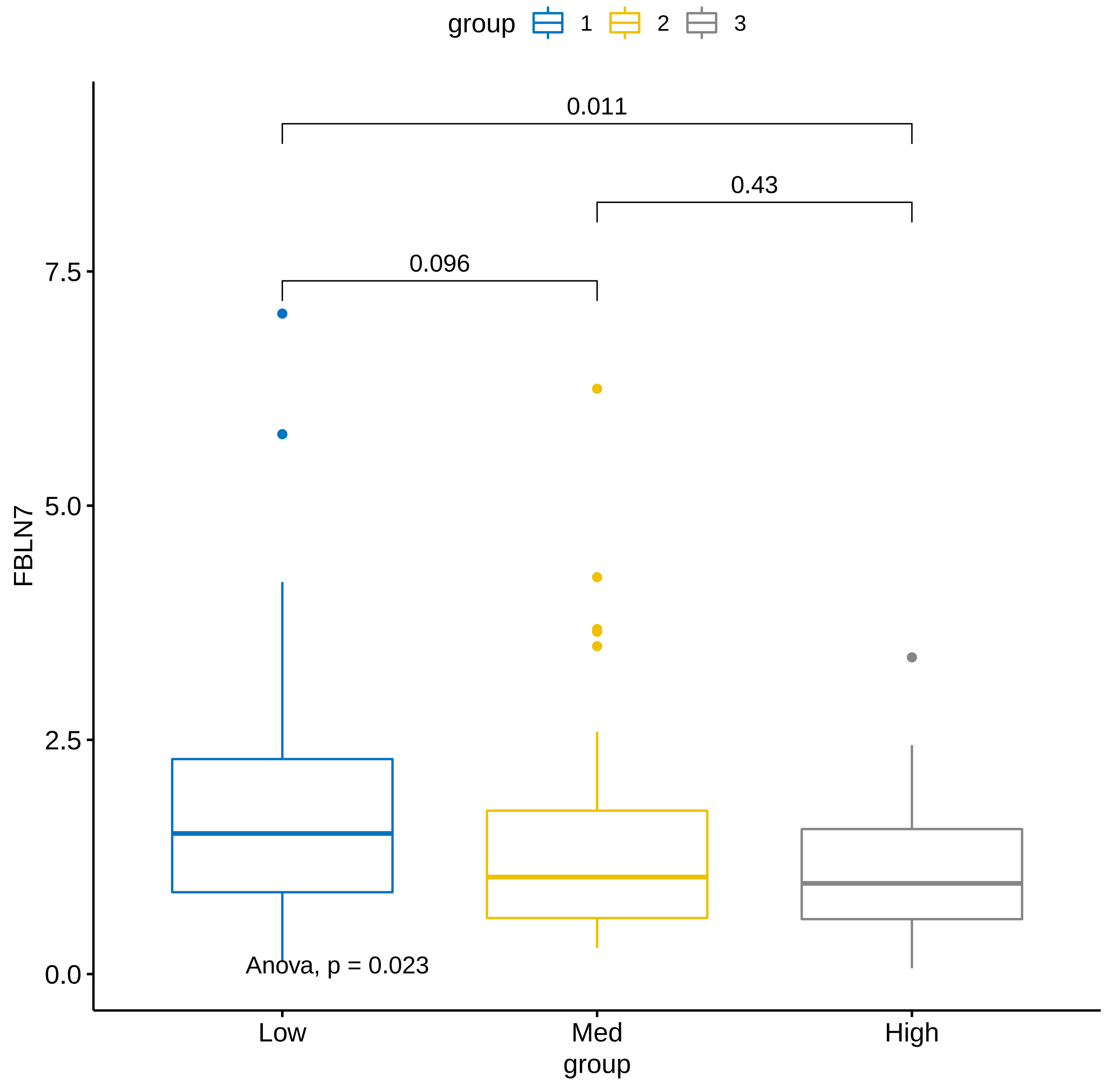
group
1
2
3
0.011
0.43
0.096
7.5
5.0
2.5
0.0
FBLN7
Anova, p = 0.023
Low
Med
High
group

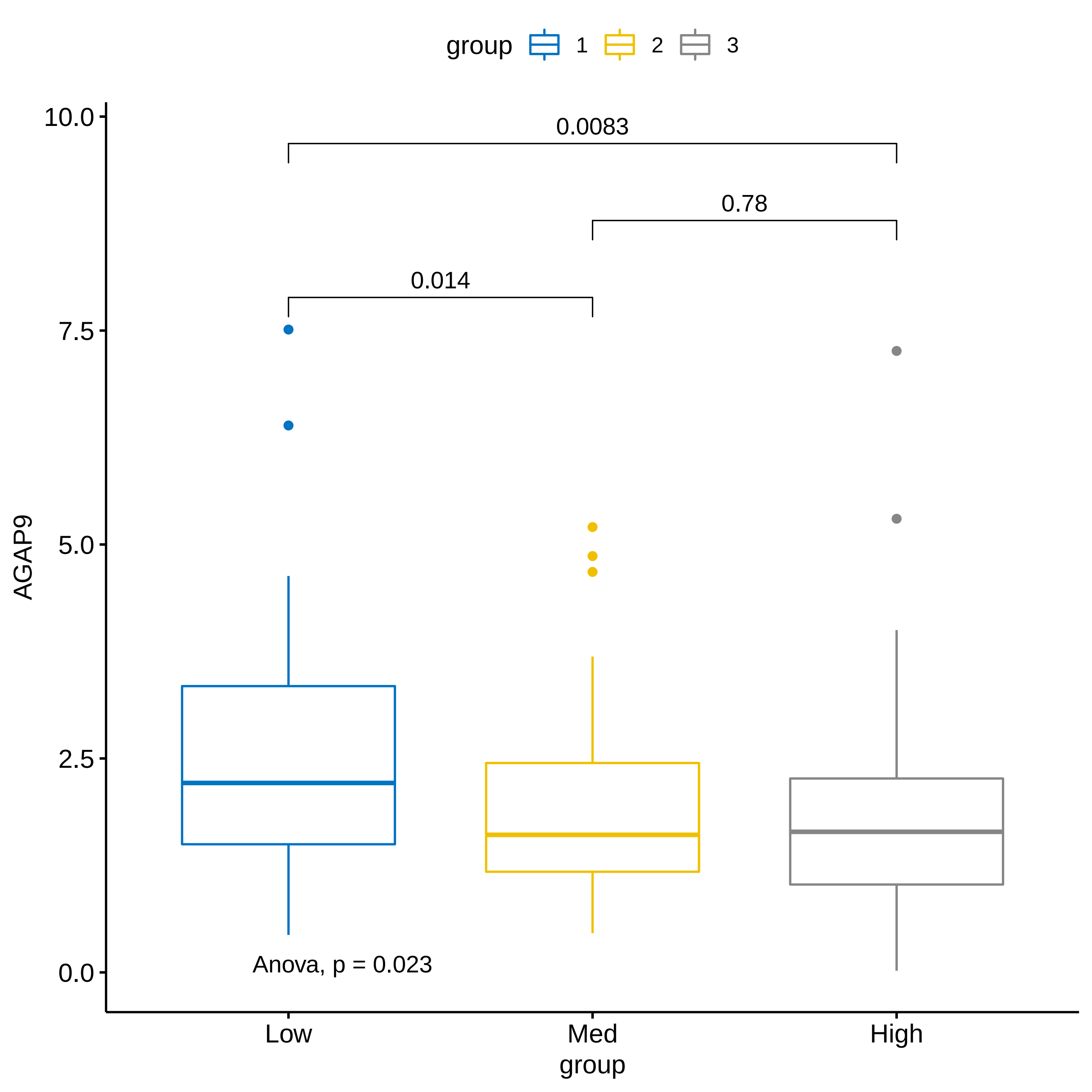
group 1 2 3
10.0
7.5
5.0
2.5
0.0
AGAP9
0.0083
0.78
0.014
Anova, p = 0.023
Low
Med
High
group

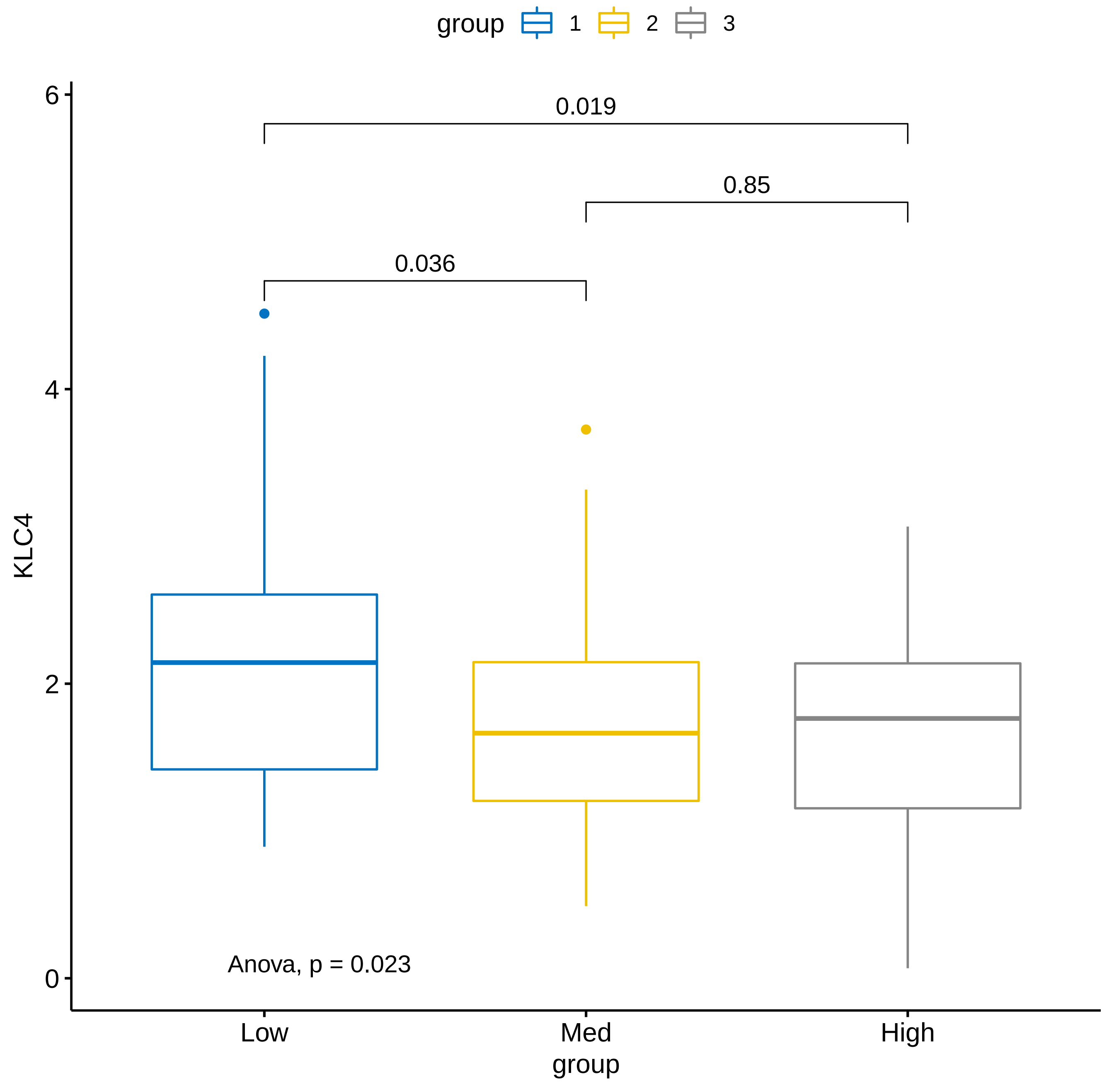

group 1 2 3
0.019
0.85
0.036
KLC4
6
4
2
0
Anova, p = 0.023
Low
Med
High
group

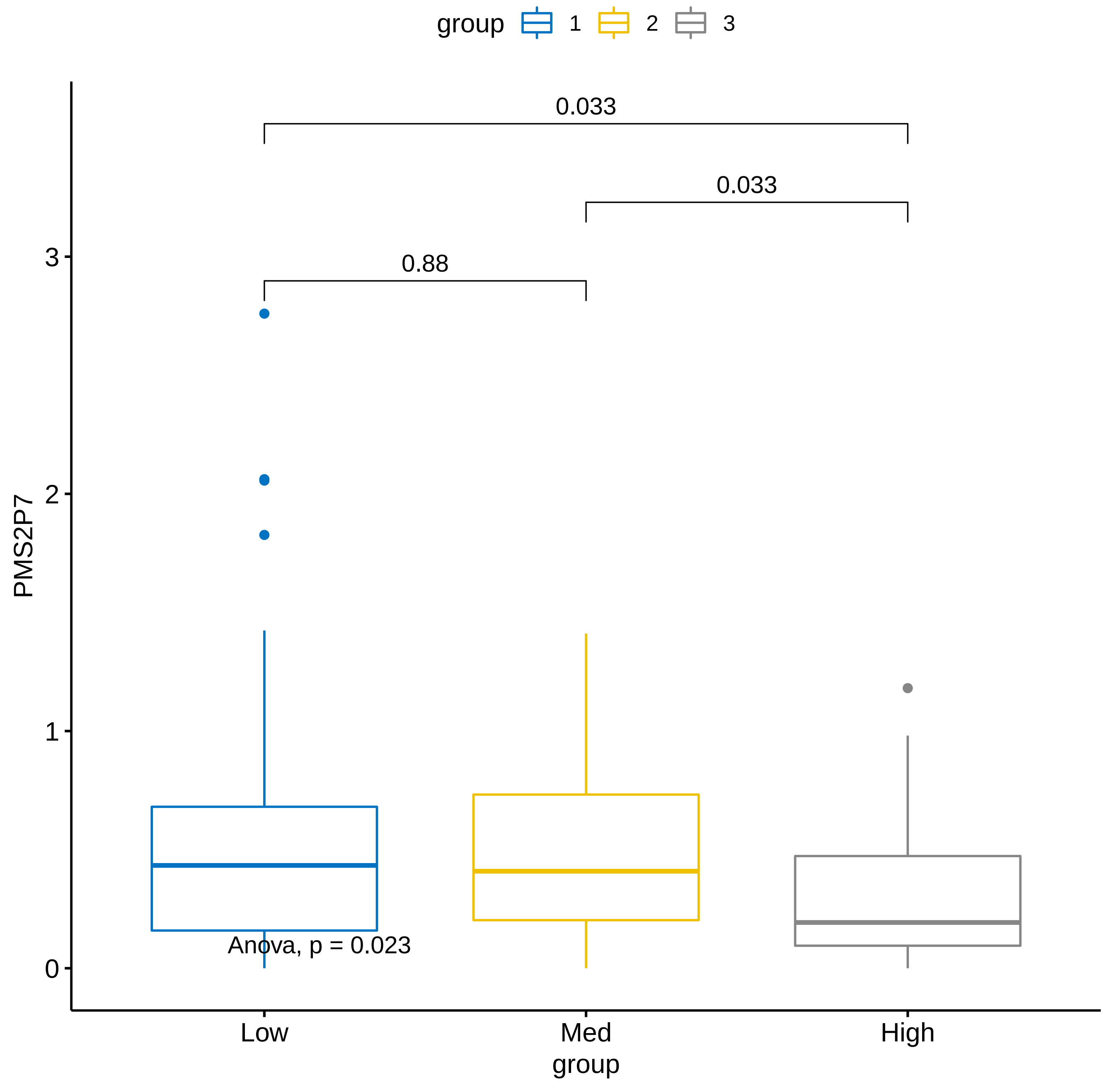

group 1 2 3
0.033
0.033
0.88
3
2
1
0
PMS2P7
Anova, p = 0.023
Low
Med
High
group

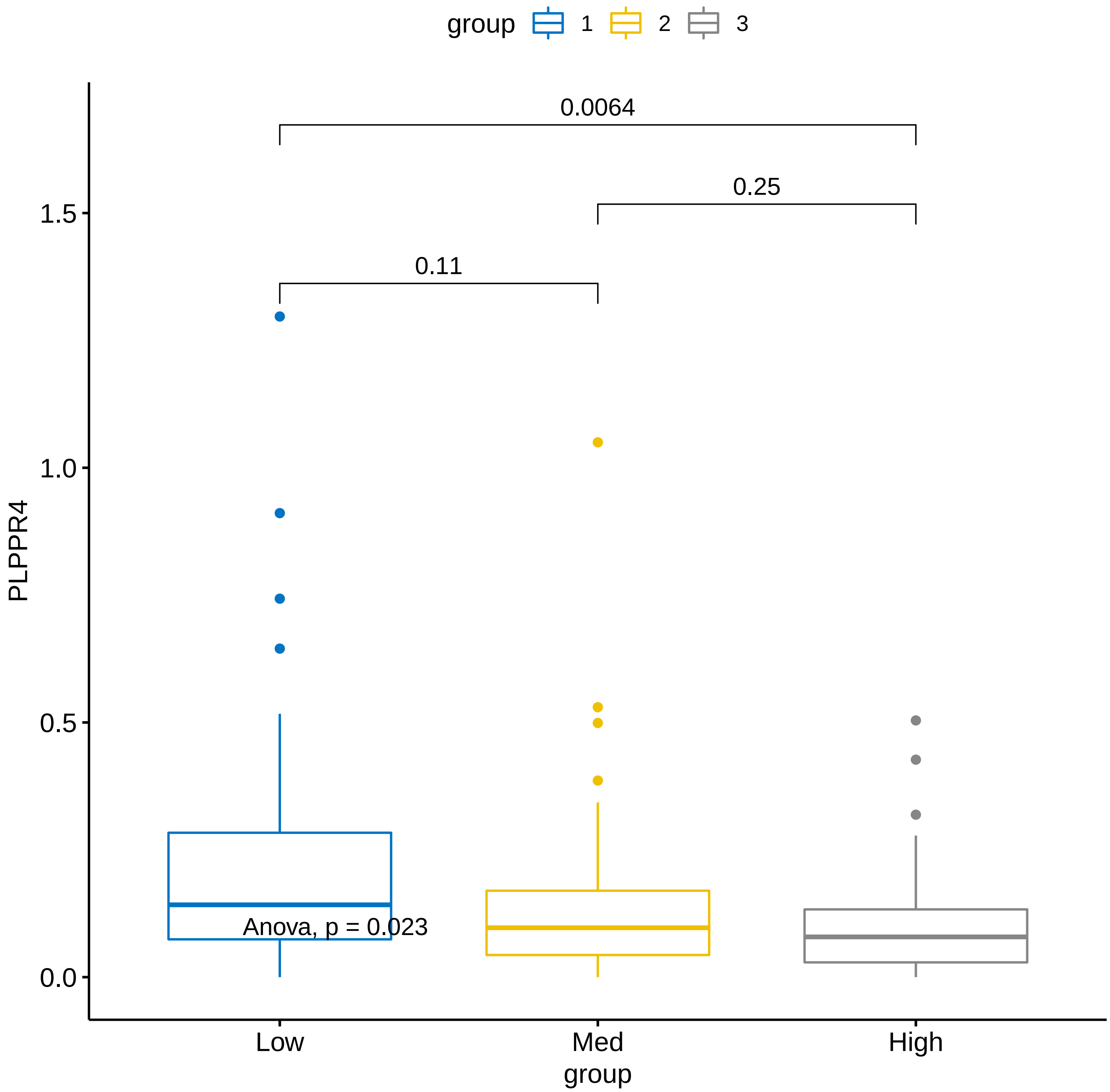

group 1 2 3
0.0064
0.25
0.11
1.5
1.0
0.5
0.0
PLPPR4
Anova, p = 0.023
Low
Med
High
group

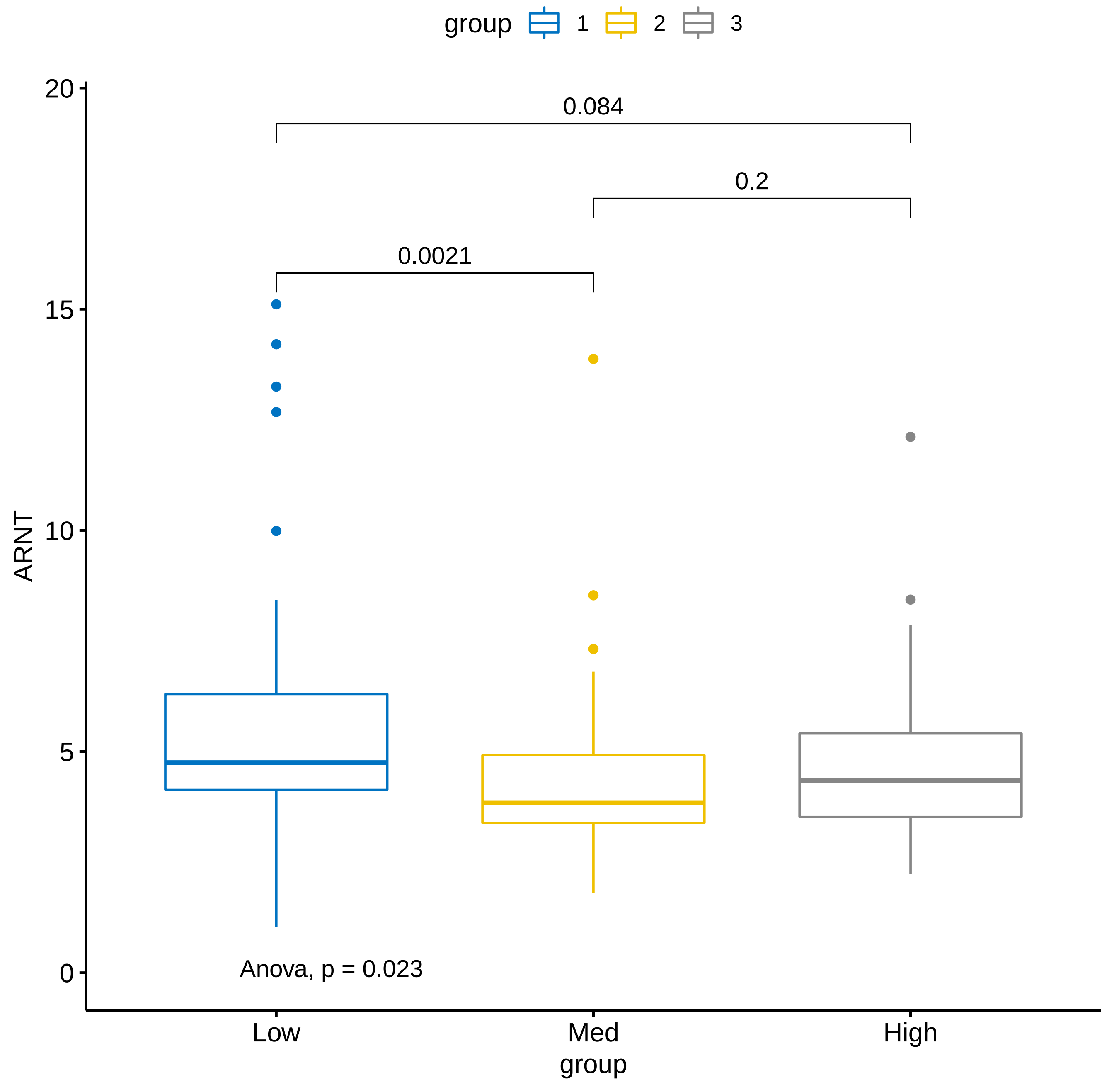

group 1 2 3
20
15
10
5
0
ARNT
0.084
0.2
0.0021
Anova, p = 0.023
Low
Med
High
group

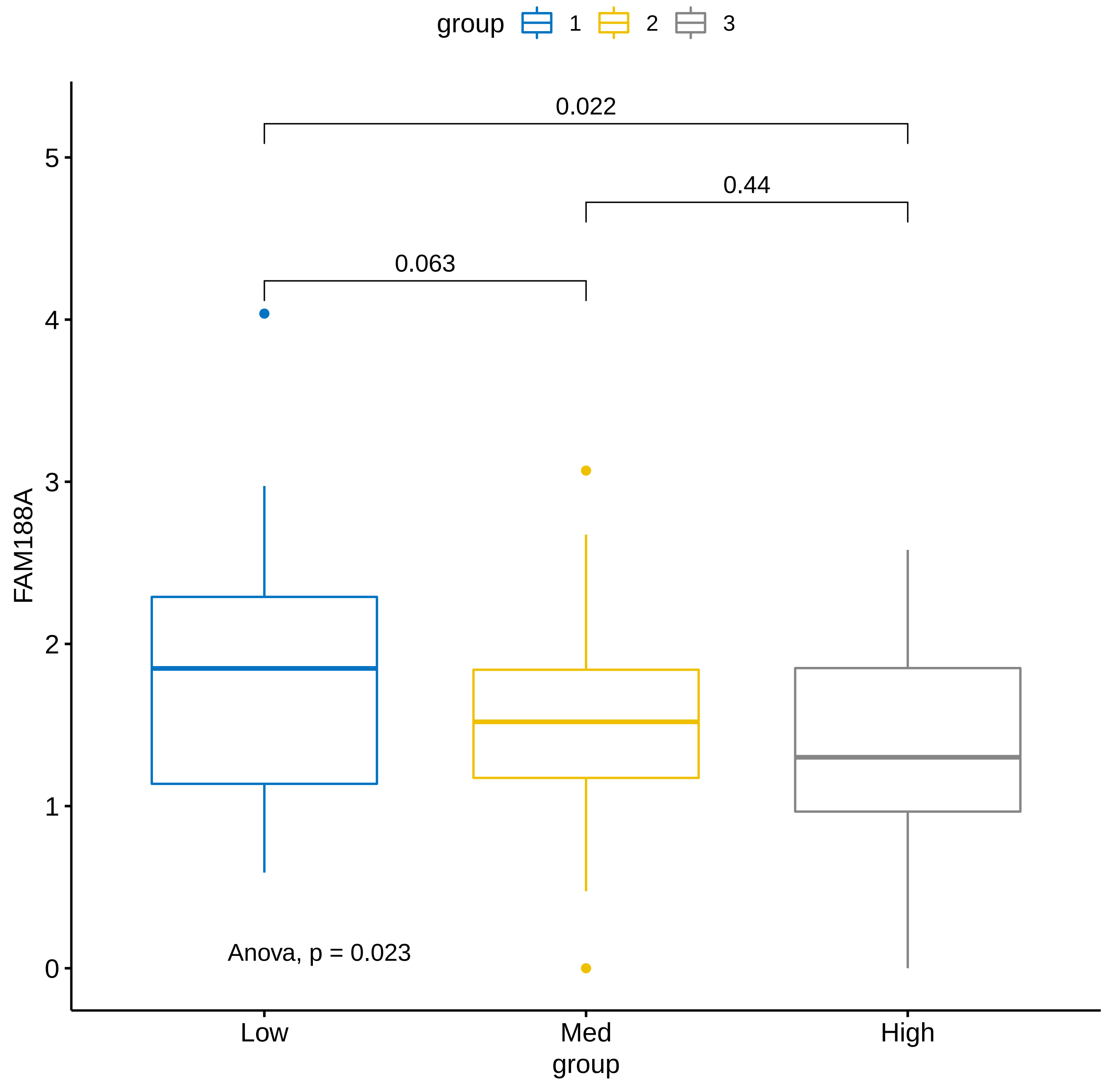
group 1 2 3
0.022
0.44
0.063
FAM188A
0
1
2
3
4
5
Anova, p = 0.023
Low
Med
High
group

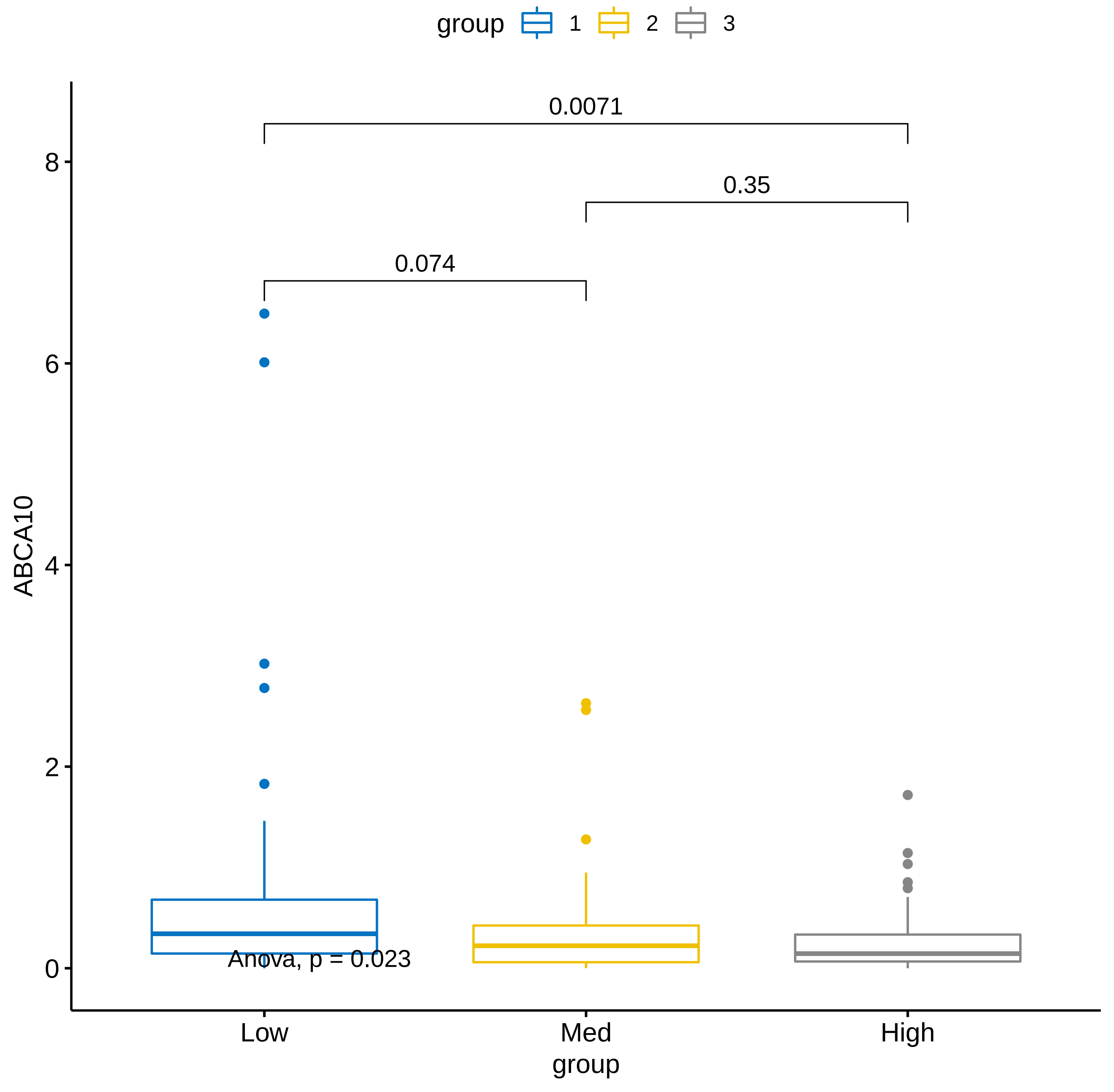

group
1
2
3
0.0071
0.35
0.074
ABCA10
8
6
4
2
0
Anova, p = 0.023
Low
Med
High
group

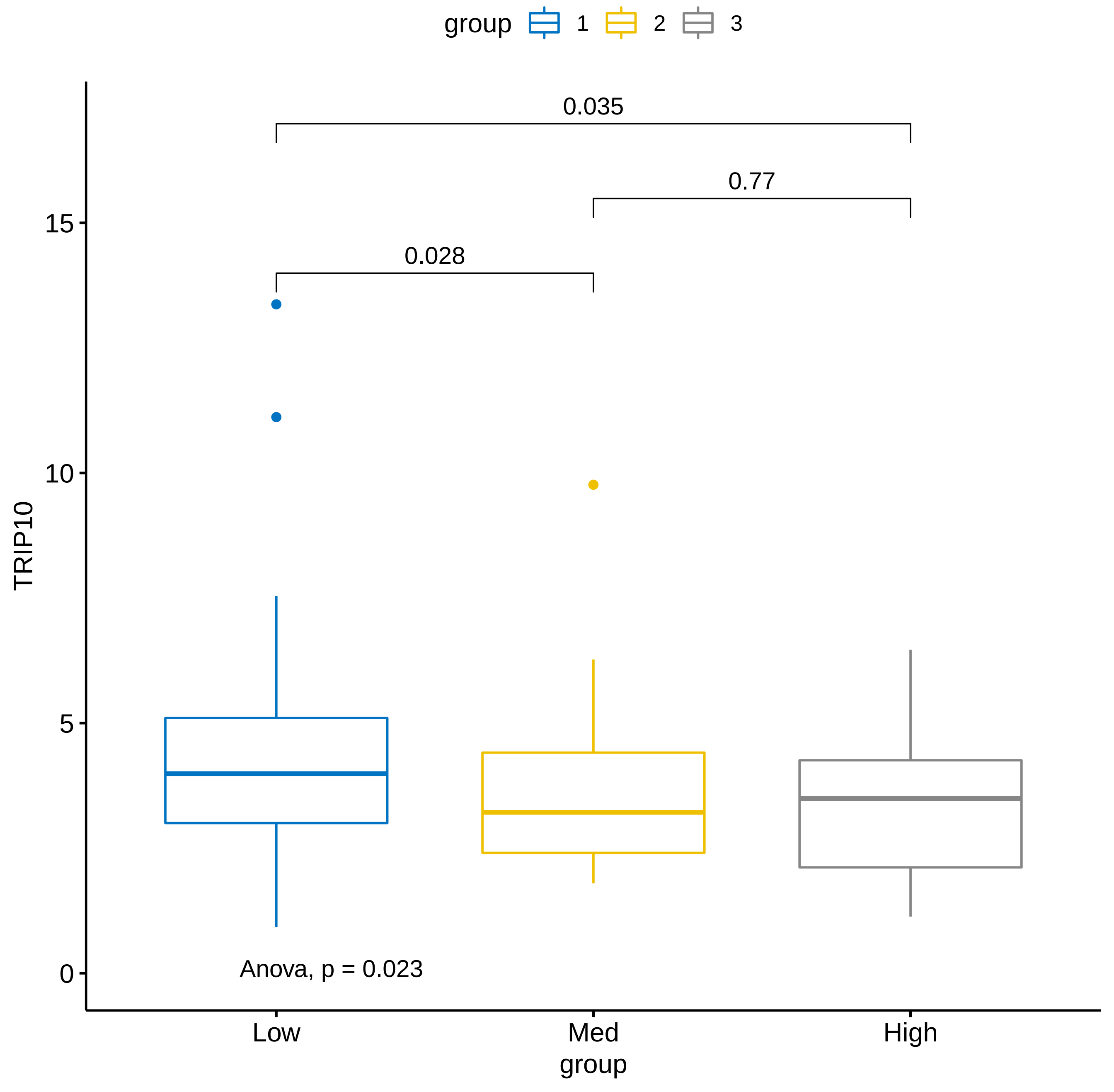

group
1
2
3
0.035
0.77
0.028
TRIP10
15
10
5
0
Anova, p = 0.023
Low
Med
High
group

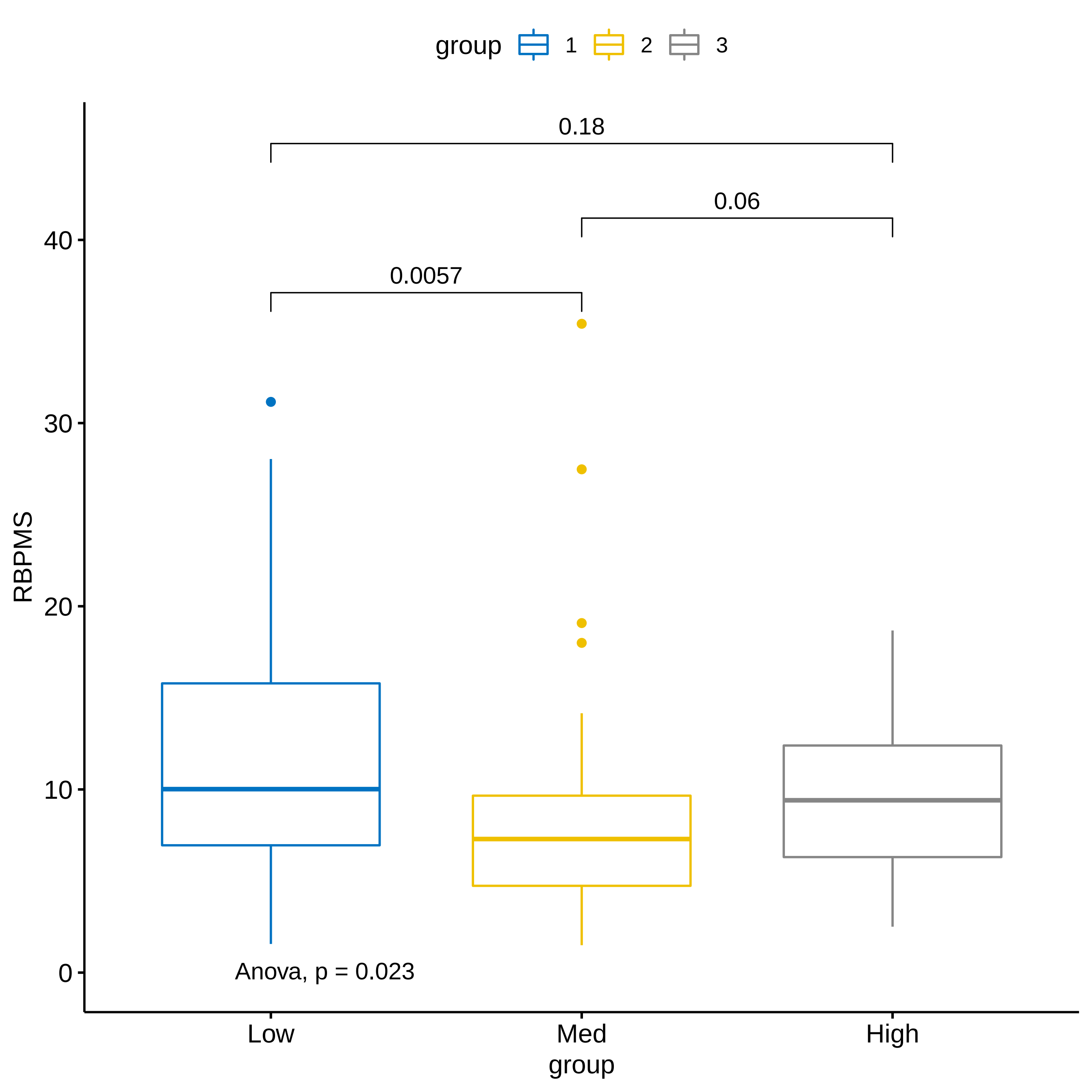
group
1
2
3
0.18
0.06
0.0057
40
30
20
10
0
RBPMS
Anova, p = 0.023
Low
Med
High
group

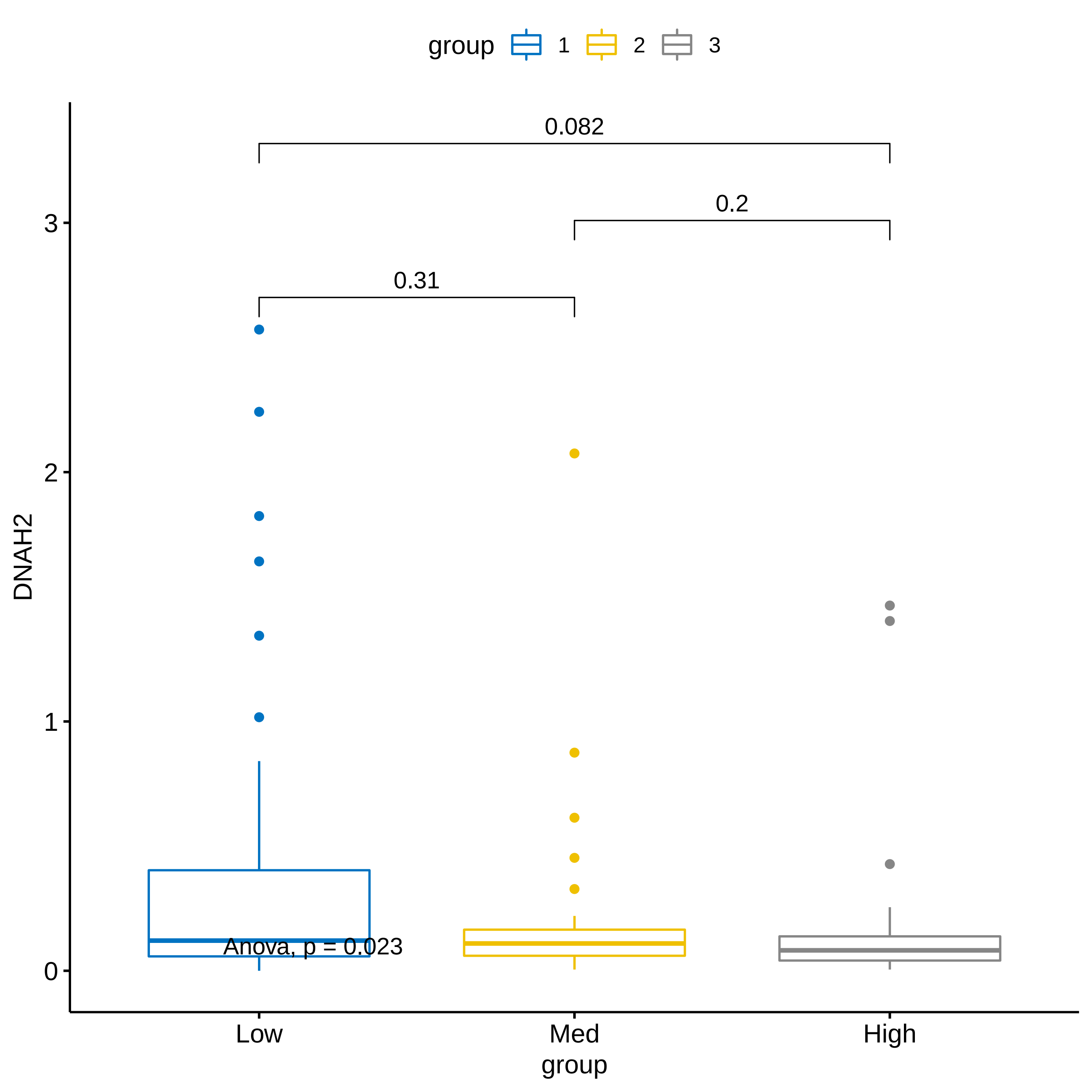

group
1
2
3
0.082
0.2
0.31
Anova, p = 0.023
DNAH2
0
1
2
3
Low
Med
High
group

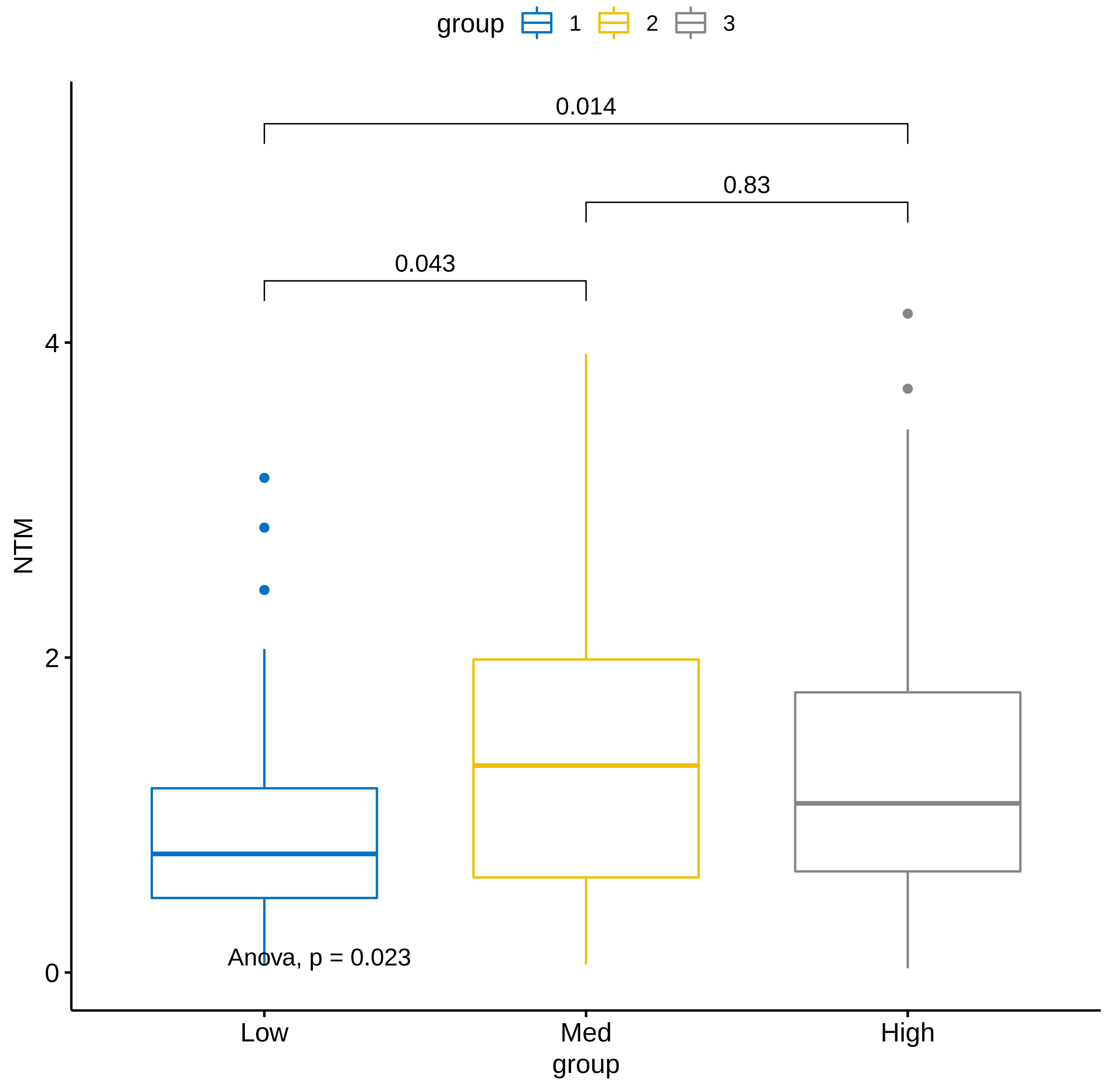

group
1
2
3
0.014
0.83
0.043
4
2
0
NTM
Anova, p = 0.023
Low
Med
High
group

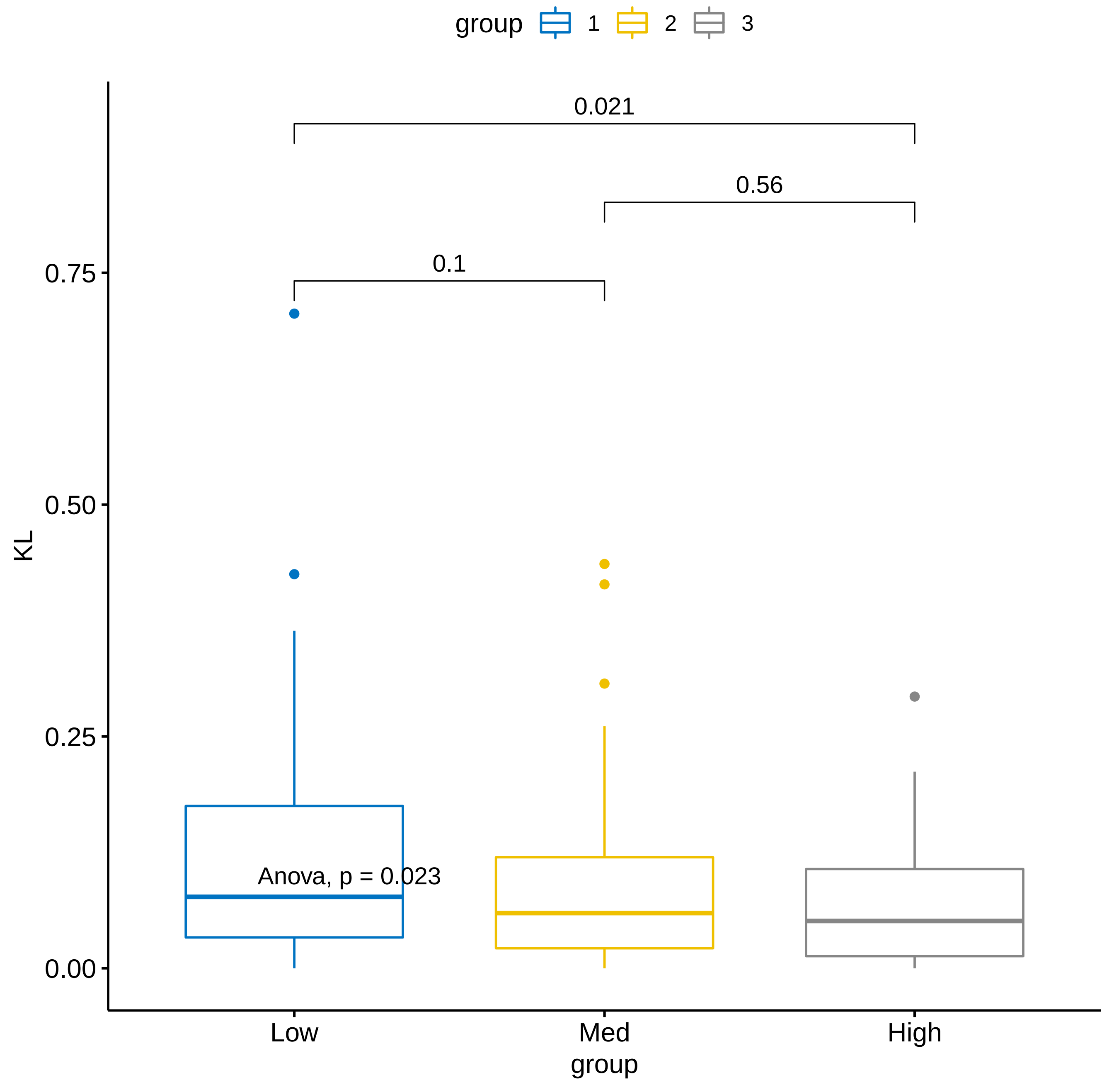
group 1 2 3
0.021
0.56
0.1
0.75
0.50
0.25
0.00
KL
Anova, p = 0.023
Low
Med
High
group

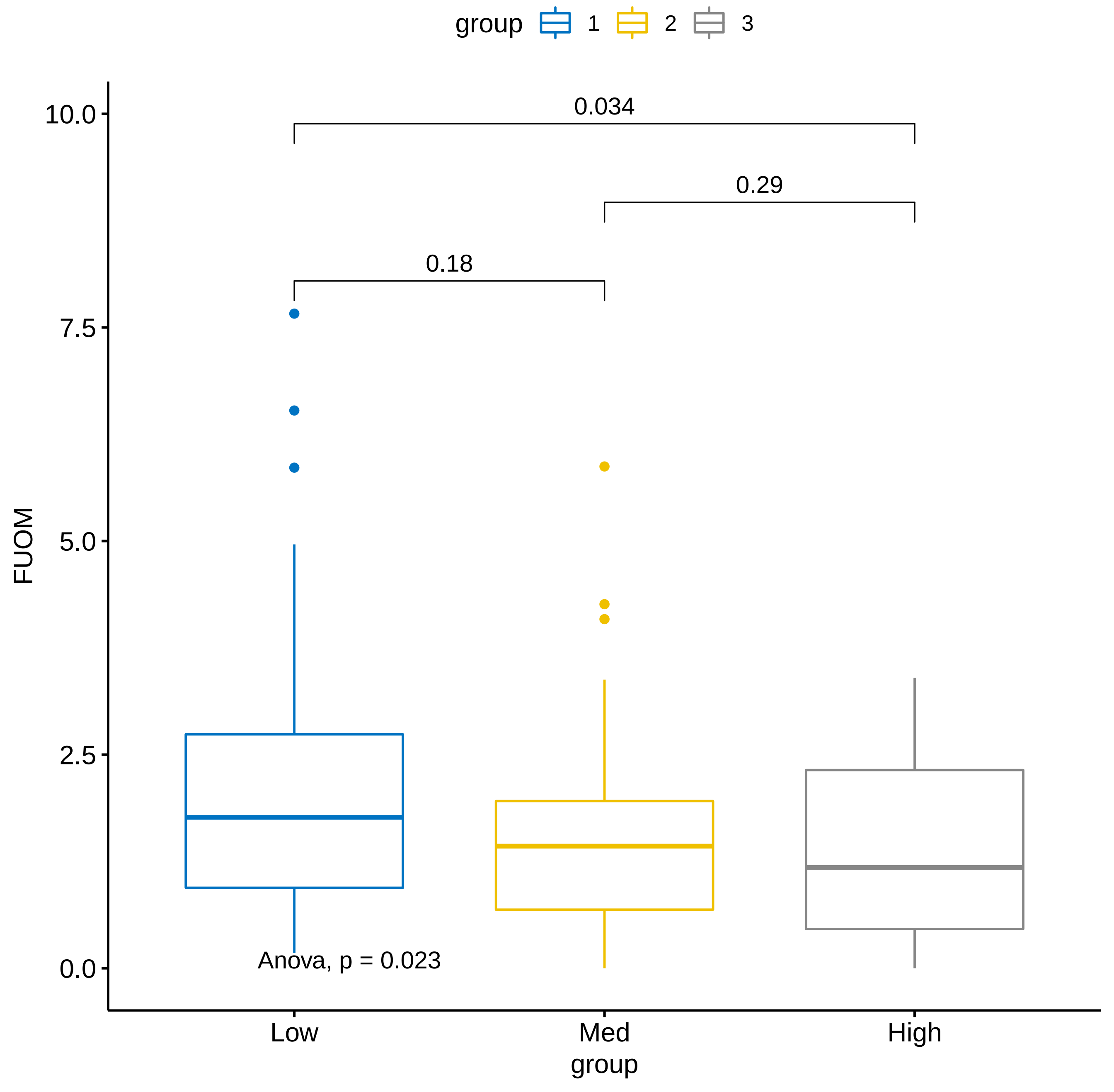

group
1
2
3
0.034
0.29
0.18
10.0
7.5
5.0
2.5
0.0
FUOM
Anova, p = 0.023
Low
Med
High
group

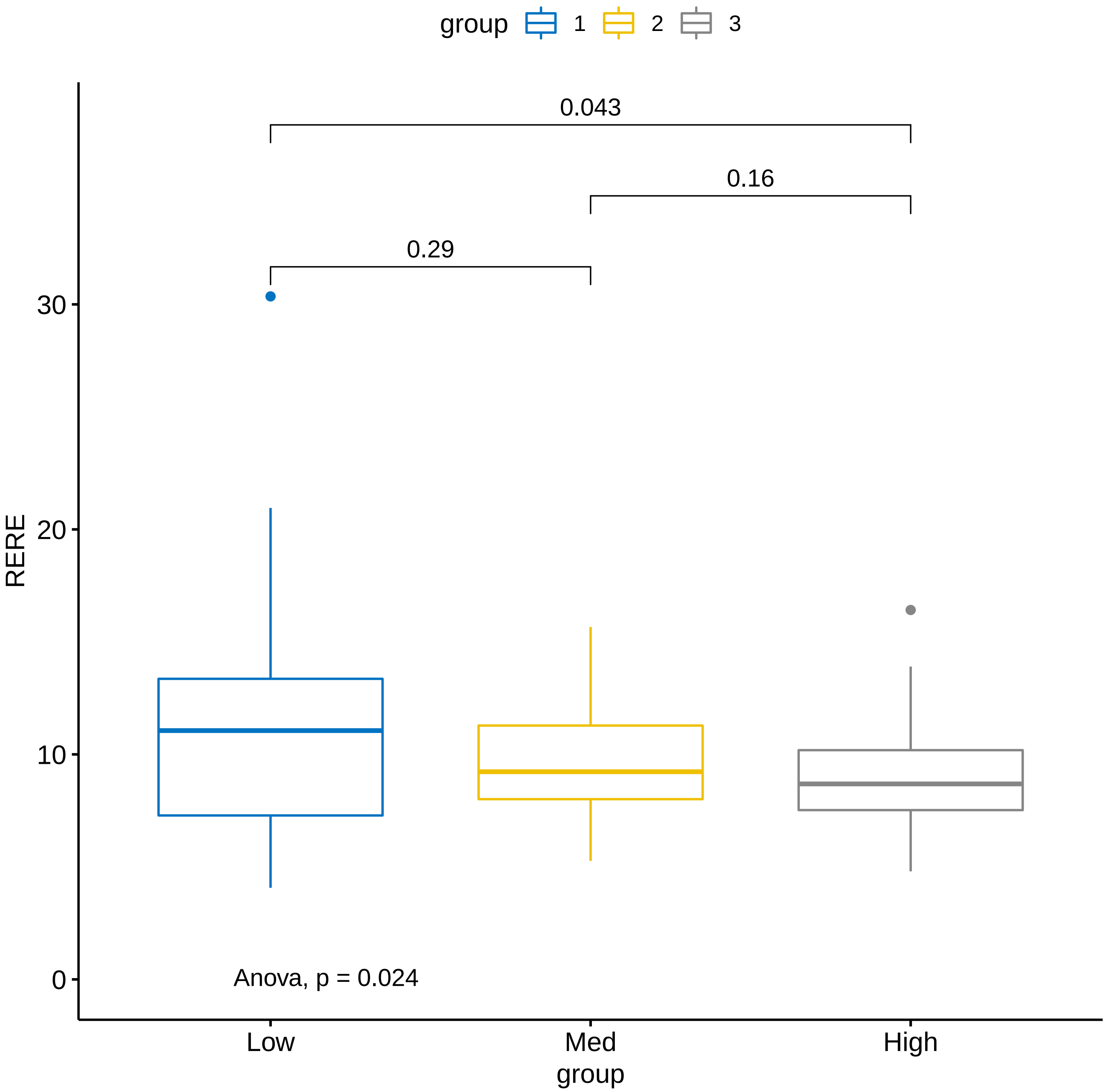
group
1
2
3
0.043
0.16
0.29
30
20
10
0
RERE
Anova, p = 0.024
Low
Med
High
group

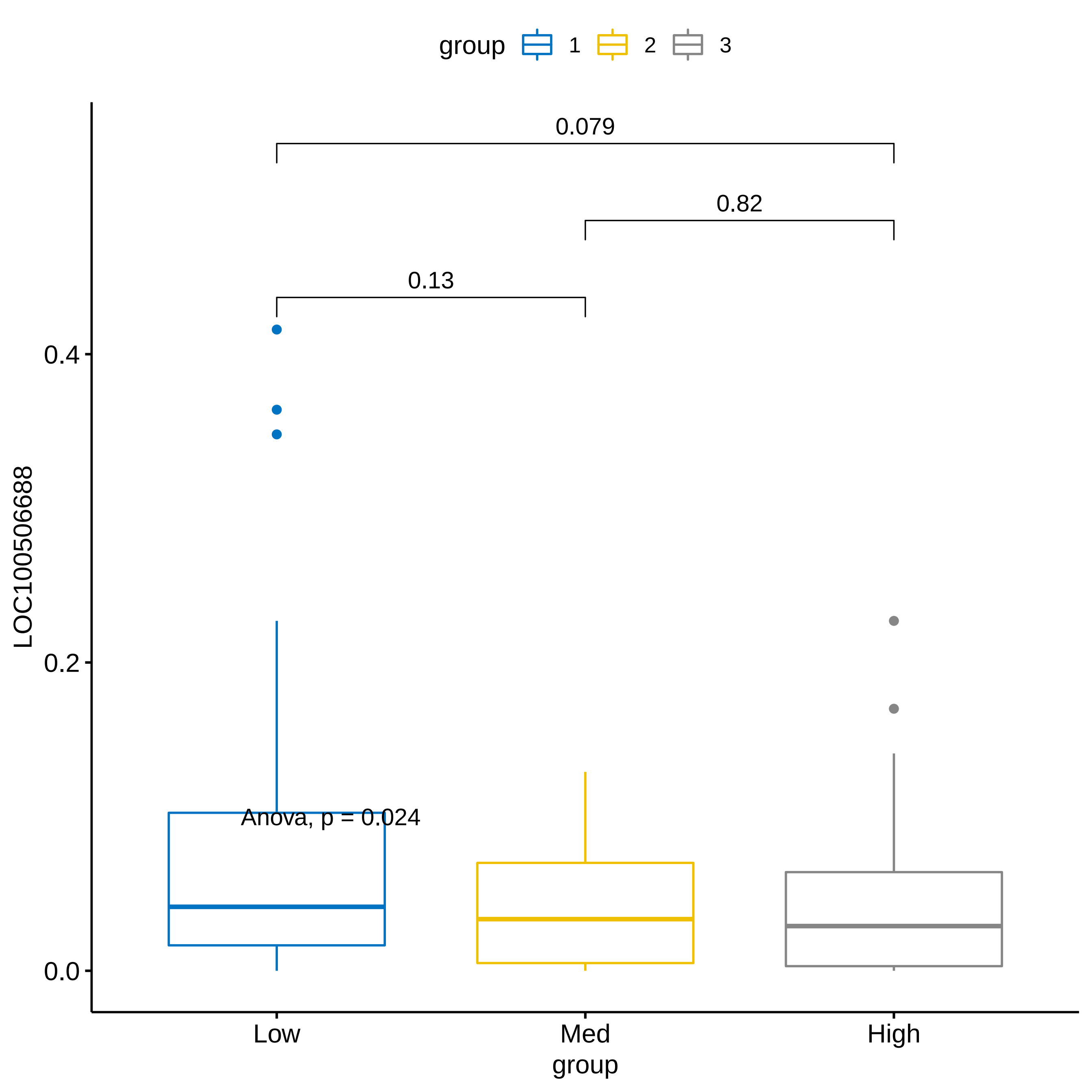
group
1
2
3
0.079
0.82
0.13
0.4
0.2
0.0
LOC100506688
Anova, p = 0.024
Low
Med
High
group

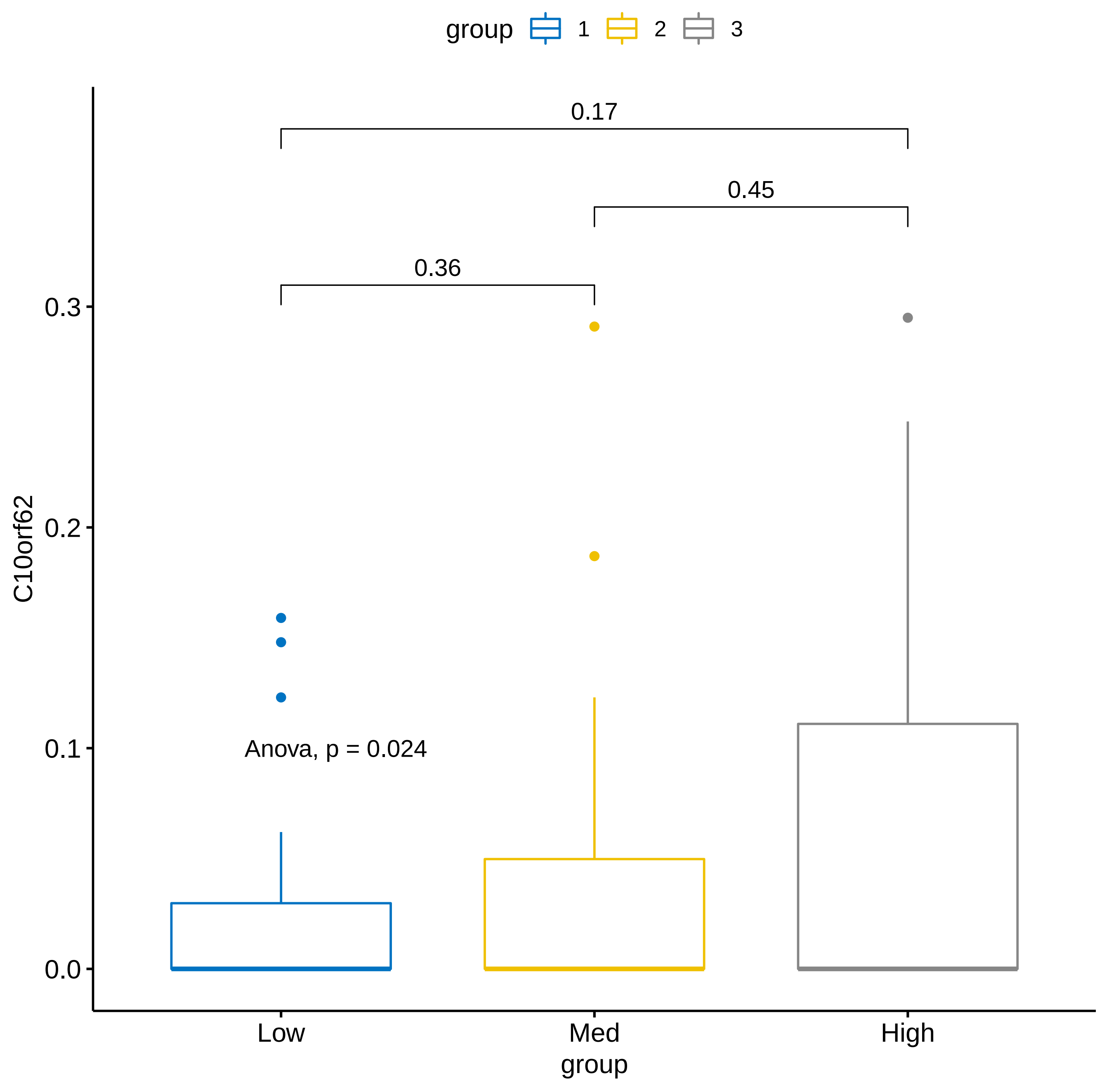

group 1 2 3
0.17
0.45
0.36
Anova, p = 0.024
C10orf62
0.0
0.1
0.2
0.3
Low
Med
High
group

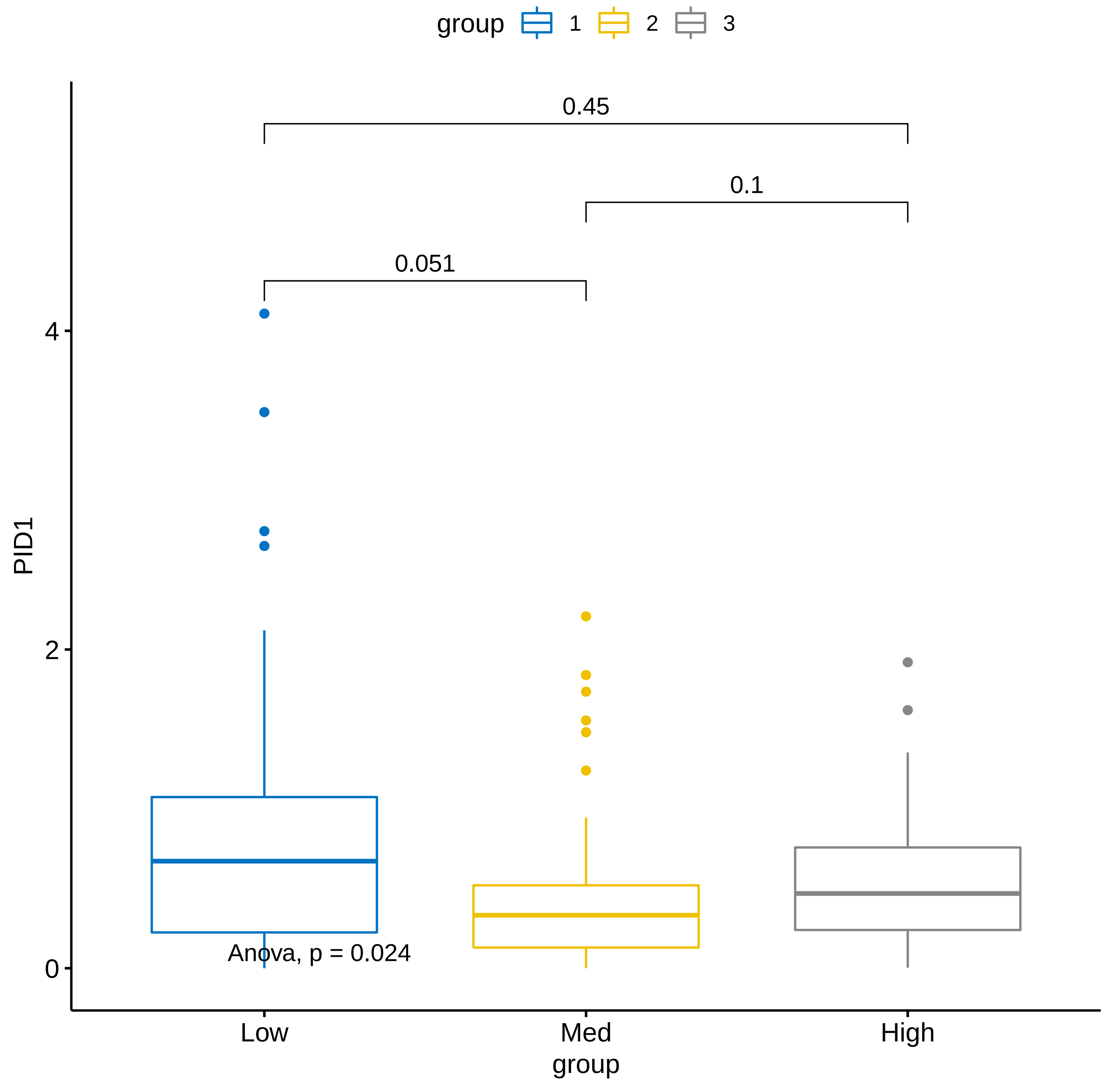

group
1
2
3
0.45
0.1
0.051
4
2
0
PID1
Anova, p = 0.024
Low
Med
High
group

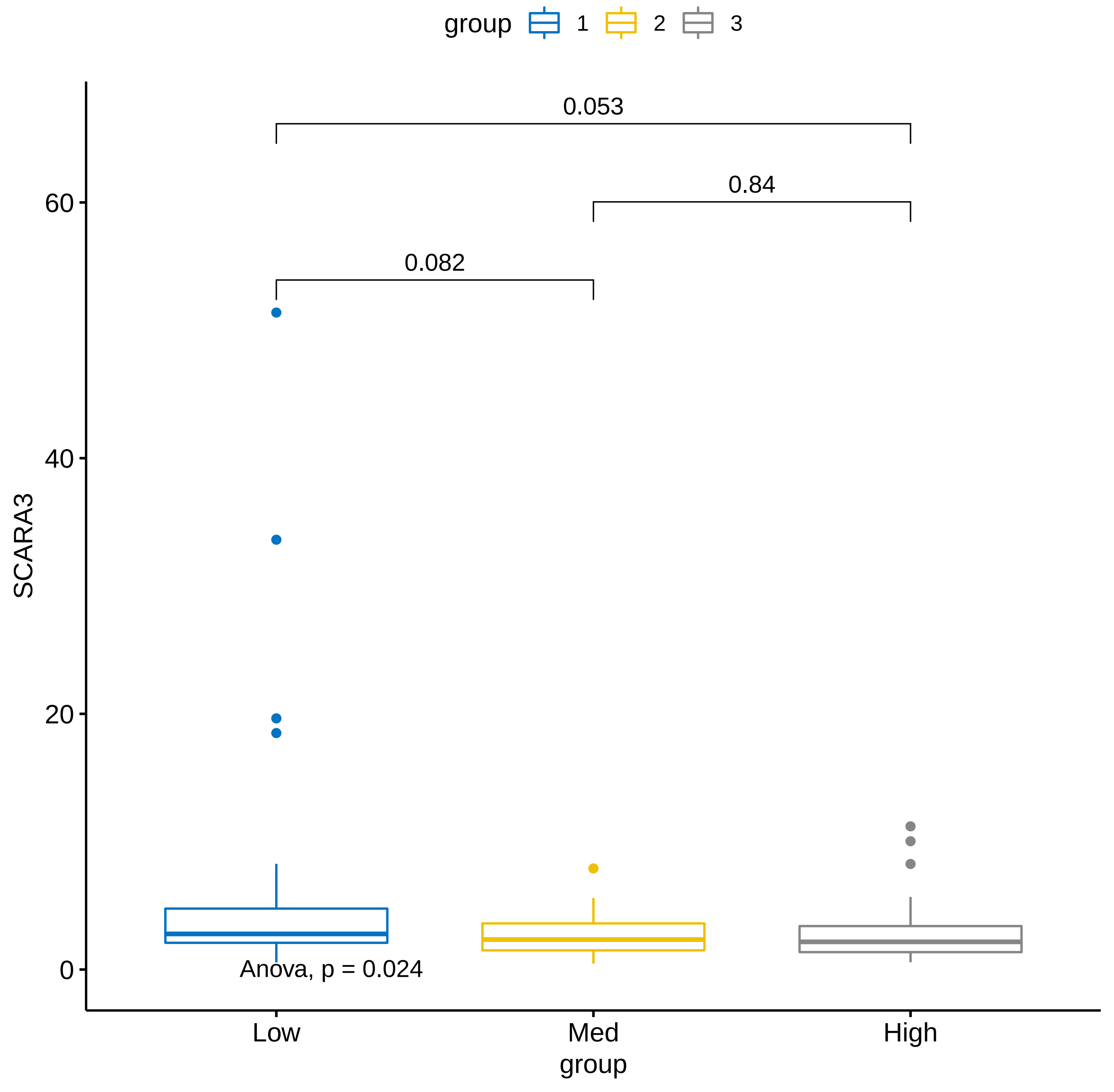
group
1
2
3
0.053
0.84
0.082
60
40
20
0
SCARA3
Anova, p = 0.024
Low
Med
High
group

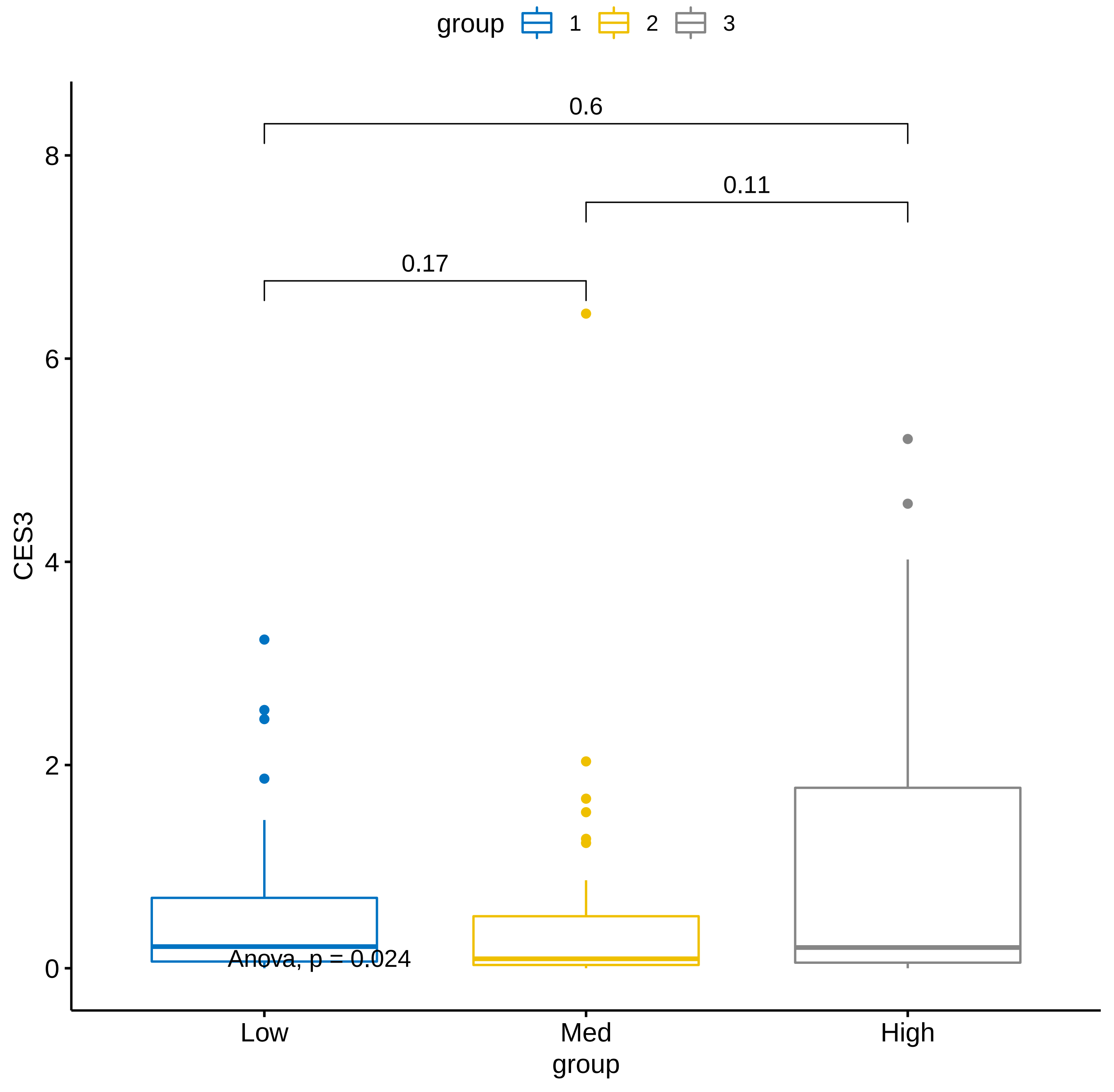
group 1 2 3
0.6
0.11
0.17
CES3
8
6
4
2
0
Anova, p = 0.024
Low
Med
High
group

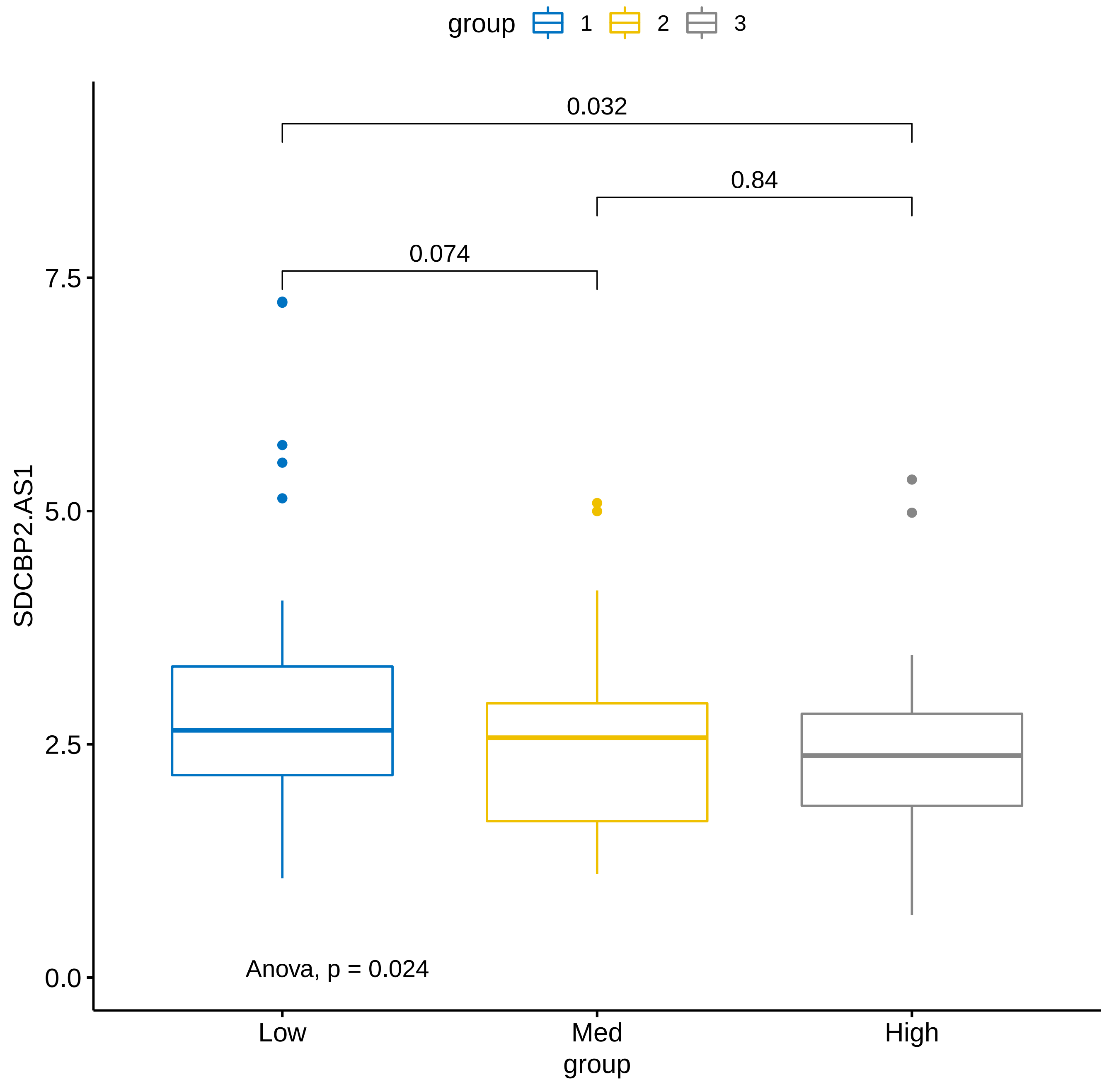
group
1
2
3
0.032
0.84
0.074
7.5
5.0
2.5
0.0
SDCBP2.AS1
Anova, p = 0.024
Low
Med
High
group

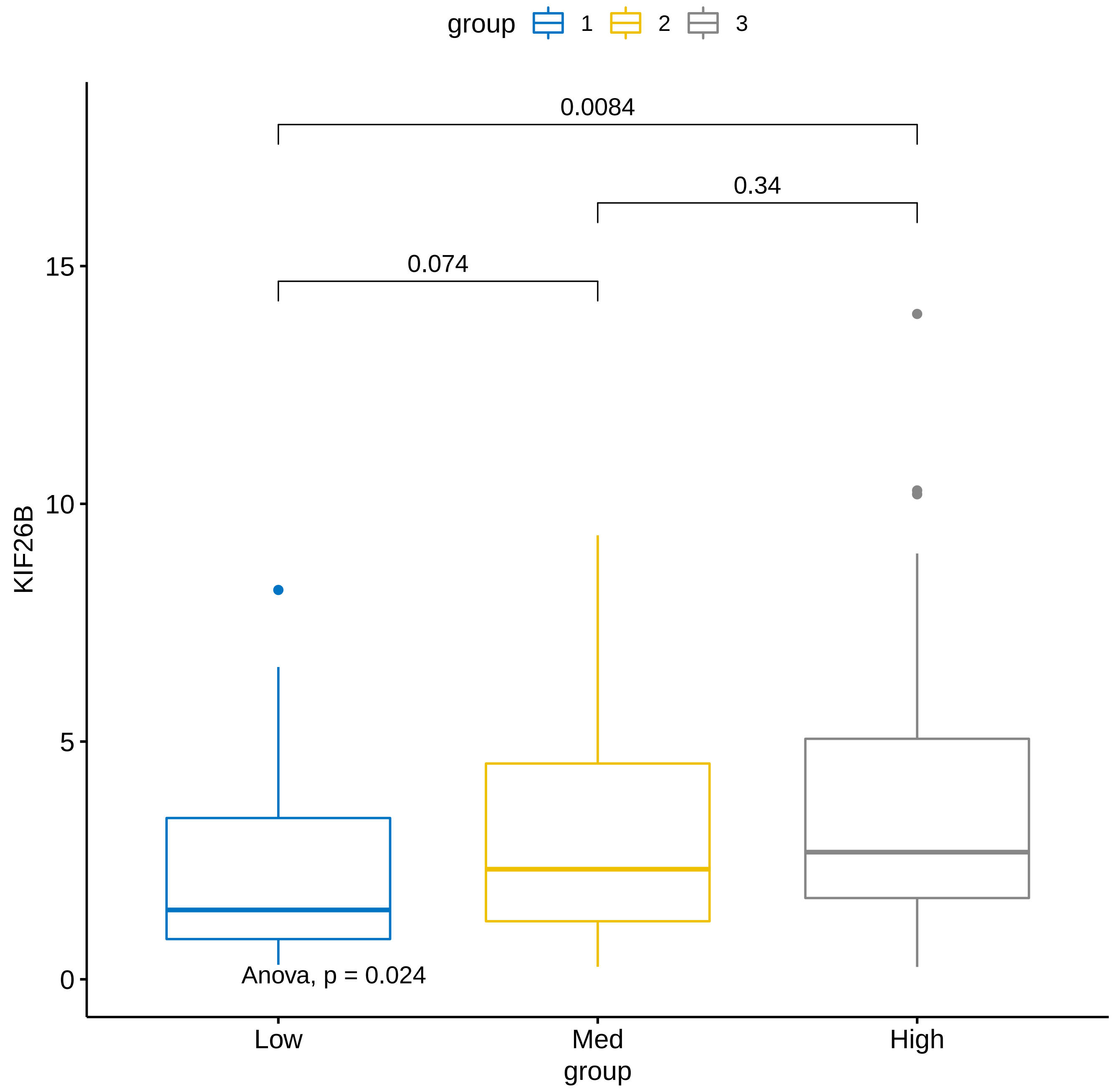

group
1
2
3
0.0084
0.34
0.074
15
10
5
0
KIF26B
Anova, p = 0.024
Low
Med
High
group

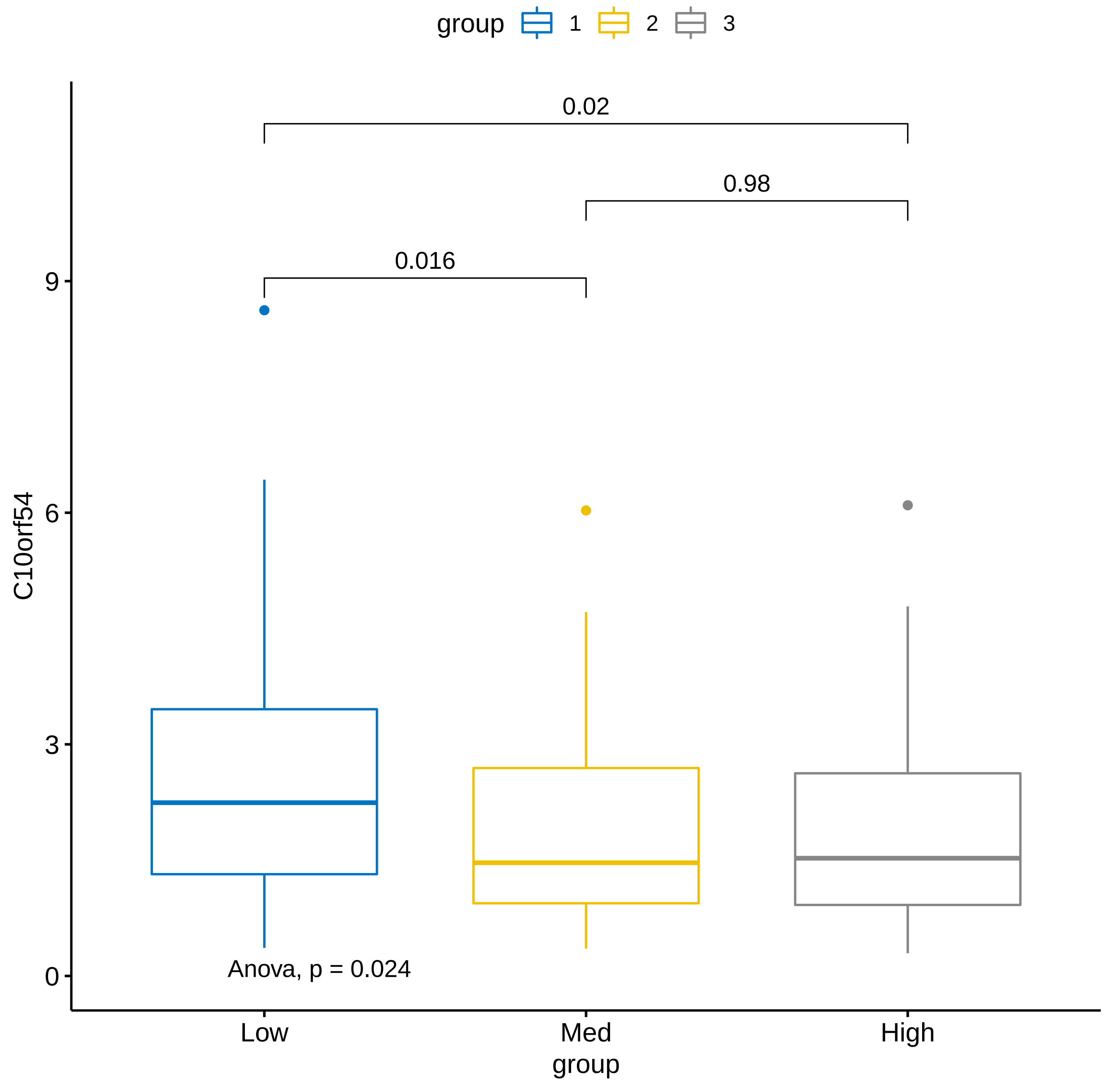

group 1 2 3
0.02
0.98
0.016
Anova, p = 0.024
9
6
3
0
C10orf54
Low
Med
High
group

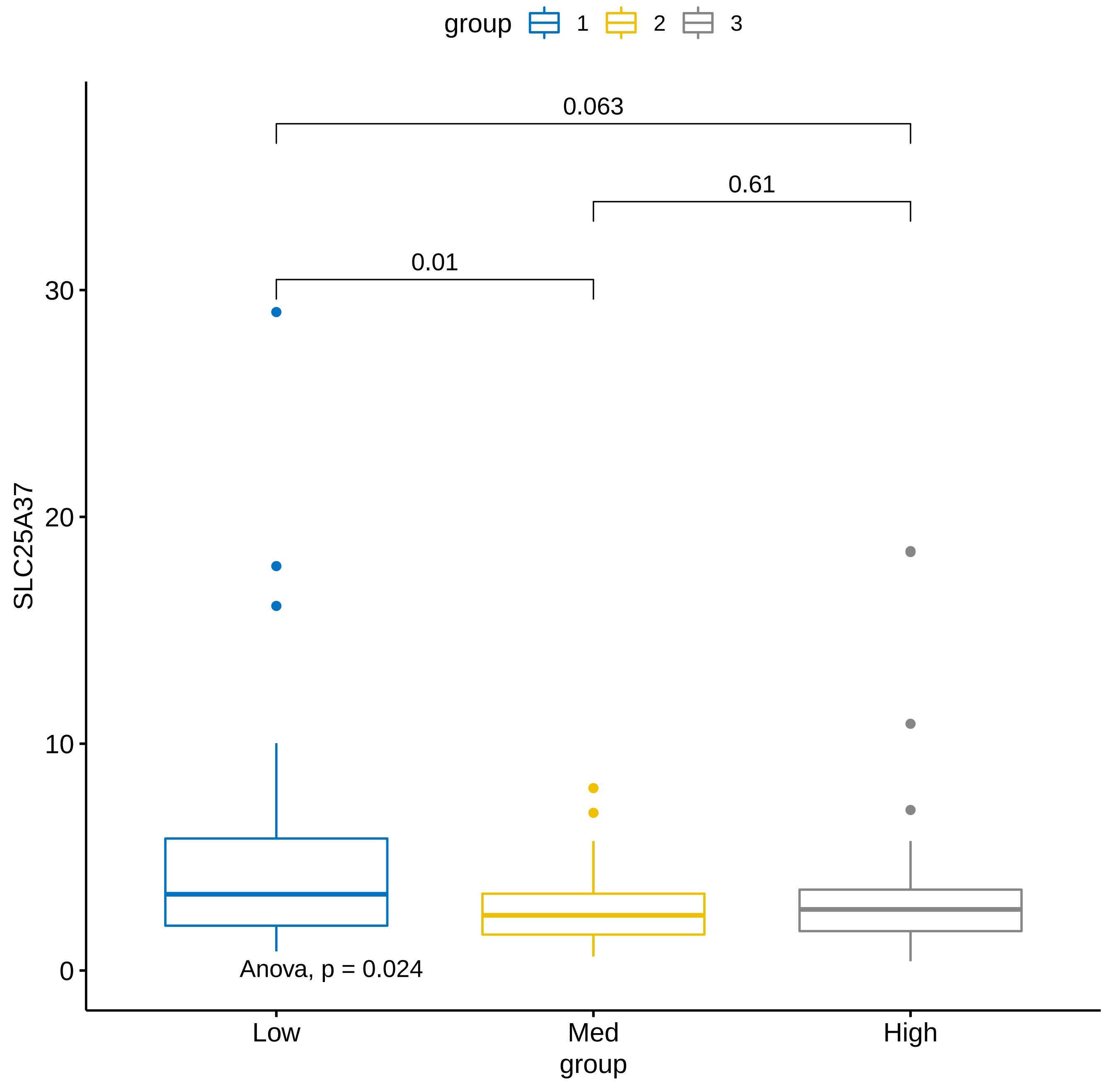
group
1
2
3
0.063
0.61
0.01
30
20
10
0
SLC25A37
Anova, p = 0.024
Low
Med
High
group

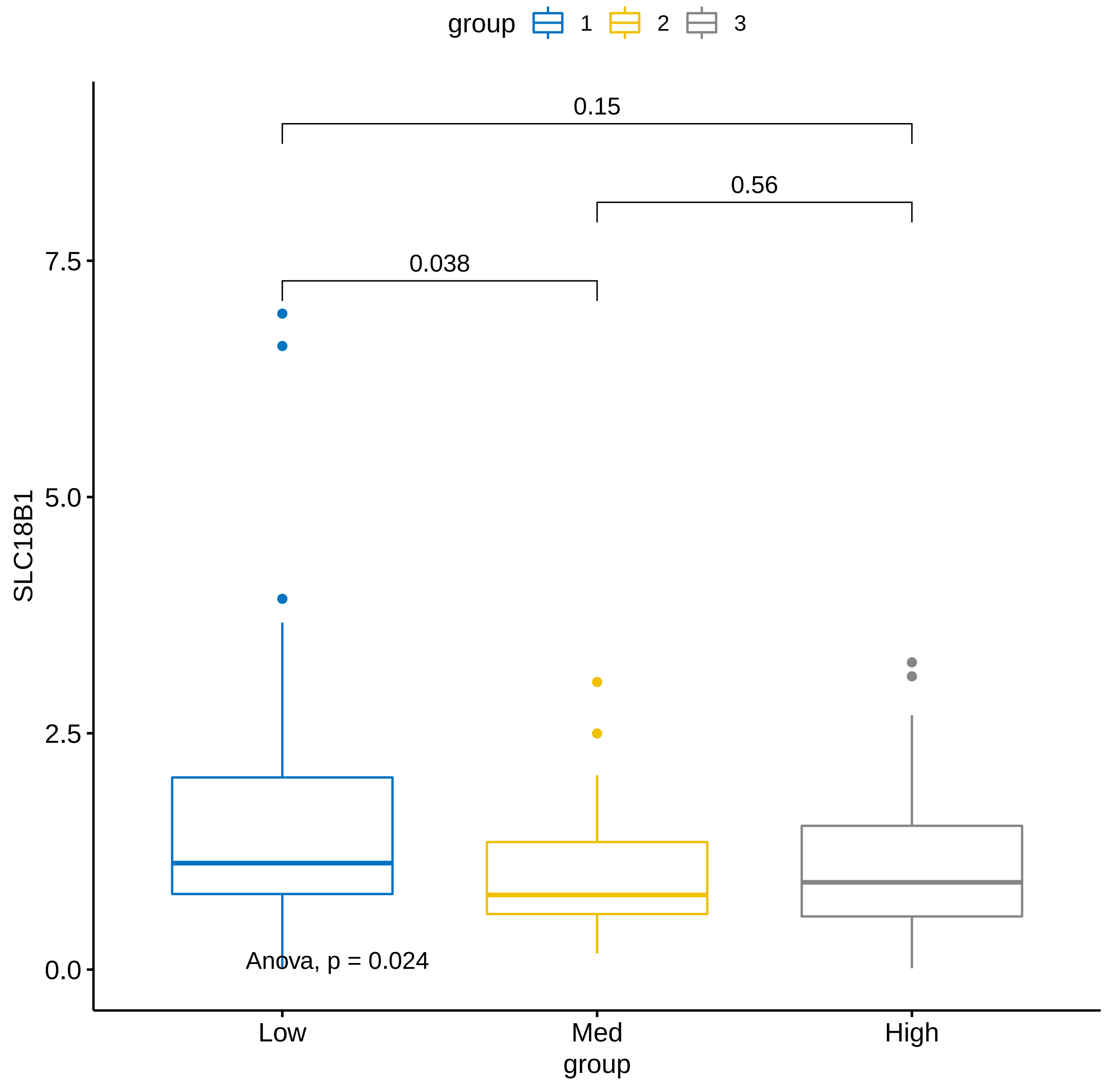

group
1
2
3
0.15
0.56
0.038
7.5
5.0
2.5
0.0
SLC18B1
Anova, p = 0.024
Low
Med
High
group

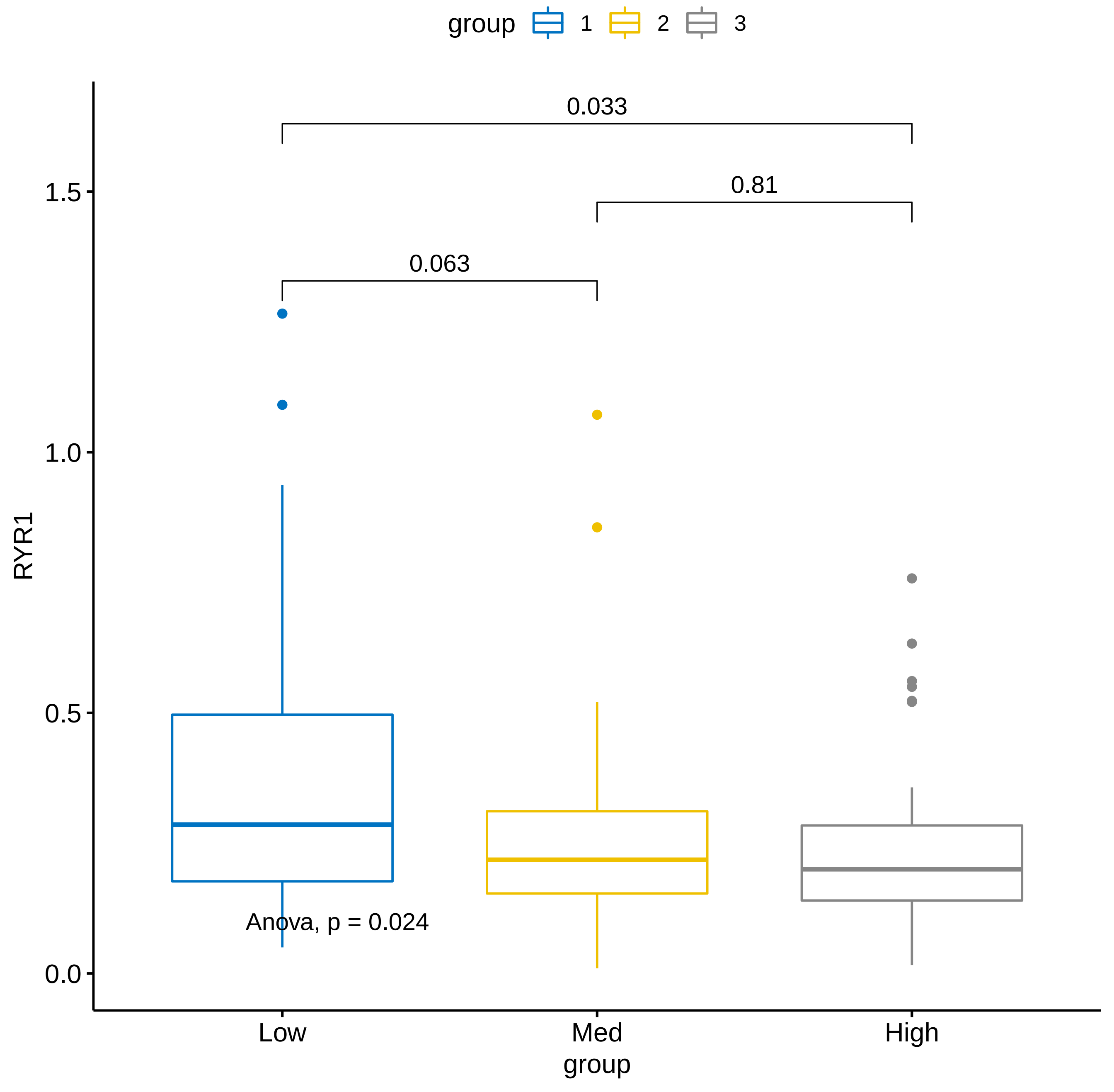
group
1
2
3
0.033
0.81
0.063
1.5
1.0
0.5
0.0
RYR1
Anova, p = 0.024
Low
Med
High
group

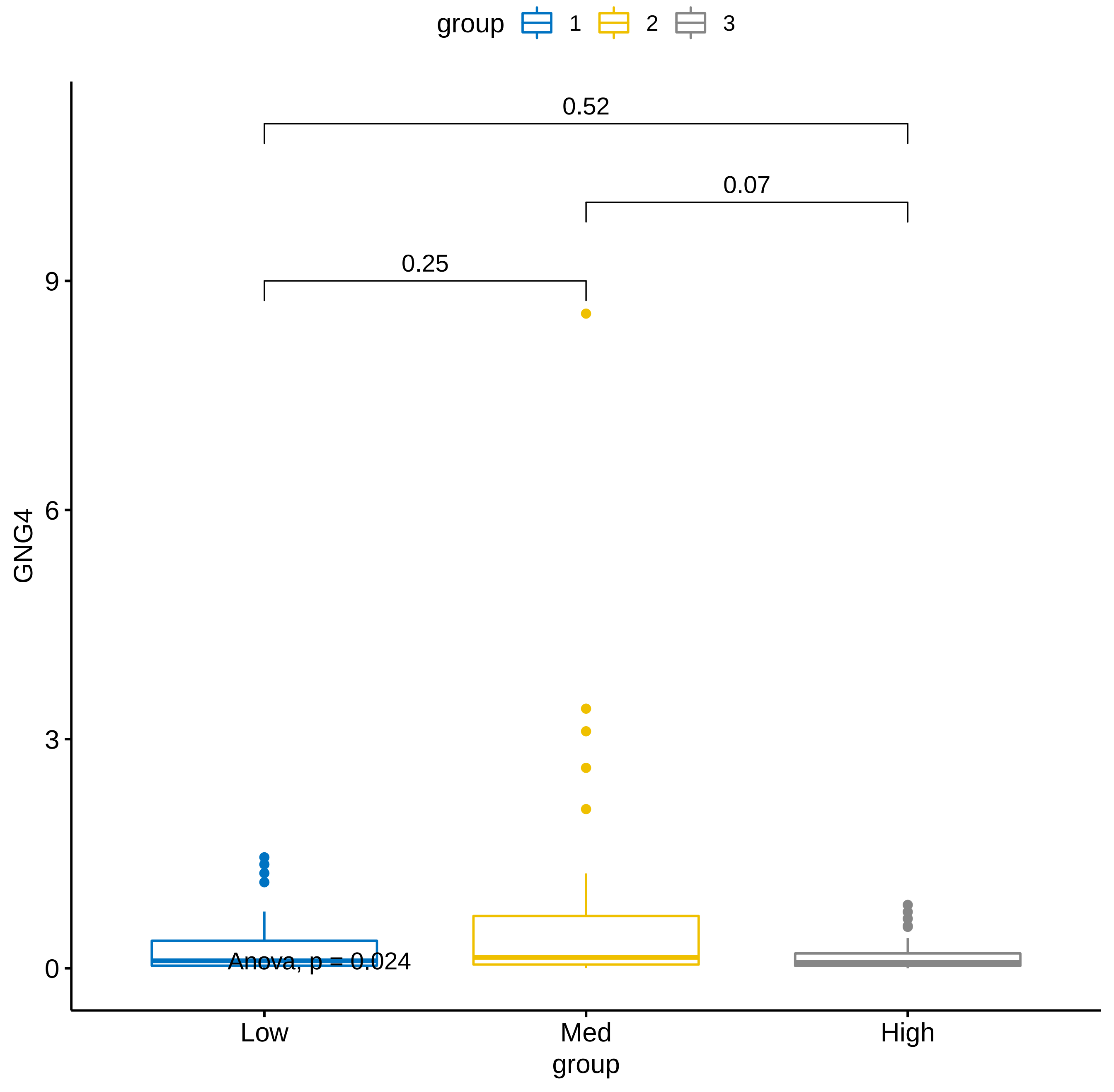
group
1
2
3
0.52
0.07
0.25
GNG4
9
6
3
0
Anova, p = 0.024
Low
Med
High
group

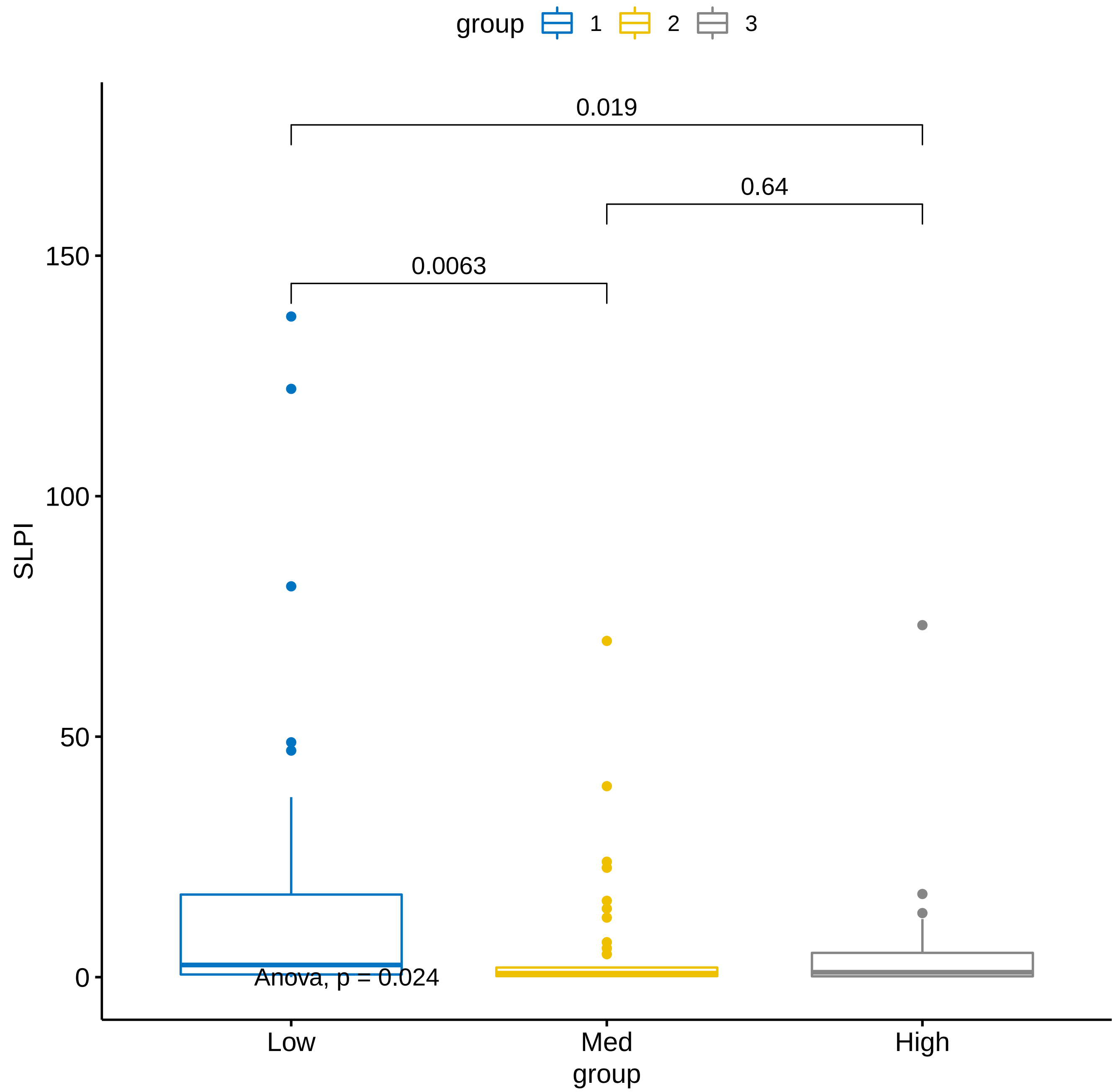

group
1
2
3
0.019
0.64
0.0063
150
100
50
0
SLPI
Anova, p = 0.024
Low
Med
High
group

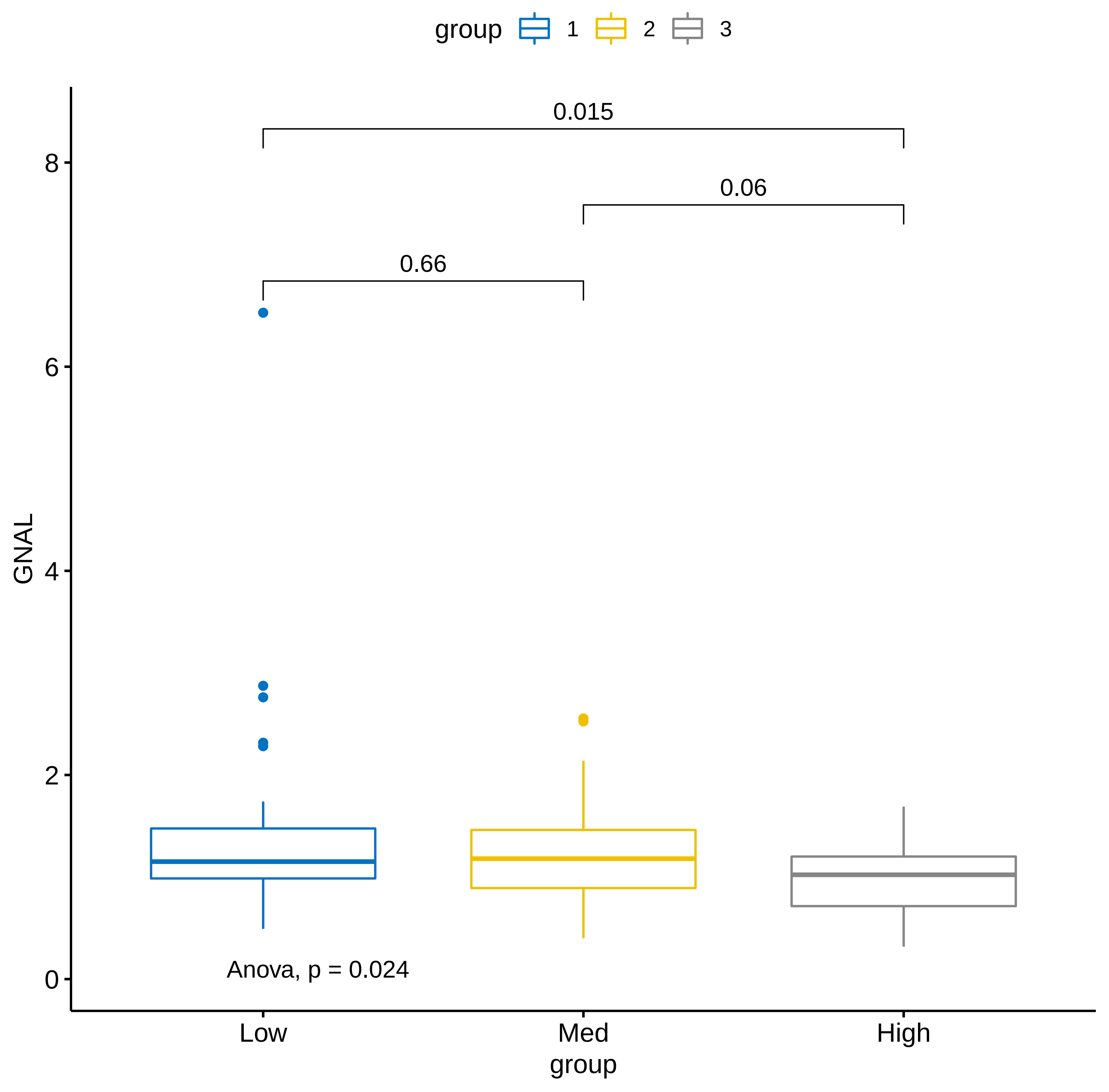

group 1 2 3
0.015
0.06
0.66
GNAL
8
6
4
2
0
Anova, p = 0.024
Low
Med
High
group

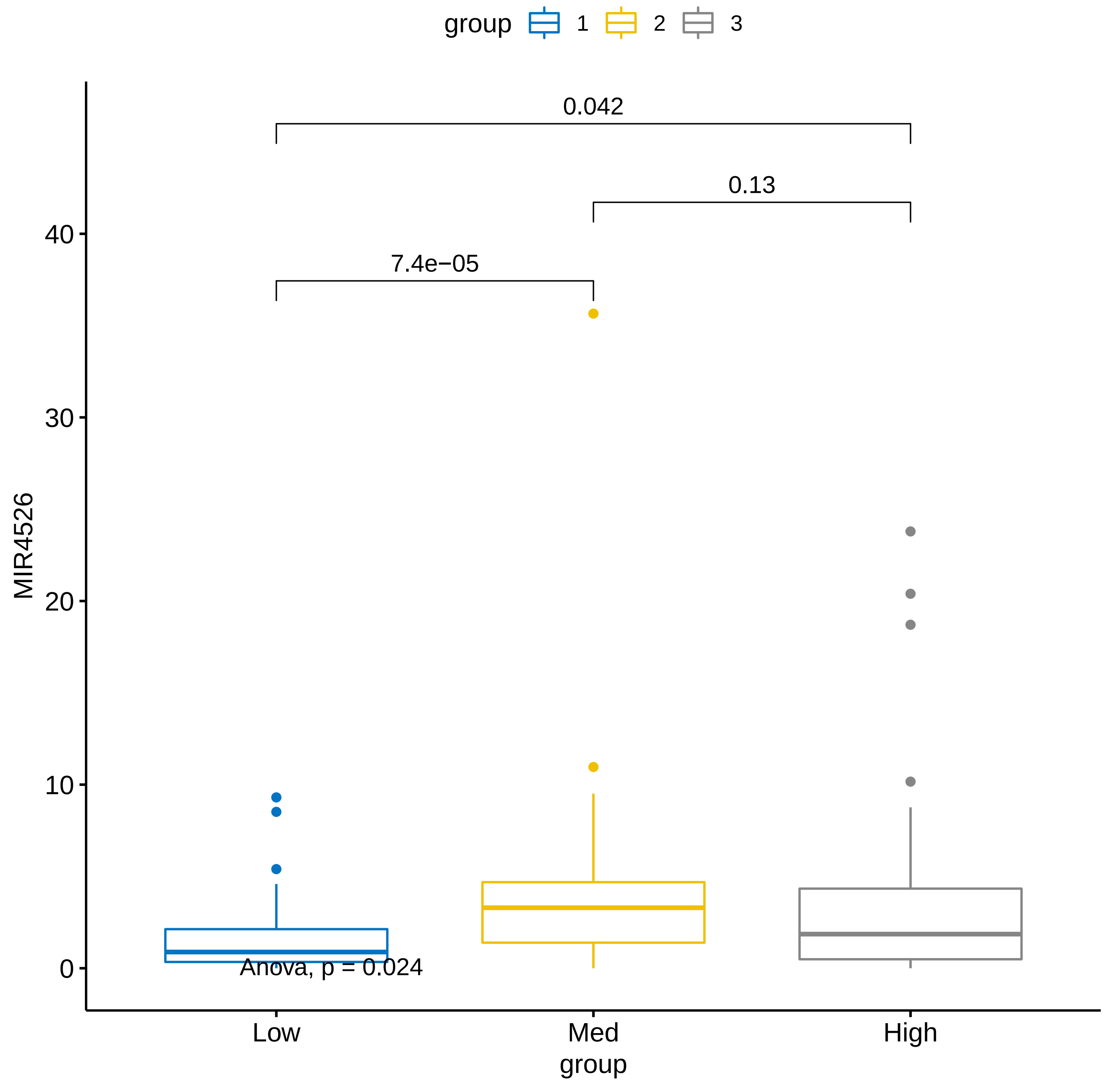

group
1
2
3
0.042
0.13
7.4e−05
40
30
20
10
0
MIR4526
Anova, p = 0.024
Low
Med
High
group

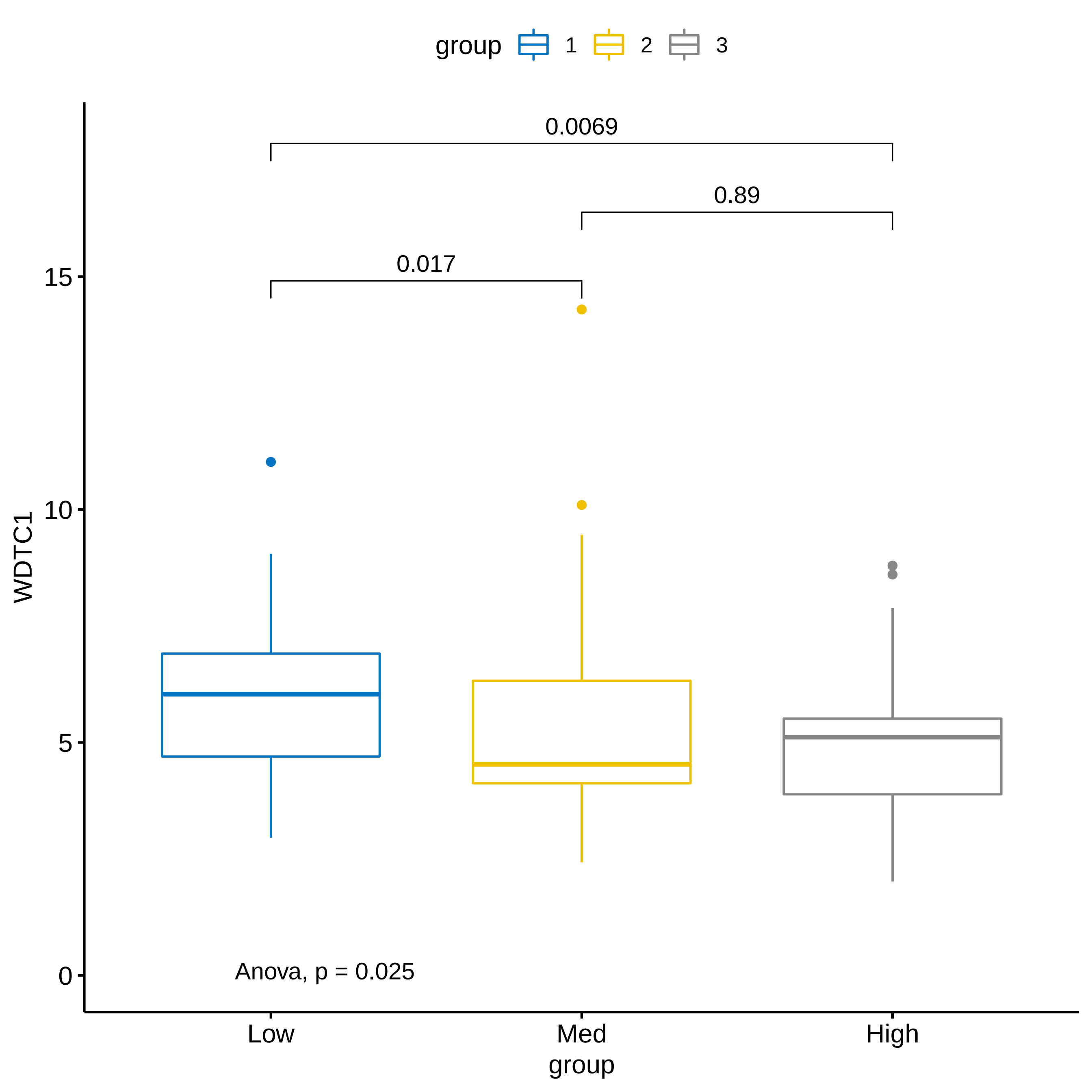

group
1
2
3
0.0069
0.89
0.017
15
10
5
0
WDTC1
Anova, p = 0.025
Low
Med
High
group

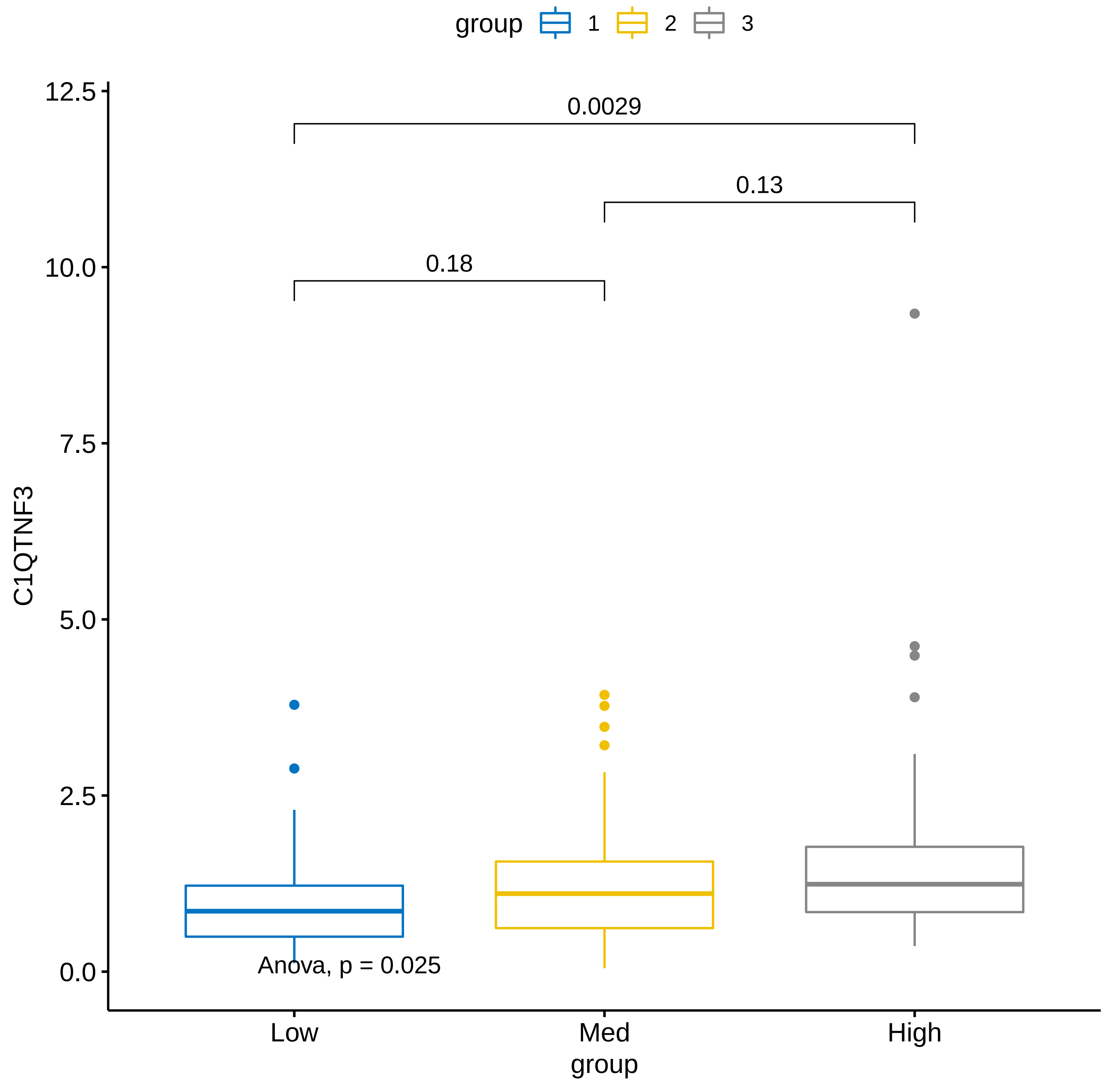

group
1
2
3
12.5
10.0
7.5
5.0
2.5
0.0
C1QTNF3
0.0029
0.13
0.18
Anova, p = 0.025
Low
Med
High
group

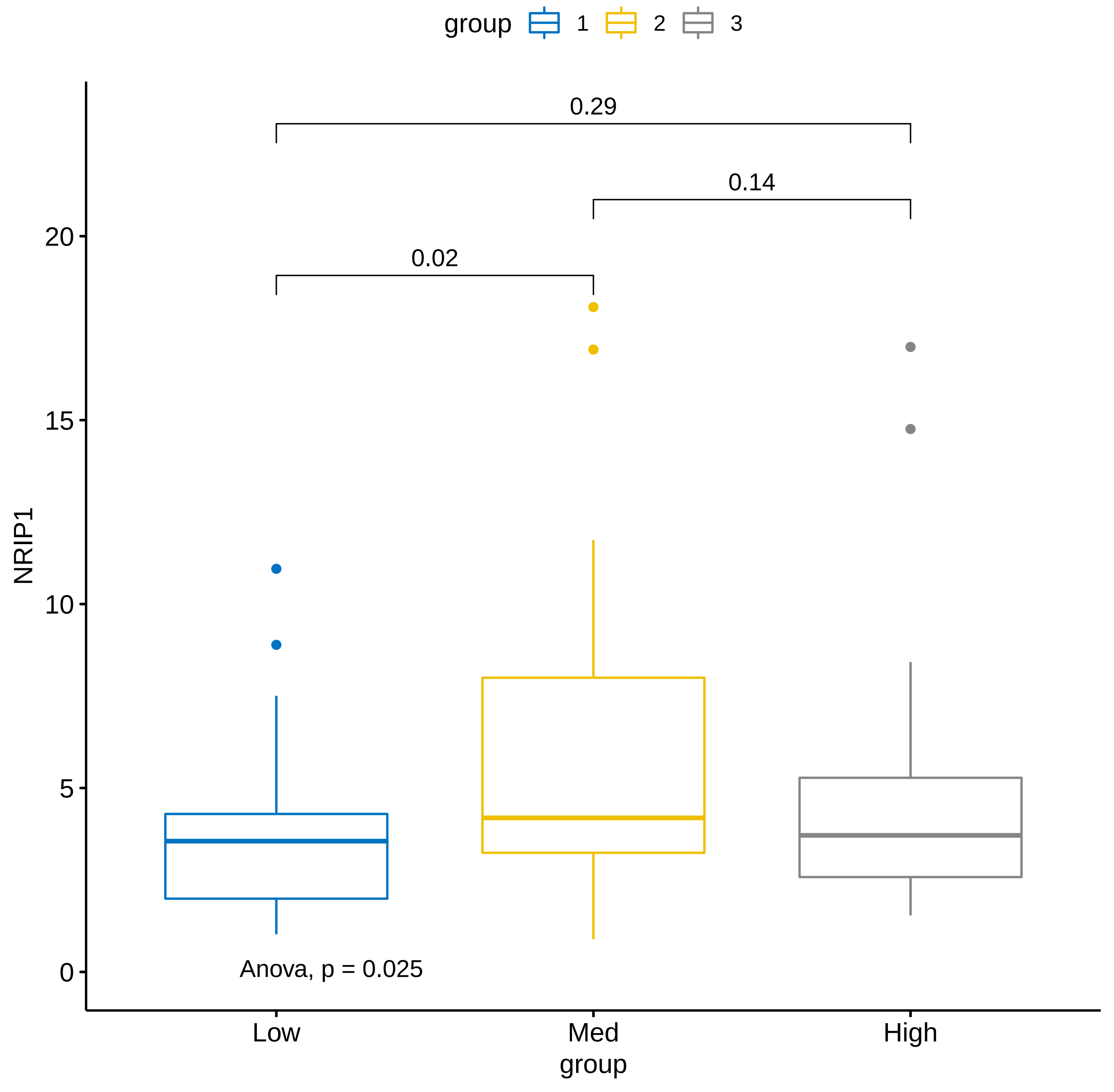
group 1 2 3
0.29
0.14
0.02
NRIP1
20
15
10
5
0
Anova, p = 0.025
Low
Med
High
group

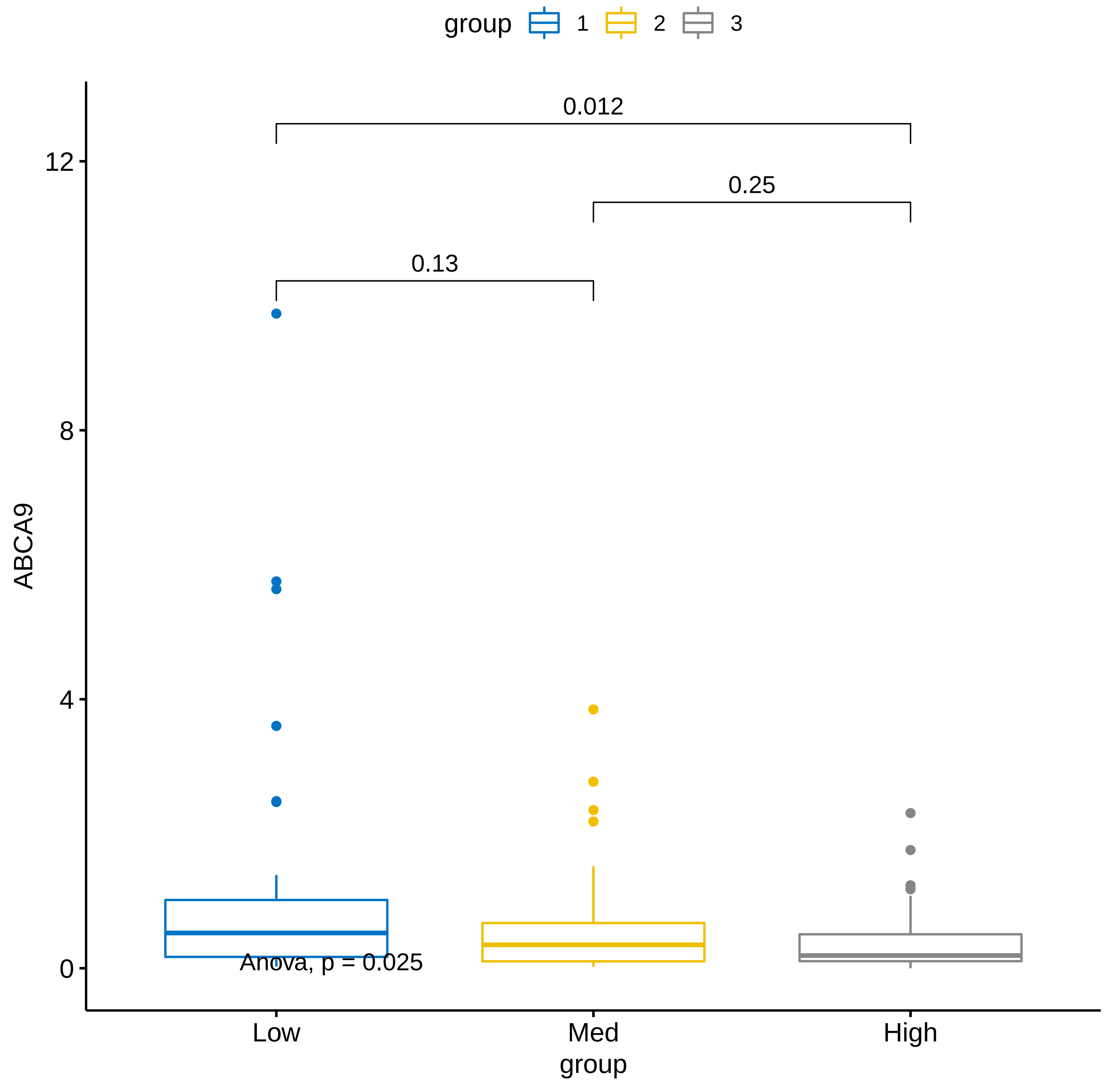
group
1
2
3
0.012
0.25
0.13
12
8
4
0
ABCA9
Anova, p = 0.025
Low
Med
High
group

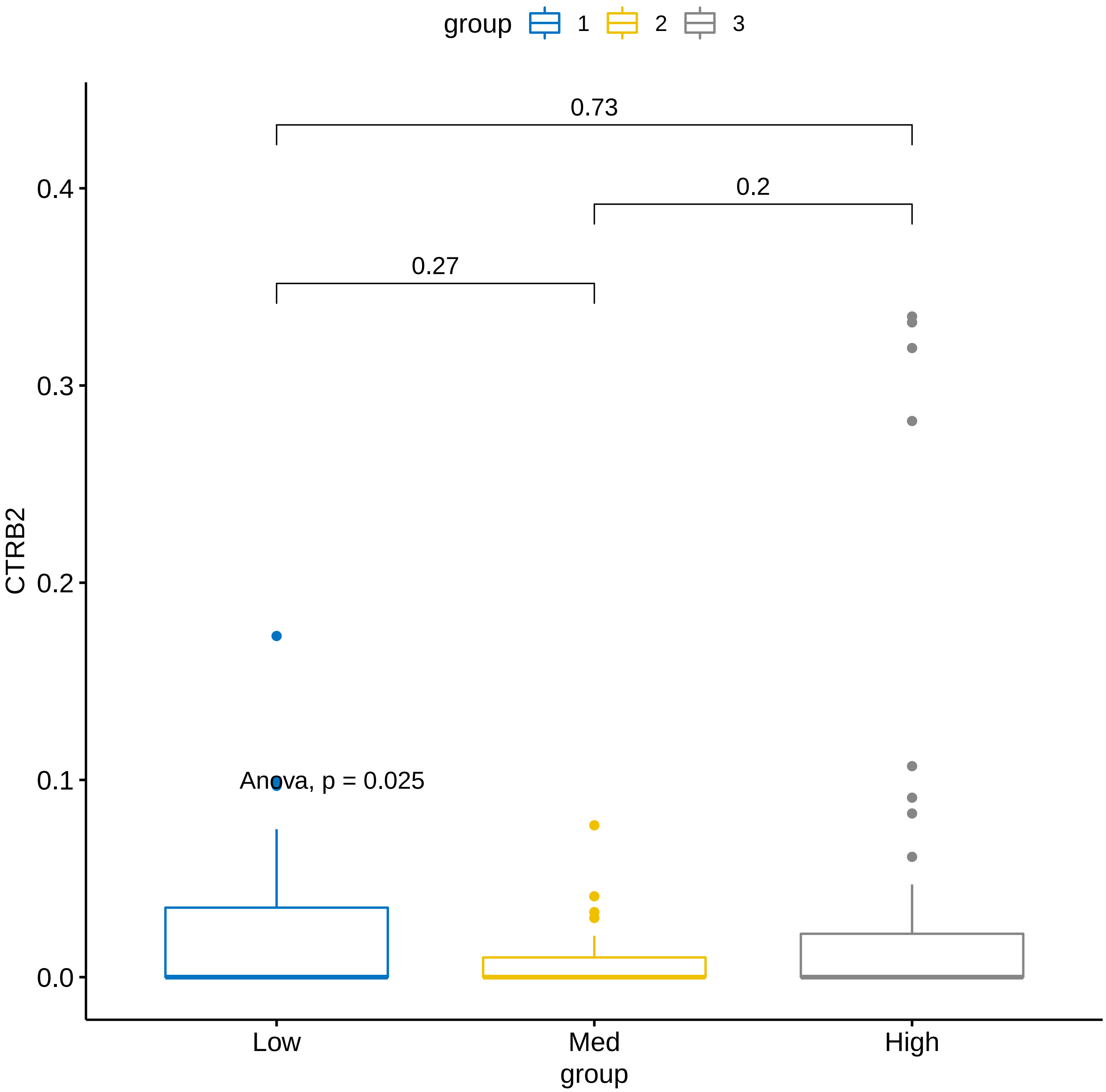
group
1
2
3
0.73
0.2
0.27
0.4
0.3
0.2
0.1
0.0
CTRB2
Anova, p = 0.025
Low
Med
High
group

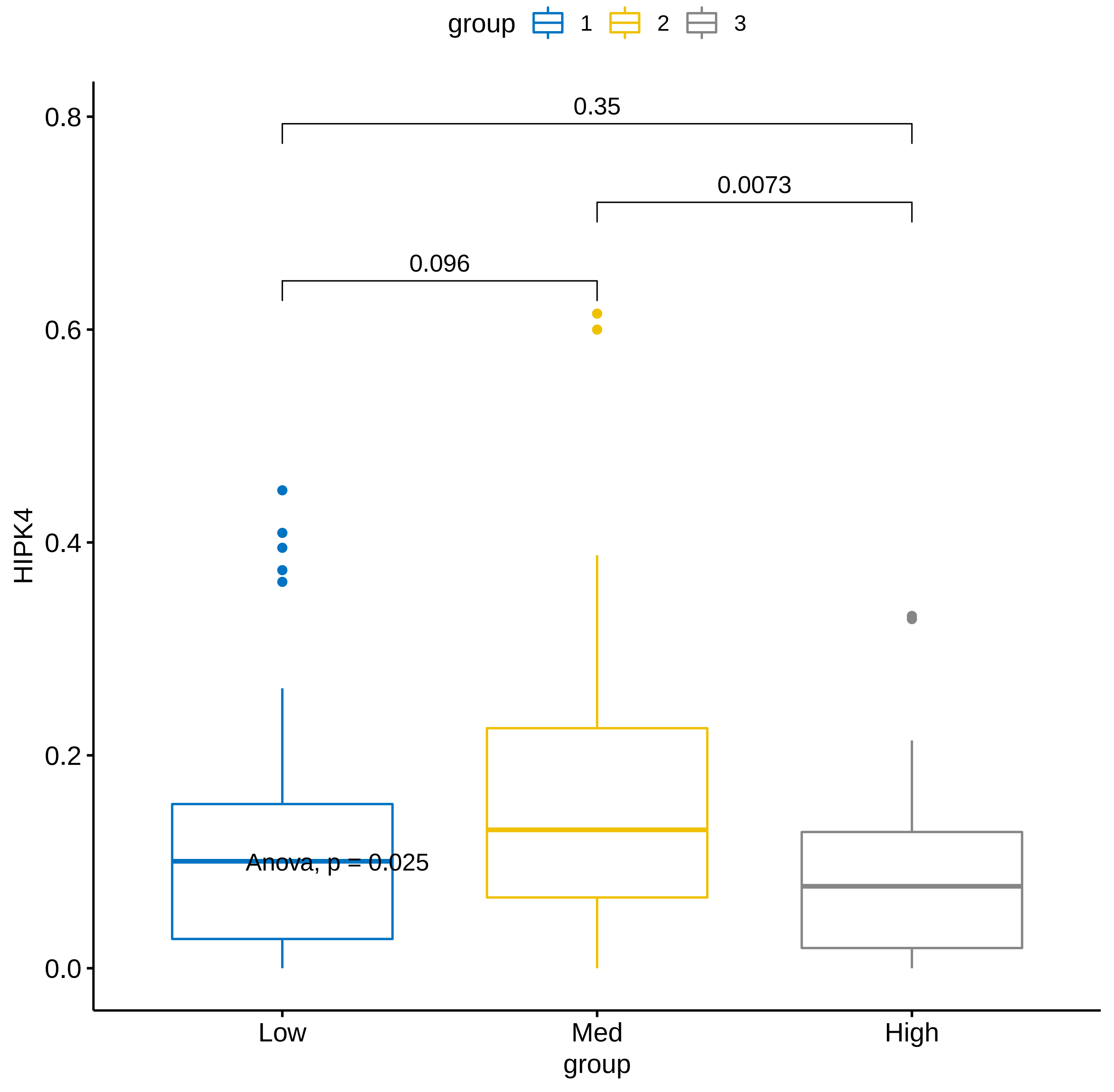

group
1
2
3
0.35
0.0073
0.096
Anova, p = 0.025
HIPK4
0.0
0.2
0.4
0.6
0.8
Low
Med
High
group

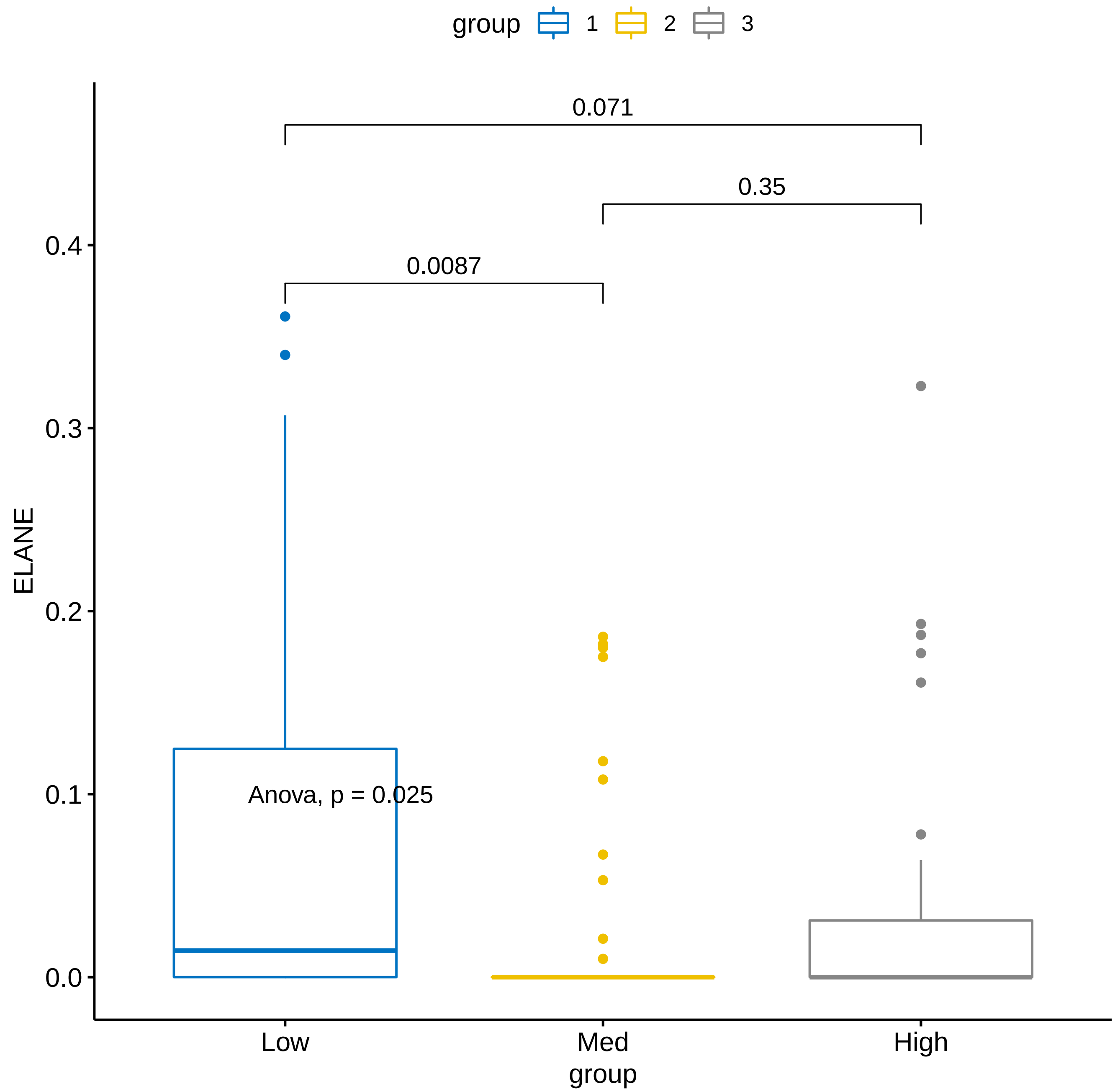

group 1 2 3
0.071
0.35
0.0087
Anova, p = 0.025
ELANE
0.4
0.3
0.2
0.1
0.0
Low
Med
High
group

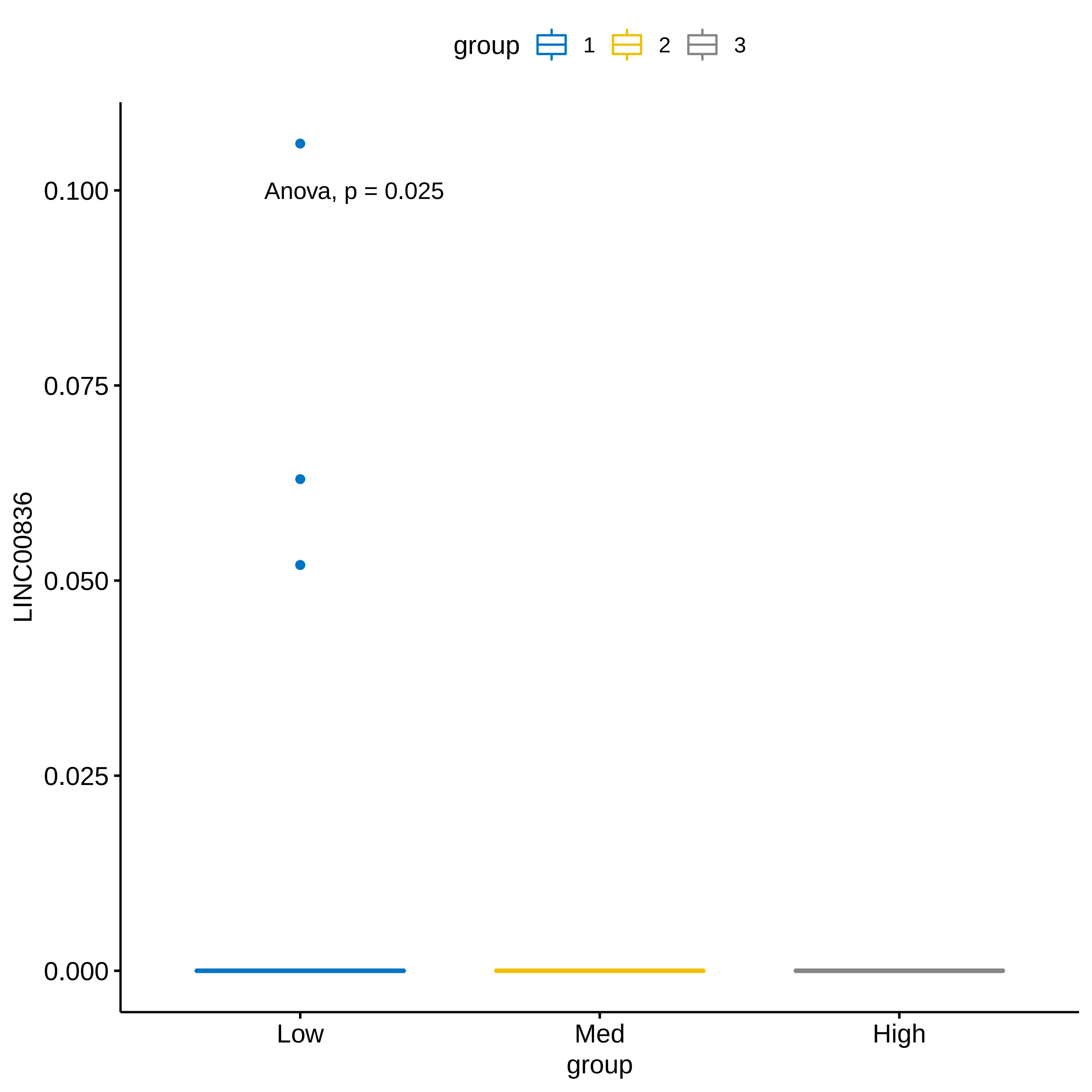

group 1 2 3
Anova, p = 0.025
0.100
0.075
0.050
0.025
0.000
LINC00836
Low
Med
High
group

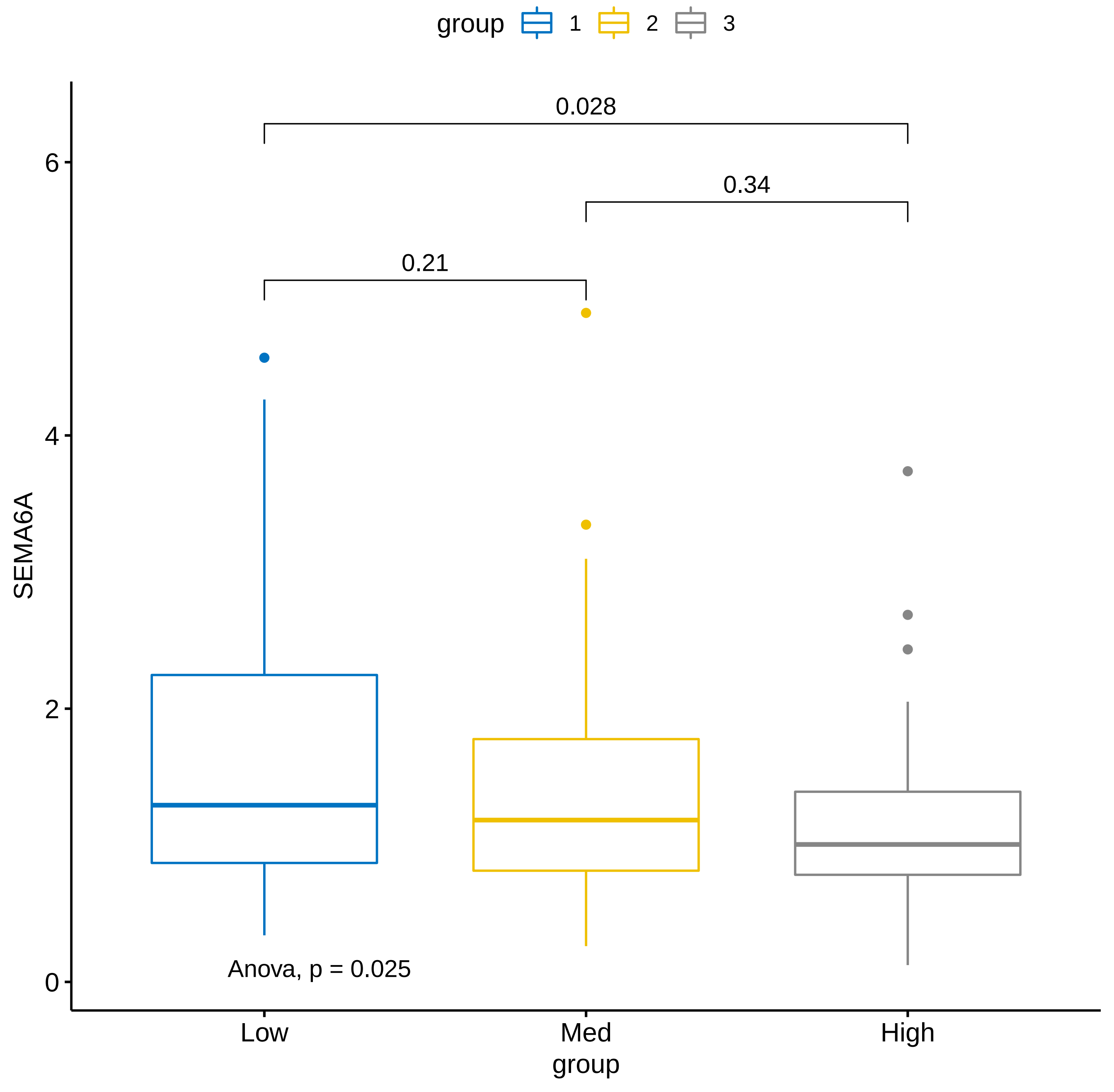

group 1 2 3
0.028
0.34
0.21
6
4
2
0
SEMA6A
Anova, p = 0.025
Low
Med
High
group

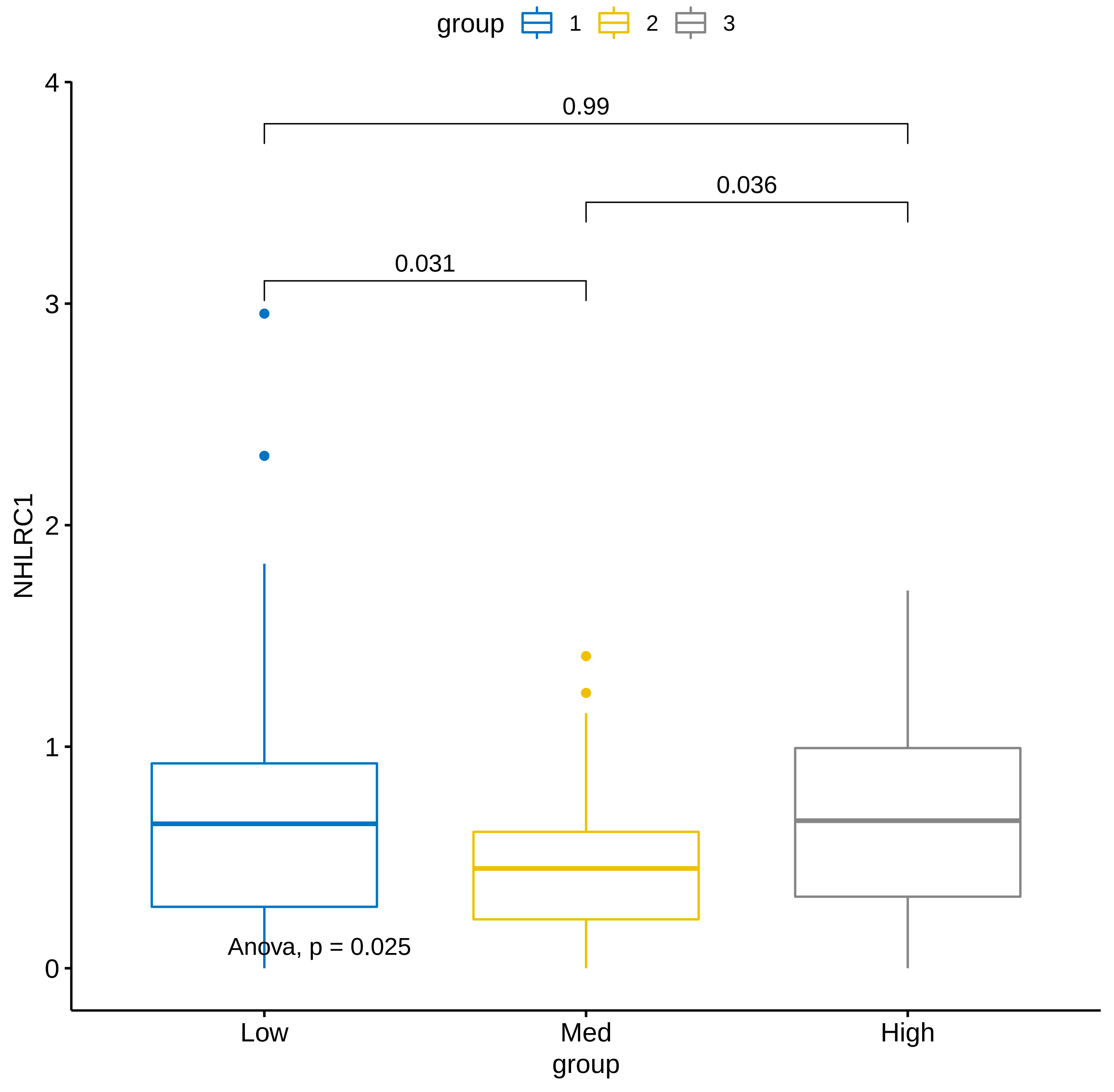

group
1
2
3
0.99
0.036
0.031
NHLRC1
4
3
2
1
0
Anova, p = 0.025
Low
Med
High
group

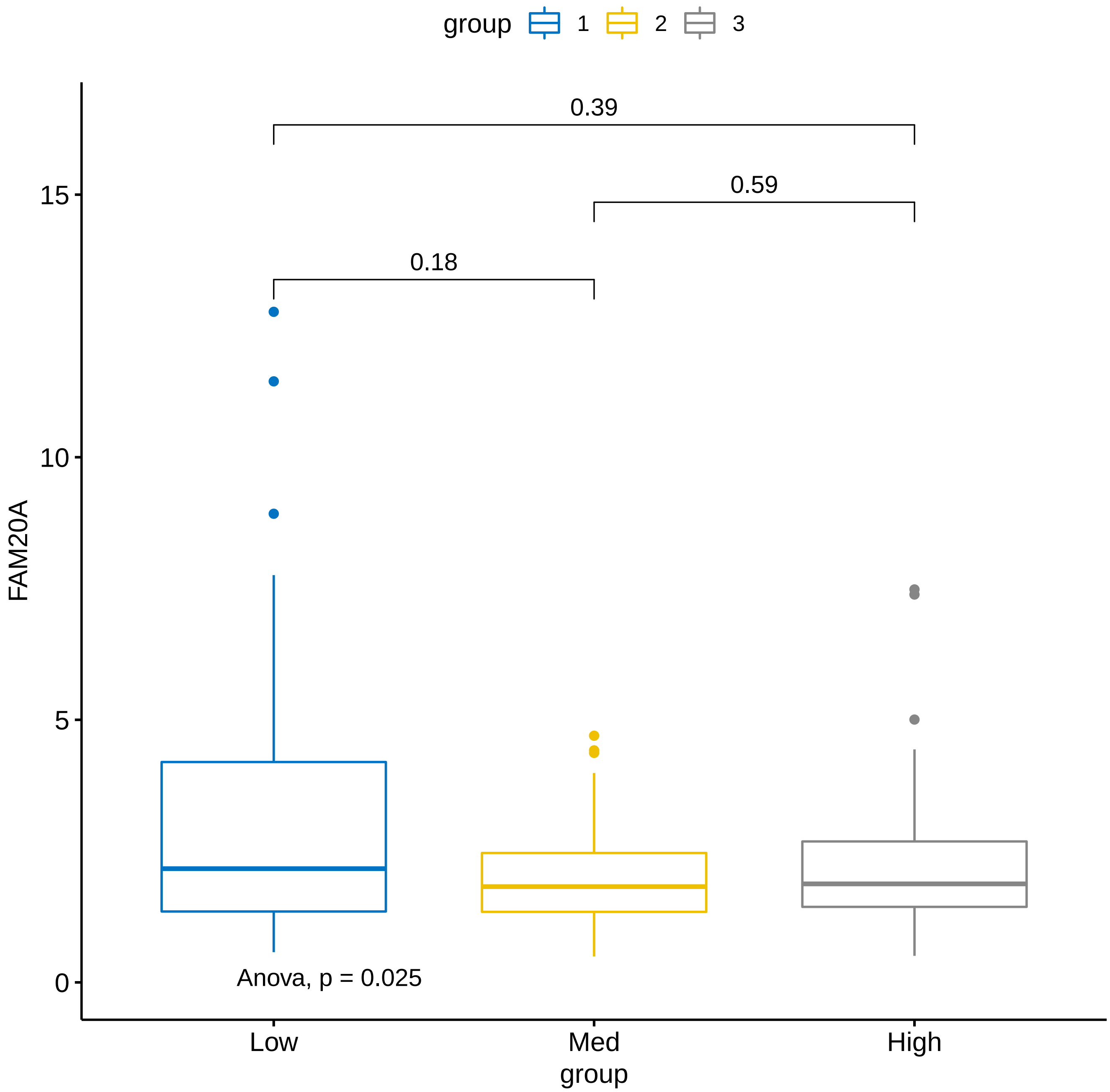
group
1
2
3
0.39
0.59
0.18
15
10
5
0
FAM20A
Anova, p = 0.025
Low
Med
High
group

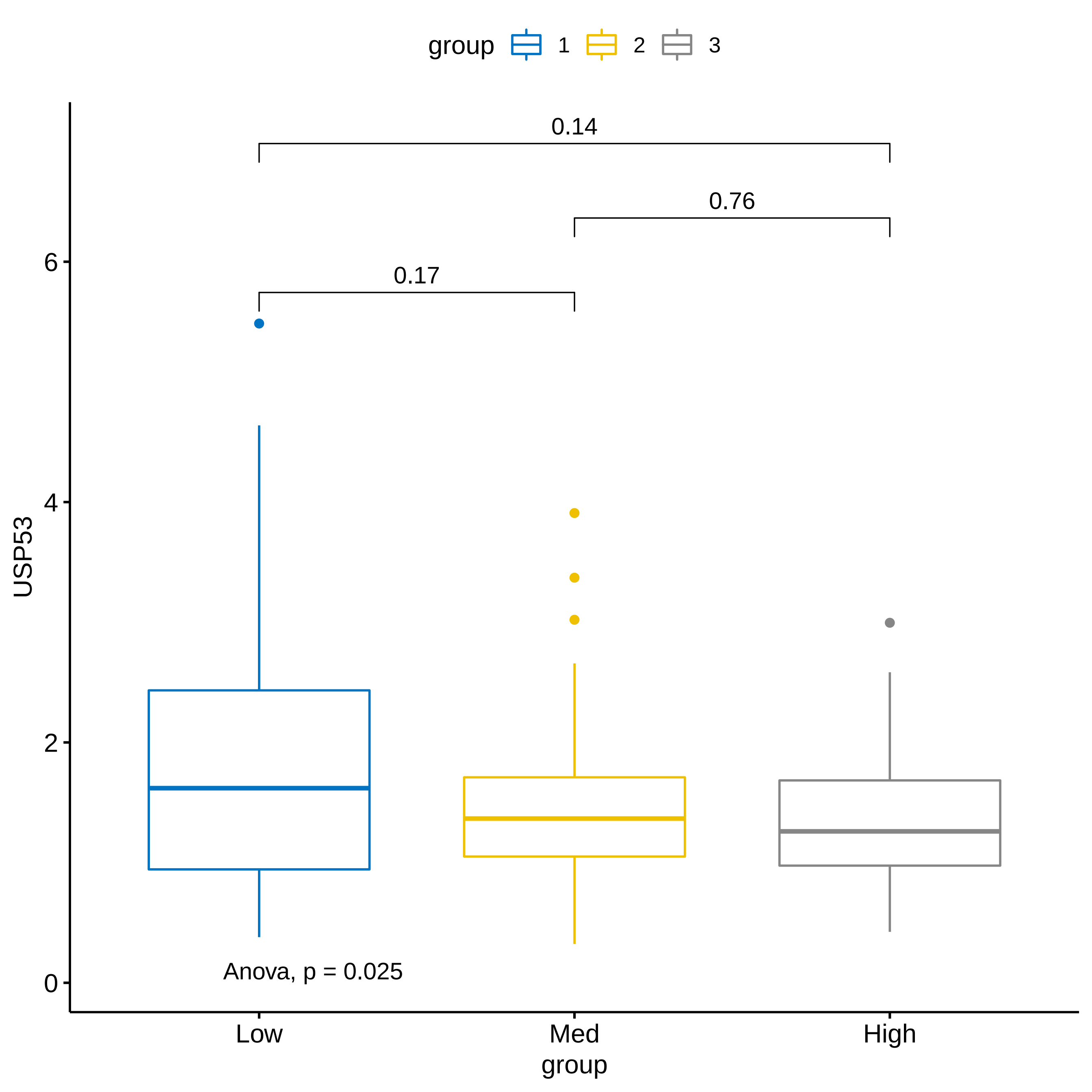
group
1
2
3
0.14
0.76
0.17
USP53
6
4
2
0
Anova, p = 0.025
Low
Med
High
group

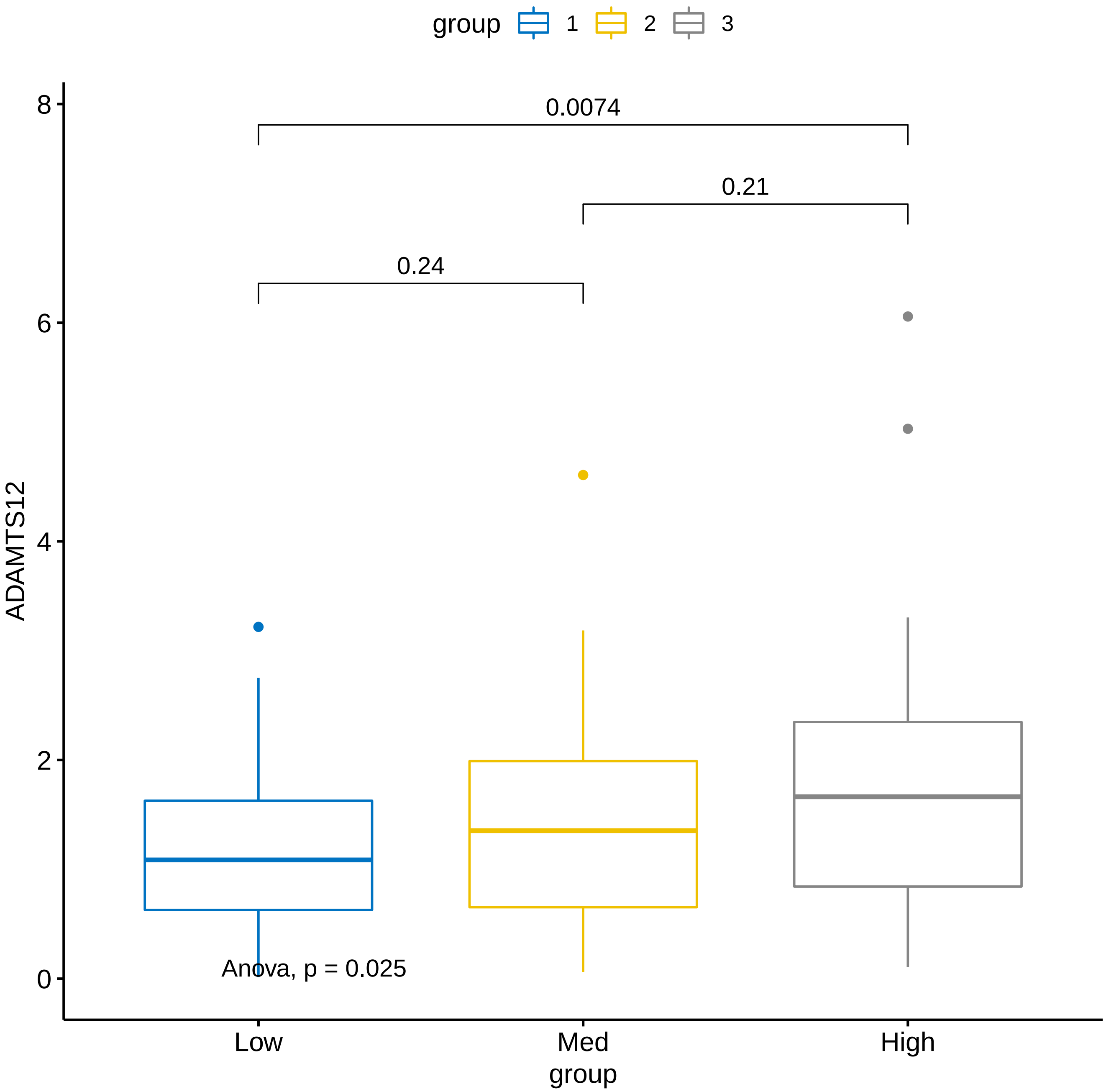
group 1 2 3
0.0074
0.21
0.24
ADAMTS12
8
6
4
2
0
Anova, p = 0.025
Low
Med
High
group

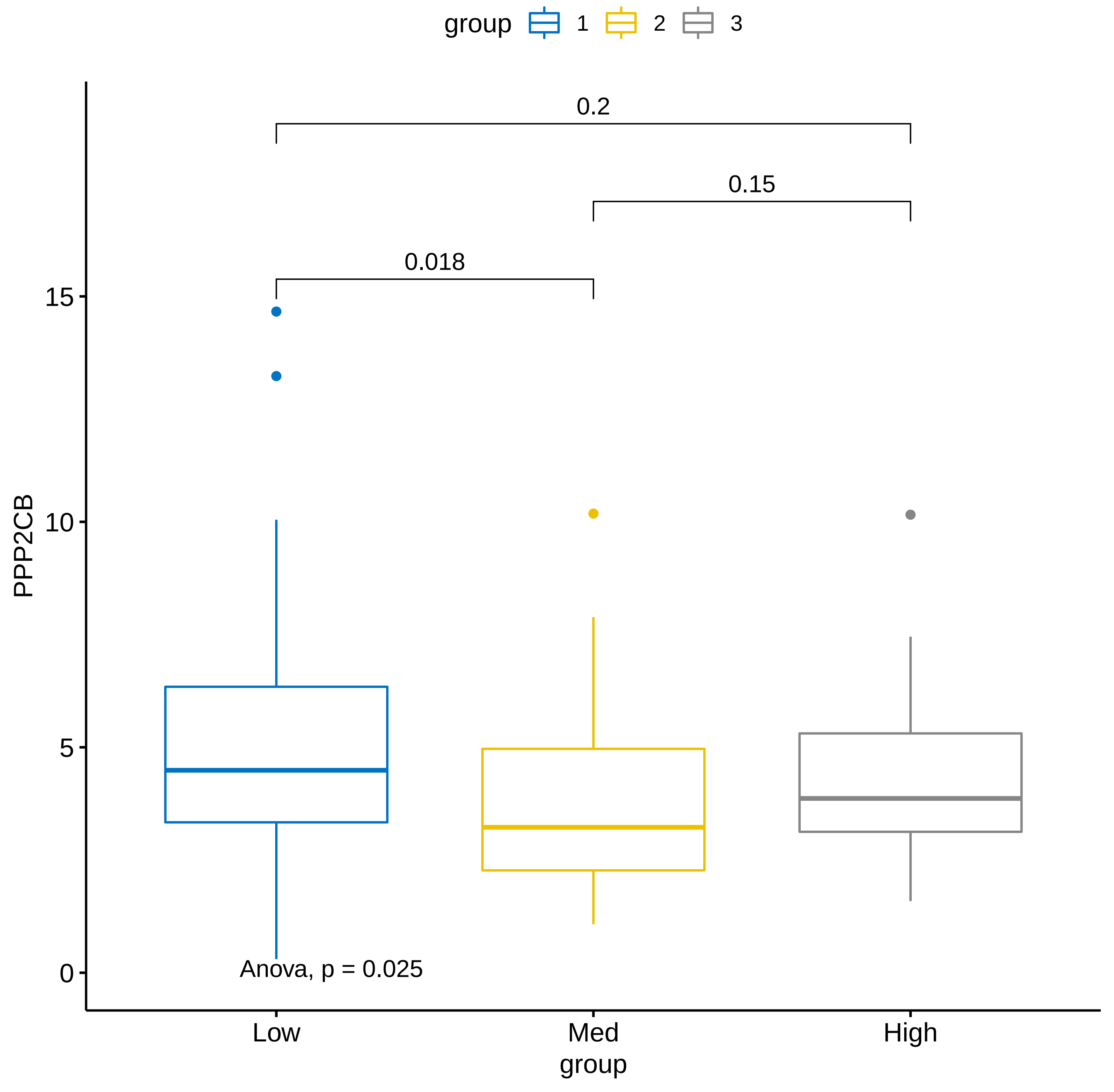

group
1
2
3
0.2
0.15
0.018
15
10
5
0
PPP2CB
Anova, p = 0.025
Low
Med
High
group

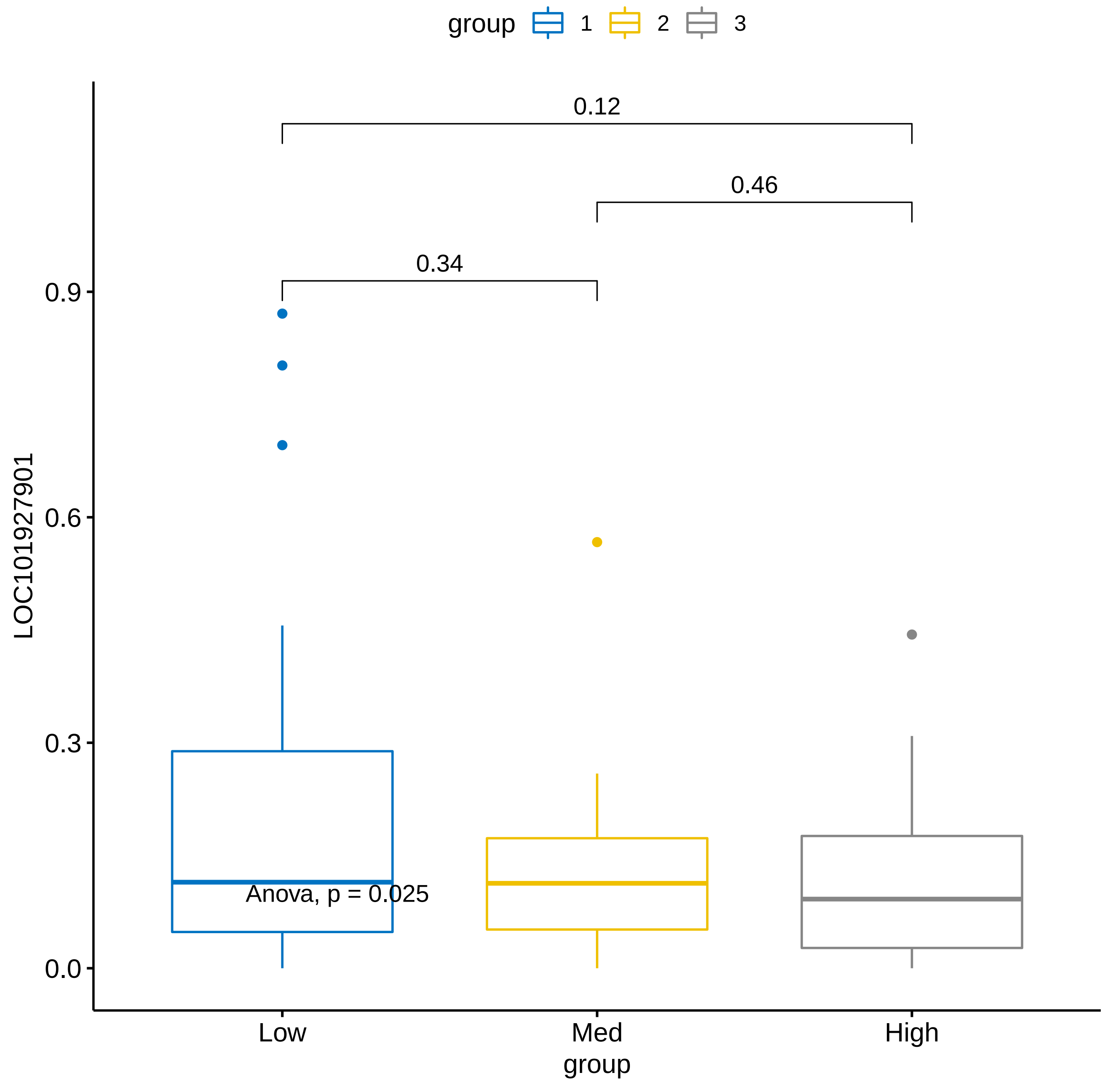

group
1
2
3
0.12
0.46
0.34
0.9
0.6
0.3
0.0
LOC101927901
Anova, p = 0.025
Low
Med
High
group

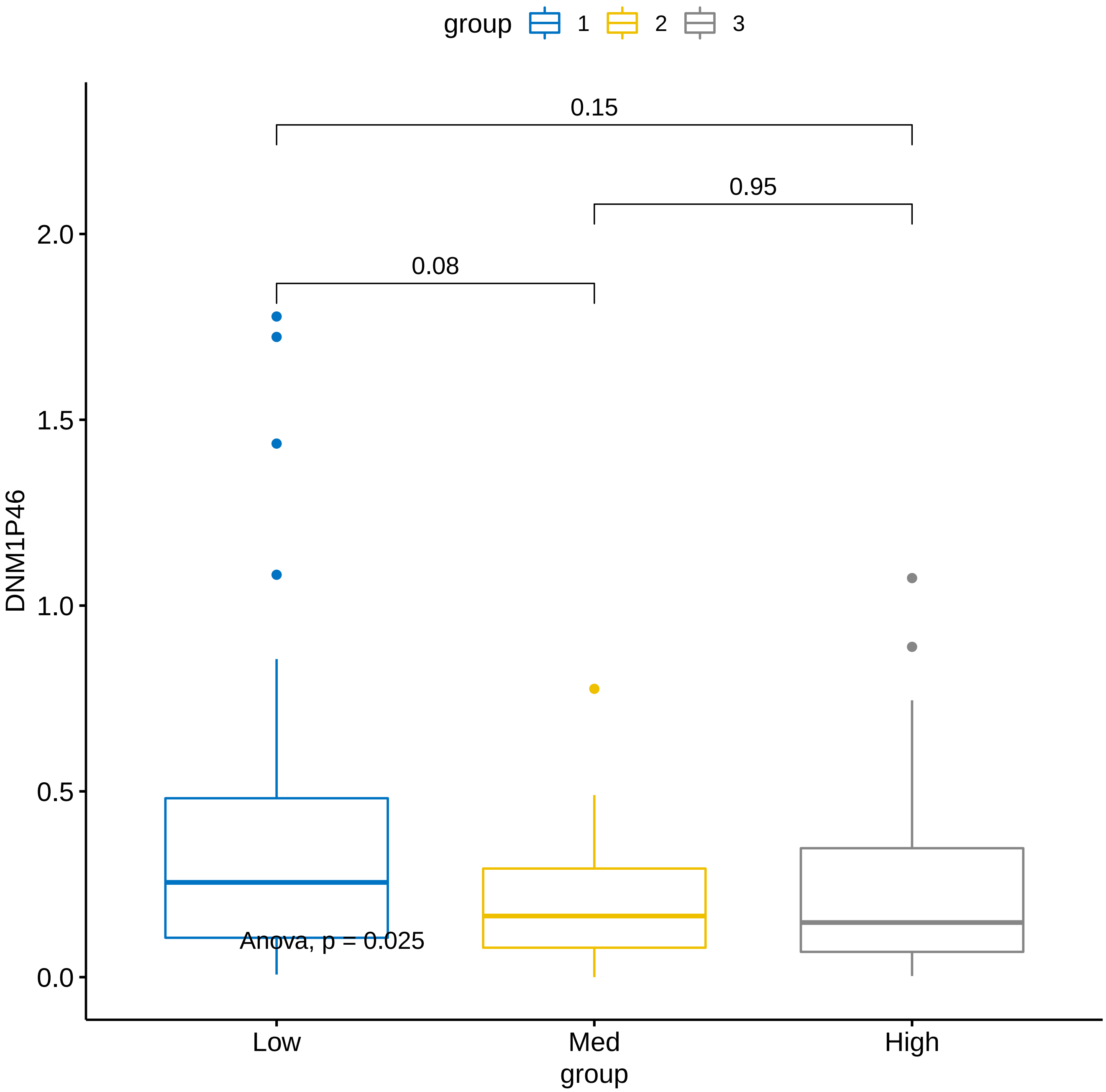
group 1 2 3
0.15
0.95
0.08
2.0
1.5
1.0
0.5
0.0
DNM1P46
Anova, p = 0.025
Low
Med
High
group

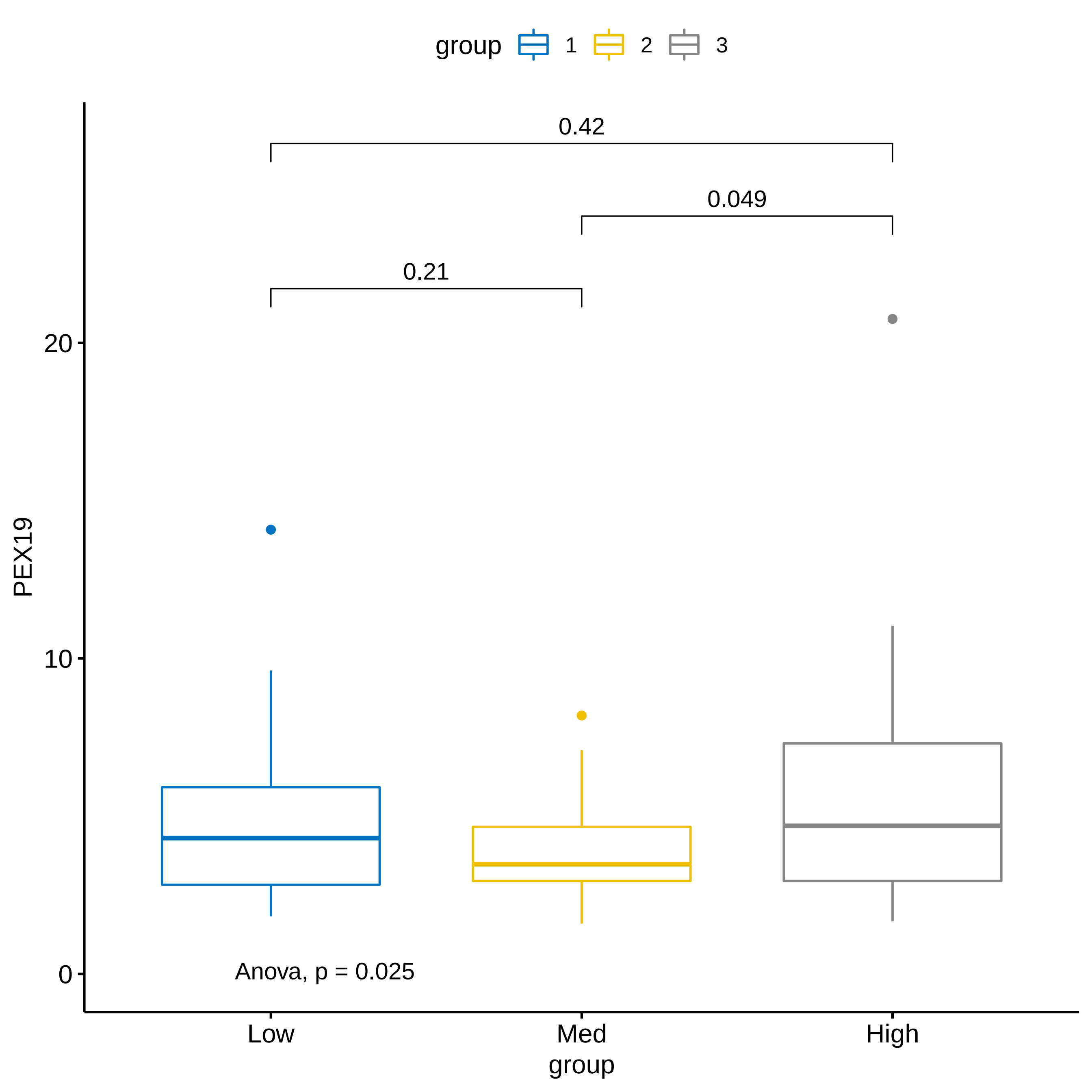

group 1 2 3
0.42
0.049
0.21
20
10
0
PEX19
Anova, p = 0.025
Low
Med
High
group

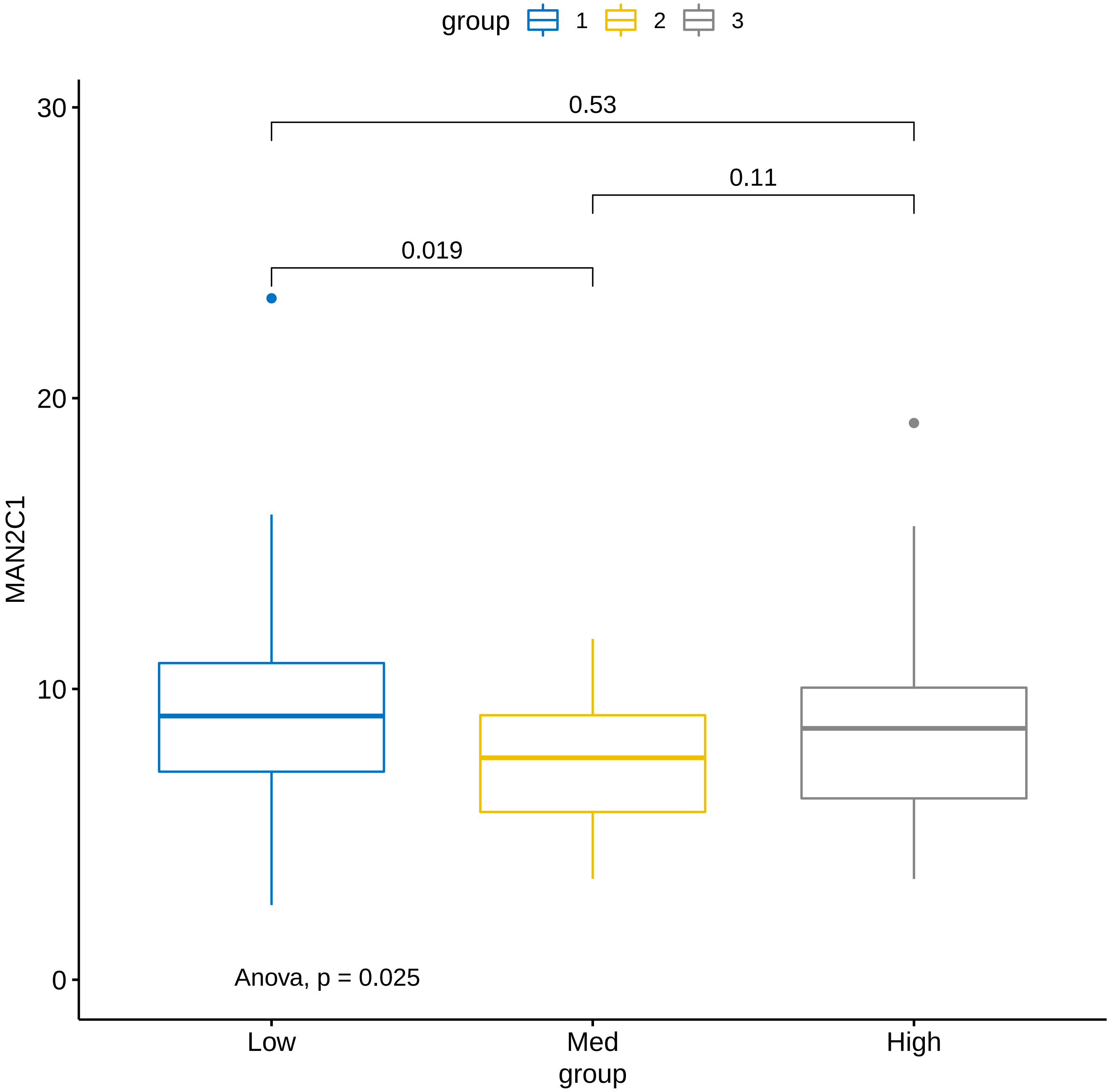
group
1
2
3
0.53
0.11
0.019
30
20
10
0
MAN2C1
Anova, p = 0.025
Low
Med
High
group

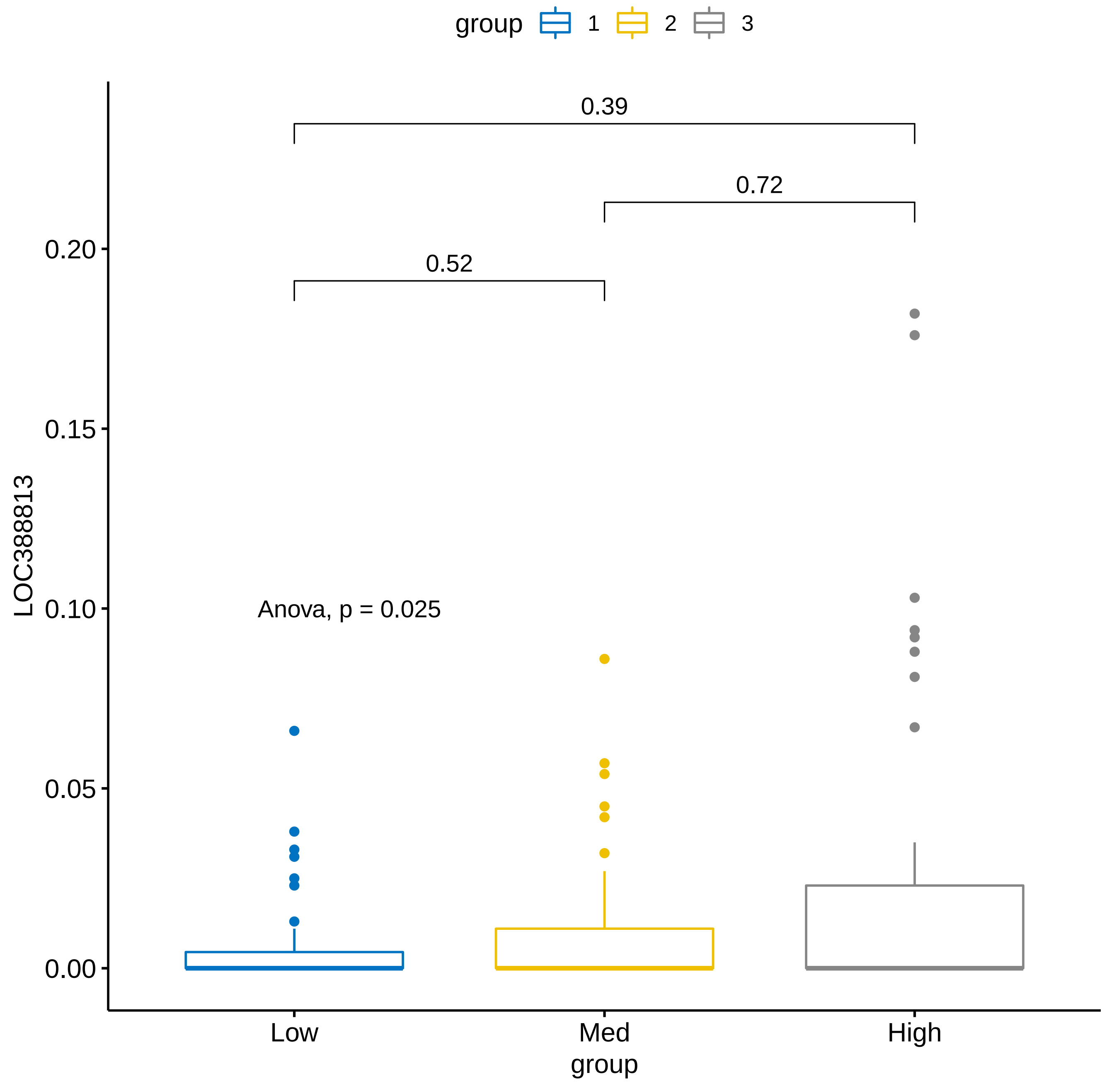
group 1 2 3
0.39
0.72
0.52
0.20
0.15
0.10
0.05
0.00
LOC388813
Anova, p = 0.025
Low
Med
High
group

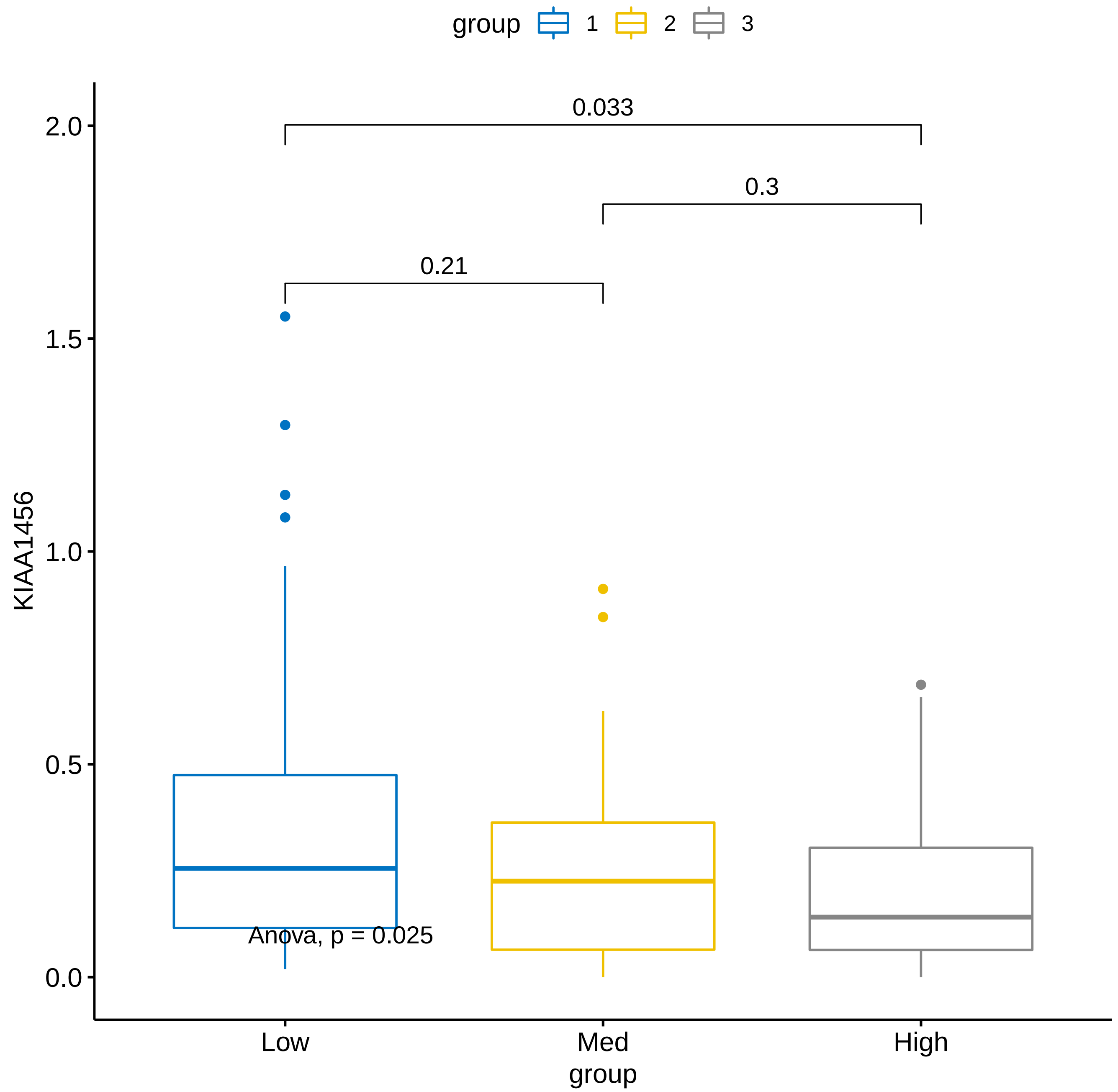

group 1 2 3
0.033
0.3
0.21
KIAA1456
2.0
1.5
1.0
0.5
0.0
Anova, p = 0.025
Low
Med
High
group

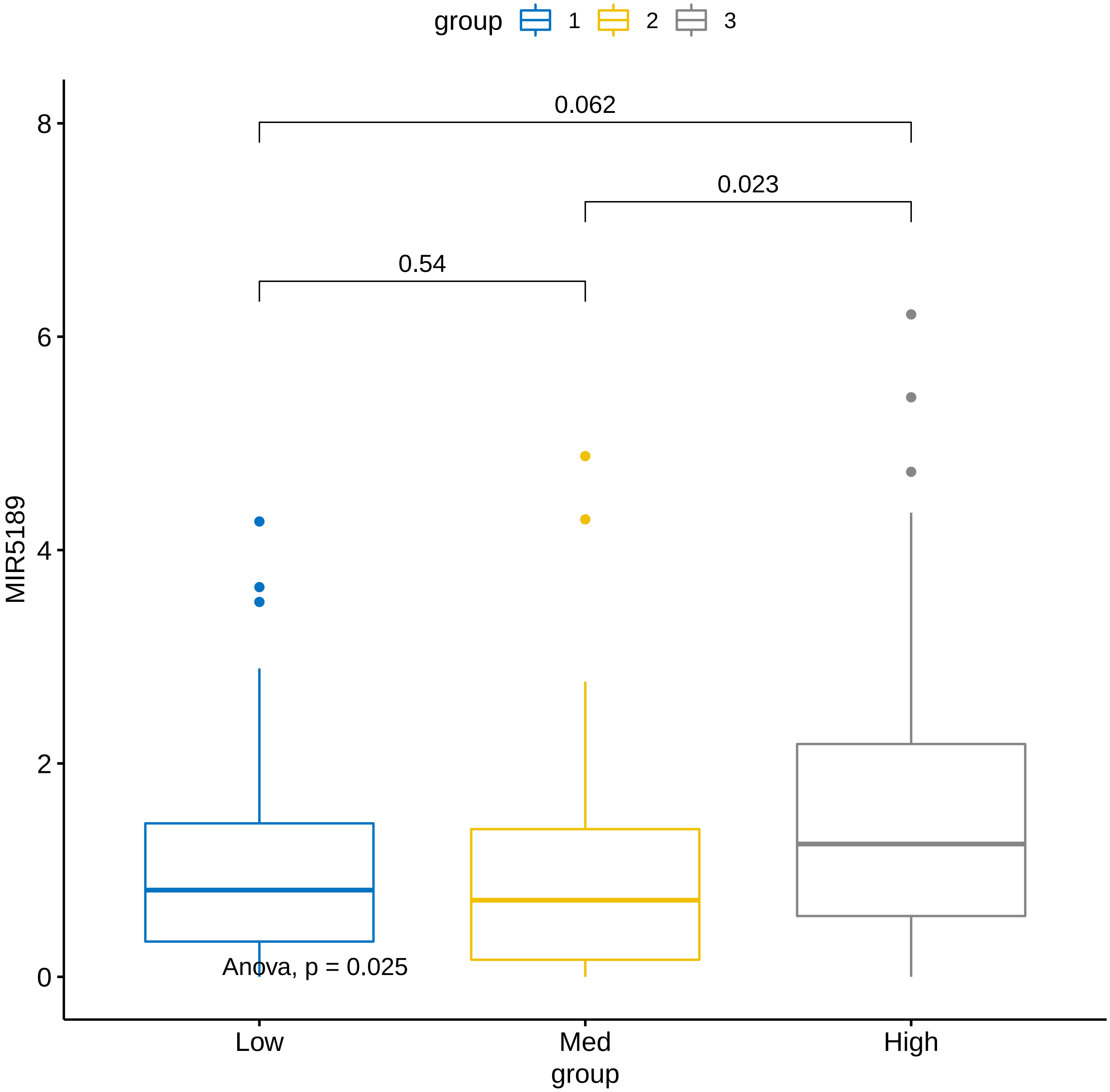
group
1
2
3
0.062
0.023
0.54
8
6
4
2
0
MIR5189
Anova, p = 0.025
Low
Med
High
group

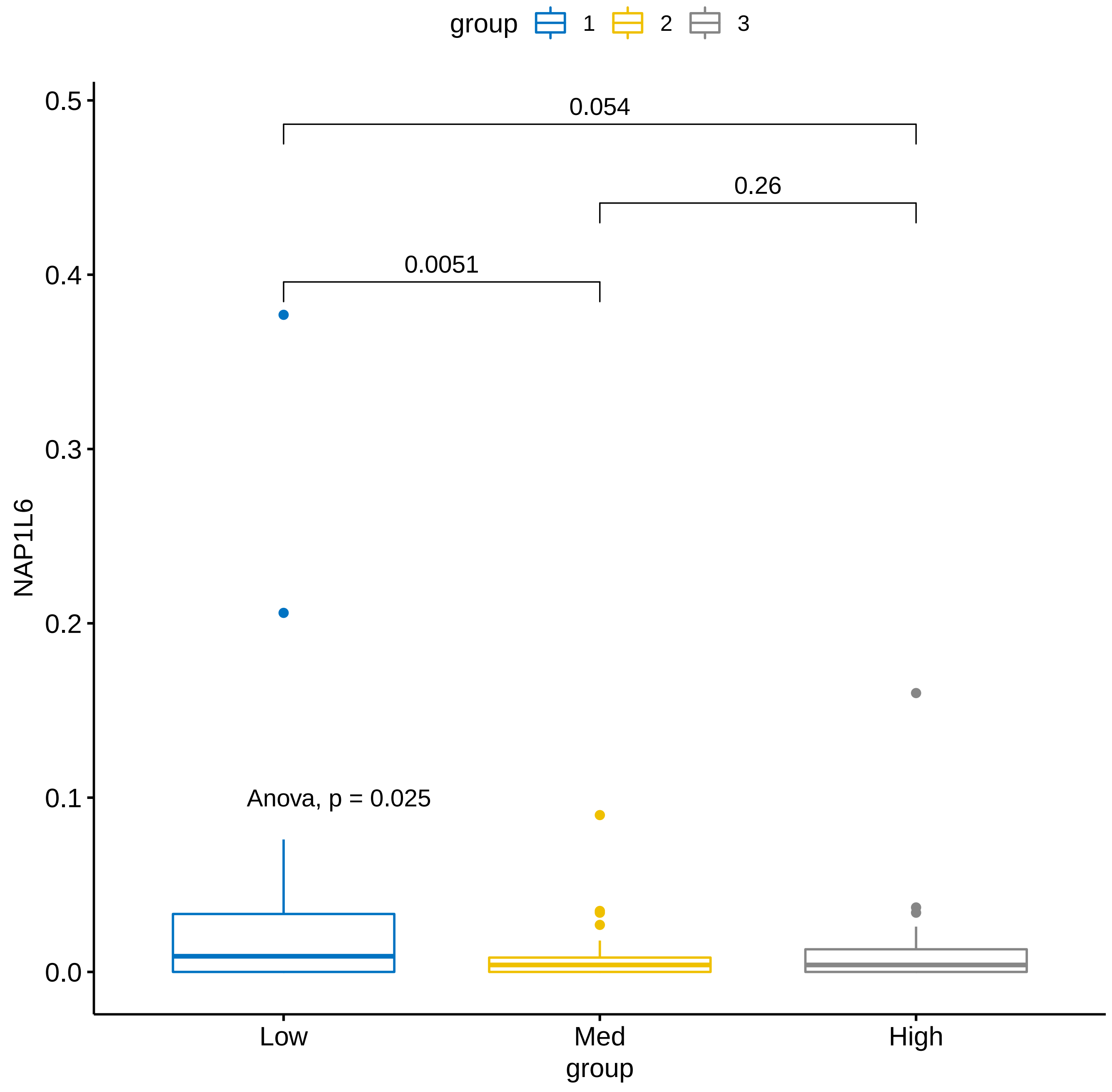
group
1
2
3
0.054
0.26
0.0051
Anova, p = 0.025
NAP1L6
0.5
0.4
0.3
0.2
0.1
0.0
Low
Med
High
group

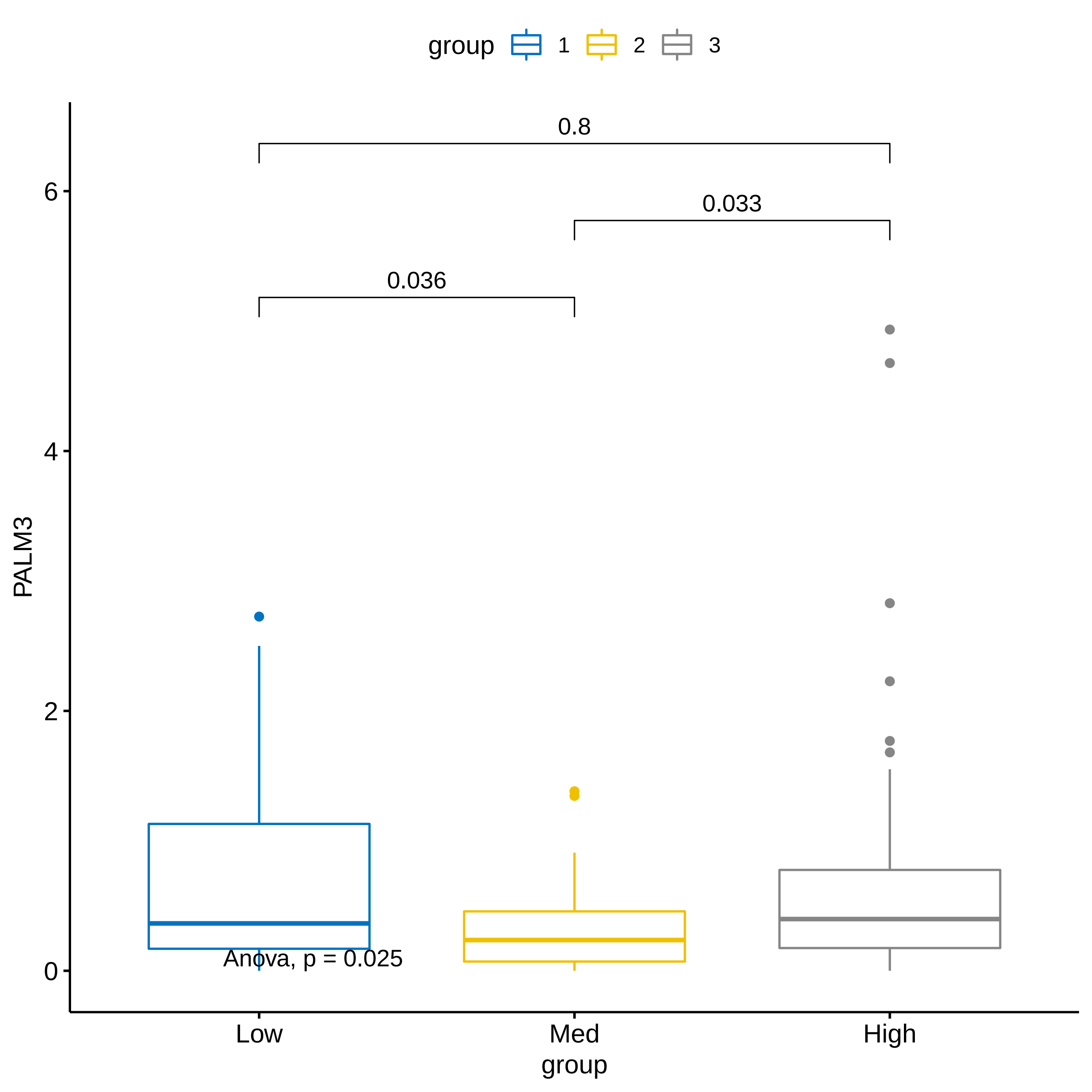

group 1 2 3
0.8
0.033
0.036
6
4
2
0
PALM3
Anova, p = 0.025
Low
Med
High
group

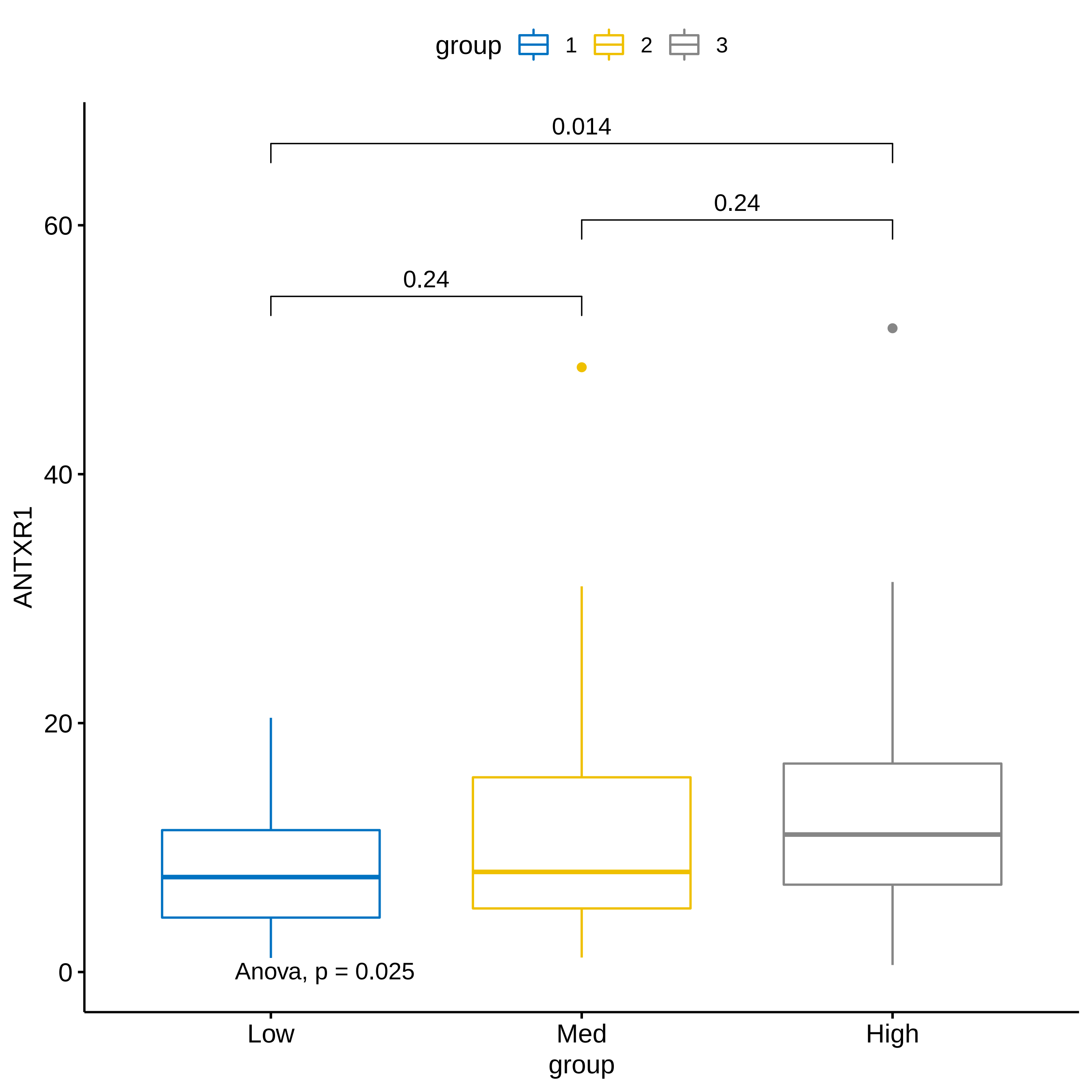
group
1
2
3
0.014
0.24
0.24
60
40
20
0
ANTXR1
Anova, p = 0.025
Low
Med
High
group

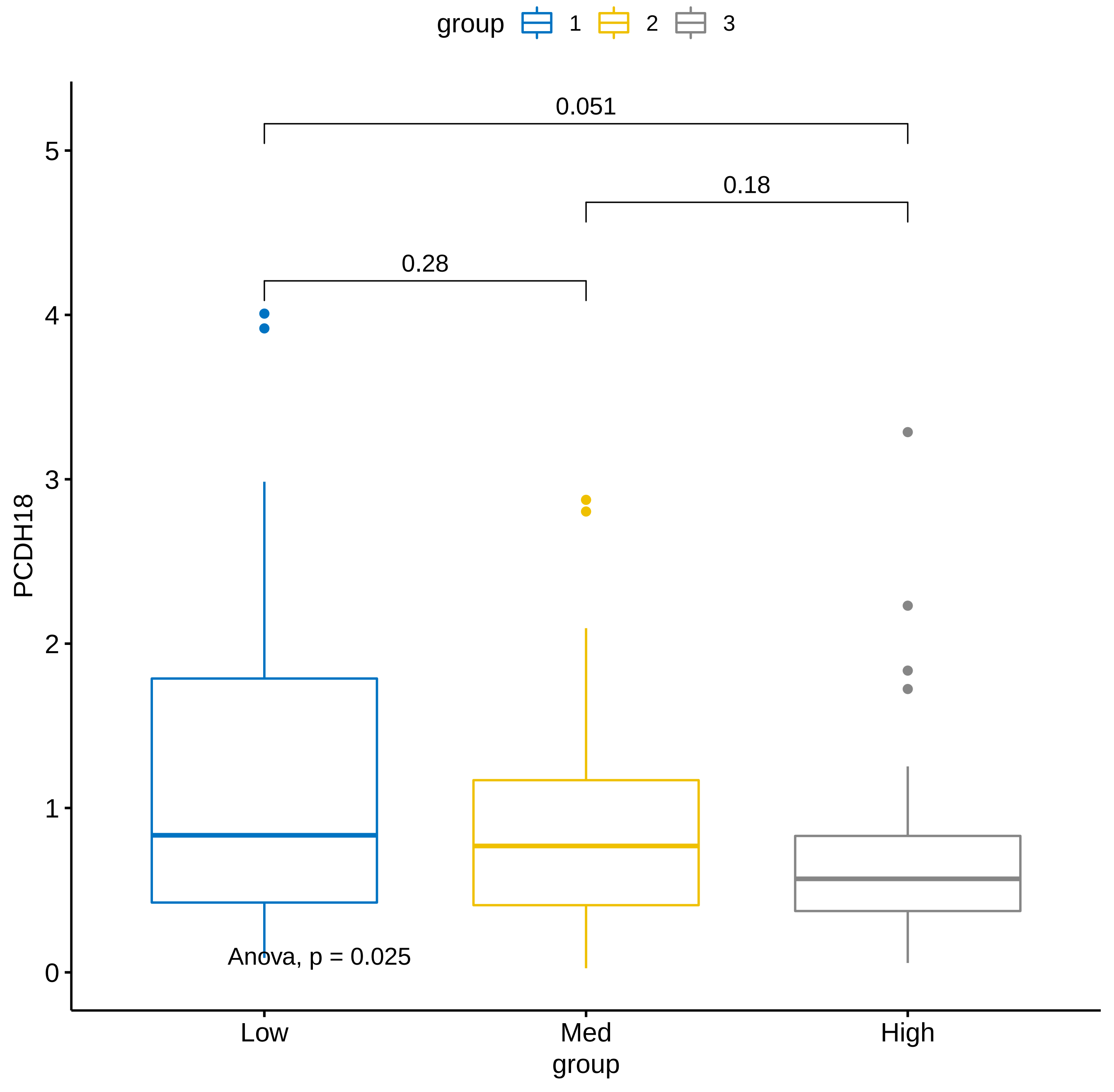
group 1 2 3
0.051
0.18
0.28
5
4
3
2
1
0
PCDH18
Anova, p = 0.025
Low
Med
High
group

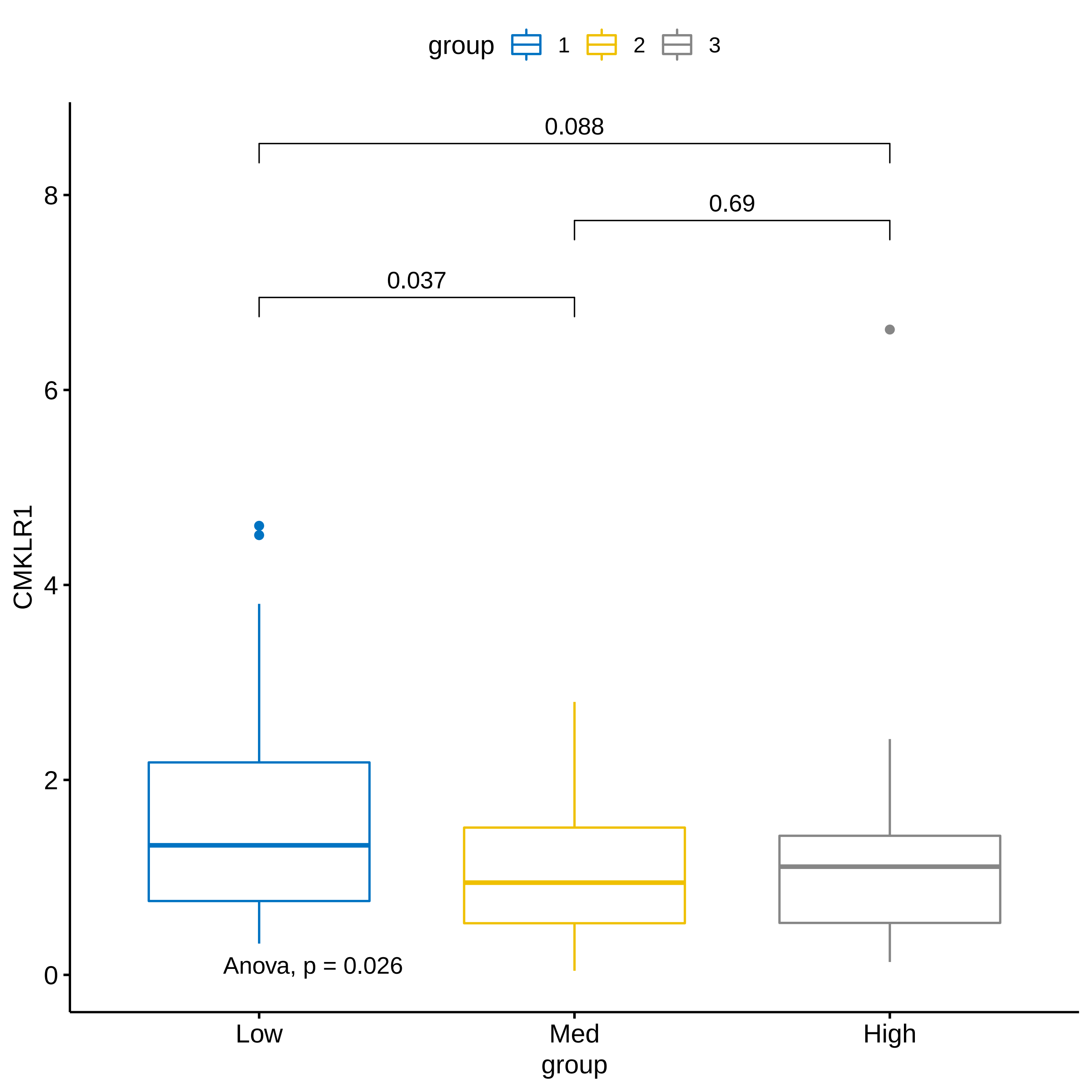
group
1
2
3
0.088
0.69
0.037
8
6
4
2
0
CMKLR1
Anova, p = 0.026
Low
Med
High
group

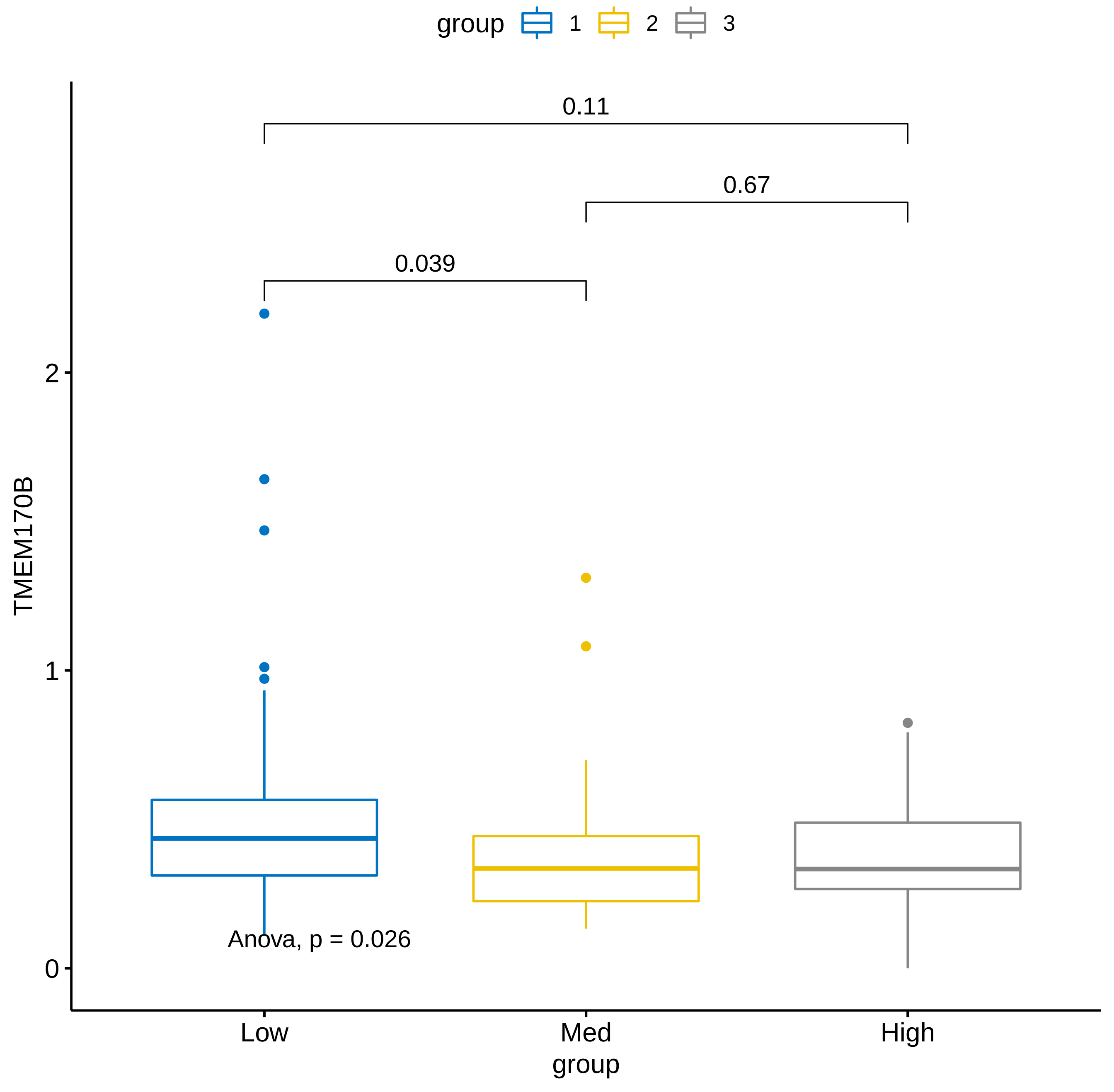
group
1
2
3
0.11
0.67
0.039
TMEM170B
2
1
0
Anova, p = 0.026
Low
Med
High
group

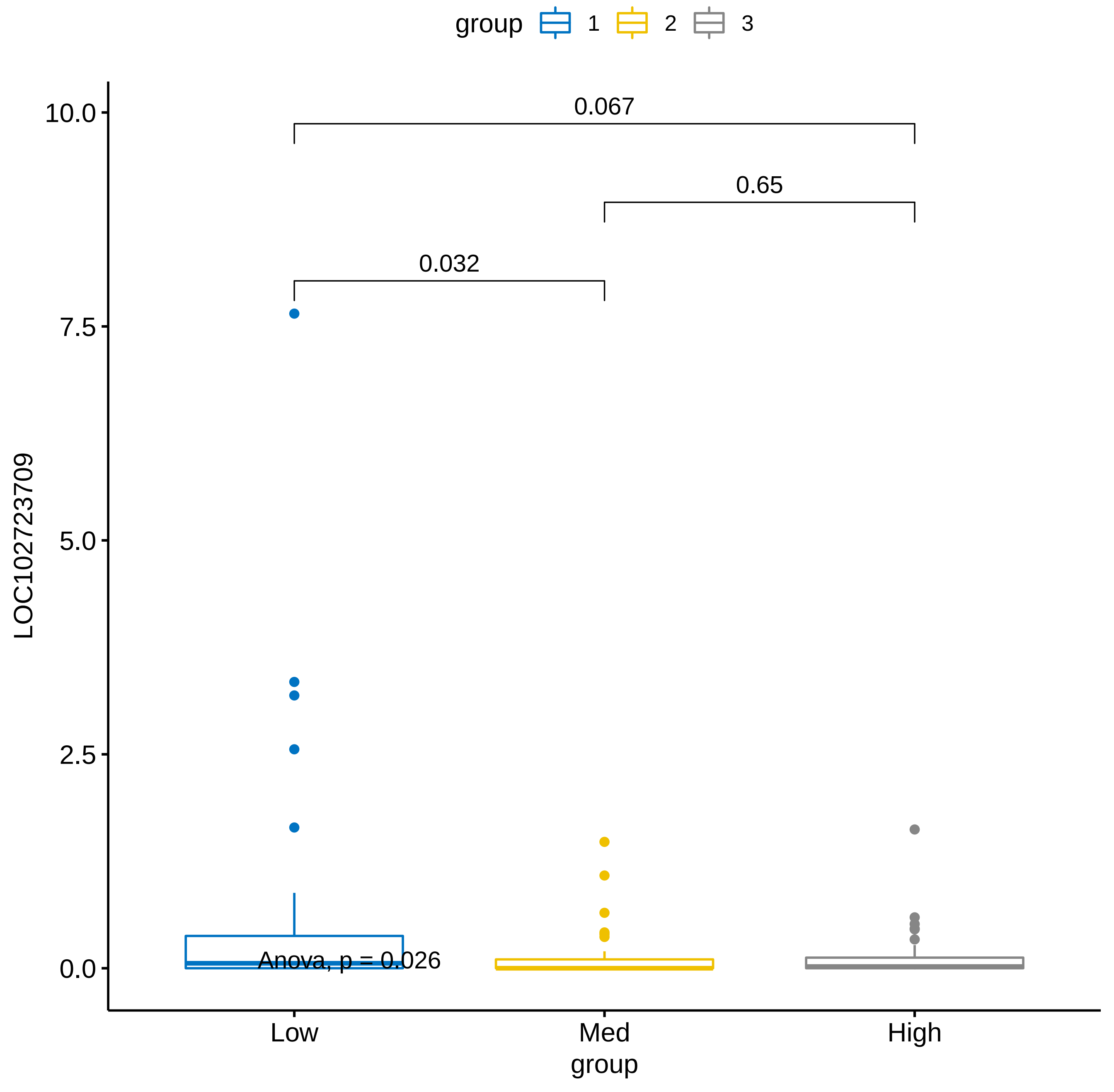
group
1
2
3
0.067
0.65
0.032
10.0
7.5
5.0
2.5
0.0
LOC102723709
Anova, p = 0.026
Low
Med
High
group

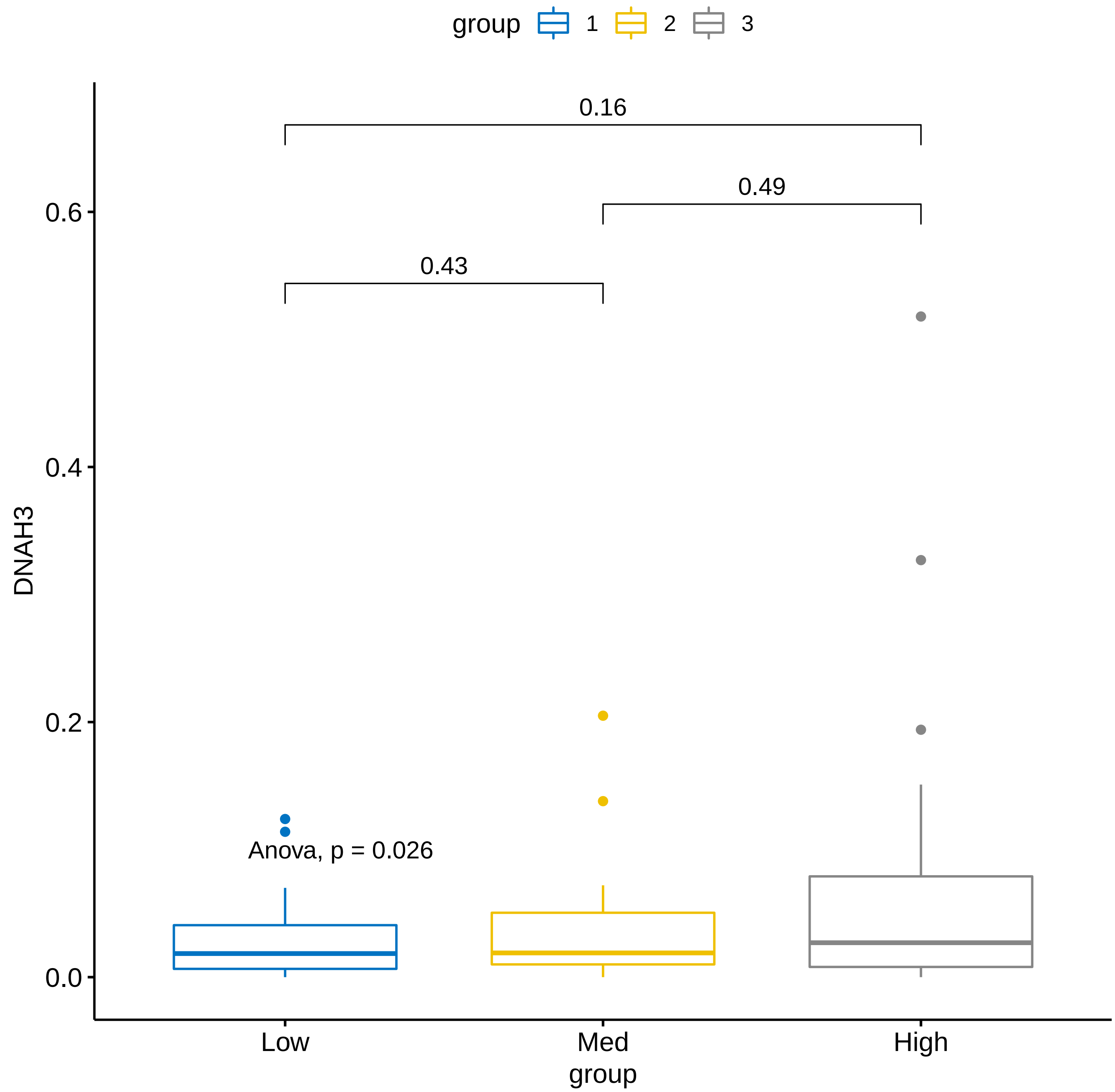
group 1 2 3
0.16
0.49
0.43
Anova, p = 0.026
0.6
0.4
0.2
0.0
DNAH3
Low
Med
High
group

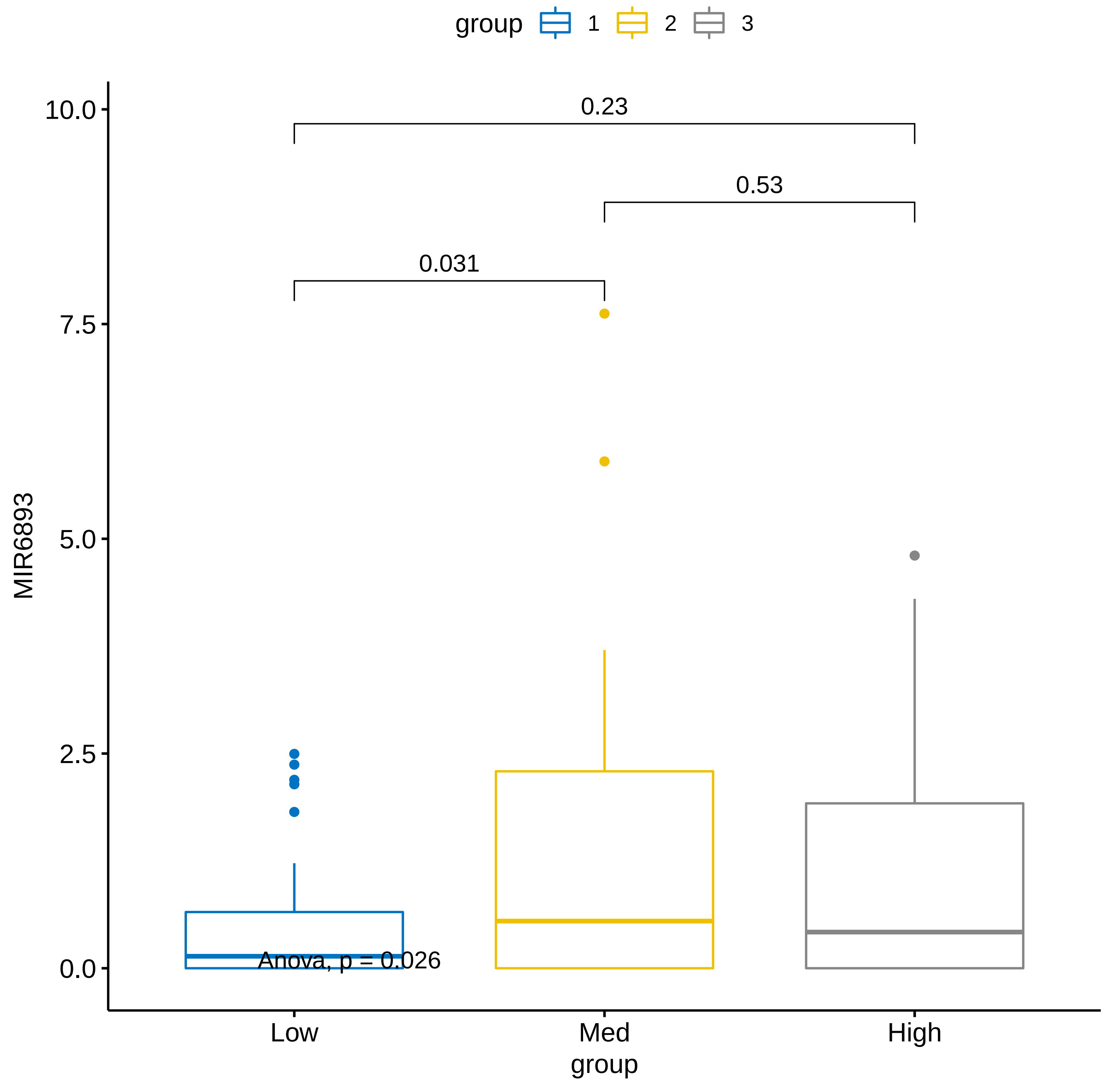

group
1
2
3
0.23
0.53
0.031
10.0
7.5
5.0
2.5
0.0
MIR6893
Anova, p = 0.026
Low
Med
High
group

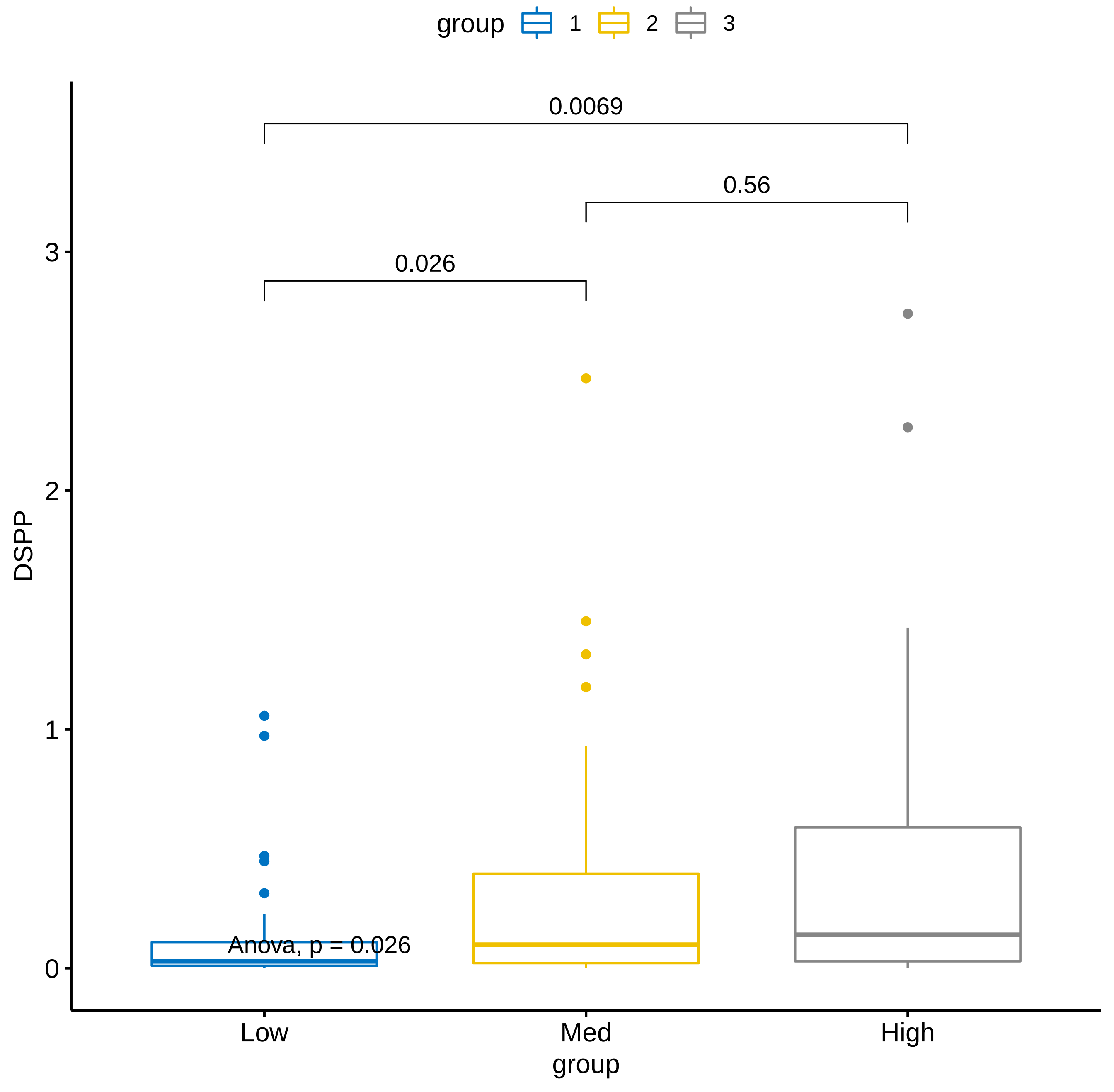
group
1
2
3
0.0069
0.56
0.026
3
2
1
0
DSPP
Anova, p = 0.026
Low
Med
High
group

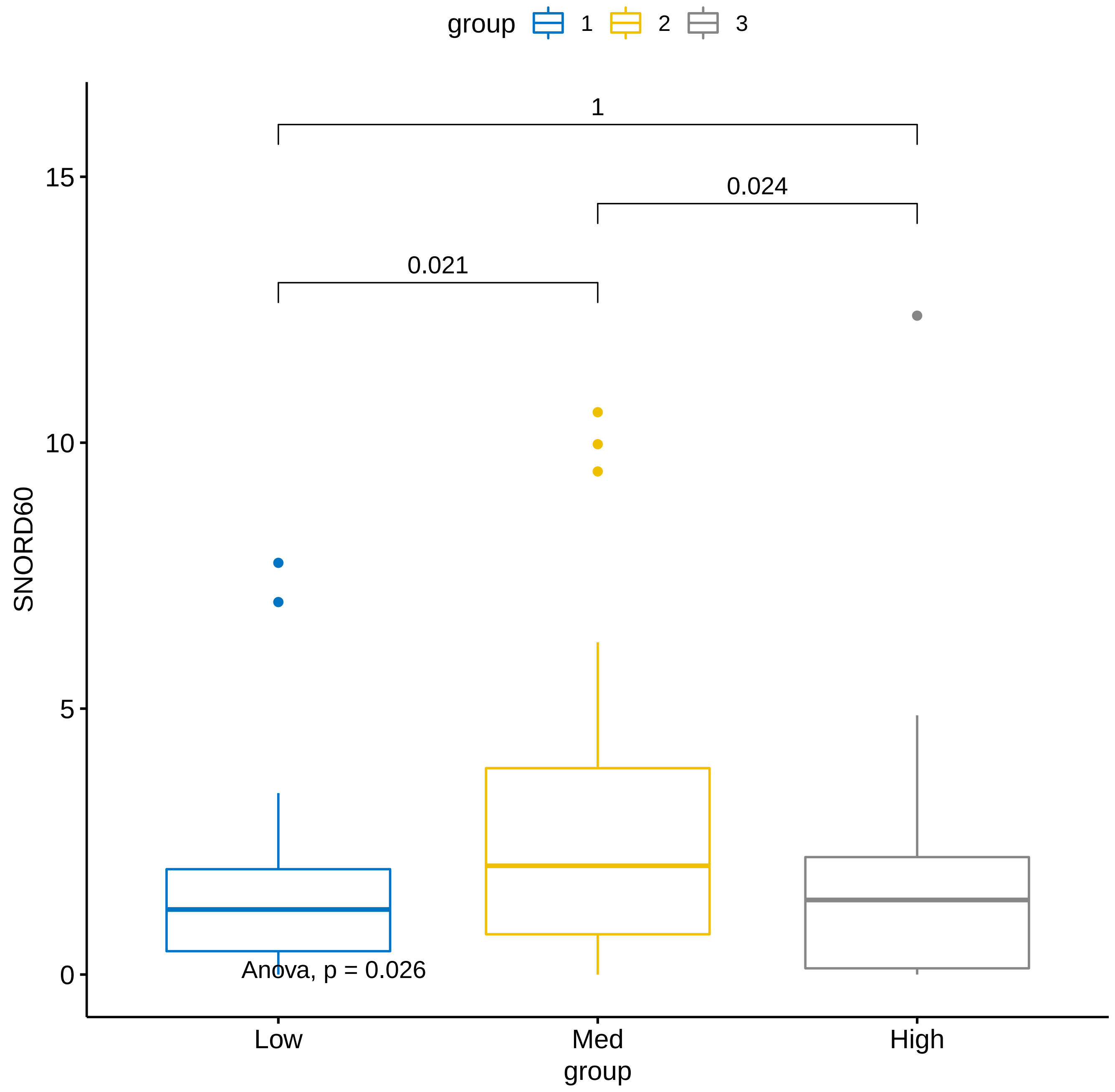
group 1 2 3
1
0.024
0.021
15
10
5
0
SNORD60
Anova, p = 0.026
Low
Med
High
group

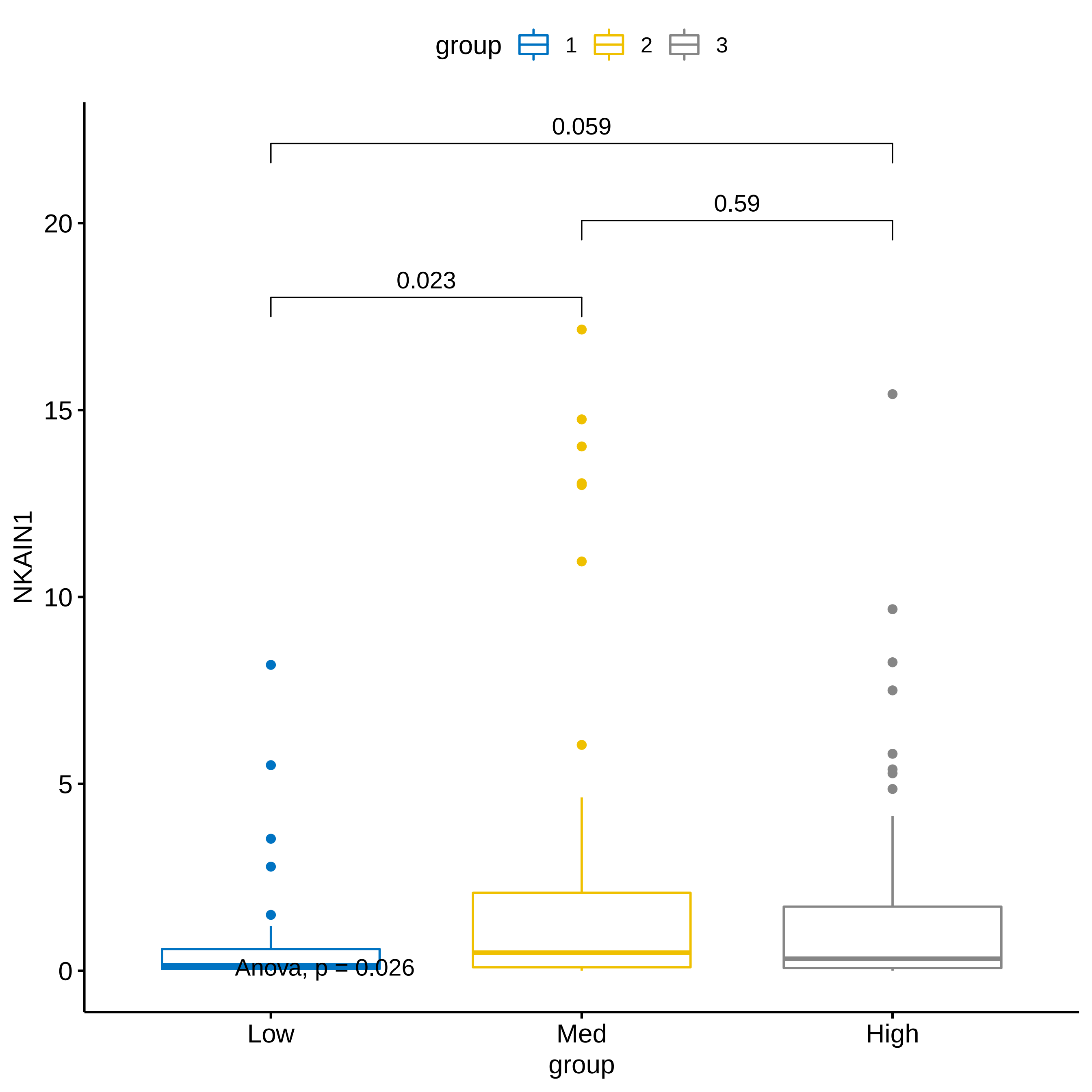

group 1 2 3
0.059
0.59
0.023
NKAIN1
20
15
10
5
0
Anova, p = 0.026
Low
Med
High
group

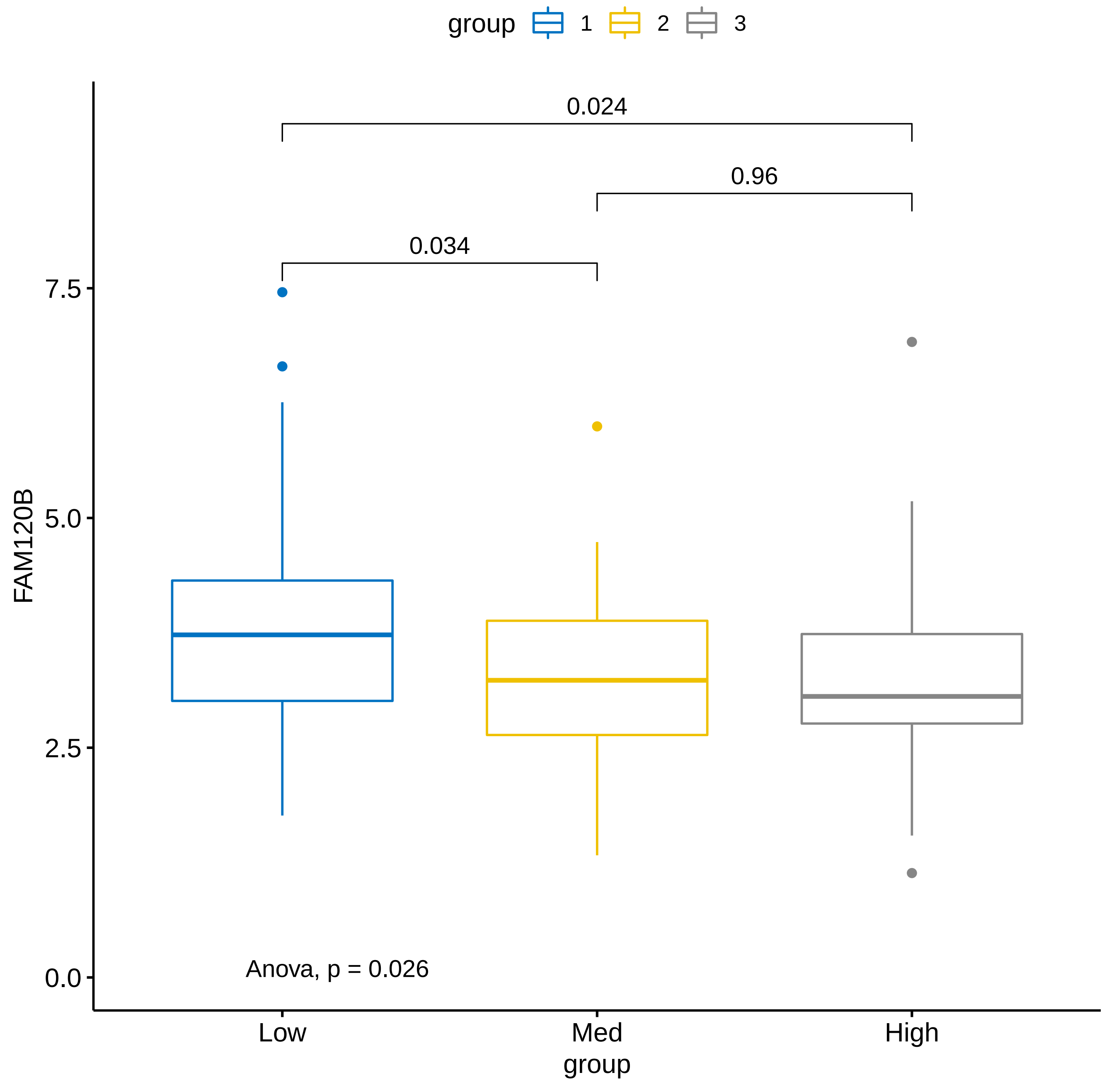

group
1
2
3
0.024
0.96
0.034
7.5
5.0
2.5
0.0
FAM120B
Anova, p = 0.026
Low
Med
High
group

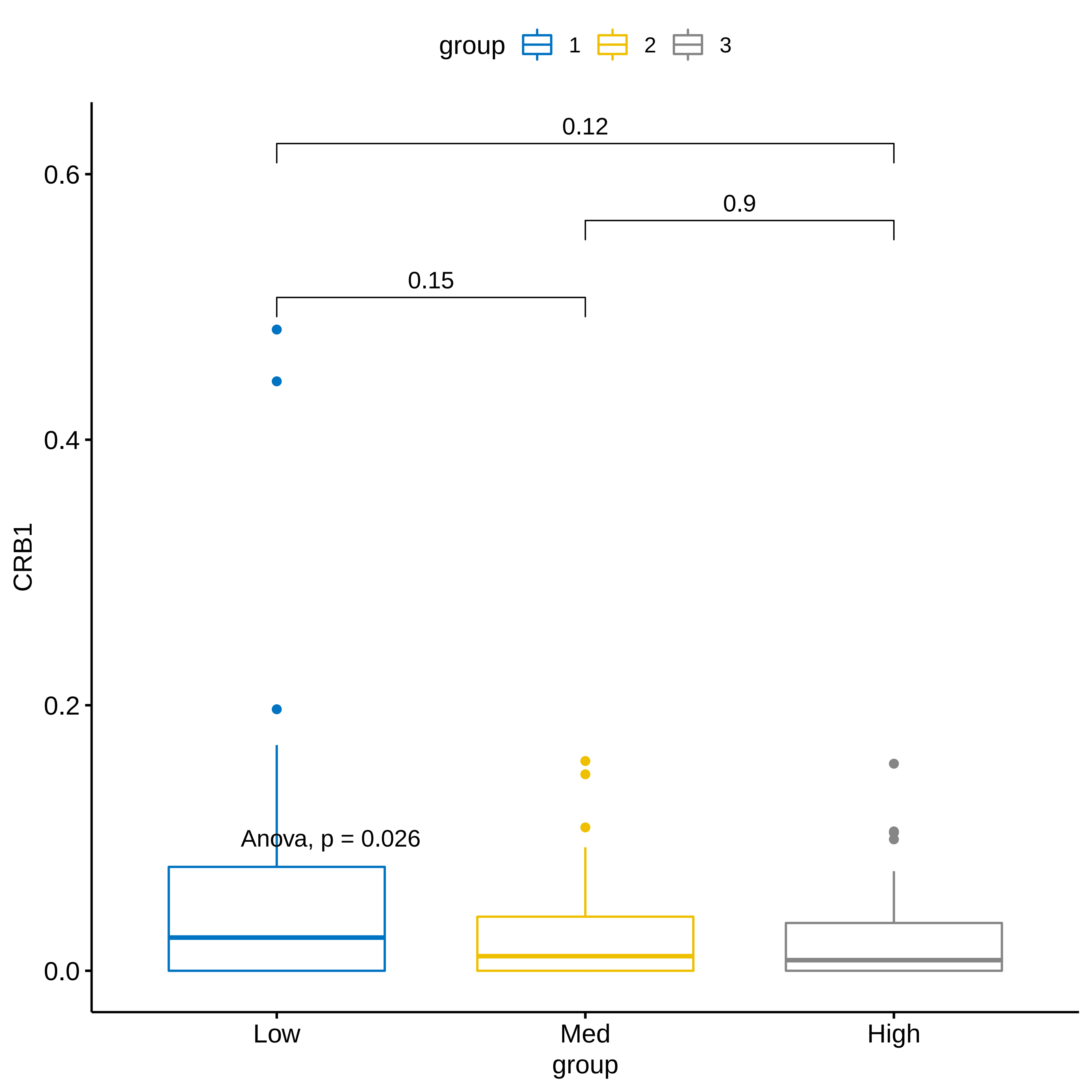
group
1
2
3
0.12
0.9
0.15
0.6
0.4
0.2
0.0
CRB1
Anova, p = 0.026
Low
Med
High
group

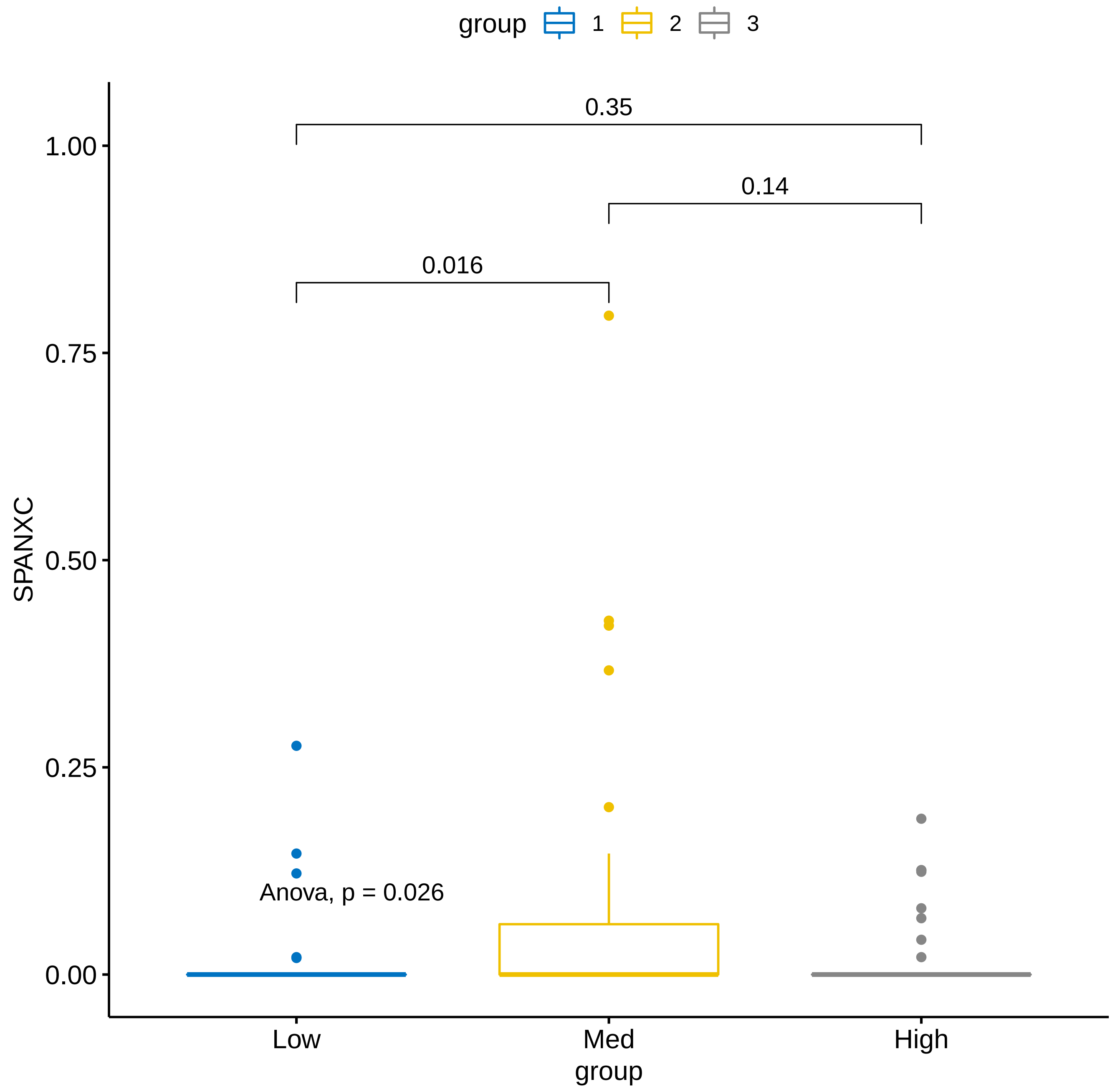

group 1 2 3
0.35
0.14
0.016
1.00
0.75
0.50
0.25
0.00
SPANXC
Anova, p = 0.026
Low
Med
High
group

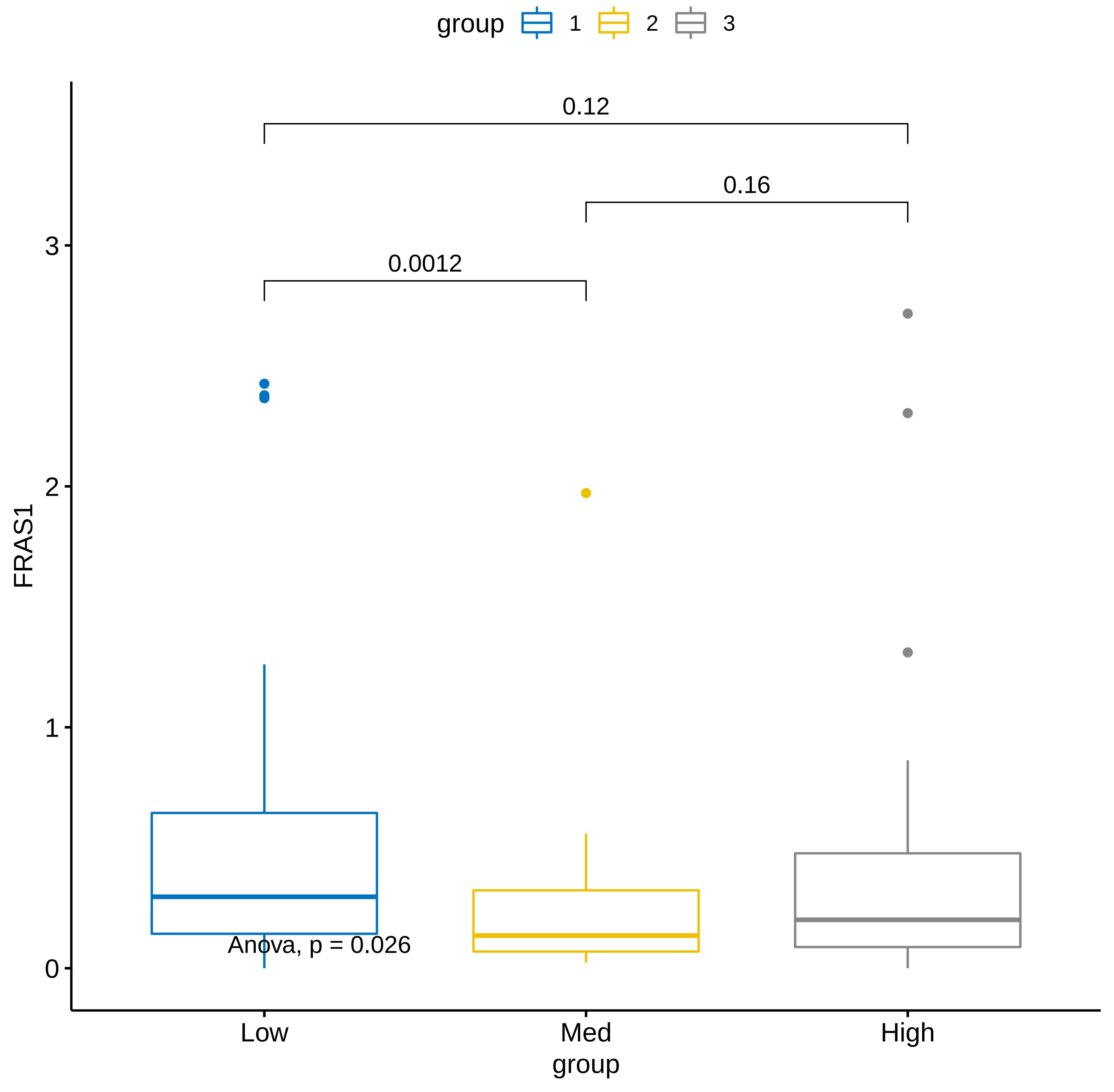

group
1
2
3
0.12
0.16
0.0012
3
2
1
0
FRAS1
Anova, p = 0.026
Low
Med
High
group

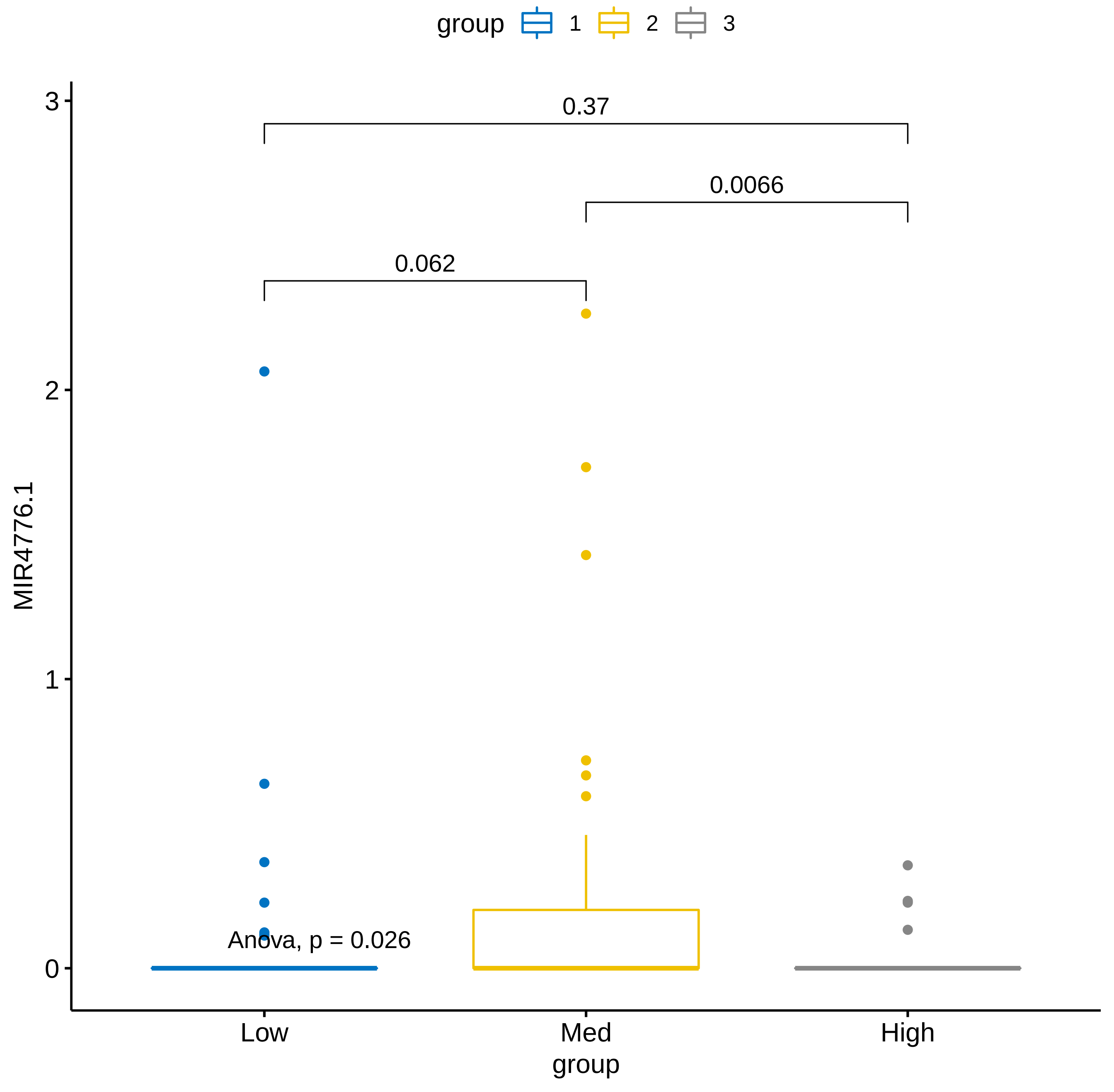

group 1 2 3
0.37
0.0066
0.062
Anova, p = 0.026
MIR4776.1
3
2
1
0
Low
Med
High
group

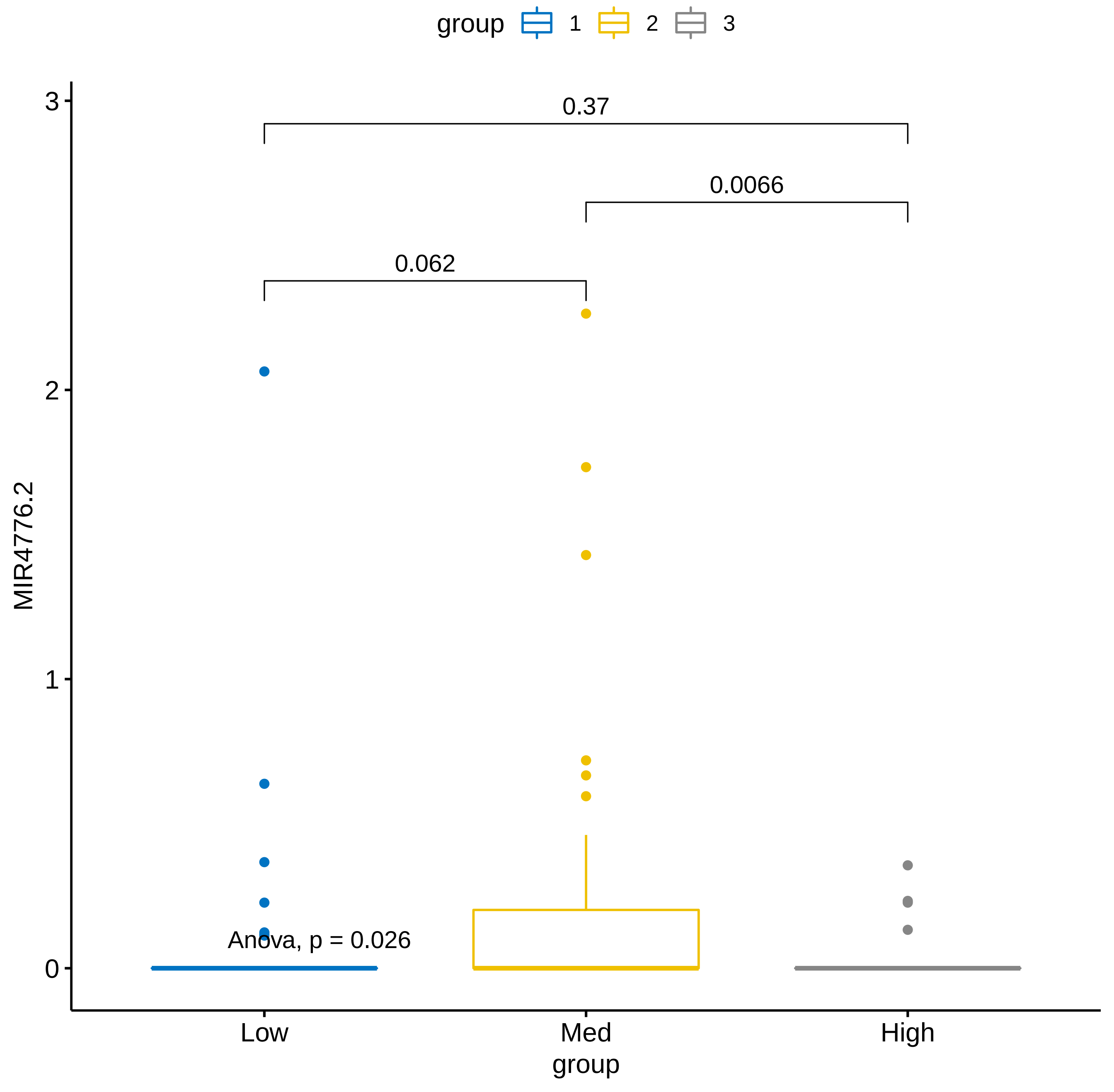
group
1
2
3
0.37
0.0066
0.062
Anova, p = 0.026
MIR4776.2
0
1
2
3
Low
Med
High
group

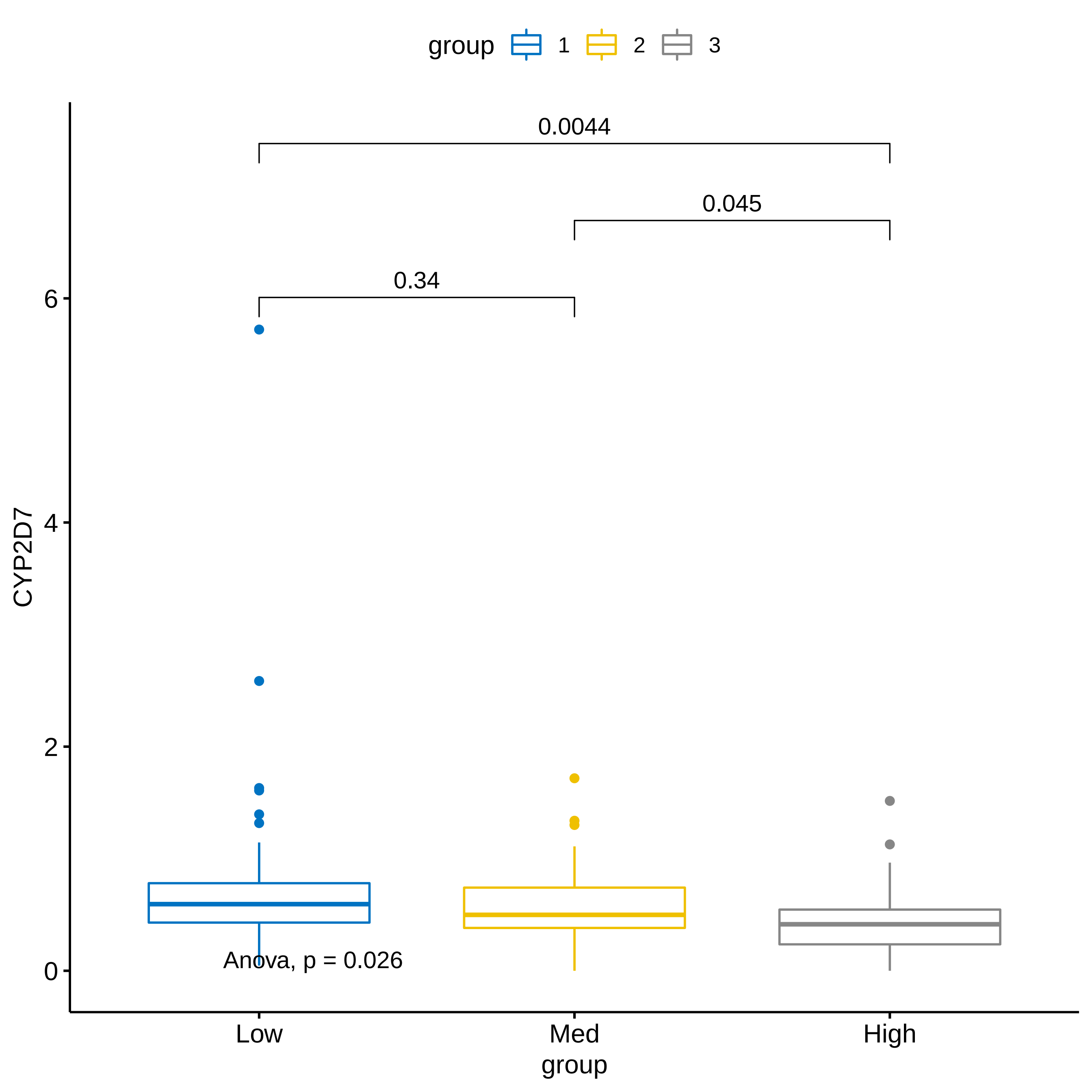
group
1
2
3
0.0044
0.045
0.34
6
4
2
0
CYP2D7
Anova, p = 0.026
Low
Med
High
group

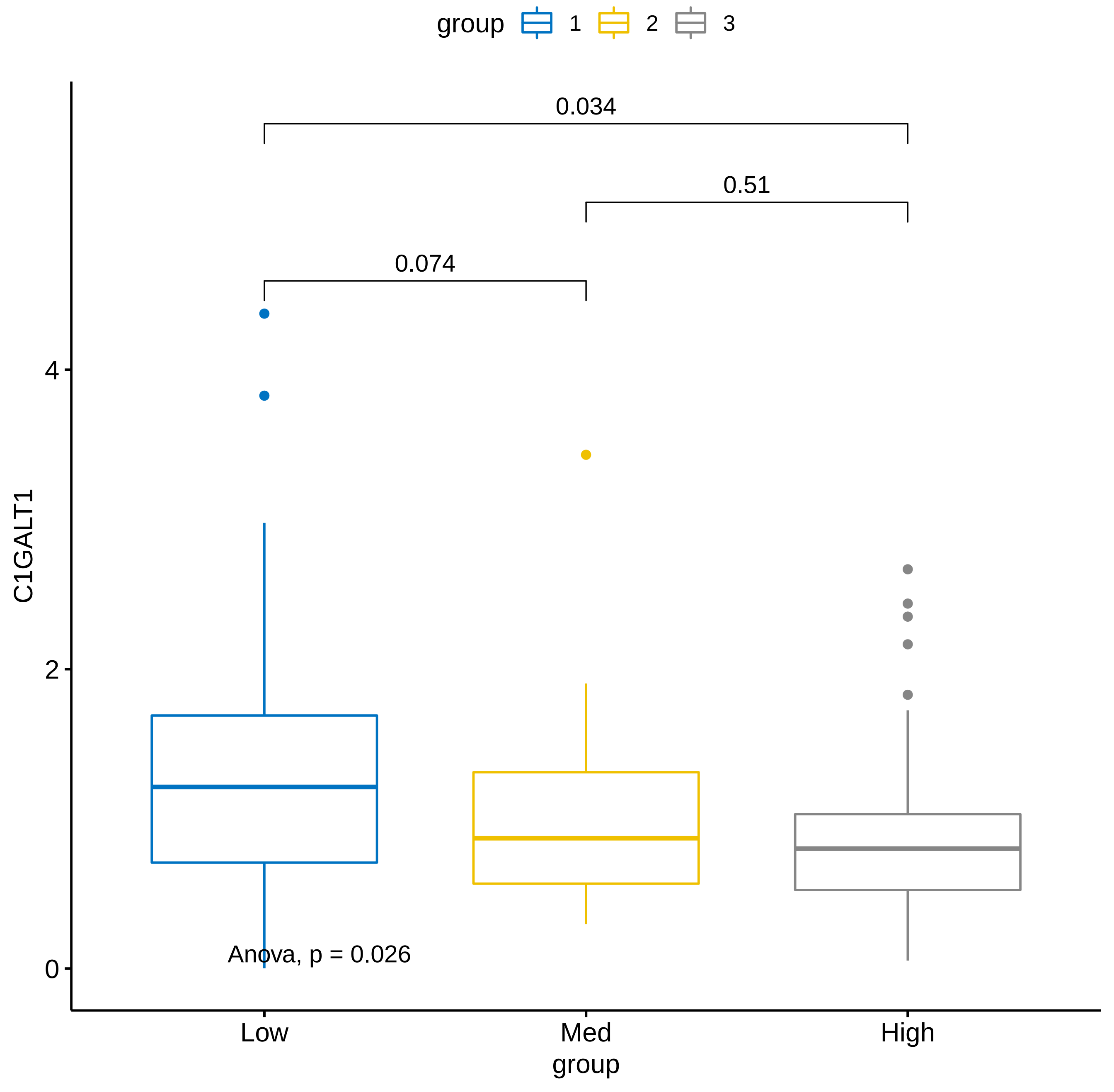

group 1 2 3
0.034
0.51
0.074
4
2
0
C1GALT1
Anova, p = 0.026
Low
Med
High
group

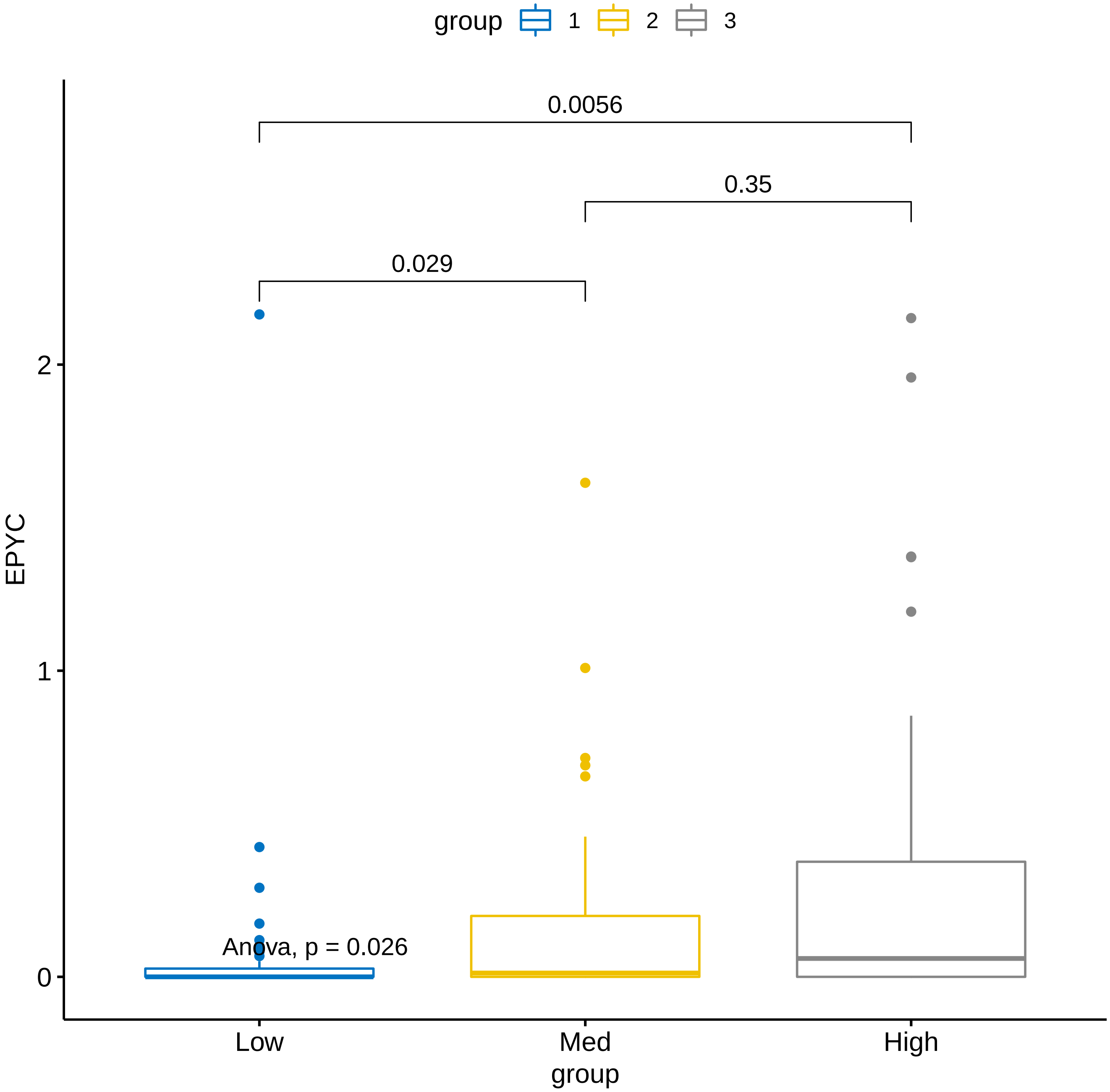
group
1
2
3
0.0056
0.35
0.029
Anova, p = 0.026
EPYC
2
1
0
Low
Med
High
group

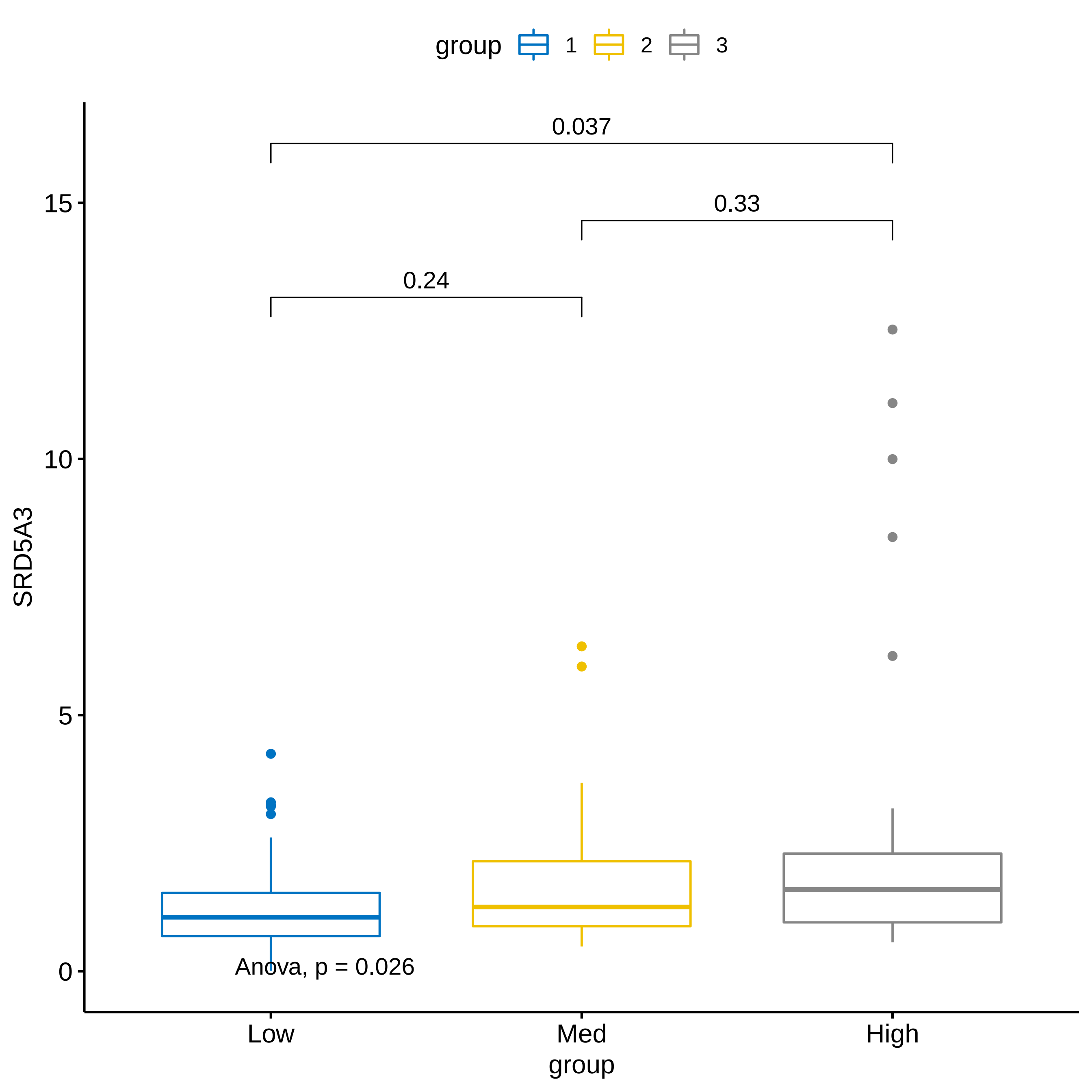

group 1 2 3
0.037
0.33
0.24
15
10
5
0
SRD5A3
Anova, p = 0.026
Low
Med
High
group

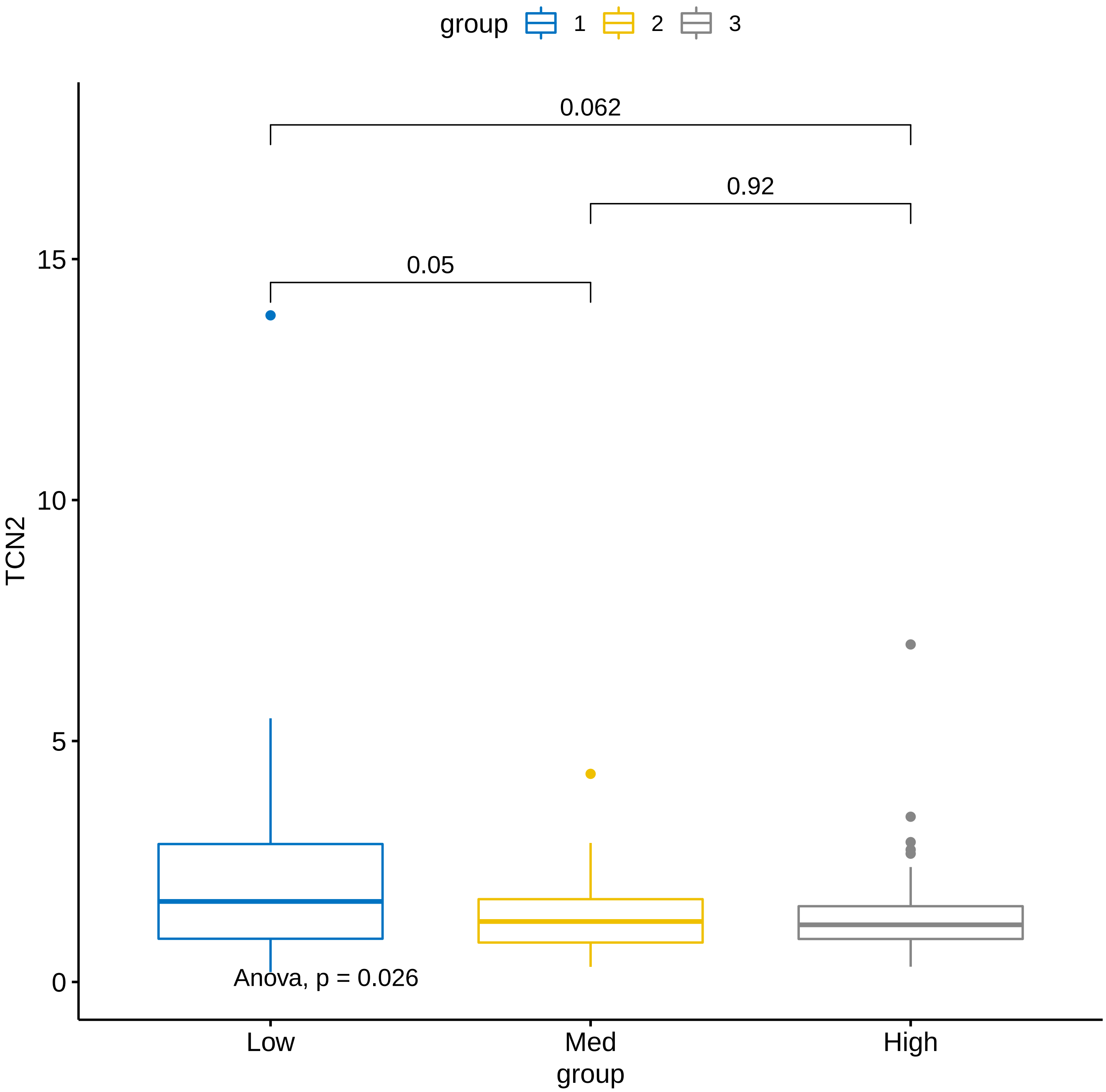
group
1
2
3
0.062
0.92
0.05
TCN2
15
10
5
0
Anova, p = 0.026
Low
Med
High
group

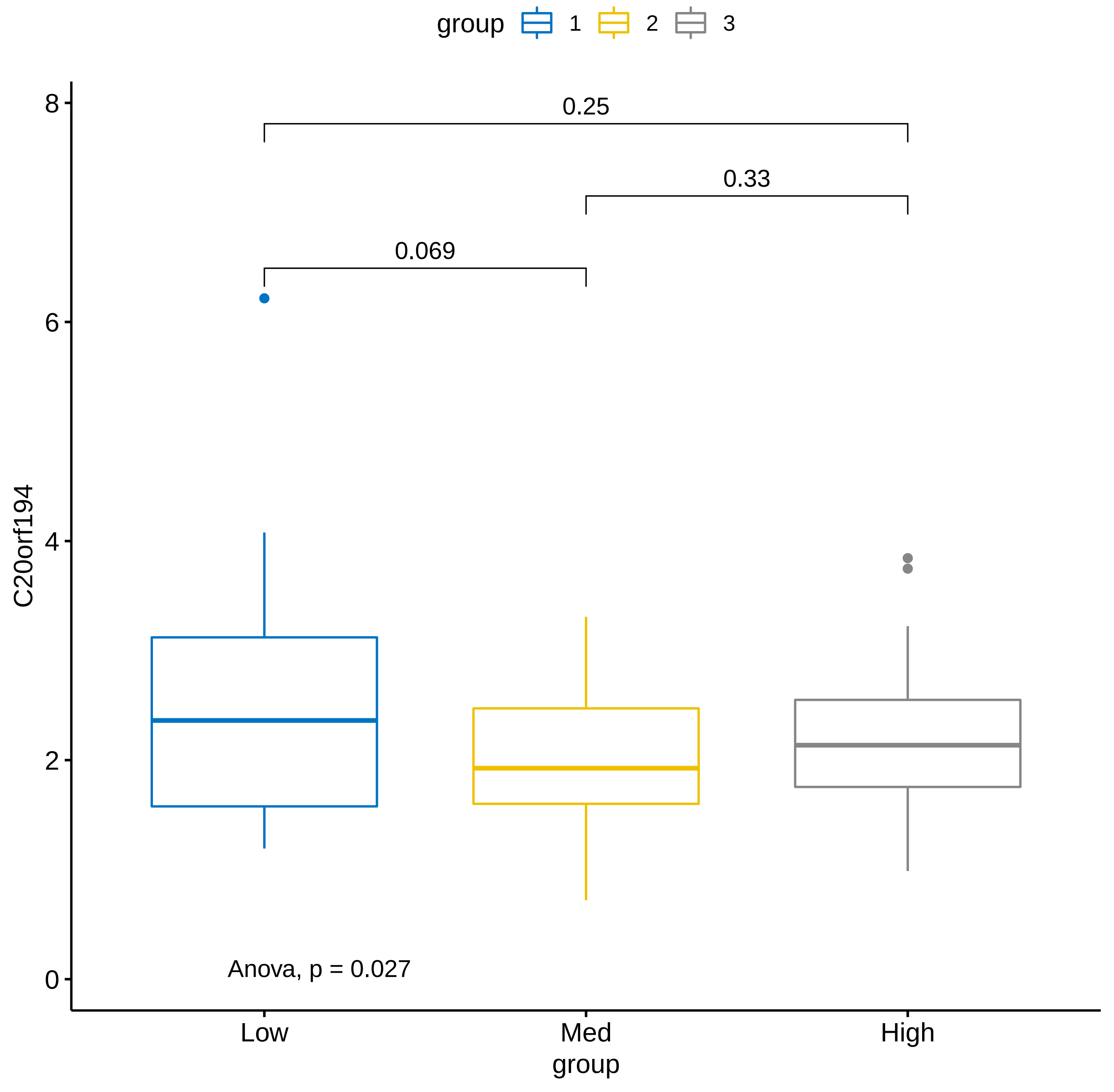

group 1 2 3
0.25
0.33
0.069
C20orf194
8
6
4
2
0
Anova, p = 0.027
Low
Med
High
group

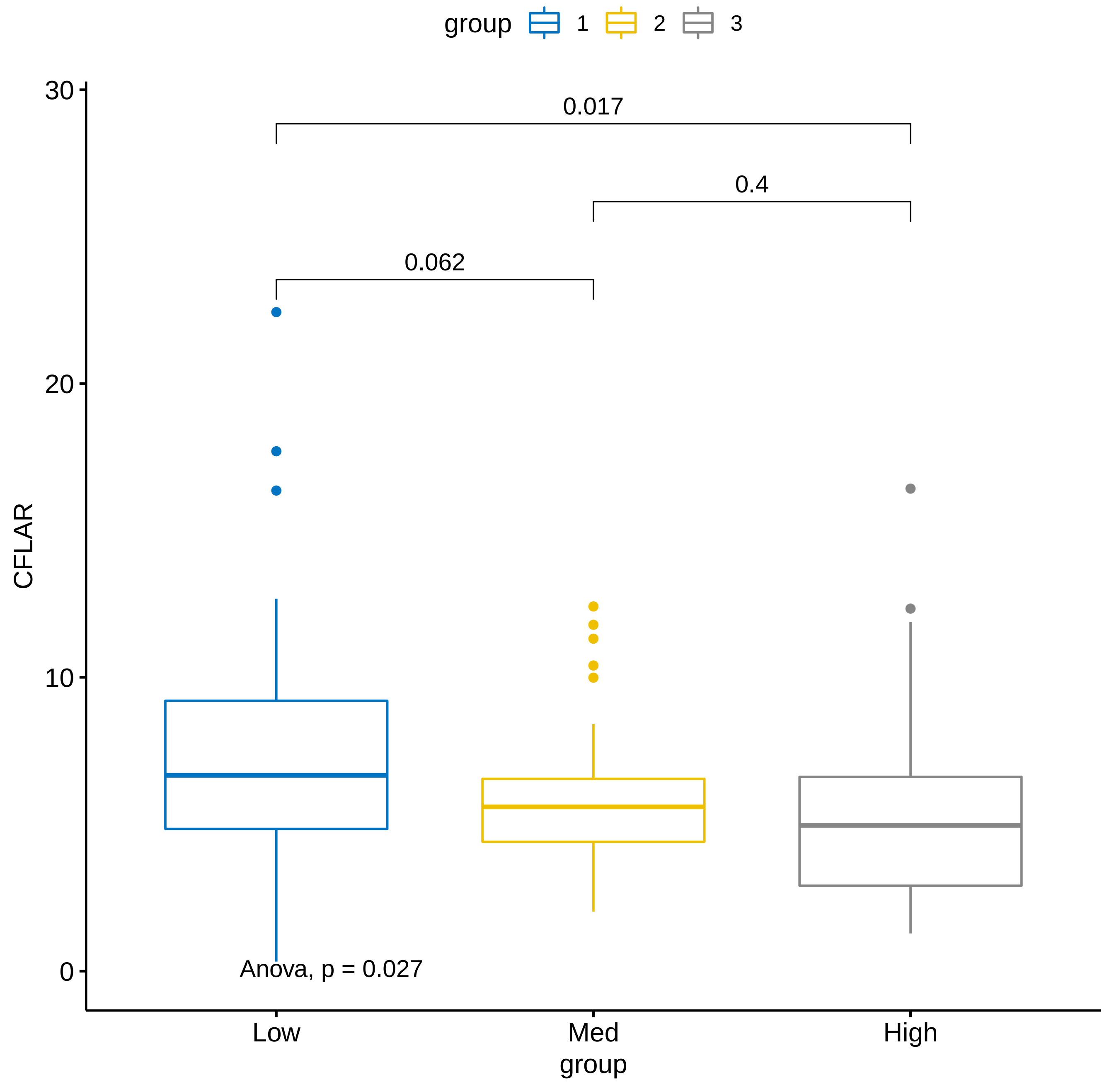
group 1 2 3
30
20
10
0
CFLAR
0.017
0.4
0.062
Anova, p = 0.027
Low
Med
High
group

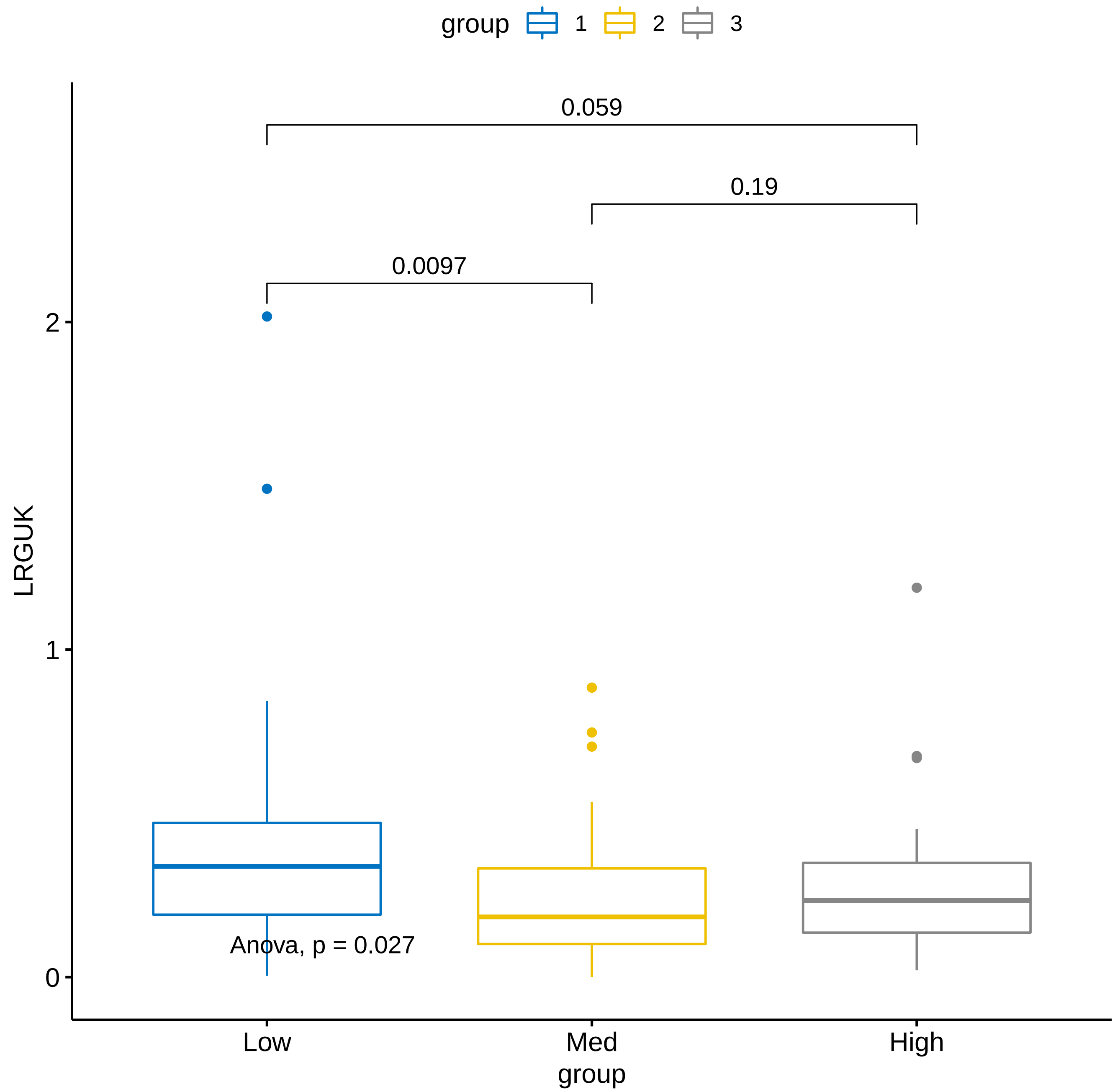
group
1
2
3
0.059
0.19
0.0097
2
1
0
LRGUK
Anova, p = 0.027
Low
Med
High
group

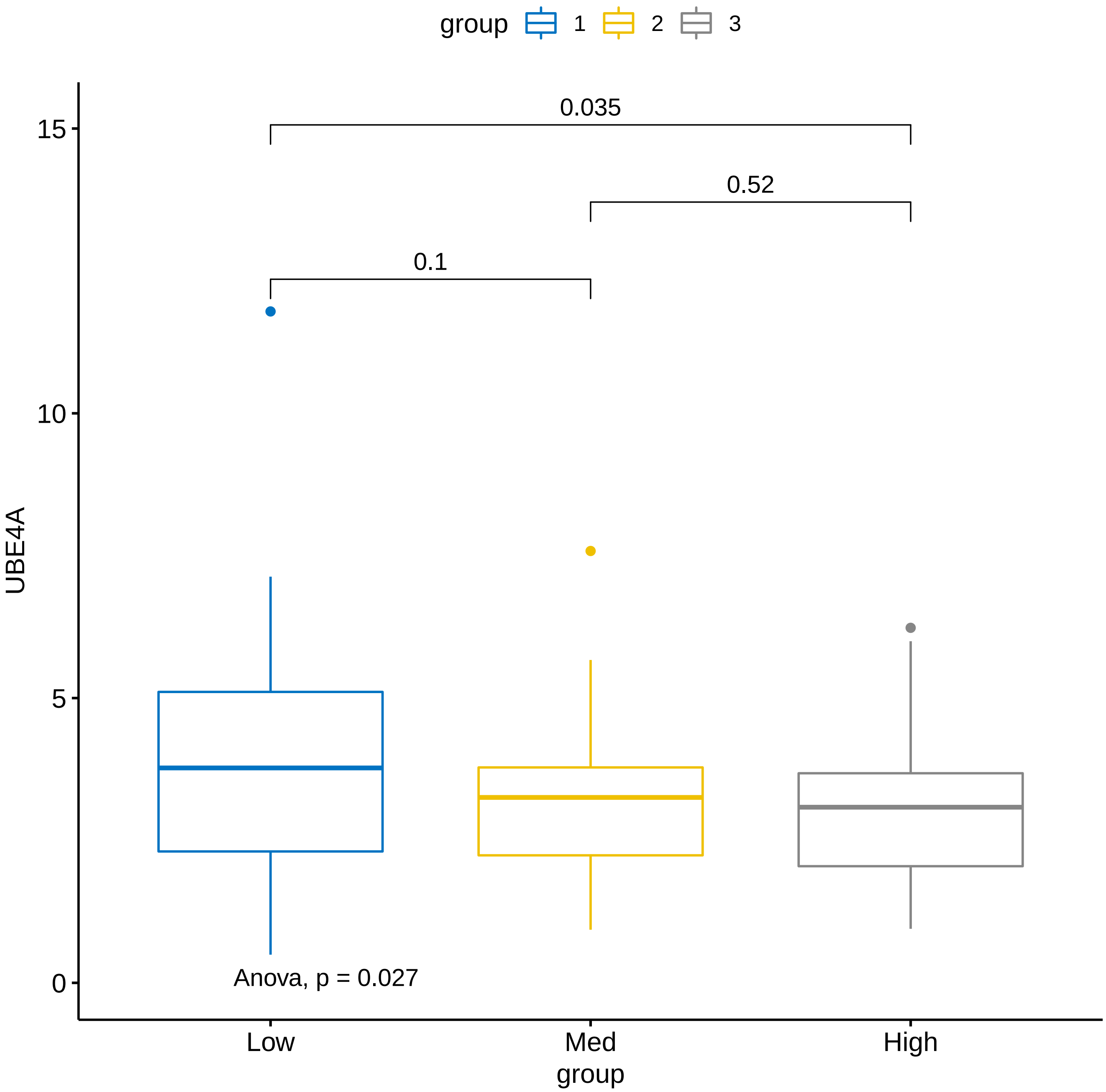
group
1
2
3
0.035
0.52
0.1
15
10
5
0
UBE4A
Anova, p = 0.027
Low
Med
High
group

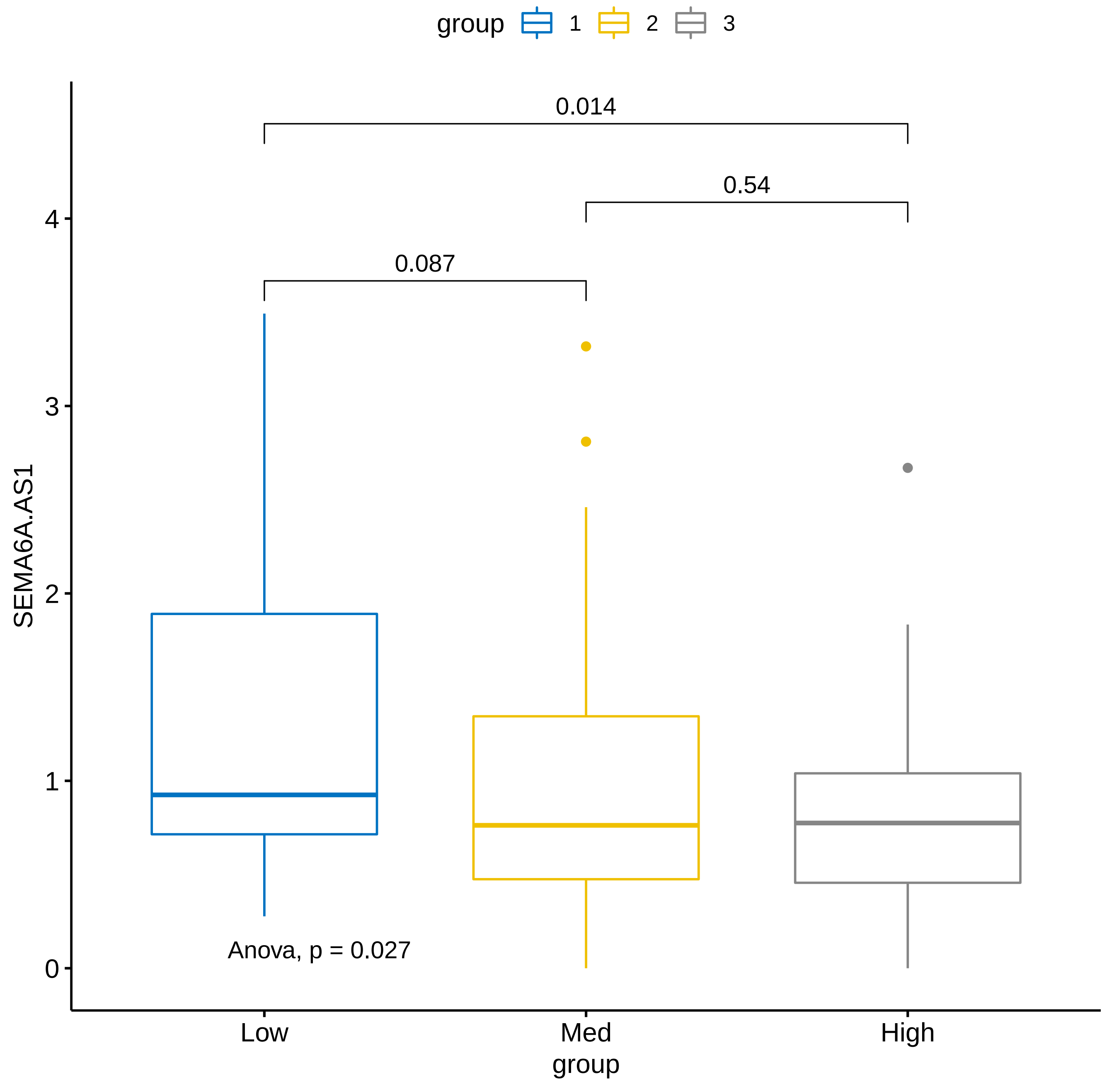

group
1
2
3
0.014
0.54
0.087
4
3
2
1
0
SEMA6A.AS1
Anova, p = 0.027
Low
Med
High
group

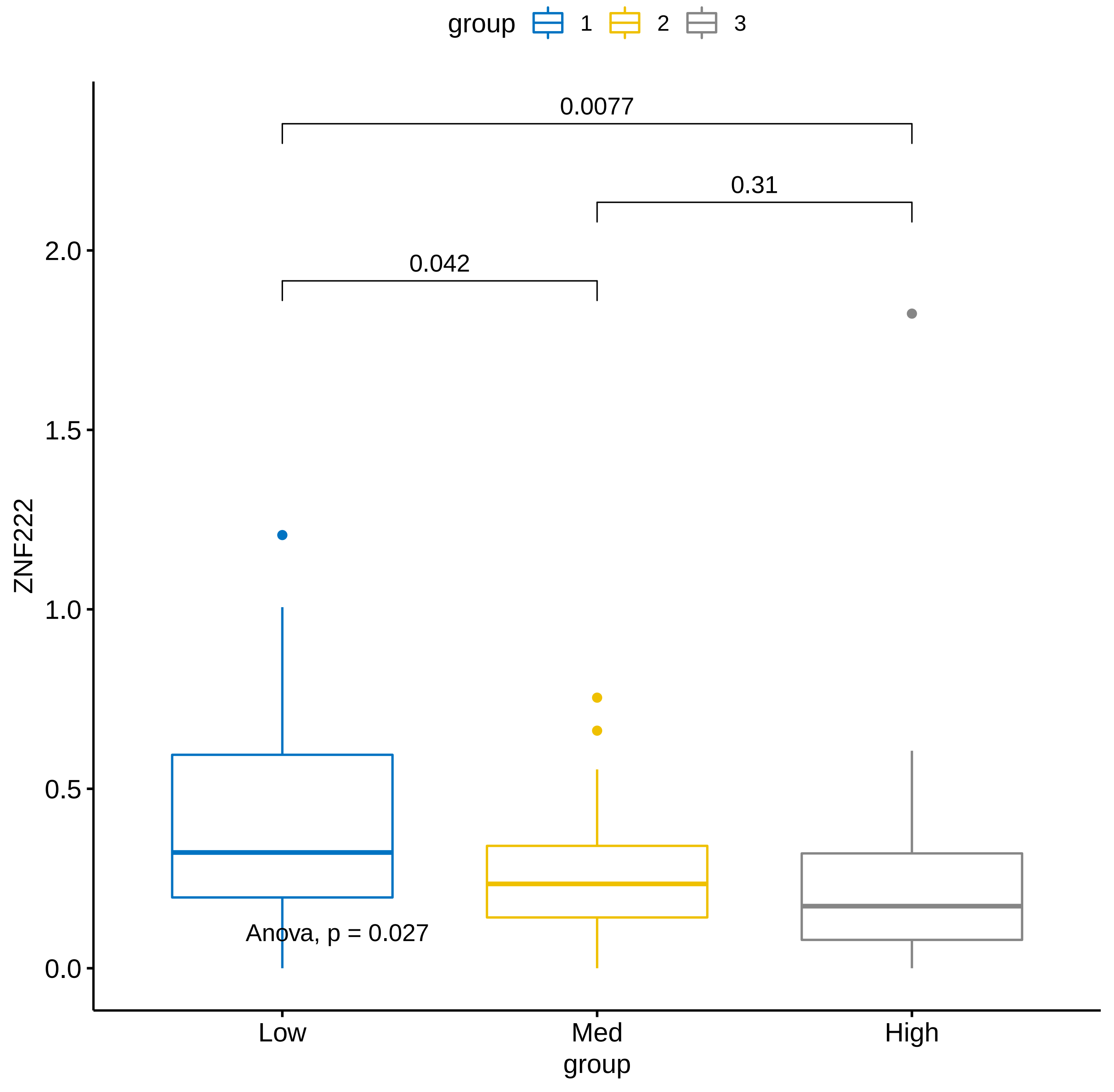
group 1 2 3
0.0077
0.31
0.042
2.0
1.5
1.0
0.5
0.0
ZNF222
Anova, p = 0.027
Low
Med
High
group

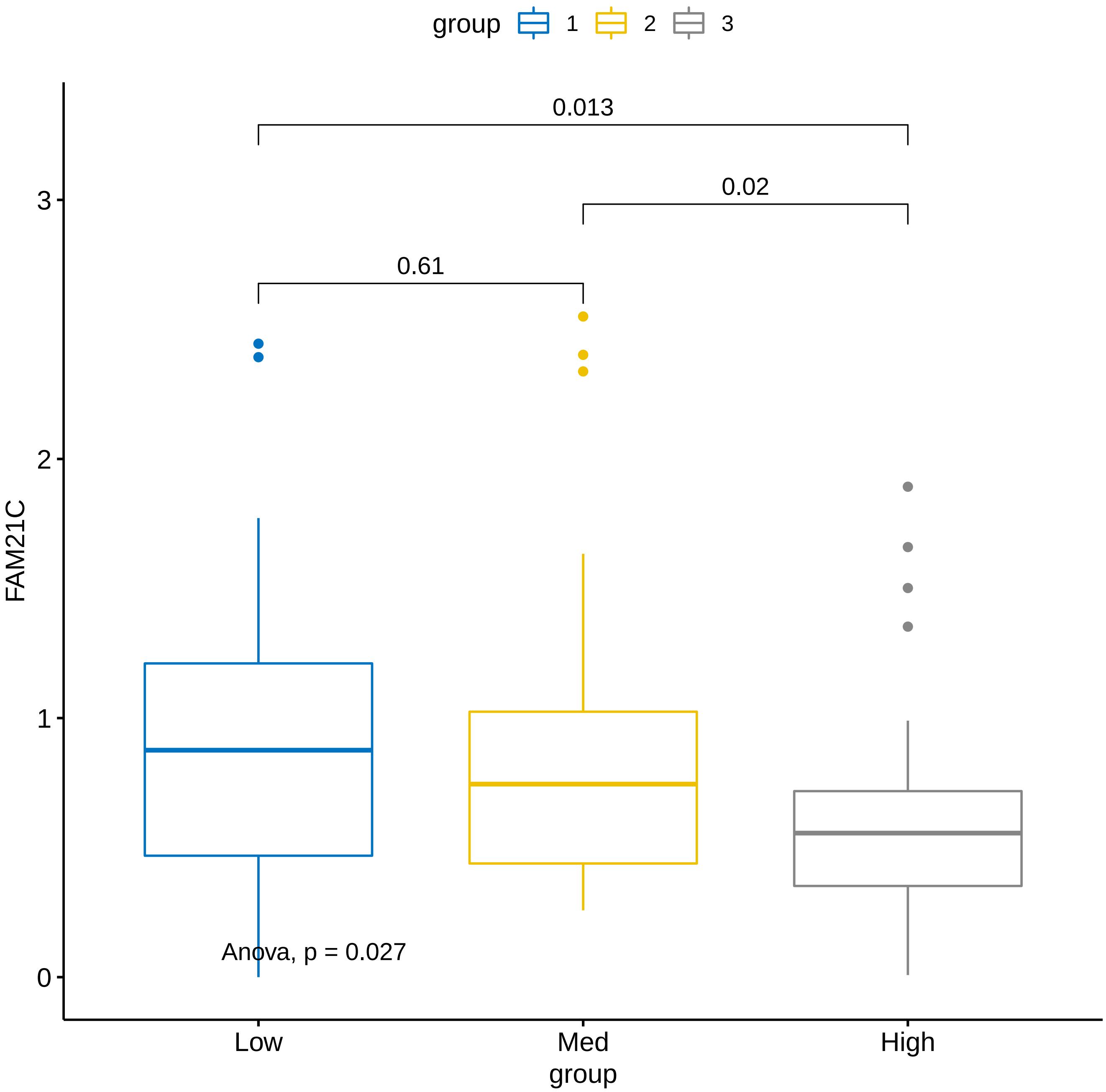
group
1
2
3
0.013
0.02
0.61
Anova, p = 0.027
FAM21C
0
1
2
3
Low
Med
High
group

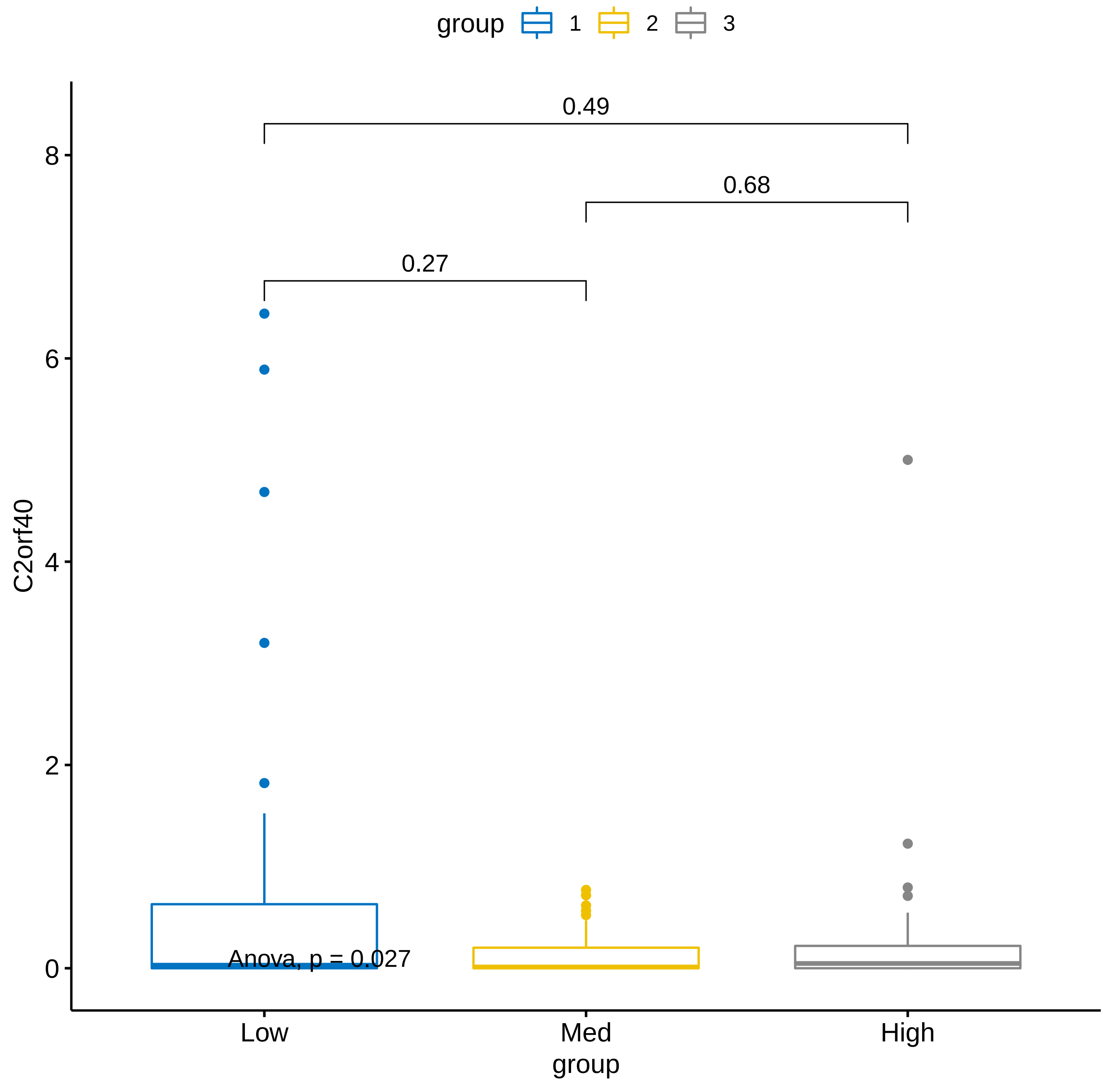

group
1
2
3
0.49
0.68
0.27
C2orf40
0
2
4
6
8
Anova, p = 0.027
Low
Med
High
group

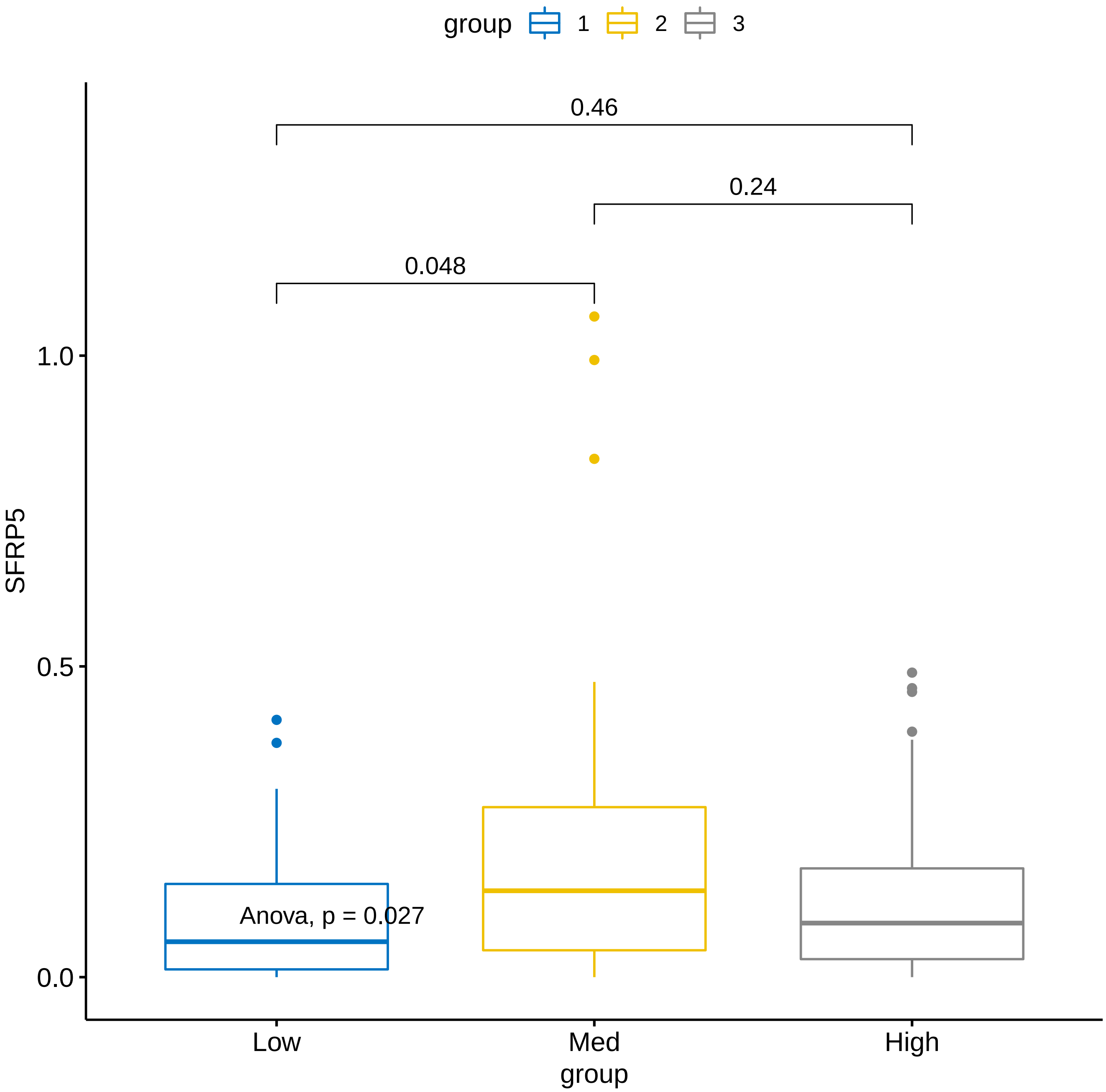

group 1 2 3
0.46
0.24
0.048
1.0
0.5
0.0
SFRP5
Anova, p = 0.027
Low
Med
High
group

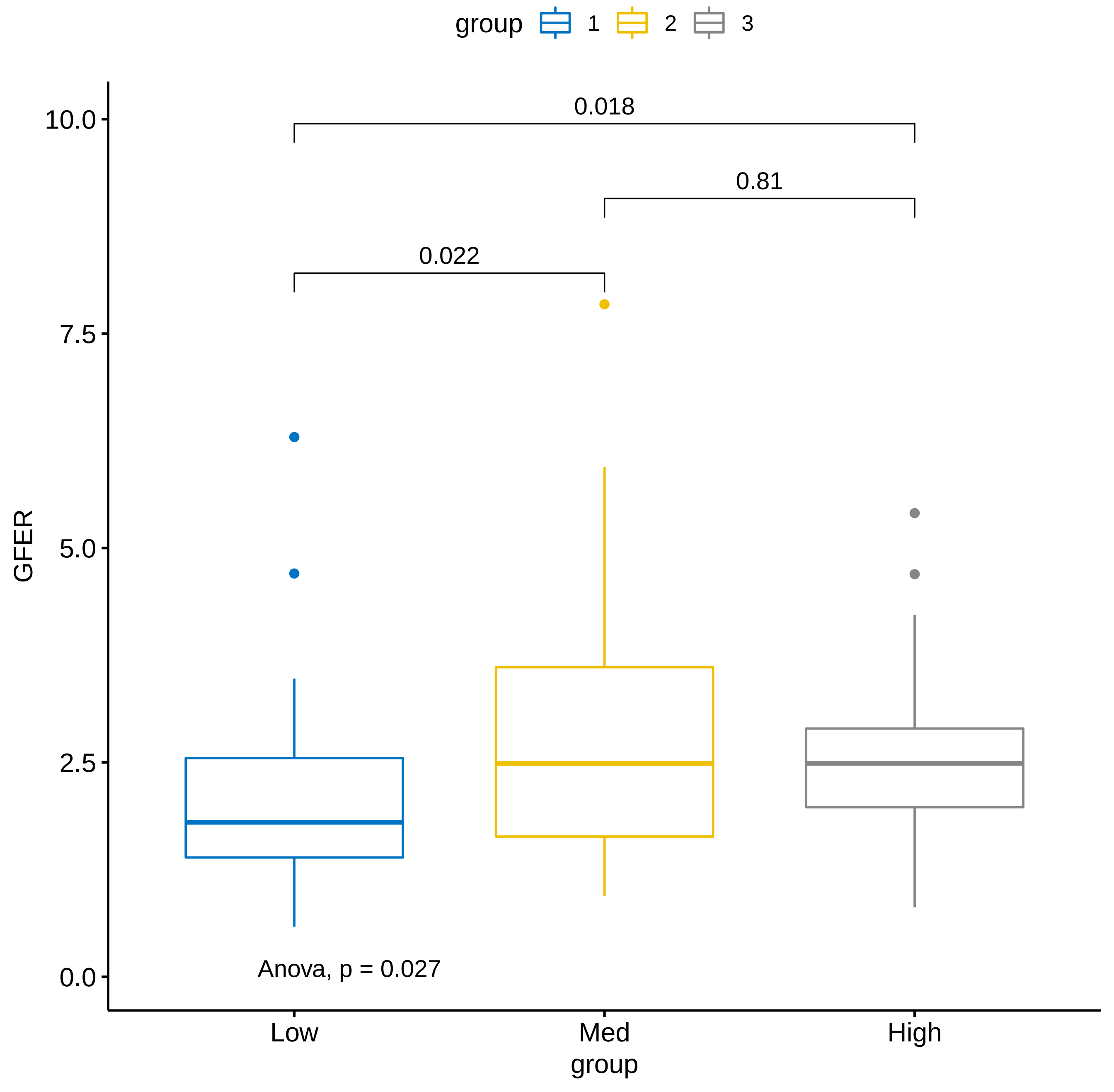

group 1 2 3
0.018
0.81
0.022
10.0
7.5
5.0
2.5
0.0
GFER
Anova, p = 0.027
Low
Med
High
group

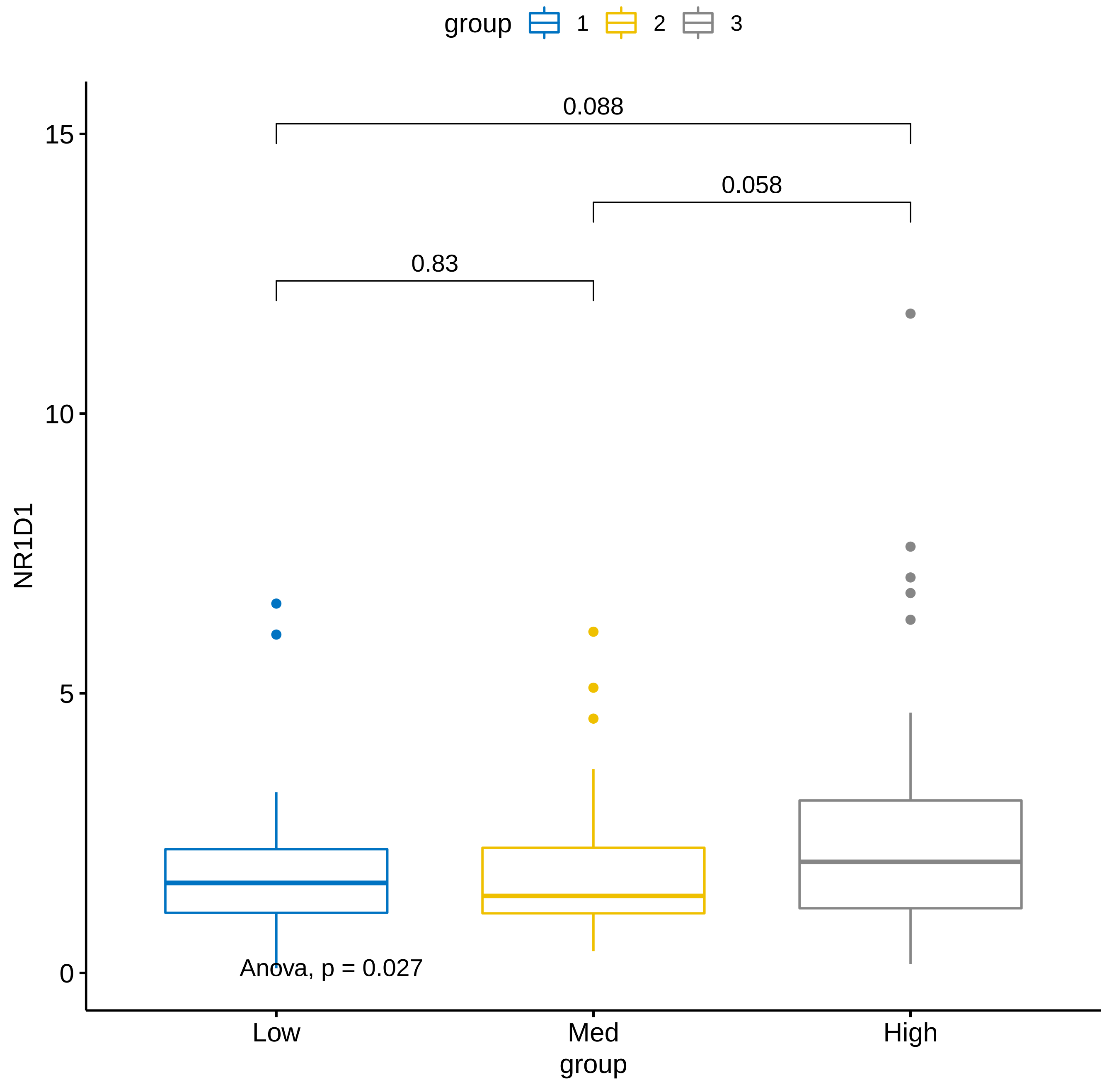
group
1
2
3
0.088
0.058
0.83
15
10
5
0
NR1D1
Anova, p = 0.027
Low
Med
High
group

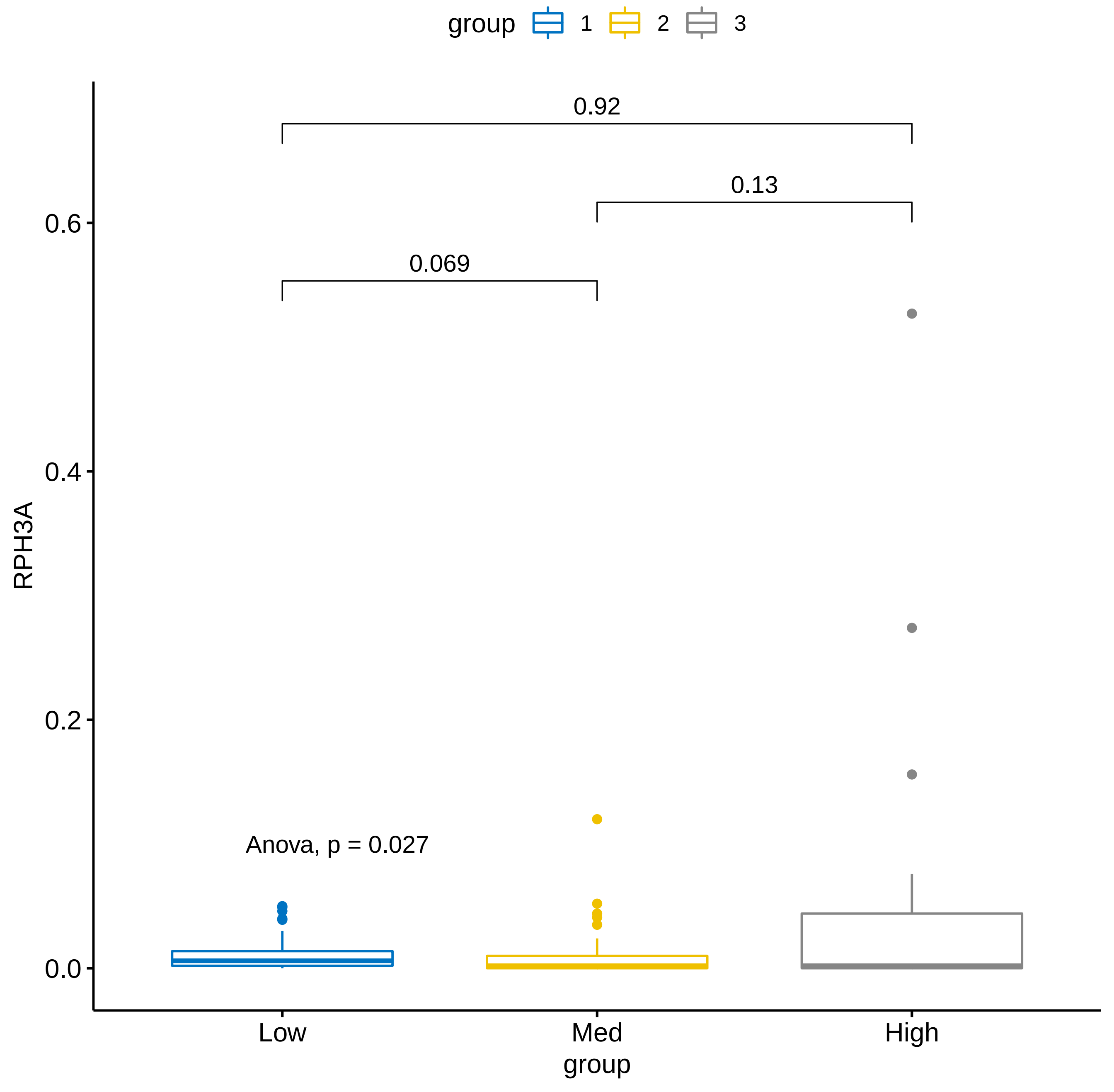

group
1
2
3
0.92
0.13
0.069
Anova, p = 0.027
RPH3A
0.6
0.4
0.2
0.0
Low
Med
High
group

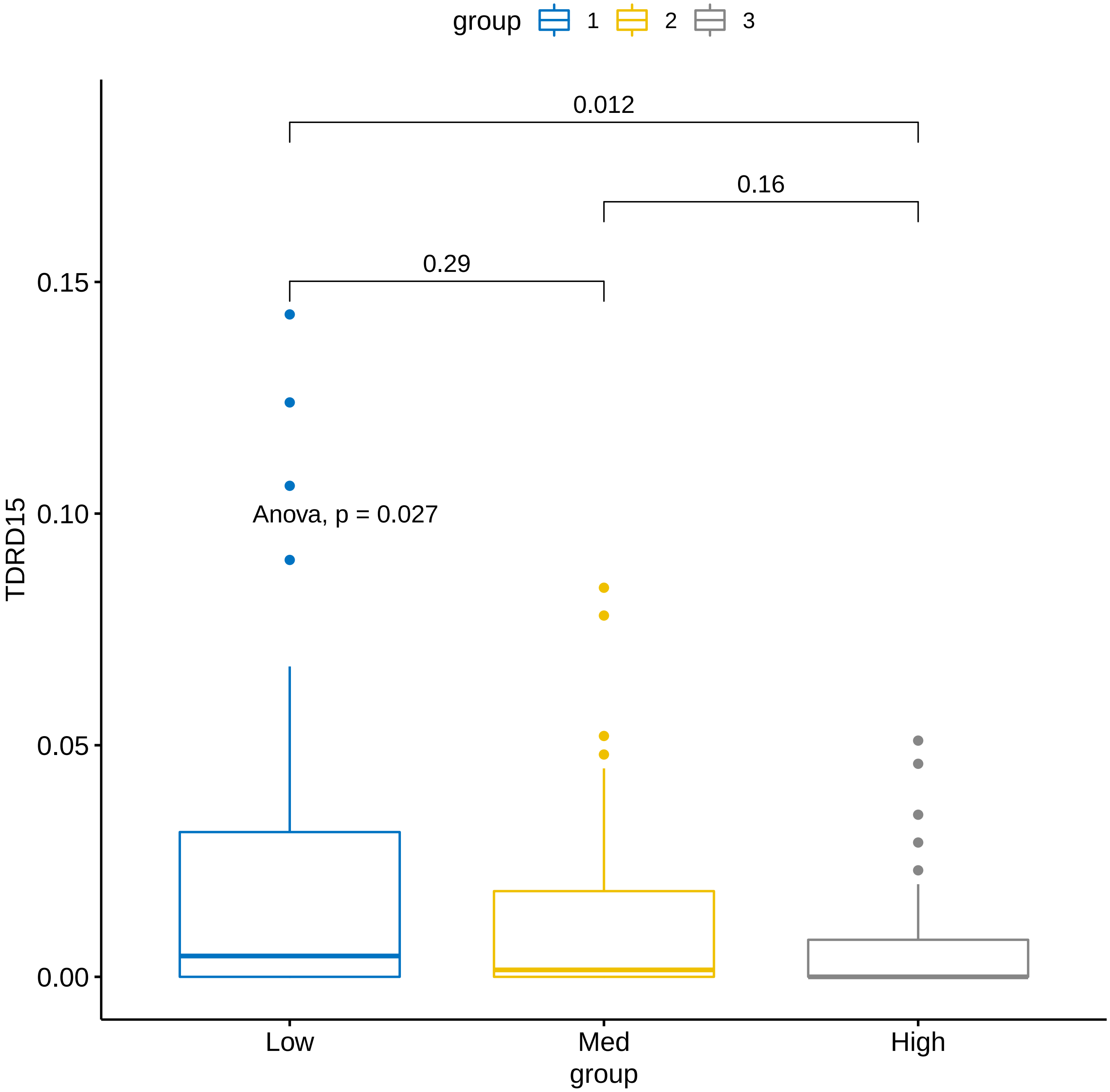
group
1
2
3
0.012
0.16
0.29
Anova, p = 0.027
TDRD15
0.15
0.10
0.05
0.00
Low
Med
High
group

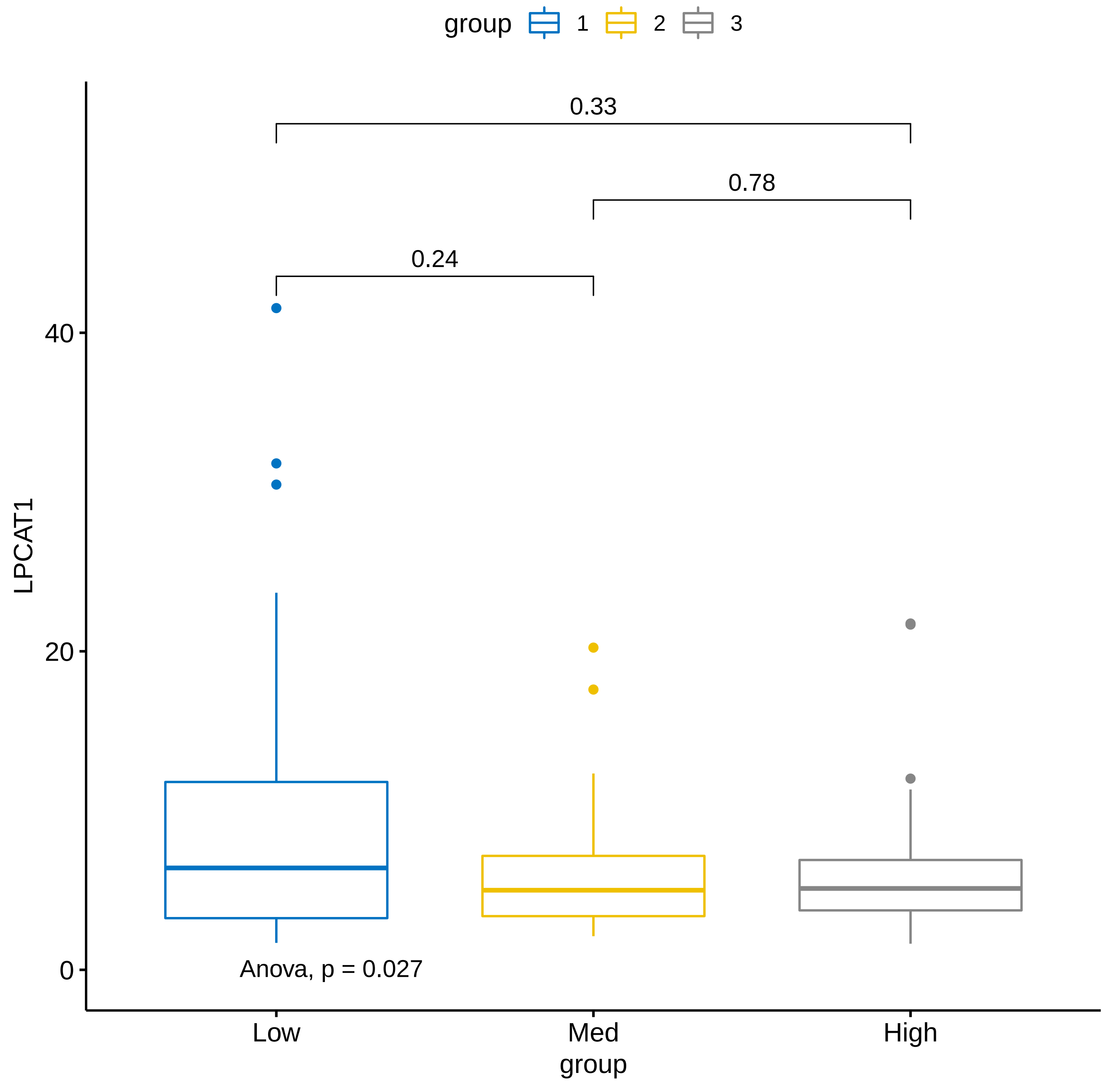

group 1 2 3
0.33
0.78
0.24
40
20
0
LPCAT1
Anova, p = 0.027
Low
Med
High
group

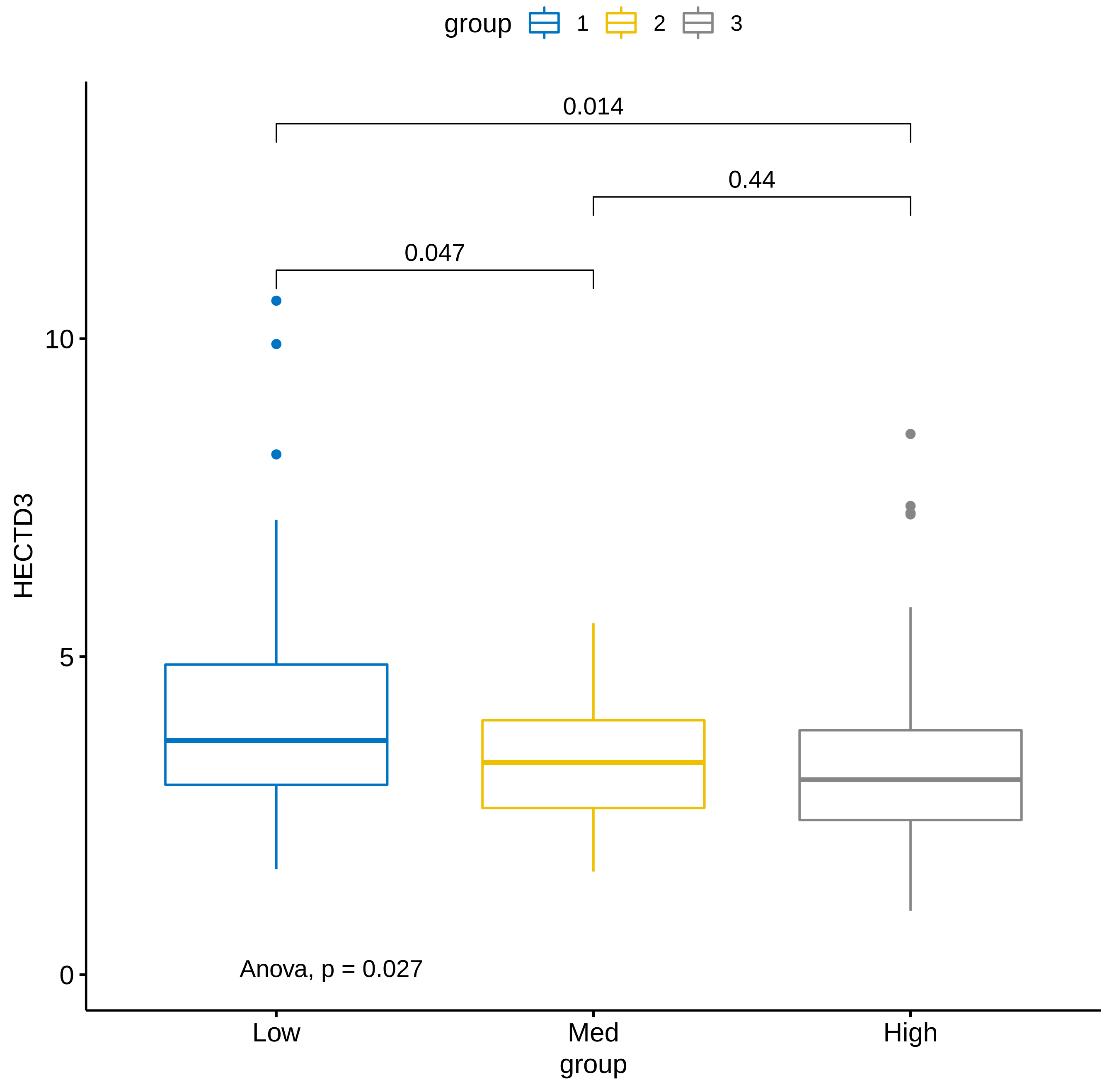
group
1
2
3
0.014
0.44
0.047
10
5
0
HECTD3
Anova, p = 0.027
Low
Med
High
group

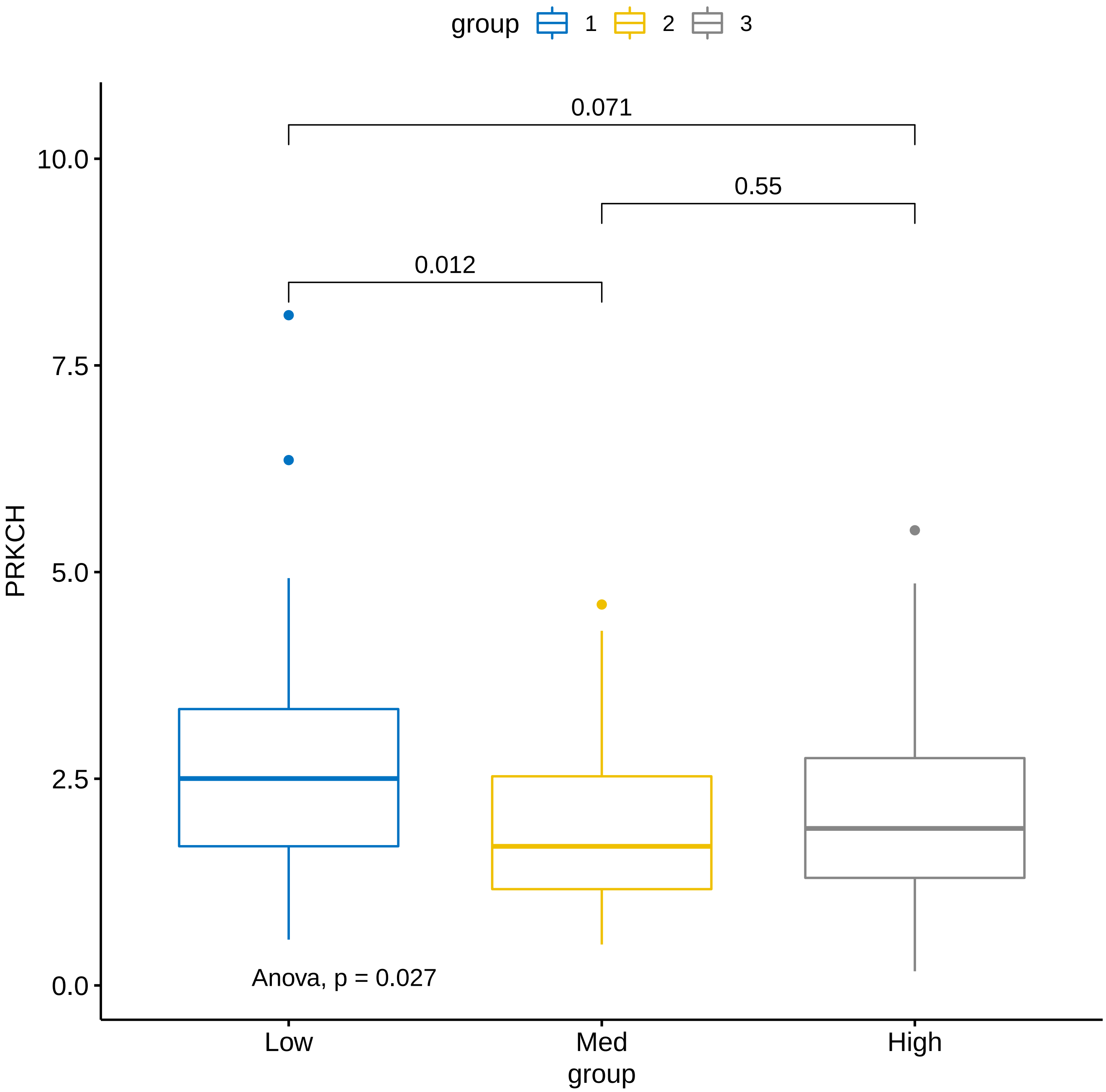
group
1
2
3
0.071
0.55
0.012
10.0
7.5
5.0
2.5
0.0
PRKCH
Anova, p = 0.027
Low
Med
High
group

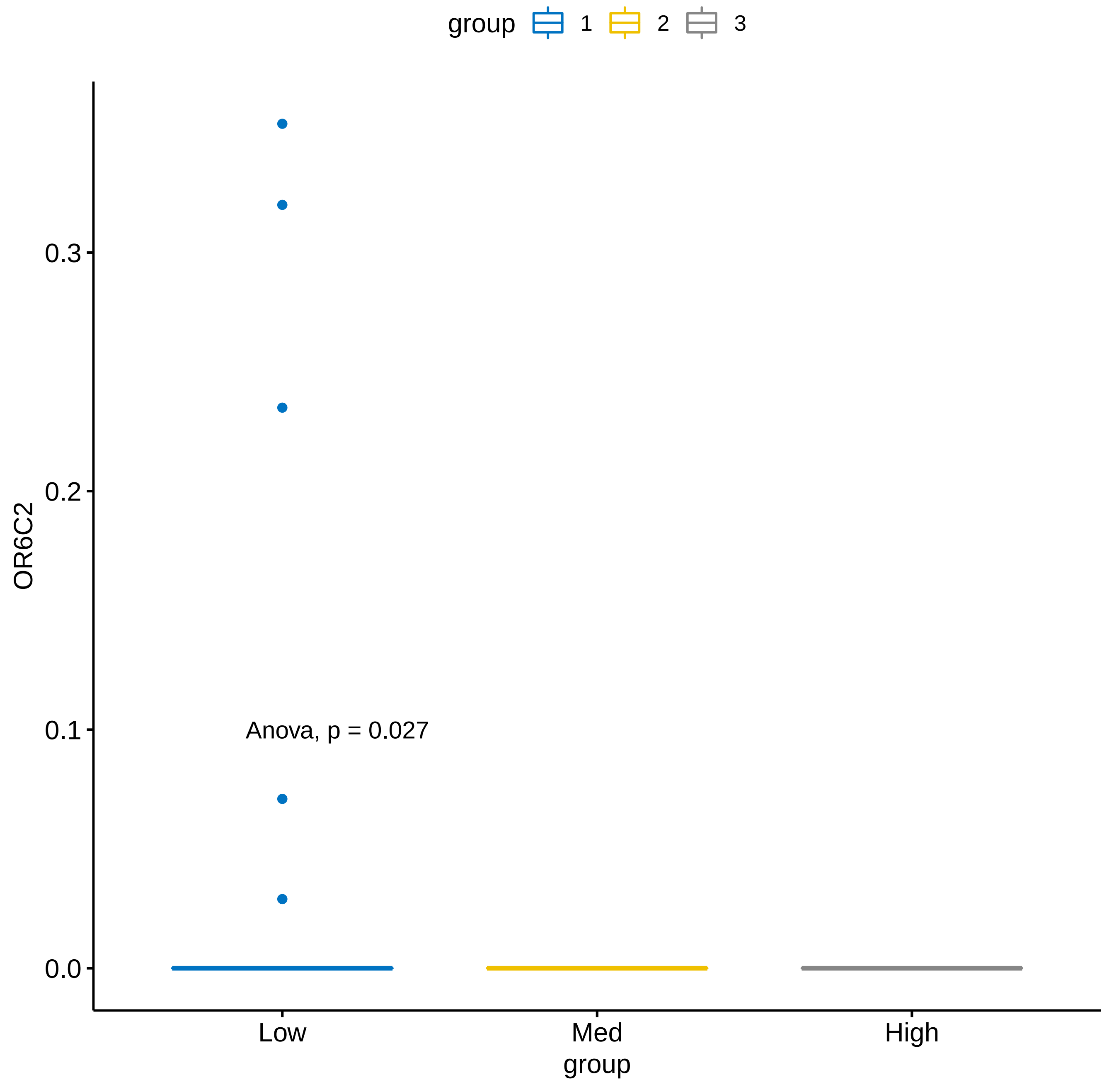

group 1 2 3
OR6C2
0.3
0.2
0.1
0.0
Anova, p = 0.027
Low
Med
High
group

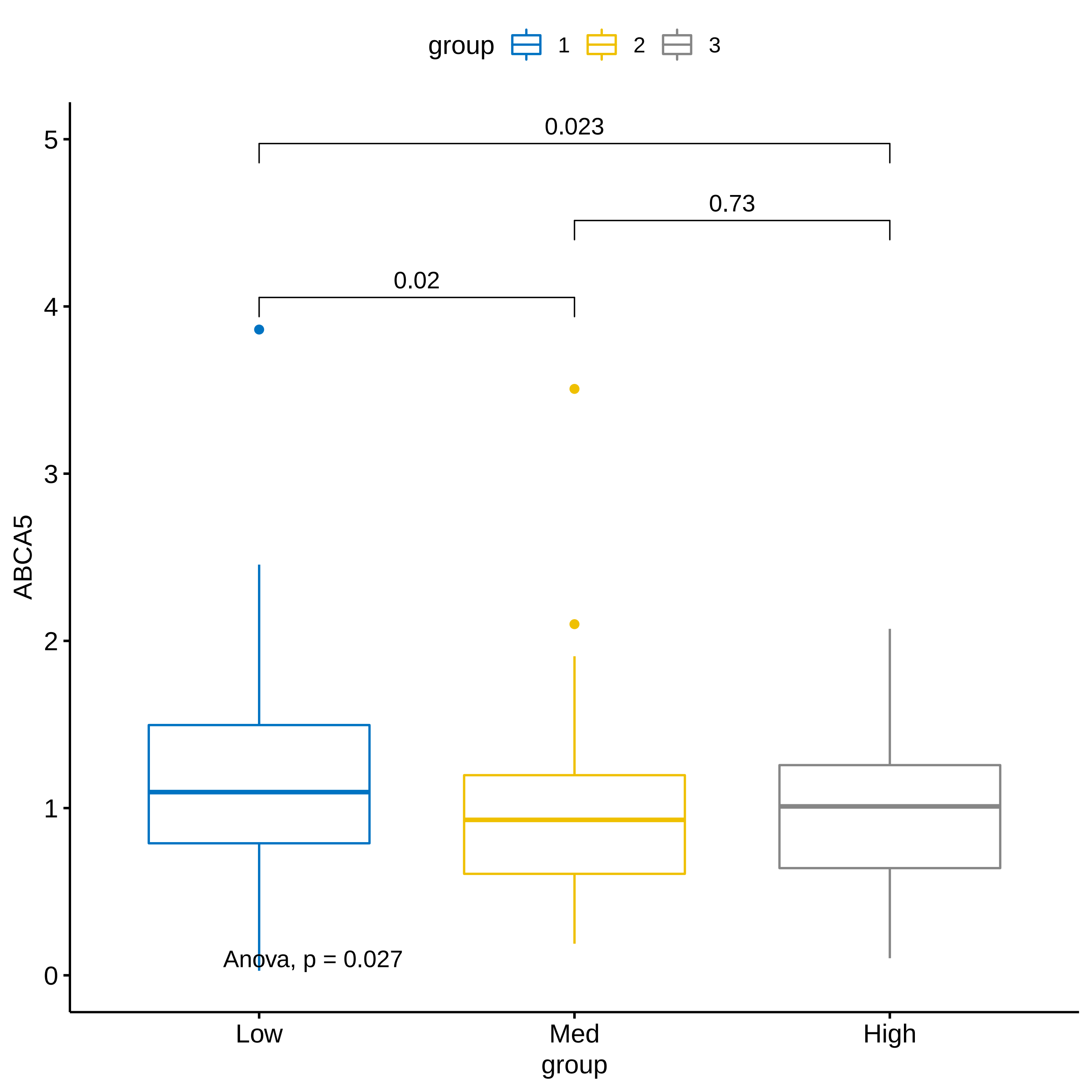
group
1
2
3
0.023
0.73
0.02
5
4
3
2
1
0
ABCA5
Anova, p = 0.027
Low
Med
High
group

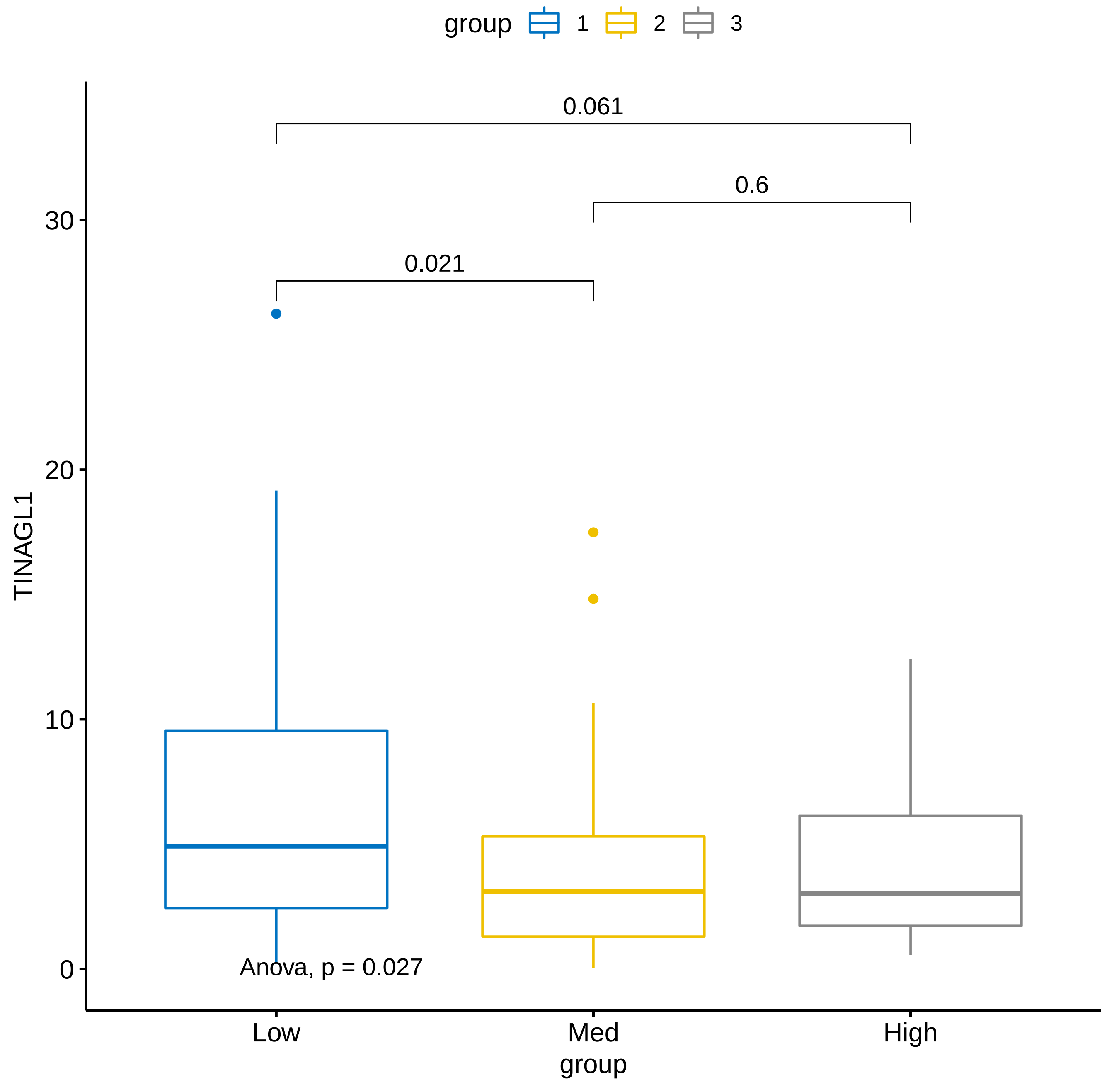

group 1 2 3
0.061
0.6
0.021
30
20
10
0
TINAGL1
Anova, p = 0.027
Low
Med
High
group

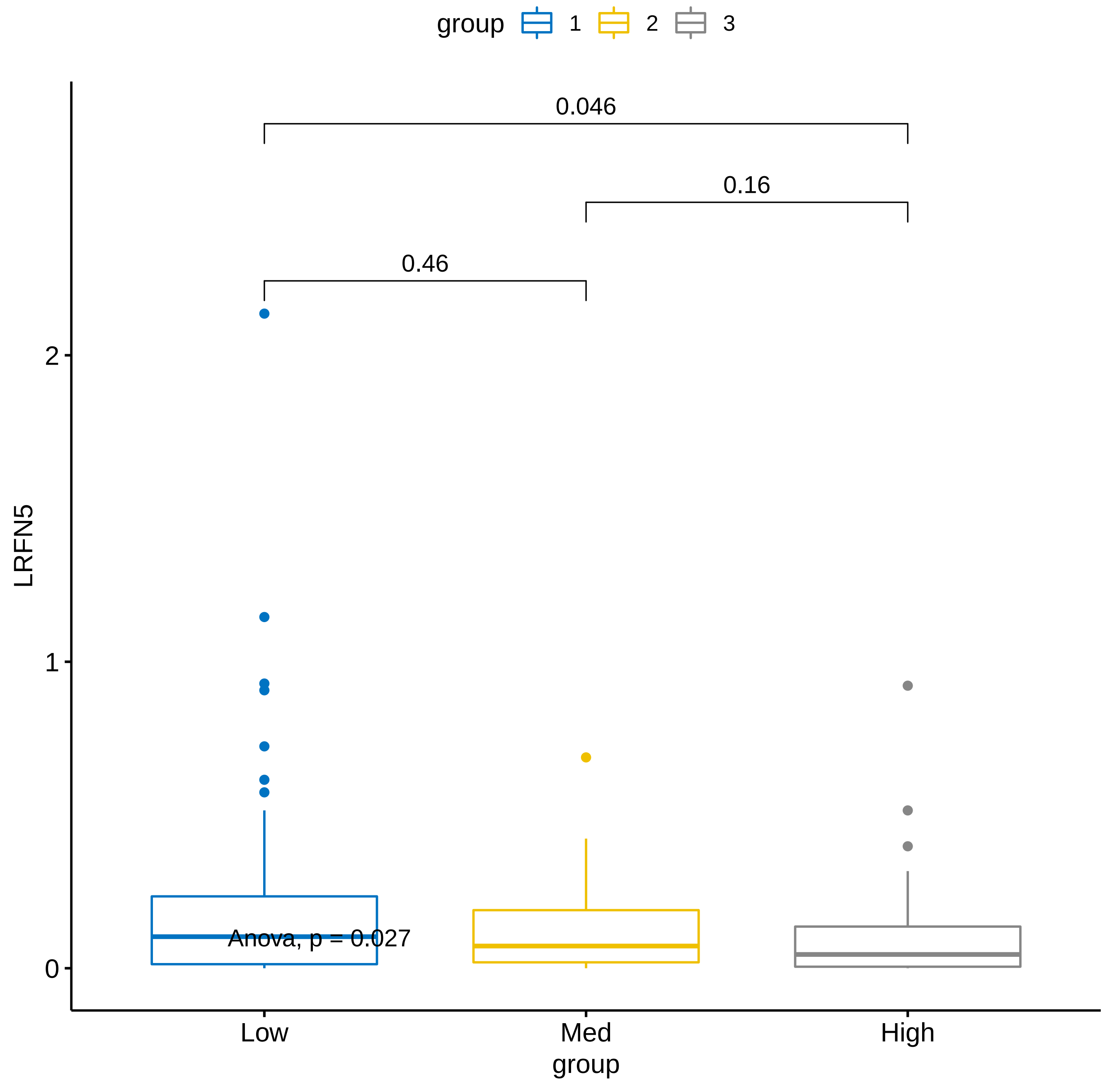
group 1 2 3
0.046
0.16
0.46
Anova, p = 0.027
LRFN5
2
1
0
Low
Med
High
group

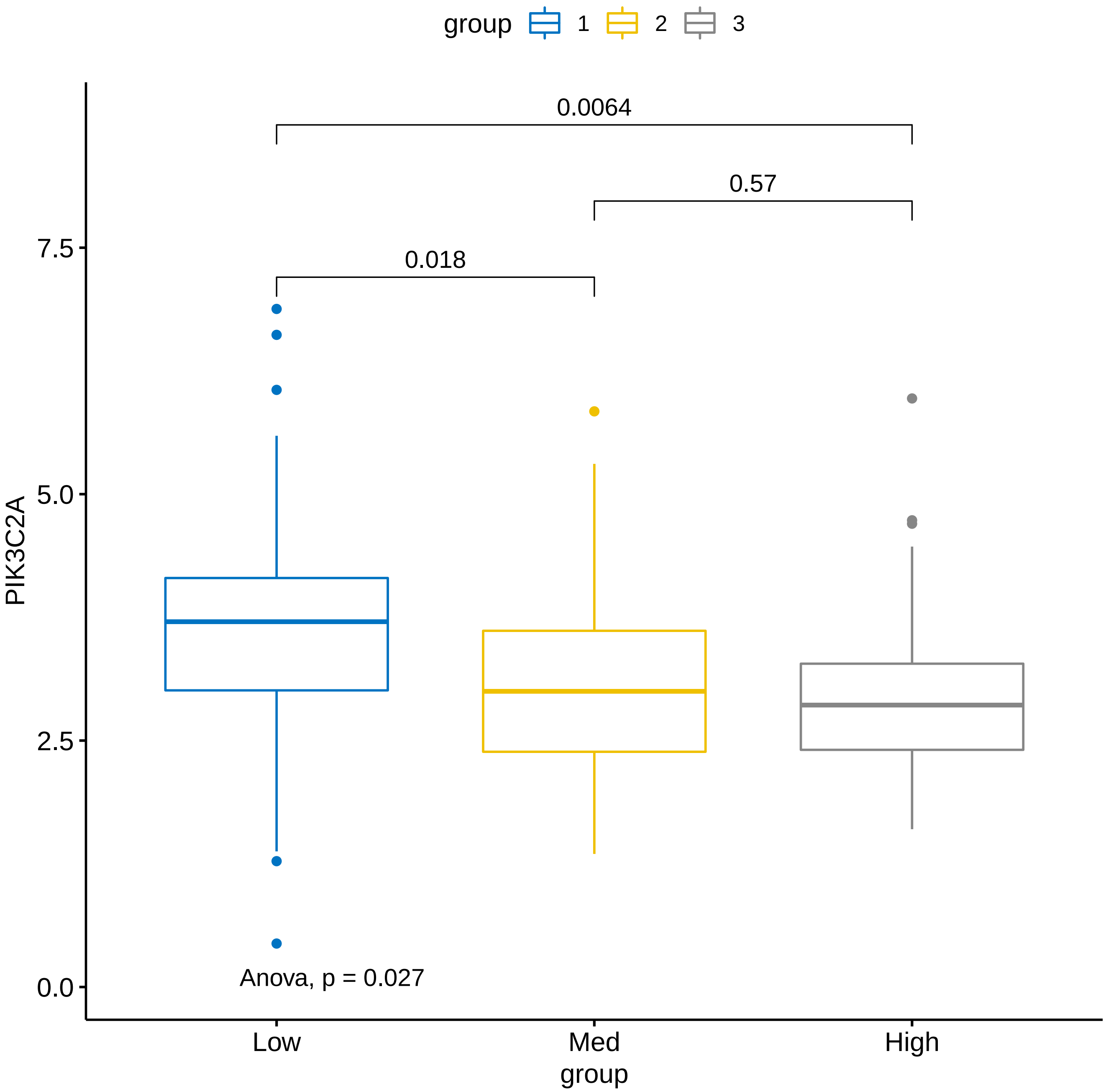

group 1 2 3
0.0064
0.57
0.018
7.5
5.0
2.5
0.0
PIK3C2A
Anova, p = 0.027
Low
Med
High
group

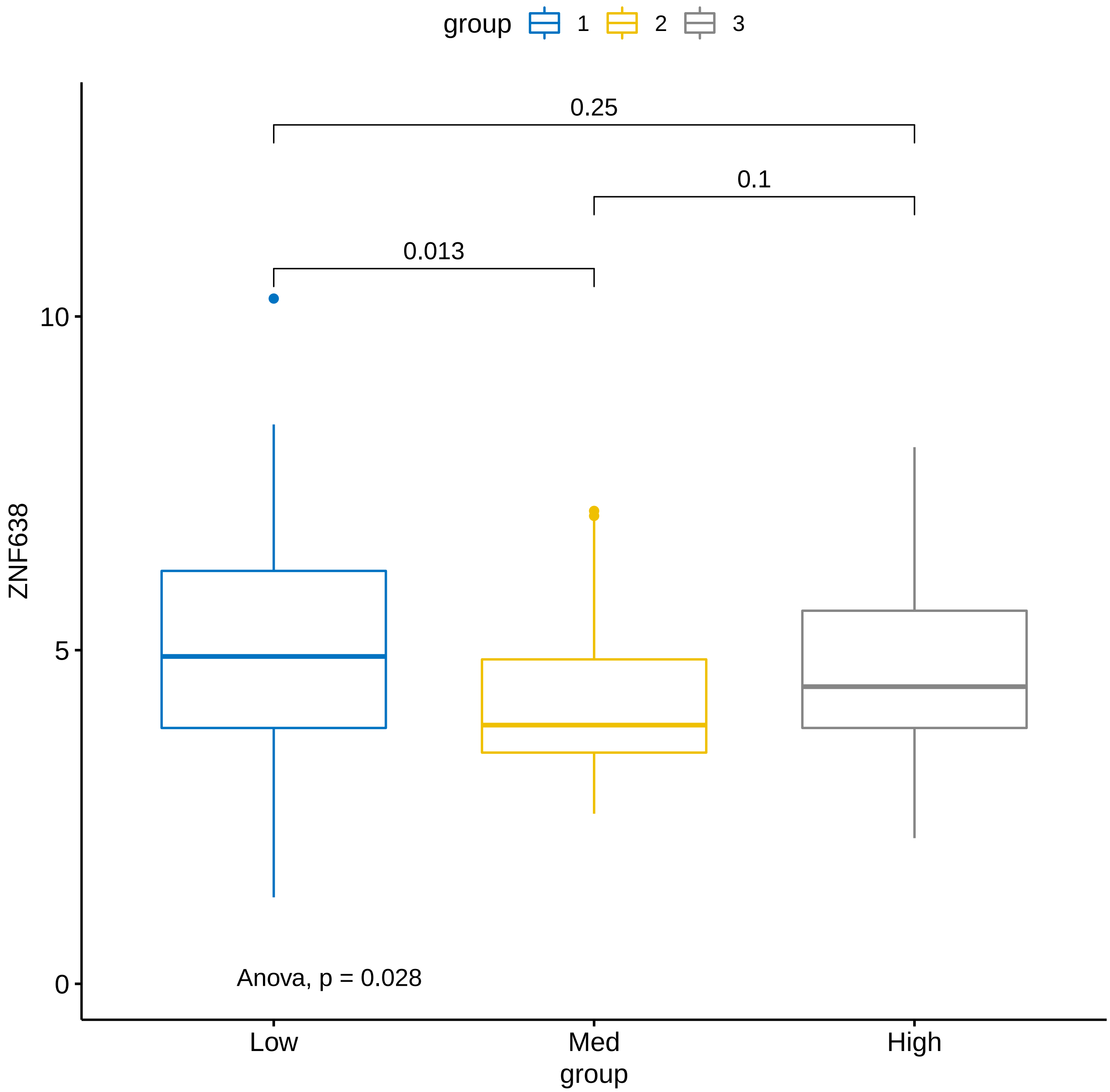
group
1
2
3
0.25
0.1
0.013
10
5
0
ZNF638
Anova, p = 0.028
Low
Med
High
group

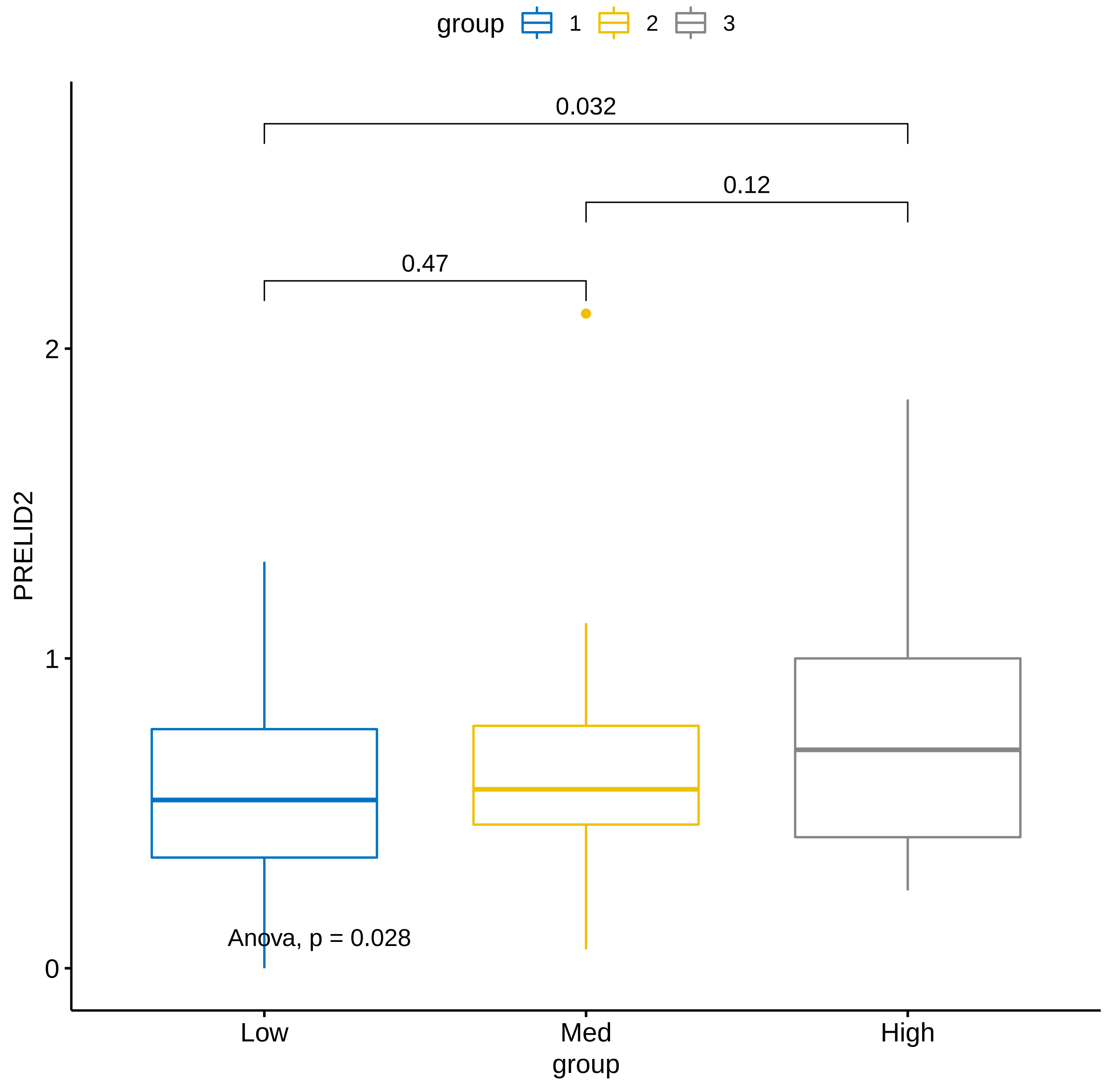
group 1 2 3
0.032
0.12
0.47
2
1
0
PRELID2
Anova, p = 0.028
Low
Med
High
group

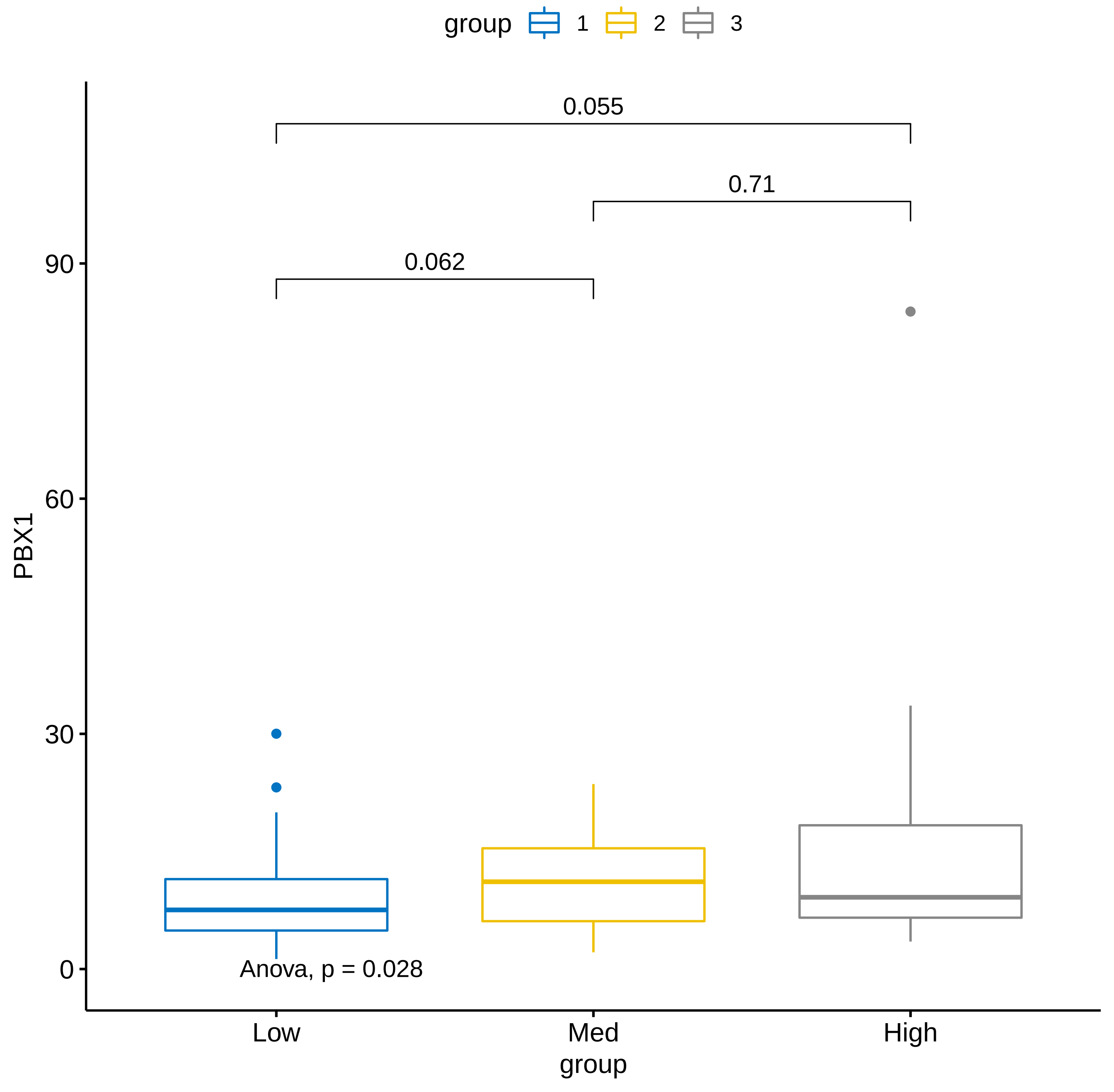
group
1
2
3
0.055
0.71
0.062
90
60
30
0
PBX1
Anova, p = 0.028
Low
Med
High
group

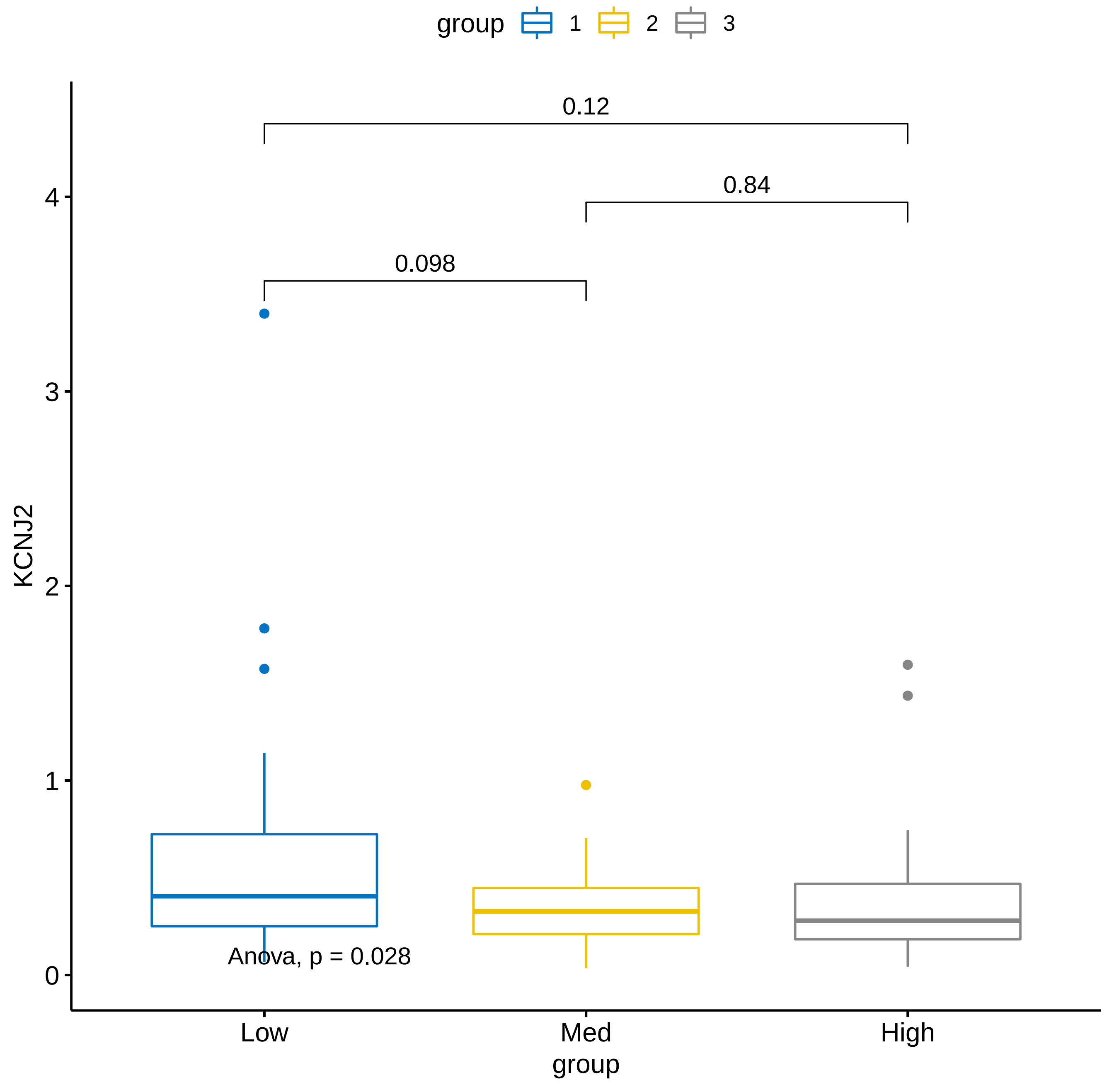
group
1
2
3
0.12
0.84
0.098
KCNJ2
4
3
2
1
0
Anova, p = 0.028
Low
Med
High
group

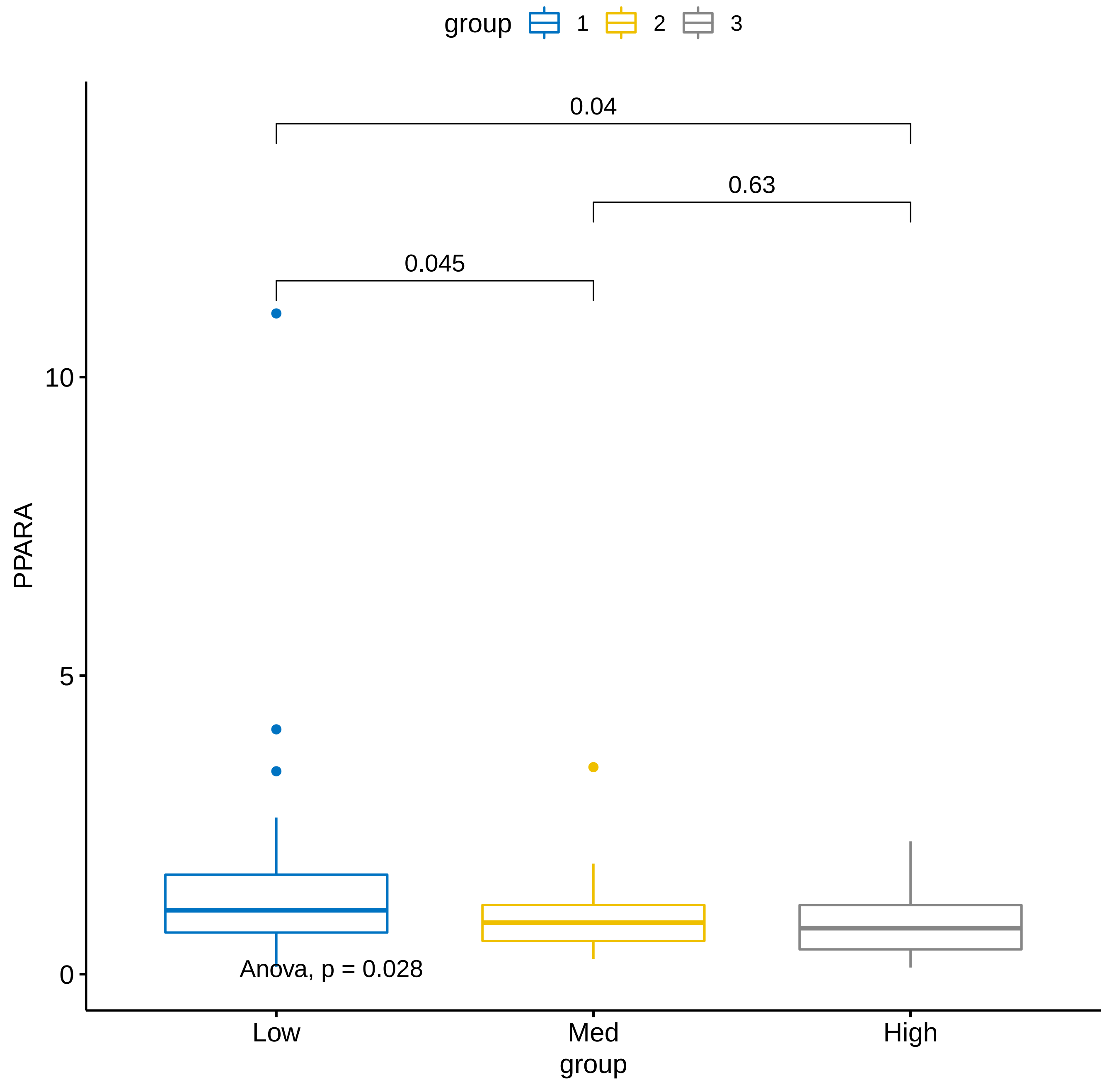

group 1 2 3
0.04
0.63
0.045
10
5
0
PPARA
Anova, p = 0.028
Low
Med
High
group

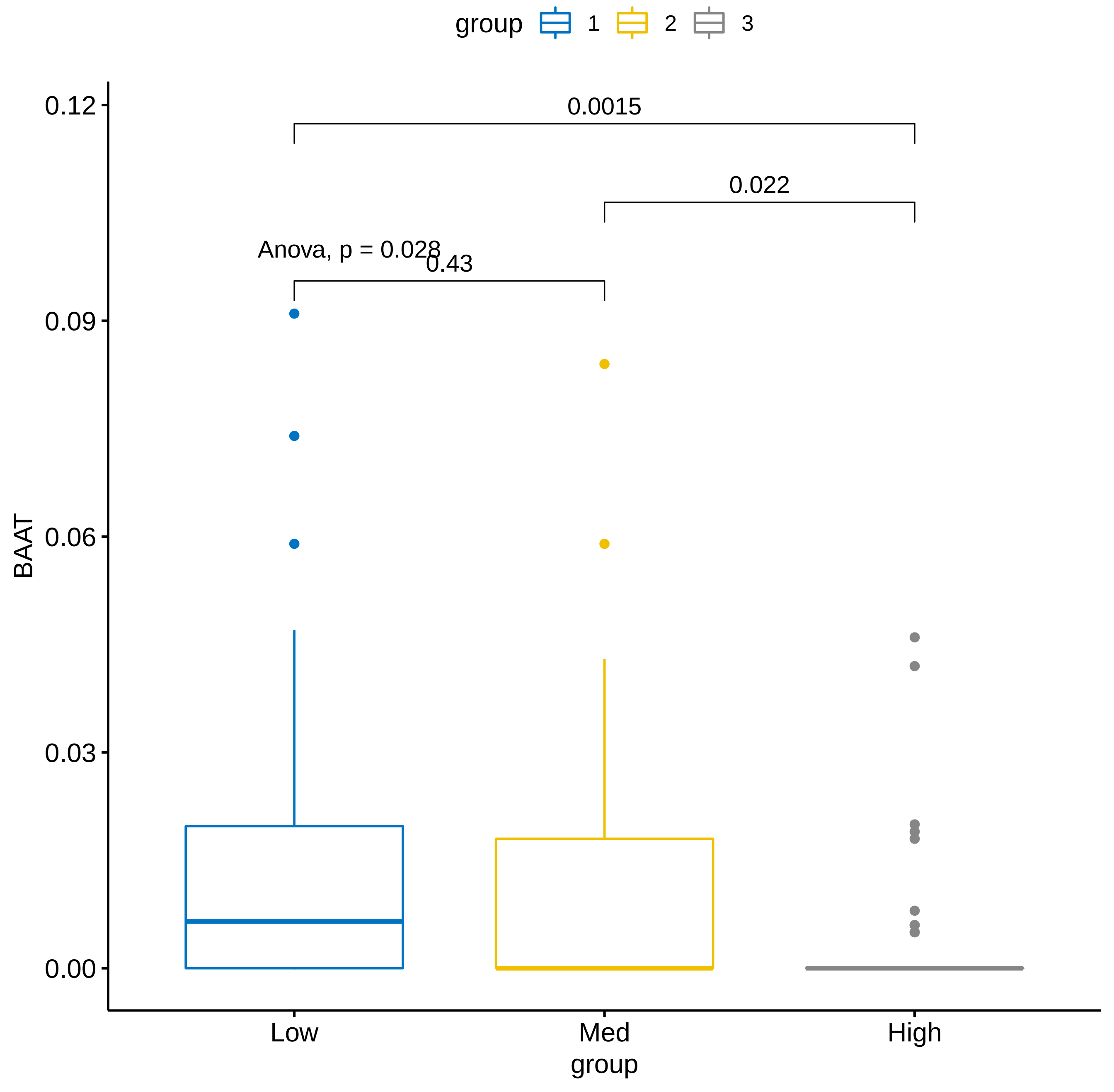
group 1 2 3
0.0015
0.022
Anova, p = 0.028
0.43
0.12
0.09
0.06
0.03
0.00
BAAT
Low
Med
High
group

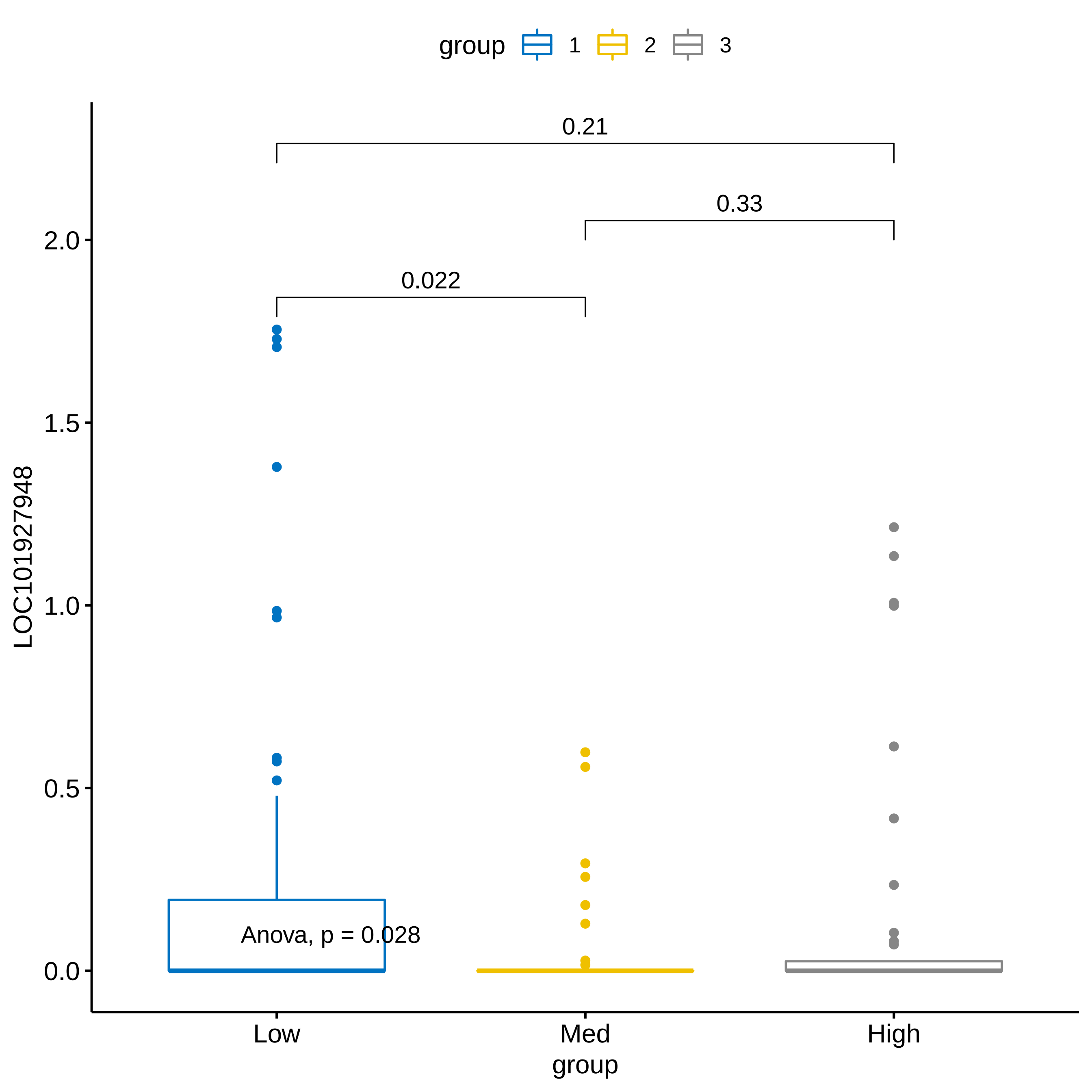

group 1 2 3
0.21
0.33
0.022
2.0
1.5
1.0
0.5
0.0
LOC101927948
Anova, p = 0.028
Low
Med
High
group

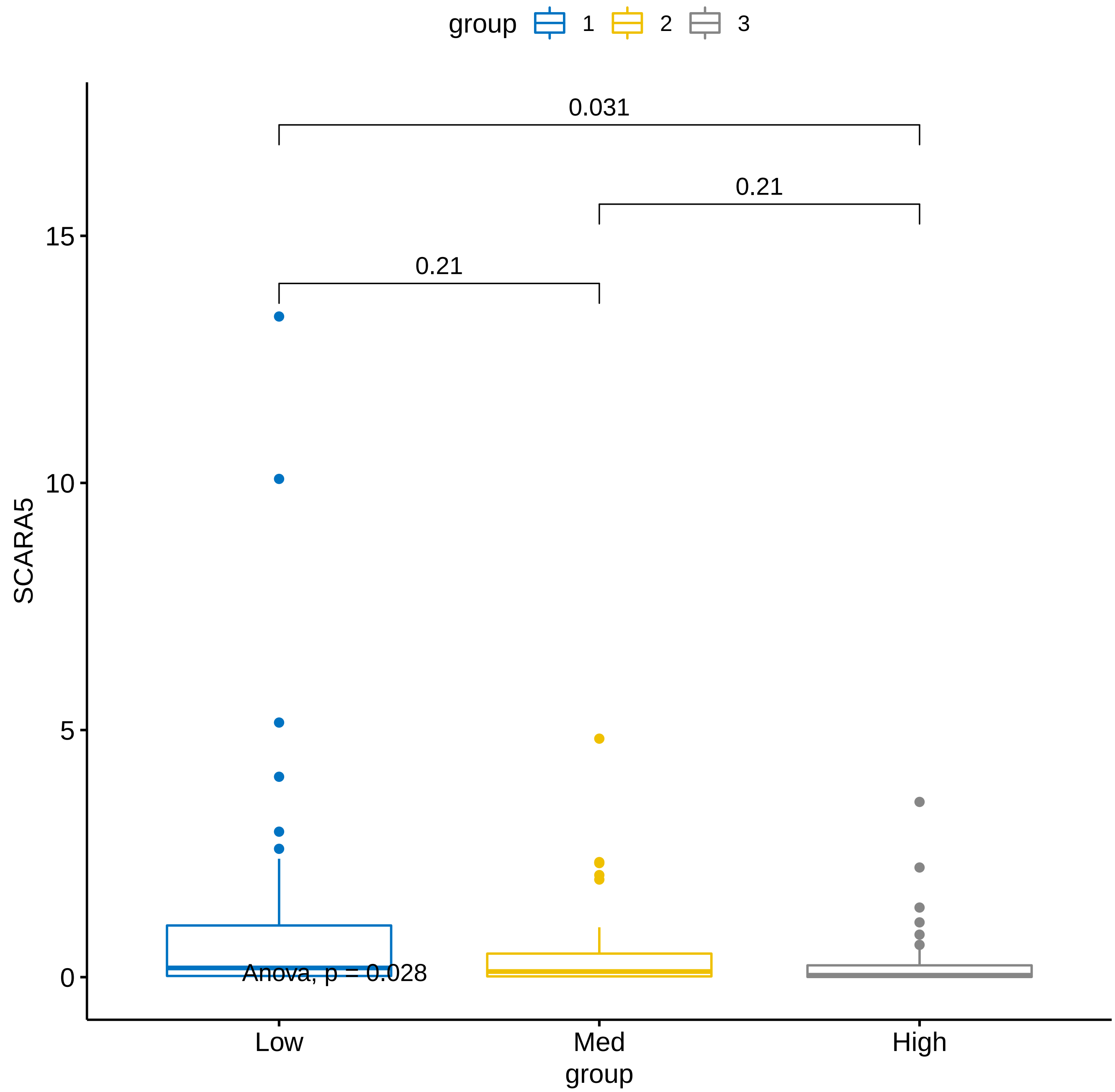

group 1 2 3
0.031
0.21
0.21
15
10
5
0
SCARA5
Anova, p = 0.028
Low
Med
High
group

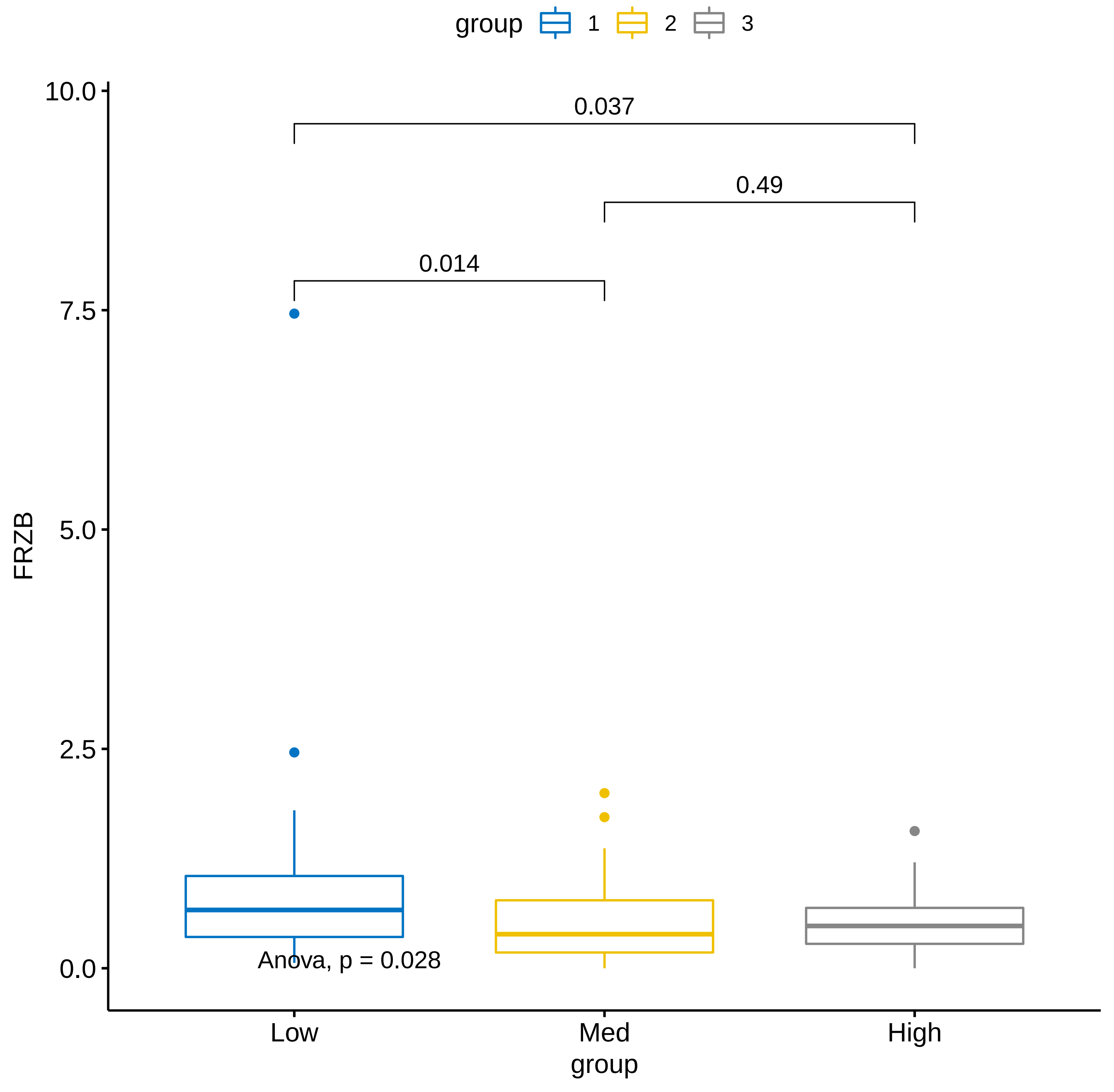

group
1
2
3
10.0
7.5
5.0
2.5
0.0
FRZB
0.037
0.49
0.014
Anova, p = 0.028
Low
Med
High
group

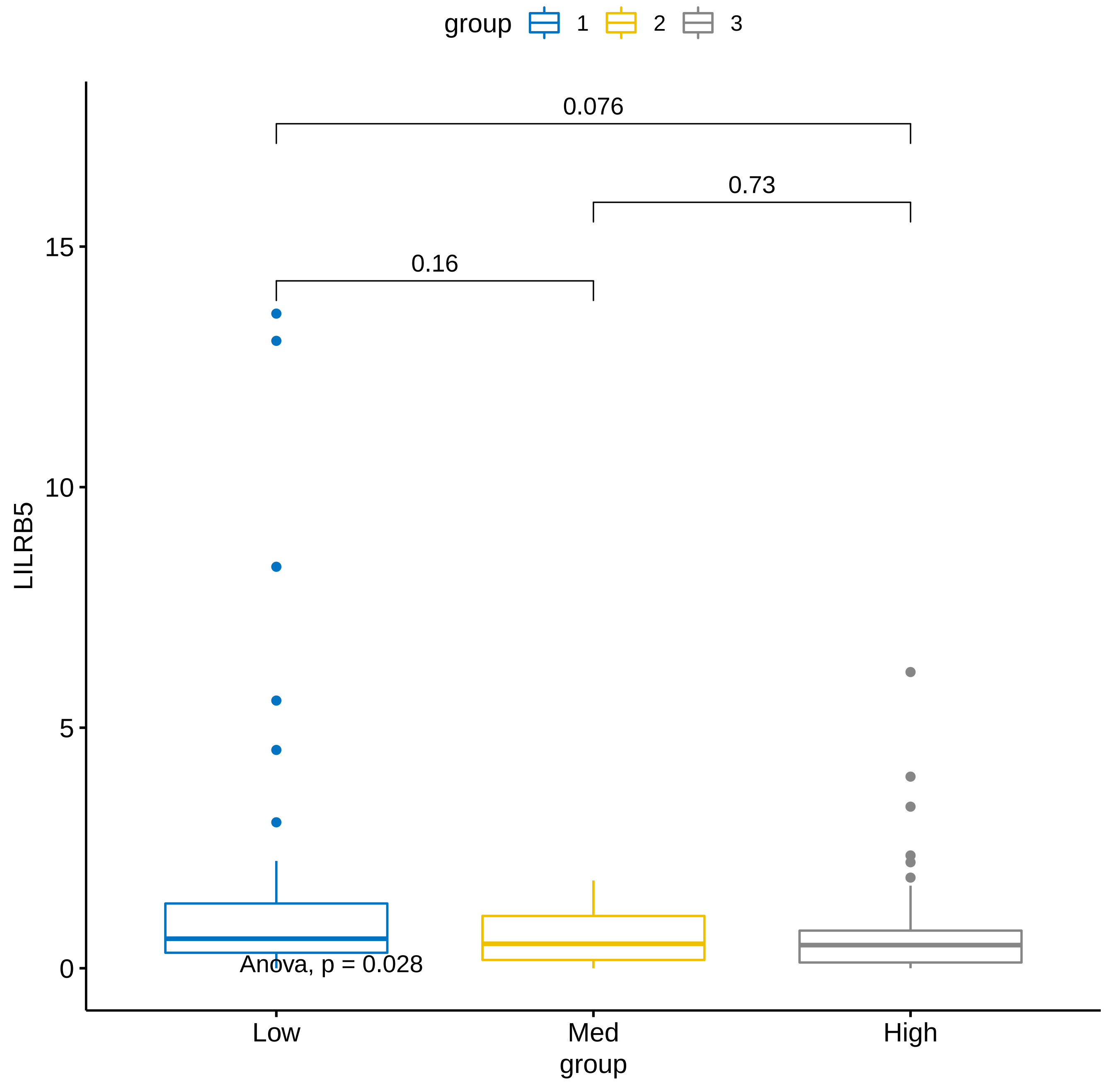
group
1
2
3
0.076
0.73
0.16
15
10
5
0
LILRB5
Anova, p = 0.028
Low
Med
High
group

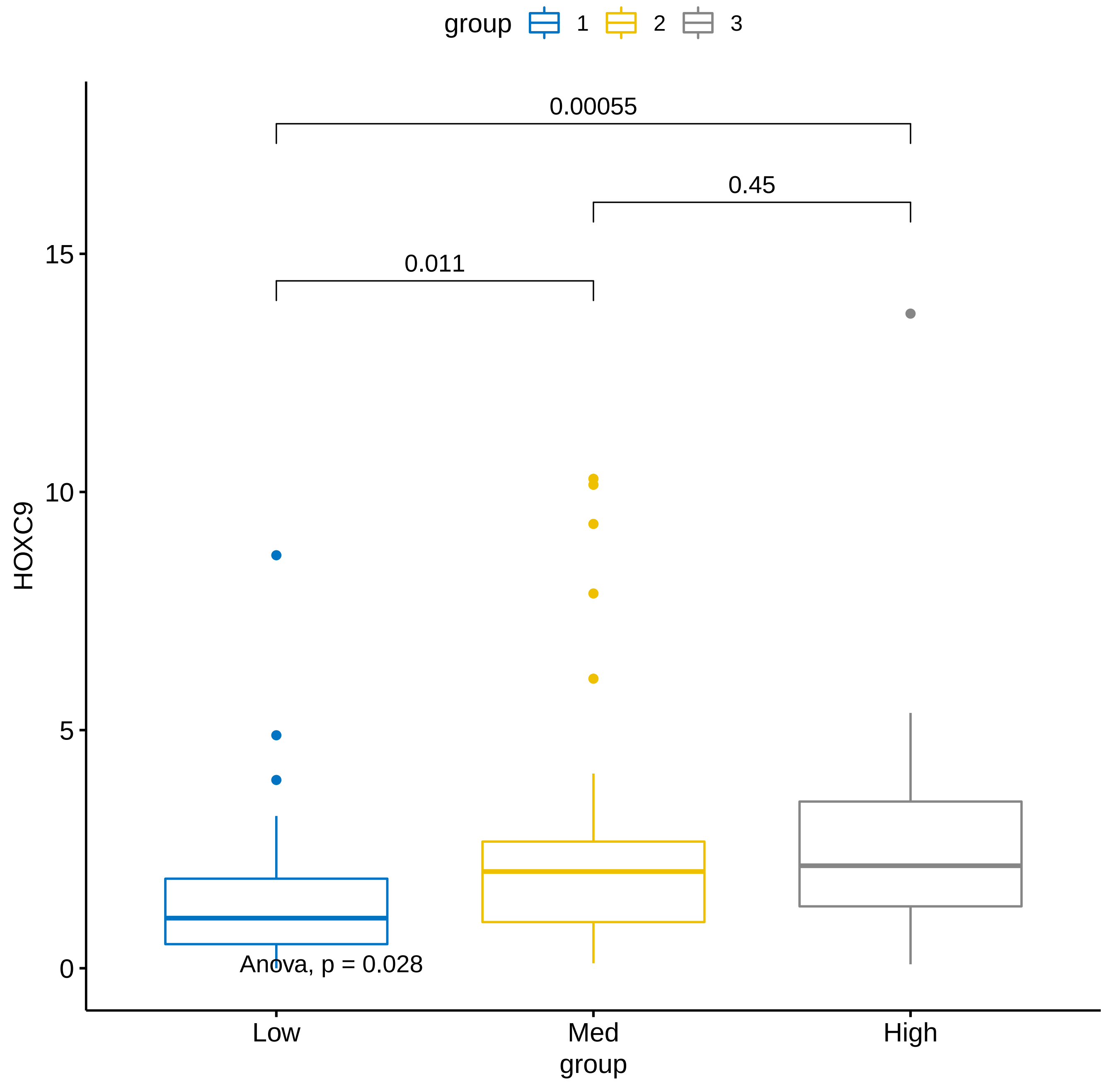
group
1
2
3
0.00055
0.45
0.011
15
10
5
0
HOXC9
Anova, p = 0.028
Low
Med
High
group

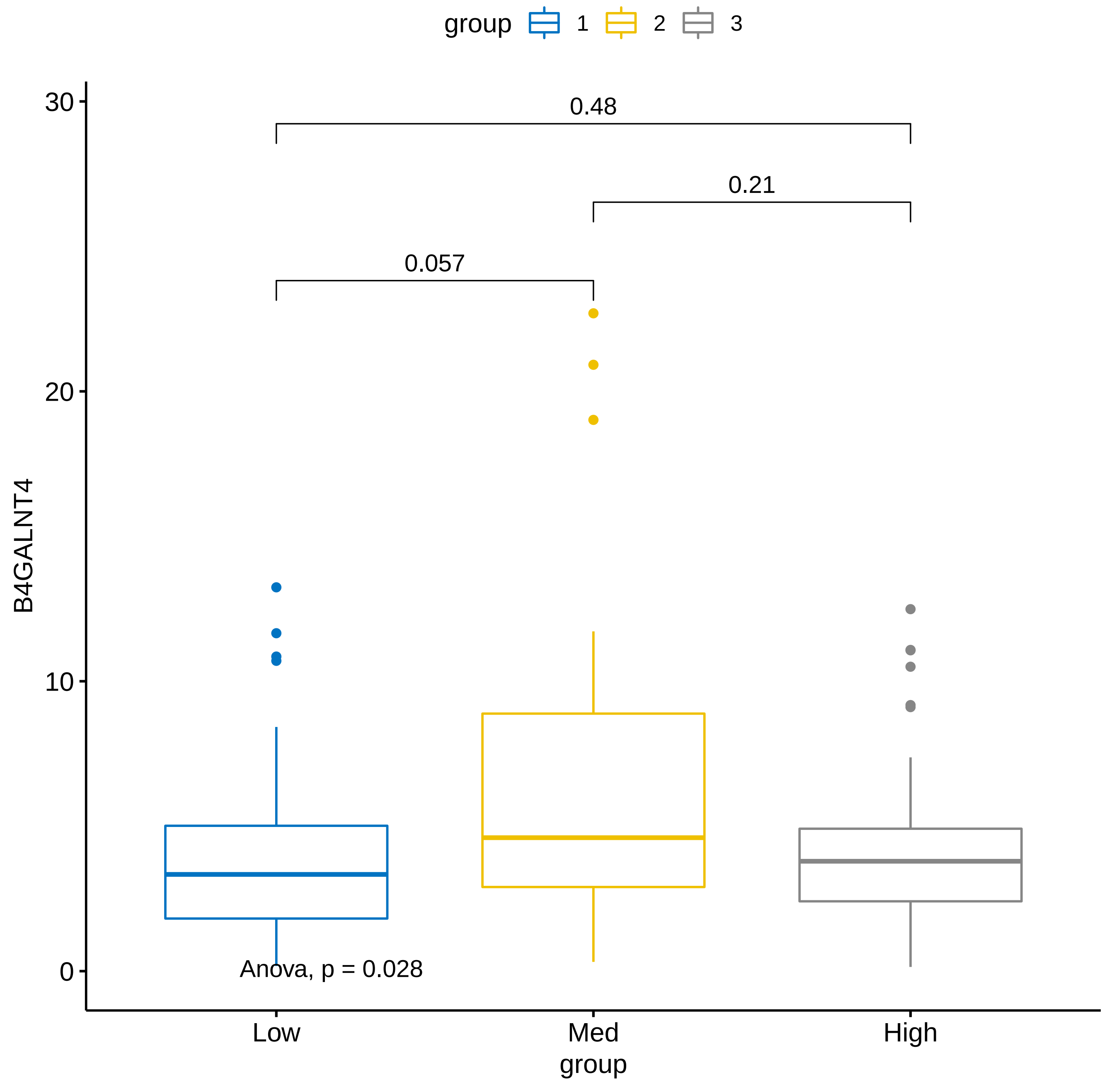
group 1 2 3
0.48
0.21
0.057
30
20
10
0
B4GALNT4
Anova, p = 0.028
Low
Med
High
group

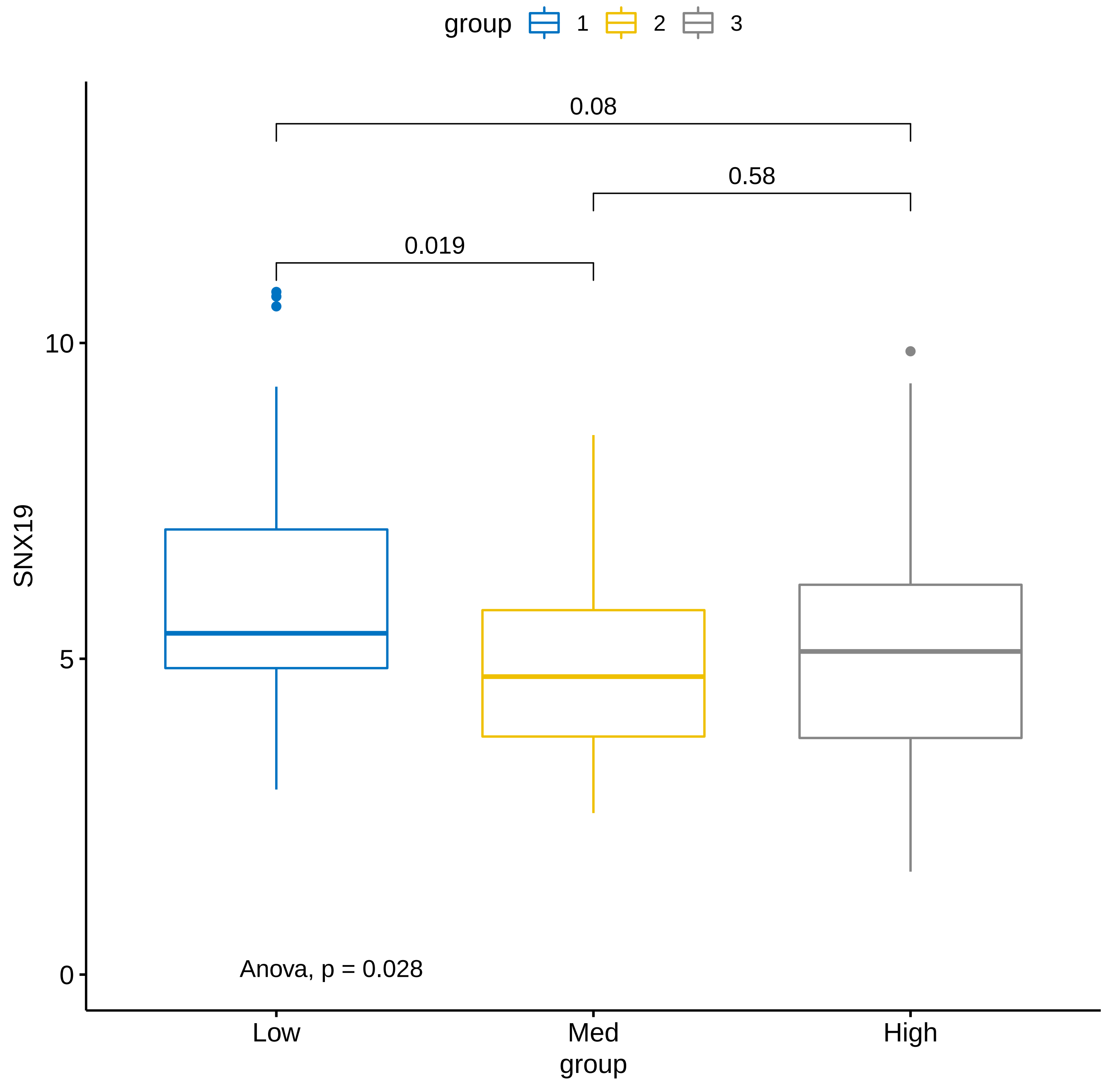
group
1
2
3
0.08
0.58
0.019
10
5
0
SNX19
Anova, p = 0.028
Low
Med
High
group

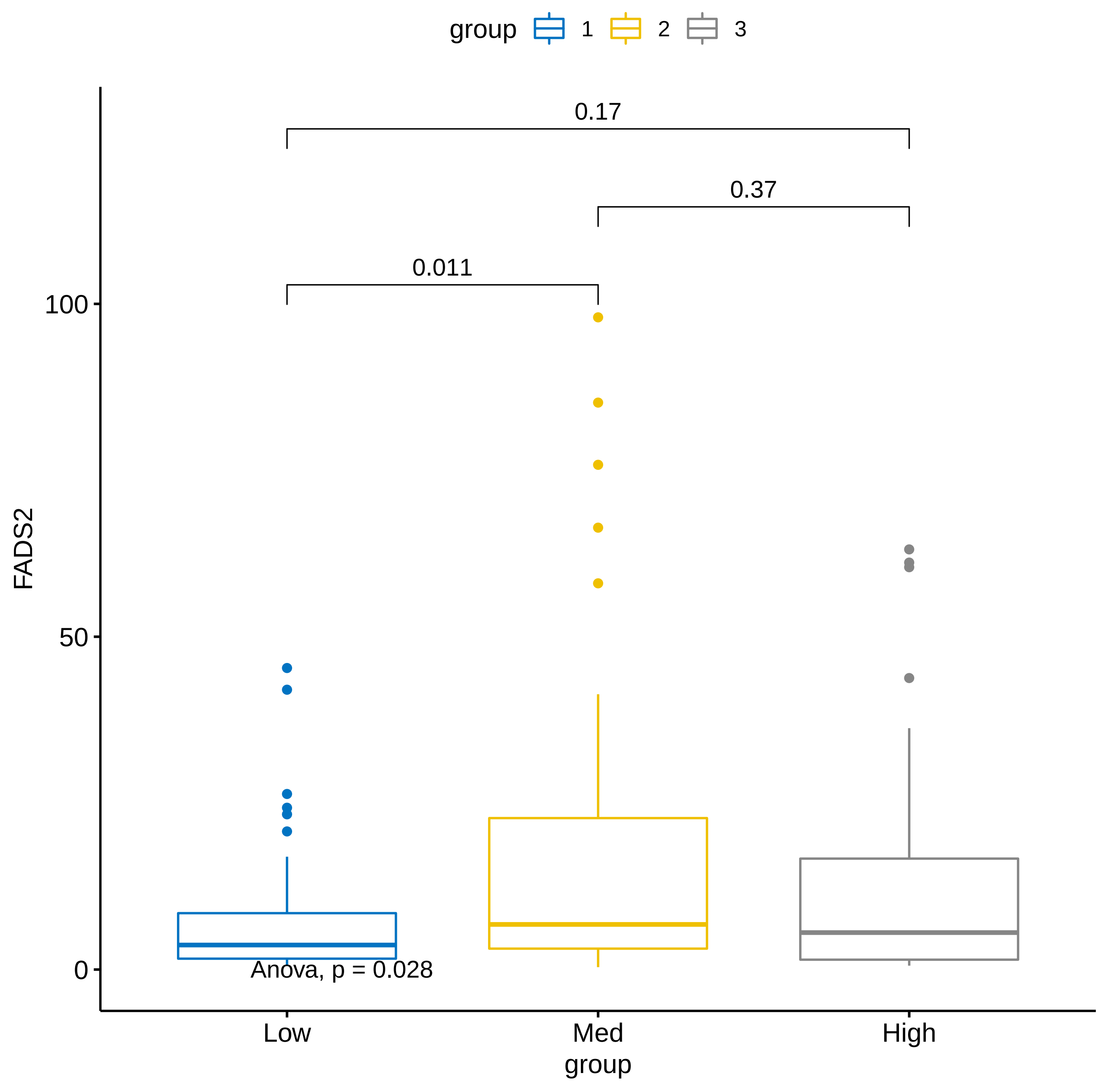
group
1
2
3
0.17
0.37
0.011
100
50
0
FADS2
Anova, p = 0.028
Low
Med
High
group

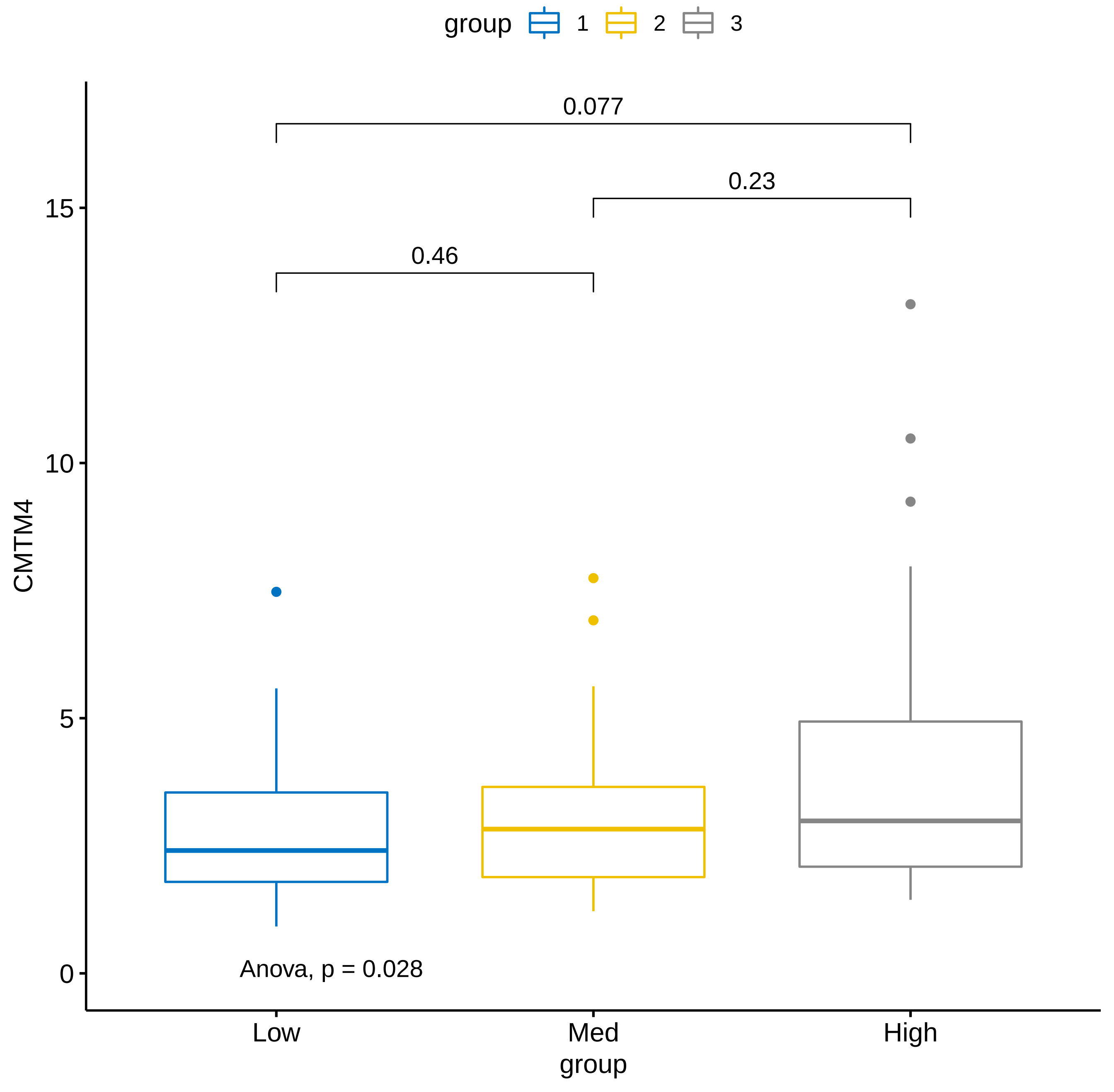
group
1
2
3
0.077
0.23
0.46
15
10
5
0
CMTM4
Anova, p = 0.028
Low
Med
High
group

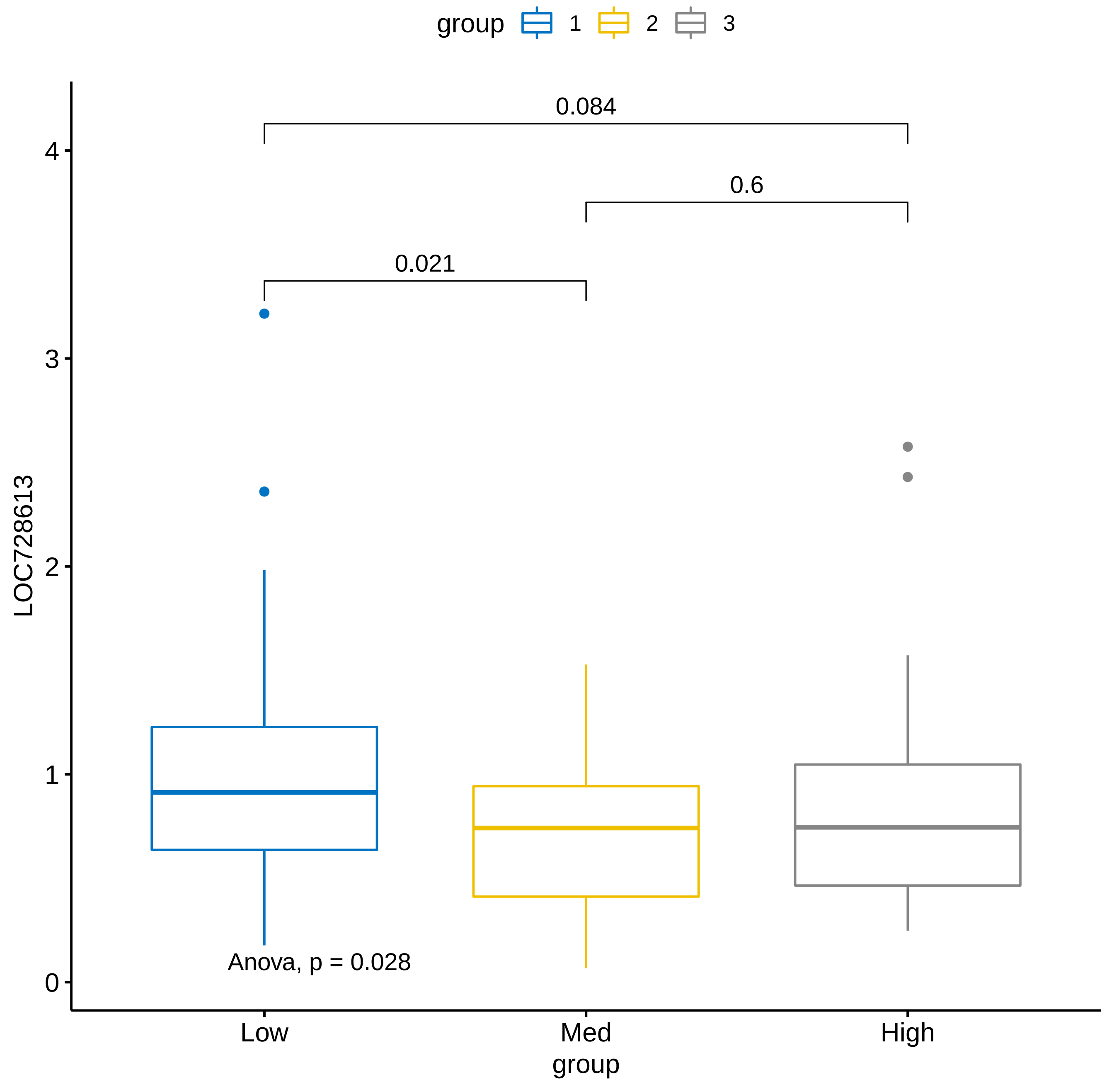
group
1
2
3
0.084
0.6
0.021
4
3
2
1
0
LOC728613
Anova, p = 0.028
Low
Med
High
group

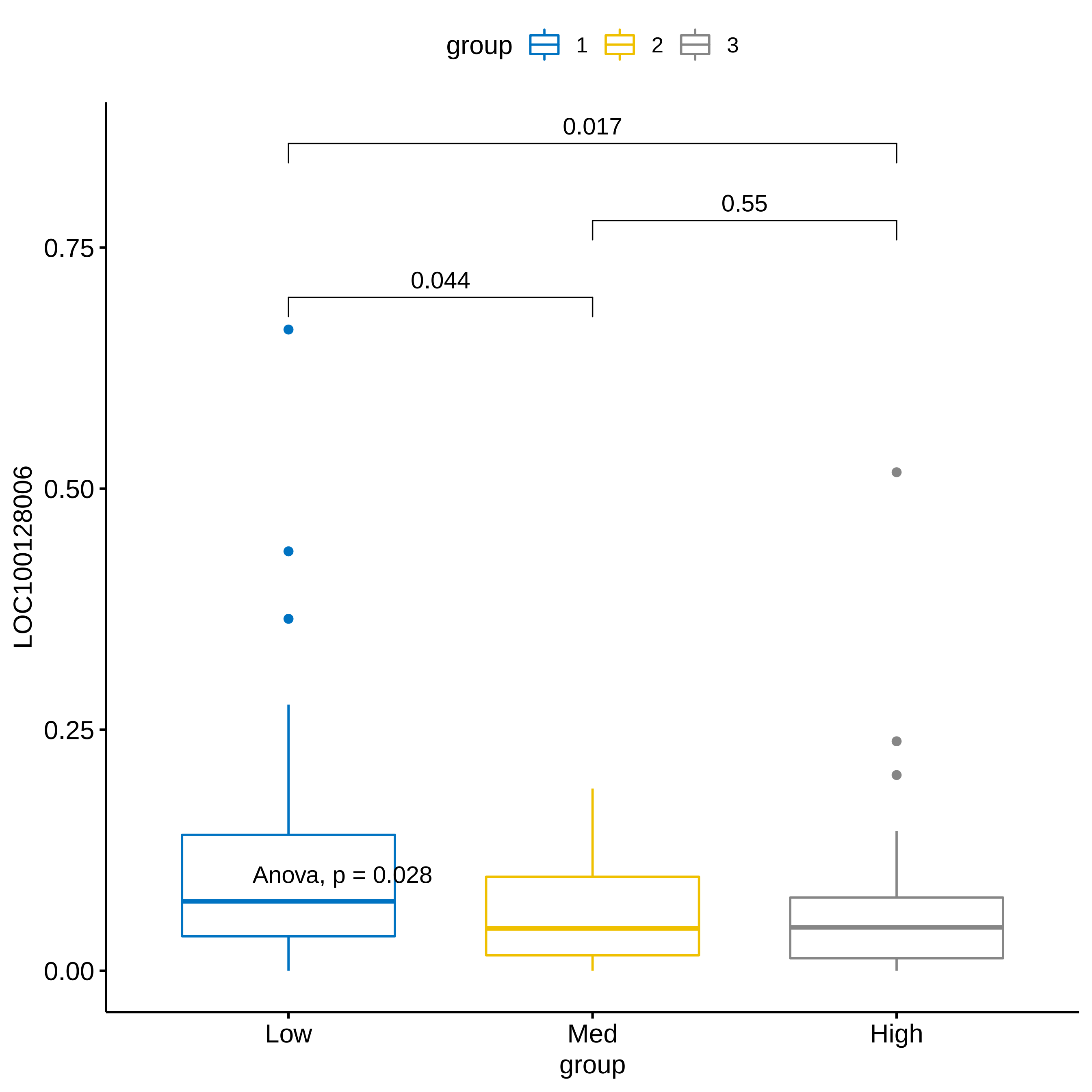

group 1 2 3
0.017
0.55
0.044
0.75
0.50
0.25
0.00
LOC100128006
Anova, p = 0.028
Low
Med
High
group

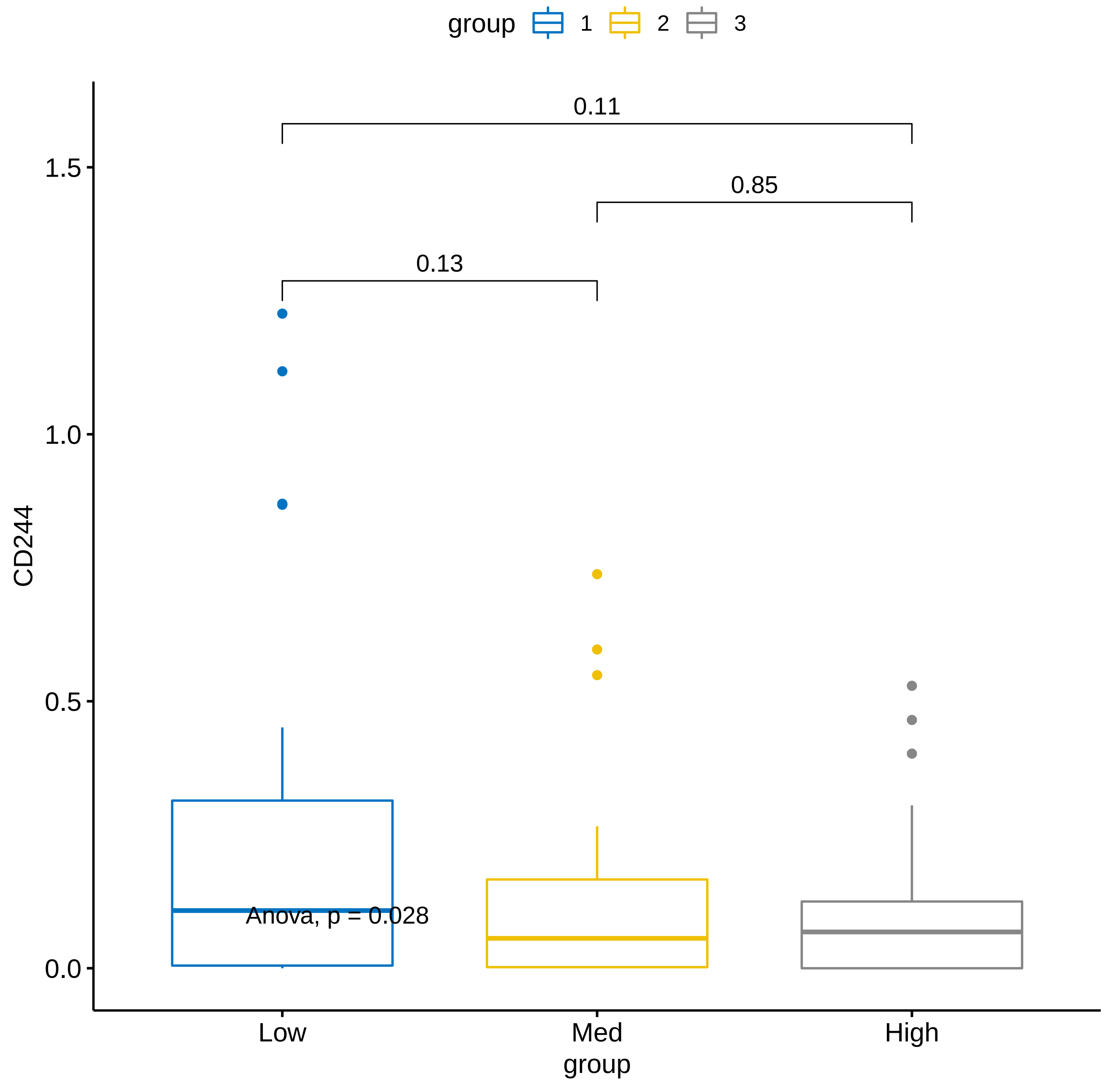
group
1
2
3
0.11
0.85
0.13
1.5
1.0
0.5
0.0
CD244
Anova, p = 0.028
Low
Med
High
group

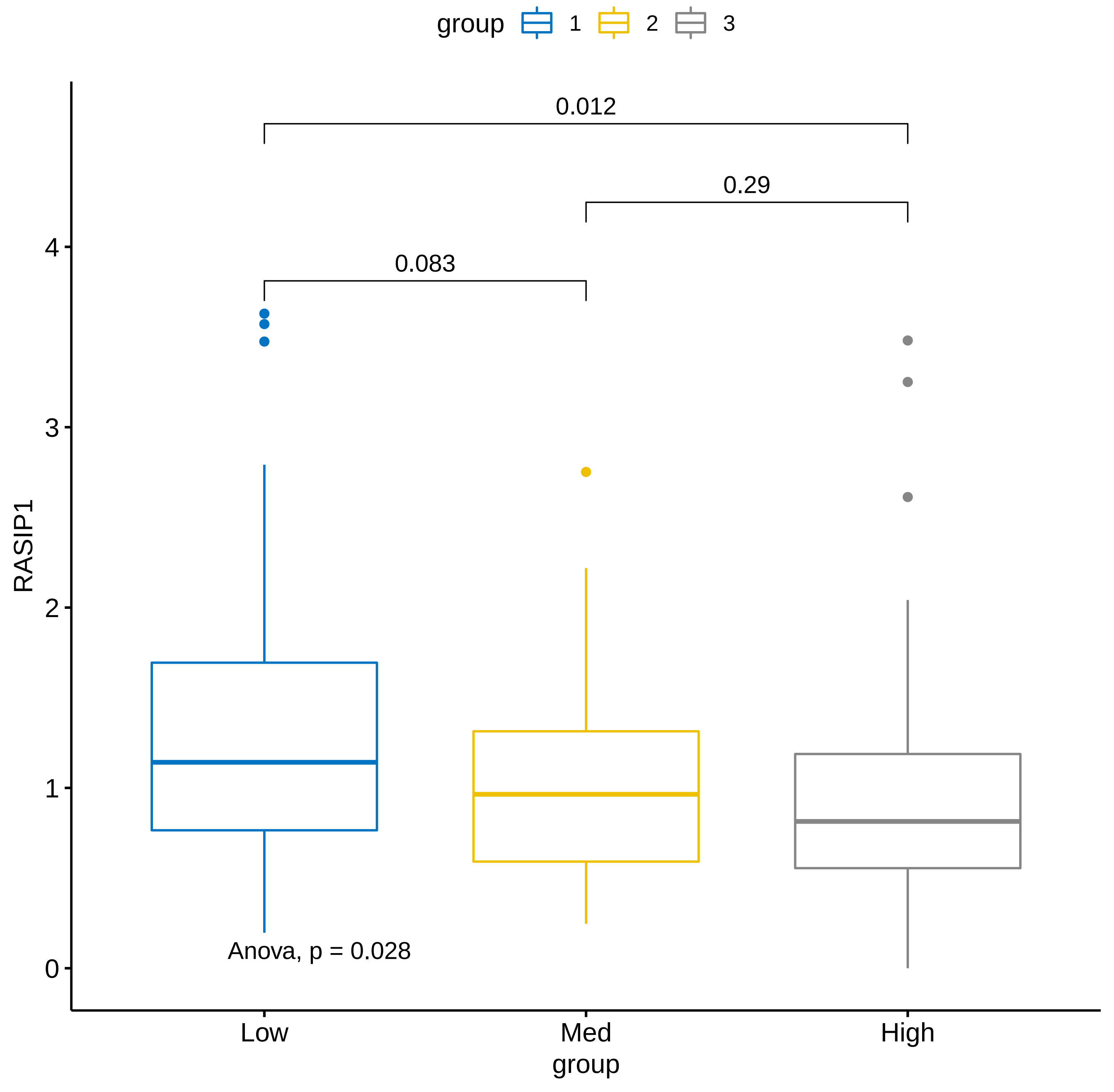
group 1 2 3
0.012
0.29
0.083
4
3
2
1
0
RASIP1
Anova, p = 0.028
Low
Med
High
group

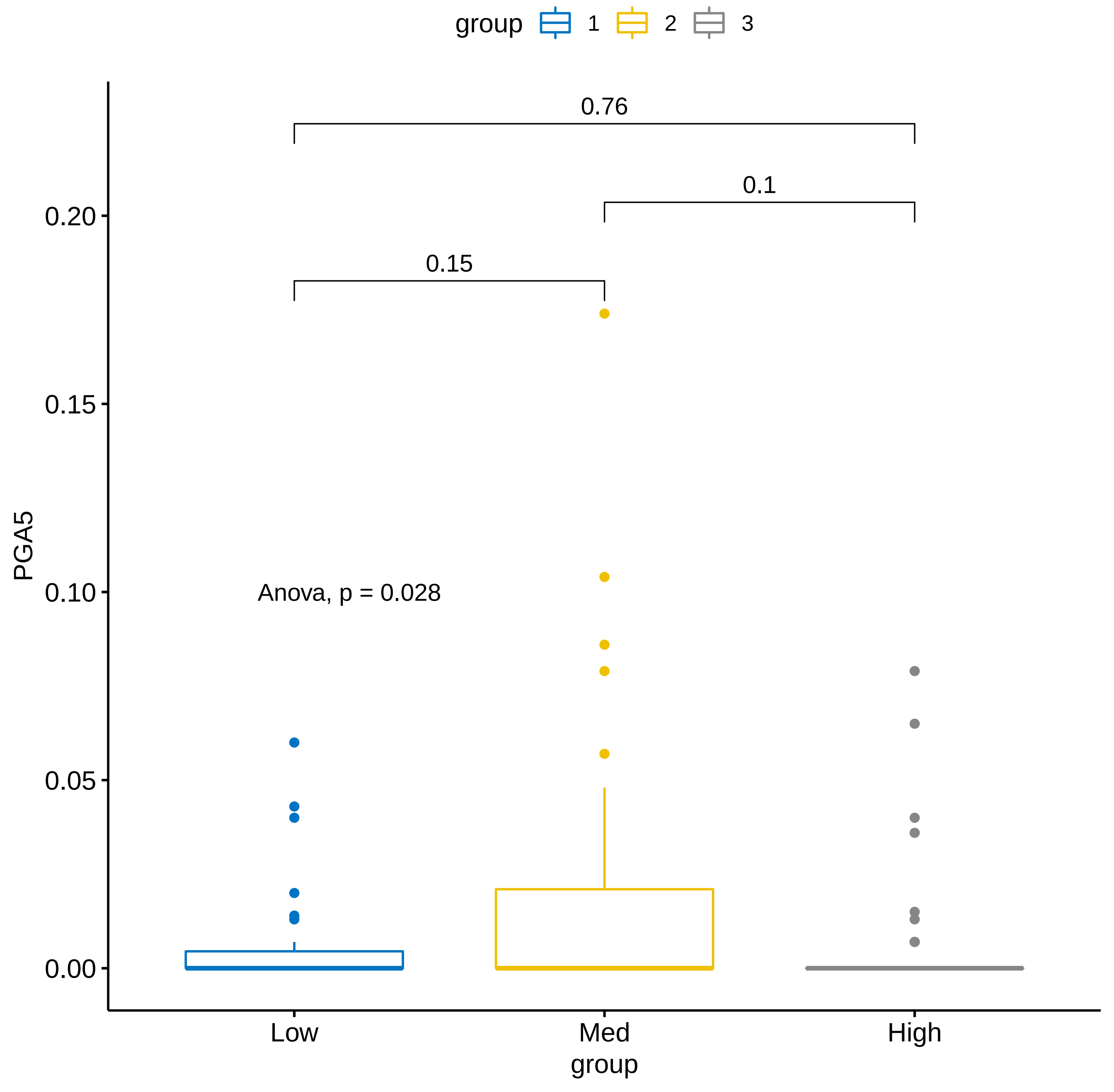

group 1 2 3
0.76
0.1
0.15
Anova, p = 0.028
PGA5
0.20
0.15
0.10
0.05
0.00
Low
Med
High
group

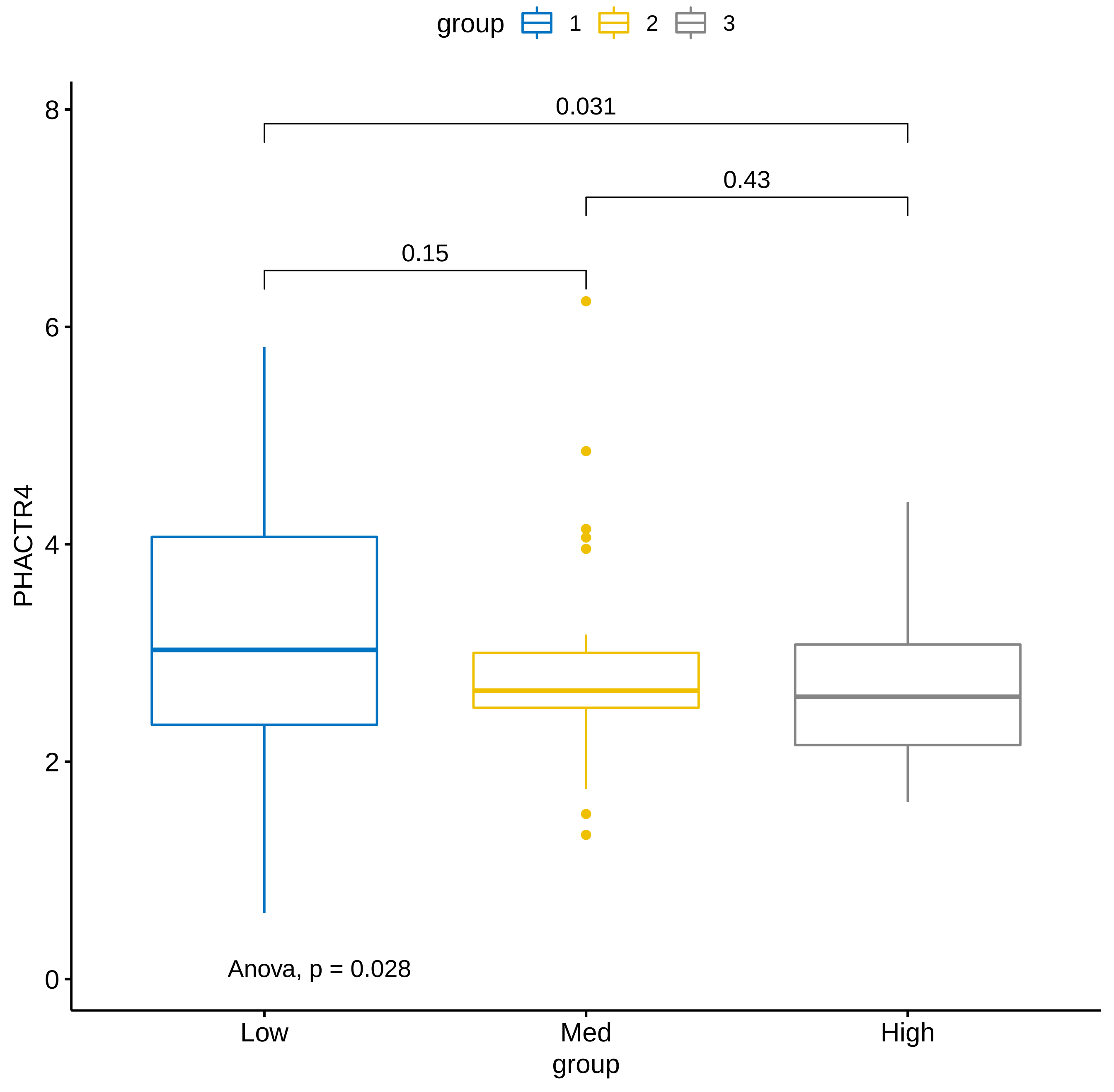

group 1 2 3
0.031
0.43
0.15
8
6
4
2
0
PHACTR4
Anova, p = 0.028
Low
Med
High
group

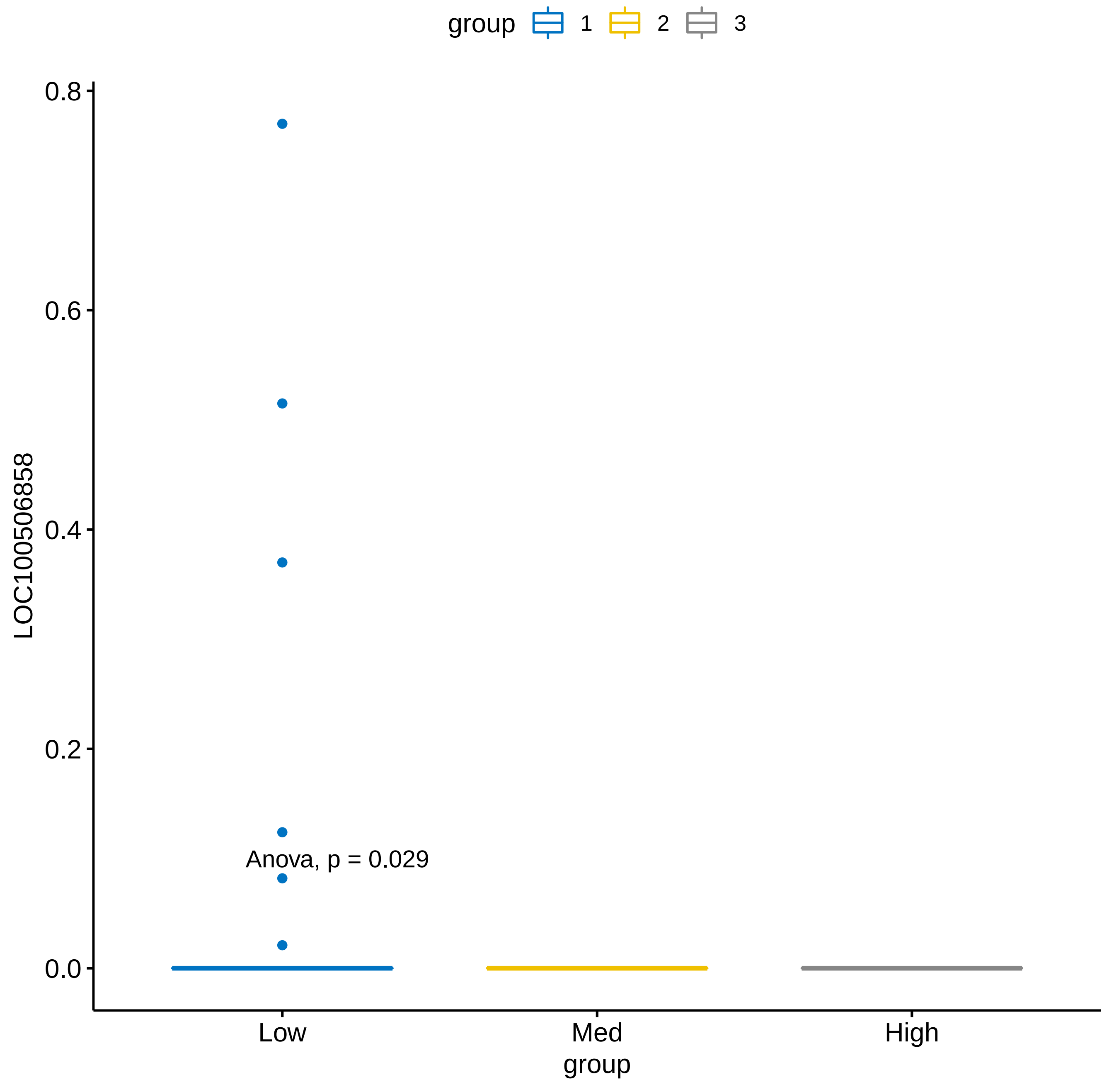

group 1 2 3
LOC100506858
0.8
0.6
0.4
0.2
0.0
Anova, p = 0.029
Low
Med
High
group

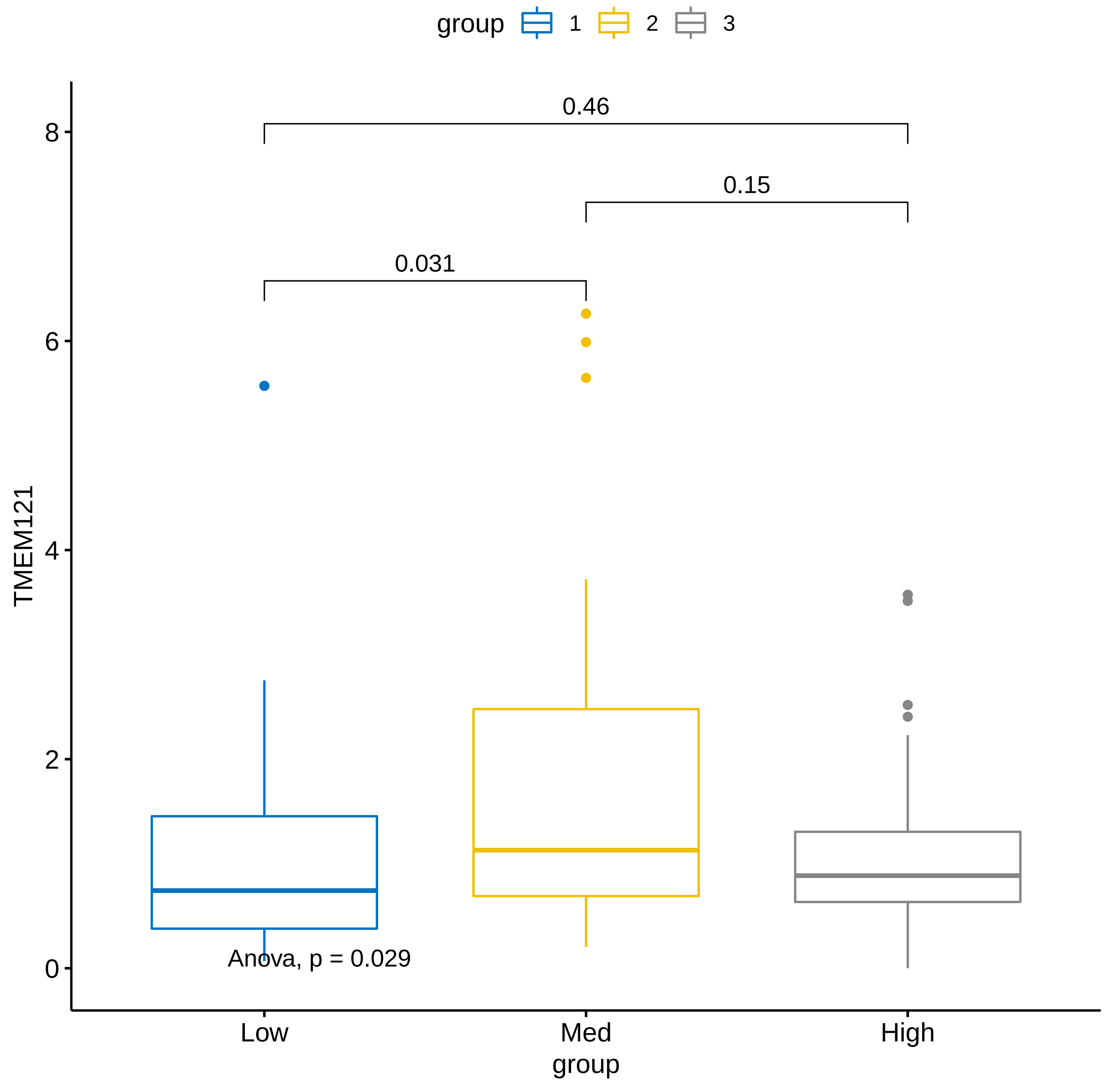

group
1
2
3
0.46
0.15
0.031
8
6
4
2
0
TMEM121
Anova, p = 0.029
Low
Med
High
group

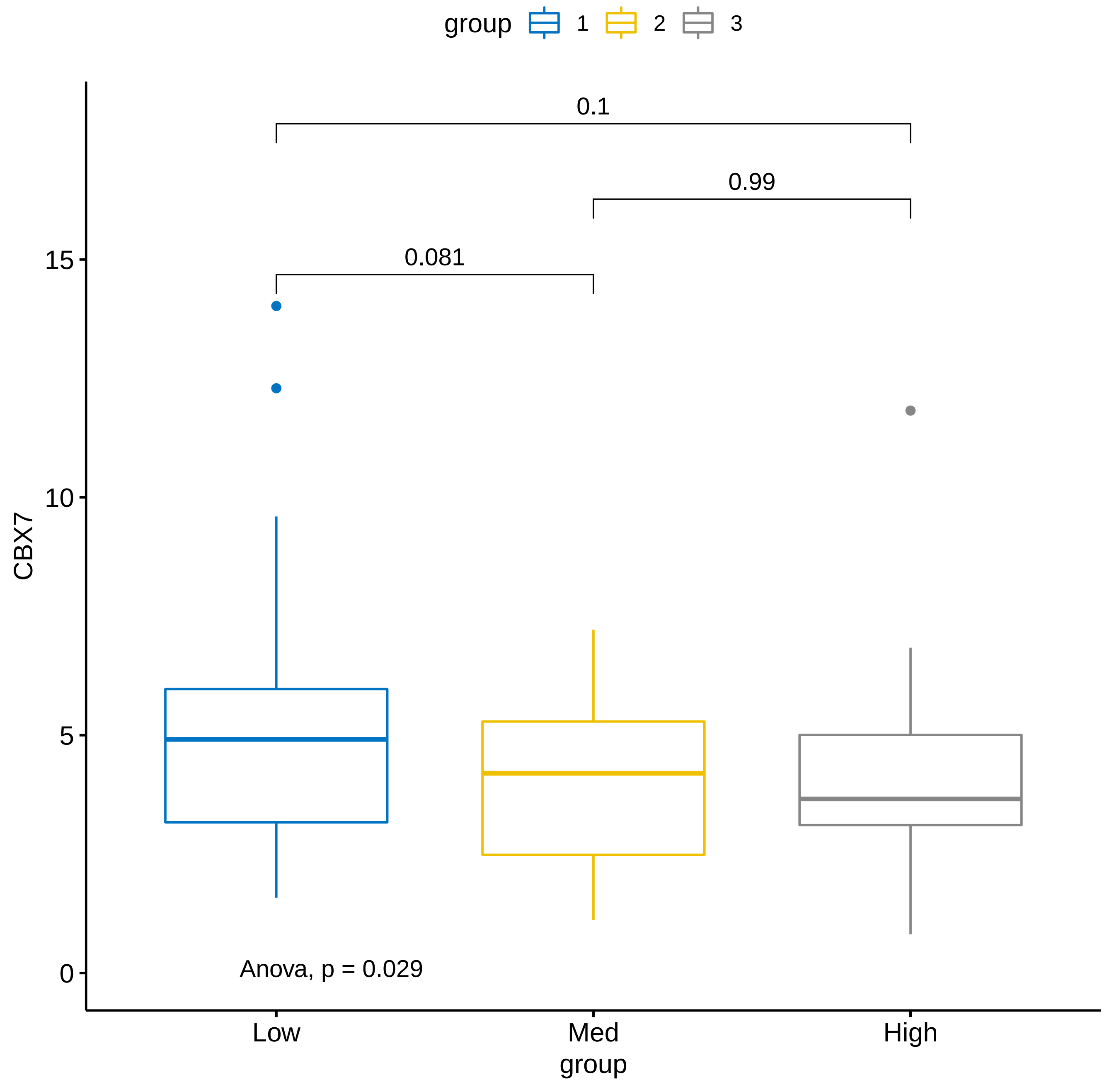
group
1
2
3
0.1
0.99
0.081
15
10
5
0
CBX7
Anova, p = 0.029
Low
Med
High
group

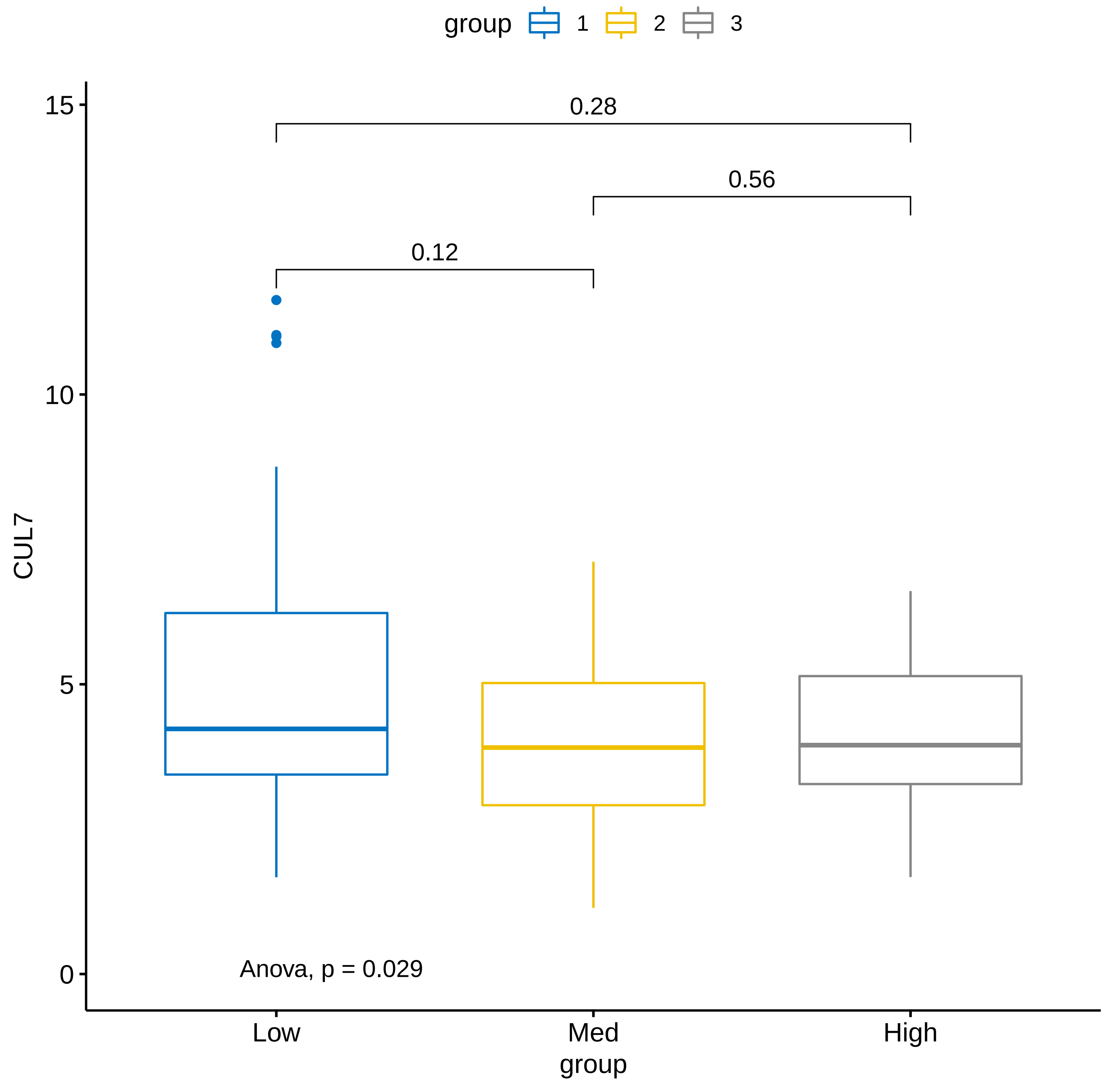

group 1 2 3
0.28
0.56
0.12
15
10
5
0
CUL7
Anova, p = 0.029
Low
Med
High
group

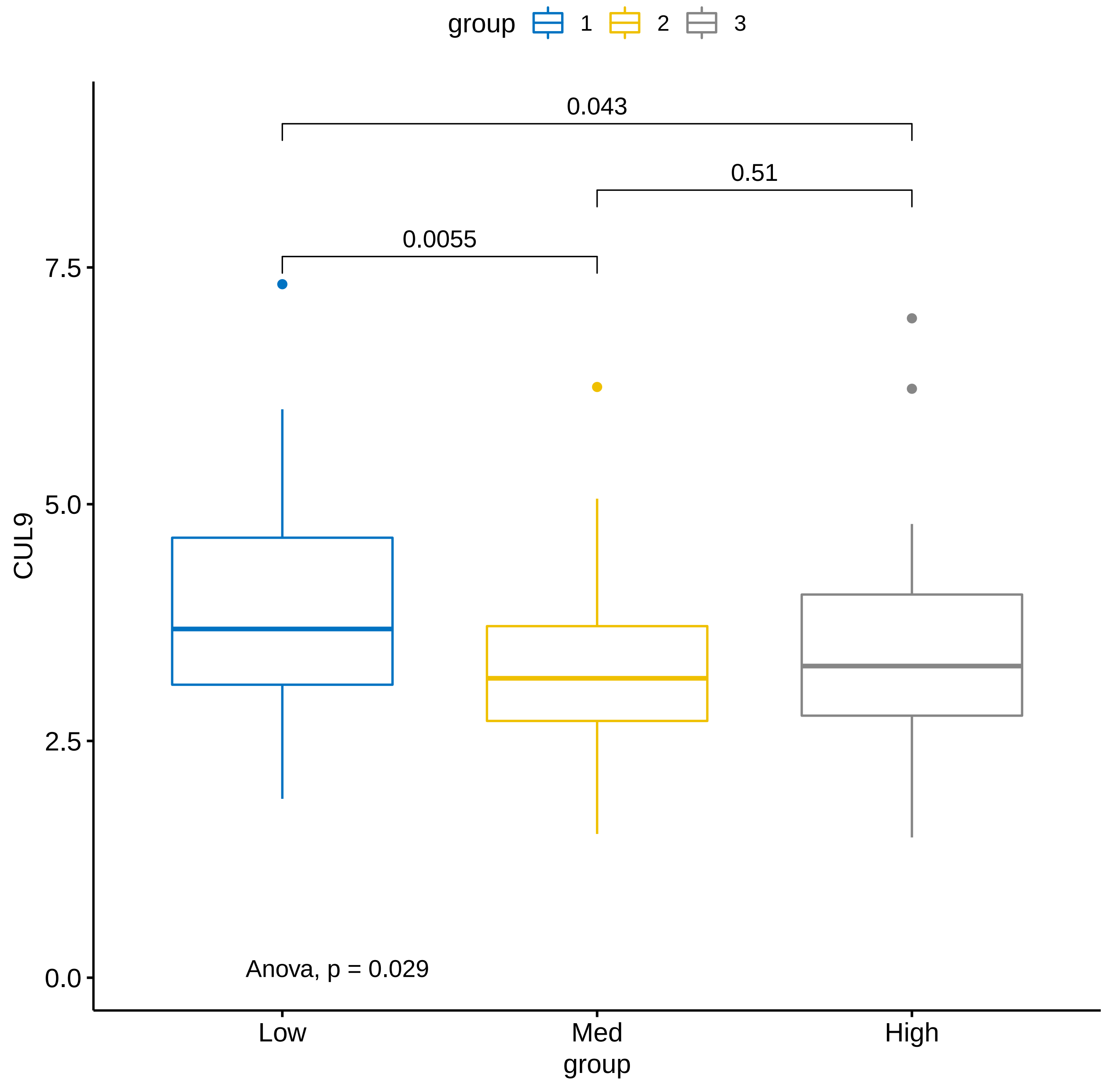
group
1
2
3
0.043
0.51
0.0055
7.5
5.0
2.5
0.0
CUL9
Anova, p = 0.029
Low
Med
High
group

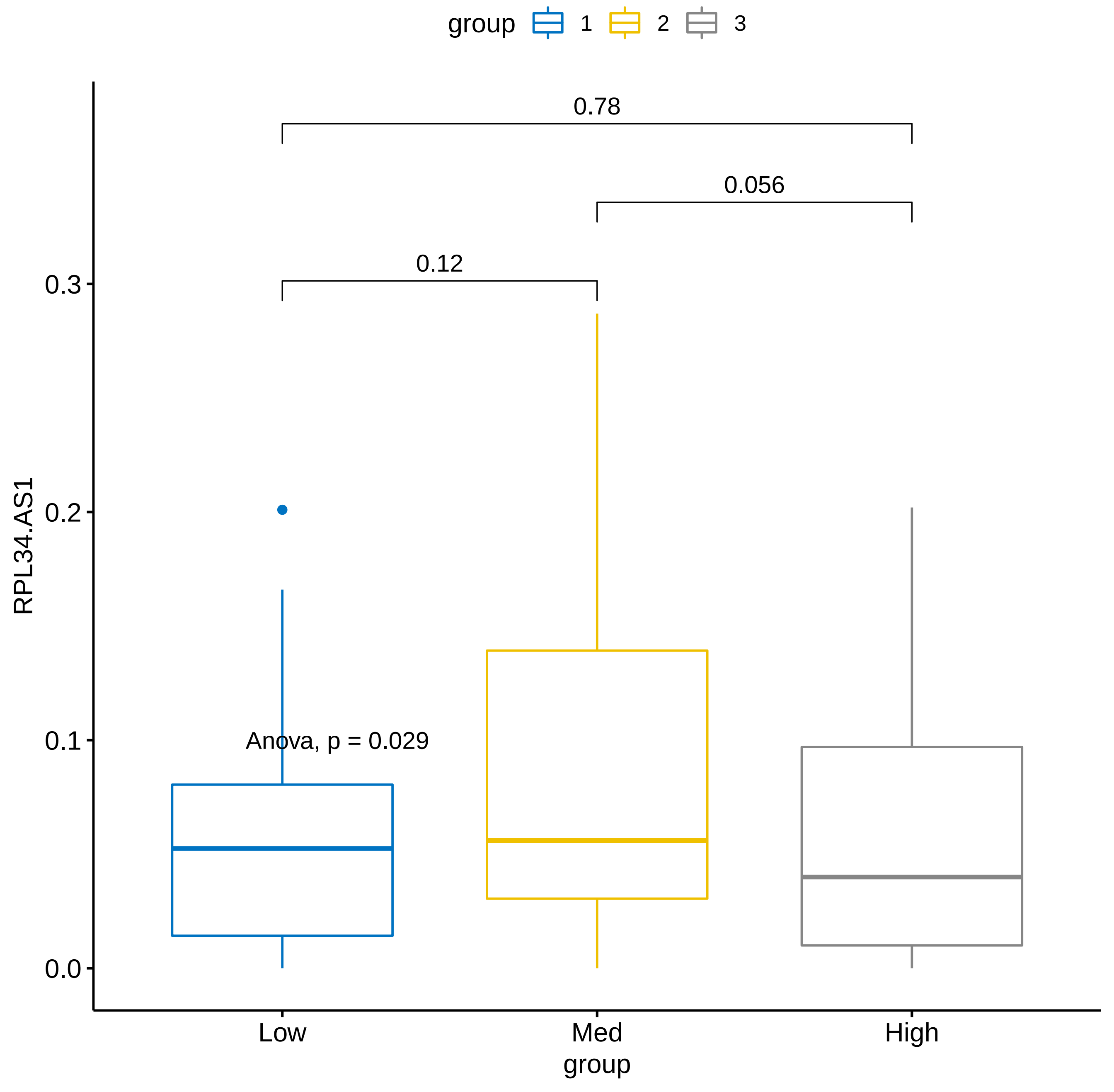

group
1
2
3
0.78
0.056
0.12
Anova, p = 0.029
RPL34.AS1
0.3
0.2
0.1
0.0
Low
Med
High
group

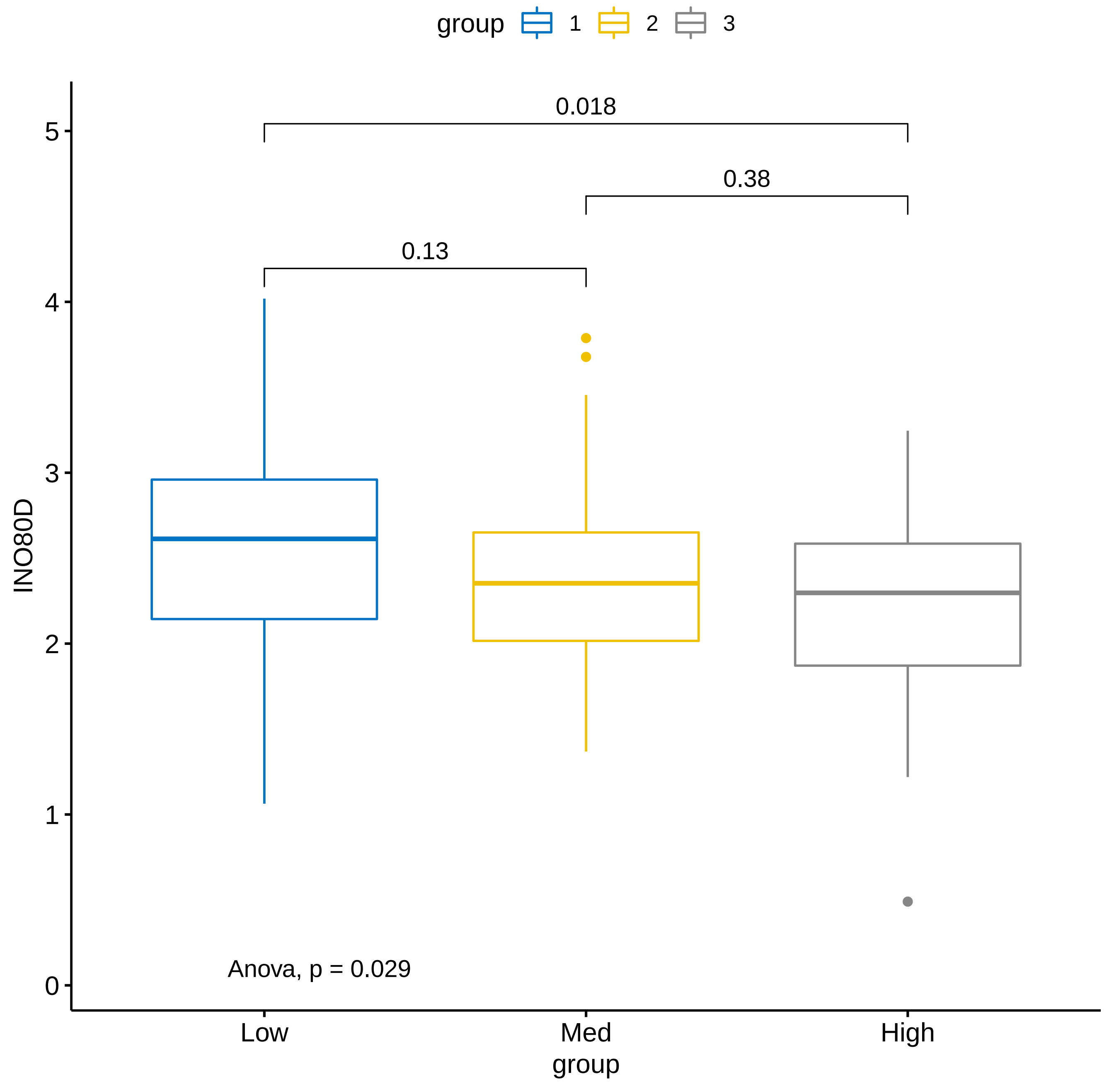
group
1
2
3
0.018
0.38
0.13
5
4
3
2
1
0
INO80D
Anova, p = 0.029
Low
Med
High
group

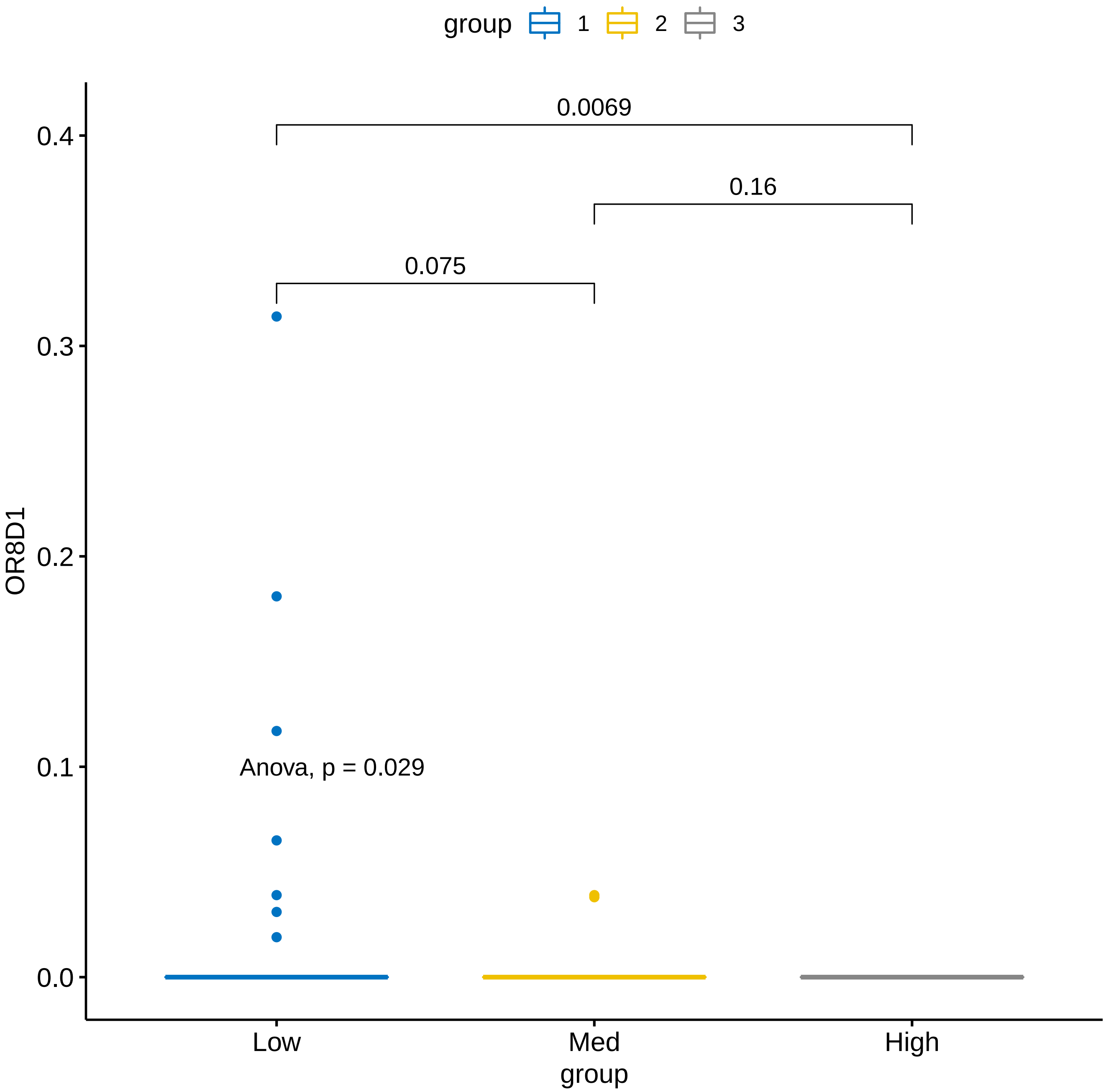

group
1
2
3
0.0069
0.16
0.075
Anova, p = 0.029
OR8D1
0.4
0.3
0.2
0.1
0.0
Low
Med
High
group

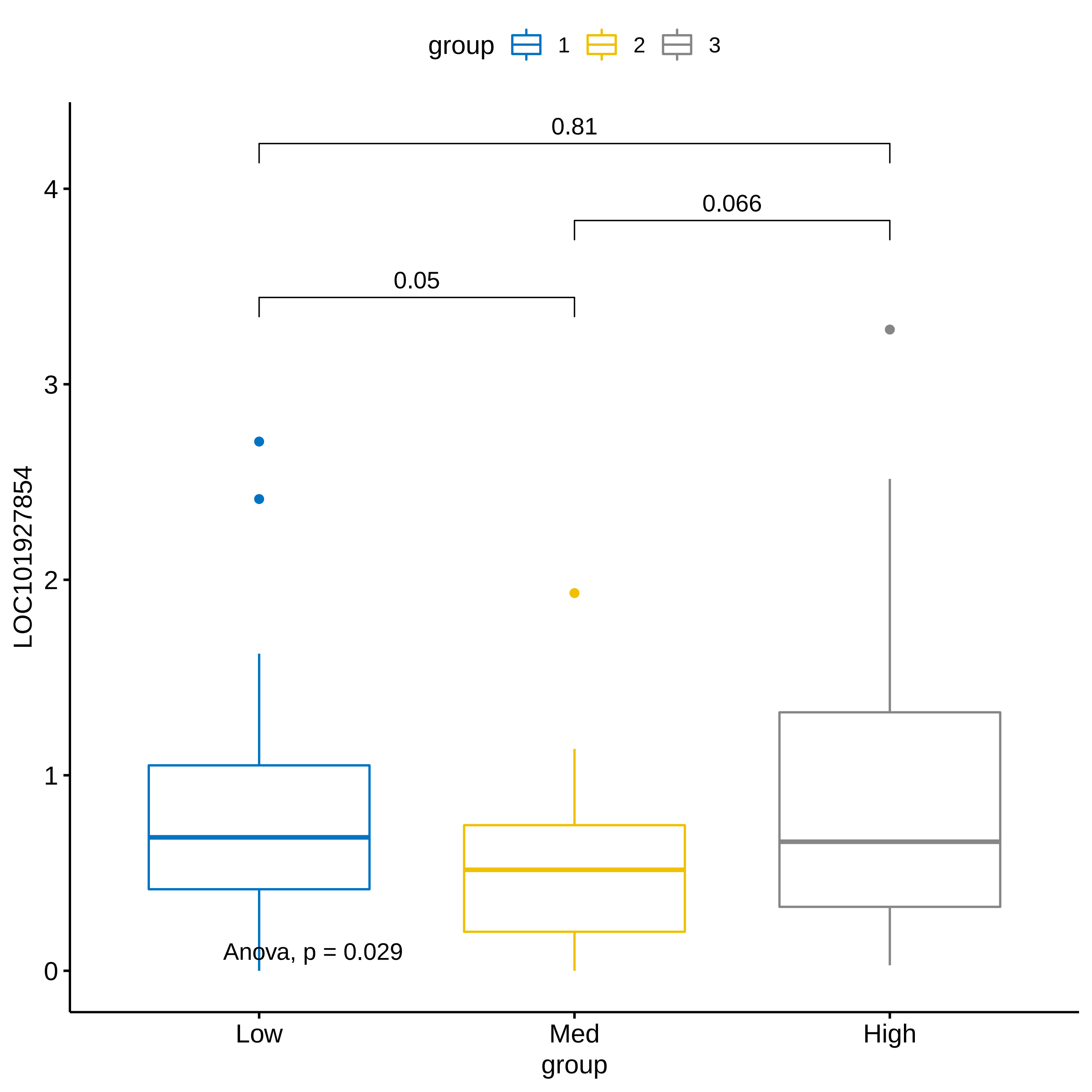
group
1
2
3
0.81
0.066
0.05
Anova, p = 0.029
LOC101927854
0
1
2
3
4
Low
Med
High
group

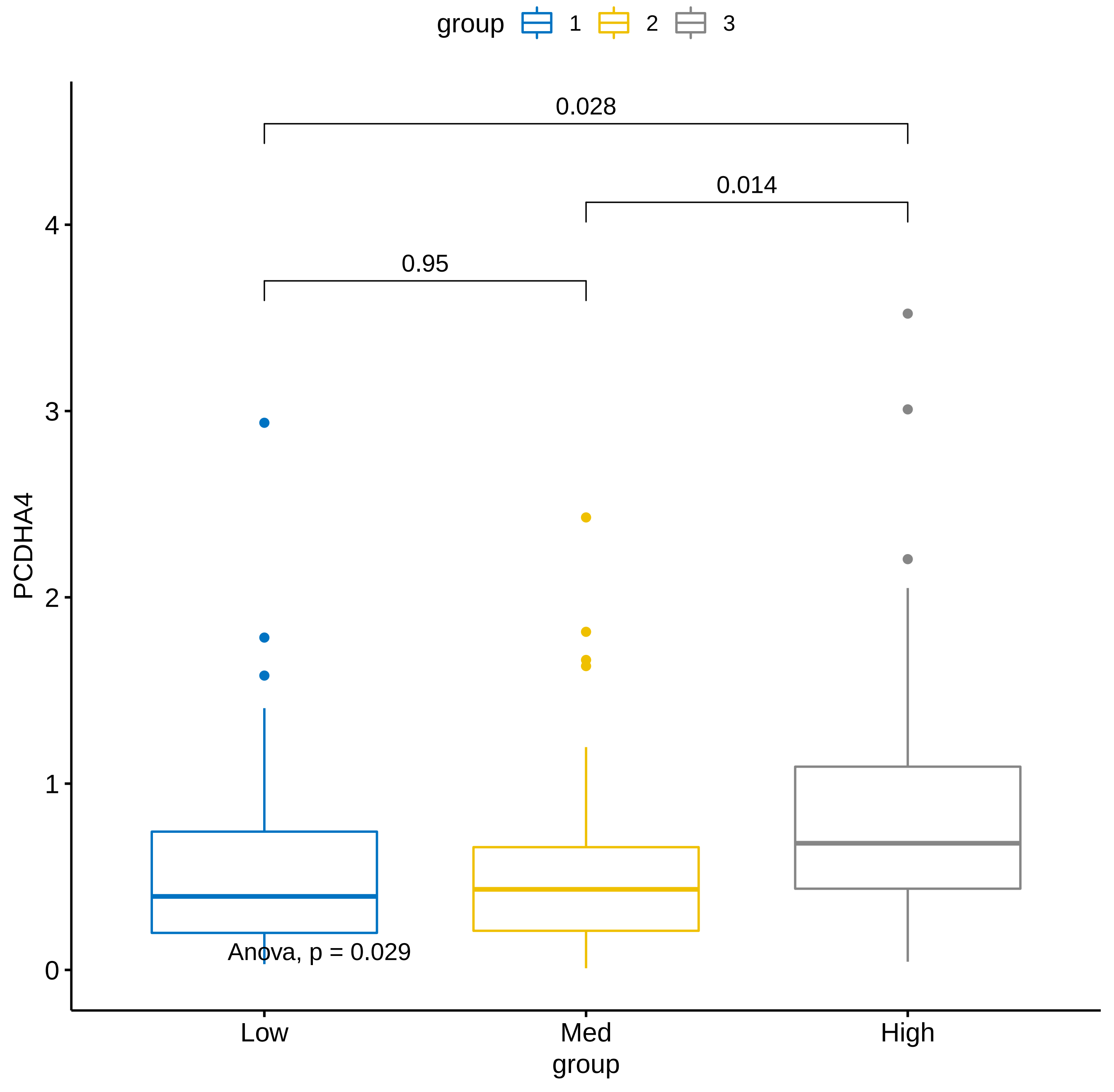

group
1
2
3
0.028
0.014
0.95
PCDHA4
4
3
2
1
0
Anova, p = 0.029
Low
Med
High
group

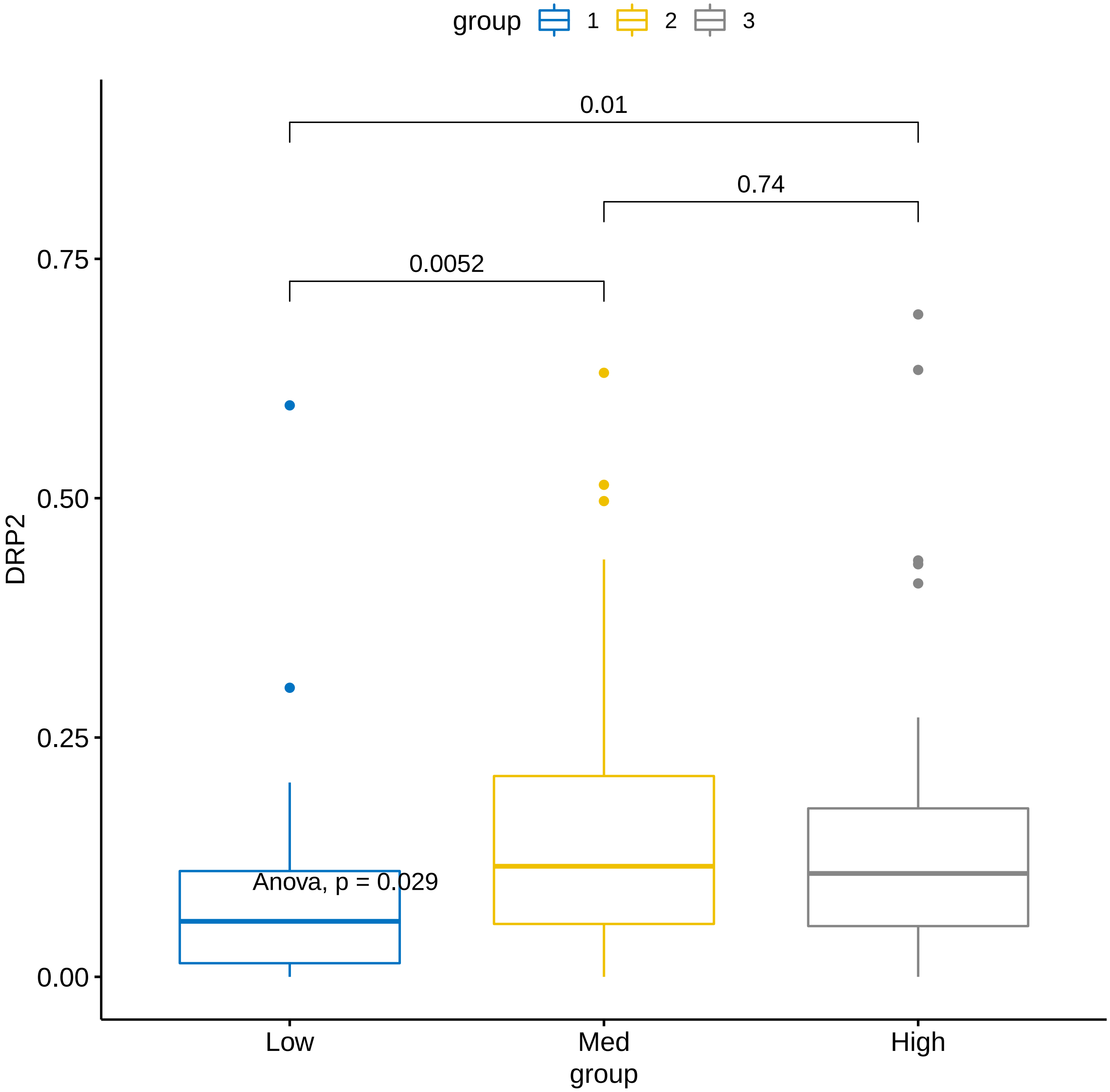

group 1 2 3
0.01
0.74
0.0052
0.75
0.50
0.25
0.00
DRP2
Anova, p = 0.029
Low
Med
High
group

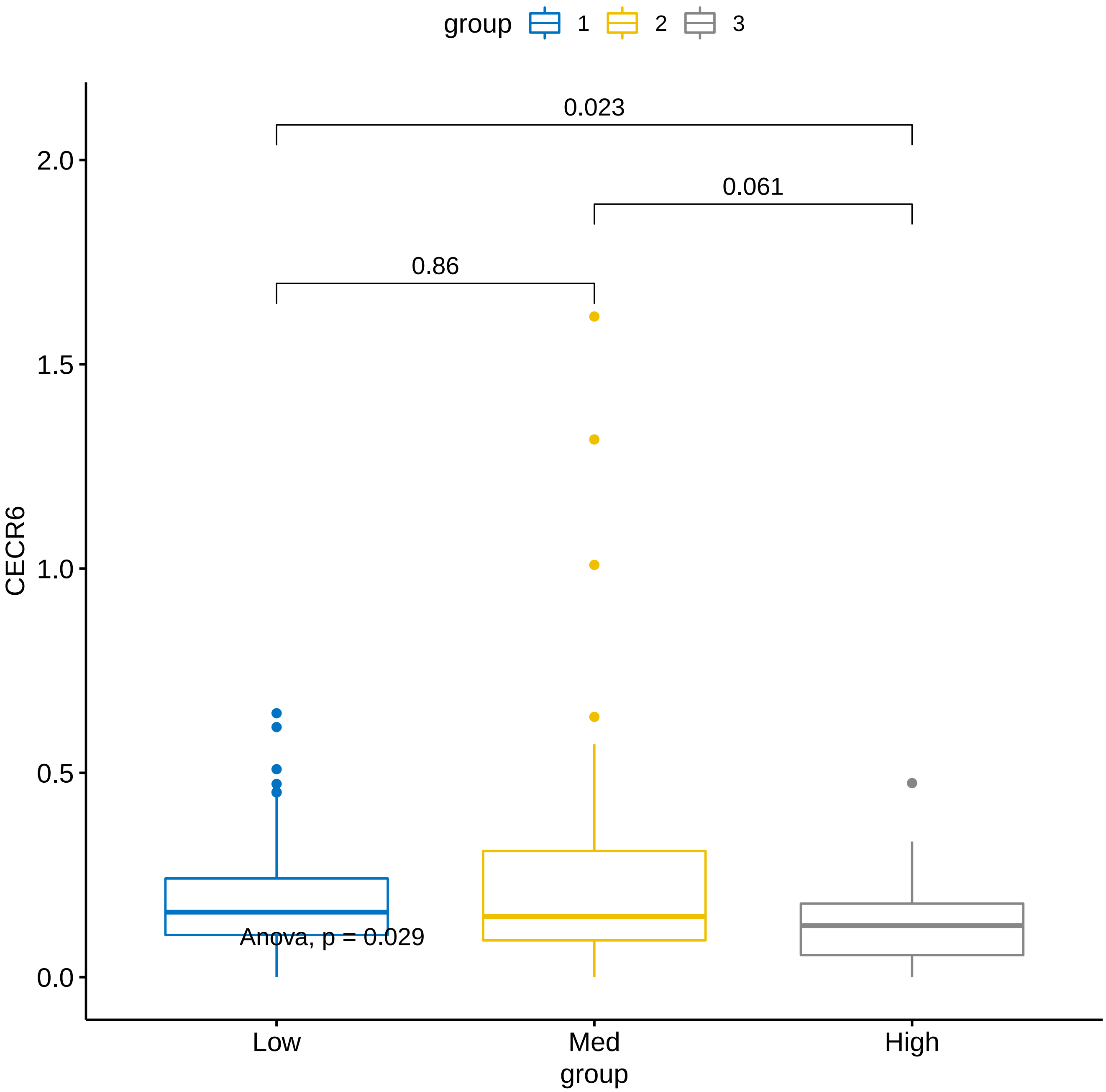
group
1
2
3
0.023
0.061
0.86
2.0
1.5
1.0
0.5
0.0
CECR6
Anova, p = 0.029
Low
Med
High
group

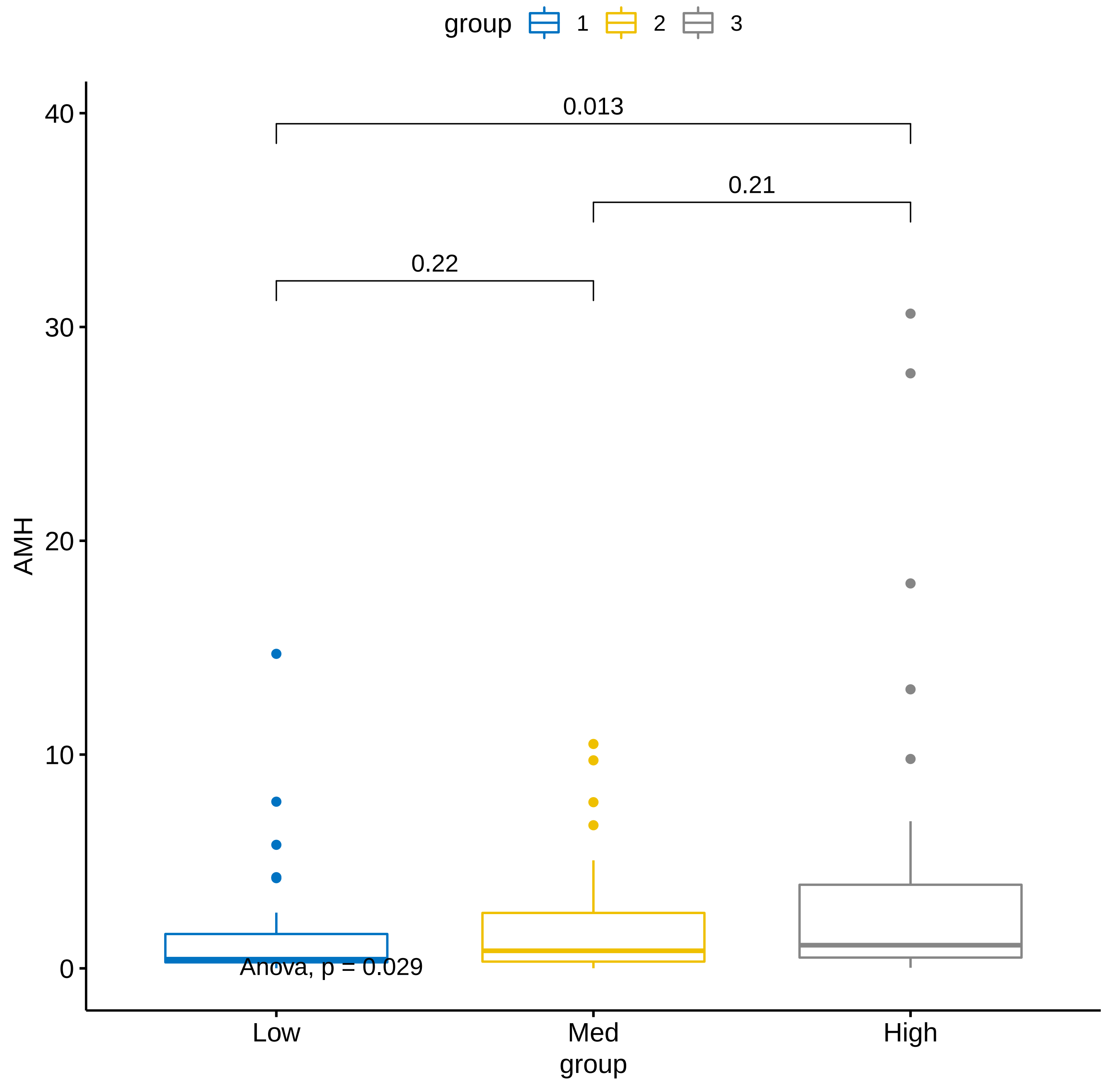

group
1
2
3
0.013
0.21
0.22
40
30
20
10
0
AMH
Anova, p = 0.029
Low
Med
High
group

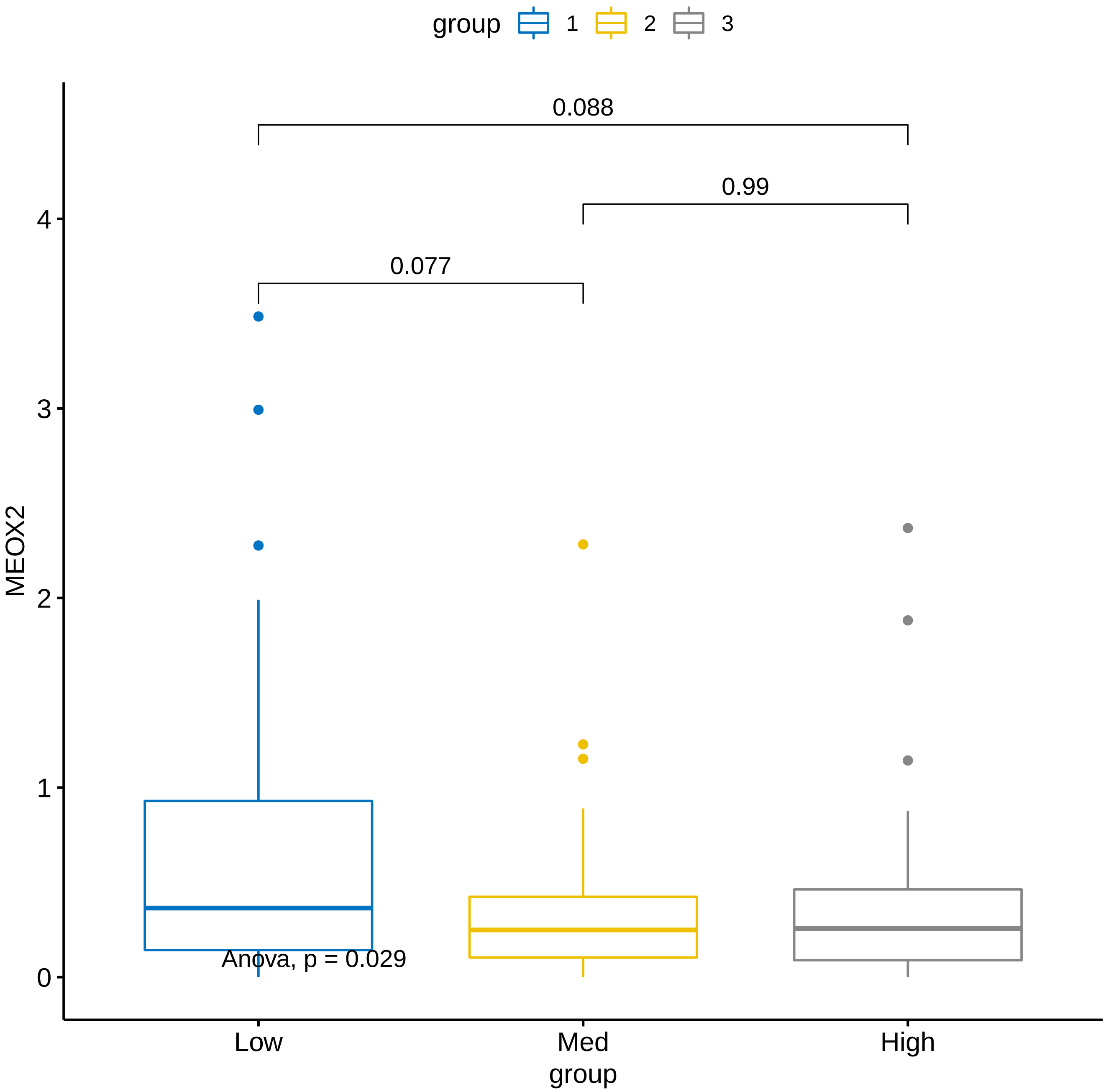
group
1
2
3
0.088
0.99
0.077
MEOX2
4
3
2
1
0
Anova, p = 0.029
Low
Med
High
group

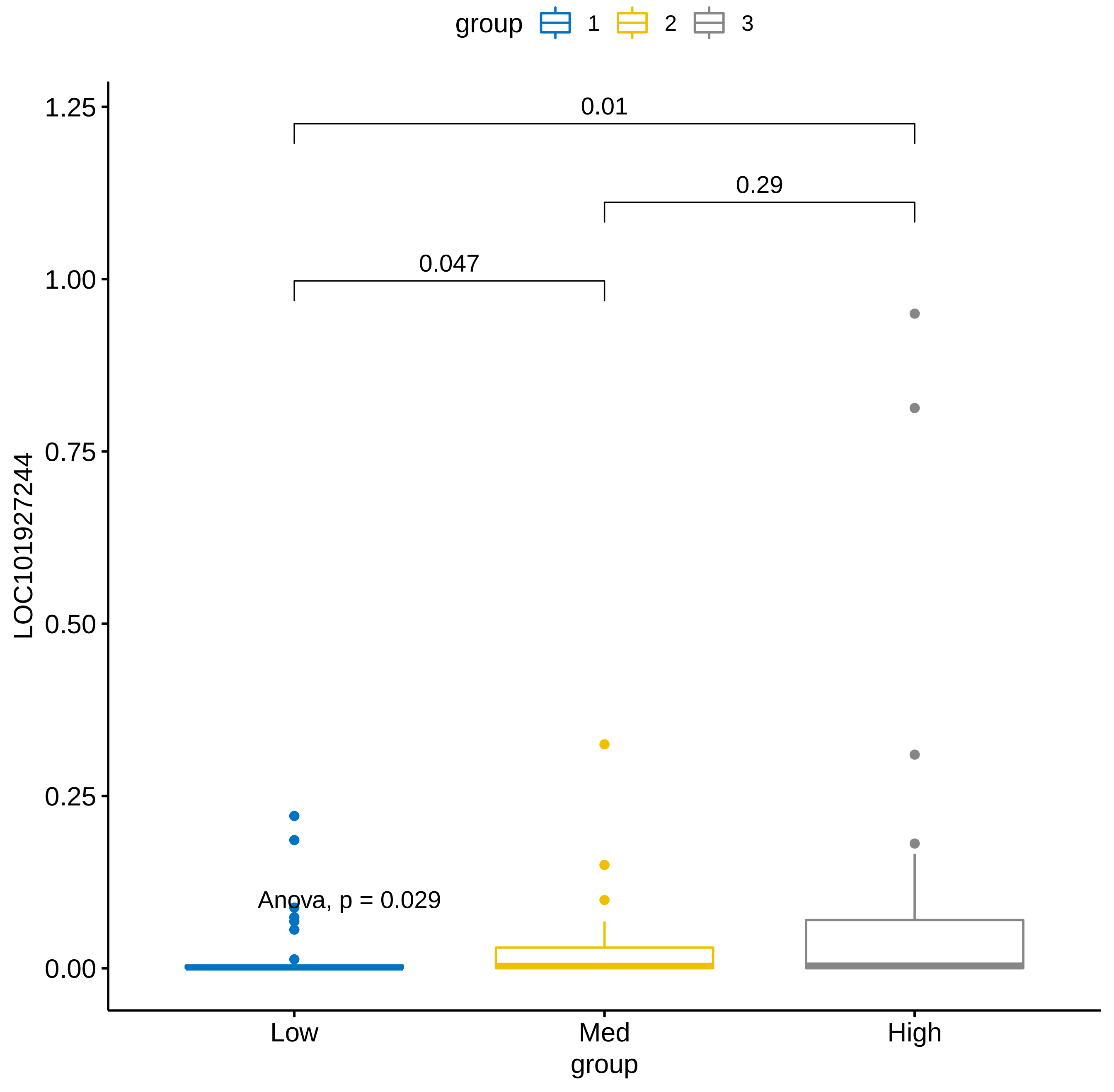

group 1 2 3
0.01
0.29
0.047
Anova, p = 0.029
1.25
1.00
0.75
0.50
0.25
0.00
LOC101927244
Low
Med
High
group

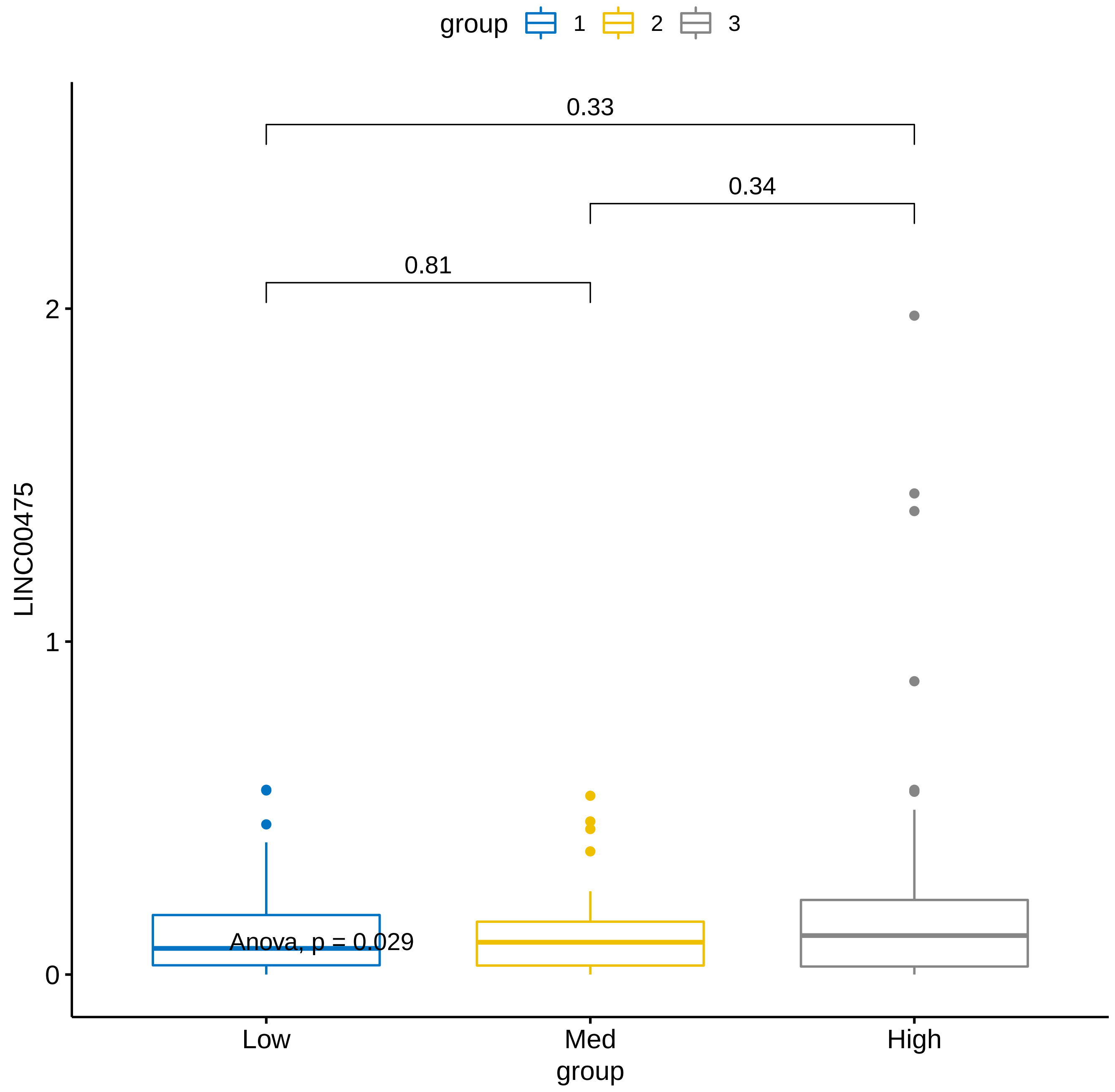
group 1 2 3
0.33
0.34
0.81
LINC00475
2
1
0
Anova, p = 0.029
Low
Med
High
group

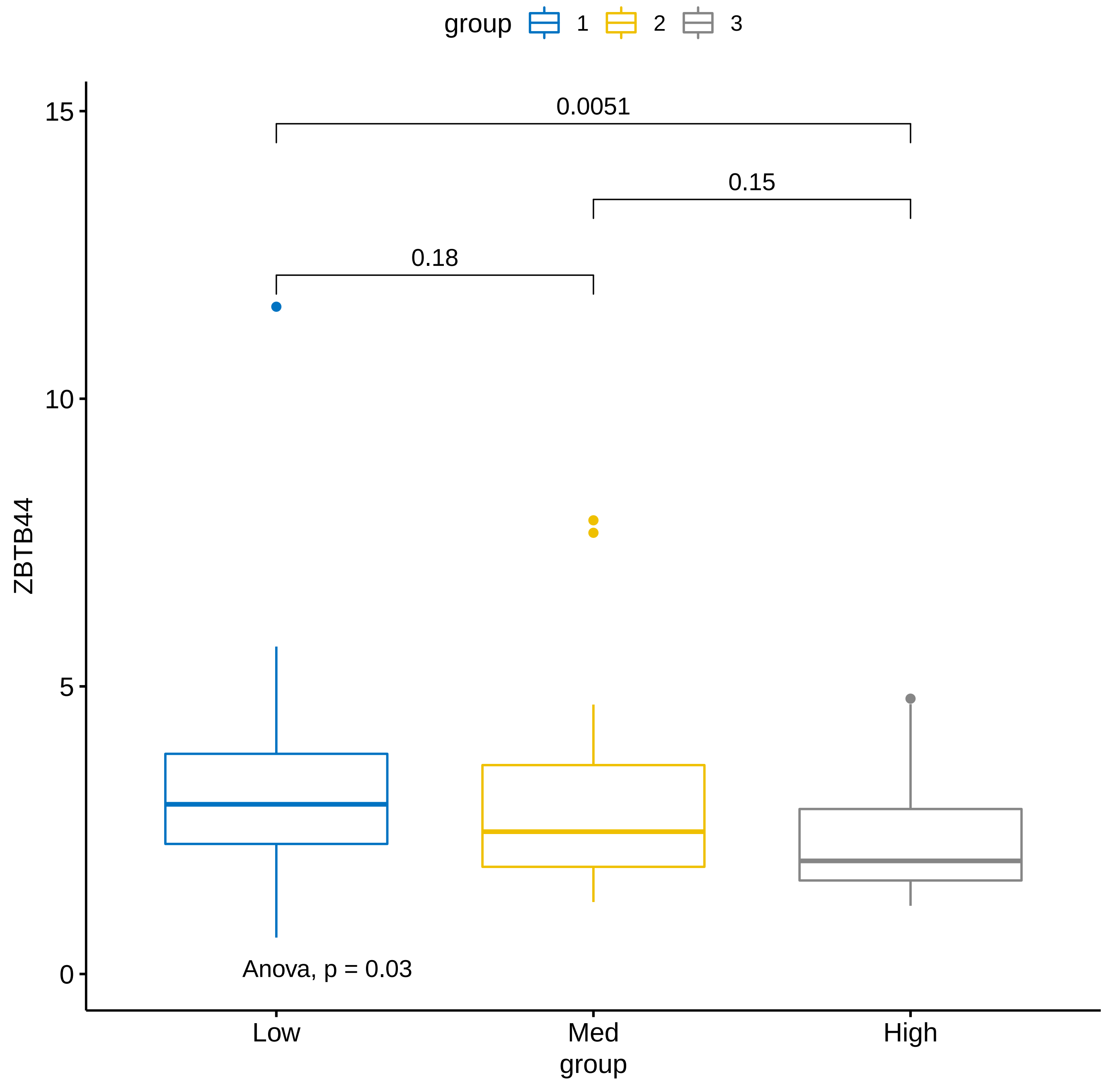

group 1 2 3
0.0051
0.15
0.18
15
10
5
0
ZBTB44
Anova, p = 0.03
Low
Med
High
group

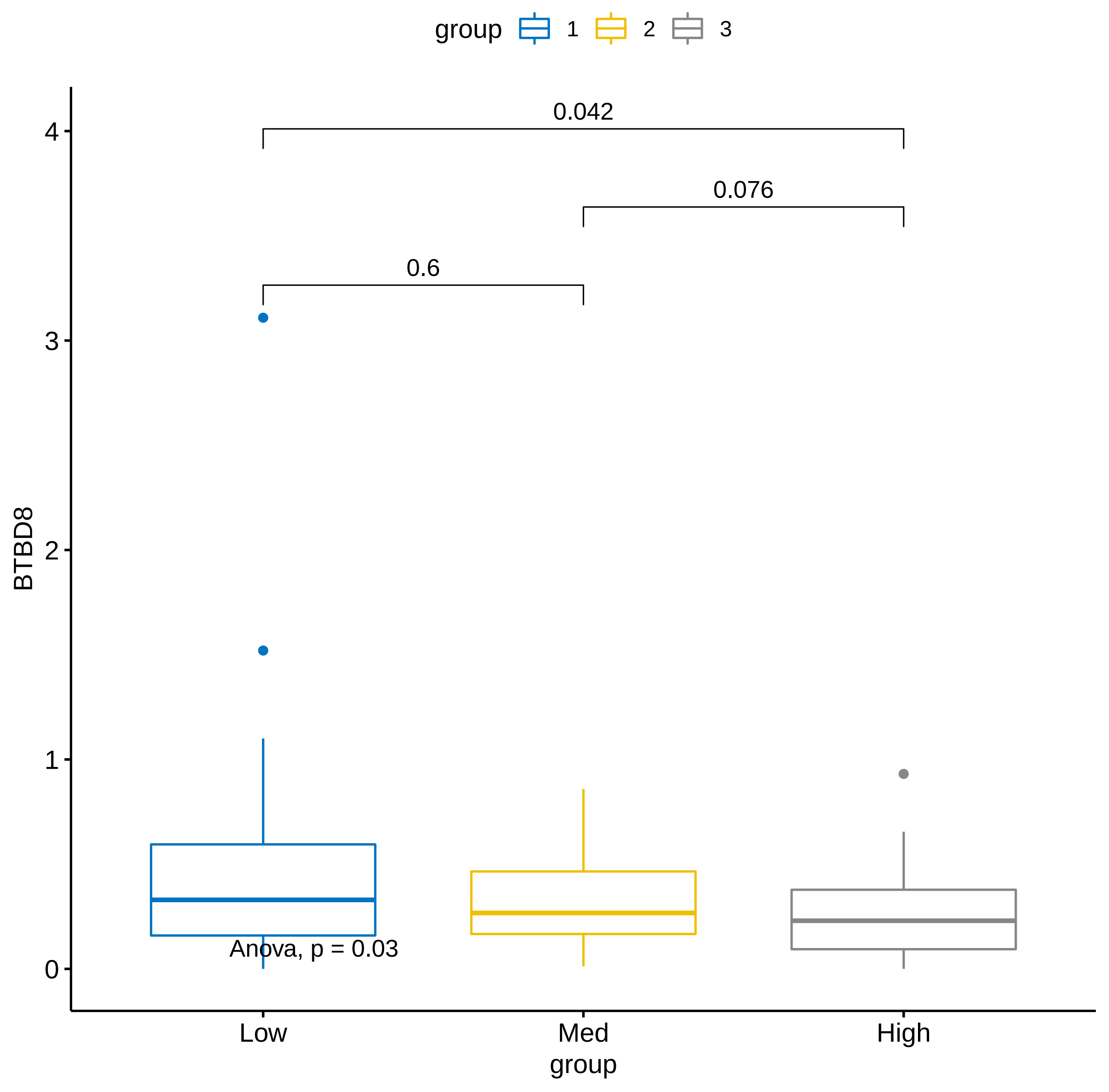
group
1
2
3
0.042
0.076
0.6
BTBD8
4
3
2
1
0
Anova, p = 0.03
Low
Med
High
group

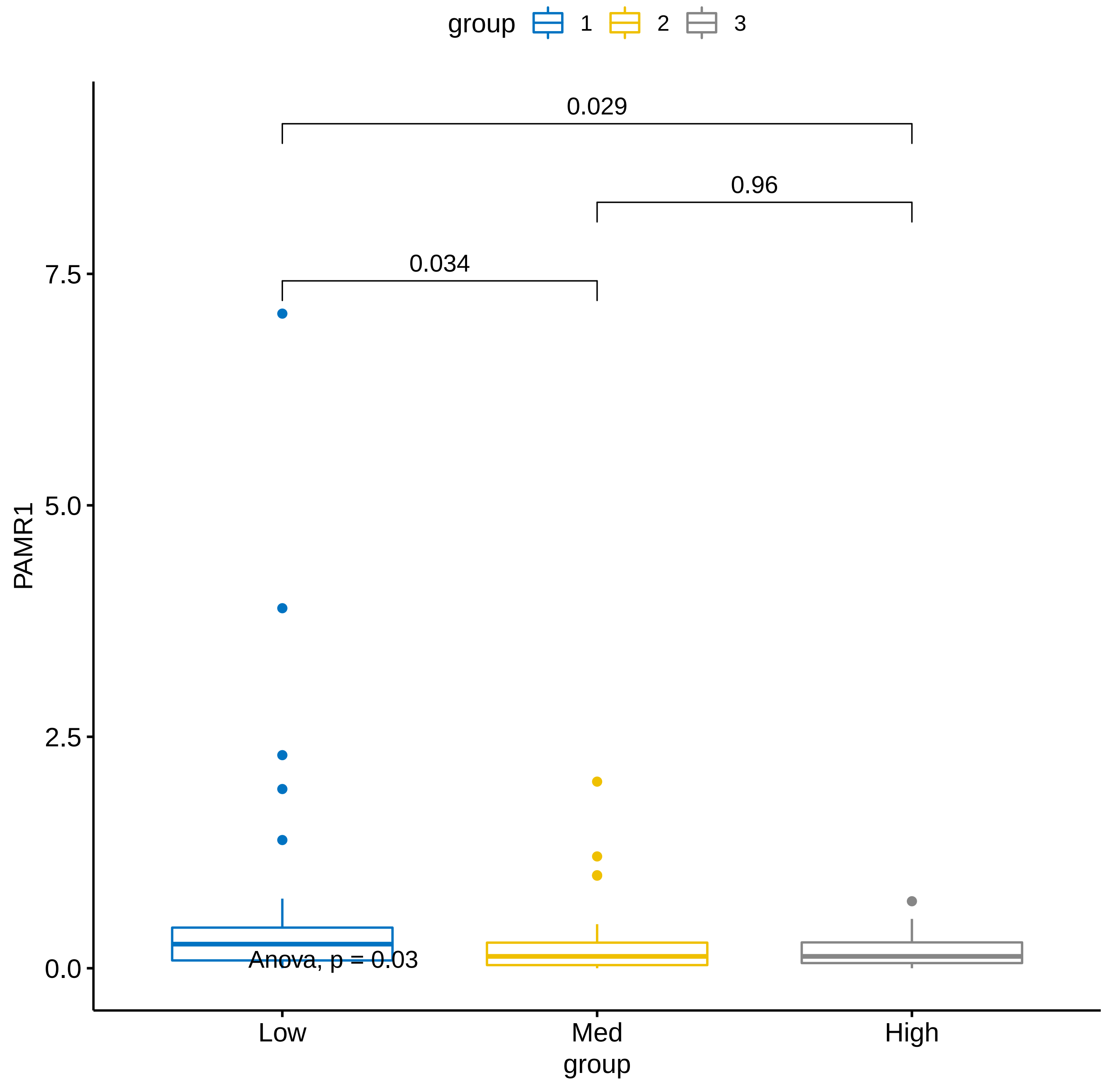
group
1
2
3
0.029
0.96
0.034
7.5
5.0
2.5
0.0
PAMR1
Anova, p = 0.03
Low
Med
High
group

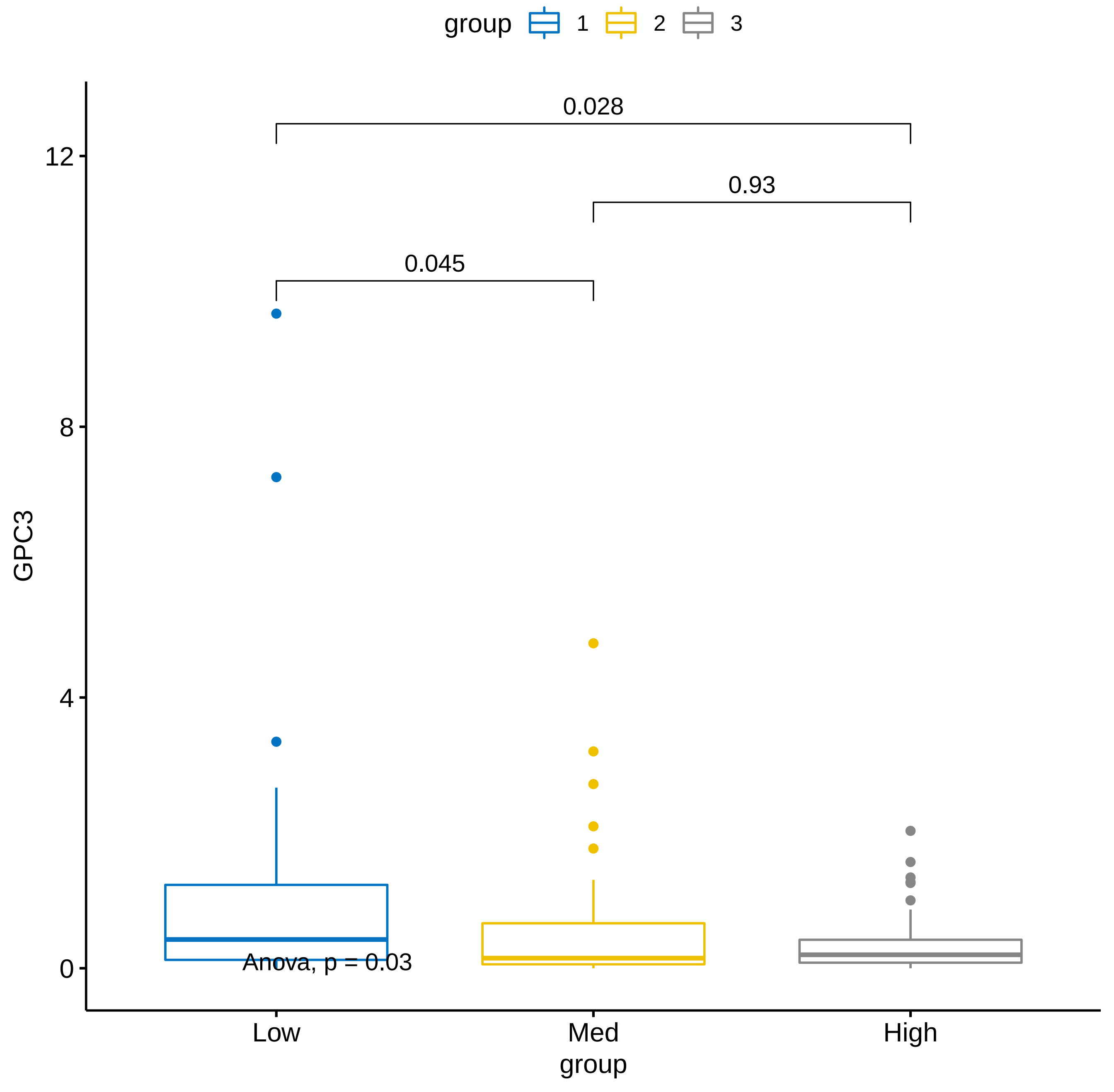
group
1
2
3
0.028
0.93
0.045
12
8
4
0
GPC3
Anova, p = 0.03
Low
Med
High
group

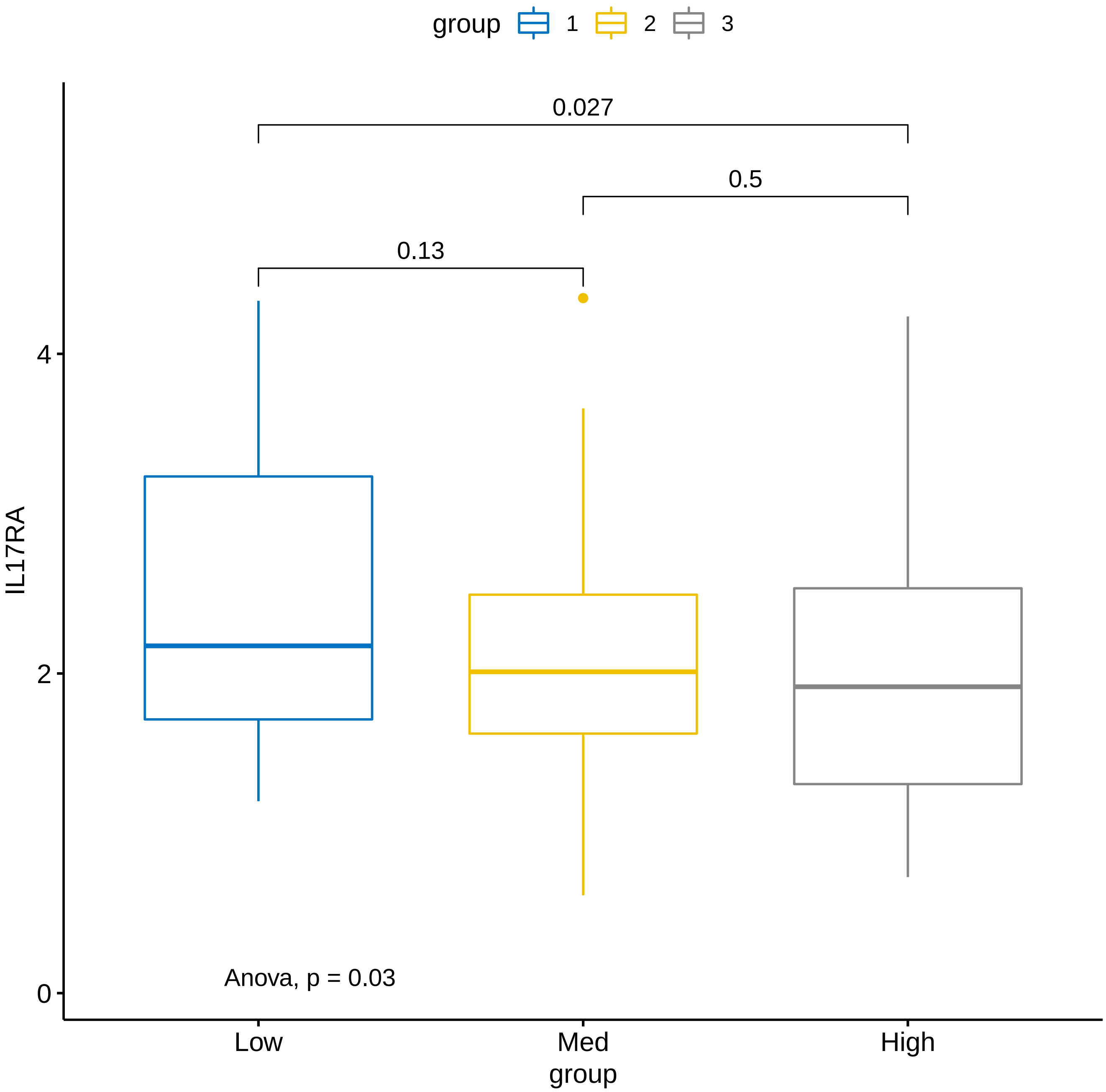

group
1
2
3
0.027
0.5
0.13
IL17RA
4
2
0
Anova, p = 0.03
Low
Med
High
group

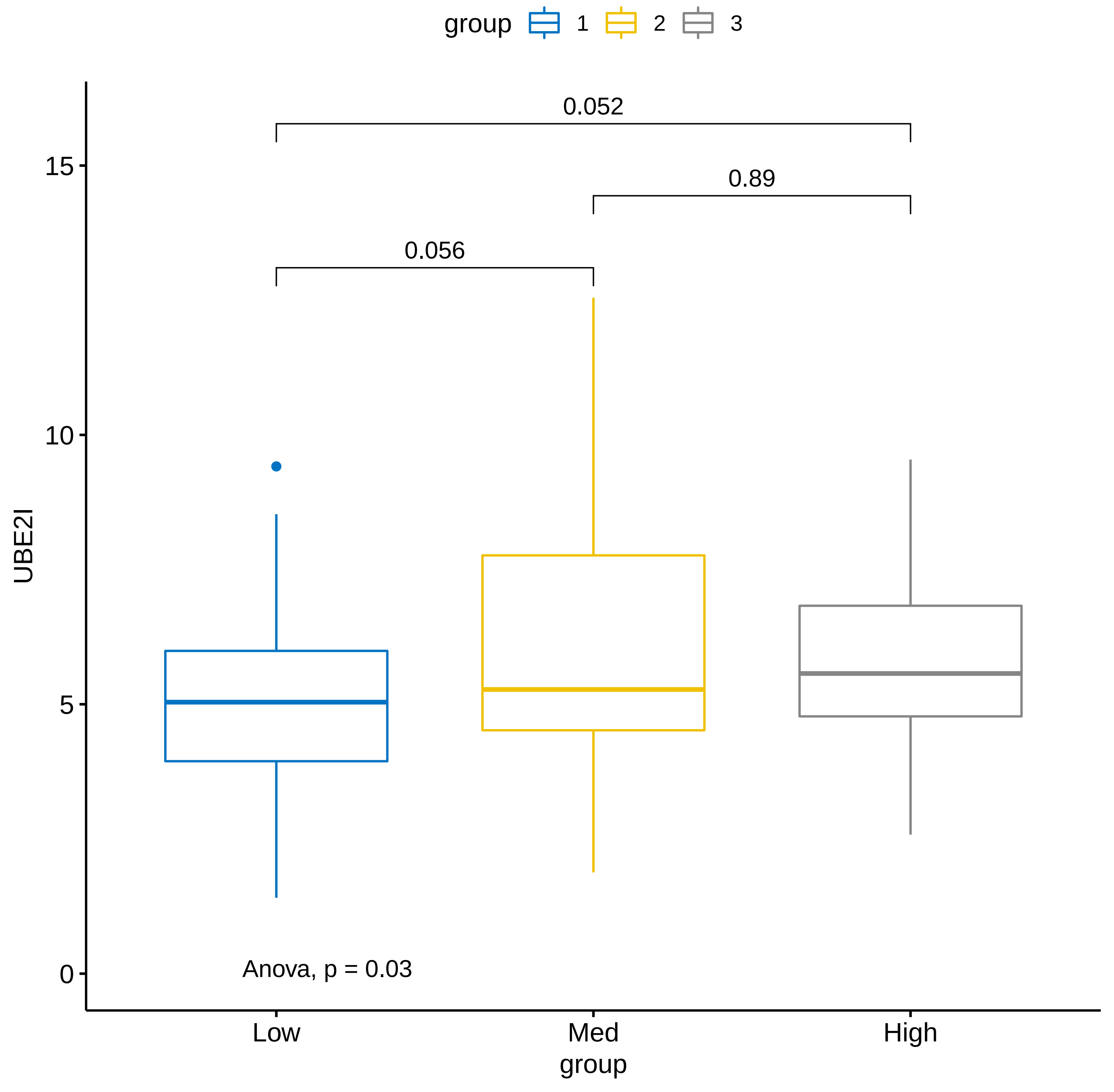
group
1
2
3
0.052
0.89
0.056
15
10
5
0
UBE2I
Anova, p = 0.03
Low
Med
High
group

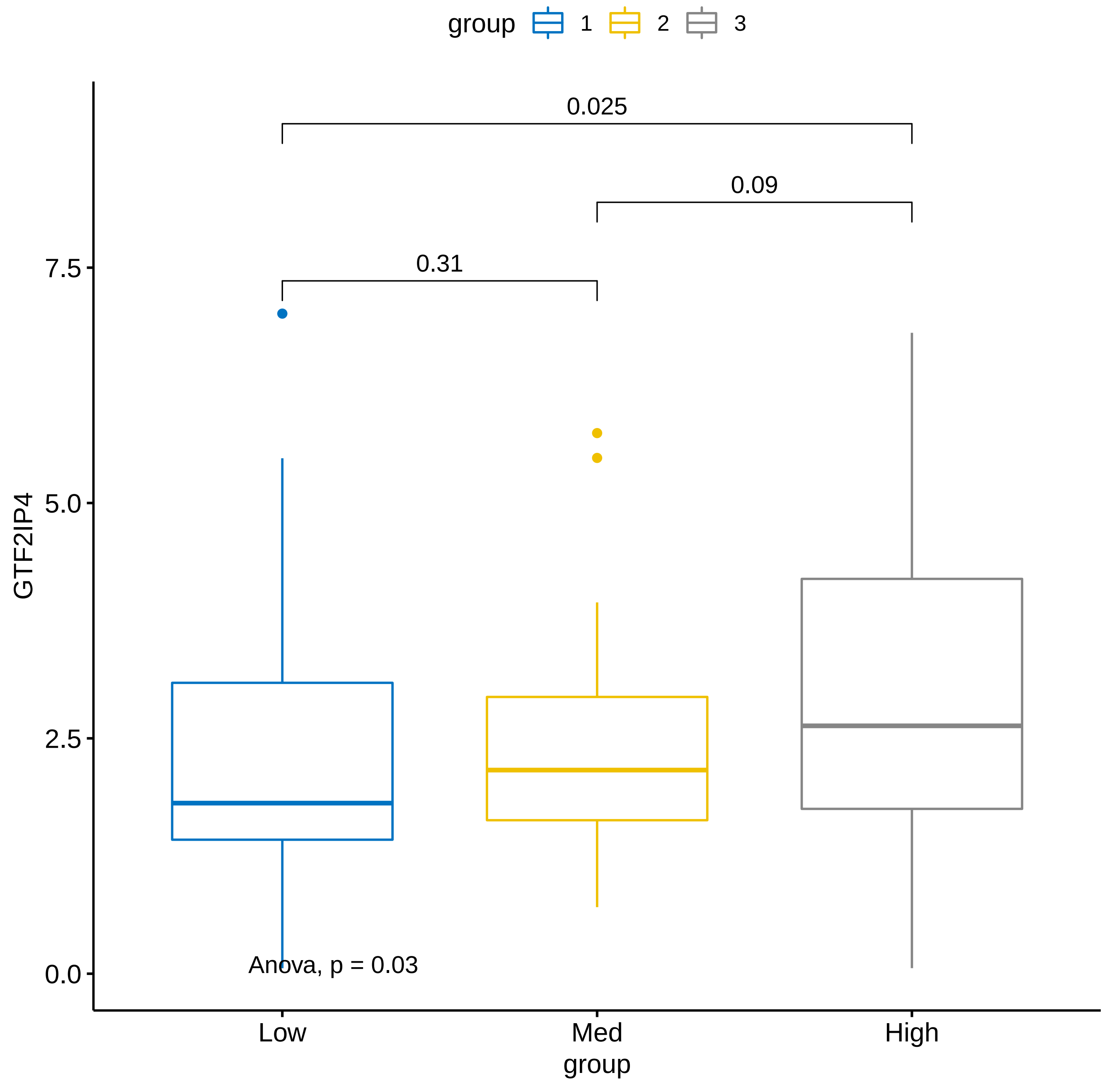
group
1
2
3
0.025
0.09
0.31
7.5
5.0
2.5
0.0
GTF2IP4
Anova, p = 0.03
Low
Med
High
group

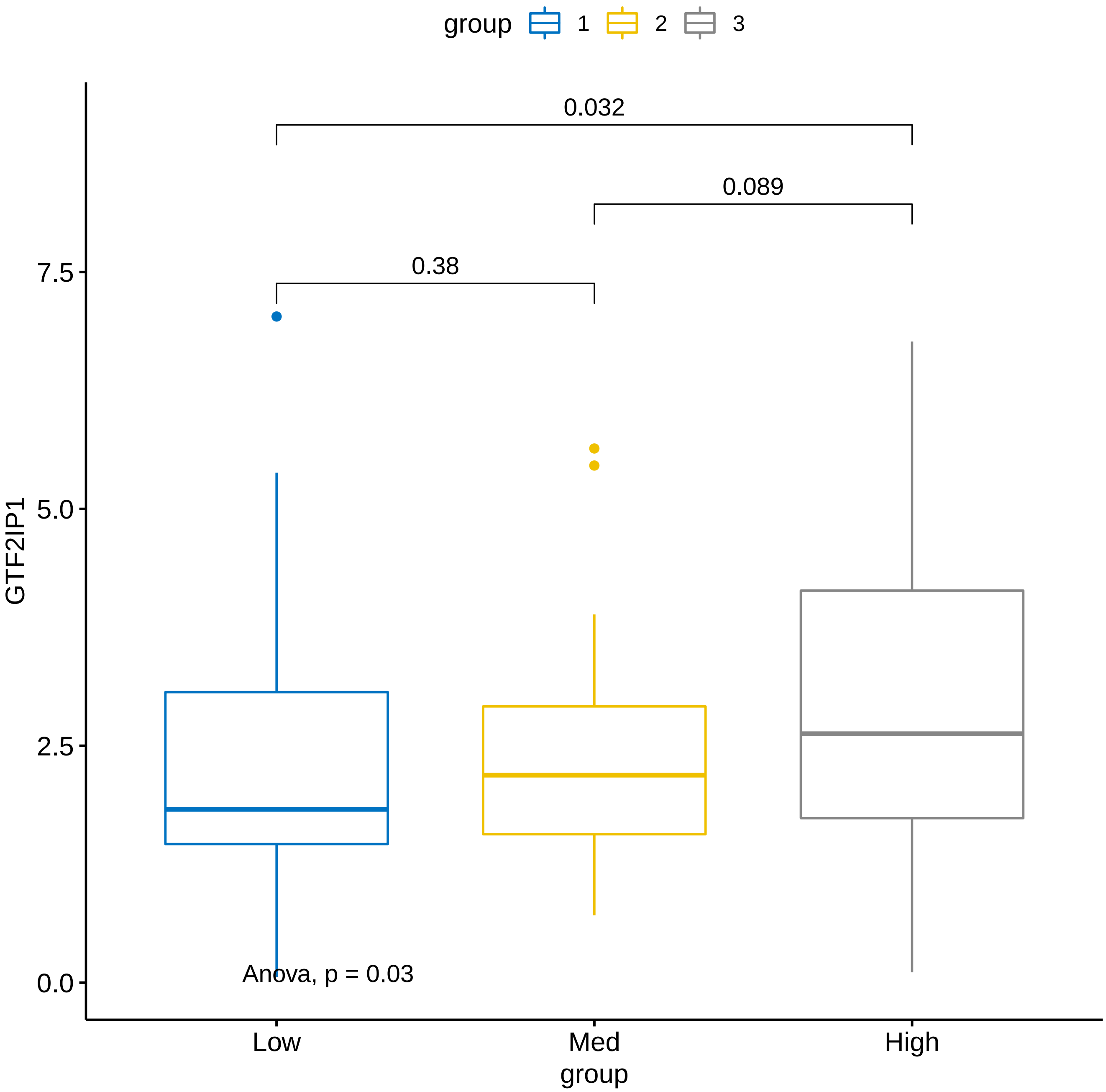
group
1
2
3
0.032
0.089
0.38
7.5
5.0
2.5
0.0
GTF2IP1
Anova, p = 0.03
Low
Med
High
group

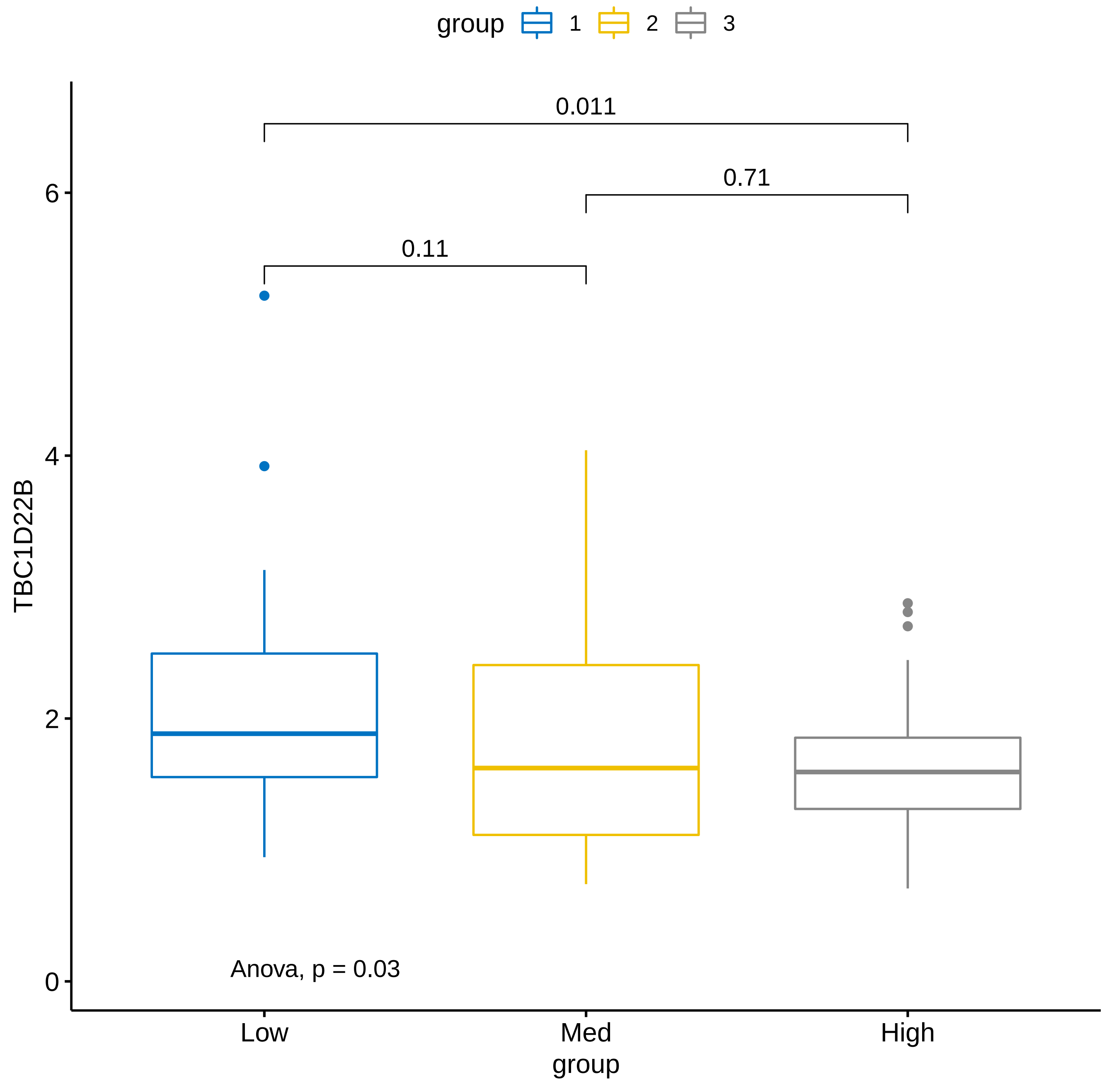
group
1
2
3
0.011
0.71
0.11
TBC1D22B
6
4
2
0
Anova, p = 0.03
Low
Med
High
group

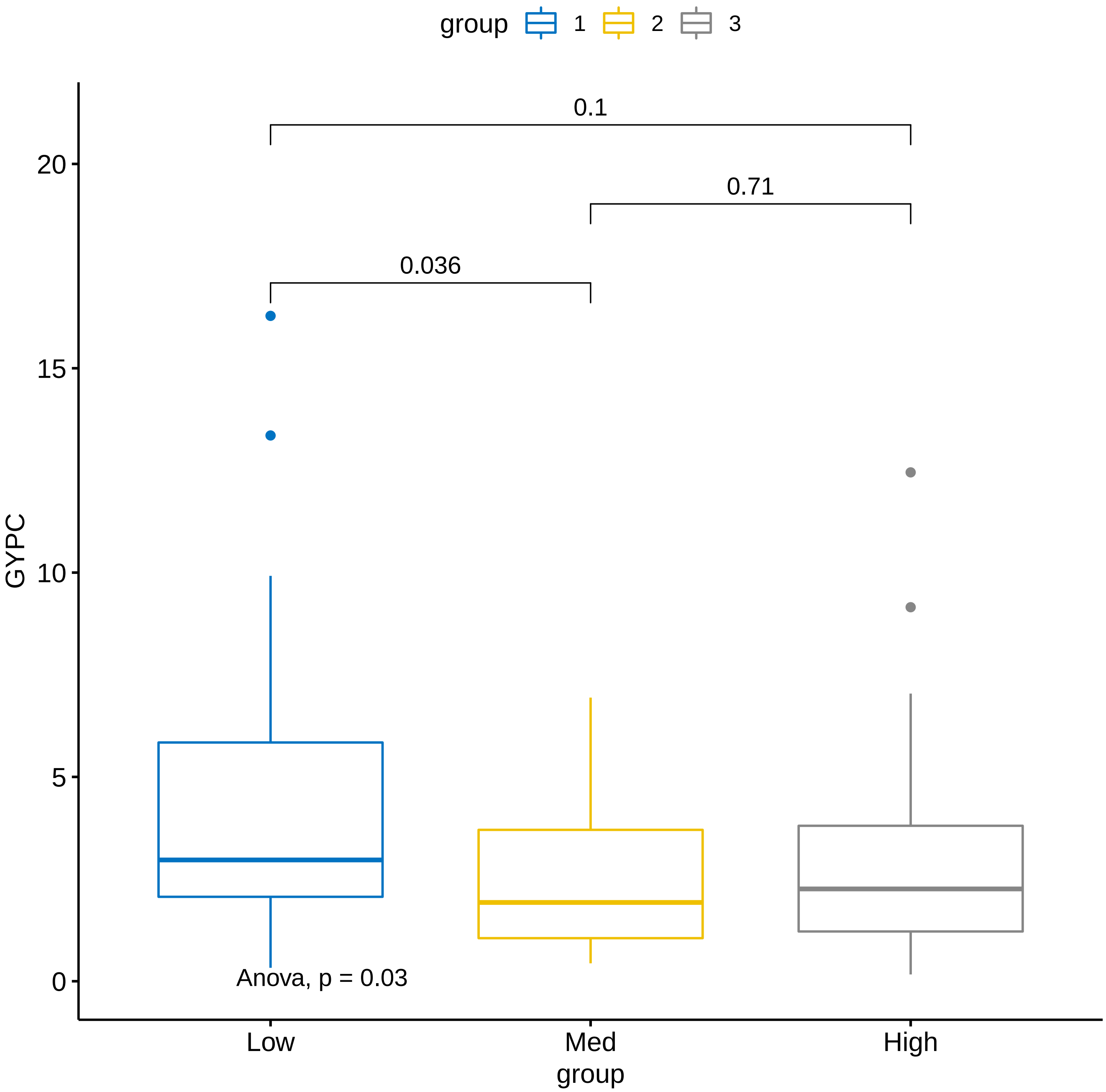

group 1 2 3
0.1
0.71
0.036
GYPC
20
15
10
5
0
Anova, p = 0.03
Low
Med
High
group

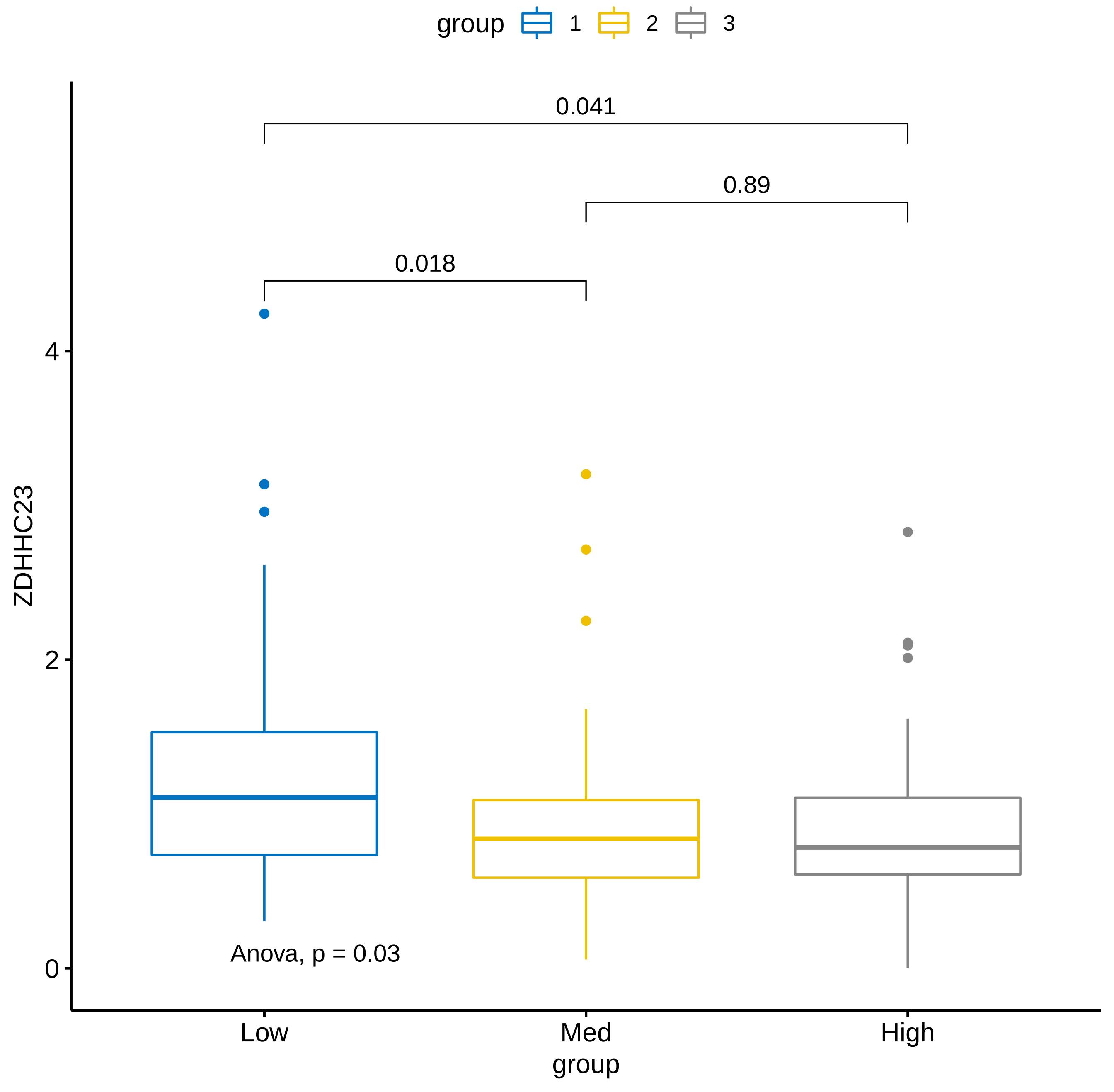
group
1
2
3
0.041
0.89
0.018
ZDHHC23
4
2
0
Anova, p = 0.03
Low
Med
High
group

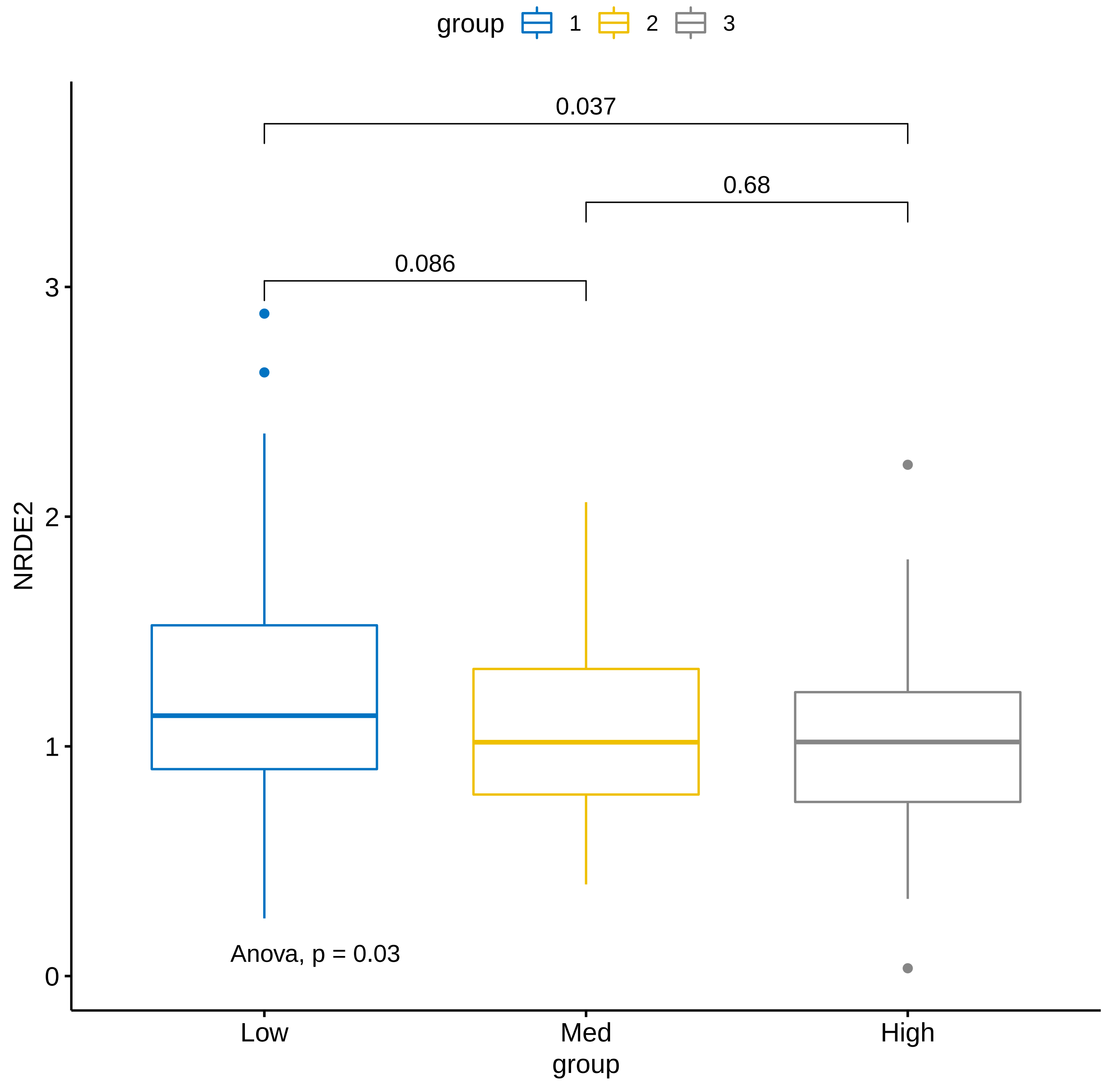
group 1 2 3
0.037
0.68
0.086
3
2
1
0
NRDE2
Anova, p = 0.03
Low
Med
High
group

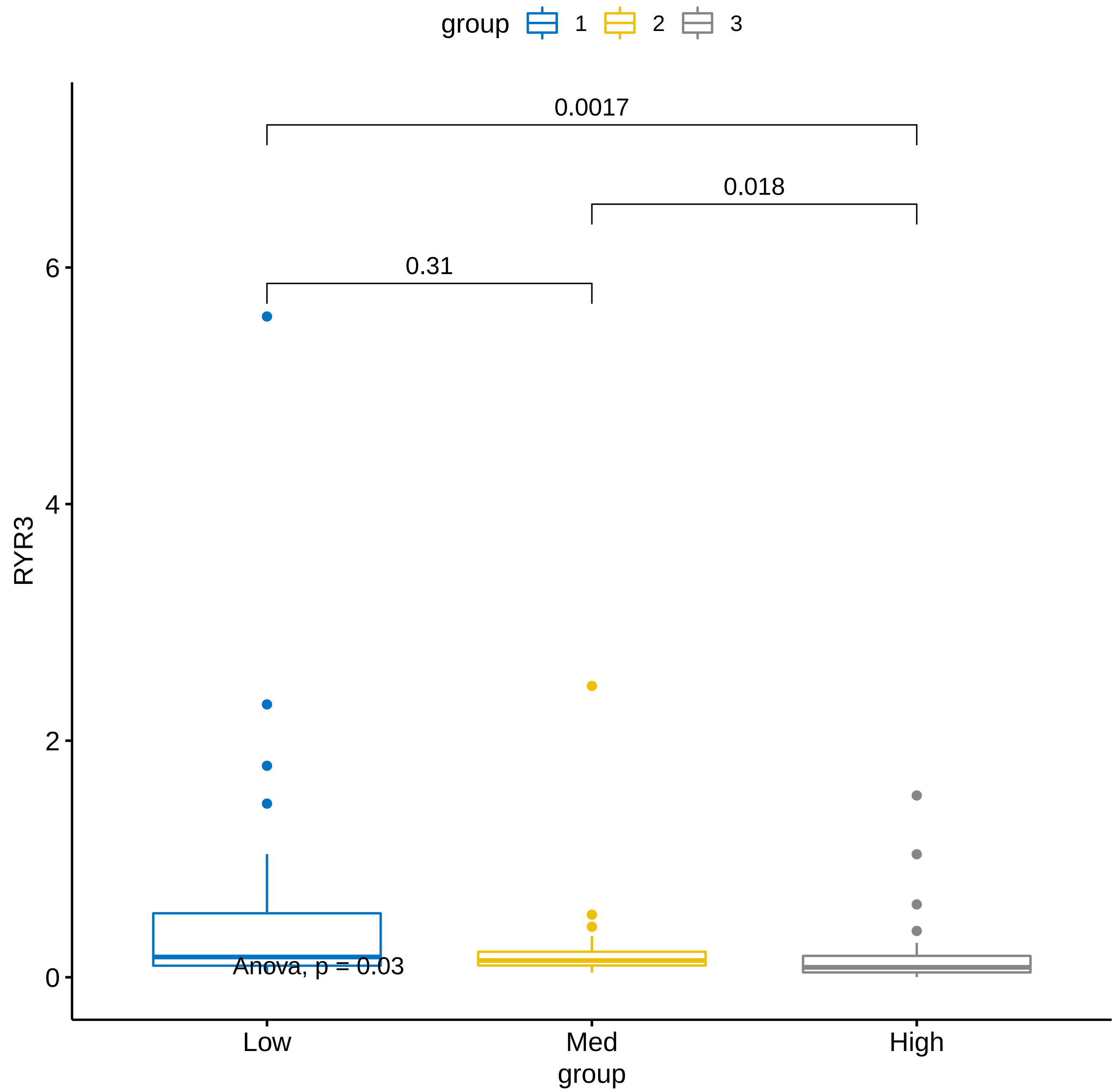

group 1 2 3
0.0017
0.018
0.31
RYR3
6
4
2
0
Anova, p = 0.03
Low
Med
High
group

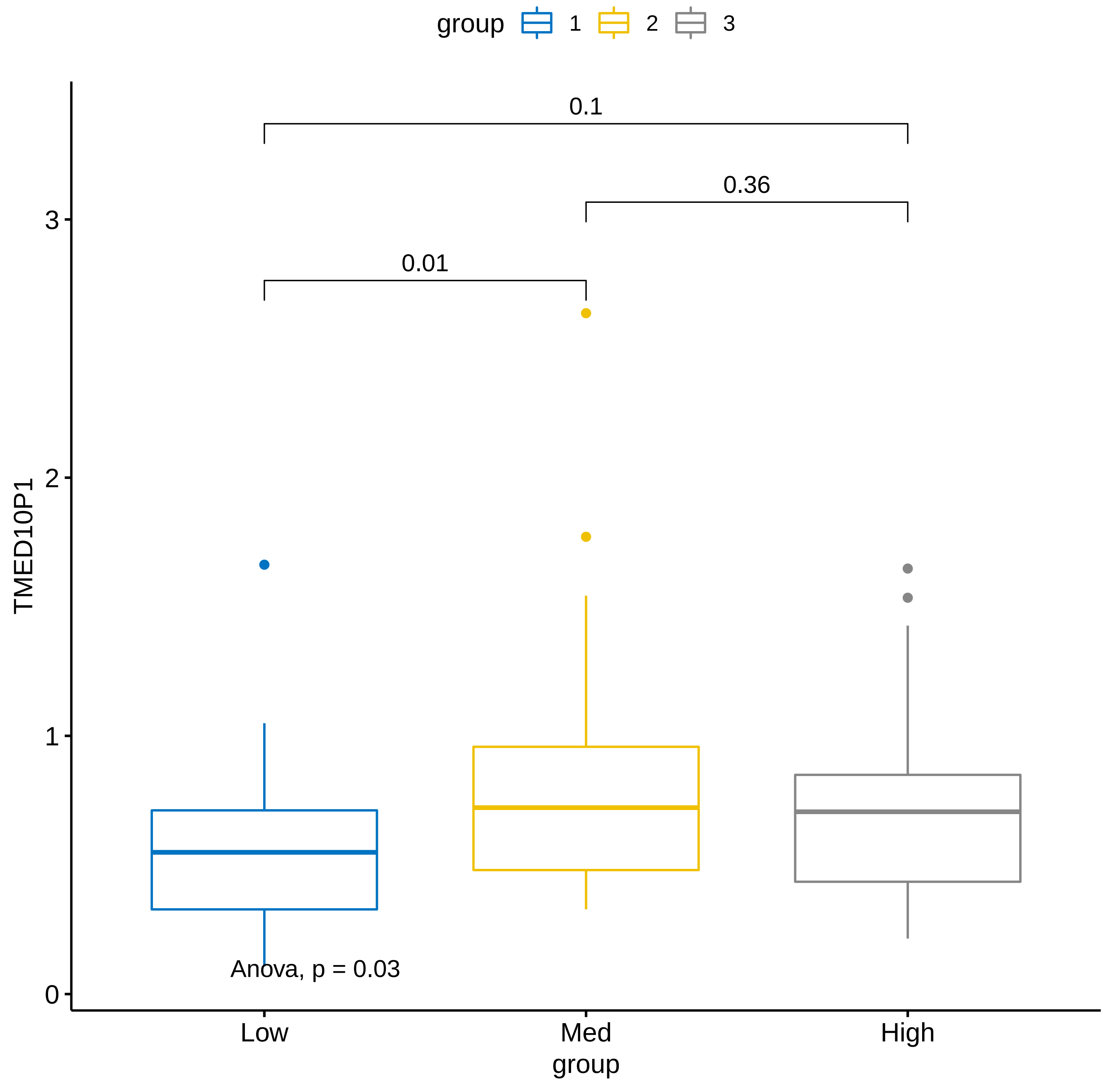

group 1 2 3
0.1
0.36
0.01
3
2
1
0
TMED10P1
Anova, p = 0.03
Low
Med
High
group

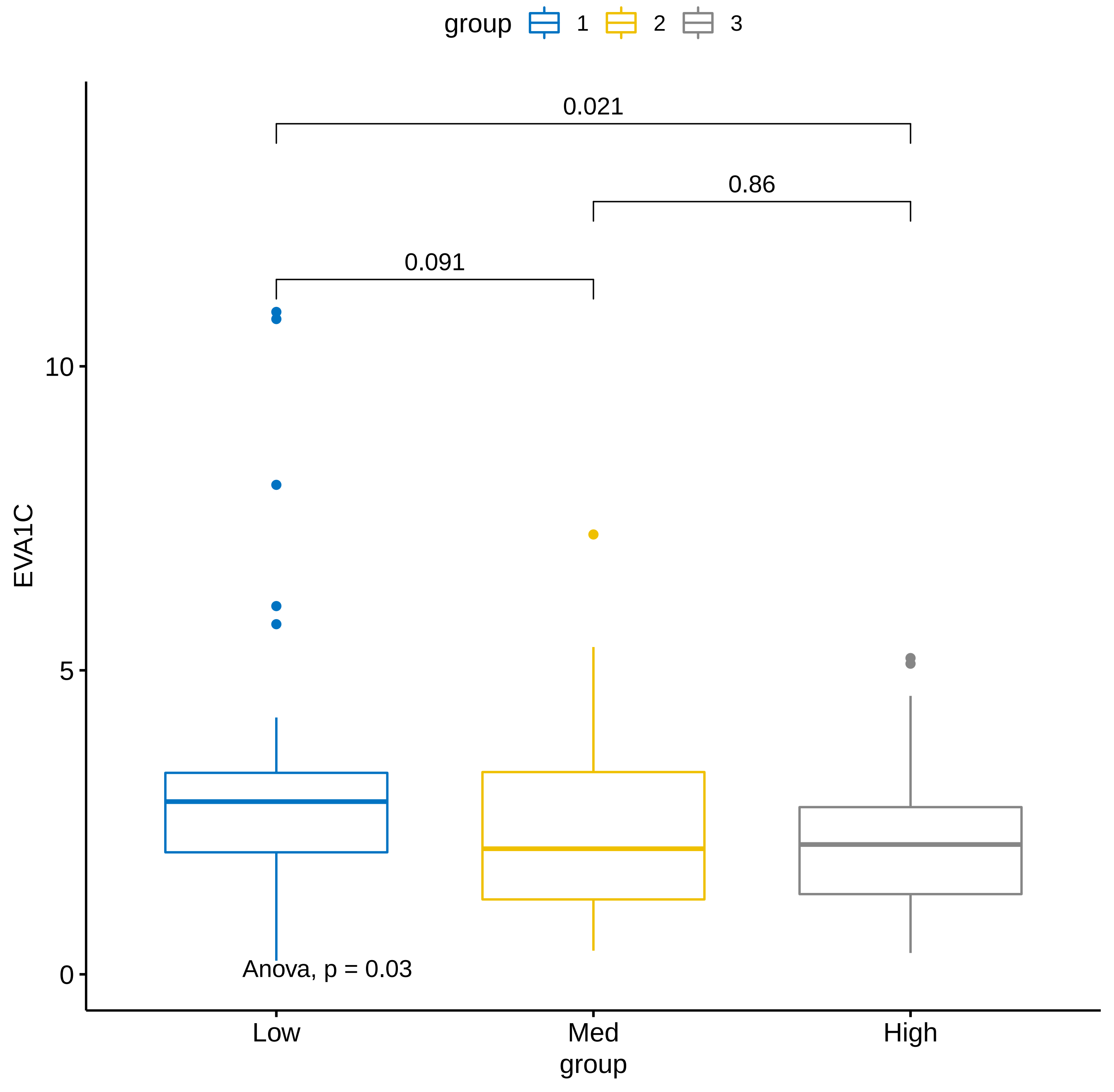
group
1
2
3
0.021
0.86
0.091
10
5
0
EVA1C
Anova, p = 0.03
Low
Med
High
group

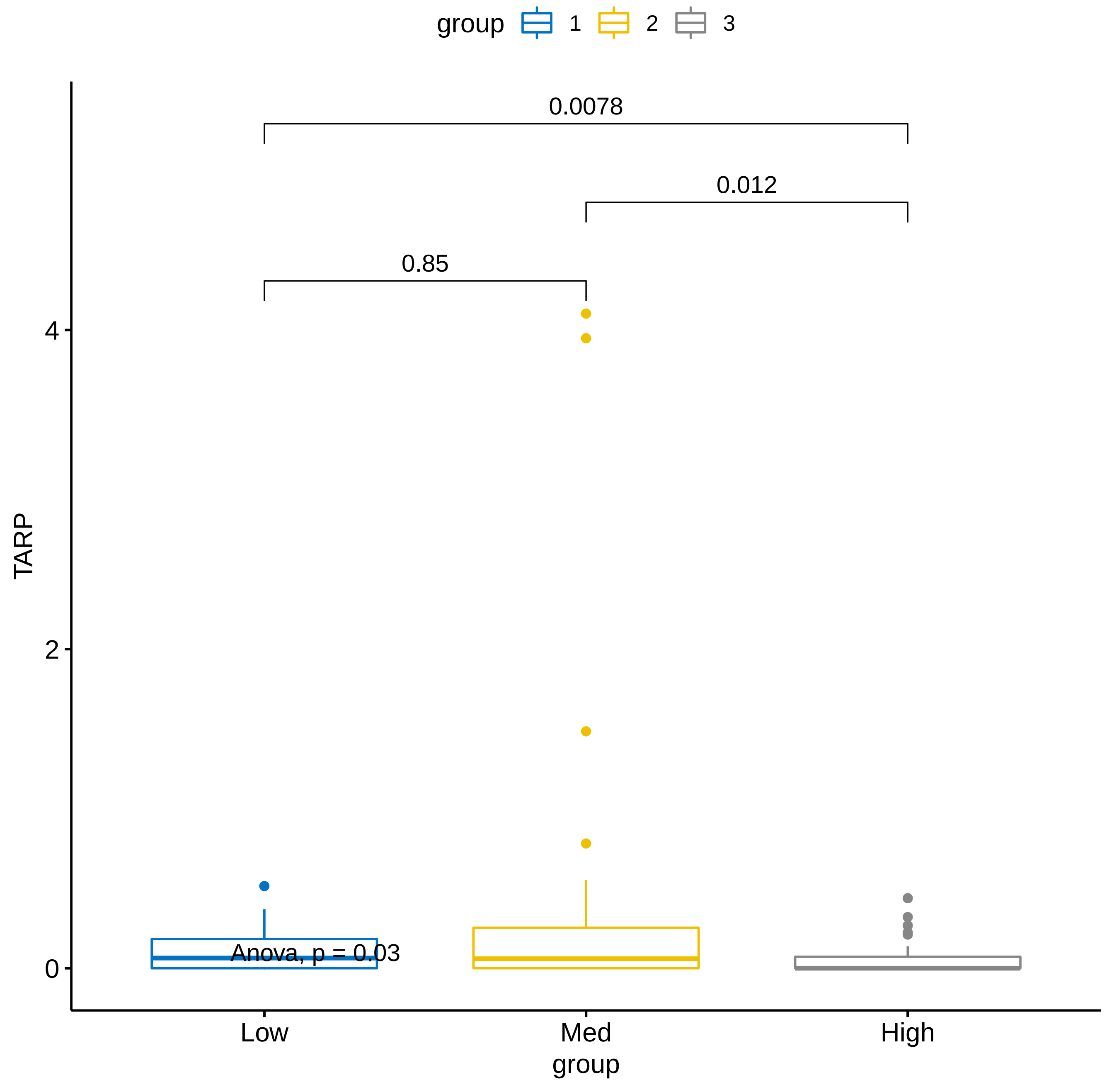

group
1
2
3
0.0078
0.012
0.85
4
2
0
TARP
Anova, p = 0.03
Low
Med
High
group

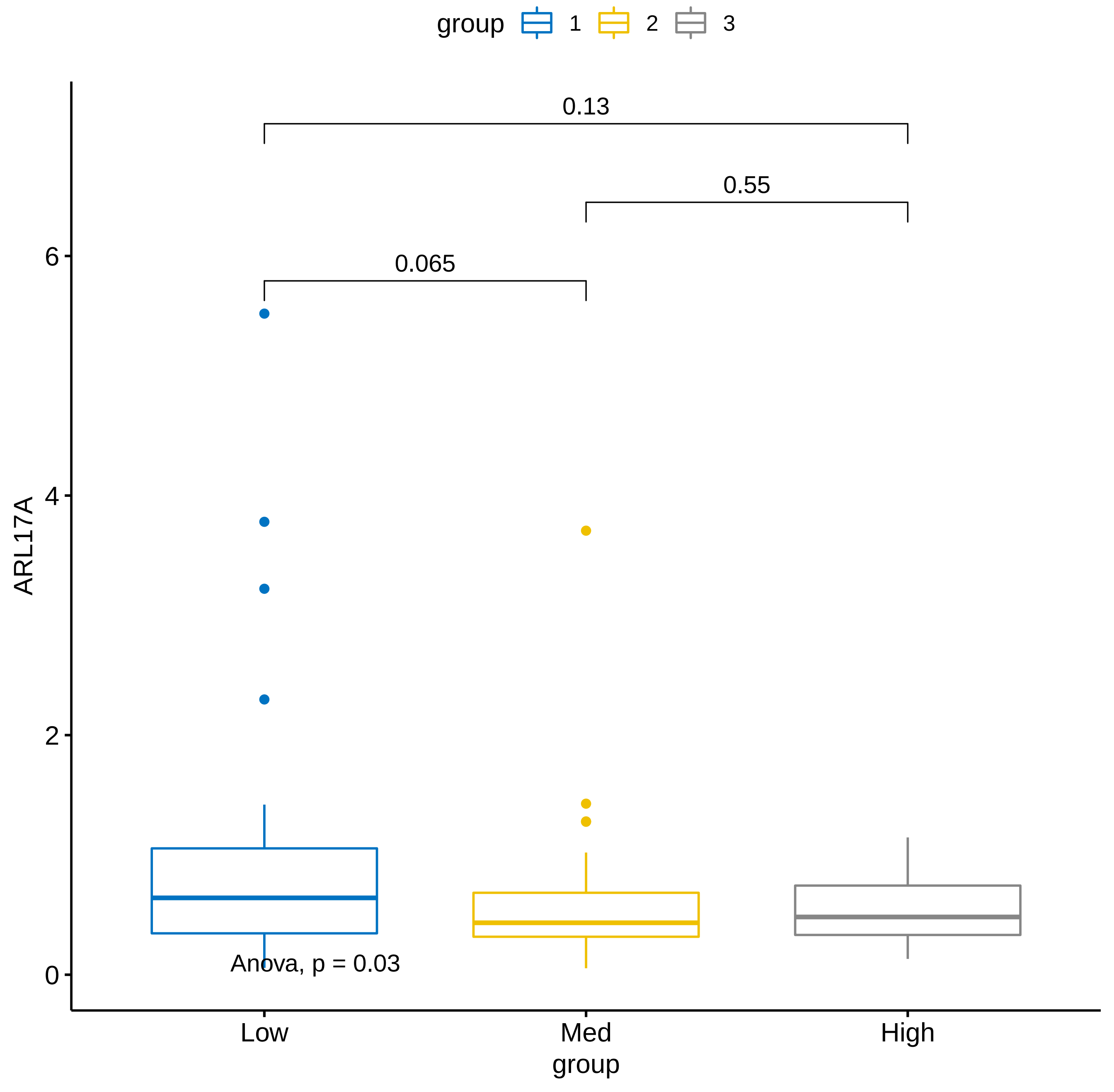

group
1
2
3
0.13
0.55
0.065
ARL17A
6
4
2
0
Anova, p = 0.03
Low
Med
High
group

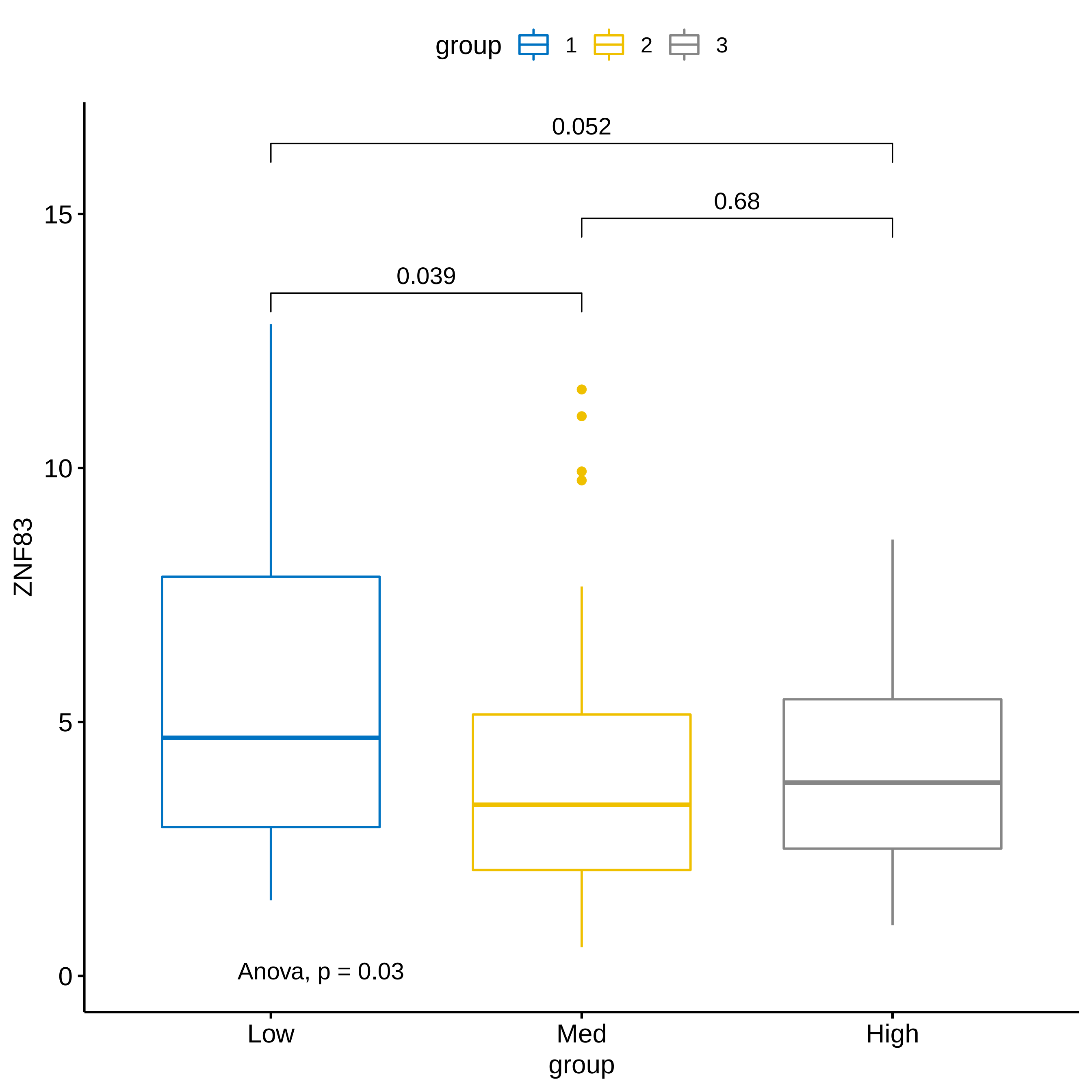
group
1
2
3
0.052
0.68
0.039
15
10
5
0
ZNF83
Anova, p = 0.03
Low
Med
High
group

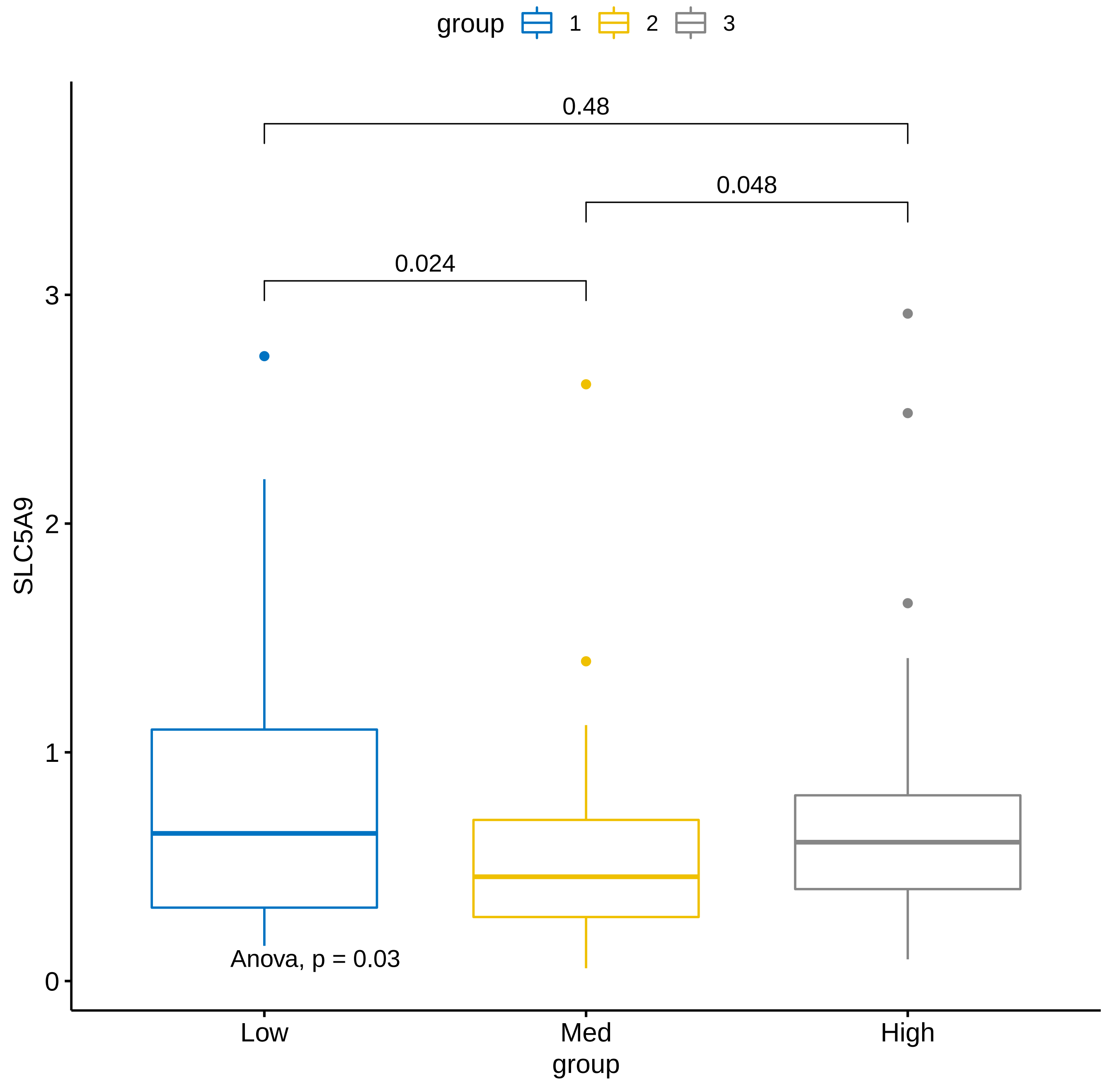
group
1
2
3
0.48
0.048
0.024
3
2
1
0
SLC5A9
Anova, p = 0.03
Low
Med
High
group

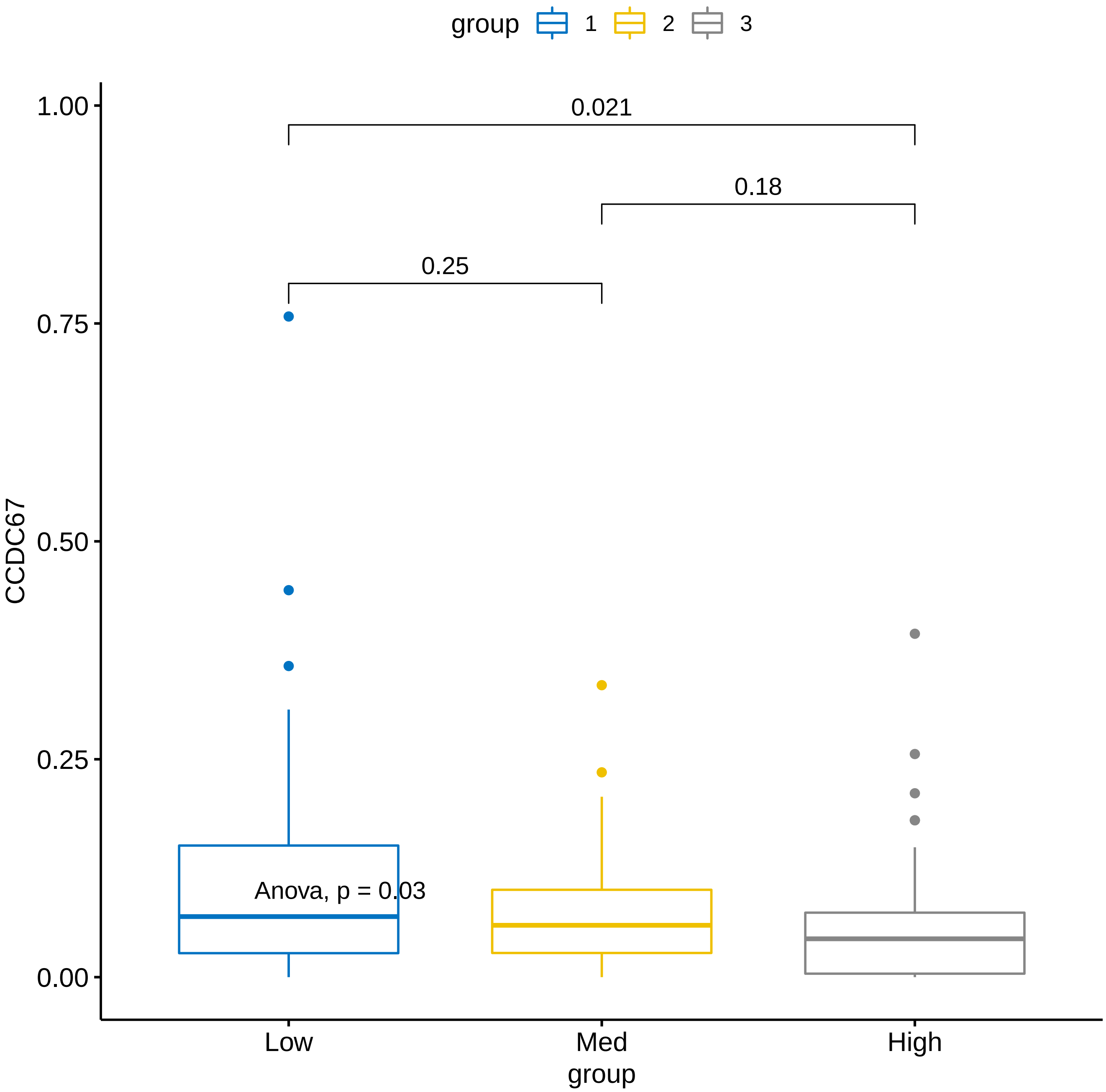

group 1 2 3
1.00
0.75
0.50
0.25
0.00
CCDC67
0.021
0.18
0.25
Anova, p = 0.03
Low
Med
High
group

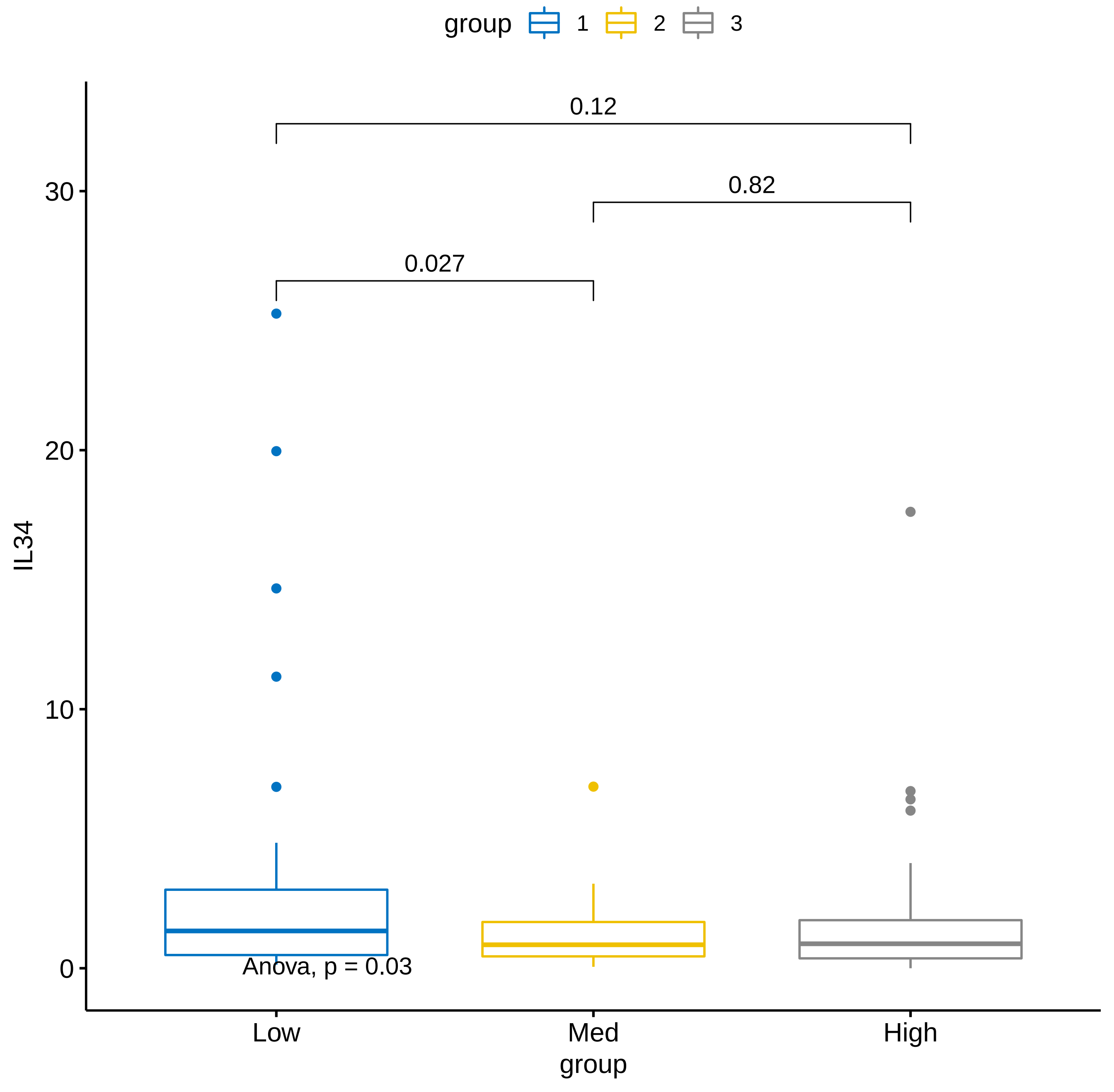

group 1 2 3
0.12
0.82
0.027
30
20
10
0
IL34
Anova, p = 0.03
Low
Med
High
group

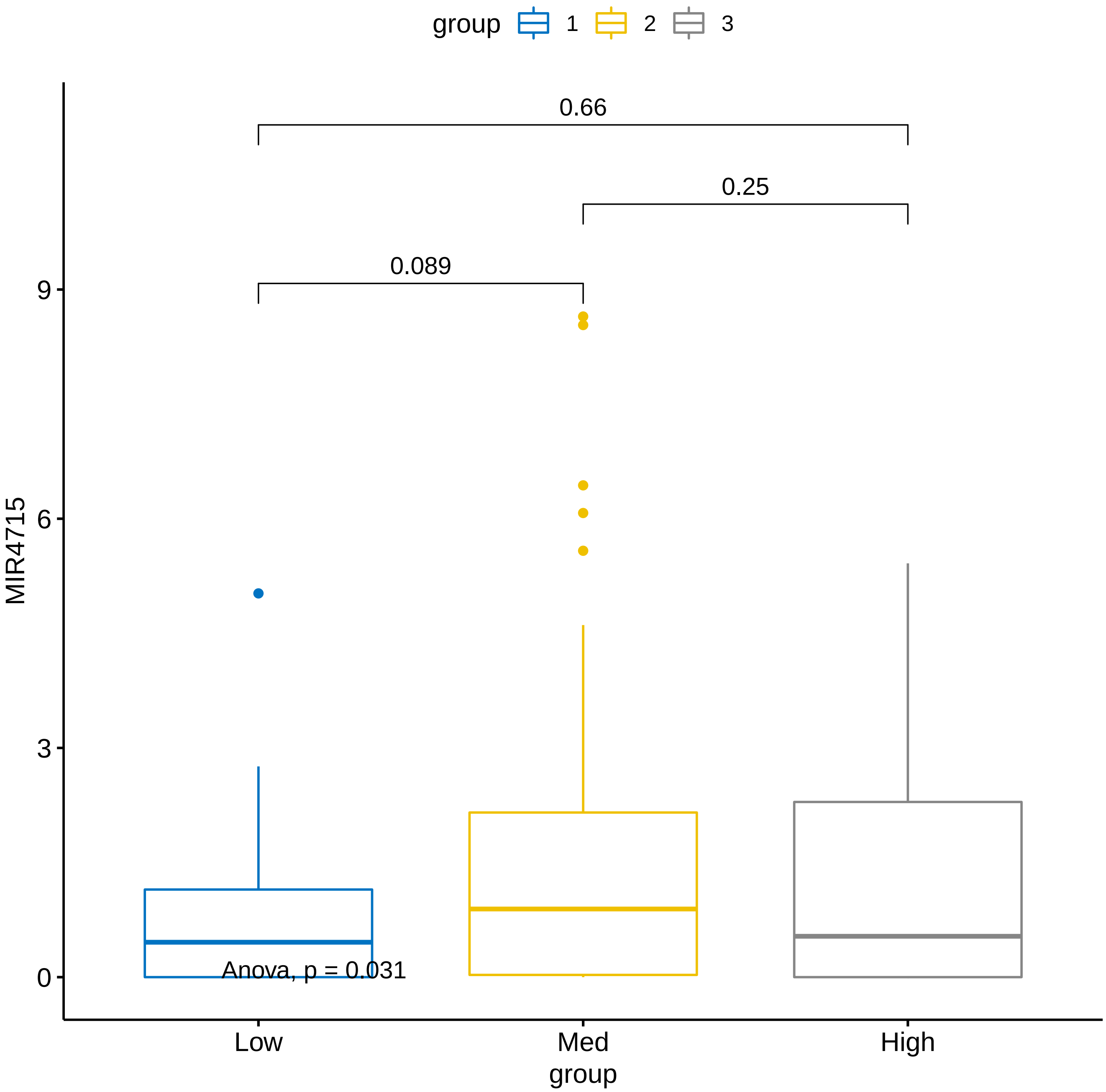
group
1
2
3
0.66
0.25
0.089
9
6
3
0
MIR4715
Anova, p = 0.031
Low
Med
High
group

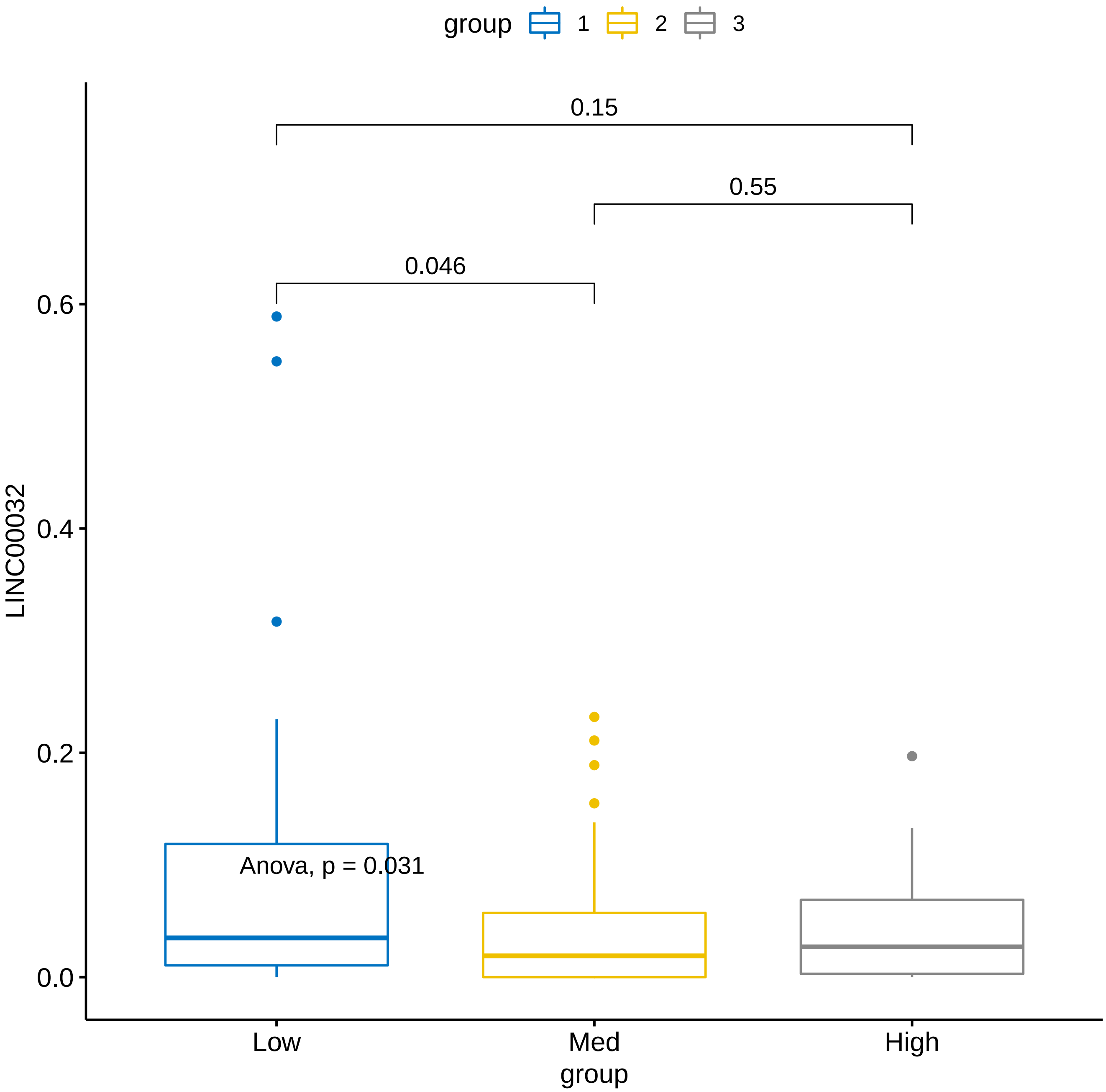
group
1
2
3
0.15
0.55
0.046
Anova, p = 0.031
LINC00032
0.6
0.4
0.2
0.0
Low
Med
High
group

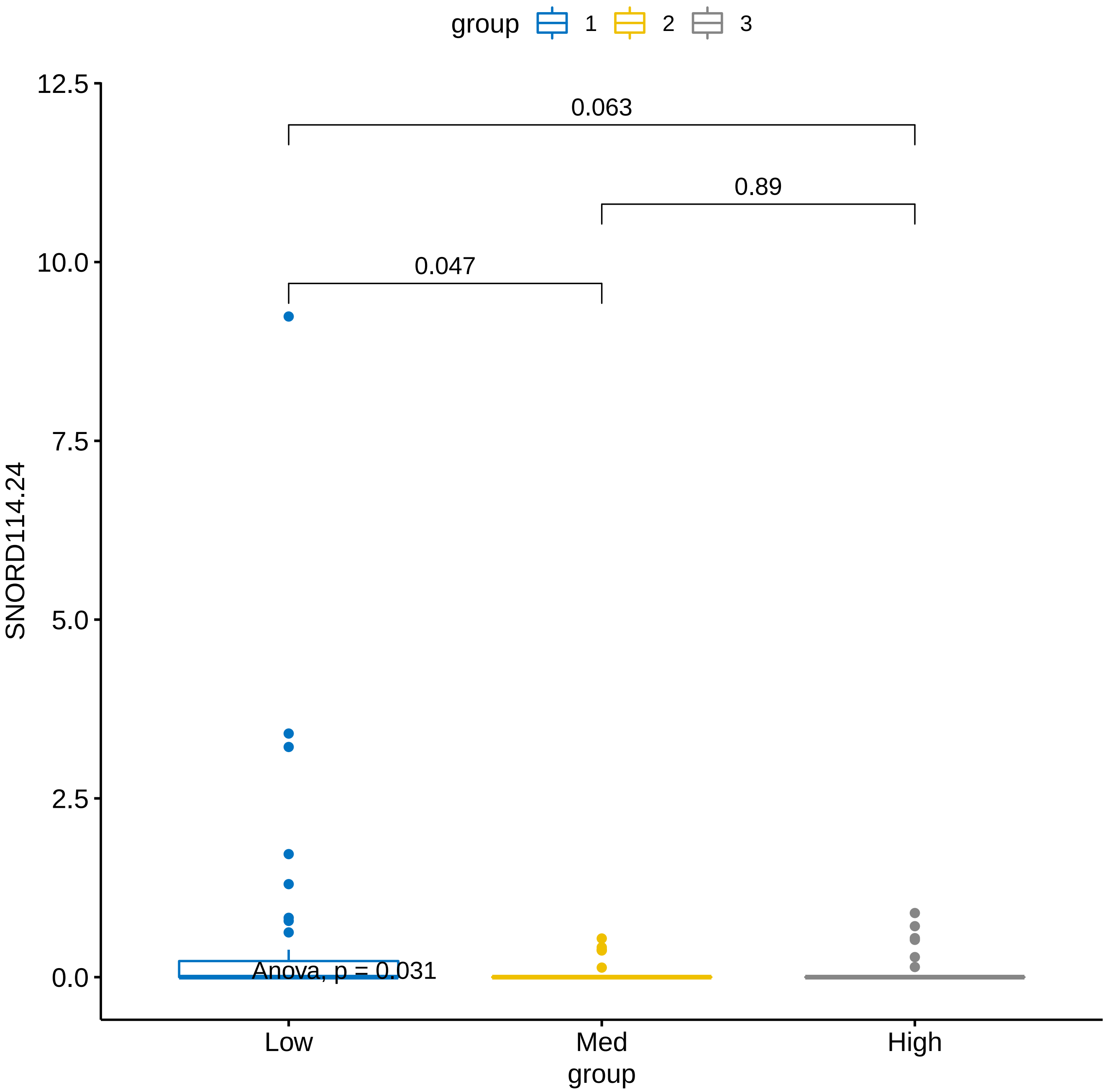
group
1
2
3
12.5
10.0
7.5
5.0
2.5
0.0
SNORD114.24
0.063
0.89
0.047
Anova, p = 0.031
Low
Med
High
group

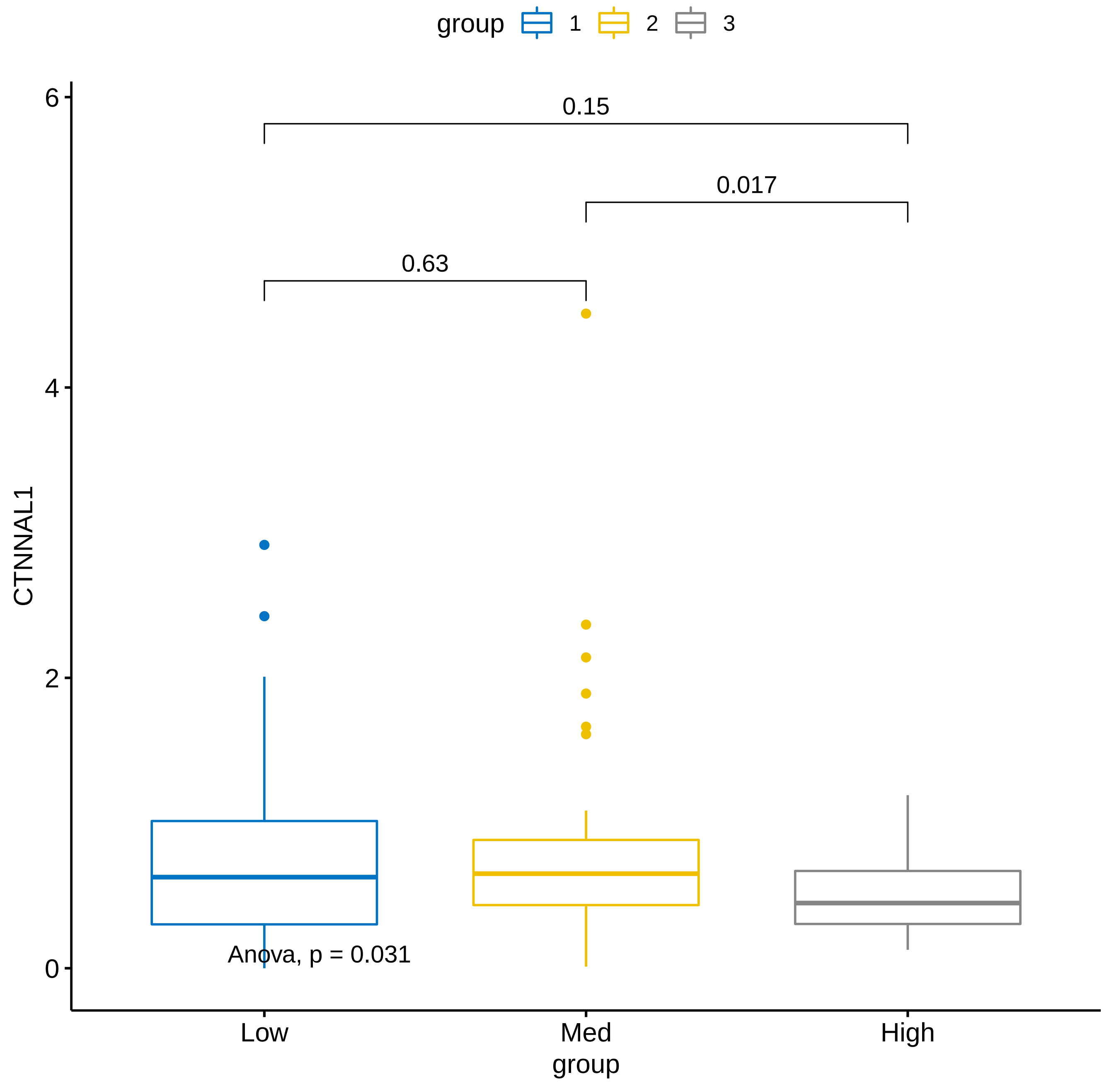

group
1
2
3
0.15
0.017
0.63
6
4
2
0
CTNNAL1
Anova, p = 0.031
Low
Med
High
group

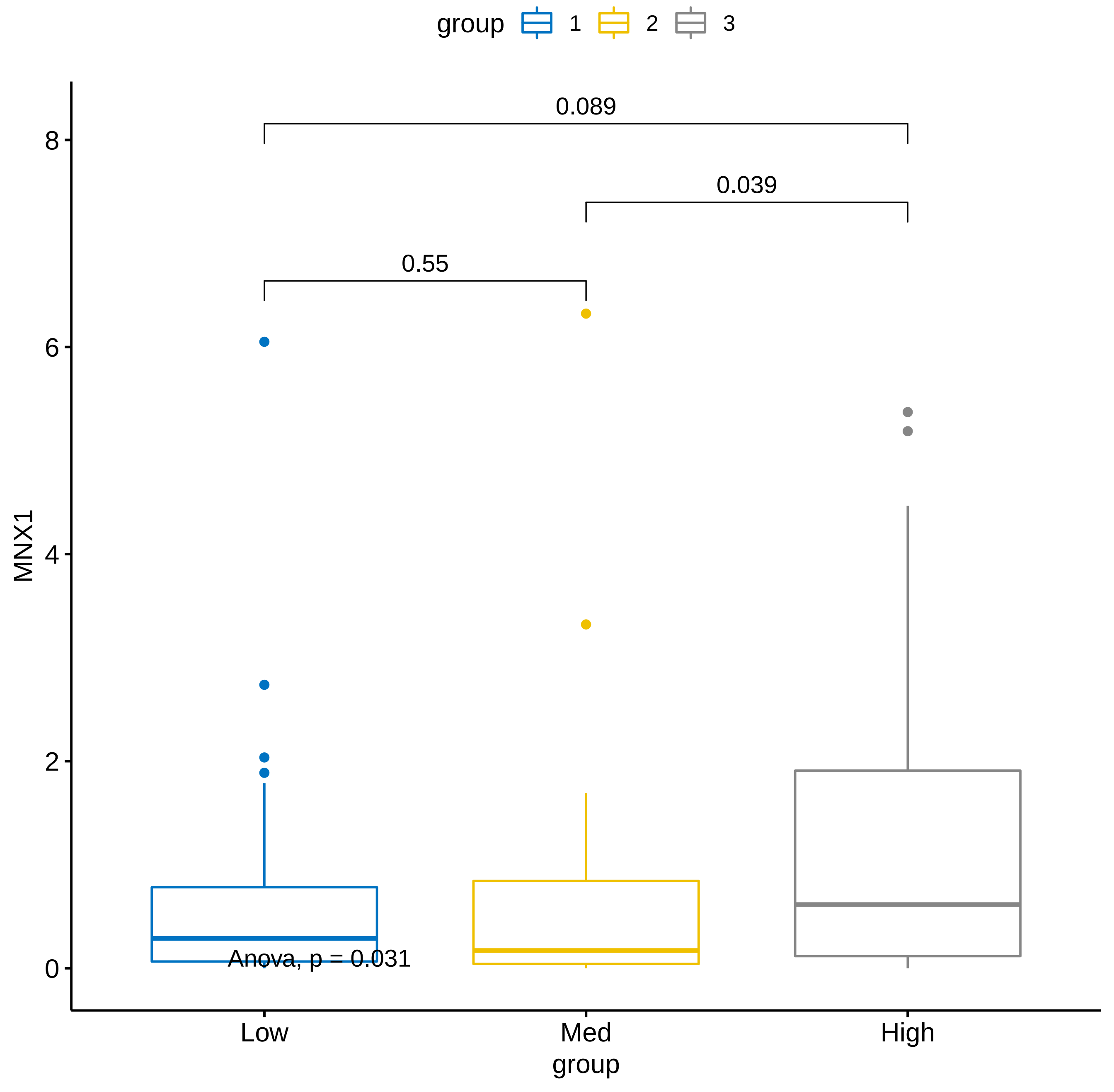

group 1 2 3
0.089
0.039
0.55
MNX1
8
6
4
2
0
Anova, p = 0.031
Low
Med
High
group

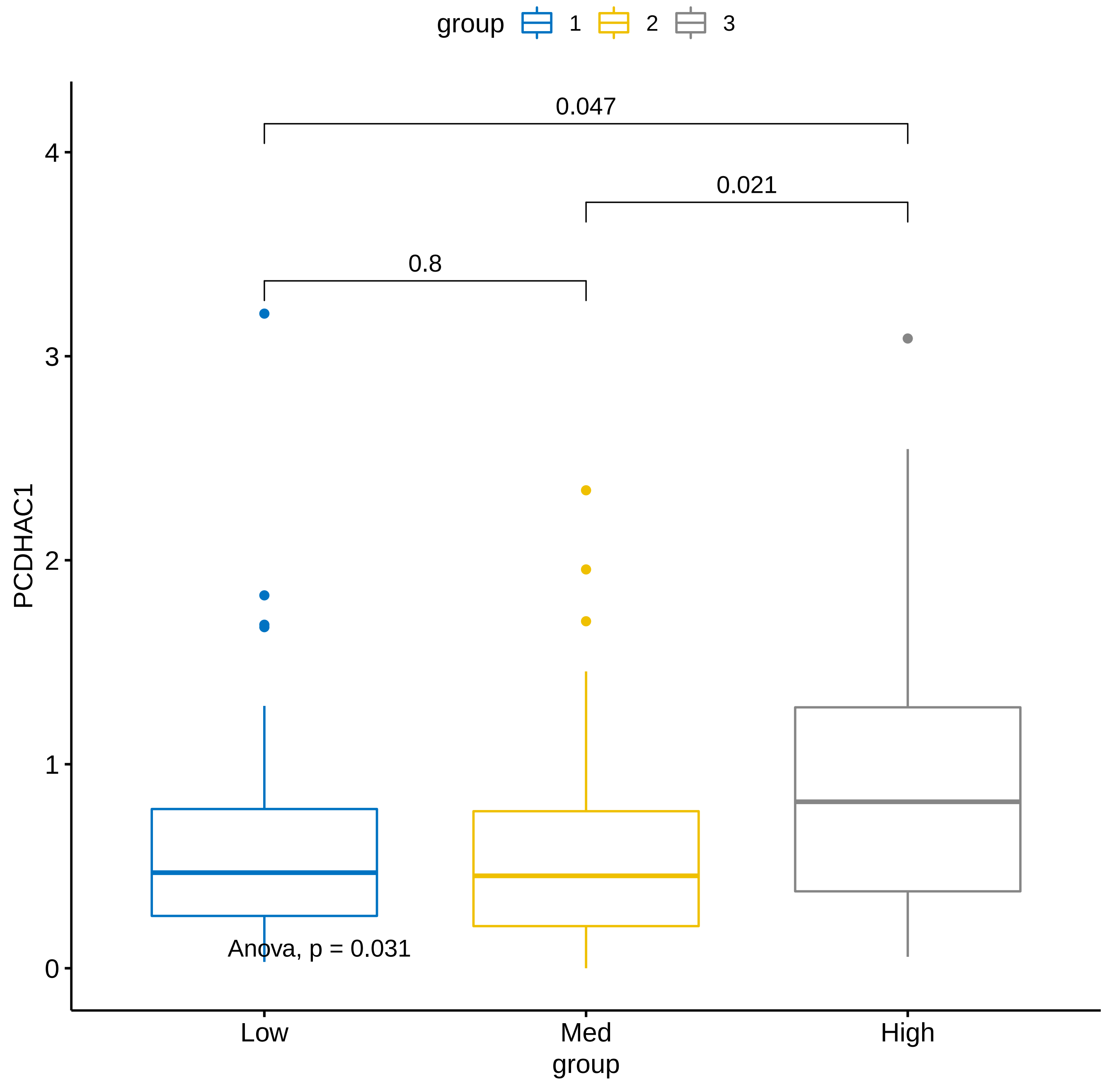

group 1 2 3
0.047
0.021
0.8
PCDHAC1
4
3
2
1
0
Anova, p = 0.031
Low
Med
High
group

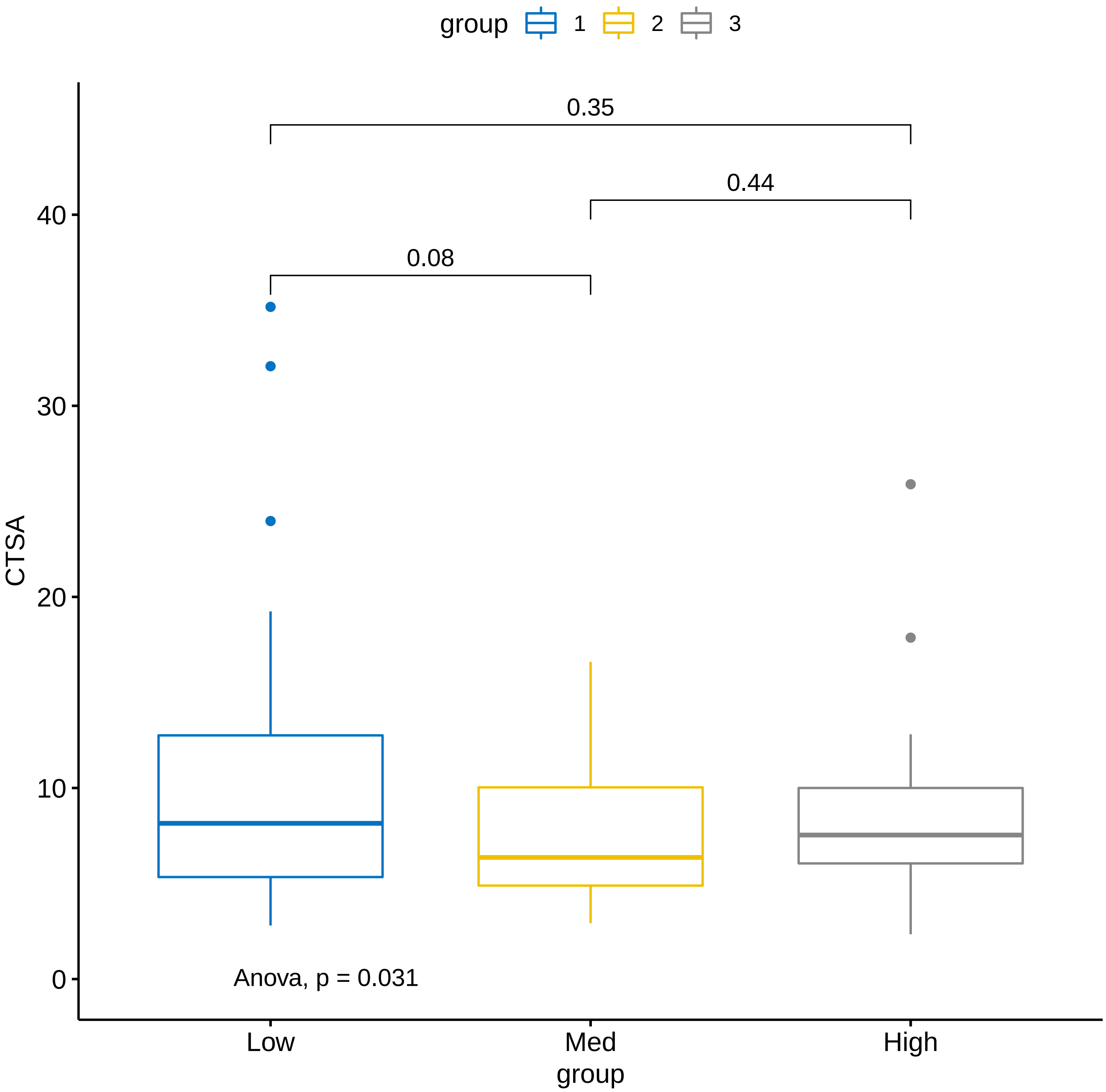
group
1
2
3
0.35
0.44
0.08
40
30
20
10
0
CTSA
Anova, p = 0.031
Low
Med
High
group

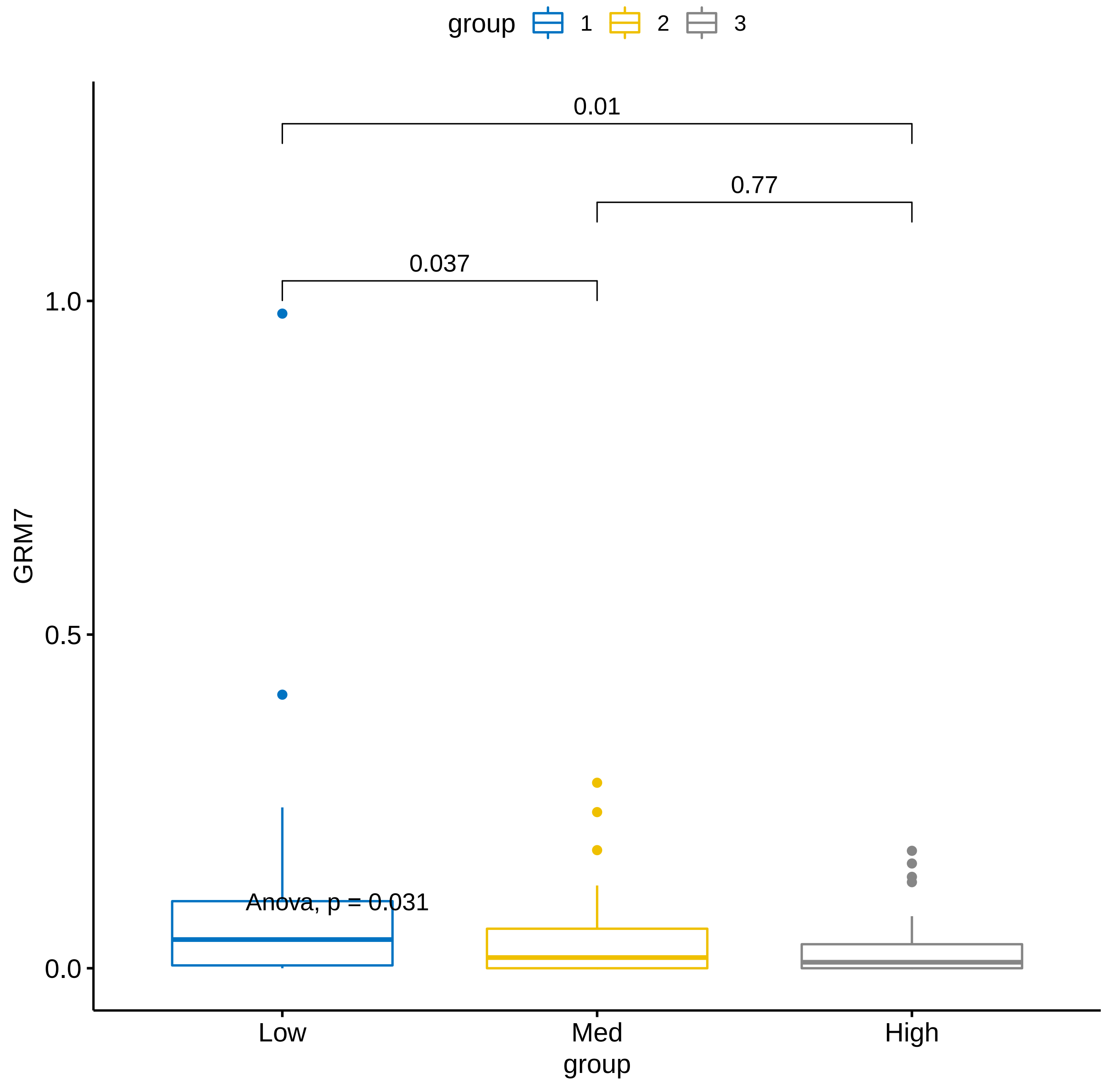
group 1 2 3
0.01
0.77
0.037
1.0
0.5
0.0
GRM7
Anova, p = 0.031
Low
Med
High
group

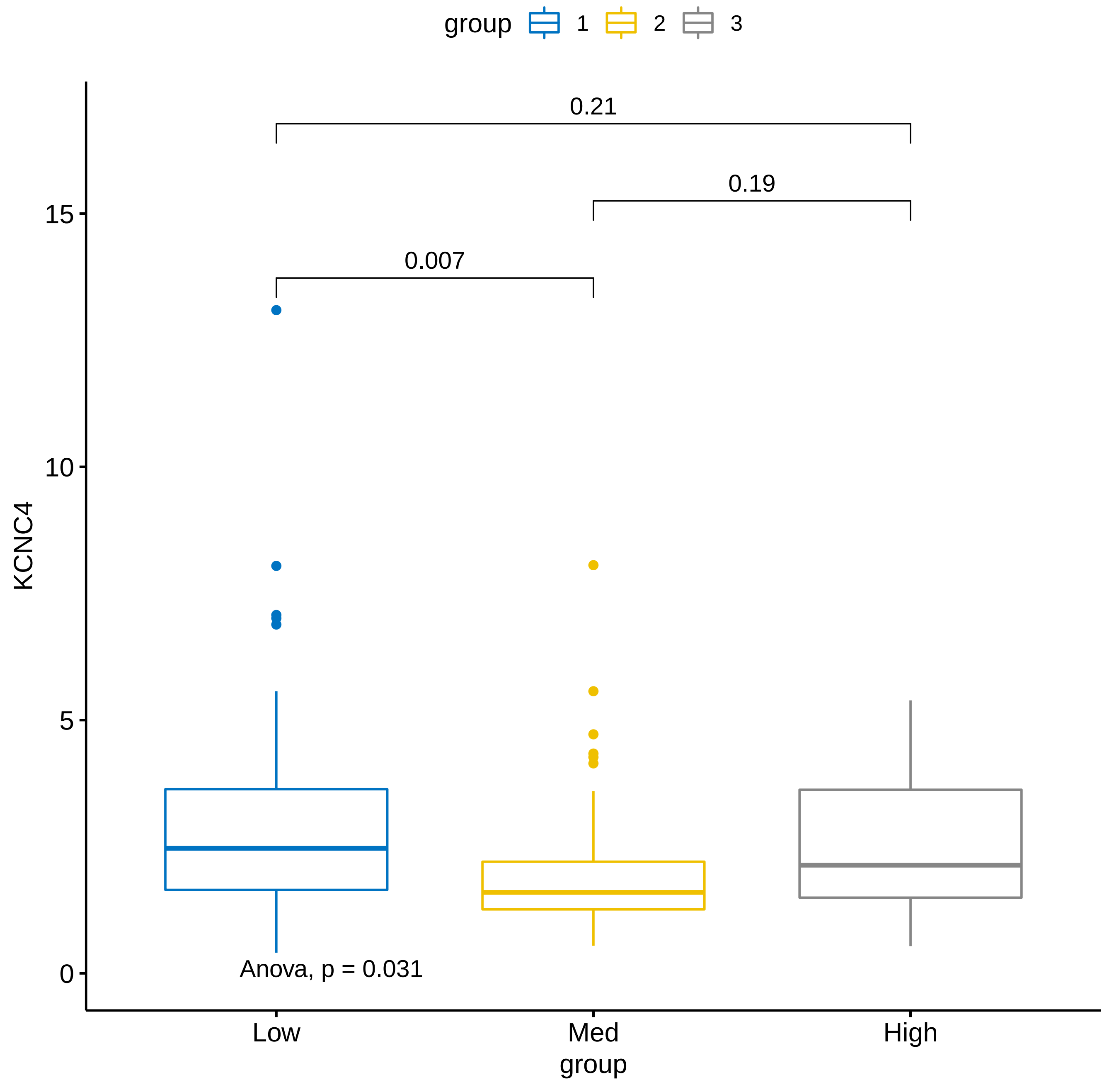
group
1
2
3
0.21
0.19
0.007
15
10
5
0
KCNC4
Anova, p = 0.031
Low
Med
High
group

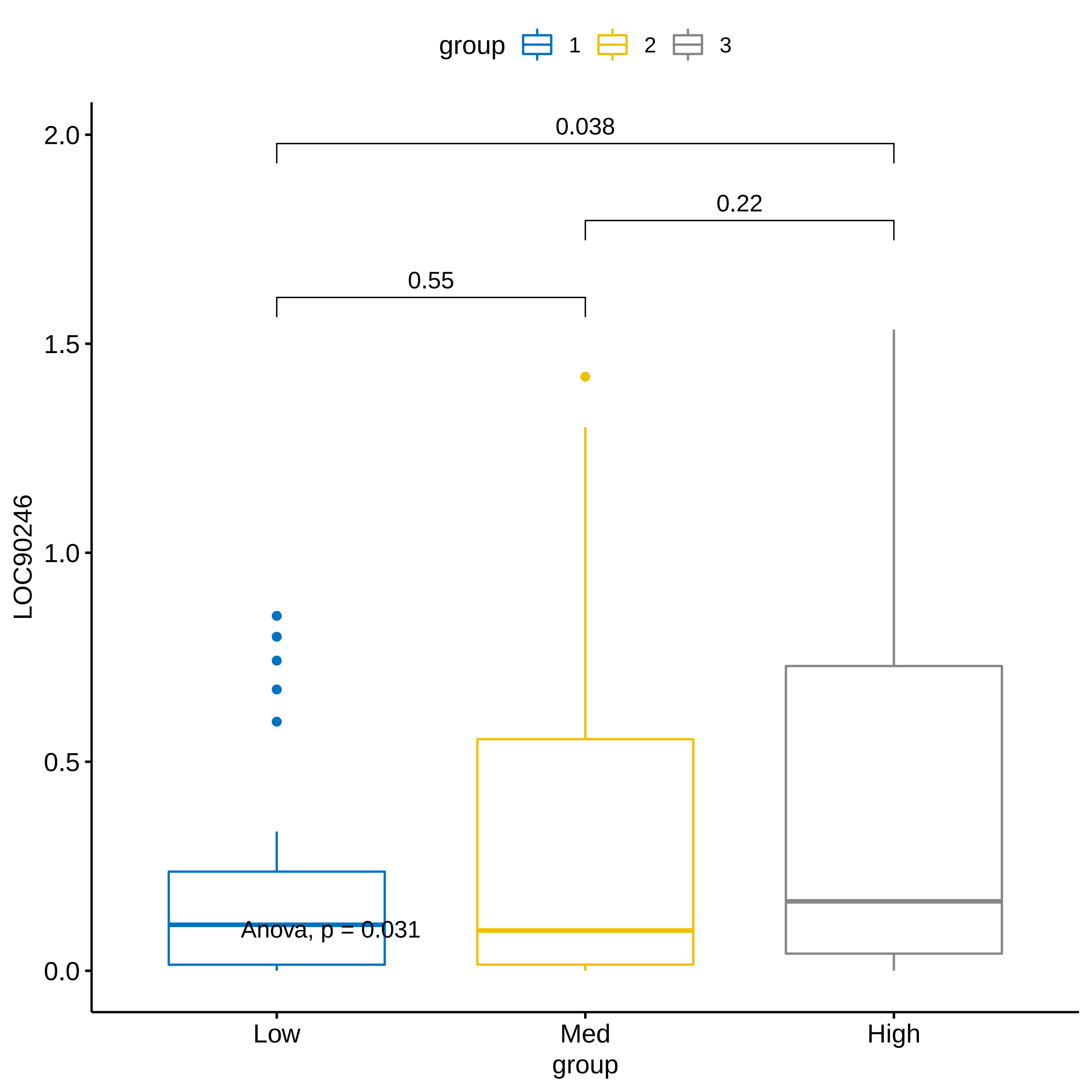

group
1
2
3
0.038
0.22
0.55
2.0
1.5
1.0
0.5
0.0
LOC90246
Anova, p = 0.031
Low
Med
High
group

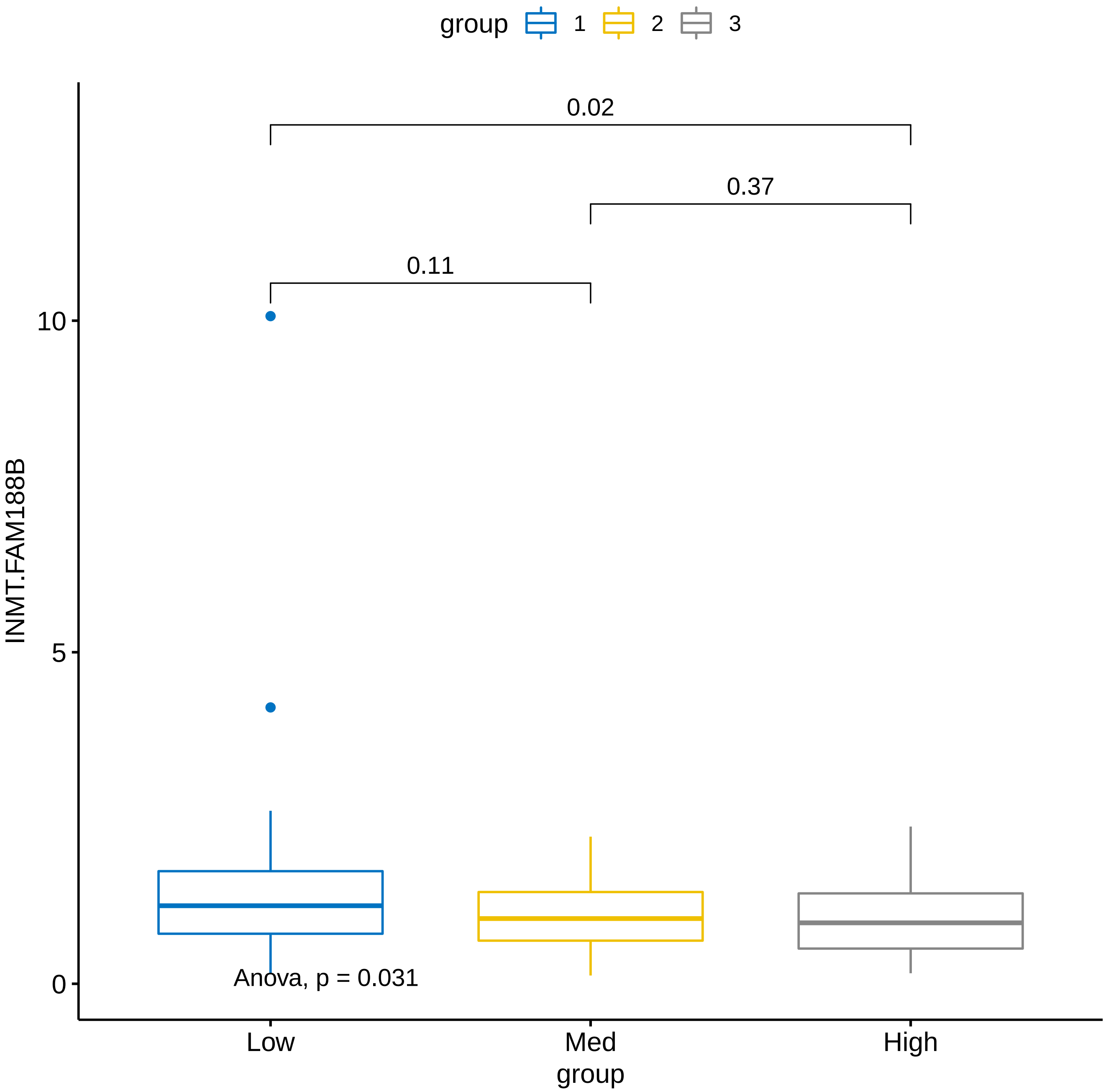
group 1 2 3
0.02
0.37
0.11
10
5
0
INMT.FAM188B
Anova, p = 0.031
Low
Med
High
group

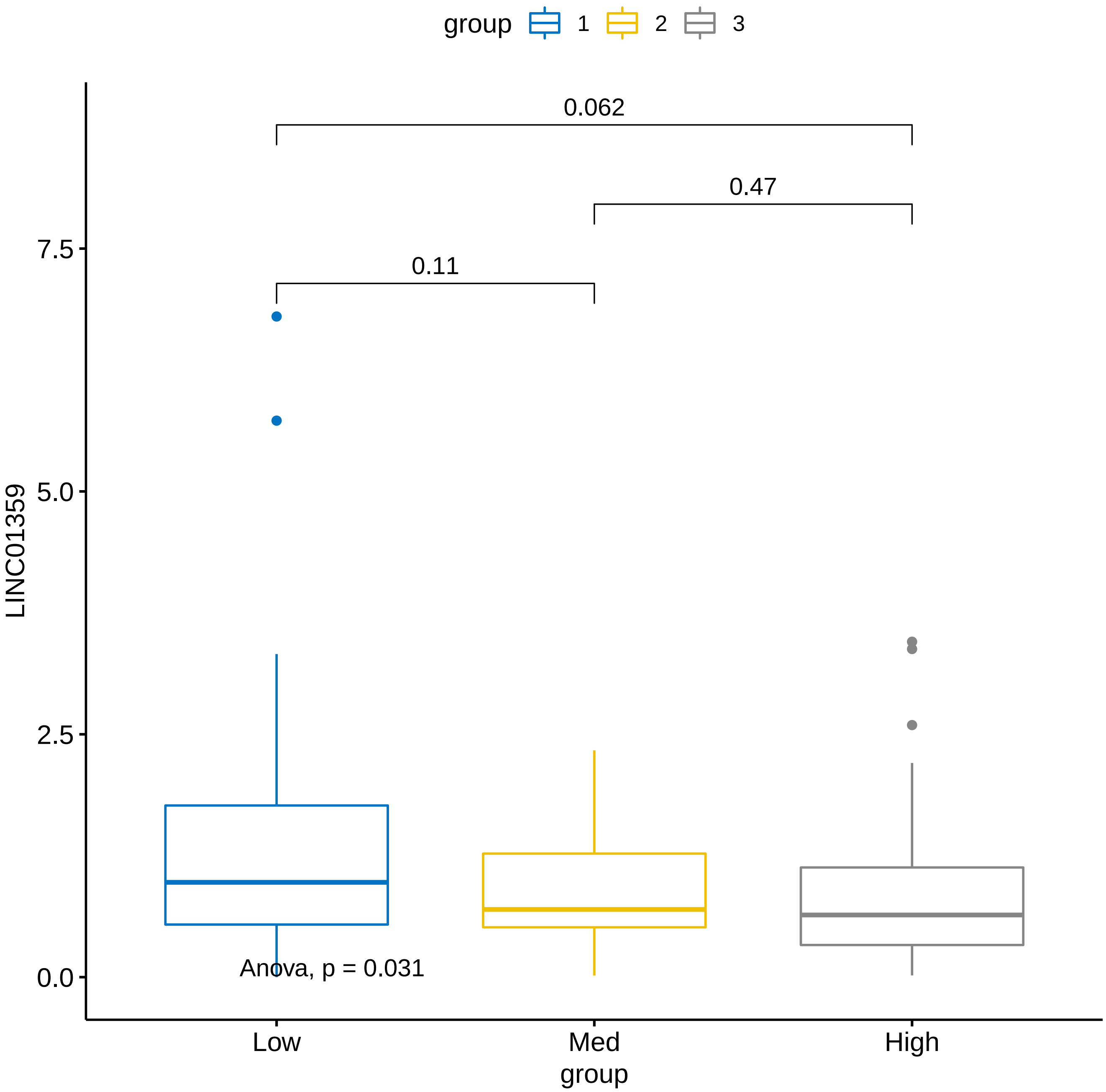

group 1 2 3
0.062
0.47
0.11
7.5
5.0
2.5
0.0
LINC01359
Anova, p = 0.031
Low
Med
High
group

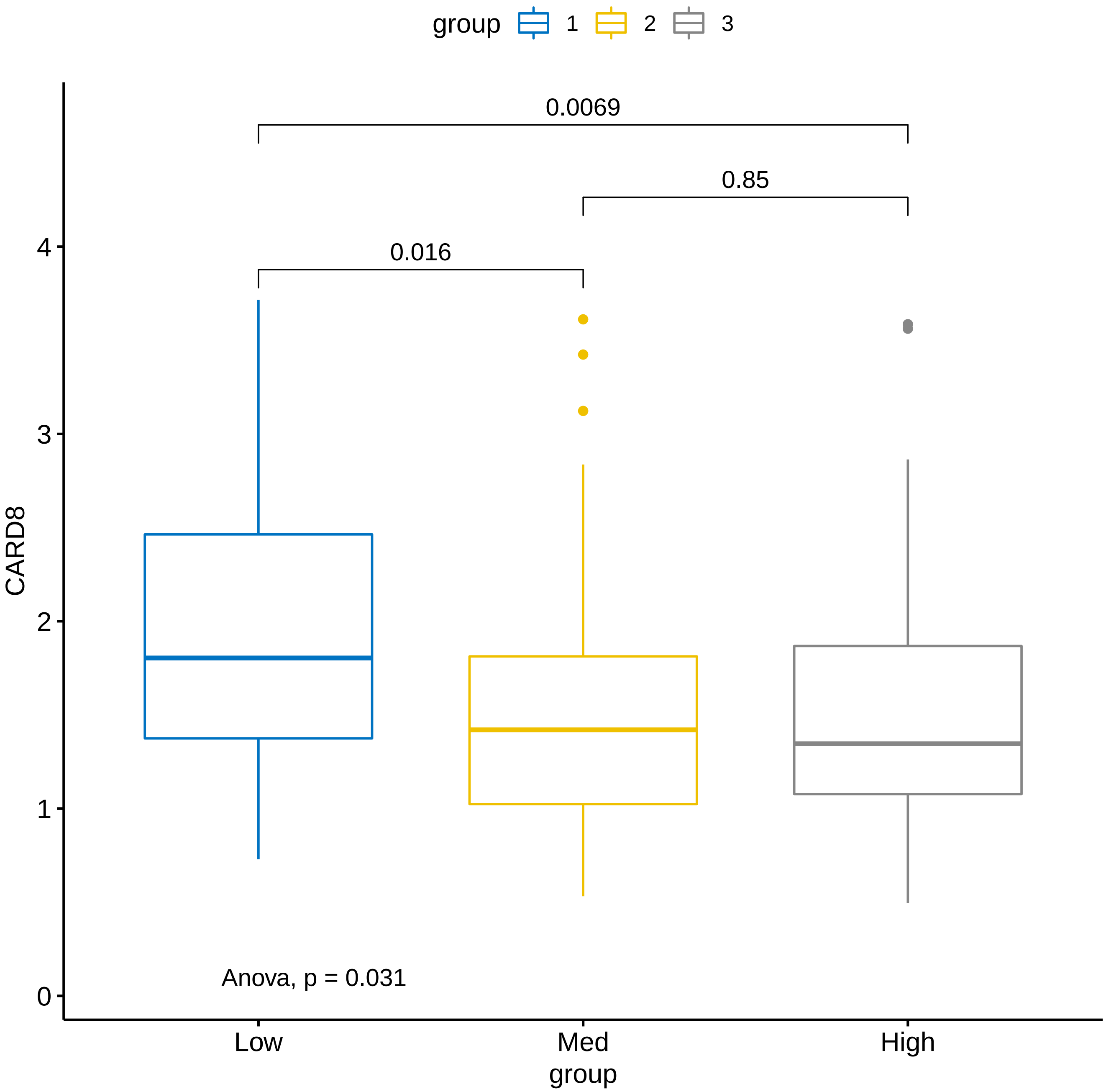

group 1 2 3
0.0069
0.85
0.016
CARD8
4
3
2
1
0
Anova, p = 0.031
Low
Med
High
group

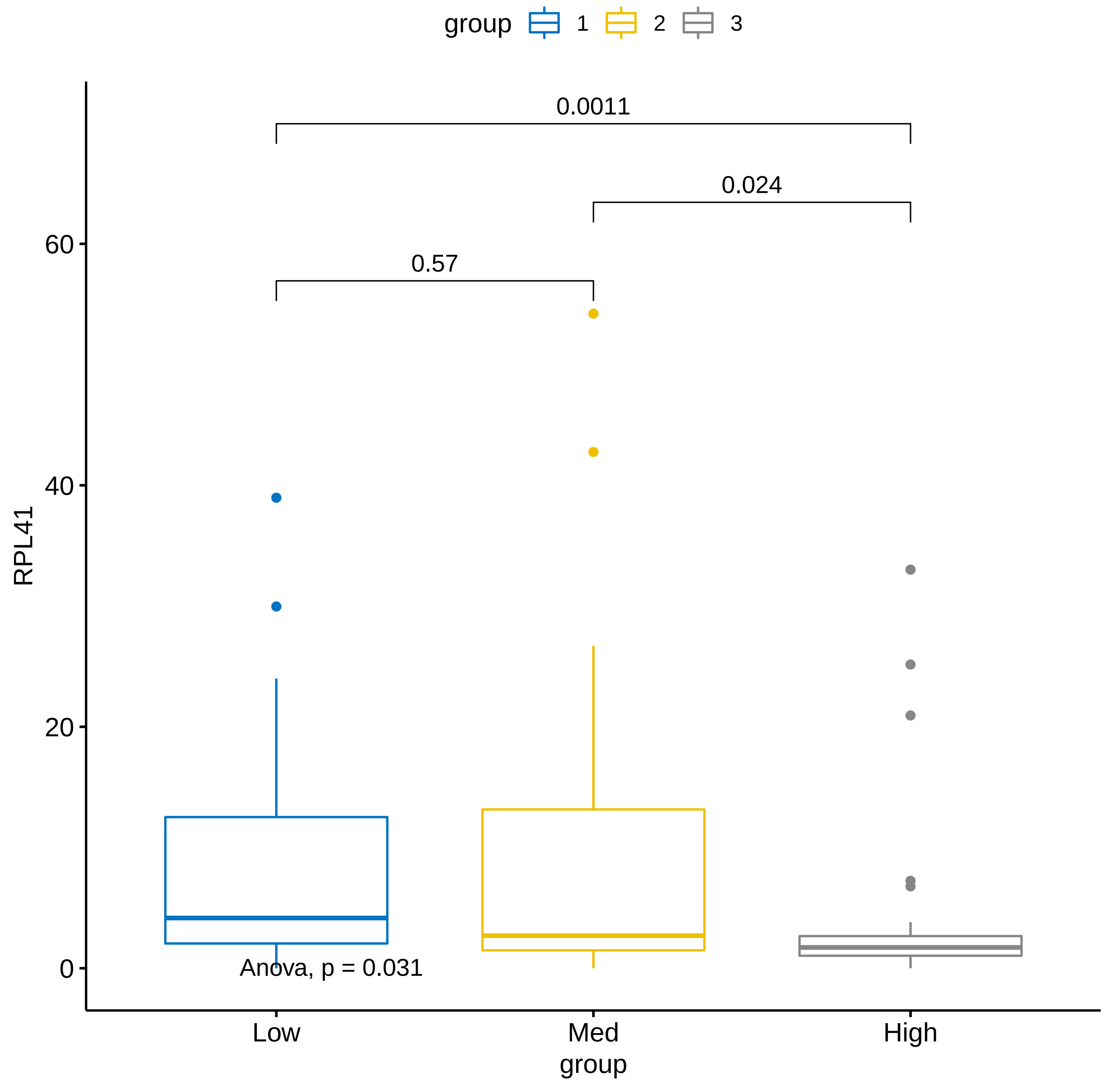

group 1 2 3
0.0011
0.024
0.57
60
40
20
0
RPL41
Anova, p = 0.031
Low
Med
High
group

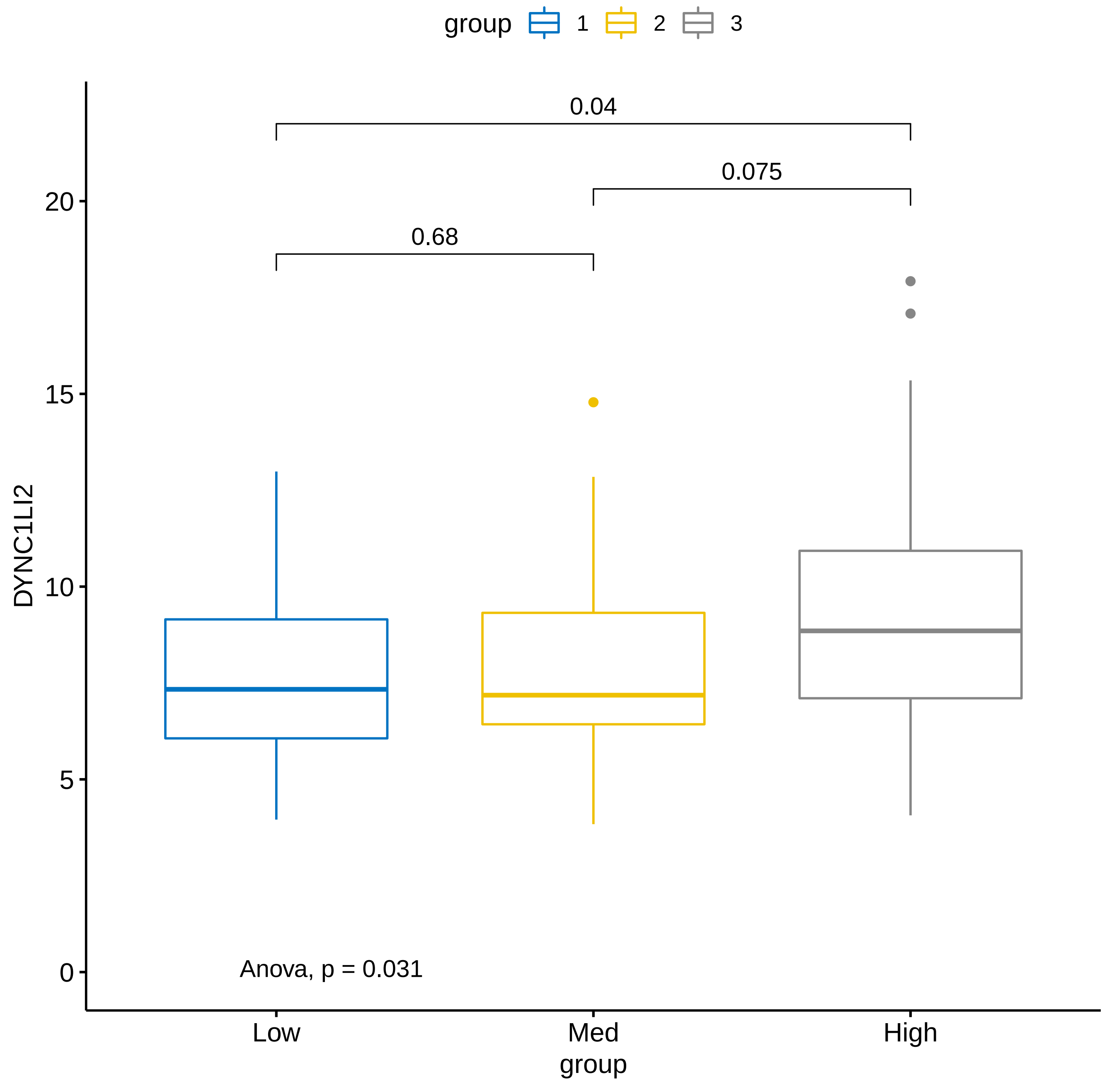
group
1
2
3
0.04
0.075
0.68
DYNC1LI2
20
15
10
5
0
Anova, p = 0.031
Low
Med
High
group

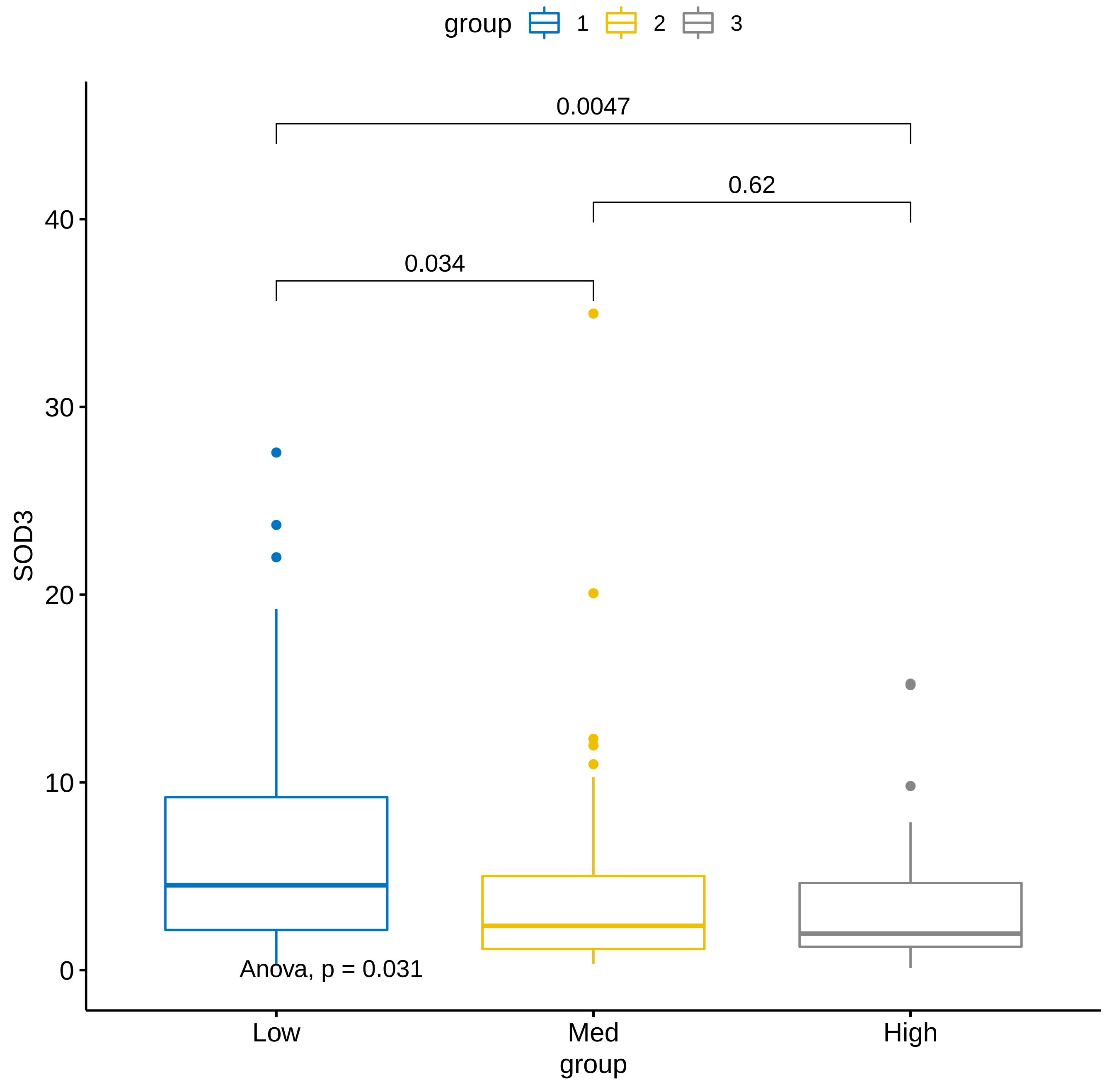

group 1 2 3
0.0047
0.62
0.034
40
30
20
10
0
SOD3
Anova, p = 0.031
Low
Med
High
group

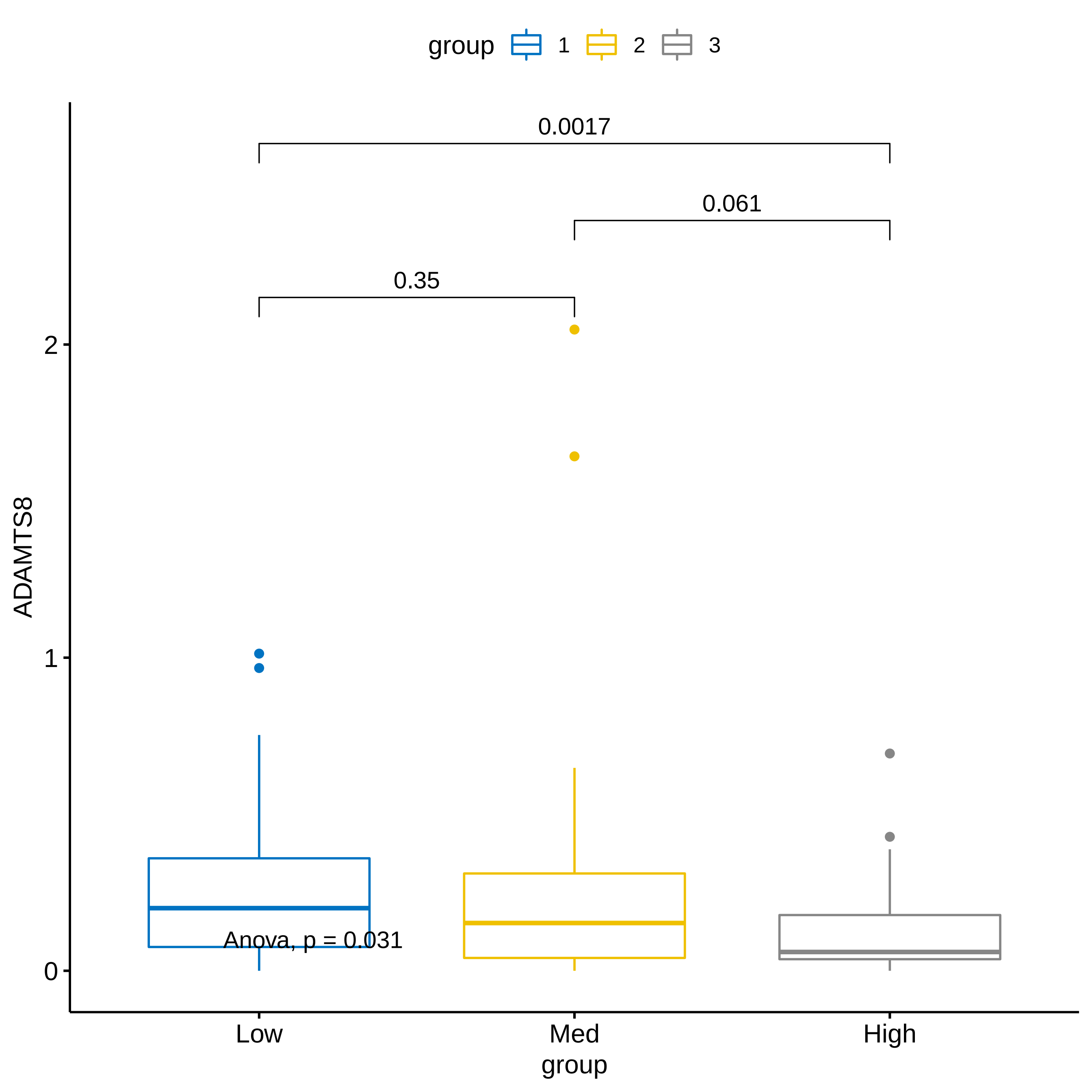

group
1
2
3
0.0017
0.061
0.35
ADAMTS8
2
1
0
Anova, p = 0.031
Low
Med
High
group

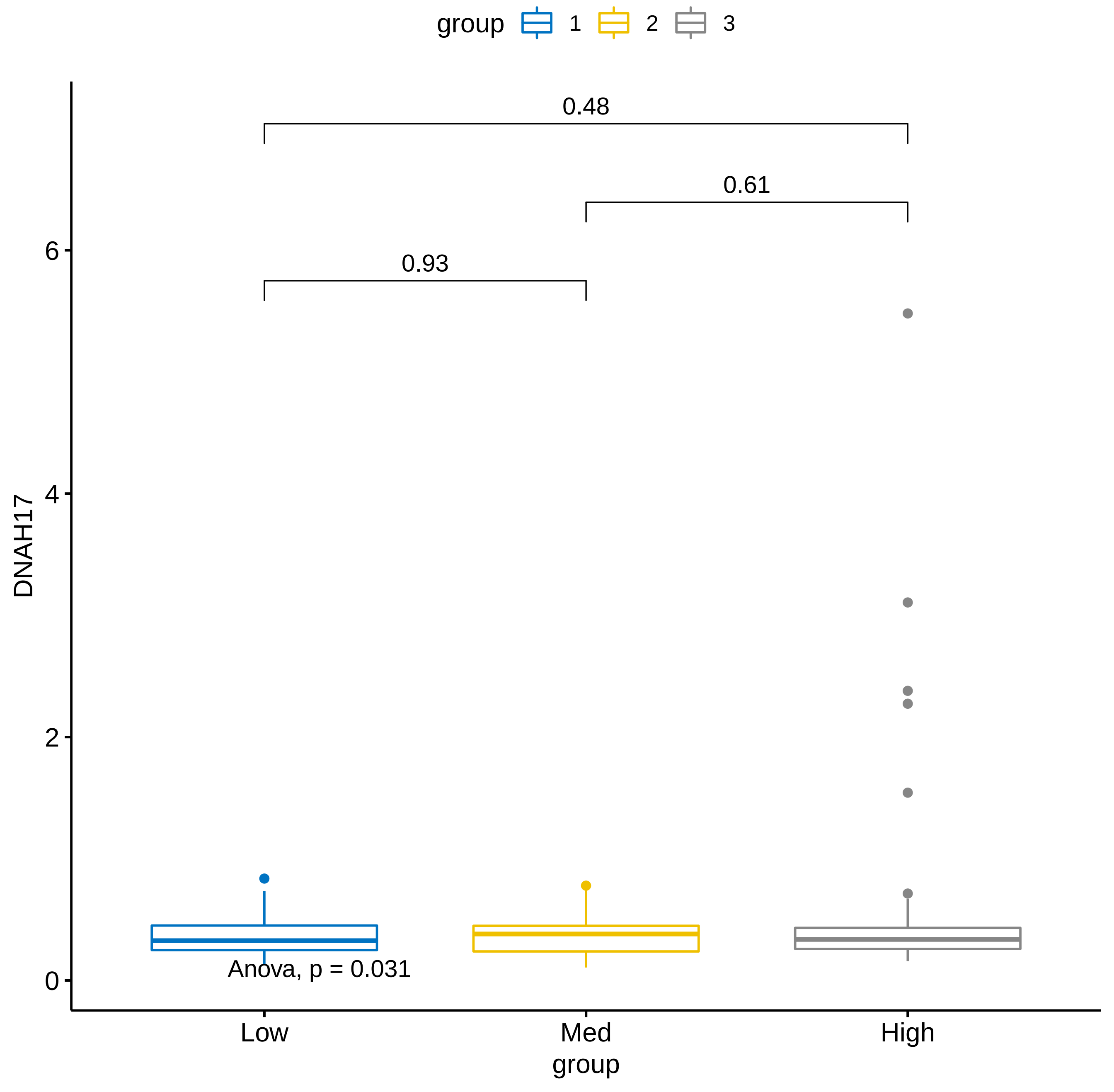
group
1
2
3
0.48
0.61
0.93
DNAH17
6
4
2
0
Anova, p = 0.031
Low
Med
High
group

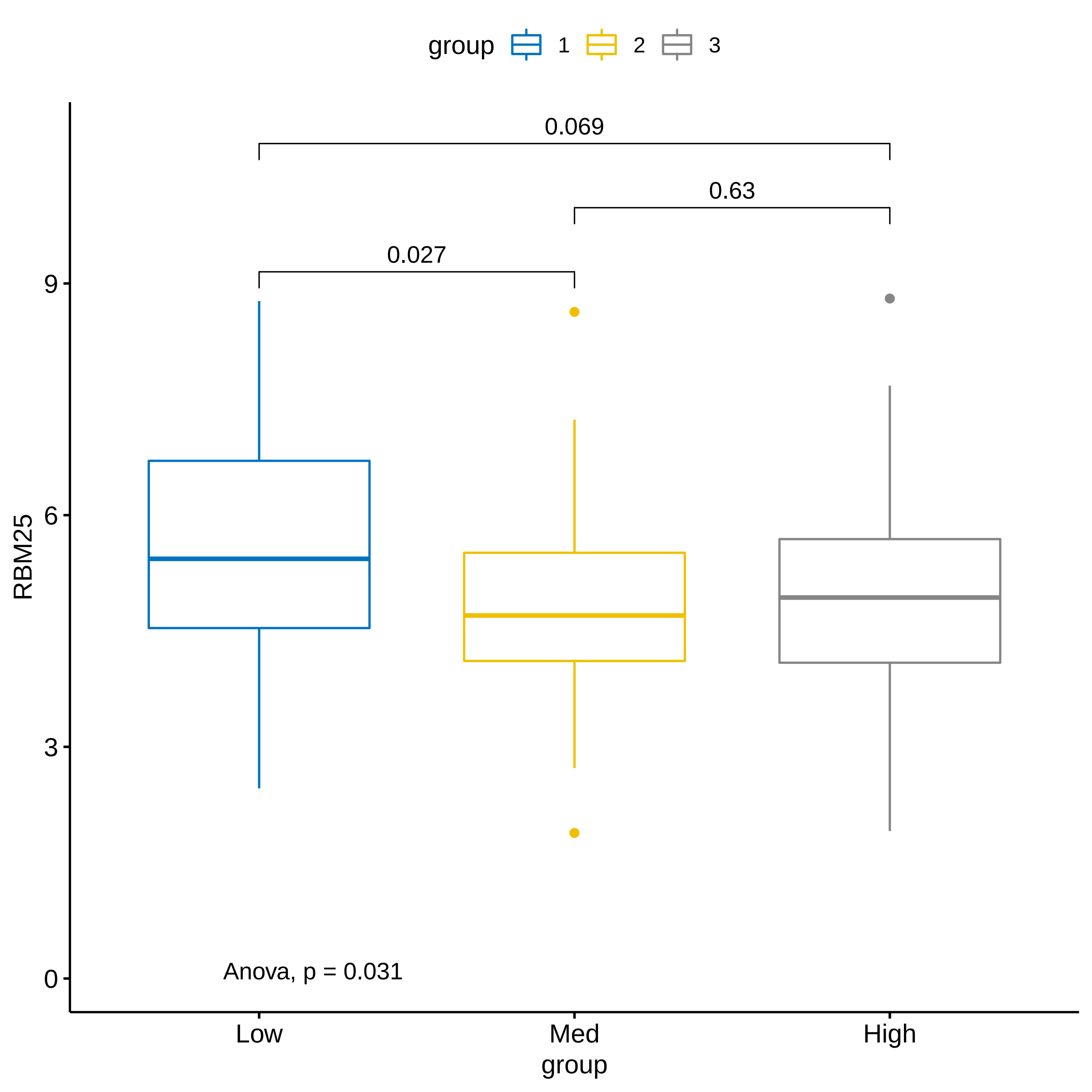
group
1
2
3
0.069
0.63
0.027
9
6
3
0
RBM25
Anova, p = 0.031
Low
Med
High
group

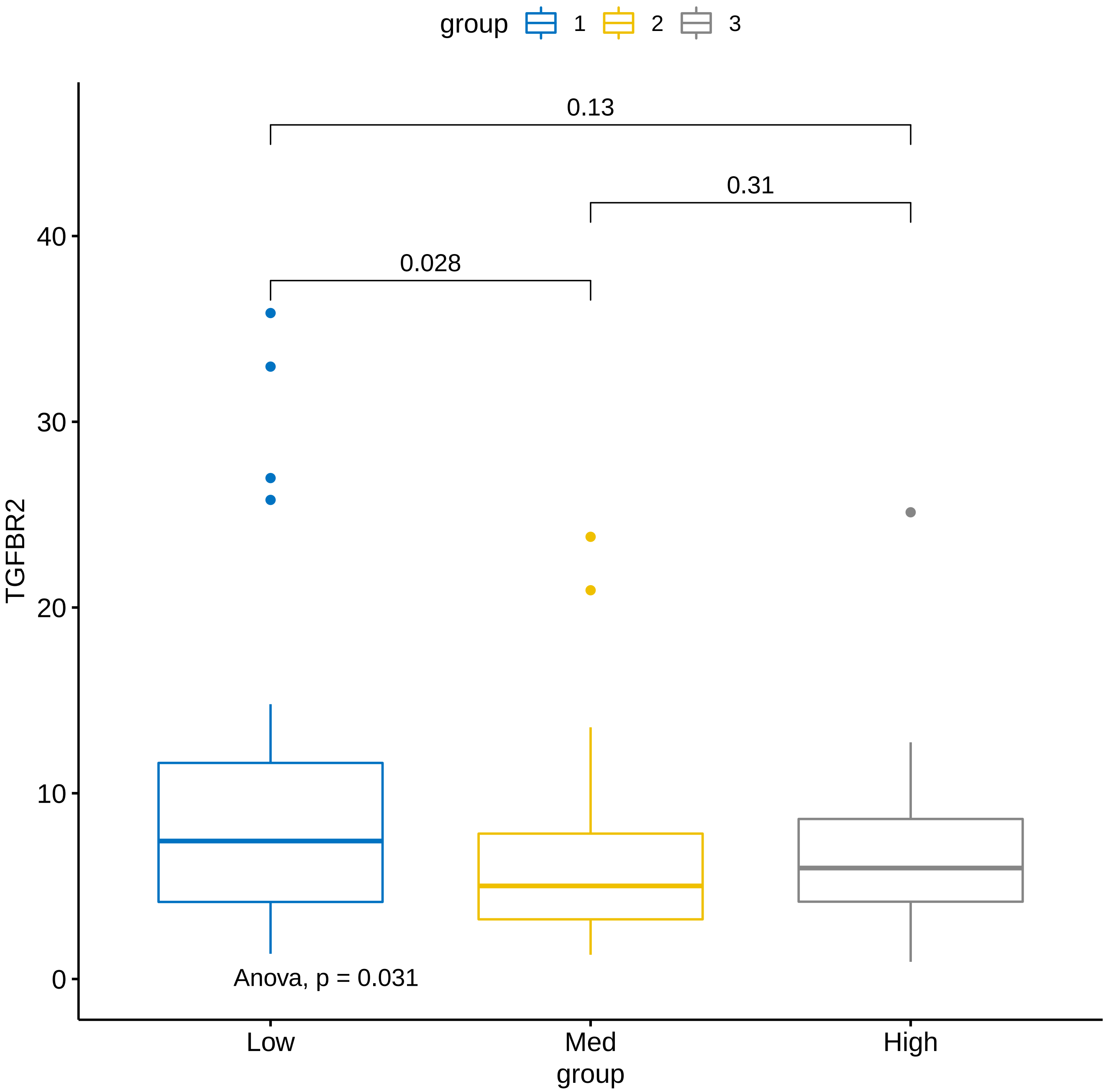

group
1
2
3
0.13
0.31
0.028
40
30
20
10
0
TGFBR2
Anova, p = 0.031
Low
Med
High
group

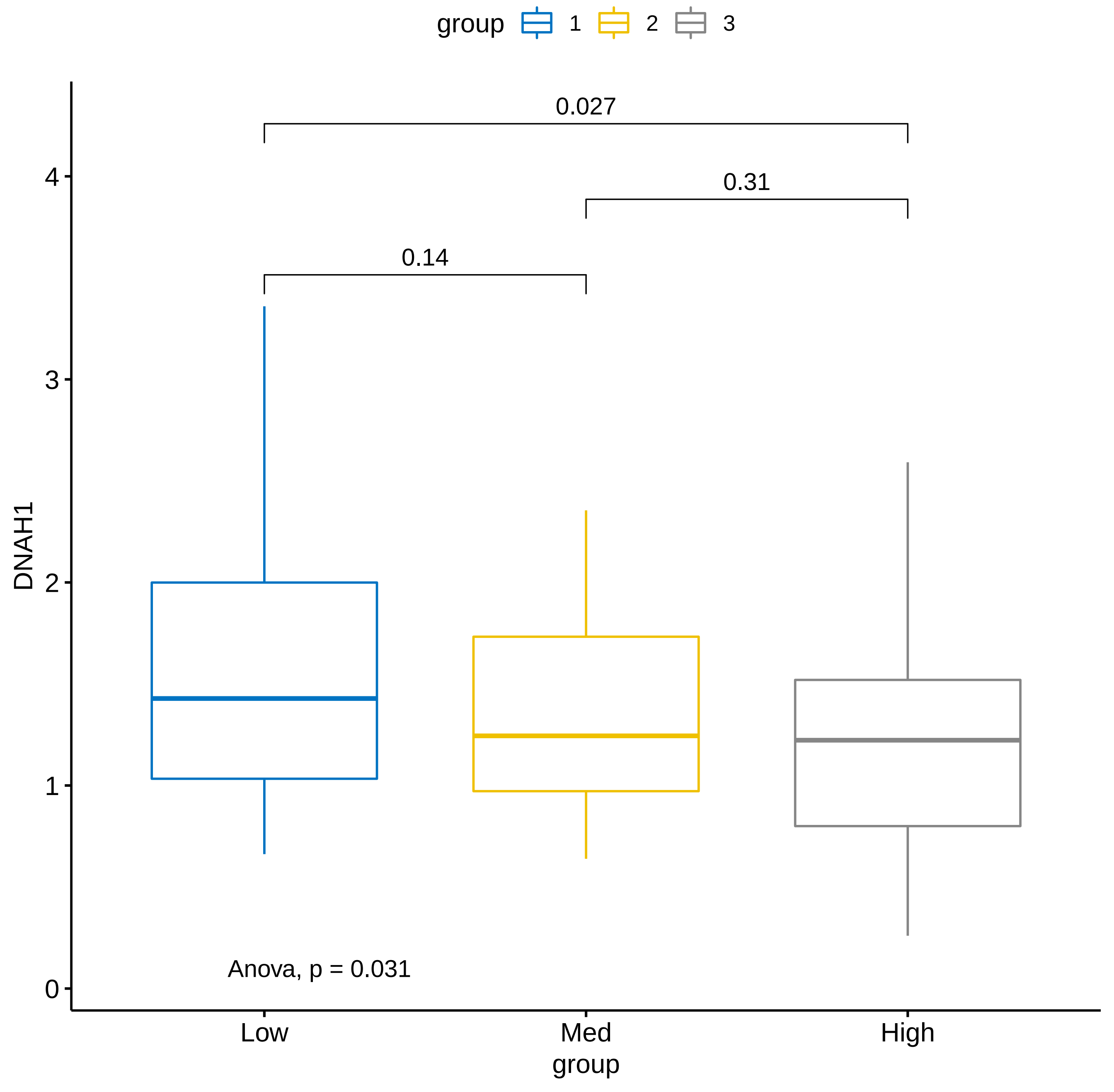
group
1
2
3
0.027
0.31
0.14
DNAH1
4
3
2
1
0
Anova, p = 0.031
Low
Med
High
group

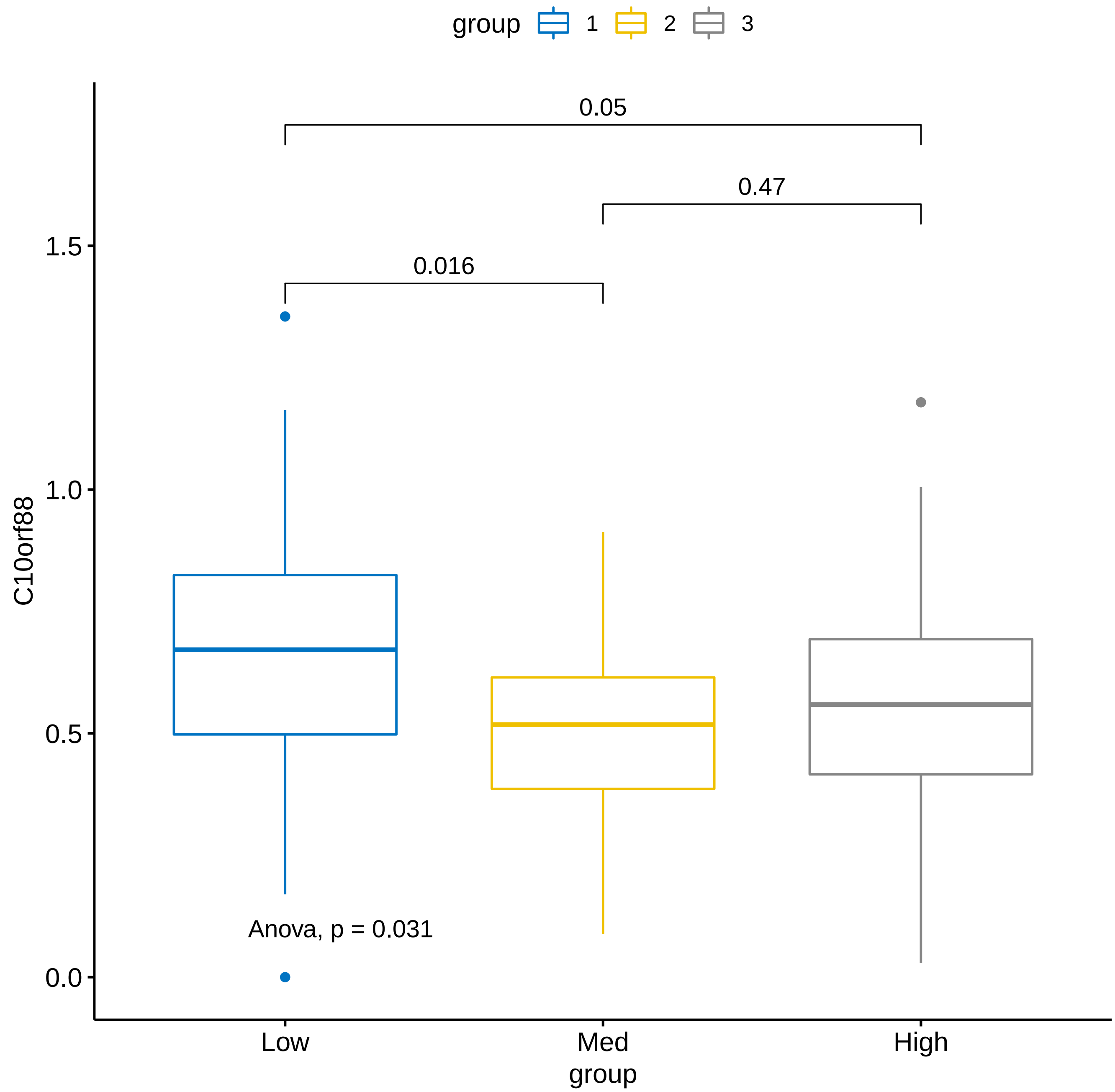
group
1
2
3
0.05
0.47
0.016
1.5
1.0
0.5
0.0
C10orf88
Anova, p = 0.031
Low
Med
High
group

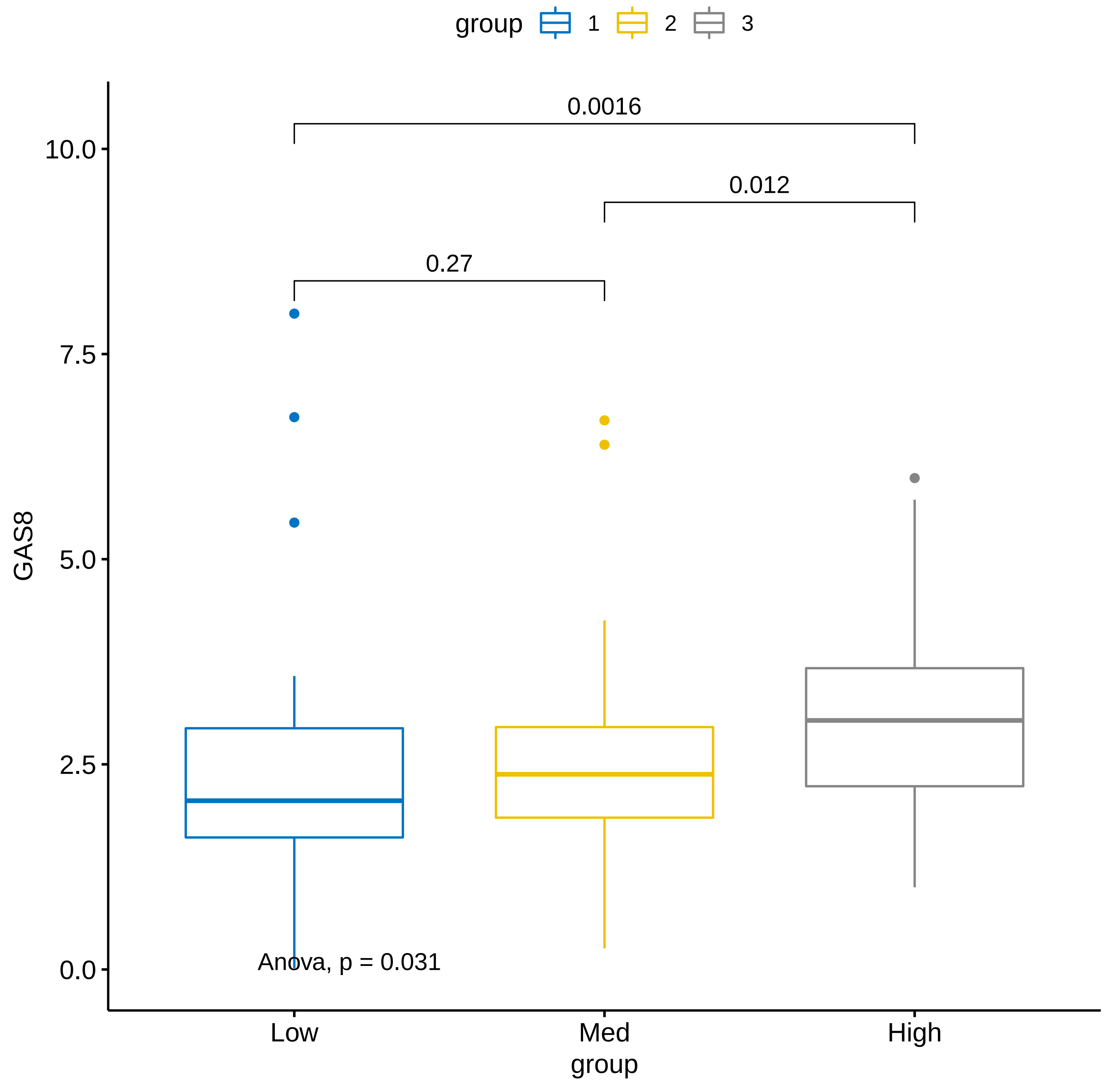
group 1 2 3
0.0016
0.012
0.27
10.0
7.5
5.0
2.5
0.0
GAS8
Anova, p = 0.031
Low
Med
High
group

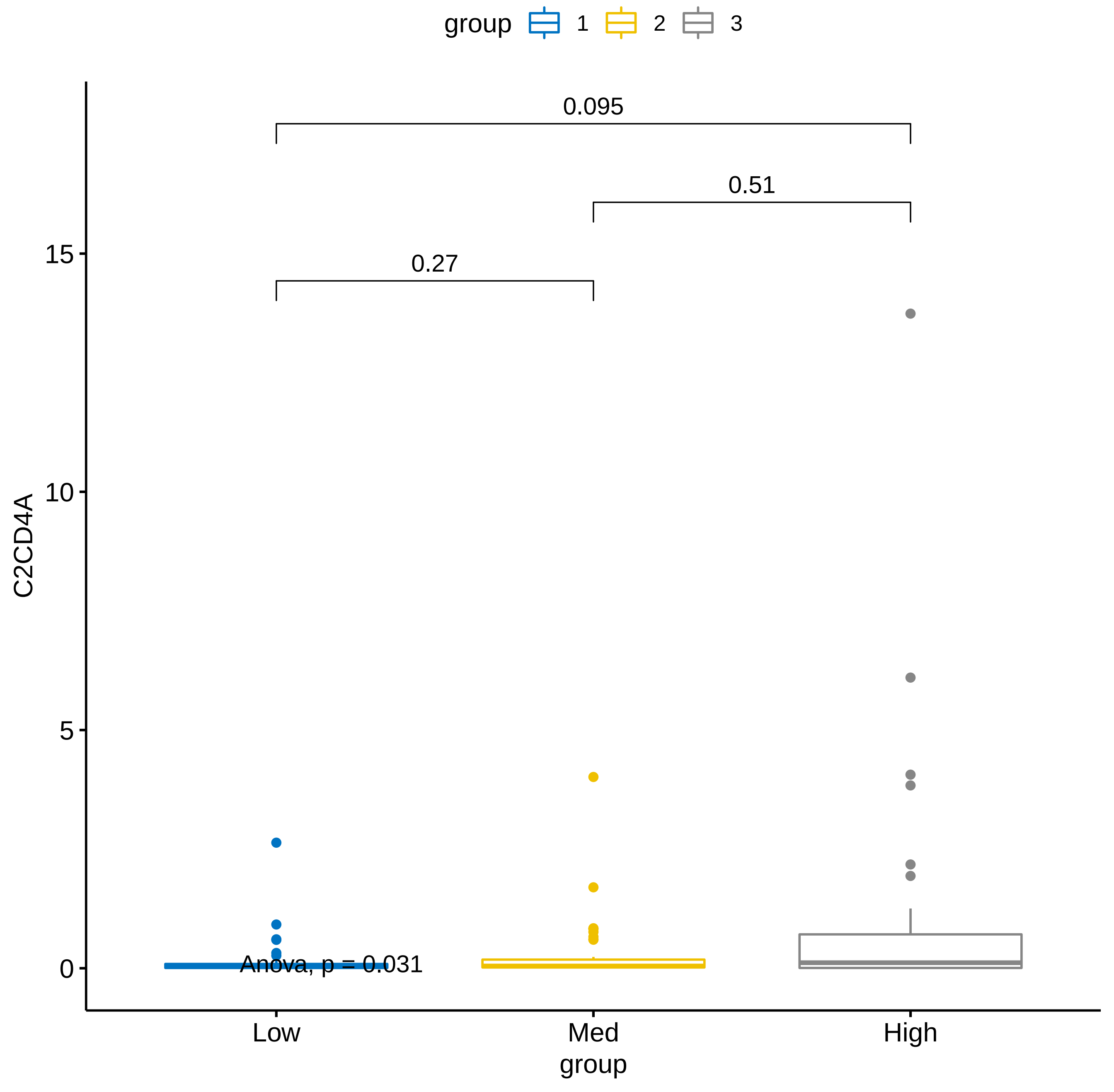

group
1
2
3
0.095
0.51
0.27
15
10
5
0
C2CD4A
Anova, p = 0.031
Low
Med
High
group

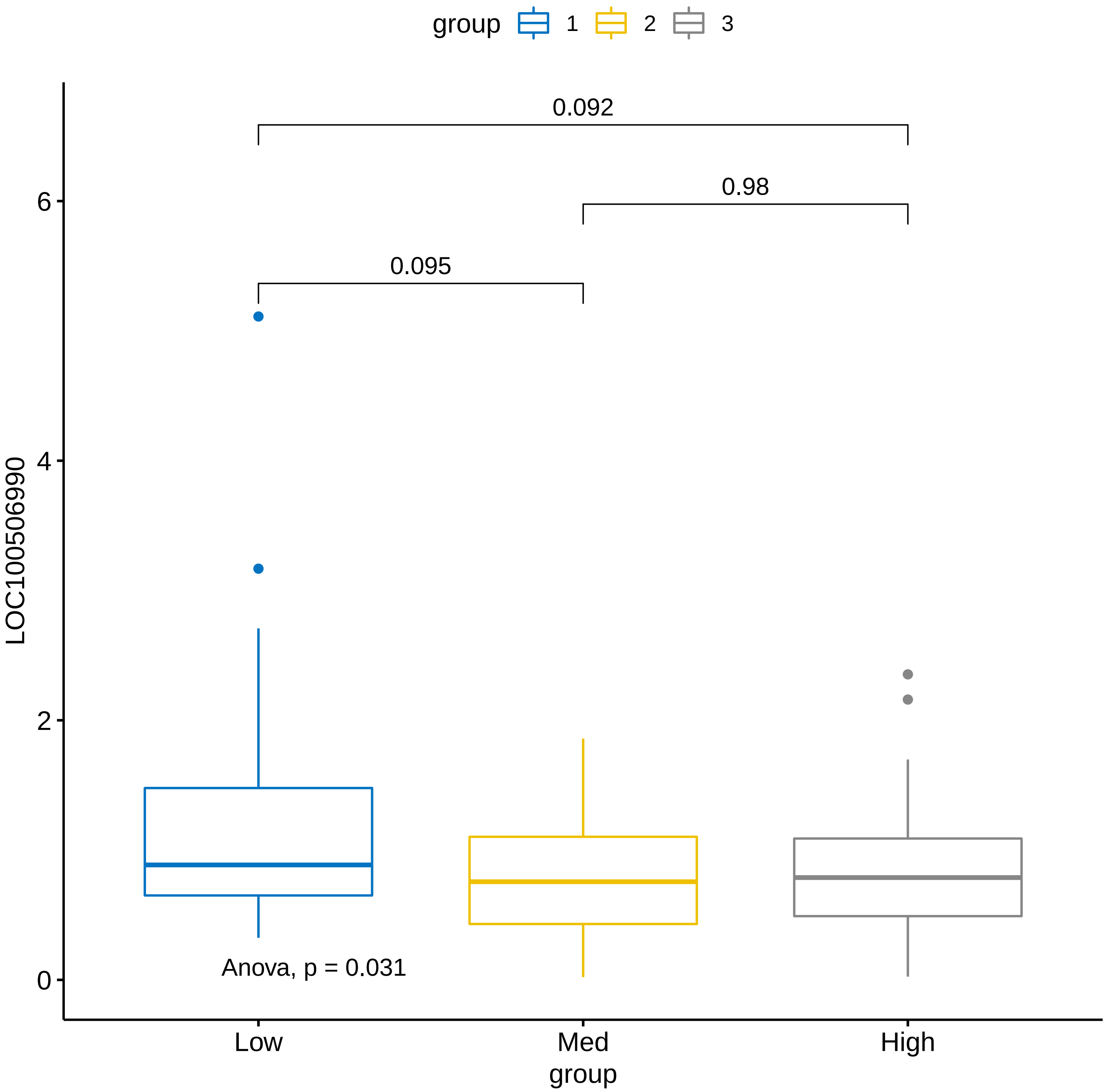
group
1
2
3
0.092
0.98
0.095
LOC100506990
6
4
2
0
Anova, p = 0.031
Low
Med
High
group

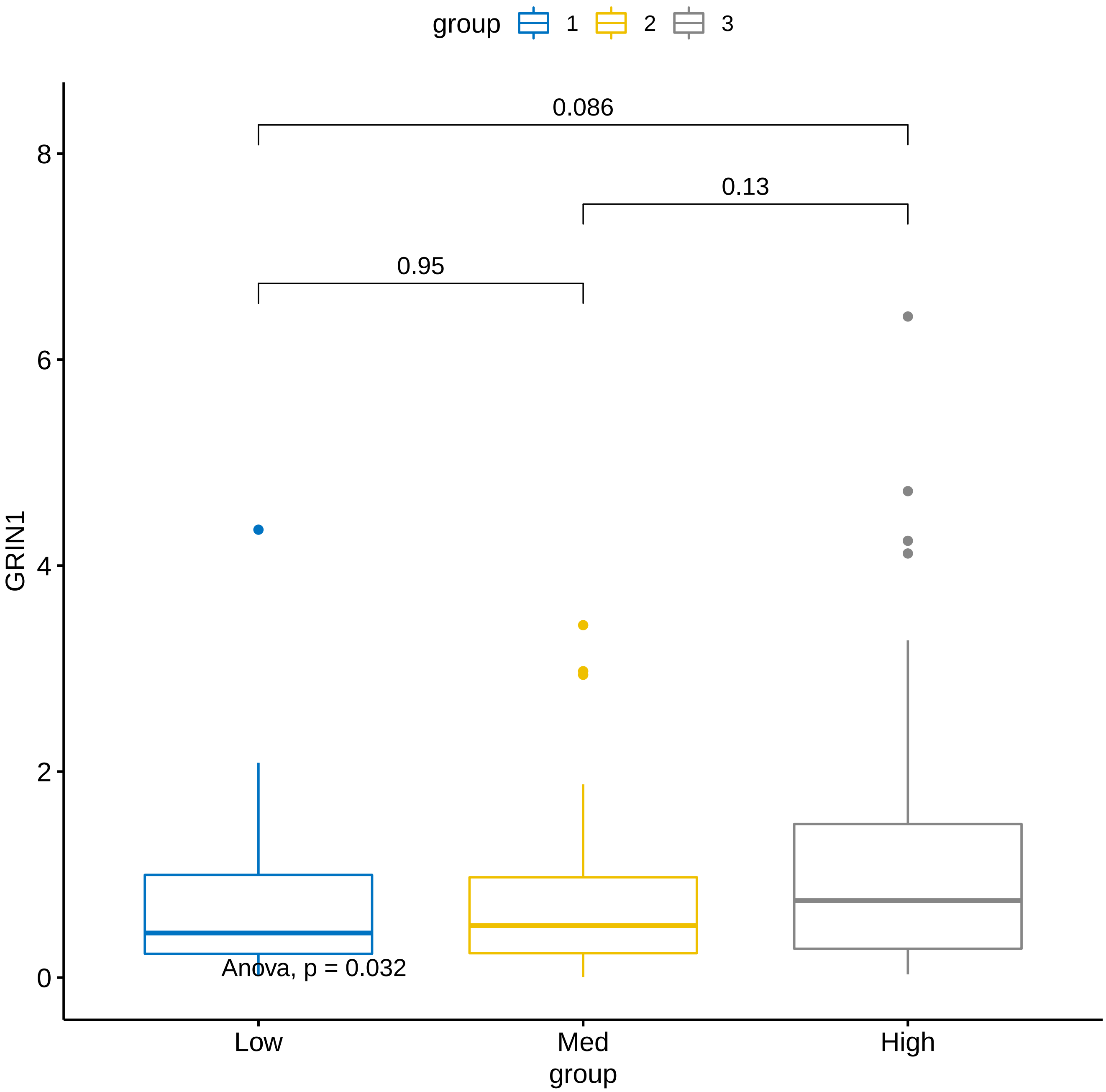

group
1
2
3
0.086
0.13
0.95
8
6
4
2
0
GRIN1
Anova, p = 0.032
Low
Med
High
group

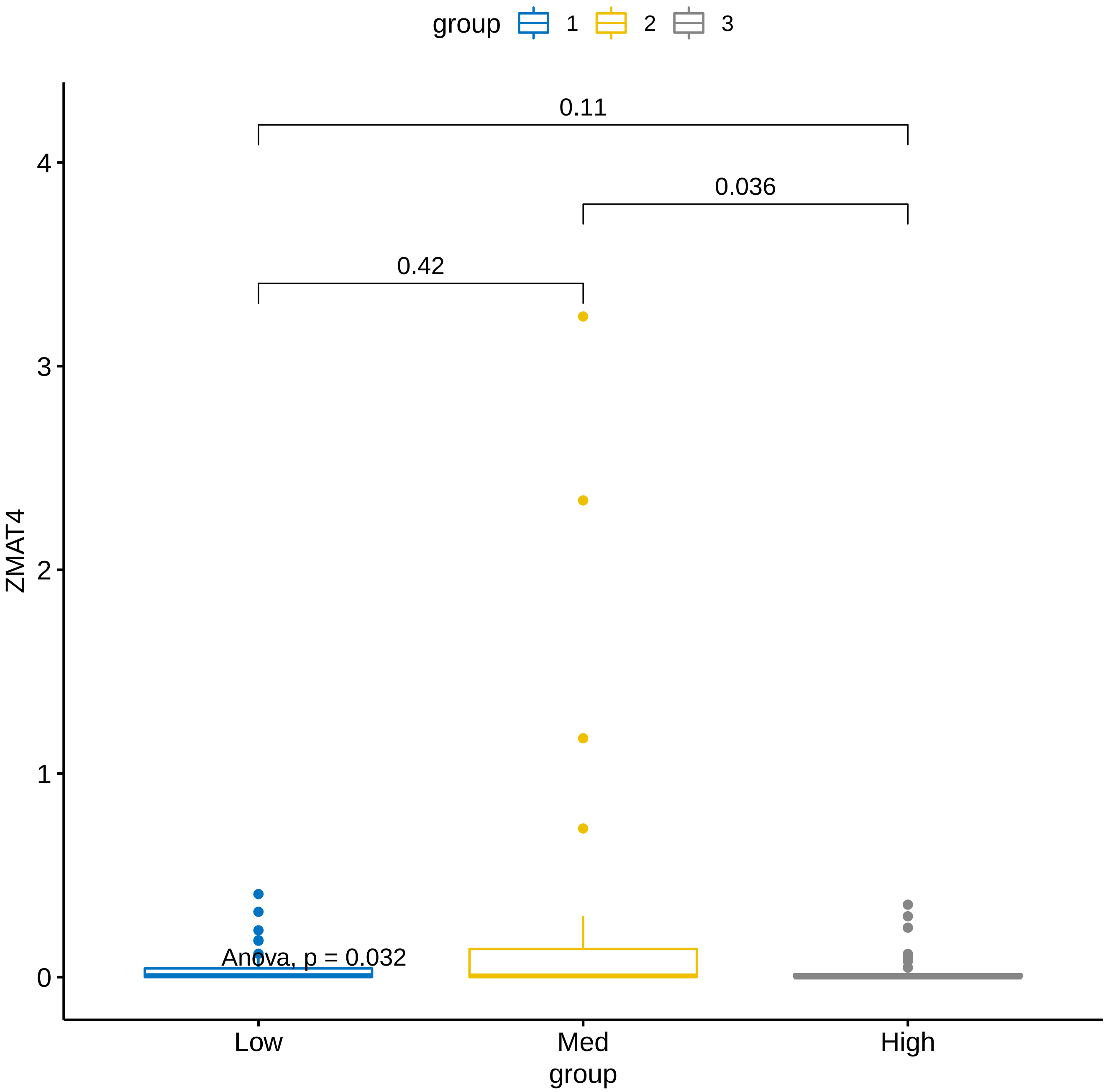
group 1 2 3
0.11
0.036
0.42
4
3
2
1
0
ZMAT4
Anova, p = 0.032
Low
Med
High
group

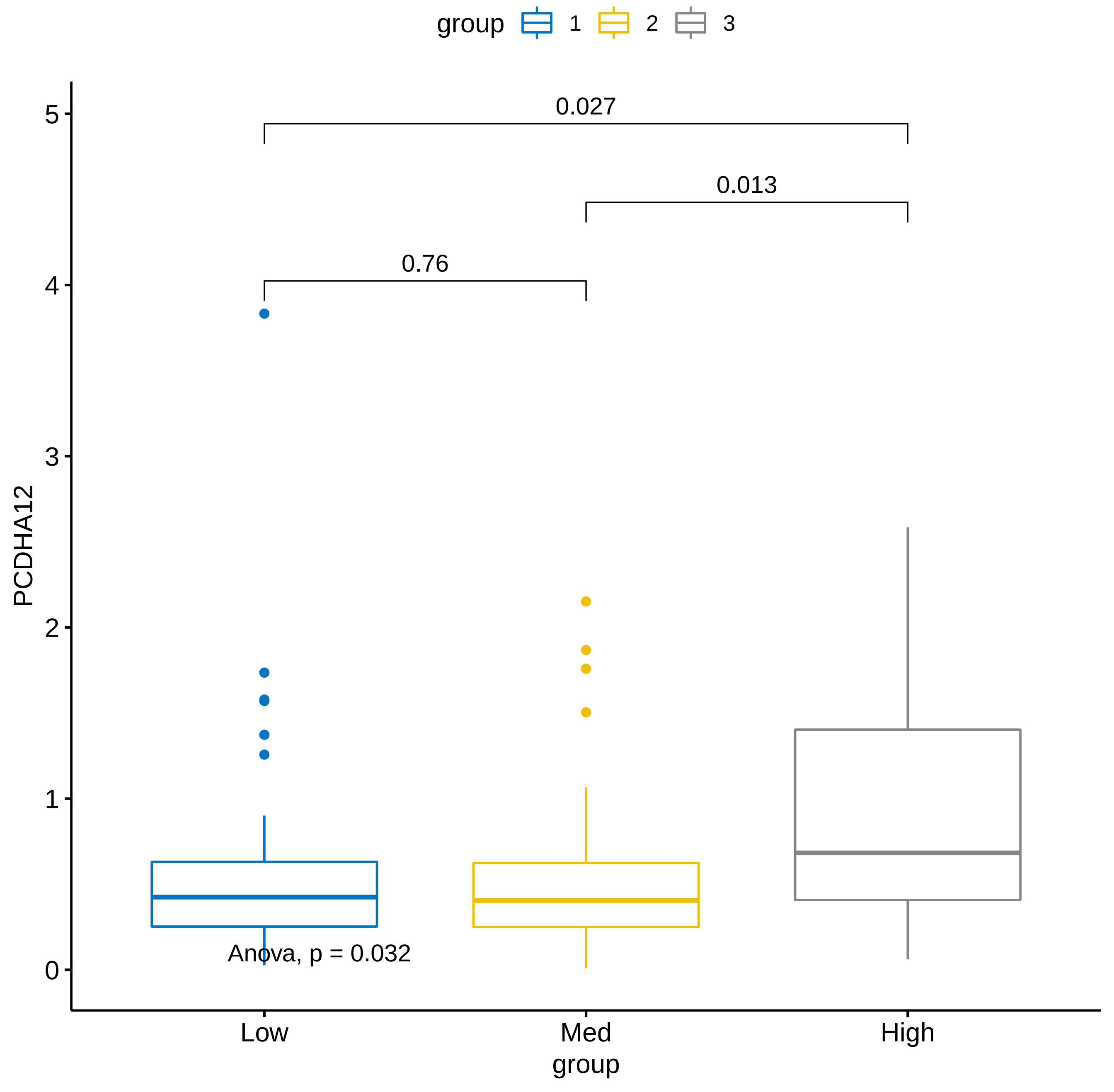
group 1 2 3
0.027
0.013
0.76
PCDHA12
5
4
3
2
1
0
Anova, p = 0.032
Low
Med
High
group

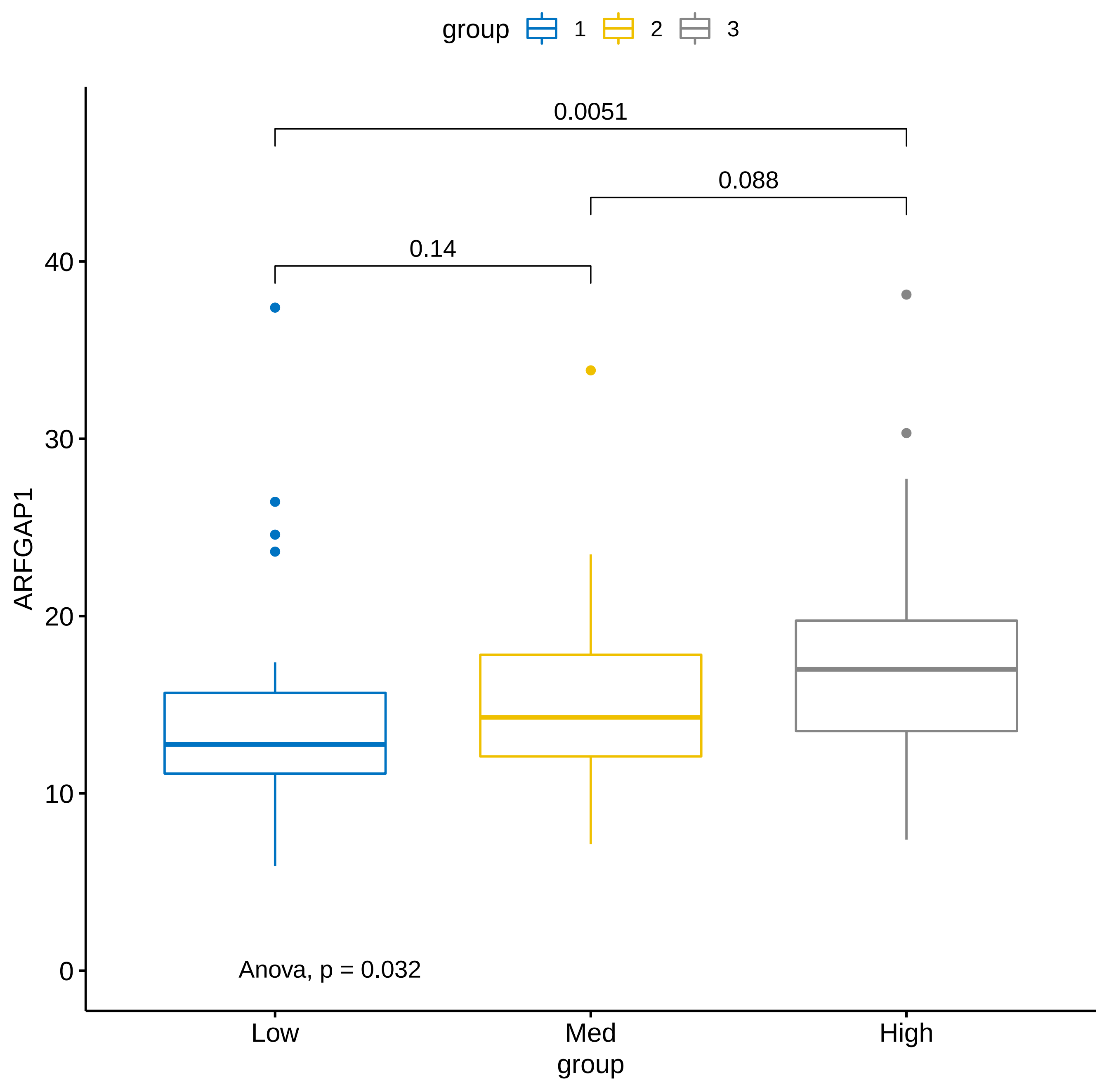
group
1
2
3
0.0051
0.088
0.14
40
30
20
10
0
ARFGAP1
Anova, p = 0.032
Low
Med
High
group

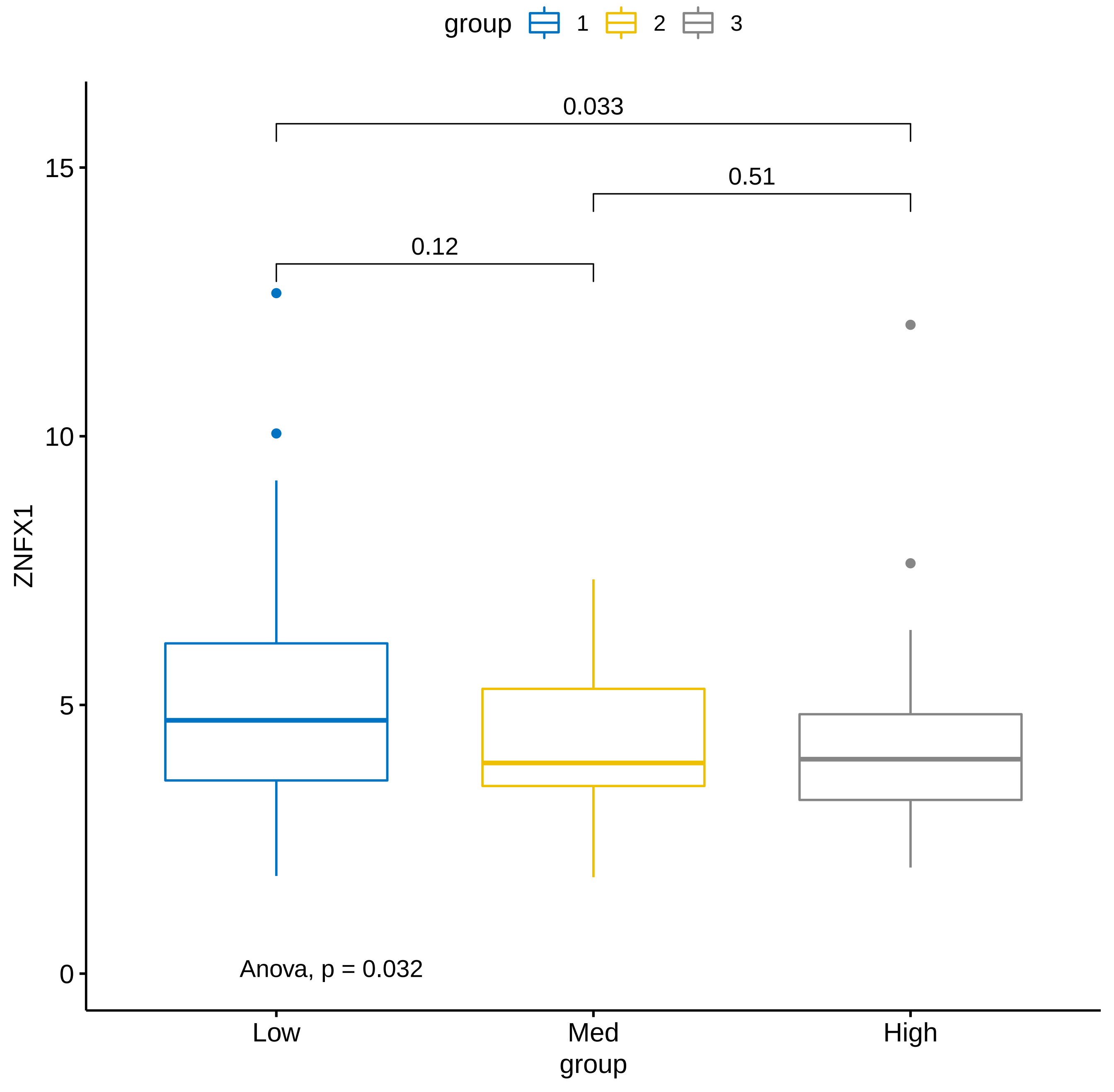
group 1 2 3
0.033
0.51
0.12
ZNFX1
15
10
5
0
Anova, p = 0.032
Low
Med
High
group

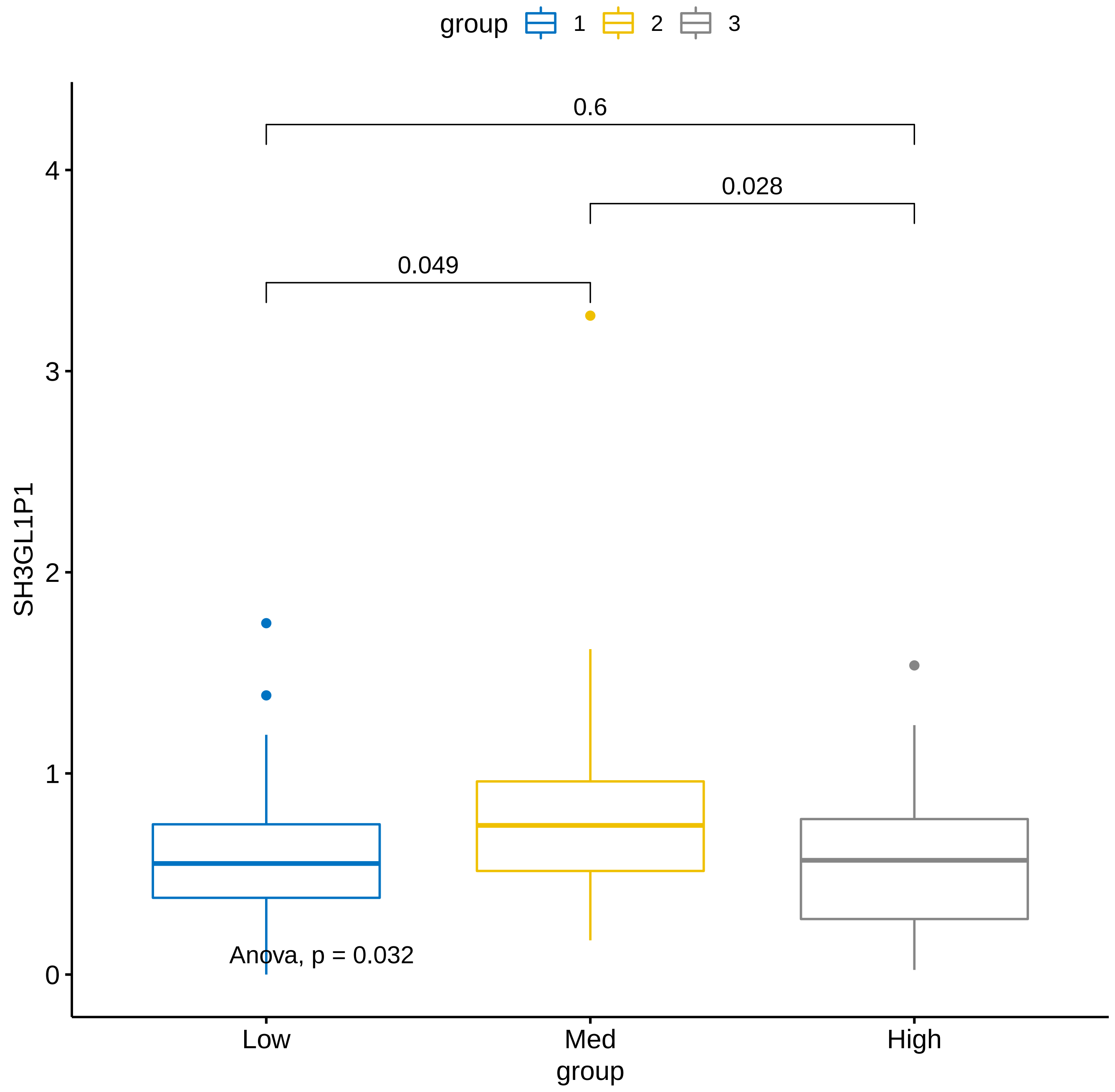
group 1 2 3
0.6
0.028
0.049
Anova, p = 0.032
SH3GL1P1
0
1
2
3
4
Low
Med
High
group

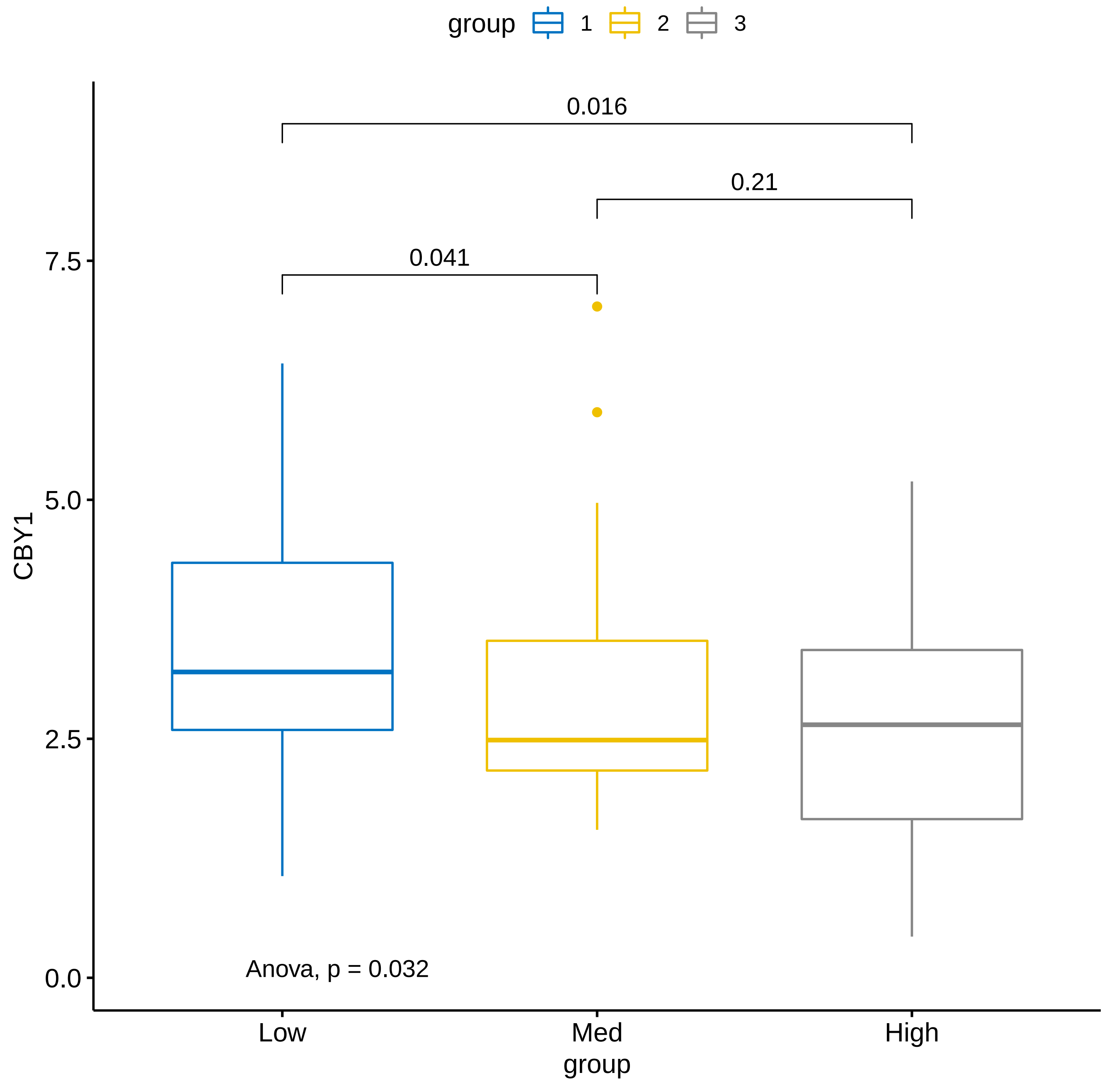
group
1
2
3
0.016
0.21
0.041
7.5
5.0
2.5
0.0
CBY1
Anova, p = 0.032
Low
Med
High
group

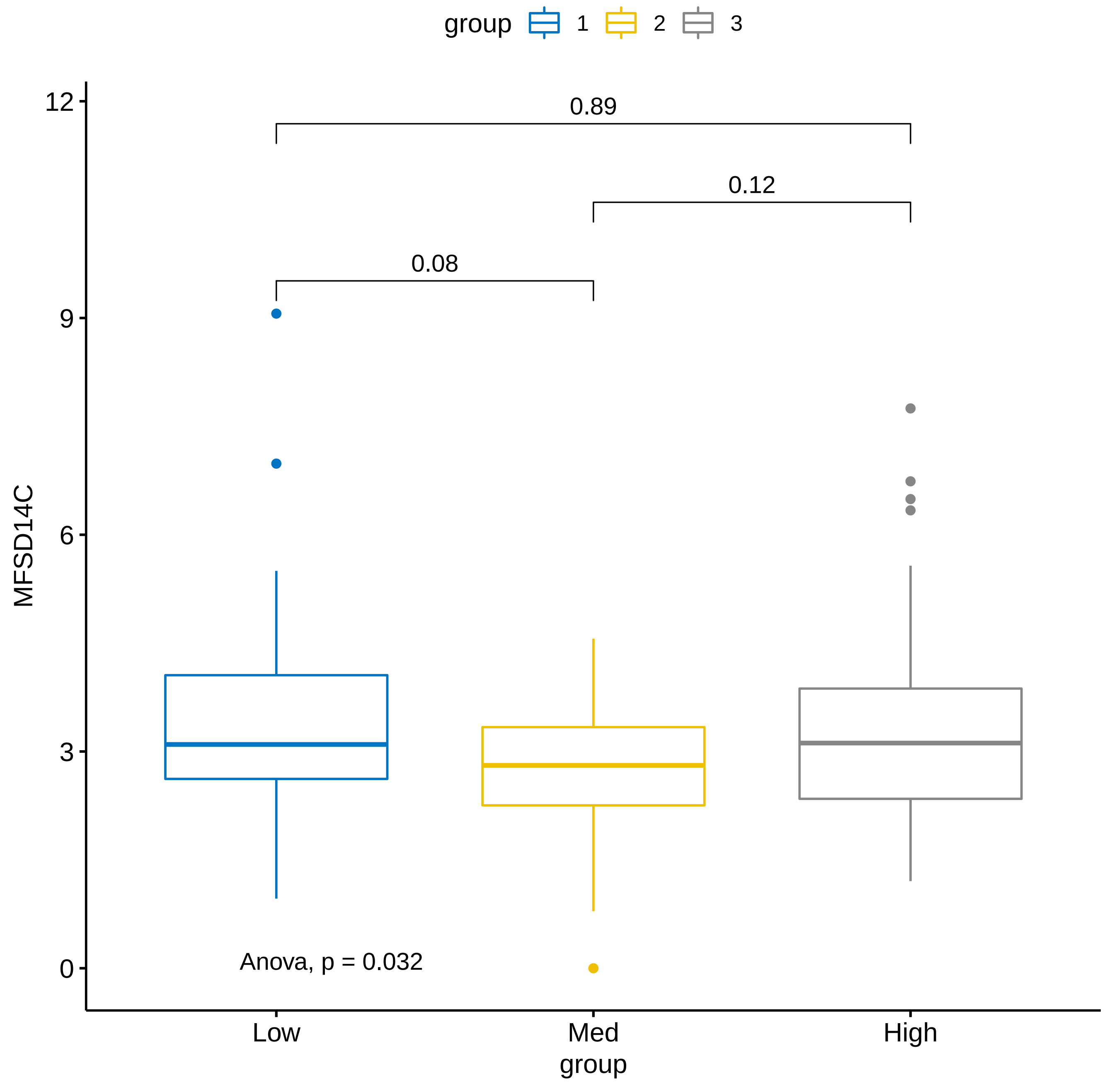
group
1
2
3
0.89
0.12
0.08
12
9
6
3
0
MFSD14C
Anova, p = 0.032
Low
Med
High
group

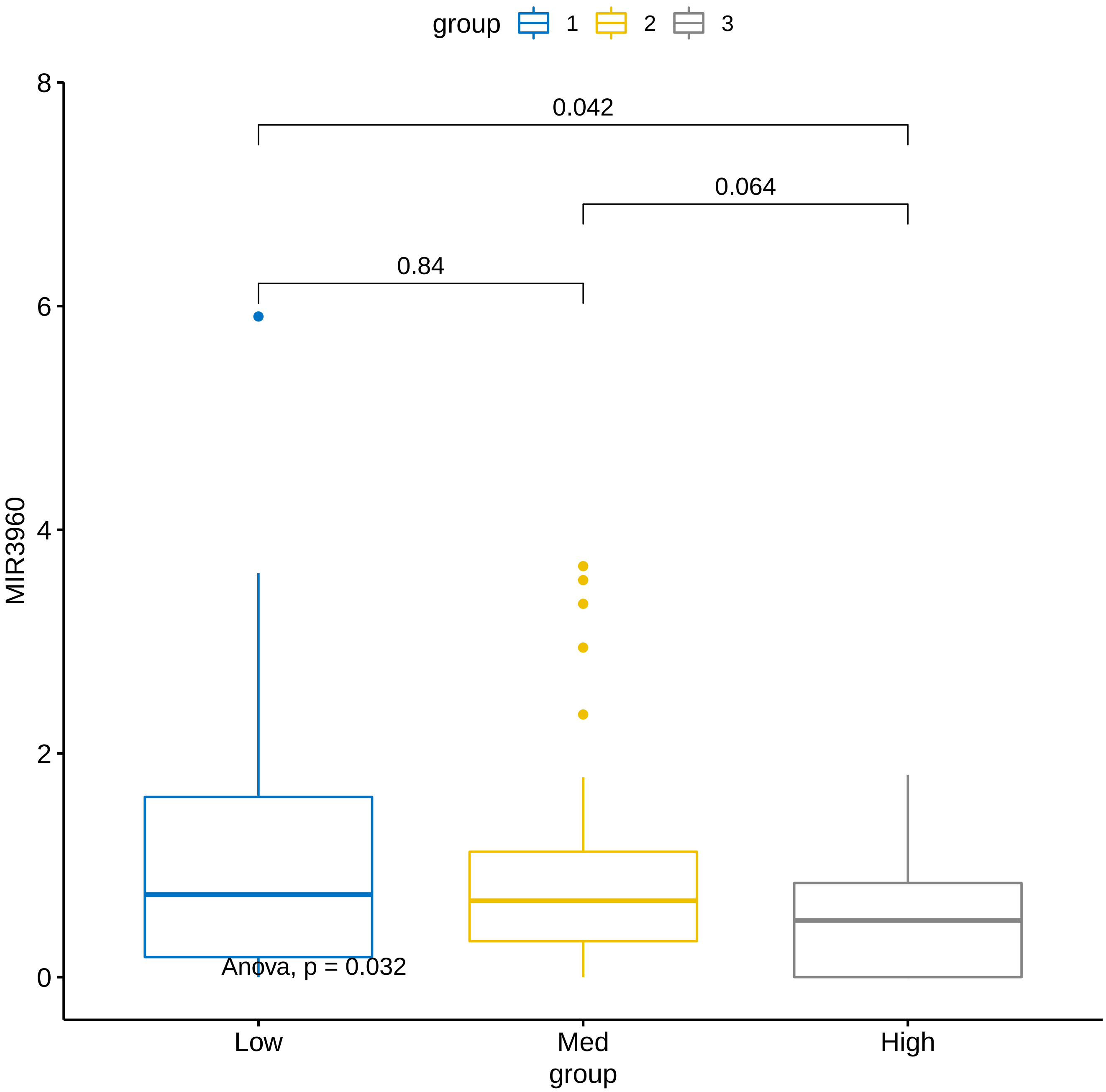
group
1
2
3
0.042
0.064
0.84
8
6
4
2
0
MIR3960
Anova, p = 0.032
Low
Med
High
group

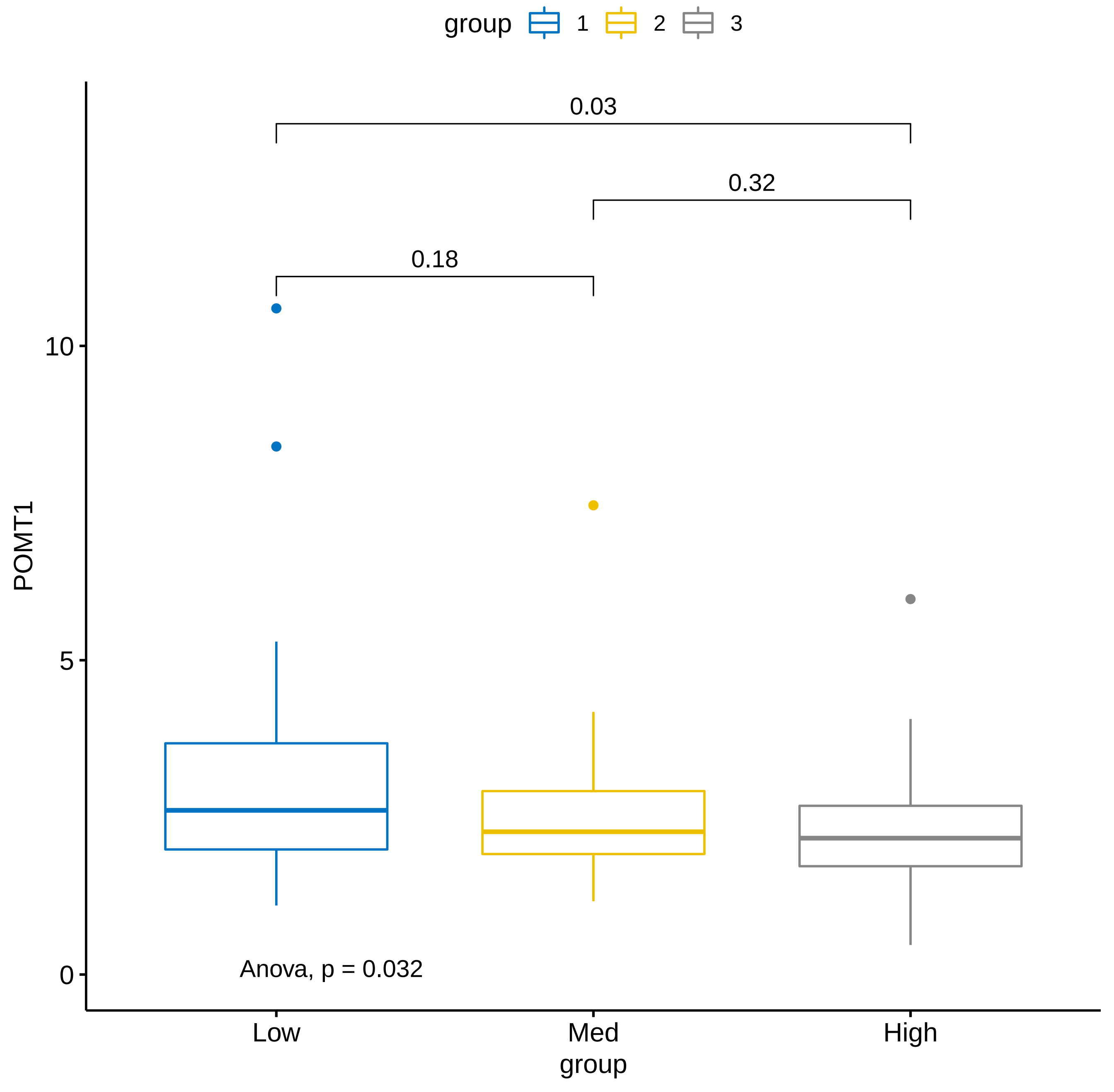

group
1
2
3
0.03
0.32
0.18
10
5
0
POMT1
Anova, p = 0.032
Low
Med
High
group

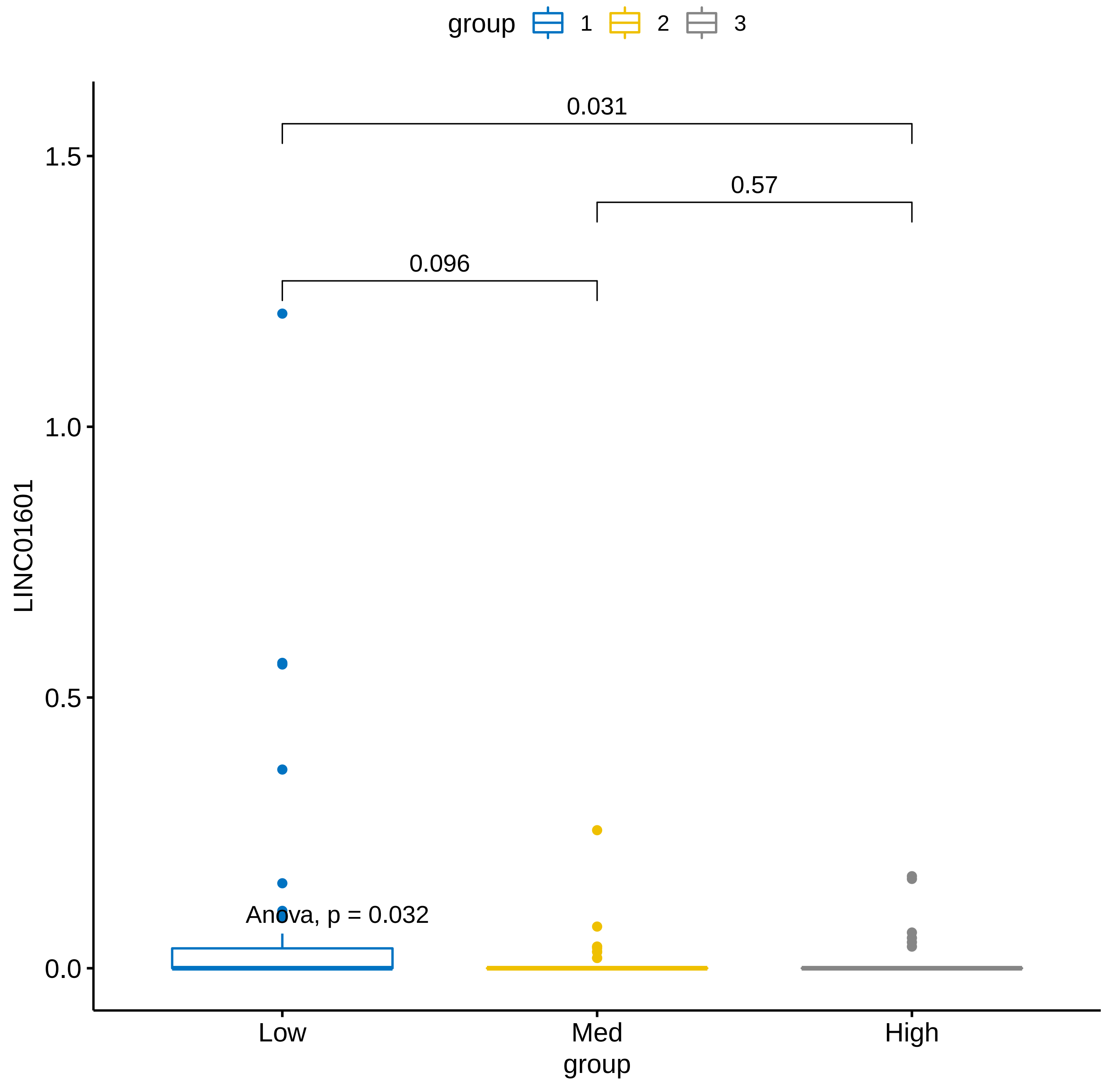

group
1
2
3
0.031
0.57
0.096
Anova, p = 0.032
1.5
1.0
0.5
0.0
LINC01601
Low
Med
High
group

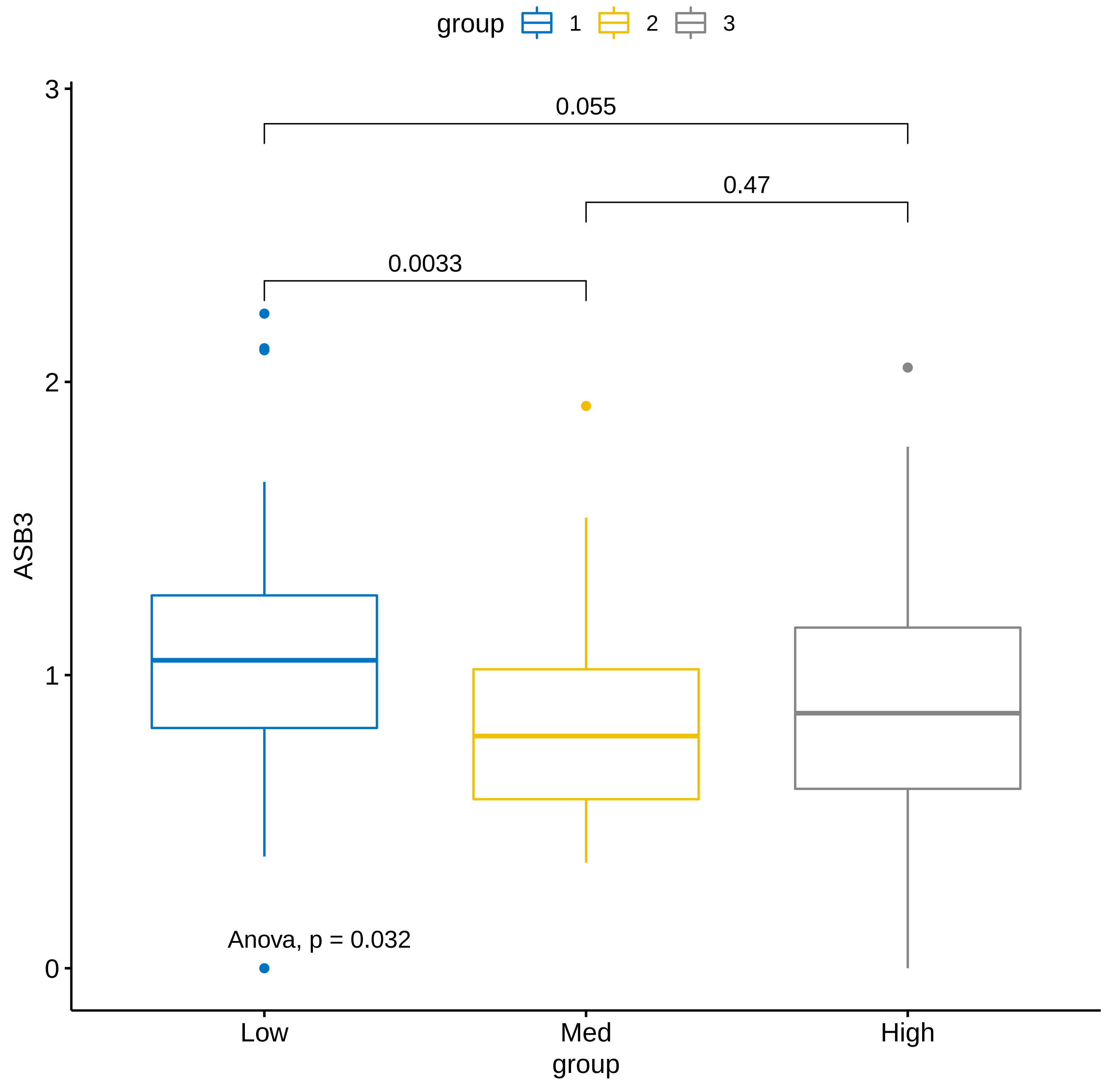
group
1
2
3
ASB3
0.055
0.47
0.0033
Anova, p = 0.032
0
1
2
3
Low
Med
High
group

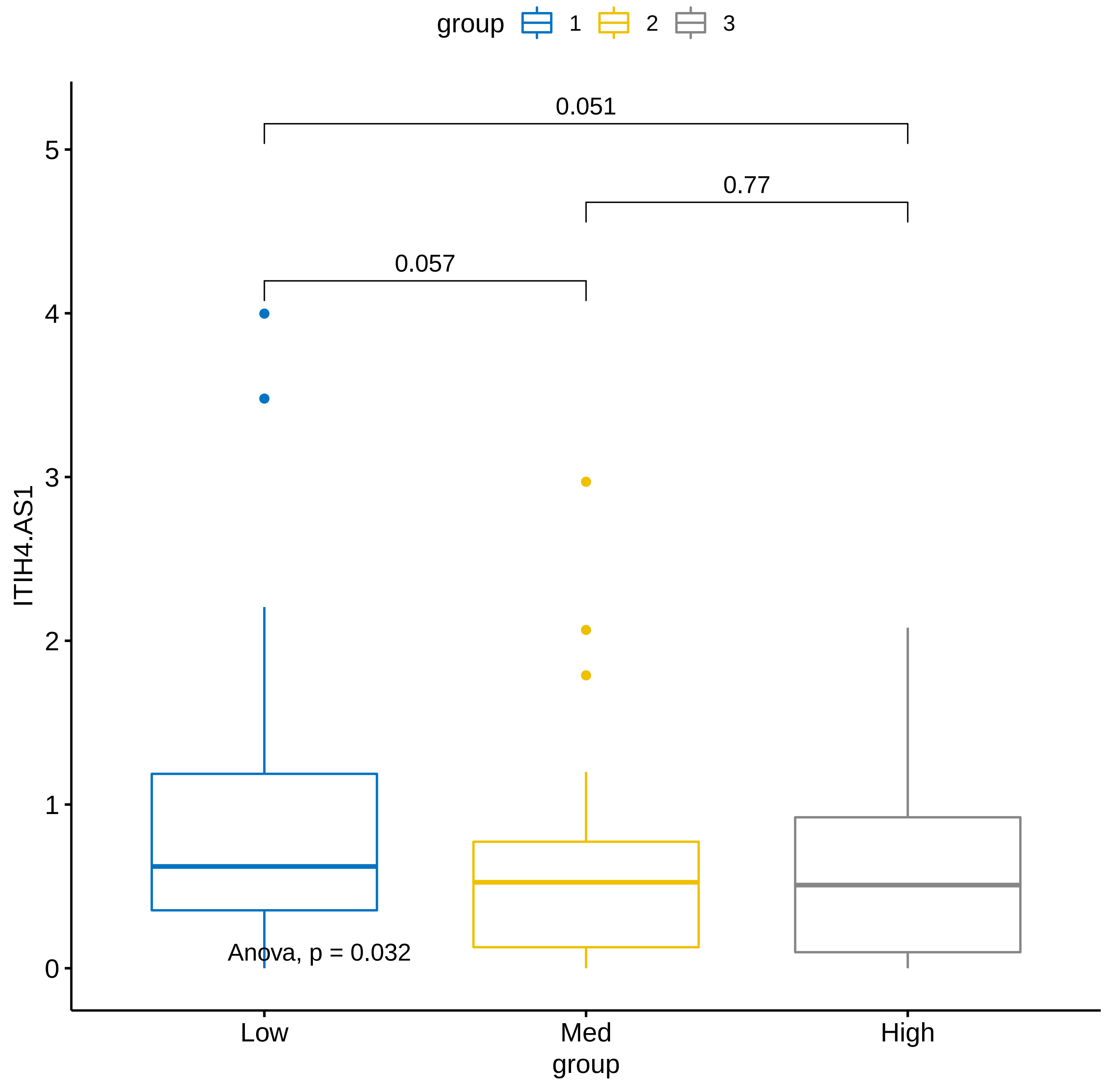

group
1
2
3
0.051
0.77
0.057
ITIH4.AS1
5
4
3
2
1
0
Anova, p = 0.032
Low
Med
High
group

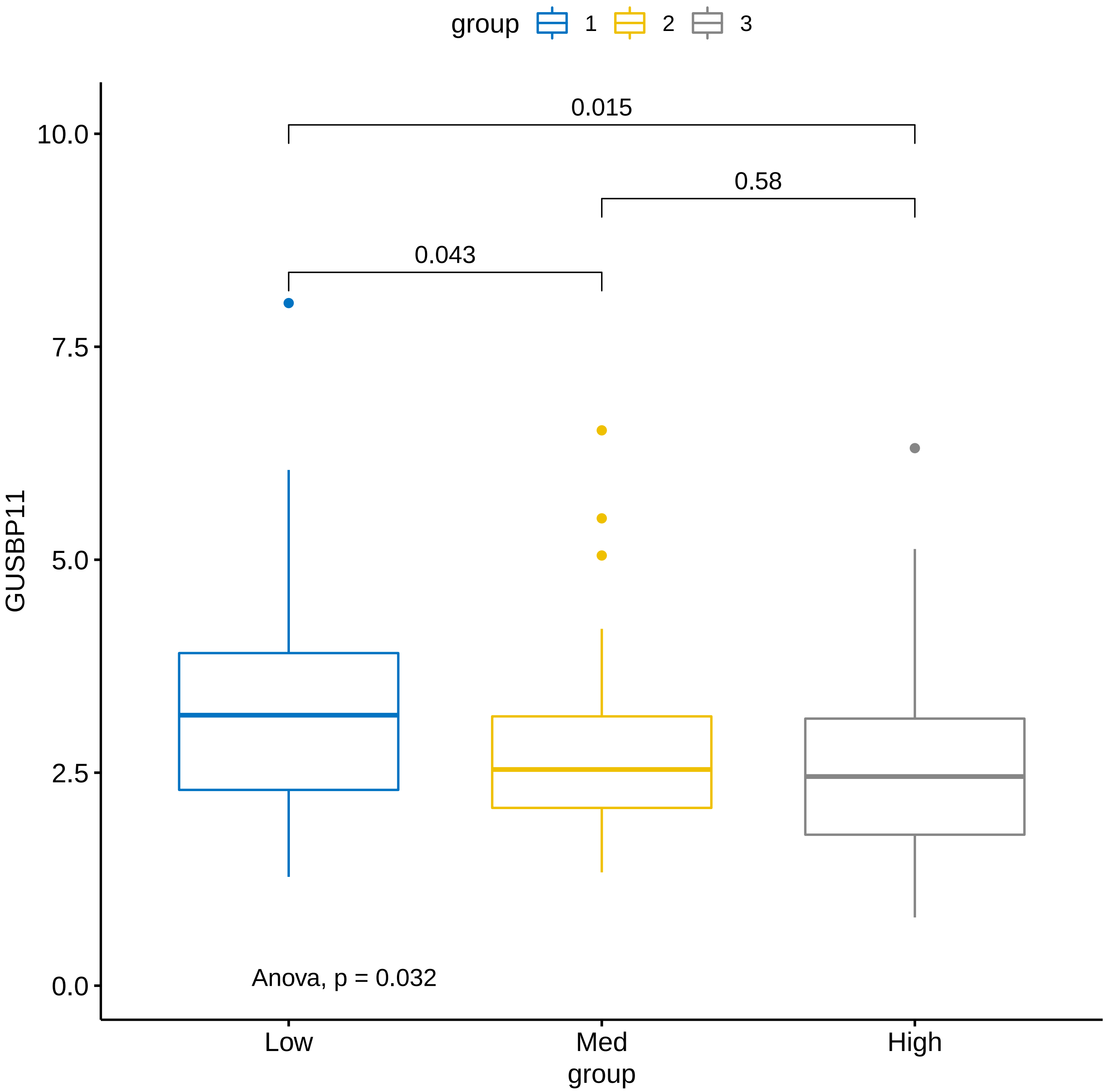
group 1 2 3
0.015
0.58
0.043
10.0
7.5
5.0
2.5
0.0
GUSBP11
Anova, p = 0.032
Low
Med
High
group

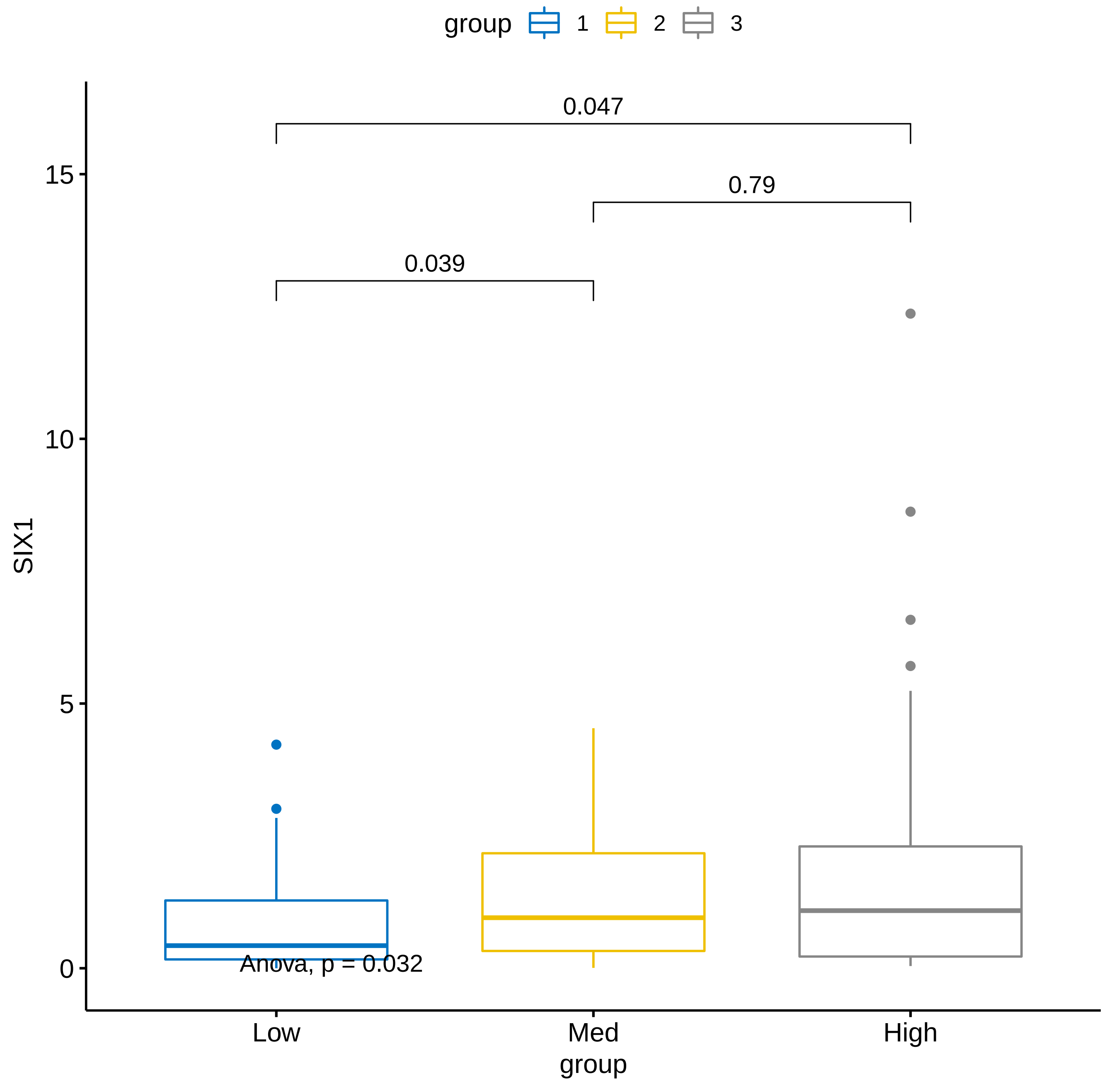

group 1 2 3
0.047
0.79
0.039
15
10
5
0
SIX1
Anova, p = 0.032
Low
Med
High
group

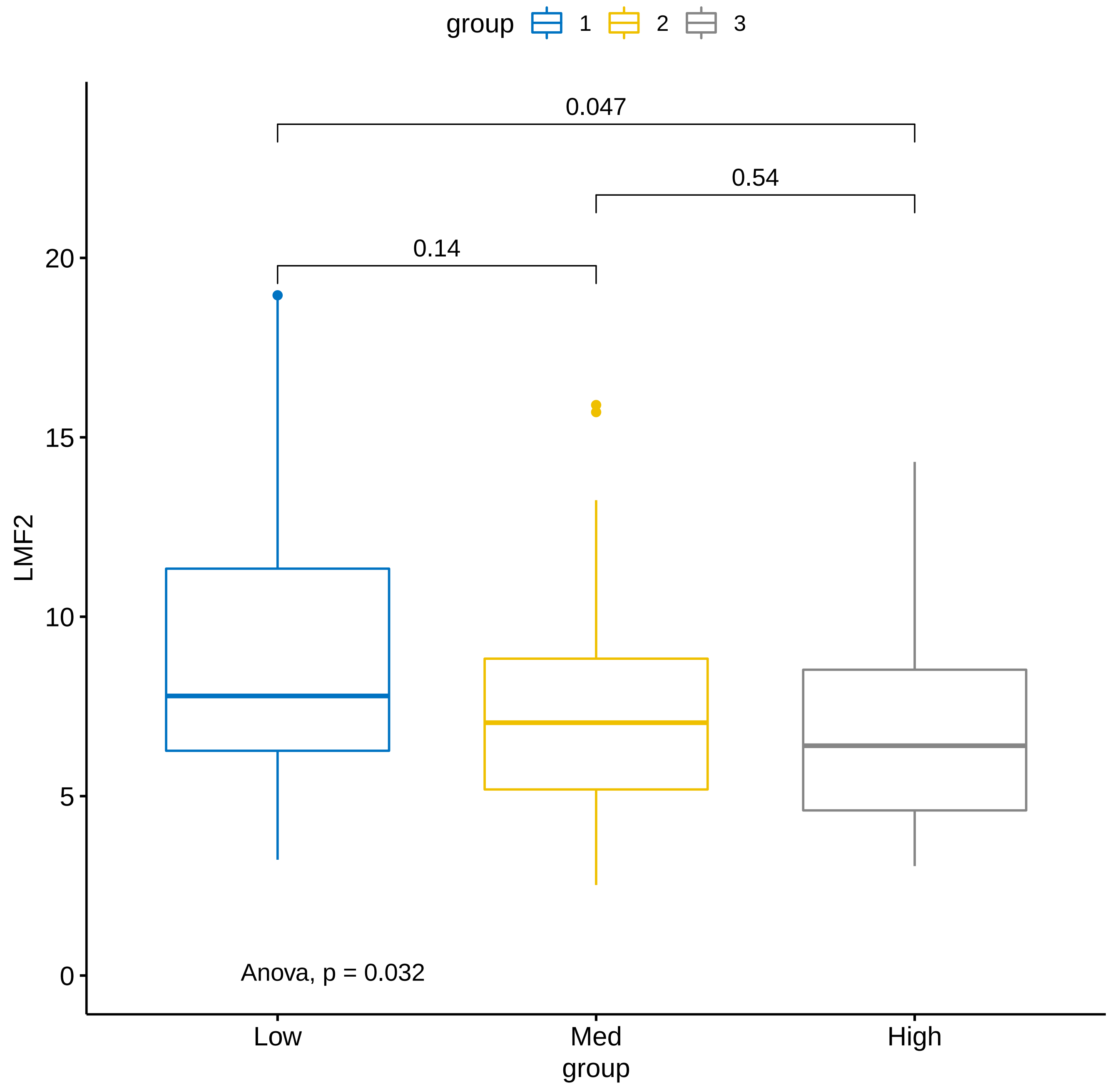

group
1
2
3
0.047
0.54
0.14
20
15
10
5
0
LMF2
Anova, p = 0.032
Low
Med
High
group

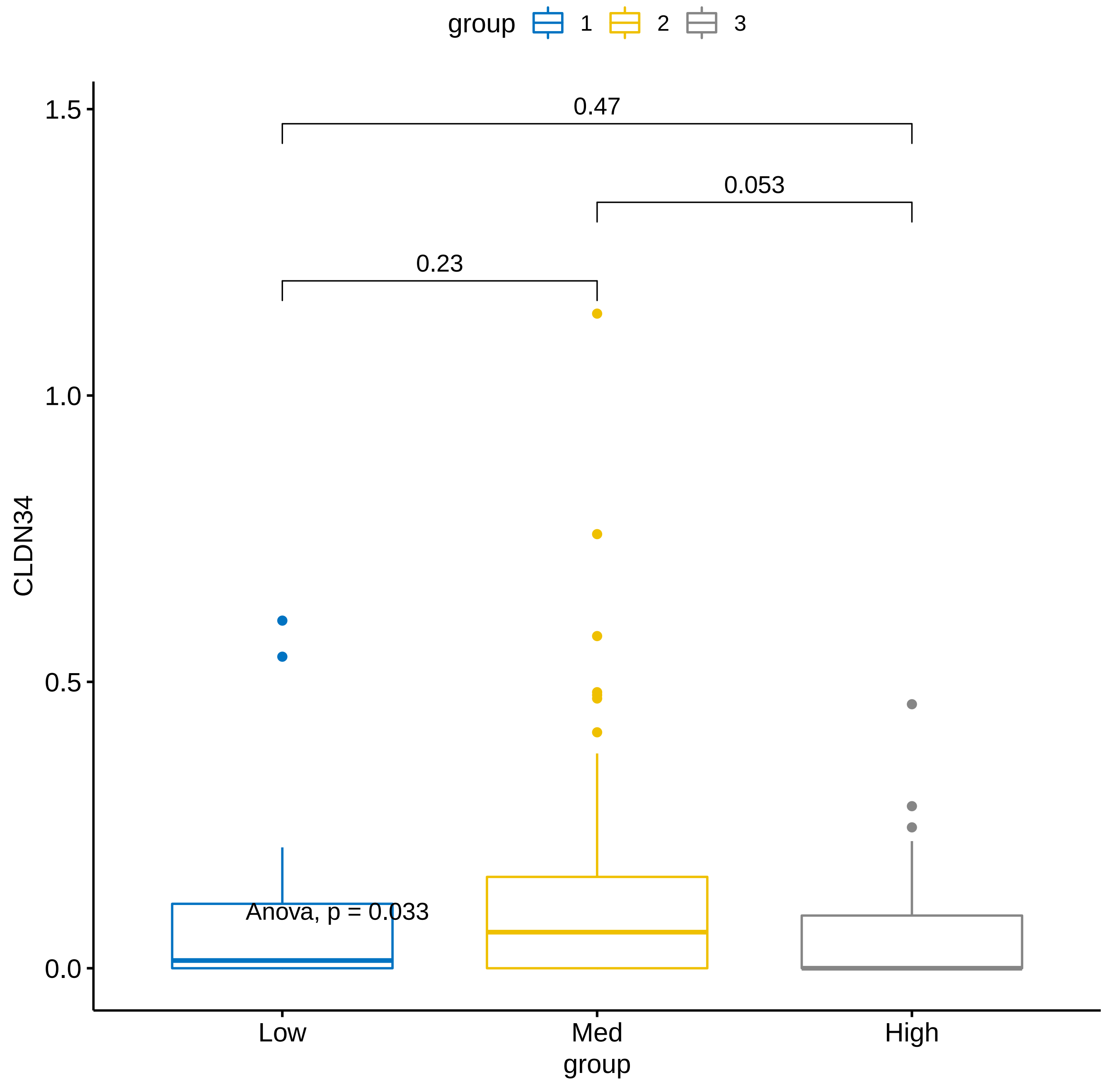

group
1
2
3
0.47
0.053
0.23
1.5
1.0
0.5
0.0
CLDN34
Anova, p = 0.033
Low
Med
High
group

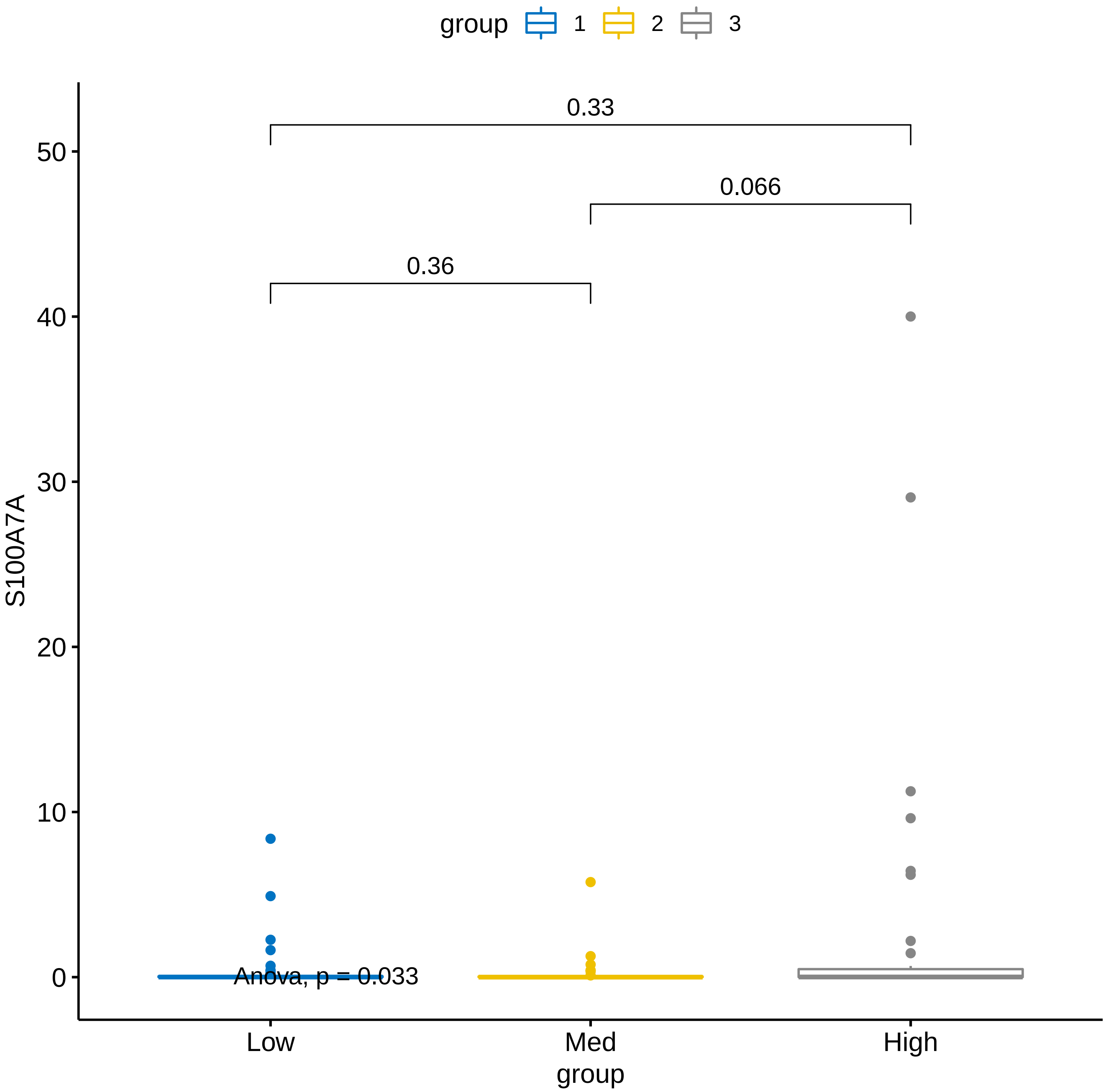

group
1
2
3
0.33
0.066
0.36
S100A7A
50
40
30
20
10
0
Anova, p = 0.033
Low
Med
High
group

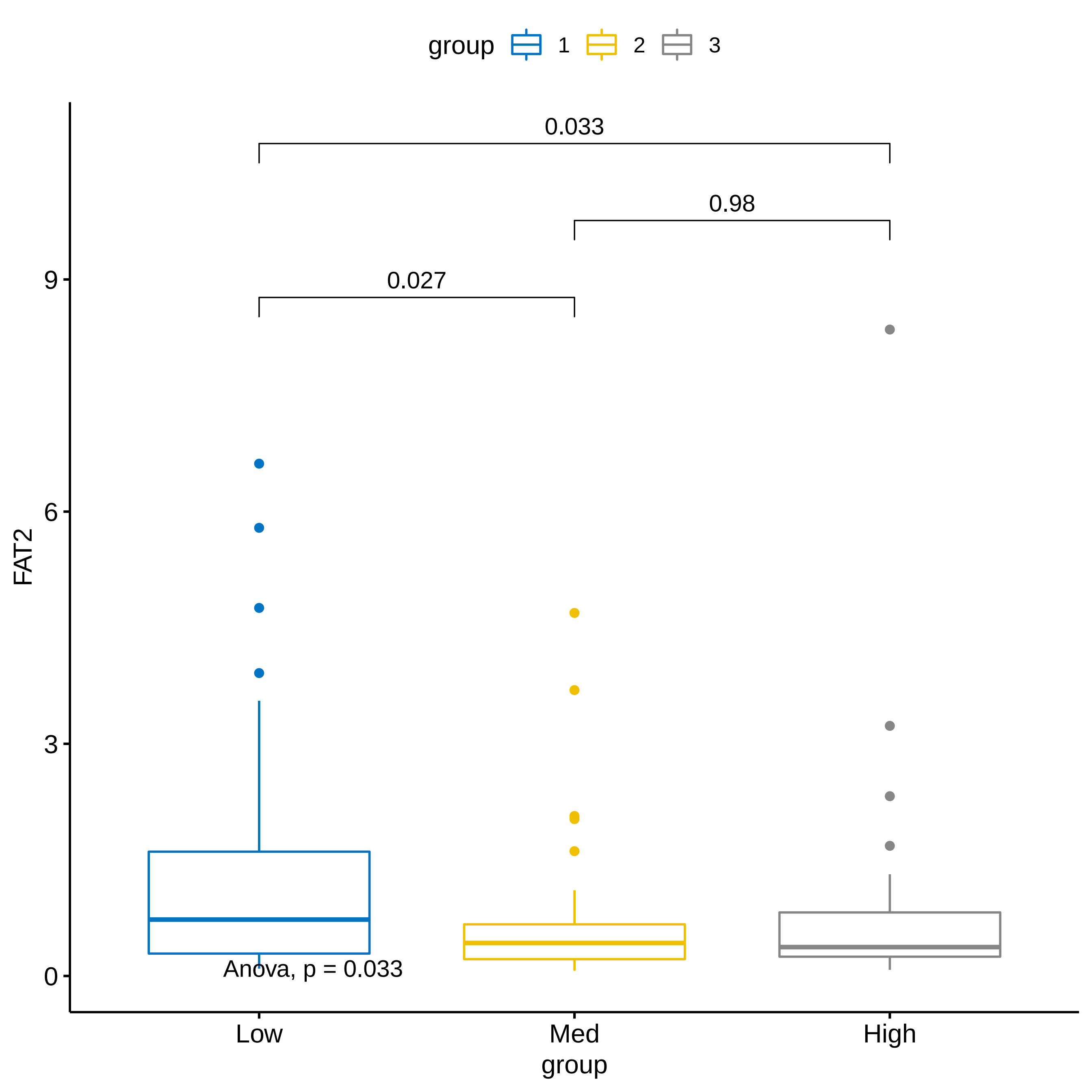
group
1
2
3
0.033
0.98
0.027
9
6
3
0
FAT2
Anova, p = 0.033
Low
Med
High
group

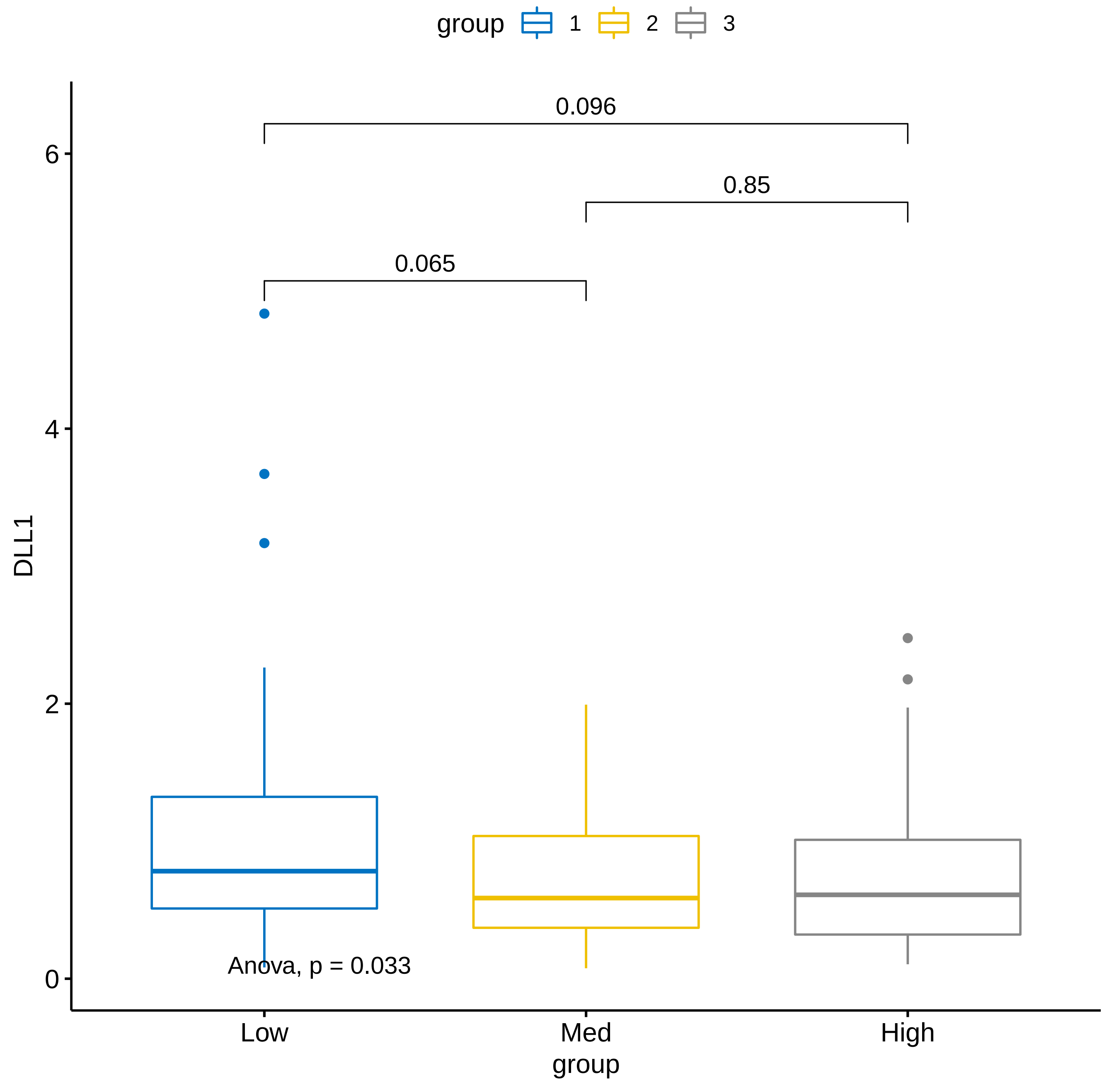
group 1 2 3
0.096
0.85
0.065
DLL1
6
4
2
0
Anova, p = 0.033
Low
Med
High
group

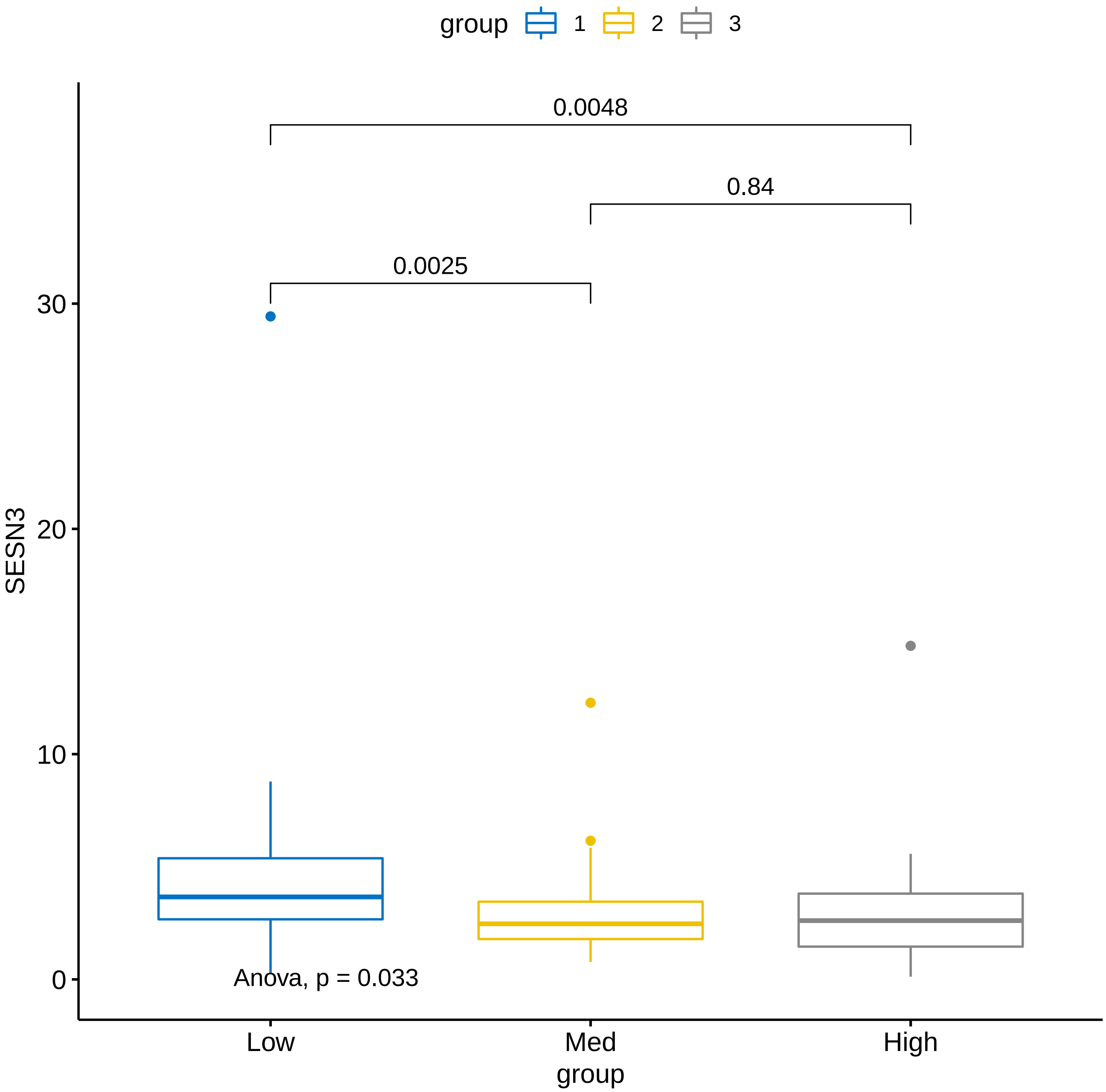
group
1
2
3
0.0048
0.84
0.0025
30
20
10
0
SESN3
Anova, p = 0.033
Low
Med
High
group

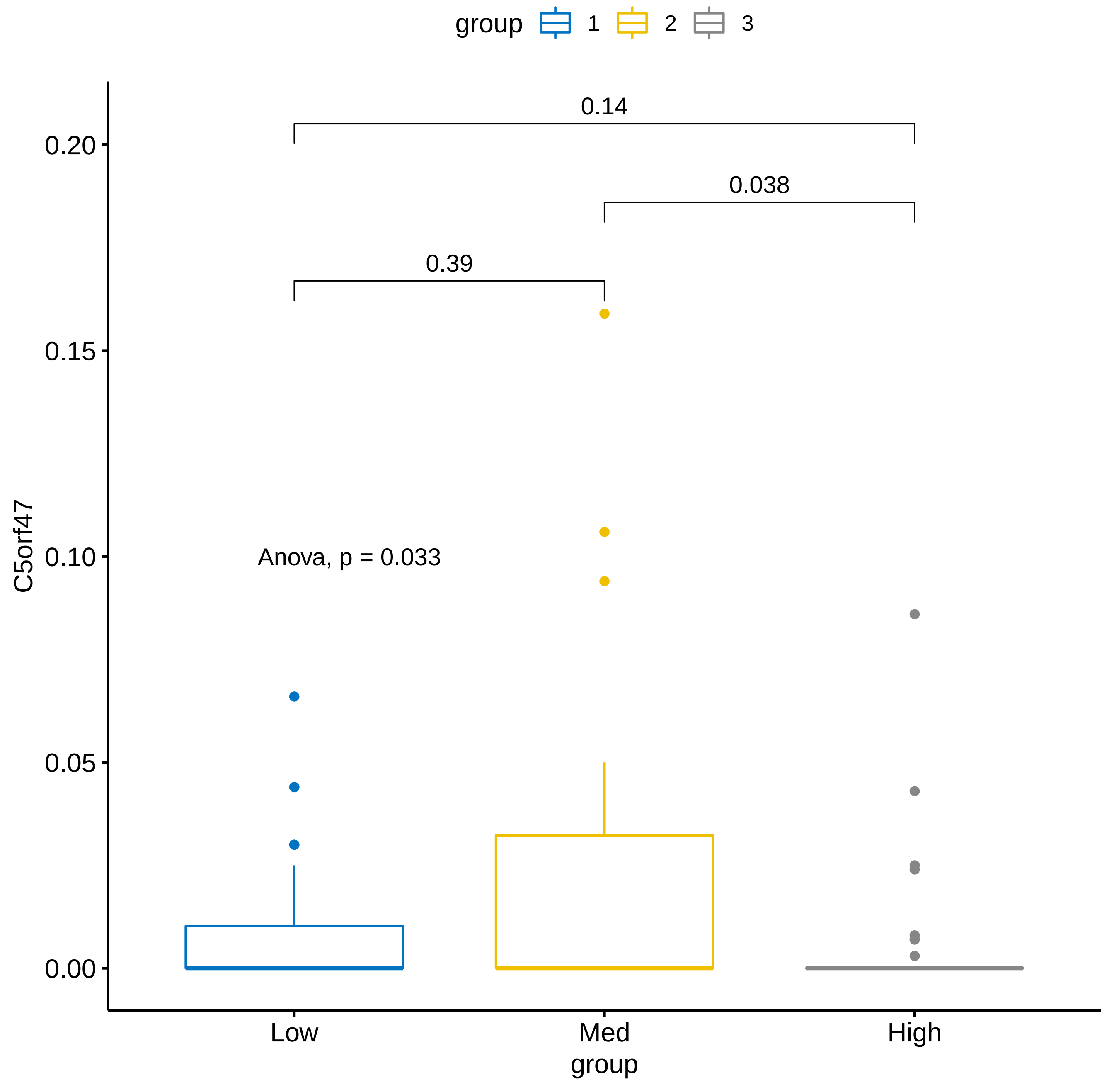

group
1
2
3
0.14
0.038
0.39
Anova, p = 0.033
C5orf47
0.20
0.15
0.10
0.05
0.00
Low
Med
High
group

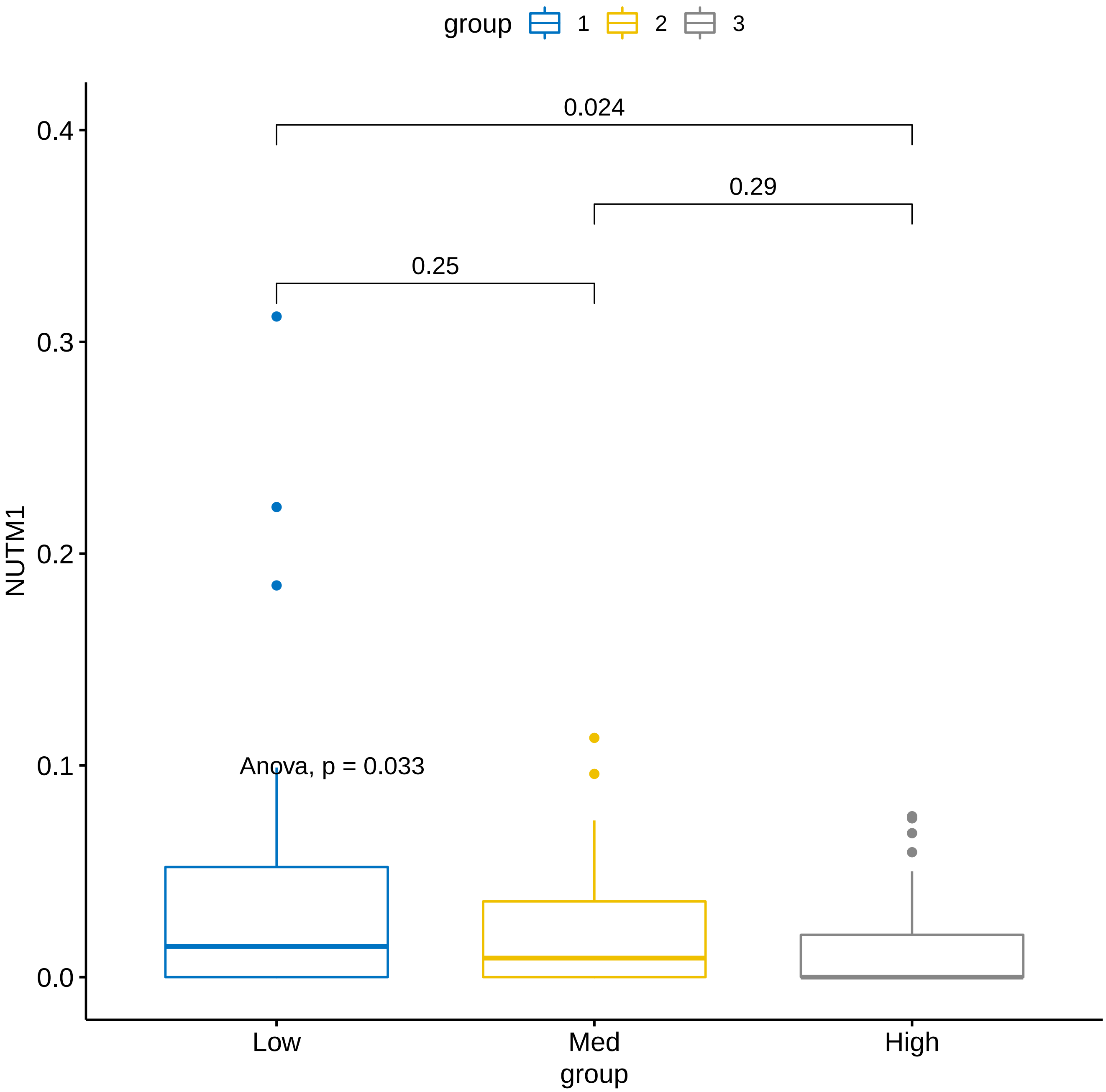

group
1
2
3
0.024
0.29
0.25
Anova, p = 0.033
NUTM1
0.4
0.3
0.2
0.1
0.0
Low
Med
High
group

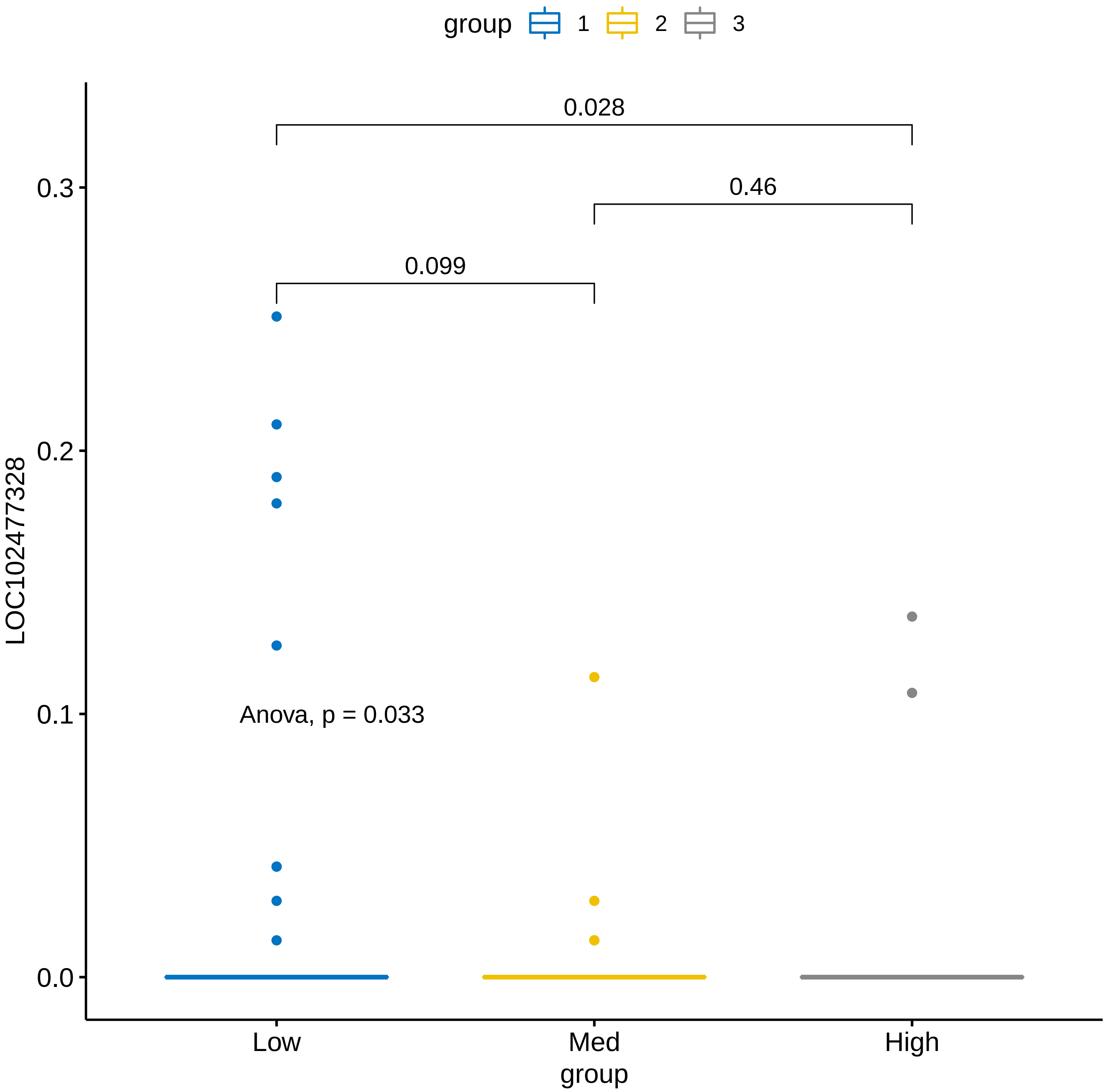
group
1
2
3
0.028
0.46
0.099
0.3
0.2
0.1
0.0
LOC102477328
Anova, p = 0.033
Low
Med
High
group

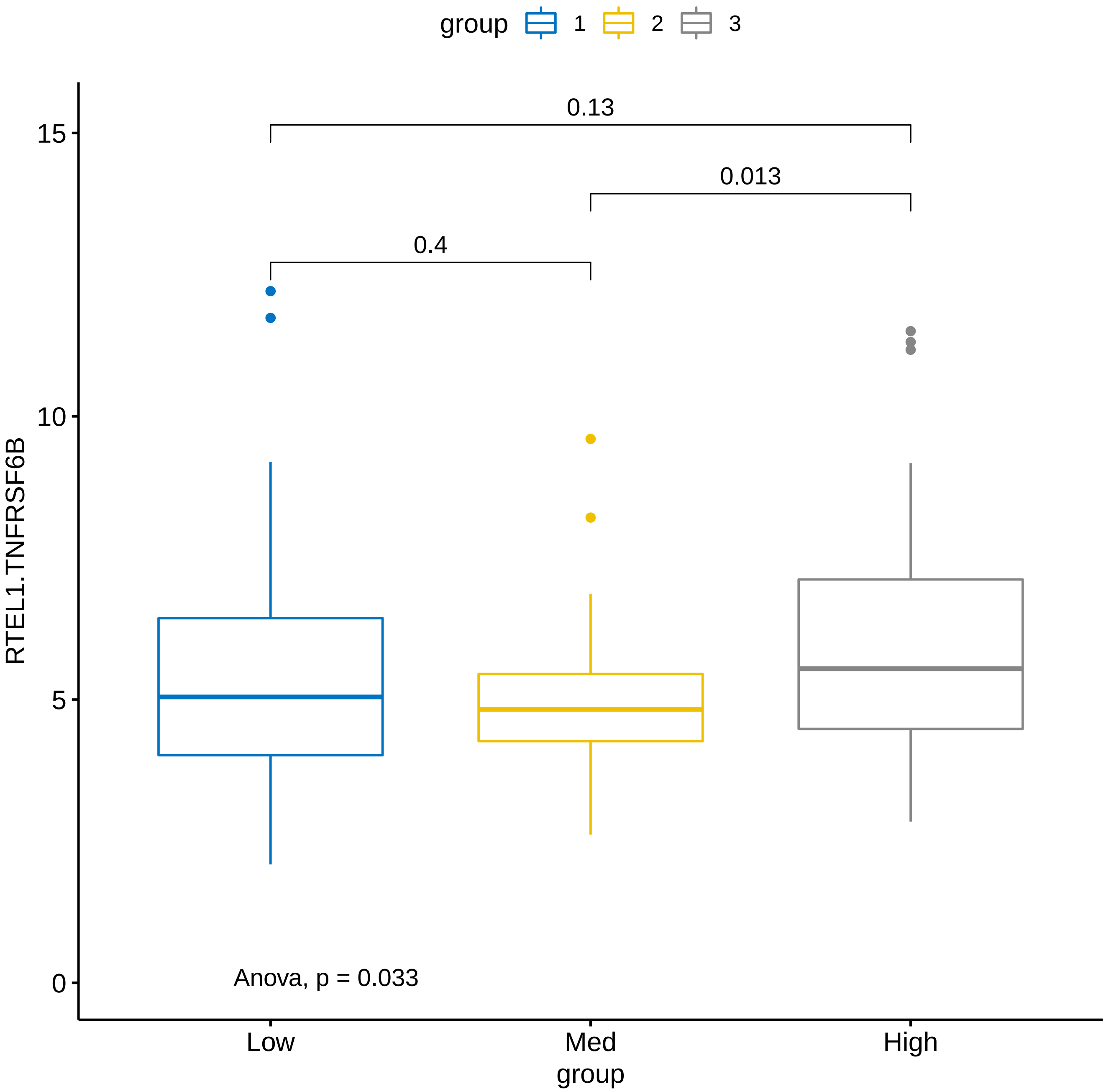
group 1 2 3
0.13
0.013
0.4
15
10
5
0
RTEL1.TNFRSF6B
Anova, p = 0.033
Low
Med
High
group

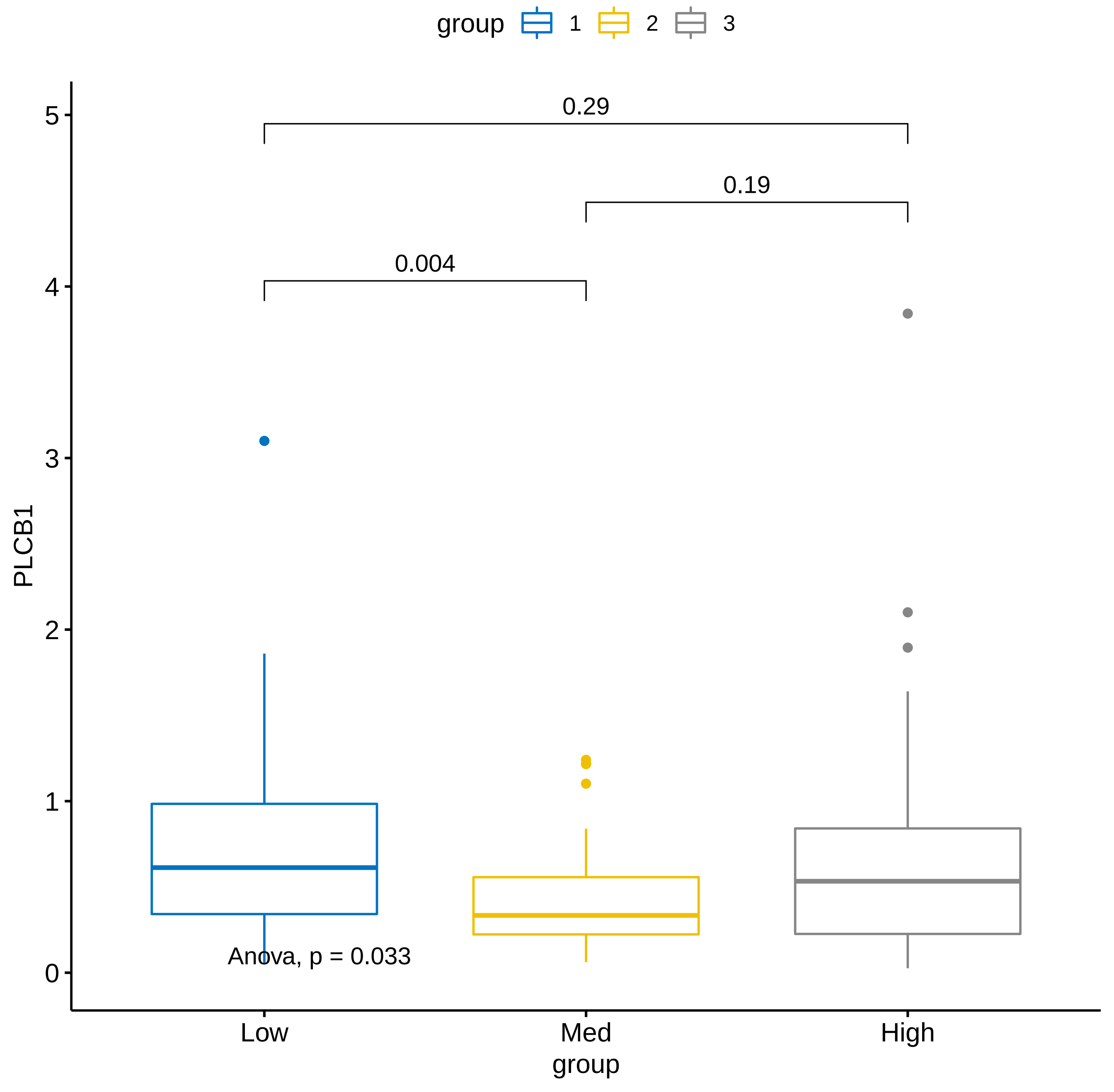
group
1
2
3
0.29
0.19
0.004
5
4
3
2
1
0
PLCB1
Anova, p = 0.033
Low
Med
High
group

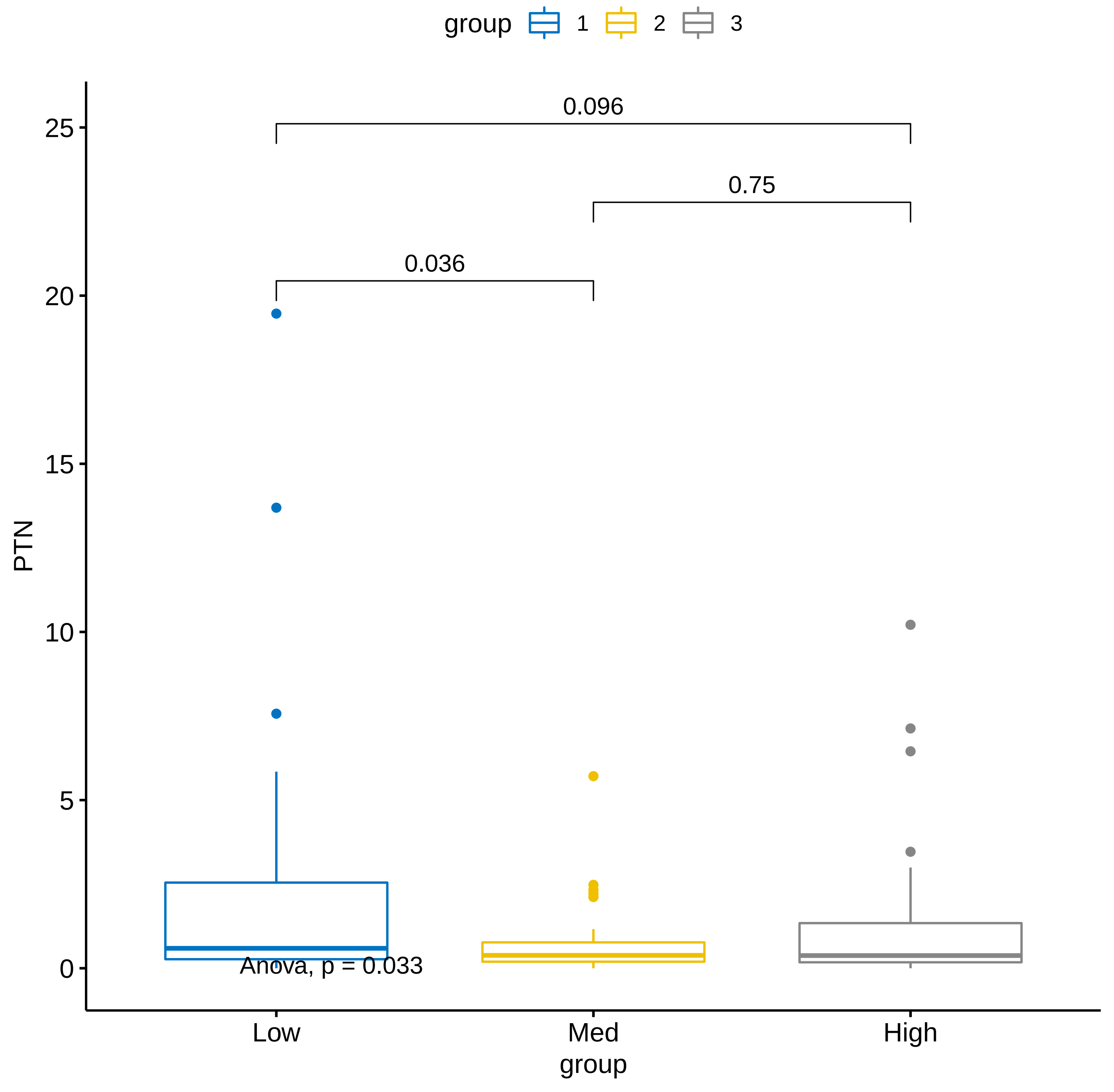
group
1
2
3
0.096
0.75
0.036
25
20
15
10
5
0
PTN
Anova, p = 0.033
Low
Med
High
group

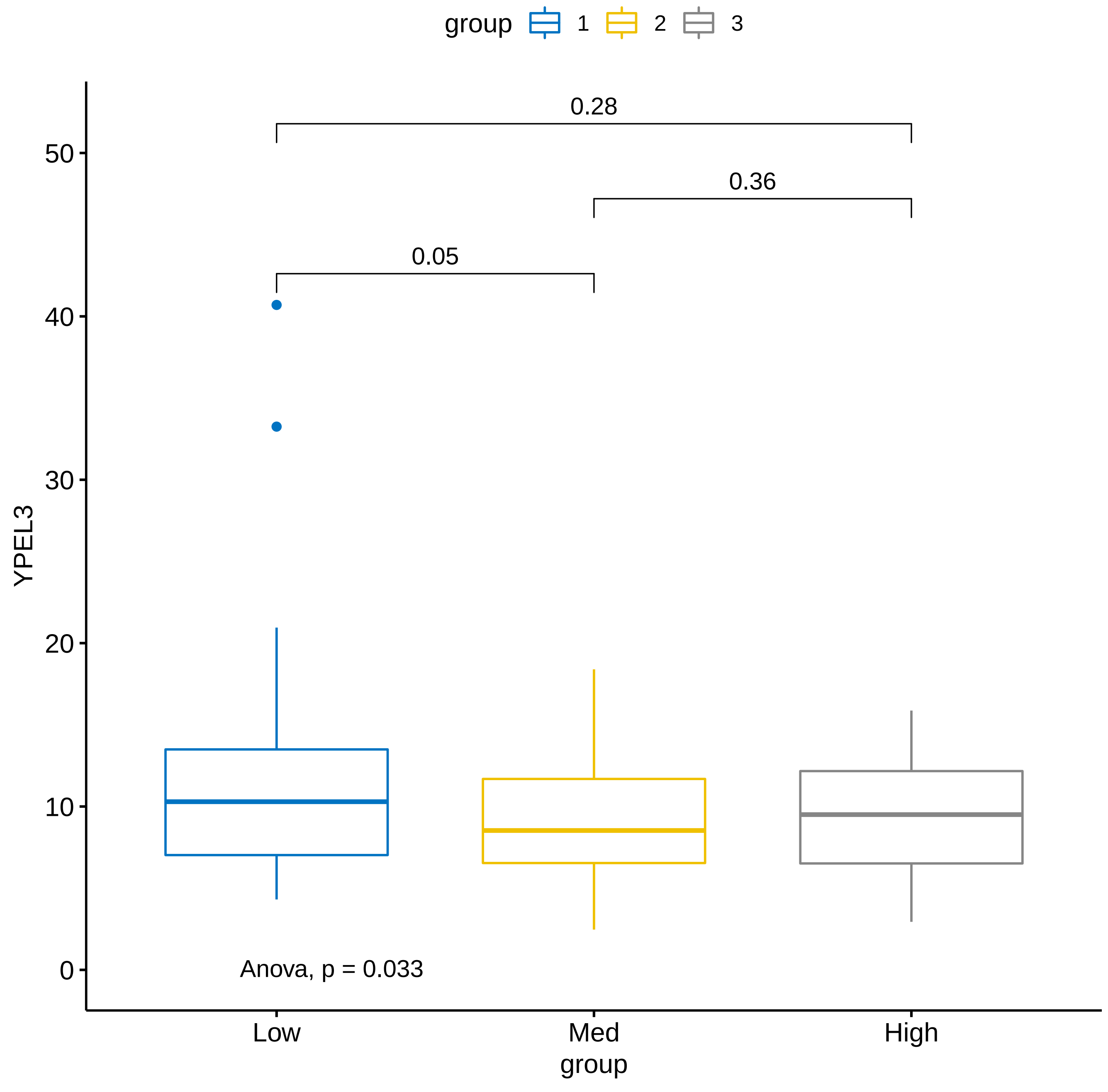

group
1
2
3
0.28
0.36
0.05
50
40
30
20
10
0
YPEL3
Anova, p = 0.033
Low
Med
High
group

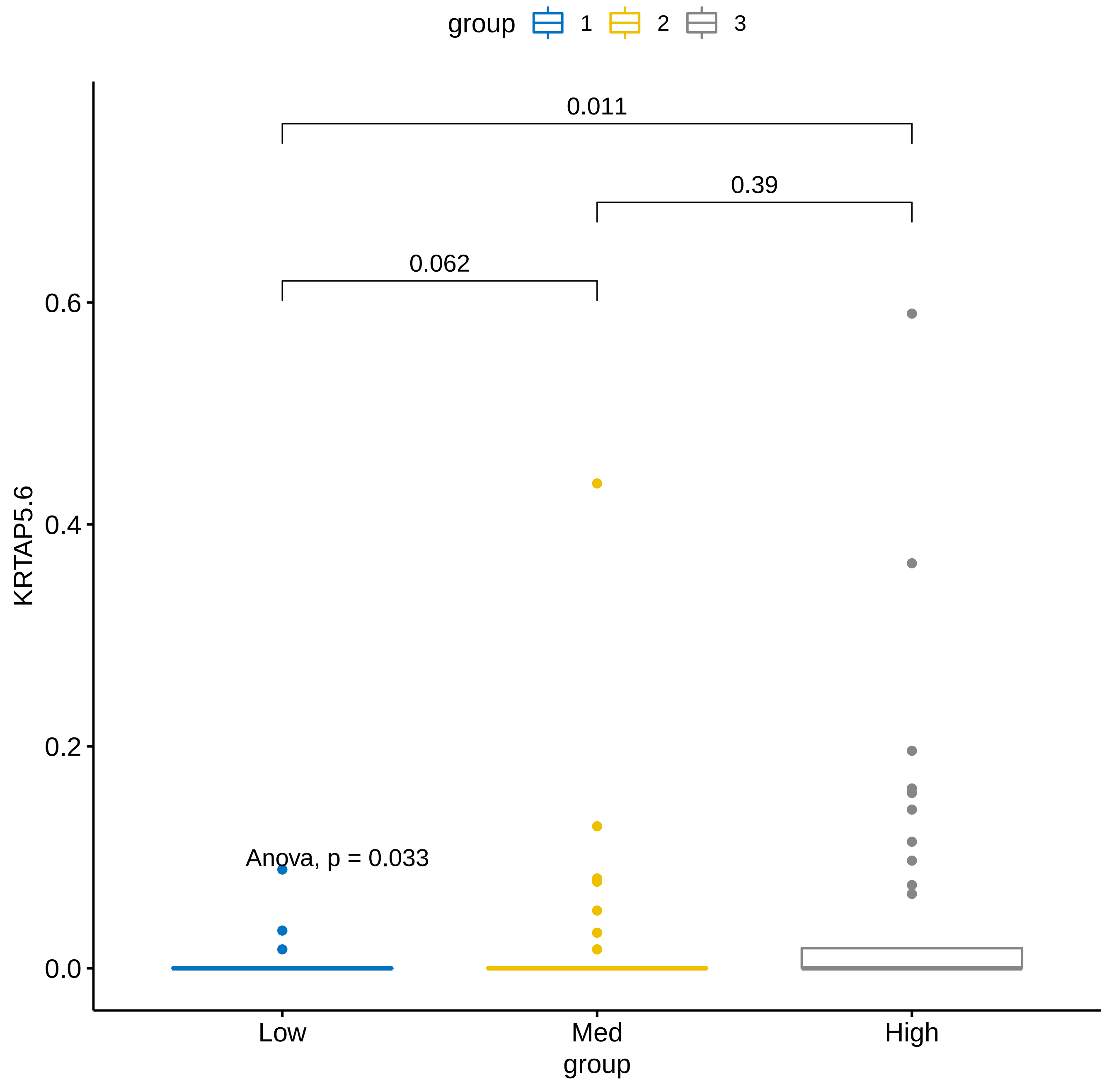
group
1
2
3
0.011
0.39
0.062
0.6
0.4
0.2
0.0
KRTAP5.6
Anova, p = 0.033
Low
Med
High
group

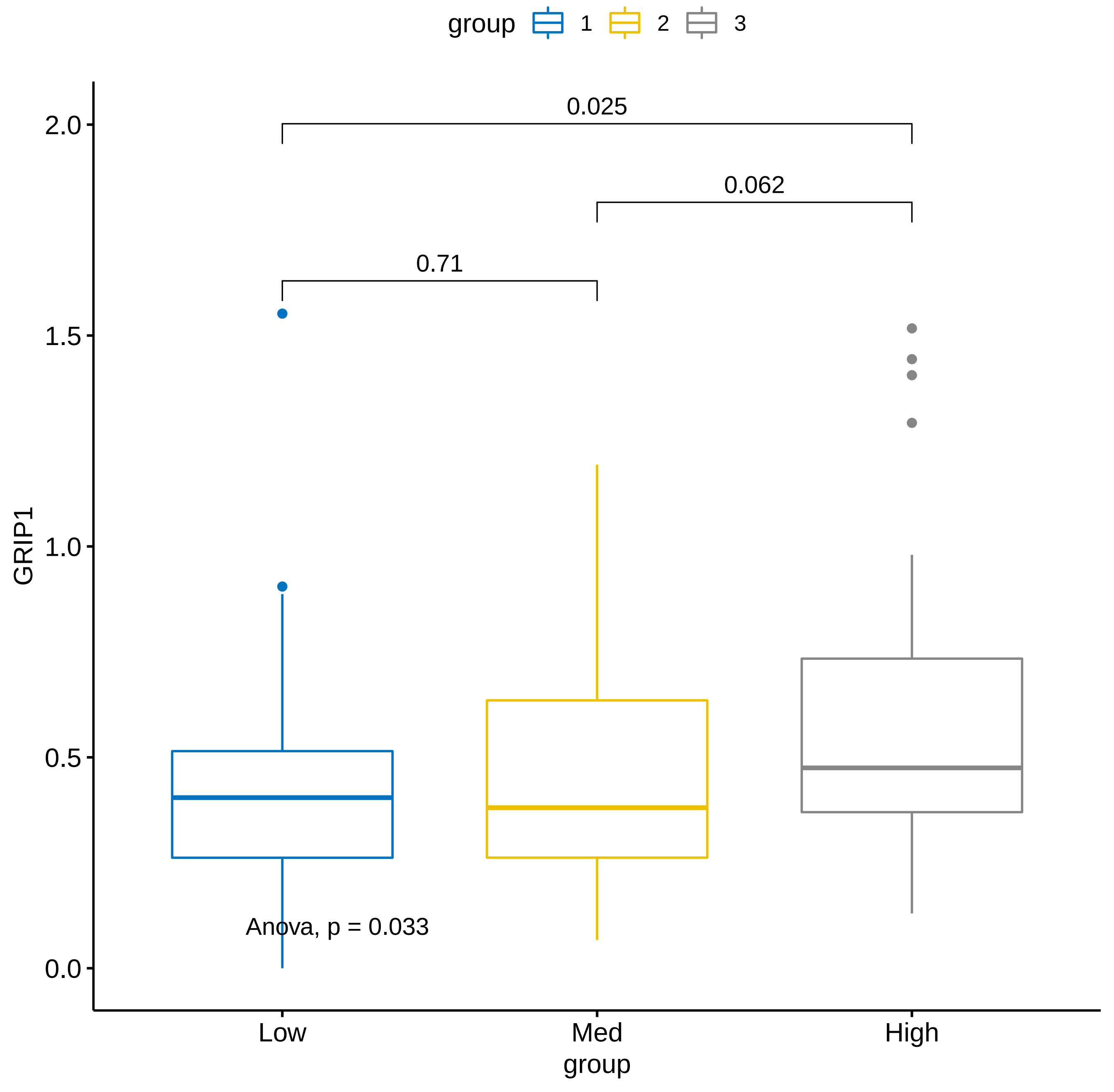

group 1 2 3
0.025
0.062
0.71
2.0
1.5
1.0
0.5
0.0
GRIP1
Anova, p = 0.033
Low
Med
High
group

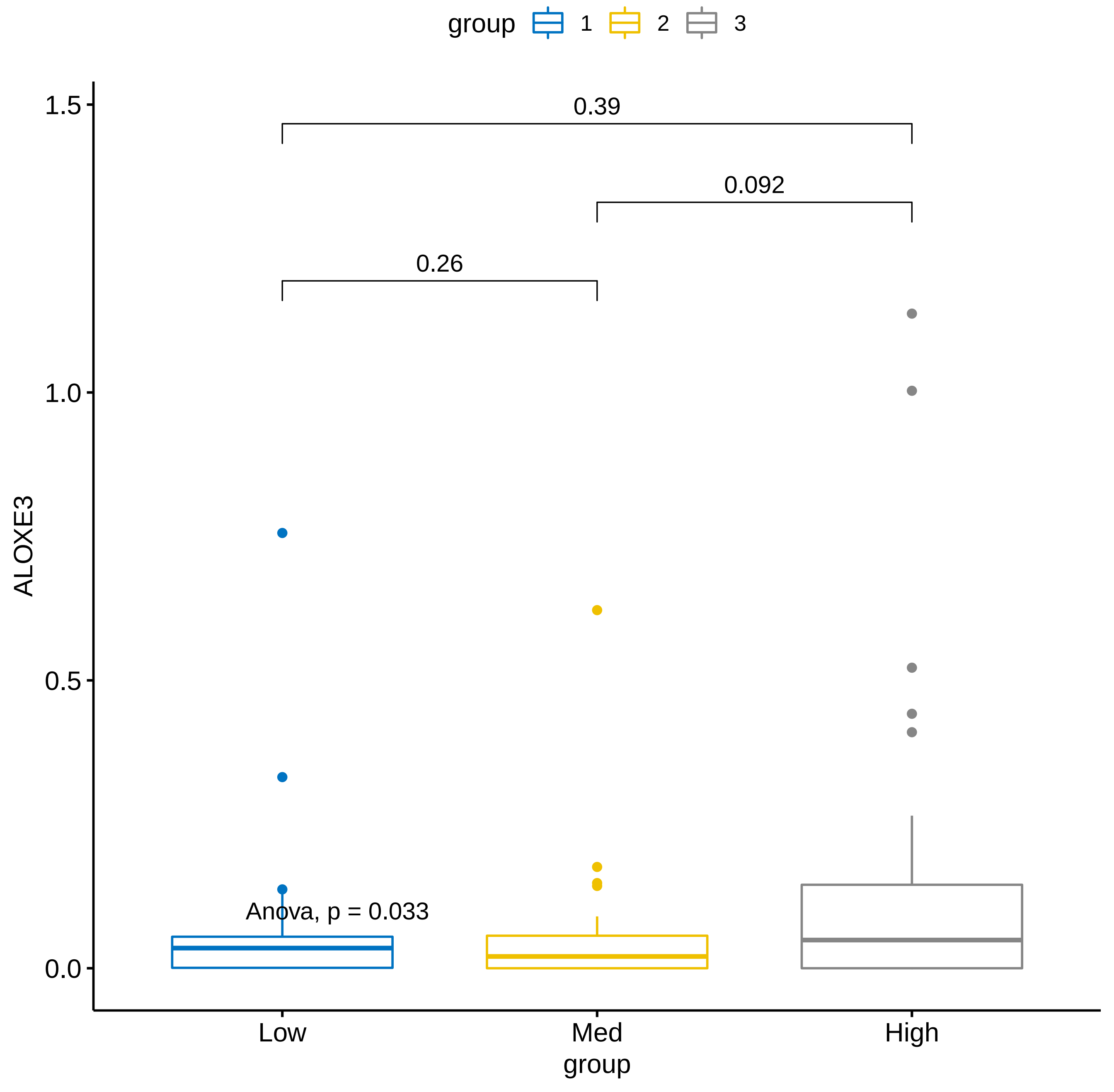

group
1
2
3
0.39
0.092
0.26
Anova, p = 0.033
1.5
1.0
0.5
0.0
ALOXE3
Low
Med
High
group

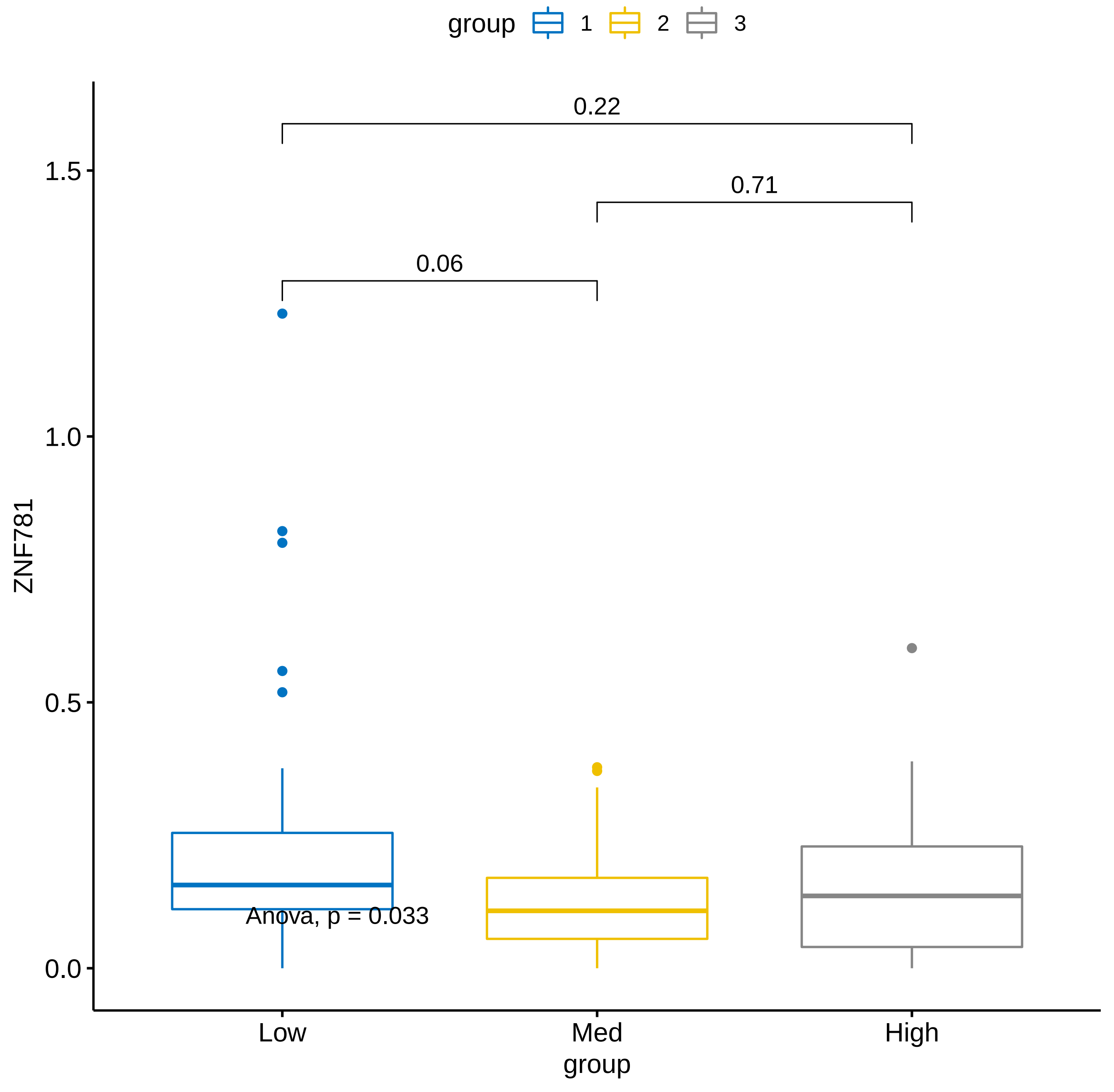
group 1 2 3
0.22
0.71
0.06
1.5
1.0
0.5
0.0
ZNF781
Anova, p = 0.033
Low
Med
High
group

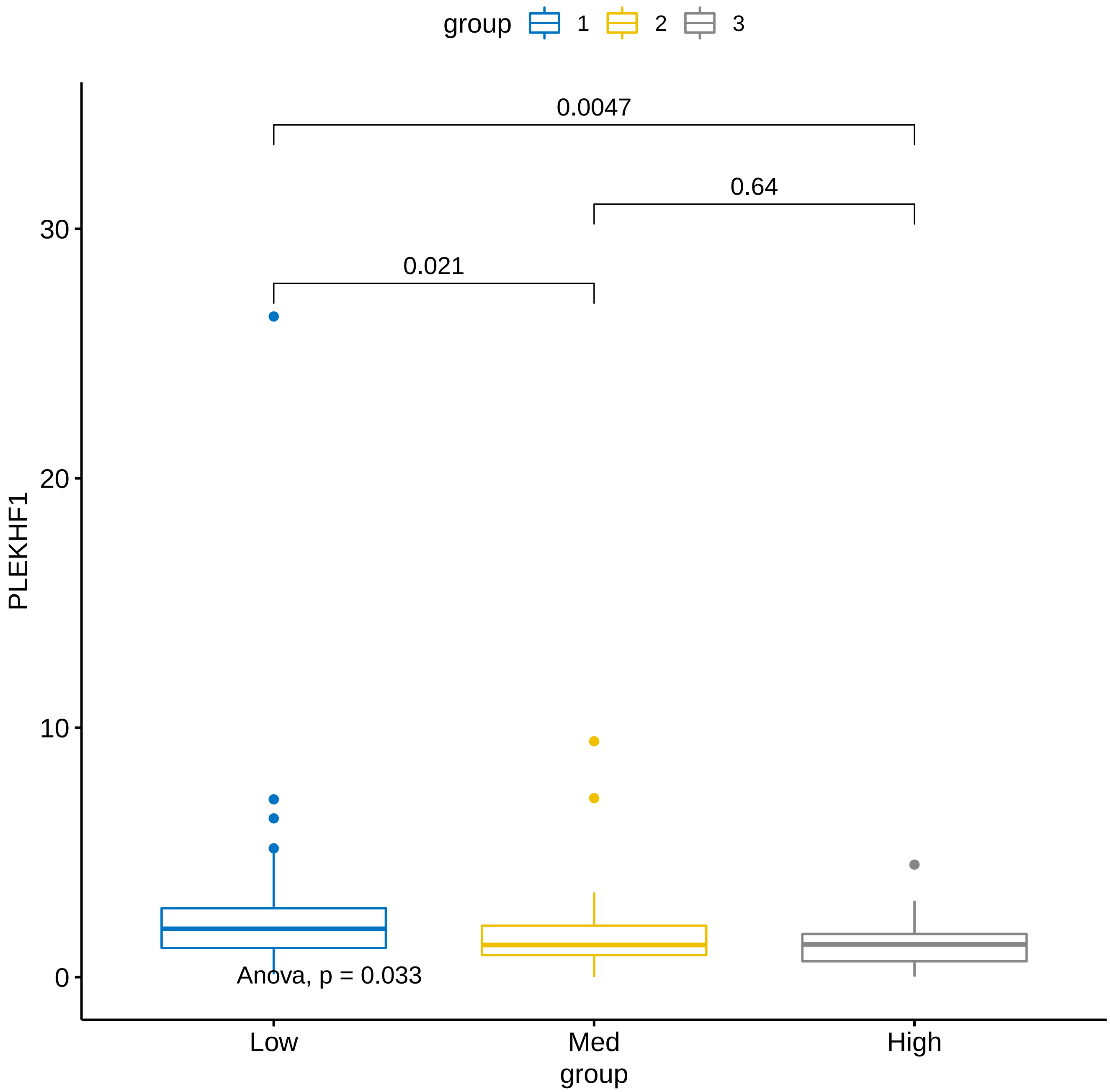

group 1 2 3
0.0047
0.64
0.021
30
20
10
0
PLEKHF1
Anova, p = 0.033
Low
Med
High
group

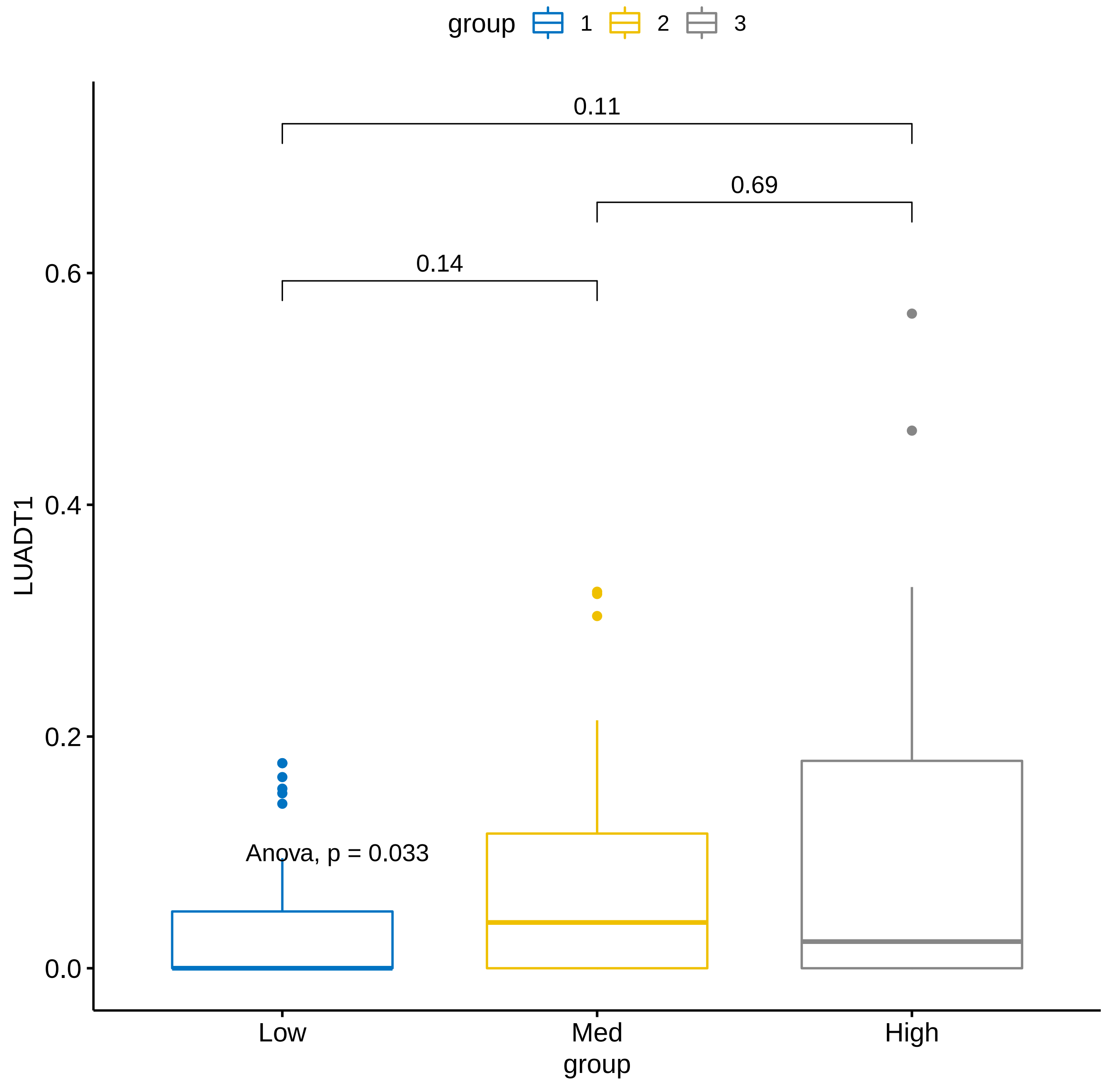

group
1
2
3
0.11
0.69
0.14
0.6
0.4
0.2
0.0
LUADT1
Anova, p = 0.033
Low
Med
High
group

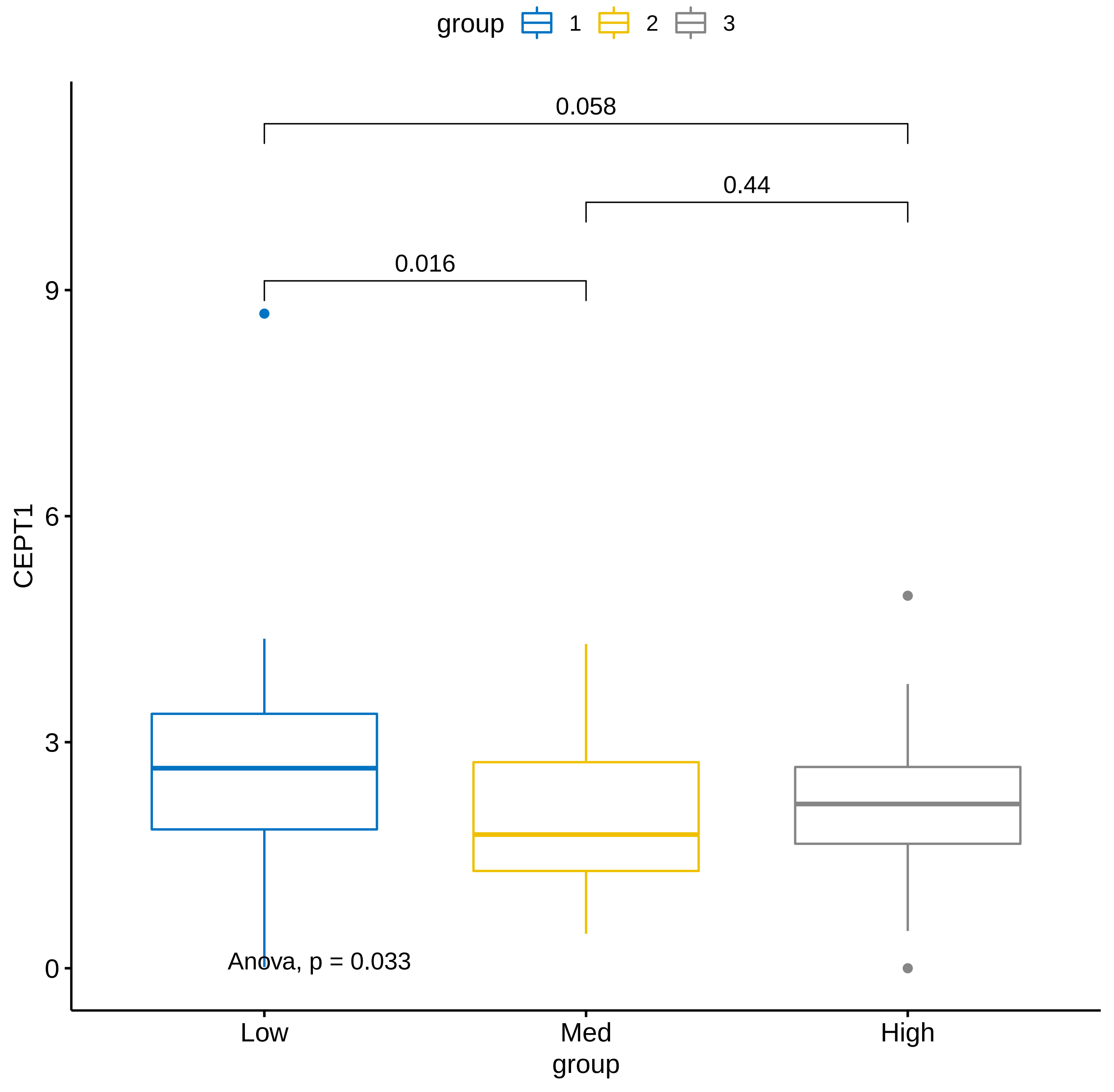
group
1
2
3
0.058
0.44
0.016
9
6
3
0
CEPT1
Anova, p = 0.033
Low
Med
High
group

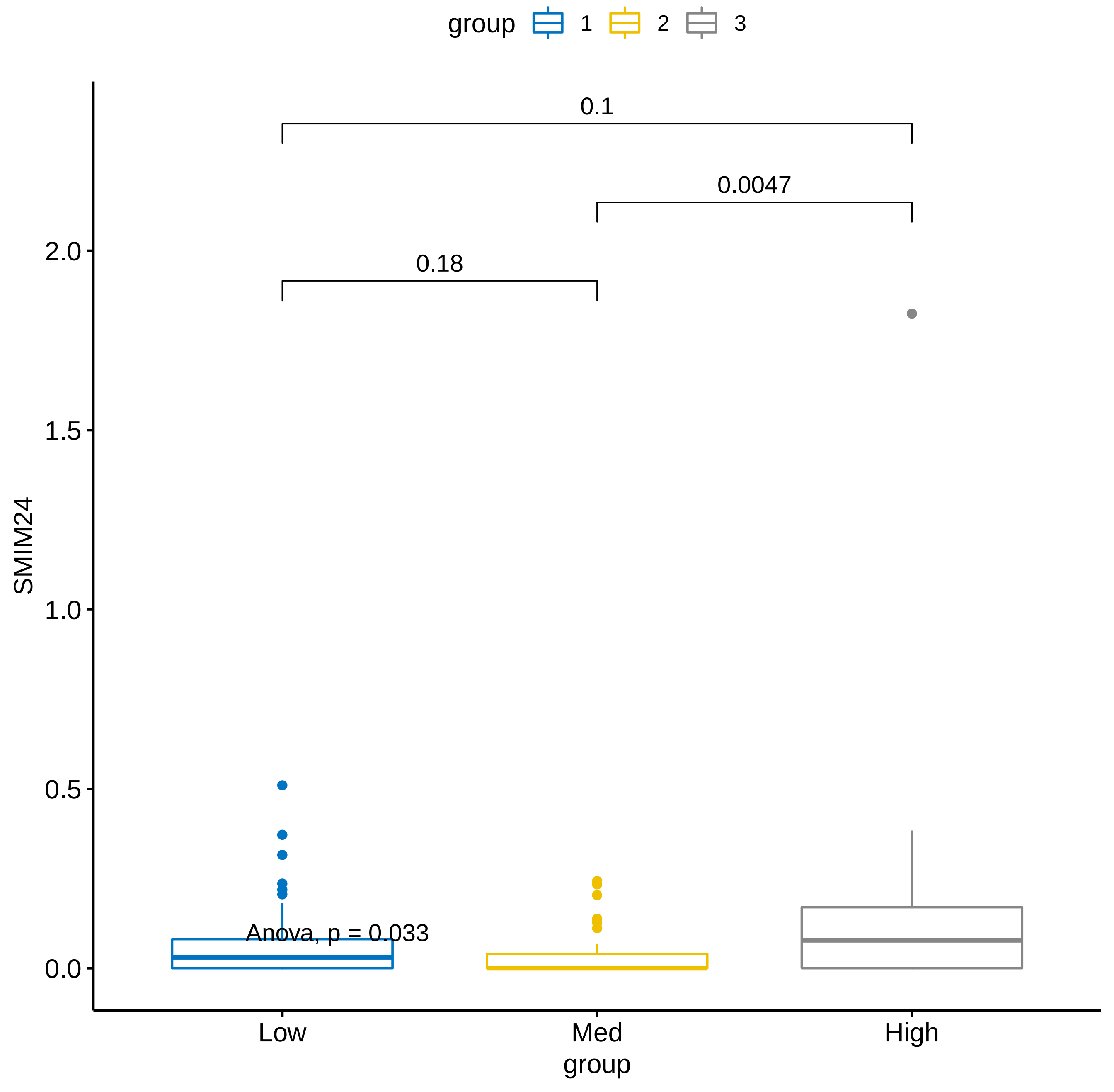

group 1 2 3
0.1
0.0047
0.18
2.0
1.5
1.0
0.5
0.0
SMIM24
Anova, p = 0.033
Low
Med
High
group

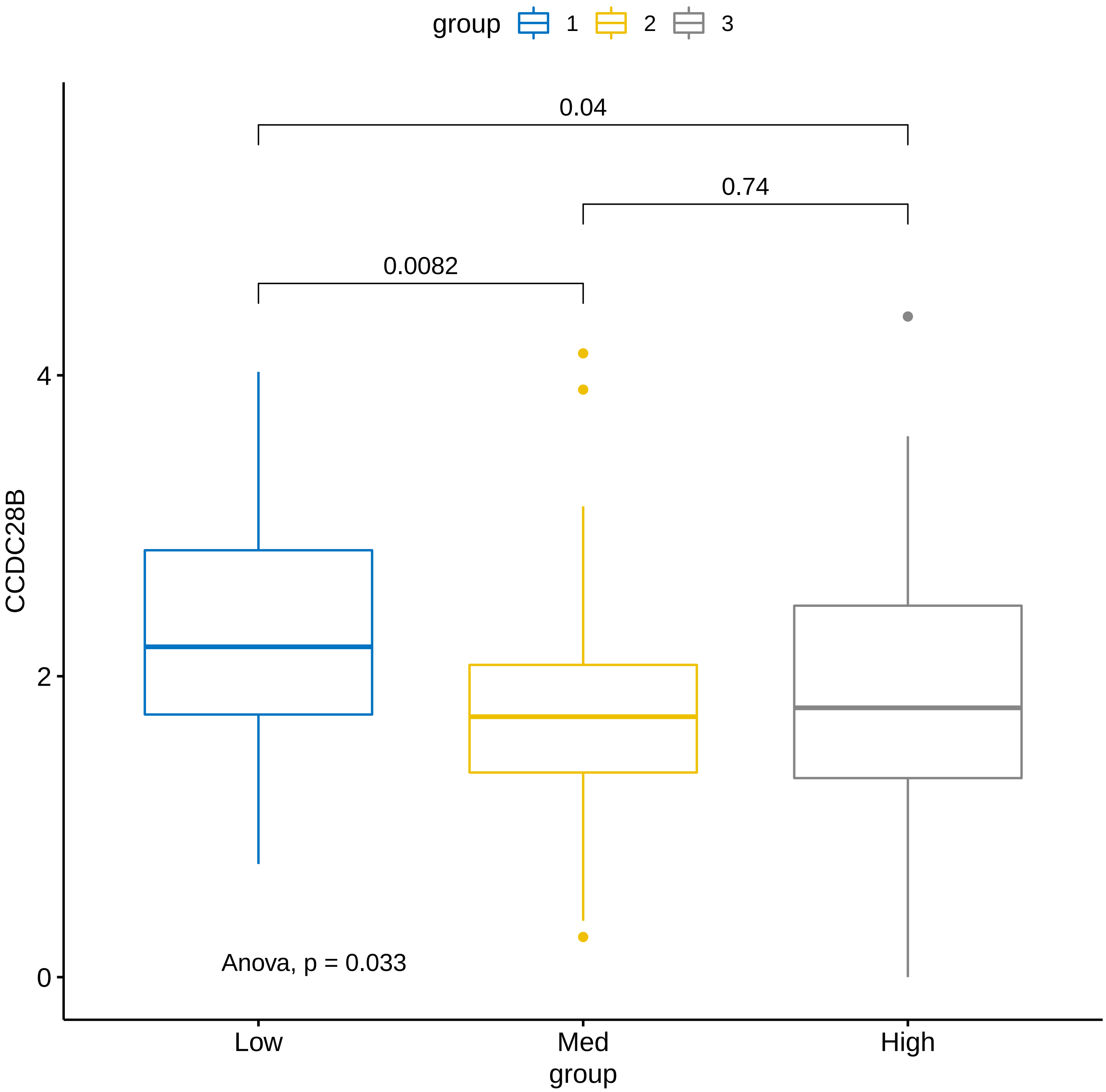

group 1 2 3
0.04
0.74
0.0082
CCDC28B
4
2
0
Anova, p = 0.033
Low
Med
High
group

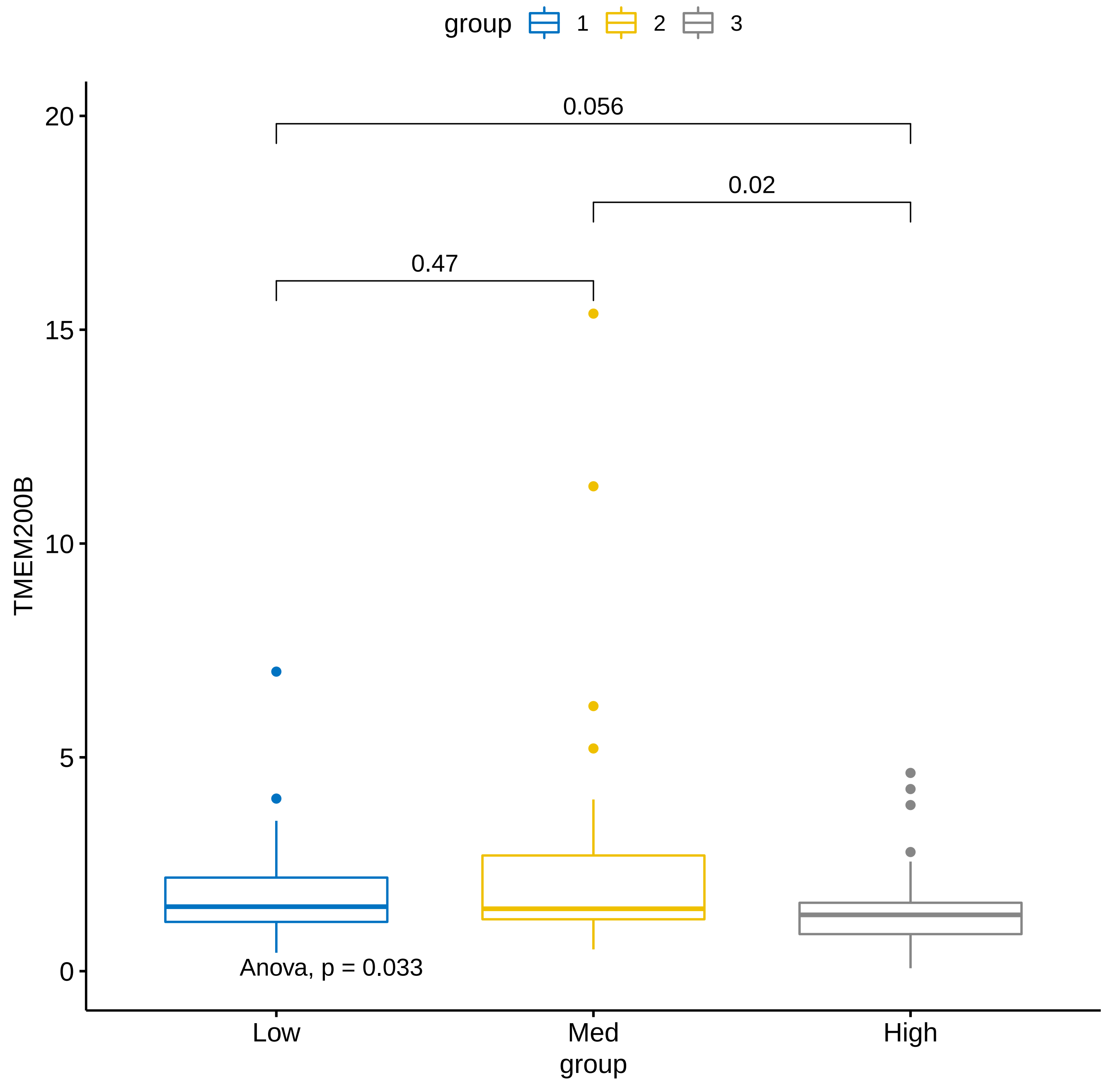
group 1 2 3
0.056
0.02
0.47
20
15
10
5
0
TMEM200B
Anova, p = 0.033
Low
Med
High
group

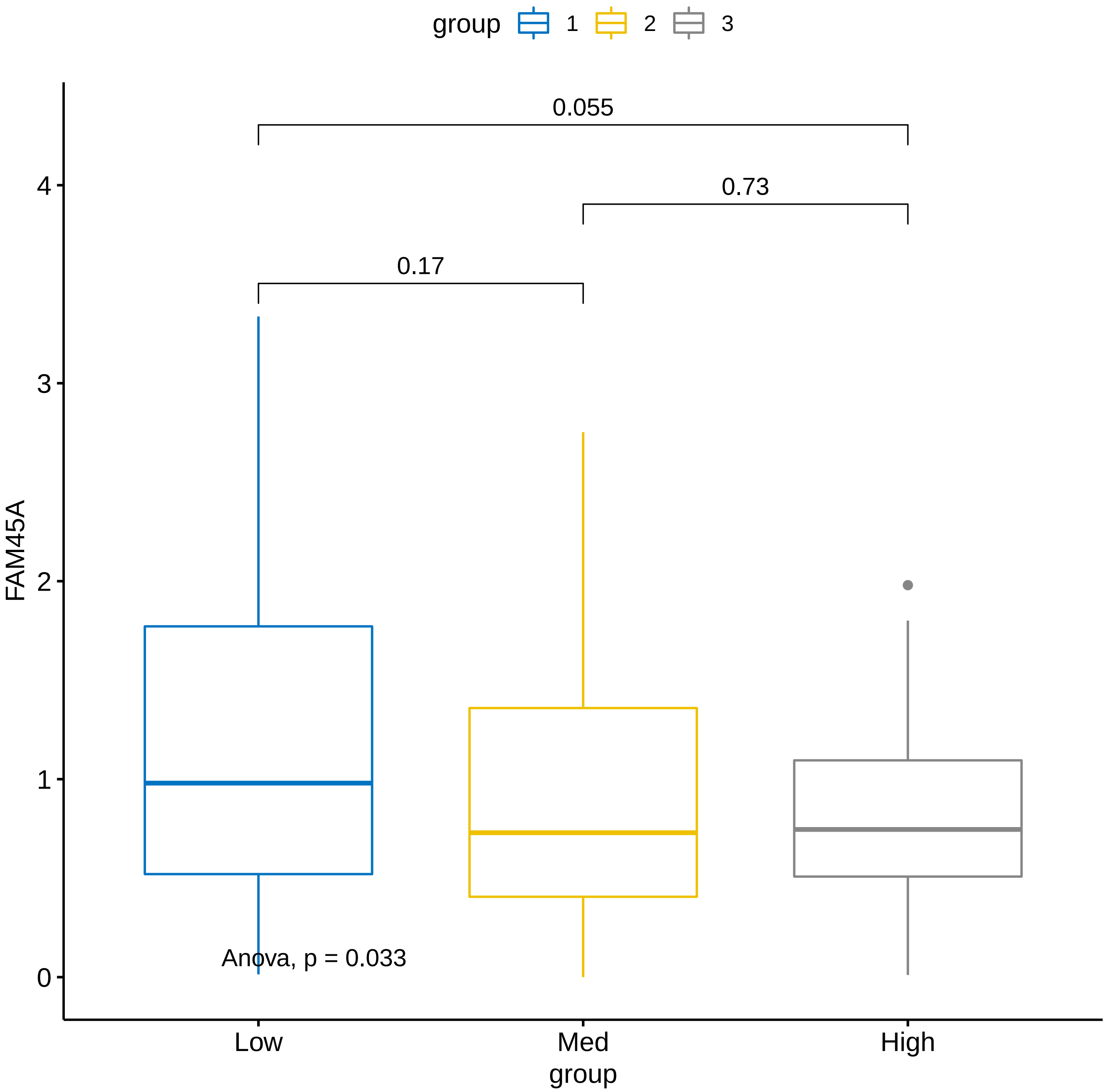
group
1
2
3
0.055
0.73
0.17
Anova, p = 0.033
FAM45A
0
1
2
3
4
Low
Med
High
group

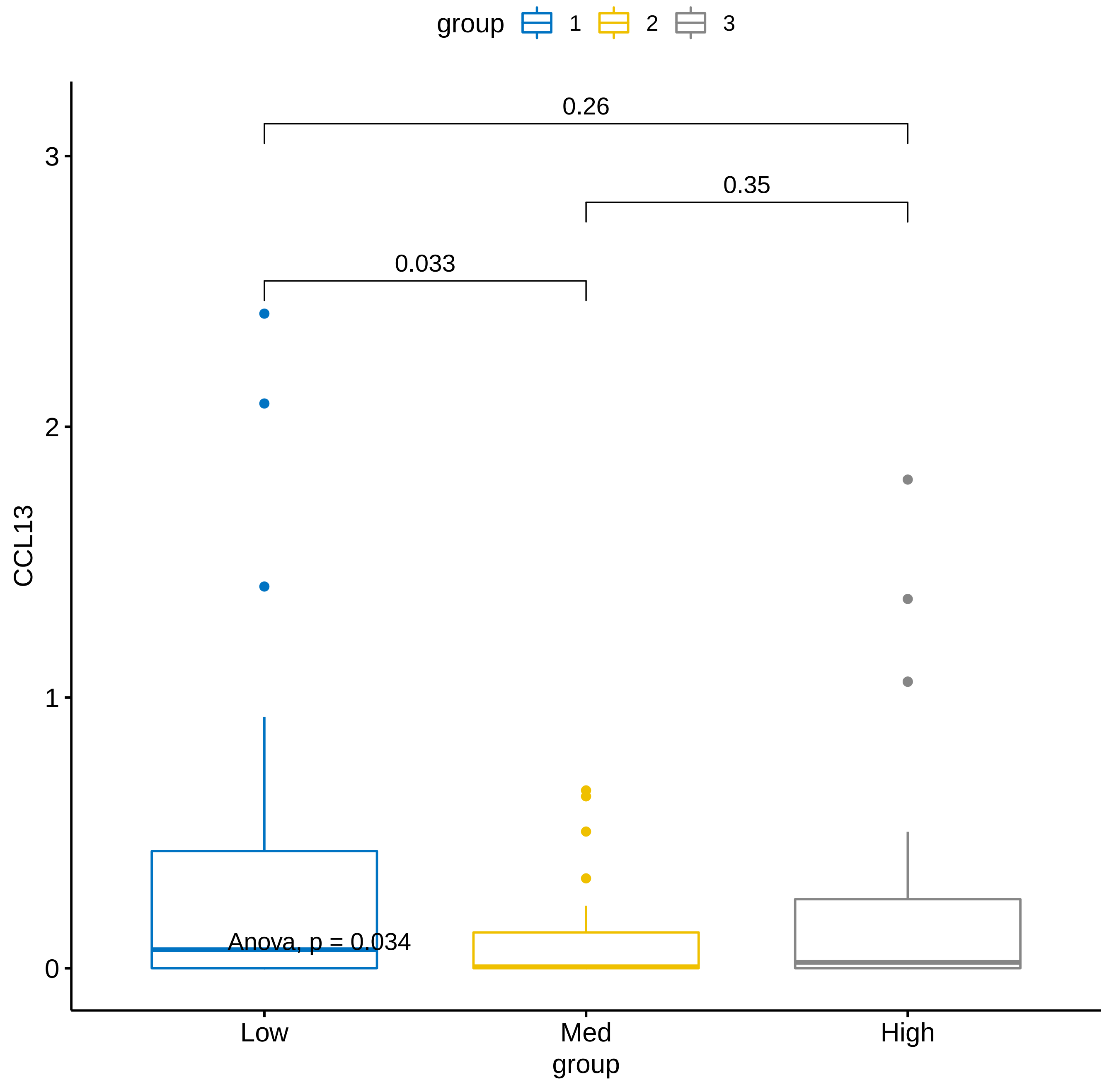

group 1 2 3
0.26
0.35
0.033
3
2
1
0
CCL13
Anova, p = 0.034
Low
Med
High
group

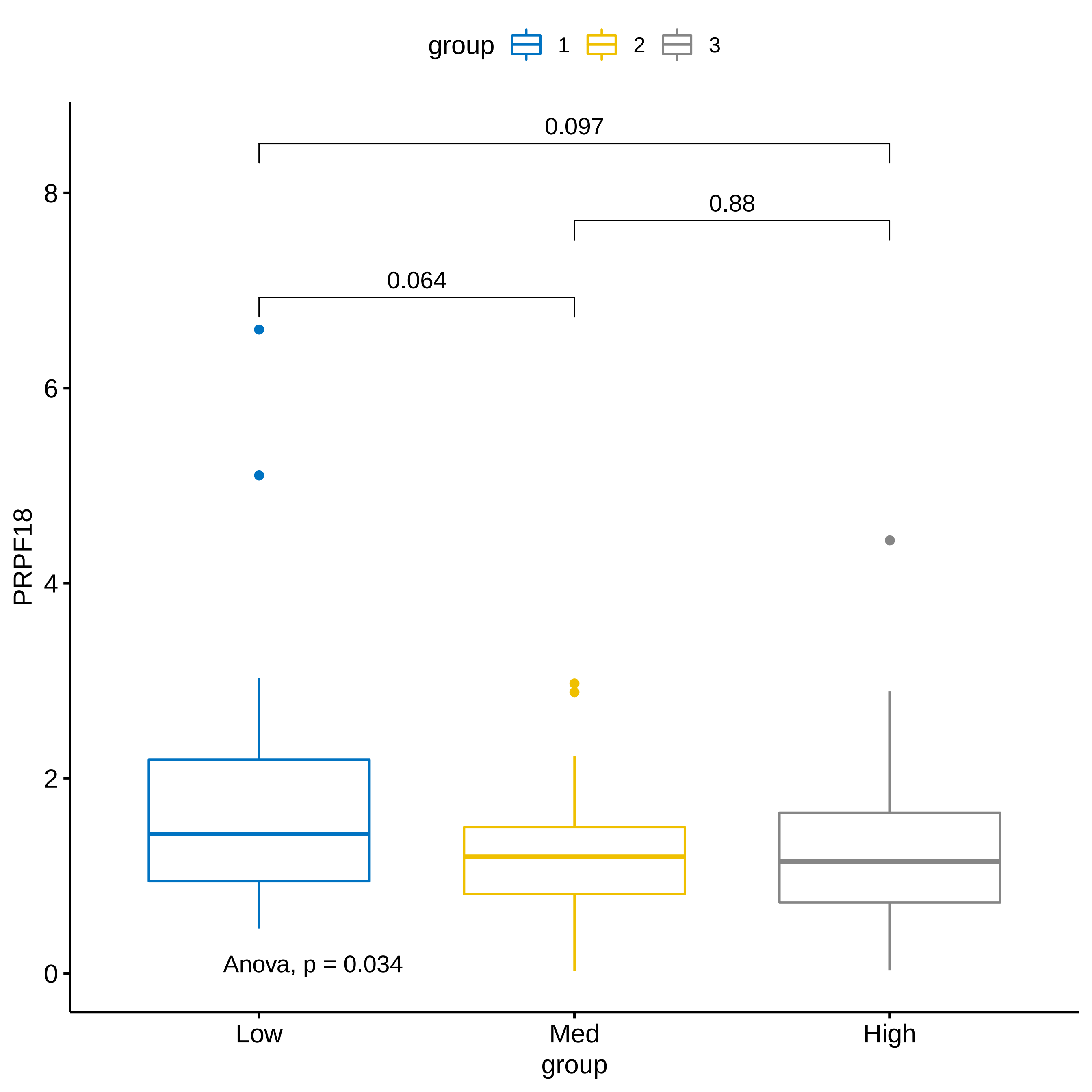

group 1 2 3
0.097
0.88
0.064
8
6
4
2
0
PRPF18
Anova, p = 0.034
Low
Med
High
group

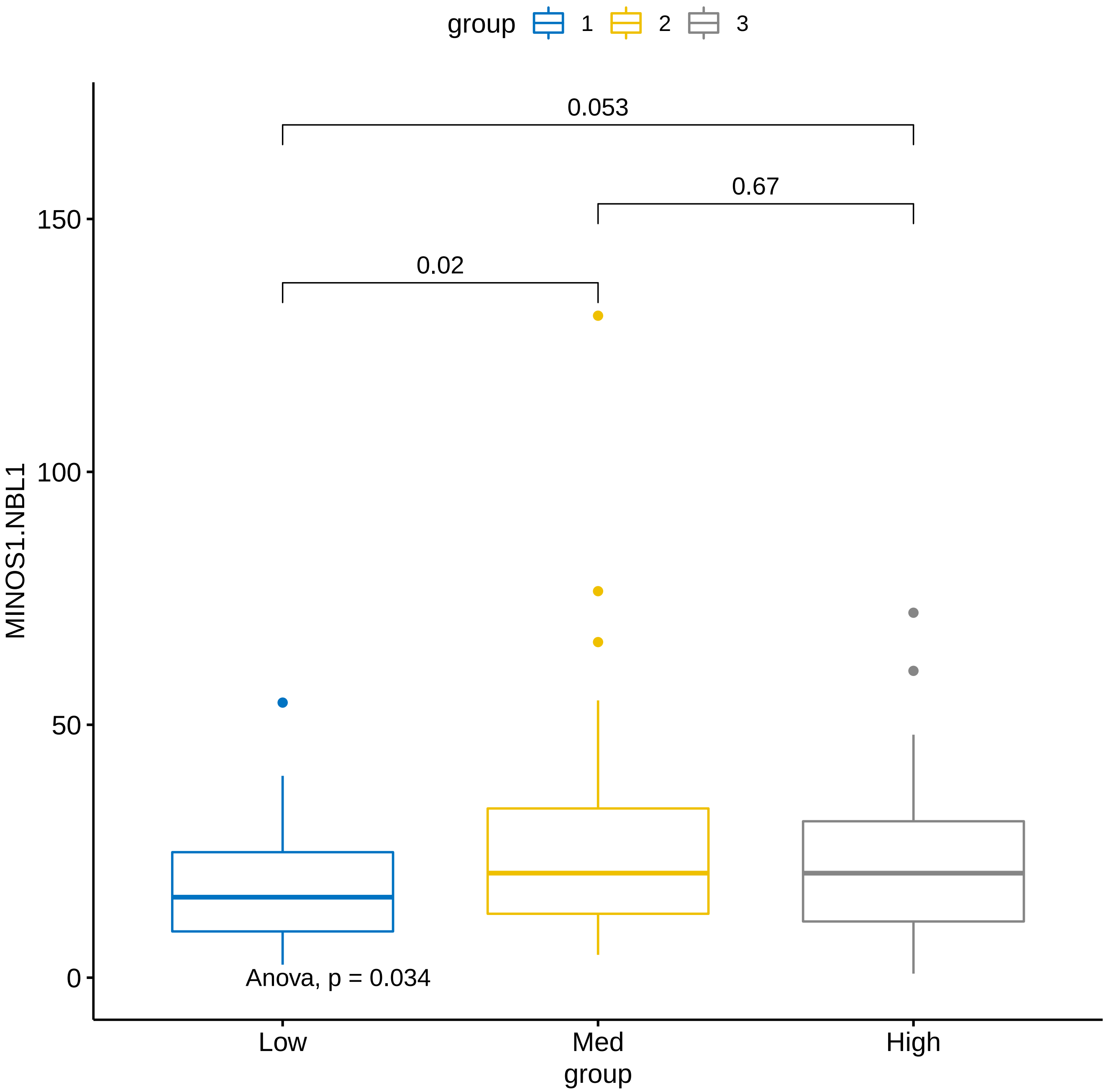

group
1
2
3
0.053
0.67
0.02
150
100
50
0
MINOS1.NBL1
Anova, p = 0.034
Low
Med
High
group

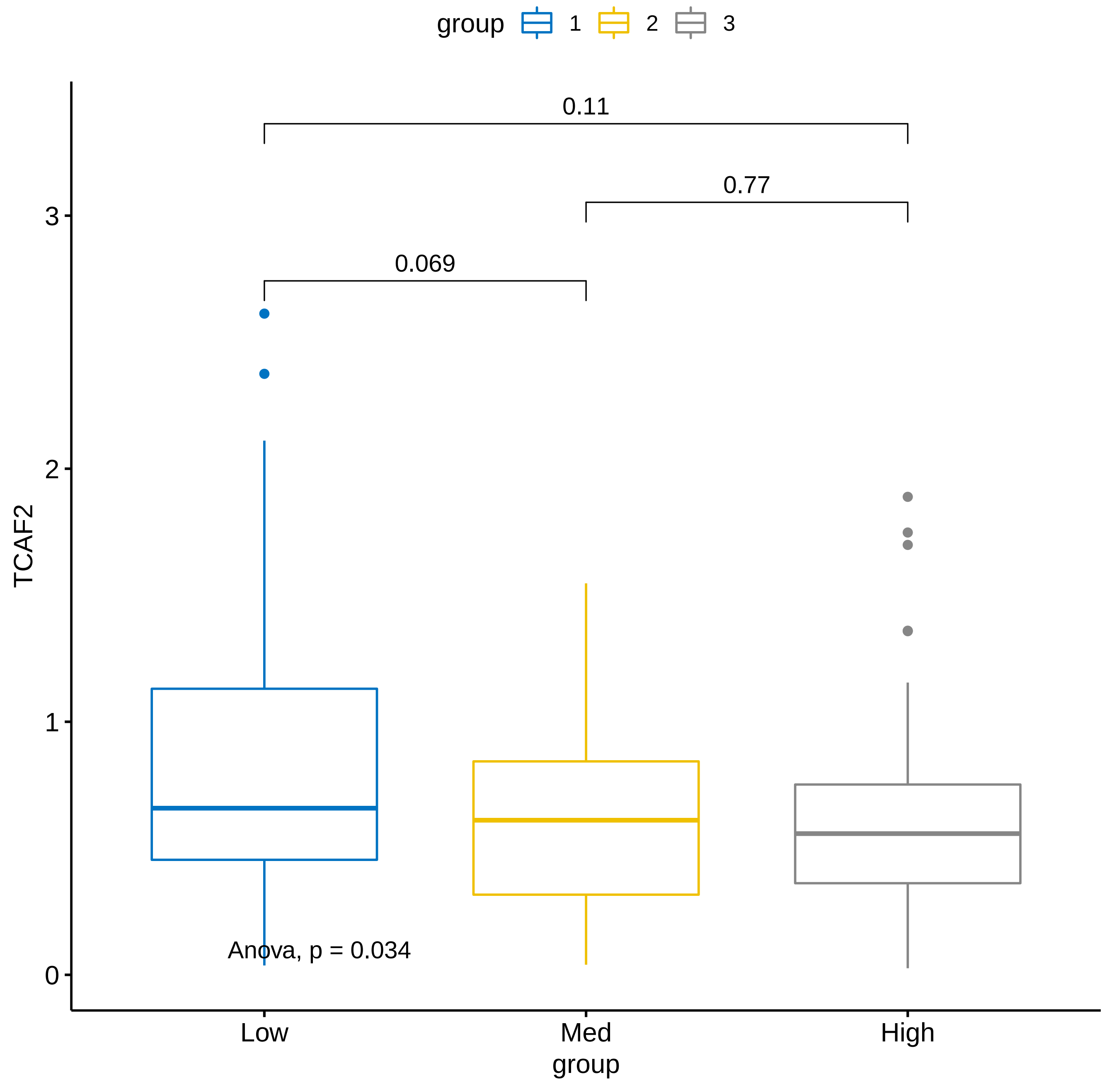
group 1 2 3
0.11
0.77
0.069
TCAF2
3
2
1
0
Anova, p = 0.034
Low
Med
High
group

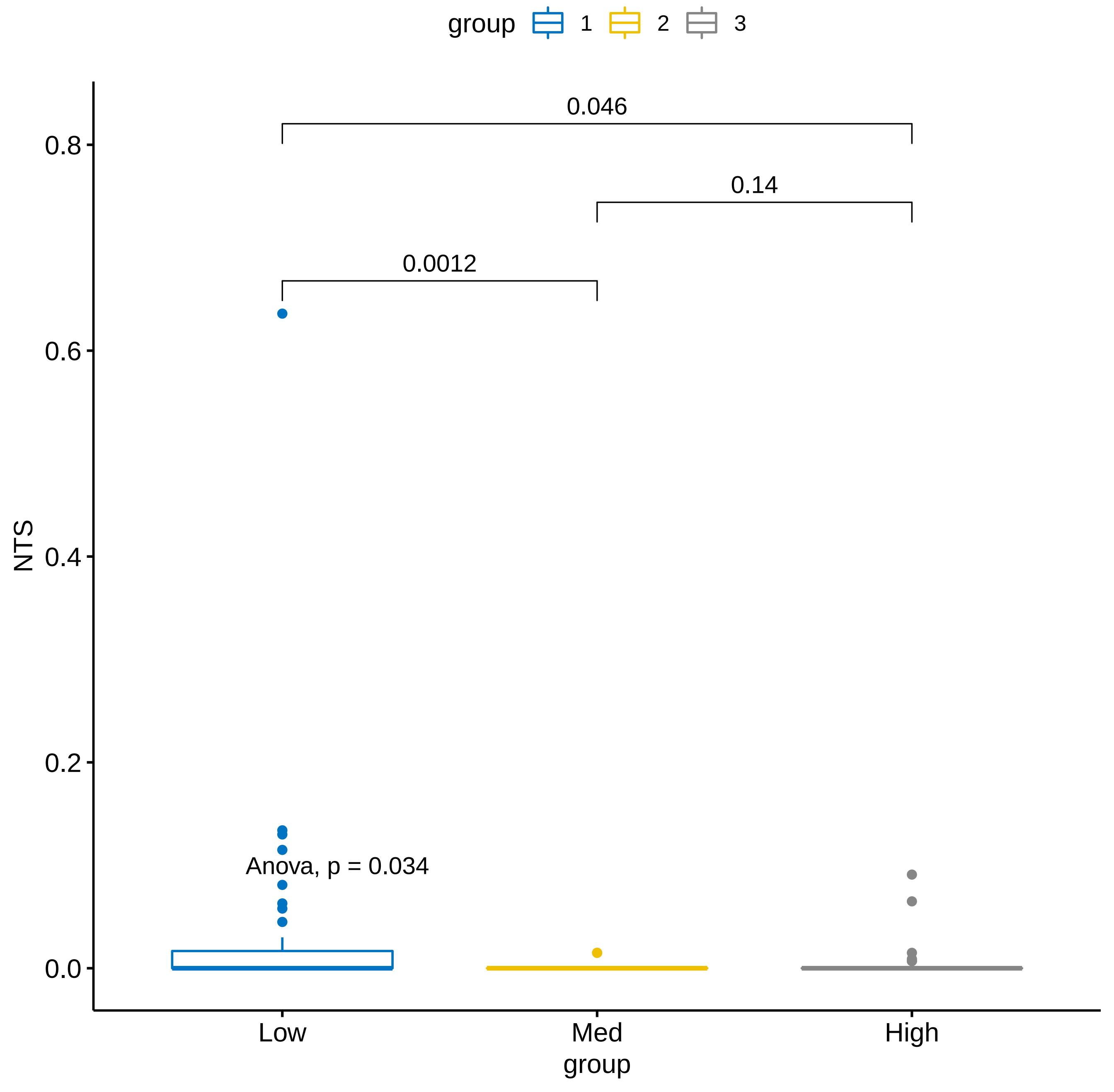

group
1
2
3
0.046
0.14
0.0012
Anova, p = 0.034
NTS
0.8
0.6
0.4
0.2
0.0
Low
Med
High
group

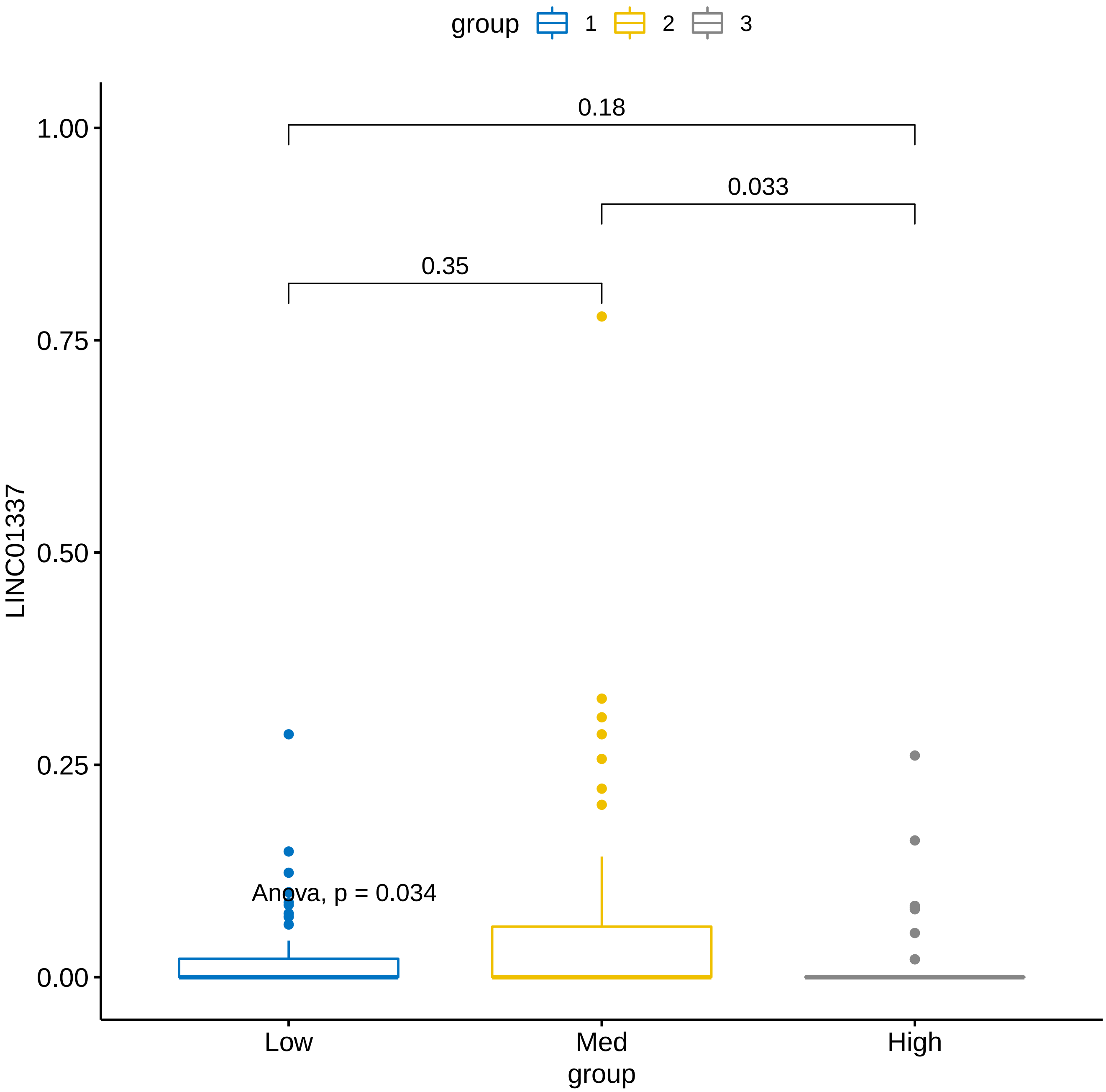

group 1 2 3
0.18
0.033
0.35
1.00
0.75
0.50
0.25
0.00
LINC01337
Anova, p = 0.034
Low
Med
High
group

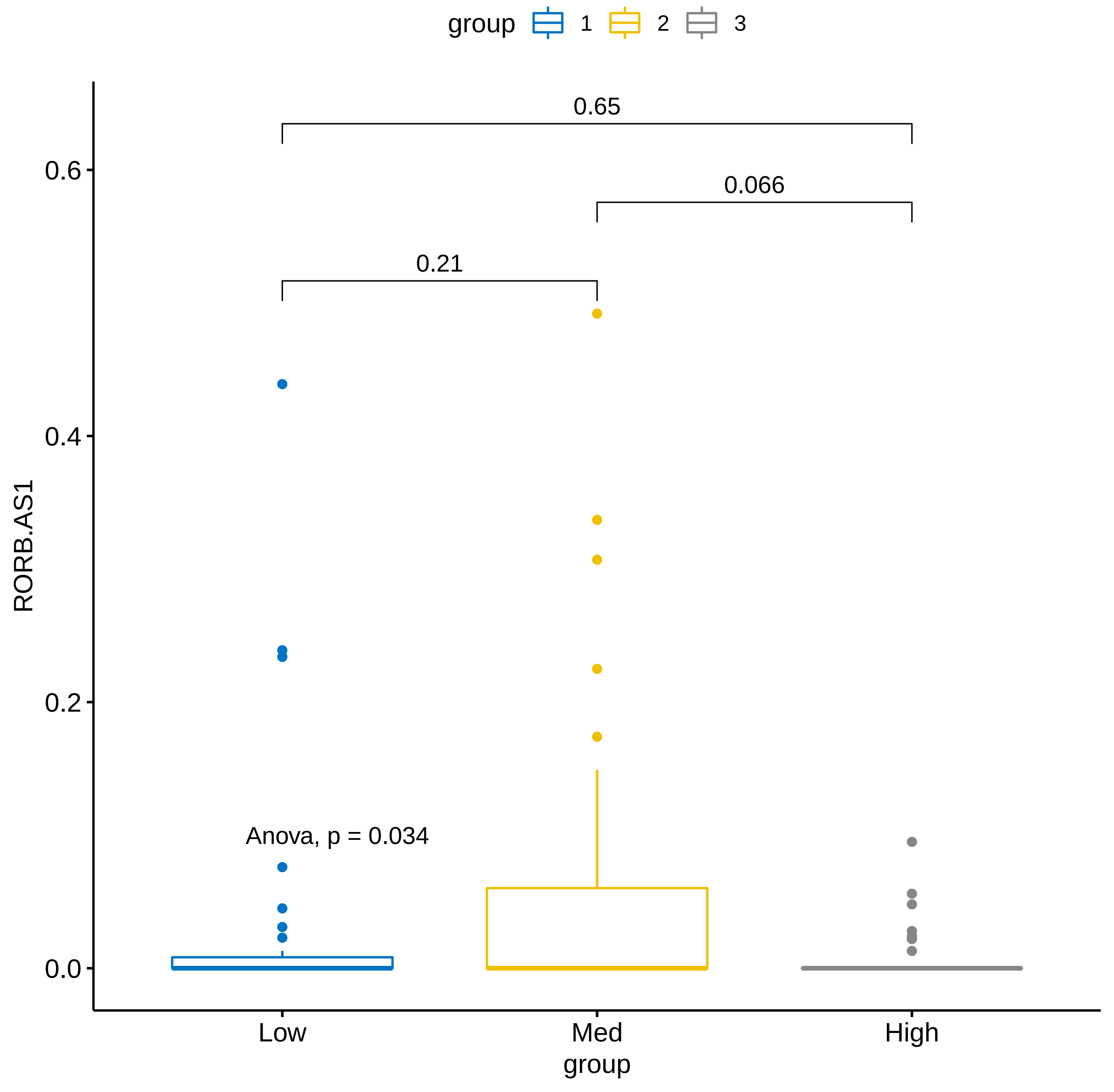
group 1 2 3
0.65
0.066
0.21
Anova, p = 0.034
0.6
0.4
0.2
0.0
RORB.AS1
Low
Med
High
group

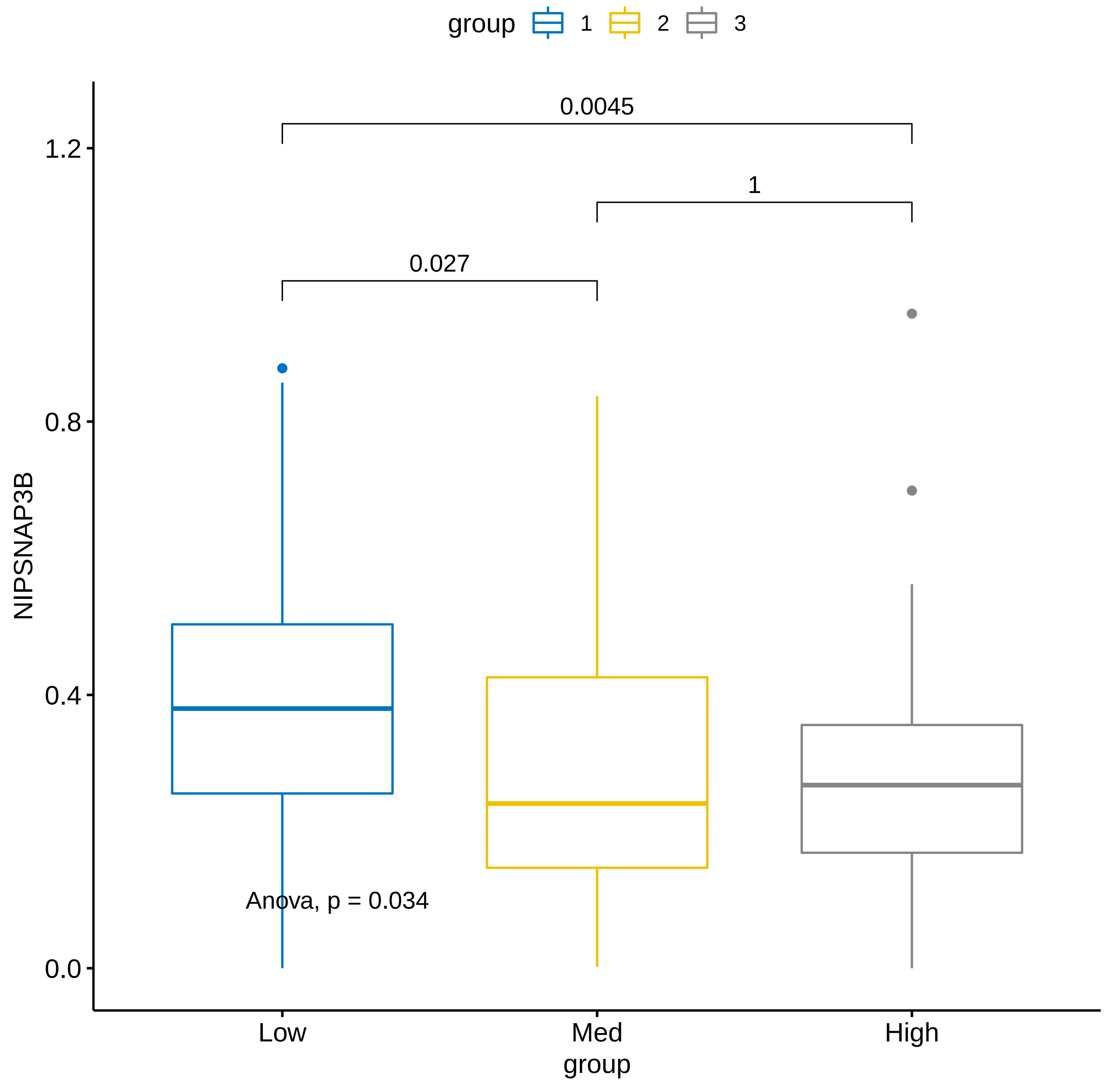
group
1
2
3
0.0045
1
0.027
Anova, p = 0.034
1.2
0.8
0.4
0.0
NIPSNAP3B
Low
Med
High
group

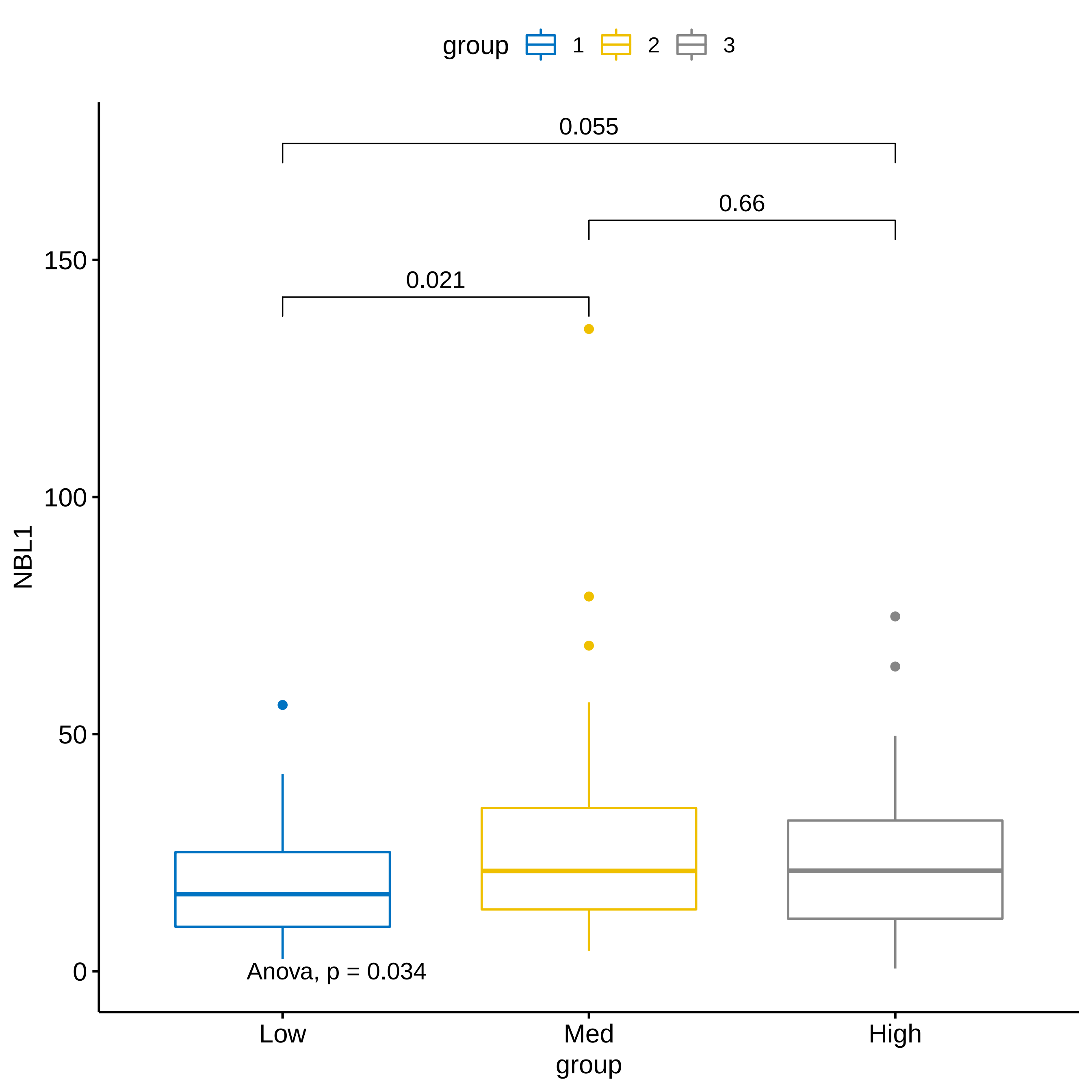

group
1
2
3
0.055
0.66
0.021
150
100
50
0
NBL1
Anova, p = 0.034
Low
Med
High
group

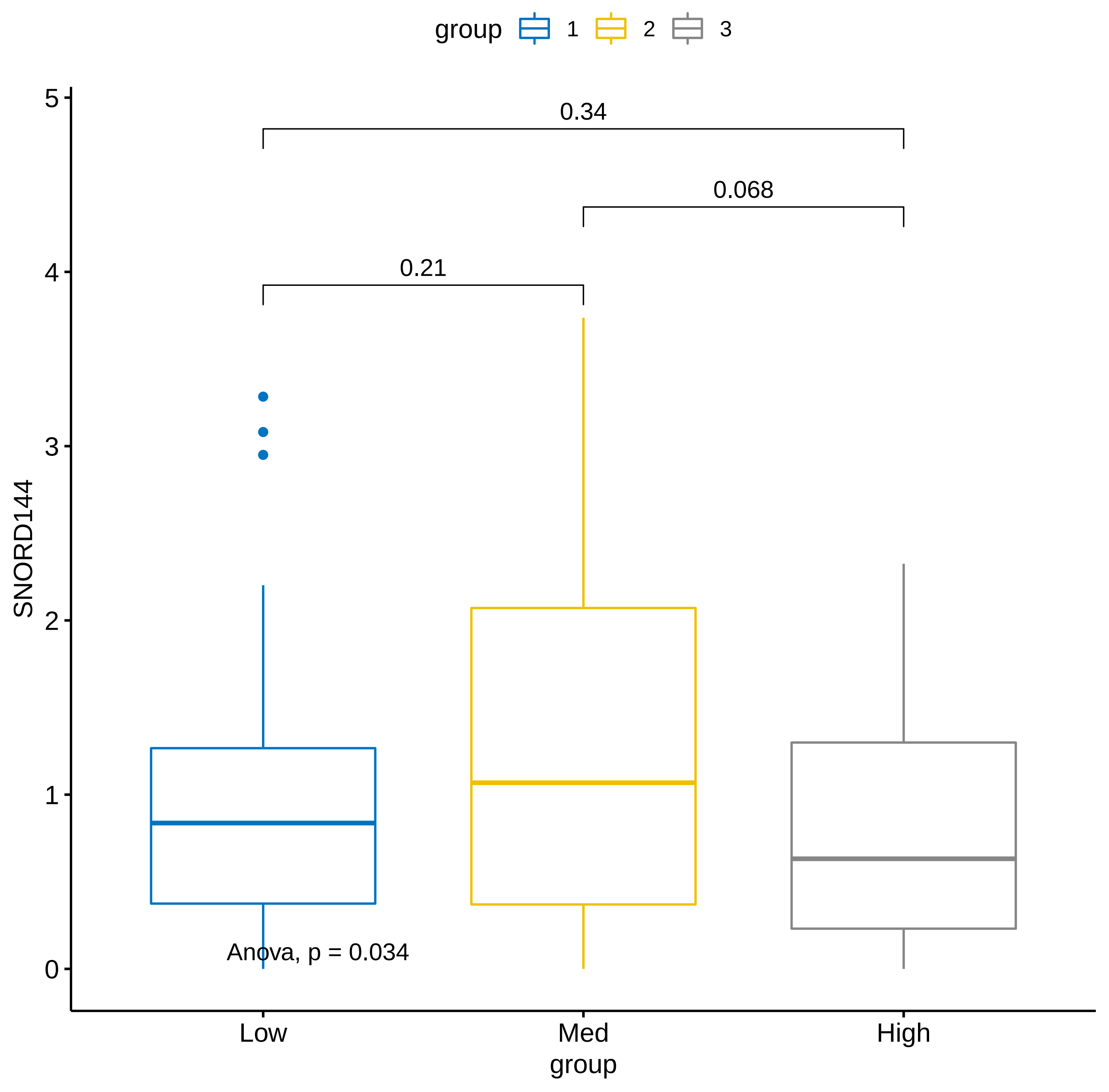
group
1
2
3
0.34
0.068
0.21
Anova, p = 0.034
SNORD144
5
4
3
2
1
0
Low
Med
High
group

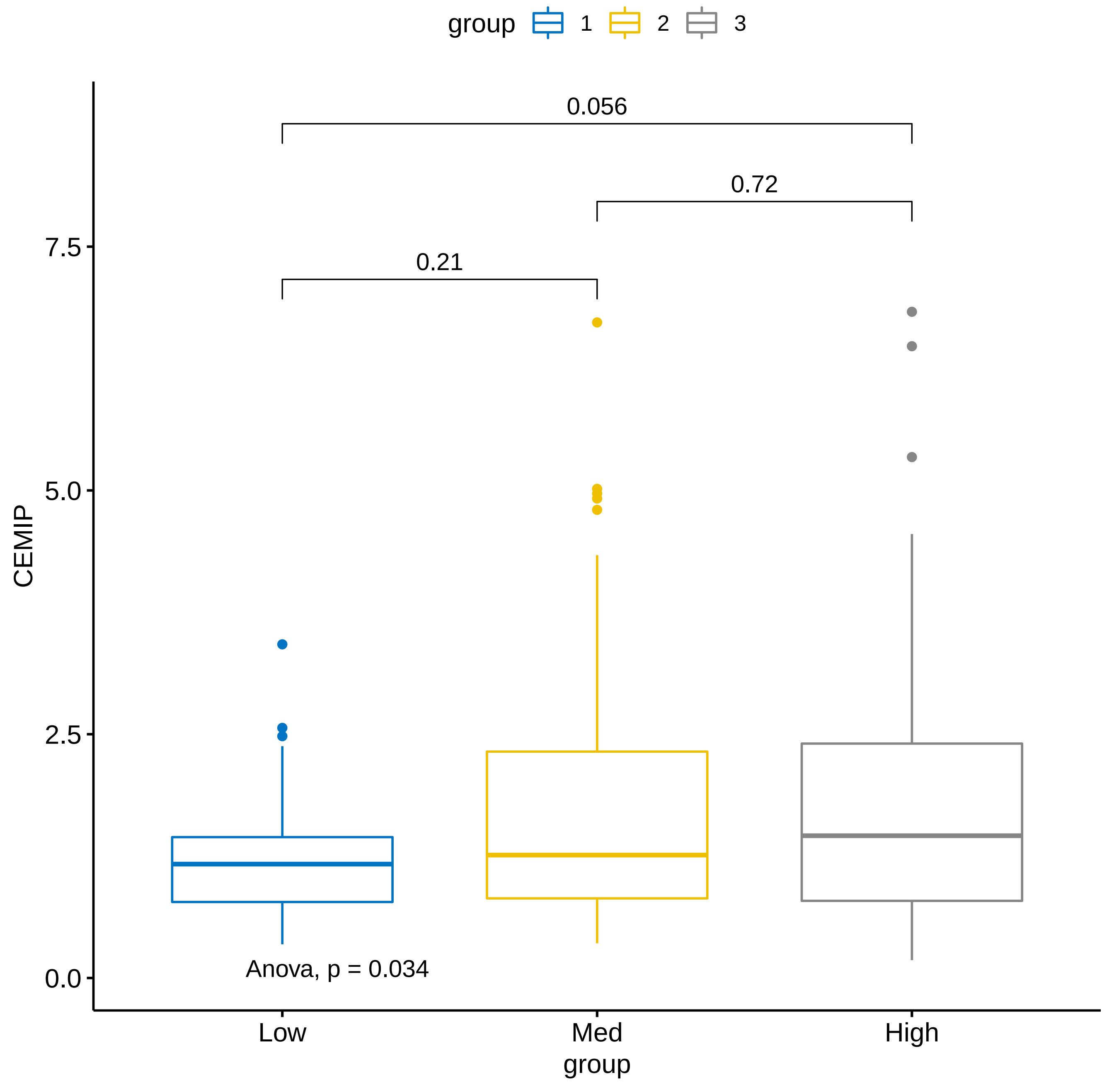
group 1 2 3
0.056
0.72
0.21
7.5
5.0
2.5
0.0
CEMIP
Anova, p = 0.034
Low
Med
High
group

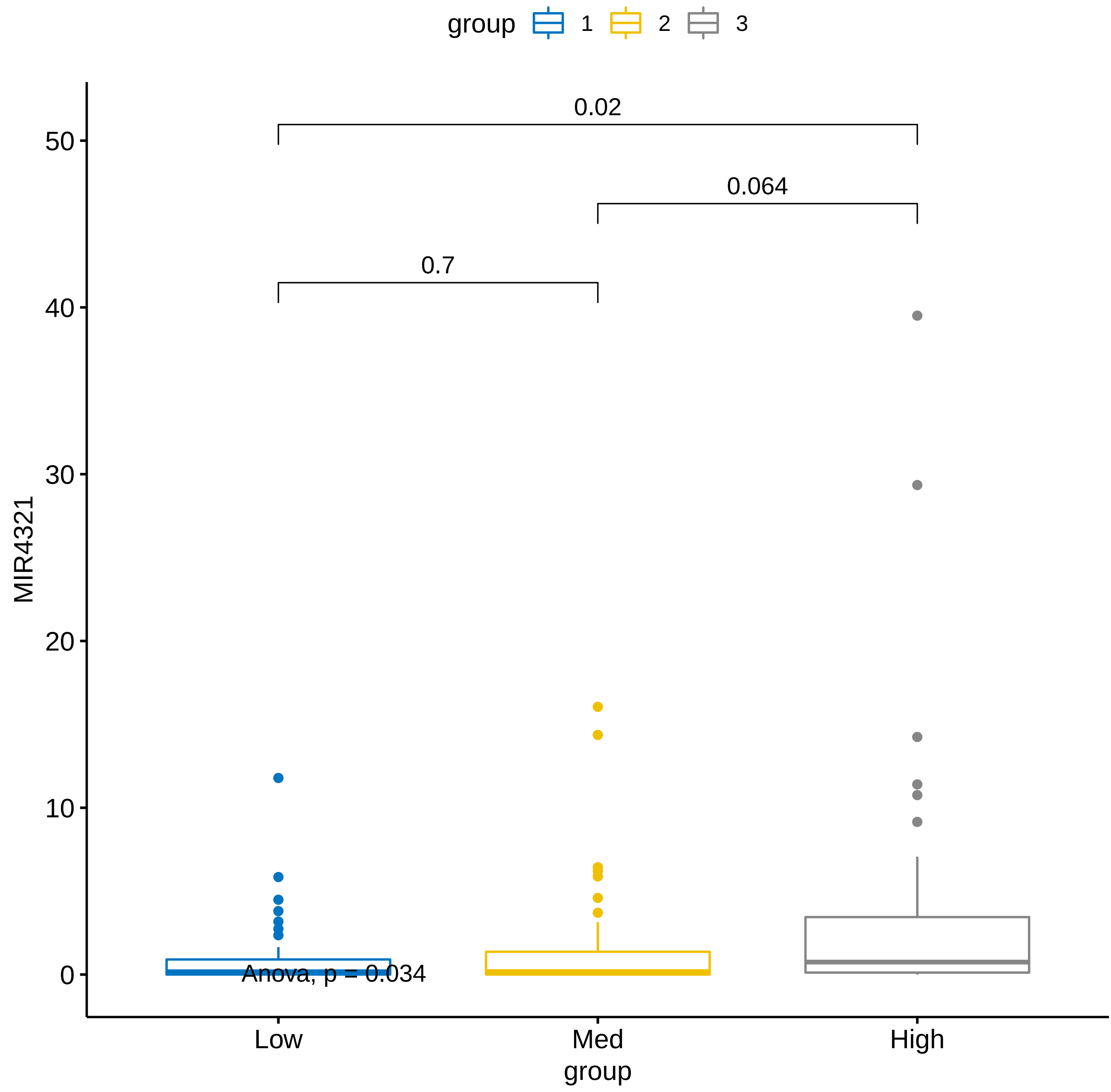

group 1 2 3
0.02
0.064
0.7
50
40
30
20
10
0
MIR4321
Anova, p = 0.034
Low
Med
High
group

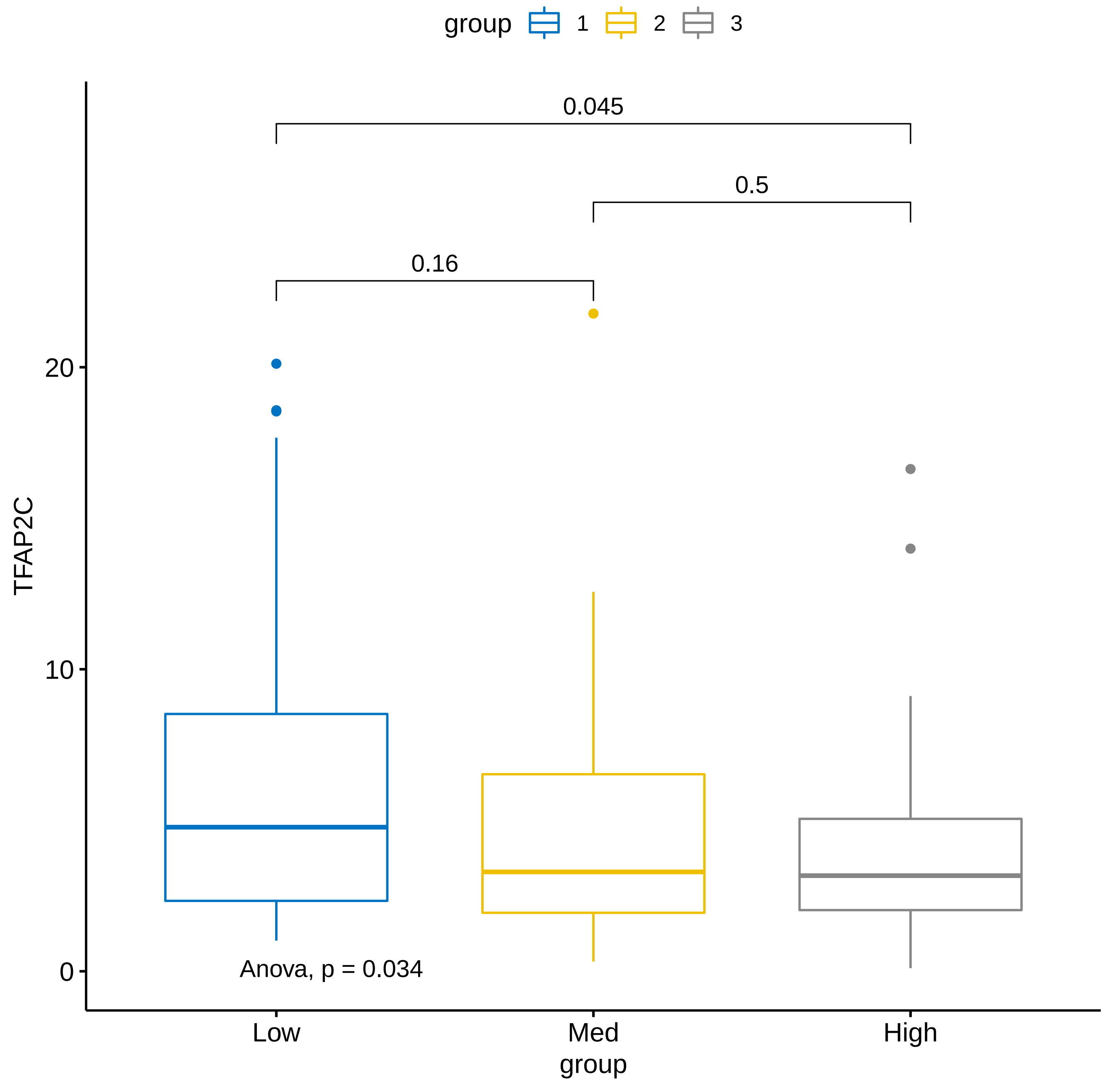

group
1
2
3
0.045
0.5
0.16
TFAP2C
20
10
0
Anova, p = 0.034
Low
Med
High
group

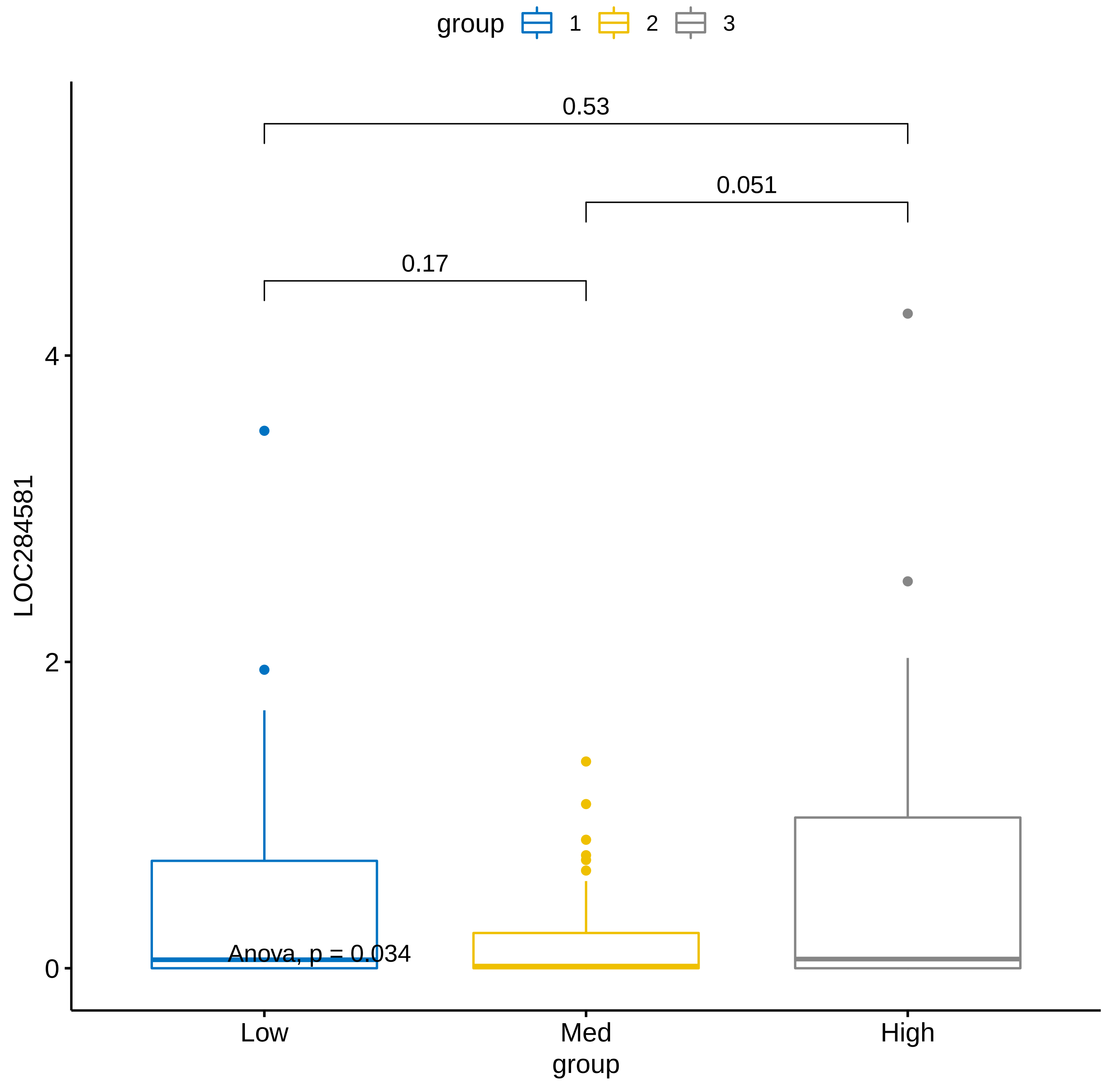

group
1
2
3
0.53
0.051
0.17
4
2
0
LOC284581
Anova, p = 0.034
Low
Med
High
group

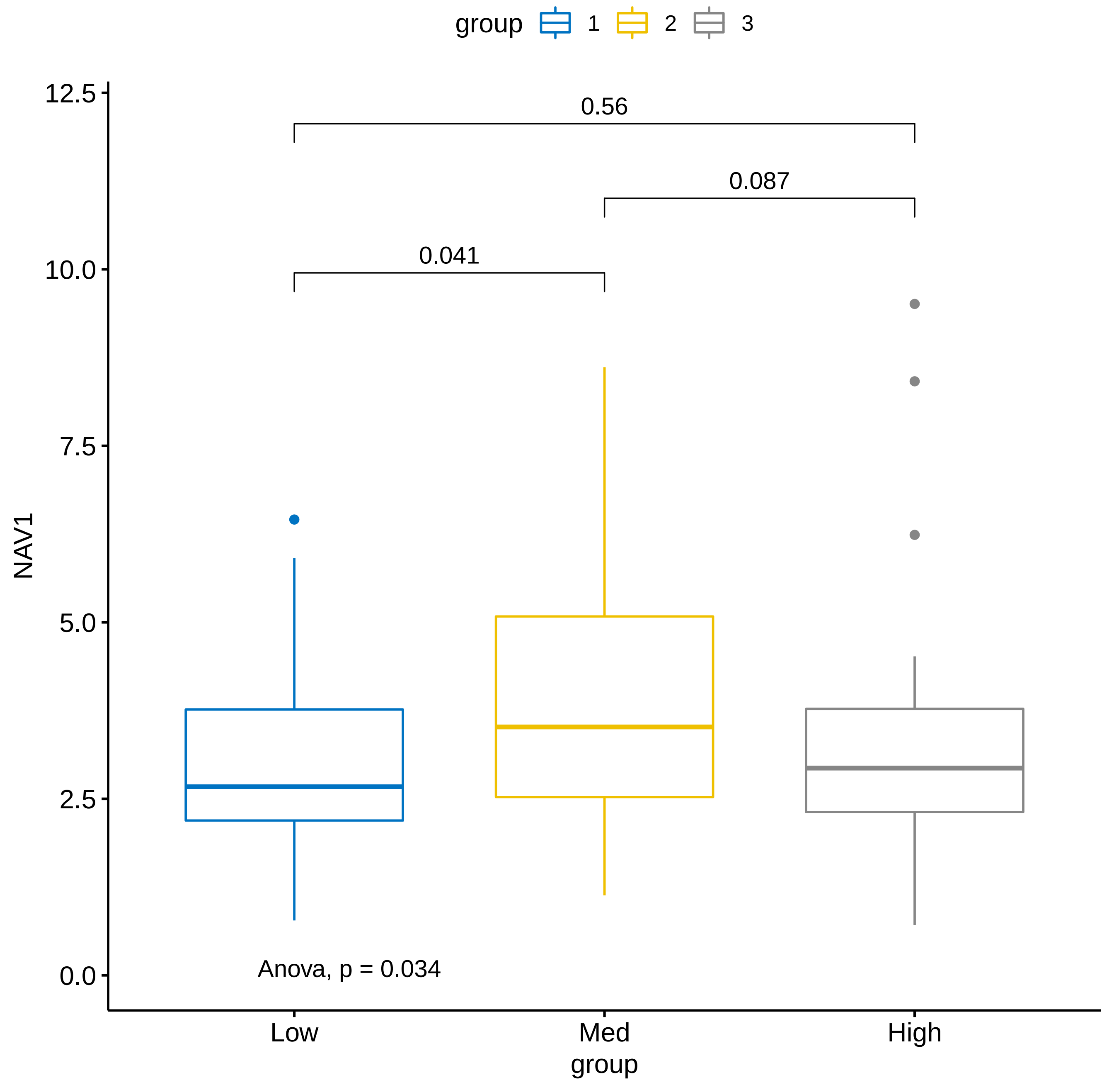

group 1 2 3
0.56
0.087
0.041
12.5
10.0
7.5
5.0
2.5
0.0
NAV1
Anova, p = 0.034
Low
Med
High
group

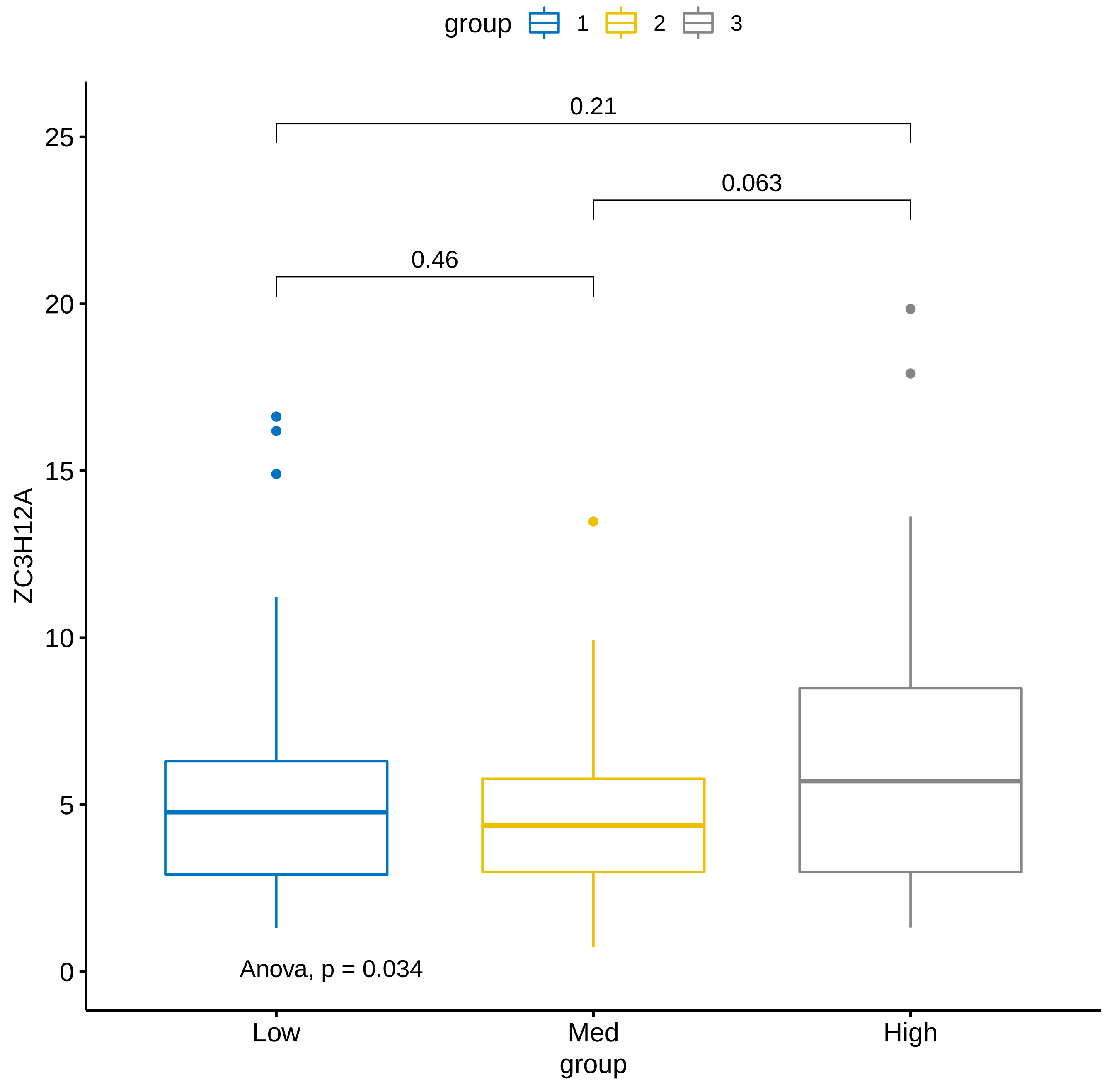
group
1
2
3
0.21
0.063
0.46
25
20
15
10
5
0
ZC3H12A
Anova, p = 0.034
Low
Med
High
group

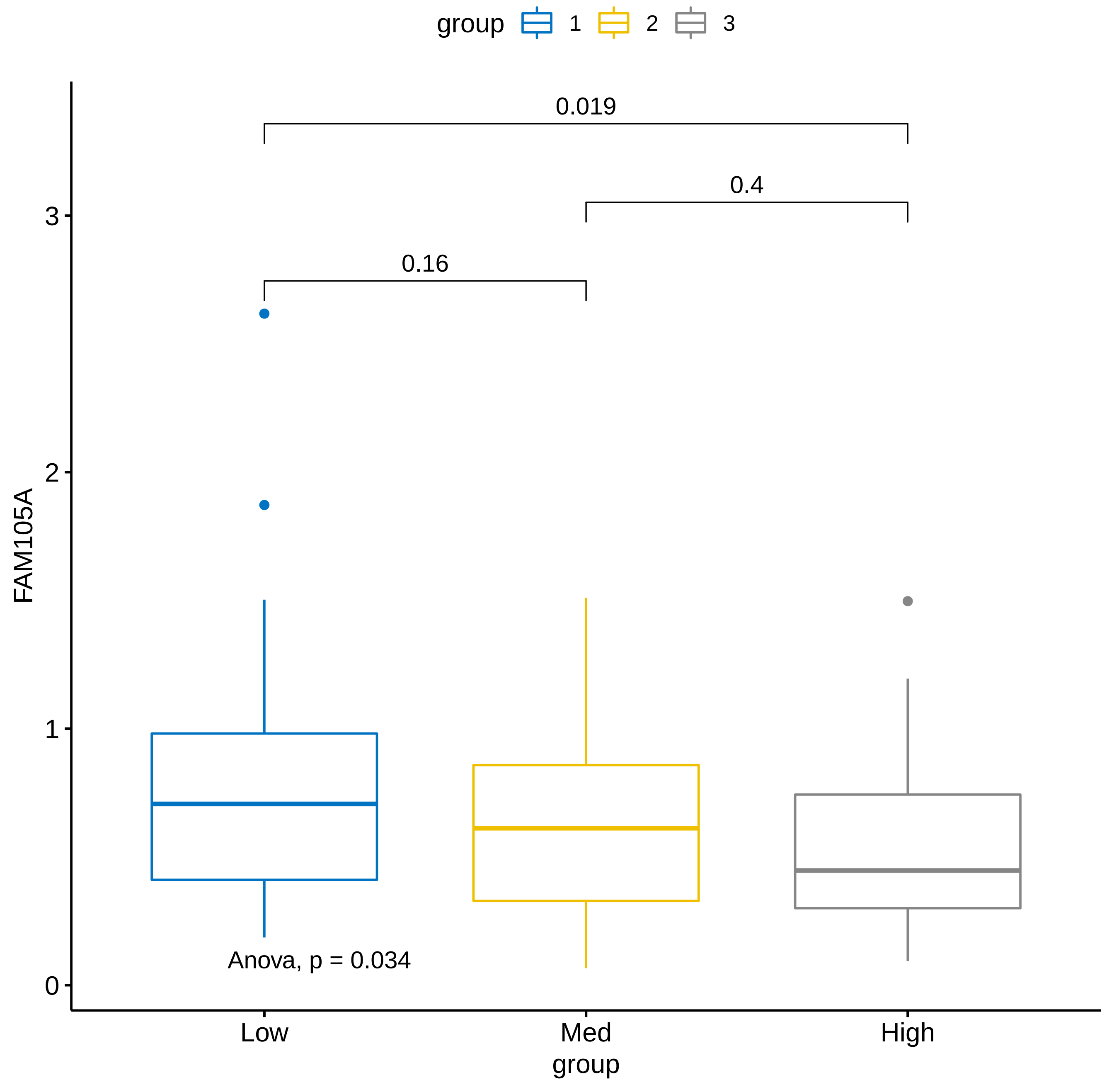

group 1 2 3
0.019
0.4
0.16
Anova, p = 0.034
FAM105A
3
2
1
0
Low
Med
High
group

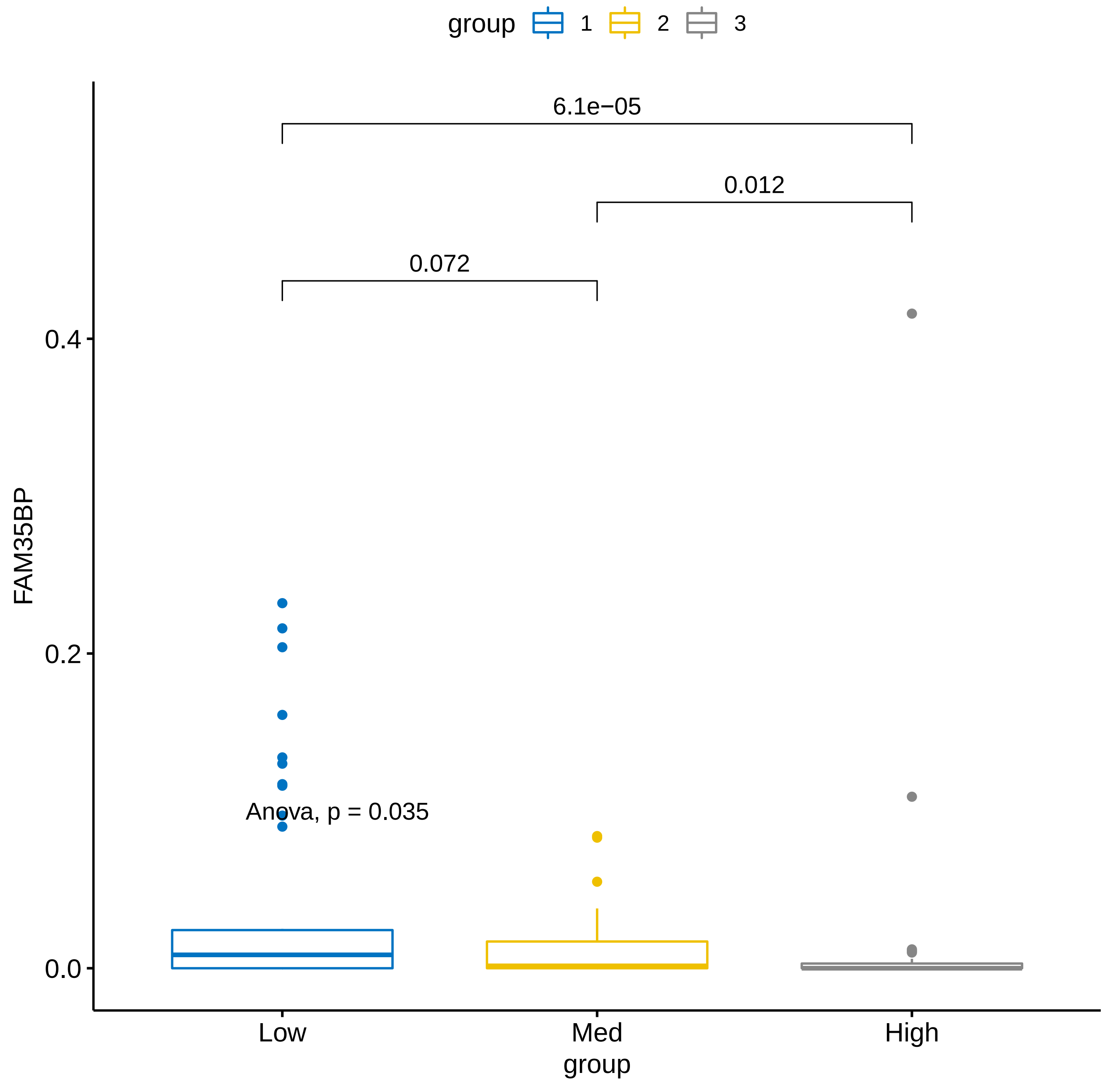

group 1 2 3
6.1e−05
0.012
0.072
0.4
0.2
0.0
FAM35BP
Anova, p = 0.035
Low
Med
High
group

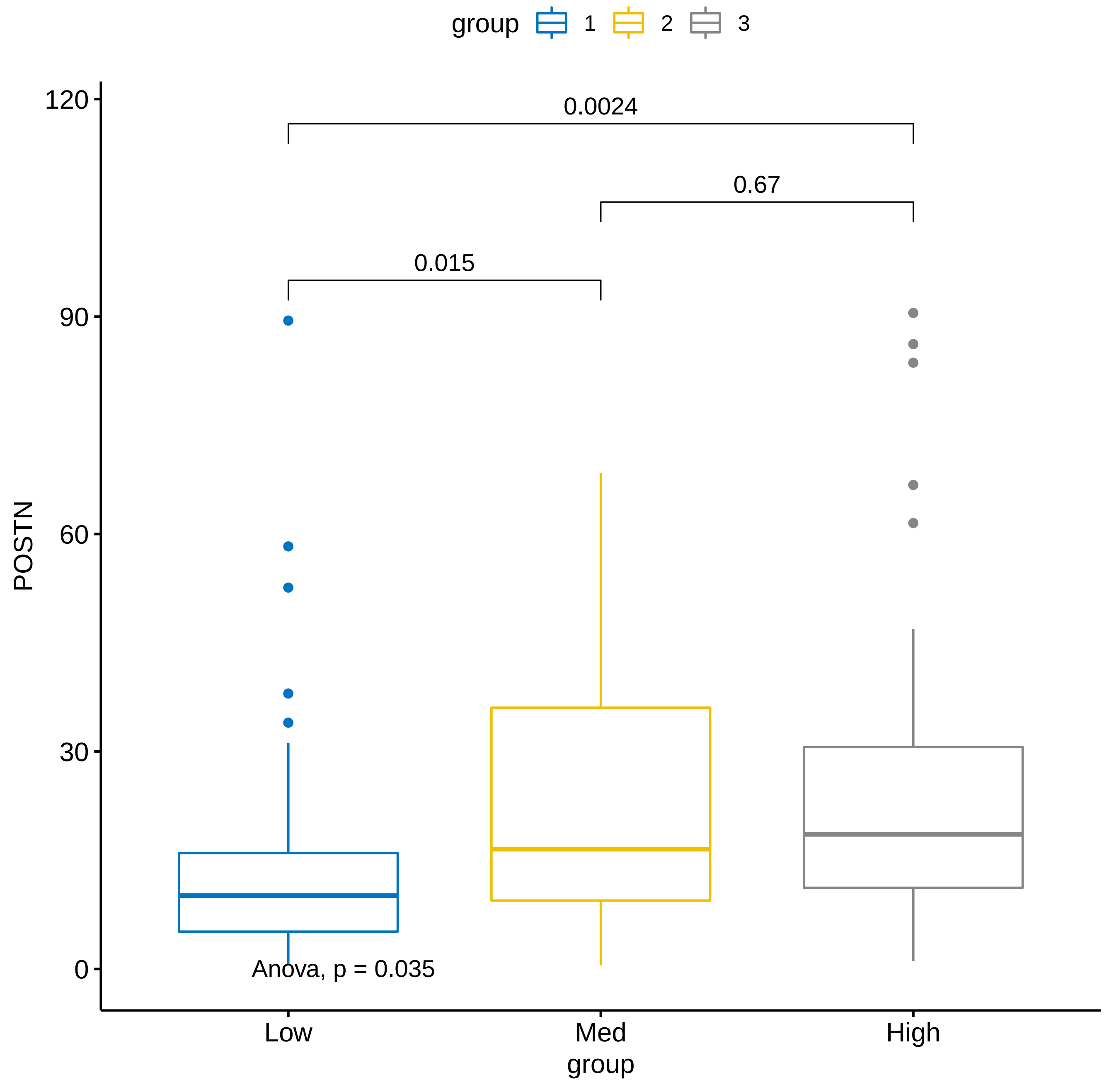
group
1
2
3
0.0024
0.67
0.015
120
90
60
30
0
POSTN
Anova, p = 0.035
Low
Med
High
group

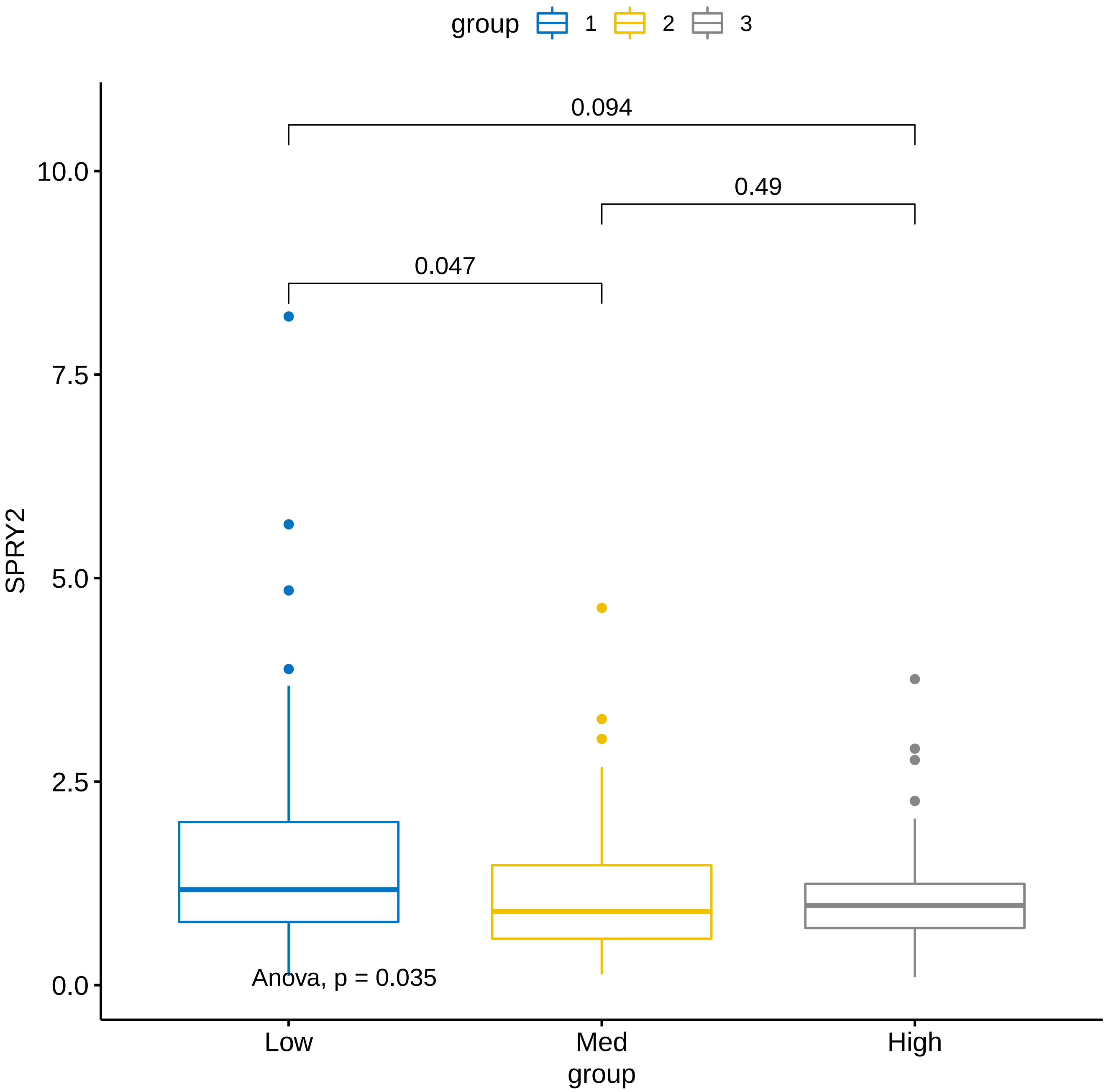
group
1
2
3
0.094
0.49
0.047
10.0
7.5
5.0
2.5
0.0
SPRY2
Anova, p = 0.035
Low
Med
High
group

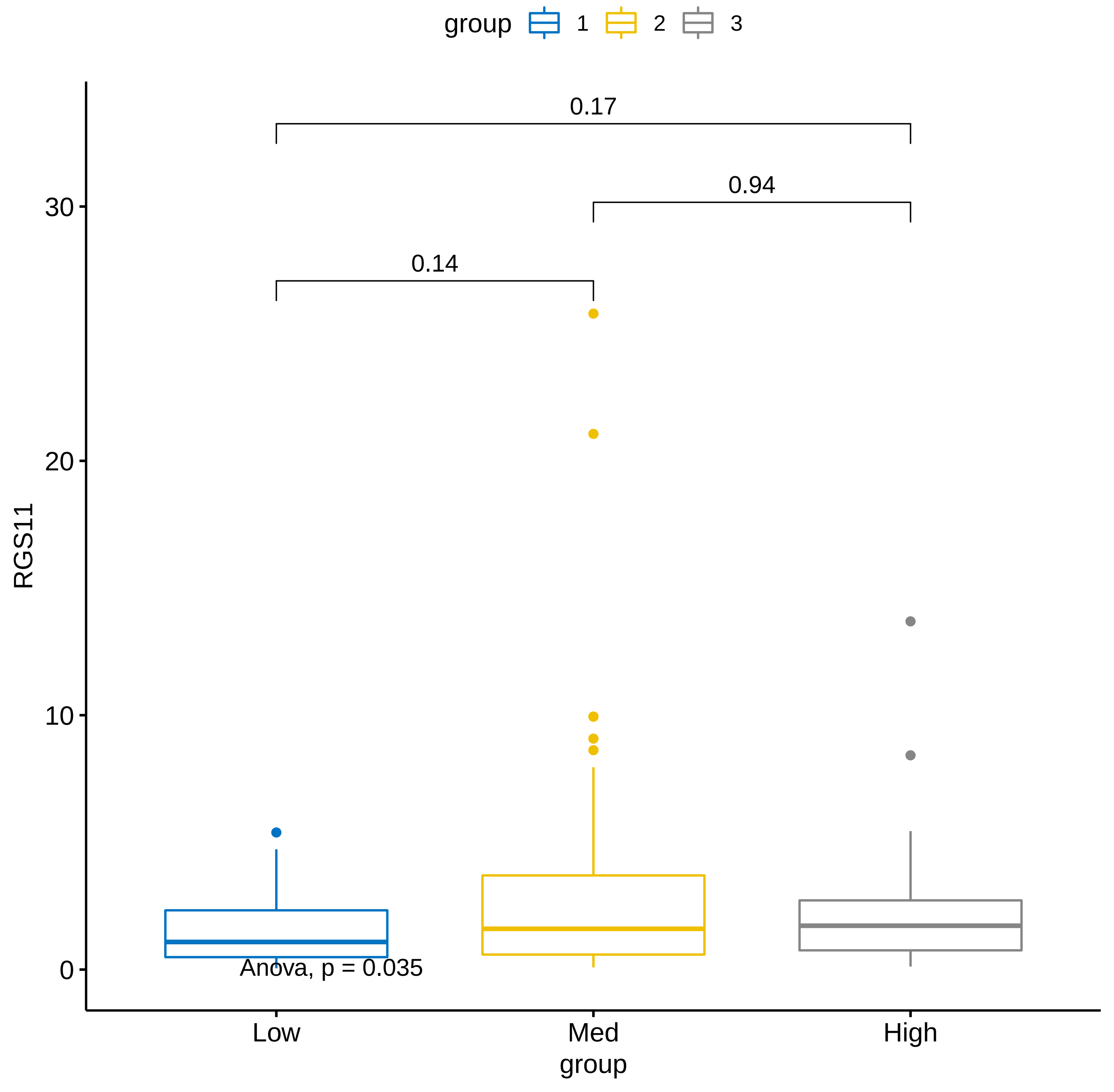

group 1 2 3
0.17
0.94
0.14
30
20
10
0
RGS11
Anova, p = 0.035
Low
Med
High
group

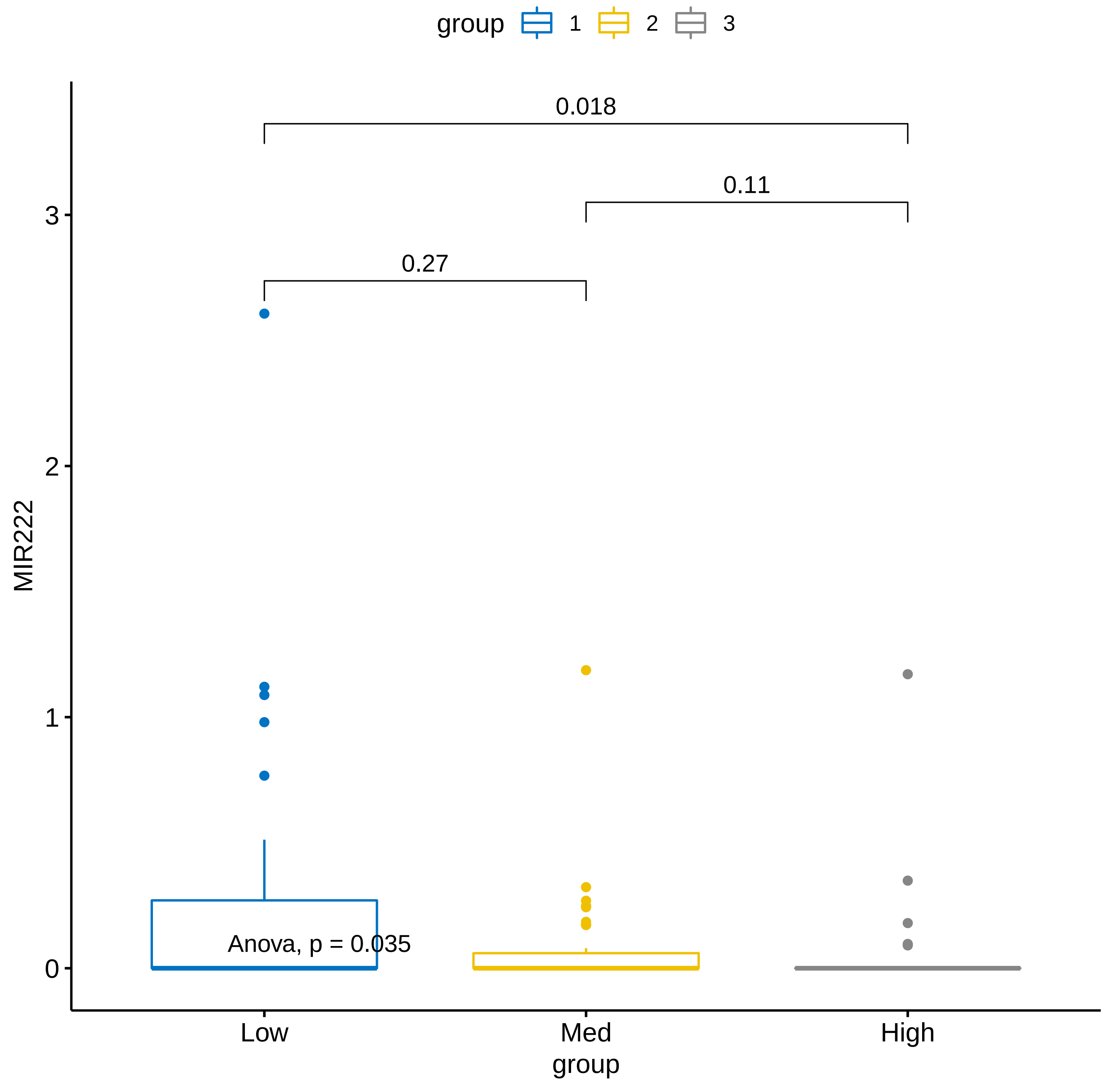
group 1 2 3
0.018
0.11
0.27
3
2
1
0
MIR222
Anova, p = 0.035
Low
Med
High
group

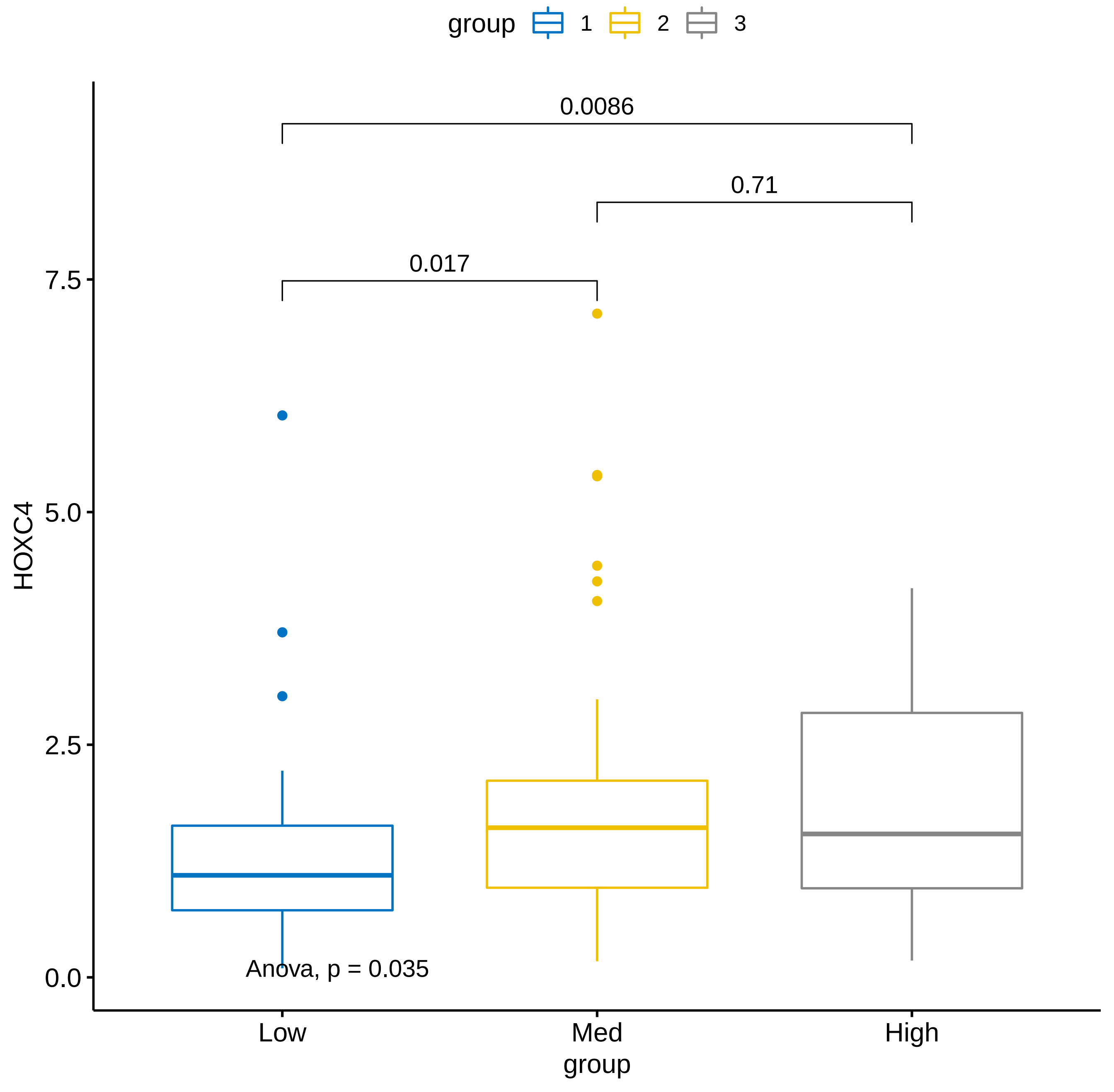
group
1
2
3
0.0086
0.71
0.017
7.5
5.0
2.5
0.0
HOXC4
Anova, p = 0.035
Low
Med
High
group

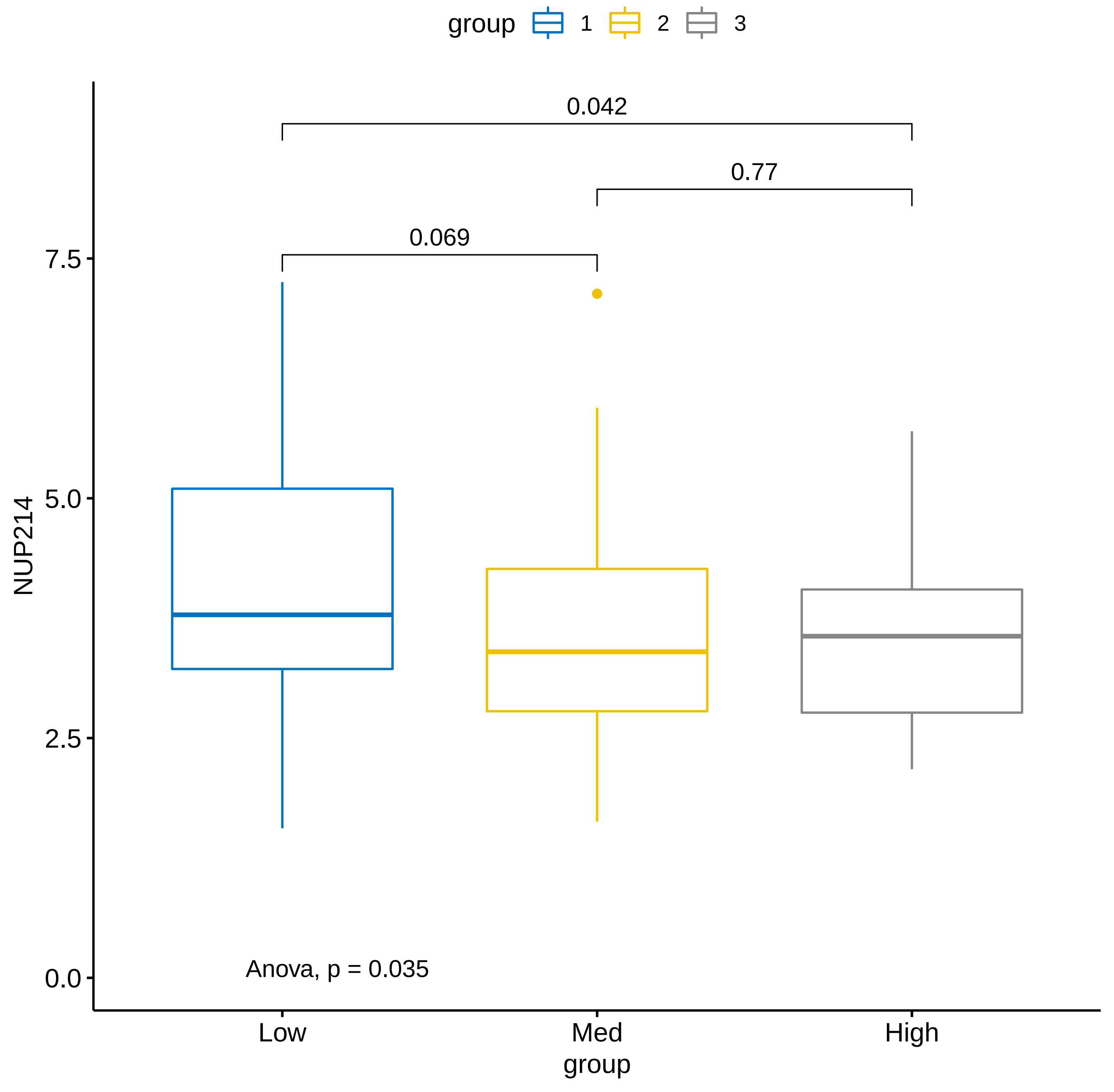

group 1 2 3
0.042
0.77
0.069
7.5
5.0
2.5
0.0
NUP214
Anova, p = 0.035
Low
Med
High
group

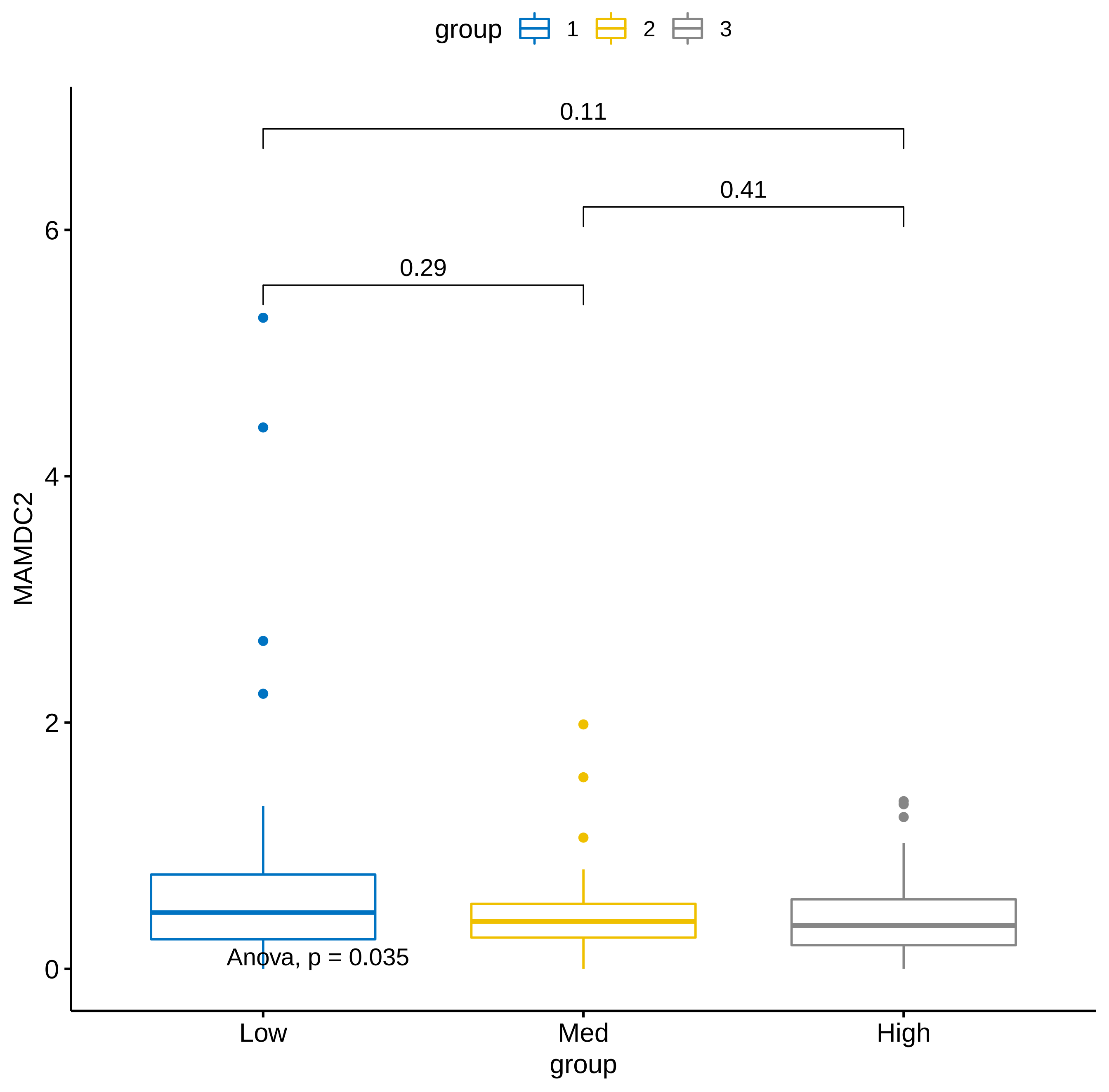

group
1
2
3
0.11
0.41
0.29
6
4
2
0
MAMDC2
Anova, p = 0.035
Low
Med
High
group

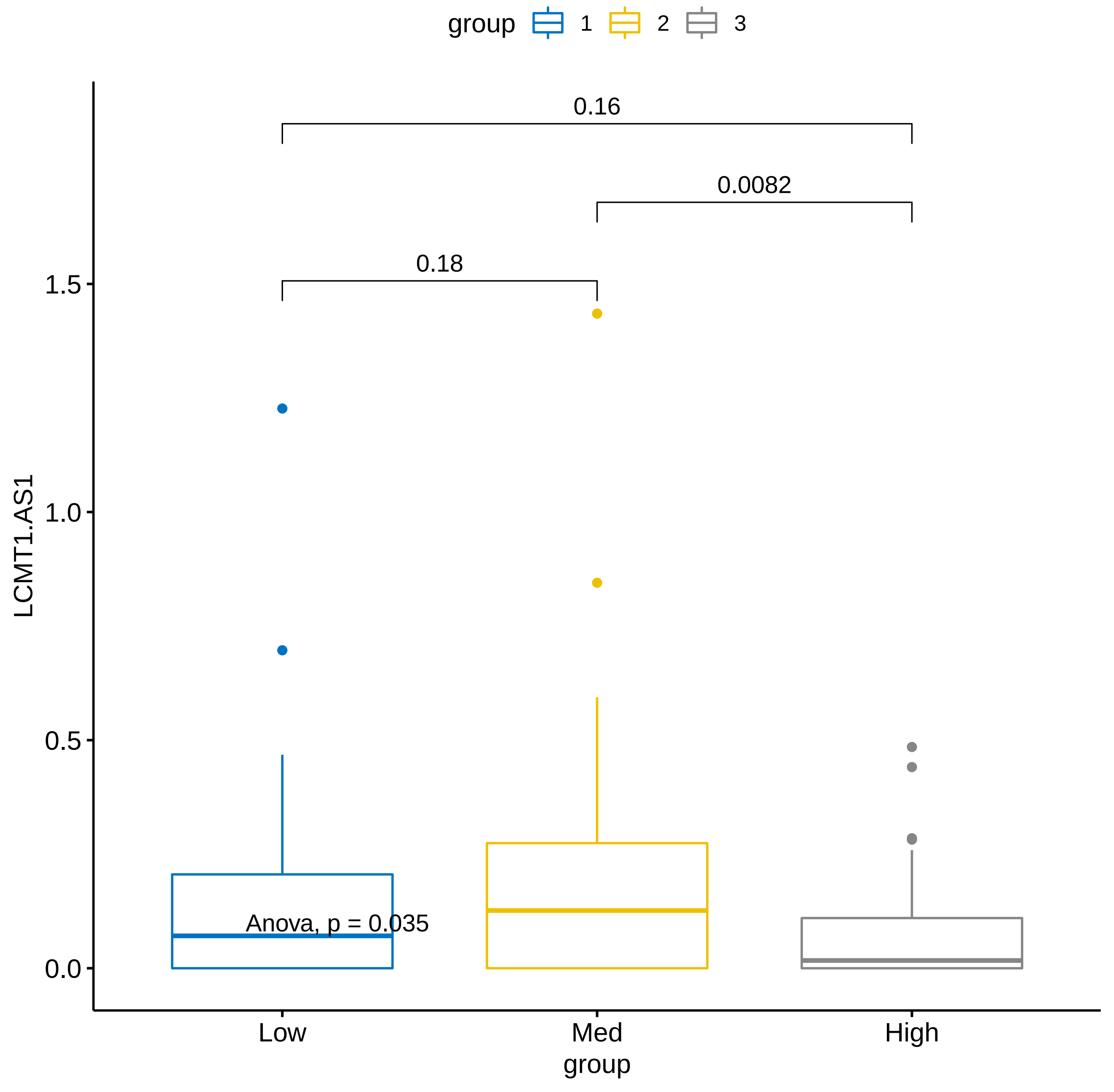
group
1
2
3
0.16
0.0082
0.18
1.5
1.0
0.5
0.0
LCMT1.AS1
Anova, p = 0.035
Low
Med
High
group

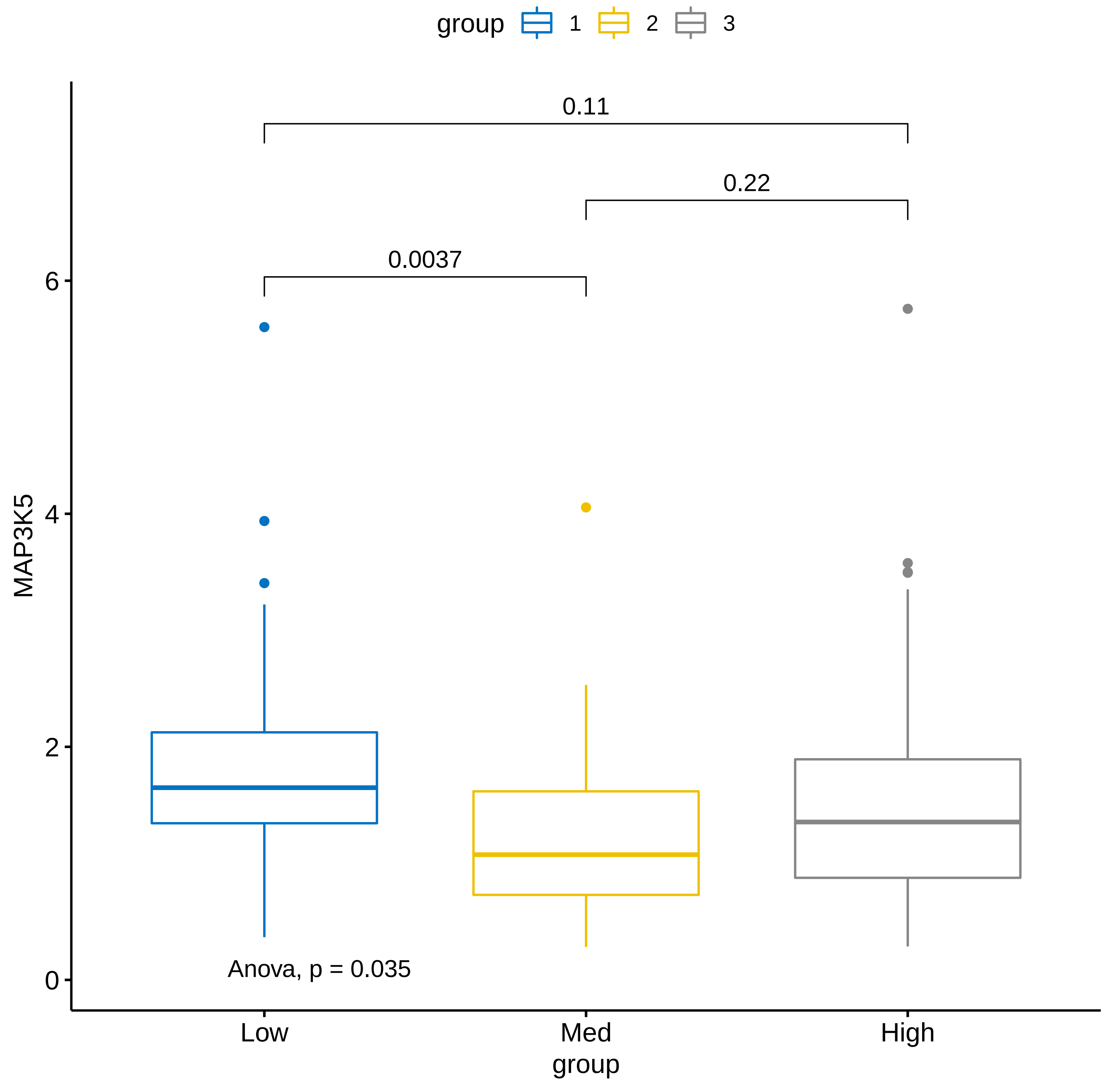
group
1
2
3
0.11
0.22
0.0037
MAP3K5
6
4
2
0
Anova, p = 0.035
Low
Med
High
group

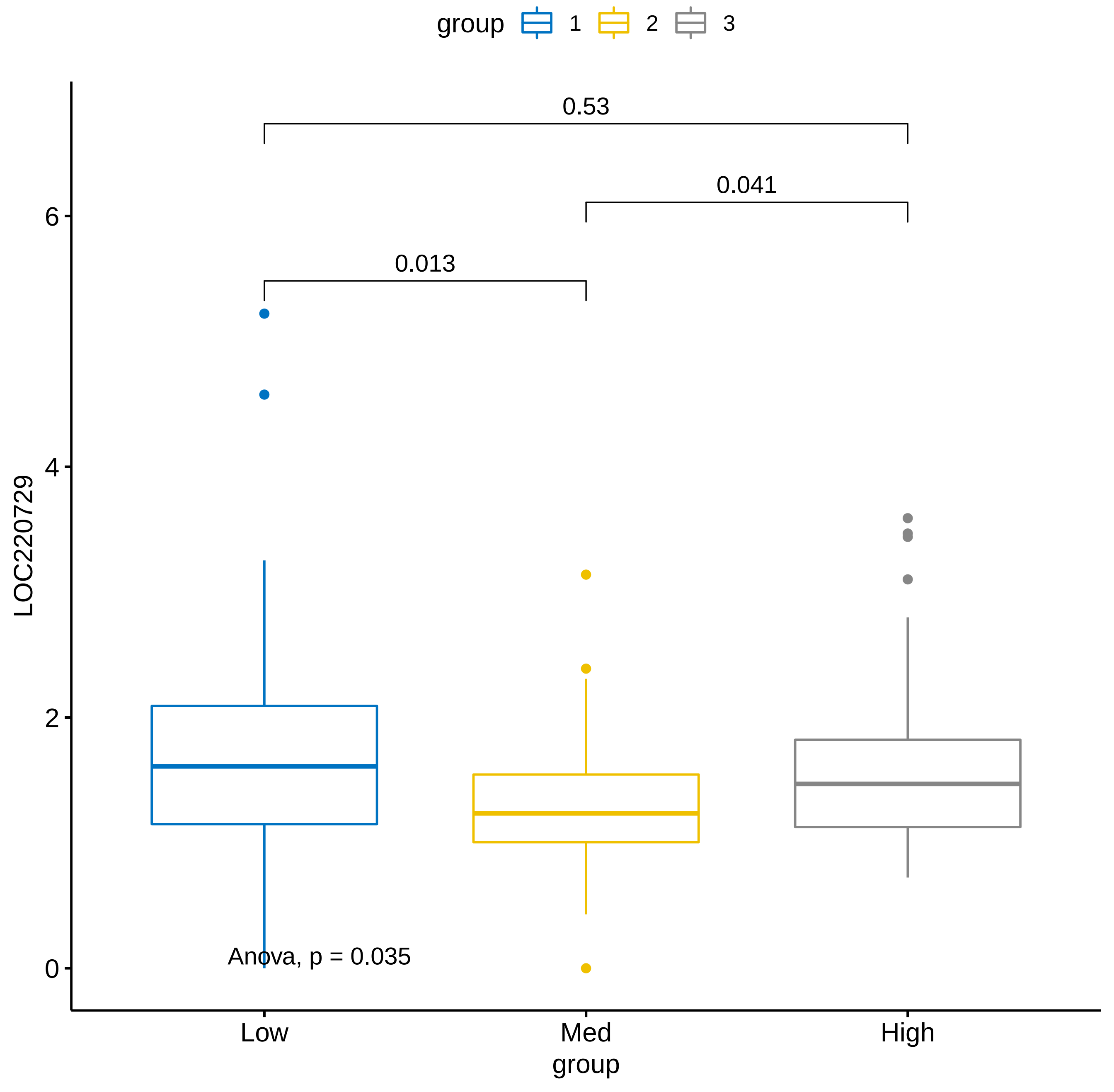

group 1 2 3
0.53
0.041
0.013
6
4
2
0
LOC220729
Anova, p = 0.035
Low
Med
High
group

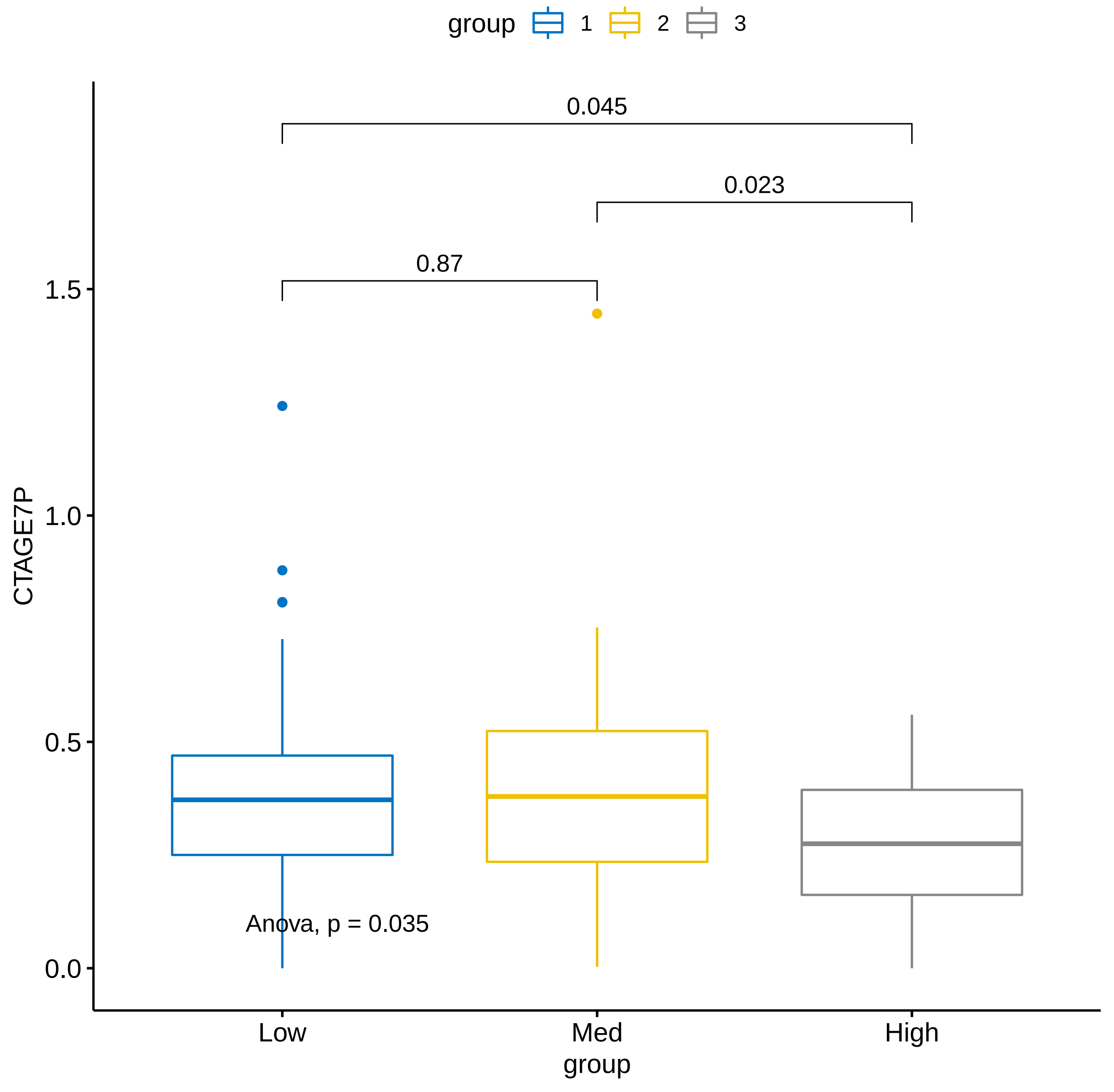
group
1
2
3
0.045
0.023
0.87
CTAGE7P
1.5
1.0
0.5
0.0
Anova, p = 0.035
Low
Med
High
group

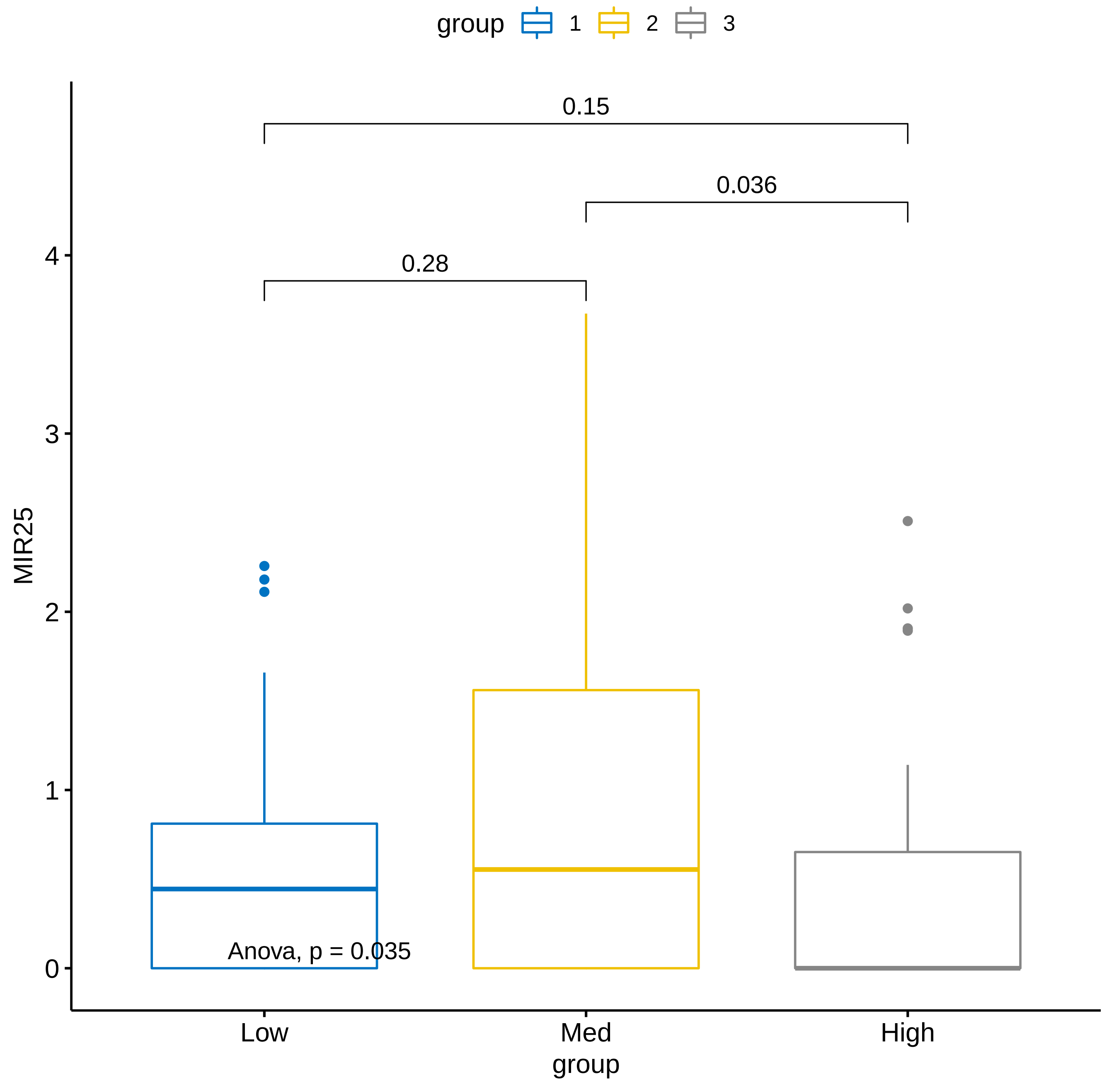
group
1
2
3
0.15
0.036
0.28
MIR25
4
3
2
1
0
Anova, p = 0.035
Low
Med
High
group

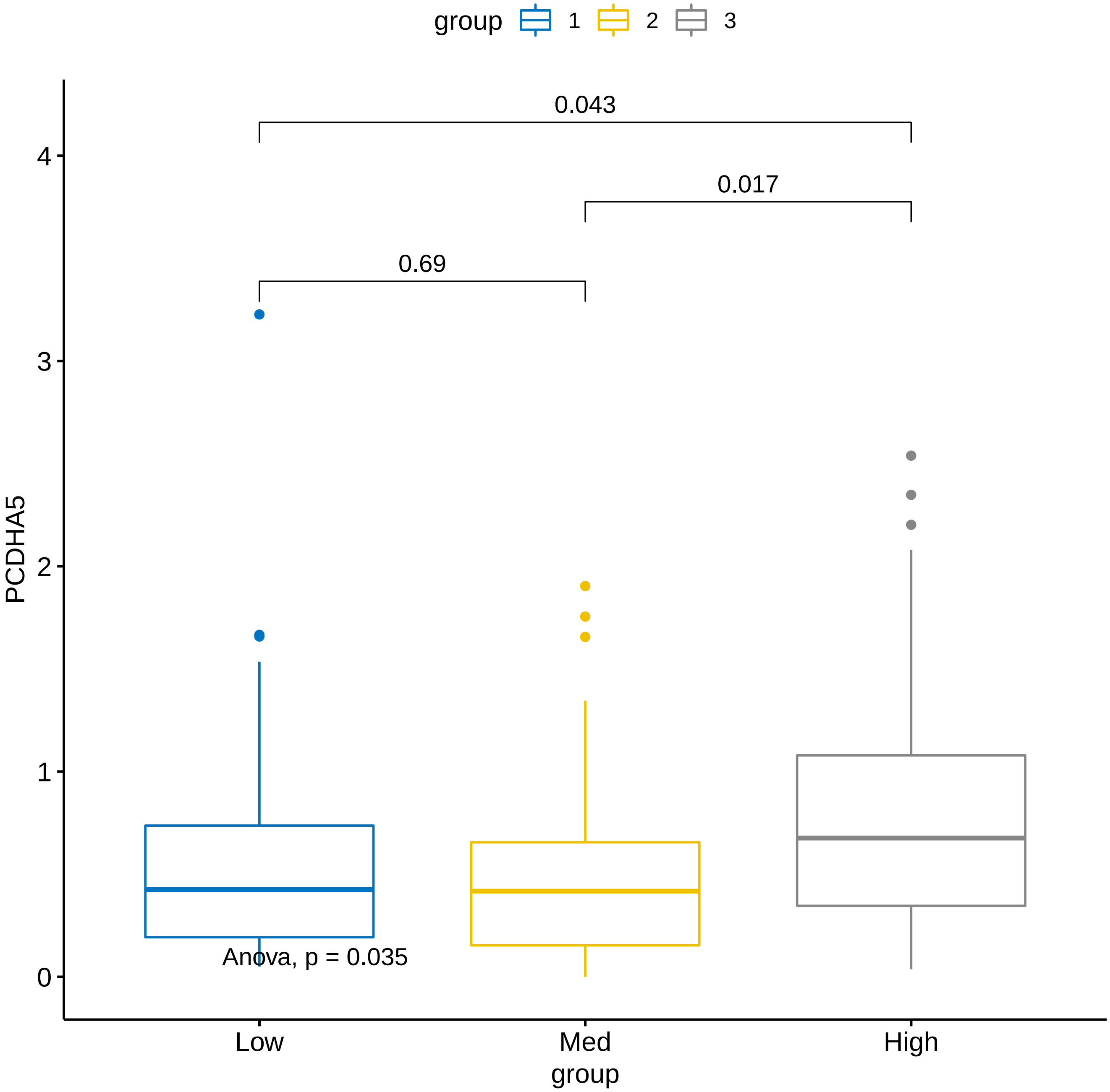
group
1
2
3
0.043
0.017
0.69
Anova, p = 0.035
PCDHA5
4
3
2
1
0
Low
Med
High
group

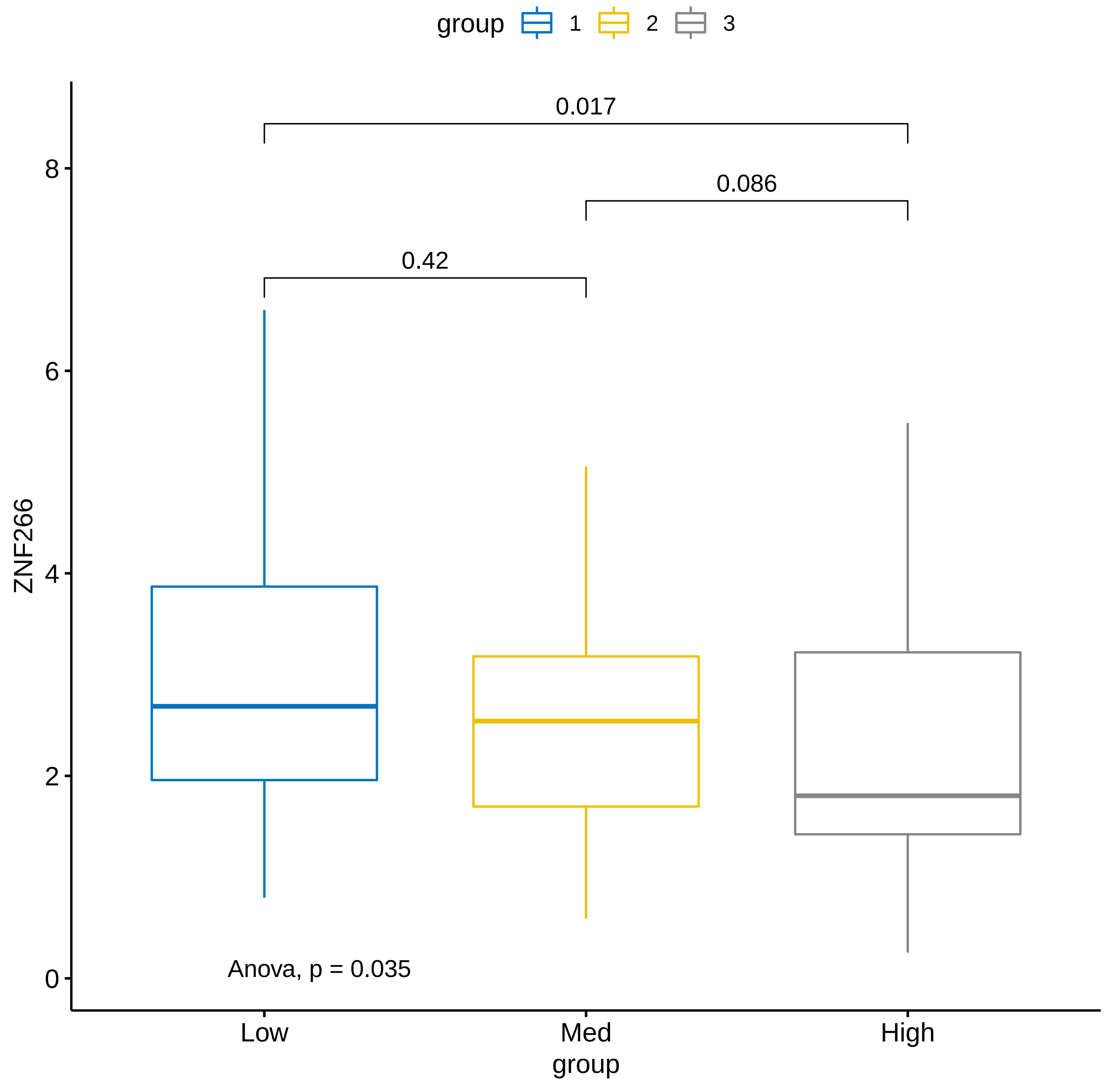

group 1 2 3
0.017
0.086
0.42
8
6
4
2
0
ZNF266
Anova, p = 0.035
Low
Med
High
group

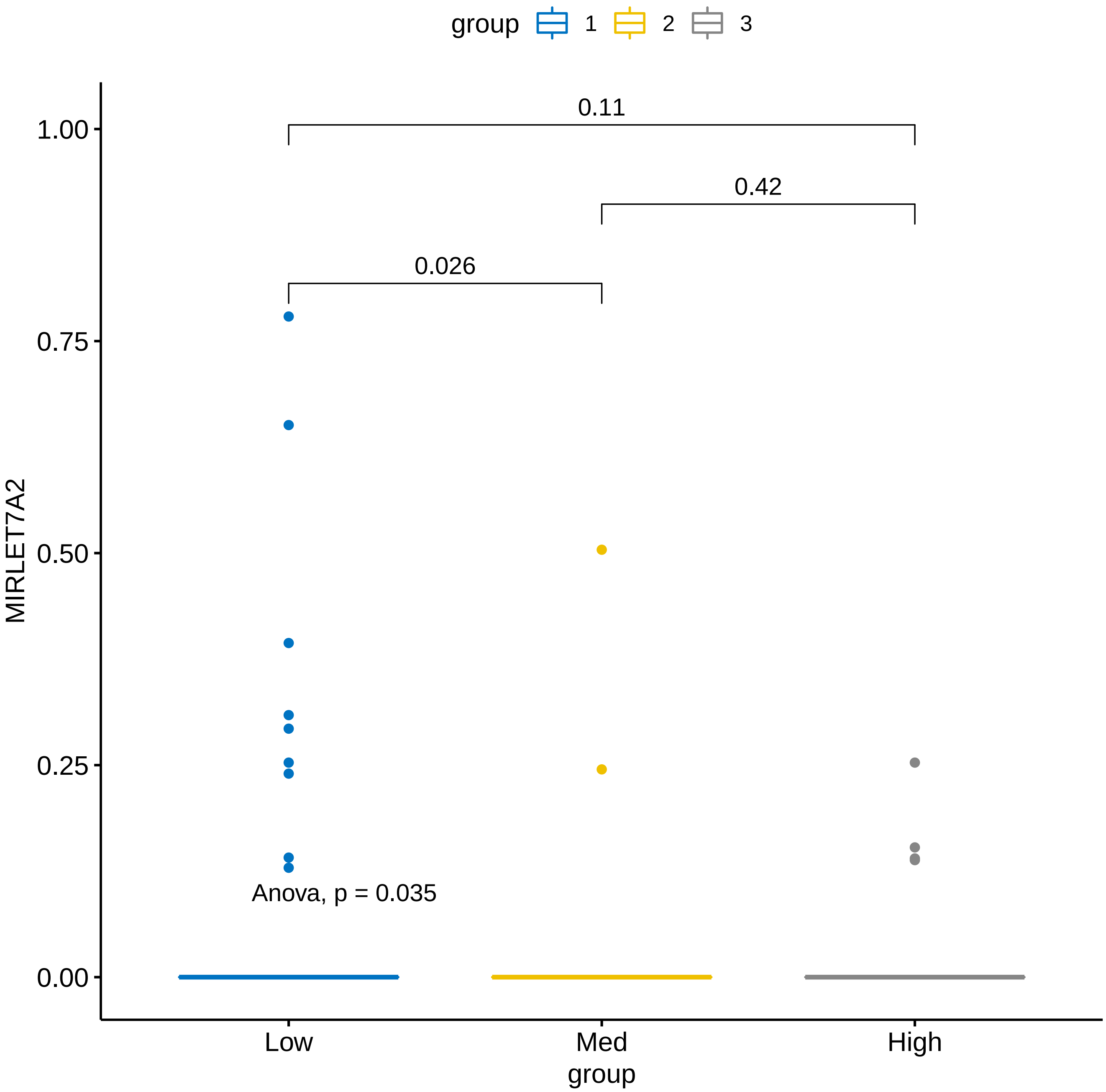

group
1
2
3
0.11
0.42
0.026
1.00
0.75
0.50
0.25
0.00
MIRLET7A2
Anova, p = 0.035
Low
Med
High
group

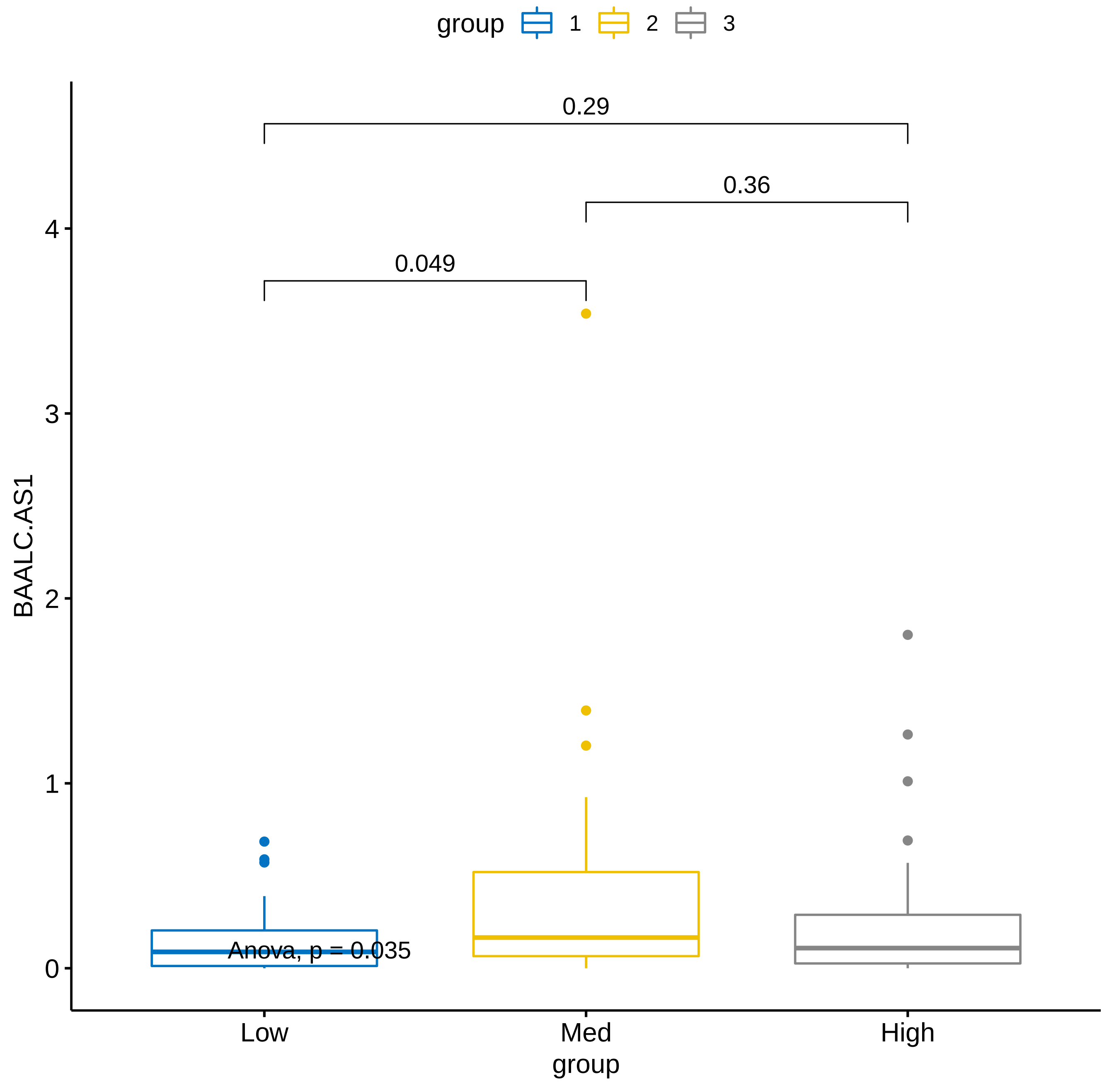
group
1
2
3
0.29
0.36
0.049
BAALC.AS1
4
3
2
1
0
Anova, p = 0.035
Low
Med
High
group

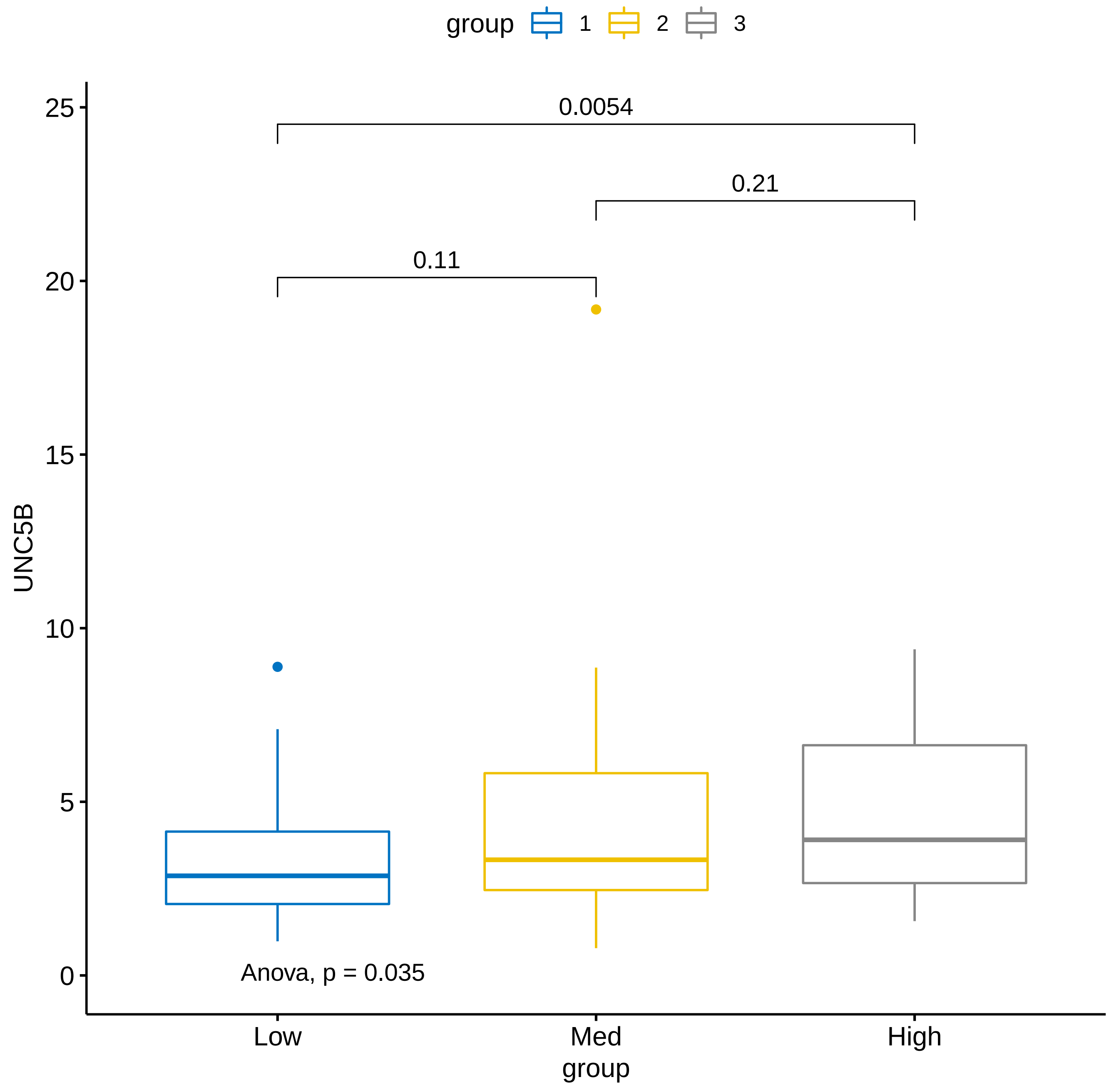
group 1 2 3
0.0054
0.21
0.11
UNC5B
25
20
15
10
5
0
Anova, p = 0.035
Low
Med
High
group

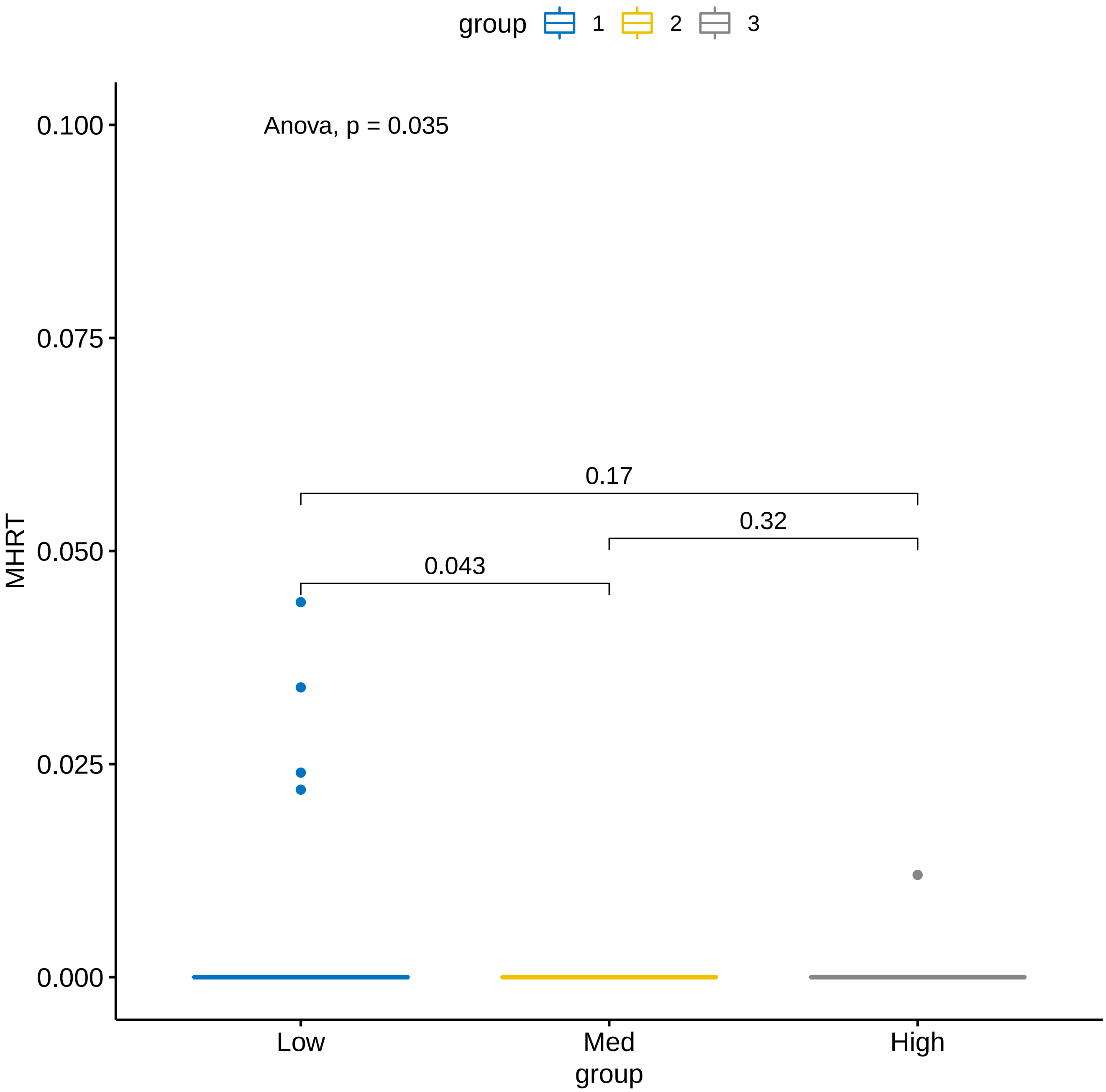
group
1
2
3
Anova, p = 0.035
0.17
0.32
0.043
0.100
0.075
0.050
0.025
0.000
MHRT
Low
Med
High
group

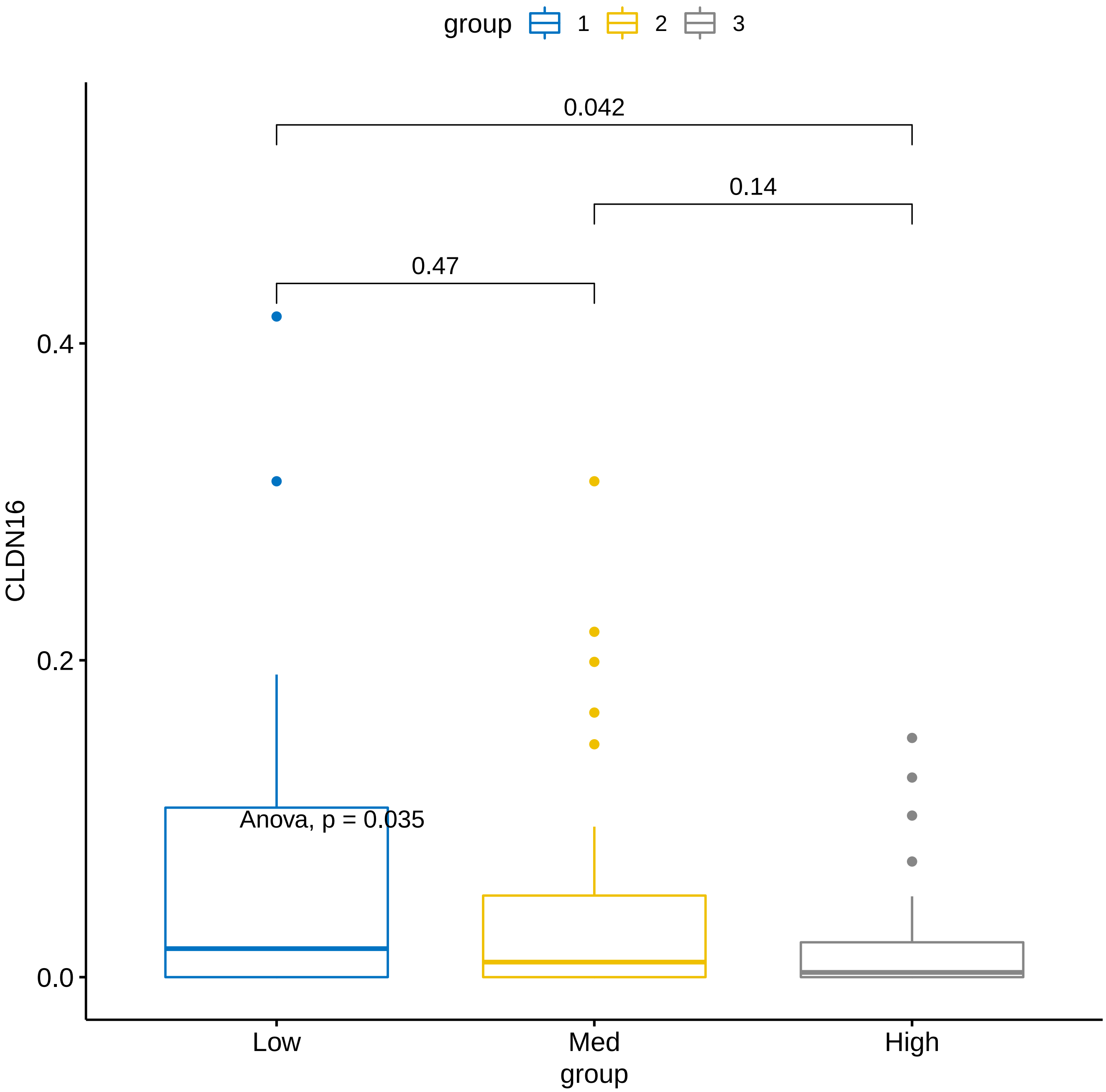
group
1
2
3
0.042
0.14
0.47
0.4
0.2
0.0
CLDN16
Anova, p = 0.035
Low
Med
High
group

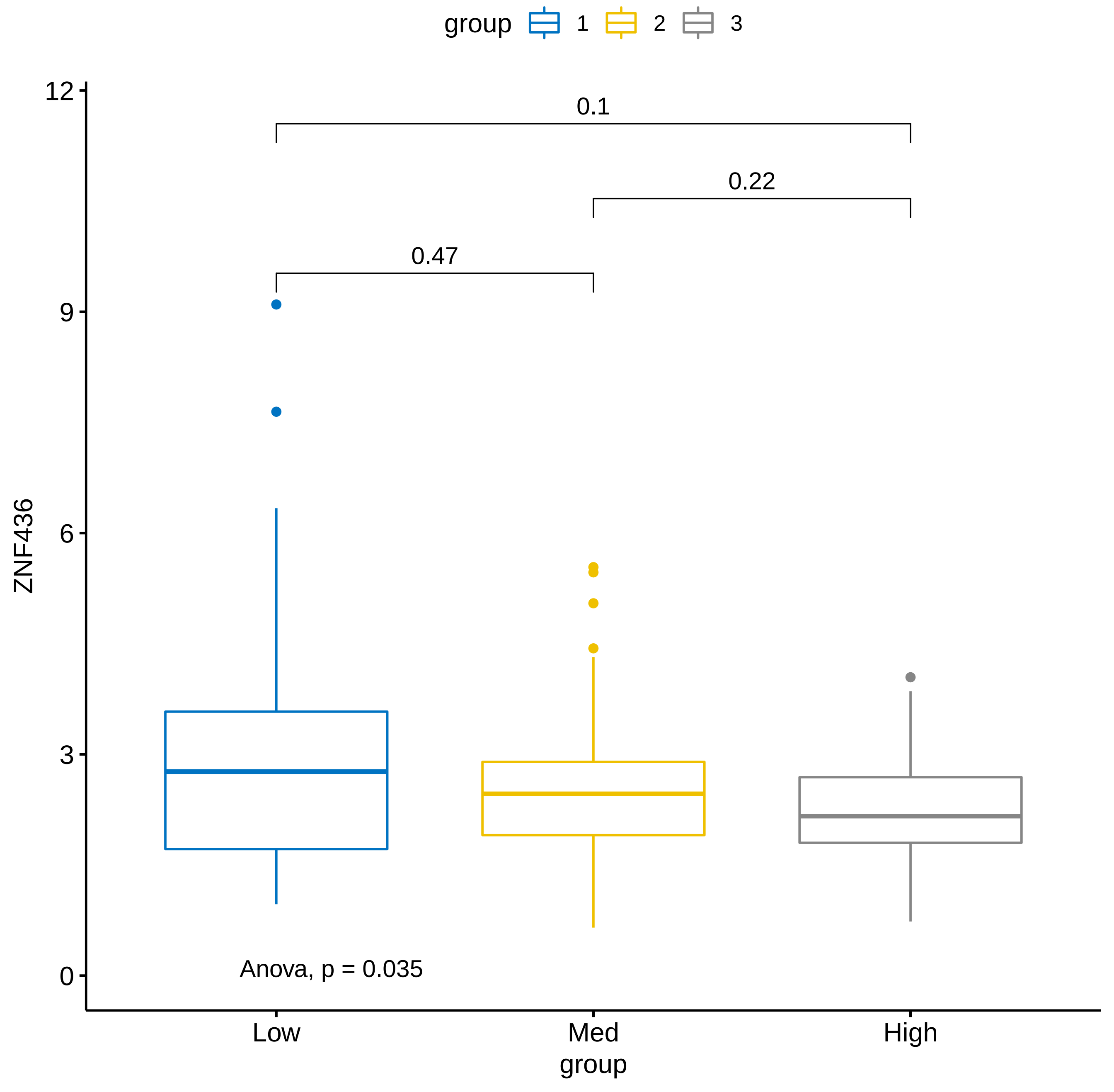

group
1
2
3
12
9
6
3
0
ZNF436
0.1
0.22
0.47
Anova, p = 0.035
Low
Med
High
group

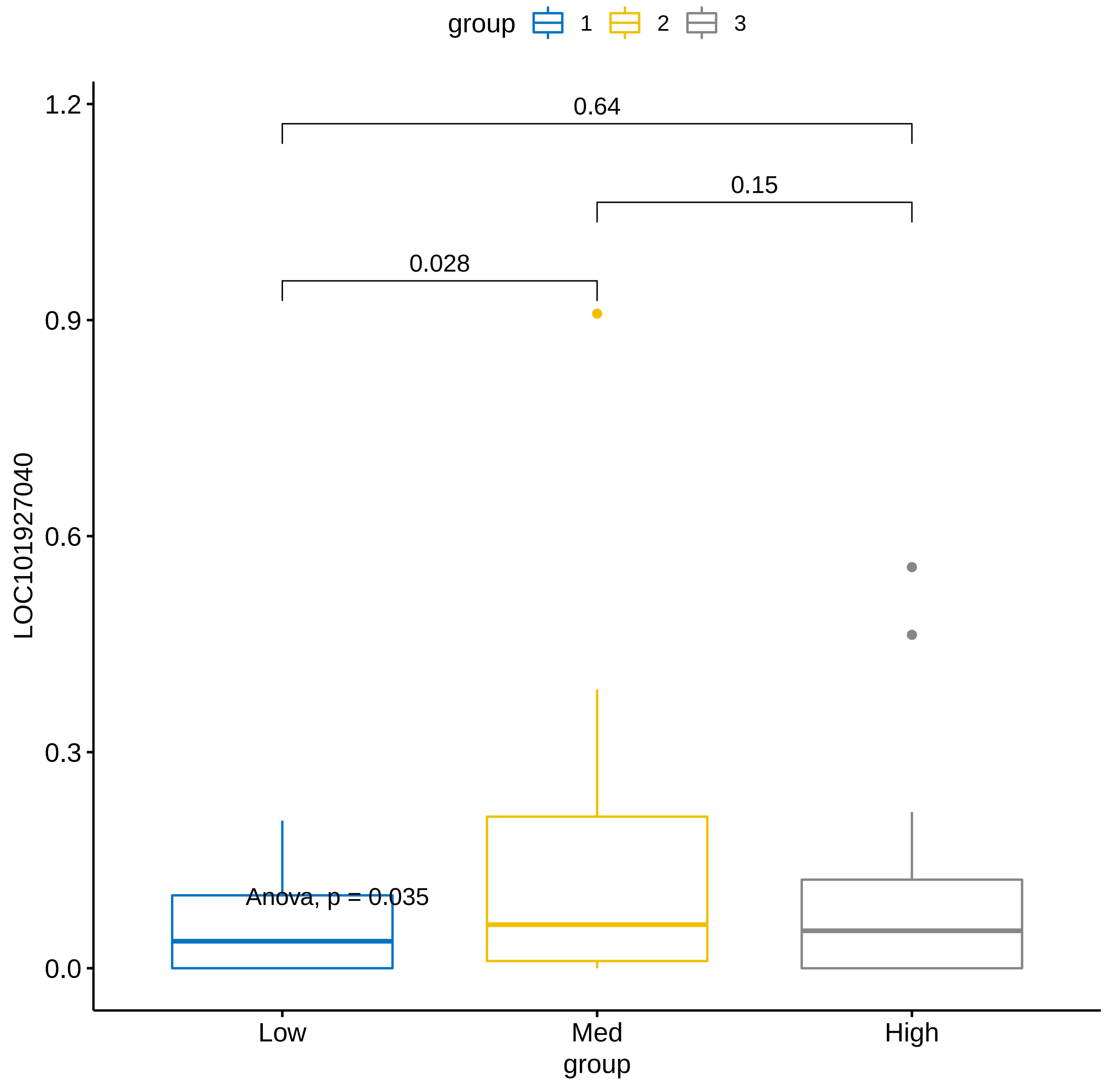
group
1
2
3
0.64
0.15
0.028
Anova, p = 0.035
1.2
0.9
0.6
0.3
0.0
LOC101927040
Low
Med
High
group

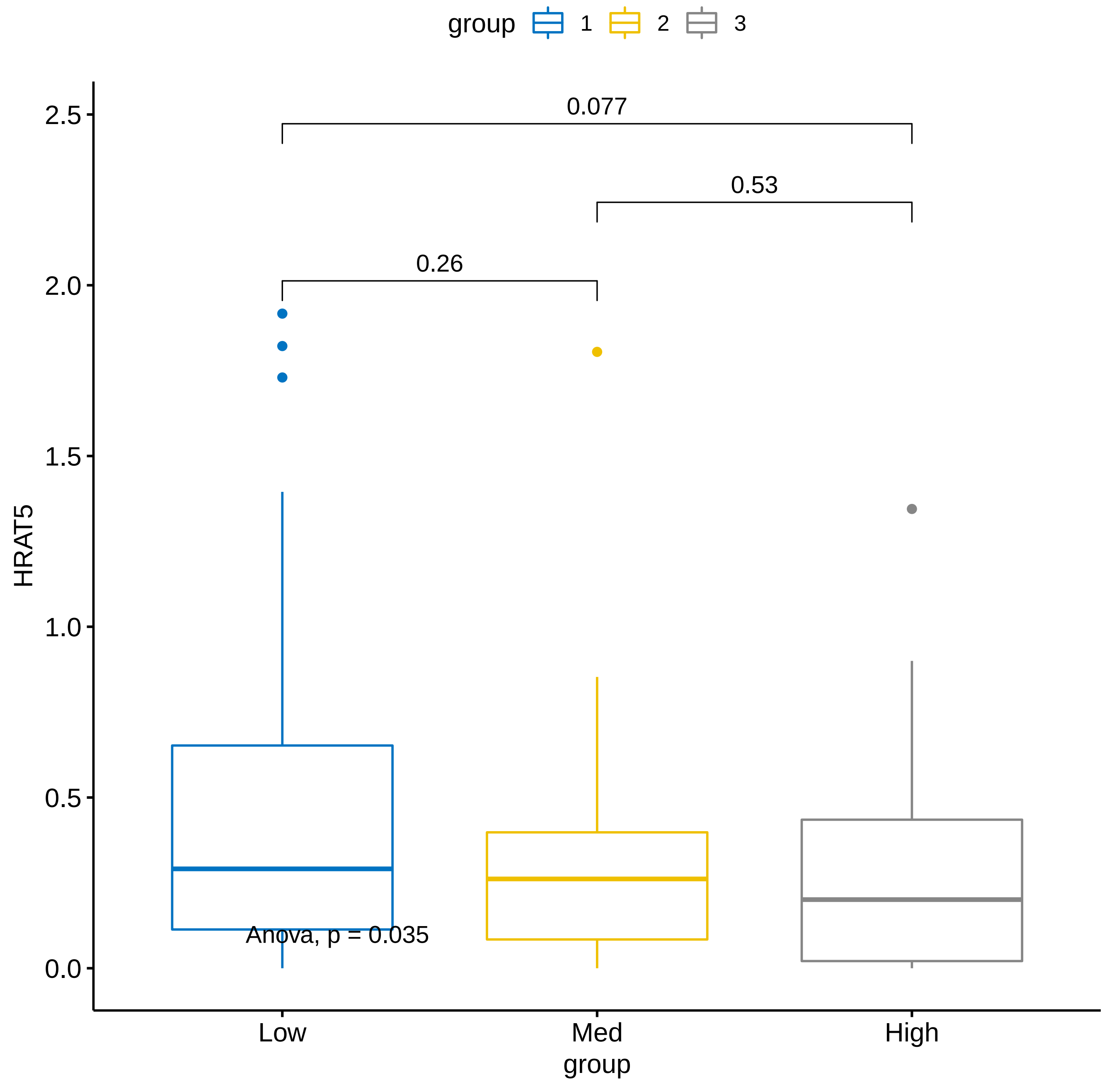
group
1
2
3
0.077
0.53
0.26
2.5
2.0
1.5
1.0
0.5
0.0
HRAT5
Anova, p = 0.035
Low
Med
High
group

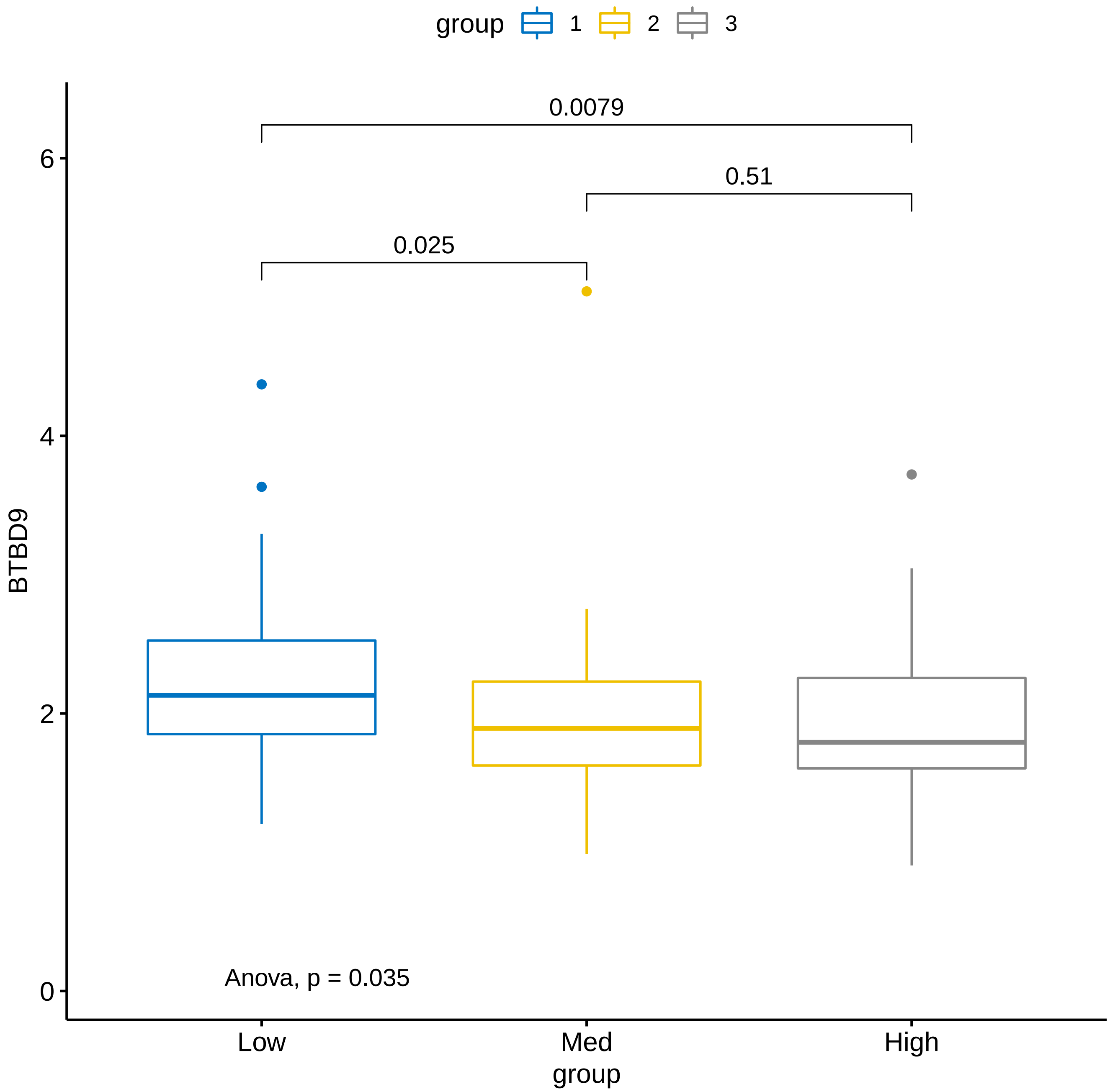
group 1 2 3
0.0079
0.51
0.025
6
4
2
0
BTBD9
Anova, p = 0.035
Low
Med
High
group

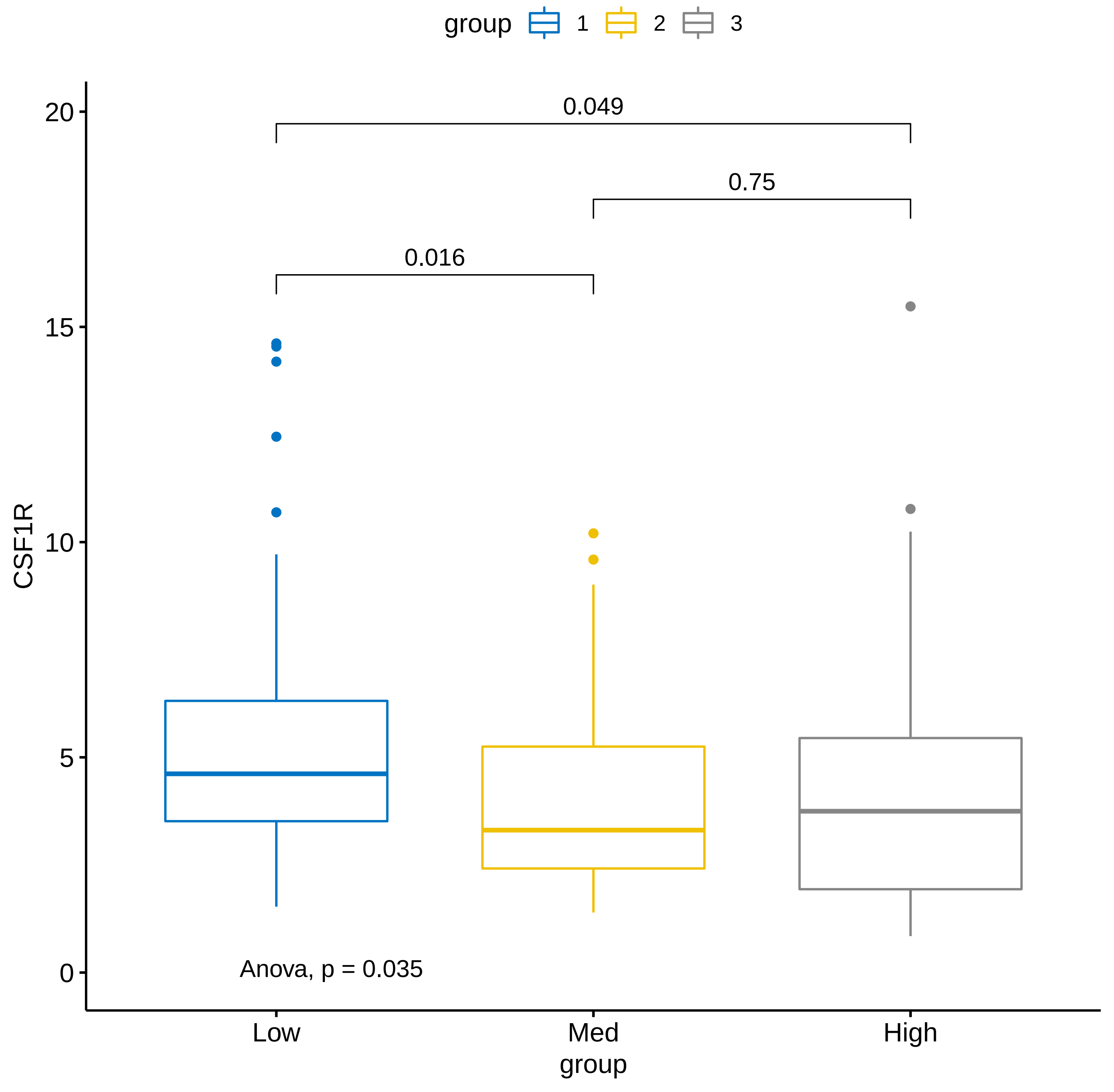
group
1
2
3
0.049
0.75
0.016
20
15
10
5
0
CSF1R
Anova, p = 0.035
Low
Med
High
group

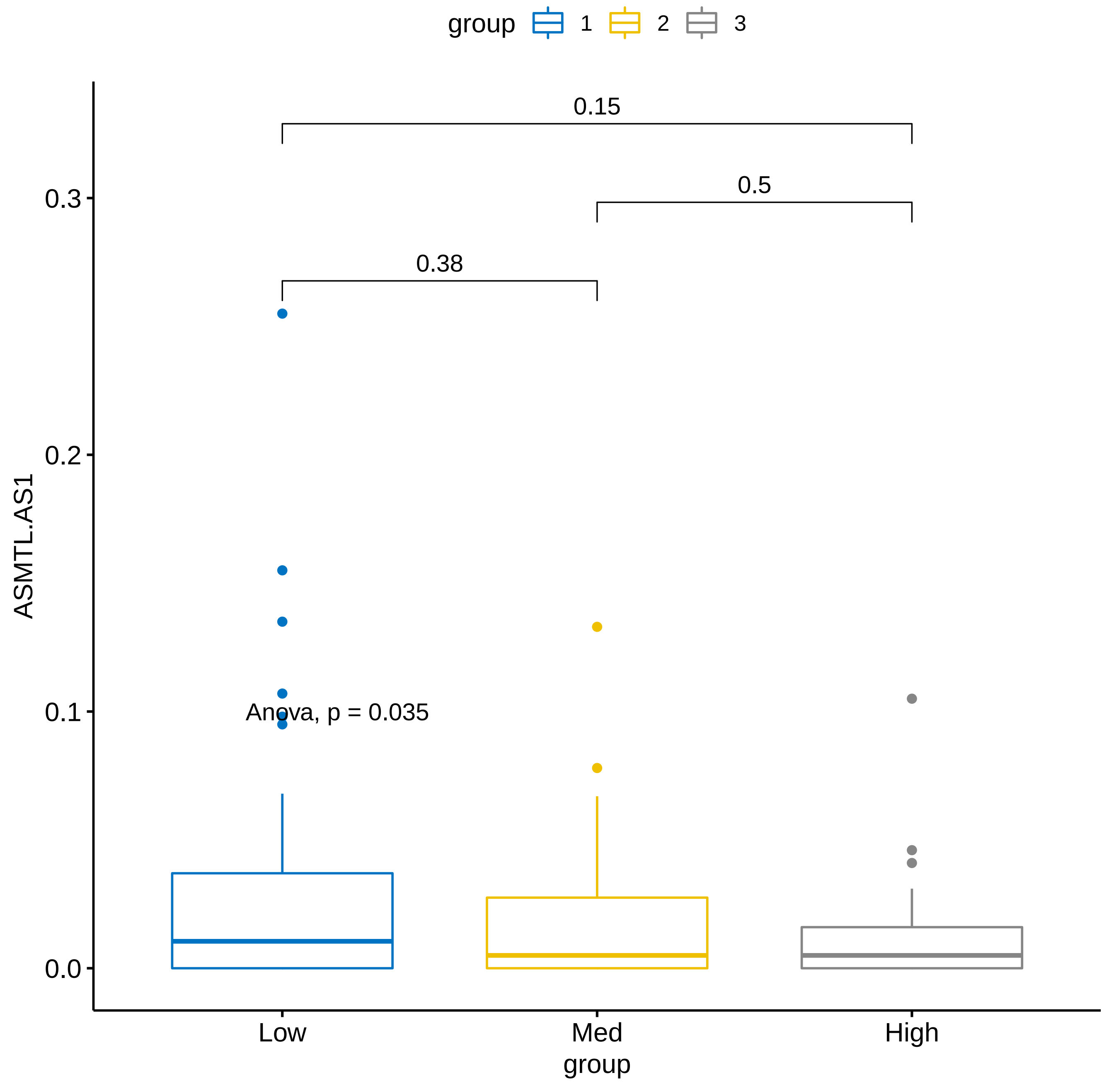
group 1 2 3
0.15
0.5
0.38
Anova, p = 0.035
ASMTL.AS1
0.3
0.2
0.1
0.0
Low
Med
High
group

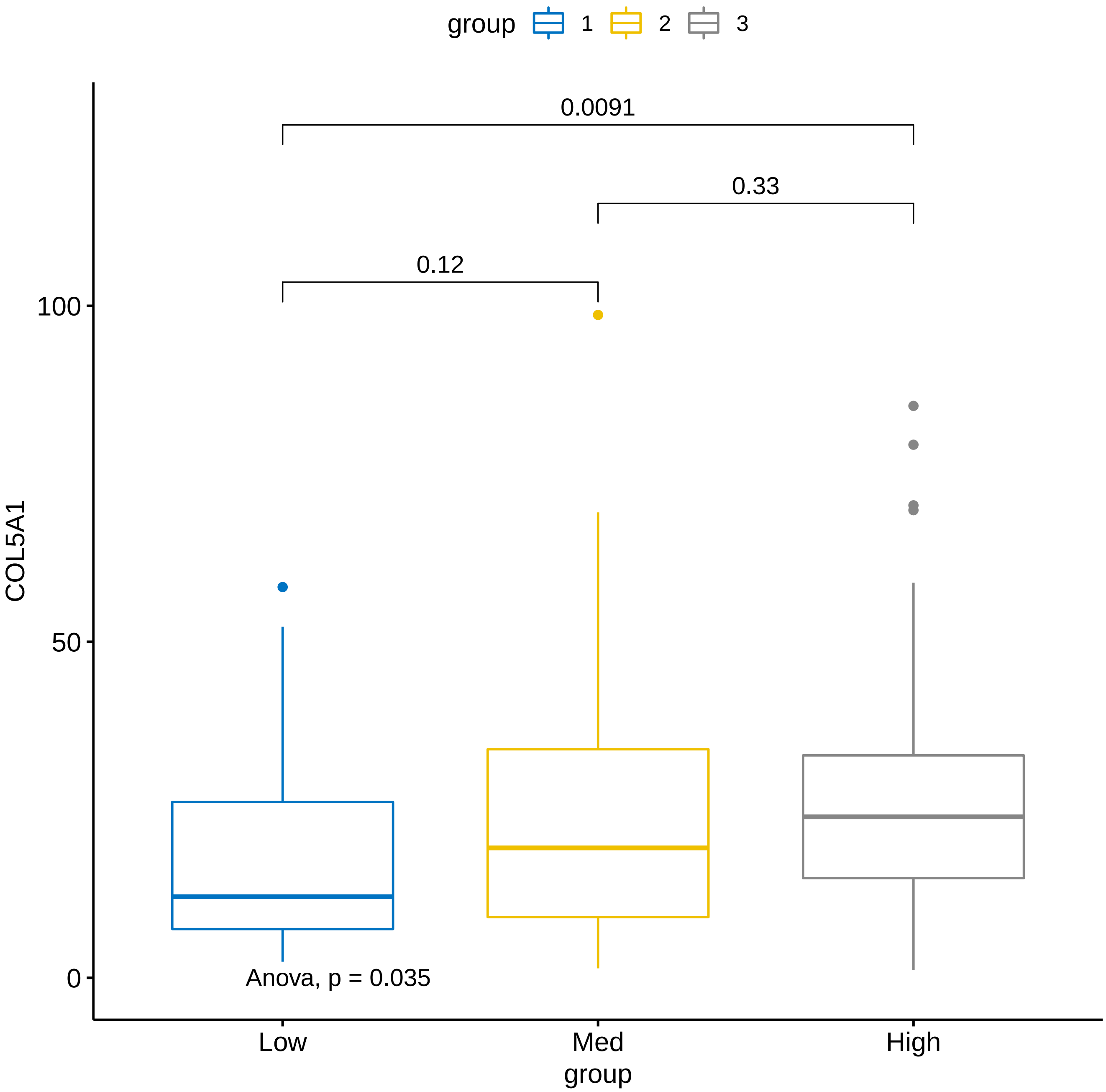
group
1
2
3
0.0091
0.33
0.12
100
50
0
COL5A1
Anova, p = 0.035
Low
Med
High
group

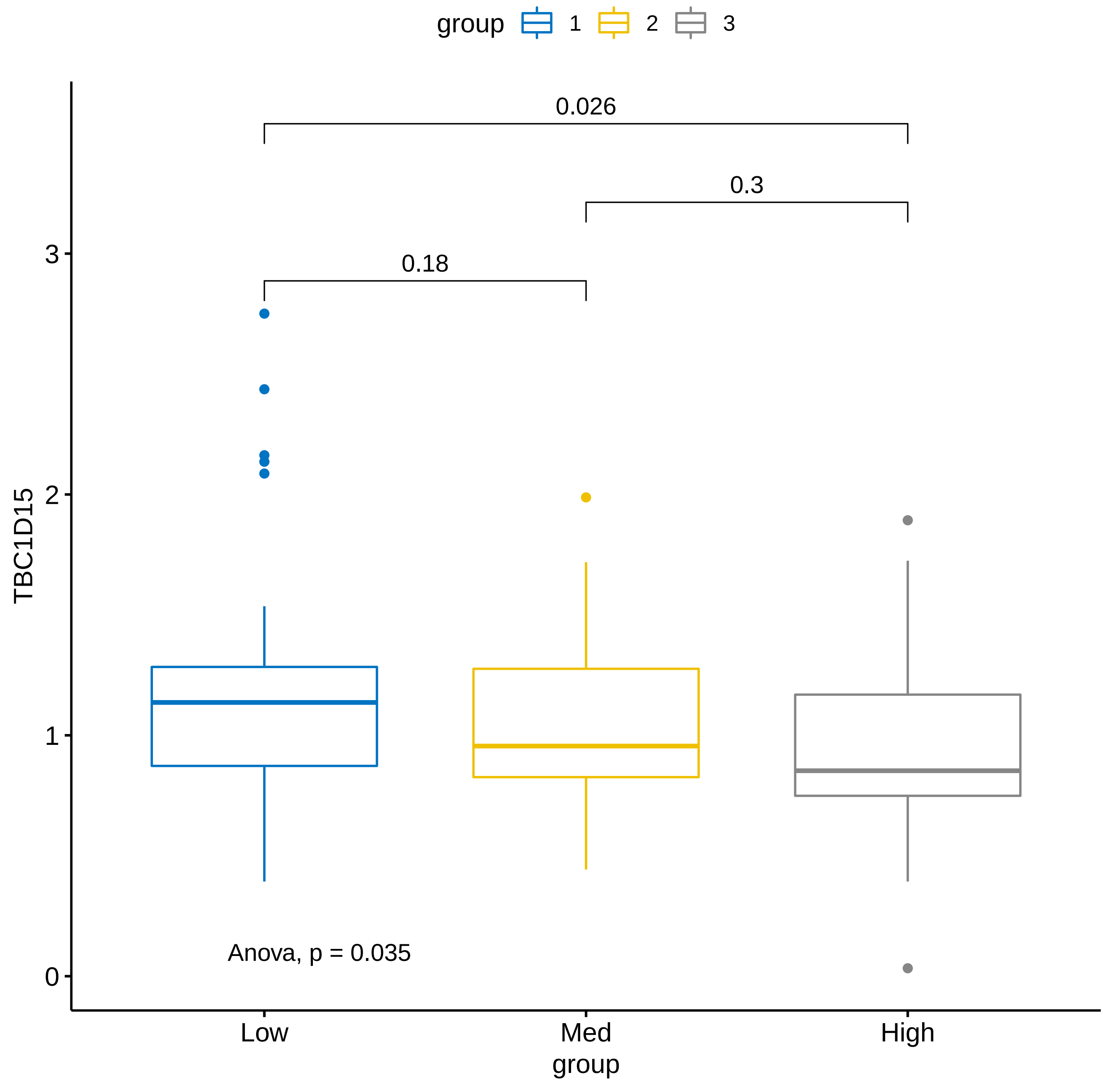

group 1 2 3
0.026
0.3
0.18
3
2
1
0
TBC1D15
Anova, p = 0.035
Low
Med
High
group

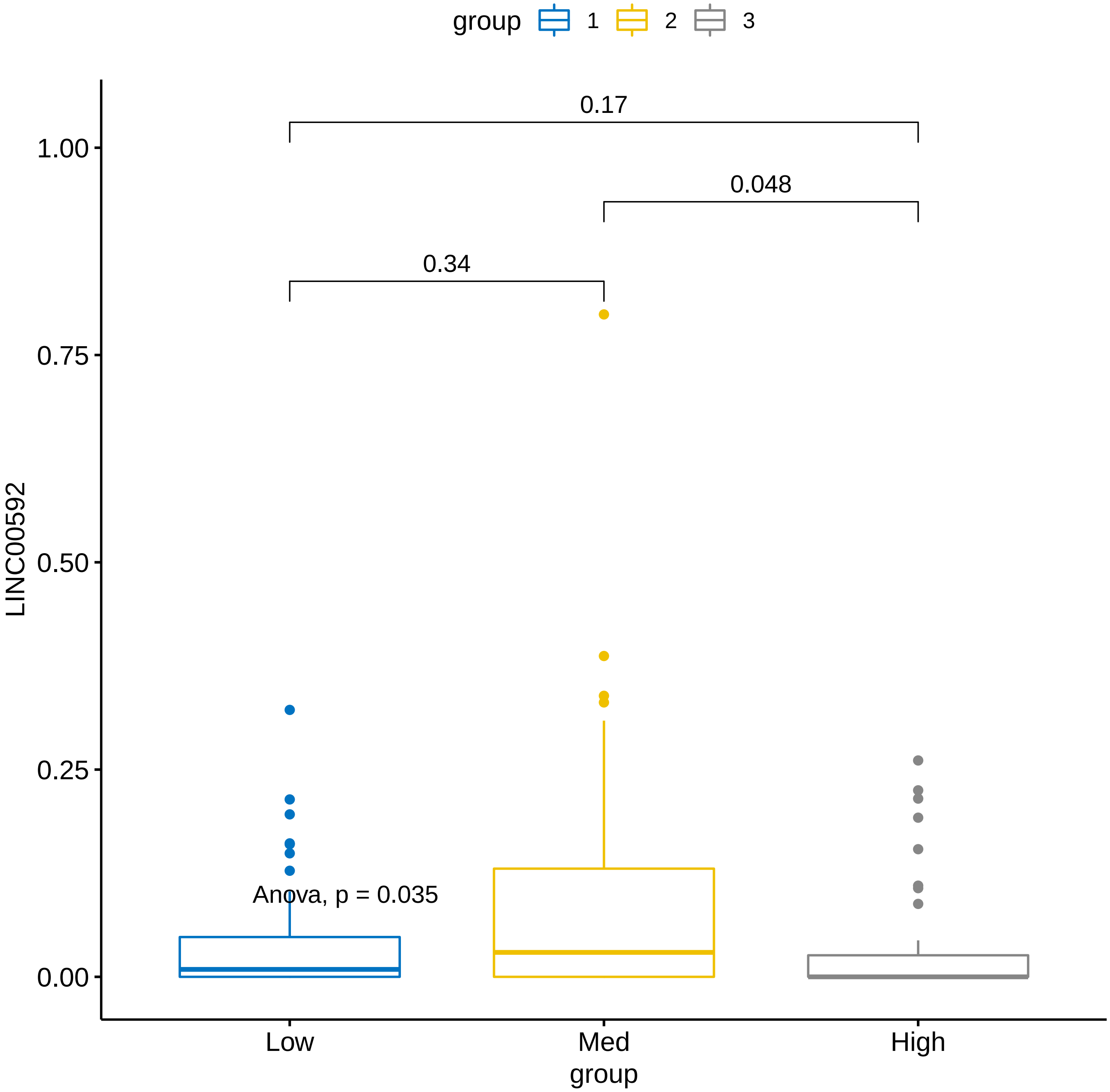

group
1
2
3
0.17
0.048
0.34
1.00
0.75
0.50
0.25
0.00
LINC00592
Anova, p = 0.035
Low
Med
High
group

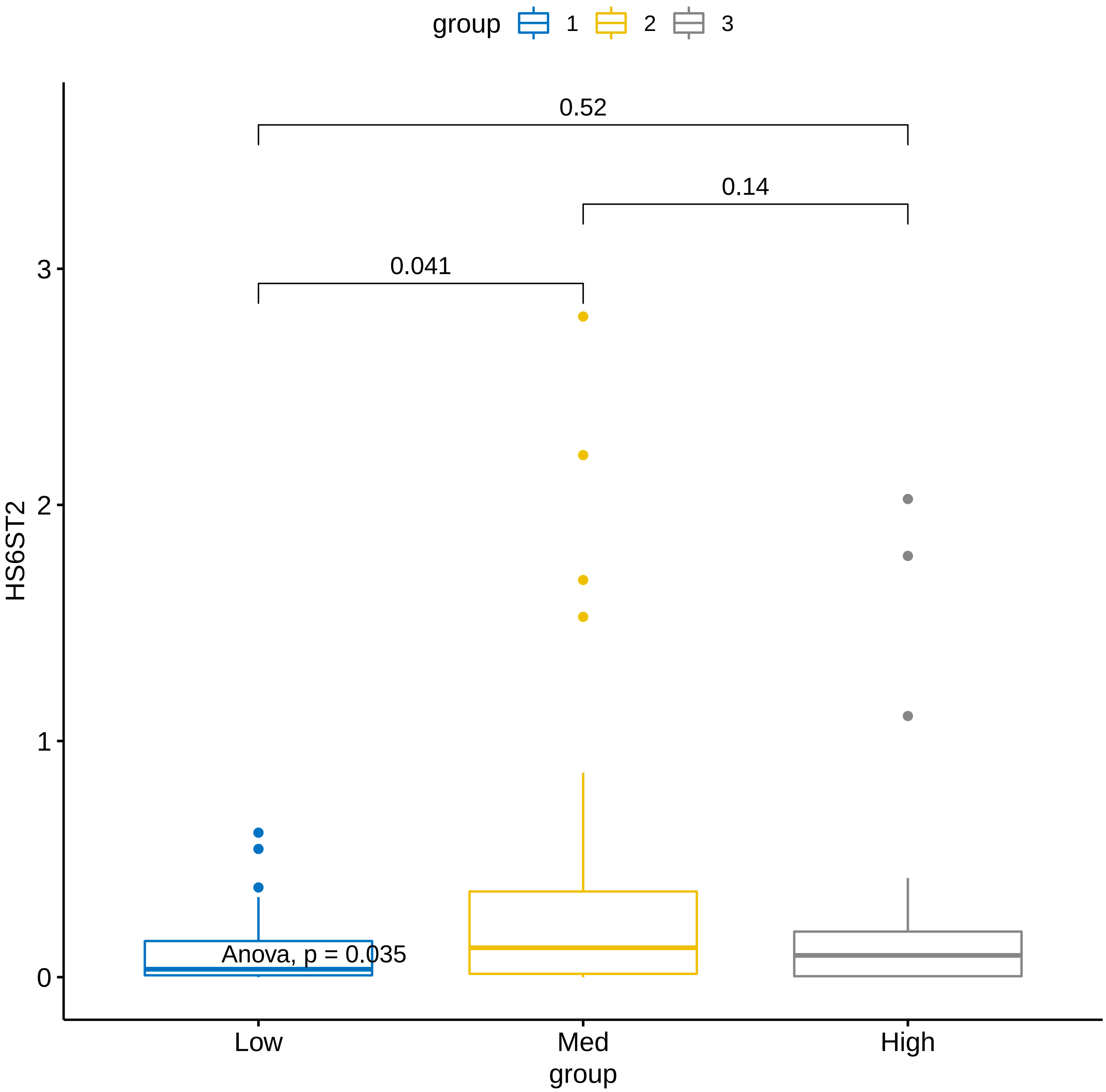
group 1 2 3
0.52
0.14
0.041
HS6ST2
3
2
1
0
Anova, p = 0.035
Low
Med
High
group

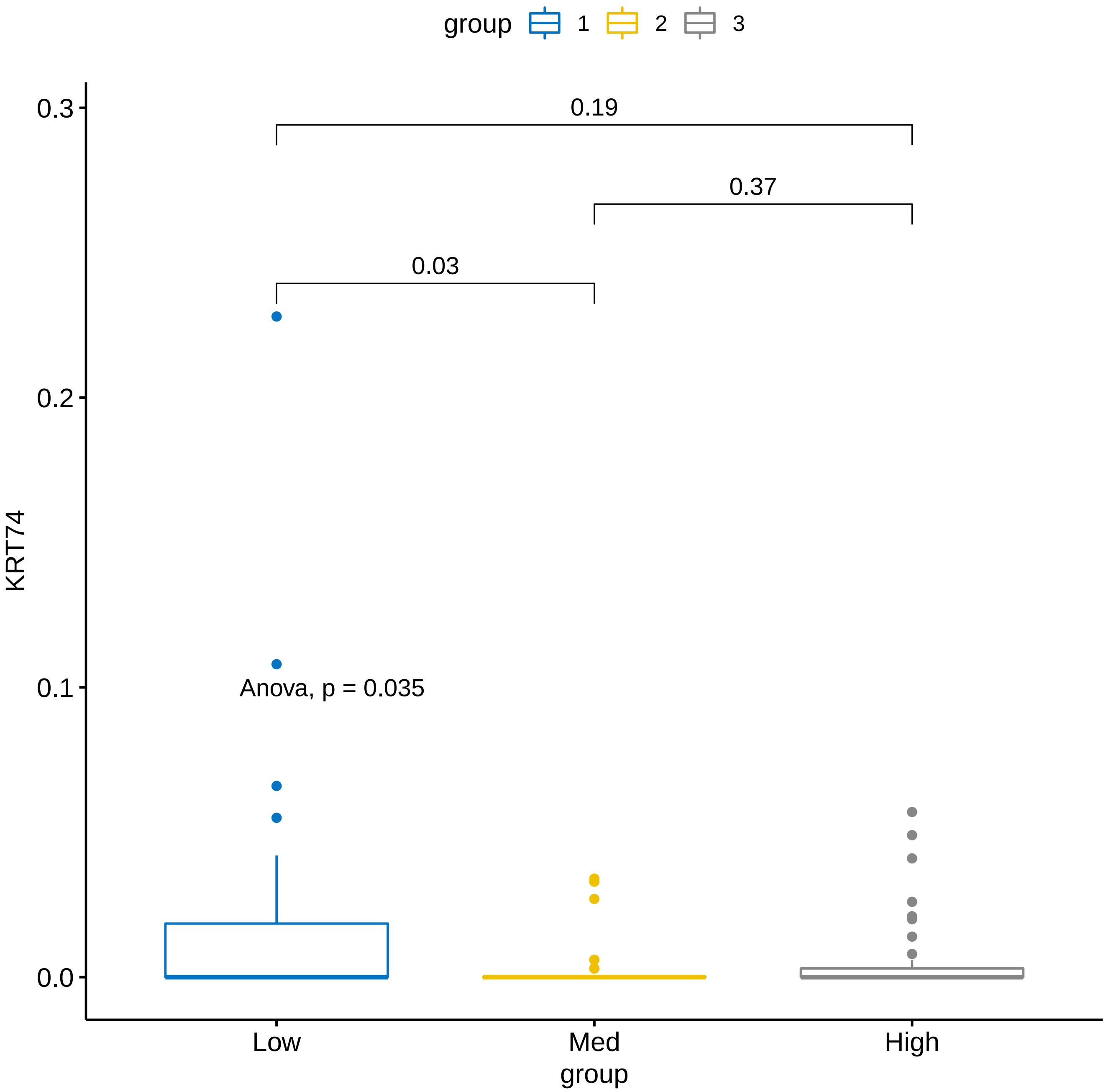
group
1
2
3
0.19
0.37
0.03
Anova, p = 0.035
KRT74
0.3
0.2
0.1
0.0
Low
Med
High
group

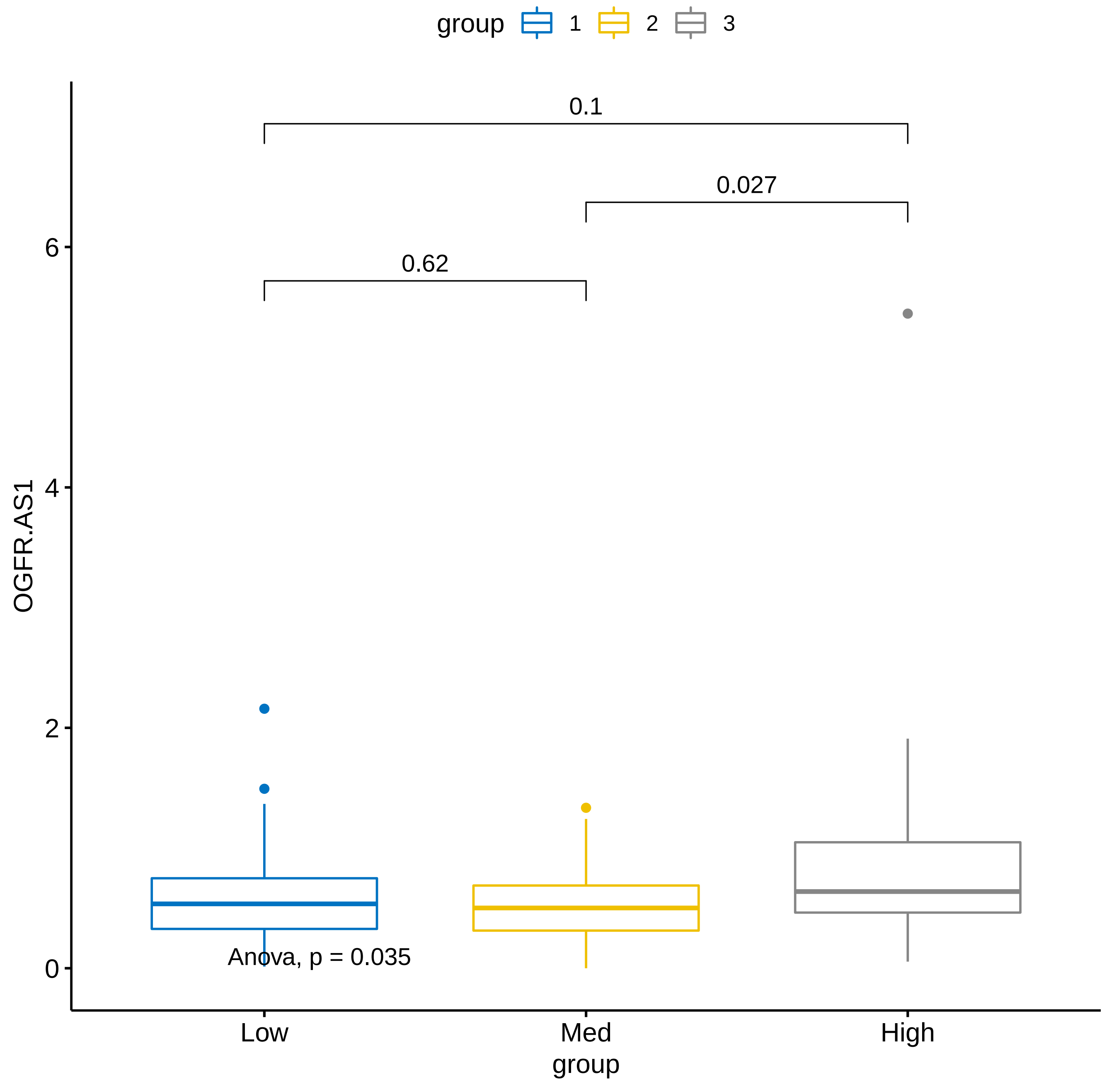
group
1
2
3
0.1
0.027
0.62
6
4
2
0
OGFR.AS1
Anova, p = 0.035
Low
Med
High
group

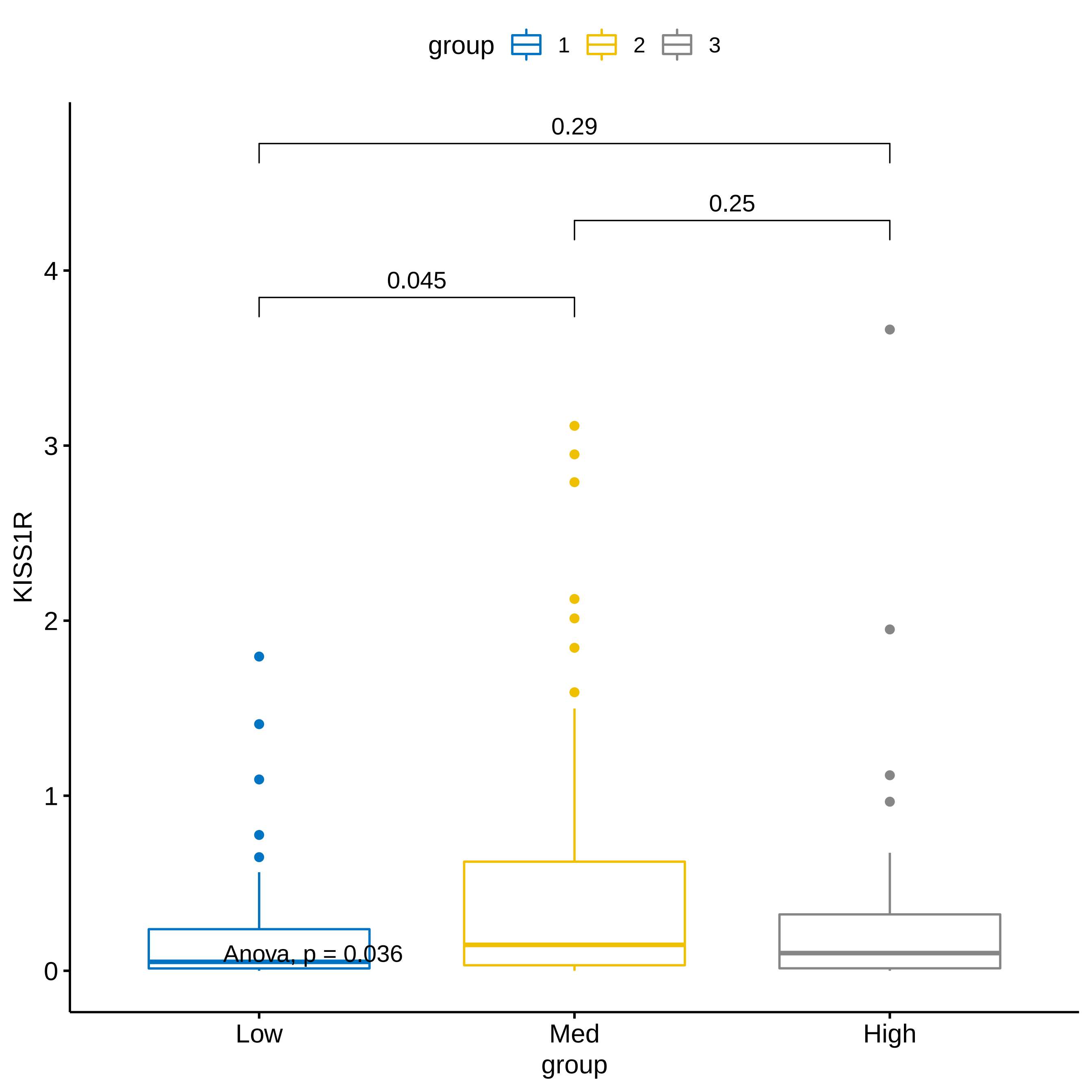
group
1
2
3
0.29
0.25
0.045
KISS1R
4
3
2
1
0
Anova, p = 0.036
Low
Med
High
group

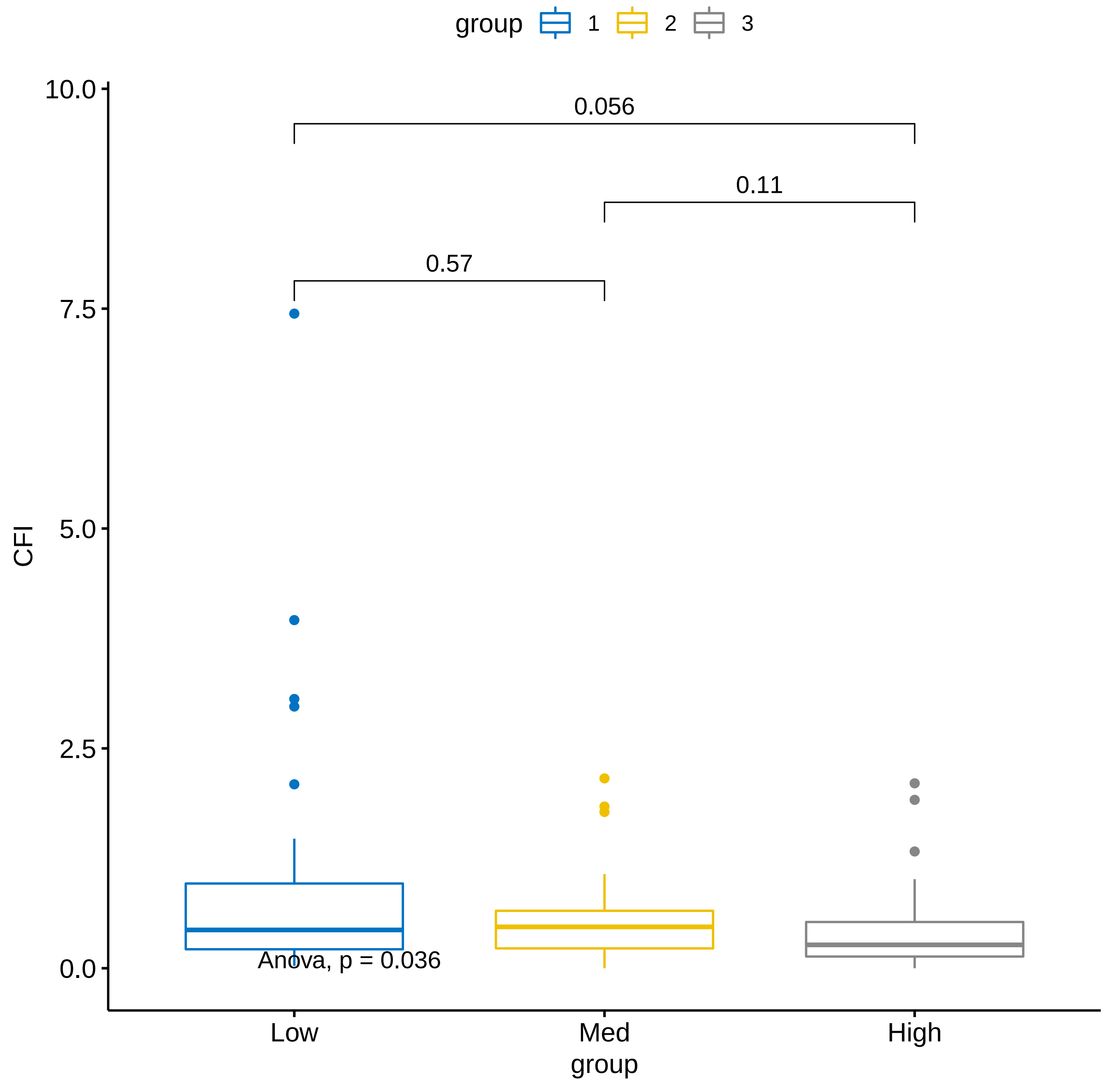

group
1
2
3
10.0
7.5
5.0
2.5
0.0
CFI
0.056
0.11
0.57
Anova, p = 0.036
Low
Med
High
group

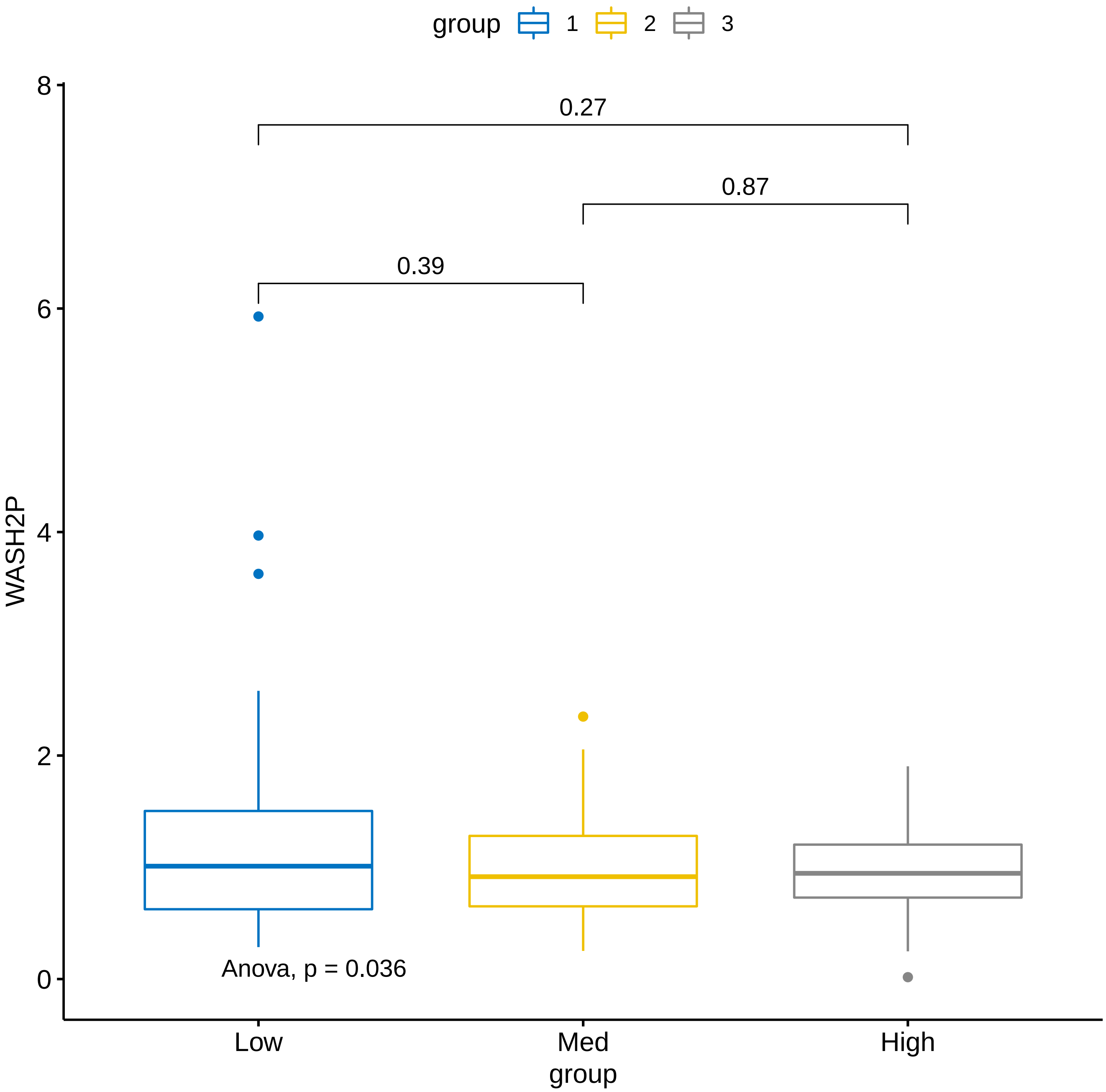

group
1
2
3
8
6
4
2
0
WASH2P
0.27
0.87
0.39
Anova, p = 0.036
Low
Med
High
group

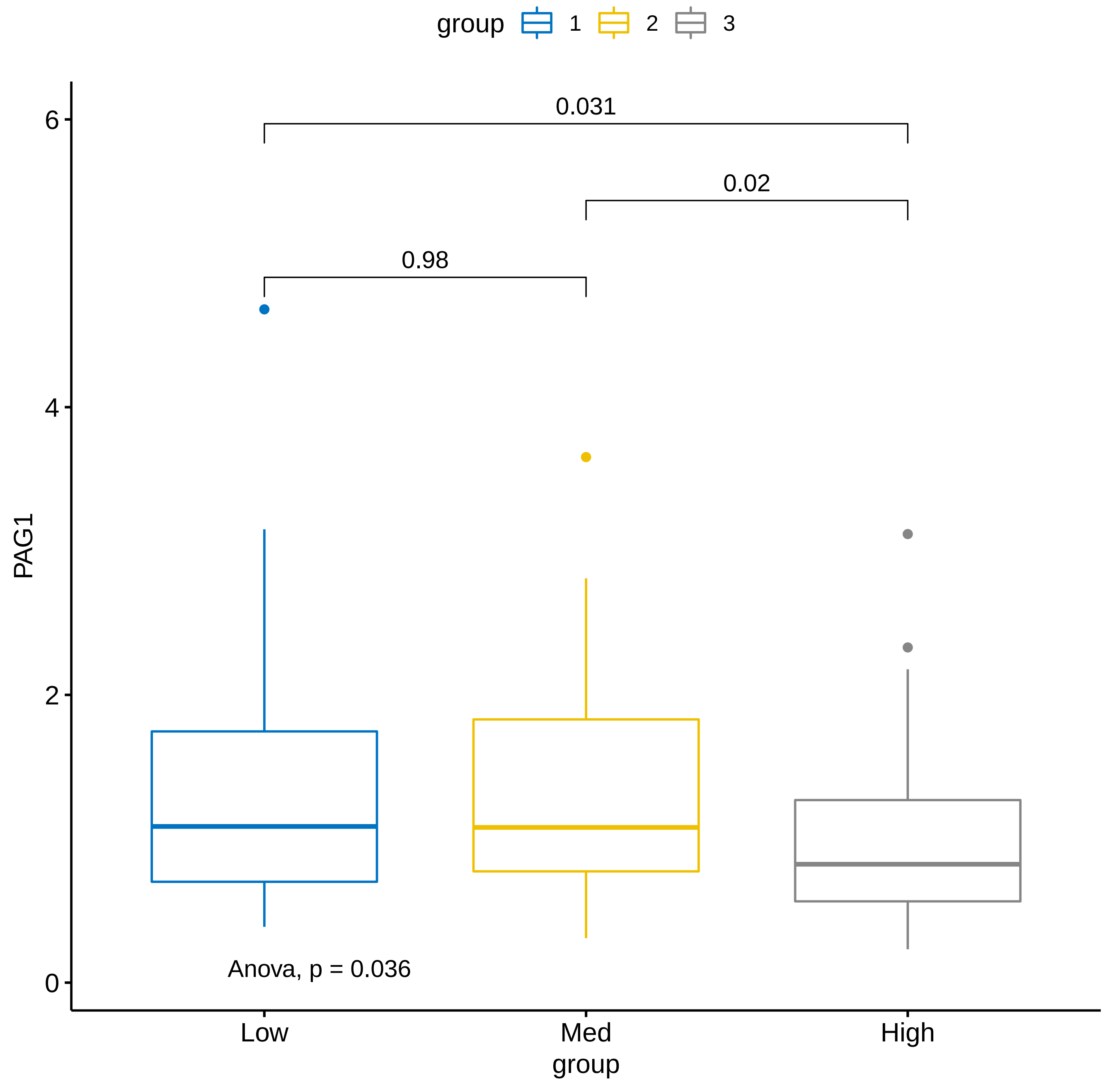
group 1 2 3
0.031
0.02
0.98
PAG1
6
4
2
0
Anova, p = 0.036
Low
Med
High
group

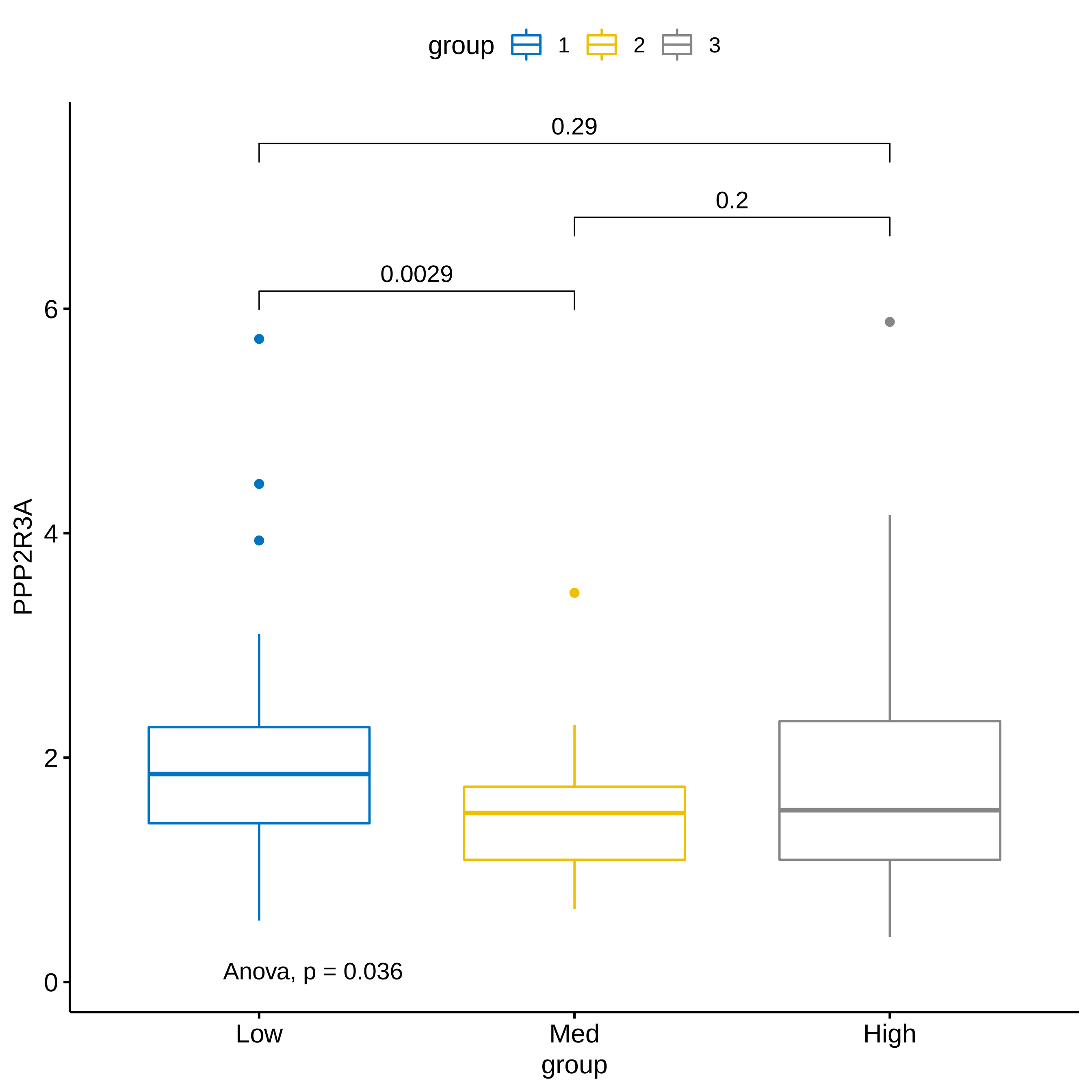

group 1 2 3
0.29
0.2
0.0029
PPP2R3A
6
4
2
0
Anova, p = 0.036
Low
Med
High
group

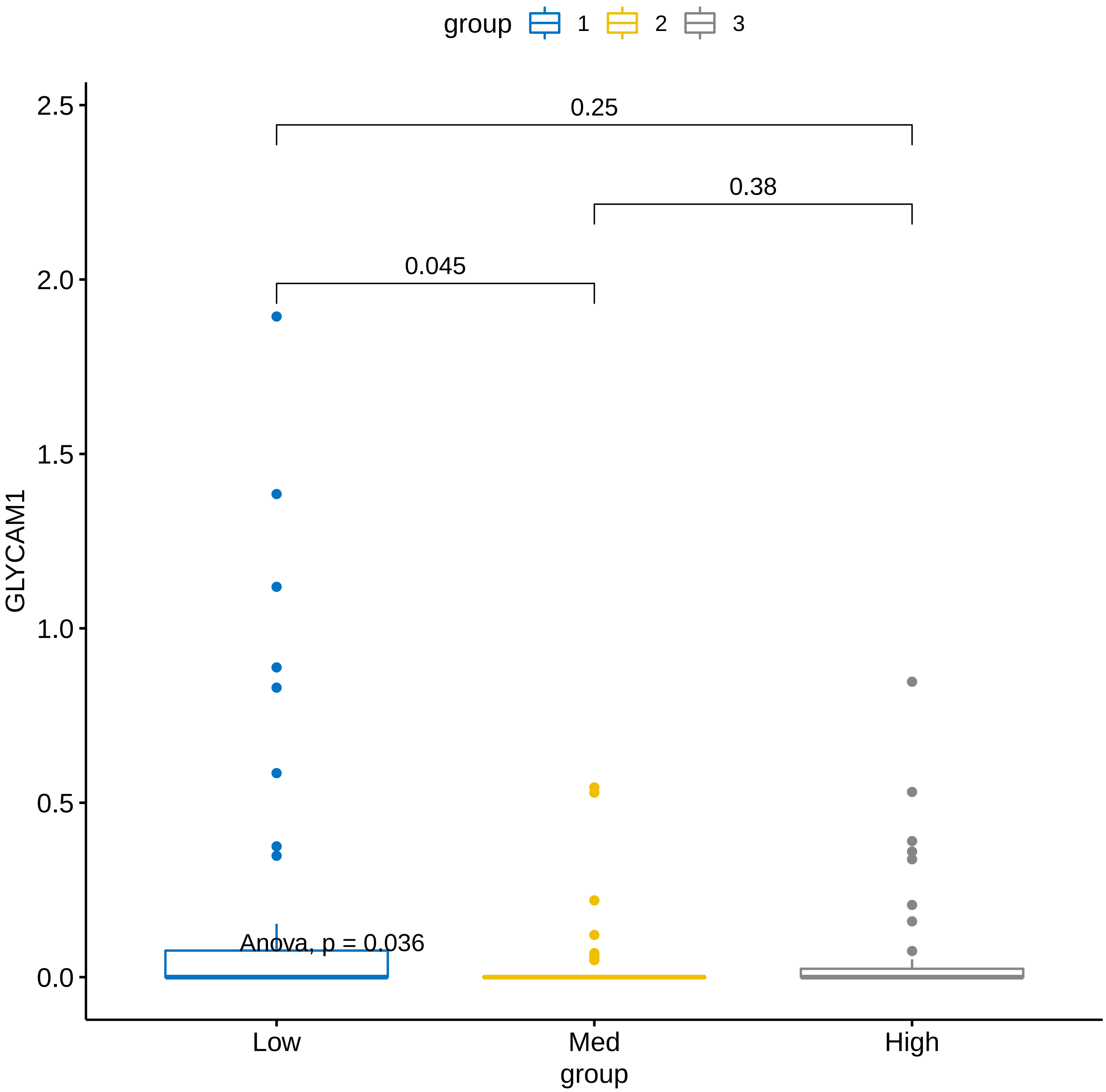

group 1 2 3
2.5
2.0
1.5
1.0
0.5
0.0
GLYCAM1
0.25
0.38
0.045
Anova, p = 0.036
Low
Med
High
group

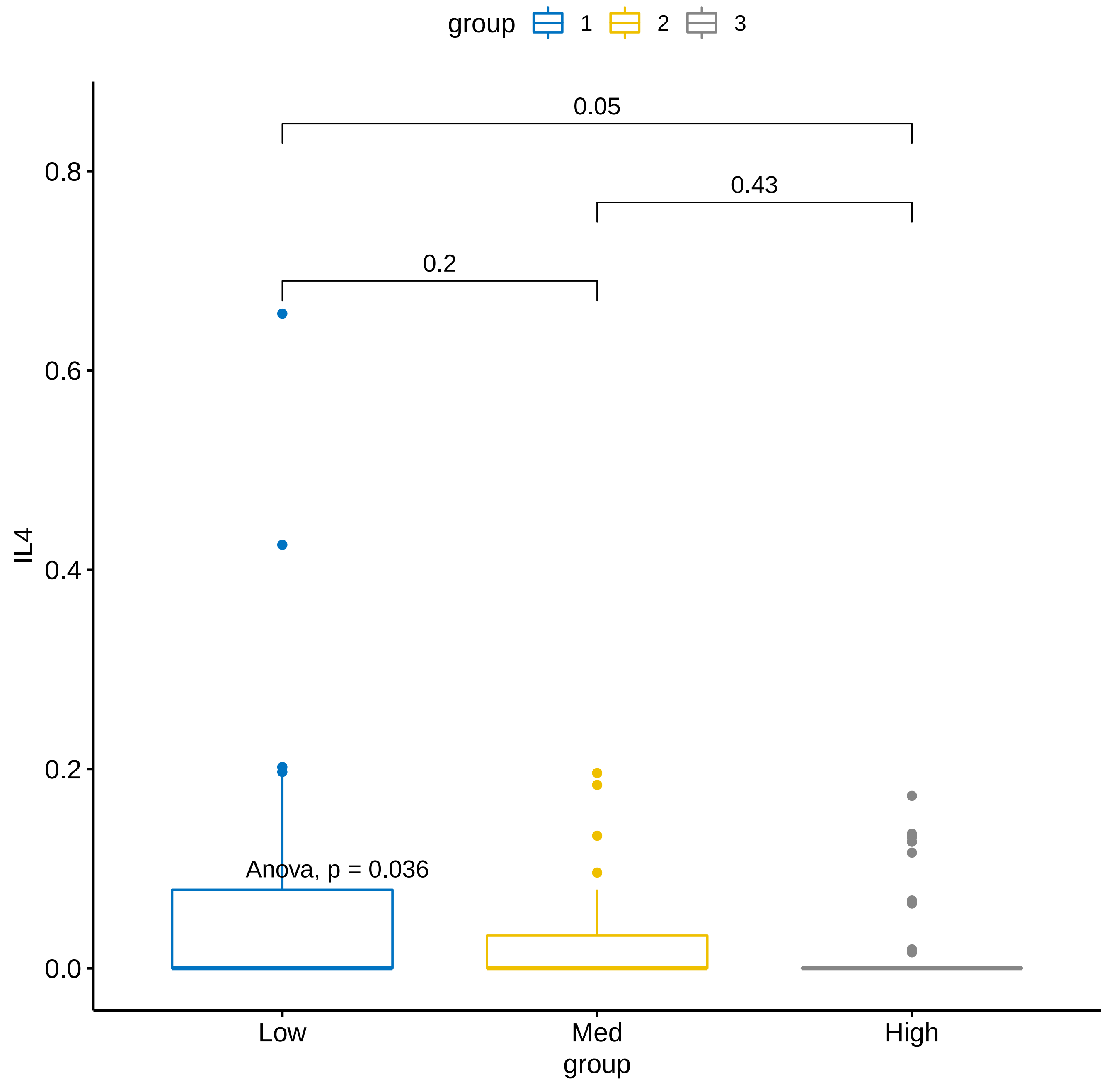

group
1
2
3
0.05
0.43
0.2
Anova, p = 0.036
IL4
0.8
0.6
0.4
0.2
0.0
Low
Med
High
group

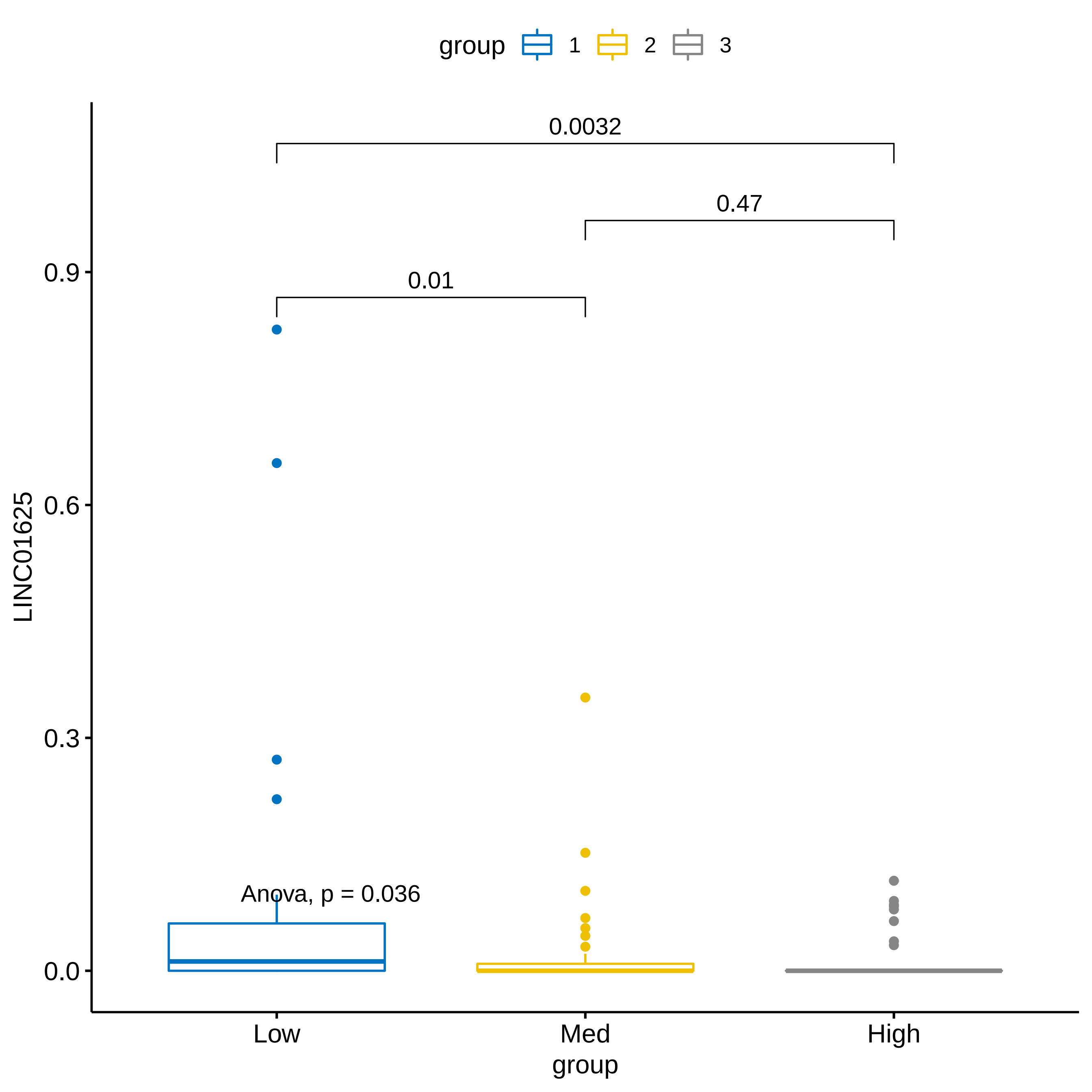

group 1 2 3
0.0032
0.47
0.01
0.9
0.6
0.3
0.0
LINC01625
Anova, p = 0.036
Low
Med
High
group

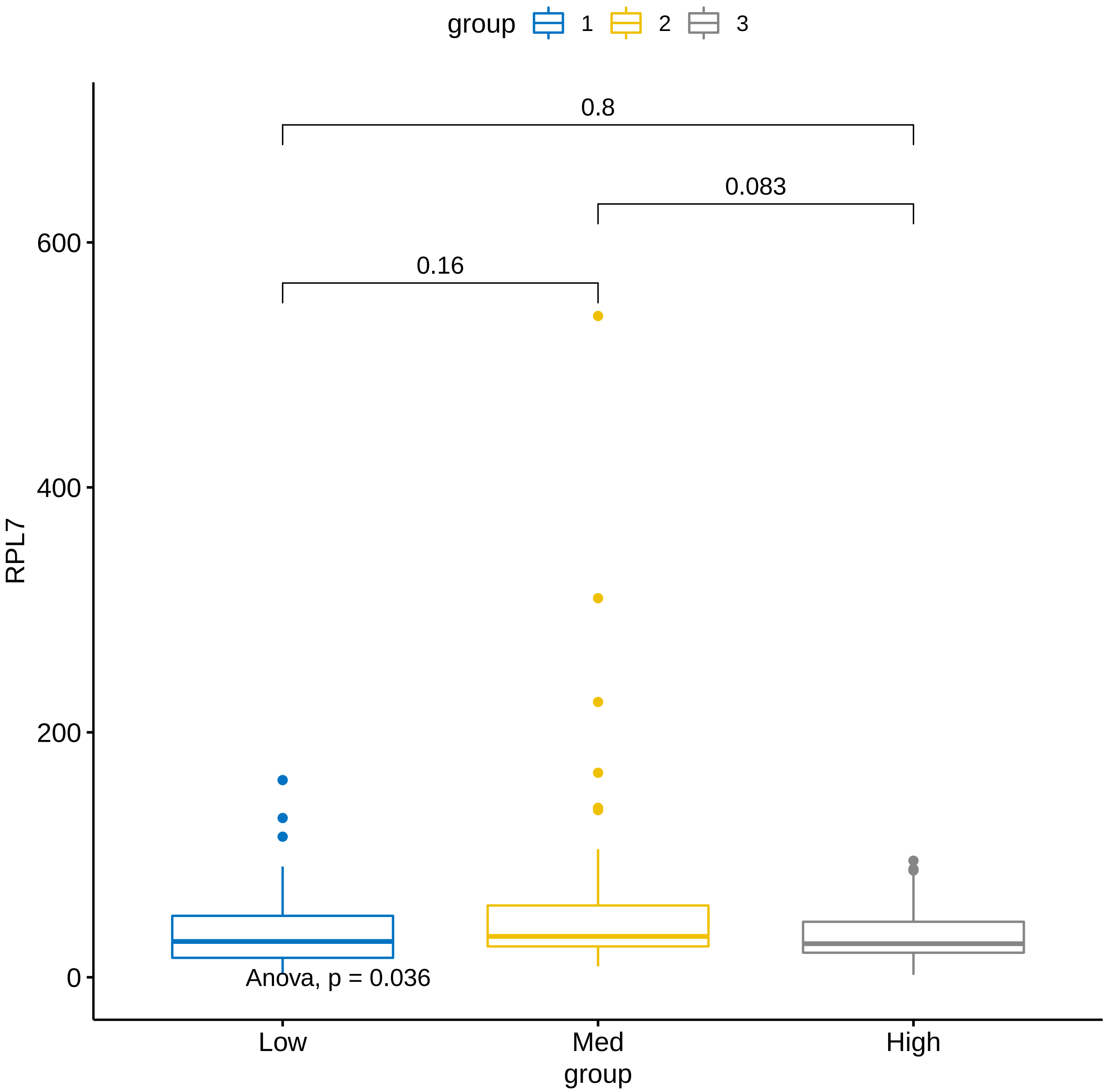
group 1 2 3
0.8
0.083
0.16
600
400
200
0
RPL7
Anova, p = 0.036
Low
Med
High
group

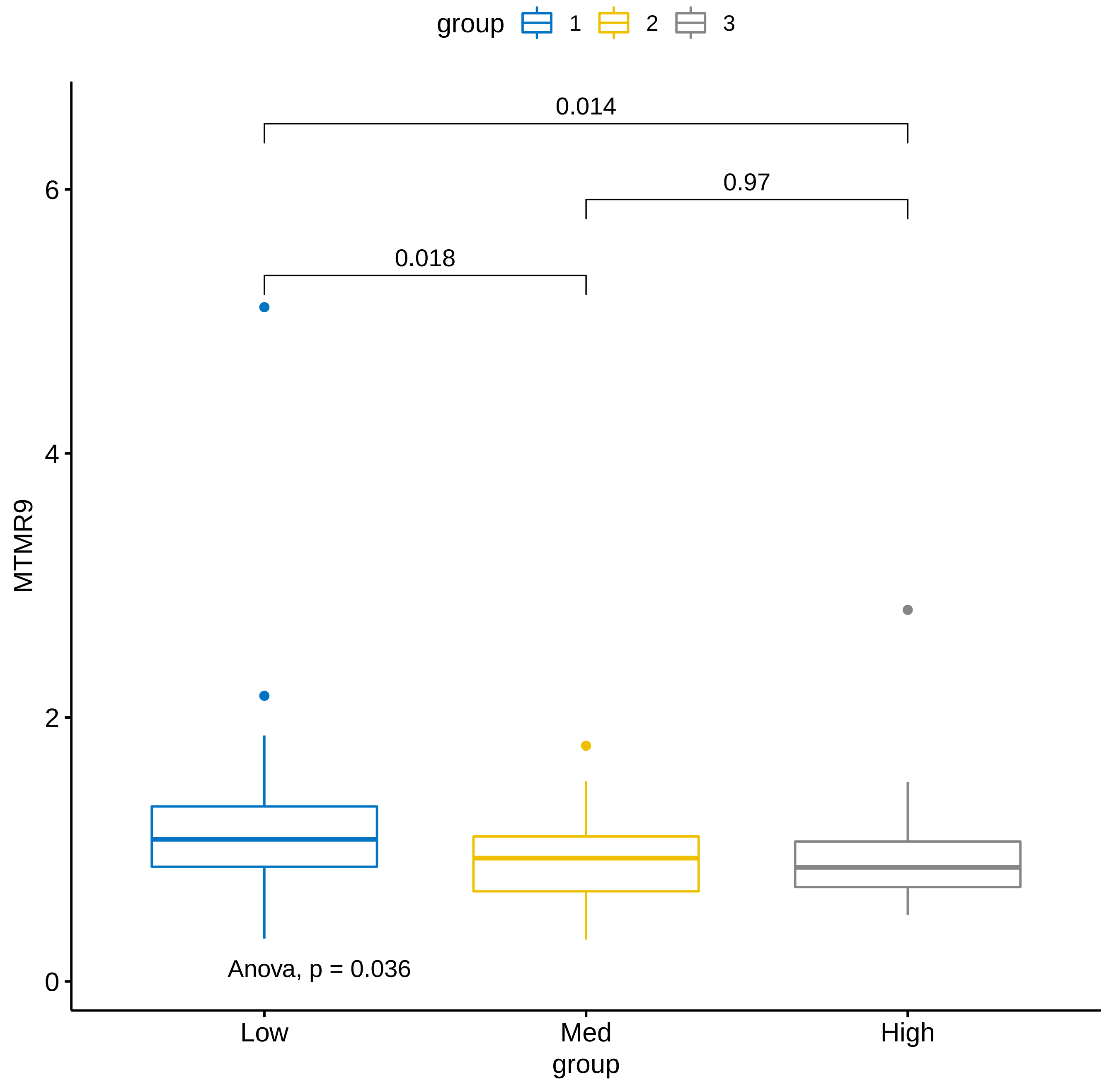
group
1
2
3
0.014
0.97
0.018
6
4
2
0
MTMR9
Anova, p = 0.036
Low
Med
High
group

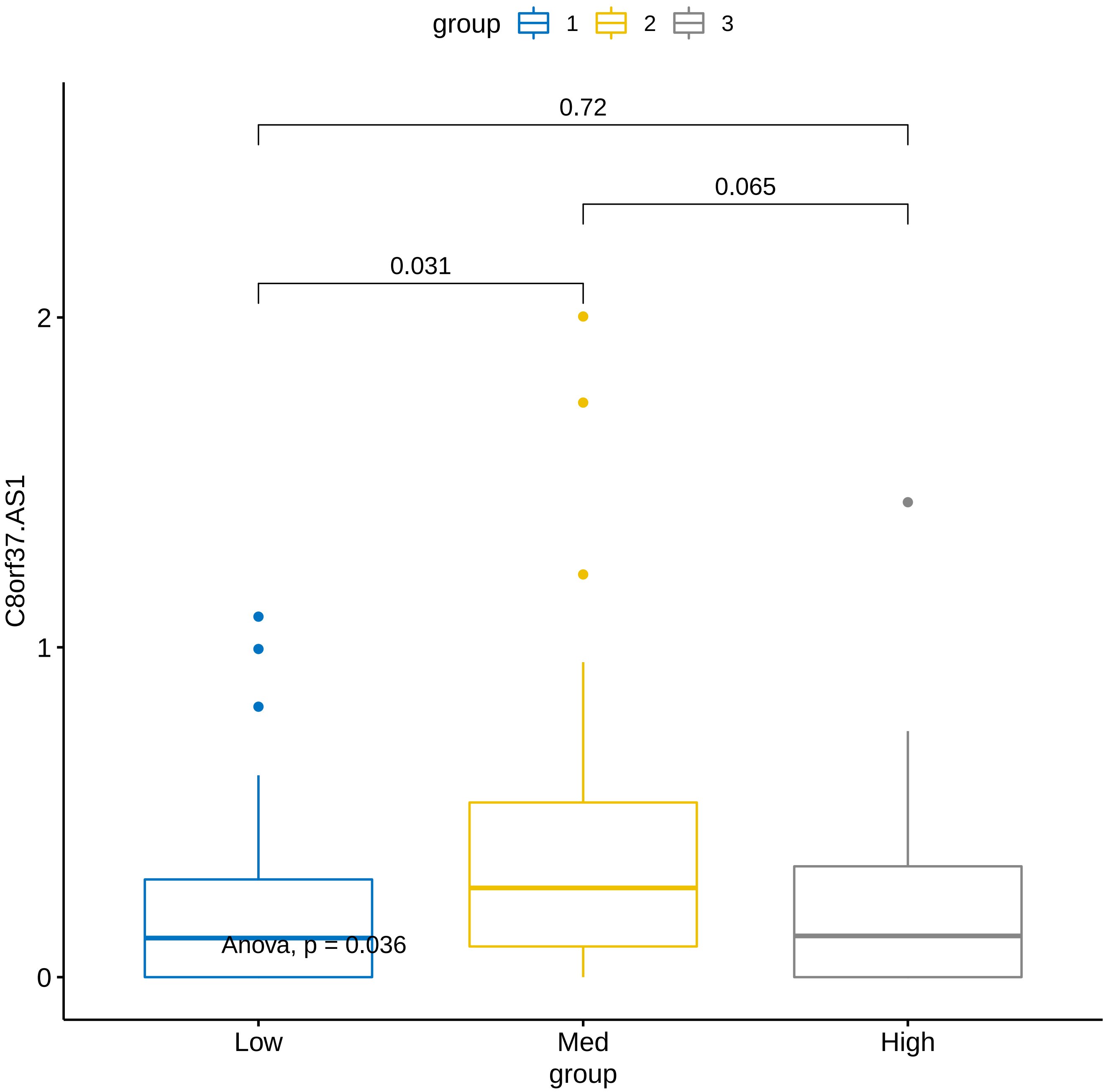

group 1 2 3
0.72
0.065
0.031
C8orf37.AS1
2
1
0
Anova, p = 0.036
Low
Med
High
group

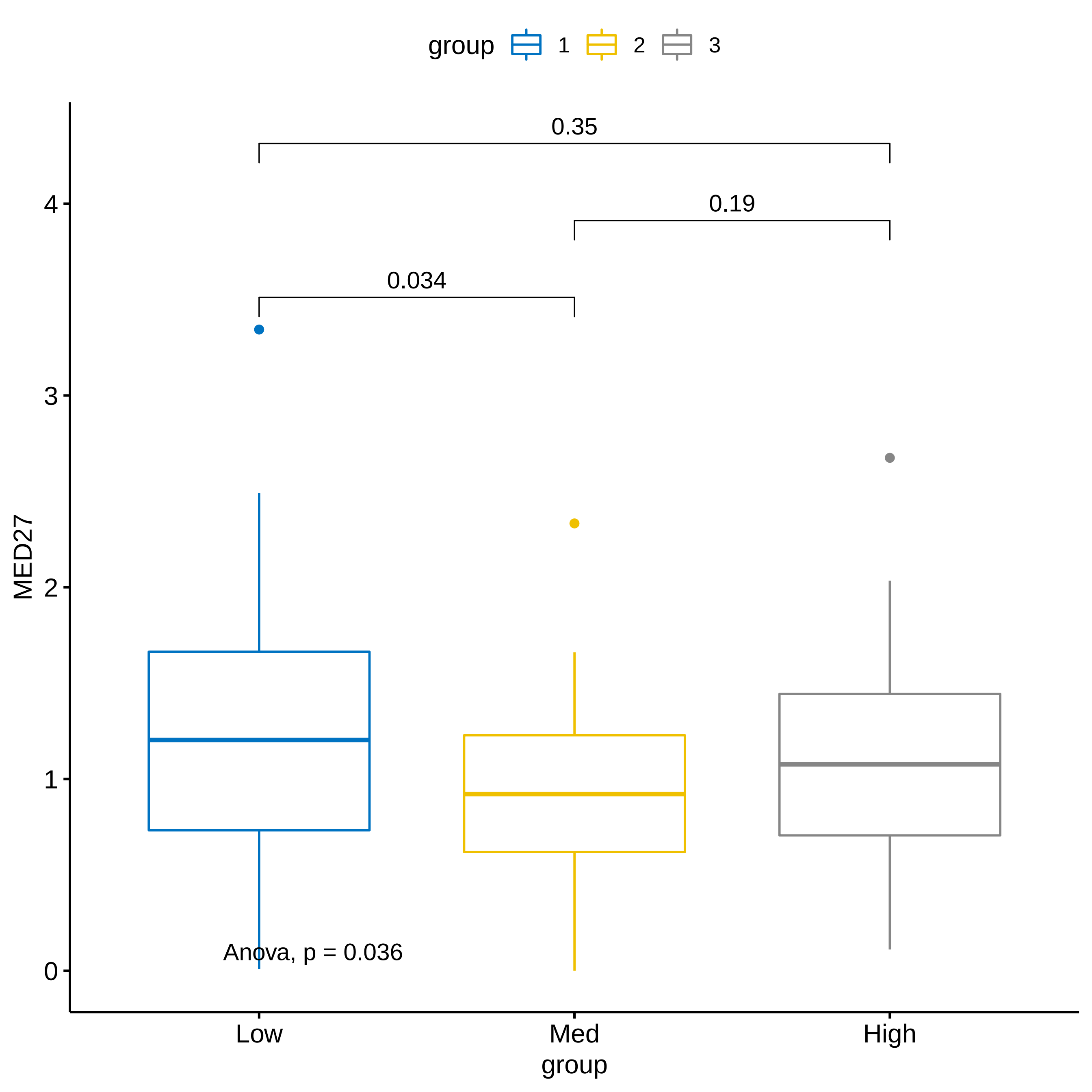

group
1
2
3
0.35
0.19
0.034
4
3
2
1
0
MED27
Anova, p = 0.036
Low
Med
High
group

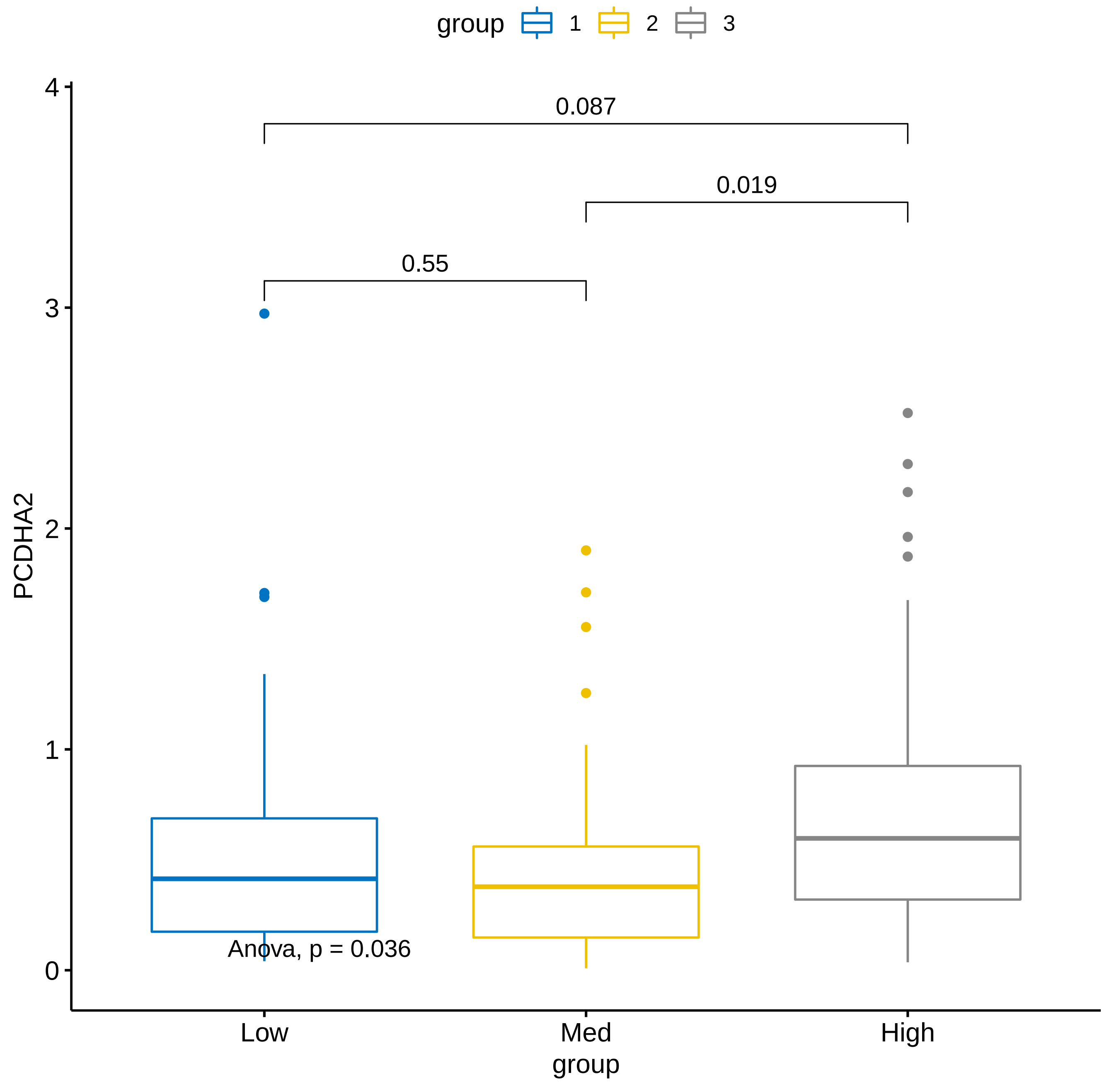
group
1
2
3
4
3
2
1
0
PCDHA2
0.087
0.019
0.55
Anova, p = 0.036
Low
Med
High
group

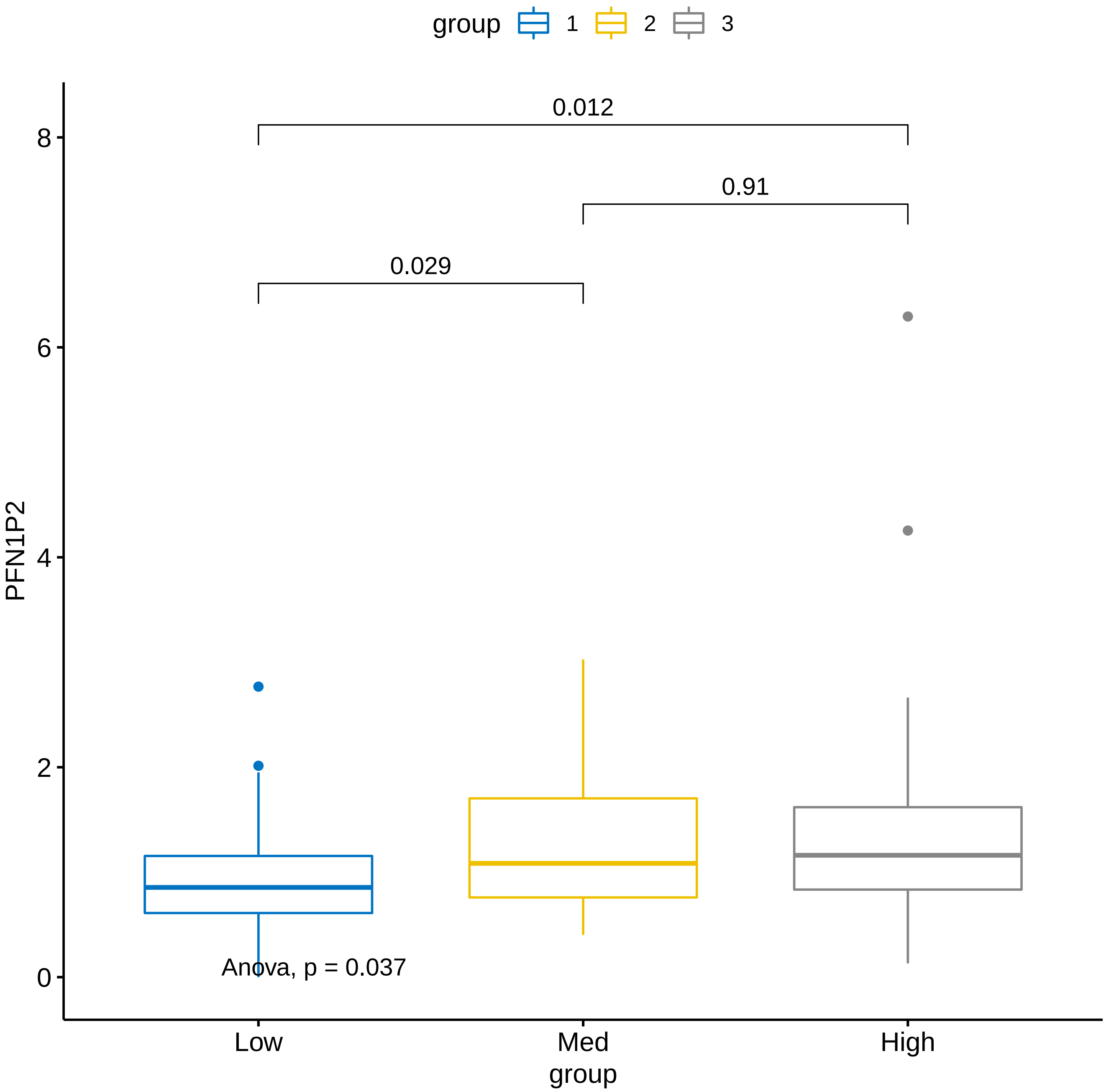
group
1
2
3
0.012
0.91
0.029
8
6
4
2
0
PFN1P2
Anova, p = 0.037
Low
Med
High
group

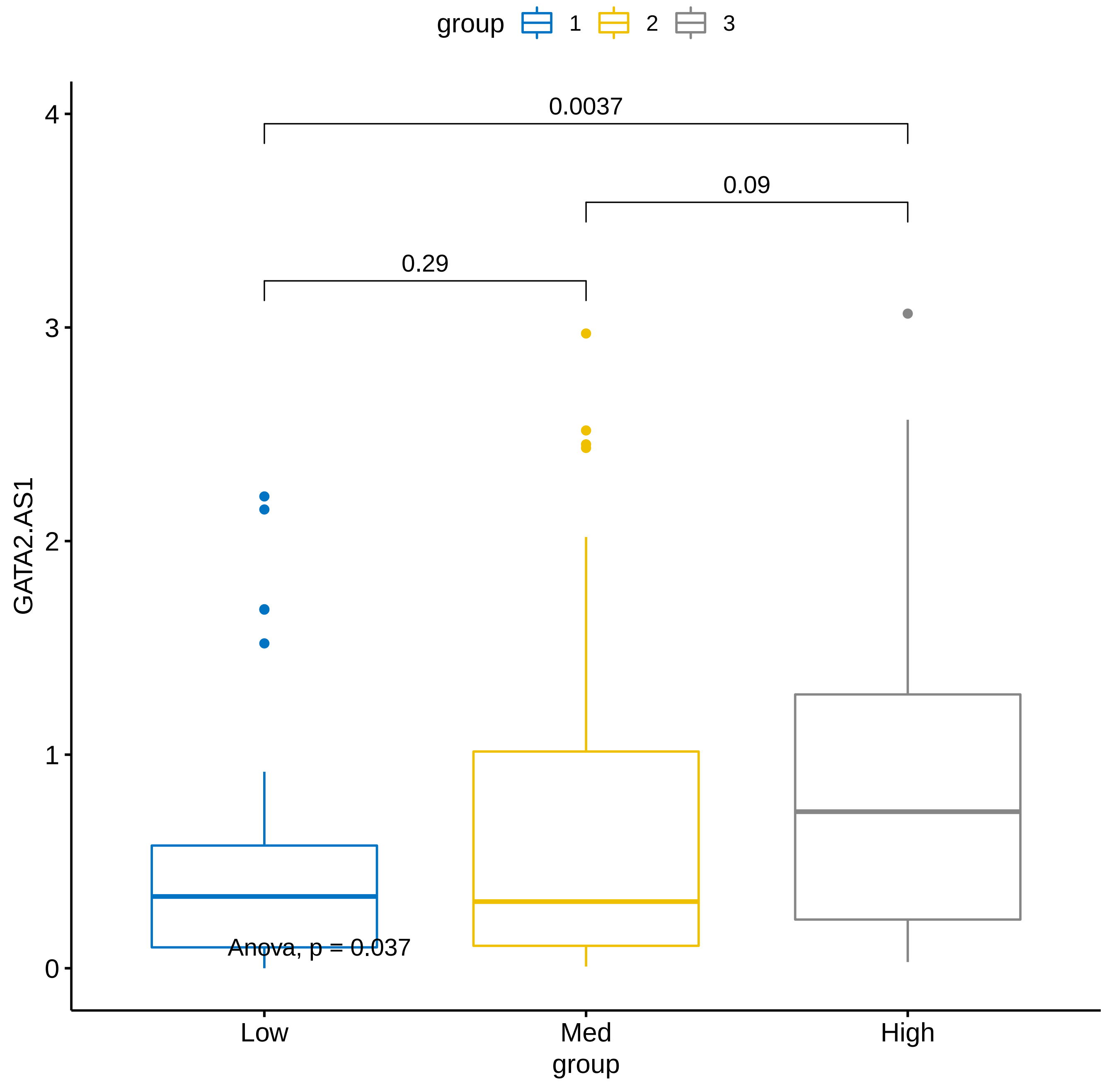
group
1
2
3
0.0037
0.09
0.29
4
3
2
1
0
GATA2.AS1
Anova, p = 0.037
Low
Med
High
group

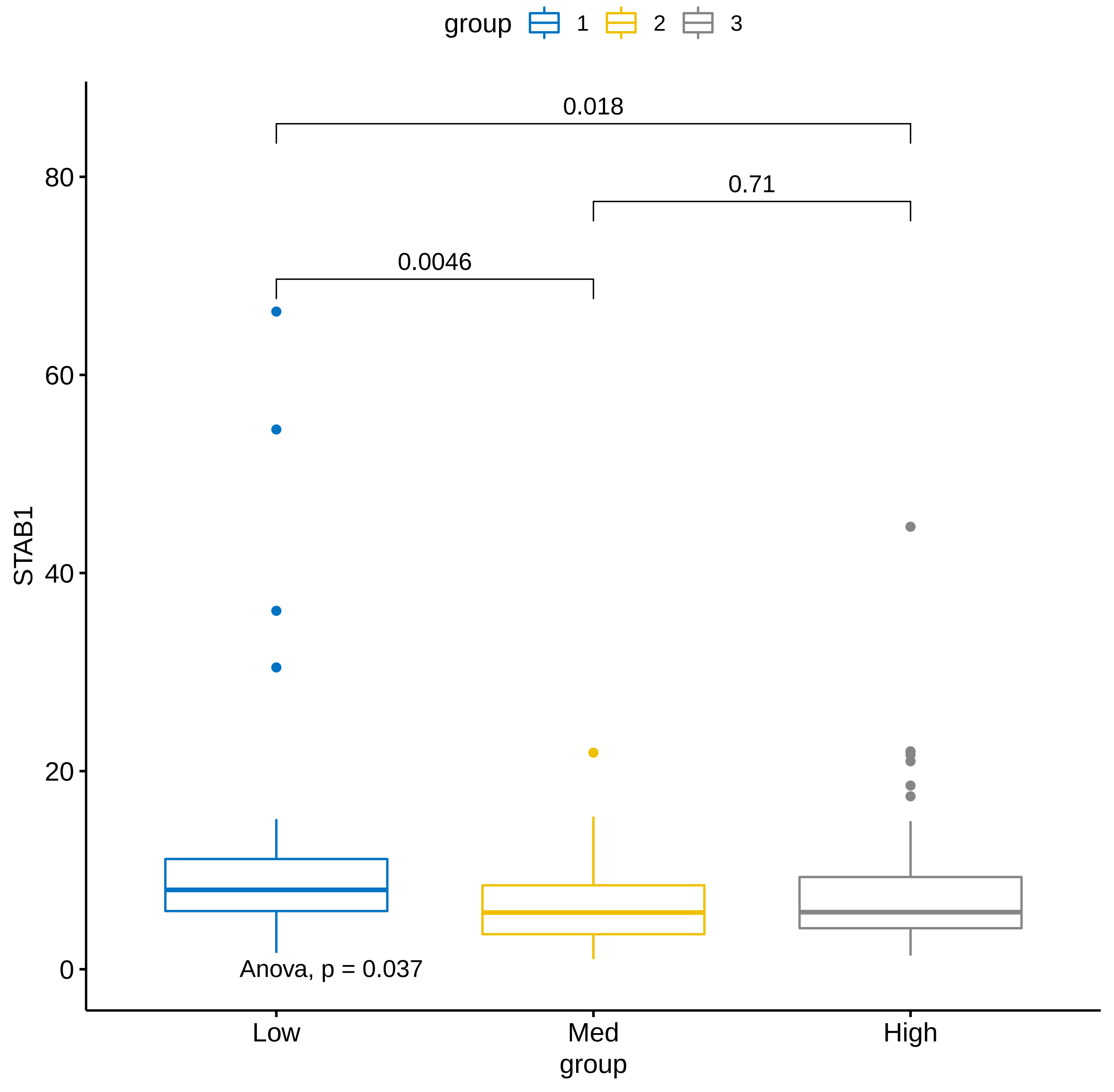
group
1
2
3
0.018
0.71
0.0046
80
60
40
20
0
STAB1
Anova, p = 0.037
Low
Med
High
group

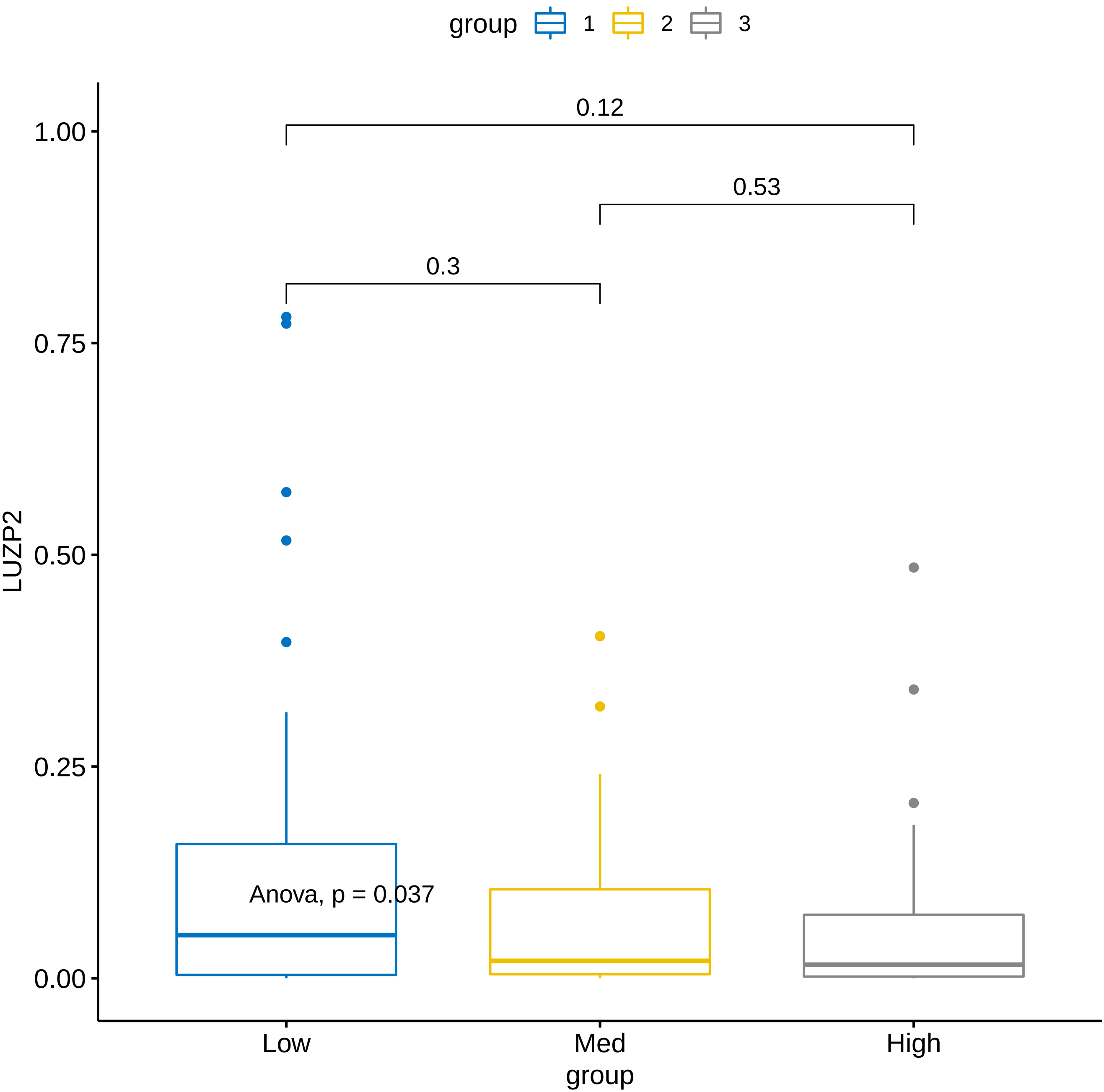

group 1 2 3
0.12
0.53
0.3
1.00
0.75
0.50
0.25
0.00
LUZP2
Anova, p = 0.037
Low
Med
High
group

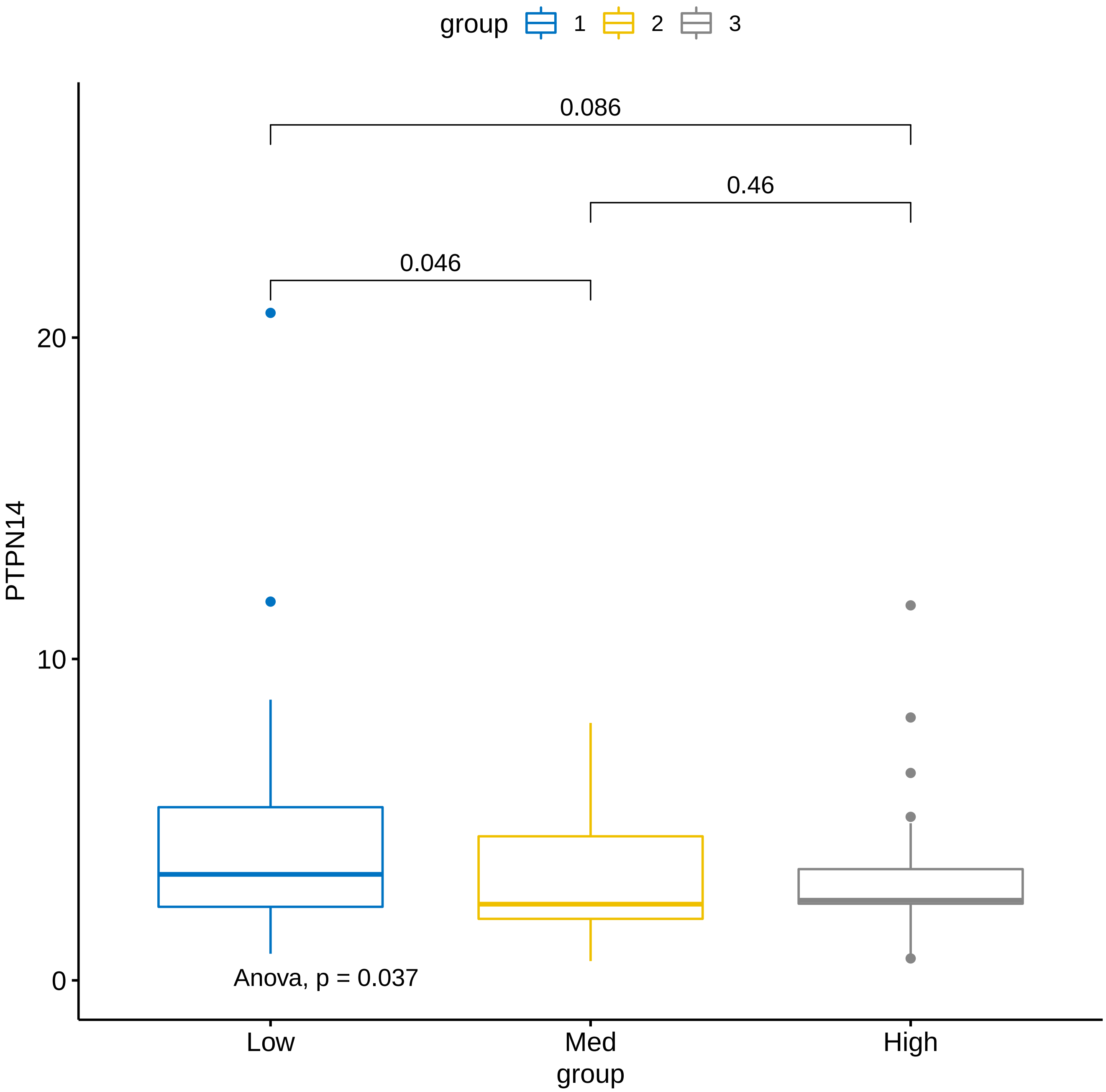
group
1
2
3
0.086
0.46
0.046
20
10
0
PTPN14
Anova, p = 0.037
Low
Med
High
group

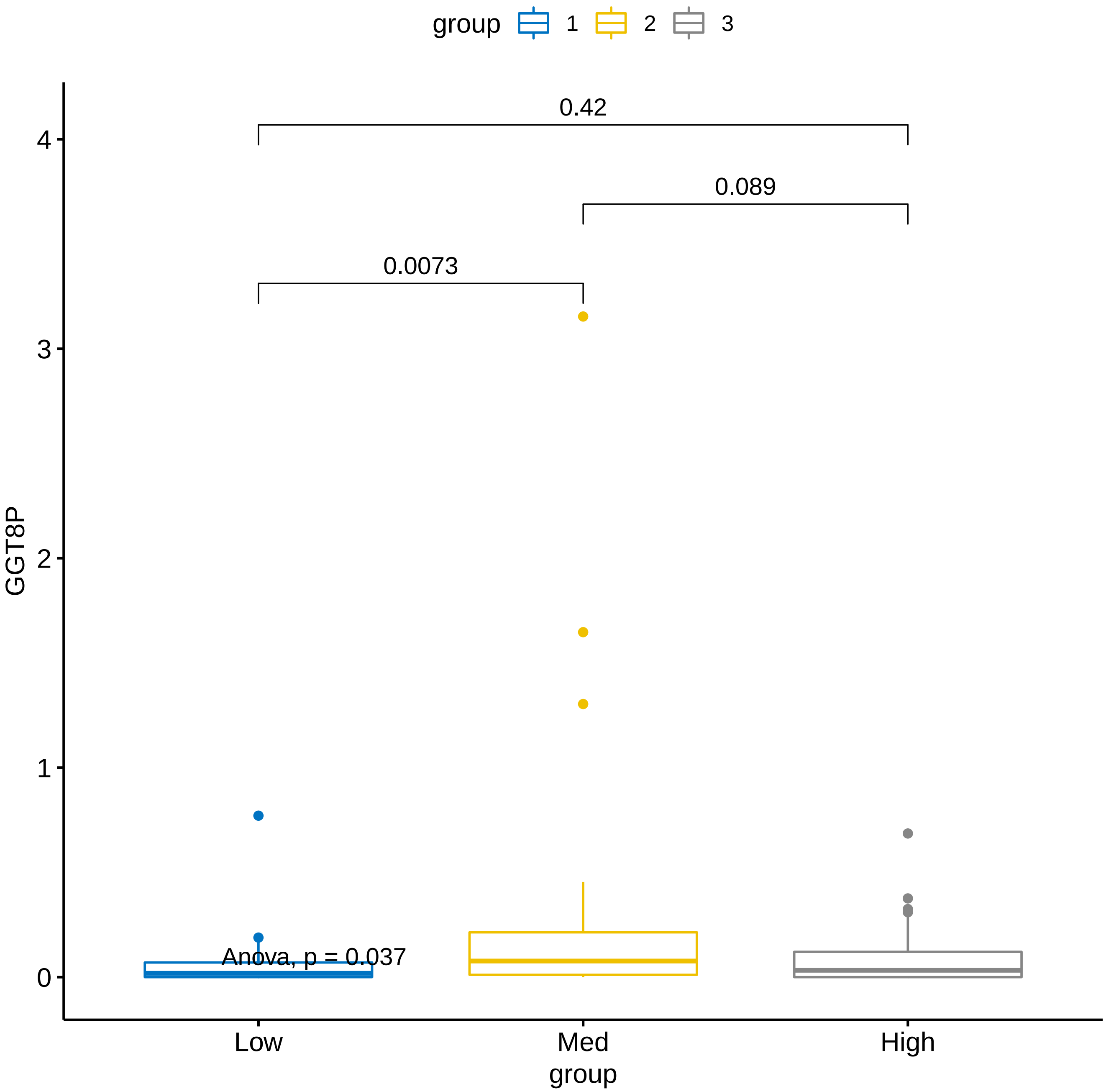

group 1 2 3
0.42
0.089
0.0073
Anova, p = 0.037
GGT8P
0
1
2
3
4
Low
Med
High
group

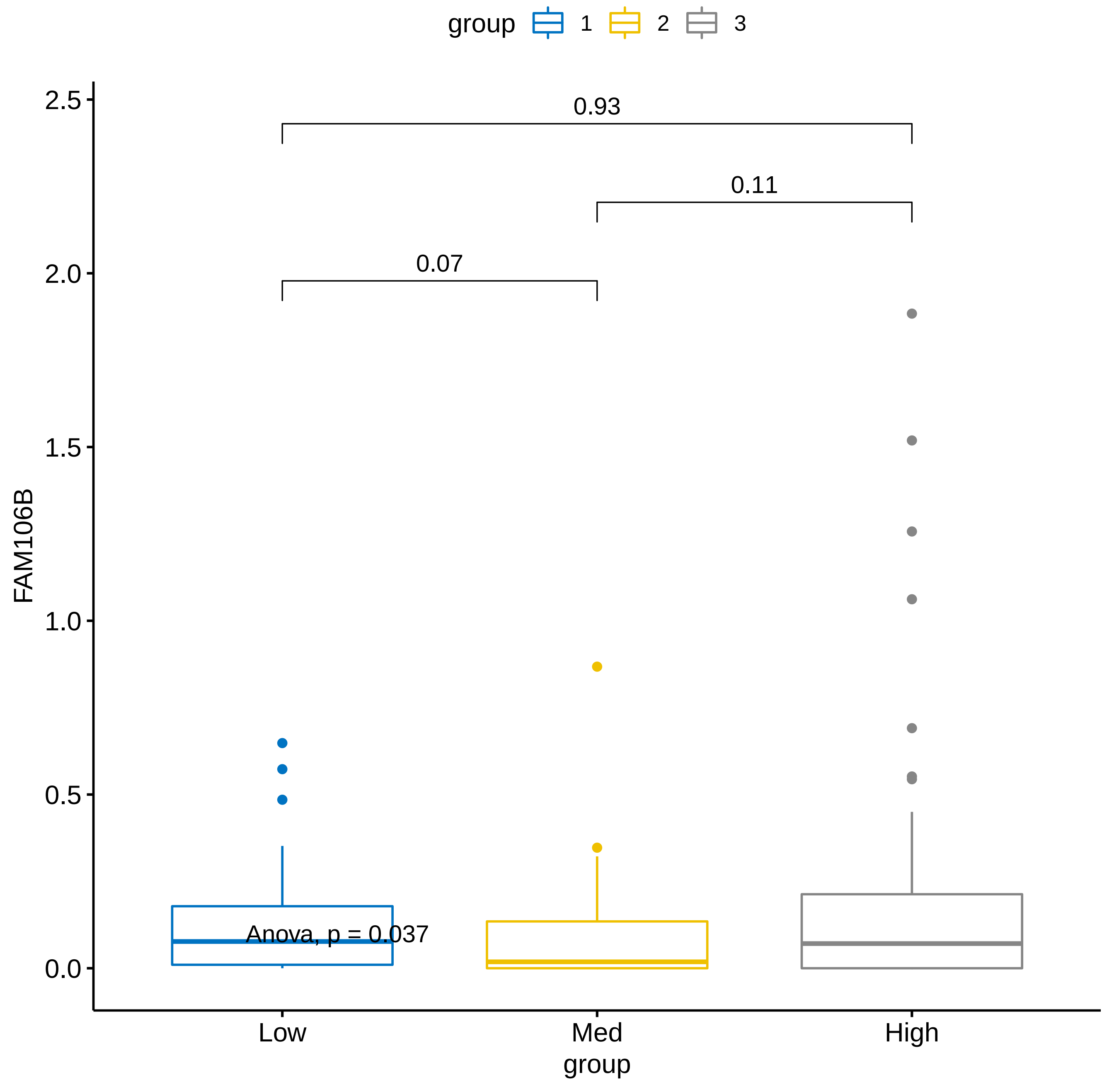

group 1 2 3
0.93
0.11
0.07
2.5
2.0
1.5
1.0
0.5
0.0
FAM106B
Anova, p = 0.037
Low
Med
High
group

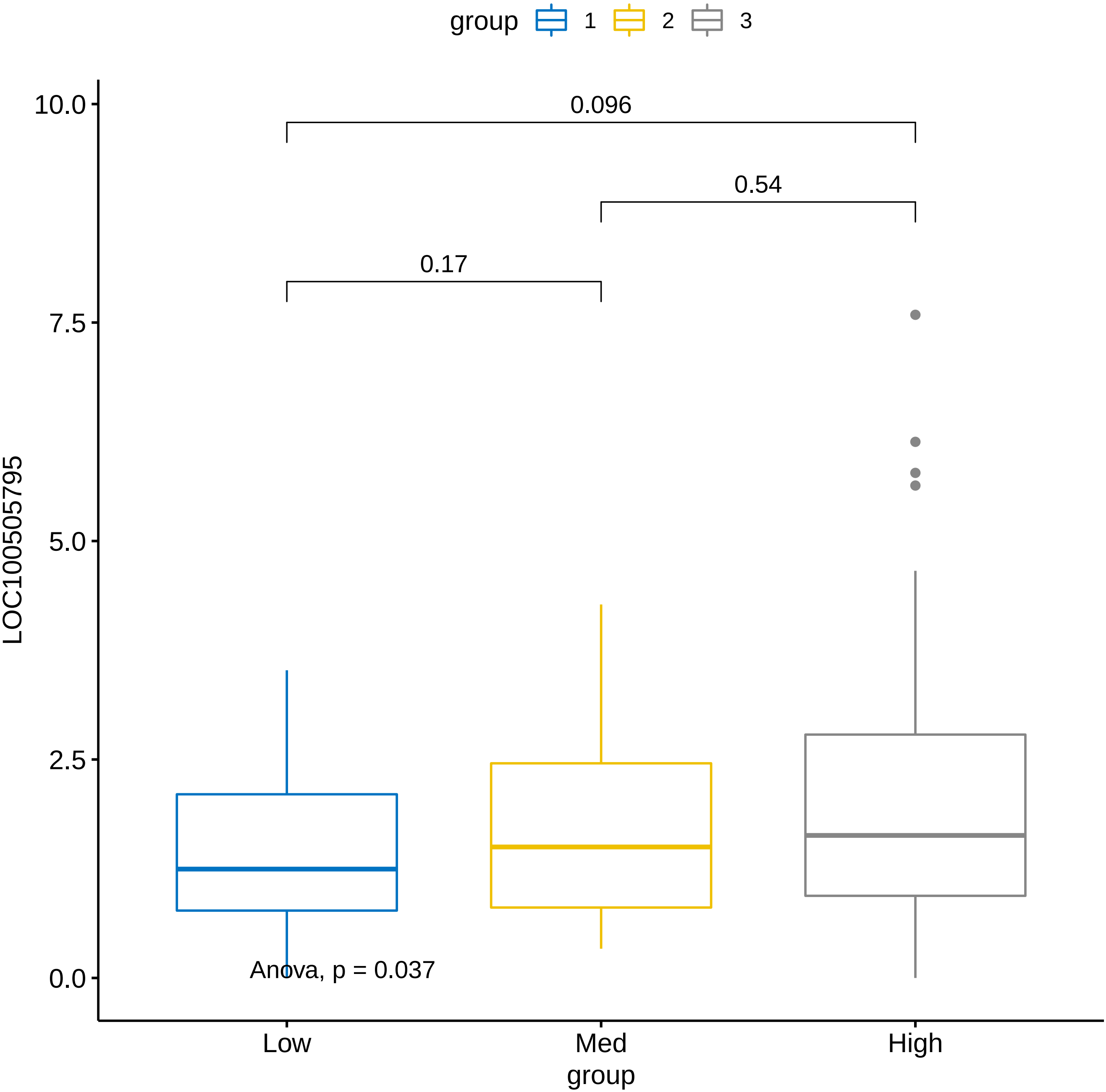
group
1
2
3
10.0
7.5
5.0
2.5
0.0
0.096
0.54
0.17
LOC100505795
Anova, p = 0.037
Low
Med
High
group

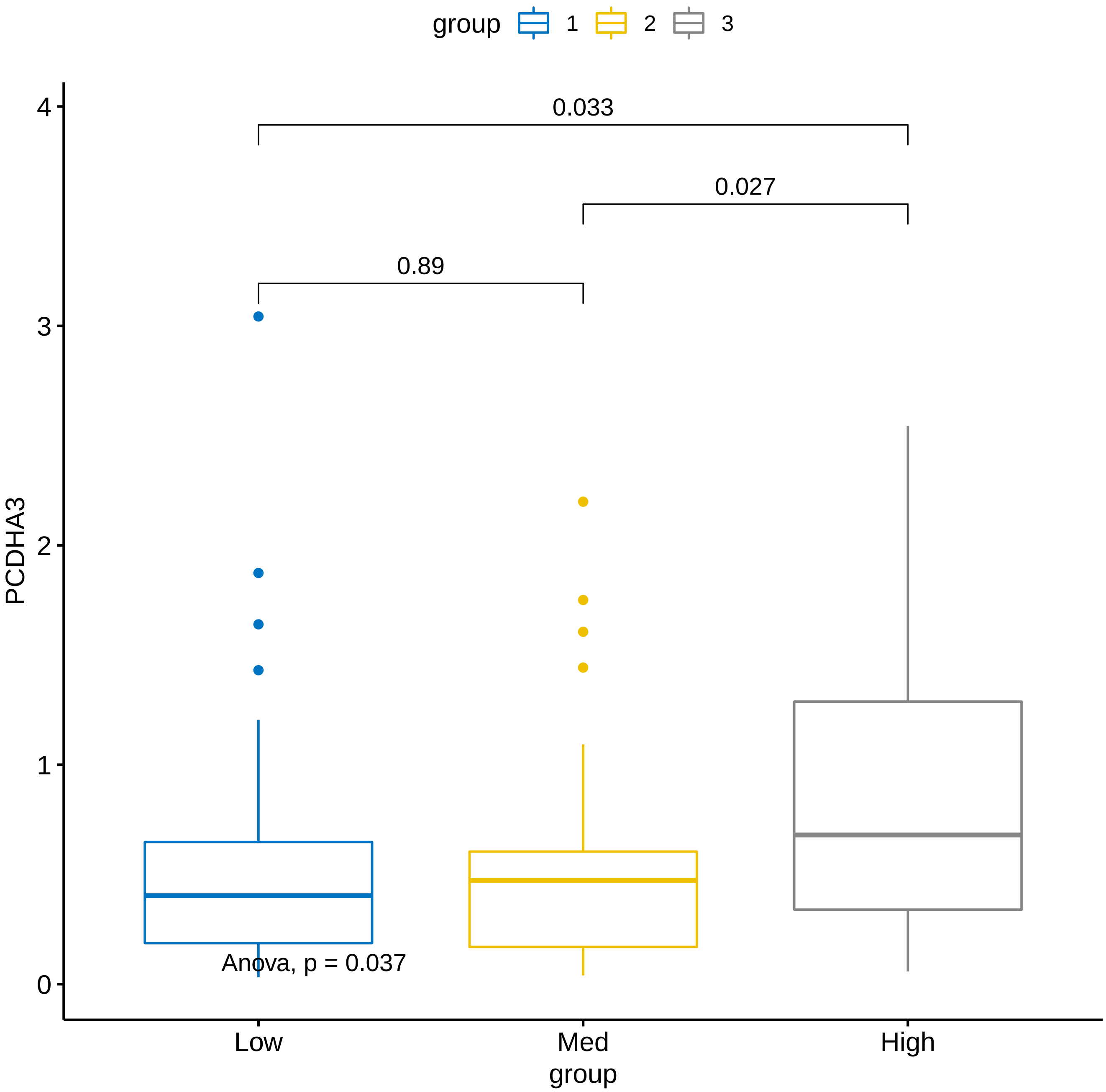
group
1
2
3
0.033
0.027
0.89
PCDHA3
4
3
2
1
0
Anova, p = 0.037
Low
Med
High
group

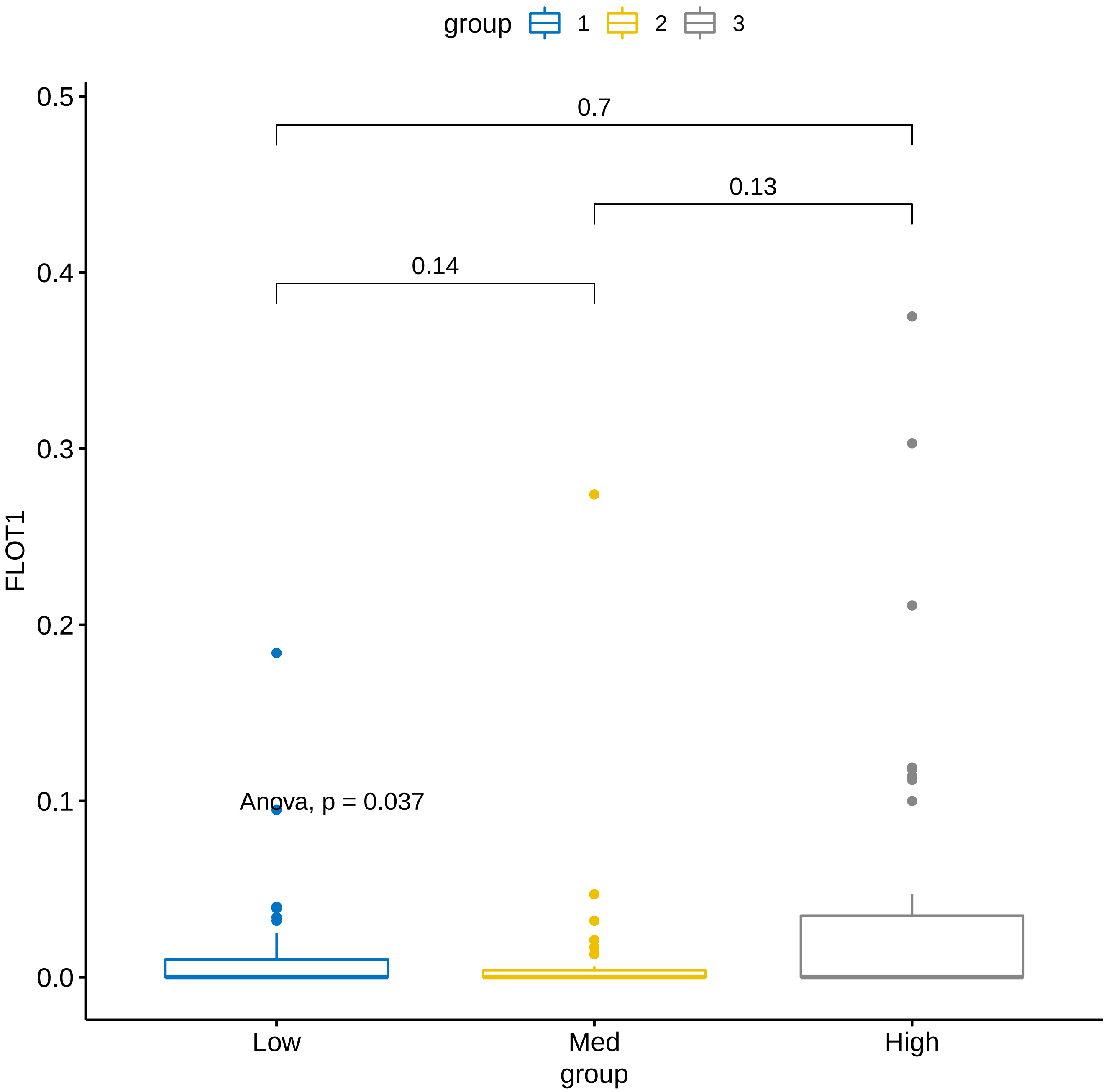

group 1 2 3
0.7
0.13
0.14
Anova, p = 0.037
FLOT1
0.0
0.1
0.2
0.3
0.4
0.5
Low
Med
High
group

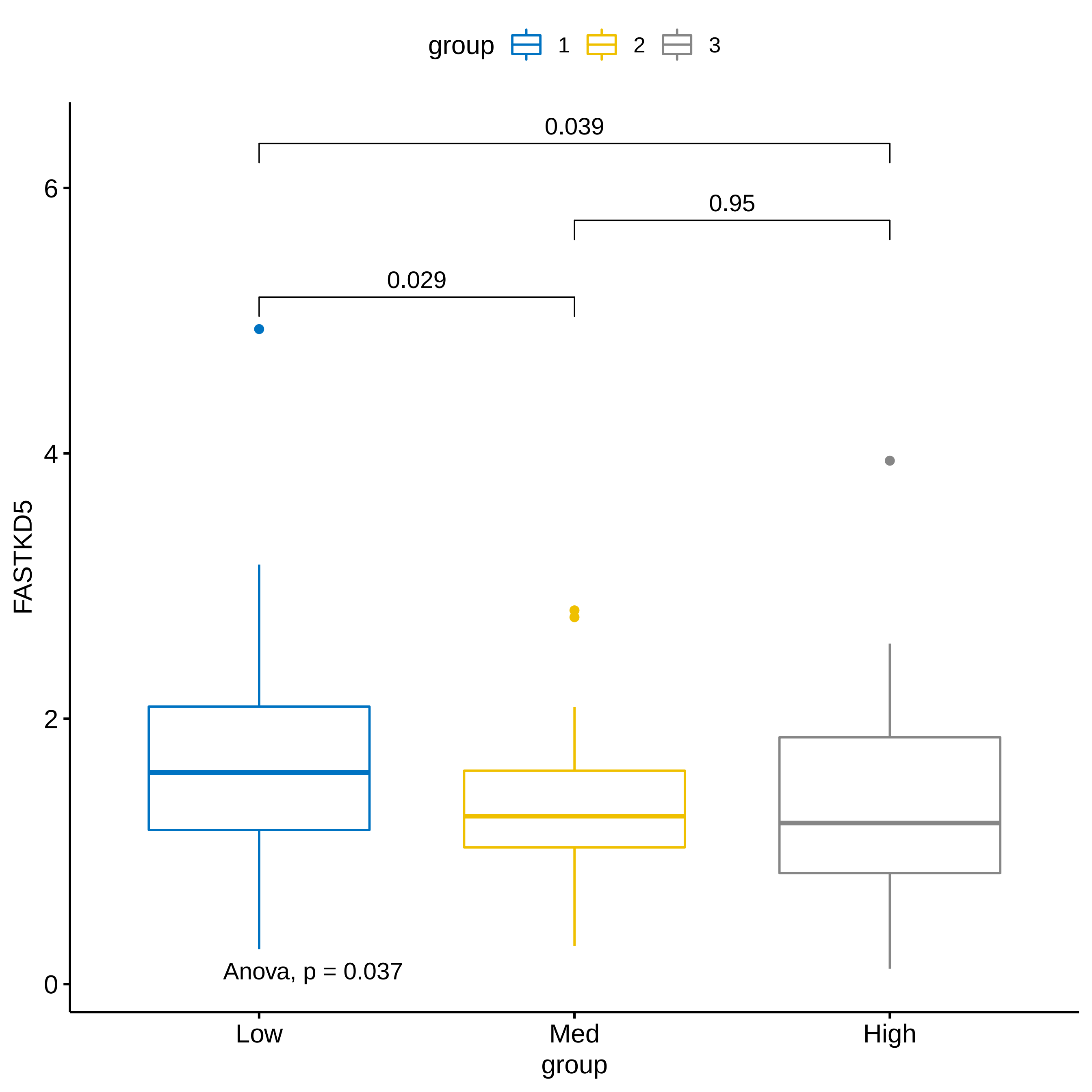

group
1
2
3
0.039
0.95
0.029
6
4
2
0
FASTKD5
Anova, p = 0.037
Low
Med
High
group

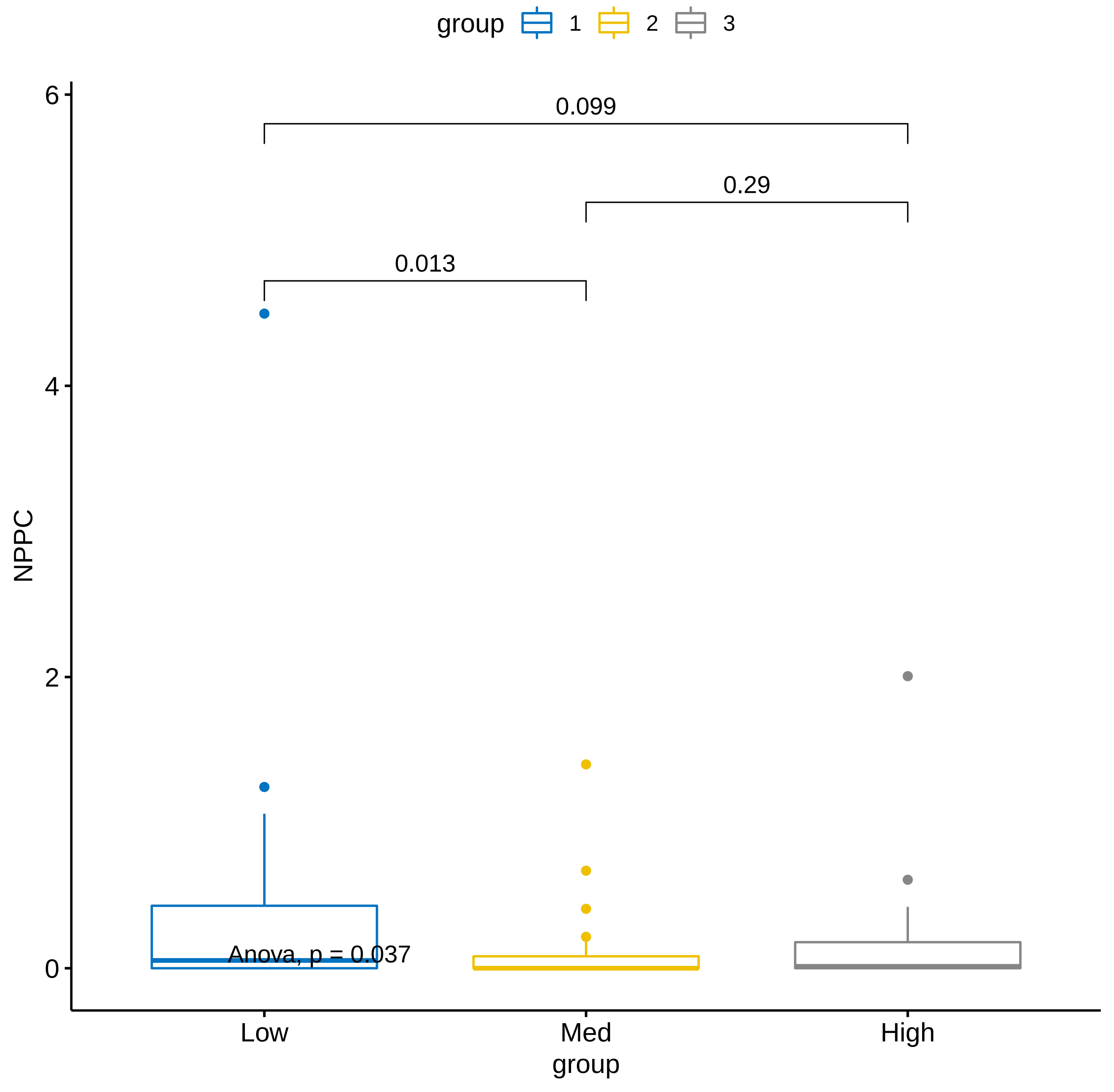

group 1 2 3
0.099
0.29
0.013
NPPC
6
4
2
0
Anova, p = 0.037
Low
Med
High
group

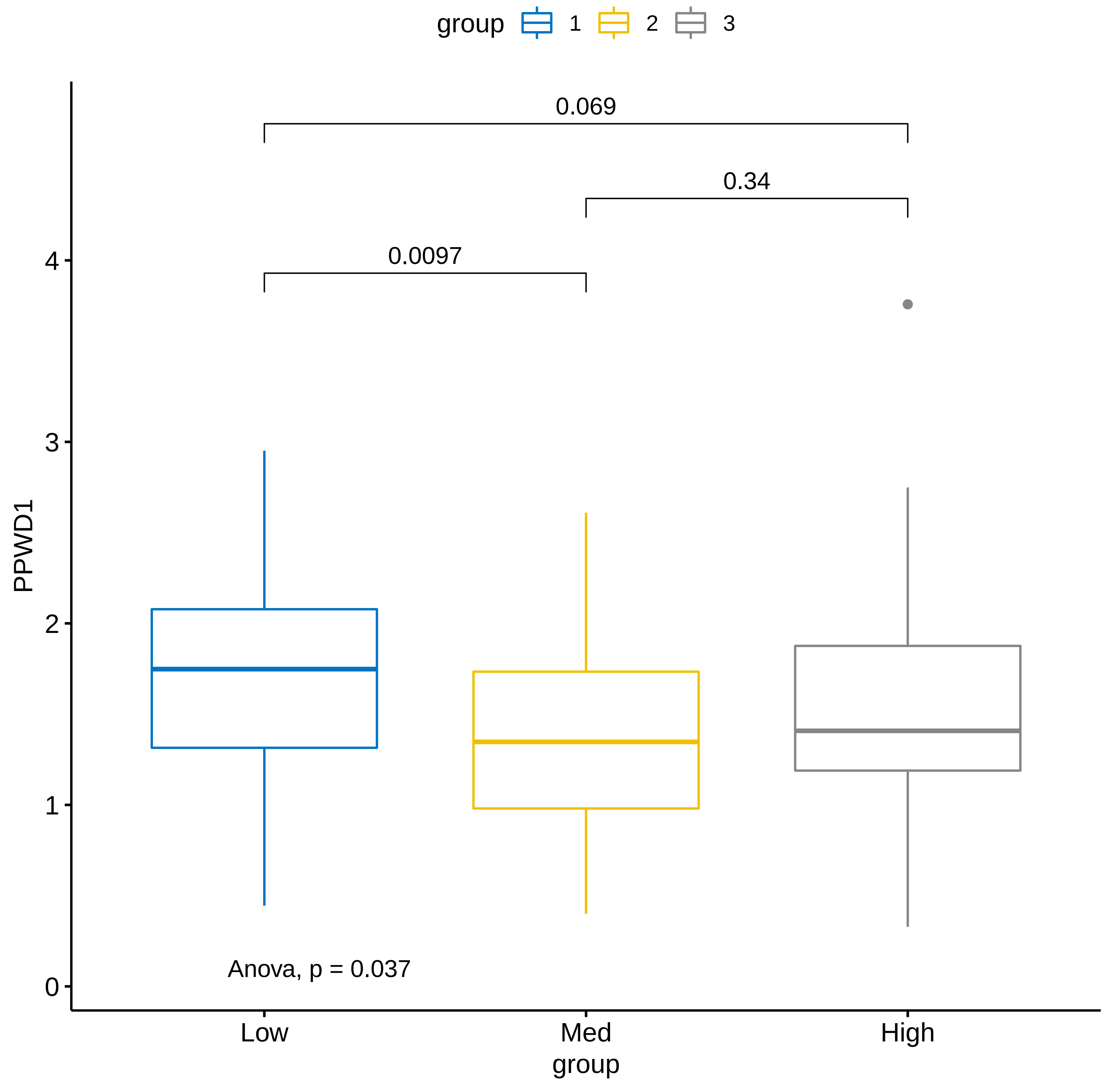
group 1 2 3
0.069
0.34
0.0097
PPWD1
4
3
2
1
0
Anova, p = 0.037
Low
Med
High
group

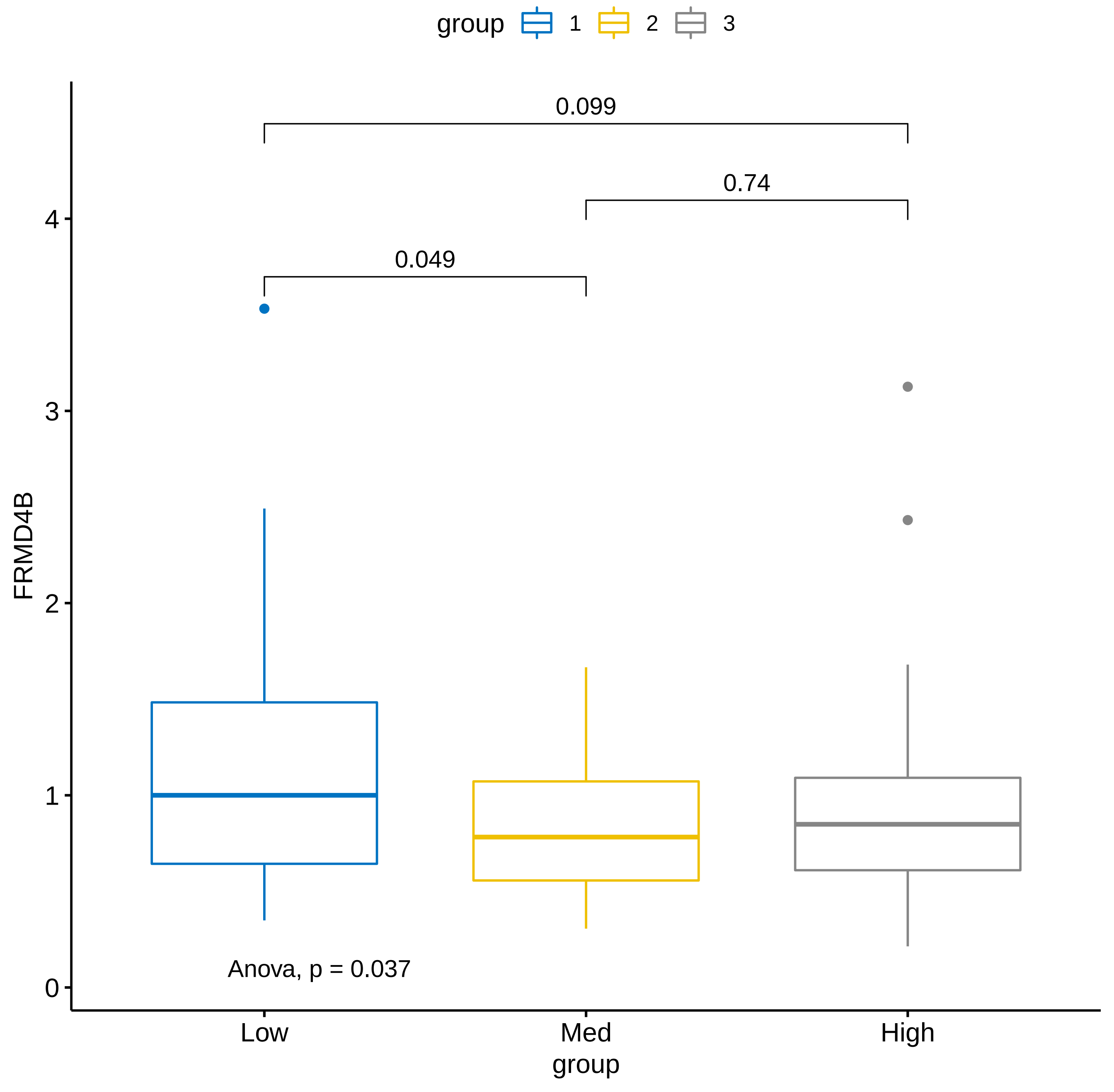
group
1
2
3
0.099
0.74
0.049
FRMD4B
4
3
2
1
0
Anova, p = 0.037
Low
Med
High
group

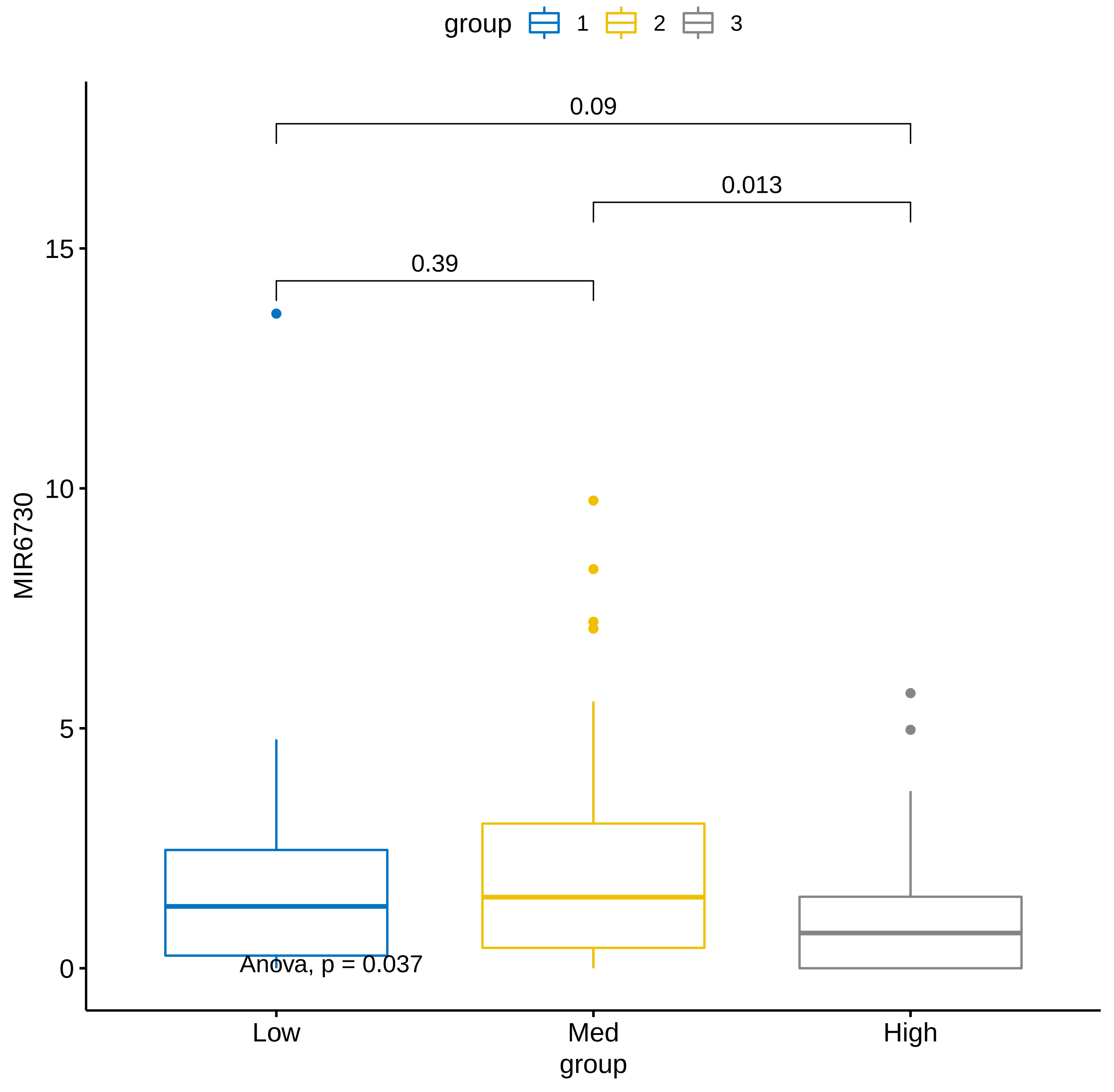

group
1
2
3
0.09
0.013
0.39
15
10
5
0
MIR6730
Anova, p = 0.037
Low
Med
High
group

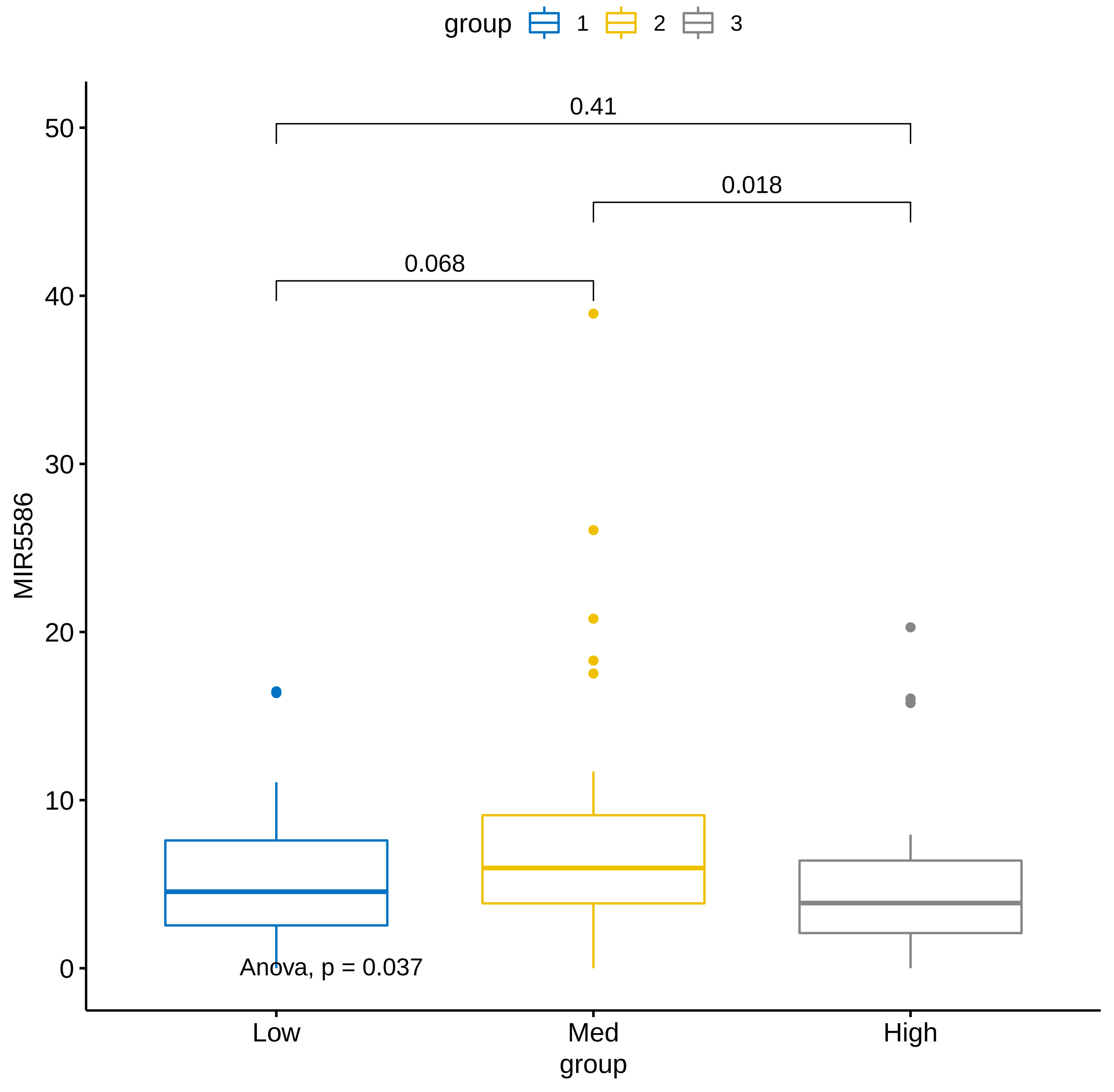
group
1
2
3
0.41
0.018
0.068
50
40
30
20
10
0
MIR5586
Anova, p = 0.037
Low
Med
High
group

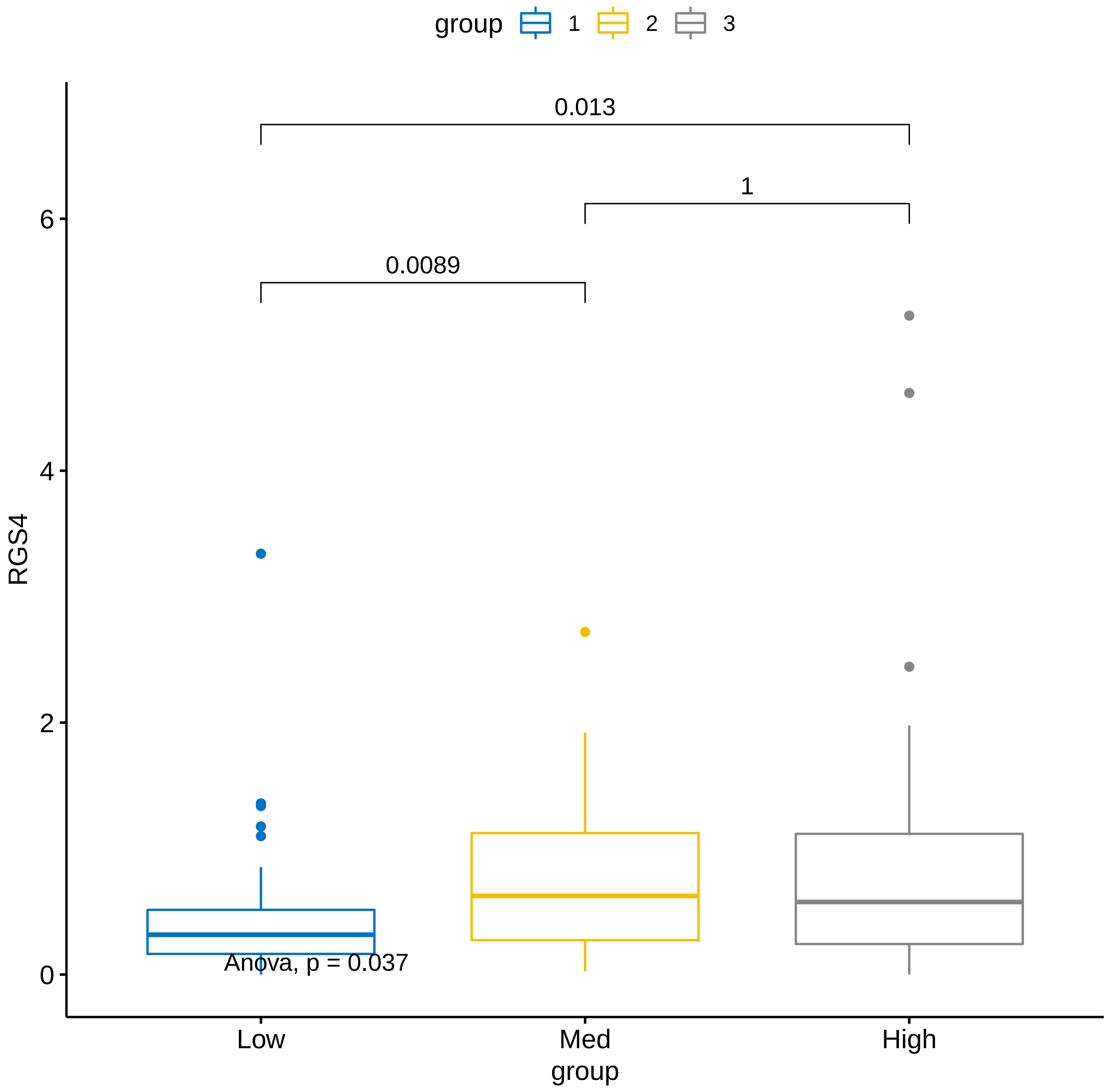
group 1 2 3
0.013
1
0.0089
RGS4
6
4
2
0
Anova, p = 0.037
Low
Med
High
group

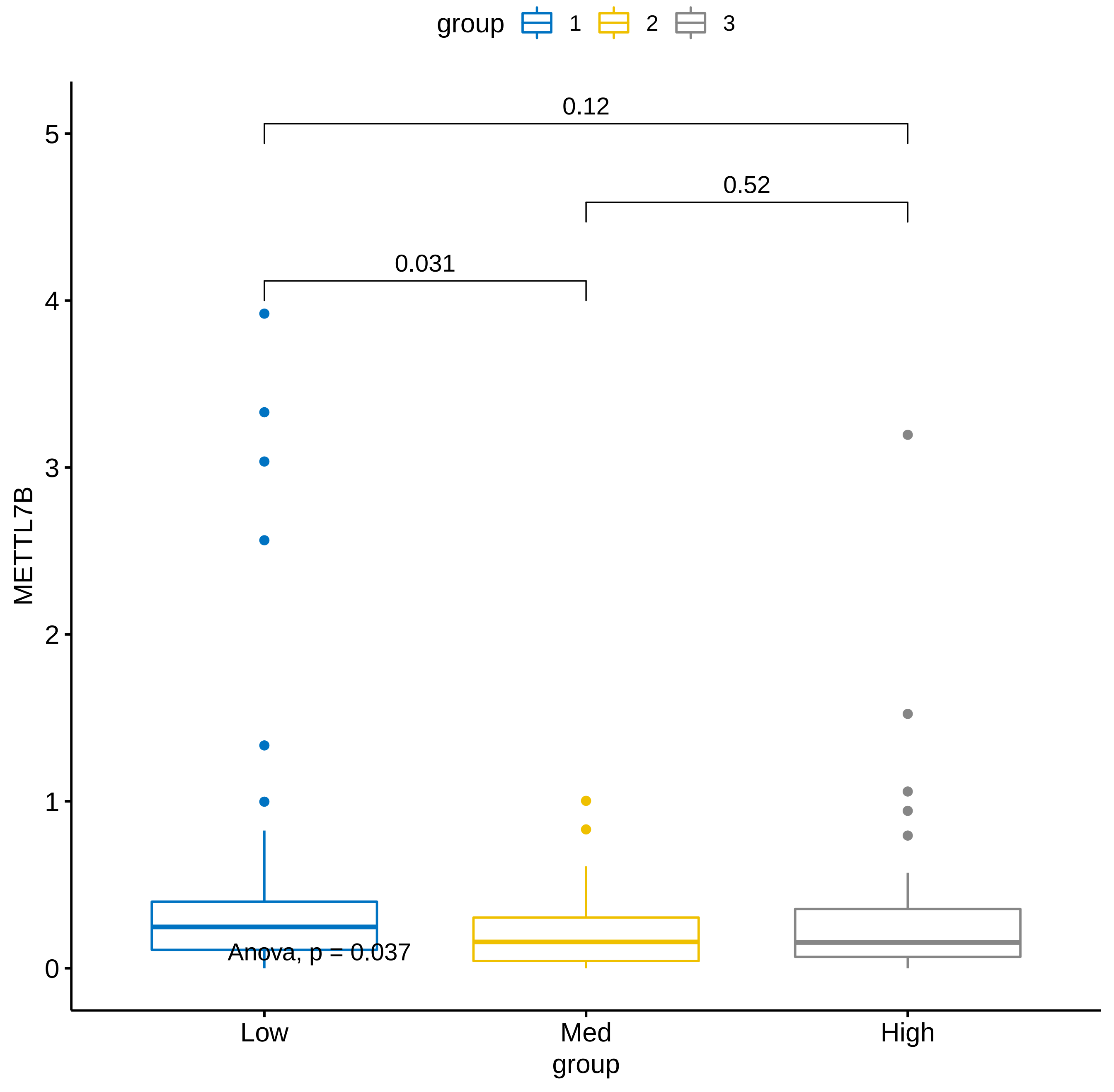

group 1 2 3
0.12
0.52
0.031
METTL7B
5
4
3
2
1
0
Anova, p = 0.037
Low
Med
High
group

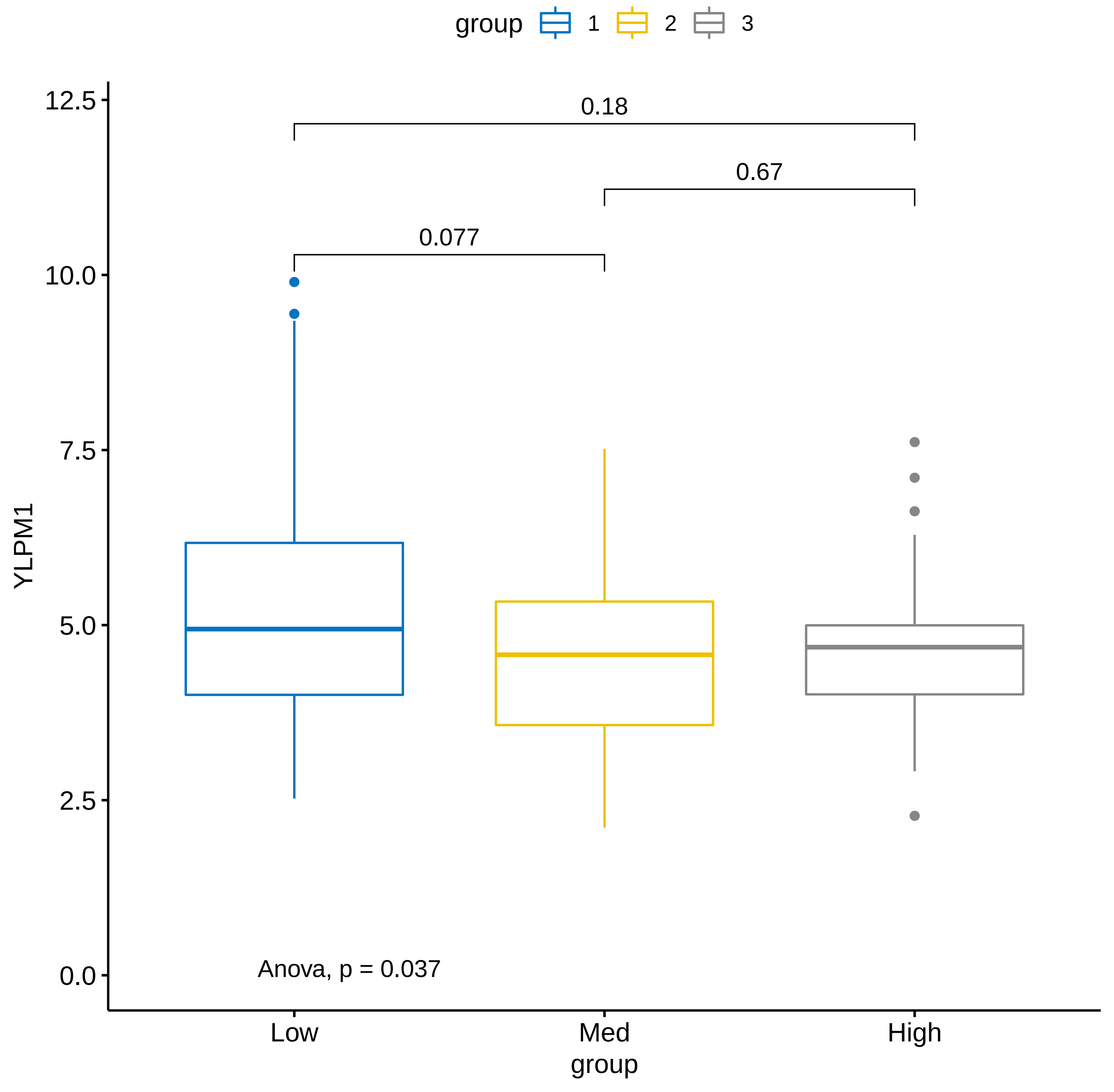
group 1 2 3
0.18
0.67
0.077
12.5
10.0
7.5
5.0
2.5
0.0
YLPM1
Anova, p = 0.037
Low
Med
High
group

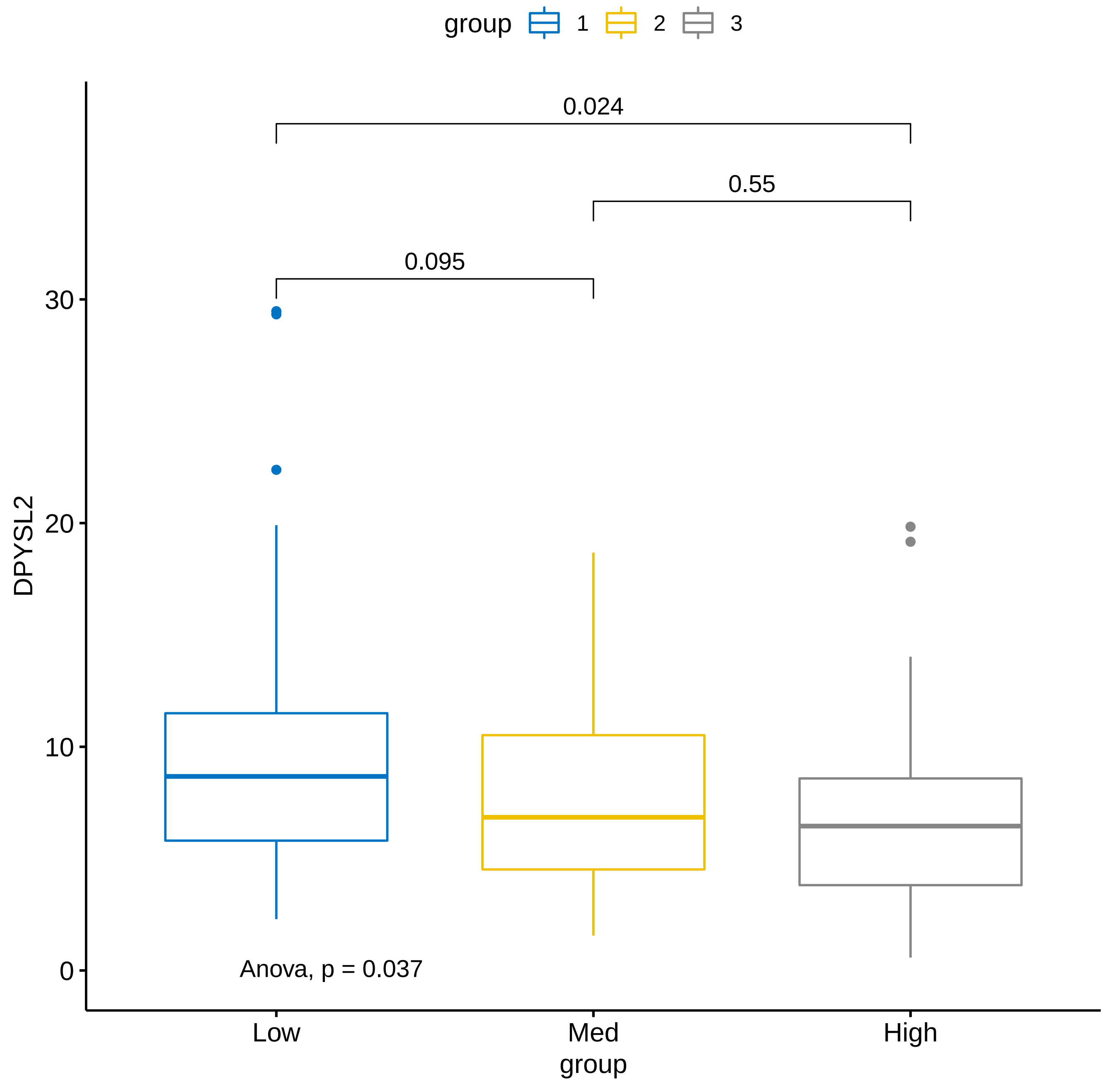
group
1
2
3
0.024
0.55
0.095
30
20
10
0
DPYSL2
Anova, p = 0.037
Low
Med
High
group

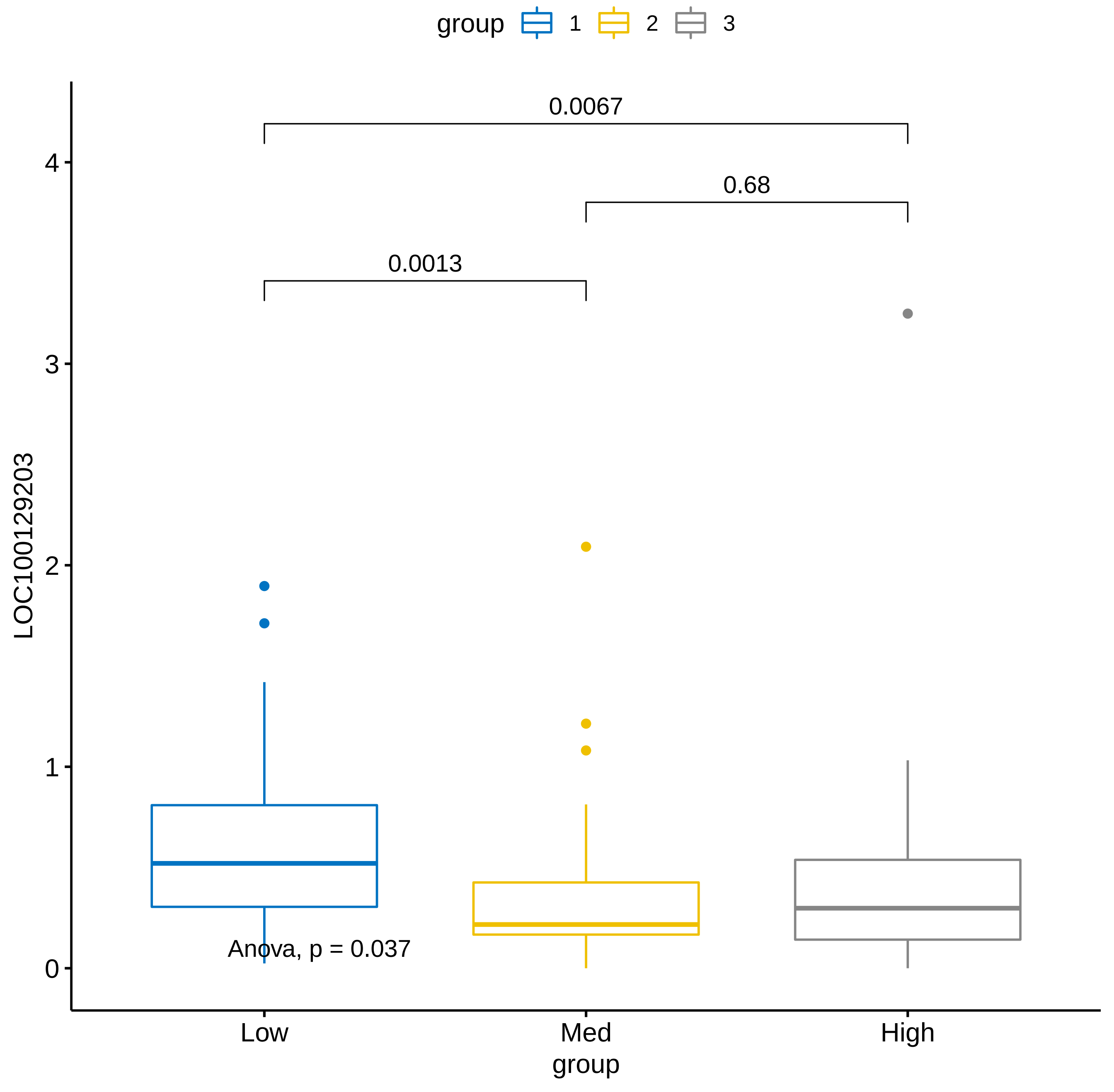

group
1
2
3
0.0067
0.68
0.0013
4
3
2
1
0
LOC100129203
Anova, p = 0.037
Low
Med
High
group

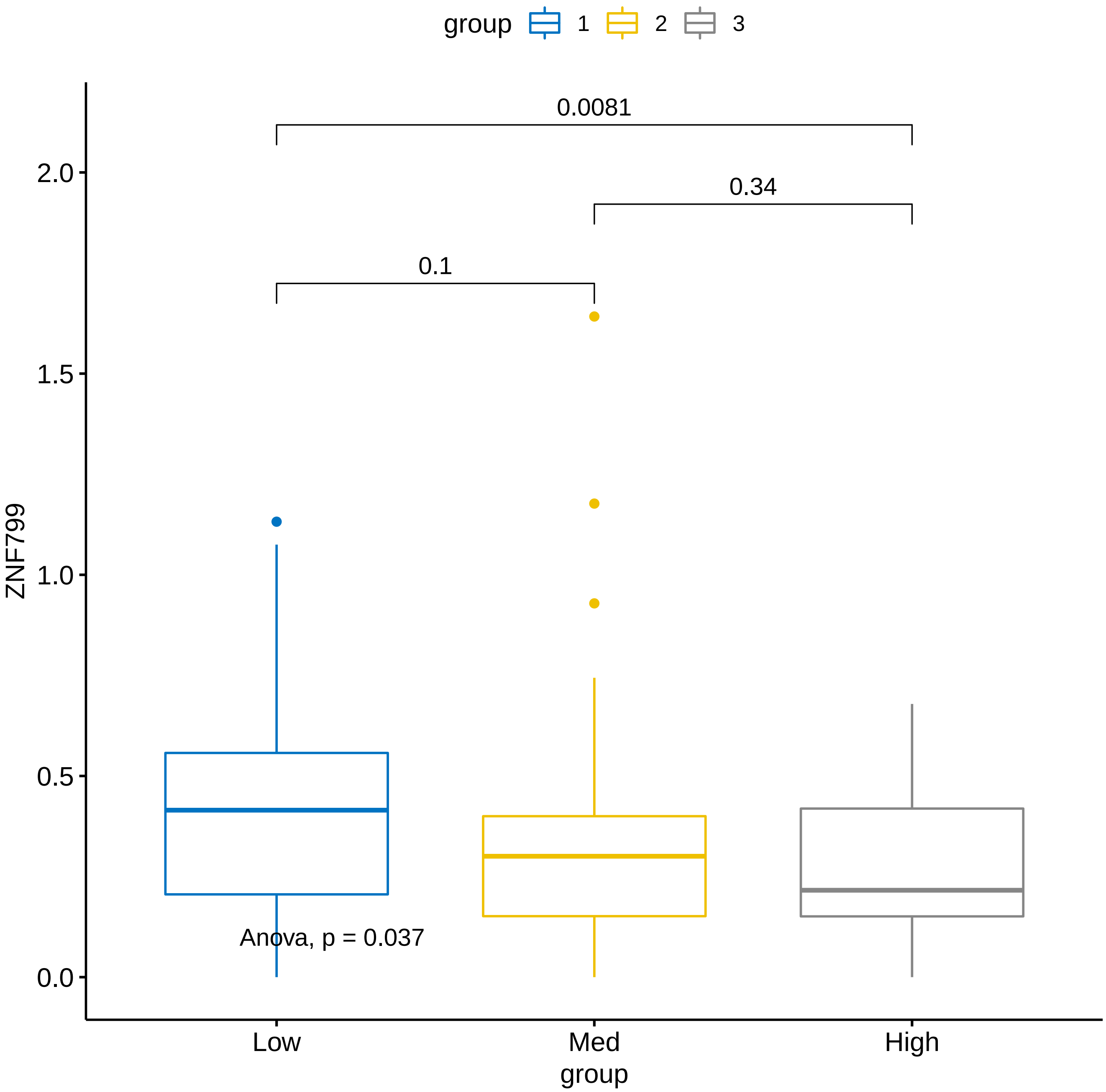

group 1 2 3
0.0081
0.34
0.1
2.0
1.5
1.0
0.5
0.0
ZNF799
Anova, p = 0.037
Low
Med
High
group

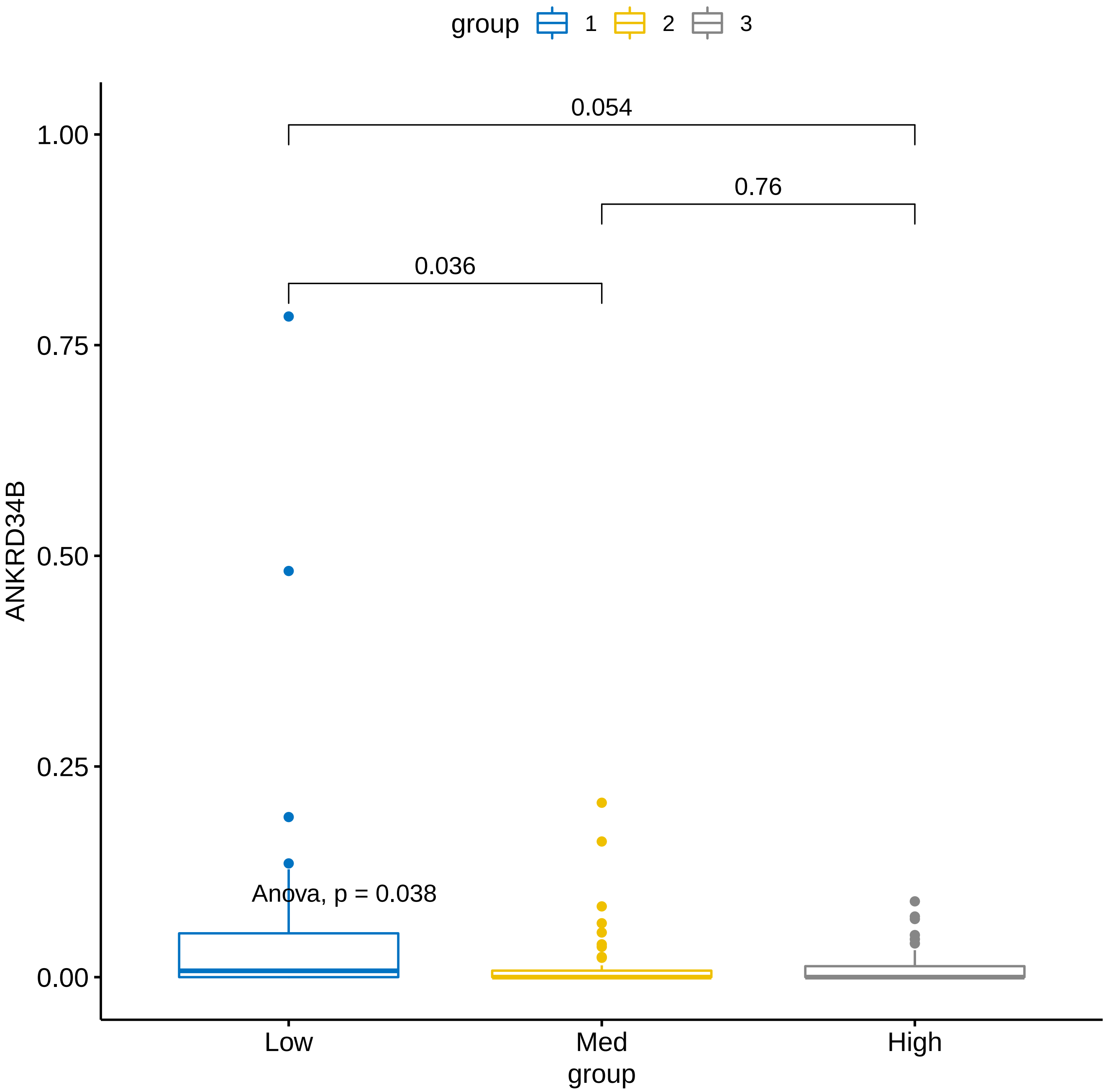

group 1 2 3
0.054
0.76
0.036
1.00
0.75
0.50
0.25
0.00
ANKRD34B
Anova, p = 0.038
Low
Med
High
group

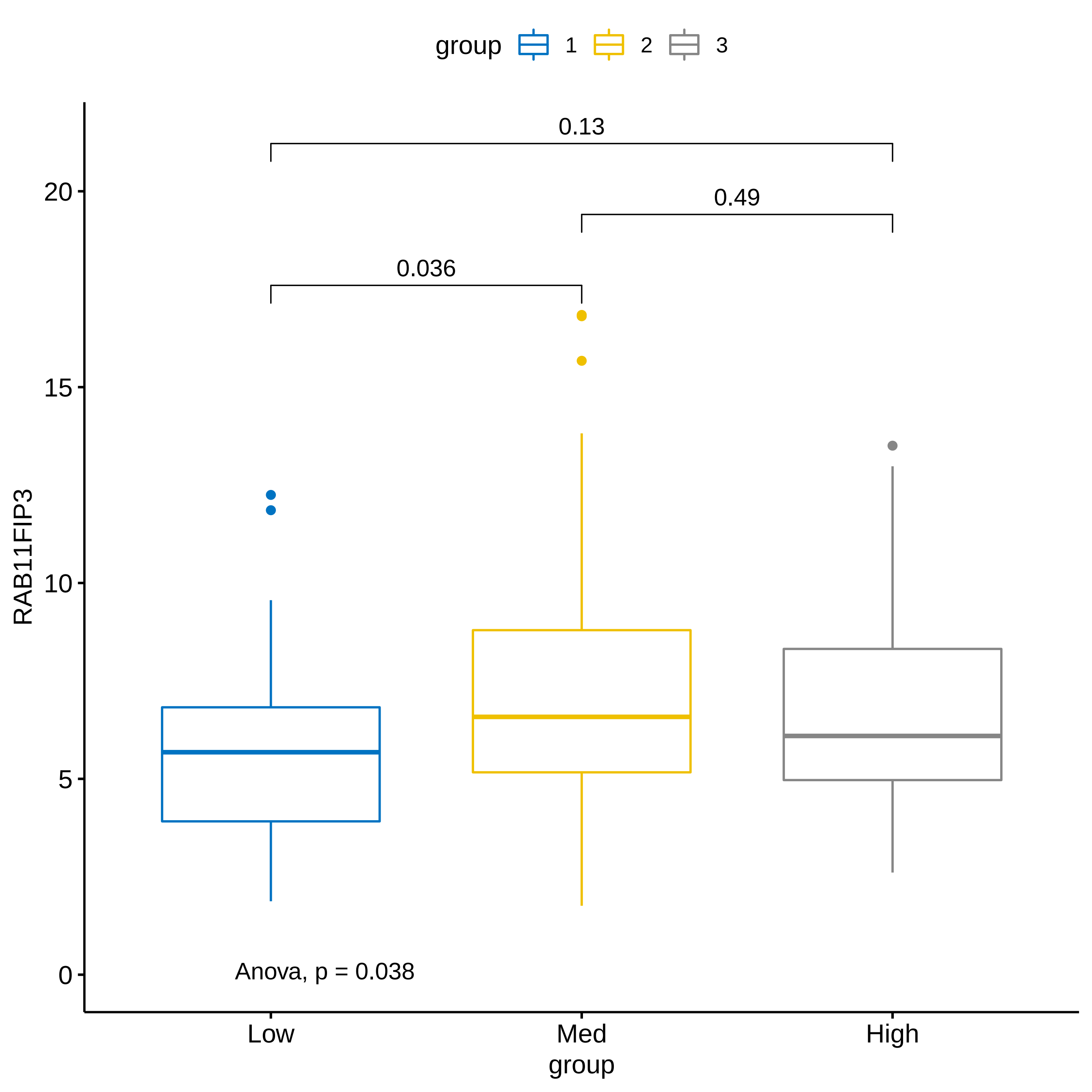

group 1 2 3
0.13
0.49
0.036
RAB11FIP3
20
15
10
5
0
Anova, p = 0.038
Low
Med
High
group

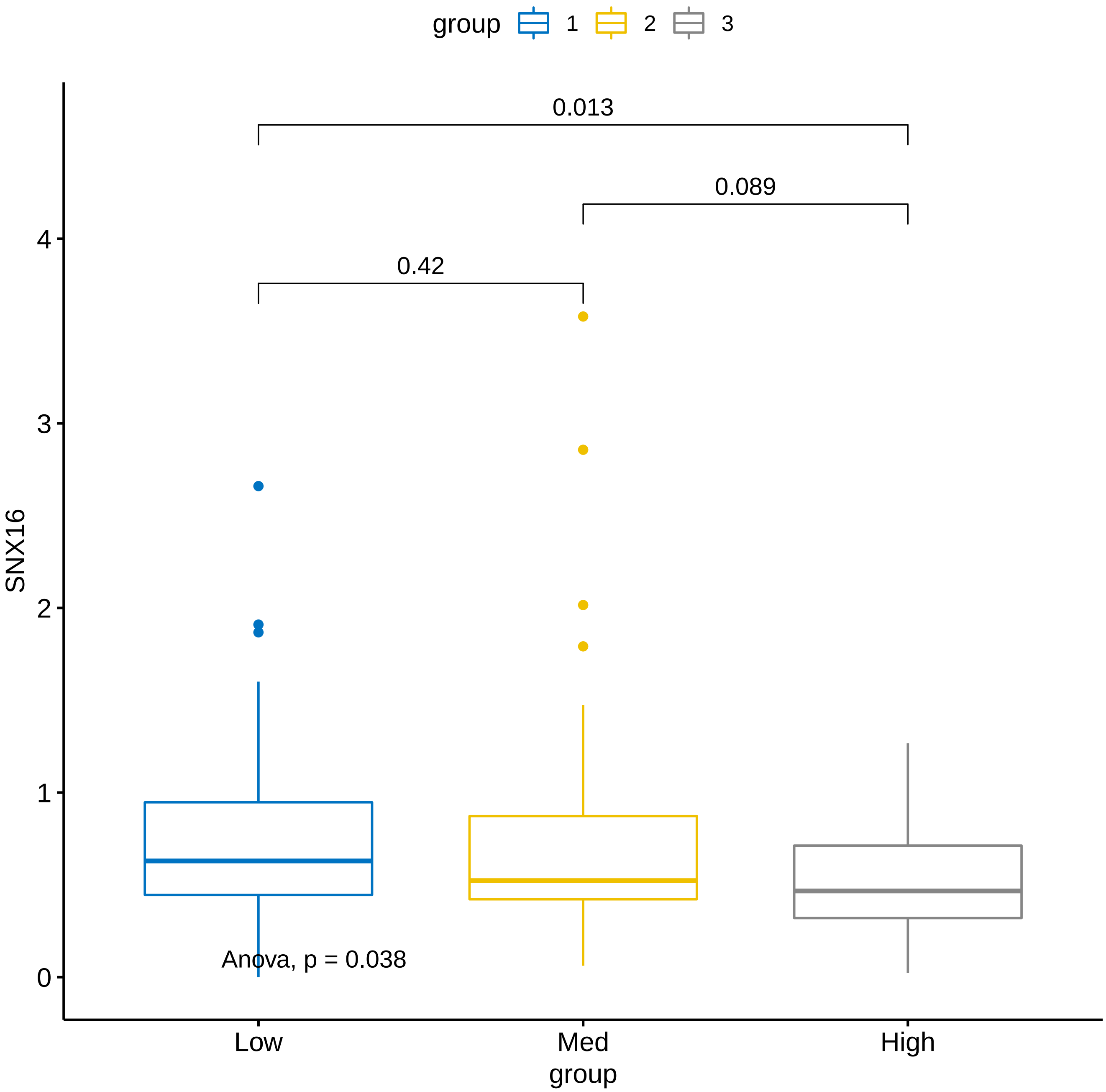

group 1 2 3
0.013
0.089
0.42
Anova, p = 0.038
SNX16
0
1
2
3
4
Low
Med
High
group

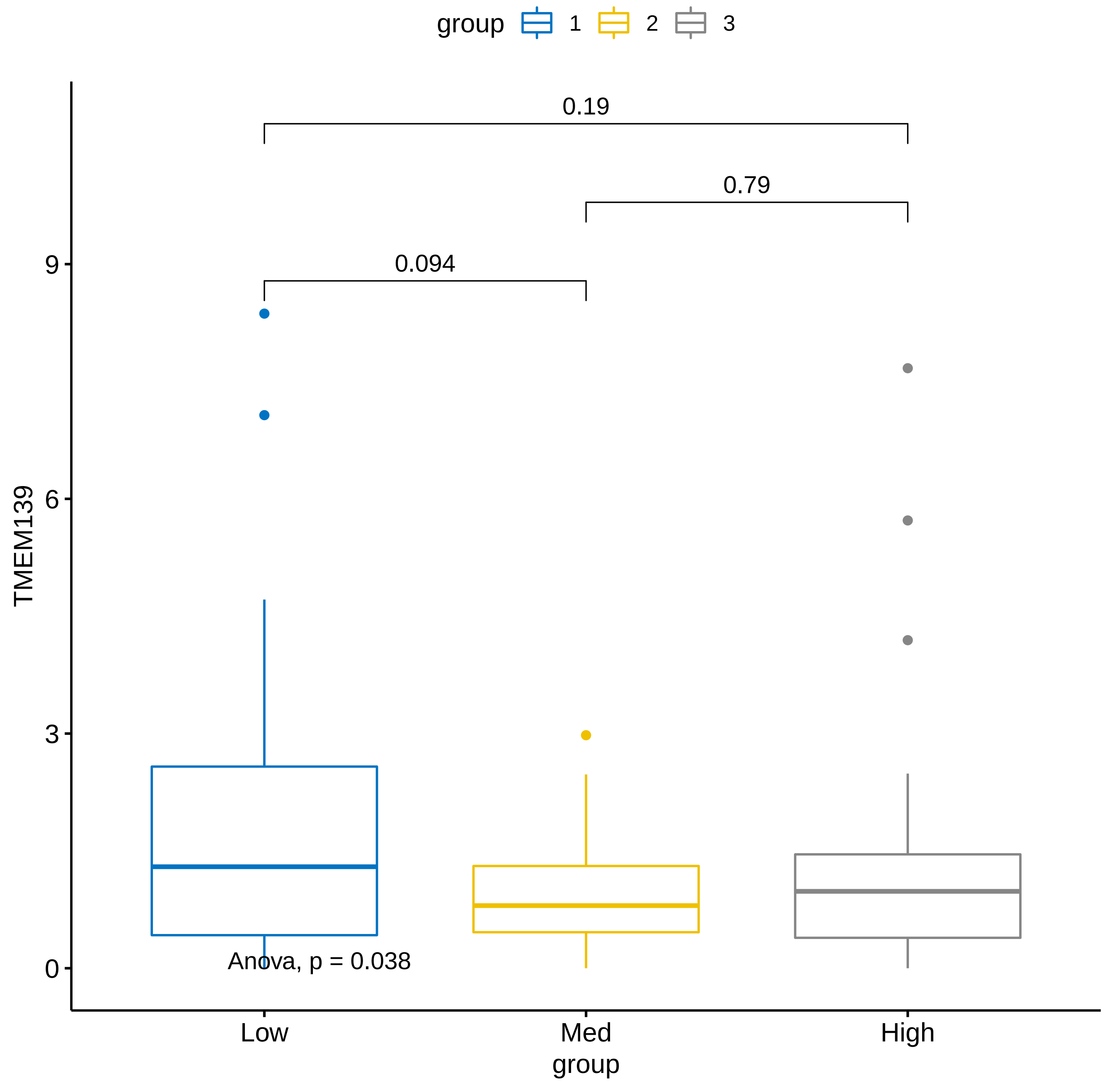
group 1 2 3
0.19
0.79
0.094
9
6
3
0
TMEM139
Anova, p = 0.038
Low
Med
High
group

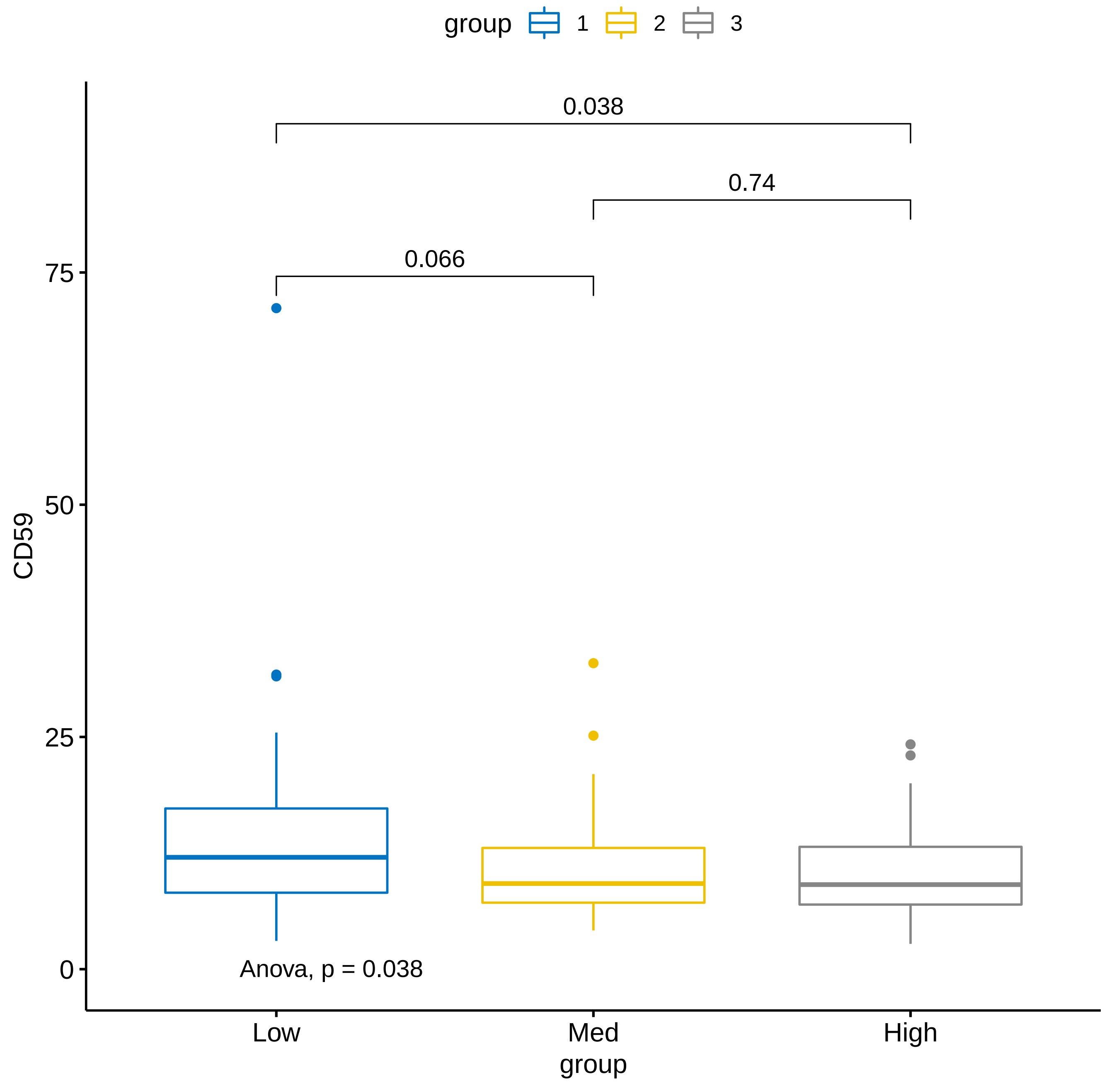

group
1
2
3
0.038
0.74
0.066
75
50
25
0
CD59
Anova, p = 0.038
Low
Med
High
group

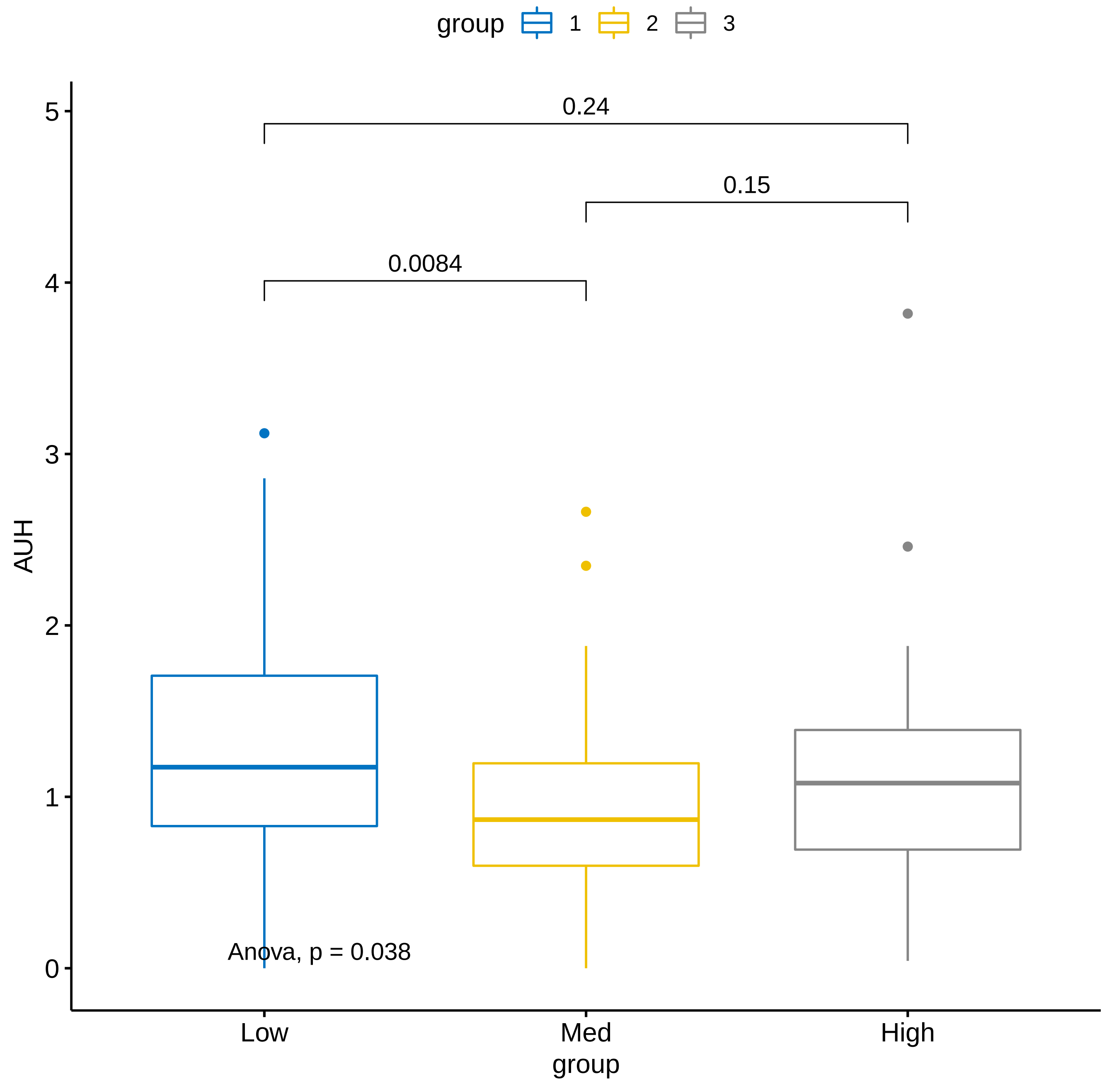

group 1 2 3
0.24
0.15
0.0084
AUH
5
4
3
2
1
0
Anova, p = 0.038
Low
Med
High
group

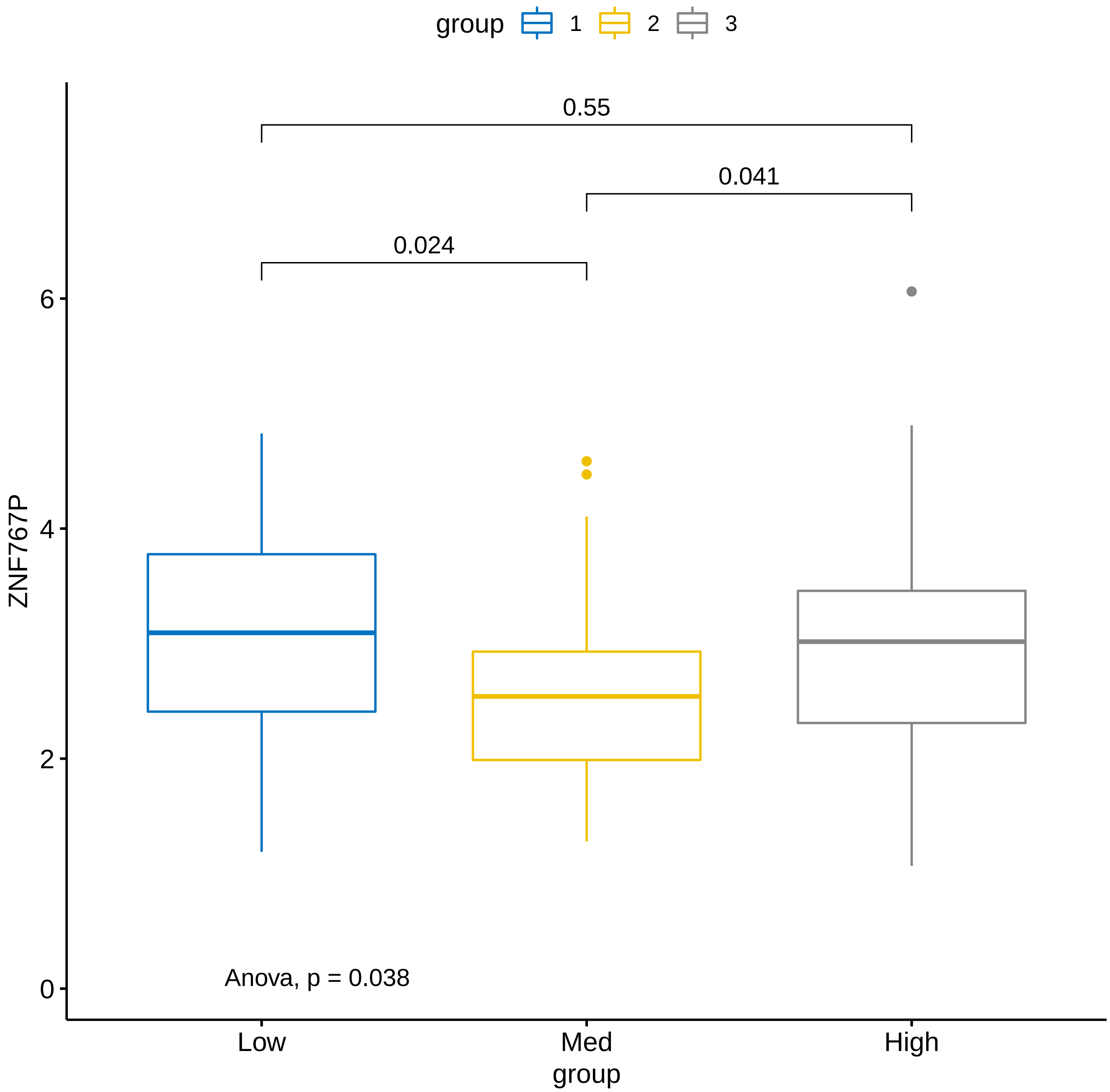

group 1 2 3
0.55
0.041
0.024
ZNF767P
6
4
2
0
Anova, p = 0.038
Low
Med
High
group

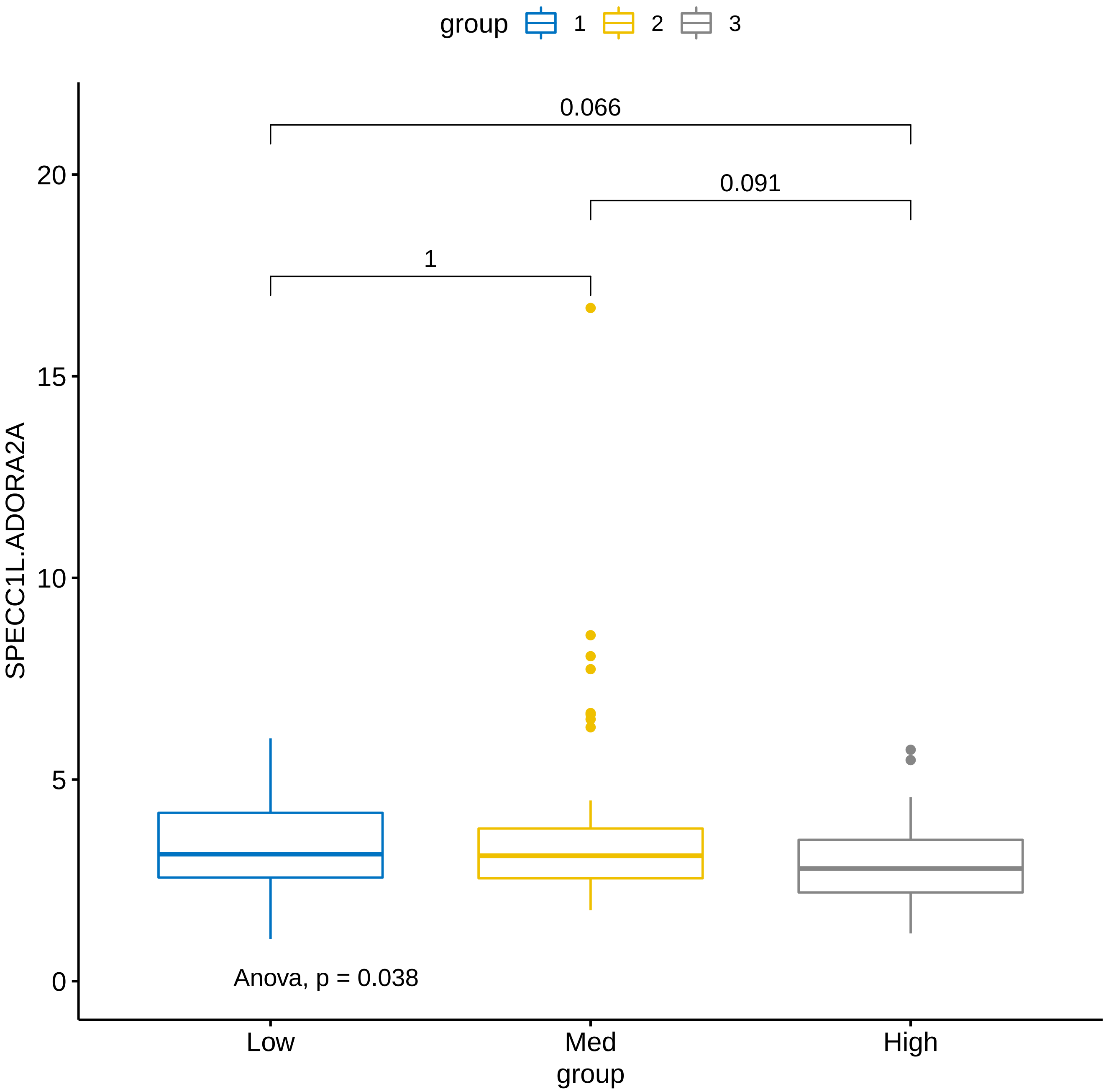

group
1
2
3
0.066
0.091
1
20
15
10
5
0
SPECC1L.ADORA2A
Anova, p = 0.038
Low
Med
High
group

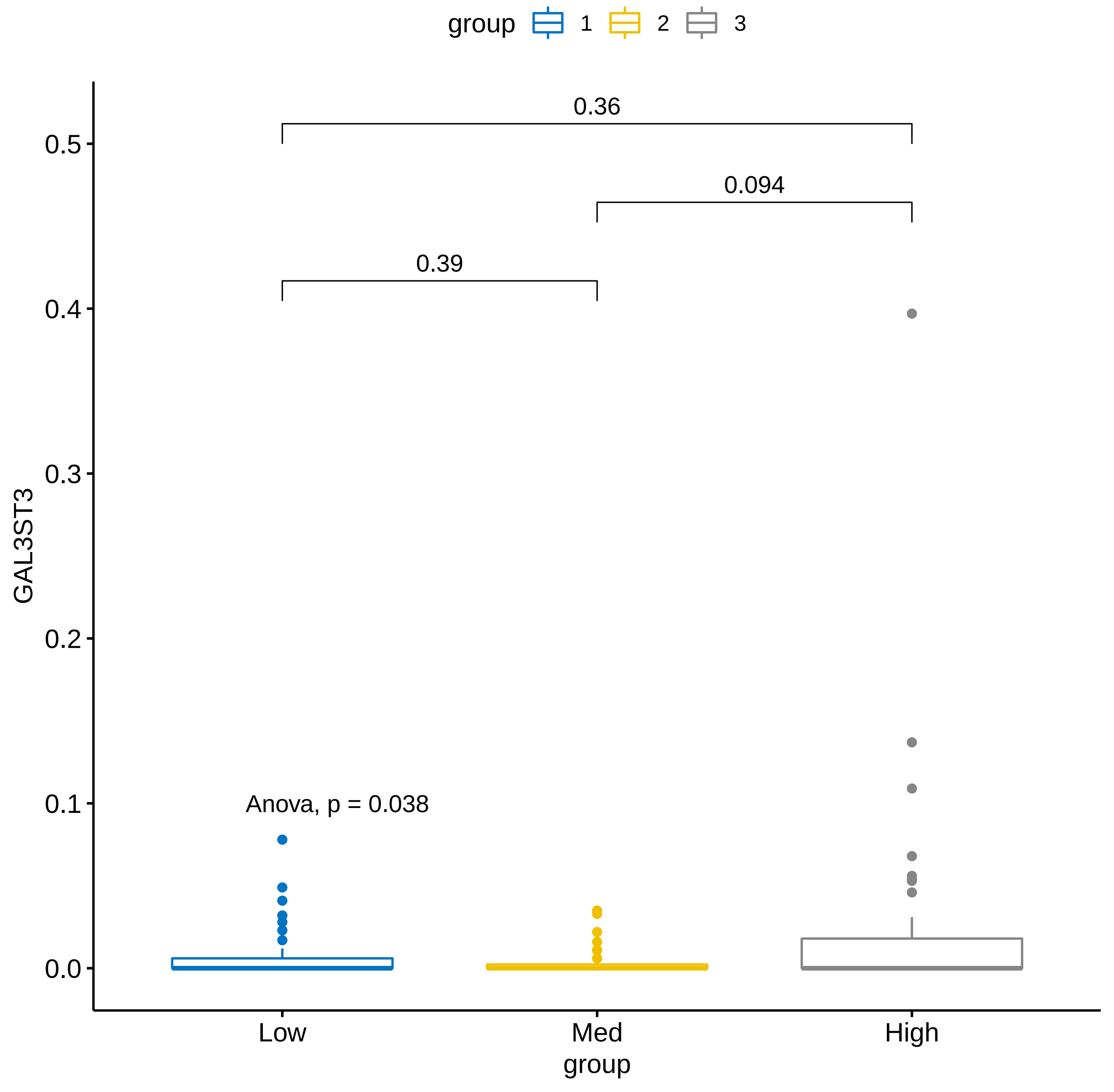

group
1
2
3
0.36
0.094
0.39
Anova, p = 0.038
GAL3ST3
0.5
0.4
0.3
0.2
0.1
0.0
Low
Med
High
group

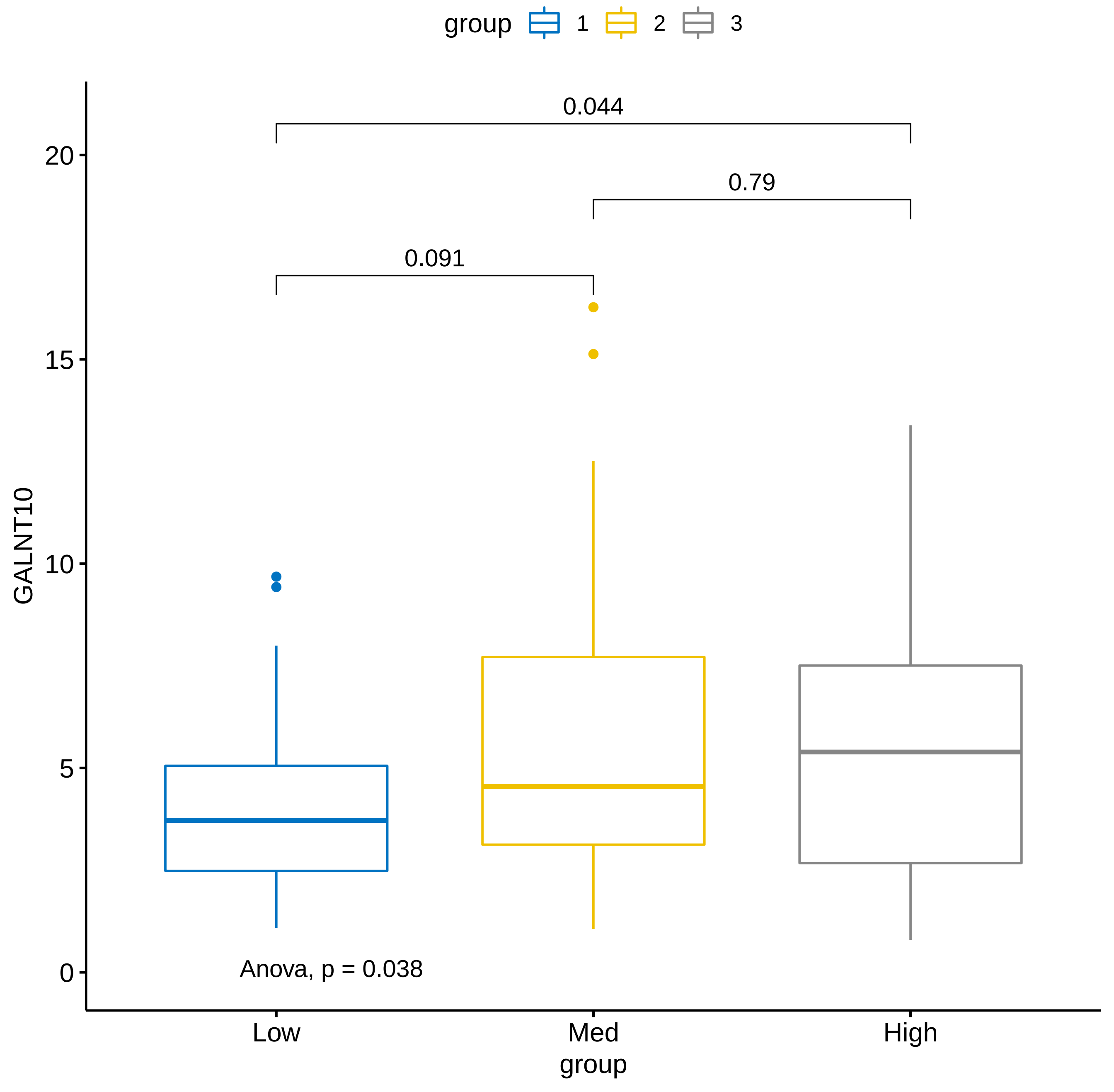

group 1 2 3
0.044
0.79
0.091
GALNT10
20
15
10
5
0
Anova, p = 0.038
Low
Med
High
group

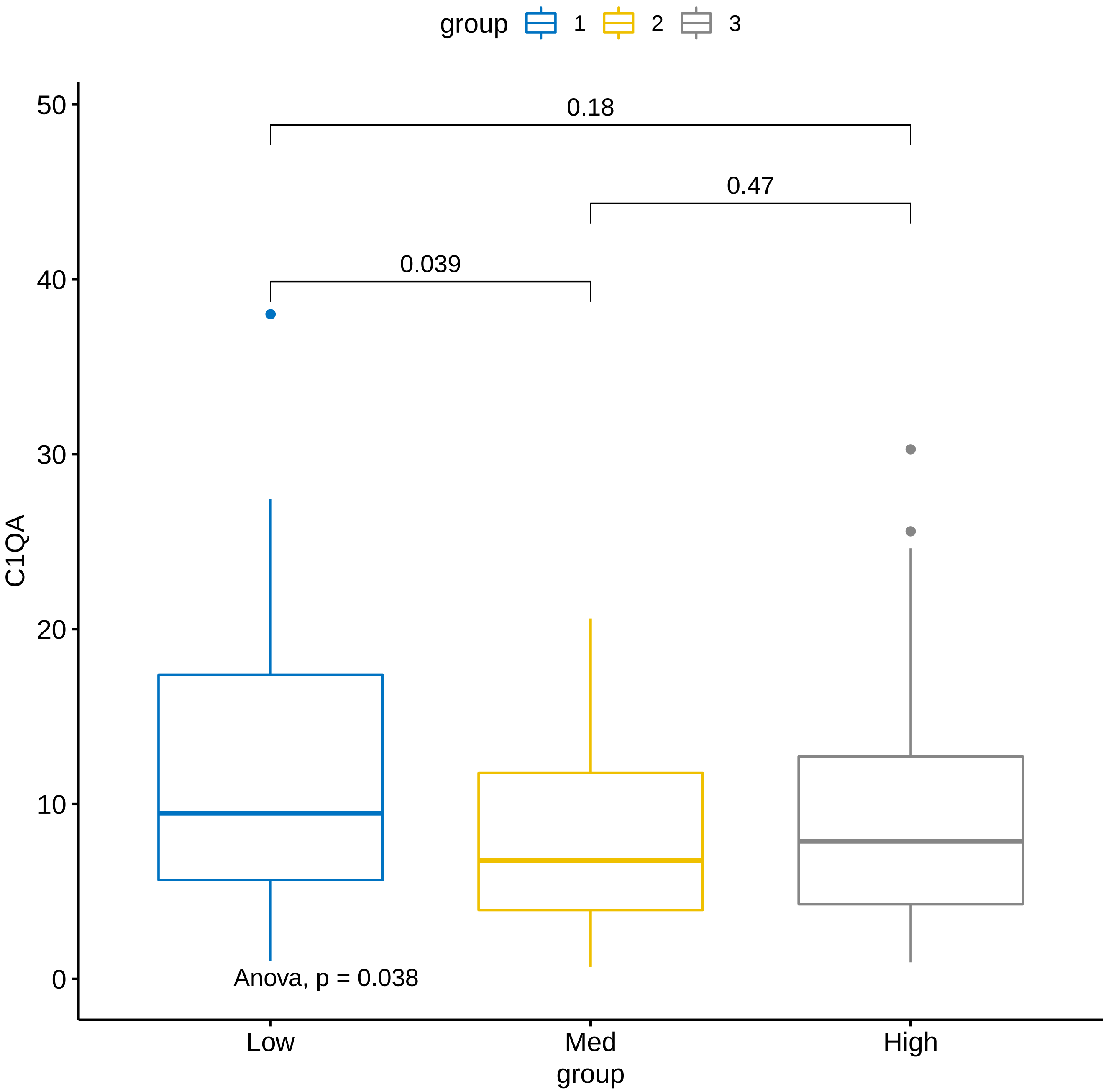
group
1
2
3
0.18
0.47
0.039
50
40
30
20
10
0
C1QA
Anova, p = 0.038
Low
Med
High
group

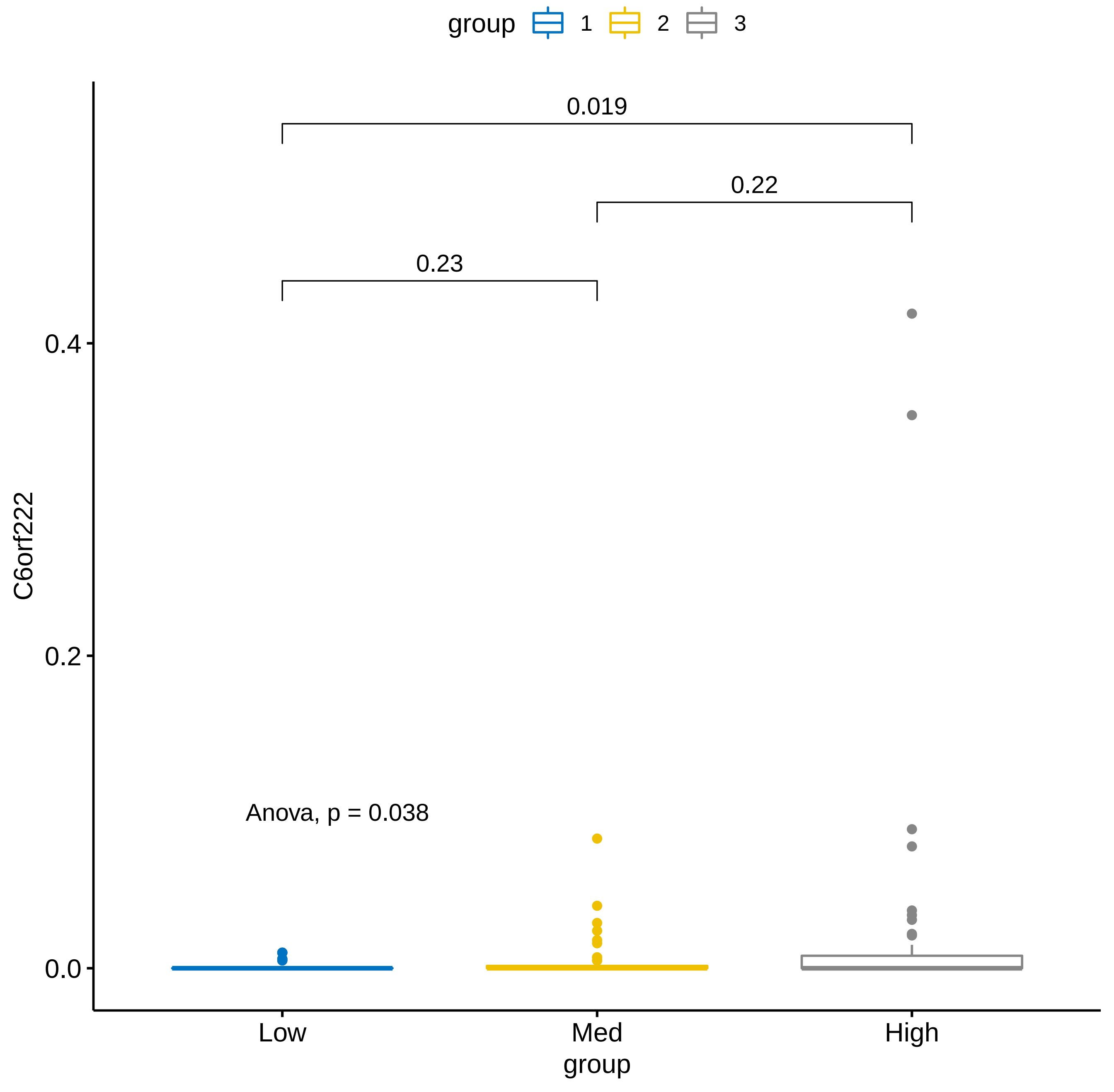
group
1
2
3
0.019
0.22
0.23
0.4
0.2
0.0
C6orf222
Anova, p = 0.038
Low
Med
High
group

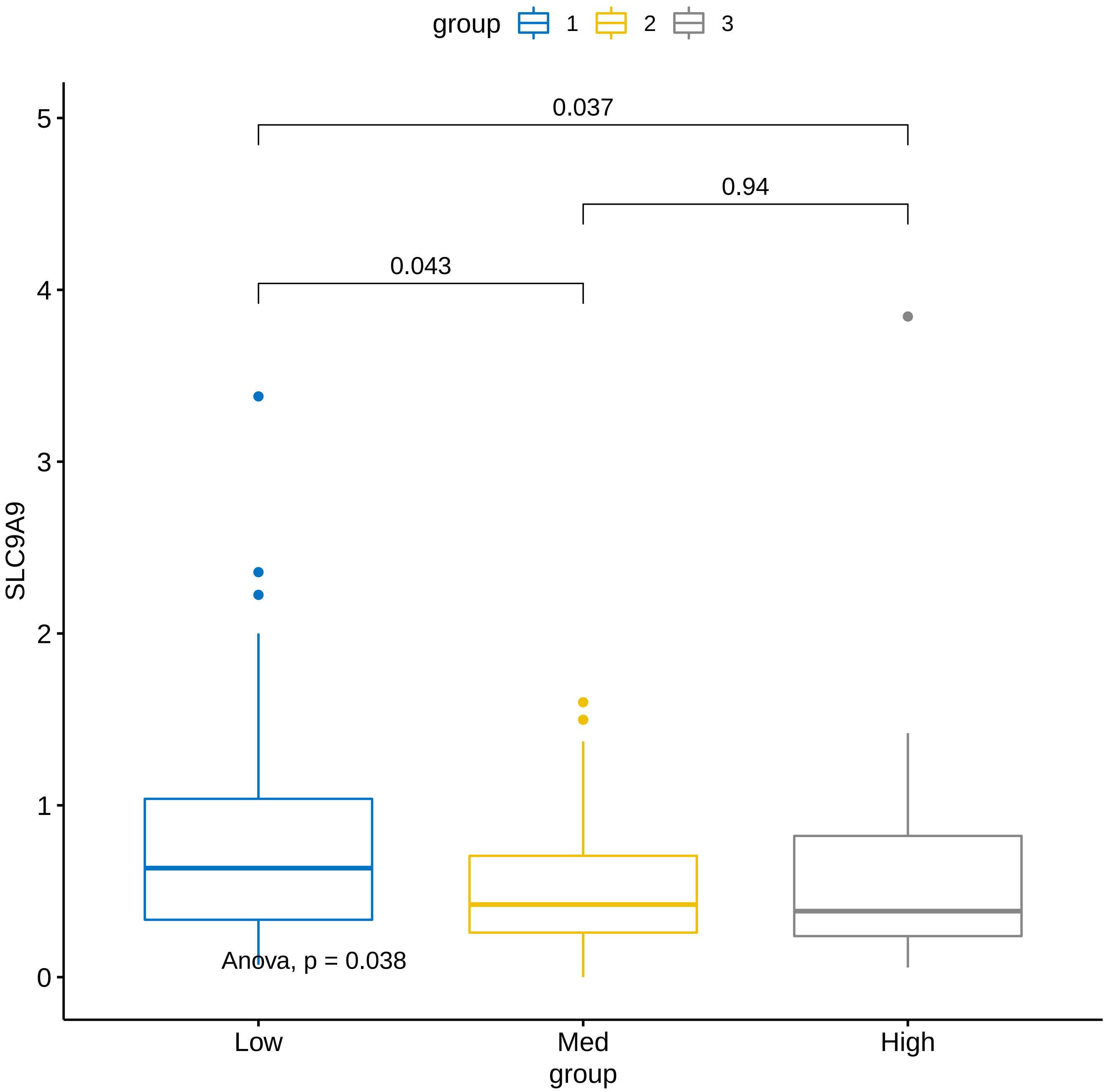

group
1
2
3
0.037
0.94
0.043
Anova, p = 0.038
SLC9A9
5
4
3
2
1
0
Low
Med
High
group

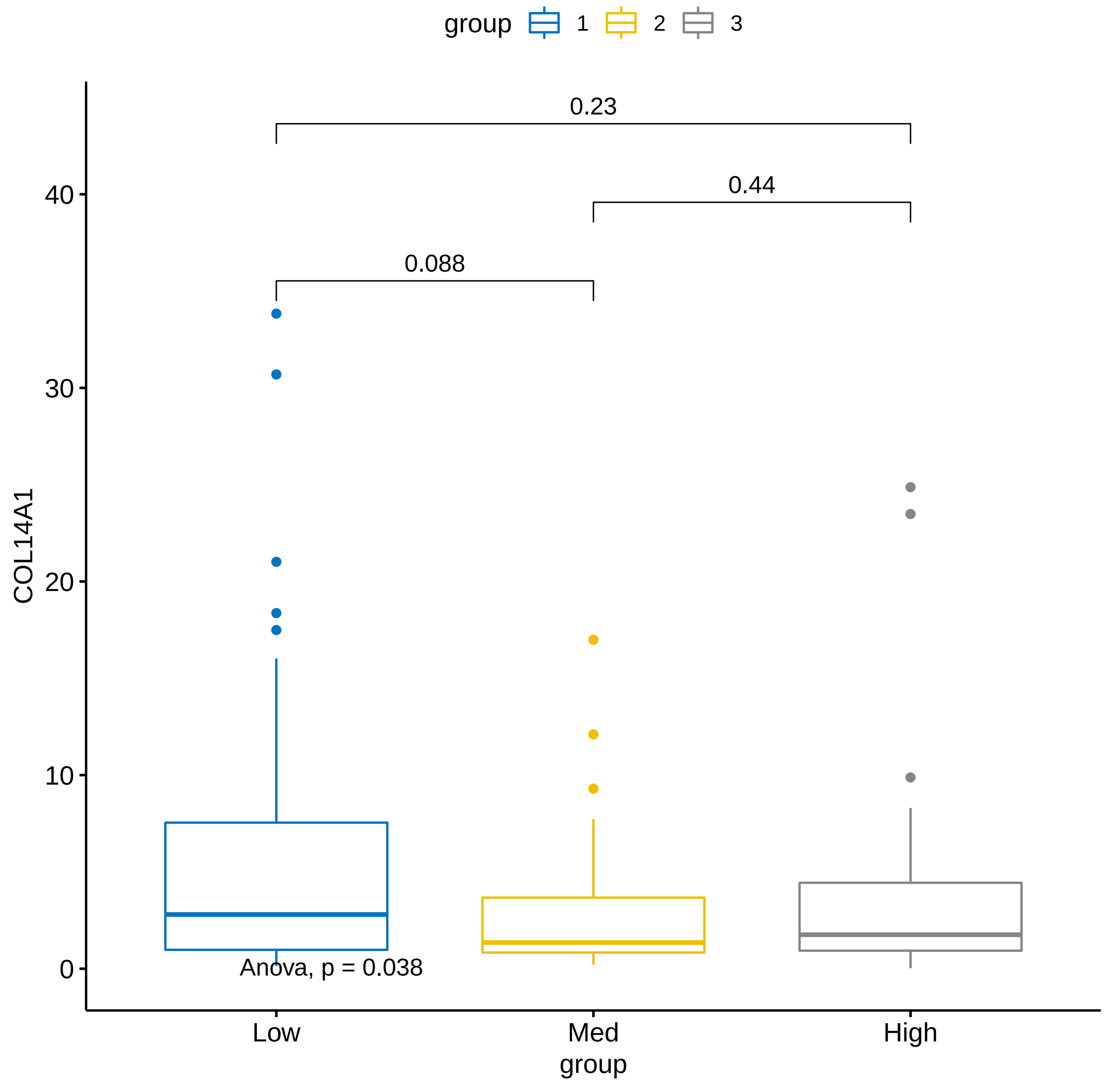

group
1
2
3
0.23
0.44
0.088
40
30
20
10
0
COL14A1
Anova, p = 0.038
Low
Med
High
group

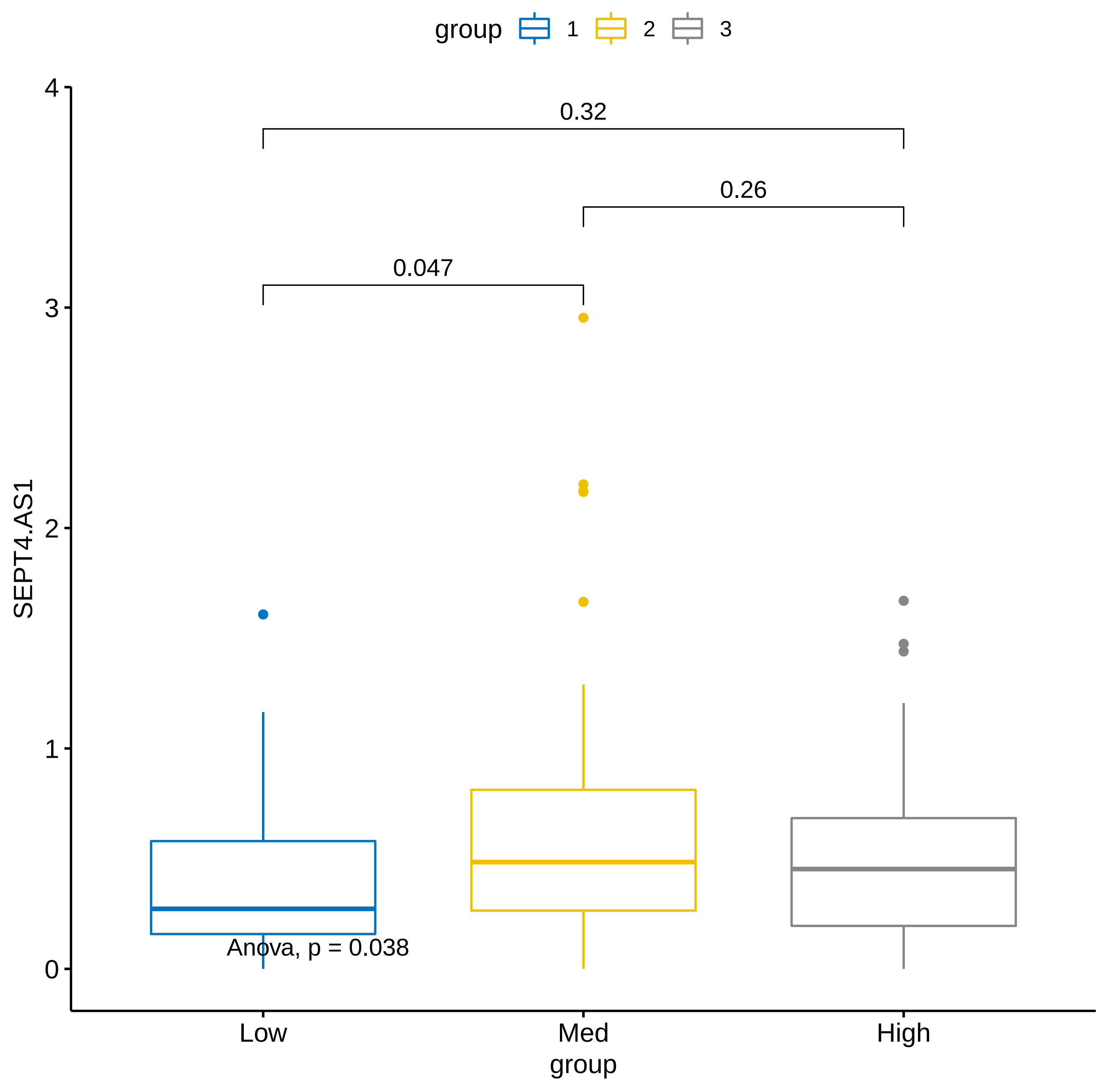
group 1 2 3
0.32
0.26
0.047
Anova, p = 0.038
SEPT4.AS1
0
1
2
3
4
Low
Med
High
group

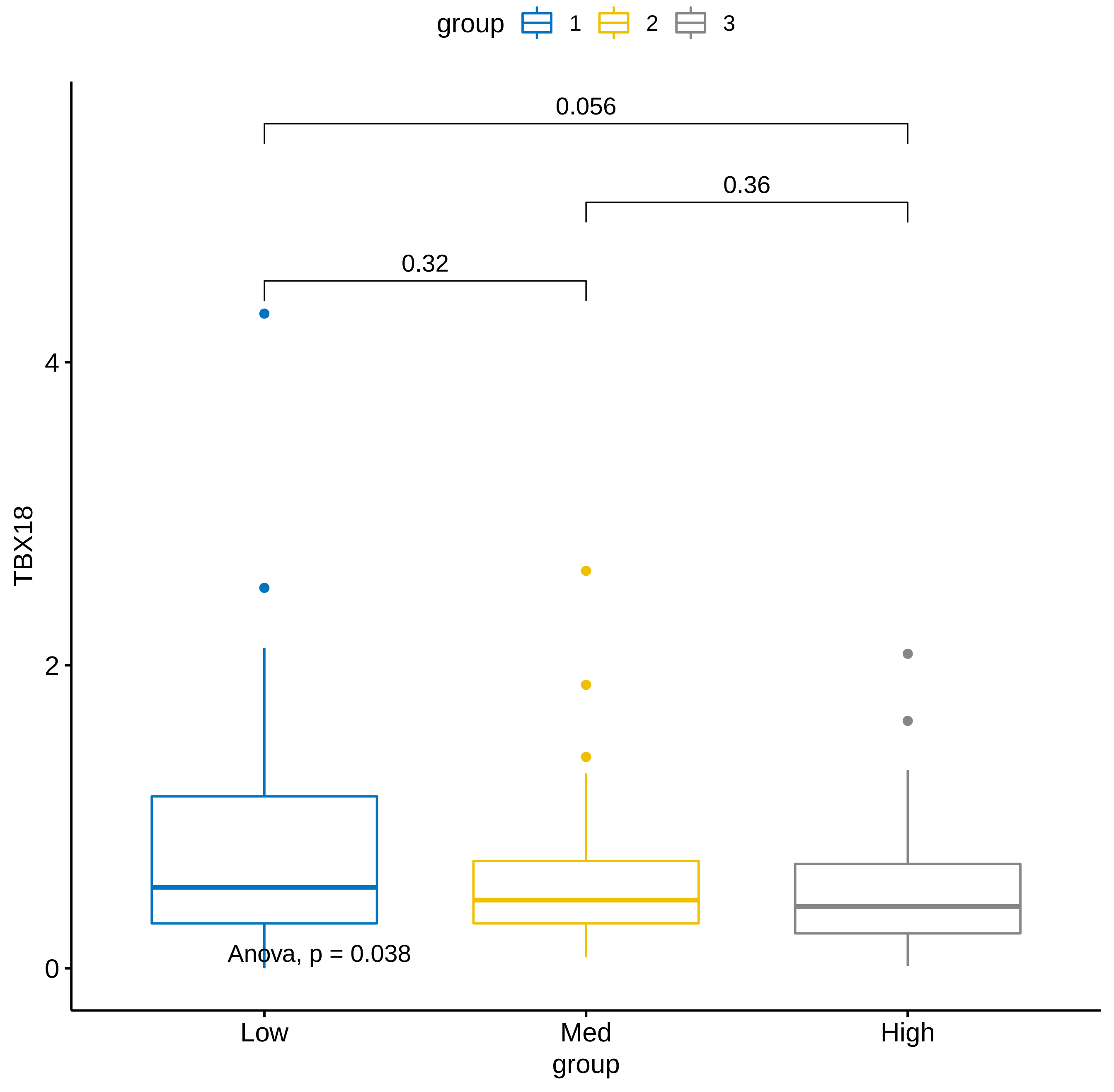
group 1 2 3
0.056
0.36
0.32
4
2
0
TBX18
Anova, p = 0.038
Low
Med
High
group

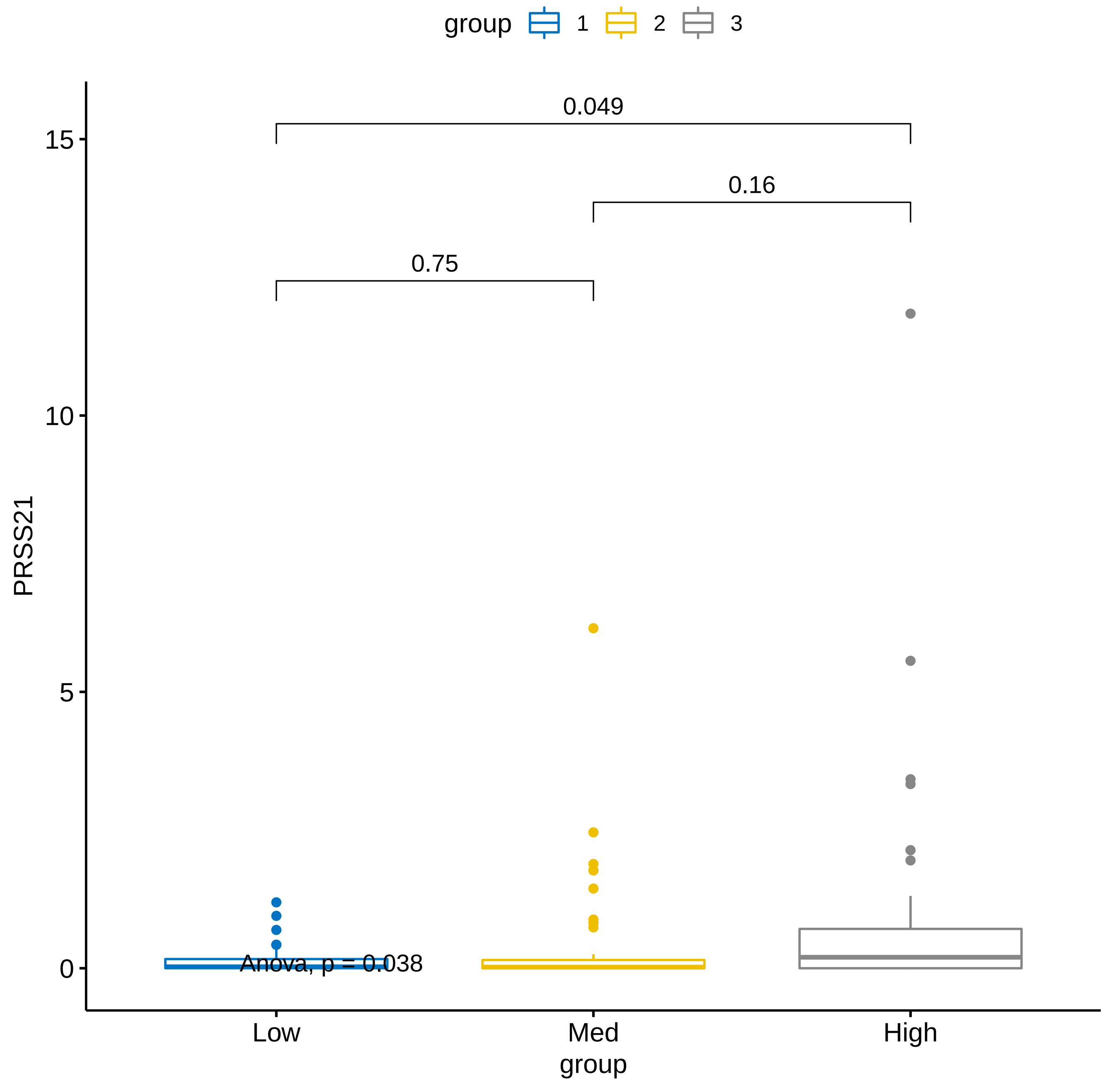
group
1
2
3
0.049
0.16
0.75
15
10
5
0
PRSS21
Anova, p = 0.038
Low
Med
High
group

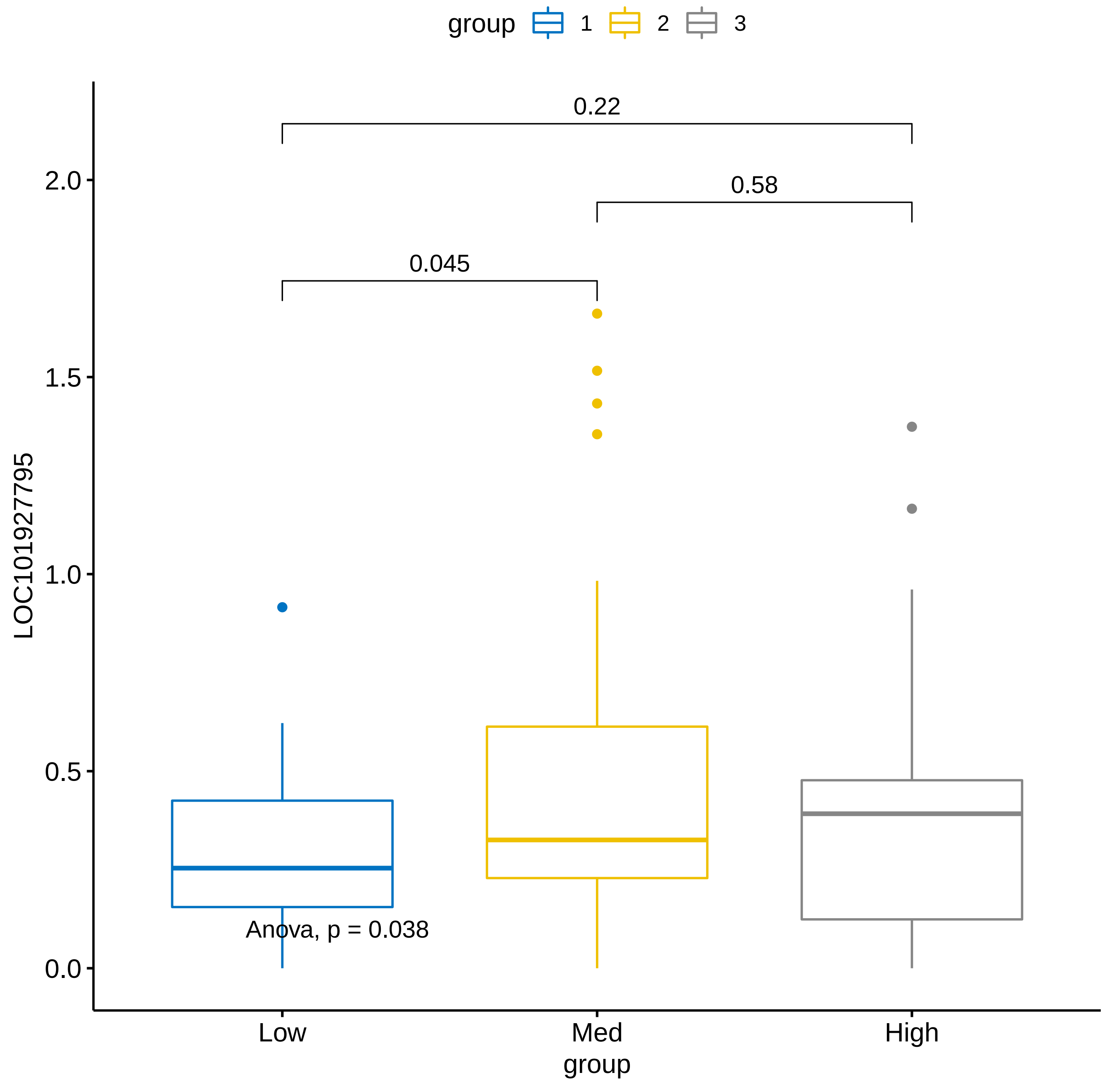
group
1
2
3
0.22
0.58
0.045
2.0
1.5
1.0
0.5
0.0
LOC101927795
Anova, p = 0.038
Low
Med
High
group

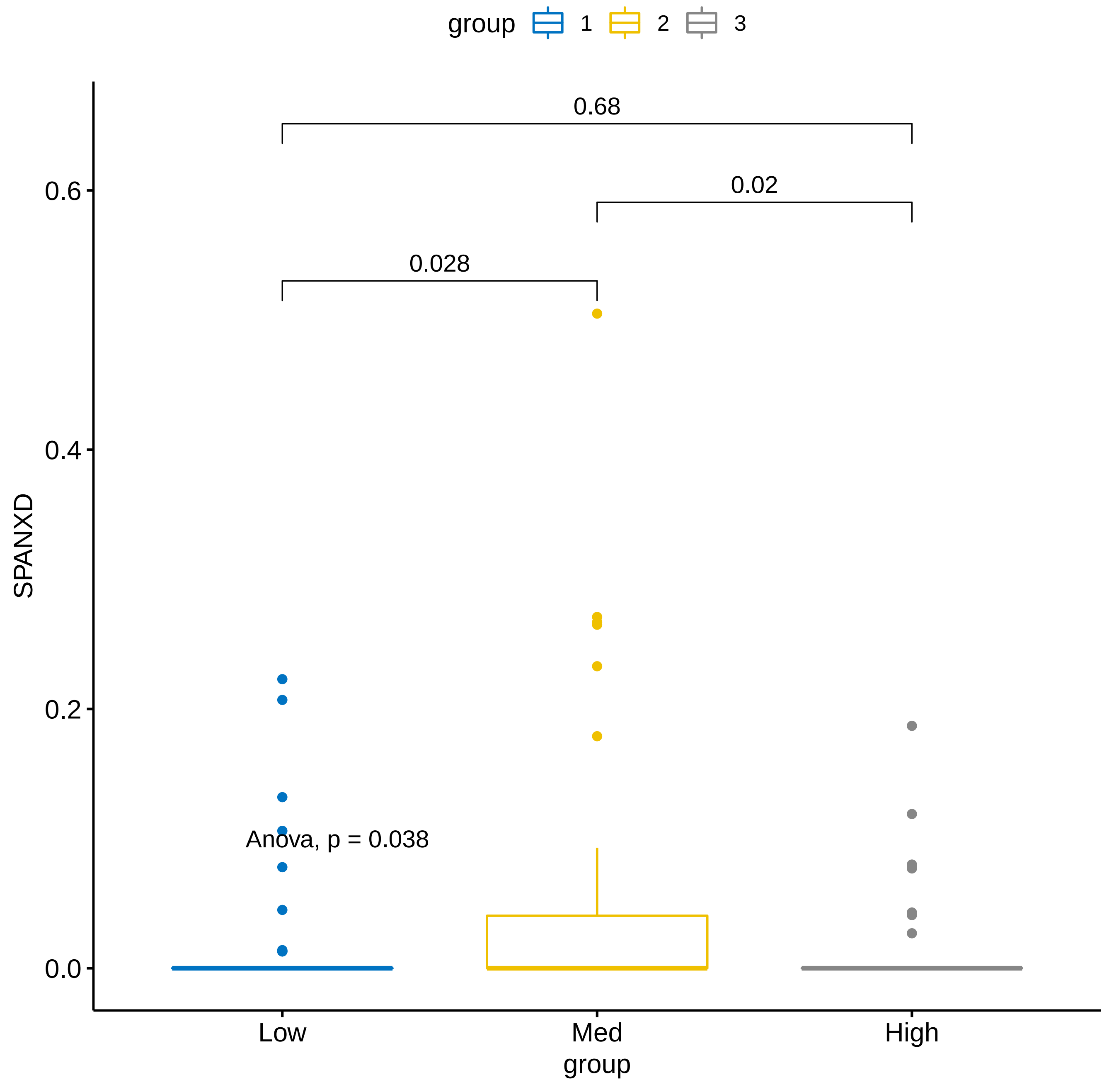
group
1
2
3
0.68
0.02
0.028
0.6
0.4
0.2
0.0
SPANXD
Anova, p = 0.038
Low
Med
High
group

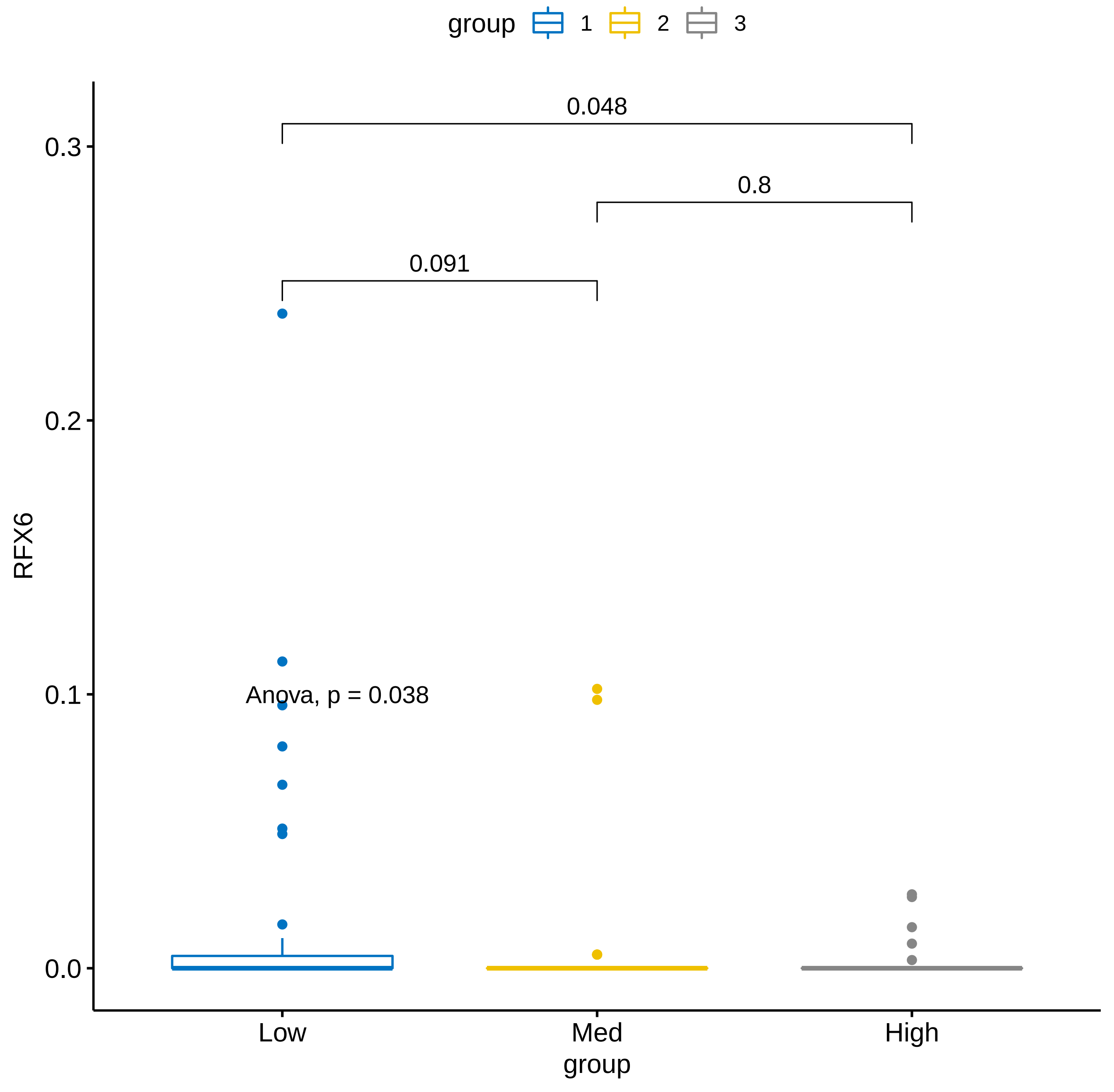

group 1 2 3
0.048
0.8
0.091
Anova, p = 0.038
RFX6
0.3
0.2
0.1
0.0
Low
Med
High
group

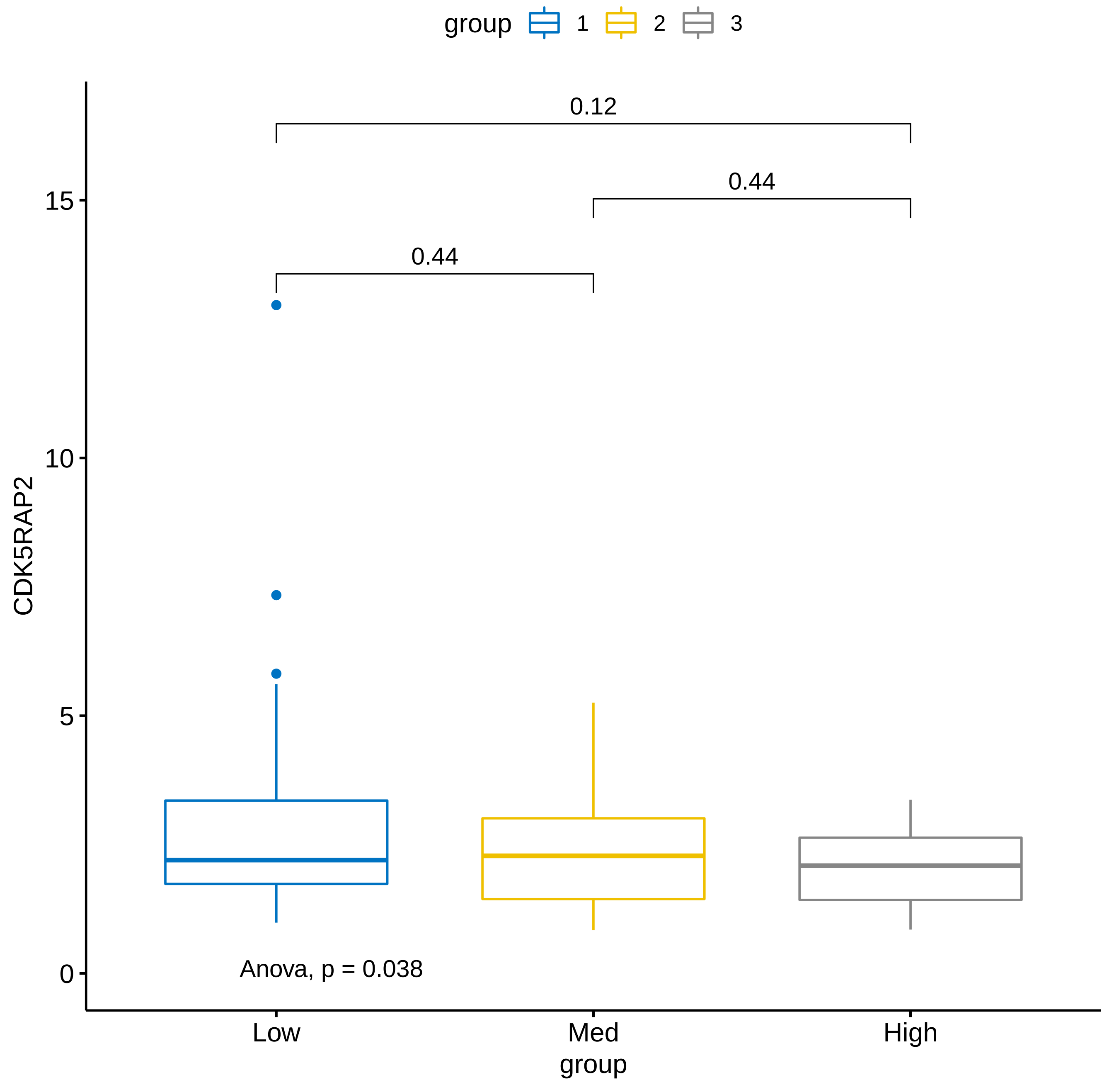

group
1
2
3
0.12
0.44
0.44
15
10
5
0
CDK5RAP2
Anova, p = 0.038
Low
Med
High
group

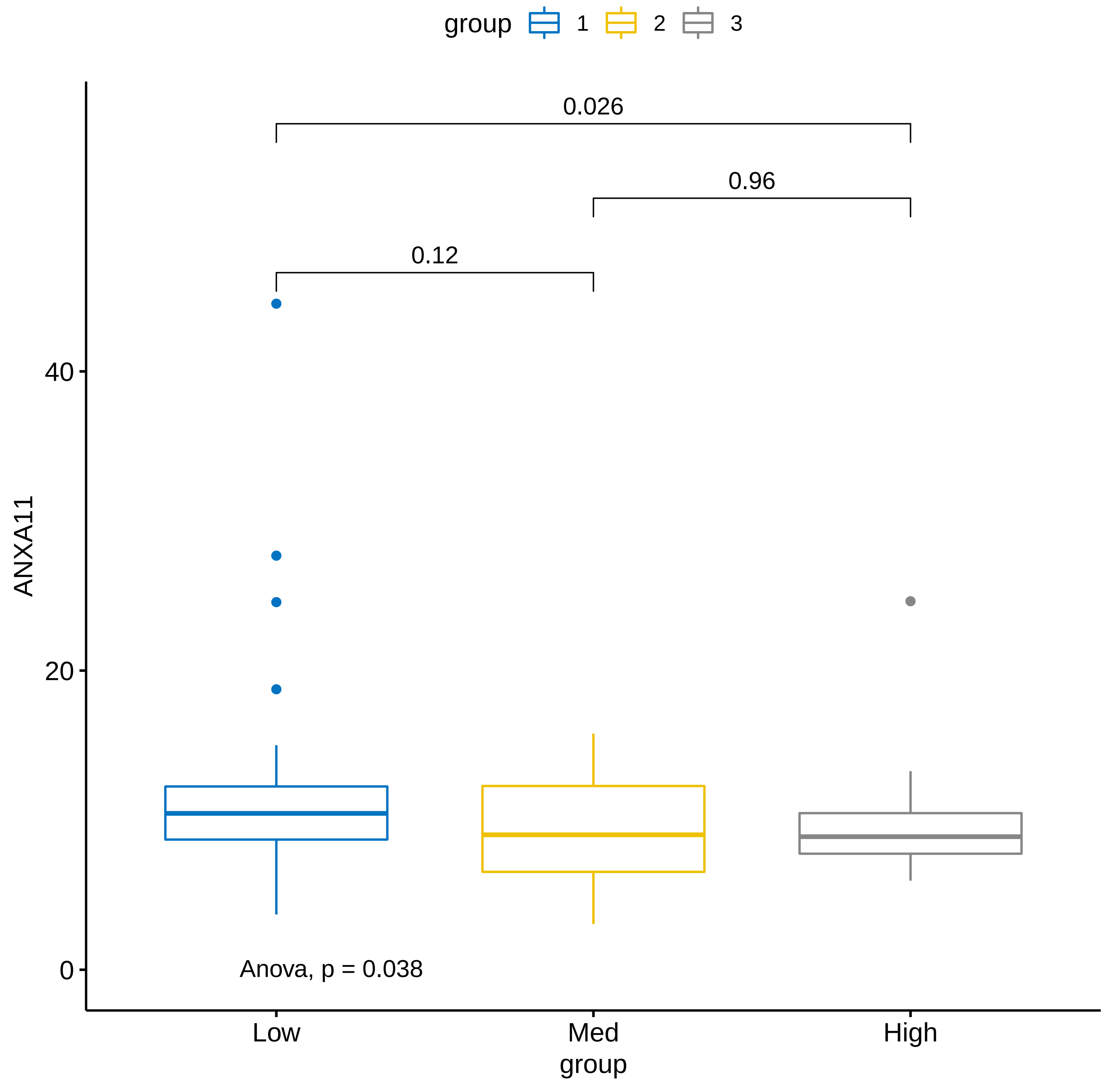

group
1
2
3
0.026
0.96
0.12
40
20
0
ANXA11
Anova, p = 0.038
Low
Med
High
group

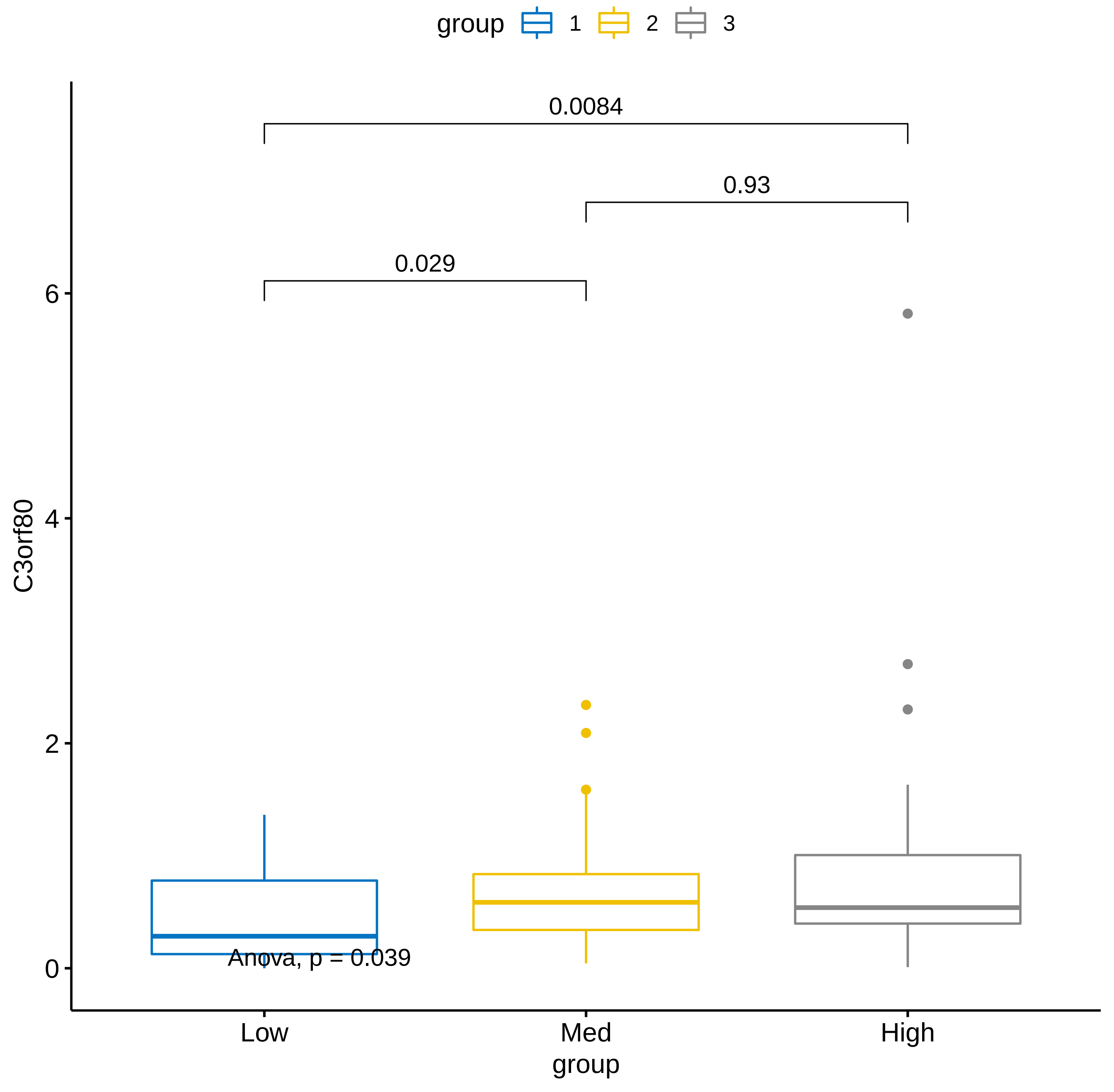

group
1
2
3
0.0084
0.93
0.029
6
4
2
0
C3orf80
Anova, p = 0.039
Low
Med
High
group

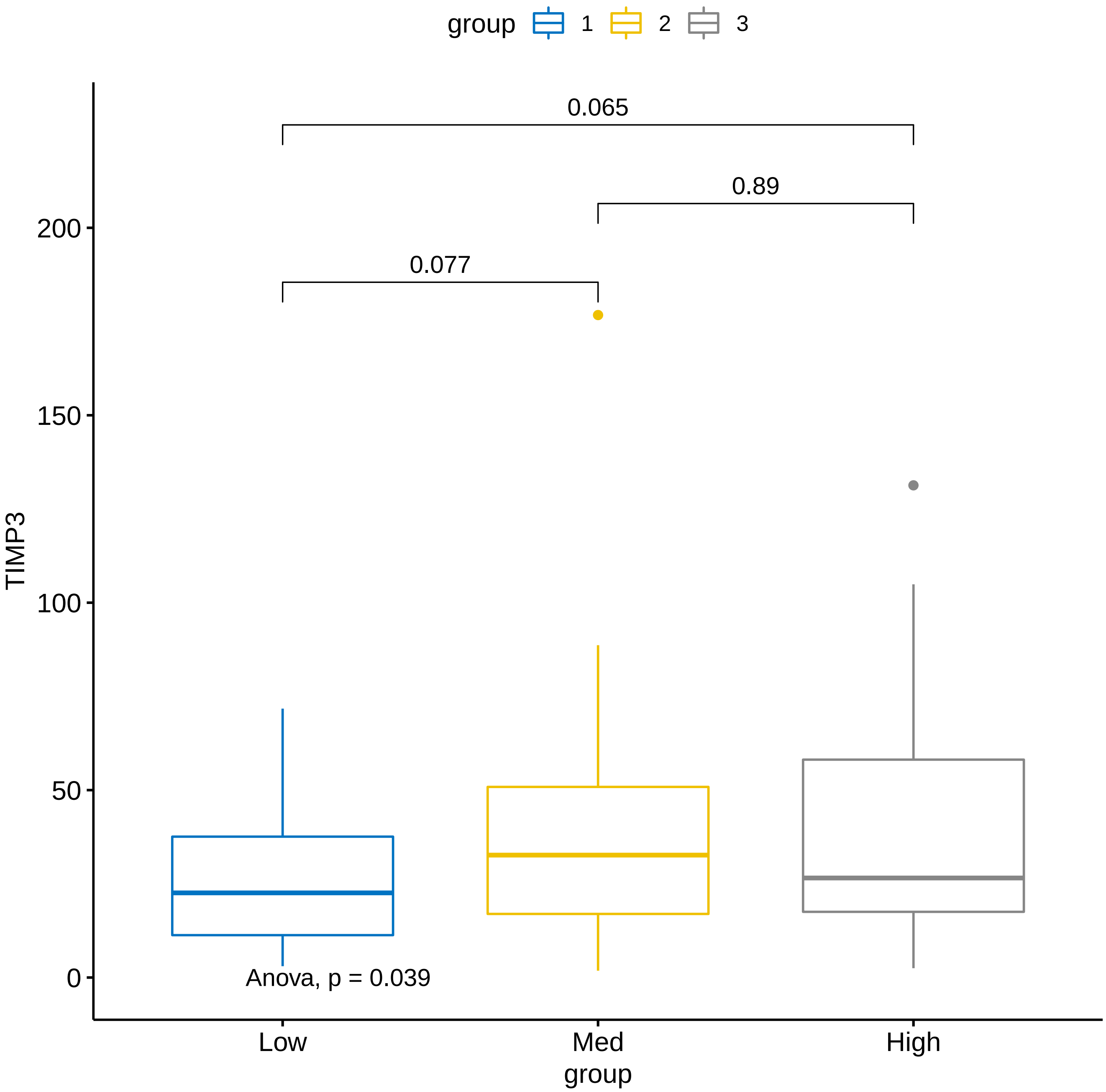

group 1 2 3
0.065
0.89
0.077
200
150
100
50
0
TIMP3
Anova, p = 0.039
Low
Med
High
group

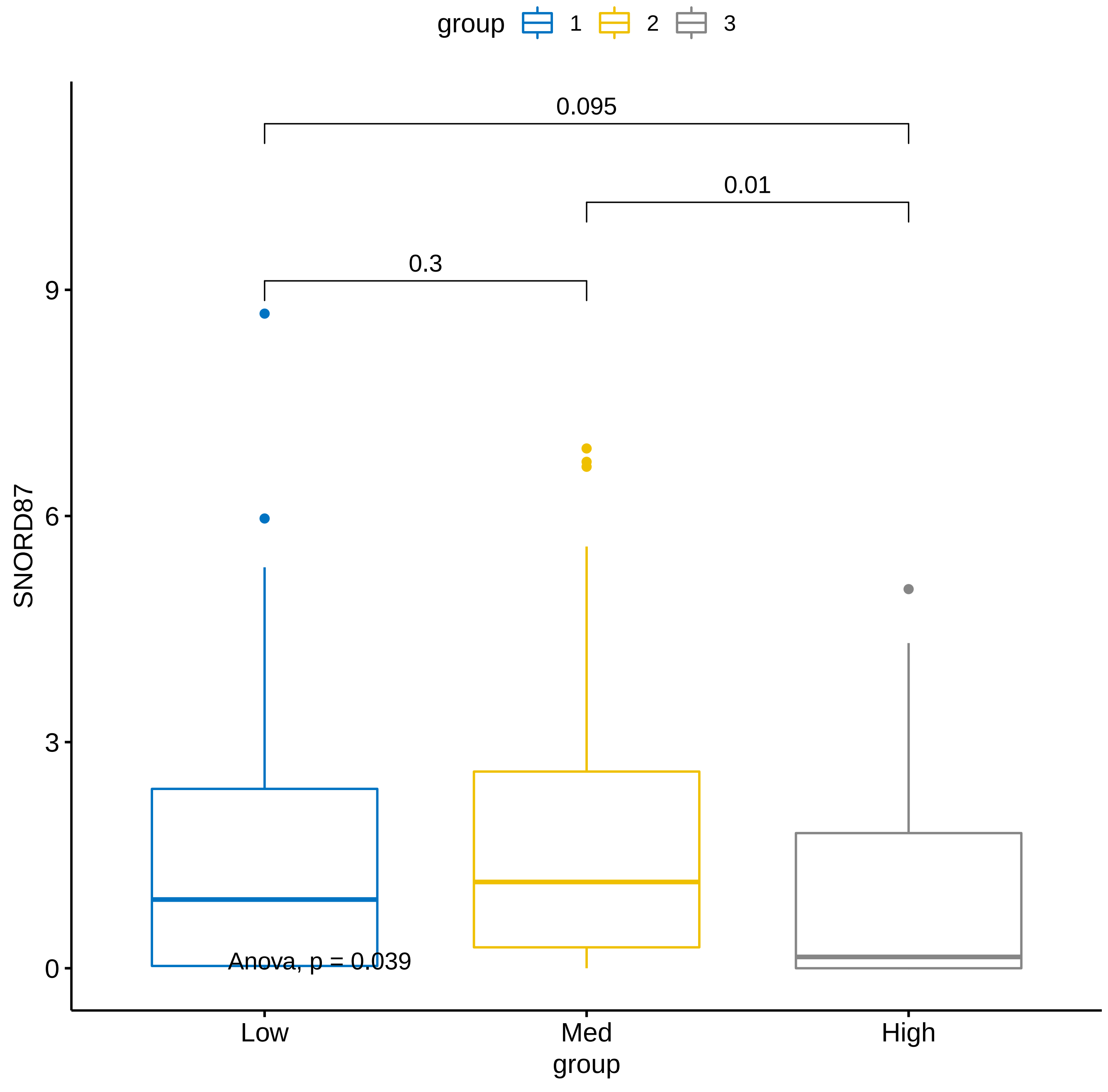

group 1 2 3
0.095
0.01
0.3
9
6
3
0
SNORD87
Anova, p = 0.039
Low
Med
High
group

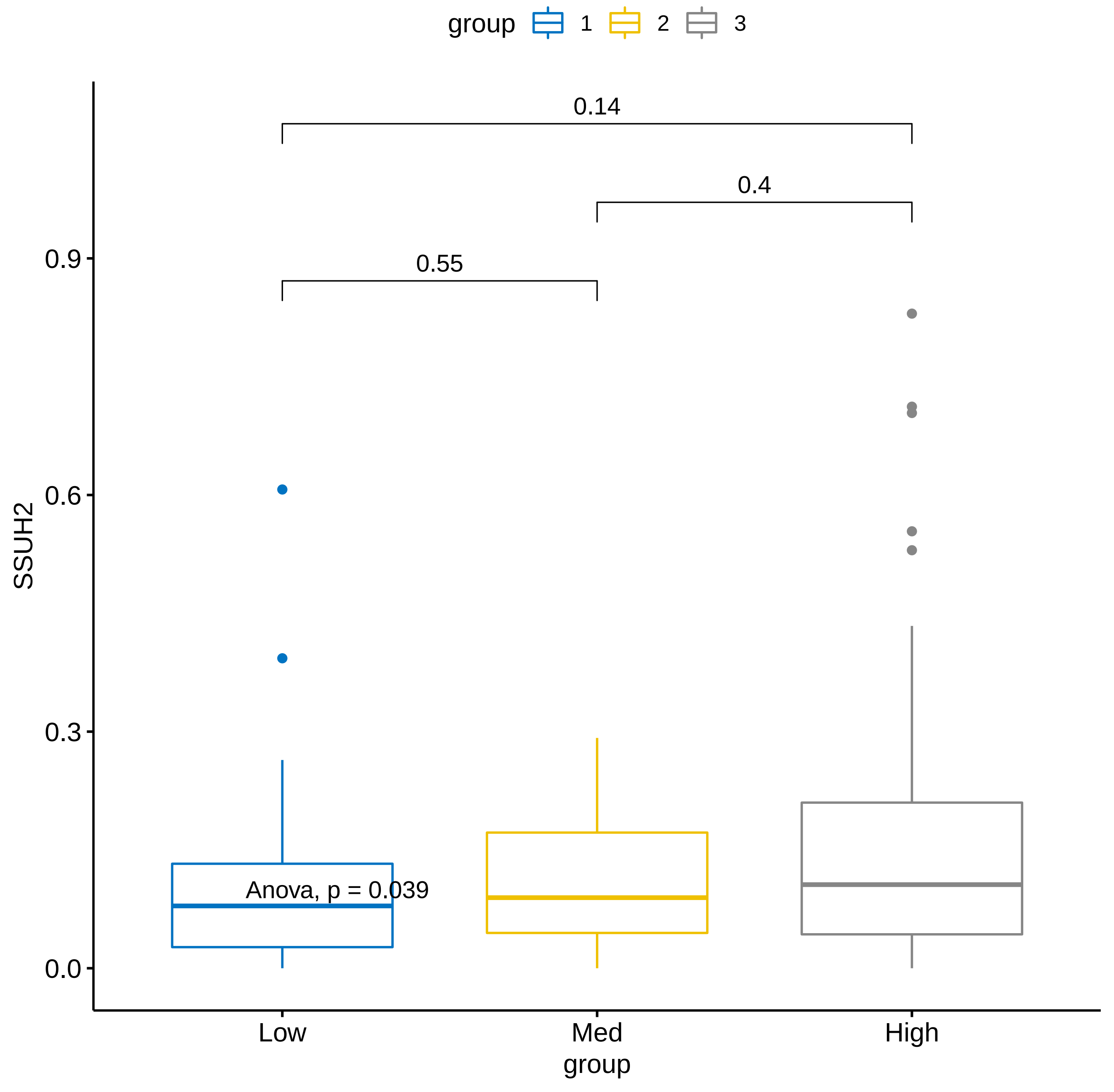

group 1 2 3
0.14
0.4
0.55
0.9
0.6
0.3
0.0
SSUH2
Anova, p = 0.039
Low
Med
High
group

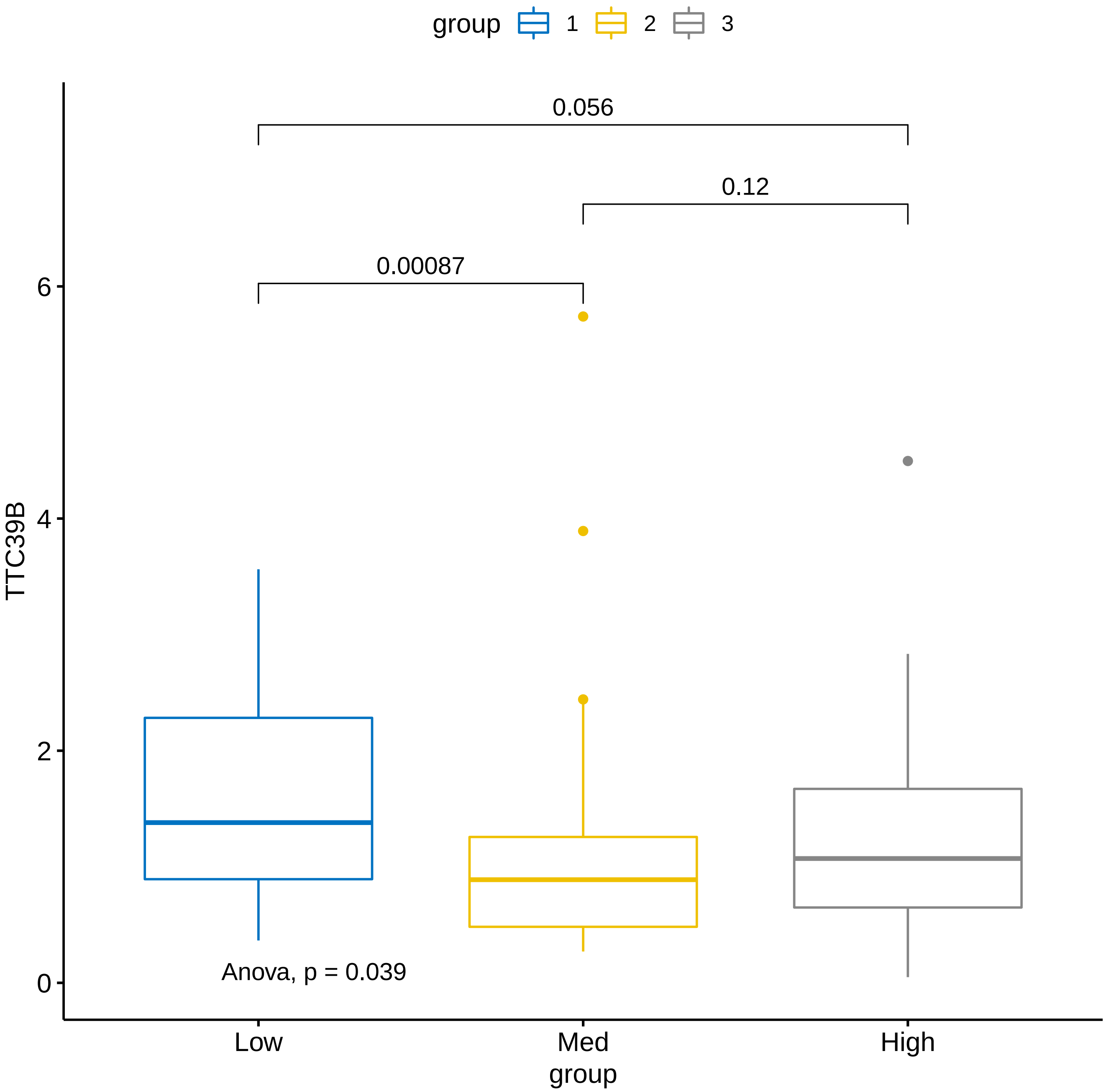
group
1
2
3
0.056
0.12
0.00087
TTC39B
6
4
2
0
Anova, p = 0.039
Low
Med
High
group

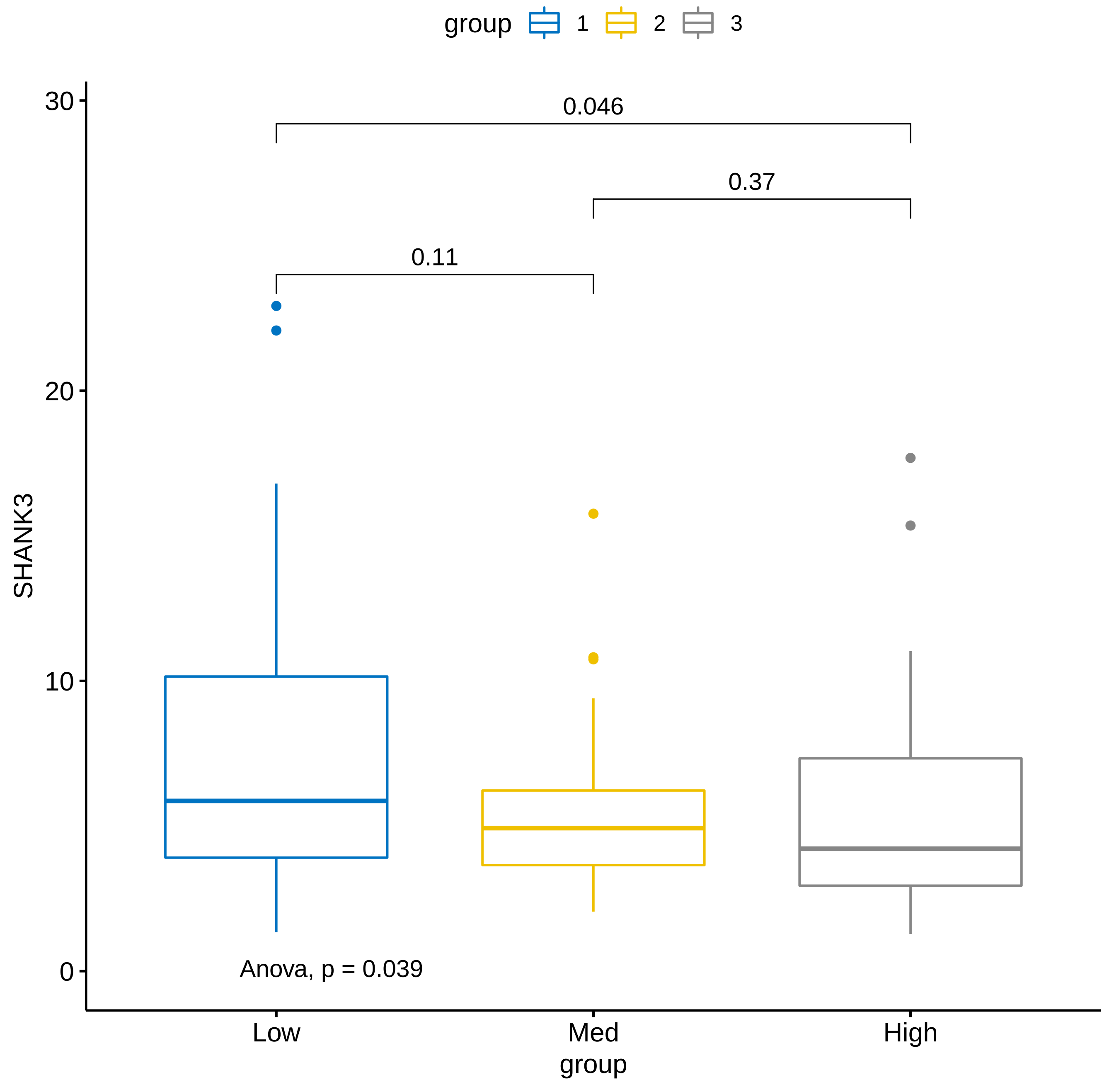

group
1
2
3
0.046
0.37
0.11
30
20
10
0
SHANK3
Anova, p = 0.039
Low
Med
High
group

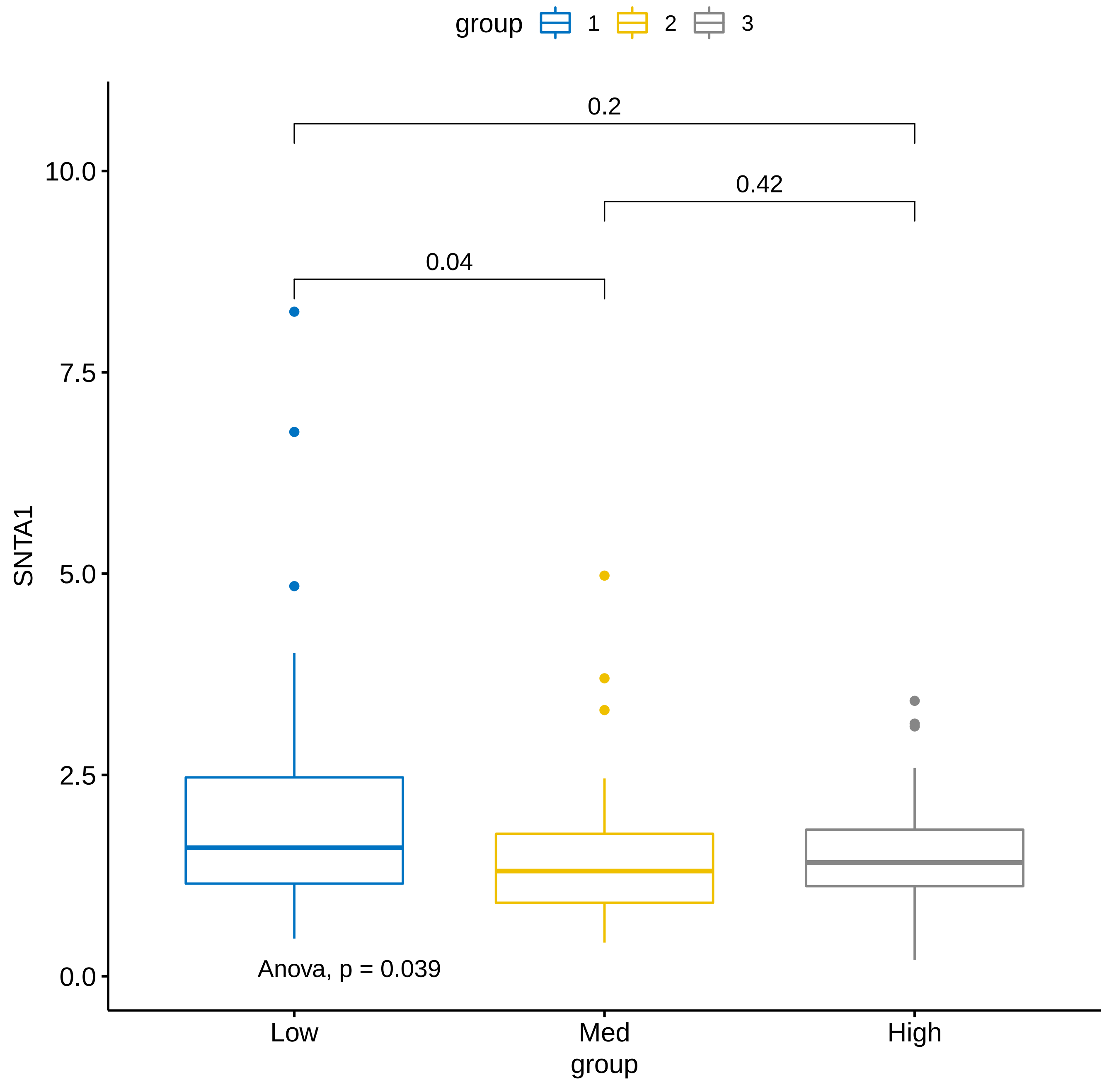
group
1
2
3
0.2
0.42
0.04
10.0
7.5
5.0
2.5
0.0
SNTA1
Anova, p = 0.039
Low
Med
High
group

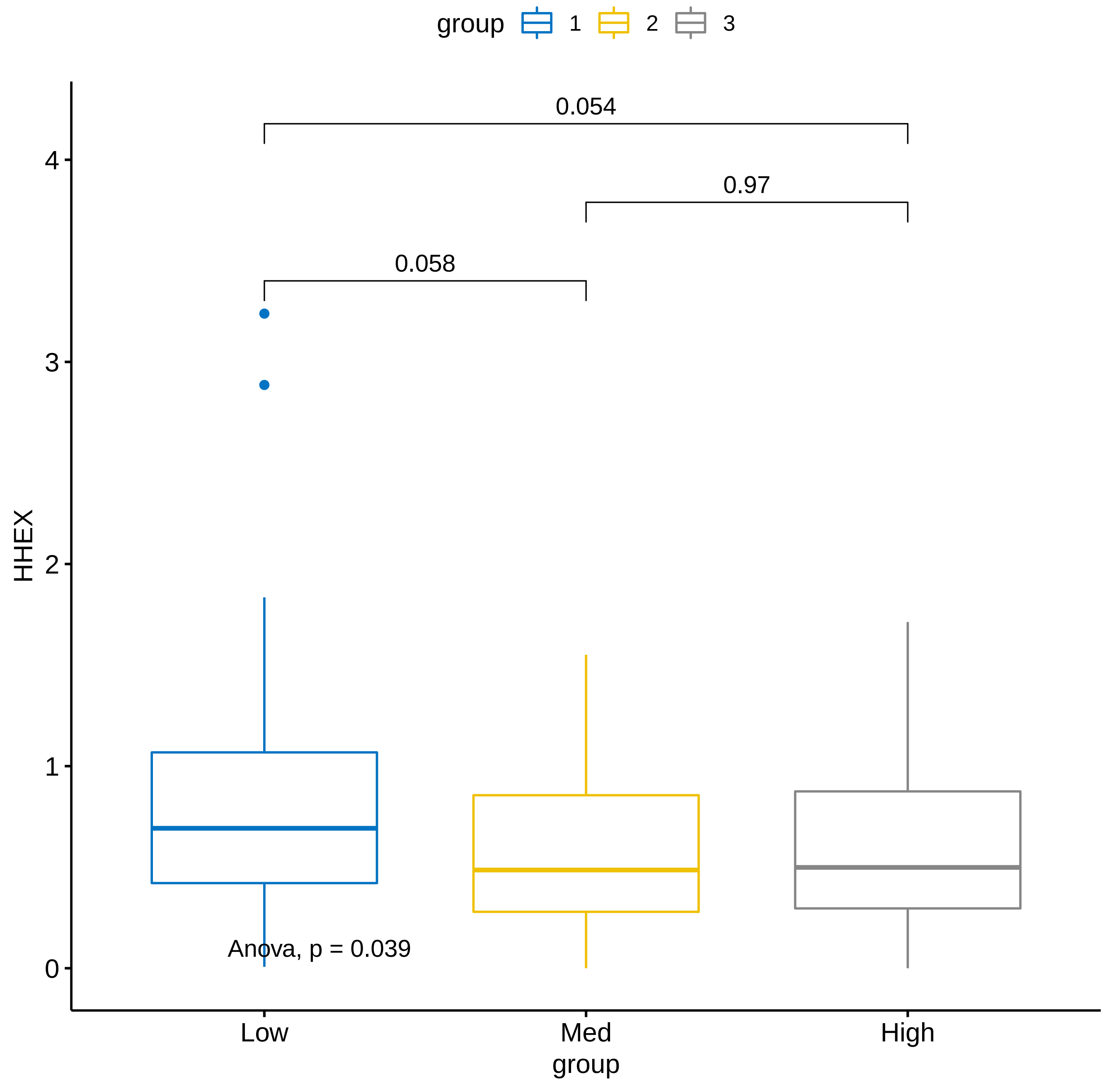
group
1
2
3
0.054
0.97
0.058
4
3
2
1
0
HHEX
Anova, p = 0.039
Low
Med
High
group

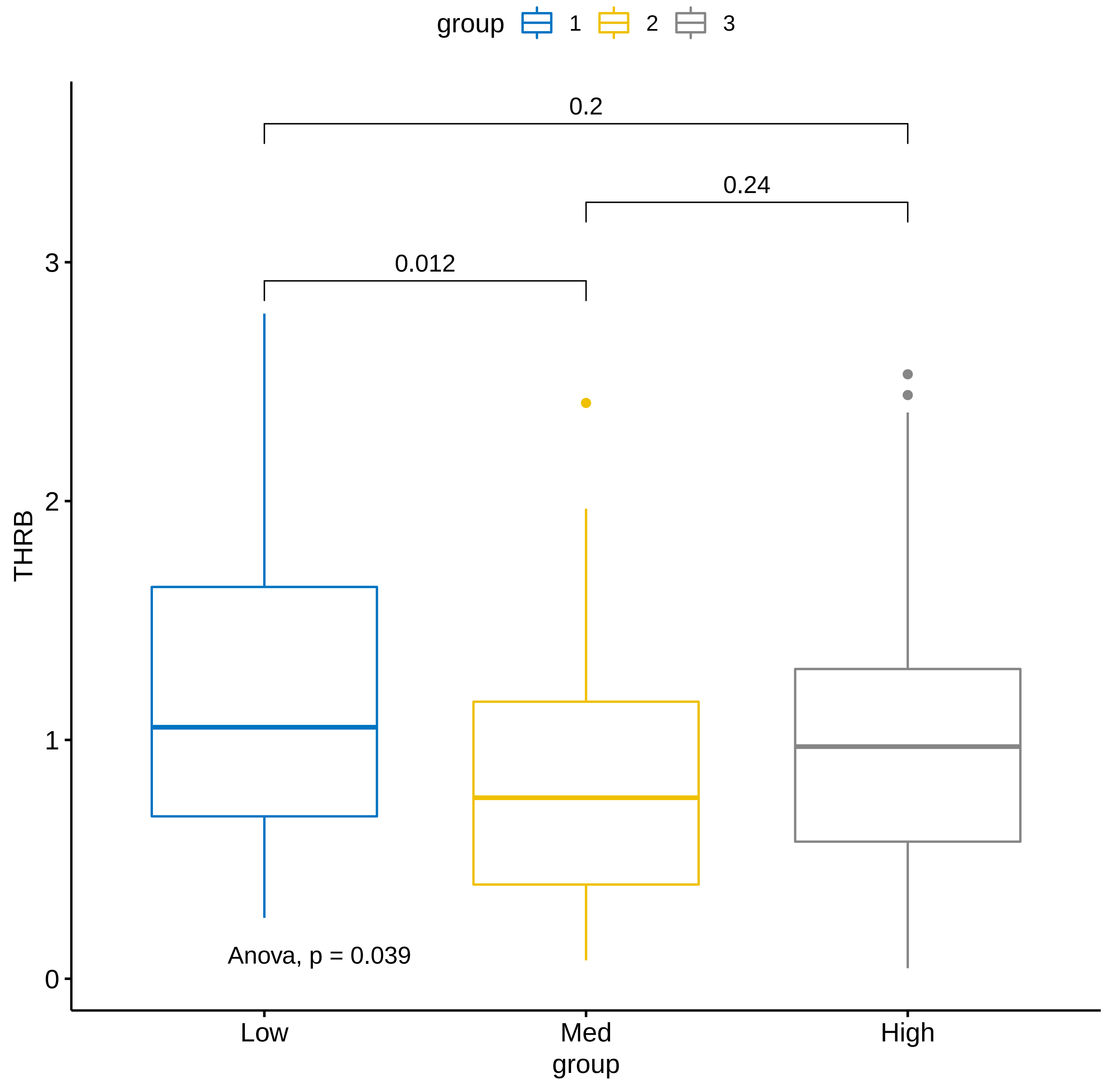
group 1 2 3
0.2
0.24
0.012
3
2
1
0
THRB
Anova, p = 0.039
Low
Med
High
group

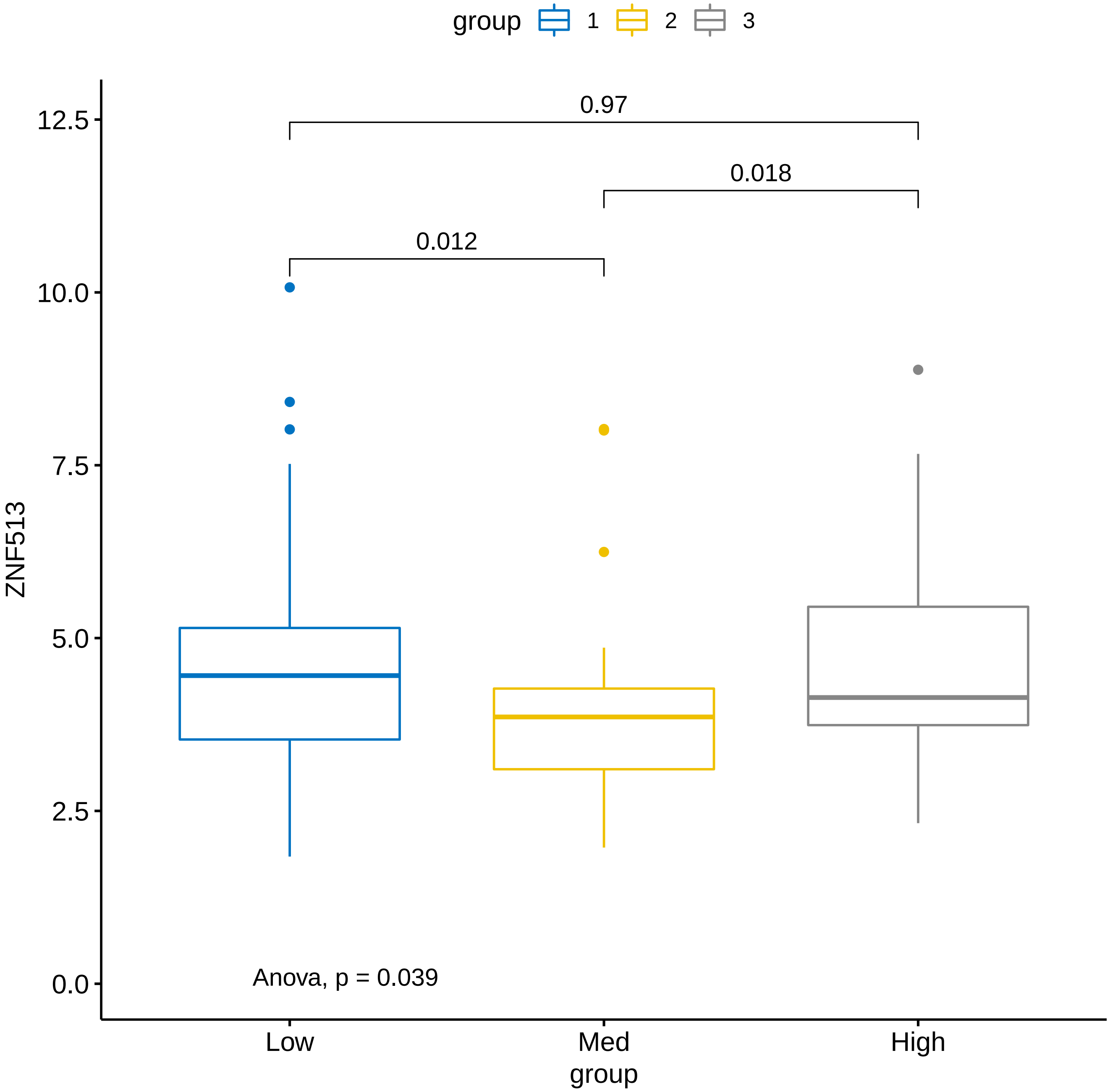

group
1
2
3
12.5
10.0
7.5
5.0
2.5
0.0
ZNF513
0.97
0.018
0.012
Anova, p = 0.039
Low
Med
High
group

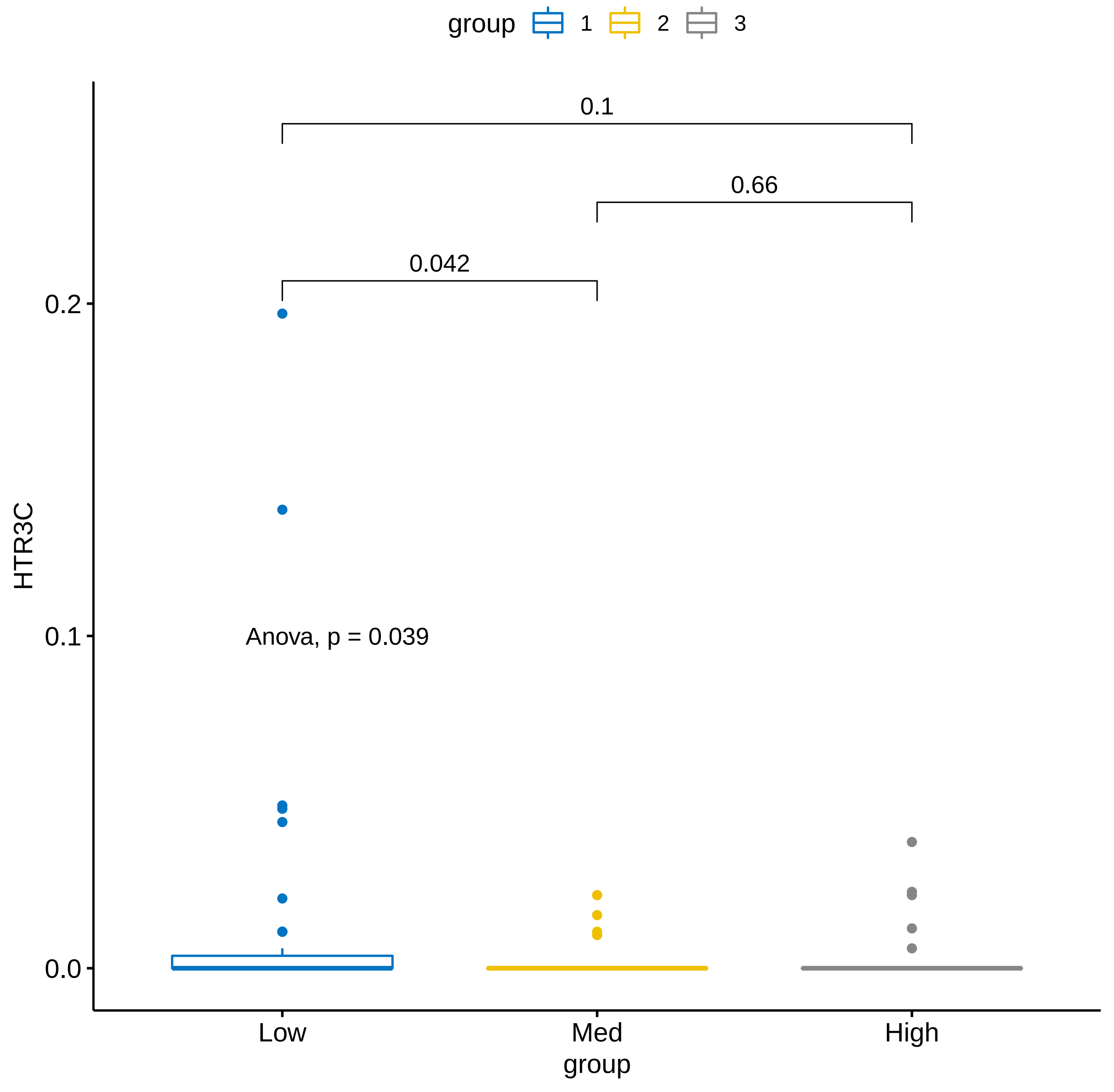
group
1
2
3
0.1
0.66
0.042
0.2
0.1
0.0
HTR3C
Anova, p = 0.039
Low
Med
High
group

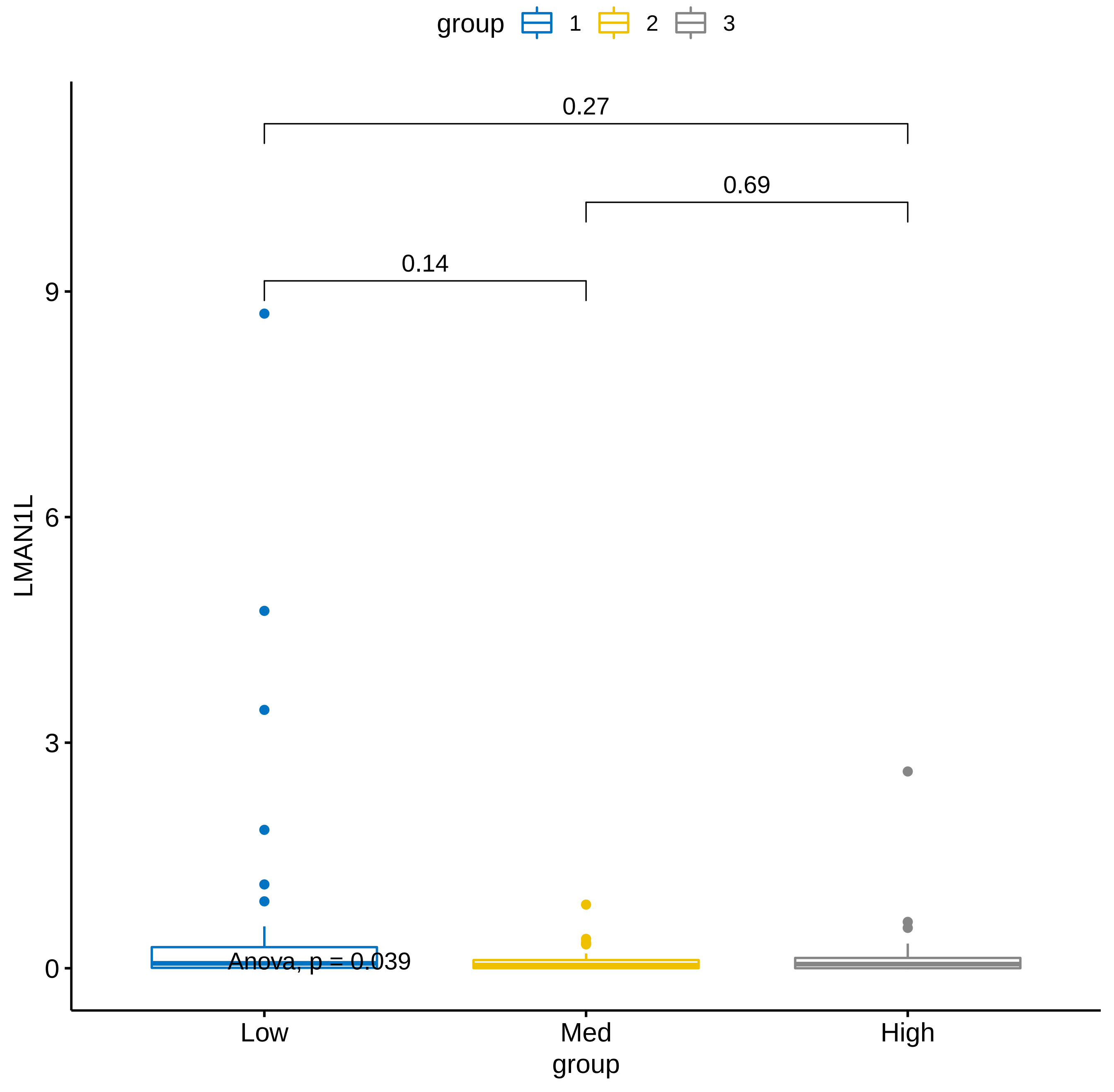
group
1
2
3
0.27
0.69
0.14
9
6
3
0
LMAN1L
Anova, p = 0.039
Low
Med
High
group

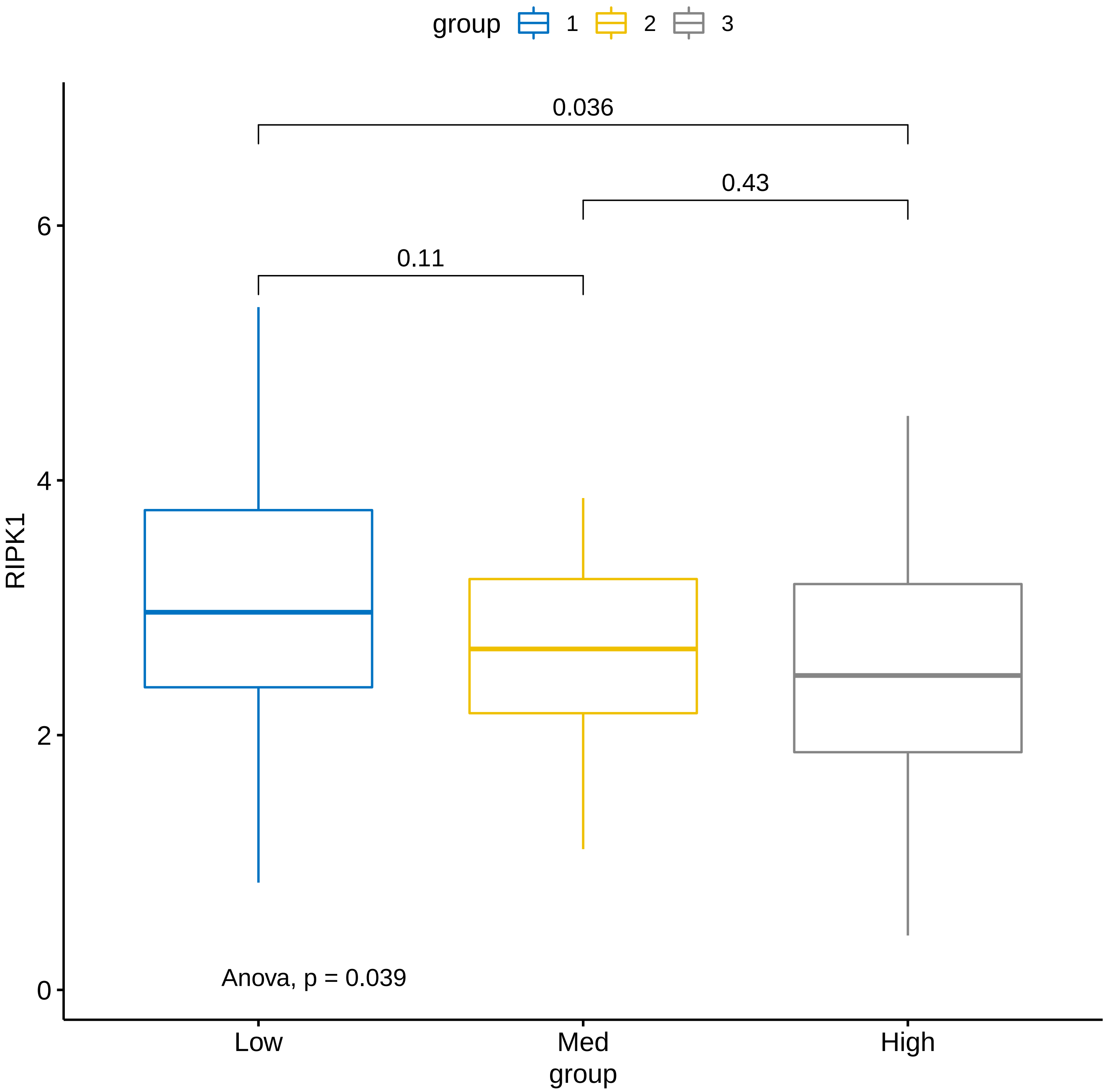
group
1
2
3
0.036
0.43
0.11
6
4
2
0
RIPK1
Anova, p = 0.039
Low
Med
High
group

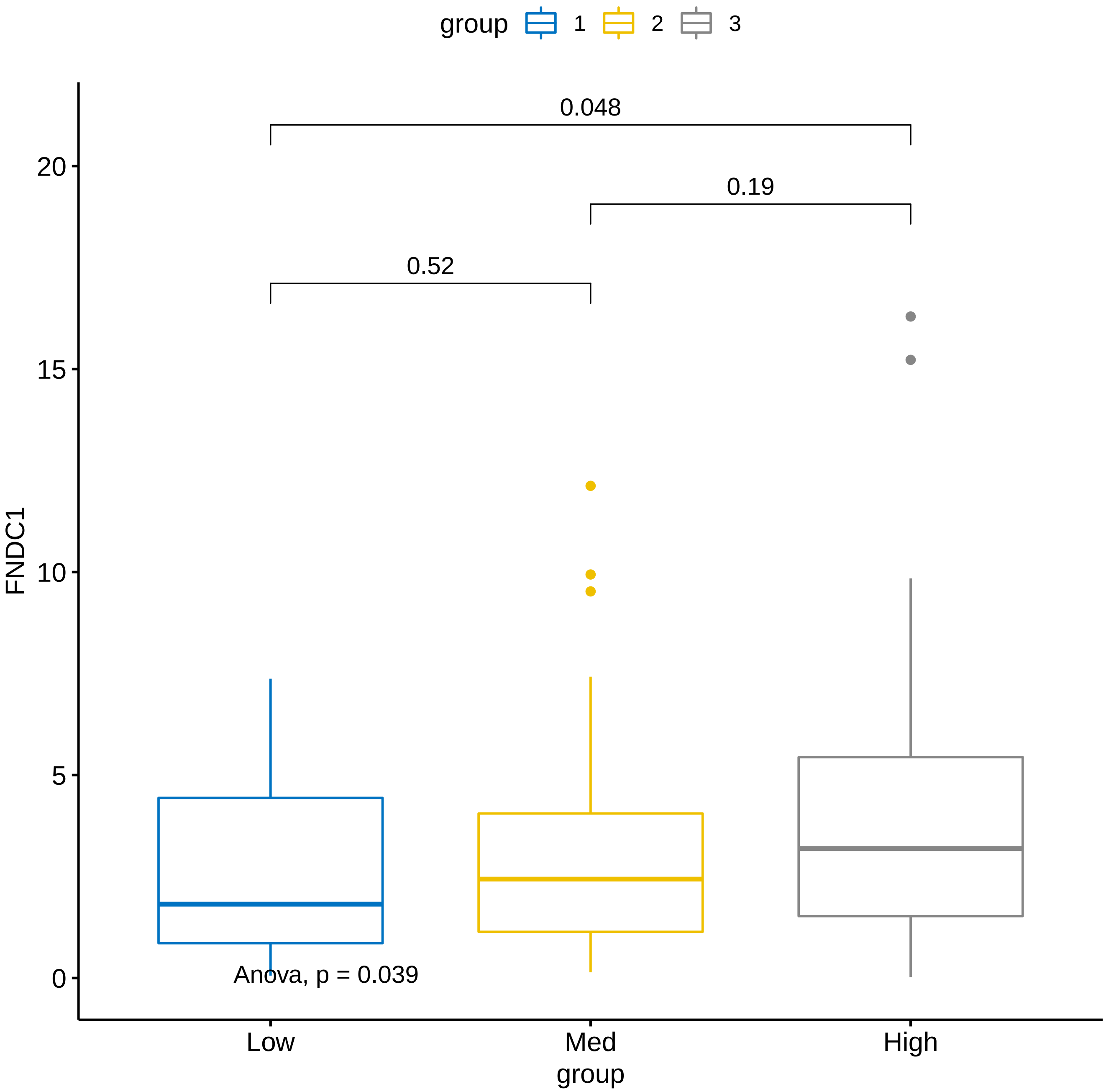
group 1 2 3
0.048
0.19
0.52
20
15
10
5
0
FNDC1
Anova, p = 0.039
Low
Med
High
group

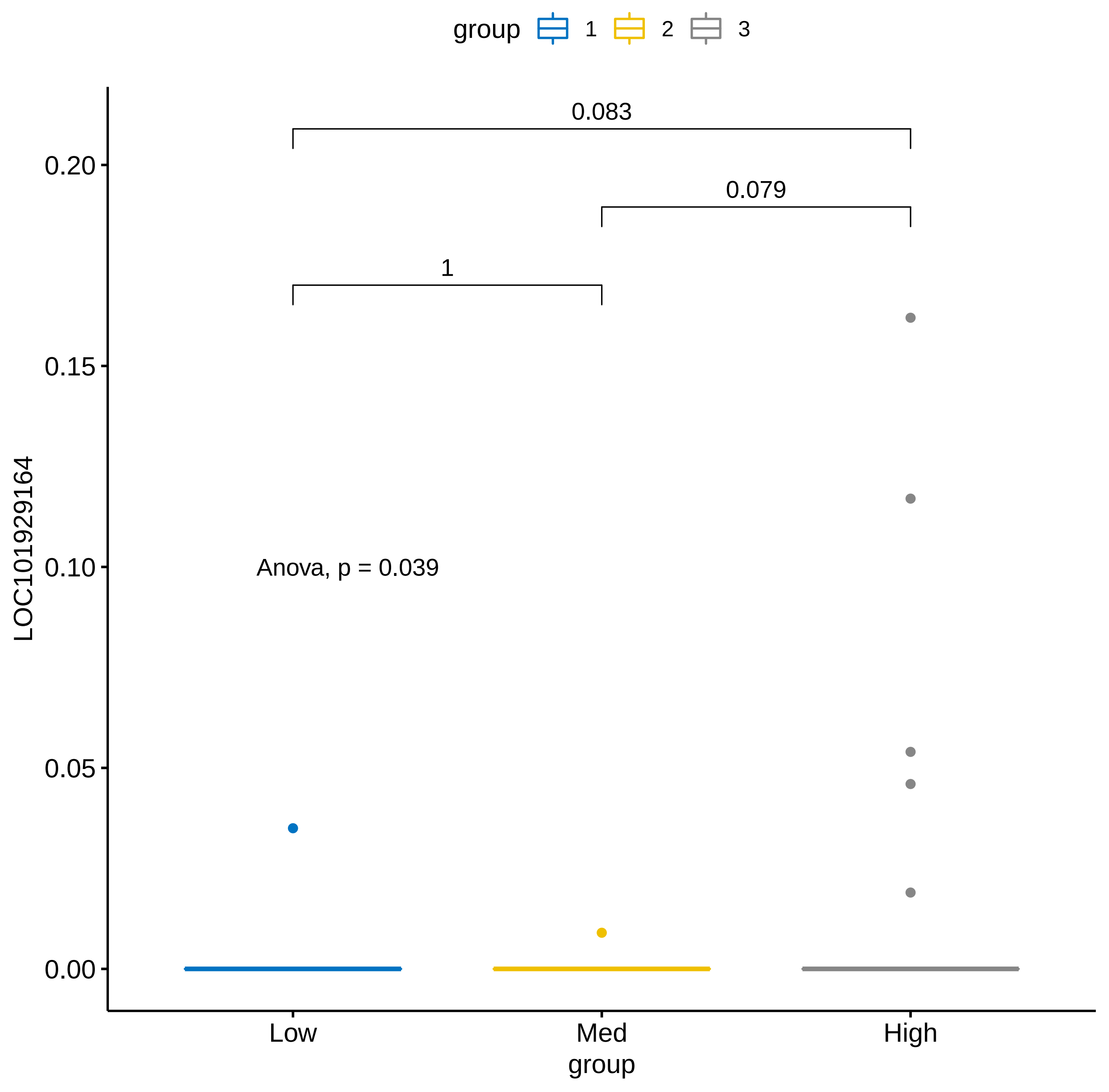

group
1
2
3
0.083
0.079
1
Anova, p = 0.039
0.20
0.15
0.10
0.05
0.00
LOC101929164
Low
Med
High
group

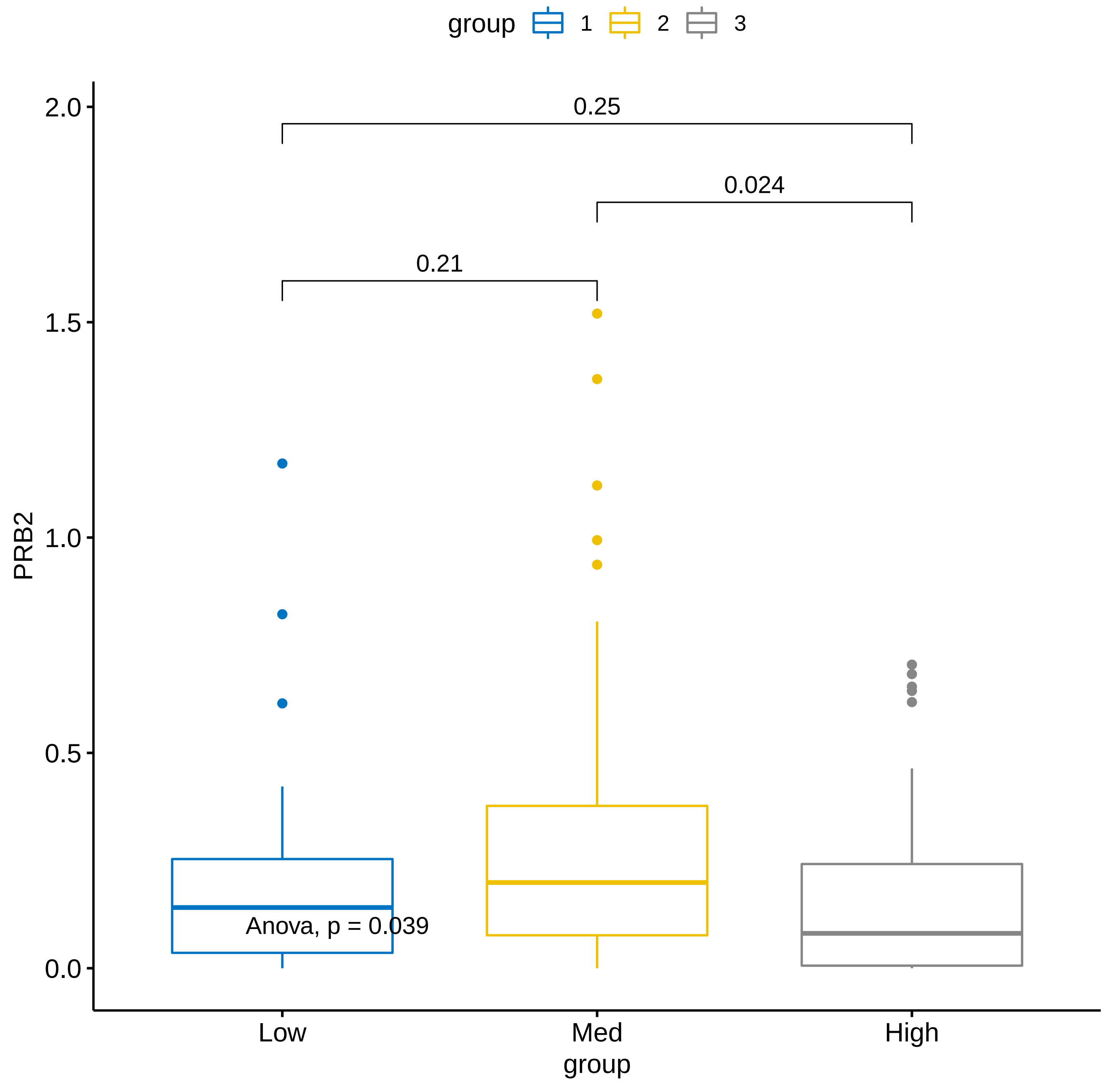

group 1 2 3
PRB2
2.0
1.5
1.0
0.5
0.0
0.25
0.024
0.21
Anova, p = 0.039
Low
Med
High
group

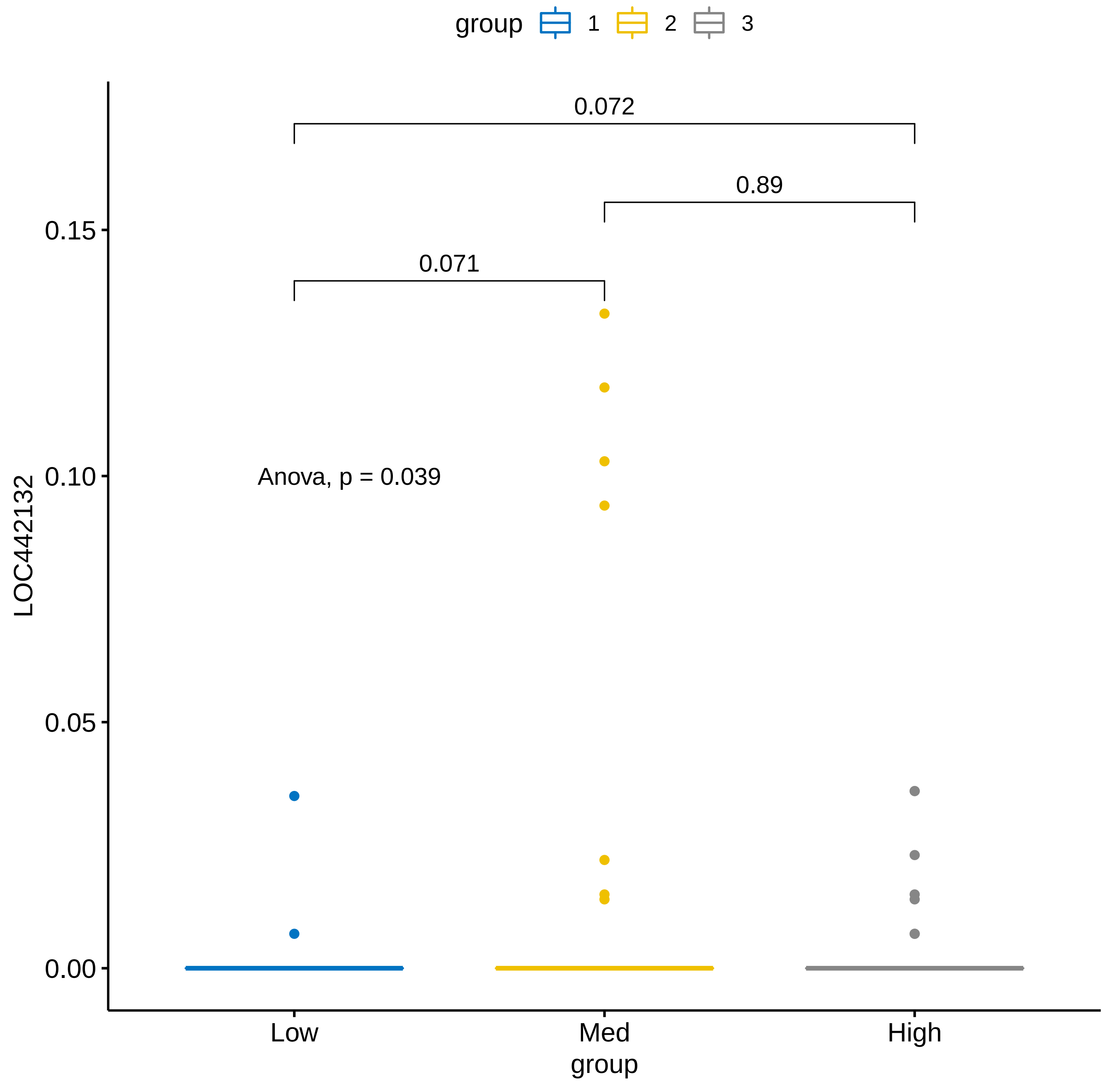
group
1
2
3
0.072
0.89
0.071
Anova, p = 0.039
0.15
0.10
0.05
0.00
LOC442132
Low
Med
High
group

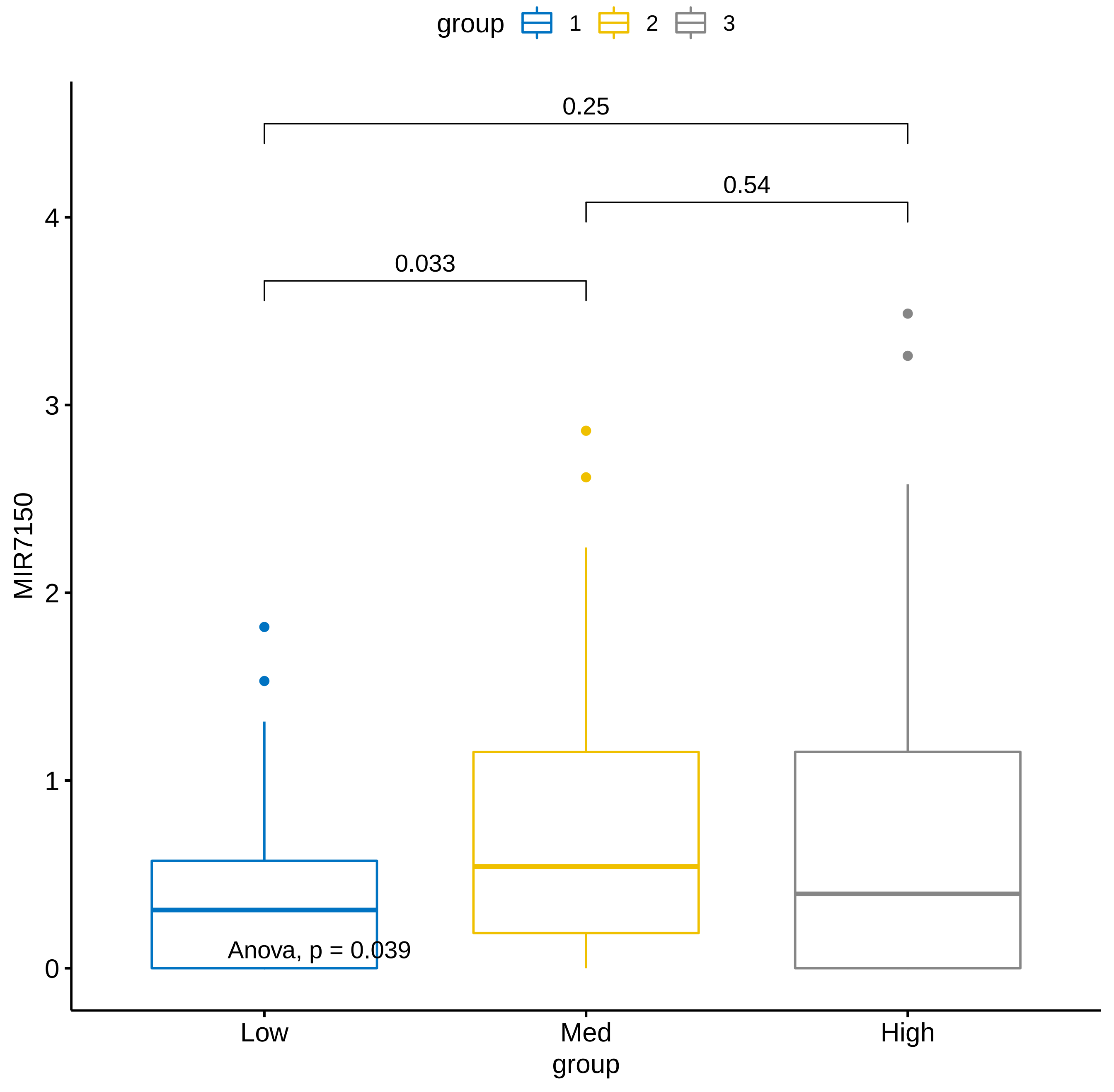
group
1
2
3
0.25
0.54
0.033
MIR7150
4
3
2
1
0
Anova, p = 0.039
Low
Med
High
group

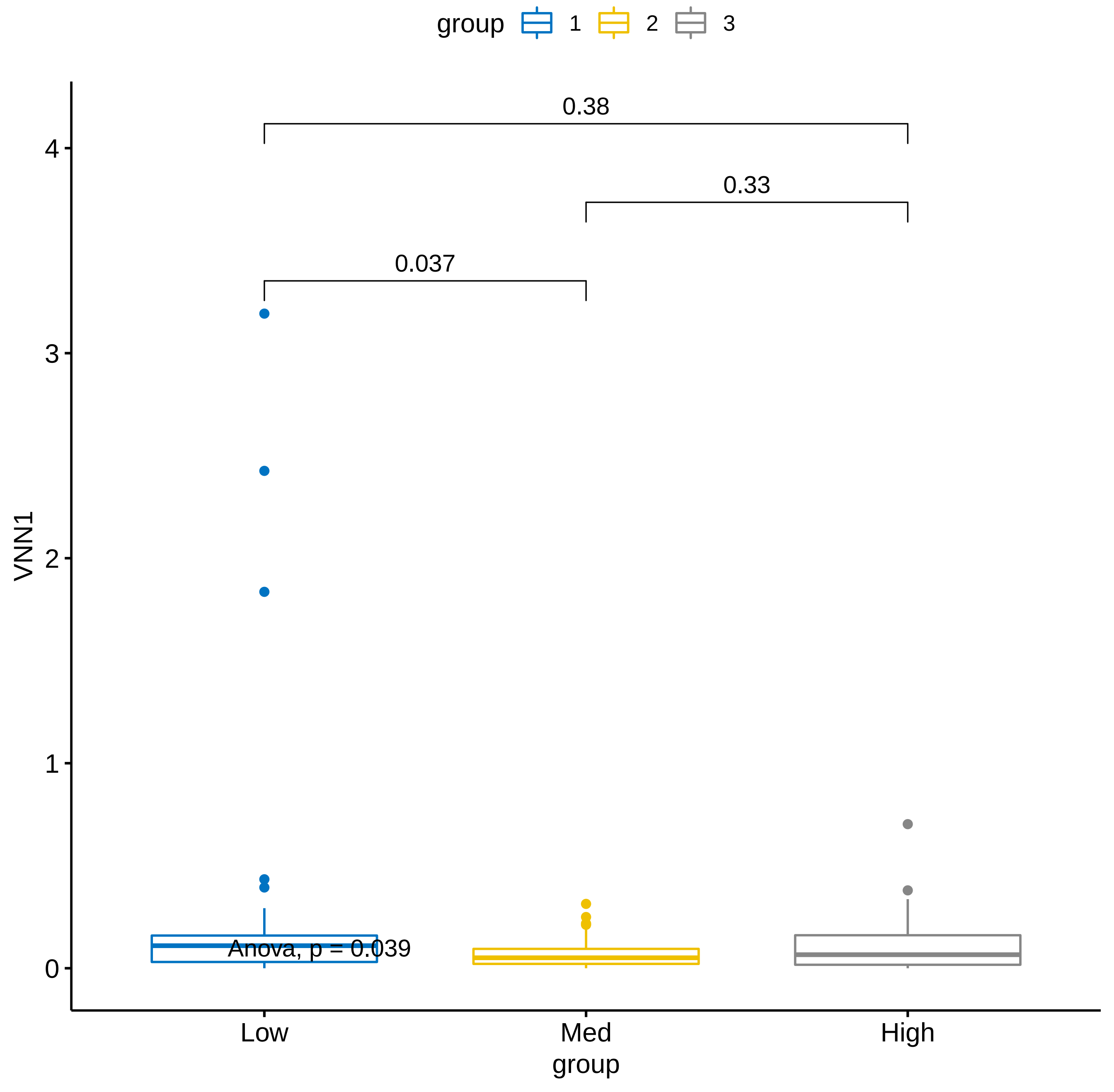

group 1 2 3
0.38
0.33
0.037
4
3
2
1
0
VNN1
Anova, p = 0.039
Low
Med
High
group

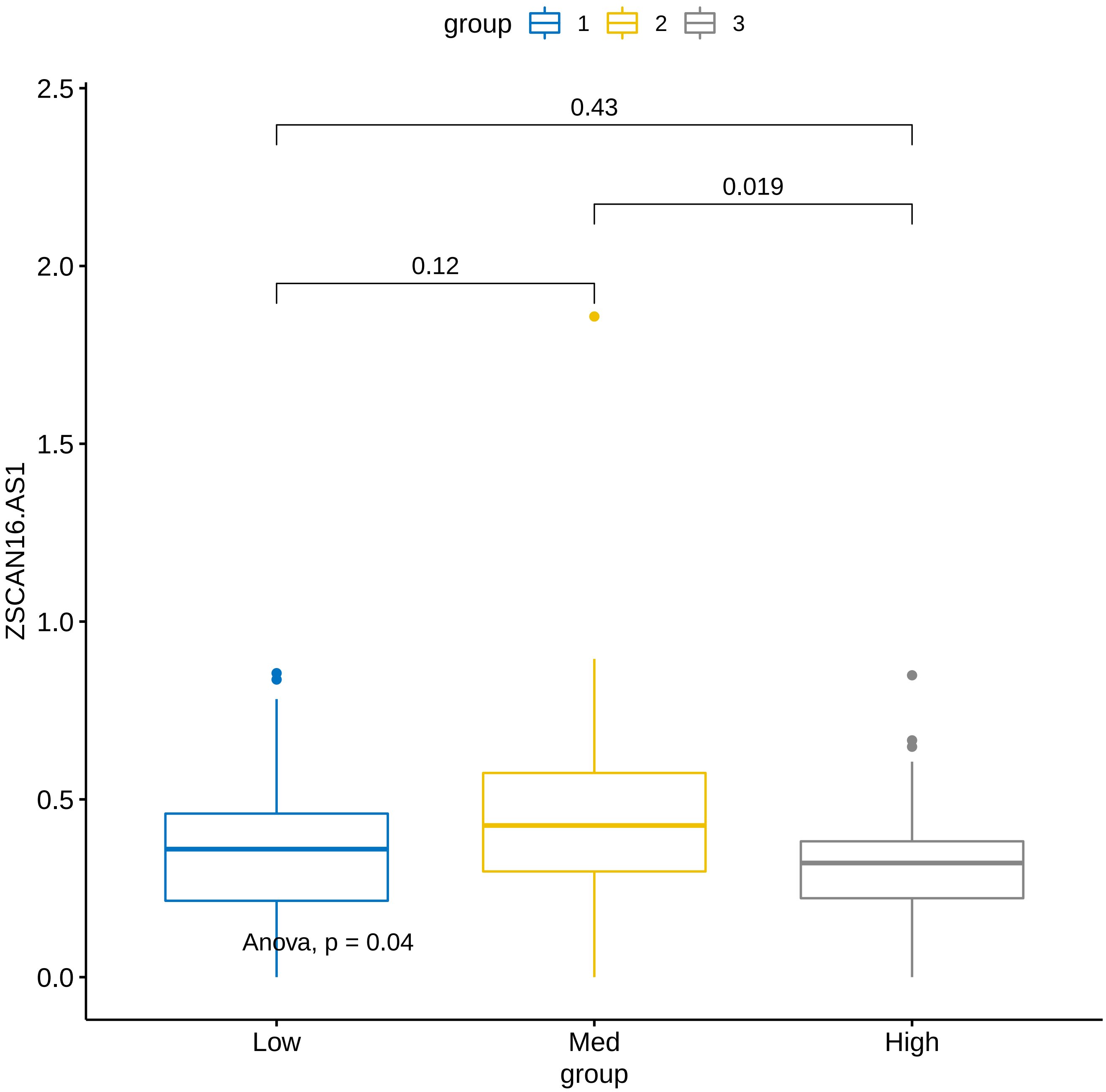
group 1 2 3
0.43
0.019
0.12
Anova, p = 0.04
ZSCAN16.AS1
2.5
2.0
1.5
1.0
0.5
0.0
Low
Med
High
group

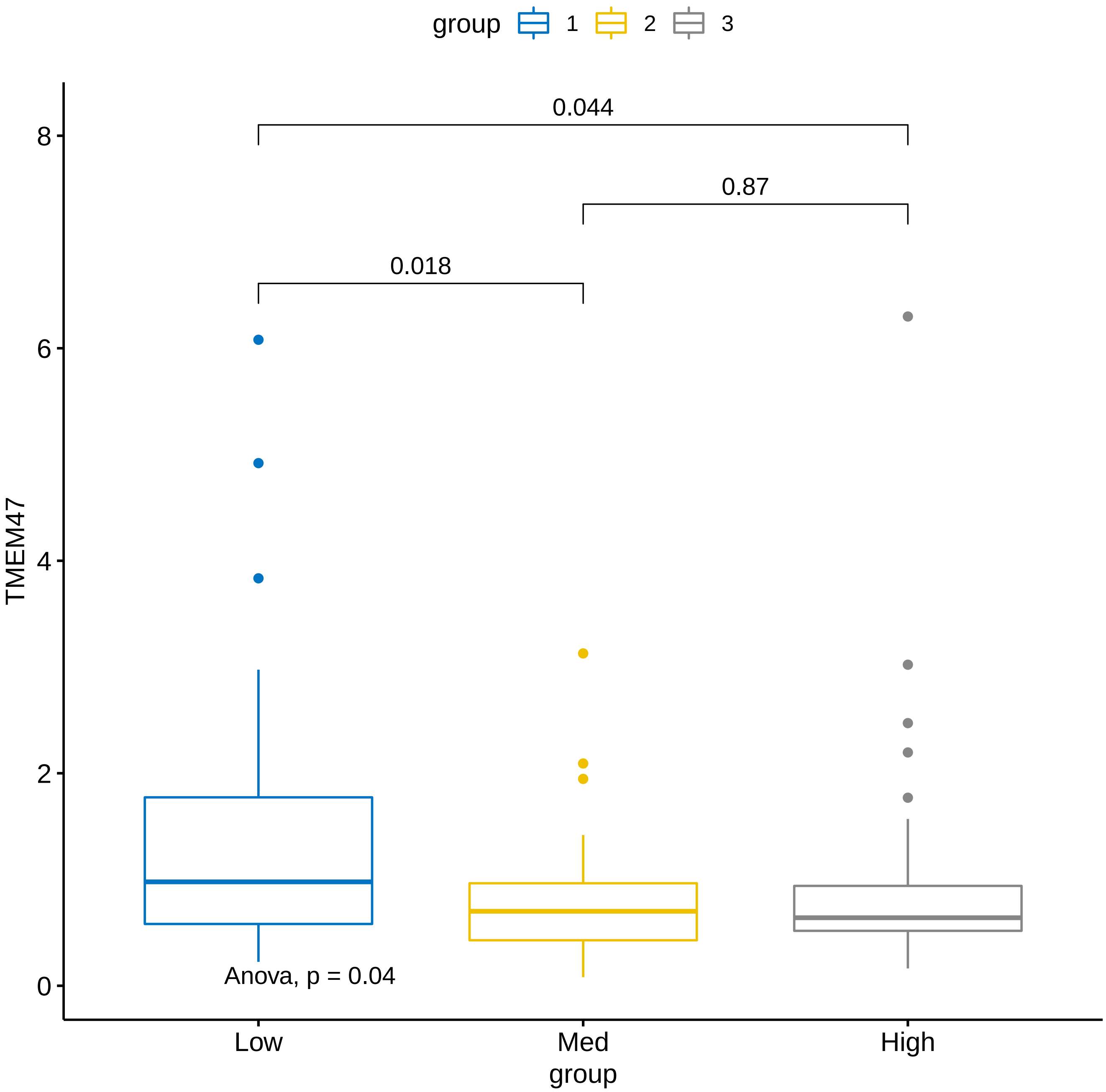
group
1
2
3
0.044
0.87
0.018
8
6
4
2
0
TMEM47
Anova, p = 0.04
Low
Med
High
group

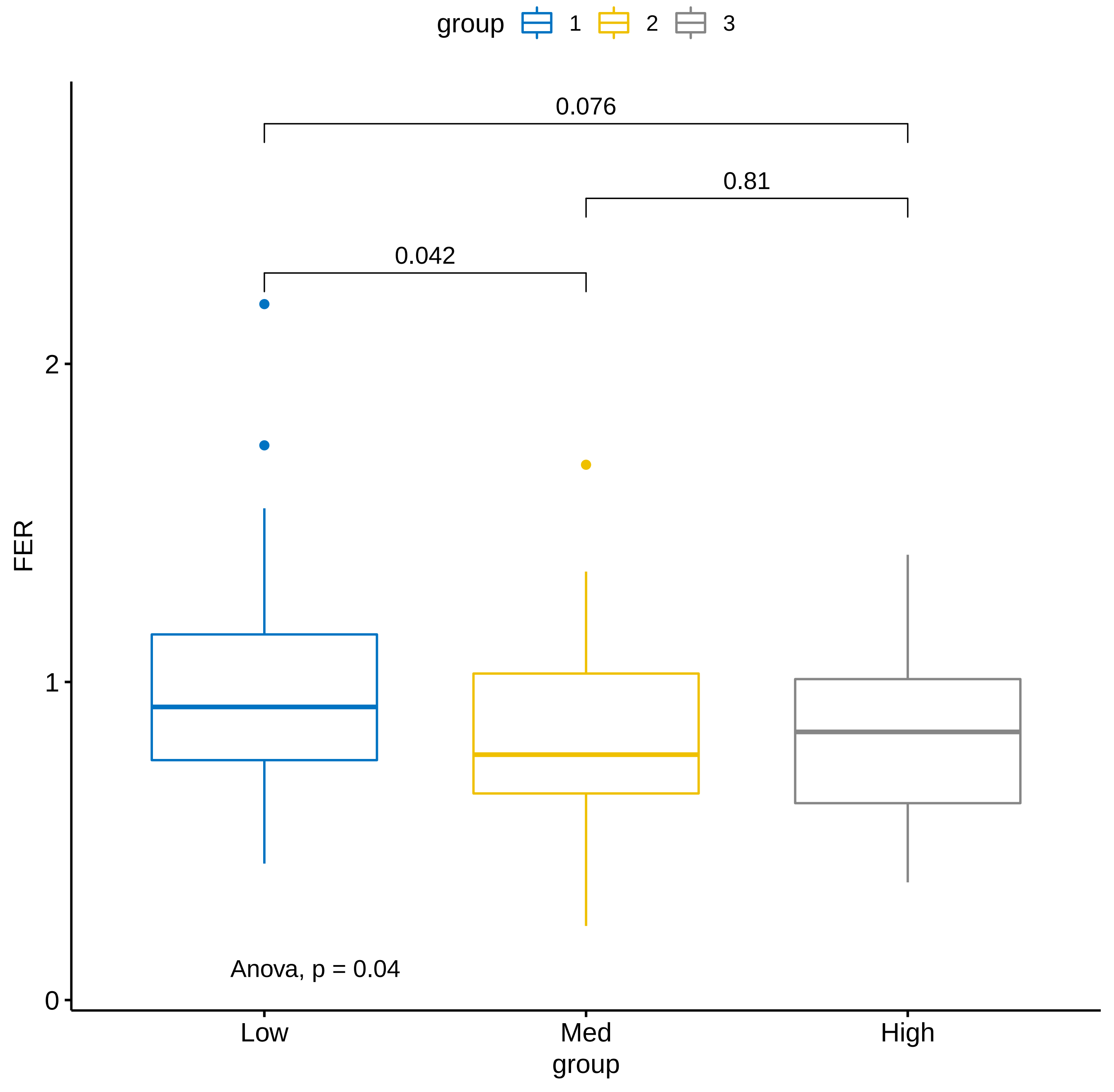
group 1 2 3
0.076
0.81
0.042
2
1
0
FER
Anova, p = 0.04
Low
Med
High
group

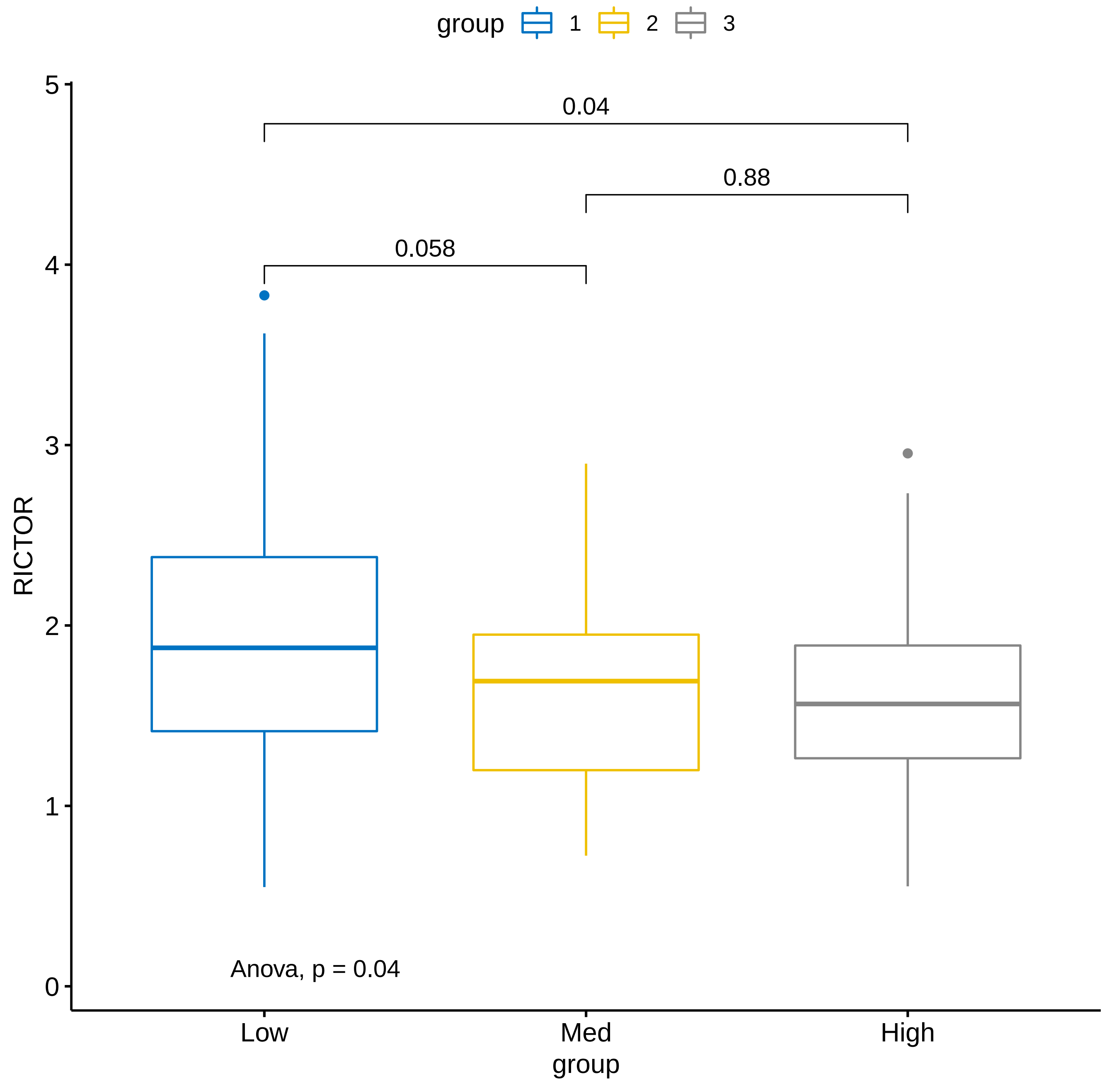
group
1
2
3
0.04
0.88
0.058
5
4
3
2
1
0
RICTOR
Anova, p = 0.04
Low
Med
High
group

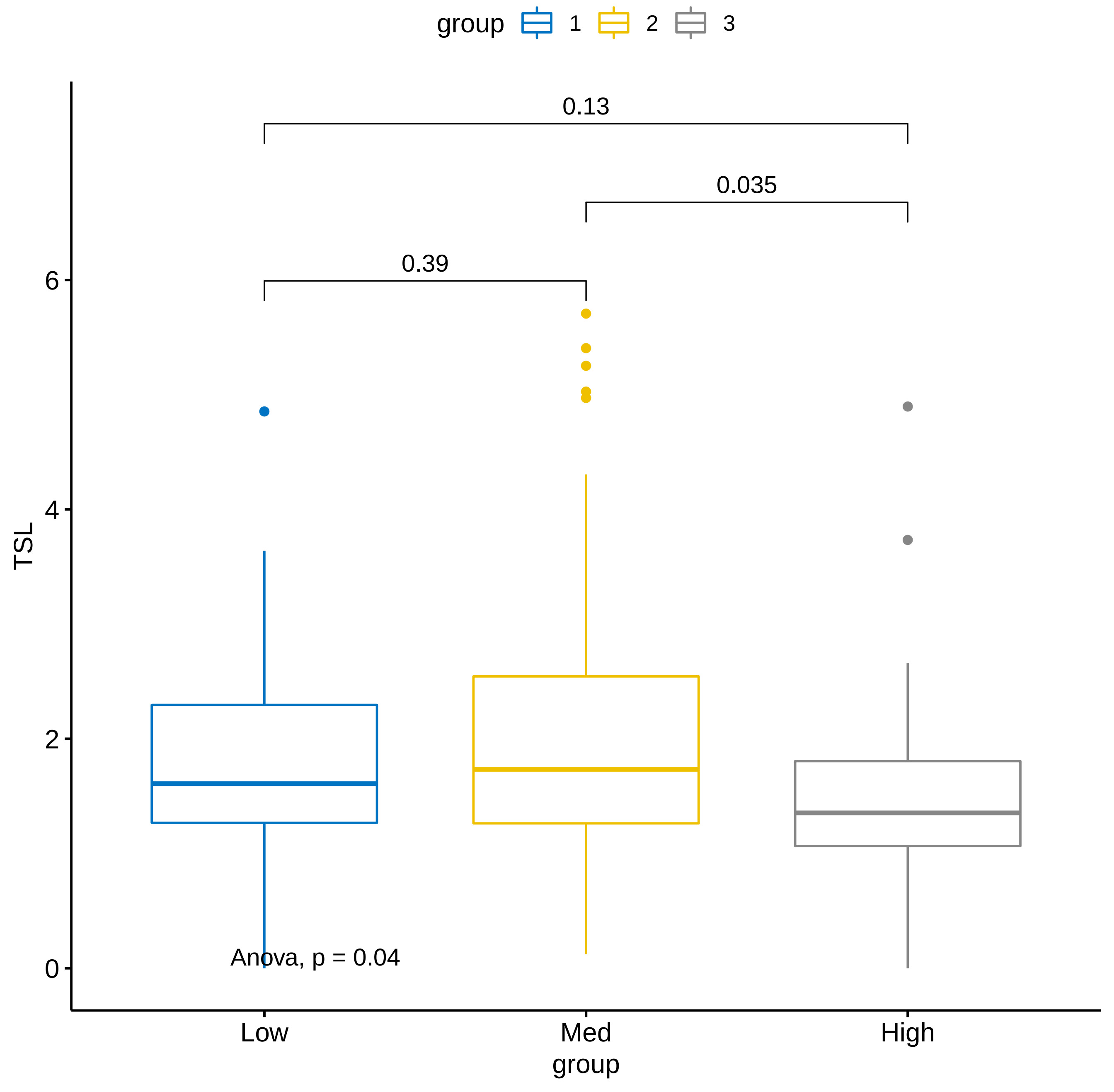
group
1
2
3
0.13
0.035
0.39
TSL
6
4
2
0
Anova, p = 0.04
Low
Med
High
group

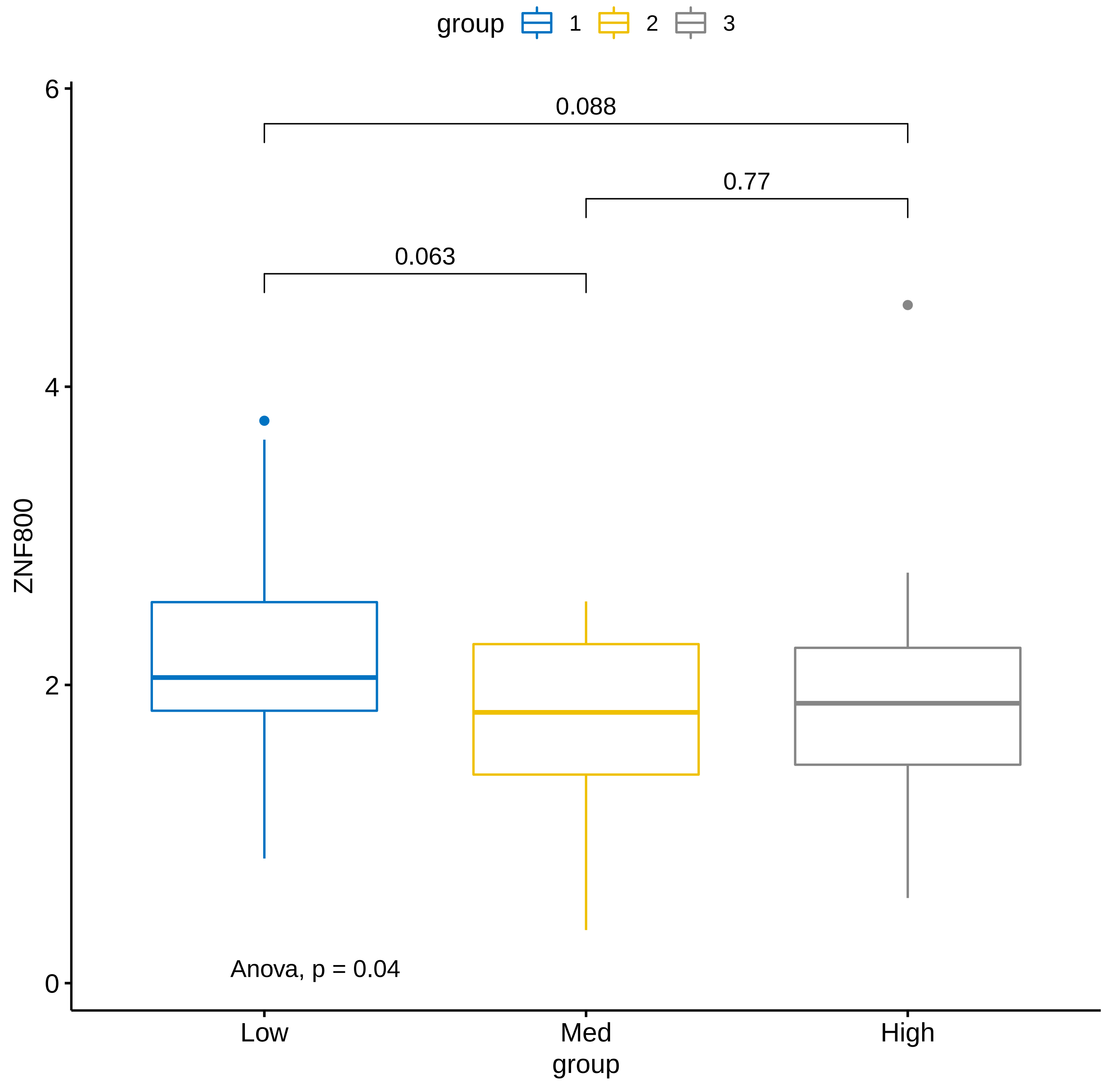
group 1 2 3
0.088
0.77
0.063
Anova, p = 0.04
6
4
2
0
ZNF800
Low
Med
High
group

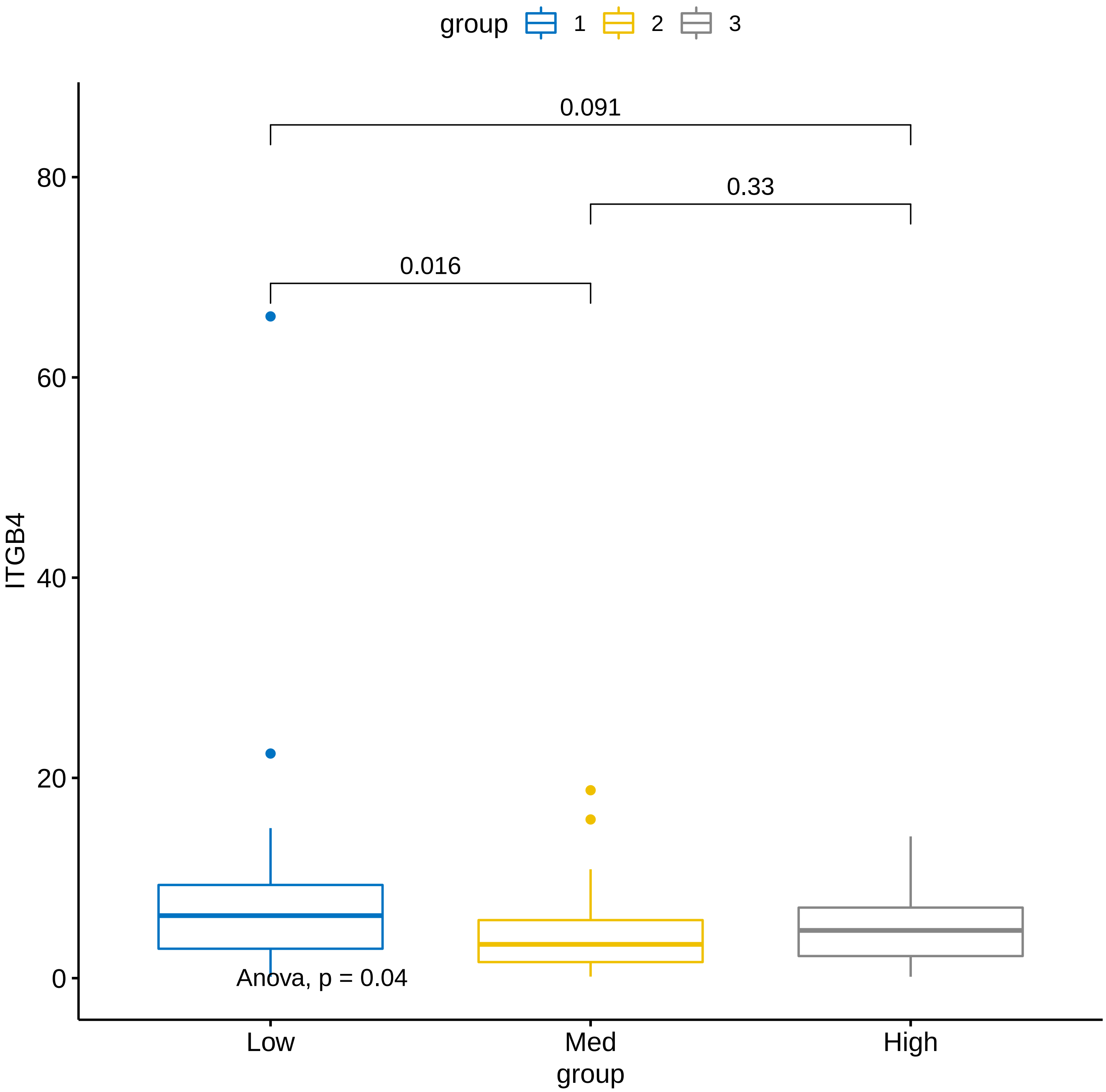
group
1
2
3
0.091
0.33
0.016
80
60
40
20
0
ITGB4
Anova, p = 0.04
Low
Med
High
group

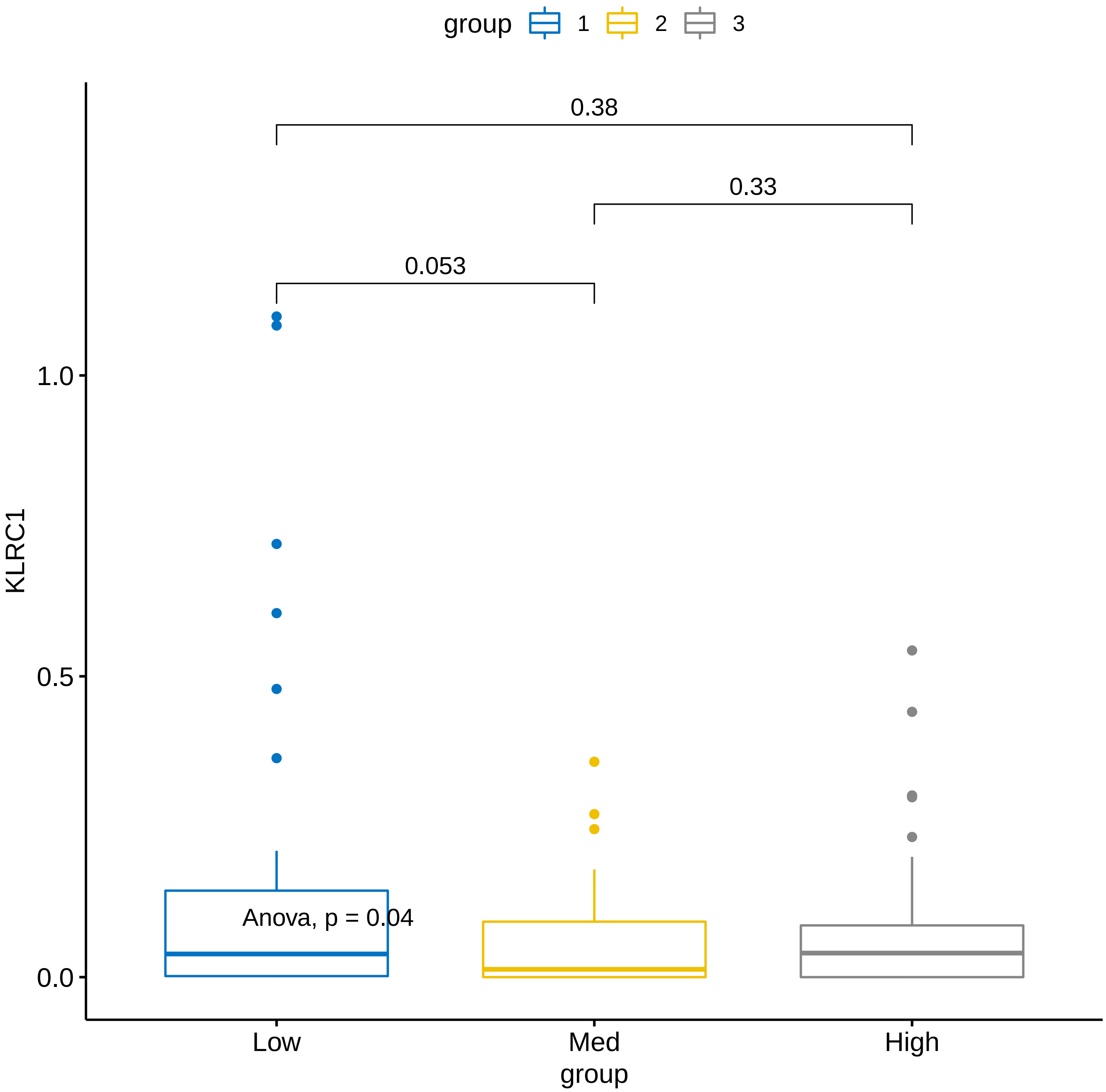
group 1 2 3
0.38
0.33
0.053
1.0
0.5
0.0
KLRC1
Anova, p = 0.04
Low
Med
High
group

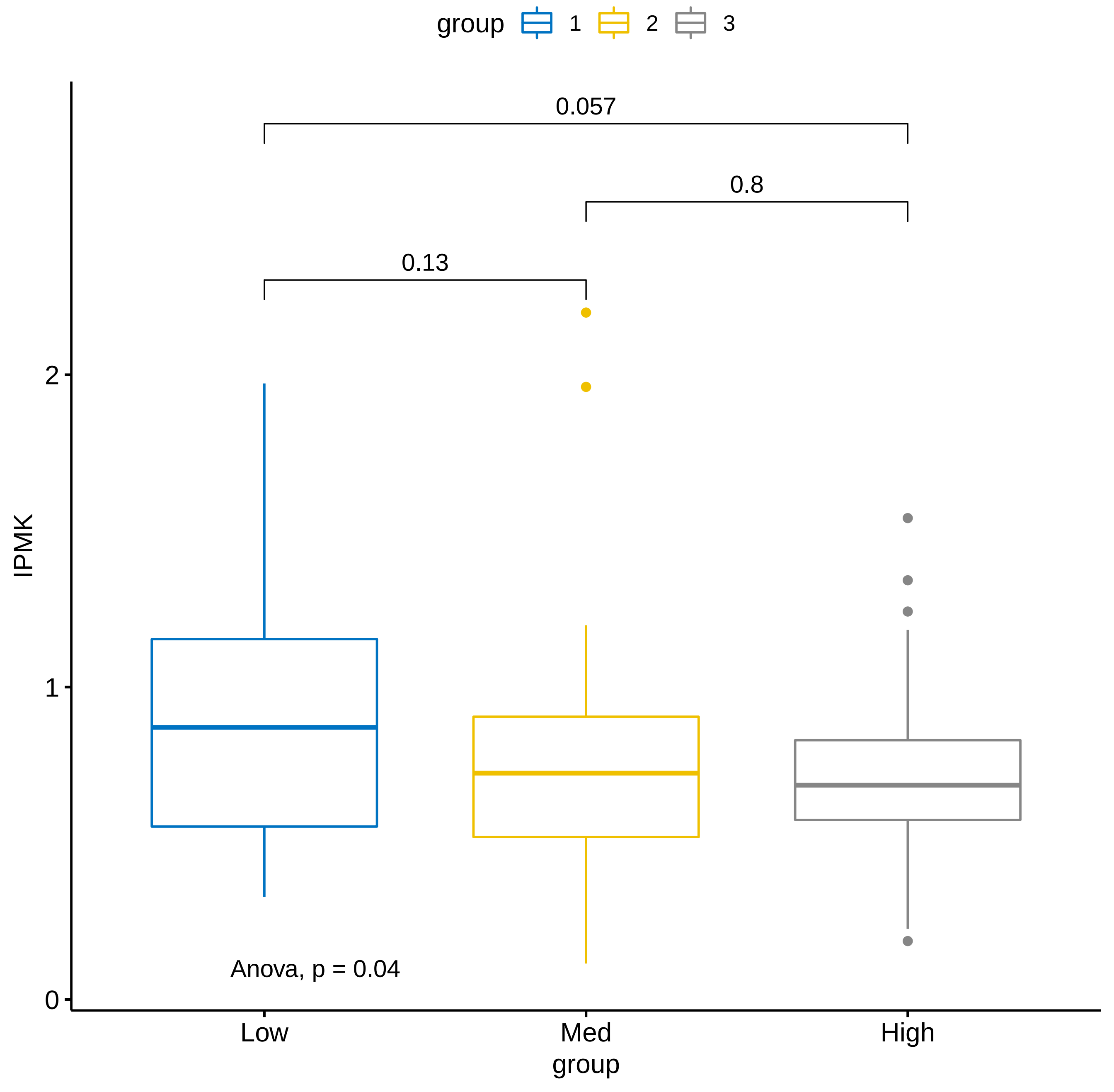

group
1
2
3
0.057
0.8
0.13
2
1
0
IPMK
Anova, p = 0.04
Low
Med
High
group

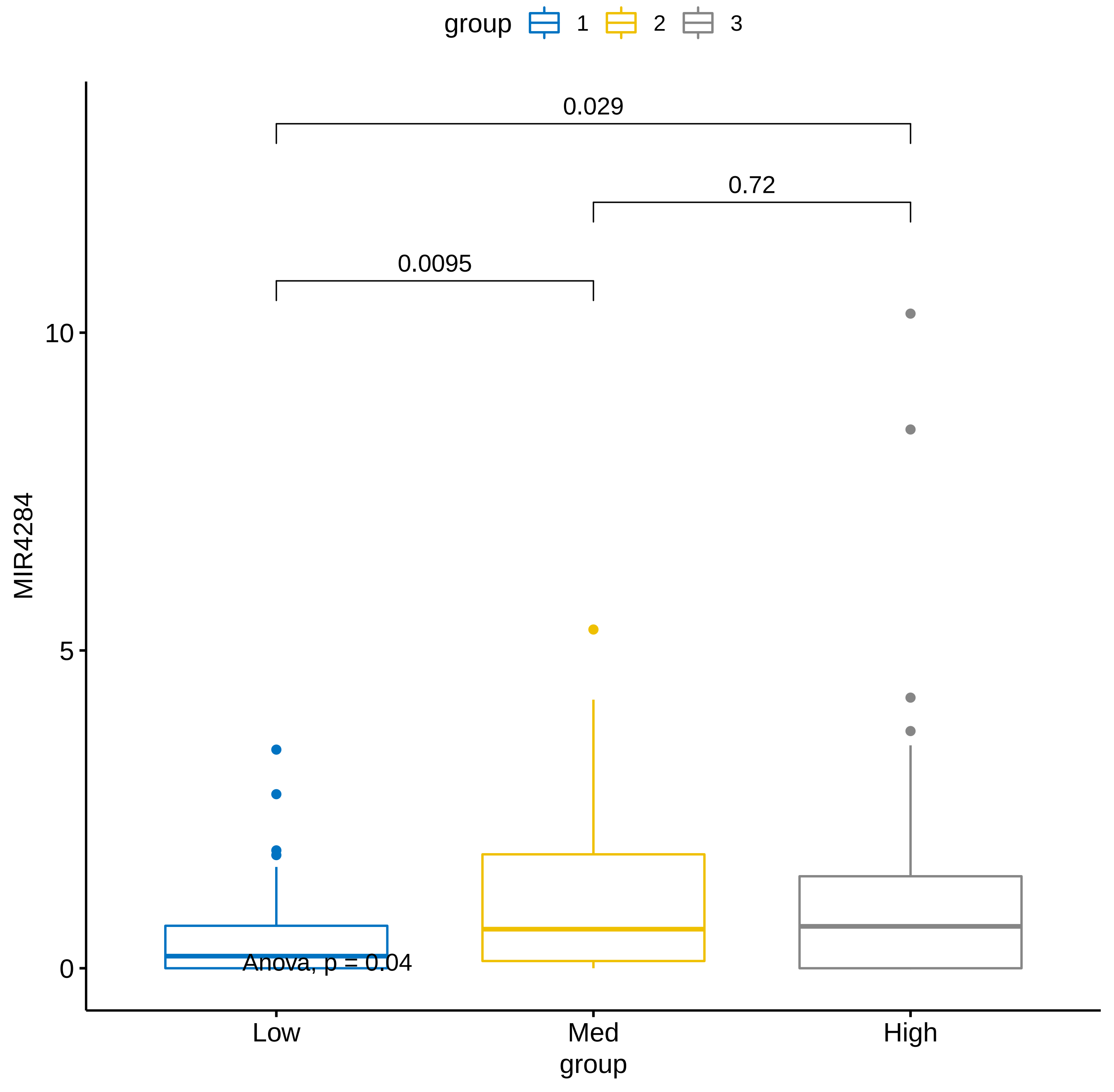
group 1 2 3
0.029
0.72
0.0095
10
5
0
MIR4284
Anova, p = 0.04
Low
Med
High
group

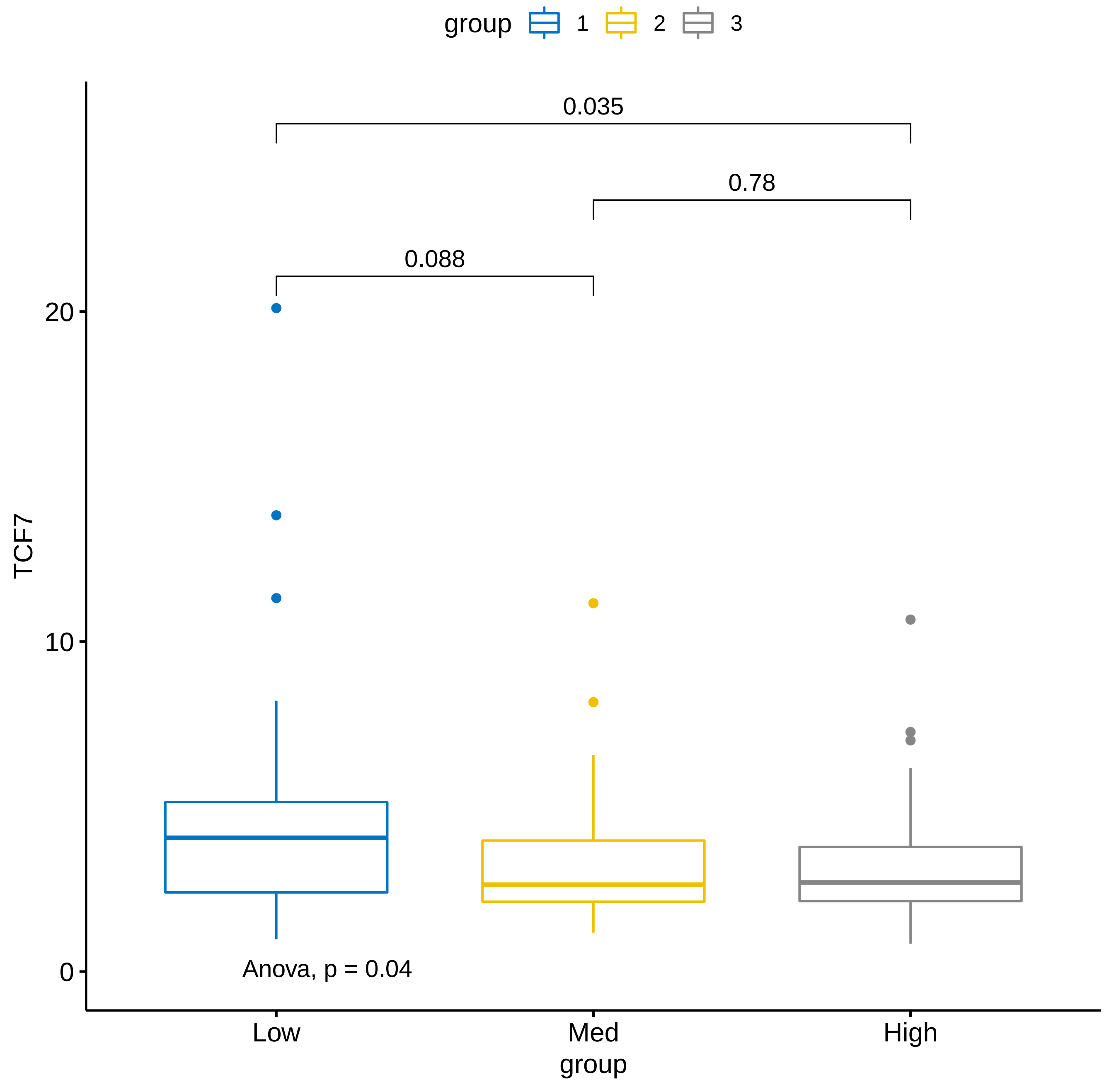

group 1 2 3
0.035
0.78
0.088
20
10
0
TCF7
Anova, p = 0.04
Low
Med
High
group

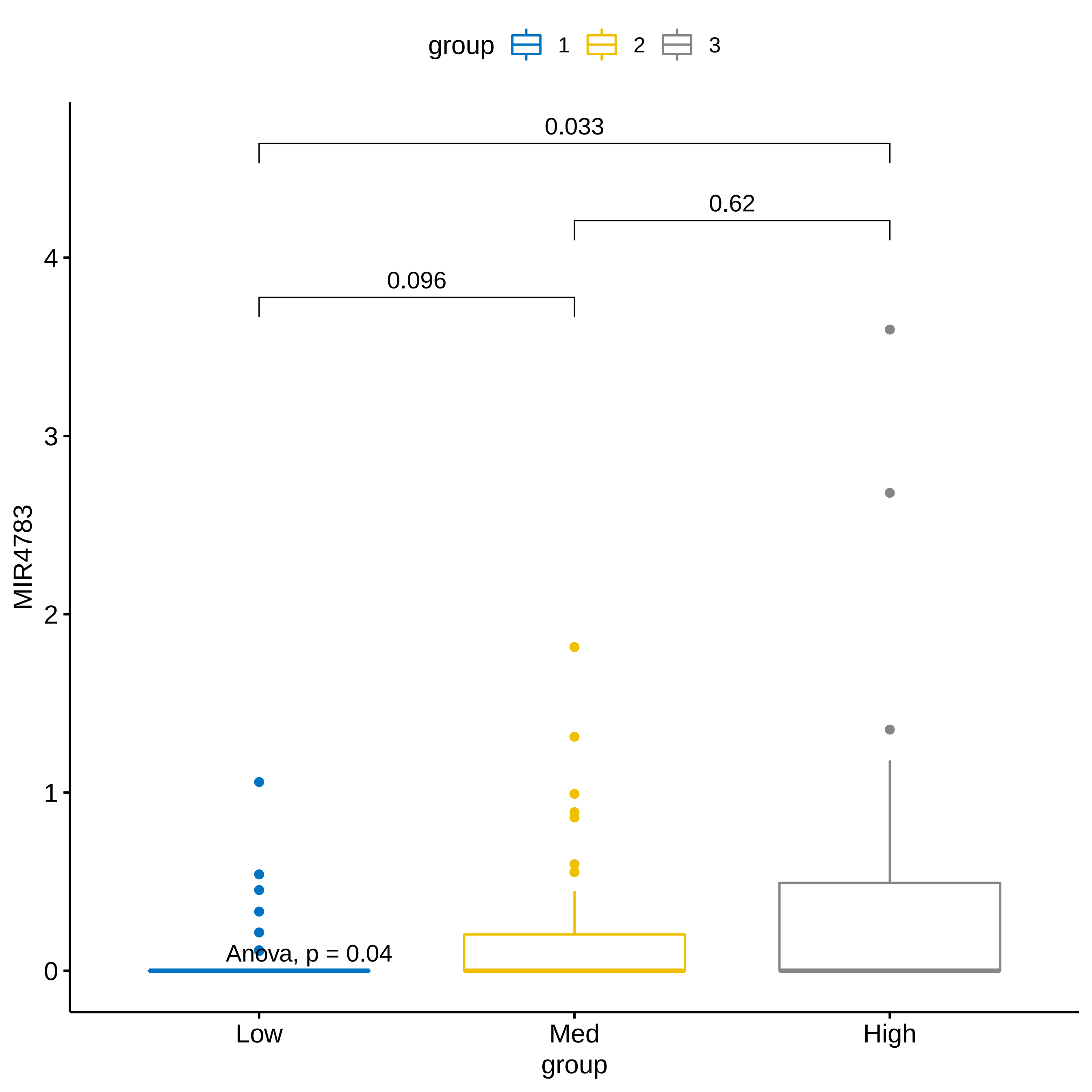

group
1
2
3
0.033
0.62
0.096
4
3
2
1
0
MIR4783
Anova, p = 0.04
Low
Med
High
group

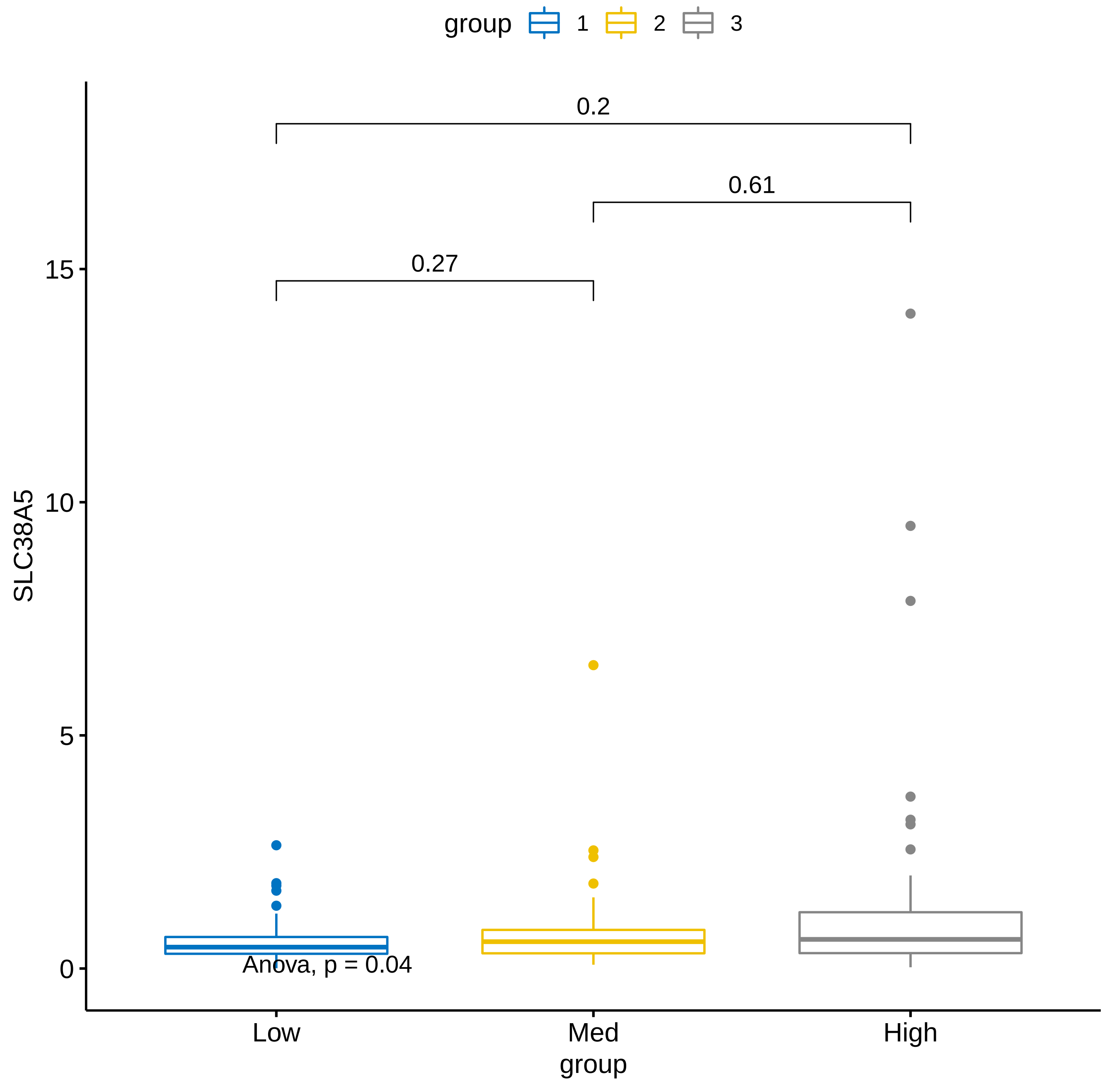
group 1 2 3
0.2
0.61
0.27
15
10
5
0
SLC38A5
Anova, p = 0.04
Low
Med
High
group

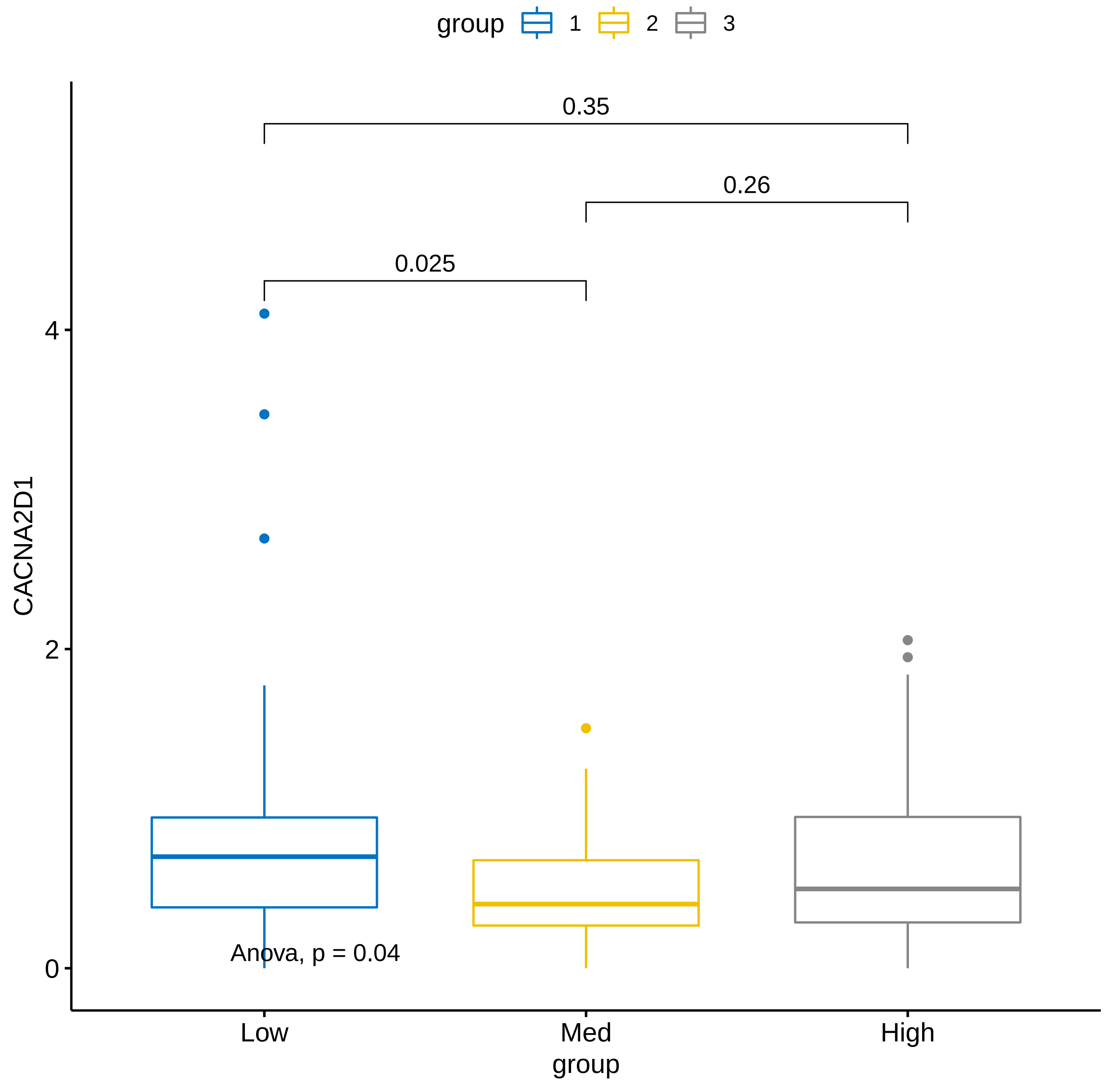

group
1
2
3
0.35
0.26
0.025
4
2
0
CACNA2D1
Anova, p = 0.04
Low
Med
High
group

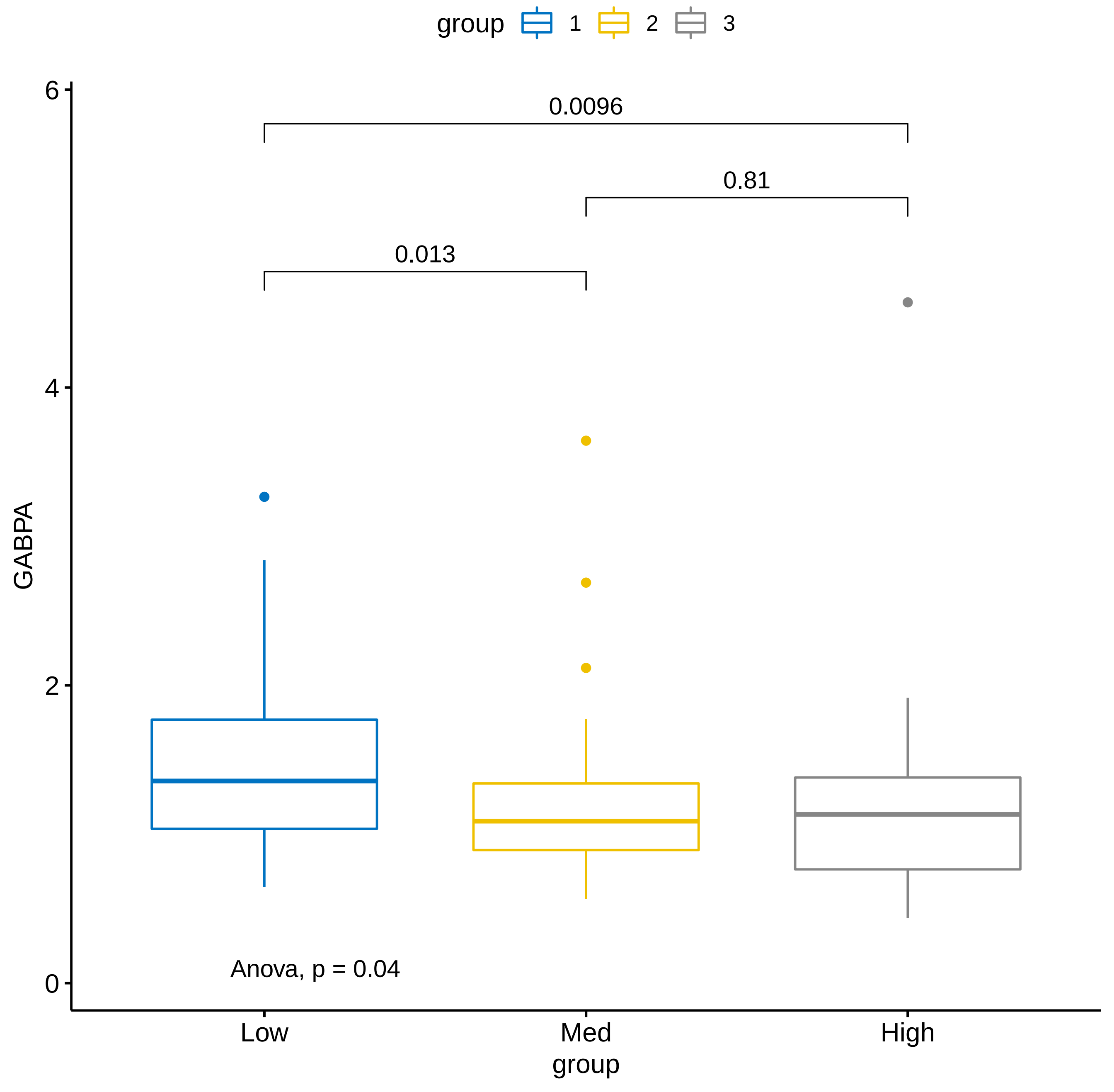
group
1
2
3
6
4
2
0
GABPA
0.0096
0.81
0.013
Anova, p = 0.04
Low
Med
High
group

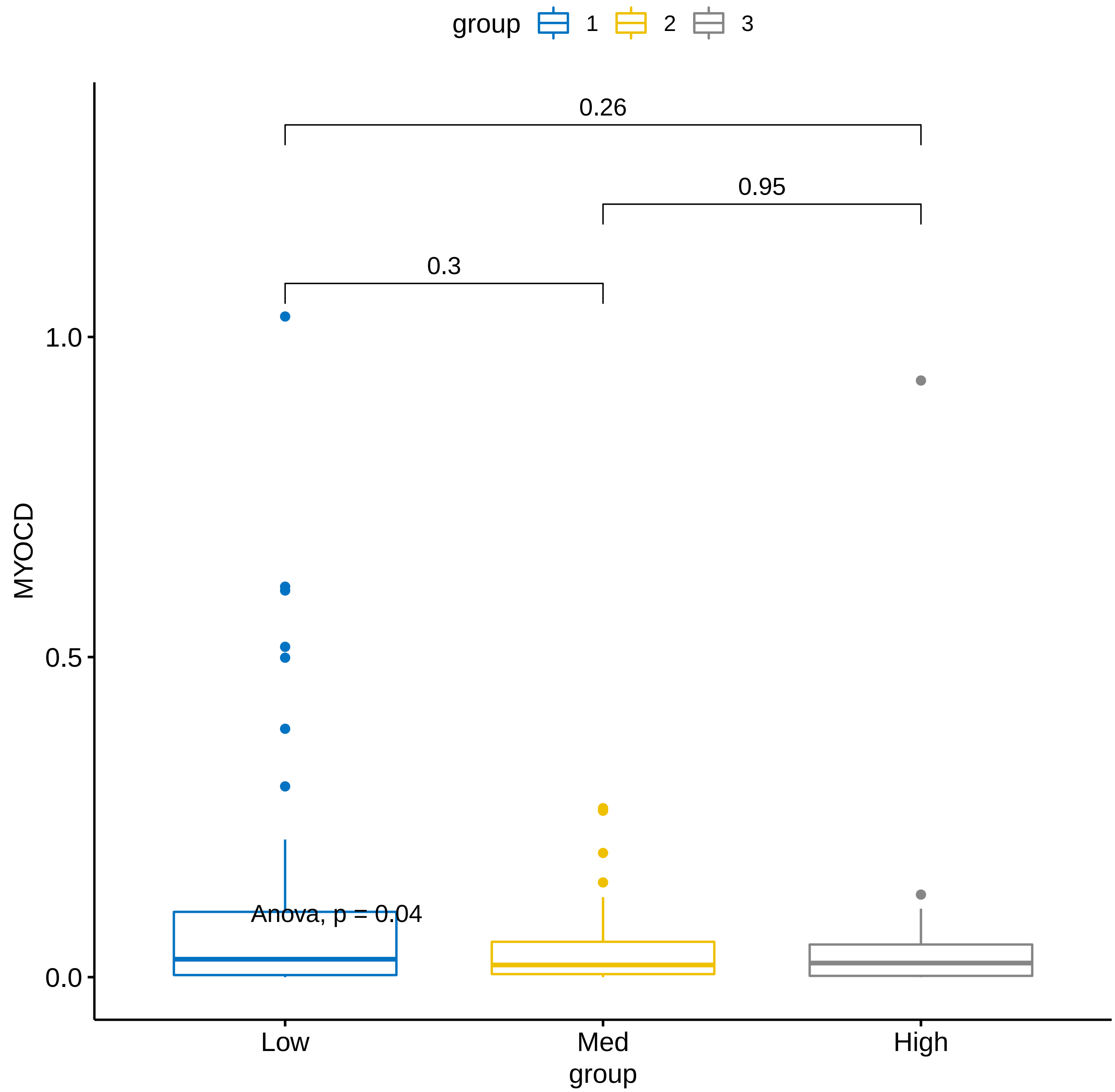

group
1
2
3
0.26
0.95
0.3
1.0
0.5
0.0
MYOCD
Anova, p = 0.04
Low
Med
High
group

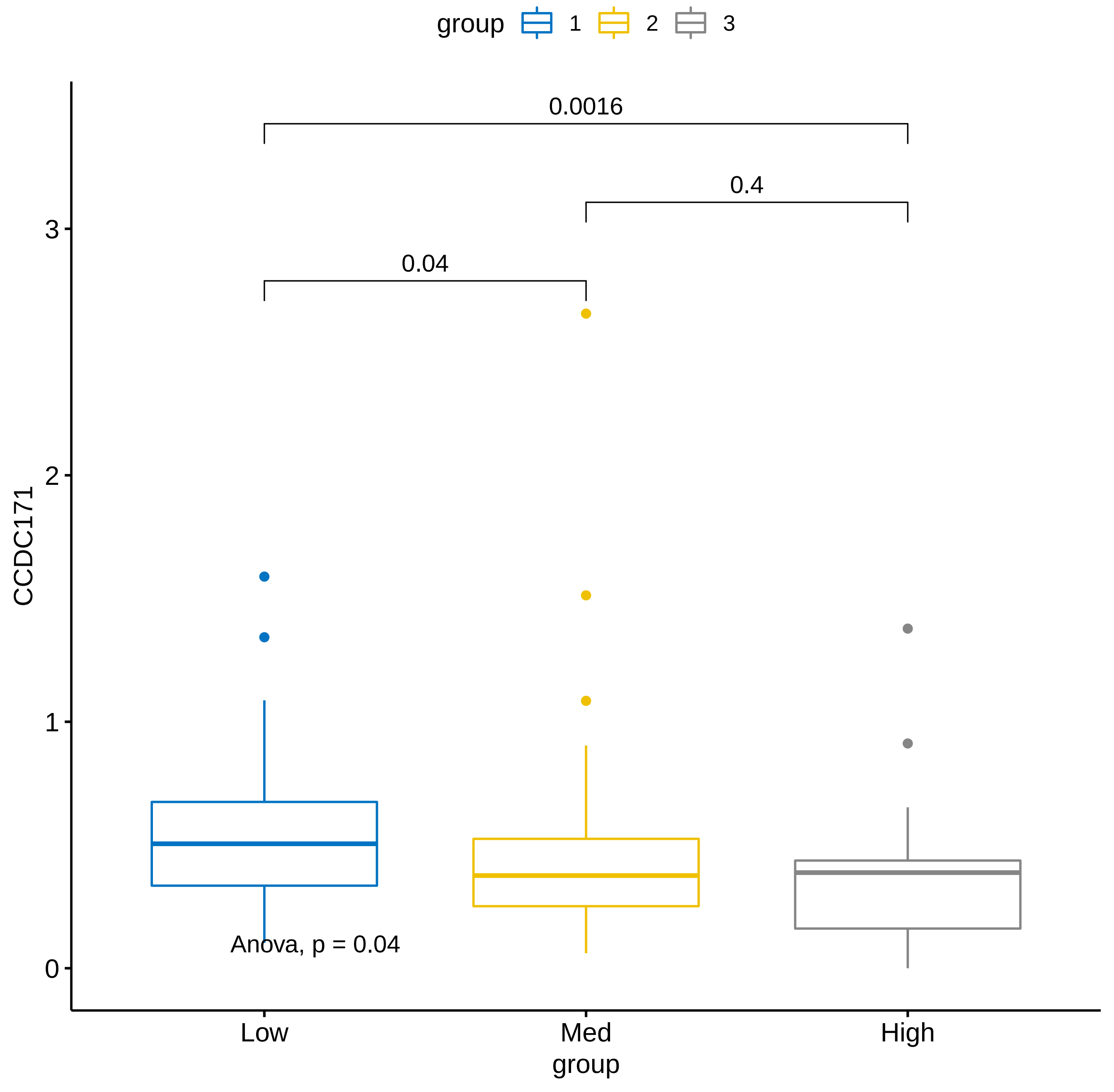

group 1 2 3
0.0016
0.4
0.04
3
2
1
0
CCDC171
Anova, p = 0.04
Low
Med
High
group

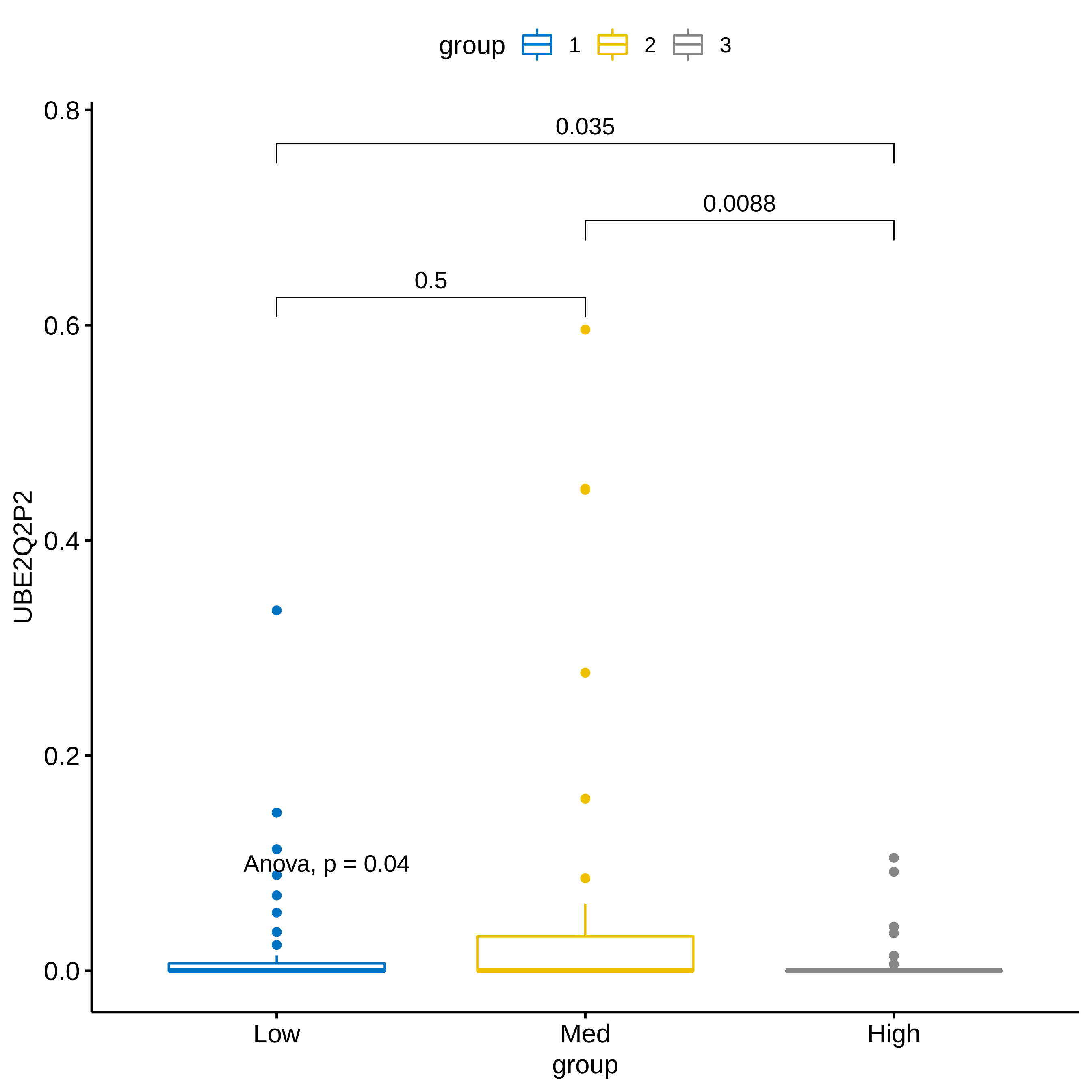
group
1
2
3
0.8
0.6
0.4
0.2
0.0
UBE2Q2P2
0.035
0.0088
0.5
Anova, p = 0.04
Low
Med
High
group

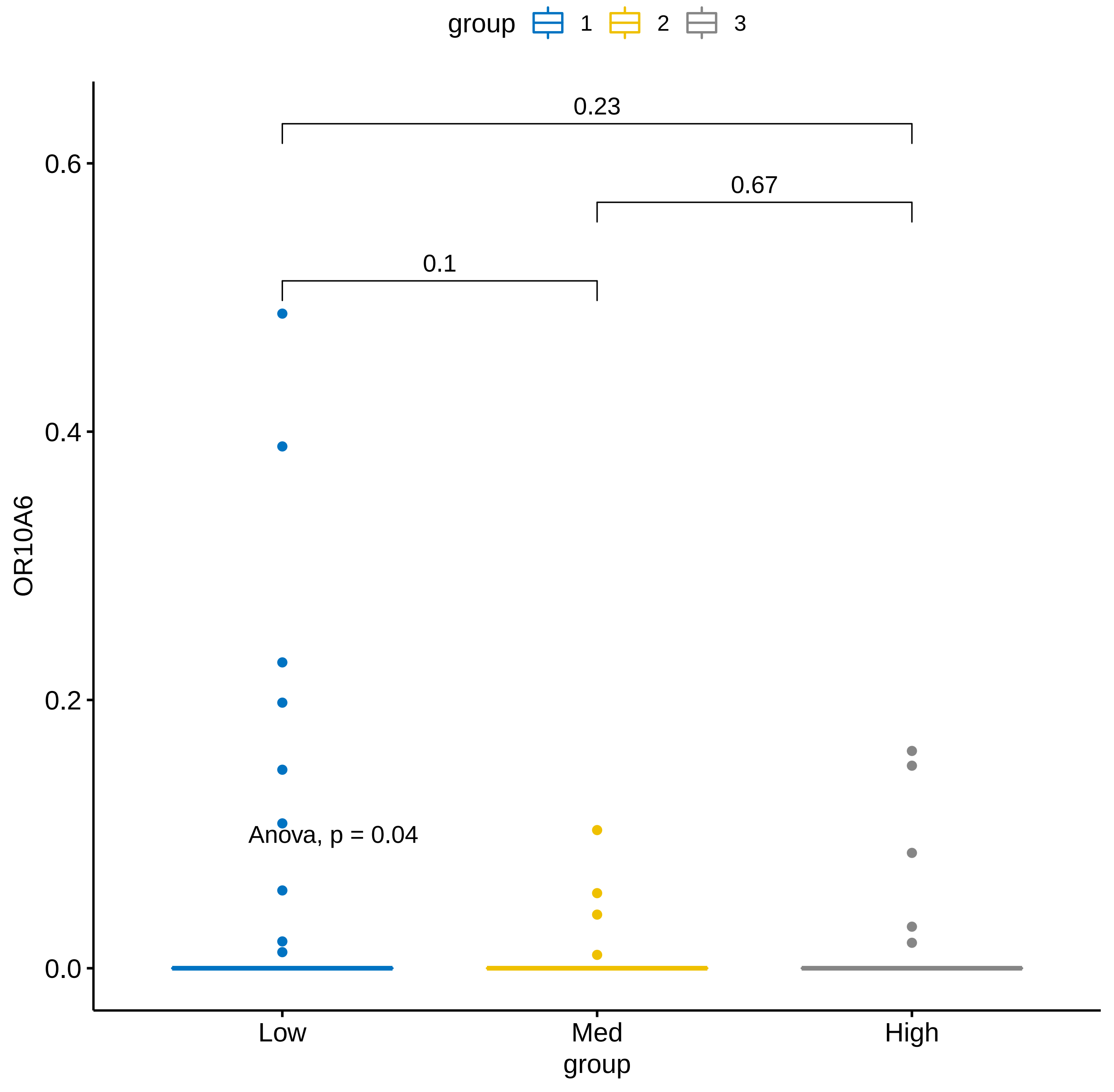
group
1
2
3
0.23
0.67
0.1
0.6
0.4
0.2
0.0
OR10A6
Anova, p = 0.04
Low
Med
High
group

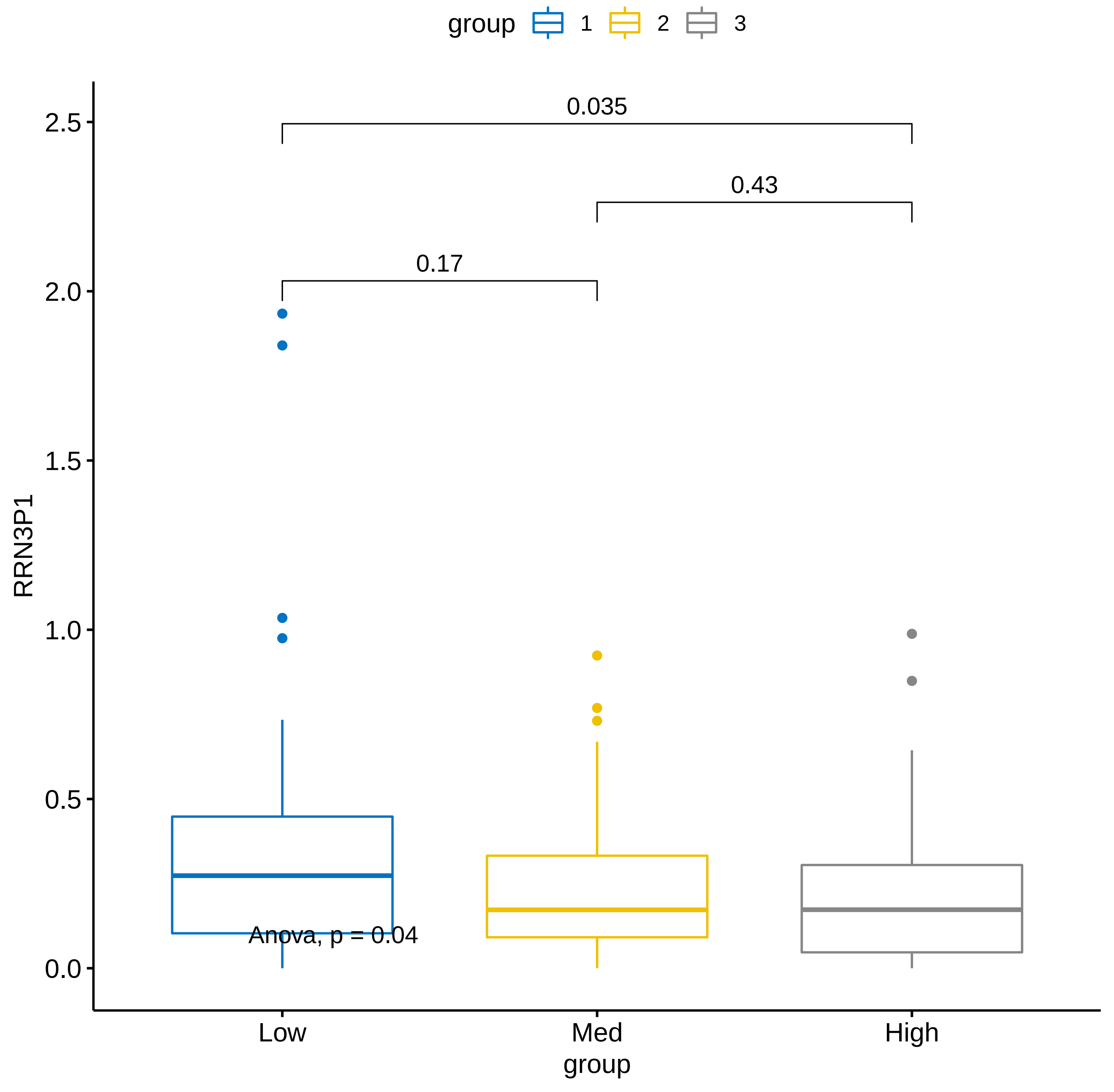

group
1
2
3
0.035
0.43
0.17
2.5
2.0
1.5
1.0
0.5
0.0
RRN3P1
Anova, p = 0.04
Low
Med
High
group

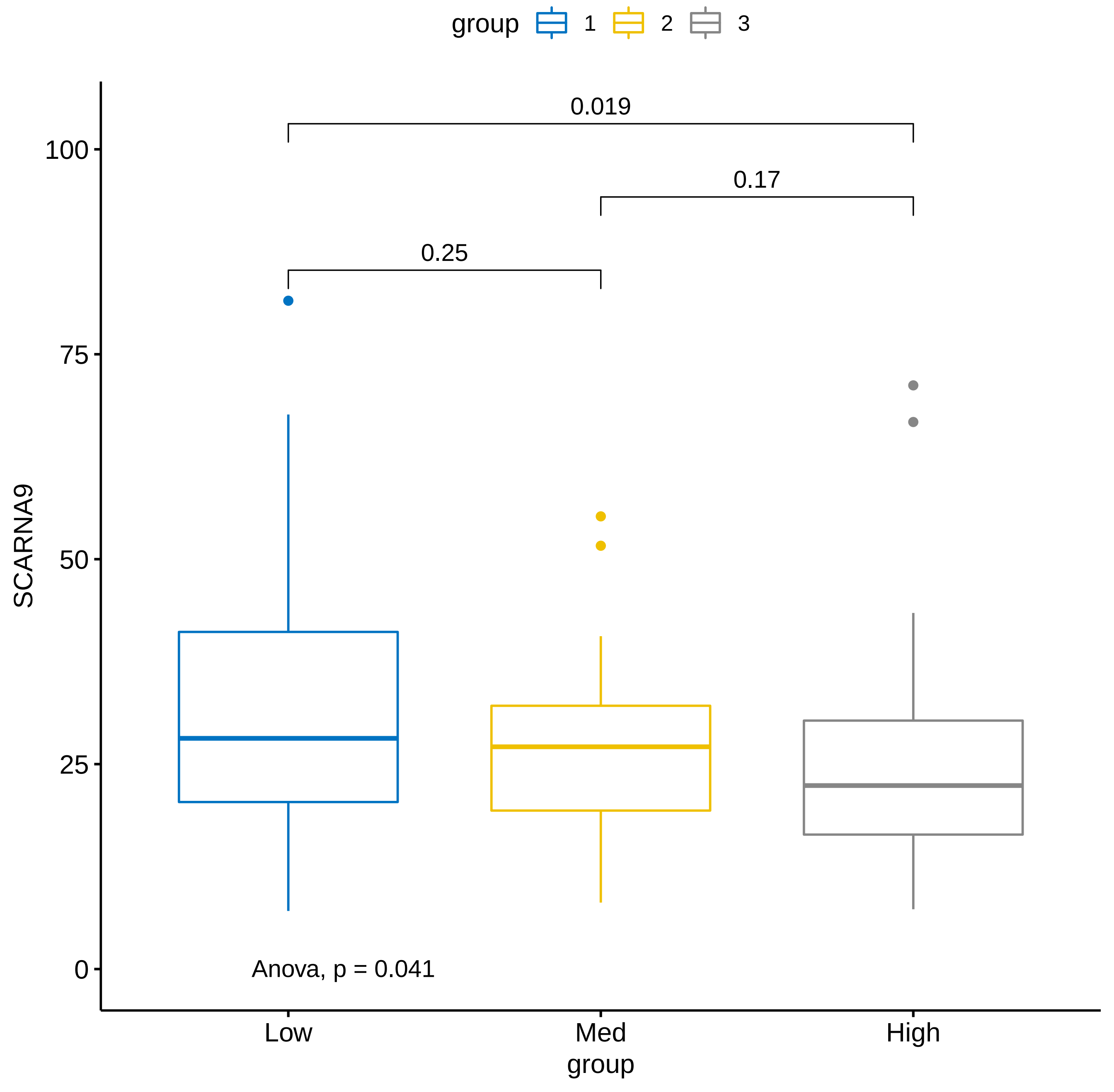
group
1
2
3
0.019
0.17
0.25
100
75
50
25
0
SCARNA9
Anova, p = 0.041
Low
Med
High
group

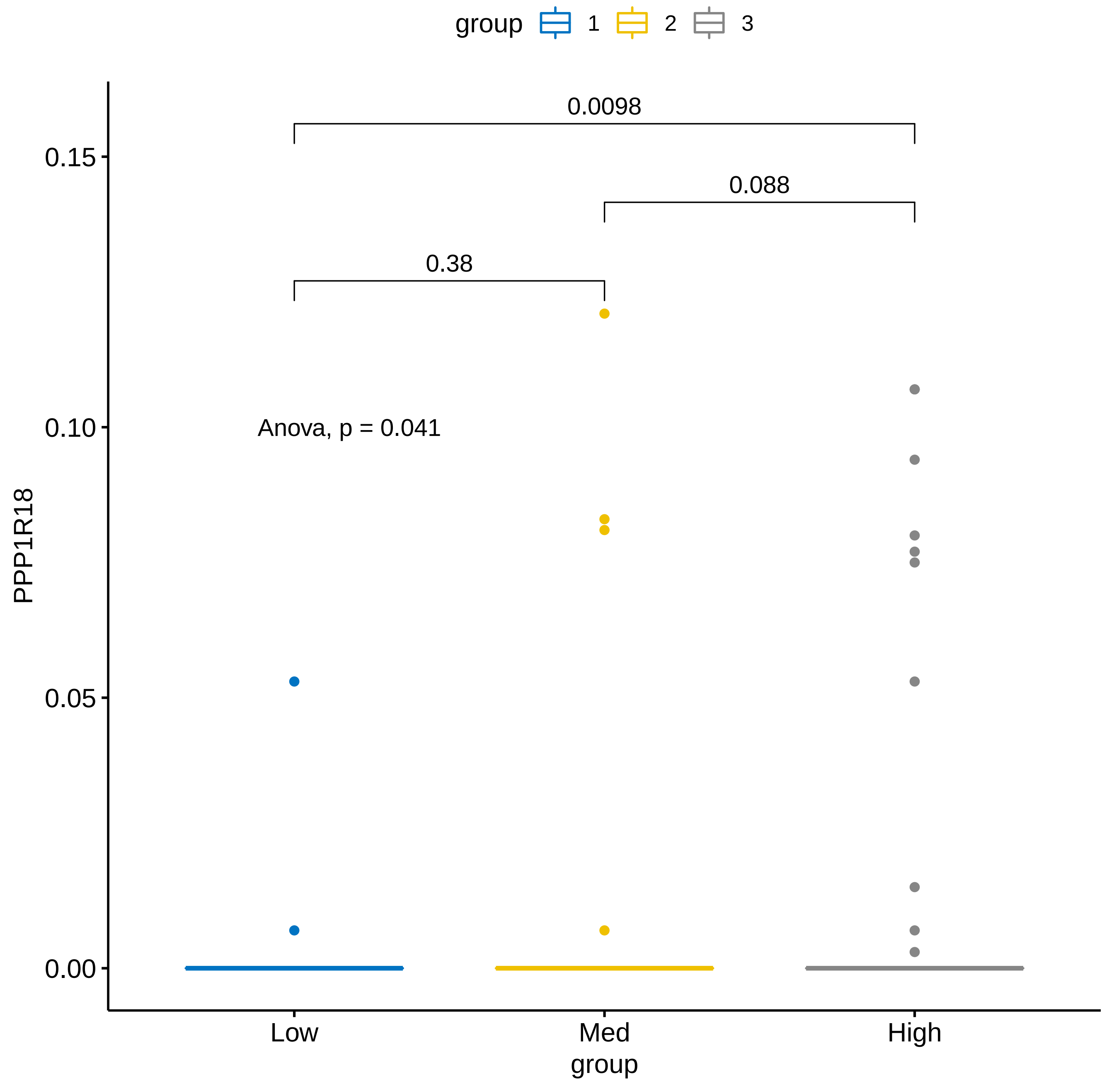

group
1
2
3
0.0098
0.088
0.38
Anova, p = 0.041
0.15
0.10
0.05
0.00
PPP1R18
Low
Med
High
group

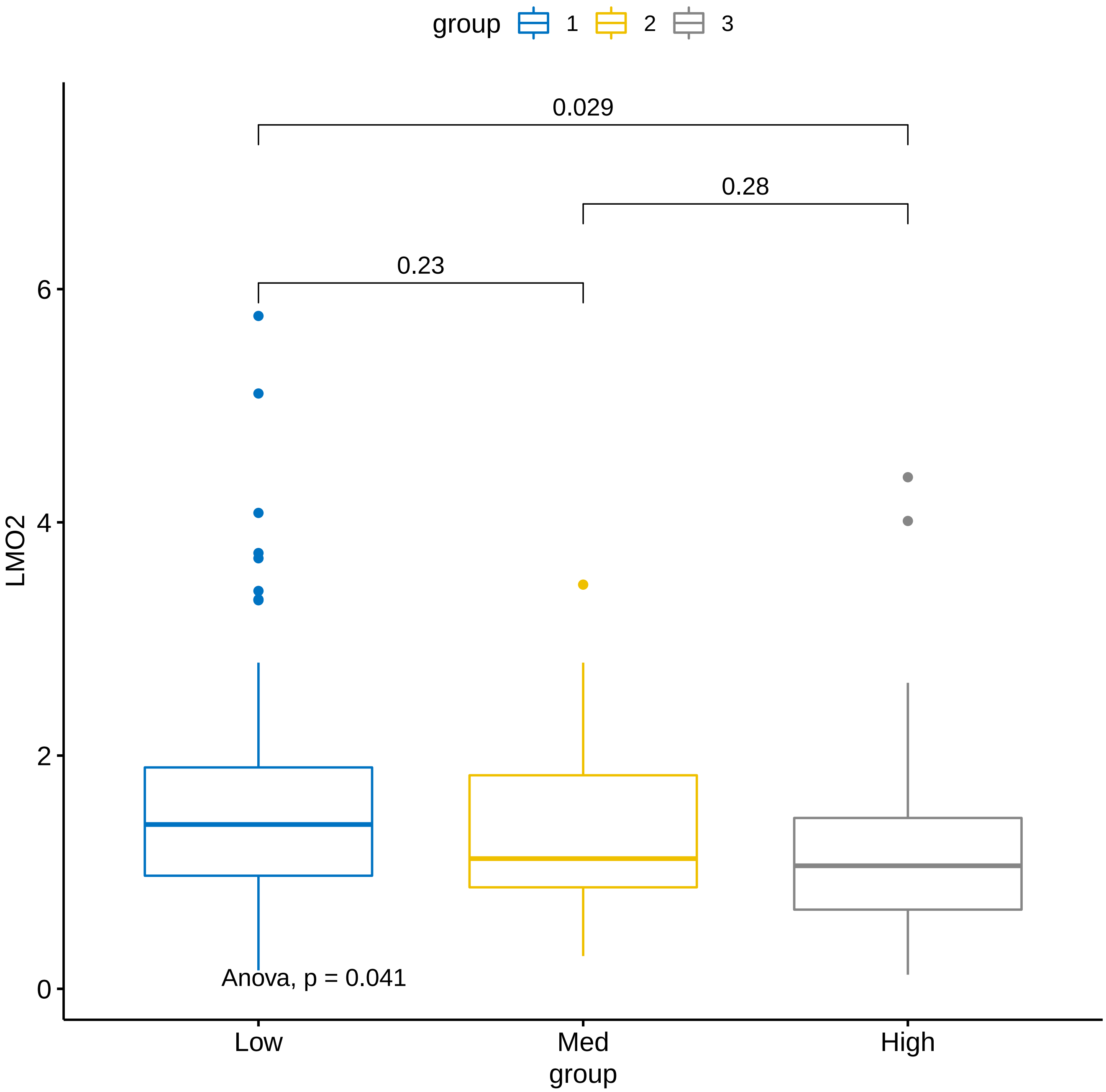

group
1
2
3
0.029
0.28
0.23
6
4
2
0
LMO2
Anova, p = 0.041
Low
Med
High
group

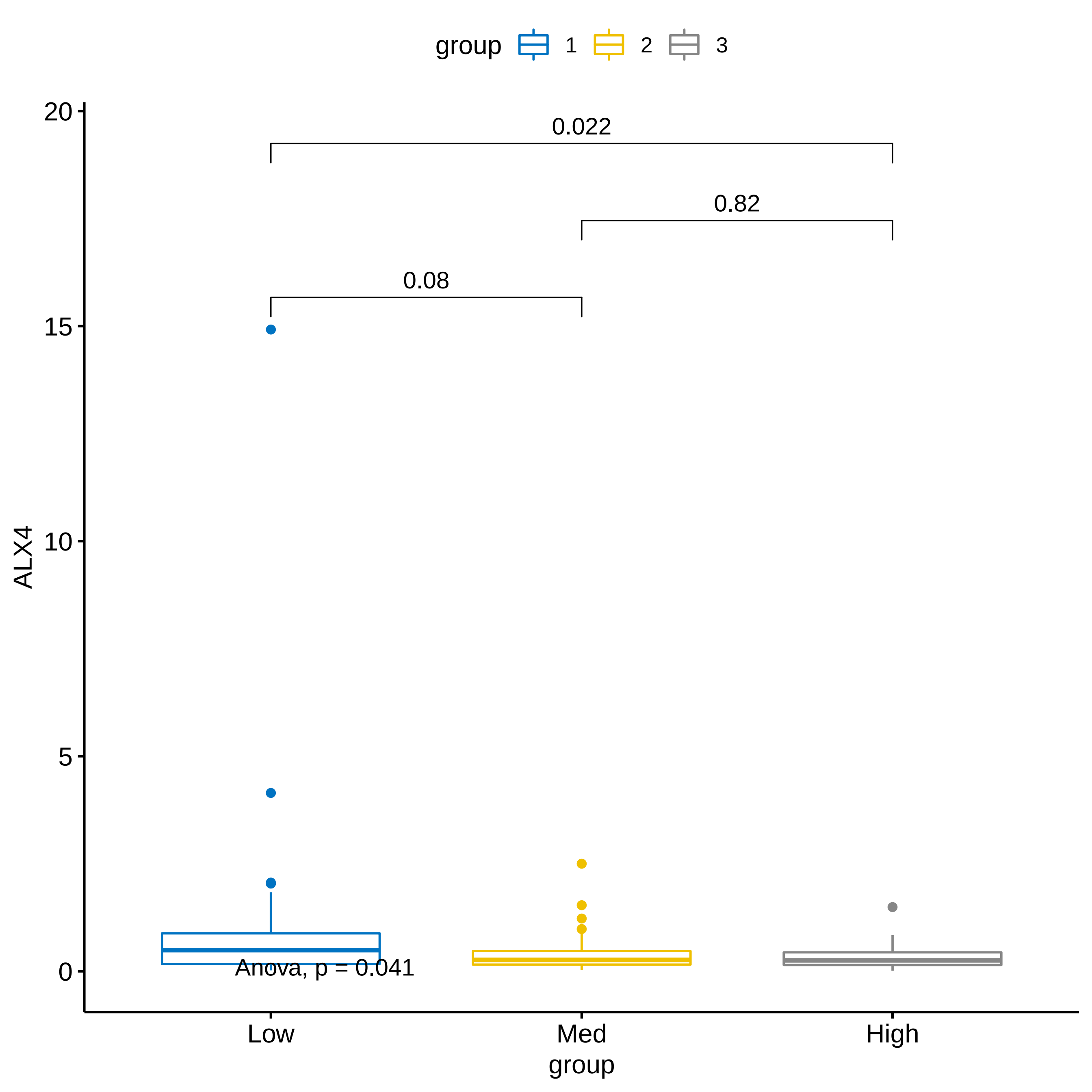

group 1 2 3
0.022
0.82
0.08
20
15
10
5
0
ALX4
Anova, p = 0.041
Low
Med
High
group

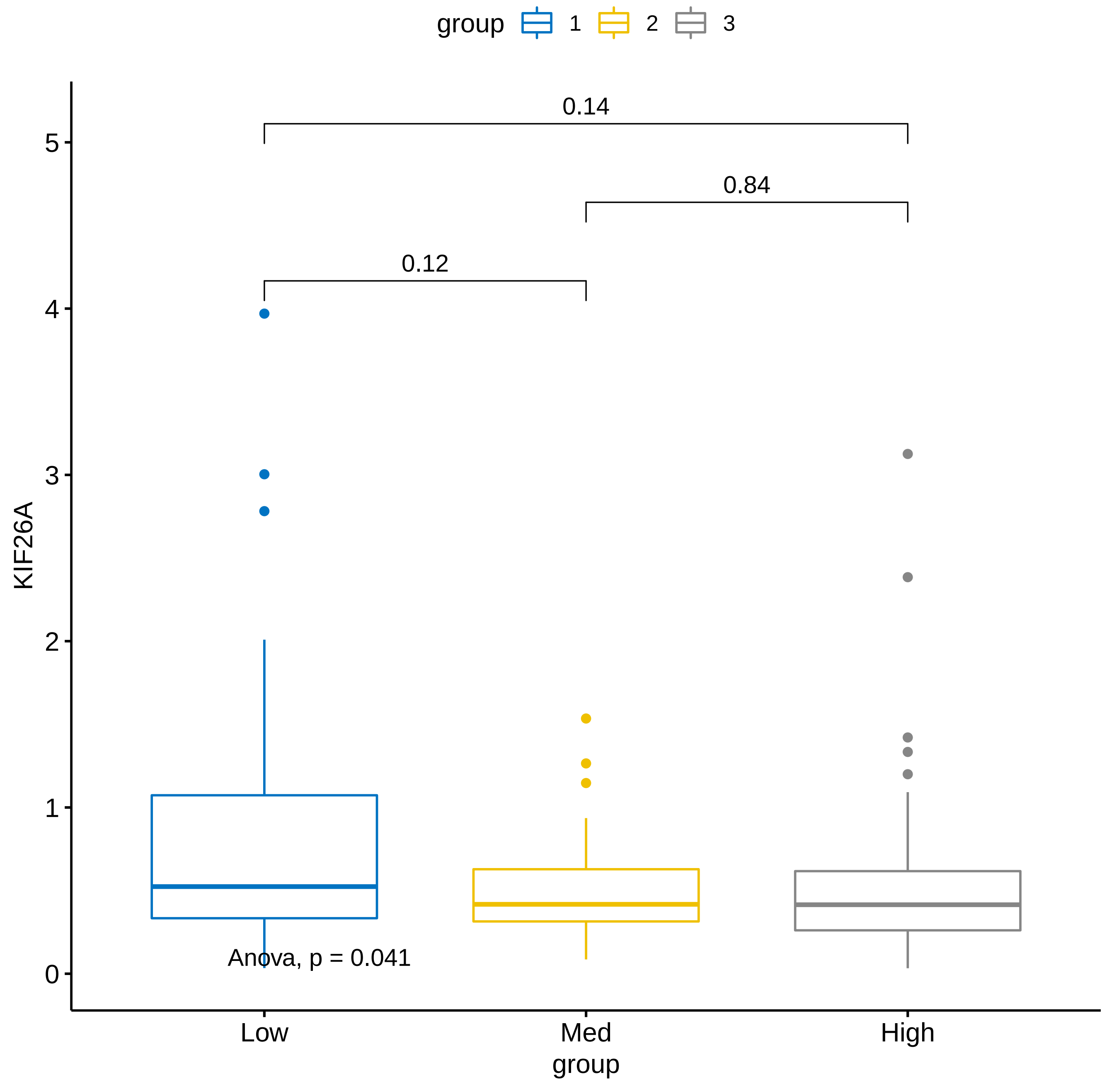

group
1
2
3
0.14
0.84
0.12
5
4
3
2
1
0
KIF26A
Anova, p = 0.041
Low
Med
High
group

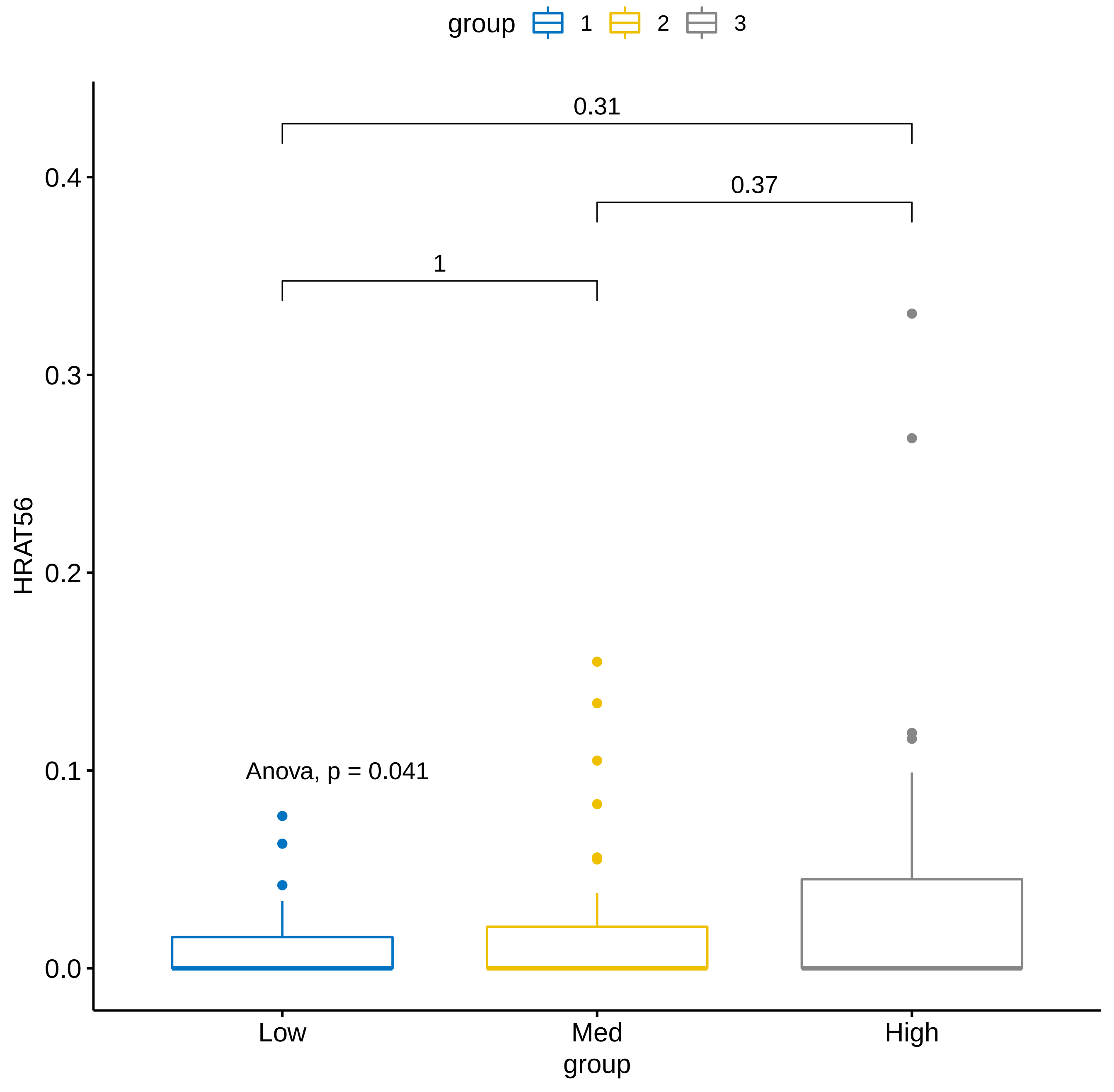
group
1
2
3
0.31
0.37
1
Anova, p = 0.041
HRAT56
0.4
0.3
0.2
0.1
0.0
Low
Med
High
group

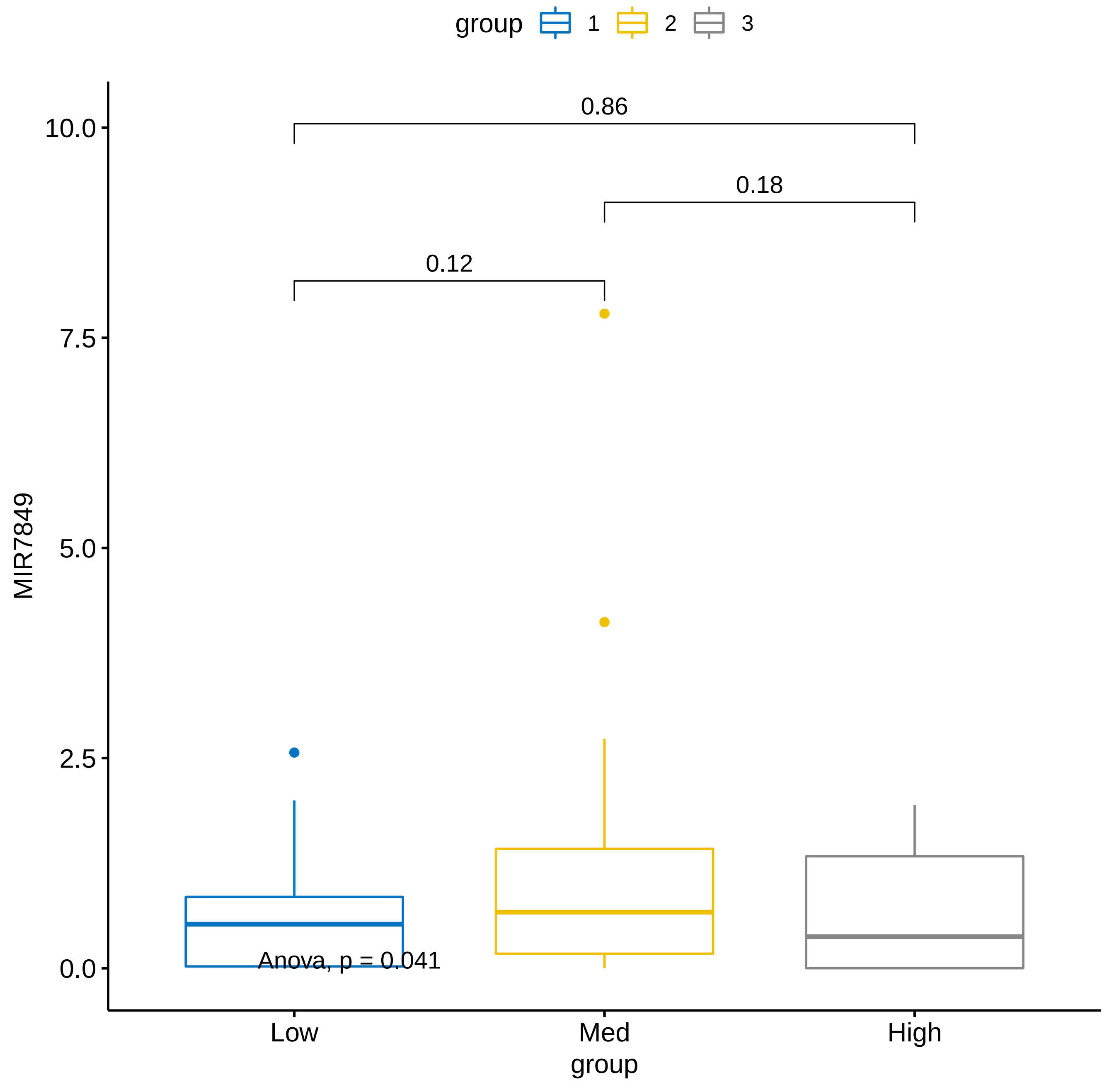
group 1 2 3
0.86
0.18
0.12
10.0
7.5
5.0
2.5
0.0
MIR7849
Anova, p = 0.041
Low
Med
High
group

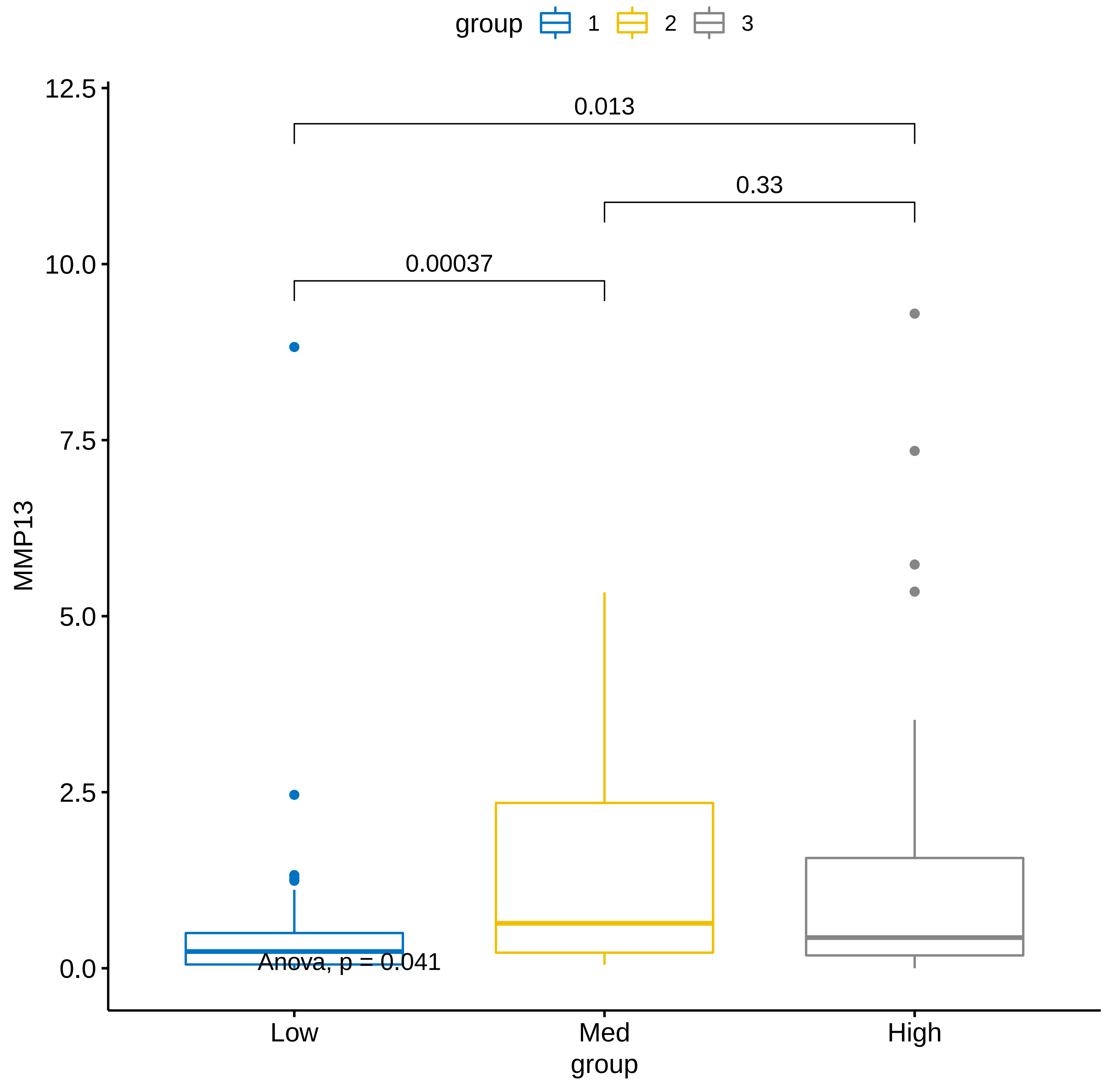
group
1
2
3
12.5
10.0
7.5
5.0
2.5
0.0
MMP13
0.013
0.33
0.00037
Anova, p = 0.041
Low
Med
High
group

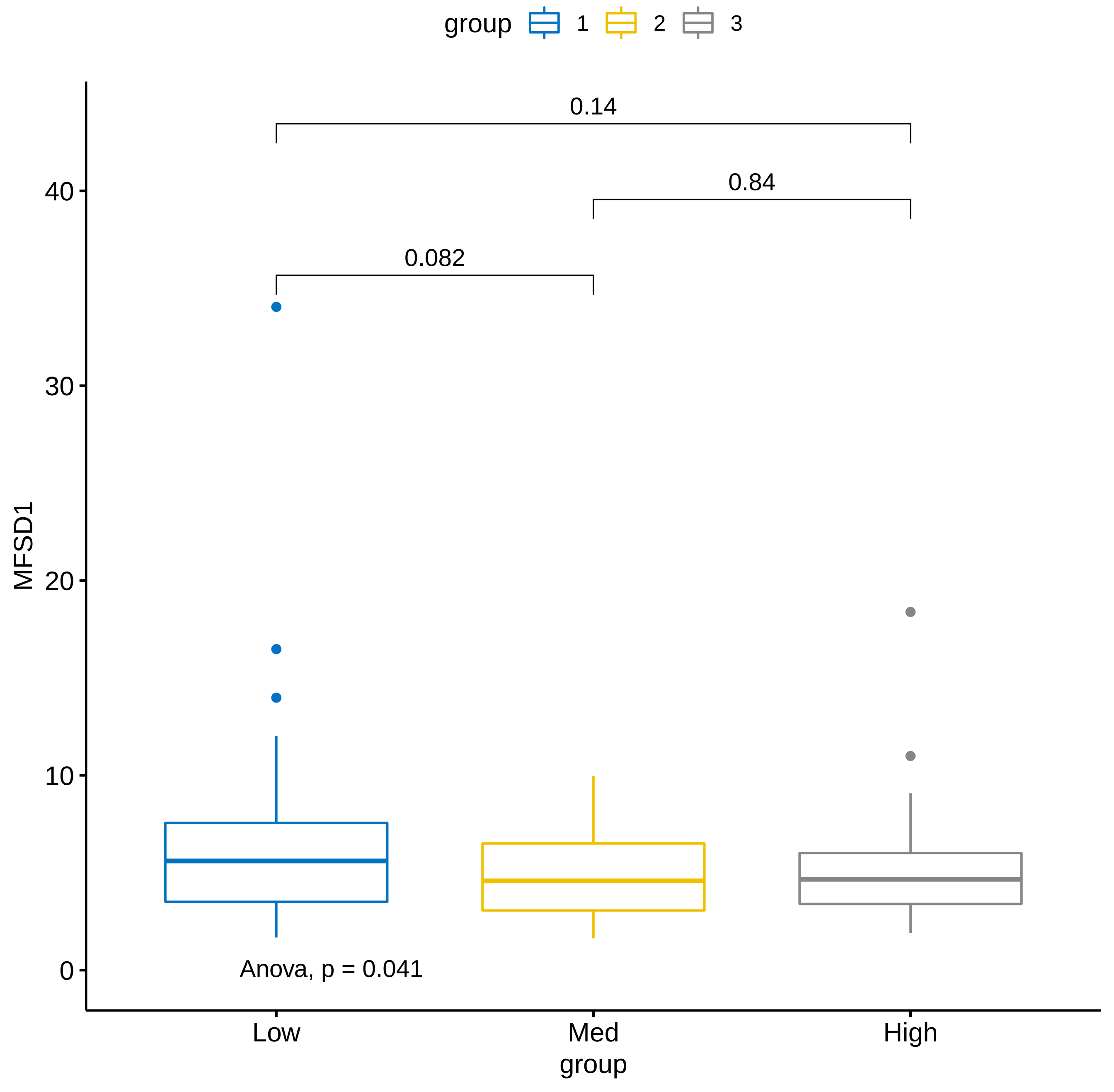

group 1 2 3
0.14
0.84
0.082
40
30
20
10
0
MFSD1
Anova, p = 0.041
Low
Med
High
group

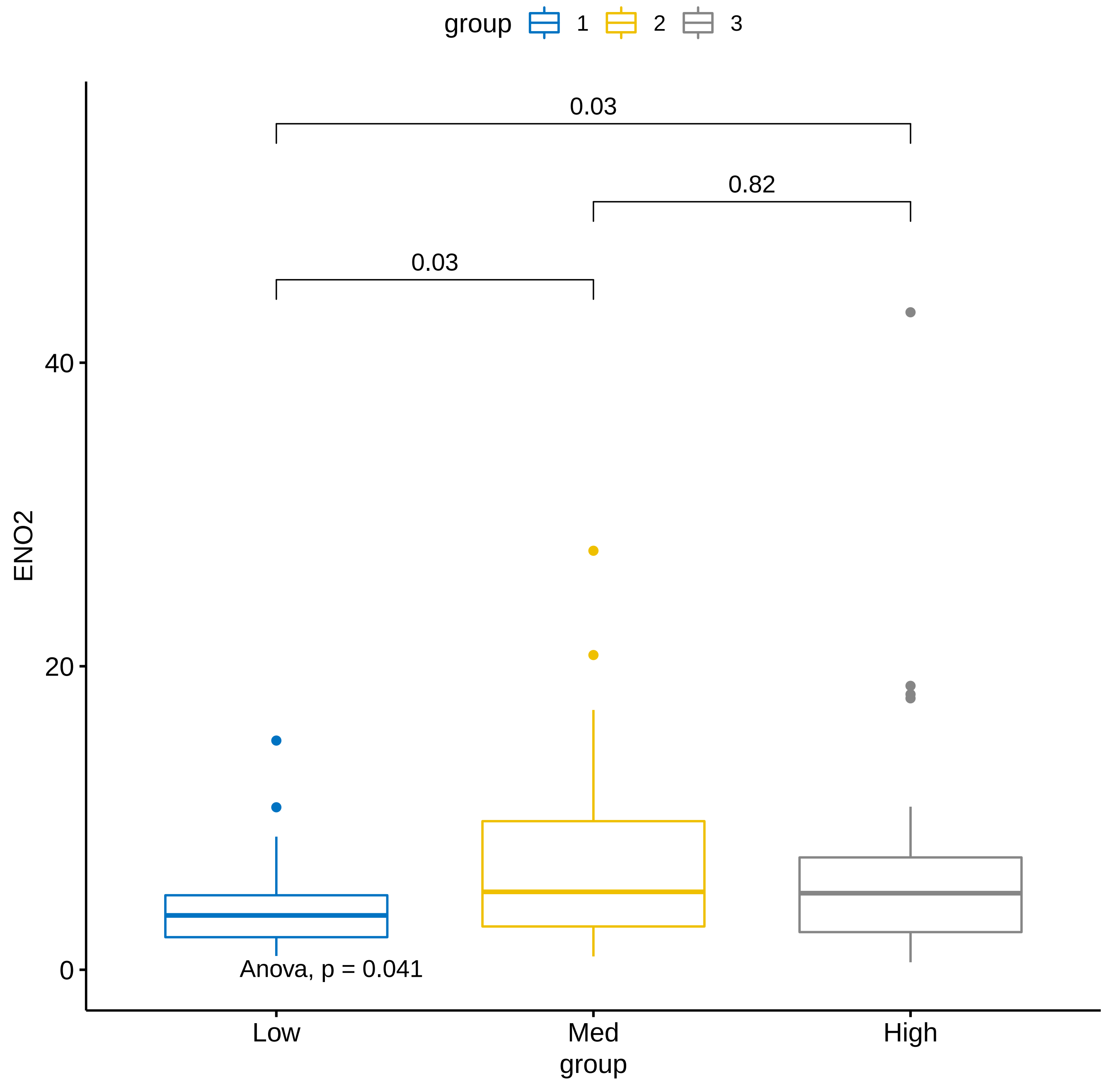
group
1
2
3
0.03
0.82
0.03
40
20
0
ENO2
Anova, p = 0.041
Low
Med
High
group

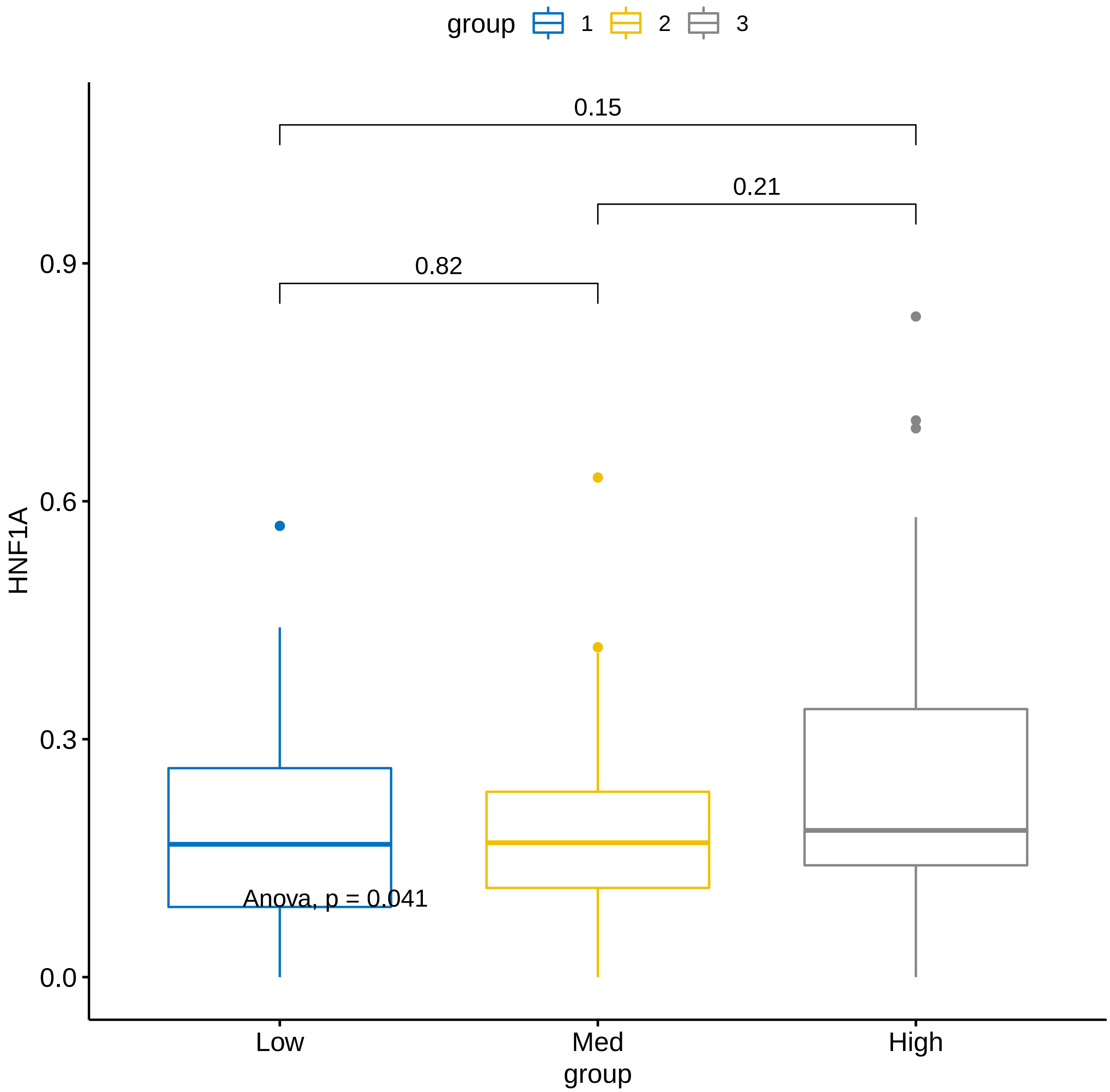

group 1 2 3
0.15
0.21
0.82
0.9
0.6
0.3
0.0
HNF1A
Anova, p = 0.041
Low
Med
High
group

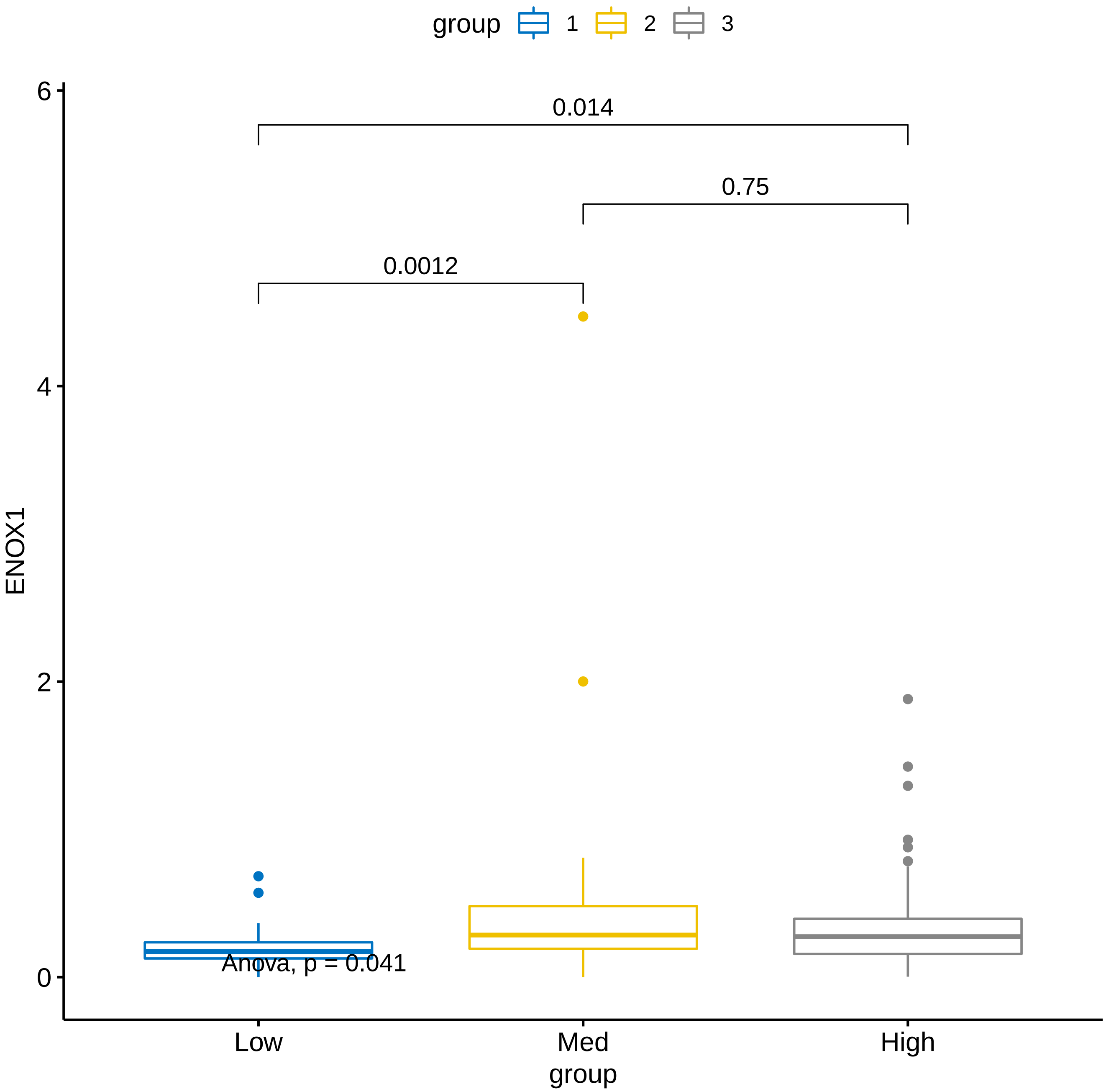
group
1
2
3
6
4
2
0
ENOX1
0.014
0.75
0.0012
Anova, p = 0.041
Low
Med
High
group

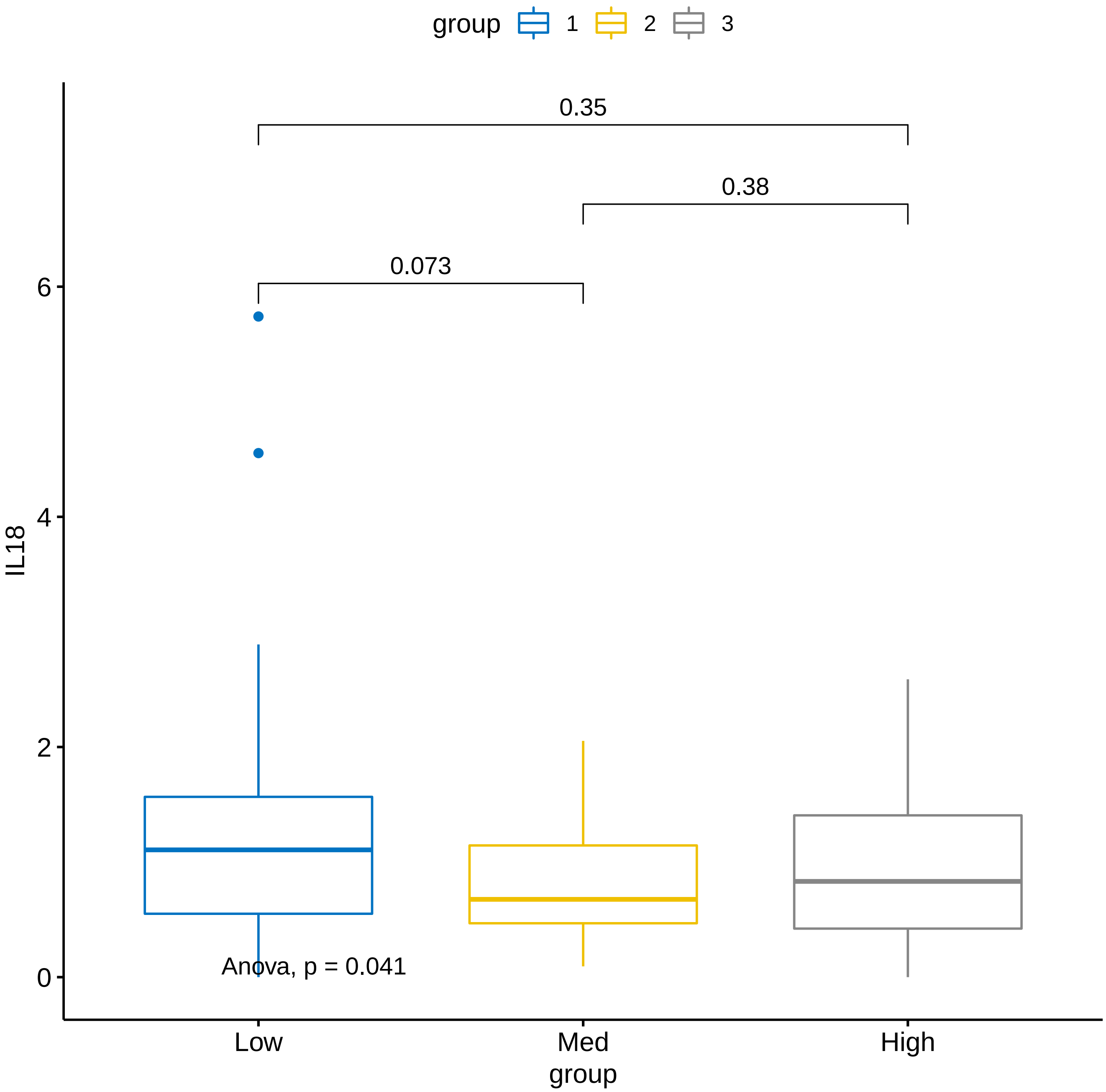

group 1 2 3
0.35
0.38
0.073
IL18
6
4
2
0
Anova, p = 0.041
Low
Med
High
group

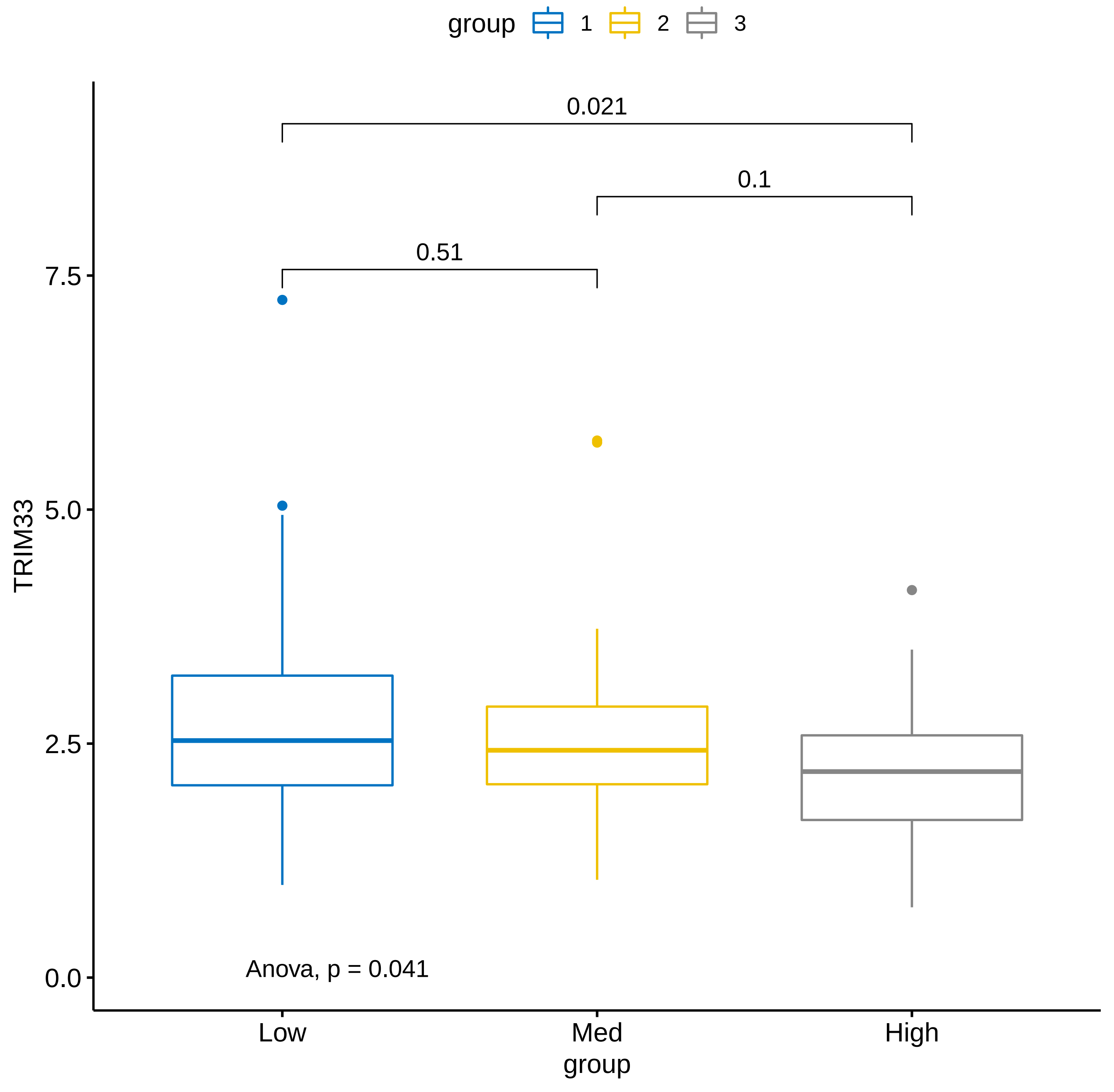
group 1 2 3
0.021
0.1
0.51
7.5
5.0
2.5
0.0
TRIM33
Anova, p = 0.041
Low
Med
High
group

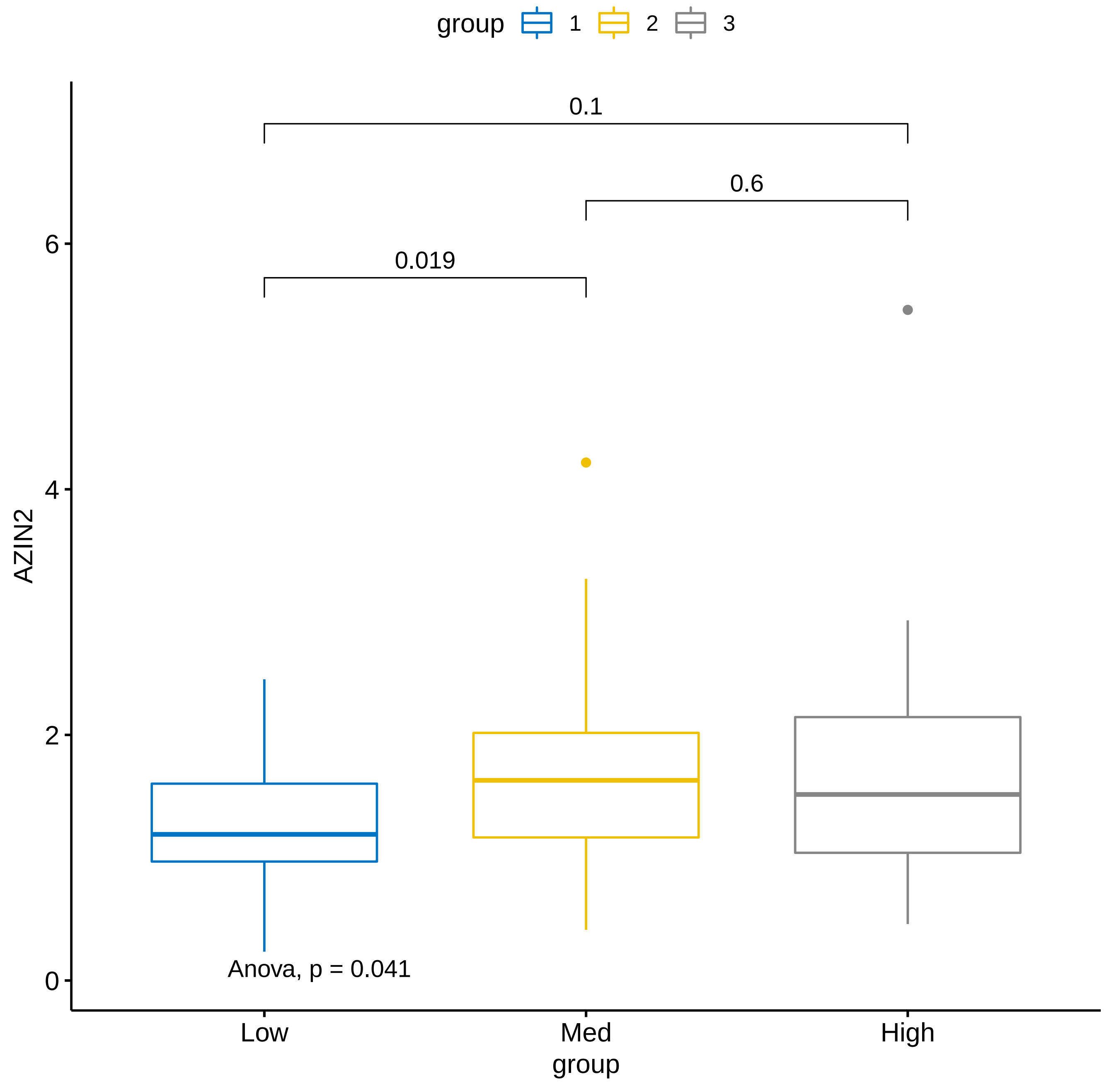
group
1
2
3
0.1
0.6
0.019
AZIN2
6
4
2
0
Anova, p = 0.041
Low
Med
High
group

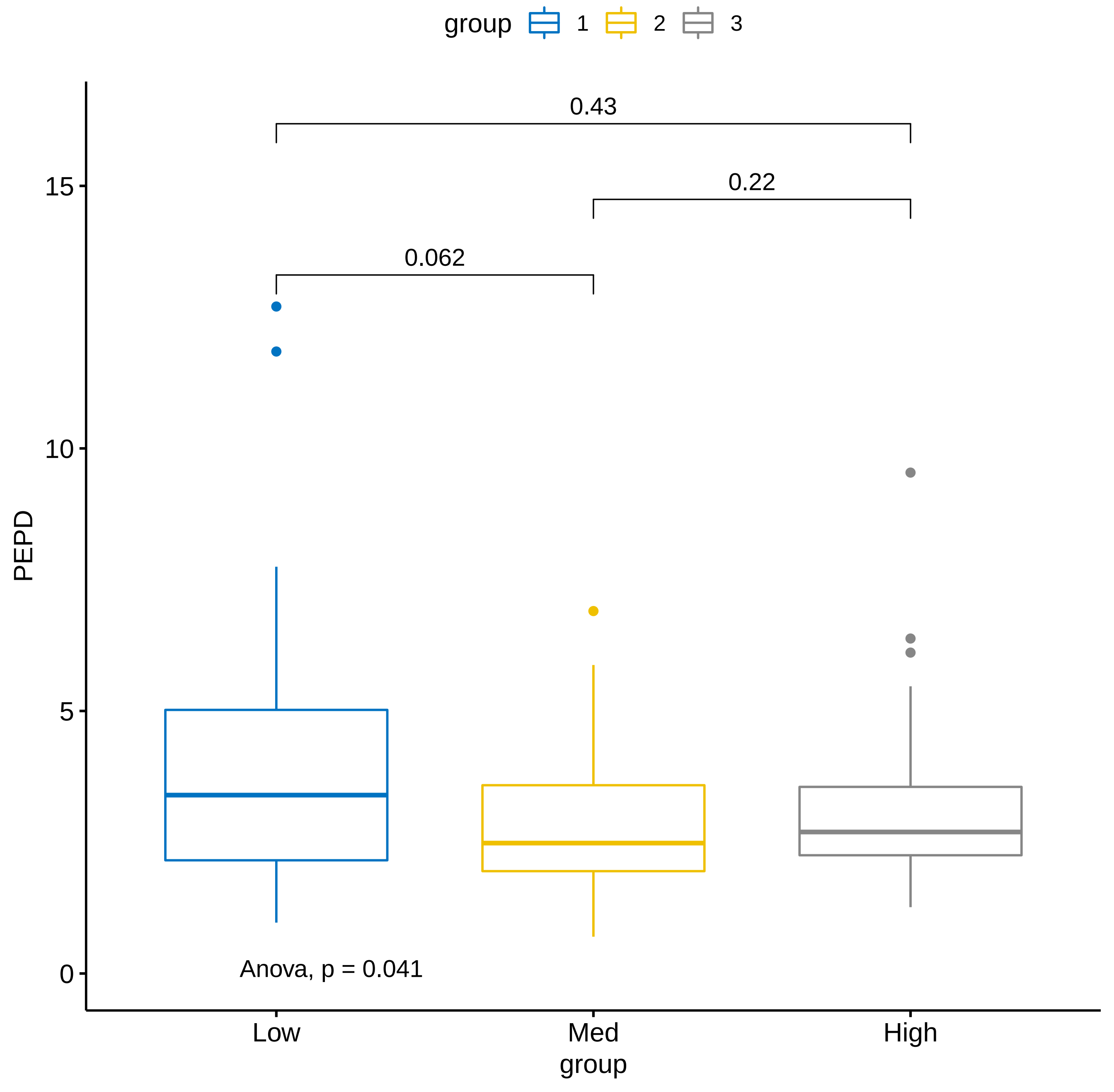
group
1
2
3
0.43
0.22
0.062
15
10
5
0
PEPD
Anova, p = 0.041
Low
Med
High
group

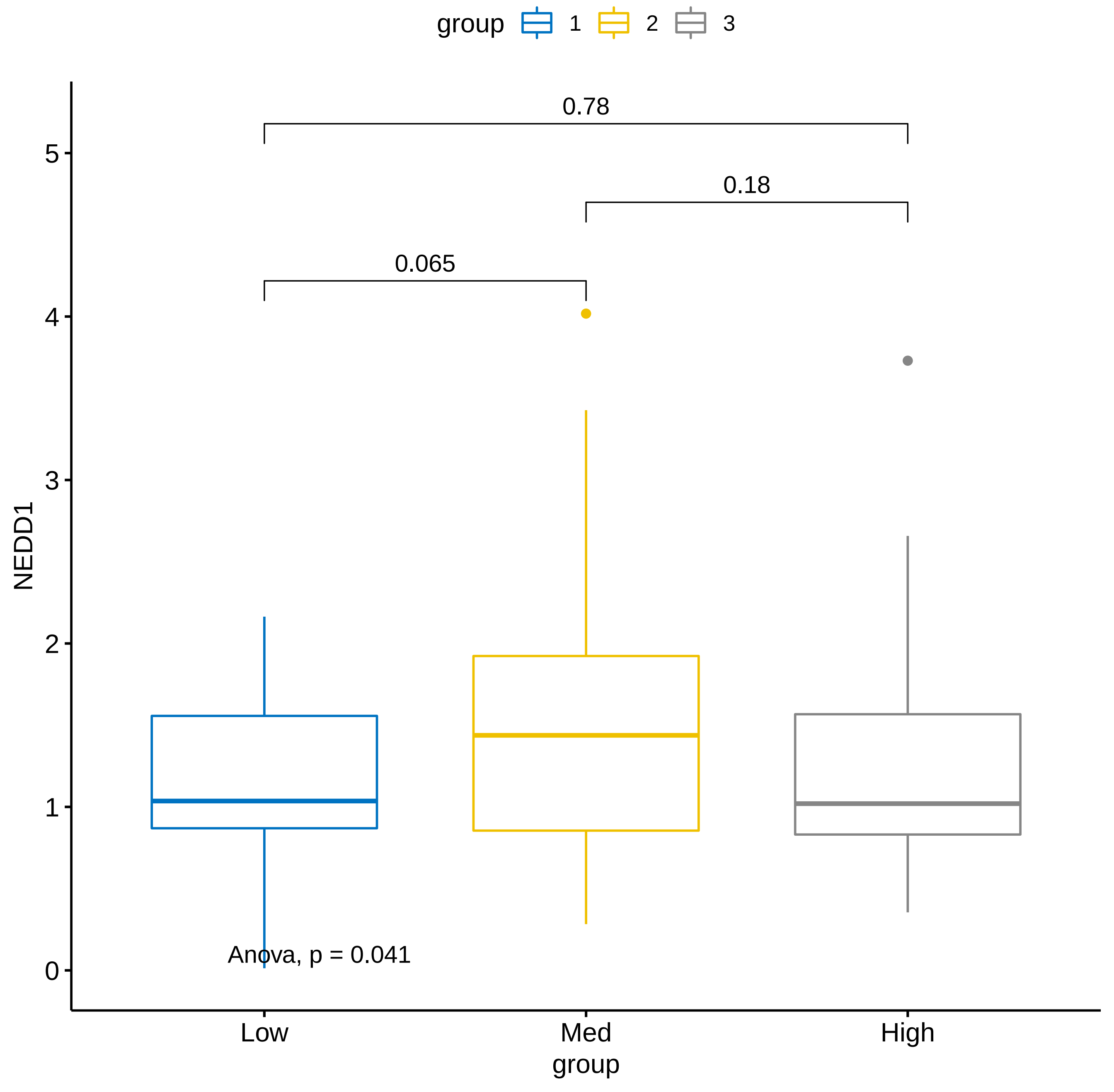

group 1 2 3
0.78
0.18
0.065
NEDD1
5
4
3
2
1
0
Anova, p = 0.041
Low
Med
High
group

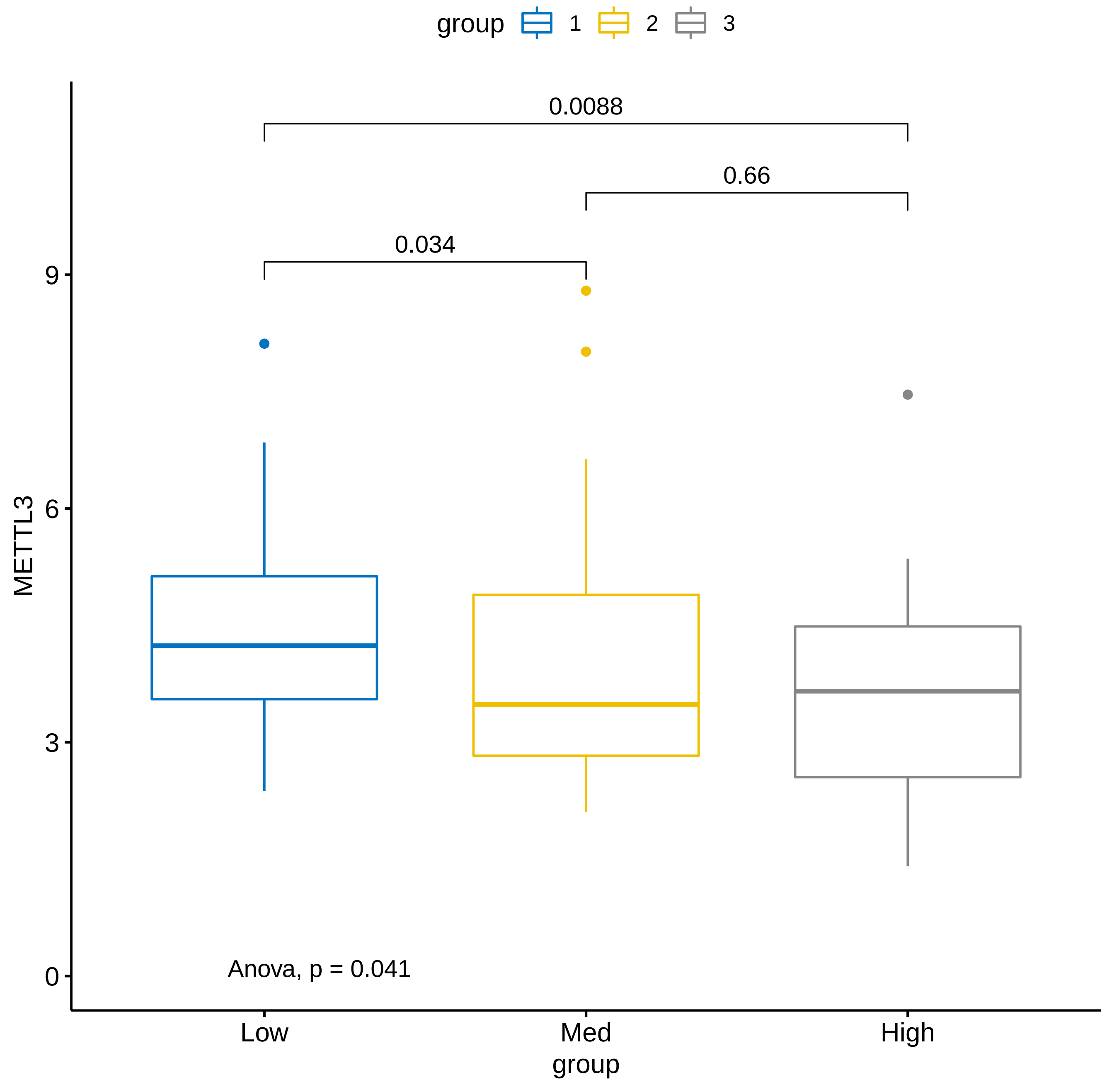

group
1
2
3
0.0088
0.66
0.034
METTL3
9
6
3
0
Anova, p = 0.041
Low
Med
High
group

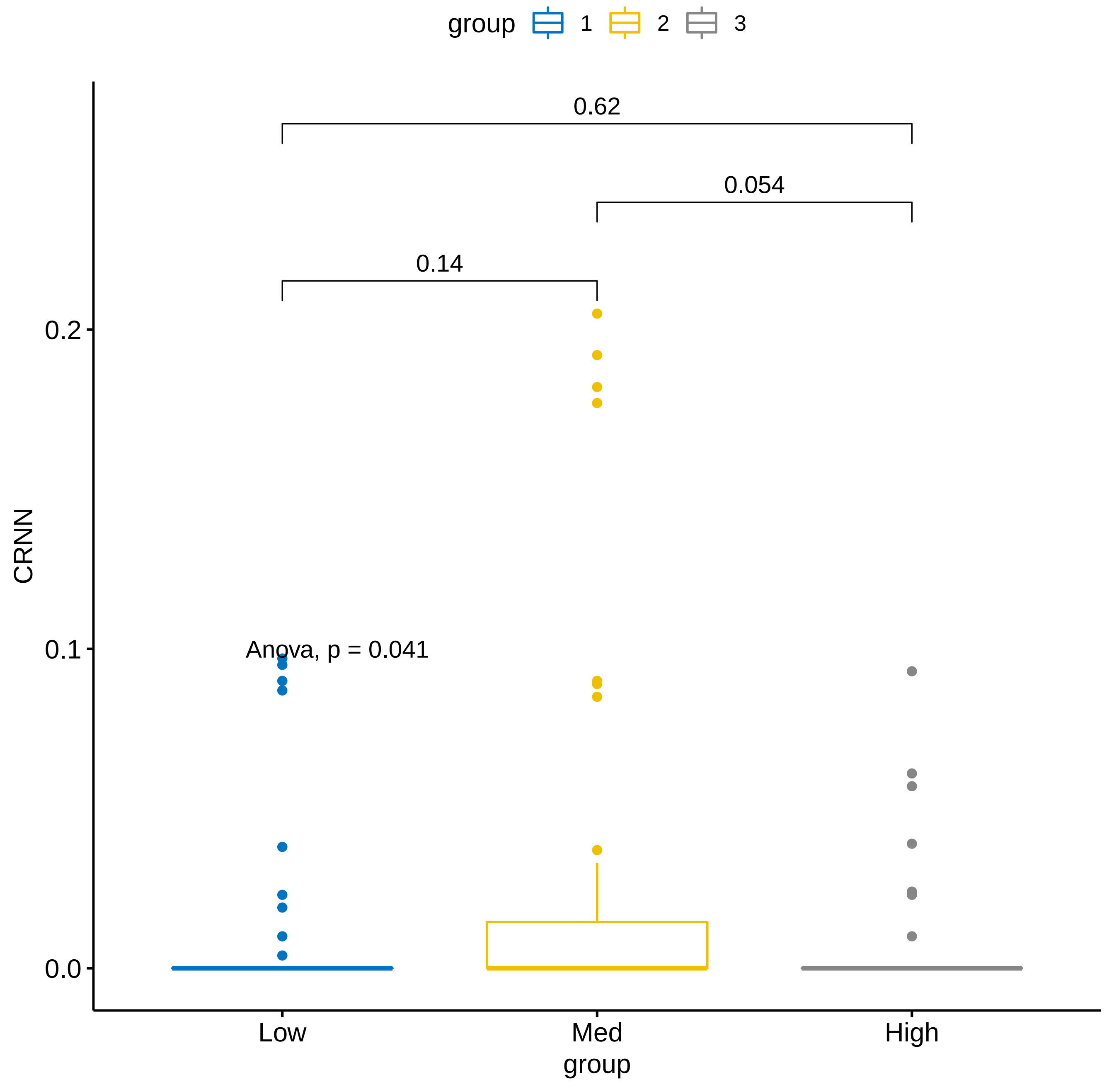

group 1 2 3
0.62
0.054
0.14
0.2
0.1
0.0
CRNN
Anova, p = 0.041
Low
Med
High
group

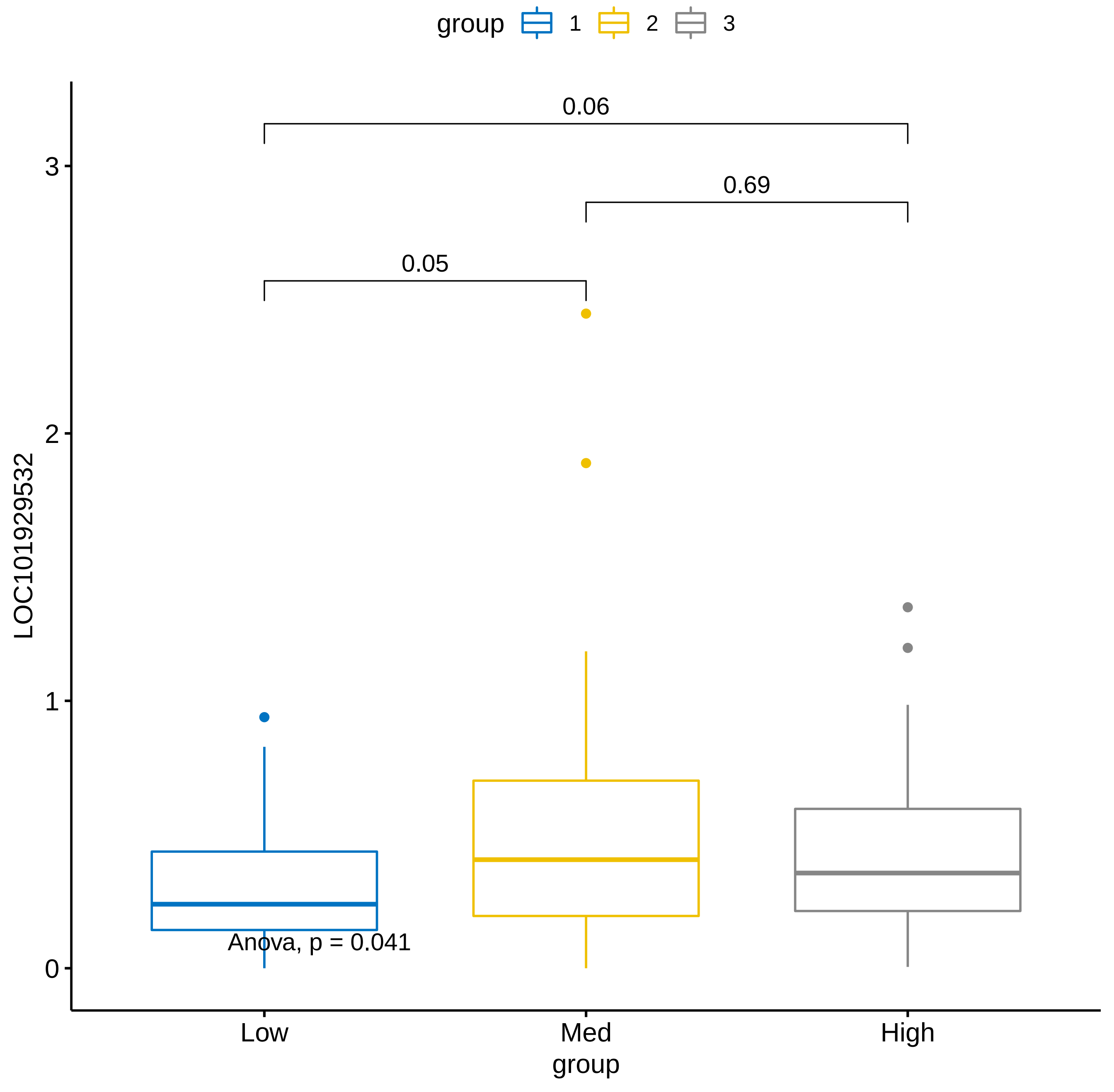

group 1 2 3
0.06
0.69
0.05
3
2
1
0
LOC101929532
Anova, p = 0.041
Low
Med
High
group

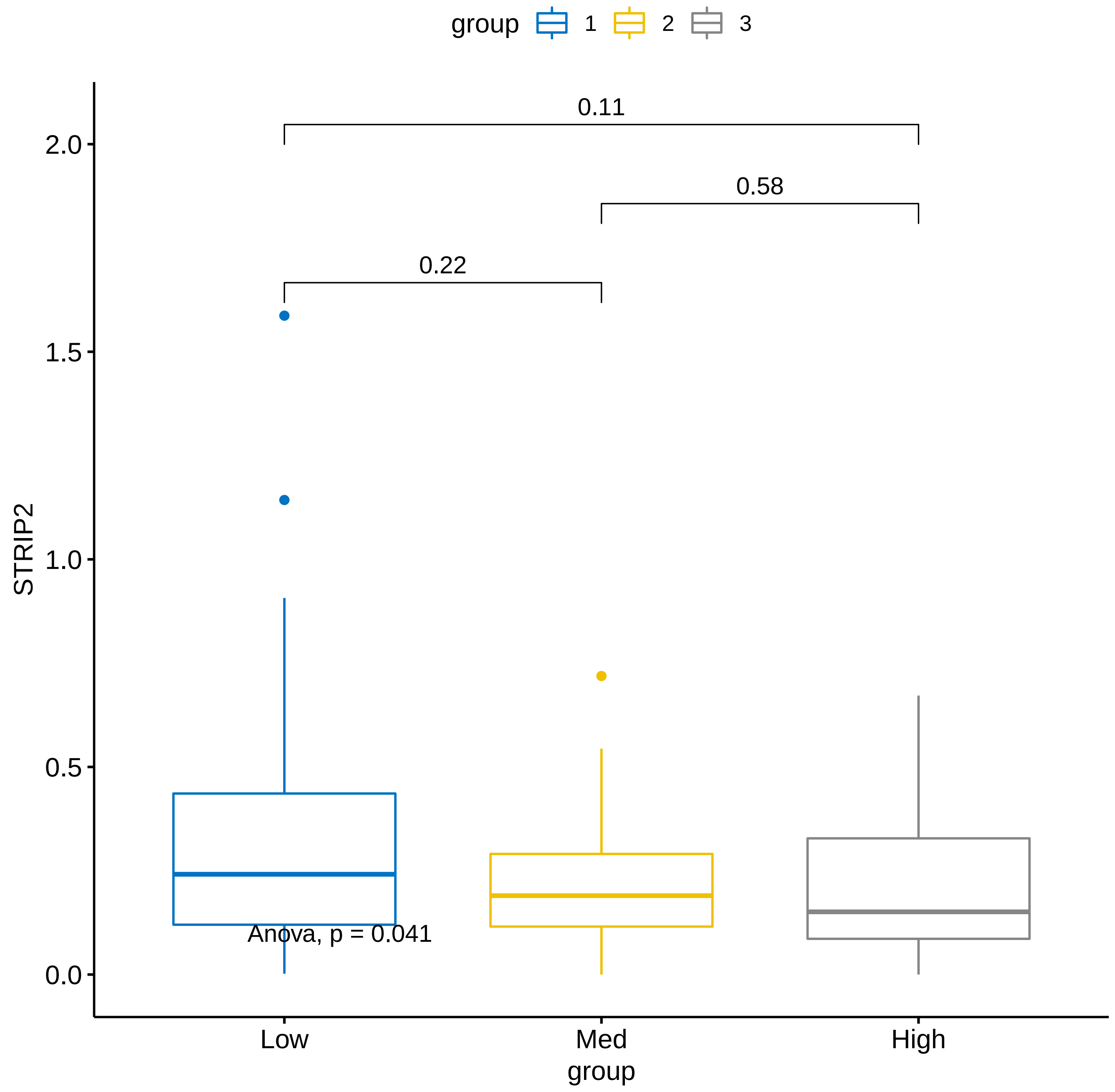
group
1
2
3
0.11
0.58
0.22
2.0
1.5
1.0
0.5
0.0
STRIP2
Anova, p = 0.041
Low
Med
High
group

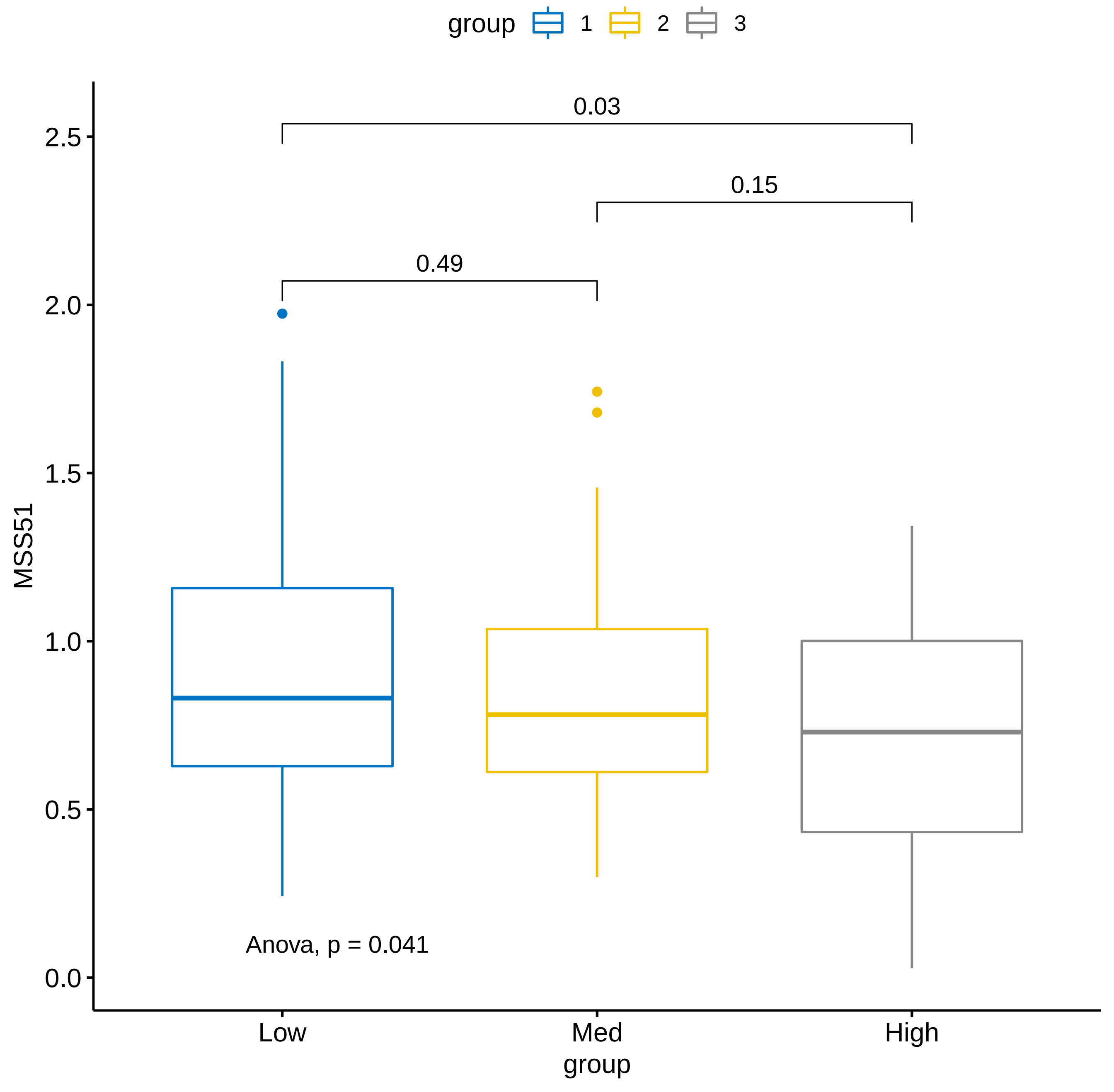
group
1
2
3
0.03
0.15
0.49
2.5
2.0
1.5
1.0
0.5
0.0
MSS51
Anova, p = 0.041
Low
Med
High
group

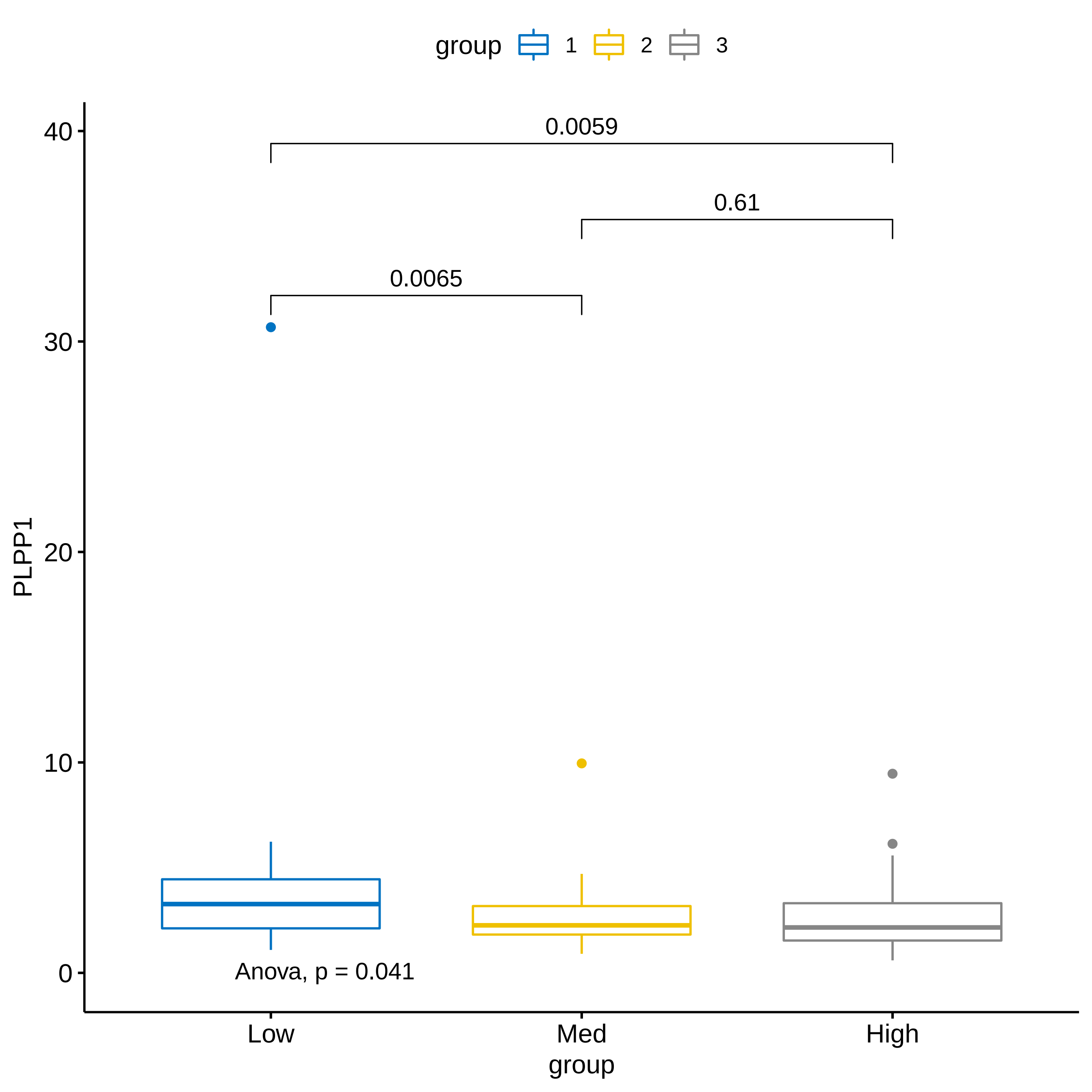
group
1
2
3
0.0059
0.61
0.0065
40
30
20
10
0
PLPP1
Anova, p = 0.041
Low
Med
High
group

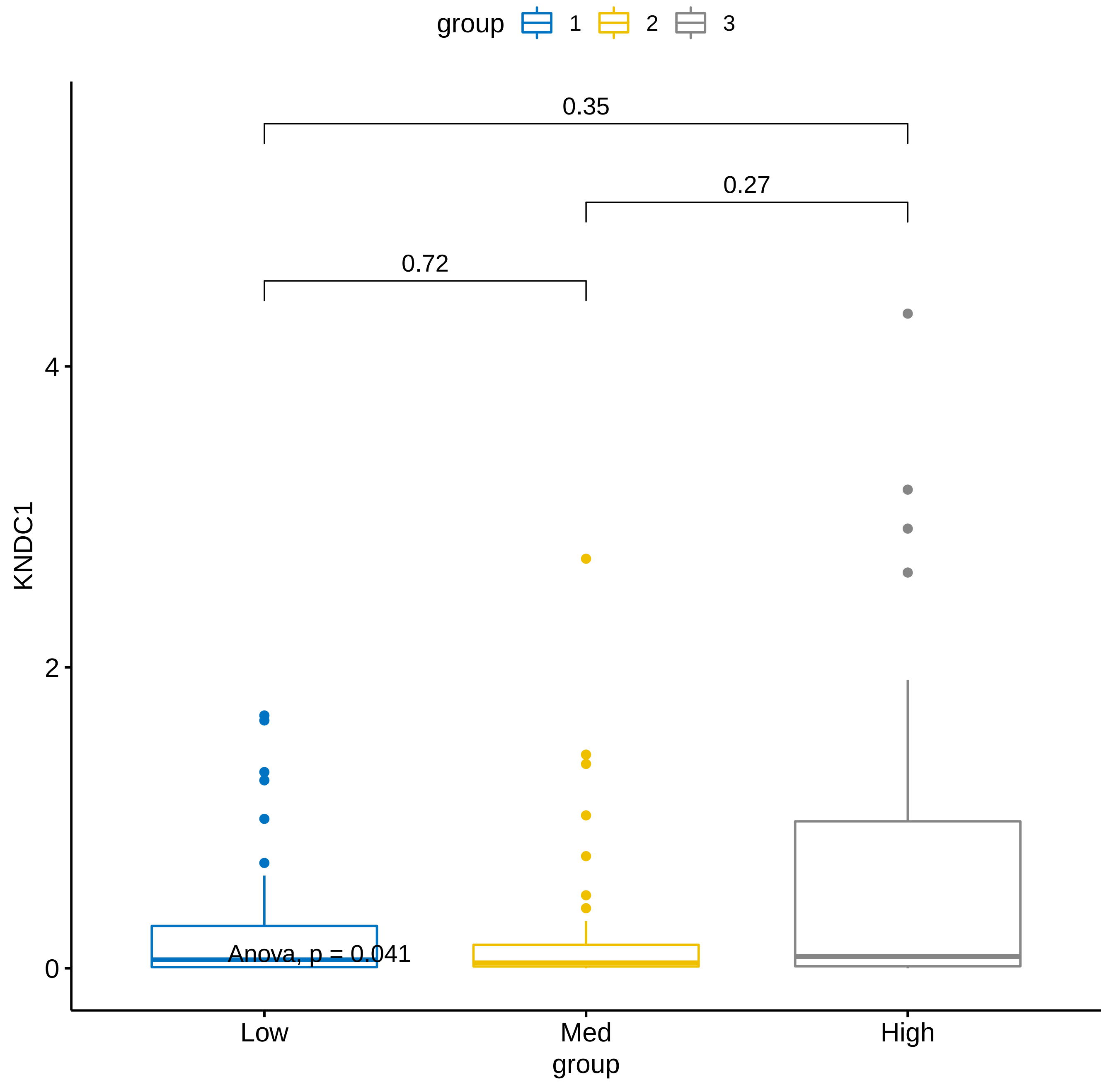

group 1 2 3
0.35
0.27
0.72
KNDC1
4
2
0
Anova, p = 0.041
Low
Med
High
group

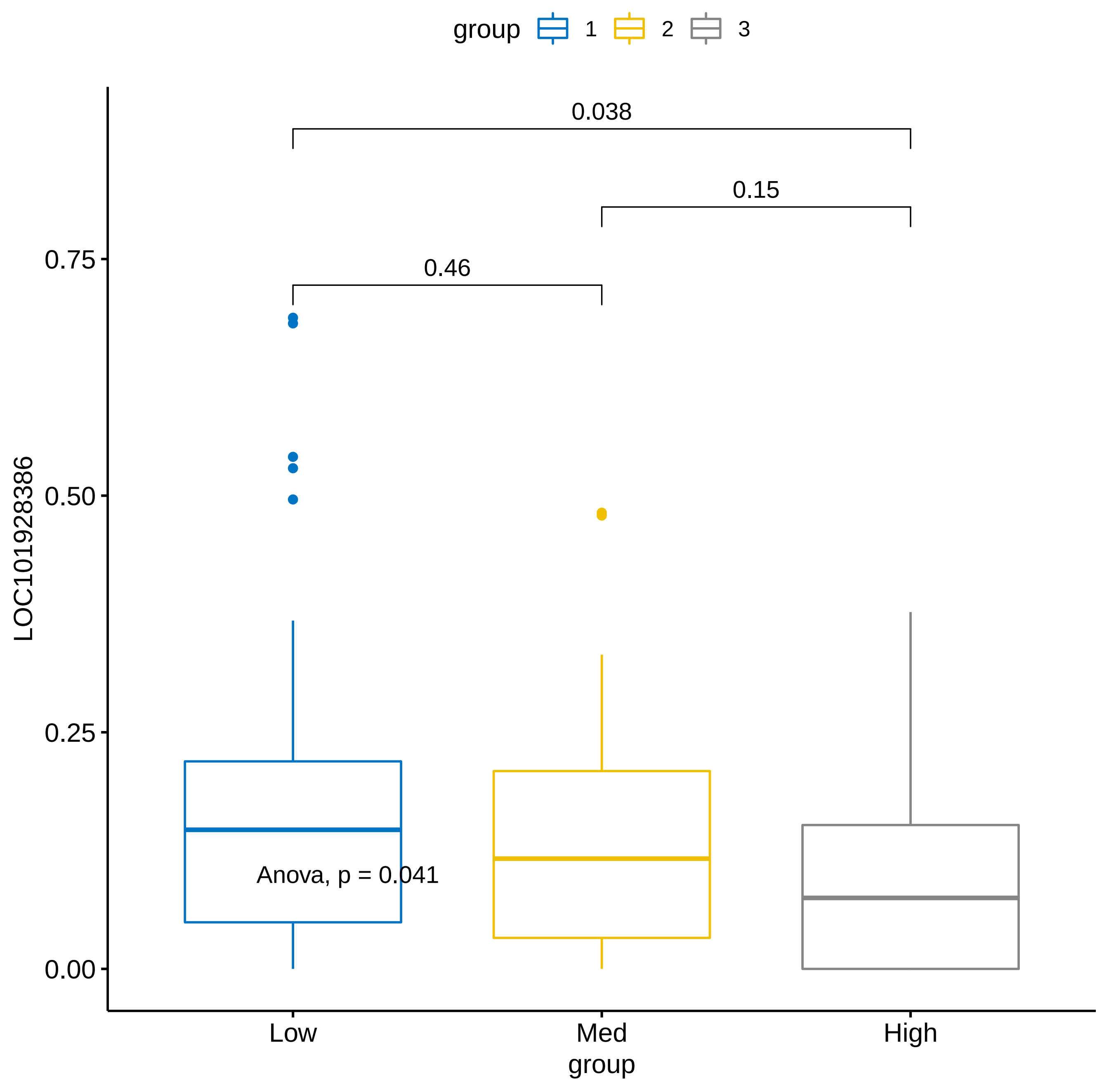
group
1
2
3
0.038
0.15
0.46
0.75
0.50
0.25
0.00
LOC101928386
Anova, p = 0.041
Low
Med
High
group

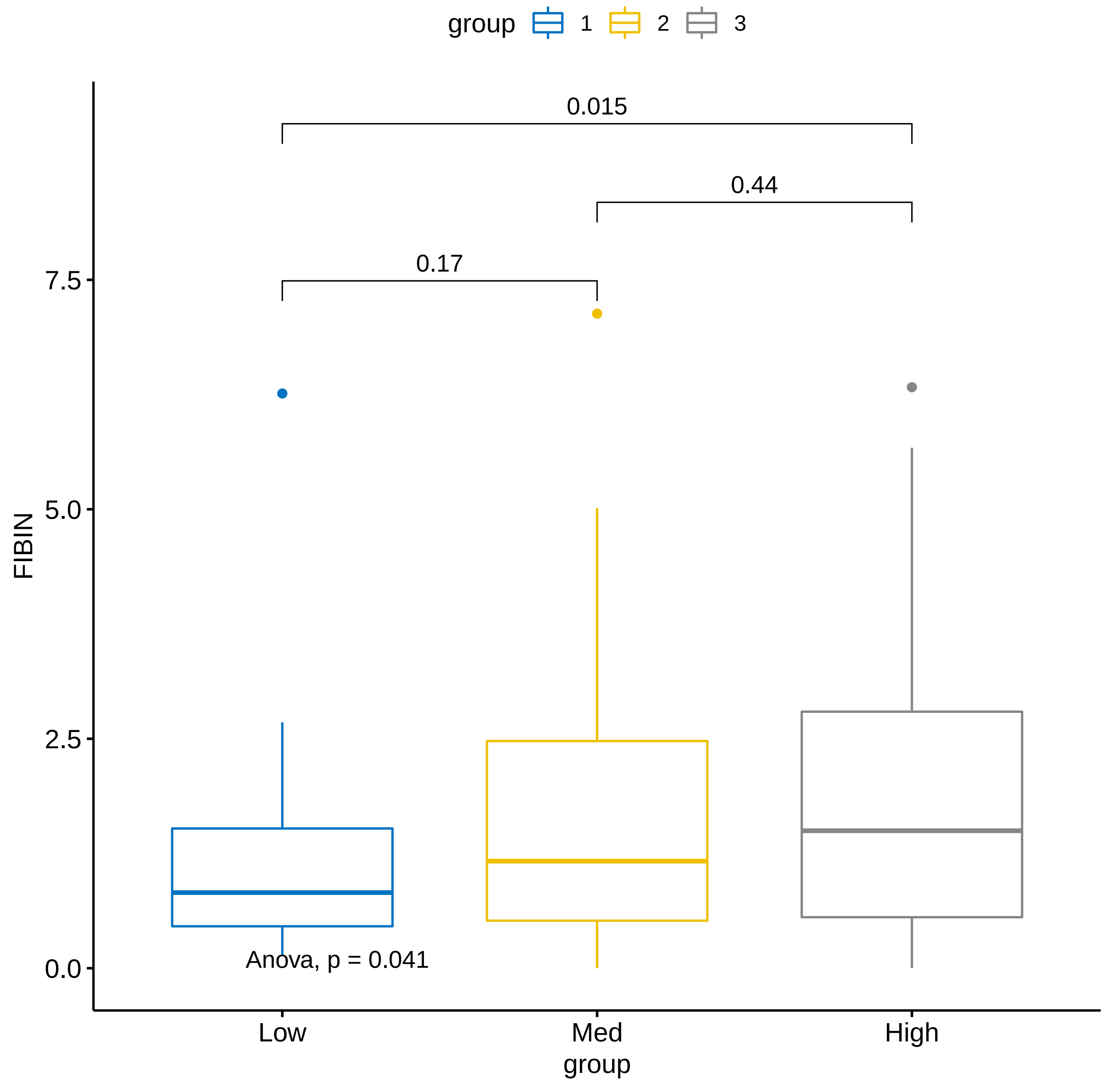

group 1 2 3
0.015
0.44
0.17
7.5
5.0
2.5
0.0
FIBIN
Anova, p = 0.041
Low
Med
High
group

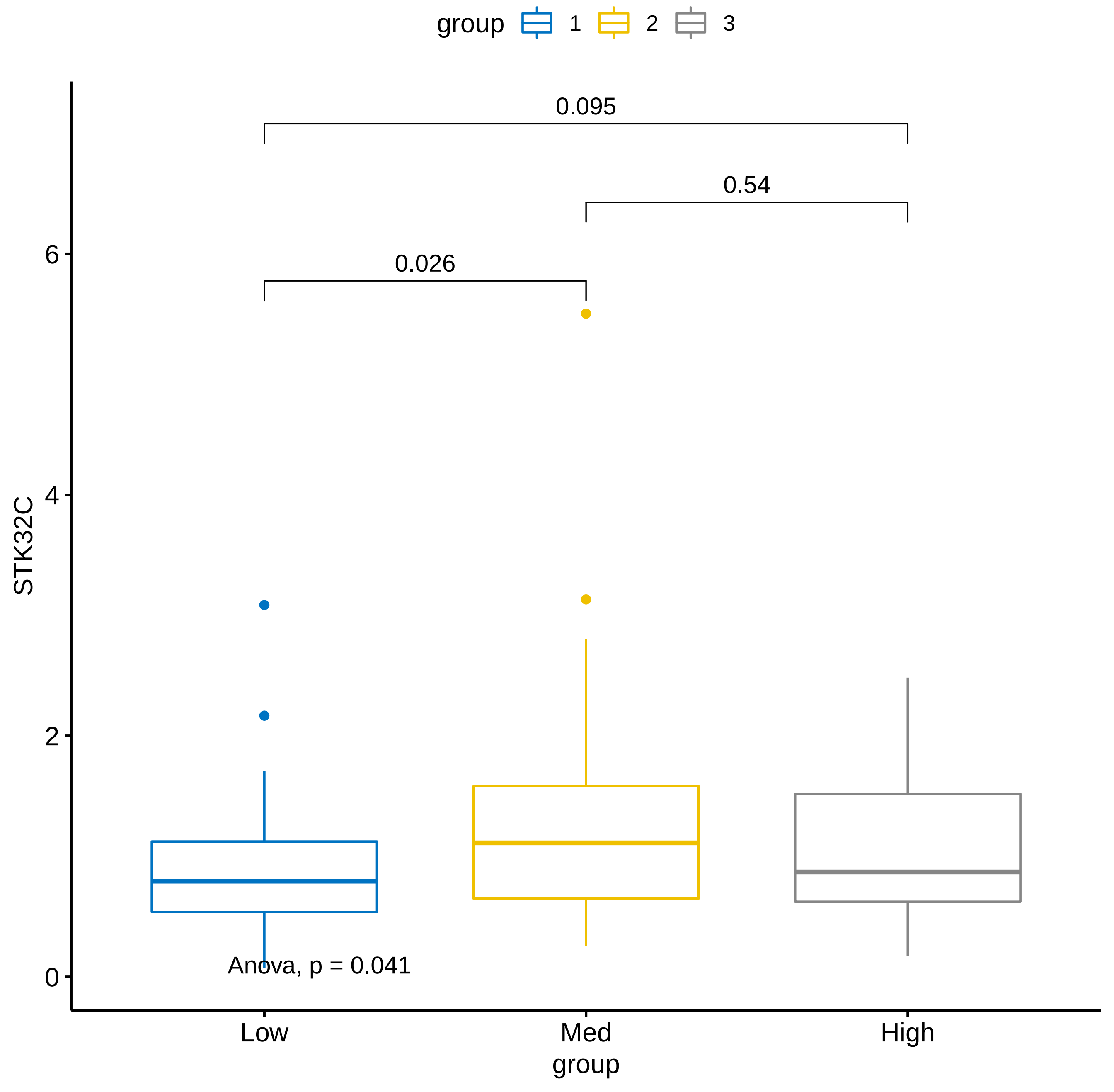
group
1
2
3
0.095
0.54
0.026
6
4
2
0
STK32C
Anova, p = 0.041
Low
Med
High
group

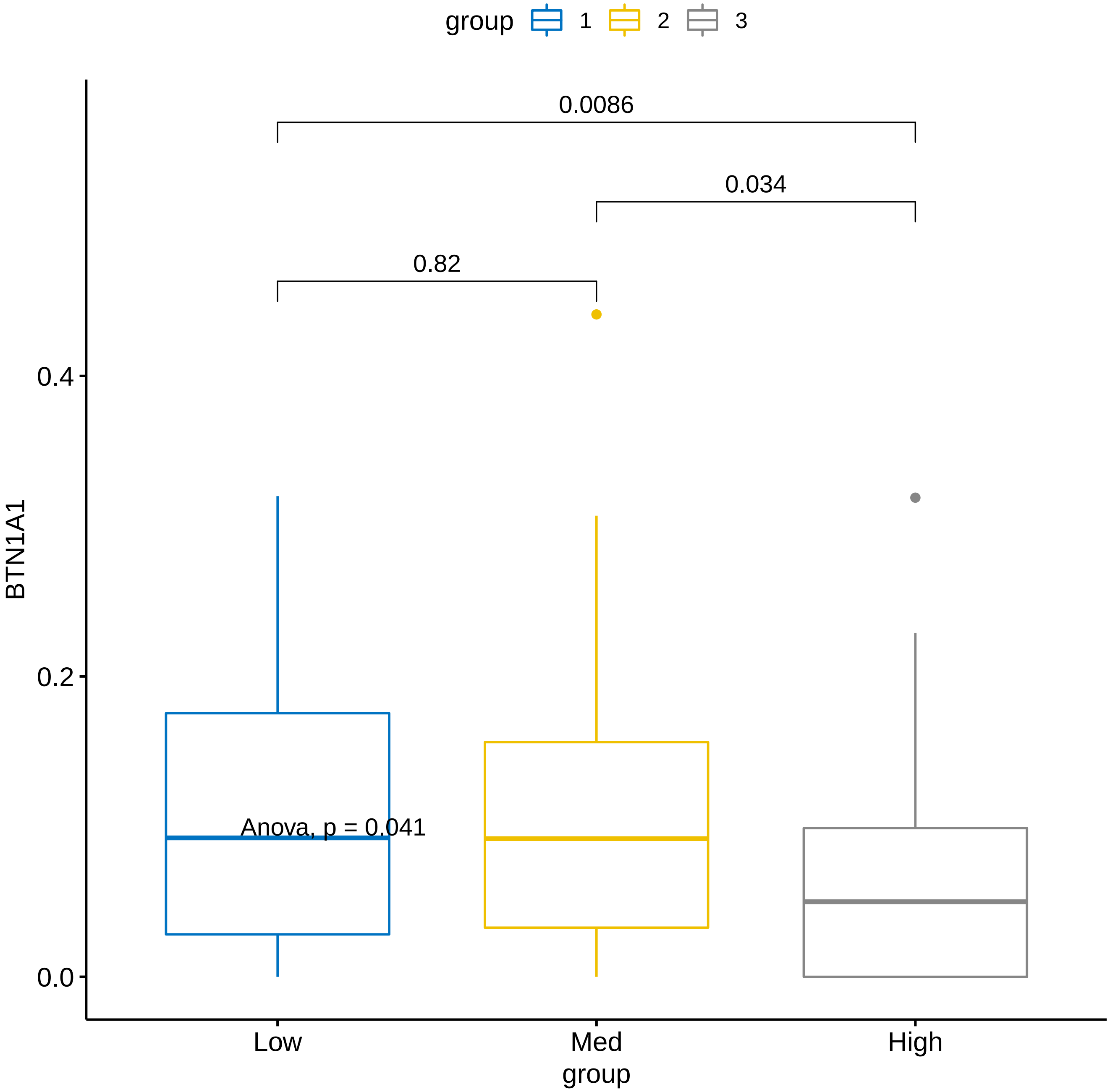
group
1
2
3
0.0086
0.034
0.82
Anova, p = 0.041
BTN1A1
0.4
0.2
0.0
Low
Med
High
group

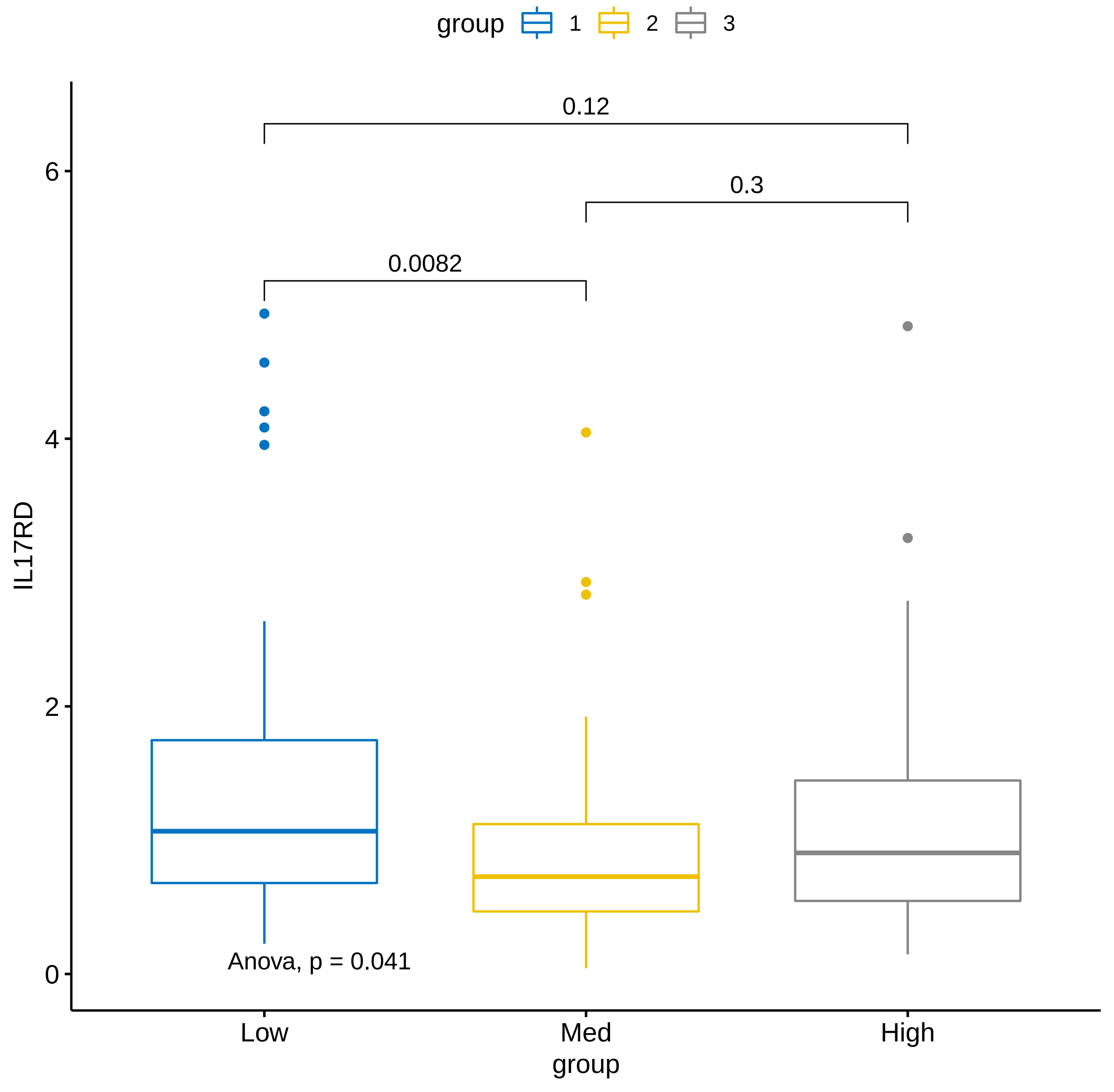
group
1
2
3
0.12
0.3
0.0082
6
4
2
0
IL17RD
Anova, p = 0.041
Low
Med
High
group

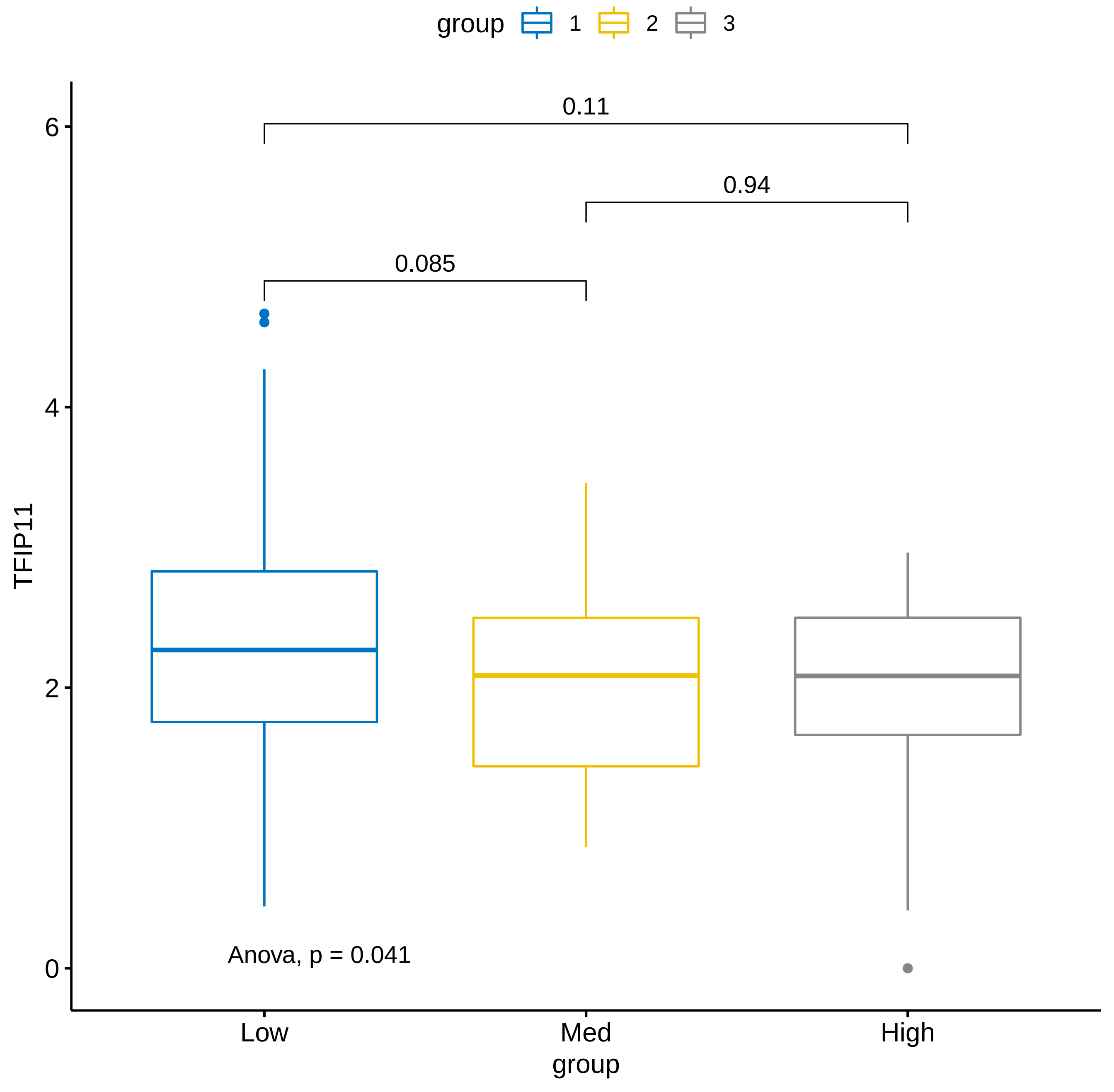

group 1 2 3
0.11
0.94
0.085
6
4
2
0
TFIP11
Anova, p = 0.041
Low
Med
High
group

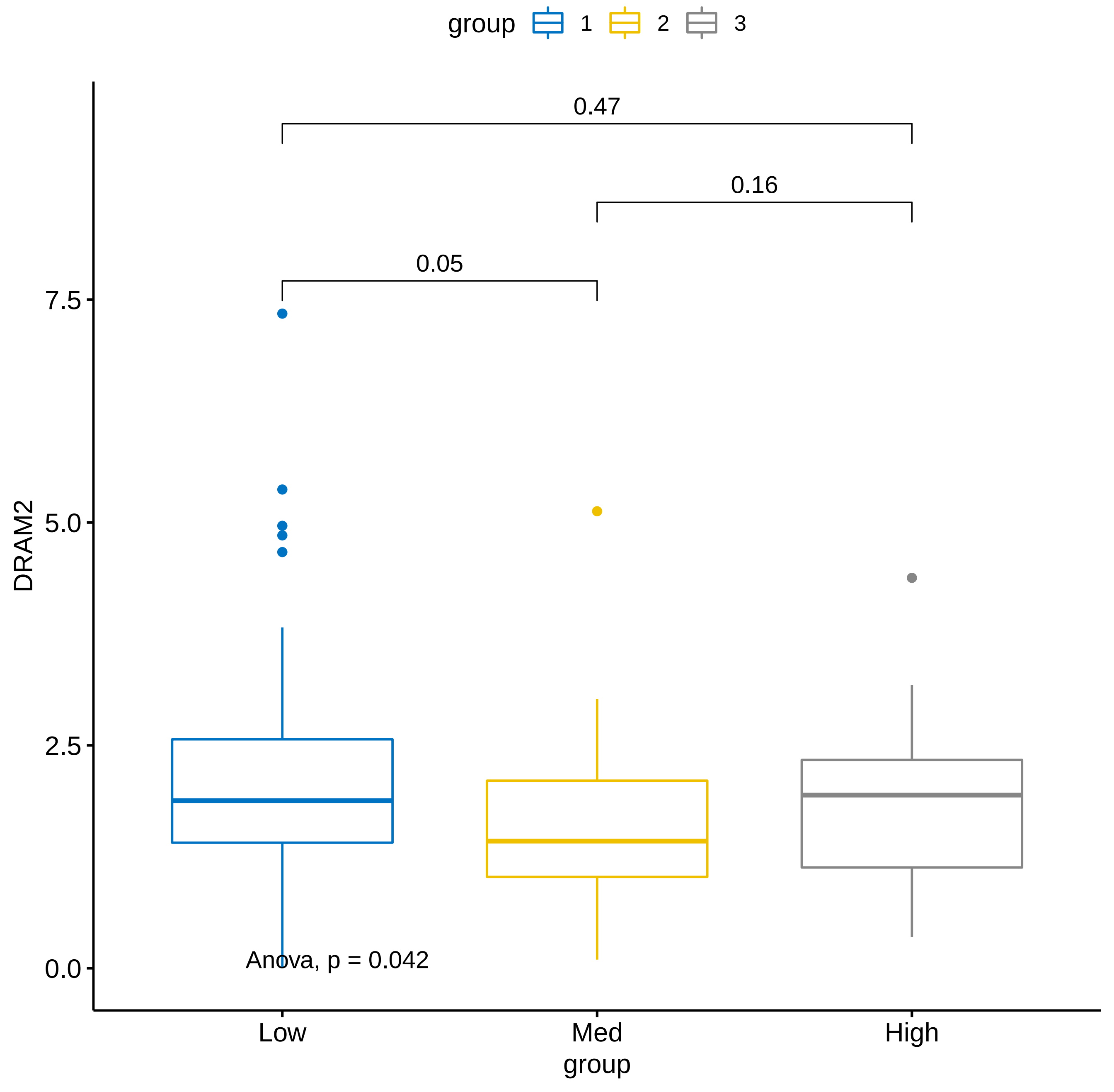
group
1
2
3
0.47
0.16
0.05
7.5
5.0
2.5
0.0
DRAM2
Anova, p = 0.042
Low
Med
High
group

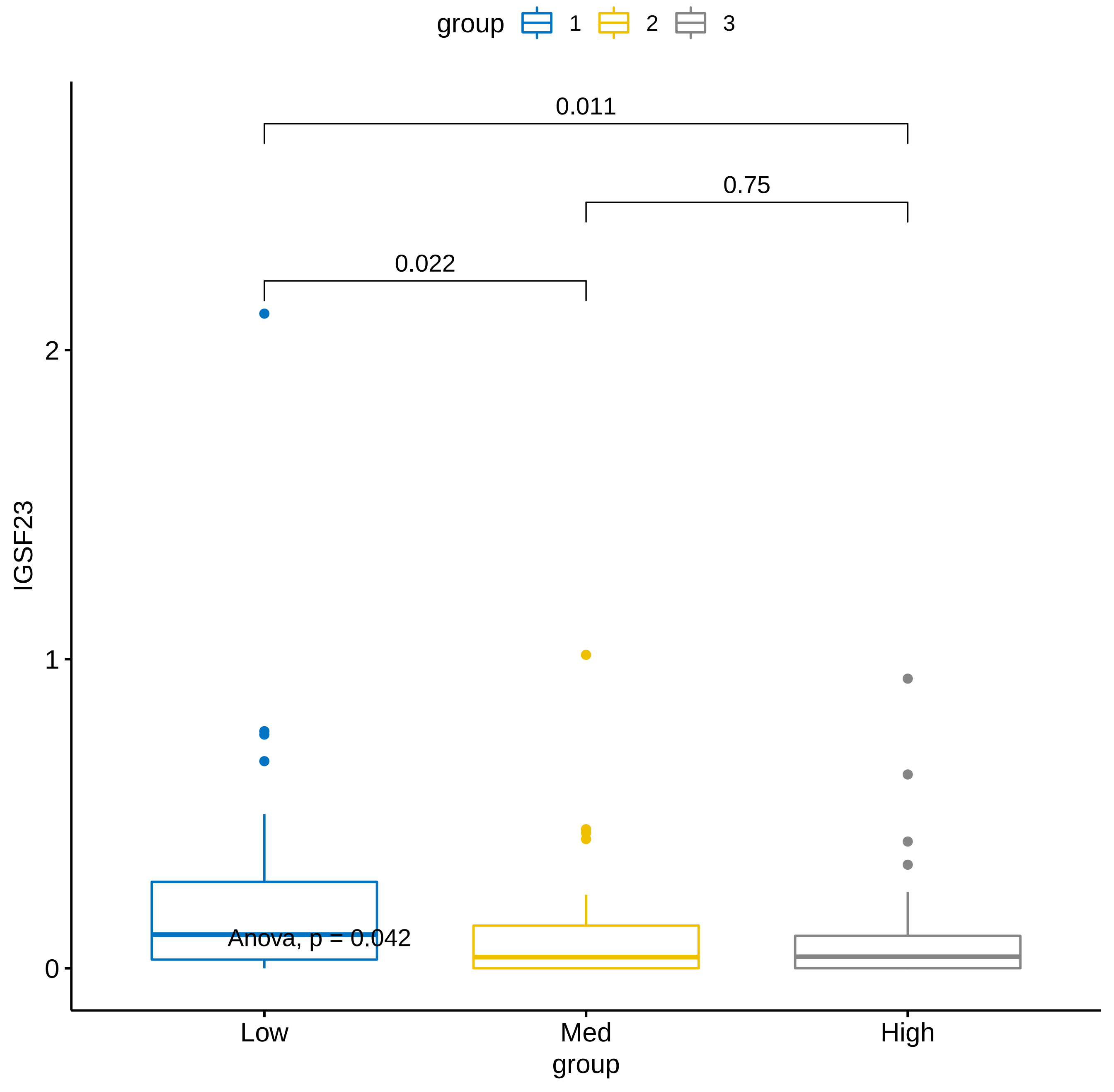
group
1
2
3
0.011
0.75
0.022
IGSF23
2
1
0
Anova, p = 0.042
Low
Med
High
group

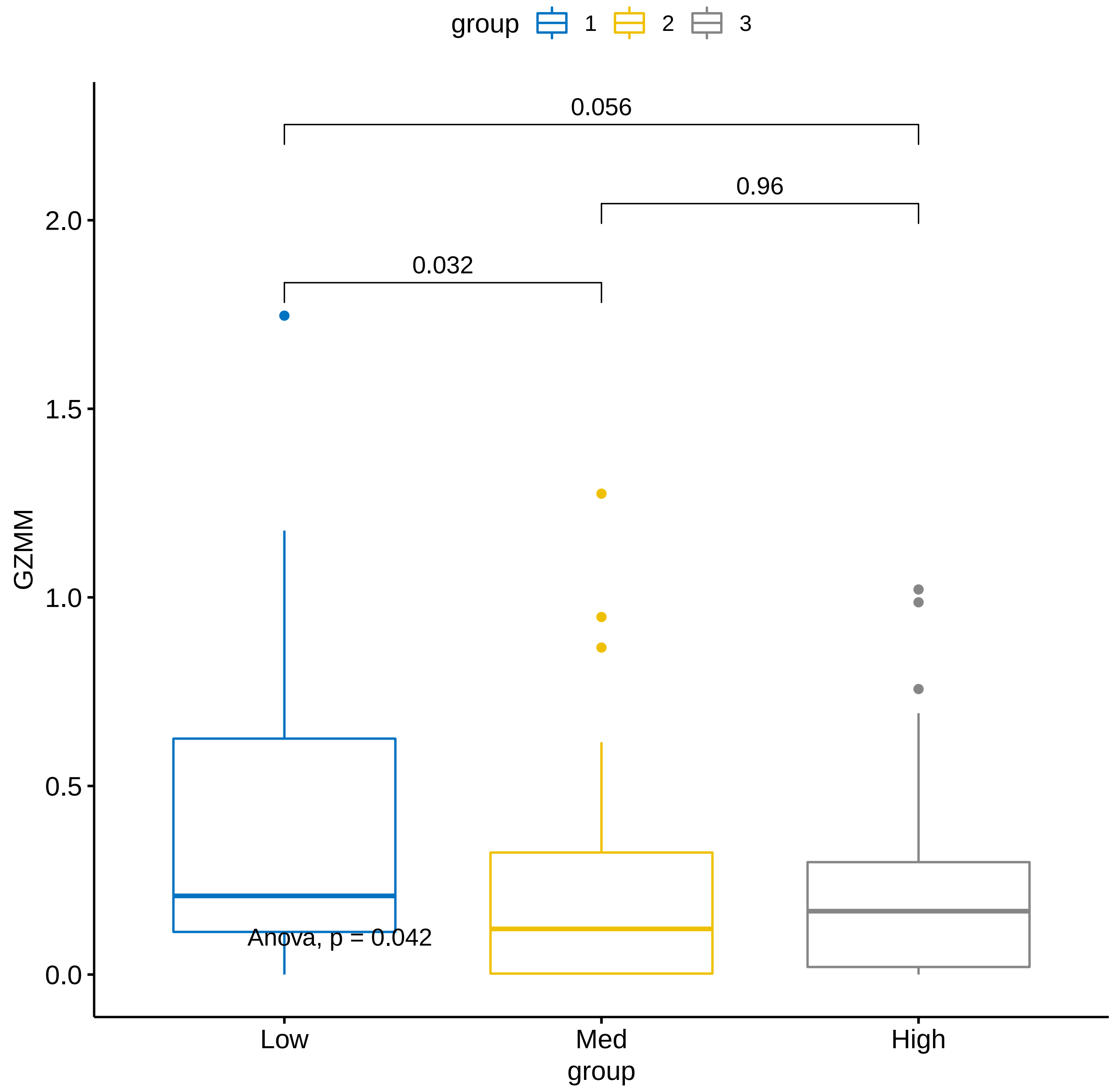

group 1 2 3
0.056
0.96
0.032
2.0
1.5
1.0
0.5
0.0
GZMM
Anova, p = 0.042
Low
Med
High
group

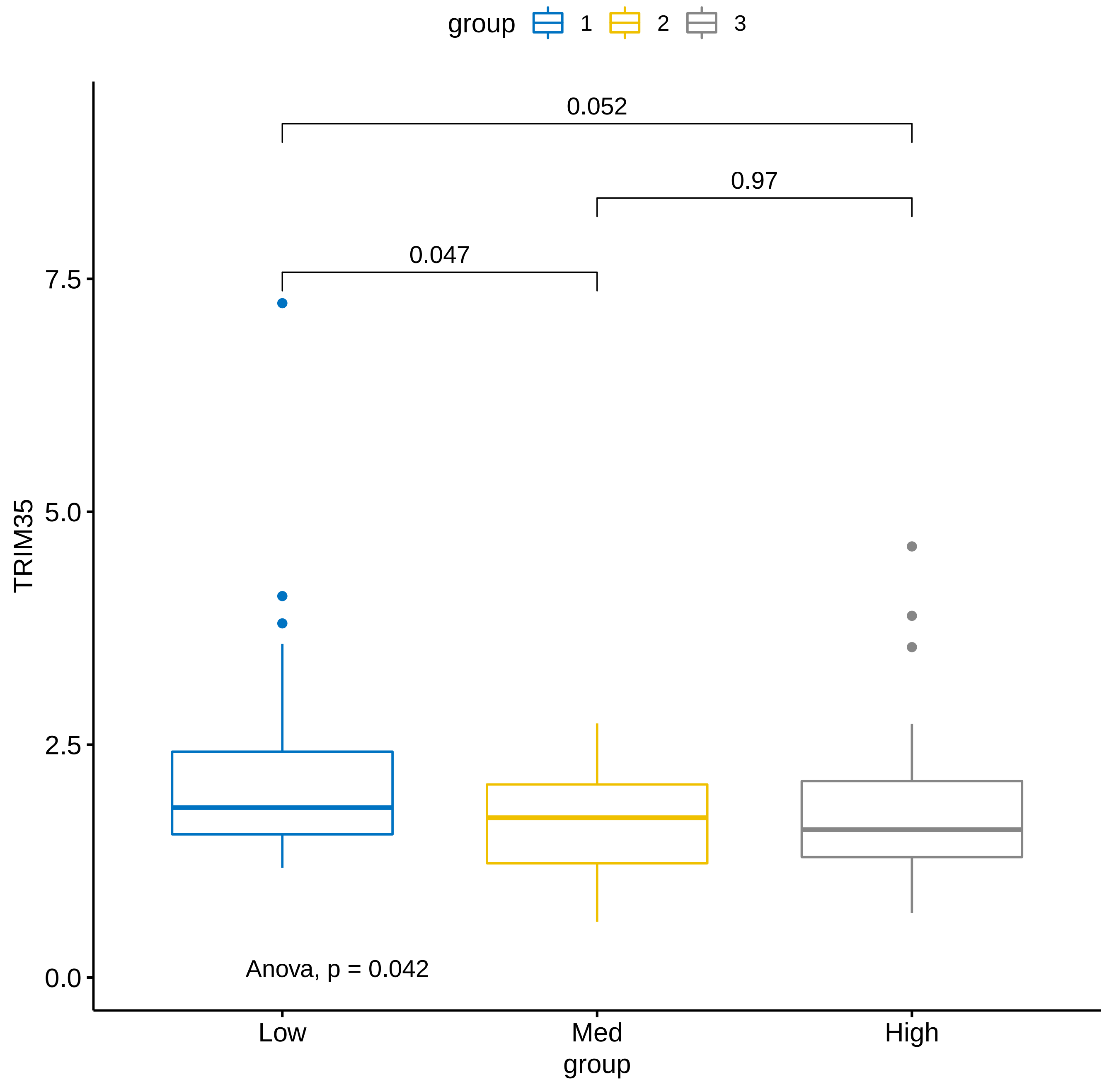
group 1 2 3
0.052
0.97
0.047
TRIM35
7.5
5.0
2.5
0.0
Anova, p = 0.042
Low
Med
High
group

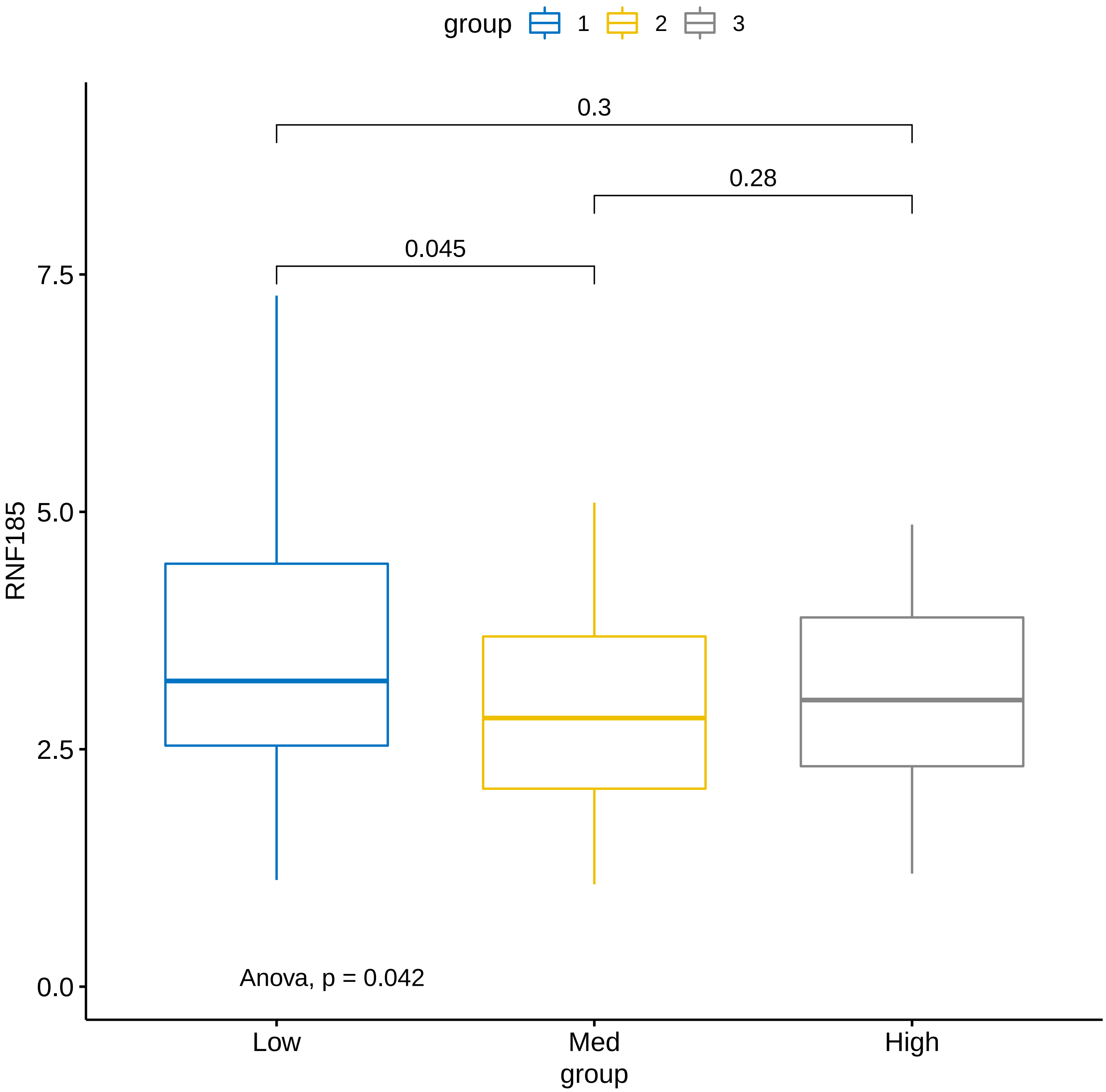
group
1
2
3
0.3
0.28
0.045
7.5
5.0
2.5
0.0
RNF185
Anova, p = 0.042
Low
Med
High
group

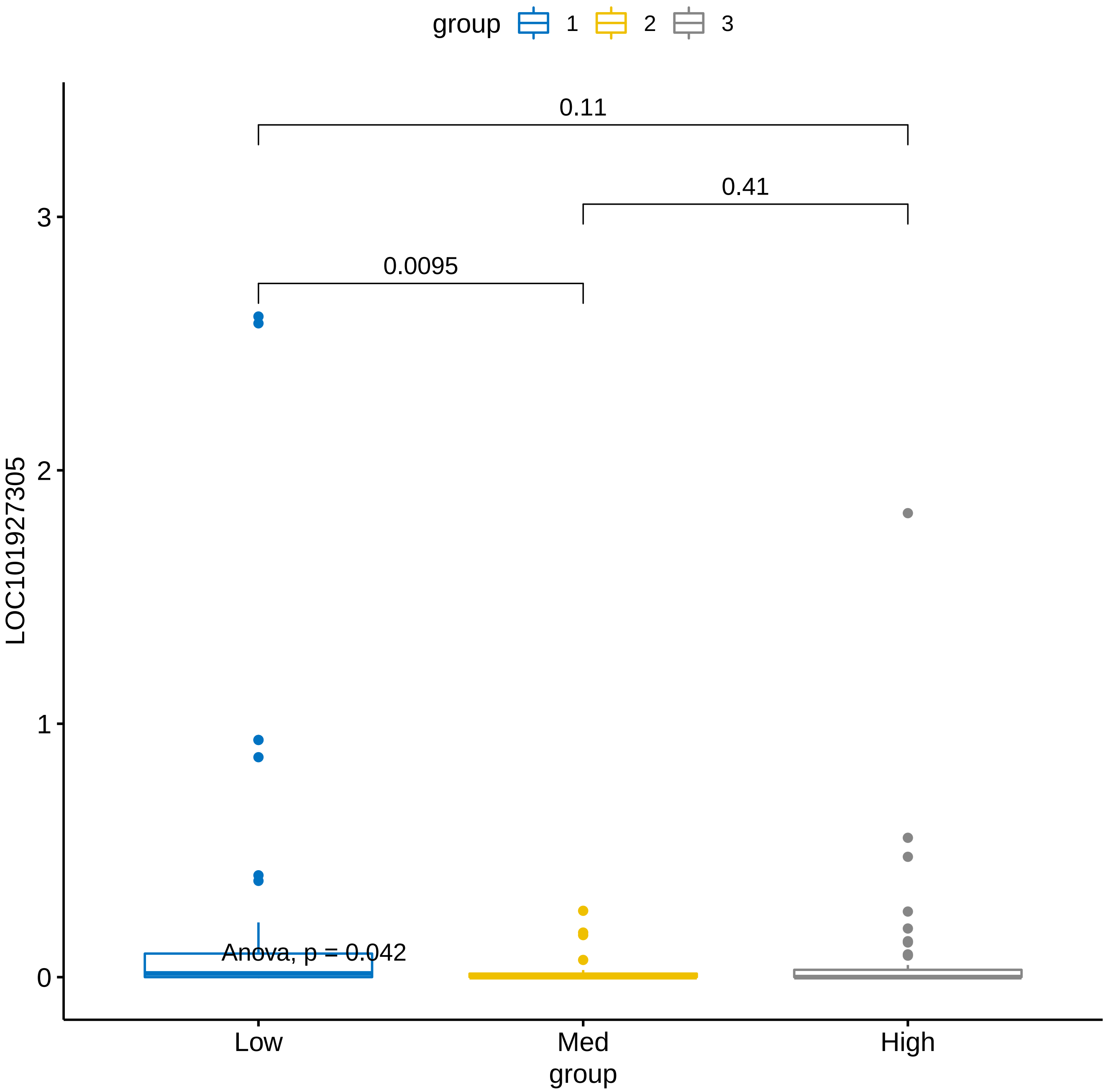

group 1 2 3
0.11
0.41
0.0095
Anova, p = 0.042
LOC101927305
0
1
2
3
Low
Med
High
group

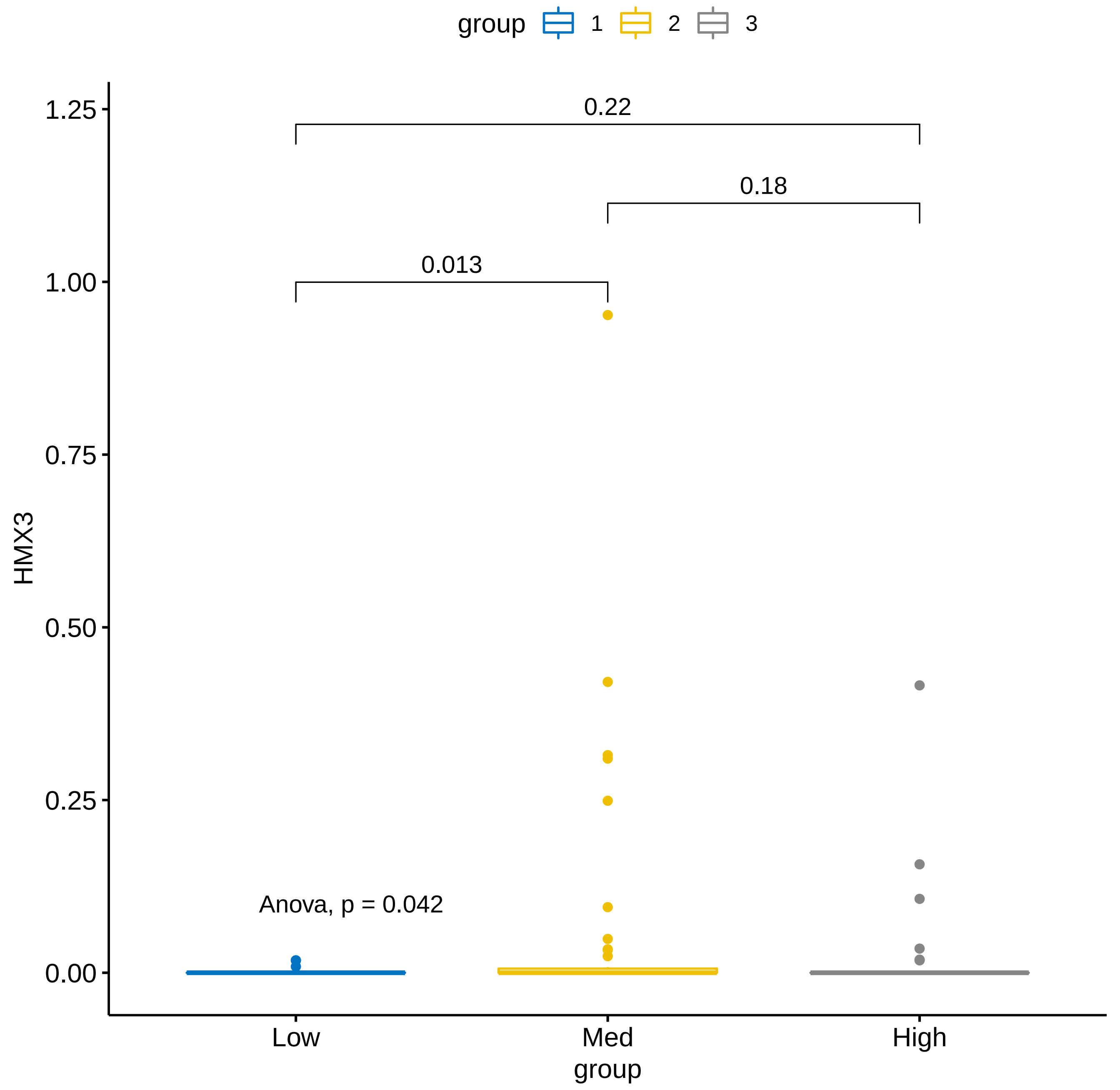

group 1 2 3
0.22
0.18
0.013
1.25
1.00
0.75
0.50
0.25
0.00
HMX3
Anova, p = 0.042
Low
Med
High
group

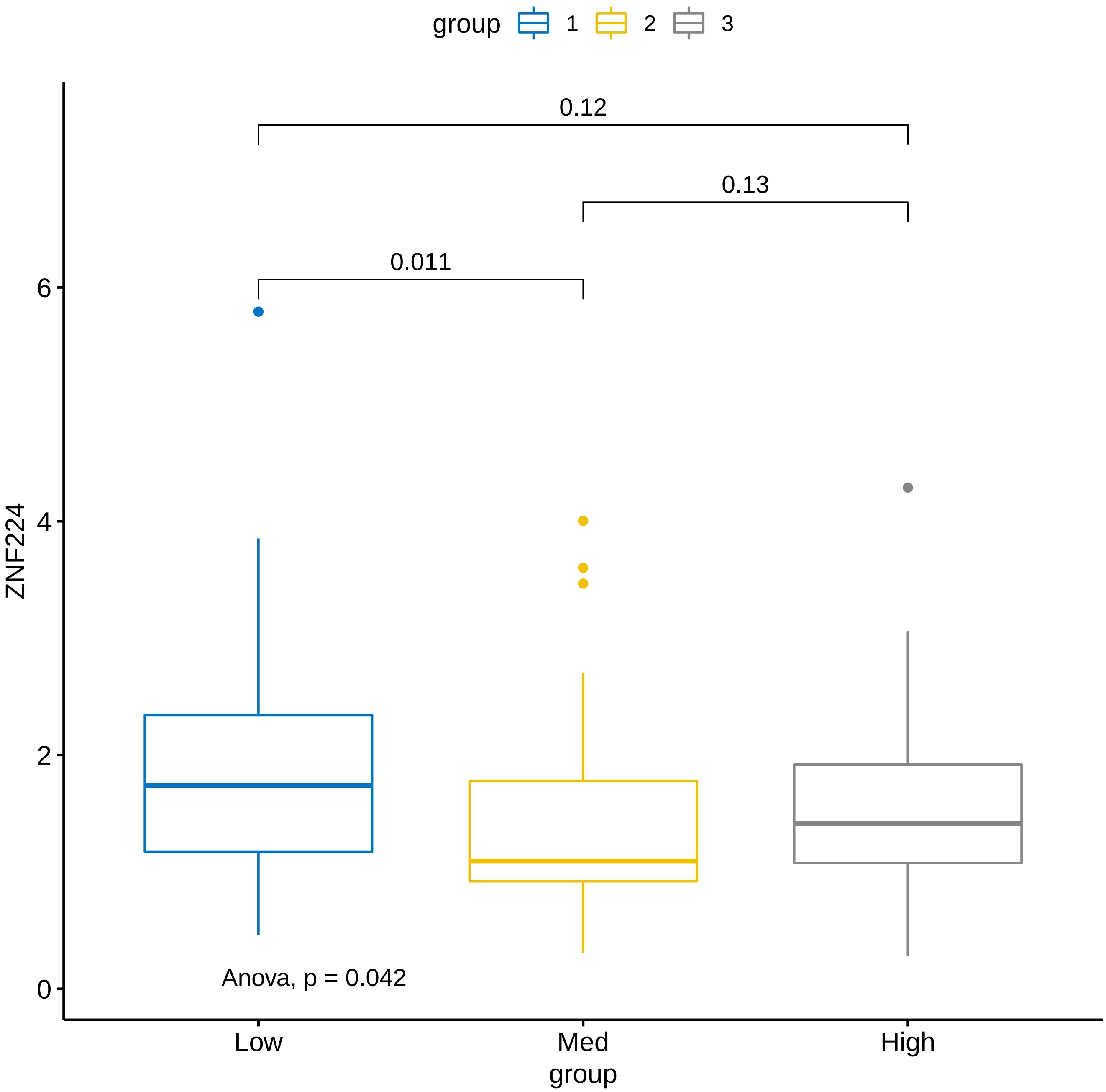

group
1
2
3
0.12
0.13
0.011
6
4
2
0
ZNF224
Anova, p = 0.042
Low
Med
High
group

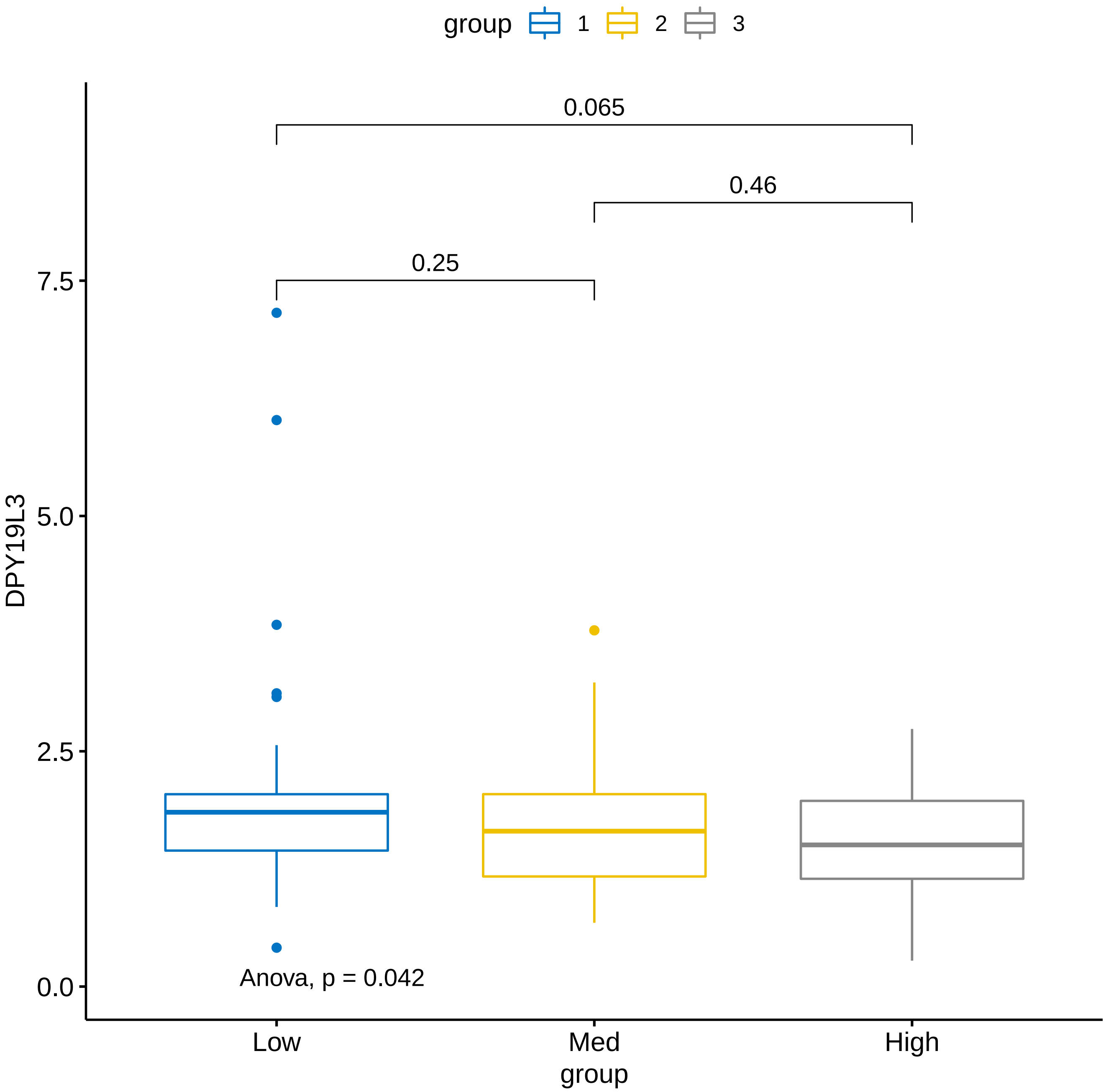
group
1
2
3
0.065
0.46
0.25
7.5
5.0
2.5
0.0
DPY19L3
Anova, p = 0.042
Low
Med
High
group

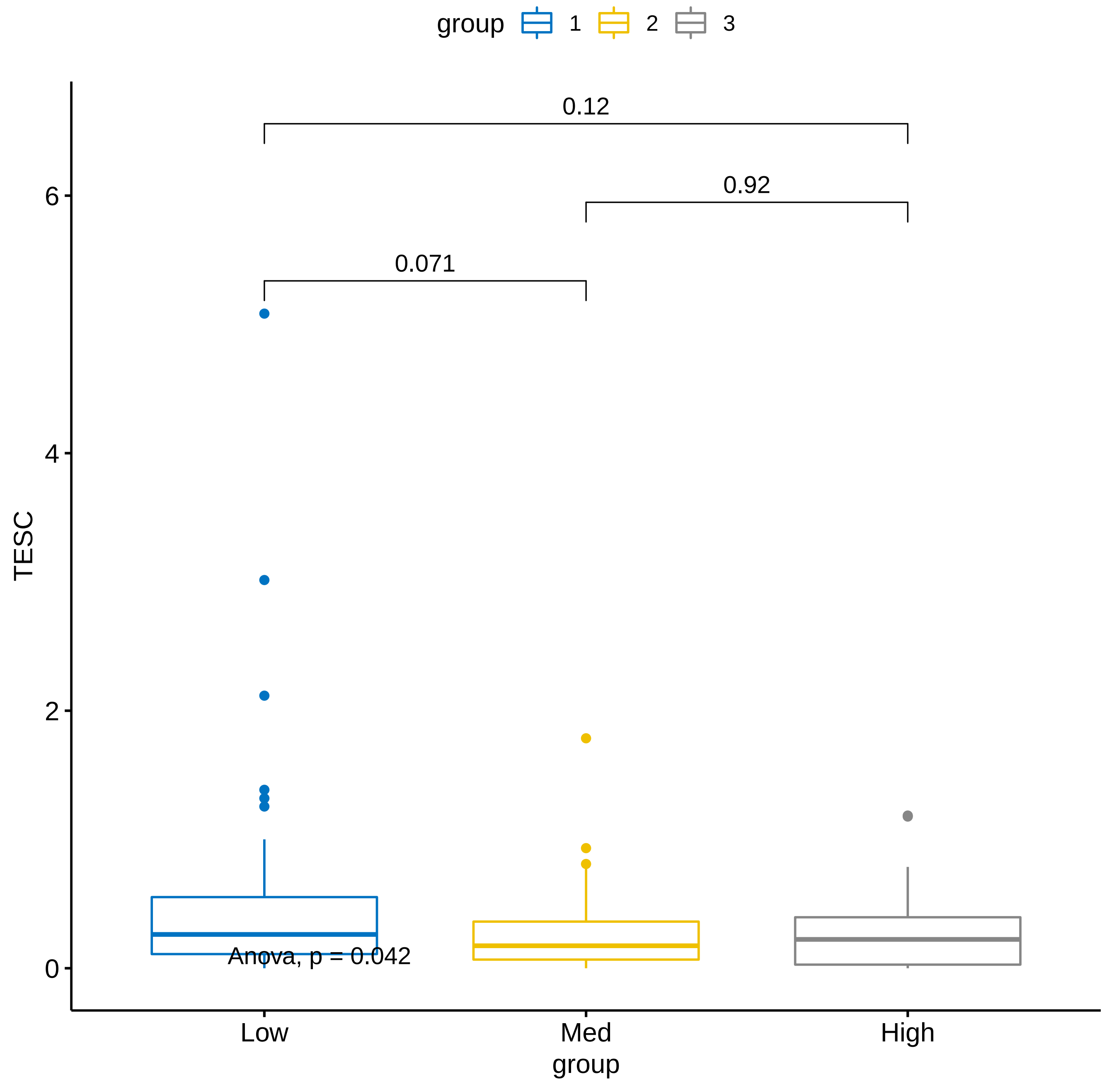

group
1
2
3
0.12
0.92
0.071
6
4
2
0
TESC
Anova, p = 0.042
Low
Med
High
group

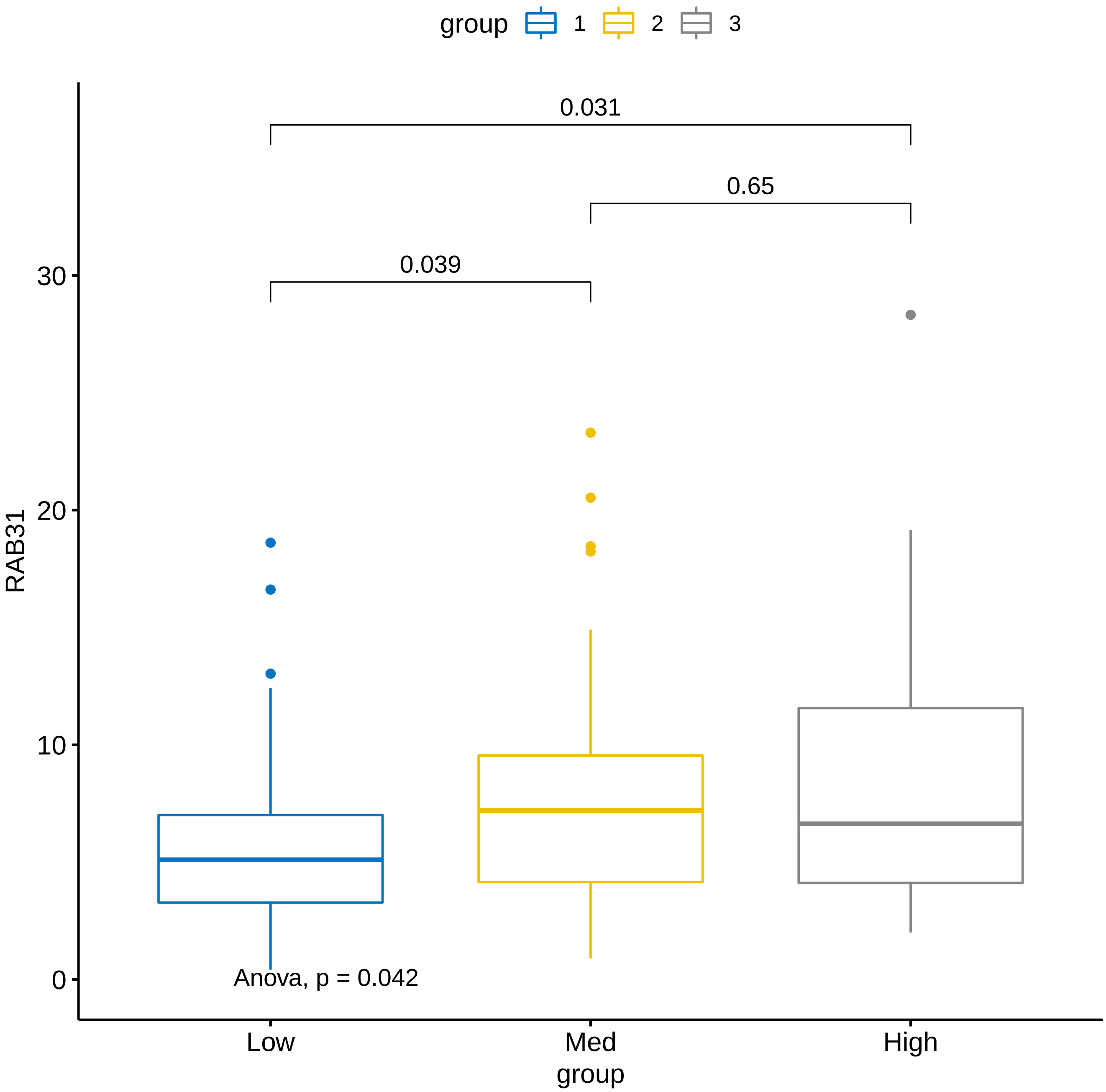
group
1
2
3
0.031
0.65
0.039
30
20
10
0
RAB31
Anova, p = 0.042
Low
Med
High
group

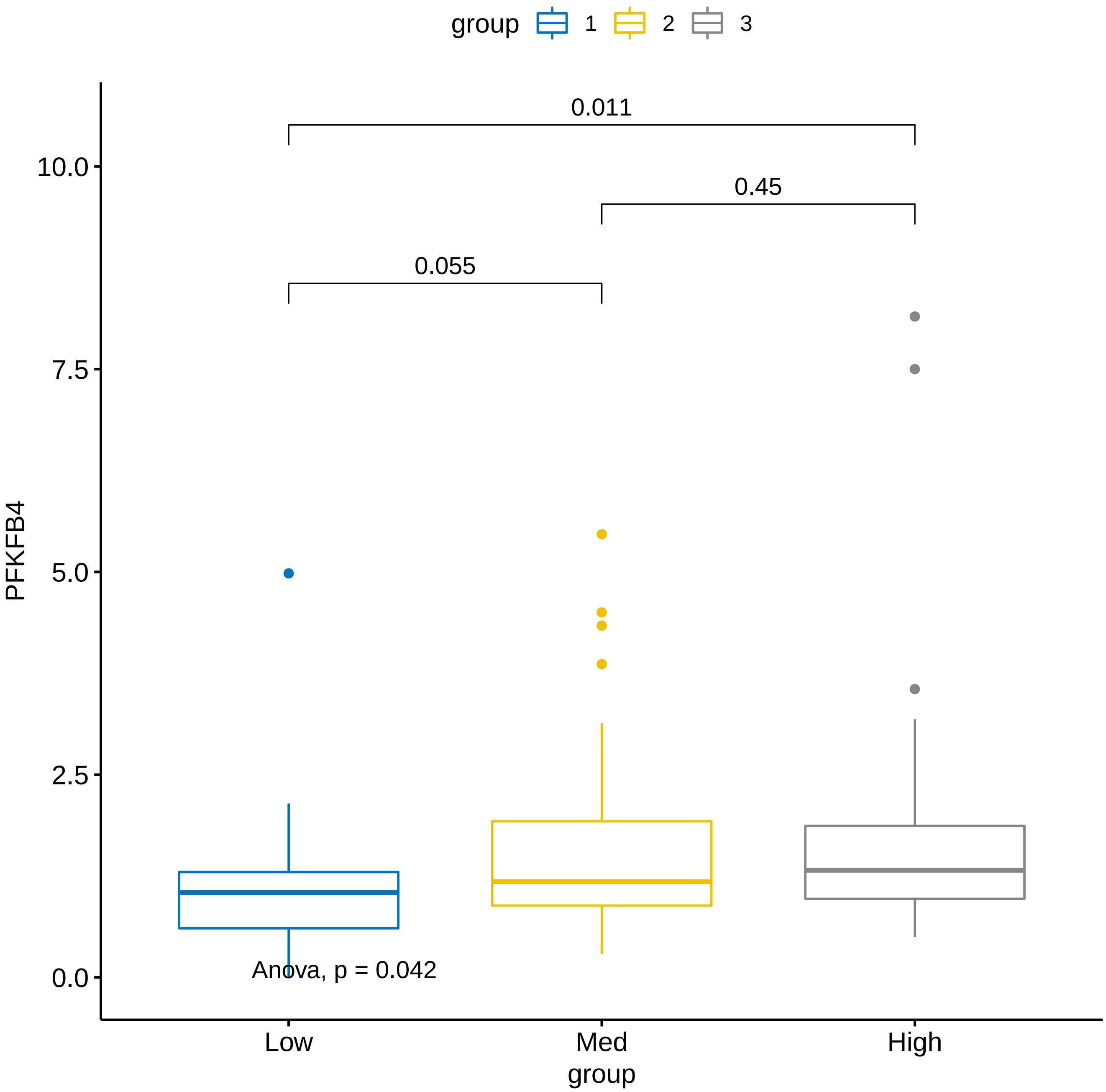

group
1
2
3
0.011
0.45
0.055
10.0
7.5
5.0
2.5
0.0
PFKFB4
Anova, p = 0.042
Low
Med
High
group

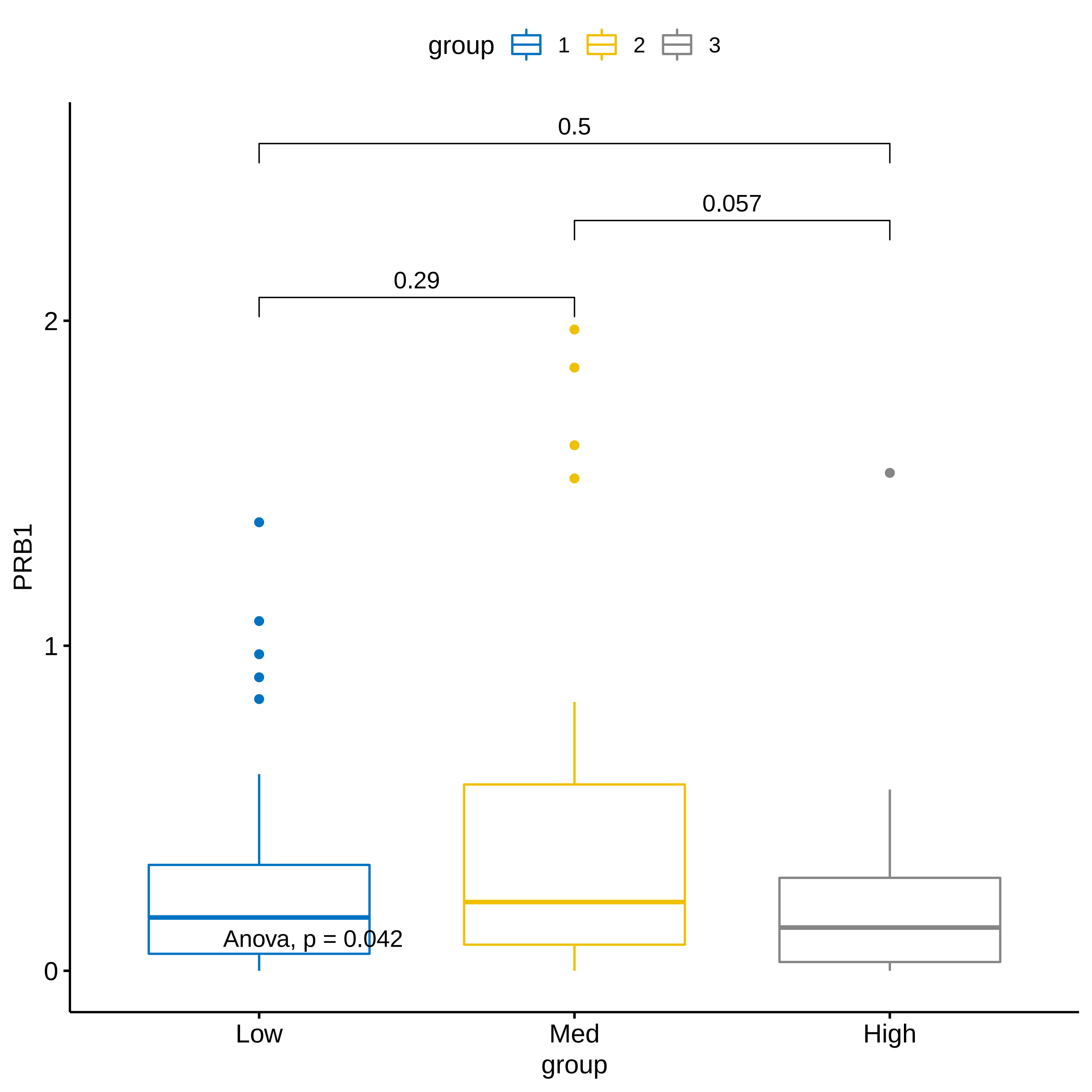

group 1 2 3
0.5
0.057
0.29
PRB1
2
1
0
Anova, p = 0.042
Low
Med
High
group

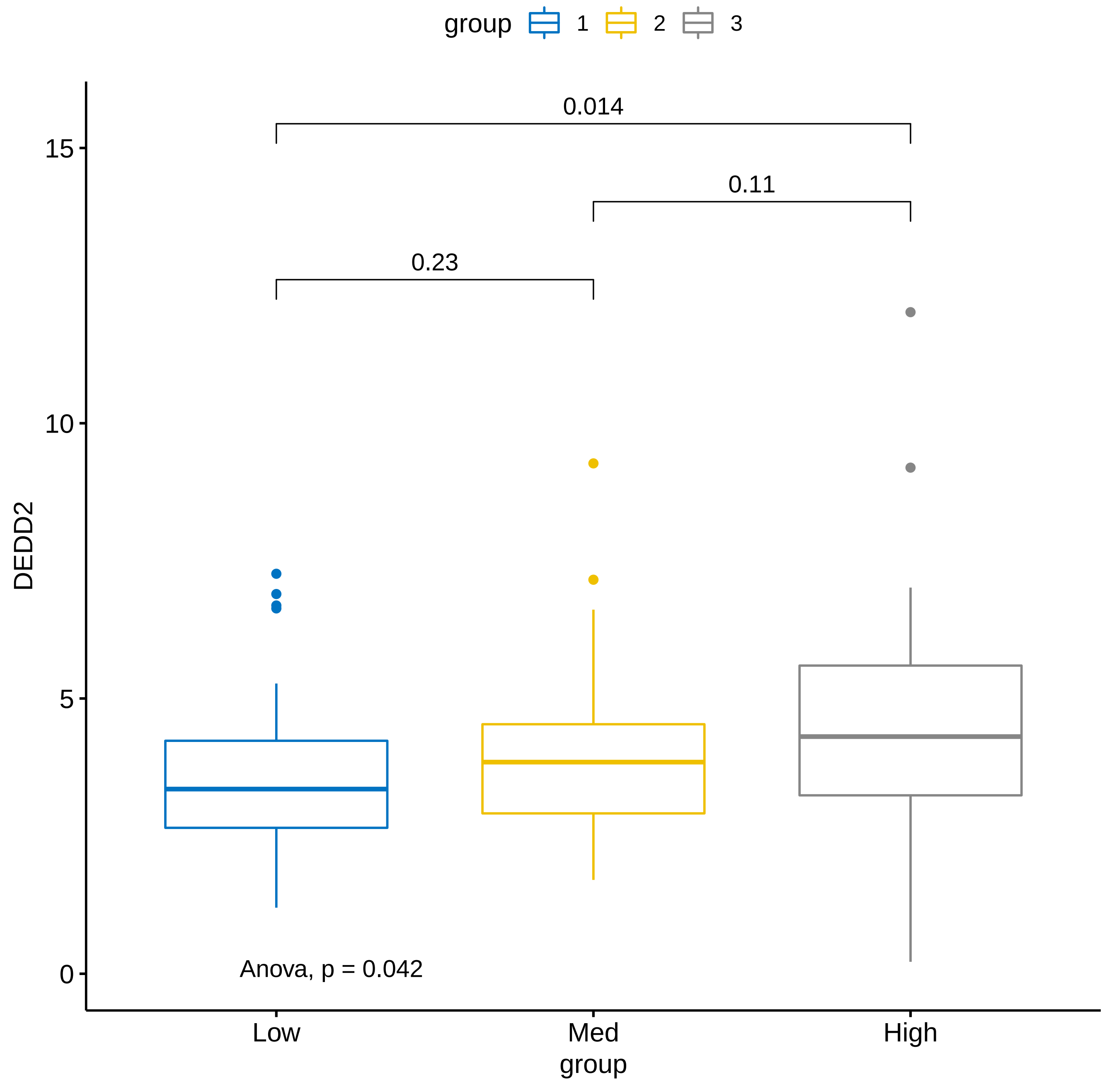
group 1 2 3
0.014
0.11
0.23
15
10
5
0
DEDD2
Anova, p = 0.042
Low
Med
High
group

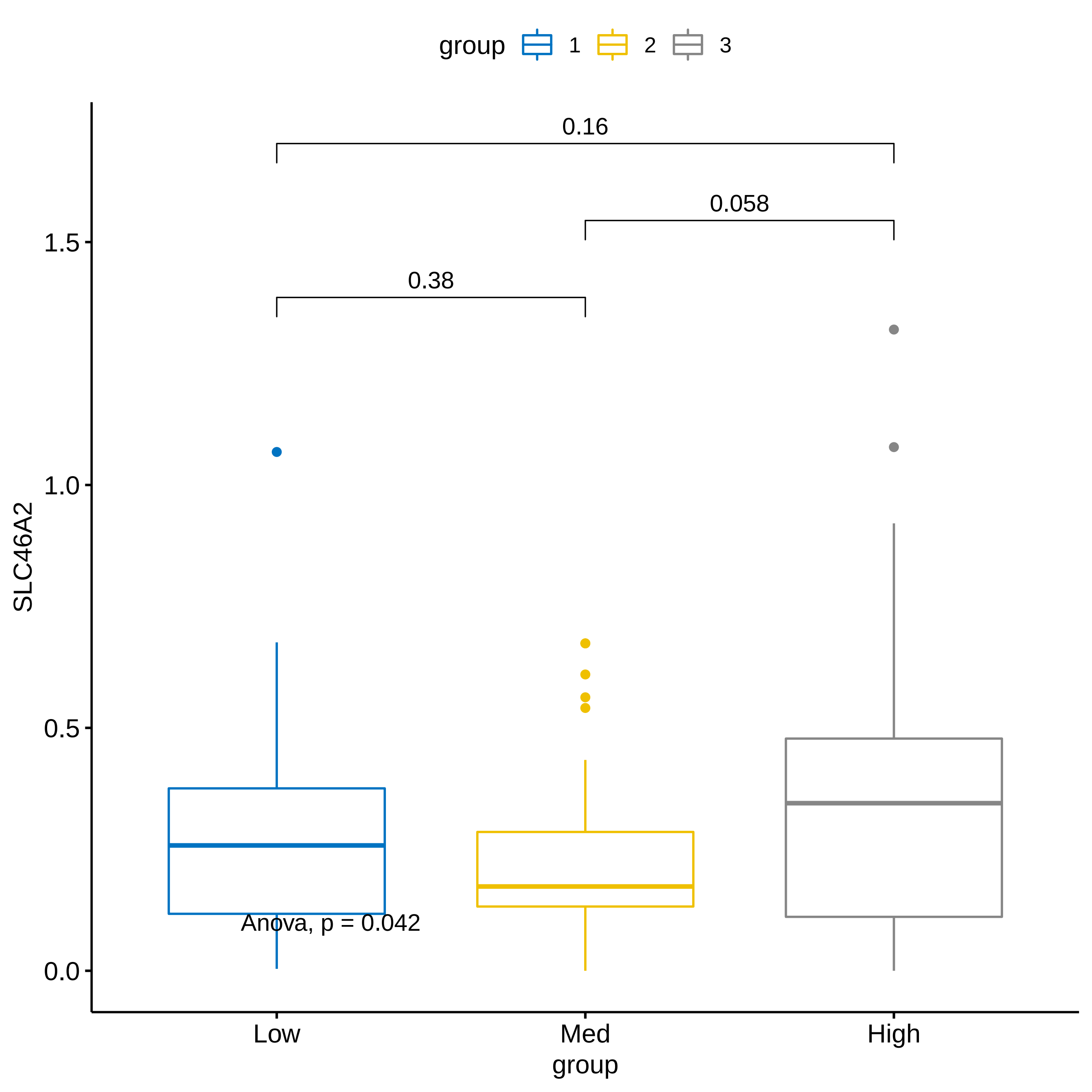
group
1
2
3
0.16
0.058
0.38
1.5
1.0
0.5
0.0
SLC46A2
Anova, p = 0.042
Low
Med
High
group

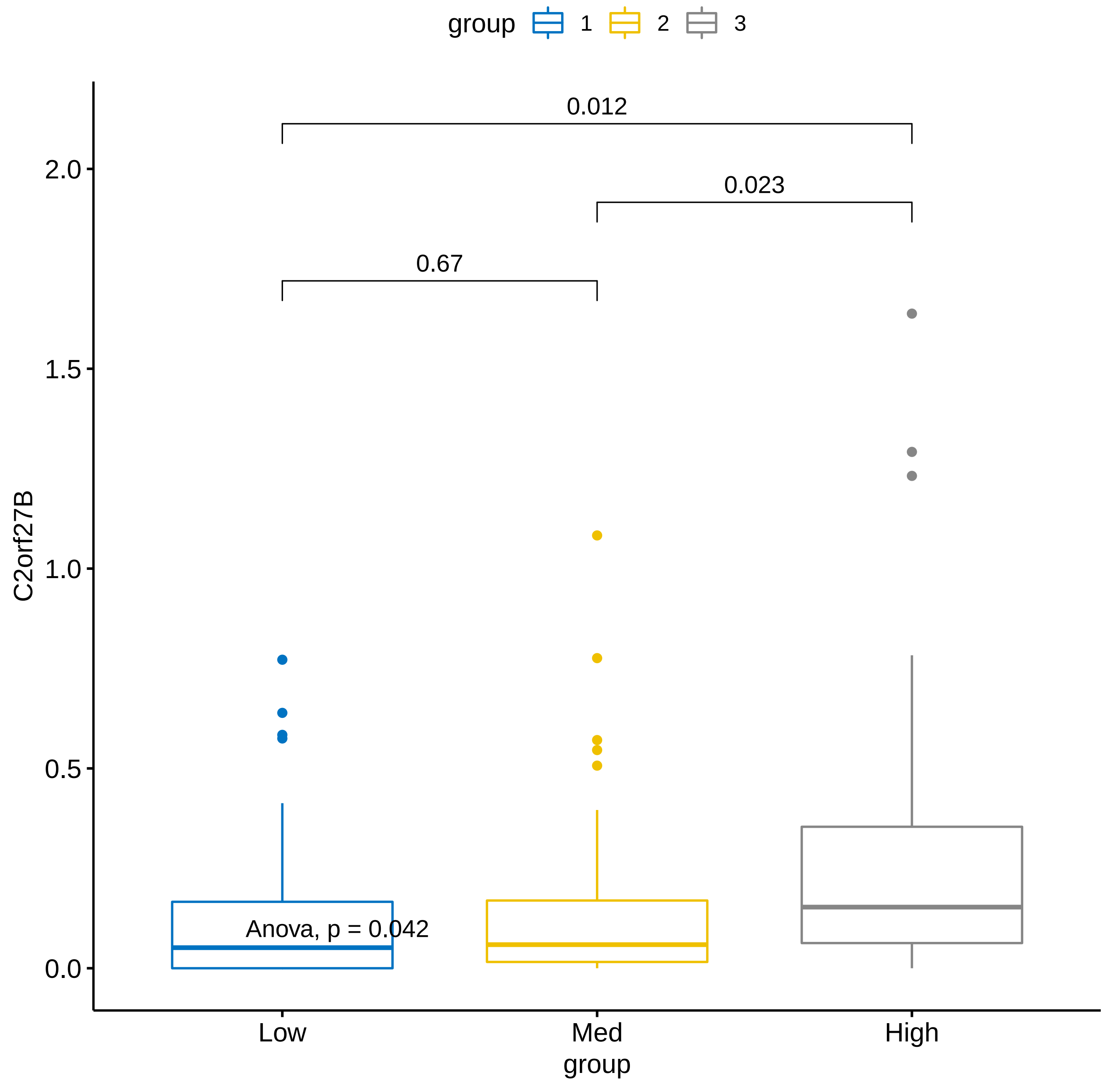

group
1
2
3
0.012
0.023
0.67
2.0
1.5
1.0
0.5
0.0
C2orf27B
Anova, p = 0.042
Low
Med
High
group

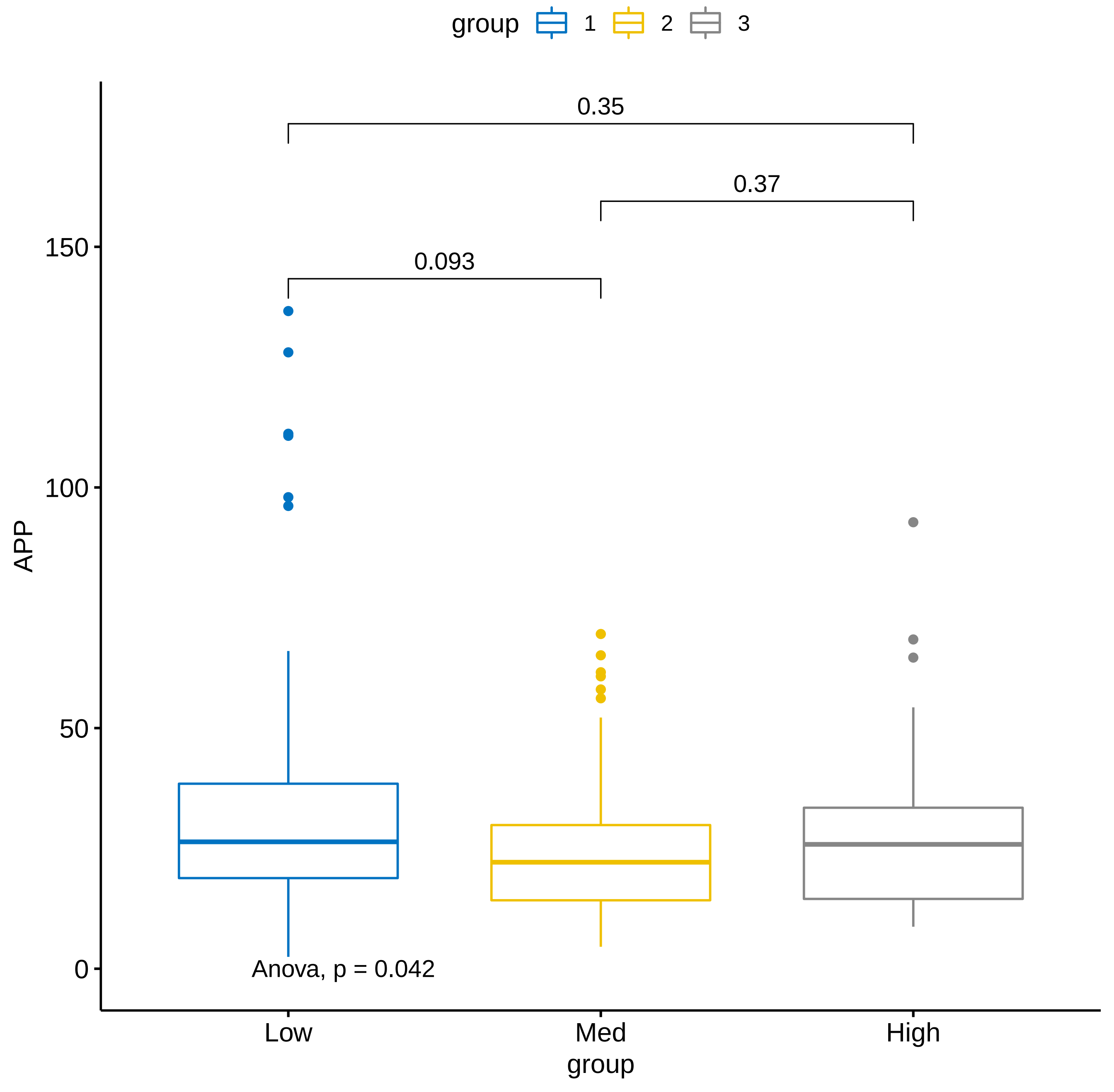

group 1 2 3
0.35
0.37
0.093
150
100
50
0
APP
Anova, p = 0.042
Low
Med
High
group

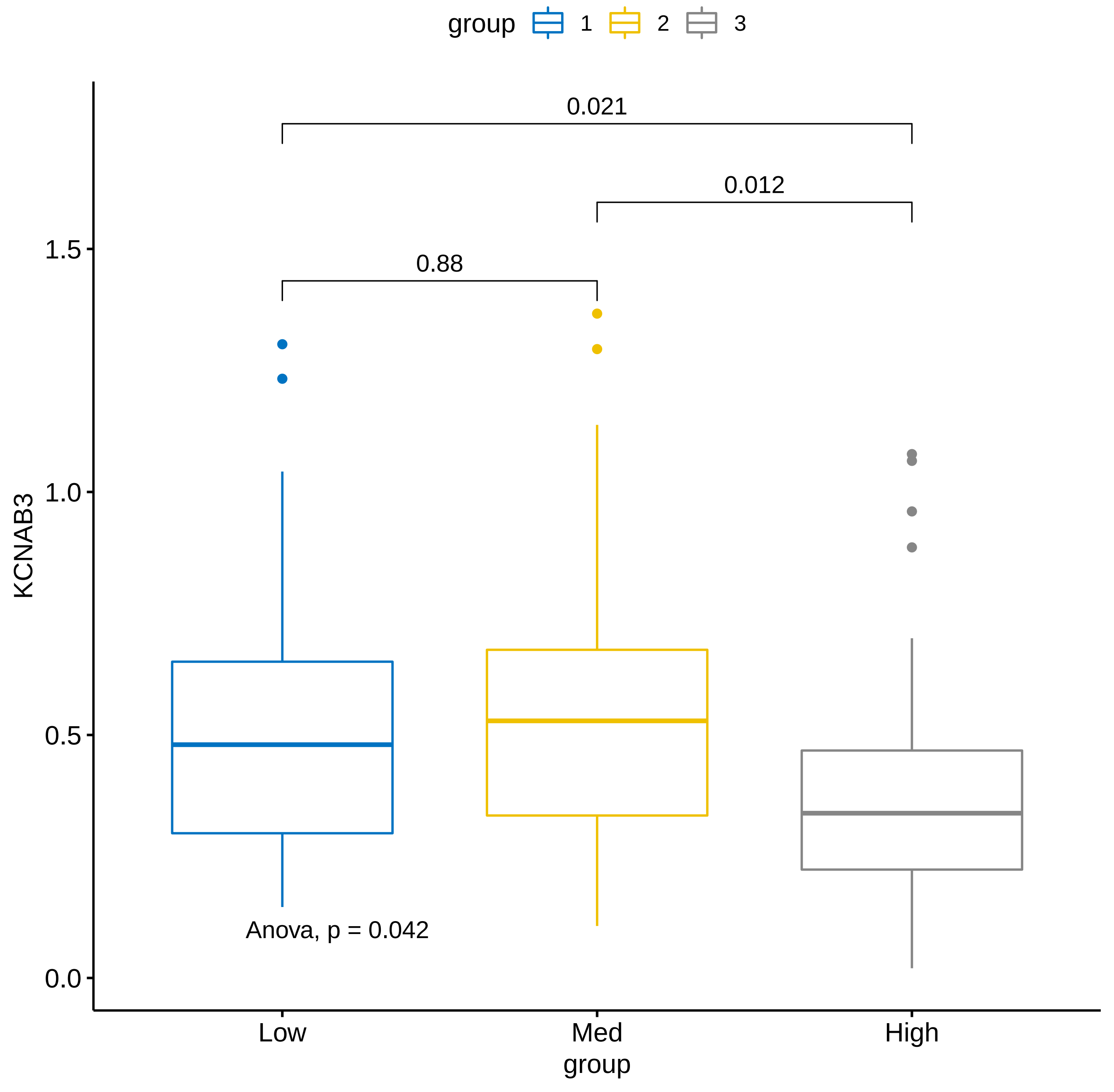

group
1
2
3
0.021
0.012
0.88
1.5
1.0
0.5
0.0
KCNAB3
Anova, p = 0.042
Low
Med
High
group

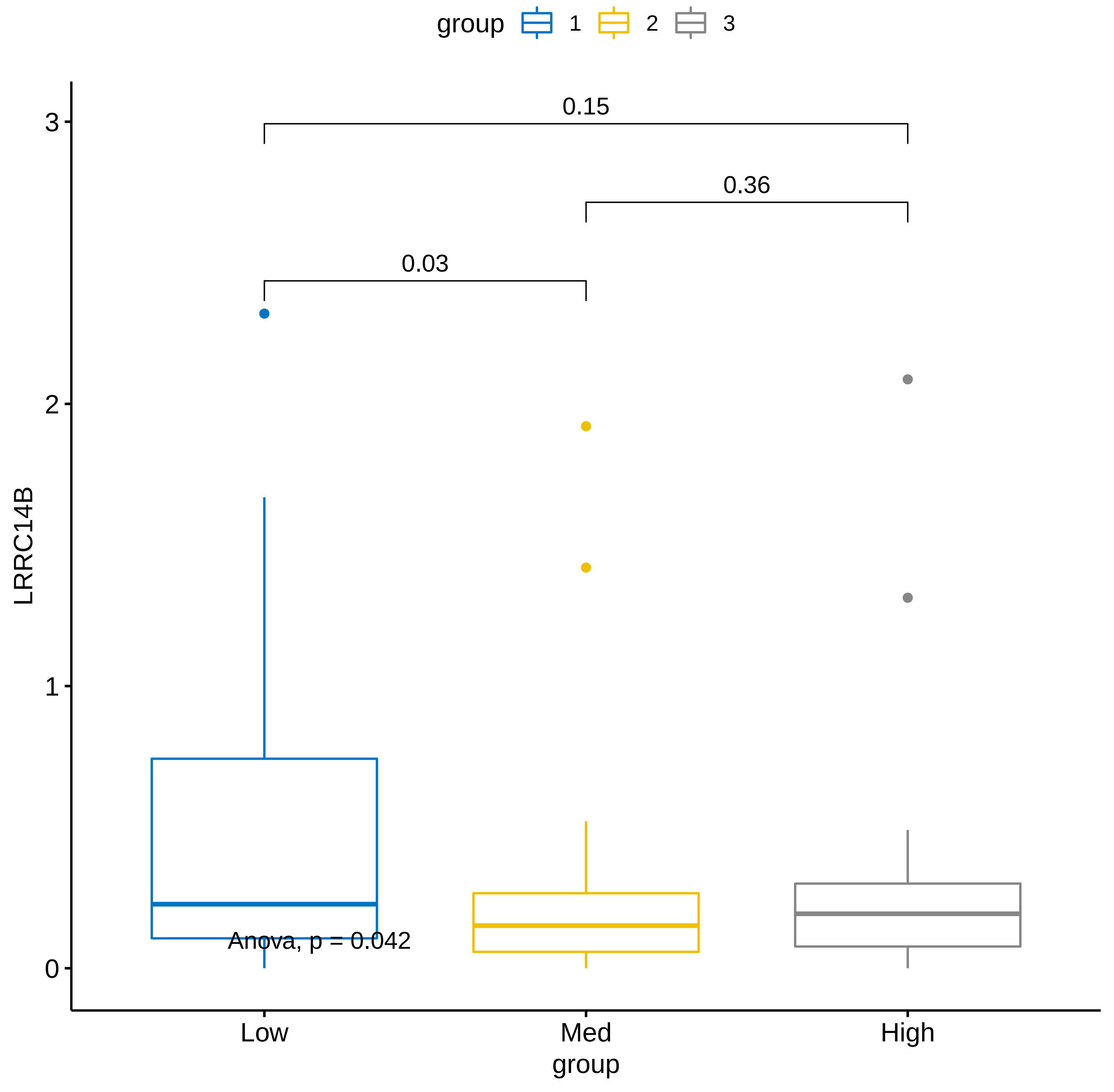

group 1 2 3
0.15
0.36
0.03
3
2
1
0
LRRC14B
Anova, p = 0.042
Low
Med
High
group

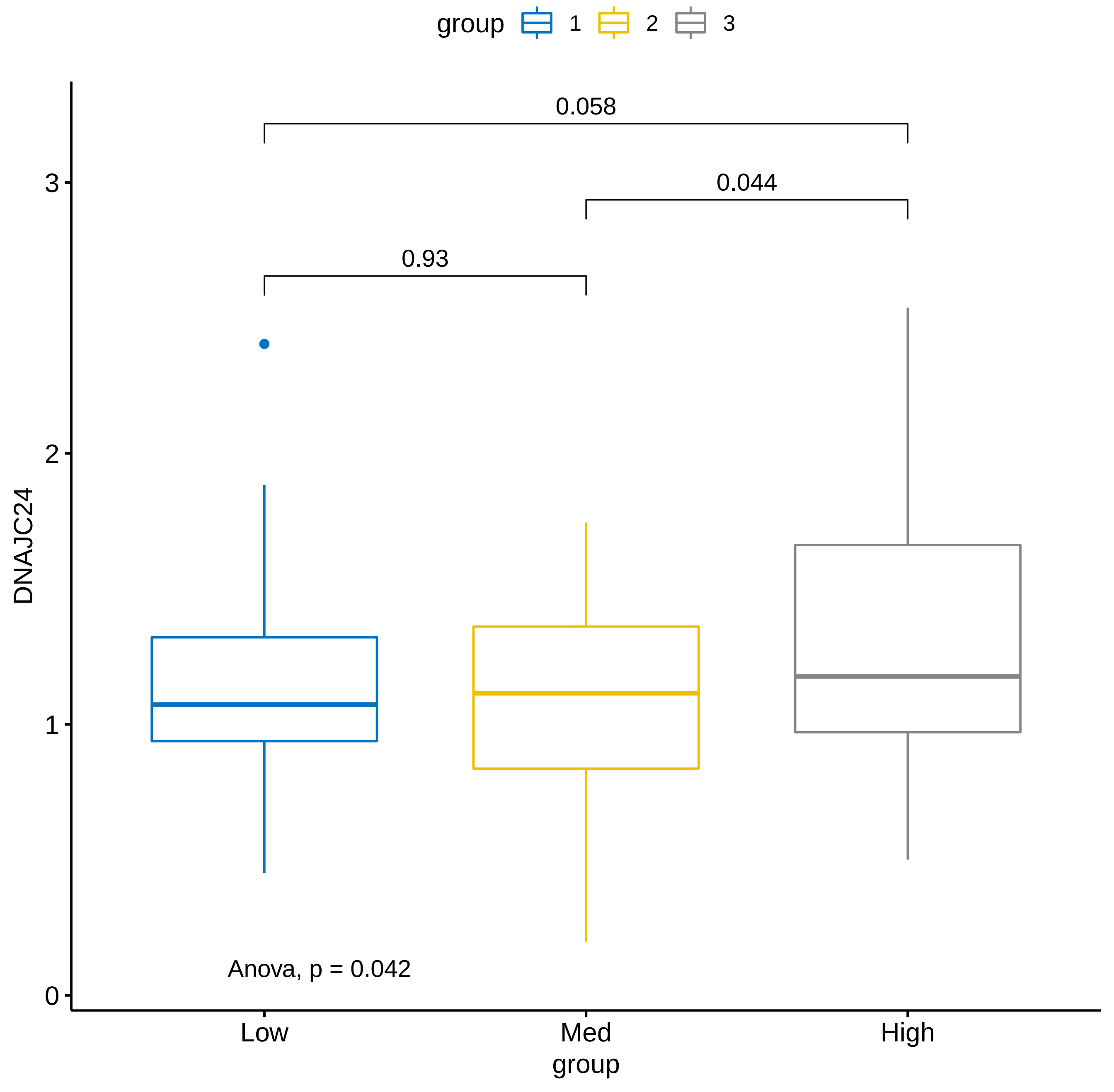
group
1
2
3
0.058
0.044
0.93
3
2
1
0
DNAJC24
Anova, p = 0.042
Low
Med
High
group

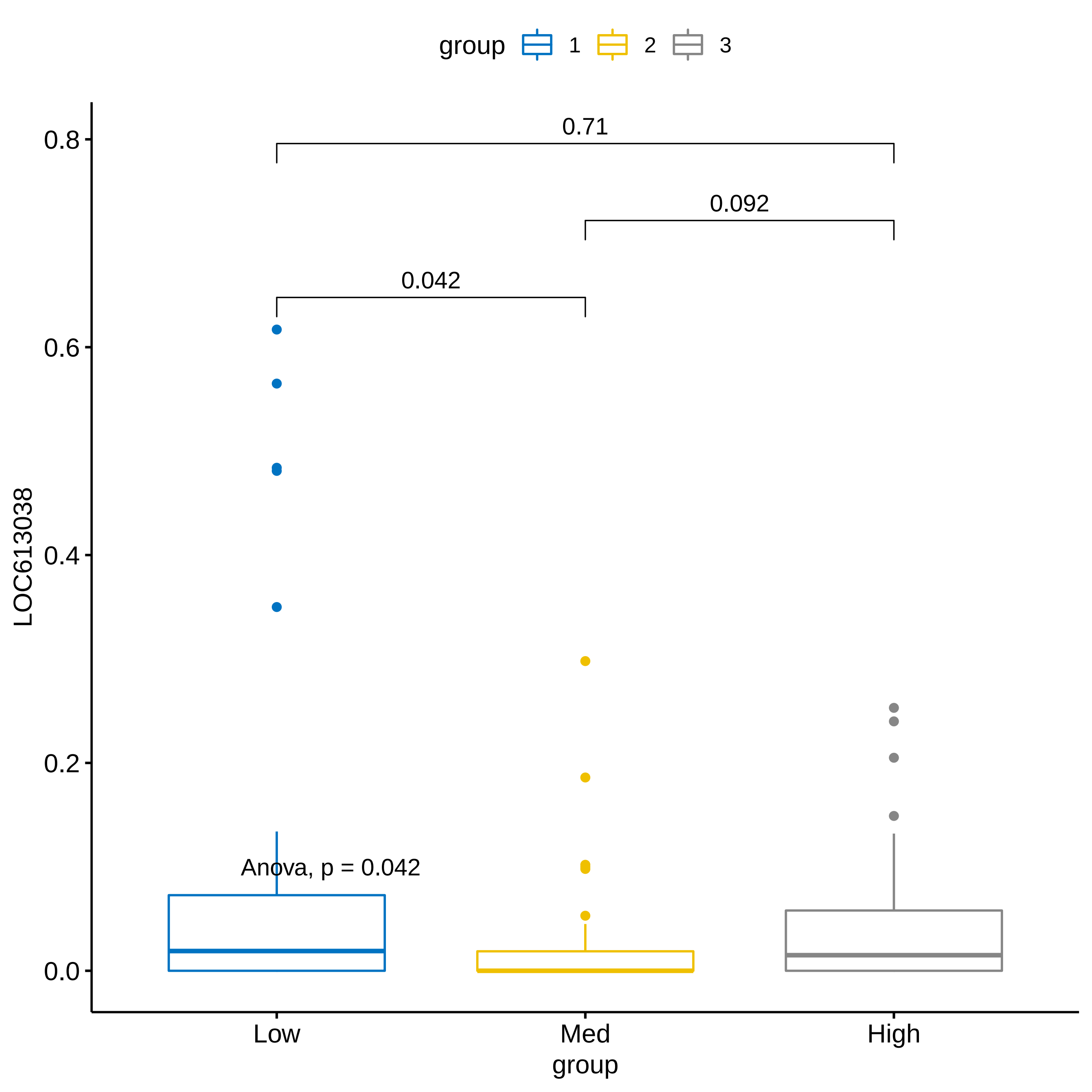
group
1
2
3
0.71
0.092
0.042
Anova, p = 0.042
LOC613038
0.0
0.2
0.4
0.6
0.8
Low
Med
High
group

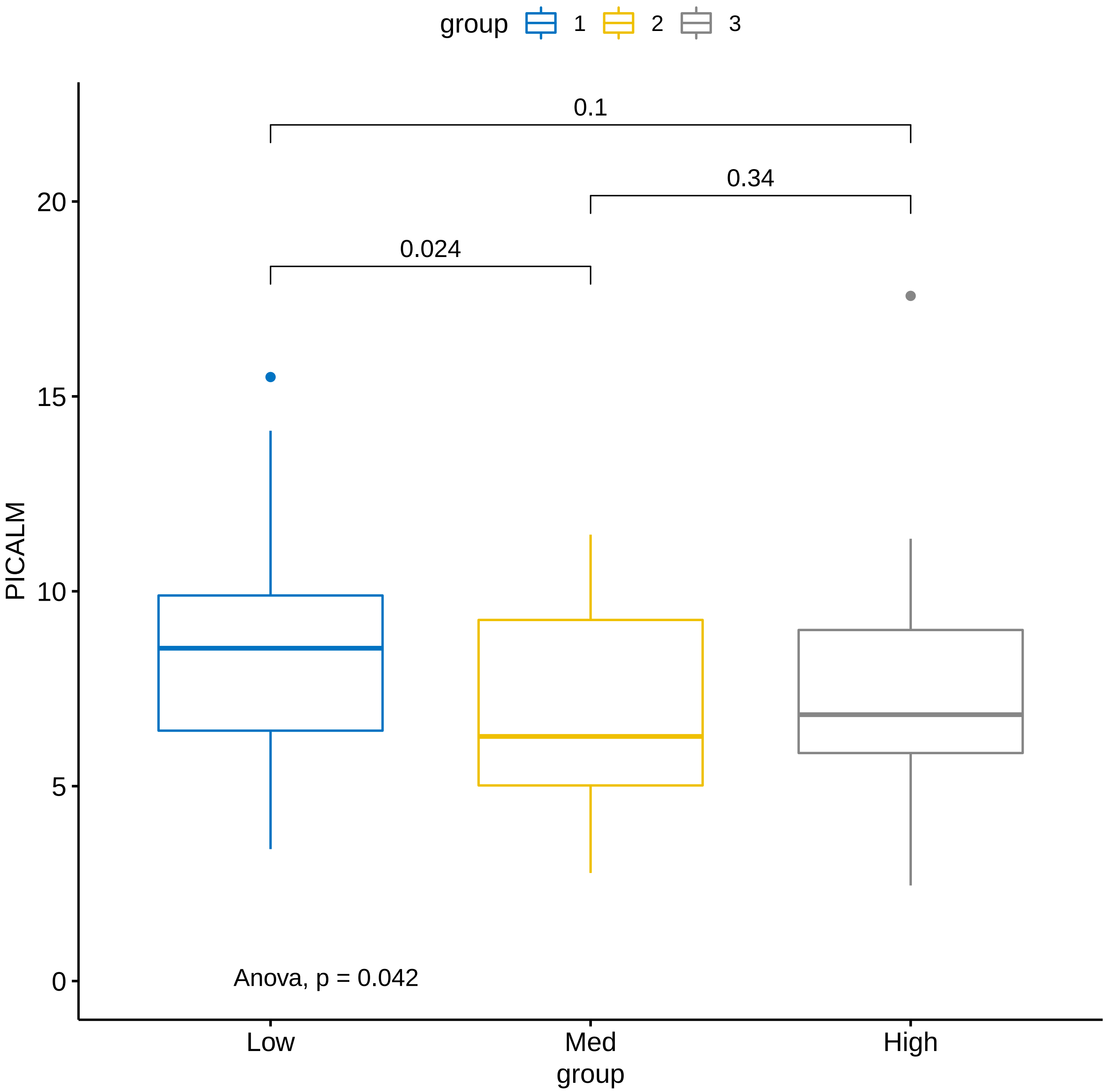

group 1 2 3
0.1
0.34
0.024
20
15
10
5
0
PICALM
Anova, p = 0.042
Low
Med
High
group

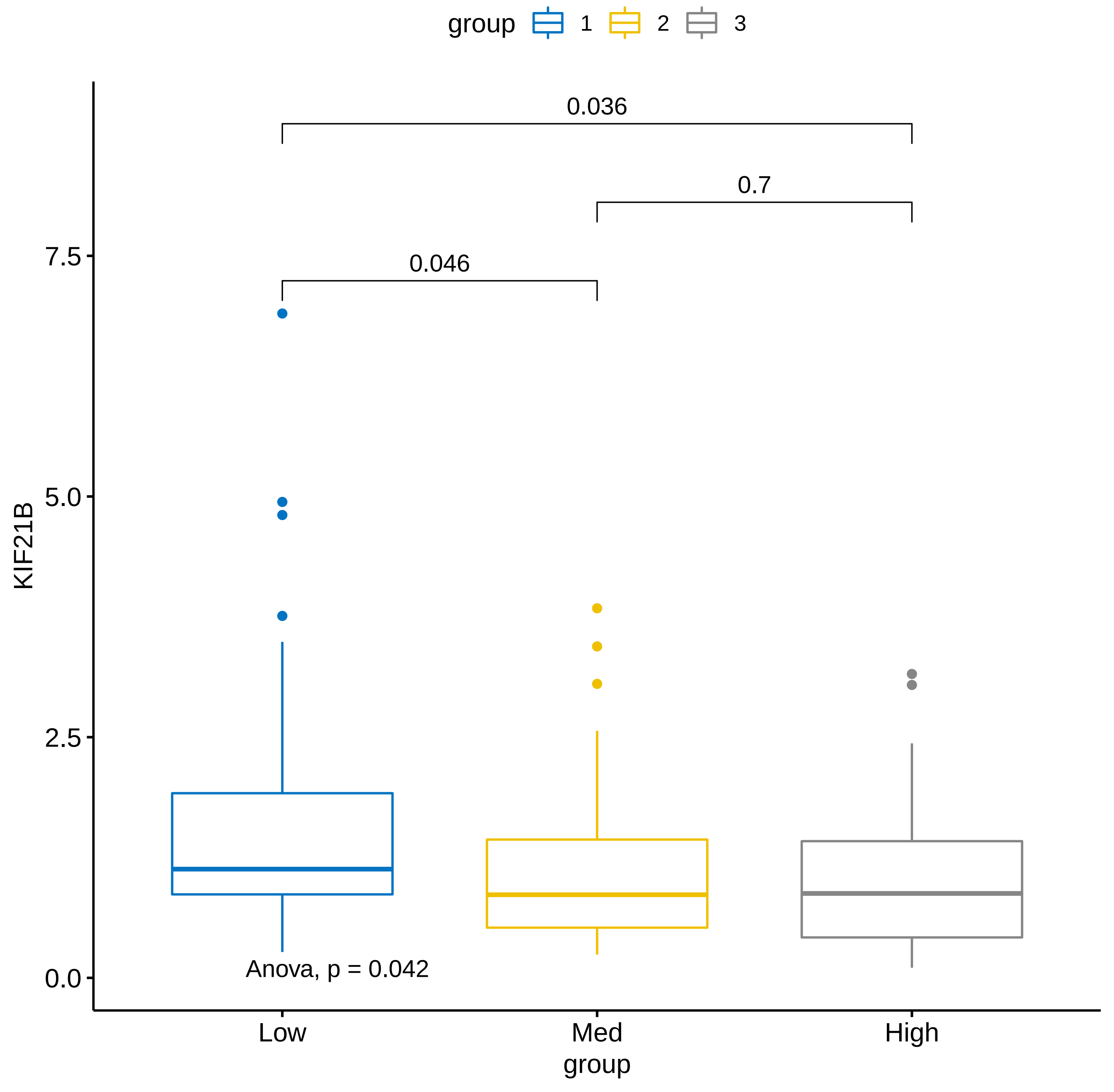
group
1
2
3
0.036
0.7
0.046
7.5
5.0
2.5
0.0
KIF21B
Anova, p = 0.042
Low
Med
High
group

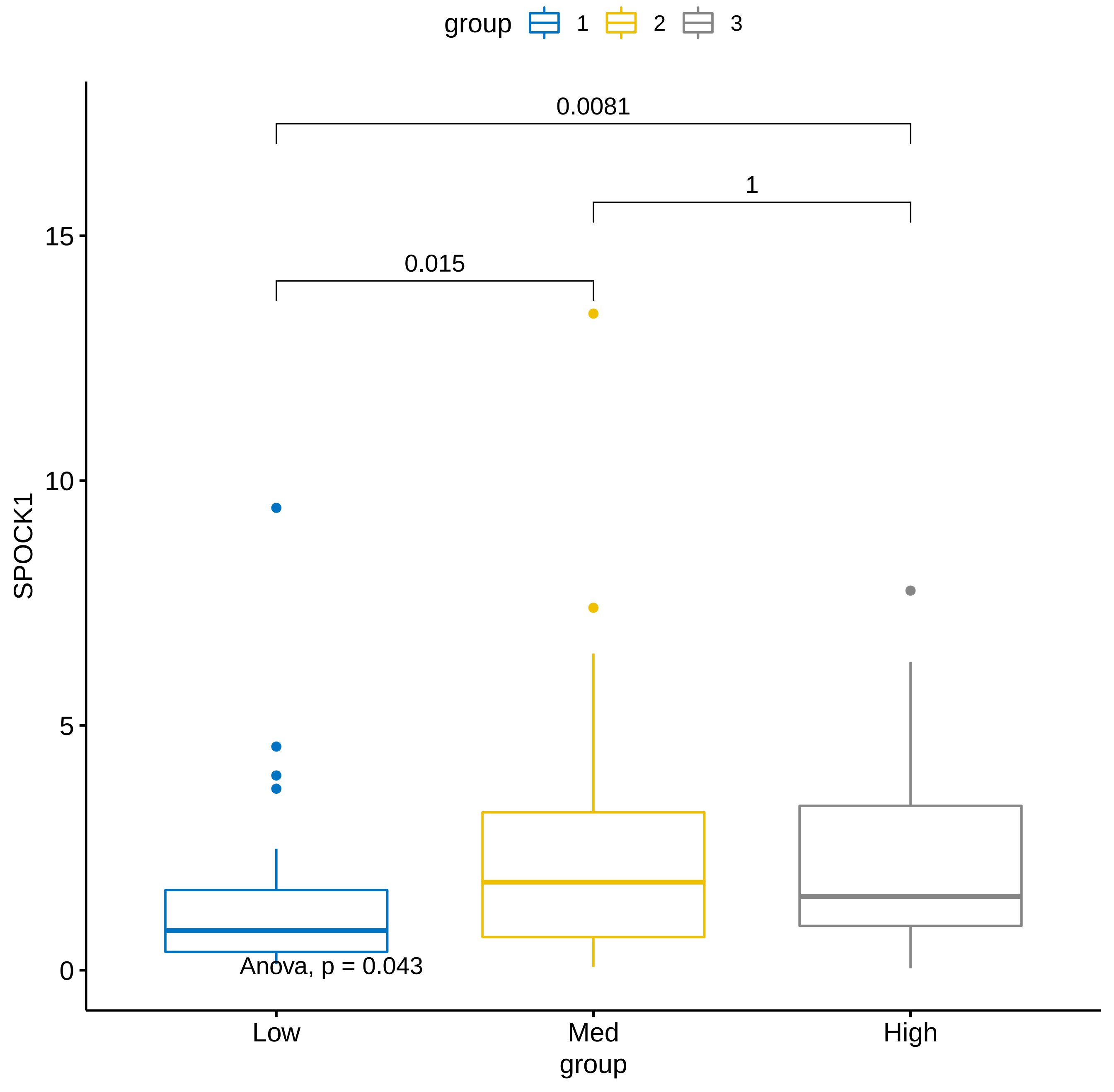
group
1
2
3
0.0081
1
0.015
15
10
5
0
SPOCK1
Anova, p = 0.043
Low
Med
High
group

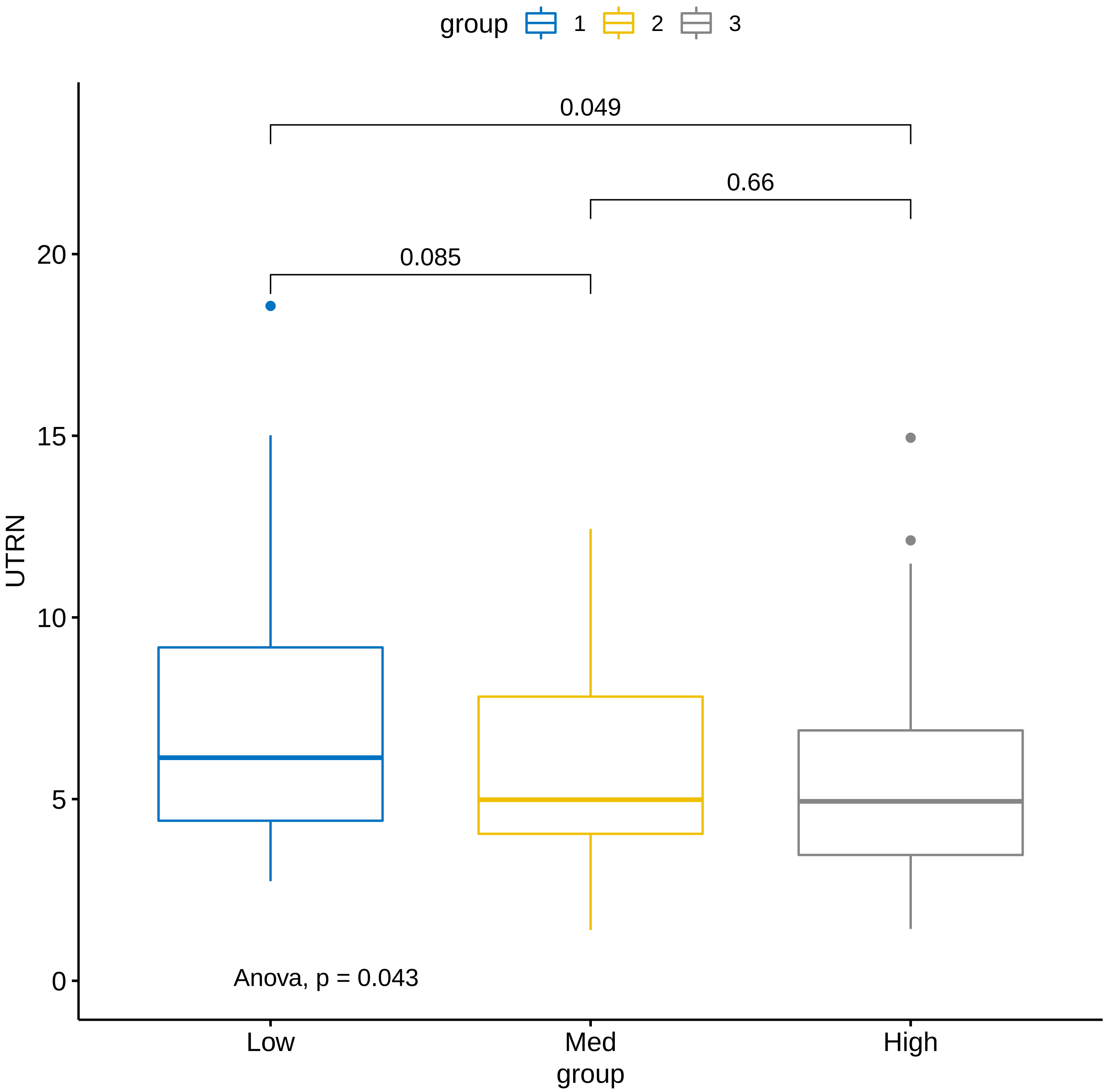
group
1
2
3
0.049
0.66
0.085
20
15
10
5
0
UTRN
Anova, p = 0.043
Low
Med
High
group

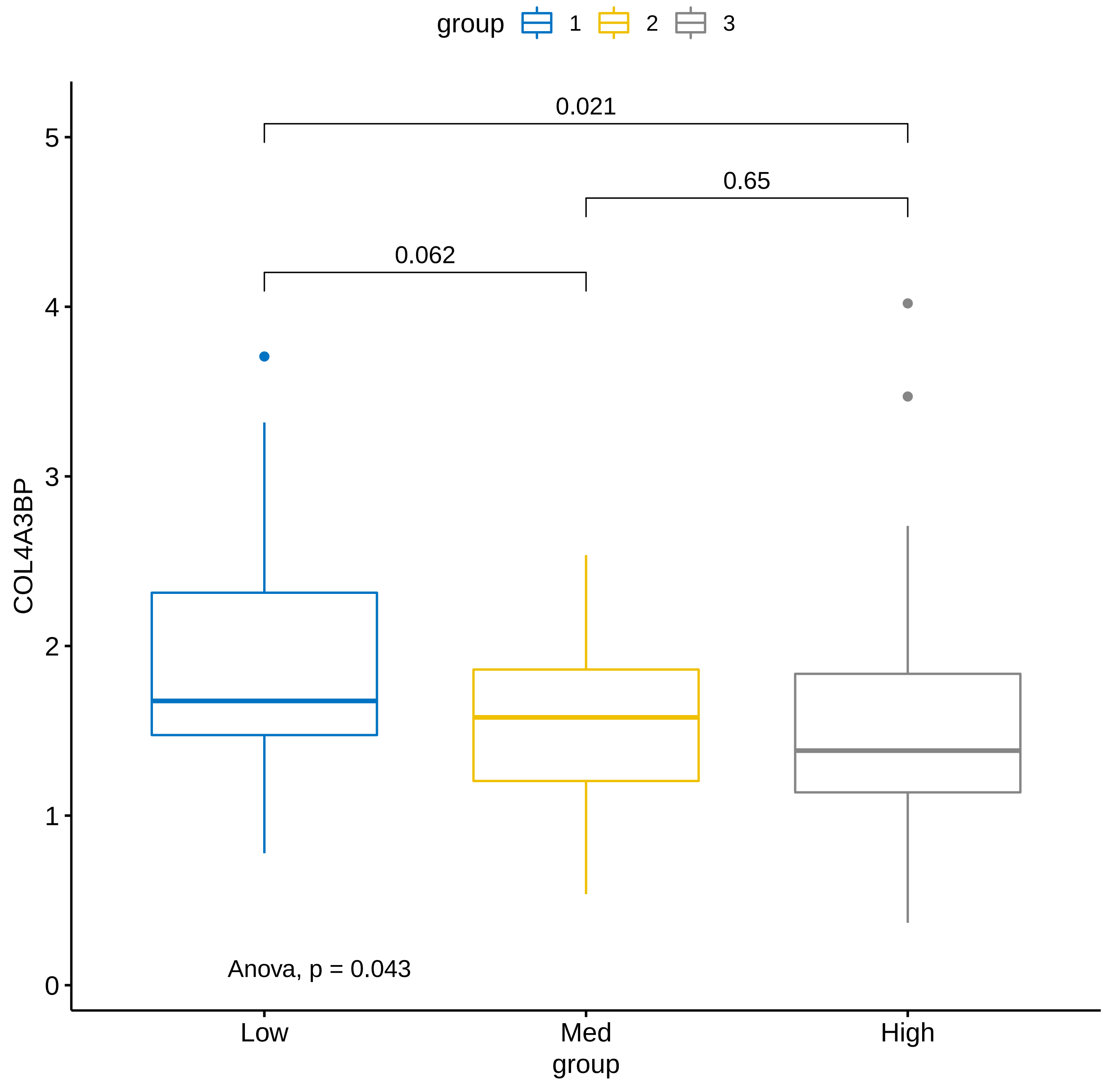

group
1
2
3
0.021
0.65
0.062
COL4A3BP
5
4
3
2
1
0
Anova, p = 0.043
Low
Med
High
group

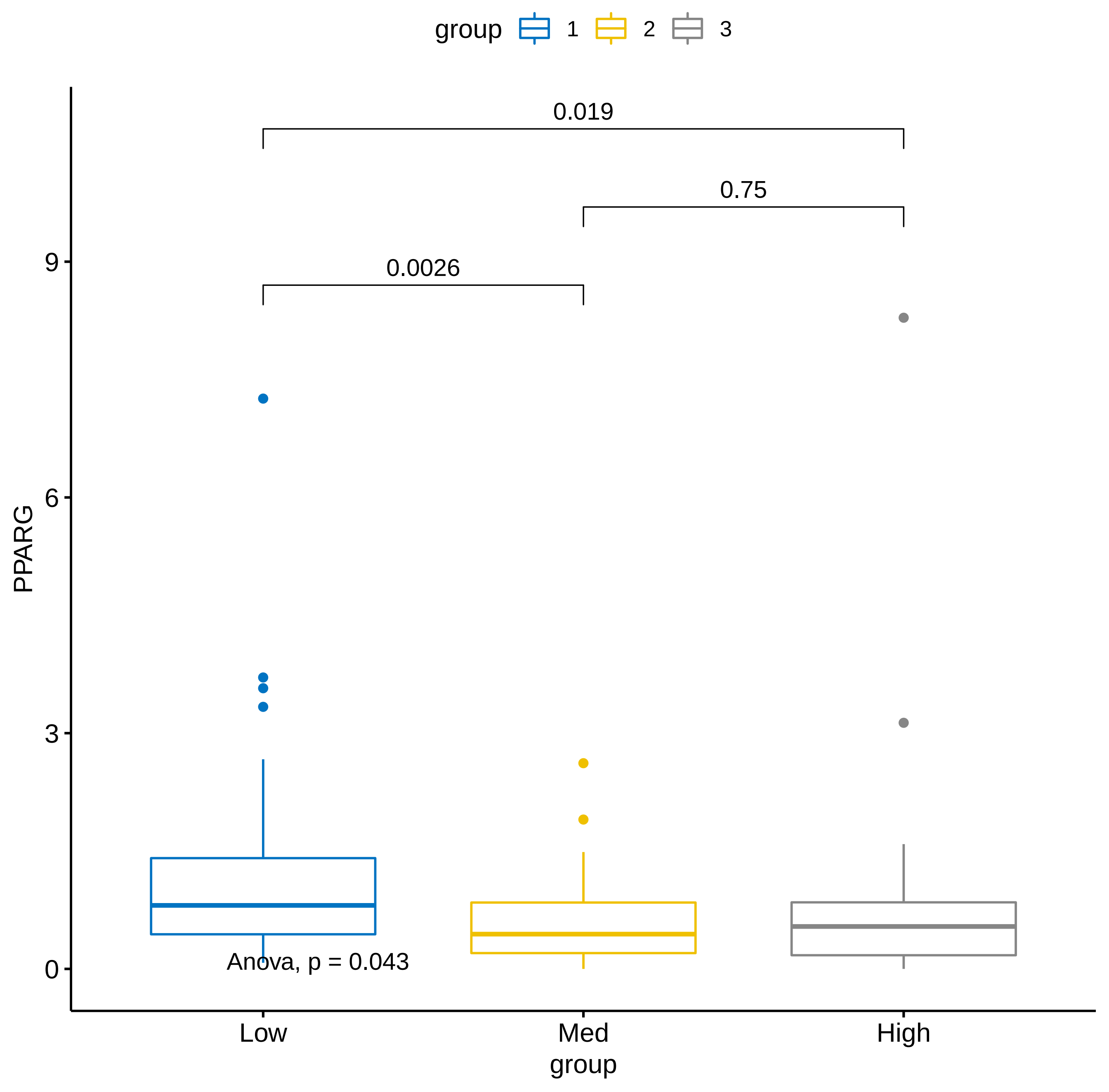
group
1
2
3
0.019
0.75
0.0026
PPARG
9
6
3
0
Anova, p = 0.043
Low
Med
High
group

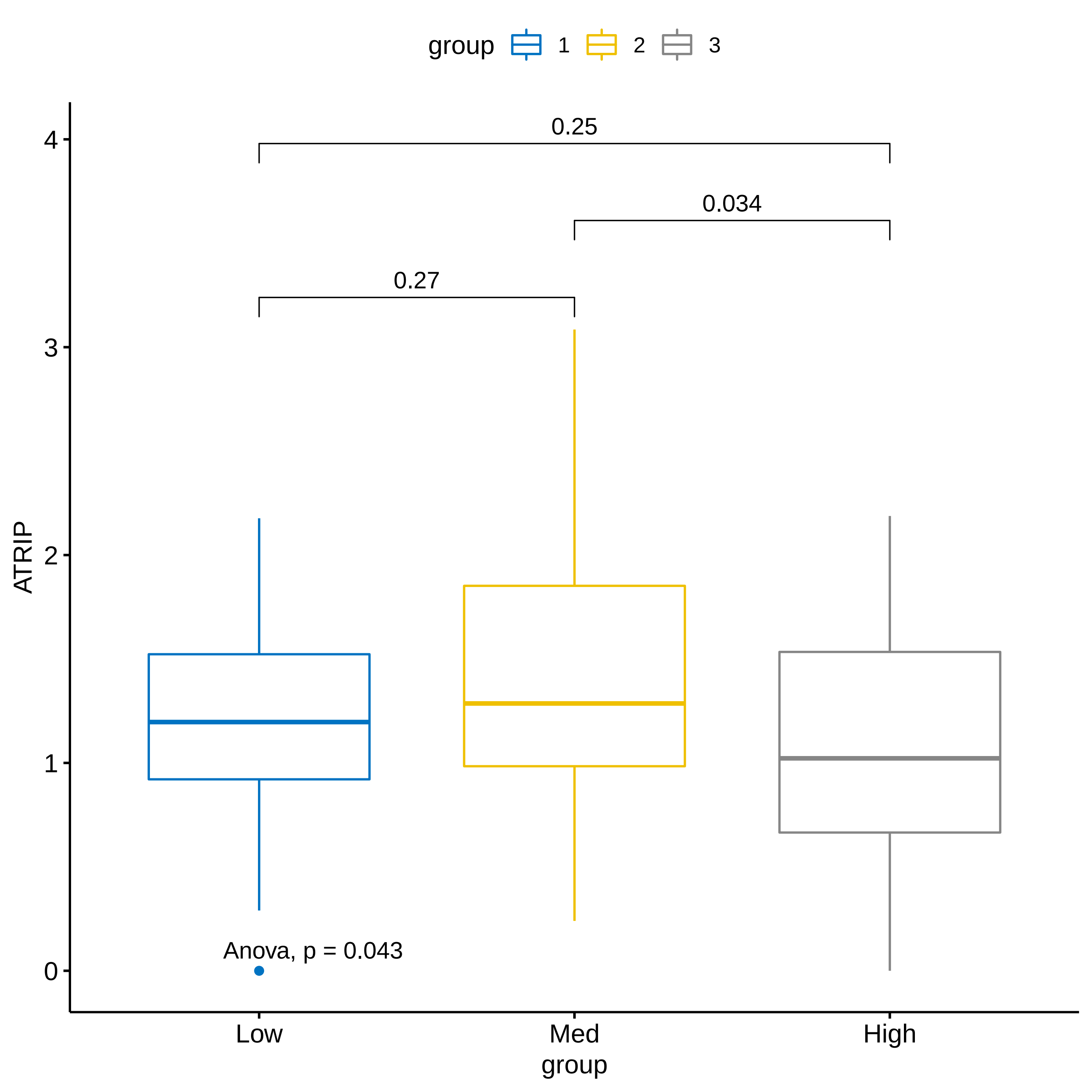
group
1
2
3
0.25
0.034
0.27
4
3
2
1
0
ATRIP
Anova, p = 0.043
Low
Med
High
group

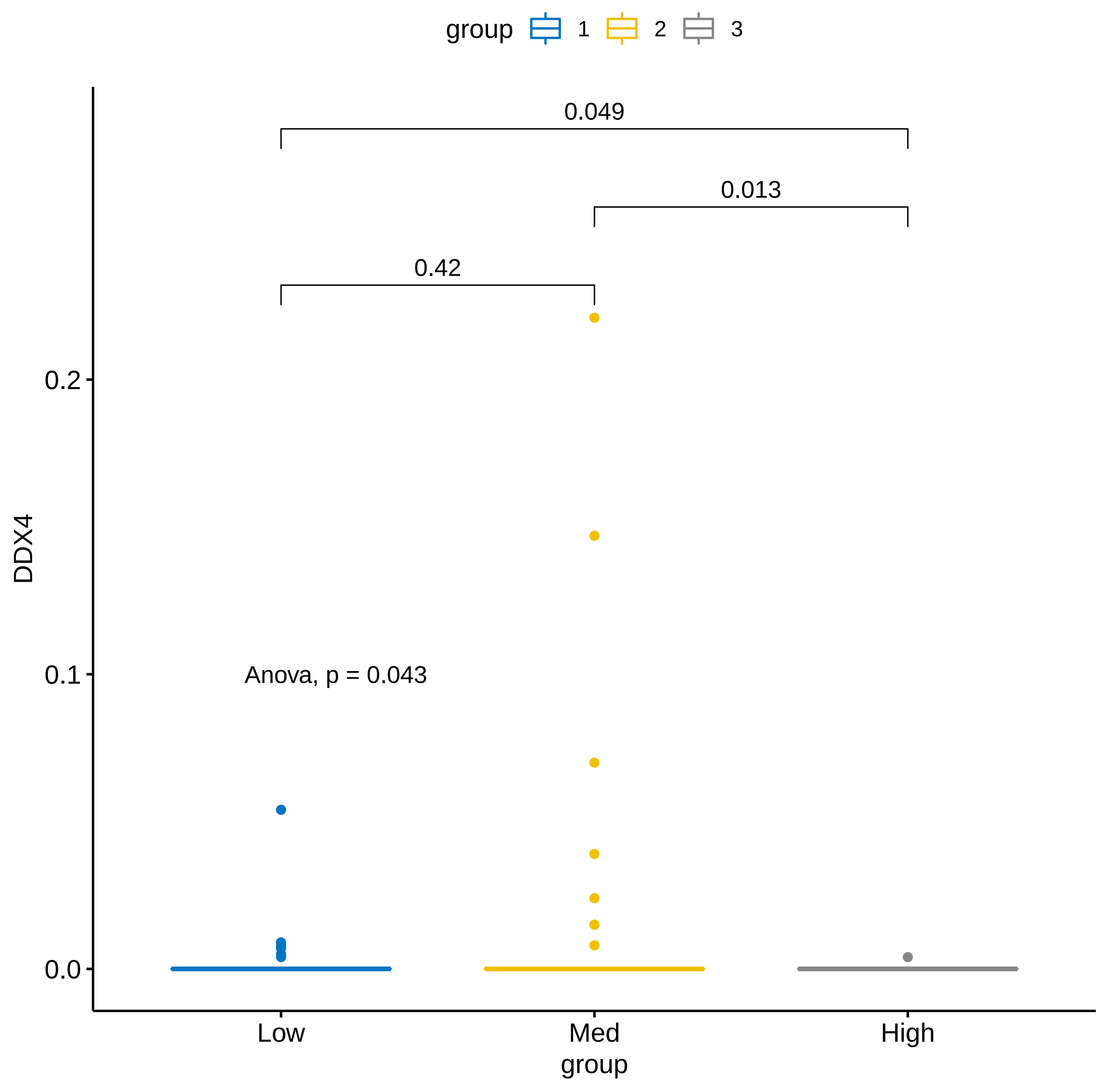
group 1 2 3
0.049
0.013
0.42
Anova, p = 0.043
0.2
0.1
0.0
DDX4
Low
Med
High
group

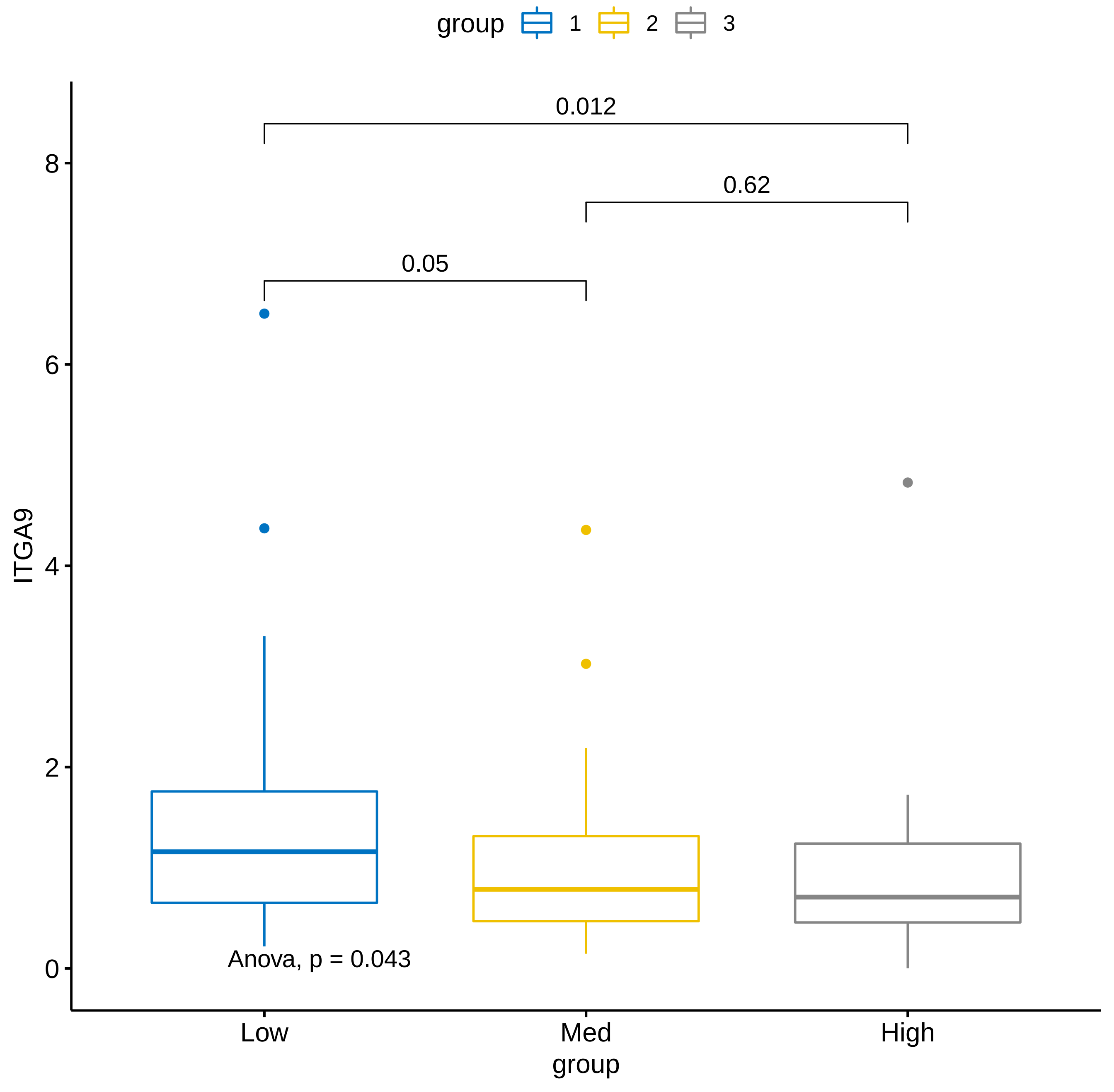

group
1
2
3
0.012
0.62
0.05
8
6
4
2
0
ITGA9
Anova, p = 0.043
Low
Med
High
group

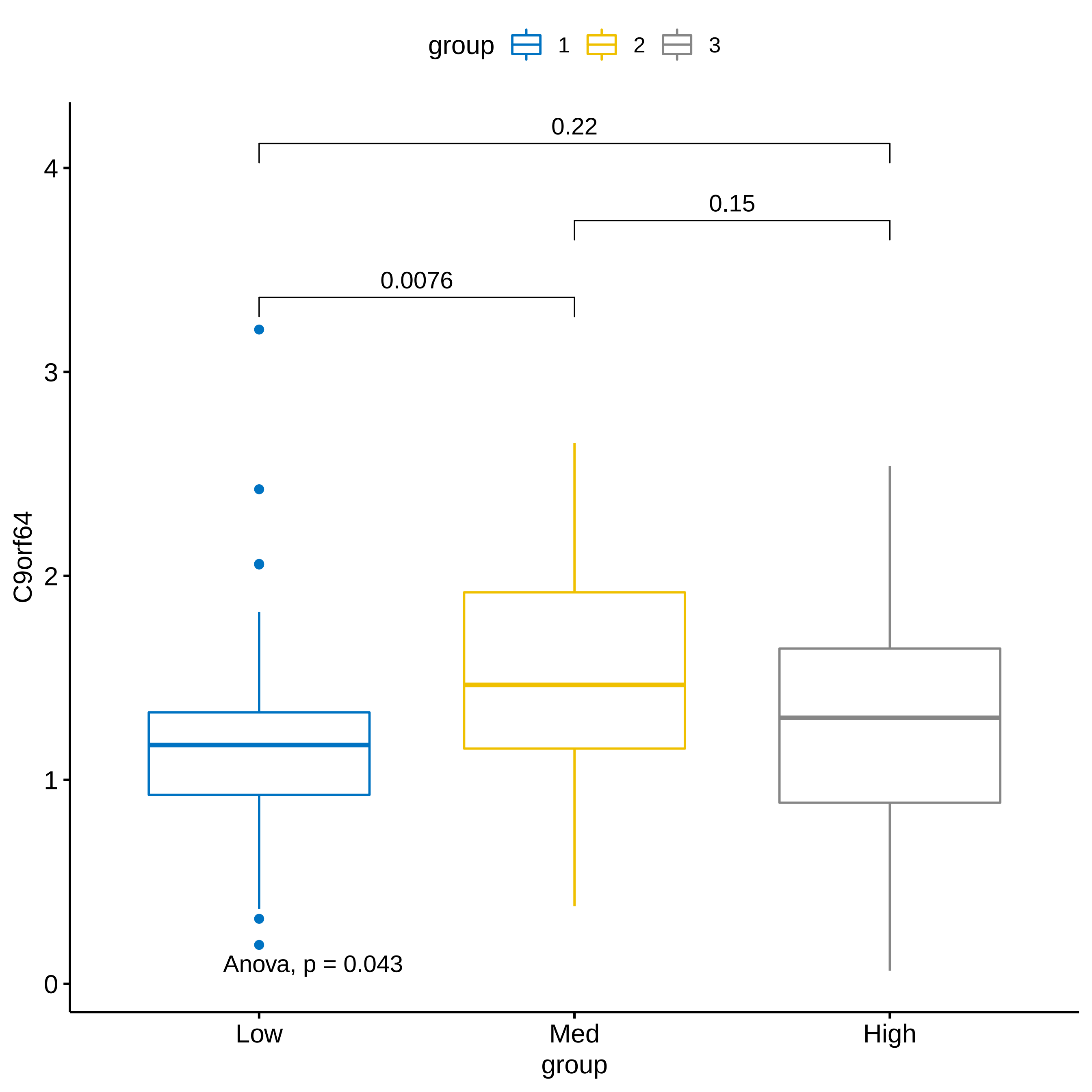
group
1
2
3
0.22
0.15
0.0076
C9orf64
4
3
2
1
0
Anova, p = 0.043
Low
Med
High
group

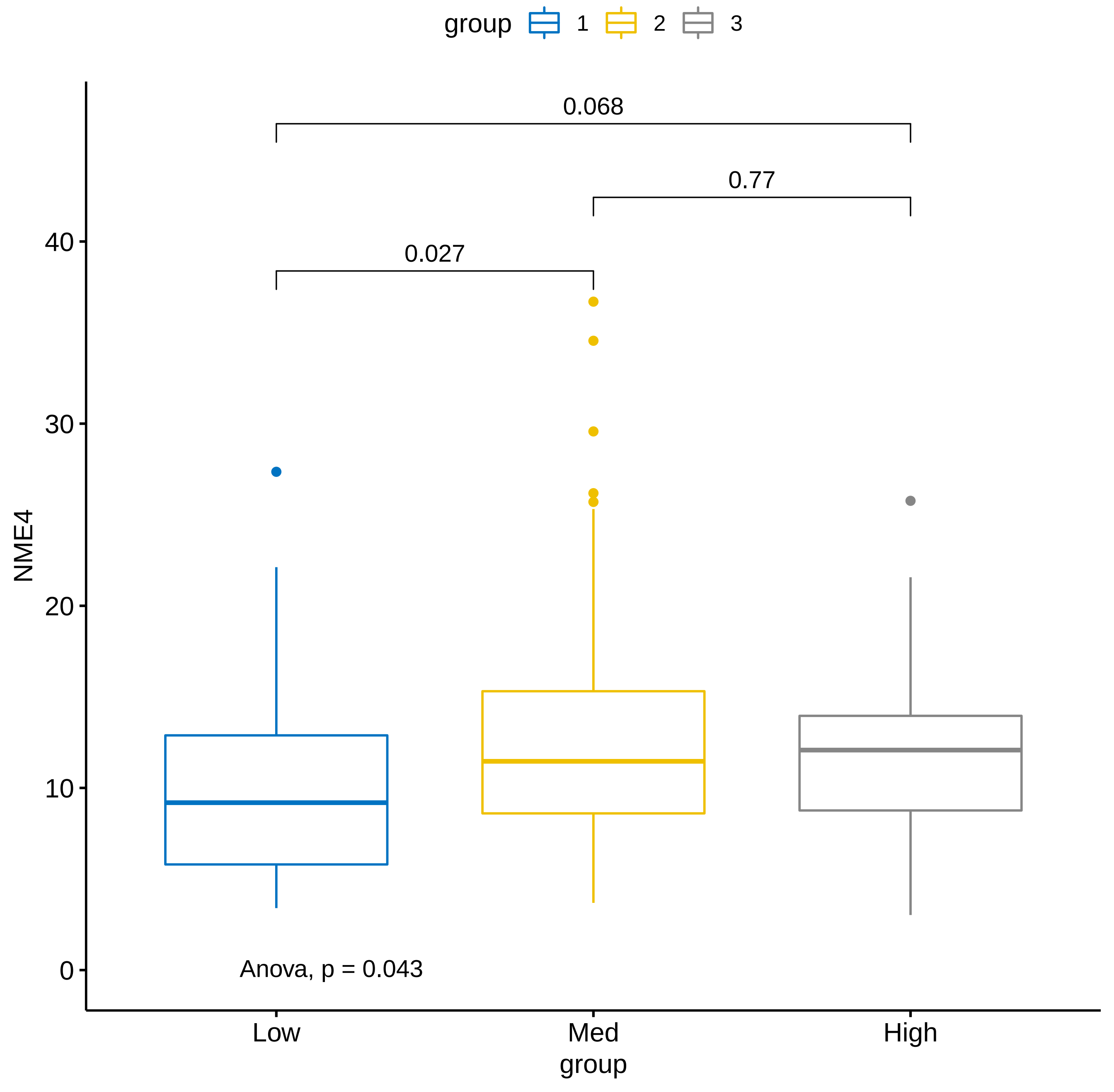
group 1 2 3
0.068
0.77
0.027
NME4
40
30
20
10
0
Anova, p = 0.043
Low
Med
High
group

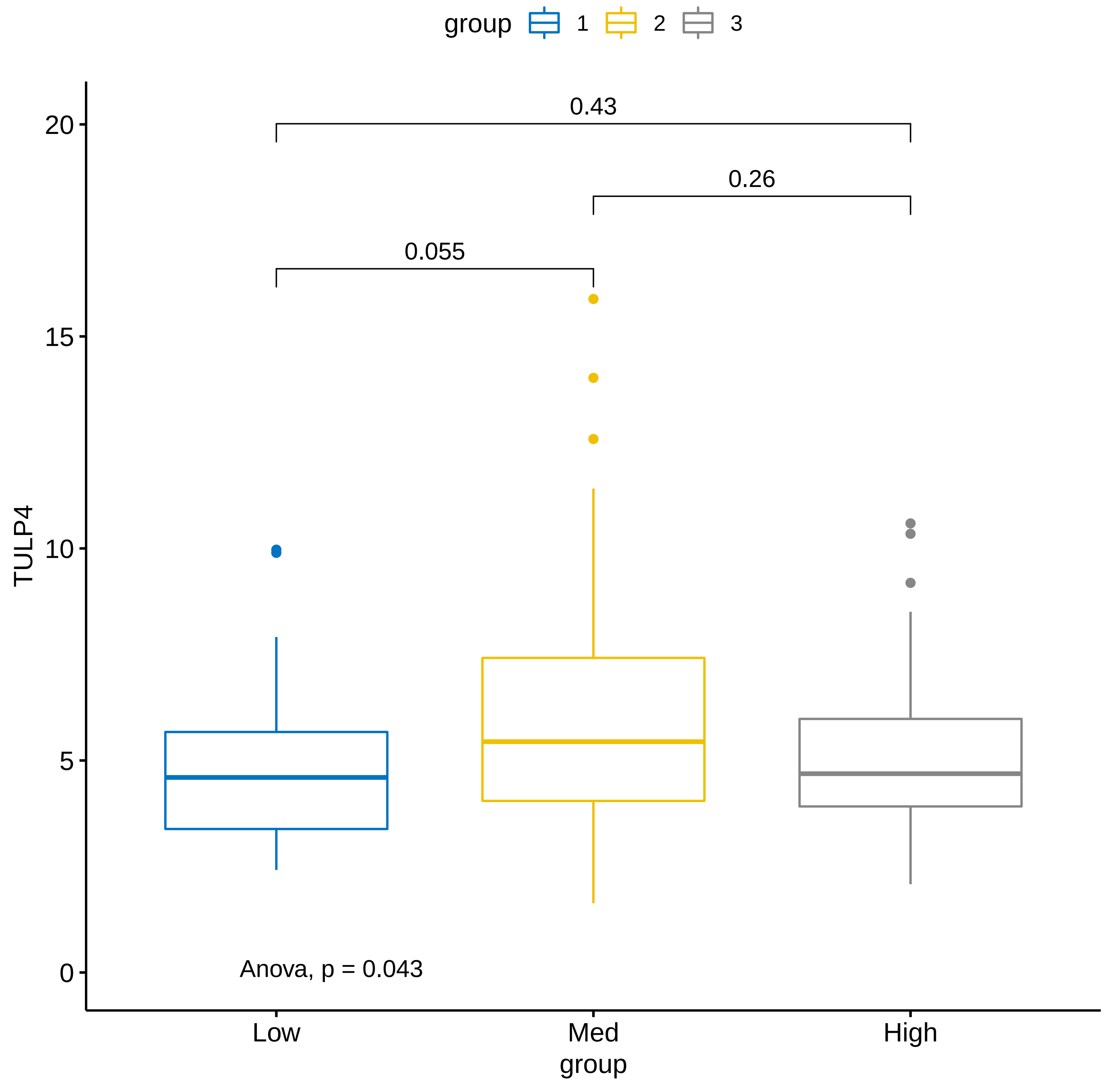

group 1 2 3
0.43
0.26
0.055
TULP4
20
15
10
5
0
Anova, p = 0.043
Low
Med
High
group

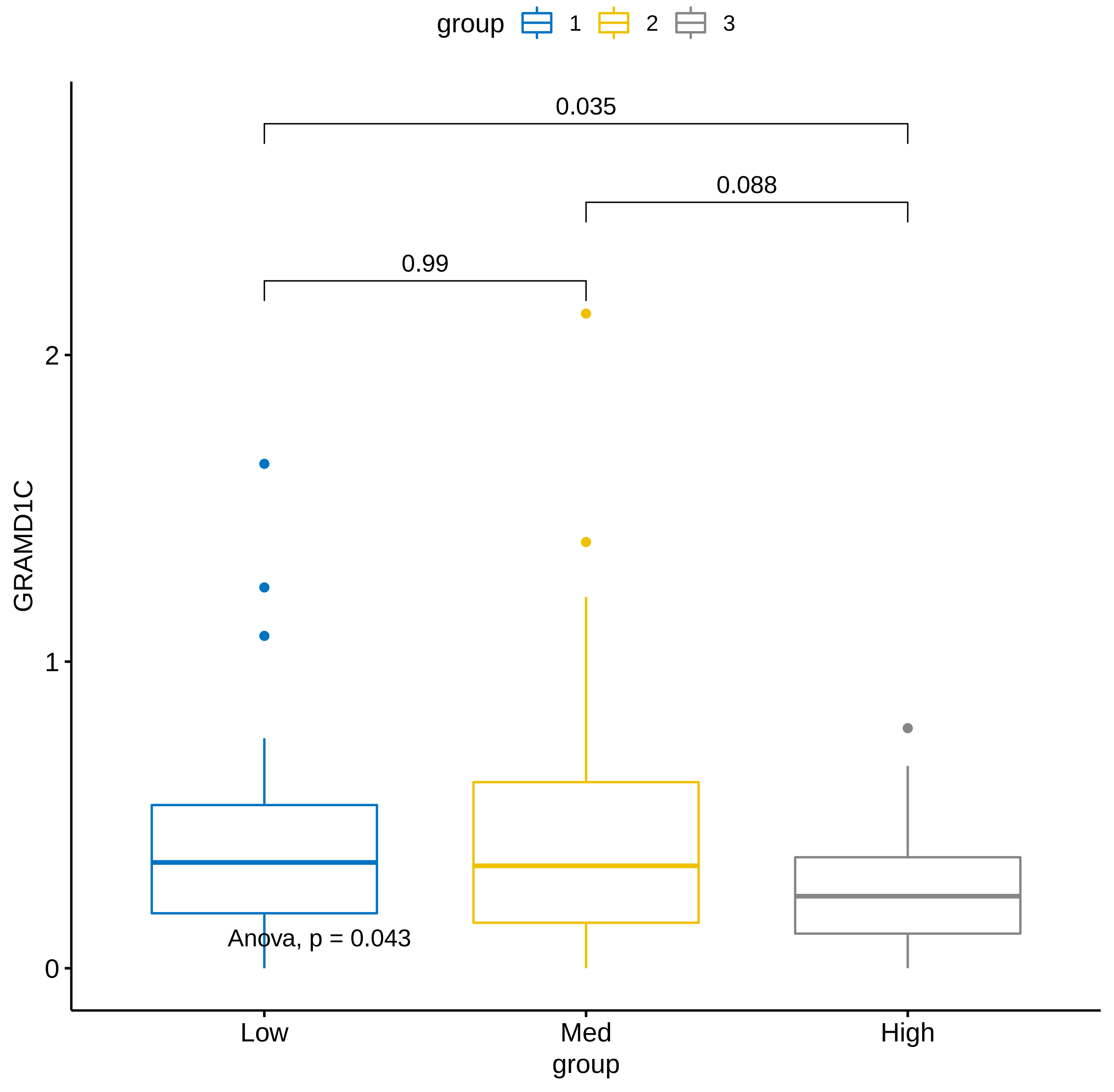
group
1
2
3
0.035
0.088
0.99
GRAMD1C
2
1
0
Anova, p = 0.043
Low
Med
High
group

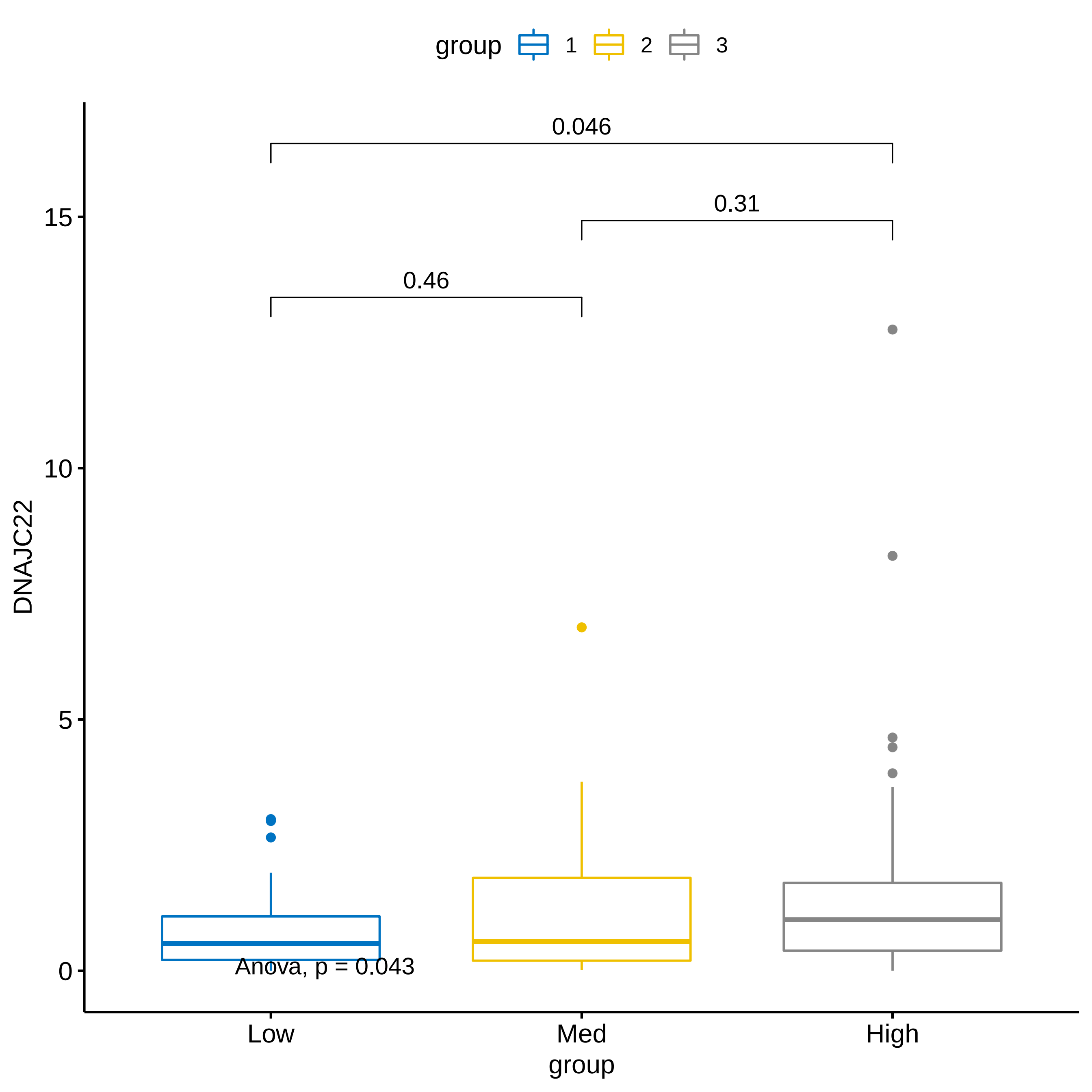
group
1
2
3
0.046
0.31
0.46
15
10
5
0
DNAJC22
Anova, p = 0.043
Low
Med
High
group

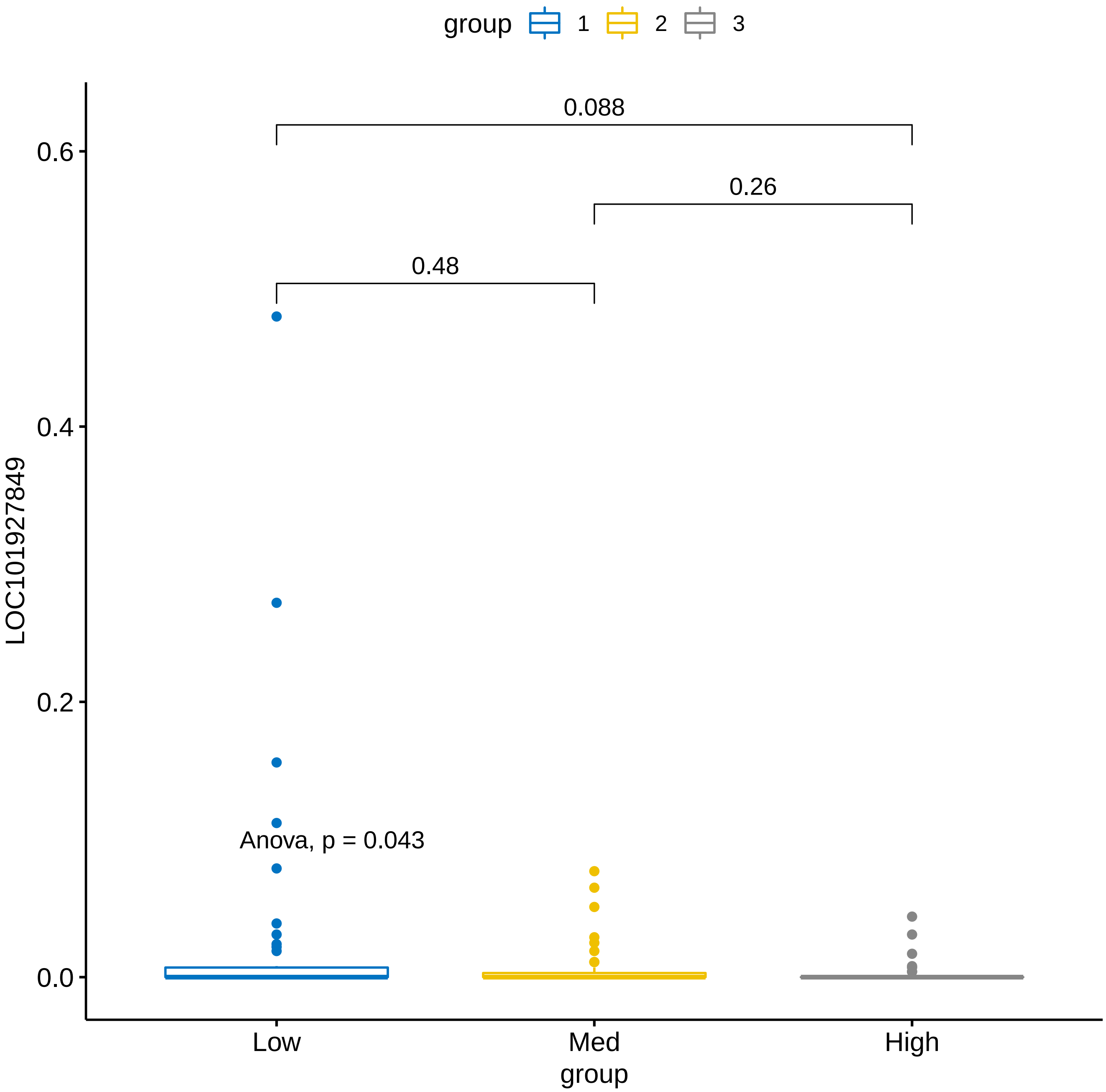
group
1
2
3
0.088
0.26
0.48
Anova, p = 0.043
0.6
0.4
0.2
0.0
LOC101927849
Low
Med
High
group

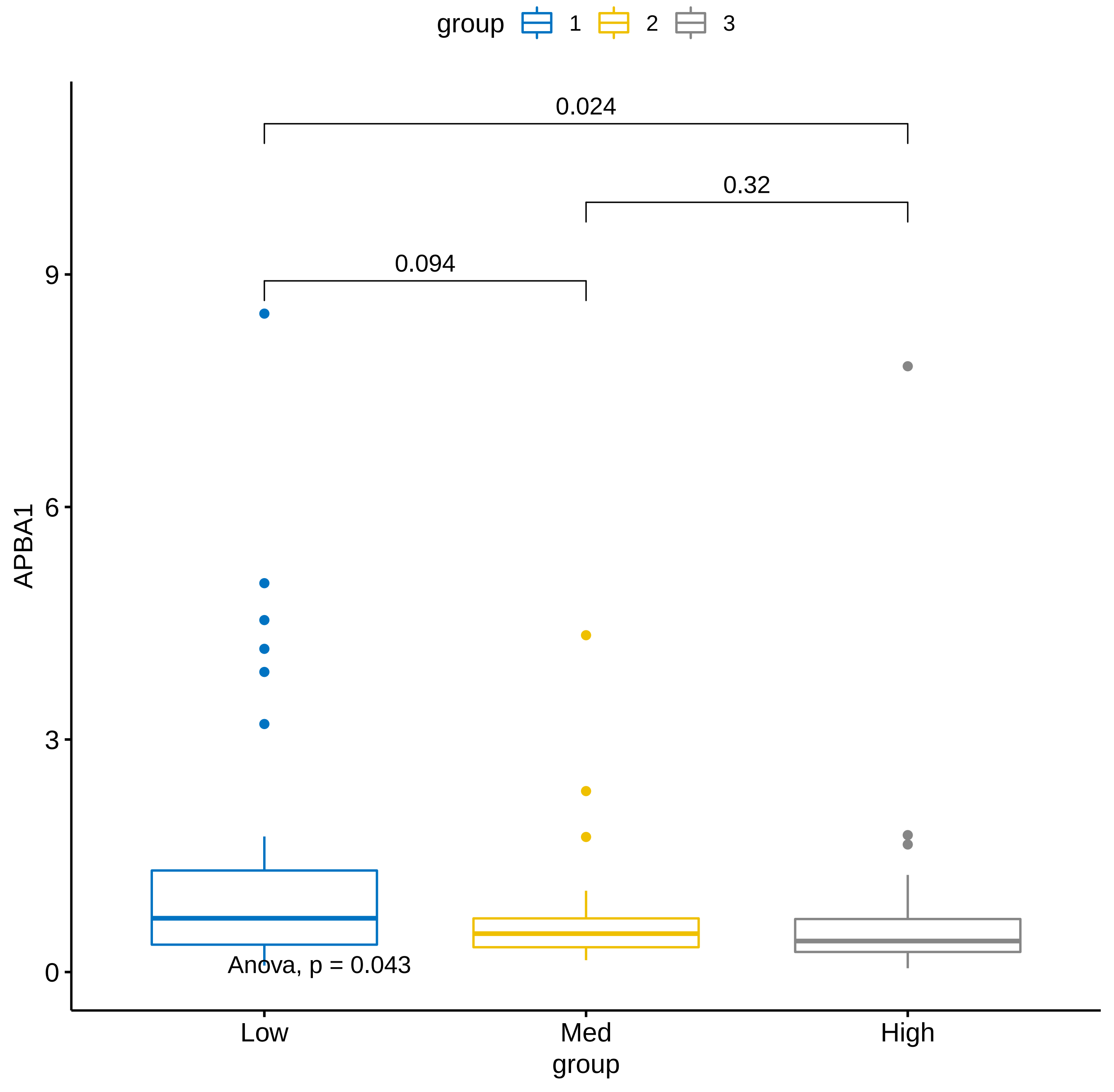

group
1
2
3
0.024
0.32
0.094
APBA1
9
6
3
0
Anova, p = 0.043
Low
Med
High
group

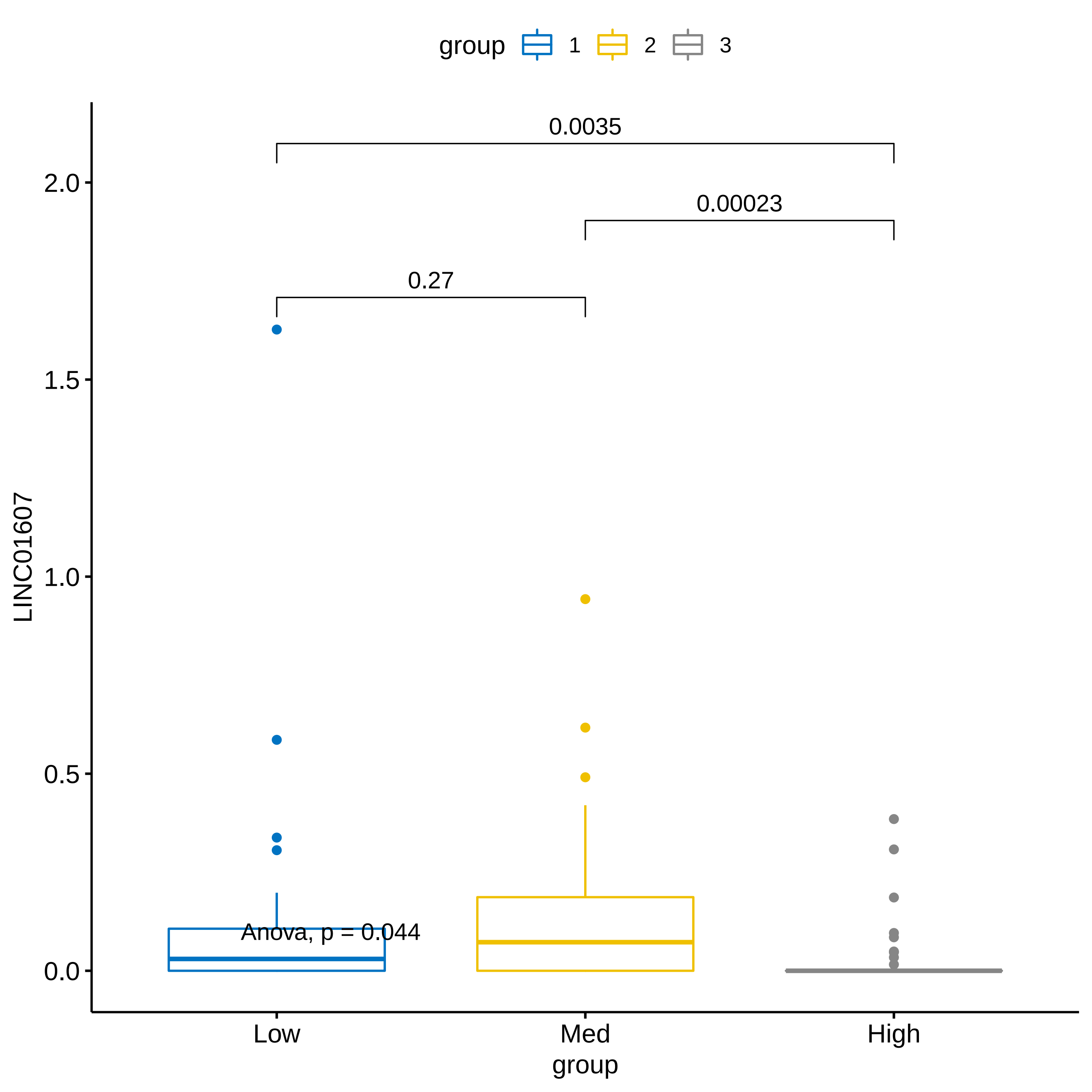
group 1 2 3
0.0035
0.00023
0.27
2.0
1.5
1.0
0.5
0.0
LINC01607
Anova, p = 0.044
Low
Med
High
group

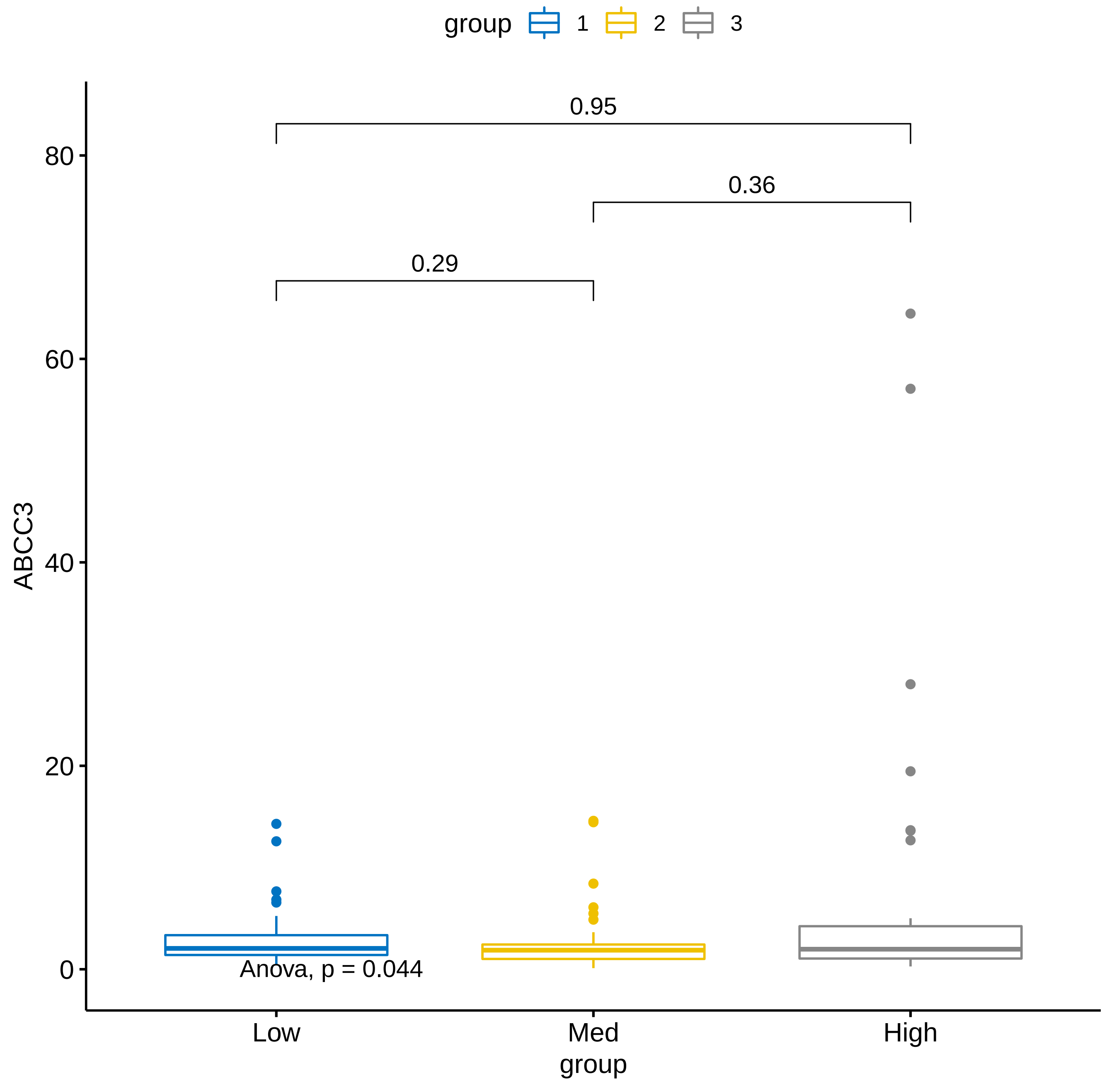
group
1
2
3
0.95
0.36
0.29
80
60
40
20
0
ABCC3
Anova, p = 0.044
Low
Med
High
group

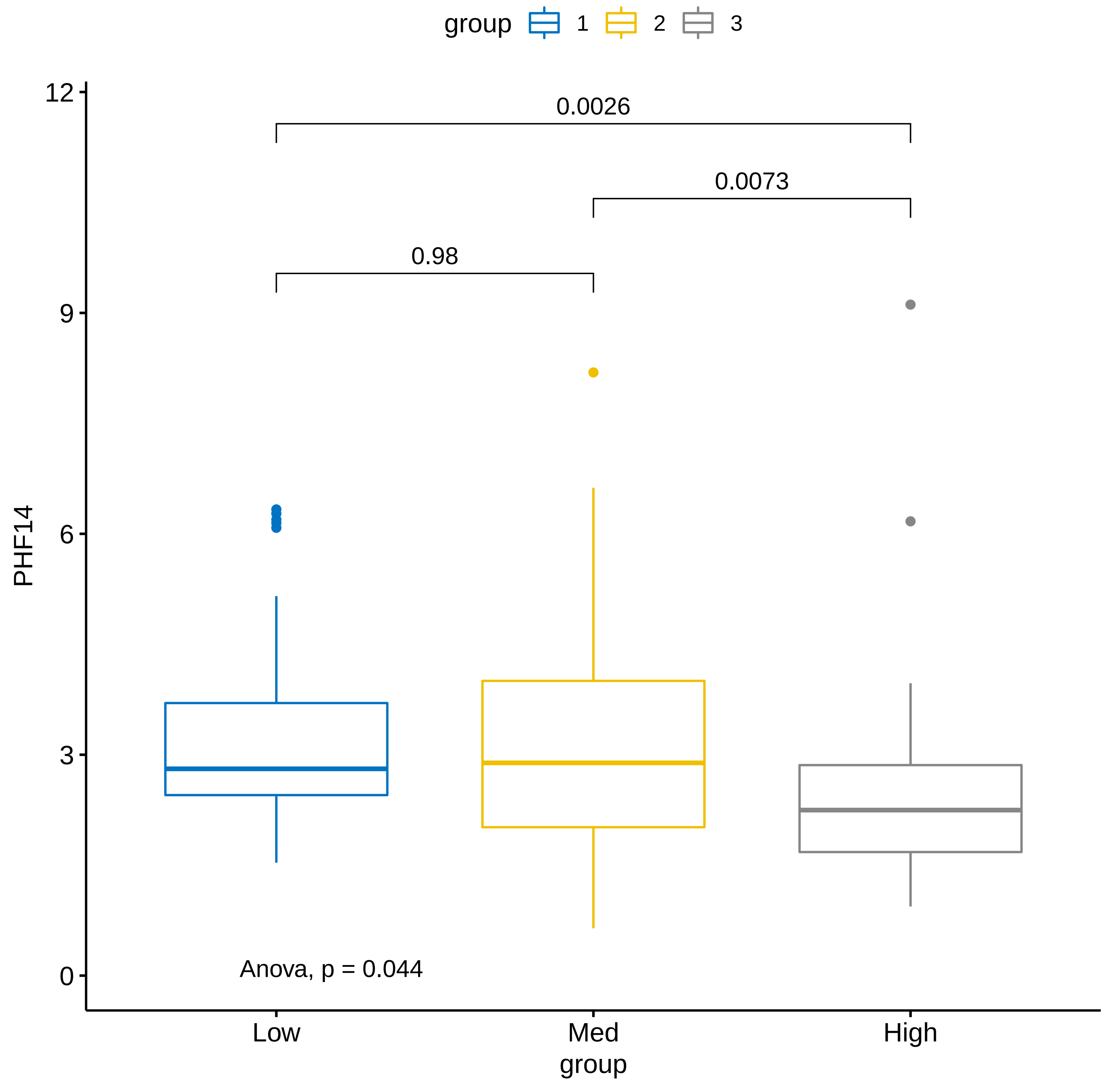
group
1
2
3
0.0026
0.0073
0.98
12
9
6
3
0
PHF14
Anova, p = 0.044
Low
Med
High
group

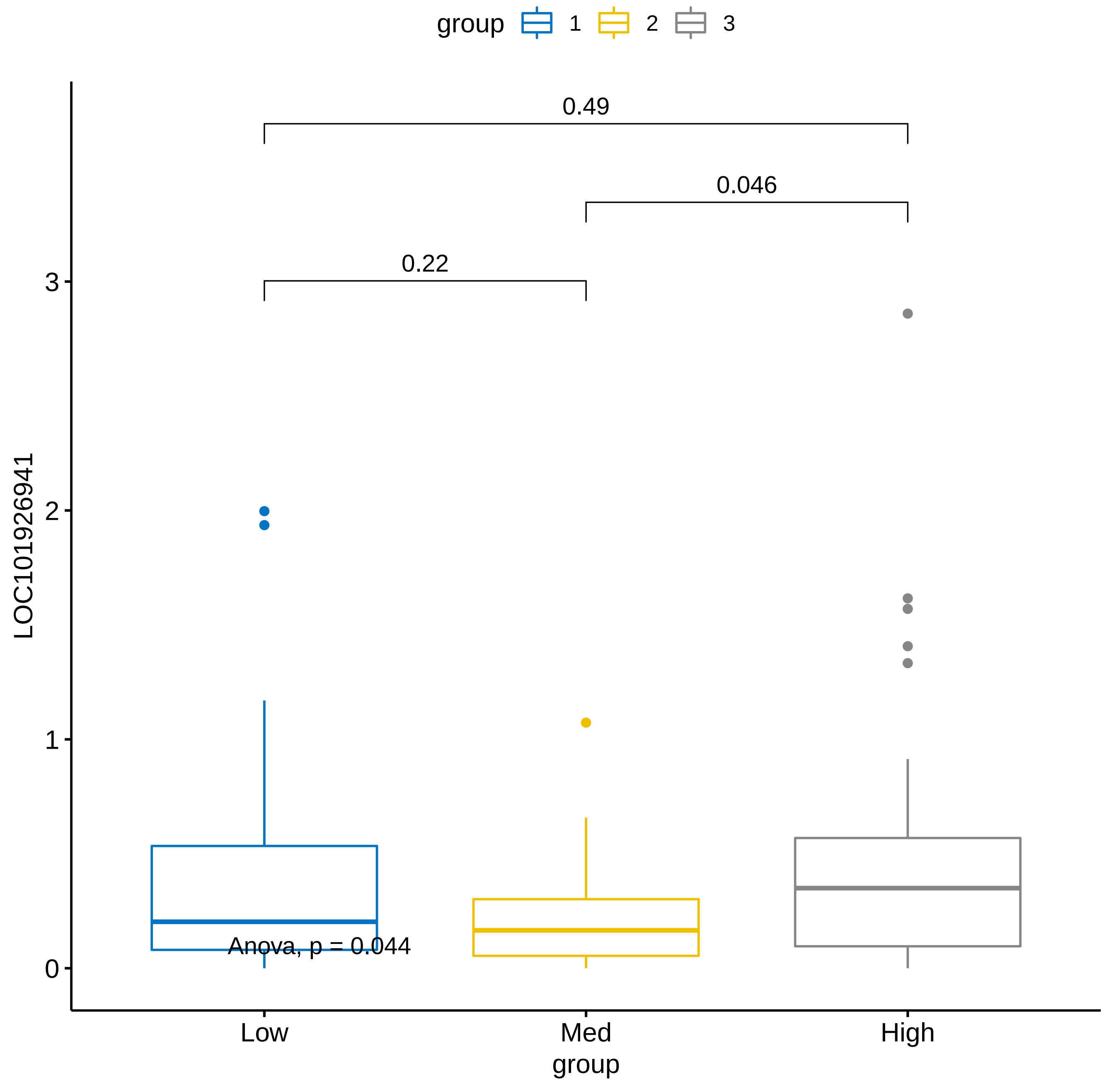
group
1
2
3
0.49
0.046
0.22
LOC101926941
3
2
1
0
Anova, p = 0.044
Low
Med
High
group

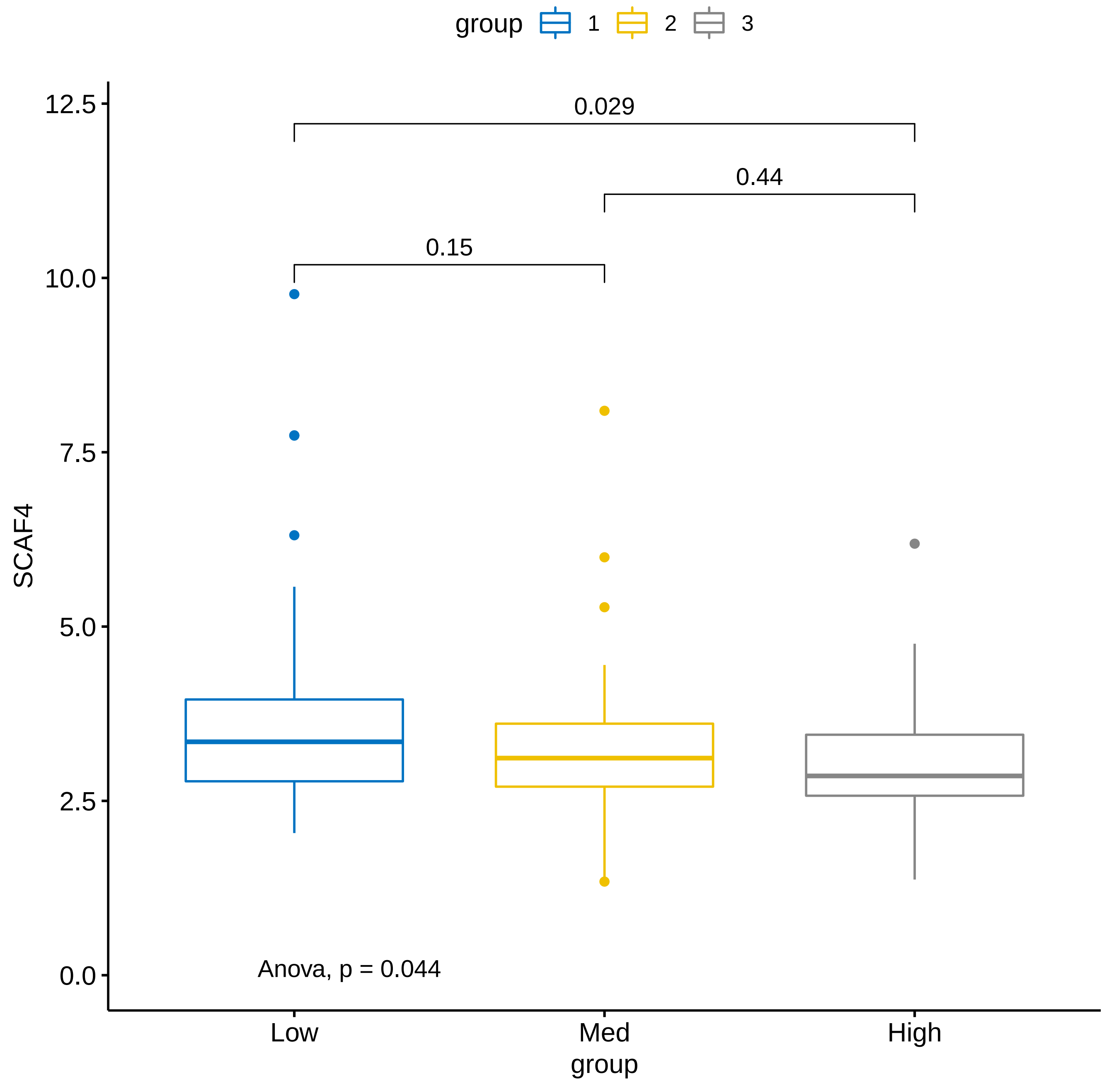
group
1
2
3
12.5
10.0
7.5
5.0
2.5
0.0
SCAF4
0.029
0.44
0.15
Anova, p = 0.044
Low
Med
High
group

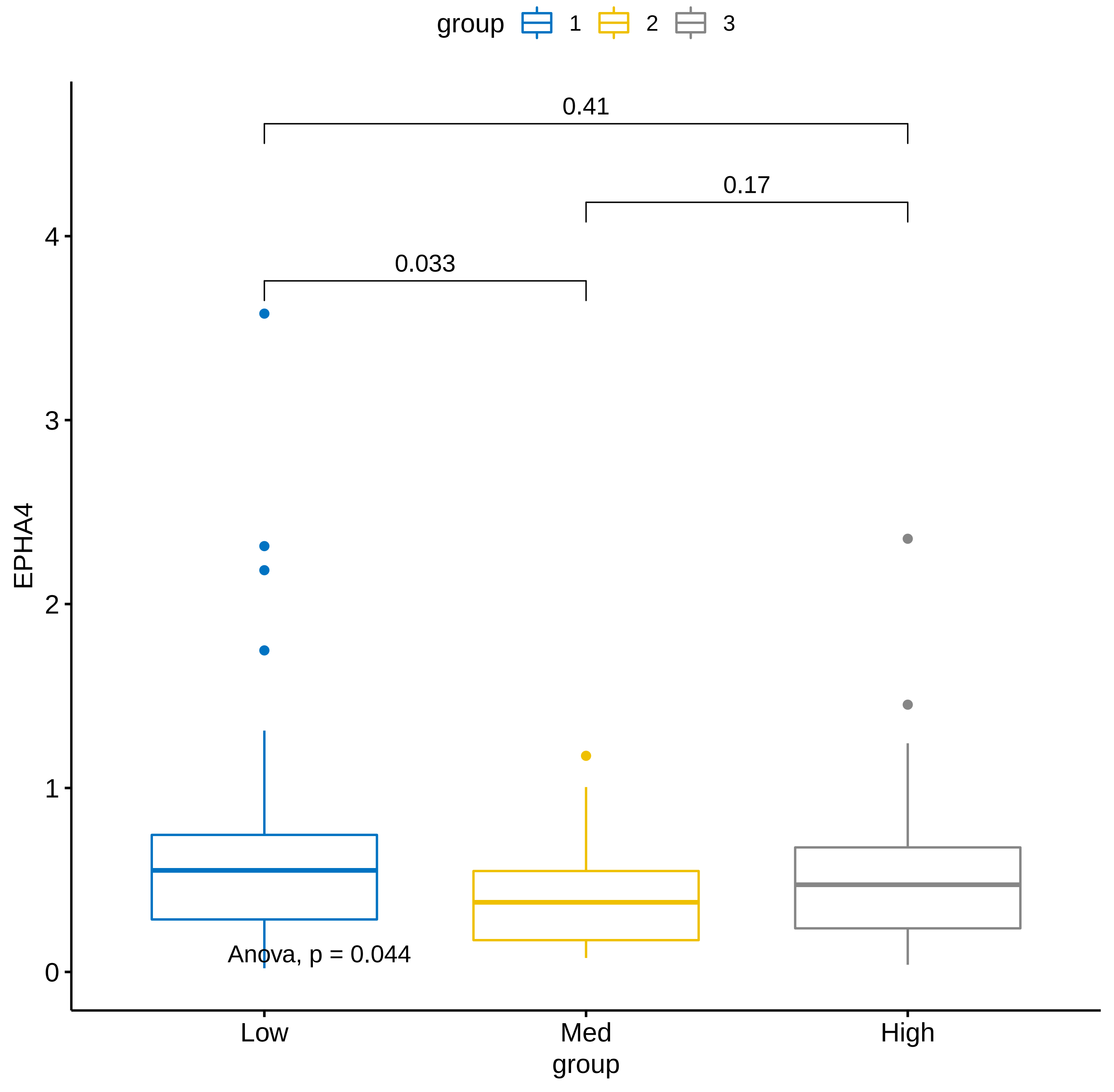

group 1 2 3
0.41
0.17
0.033
EPHA4
4
3
2
1
0
Anova, p = 0.044
Low
Med
High
group

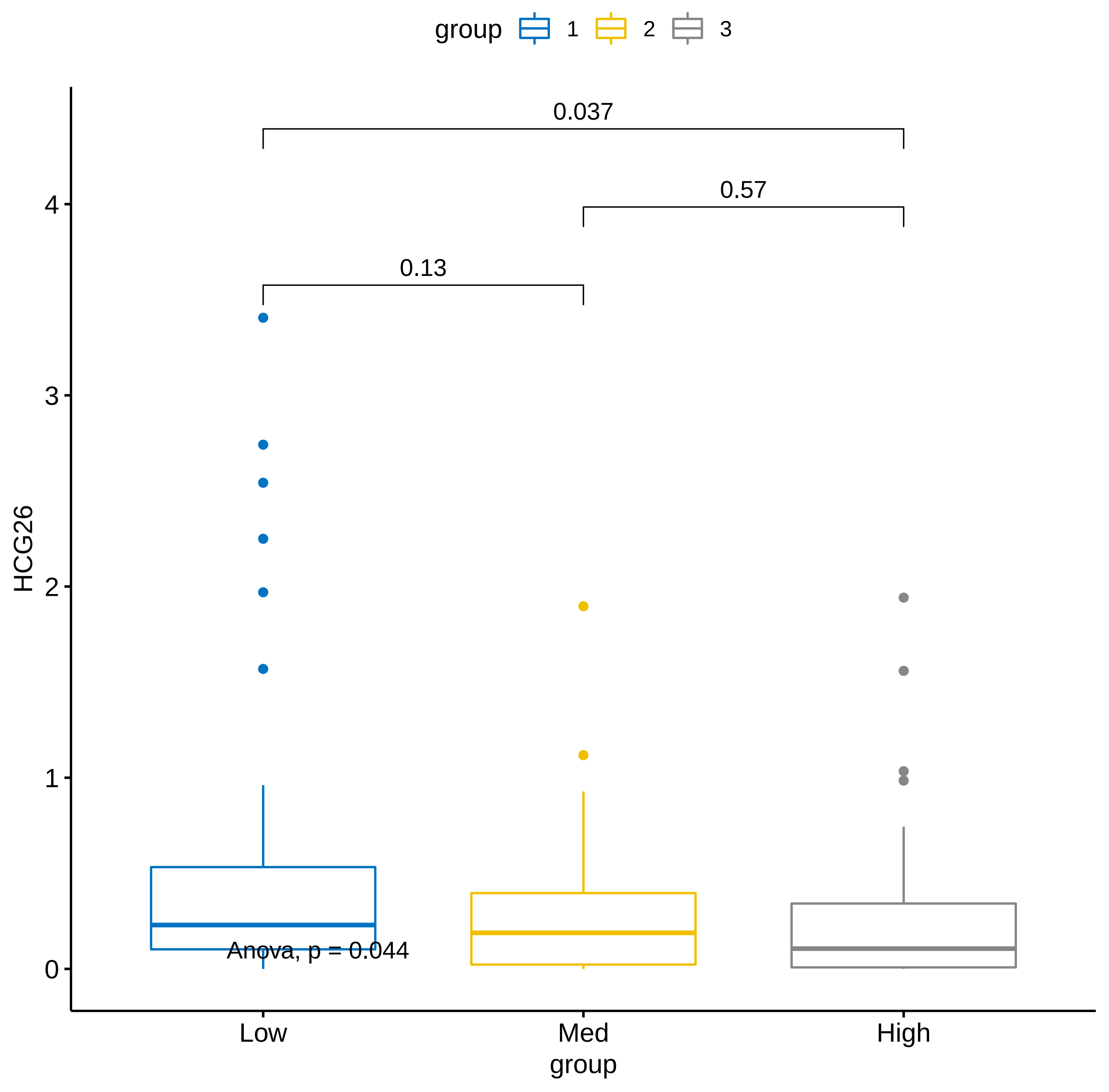

group
1
2
3
0.037
0.57
0.13
HCG26
4
3
2
1
0
Anova, p = 0.044
Low
Med
High
group

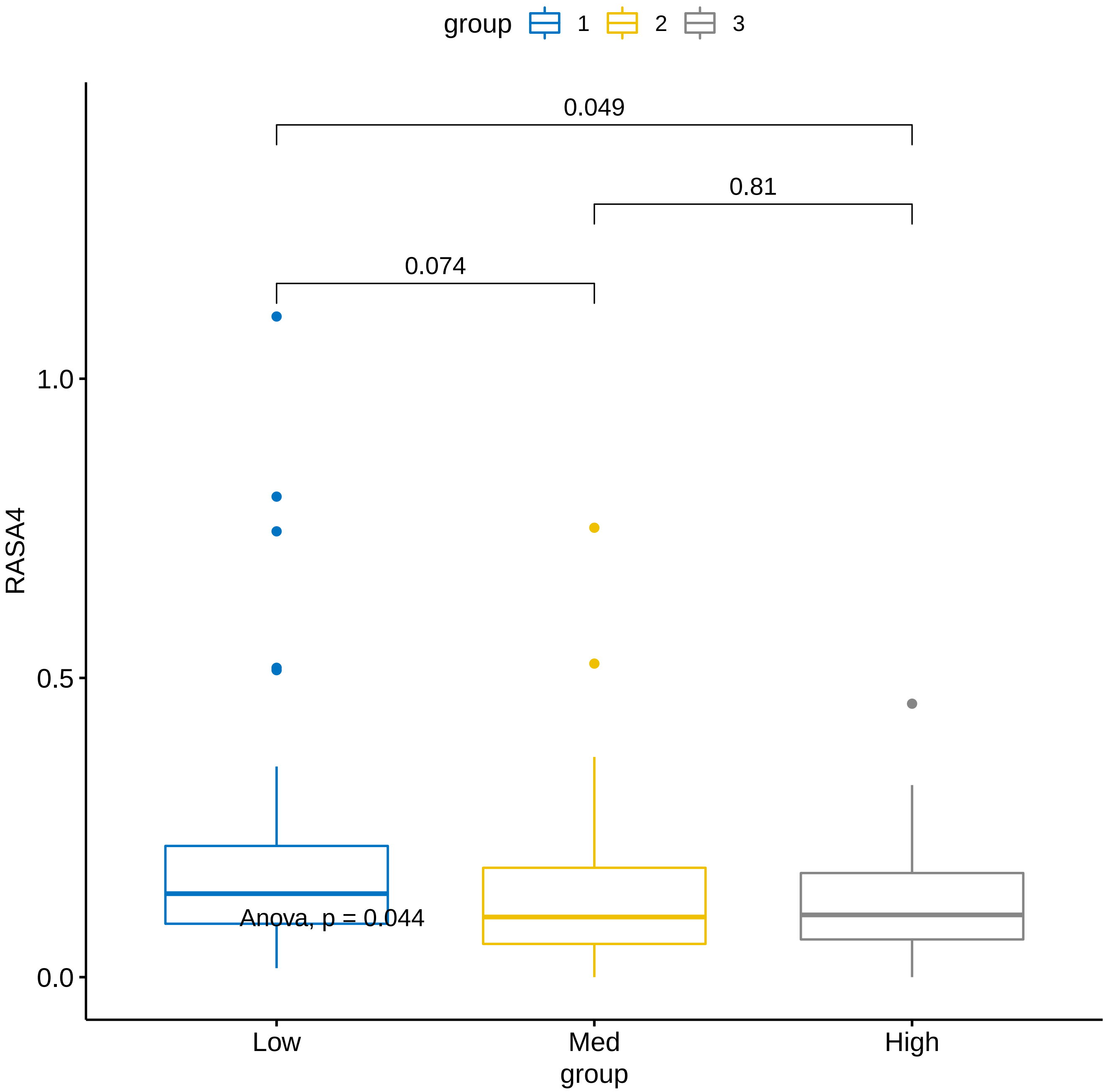
group
1
2
3
0.049
0.81
0.074
1.0
0.5
0.0
RASA4
Anova, p = 0.044
Low
Med
High
group

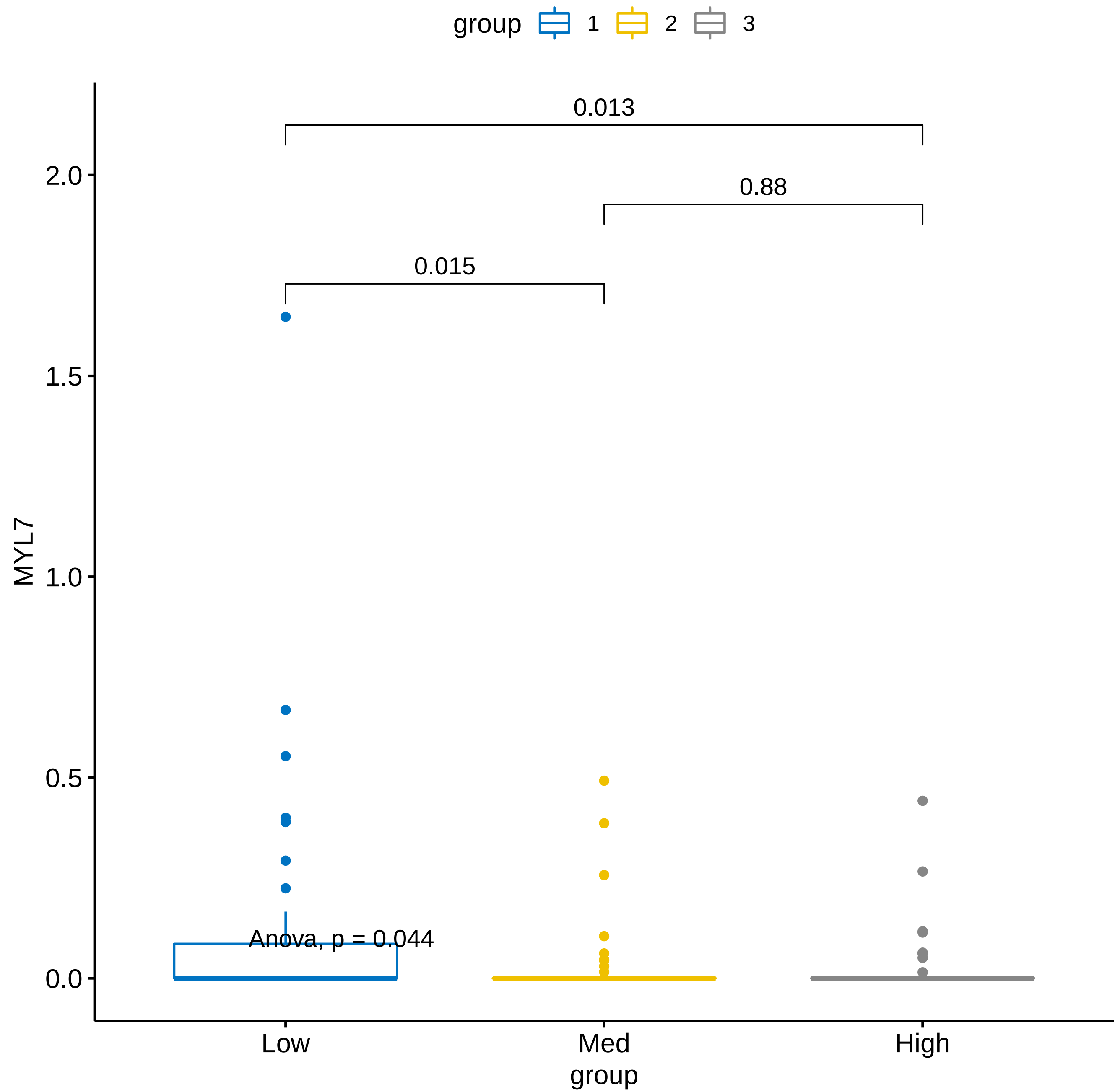
group
1
2
3
0.013
0.88
0.015
2.0
1.5
1.0
0.5
0.0
MYL7
Anova, p = 0.044
Low
Med
High
group

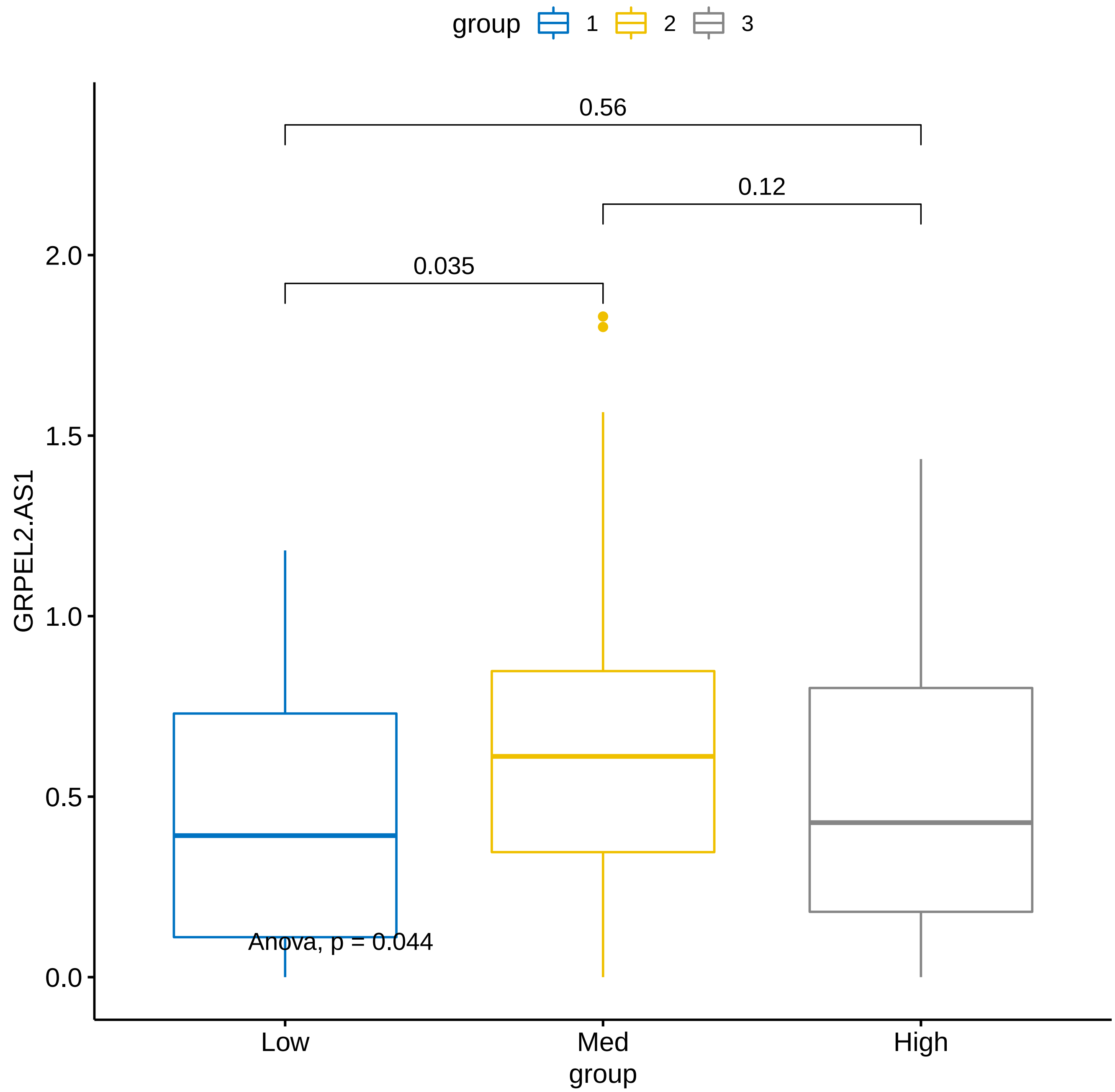
group
1
2
3
0.56
0.12
0.035
2.0
1.5
1.0
0.5
0.0
GRPEL2.AS1
Anova, p = 0.044
Low
Med
High
group

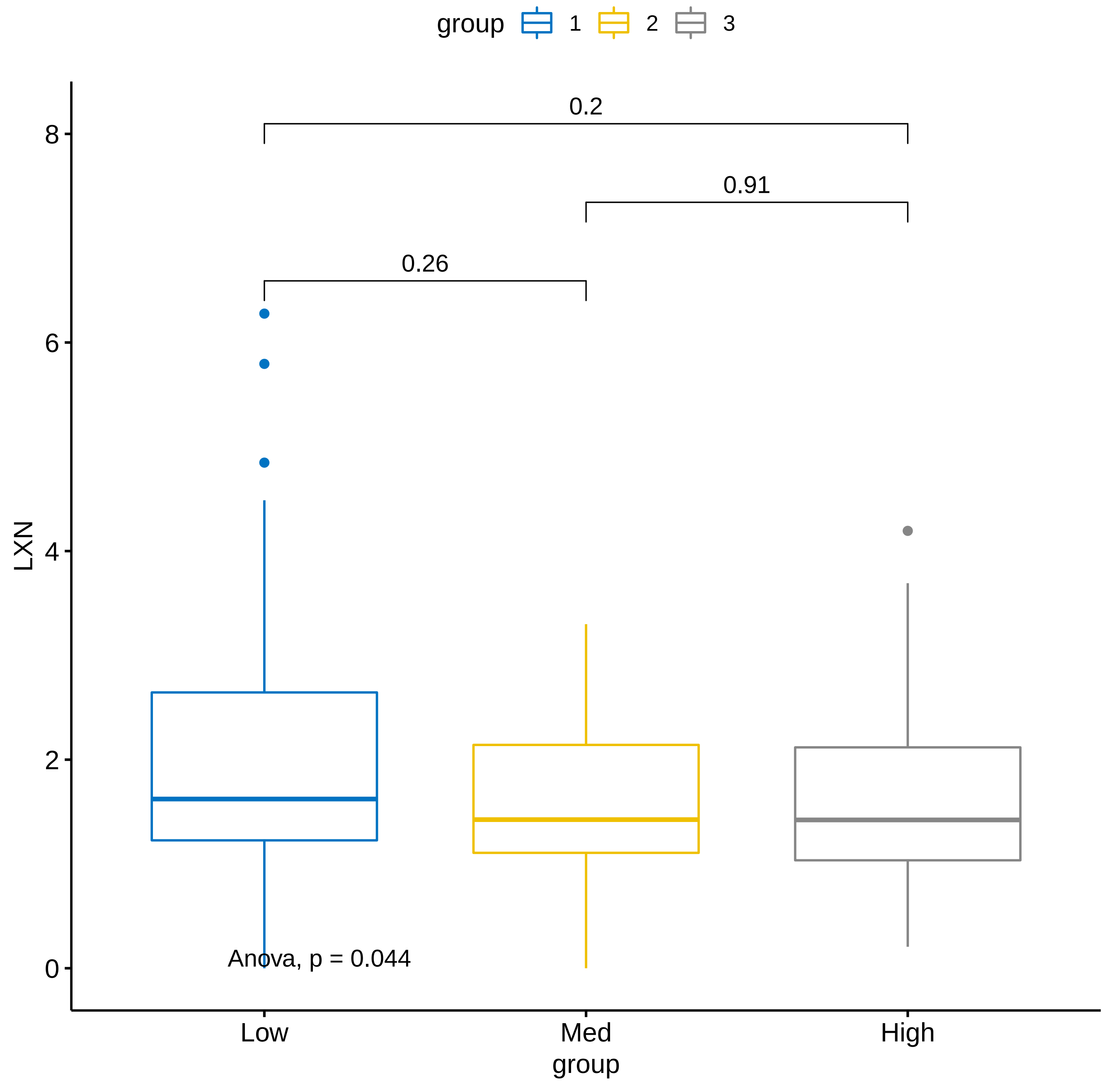
group
1
2
3
0.2
0.91
0.26
LXN
8
6
4
2
0
Anova, p = 0.044
Low
Med
High
group

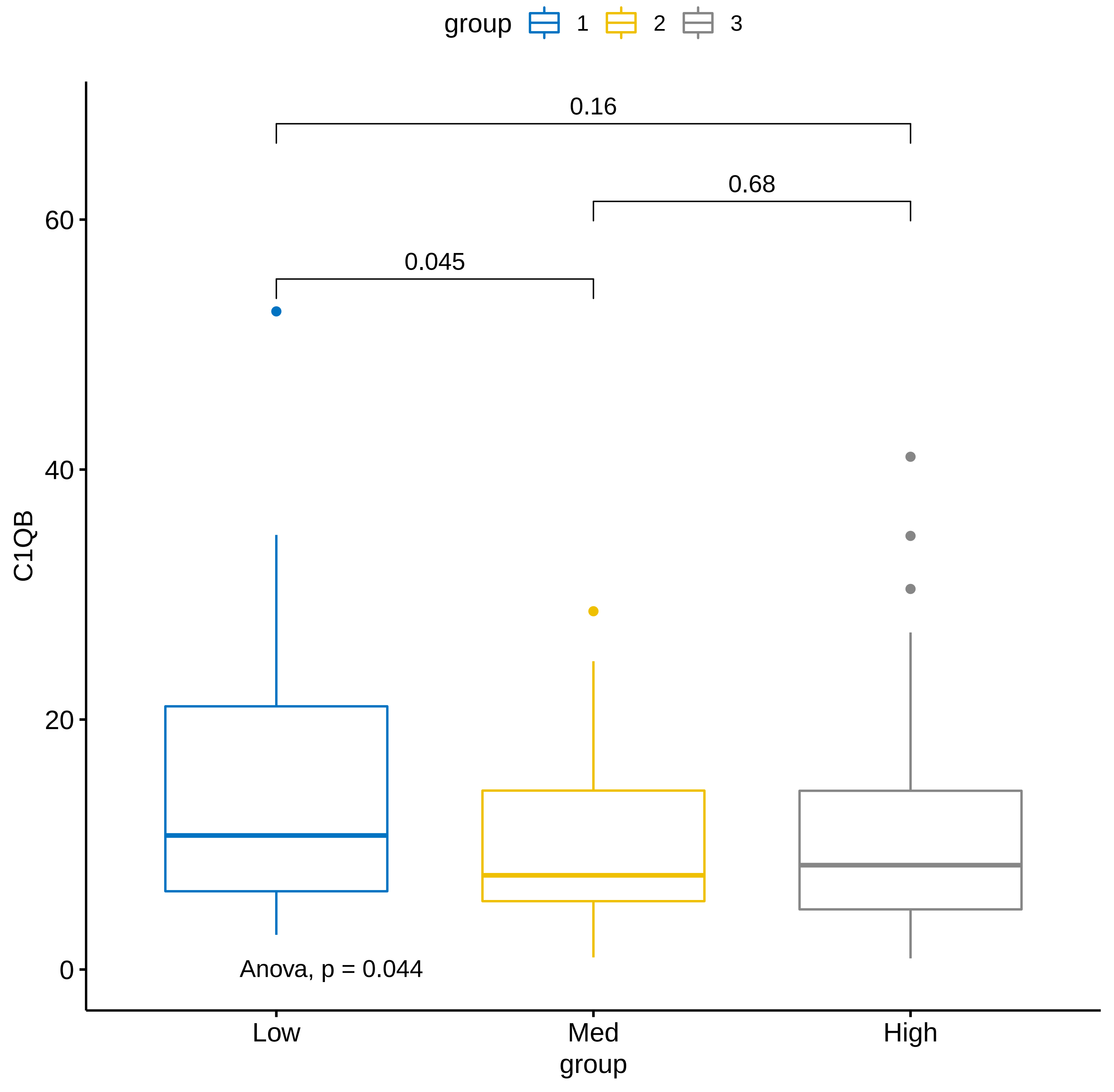

group
1
2
3
0.16
0.68
0.045
60
40
20
0
C1QB
Anova, p = 0.044
Low
Med
High
group

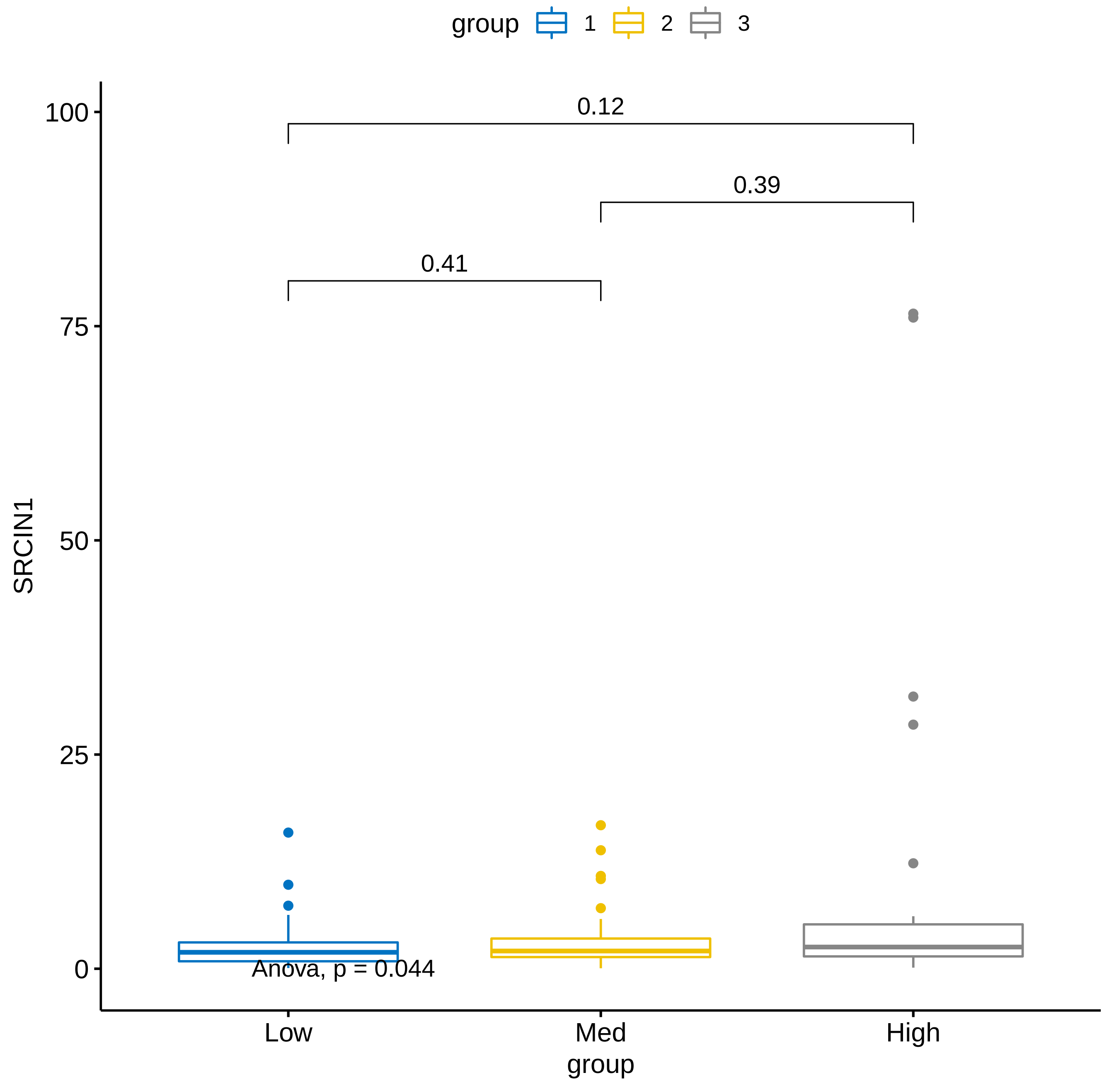
group
1
2
3
0.12
0.39
0.41
100
75
50
25
0
SRCIN1
Anova, p = 0.044
Low
Med
High
group

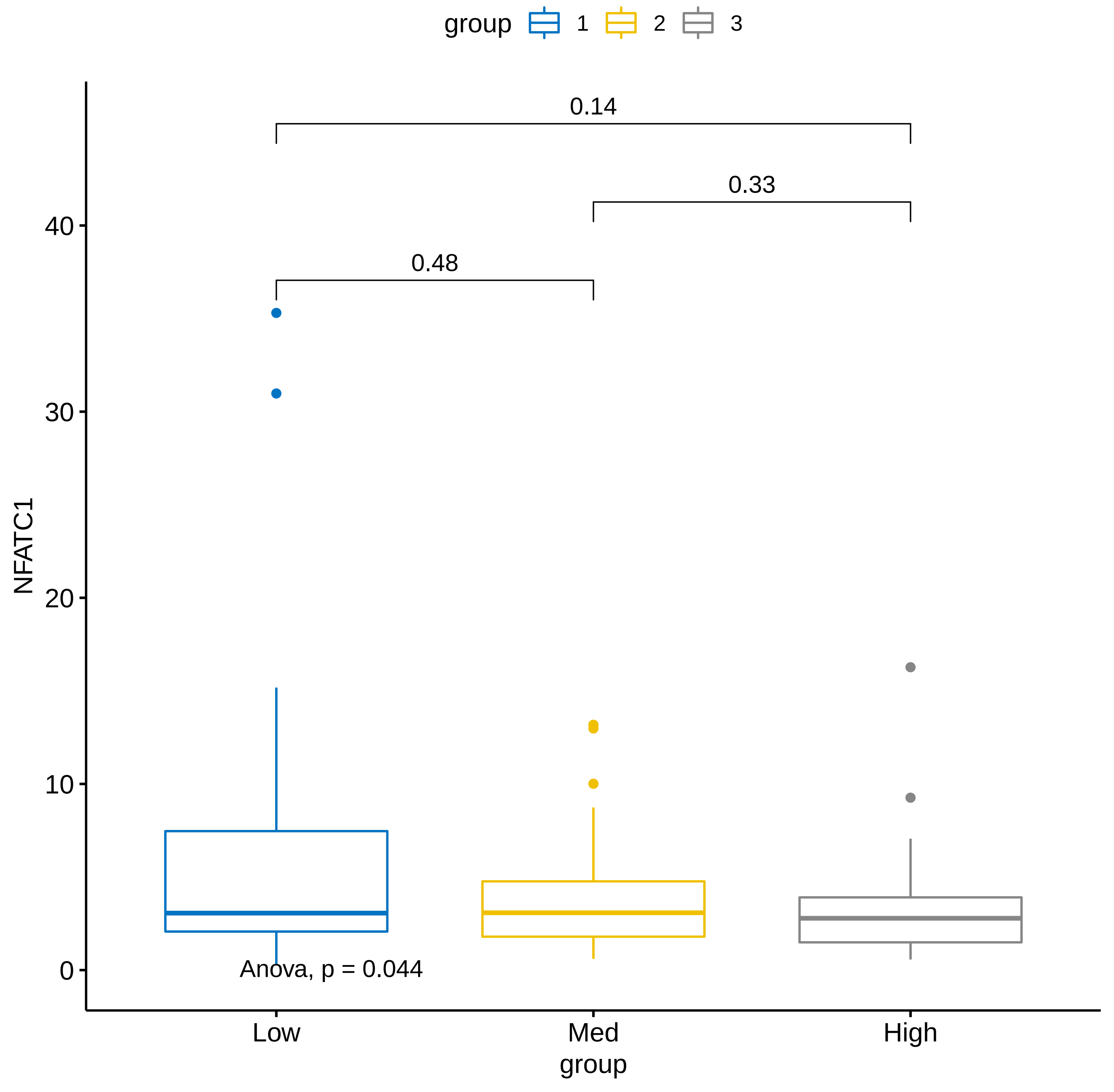

group
1
2
3
0.14
0.33
0.48
40
30
20
10
0
NFATC1
Anova, p = 0.044
Low
Med
High
group

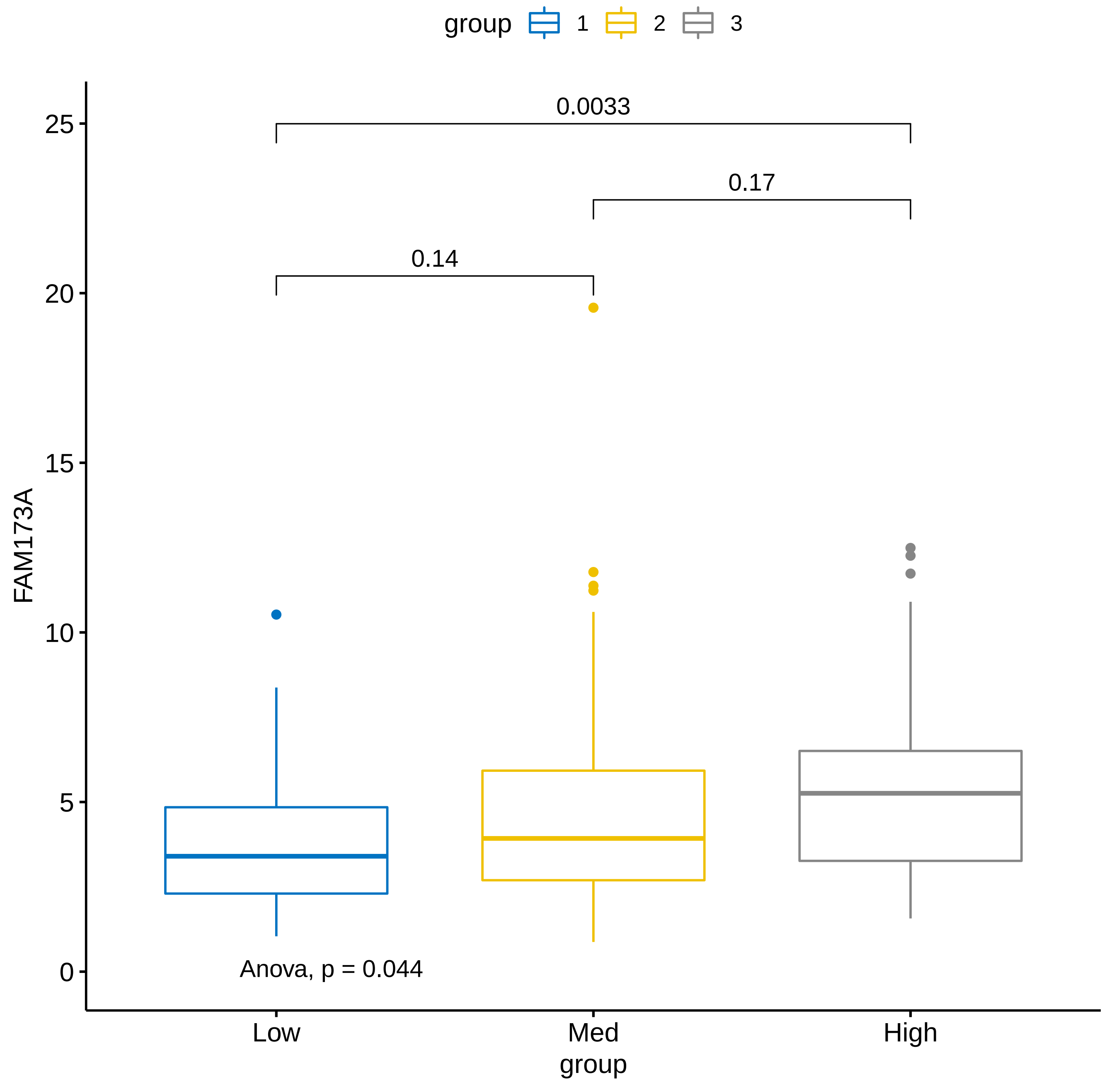
group
1
2
3
0.0033
0.17
0.14
25
20
15
10
5
0
FAM173A
Anova, p = 0.044
Low
Med
High
group

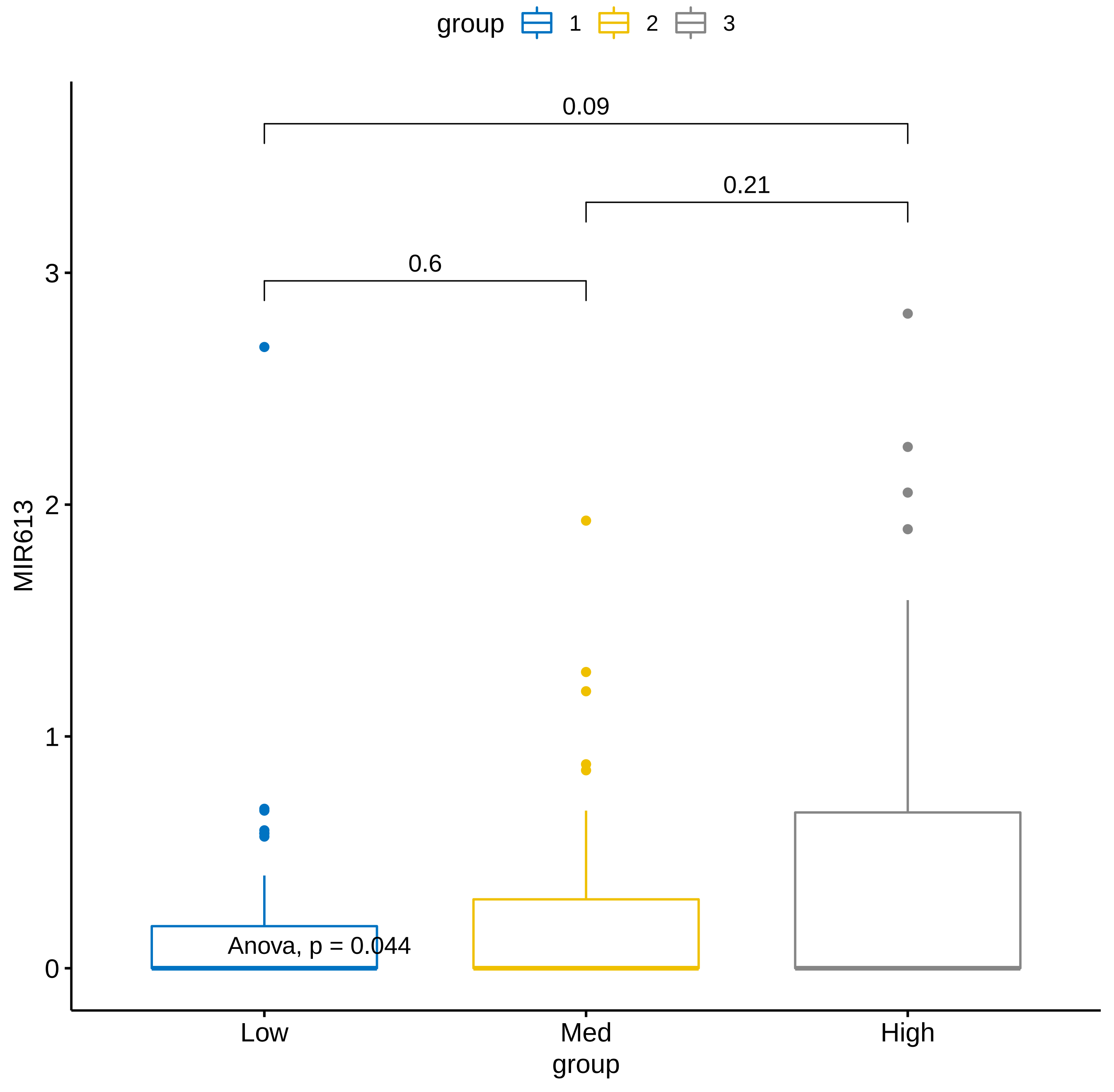
group
1
2
3
0.09
0.21
0.6
3
2
1
0
MIR613
Anova, p = 0.044
Low
Med
High
group

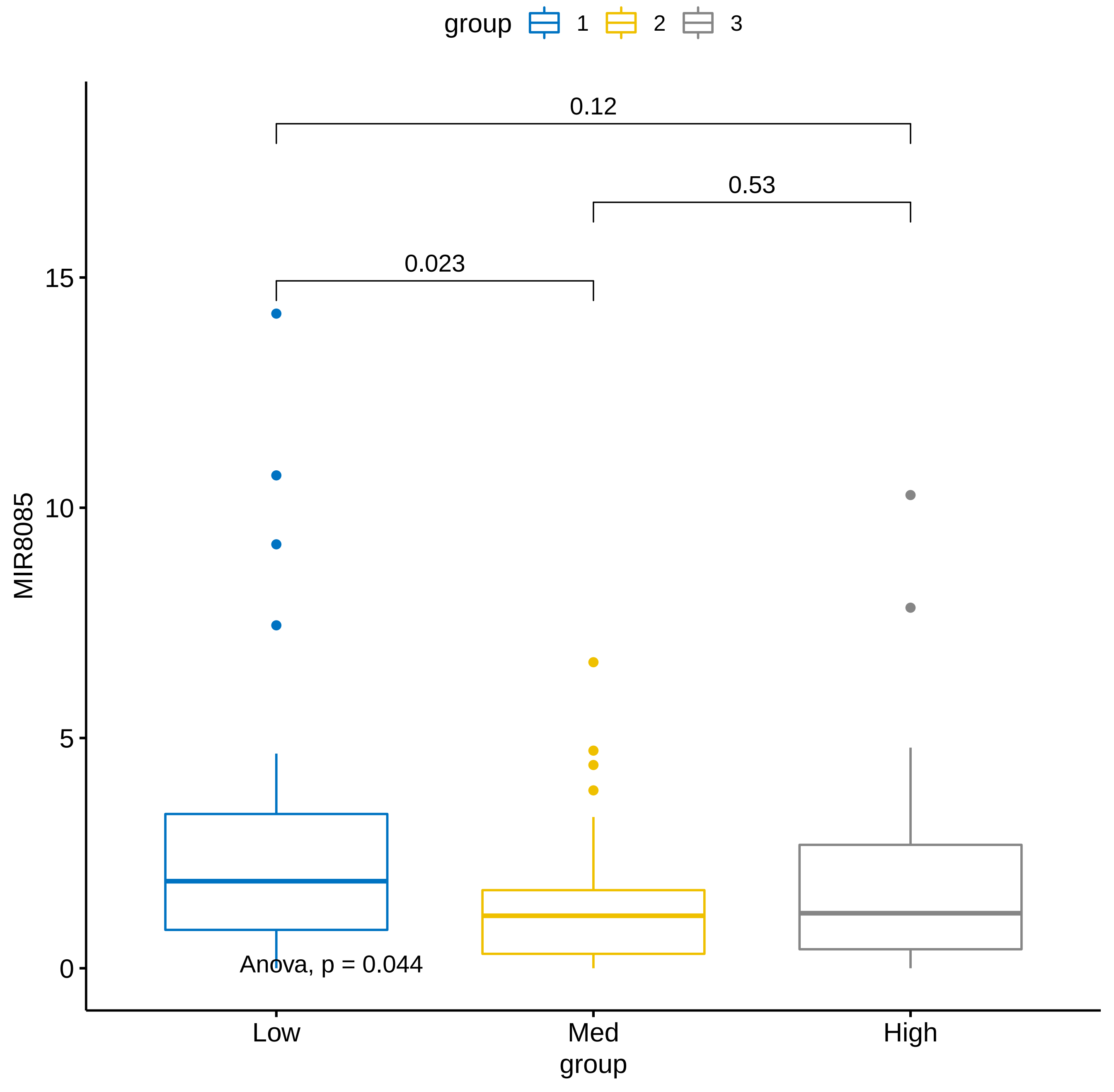
group 1 2 3
0.12
0.53
0.023
MIR8085
15
10
5
0
Anova, p = 0.044
Low
Med
High
group

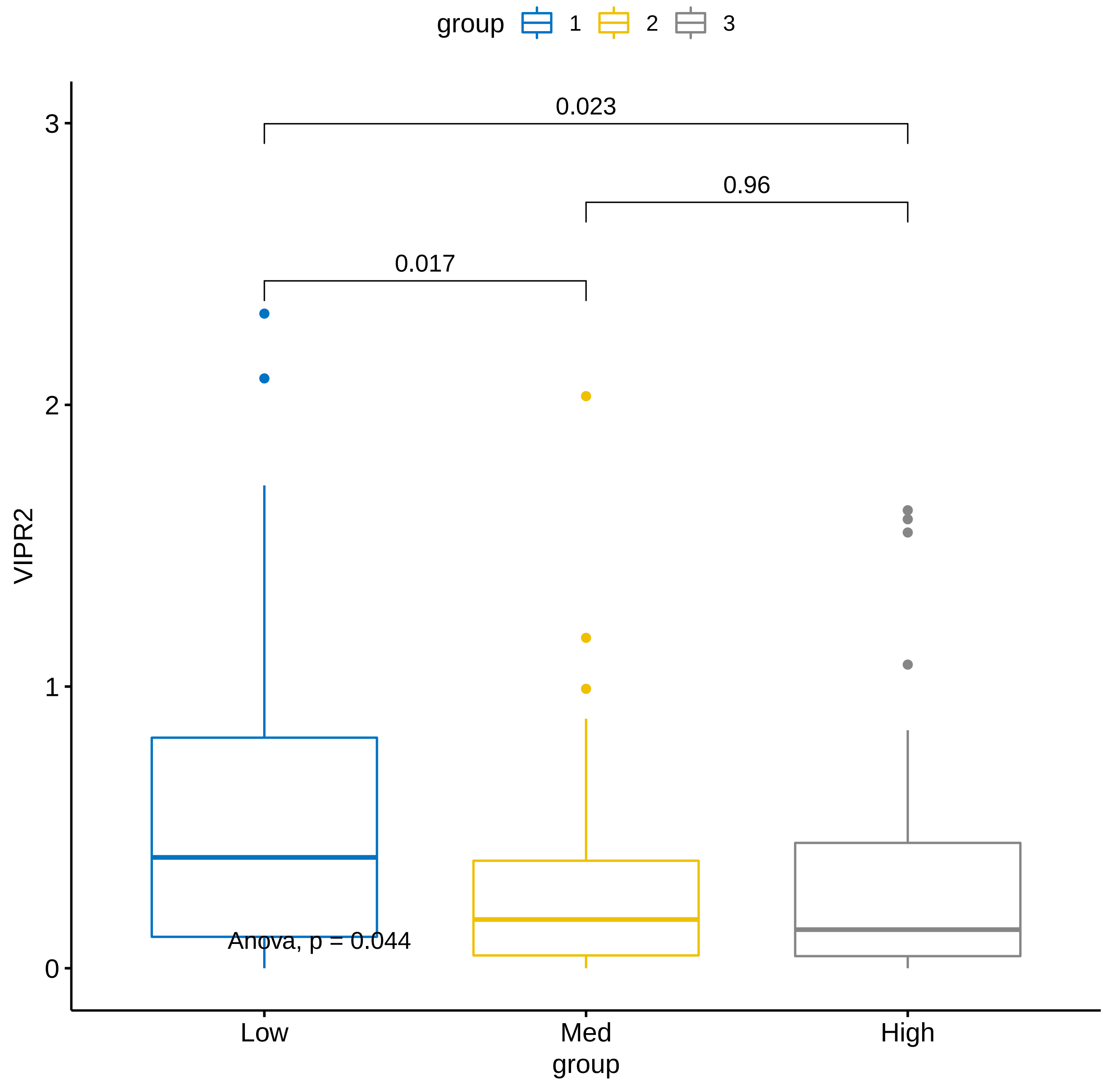
group
1
2
3
0.023
0.96
0.017
Anova, p = 0.044
VIPR2
3
2
1
0
Low
Med
High
group

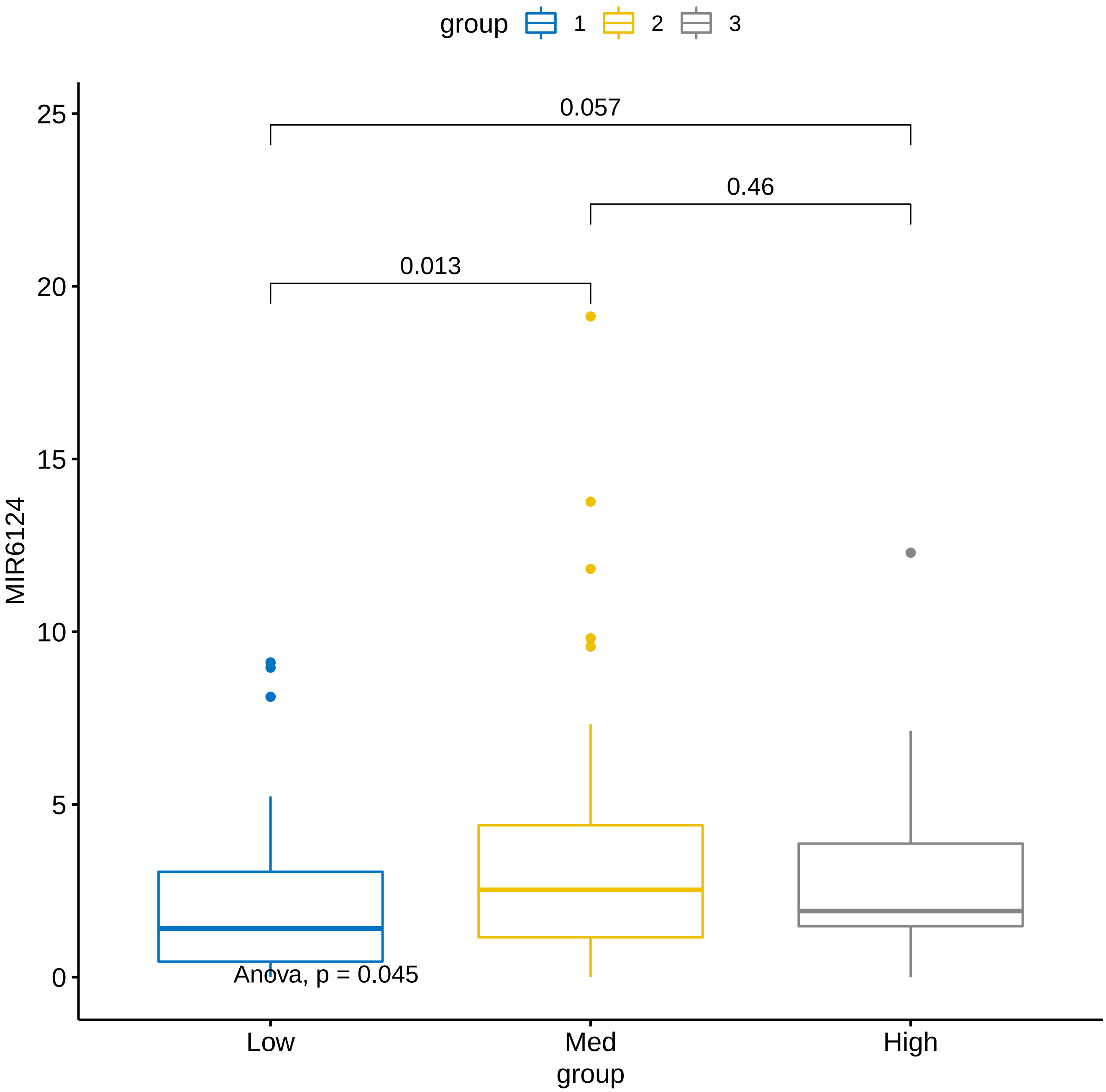

group
1
2
3
0.057
0.46
0.013
MIR6124
25
20
15
10
5
0
Anova, p = 0.045
Low
Med
High
group

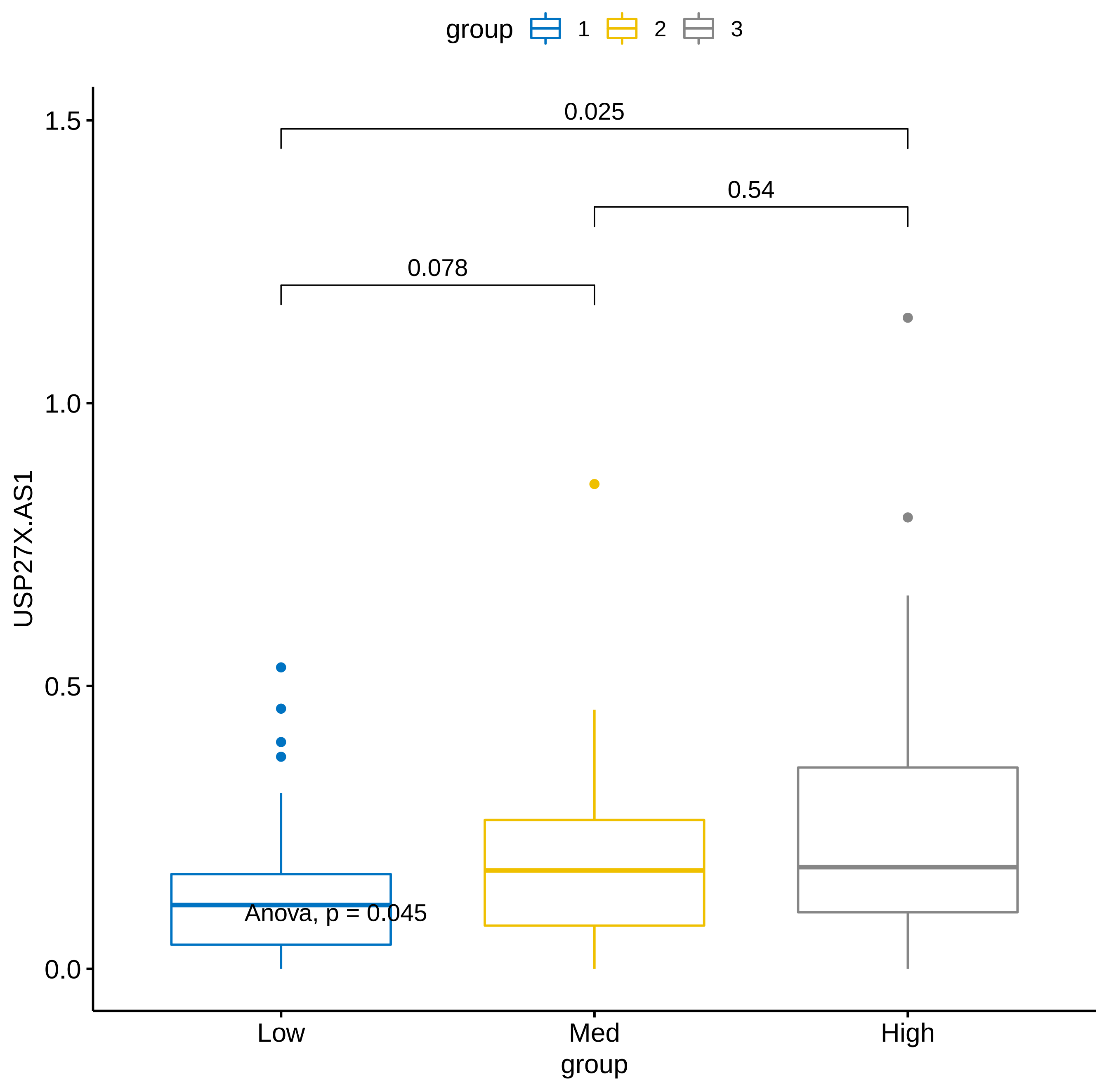
group 1 2 3
0.025
0.54
0.078
1.5
1.0
0.5
0.0
USP27X.AS1
Anova, p = 0.045
Low
Med
High
group

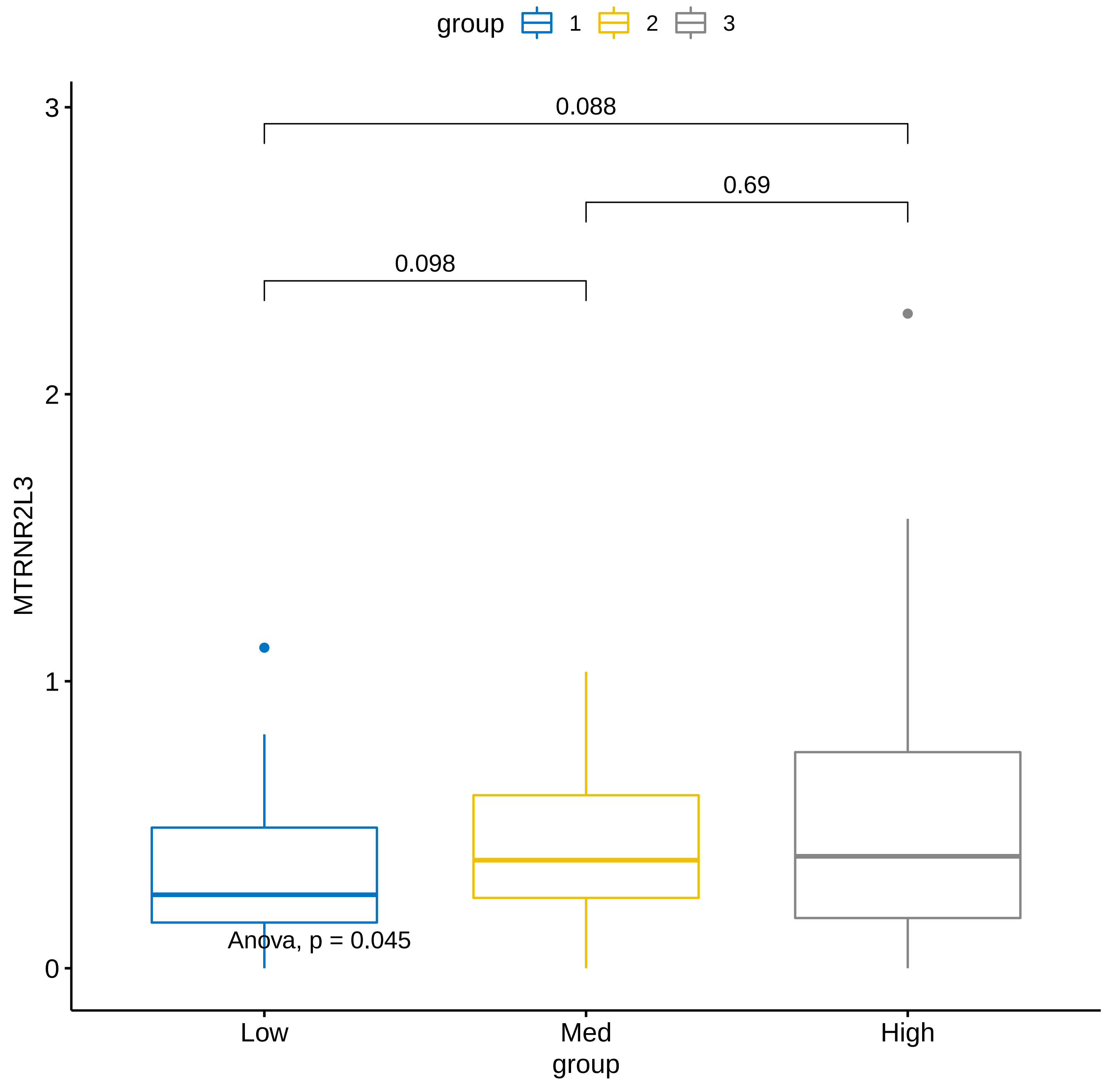
group
1
2
3
3
2
1
0
MTRNR2L3
0.088
0.69
0.098
Anova, p = 0.045
Low
Med
High
group

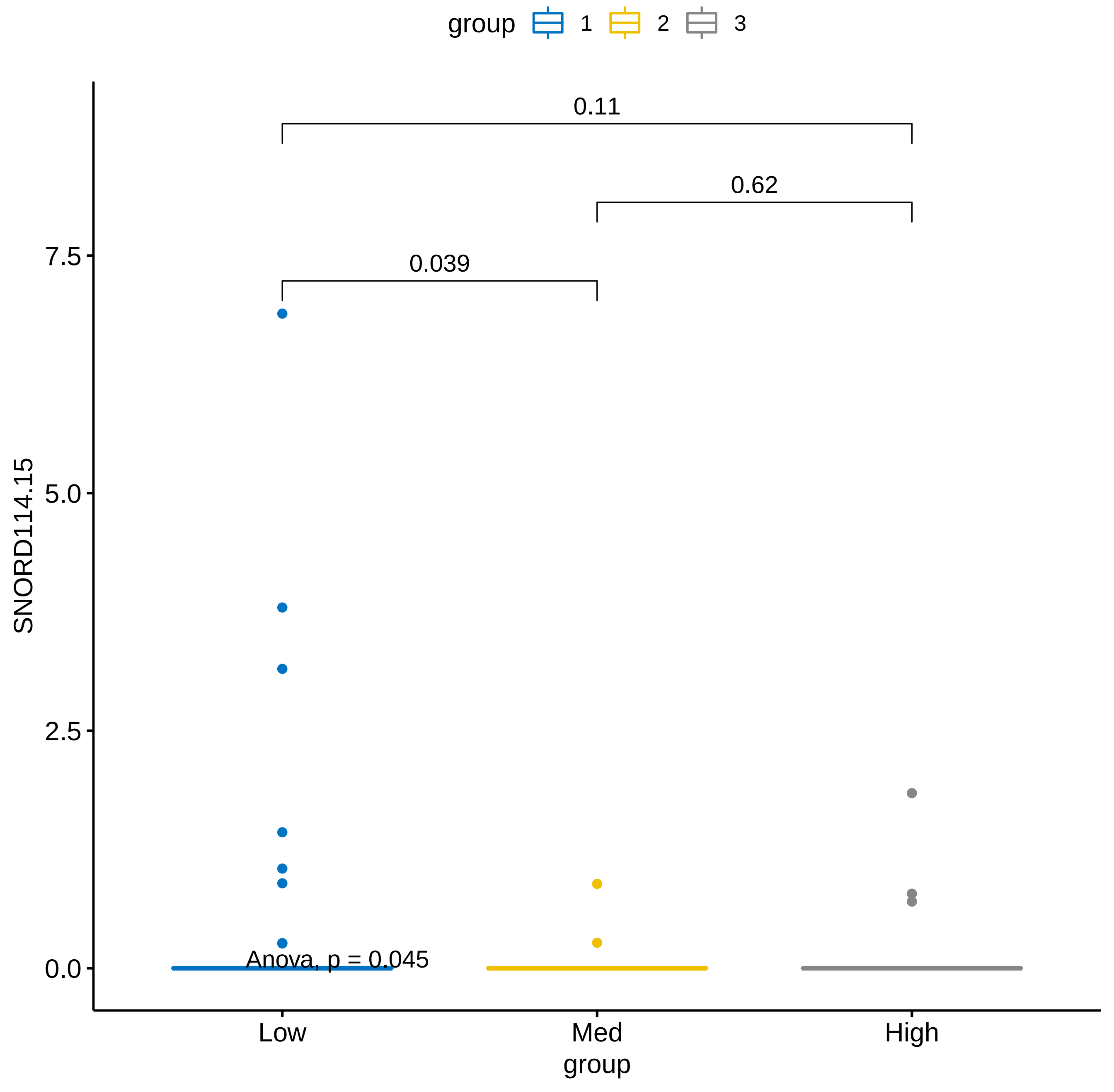

group
1
2
3
0.11
0.62
0.039
SNORD114.15
7.5
5.0
2.5
0.0
Anova, p = 0.045
Low
Med
High
group

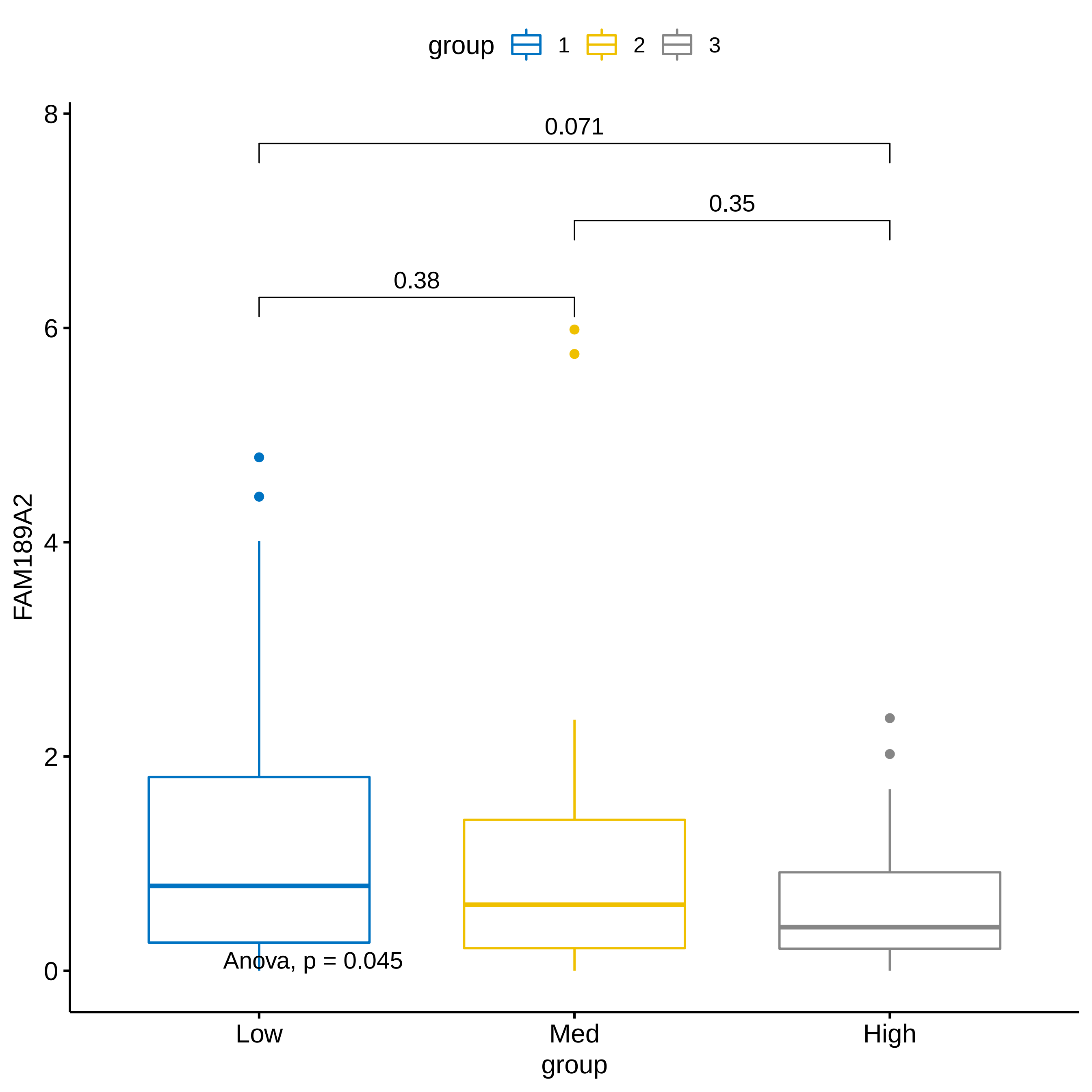
group 1 2 3
0.071
0.35
0.38
8
6
4
2
0
FAM189A2
Anova, p = 0.045
Low
Med
High
group

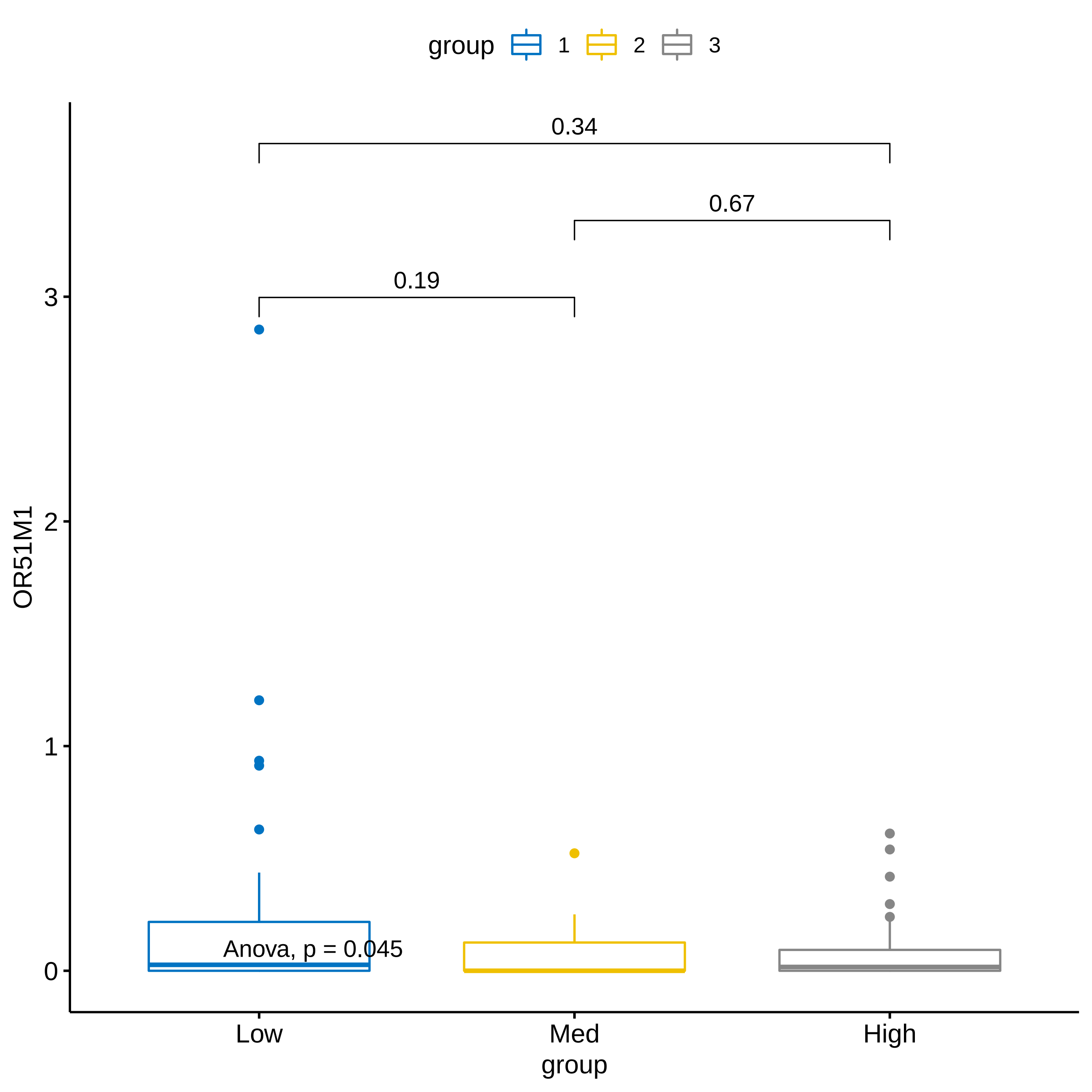

group 1 2 3
0.34
0.67
0.19
OR51M1
3
2
1
0
Anova, p = 0.045
Low
Med
High
group

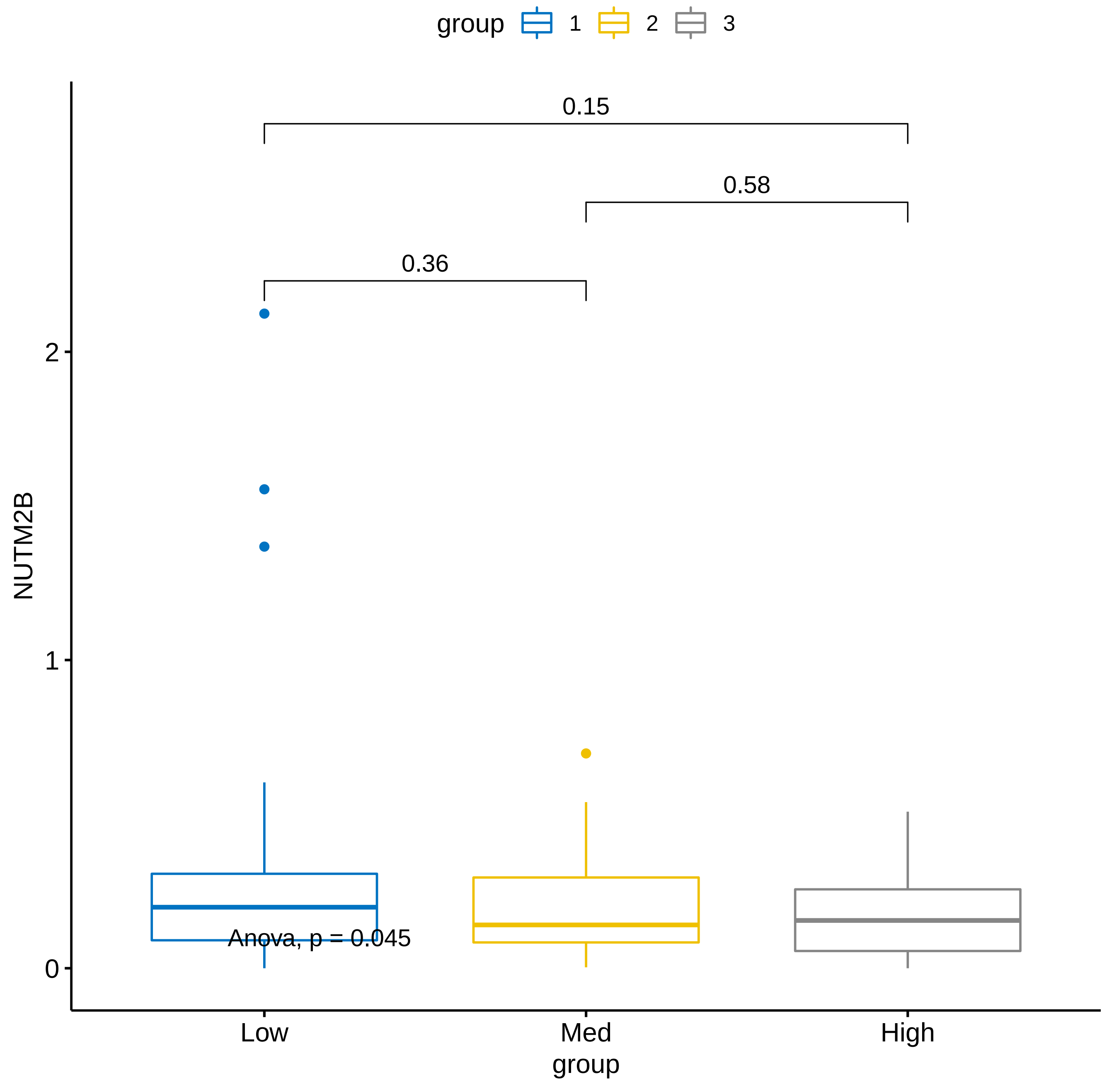
group
1
2
3
0.15
0.58
0.36
NUTM2B
2
1
0
Anova, p = 0.045
Low
Med
High
group

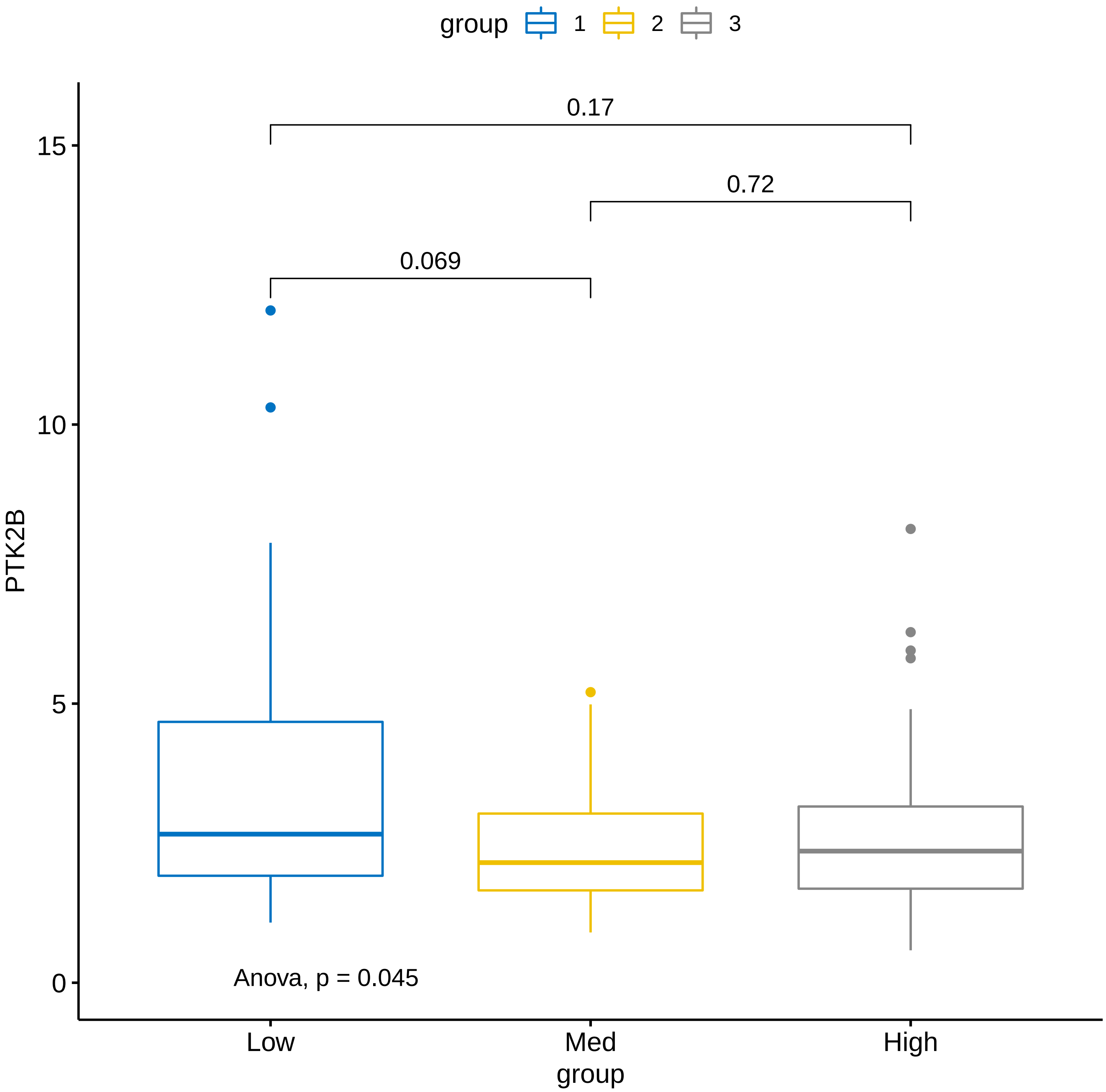

group
1
2
3
0.17
0.72
0.069
15
10
5
0
PTK2B
Anova, p = 0.045
Low
Med
High
group

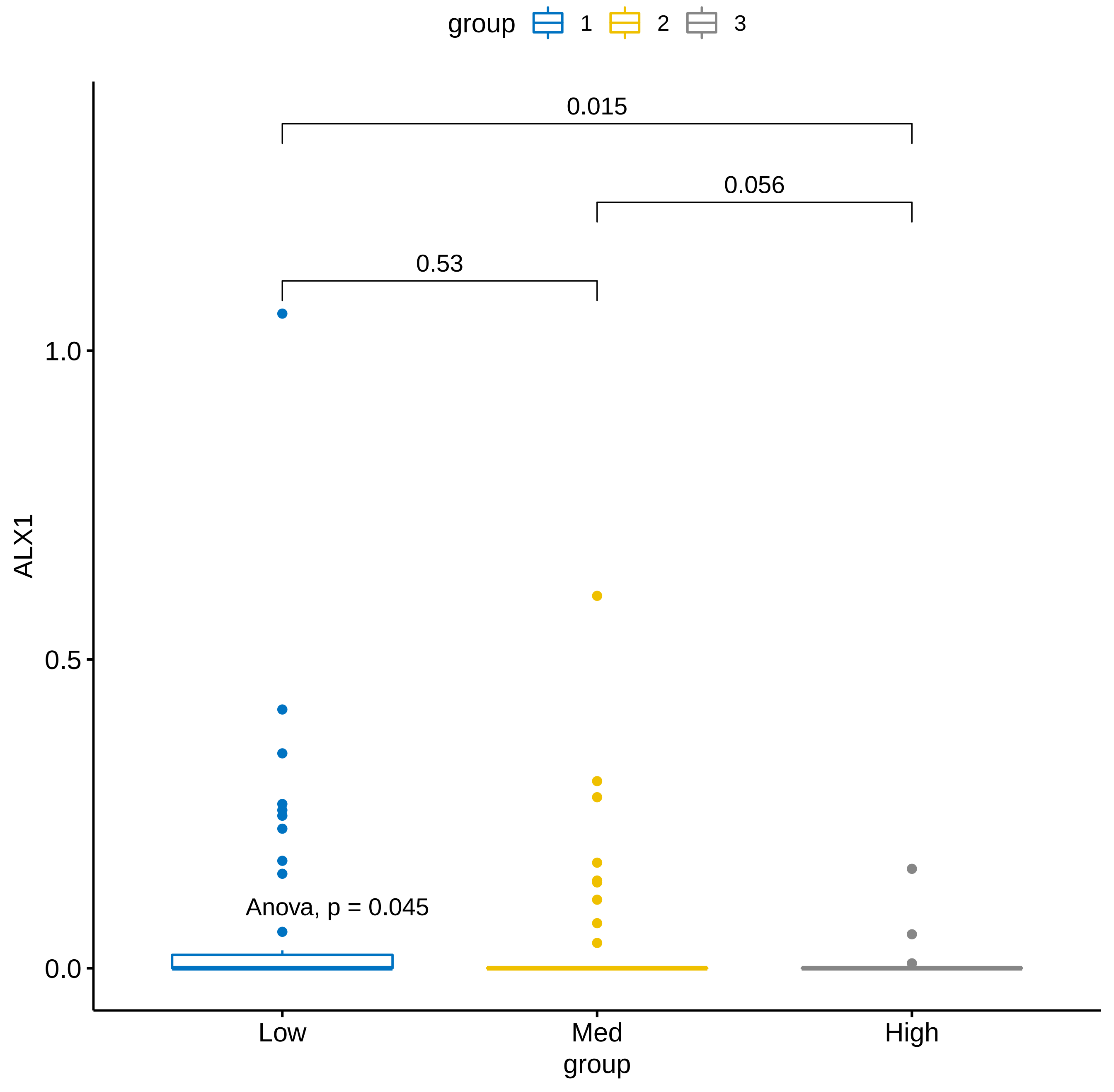

group
1
2
3
0.015
0.056
0.53
Anova, p = 0.045
ALX1
1.0
0.5
0.0
Low
Med
High
group

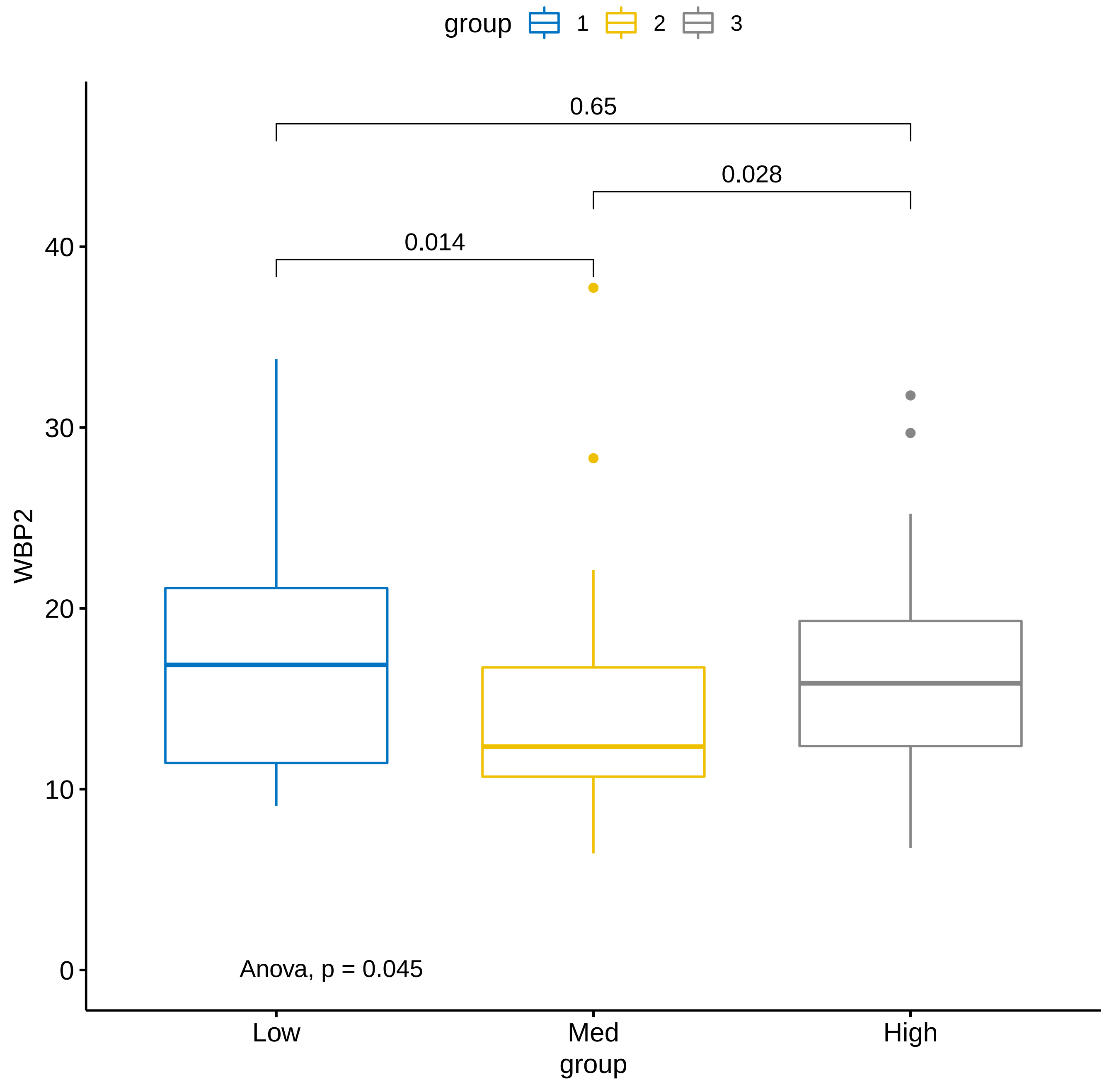
group 1 2 3
0.65
0.028
0.014
WBP2
40
30
20
10
0
Anova, p = 0.045
Low
Med
High
group

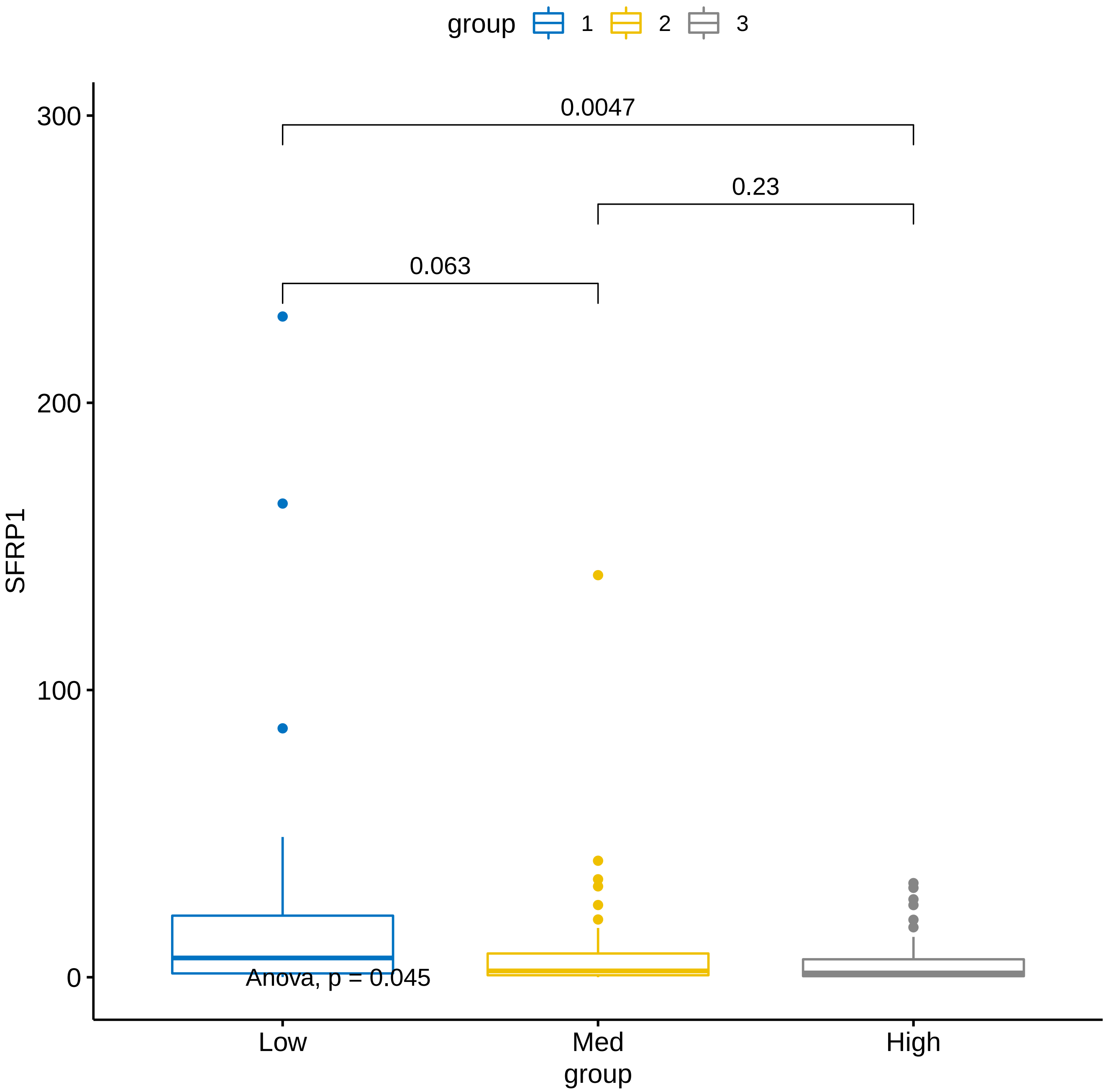

group
1
2
3
0.0047
0.23
0.063
300
200
100
0
SFRP1
Anova, p = 0.045
Low
Med
High
group

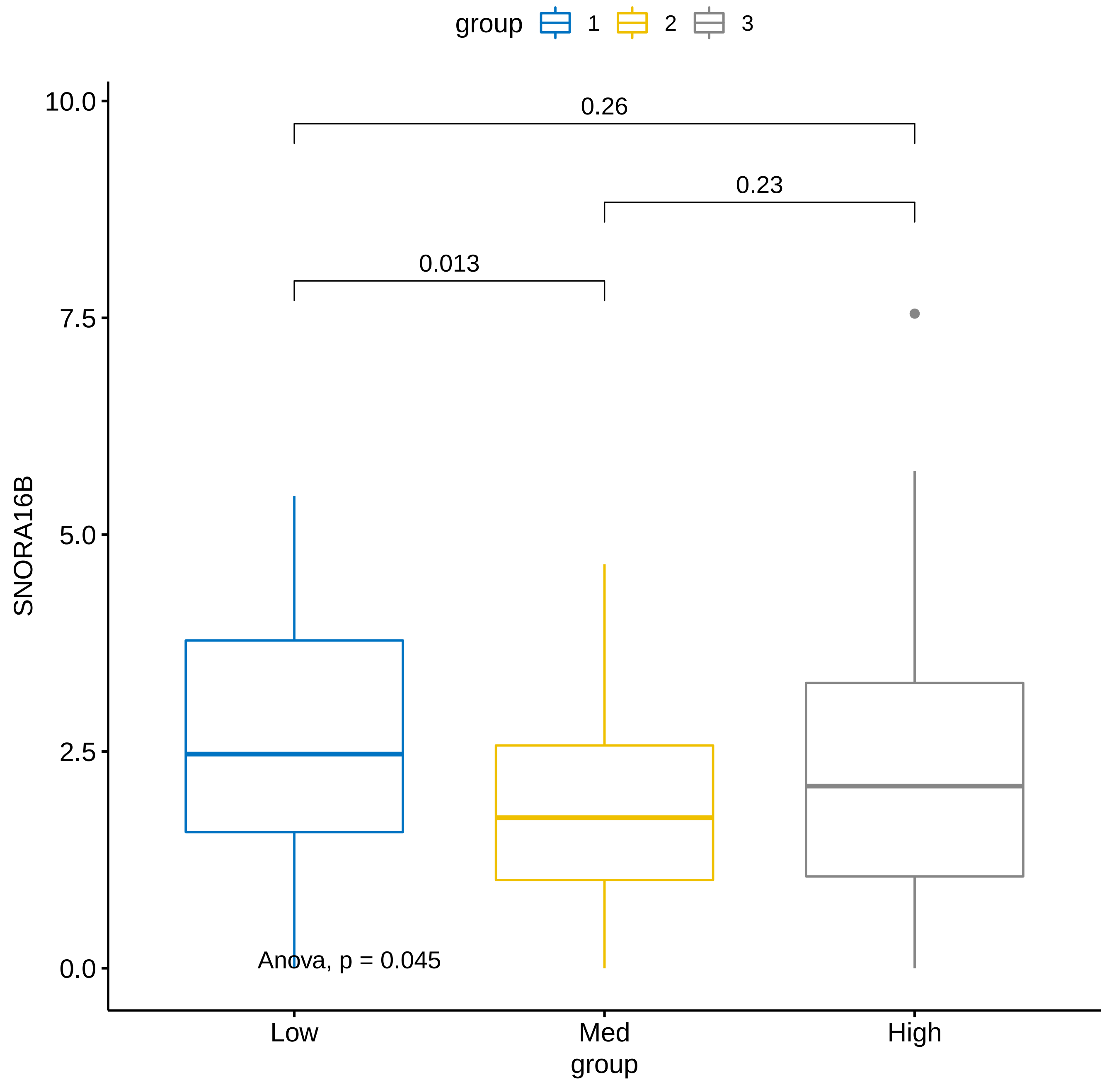
group 1 2 3
0.26
0.23
0.013
10.0
7.5
5.0
2.5
0.0
SNORA16B
Anova, p = 0.045
Low
Med
High
group

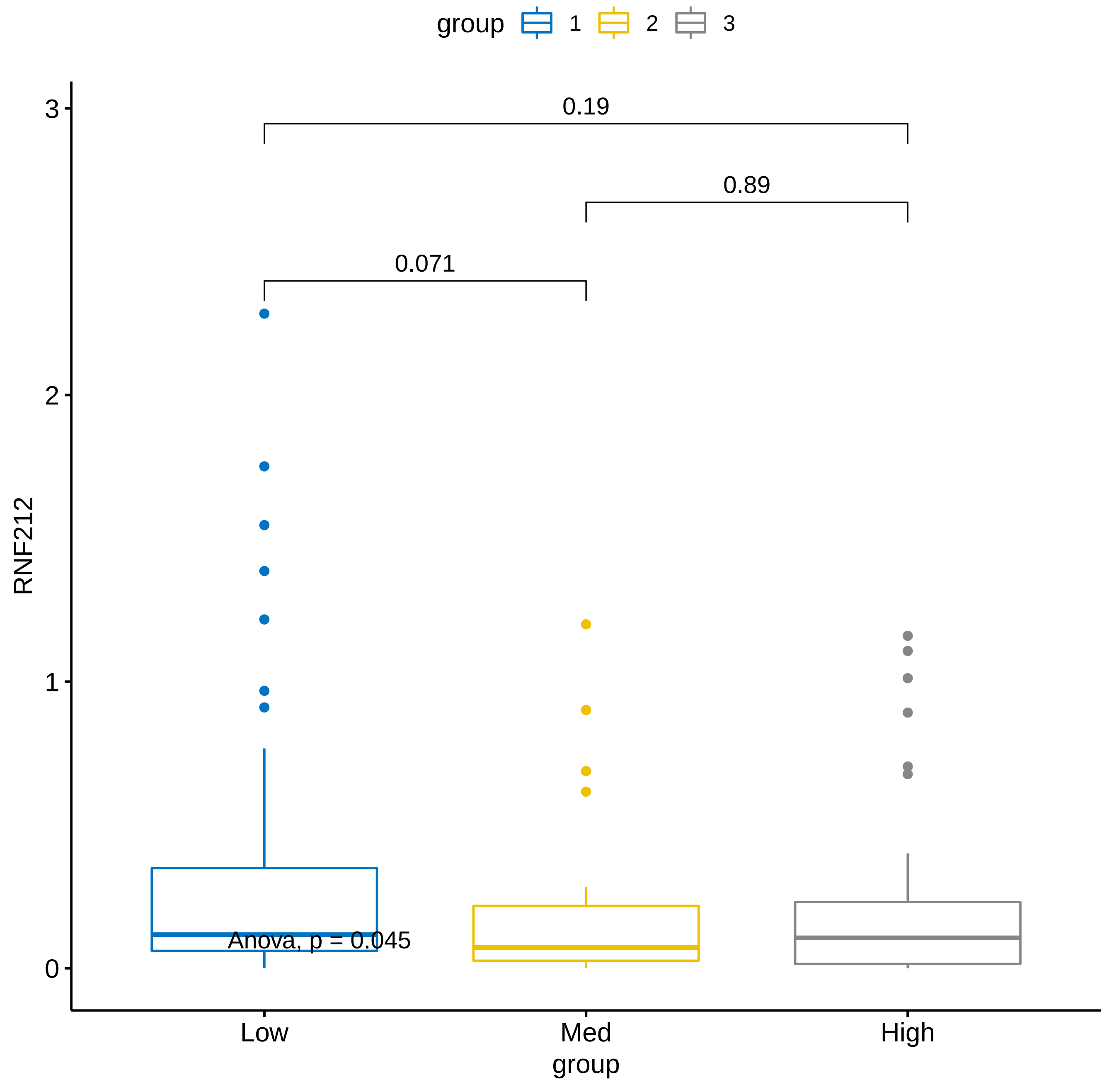
group
1
2
3
0.19
0.89
0.071
3
2
1
0
RNF212
Anova, p = 0.045
Low
Med
High
group

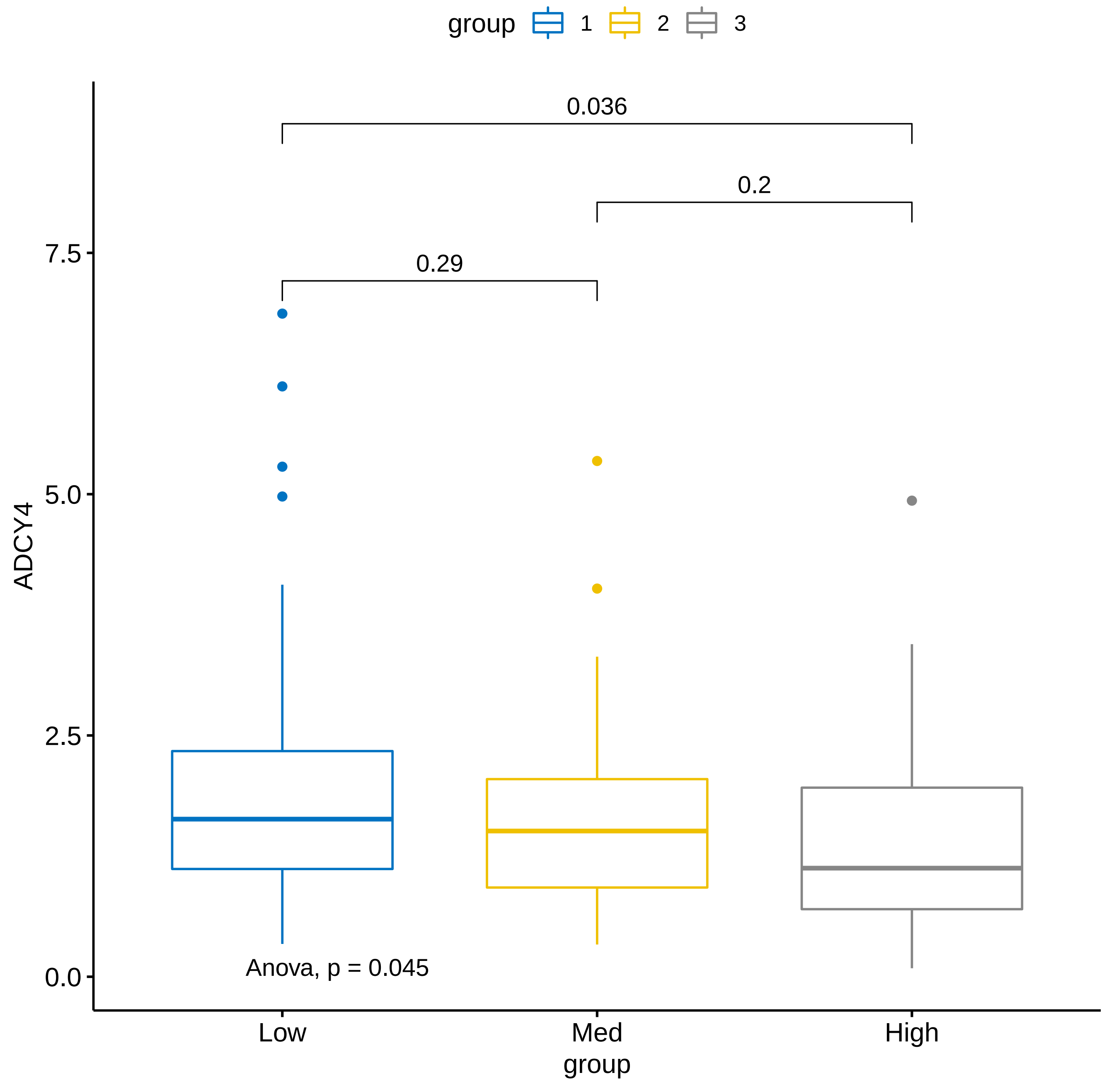

group 1 2 3
0.036
0.2
0.29
7.5
5.0
2.5
0.0
ADCY4
Anova, p = 0.045
Low
Med
High
group

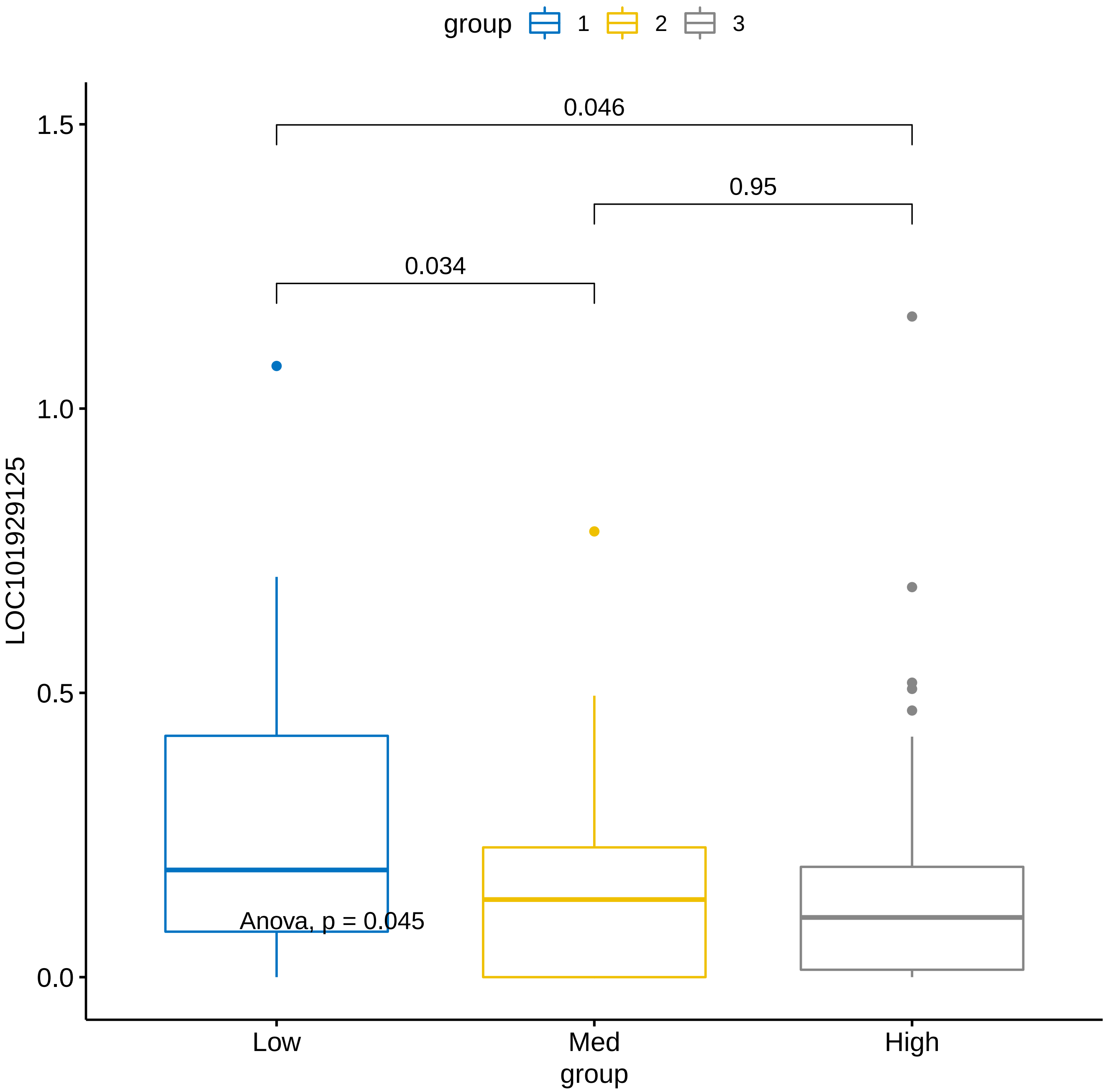

group 1 2 3
0.046
0.95
0.034
1.5
1.0
0.5
0.0
LOC101929125
Anova, p = 0.045
Low
Med
High
group

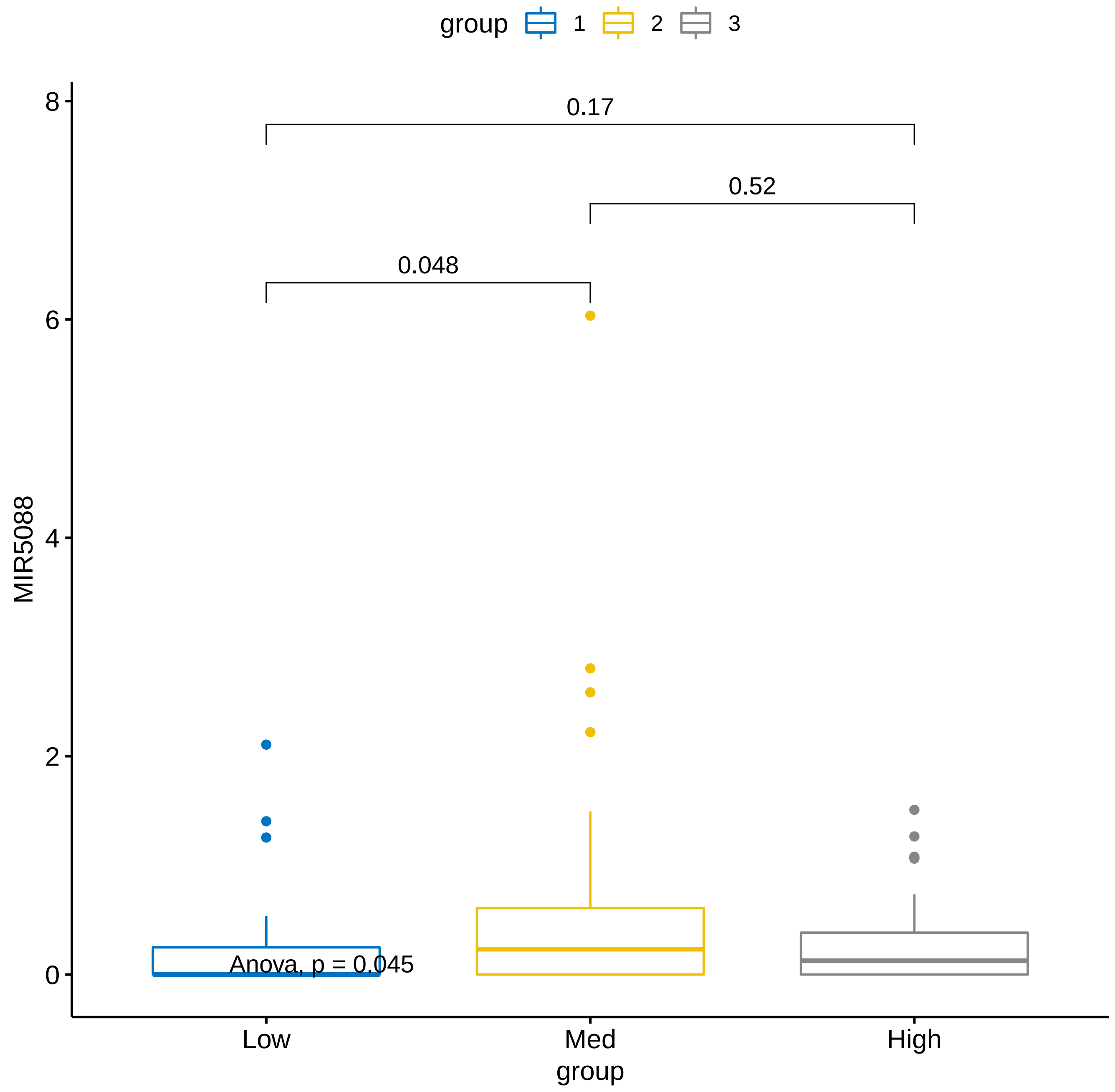
group
1
2
3
0.17
0.52
0.048
8
6
4
2
0
MIR5088
Anova, p = 0.045
Low
Med
High
group

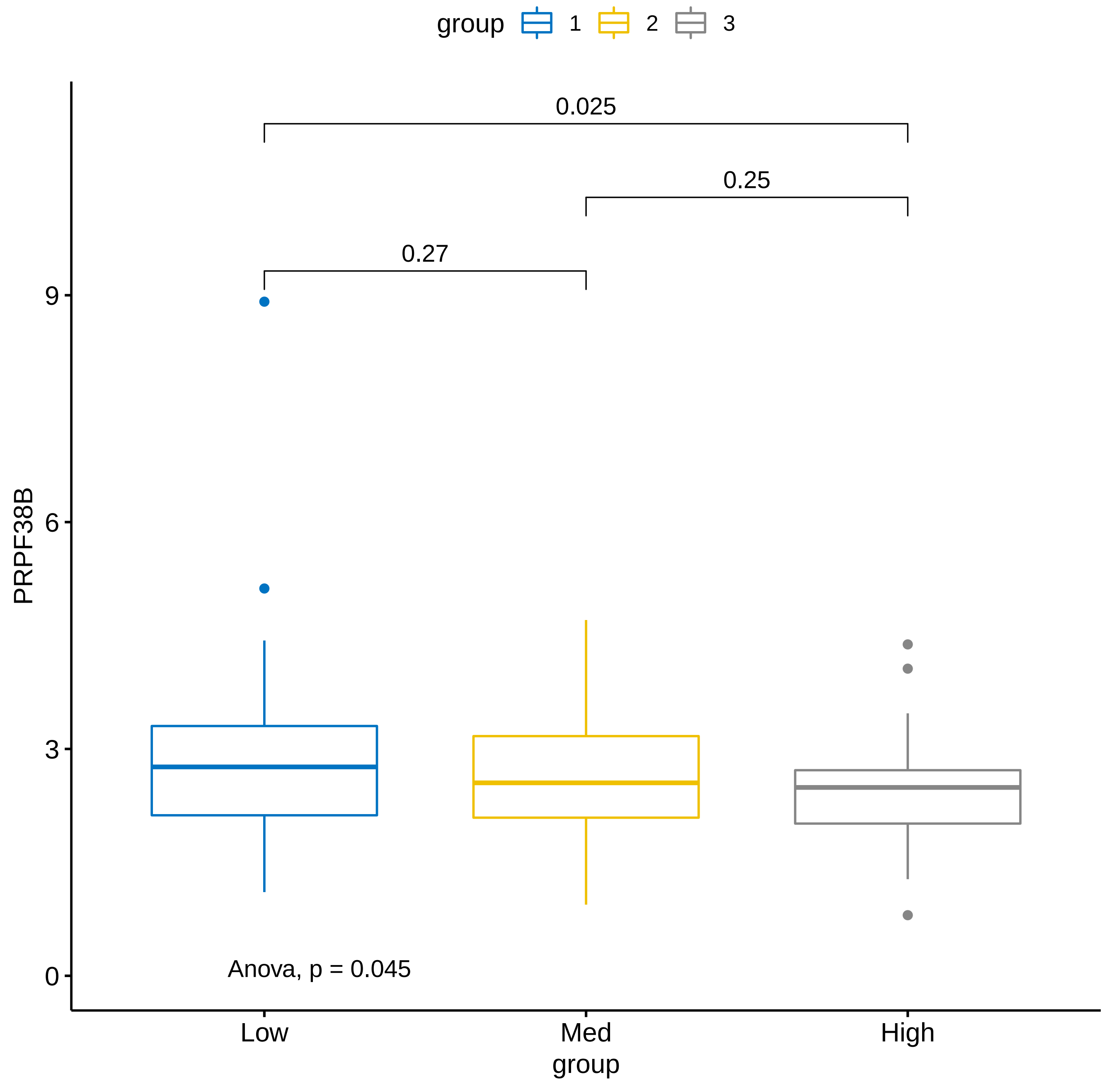
group
1
2
3
0.025
0.25
0.27
9
6
3
0
PRPF38B
Anova, p = 0.045
Low
Med
High
group

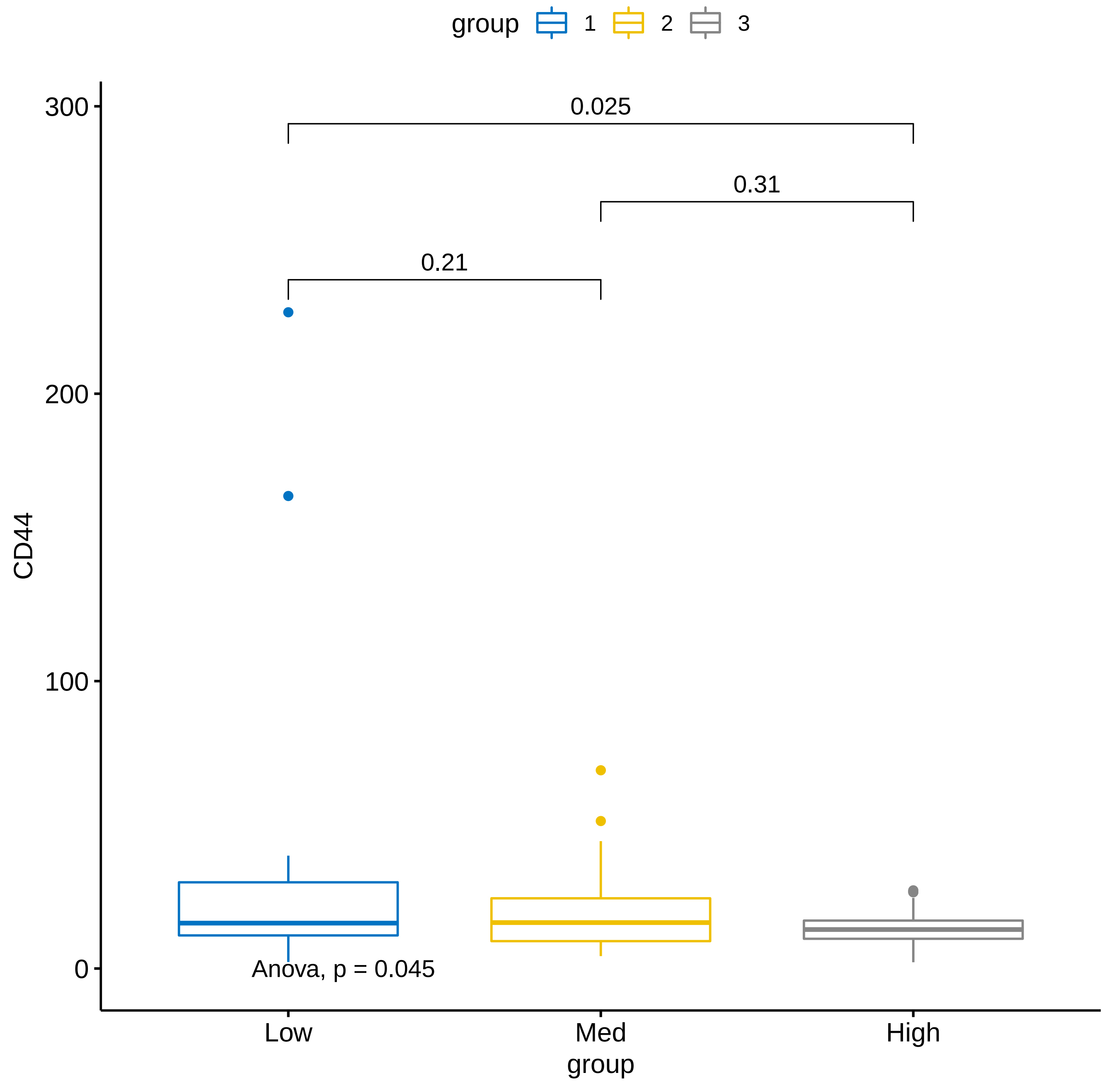

group 1 2 3
0.025
0.31
0.21
300
200
100
0
CD44
Anova, p = 0.045
Low
Med
High
group

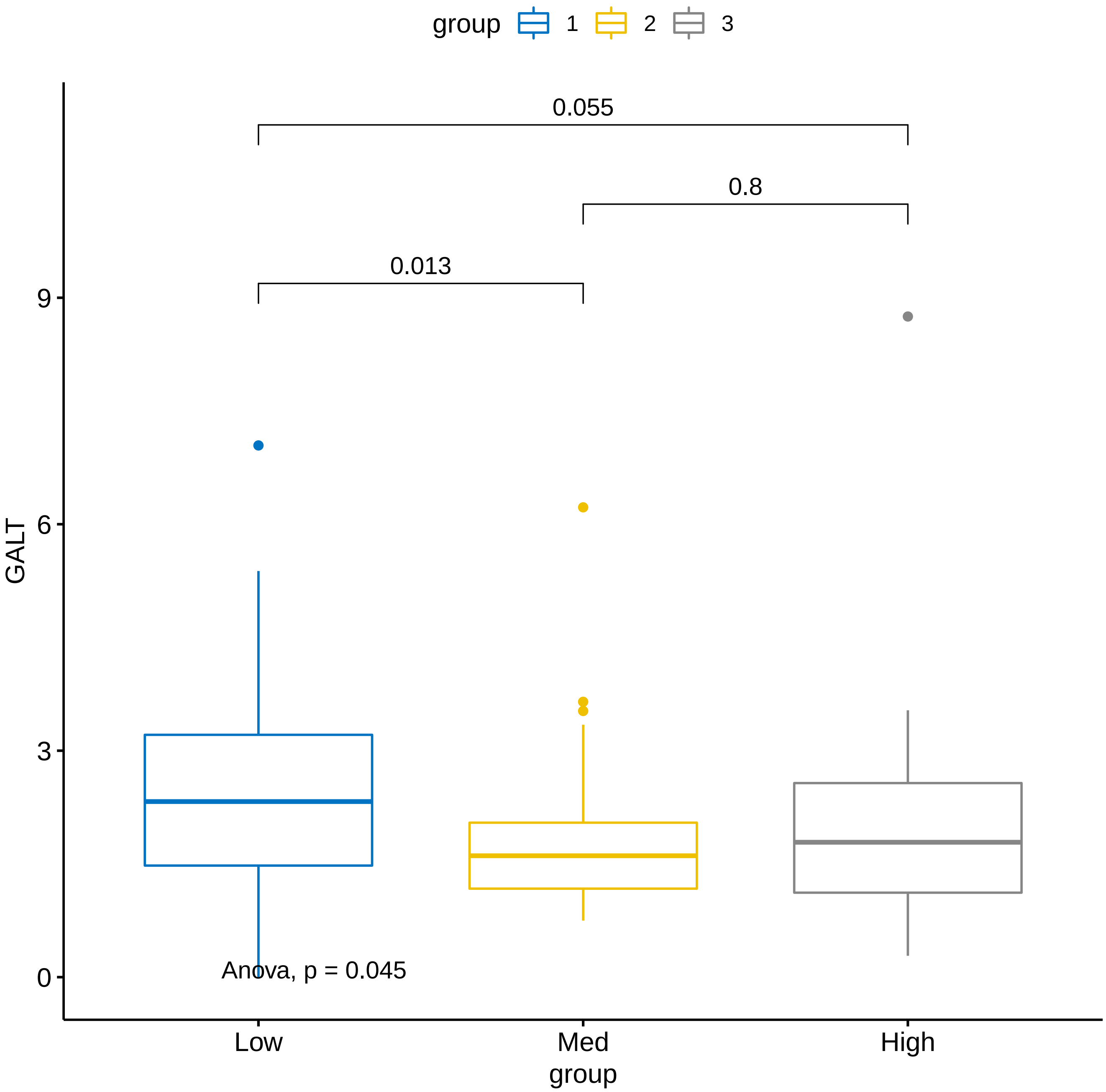
group
1
2
3
0.055
0.8
0.013
GALT
9
6
3
0
Anova, p = 0.045
Low
Med
High
group

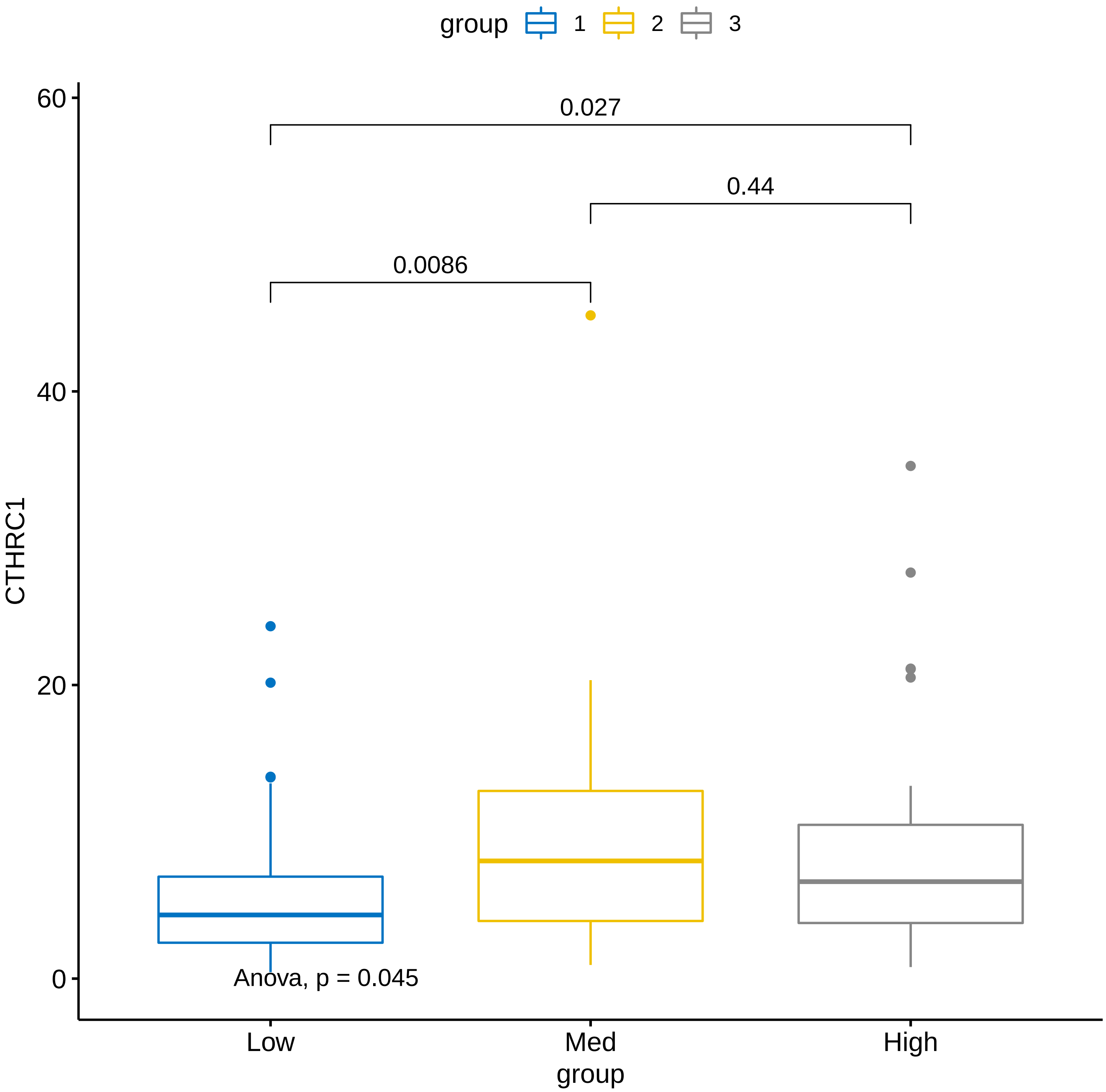

group 1 2 3
60
40
20
0
CTHRC1
0.027
0.44
0.0086
Anova, p = 0.045
Low
Med
High
group

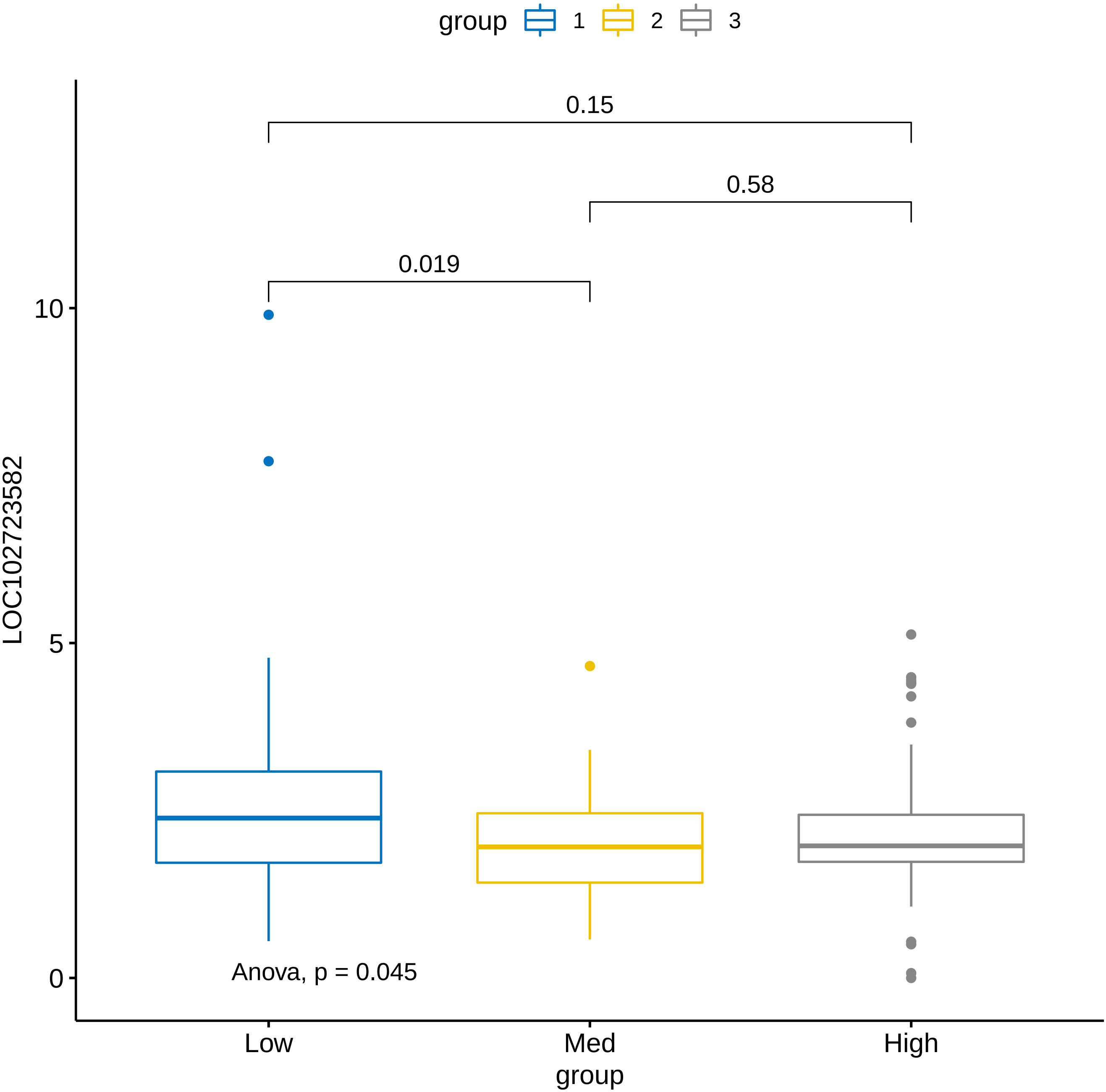

group
1
2
3
0.15
0.58
0.019
10
5
0
LOC102723582
Anova, p = 0.045
Low
Med
High
group

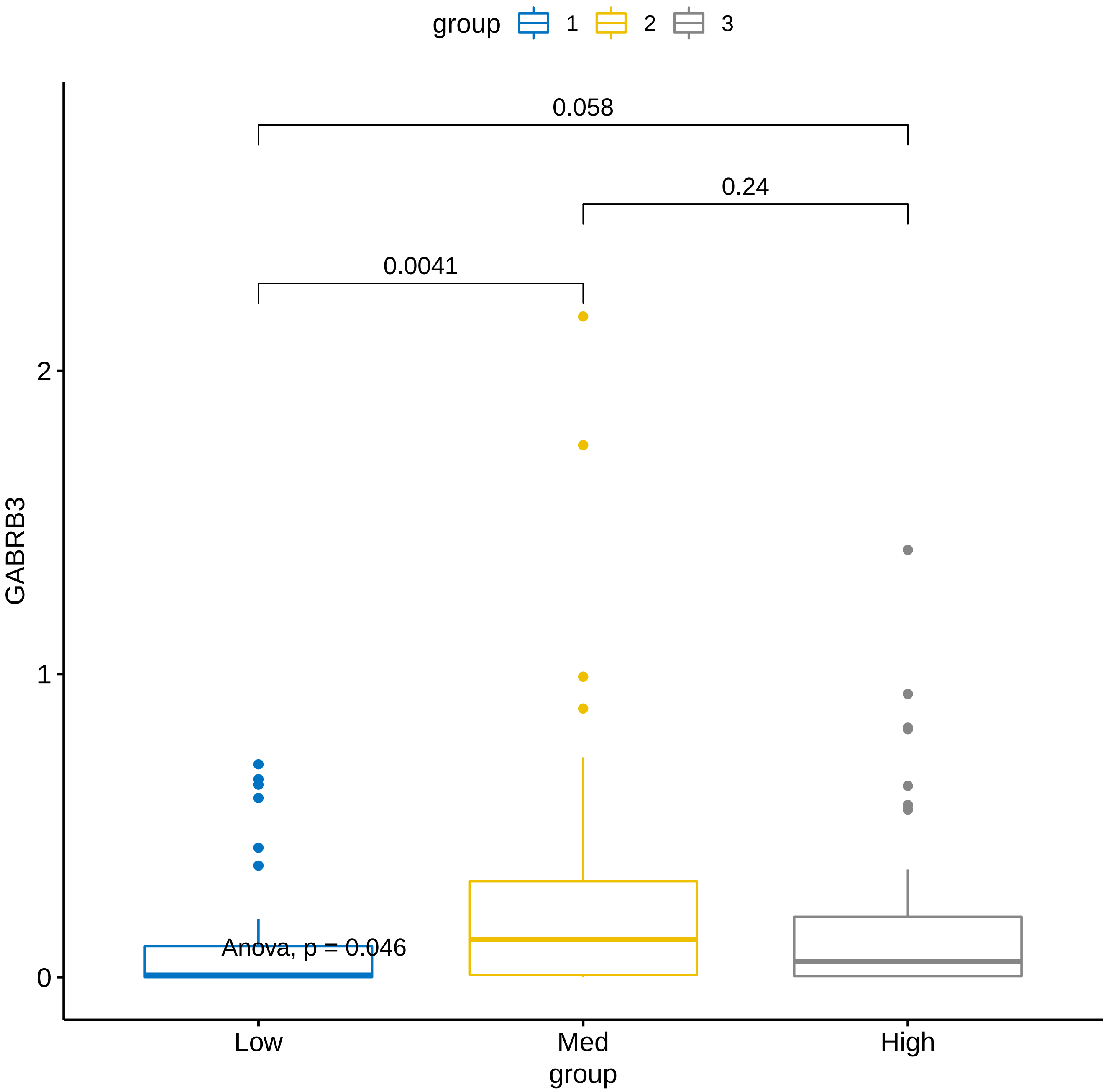
group
1
2
3
0.058
0.24
0.0041
GABRB3
2
1
0
Anova, p = 0.046
Low
Med
High
group

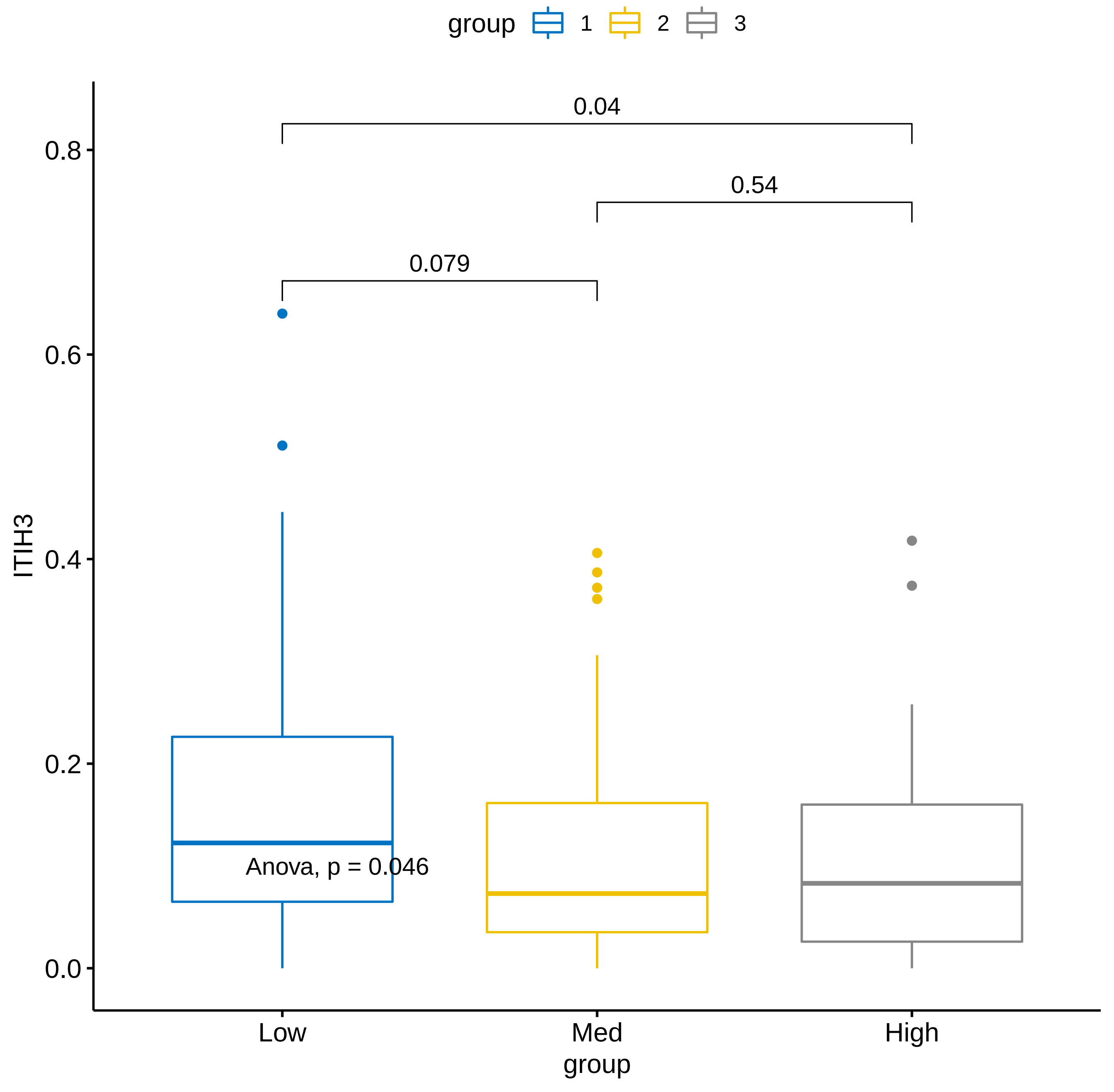
group 1 2 3
0.04
0.54
0.079
0.8
0.6
0.4
0.2
0.0
ITIH3
Anova, p = 0.046
Low
Med
High
group

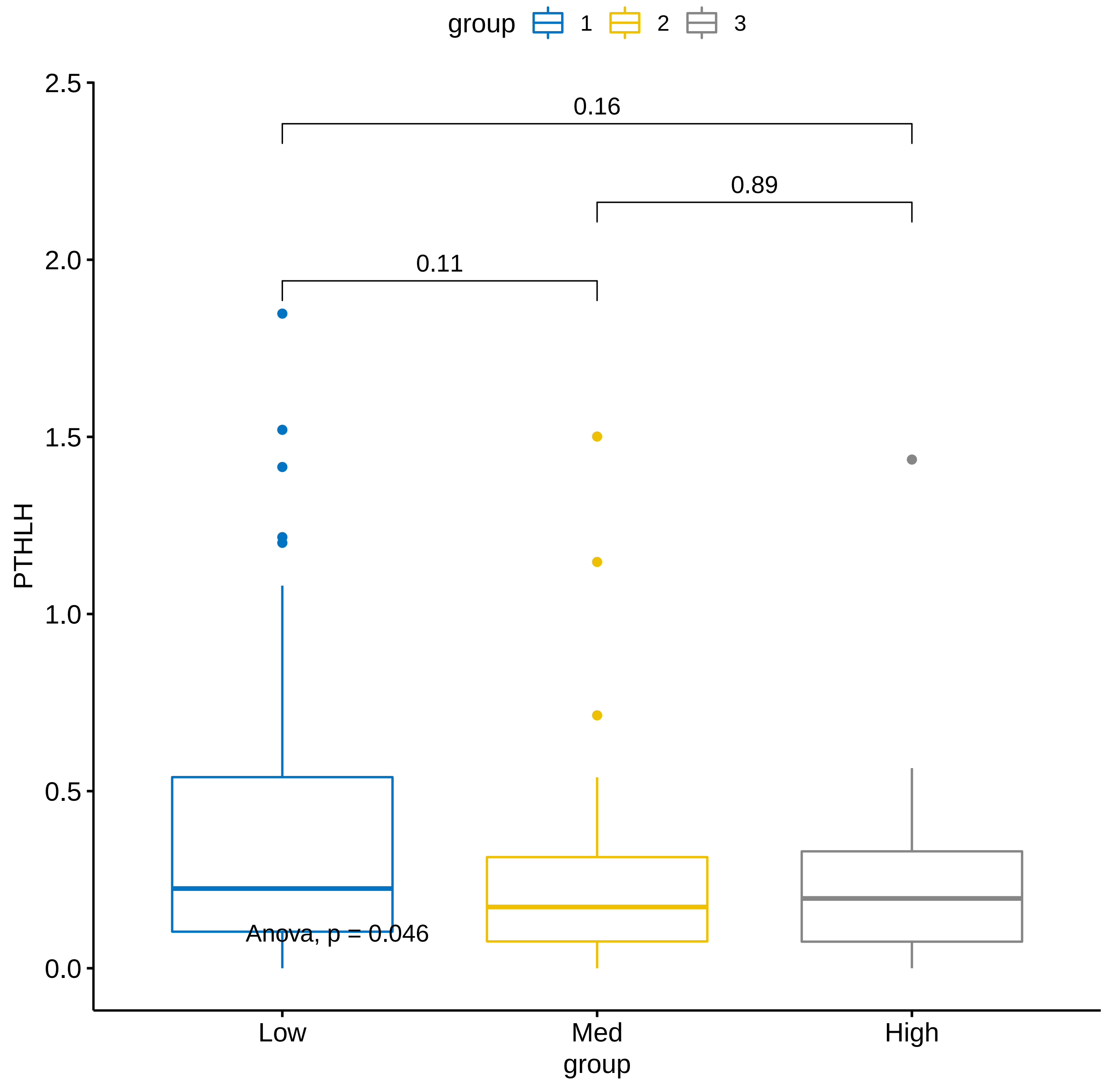

group 1 2 3
2.5
2.0
1.5
1.0
0.5
0.0
PTHLH
0.16
0.89
0.11
Anova, p = 0.046
Low
Med
High
group

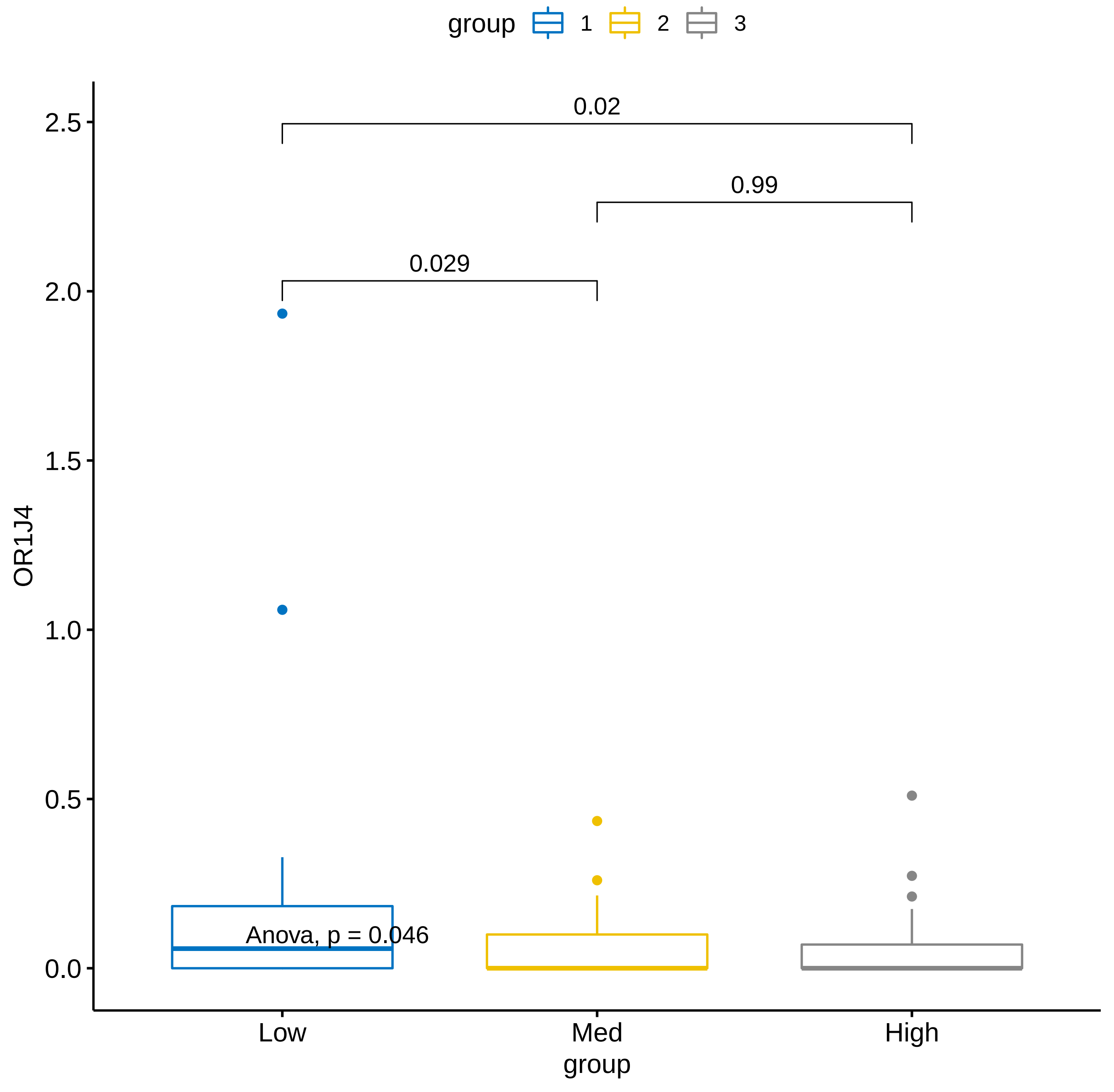

group 1 2 3
0.02
0.99
0.029
2.5
2.0
1.5
1.0
0.5
0.0
OR1J4
Anova, p = 0.046
Low
Med
High
group

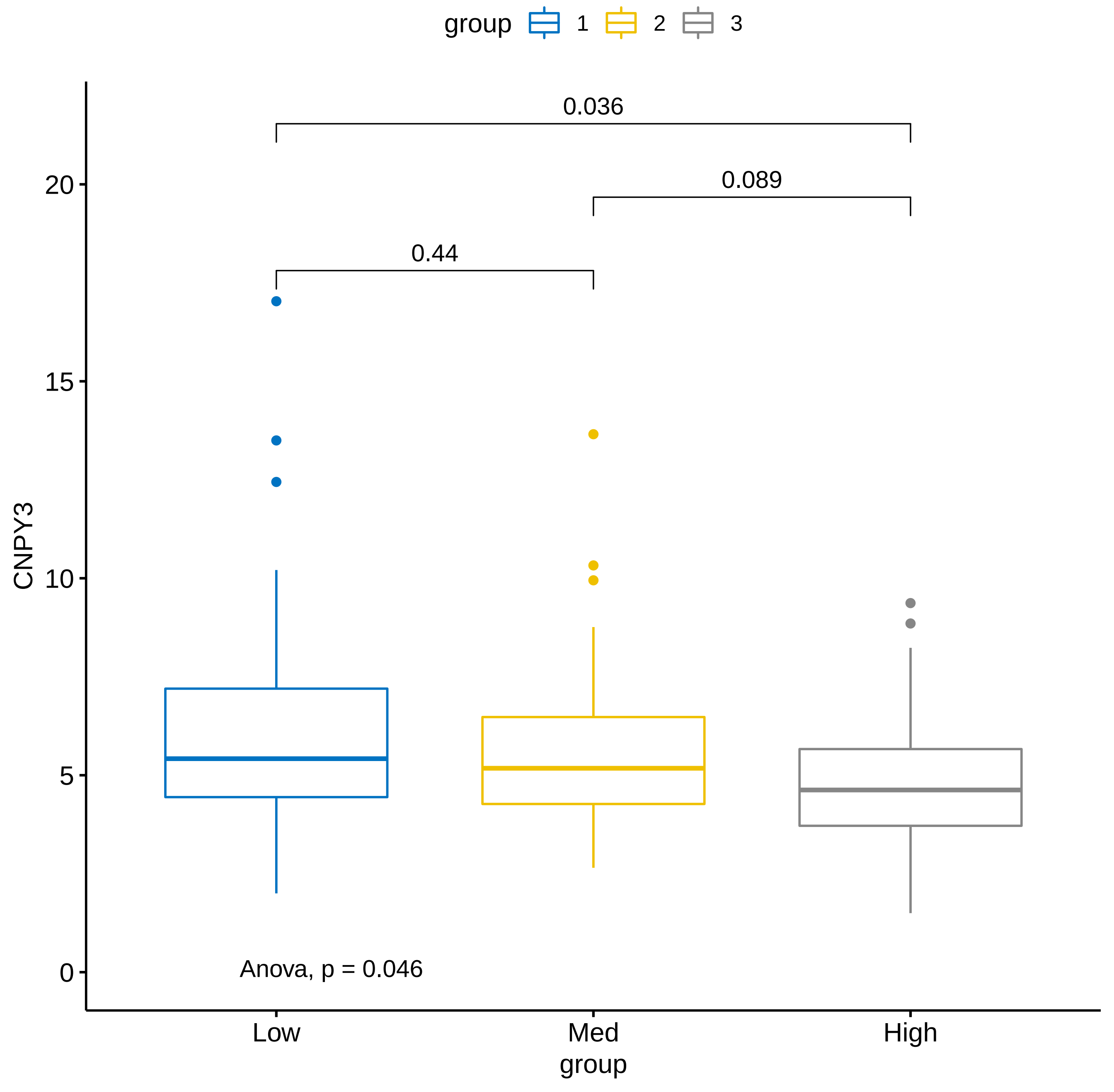
group 1 2 3
0.036
0.089
0.44
CNPY3
20
15
10
5
0
Anova, p = 0.046
Low
Med
High
group

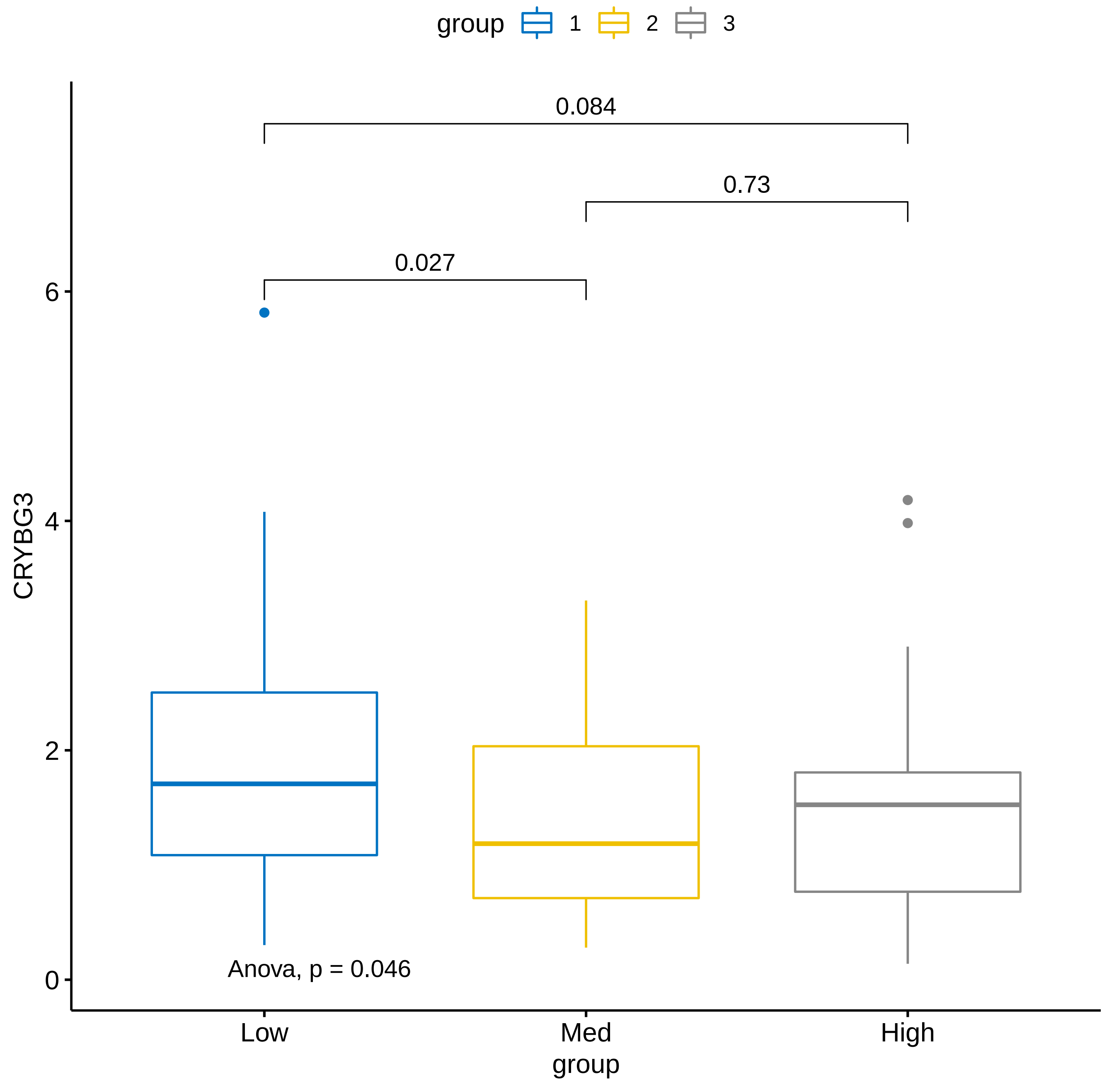
group
1
2
3
0.084
0.73
0.027
CRYBG3
6
4
2
0
Anova, p = 0.046
Low
Med
High
group

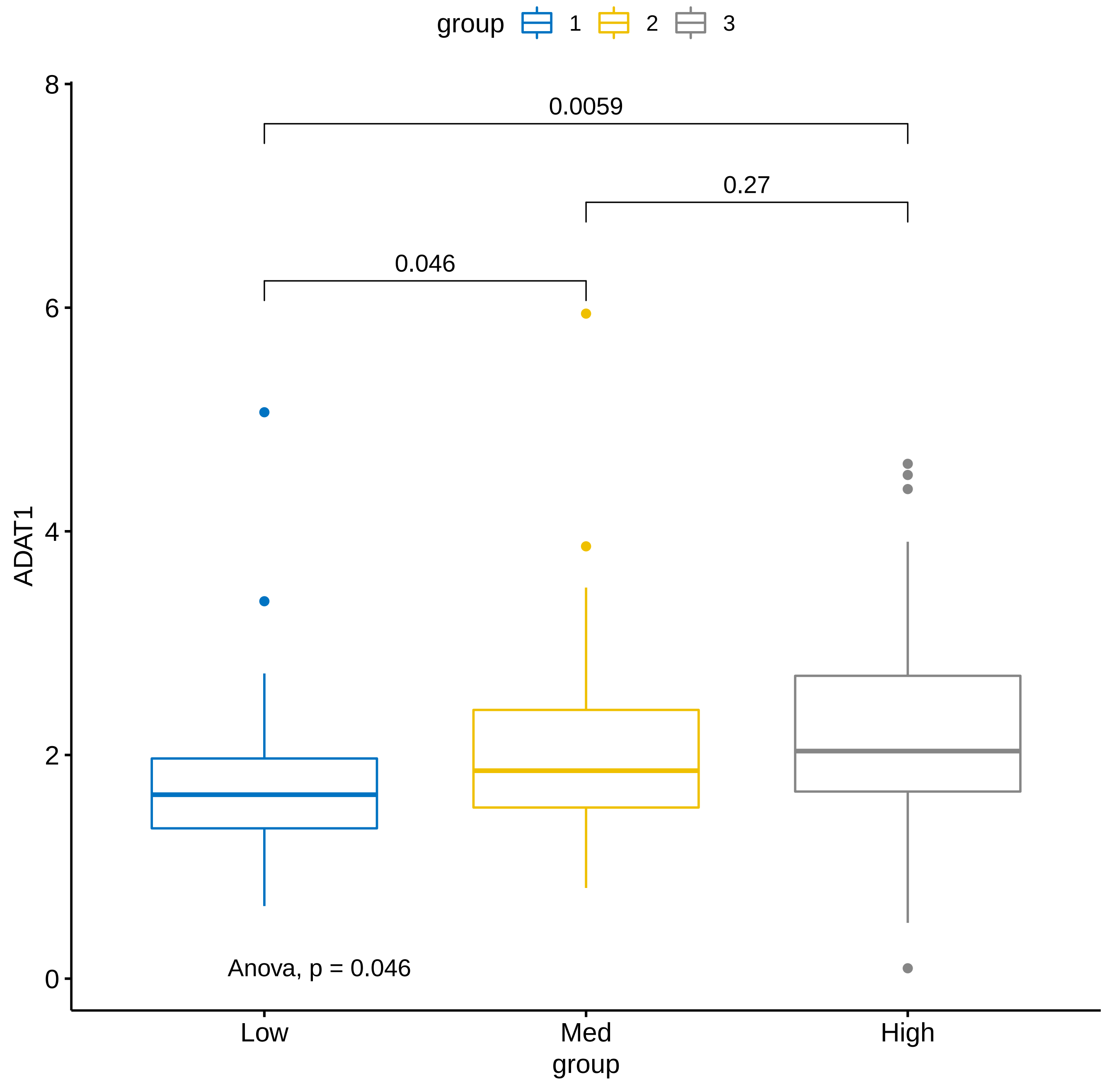

group 1 2 3
0.0059
0.27
0.046
ADAT1
8
6
4
2
0
Anova, p = 0.046
Low
Med
High
group

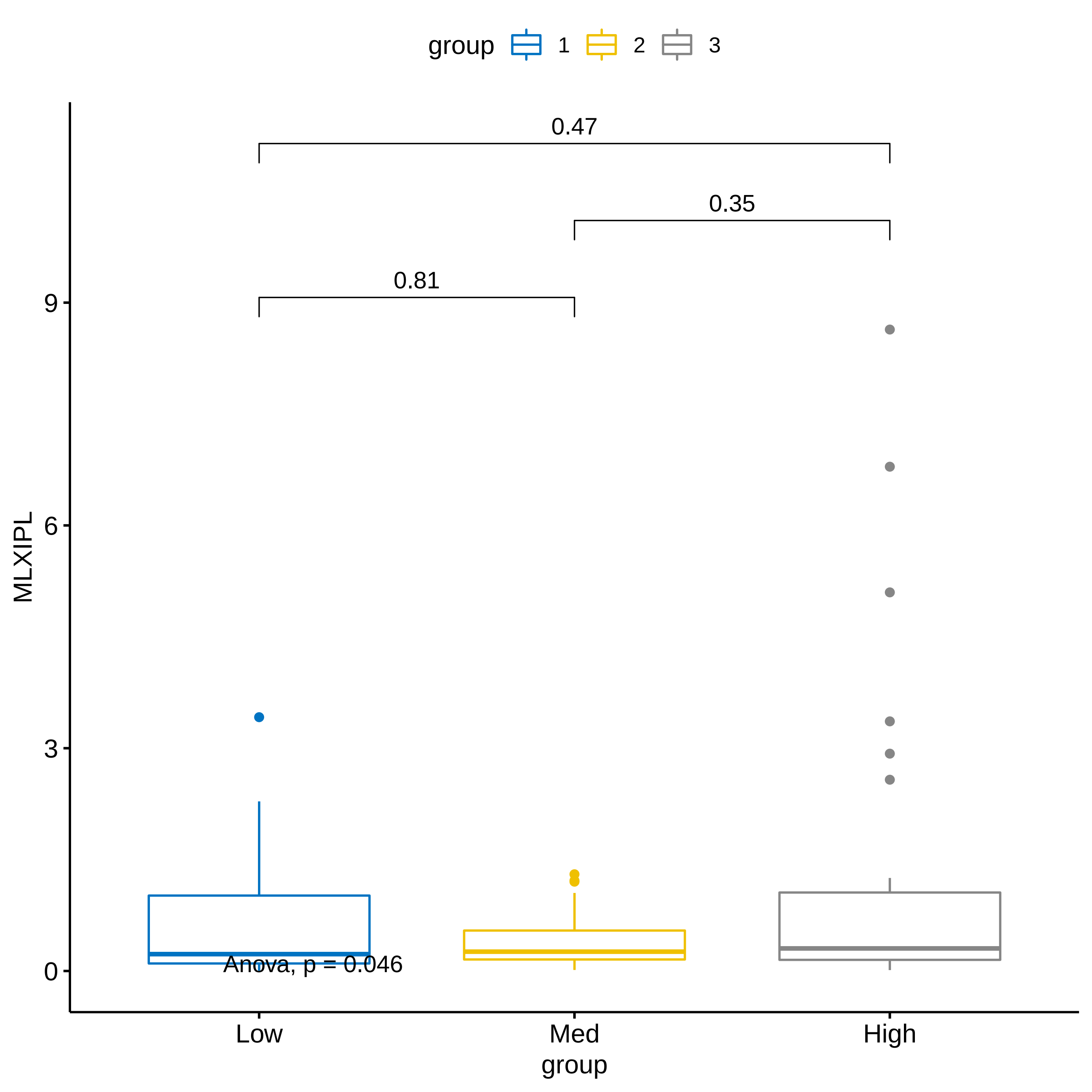
group
1
2
3
0.47
0.35
0.81
9
6
3
0
MLXIPL
Anova, p = 0.046
Low
Med
High
group

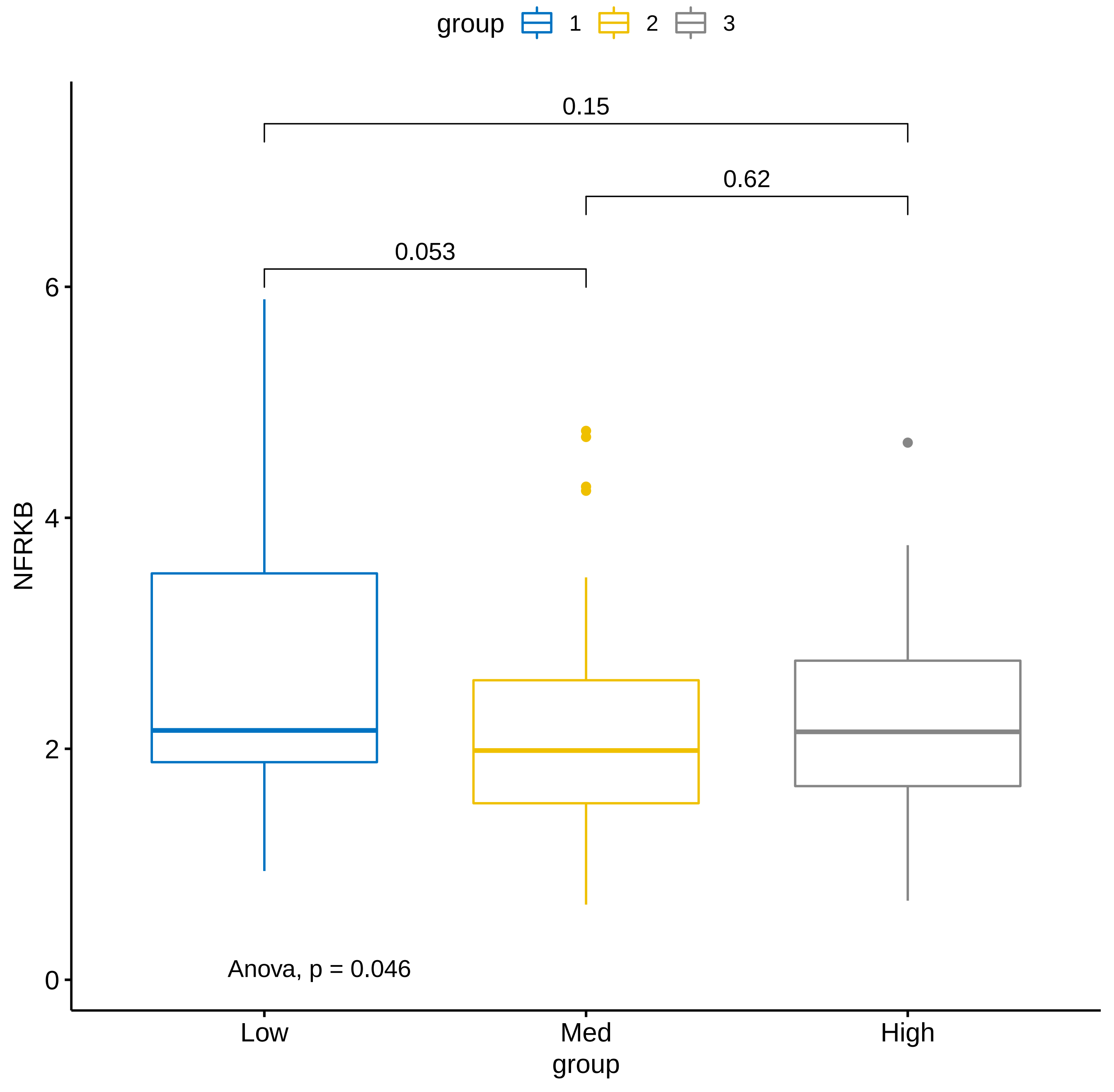
group
1
2
3
0.15
0.62
0.053
6
4
2
0
NFRKB
Anova, p = 0.046
Low
Med
High
group

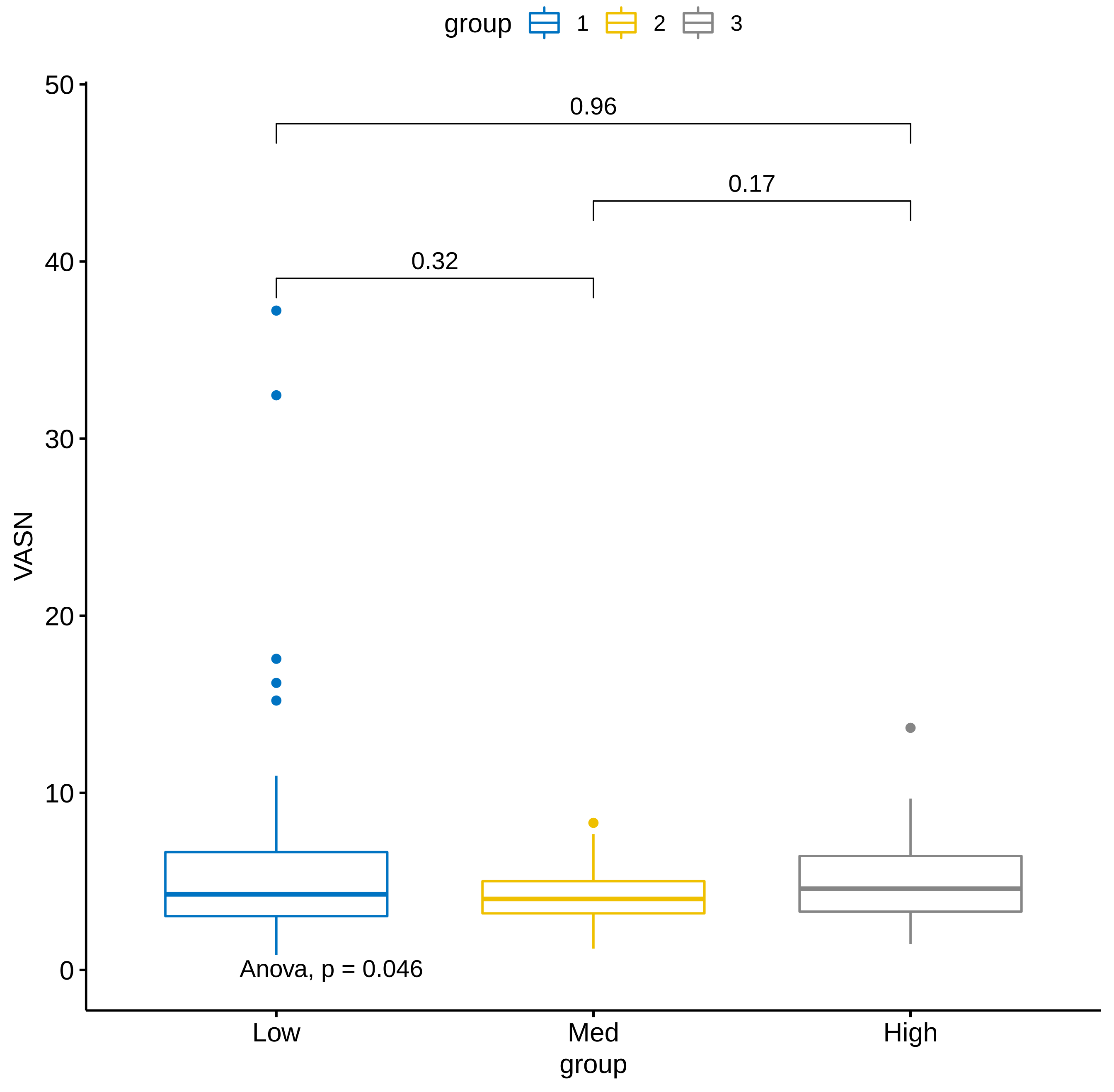

group 1 2 3
0.96
0.17
0.32
50
40
30
20
10
0
VASN
Anova, p = 0.046
Low
Med
High
group

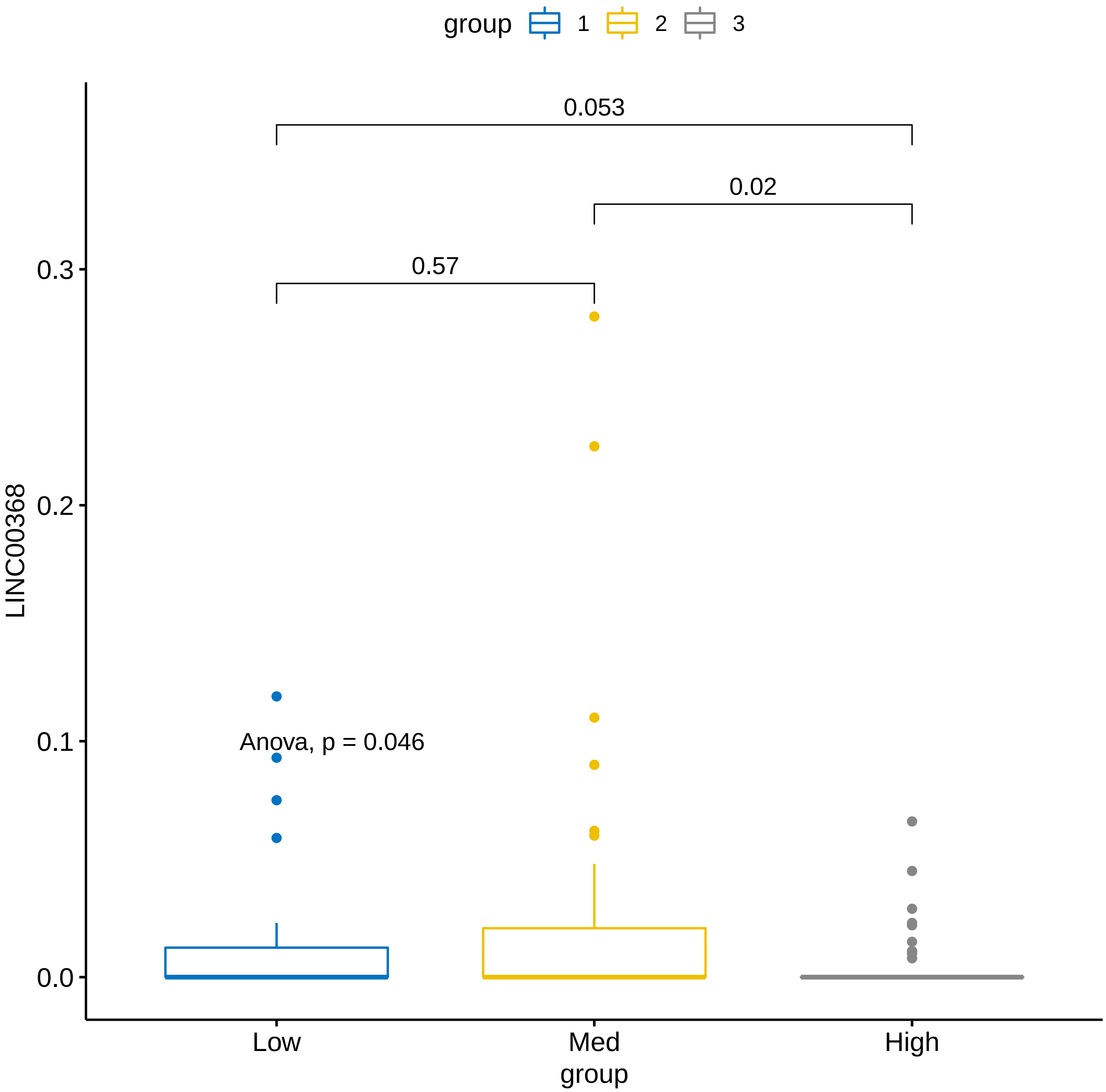

group
1
2
3
0.053
0.02
0.57
0.3
0.2
0.1
0.0
LINC00368
Anova, p = 0.046
Low
Med
High
group

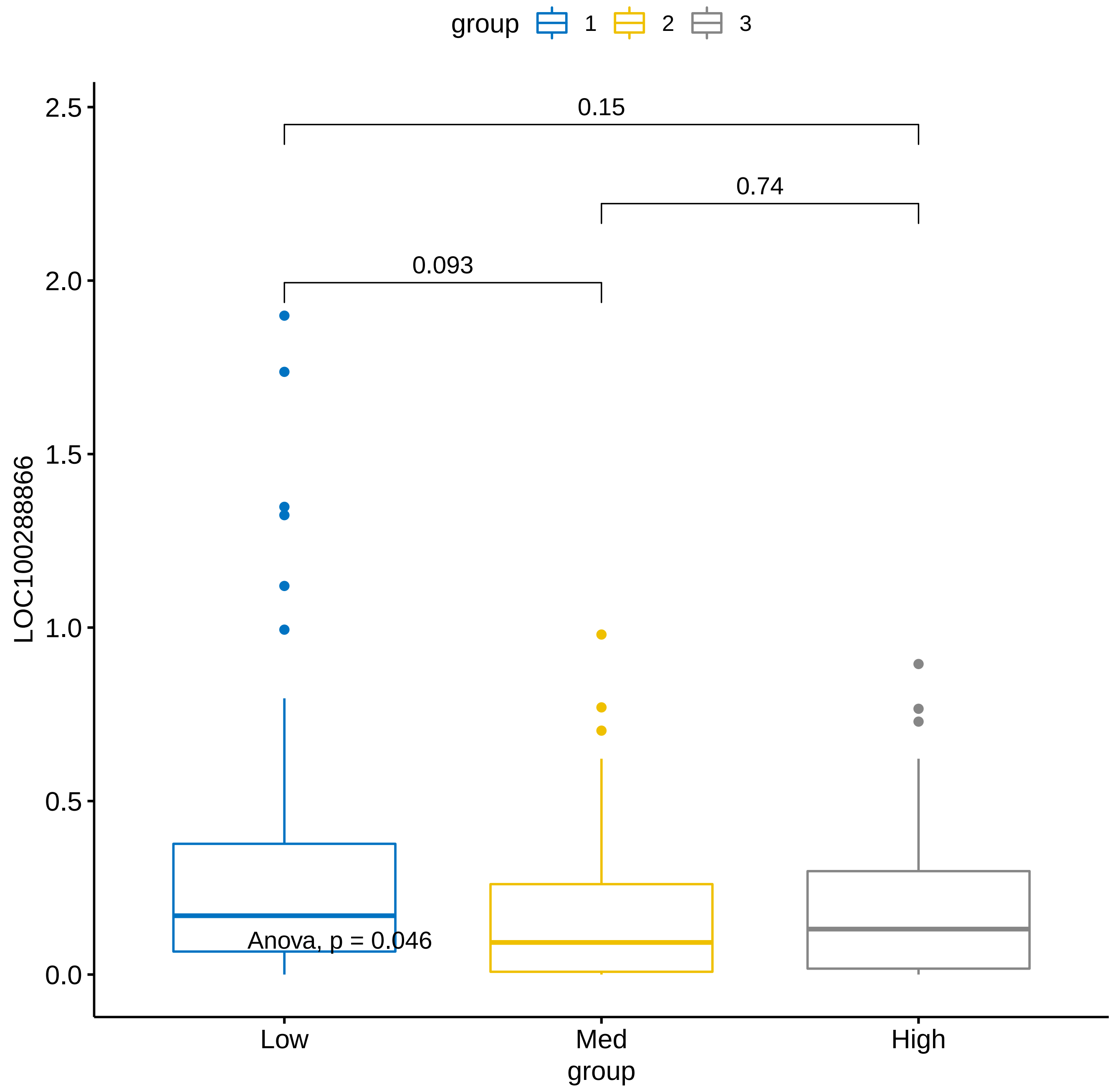
group 1 2 3
0.15
0.74
0.093
2.5
2.0
1.5
1.0
0.5
0.0
LOC100288866
Anova, p = 0.046
Low
Med
High
group

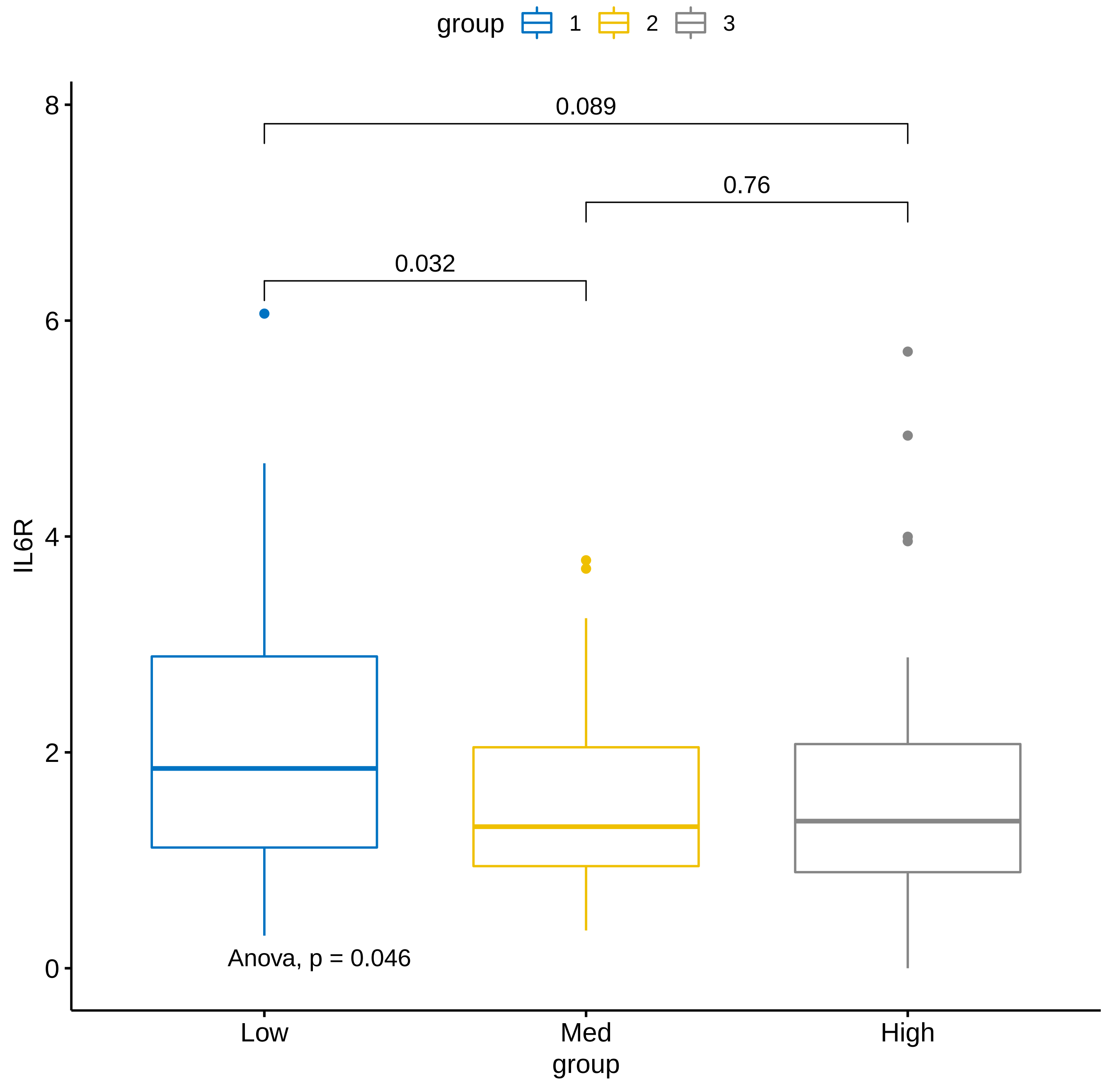
group
1
2
3
0.089
0.76
0.032
8
6
4
2
0
IL6R
Anova, p = 0.046
Low
Med
High
group

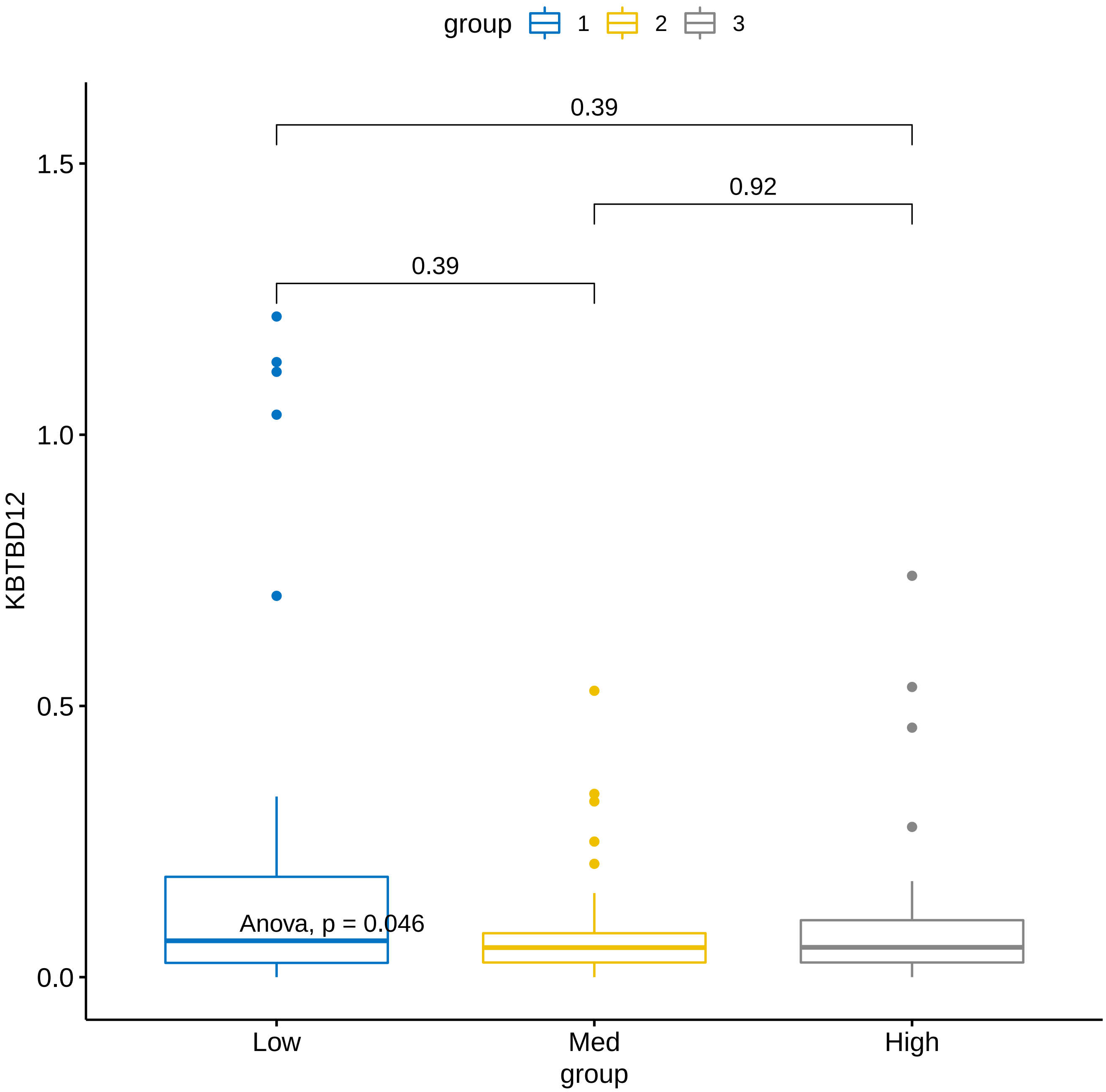

group 1 2 3
0.39
0.92
0.39
1.5
1.0
0.5
0.0
KBTBD12
Anova, p = 0.046
Low
Med
High
group

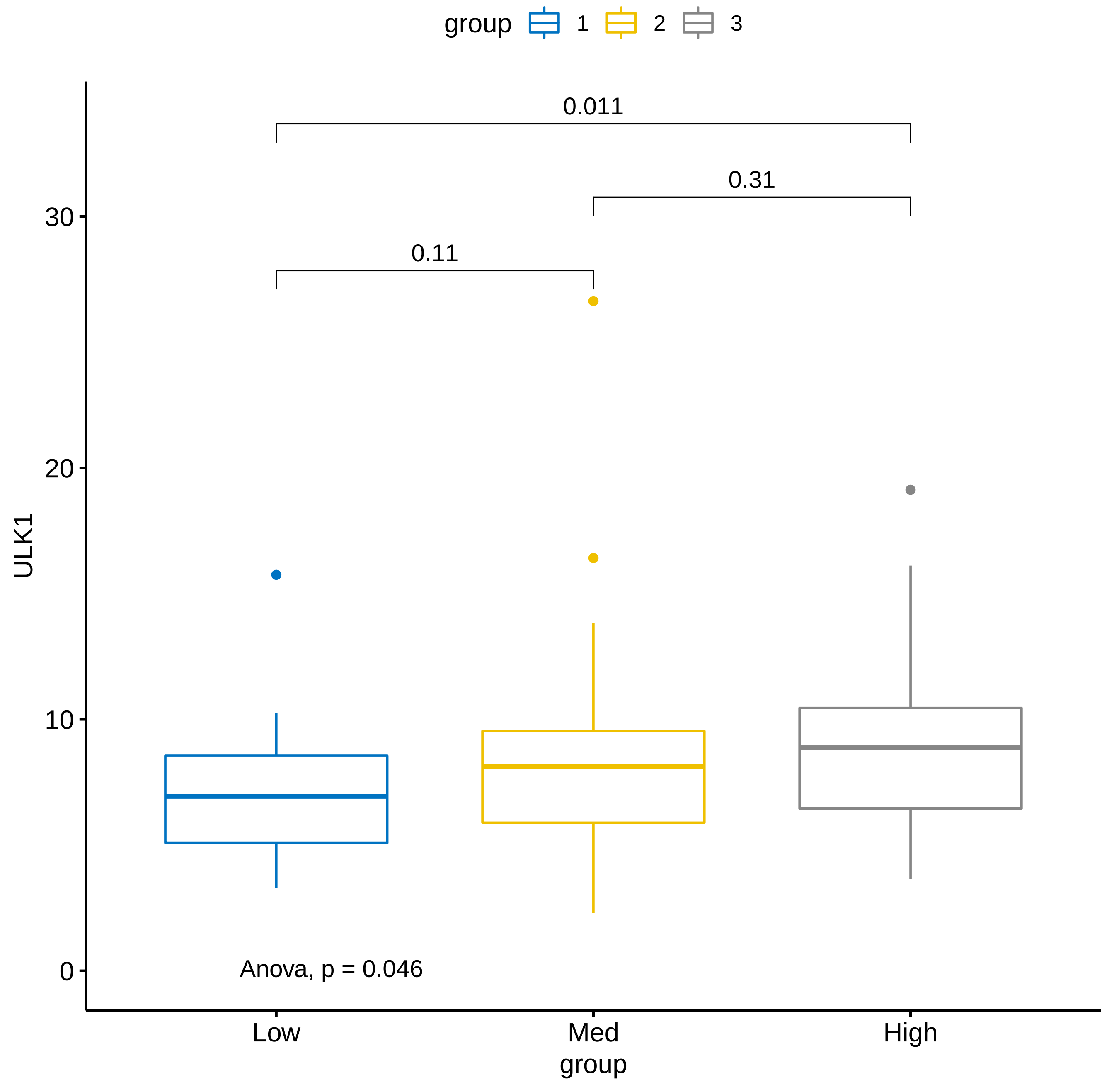
group
1
2
3
0.011
0.31
0.11
30
20
10
0
ULK1
Anova, p = 0.046
Low
Med
High
group

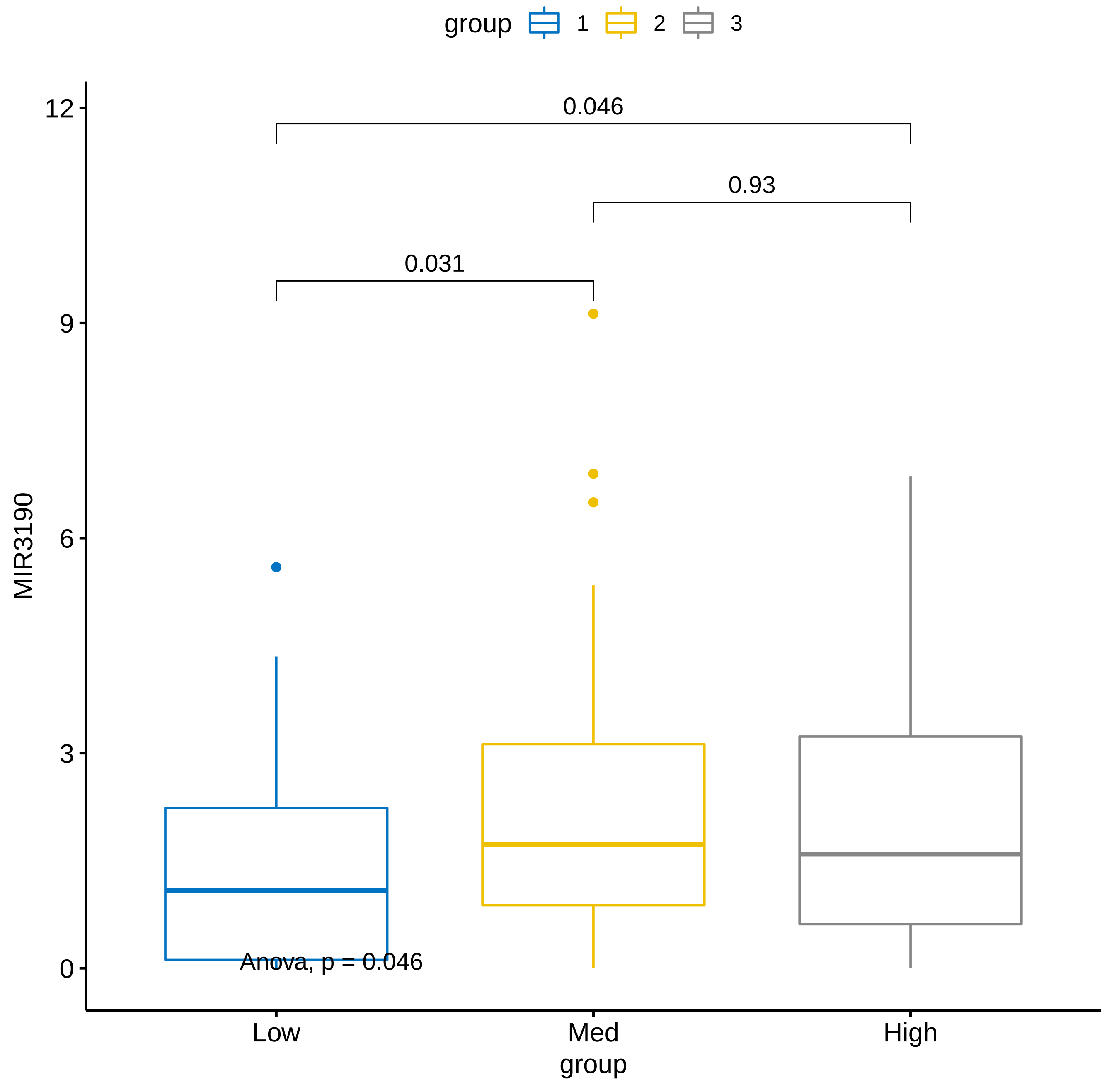
group
1
2
3
12
9
6
3
0
MIR3190
0.046
0.93
0.031
Anova, p = 0.046
Low
Med
High
group

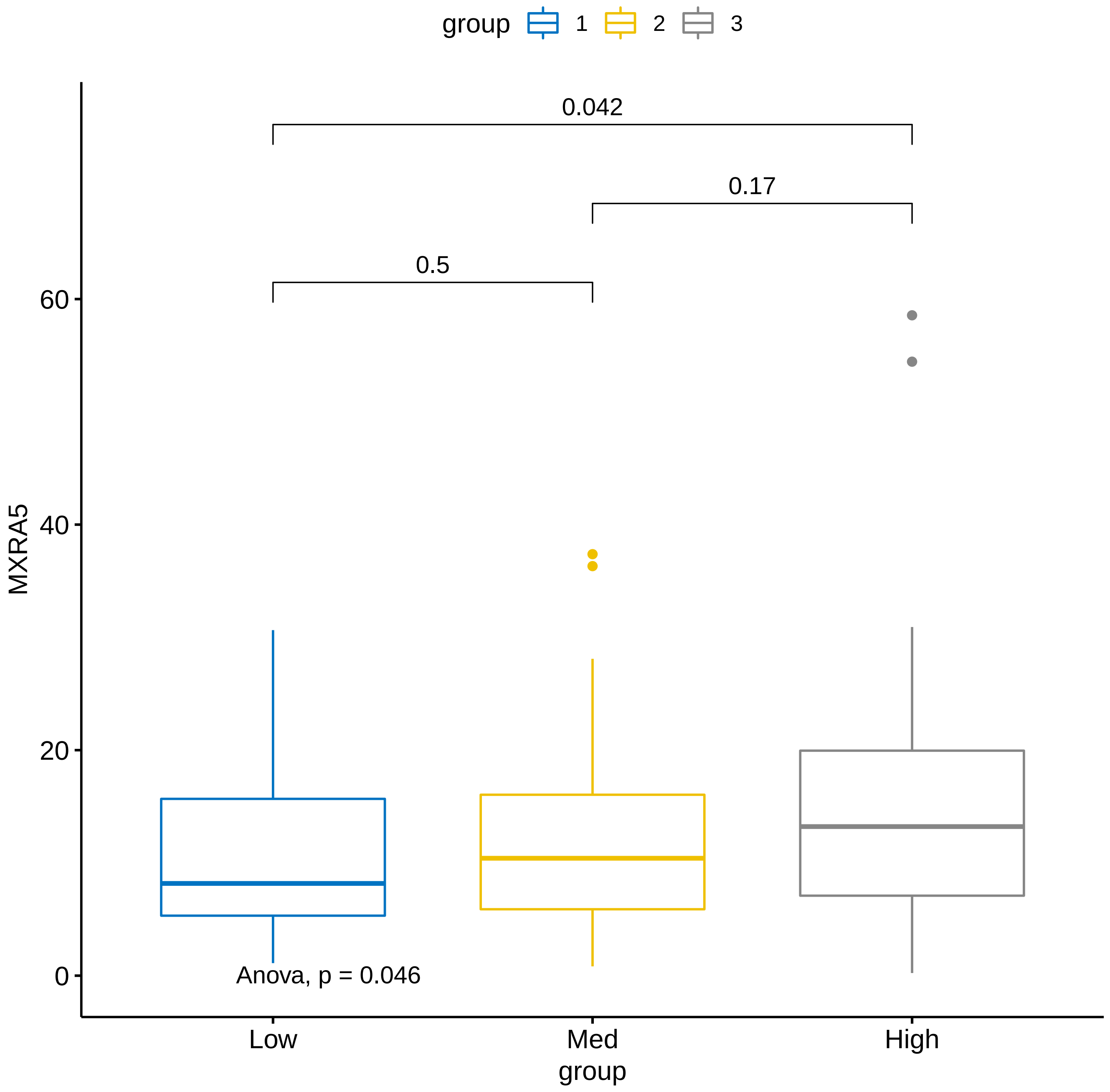
group
1
2
3
0.042
0.17
0.5
60
40
20
0
MXRA5
Anova, p = 0.046
Low
Med
High
group

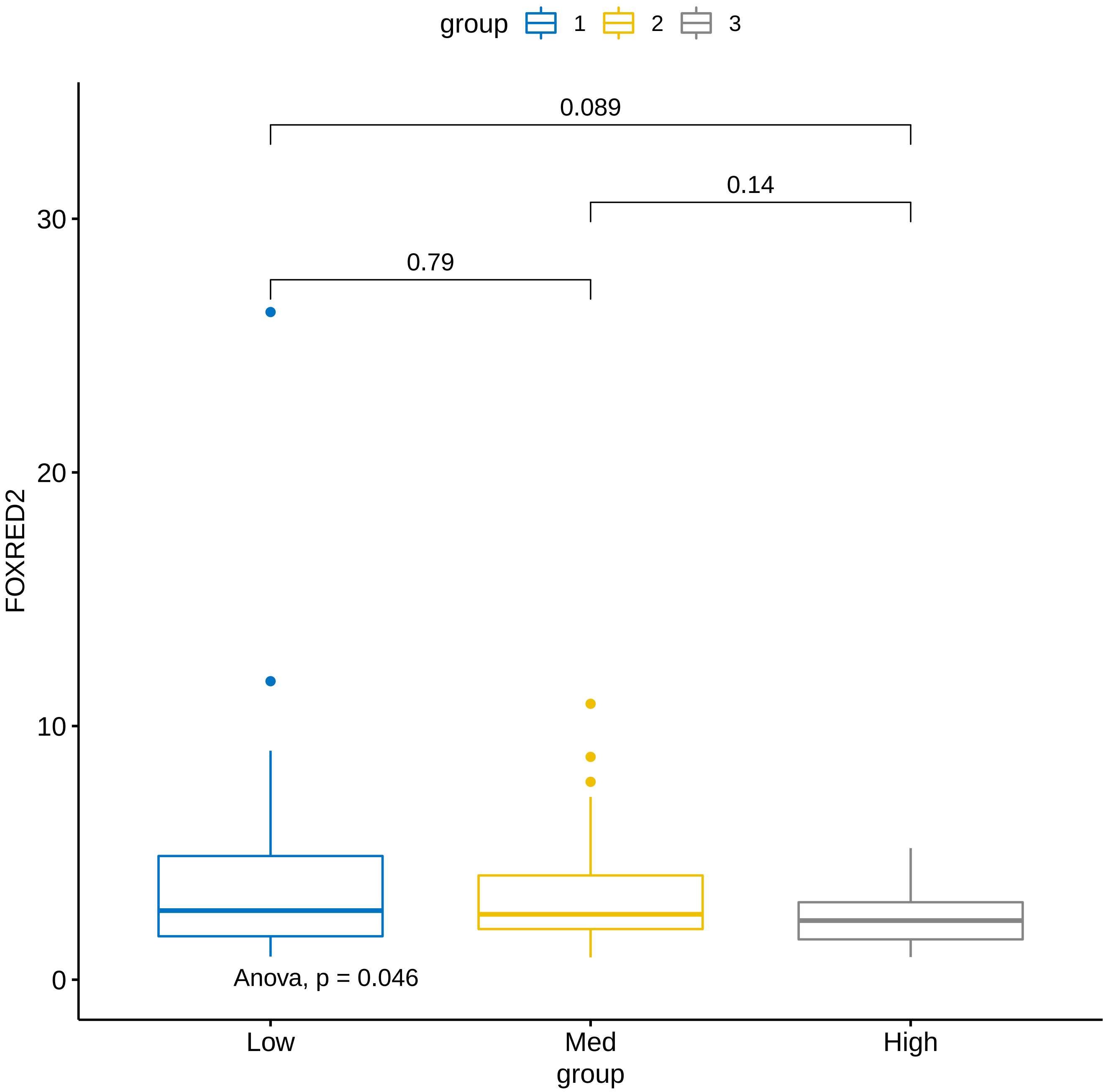
group
1
2
3
0.089
0.14
0.79
30
20
10
0
FOXRED2
Anova, p = 0.046
Low
Med
High
group

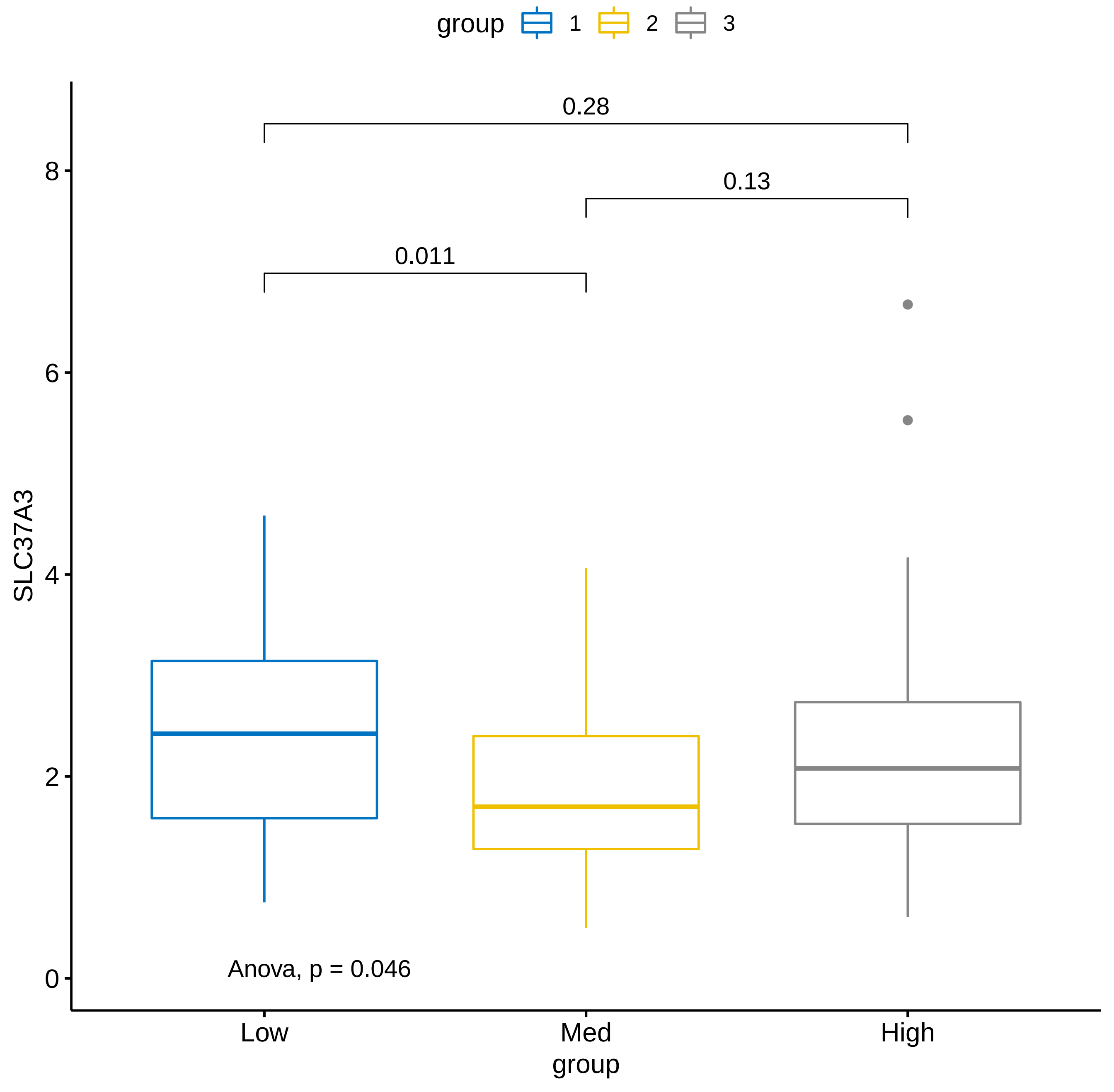
group
1
2
3
0.28
0.13
0.011
SLC37A3
8
6
4
2
0
Anova, p = 0.046
Low
Med
High
group

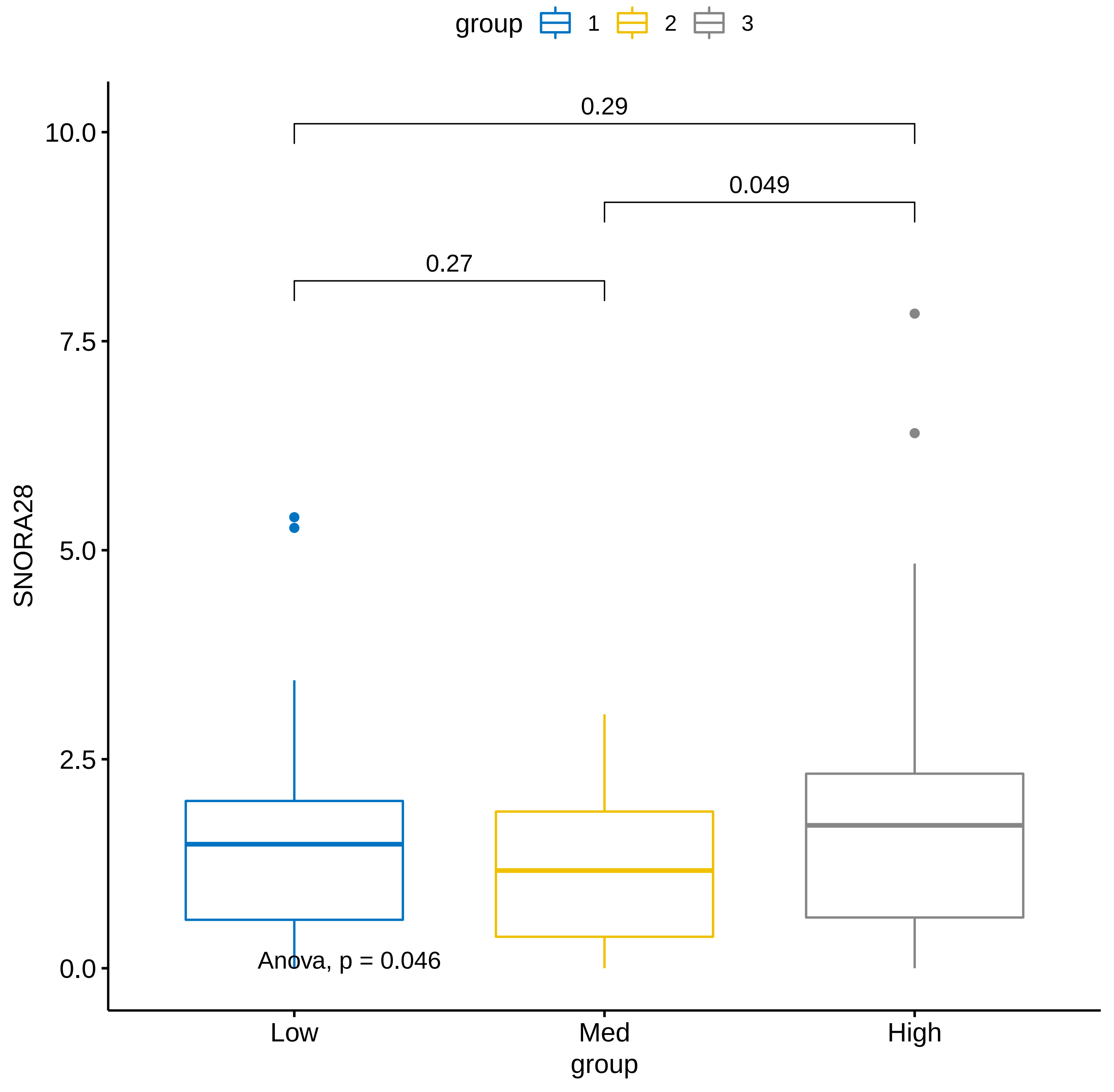
group
1
2
3
0.29
0.049
0.27
10.0
7.5
5.0
2.5
0.0
SNORA28
Anova, p = 0.046
Low
Med
High
group

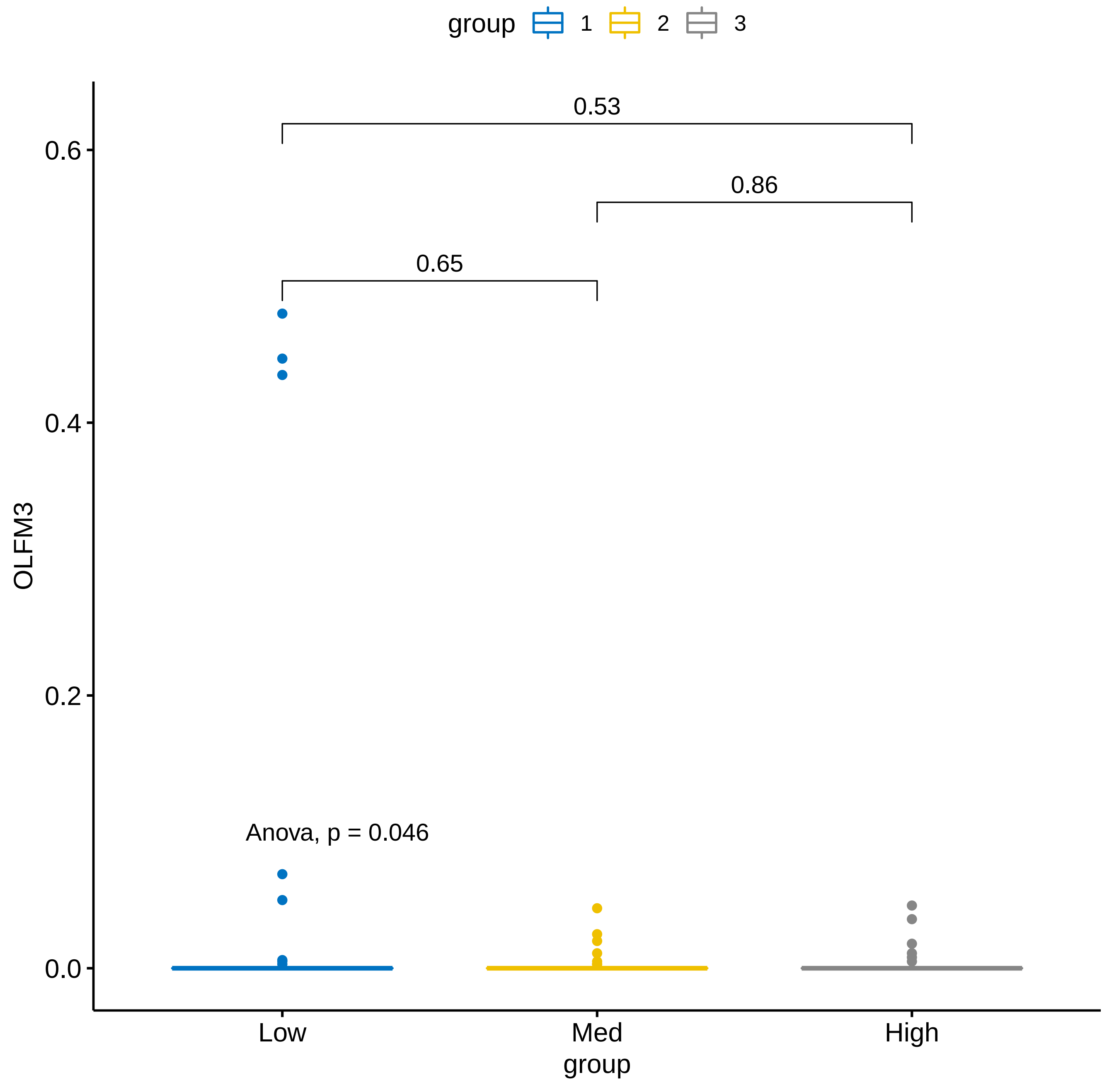

group
1
2
3
0.53
0.86
0.65
0.6
0.4
0.2
0.0
OLFM3
Anova, p = 0.046
Low
Med
High
group

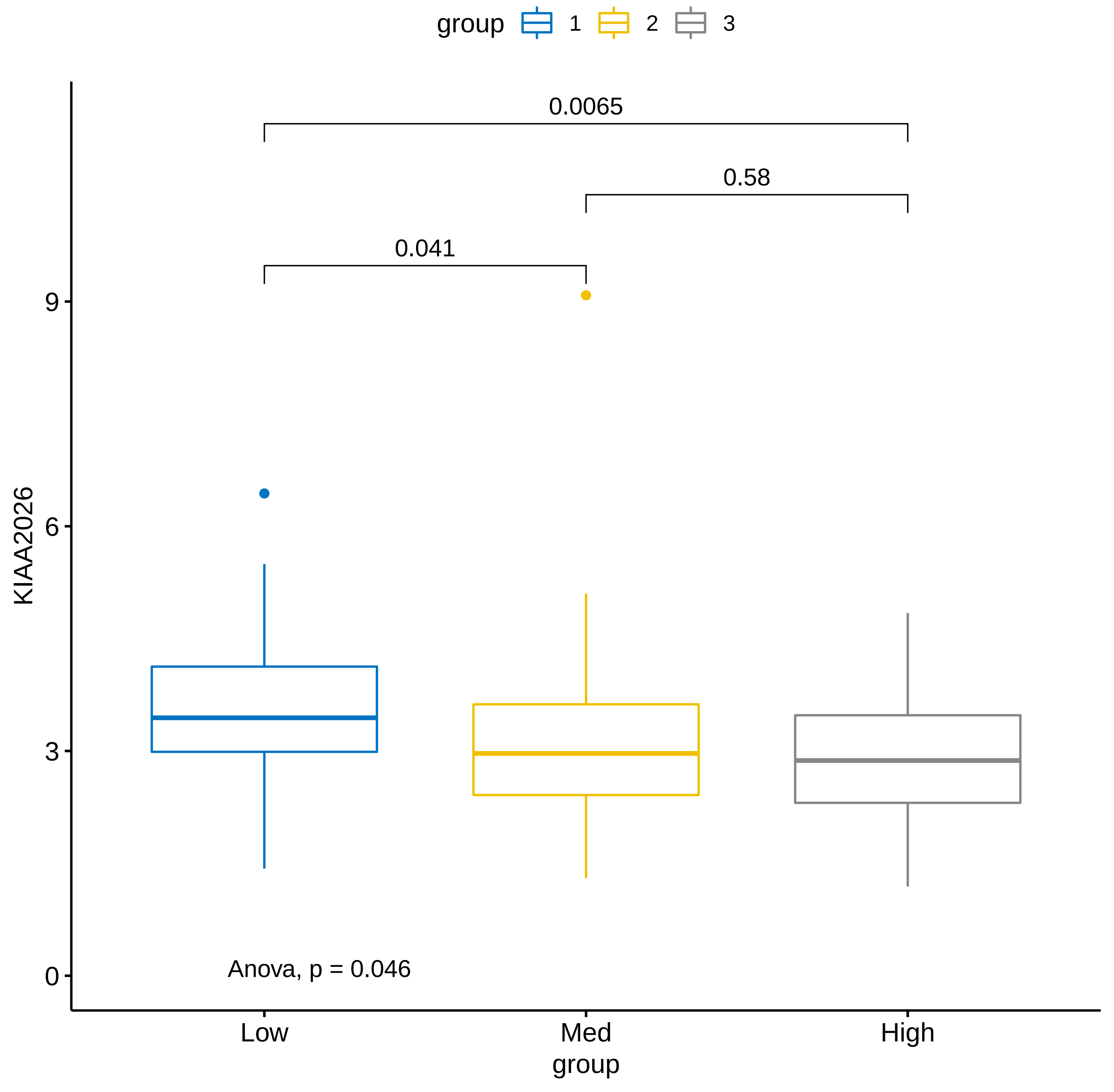
group
1
2
3
0.0065
0.58
0.041
9
6
3
0
KIAA2026
Anova, p = 0.046
Low
Med
High
group

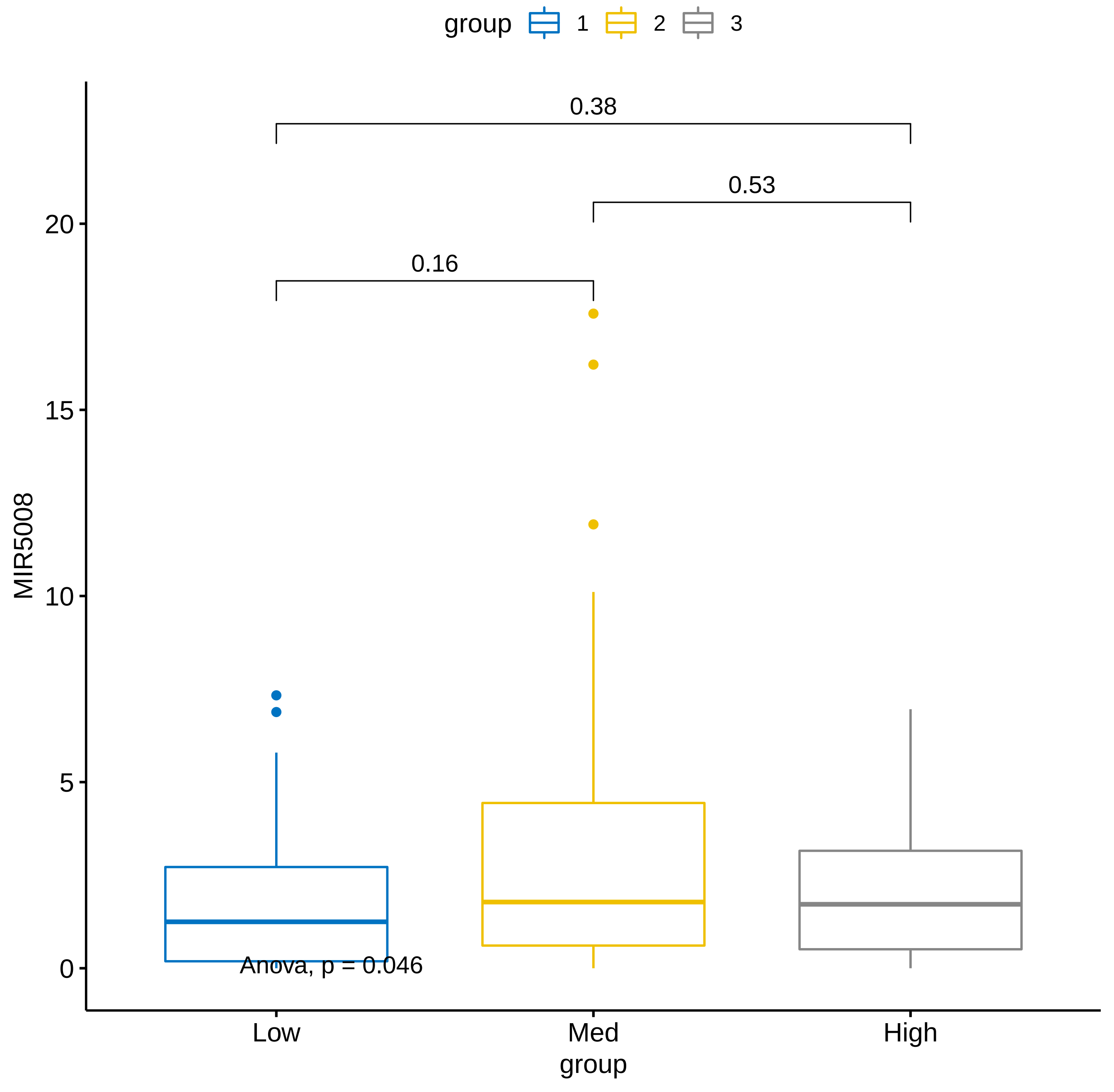

group 1 2 3
0.38
0.53
0.16
MIR5008
20
15
10
5
0
Anova, p = 0.046
Low
Med
High
group

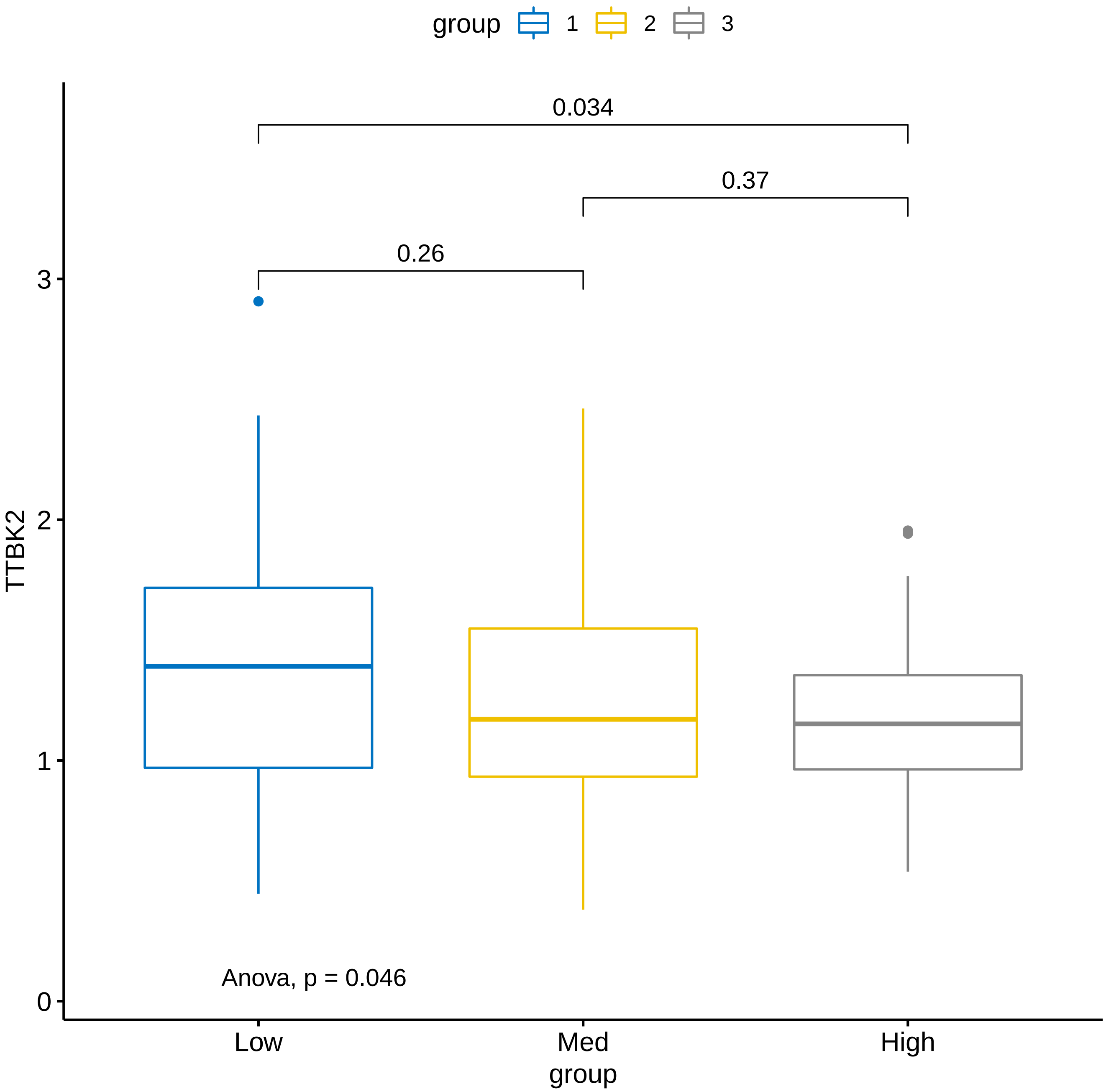
group
1
2
3
0.034
0.37
0.26
3
2
1
0
TTBK2
Anova, p = 0.046
Low
Med
High
group

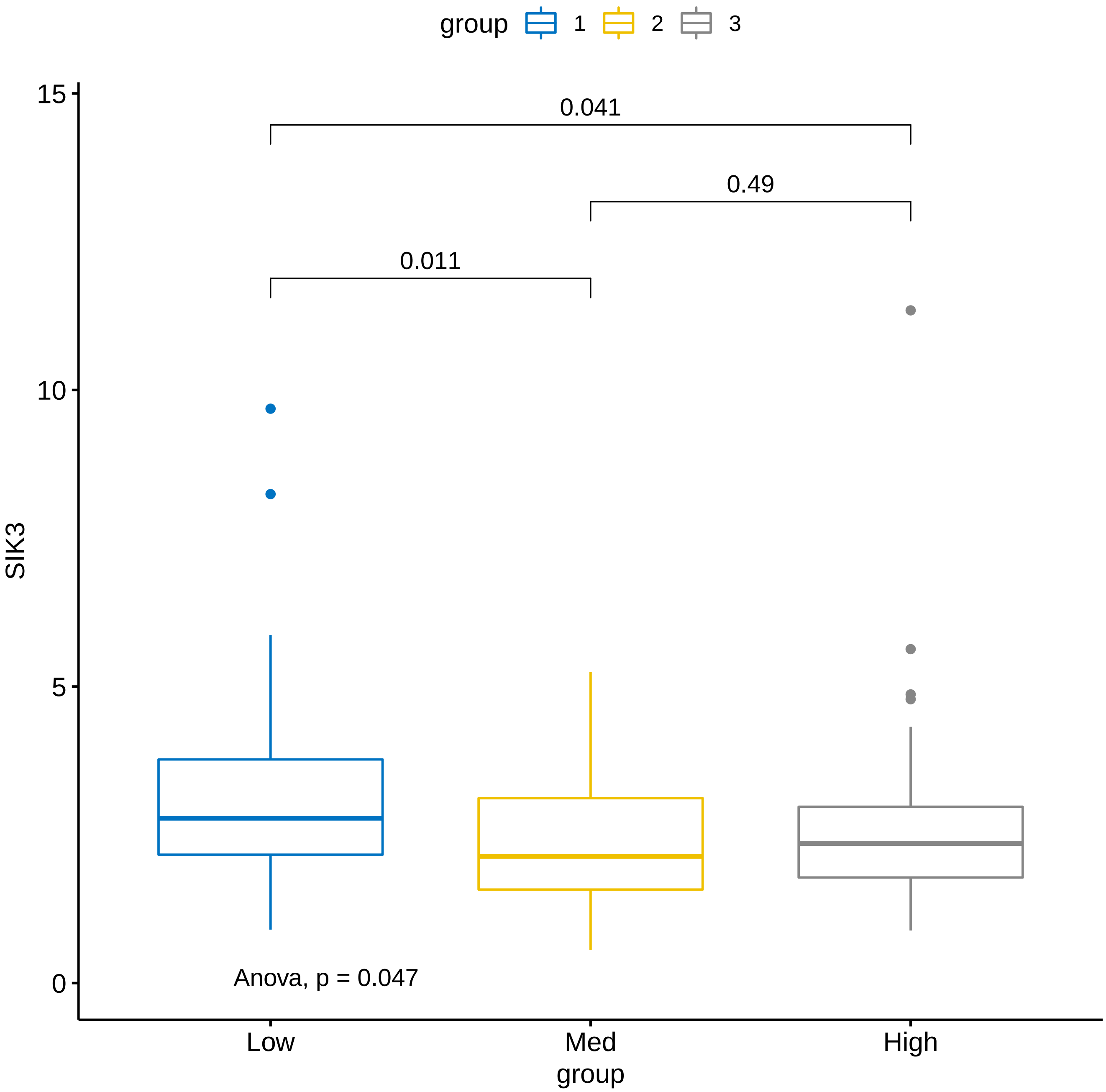
group 1 2 3
15
10
5
0
SIK3
0.041
0.49
0.011
Anova, p = 0.047
Low
Med
High
group

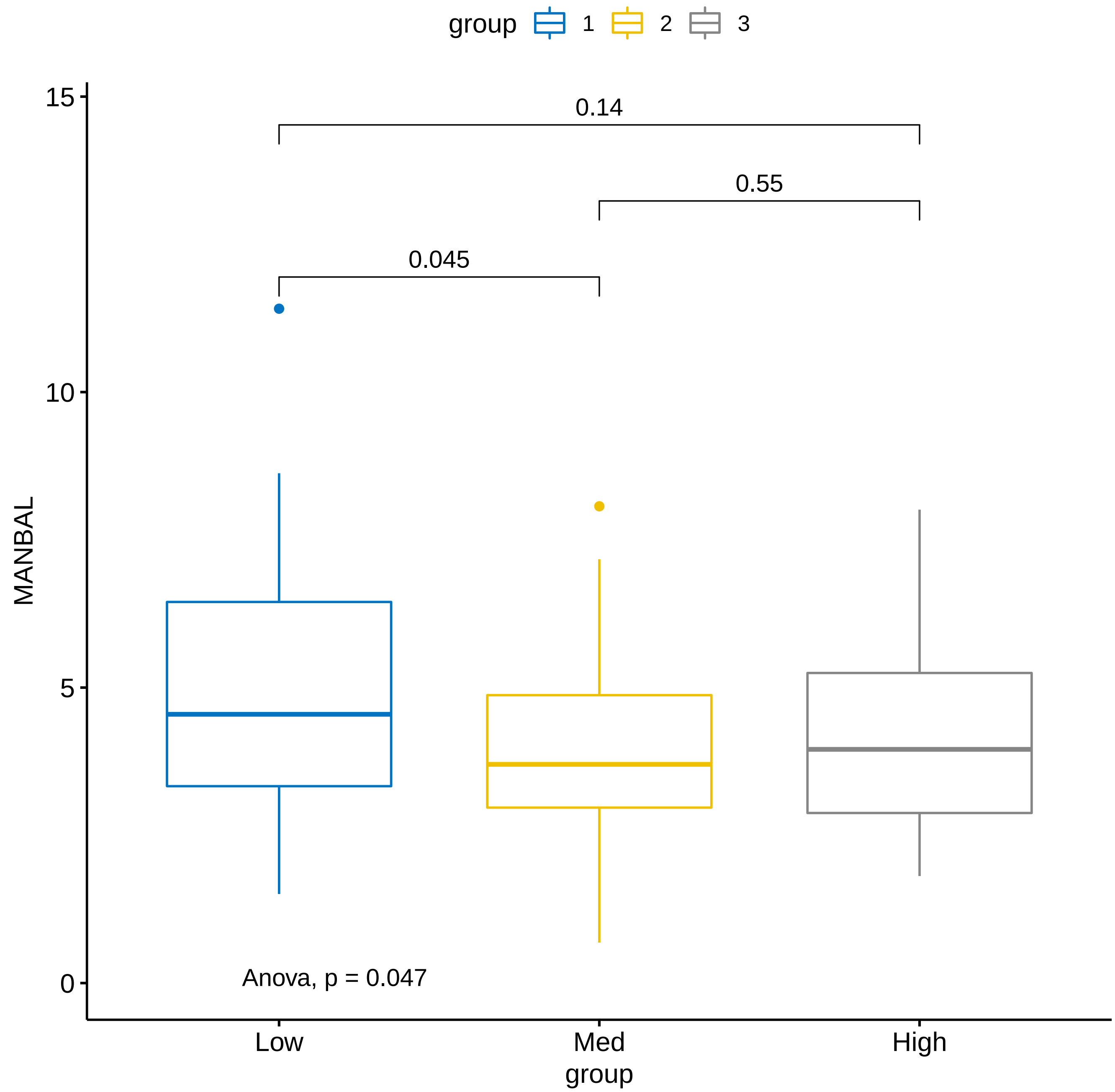
group
1
2
3
0.14
0.55
0.045
15
10
5
0
MANBAL
Anova, p = 0.047
Low
Med
High
group

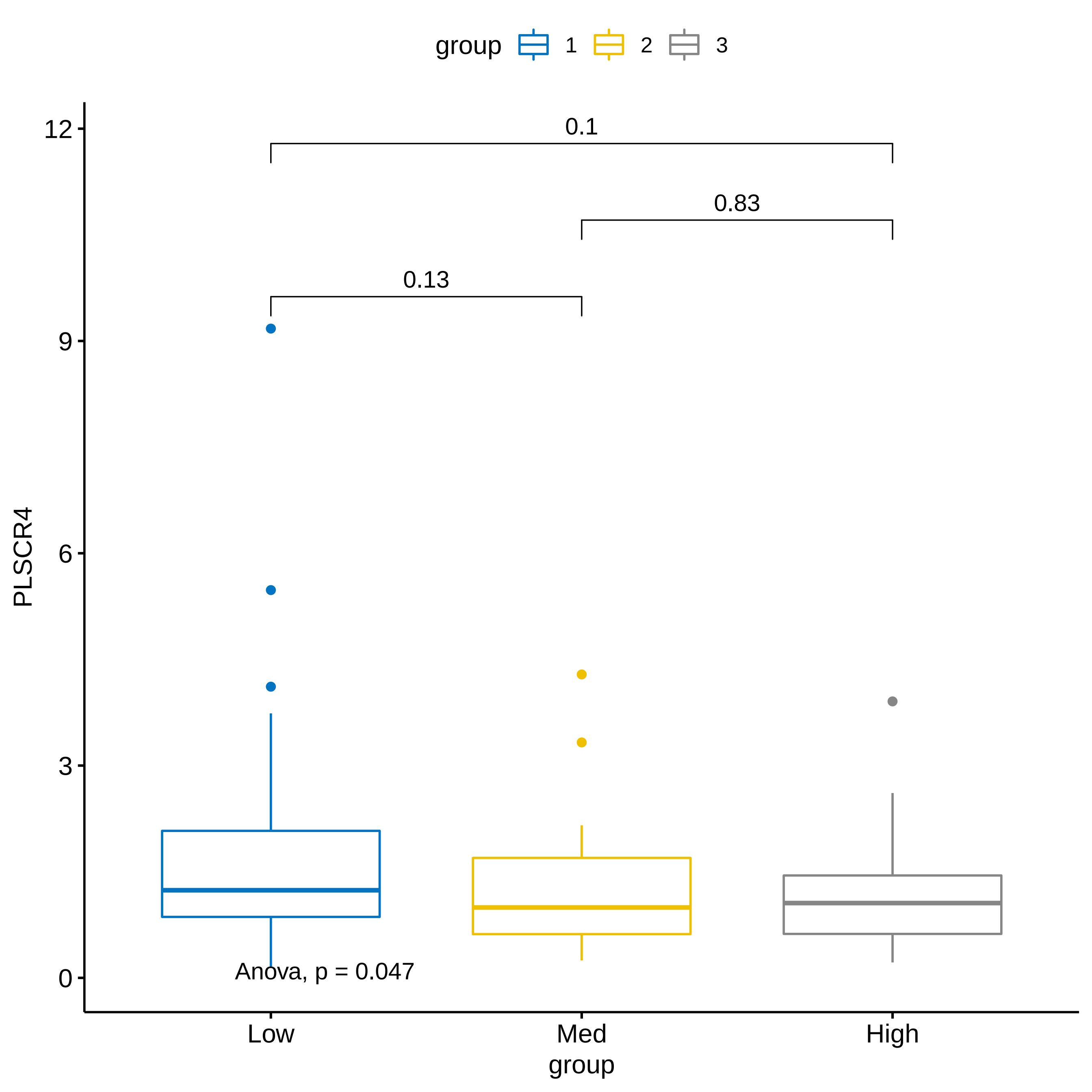

group 1 2 3
0.1
0.83
0.13
12
9
6
3
0
PLSCR4
Anova, p = 0.047
Low
Med
High
group

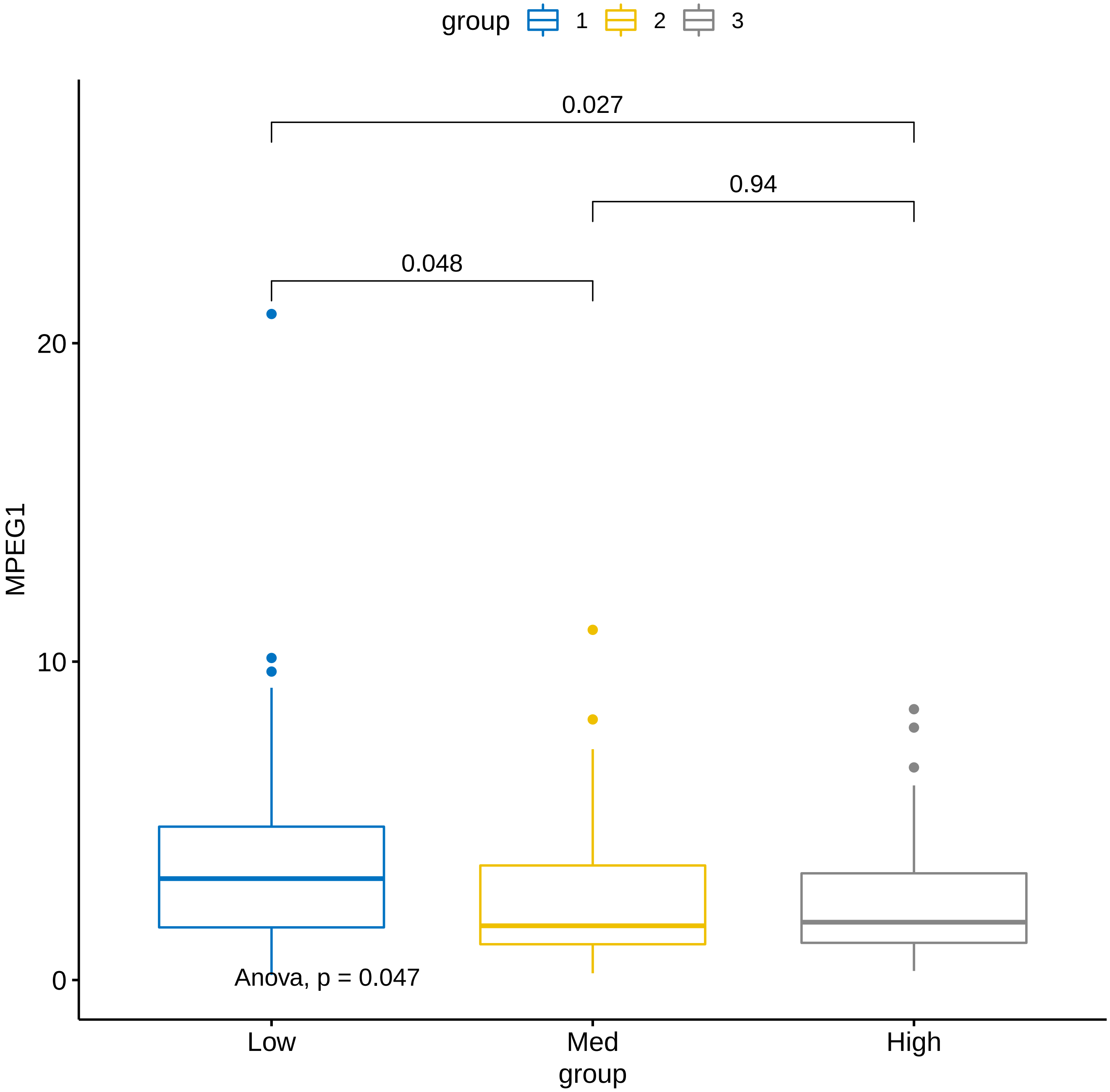

group
1
2
3
0.027
0.94
0.048
MPEG1
20
10
0
Anova, p = 0.047
Low
Med
High
group

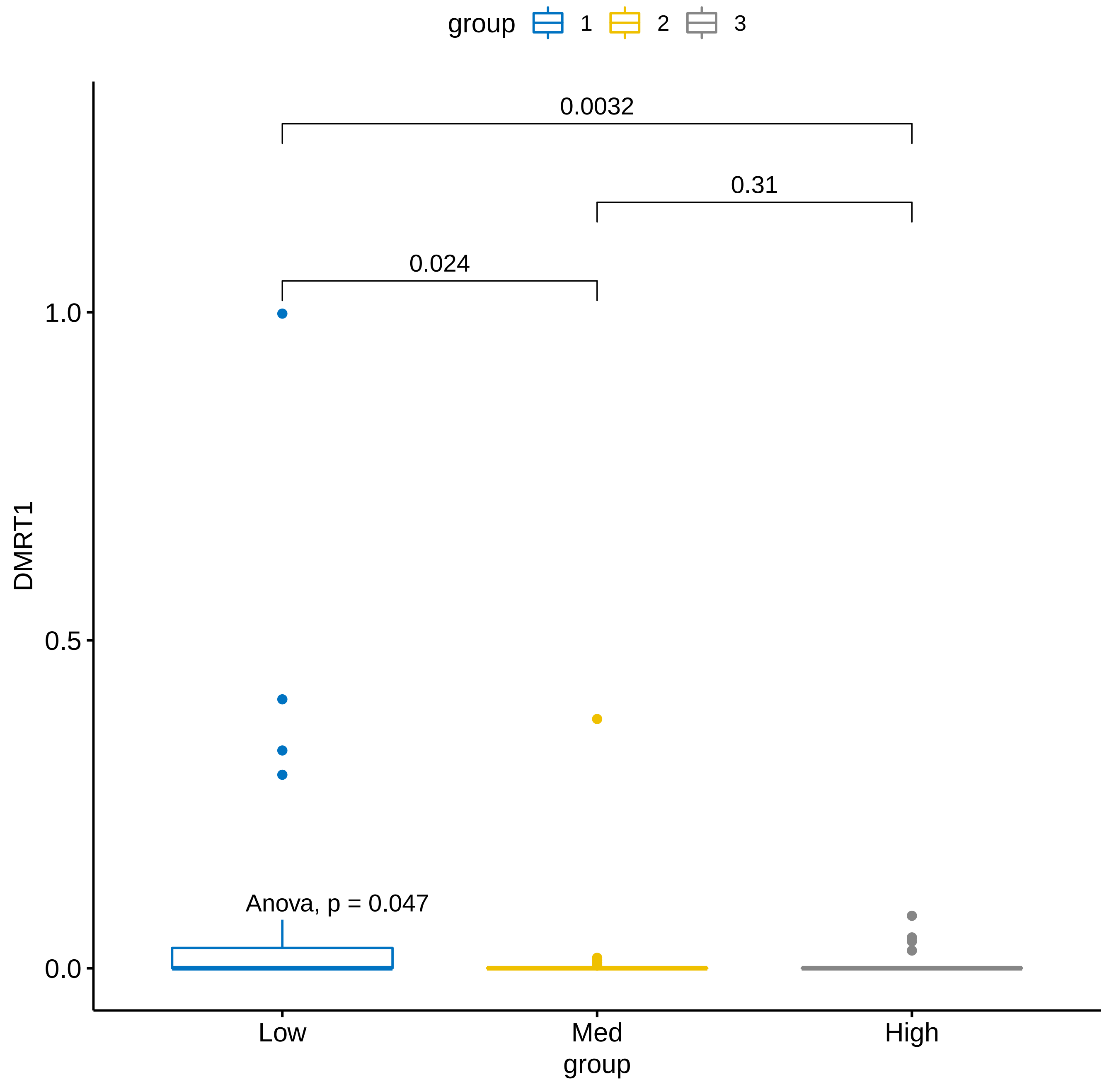

group
1
2
3
0.0032
0.31
0.024
1.0
0.5
0.0
DMRT1
Anova, p = 0.047
Low
Med
High
group

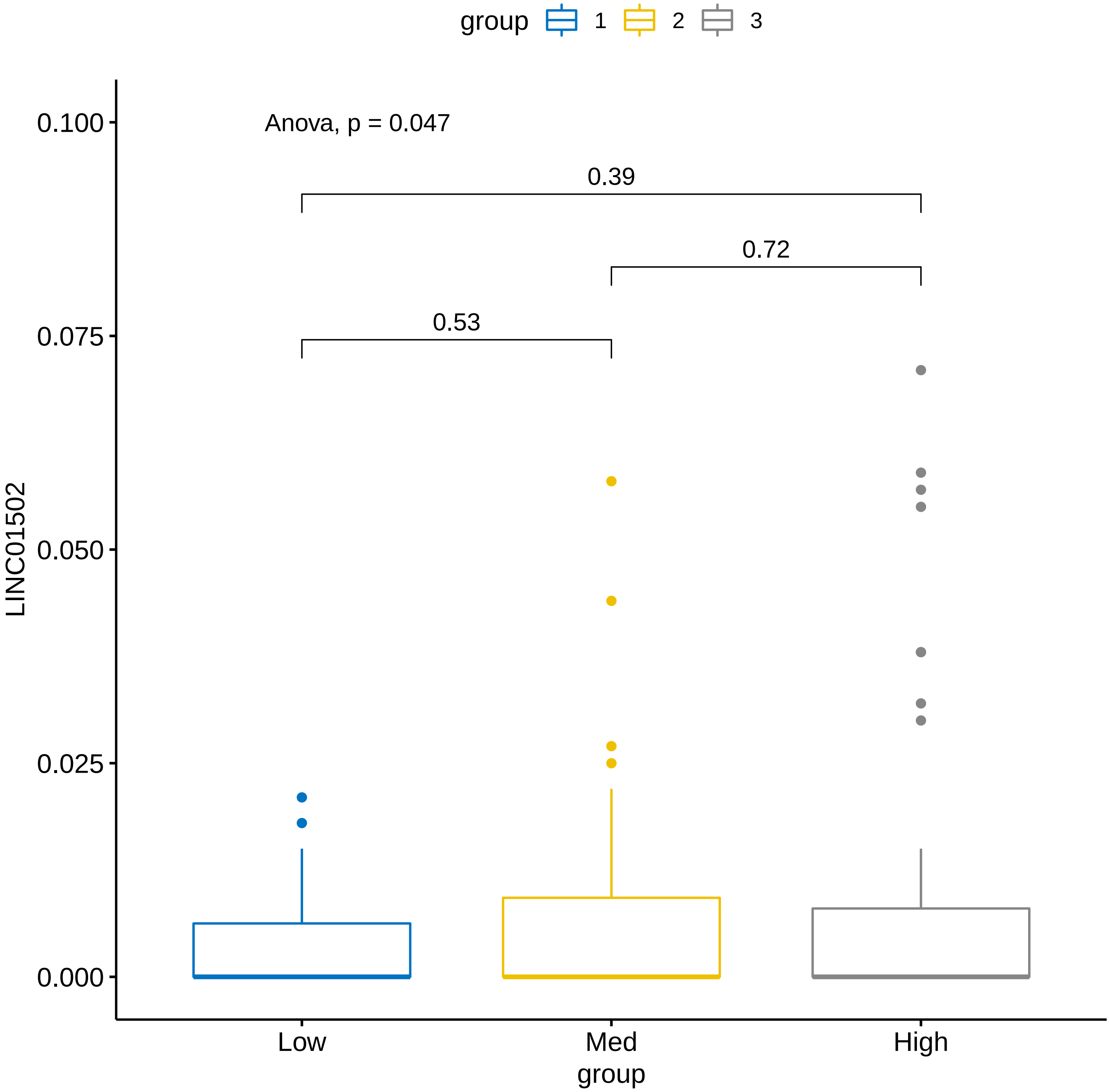
group
1
2
3
Anova, p = 0.047
0.39
0.72
0.53
0.100
0.075
0.050
0.025
0.000
LINC01502
Low
Med
High
group

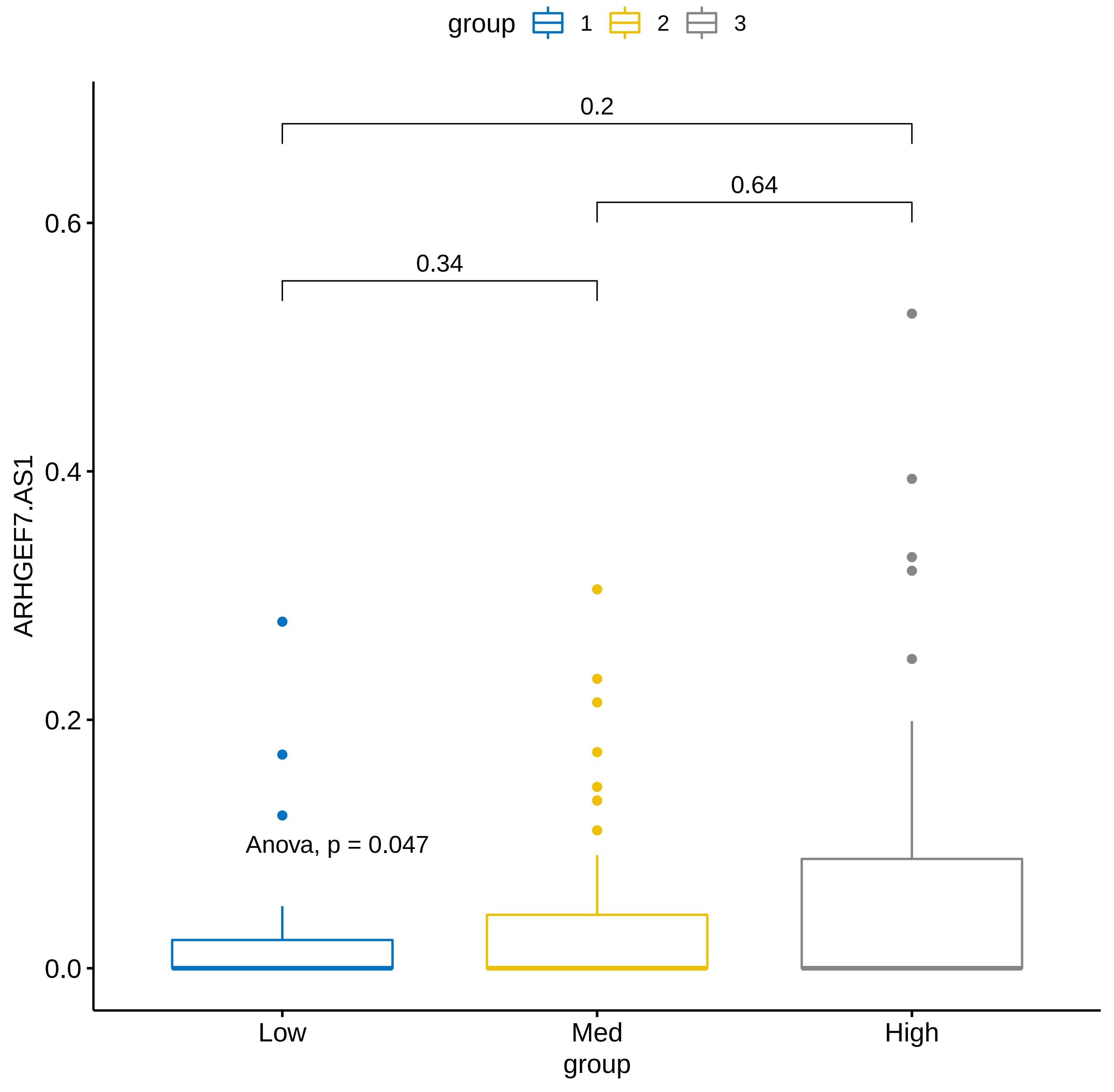
group 1 2 3
0.2
0.64
0.34
Anova, p = 0.047
ARHGEF7.AS1
0.0
0.2
0.4
0.6
Low
Med
High
group

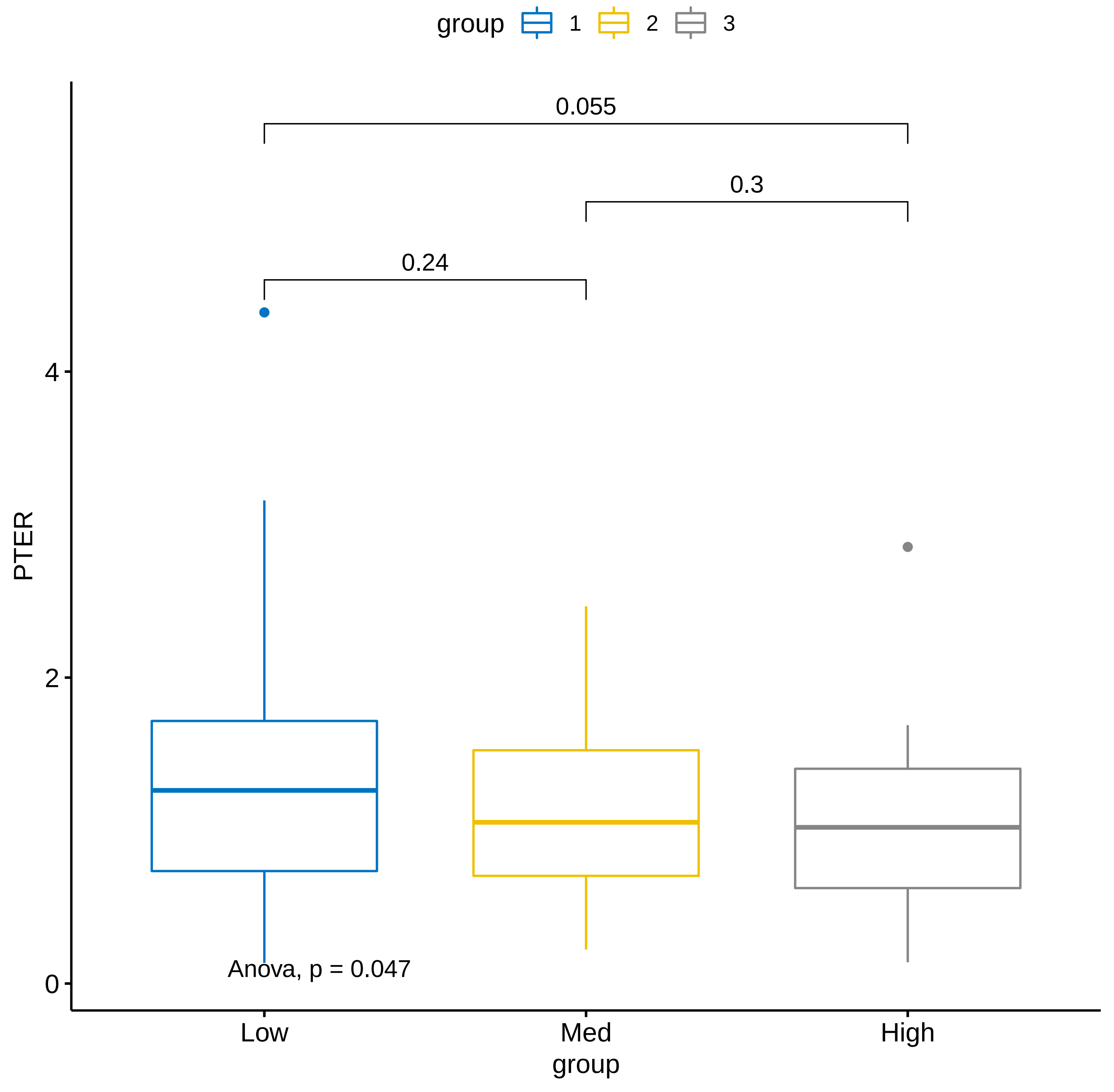
group 1 2 3
0.055
0.3
0.24
PTER
4
2
0
Anova, p = 0.047
Low
Med
High
group

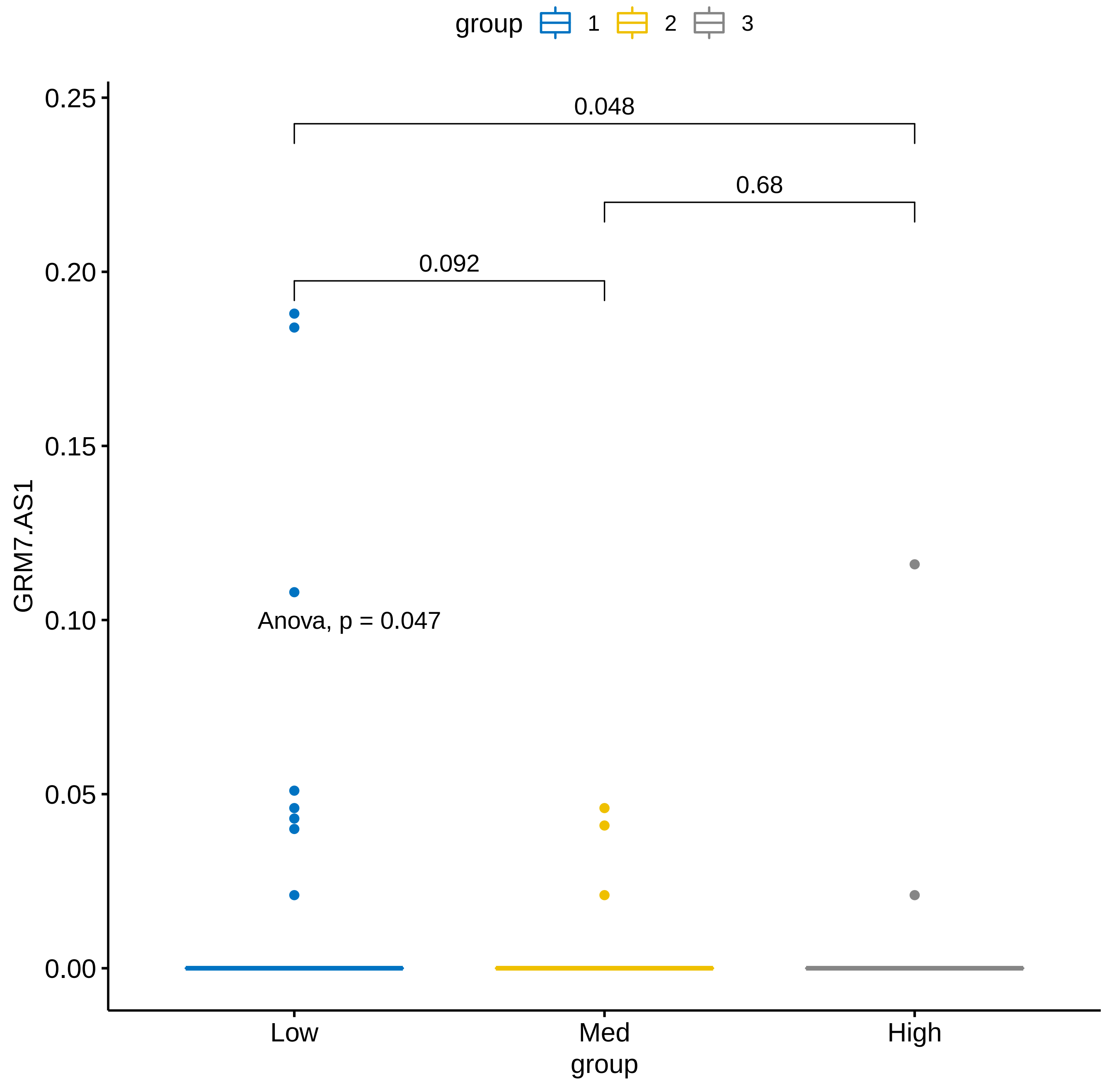
group
1
2
3
0.25
0.20
0.15
0.10
0.05
0.00
GRM7.AS1
0.048
0.68
0.092
Anova, p = 0.047
Low
Med
High
group

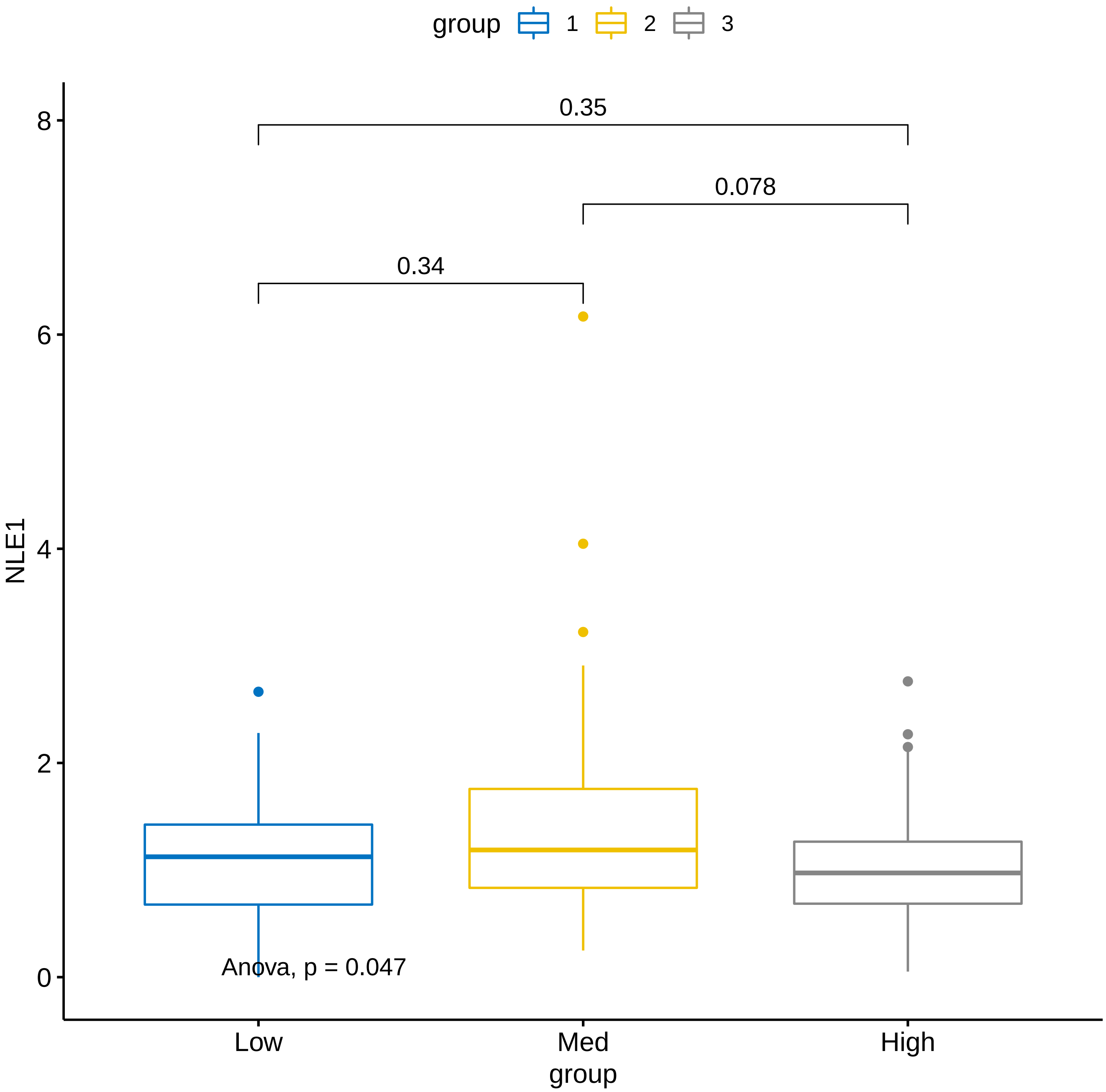

group
1
2
3
0.35
0.078
0.34
Anova, p = 0.047
NLE1
8
6
4
2
0
Low
Med
High
group

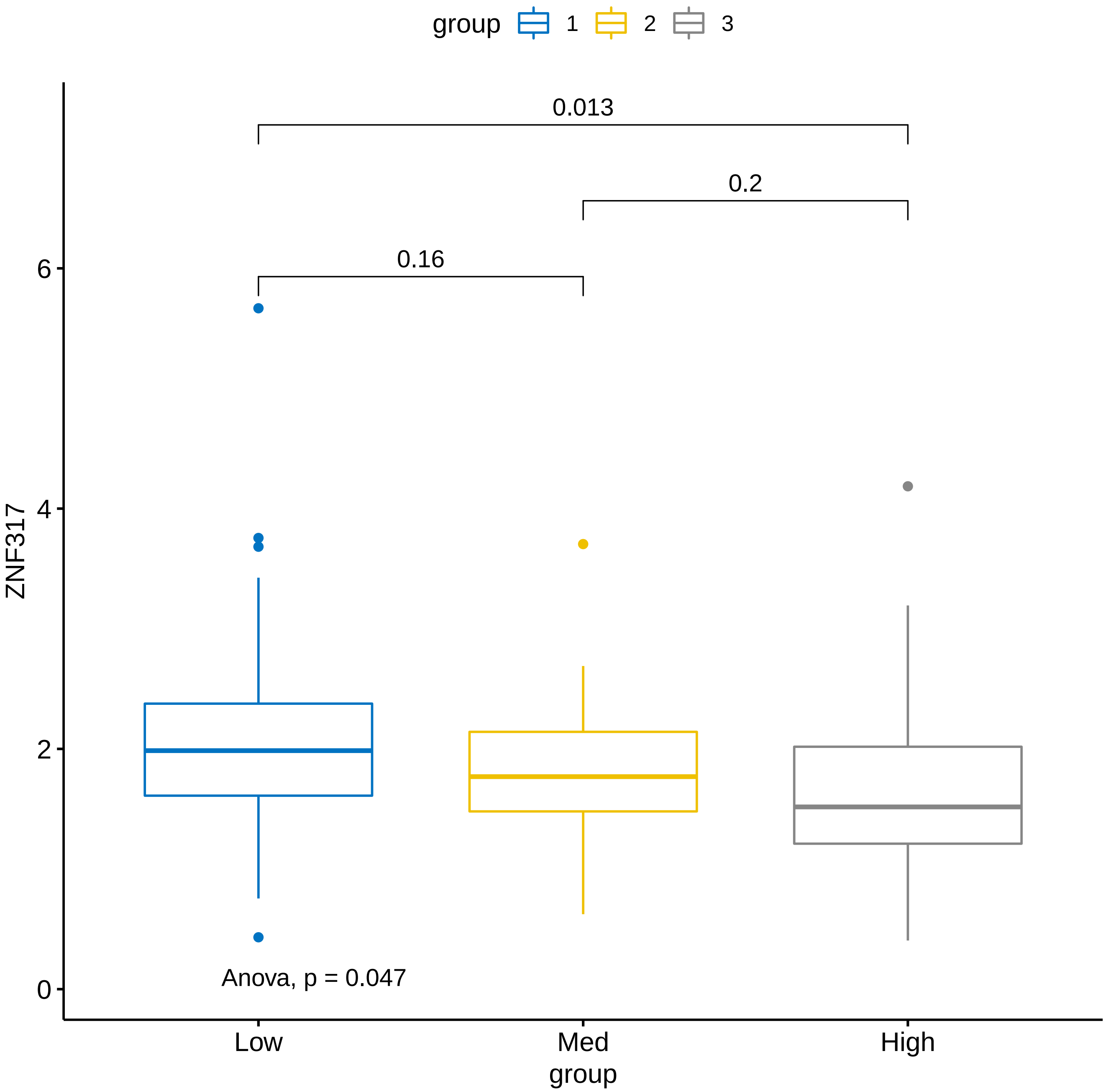
group
1
2
3
0.013
0.2
0.16
6
4
2
0
ZNF317
Anova, p = 0.047
Low
Med
High
group

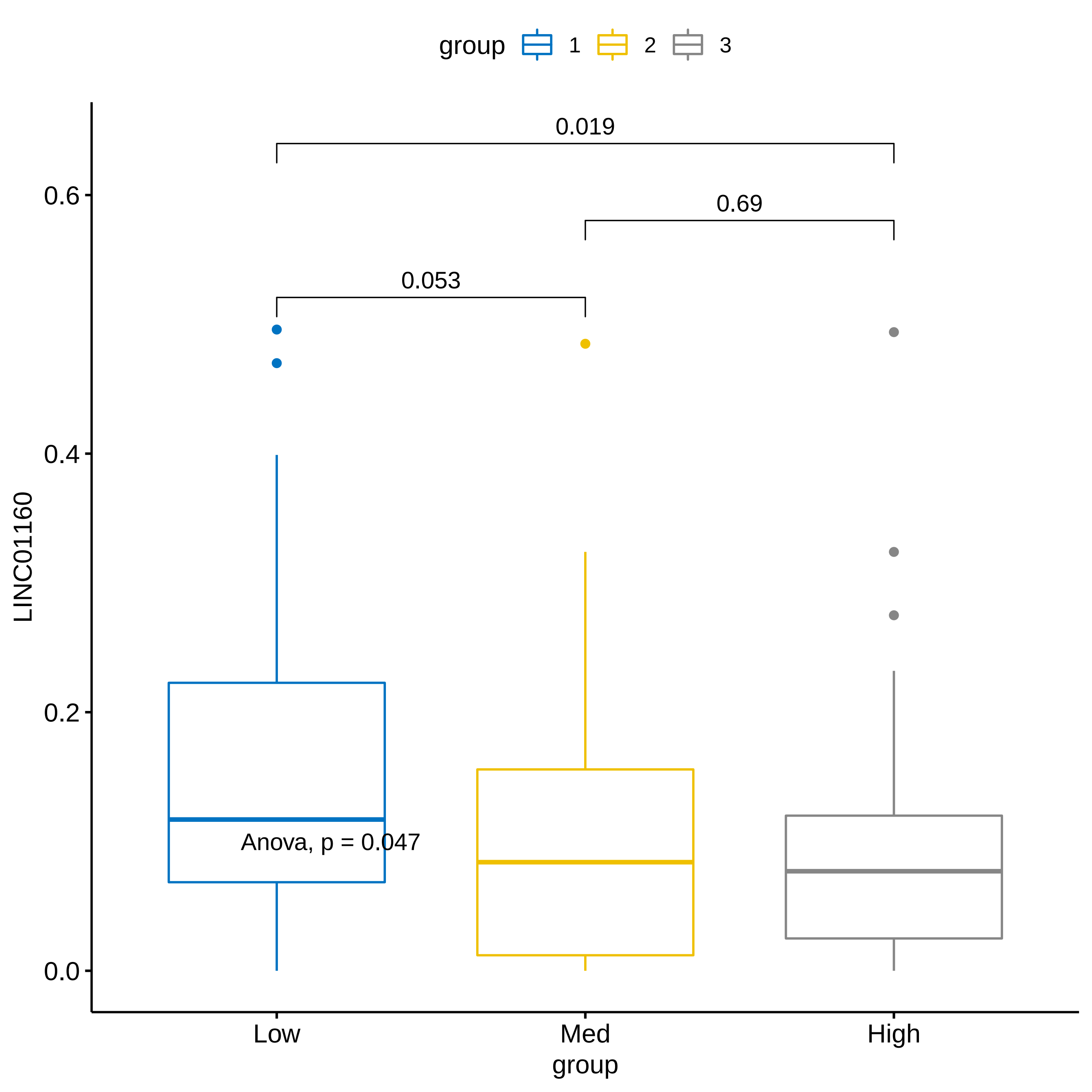
group
1
2
3
0.019
0.69
0.053
Anova, p = 0.047
LINC01160
0.0
0.2
0.4
0.6
Low
Med
High
group

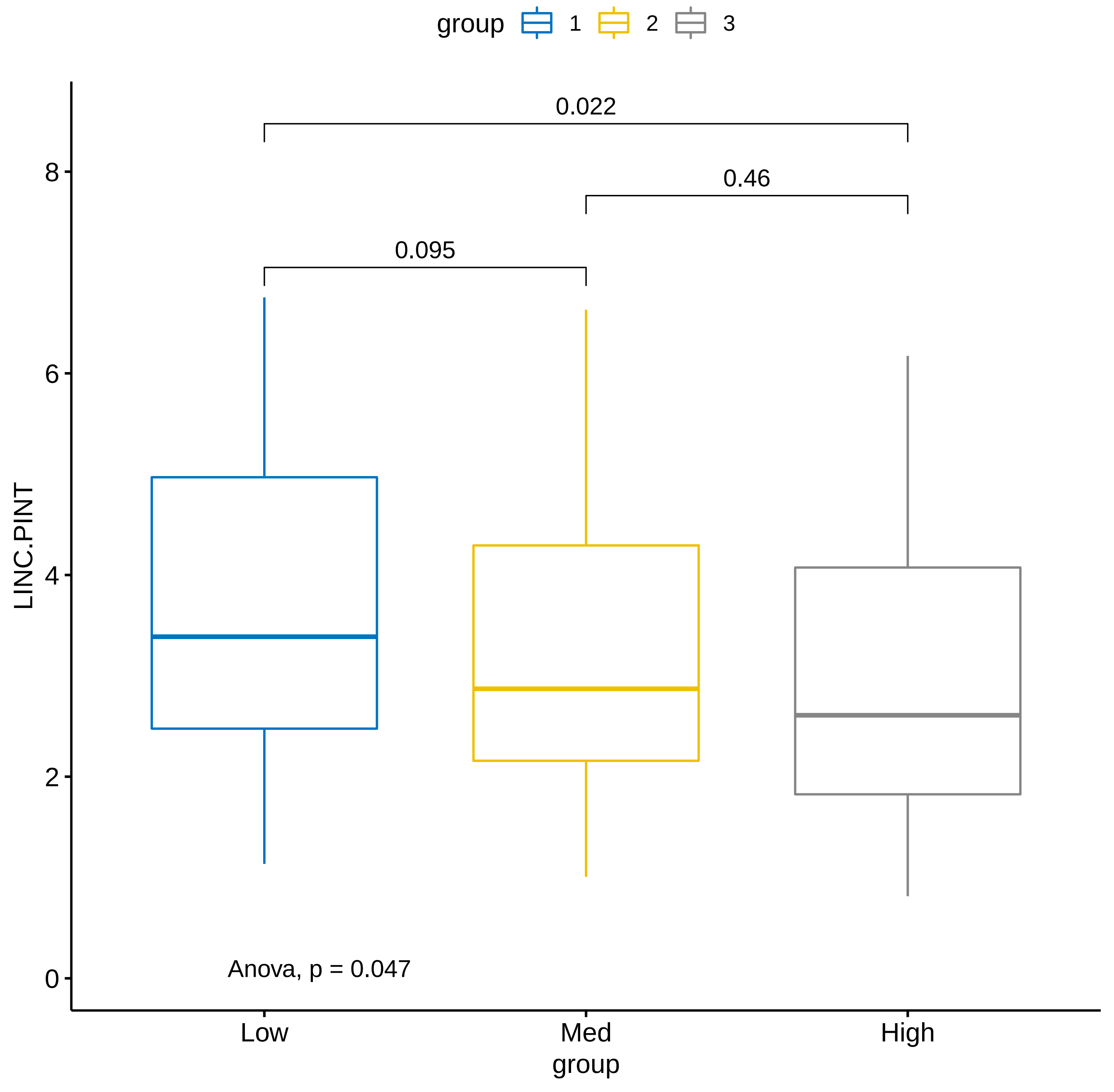

group 1 2 3
0.022
0.46
0.095
LINC.PINT
8
6
4
2
0
Anova, p = 0.047
Low
Med
High
group

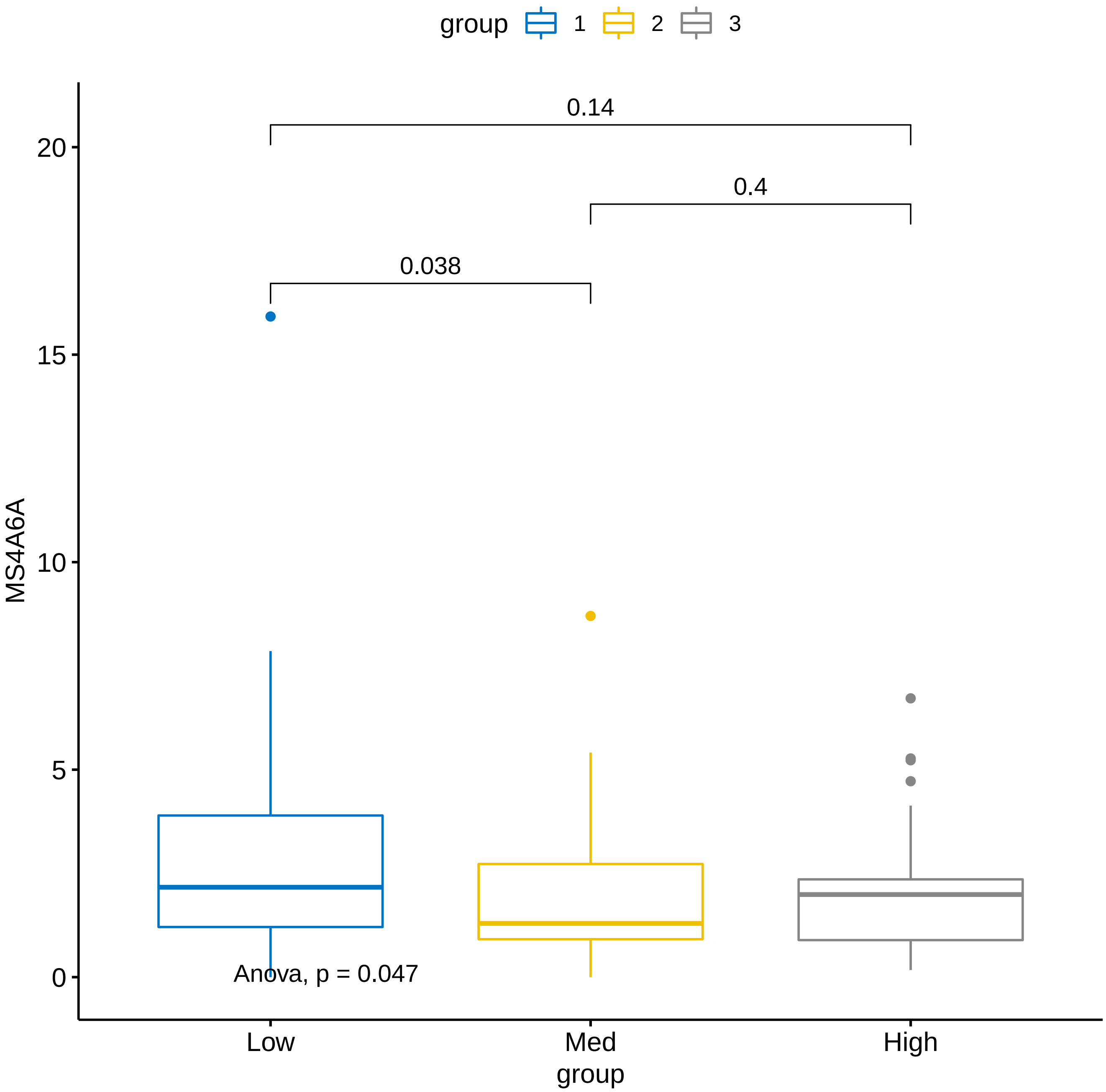
group
1
2
3
0.14
0.4
0.038
MS4A6A
20
15
10
5
0
Anova, p = 0.047
Low
Med
High
group

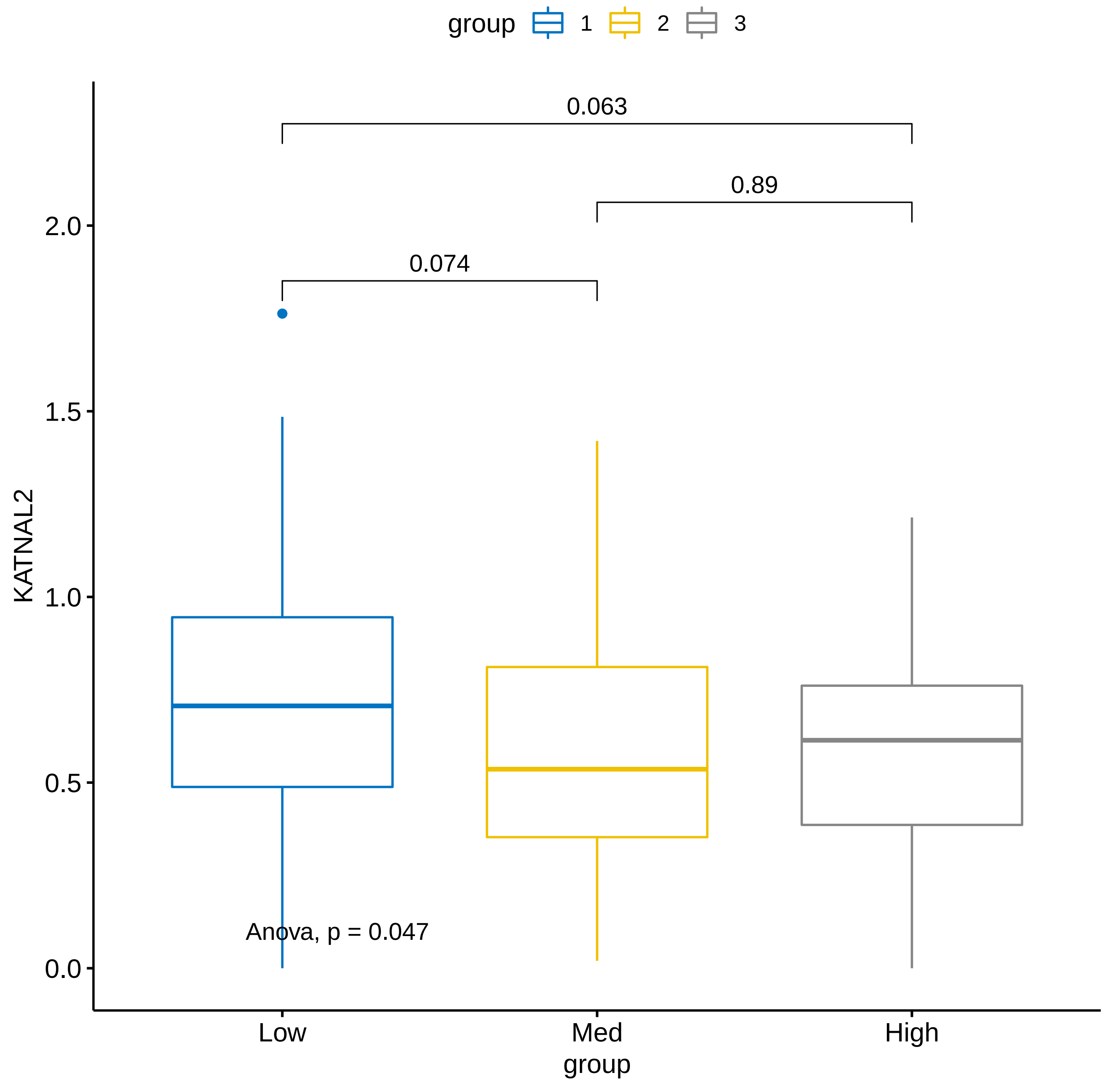
group 1 2 3
0.063
0.89
0.074
2.0
1.5
1.0
0.5
0.0
KATNAL2
Anova, p = 0.047
Low
Med
High
group

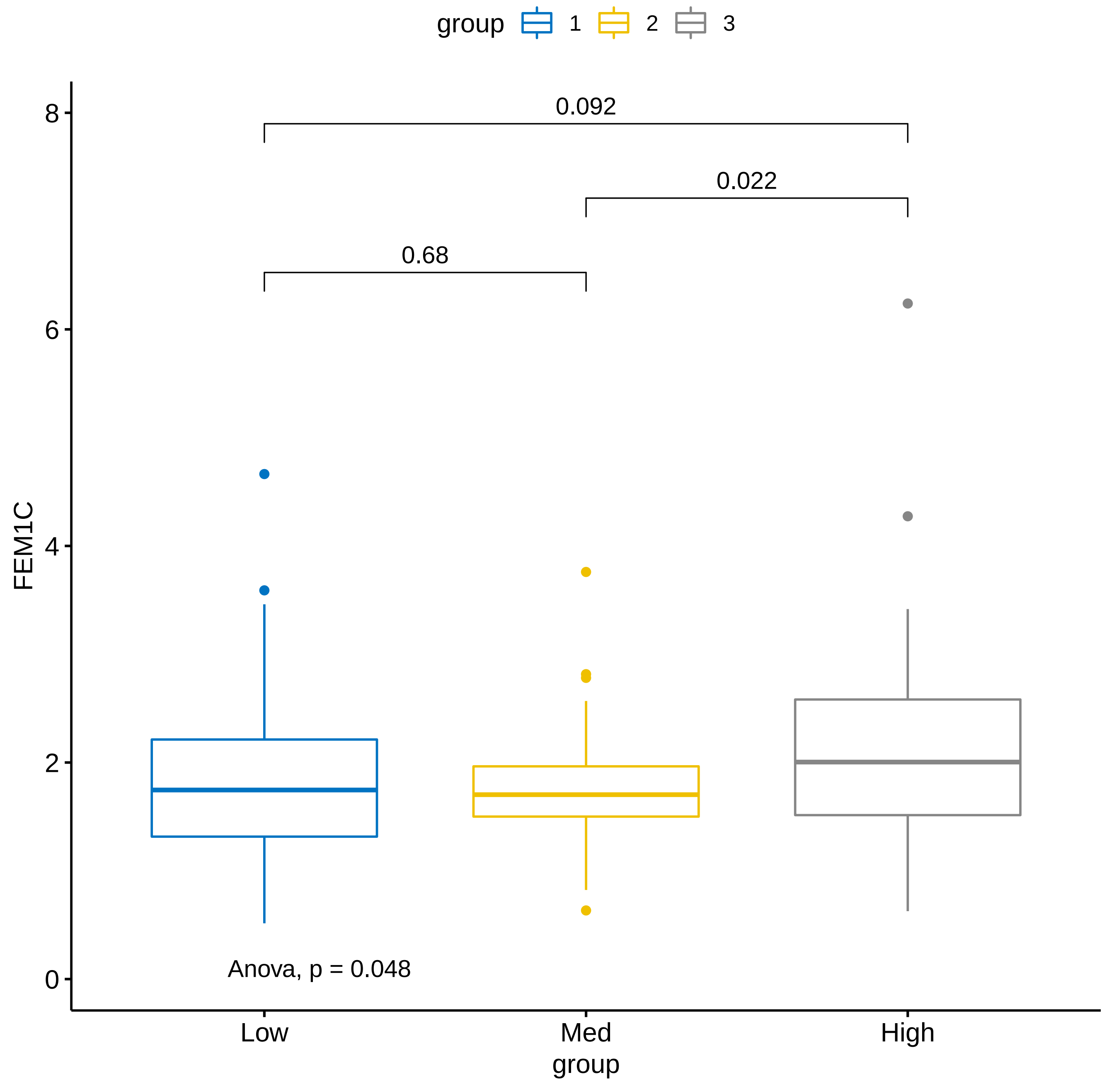

group
1
2
3
0.092
0.022
0.68
8
6
4
2
0
FEM1C
Anova, p = 0.048
Low
Med
High
group

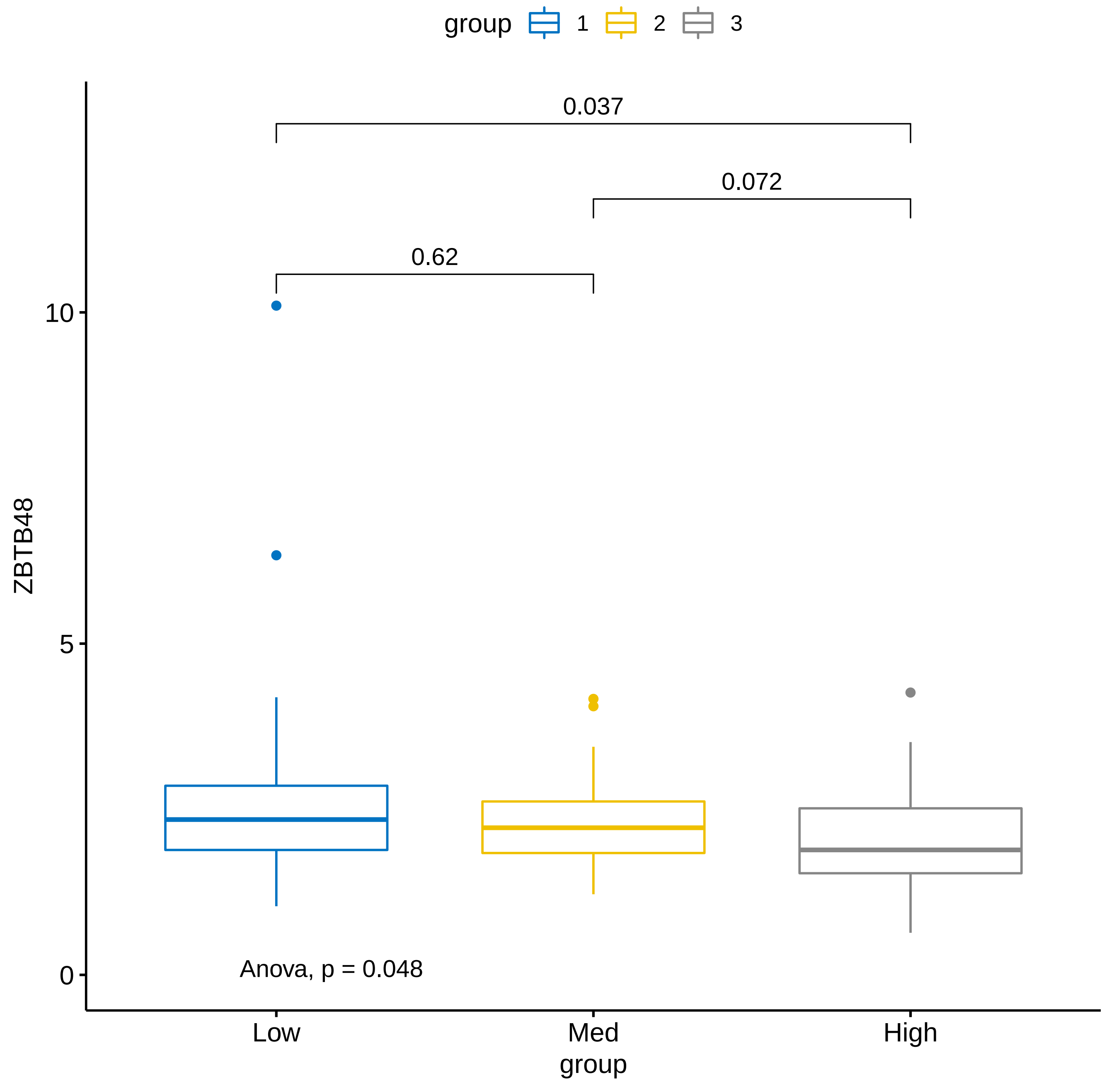

group
1
2
3
0.037
0.072
0.62
10
5
0
ZBTB48
Anova, p = 0.048
Low
Med
High
group

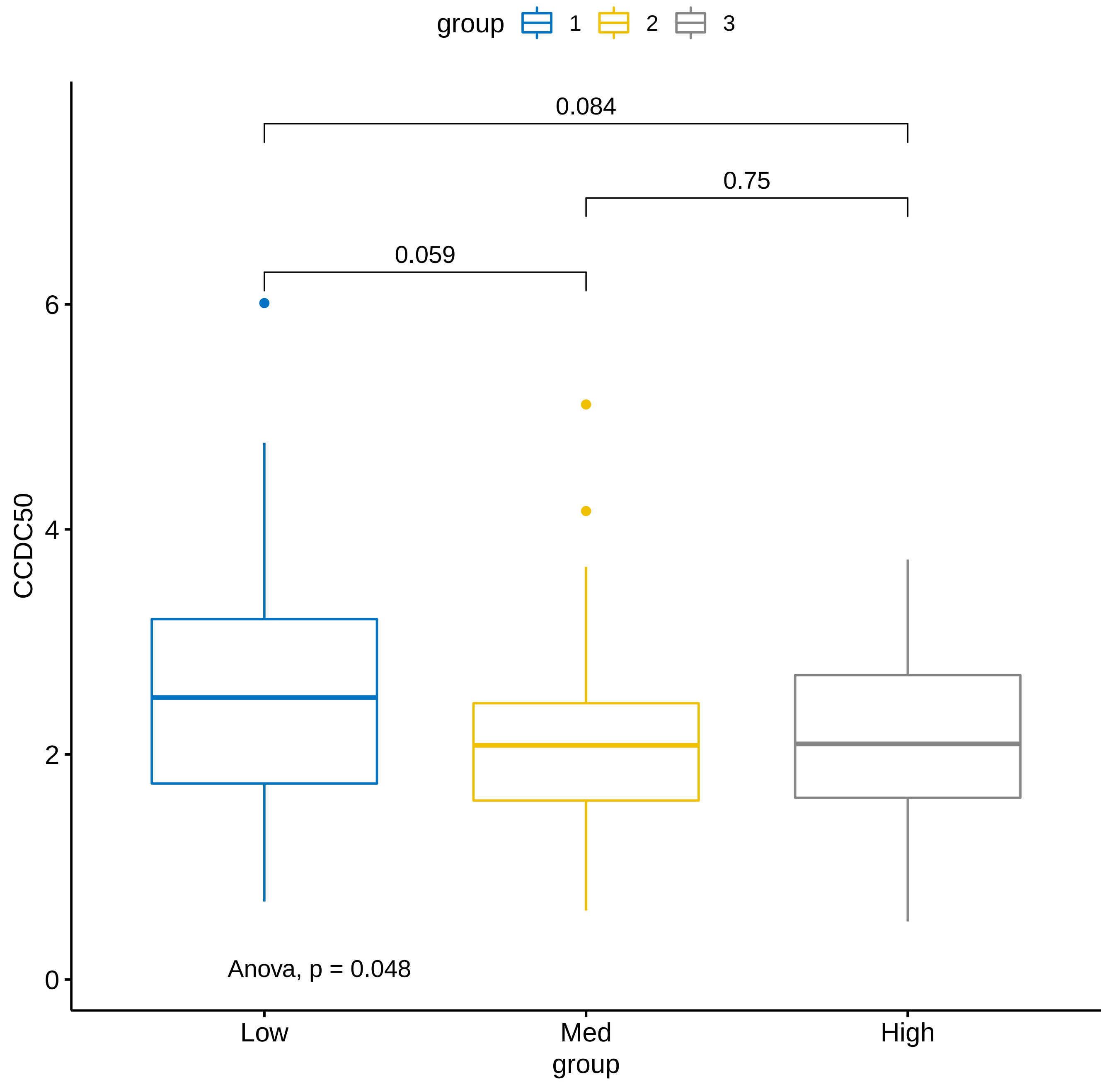

group 1 2 3
0.084
0.75
0.059
CCDC50
6
4
2
0
Anova, p = 0.048
Low
Med
High
group

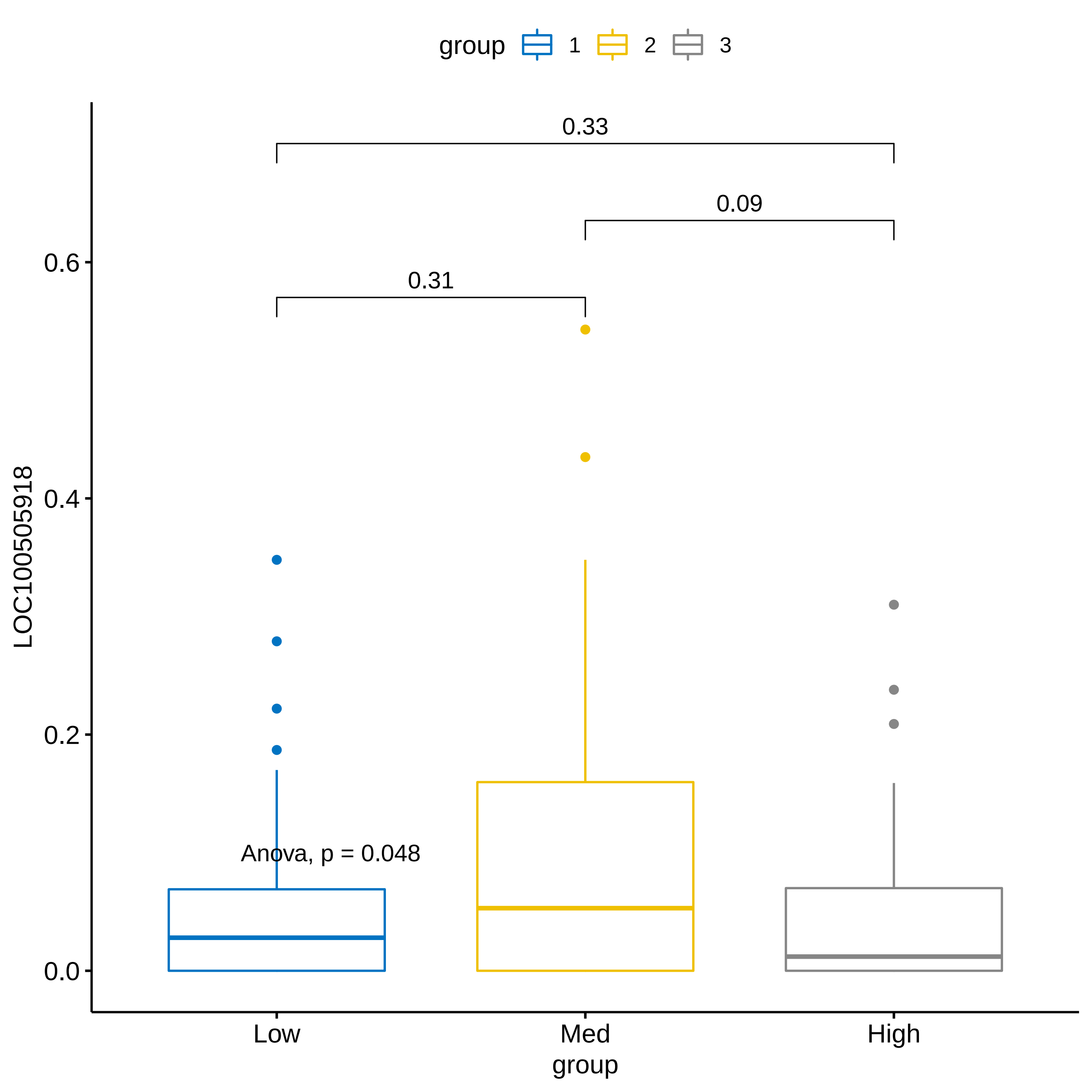
group
1
2
3
0.33
0.09
0.31
Anova, p = 0.048
0.6
0.4
0.2
0.0
LOC100505918
Low
Med
High
group

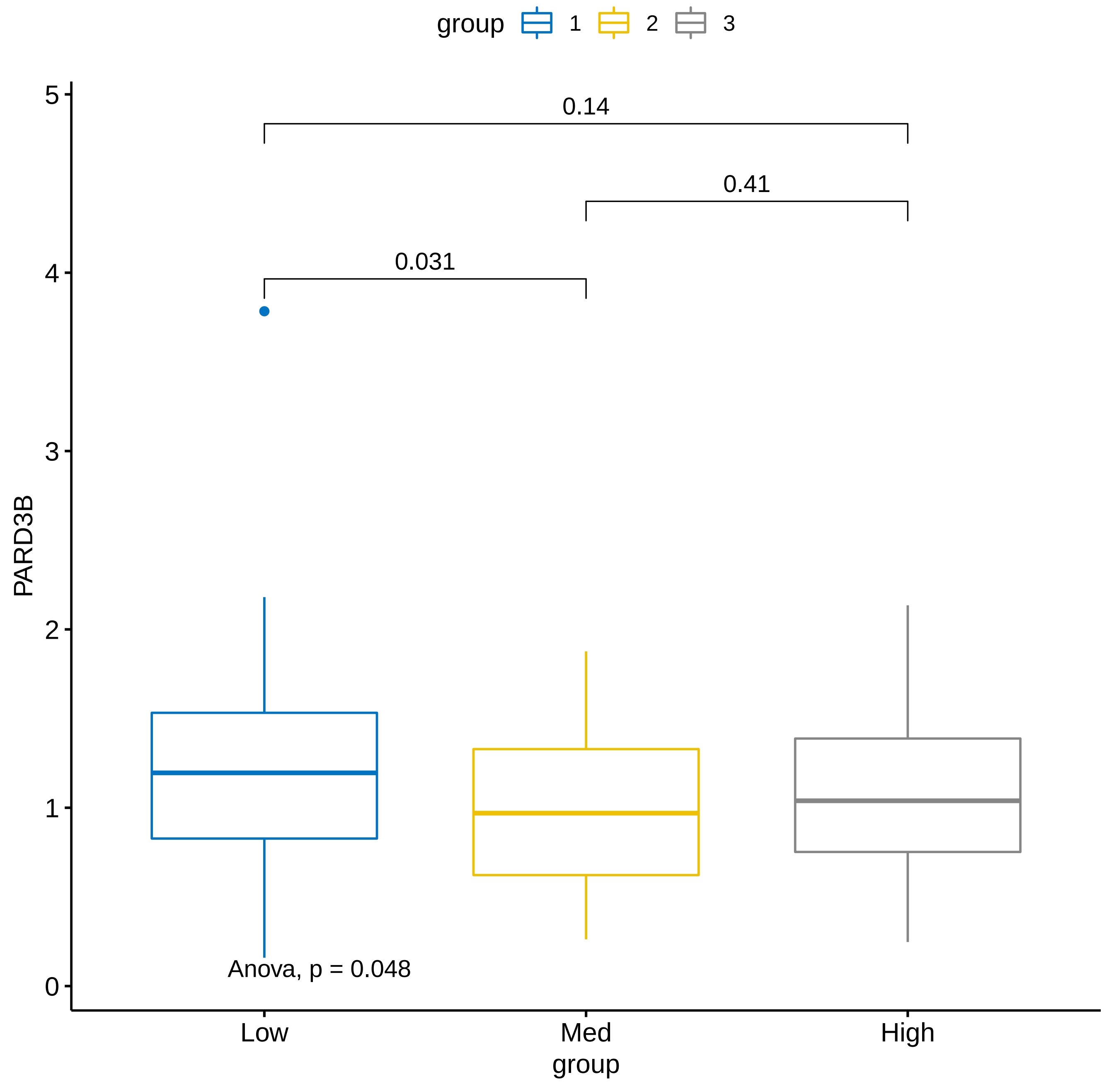
group
1
2
3
0.14
0.41
0.031
5
4
3
2
1
0
PARD3B
Anova, p = 0.048
Low
Med
High
group

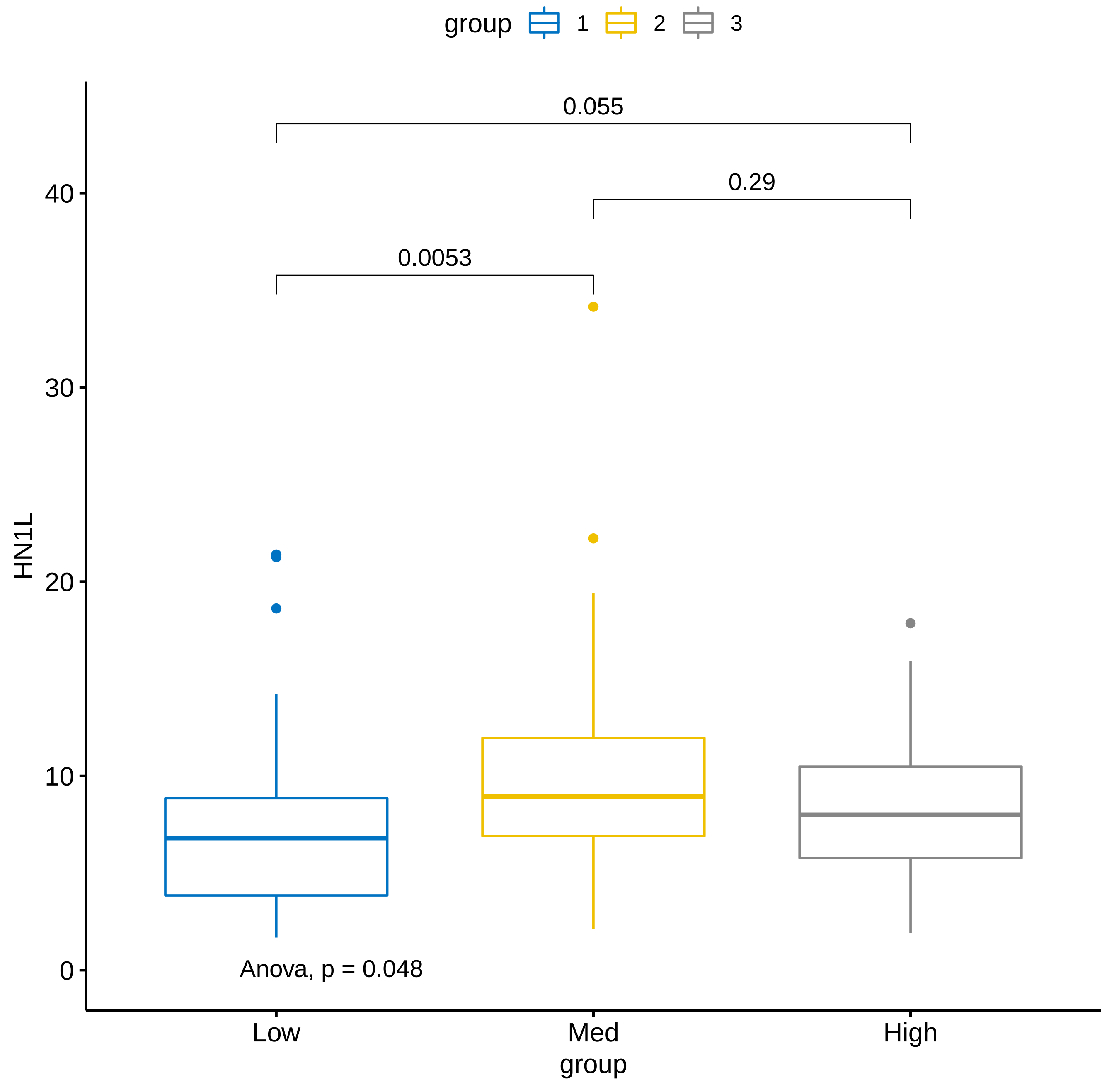
group 1 2 3
0.055
0.29
0.0053
40
30
20
10
0
HN1L
Anova, p = 0.048
Low
Med
High
group

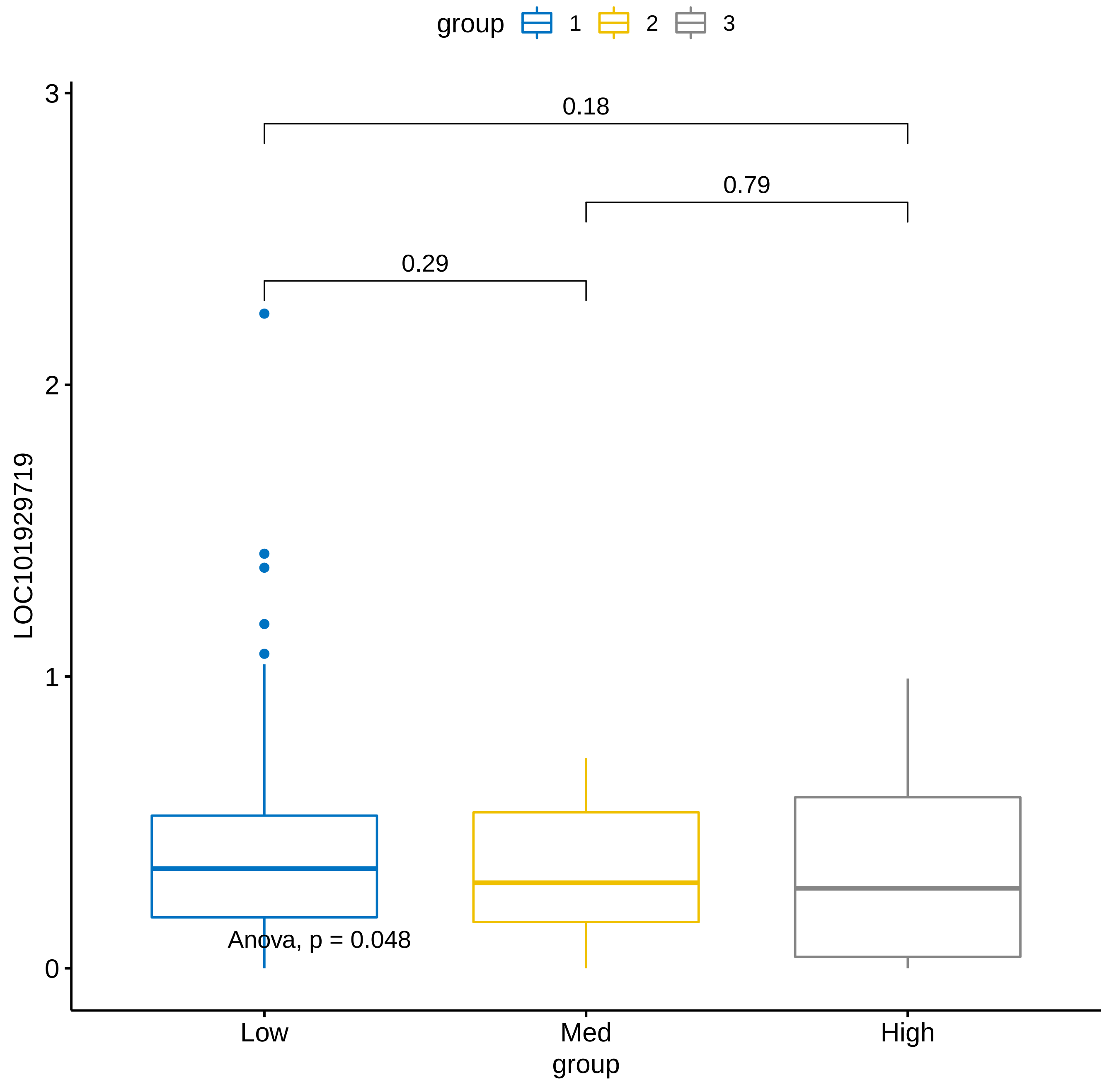
group
1
2
3
0.18
0.79
0.29
LOC101929719
3
2
1
0
Anova, p = 0.048
Low
Med
High
group

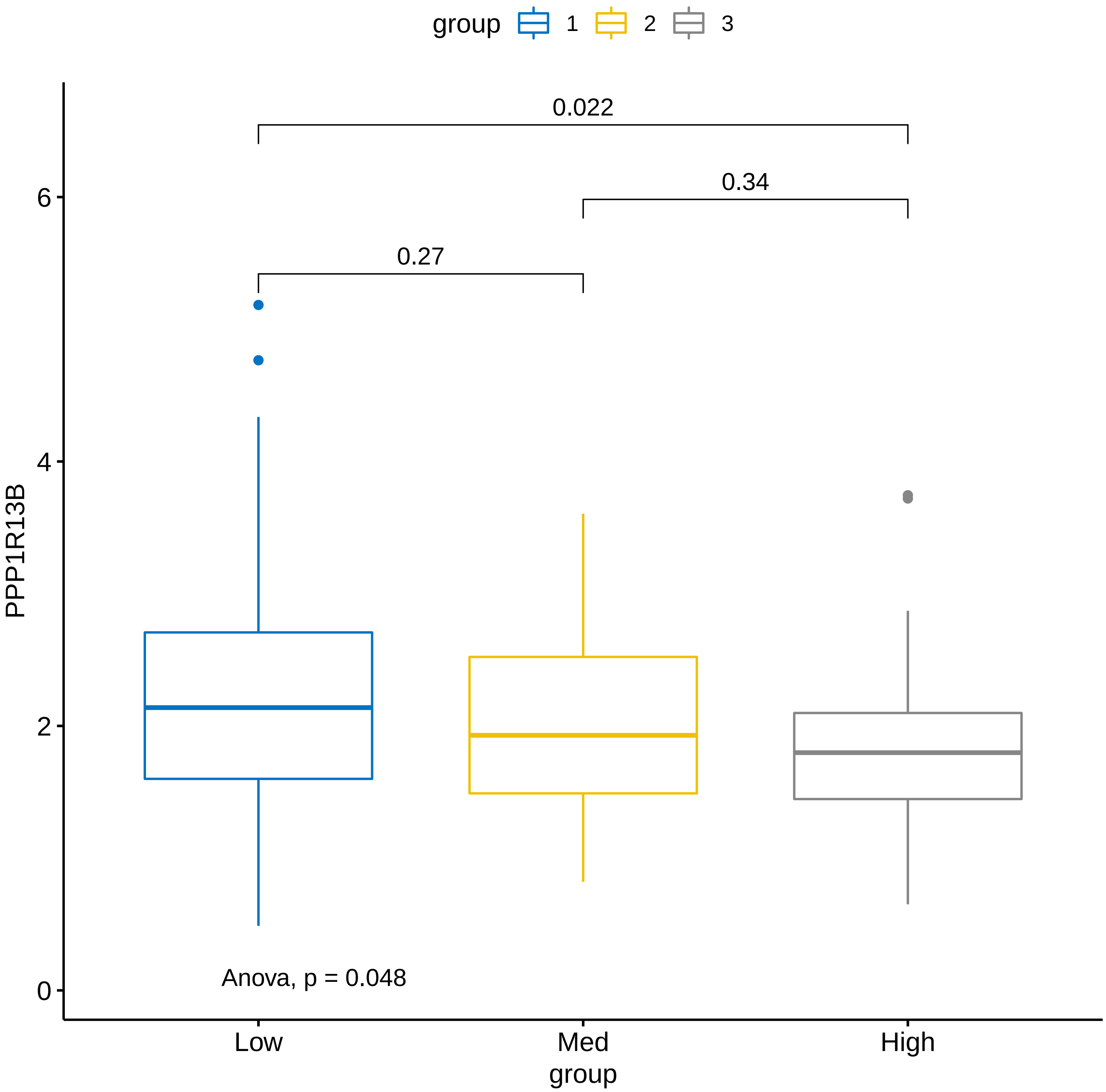

group 1 2 3
0.022
0.34
0.27
Anova, p = 0.048
PPP1R13B
0
2
4
6
Low
Med
High
group

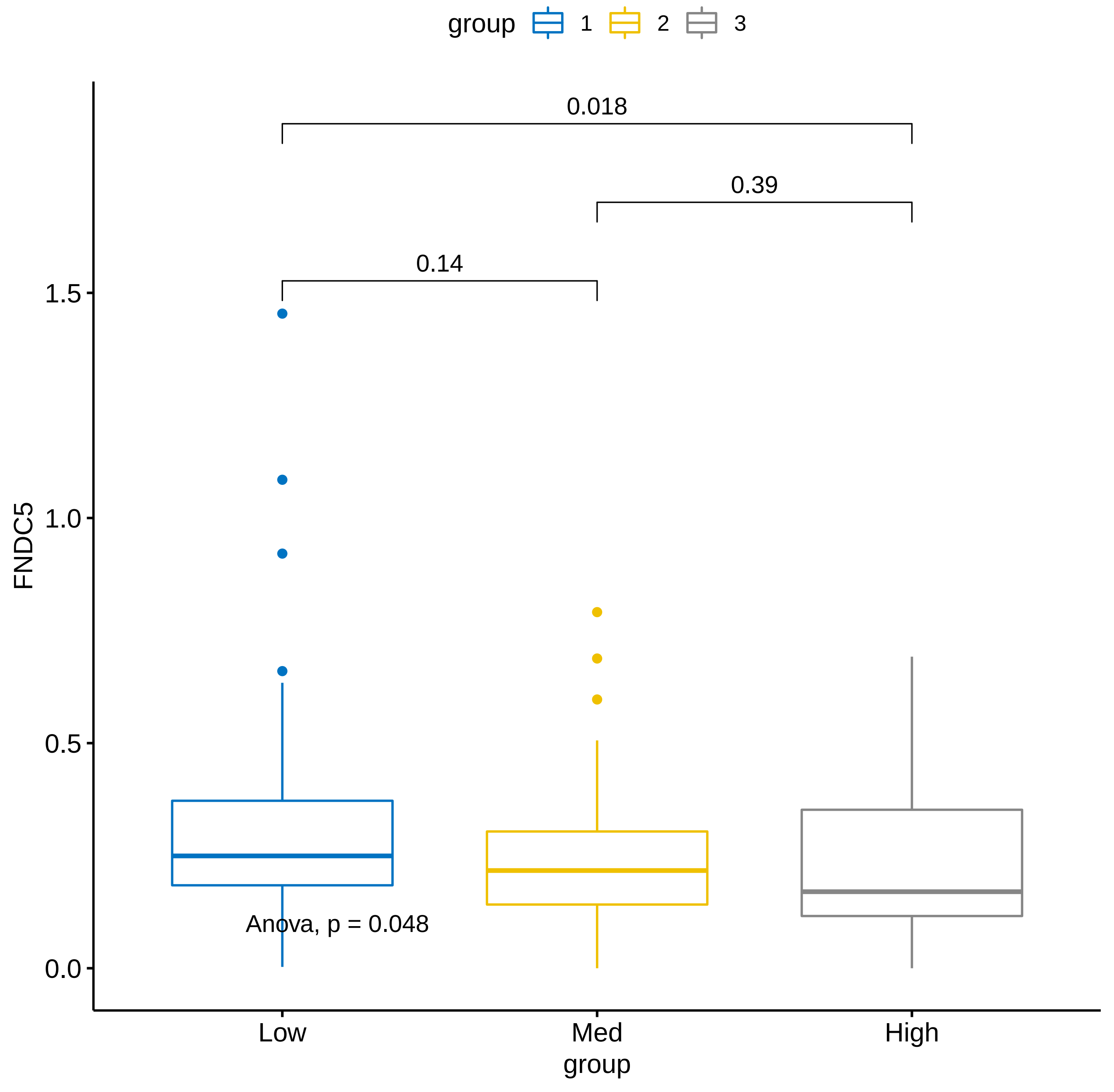
group
1
2
3
0.018
0.39
0.14
1.5
1.0
0.5
0.0
FNDC5
Anova, p = 0.048
Low
Med
High
group

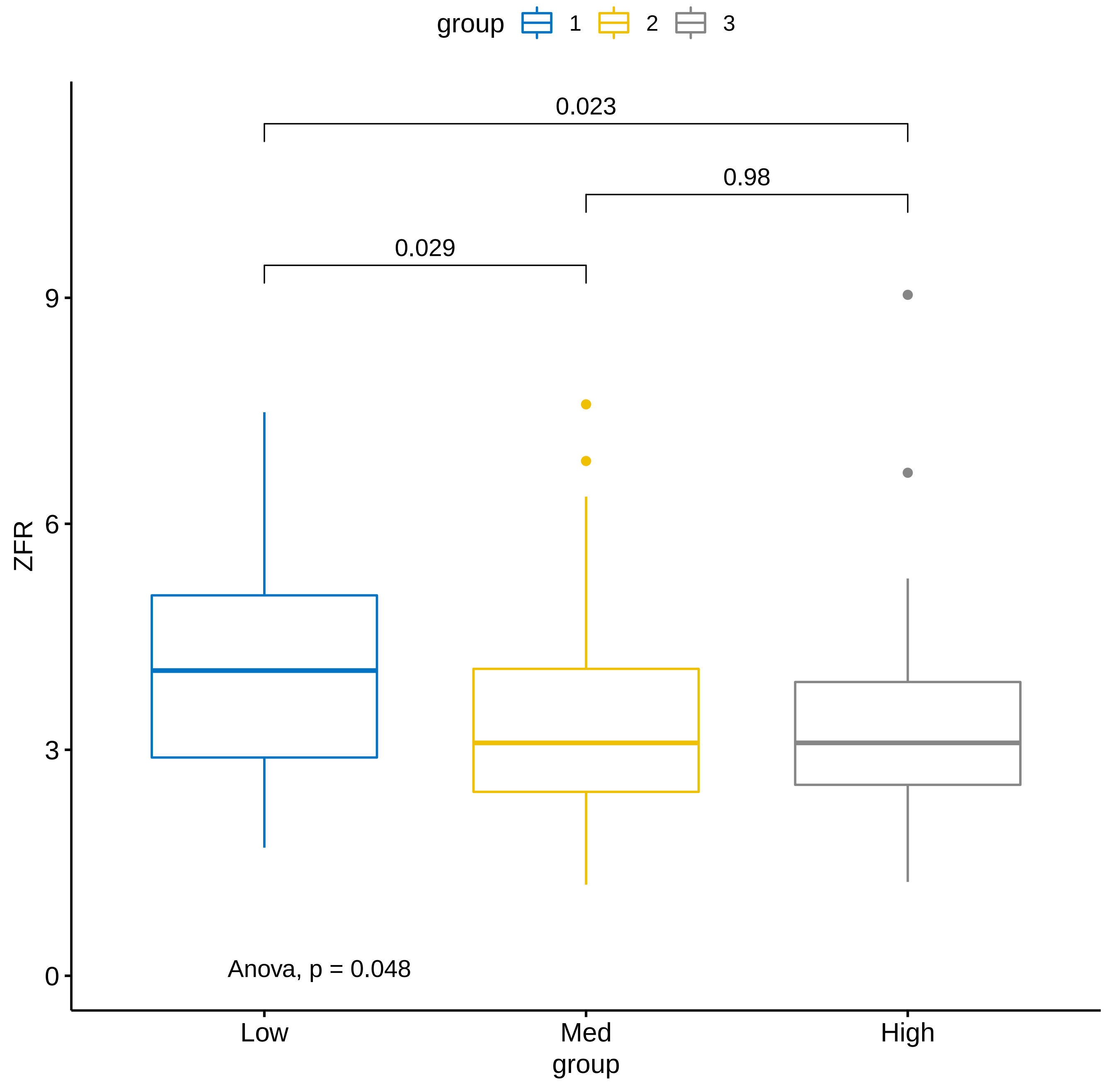

group 1 2 3
0.023
0.98
0.029
ZFR
9
6
3
0
Anova, p = 0.048
Low
Med
High
group

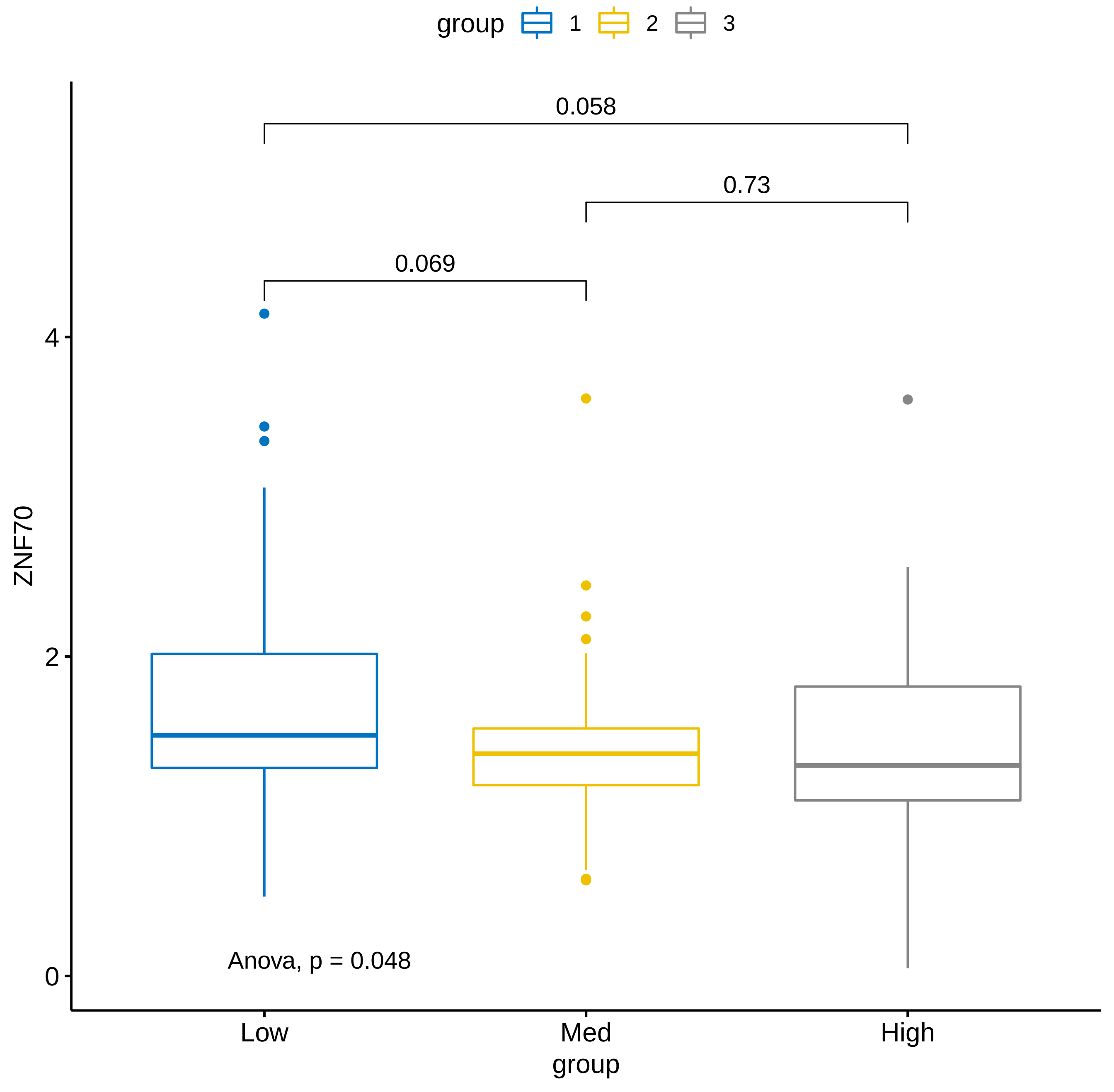

group
1
2
3
0.058
0.73
0.069
4
2
0
ZNF70
Anova, p = 0.048
Low
Med
High
group

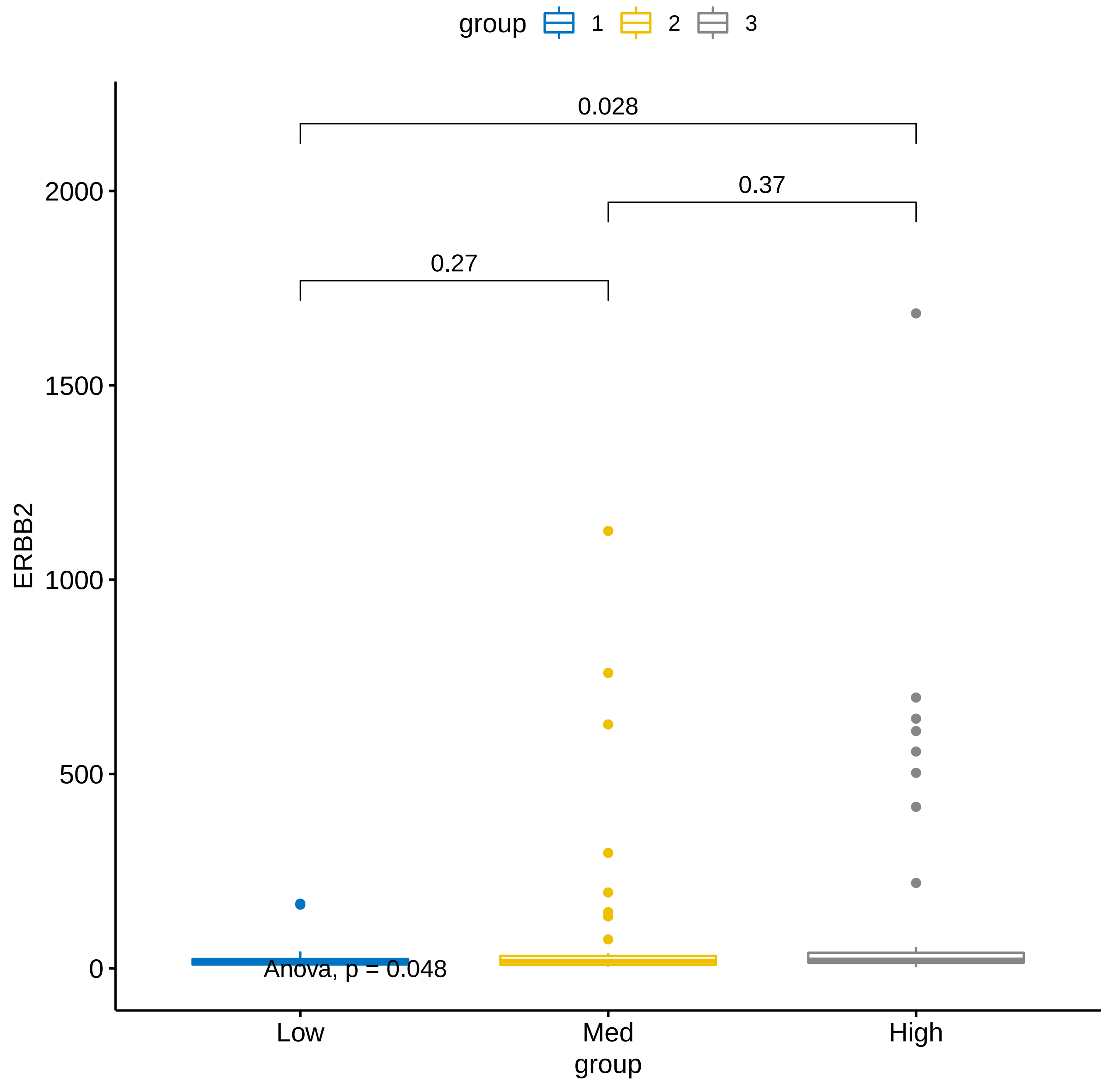

group
1
2
3
0.028
0.37
0.27
2000
1500
1000
500
0
ERBB2
Anova, p = 0.048
Low
Med
High
group

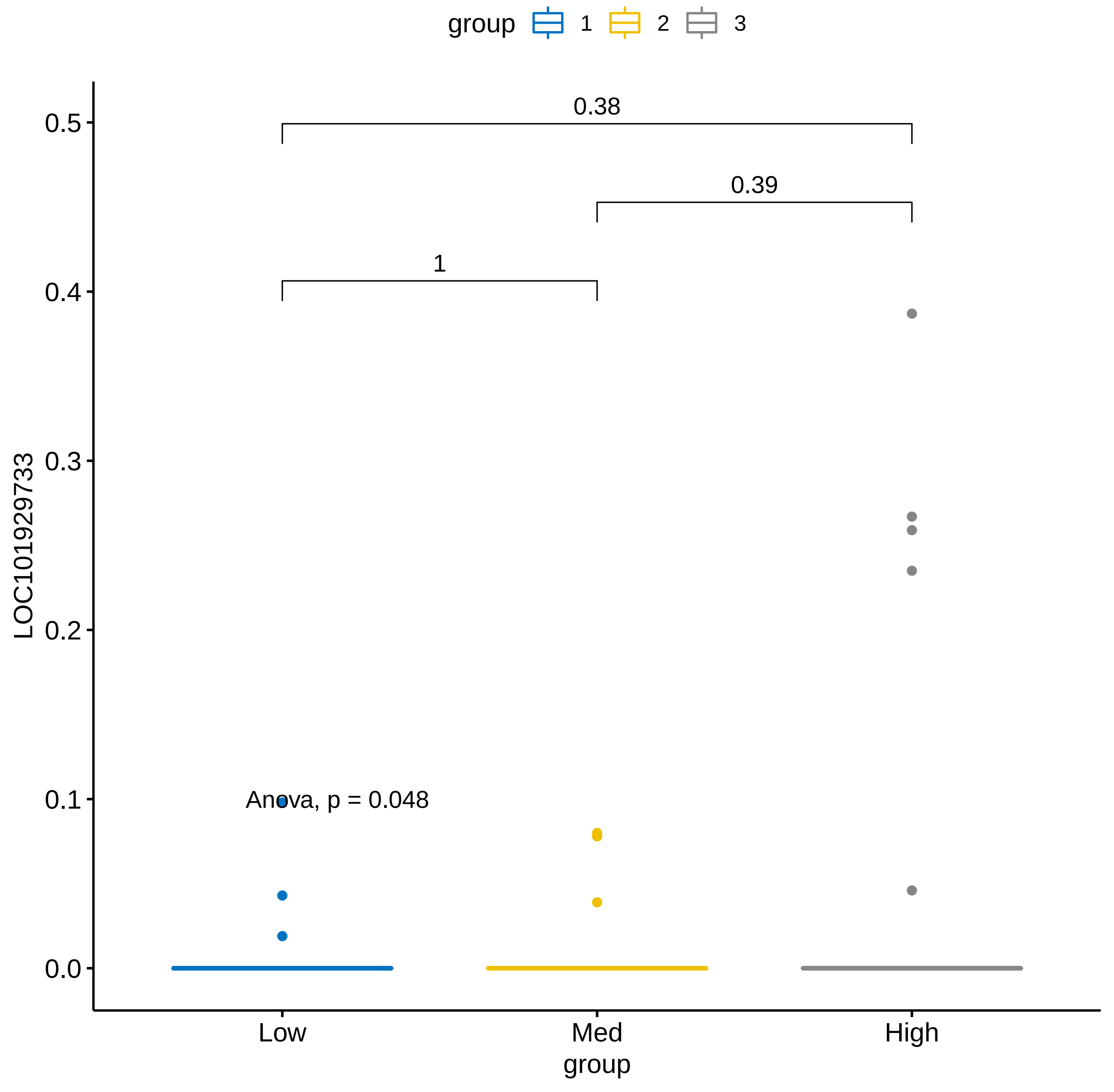

group
1
2
3
0.38
0.39
1
Anova, p = 0.048
0.5
0.4
0.3
0.2
0.1
0.0
LOC101929733
Low
Med
High
group

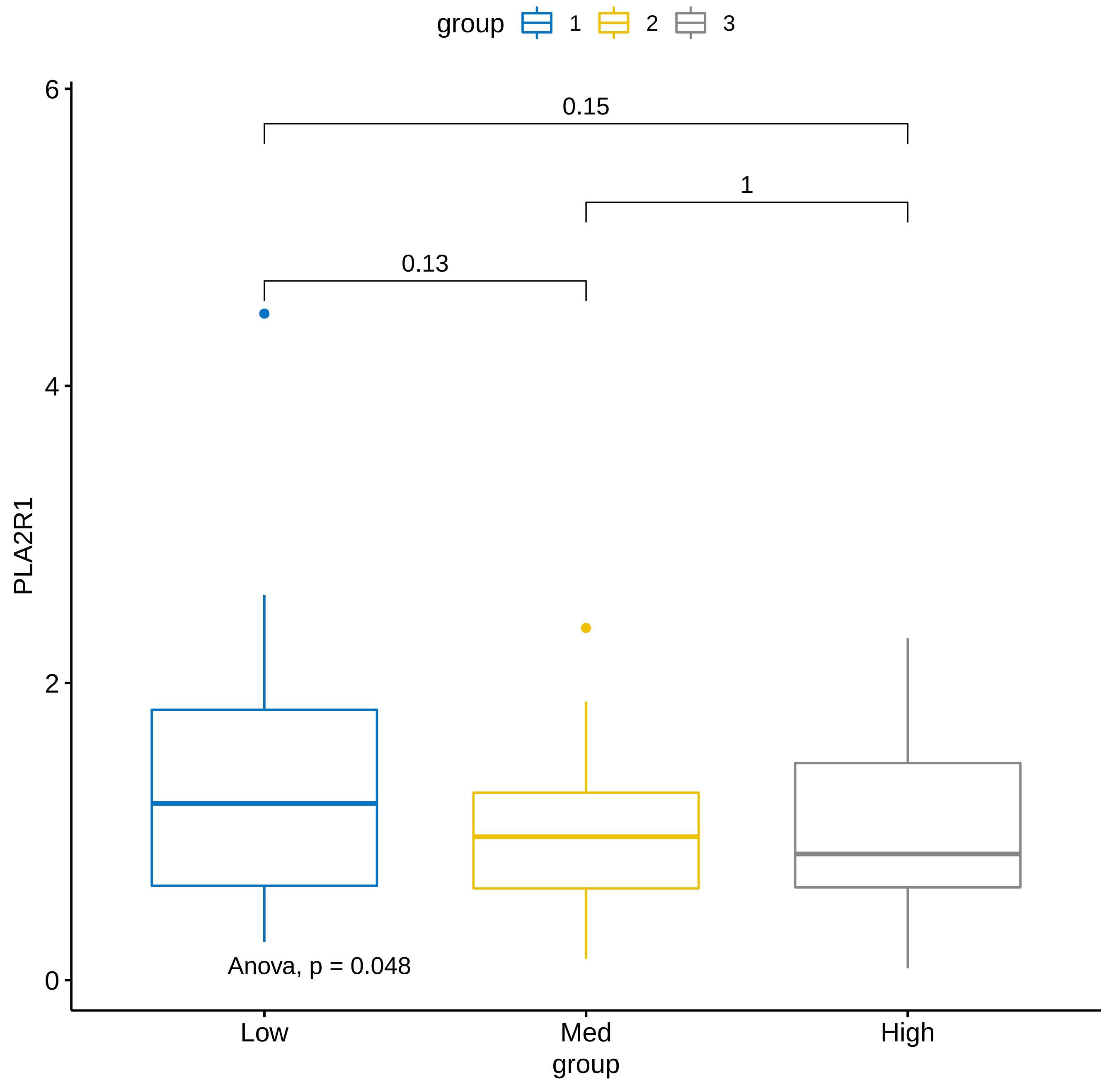

group 1 2 3
0.15
1
0.13
PLA2R1
6
4
2
0
Anova, p = 0.048
Low
Med
High
group

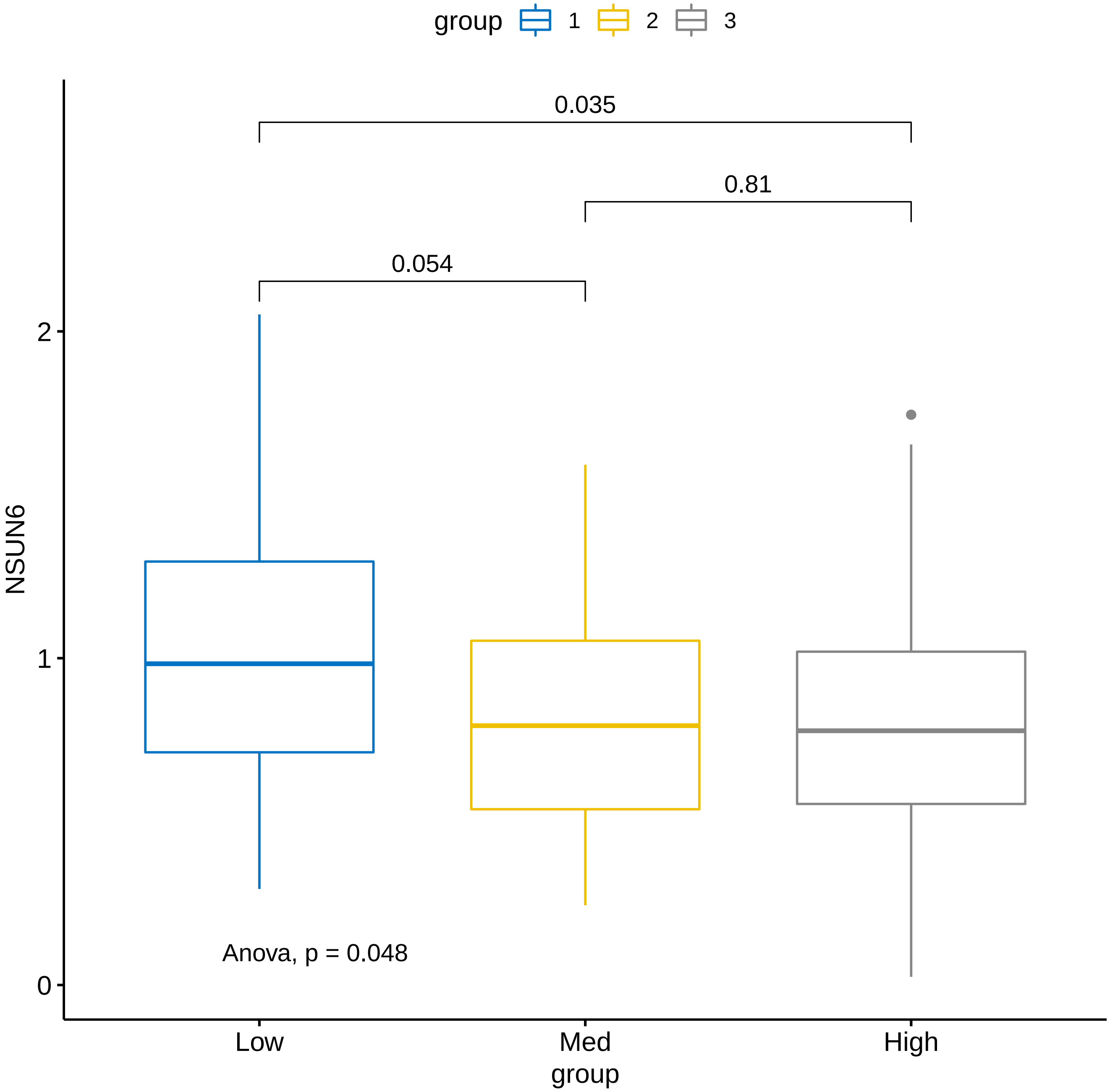

group
1
2
3
0.035
0.81
0.054
2
1
0
NSUN6
Anova, p = 0.048
Low
Med
High
group

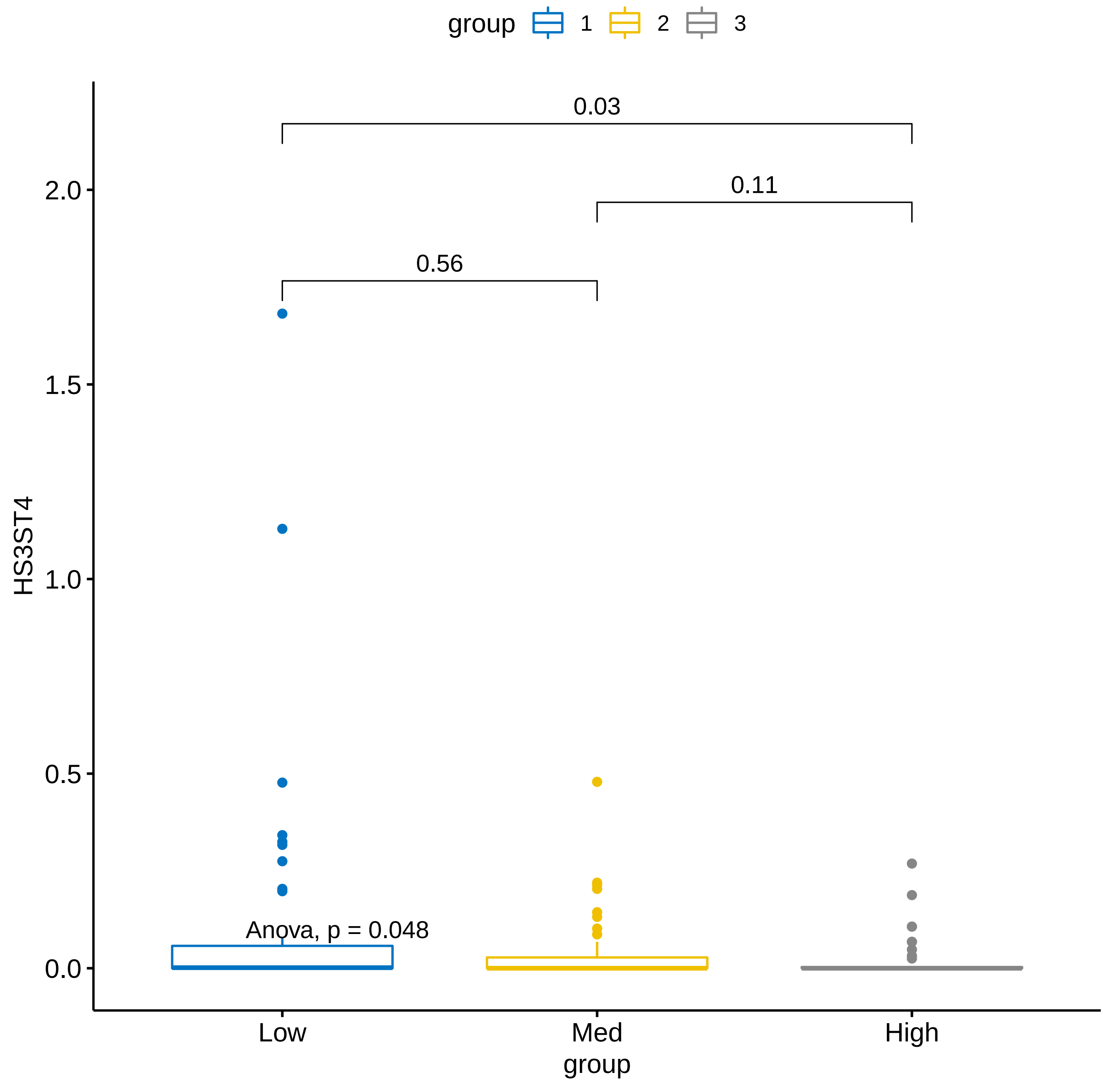

group 1 2 3
0.03
0.11
0.56
2.0
1.5
1.0
0.5
0.0
HS3ST4
Anova, p = 0.048
Low
Med
High
group

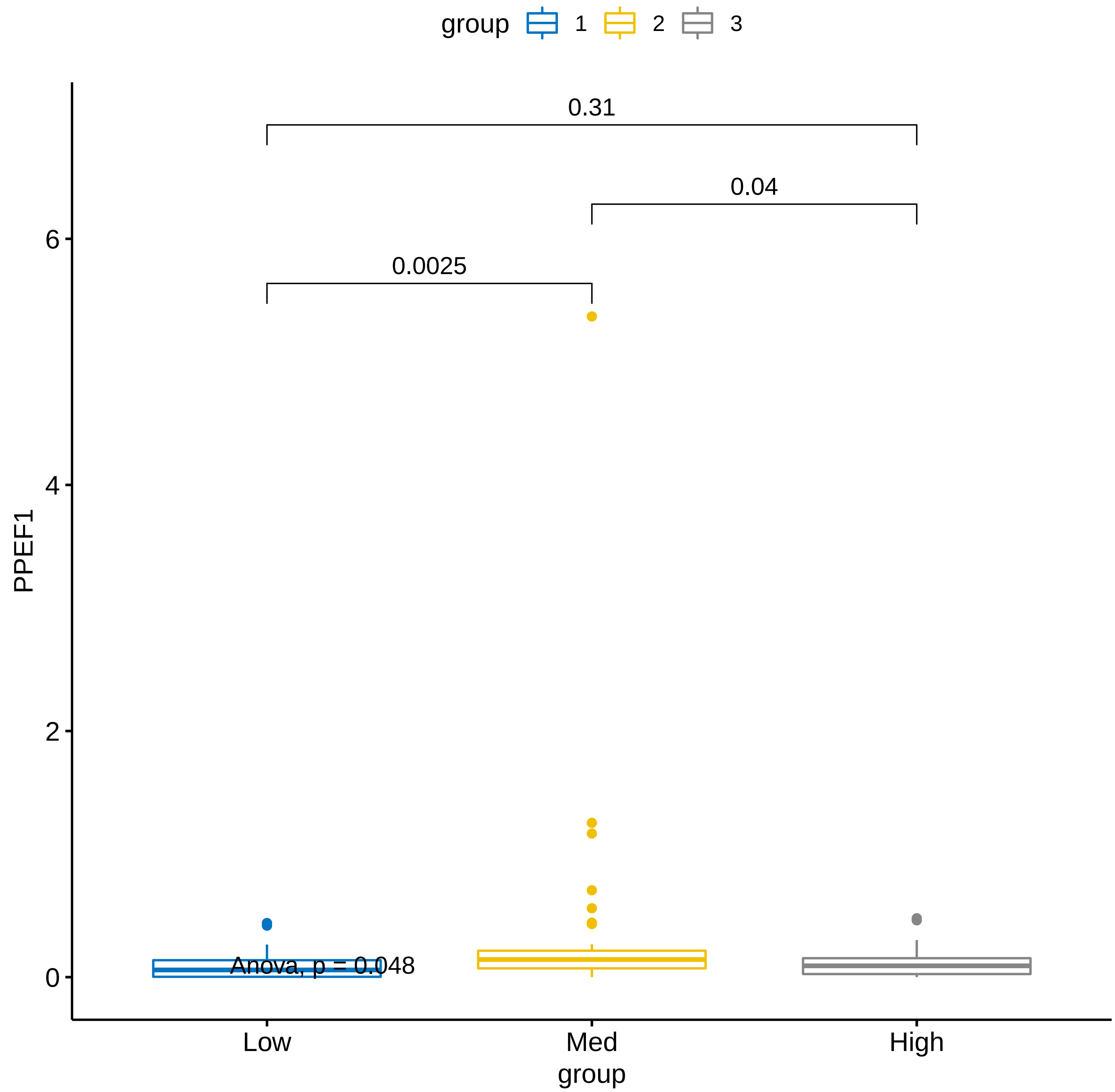

group
1
2
3
0.31
0.04
0.0025
Anova, p = 0.048
PPEF1
6
4
2
0
Low
Med
High
group

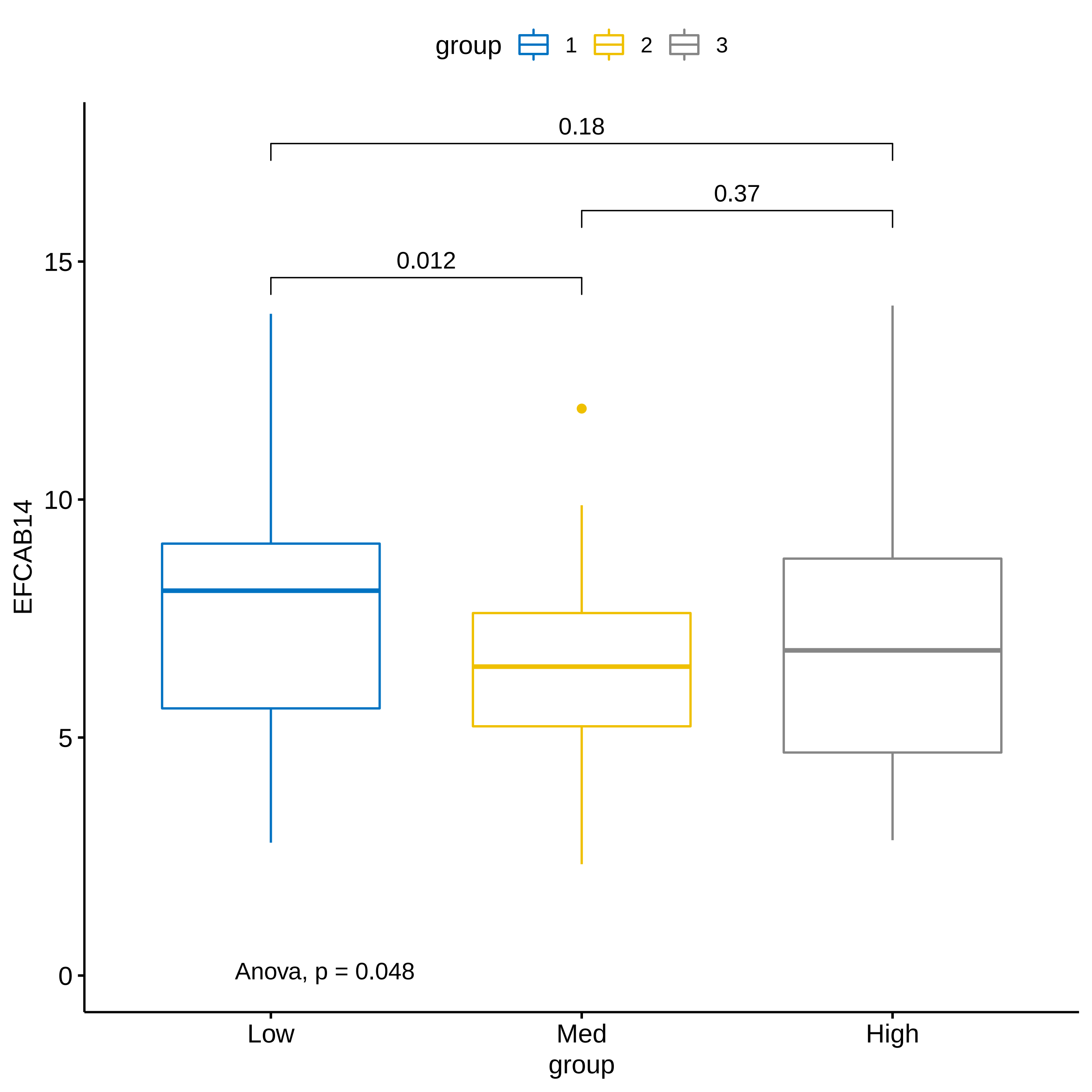
group 1 2 3
0.18
0.37
0.012
15
10
5
0
EFCAB14
Anova, p = 0.048
Low
Med
High
group

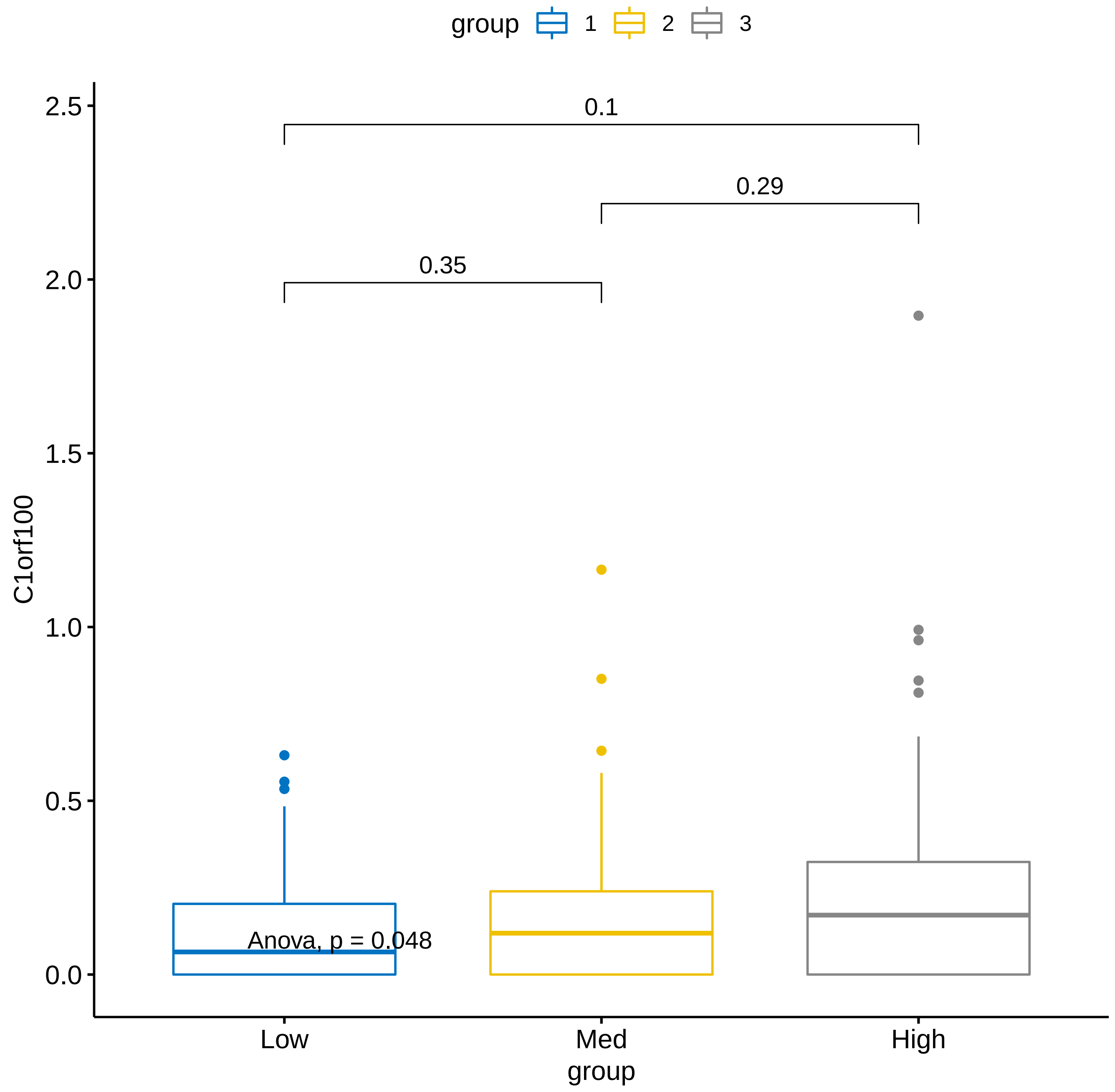
group
1
2
3
0.1
0.29
0.35
Anova, p = 0.048
C1orf100
2.5
2.0
1.5
1.0
0.5
0.0
Low
Med
High
group

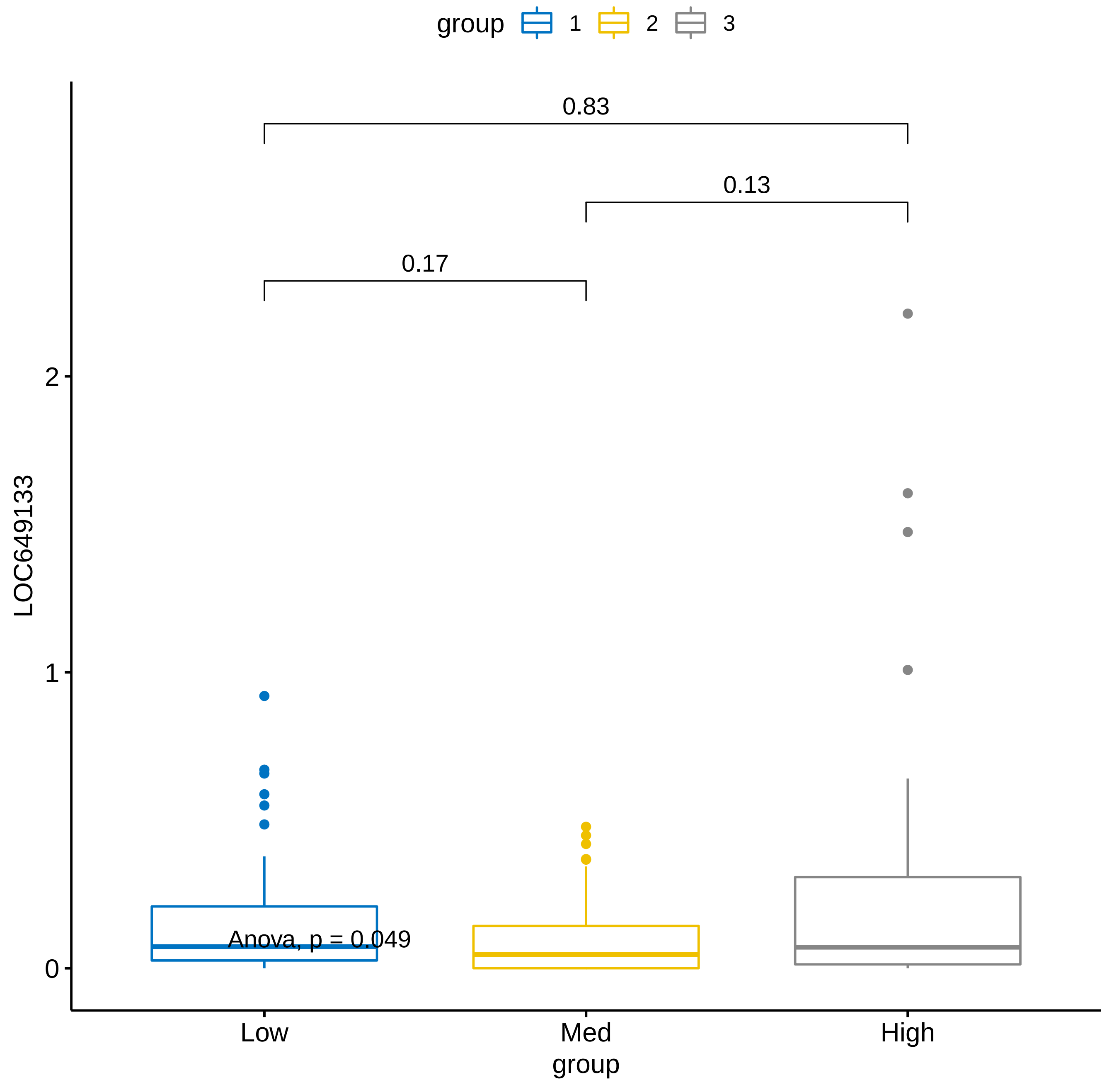

group
1
2
3
0.83
0.13
0.17
2
1
0
LOC649133
Anova, p = 0.049
Low
Med
High
group

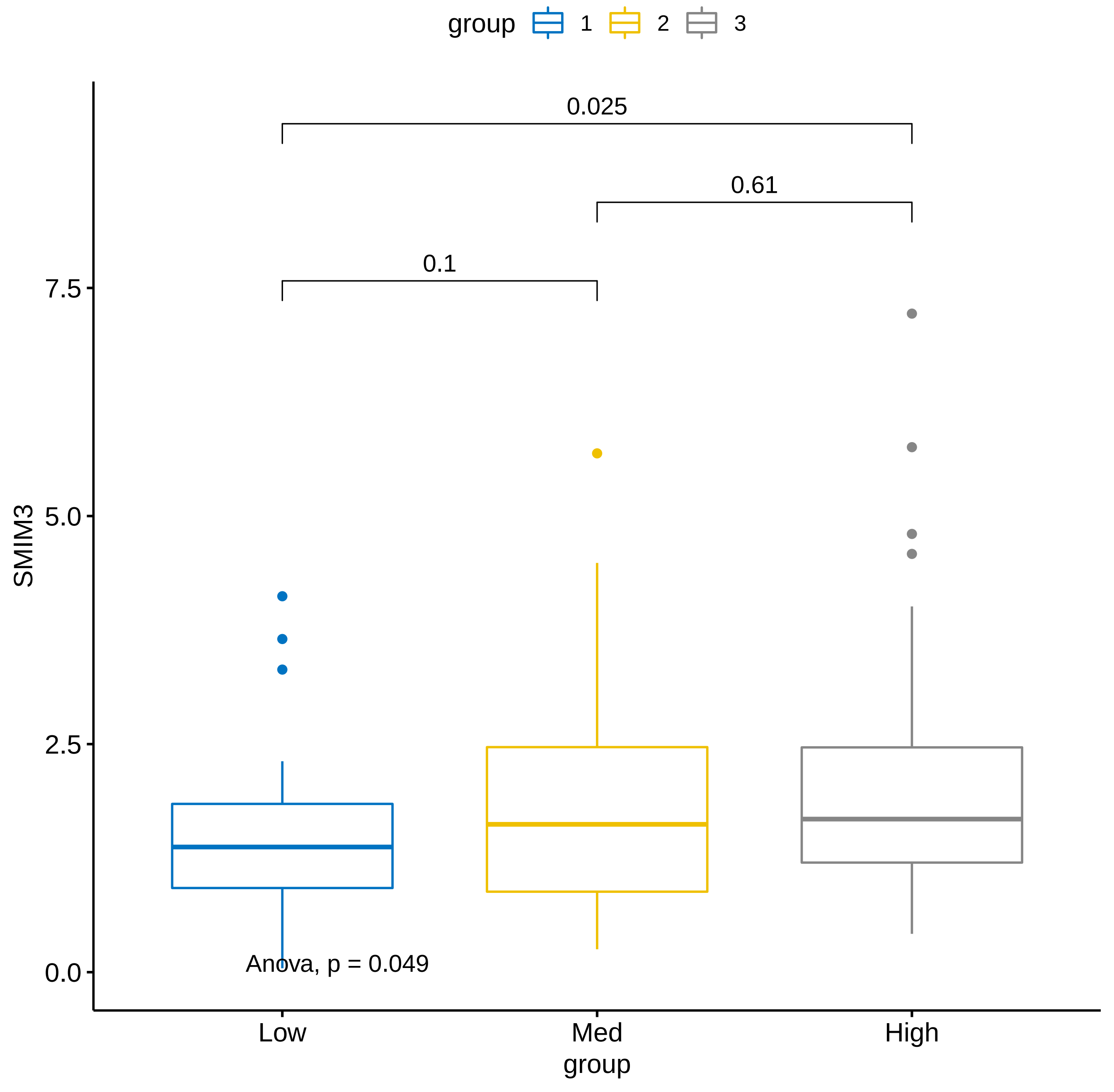

group 1 2 3
0.025
0.61
0.1
7.5
5.0
2.5
0.0
SMIM3
Anova, p = 0.049
Low
Med
High
group

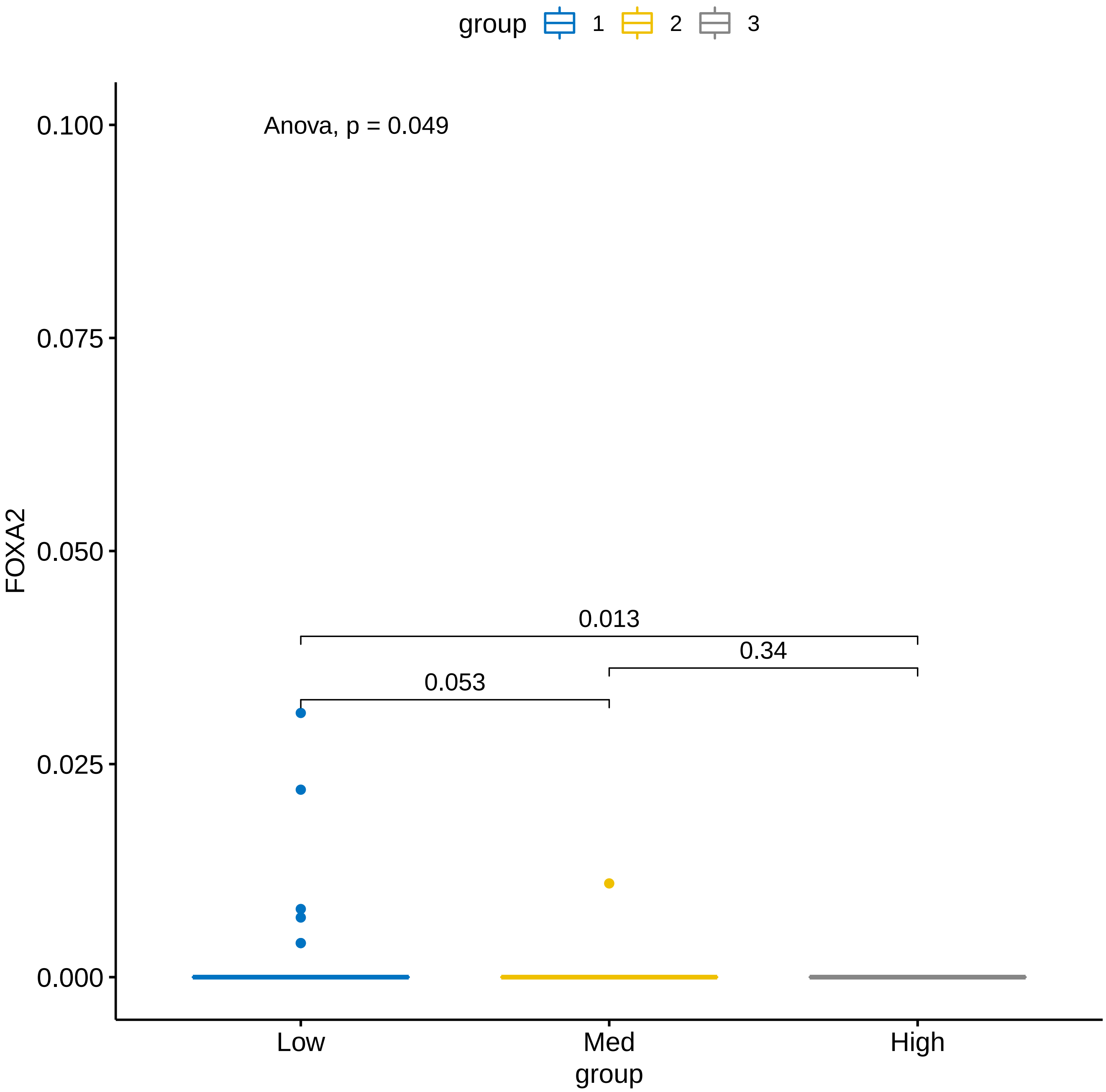
group 1 2 3
Anova, p = 0.049
0.100
0.075
0.050
0.025
0.000
FOXA2
0.013
0.34
0.053
Low
Med
High
group

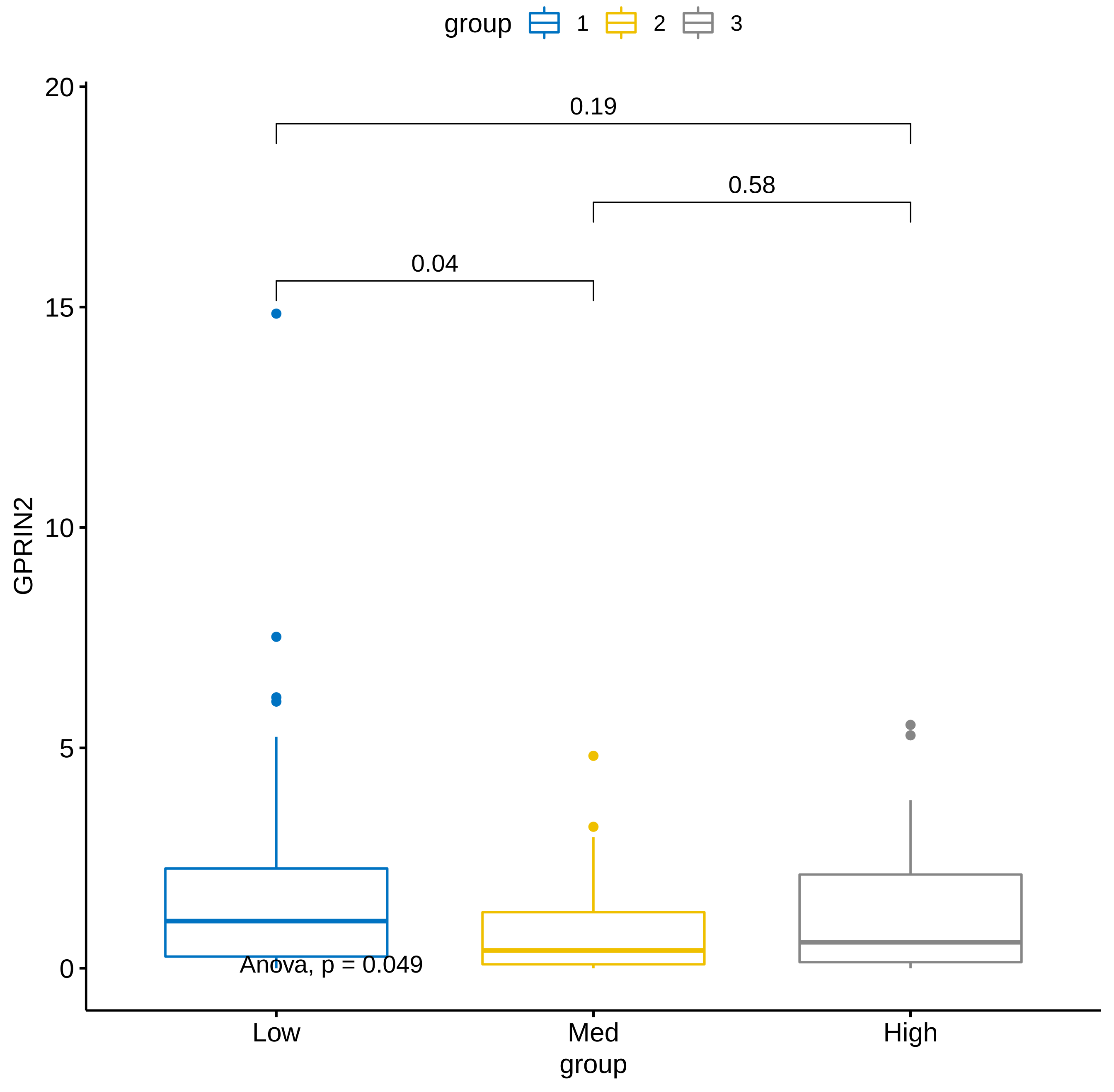
group
1
2
3
20
15
10
5
0
GPRIN2
0.19
0.58
0.04
Anova, p = 0.049
Low
Med
High
group

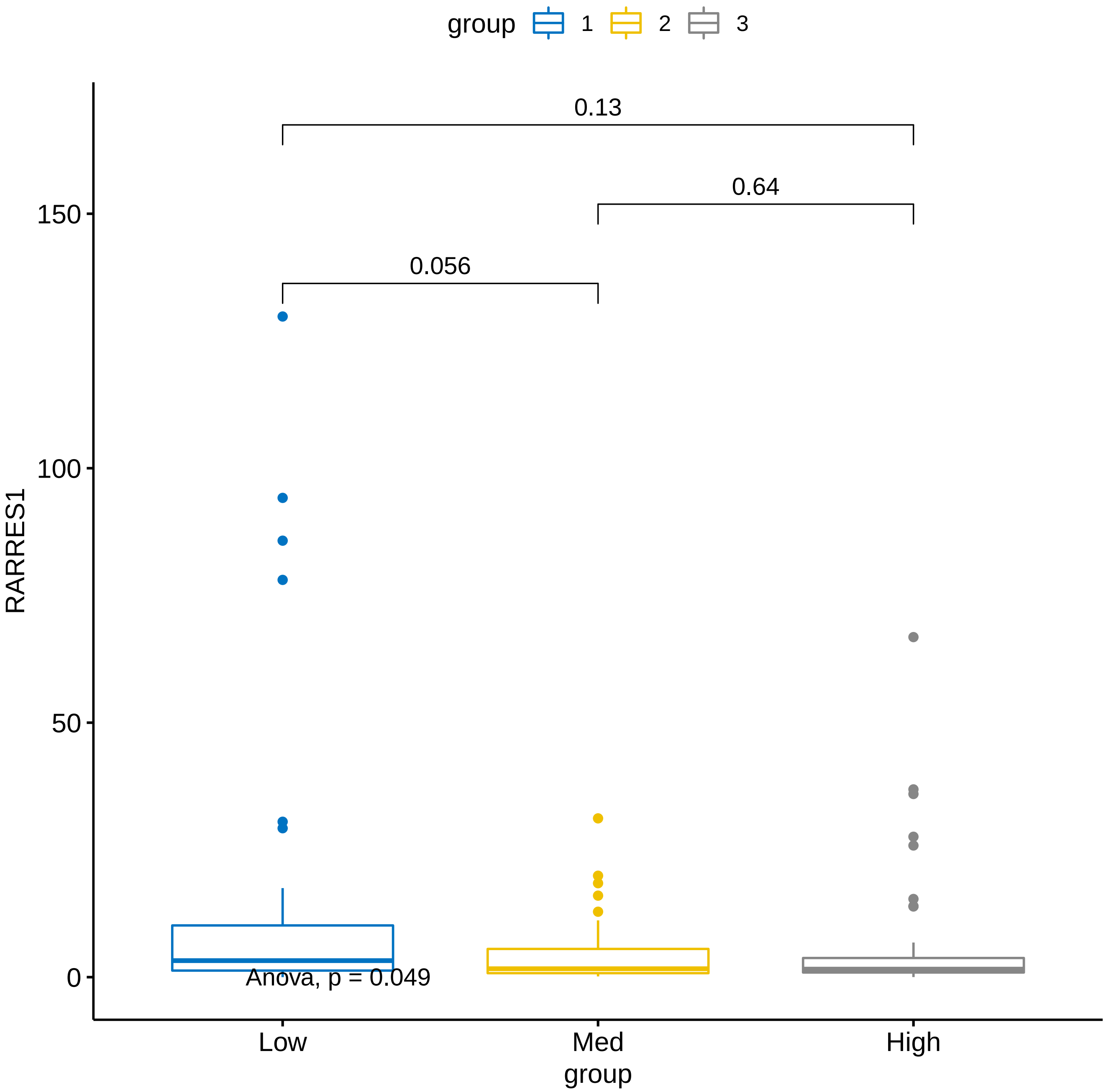

group
1
2
3
0.13
0.64
0.056
150
100
50
0
RARRES1
Anova, p = 0.049
Low
Med
High
group

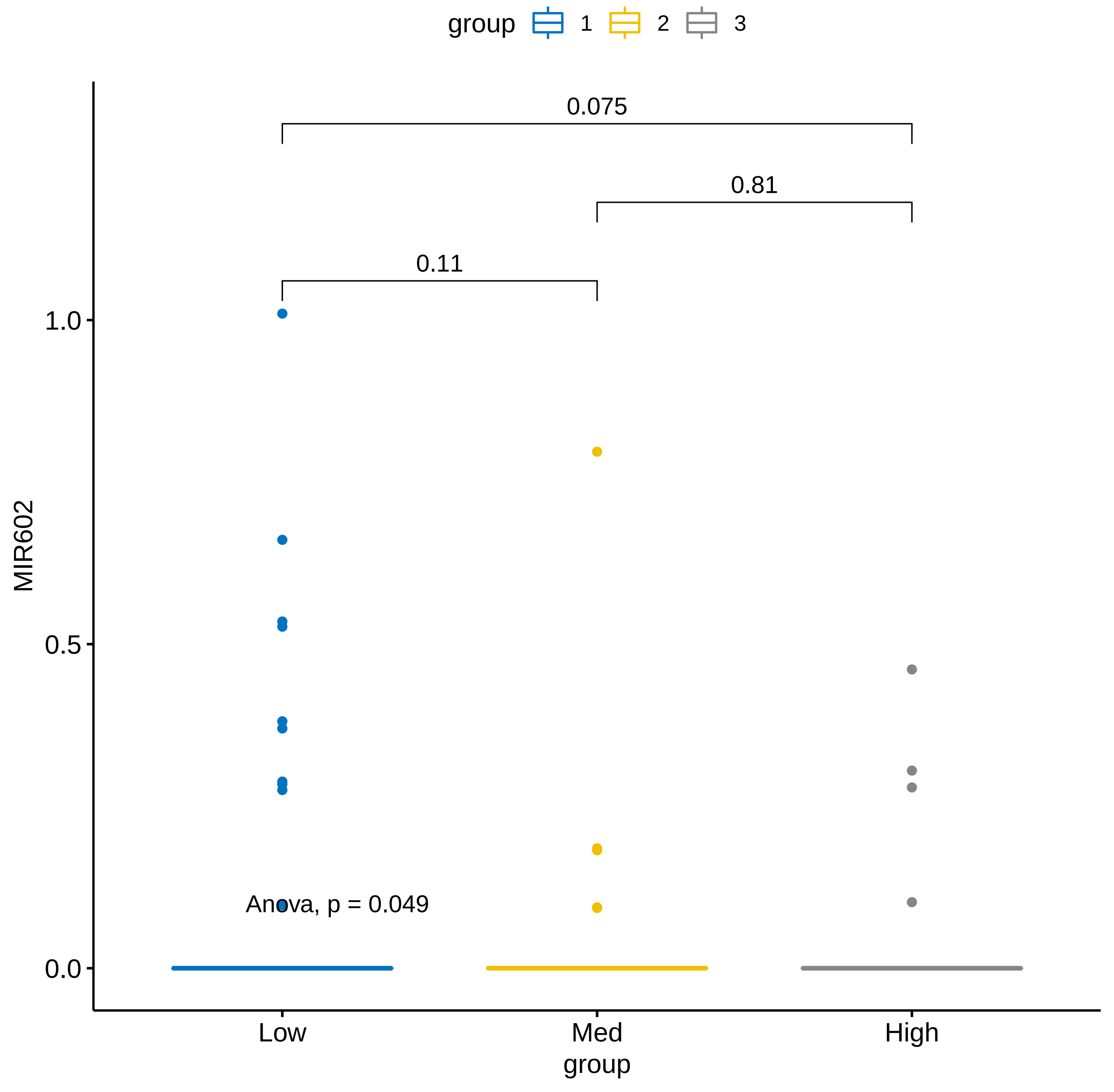

group
1
2
3
0.075
0.81
0.11
1.0
0.5
0.0
MIR602
Anova, p = 0.049
Low
Med
High
group

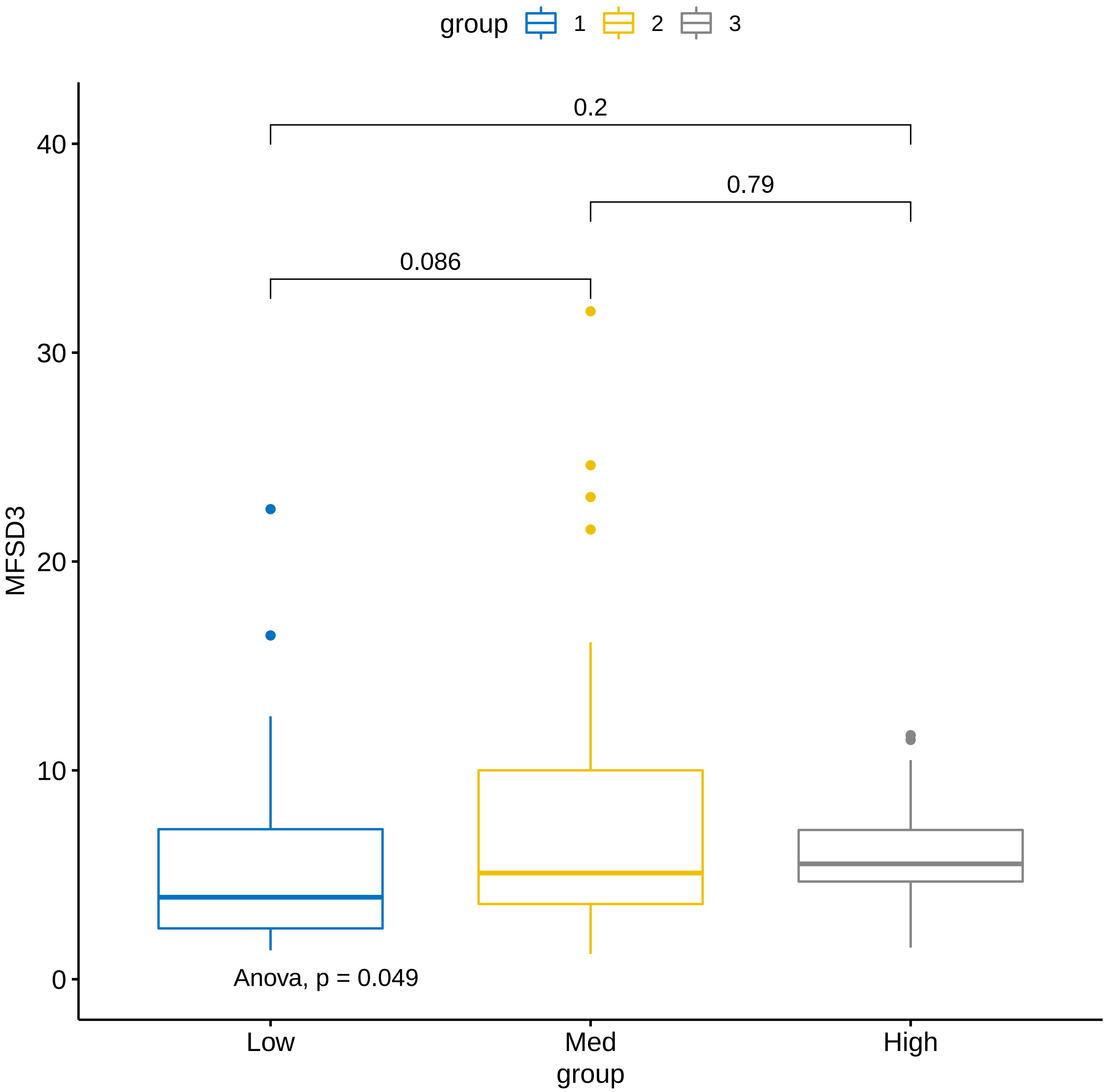

group 1 2 3
0.2
0.79
0.086
40
30
20
10
0
MFSD3
Anova, p = 0.049
Low
Med
High
group

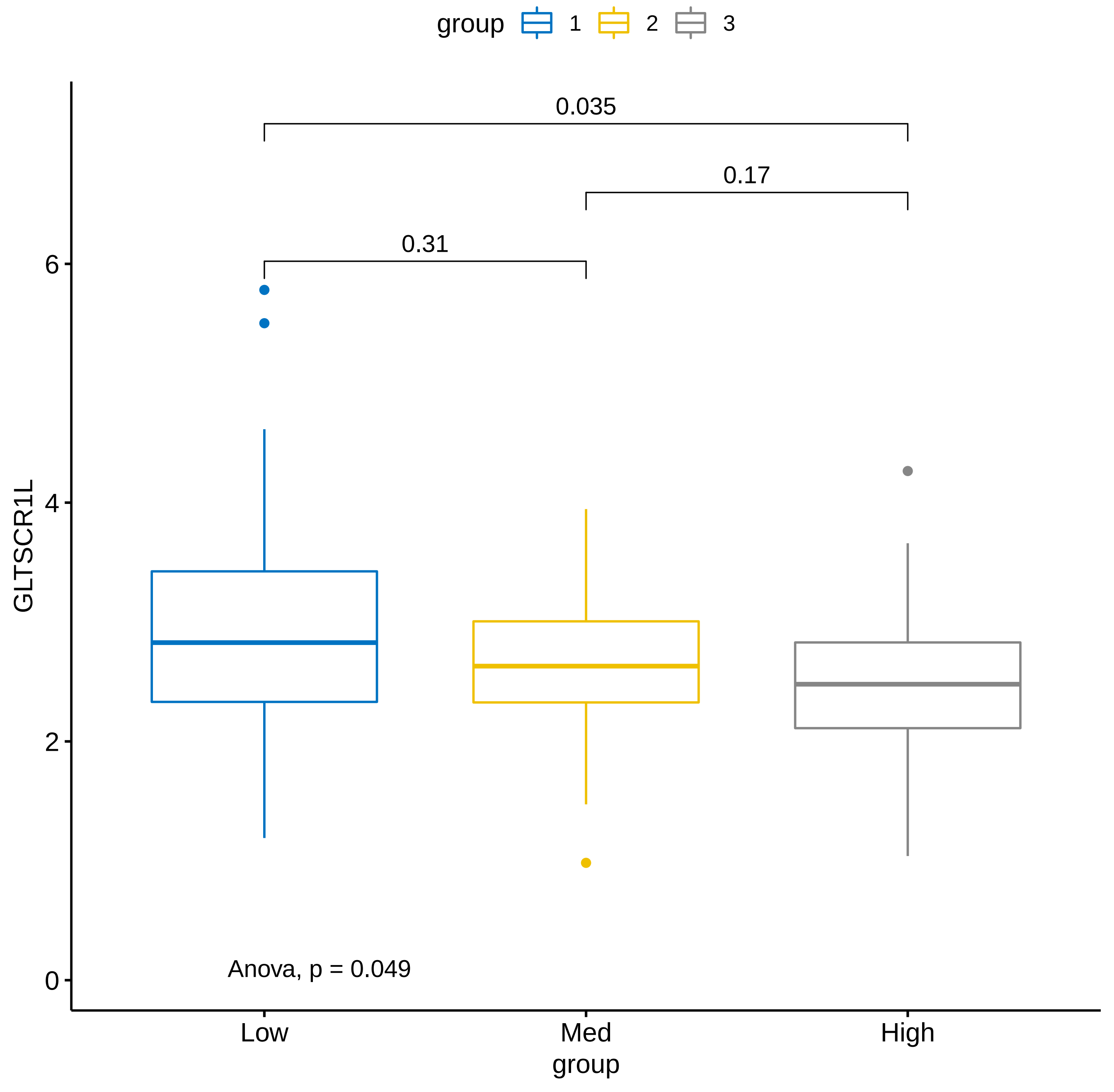
group
1
2
3
0.035
0.17
0.31
GLTSCR1L
6
4
2
0
Anova, p = 0.049
Low
Med
High
group

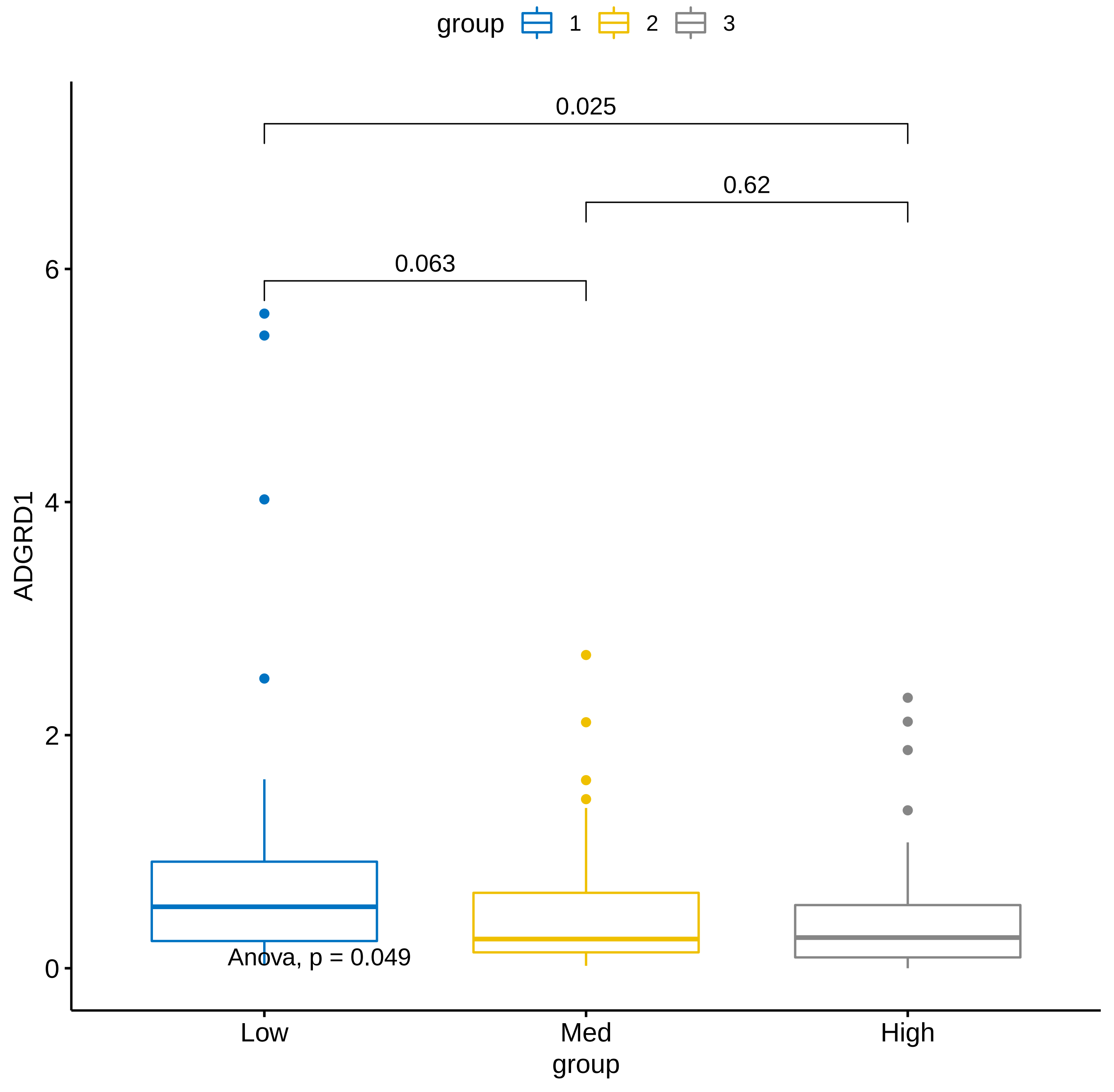
group
1
2
3
0.025
0.62
0.063
6
4
2
0
ADGRD1
Anova, p = 0.049
Low
Med
High
group

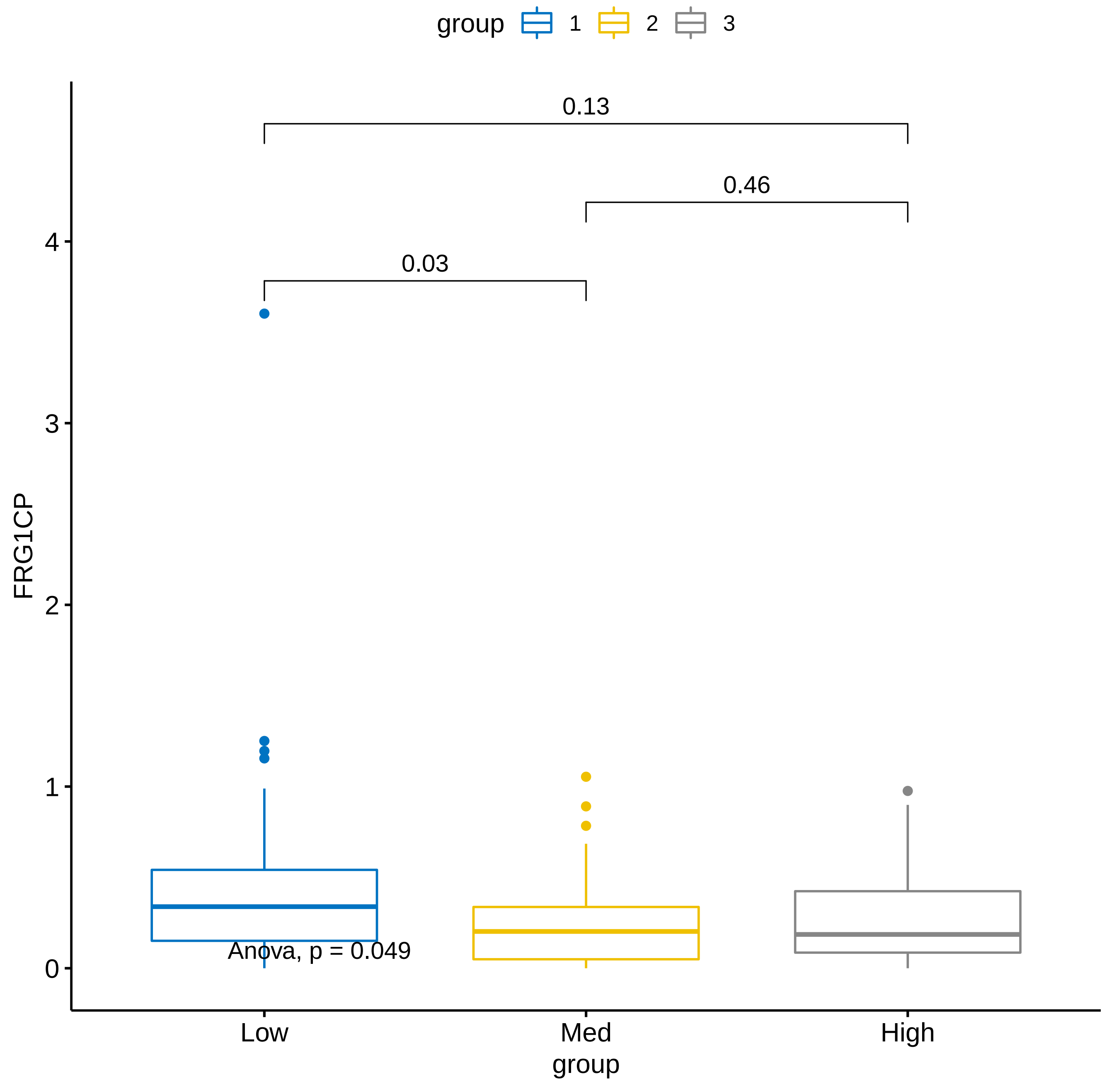

group 1 2 3
0.13
0.46
0.03
FRG1CP
4
3
2
1
0
Anova, p = 0.049
Low
Med
High
group

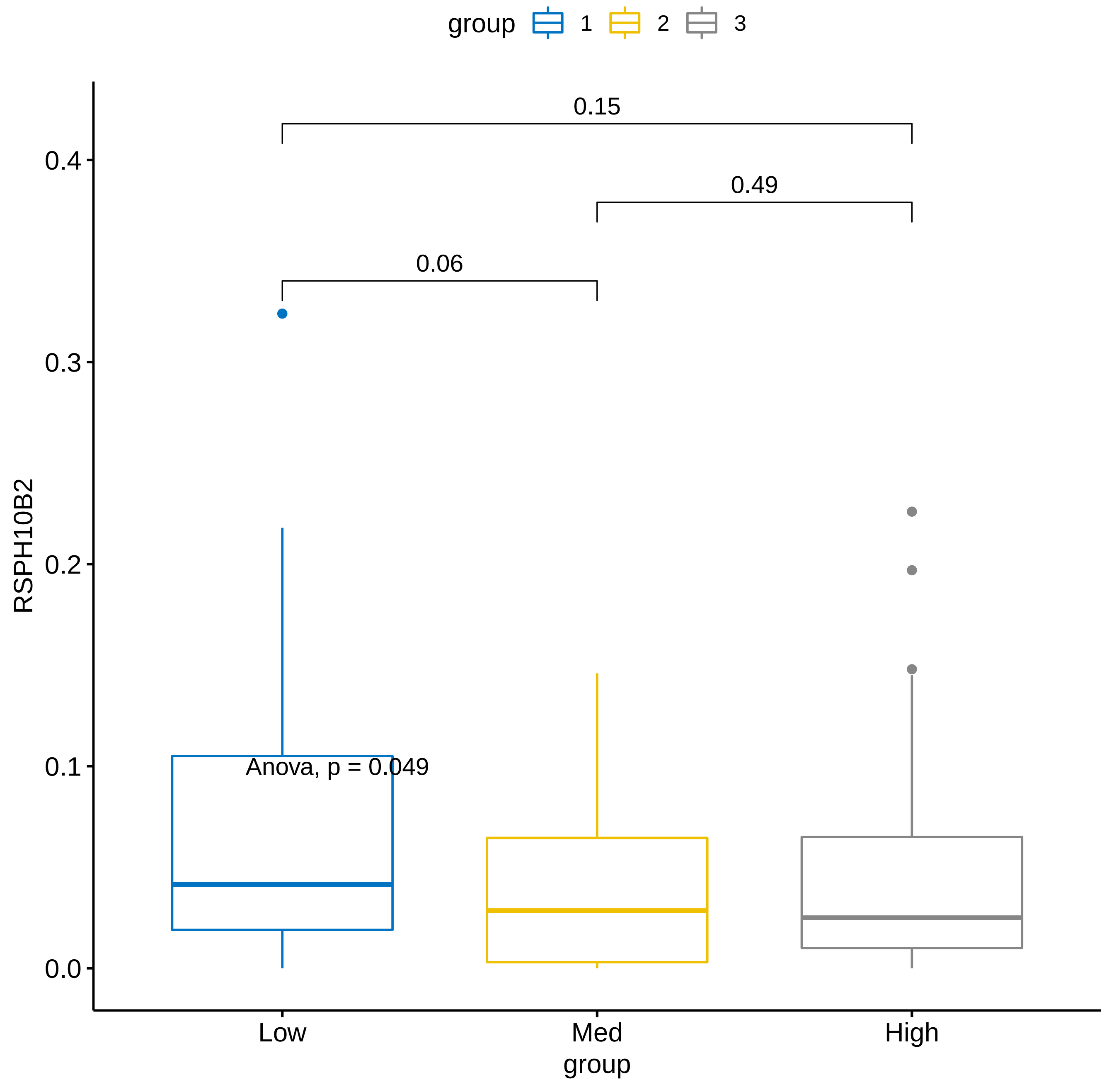
group 1 2 3
0.15
0.49
0.06
Anova, p = 0.049
RSPH10B2
0.4
0.3
0.2
0.1
0.0
Low
Med
High
group

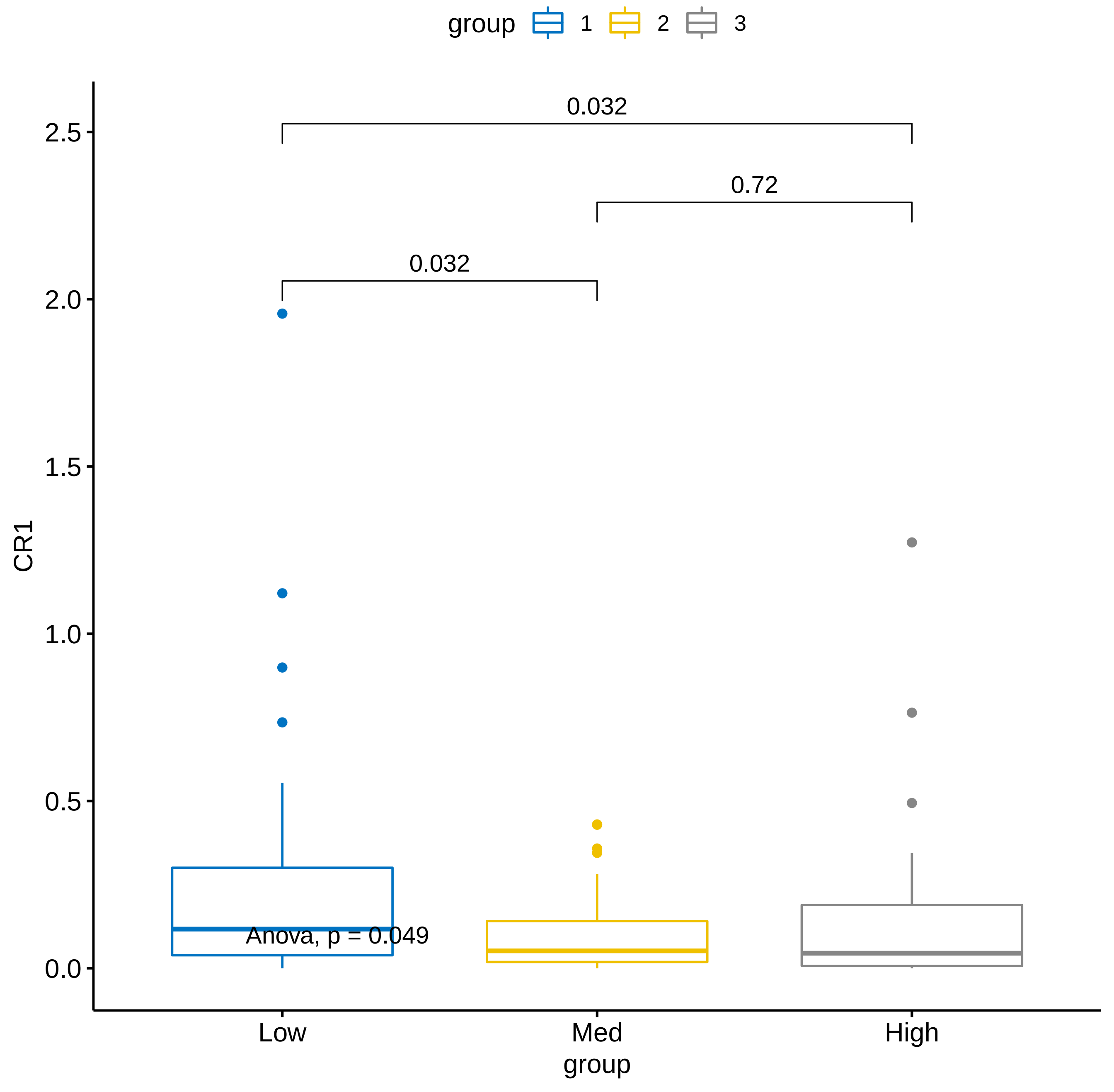
group
1
2
3
0.032
0.72
0.032
2.5
2.0
1.5
1.0
0.5
0.0
CR1
Anova, p = 0.049
Low
Med
High
group

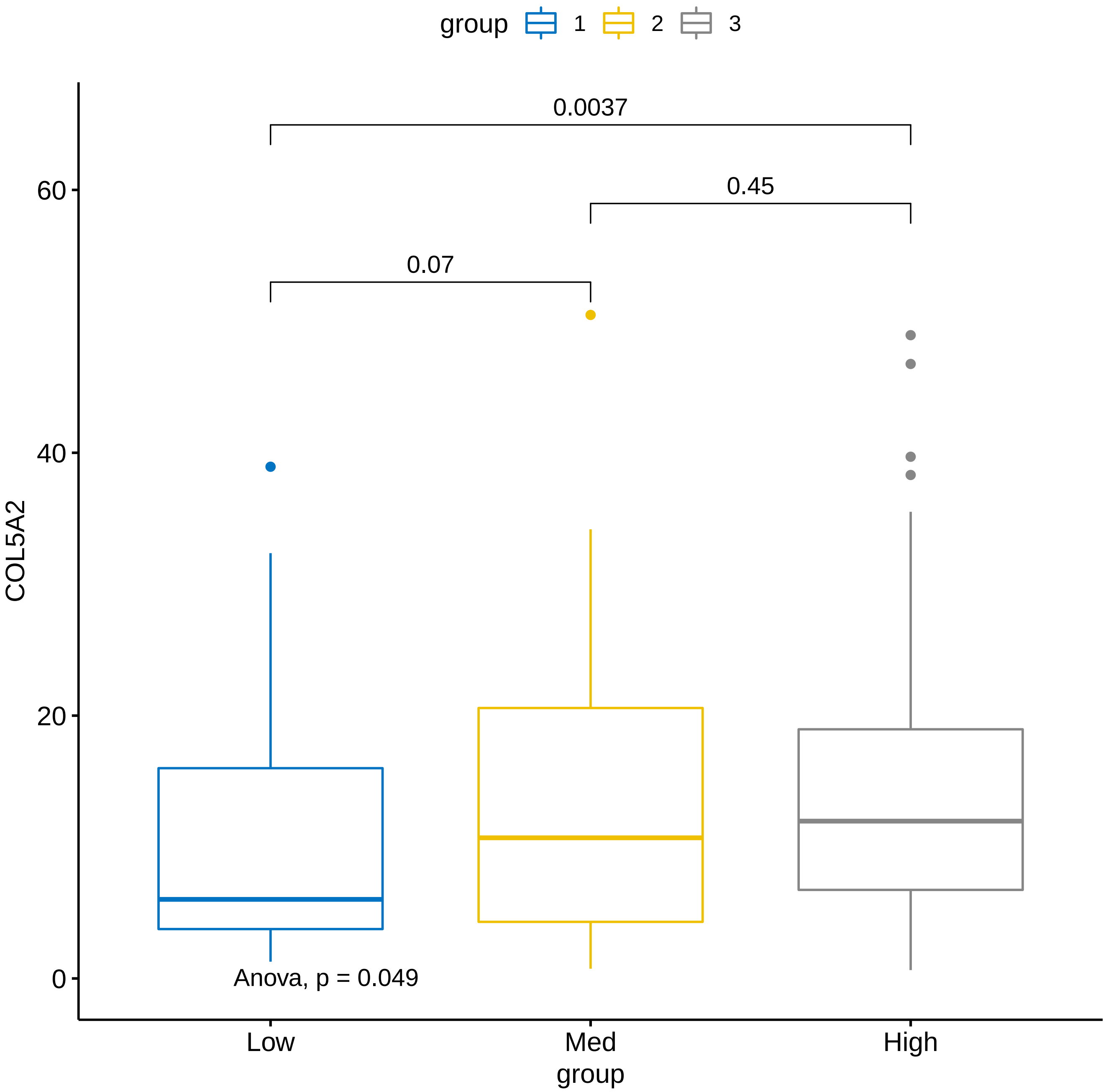
group
1
2
3
0.0037
0.45
0.07
60
40
20
0
COL5A2
Anova, p = 0.049
Low
Med
High
group

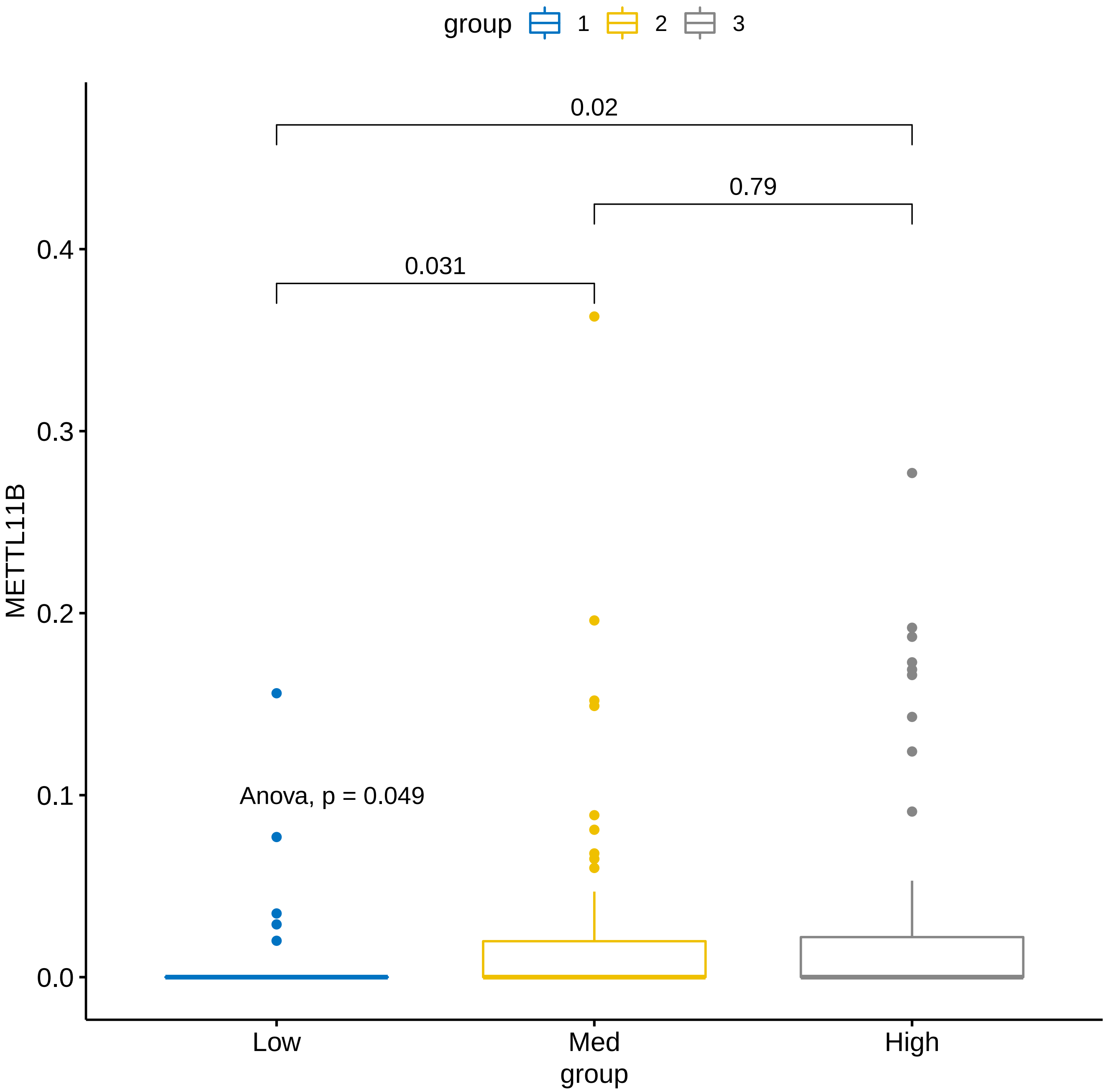

group 1 2 3
0.02
0.79
0.031
Anova, p = 0.049
METTL11B
0.0
0.1
0.2
0.3
0.4
Low
Med
High
group

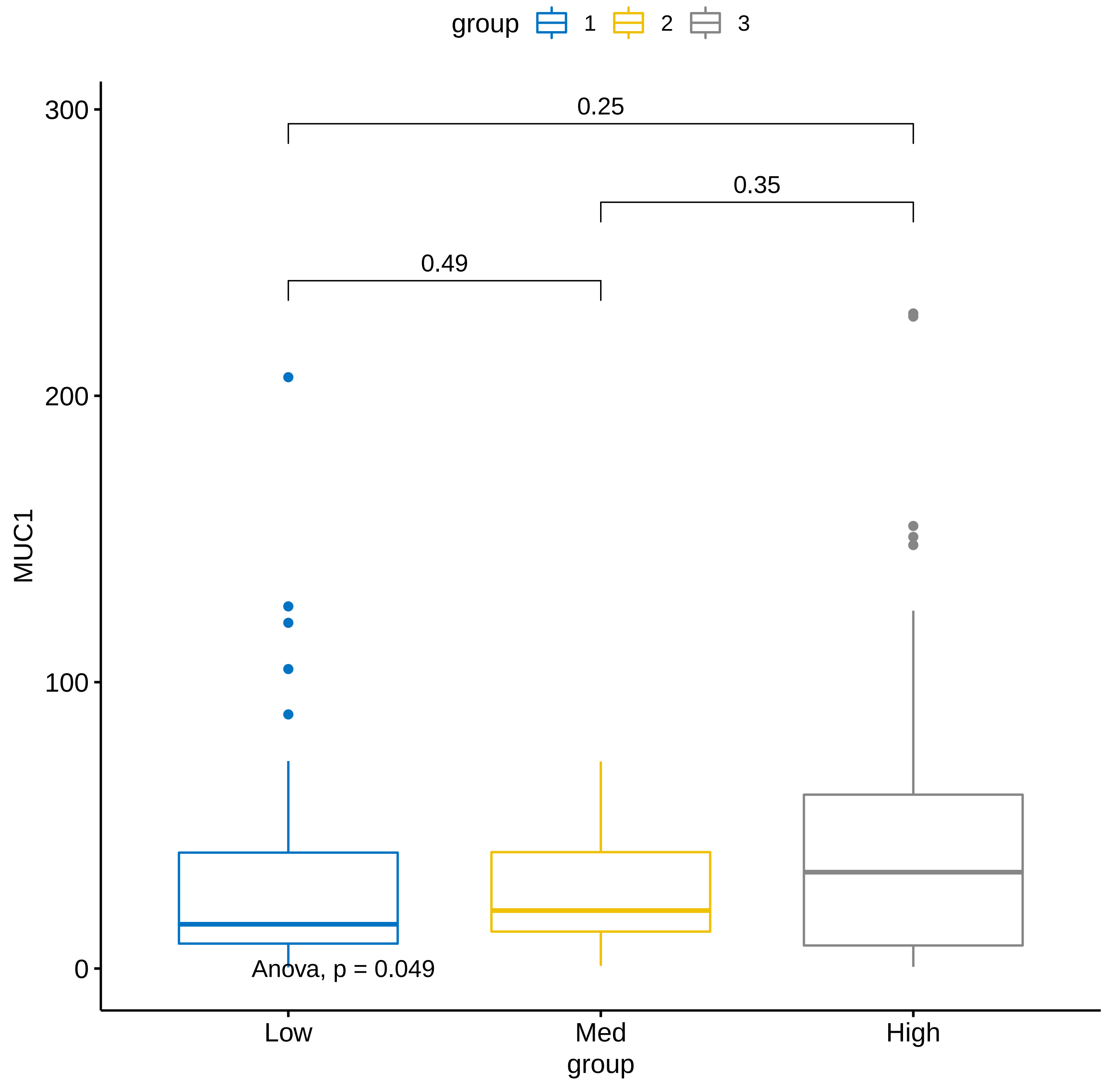
group 1 2 3
0.25
0.35
0.49
300
200
100
0
MUC1
Anova, p = 0.049
Low
Med
High
group

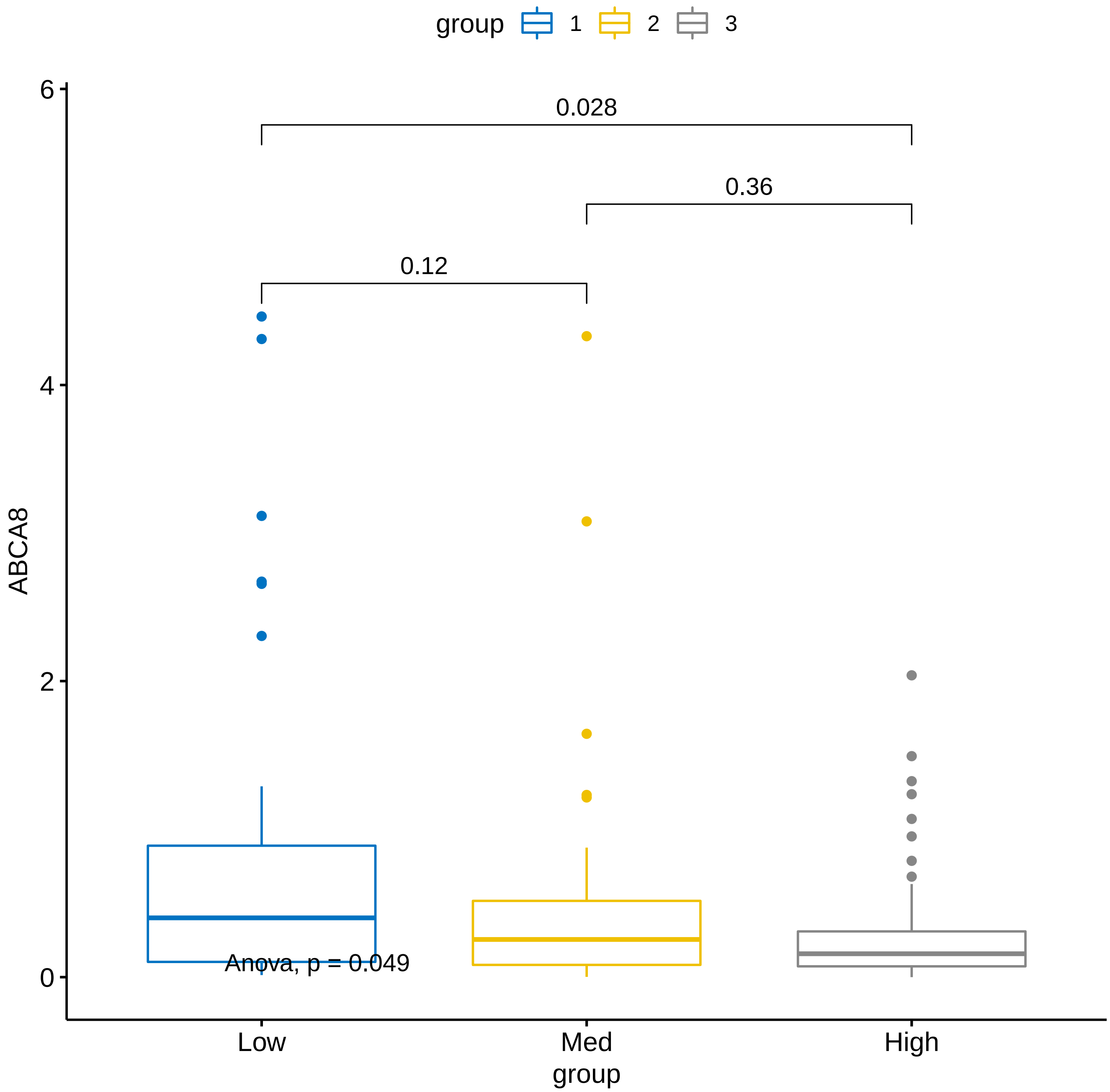
group 1 2 3
0.028
0.36
0.12
ABCA8
6
4
2
0
Anova, p = 0.049
Low
Med
High
group

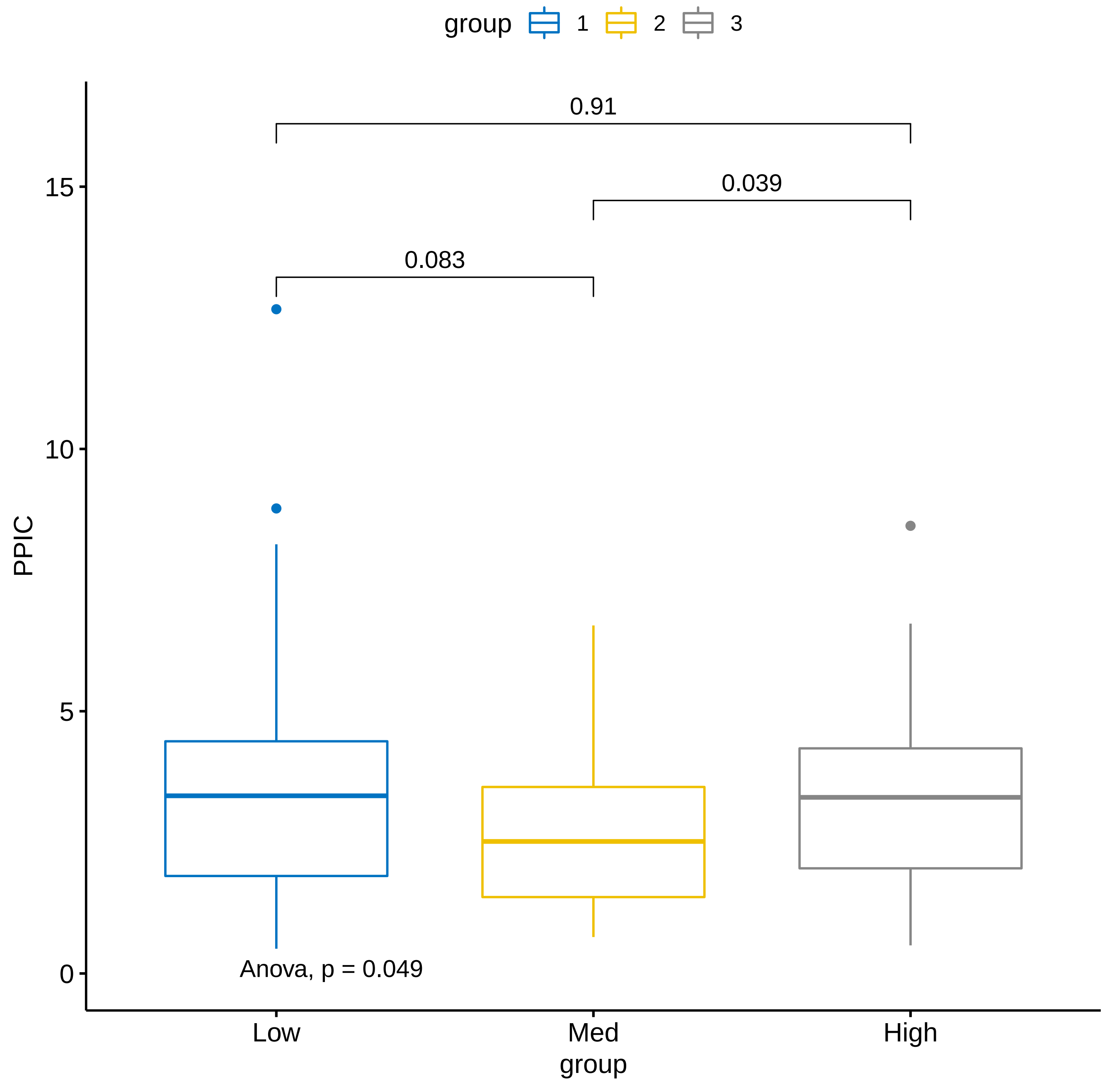

group 1 2 3
0.91
0.039
0.083
15
10
5
0
PPIC
Anova, p = 0.049
Low
Med
High
group

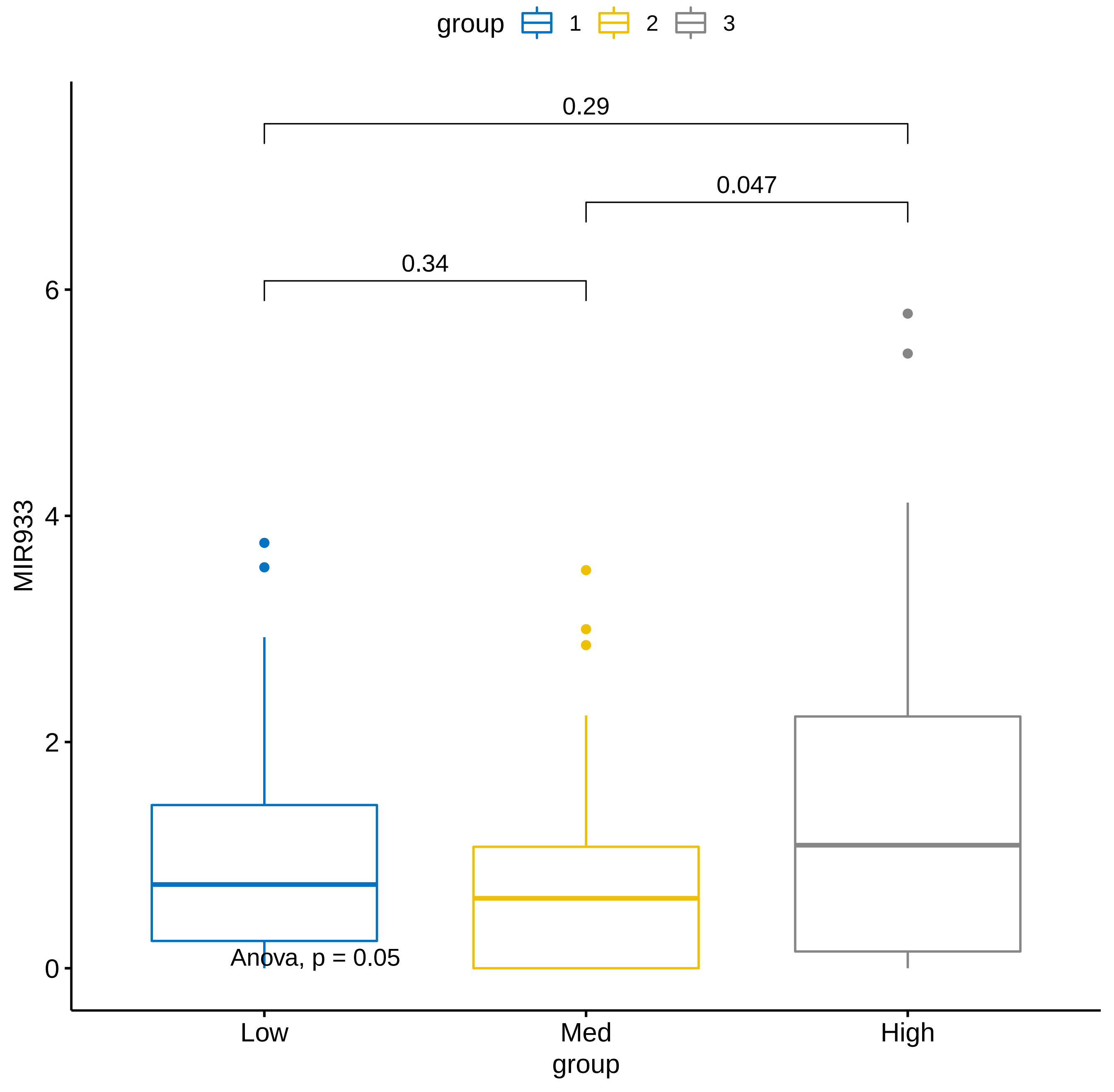

group
1
2
3
0.29
0.047
0.34
6
4
2
0
MIR933
Anova, p = 0.05
Low
Med
High
group

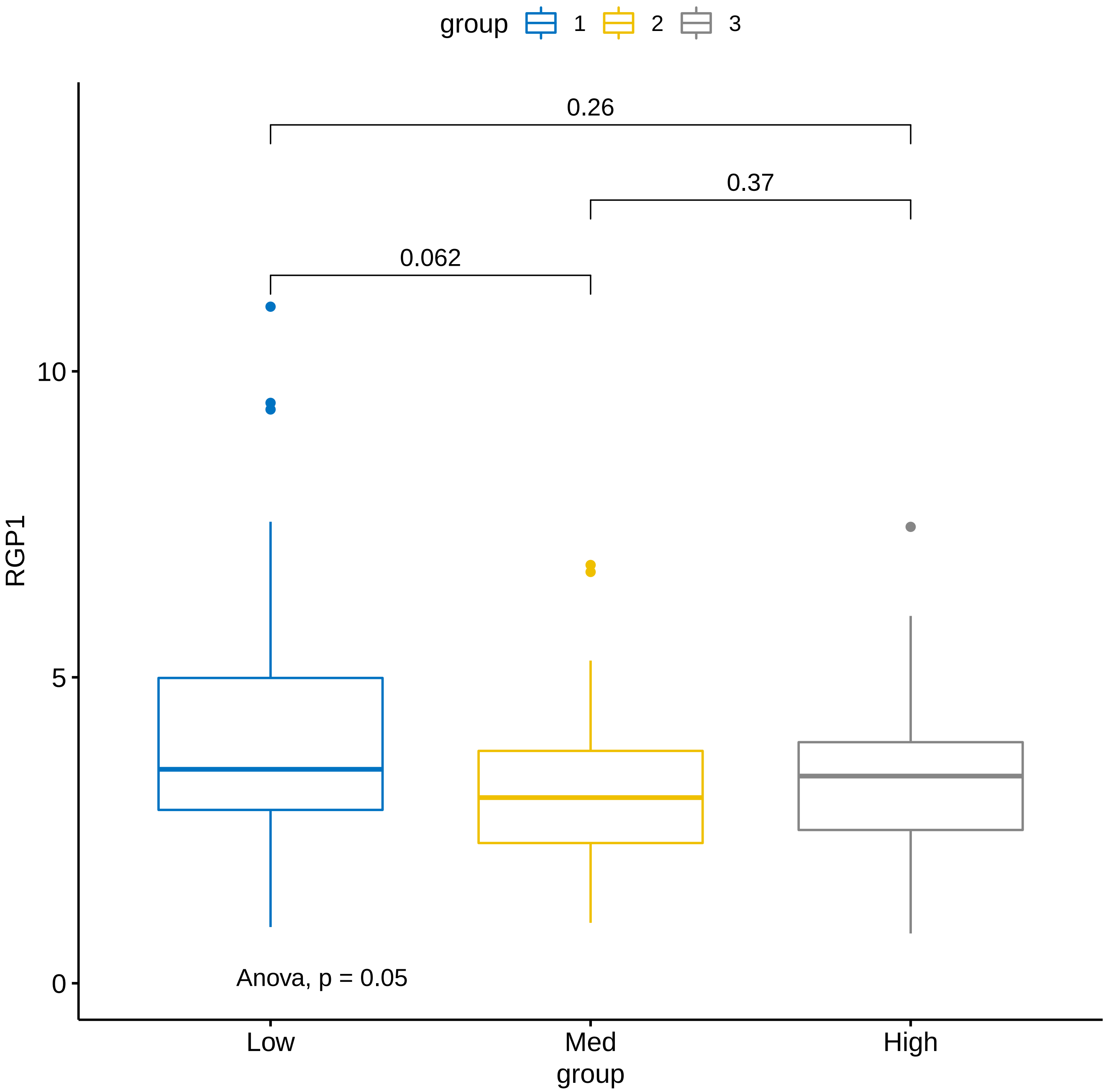
group 1 2 3
0.26
0.37
0.062
RGP1
10
5
0
Anova, p = 0.05
Low
Med
High
group

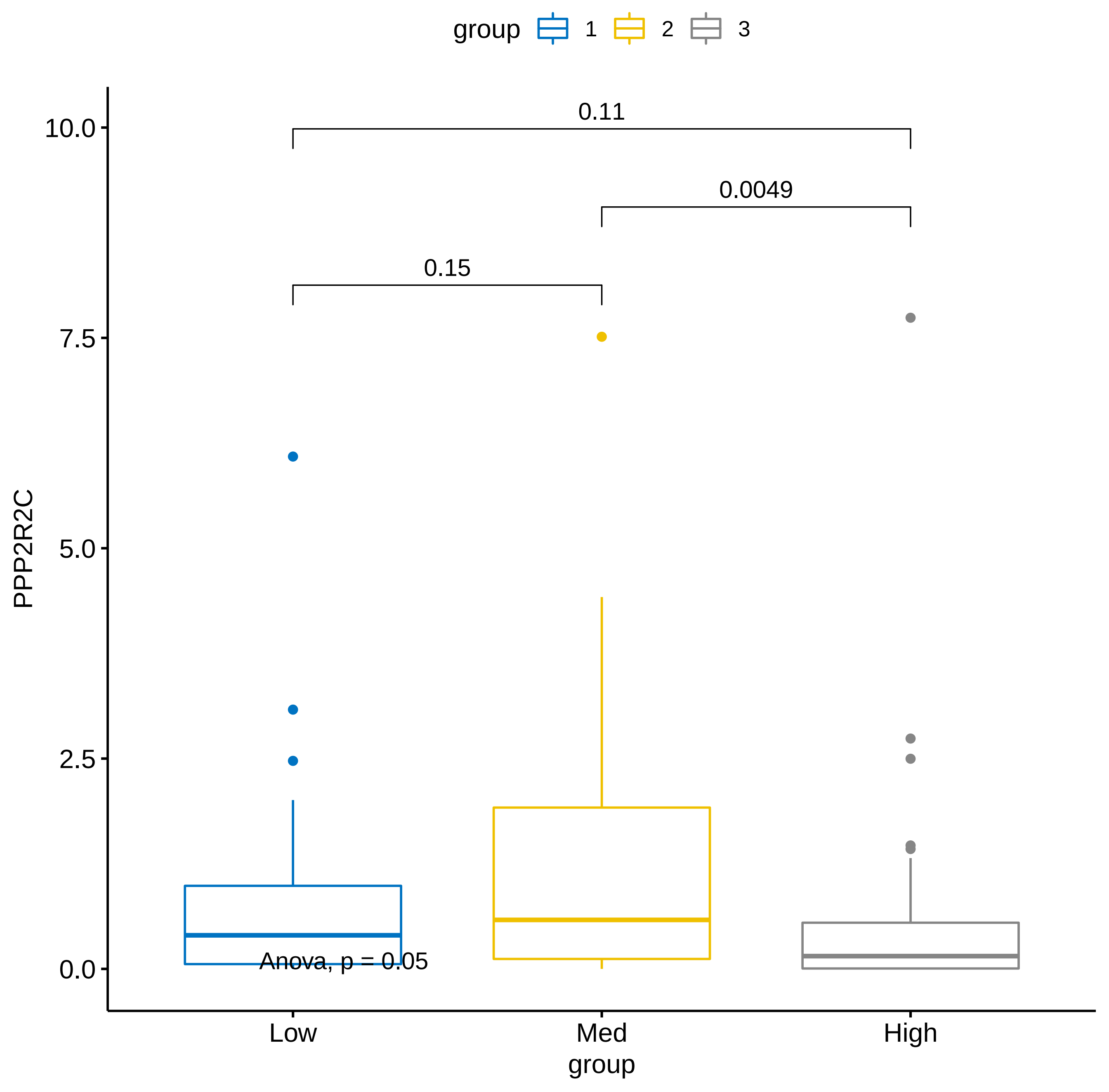

group
1
2
3
0.11
0.0049
0.15
10.0
7.5
5.0
2.5
0.0
PPP2R2C
Anova, p = 0.05
Low
Med
High
group

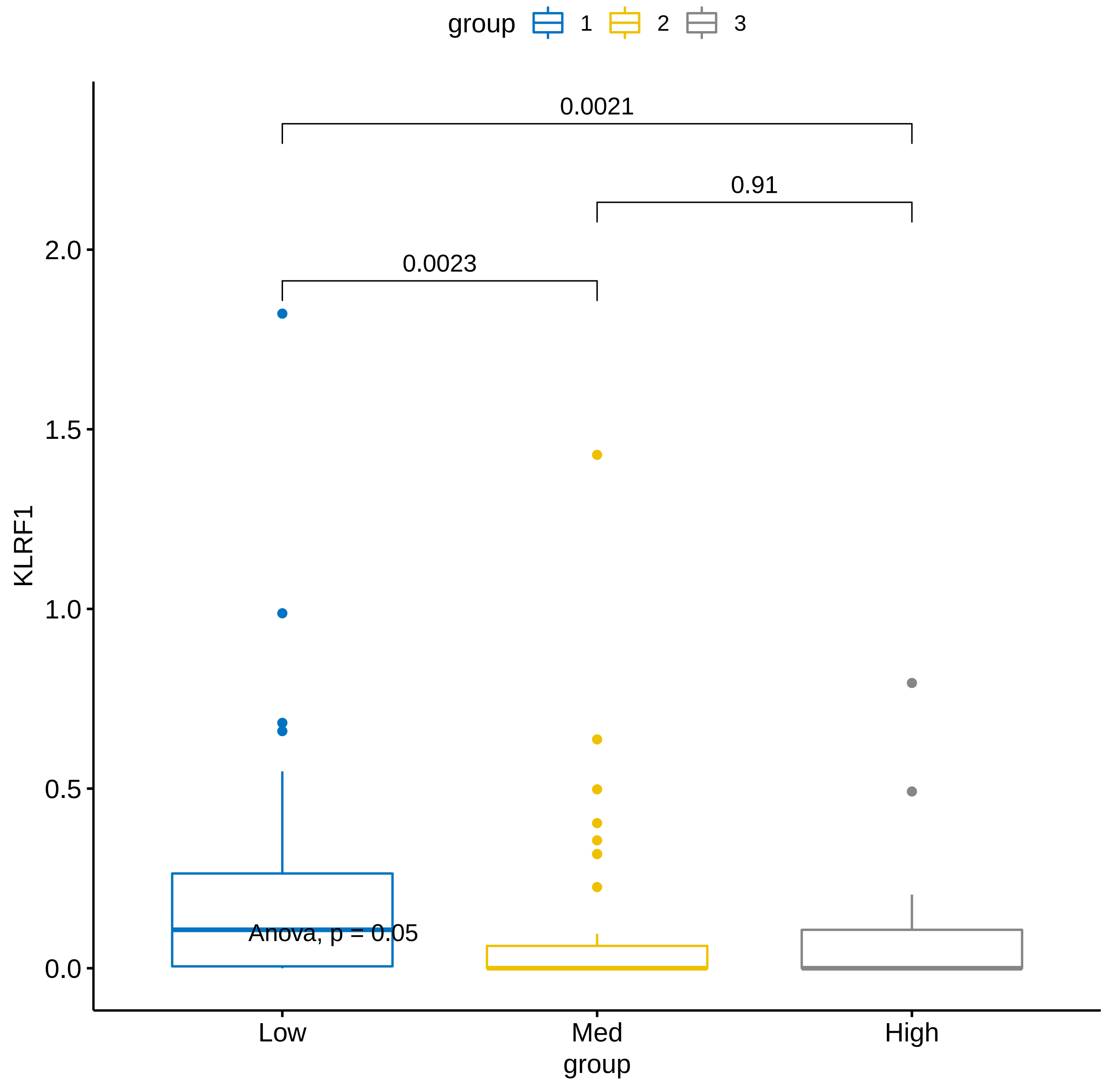
group
1
2
3
0.0021
0.91
0.0023
2.0
1.5
1.0
0.5
0.0
KLRF1
Anova, p = 0.05
Low
Med
High
group
