# Supplementary material for: Epigenetic re-wiring of breast cancer by pharmacological targeting of C-terminal binding protein
Source: Cell Death Dis. 2019 Sep 18;10(10):689. doi: 10.1038/s41419-019-1892-7 (PMC6751206; doi:10.1038/s41419-019-1892-7)
Supplement: Supplementary file 5 — Supplementary Table 4. [file 41419_2019_1892_MOESM5_ESM.pdf]

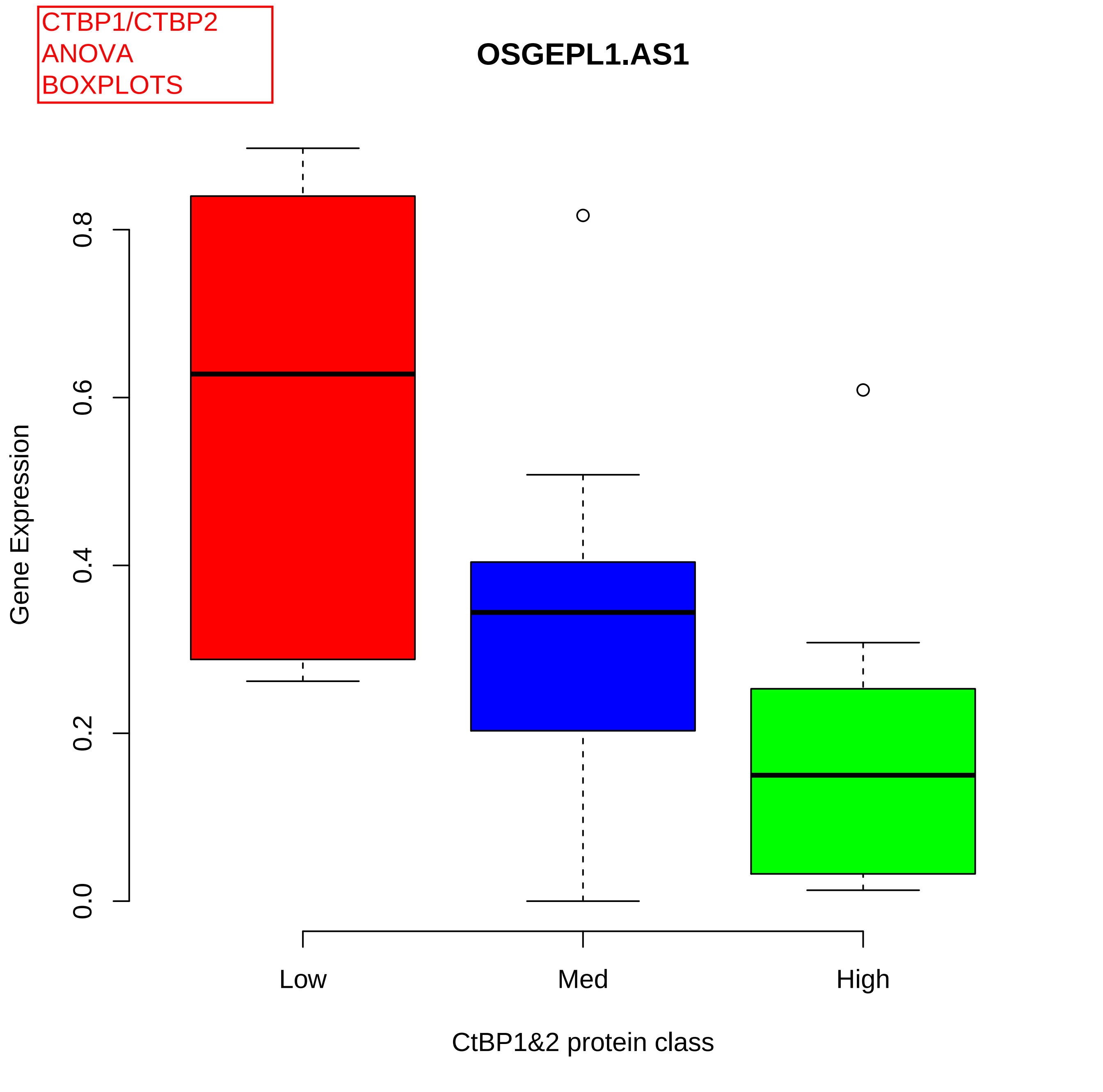
CTBP1/CTBP2
ANOVA
BOXPLOTS
OSGEPL1.AS1
Gene Expression
0.0
0.2
0.4
0.6
0.8
Low
Med
High
CtBP1&2 protein class

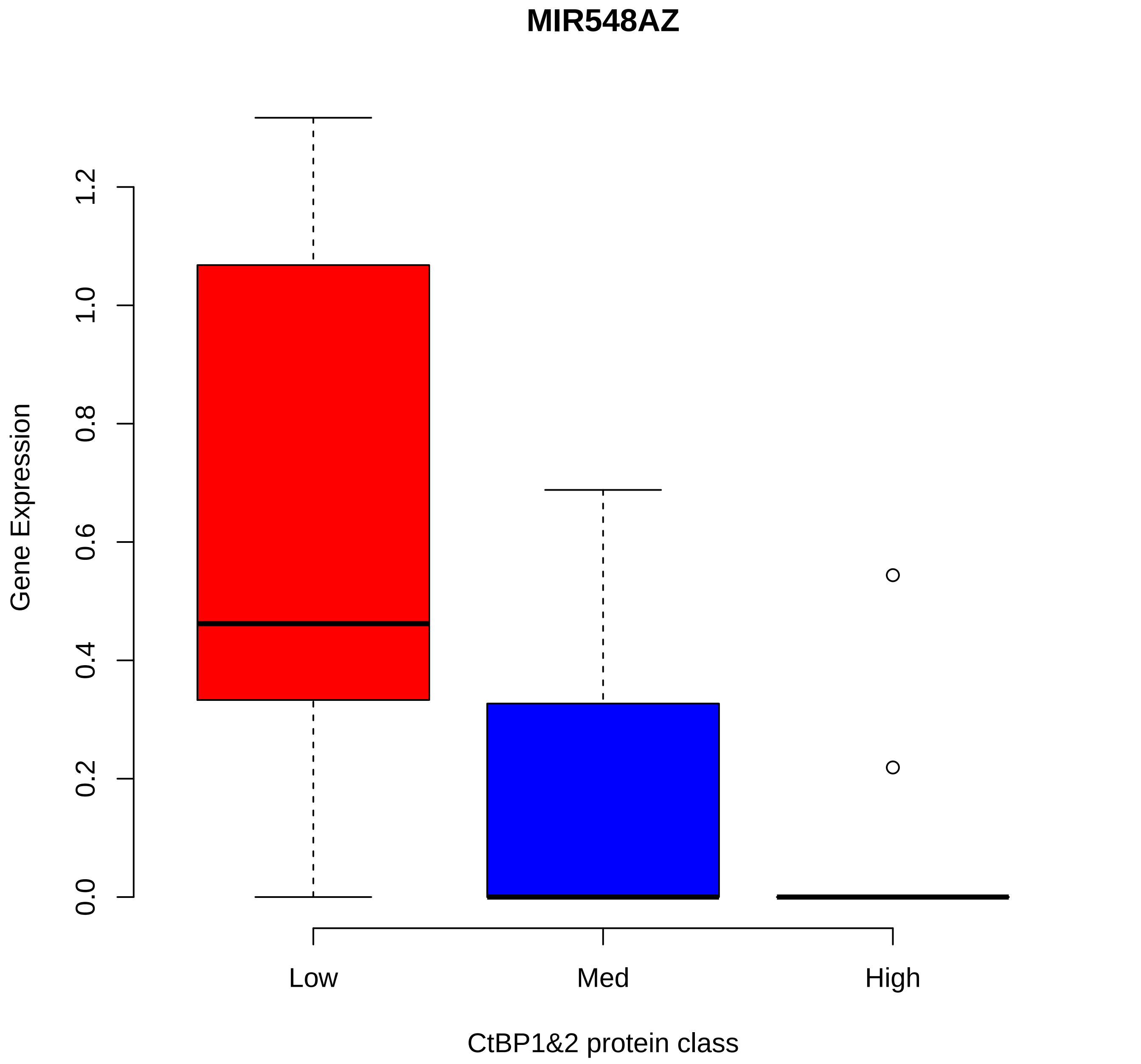
MIR548AZ
Gene Expression
0.0
0.2
0.4
0.6
0.8
1.0
1.2
Low
Med
High
CtBP1&2 protein class

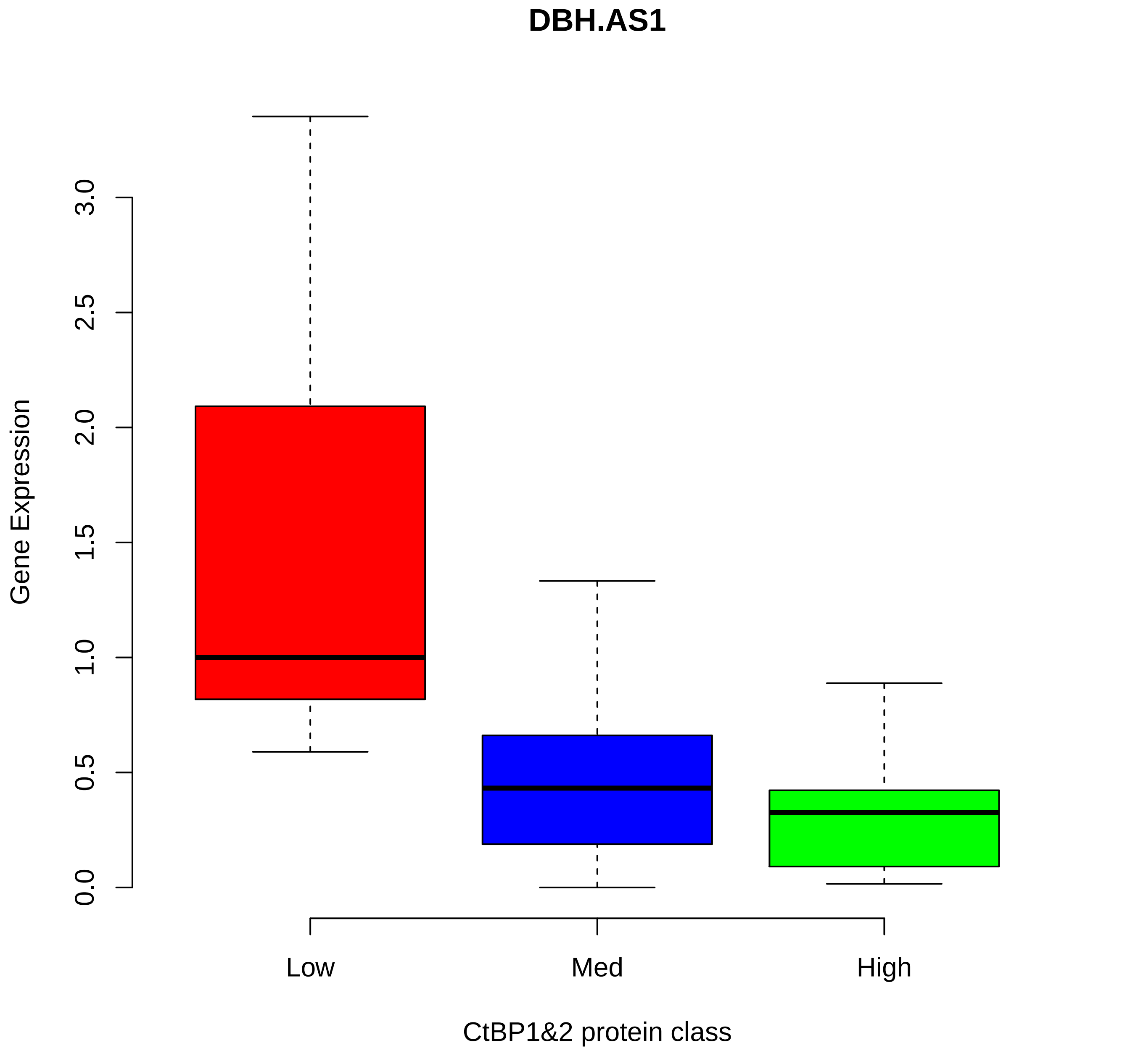

DBH.AS1
Gene Expression
0.0
0.5
1.0
1.5
2.0
2.5
3.0
Low
Med
High
CtBP1&2 protein class

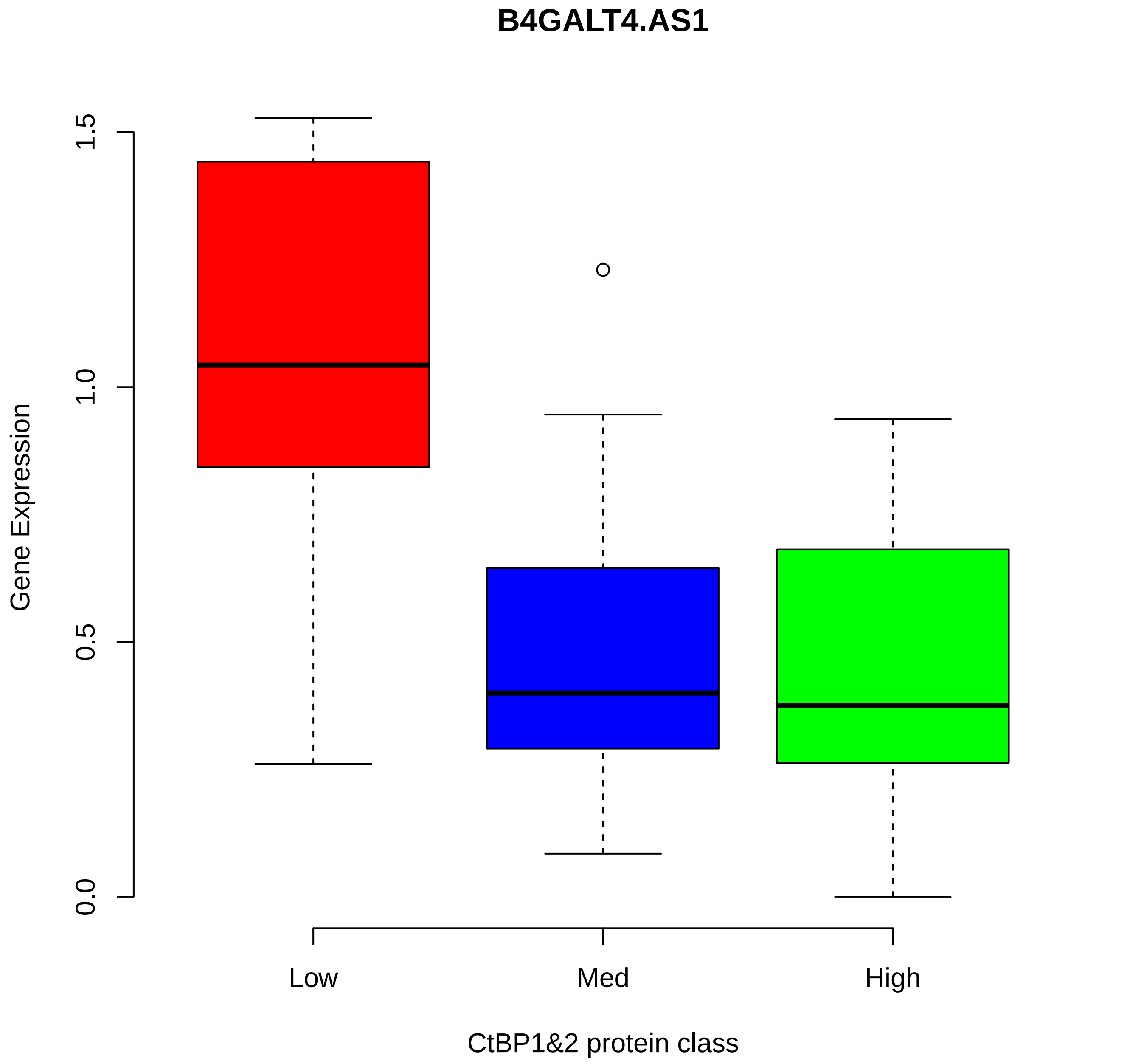

B4GALT4.AS1
Gene Expression
0.0
0.5
1.0
1.5
Low
Med
High
CtBP1&2 protein class

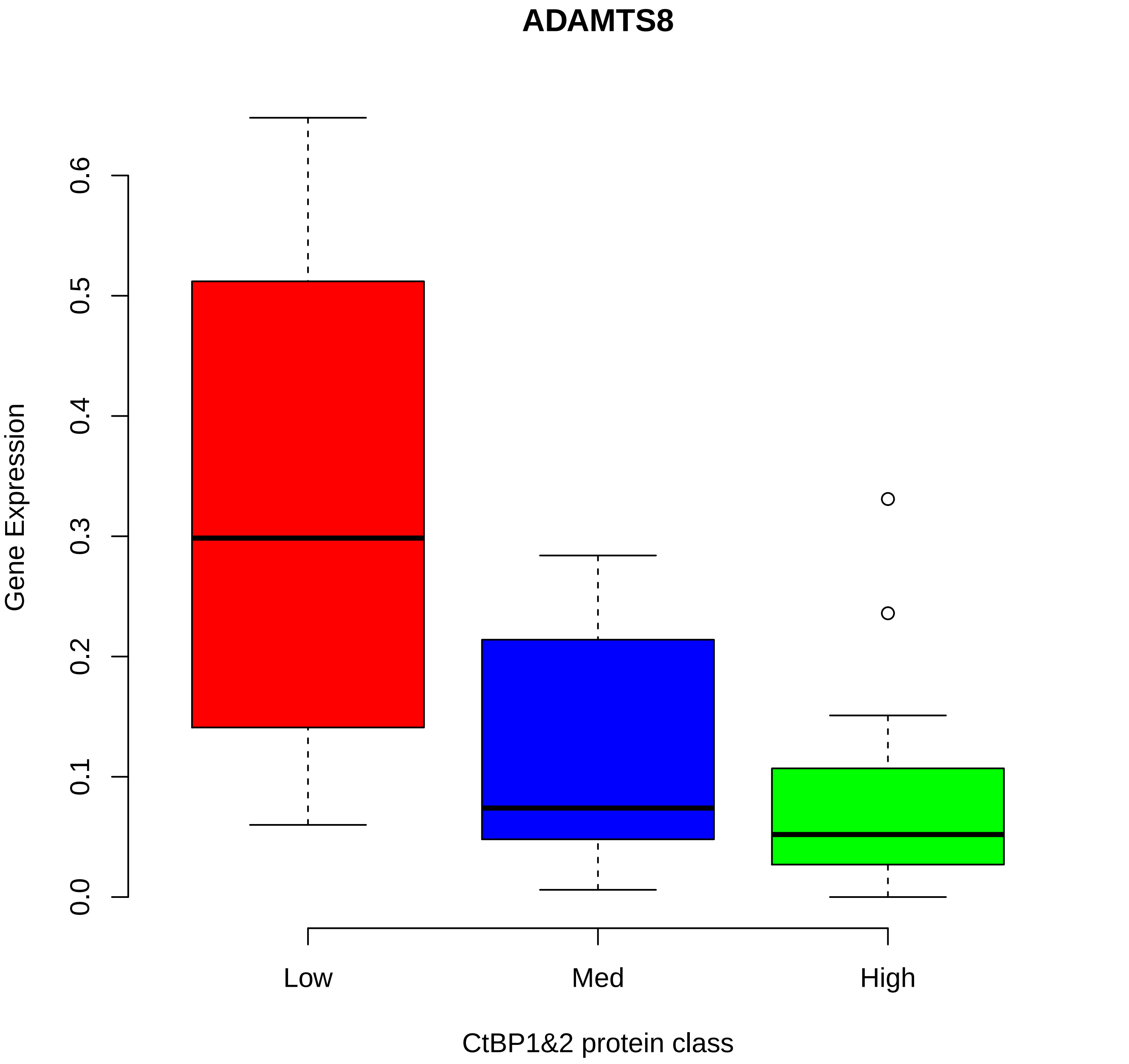
ADAMTS8
Gene Expression
0.0
0.1
0.2
0.3
0.4
0.5
0.6
Low
Med
High
CtBP1&2 protein class

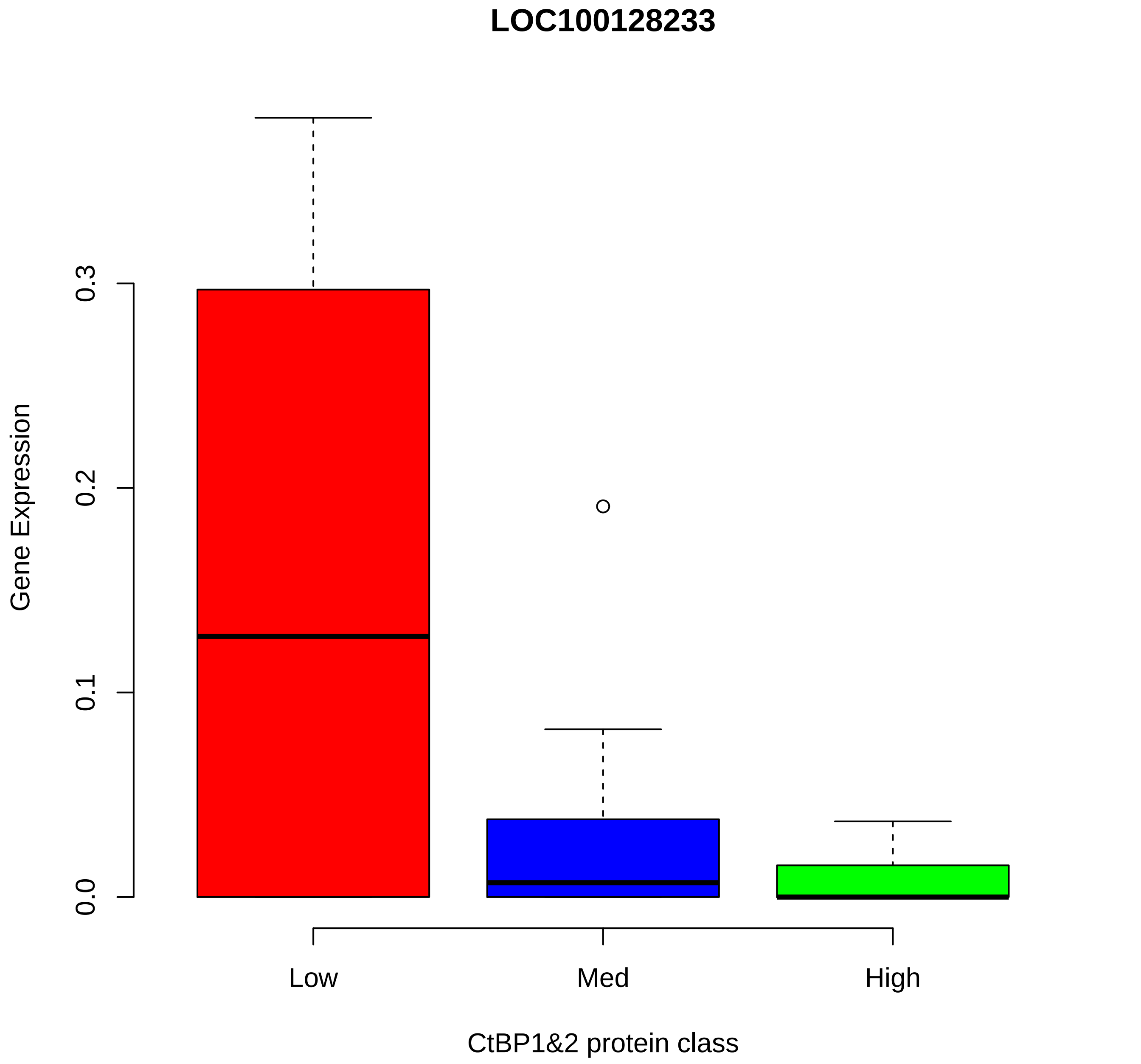
LOC100128233
Gene Expression
0.0
0.1
0.2
0.3
Low
Med
High
CtBP1&2 protein class

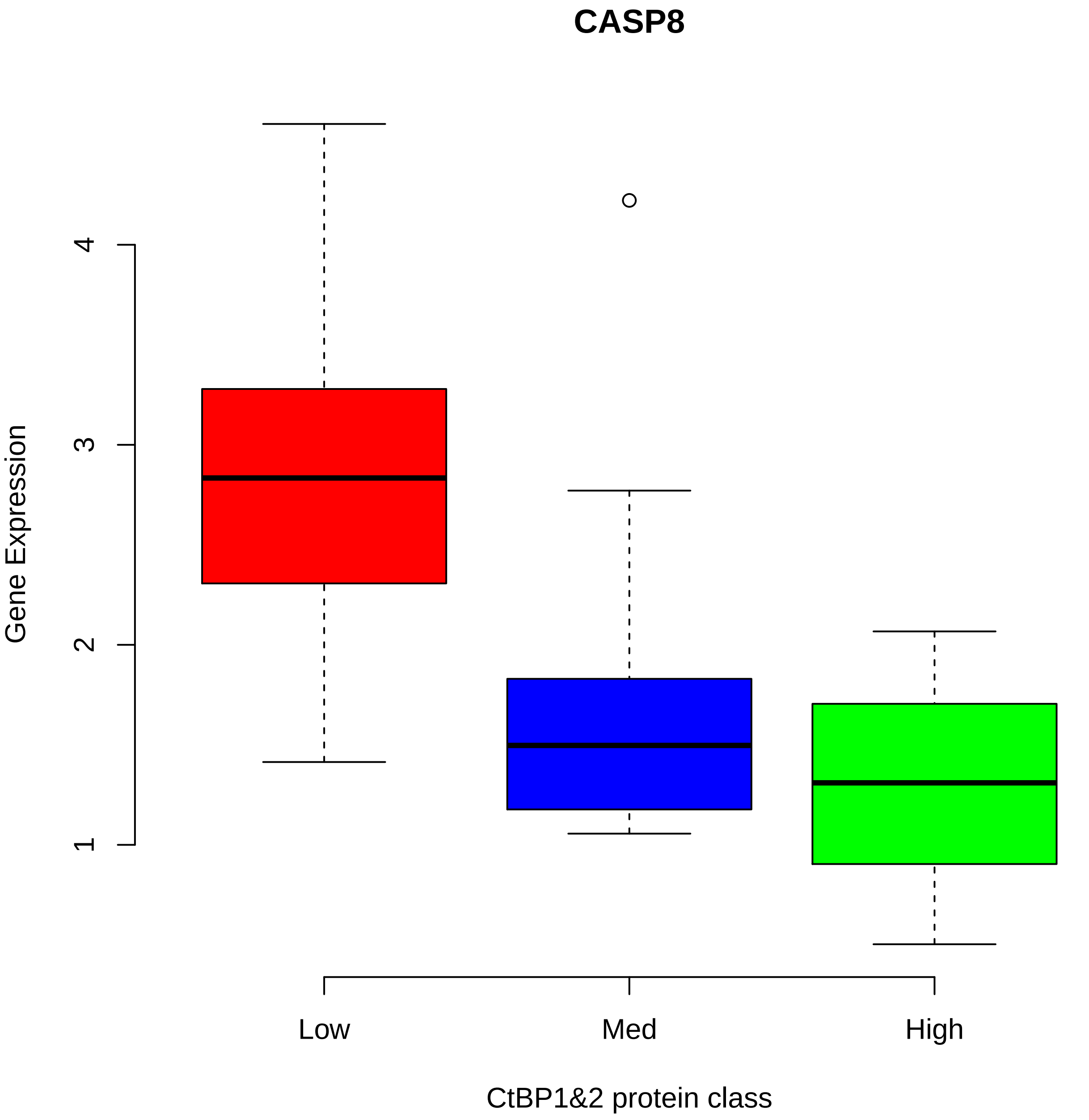
CASP8
Gene Expression
1
2
3
4
Low
Med
High
CtBP1&2 protein class

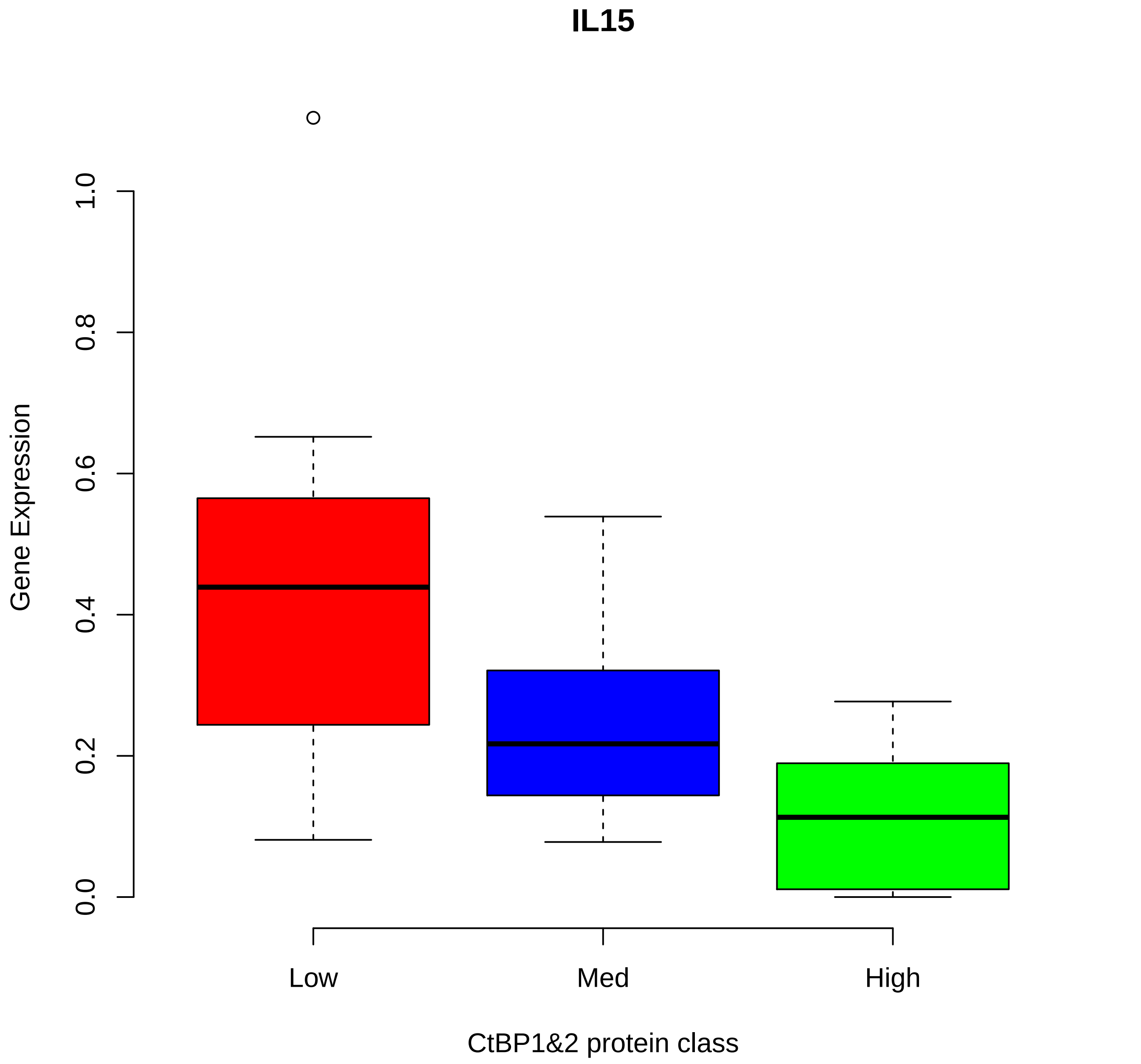

IL15
Gene Expression
0.0
0.2
0.4
0.6
0.8
1.0
Low
Med
High
CtBP1&2 protein class

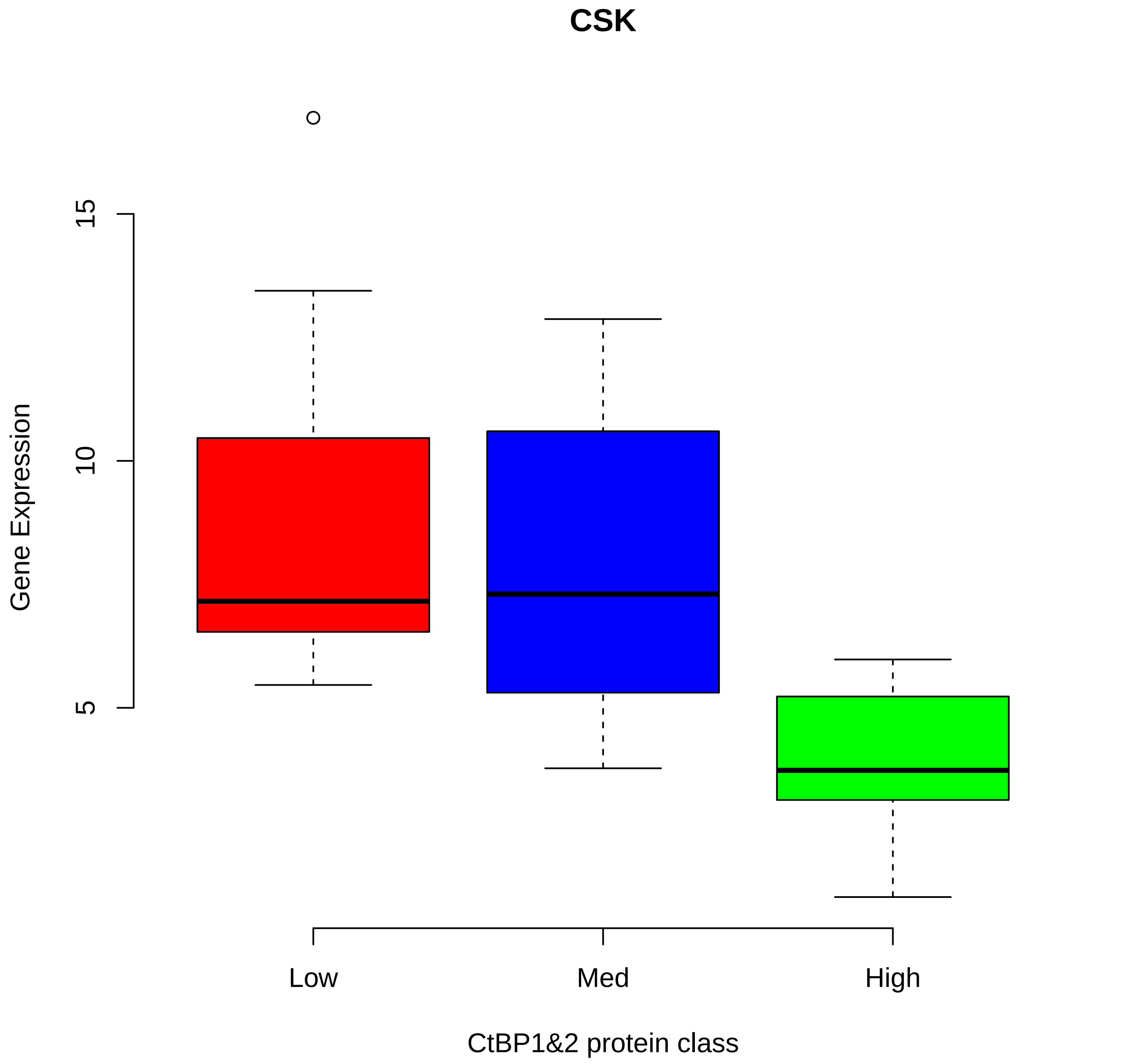

CSK
Gene Expression
15
10
5
Low
Med
High
CtBP1&2 protein class

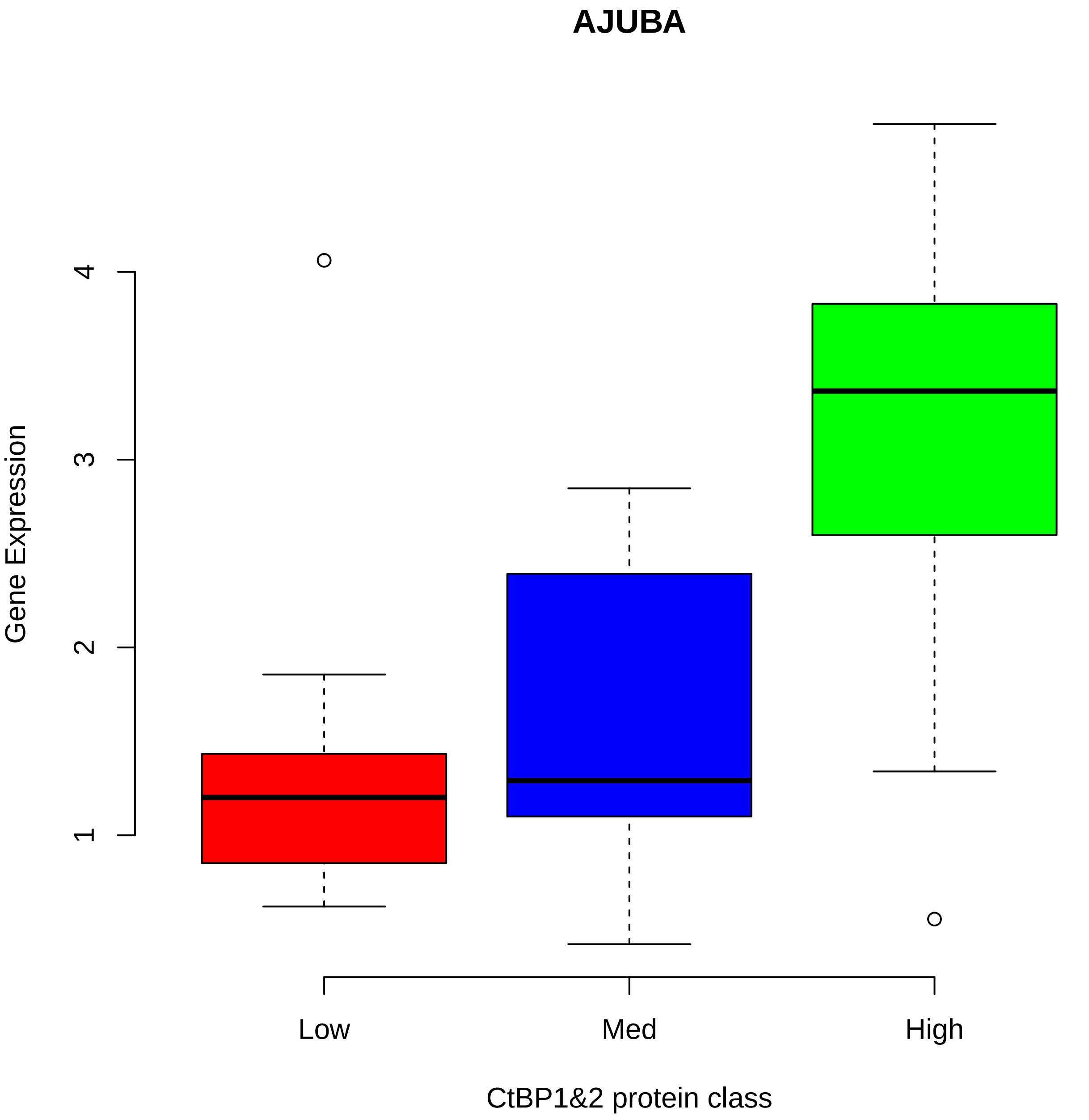

AJUBA
Gene Expression
1
2
3
4
Low
Med
High
CtBP1&2 protein class

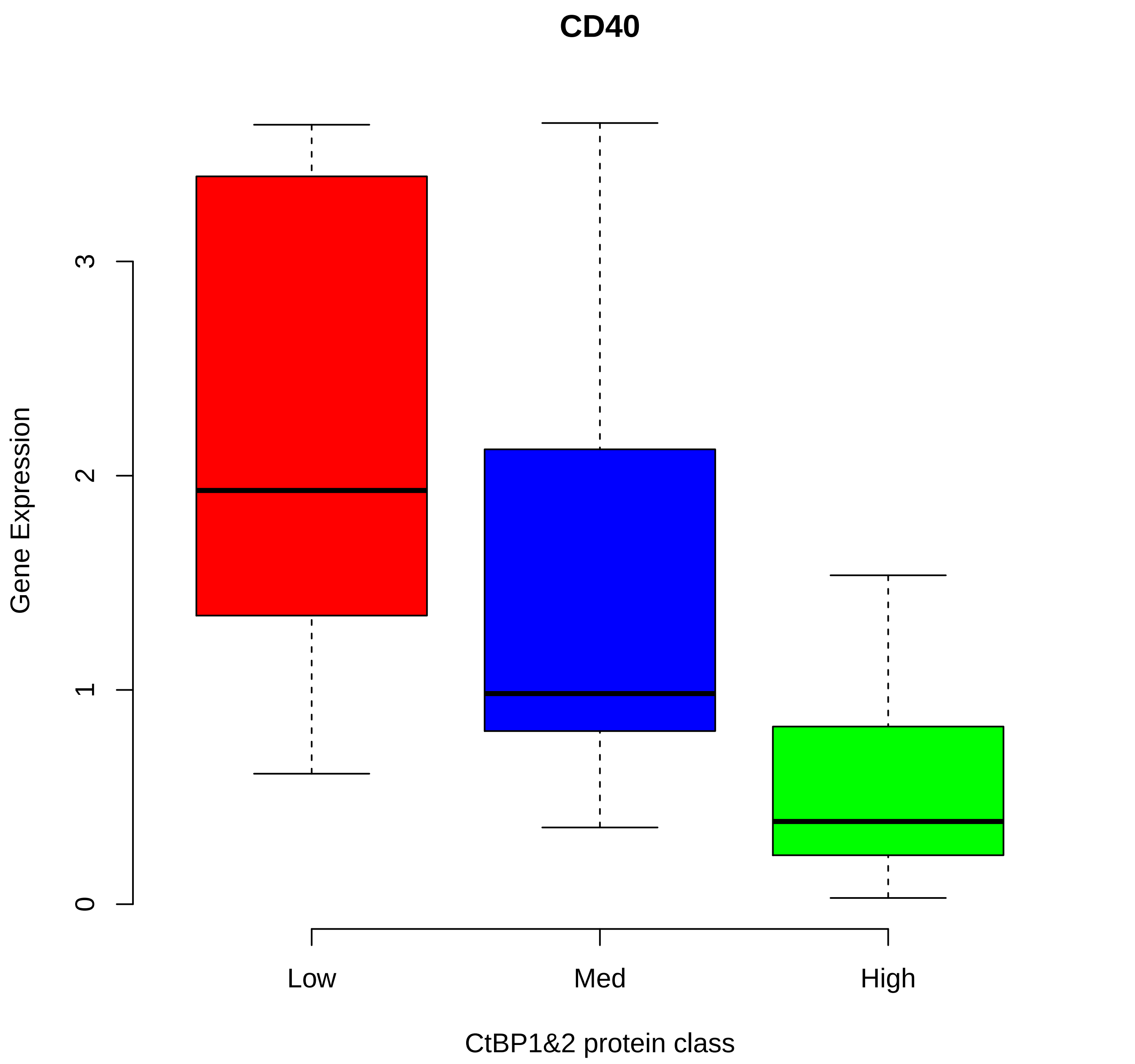
CD40
Gene Expression
0
1
2
3
Low
Med
High
CtBP1&2 protein class

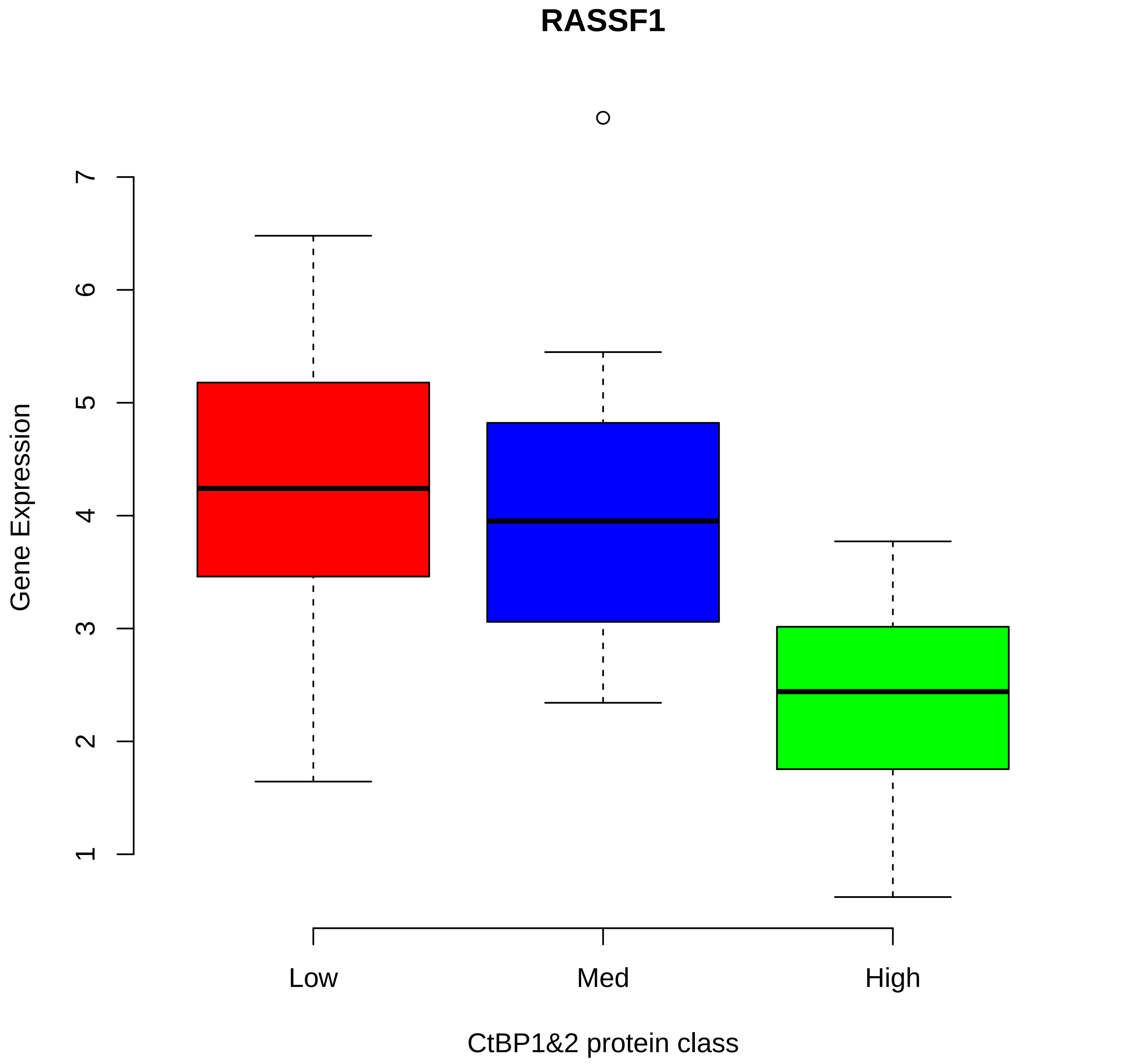

RASSF1
Gene Expression
1
2
3
4
5
6
7
Low
Med
High
CtBP1&2 protein class

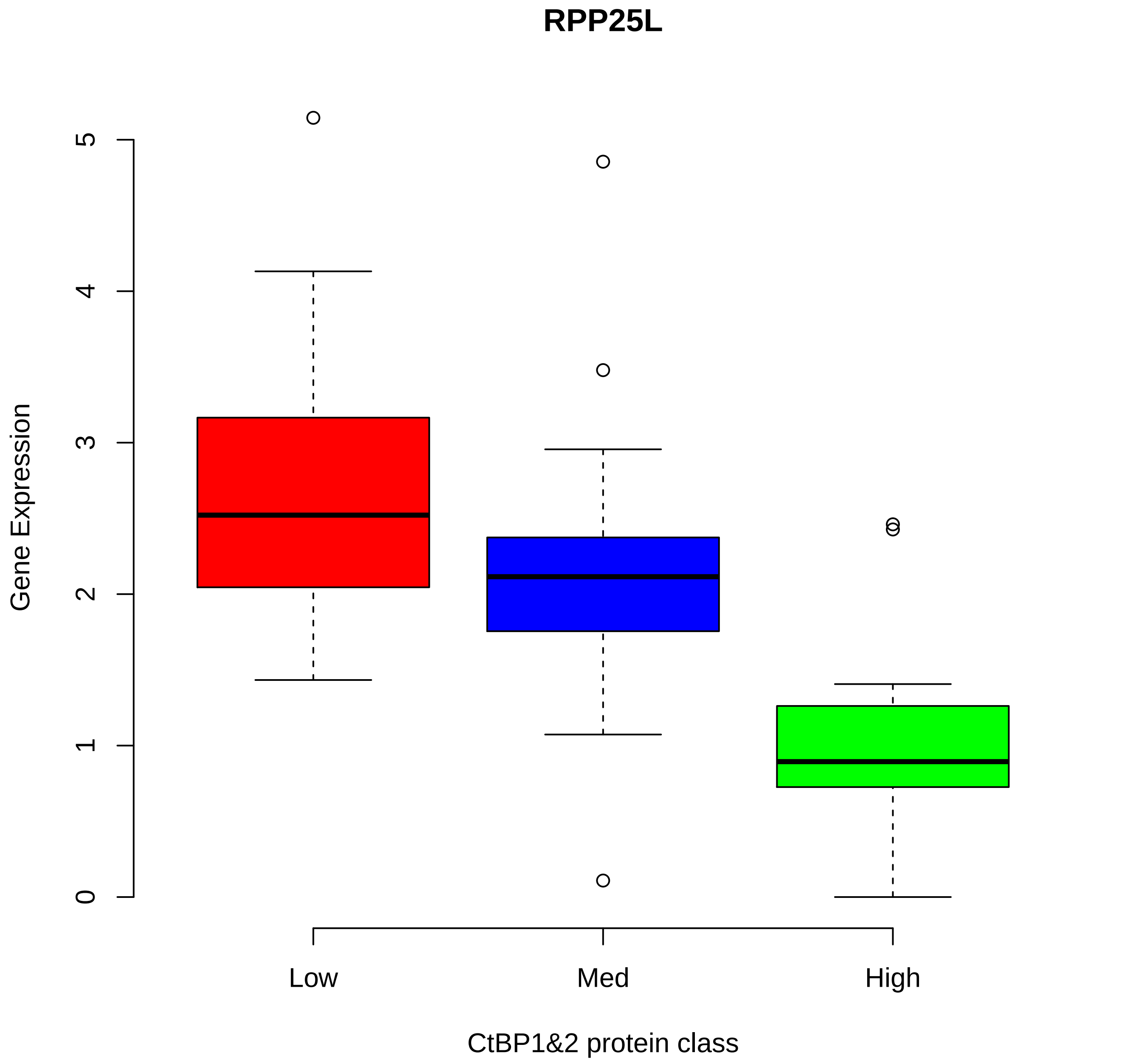
RPP25L
Gene Expression
0
1
2
3
4
5
Low
Med
High
CtBP1&2 protein class

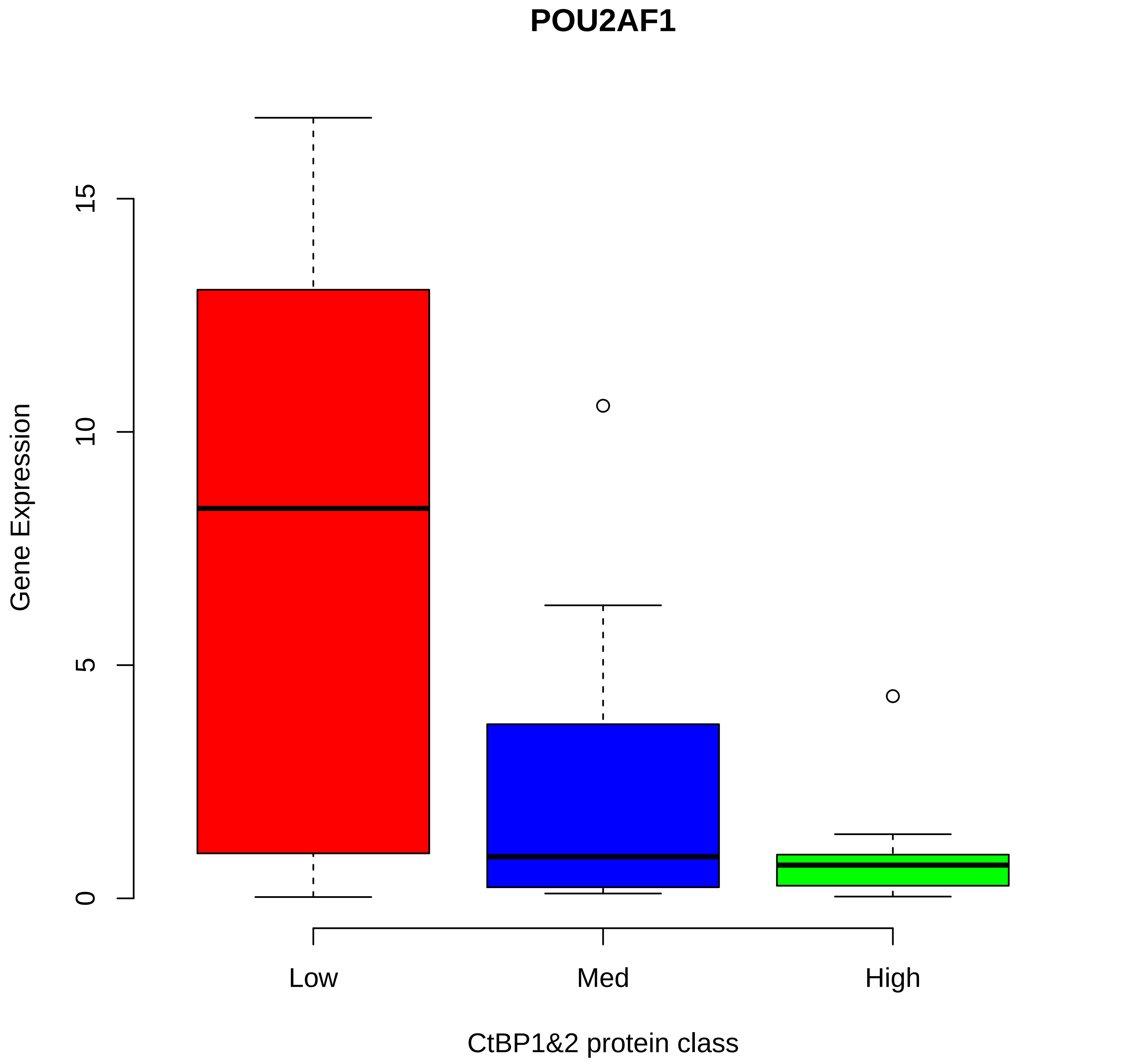
POU2AF1
Gene Expression
0
5
10
15
Low
Med
High
CtBP1&2 protein class

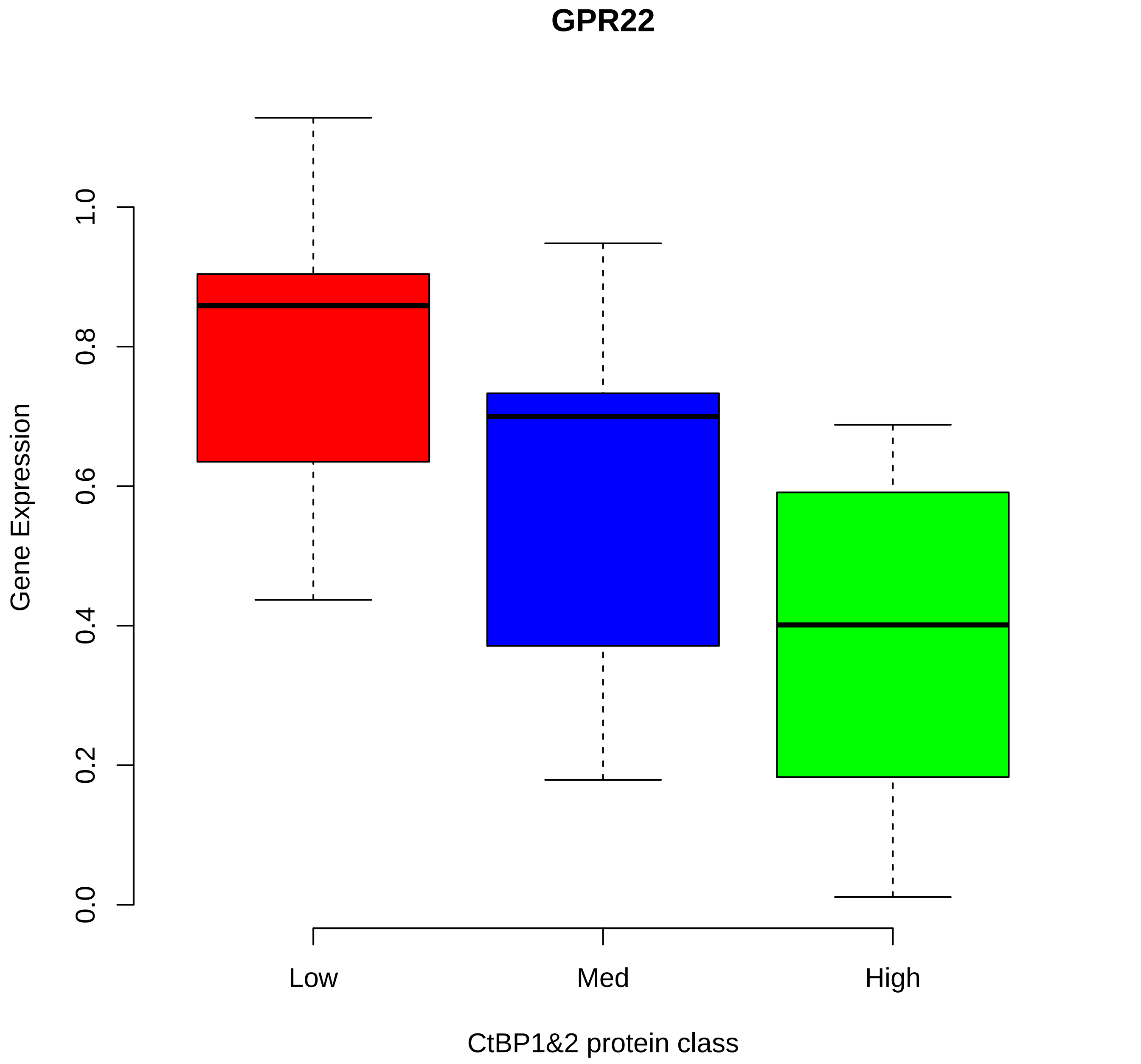

GPR22
Gene Expression
0.0
0.2
0.4
0.6
0.8
1.0
Low
Med
High
CtBP1&2 protein class

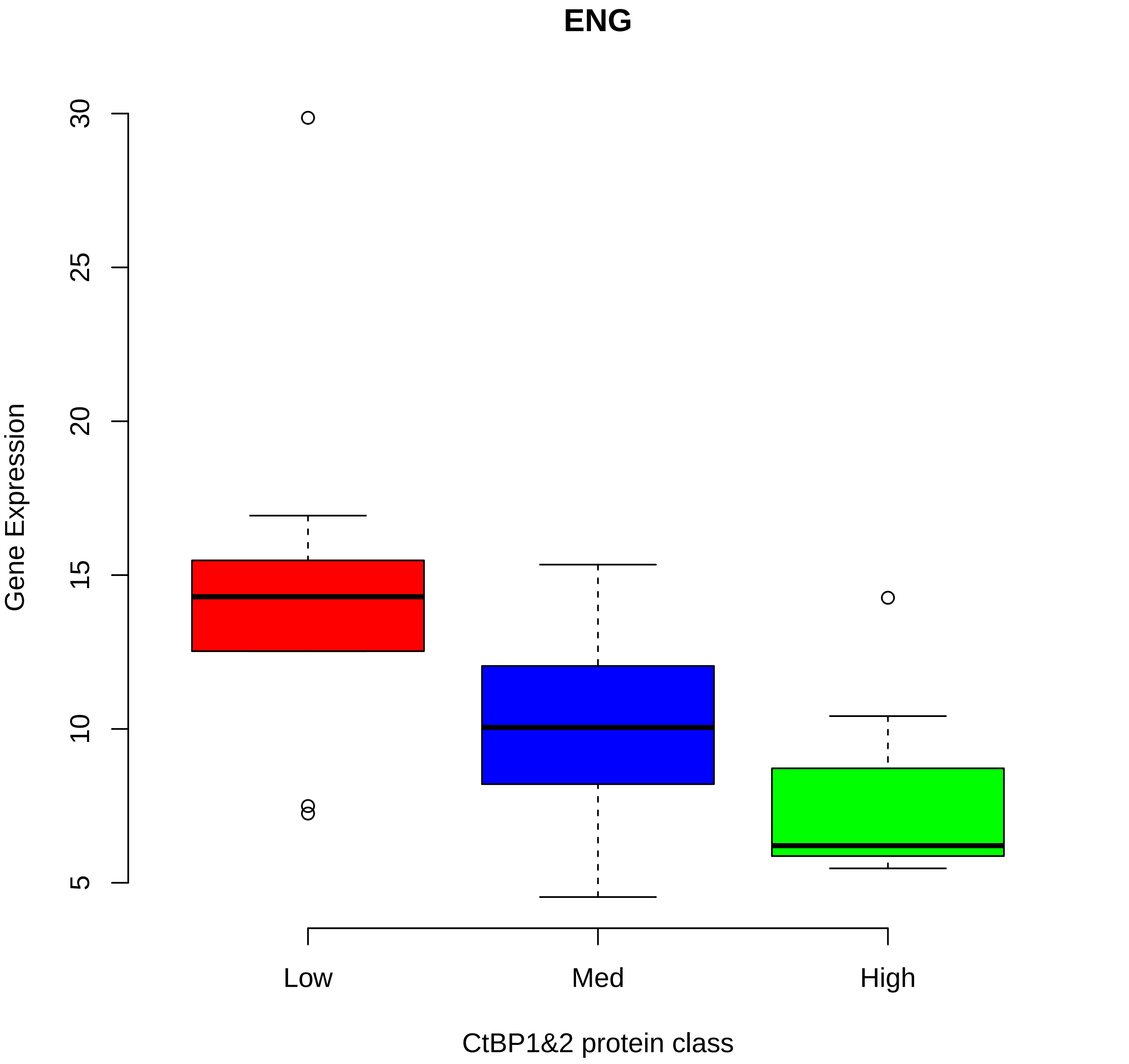

ENG
30
25
20
15
10
5
Gene Expression
Low
Med
High
CtBP1&2 protein class

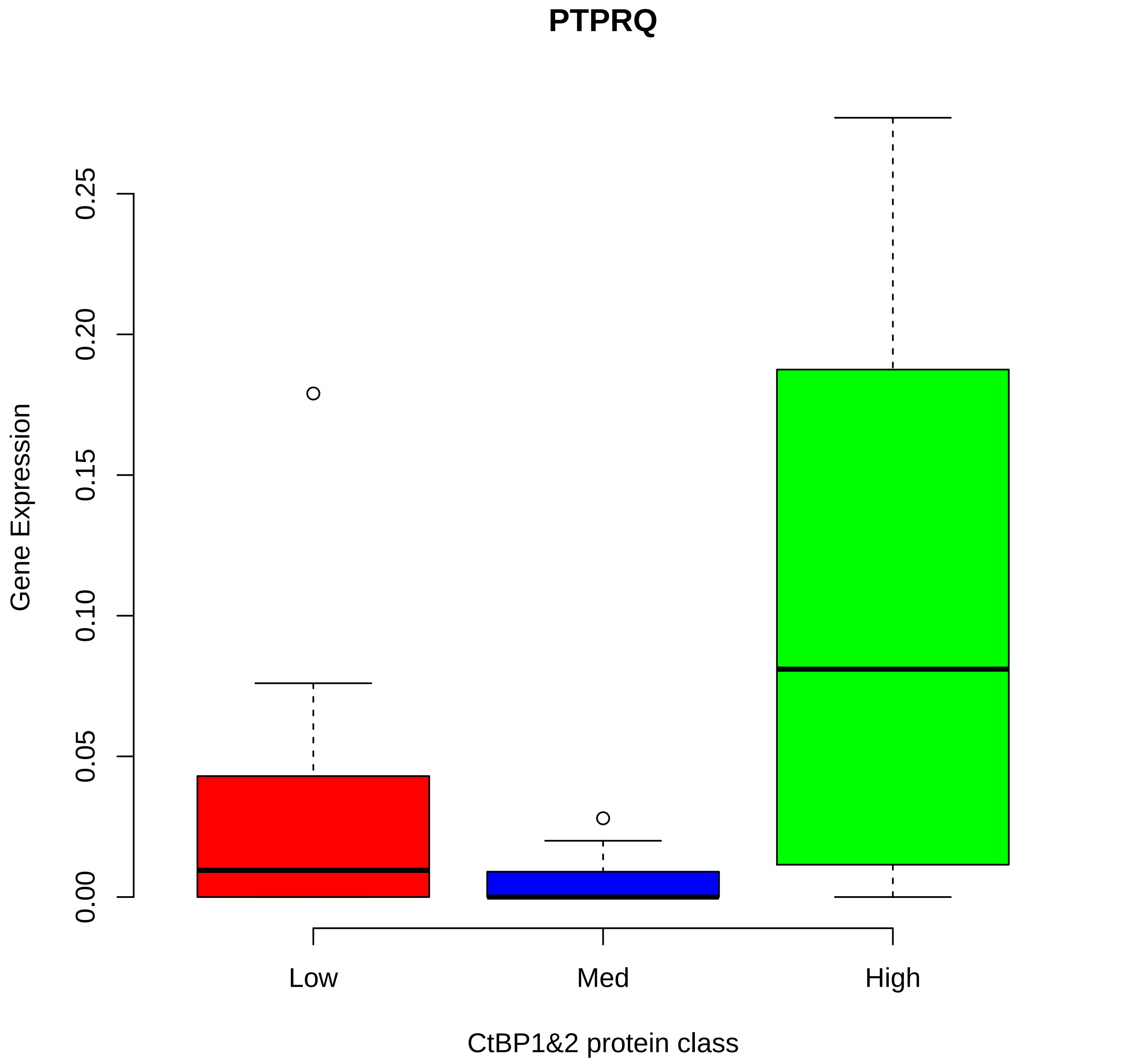
PTPRQ
Gene Expression
0.00
0.05
0.10
0.15
0.20
0.25
Low
Med
High
CtBP1&2 protein class

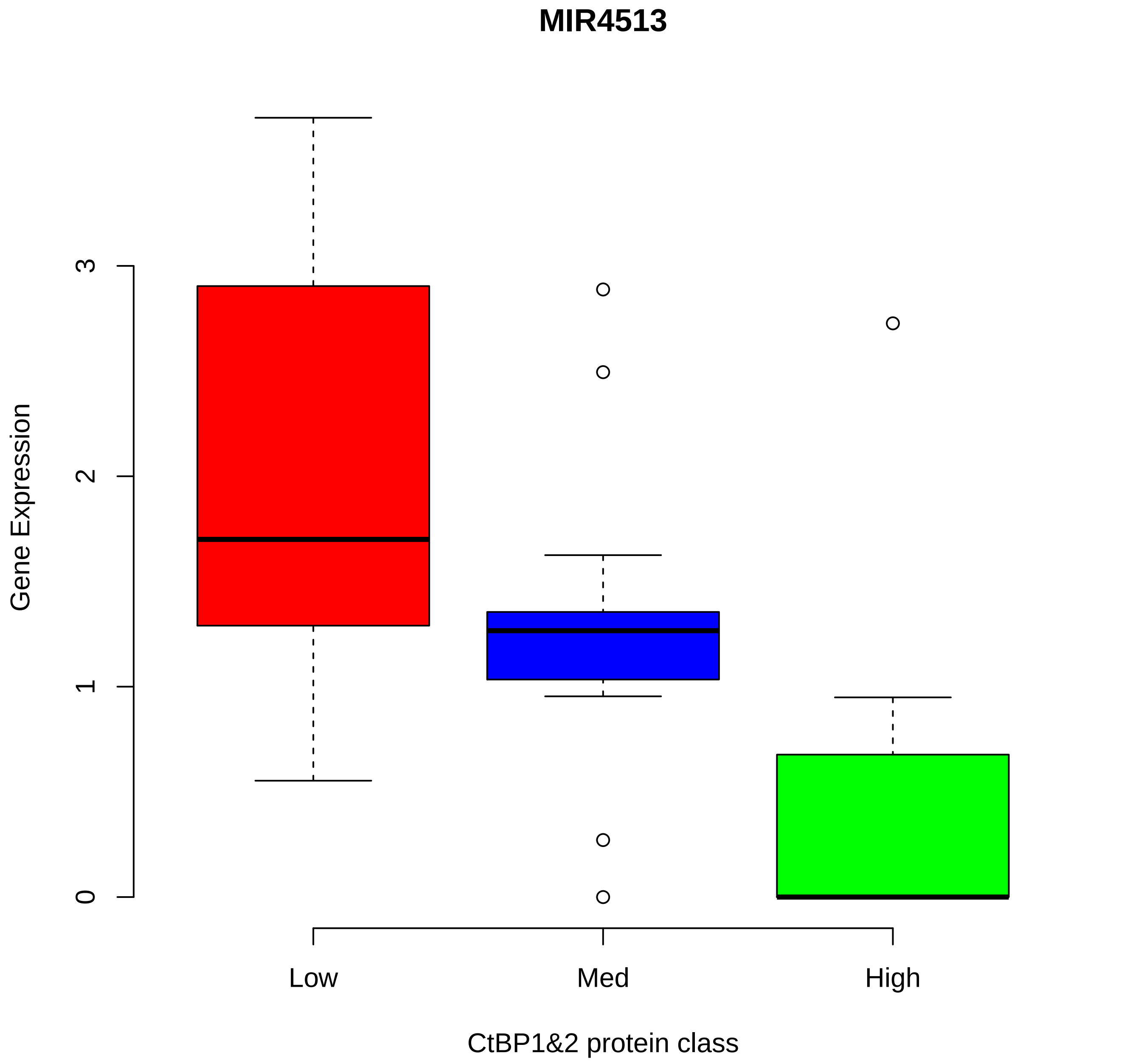
MIR4513
Gene Expression
0
1
2
3
Low
Med
High
CtBP1&2 protein class

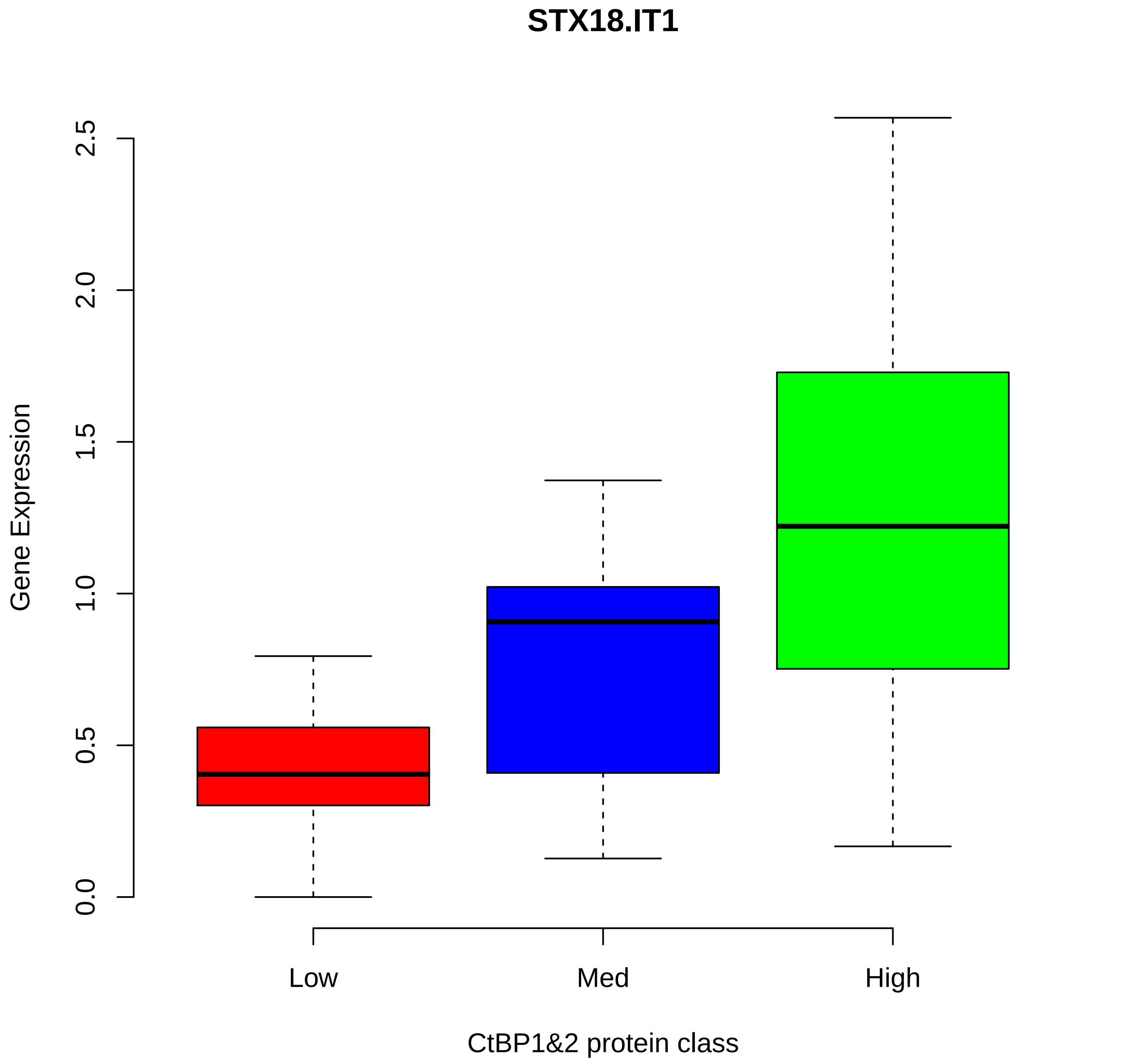

STX18.IT1
Gene Expression
0.0
0.5
1.0
1.5
2.0
2.5
Low
Med
High
CtBP1&2 protein class

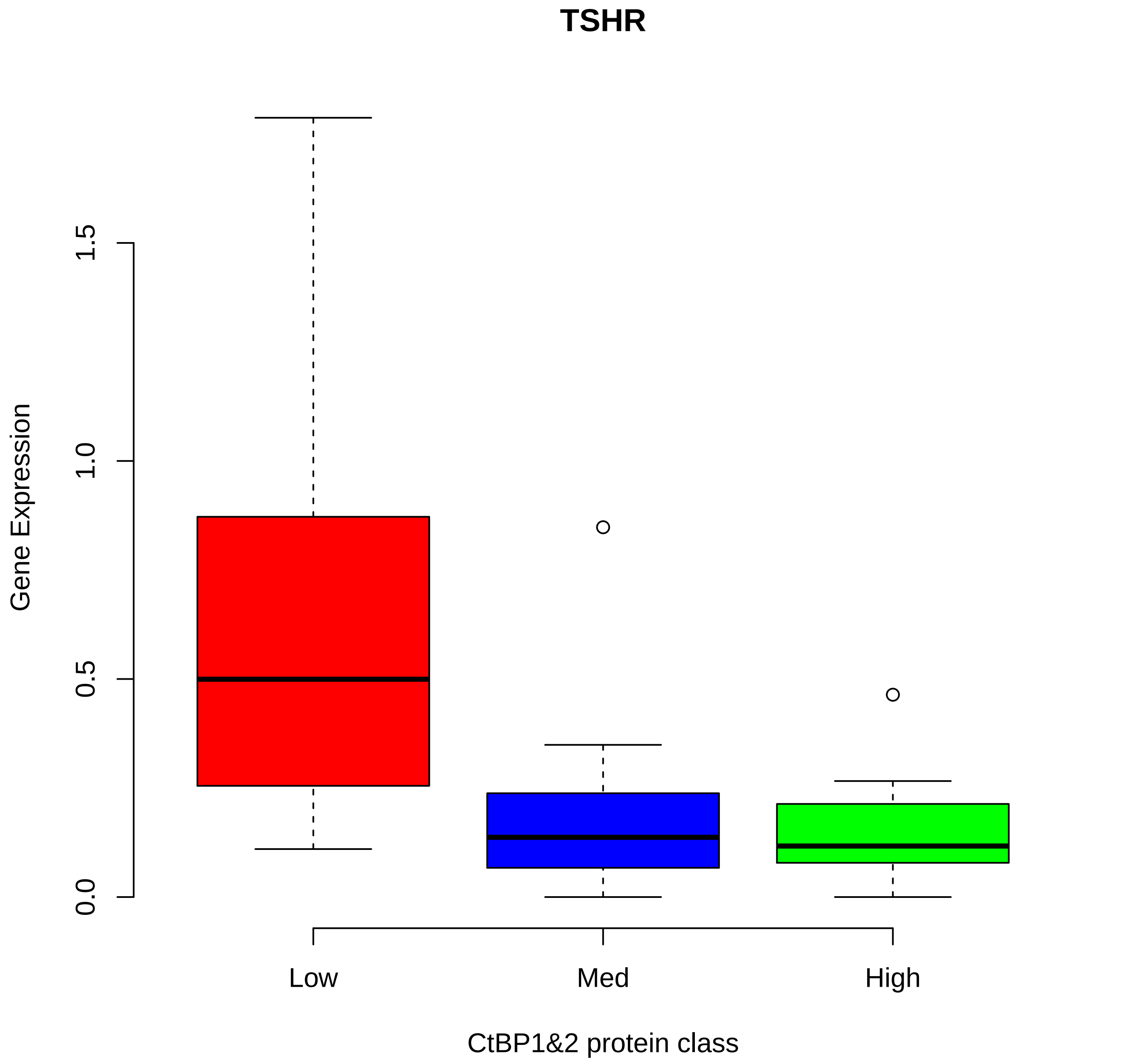
TSHR
Gene Expression
0.0
0.5
1.0
1.5
Low
Med
High
CtBP1&2 protein class

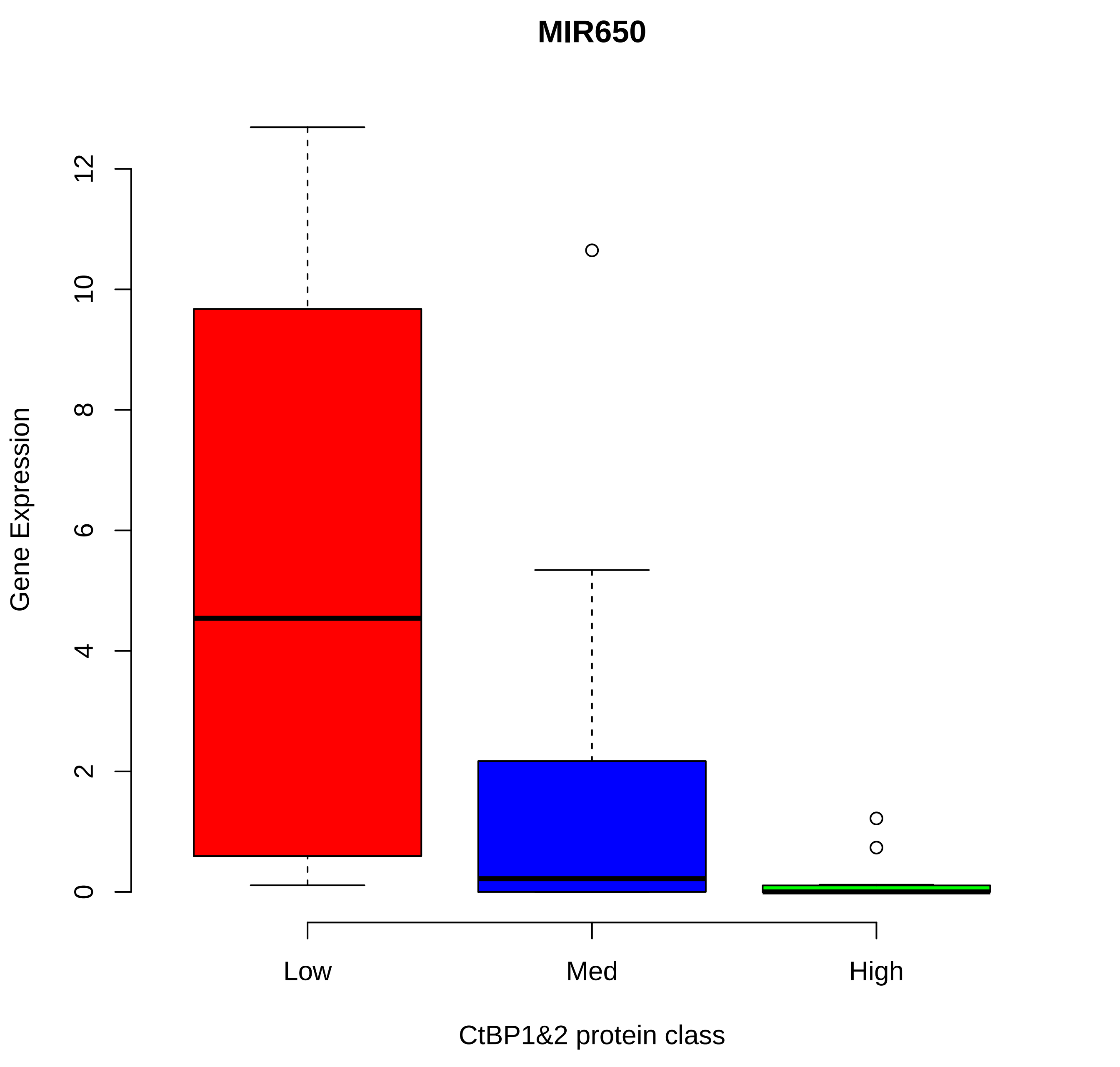
MIR650
Gene Expression
0
2
4
6
8
10
12
Low
Med
High
CtBP1&2 protein class

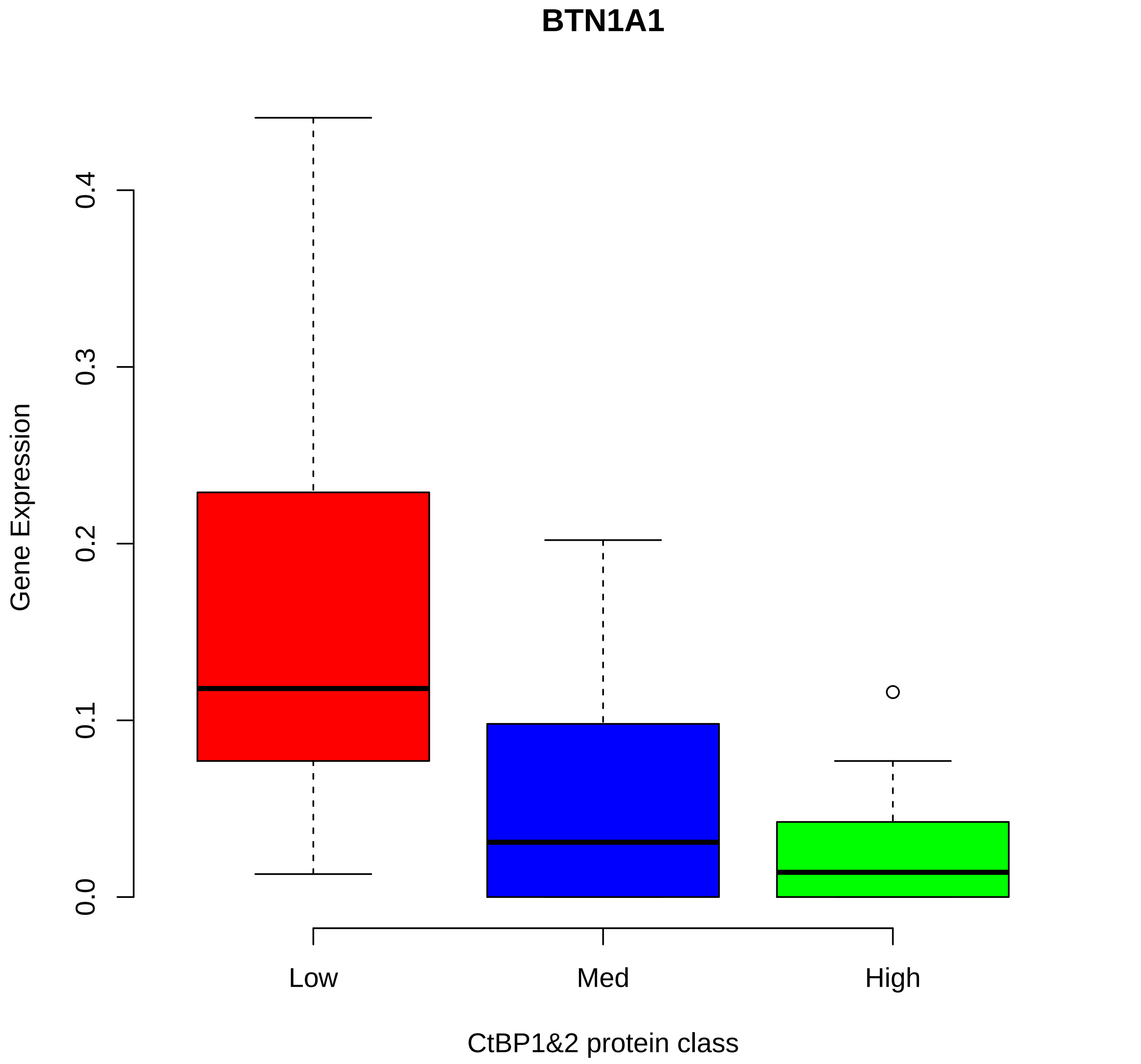

BTN1A1
Gene Expression
0.0
0.1
0.2
0.3
0.4
Low
Med
High
CtBP1&2 protein class

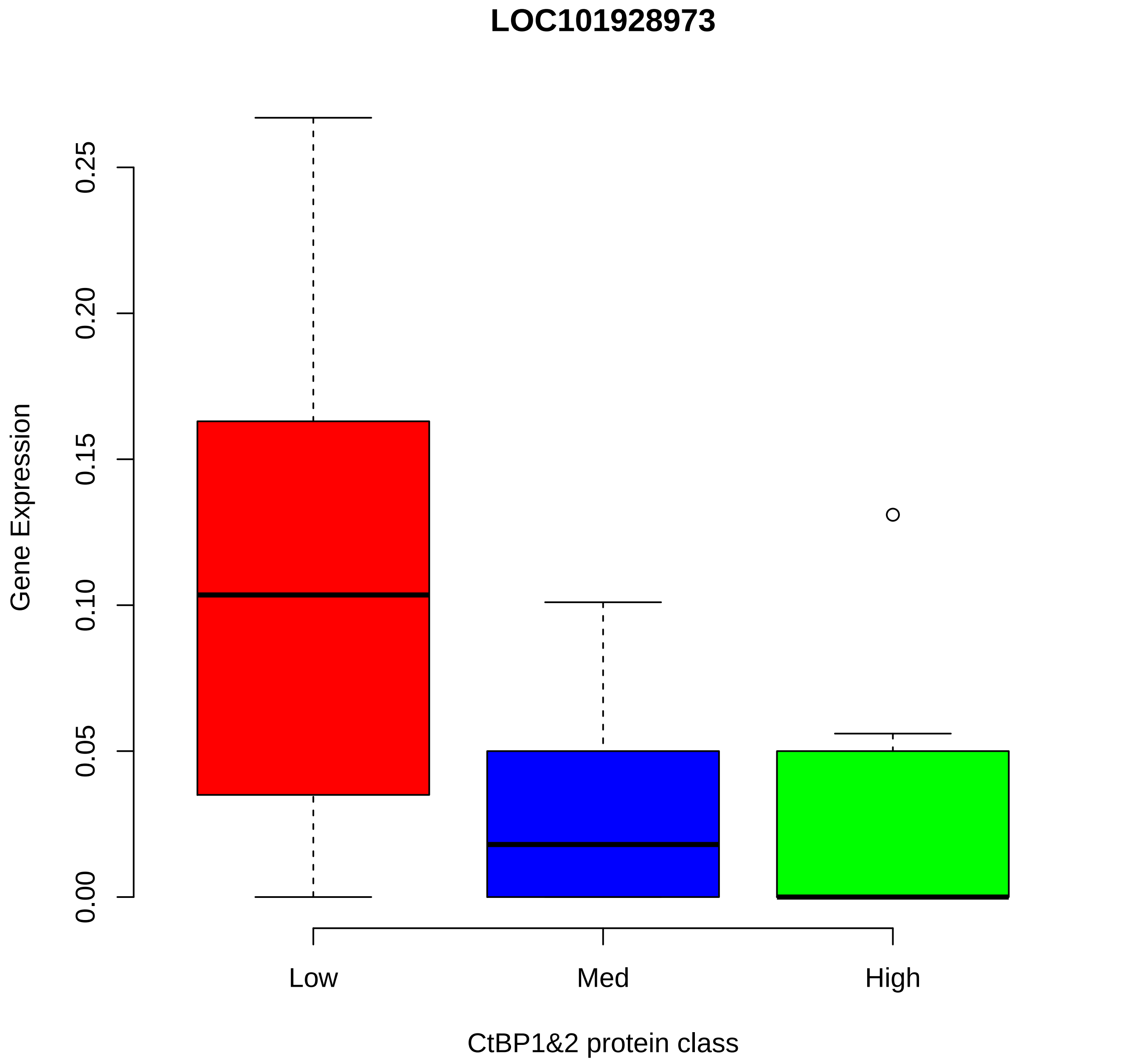
LOC101928973
Gene Expression
0.00
0.05
0.10
0.15
0.20
0.25
Low
Med
High
CtBP1&2 protein class

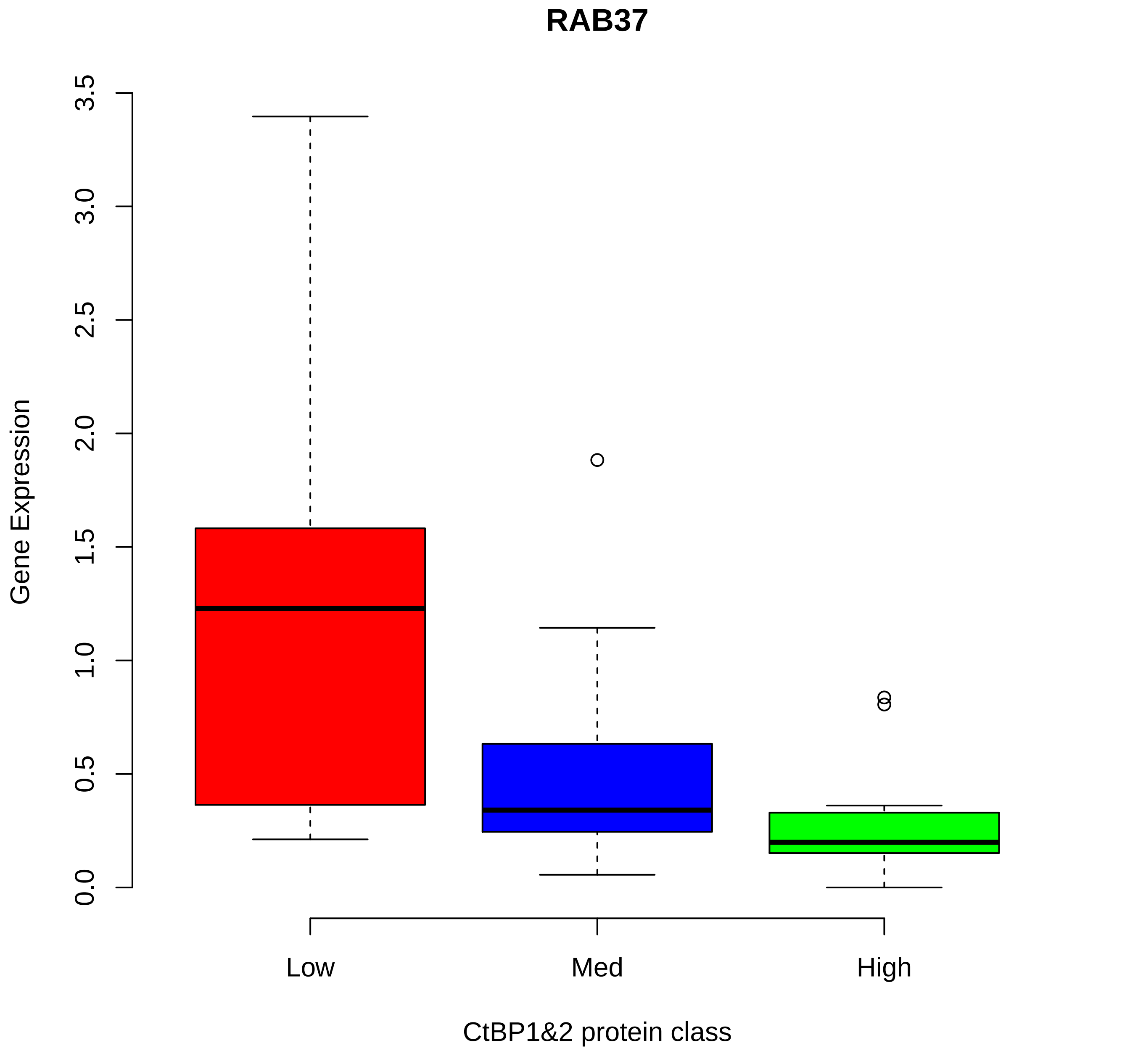
RAB37
Gene Expression
0.0
0.5
1.0
1.5
2.0
2.5
3.0
3.5
Low
Med
High
CtBP1&2 protein class

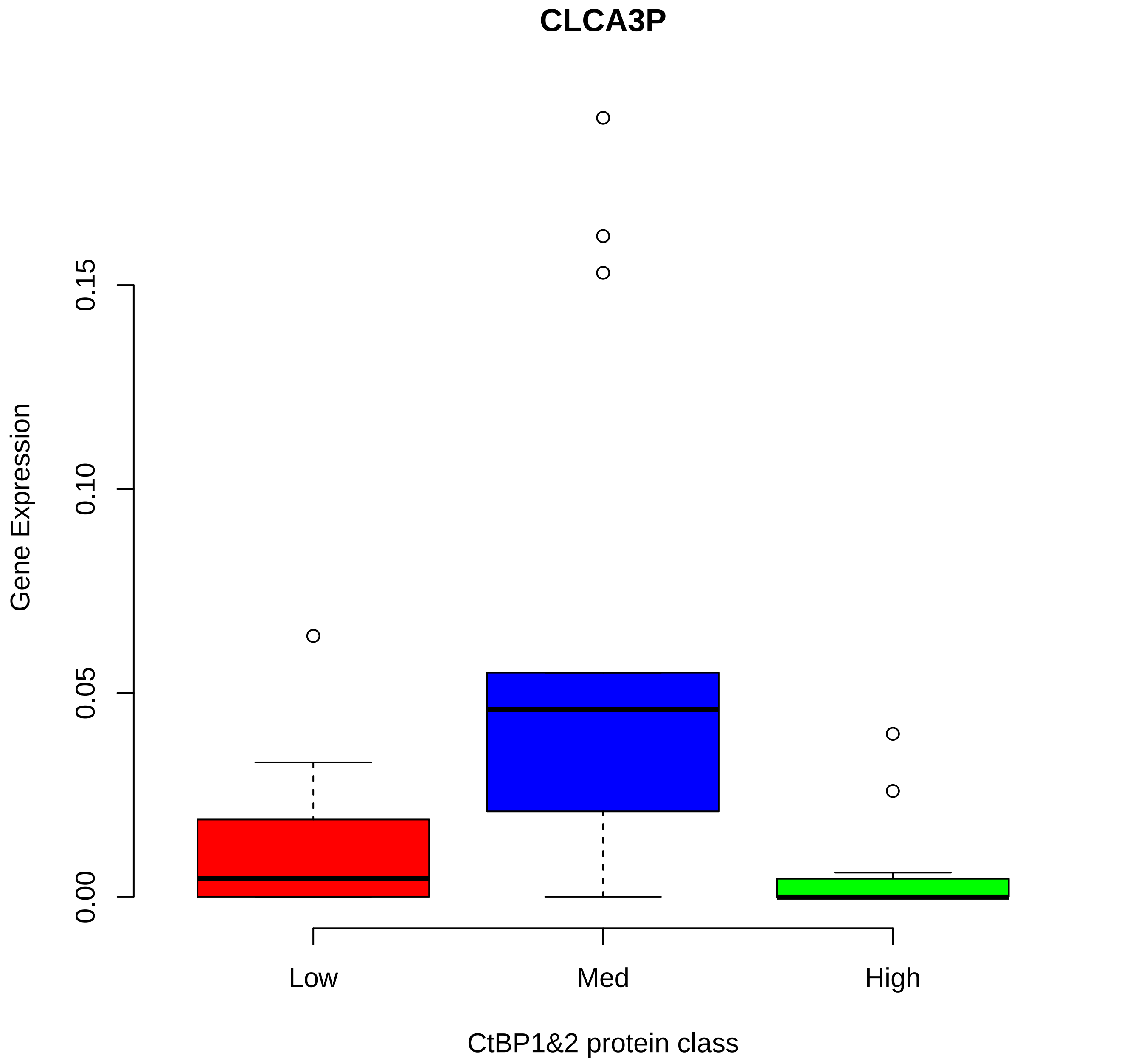

CLCA3P
Gene Expression
0.00
0.05
0.10
0.15
Low
Med
High
CtBP1&2 protein class

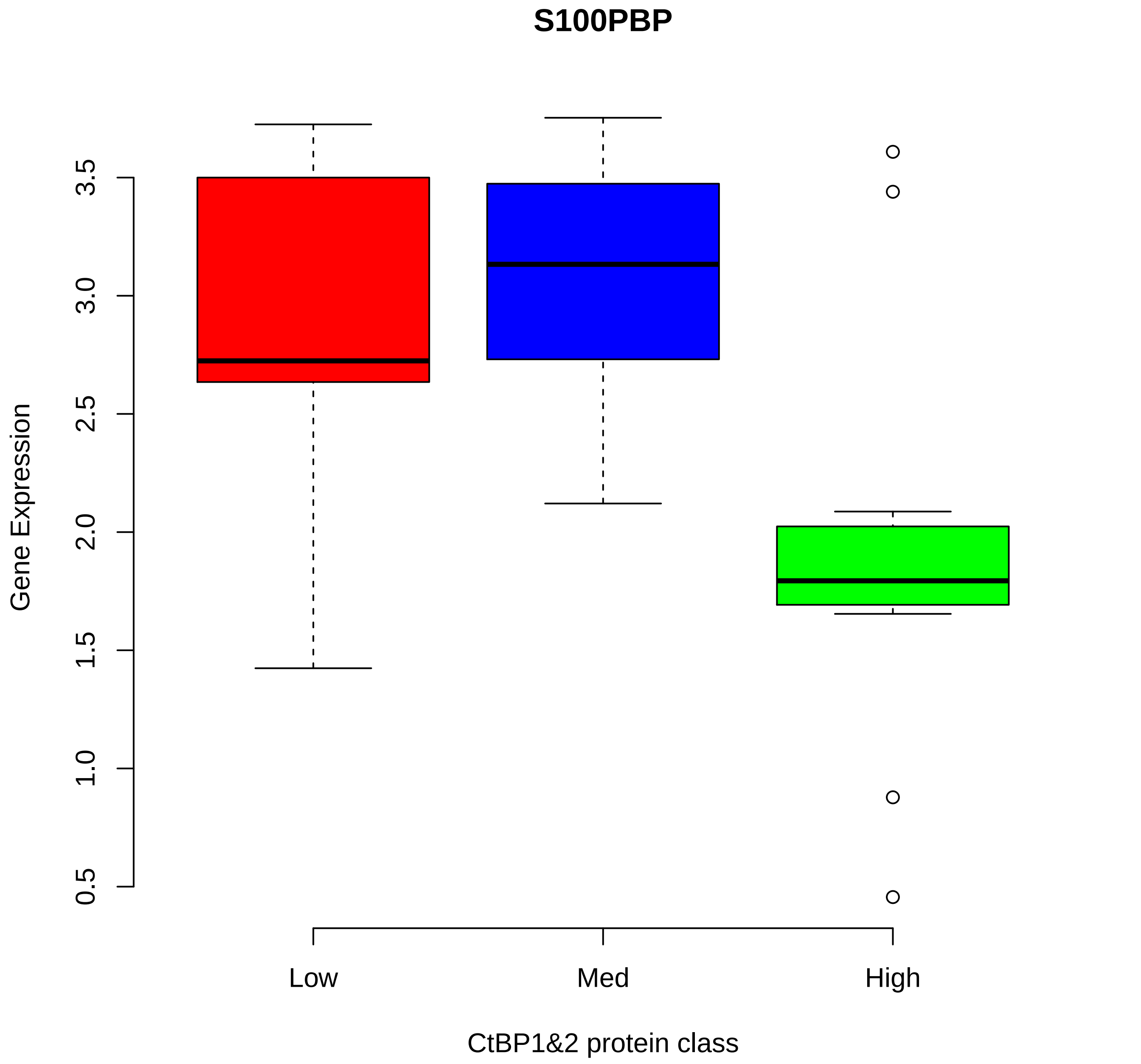
S100PBP
Gene Expression
0.5
1.0
1.5
2.0
2.5
3.0
3.5
Low
Med
High
CtBP1&2 protein class

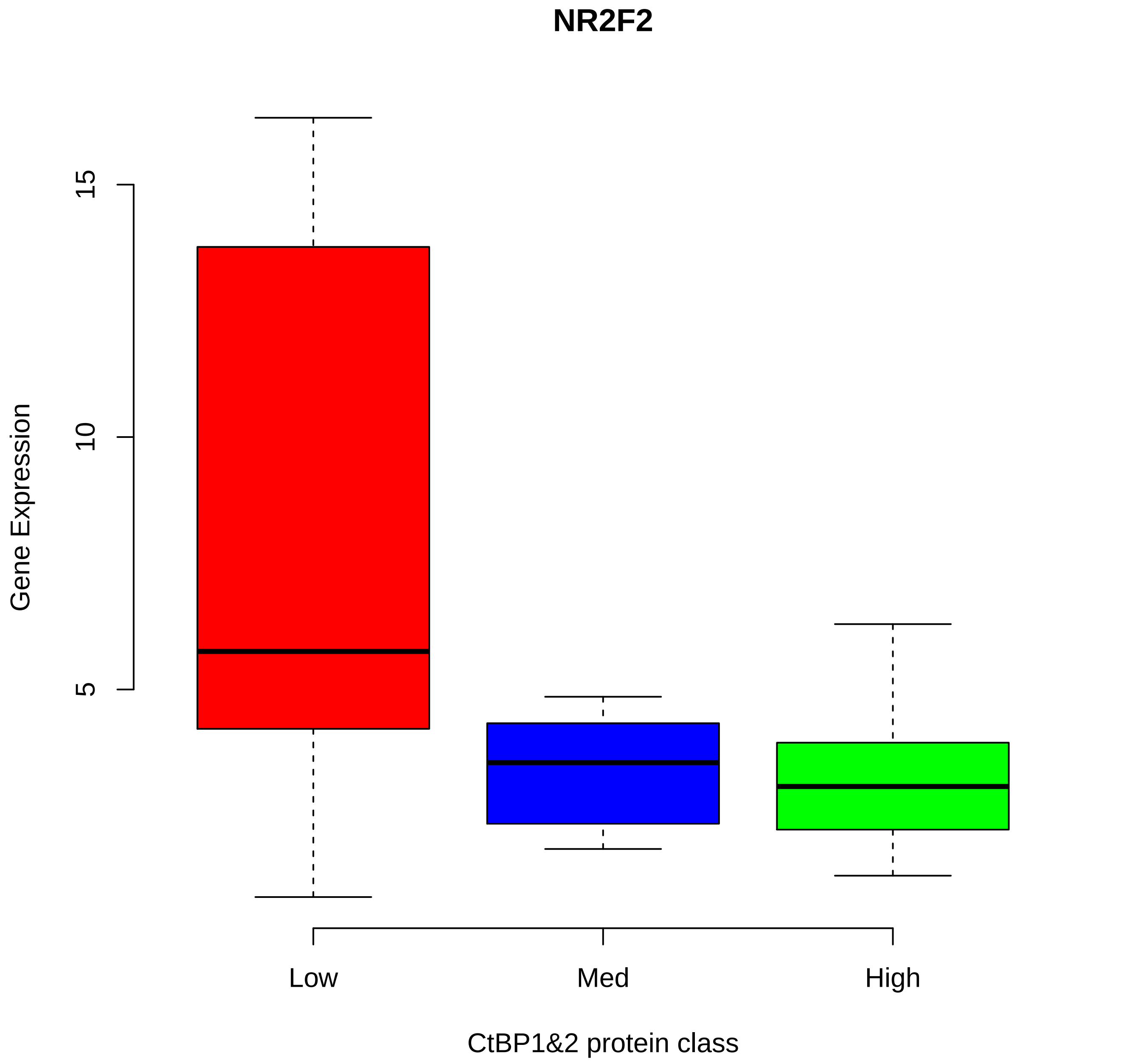
NR2F2
Gene Expression
15
10
5
Low
Med
High
CtBP1&2 protein class

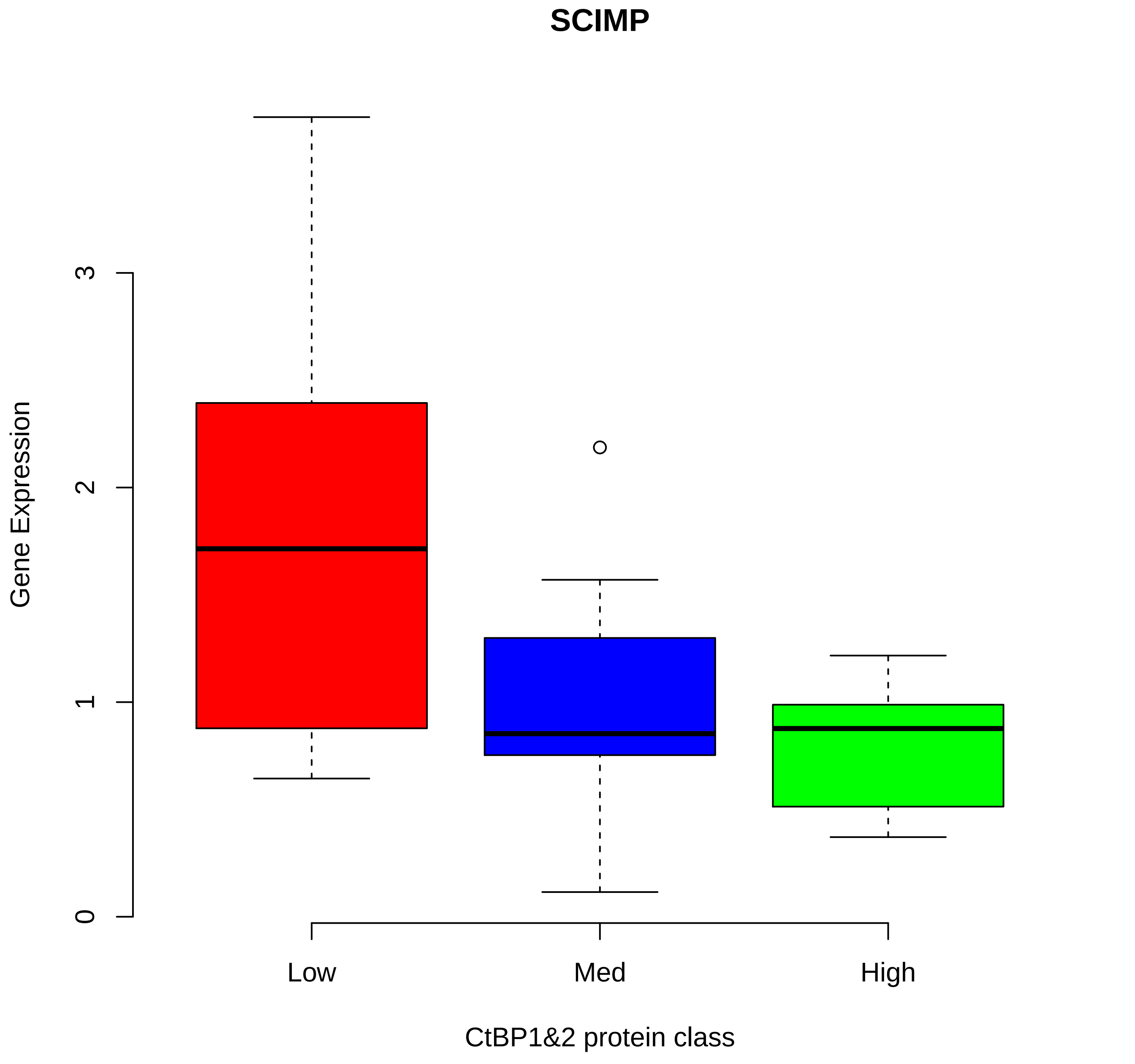
SCIMP
Gene Expression
0
1
2
3
Low
Med
High
CtBP1&2 protein class

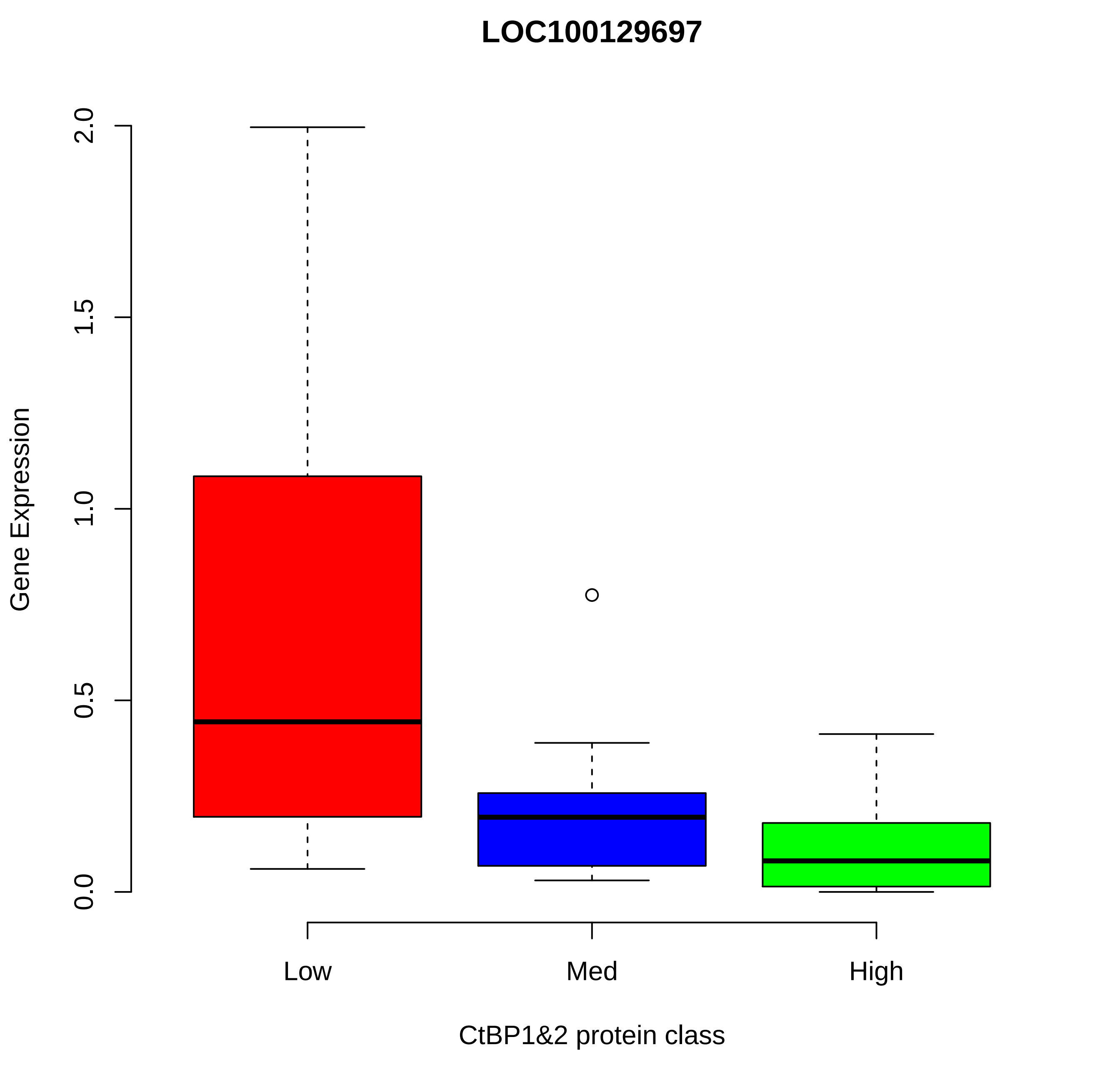

LOC100129697
Gene Expression
0.0
0.5
1.0
1.5
2.0
Low
Med
High
CtBP1&2 protein class

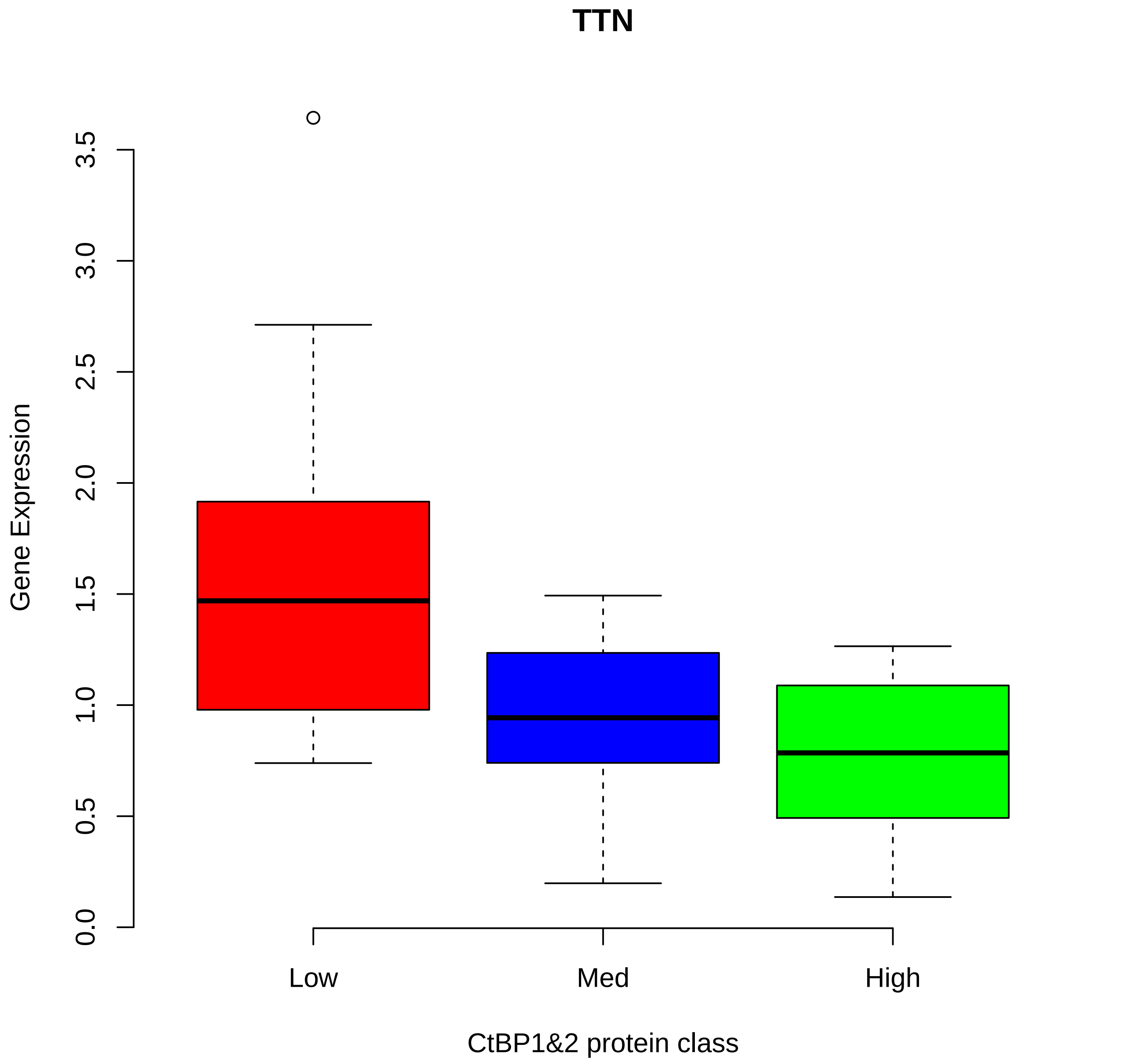
TTN
Gene Expression
0.0
0.5
1.0
1.5
2.0
2.5
3.0
3.5
Low
Med
High
CtBP1&2 protein class

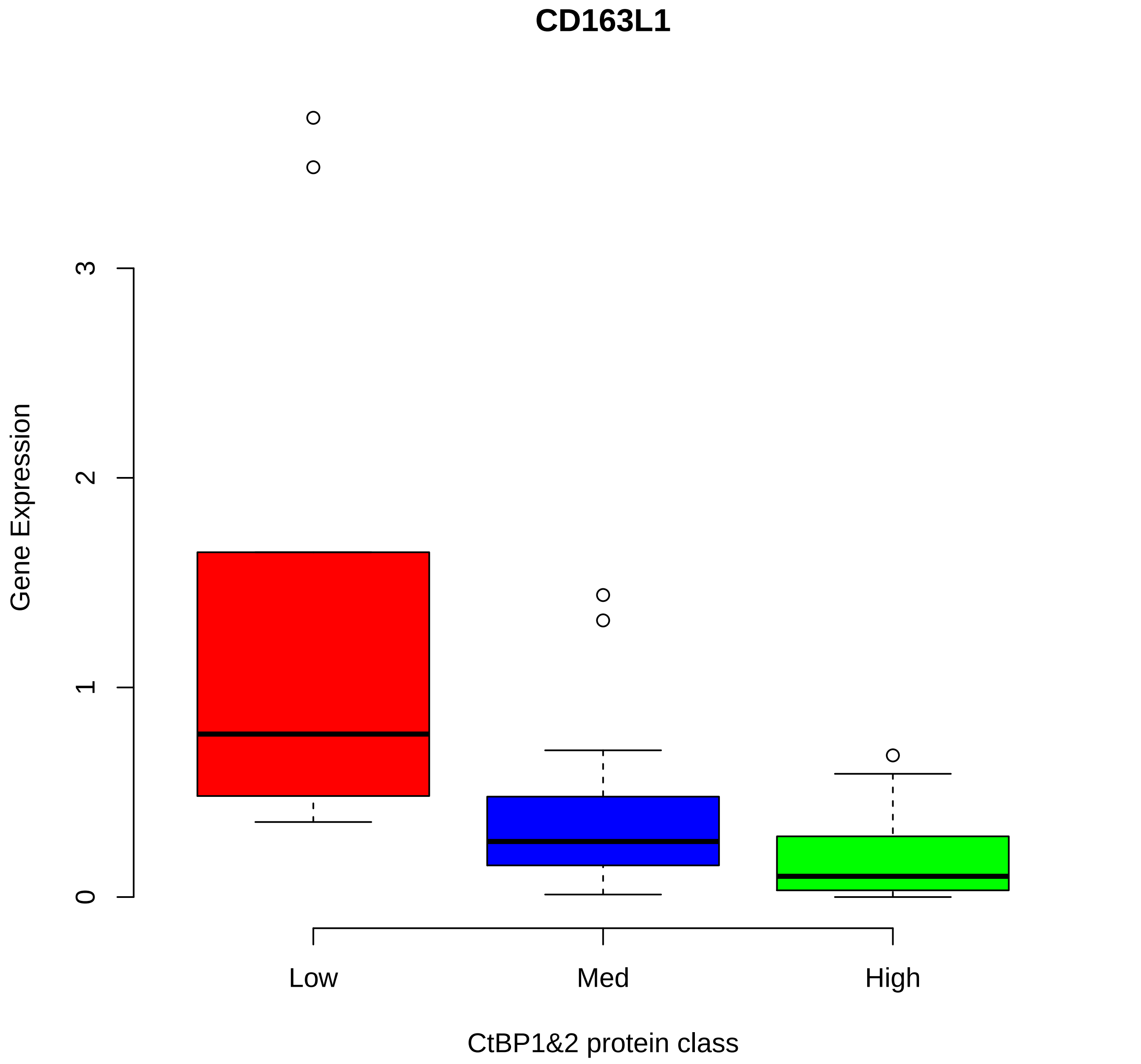
CD163L1
Gene Expression
0
1
2
3
Low
Med
High
CtBP1&2 protein class

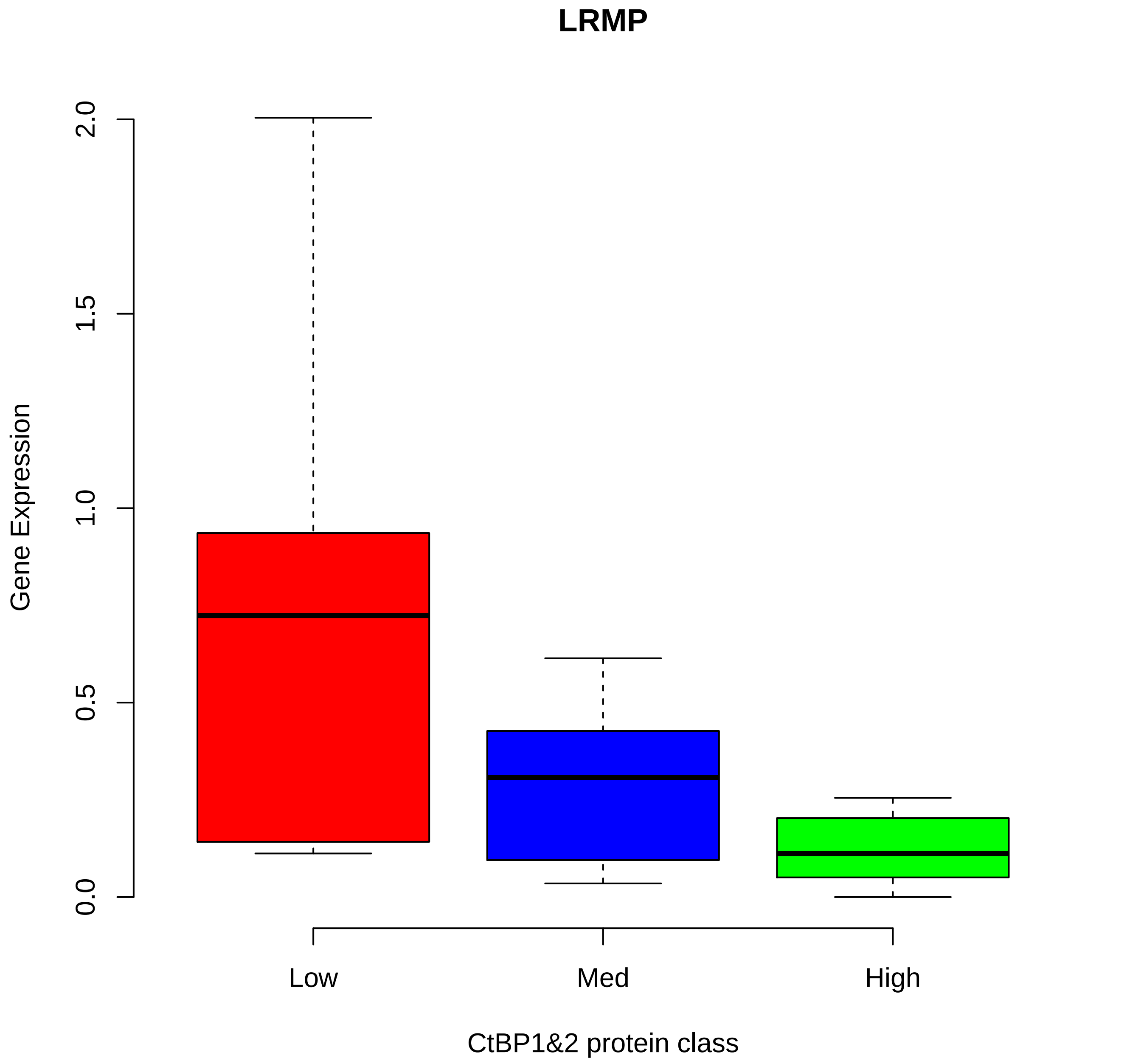

LRMP
Gene Expression
0.0
0.5
1.0
1.5
2.0
Low
Med
High
CtBP1&2 protein class

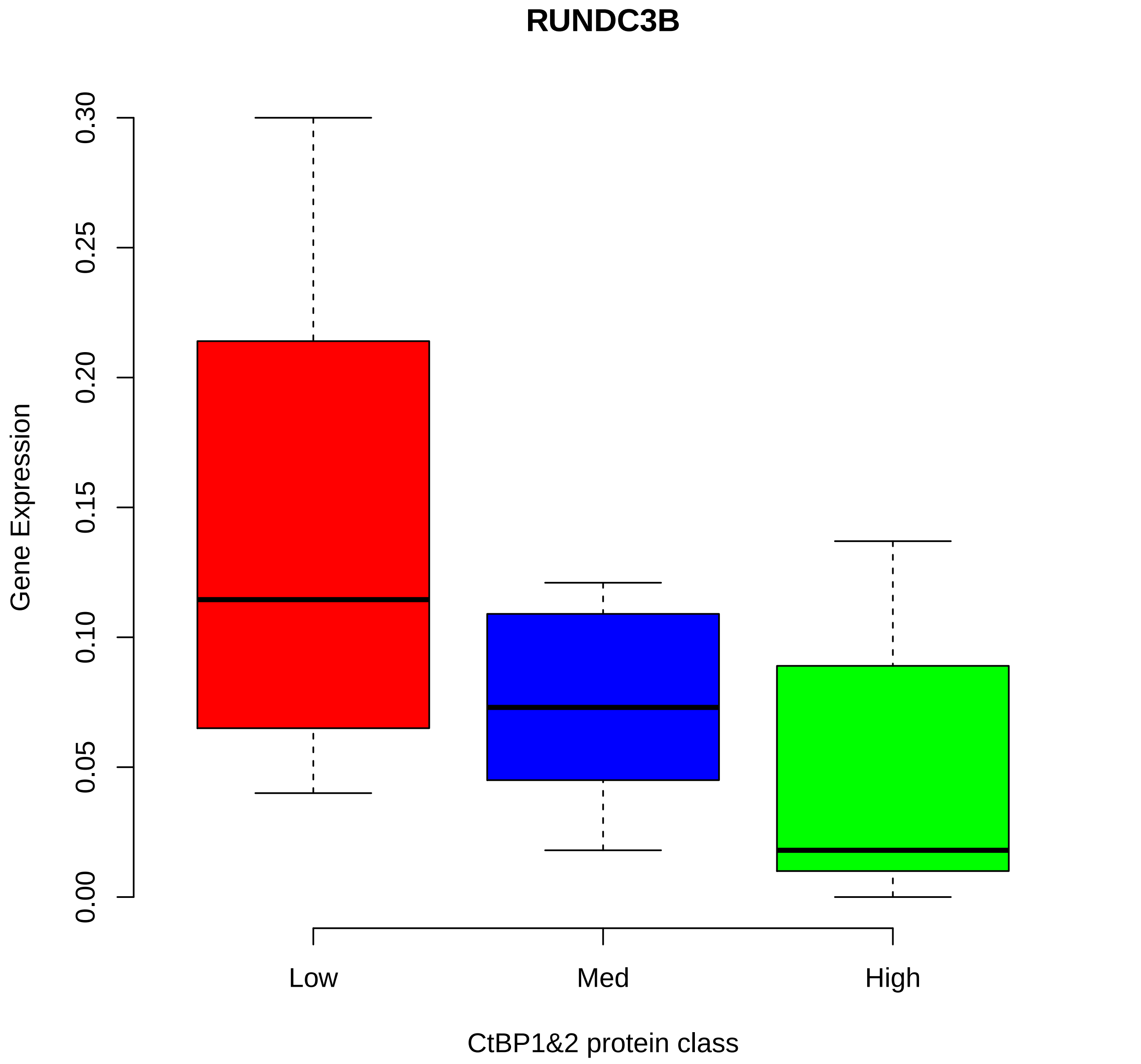
RUNDC3B
Gene Expression
0.00
0.05
0.10
0.15
0.20
0.25
0.30
Low
Med
High
CtBP1&2 protein class

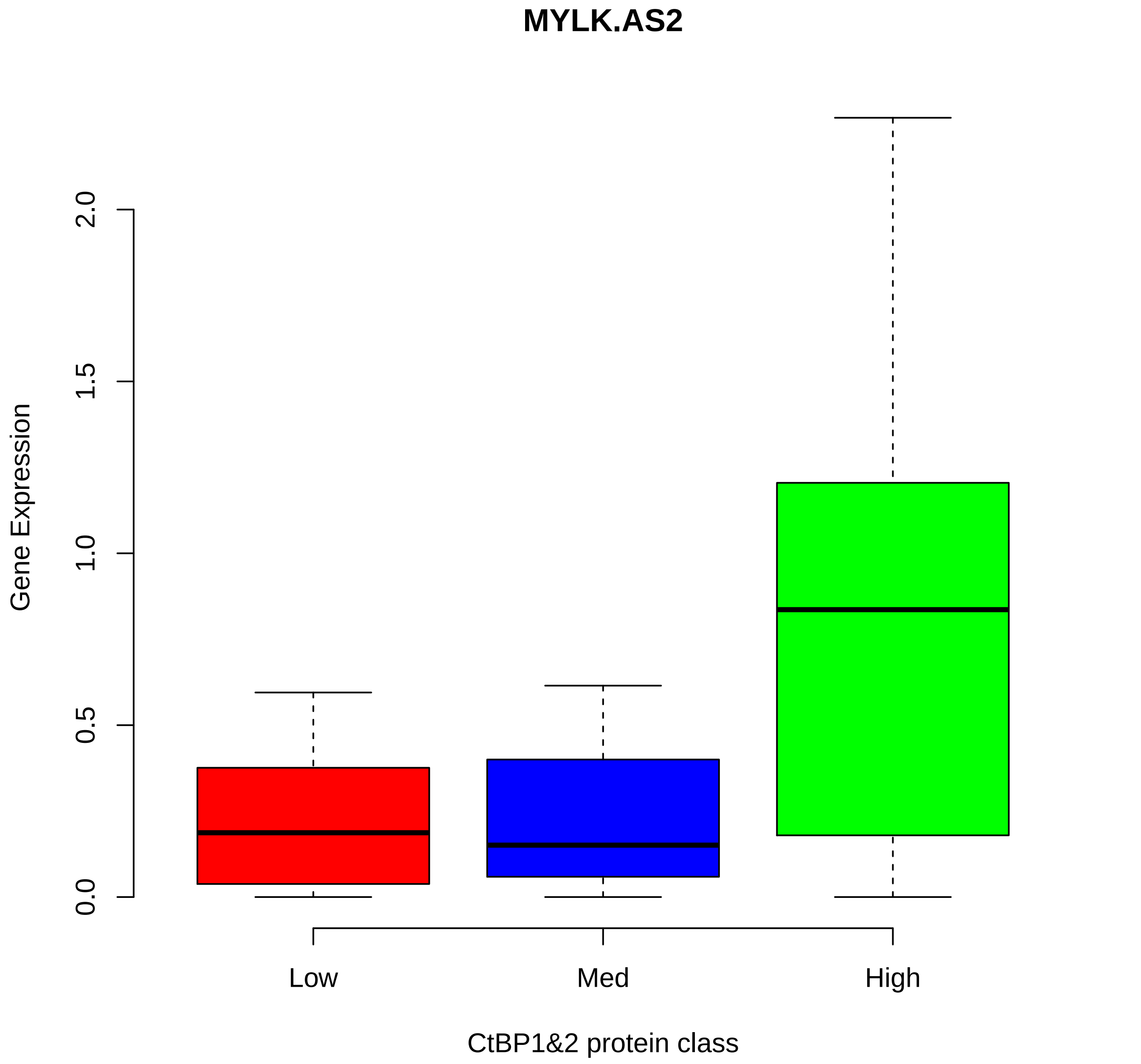

MYLK.AS2
Gene Expression
0.0
0.5
1.0
1.5
2.0
Low
Med
High
CtBP1&2 protein class

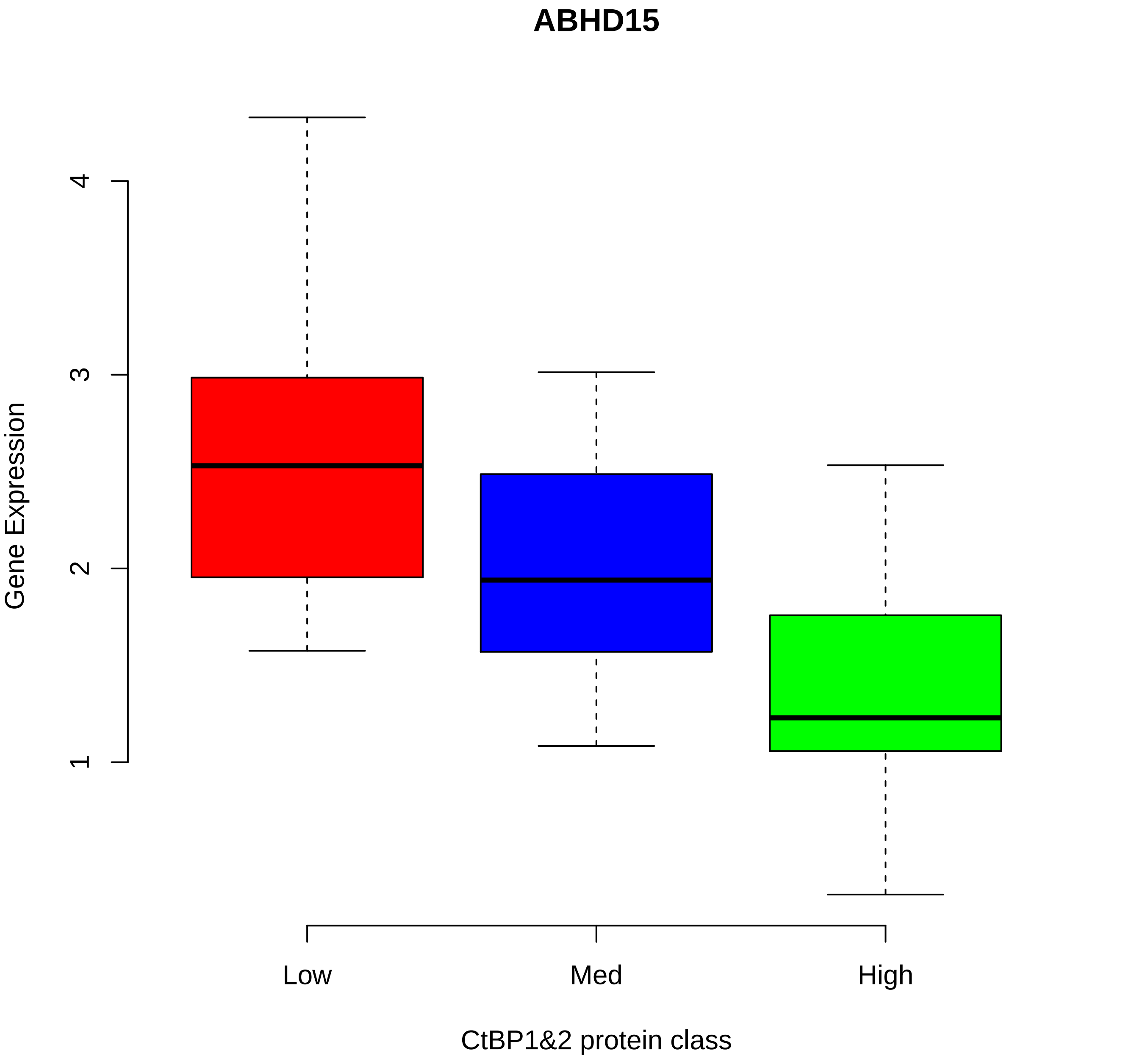
ABHD15
Gene Expression
4
3
2
1
Low
Med
High
CtBP1&2 protein class

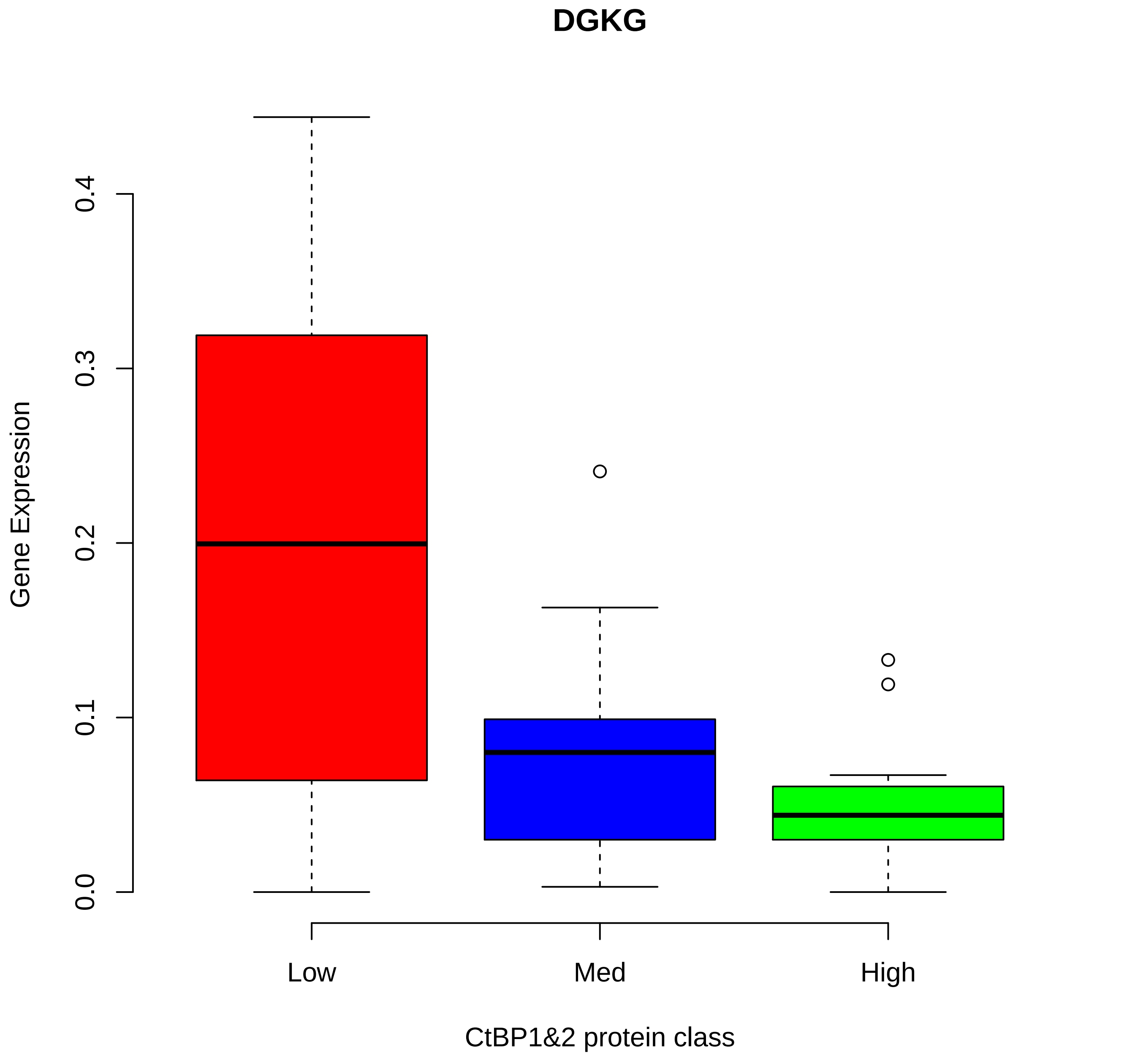
DGKG
Gene Expression
0.0
0.1
0.2
0.3
0.4
Low
Med
High
CtBP1&2 protein class

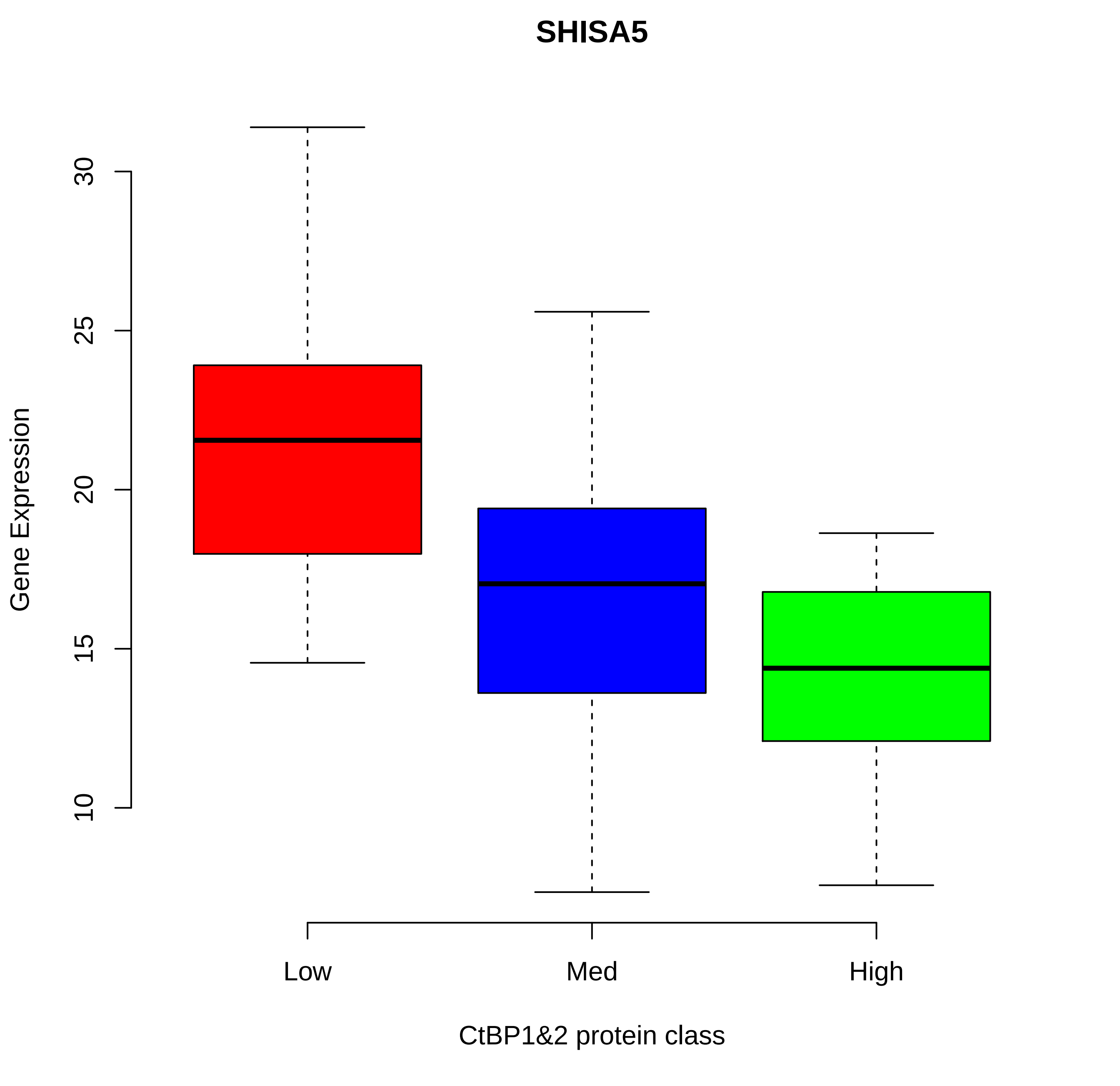
SHISA5
Gene Expression
30
25
20
15
10
Low
Med
High
CtBP1&2 protein class

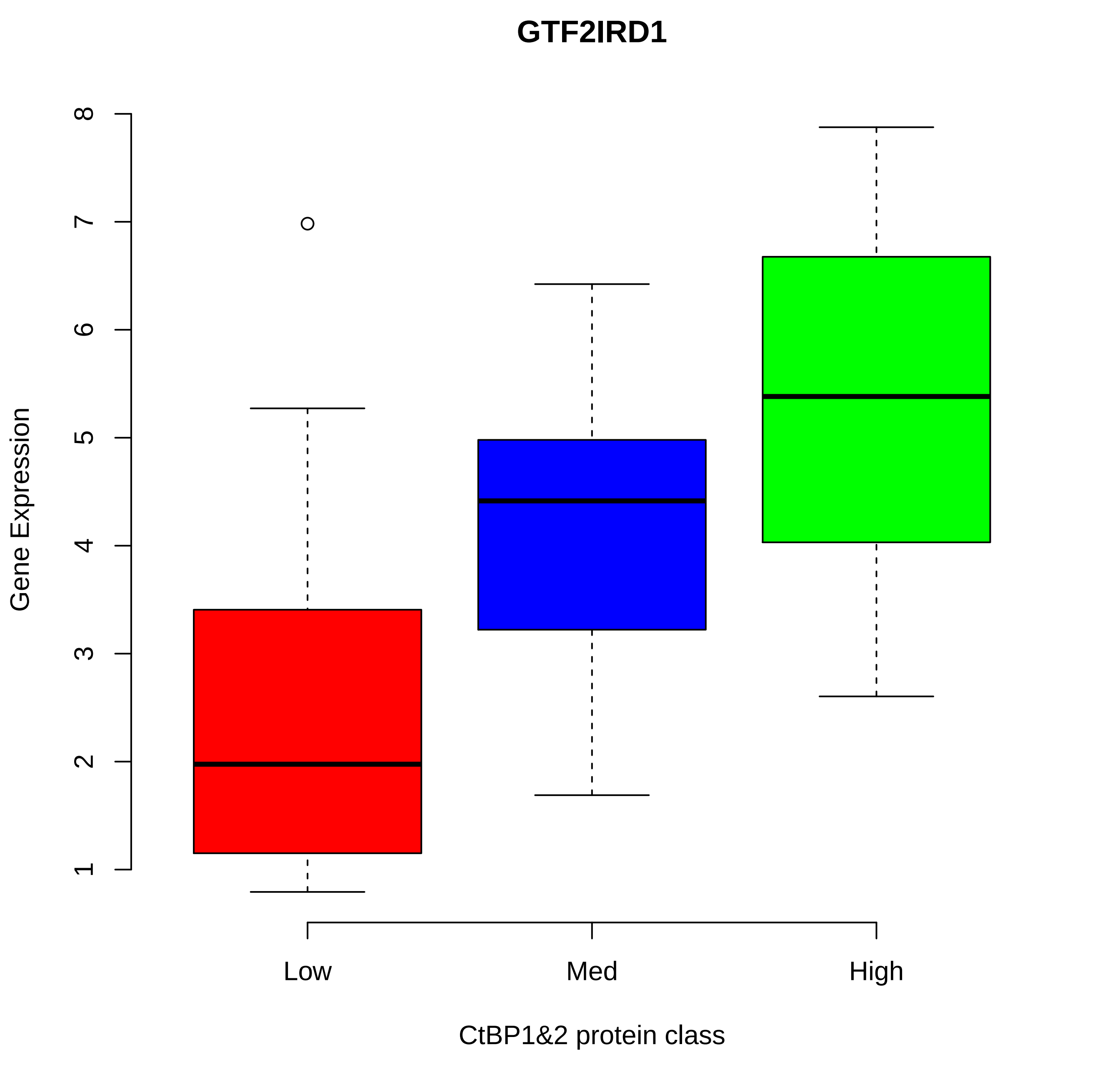

GTF2IRD1
Gene Expression
1
2
3
4
5
6
7
8
Low
Med
High
CtBP1&2 protein class

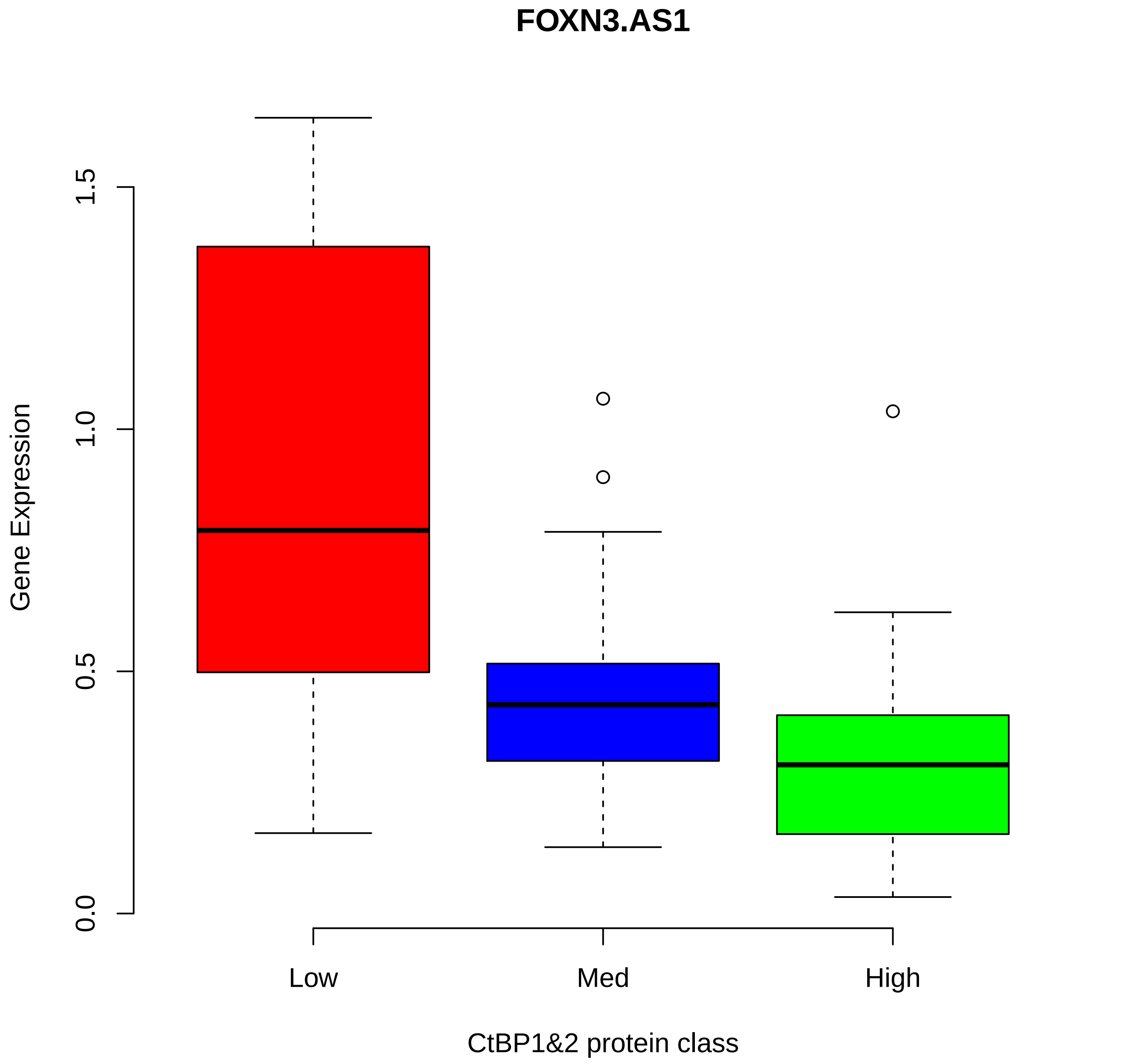
FOXN3.AS1
Gene Expression
0.0
0.5
1.0
1.5
Low
Med
High
CtBP1&2 protein class

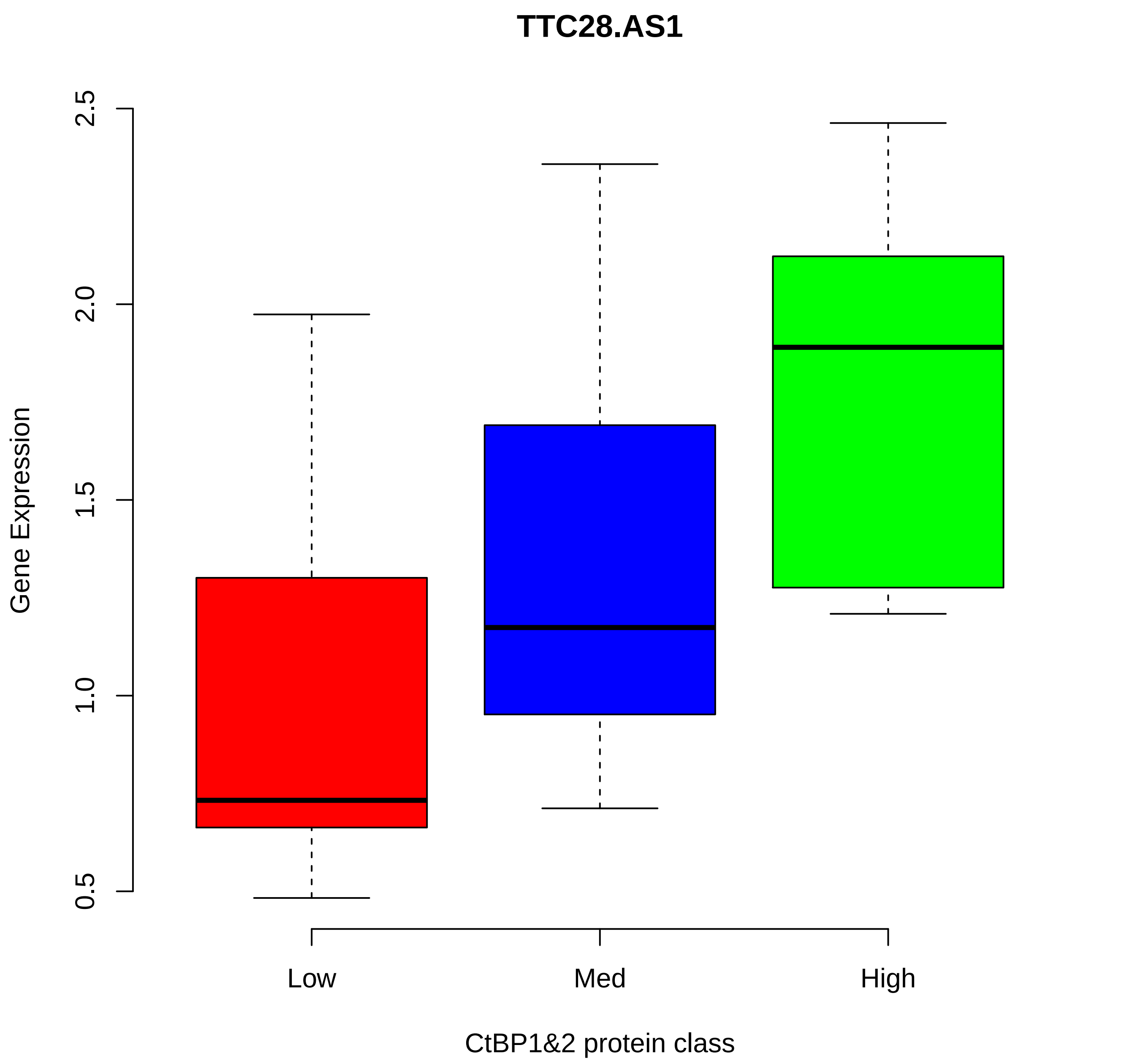

TTC28.AS1
Gene Expression
2.5
2.0
1.5
1.0
0.5
Low
Med
High
CtBP1&2 protein class

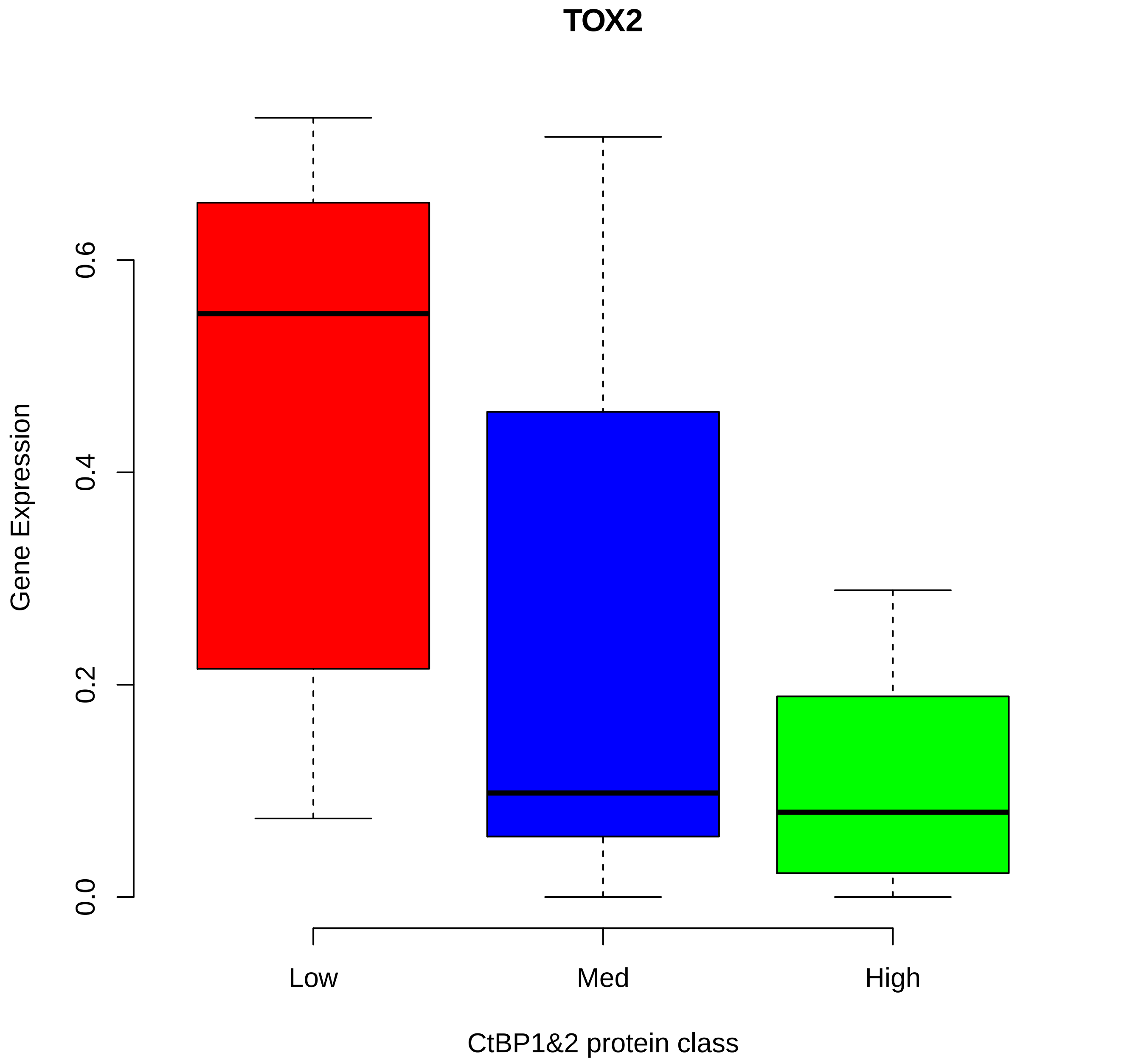
TOX2
Gene Expression
0.0
0.2
0.4
0.6
Low
Med
High
CtBP1&2 protein class

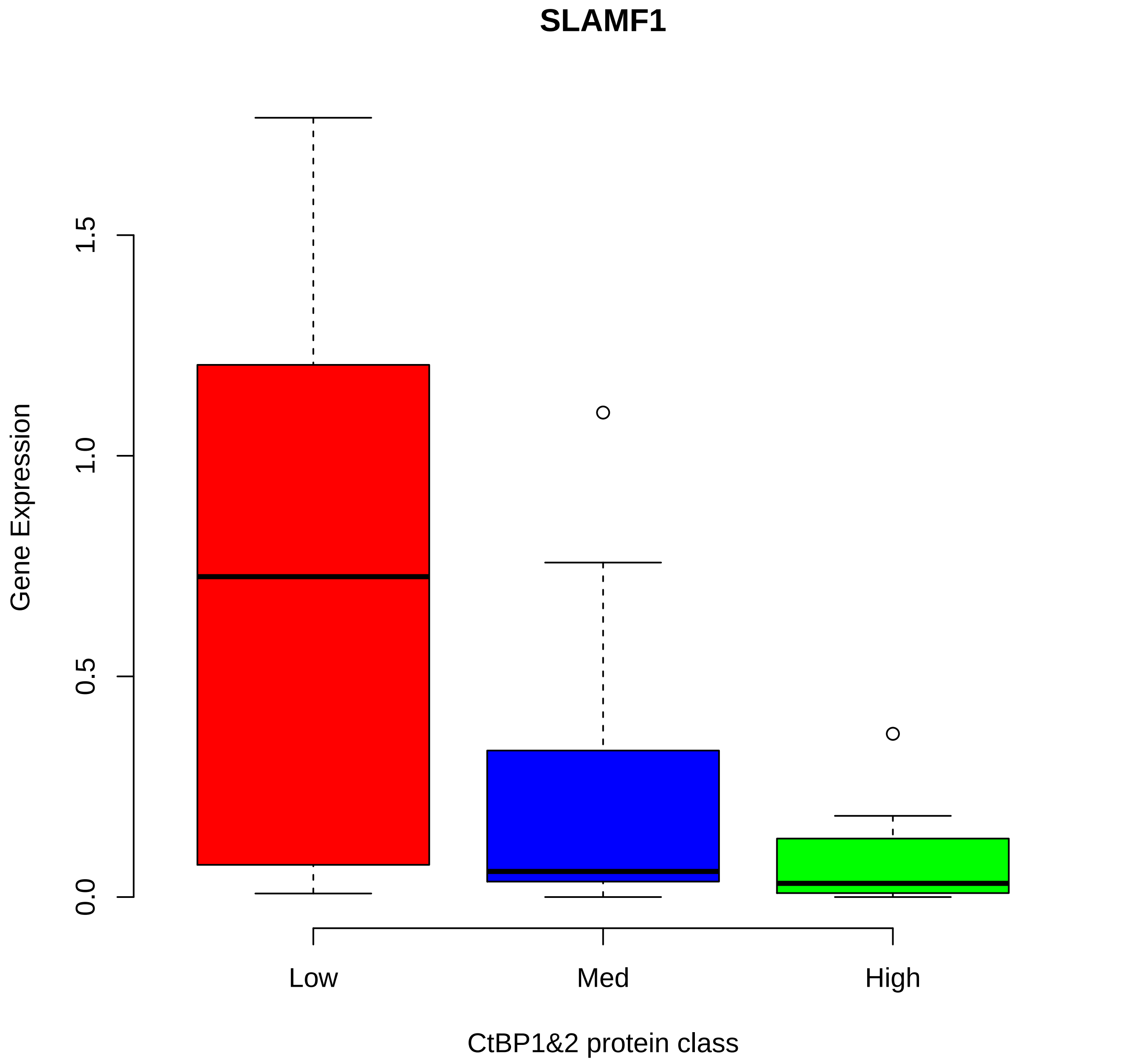

SLAMF1
Gene Expression
0.0
0.5
1.0
1.5
Low
Med
High
CtBP1&2 protein class

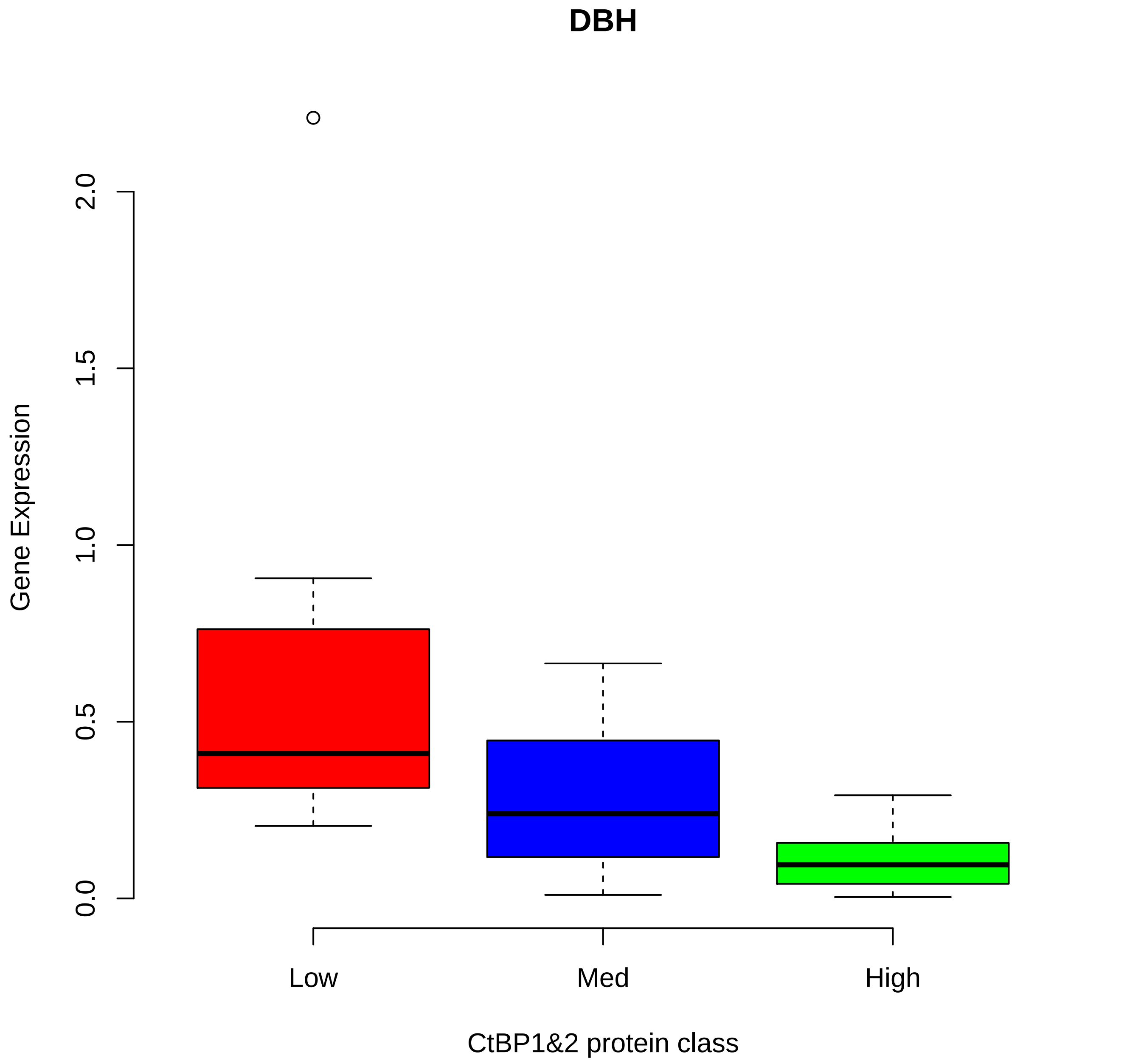
DBH
Gene Expression
0.0
0.5
1.0
1.5
2.0
Low
Med
High
CtBP1&2 protein class

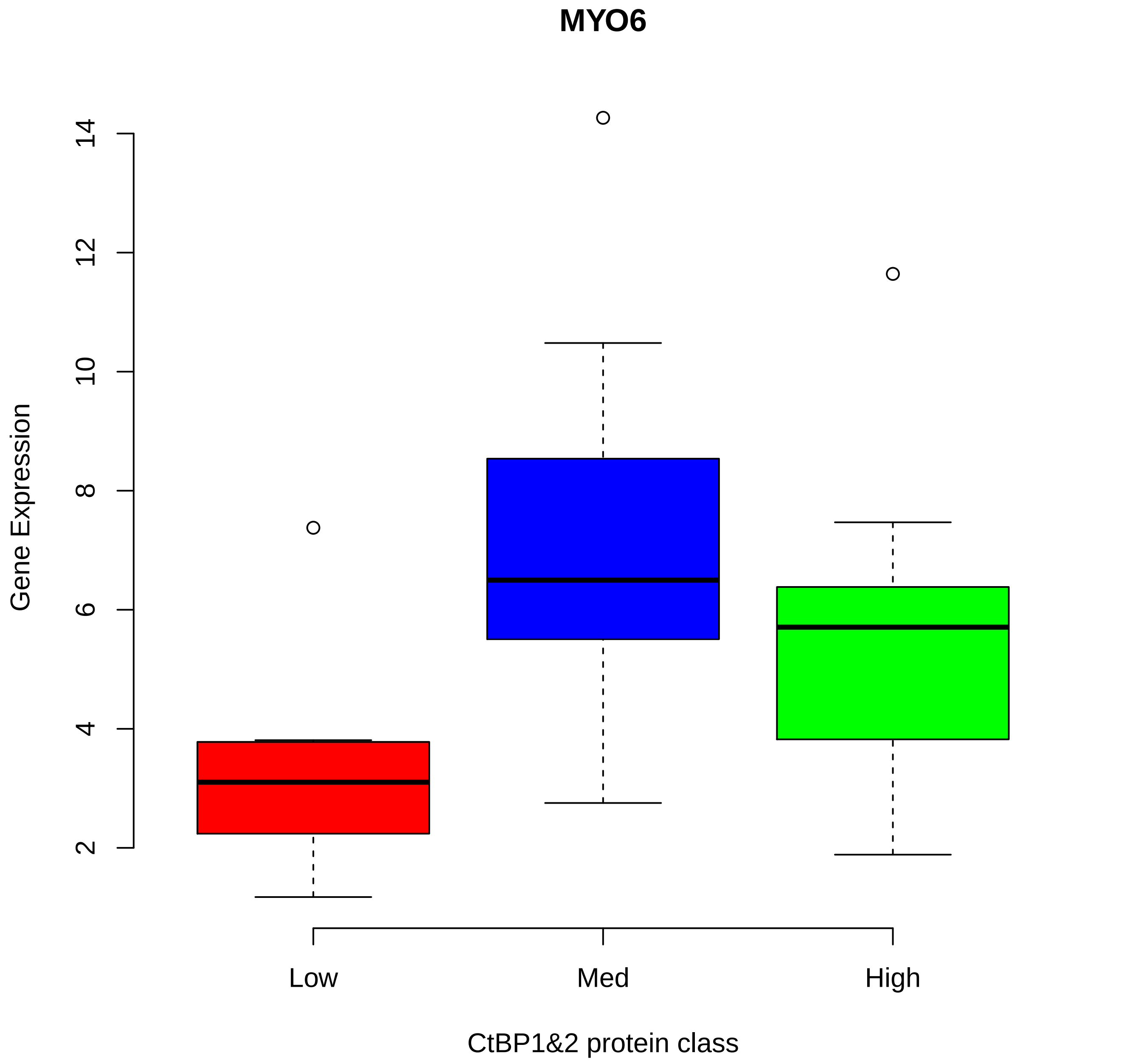
MYO6
14
12
10
8
6
4
2
Gene Expression
Low
Med
High
CtBP1&2 protein class

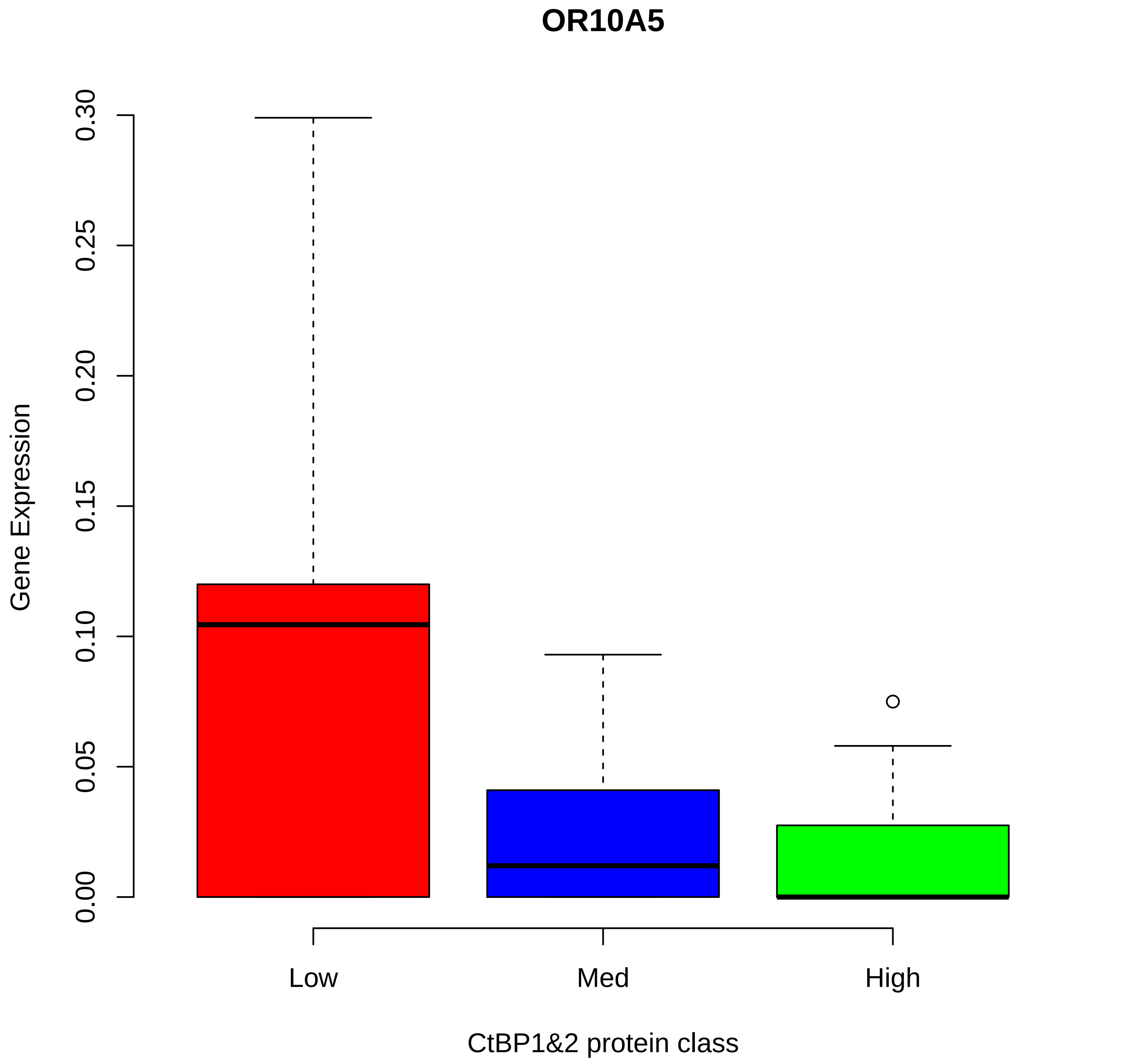

OR10A5
Gene Expression
0.00
0.05
0.10
0.15
0.20
0.25
0.30
Low
Med
High
CtBP1&2 protein class

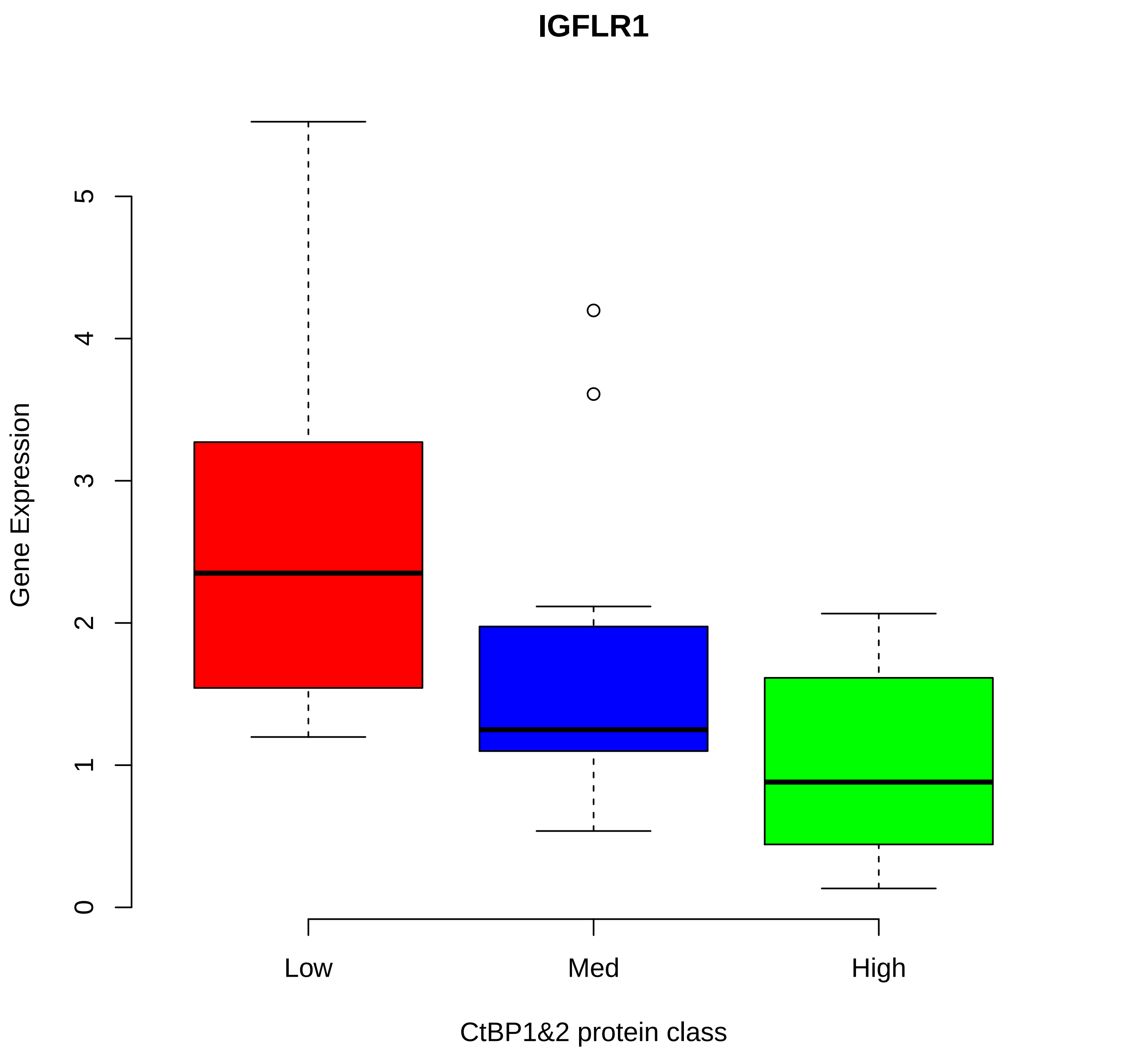
IGFLR1
Gene Expression
0
1
2
3
4
5
Low
Med
High
CtBP1&2 protein class

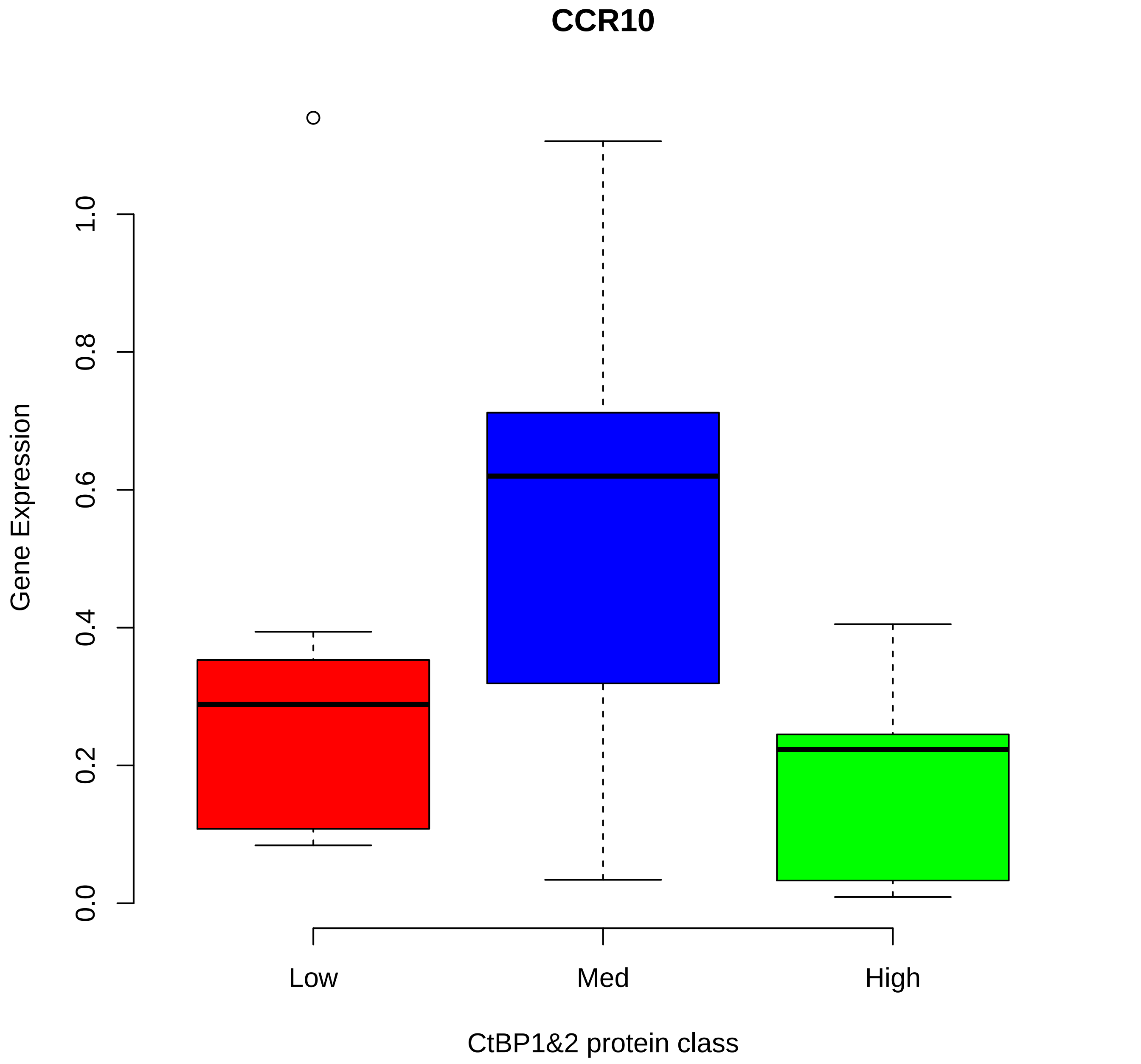

CCR10
Gene Expression
0.0
0.2
0.4
0.6
0.8
1.0
Low
Med
High
CtBP1&2 protein class

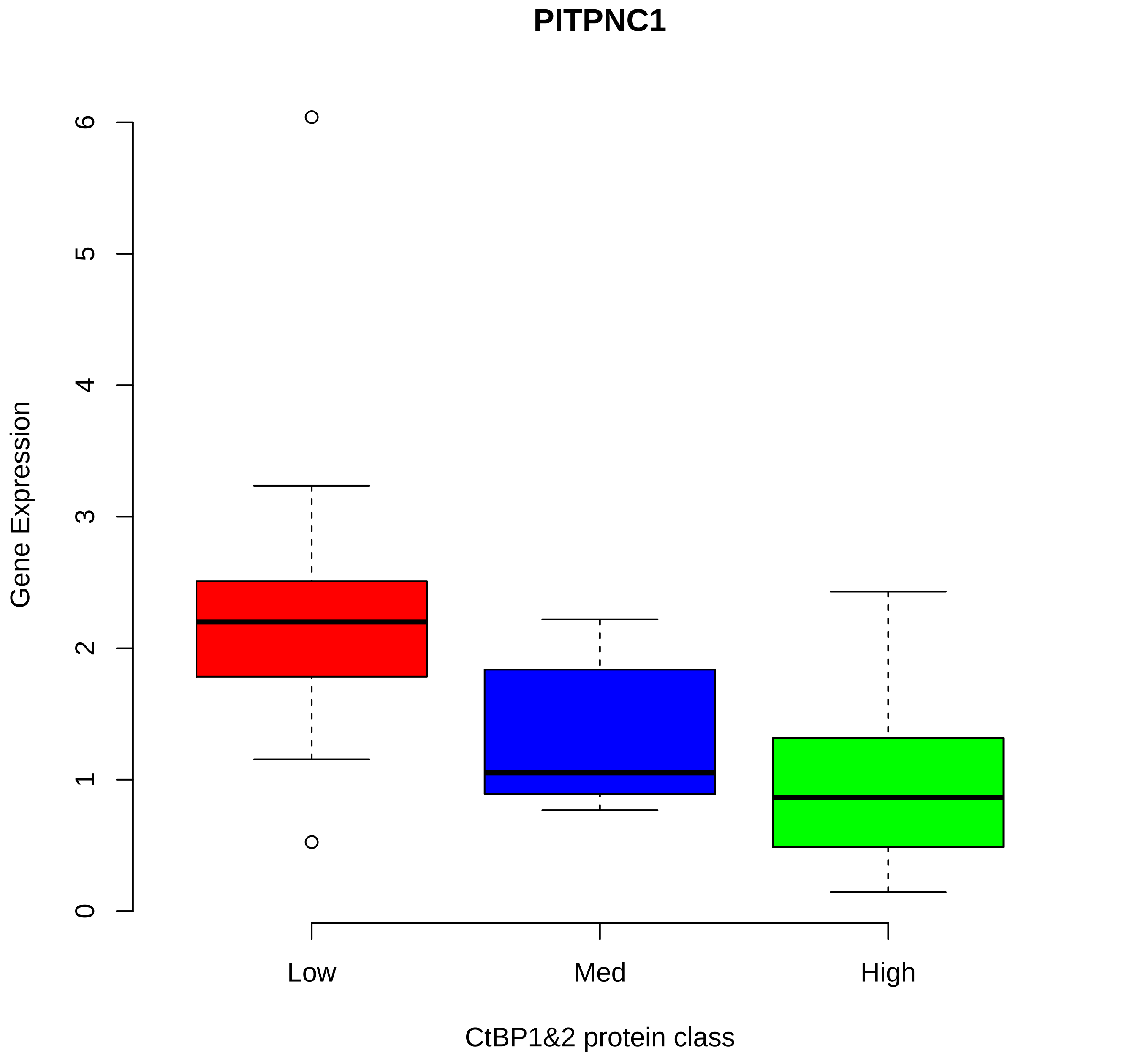
PITPNC1
Gene Expression
0
1
2
3
4
5
6
Low
Med
High
CtBP1&2 protein class

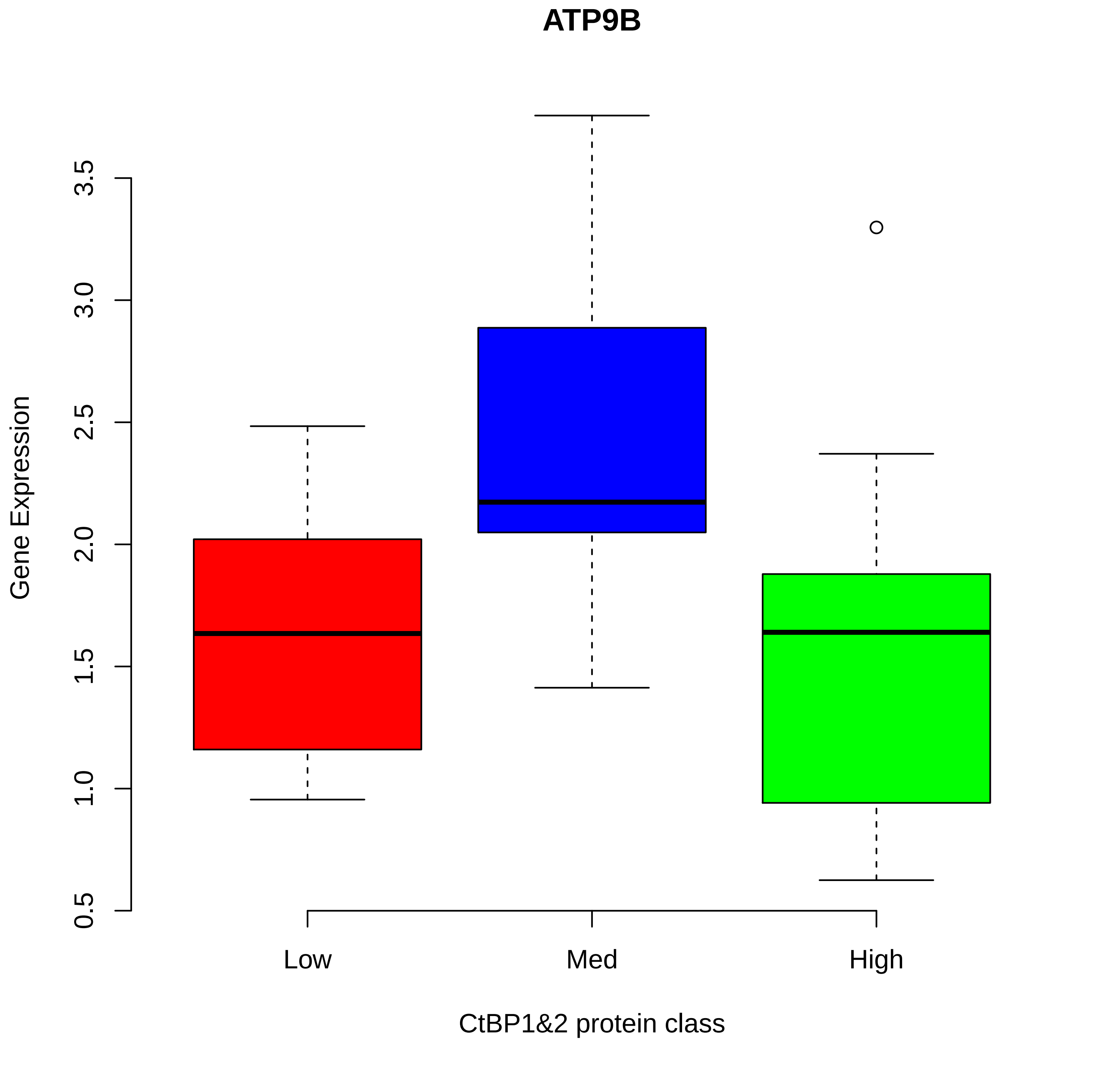
ATP9B
Gene Expression
0.5
1.0
1.5
2.0
2.5
3.0
3.5
Low
Med
High
CtBP1&2 protein class

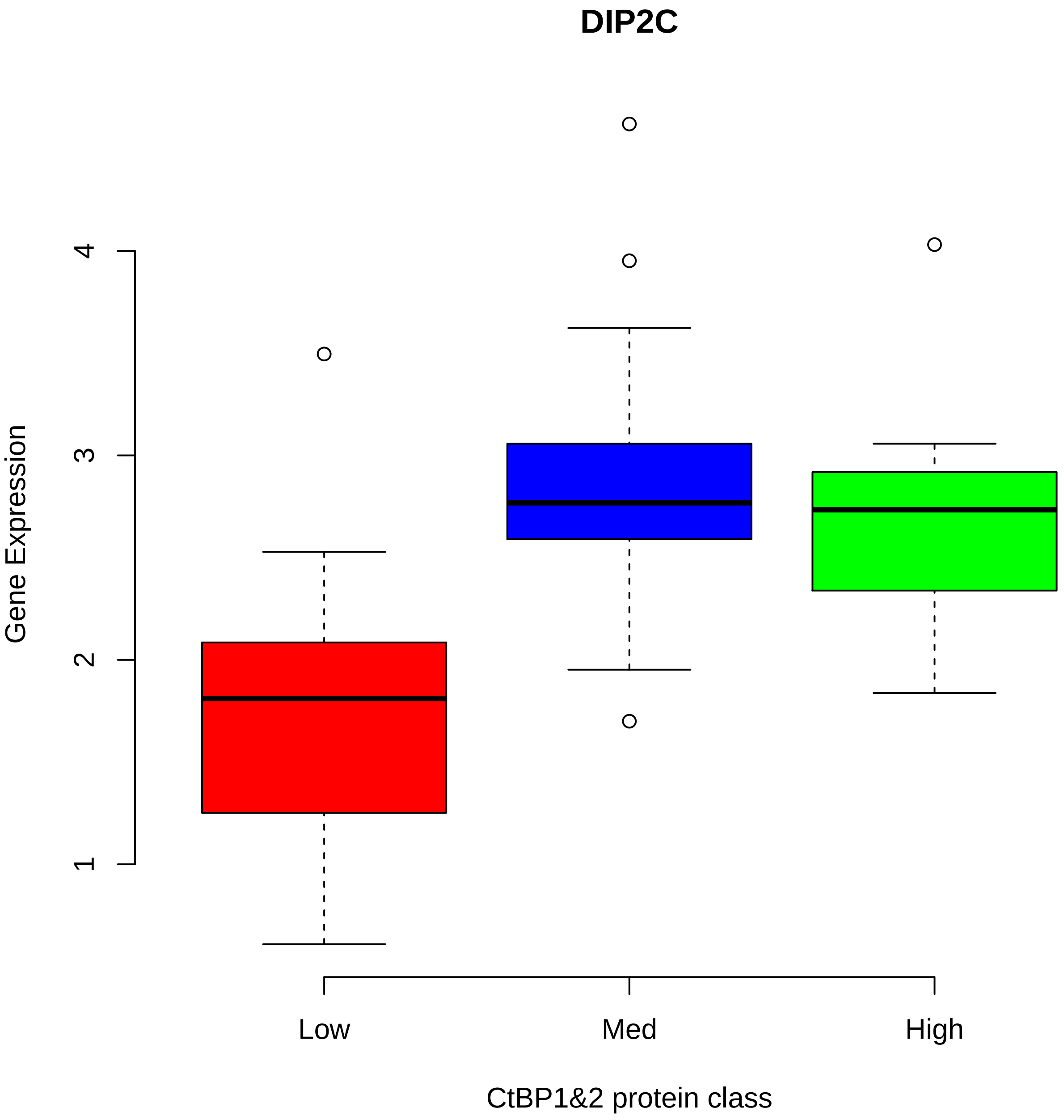

DIP2C
Gene Expression
1
2
3
4
Low
Med
High
CtBP1&2 protein class

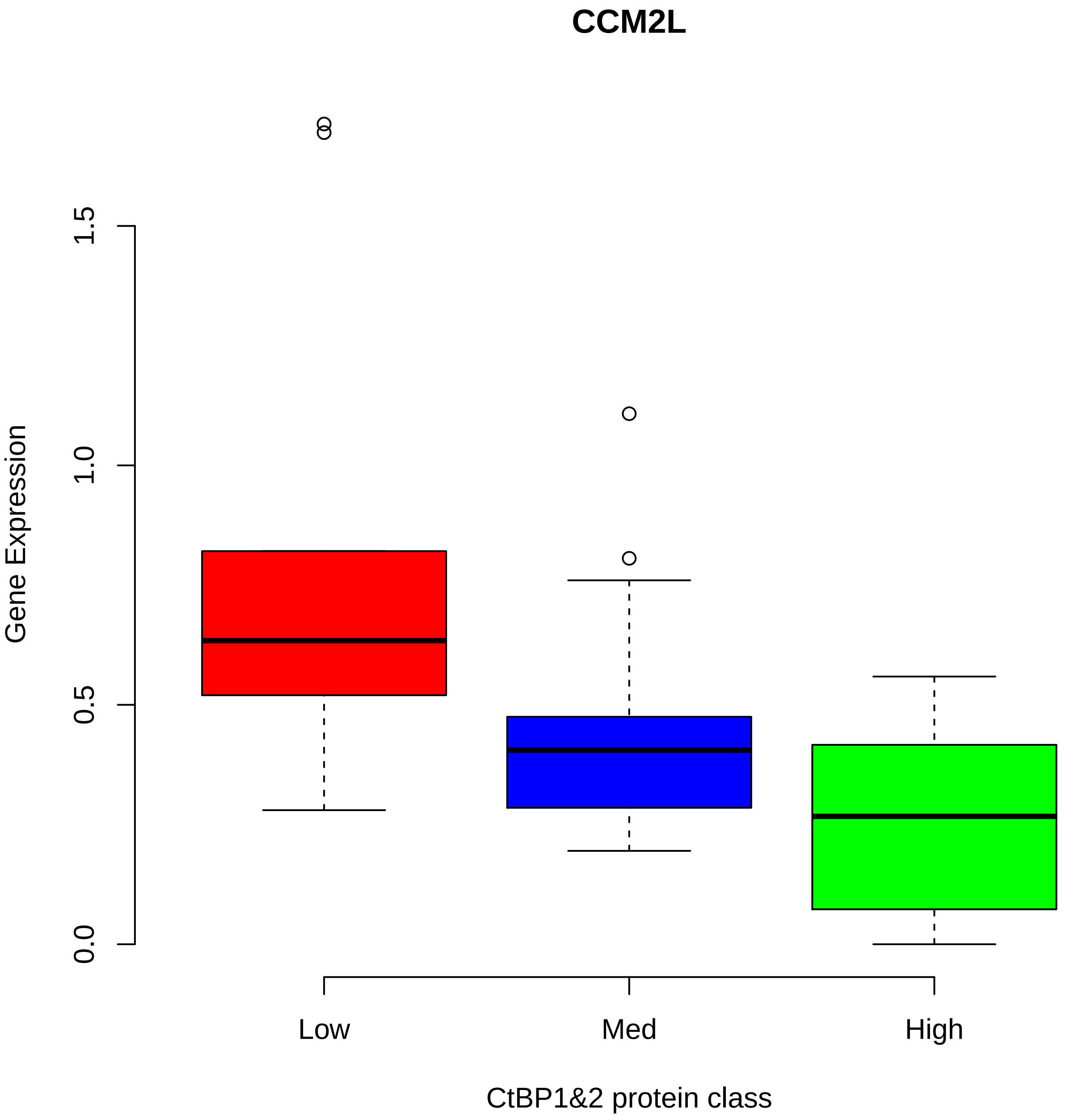
CCM2L
Gene Expression
0.0
0.5
1.0
1.5
Low
Med
High
CtBP1&2 protein class

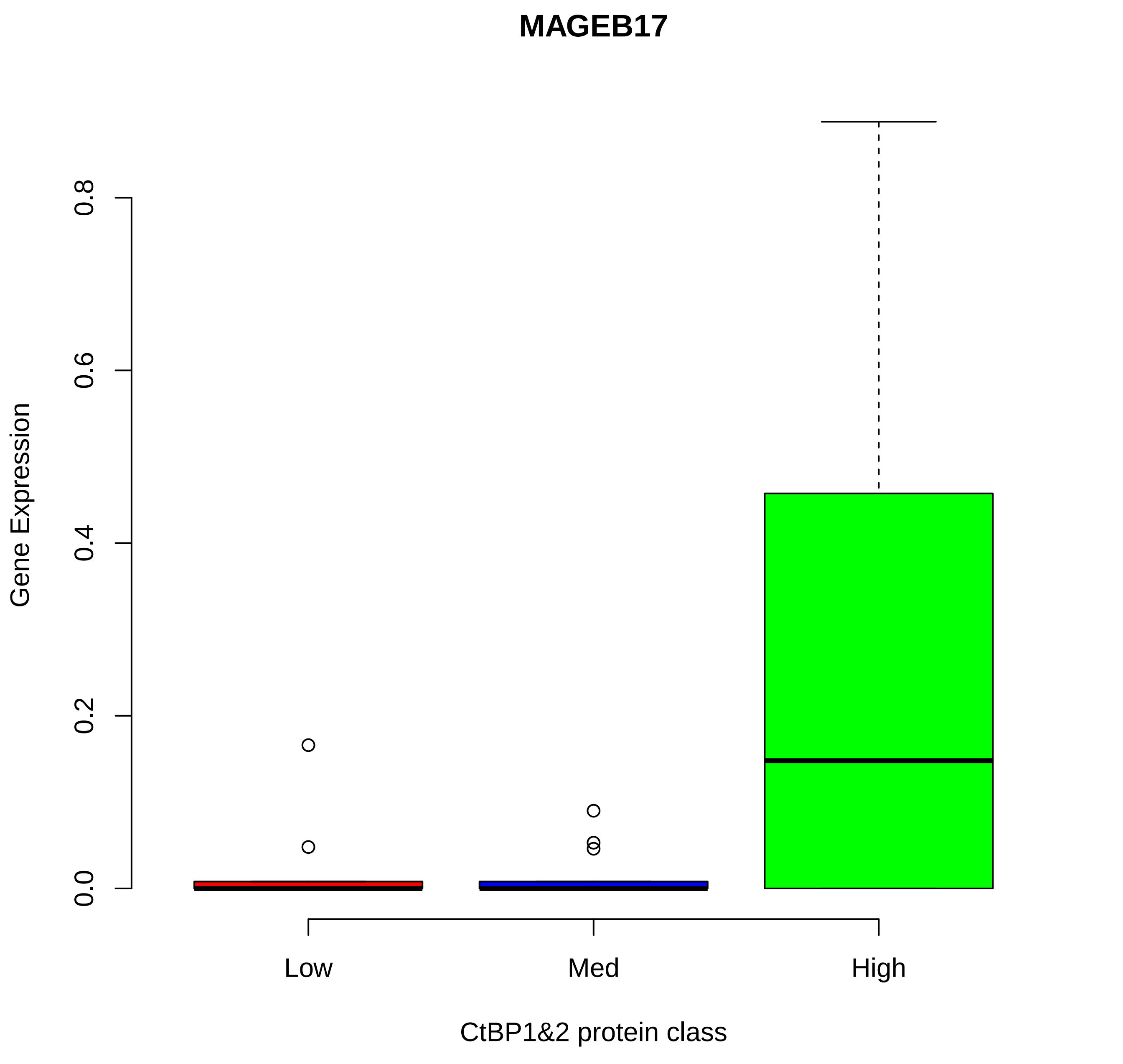
MAGEB17
Gene Expression
0.0
0.2
0.4
0.6
0.8
Low
Med
High
CtBP1&2 protein class

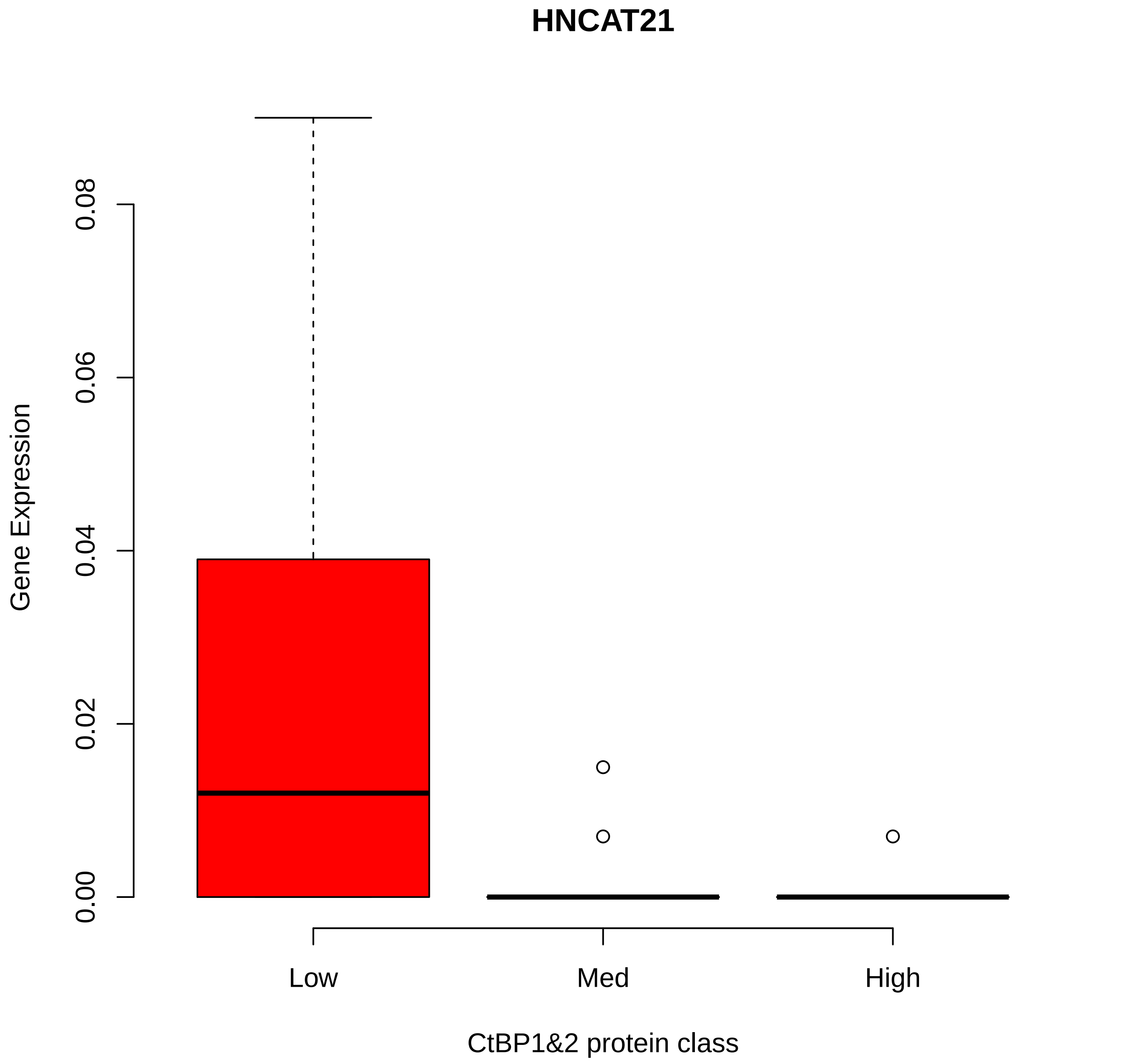

HNCAT21
Gene Expression
0.00
0.02
0.04
0.06
0.08
Low
Med
High
CtBP1&2 protein class

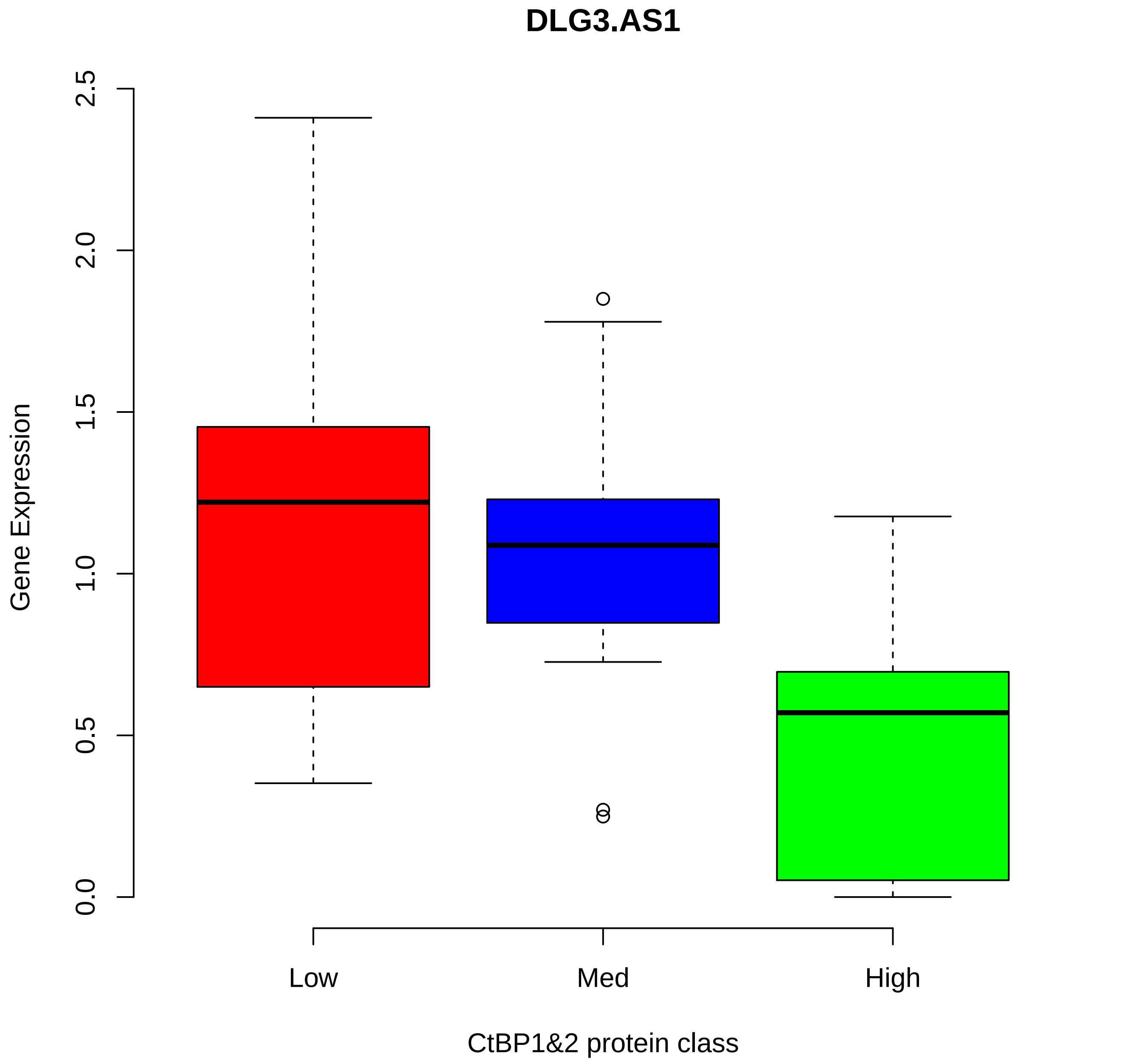

DLG3.AS1
Gene Expression
0.0
0.5
1.0
1.5
2.0
2.5
Low
Med
High
CtBP1&2 protein class

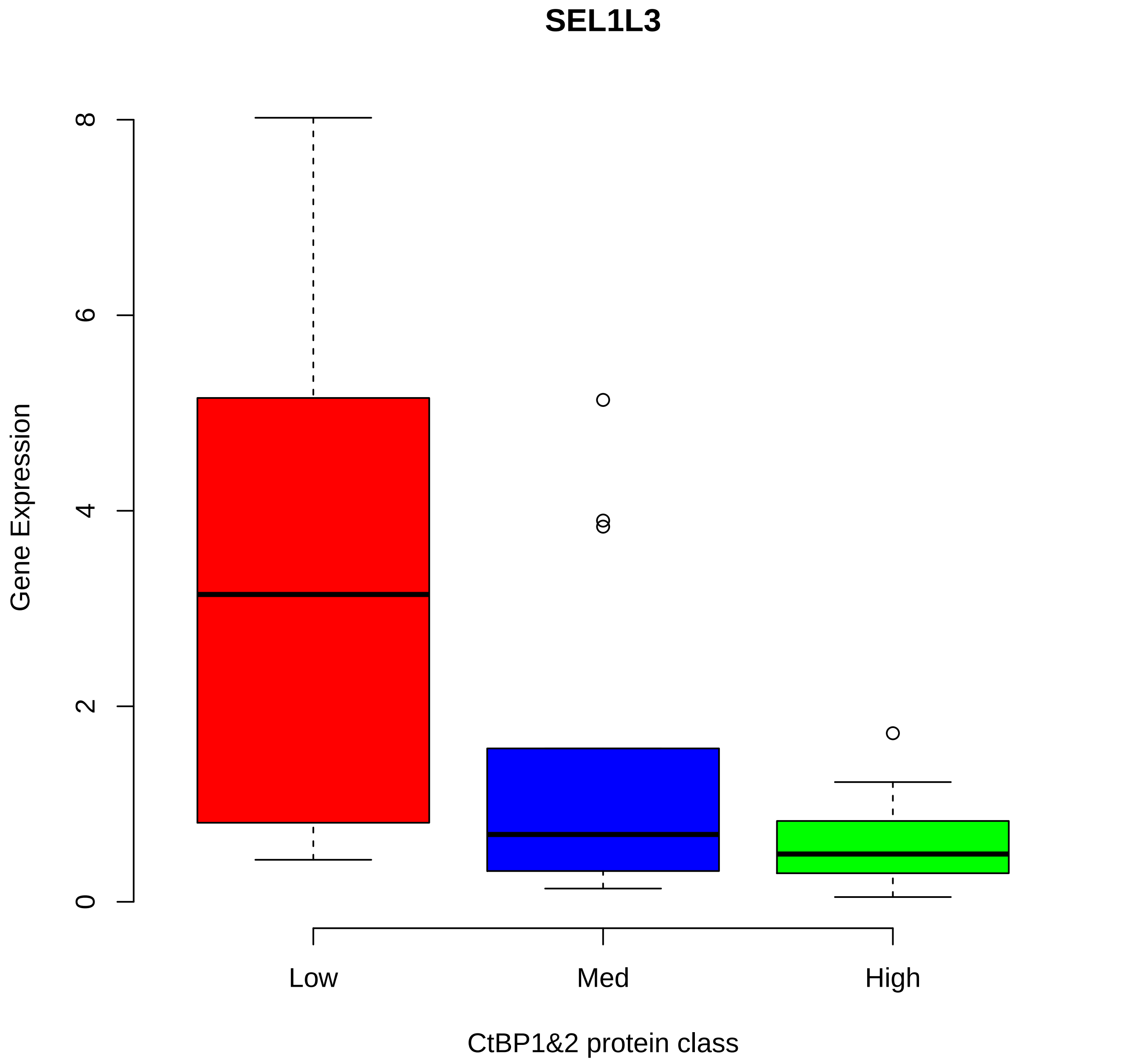
SEL1L3
Gene Expression
0
2
4
6
8
Low
Med
High
CtBP1&2 protein class

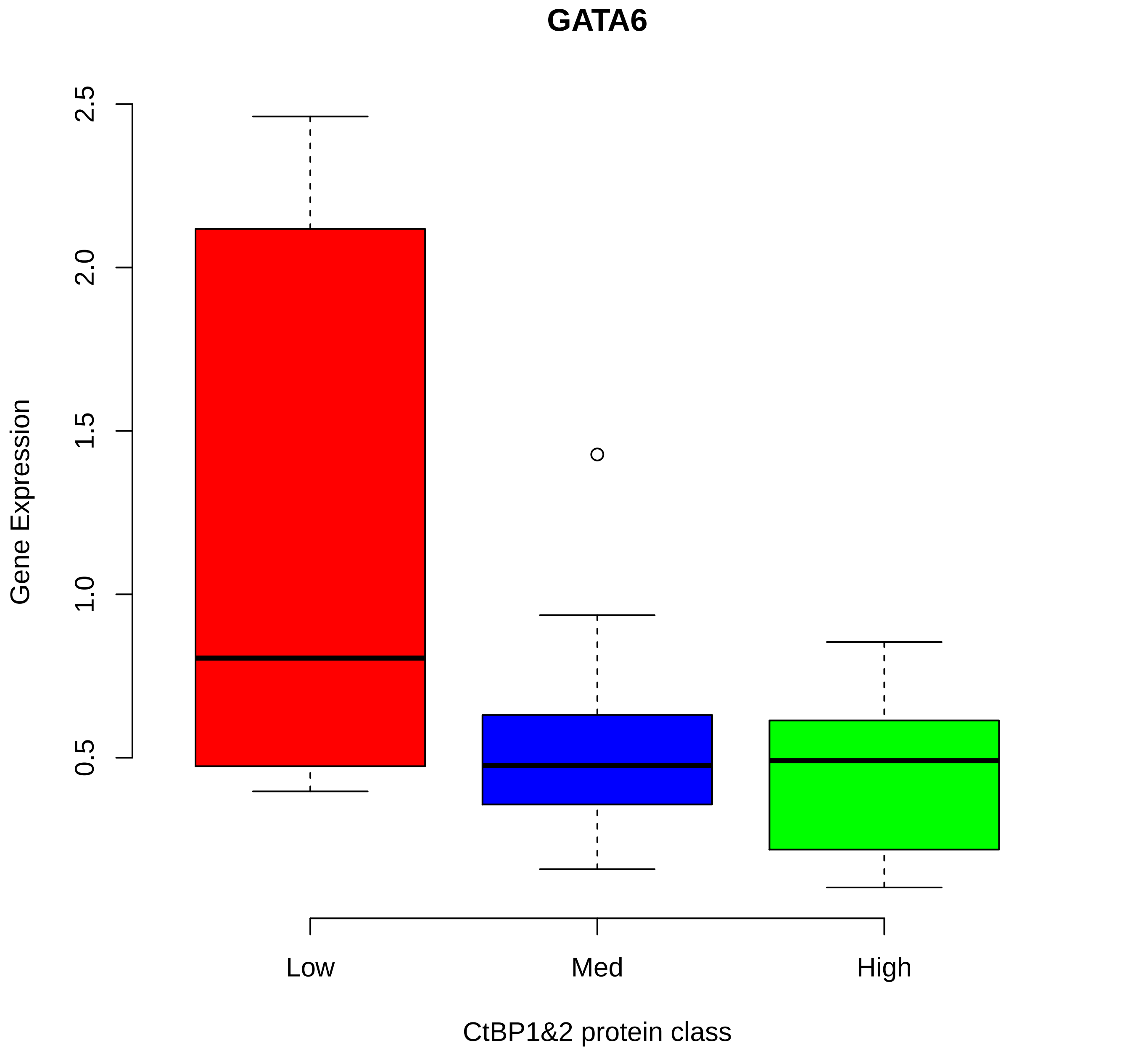
GATA6
Gene Expression
0.5
1.0
1.5
2.0
2.5
Low
Med
High
CtBP1&2 protein class

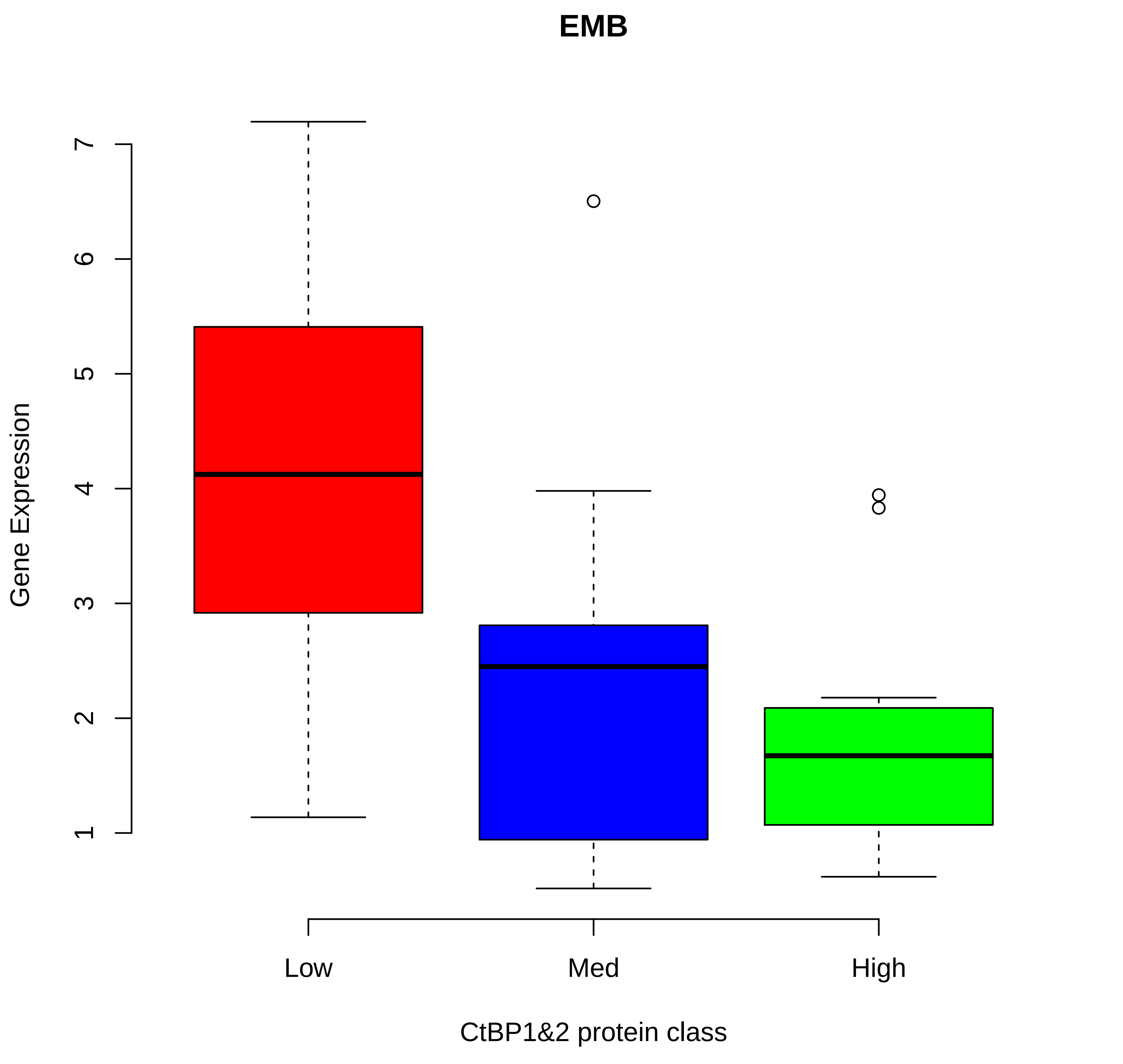

EMB
Gene Expression
1
2
3
4
5
6
7
Low
Med
High
CtBP1&2 protein class

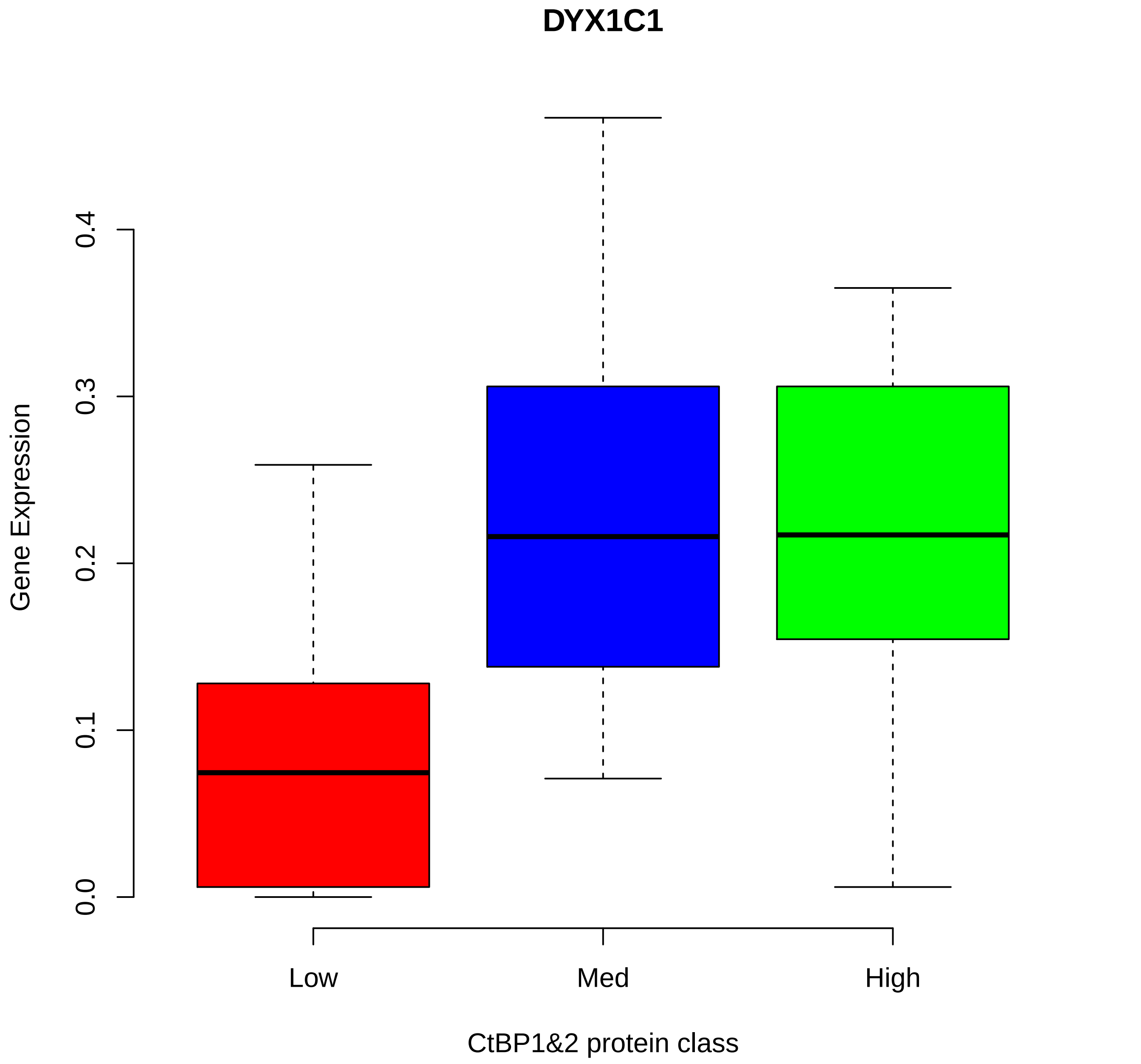

DYX1C1
Gene Expression
0.0
0.1
0.2
0.3
0.4
Low
Med
High
CtBP1&2 protein class

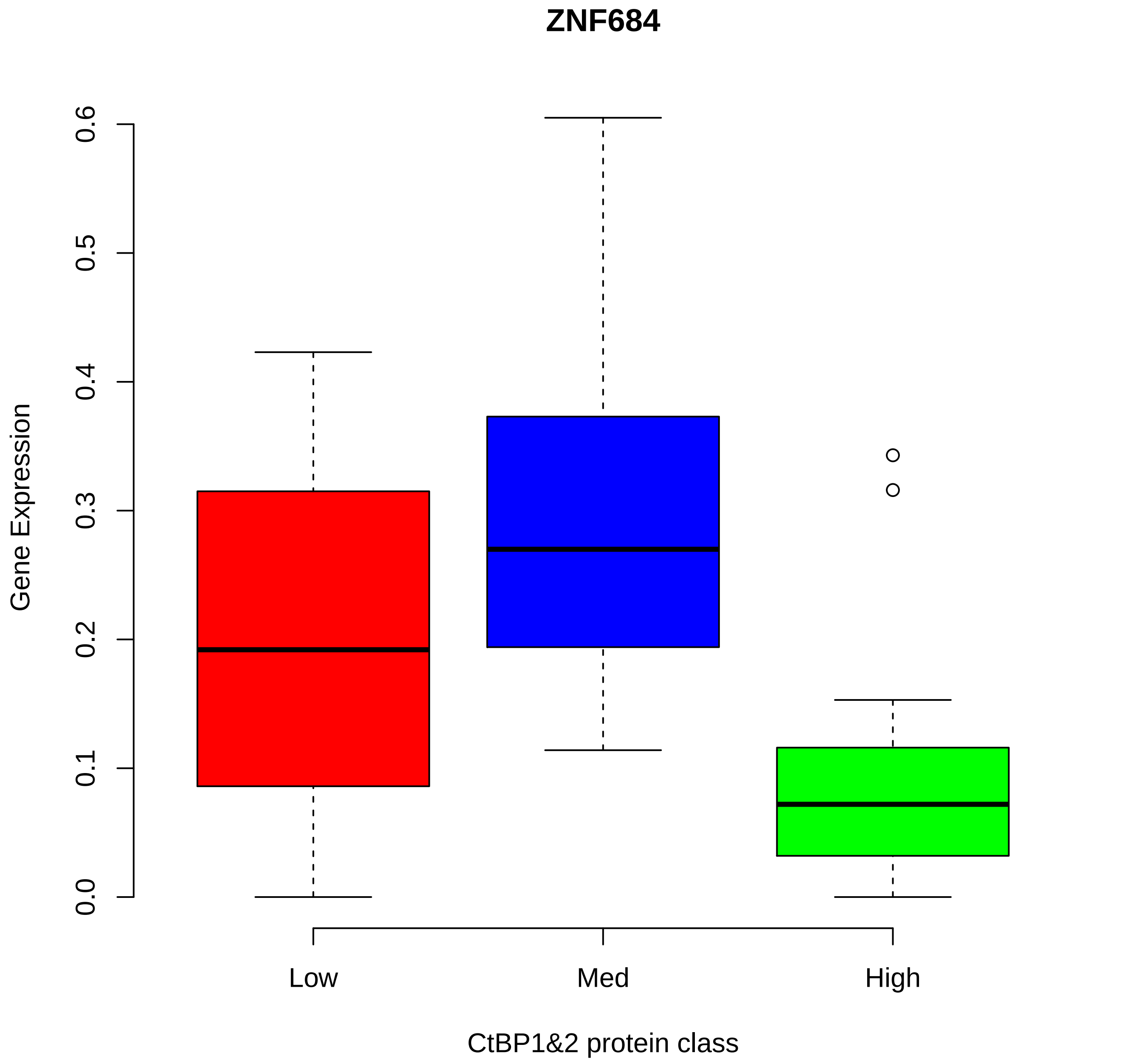
ZNF684
Gene Expression
0.0
0.1
0.2
0.3
0.4
0.5
0.6
Low
Med
High
CtBP1&2 protein class

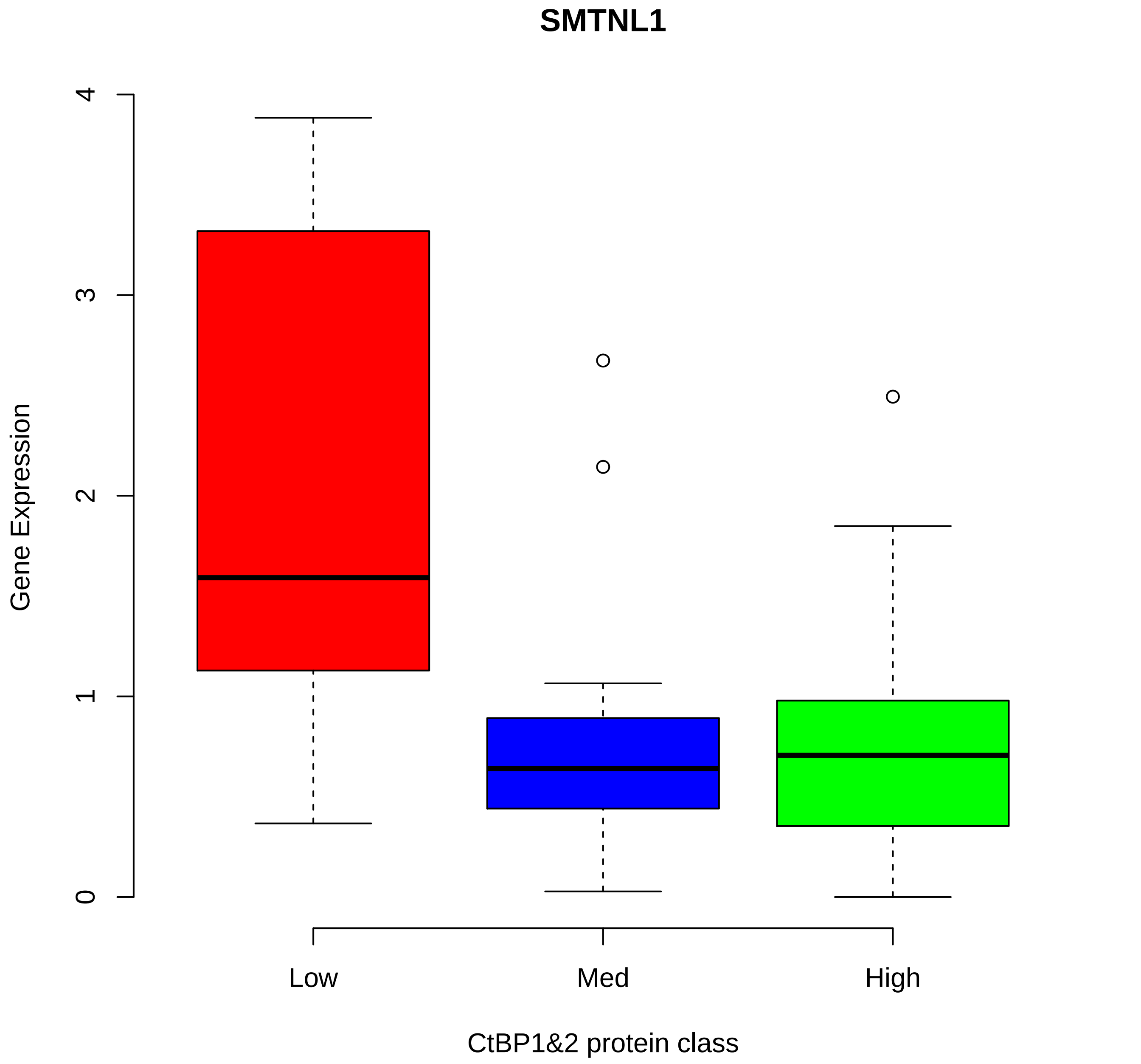
SMTNL1
Gene Expression
0
1
2
3
4
Low
Med
High
CtBP1&2 protein class

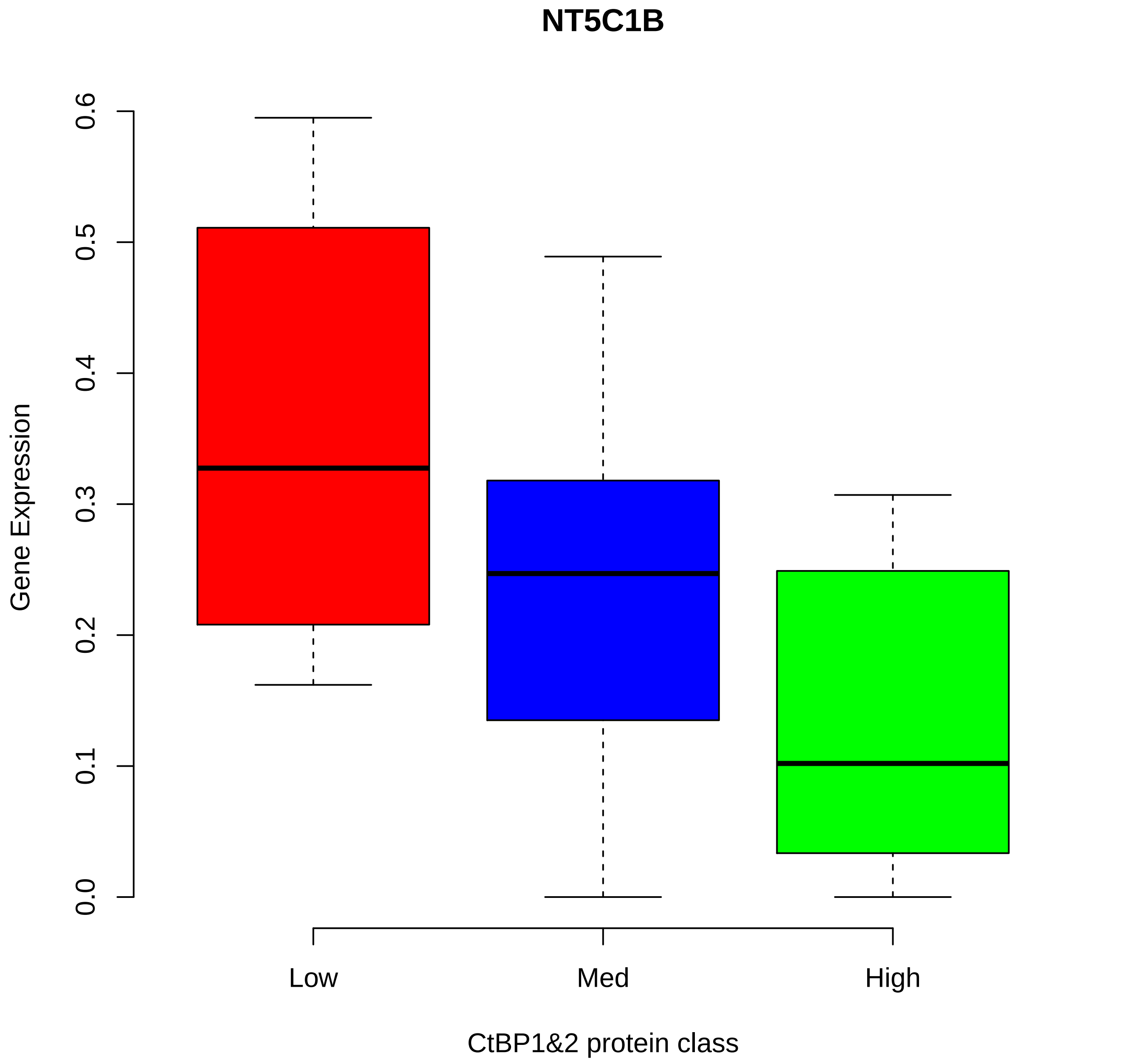

NT5C1B
Gene Expression
0.0
0.1
0.2
0.3
0.4
0.5
0.6
Low
Med
High
CtBP1&2 protein class

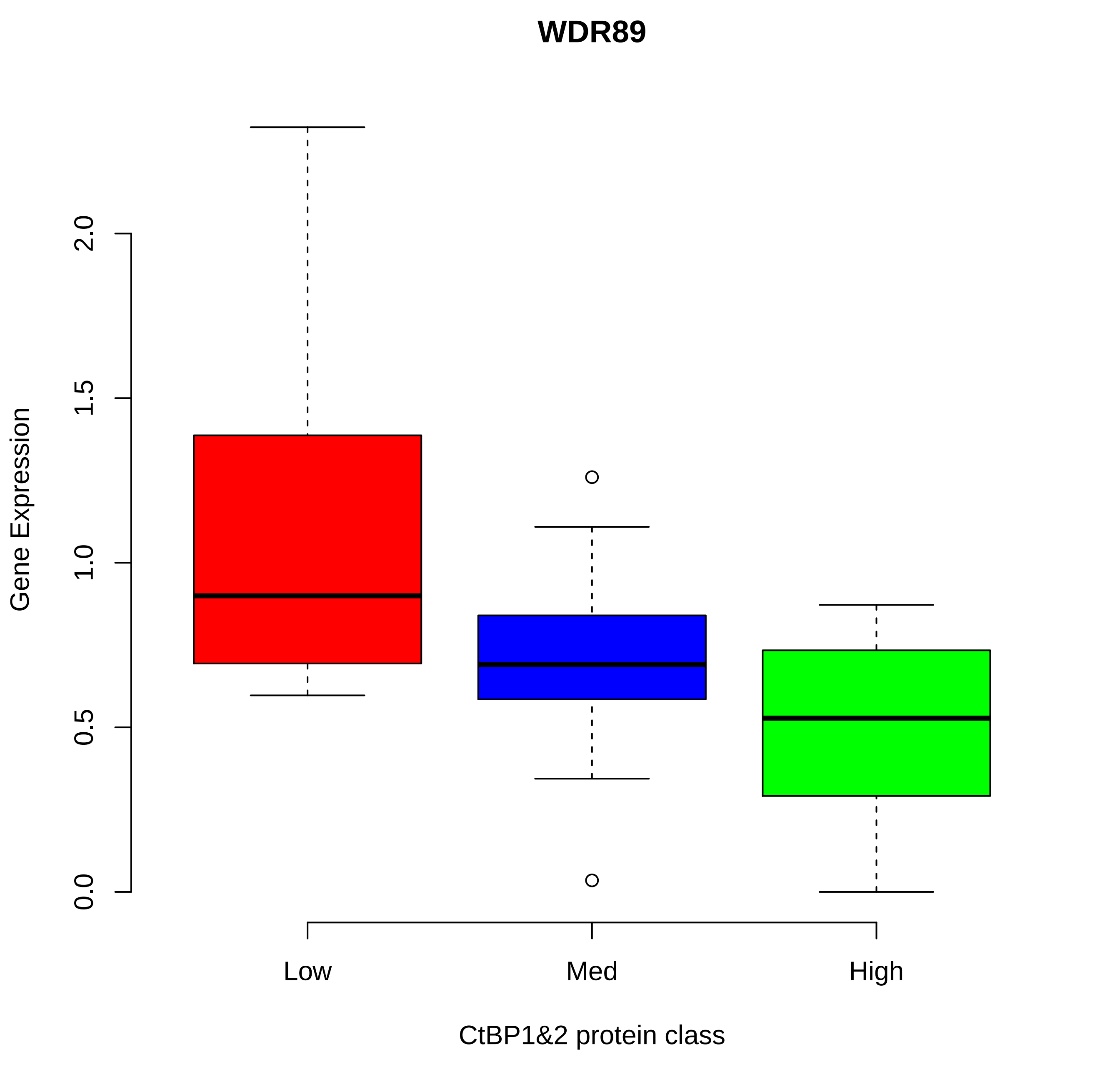

WDR89
Gene Expression
0.0
0.5
1.0
1.5
2.0
Low
Med
High
CtBP1&2 protein class

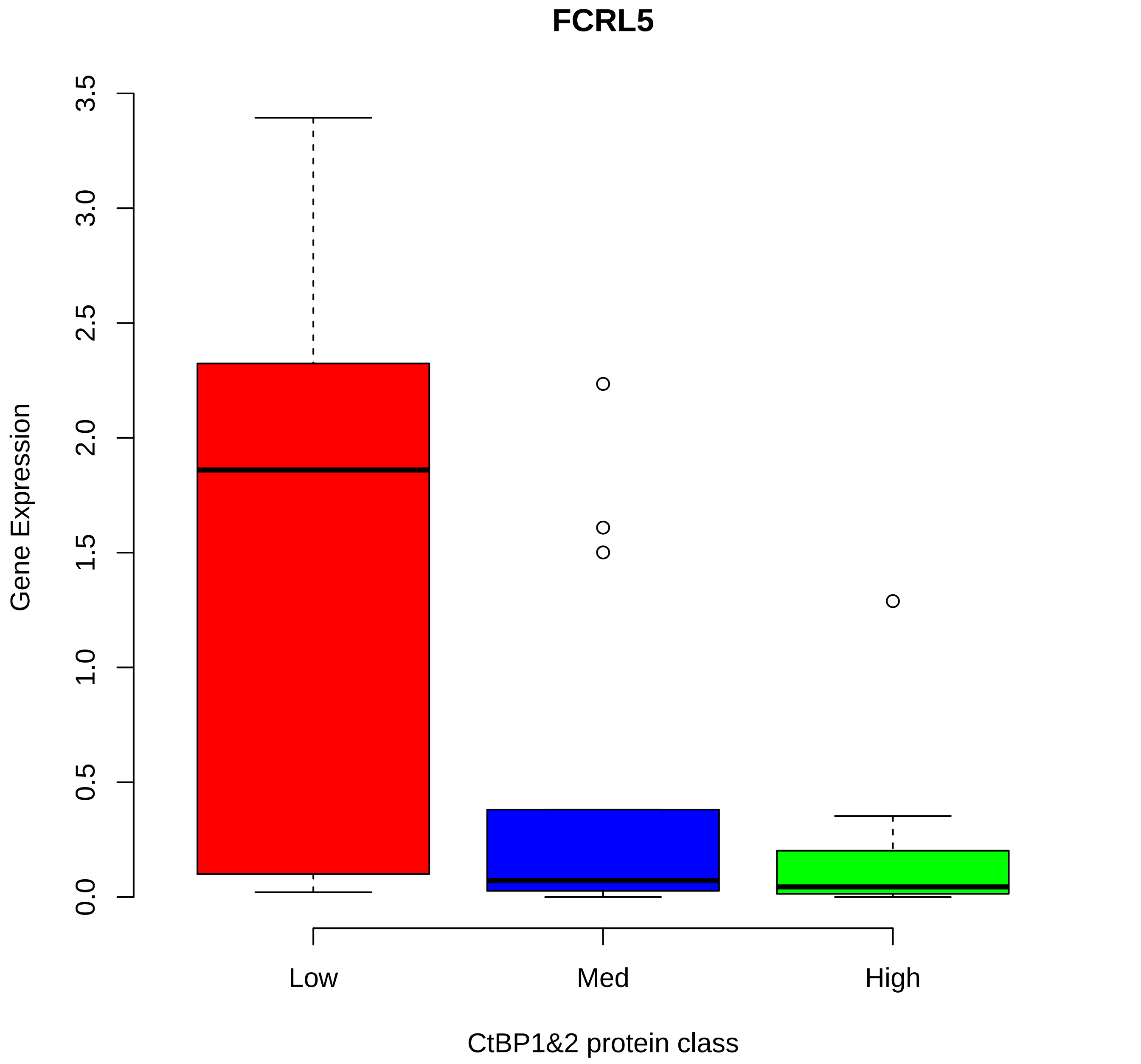

FCRL5
Gene Expression
0.0
0.5
1.0
1.5
2.0
2.5
3.0
3.5
Low
Med
High
CtBP1&2 protein class

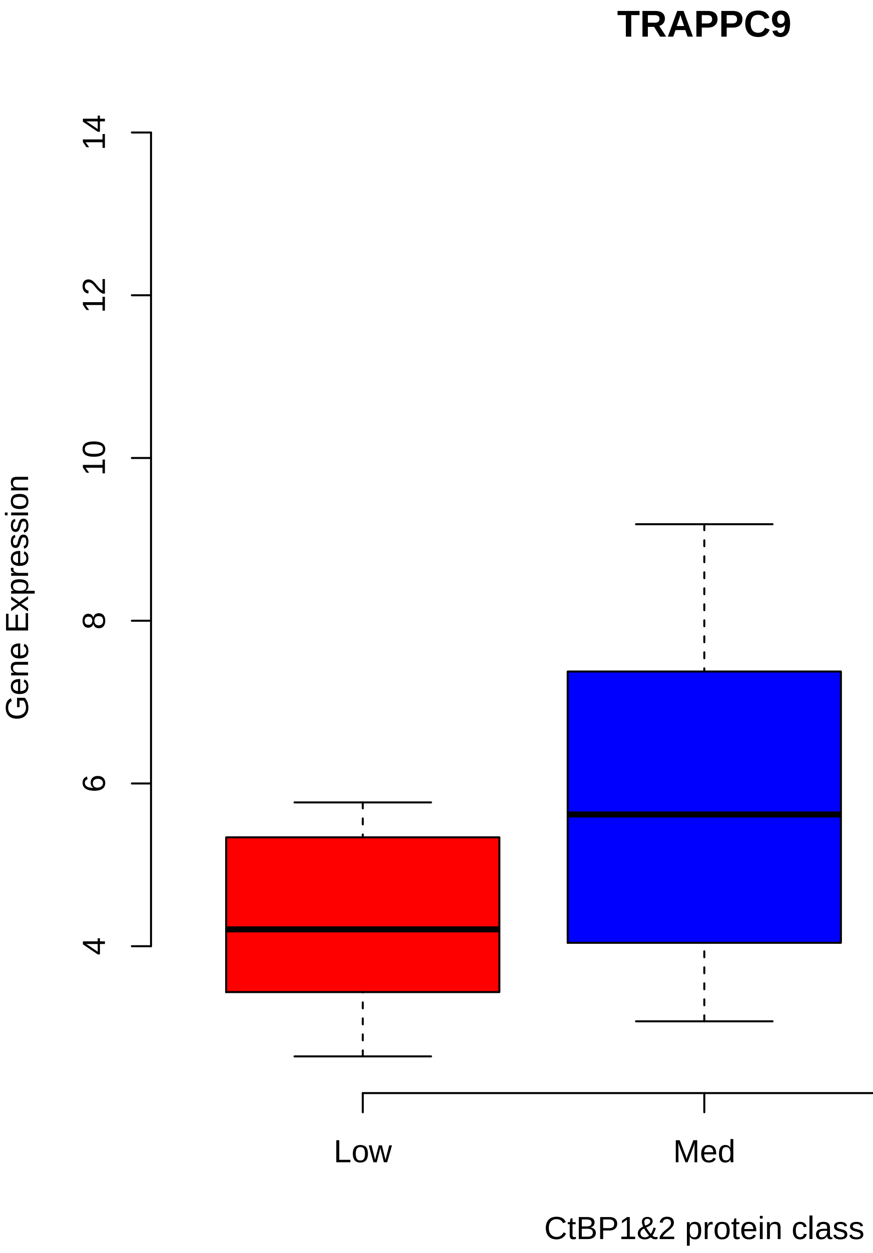
TRAPPC9
Gene Expression
4
6
8
10
12
14
Low
Med
High
CtBP1&2 protein class

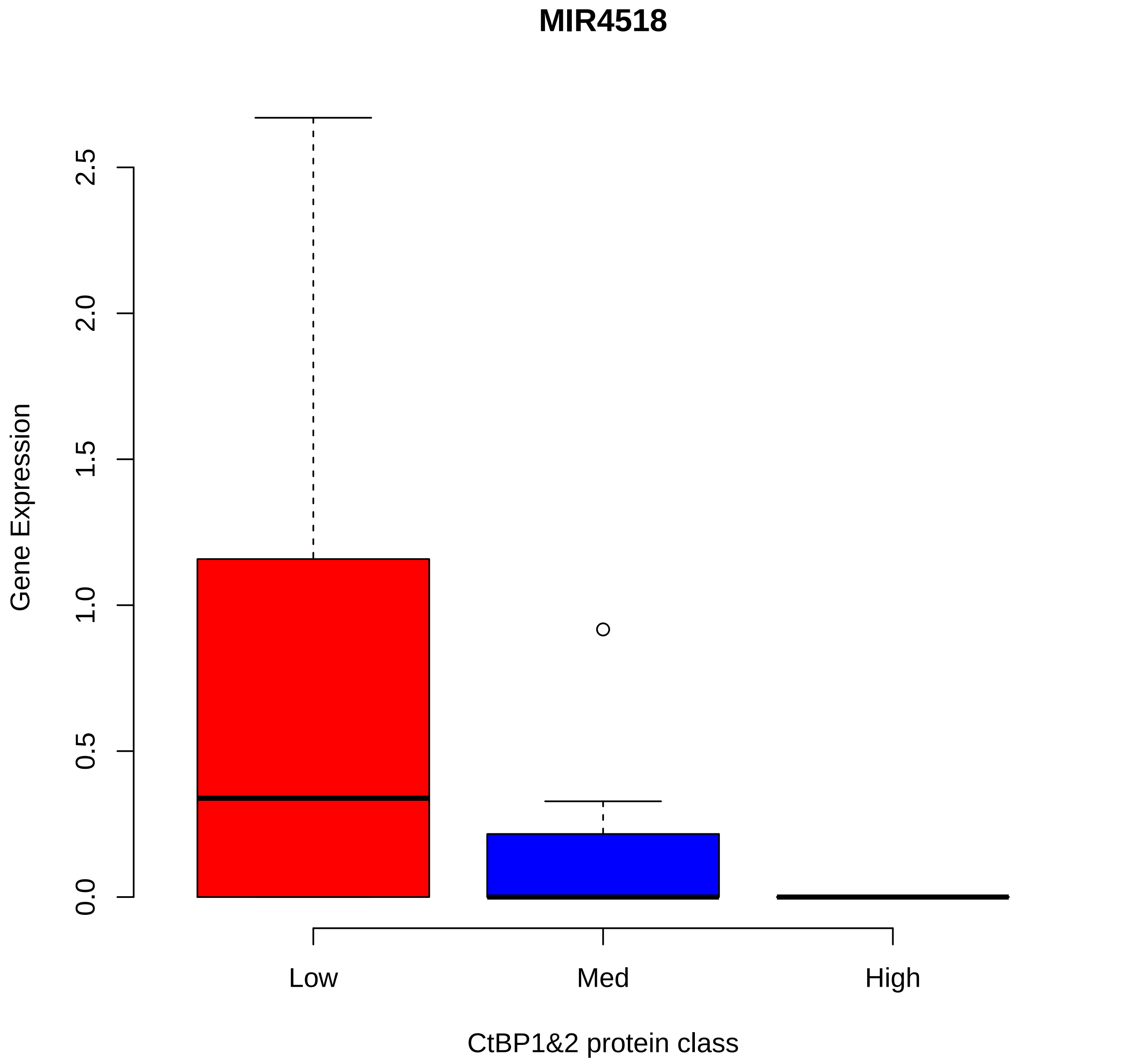
MIR4518
Gene Expression
0.0
0.5
1.0
1.5
2.0
2.5
Low
Med
High
CtBP1&2 protein class

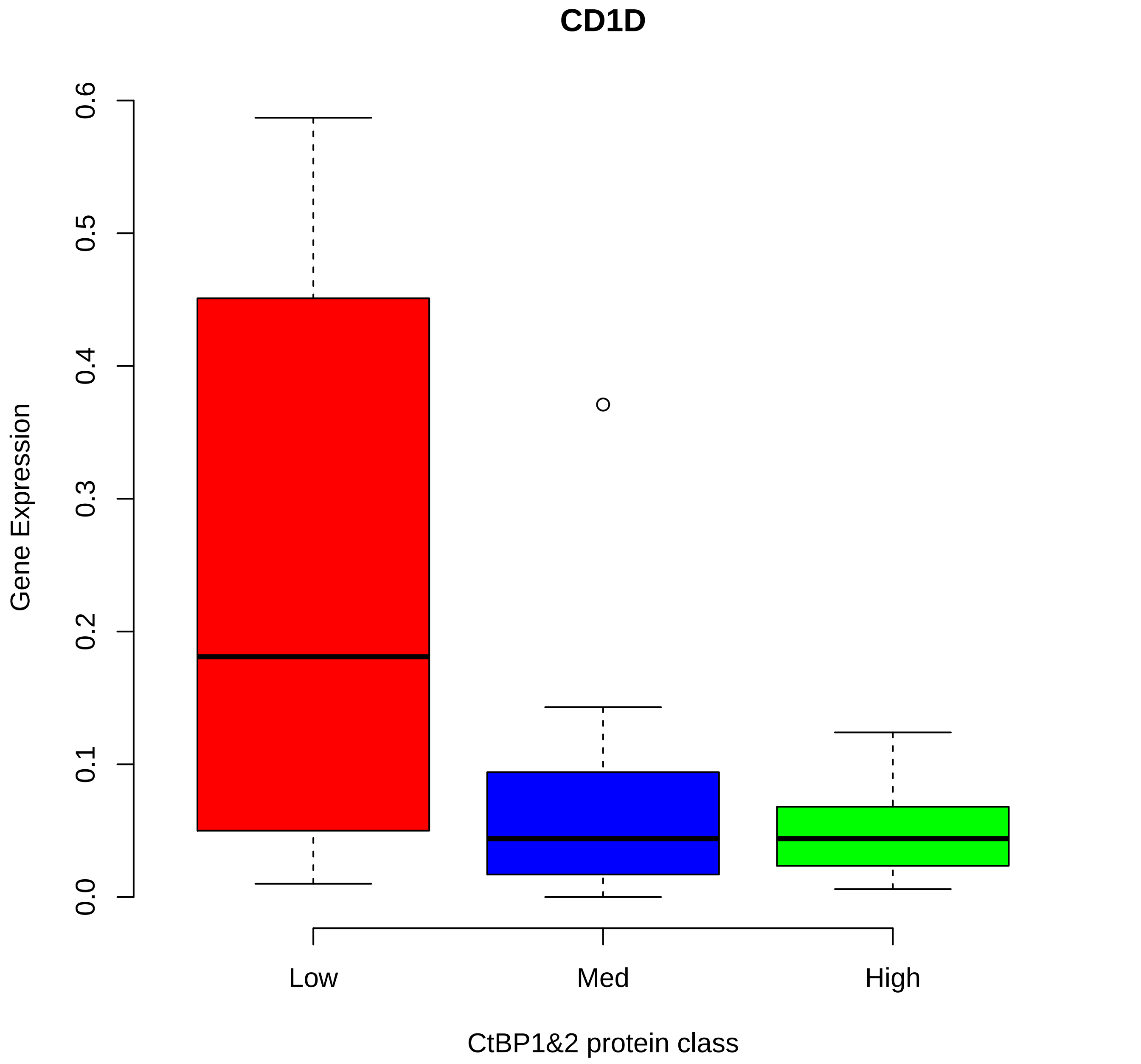

CD1D
Gene Expression
0.0
0.1
0.2
0.3
0.4
0.5
0.6
Low
Med
High
CtBP1&2 protein class

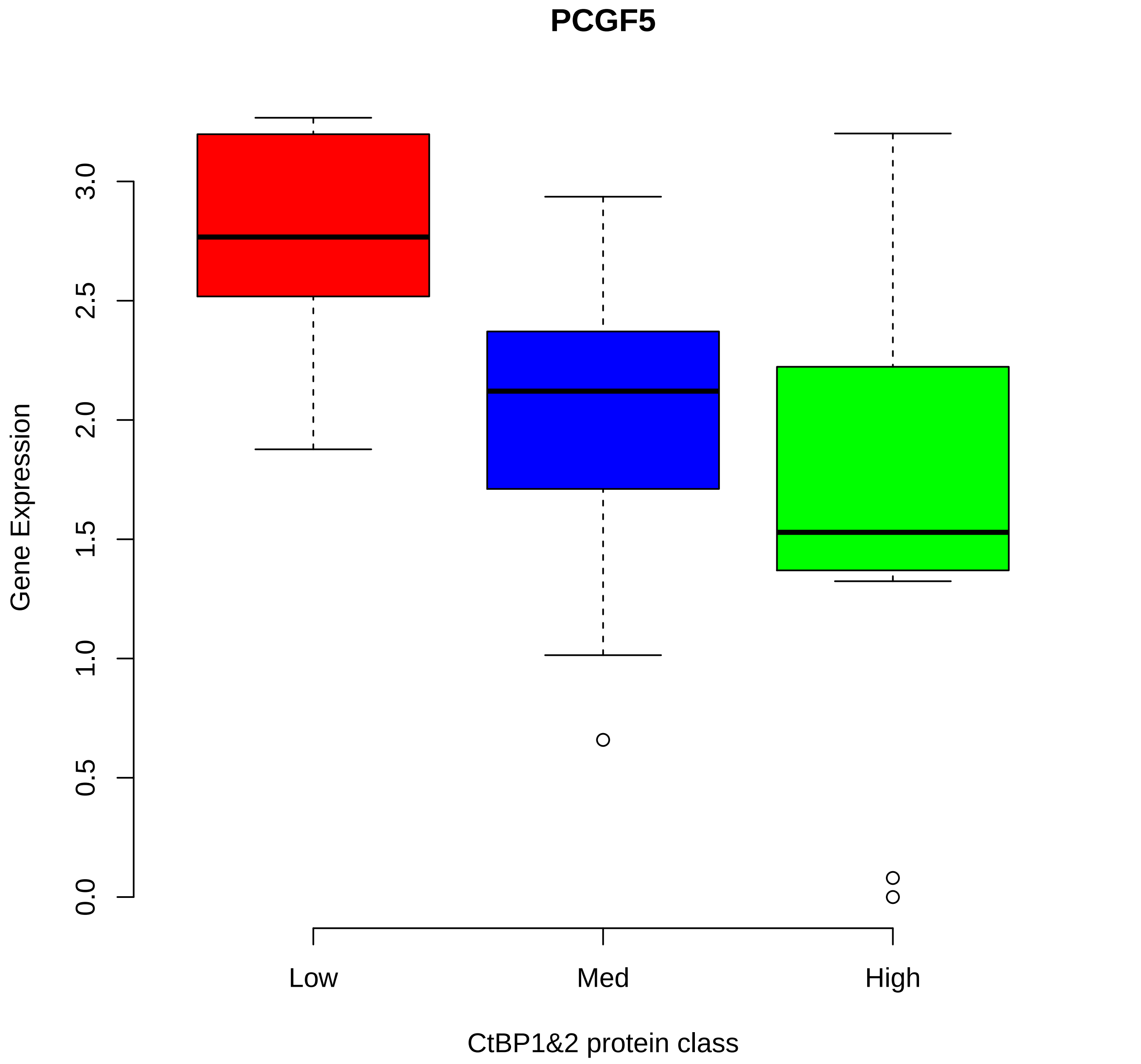
PCGF5
Gene Expression
0.0
0.5
1.0
1.5
2.0
2.5
3.0
Low
Med
High
CtBP1&2 protein class

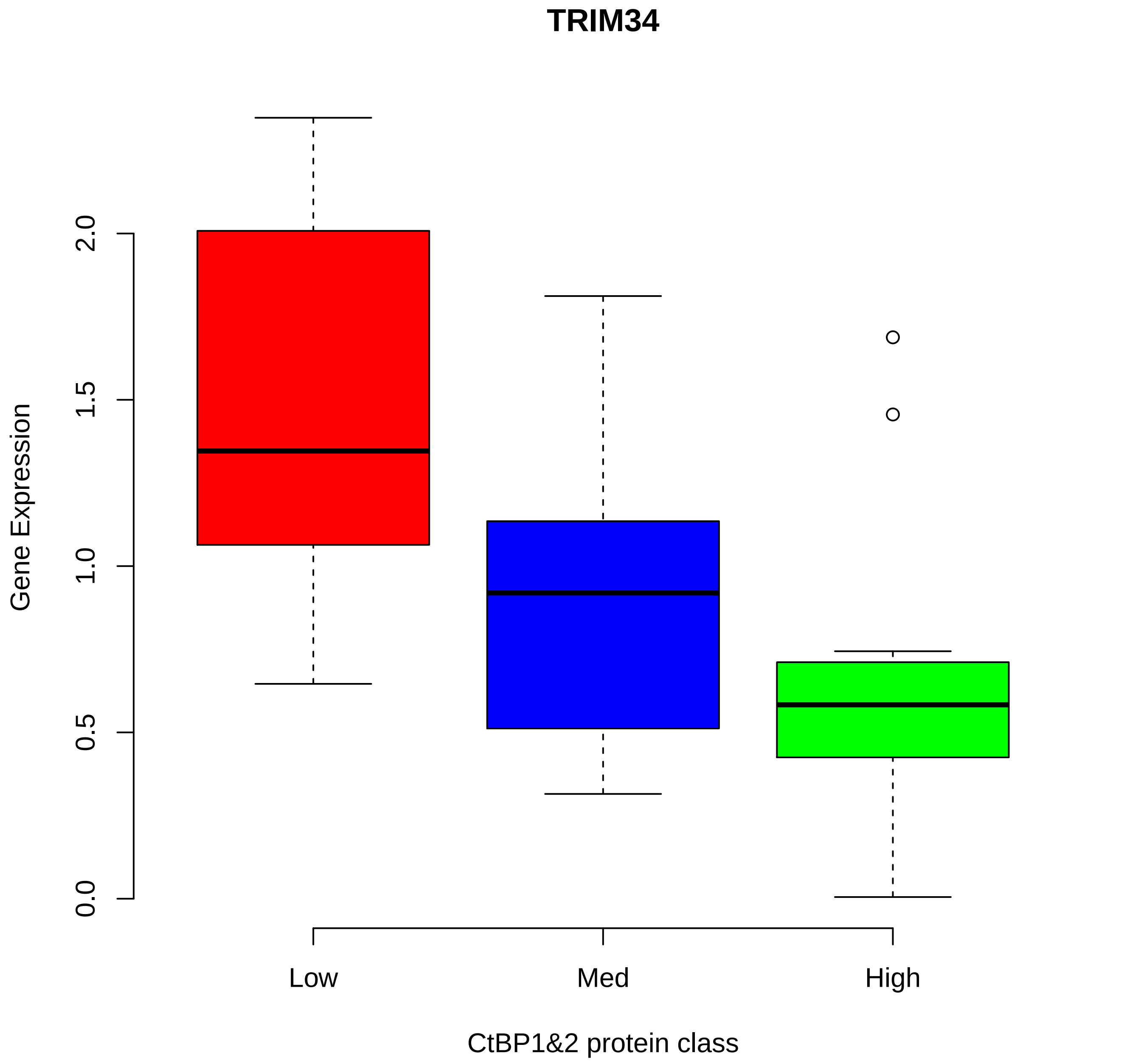
TRIM34
Gene Expression
0.0
0.5
1.0
1.5
2.0
Low
Med
High
CtBP1&2 protein class

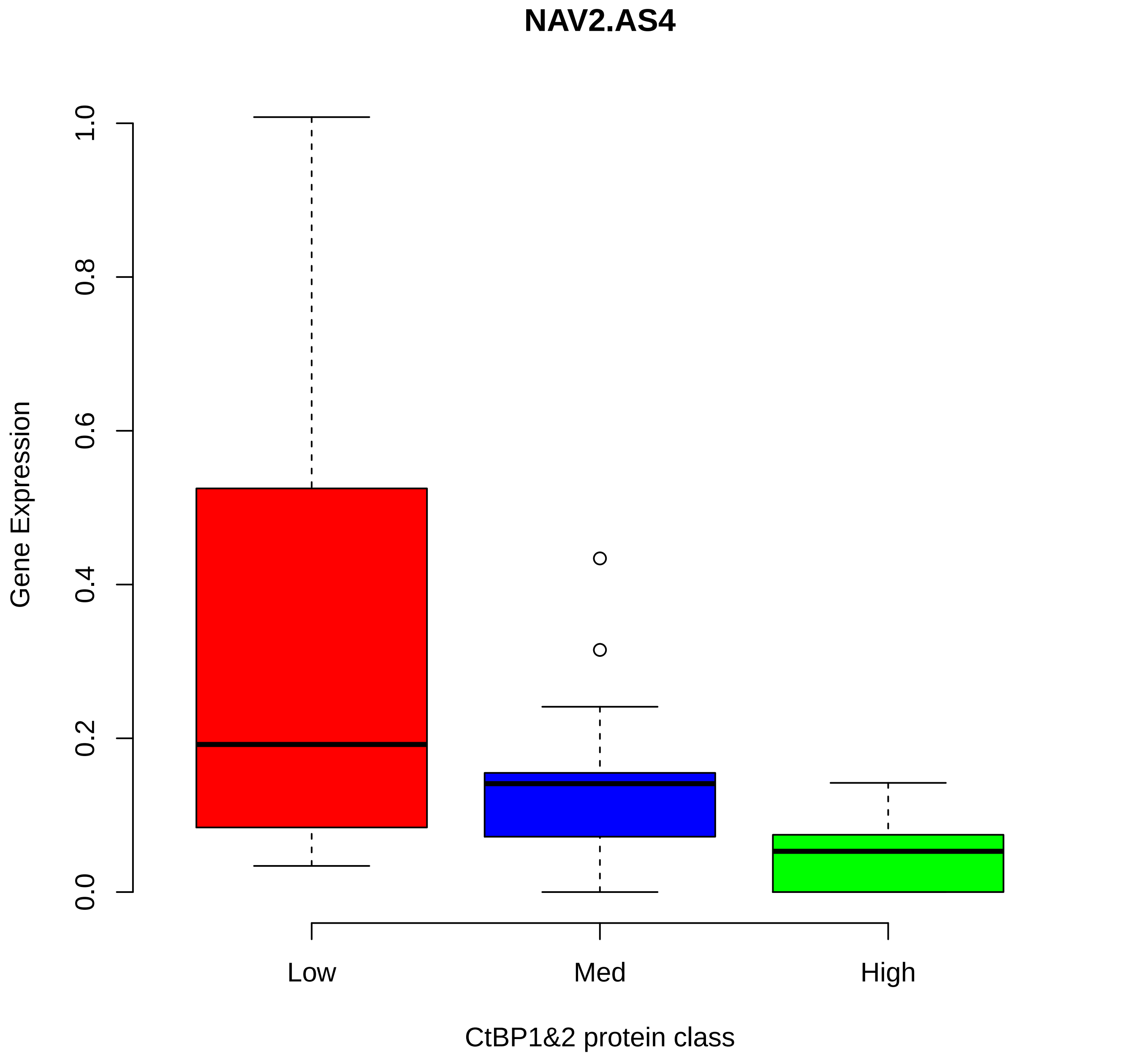
NAV2.AS4
Gene Expression
0.0
0.2
0.4
0.6
0.8
1.0
Low
Med
High
CtBP1&2 protein class

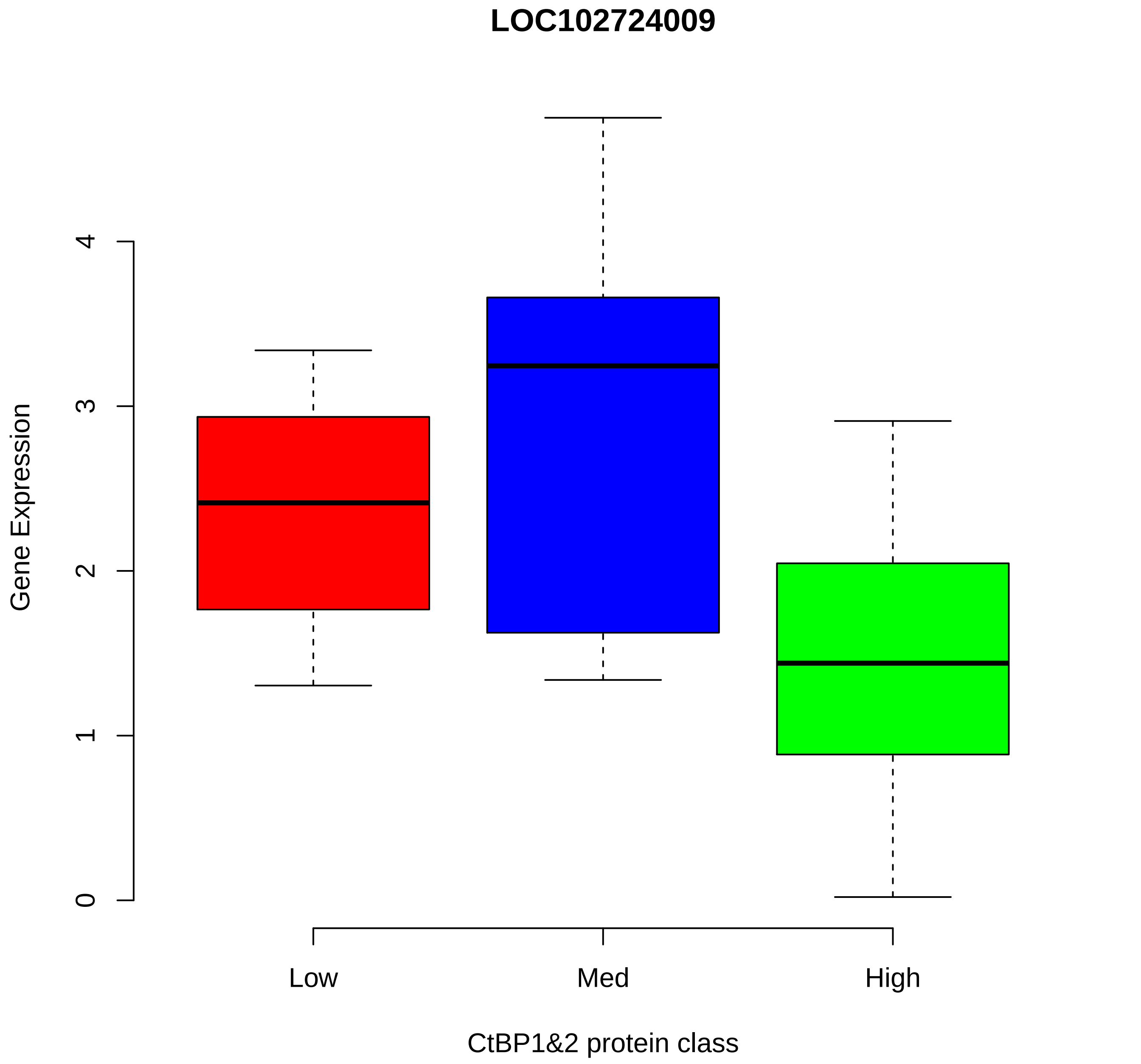

LOC102724009
Gene Expression
0
1
2
3
4
Low
Med
High
CtBP1&2 protein class

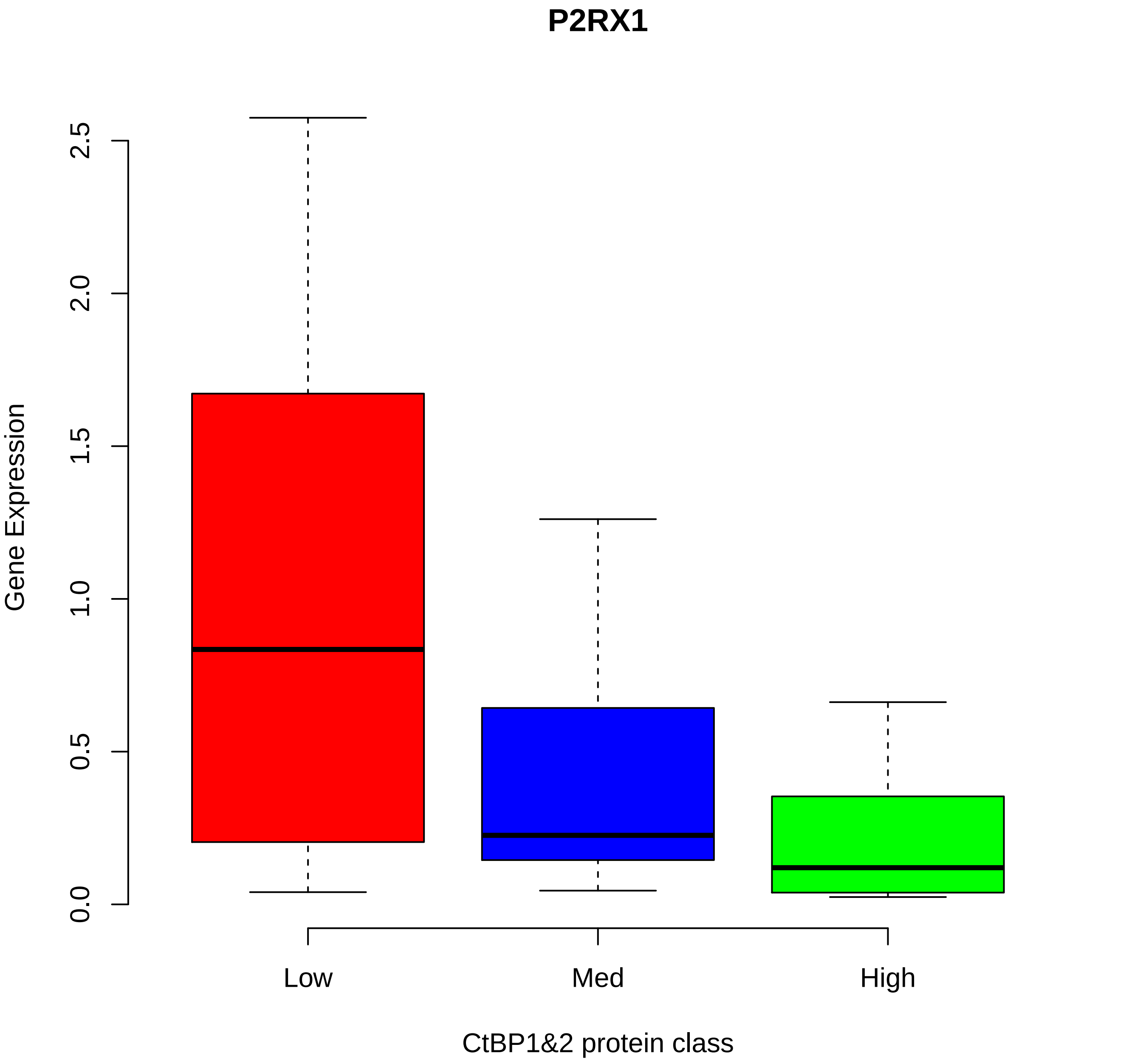
P2RX1
Gene Expression
0.0
0.5
1.0
1.5
2.0
2.5
Low
Med
High
CtBP1&2 protein class

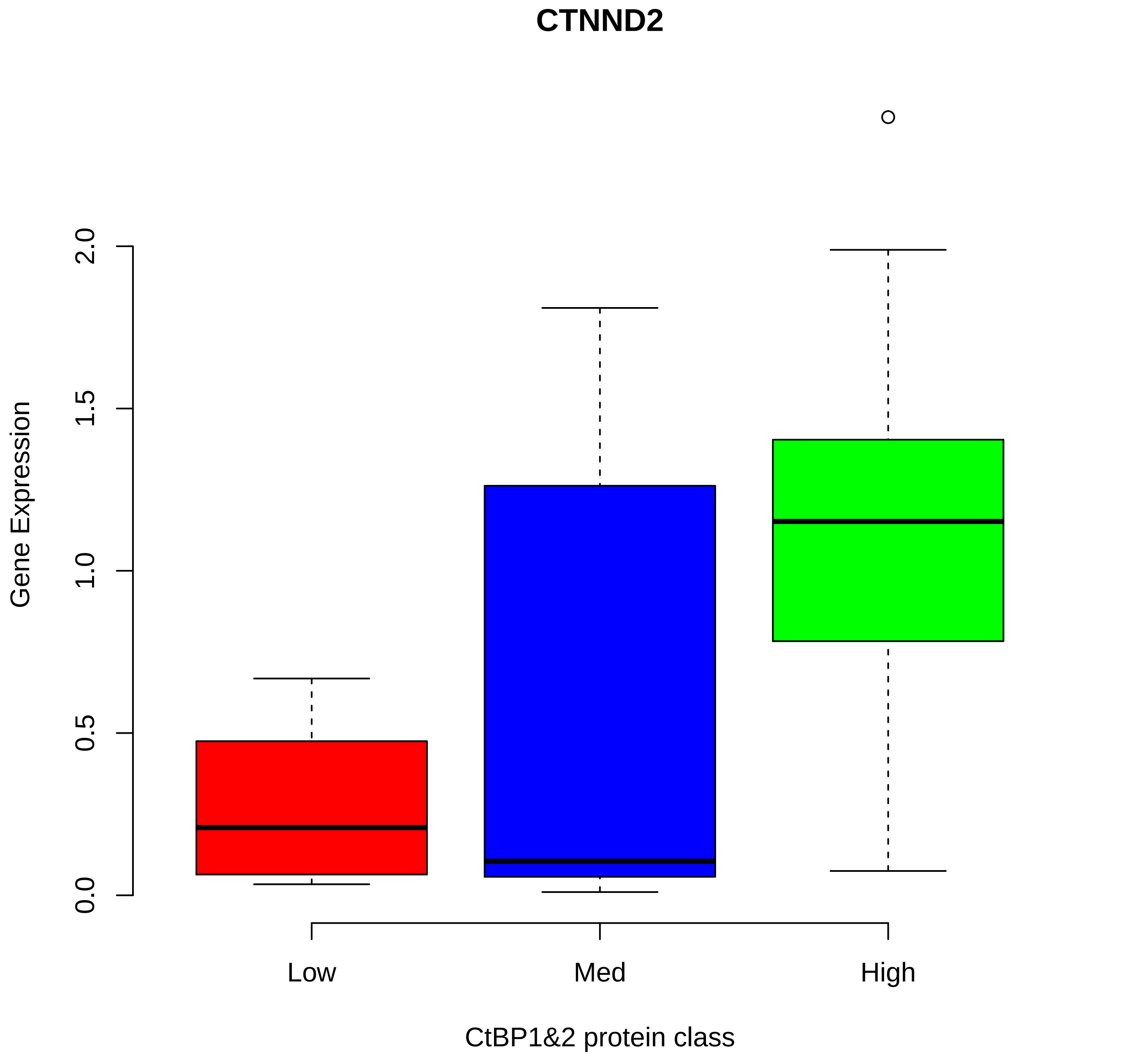
CTNND2
Gene Expression
0.0
0.5
1.0
1.5
2.0
Low
Med
High
CtBP1&2 protein class

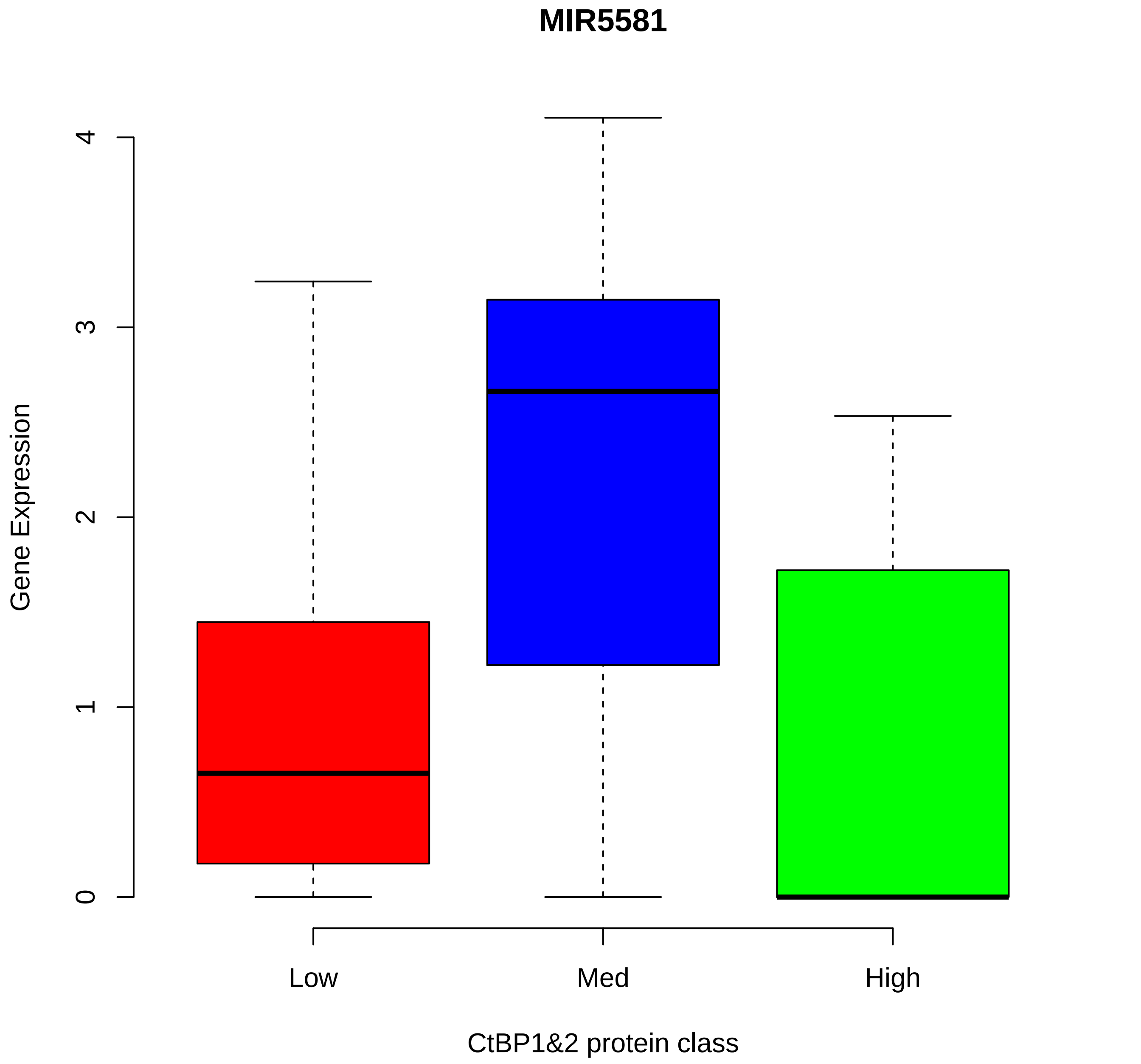
MIR5581
Gene Expression
0
1
2
3
4
Low
Med
High
CtBP1&2 protein class

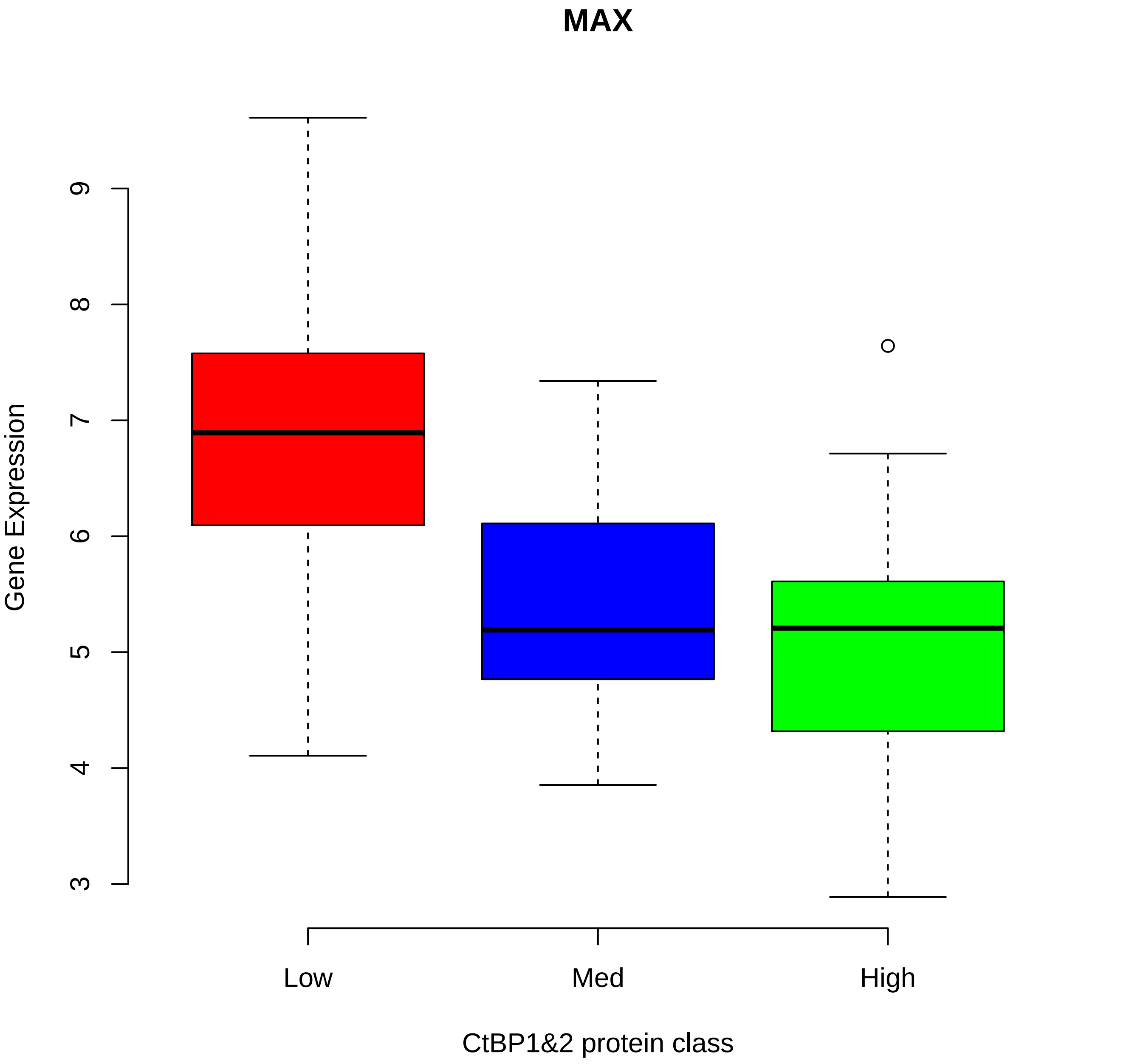

MAX
Gene Expression
3
4
5
6
7
8
9
Low
Med
High
CtBP1&2 protein class

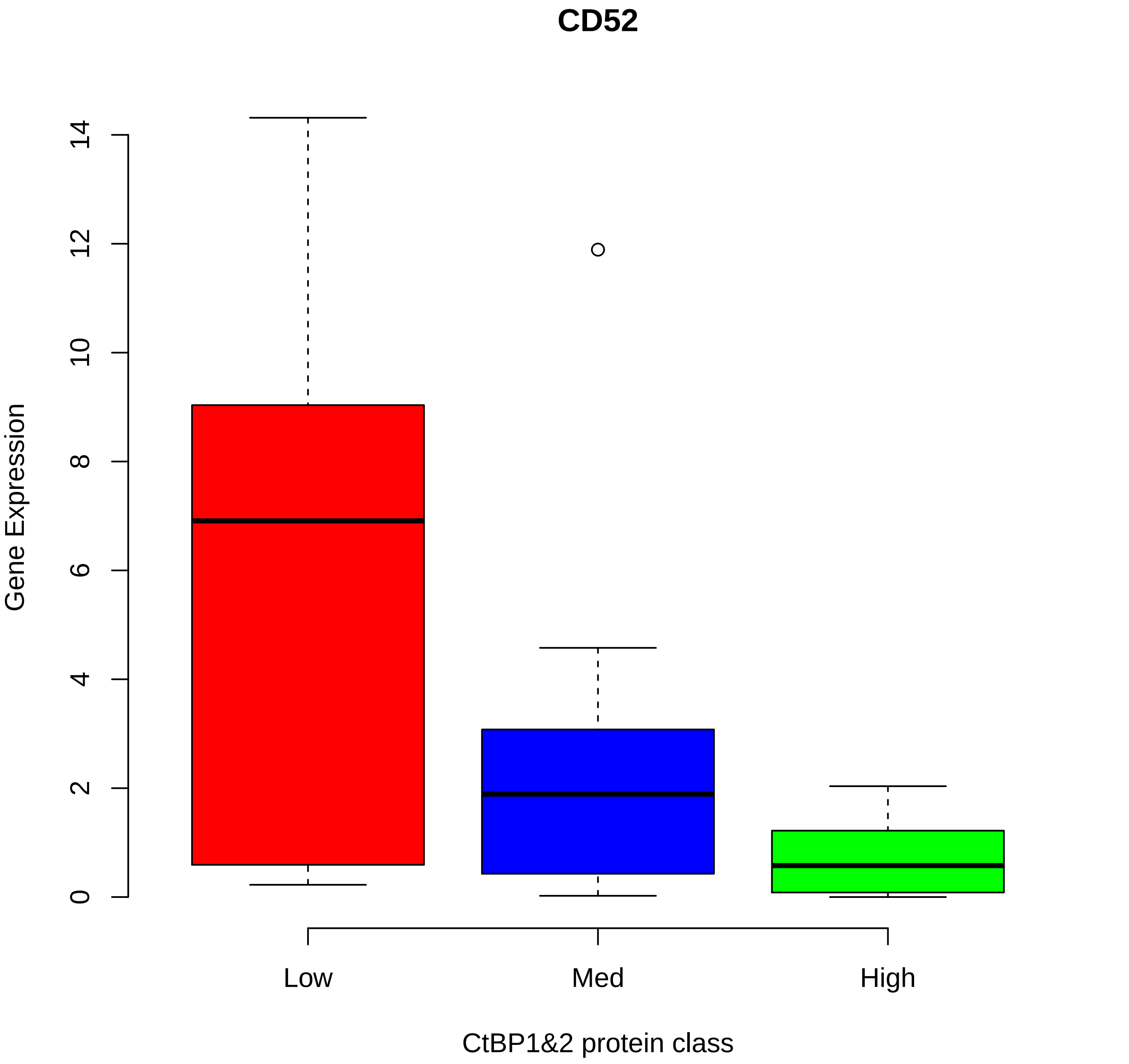
CD52
Gene Expression
0
2
4
6
8
10
12
14
Low
Med
High
CtBP1&2 protein class

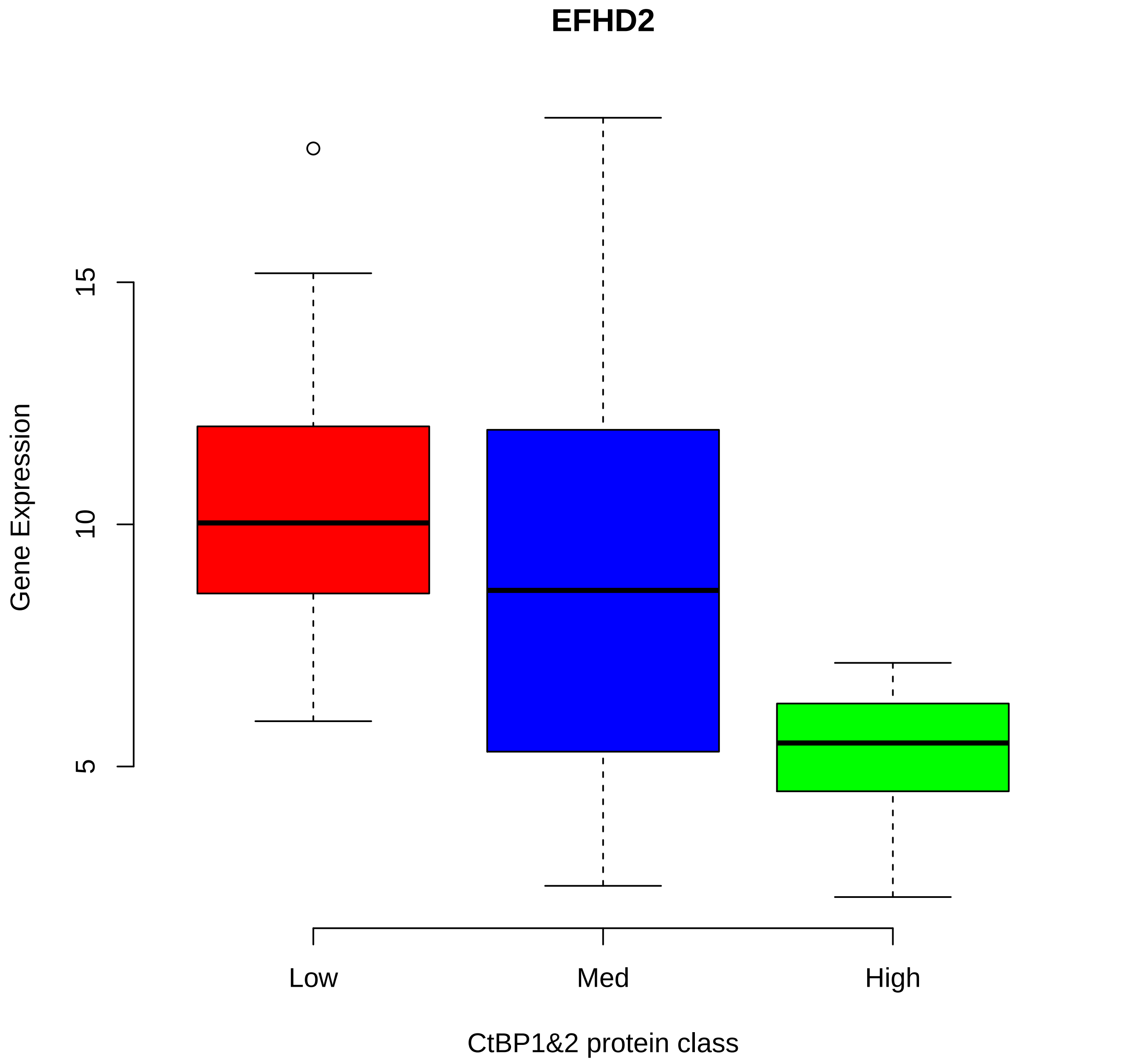
EFHD2
Gene Expression
15
10
5
Low
Med
High
CtBP1&2 protein class

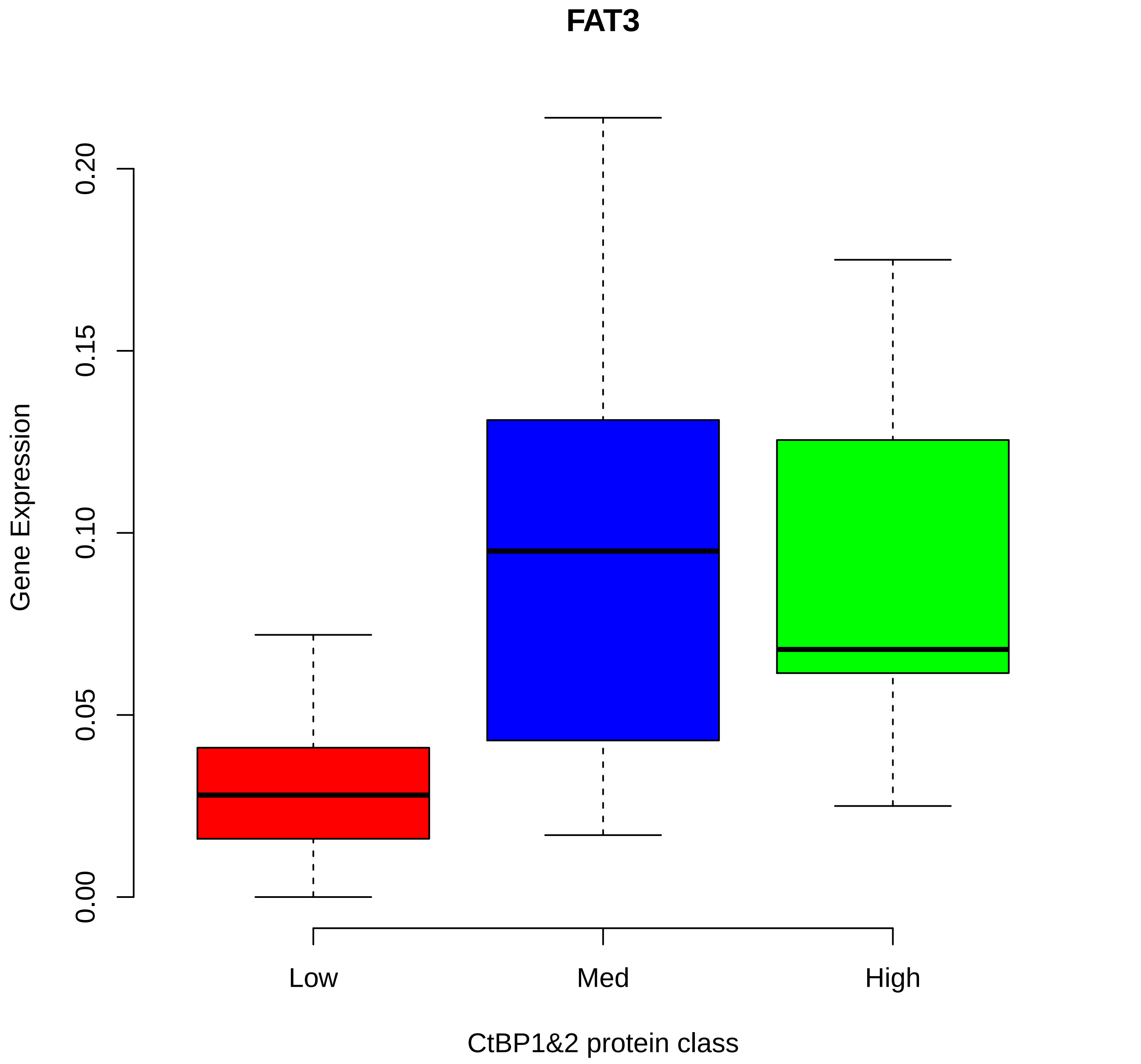
FAT3
Gene Expression
0.00
0.05
0.10
0.15
0.20
Low
Med
High
CtBP1&2 protein class

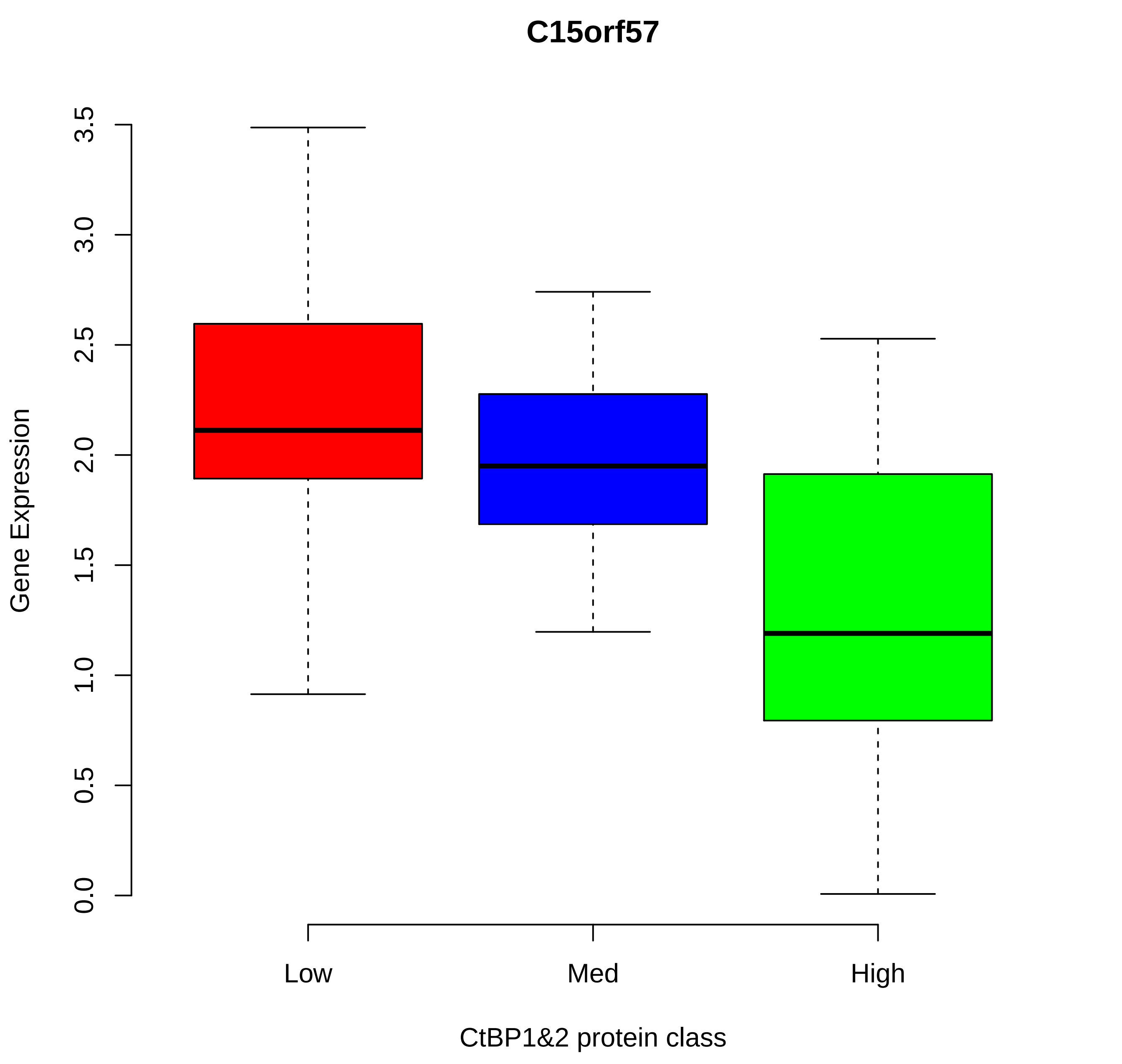
C15orf57
Gene Expression
0.0
0.5
1.0
1.5
2.0
2.5
3.0
3.5
Low
Med
High
CtBP1&2 protein class

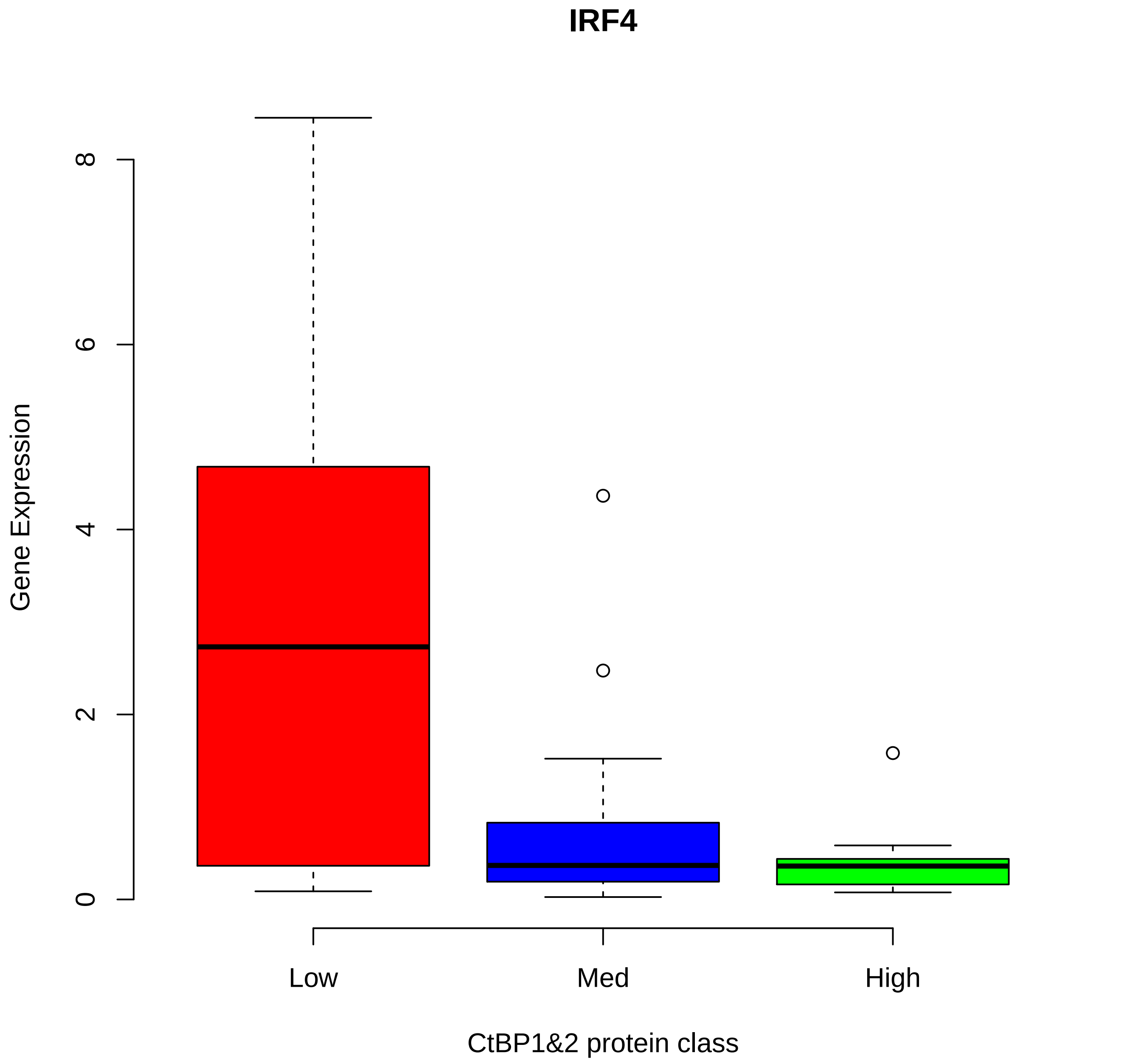

IRF4
Gene Expression
0
2
4
6
8
Low
Med
High
CtBP1&2 protein class

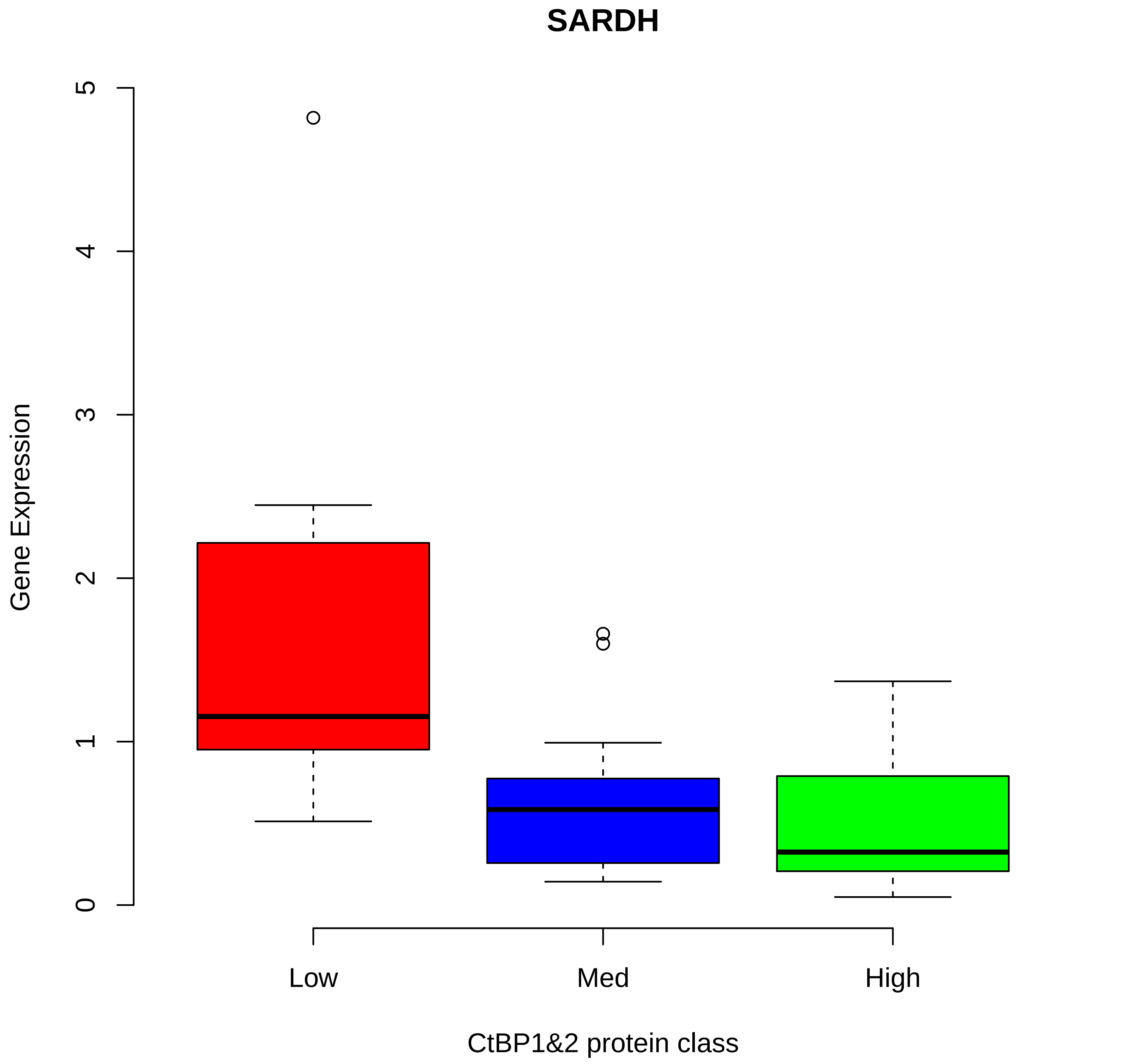
SARDH
Gene Expression
0
1
2
3
4
5
Low
Med
High
CtBP1&2 protein class

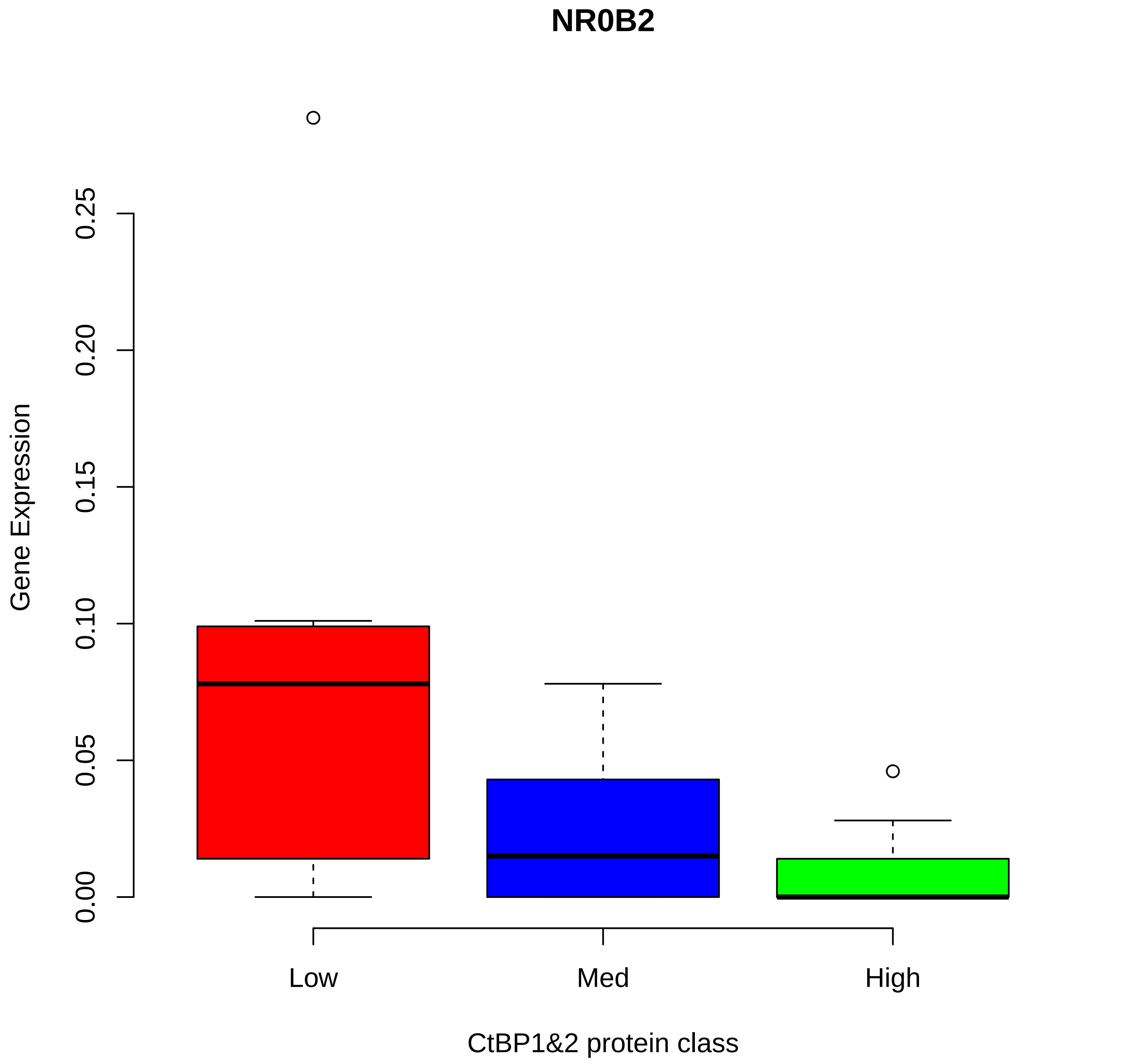
NR0B2
Gene Expression
0.00
0.05
0.10
0.15
0.20
0.25
Low
Med
High
CtBP1&2 protein class

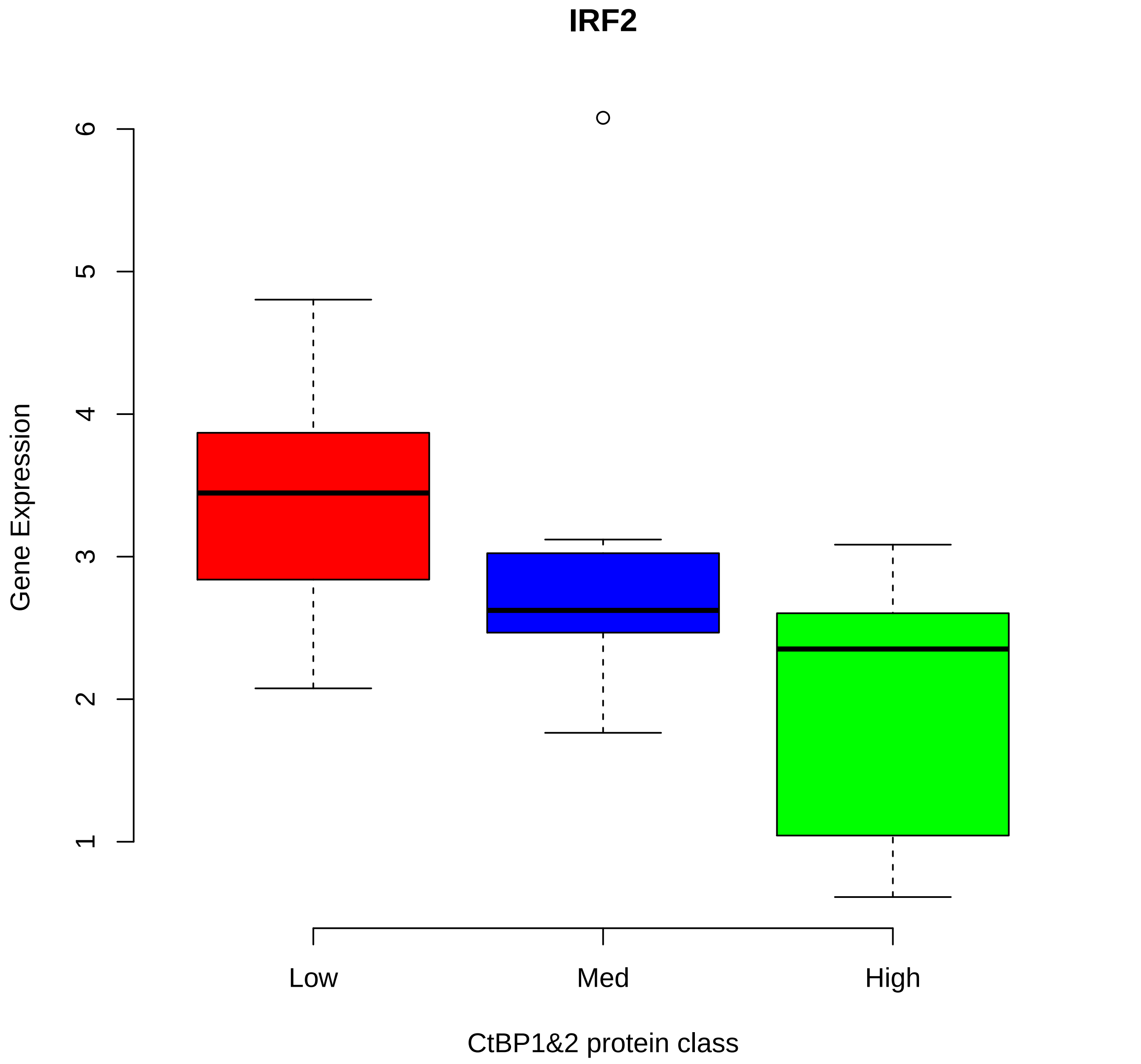
IRF2
Gene Expression
1
2
3
4
5
6
Low
Med
High
CtBP1&2 protein class

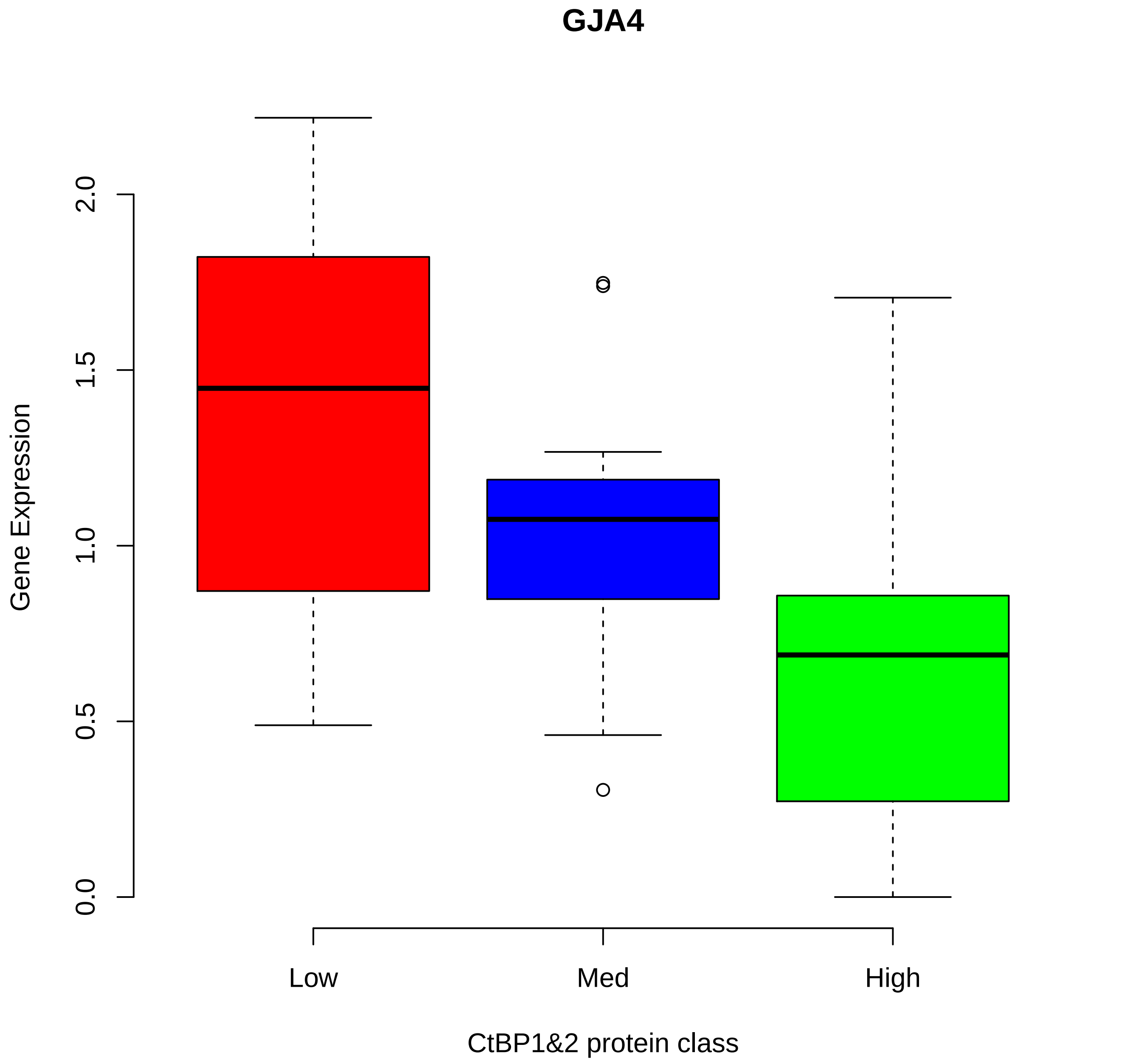

GJA4
Gene Expression
0.0
0.5
1.0
1.5
2.0
Low
Med
High
CtBP1&2 protein class

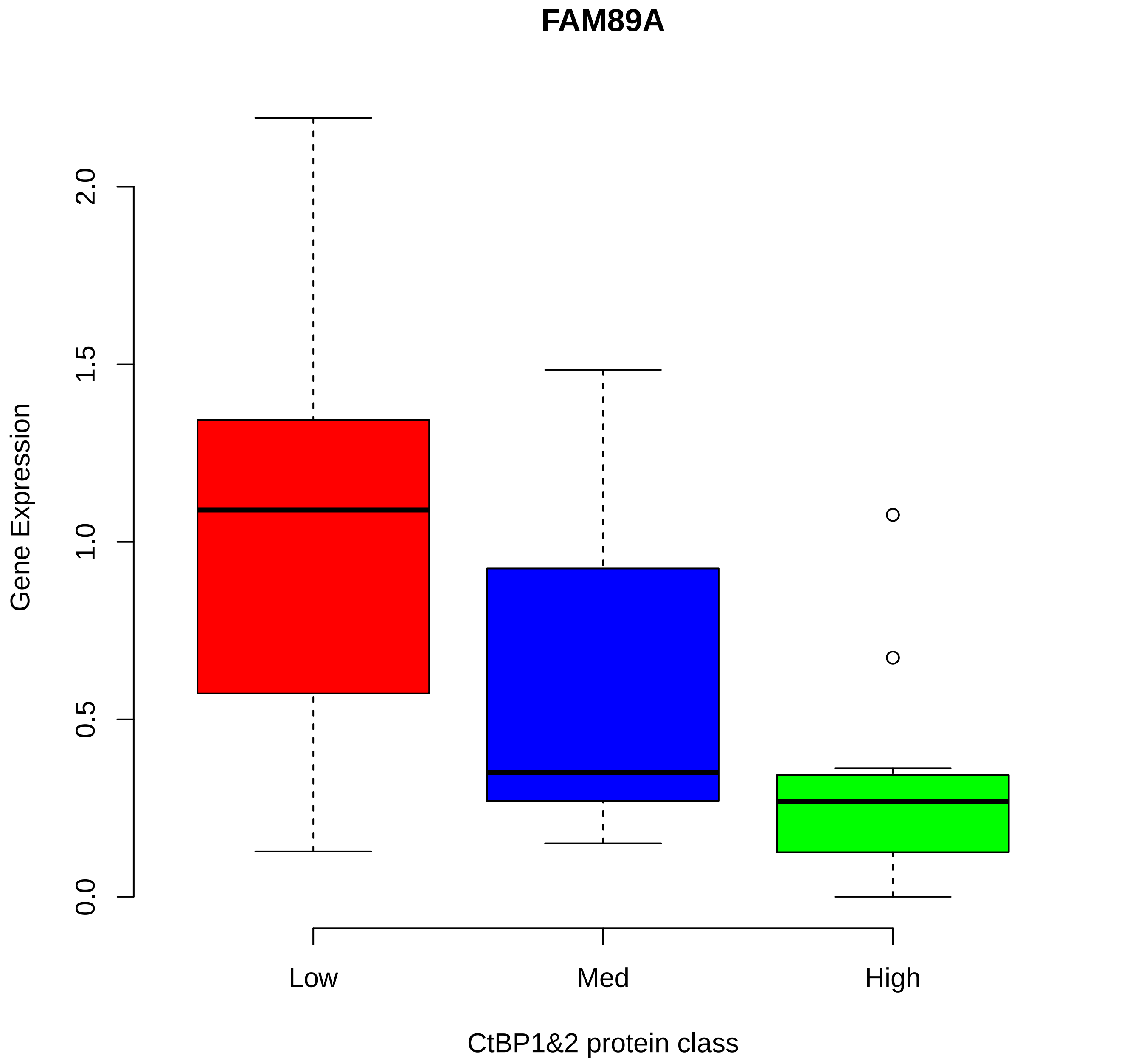
FAM89A
Gene Expression
0.0
0.5
1.0
1.5
2.0
Low
Med
High
CtBP1&2 protein class

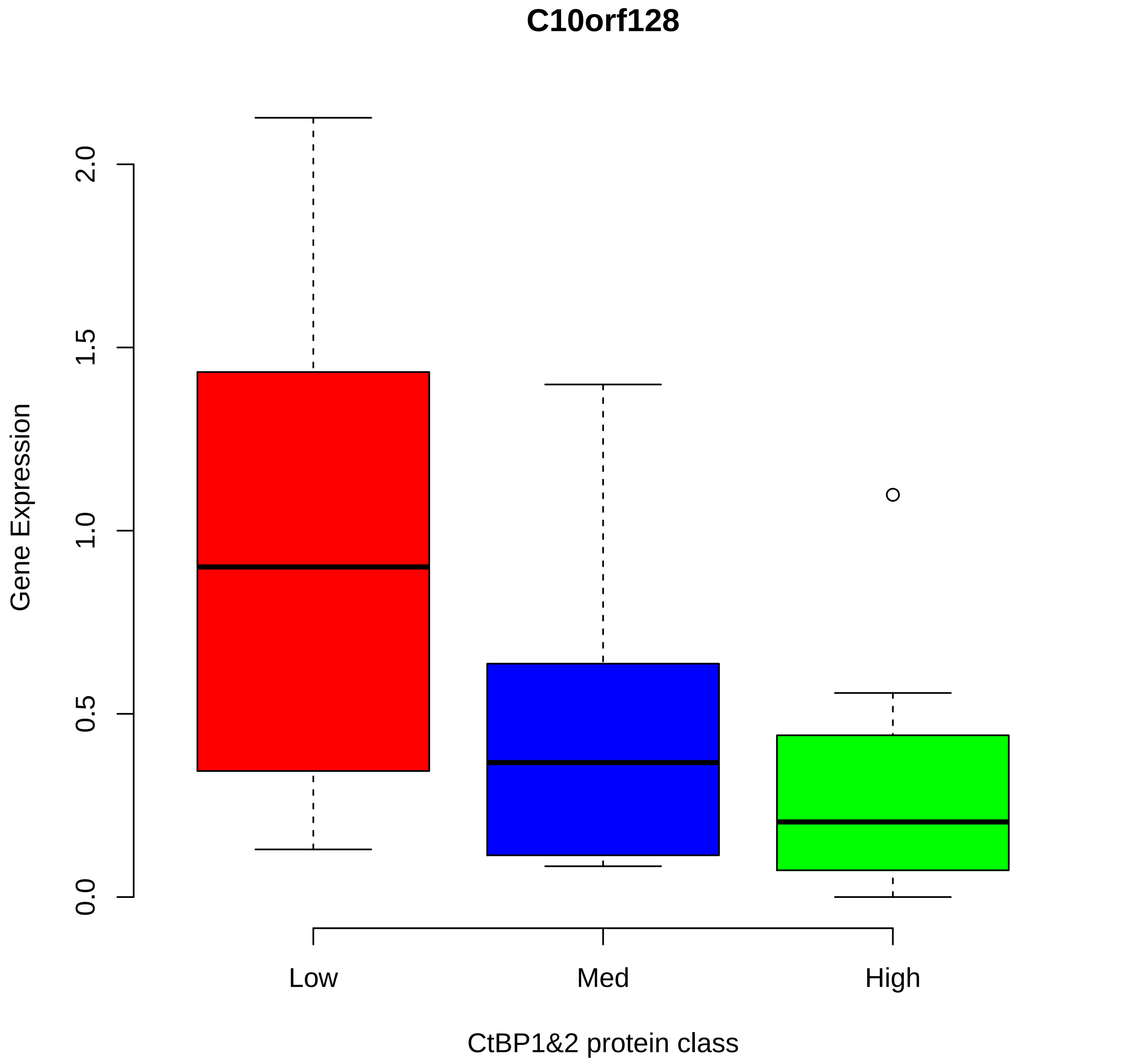

C10orf128
Gene Expression
0.0
0.5
1.0
1.5
2.0
Low
Med
High
CtBP1&2 protein class

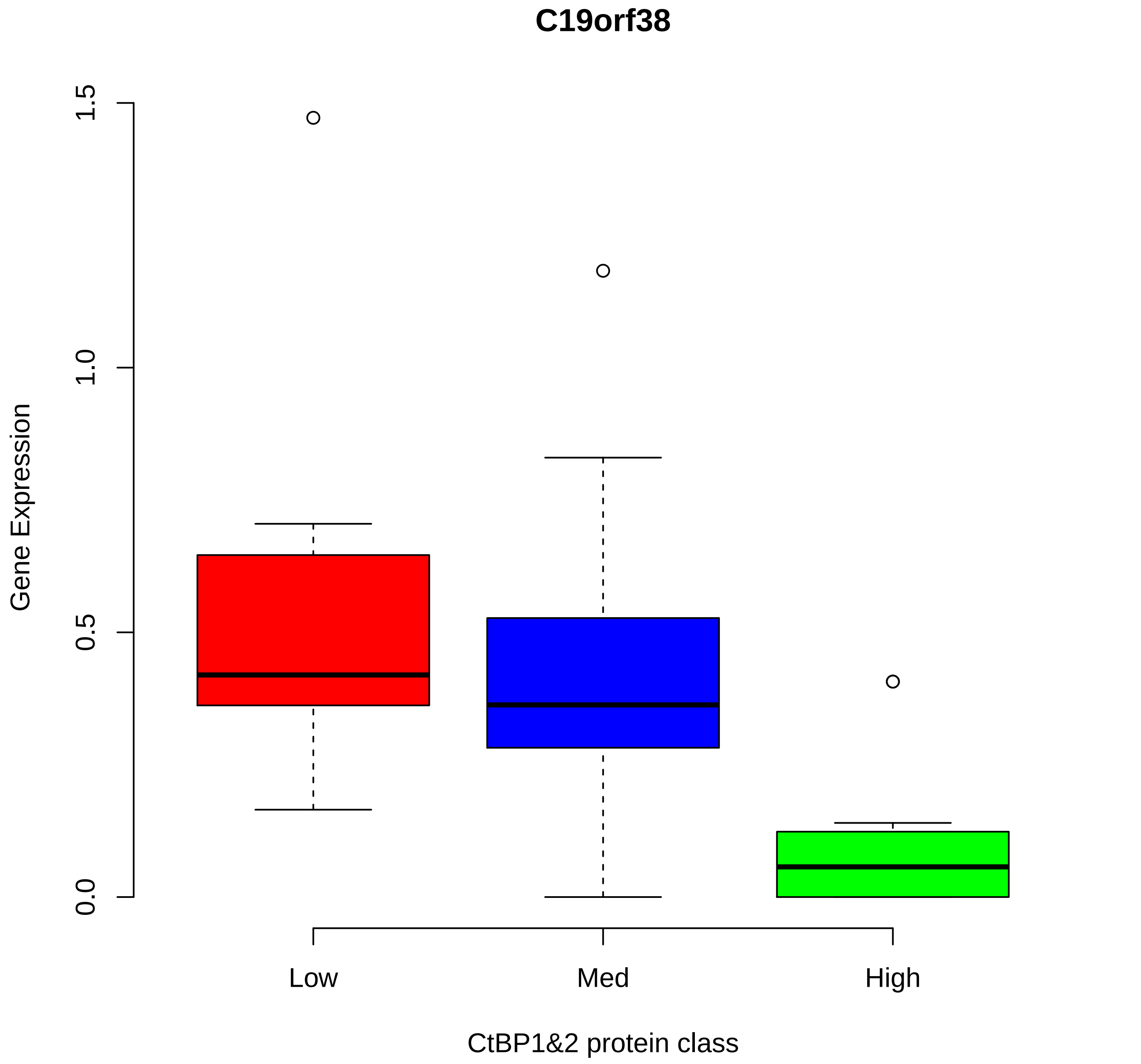

C19orf38
Gene Expression
0.0
0.5
1.0
1.5
Low
Med
High
CtBP1&2 protein class

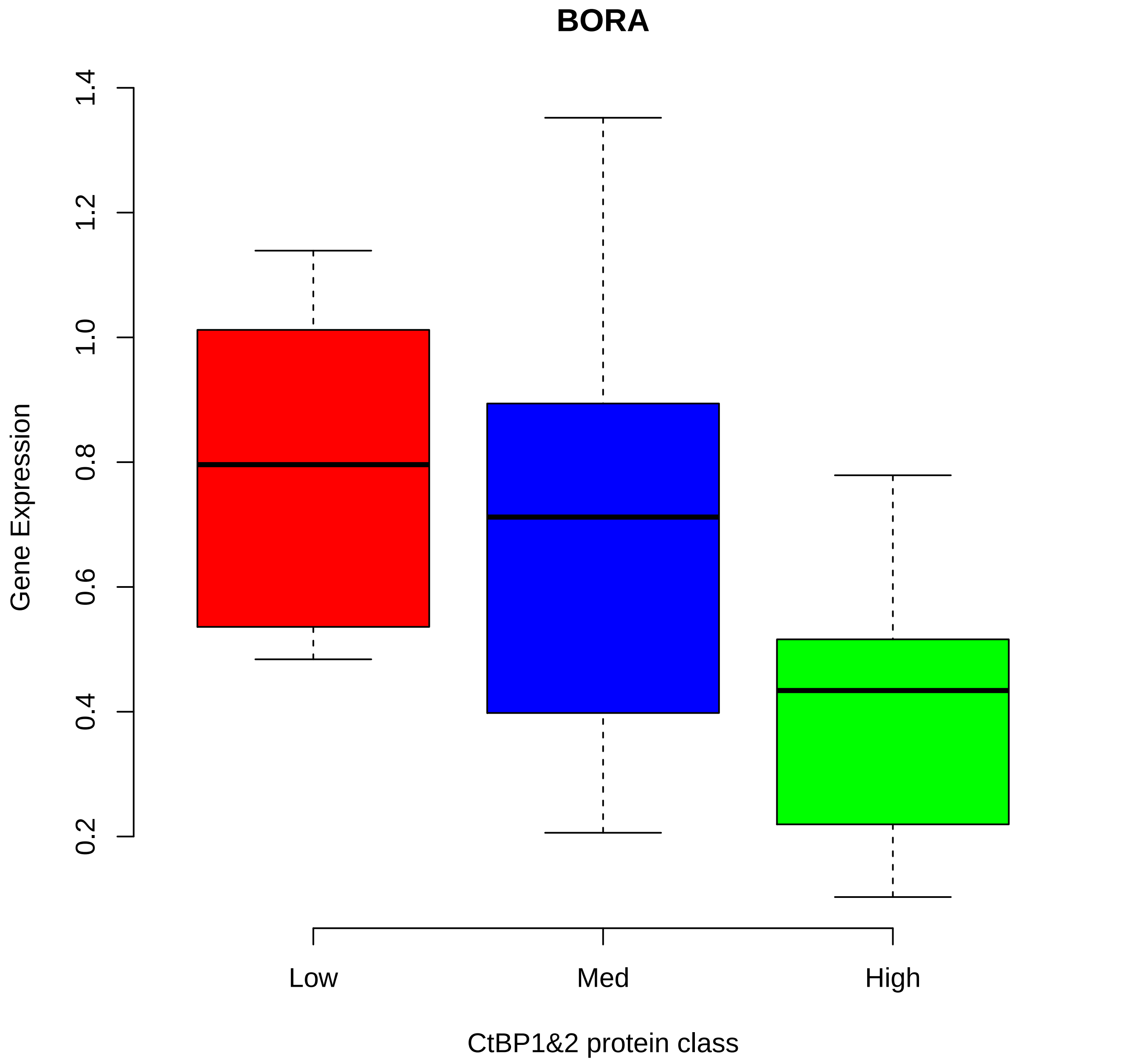
BORA
Gene Expression
0.2
0.4
0.6
0.8
1.0
1.2
1.4
Low
Med
High
CtBP1&2 protein class

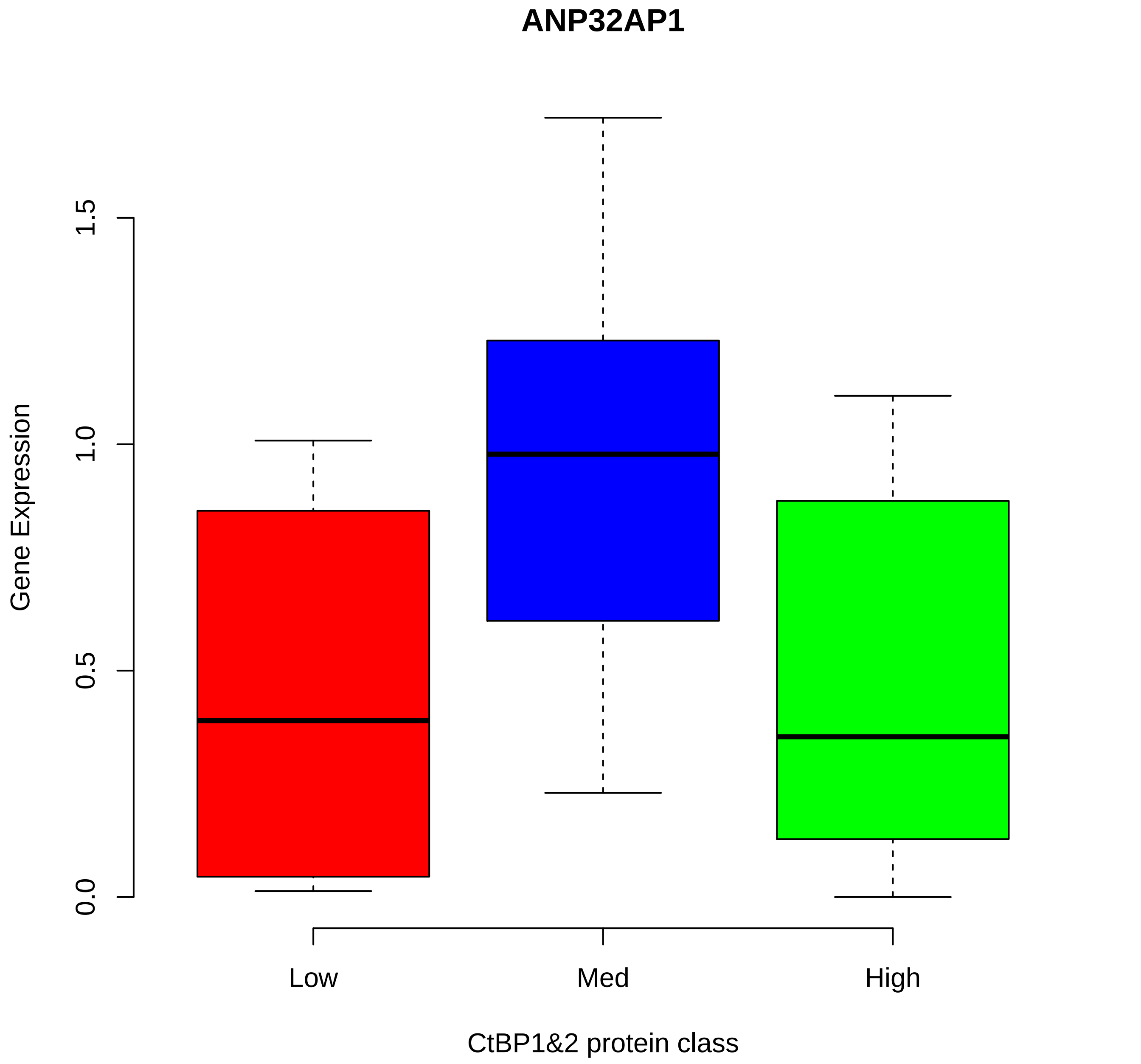

ANP32AP1
Gene Expression
0.0
0.5
1.0
1.5
Low
Med
High
CtBP1&2 protein class

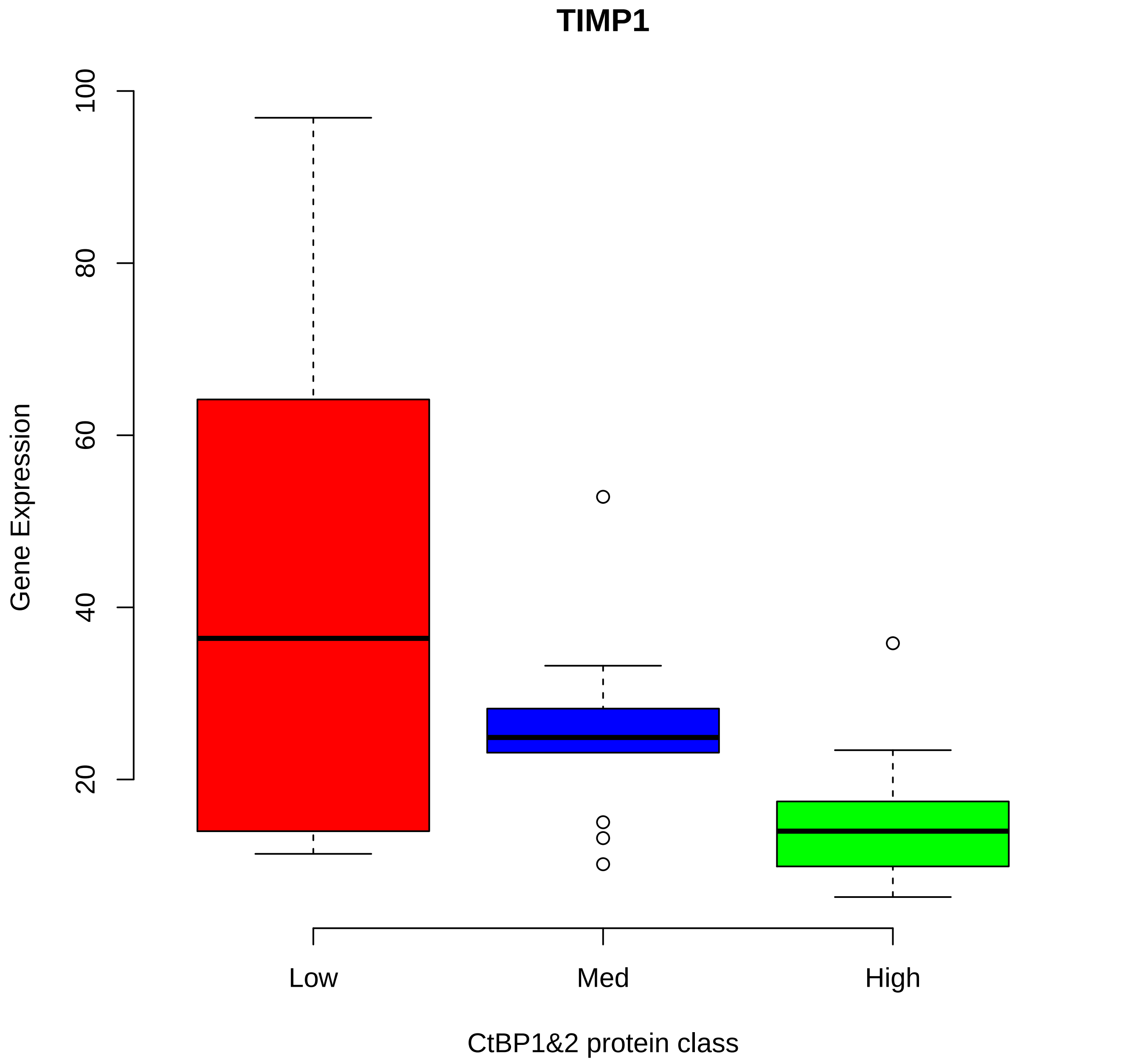

TIMP1
Gene Expression
100
80
60
40
20
Low
Med
High
CtBP1&2 protein class

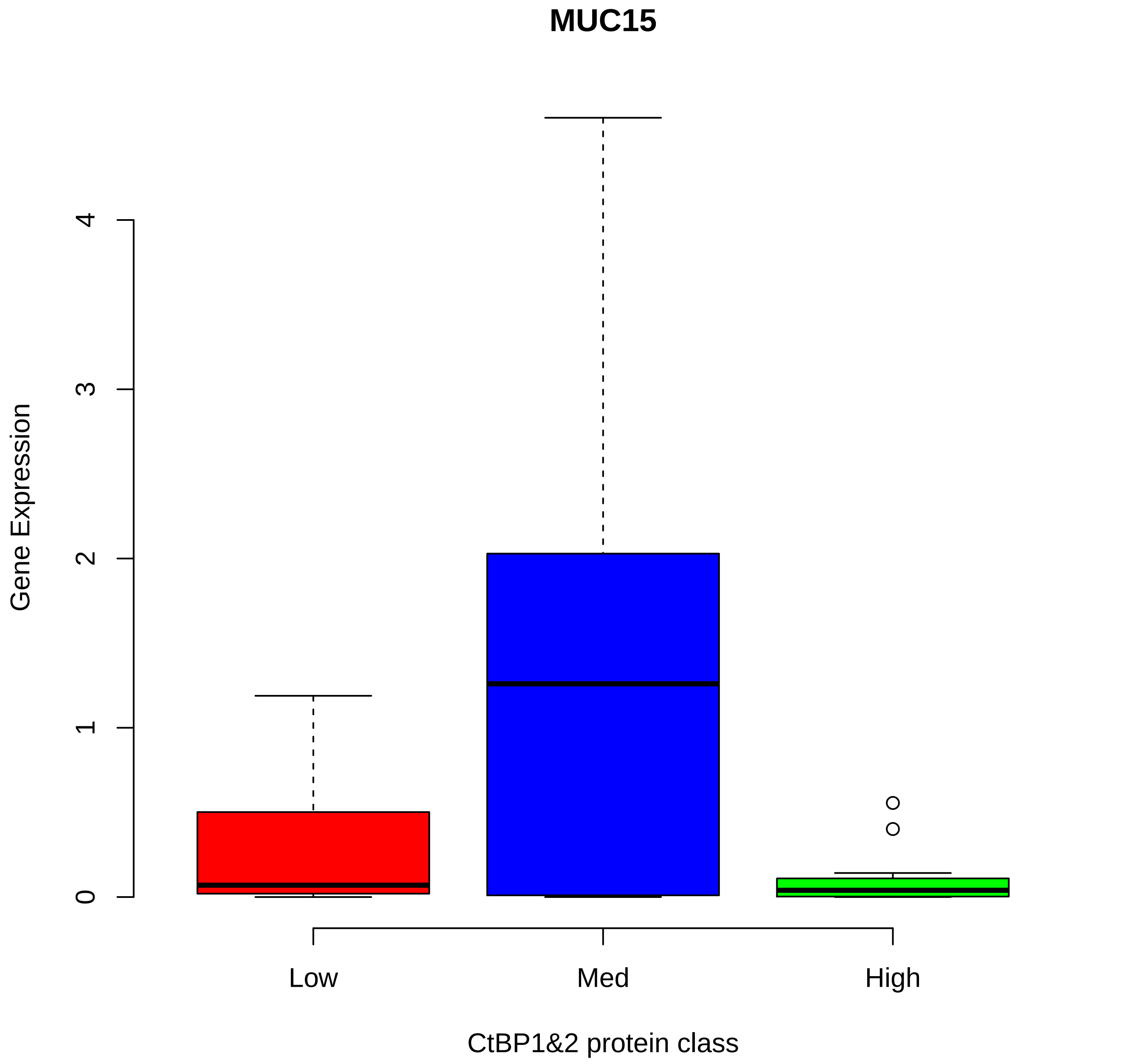
MUC15
Gene Expression
0
1
2
3
4
Low
Med
High
CtBP1&2 protein class

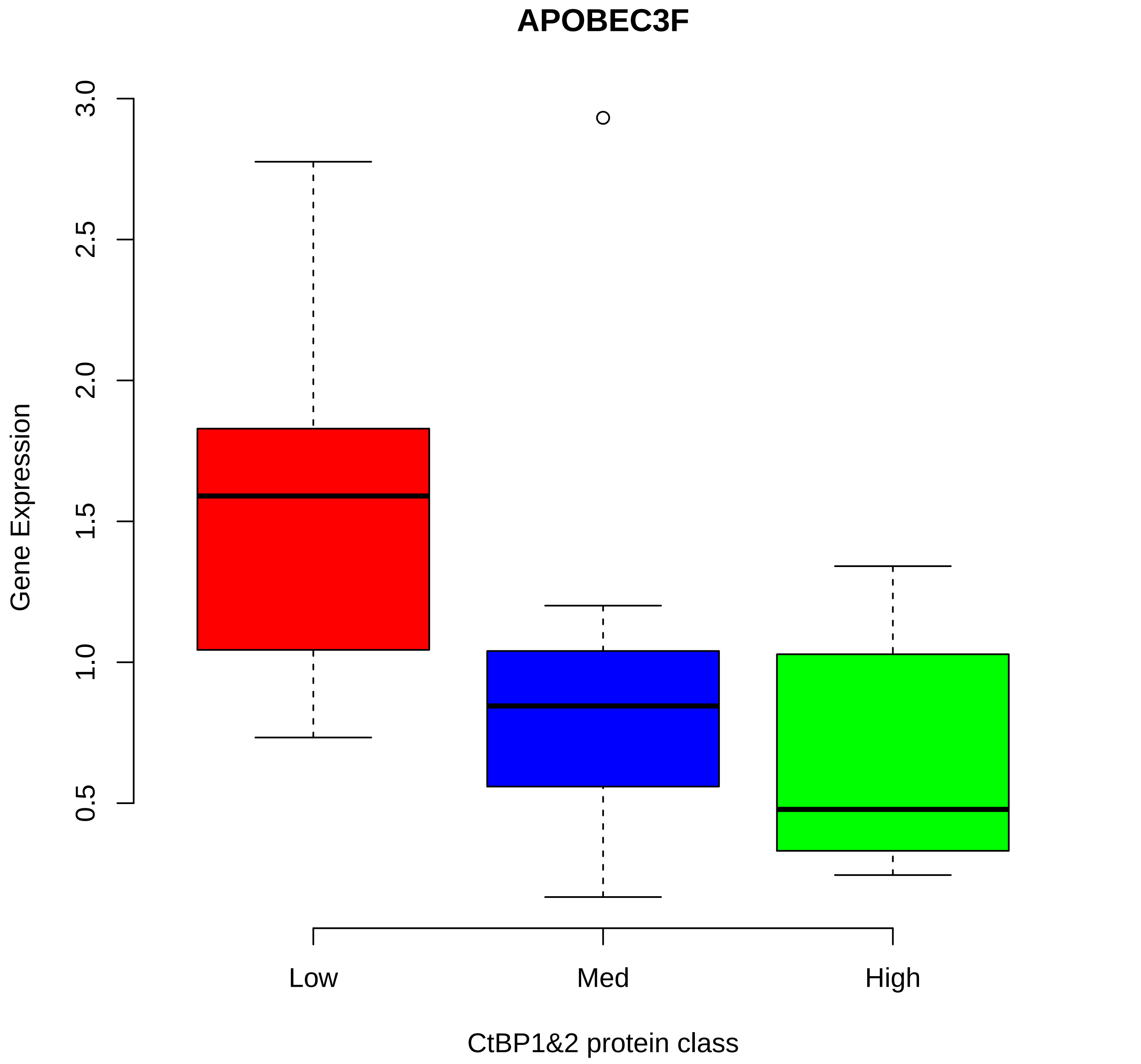

APOBEC3F
Gene Expression
3.0
2.5
2.0
1.5
1.0
0.5
Low
Med
High
CtBP1&2 protein class

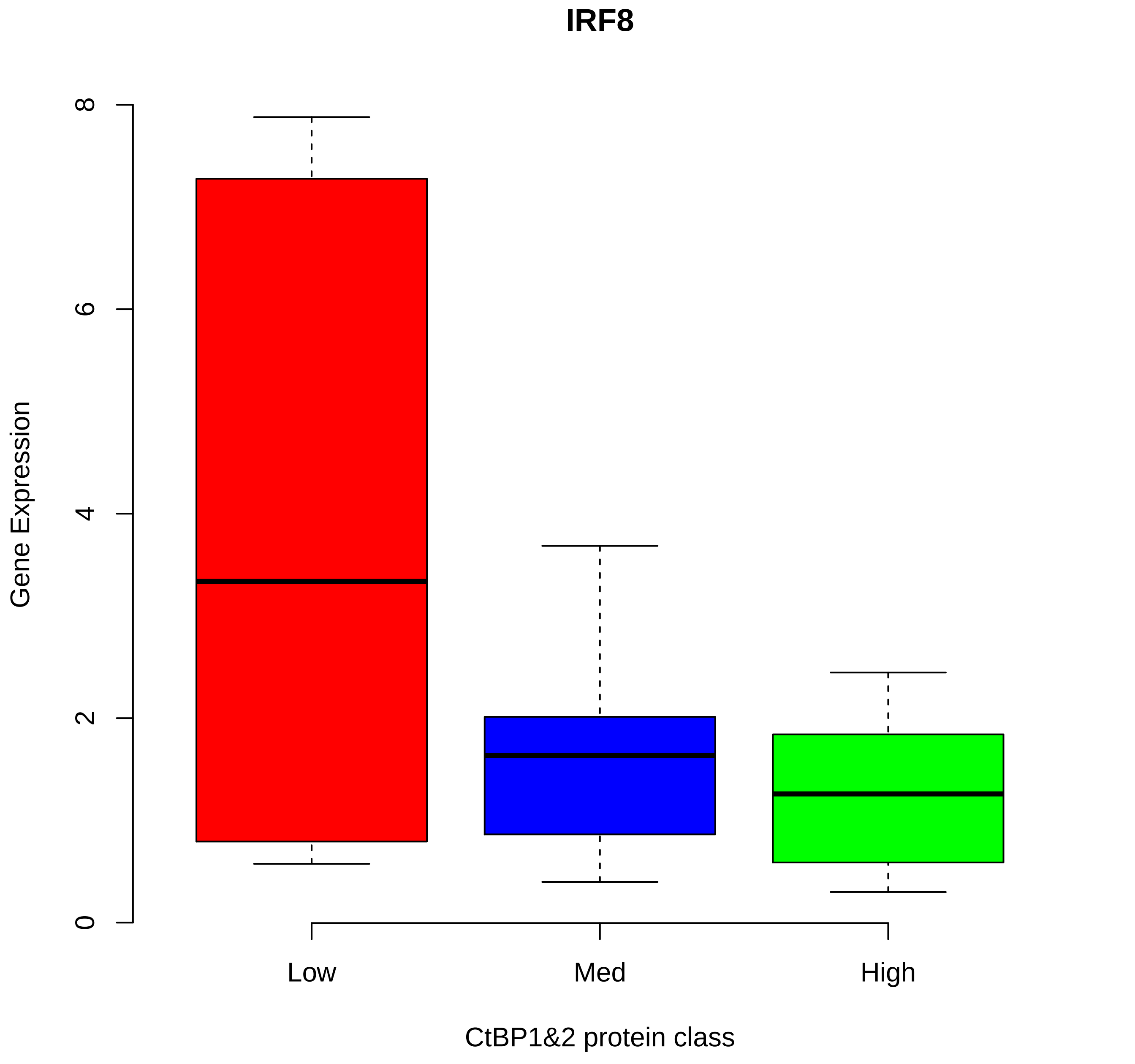
IRF8
Gene Expression
0
2
4
6
8
Low
Med
High
CtBP1&2 protein class

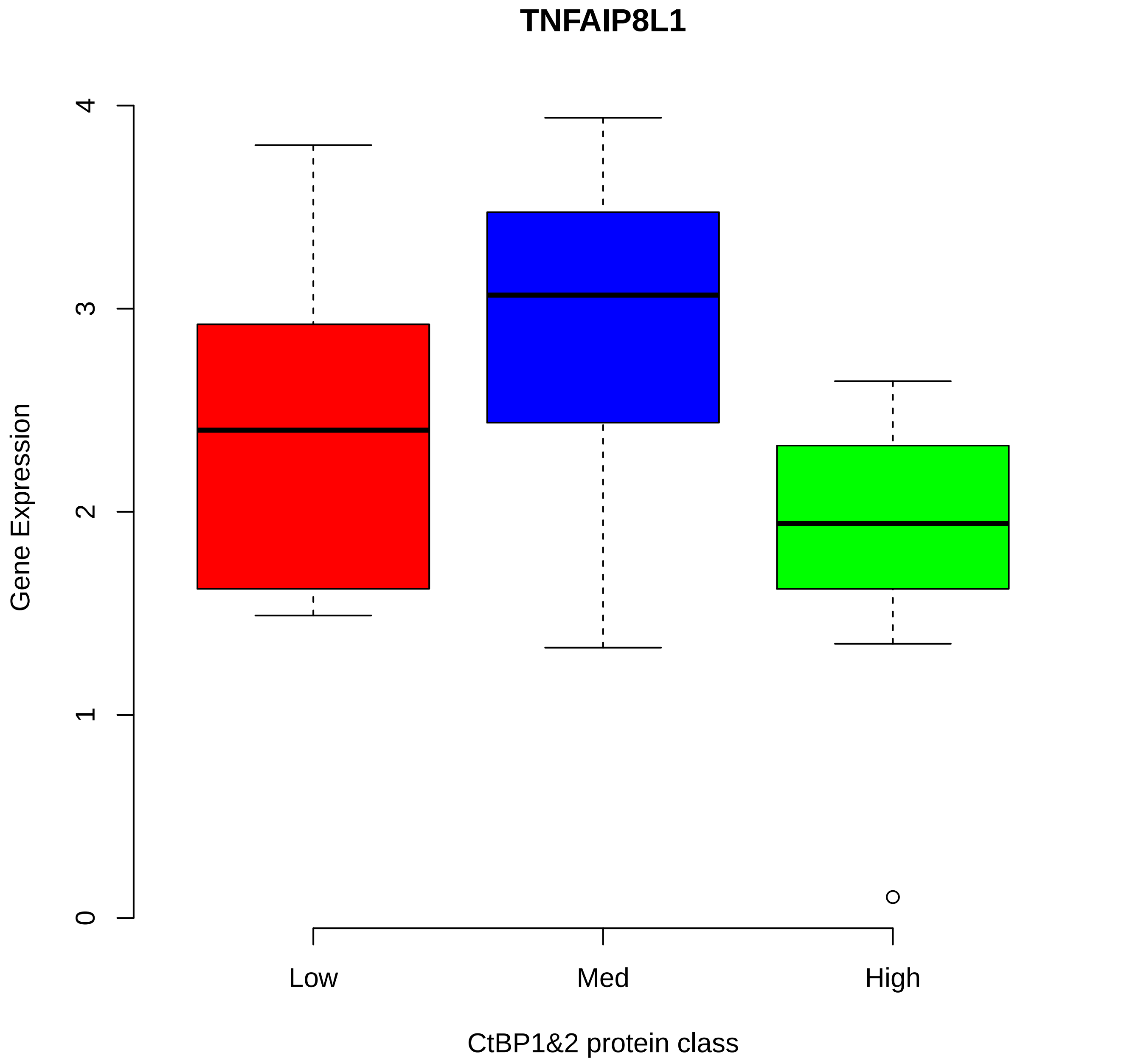

TNFAIP8L1
Gene Expression
0
1
2
3
4
Low
Med
High
CtBP1&2 protein class

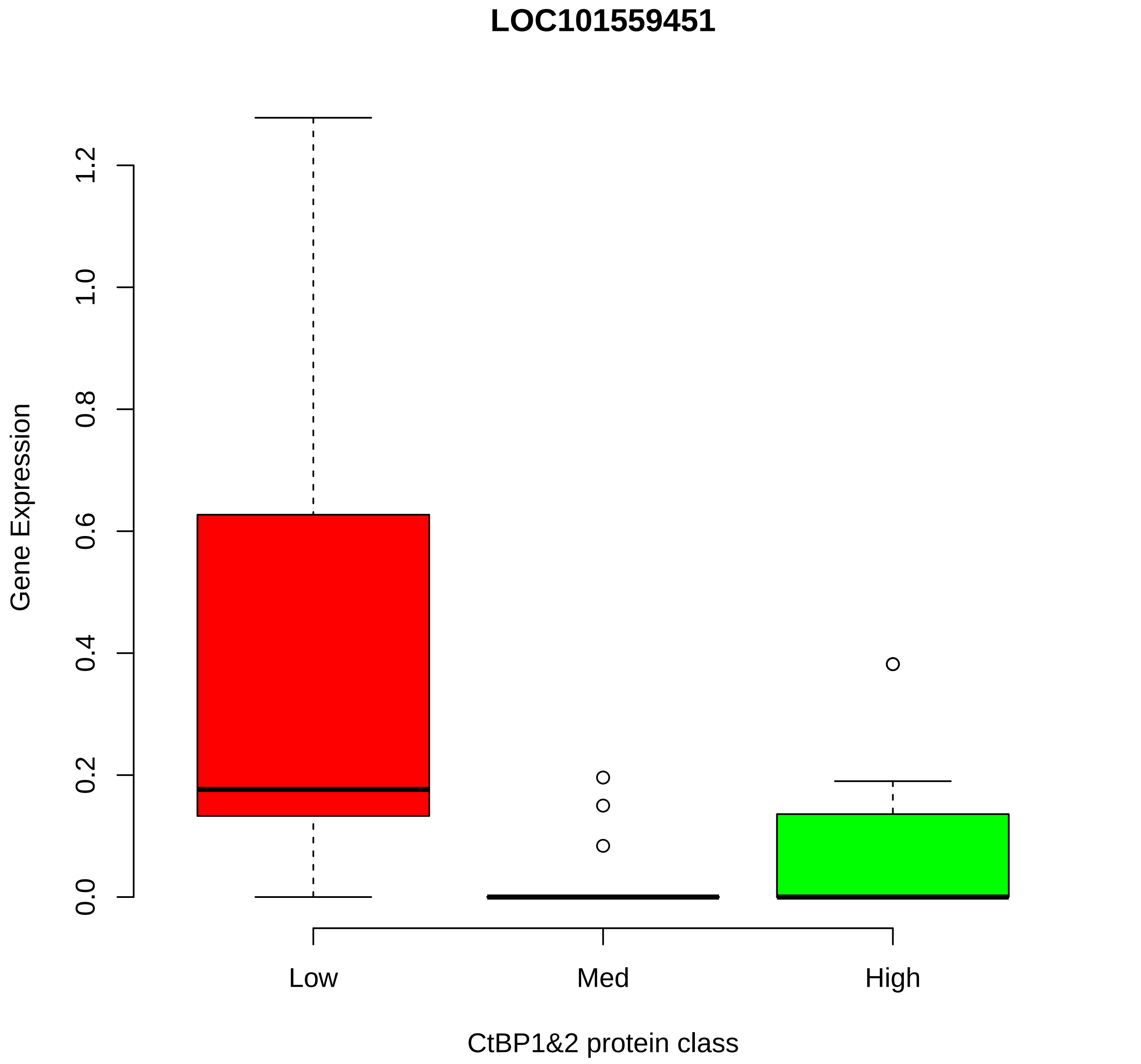

LOC101559451
Gene Expression
0.0
0.2
0.4
0.6
0.8
1.0
1.2
Low
Med
High
CtBP1&2 protein class

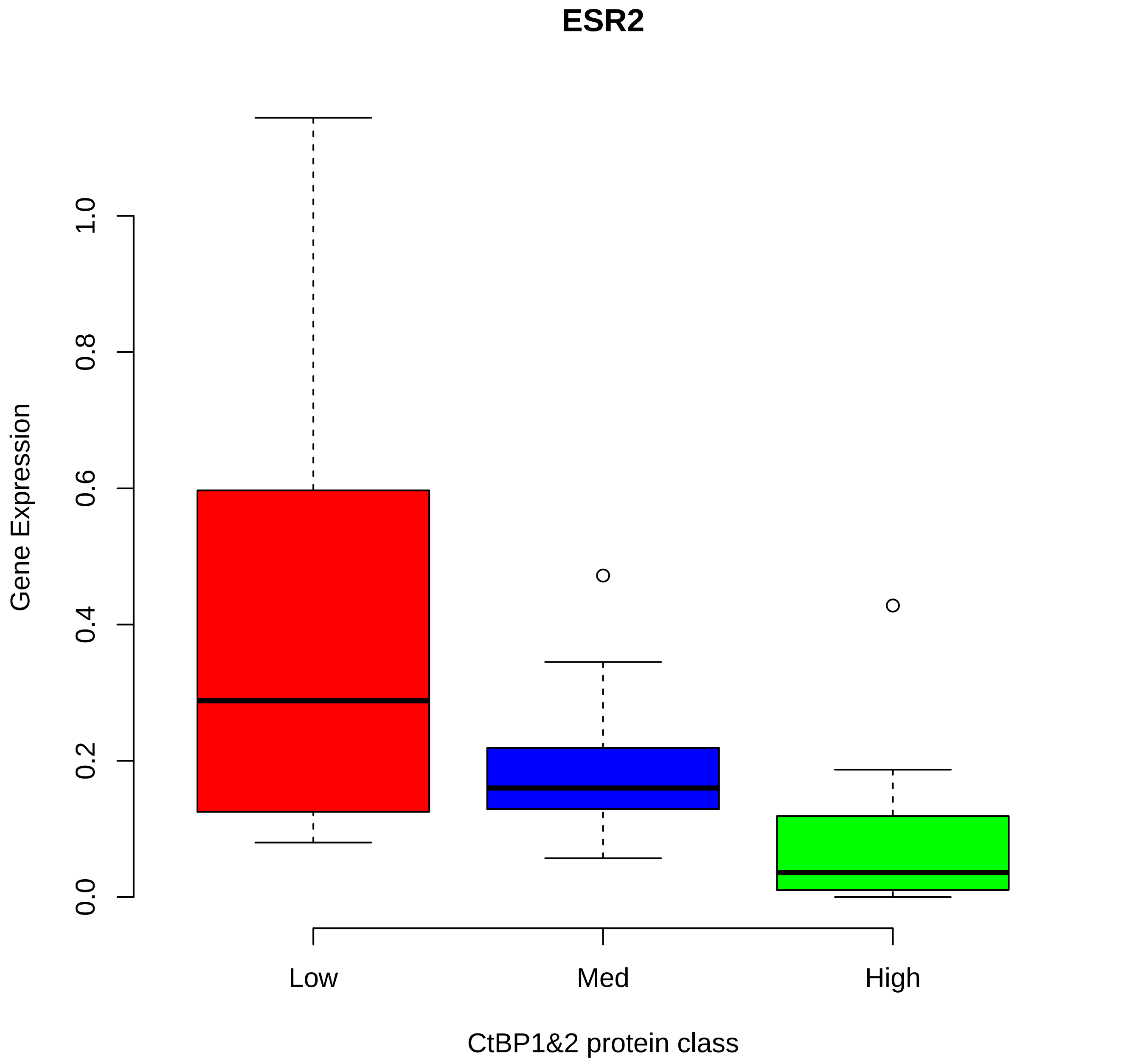
ESR2
Gene Expression
0.0
0.2
0.4
0.6
0.8
1.0
Low
Med
High
CtBP1&2 protein class

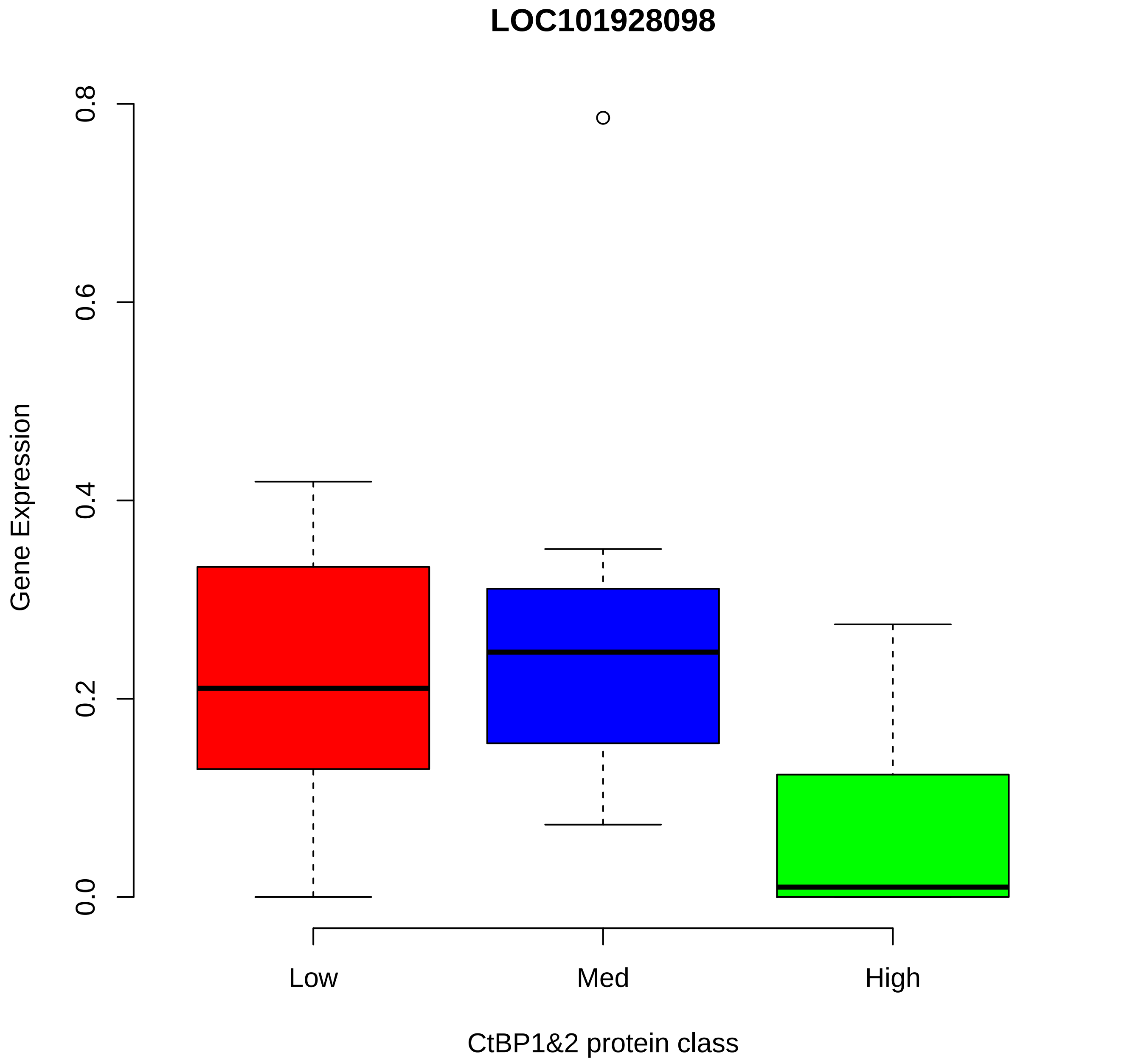

LOC101928098
Gene Expression
0.0
0.2
0.4
0.6
0.8
Low
Med
High
CtBP1&2 protein class

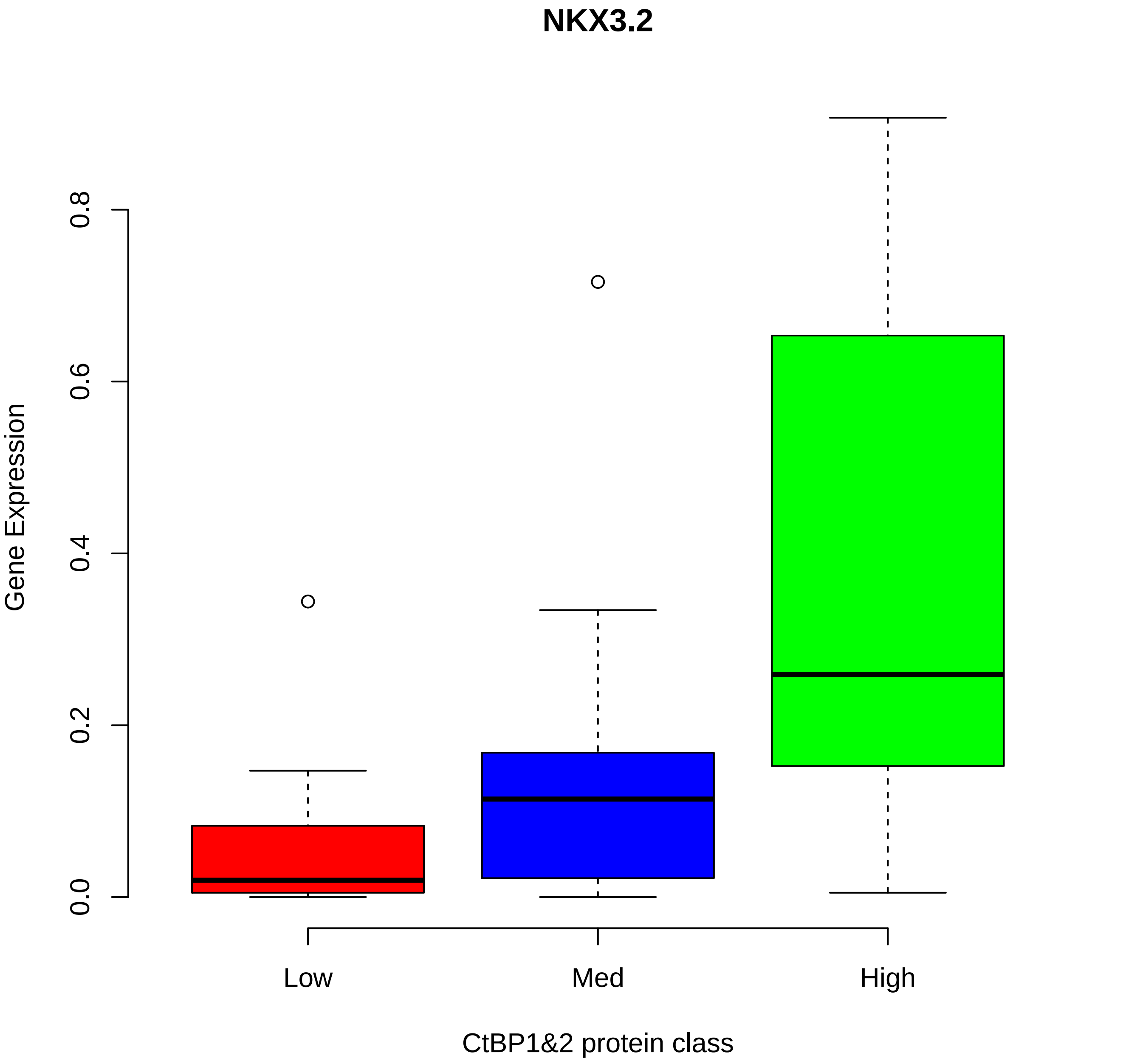

NKX3.2
Gene Expression
0.0
0.2
0.4
0.6
0.8
Low
Med
High
CtBP1&2 protein class

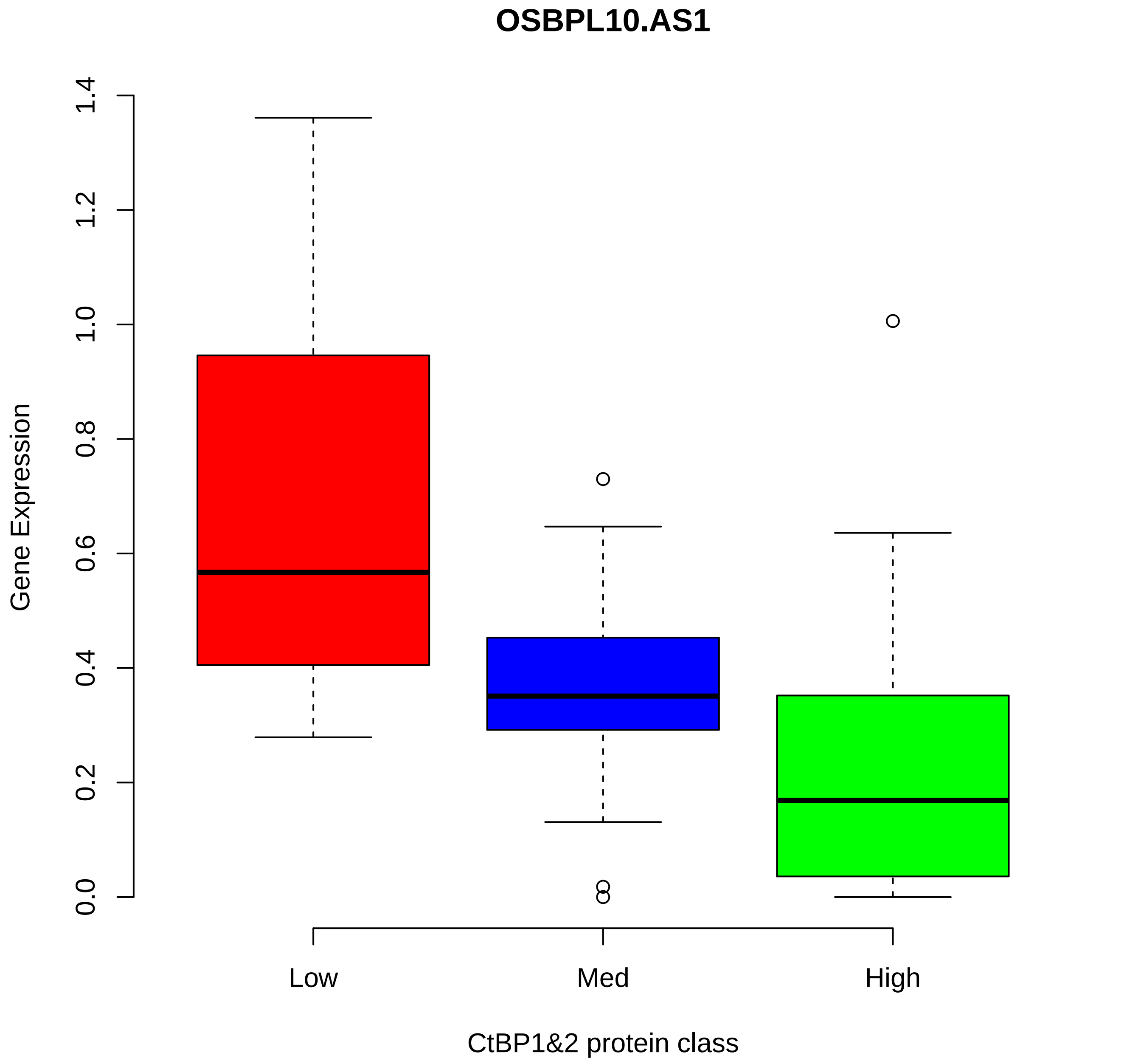

OSBPL10.AS1
Gene Expression
0.0
0.2
0.4
0.6
0.8
1.0
1.2
1.4
Low
Med
High
CtBP1&2 protein class

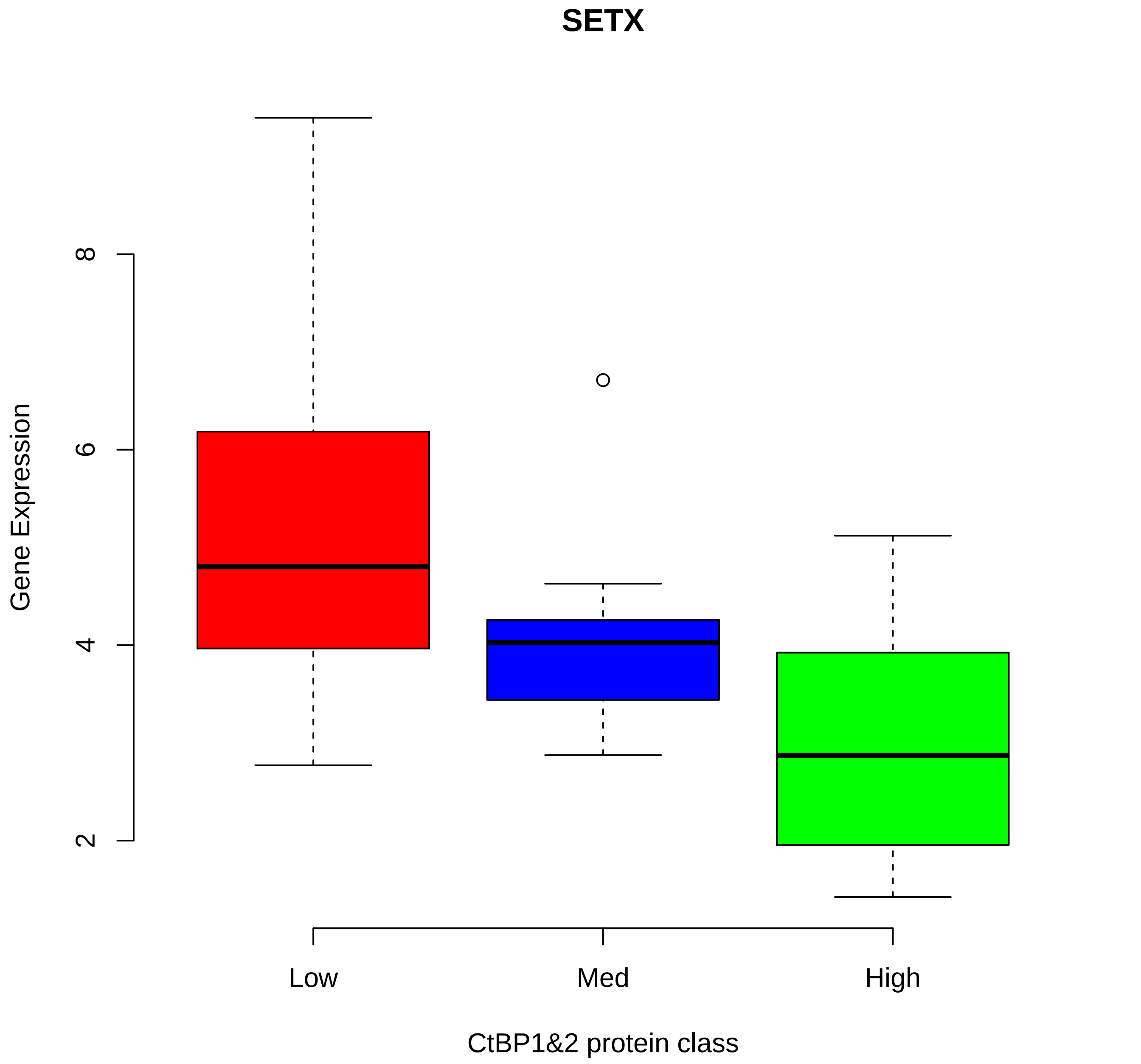

SETX
Gene Expression
2
4
6
8
Low
Med
High
CtBP1&2 protein class

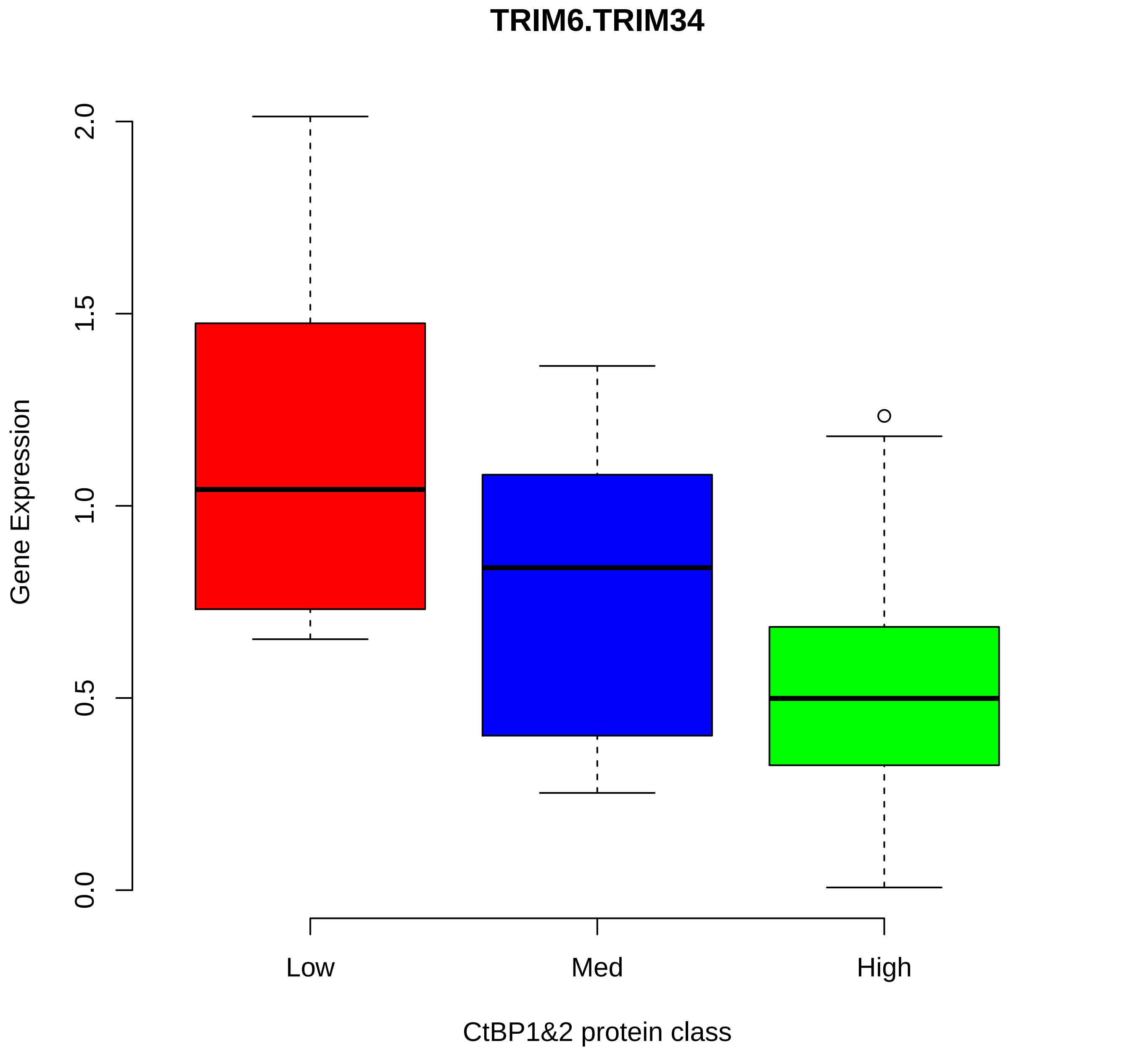

TRIM6.TRIM34
Gene Expression
0.0
0.5
1.0
1.5
2.0
Low
Med
High
CtBP1&2 protein class

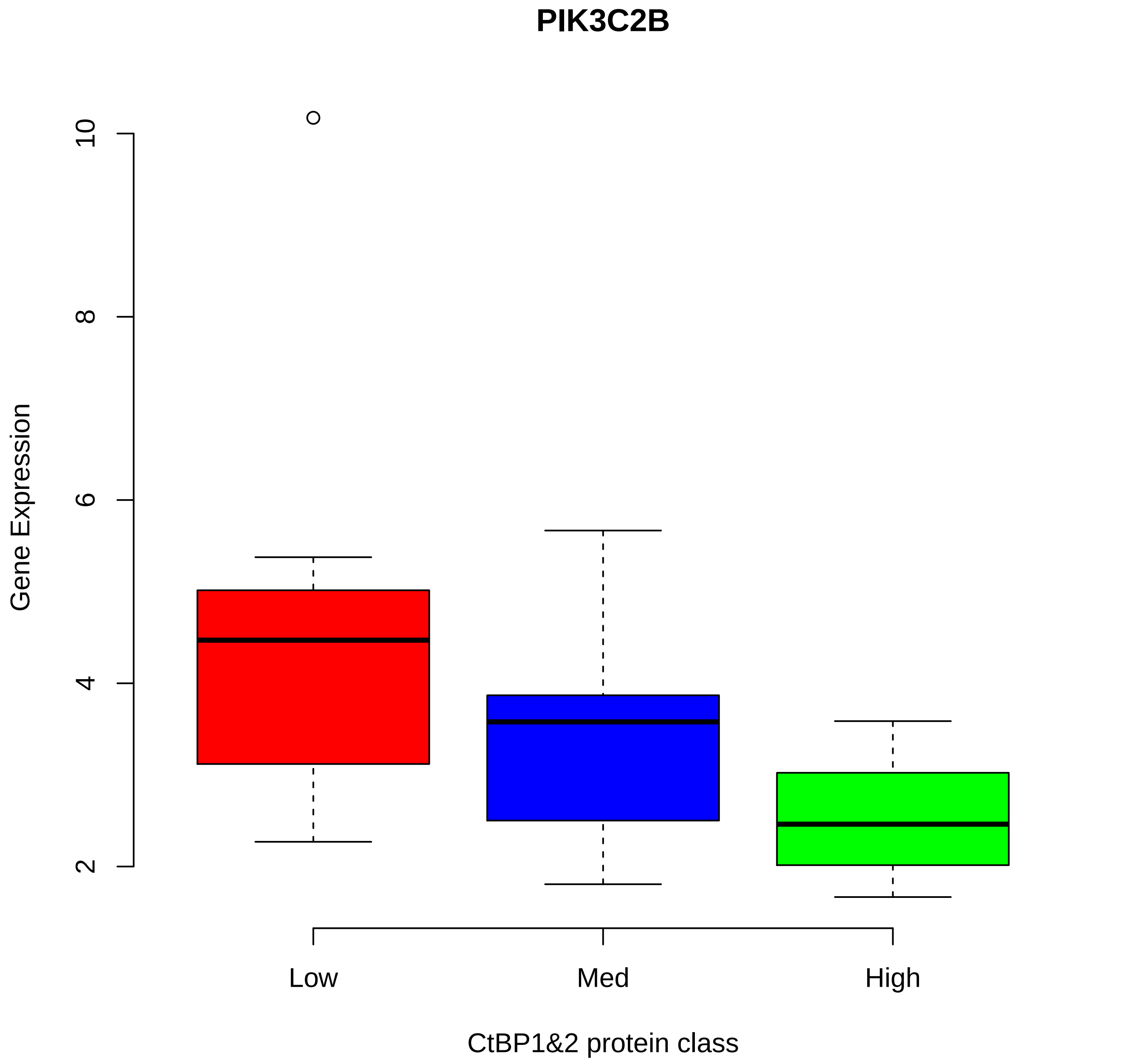

PIK3C2B
Gene Expression
10
8
6
4
2
Low
Med
High
CtBP1&2 protein class

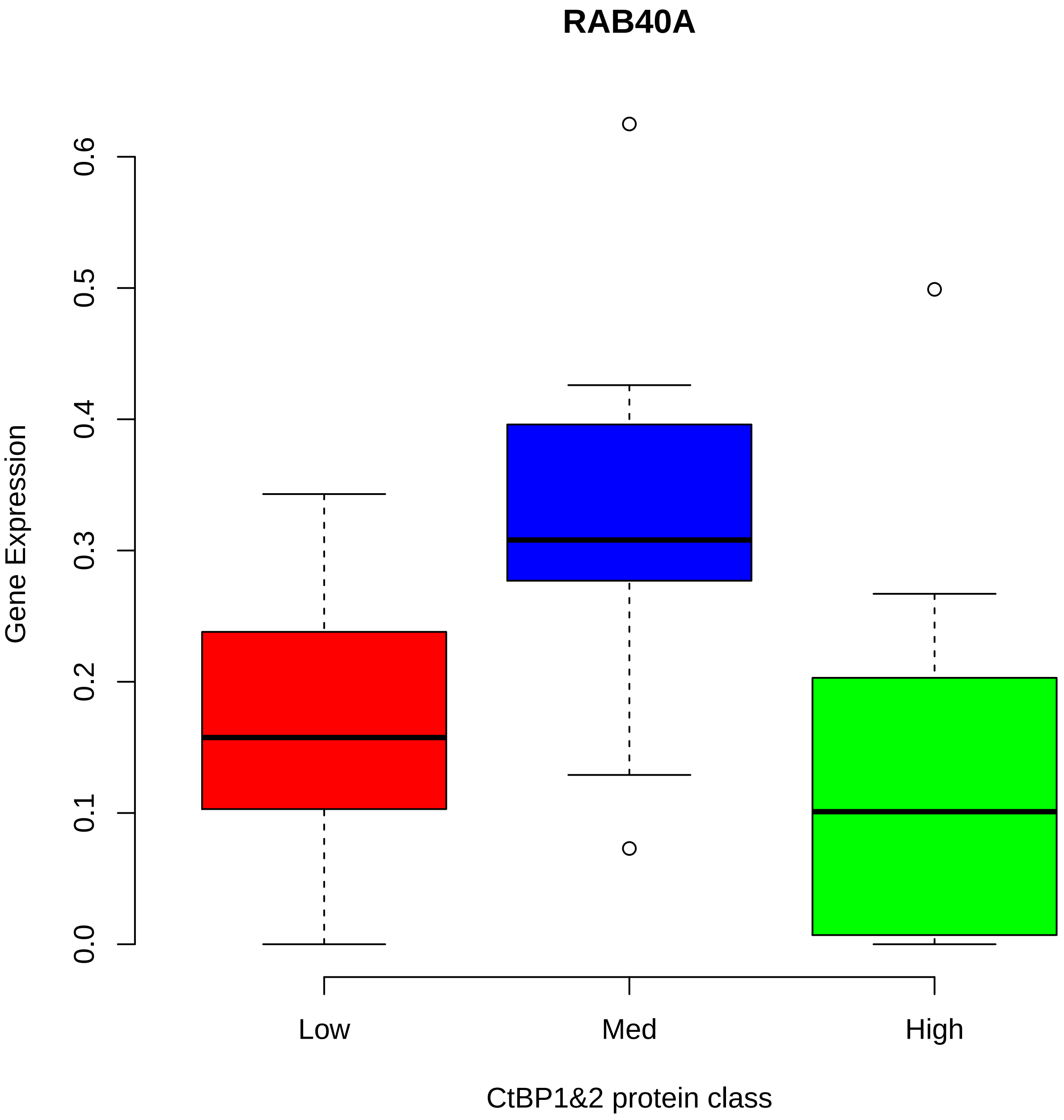
RAB40A
Gene Expression
0.0
0.1
0.2
0.3
0.4
0.5
0.6
Low
Med
High
CtBP1&2 protein class

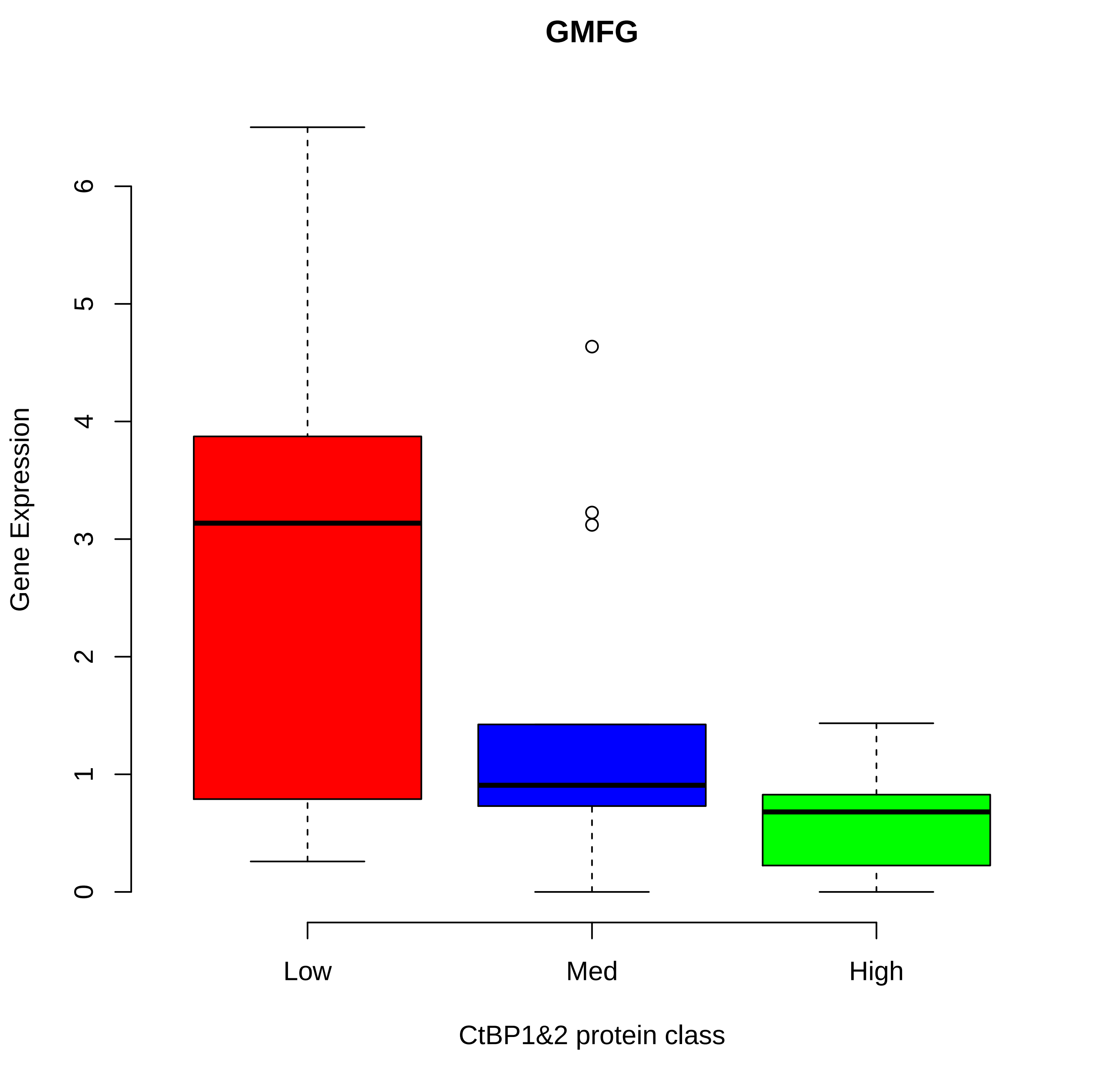
GMFG
Gene Expression
0
1
2
3
4
5
6
Low
Med
High
CtBP1&2 protein class

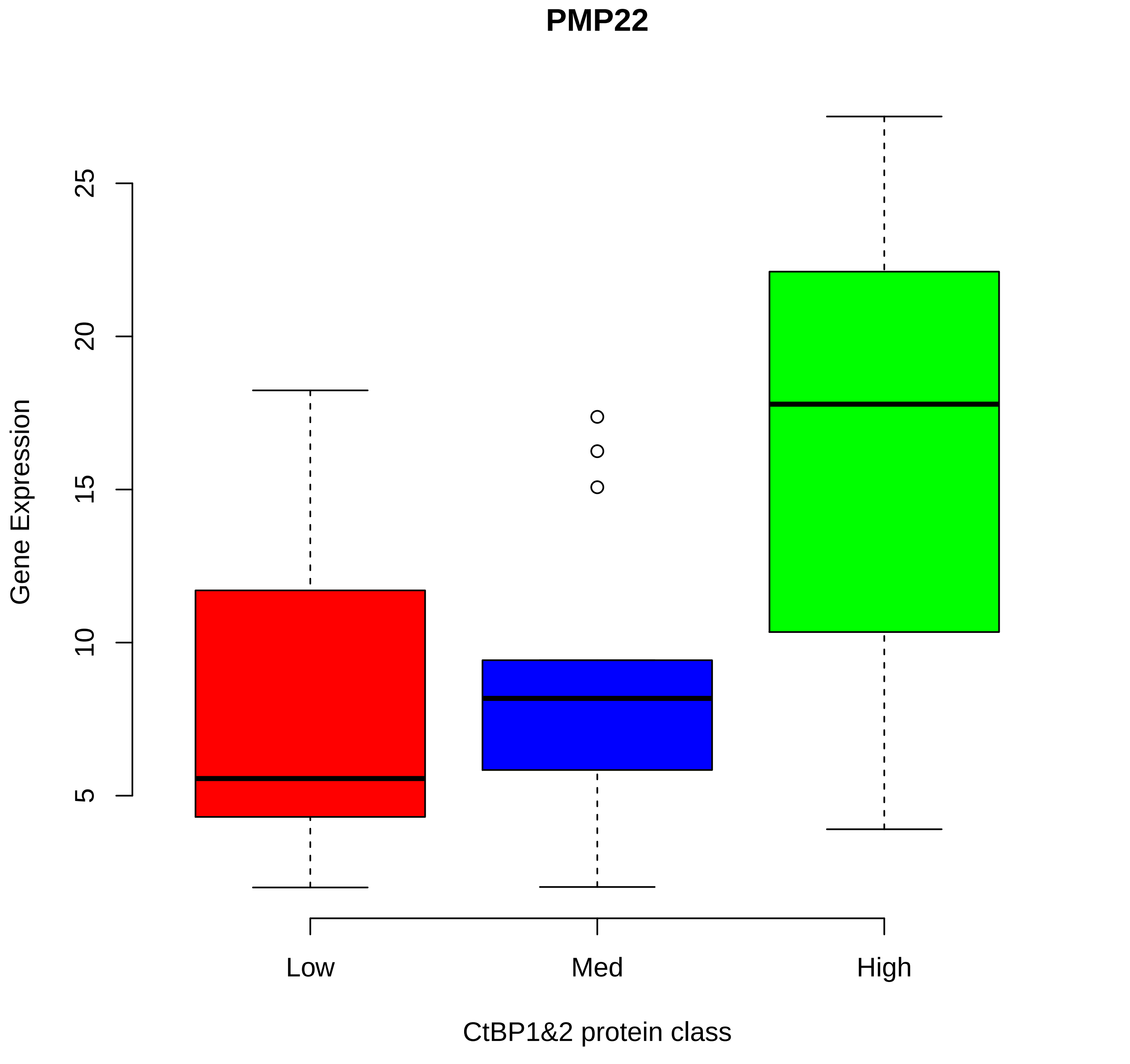

PMP22
Gene Expression
25
20
15
10
5
Low
Med
High
CtBP1&2 protein class

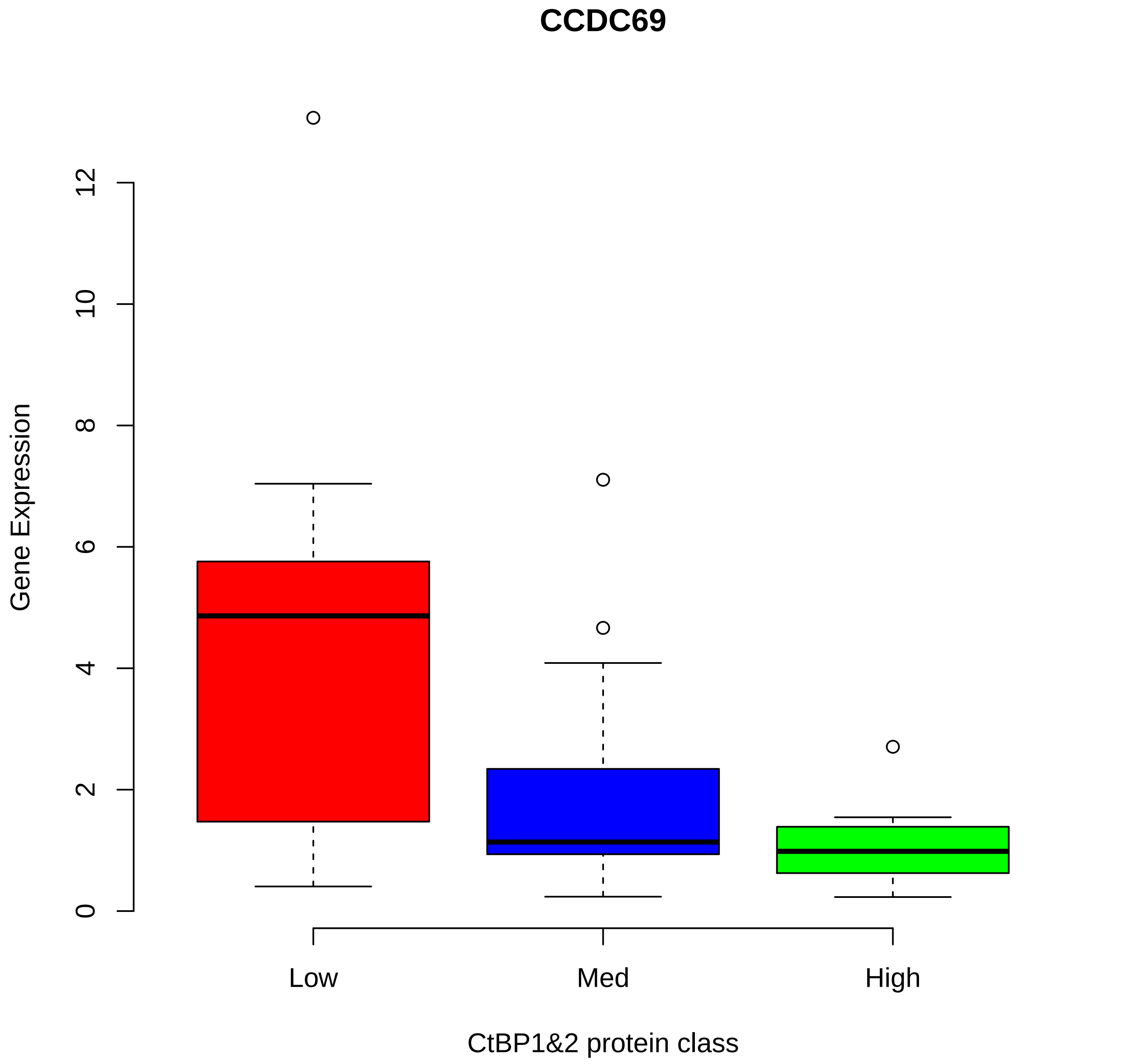

CCDC69
Gene Expression
0
2
4
6
8
10
12
Low
Med
High
CtBP1&2 protein class

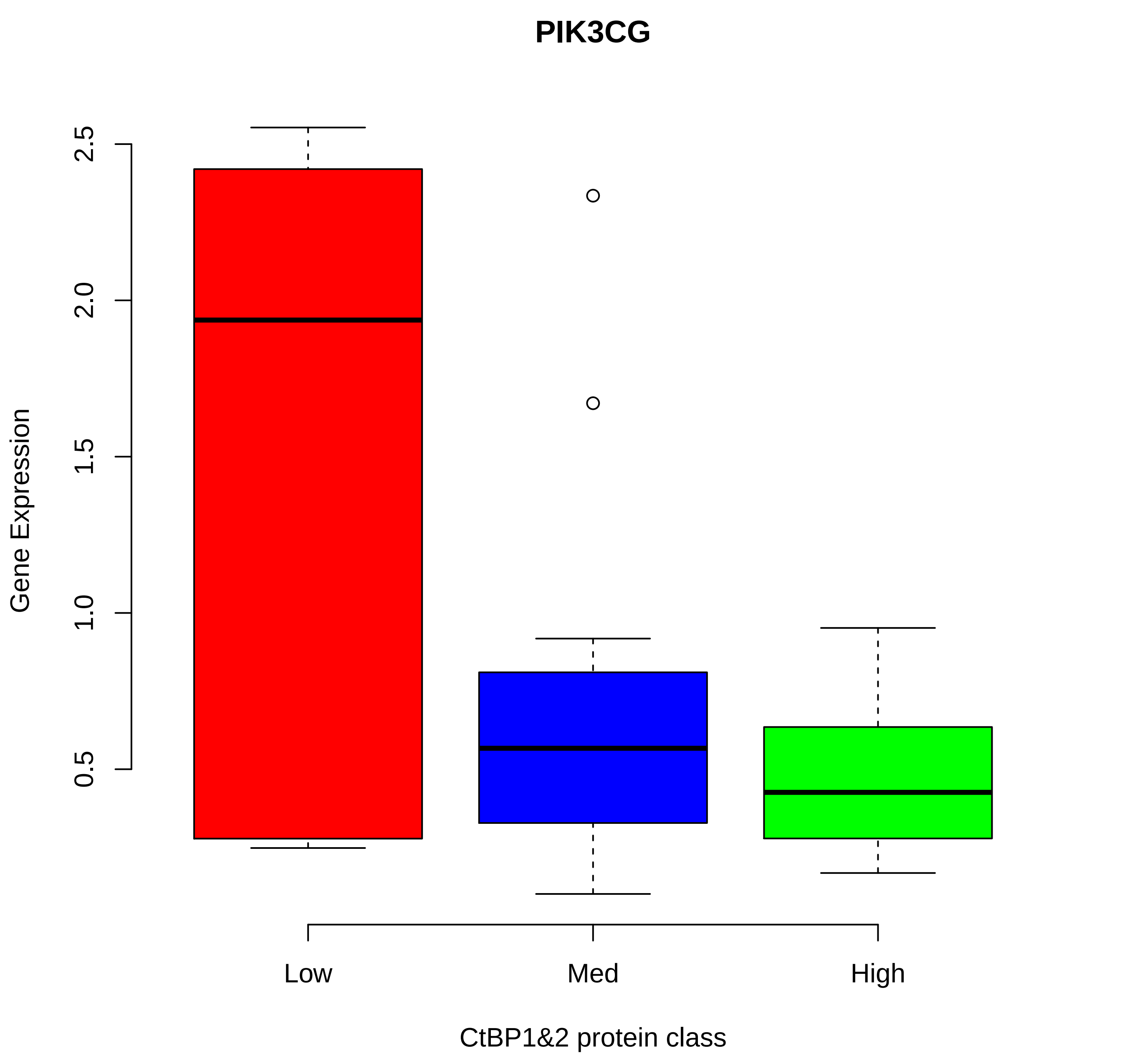

PIK3CG
Gene Expression
0.5
1.0
1.5
2.0
2.5
Low
Med
High
CtBP1&2 protein class

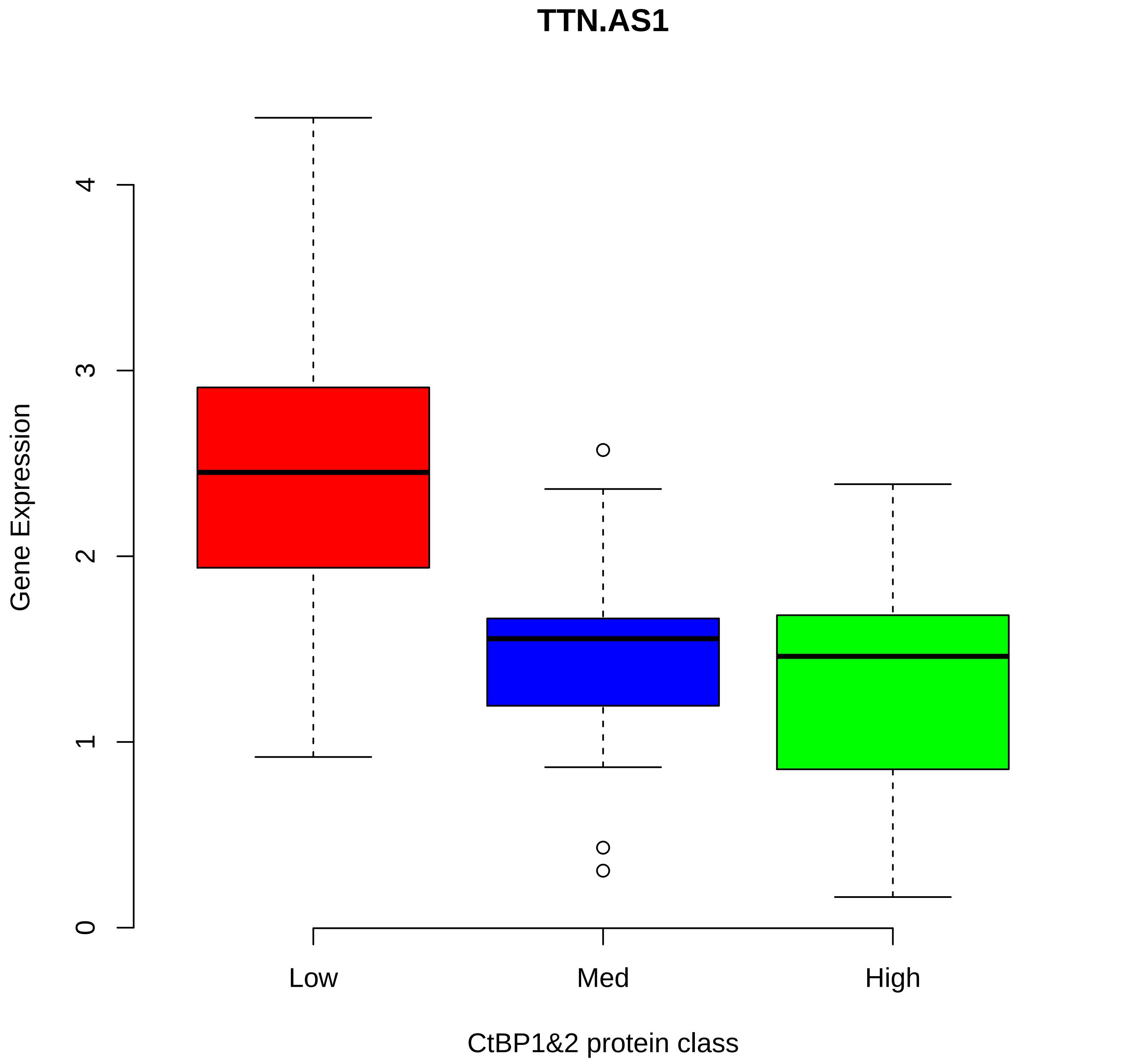
TTN.AS1
Gene Expression
0
1
2
3
4
Low
Med
High
CtBP1&2 protein class

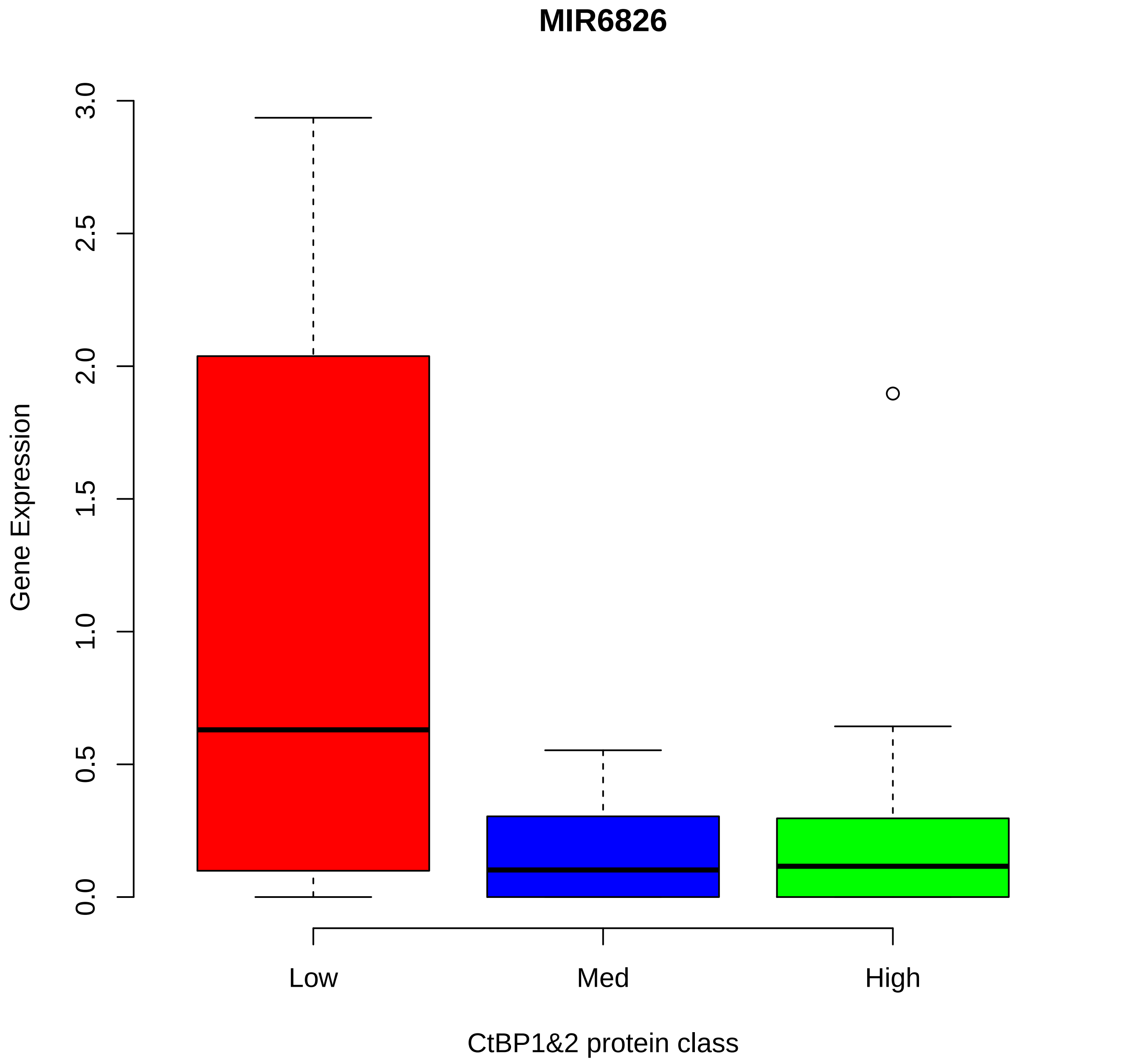
MIR6826
Gene Expression
0.0
0.5
1.0
1.5
2.0
2.5
3.0
Low
Med
High
CtBP1&2 protein class

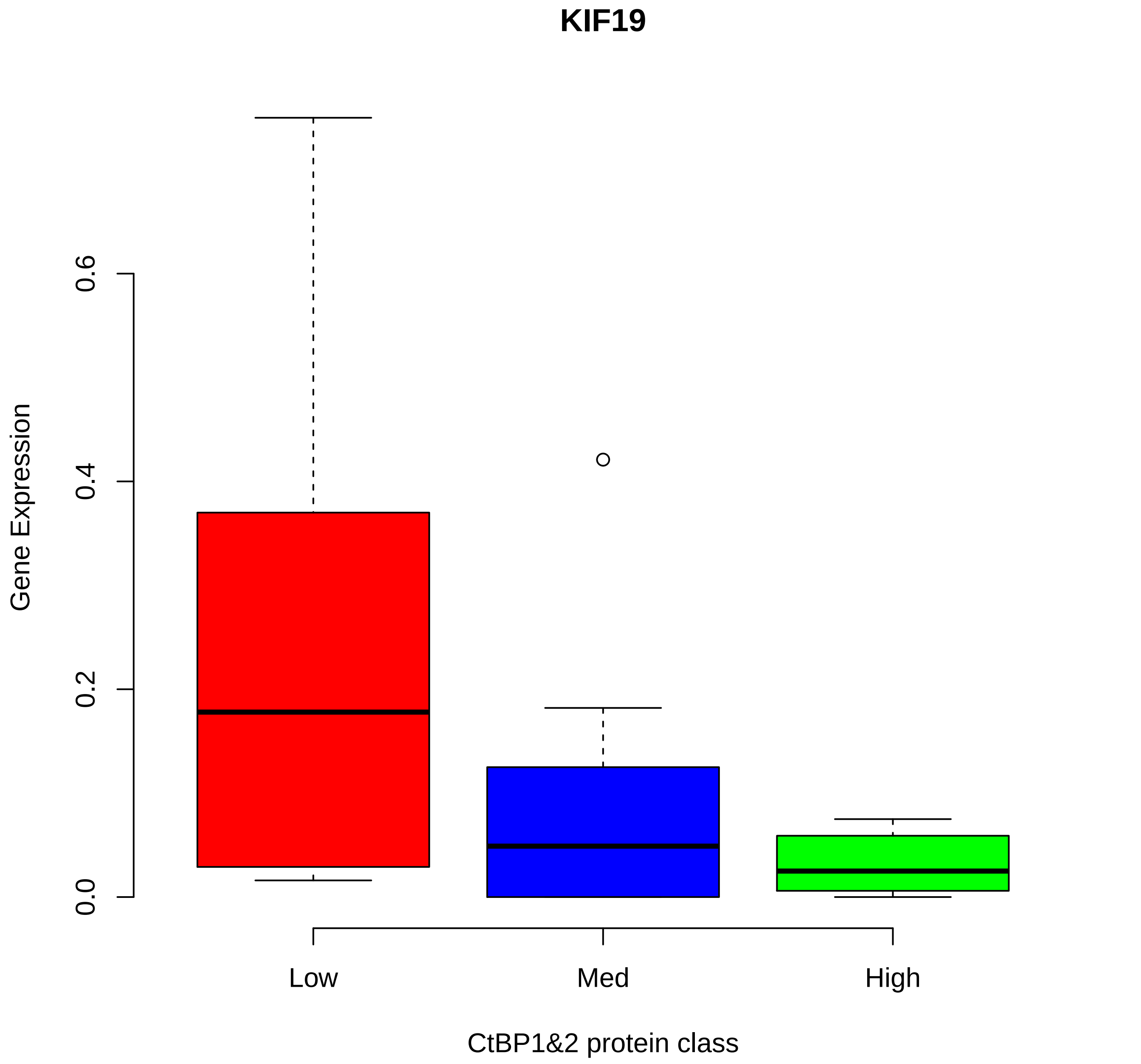

KIF19
Gene Expression
0.0
0.2
0.4
0.6
Low
Med
High
CtBP1&2 protein class

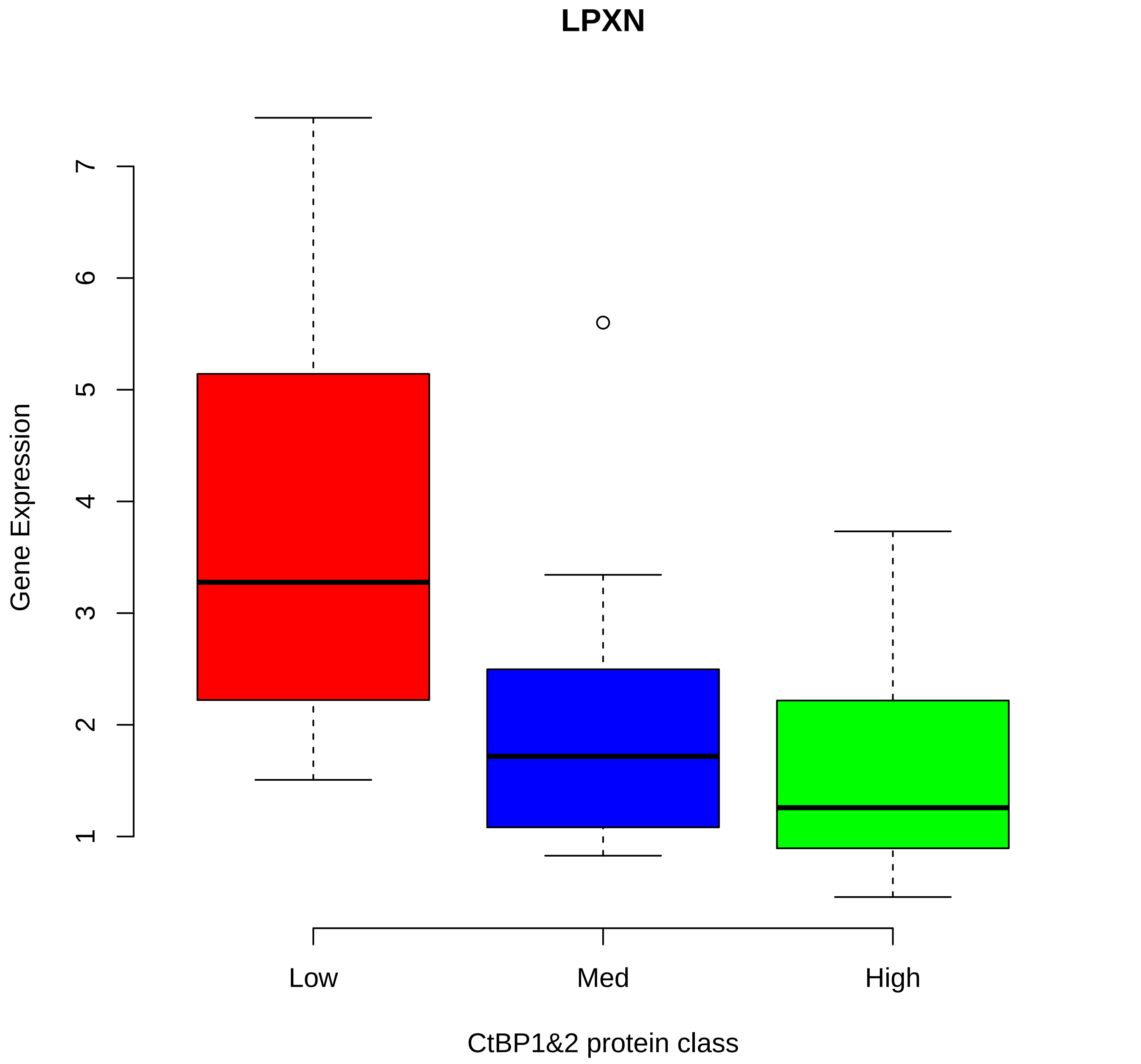
LPXN
Gene Expression
1
2
3
4
5
6
7
Low
Med
High
CtBP1&2 protein class

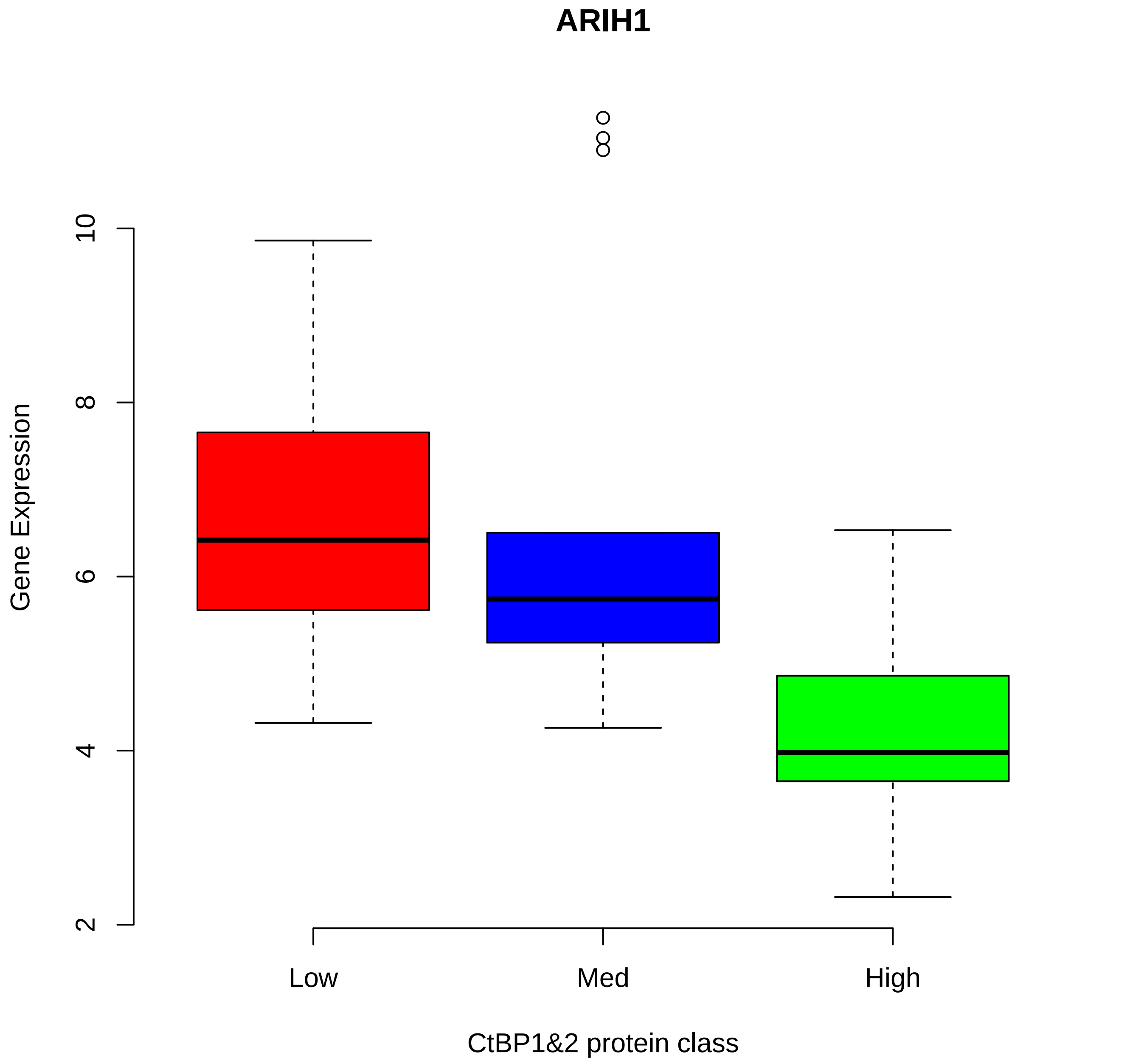

ARIH1
Gene Expression
2
4
6
8
10
Low
Med
High
CtBP1&2 protein class

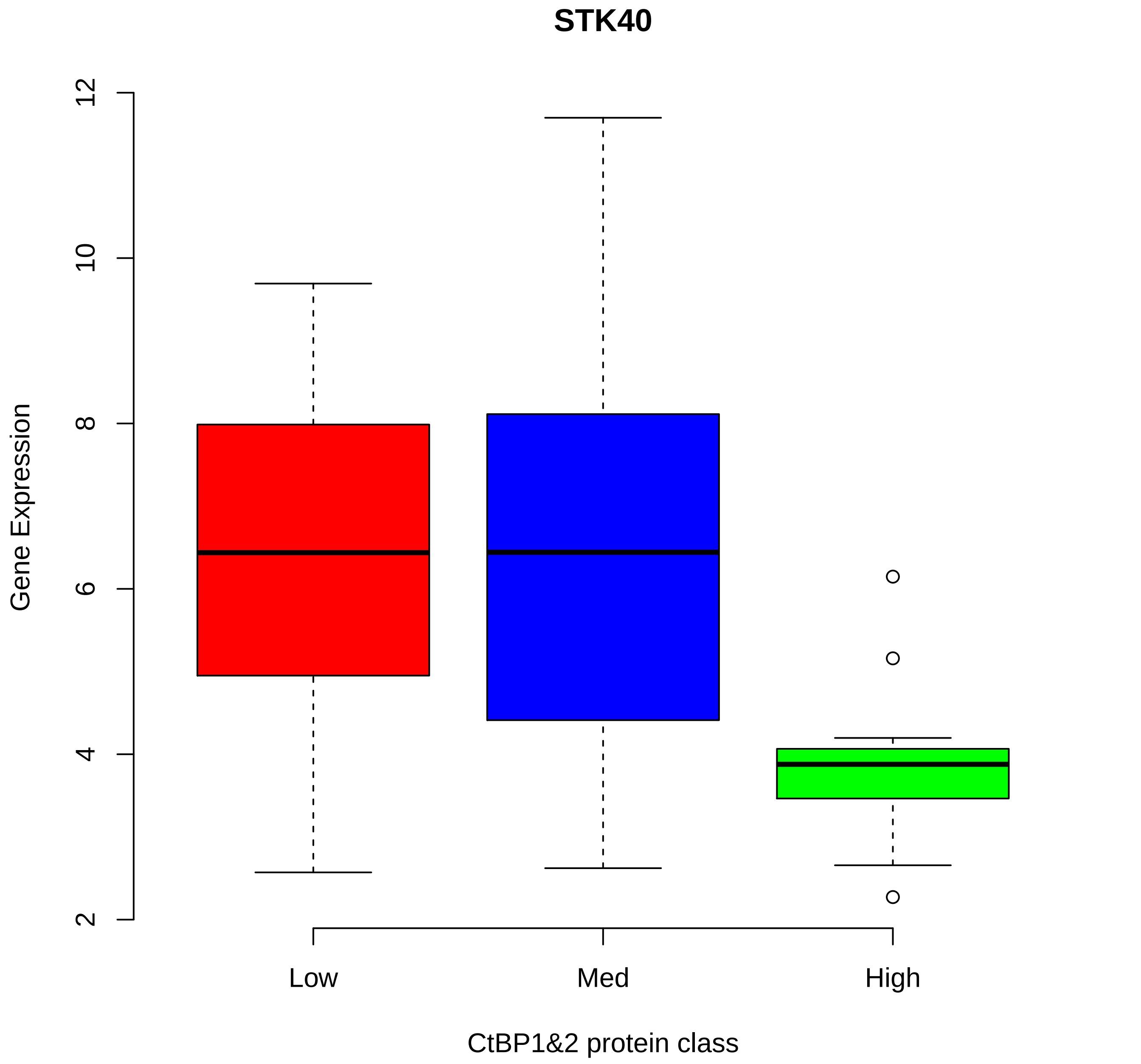

STK40
Gene Expression
2
4
6
8
10
12
Low
Med
High
CtBP1&2 protein class

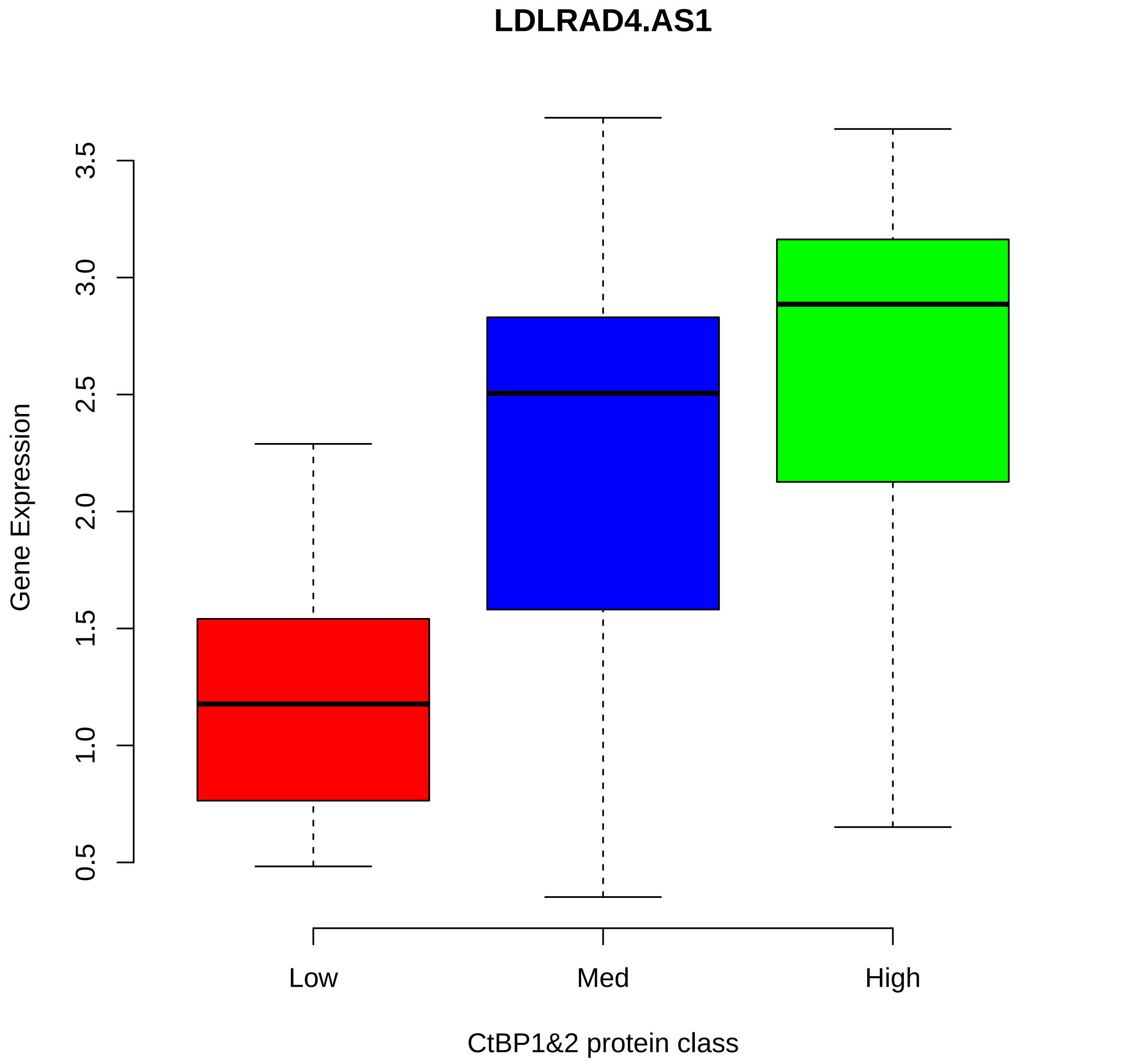
LDLRAD4.AS1
Gene Expression
0.5
1.0
1.5
2.0
2.5
3.0
3.5
Low
Med
High
CtBP1&2 protein class

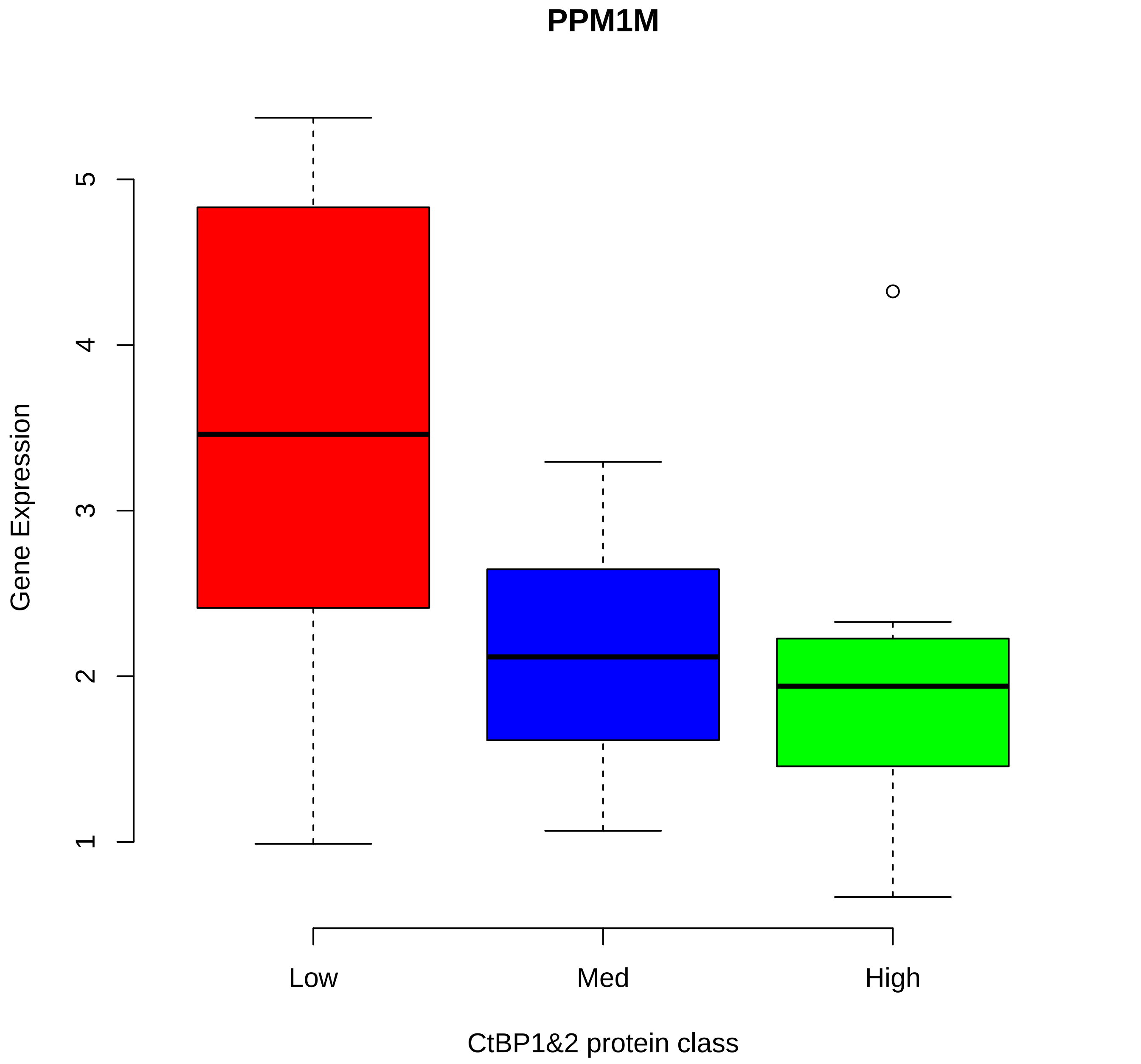
PPM1M
Gene Expression
1
2
3
4
5
Low
Med
High
CtBP1&2 protein class

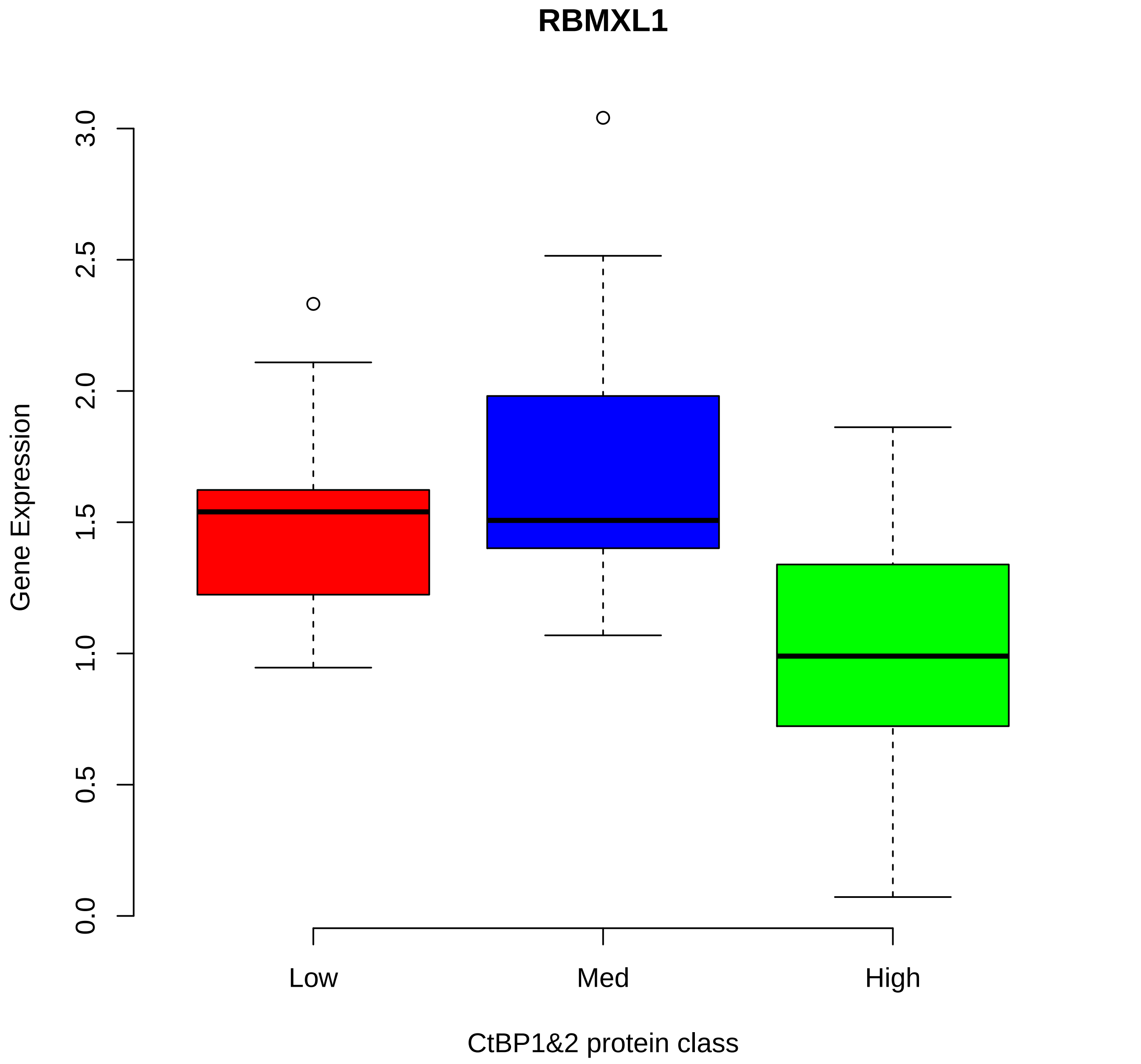
RBMXL1
Gene Expression
0.0
0.5
1.0
1.5
2.0
2.5
3.0
Low
Med
High
CtBP1&2 protein class

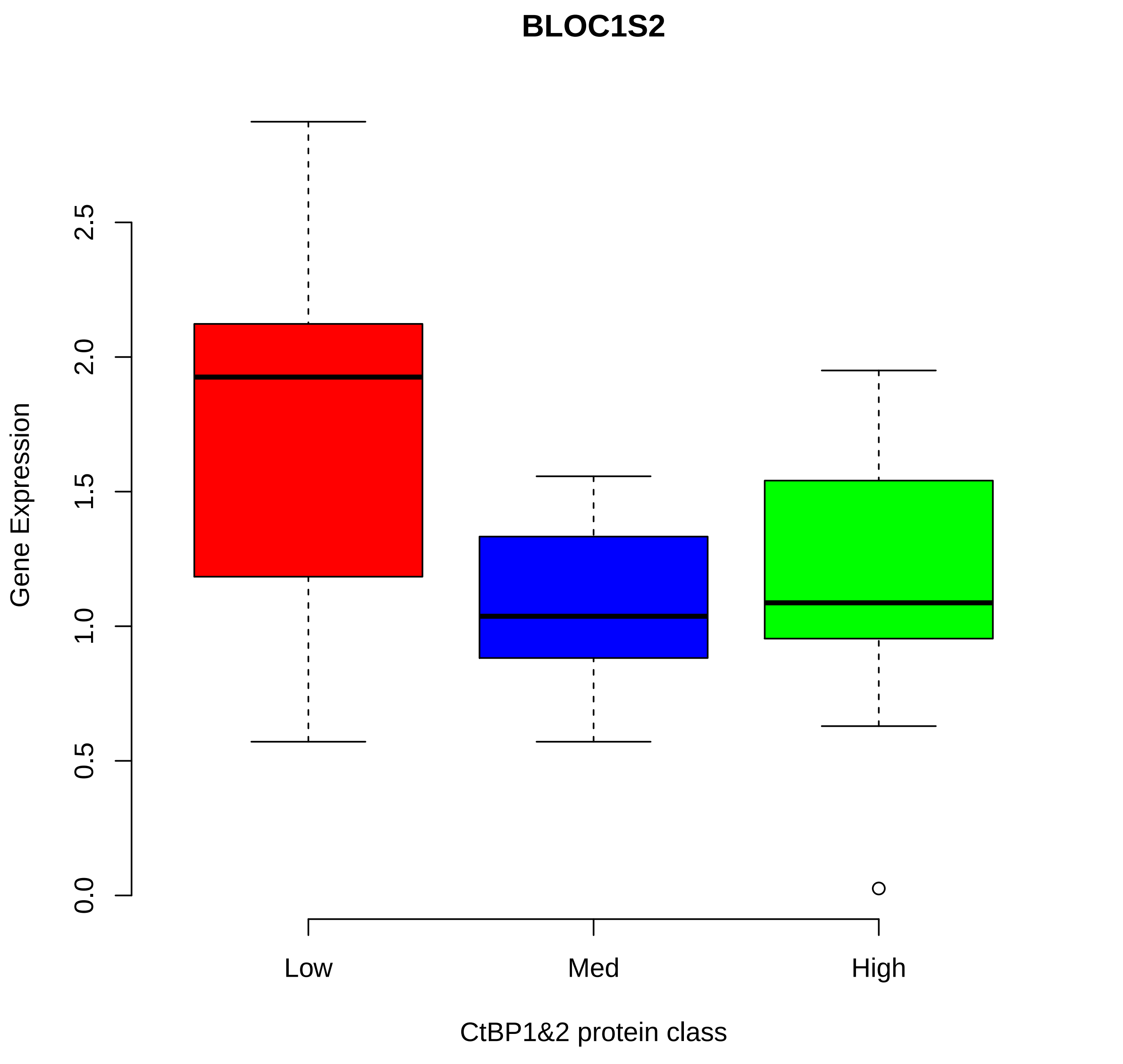
BLOC1S2
Gene Expression
0.0
0.5
1.0
1.5
2.0
2.5
Low
Med
High
CtBP1&2 protein class

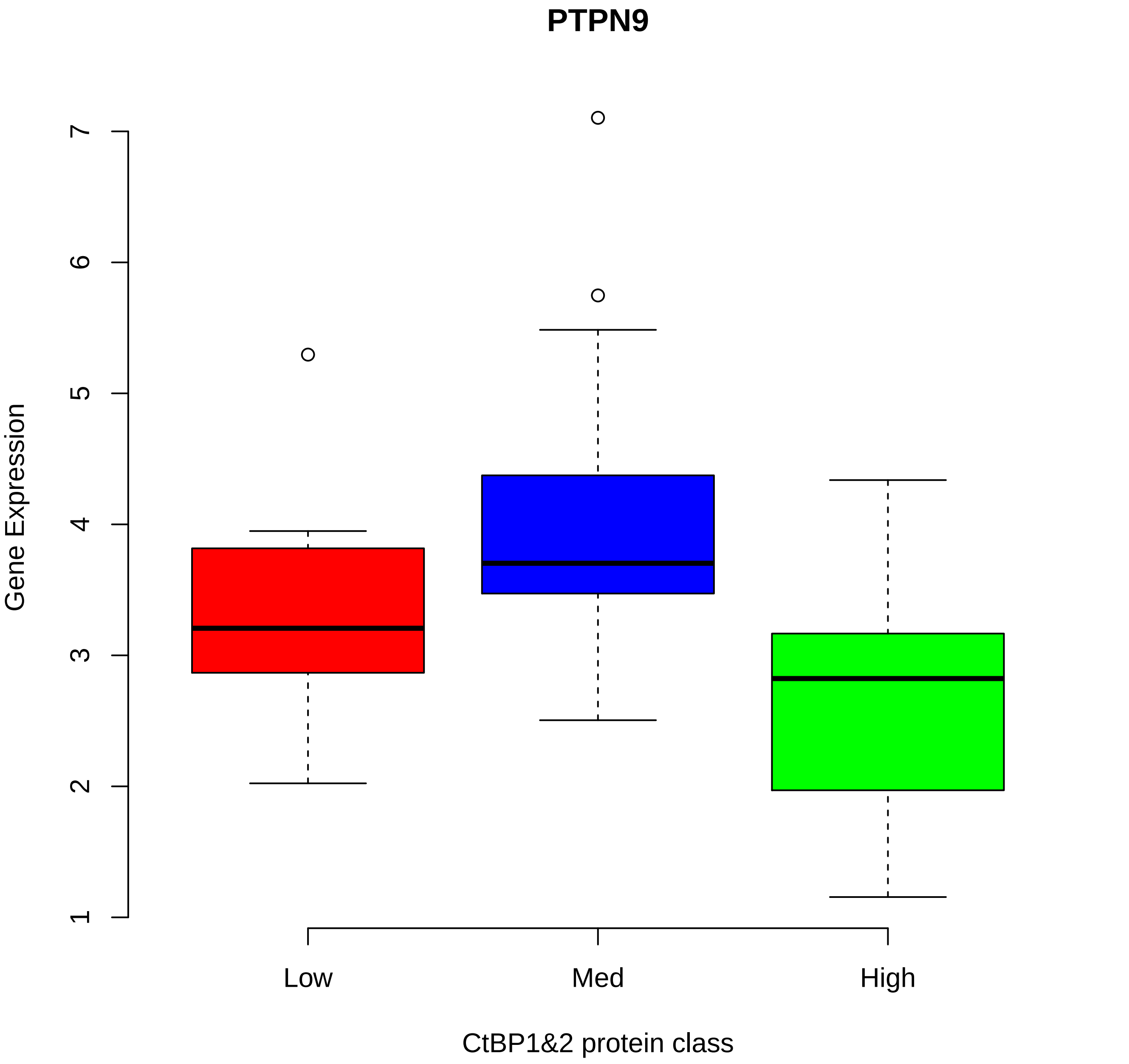

PTPN9
Gene Expression
1
2
3
4
5
6
7
Low
Med
High
CtBP1&2 protein class

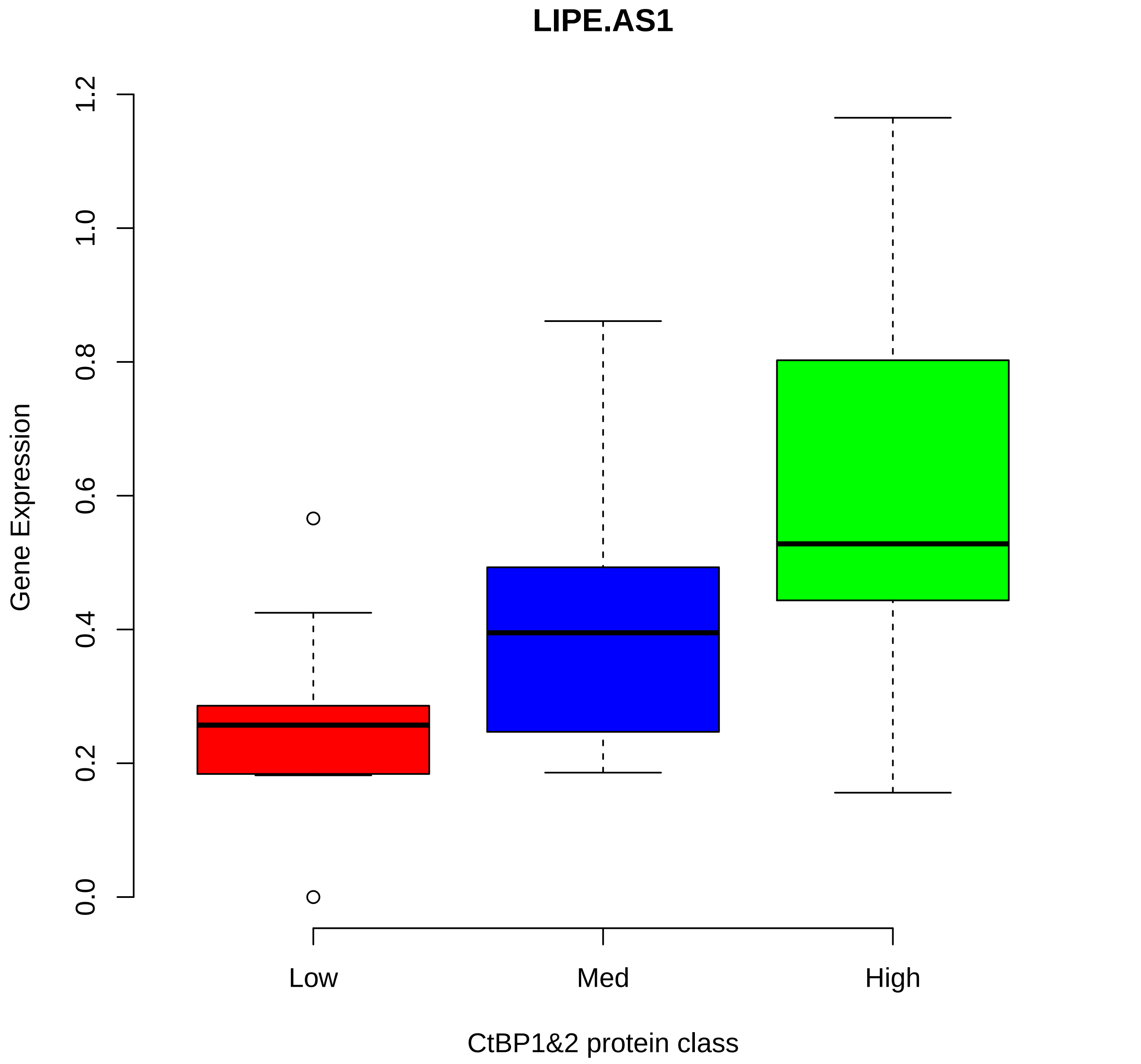
LIPE.AS1
Gene Expression
0.0
0.2
0.4
0.6
0.8
1.0
1.2
Low
Med
High
CtBP1&2 protein class

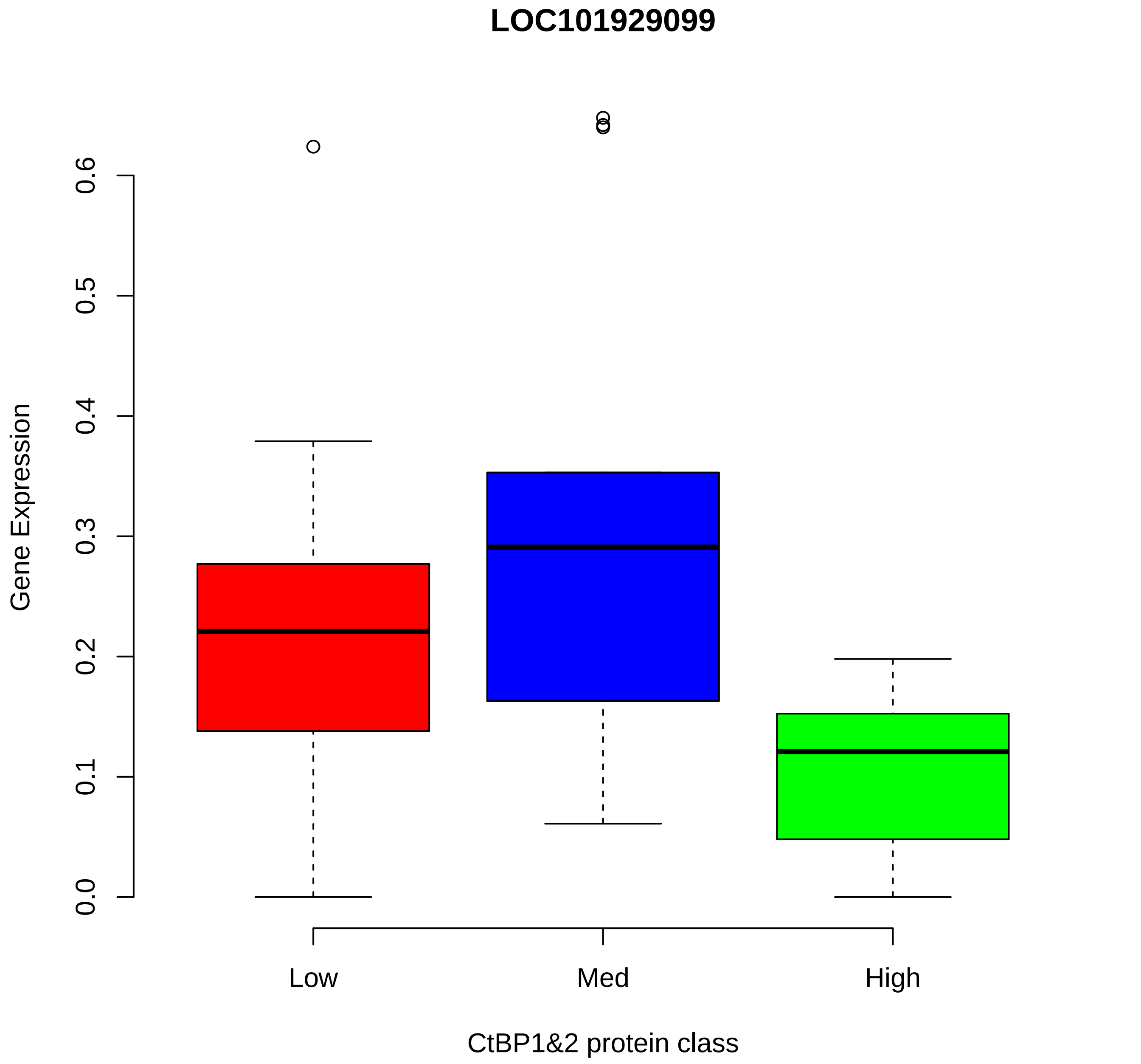

LOC101929099
Gene Expression
0.0
0.1
0.2
0.3
0.4
0.5
0.6
Low
Med
High
CtBP1&2 protein class

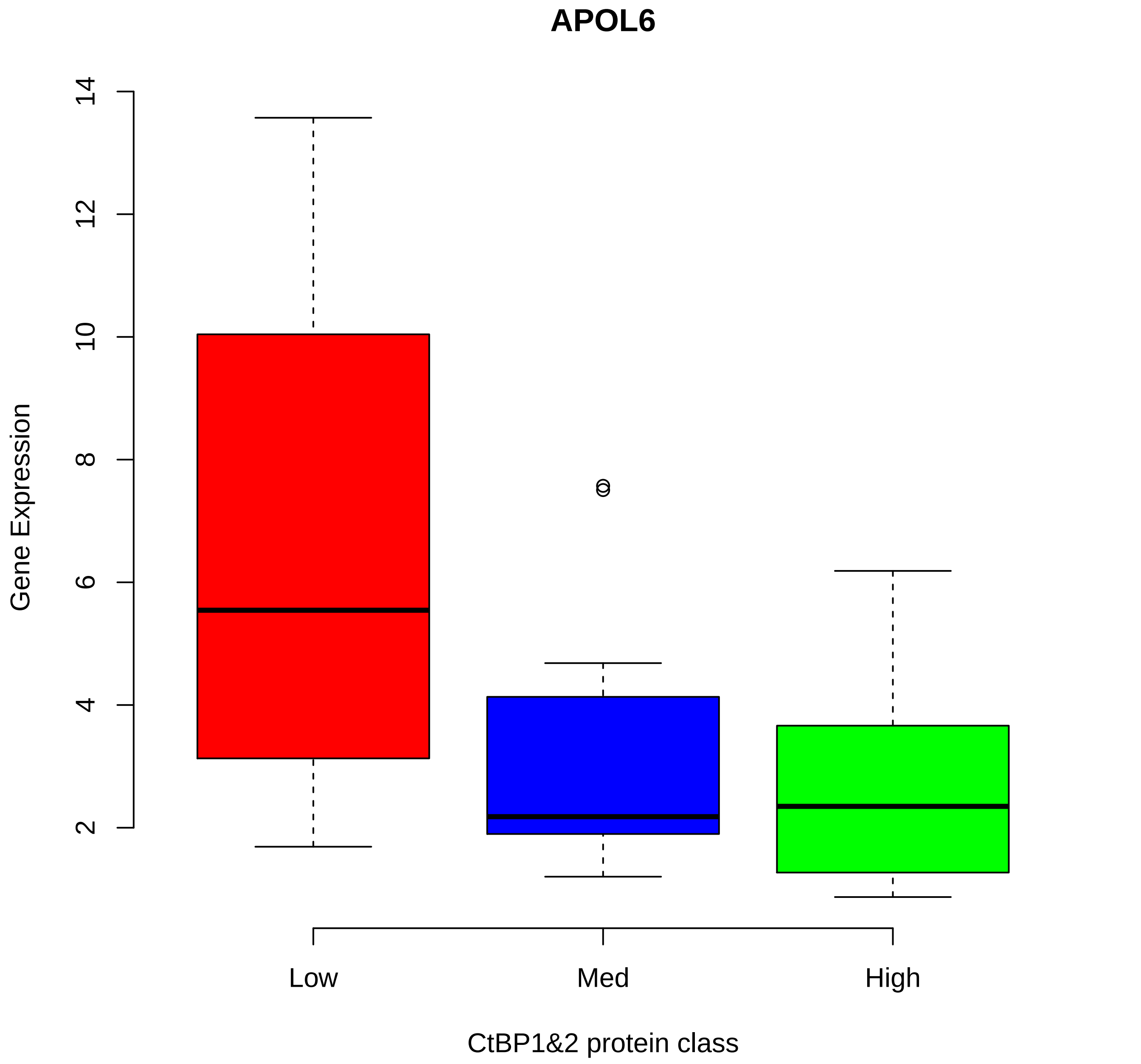
APOL6
Gene Expression
14
12
10
8
6
4
2
Low
Med
High
CtBP1&2 protein class

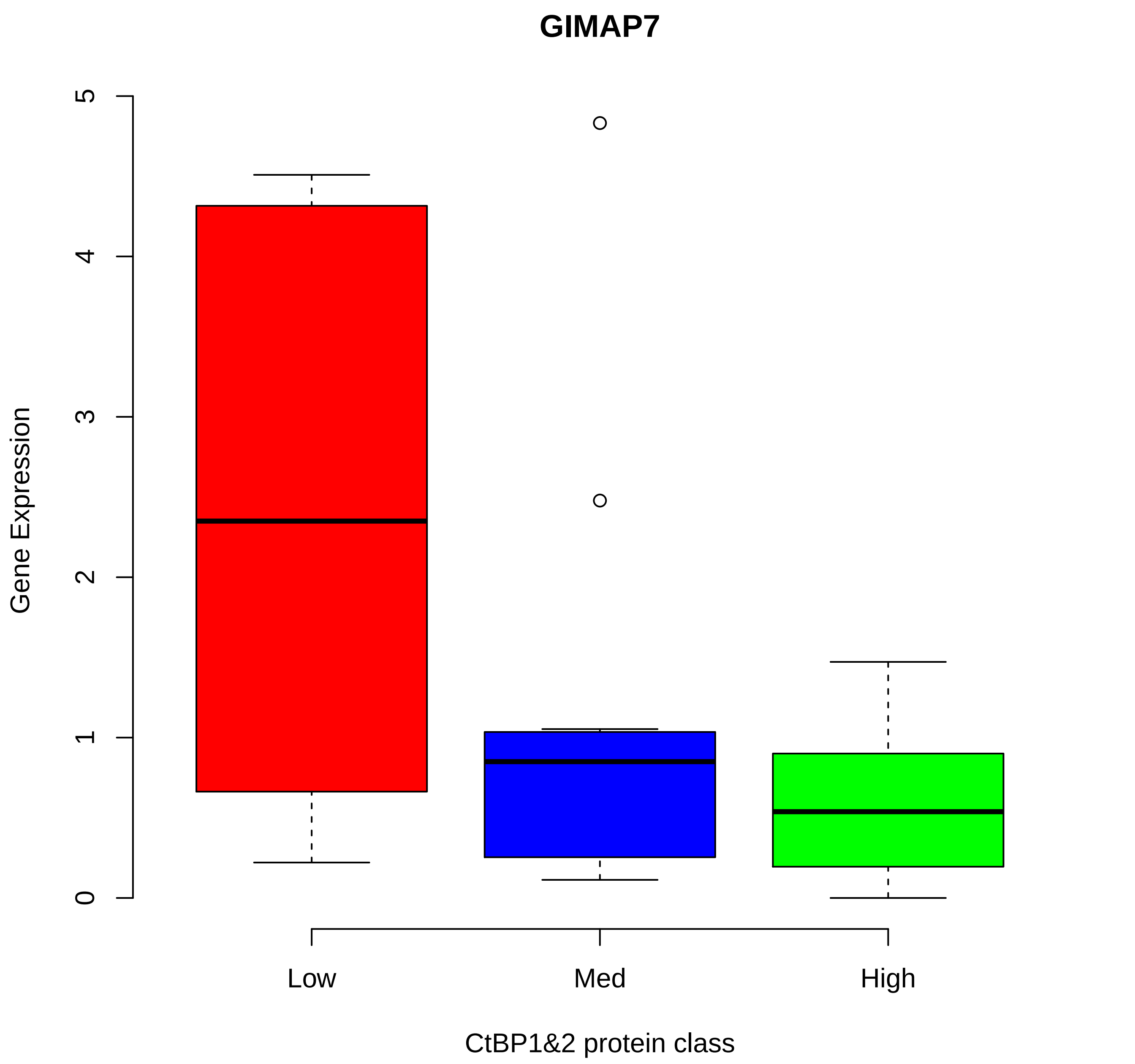
GIMAP7
Gene Expression
0
1
2
3
4
5
Low
Med
High
CtBP1&2 protein class

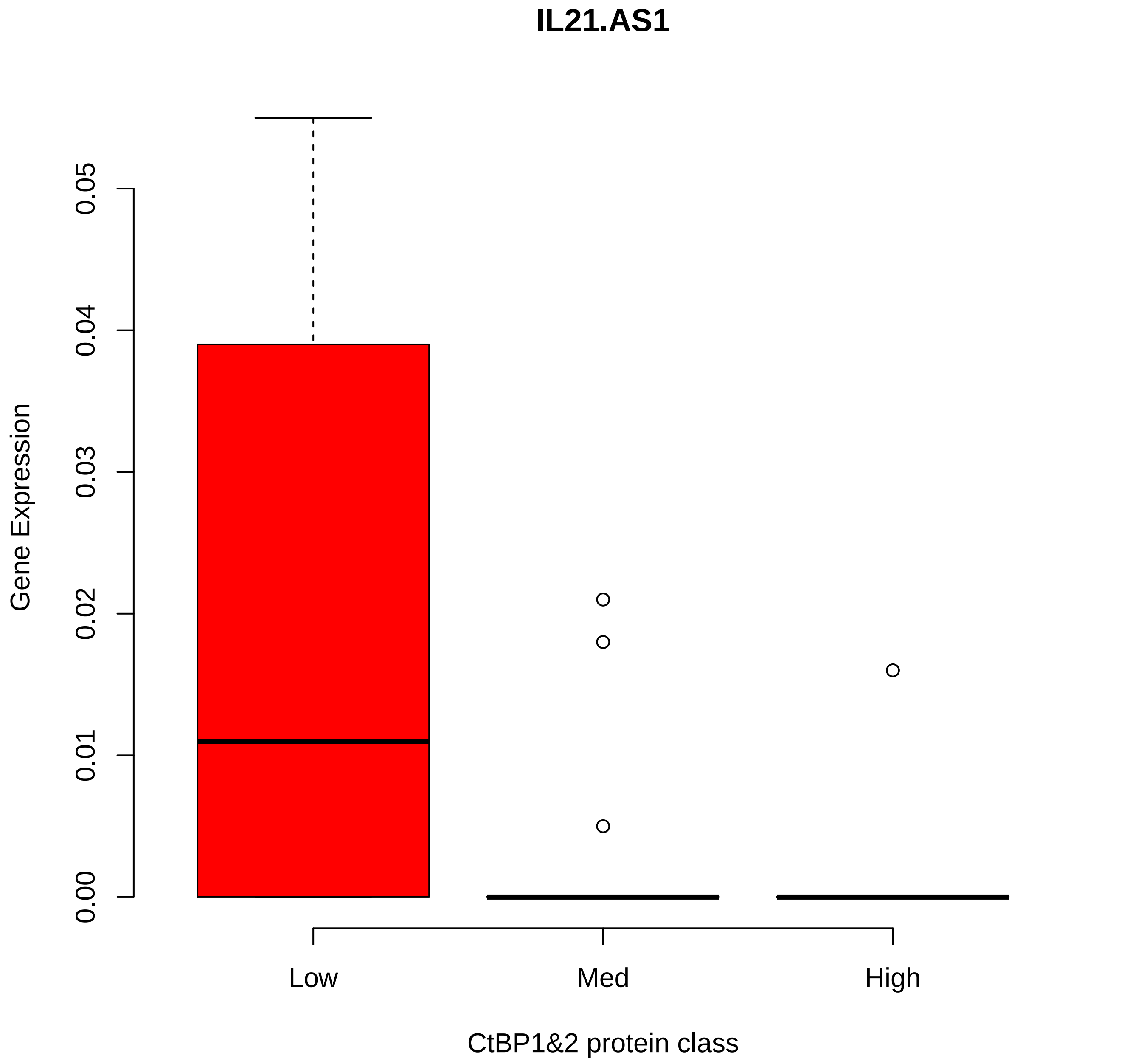

IL21.AS1
Gene Expression
0.00
0.01
0.02
0.03
0.04
0.05
Low
Med
High
CtBP1&2 protein class

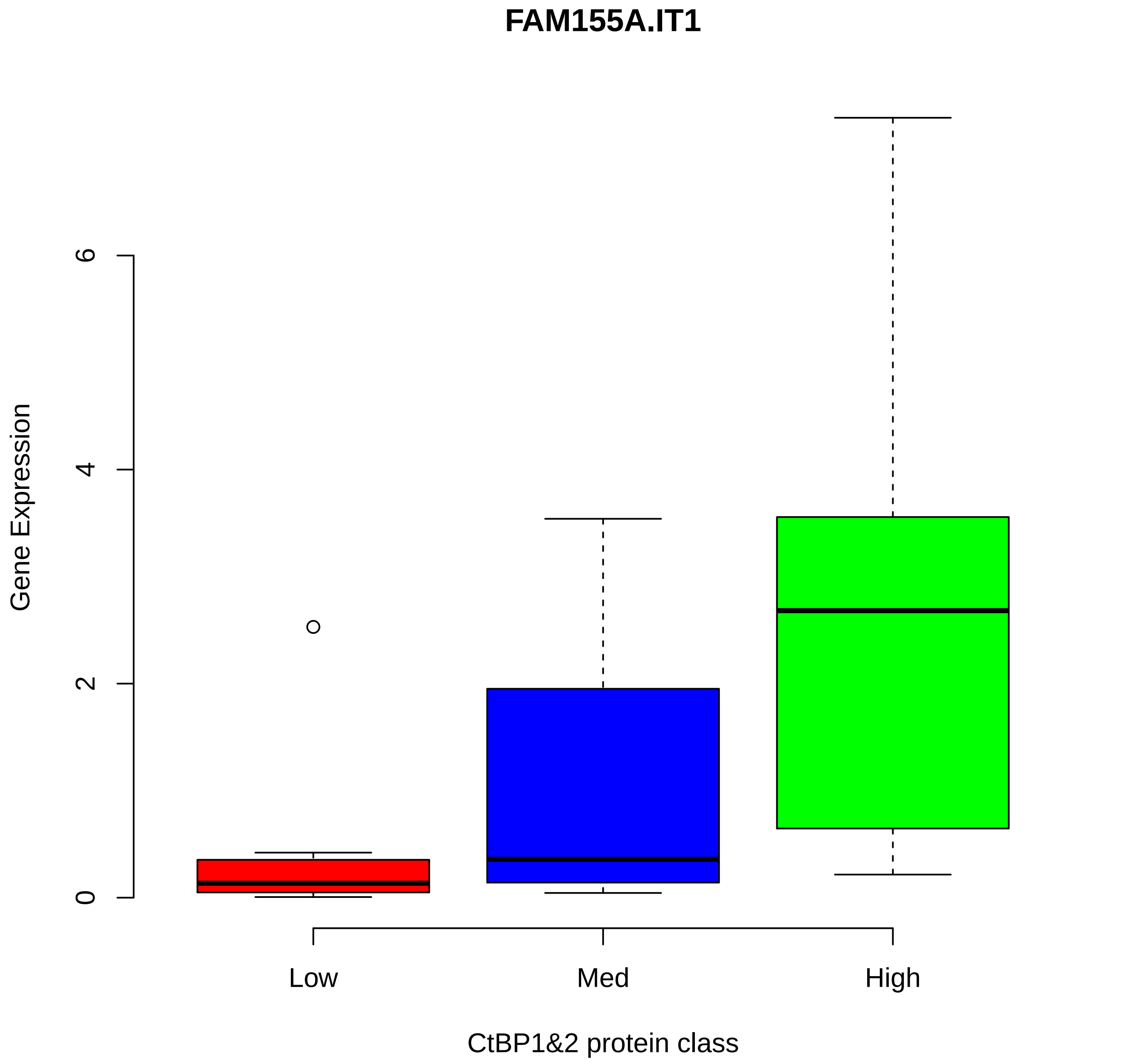
FAM155A.IT1
Gene Expression
0
2
4
6
Low
Med
High
CtBP1&2 protein class

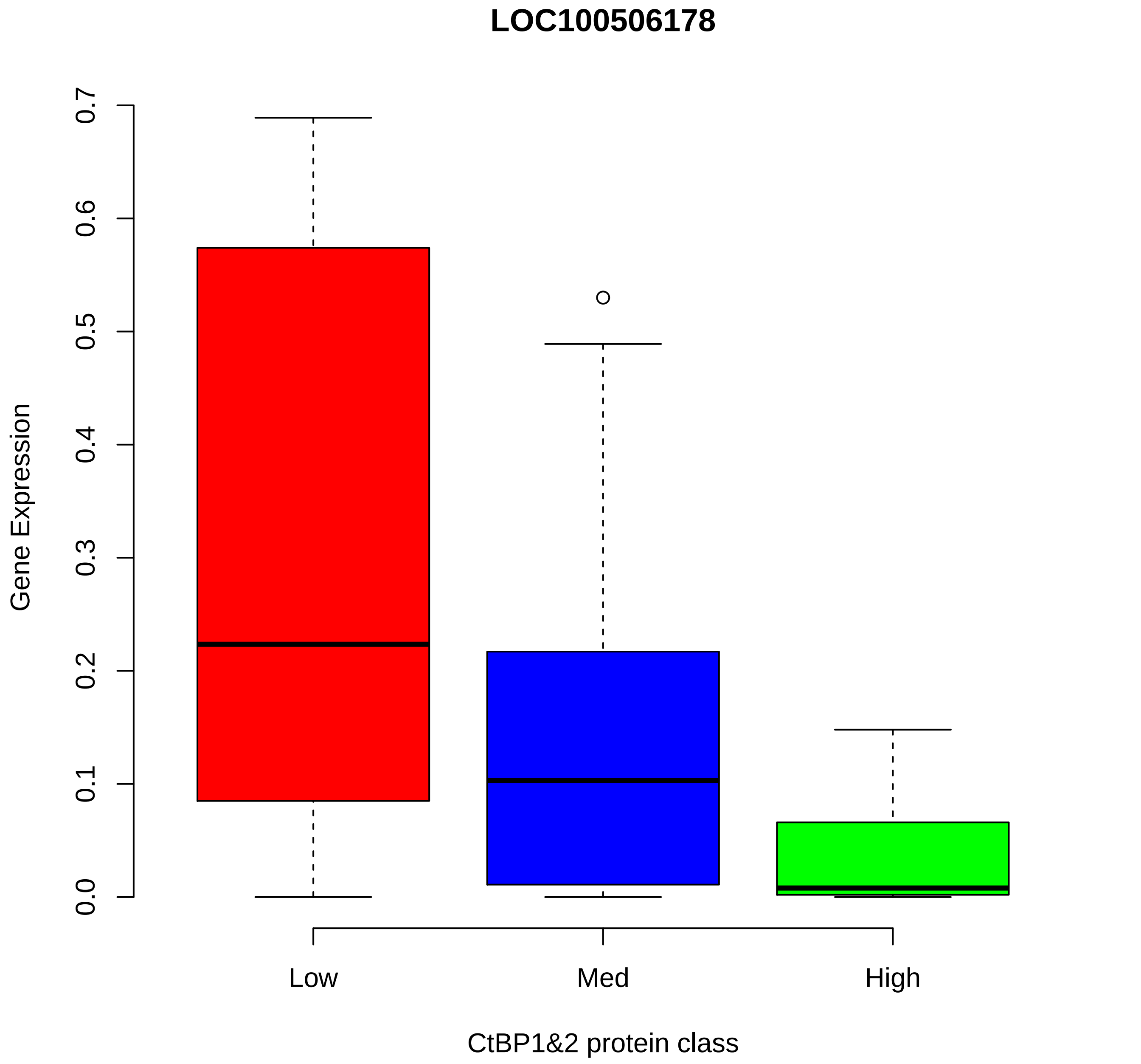

LOC100506178
Gene Expression
0.0
0.1
0.2
0.3
0.4
0.5
0.6
0.7
Low
Med
High
CtBP1&2 protein class

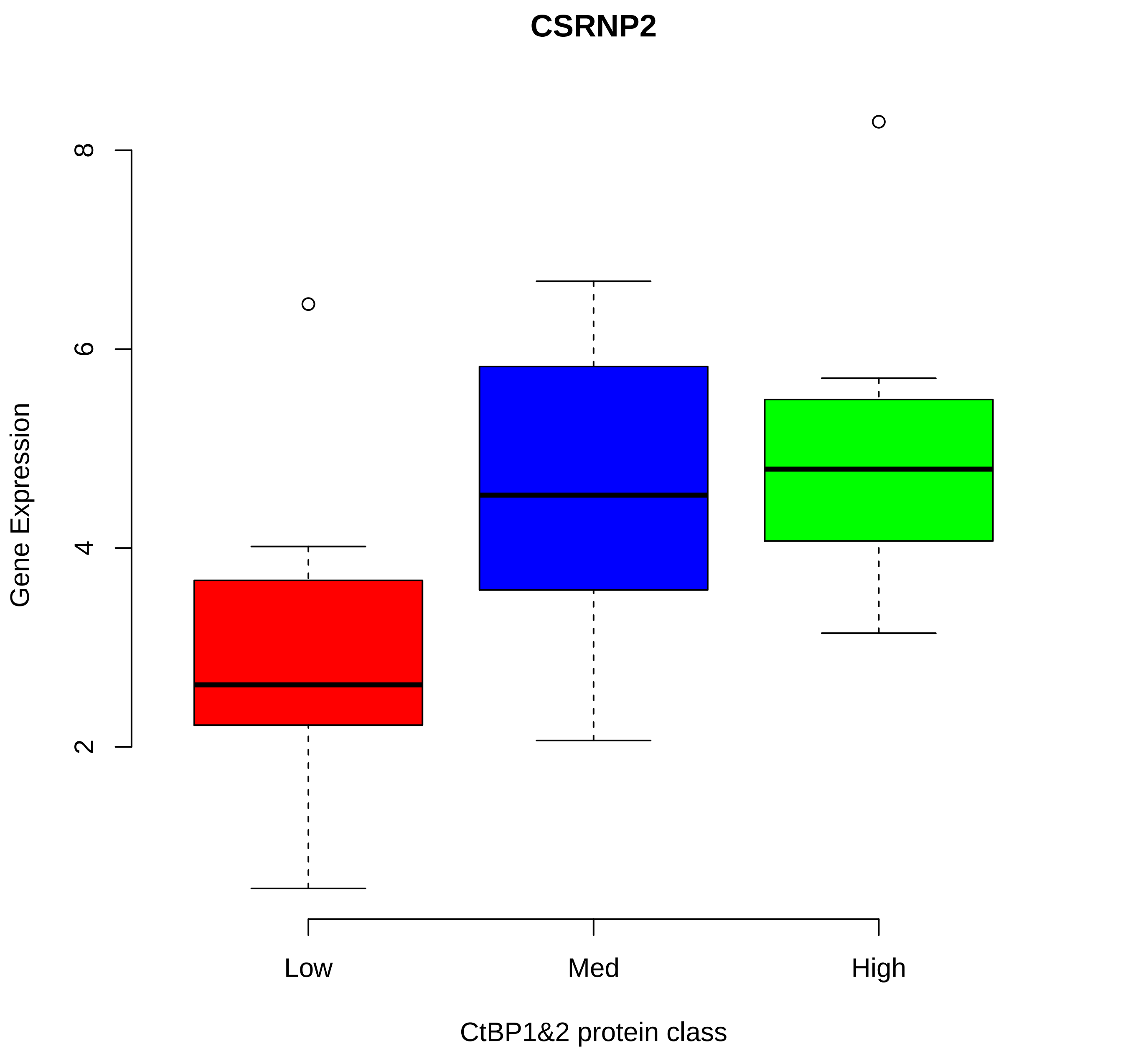

CSRNP2
8
6
4
2
Gene Expression
Low
Med
High
CtBP1&2 protein class

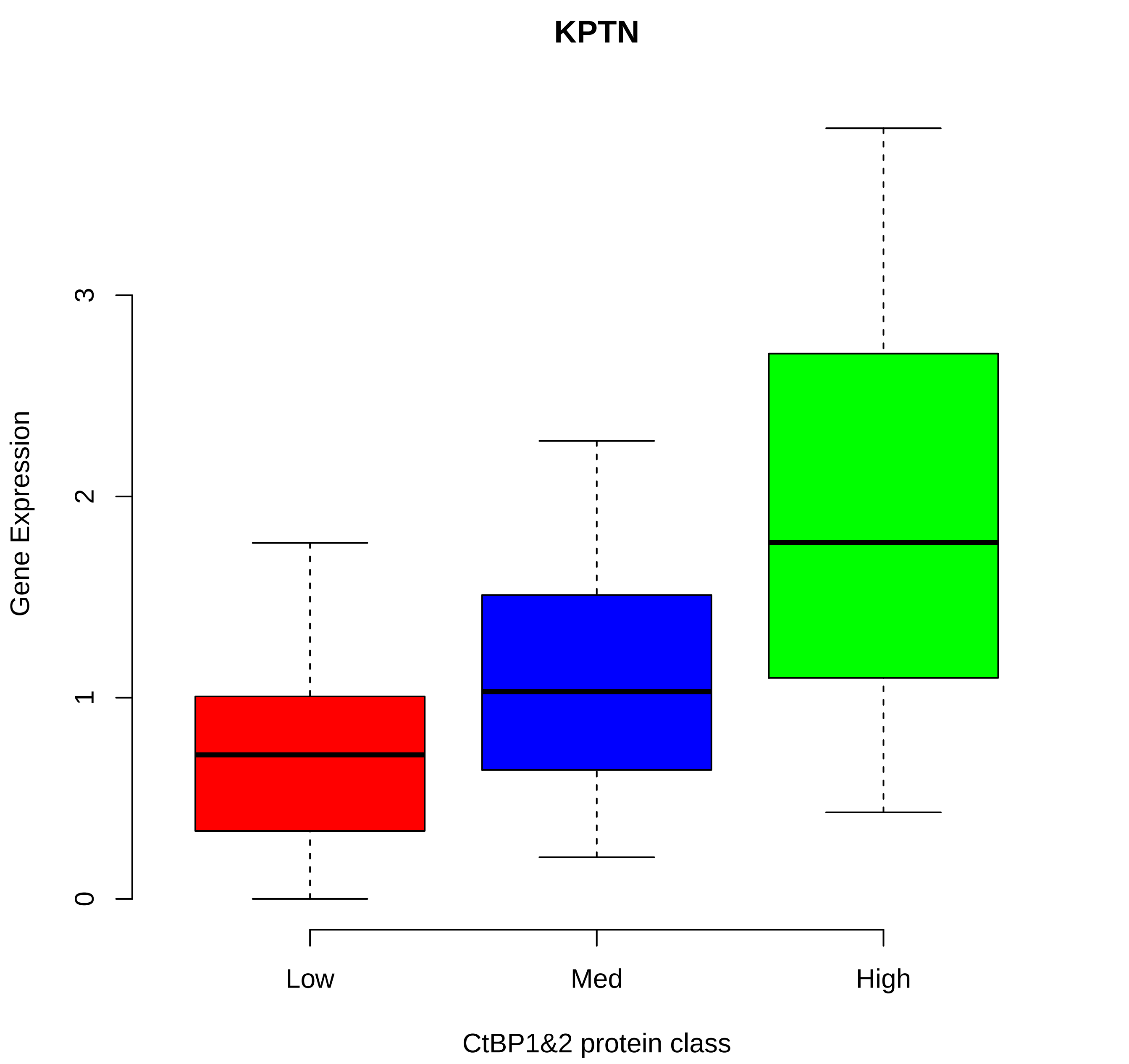
KPTN
Gene Expression
0
1
2
3
Low
Med
High
CtBP1&2 protein class

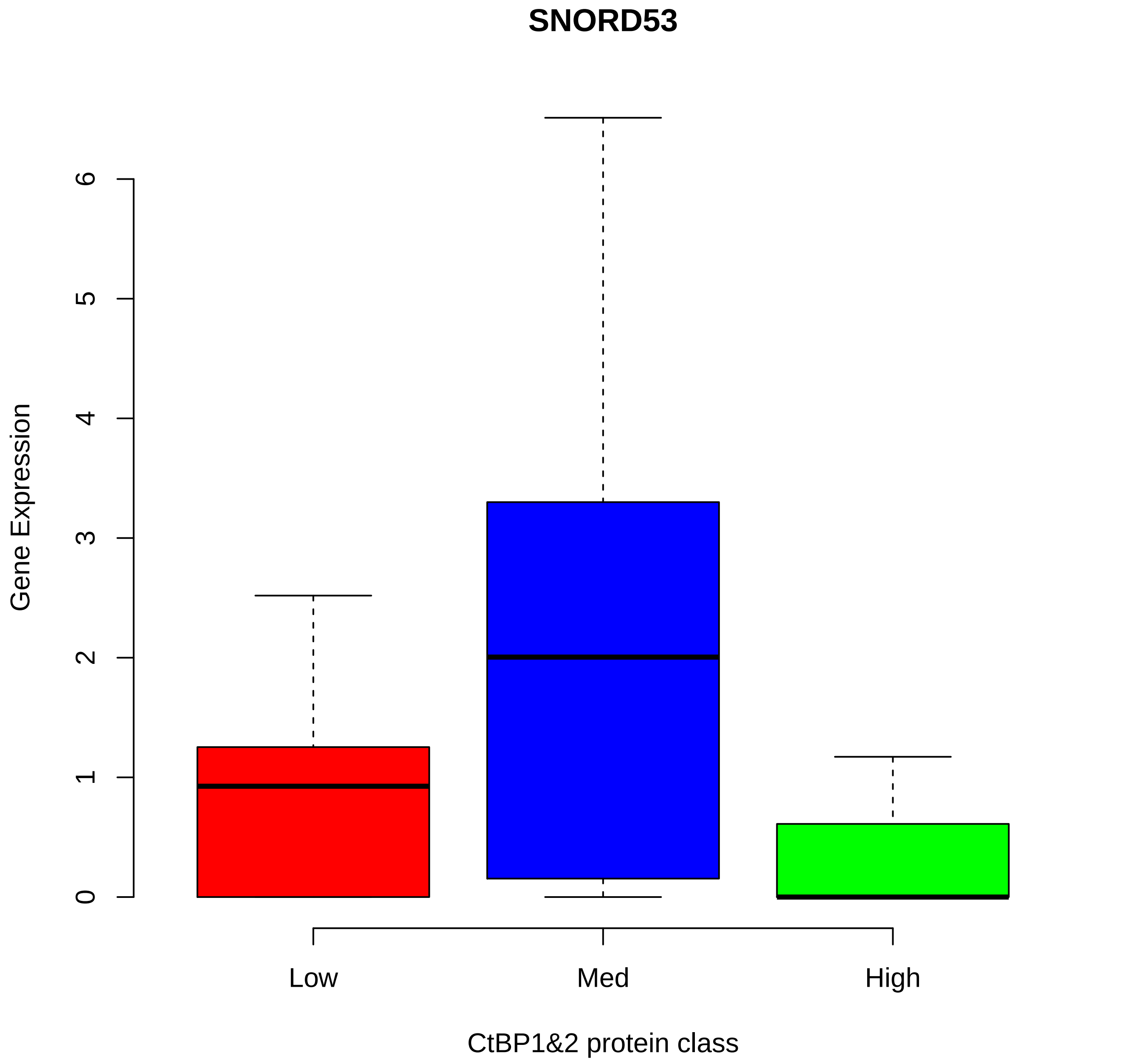

SNORD53
Gene Expression
0
1
2
3
4
5
6
Low
Med
High
CtBP1&2 protein class

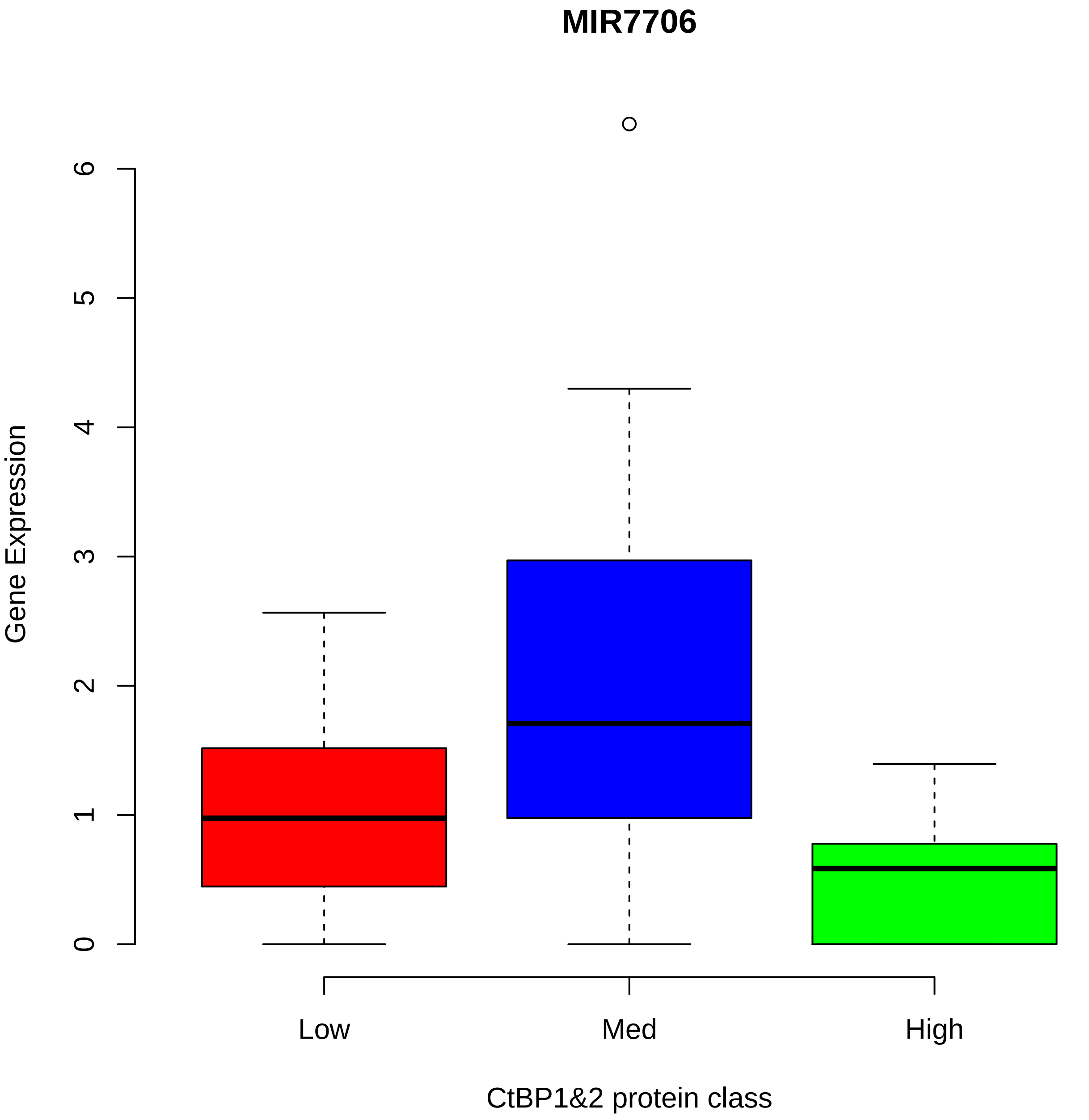

MIR7706
Gene Expression
0
1
2
3
4
5
6
Low
Med
High
CtBP1&2 protein class

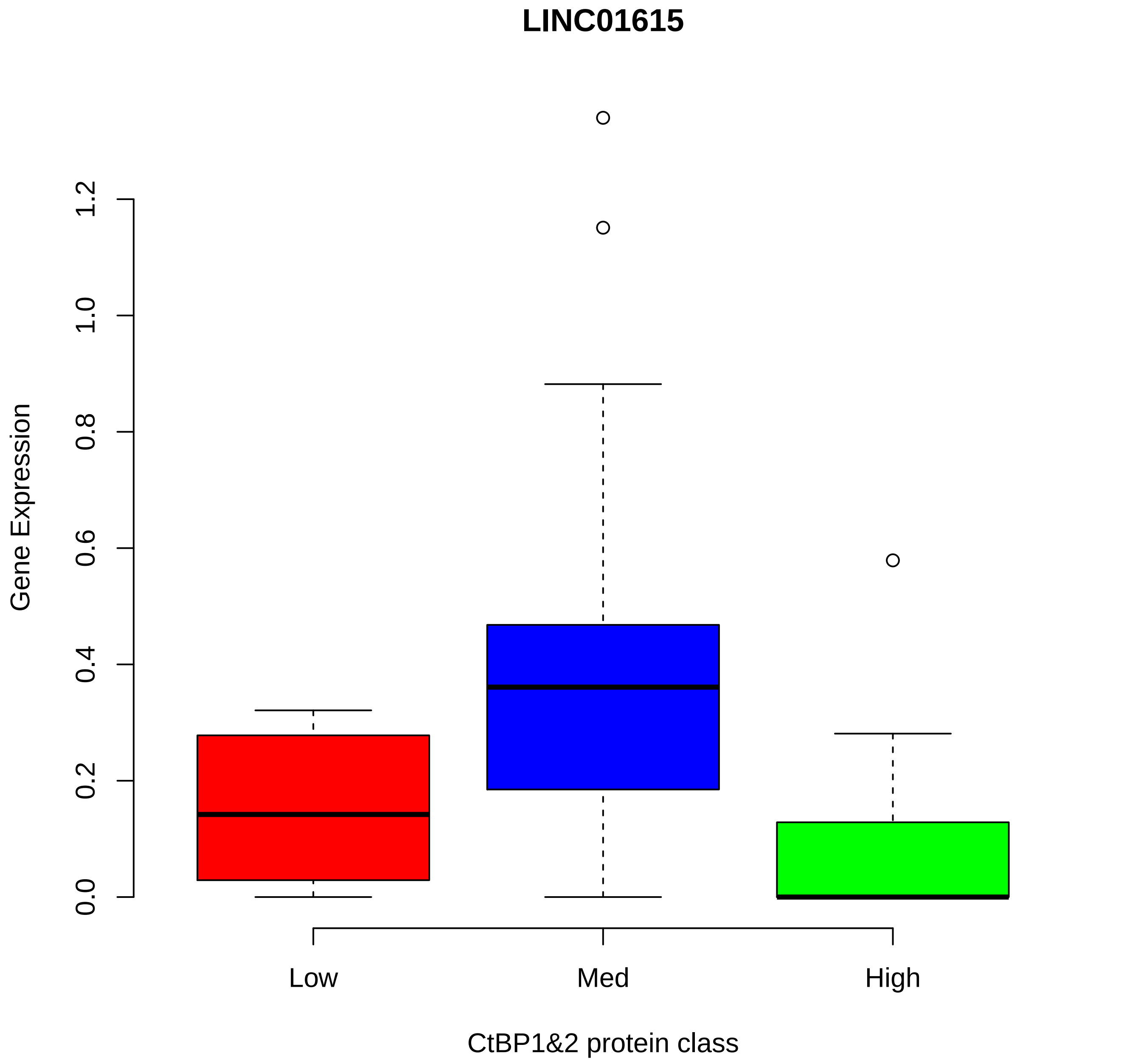

LINC01615
Gene Expression
0.0
0.2
0.4
0.6
0.8
1.0
1.2
Low
Med
High
CtBP1&2 protein class

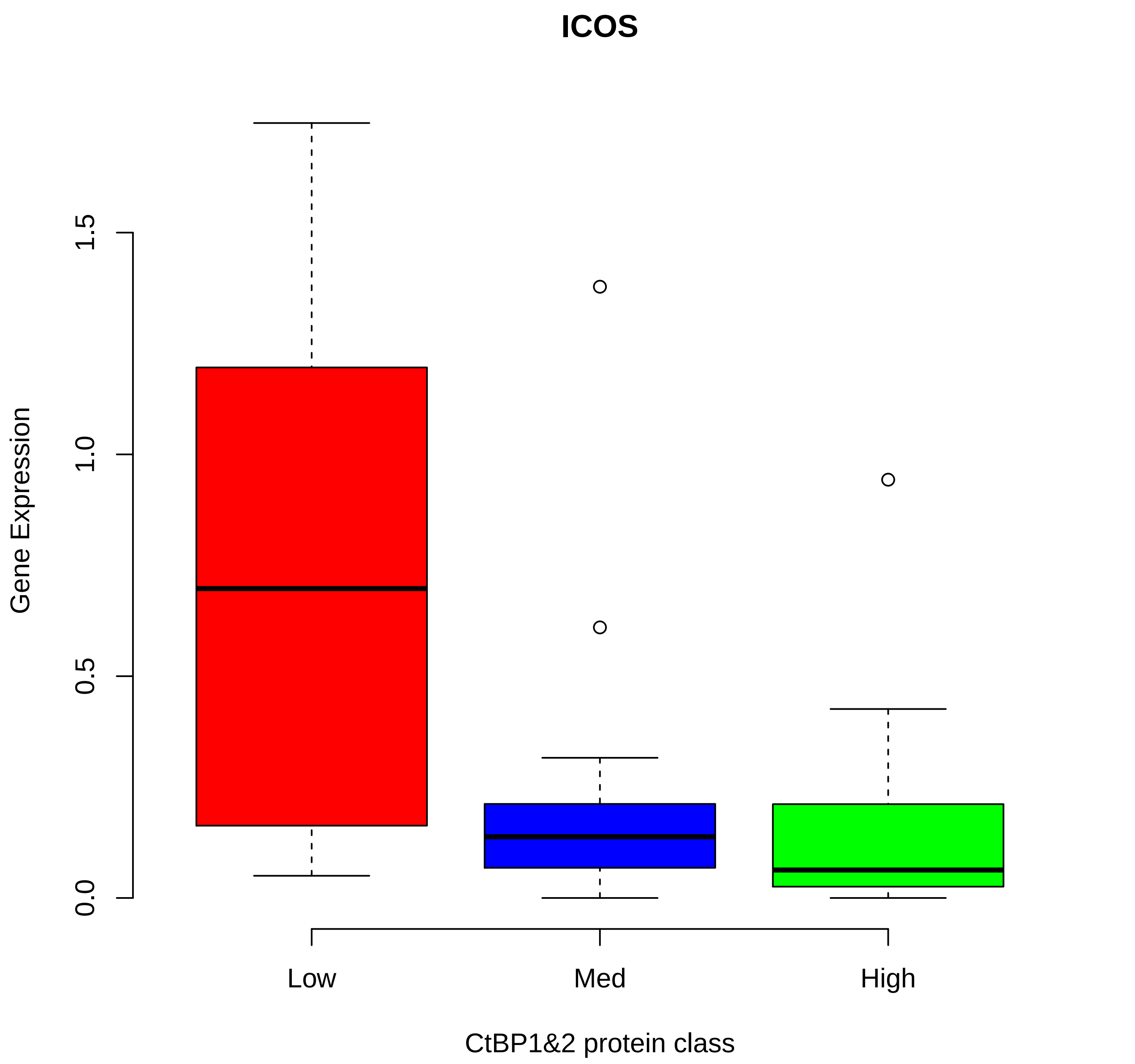
ICOS
Gene Expression
0.0
0.5
1.0
1.5
Low
Med
High
CtBP1&2 protein class

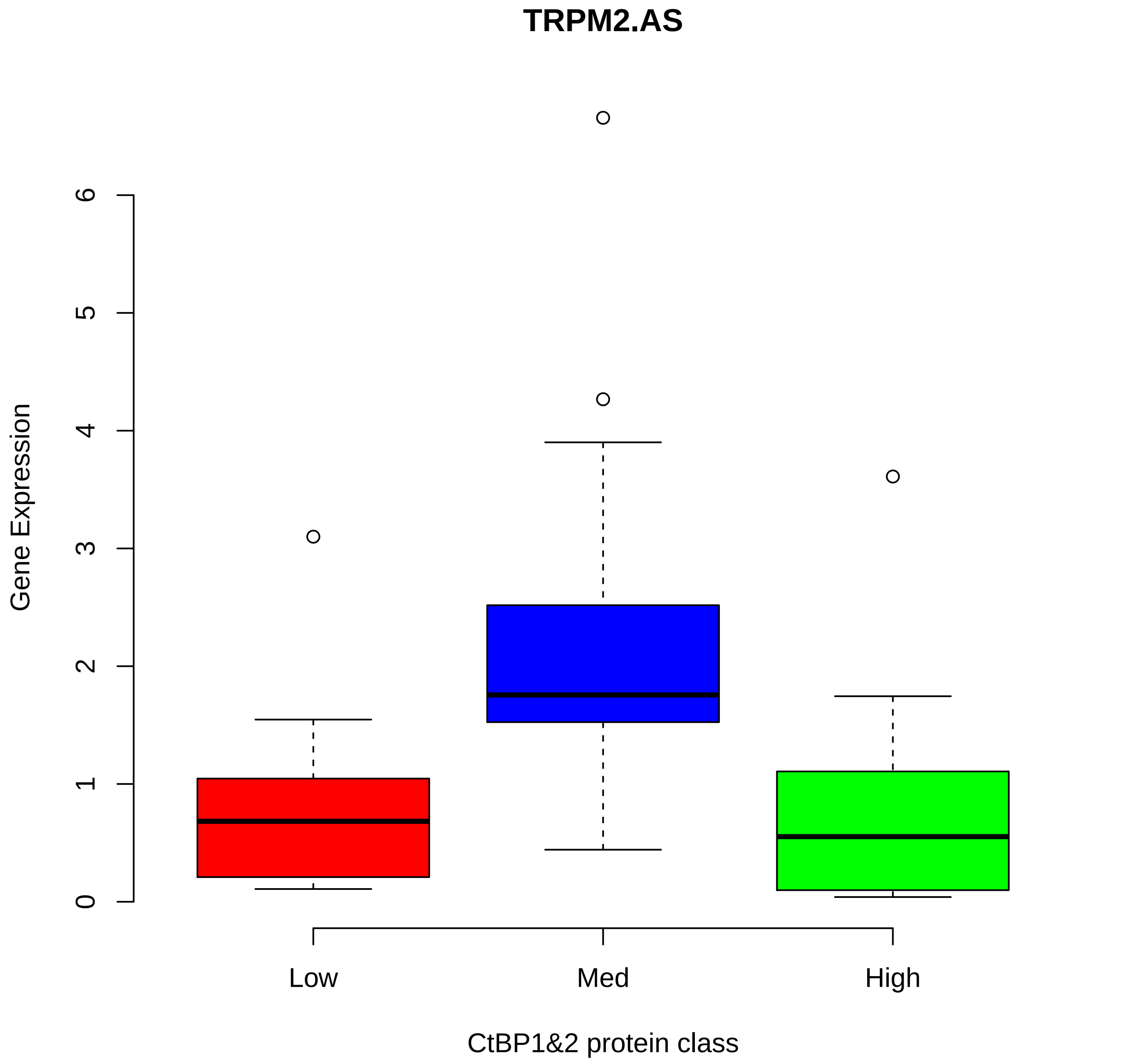

TRPM2.AS
Gene Expression
0
1
2
3
4
5
6
Low
Med
High
CtBP1&2 protein class

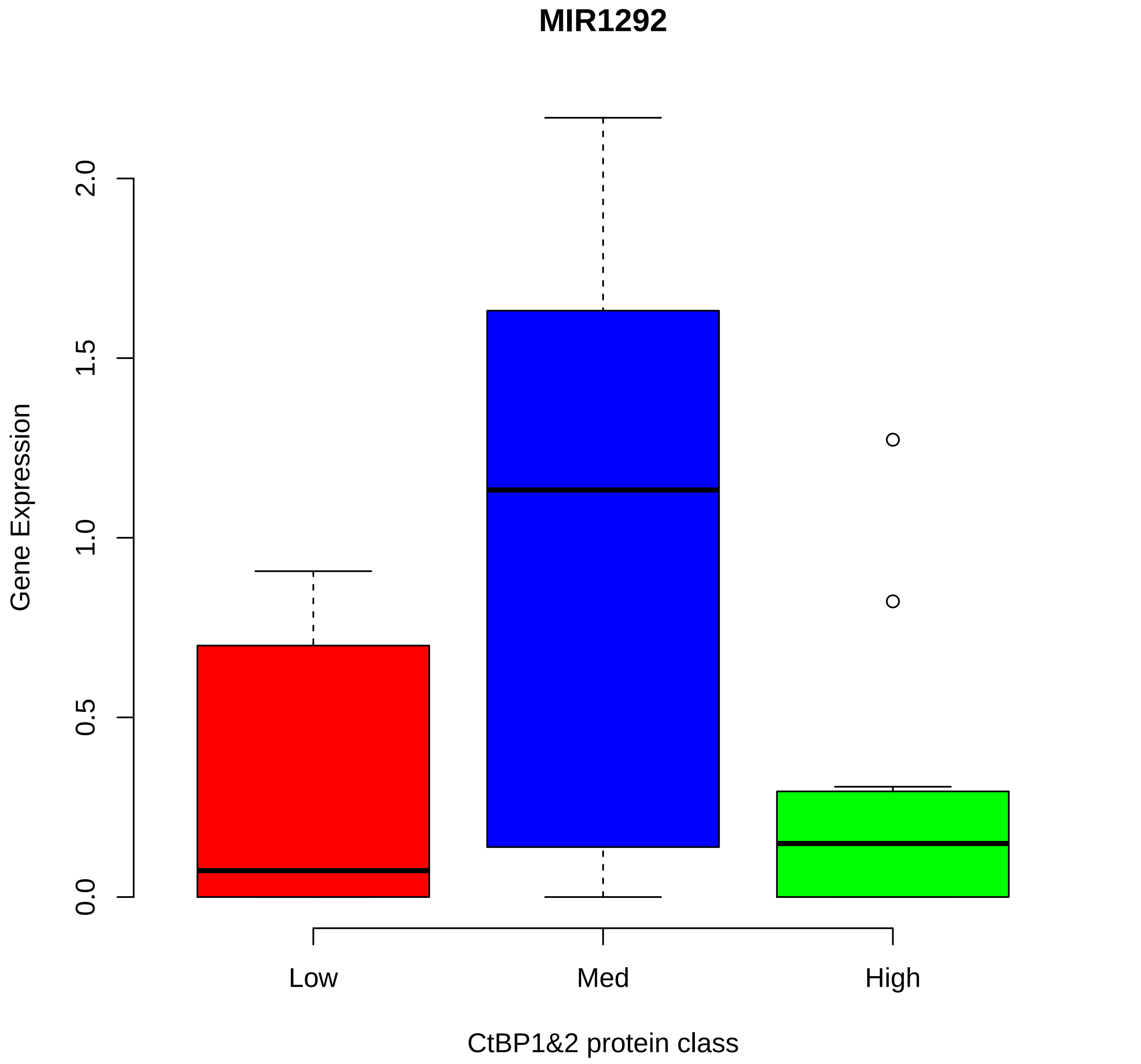

MIR1292
Gene Expression
0.0
0.5
1.0
1.5
2.0
Low
Med
High
CtBP1&2 protein class

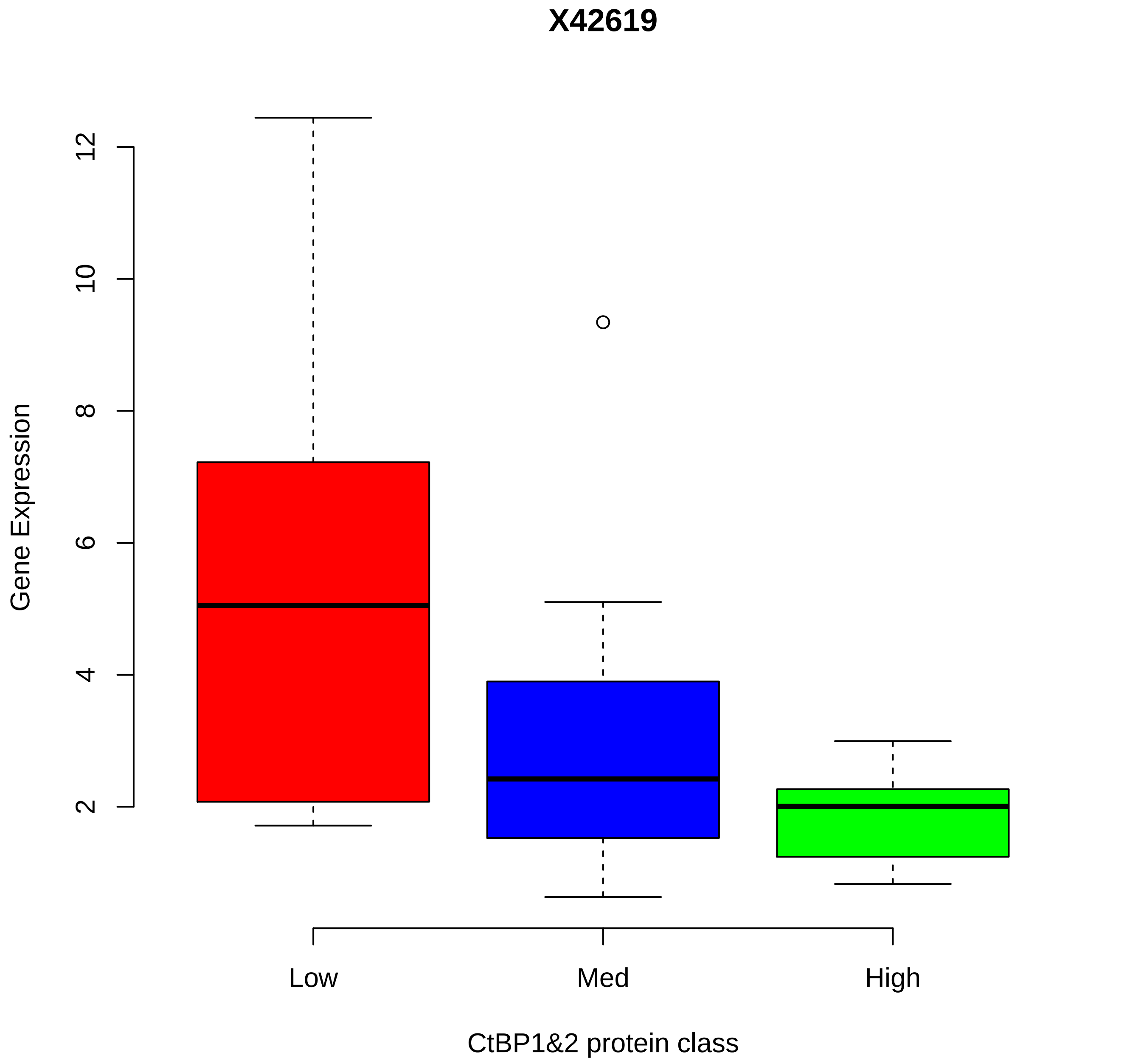
X42619
Gene Expression
2
4
6
8
10
12
Low
Med
High
CtBP1&2 protein class

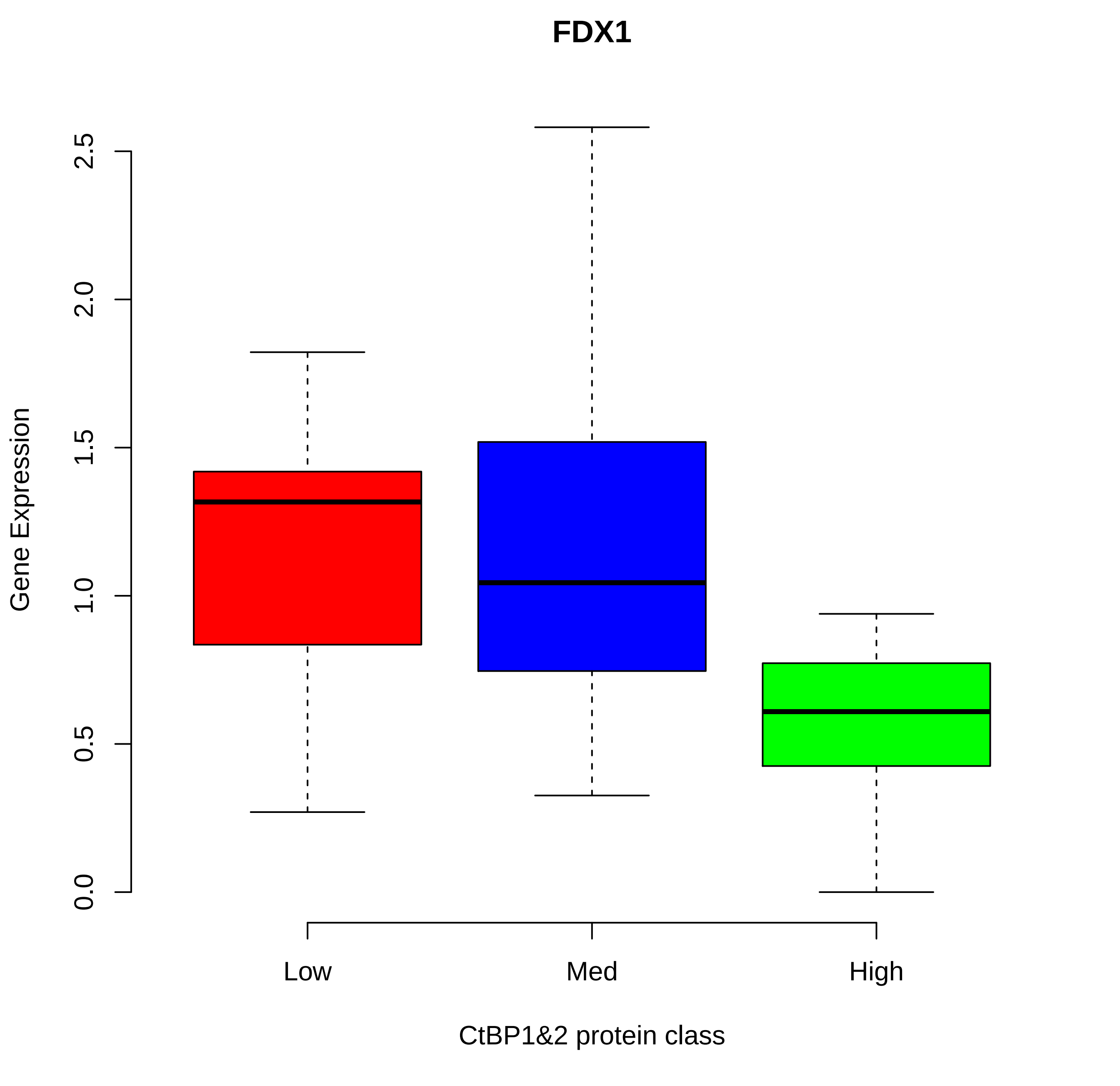
FDX1
Gene Expression
0.0
0.5
1.0
1.5
2.0
2.5
Low
Med
High
CtBP1&2 protein class

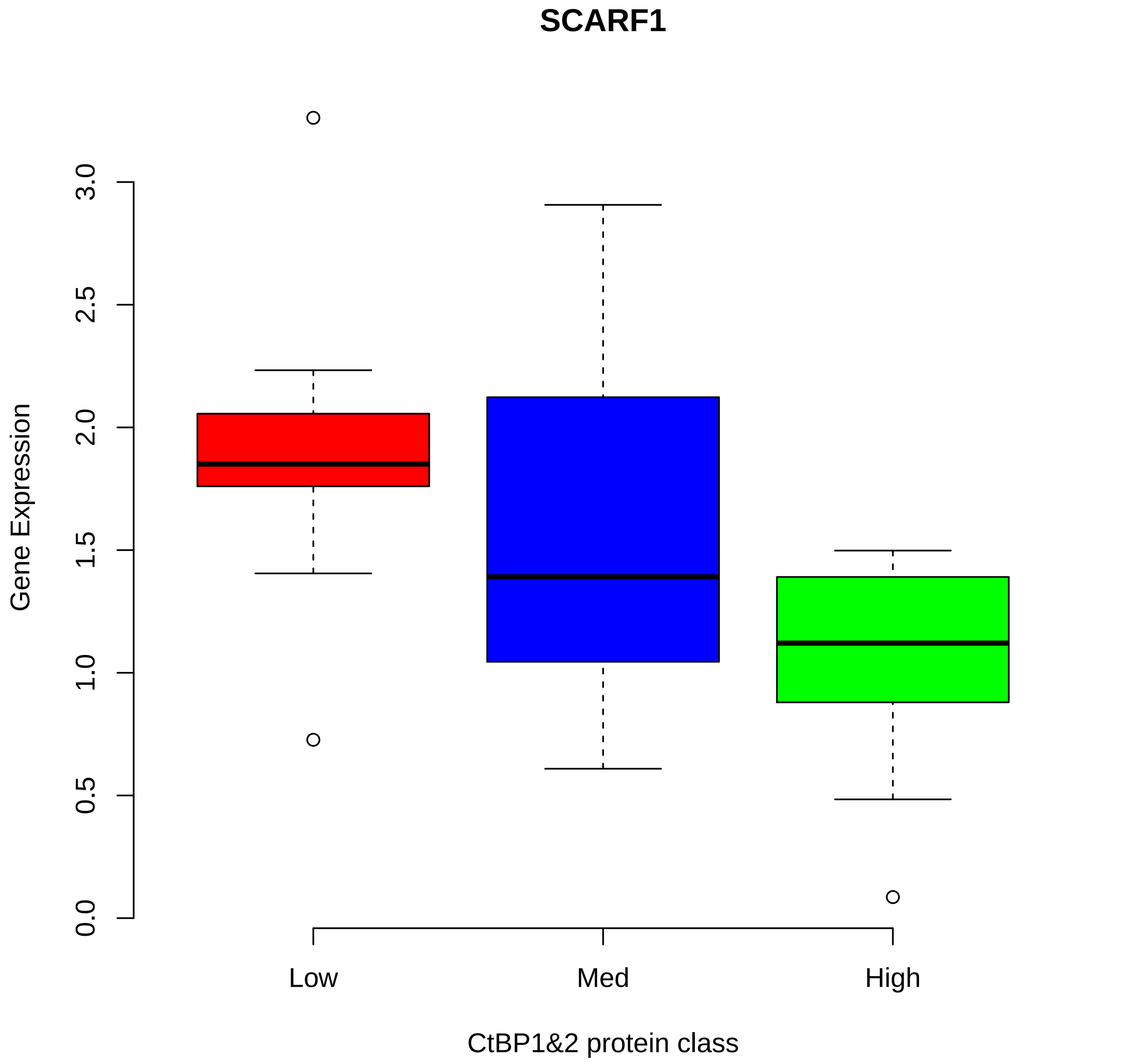
SCARF1
Gene Expression
0.0
0.5
1.0
1.5
2.0
2.5
3.0
Low
Med
High
CtBP1&2 protein class

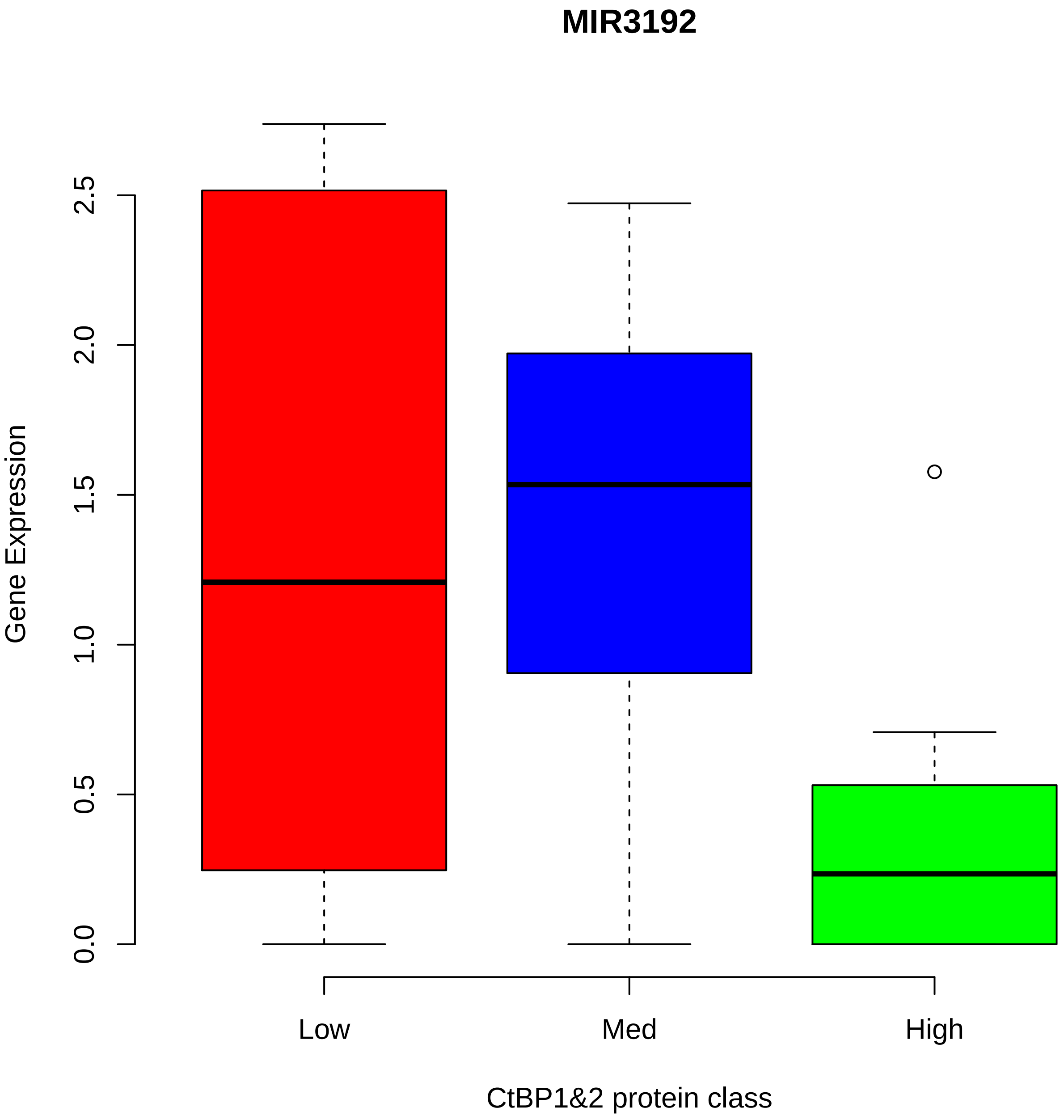
MIR3192
Gene Expression
0.0
0.5
1.0
1.5
2.0
2.5
Low
Med
High
CtBP1&2 protein class

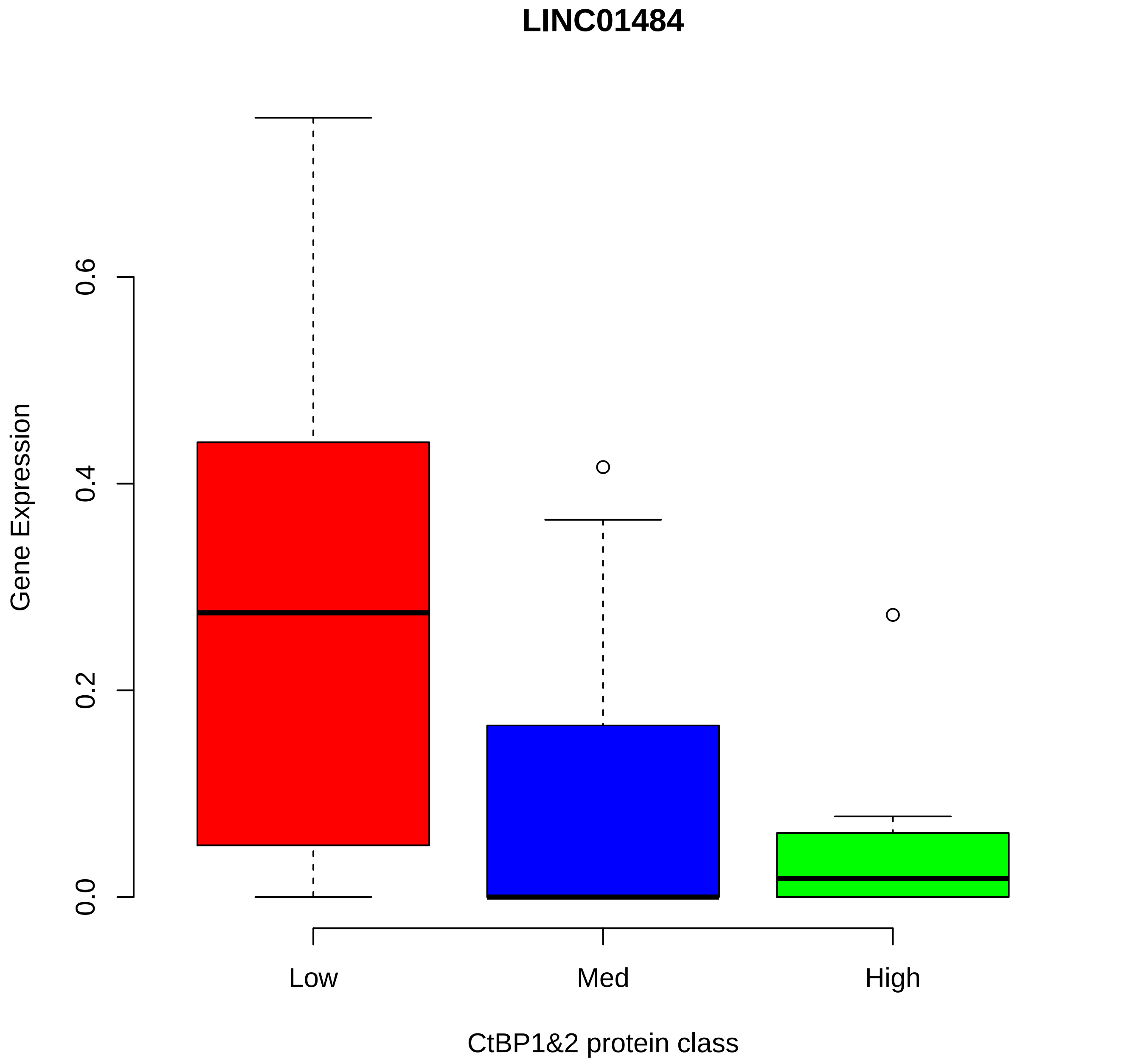
LINC01484
Gene Expression
0.0
0.2
0.4
0.6
Low
Med
High
CtBP1&2 protein class

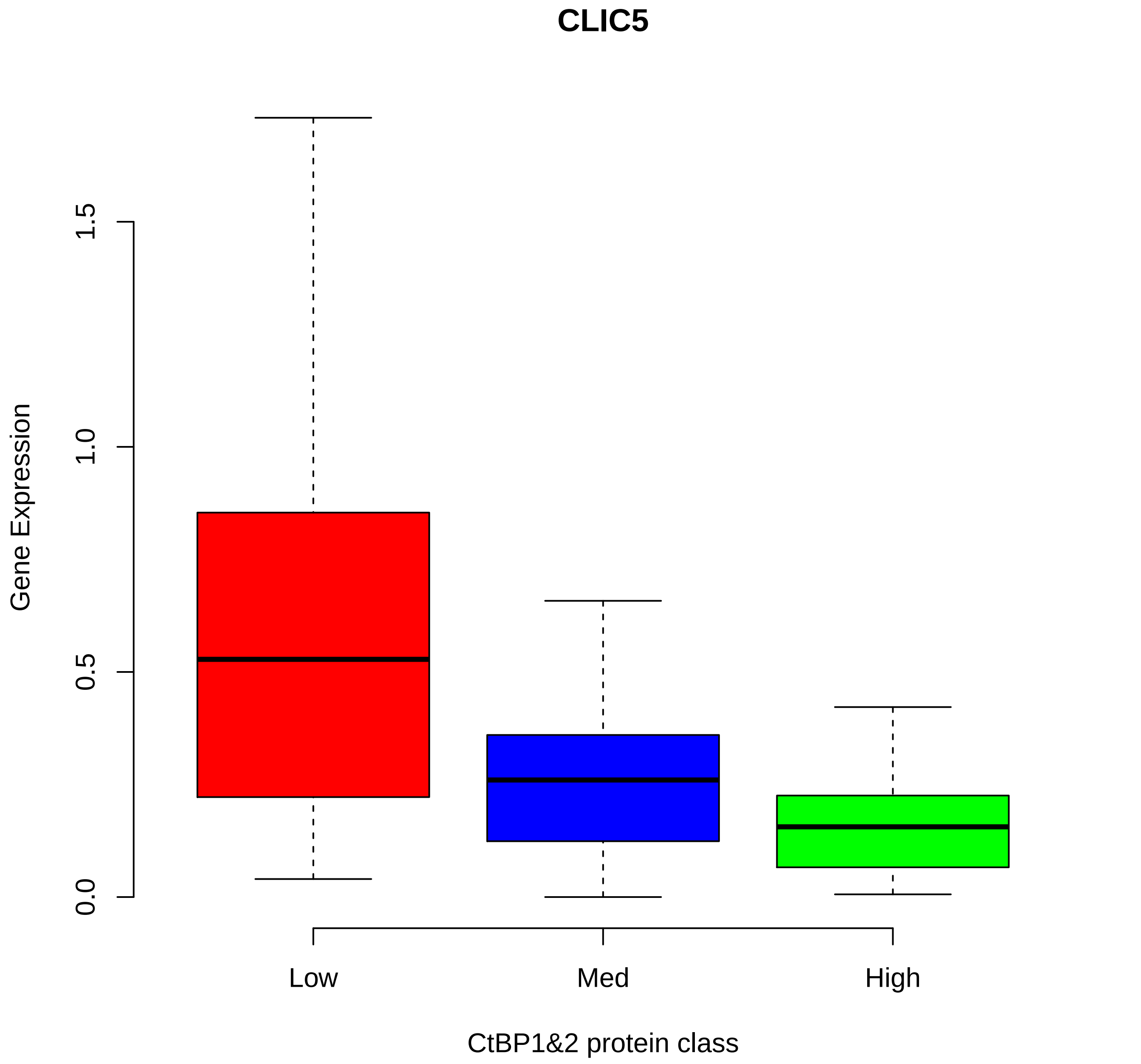
CLIC5
Gene Expression
0.0
0.5
1.0
1.5
Low
Med
High
CtBP1&2 protein class

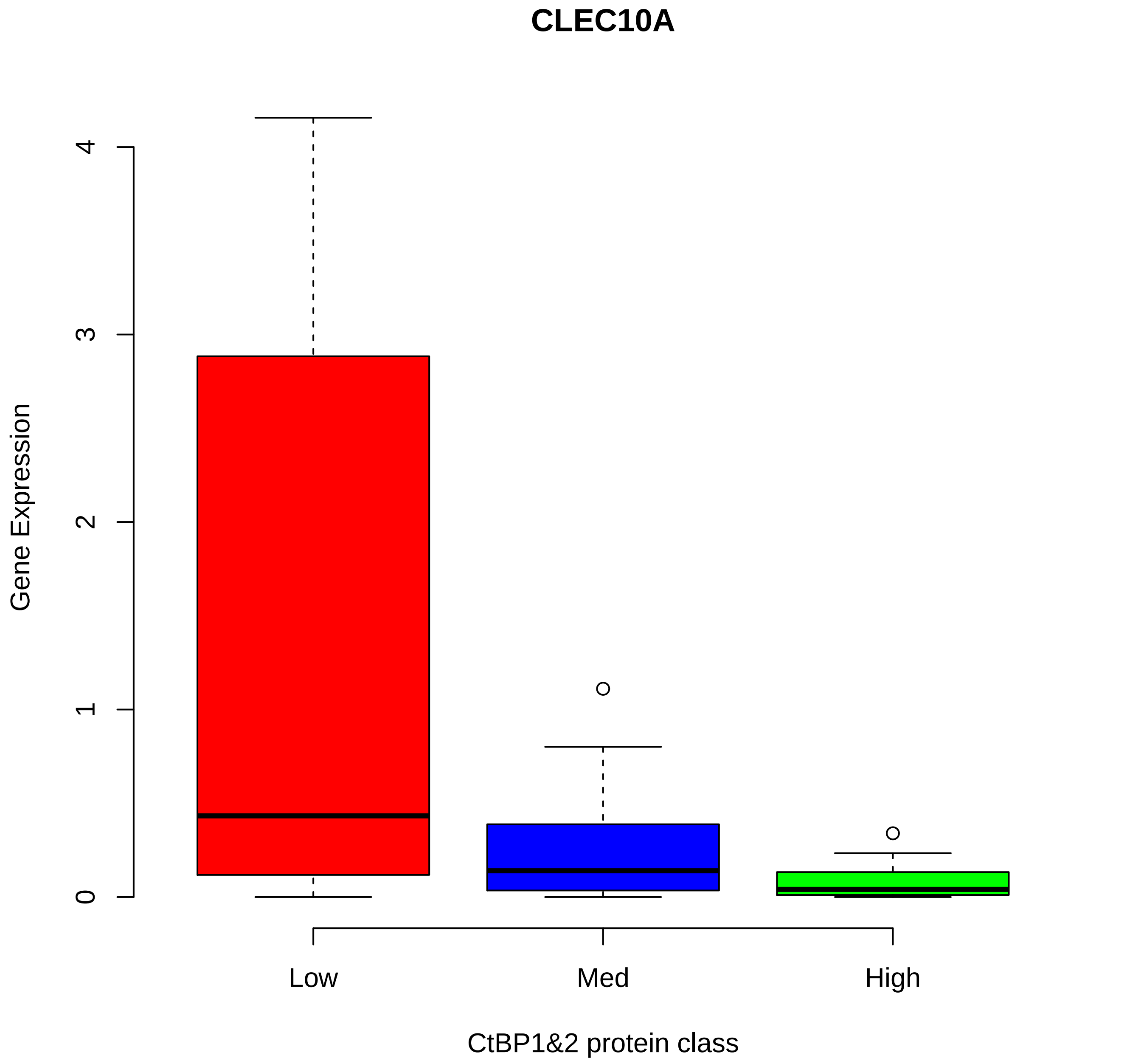

CLEC10A
Gene Expression
0
1
2
3
4
Low
Med
High
CtBP1&2 protein class

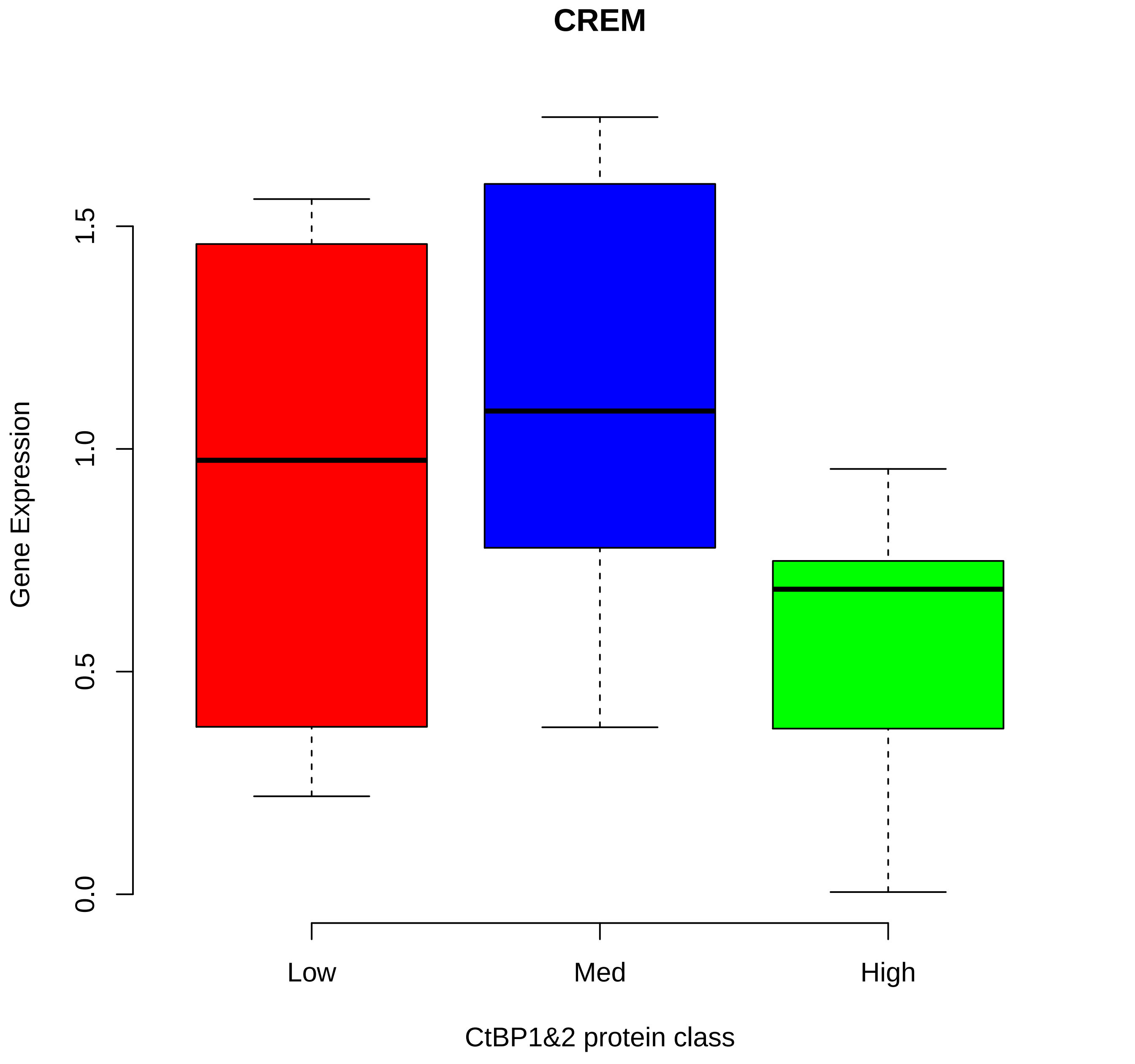
CREM
Gene Expression
0.0
0.5
1.0
1.5
Low
Med
High
CtBP1&2 protein class

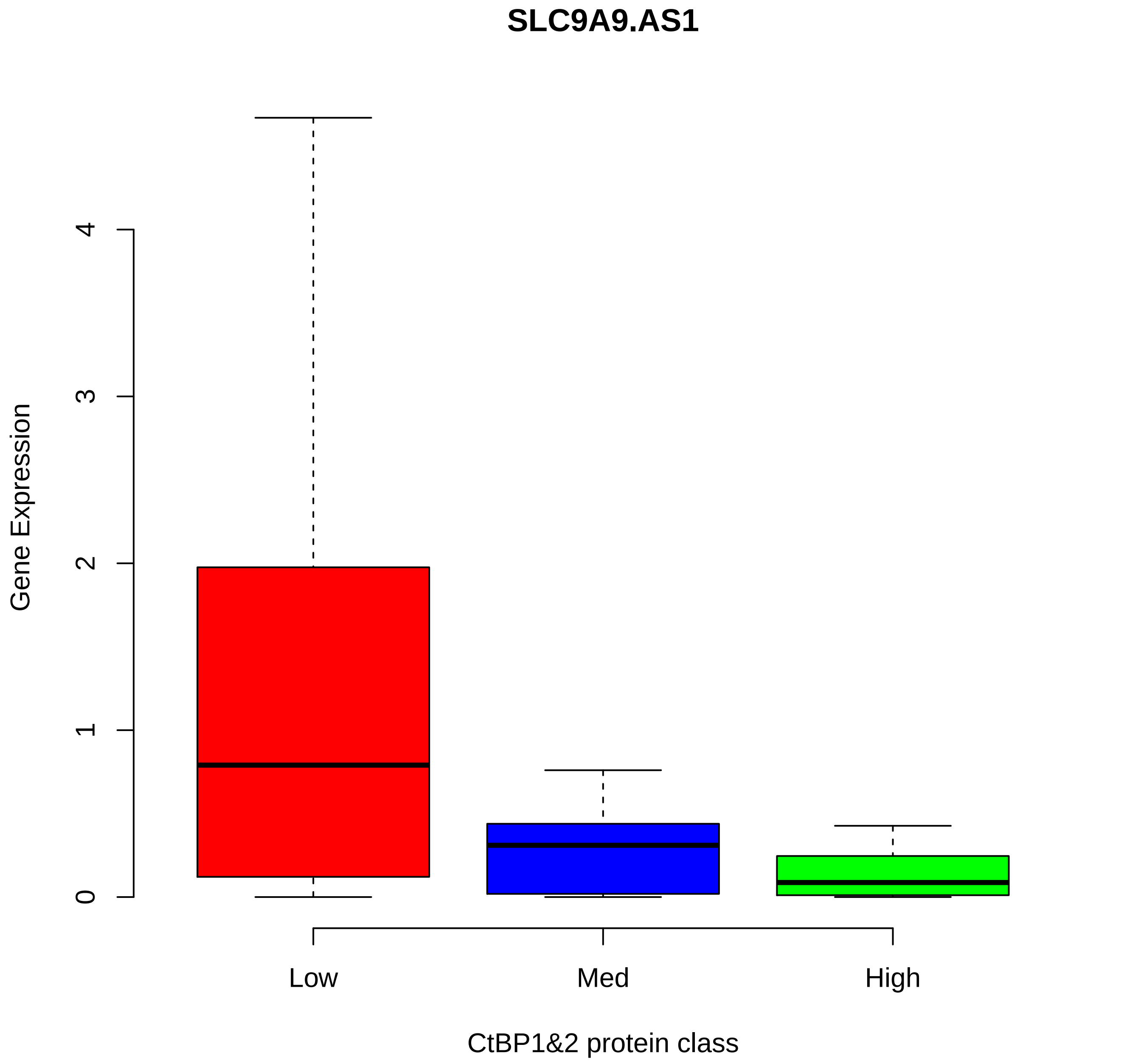

SLC9A9.AS1
Gene Expression
0
1
2
3
4
Low
Med
High
CtBP1&2 protein class

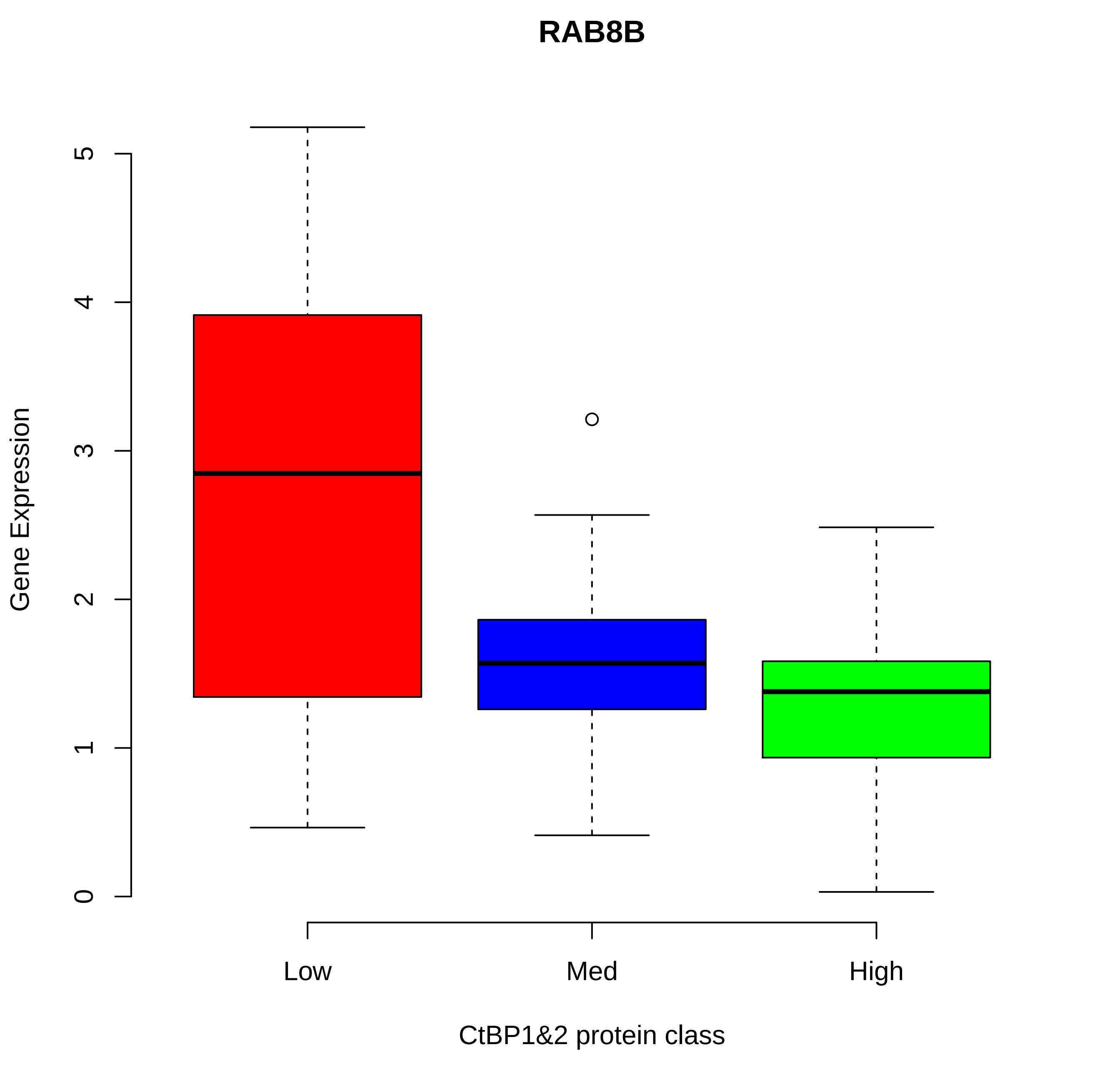

RAB8B
Gene Expression
0
1
2
3
4
5
Low
Med
High
CtBP1&2 protein class

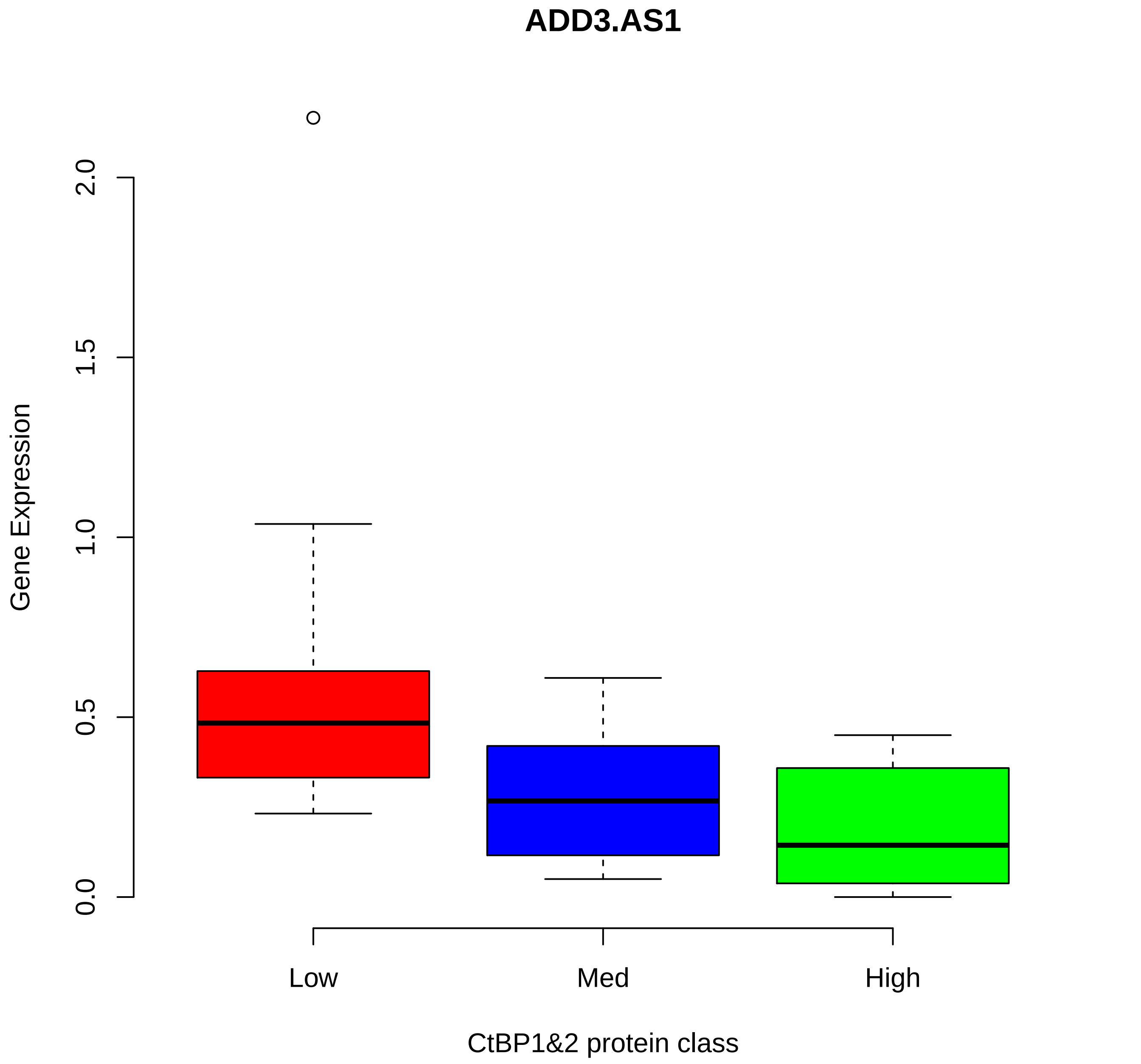
ADD3.AS1
Gene Expression
0.0
0.5
1.0
1.5
2.0
Low
Med
High
CtBP1&2 protein class

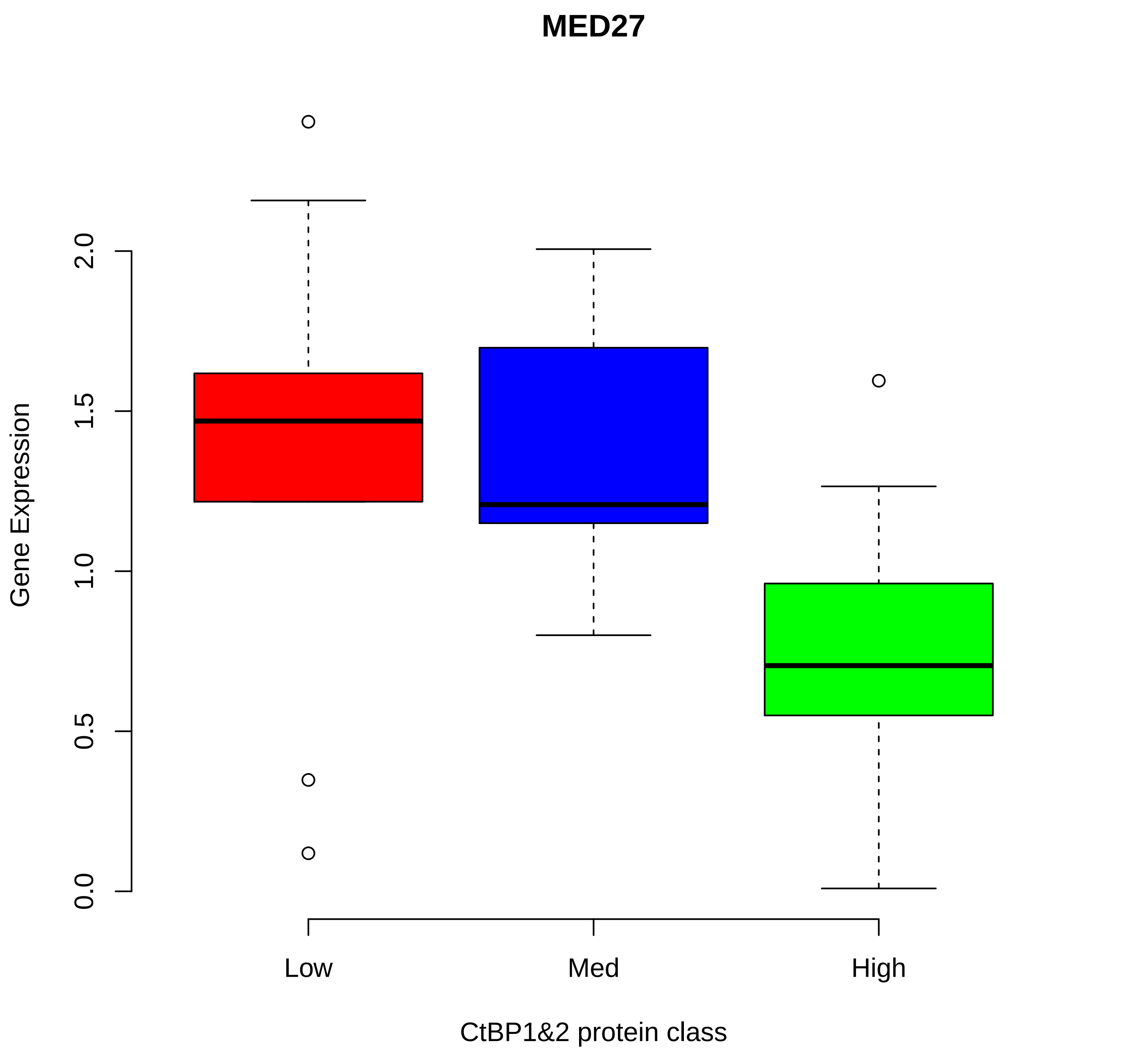
MED27
Gene Expression
0.0
0.5
1.0
1.5
2.0
Low
Med
High
CtBP1&2 protein class

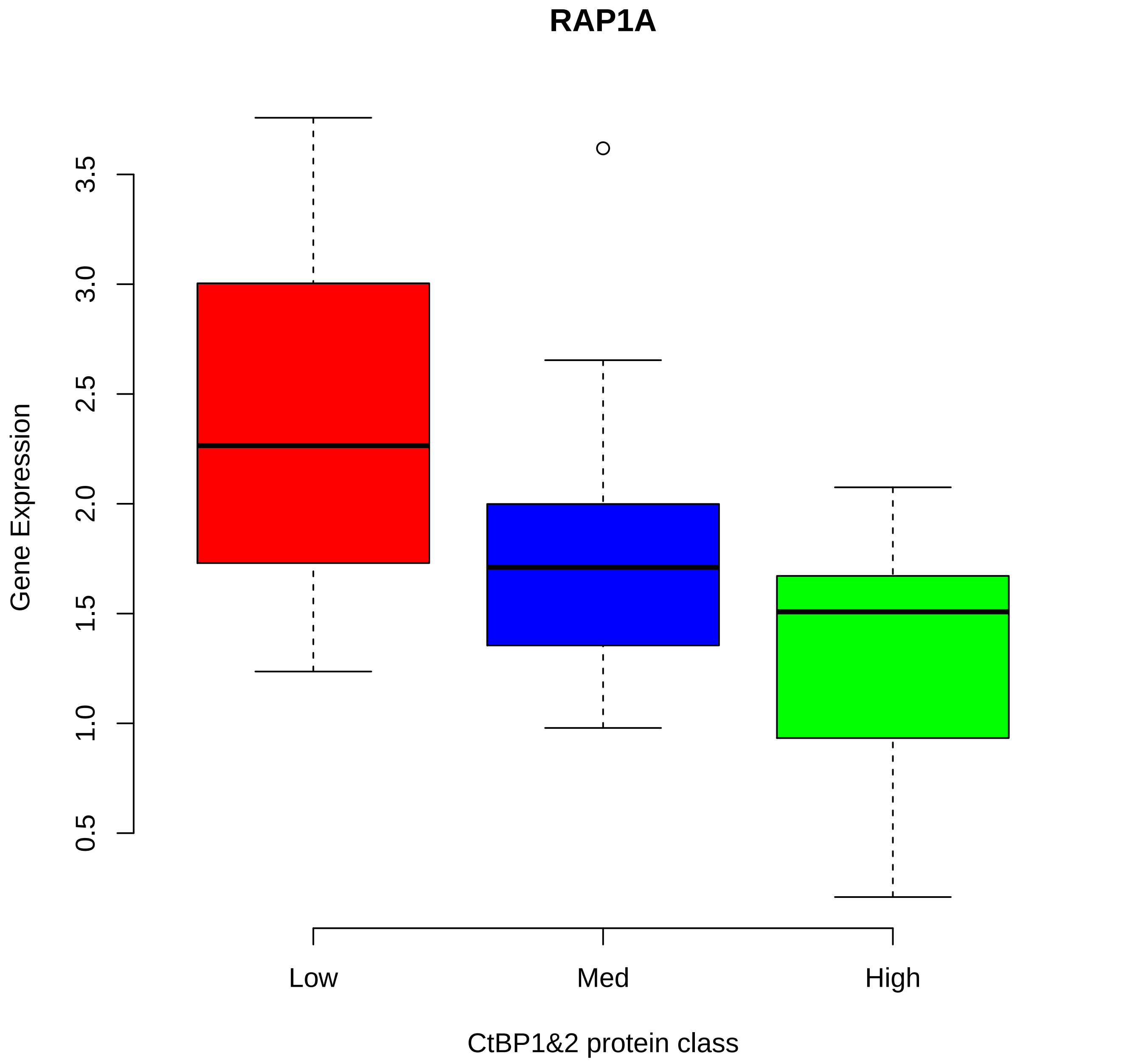
RAP1A
Gene Expression
0.5
1.0
1.5
2.0
2.5
3.0
3.5
Low
Med
High
CtBP1&2 protein class

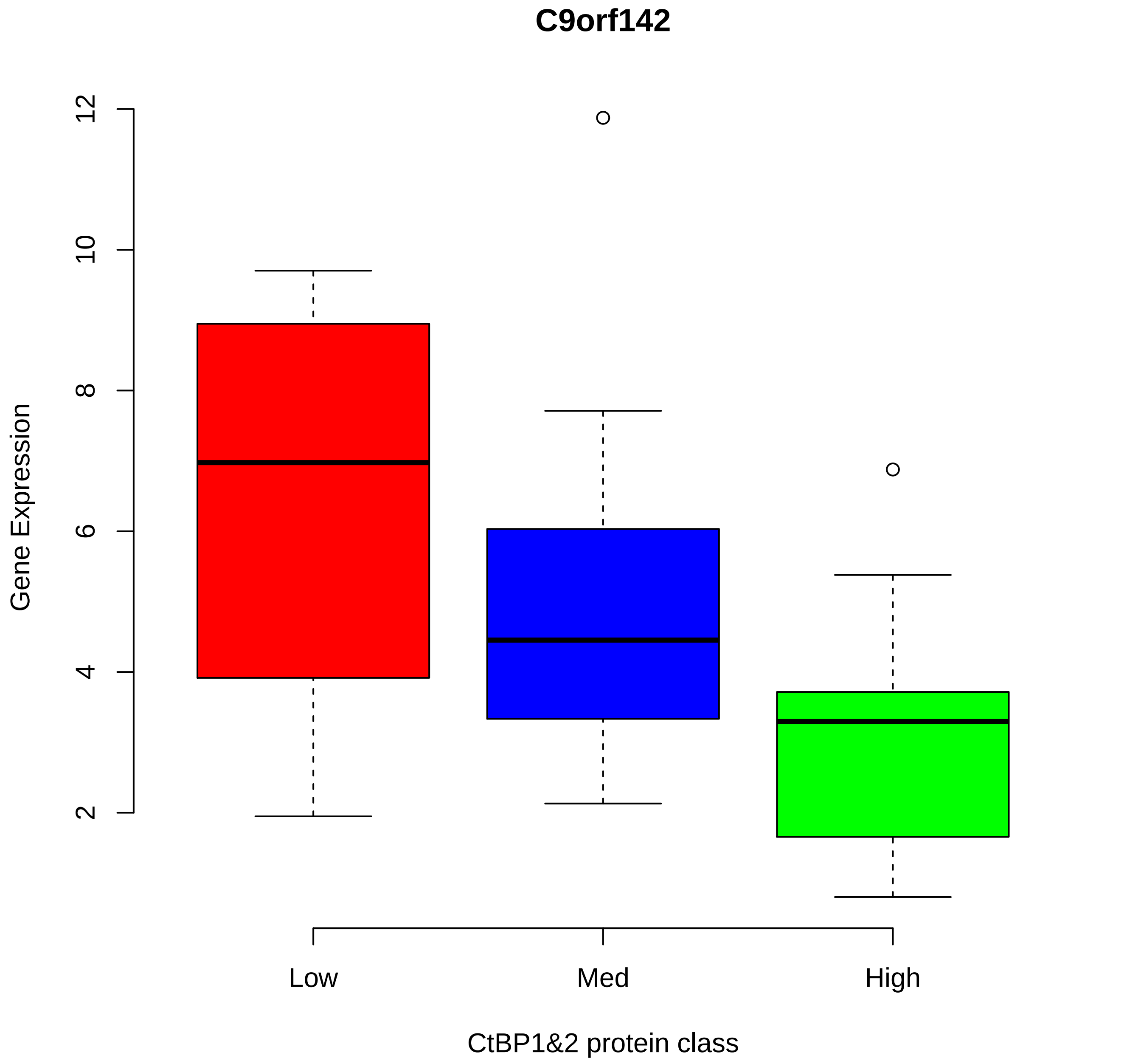
C9orf142
Gene Expression
2
4
6
8
10
12
Low
Med
High
CtBP1&2 protein class

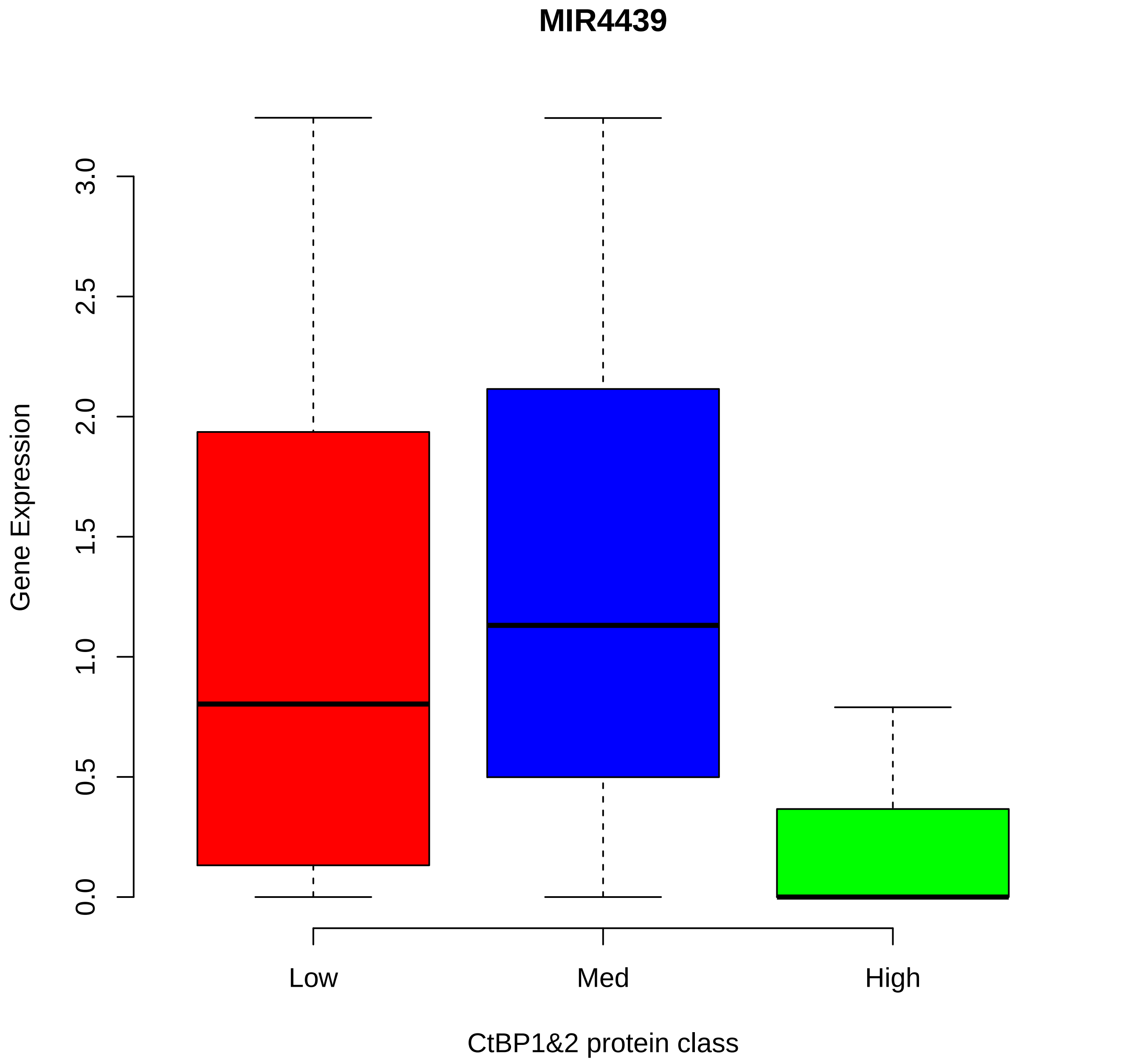

MIR4439
Gene Expression
0.0
0.5
1.0
1.5
2.0
2.5
3.0
Low
Med
High
CtBP1&2 protein class

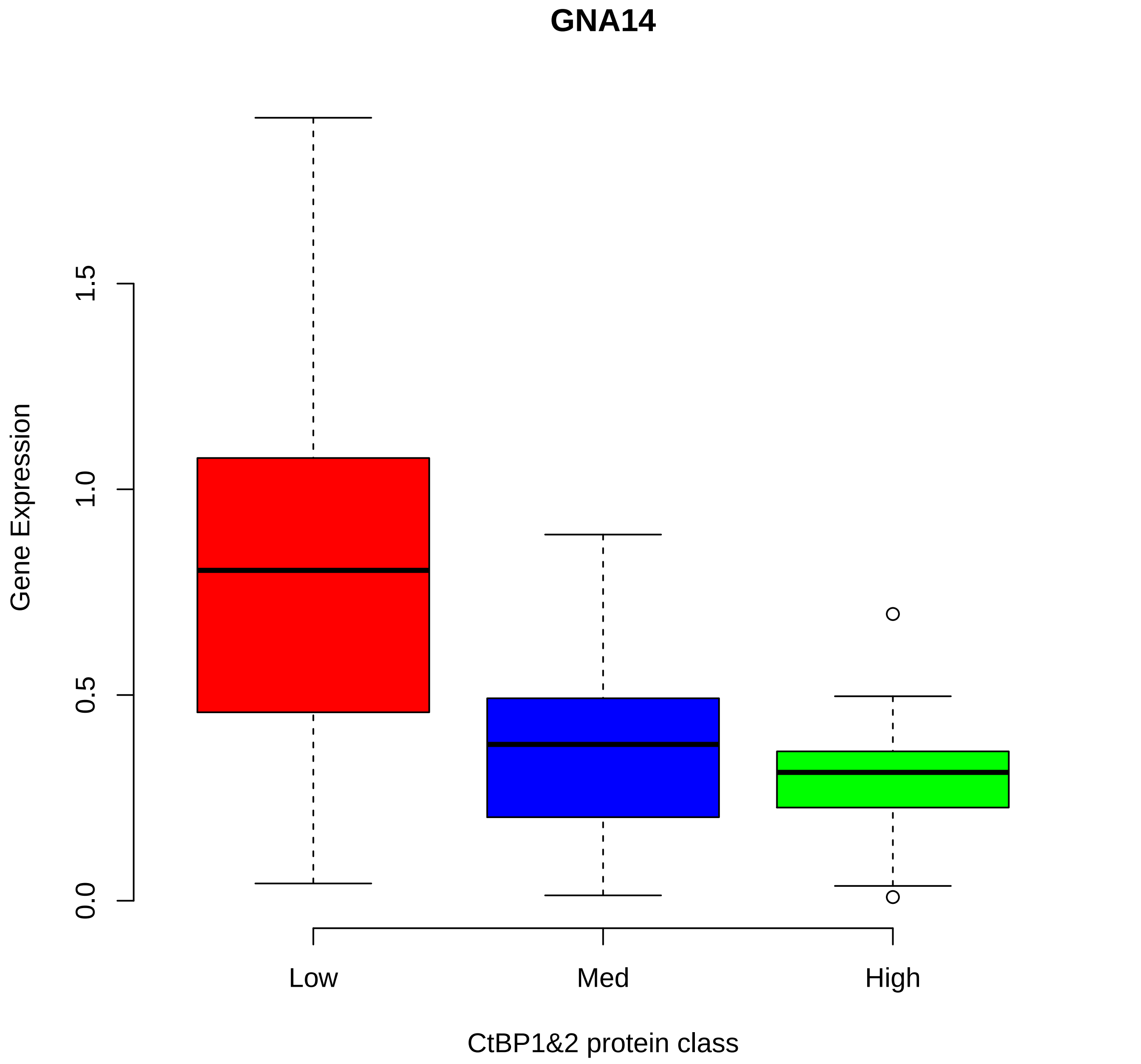
GNA14
Gene Expression
0.0
0.5
1.0
1.5
Low
Med
High
CtBP1&2 protein class

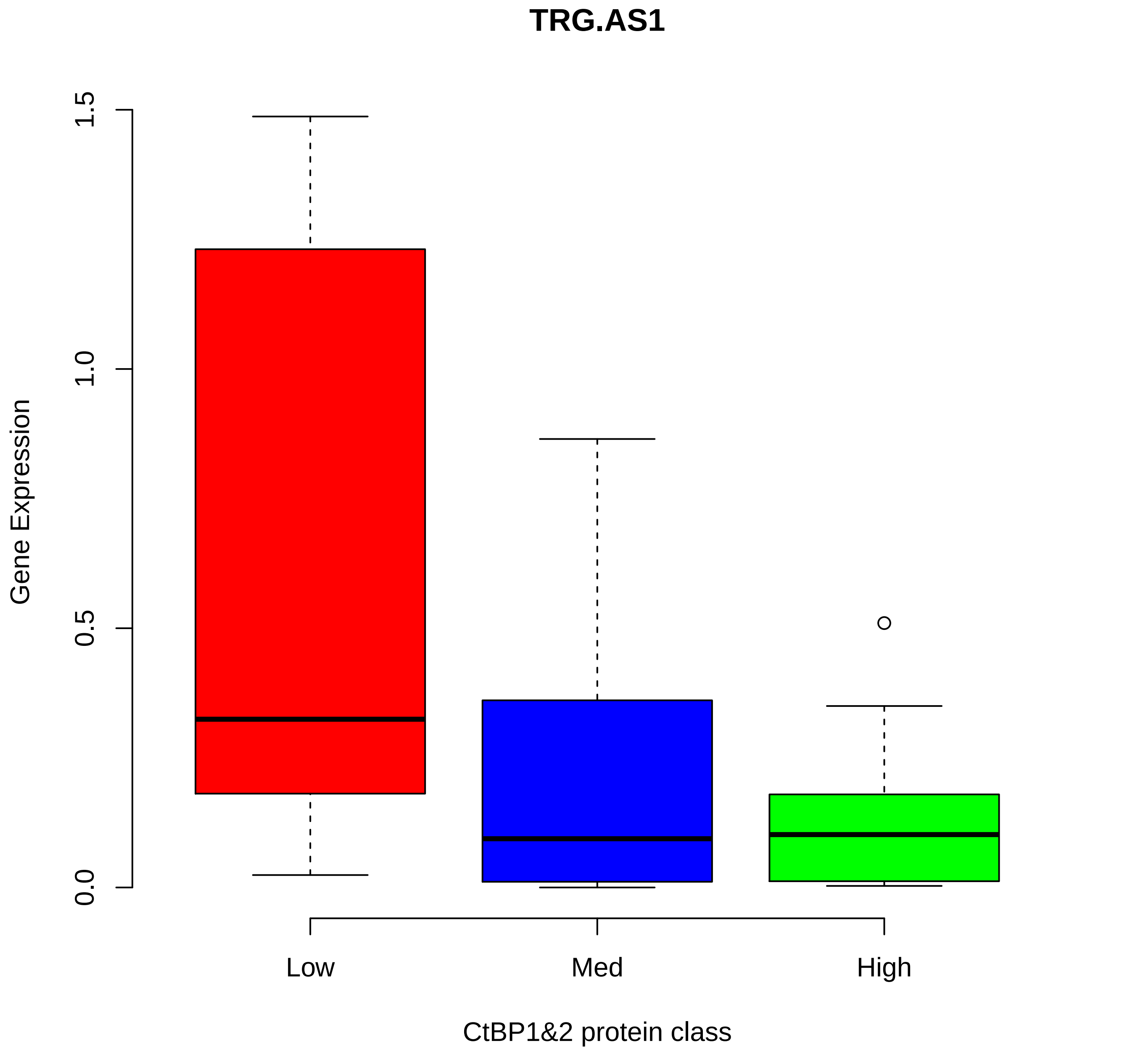
TRG.AS1
Gene Expression
0.0
0.5
1.0
1.5
Low
Med
High
CtBP1&2 protein class

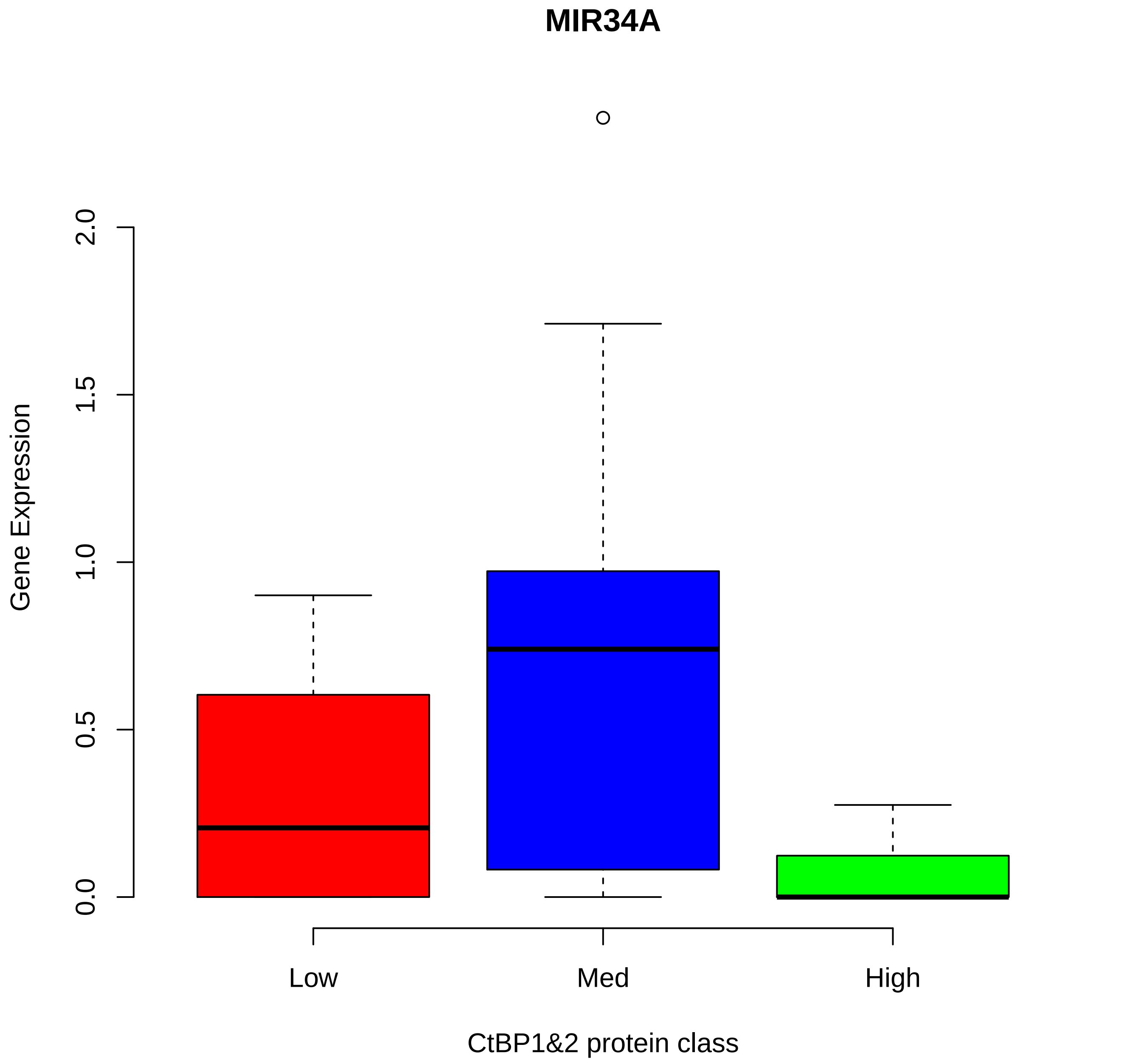
MIR34A
Gene Expression
0.0
0.5
1.0
1.5
2.0
Low
Med
High
CtBP1&2 protein class

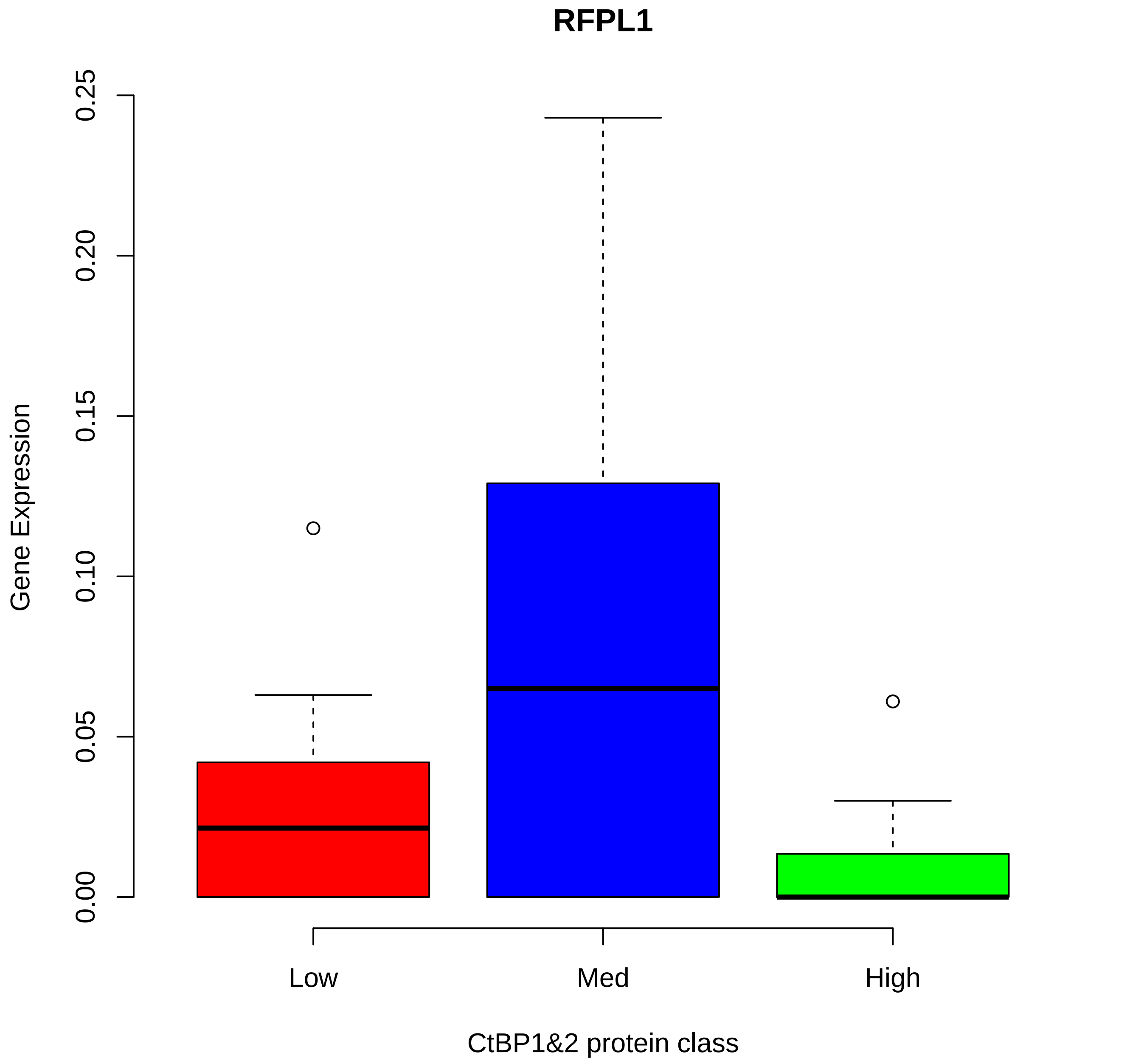
RFPL1
Gene Expression
0.00
0.05
0.10
0.15
0.20
0.25
Low
Med
High
CtBP1&2 protein class

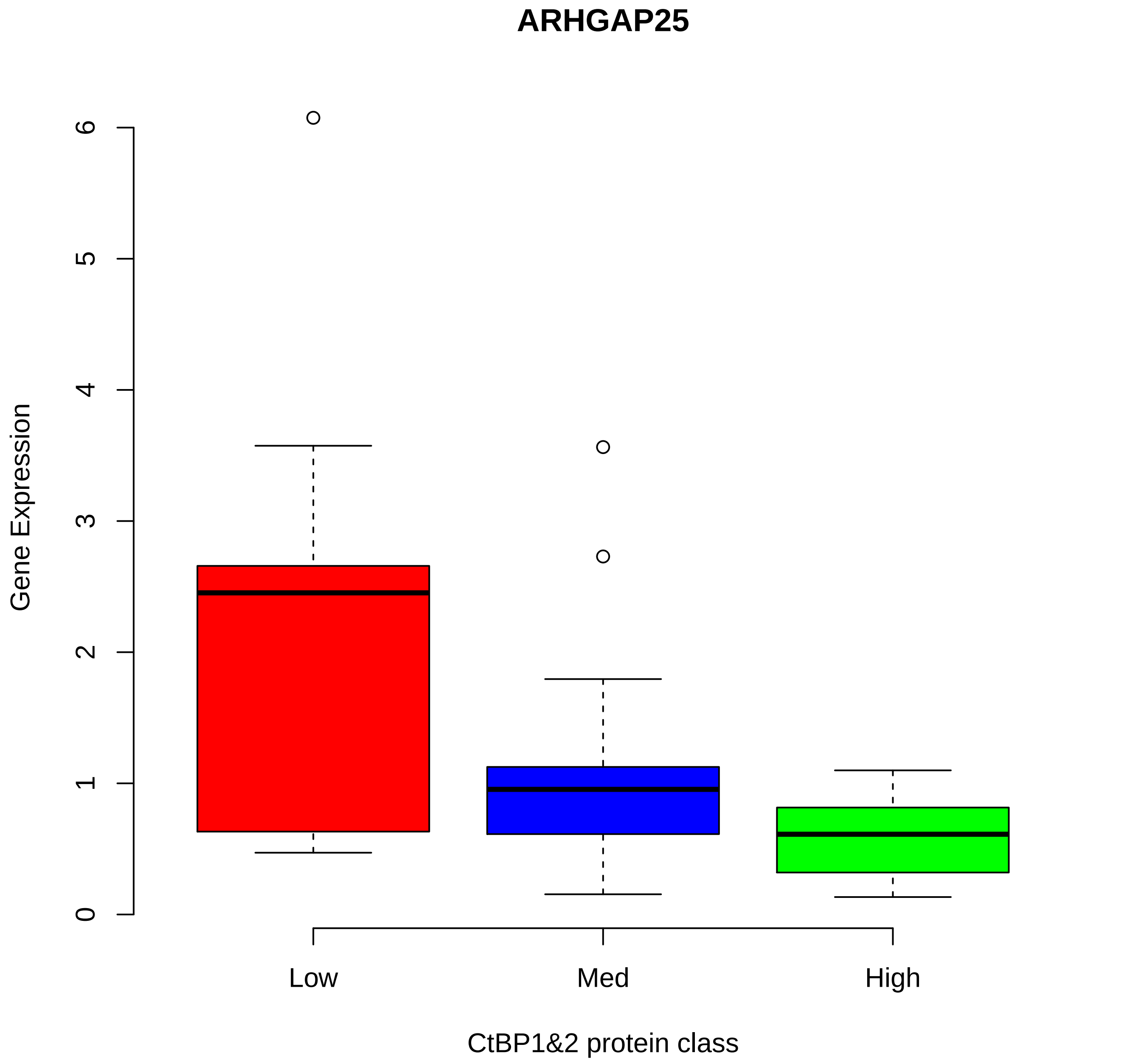
ARHGAP25
Gene Expression
0
1
2
3
4
5
6
Low
Med
High
CtBP1&2 protein class

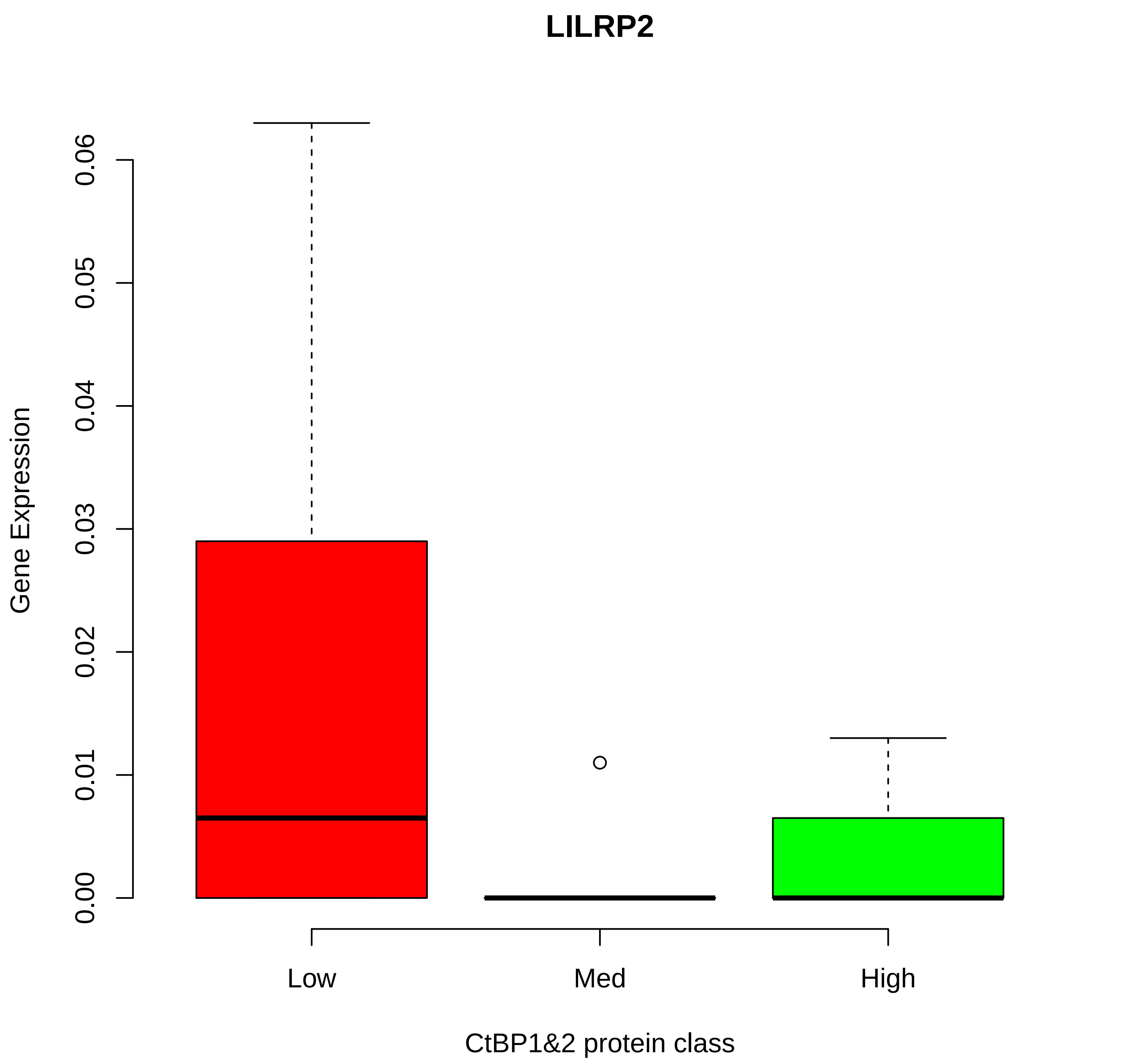

LILRP2
Gene Expression
0.00
0.01
0.02
0.03
0.04
0.05
0.06
Low
Med
High
CtBP1&2 protein class

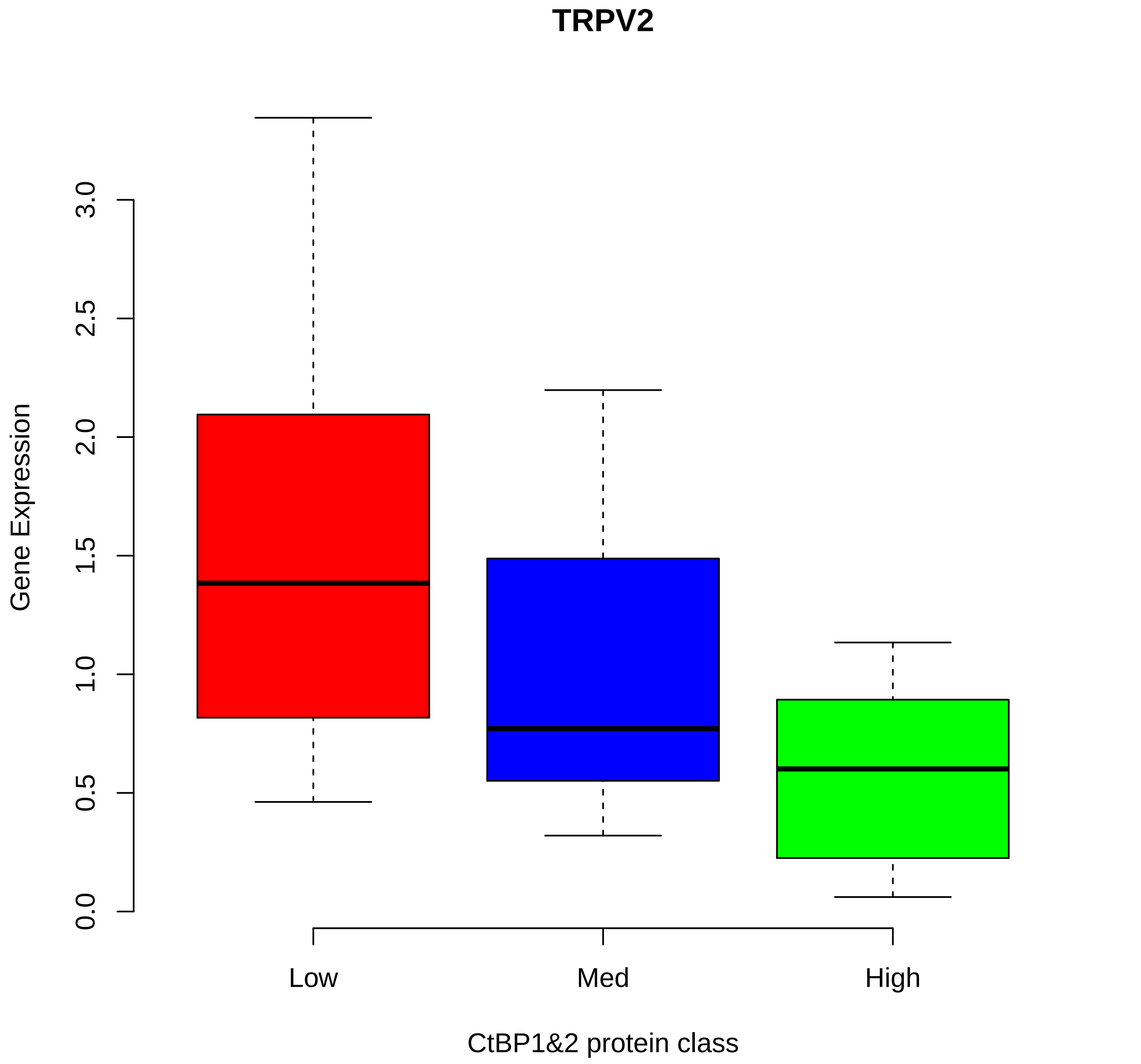

TRPV2
Gene Expression
0.0
0.5
1.0
1.5
2.0
2.5
3.0
Low
Med
High
CtBP1&2 protein class

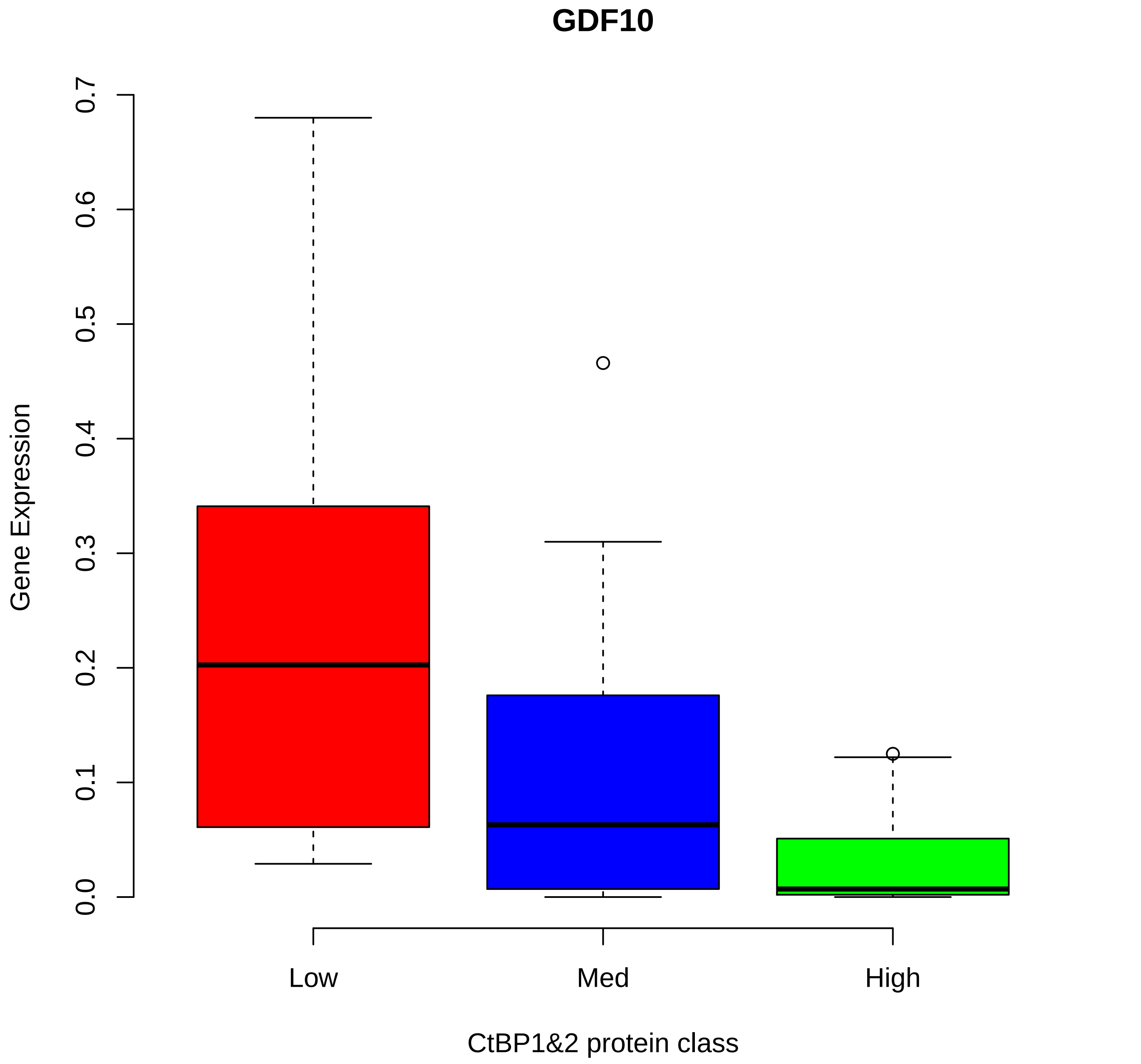
GDF10
Gene Expression
0.0
0.1
0.2
0.3
0.4
0.5
0.6
0.7
Low
Med
High
CtBP1&2 protein class

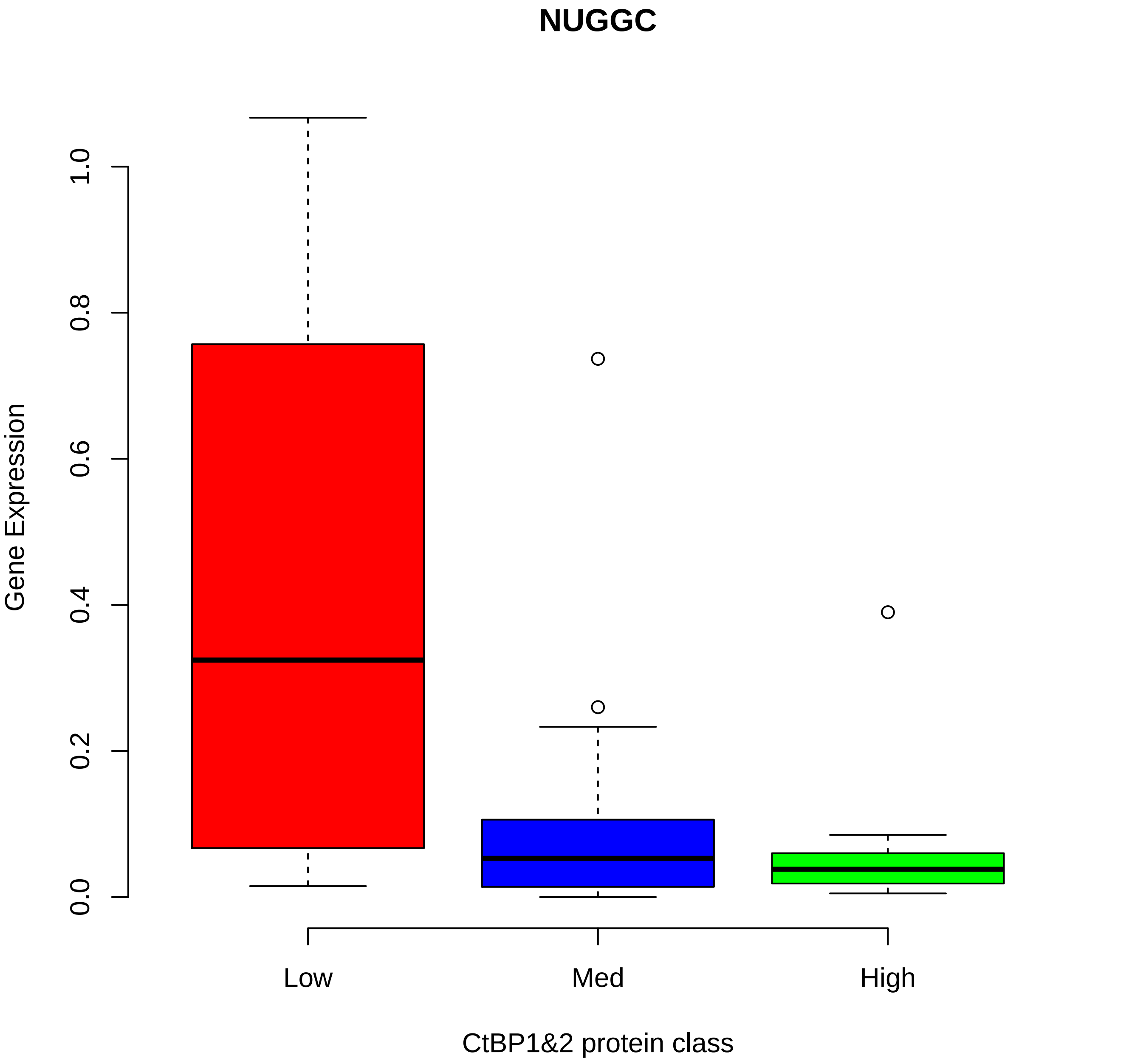

NUGGC
Gene Expression
0.0
0.2
0.4
0.6
0.8
1.0
Low
Med
High
CtBP1&2 protein class

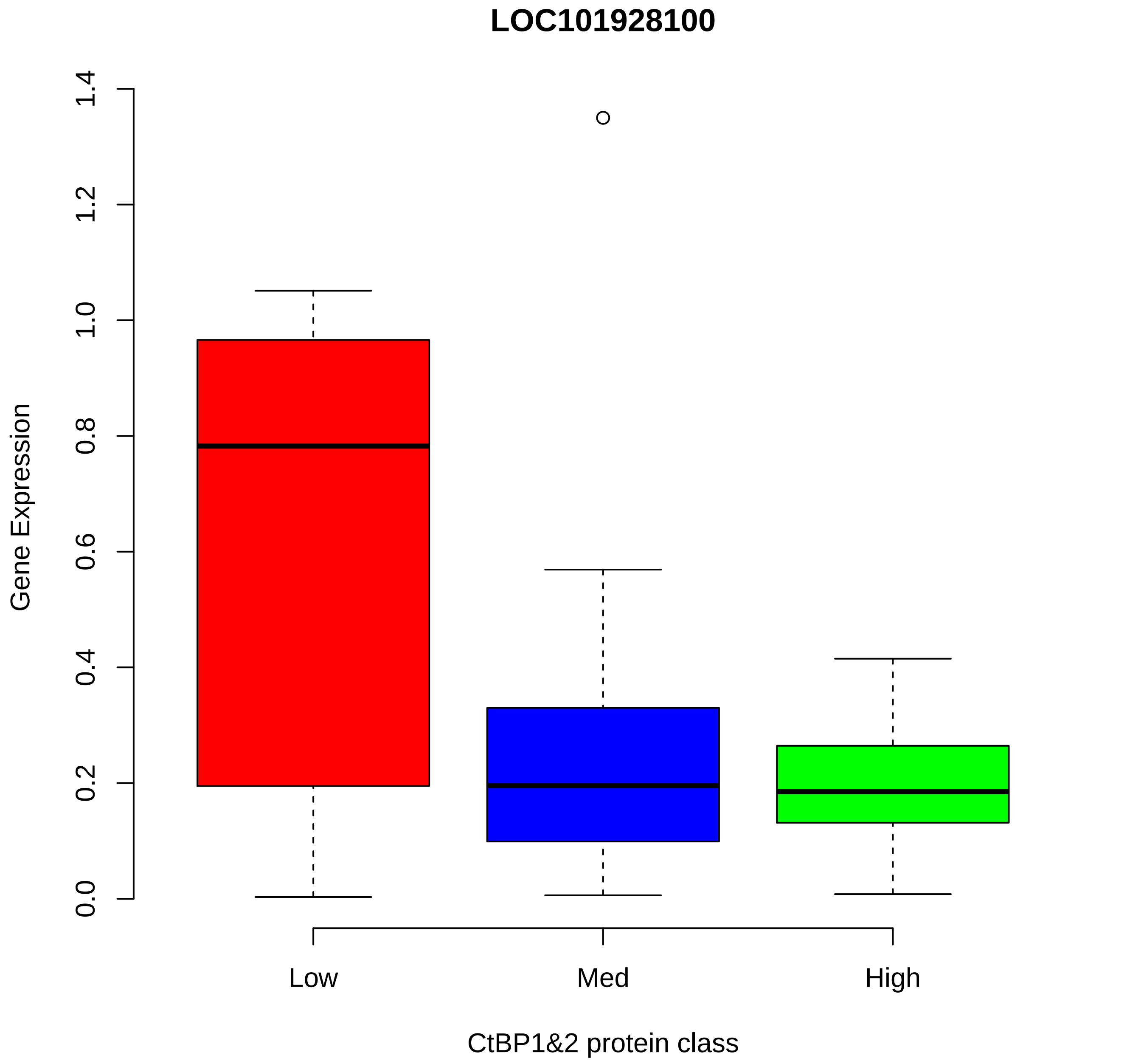
LOC101928100
Gene Expression
0.0
0.2
0.4
0.6
0.8
1.0
1.2
1.4
Low
Med
High
CtBP1&2 protein class

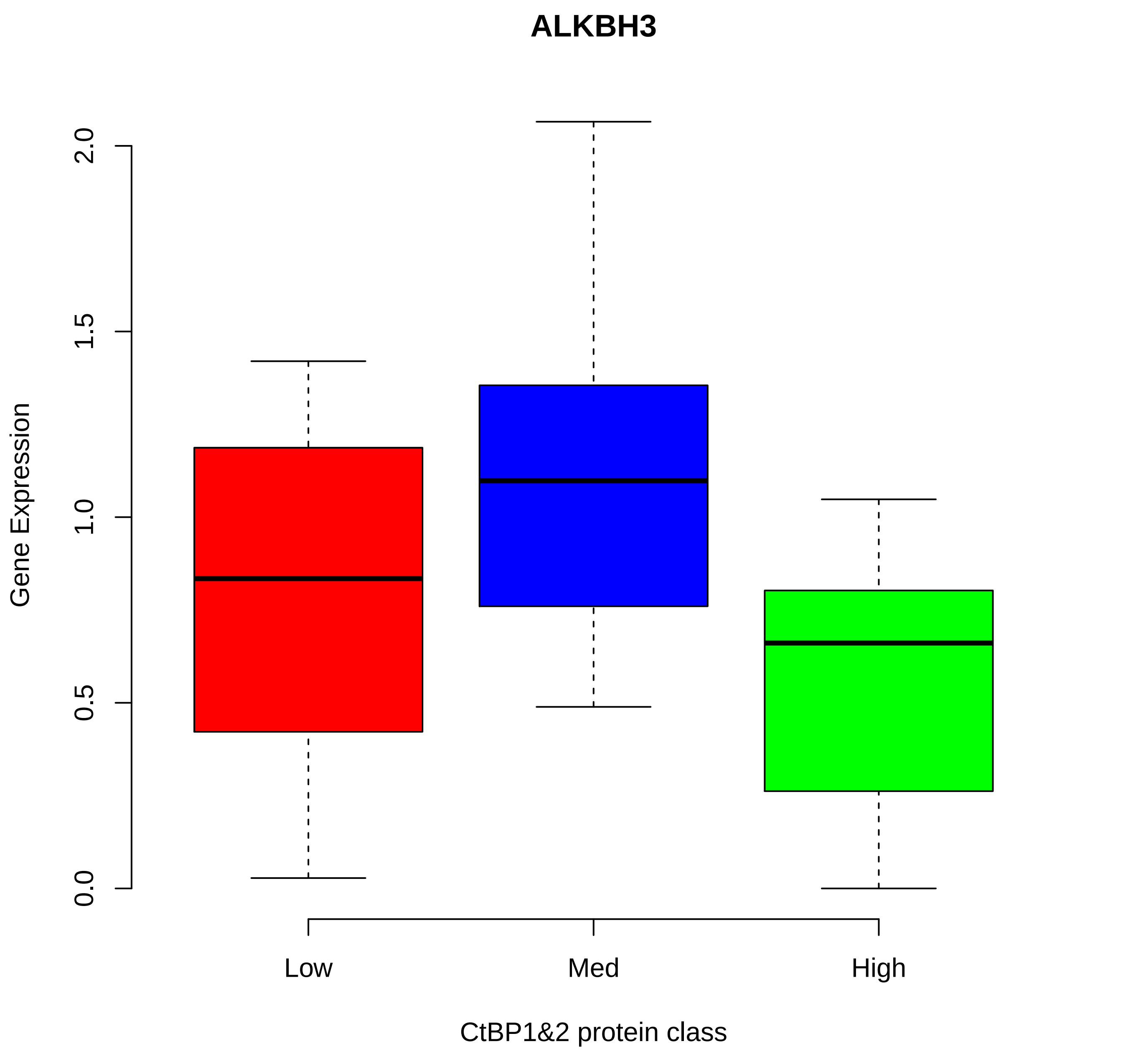
ALKBH3
Gene Expression
0.0
0.5
1.0
1.5
2.0
Low
Med
High
CtBP1&2 protein class

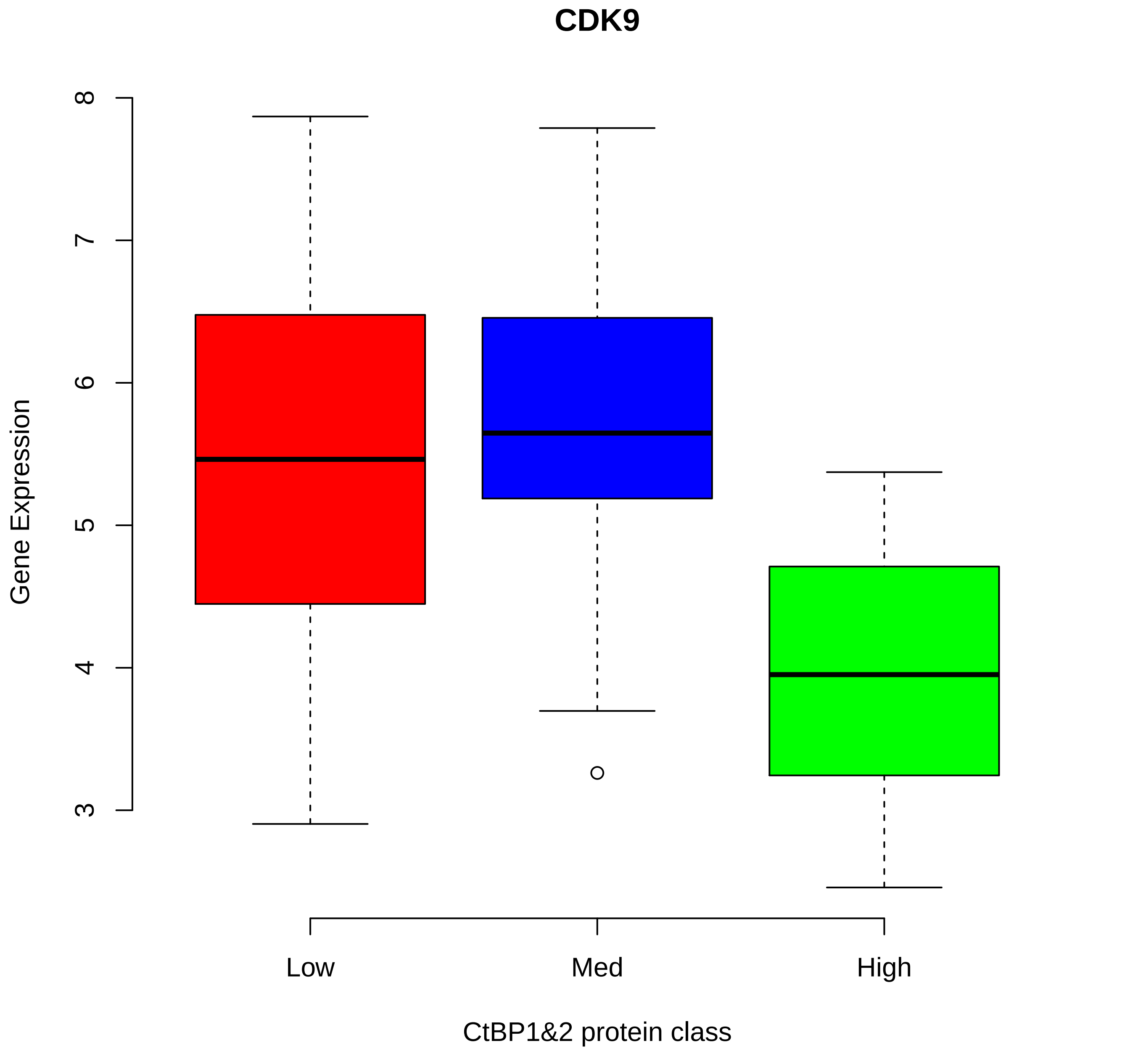
CDK9
Gene Expression
3
4
5
6
7
8
Low
Med
High
CtBP1&2 protein class

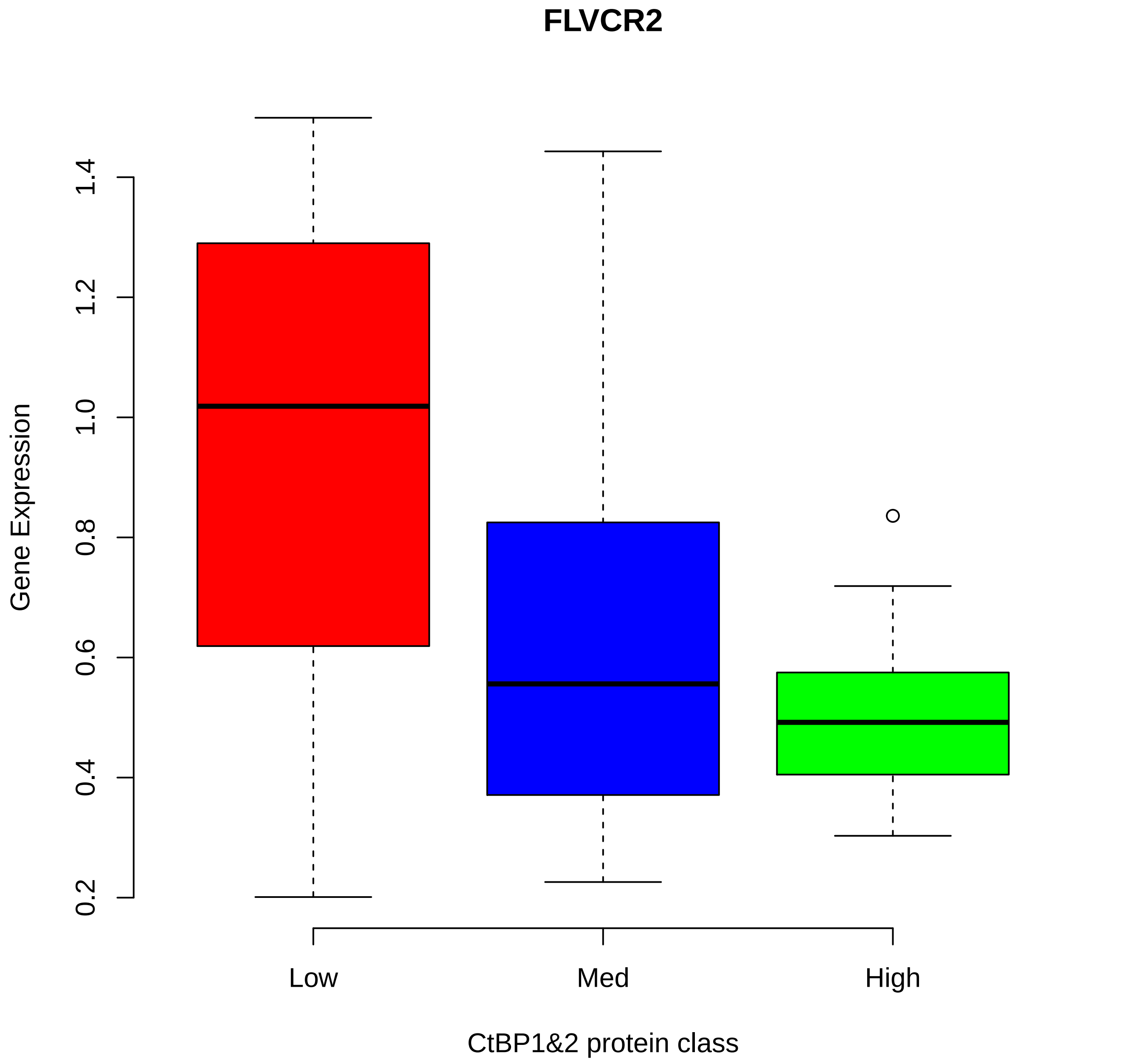
FLVCR2
Gene Expression
0.2
0.4
0.6
0.8
1.0
1.2
1.4
Low
Med
High
CtBP1&2 protein class

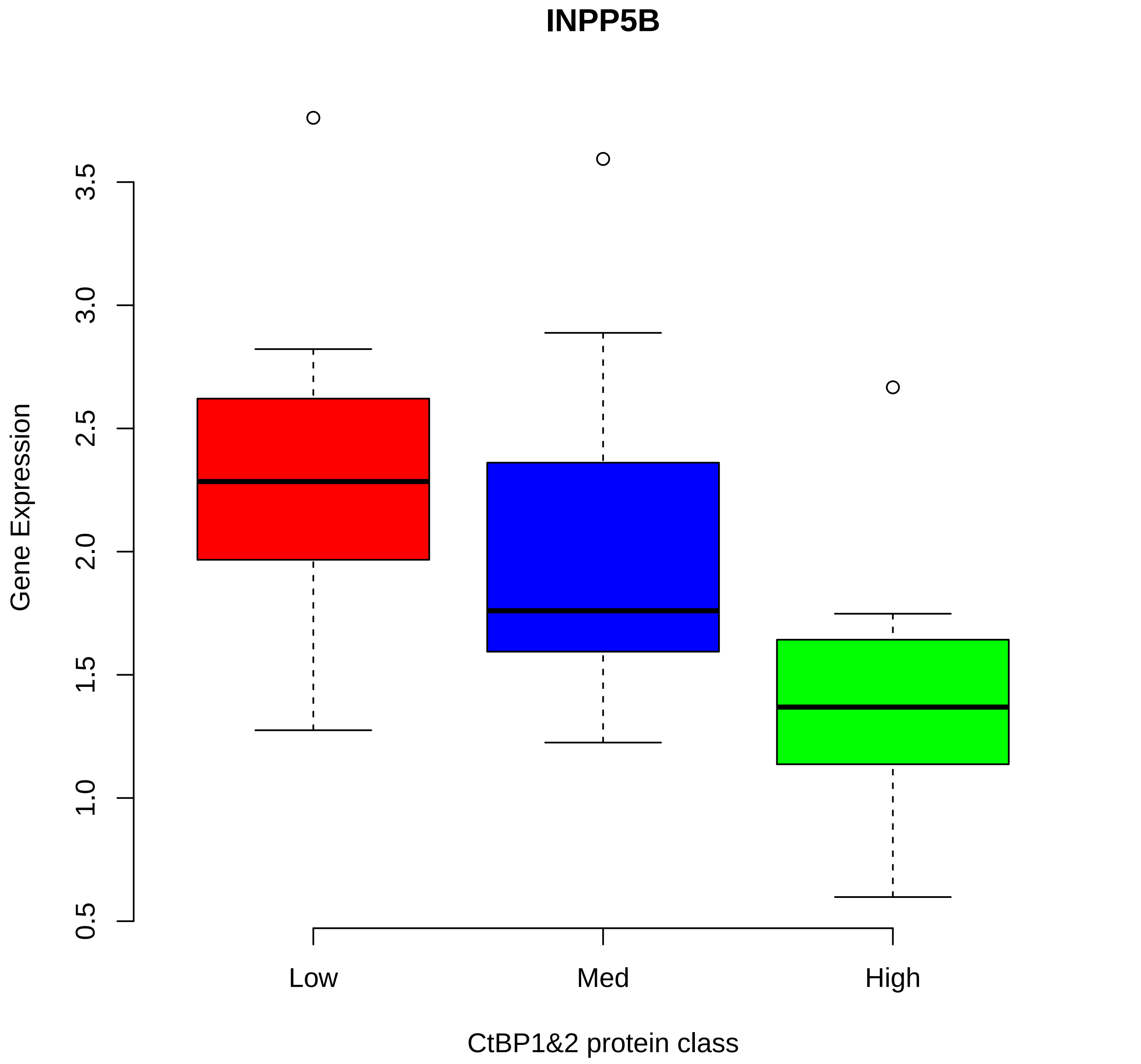

INPP5B
Gene Expression
0.5
1.0
1.5
2.0
2.5
3.0
3.5
Low
Med
High
CtBP1&2 protein class

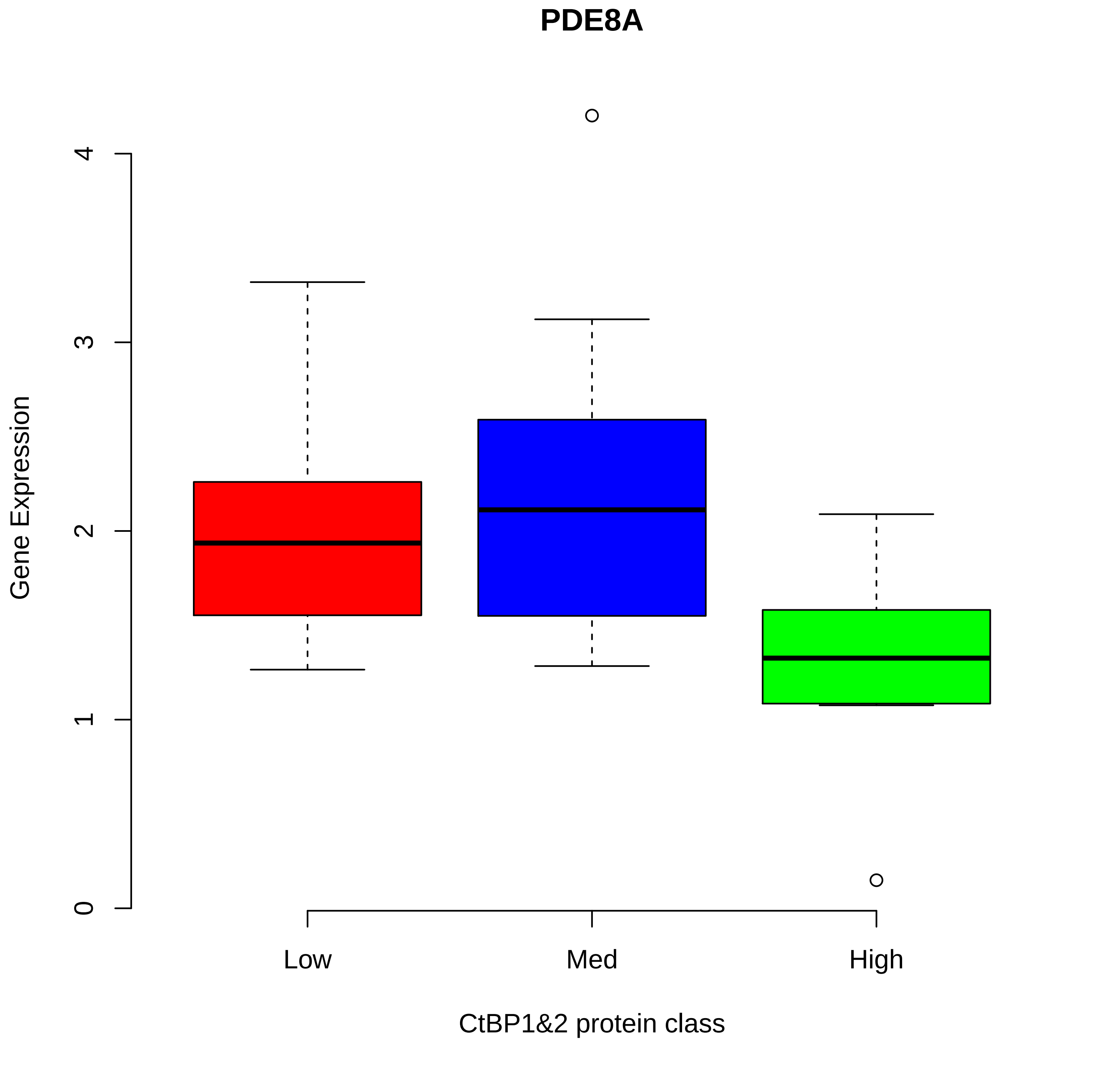

PDE8A
Gene Expression
0
1
2
3
4
Low
Med
High
CtBP1&2 protein class

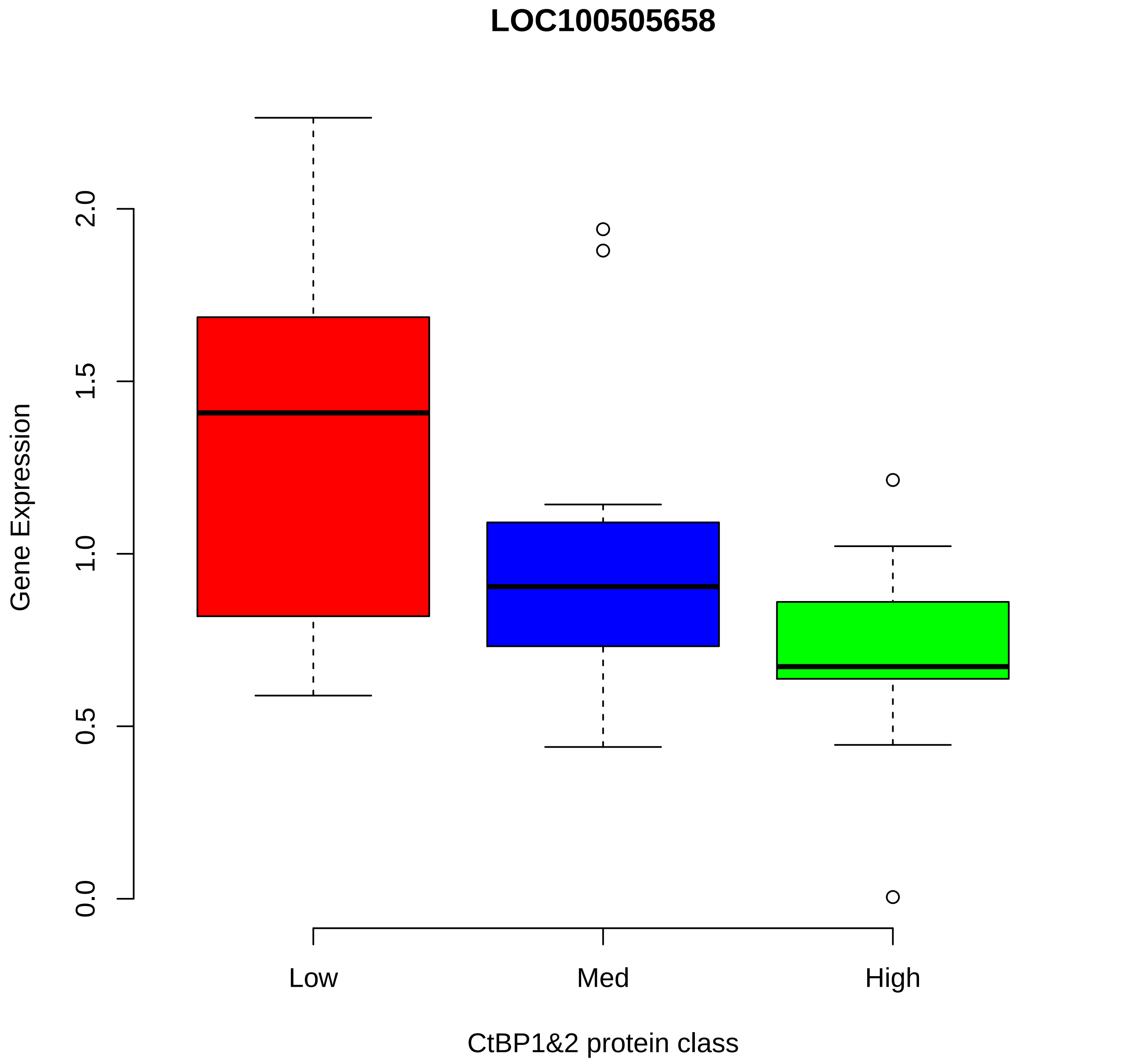

LOC100505658
Gene Expression
2.0
1.5
1.0
0.5
0.0
Low
Med
High
CtBP1&2 protein class

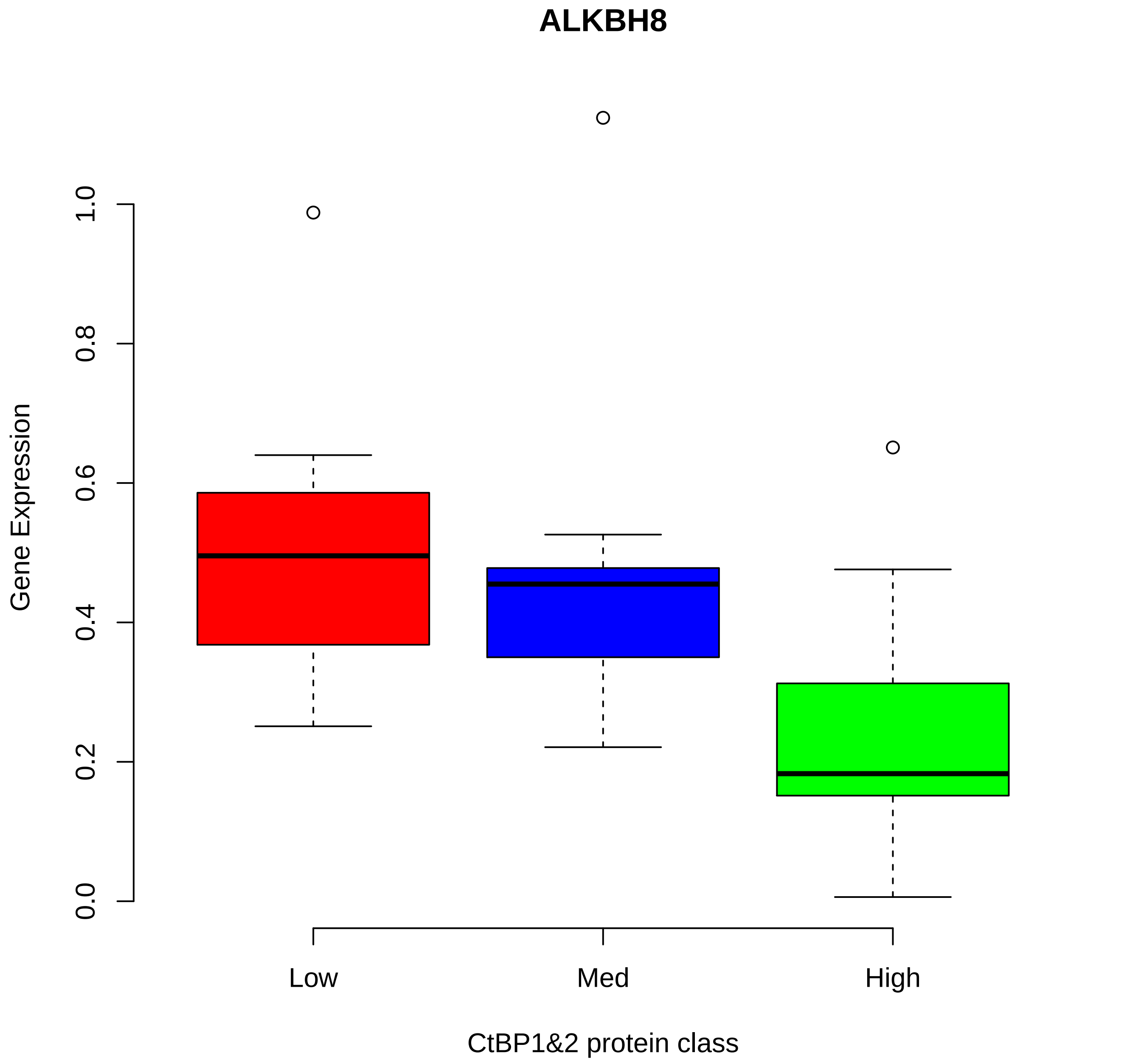

ALKBH8
Gene Expression
0.0
0.2
0.4
0.6
0.8
1.0
Low
Med
High
CtBP1&2 protein class

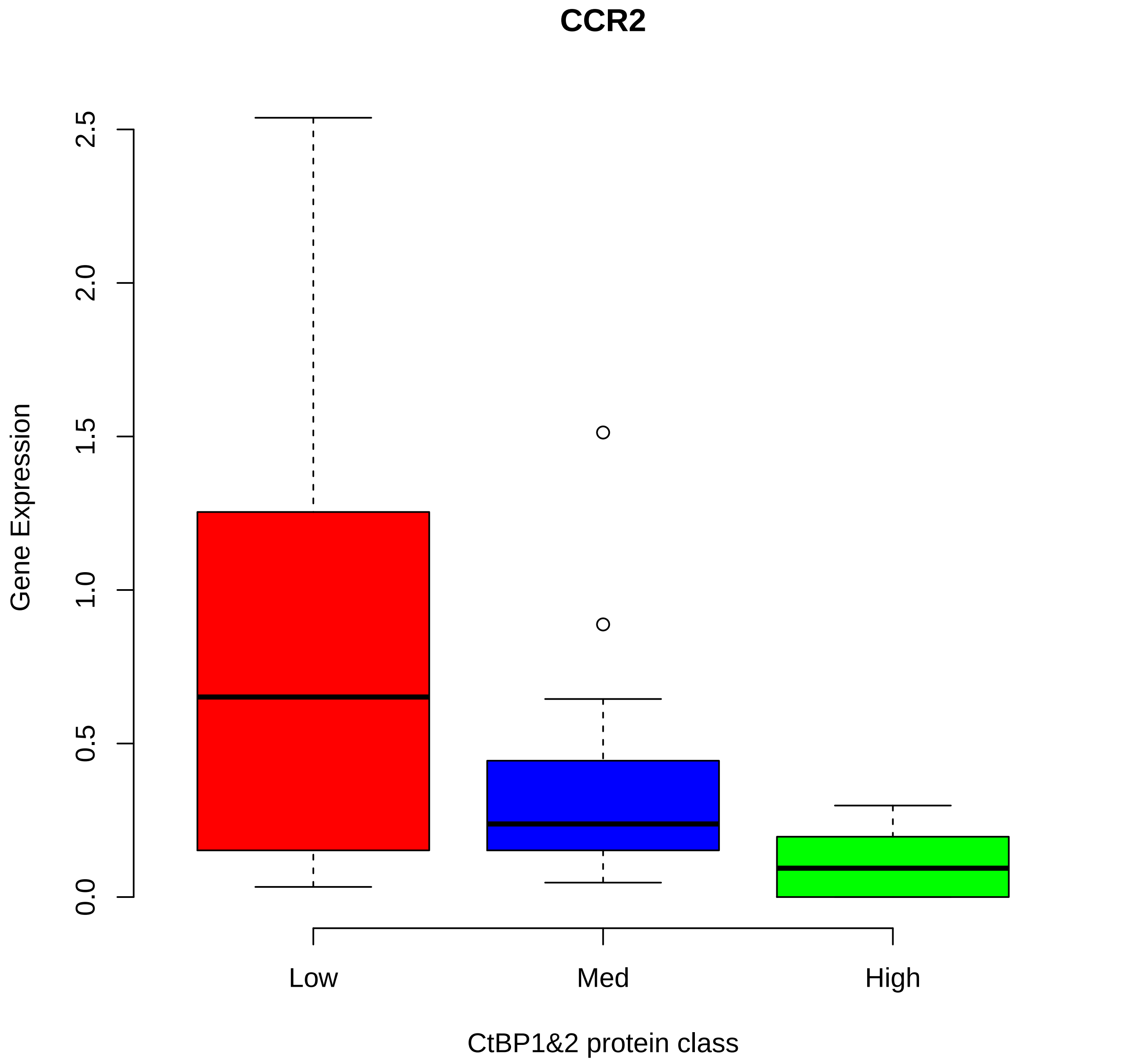
CCR2
Gene Expression
0.0
0.5
1.0
1.5
2.0
2.5
Low
Med
High
CtBP1&2 protein class

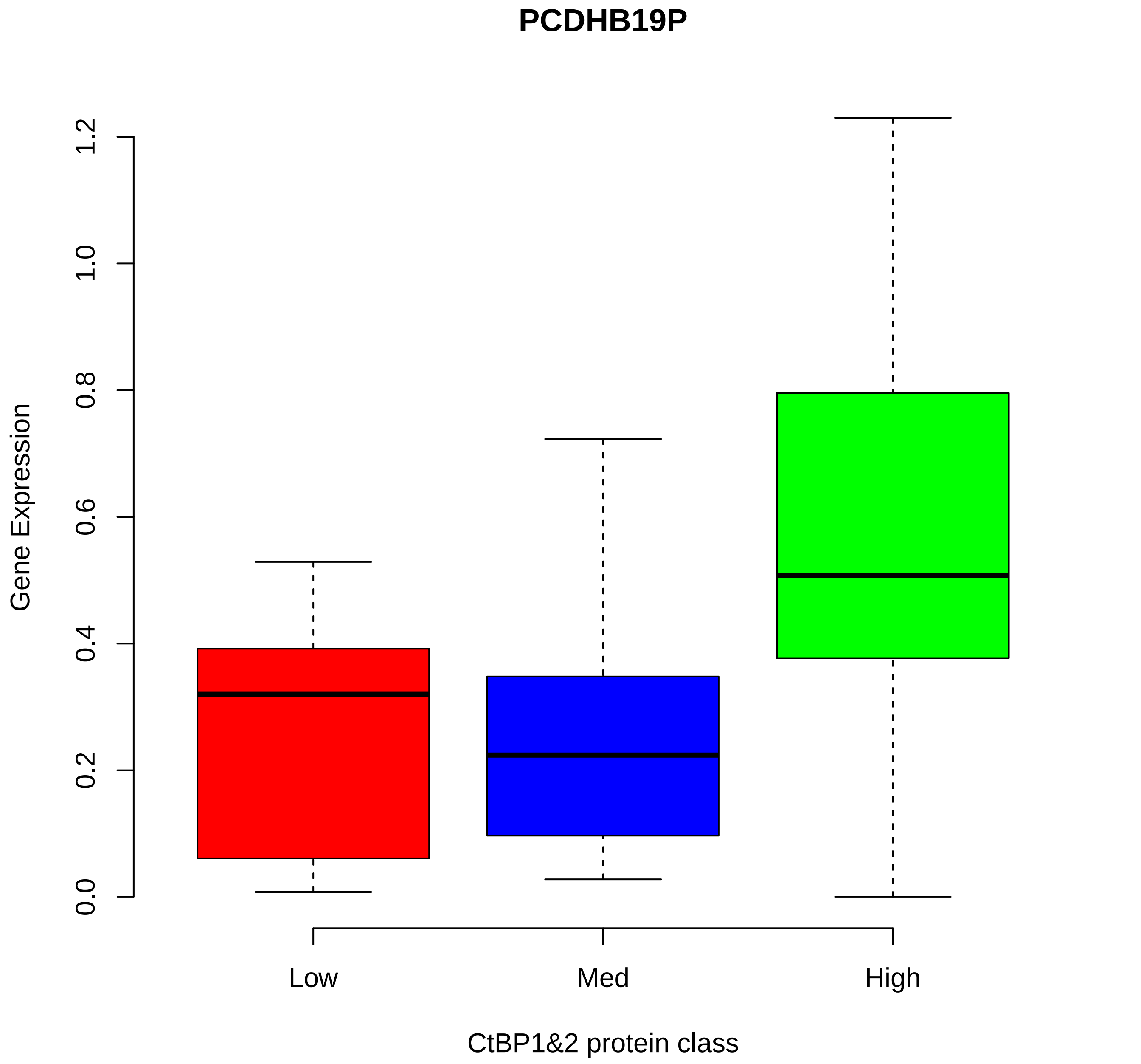

PCDHB19P
Gene Expression
0.0
0.2
0.4
0.6
0.8
1.0
1.2
Low
Med
High
CtBP1&2 protein class

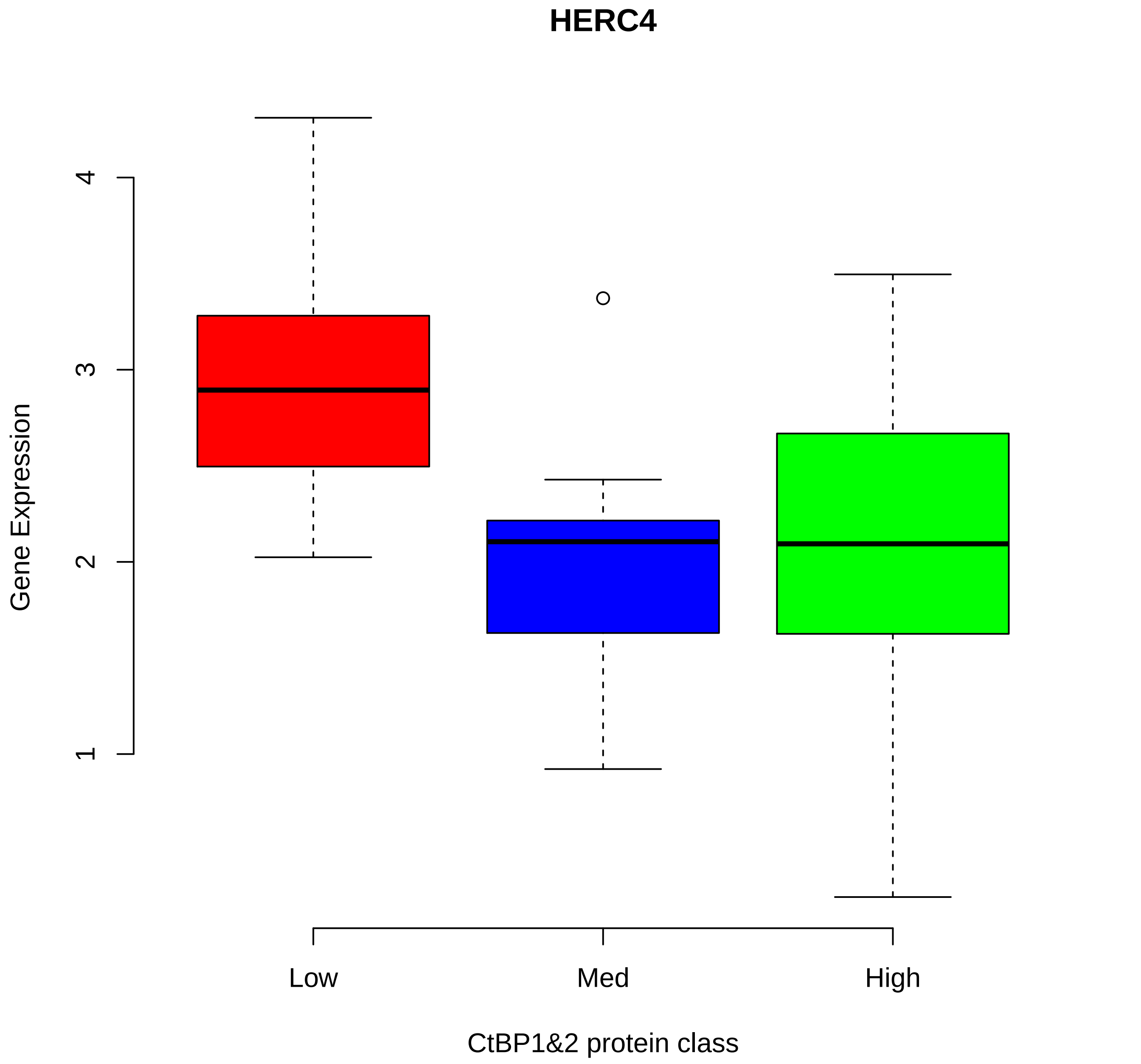
HERC4
Gene Expression
1
2
3
4
Low
Med
High
CtBP1&2 protein class

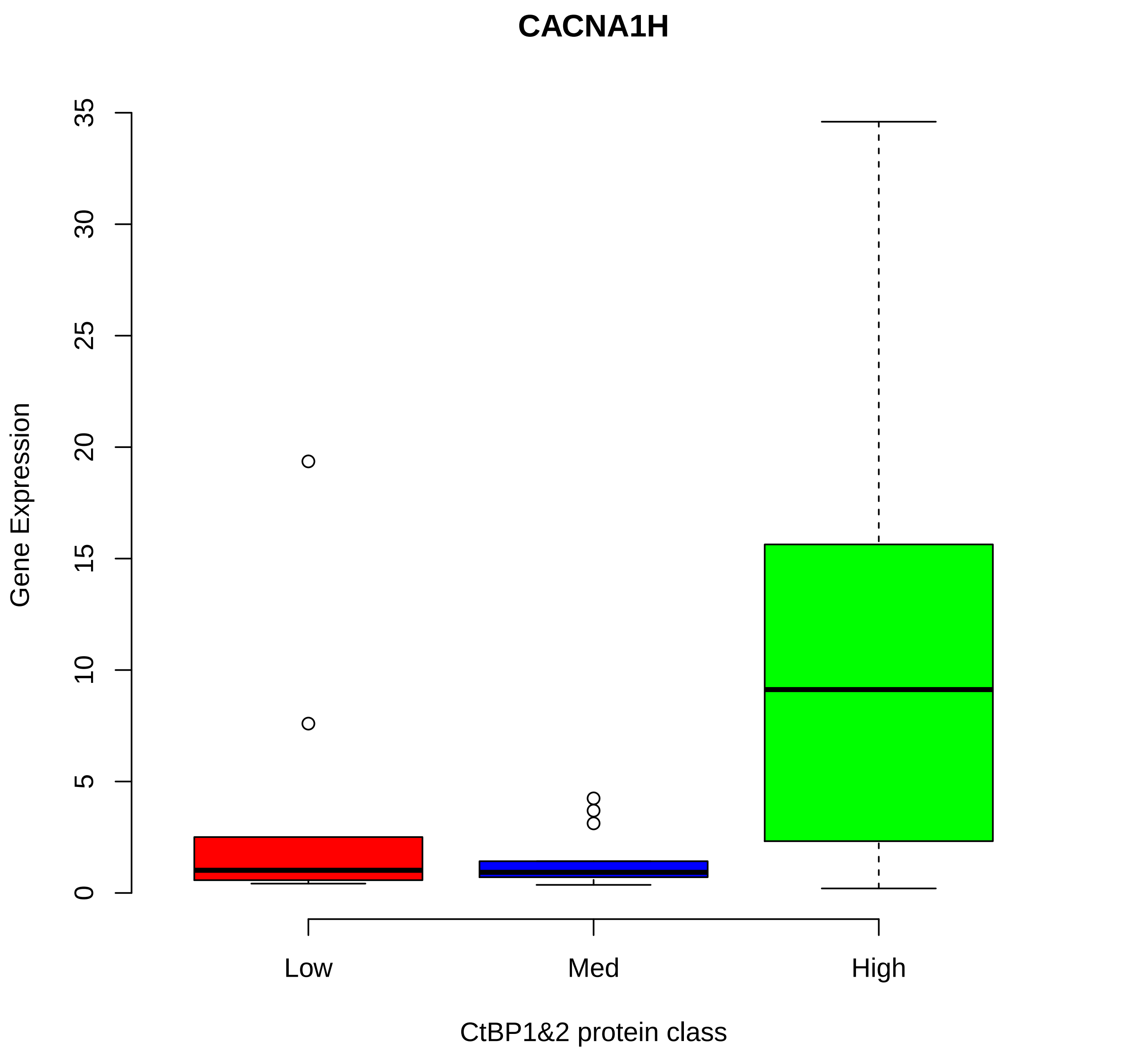
CACNA1H
Gene Expression
0
5
10
15
20
25
30
35
Low
Med
High
CtBP1&2 protein class

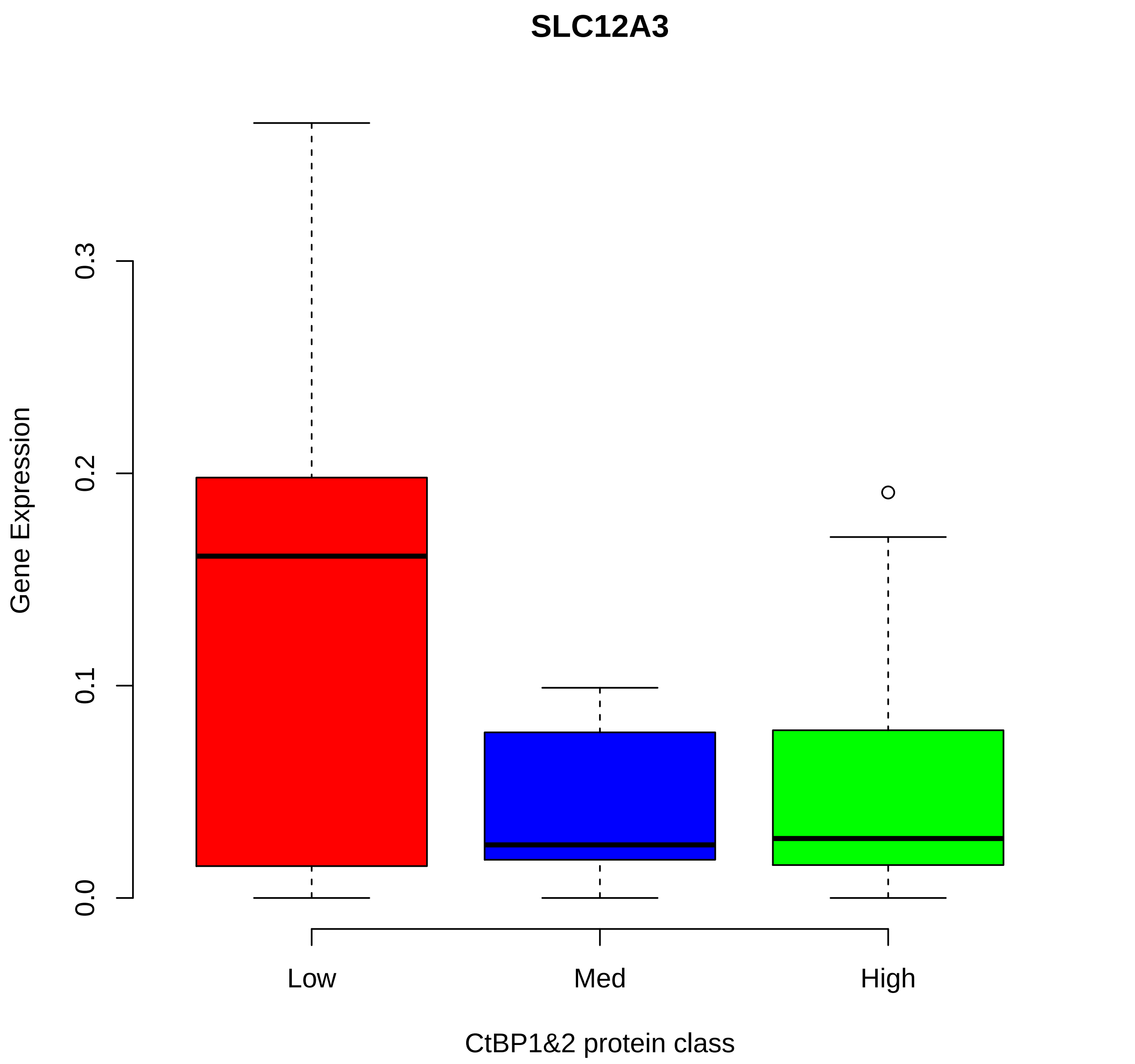
SLC12A3
Gene Expression
0.0
0.1
0.2
0.3
Low
Med
High
CtBP1&2 protein class

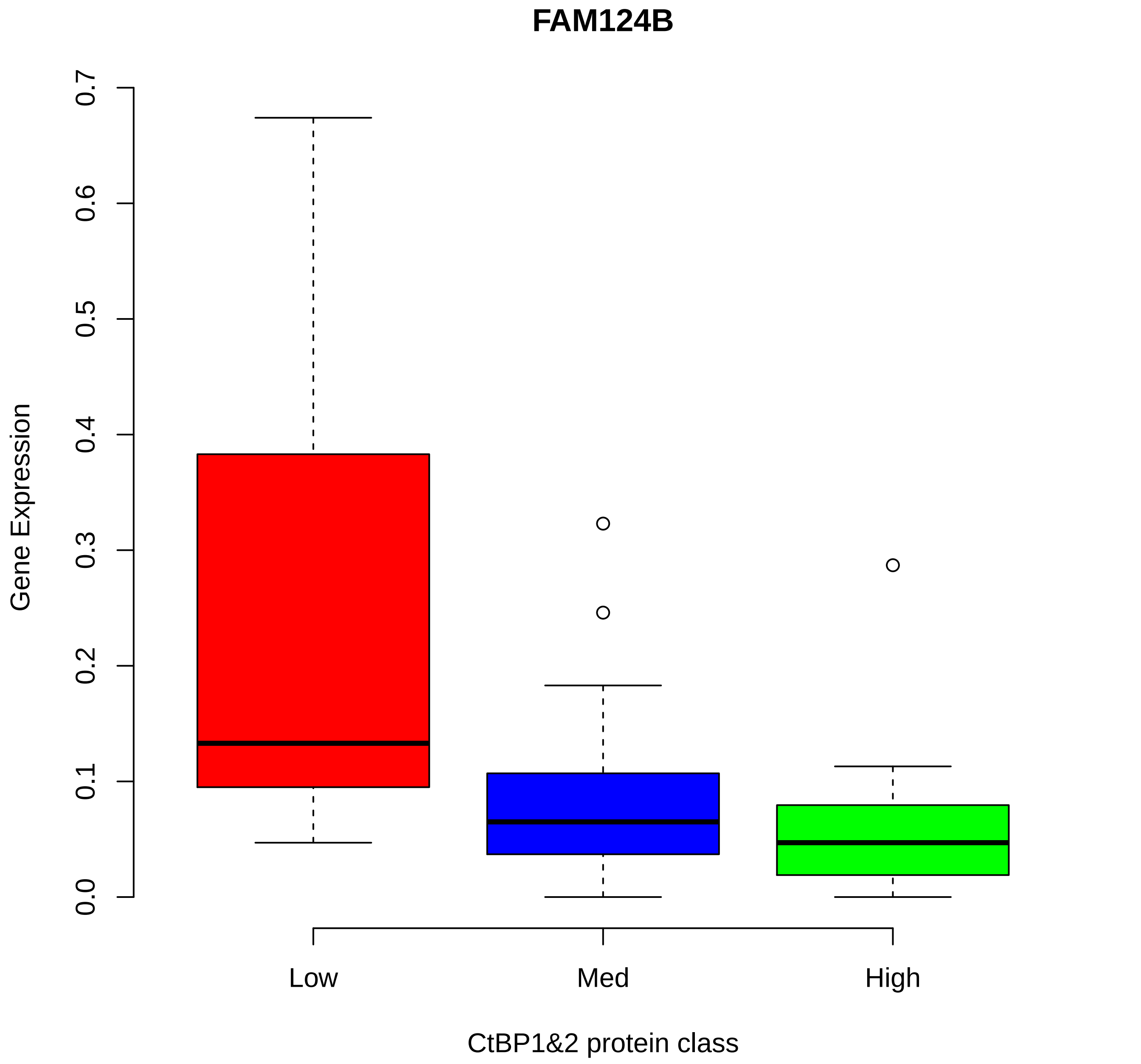
FAM124B
Gene Expression
0.0
0.1
0.2
0.3
0.4
0.5
0.6
0.7
Low
Med
High
CtBP1&2 protein class

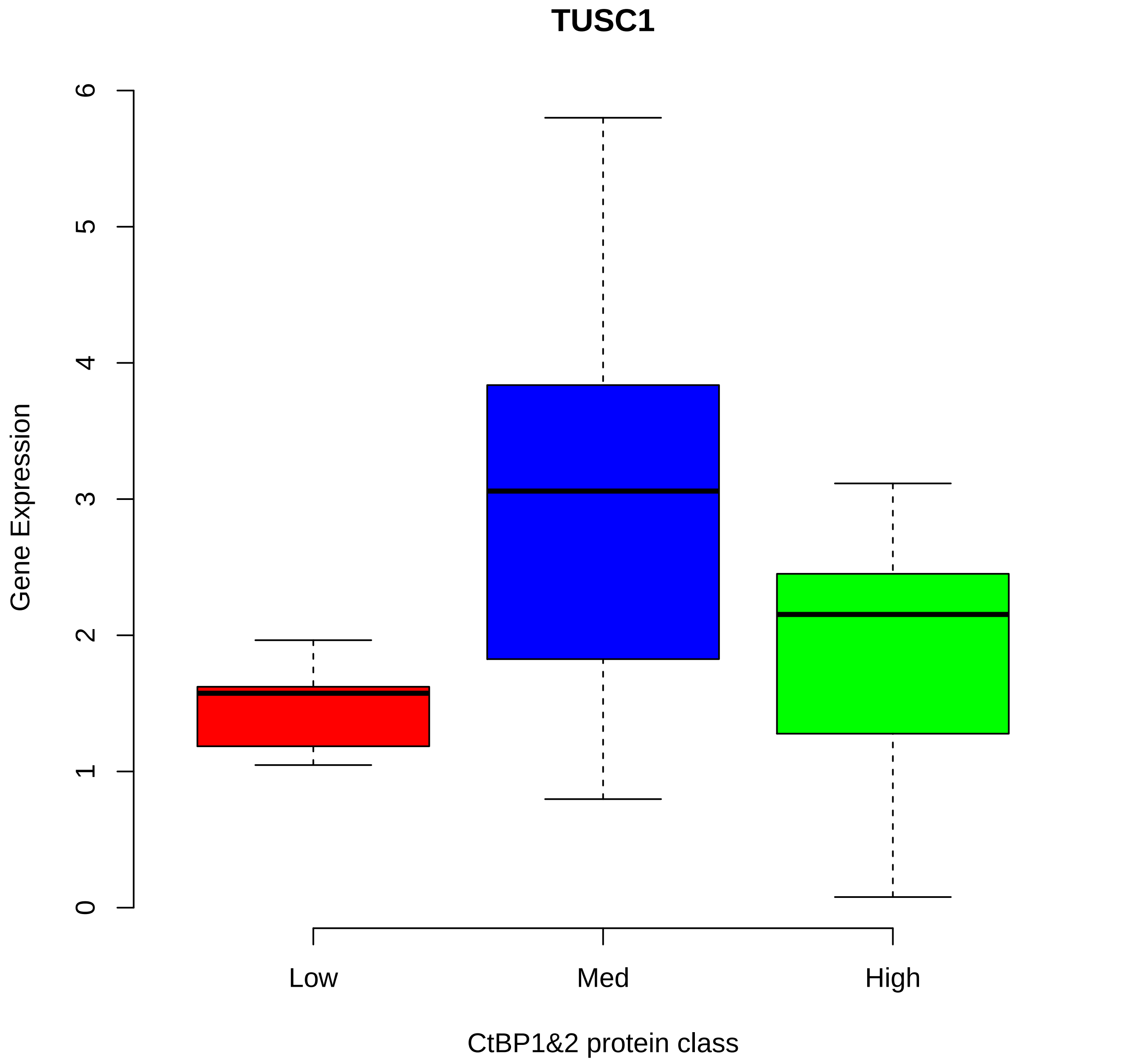

TUSC1
Gene Expression
0
1
2
3
4
5
6
Low
Med
High
CtBP1&2 protein class

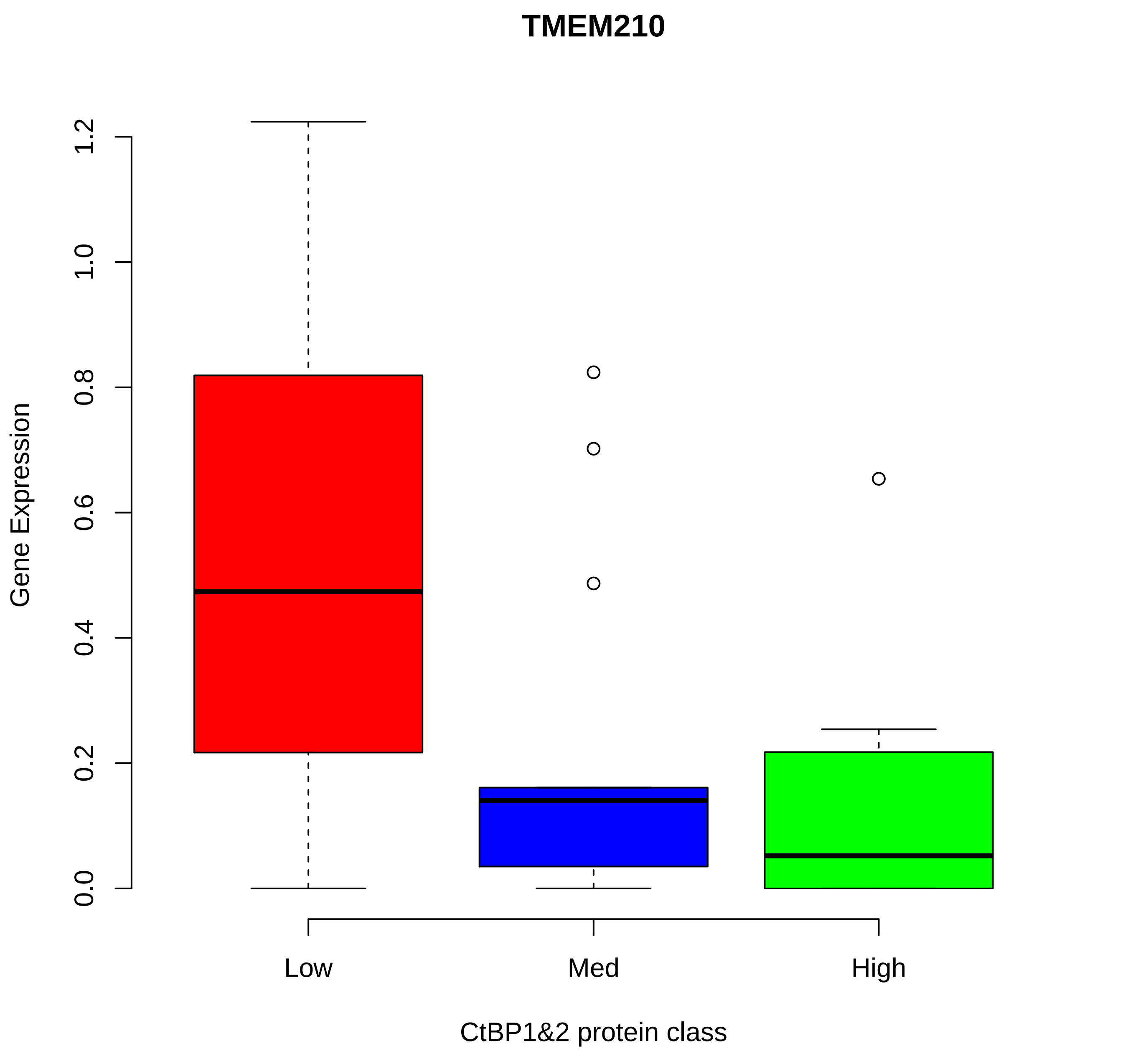
TMEM210
Gene Expression
0.0
0.2
0.4
0.6
0.8
1.0
1.2
Low
Med
High
CtBP1&2 protein class

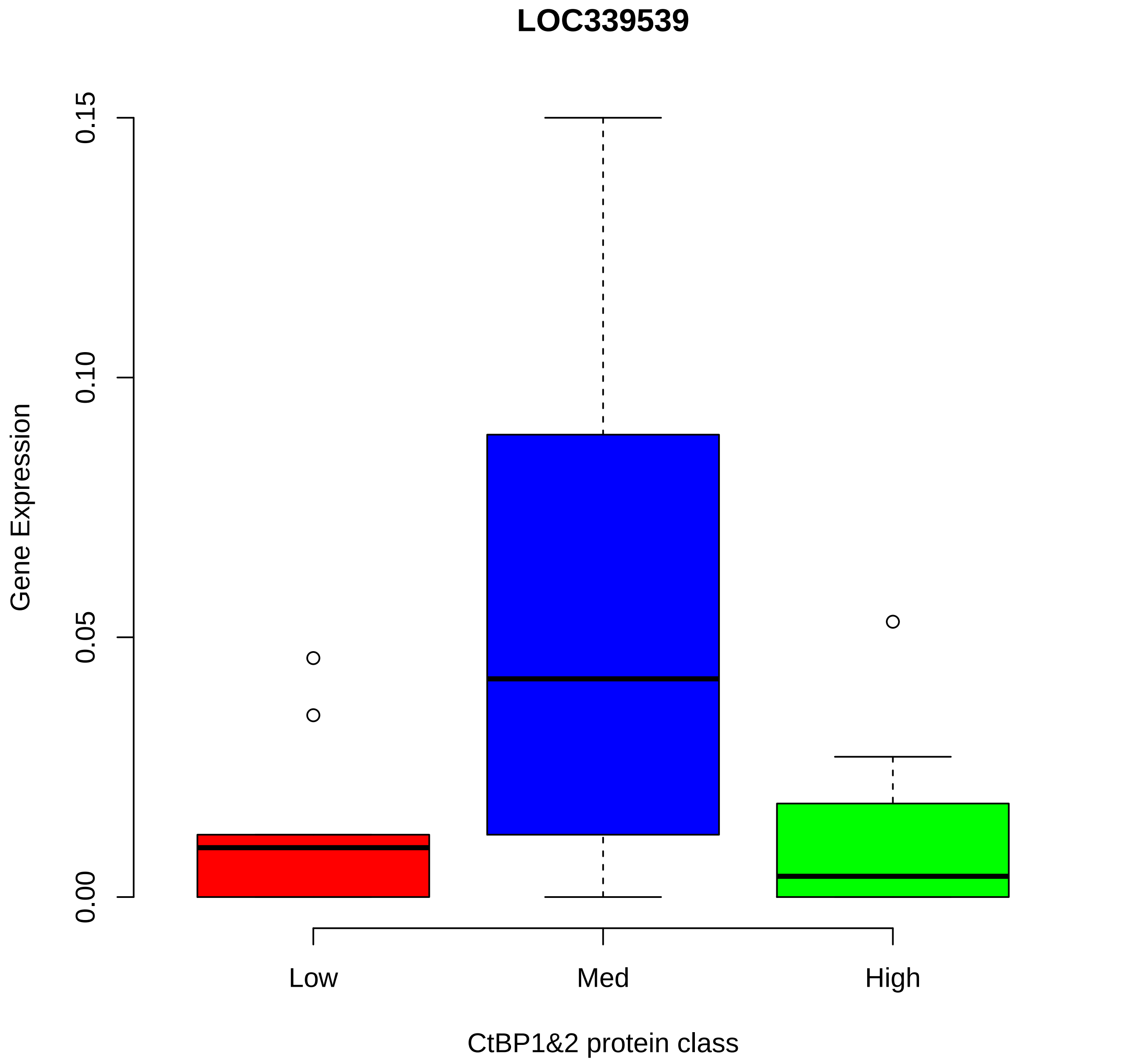

LOC339539
Gene Expression
0.00
0.05
0.10
0.15
Low
Med
High
CtBP1&2 protein class

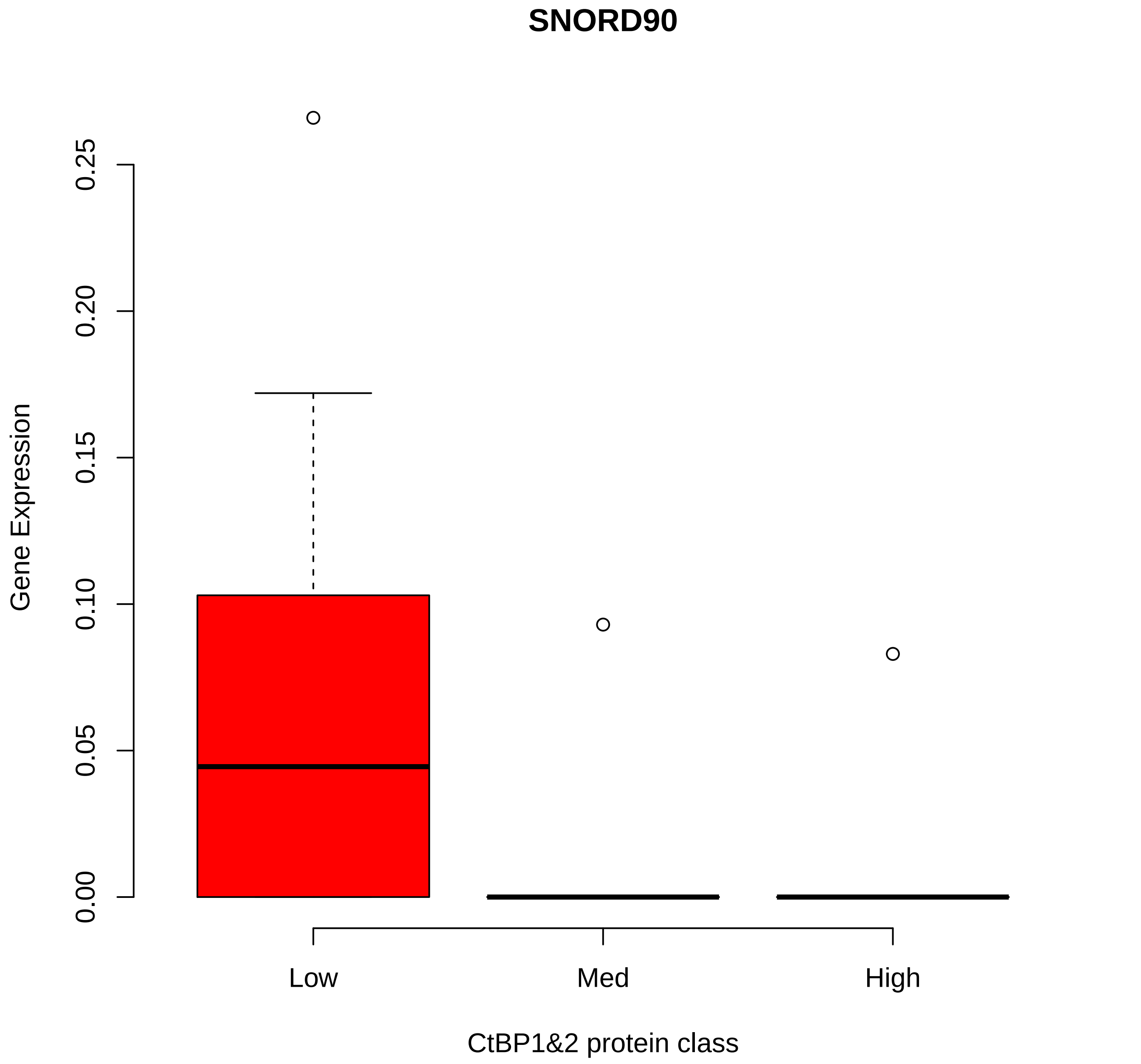

SNORD90
Gene Expression
0.00
0.05
0.10
0.15
0.20
0.25
Low
Med
High
CtBP1&2 protein class

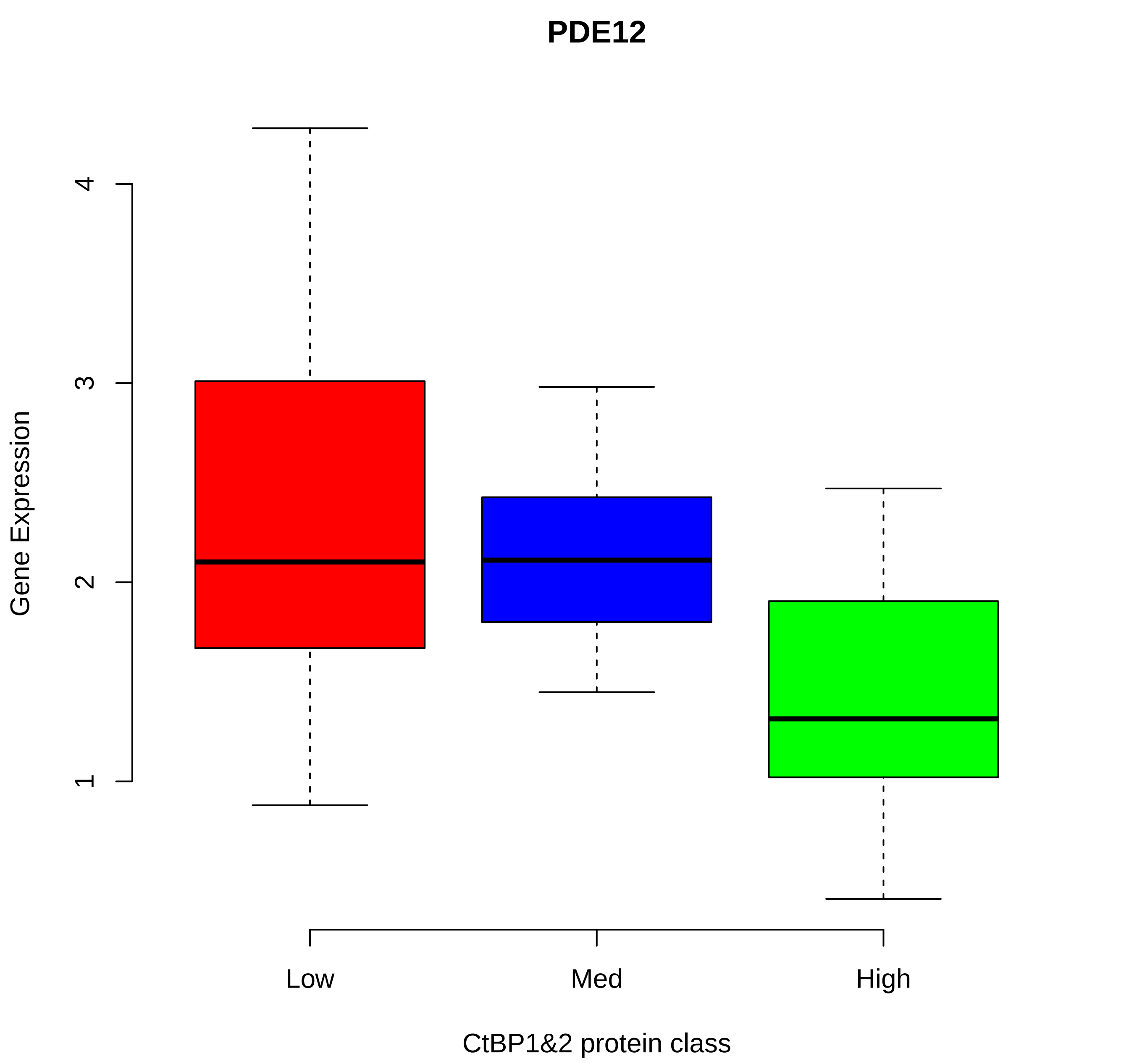

PDE12
Gene Expression
1
2
3
4
Low
Med
High
CtBP1&2 protein class

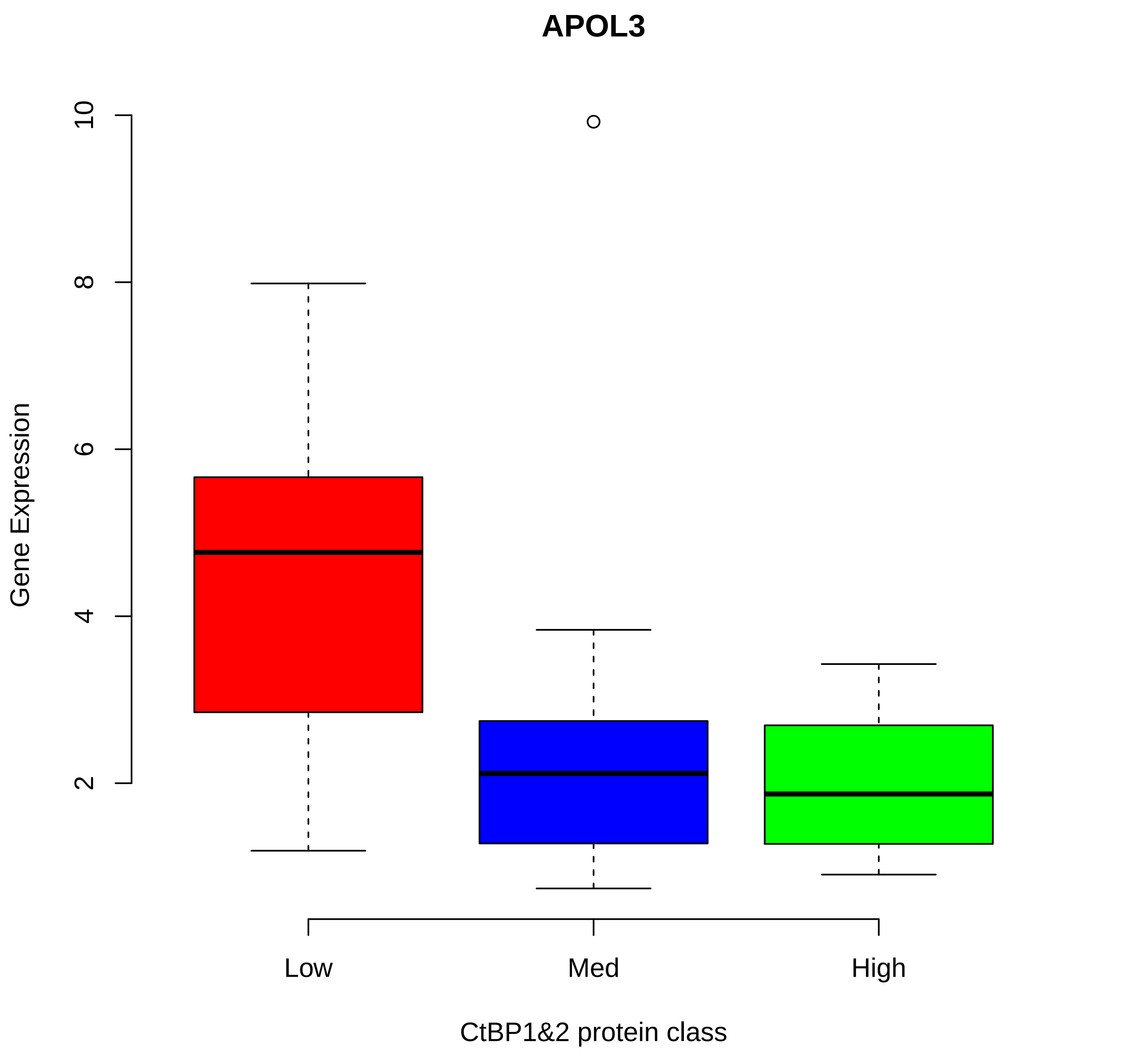
APOL3
Gene Expression
10
8
6
4
2
Low
Med
High
CtBP1&2 protein class

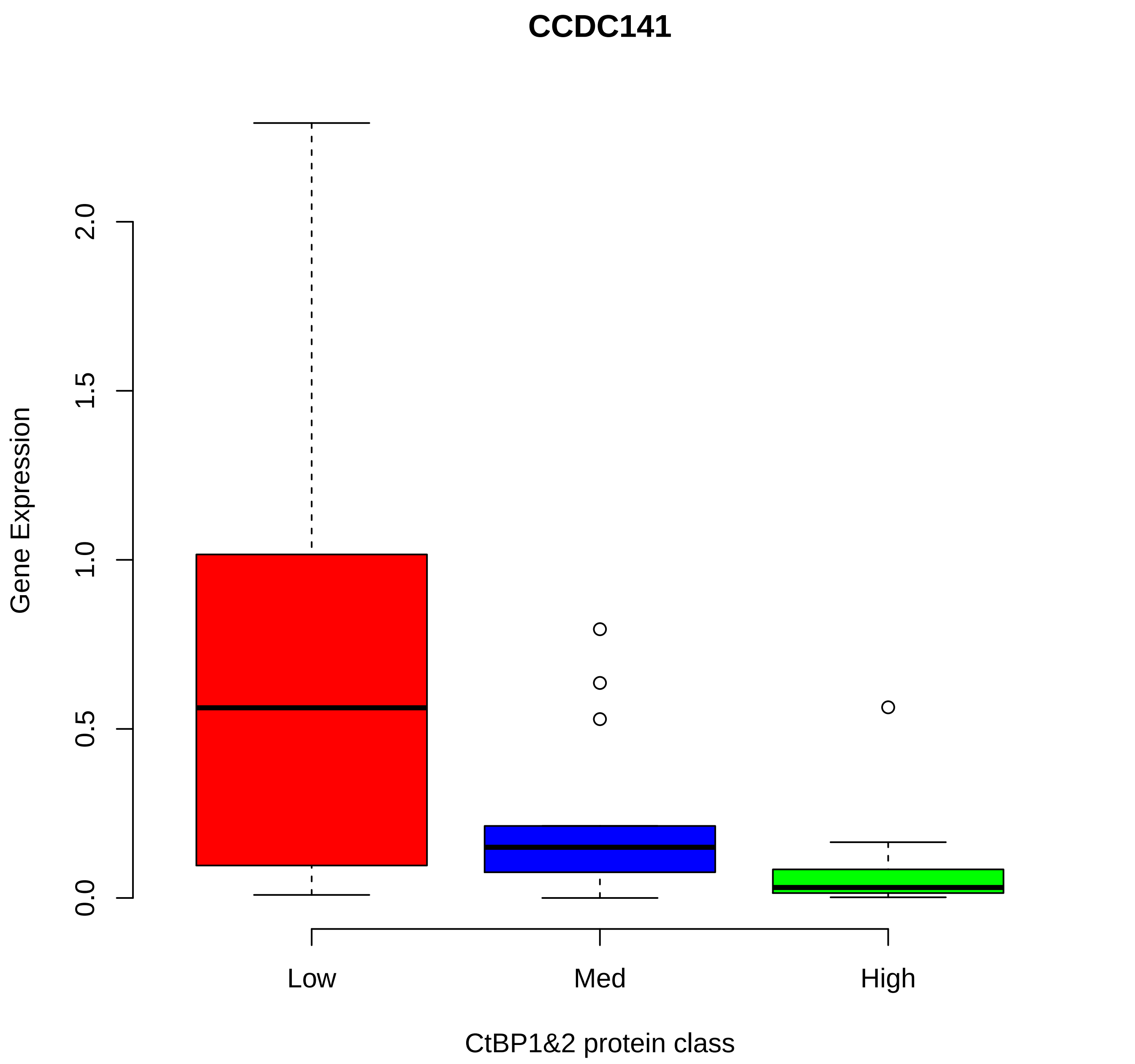
CCDC141
Gene Expression
0.0
0.5
1.0
1.5
2.0
Low
Med
High
CtBP1&2 protein class

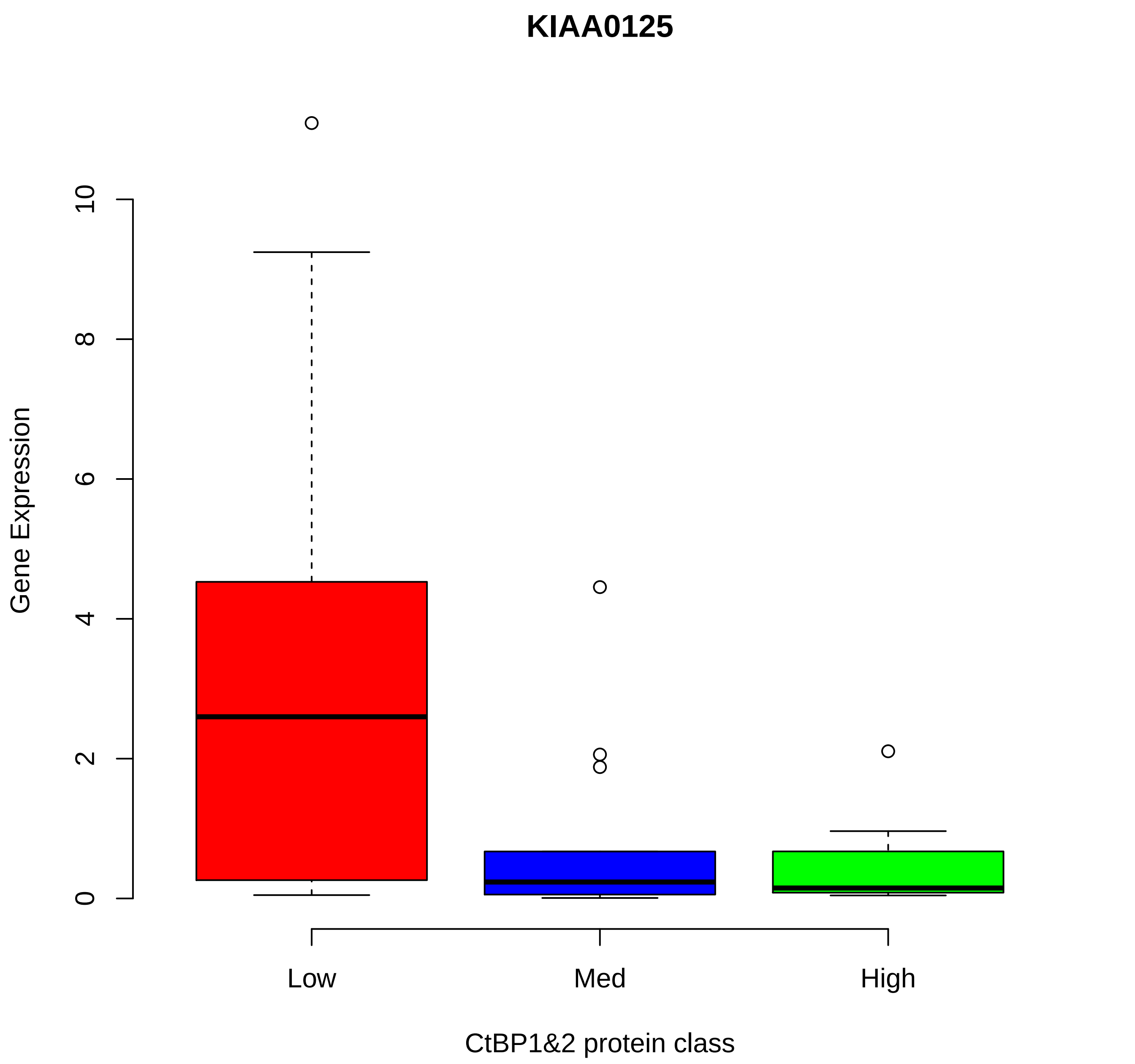

KIAA0125
Gene Expression
0
2
4
6
8
10
Low
Med
High
CtBP1&2 protein class

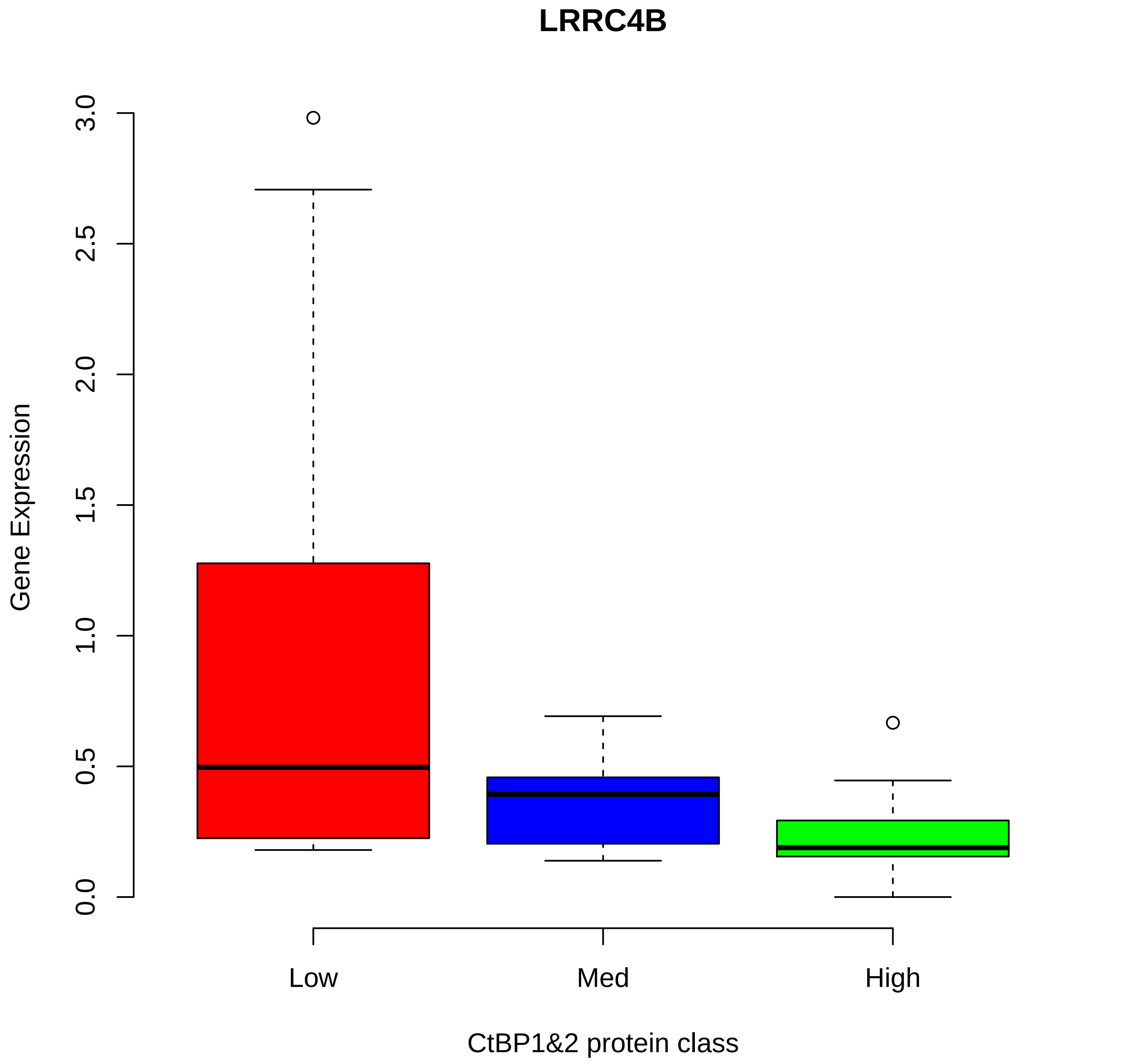
LRRC4B
Gene Expression
0.0
0.5
1.0
1.5
2.0
2.5
3.0
Low
Med
High
CtBP1&2 protein class

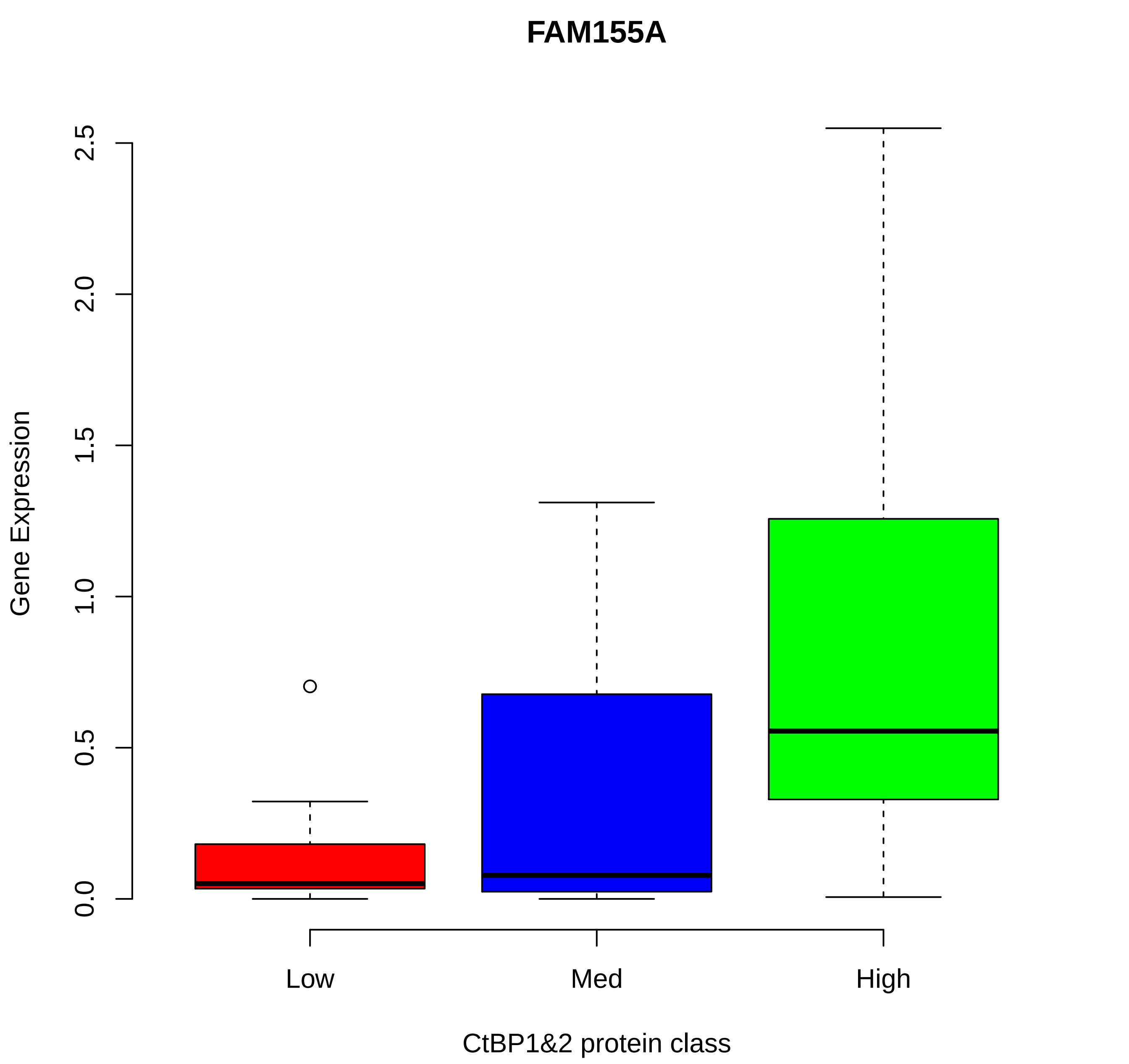

FAM155A
Gene Expression
0.0
0.5
1.0
1.5
2.0
2.5
Low
Med
High
CtBP1&2 protein class

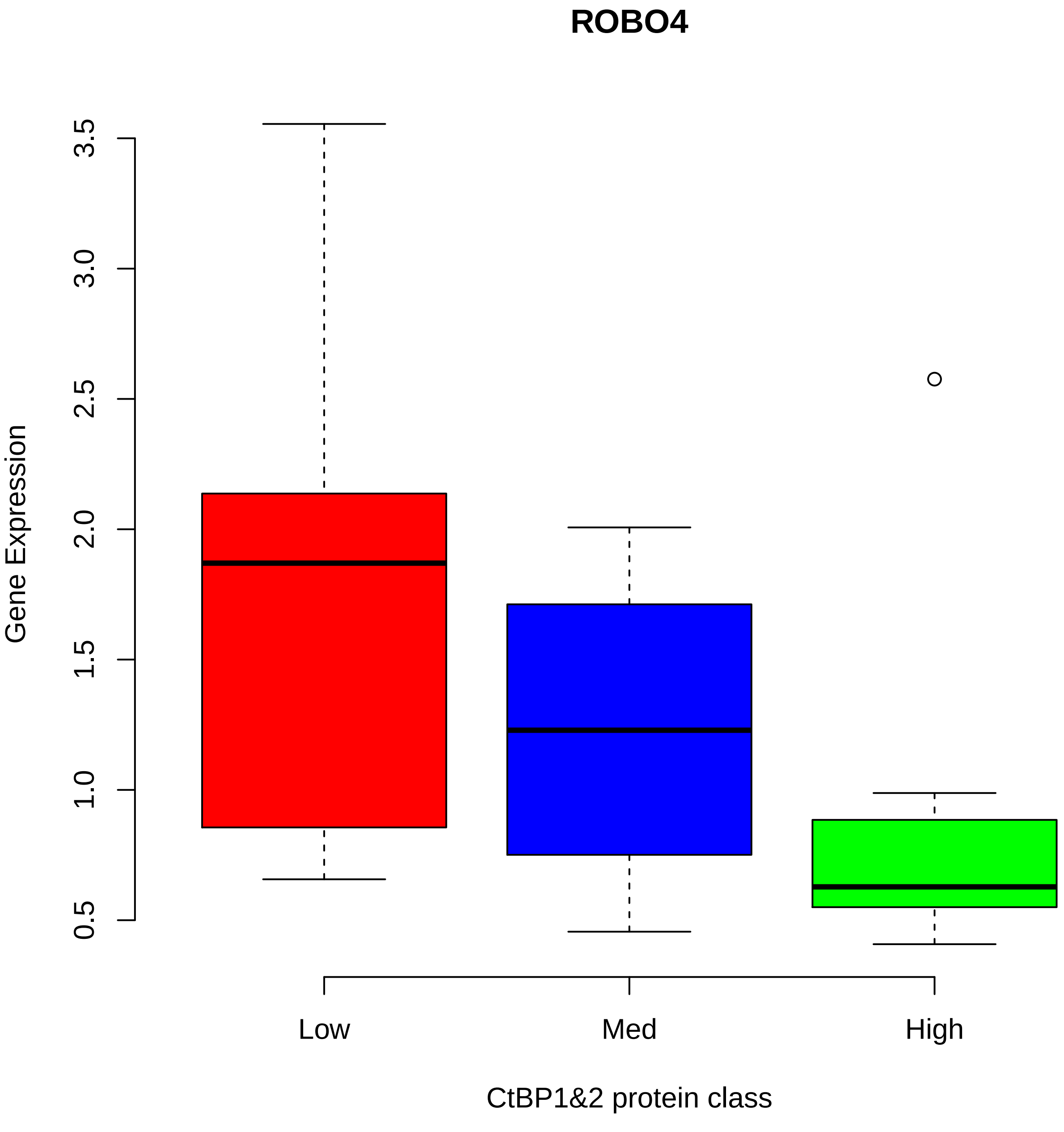

ROBO4
Gene Expression
0.5
1.0
1.5
2.0
2.5
3.0
3.5
Low
Med
High
CtBP1&2 protein class

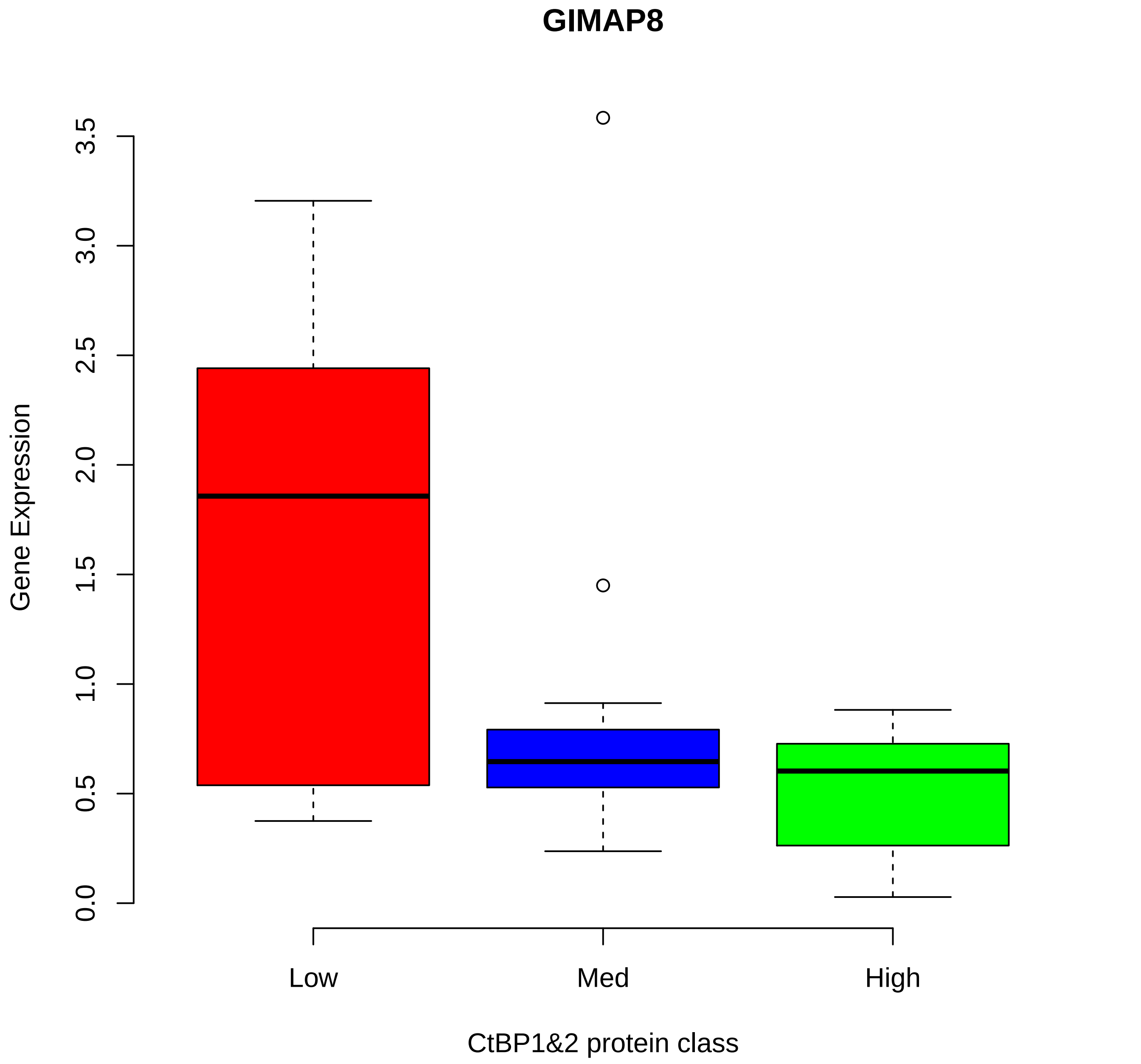

GIMAP8
Gene Expression
0.0
0.5
1.0
1.5
2.0
2.5
3.0
3.5
Low
Med
High
CtBP1&2 protein class

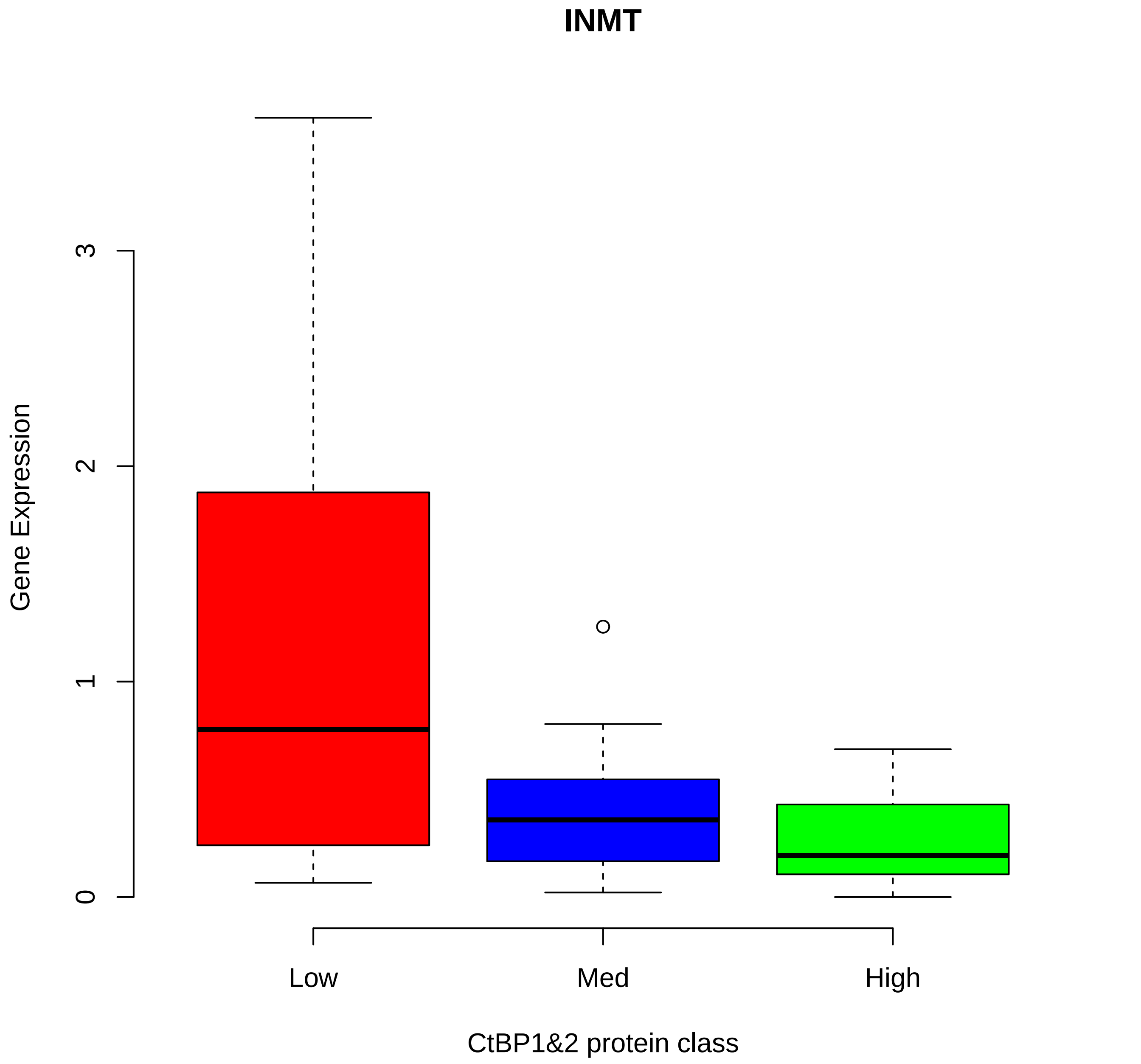

INMT
Gene Expression
0
1
2
3
Low
Med
High
CtBP1&2 protein class

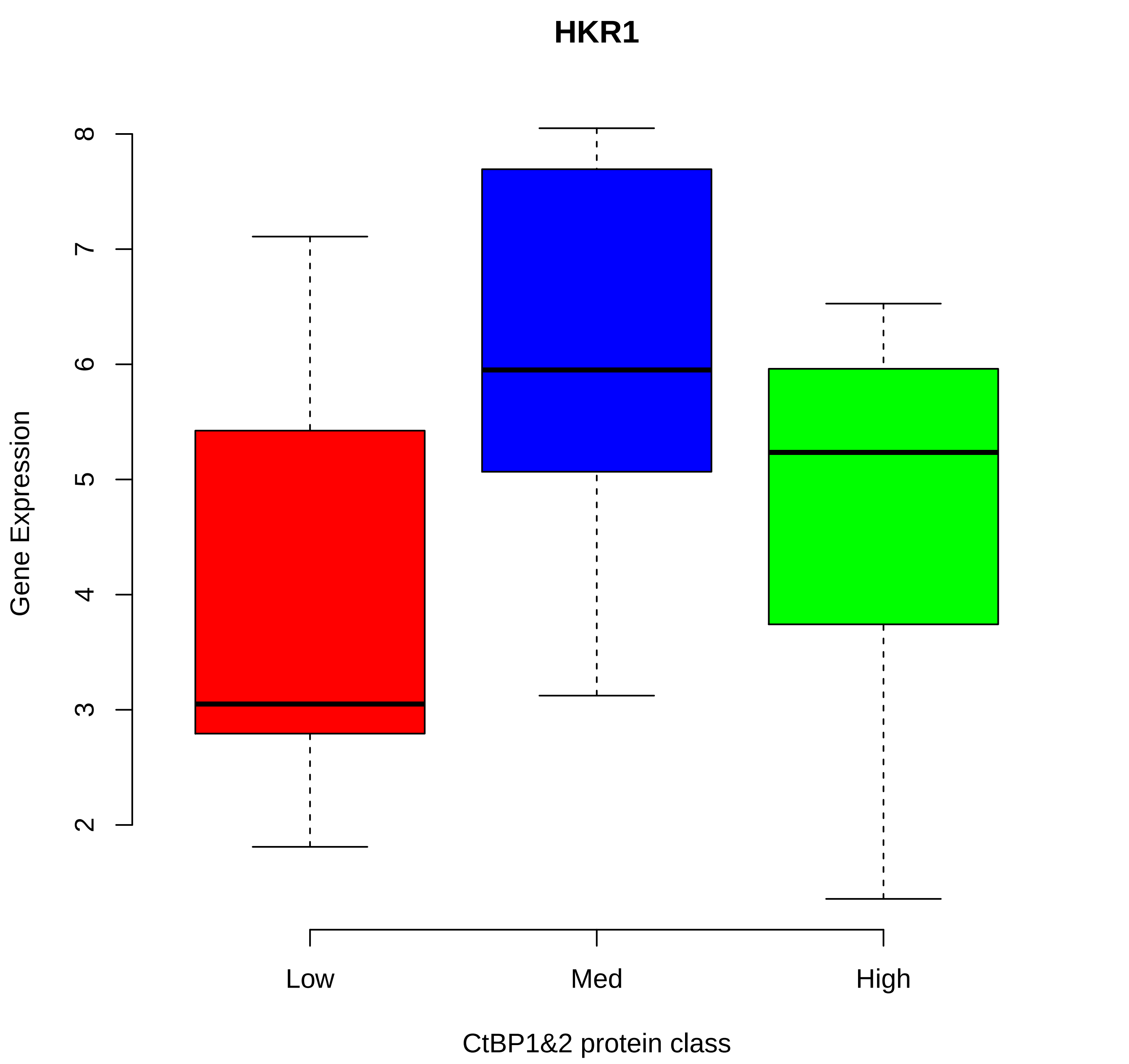

HKR1
Gene Expression
2
3
4
5
6
7
8
Low
Med
High
CtBP1&2 protein class

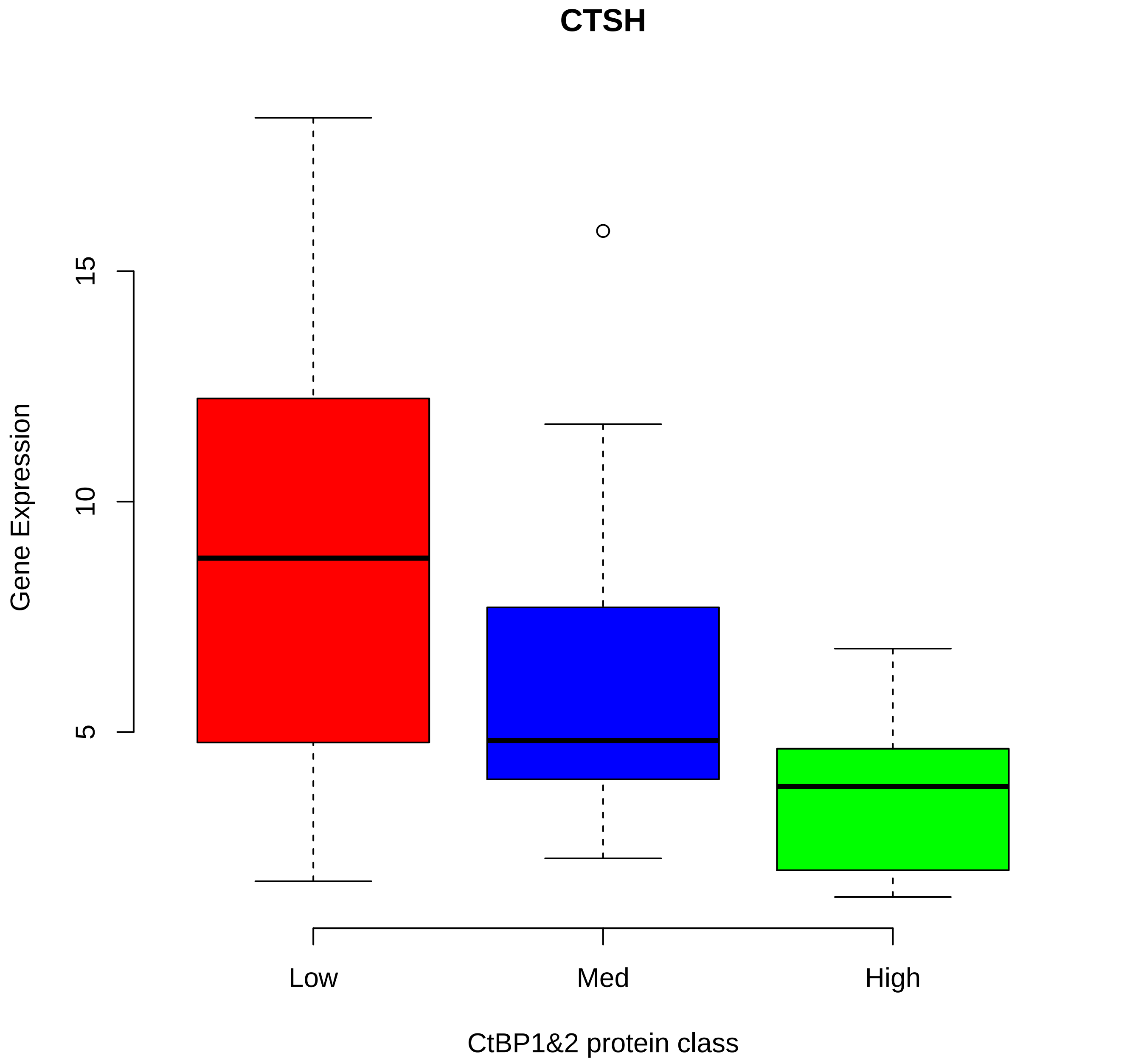

CTSH
Gene Expression
15
10
5
Low
Med
High
CtBP1&2 protein class

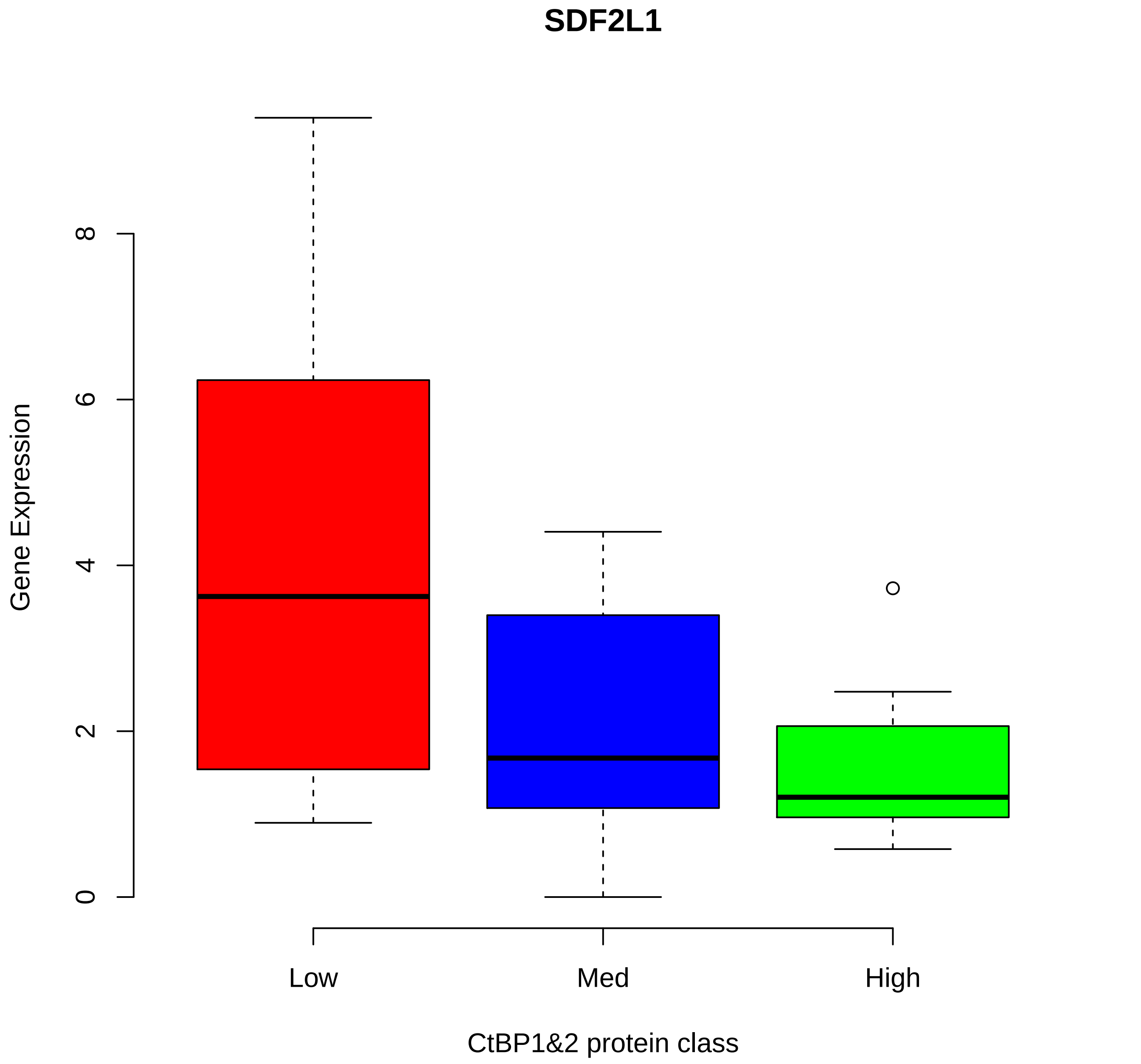

SDF2L1
Gene Expression
0
2
4
6
8
Low
Med
High
CtBP1&2 protein class

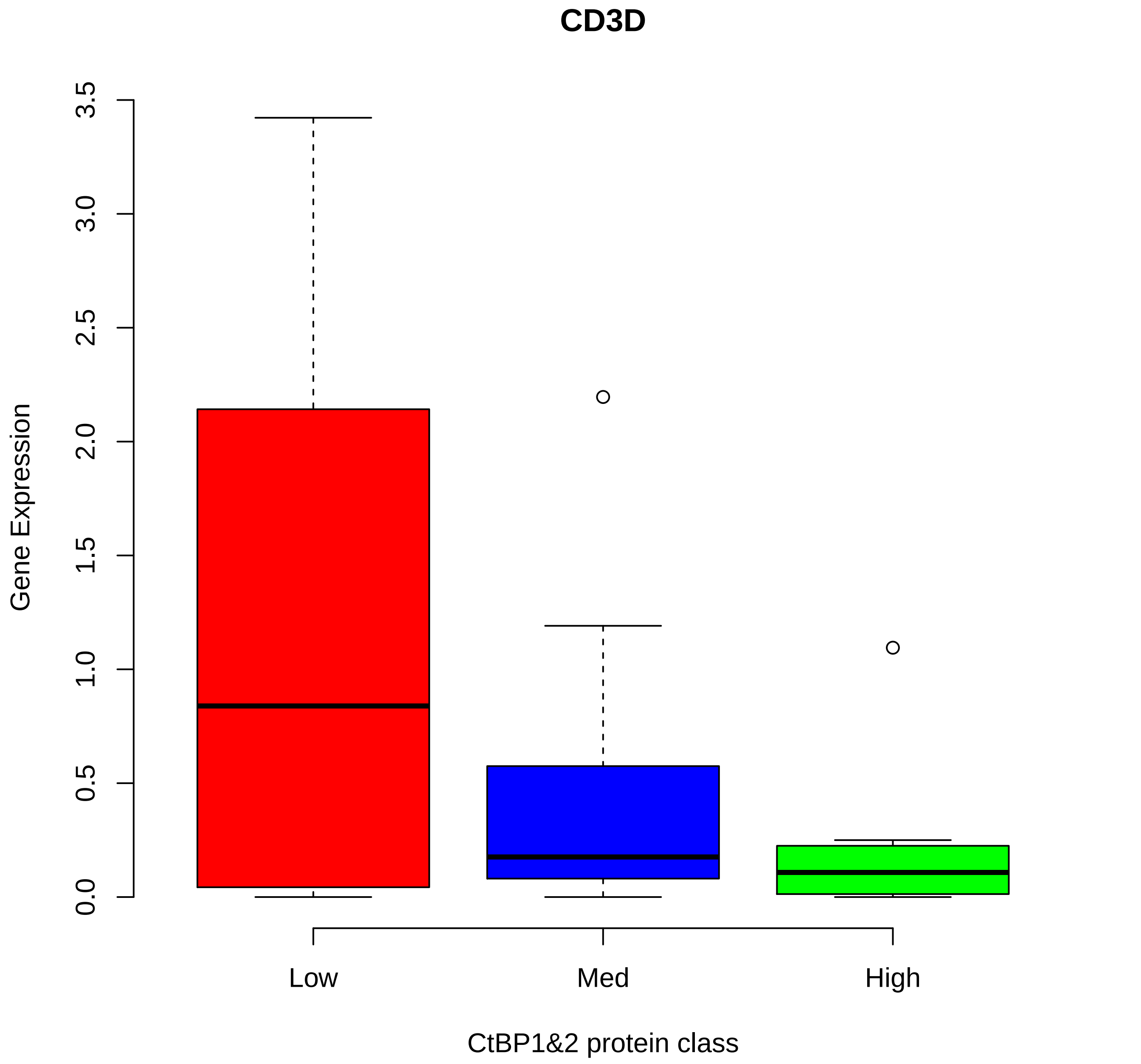

CD3D
Gene Expression
0.0
0.5
1.0
1.5
2.0
2.5
3.0
3.5
Low
Med
High
CtBP1&2 protein class

GOLGA8IP

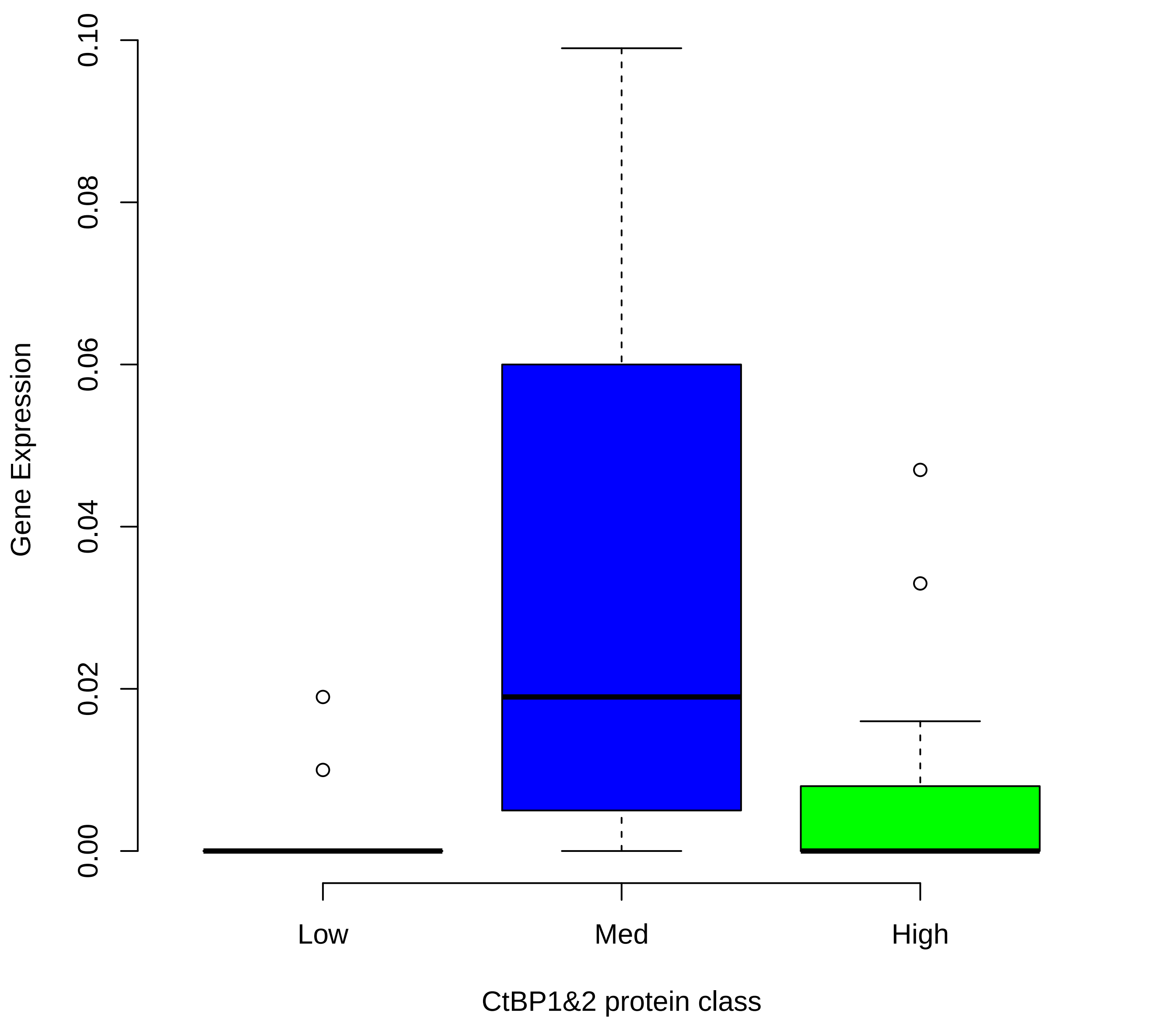

Gene Expression
0.00
0.02
0.04
0.06
0.08
0.10
Low
Med
High
CtBP1&2 protein class

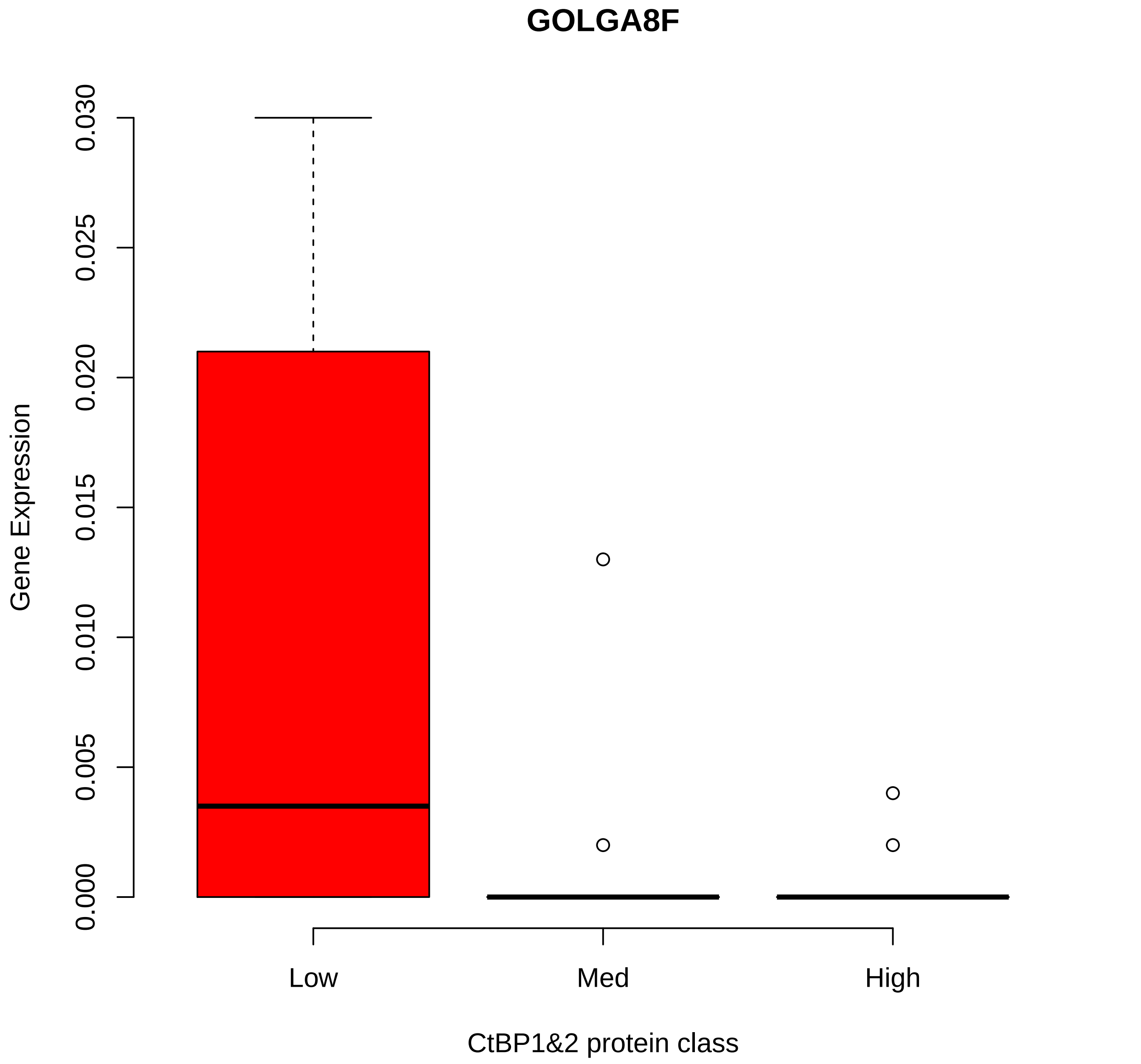
GOLGA8F
Gene Expression
0.000
0.005
0.010
0.015
0.020
0.025
0.030
Low
Med
High
CtBP1&2 protein class

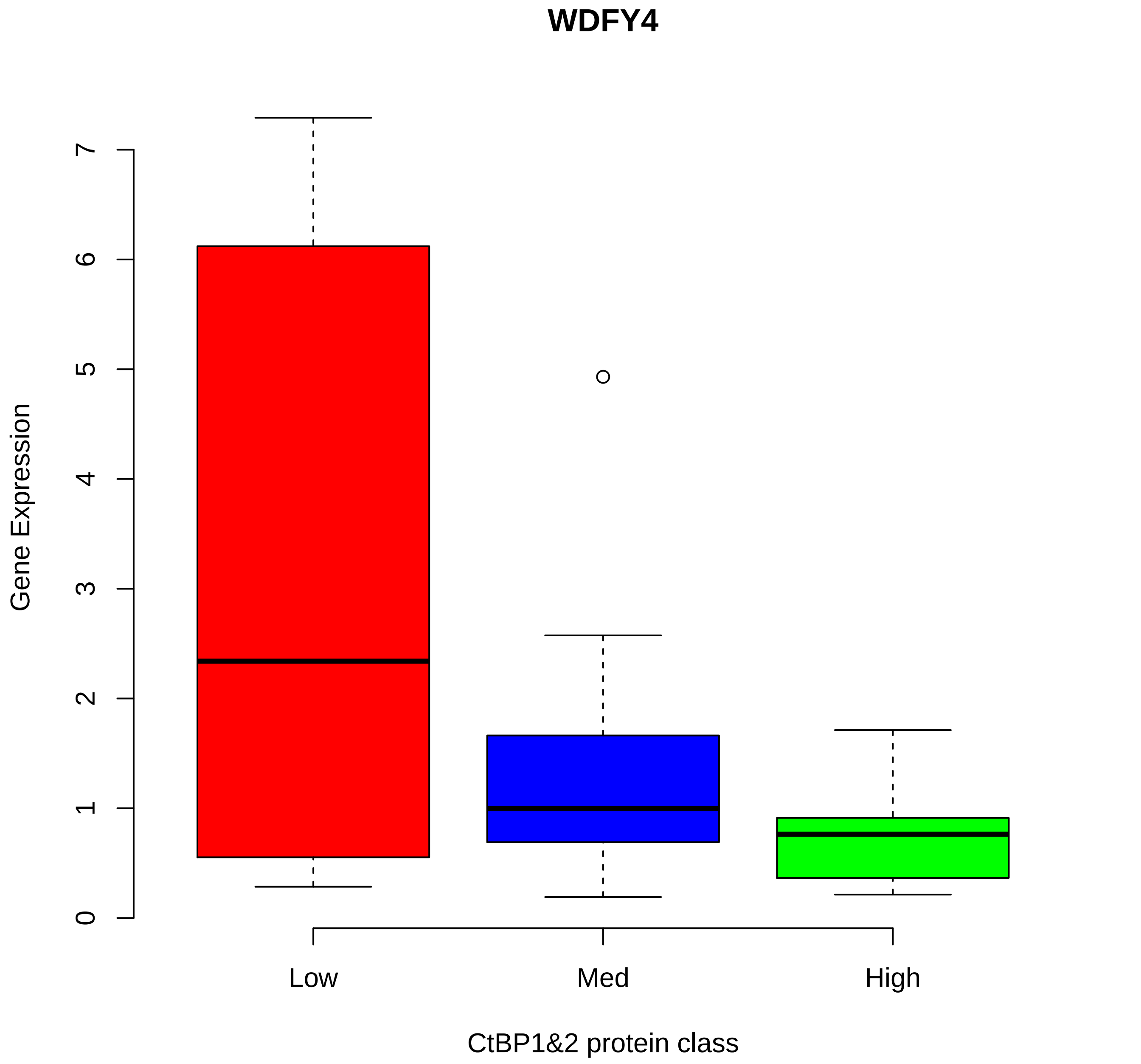

WDFY4
Gene Expression
0
1
2
3
4
5
6
7
Low
Med
High
CtBP1&2 protein class

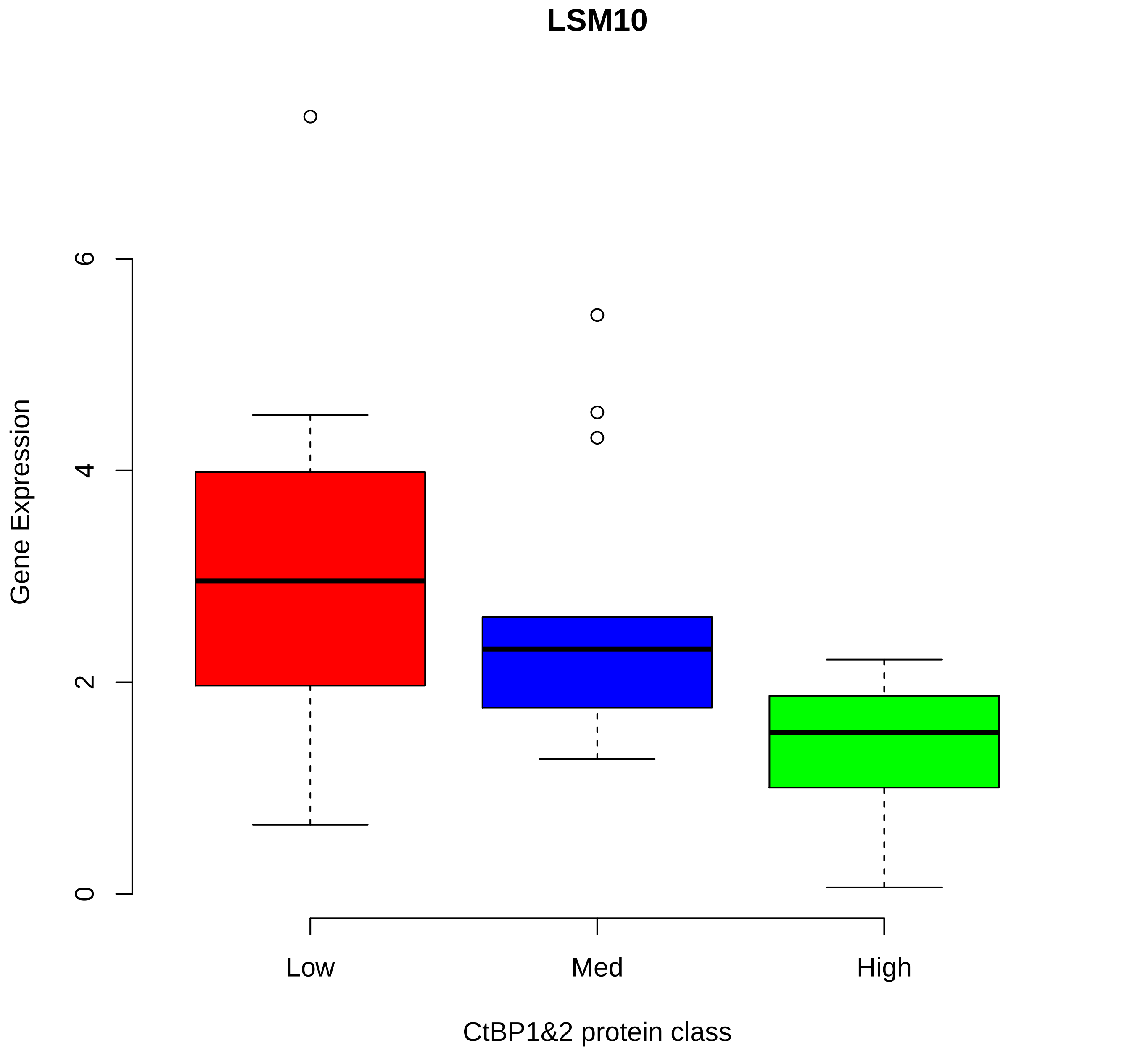

LSM10
Gene Expression
0
2
4
6
Low
Med
High
CtBP1&2 protein class

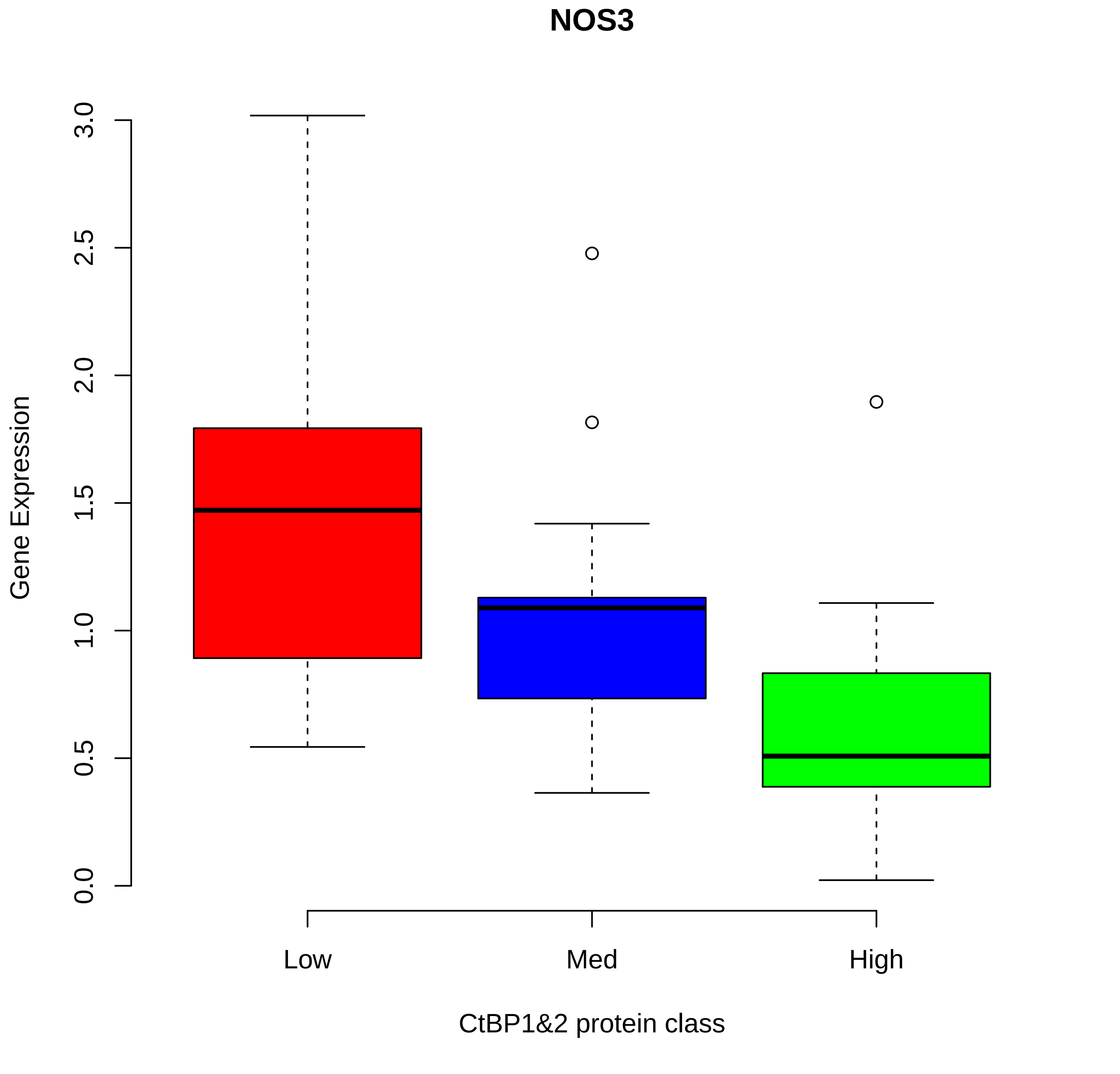
NOS3
Gene Expression
0.0
0.5
1.0
1.5
2.0
2.5
3.0
Low
Med
High
CtBP1&2 protein class

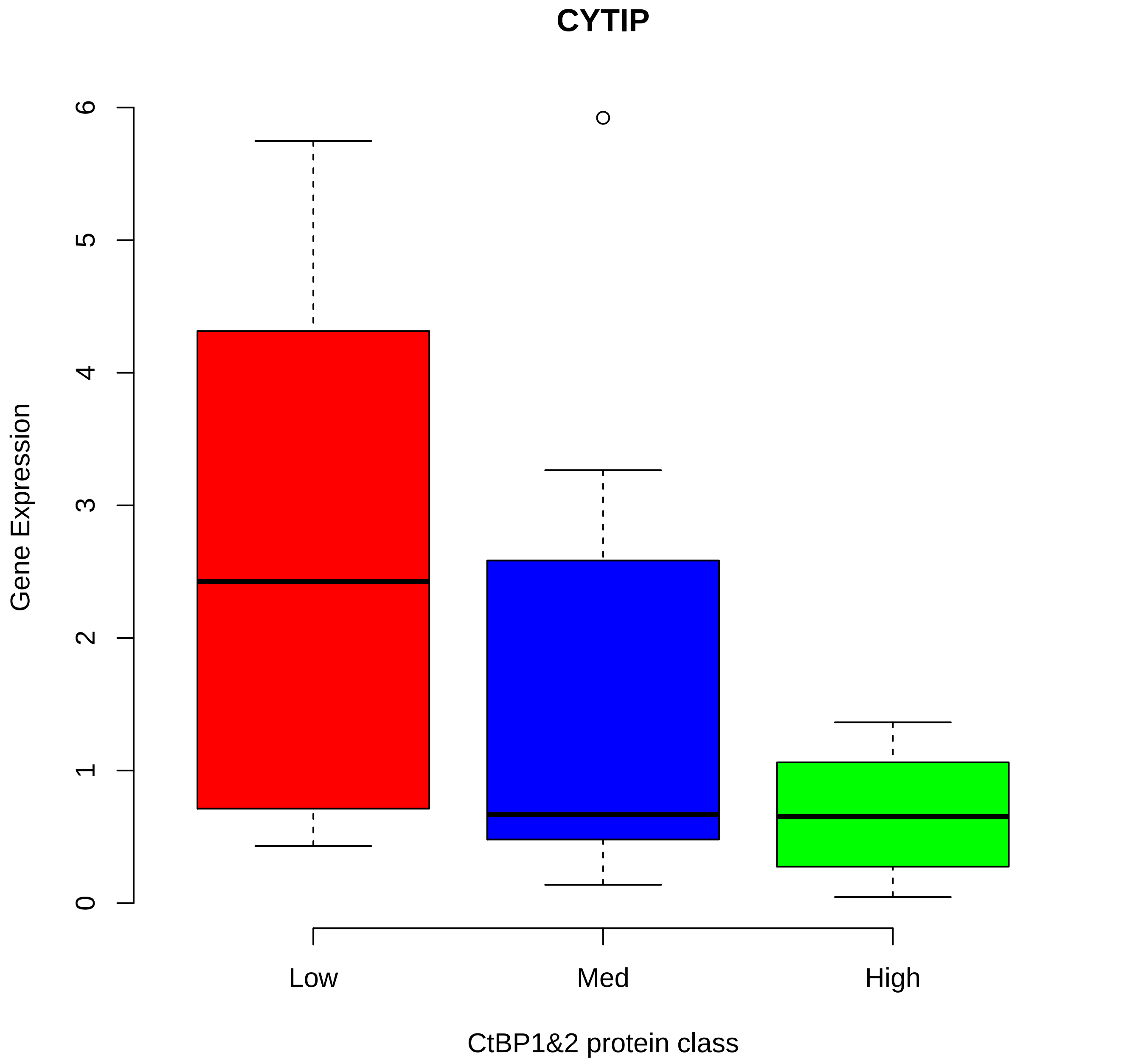

CYTIP
Gene Expression
0
1
2
3
4
5
6
Low
Med
High
CtBP1&2 protein class

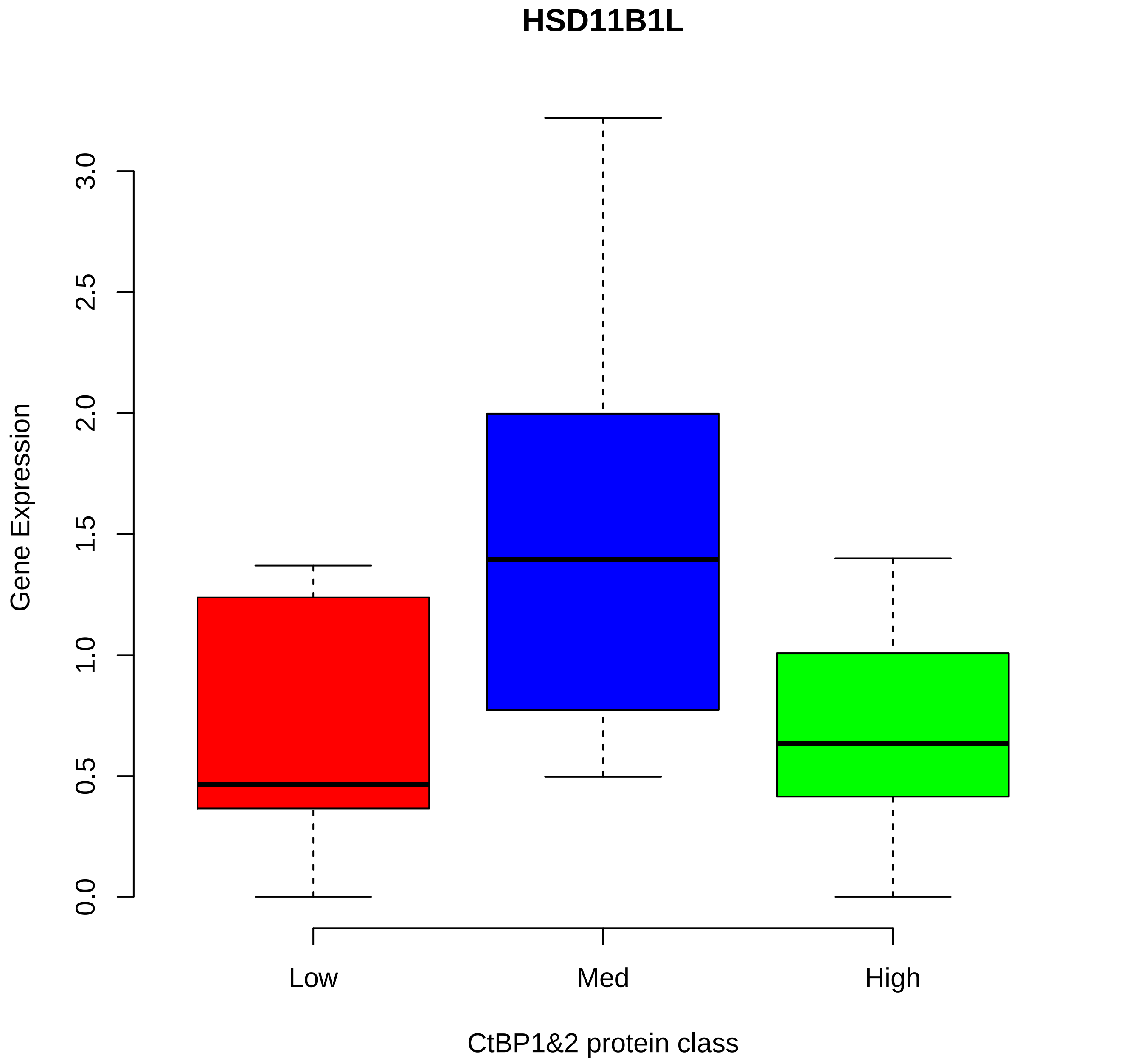

HSD11B1L
Gene Expression
0.0
0.5
1.0
1.5
2.0
2.5
3.0
Low
Med
High
CtBP1&2 protein class

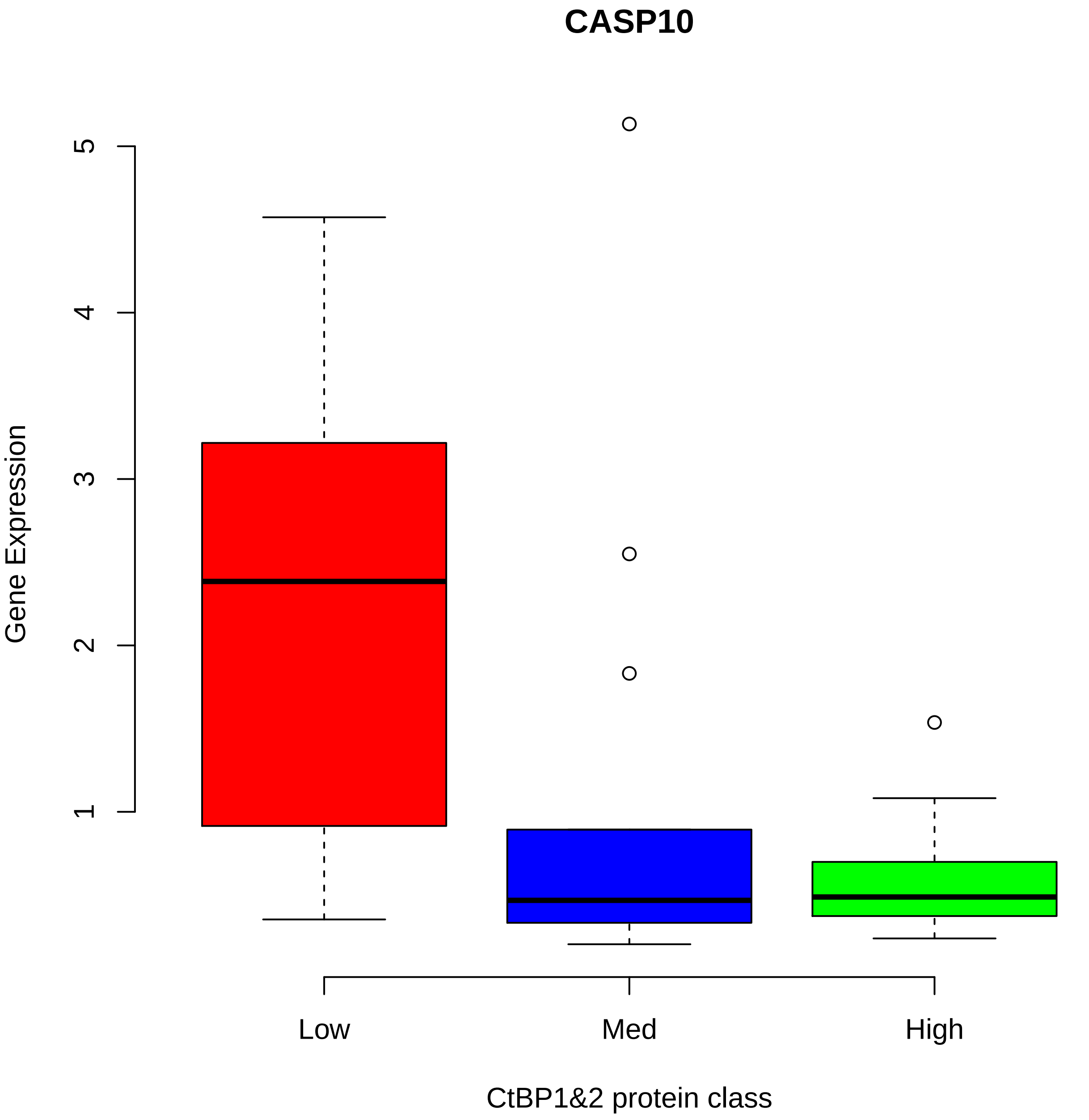
CASP10
Gene Expression
1
2
3
4
5
Low
Med
High
CtBP1&2 protein class

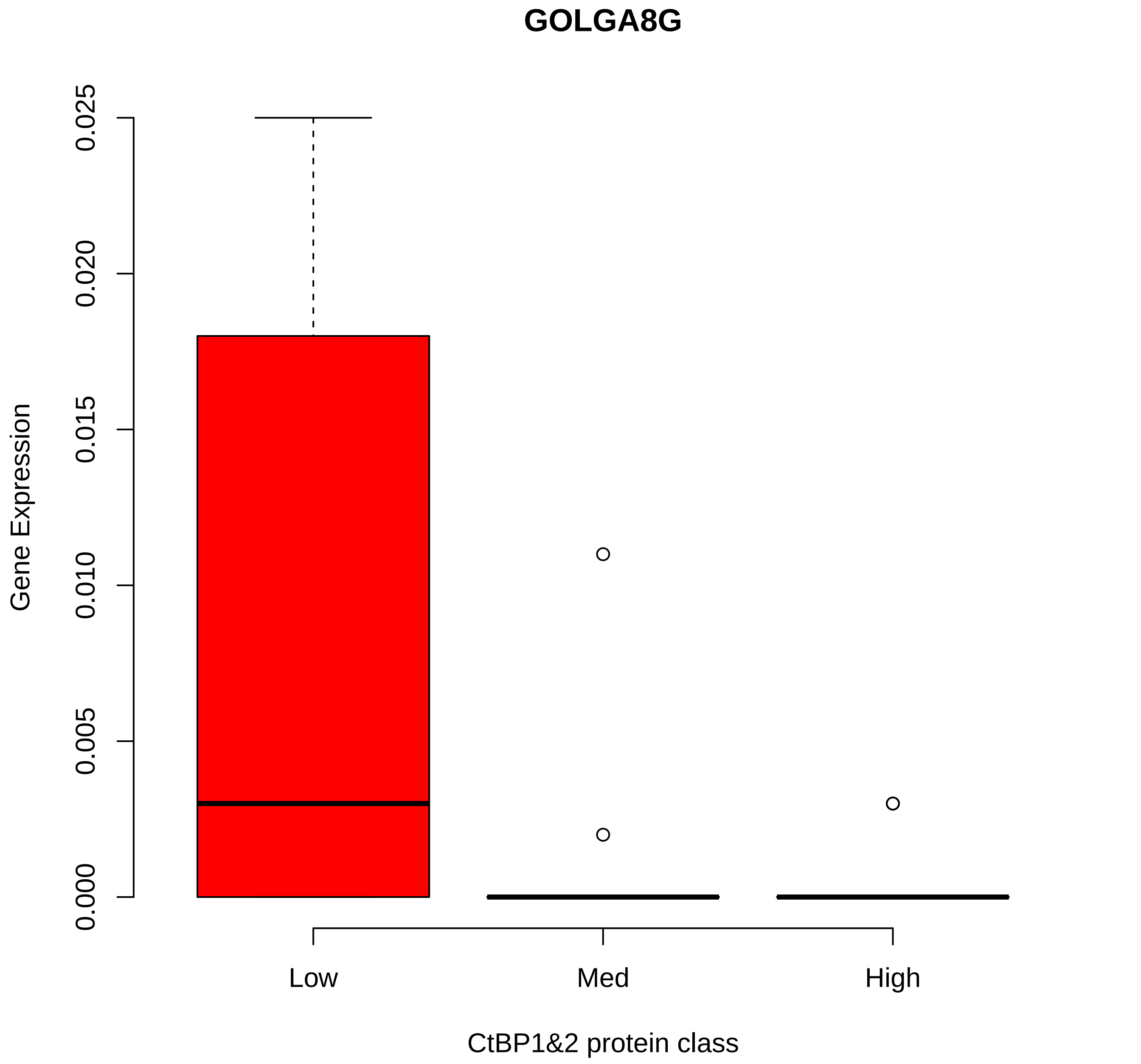

GOLGA8G
Gene Expression
0.000
0.005
0.010
0.015
0.020
0.025
Low
Med
High
CtBP1&2 protein class

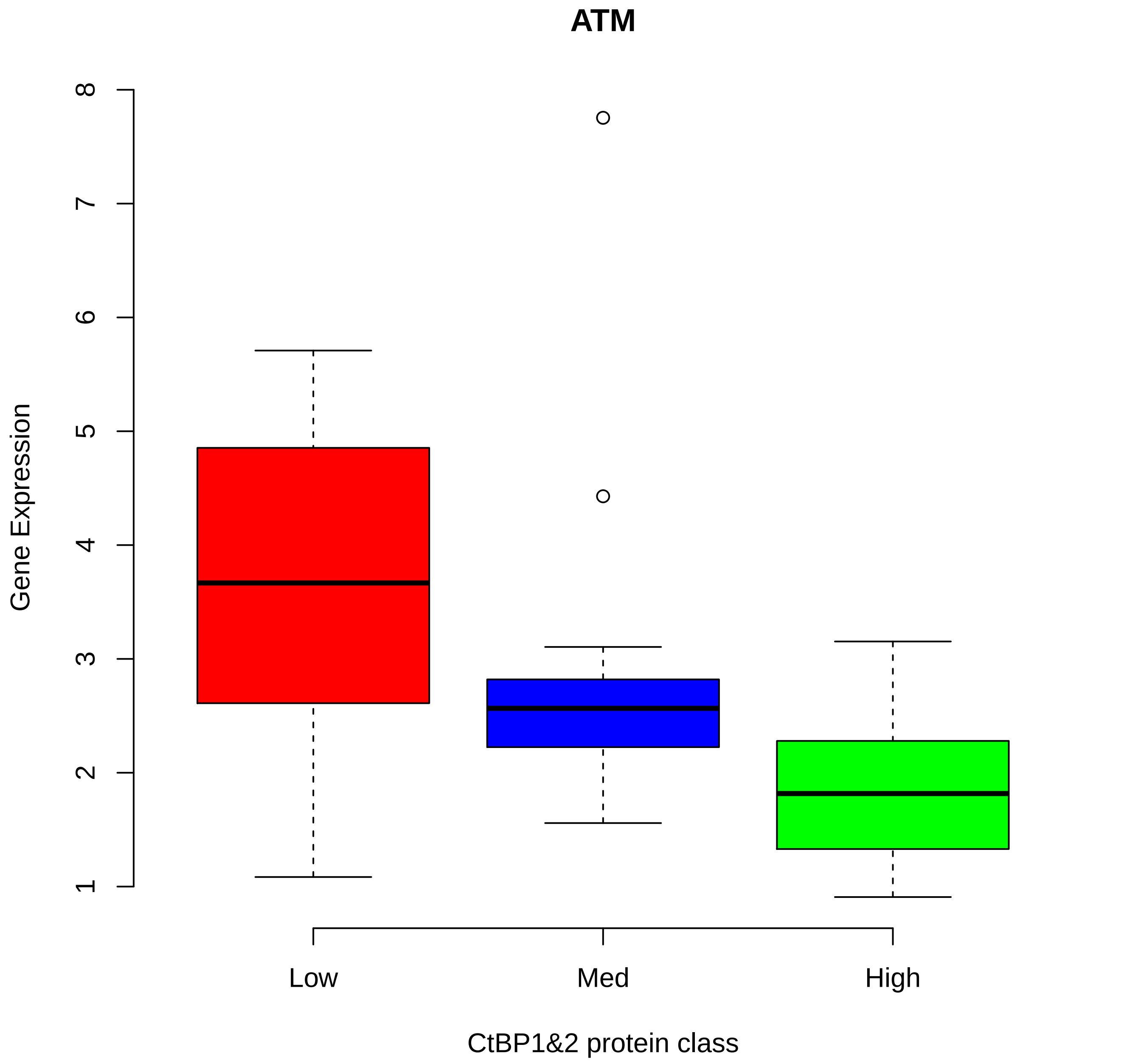
ATM
Gene Expression
1
2
3
4
5
6
7
8
Low
Med
High
CtBP1&2 protein class

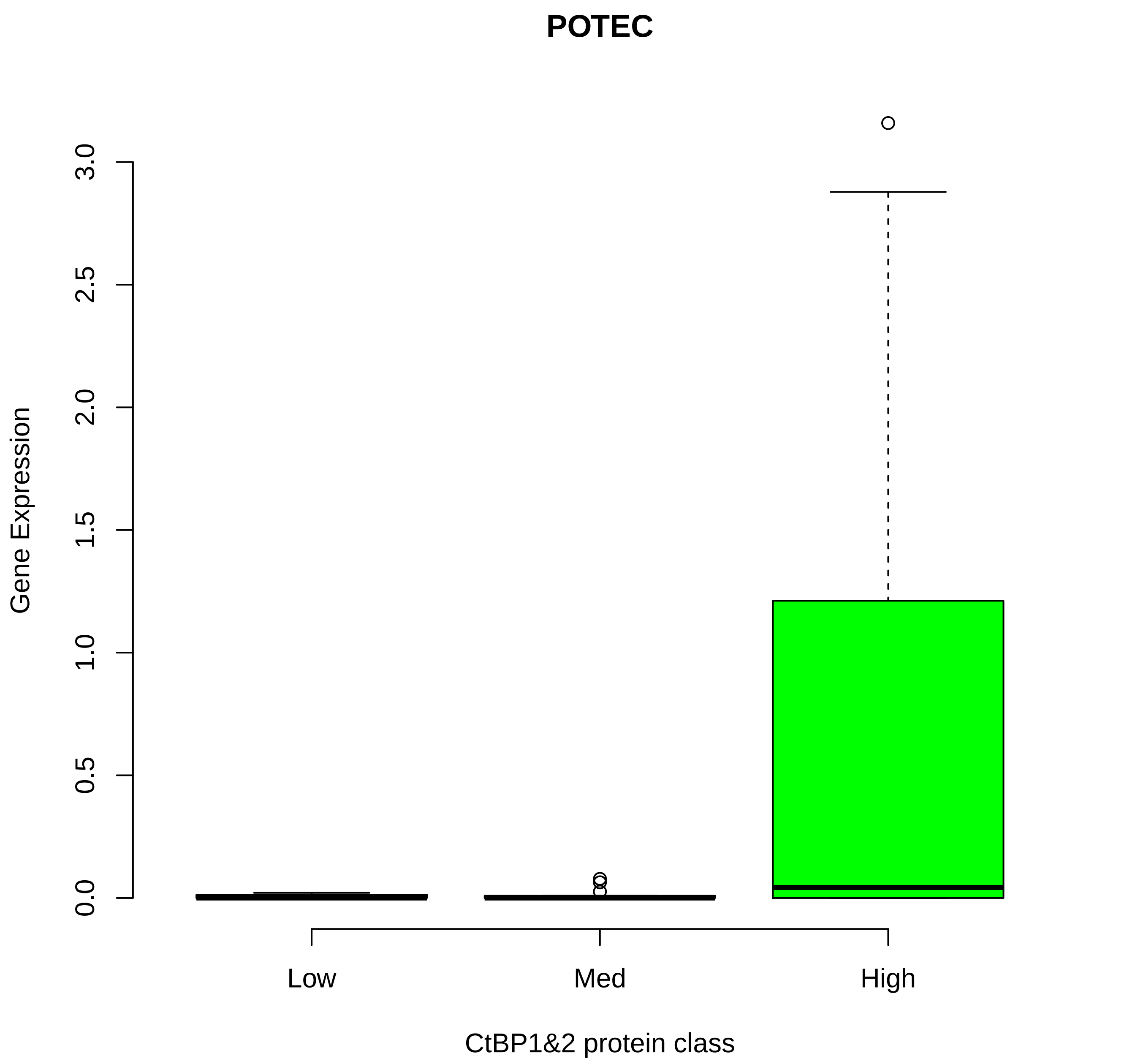
POTEC
Gene Expression
0.0
0.5
1.0
1.5
2.0
2.5
3.0
Low
Med
High
CtBP1&2 protein class

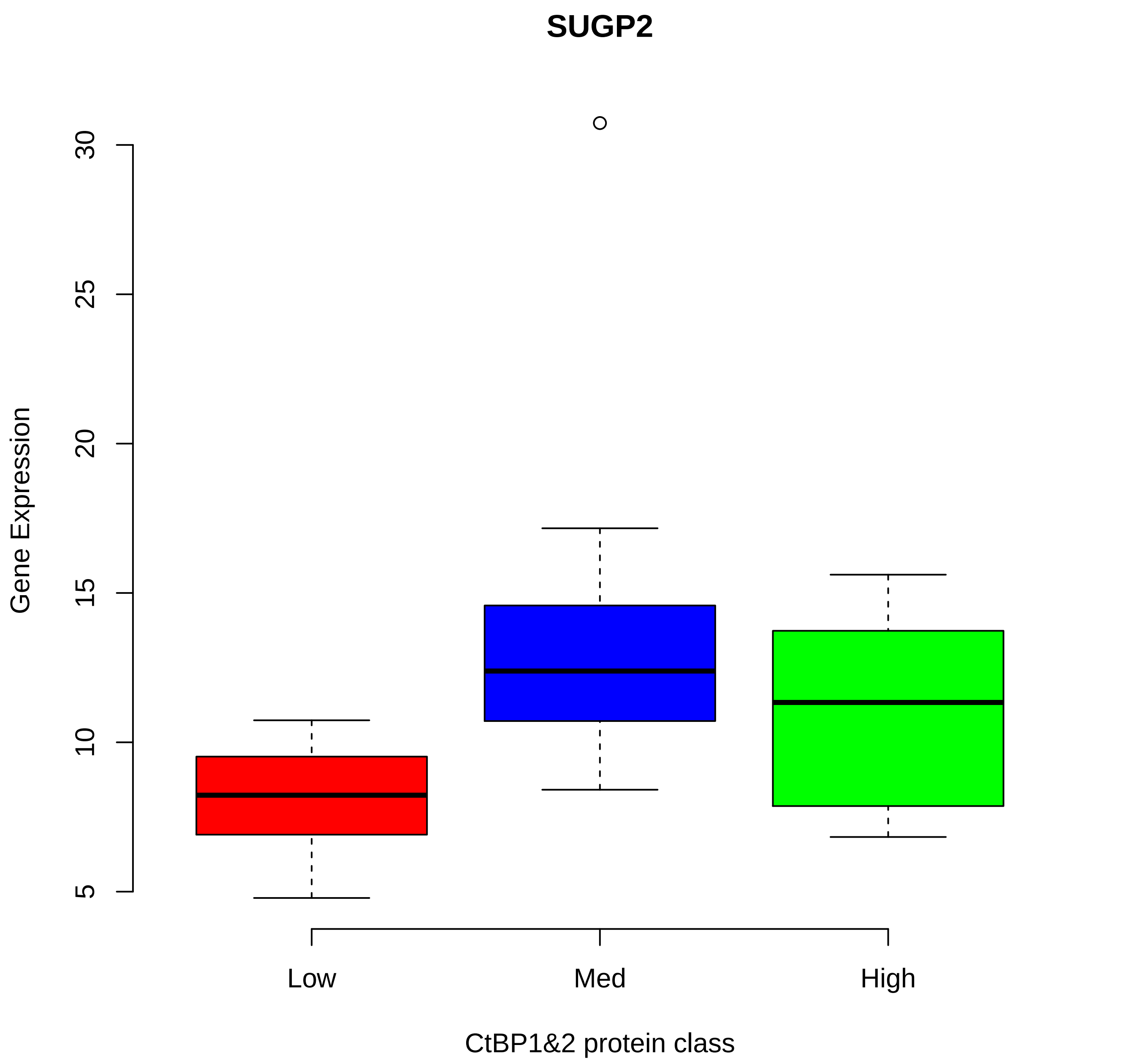

SUGP2
Gene Expression
30
25
20
15
10
5
Low
Med
High
CtBP1&2 protein class

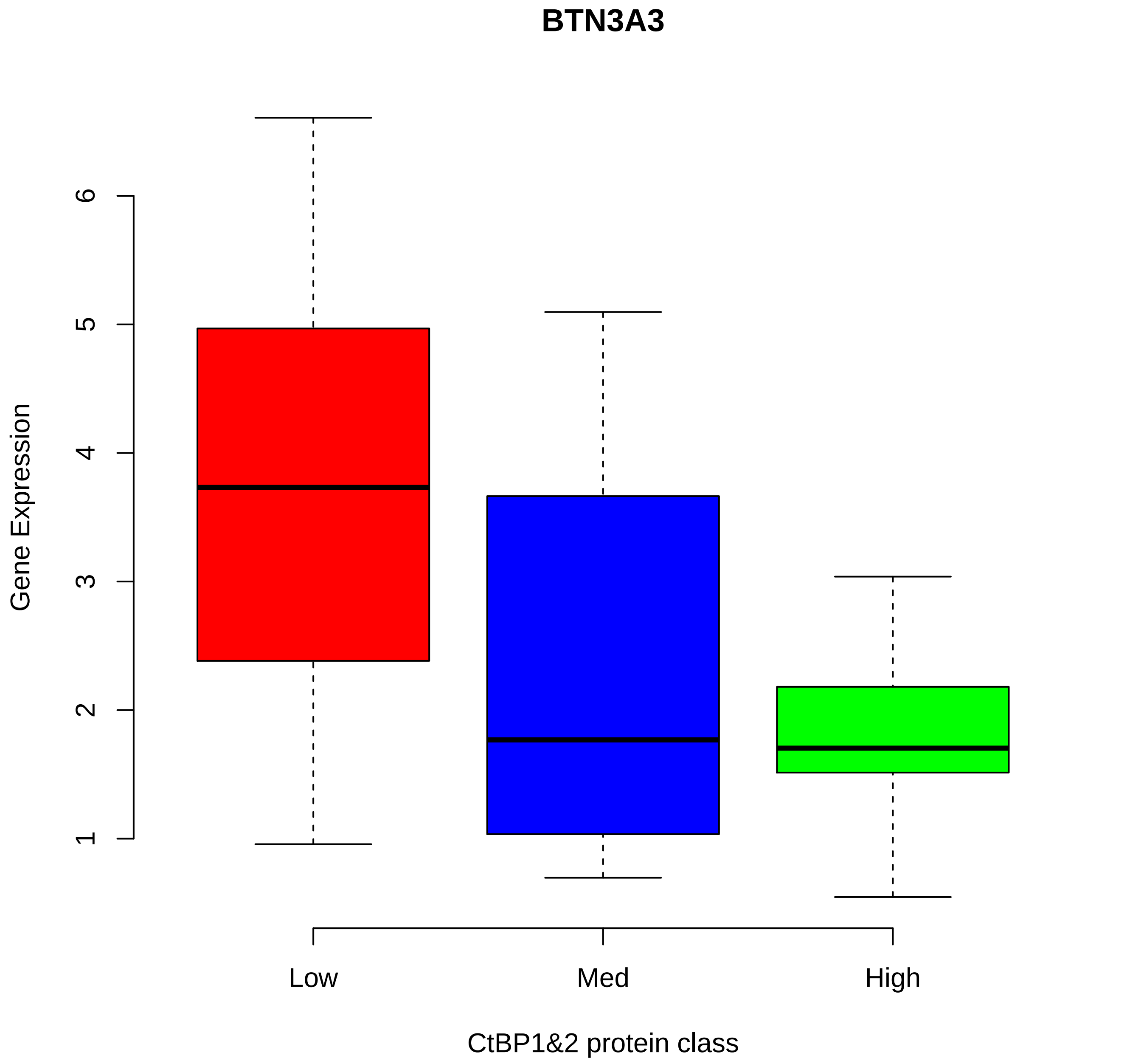
BTN3A3
Gene Expression
1
2
3
4
5
6
Low
Med
High
CtBP1&2 protein class

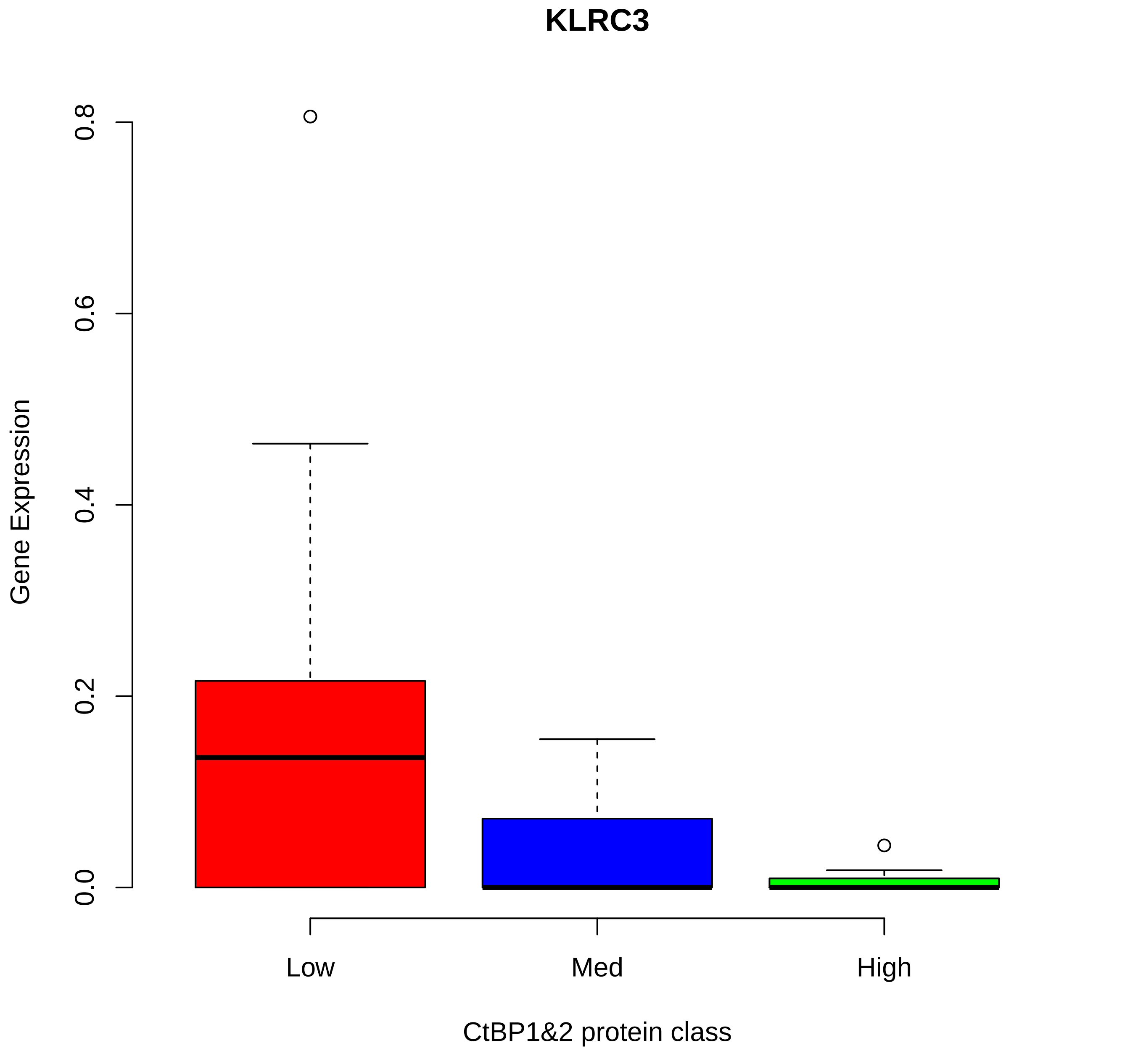
KLRC3
Gene Expression
0.0
0.2
0.4
0.6
0.8
Low
Med
High
CtBP1&2 protein class

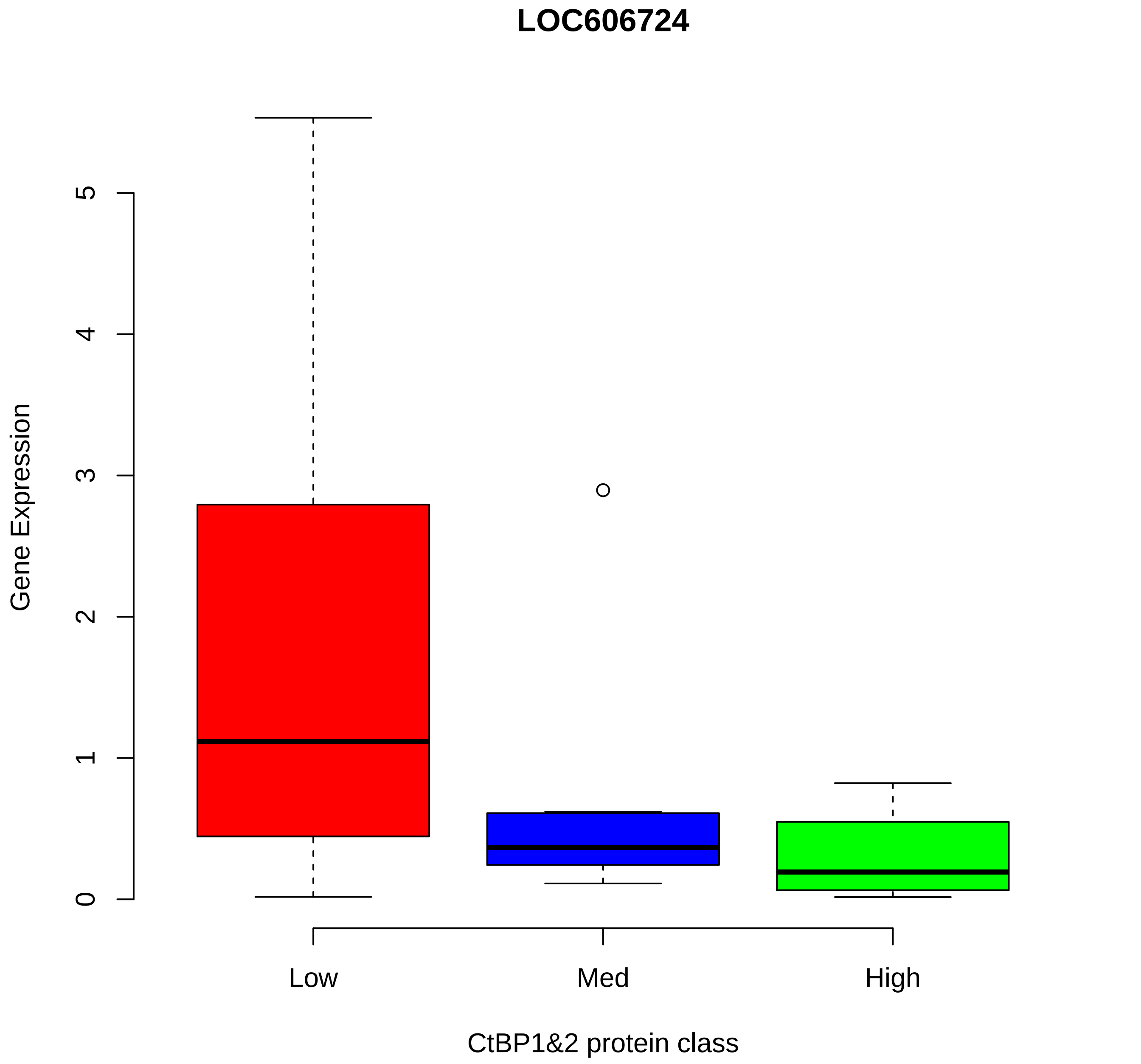

LOC606724
Gene Expression
0
1
2
3
4
5
Low
Med
High
CtBP1&2 protein class

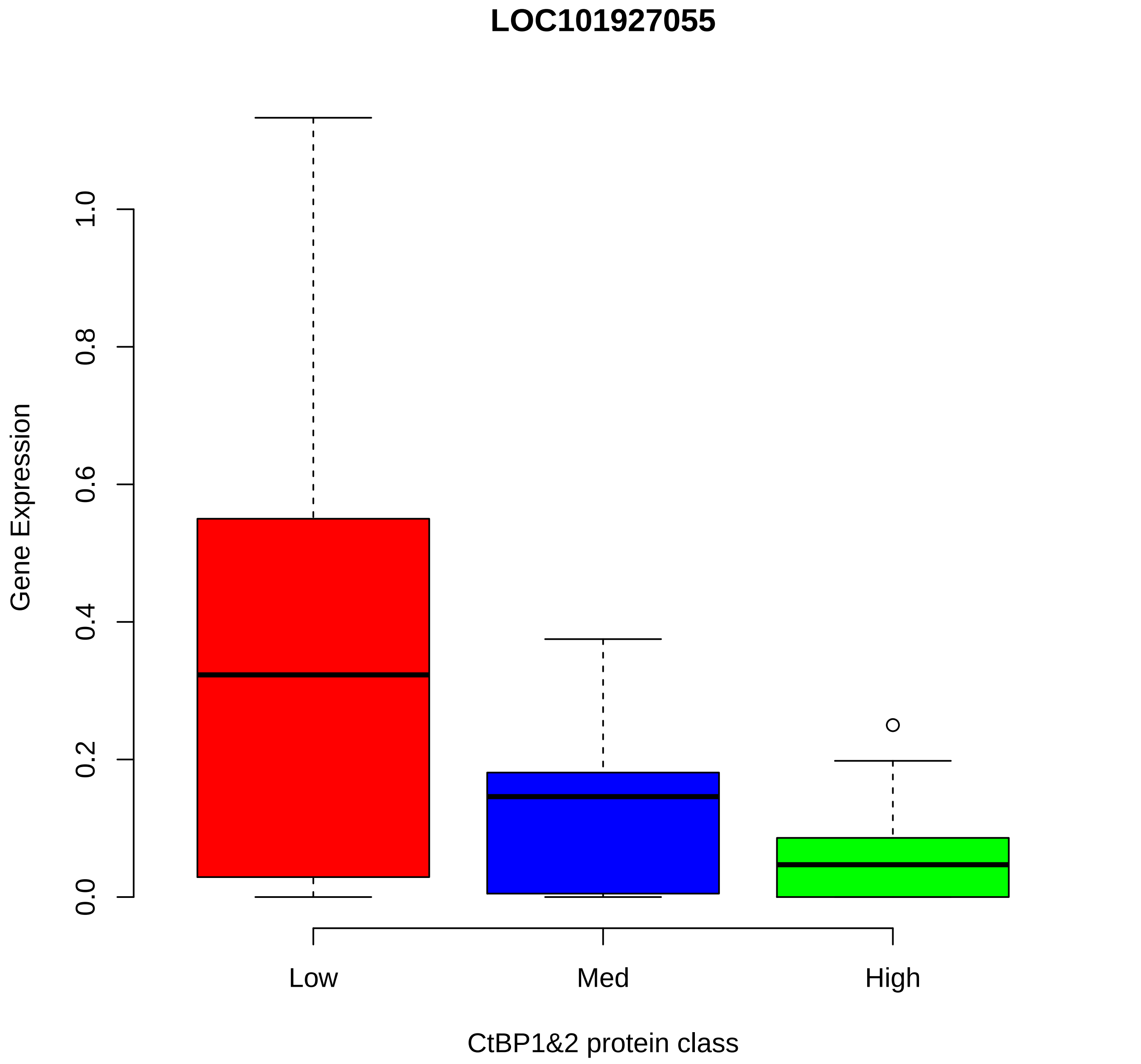

LOC101927055
Gene Expression
0.0
0.2
0.4
0.6
0.8
1.0
Low
Med
High
CtBP1&2 protein class

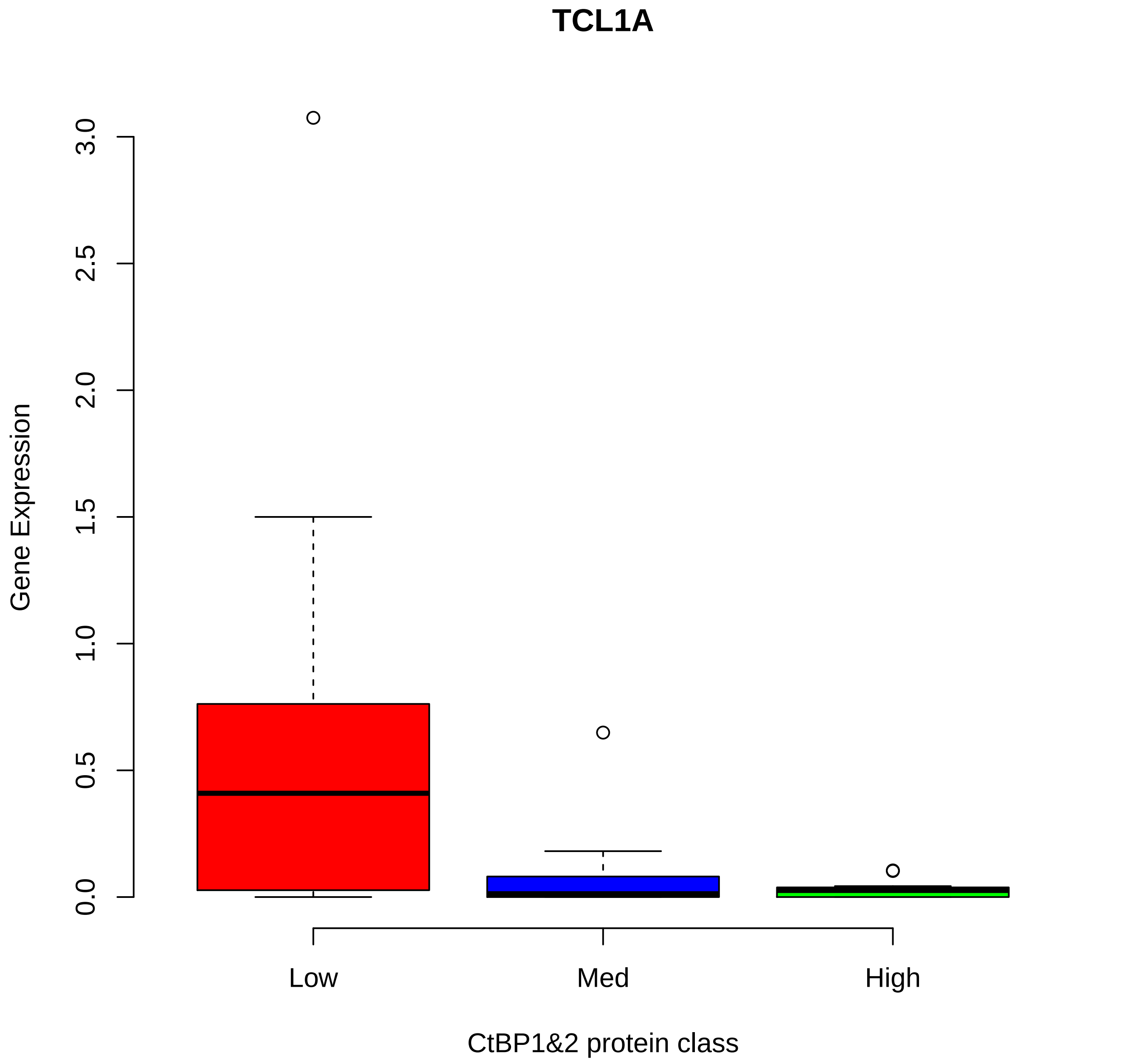

TCL1A
Gene Expression
0.0
0.5
1.0
1.5
2.0
2.5
3.0
Low
Med
High
CtBP1&2 protein class

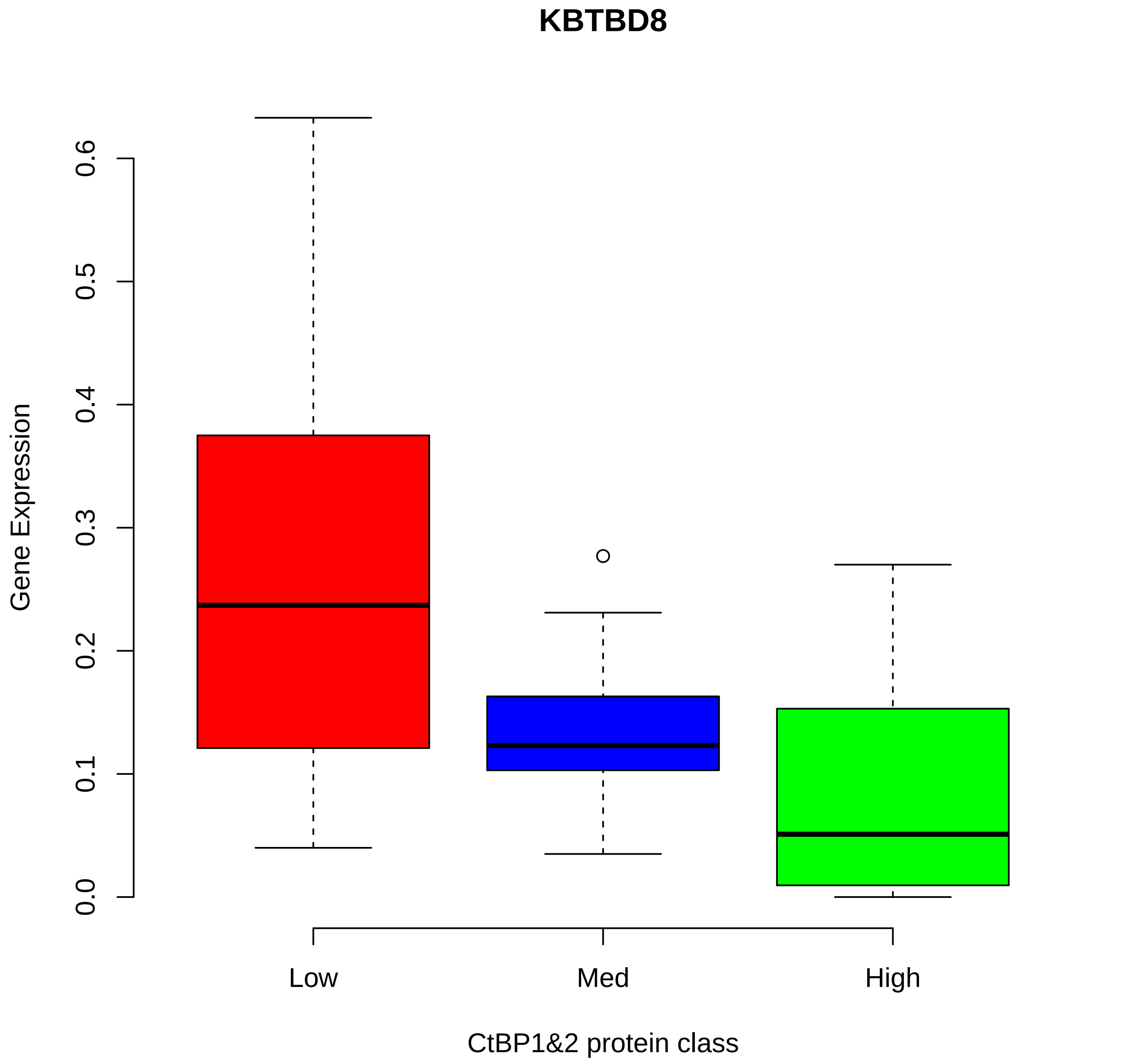
KBTBD8
Gene Expression
0.0
0.1
0.2
0.3
0.4
0.5
0.6
Low
Med
High
CtBP1&2 protein class

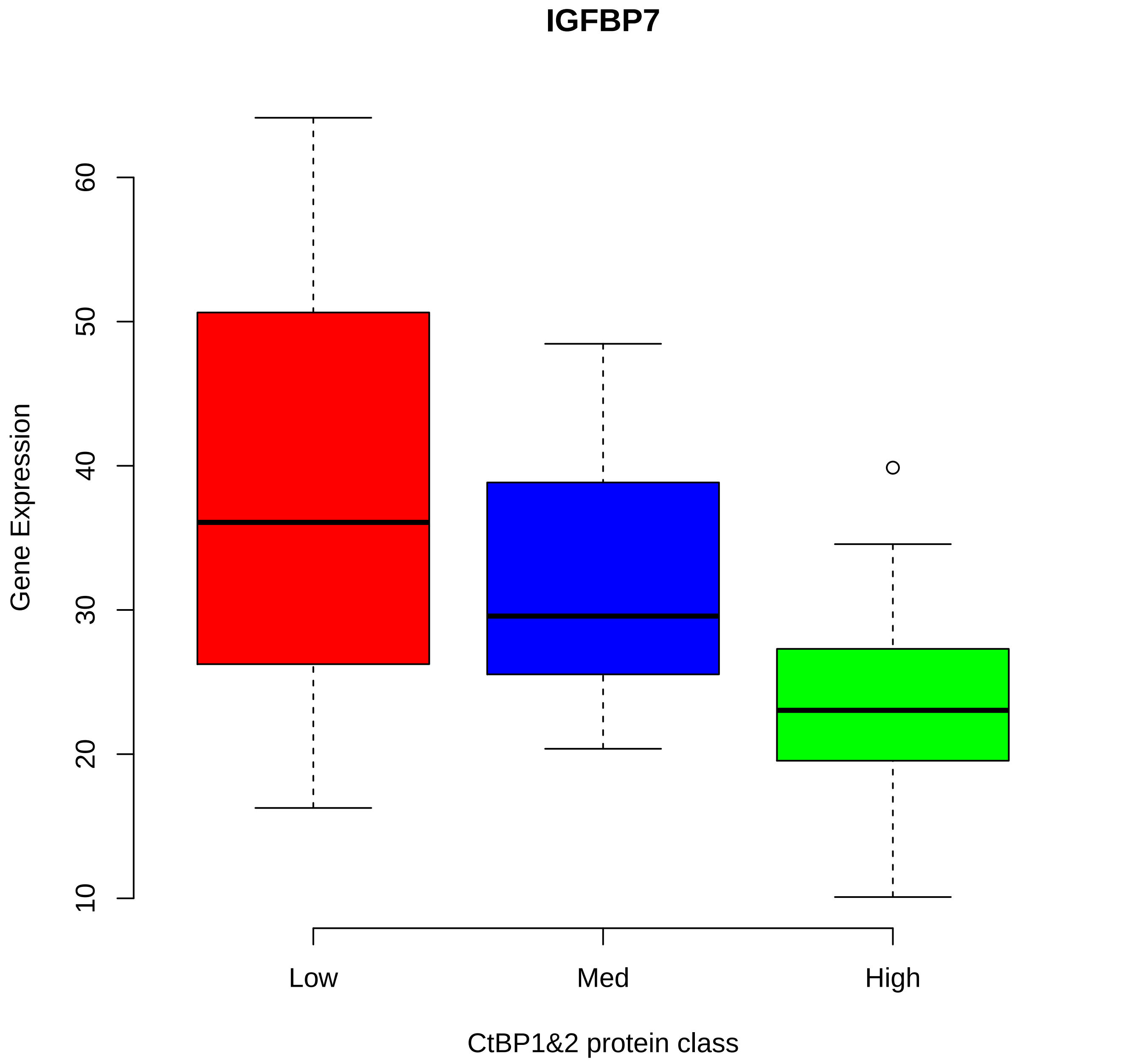

IGFBP7
Gene Expression
10
20
30
40
50
60
Low
Med
High
CtBP1&2 protein class

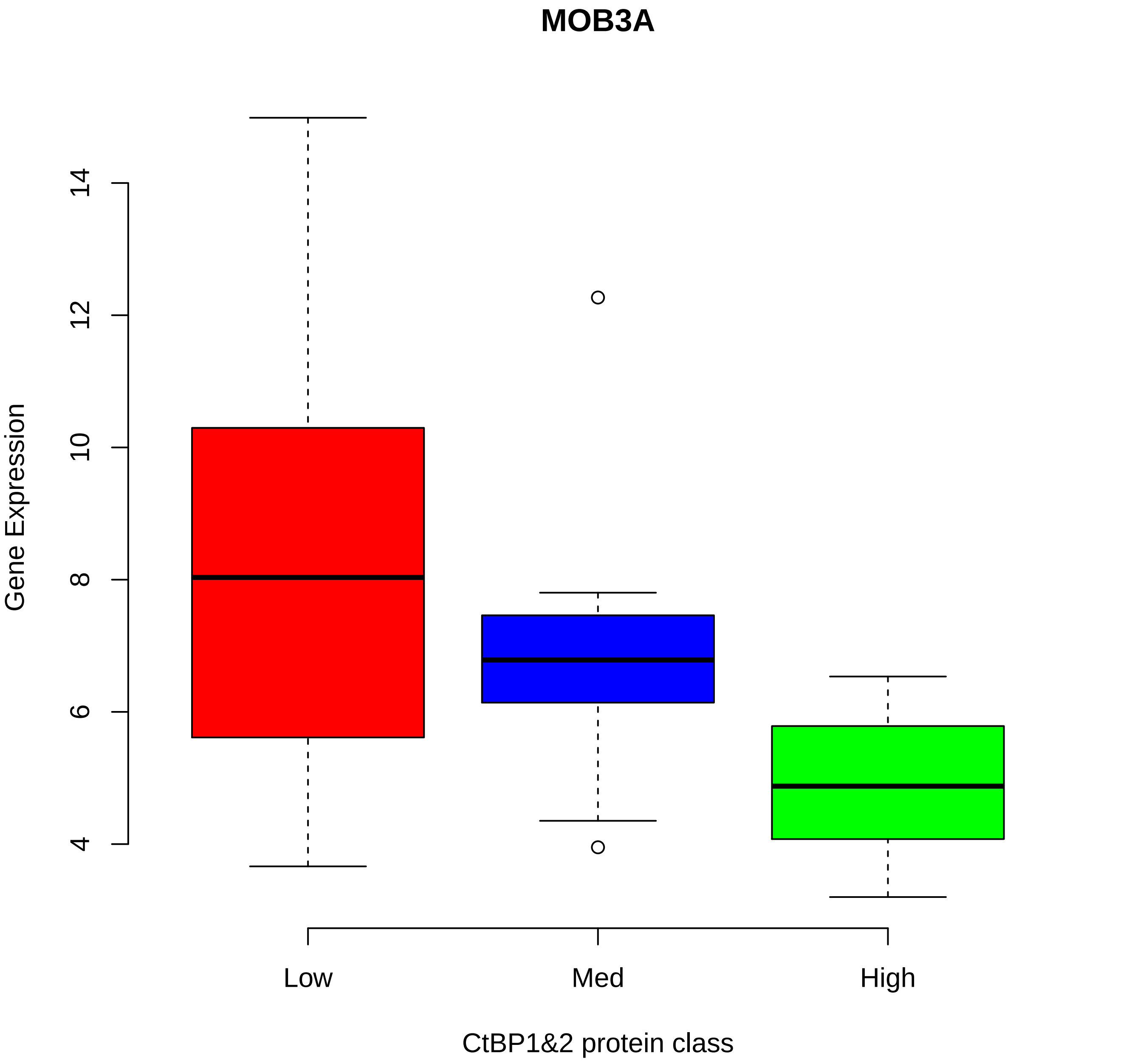
MOB3A
Gene Expression
4
6
8
10
12
14
Low
Med
High
CtBP1&2 protein class

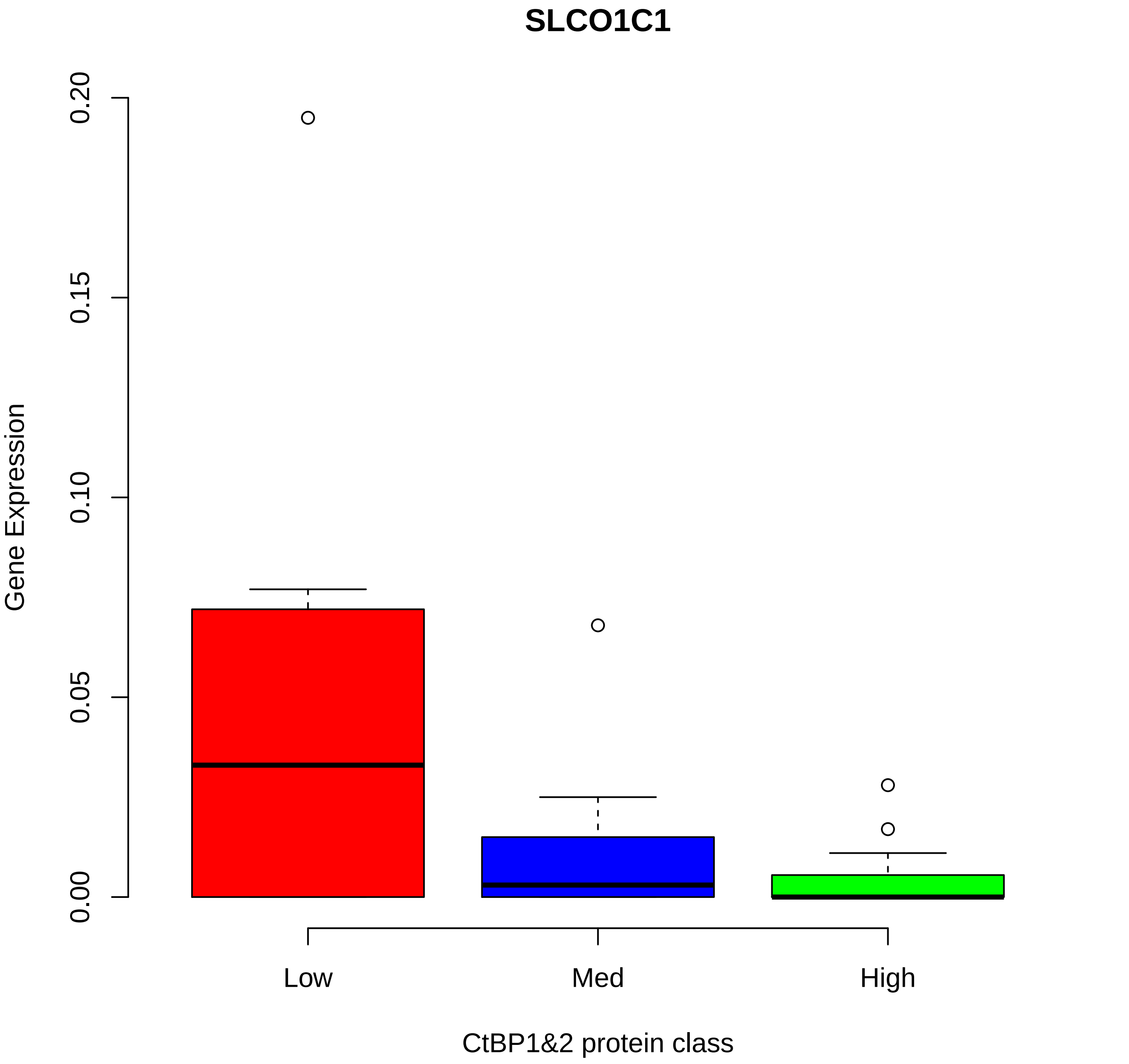
SLCO1C1
Gene Expression
0.00
0.05
0.10
0.15
0.20
Low
Med
High
CtBP1&2 protein class

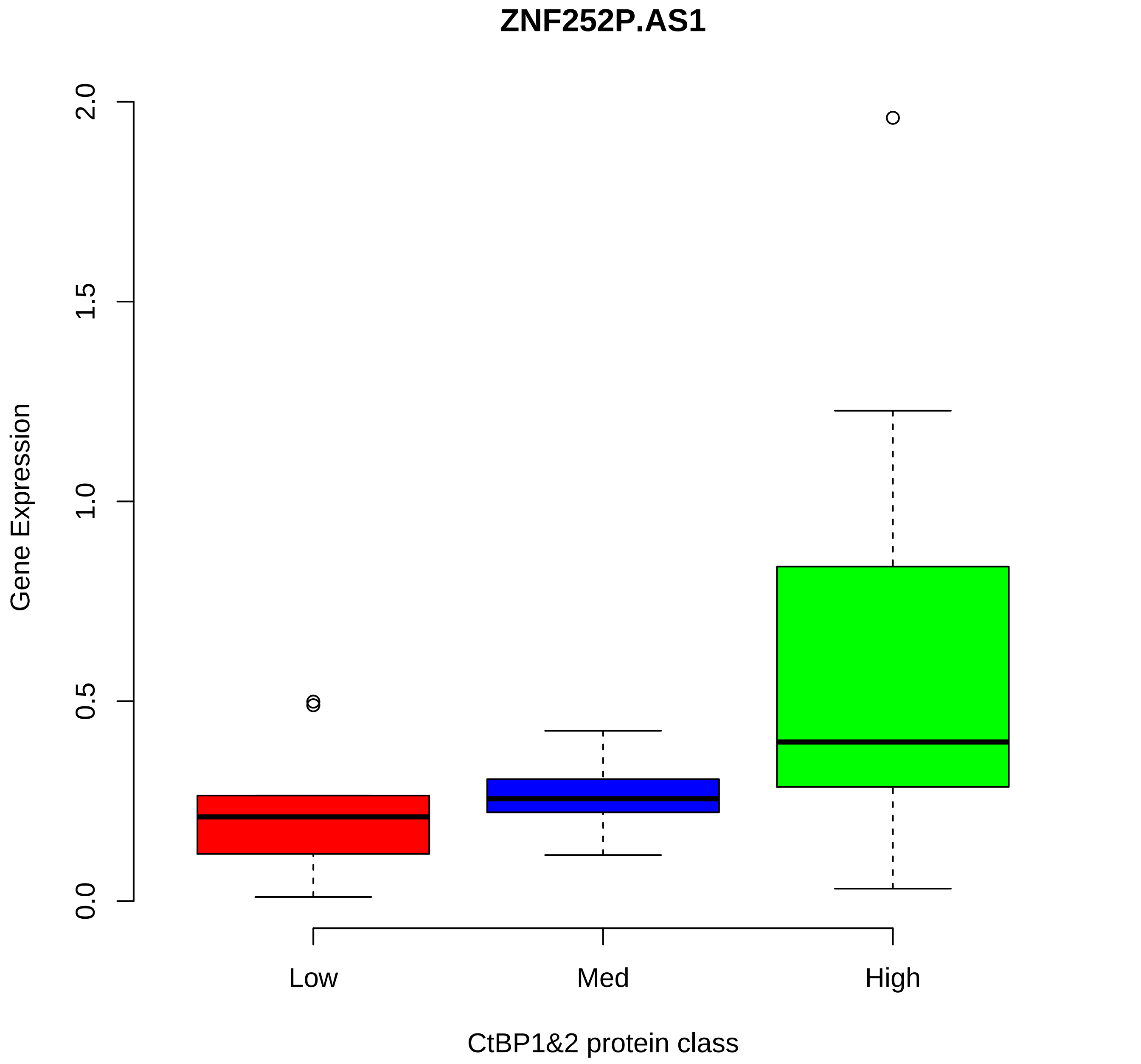

ZNF252P.AS1
Gene Expression
0.0
0.5
1.0
1.5
2.0
Low
Med
High
CtBP1&2 protein class

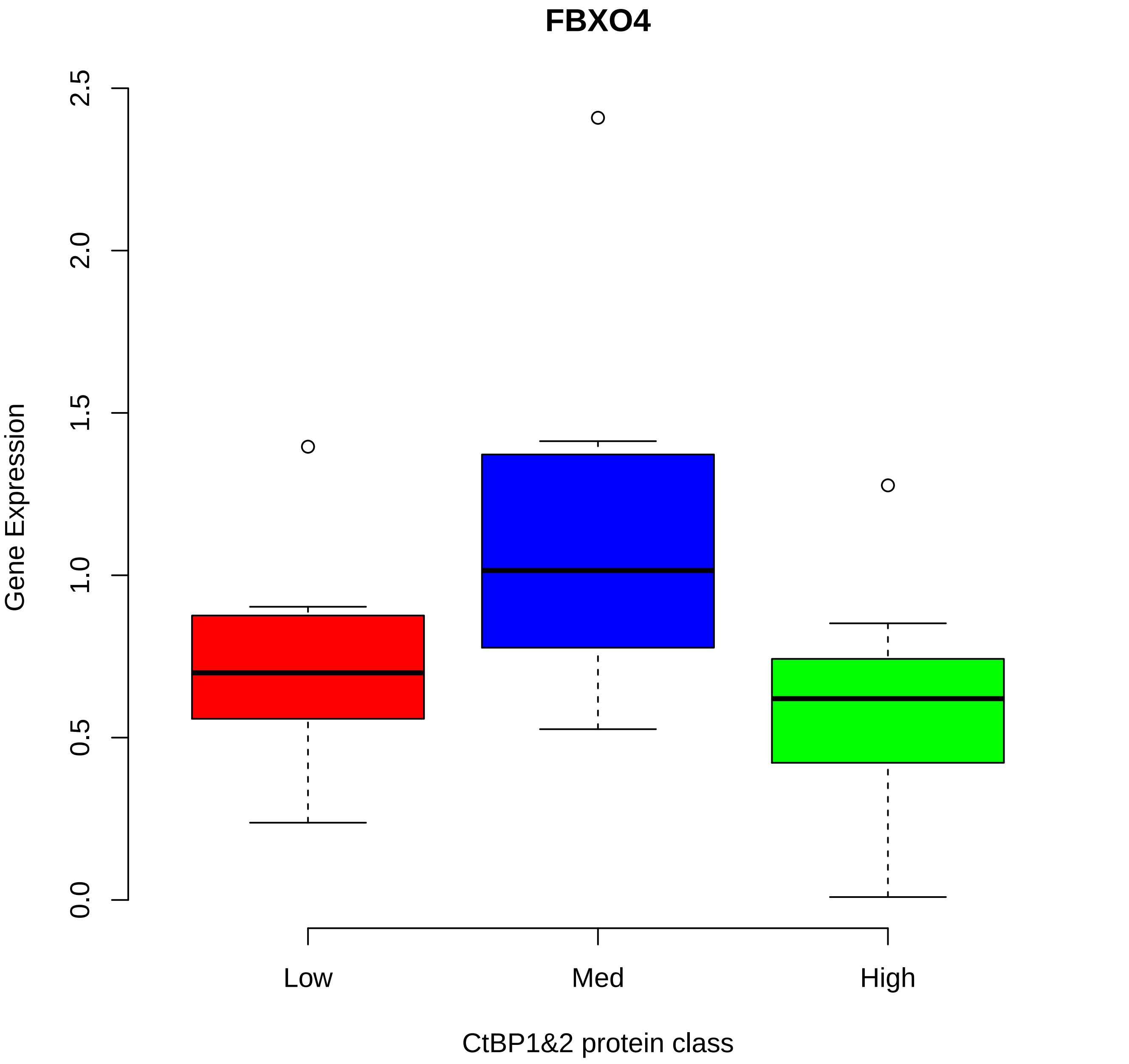

FBXO4
Gene Expression
0.0
0.5
1.0
1.5
2.0
2.5
Low
Med
High
CtBP1&2 protein class

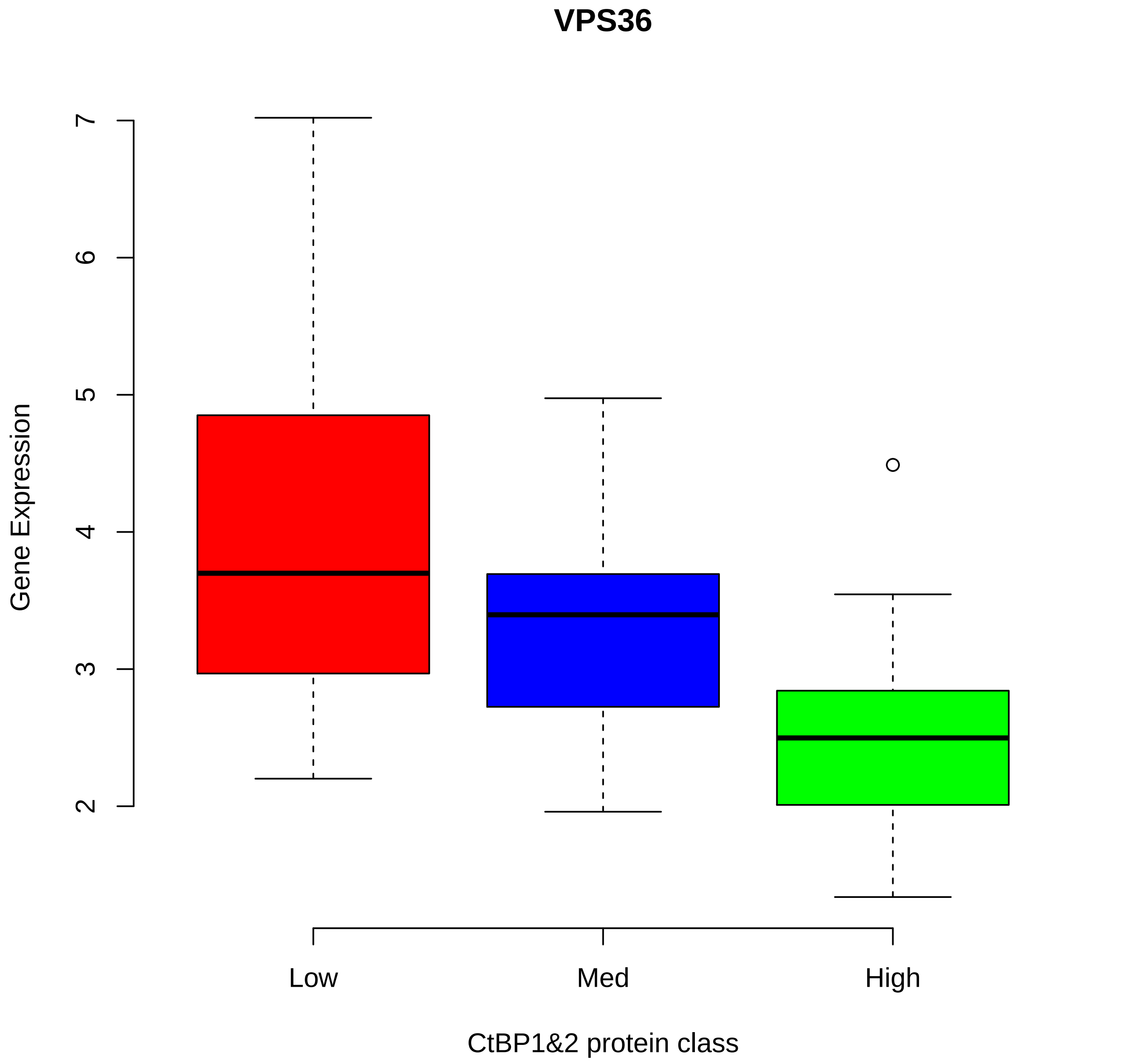

VPS36
Gene Expression
7
6
5
4
3
2
Low
Med
High
CtBP1&2 protein class

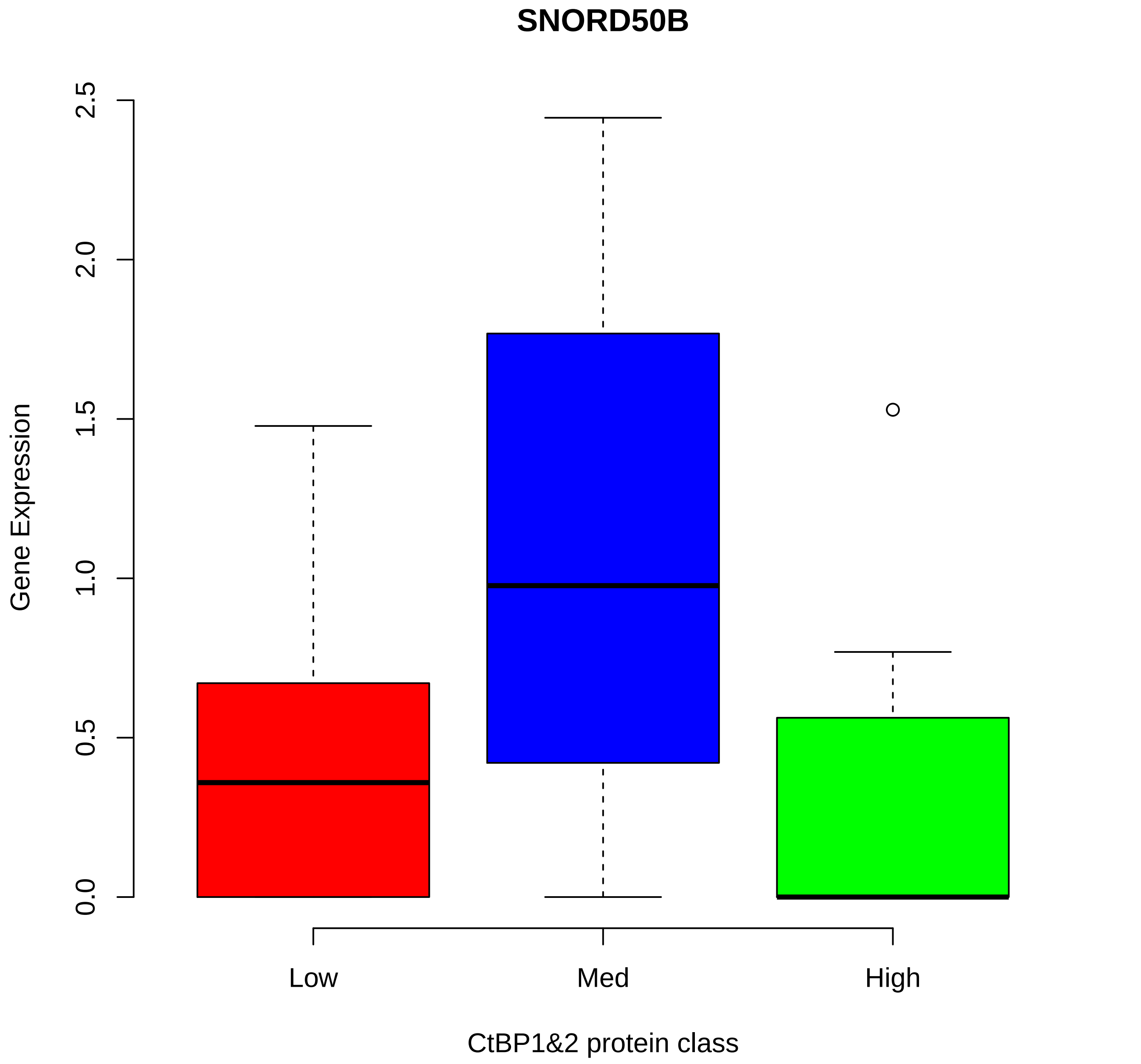

SNORD50B
Gene Expression
0.0
0.5
1.0
1.5
2.0
2.5
Low
Med
High
CtBP1&2 protein class

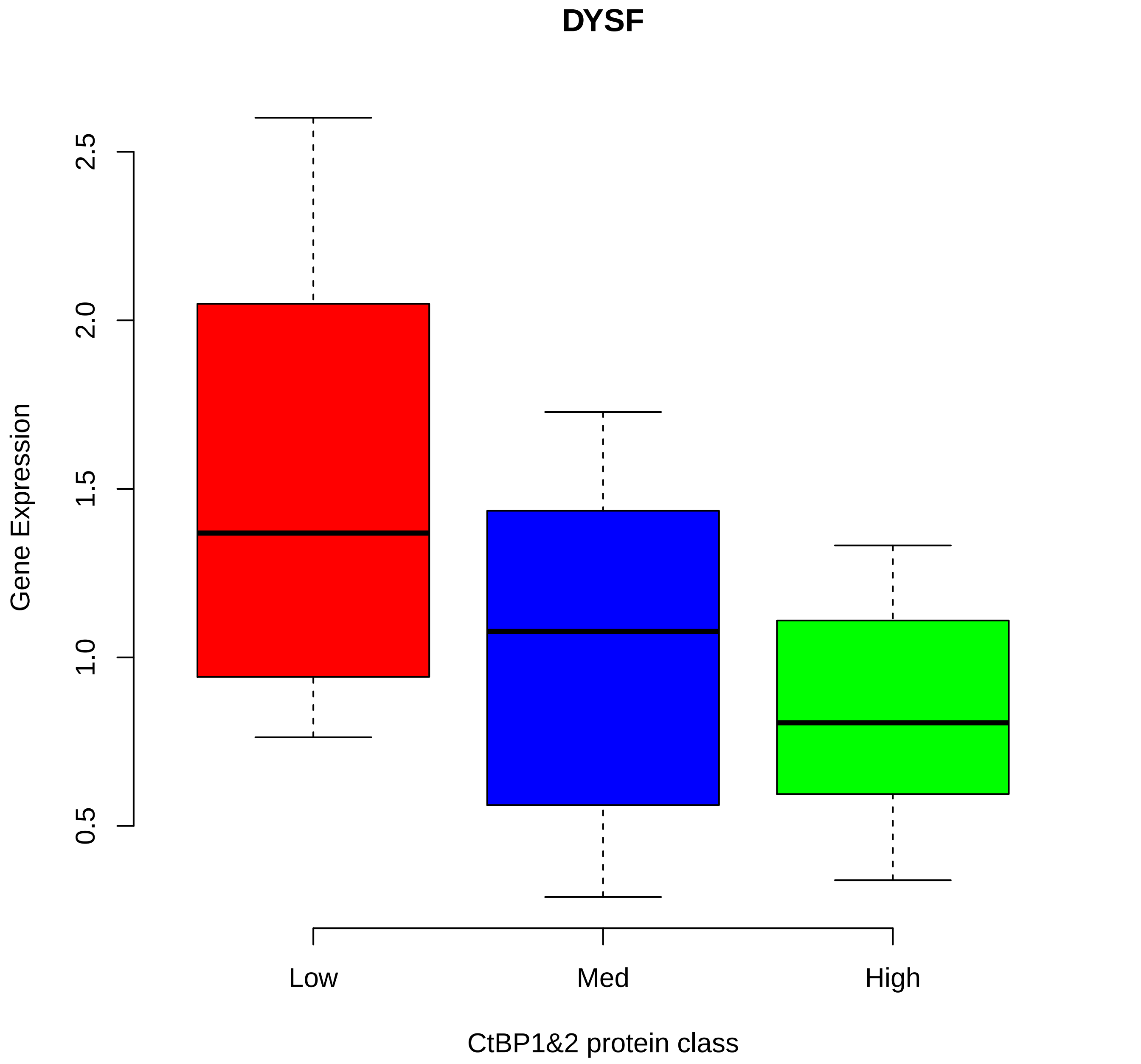

DYSF
Gene Expression
0.5
1.0
1.5
2.0
2.5
Low
Med
High
CtBP1&2 protein class

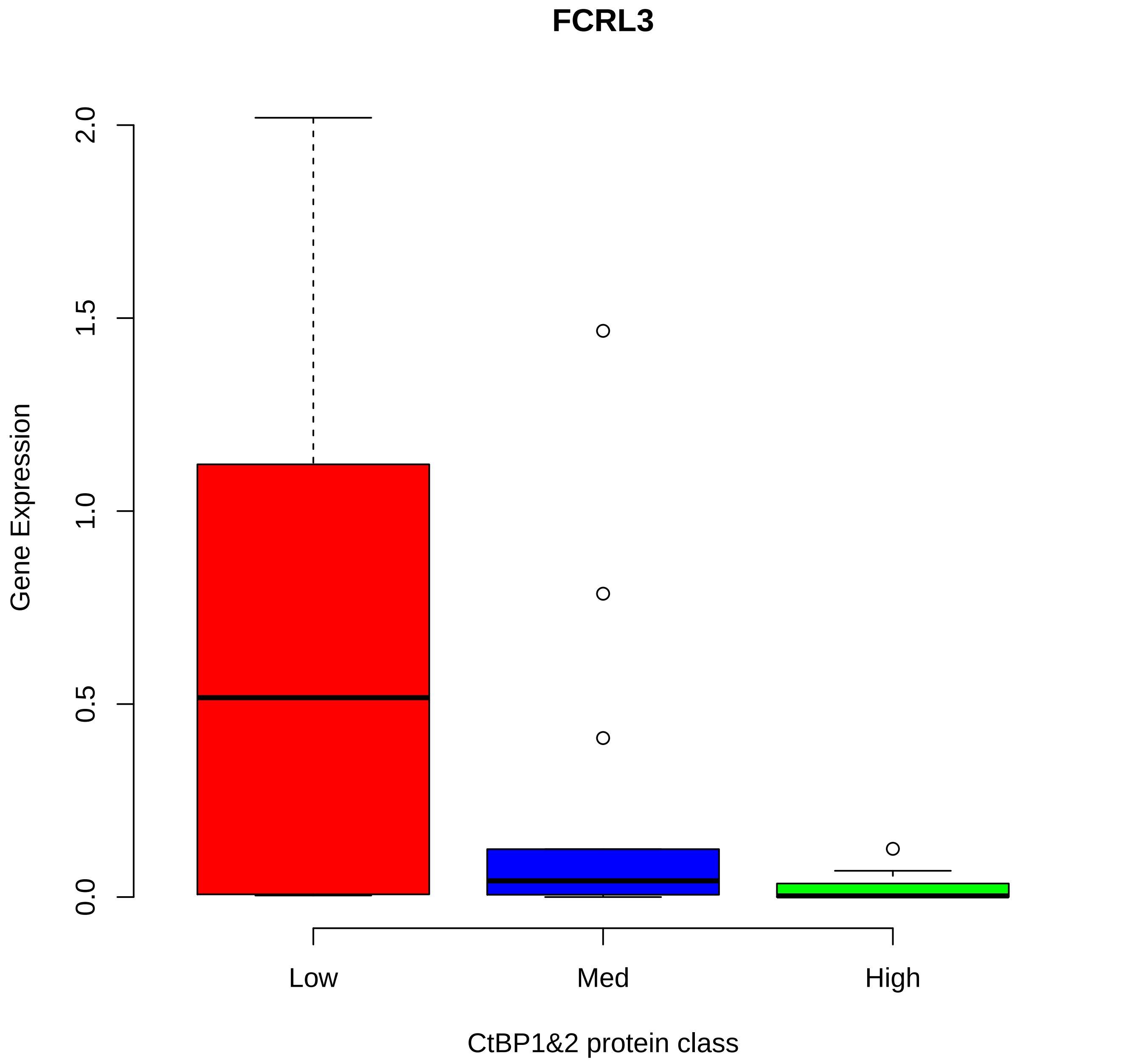

FCRL3
Gene Expression
0.0
0.5
1.0
1.5
2.0
Low
Med
High
CtBP1&2 protein class

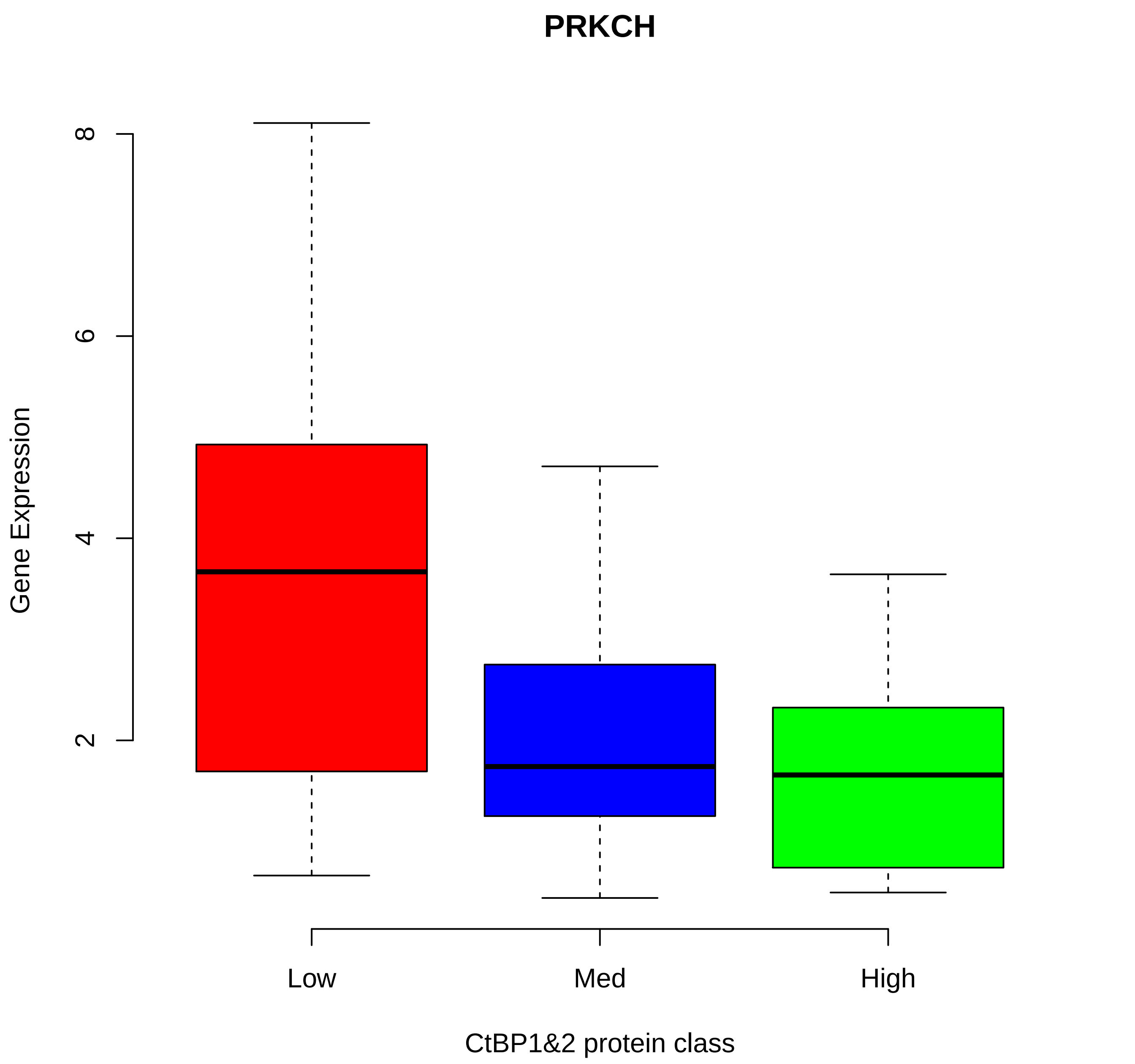

PRKCH
Gene Expression
8
6
4
2
Low
Med
High
CtBP1&2 protein class

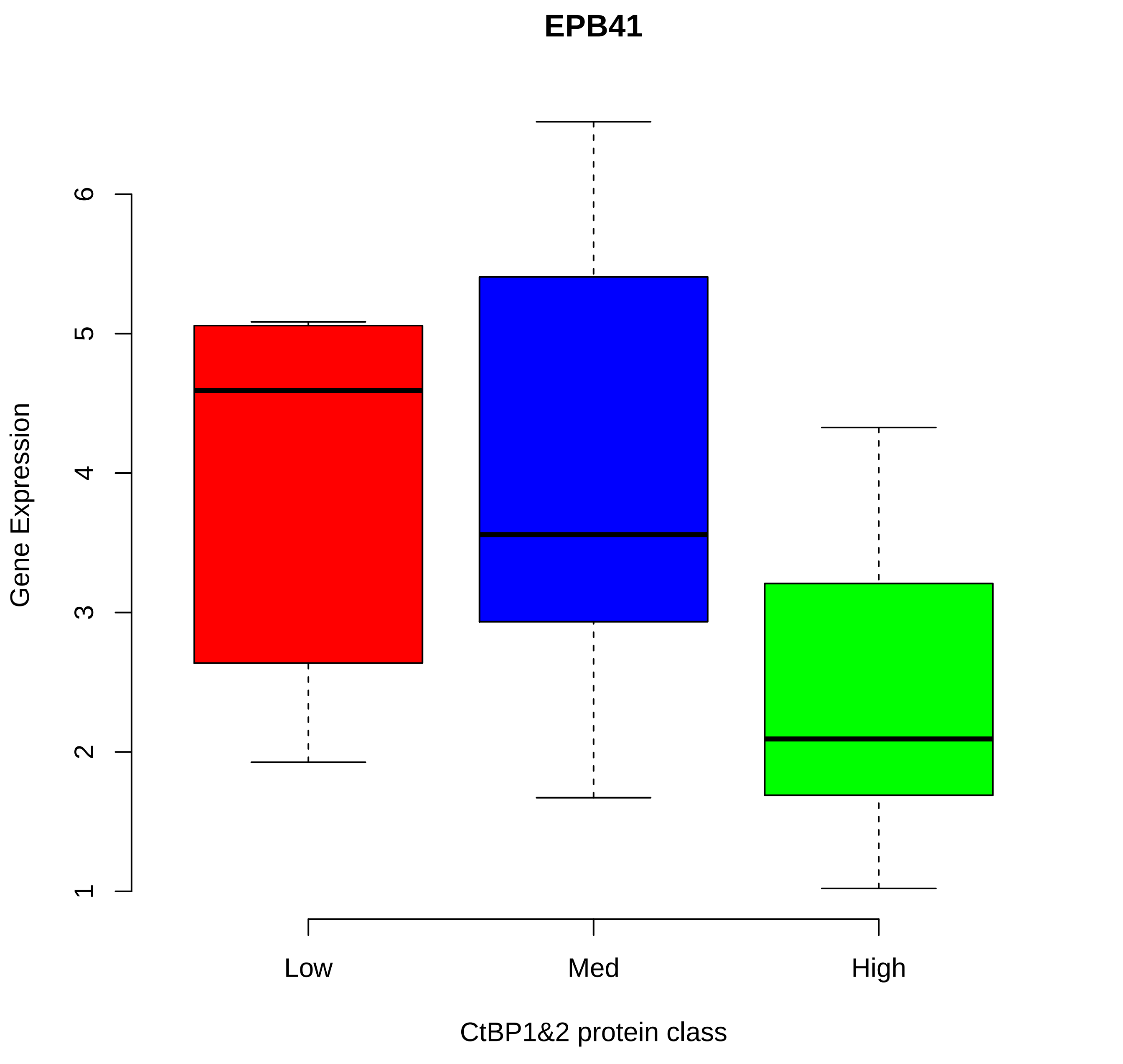

EPB41
Gene Expression
1
2
3
4
5
6
Low
Med
High
CtBP1&2 protein class

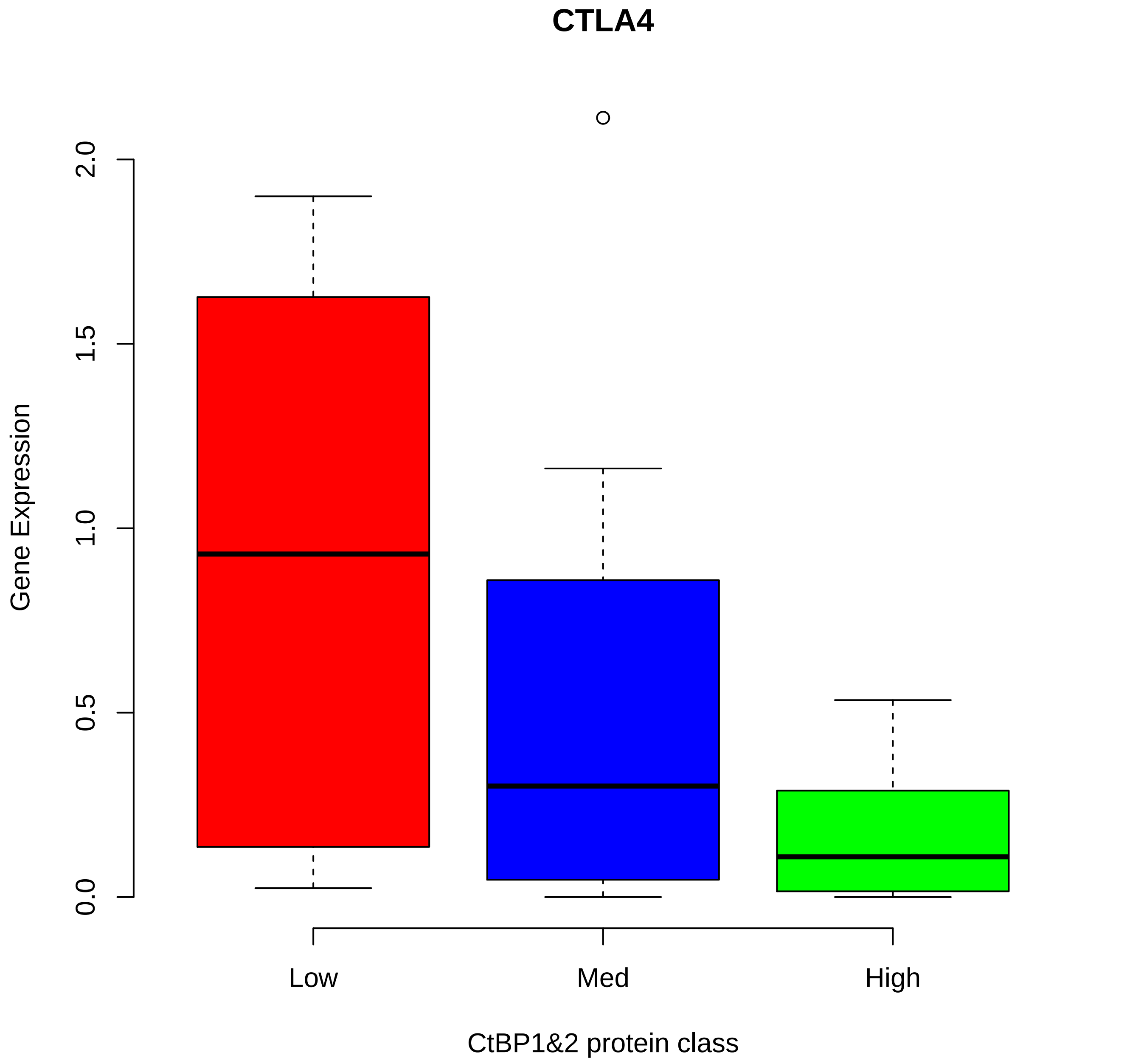

CTLA4
Gene Expression
0.0
0.5
1.0
1.5
2.0
Low
Med
High
CtBP1&2 protein class

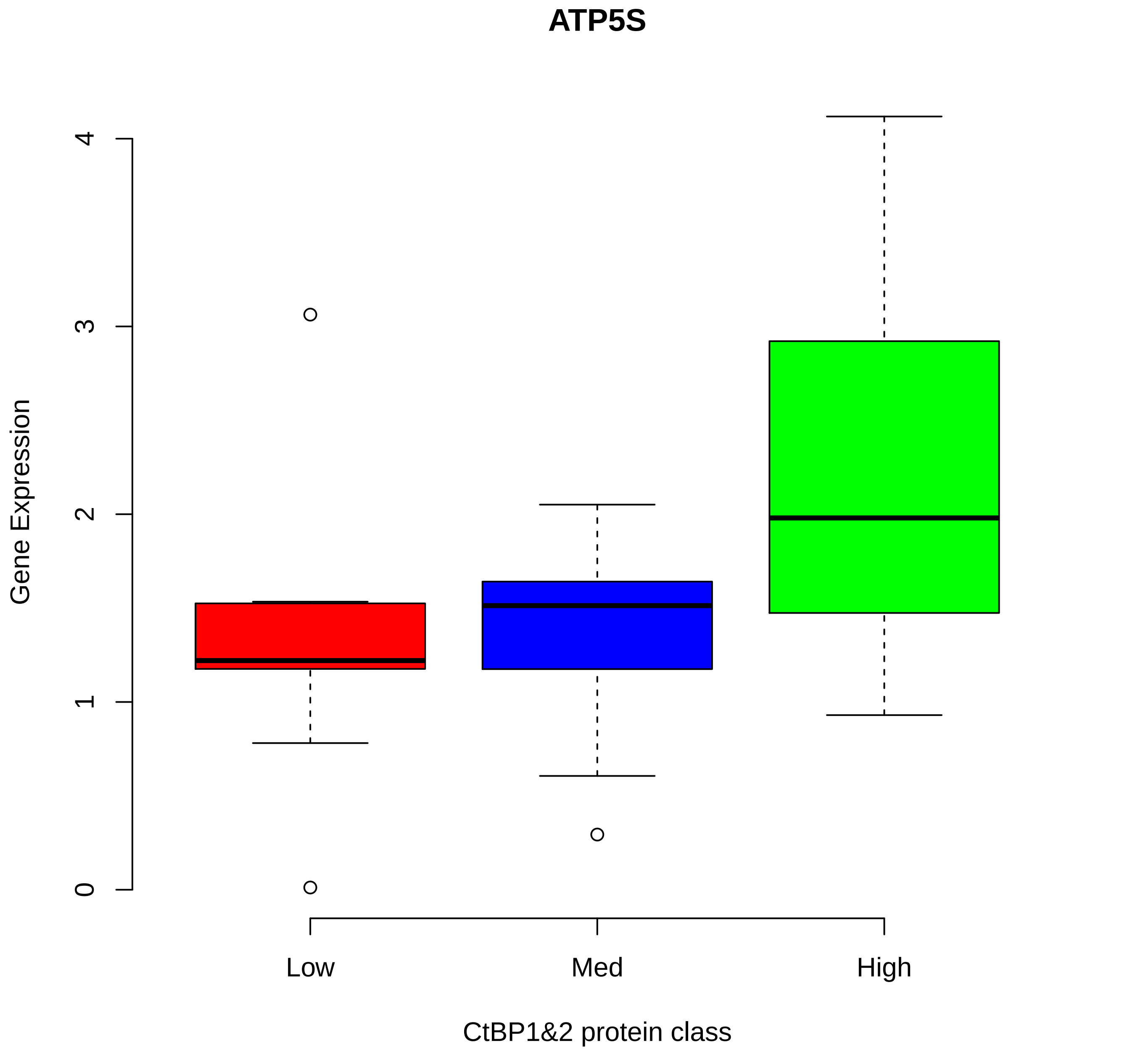

ATP5S
Gene Expression
0
1
2
3
4
Low
Med
High
CtBP1&2 protein class

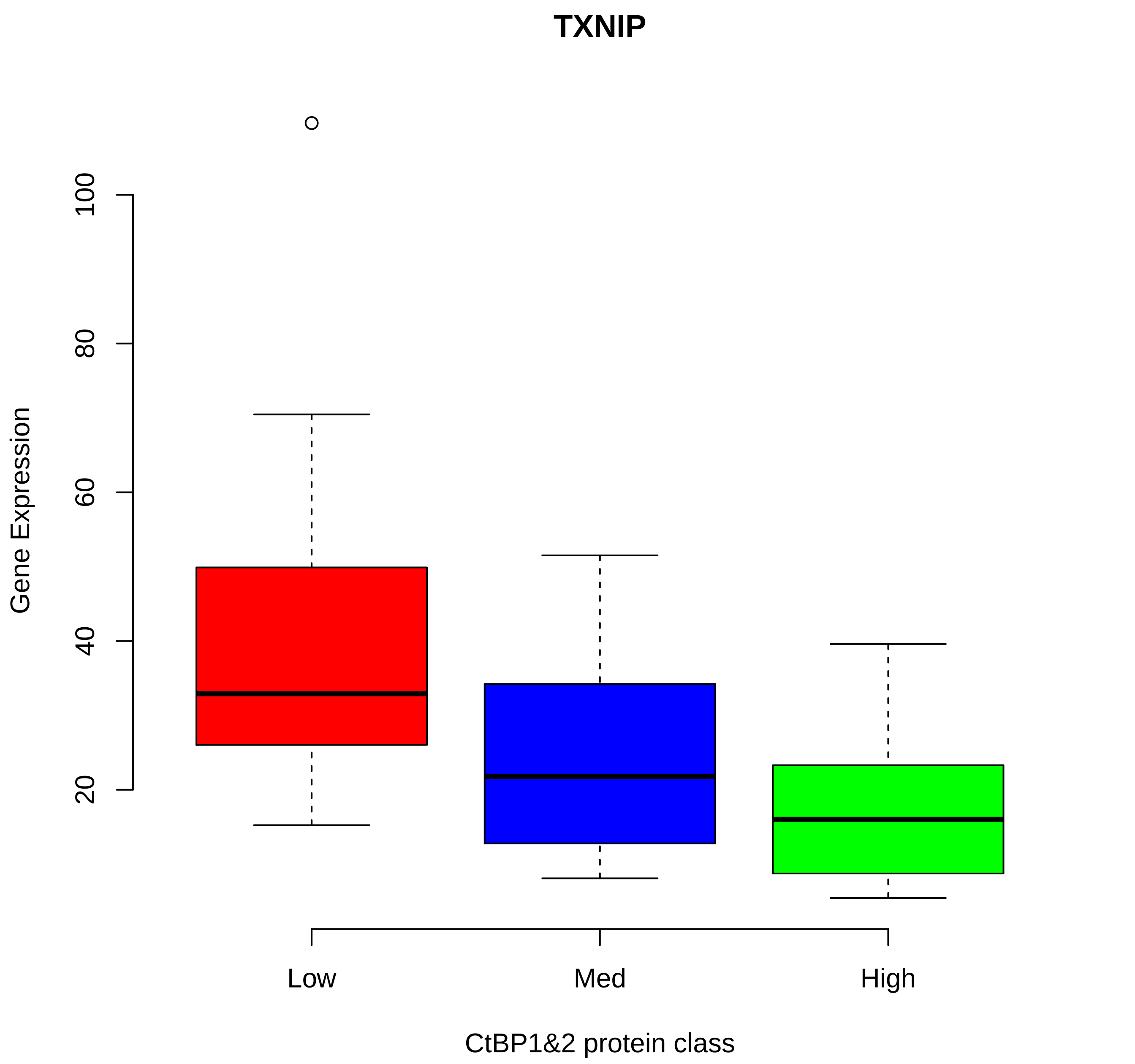
TXNIP
Gene Expression
20
40
60
80
100
Low
Med
High
CtBP1&2 protein class

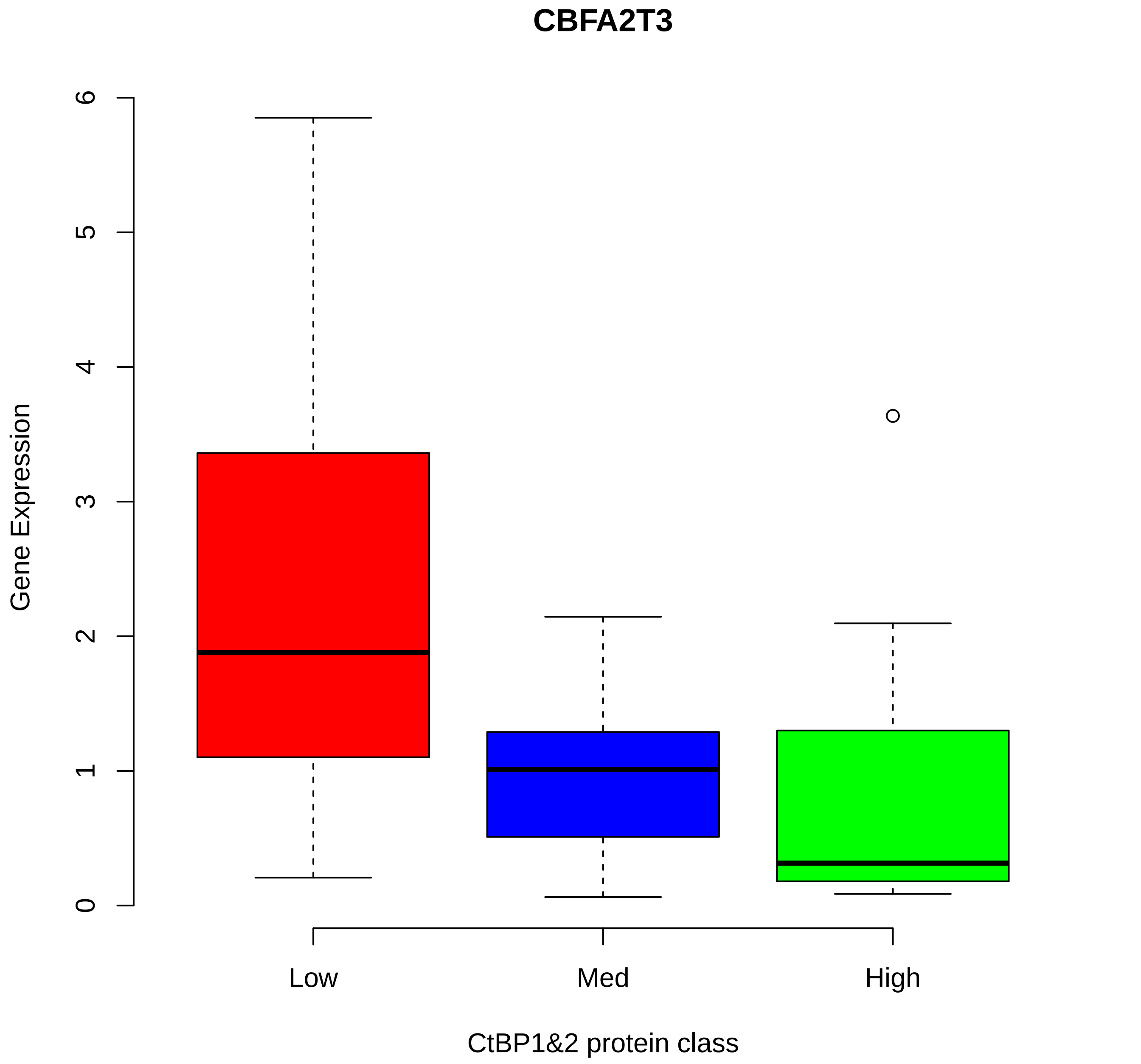
CBFA2T3
Gene Expression
0
1
2
3
4
5
6
Low
Med
High
CtBP1&2 protein class

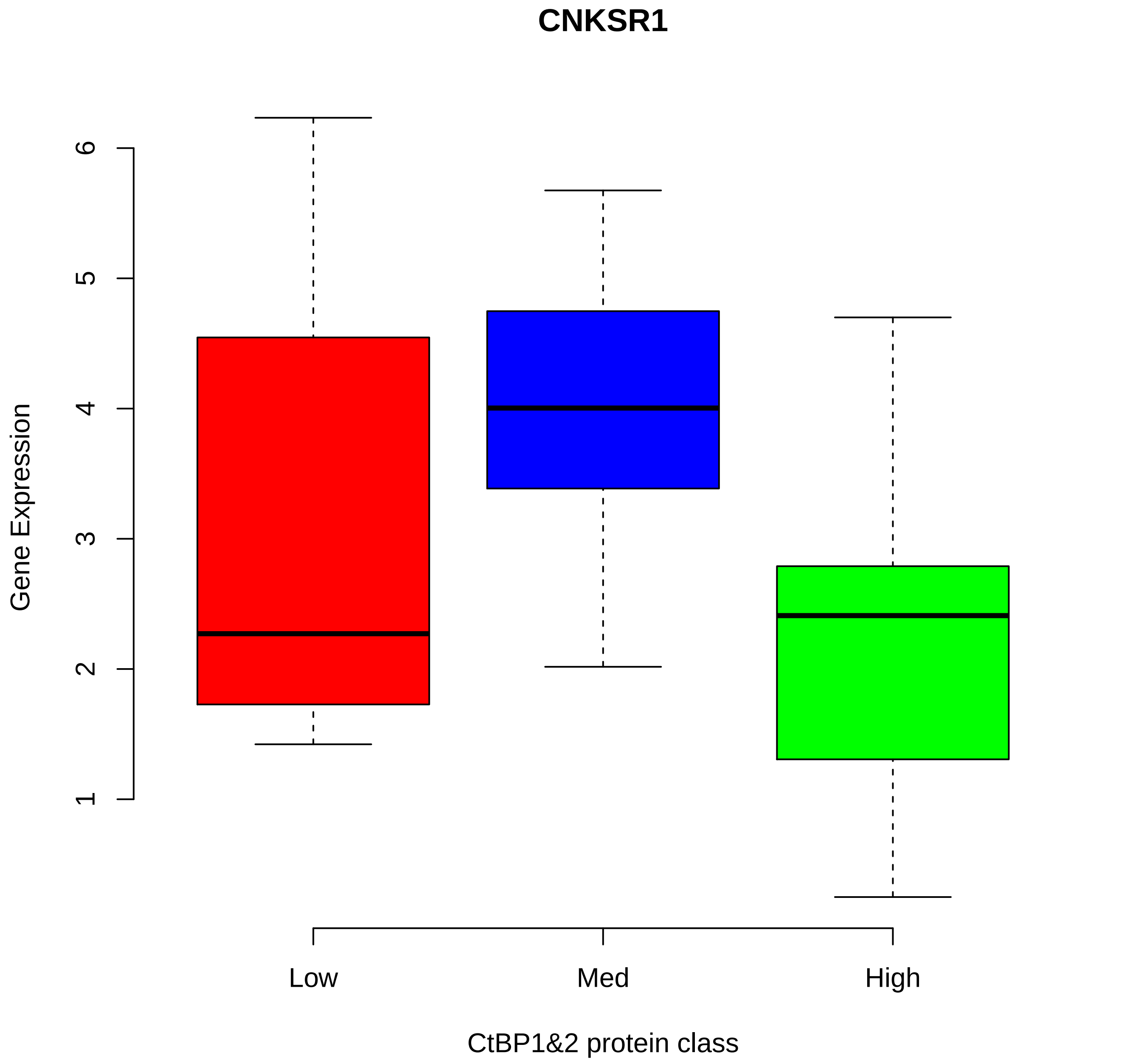

CNKSR1
Gene Expression
1
2
3
4
5
6
Low
Med
High
CtBP1&2 protein class

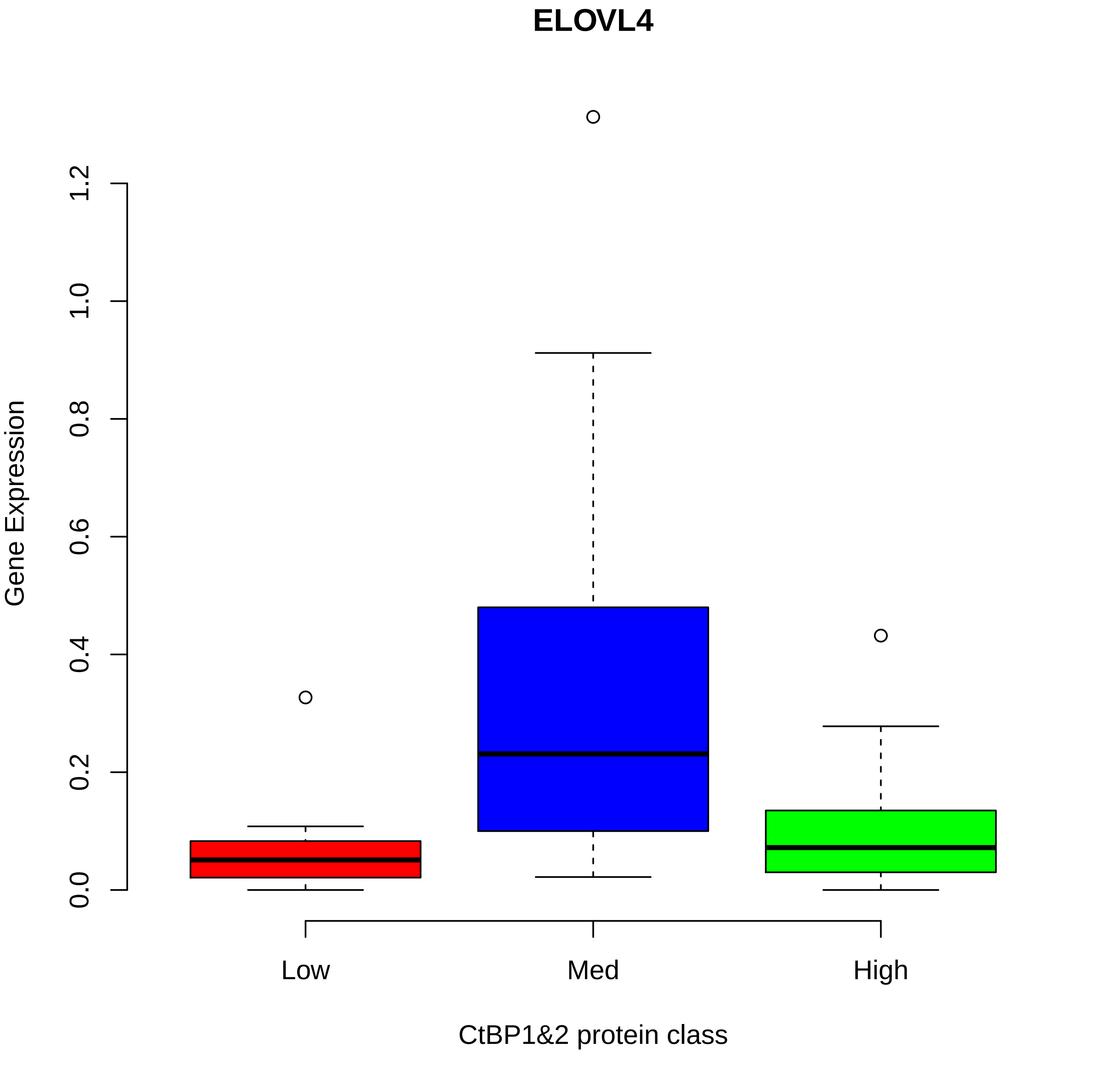
ELOVL4
Gene Expression
0.0
0.2
0.4
0.6
0.8
1.0
1.2
Low
Med
High
CtBP1&2 protein class

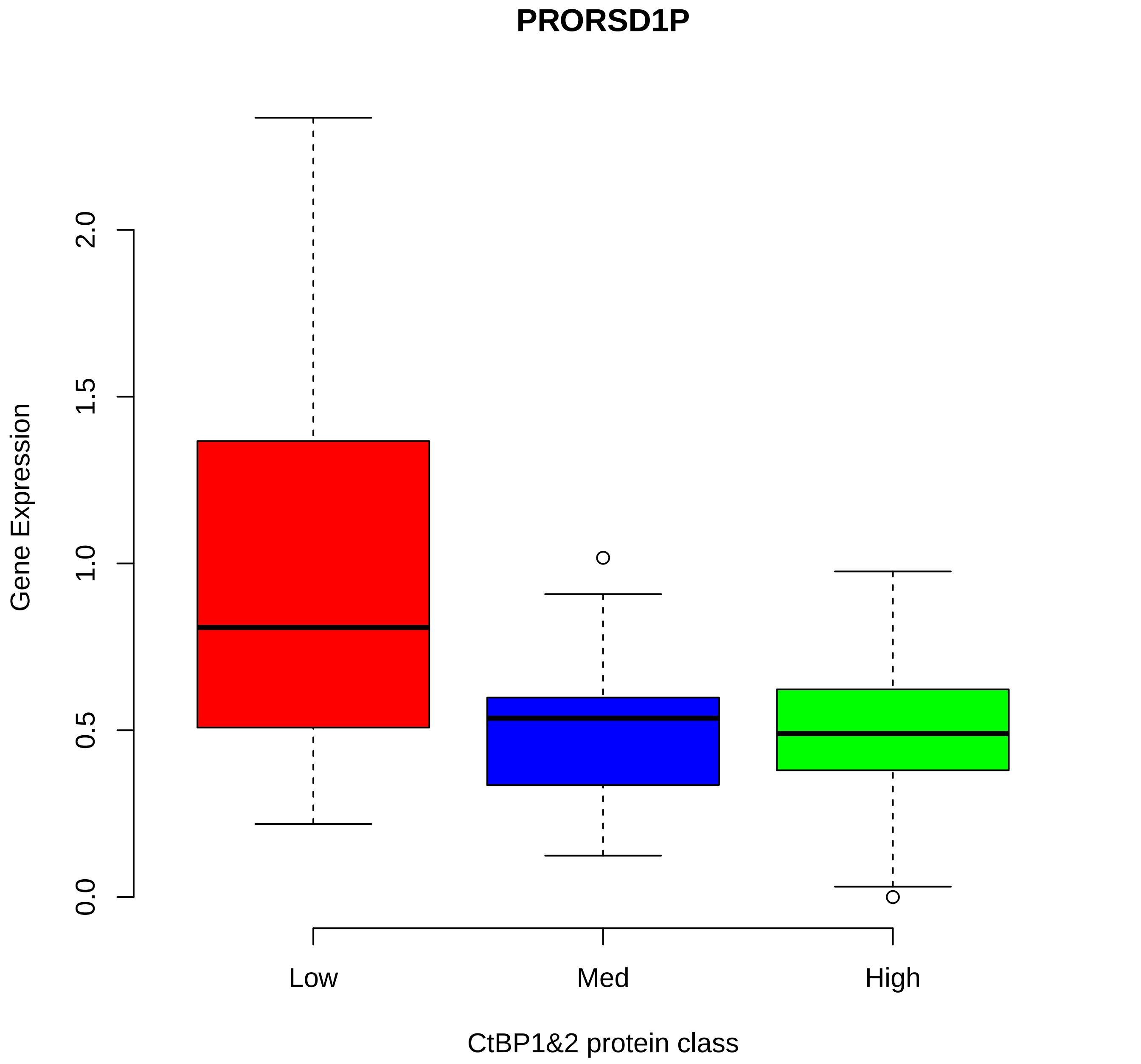
PRORSD1P
Gene Expression
0.0
0.5
1.0
1.5
2.0
Low
Med
High
CtBP1&2 protein class

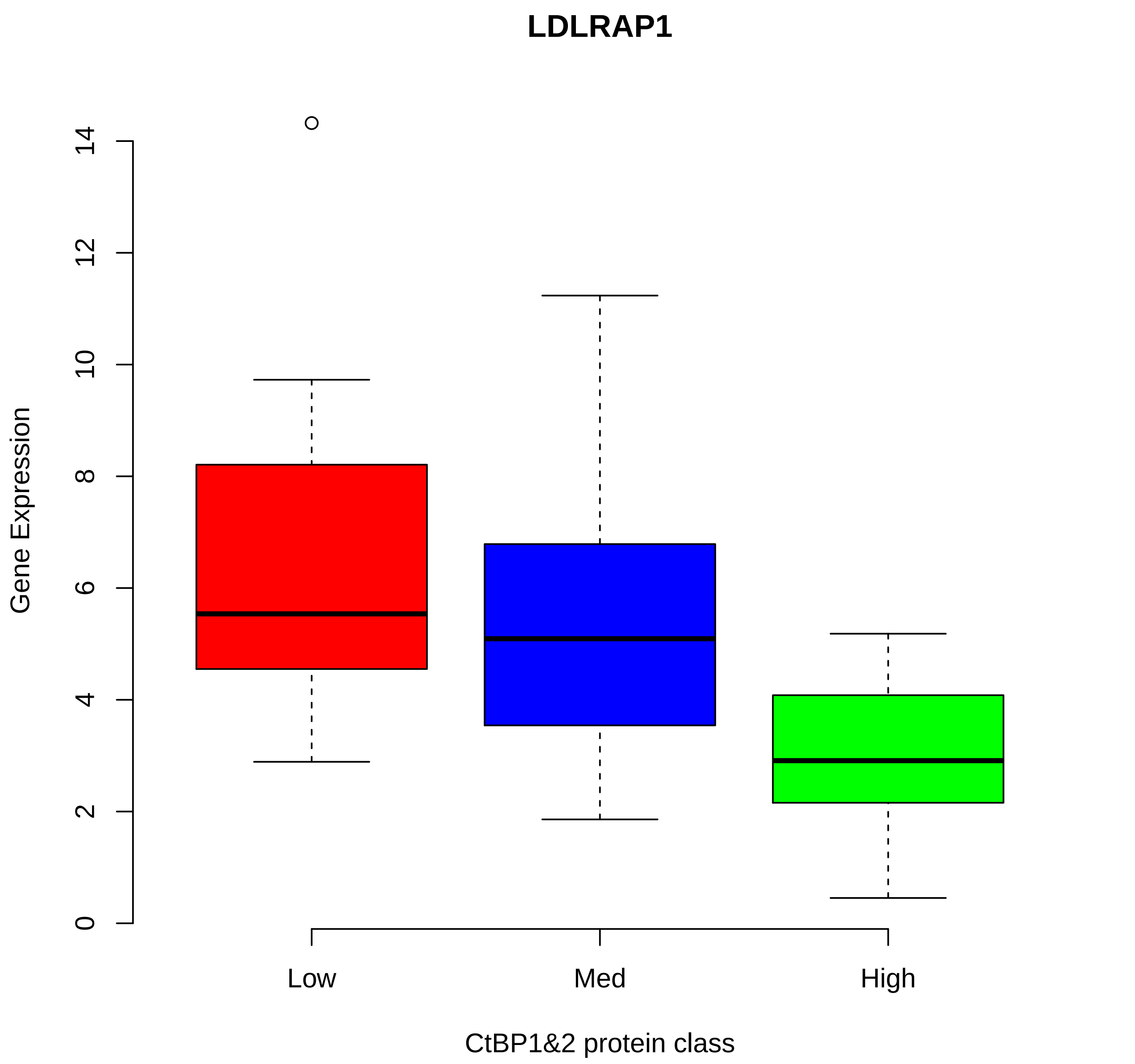

LDLRAP1
Gene Expression
0
2
4
6
8
10
12
14
Low
Med
High
CtBP1&2 protein class

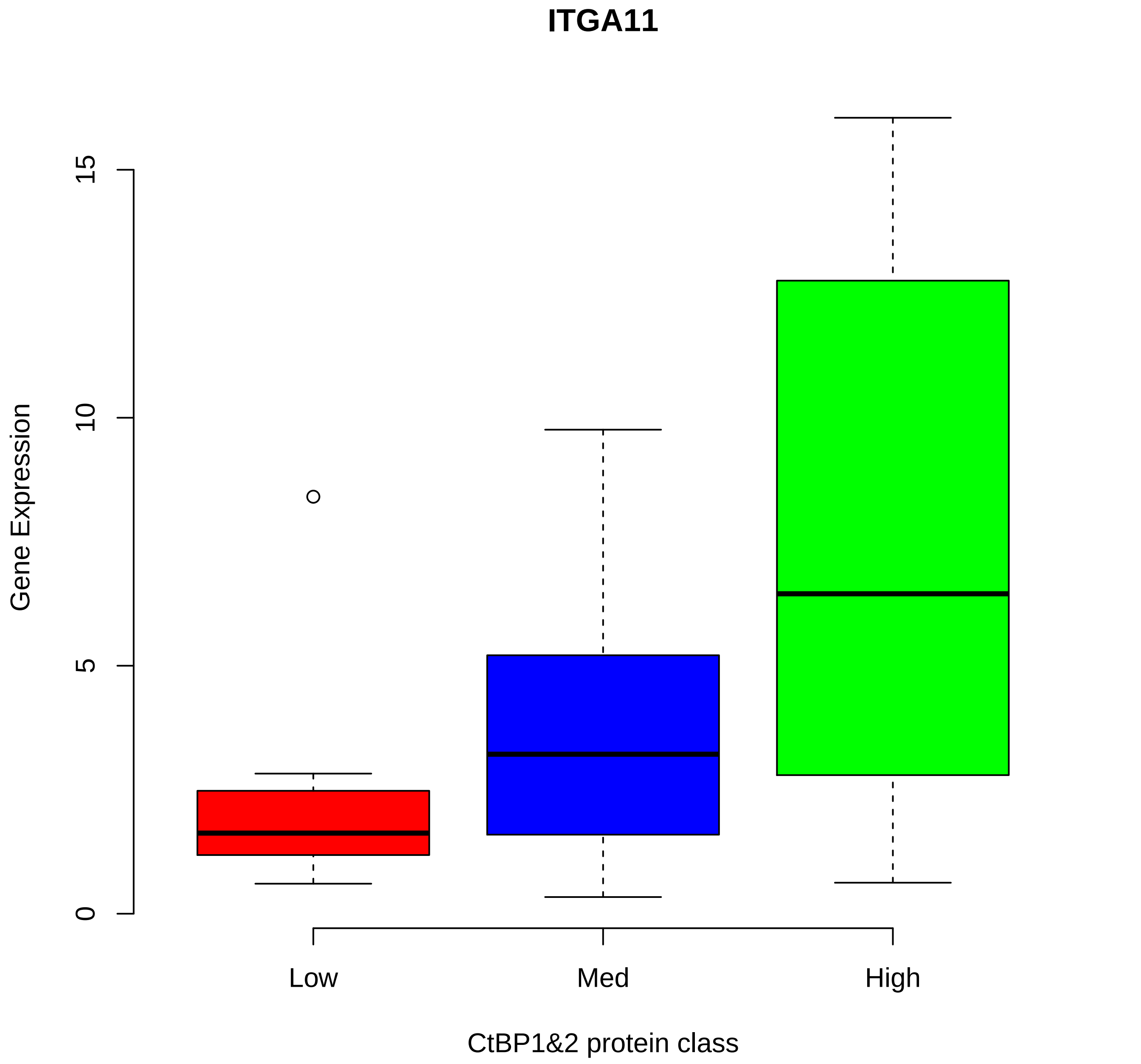
ITGA11
Gene Expression
0
5
10
15
Low
Med
High
CtBP1&2 protein class

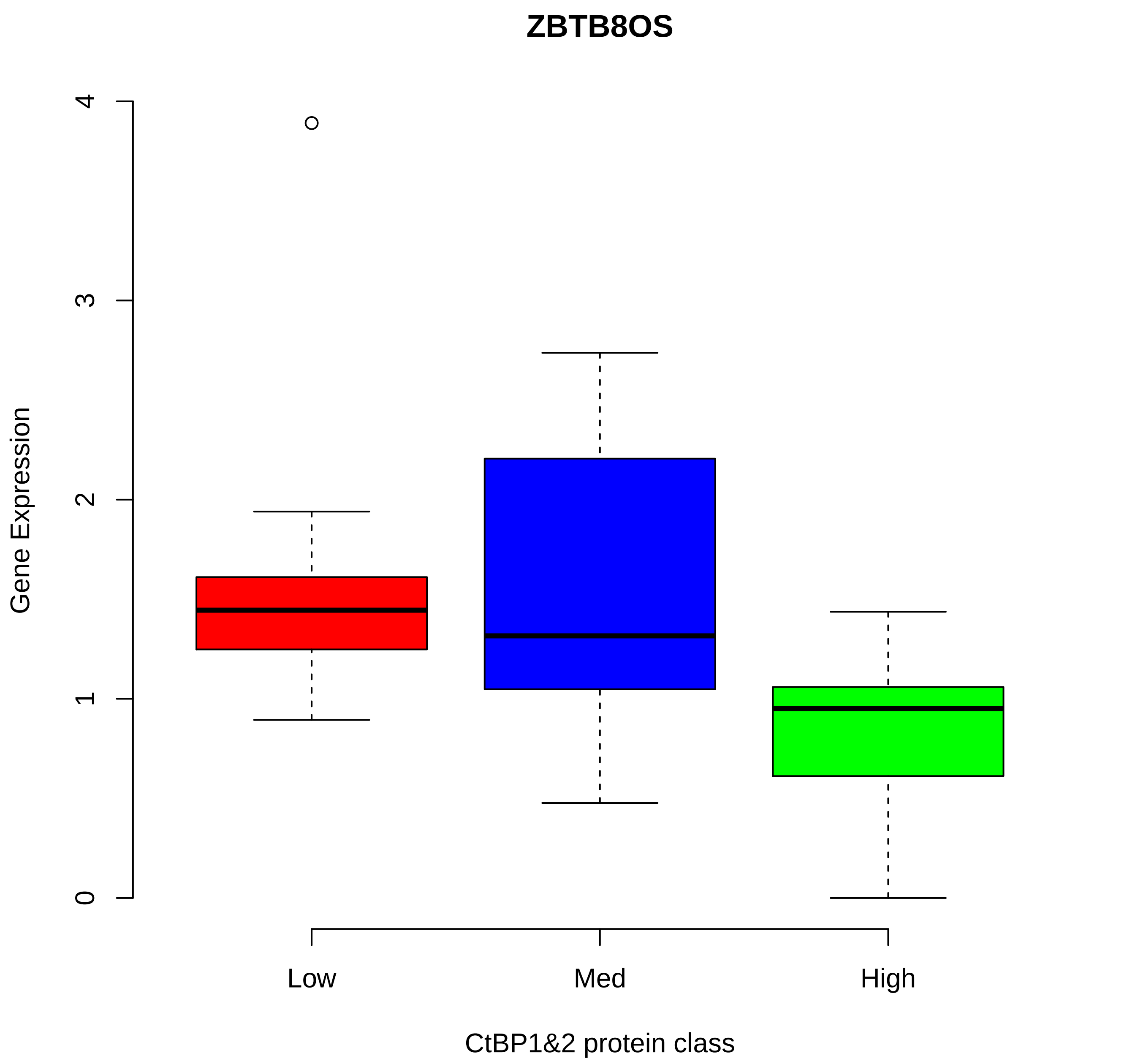

ZBTB8OS
Gene Expression
0
1
2
3
4
Low
Med
High
CtBP1&2 protein class

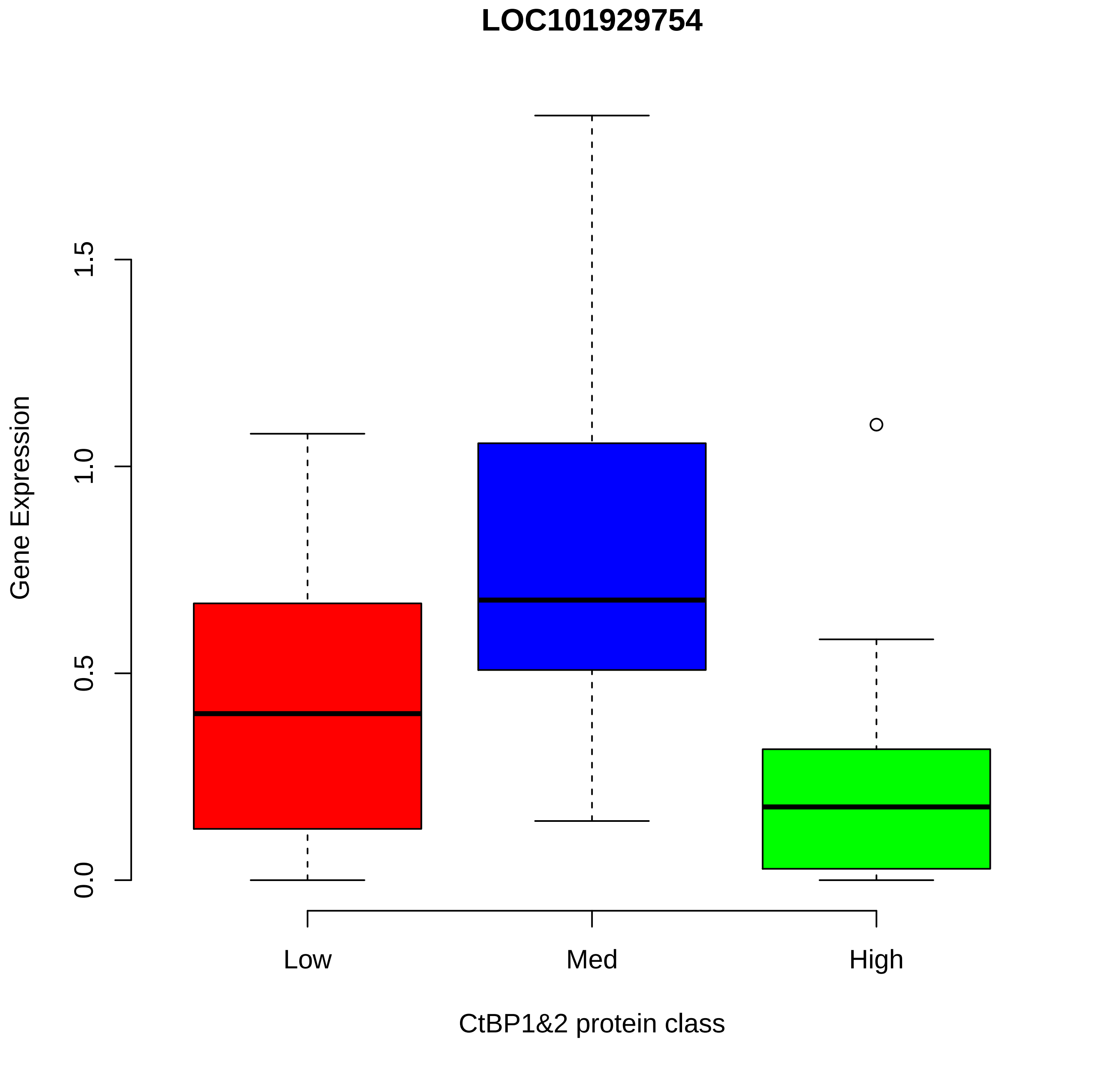
LOC101929754
Gene Expression
0.0
0.5
1.0
1.5
Low
Med
High
CtBP1&2 protein class

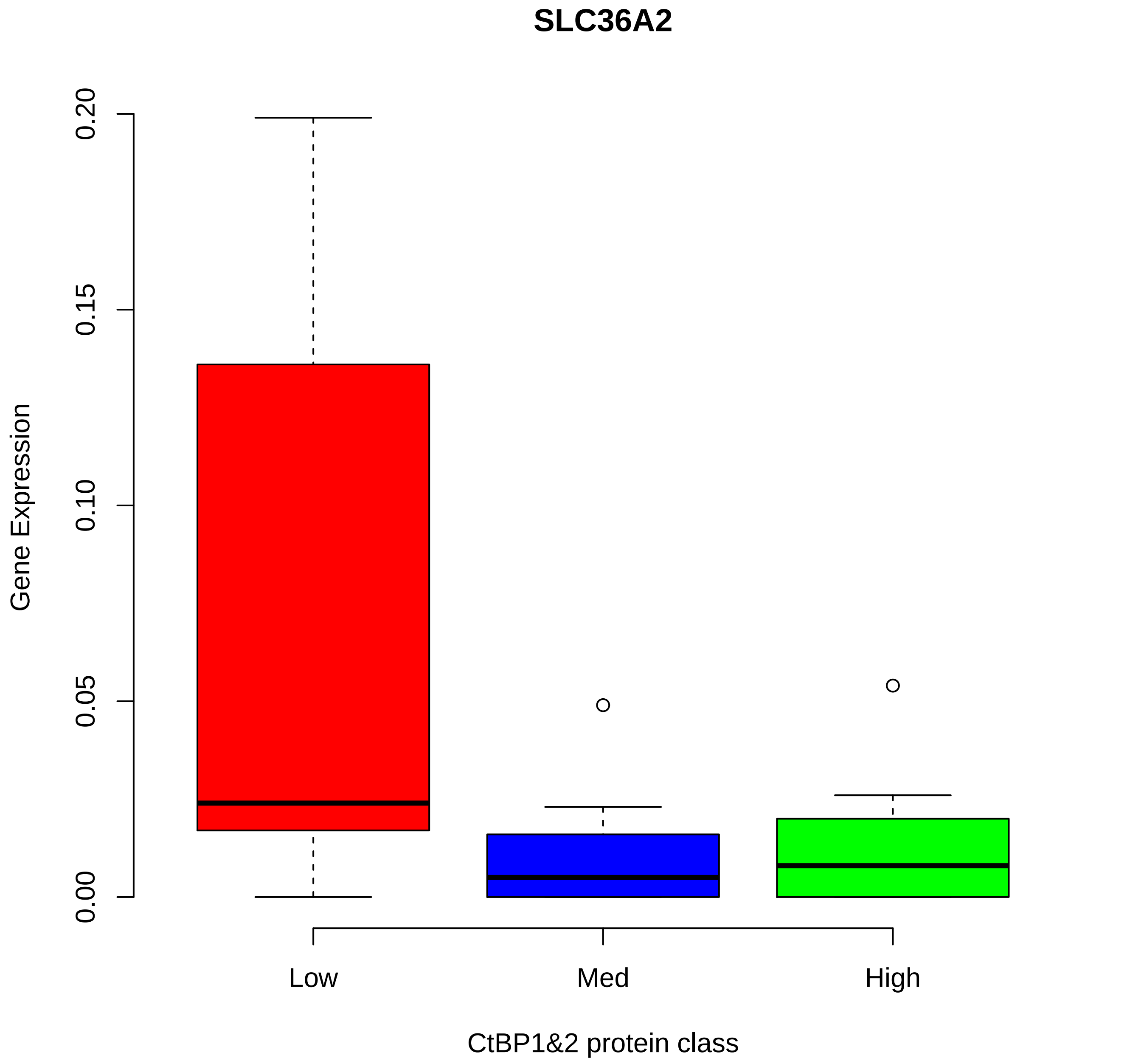

SLC36A2
Gene Expression
0.00
0.05
0.10
0.15
0.20
Low
Med
High
CtBP1&2 protein class

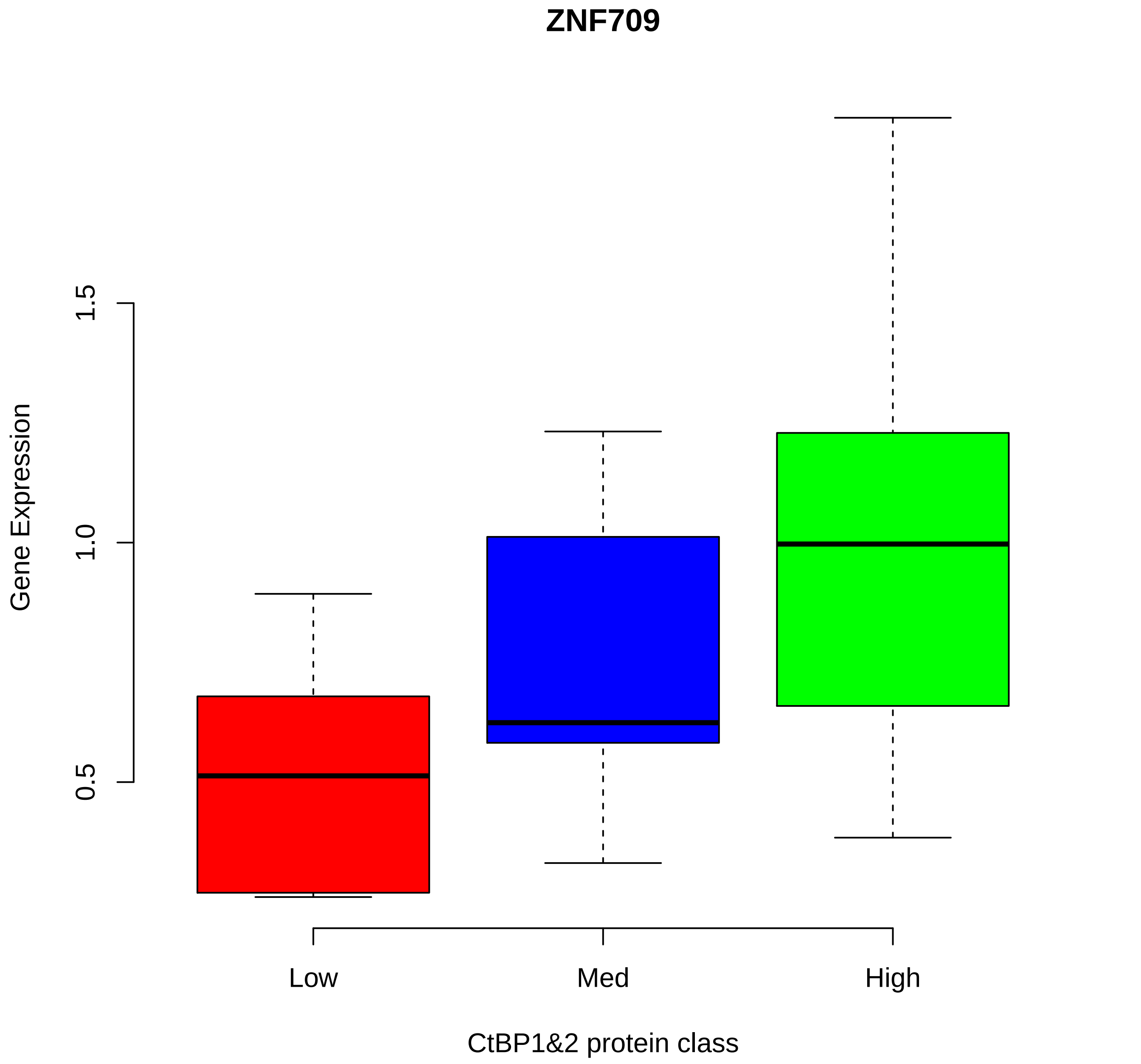
ZNF709
Gene Expression
1.5
1.0
0.5
Low
Med
High
CtBP1&2 protein class

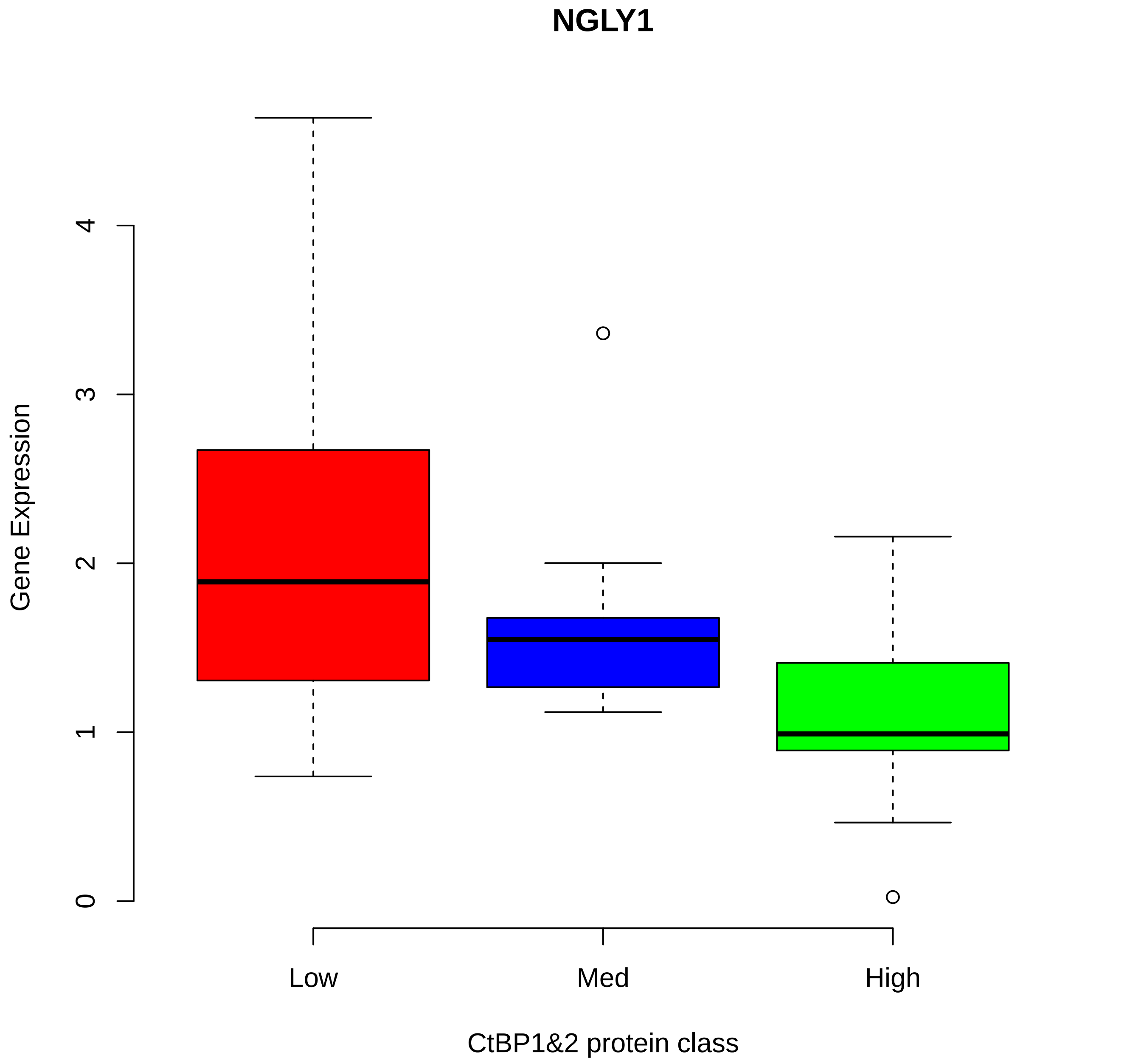

NGLY1
Gene Expression
0
1
2
3
4
Low
Med
High
CtBP1&2 protein class

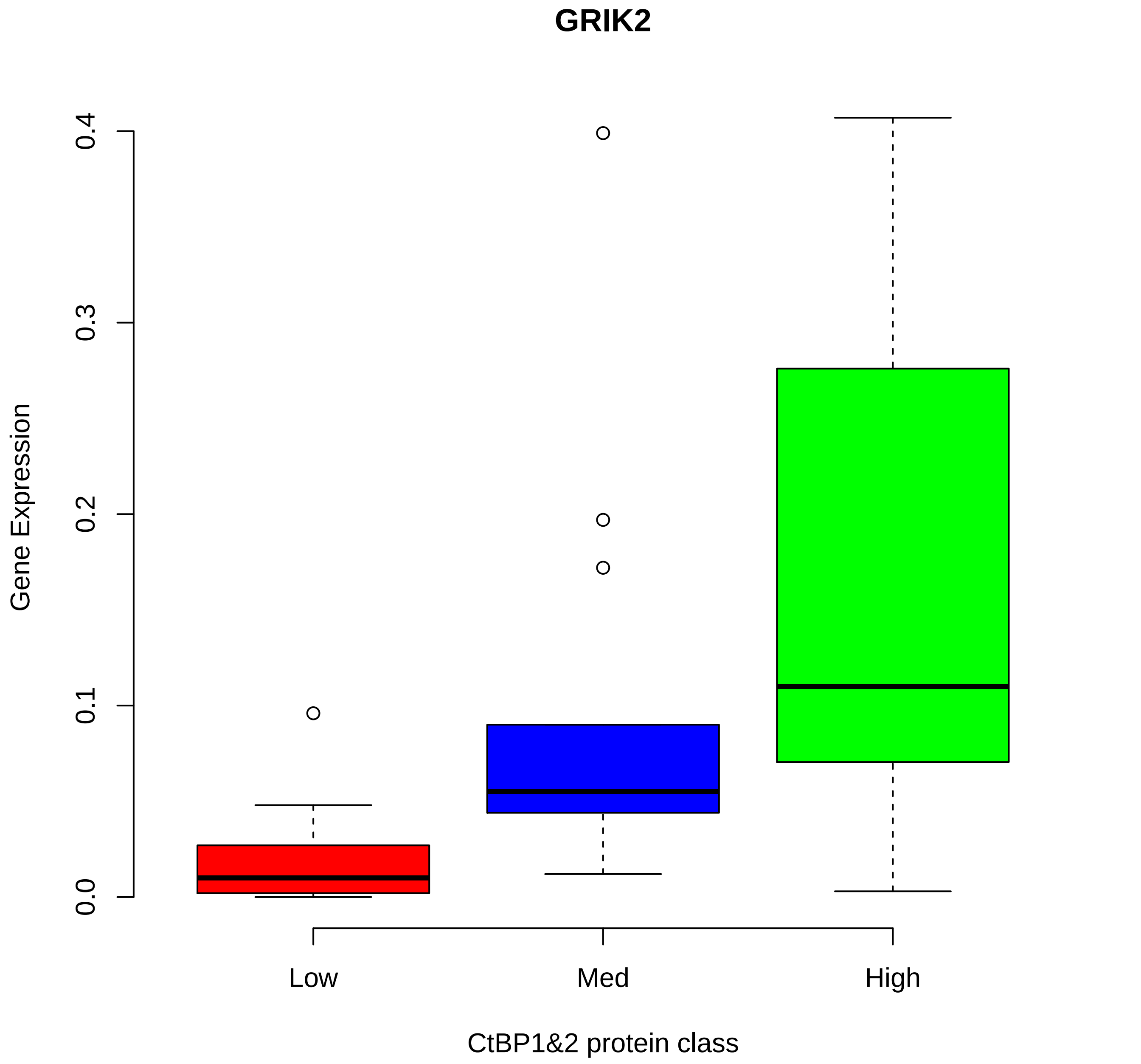

GRIK2
Gene Expression
0.0
0.1
0.2
0.3
0.4
Low
Med
High
CtBP1&2 protein class

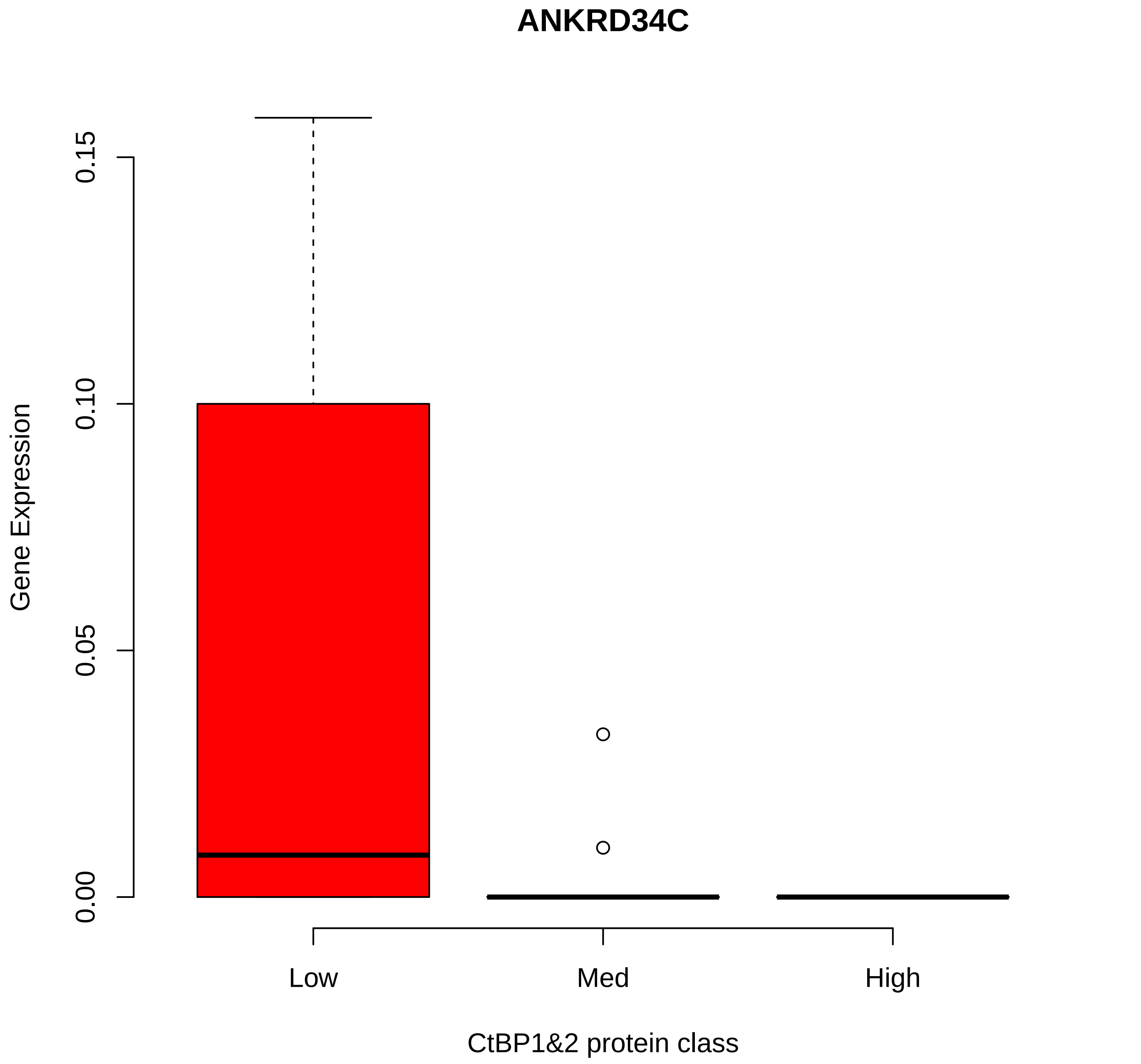
ANKRD34C
Gene Expression
0.00
0.05
0.10
0.15
Low
Med
High
CtBP1&2 protein class

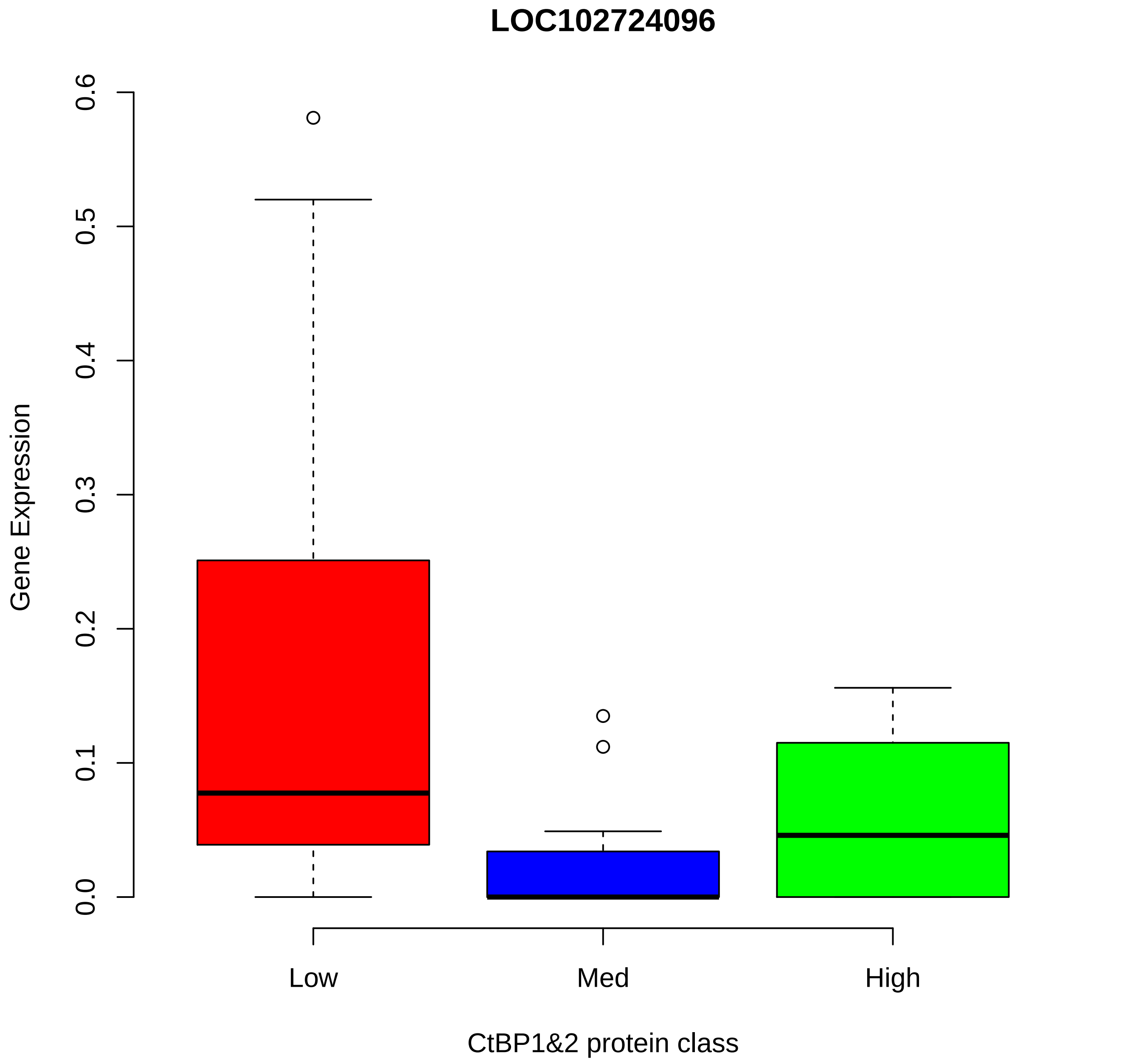
LOC102724096
Gene Expression
0.0
0.1
0.2
0.3
0.4
0.5
0.6
Low
Med
High
CtBP1&2 protein class

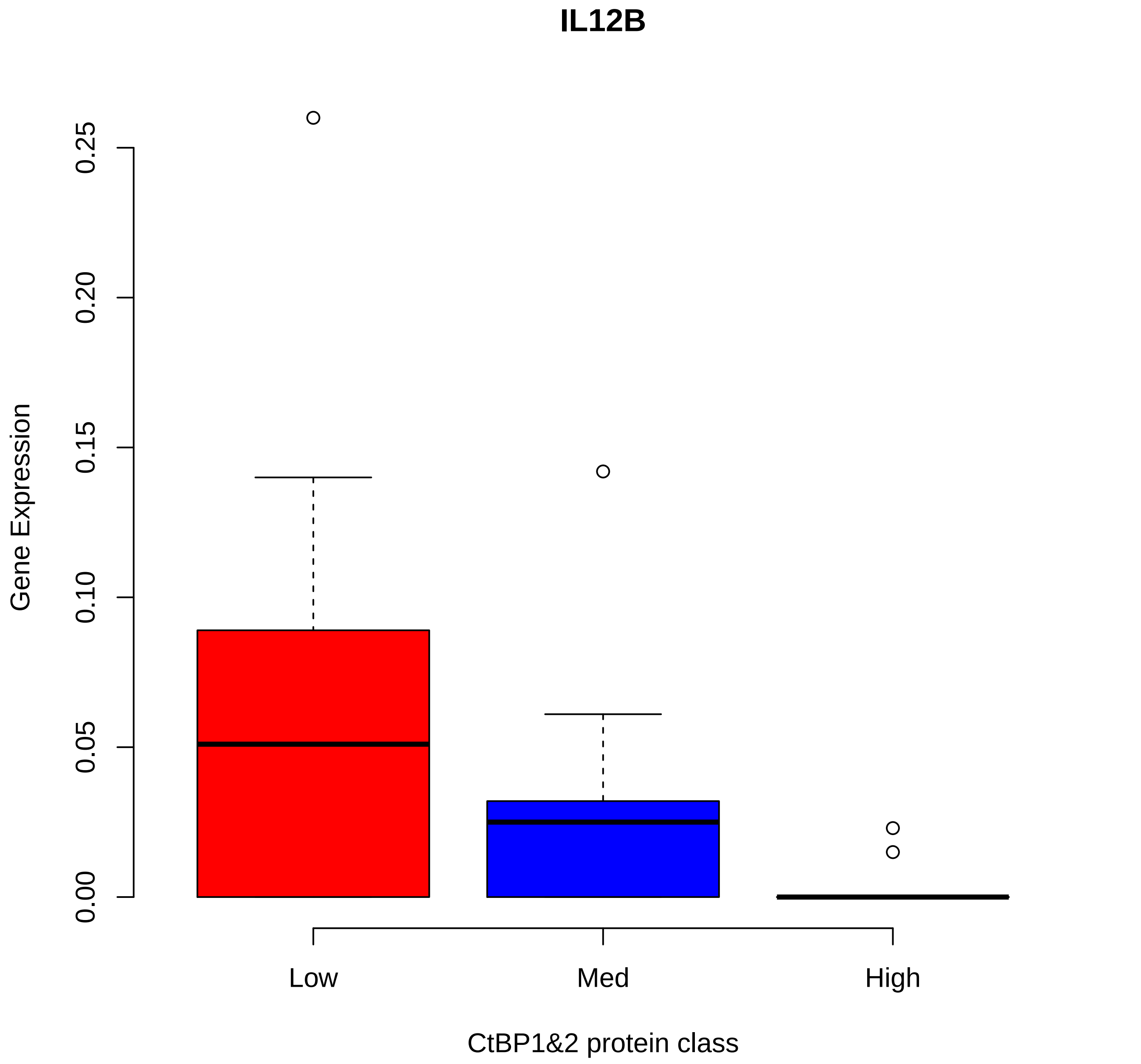

IL12B
Gene Expression
0.00
0.05
0.10
0.15
0.20
0.25
Low
Med
High
CtBP1&2 protein class

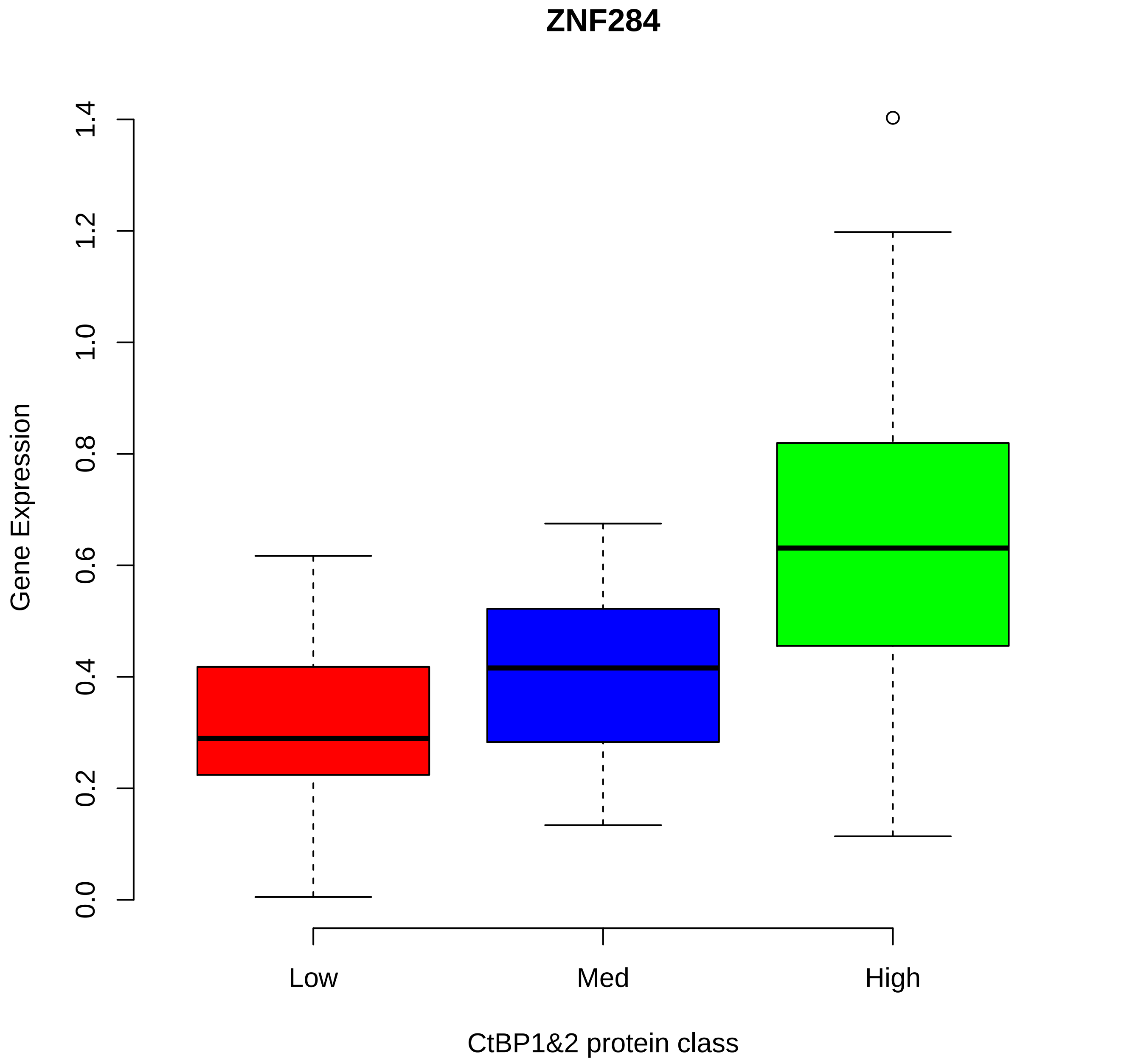
ZNF284
Gene Expression
0.0
0.2
0.4
0.6
0.8
1.0
1.2
1.4
Low
Med
High
CtBP1&2 protein class

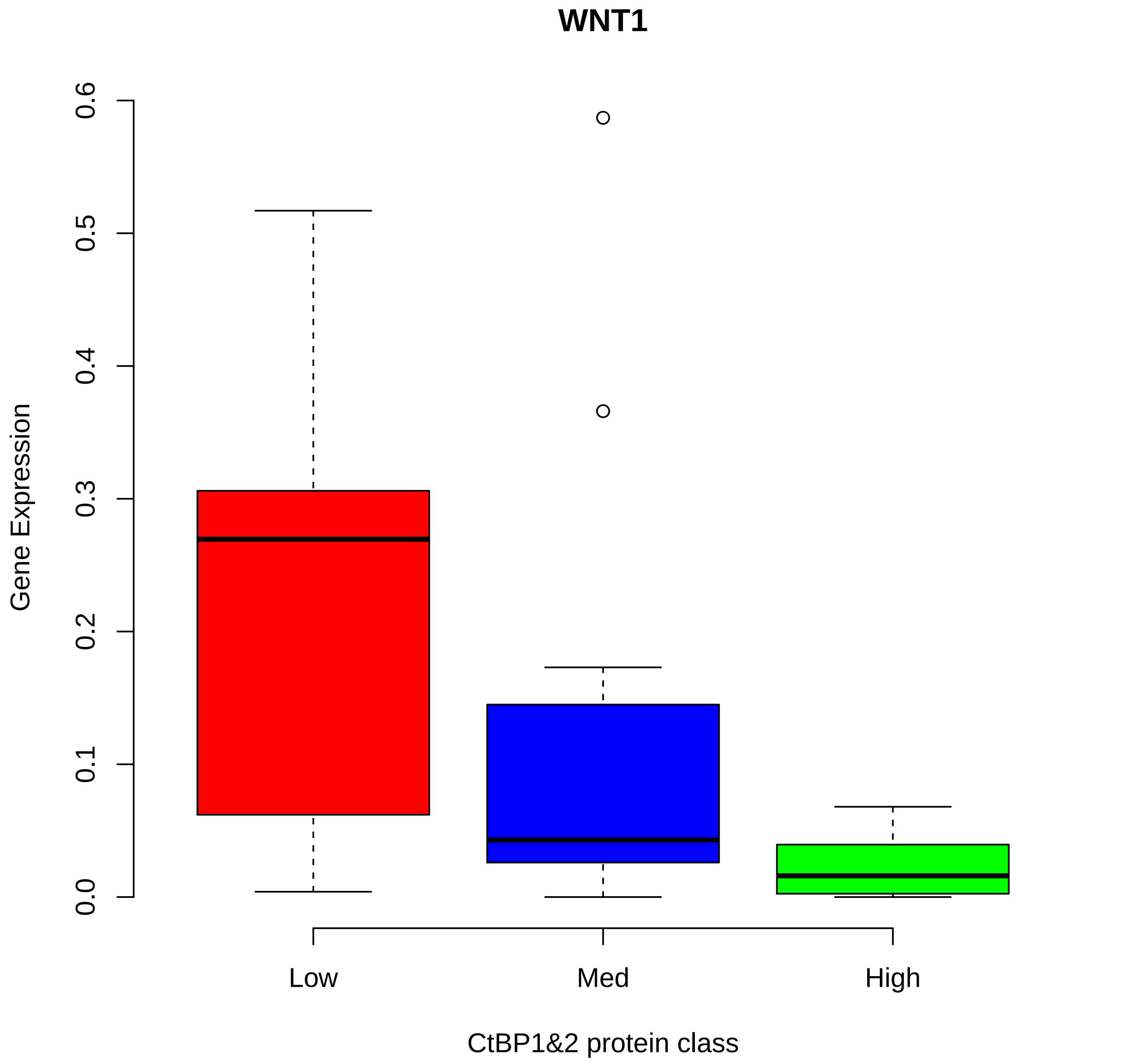
WNT1
Gene Expression
0.0
0.1
0.2
0.3
0.4
0.5
0.6
Low
Med
High
CtBP1&2 protein class

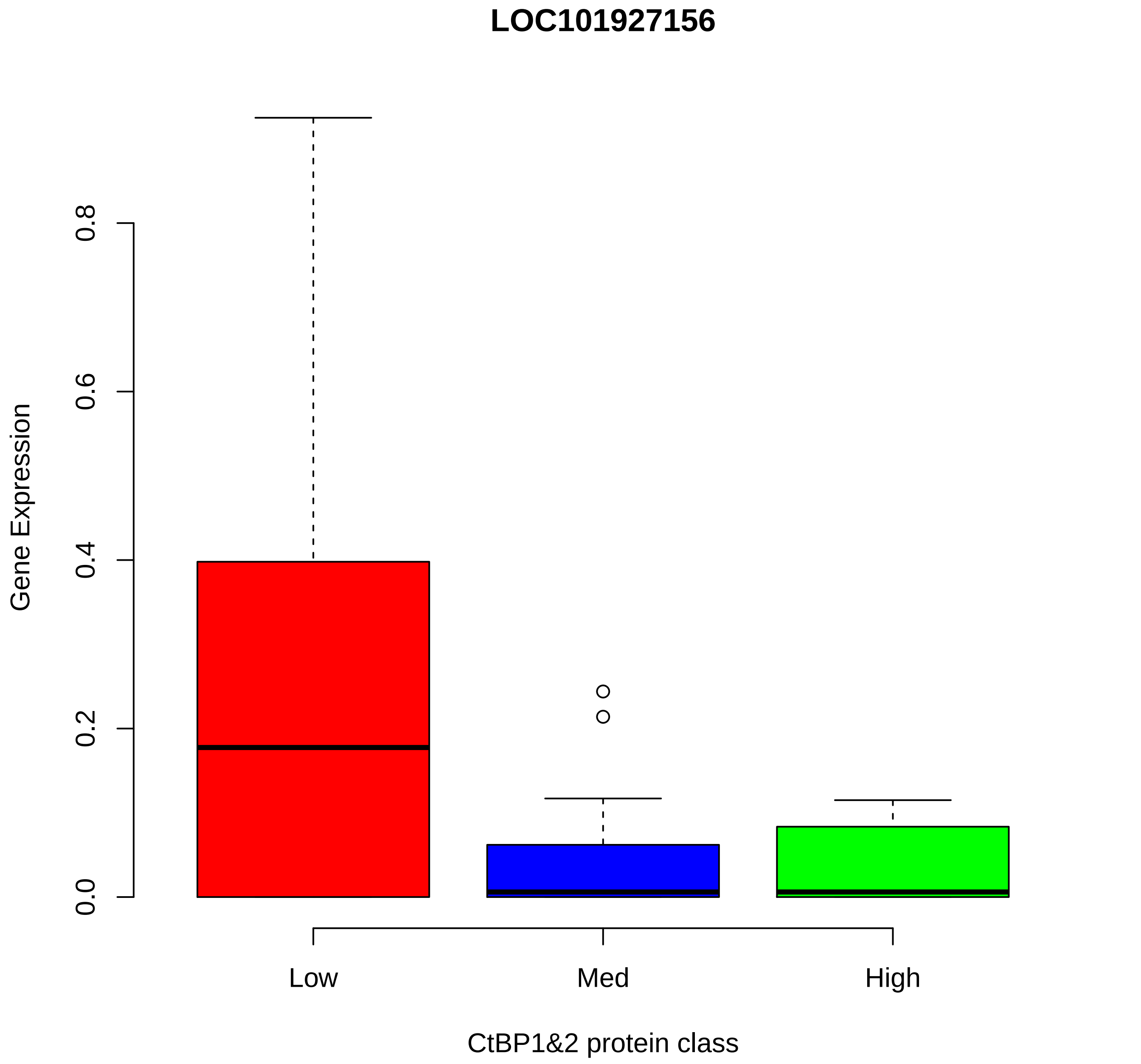

LOC101927156
Gene Expression
0.0
0.2
0.4
0.6
0.8
Low
Med
High
CtBP1&2 protein class

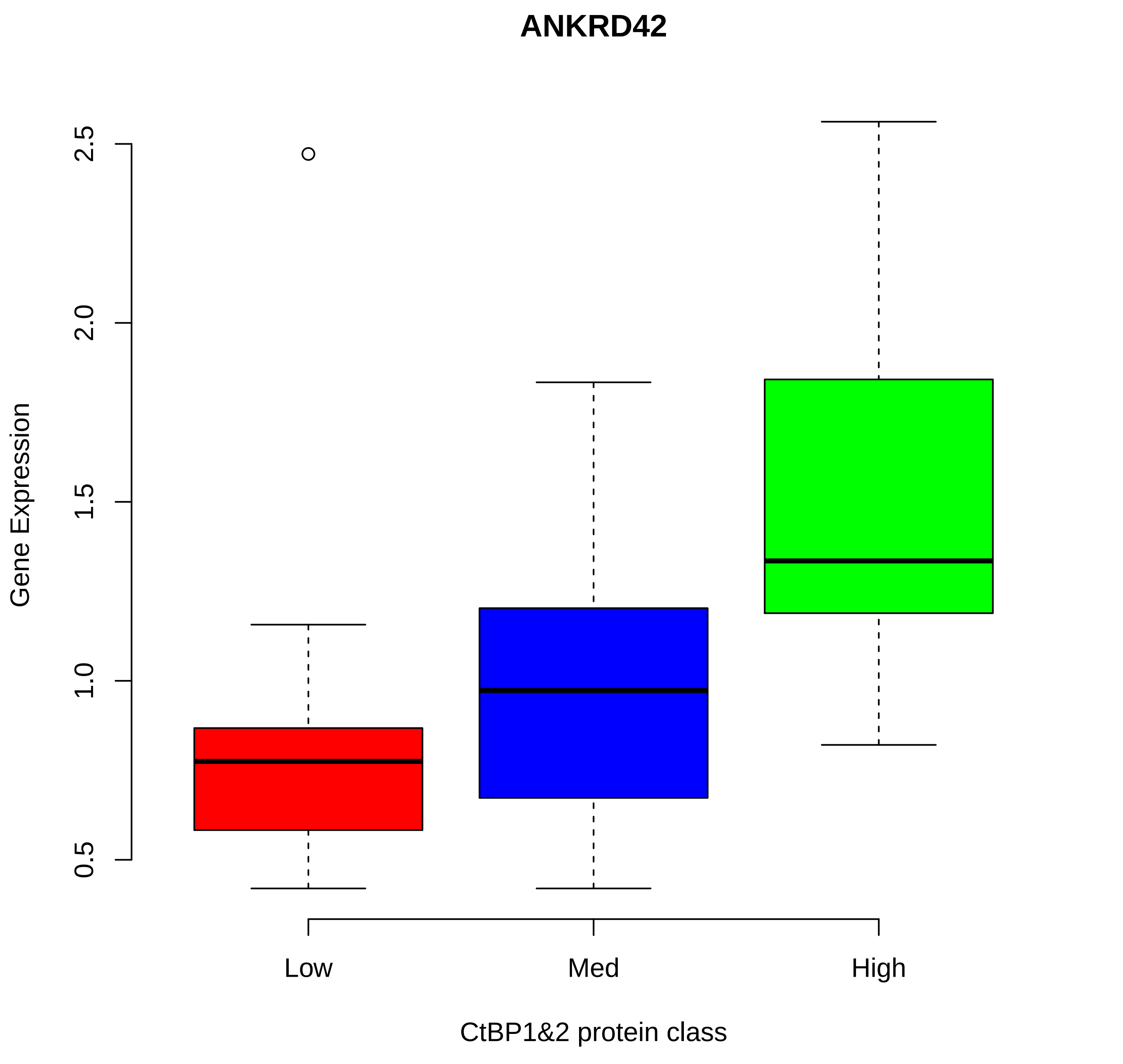
ANKRD42
Gene Expression
0.5
1.0
1.5
2.0
2.5
Low
Med
High
CtBP1&2 protein class

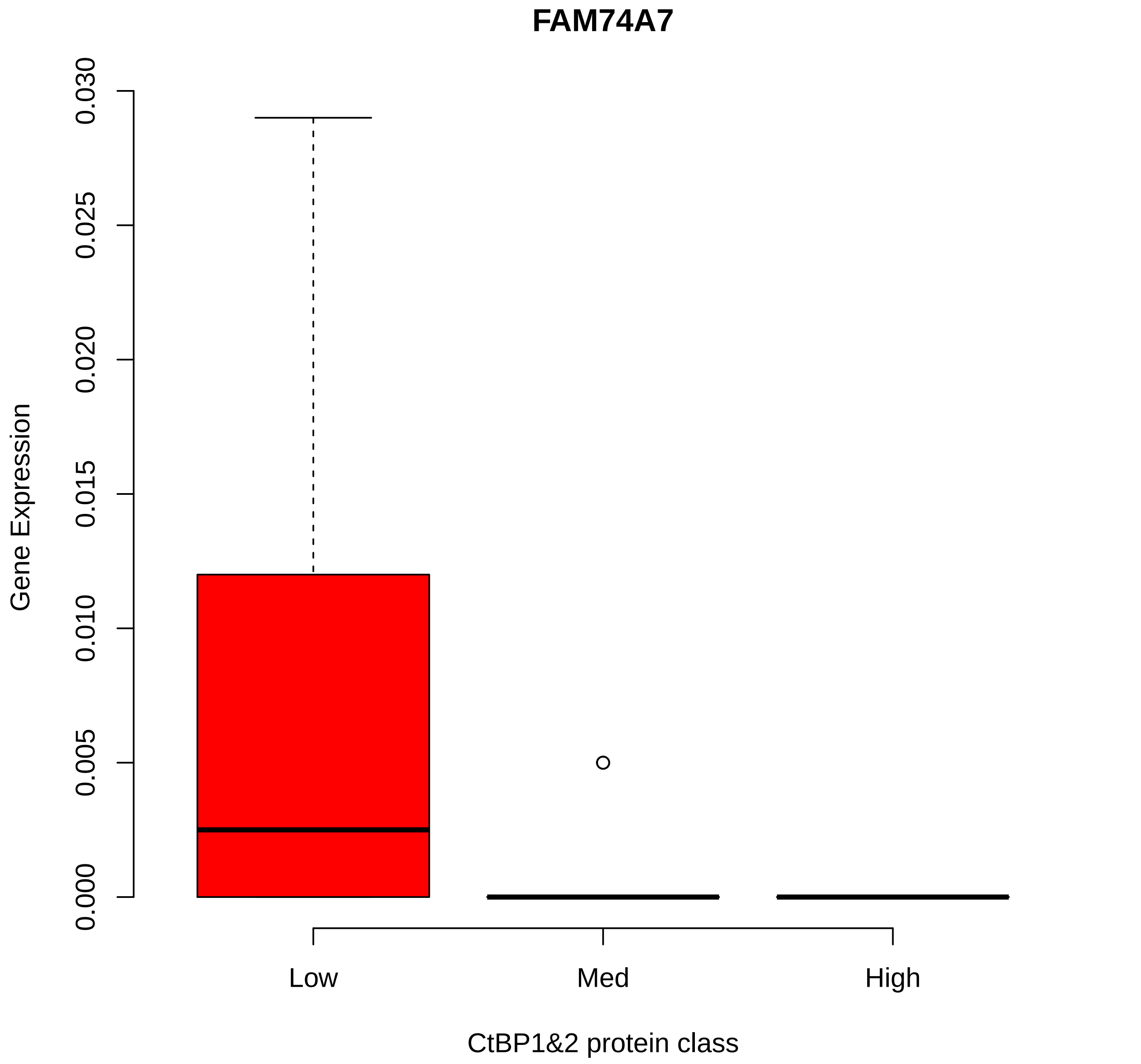

FAM74A7
Gene Expression
0.000
0.005
0.010
0.015
0.020
0.025
0.030
Low
Med
High
CtBP1&2 protein class

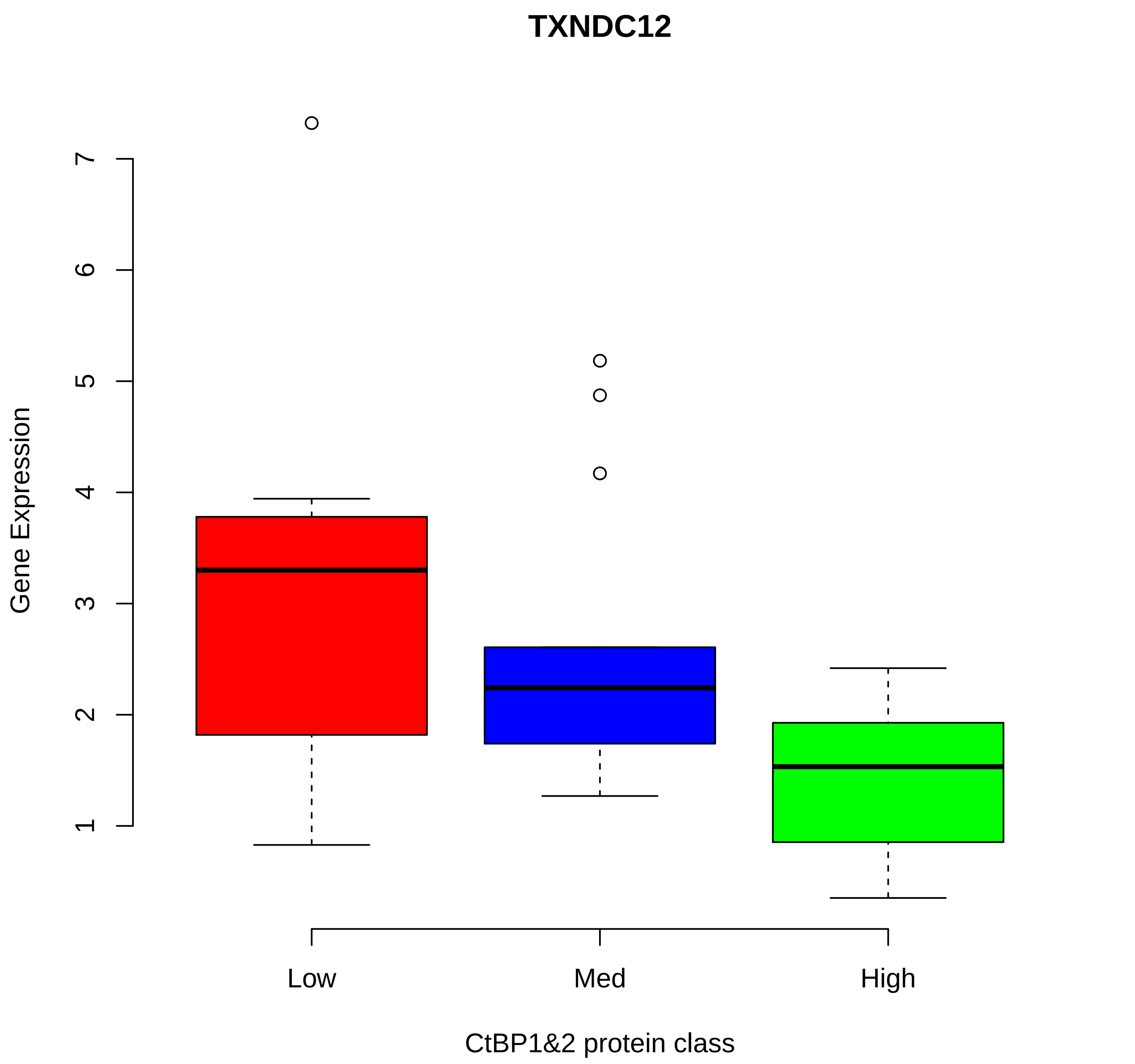
TXNDC12
Gene Expression
1
2
3
4
5
6
7
Low
Med
High
CtBP1&2 protein class

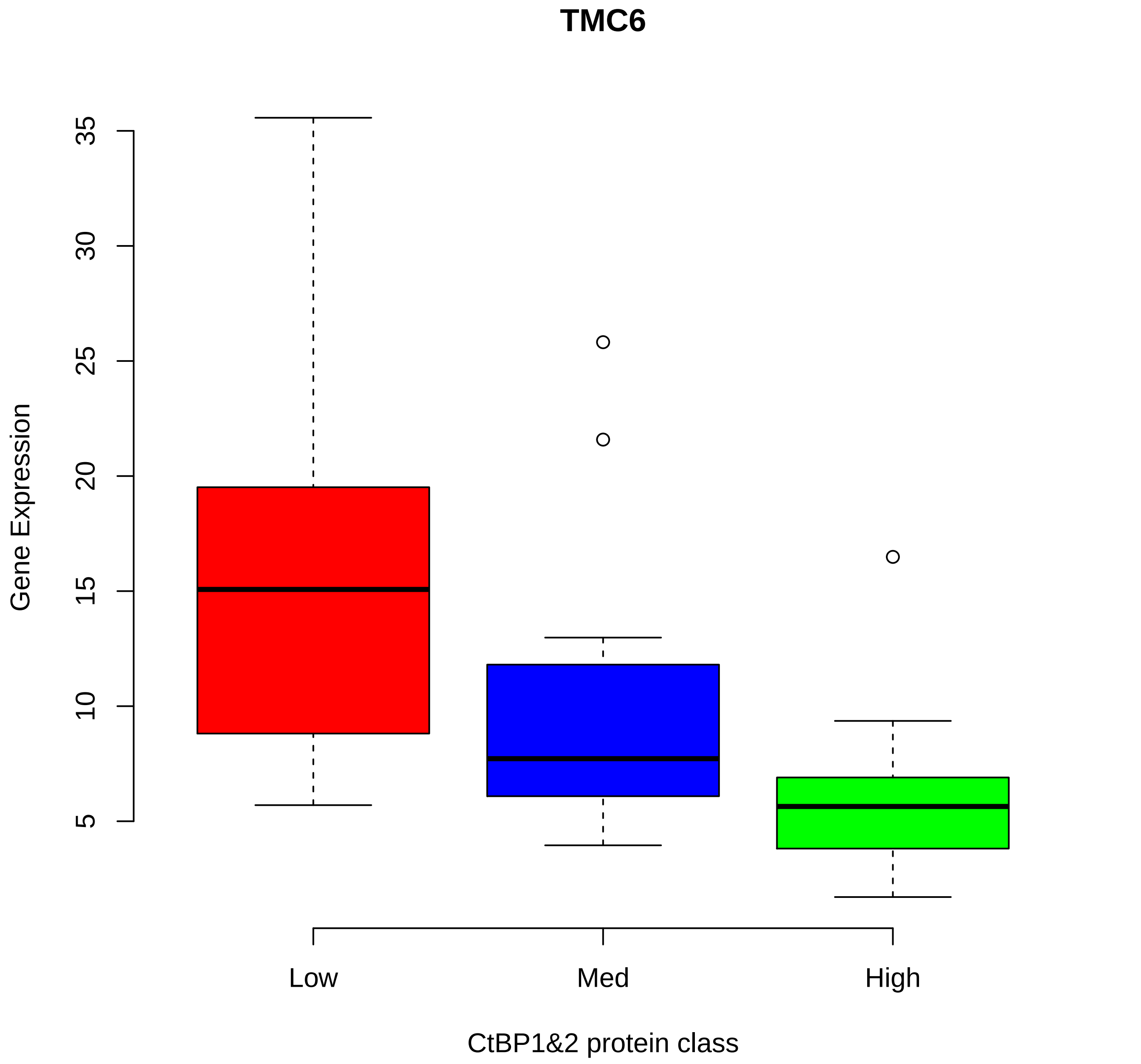

TMC6
Gene Expression
5
10
15
20
25
30
35
Low
Med
High
CtBP1&2 protein class

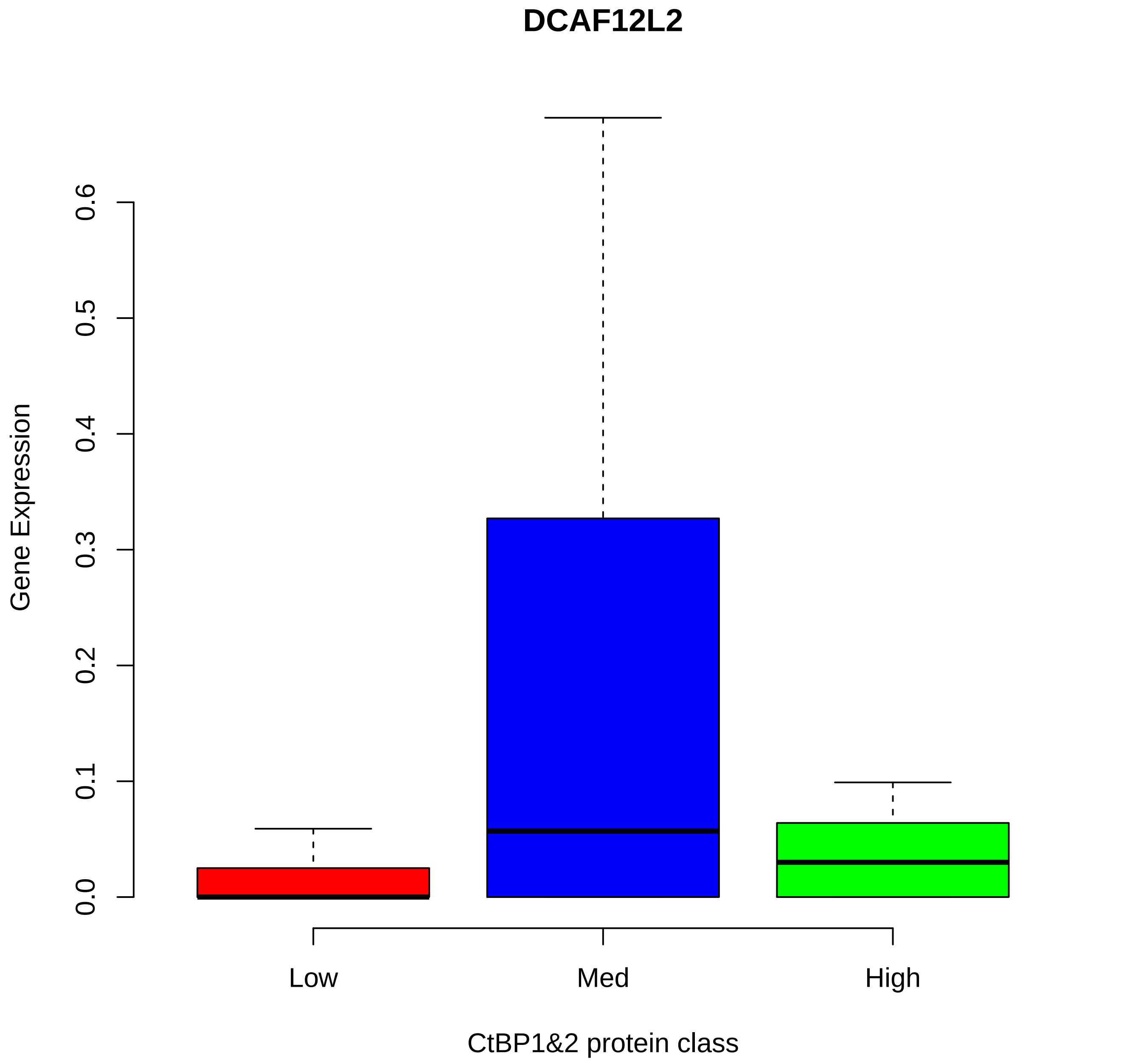

DCAF12L2
Gene Expression
0.0
0.1
0.2
0.3
0.4
0.5
0.6
Low
Med
High
CtBP1&2 protein class

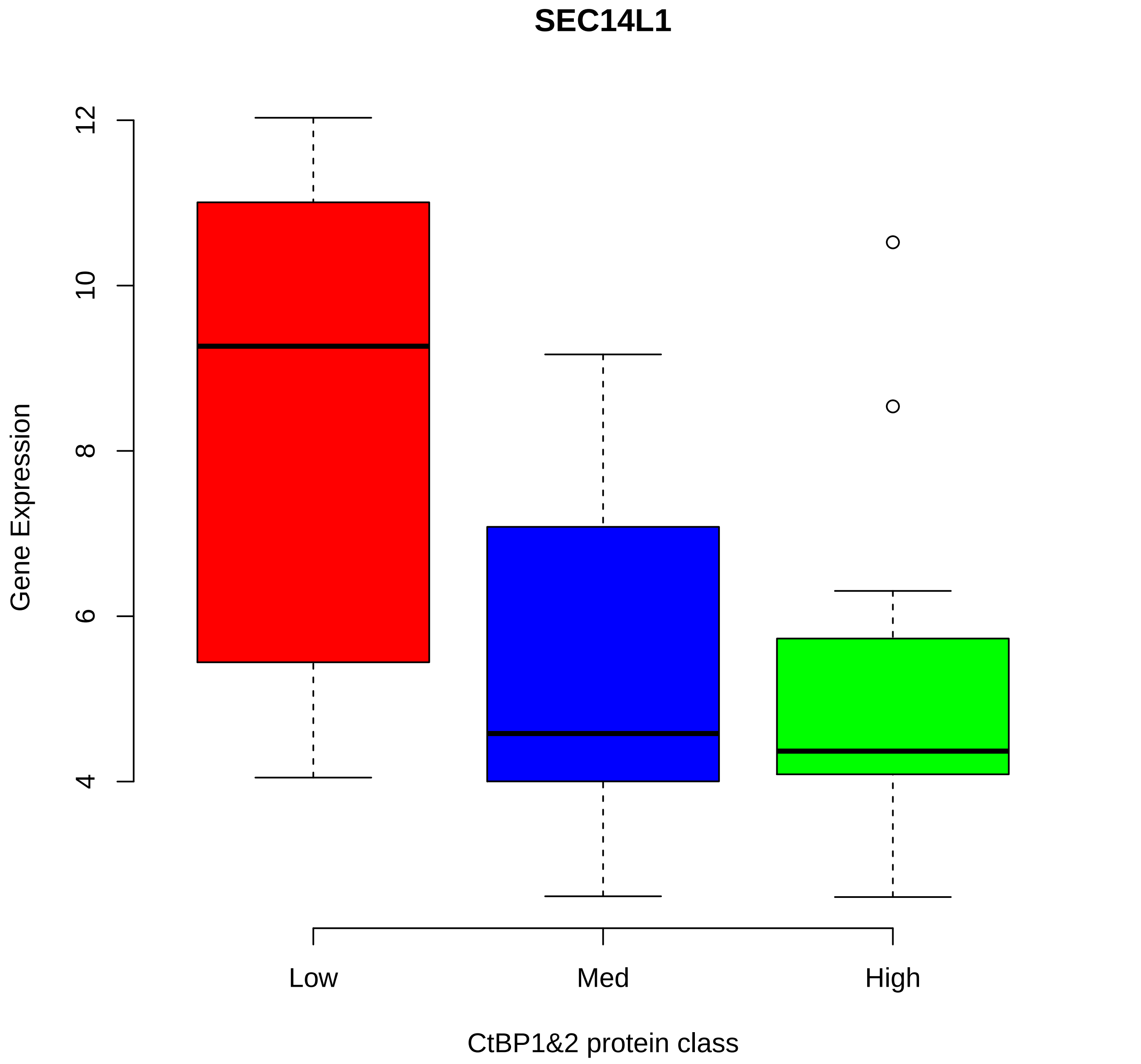

SEC14L1
Gene Expression
12
10
8
6
4
Low
Med
High
CtBP1&2 protein class

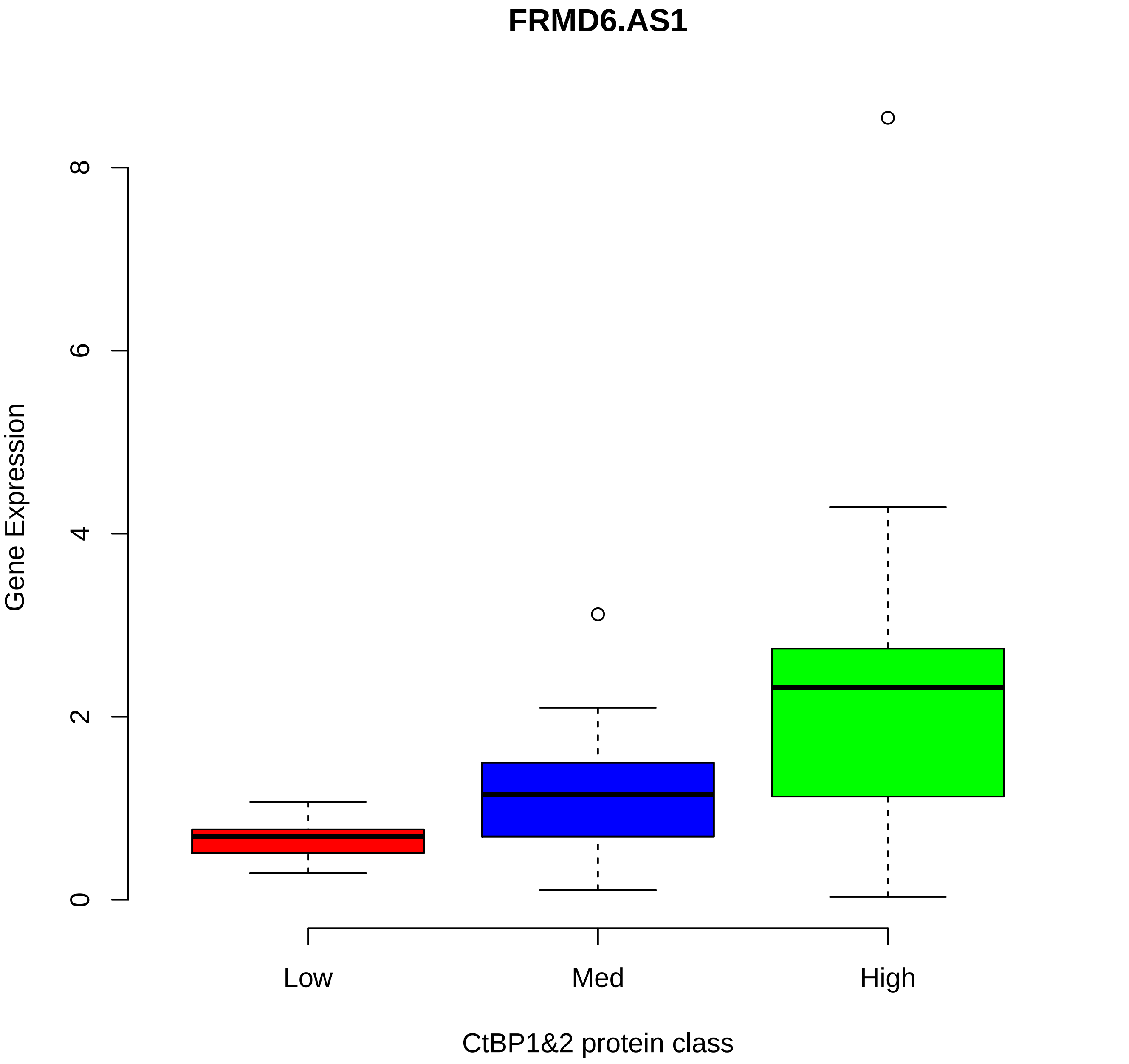
FRMD6.AS1
Gene Expression
0
2
4
6
8
Low
Med
High
CtBP1&2 protein class

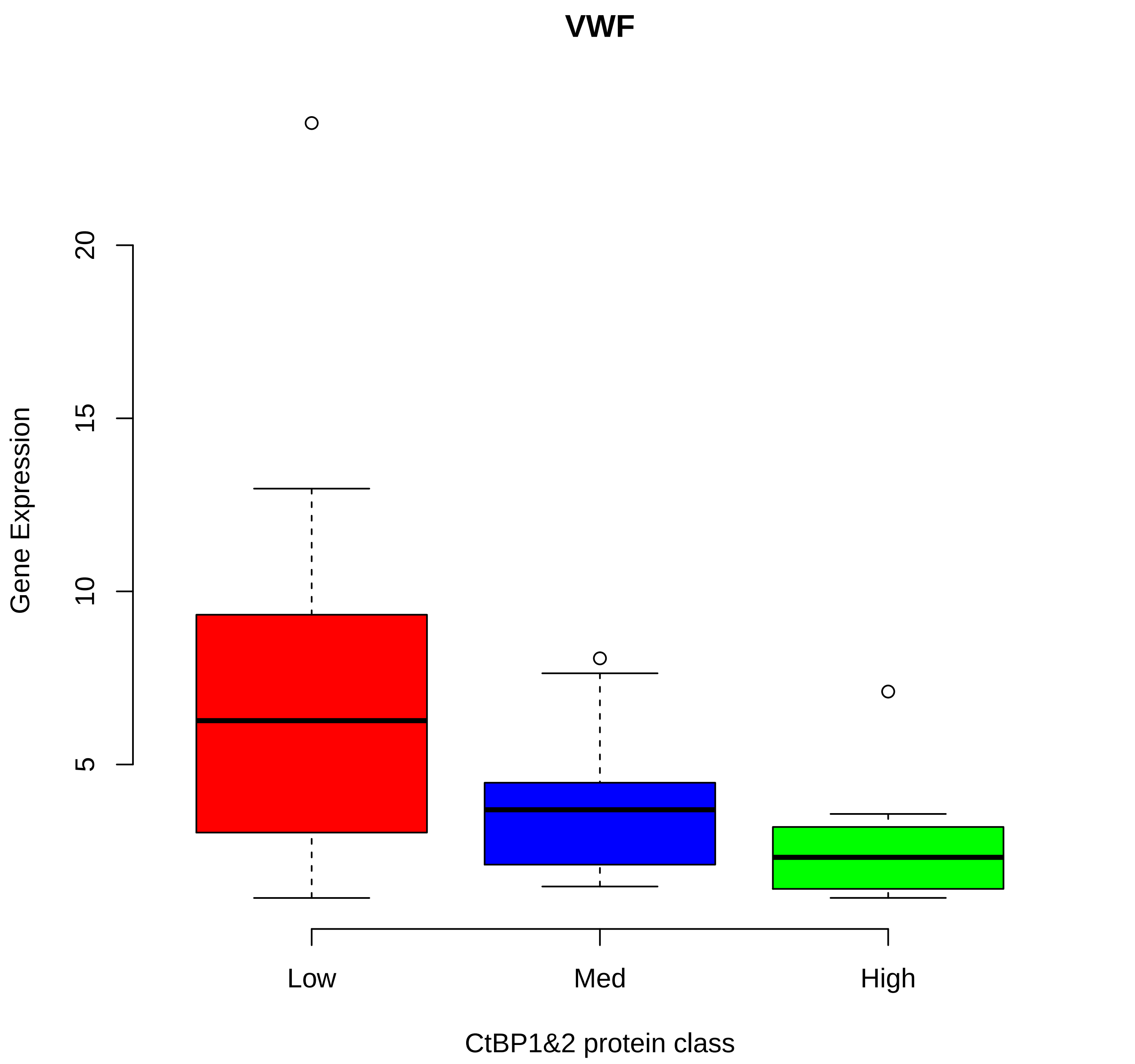

VWF
Gene Expression
20
15
10
5
Low
Med
High
CtBP1&2 protein class

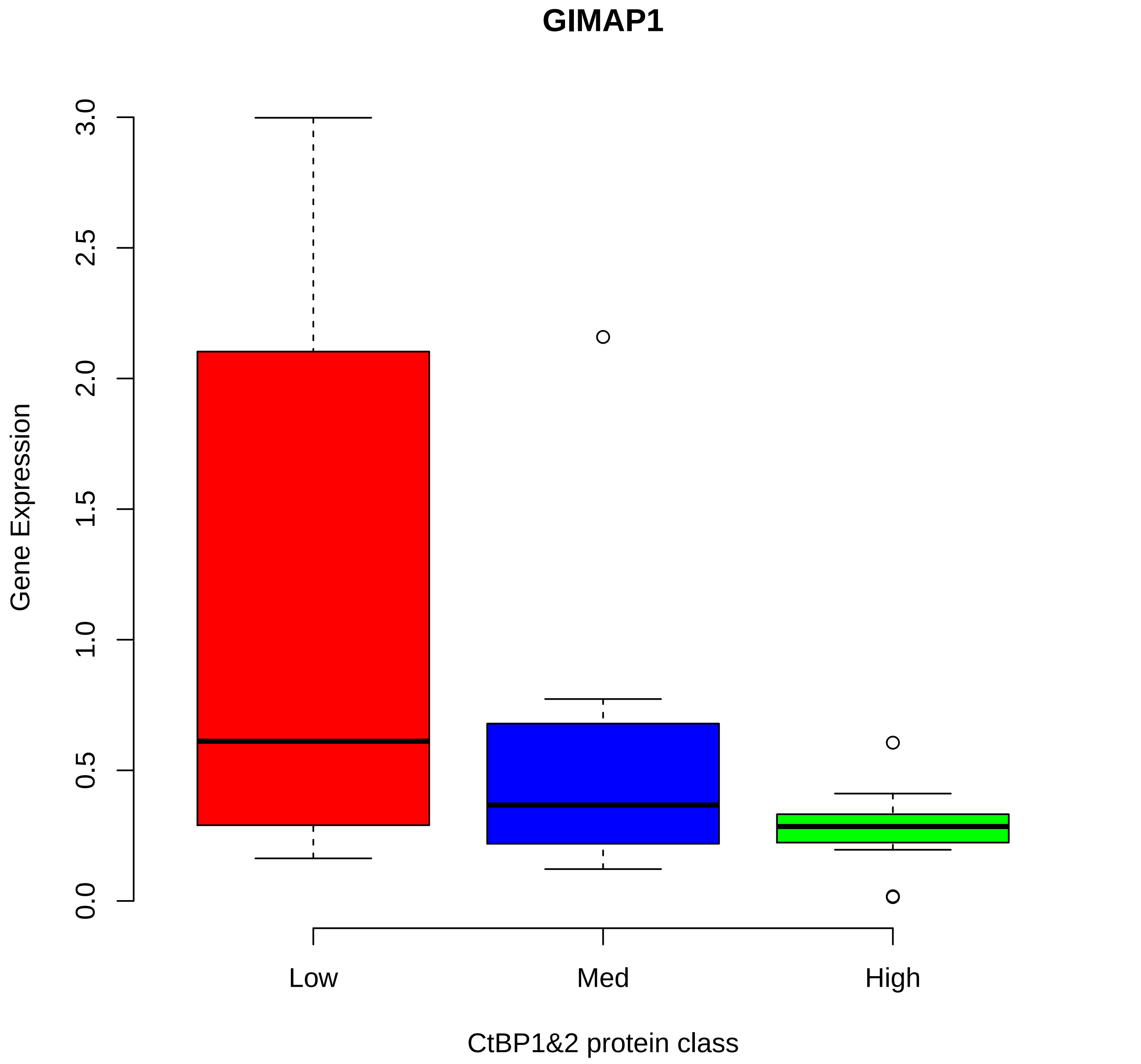
GIMAP1
Gene Expression
0.0
0.5
1.0
1.5
2.0
2.5
3.0
Low
Med
High
CtBP1&2 protein class

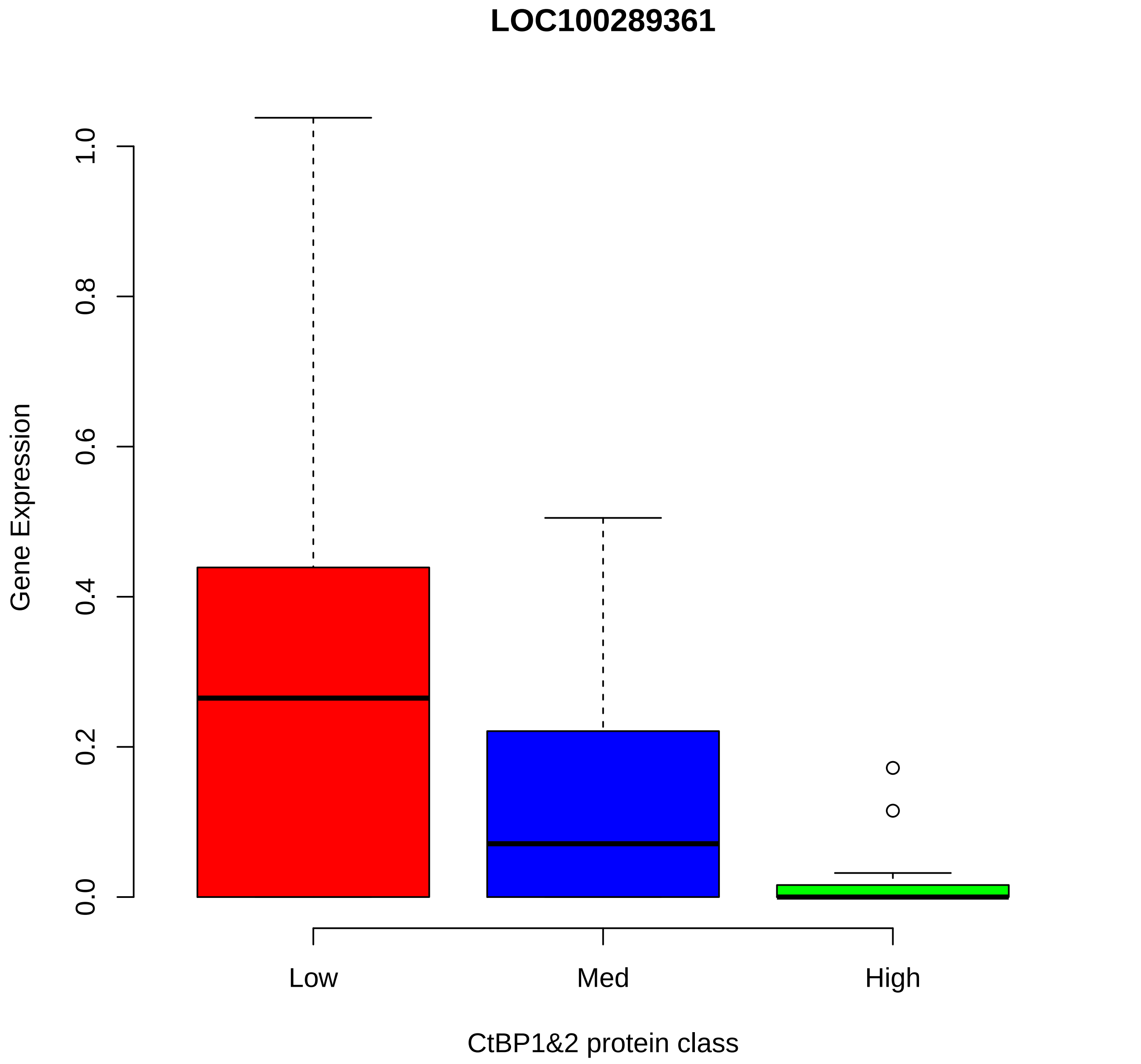
LOC100289361
Gene Expression
0.0
0.2
0.4
0.6
0.8
1.0
Low
Med
High
CtBP1&2 protein class

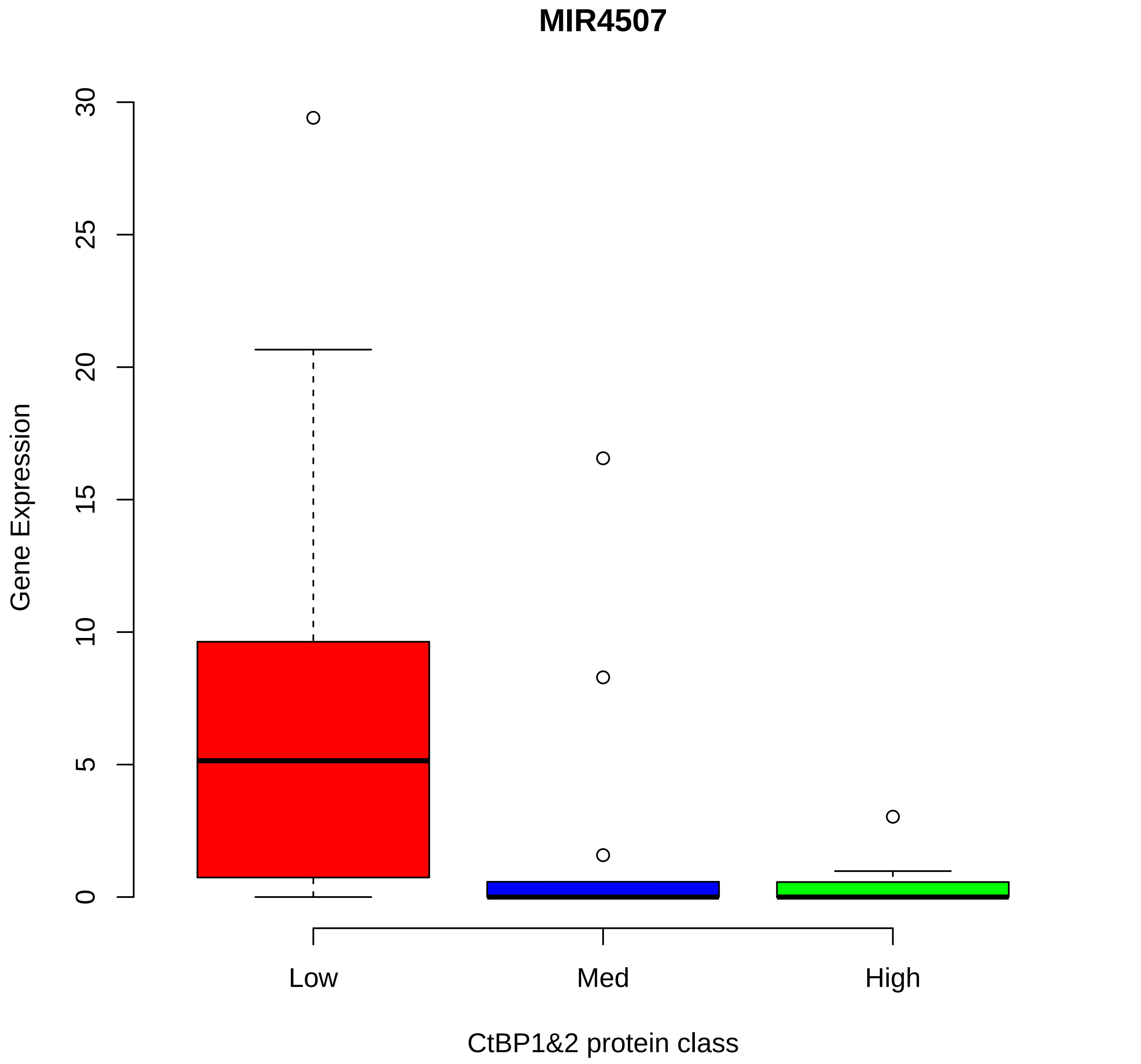

MIR4507
Gene Expression
0
5
10
15
20
25
30
Low
Med
High
CtBP1&2 protein class

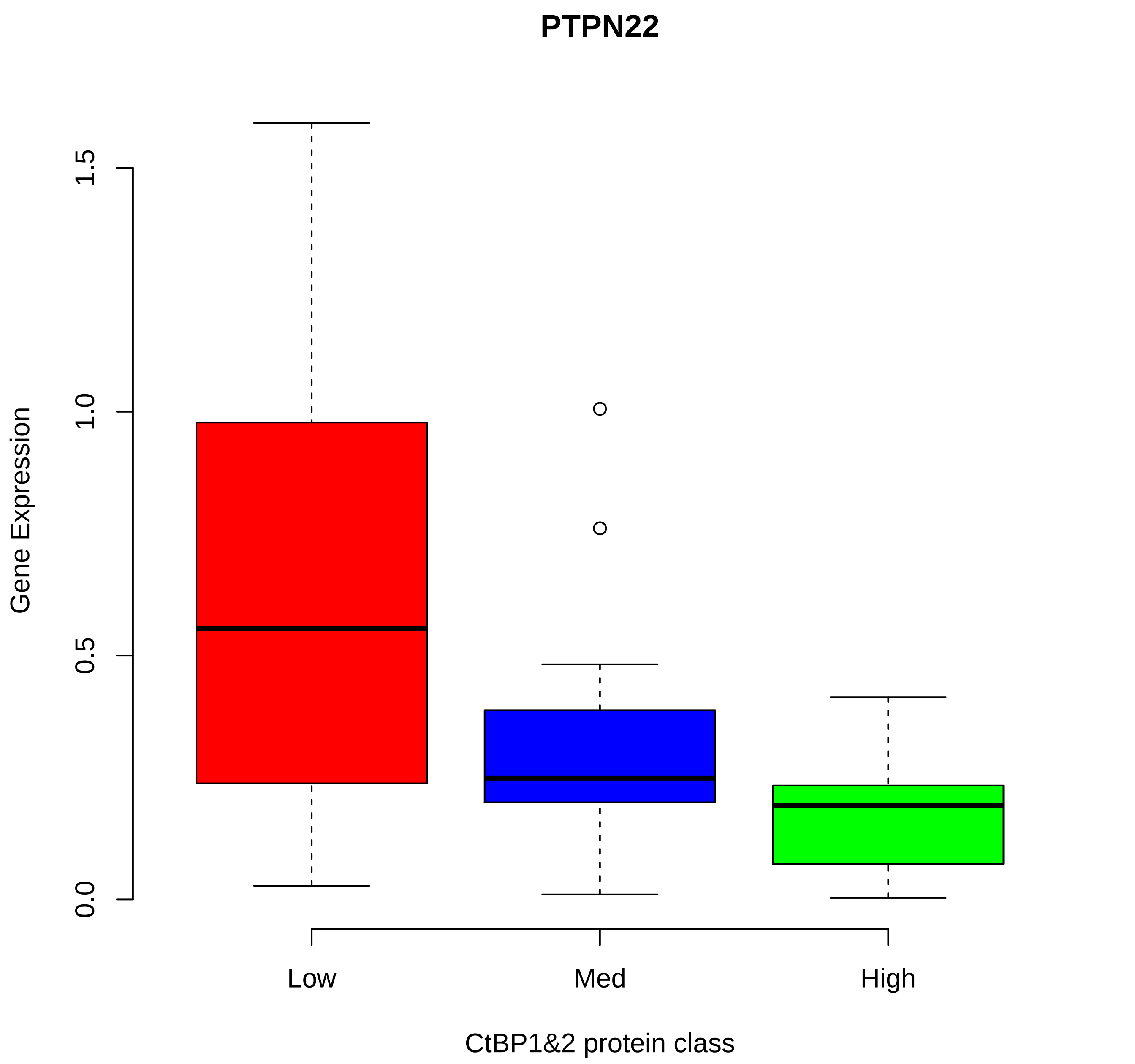

PTPN22
Gene Expression
0.0
0.5
1.0
1.5
Low
Med
High
CtBP1&2 protein class

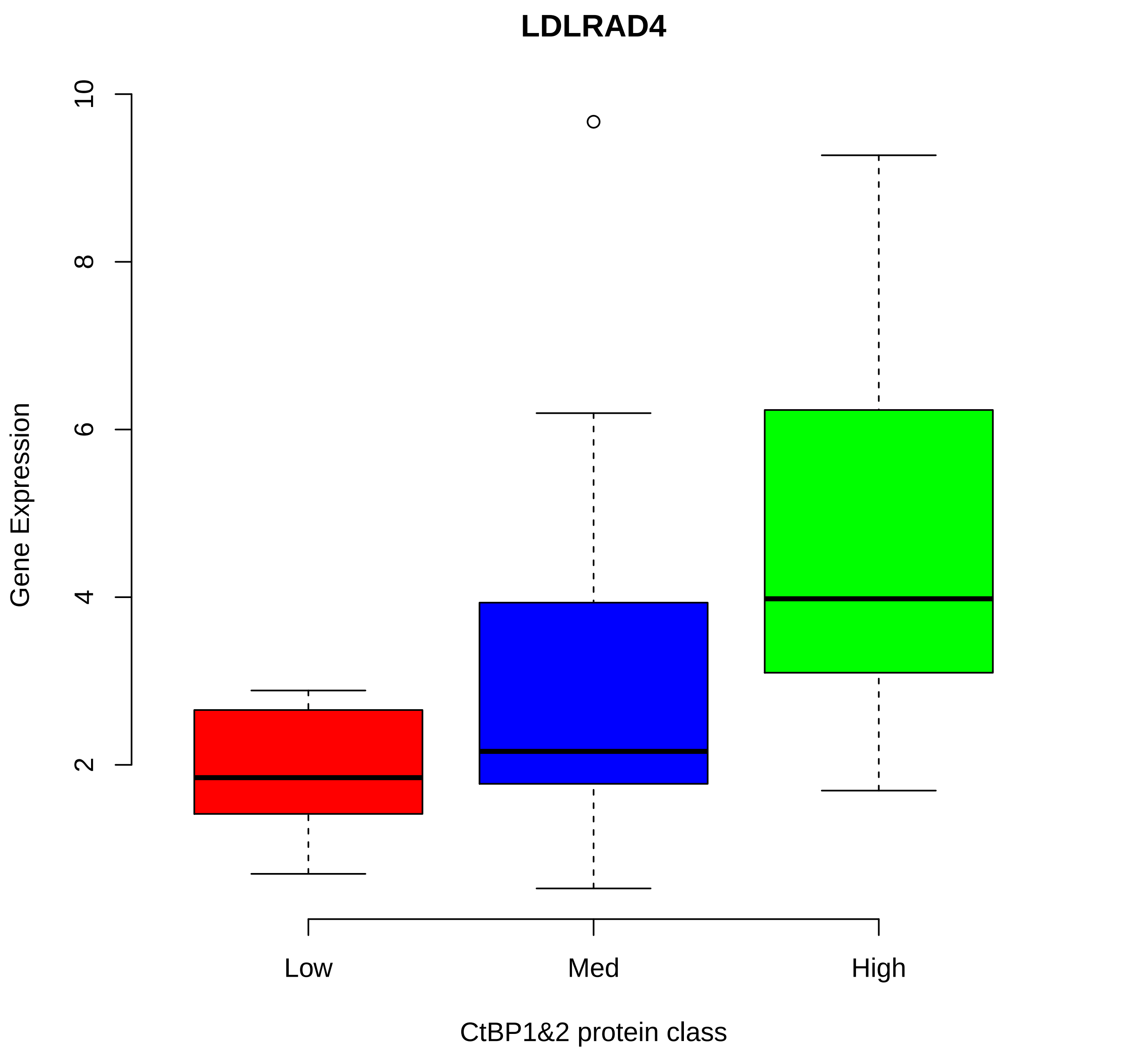

LDLRAD4
Gene Expression
10
8
6
4
2
Low
Med
High
CtBP1&2 protein class

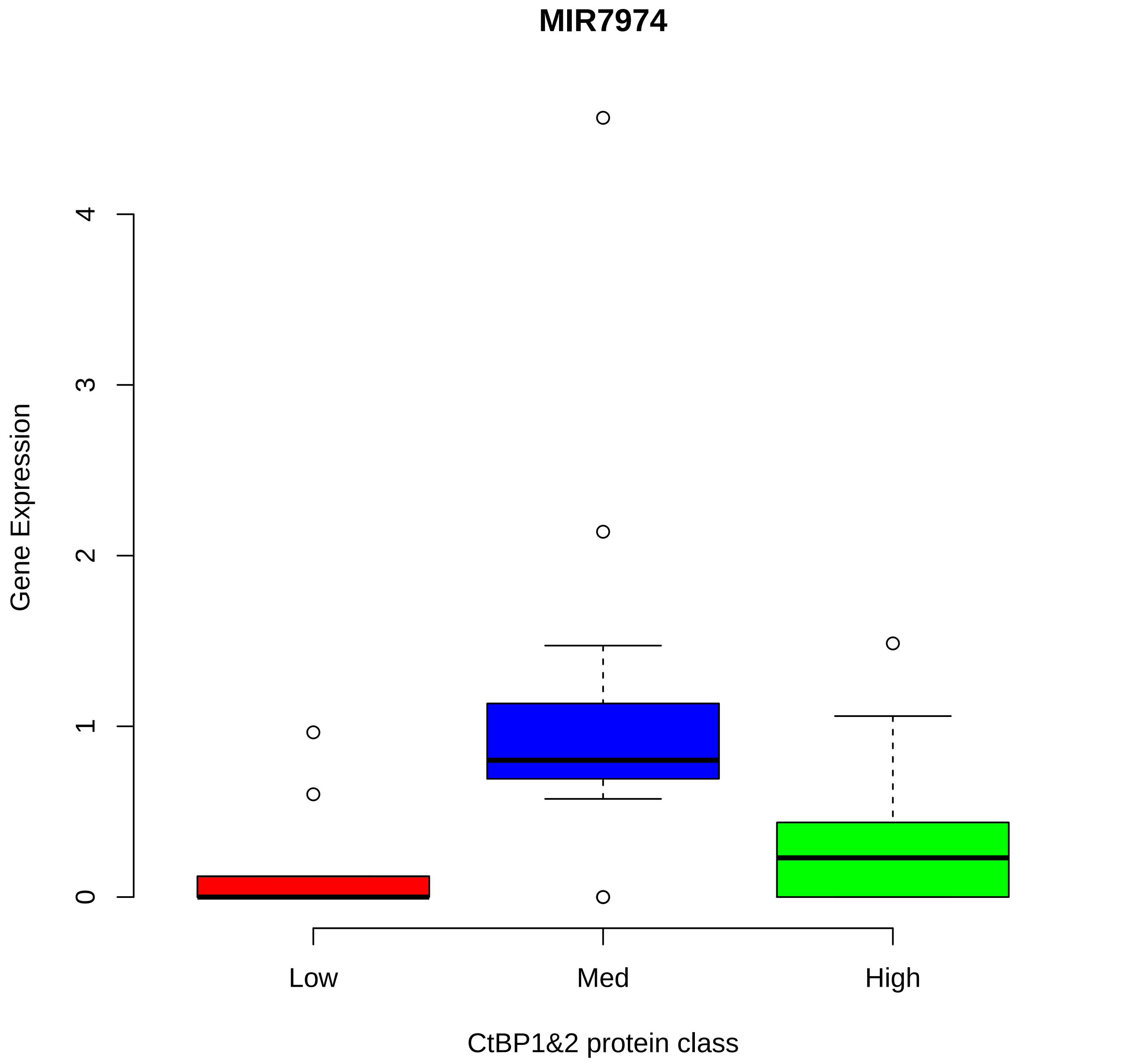

MIR7974
Gene Expression
0
1
2
3
4
Low
Med
High
CtBP1&2 protein class

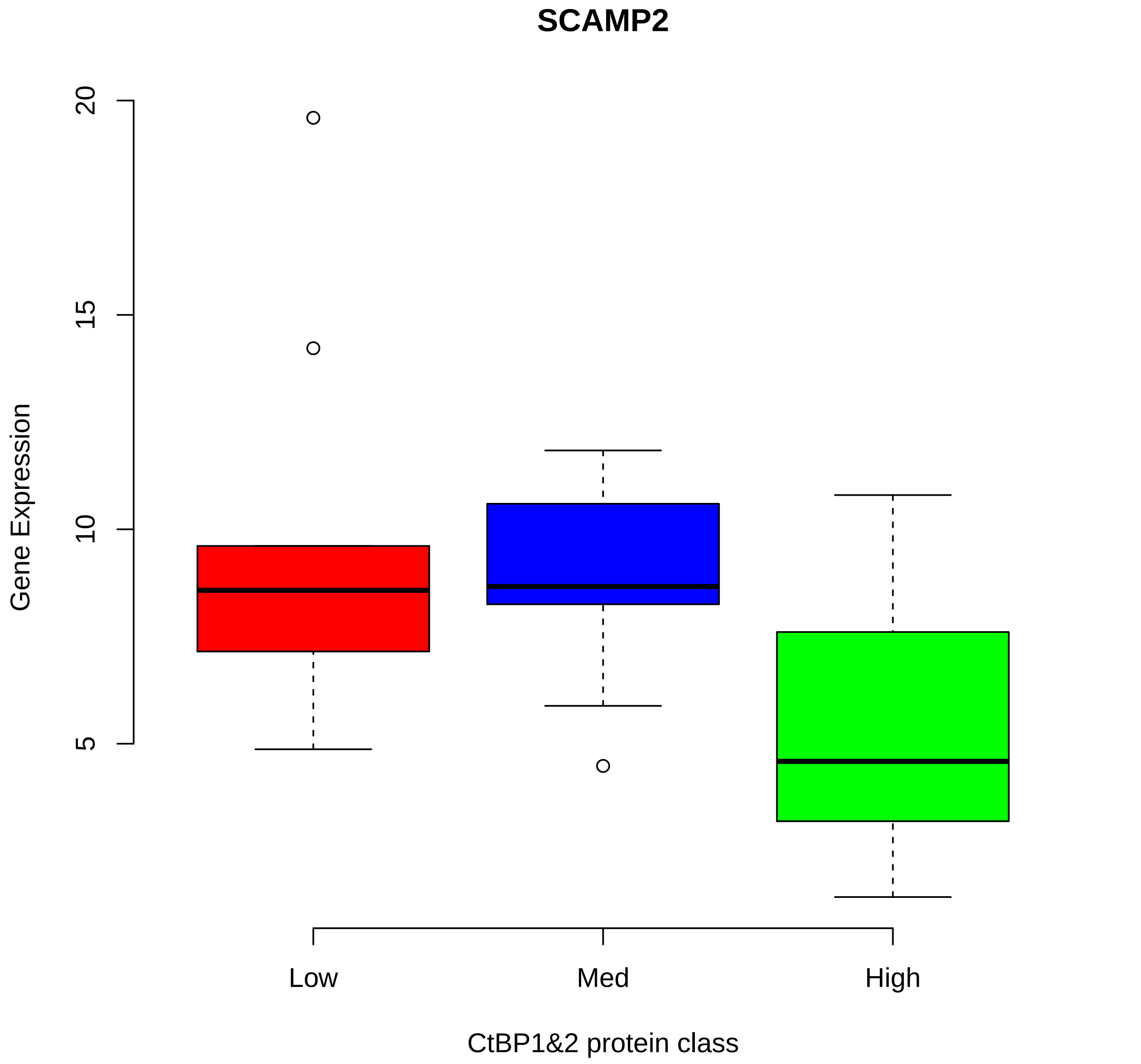
SCAMP2
Gene Expression
20
15
10
5
Low
Med
High
CtBP1&2 protein class

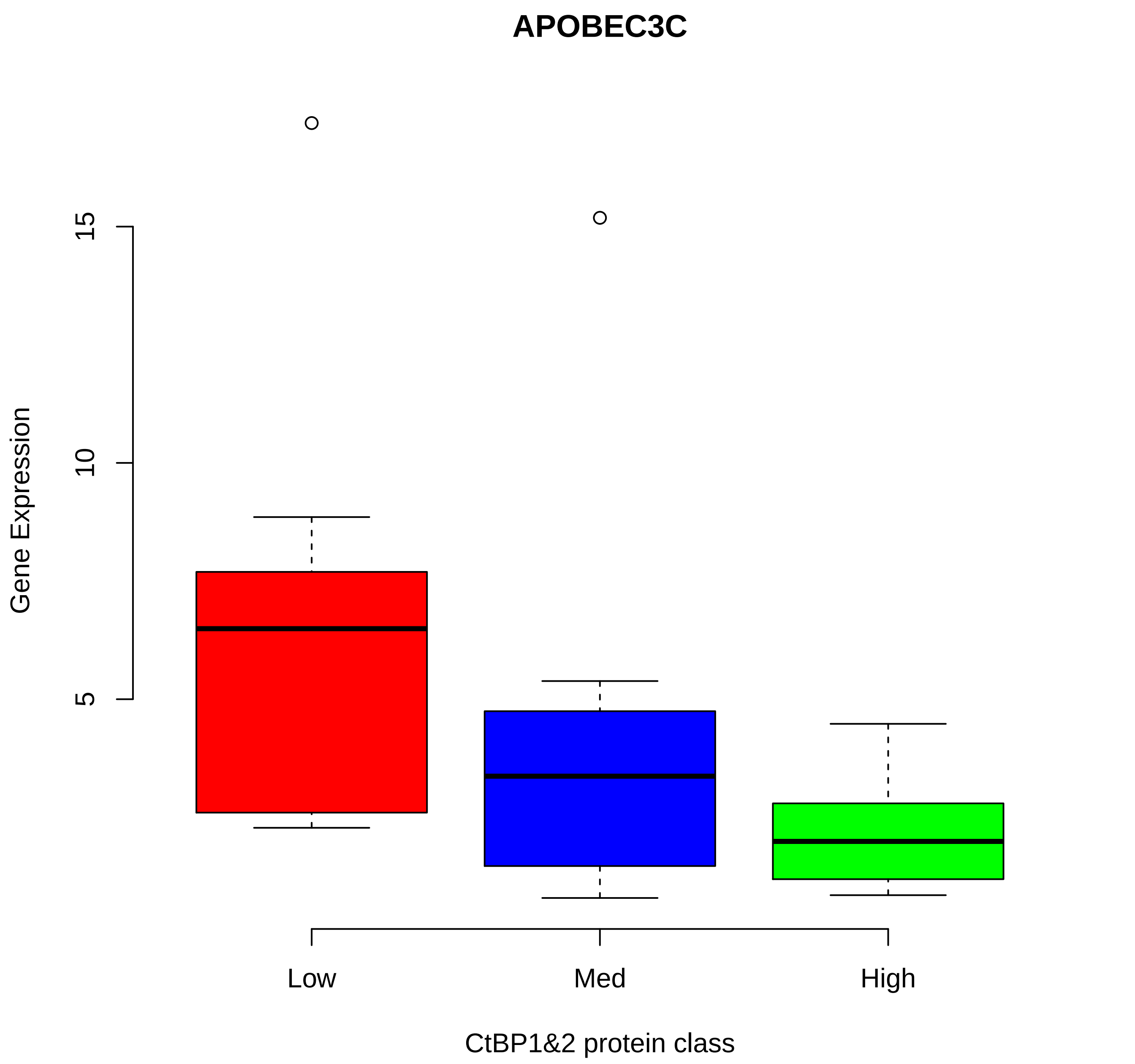
APOBEC3C
Gene Expression
15
10
5
Low
Med
High
CtBP1&2 protein class

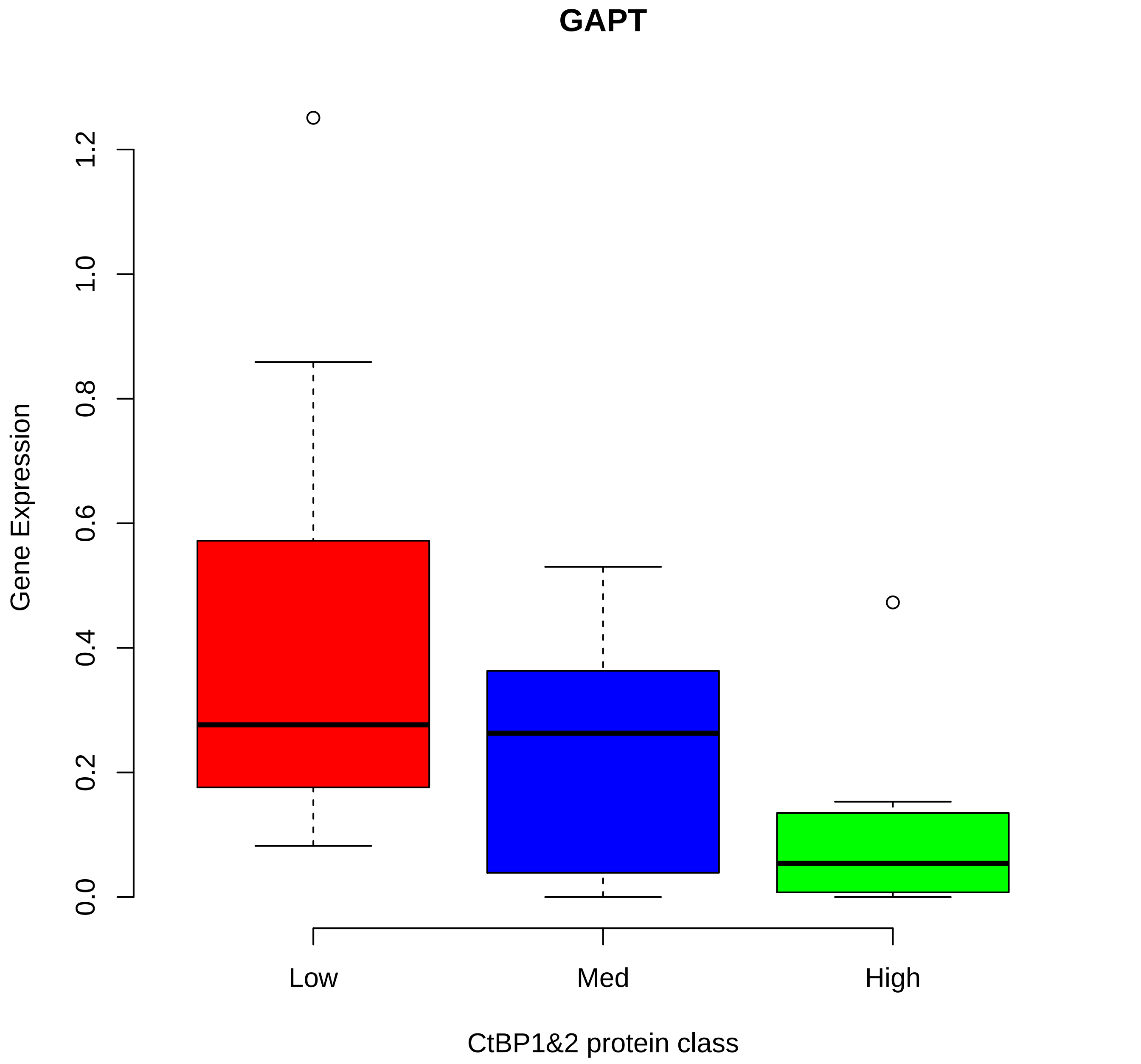

GAPT
Gene Expression
0.0
0.2
0.4
0.6
0.8
1.0
1.2
Low
Med
High
CtBP1&2 protein class

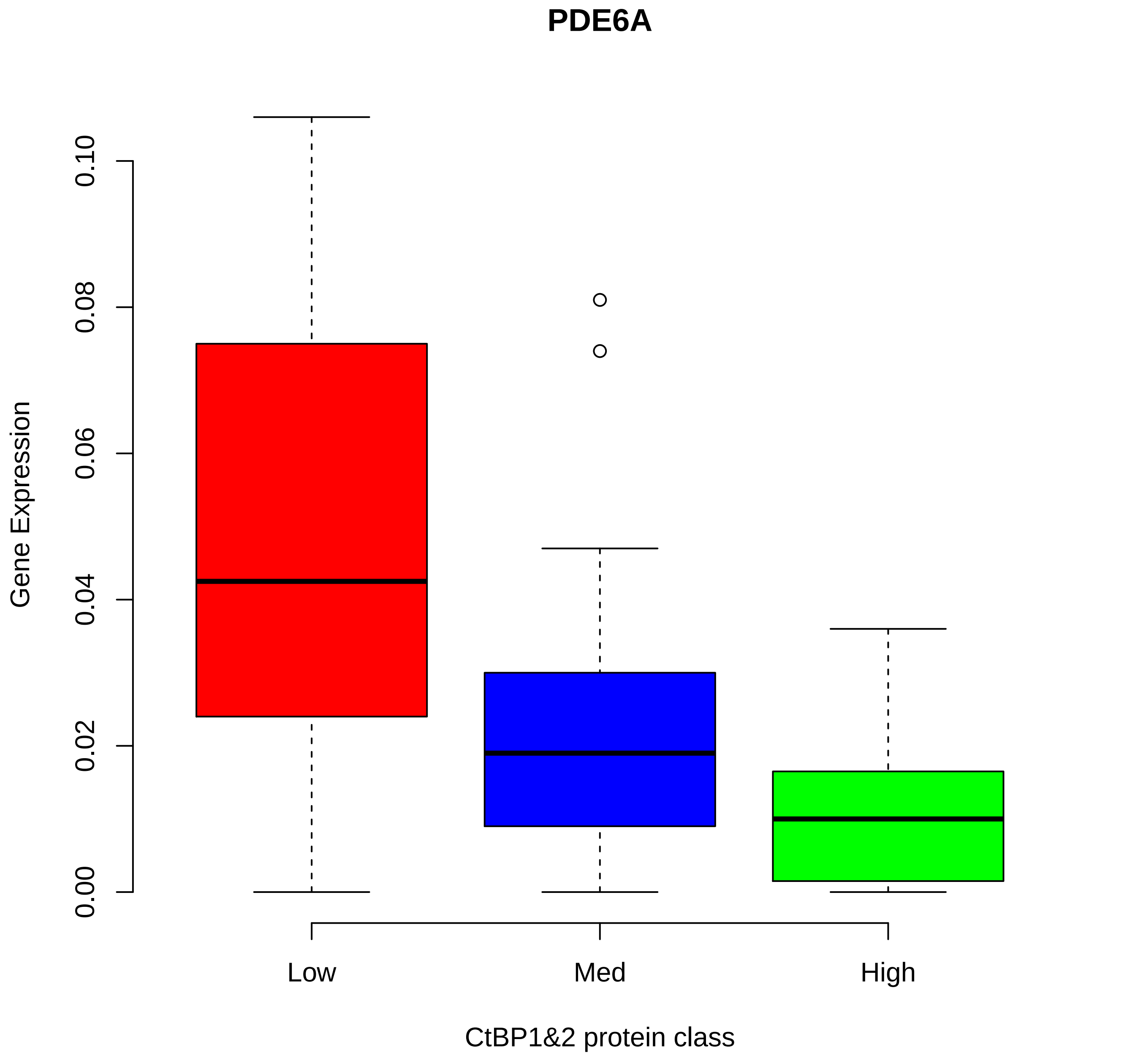

PDE6A
Gene Expression
0.00
0.02
0.04
0.06
0.08
0.10
Low
Med
High
CtBP1&2 protein class

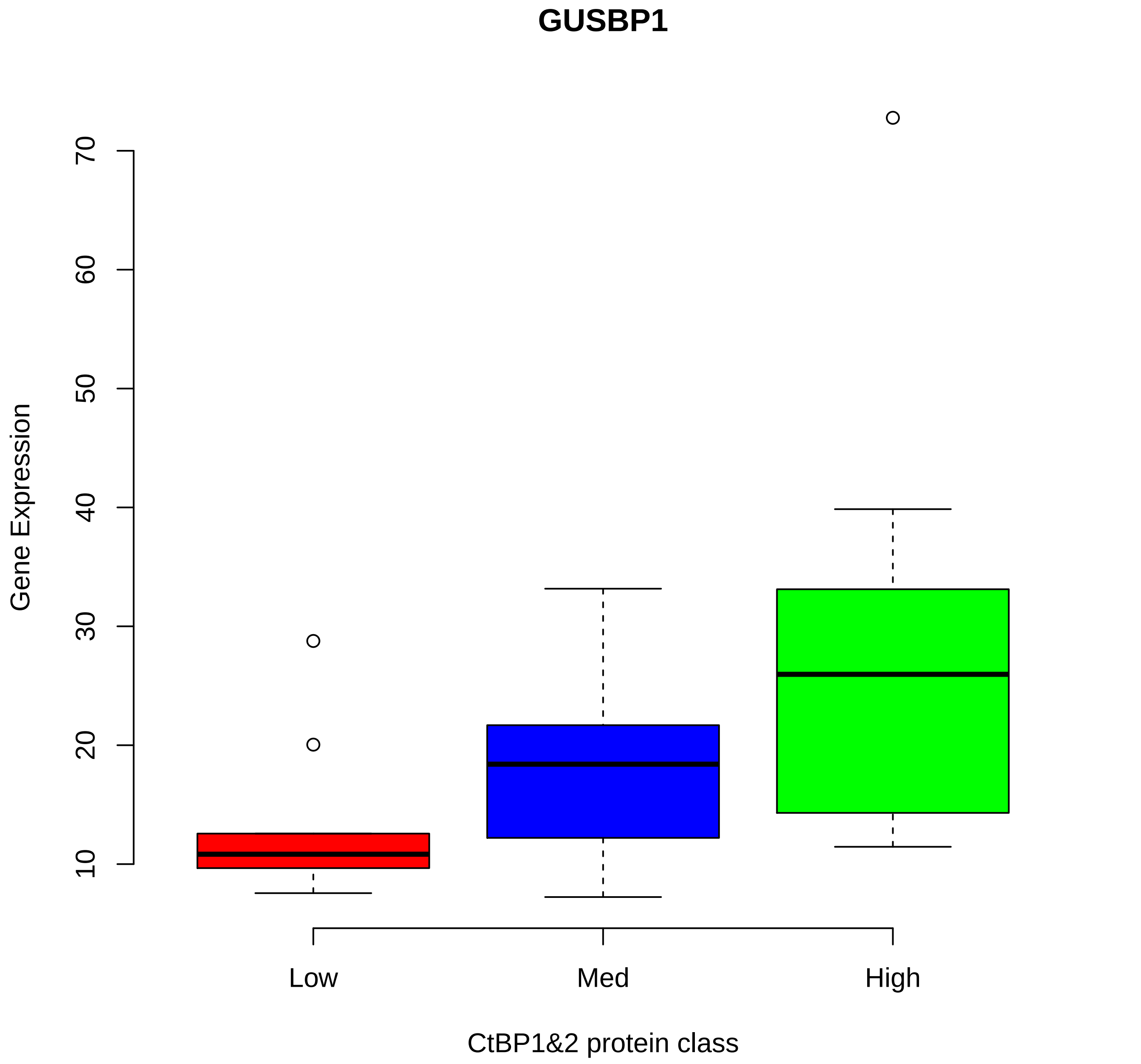

GUSBP1
Gene Expression
10
20
30
40
50
60
70
Low
Med
High
CtBP1&2 protein class

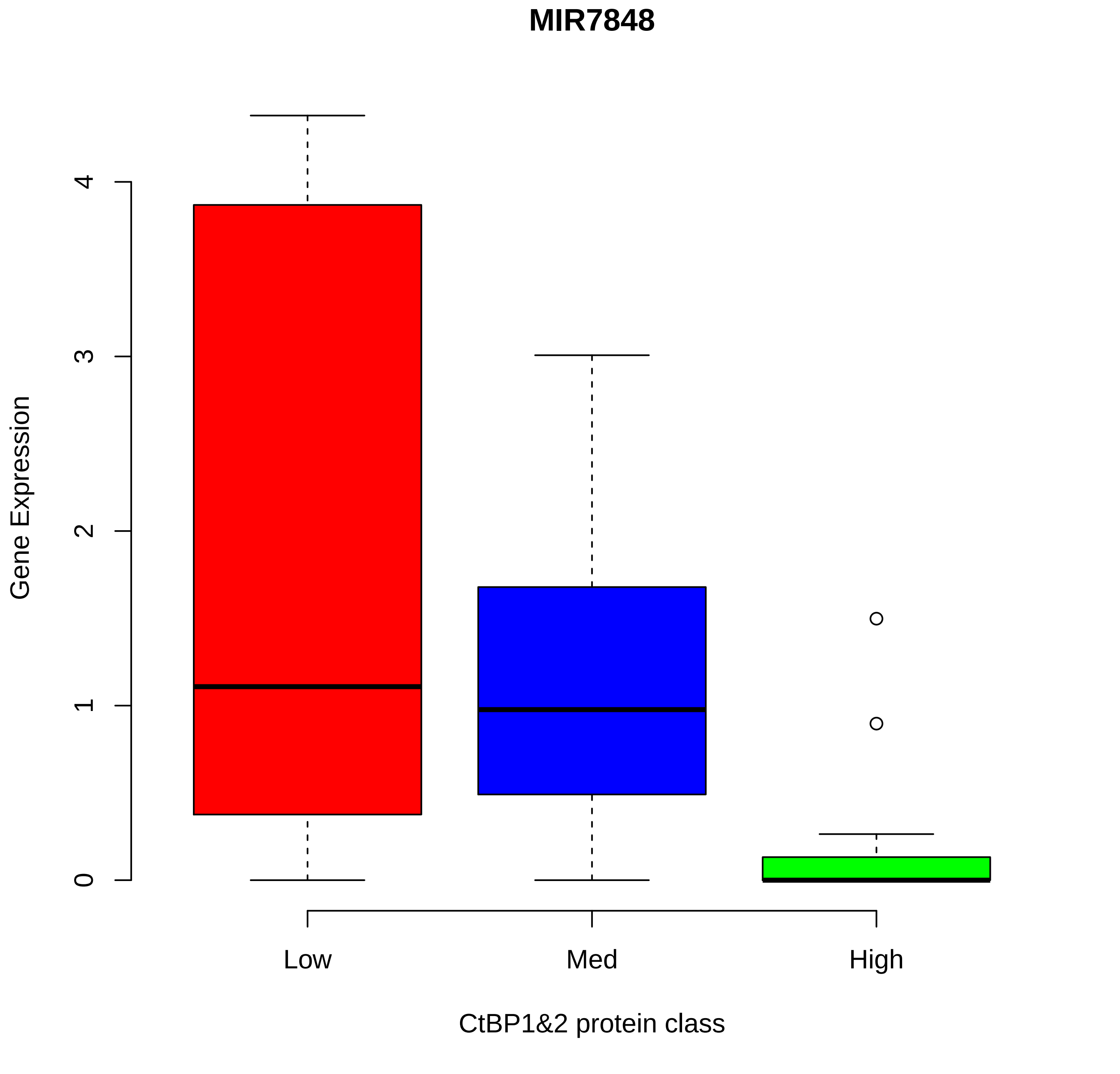

MIR7848
Gene Expression
0
1
2
3
4
Low
Med
High
CtBP1&2 protein class

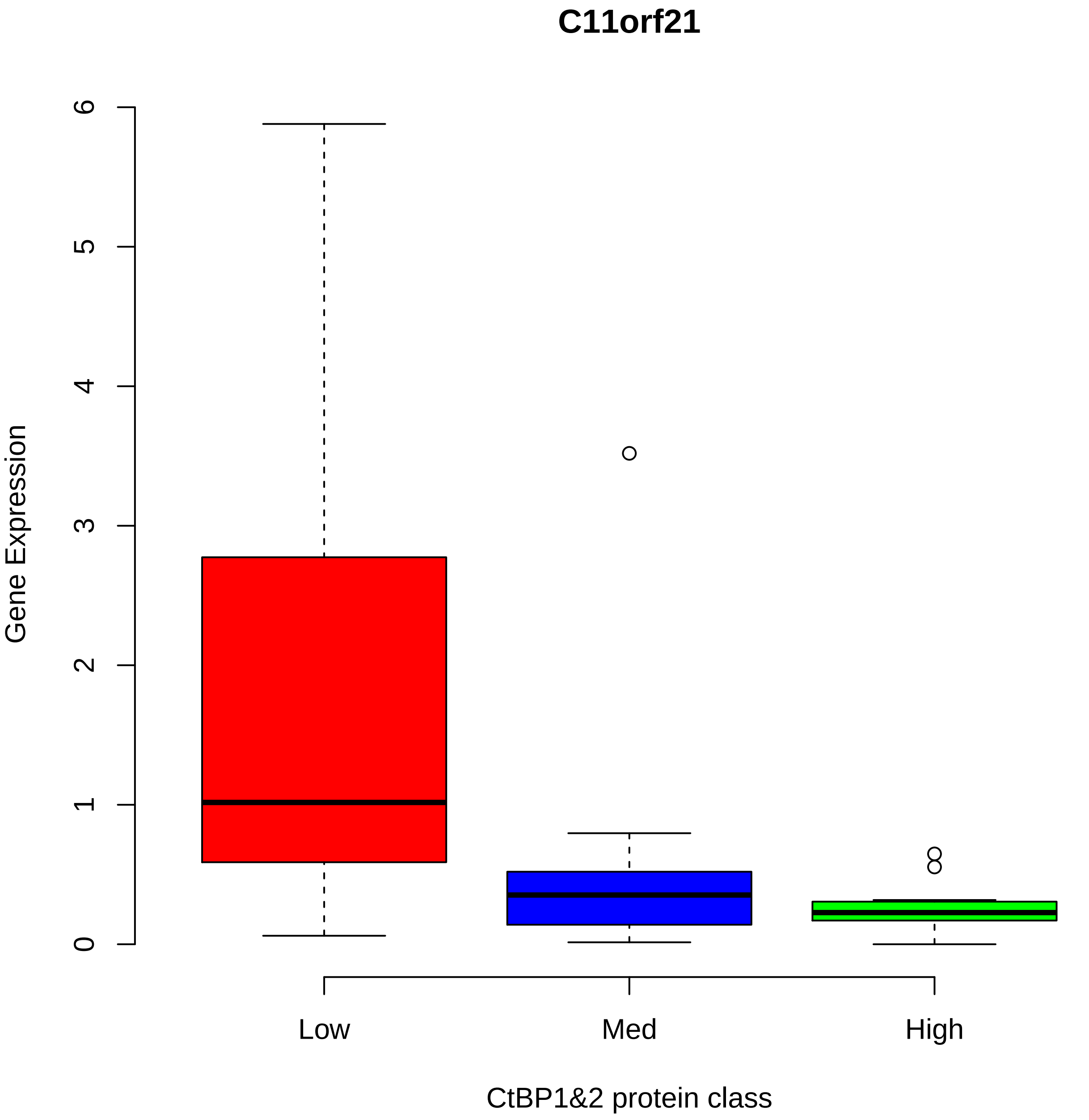

C11orf21
Gene Expression
0
1
2
3
4
5
6
Low
Med
High
CtBP1&2 protein class

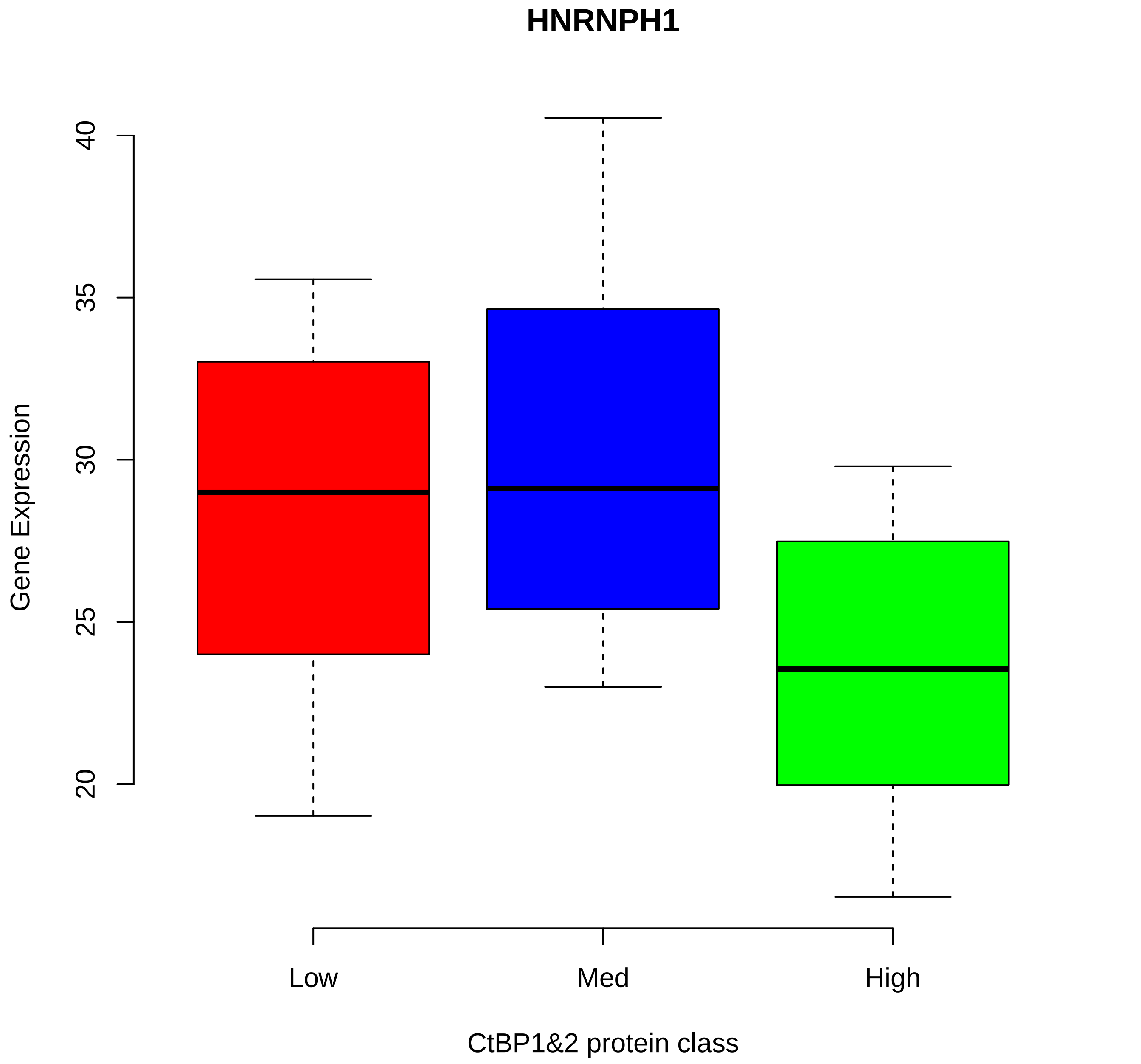

HNRNPH1
Gene Expression
40
35
30
25
20
Low
Med
High
CtBP1&2 protein class

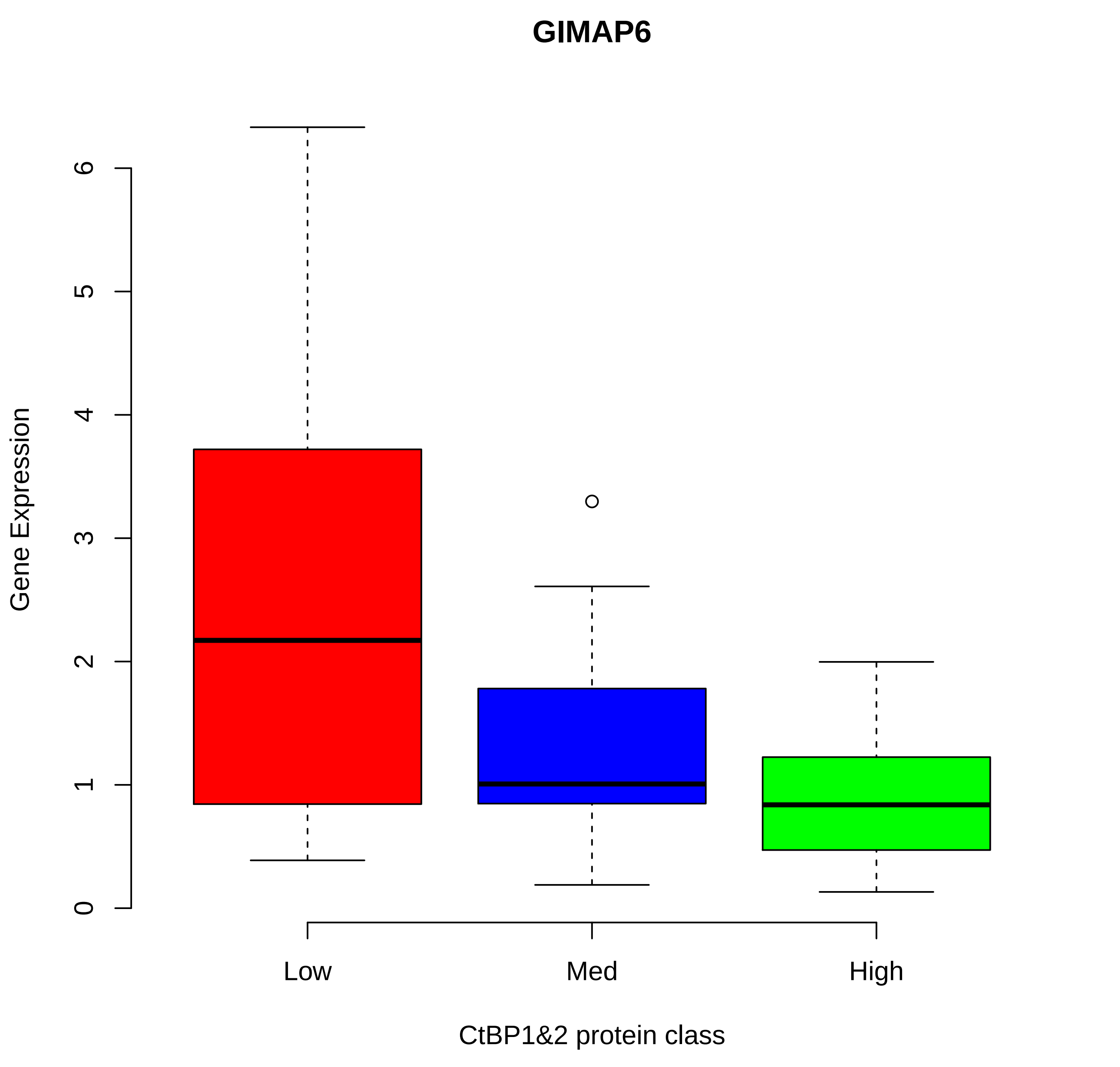

GIMAP6
Gene Expression
0
1
2
3
4
5
6
Low
Med
High
CtBP1&2 protein class

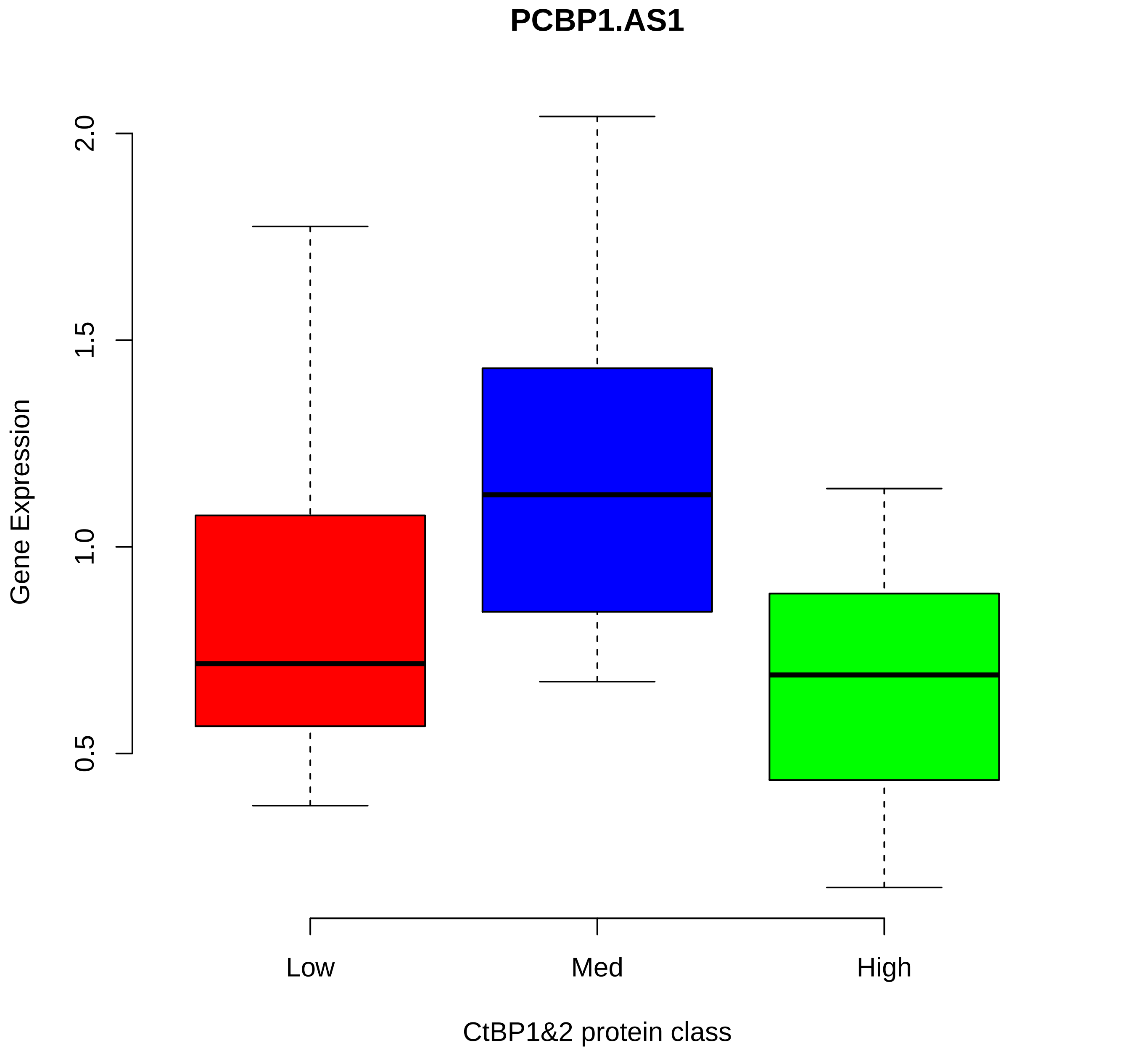
PCBP1.AS1
Gene Expression
0.5
1.0
1.5
2.0
Low
Med
High
CtBP1&2 protein class

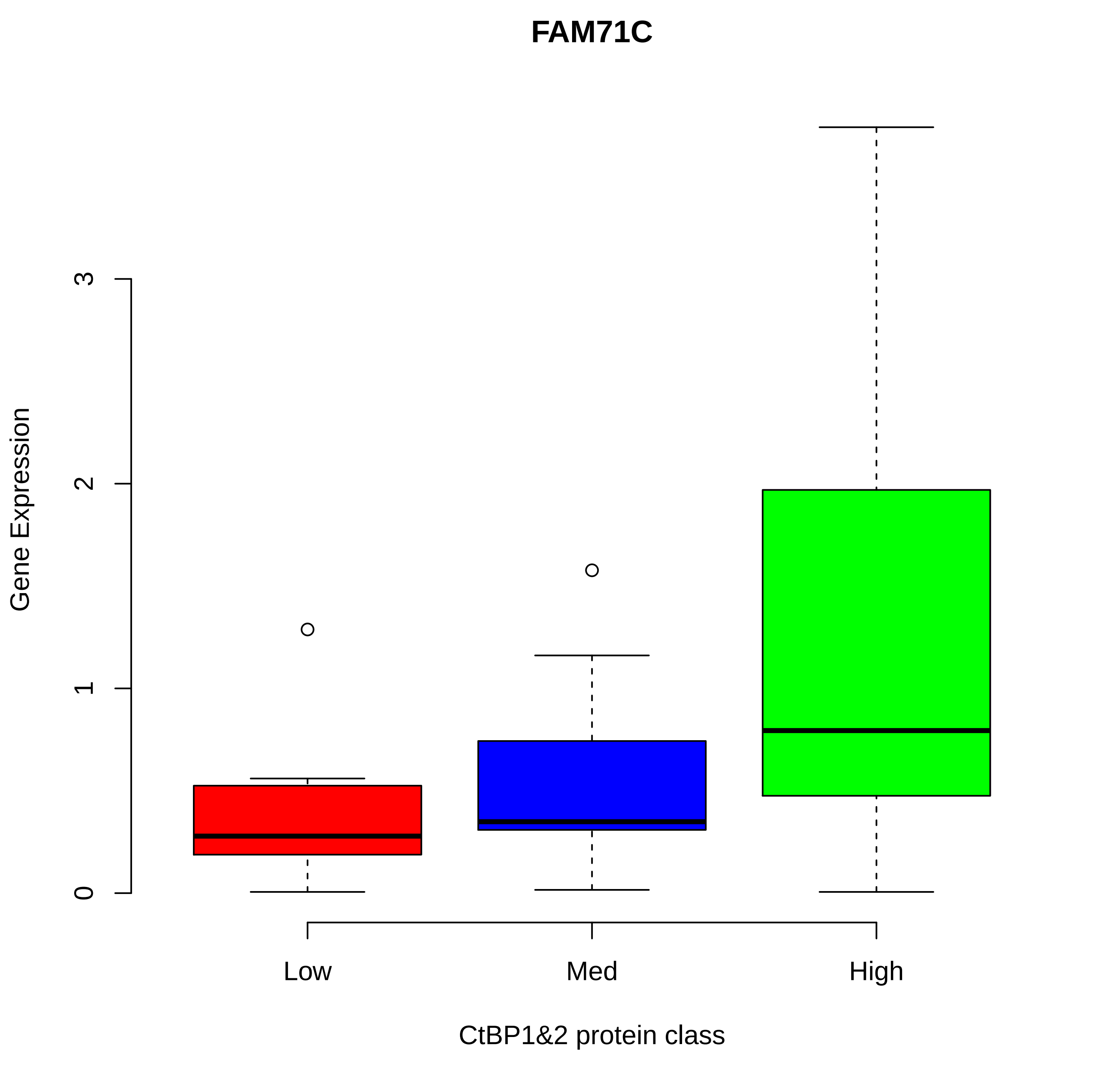

FAM71C
Gene Expression
0
1
2
3
Low
Med
High
CtBP1&2 protein class

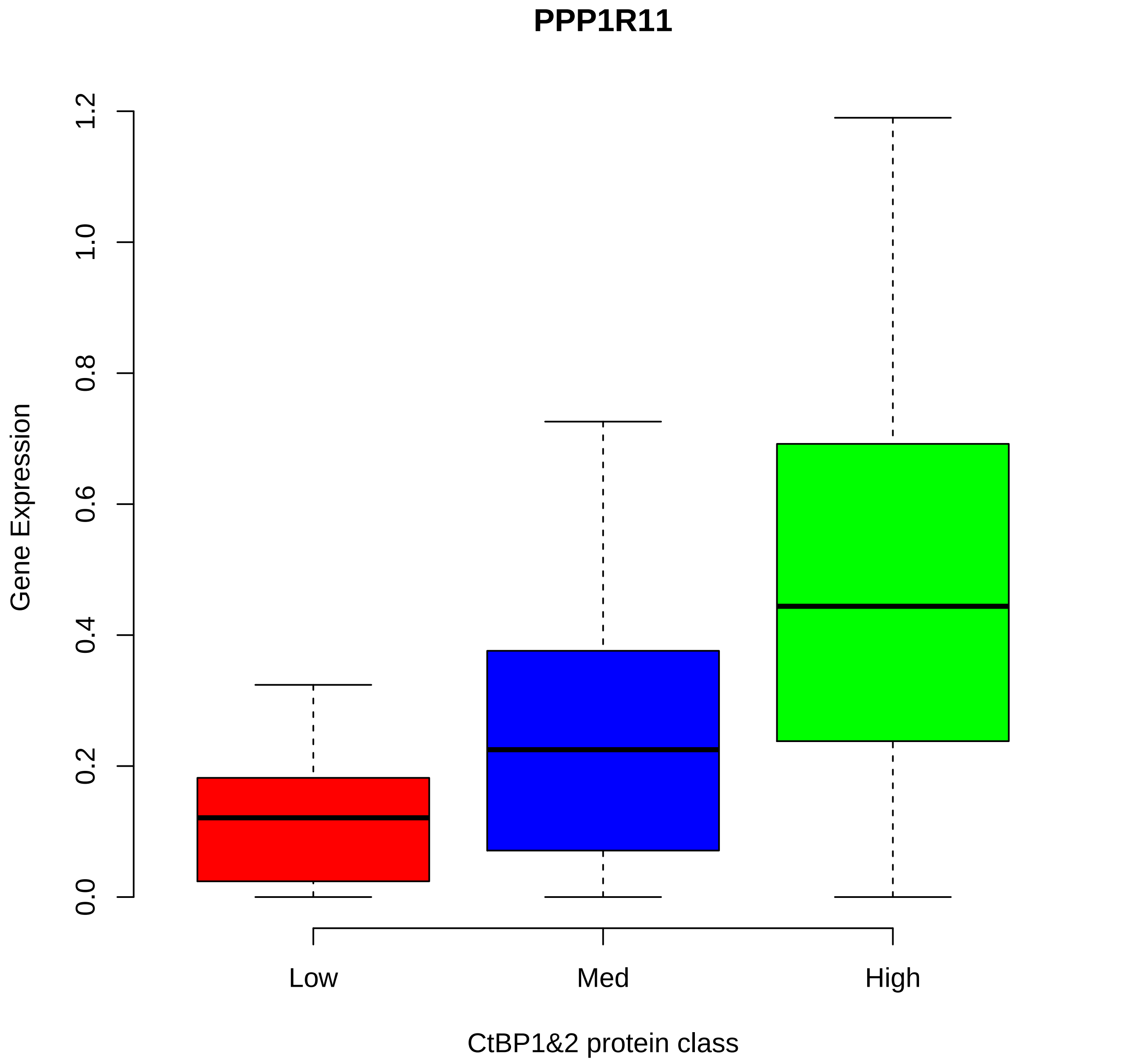

PPP1R11
Gene Expression
0.0
0.2
0.4
0.6
0.8
1.0
1.2
Low
Med
High
CtBP1&2 protein class

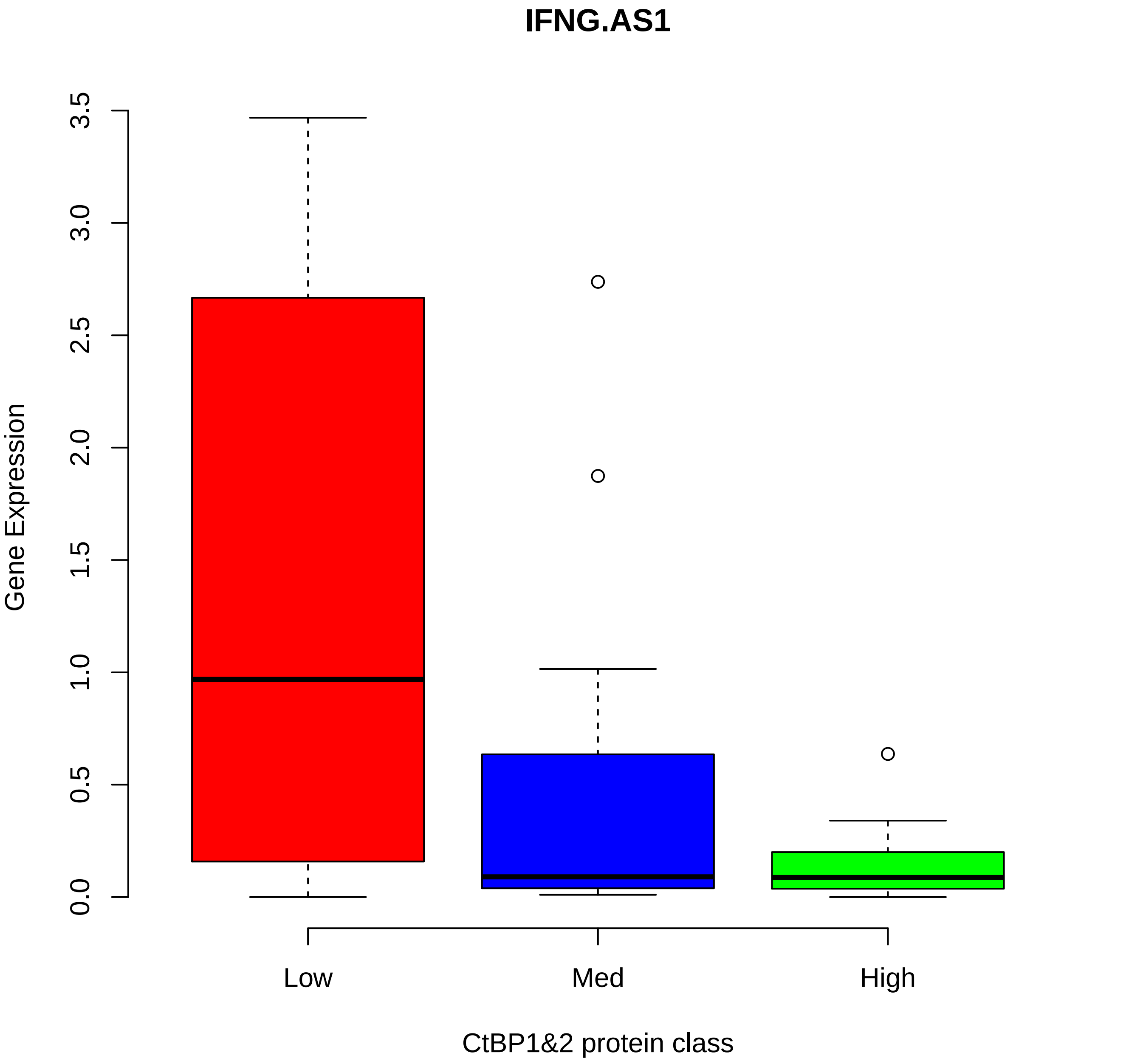

IFNG.AS1
Gene Expression
0.0
0.5
1.0
1.5
2.0
2.5
3.0
3.5
Low
Med
High
CtBP1&2 protein class

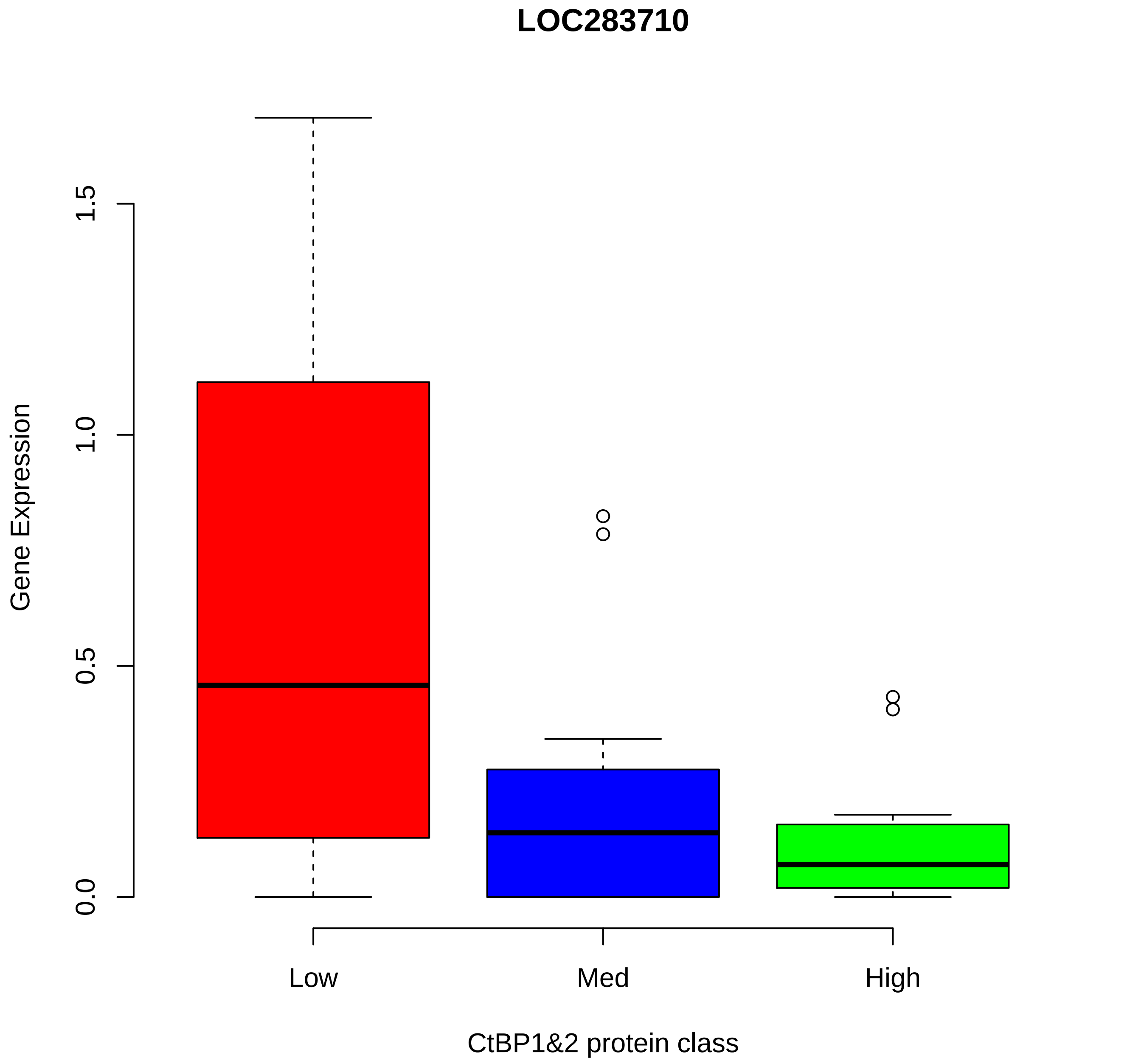

LOC283710
Gene Expression
0.0
0.5
1.0
1.5
Low
Med
High
CtBP1&2 protein class

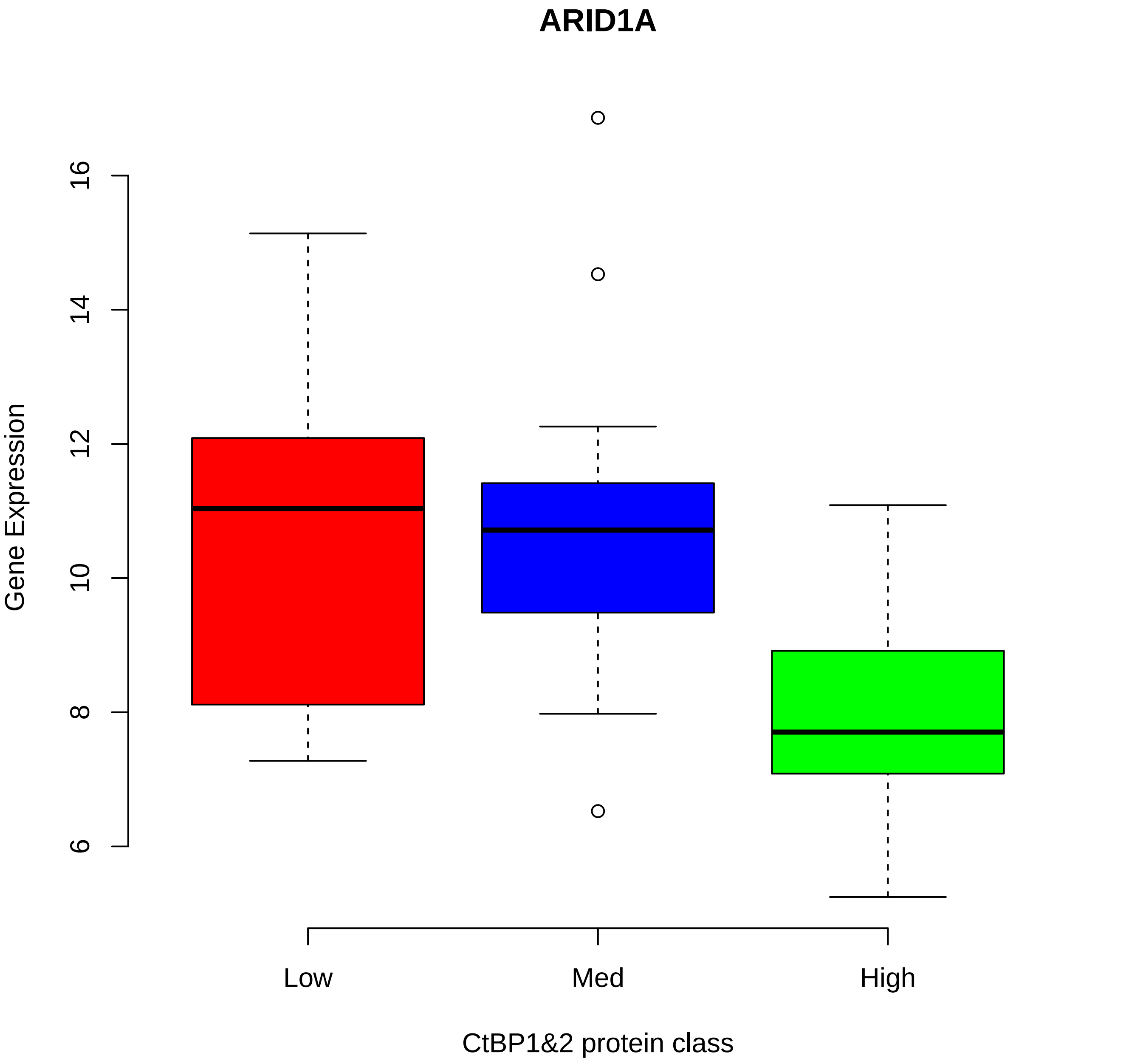

ARID1A
Gene Expression
6
8
10
12
14
16
Low
Med
High
CtBP1&2 protein class

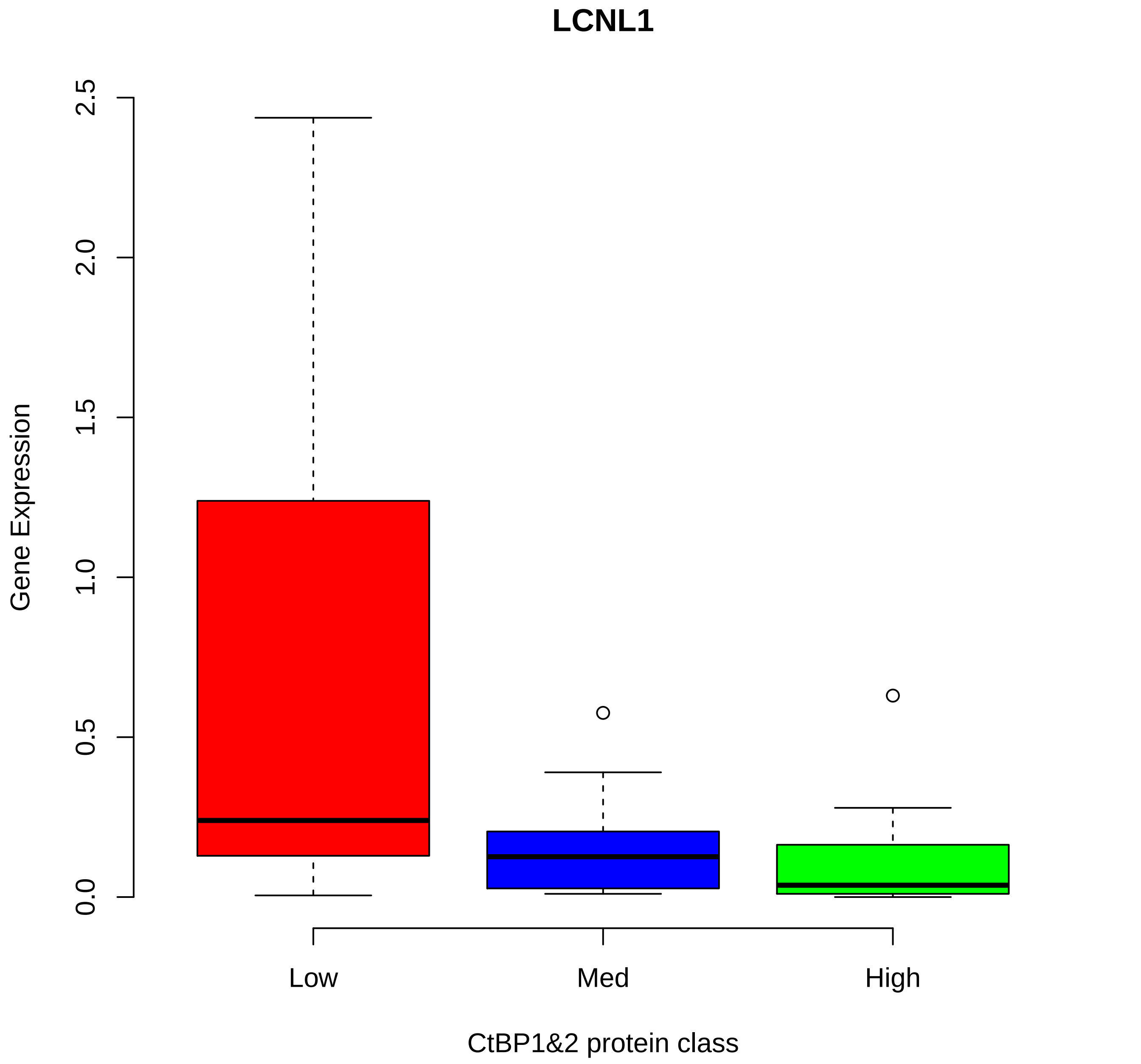
LCNL1
Gene Expression
2.5
2.0
1.5
1.0
0.5
0.0
Low
Med
High
CtBP1&2 protein class

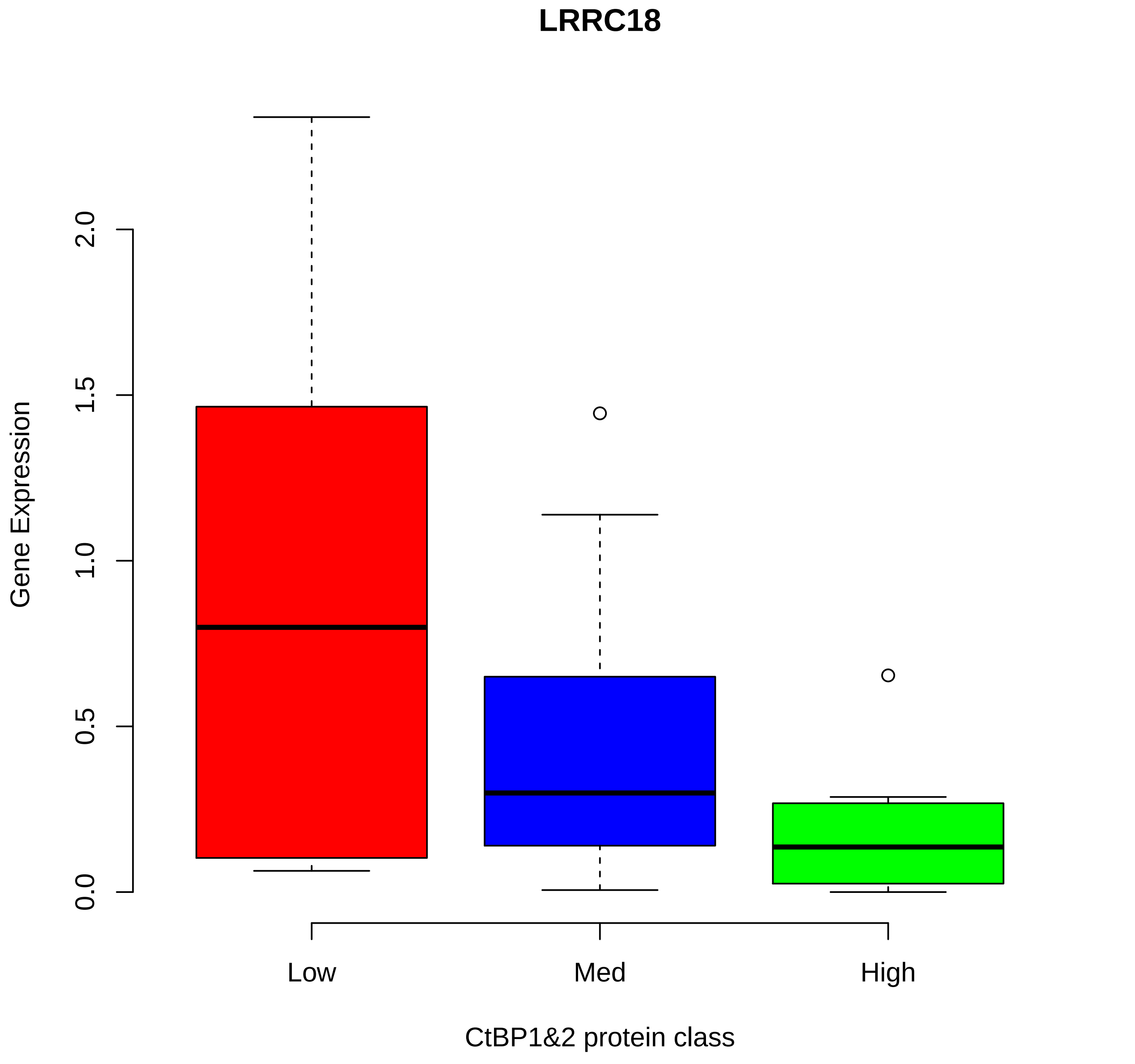

LRRC18
Gene Expression
0.0
0.5
1.0
1.5
2.0
Low
Med
High
CtBP1&2 protein class

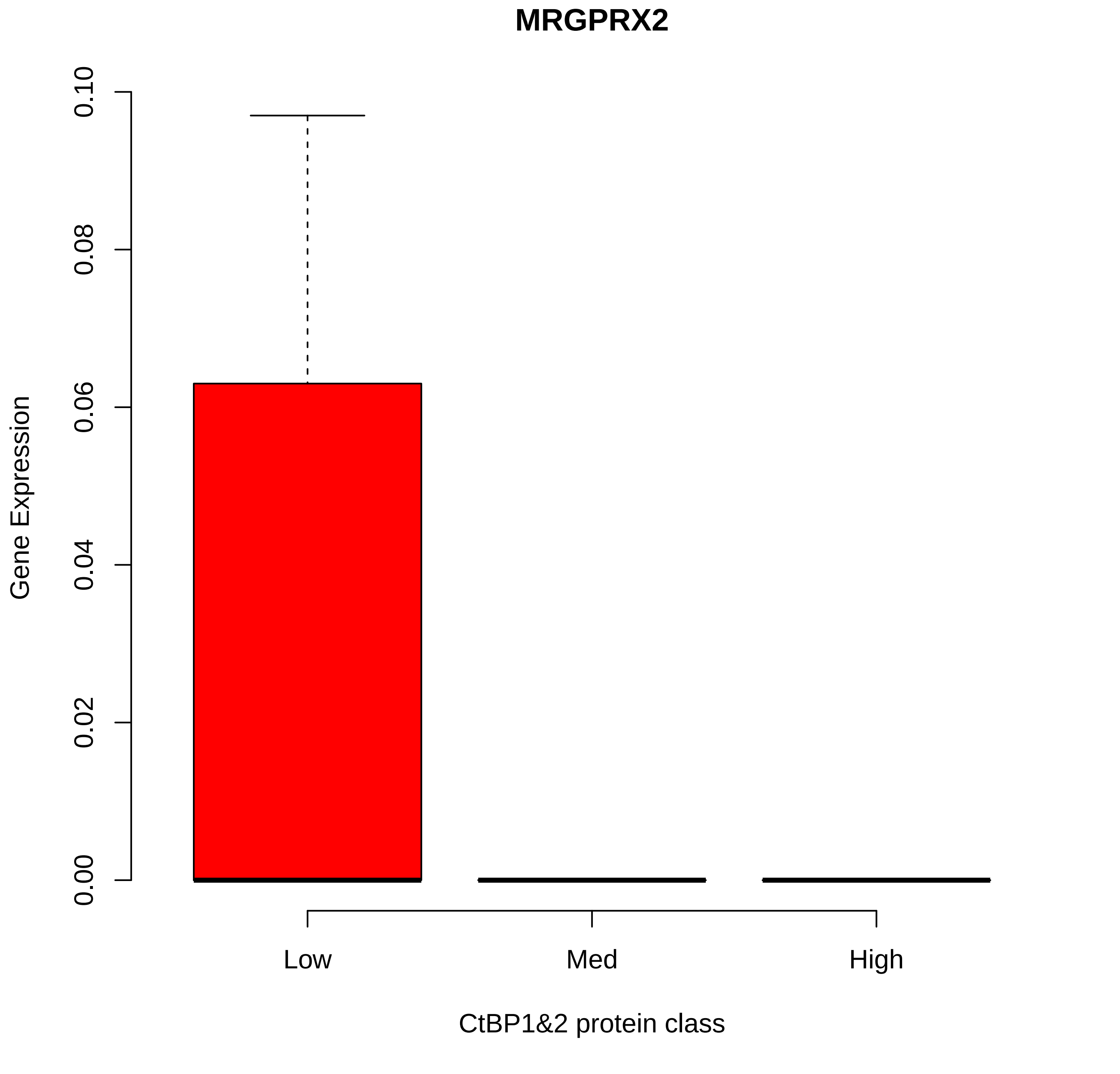
MRGPRX2
Gene Expression
0.00
0.02
0.04
0.06
0.08
0.10
Low
Med
High
CtBP1&2 protein class

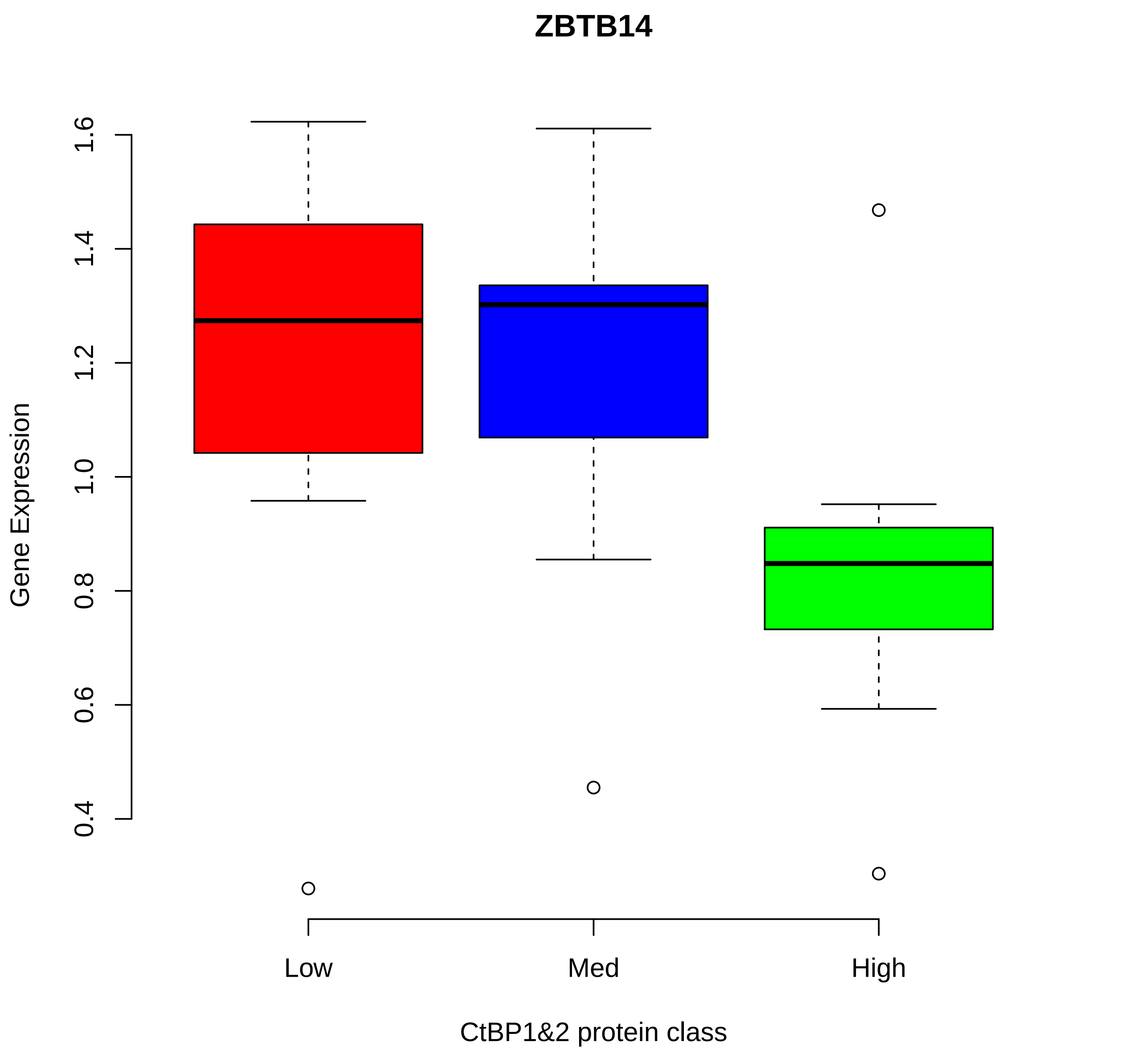

ZBTB14
Gene Expression
0.4
0.6
0.8
1.0
1.2
1.4
1.6
Low
Med
High
CtBP1&2 protein class

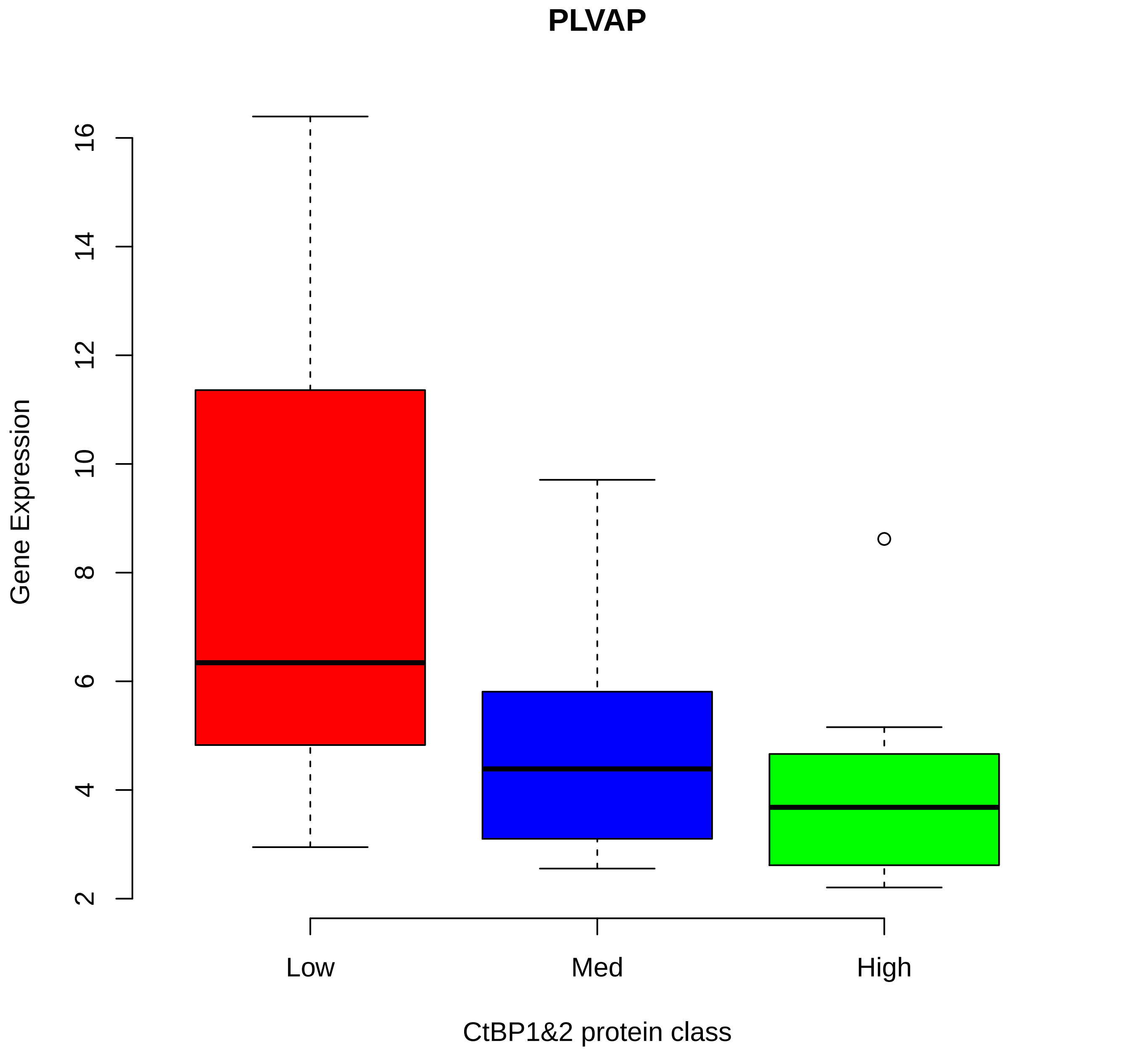
PLVAP
Gene Expression
2
4
6
8
10
12
14
16
Low
Med
High
CtBP1&2 protein class

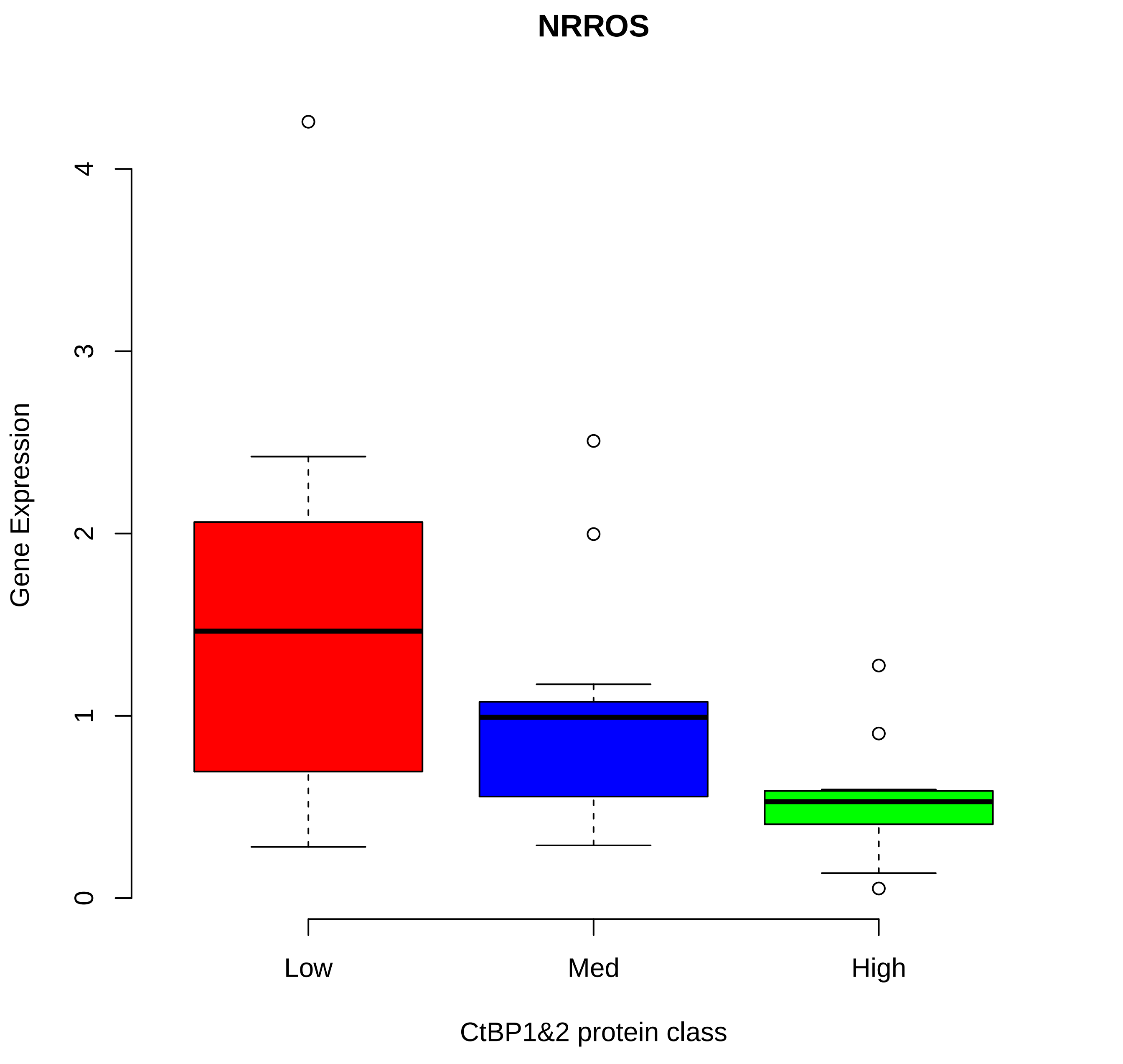

NRROS
Gene Expression
0
1
2
3
4
Low
Med
High
CtBP1&2 protein class

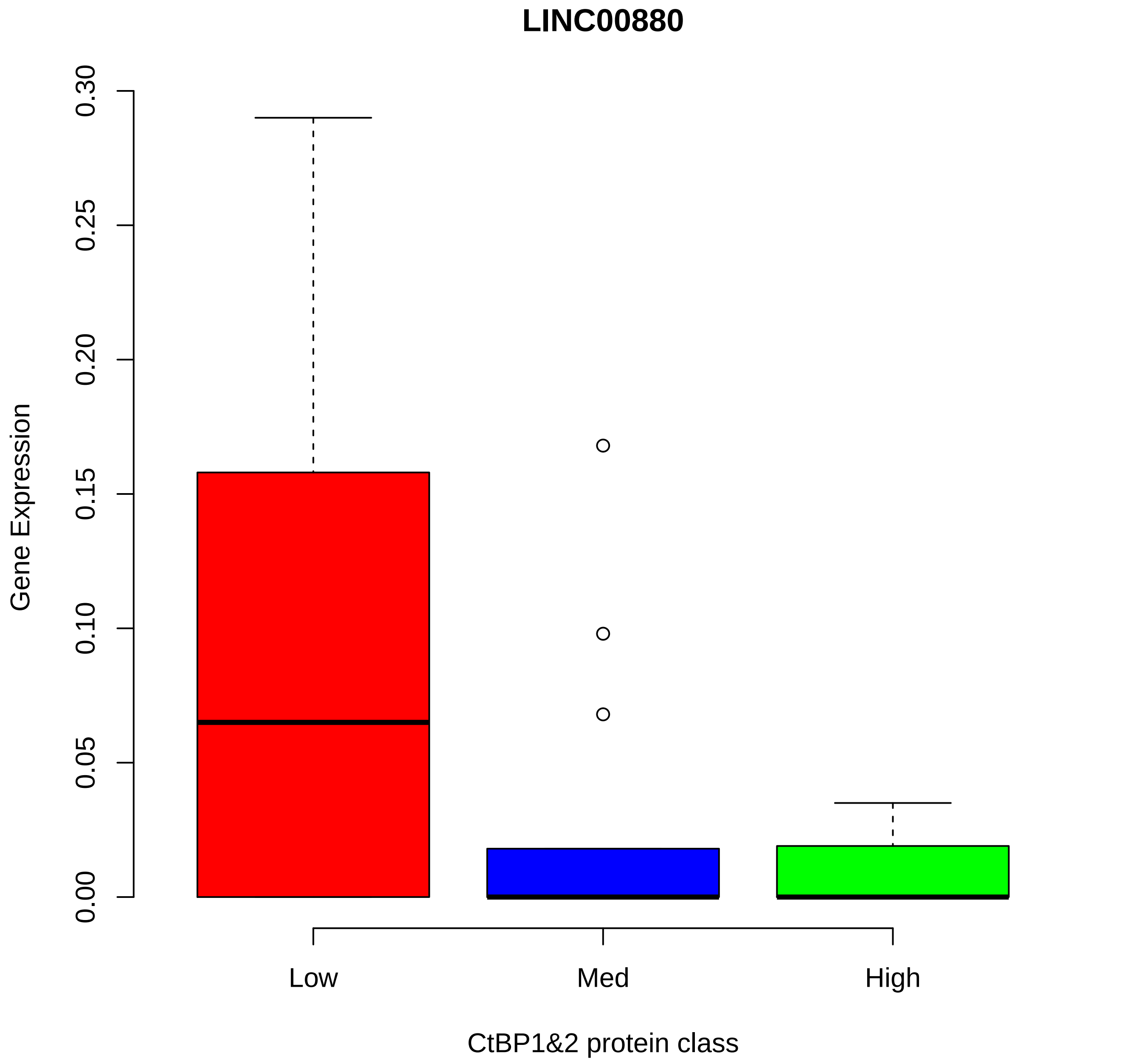
LINC00880
0.30
0.25
0.20
0.15
0.10
0.05
0.00
Gene Expression
Low
Med
High
CtBP1&2 protein class

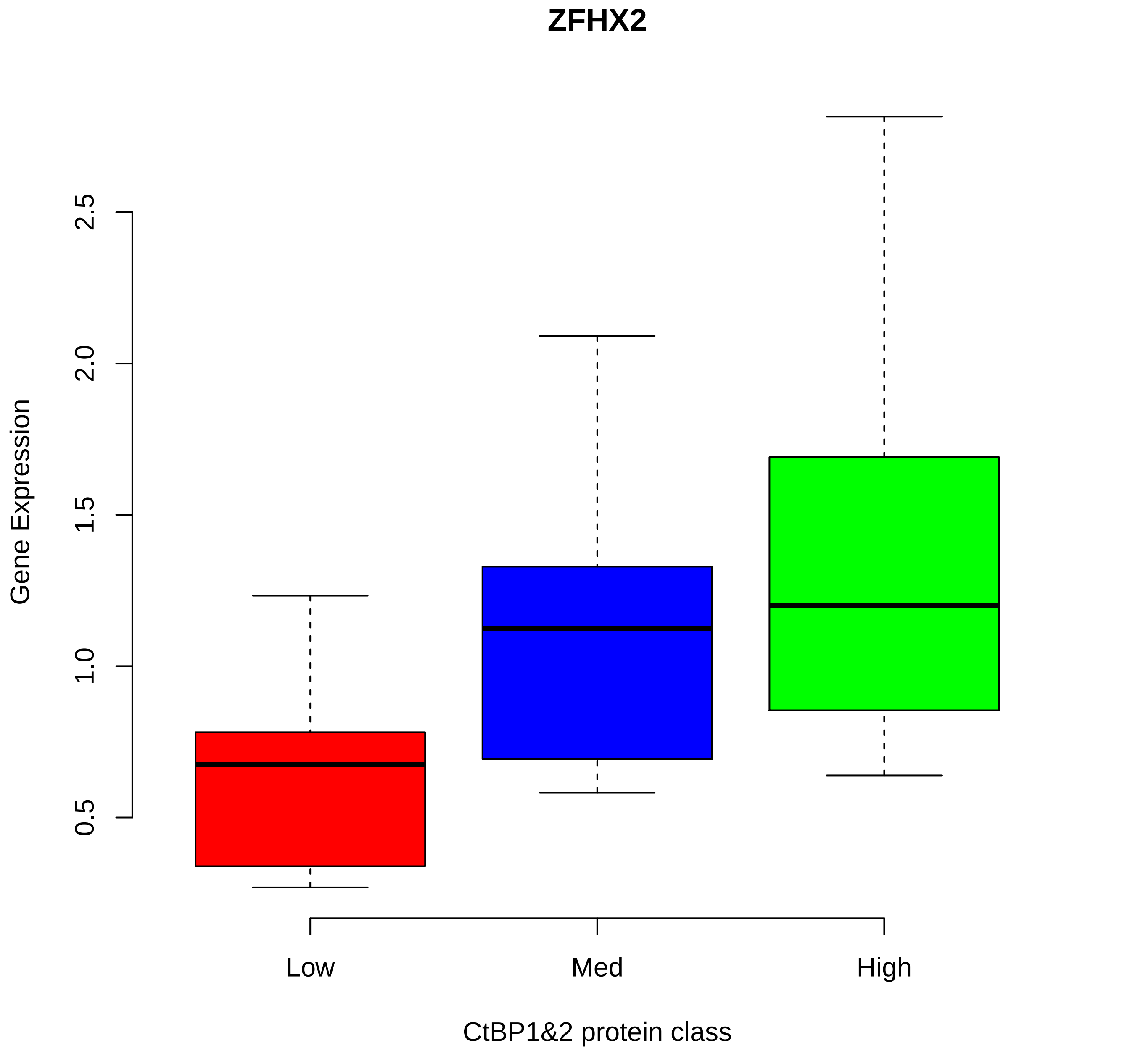
ZFHX2
Gene Expression
0.5
1.0
1.5
2.0
2.5
Low
Med
High
CtBP1&2 protein class

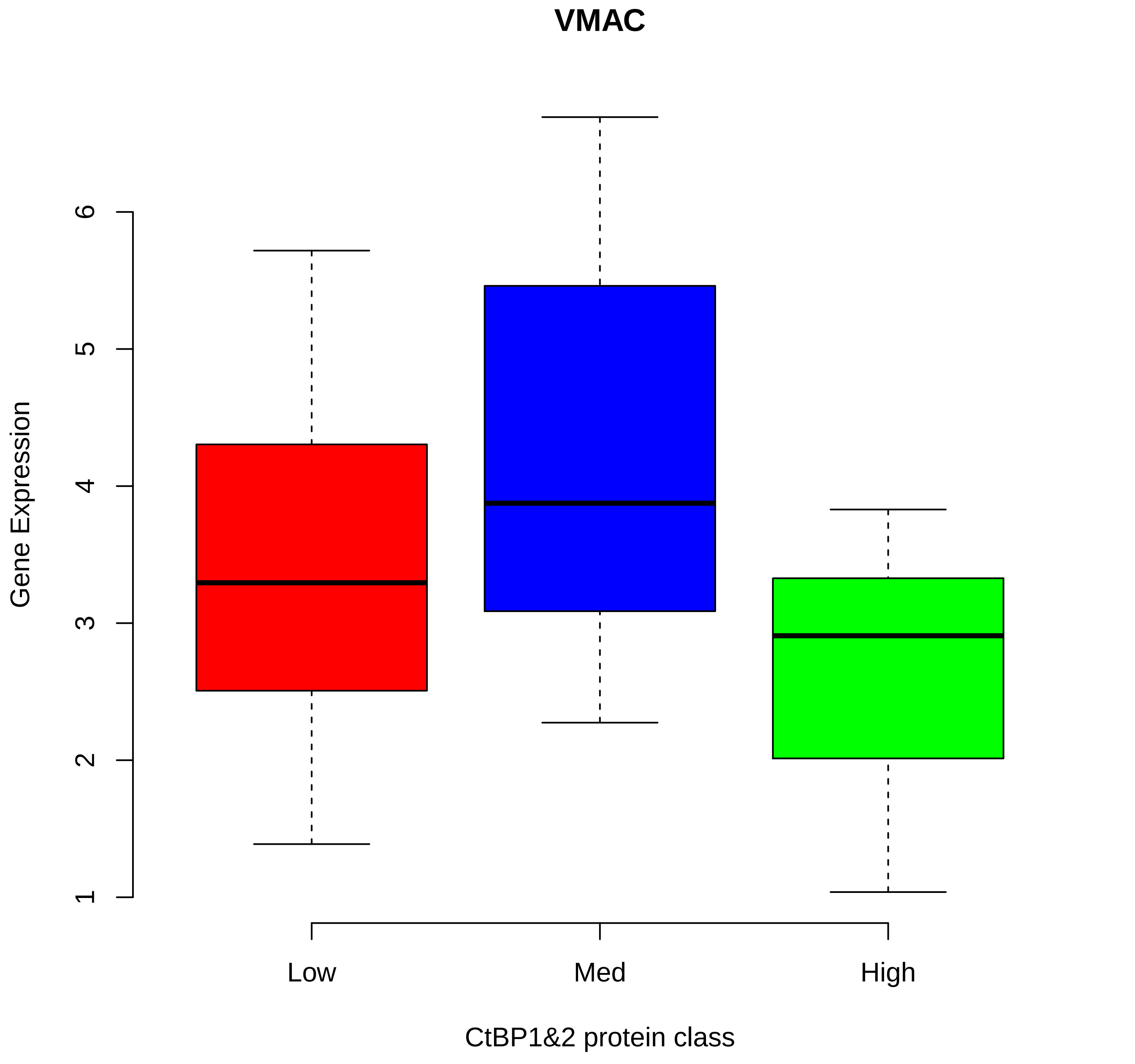

VMAC
Gene Expression
1
2
3
4
5
6
Low
Med
High
CtBP1&2 protein class

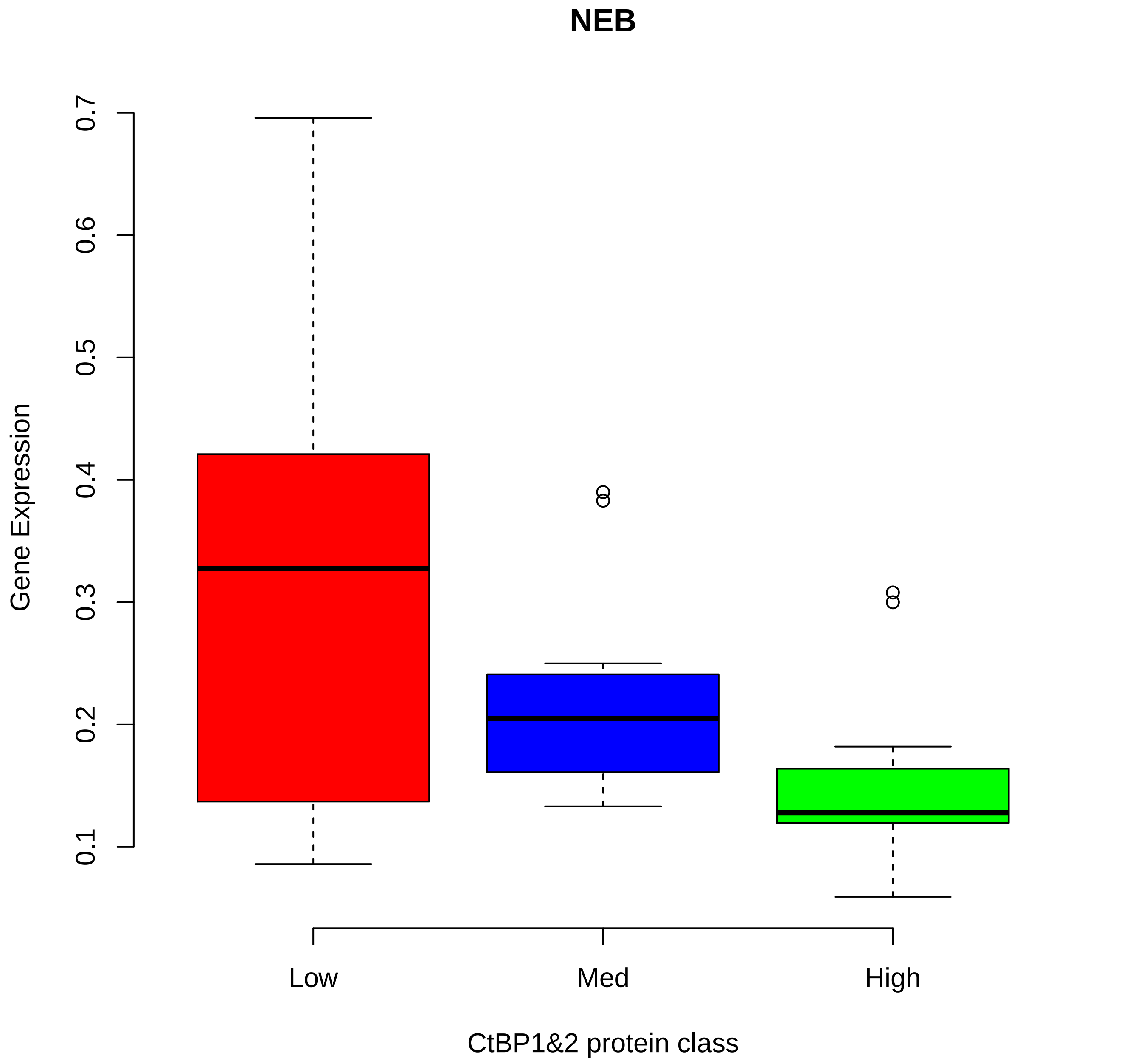

NEB
Gene Expression
0.1
0.2
0.3
0.4
0.5
0.6
0.7
Low
Med
High
CtBP1&2 protein class

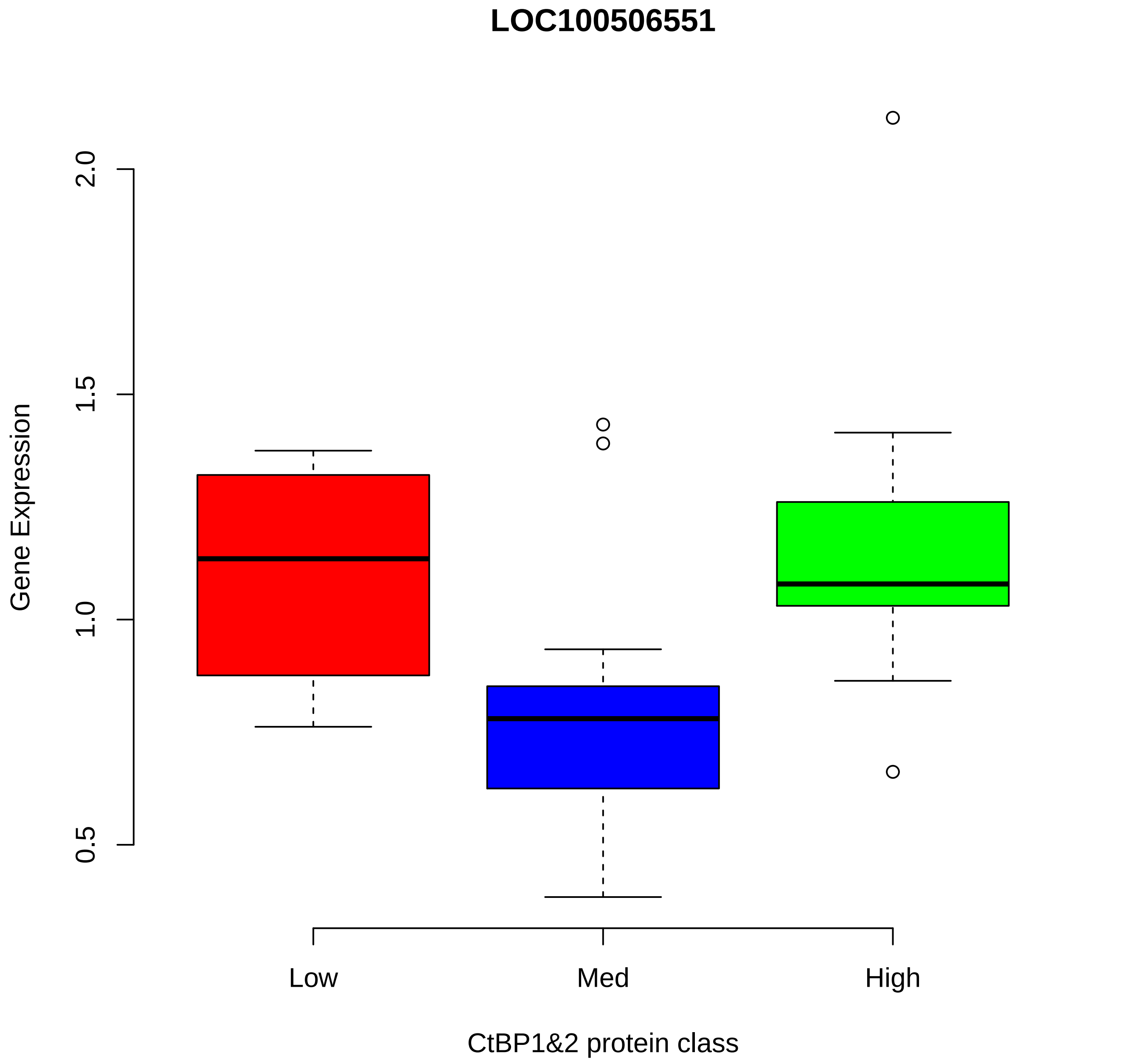
LOC100506551
Gene Expression
0.5
1.0
1.5
2.0
Low
Med
High
CtBP1&2 protein class

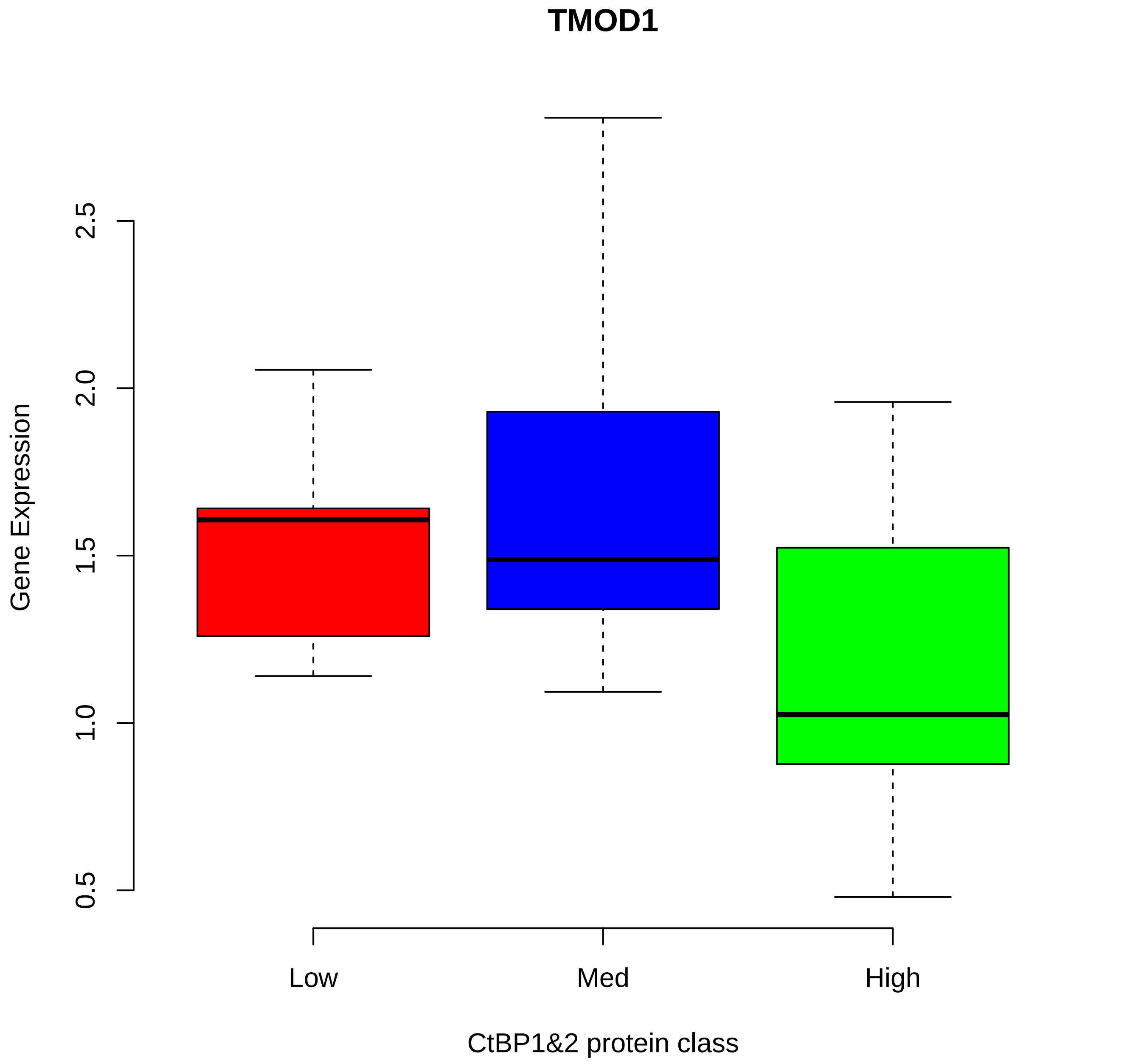
TMOD1
Gene Expression
2.5
2.0
1.5
1.0
0.5
Low
Med
High
CtBP1&2 protein class

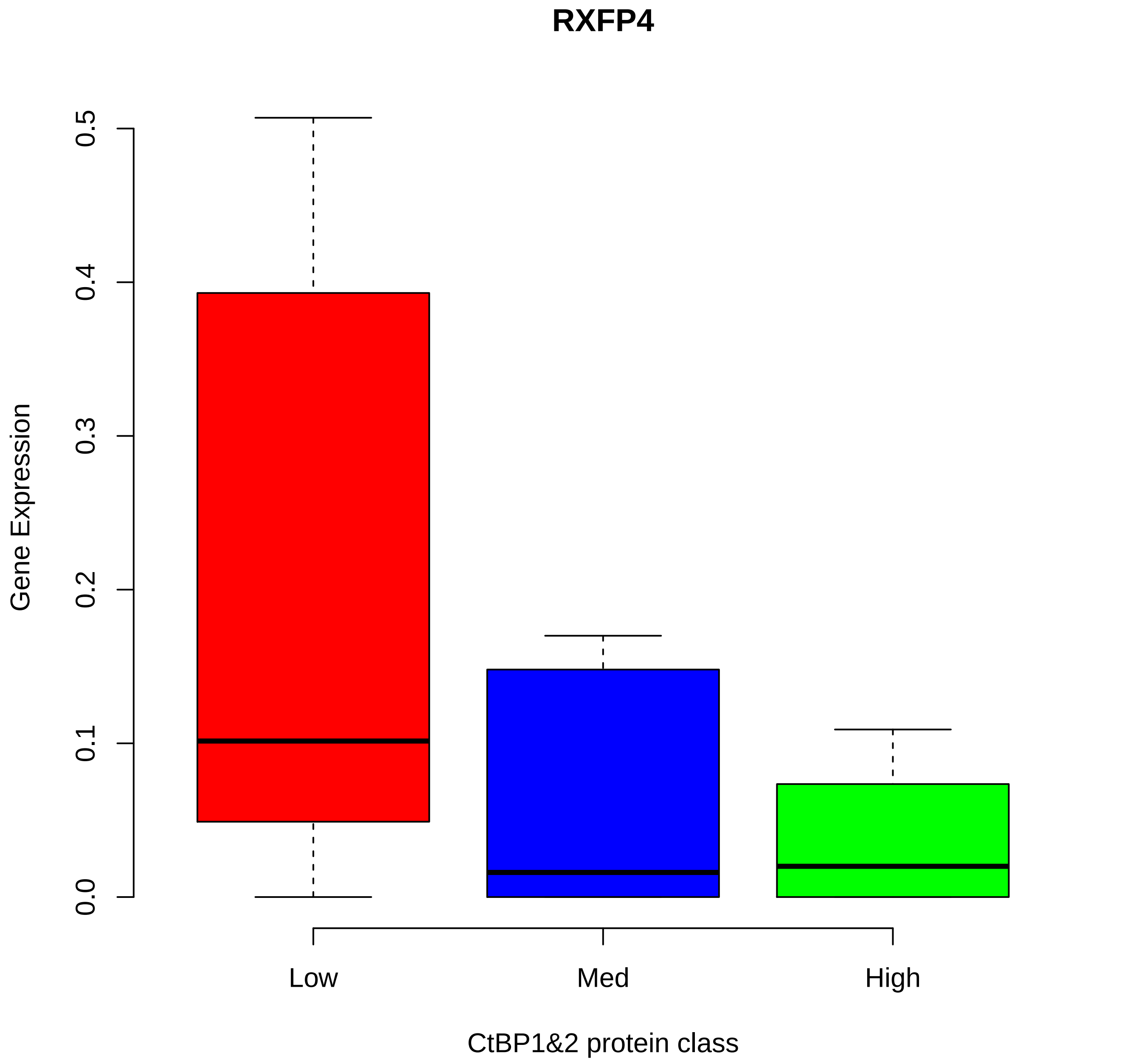

RXFP4
Gene Expression
0.0
0.1
0.2
0.3
0.4
0.5
Low
Med
High
CtBP1&2 protein class

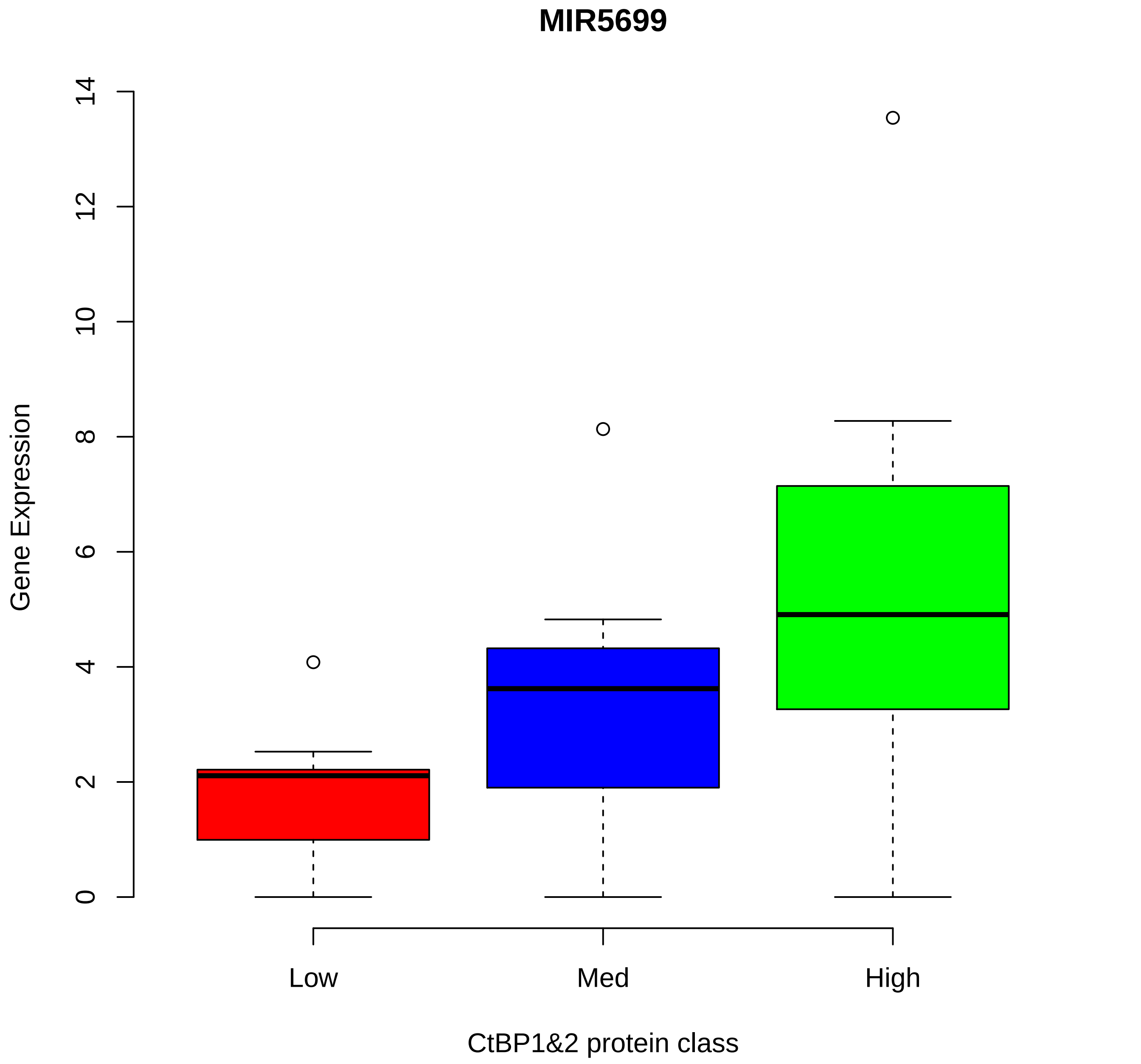

MIR5699
Gene Expression
0
2
4
6
8
10
12
14
Low
Med
High
CtBP1&2 protein class

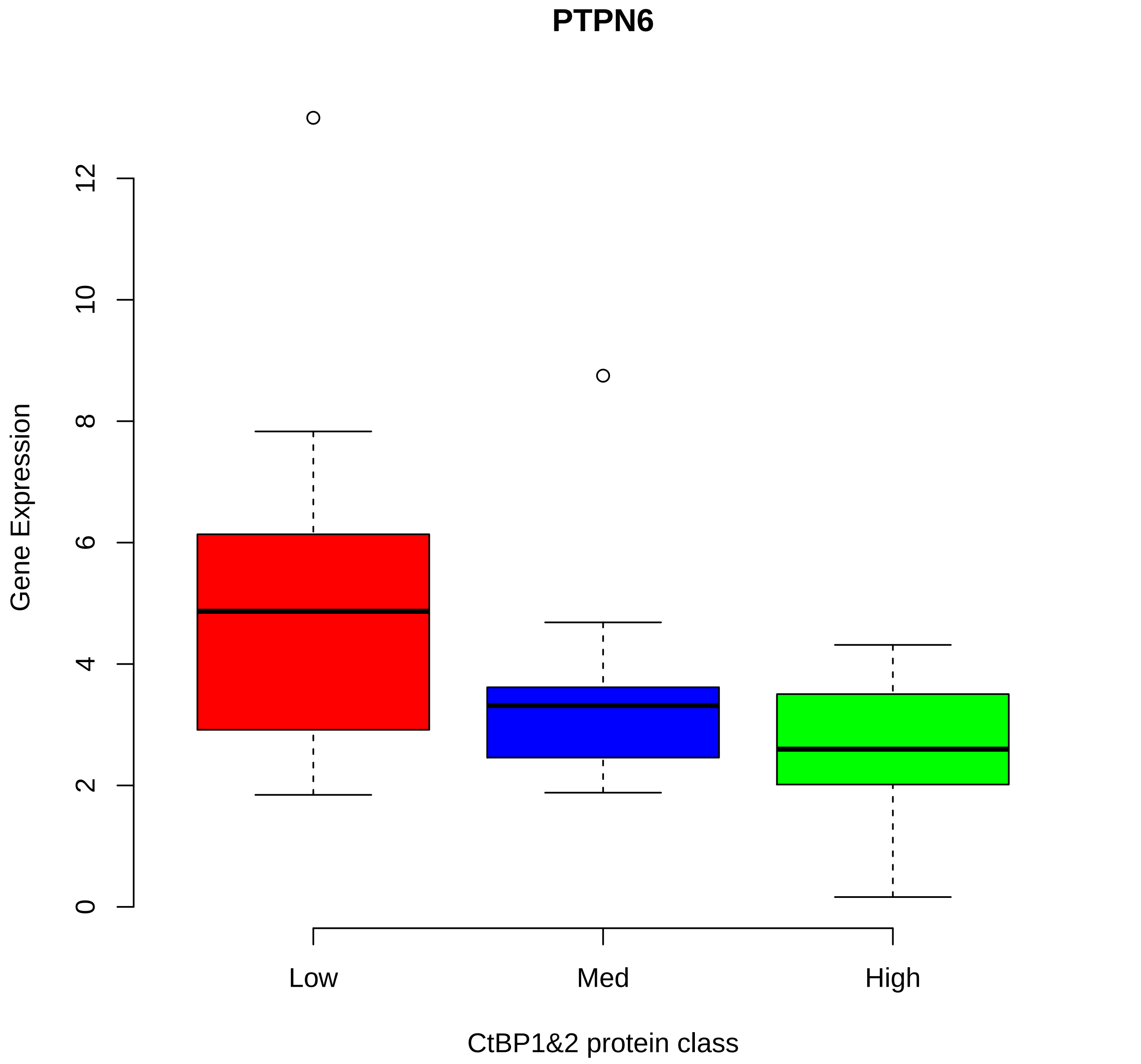

PTPN6
Gene Expression
0
2
4
6
8
10
12
Low
Med
High
CtBP1&2 protein class

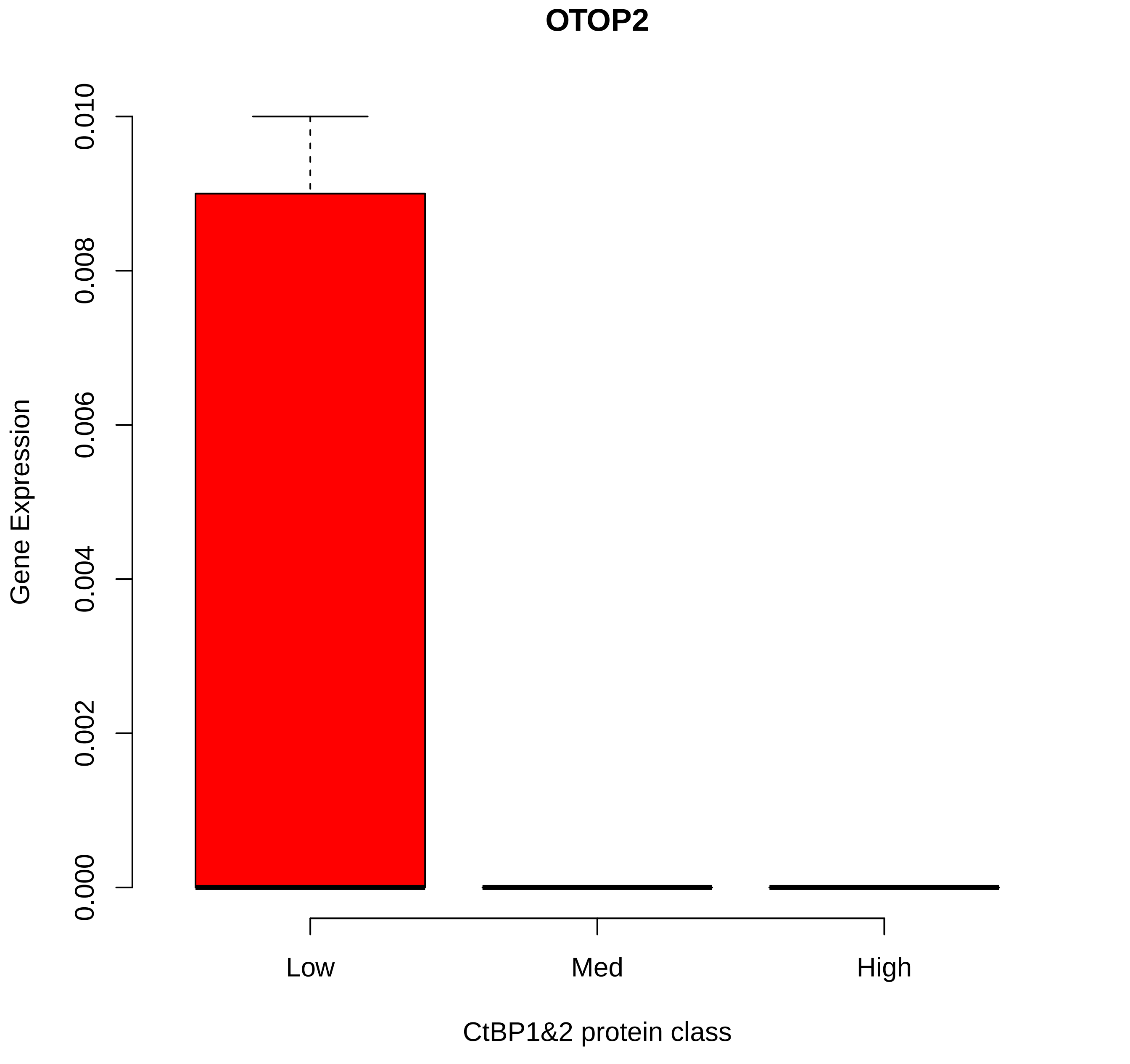

OTOP2
Gene Expression
0.000
0.002
0.004
0.006
0.008
0.010
Low
Med
High
CtBP1&2 protein class

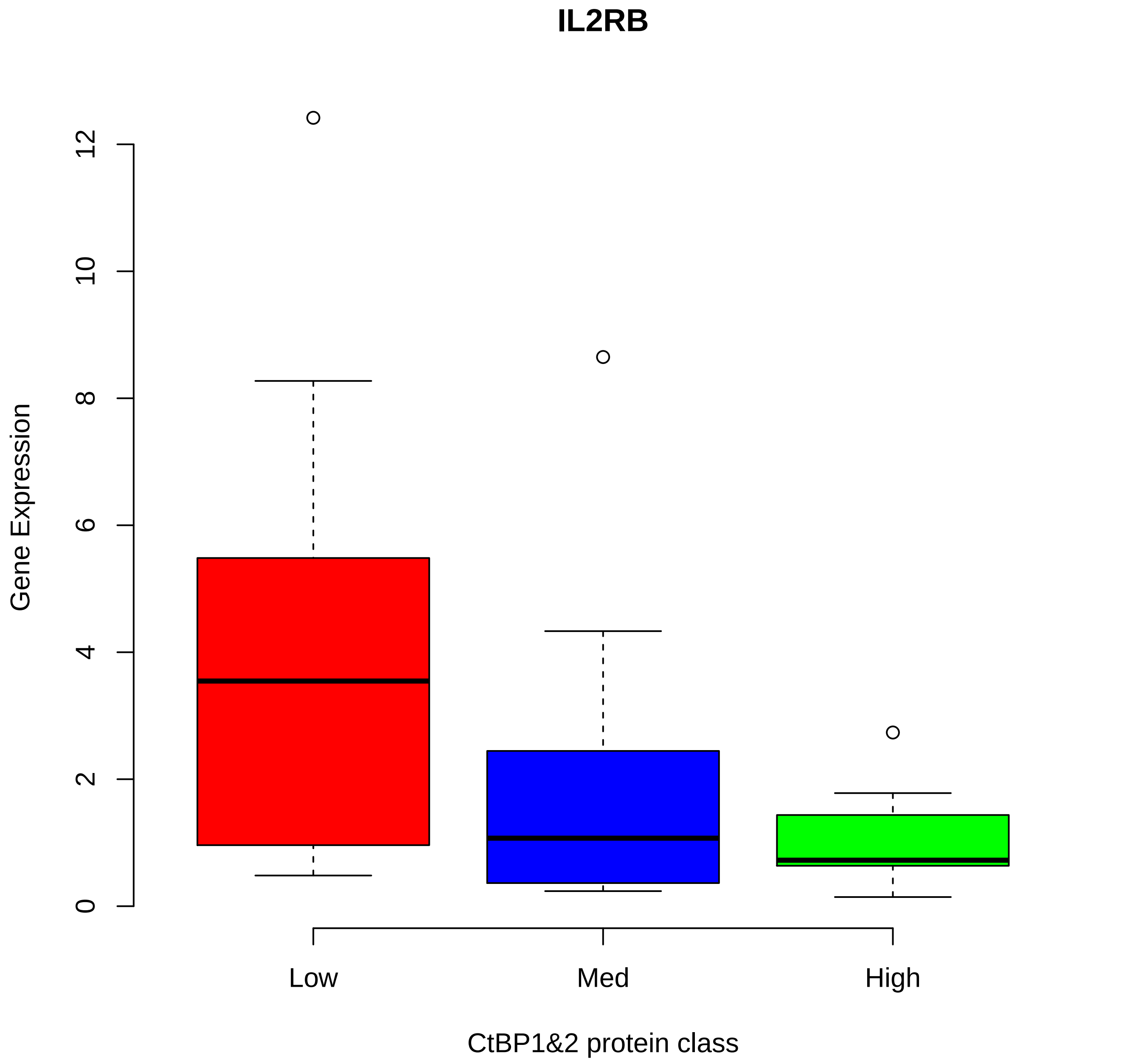

IL2RB
Gene Expression
0
2
4
6
8
10
12
Low
Med
High
CtBP1&2 protein class

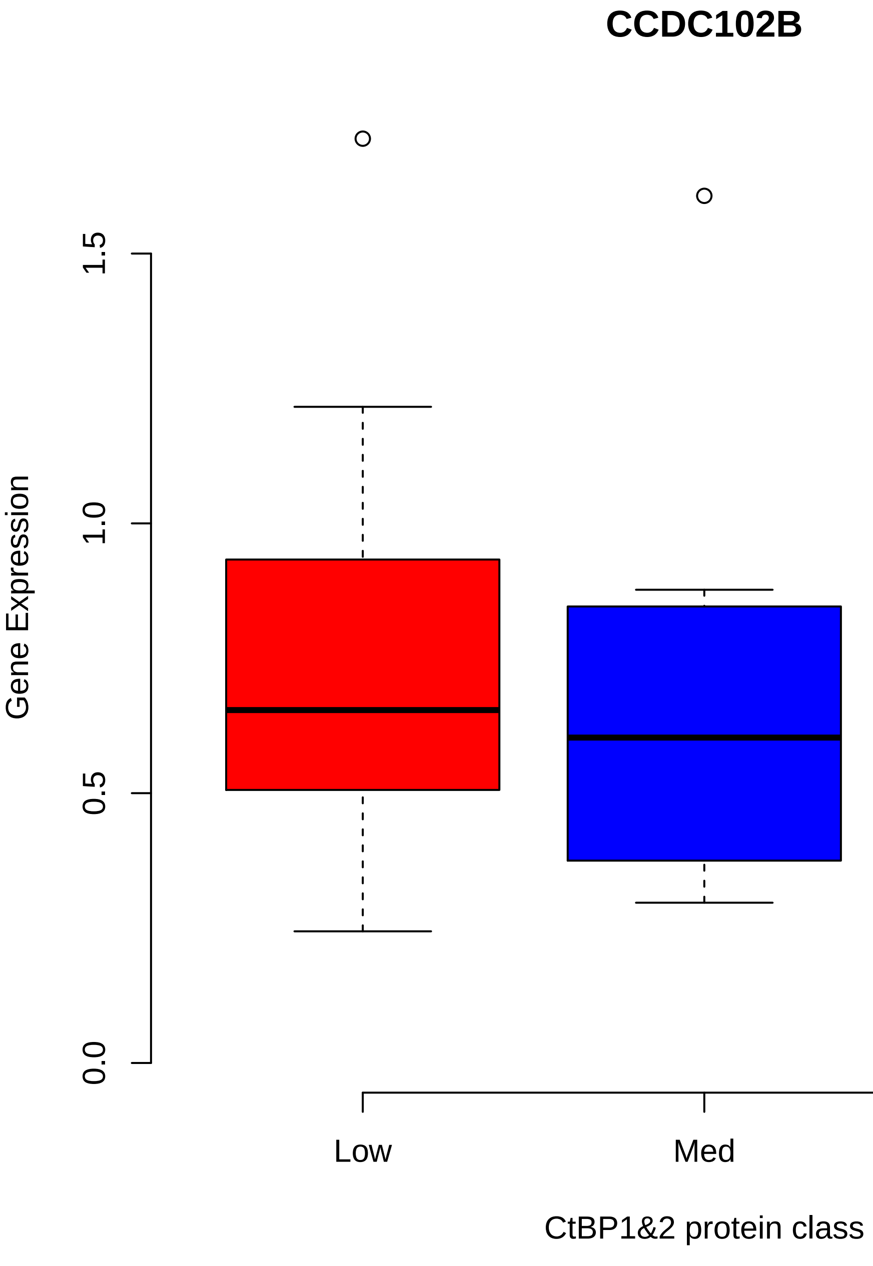
CCDC102B
Gene Expression
0.0
0.5
1.0
1.5
Low
Med
High
CtBP1&2 protein class

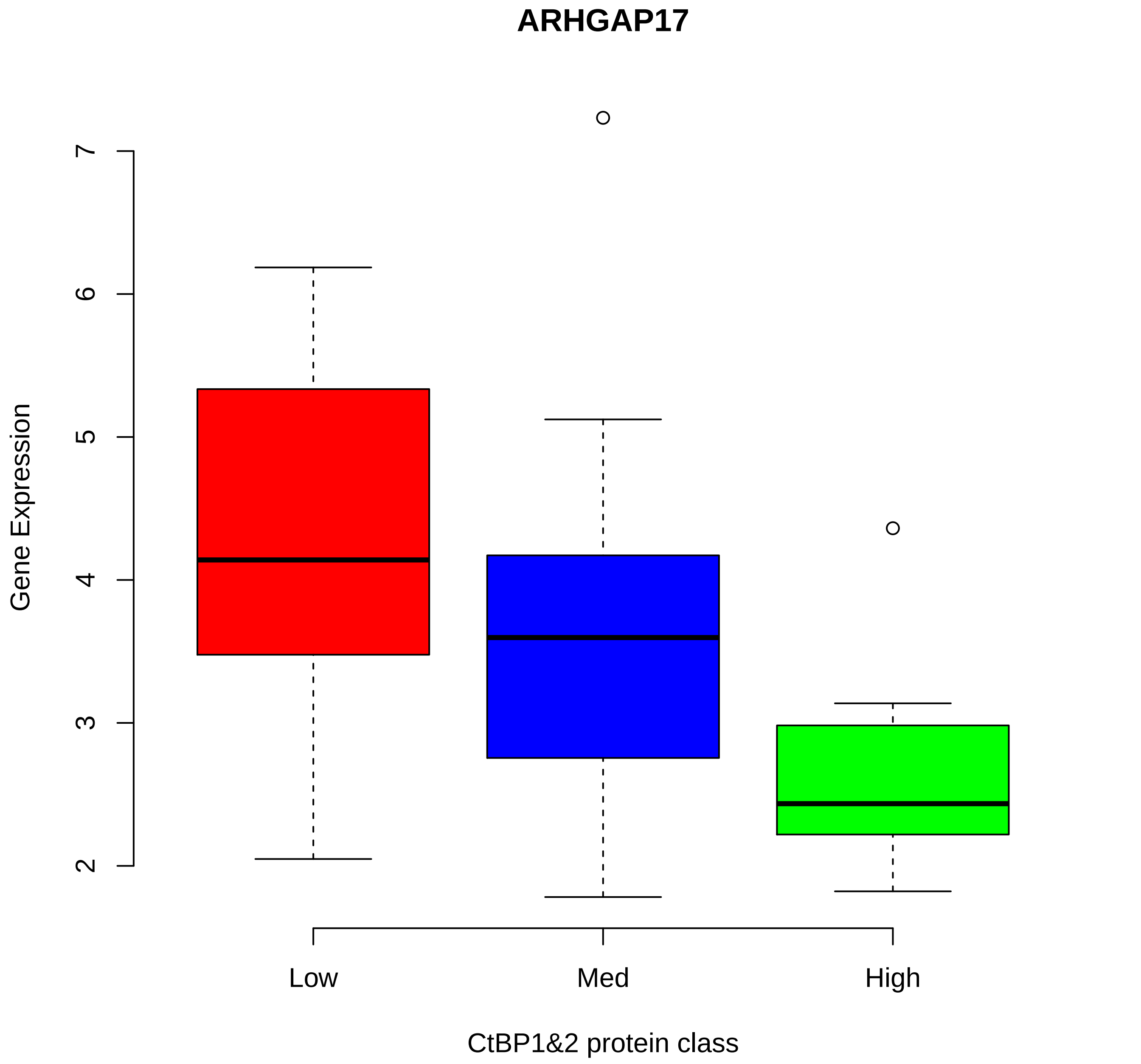

ARHGAP17
Gene Expression
2
3
4
5
6
7
Low
Med
High
CtBP1&2 protein class

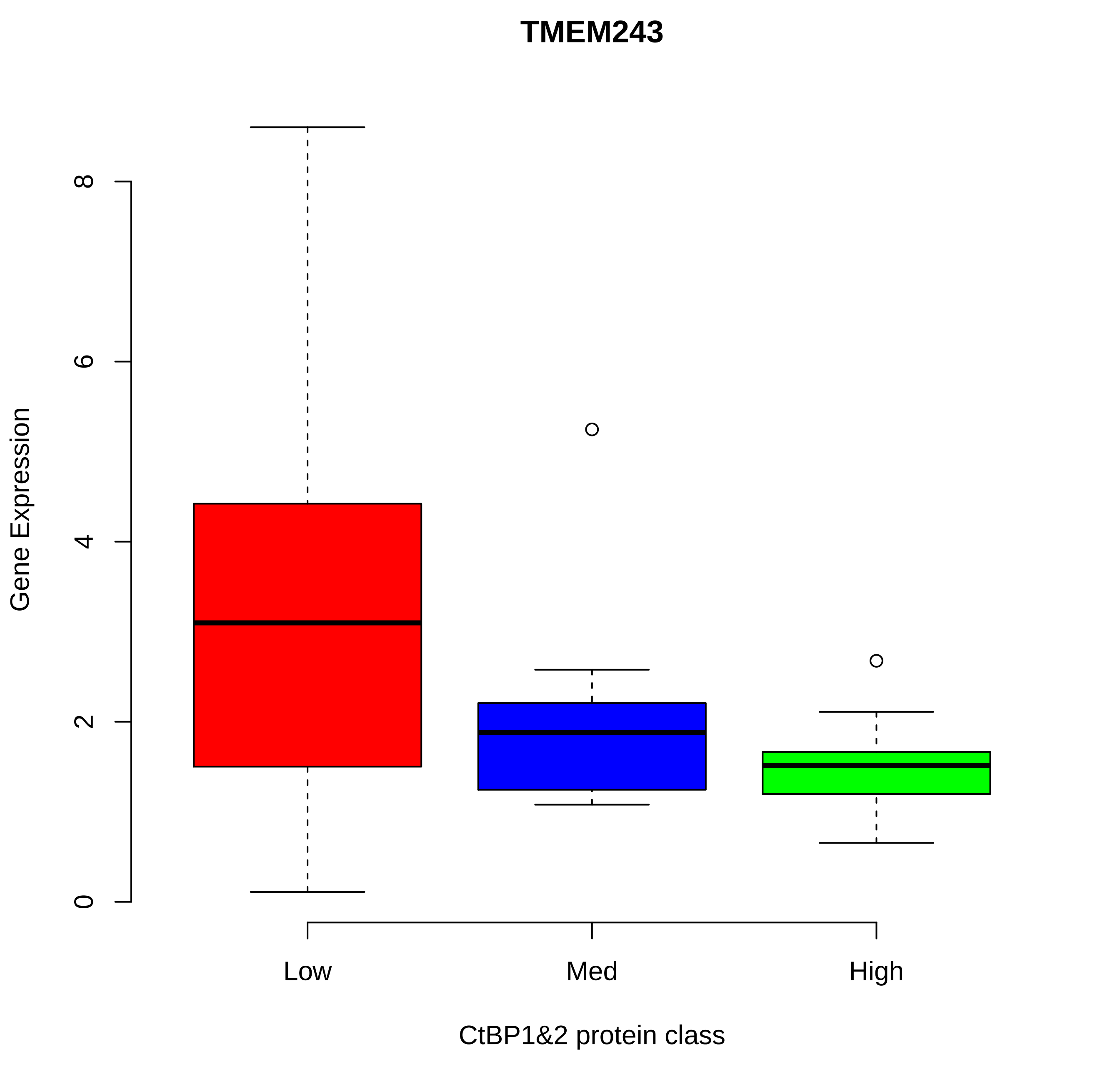
TMEM243
Gene Expression
0
2
4
6
8
Low
Med
High
CtBP1&2 protein class

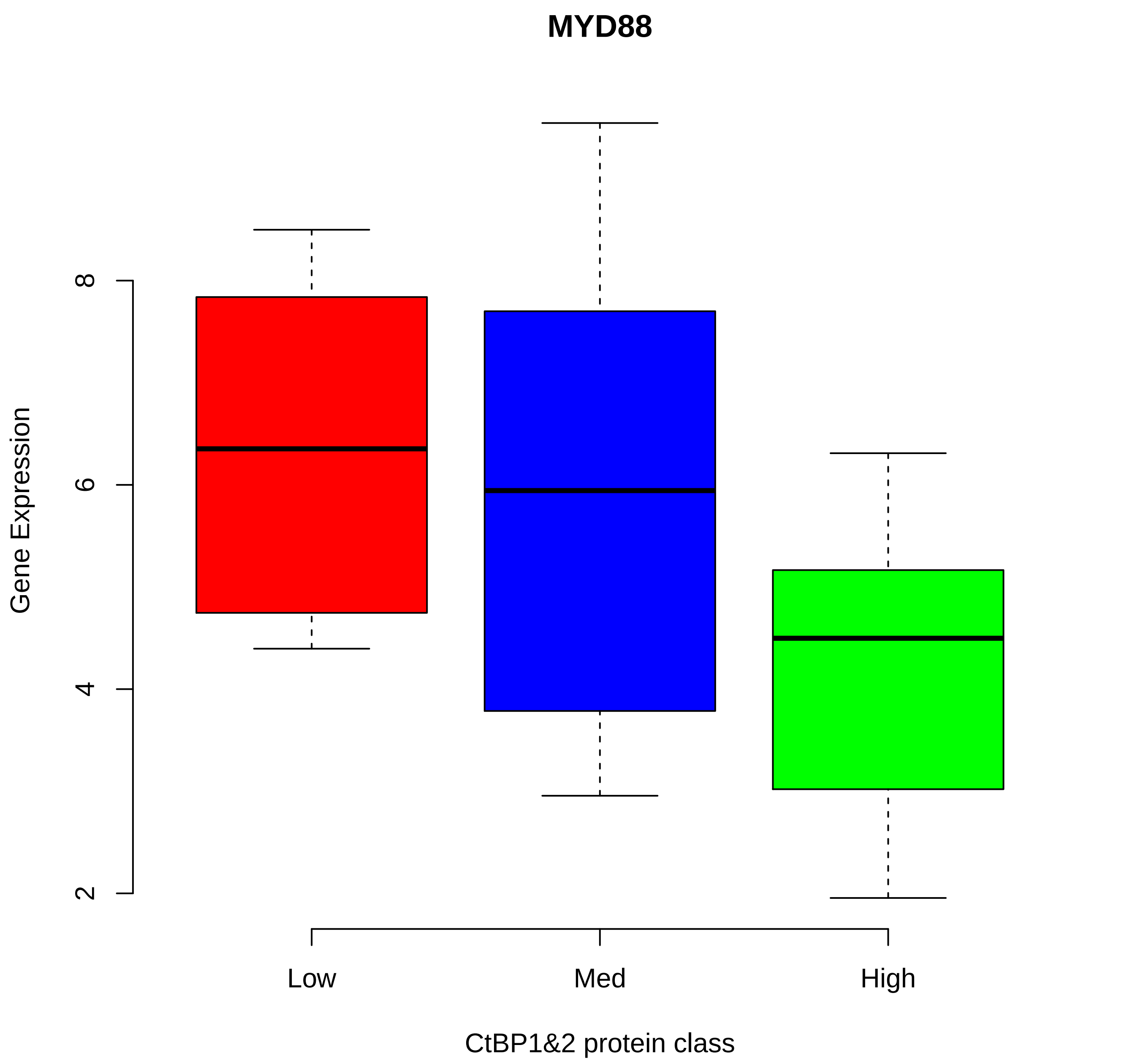
MYD88
Gene Expression
2
4
6
8
Low
Med
High
CtBP1&2 protein class

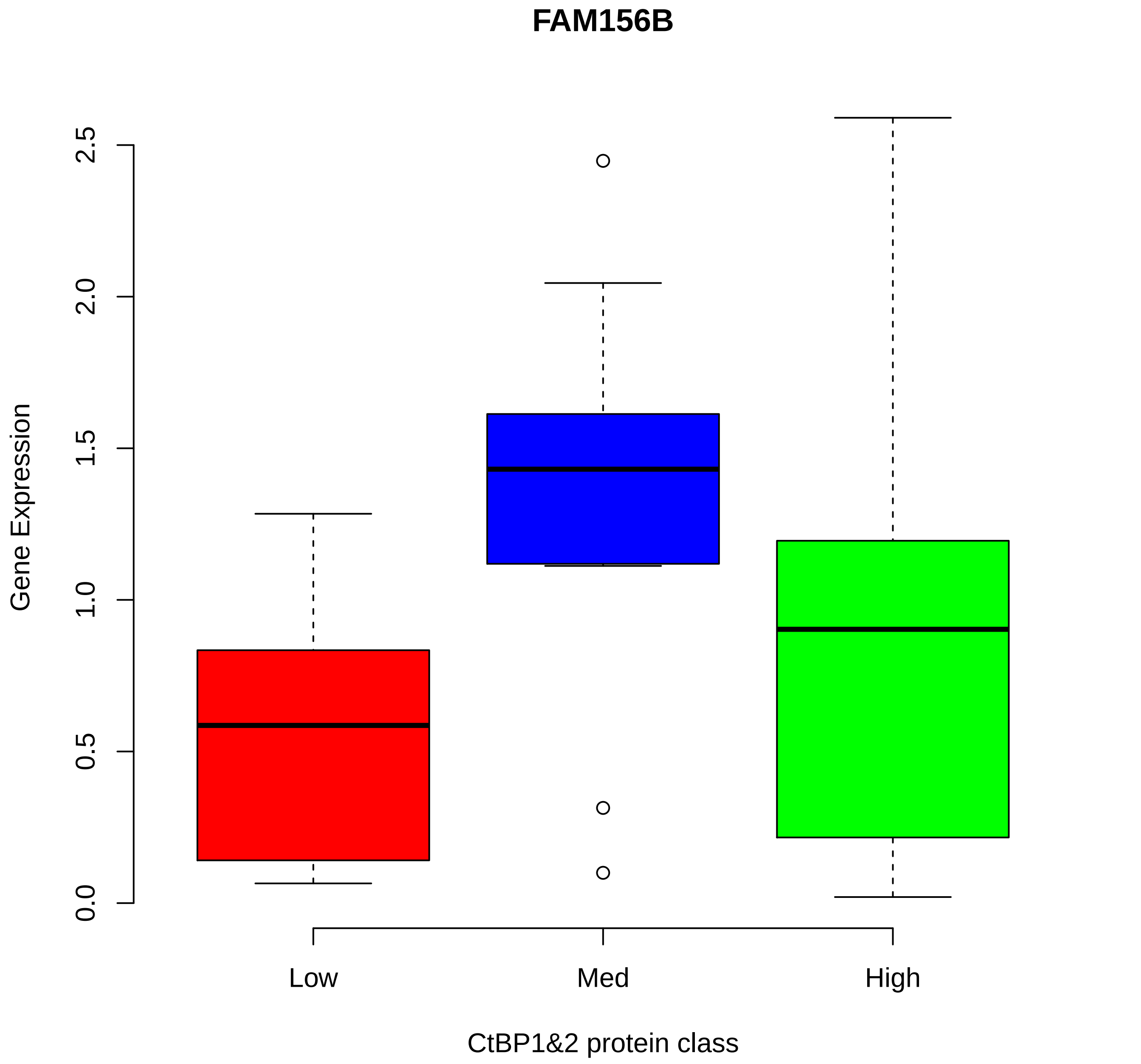

FAM156B
Gene Expression
0.0
0.5
1.0
1.5
2.0
2.5
Low
Med
High
CtBP1&2 protein class

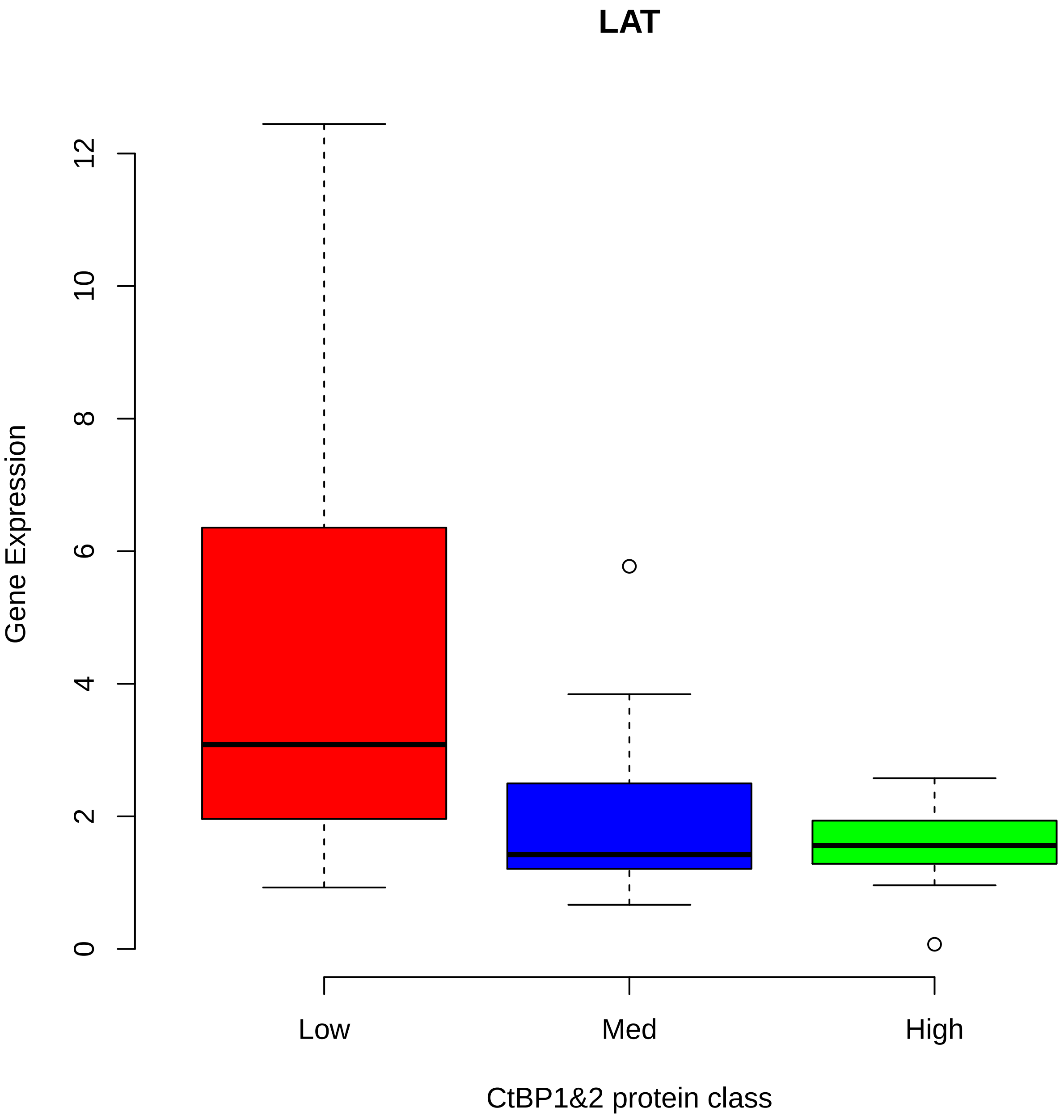

LAT
Gene Expression
0
2
4
6
8
10
12
Low
Med
High
CtBP1&2 protein class

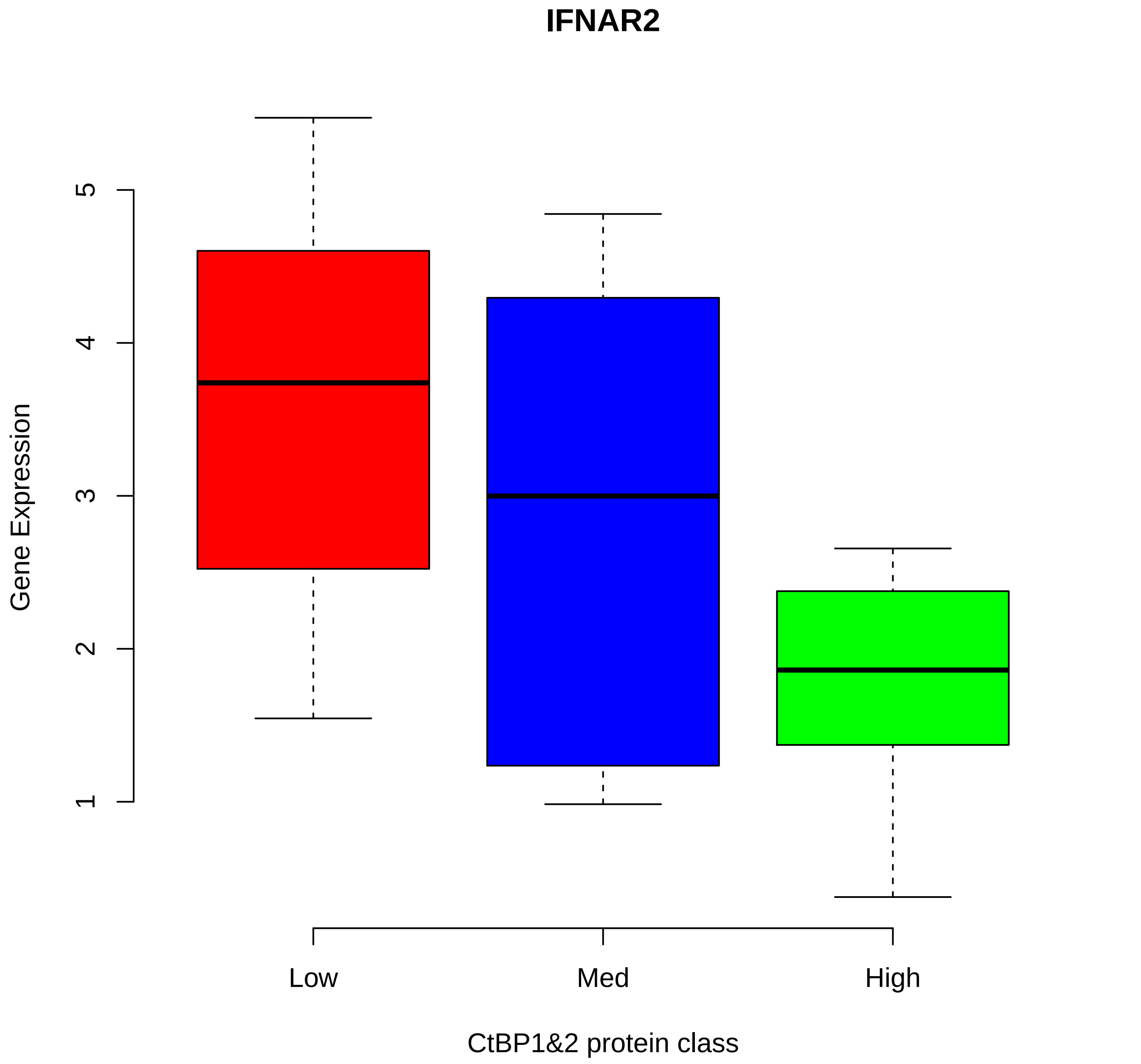
IFNAR2
Gene Expression
1
2
3
4
5
Low
Med
High
CtBP1&2 protein class

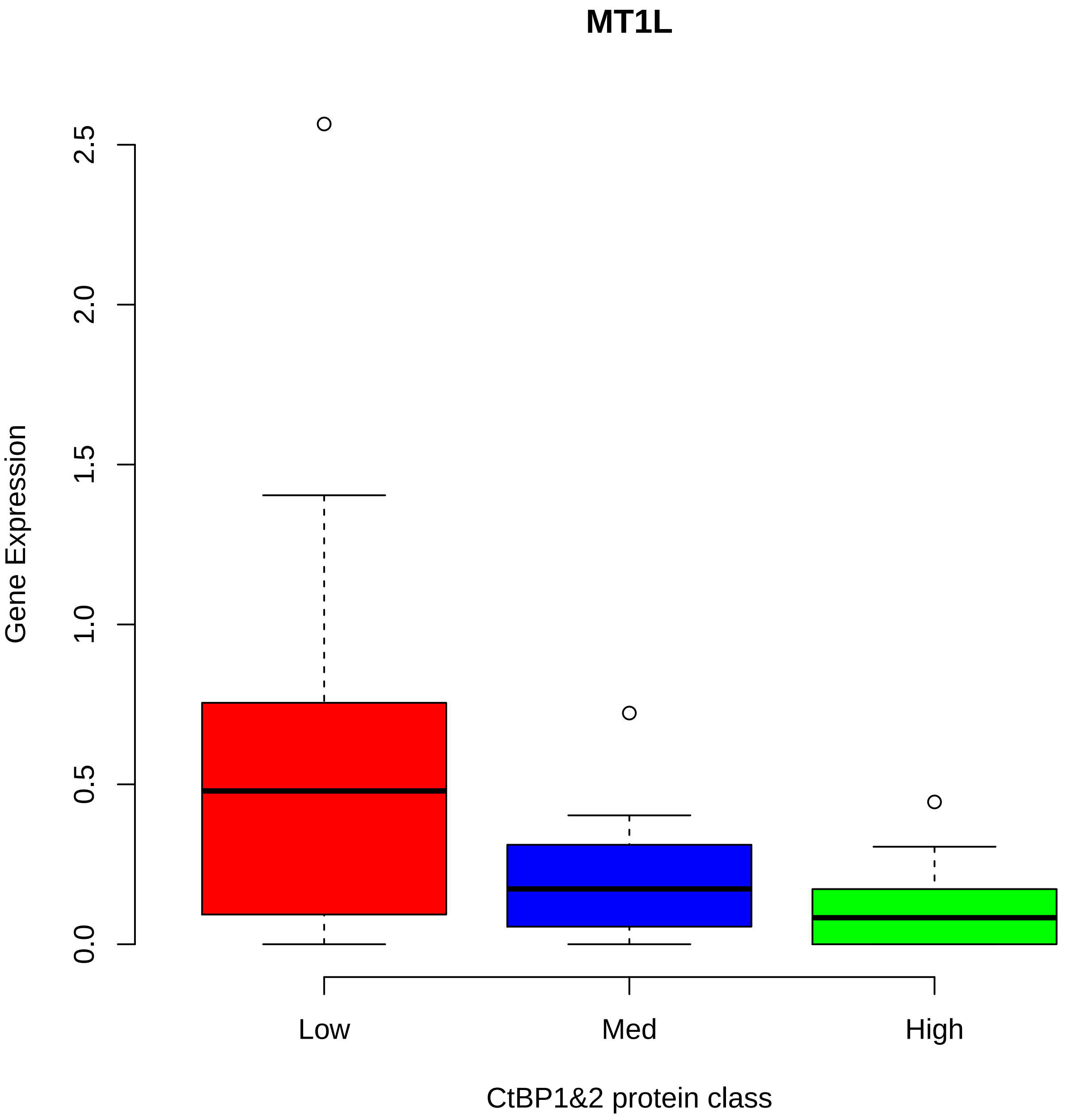

MT1L
Gene Expression
0.0
0.5
1.0
1.5
2.0
2.5
Low
Med
High
CtBP1&2 protein class

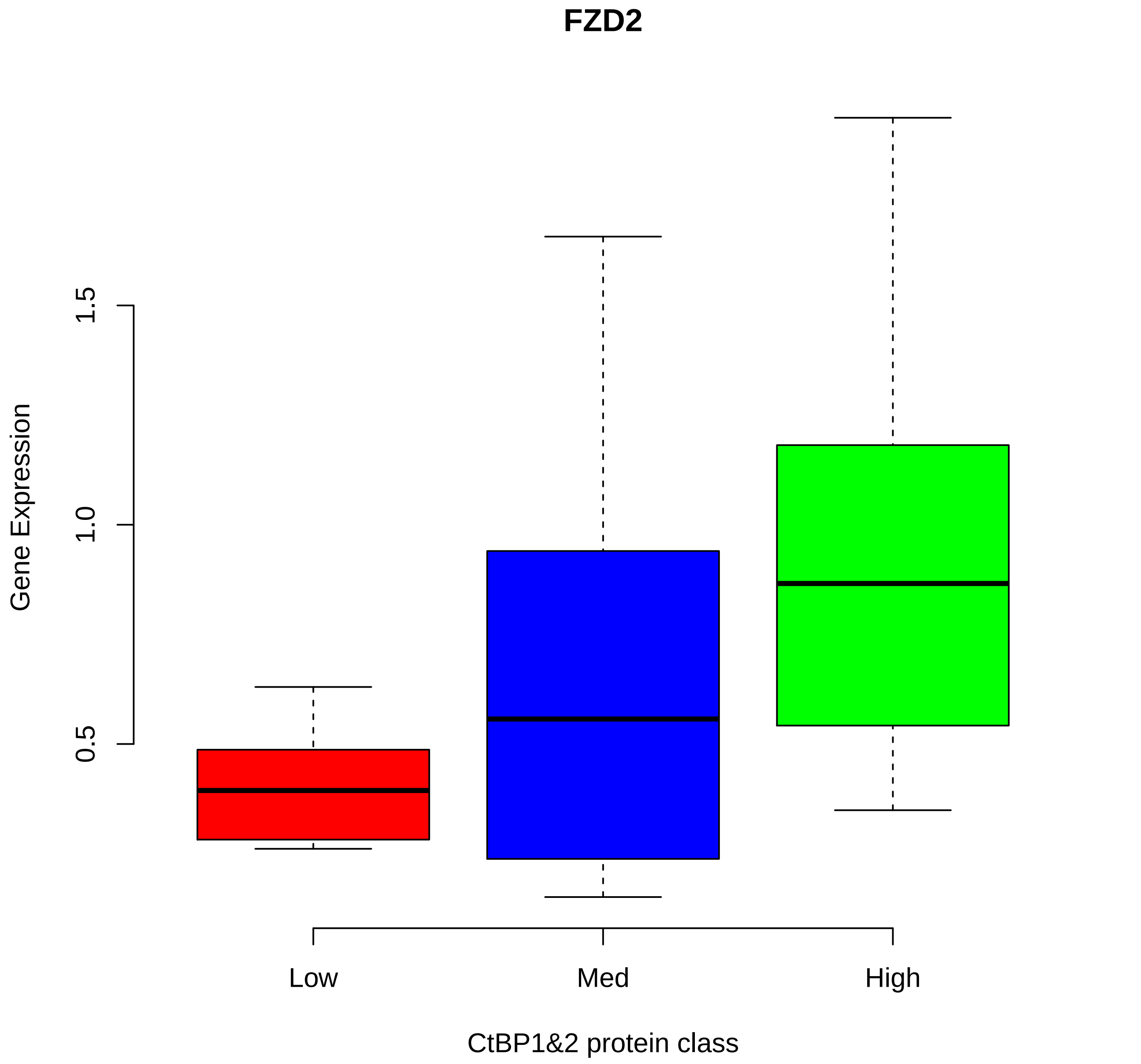
FZD2
Gene Expression
0.5
1.0
1.5
Low
Med
High
CtBP1&2 protein class

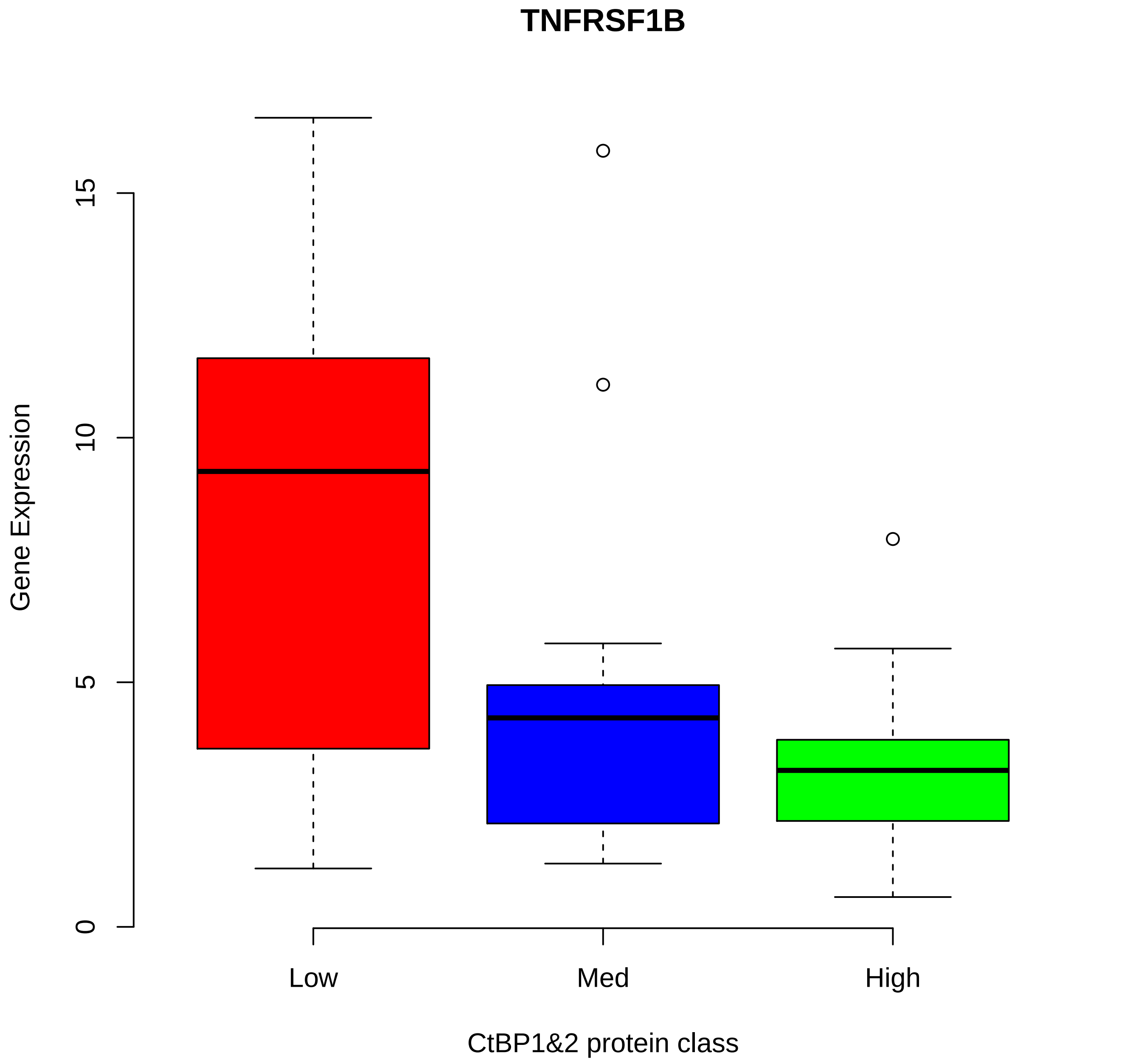

TNFRSF1B
Gene Expression
0
5
10
15
Low
Med
High
CtBP1&2 protein class

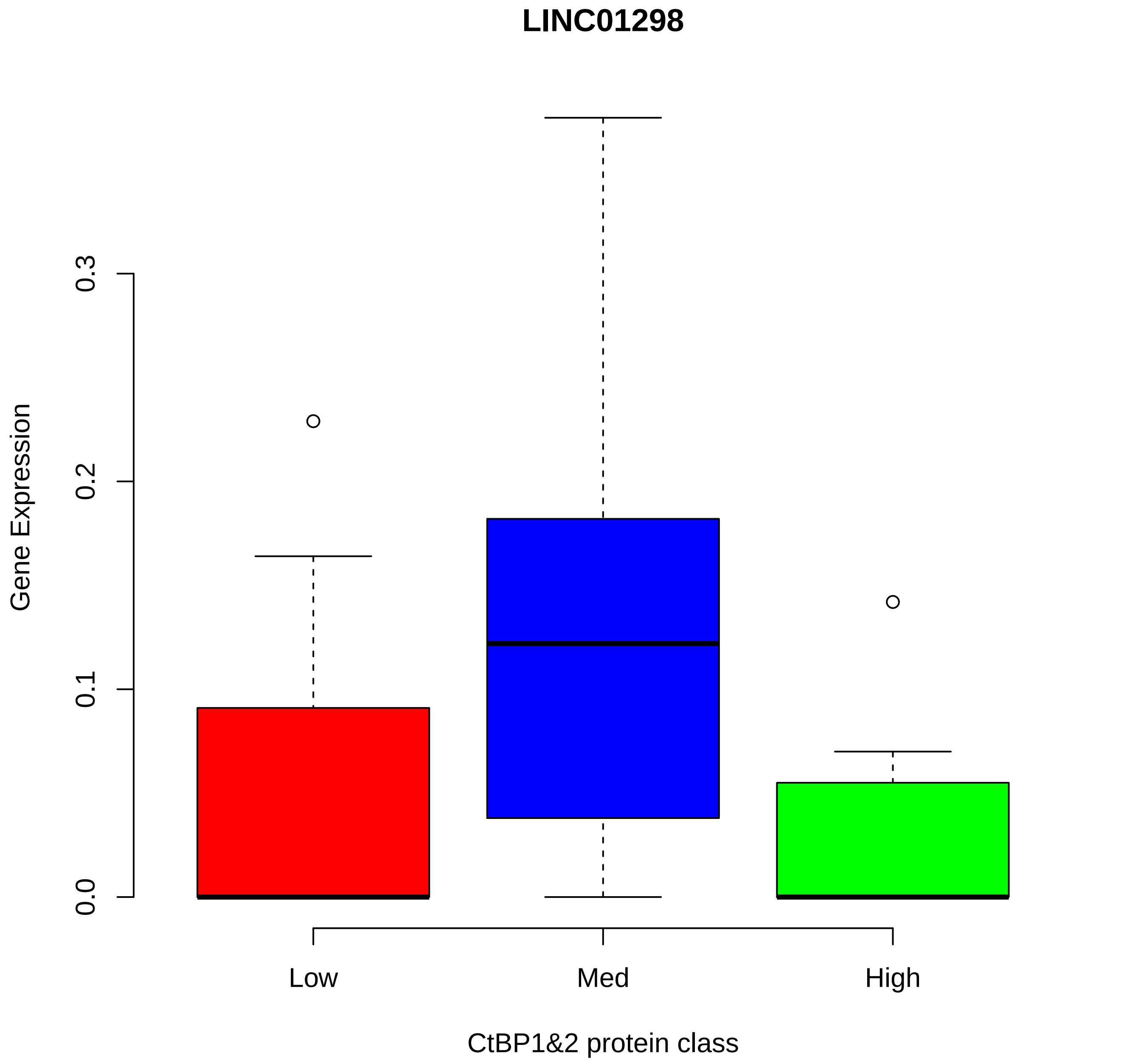

LINC01298
Gene Expression
0.0
0.1
0.2
0.3
Low
Med
High
CtBP1&2 protein class

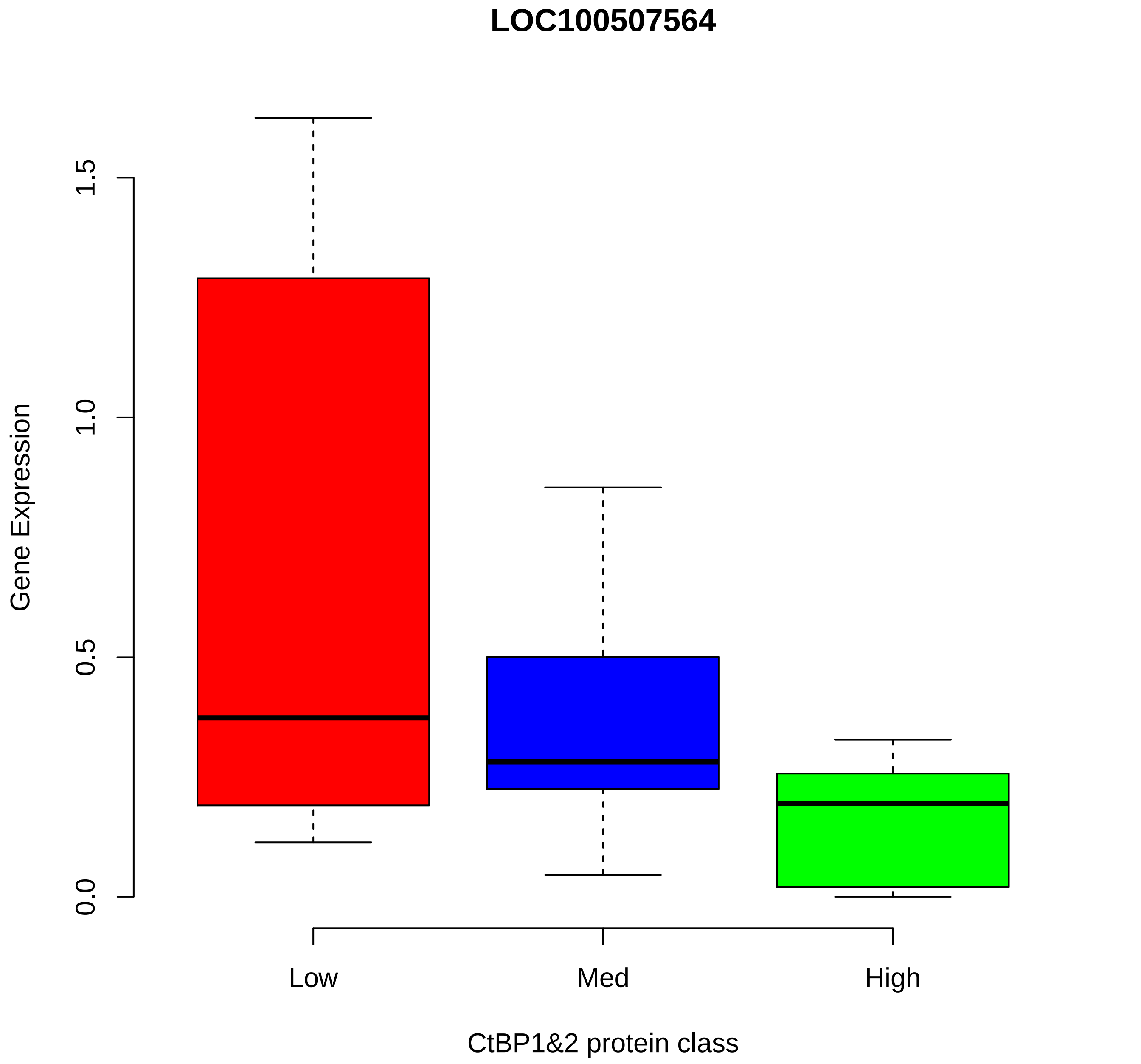
LOC100507564
Gene Expression
0.0
0.5
1.0
1.5
Low
Med
High
CtBP1&2 protein class

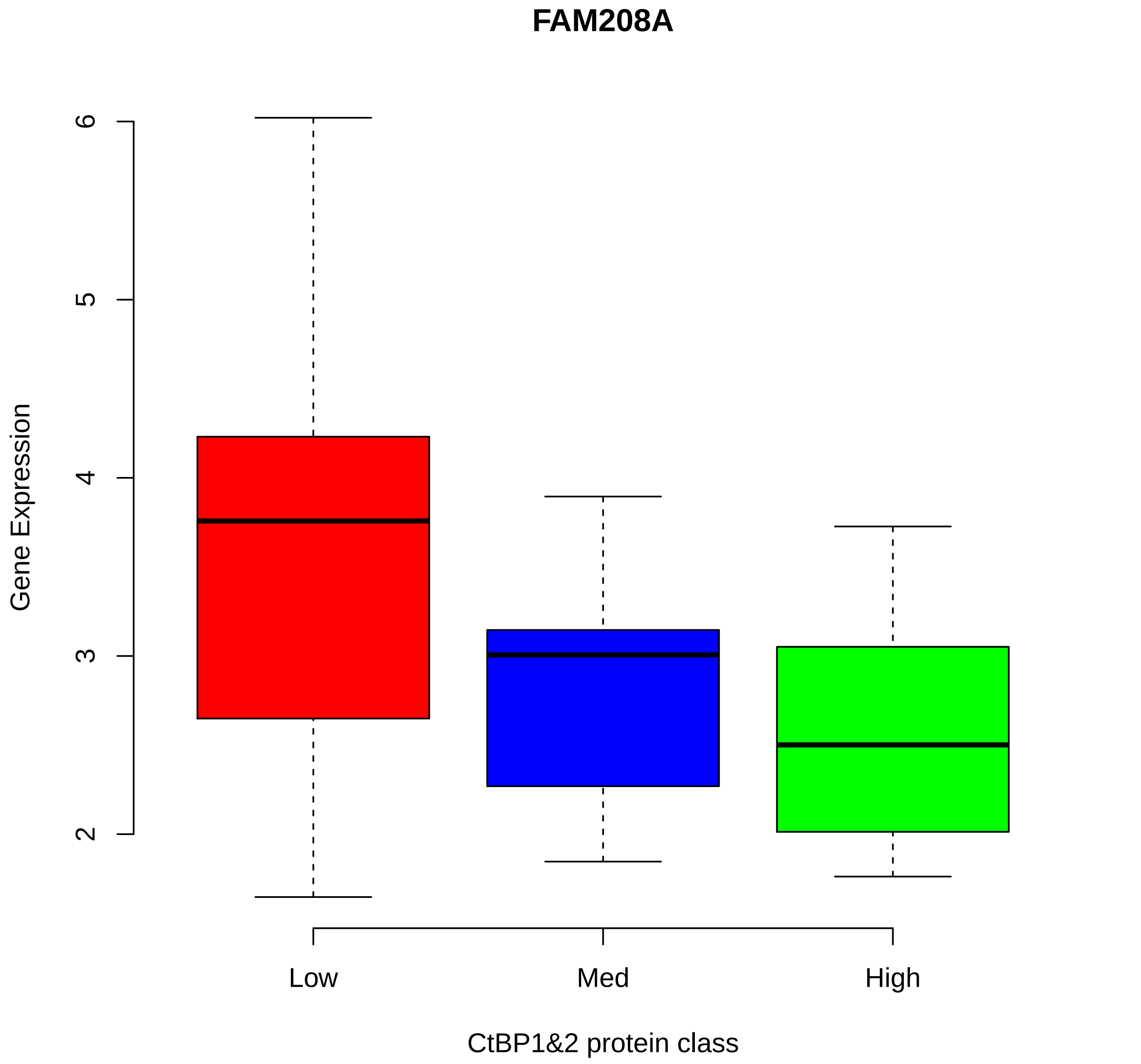
FAM208A
Gene Expression
2
3
4
5
6
Low
Med
High
CtBP1&2 protein class

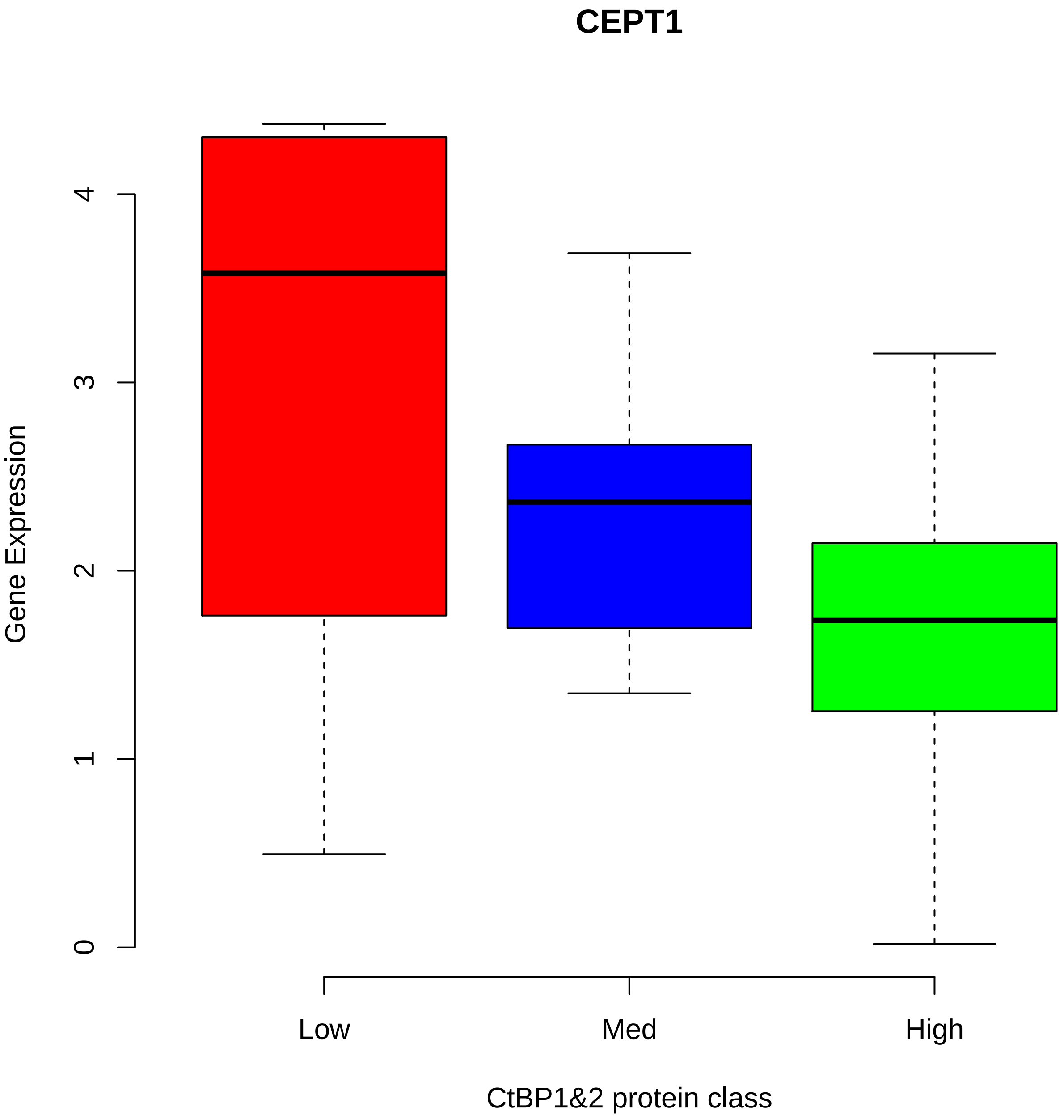
CEPT1
Gene Expression
0
1
2
3
4
Low
Med
High
CtBP1&2 protein class

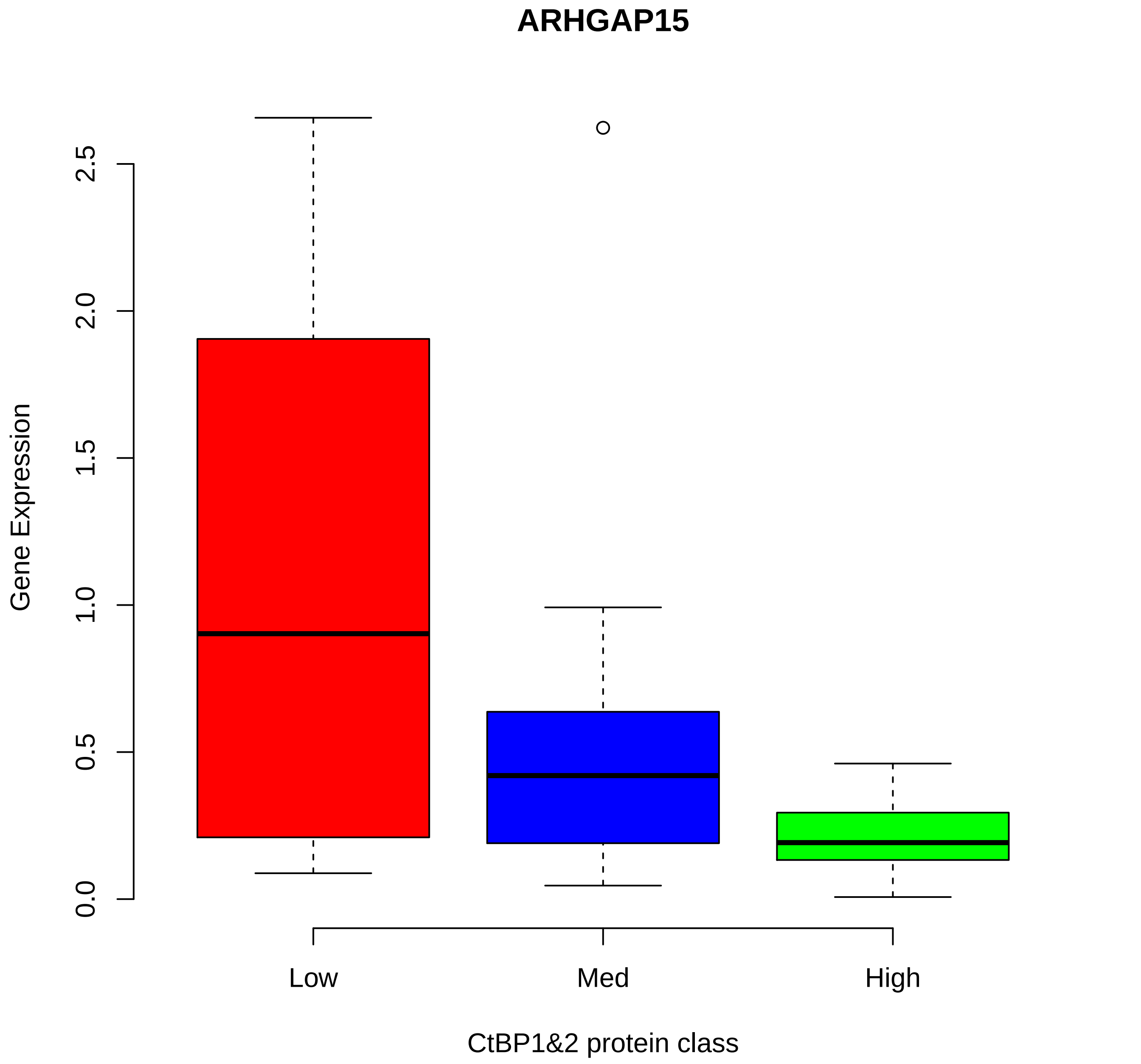
ARHGAP15
Gene Expression
0.0
0.5
1.0
1.5
2.0
2.5
Low
Med
High
CtBP1&2 protein class

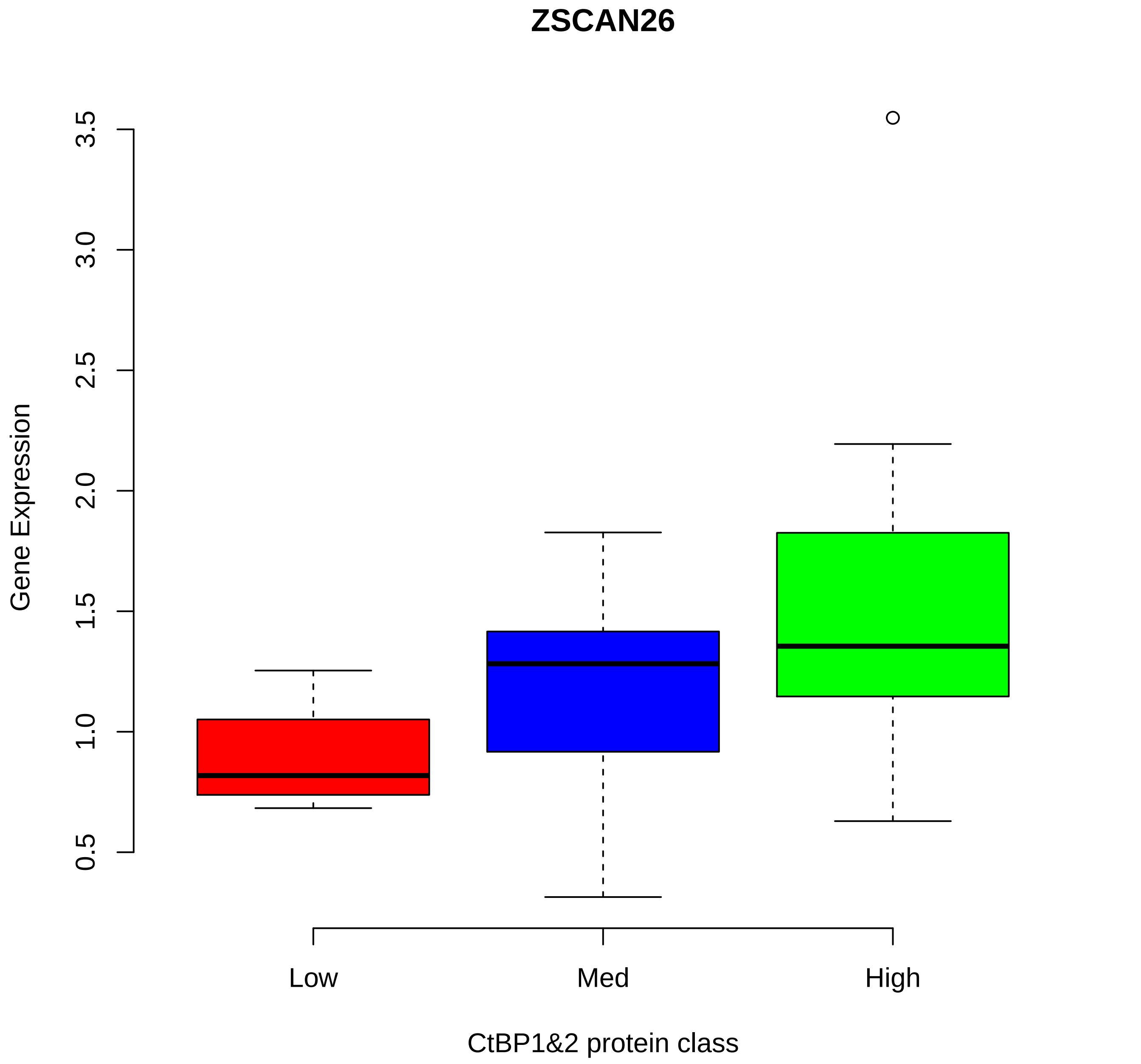
ZSCAN26
Gene Expression
0.5
1.0
1.5
2.0
2.5
3.0
3.5
Low
Med
High
CtBP1&2 protein class

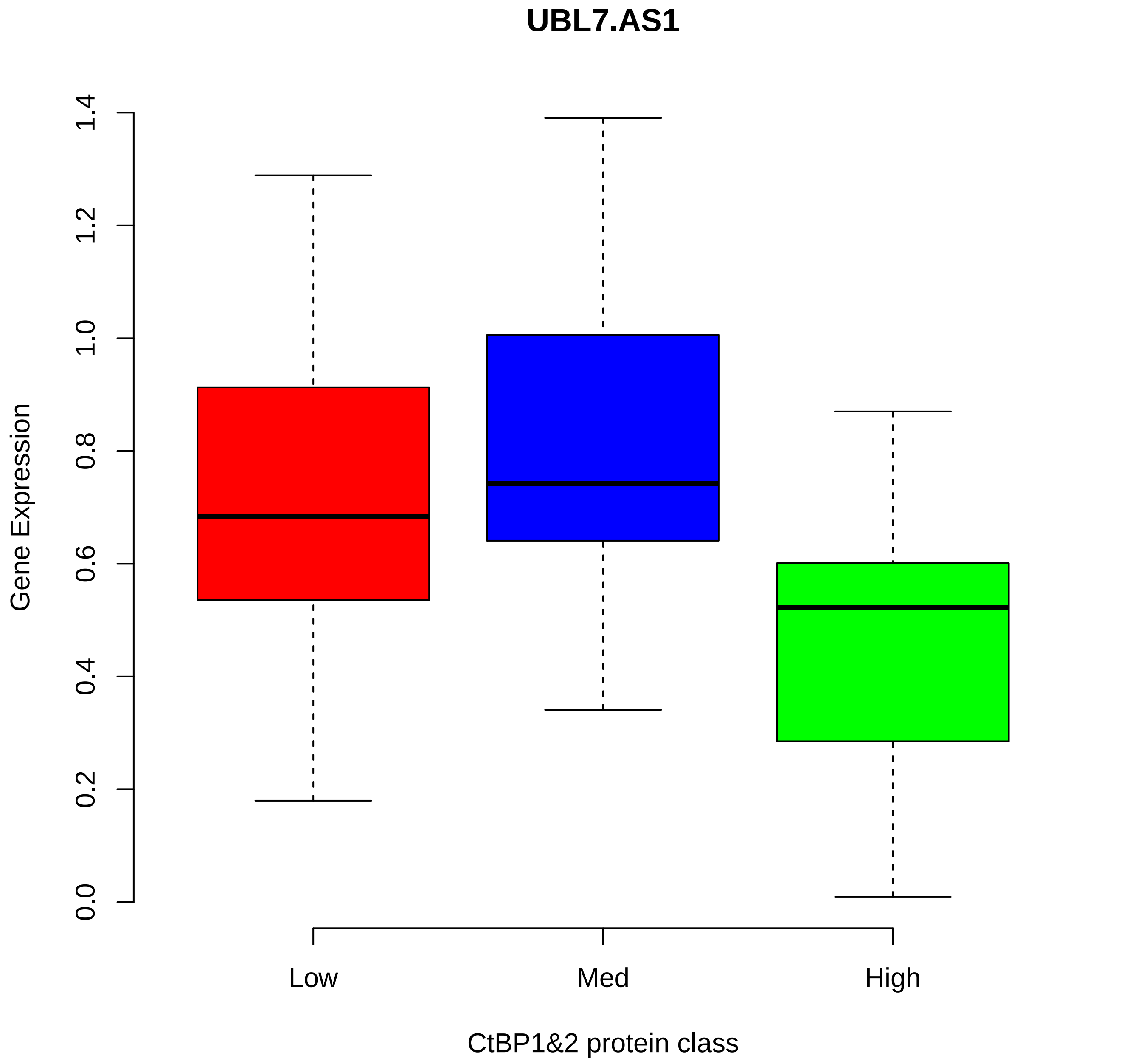
UBL7.AS1
Gene Expression
0.0
0.2
0.4
0.6
0.8
1.0
1.2
1.4
Low
Med
High
CtBP1&2 protein class

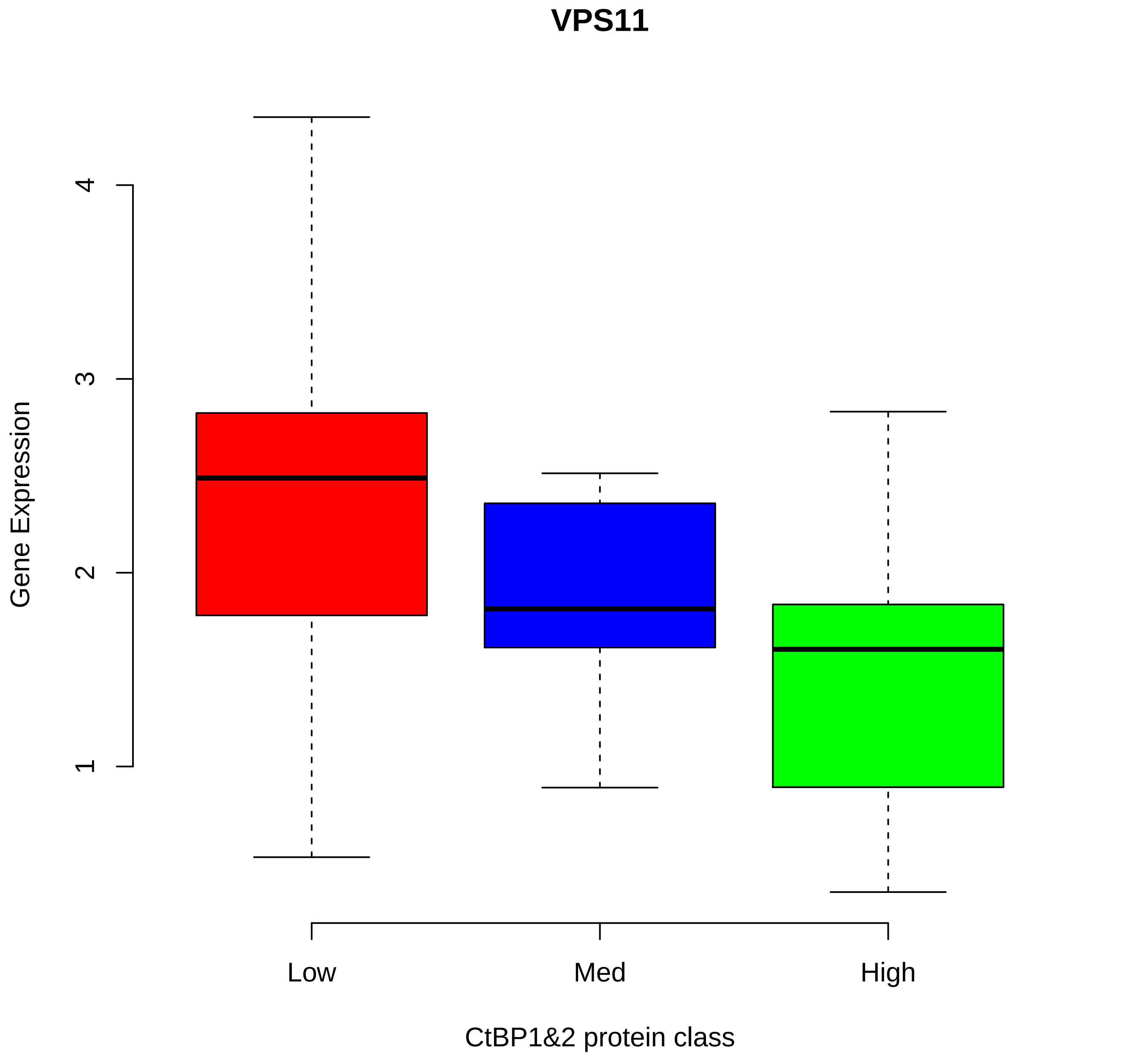
VPS11
Gene Expression
1
2
3
4
Low
Med
High
CtBP1&2 protein class

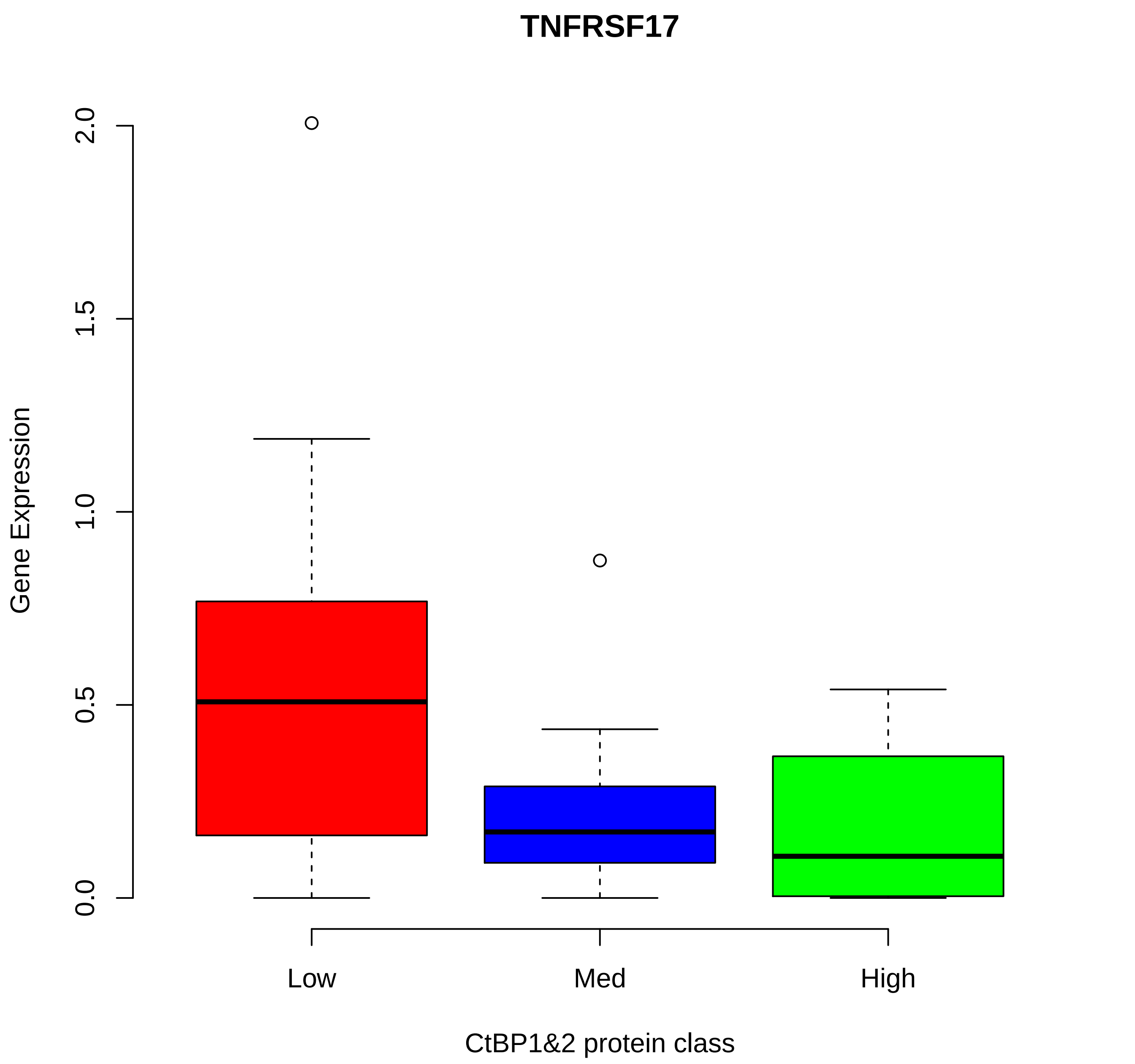
TNFRSF17
Gene Expression
0.0
0.5
1.0
1.5
2.0
Low
Med
High
CtBP1&2 protein class

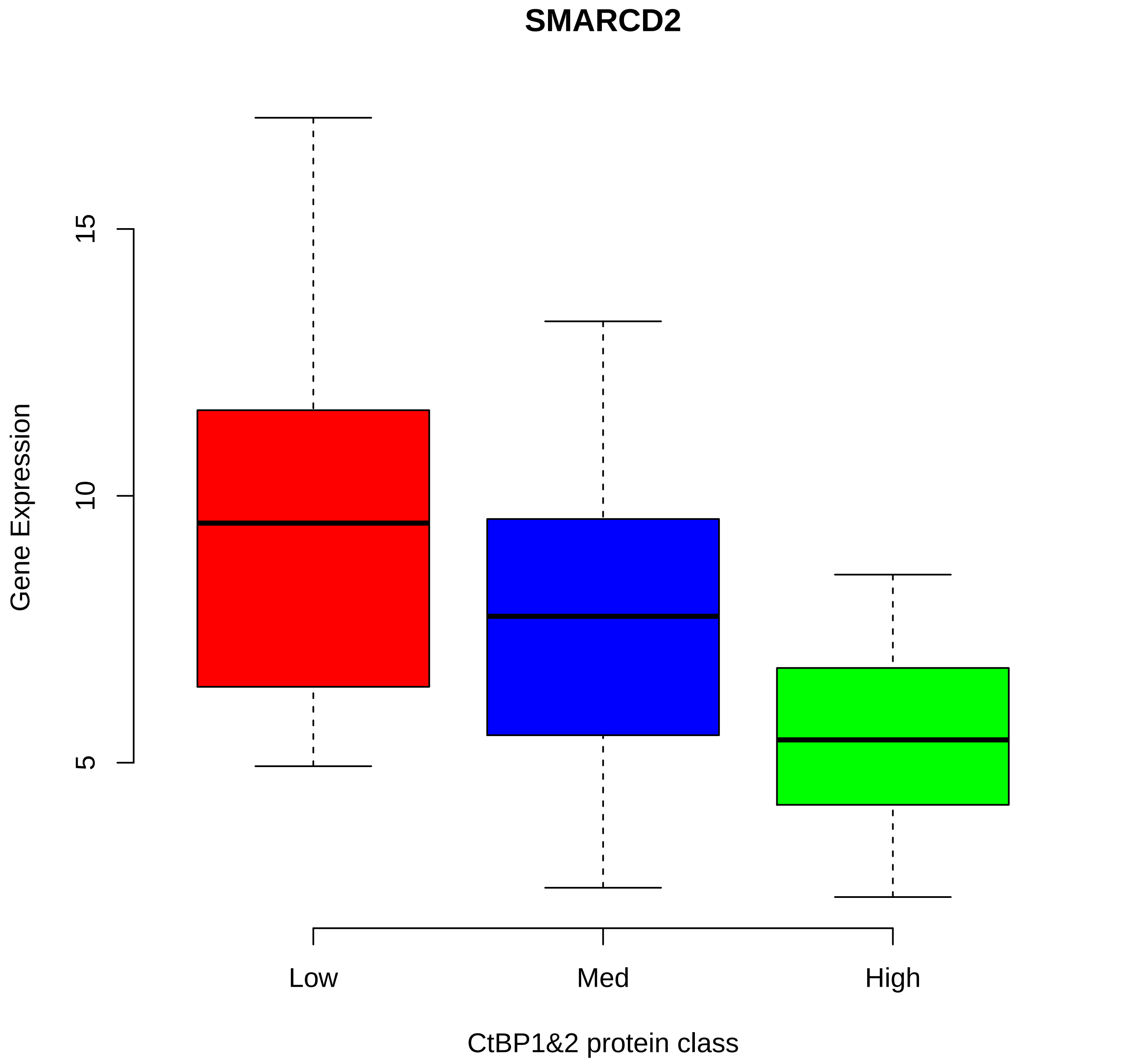
SMARCD2
Gene Expression
15
10
5
Low
Med
High
CtBP1&2 protein class

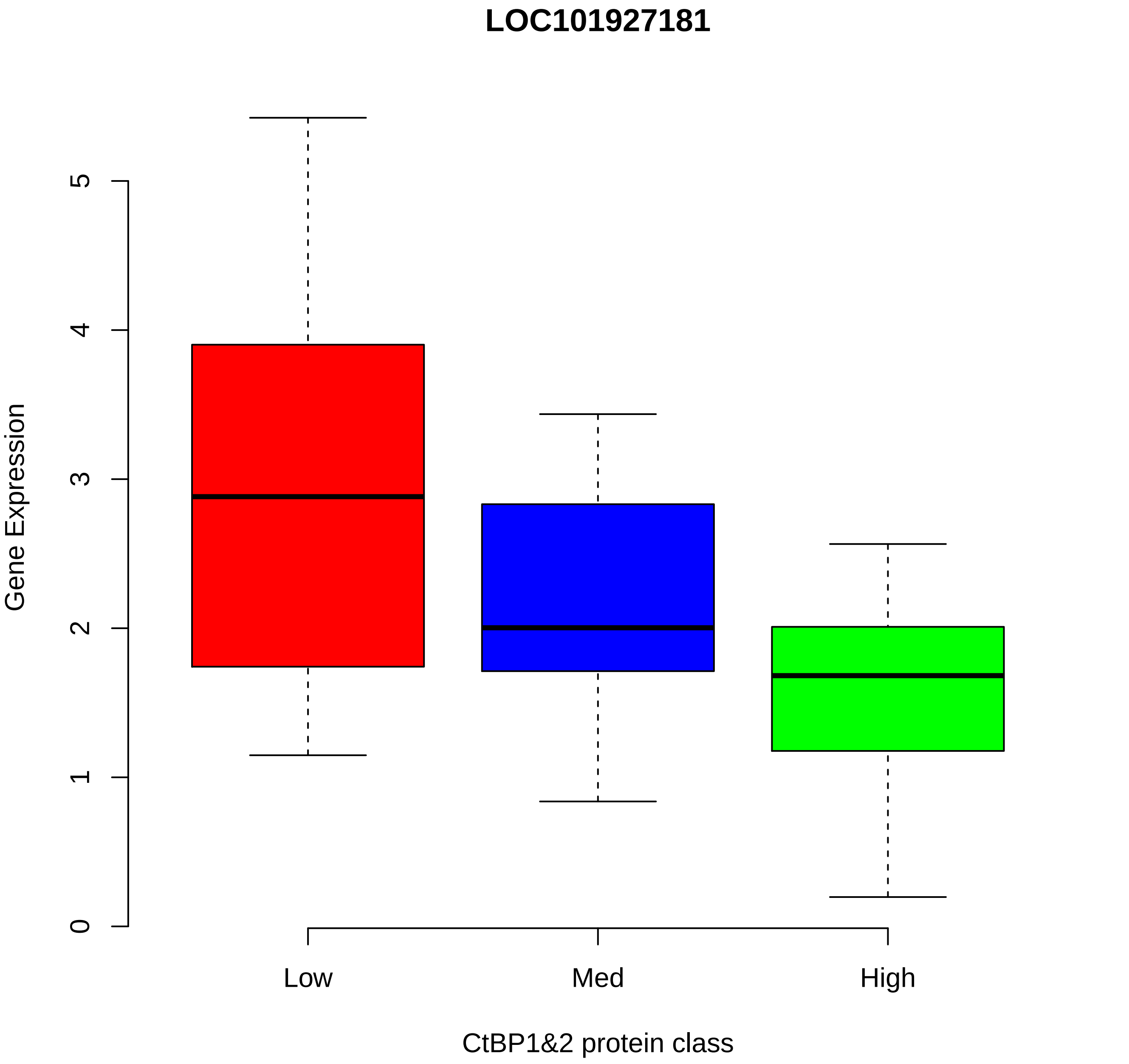
LOC101927181
Gene Expression
0
1
2
3
4
5
Low
Med
High
CtBP1&2 protein class

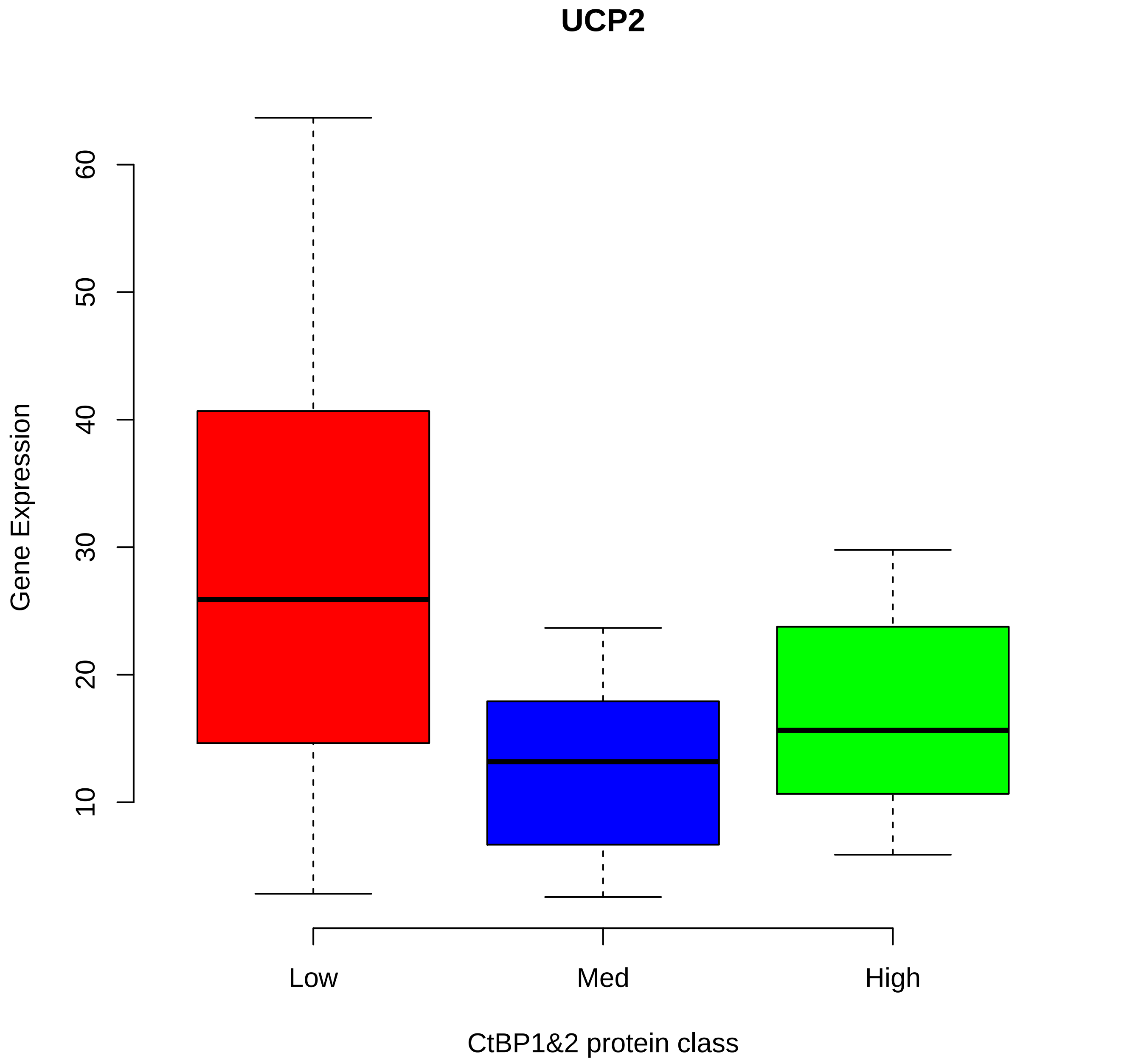

UCP2
Gene Expression
10
20
30
40
50
60
Low
Med
High
CtBP1&2 protein class

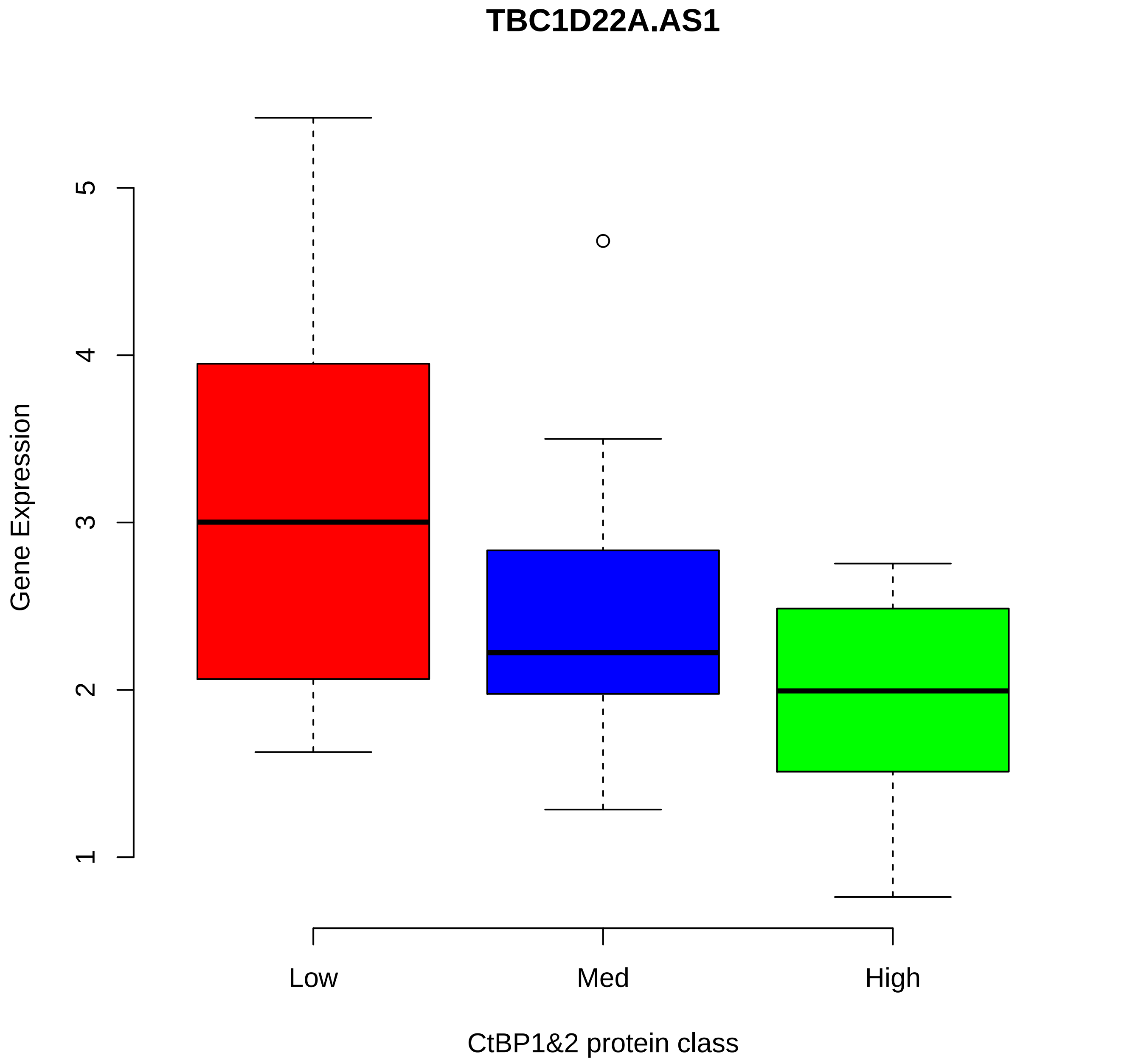
TBC1D22A.AS1
Gene Expression
1
2
3
4
5
Low
Med
High
CtBP1&2 protein class

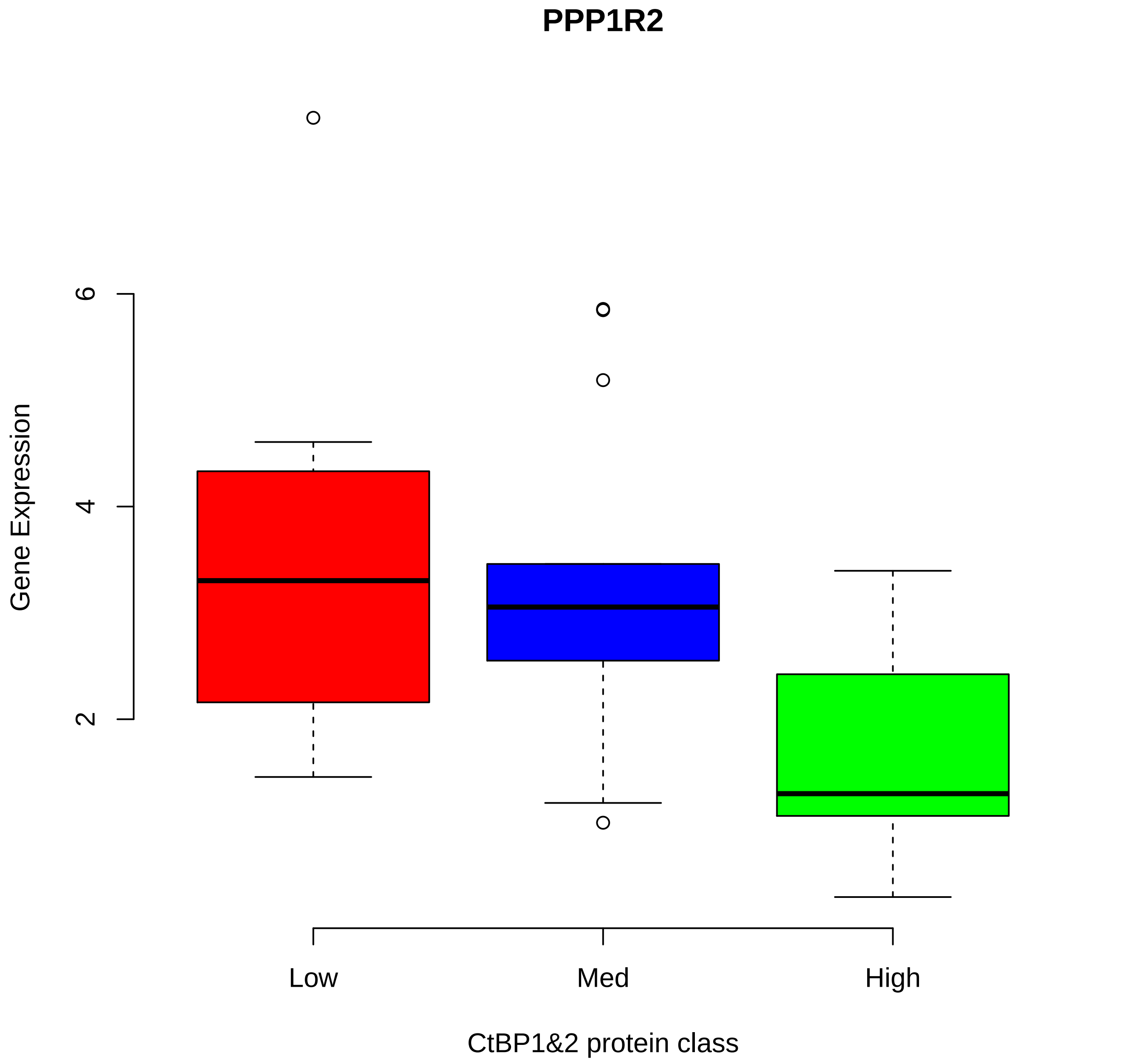

PPP1R2
Gene Expression
6
4
2
Low
Med
High
CtBP1&2 protein class

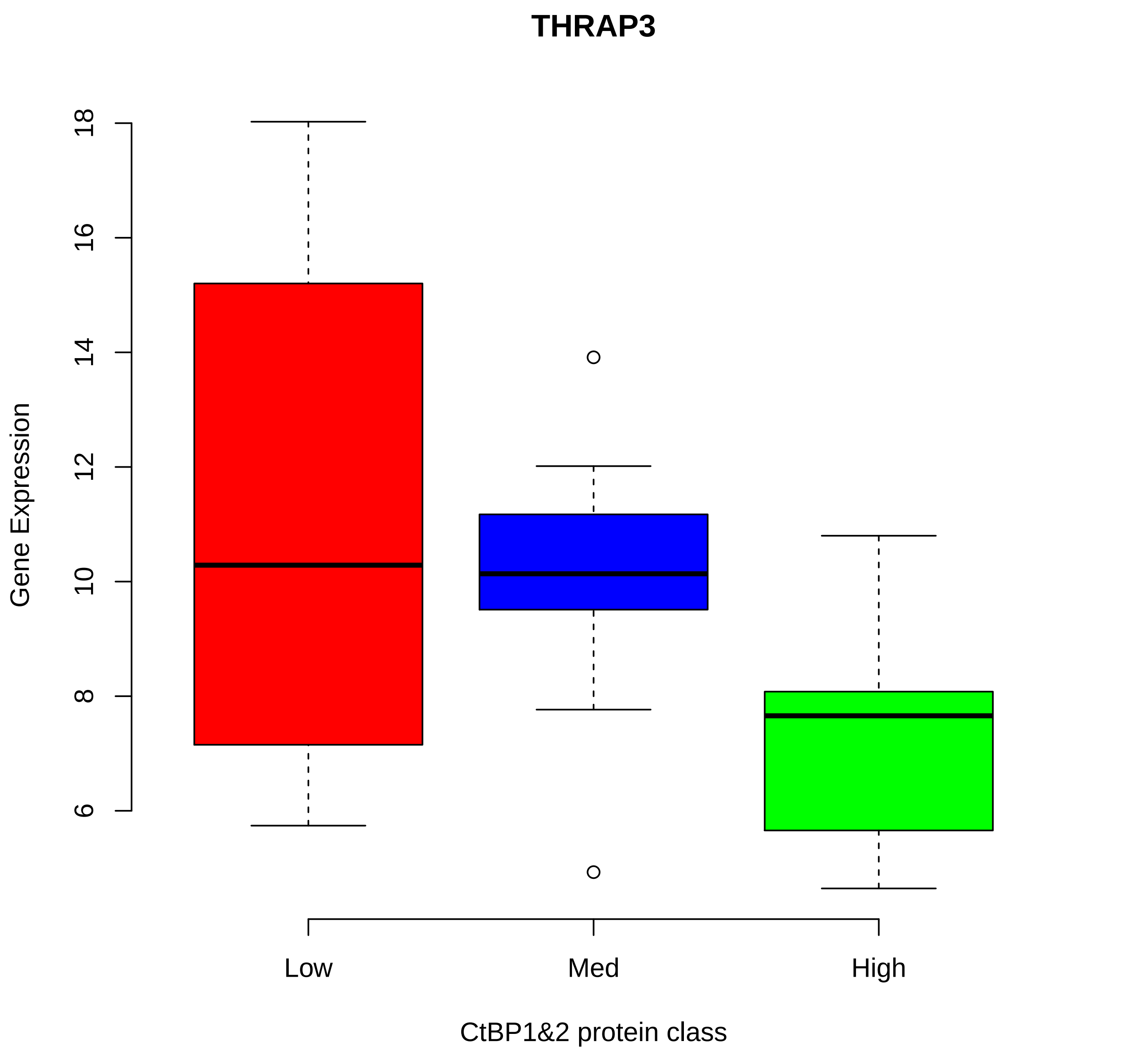

THRAP3
Gene Expression
6
8
10
12
14
16
18
Low
Med
High
CtBP1&2 protein class

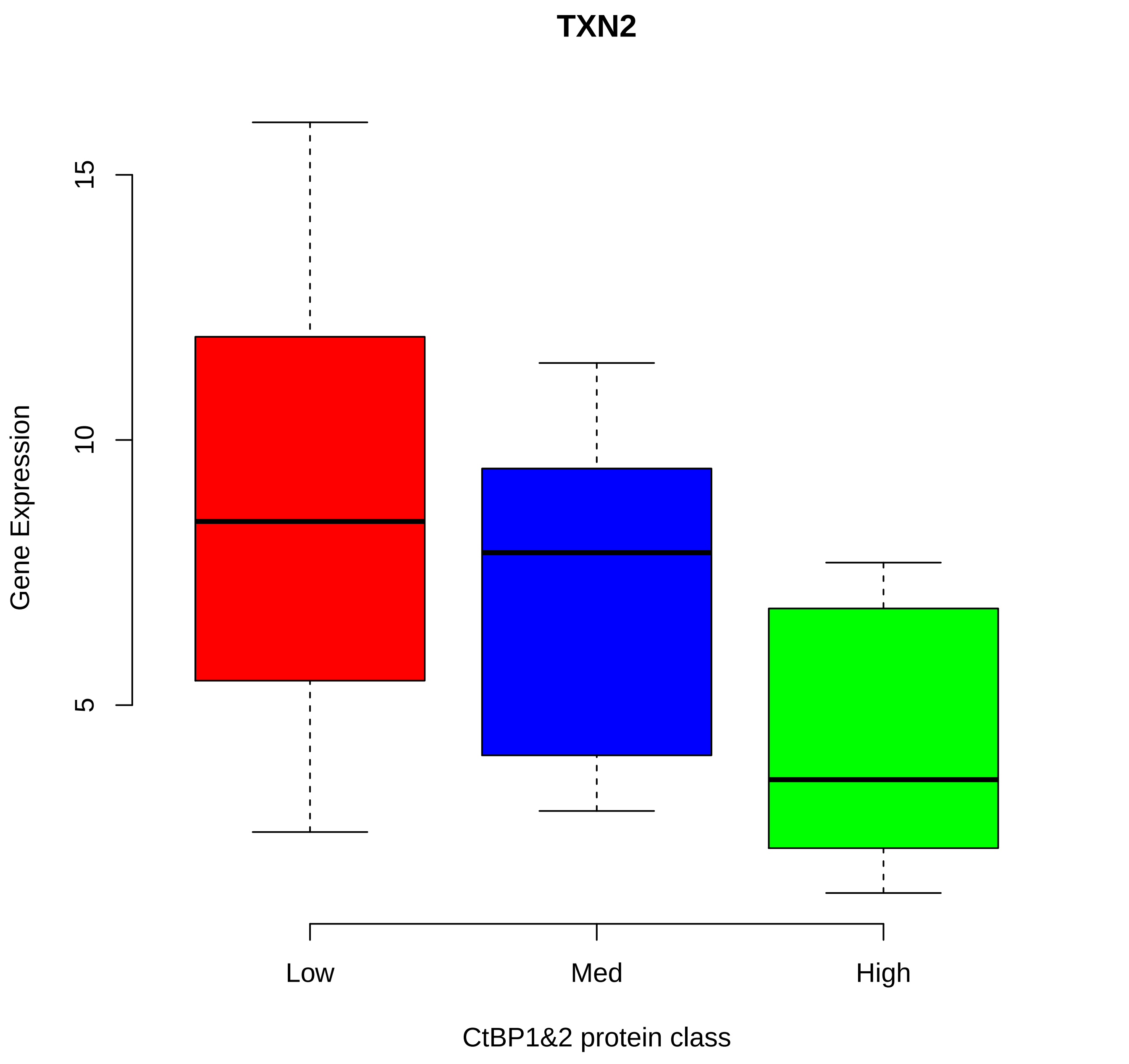
TXN2
Gene Expression
15
10
5
Low
Med
High
CtBP1&2 protein class

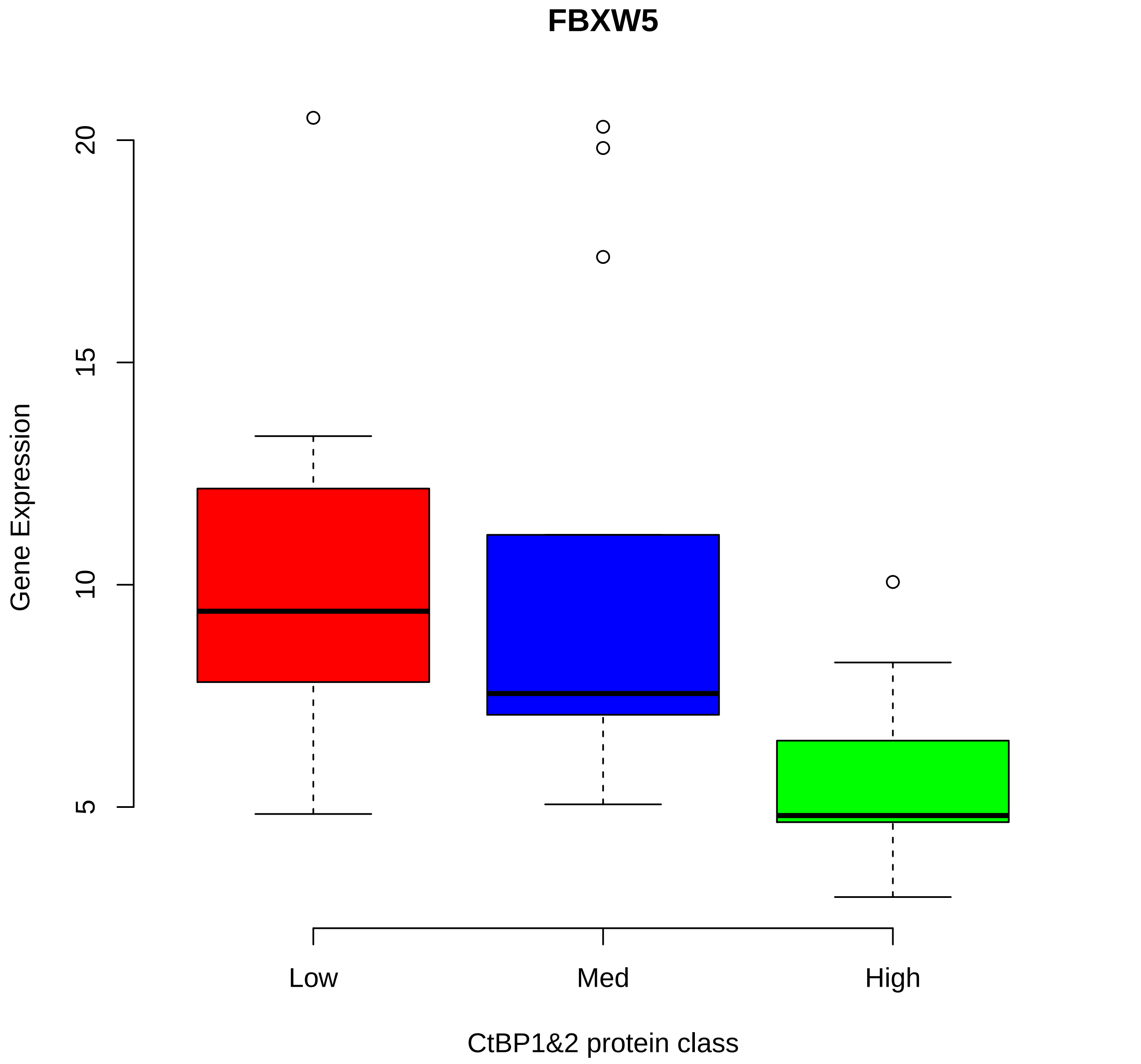
FBXW5
Gene Expression
20
15
10
5
Low
Med
High
CtBP1&2 protein class

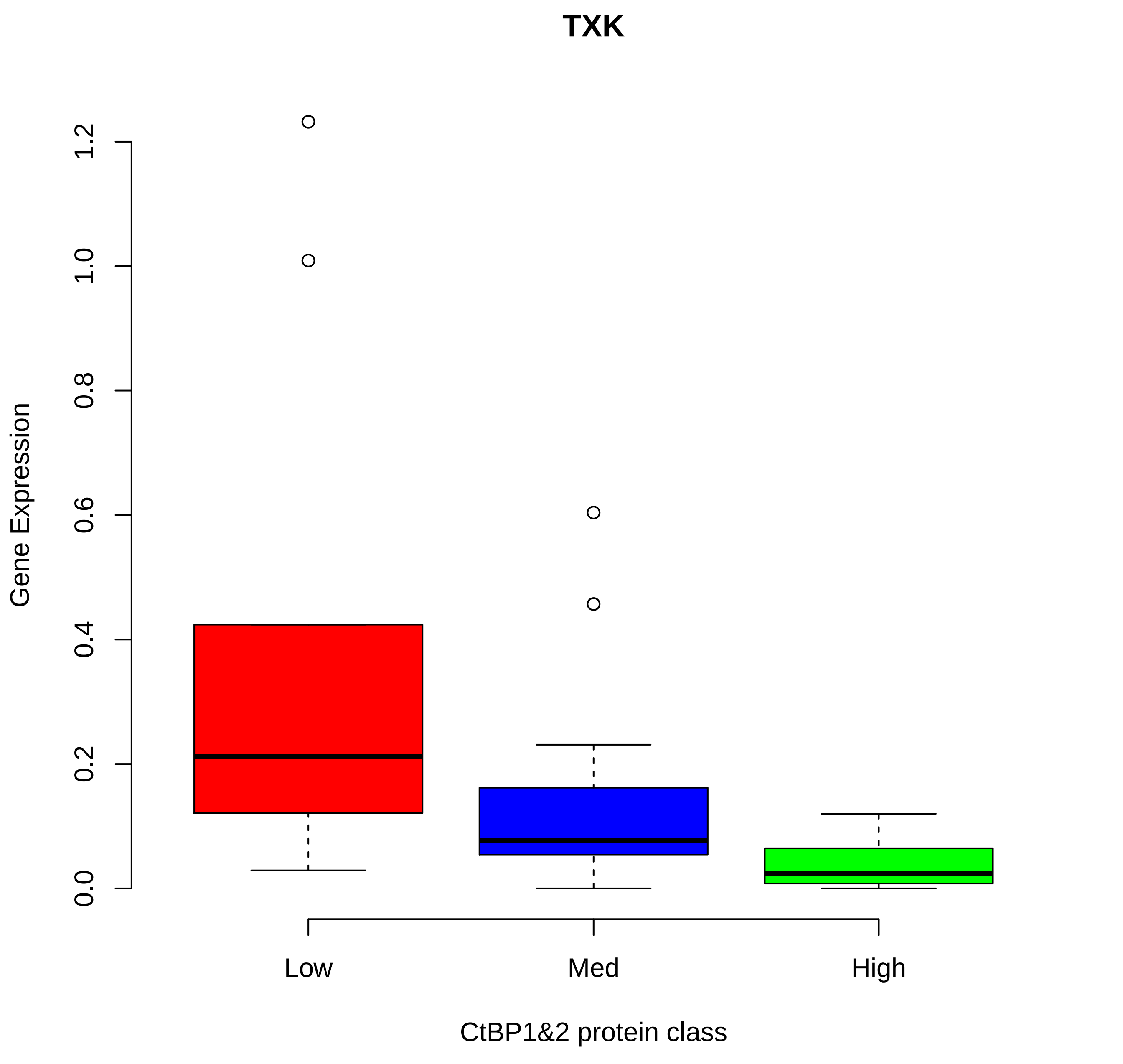

TXK
Gene Expression
0.0
0.2
0.4
0.6
0.8
1.0
1.2
Low
Med
High
CtBP1&2 protein class

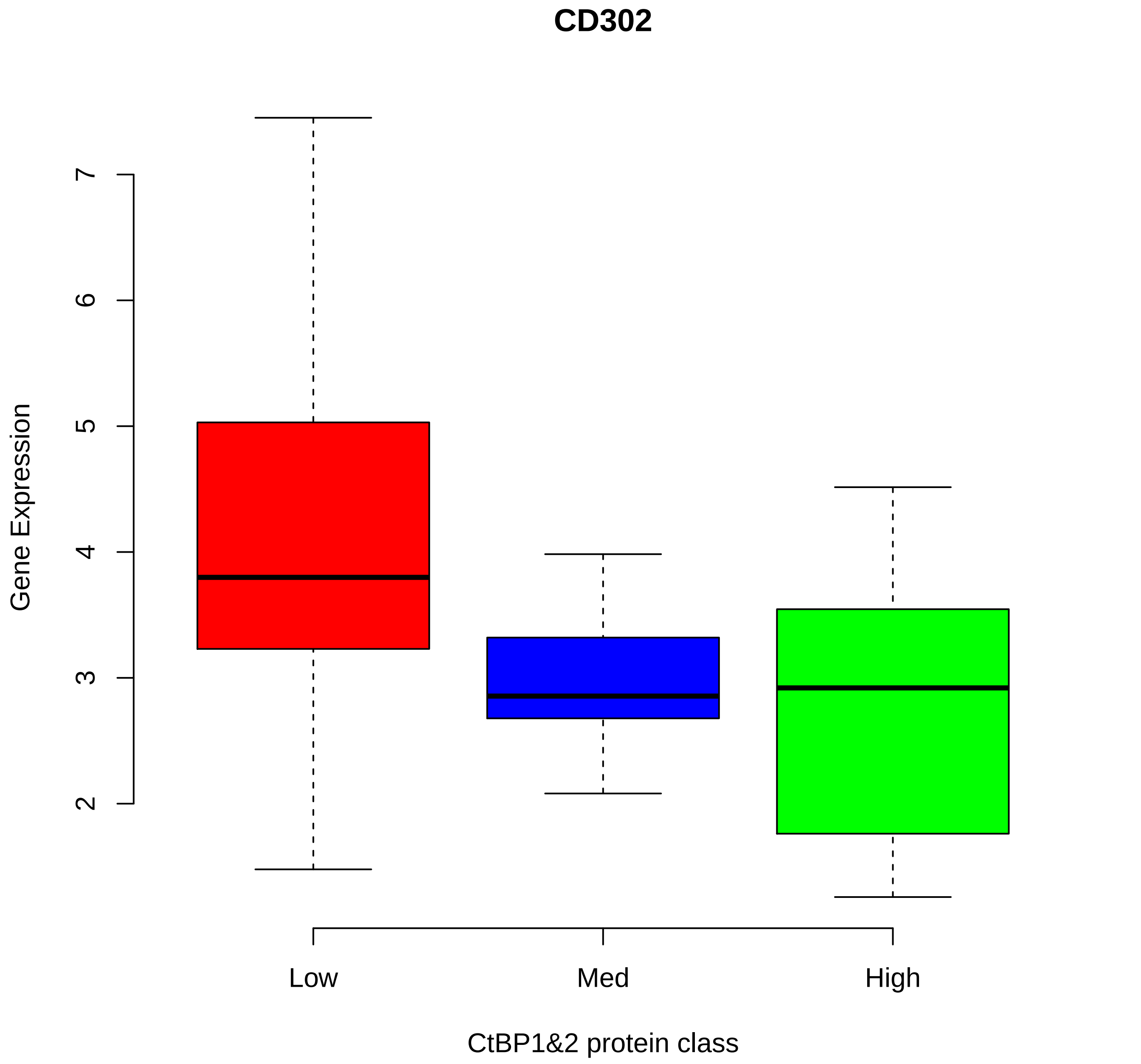

CD302
Gene Expression
7
6
5
4
3
2
Low
Med
High
CtBP1&2 protein class

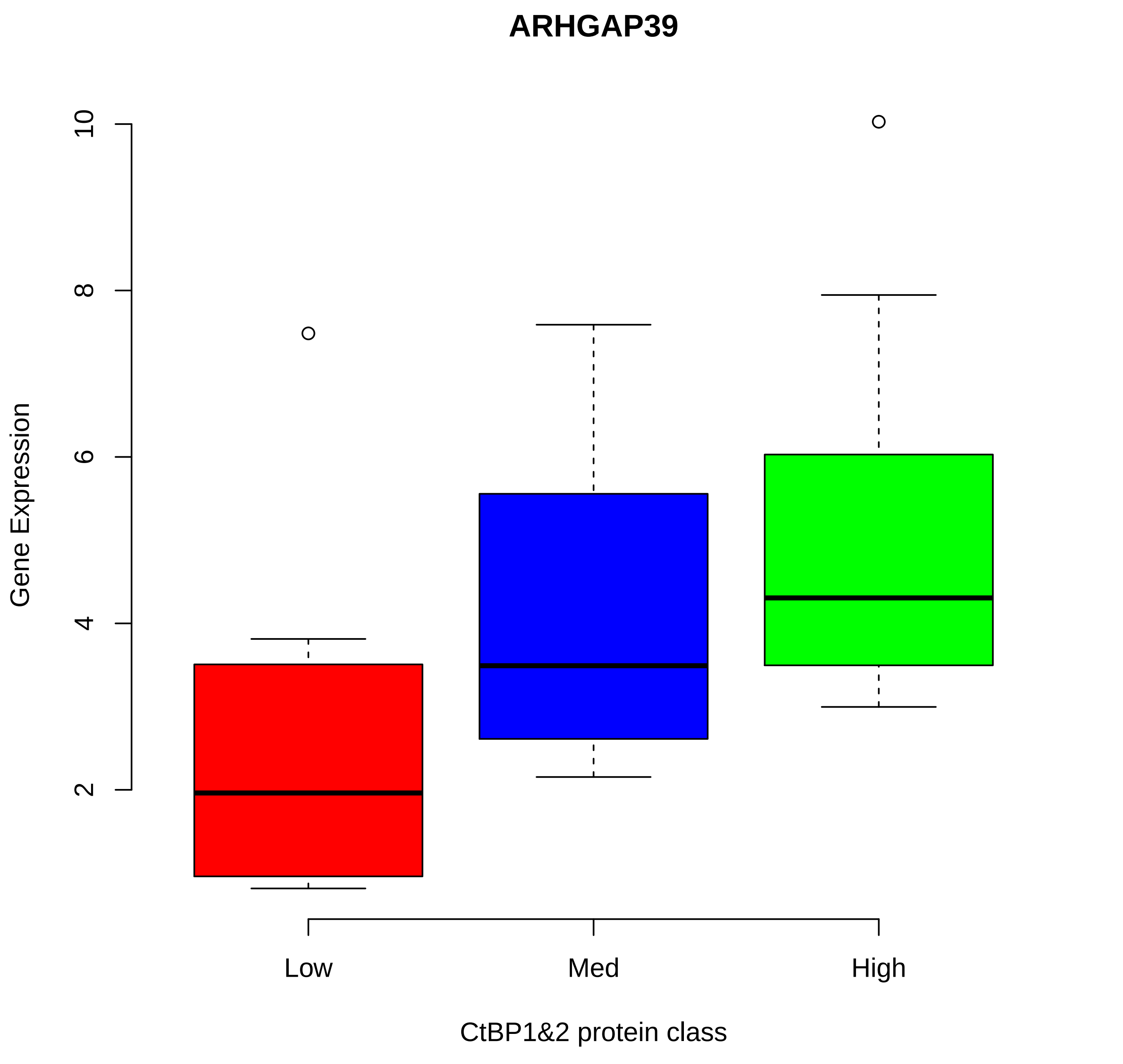
ARHGAP39
Gene Expression
10
8
6
4
2
Low
Med
High
CtBP1&2 protein class

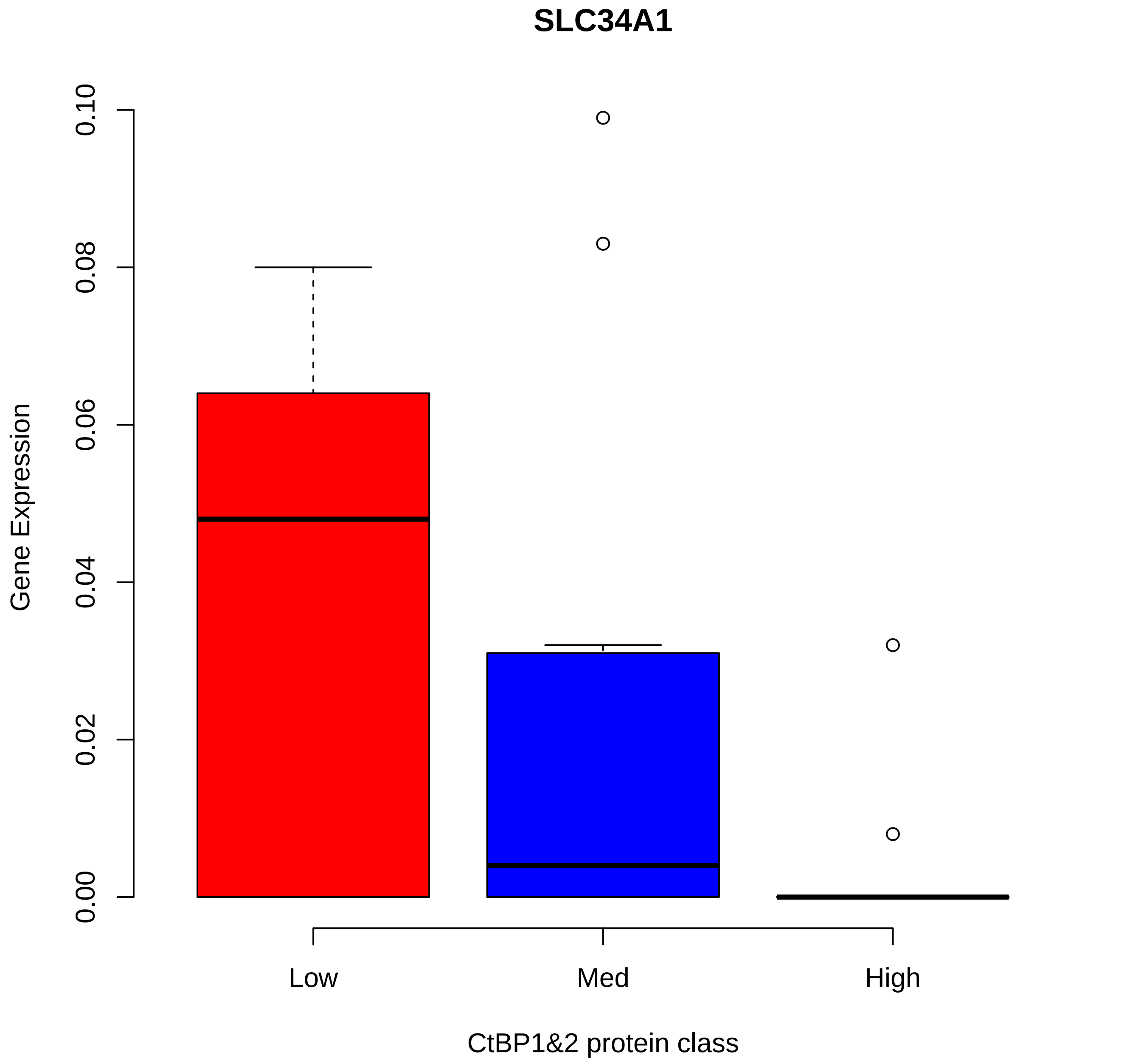

SLC34A1
Gene Expression
0.00
0.02
0.04
0.06
0.08
0.10
Low
Med
High
CtBP1&2 protein class

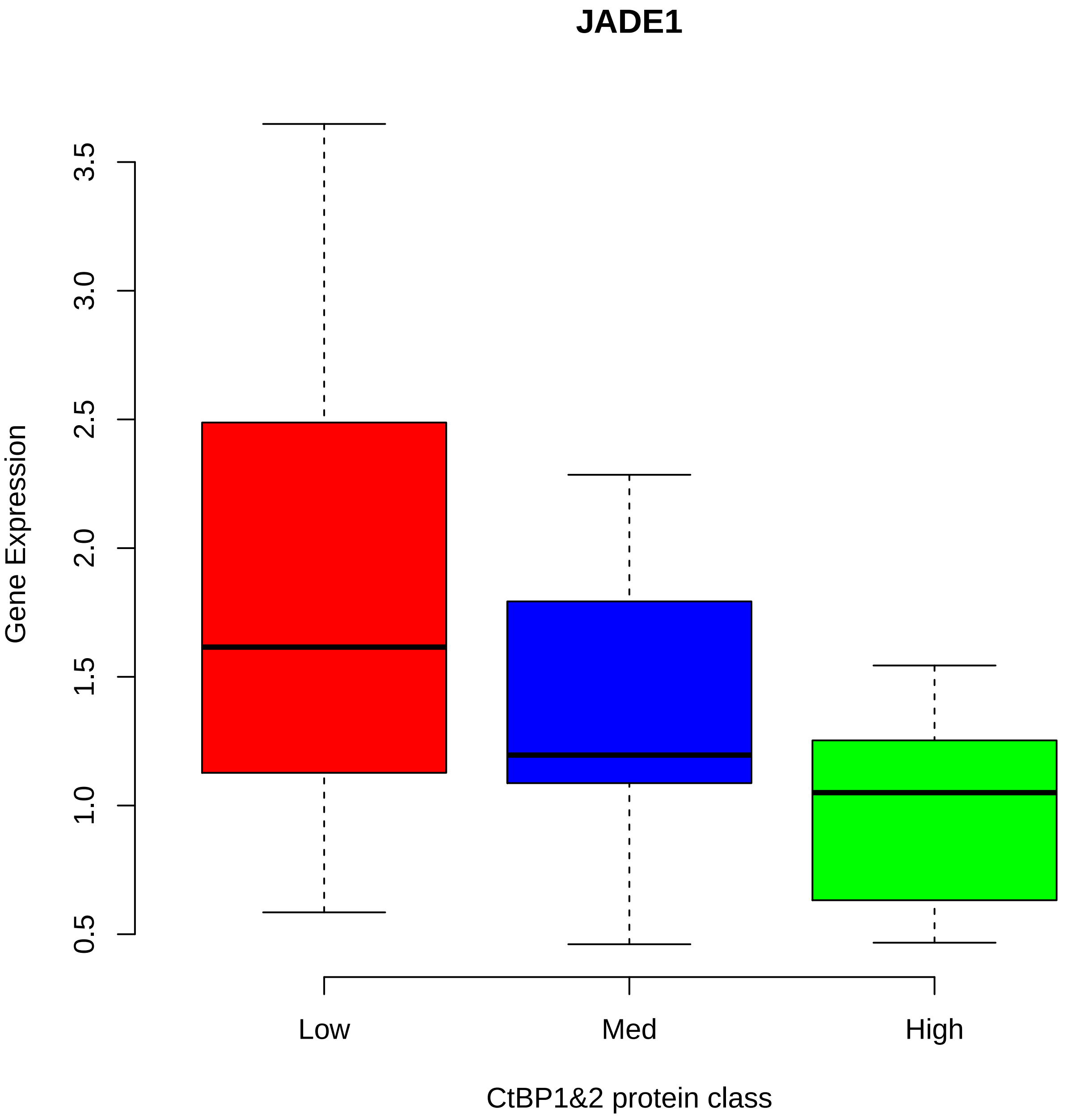
JADE1
Gene Expression
0.5
1.0
1.5
2.0
2.5
3.0
3.5
Low
Med
High
CtBP1&2 protein class

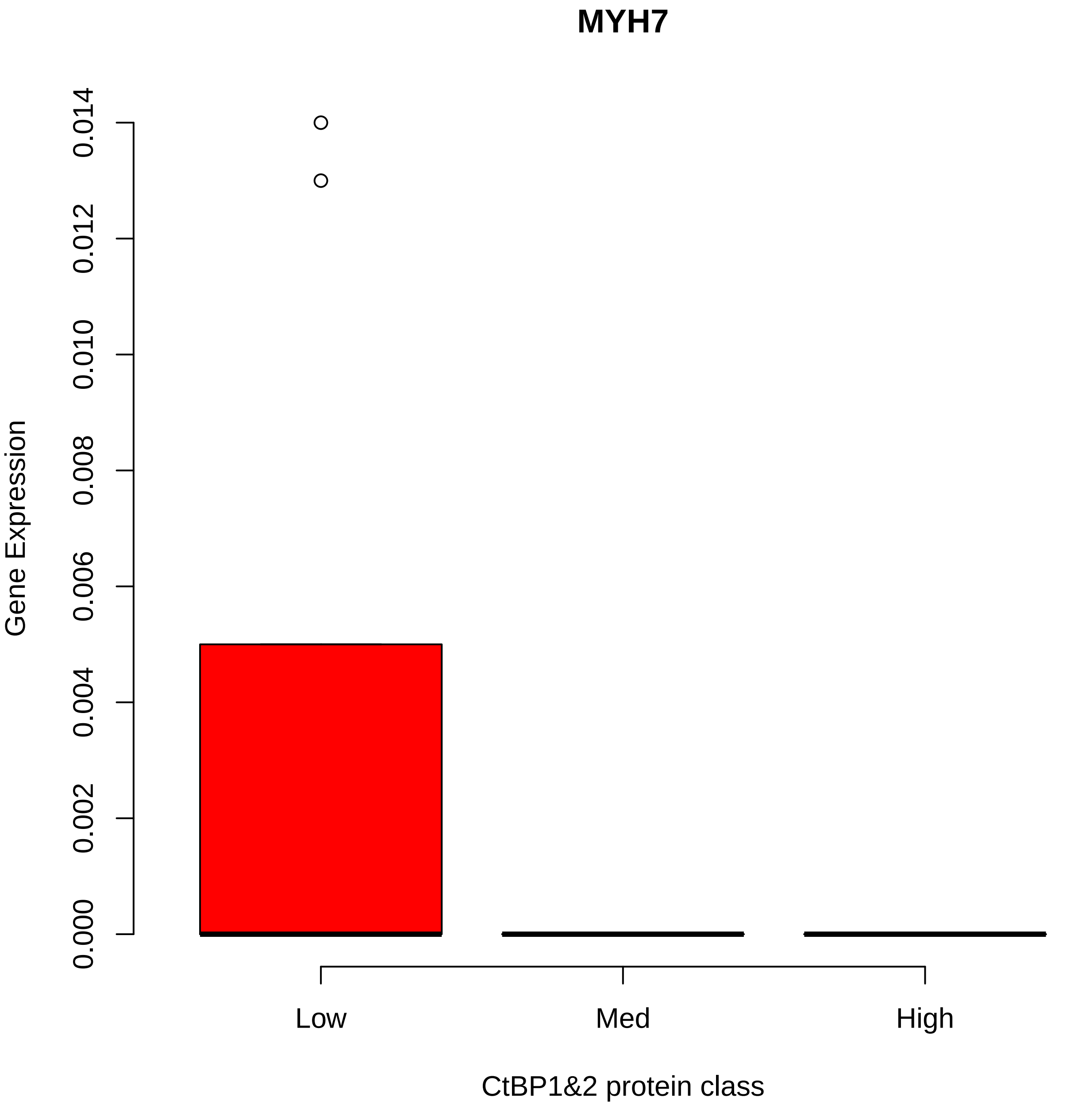

MYH7
Gene Expression
0.000
0.002
0.004
0.006
0.008
0.010
0.012
0.014
Low
Med
High
CtBP1&2 protein class

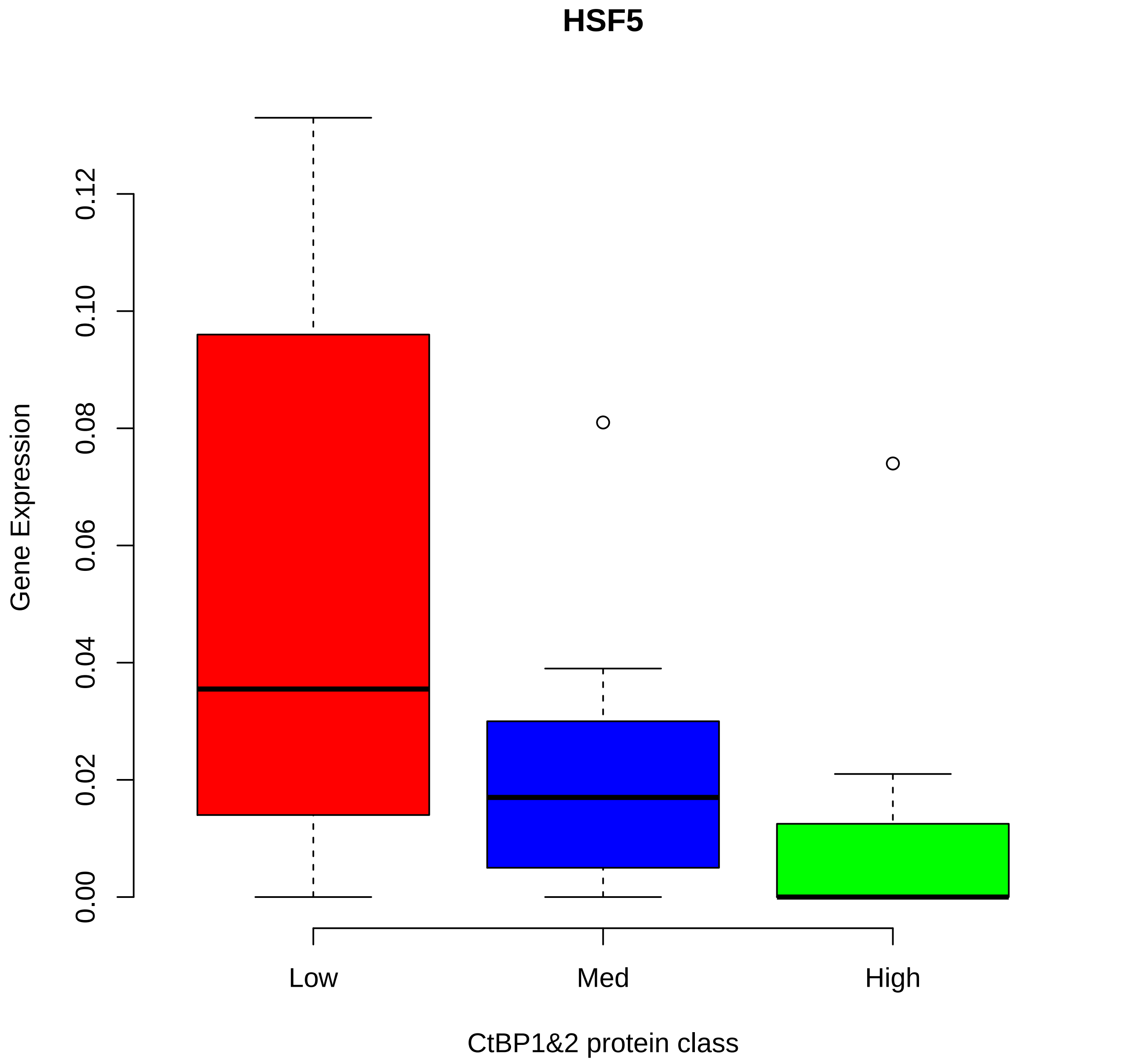
HSF5
Gene Expression
0.00
0.02
0.04
0.06
0.08
0.10
0.12
Low
Med
High
CtBP1&2 protein class

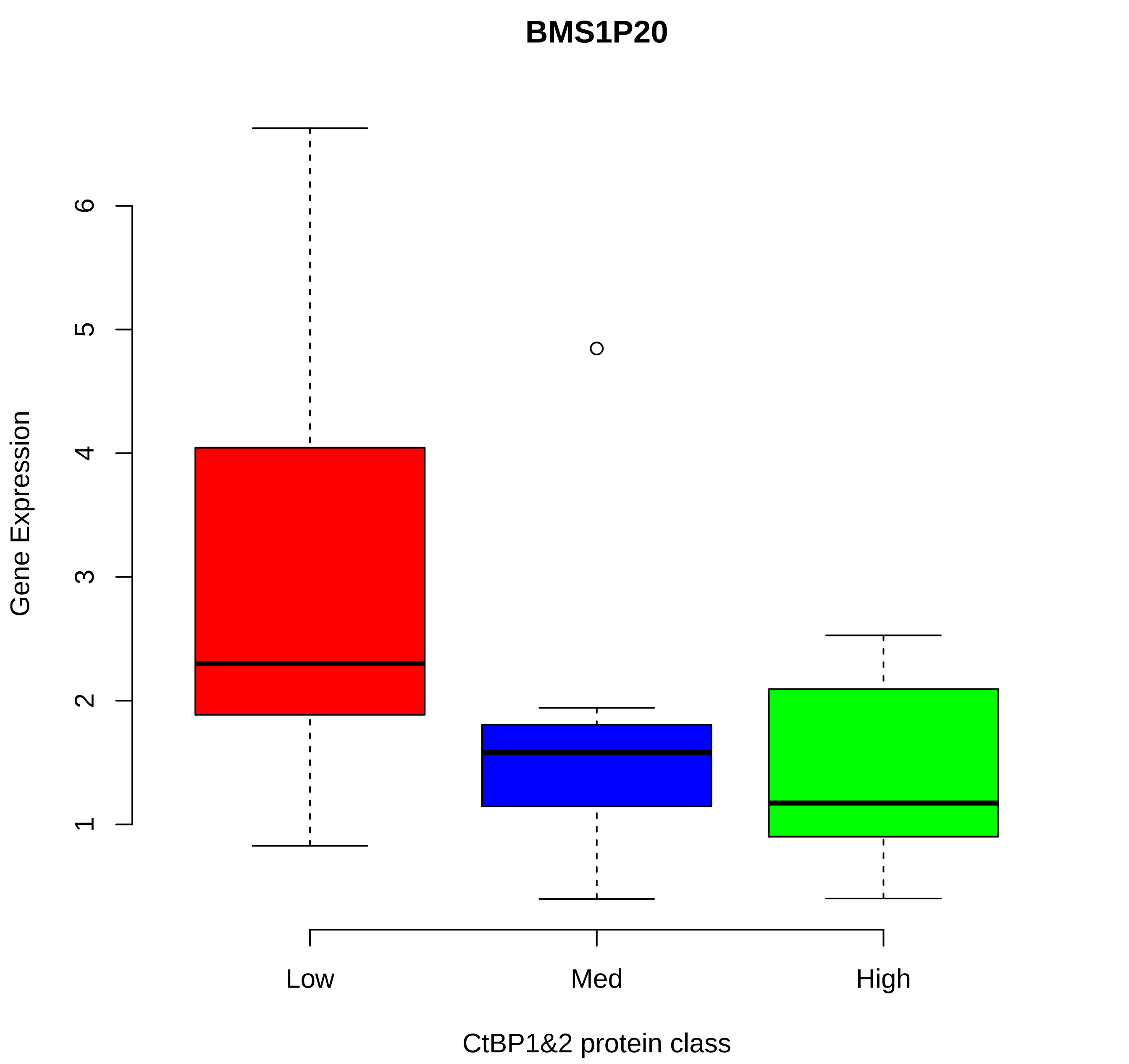

BMS1P20
Gene Expression
1
2
3
4
5
6
Low
Med
High
CtBP1&2 protein class

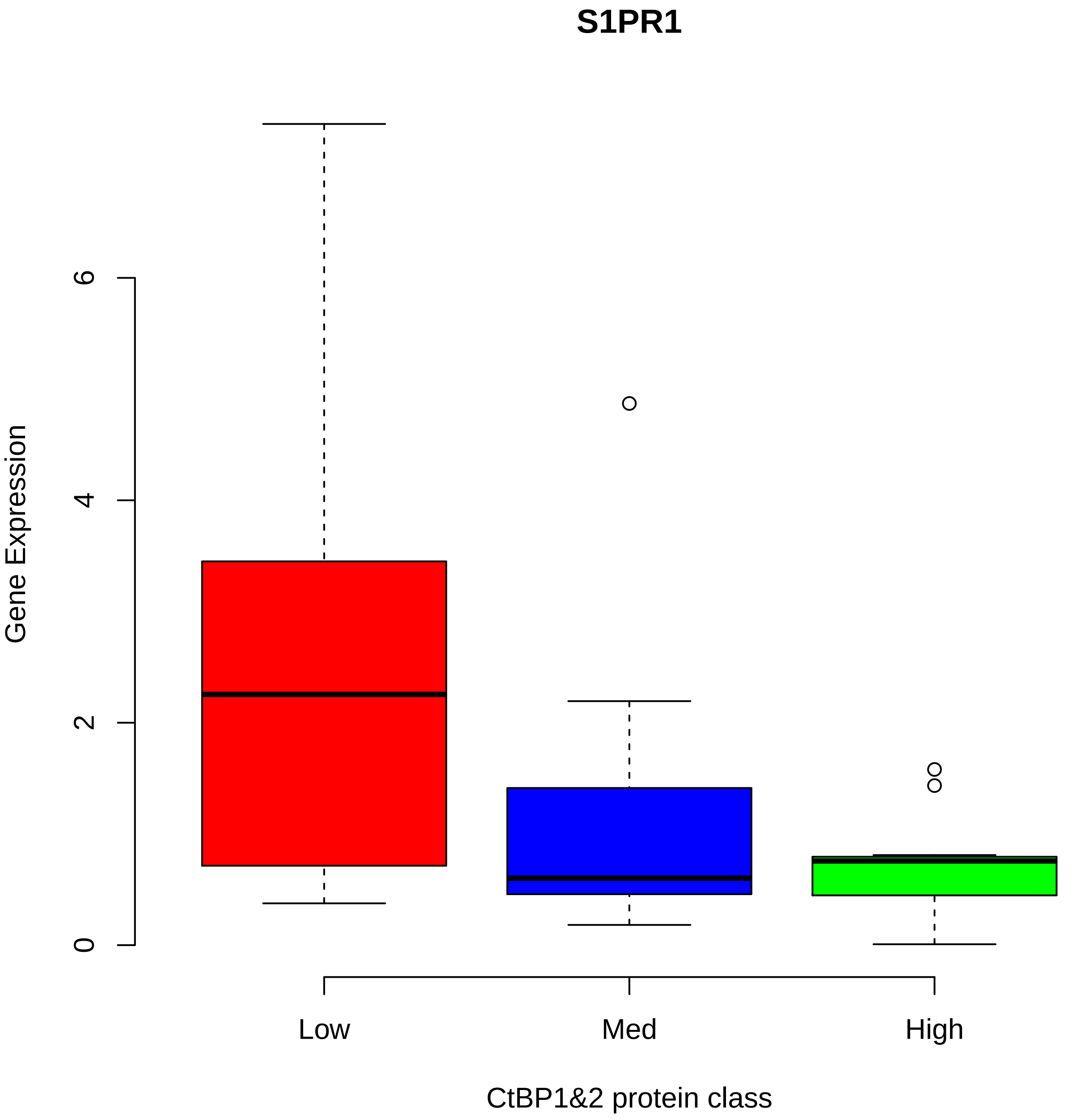
S1PR1
Gene Expression
0
2
4
6
Low
Med
High
CtBP1&2 protein class

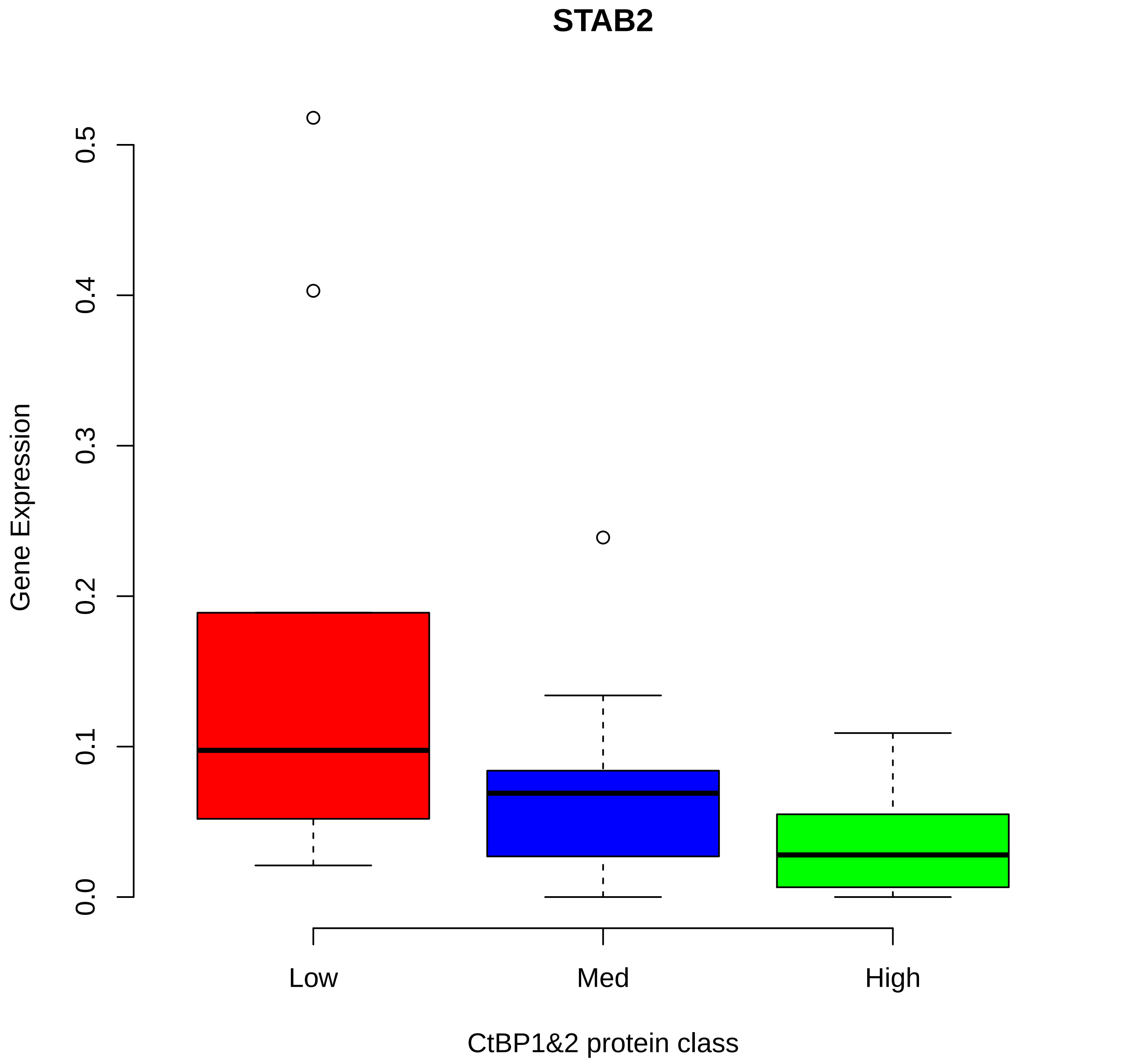

STAB2
Gene Expression
0.0
0.1
0.2
0.3
0.4
0.5
Low
Med
High
CtBP1&2 protein class

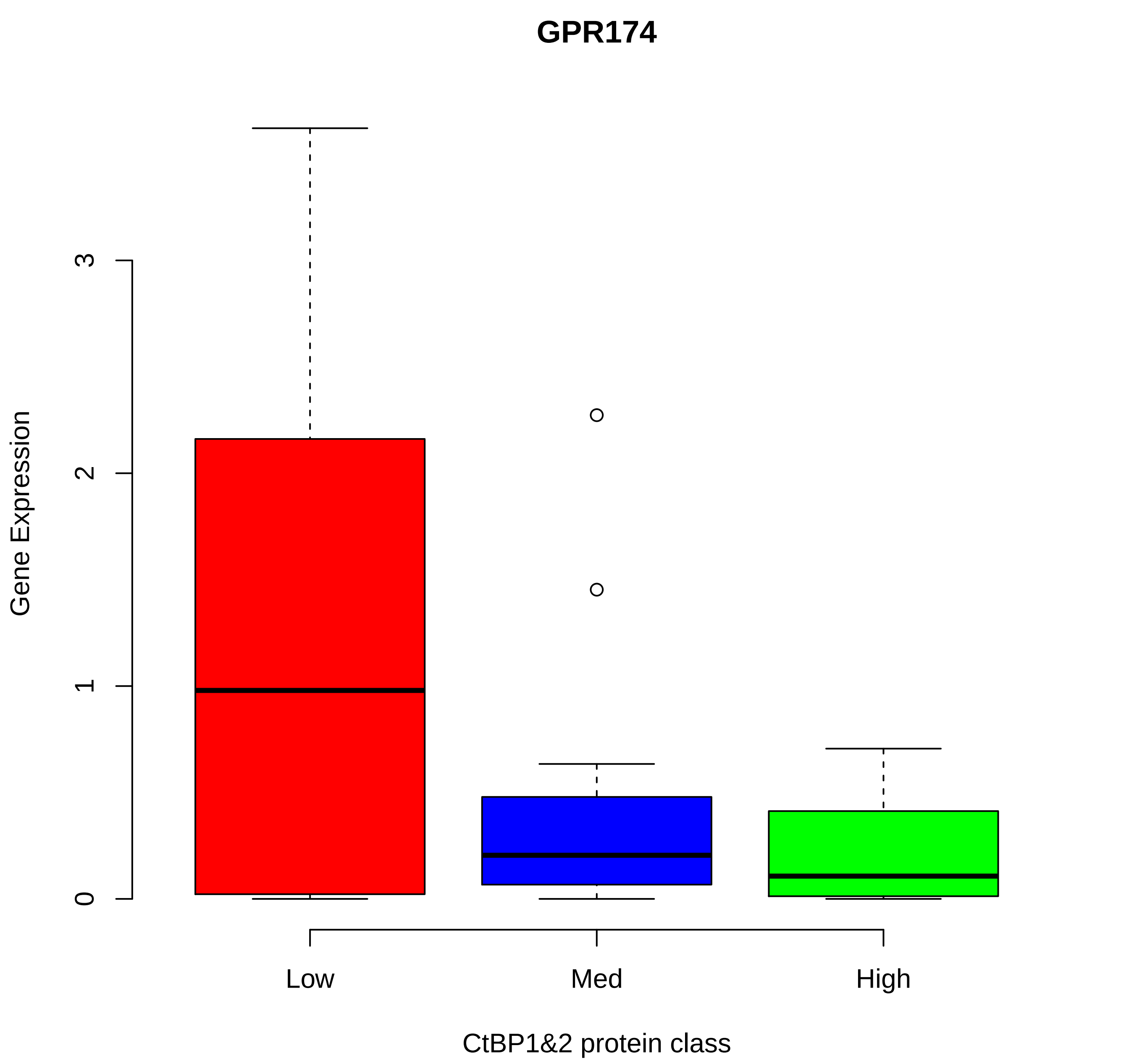

GPR174
Gene Expression
0
1
2
3
Low
Med
High
CtBP1&2 protein class

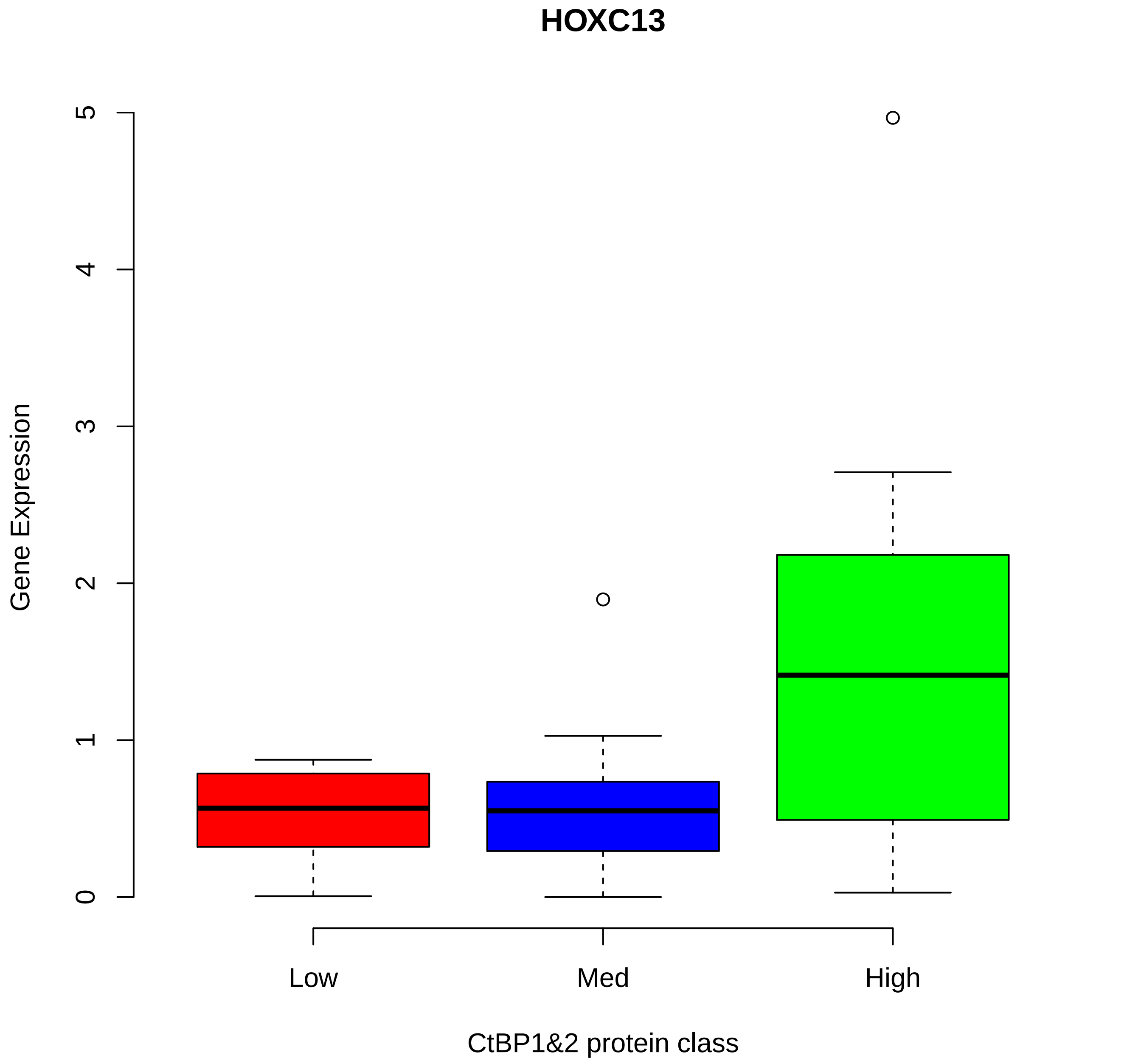

HOXC13
Gene Expression
0
1
2
3
4
5
Low
Med
High
CtBP1&2 protein class

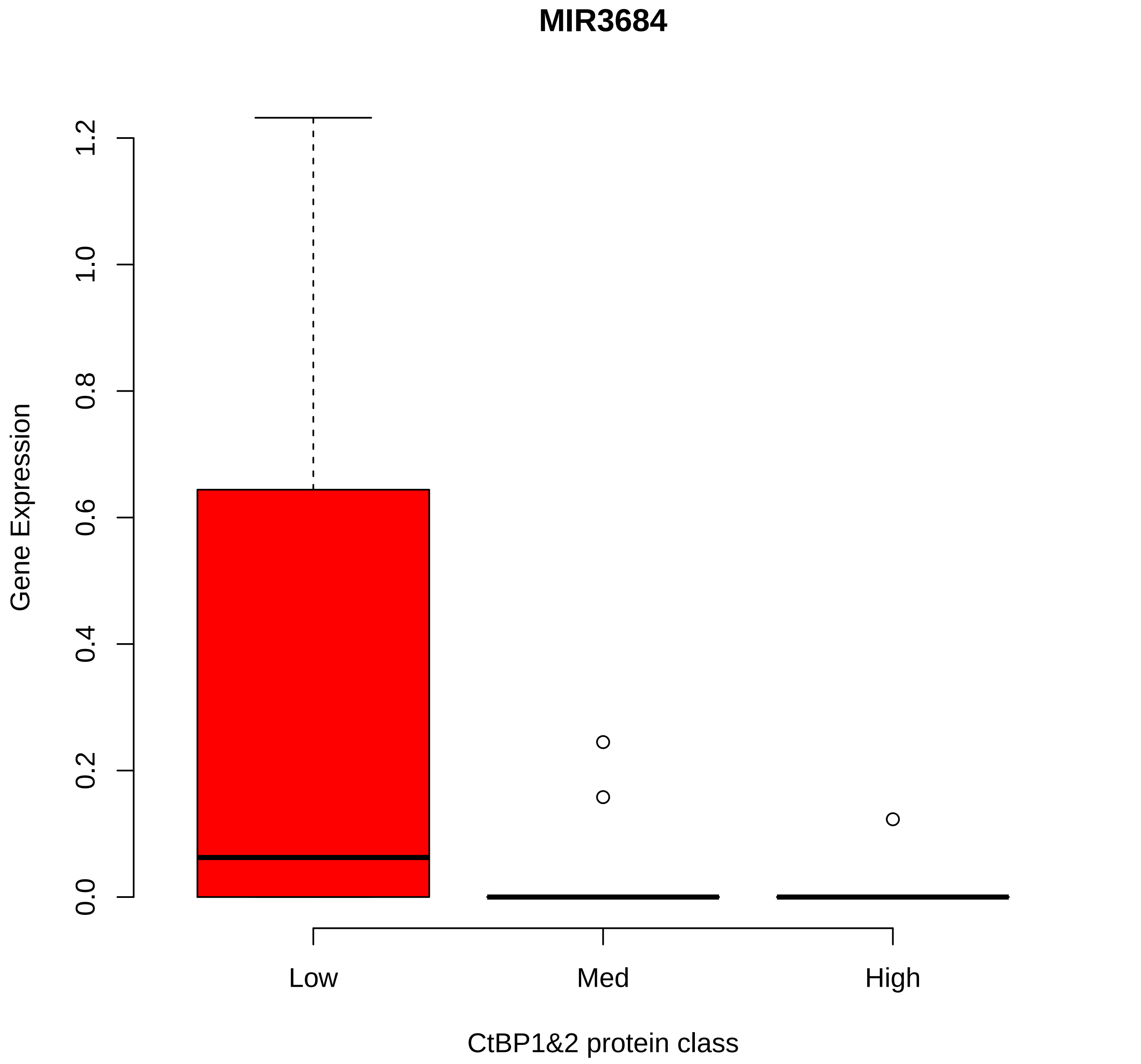
MIR3684
Gene Expression
0.0
0.2
0.4
0.6
0.8
1.0
1.2
Low
Med
High
CtBP1&2 protein class

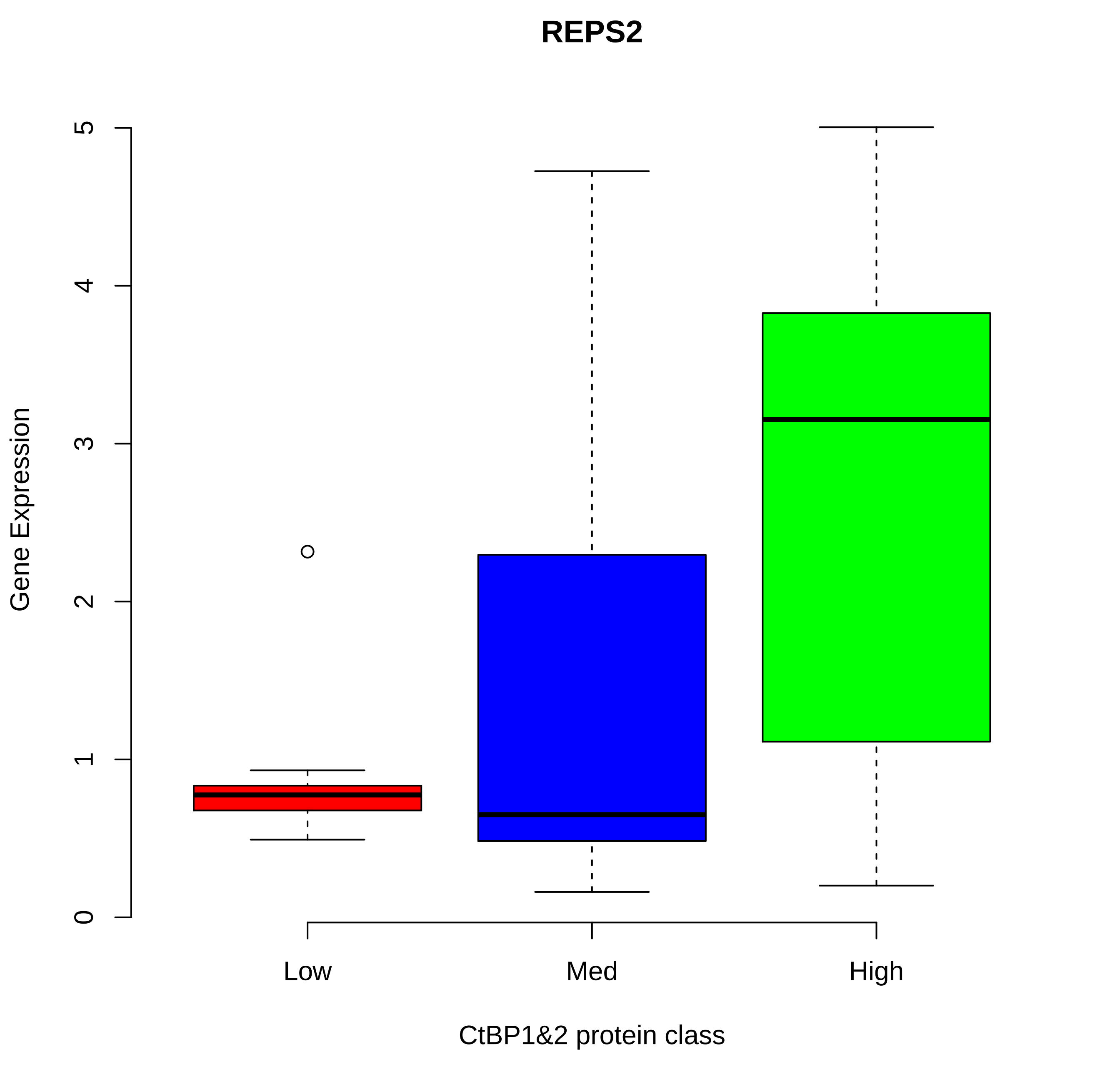

REPS2
Gene Expression
0
1
2
3
4
5
Low
Med
High
CtBP1&2 protein class

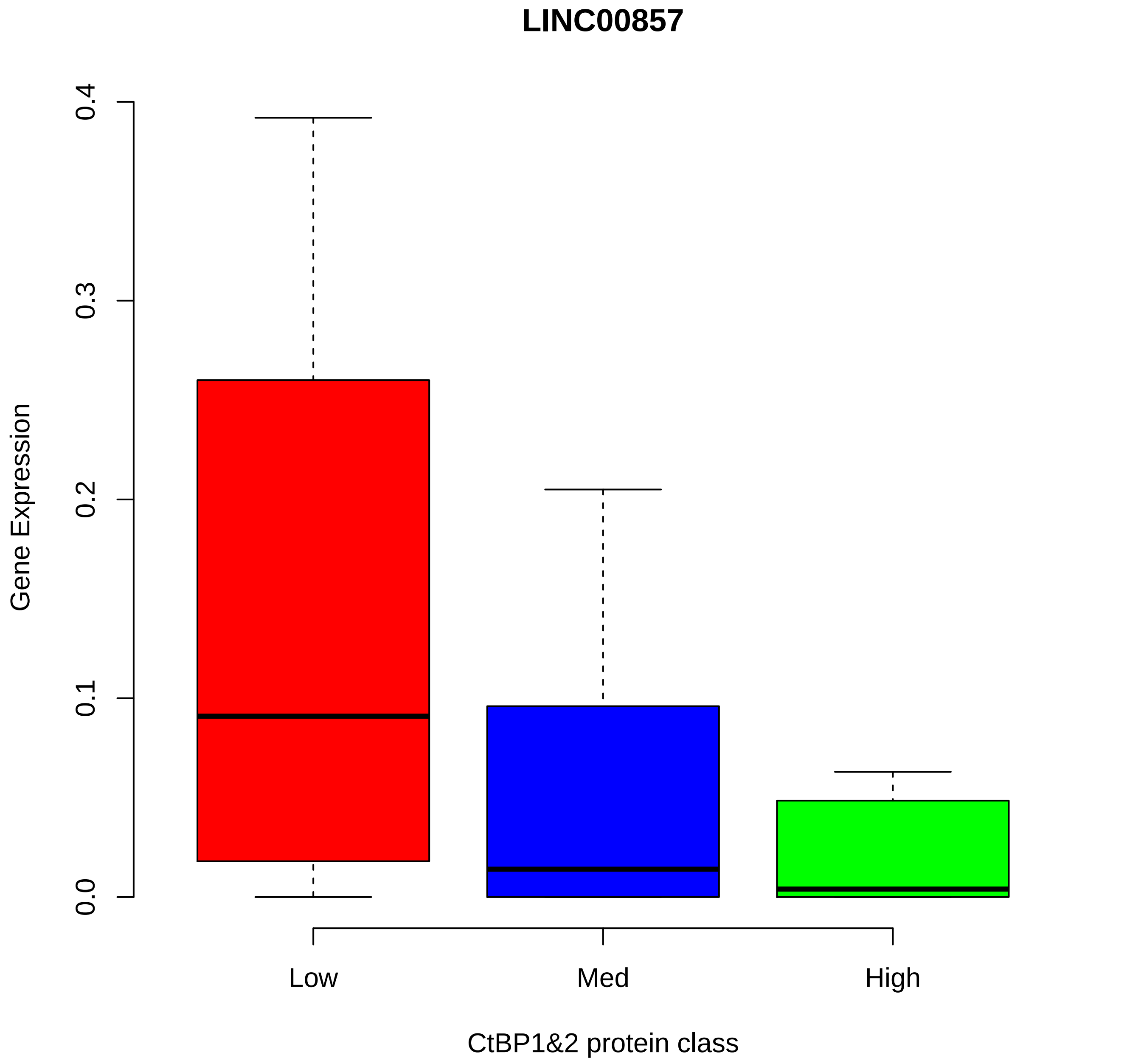
LINC00857
Gene Expression
0.0
0.1
0.2
0.3
0.4
Low
Med
High
CtBP1&2 protein class

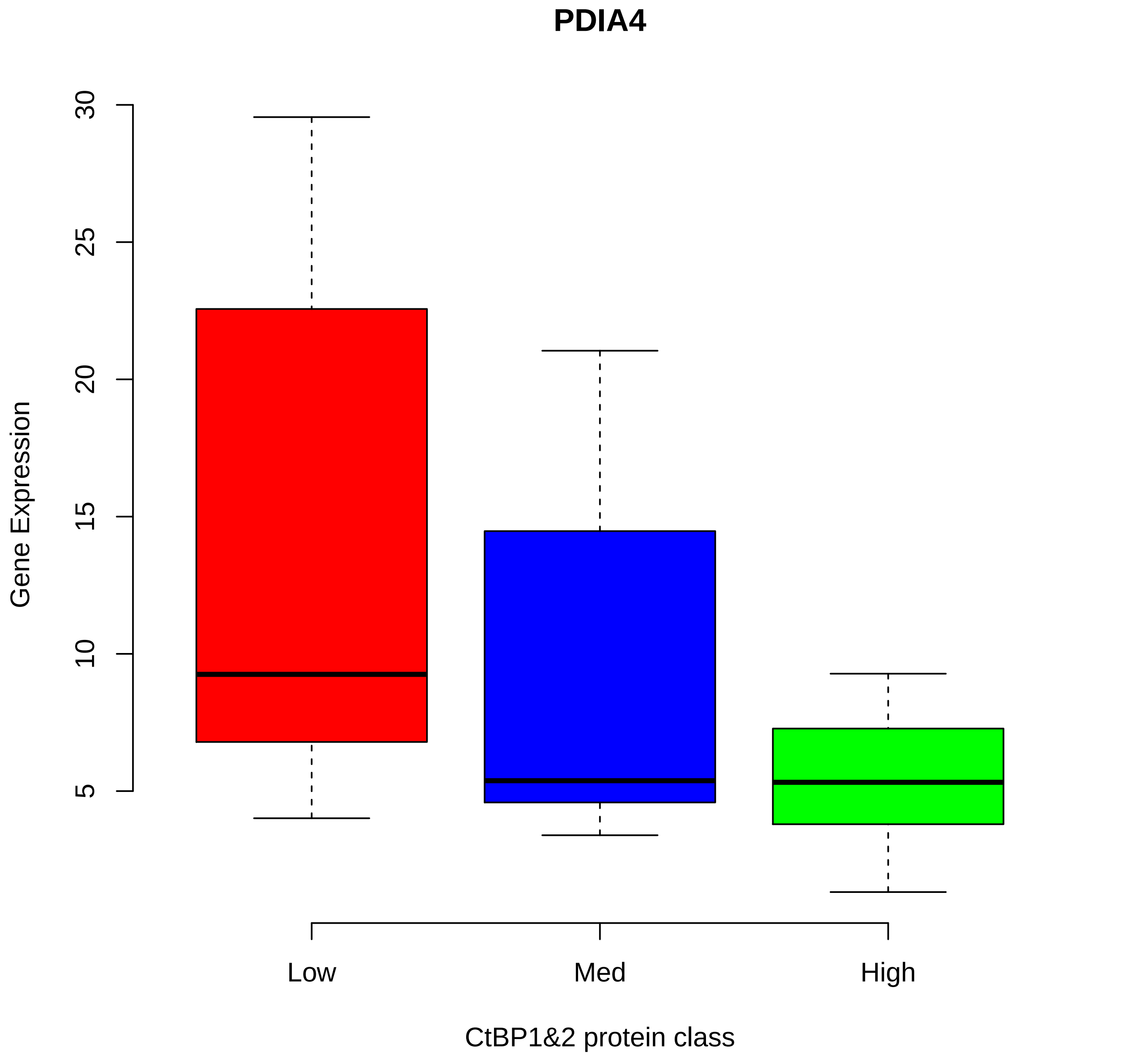
PDIA4
Gene Expression
30
25
20
15
10
5
Low
Med
High
CtBP1&2 protein class

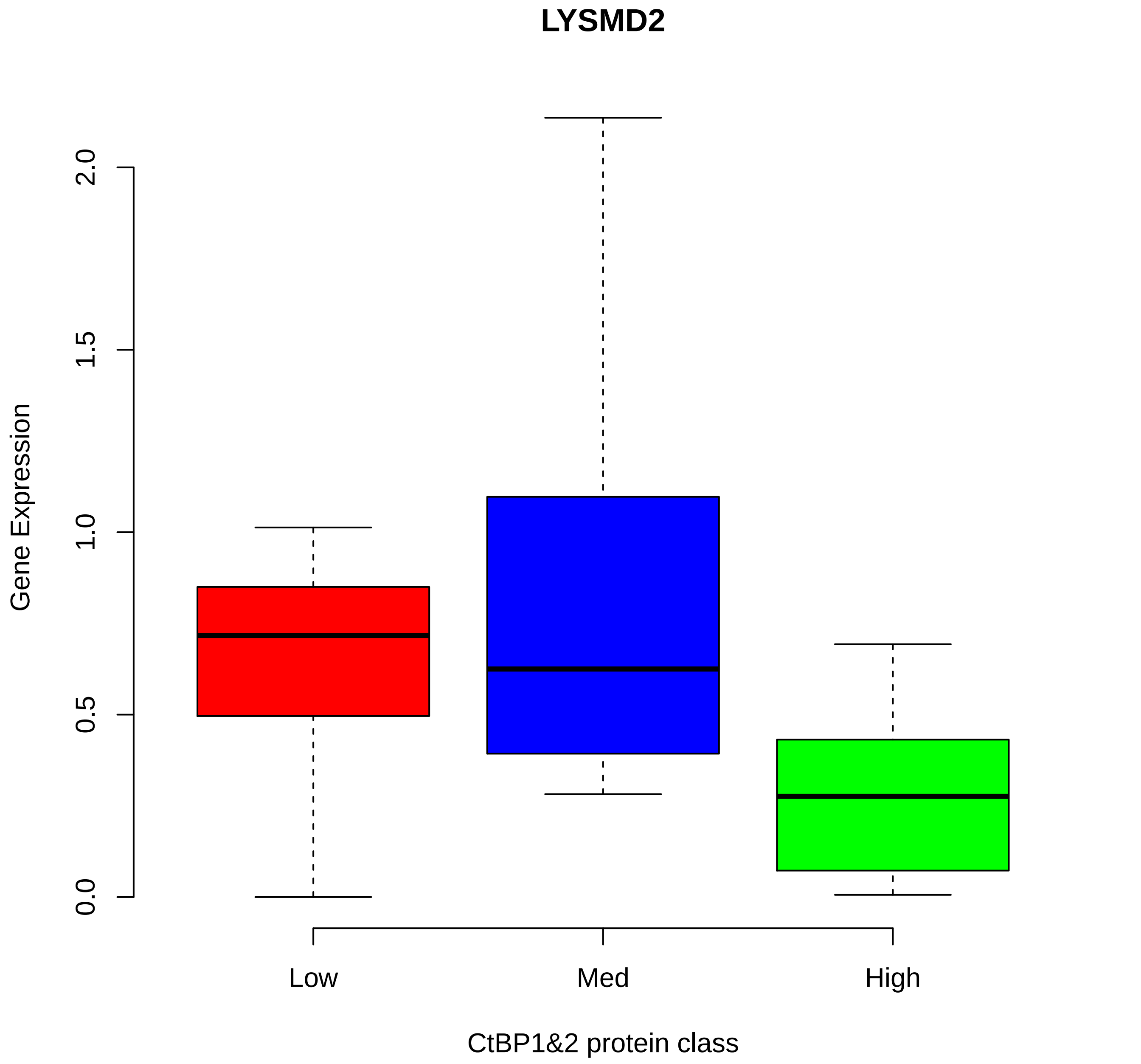

LYSMD2
Gene Expression
0.0
0.5
1.0
1.5
2.0
Low
Med
High
CtBP1&2 protein class

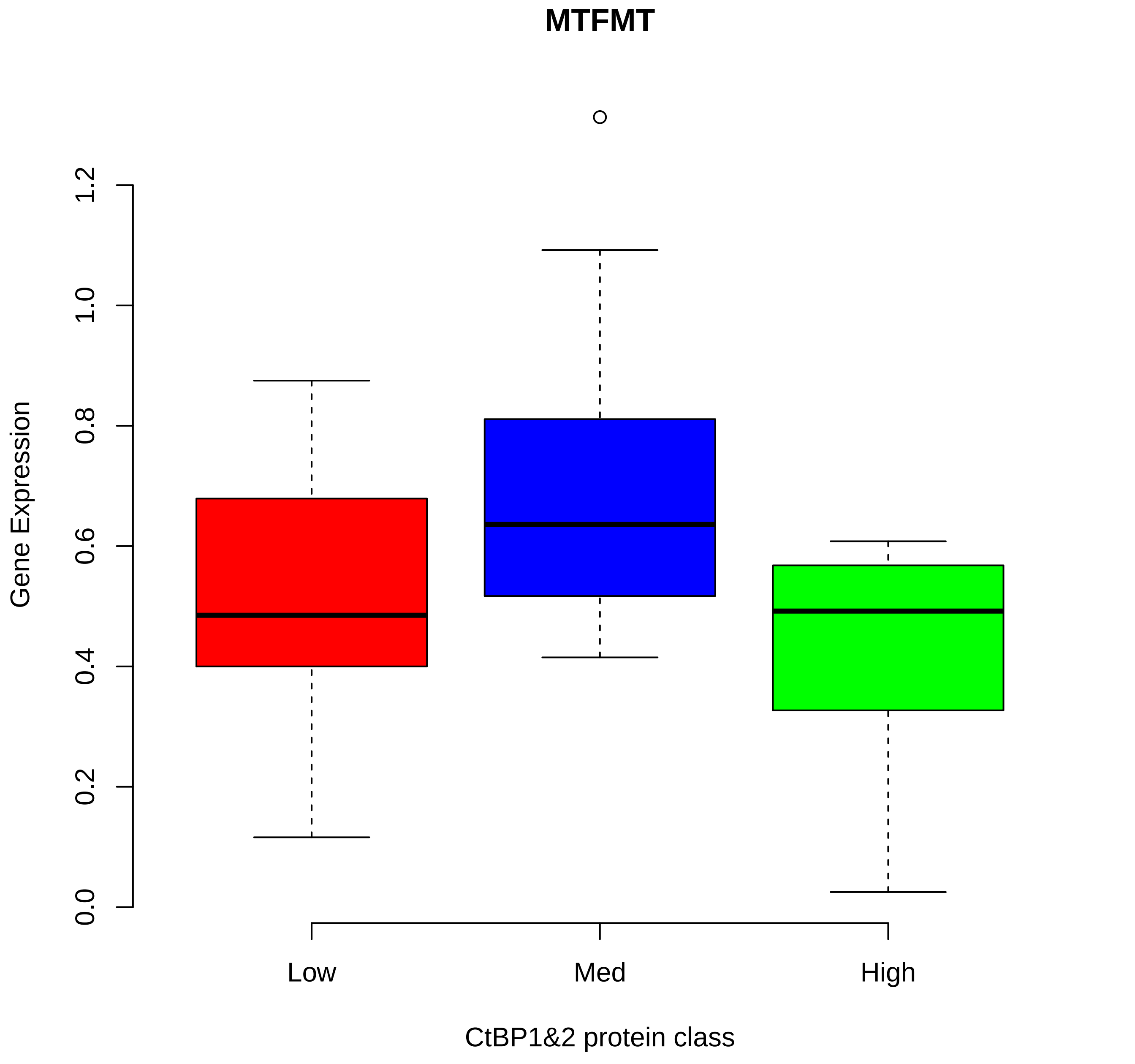

MTFMT
Gene Expression
0.0
0.2
0.4
0.6
0.8
1.0
1.2
Low
Med
High
CtBP1&2 protein class

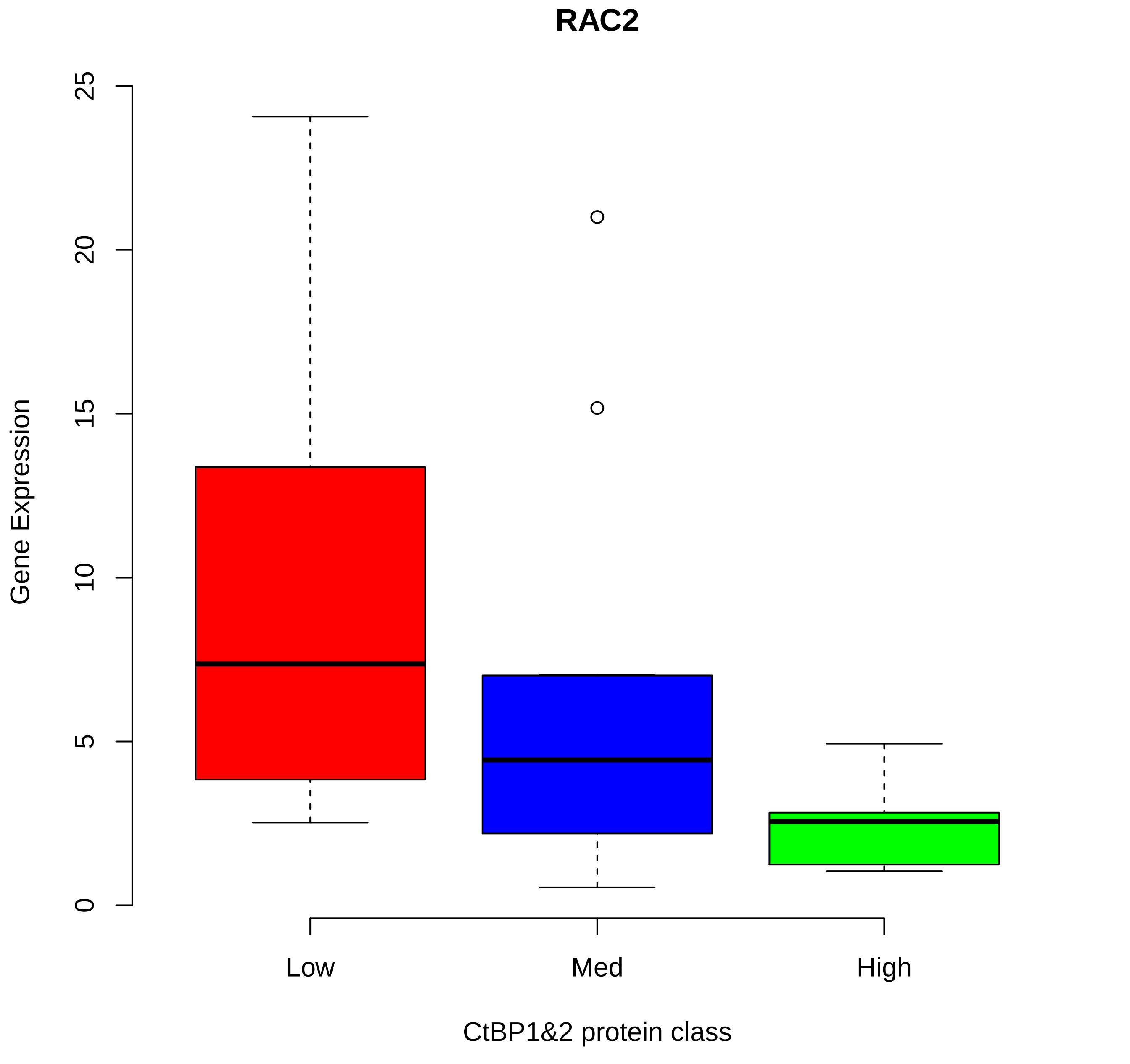

RAC2
Gene Expression
0
5
10
15
20
25
Low
Med
High
CtBP1&2 protein class

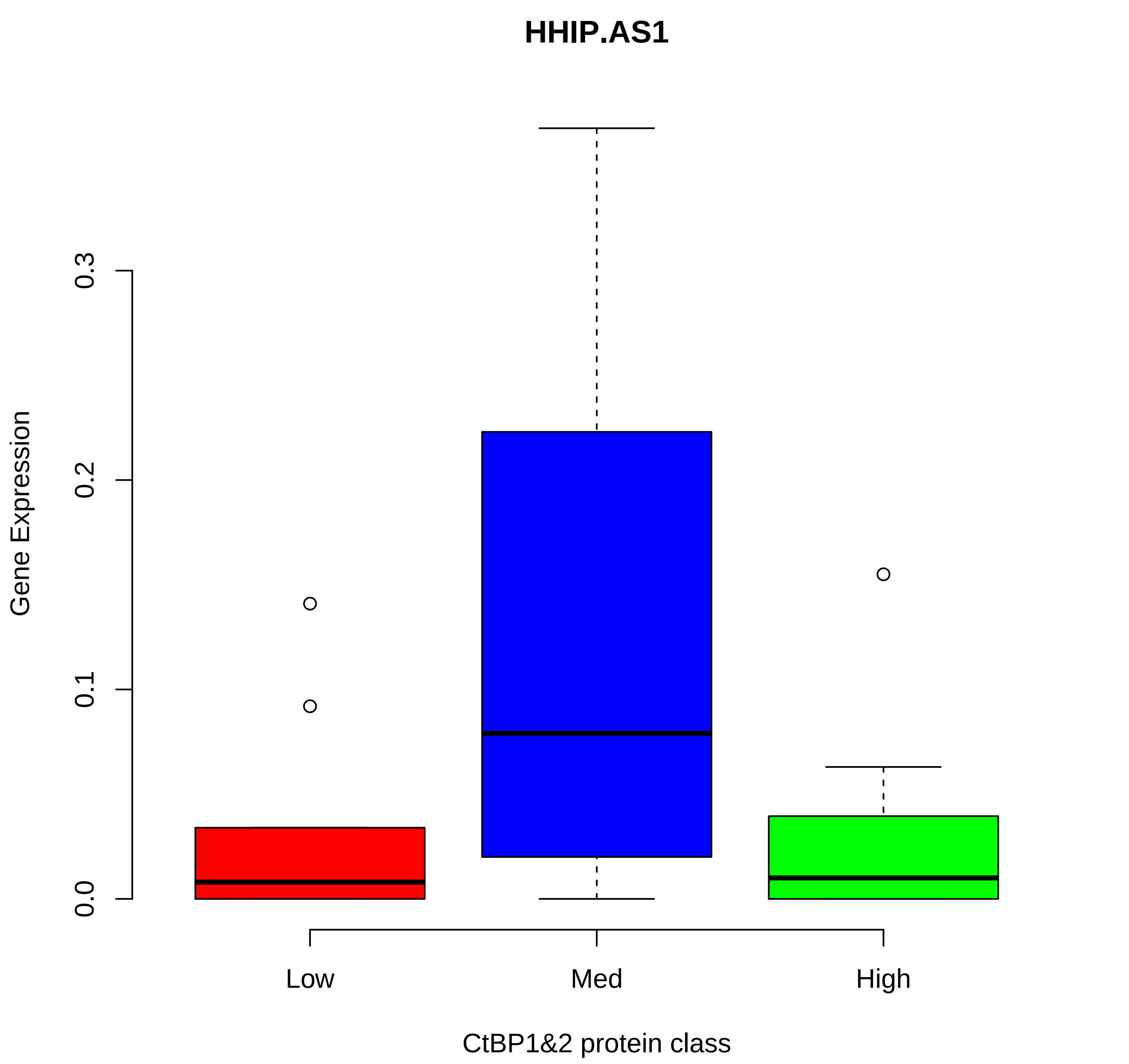
HHIP.AS1
Gene Expression
0.0
0.1
0.2
0.3
Low
Med
High
CtBP1&2 protein class

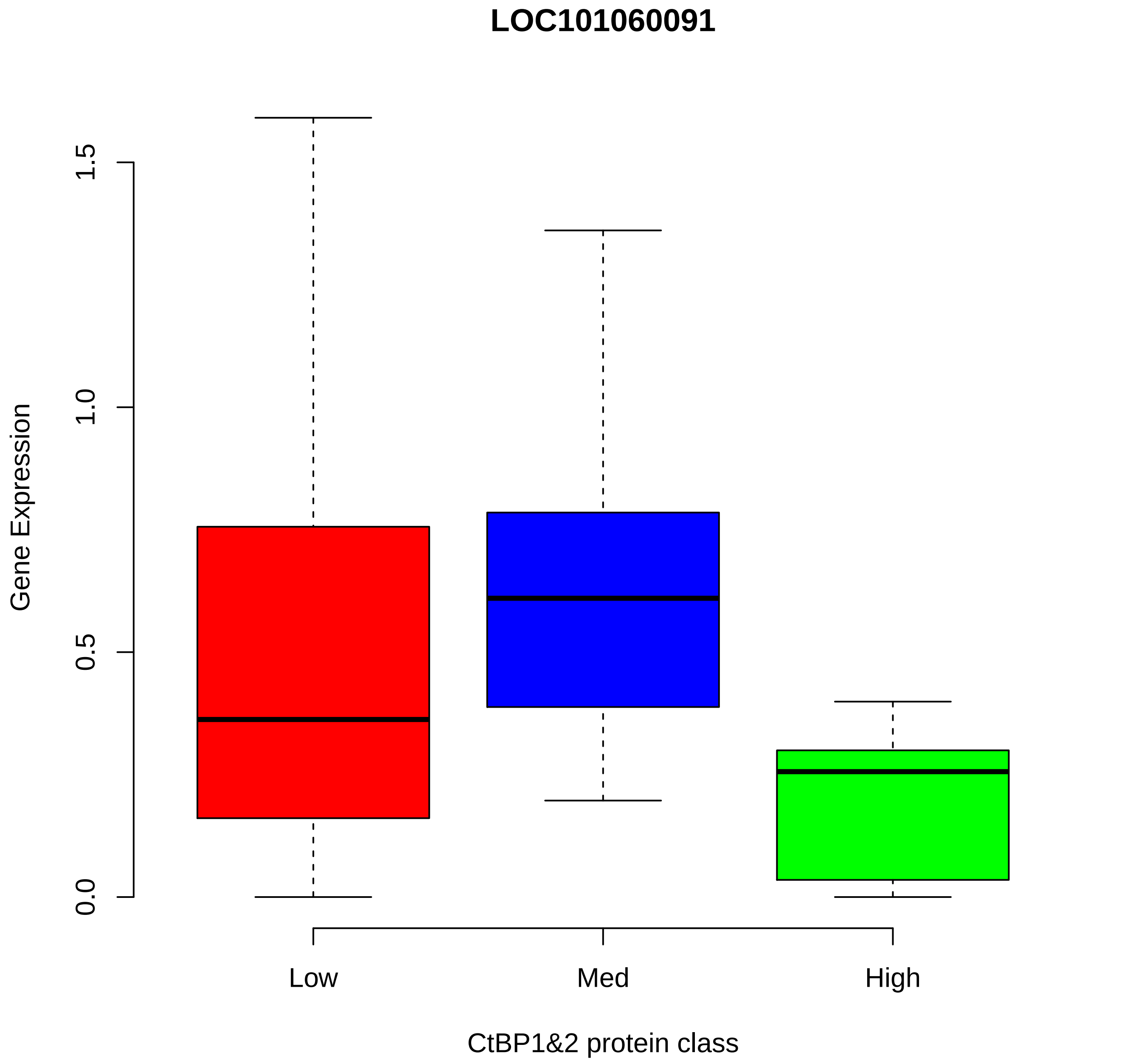

LOC101060091
Gene Expression
0.0
0.5
1.0
1.5
Low
Med
High
CtBP1&2 protein class

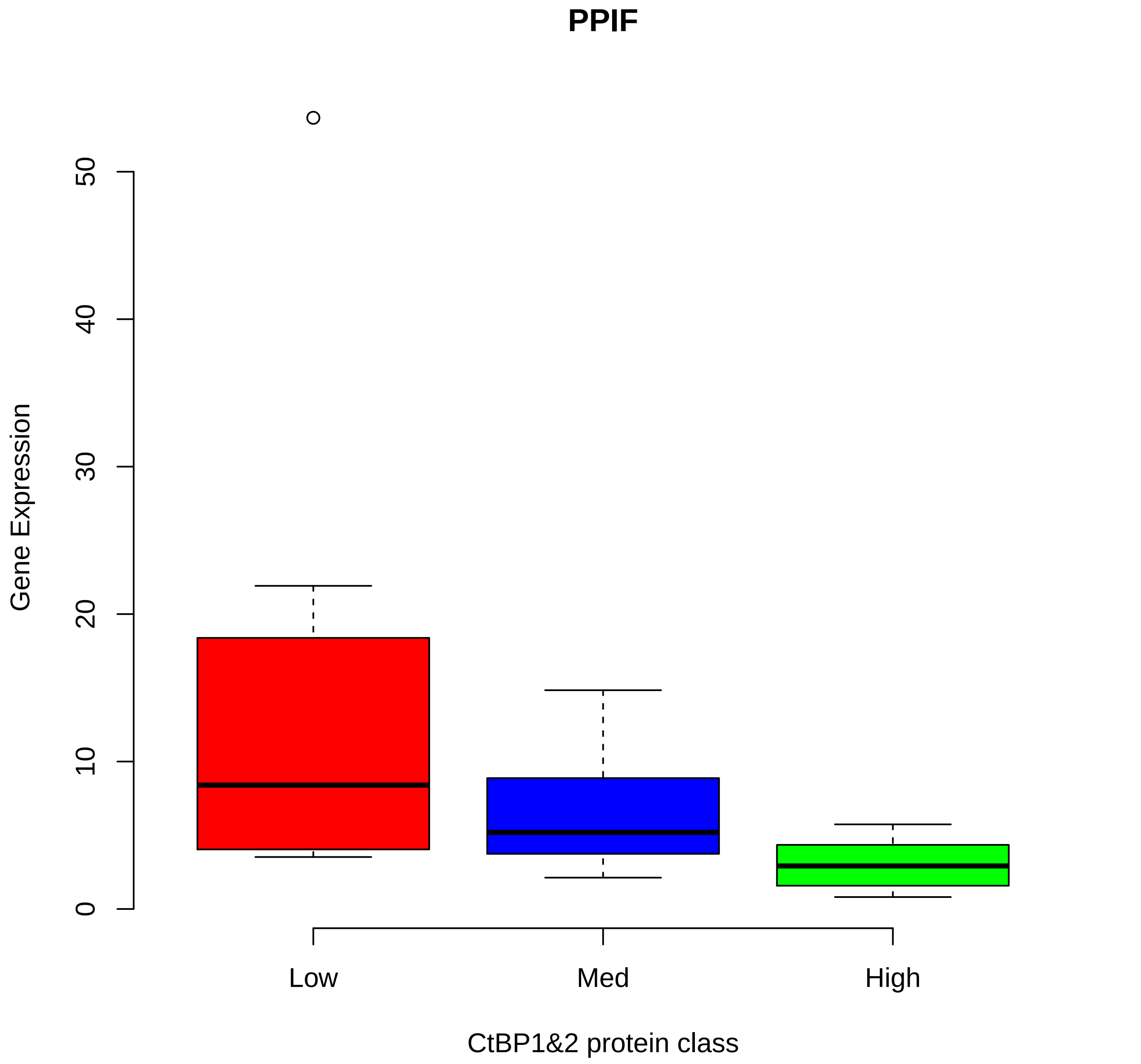
PPIF
Gene Expression
0
10
20
30
40
50
Low
Med
High
CtBP1&2 protein class

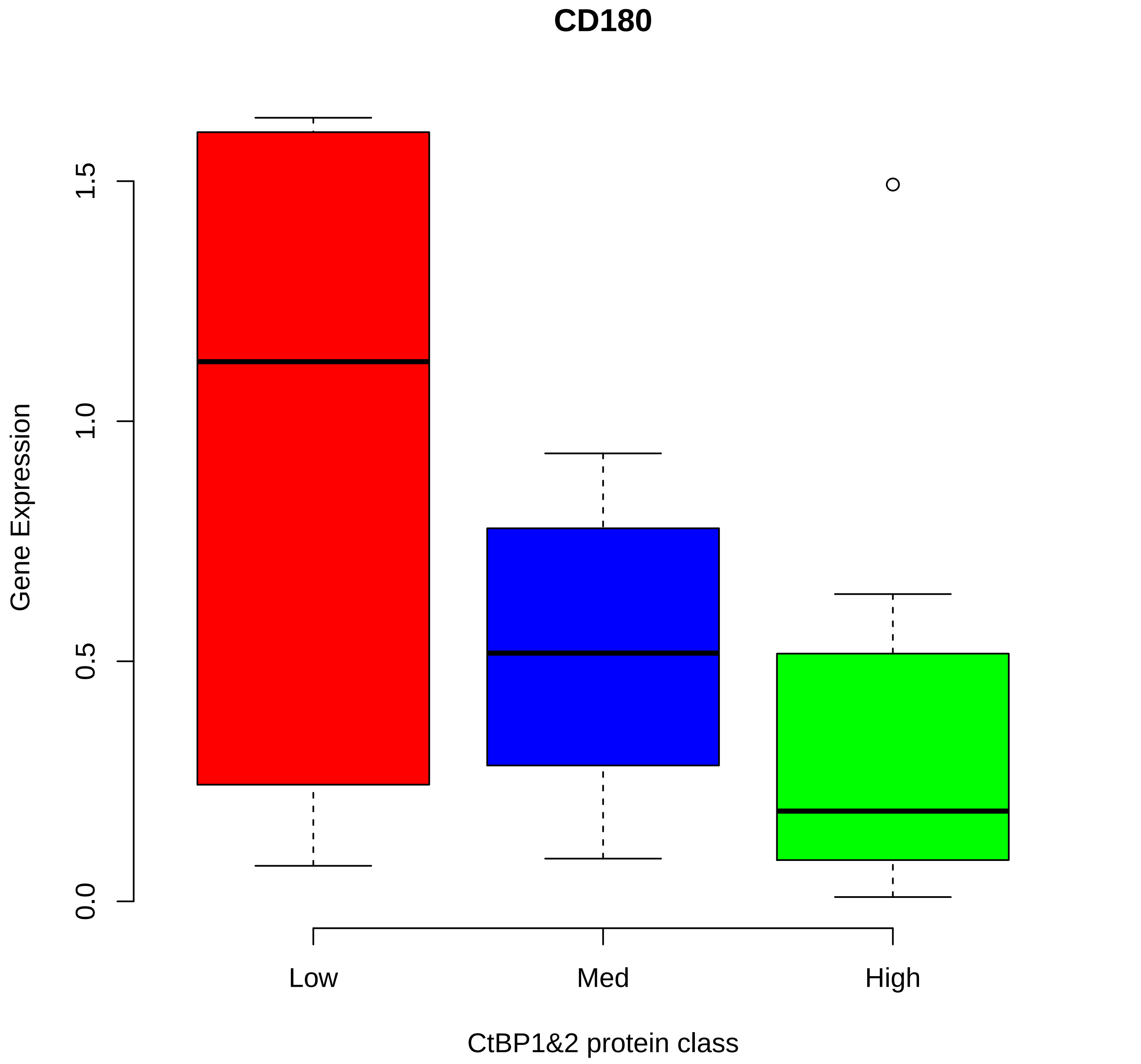
CD180
Gene Expression
0.0
0.5
1.0
1.5
Low
Med
High
CtBP1&2 protein class

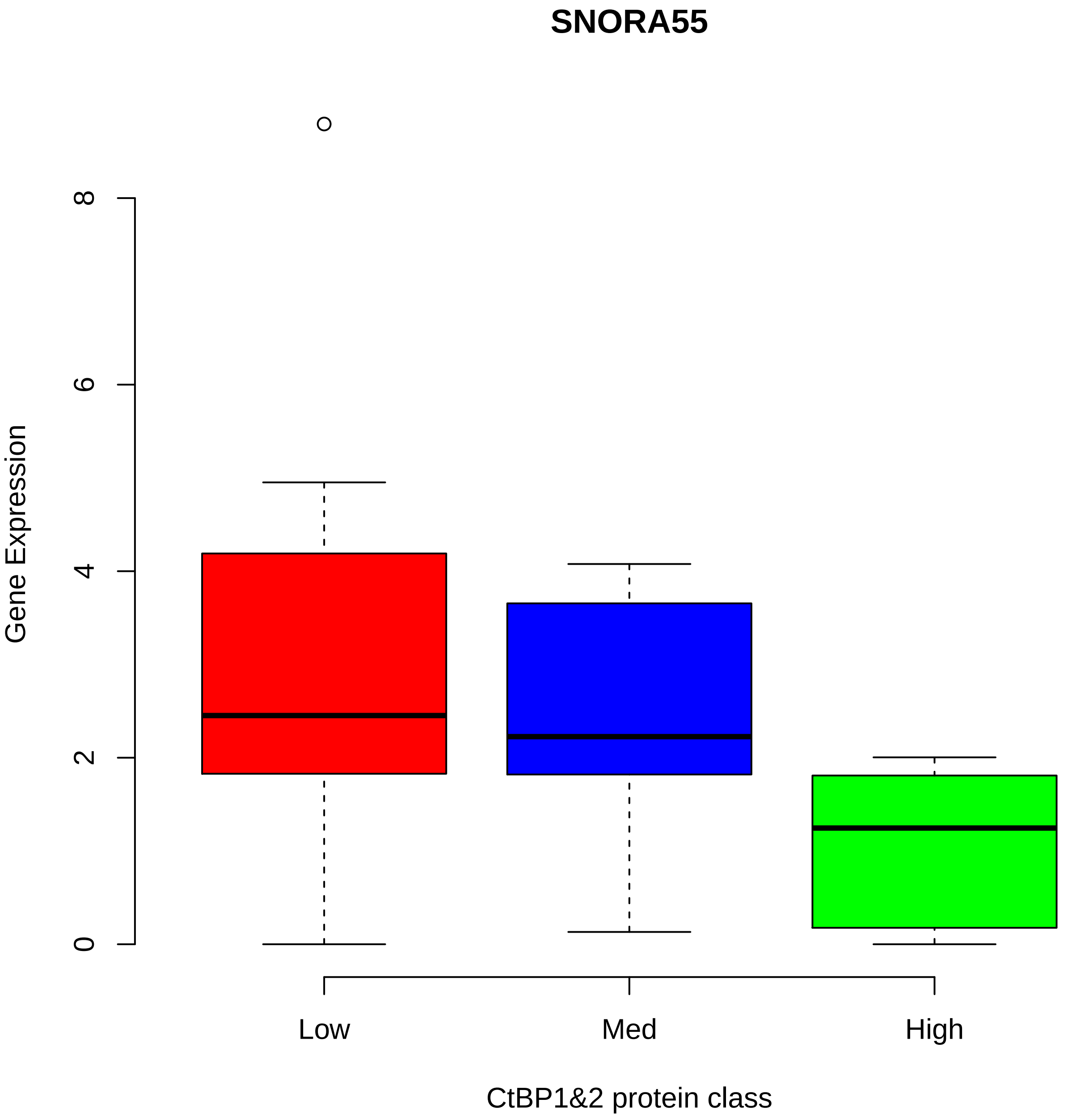

SNORA55
Gene Expression
0
2
4
6
8
Low
Med
High
CtBP1&2 protein class

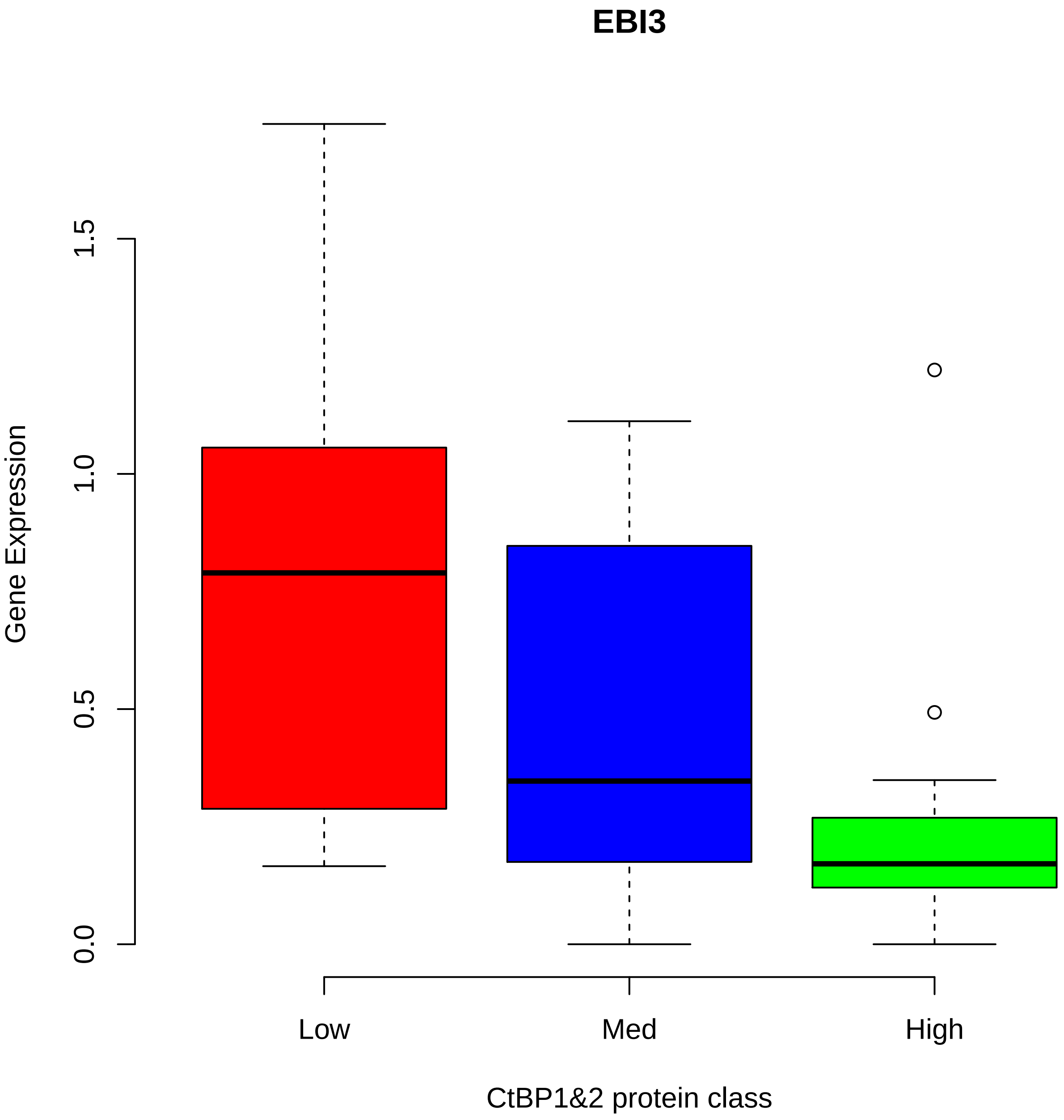
EBI3
Gene Expression
0.0
0.5
1.0
1.5
Low
Med
High
CtBP1&2 protein class

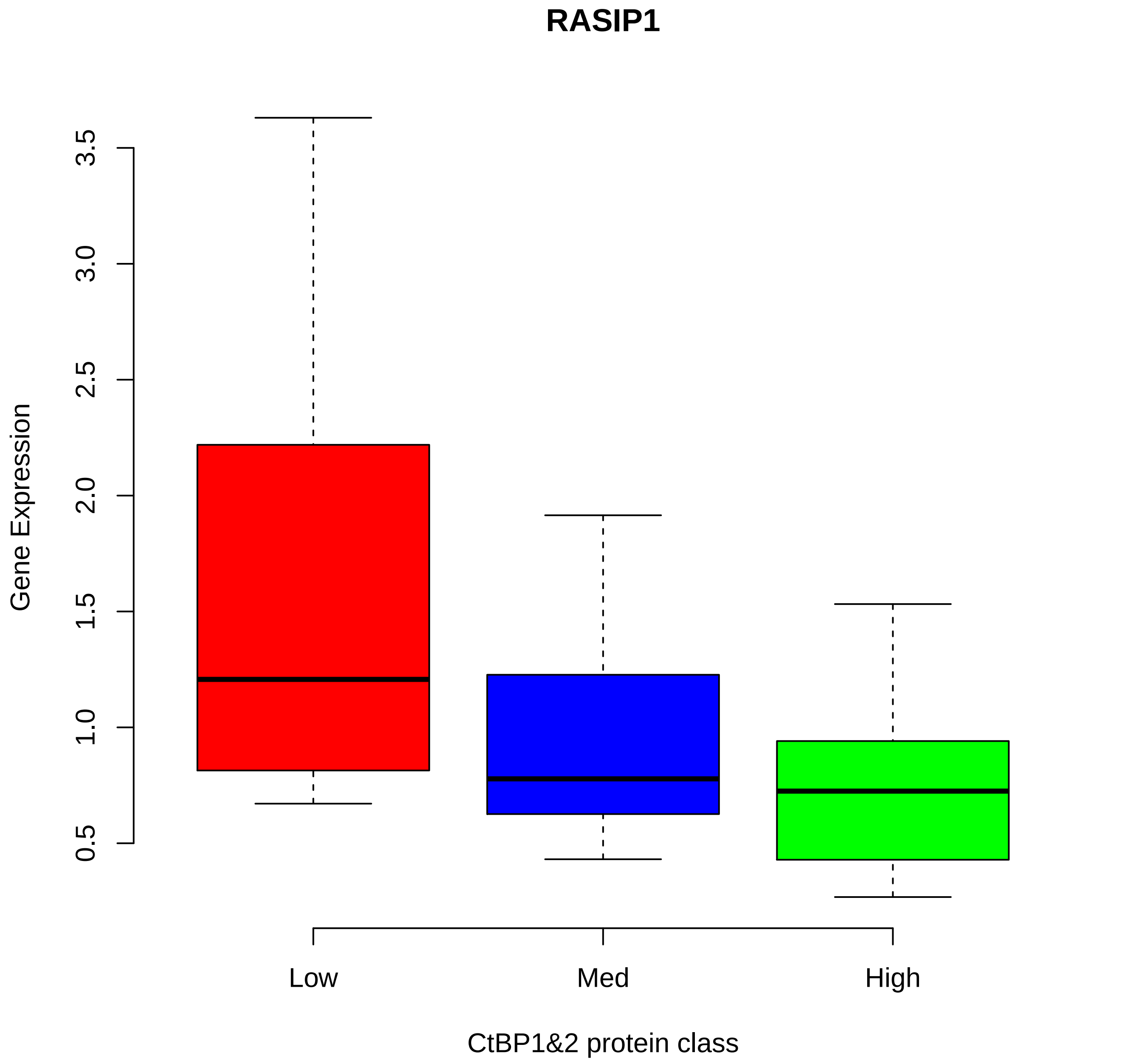
RASIP1
Gene Expression
0.5
1.0
1.5
2.0
2.5
3.0
3.5
Low
Med
High
CtBP1&2 protein class

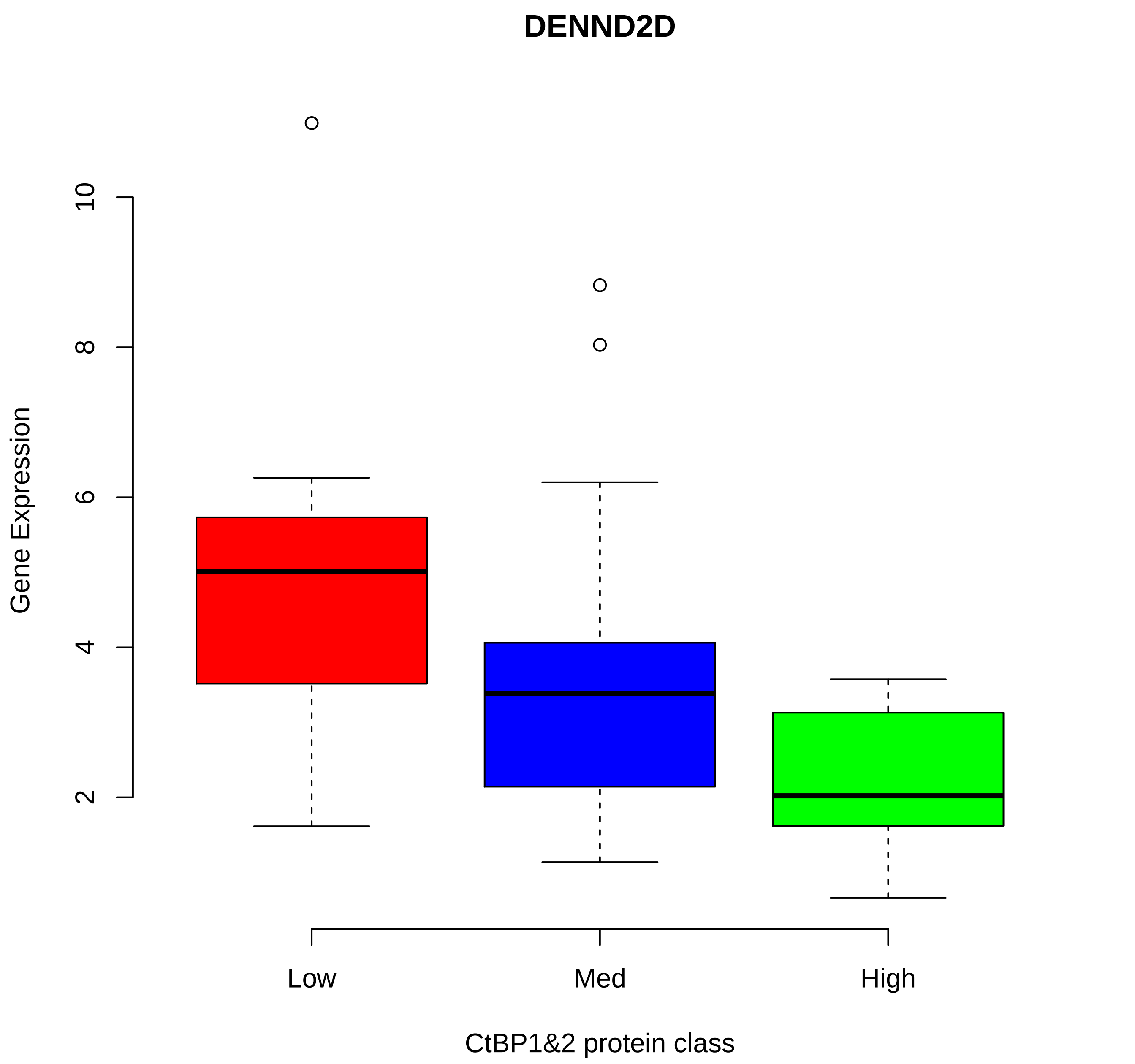

DENND2D
Gene Expression
2
4
6
8
10
Low
Med
High
CtBP1&2 protein class

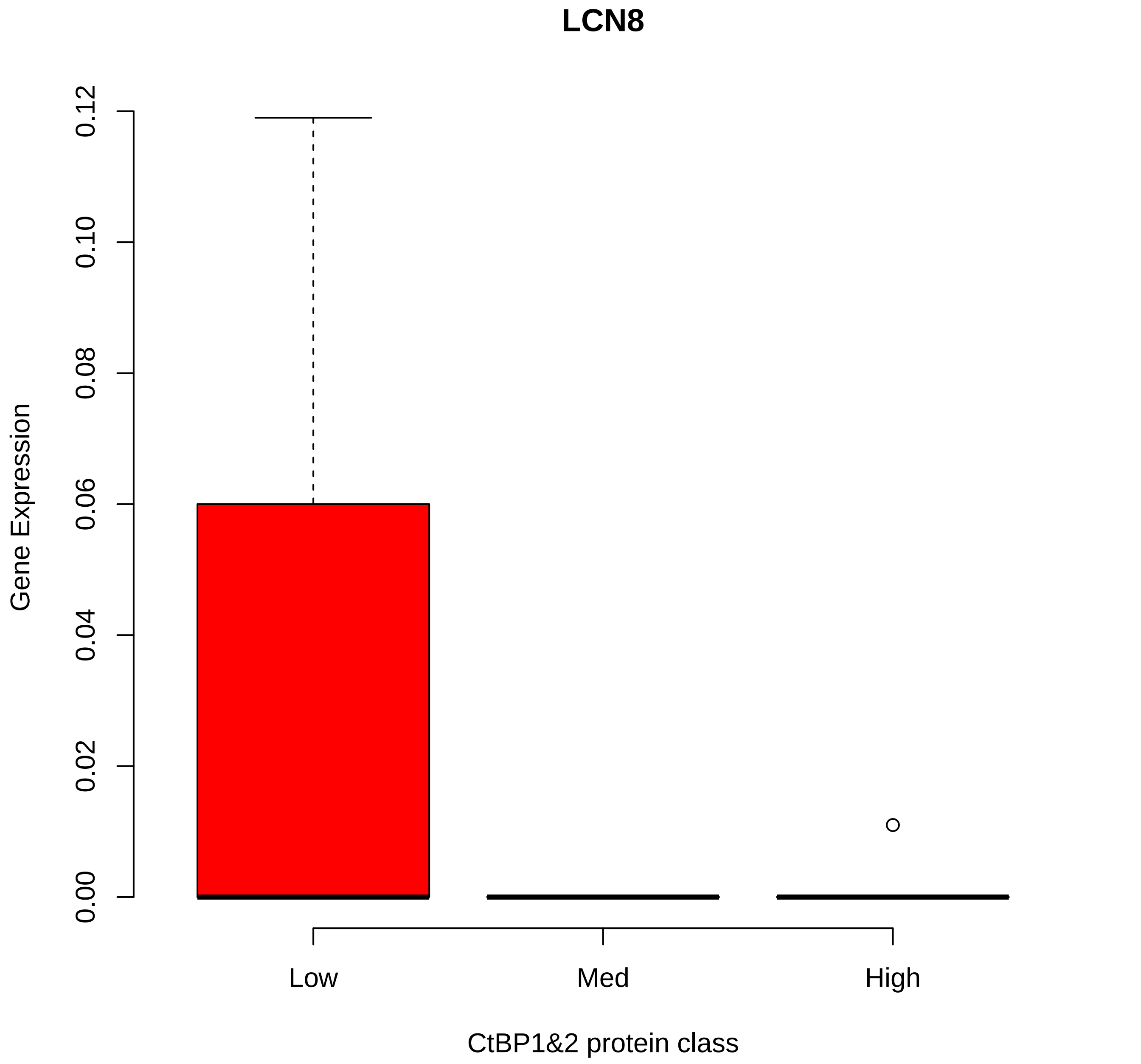
LCN8
Gene Expression
0.00
0.02
0.04
0.06
0.08
0.10
0.12
Low
Med
High
CtBP1&2 protein class

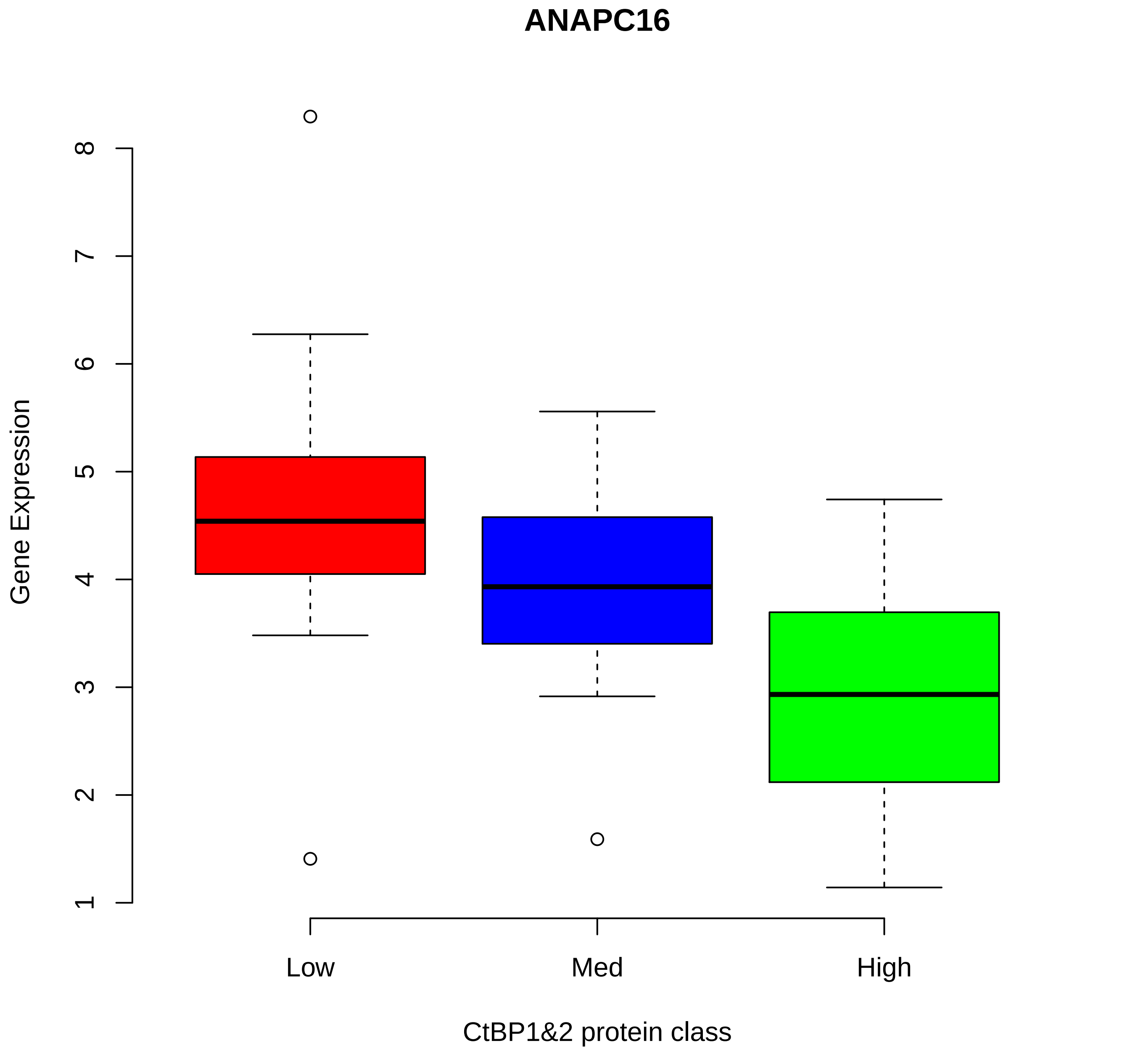
ANAPC16
Gene Expression
1
2
3
4
5
6
7
8
Low
Med
High
CtBP1&2 protein class

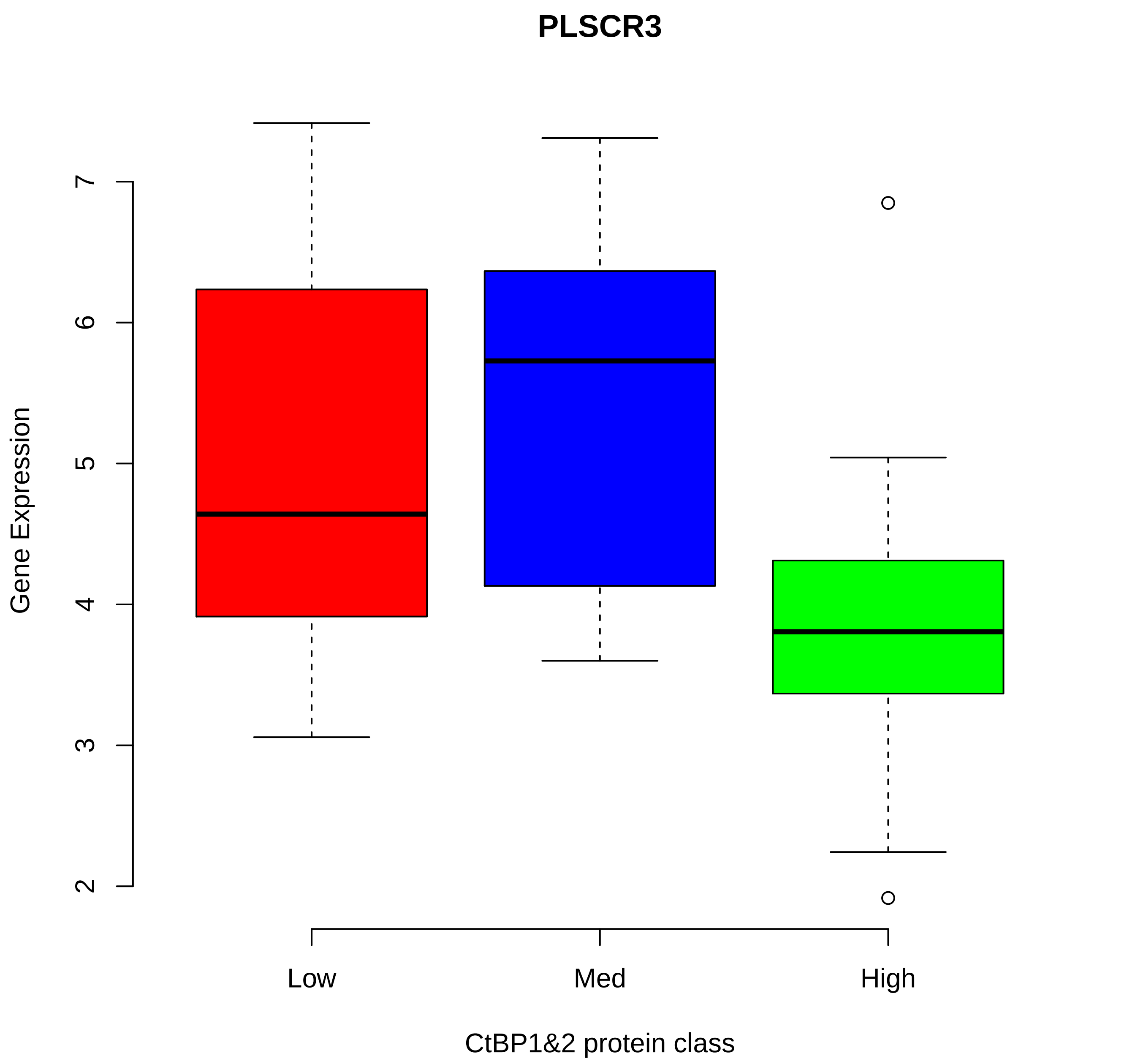

PLSCR3
Gene Expression
2
3
4
5
6
7
Low
Med
High
CtBP1&2 protein class

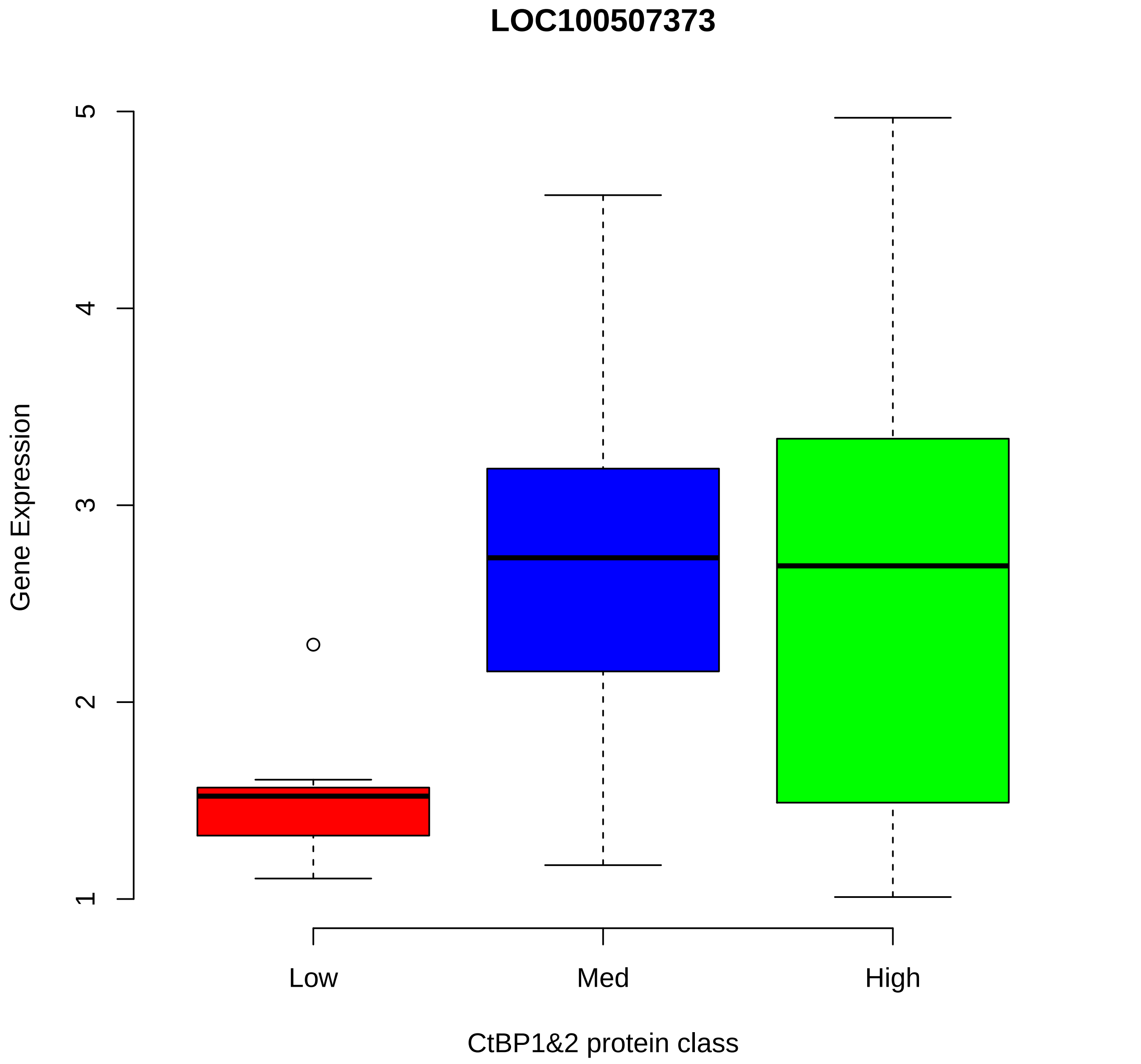

LOC100507373
Gene Expression
1
2
3
4
5
Low
Med
High
CtBP1&2 protein class

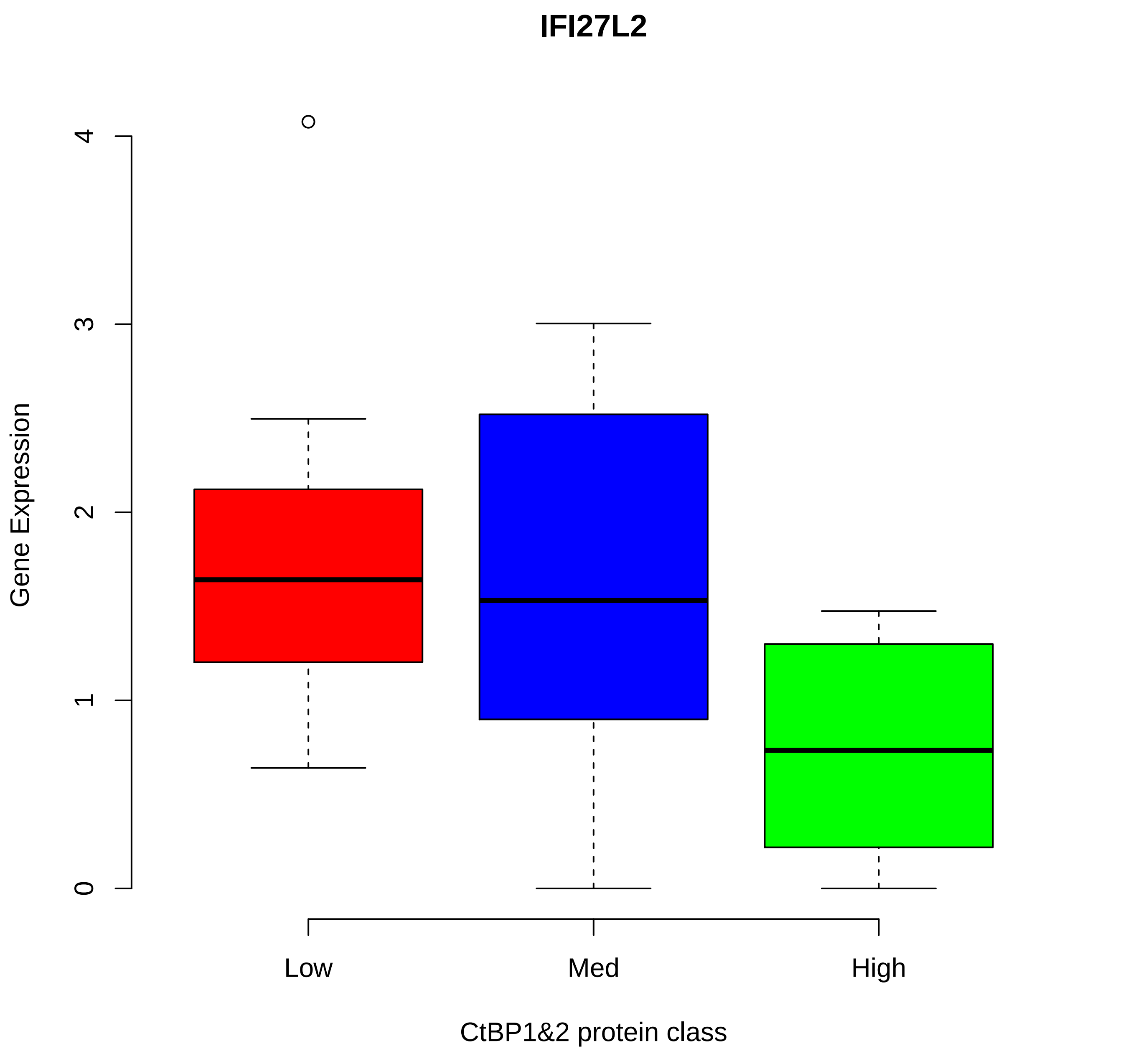
IFI27L2
Gene Expression
0
1
2
3
4
Low
Med
High
CtBP1&2 protein class

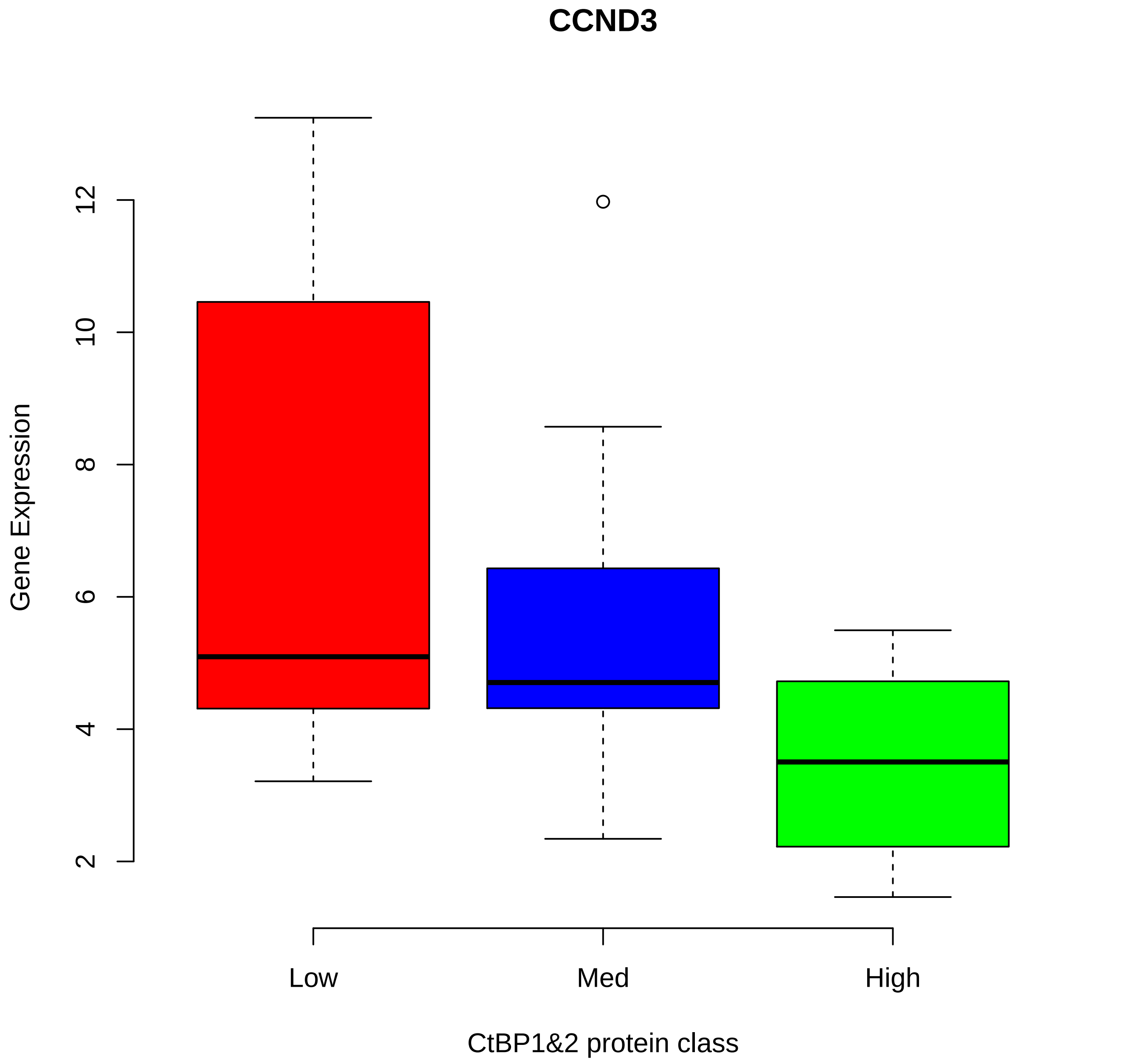

CCND3
Gene Expression
2
4
6
8
10
12
Low
Med
High
CtBP1&2 protein class

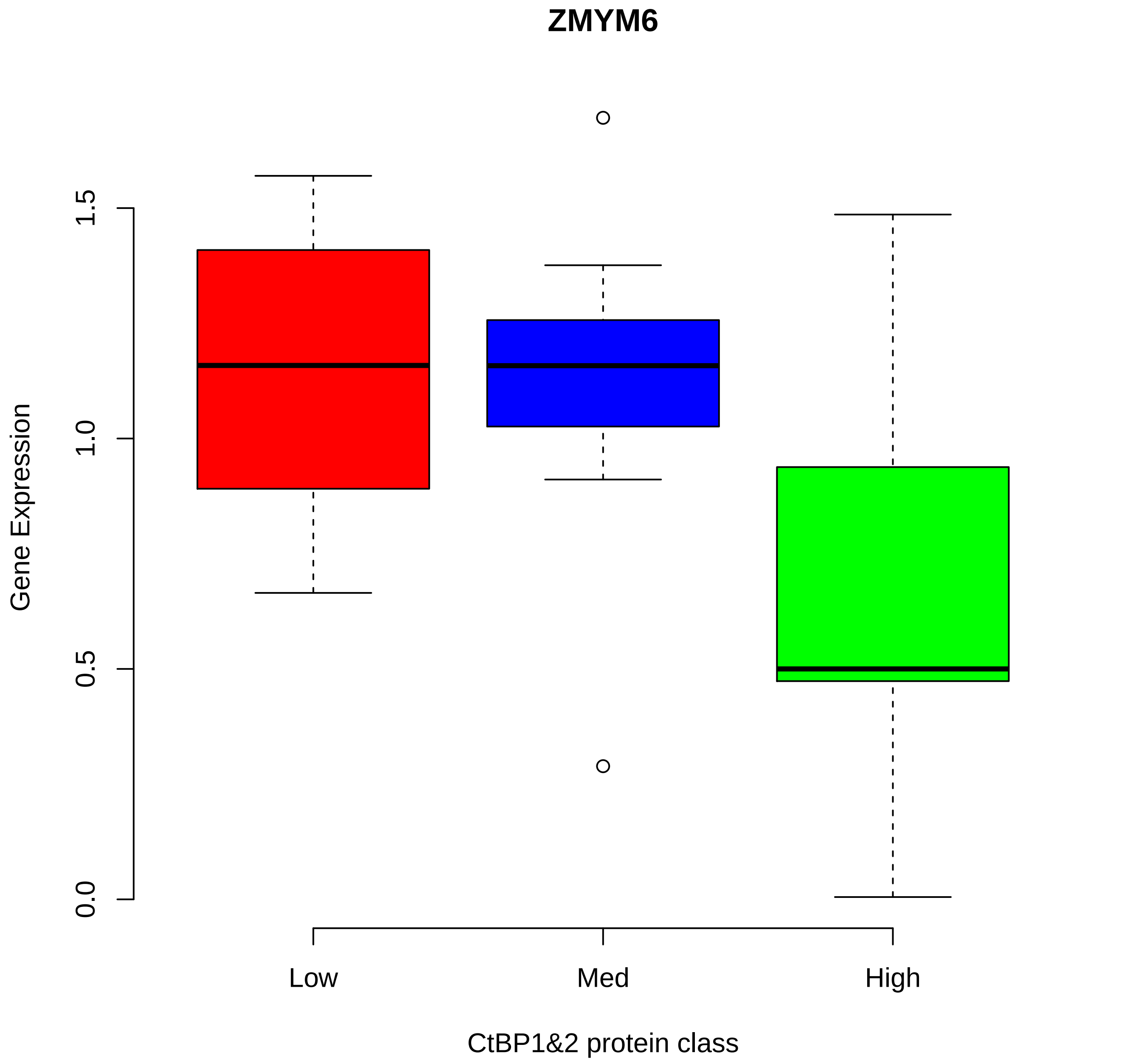

ZMYM6
Gene Expression
0.0
0.5
1.0
1.5
Low
Med
High
CtBP1&2 protein class

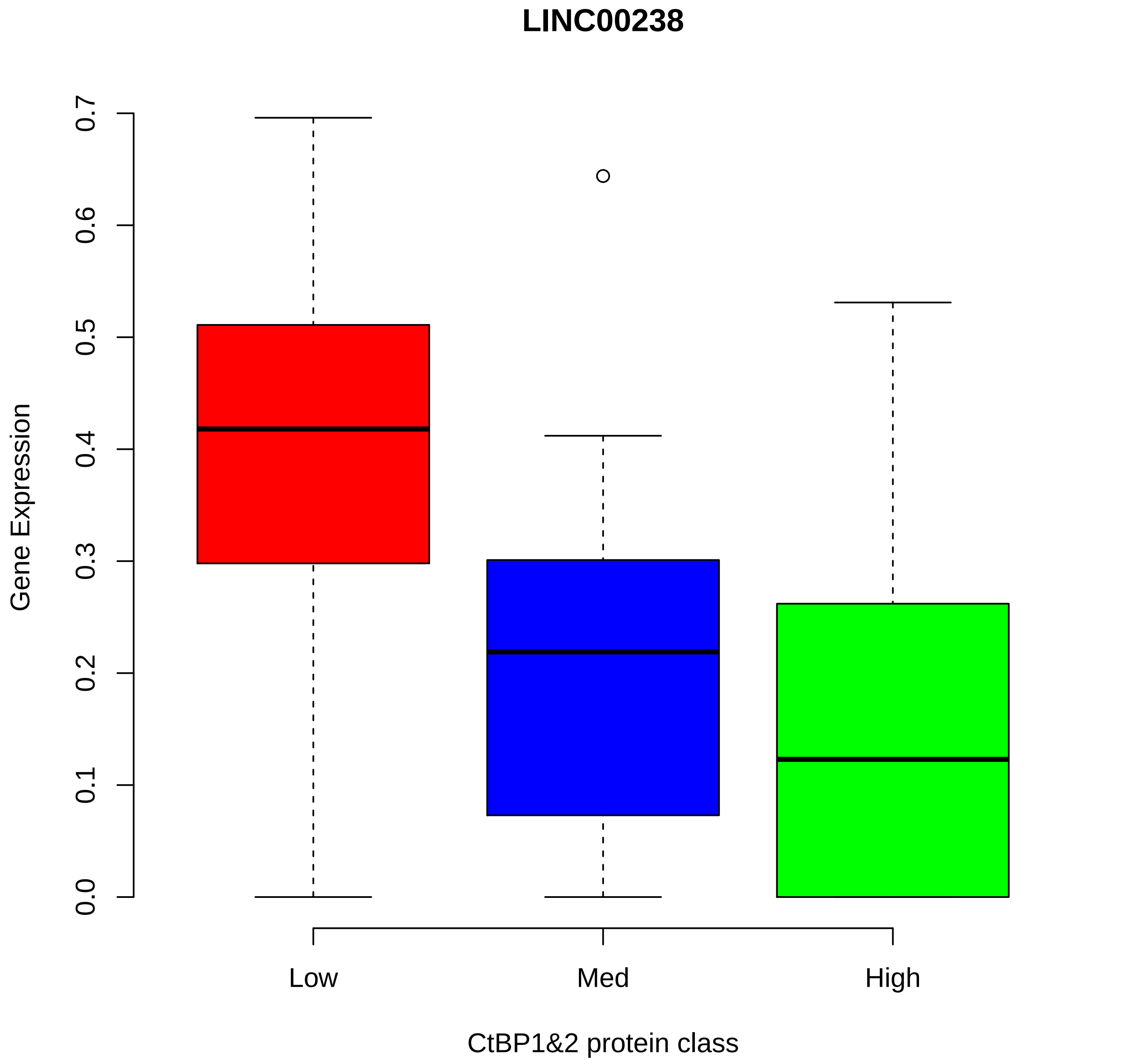

LINC00238
Gene Expression
0.0
0.1
0.2
0.3
0.4
0.5
0.6
0.7
Low
Med
High
CtBP1&2 protein class

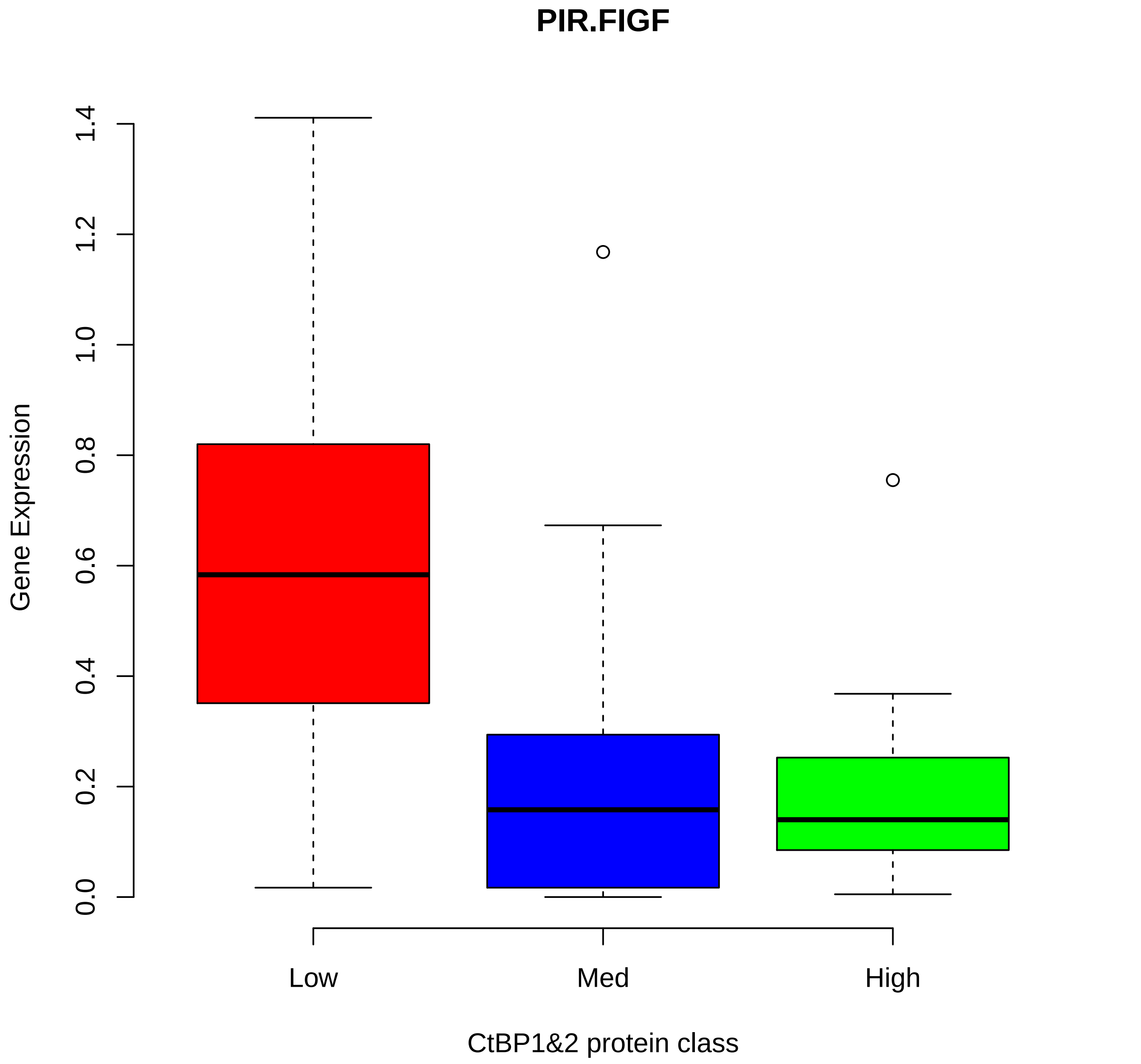
PIR.FIGF
Gene Expression
0.0
0.2
0.4
0.6
0.8
1.0
1.2
1.4
Low
Med
High
CtBP1&2 protein class

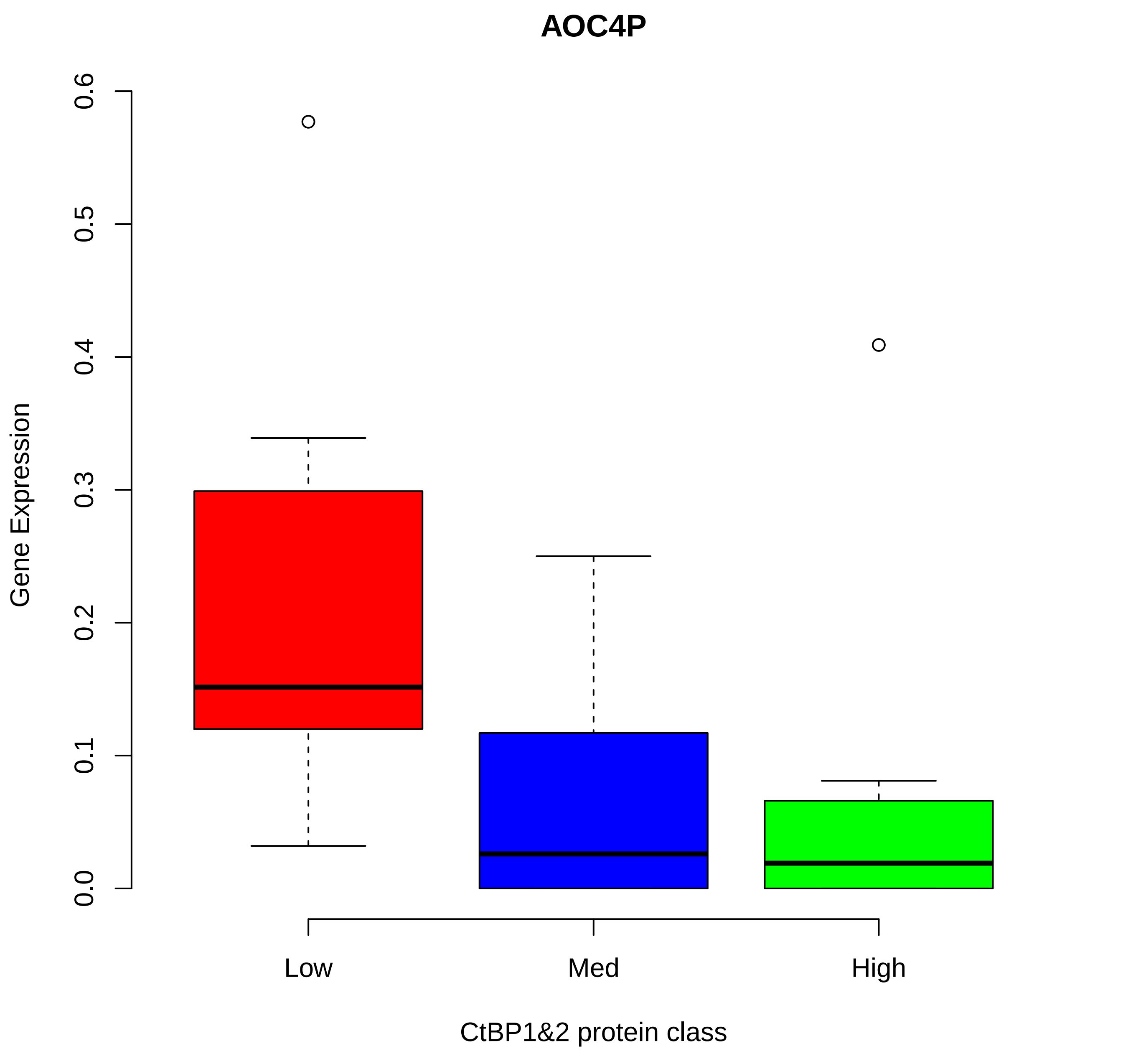

AOC4P
Gene Expression
0.0
0.1
0.2
0.3
0.4
0.5
0.6
Low
Med
High
CtBP1&2 protein class

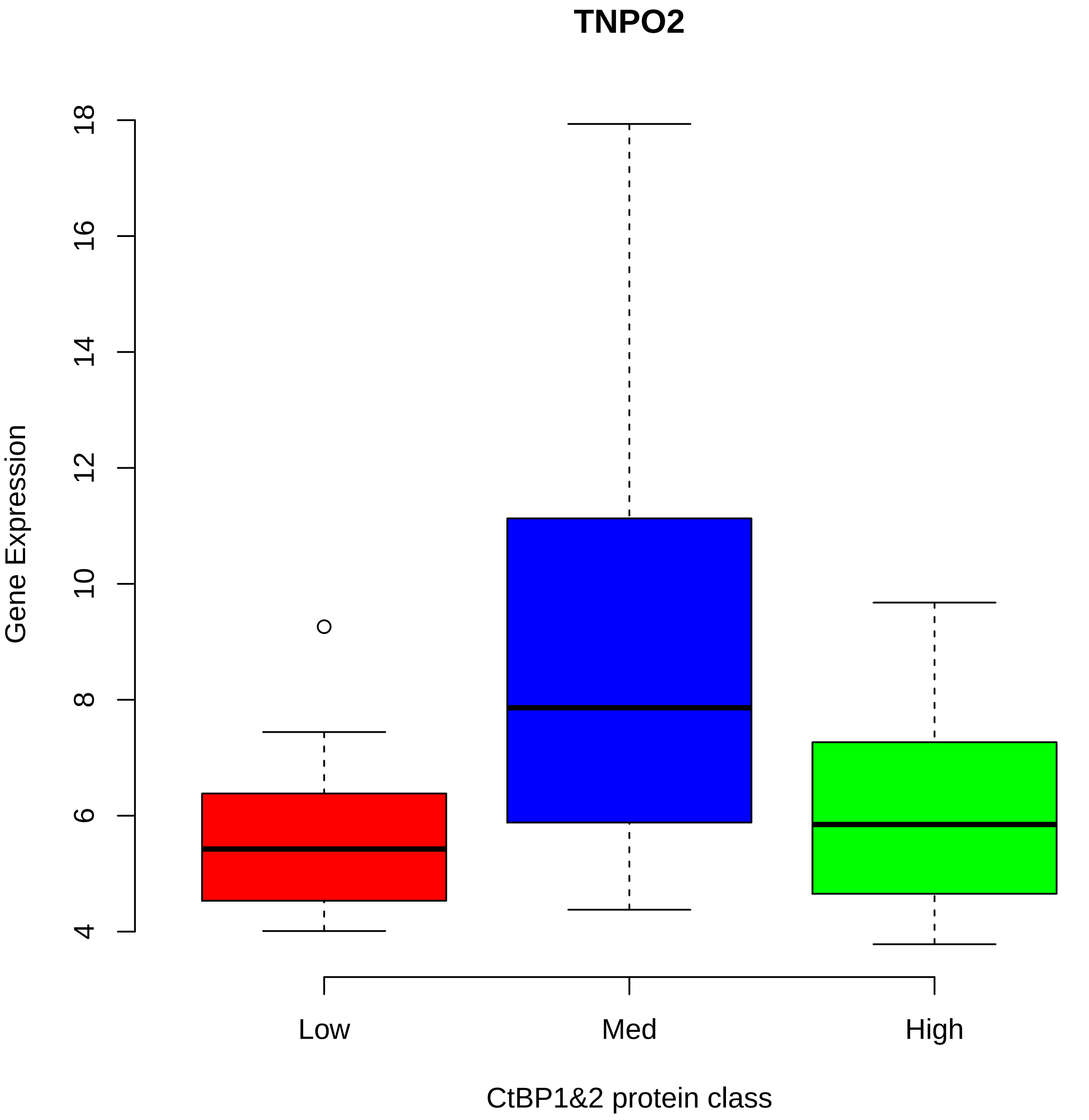

TNPO2
Gene Expression
18
16
14
12
10
8
6
4
Low
Med
High
CtBP1&2 protein class

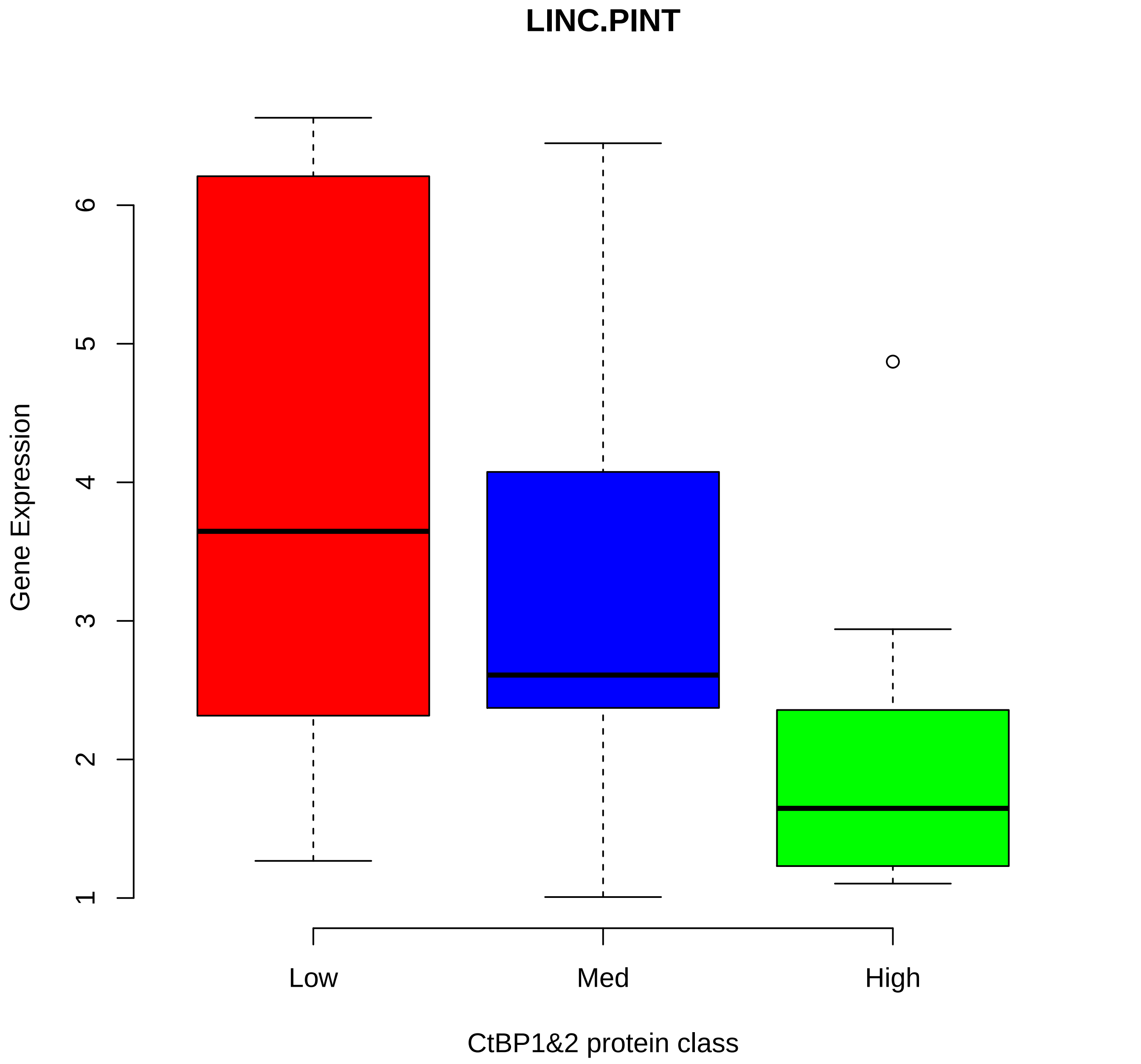

LINC.PINT
Gene Expression
1
2
3
4
5
6
Low
Med
High
CtBP1&2 protein class

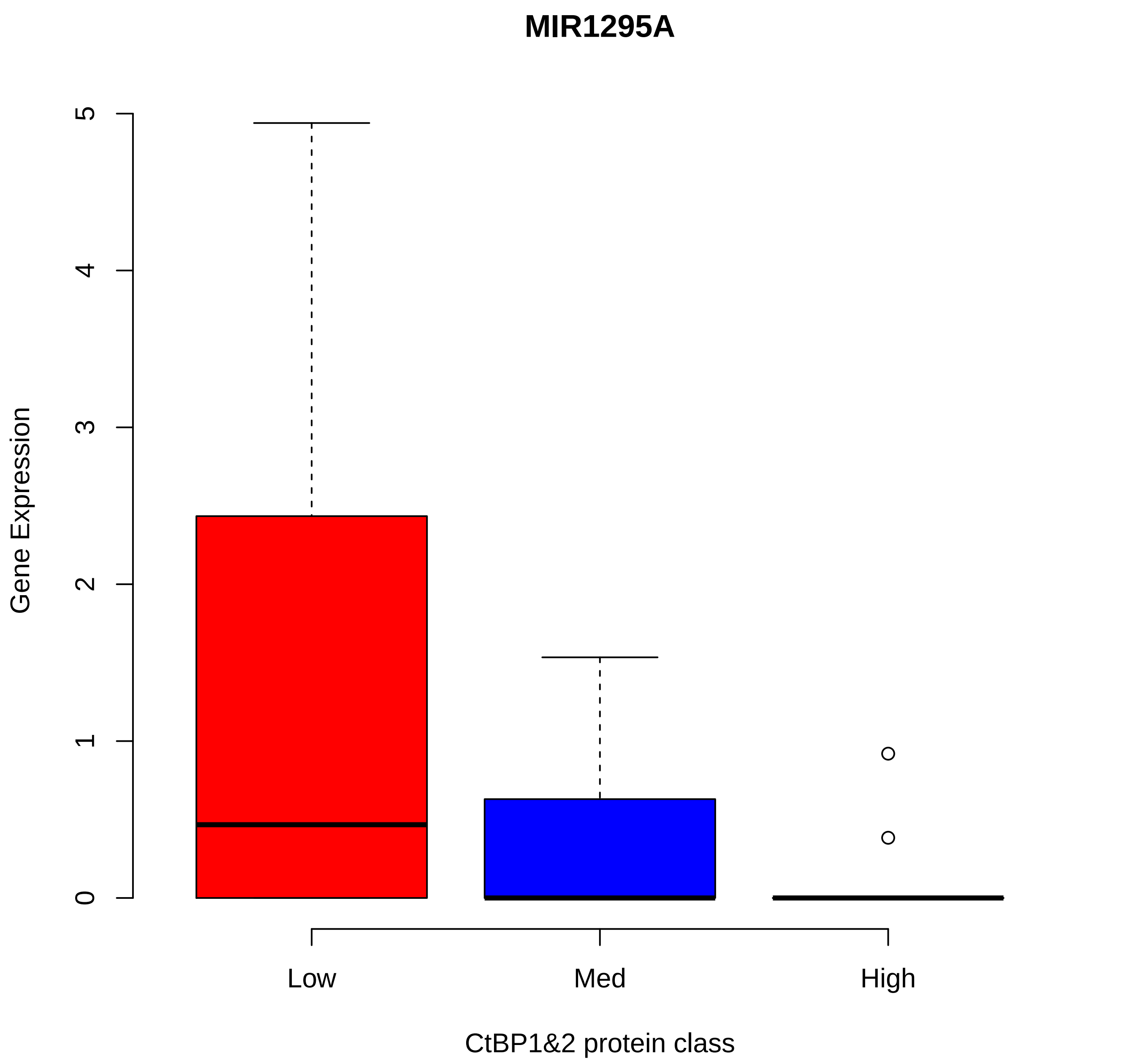
MIR1295A
Gene Expression
0
1
2
3
4
5
Low
Med
High
CtBP1&2 protein class

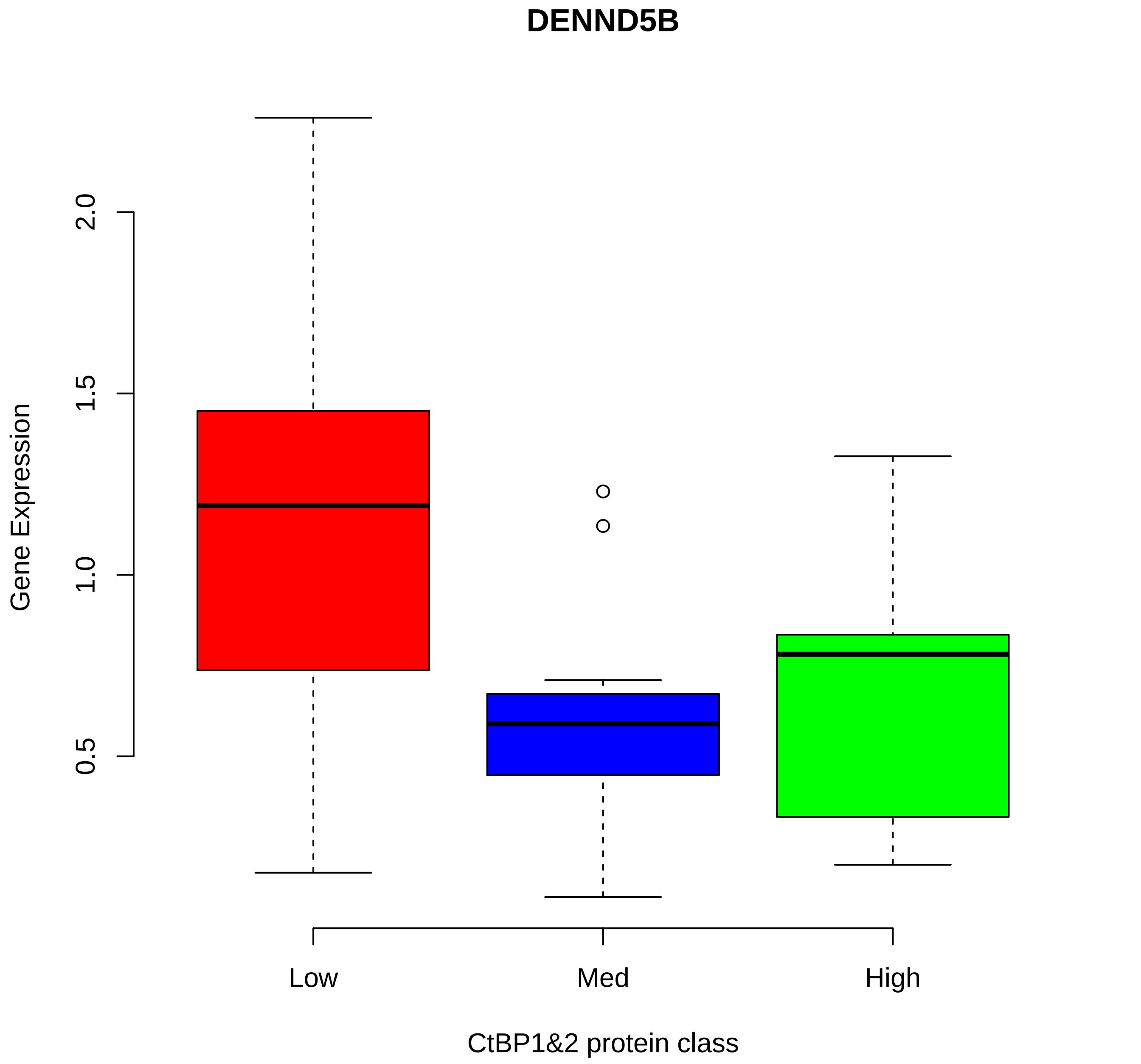
DENND5B
Gene Expression
2.0
1.5
1.0
0.5
Low
Med
High
CtBP1&2 protein class

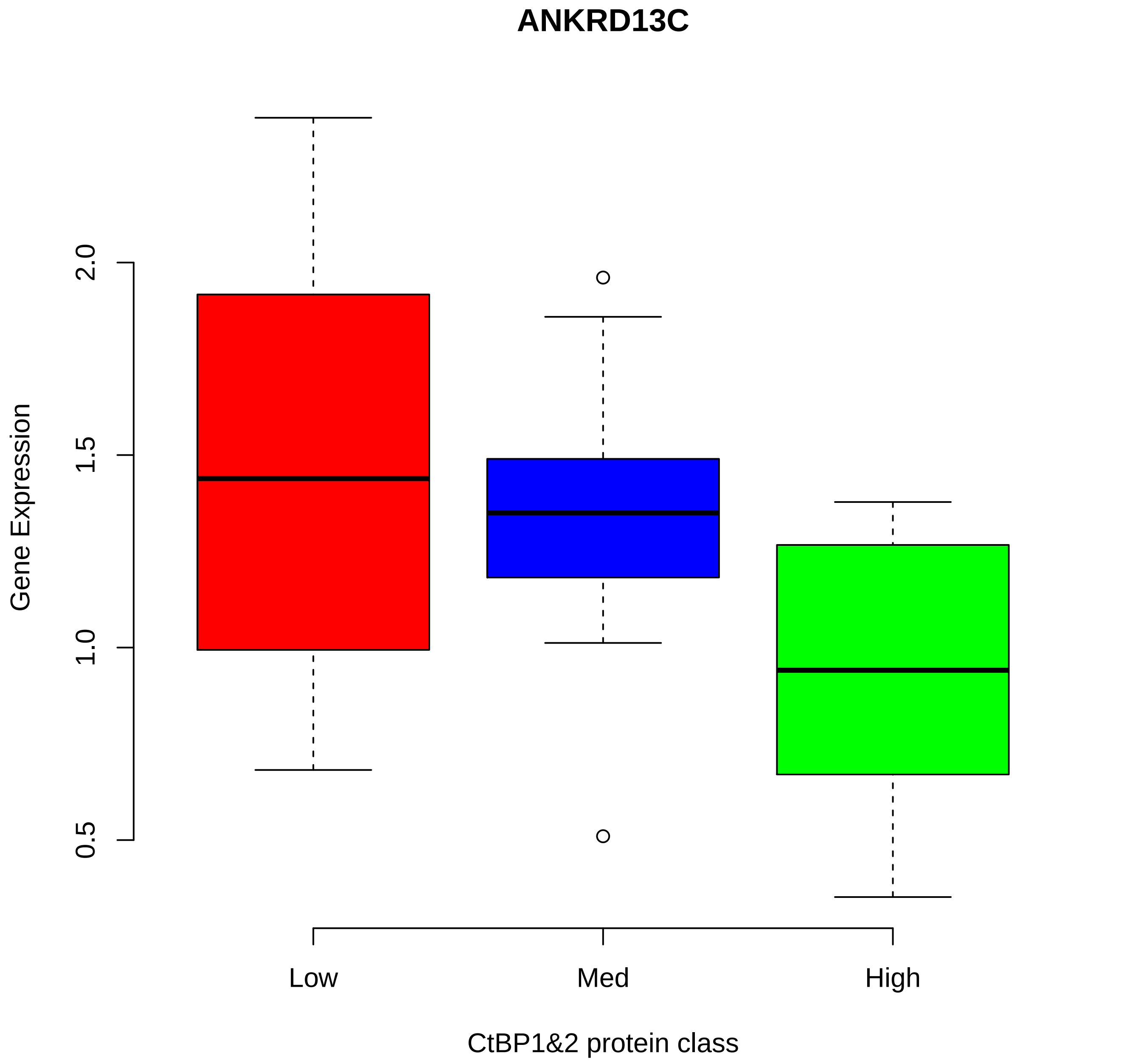
ANKRD13C
Gene Expression
2.0
1.5
1.0
0.5
Low
Med
High
CtBP1&2 protein class

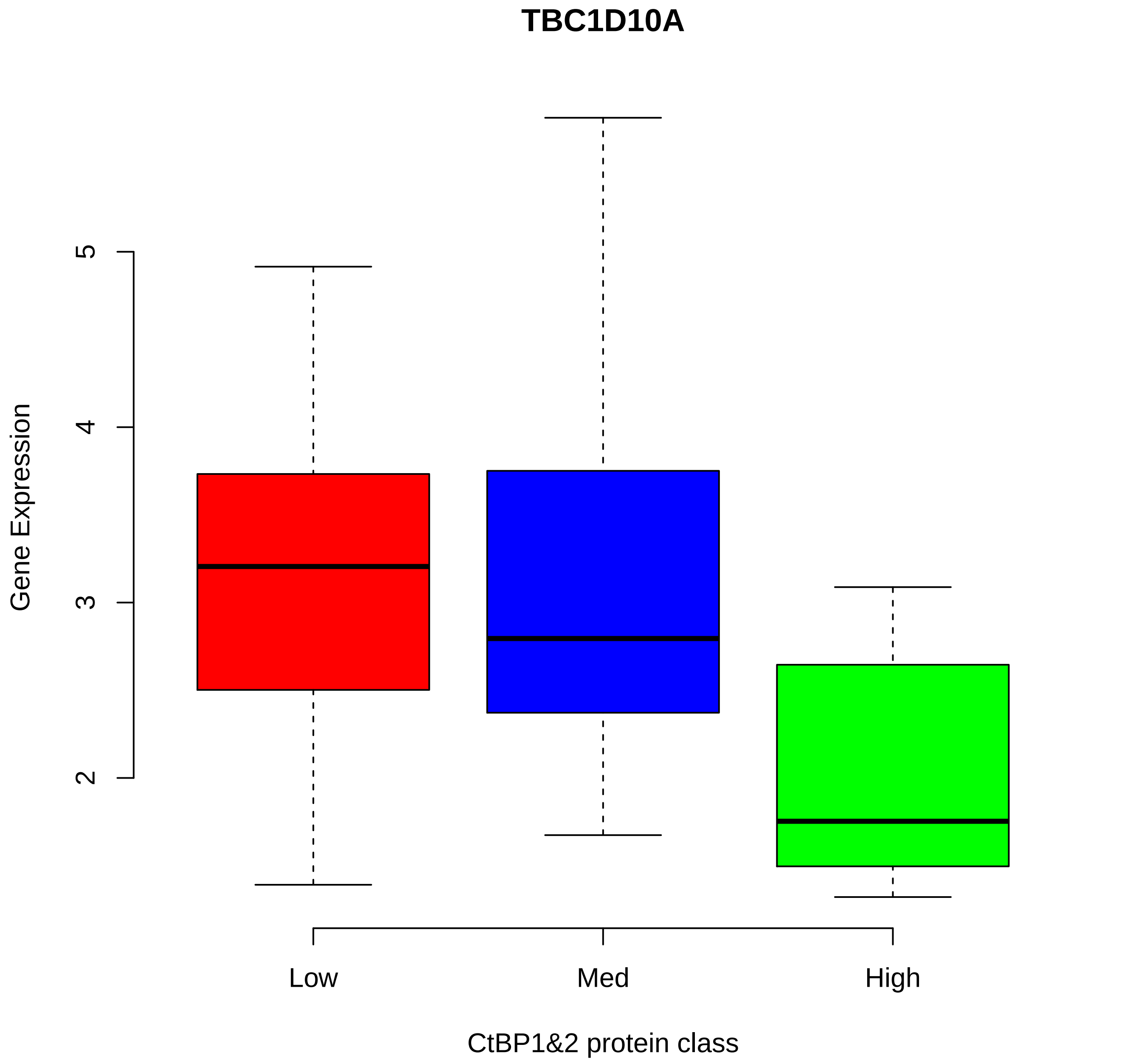

TBC1D10A
Gene Expression
5
4
3
2
Low
Med
High
CtBP1&2 protein class

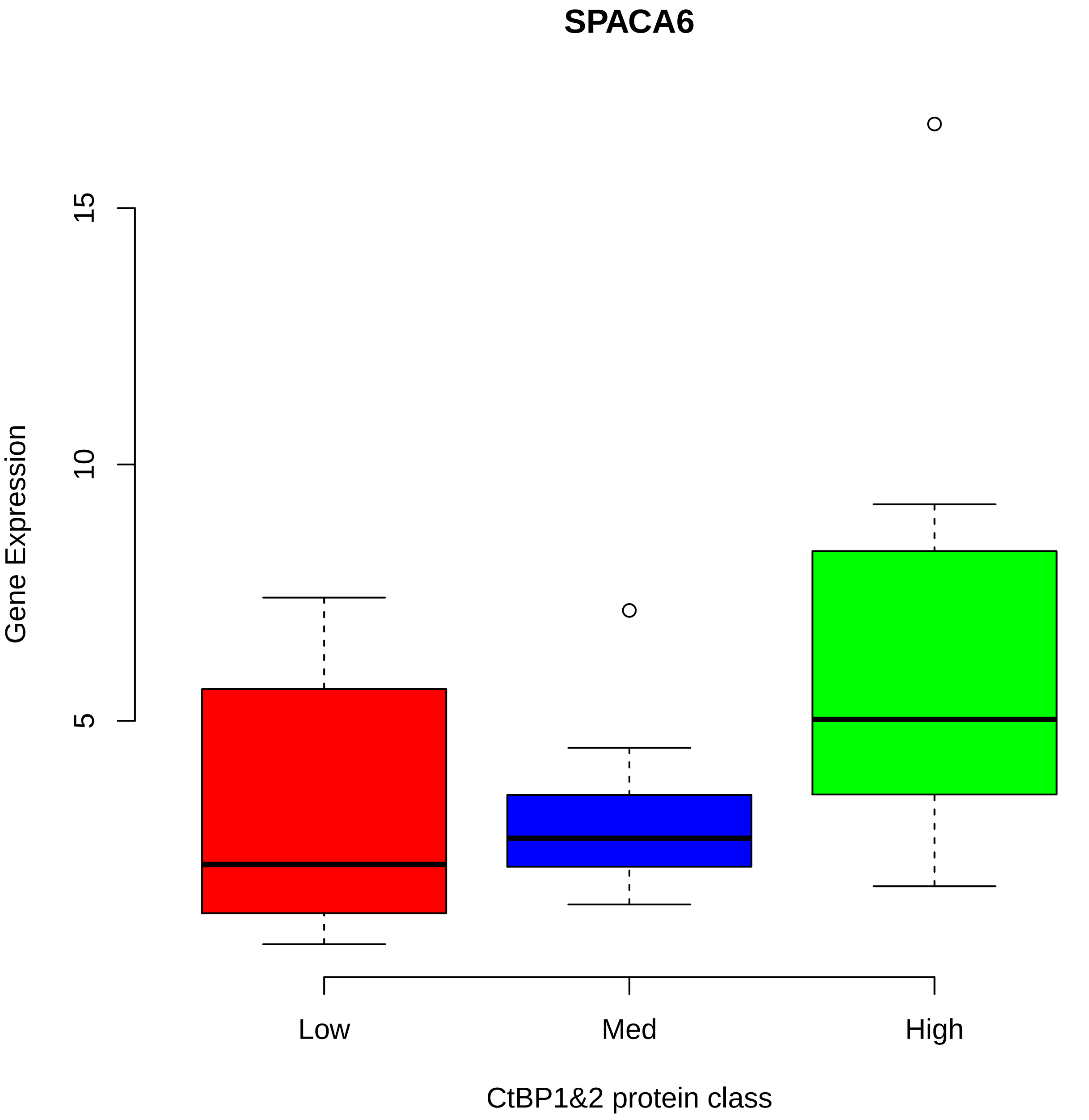
SPACA6
Gene Expression
5
10
15
Low
Med
High
CtBP1&2 protein class

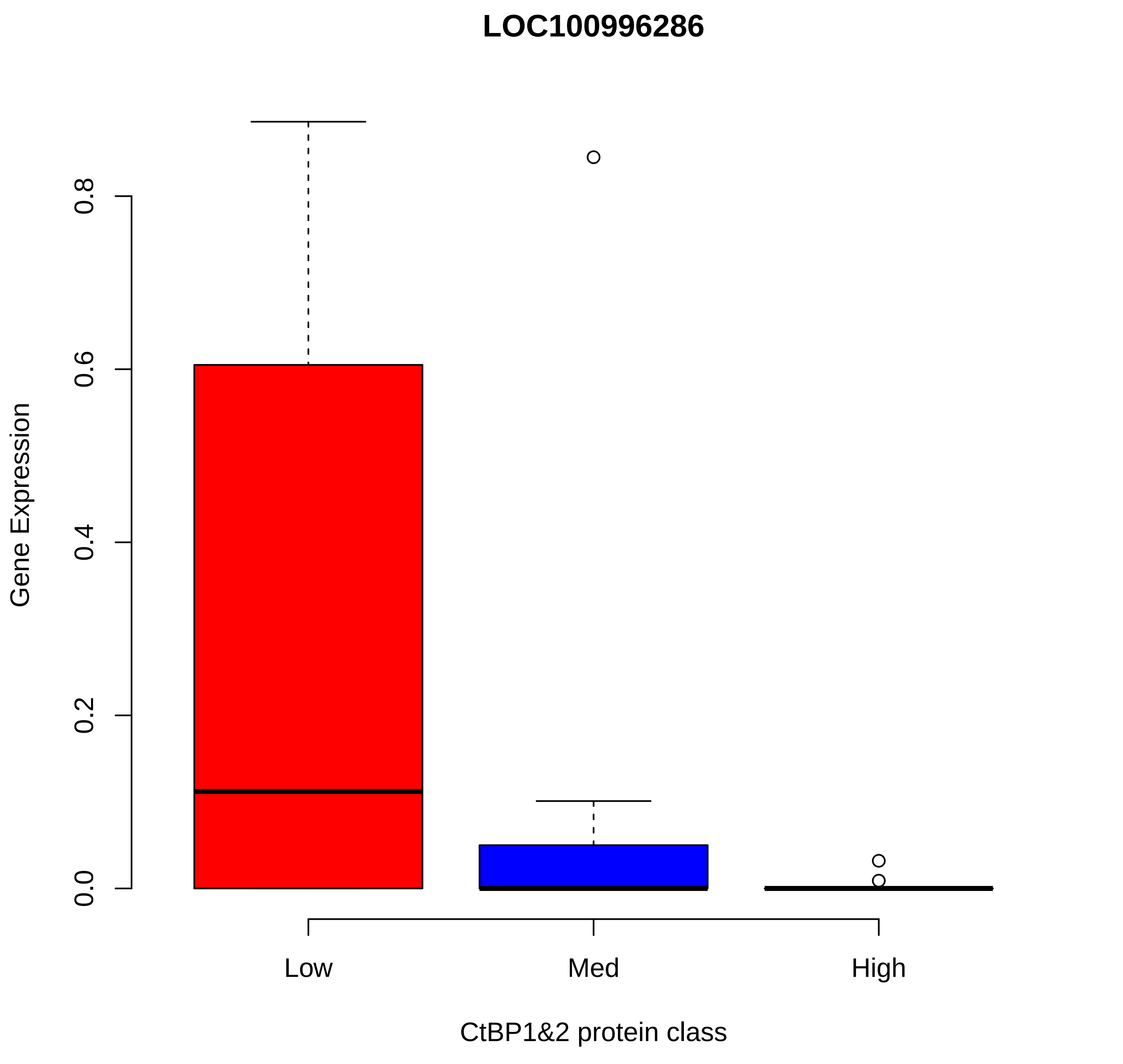
LOC100996286
Gene Expression
0.0
0.2
0.4
0.6
0.8
Low
Med
High
CtBP1&2 protein class

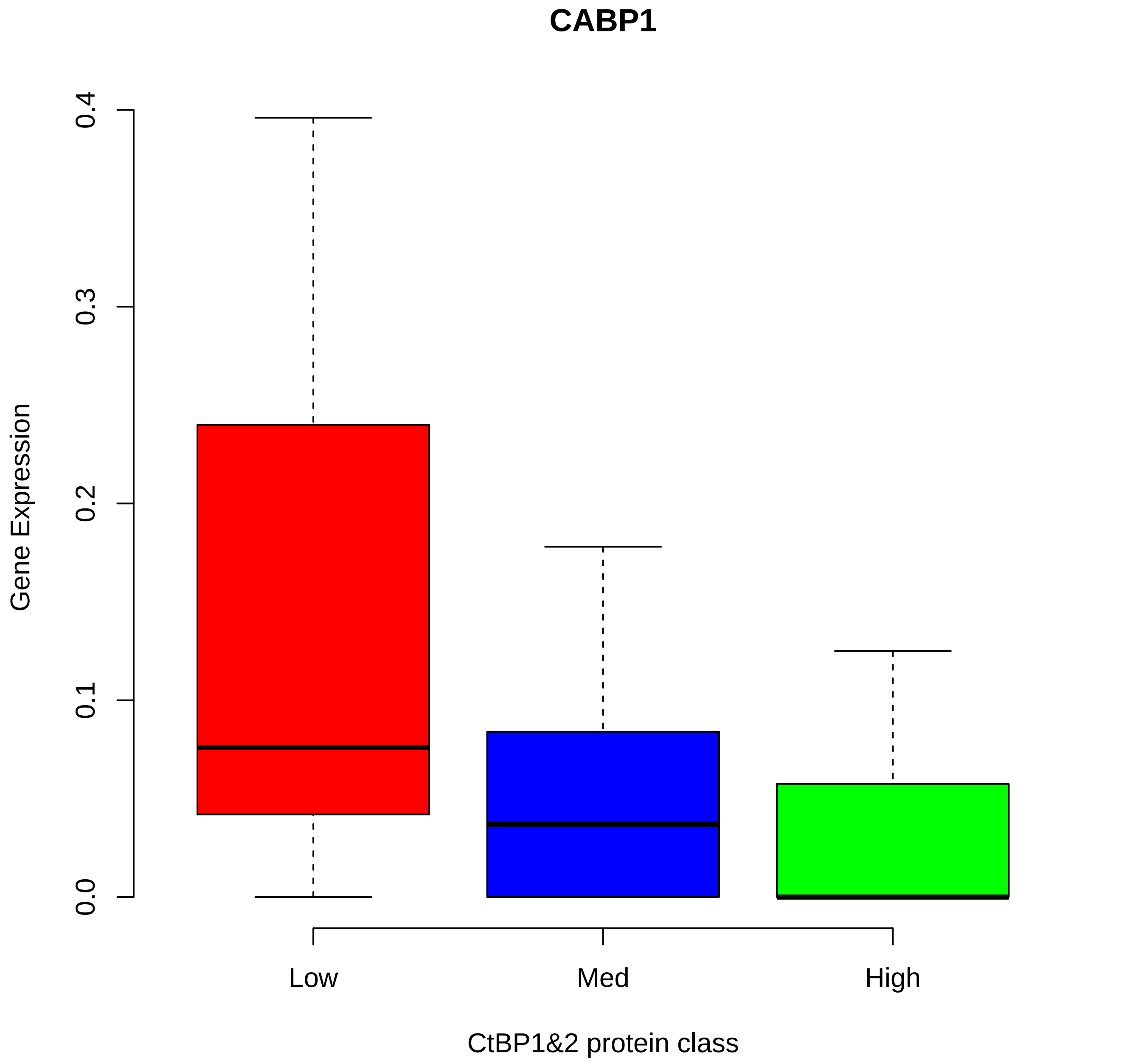

CABP1
Gene Expression
0.0
0.1
0.2
0.3
0.4
Low
Med
High
CtBP1&2 protein class

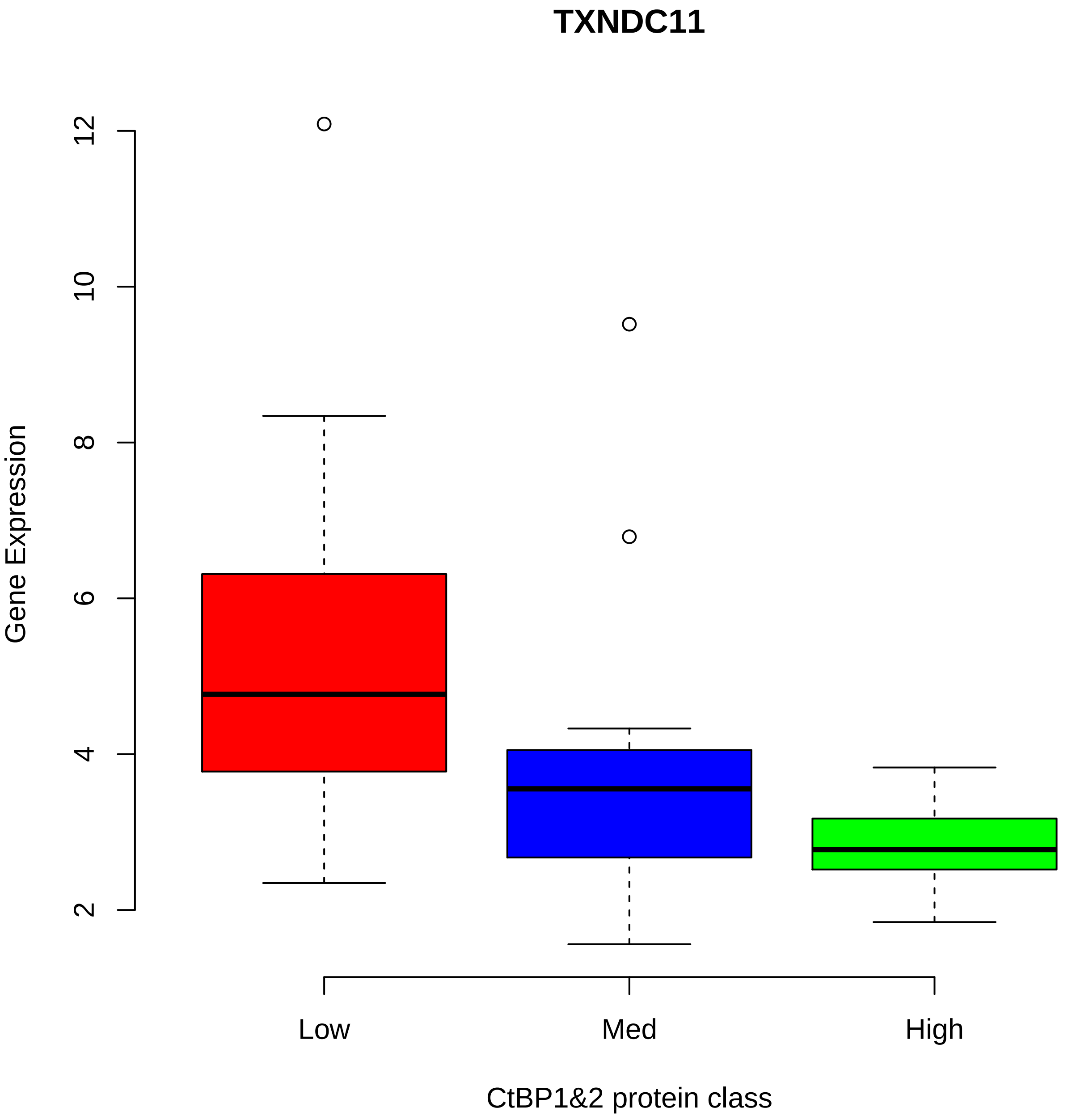
TXNDC11
Gene Expression
2
4
6
8
10
12
Low
Med
High
CtBP1&2 protein class

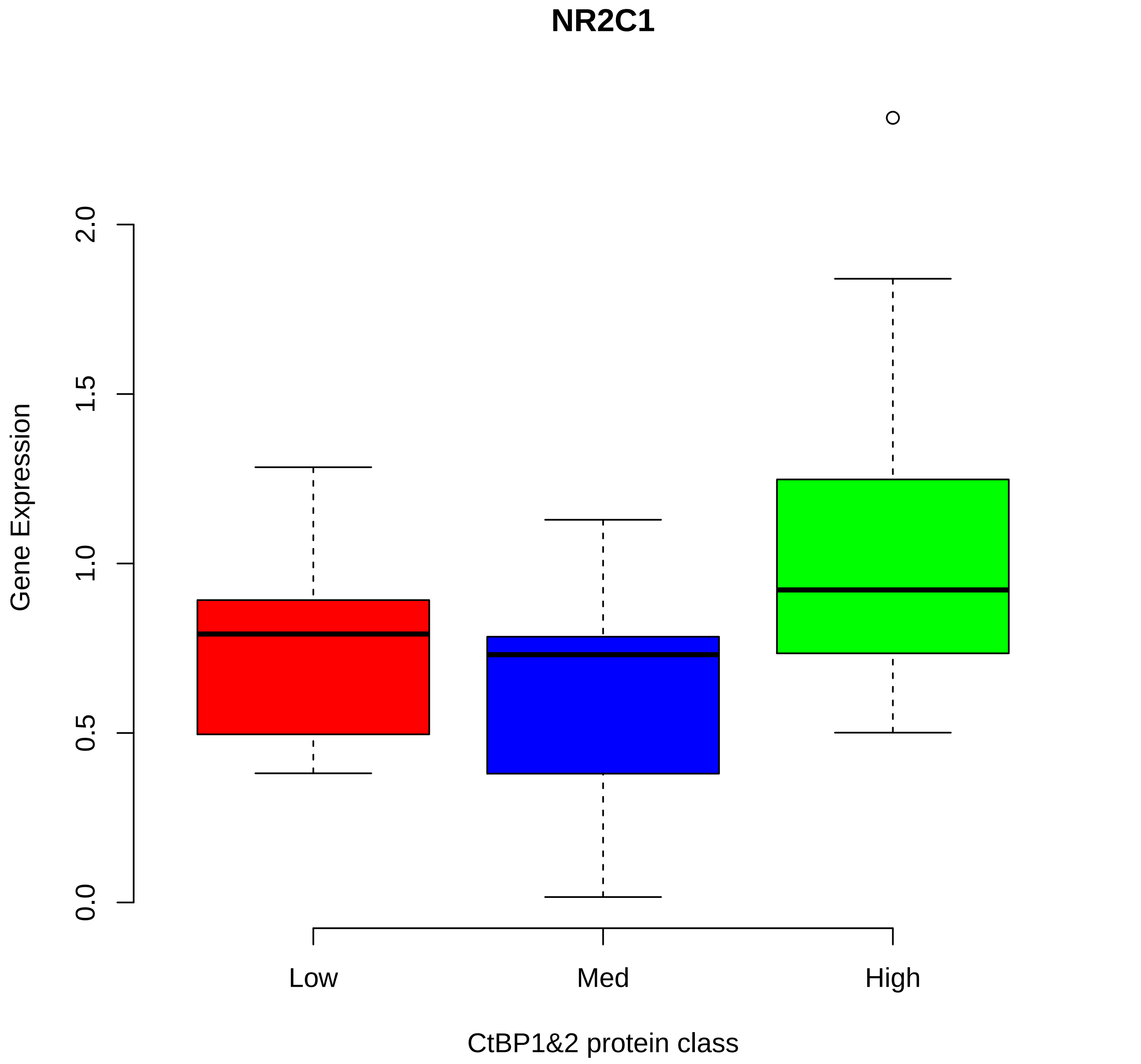
NR2C1
Gene Expression
0.0
0.5
1.0
1.5
2.0
Low
Med
High
CtBP1&2 protein class

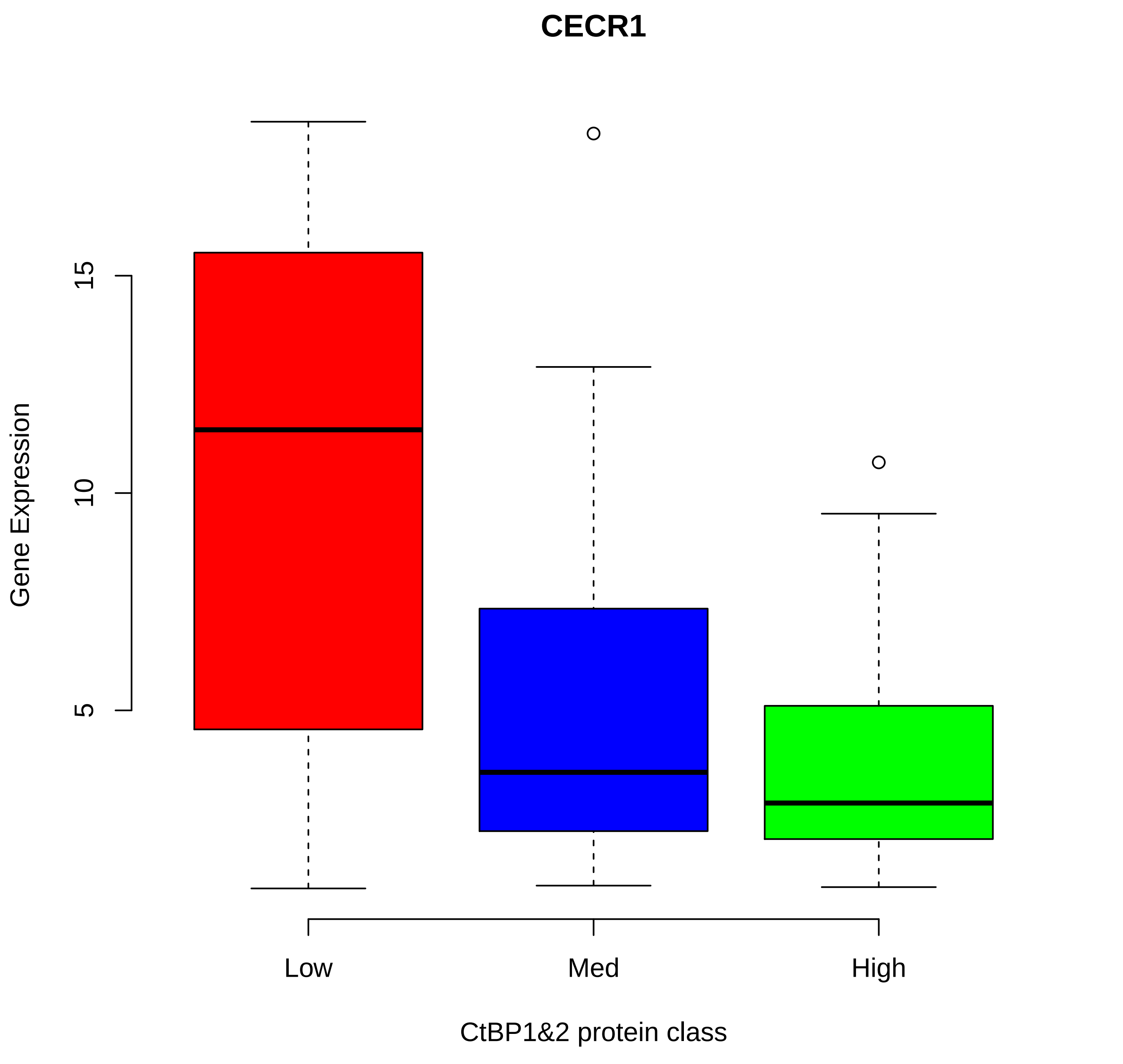

CECR1
Gene Expression
15
10
5
Low
Med
High
CtBP1&2 protein class

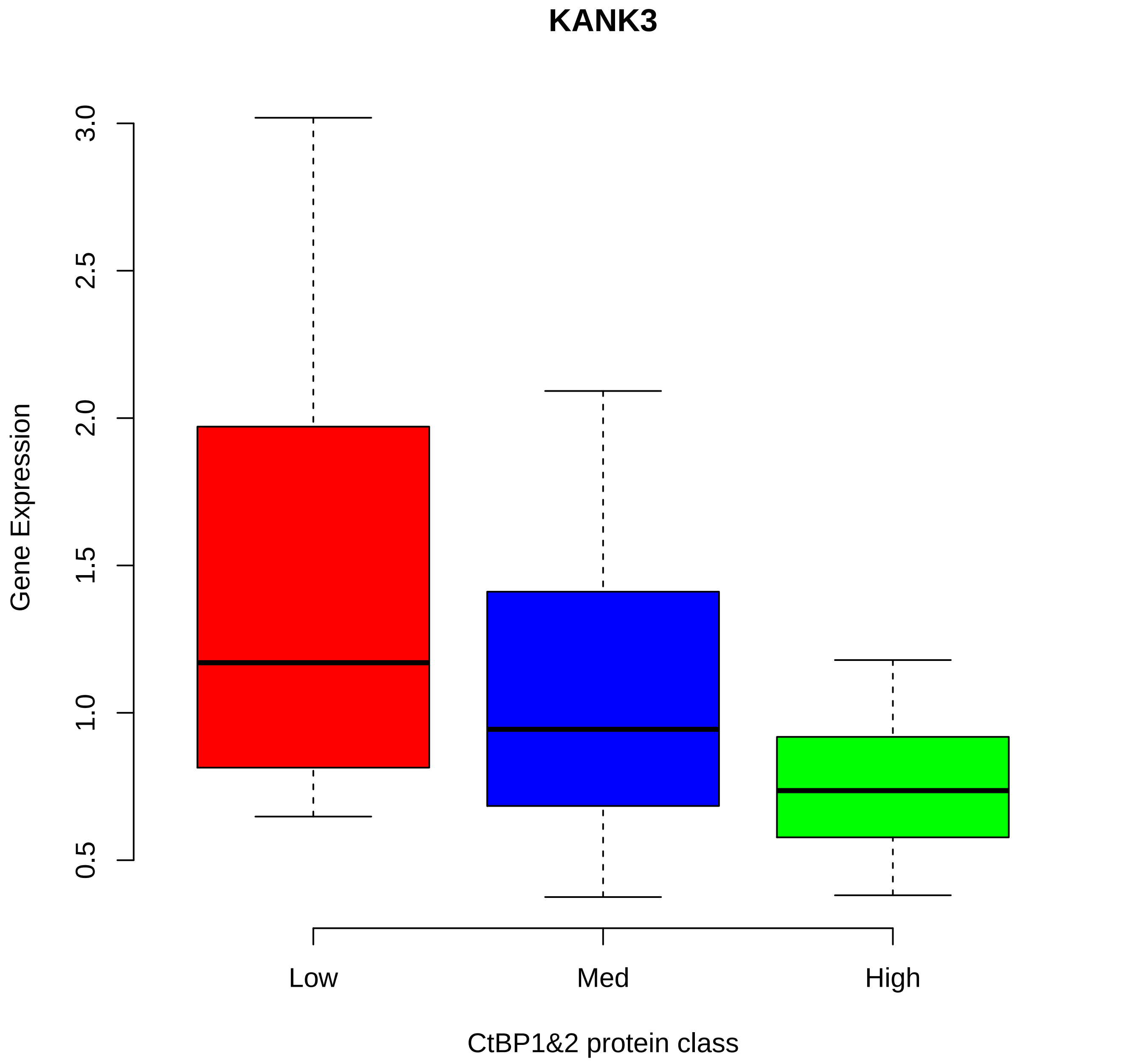

KANK3
Gene Expression
3.0
2.5
2.0
1.5
1.0
0.5
Low
Med
High
CtBP1&2 protein class

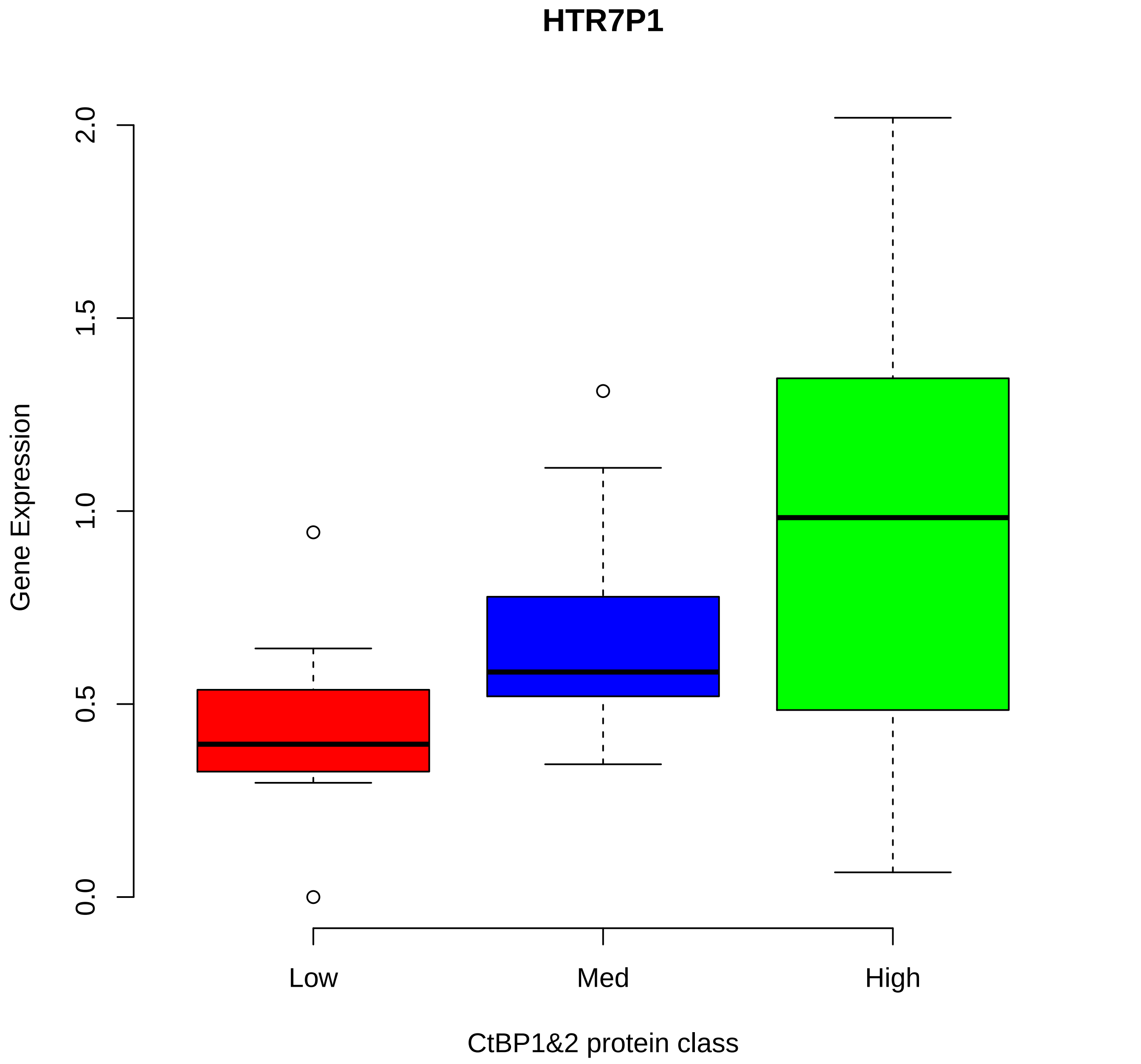

HTR7P1
Gene Expression
0.0
0.5
1.0
1.5
2.0
Low
Med
High
CtBP1&2 protein class

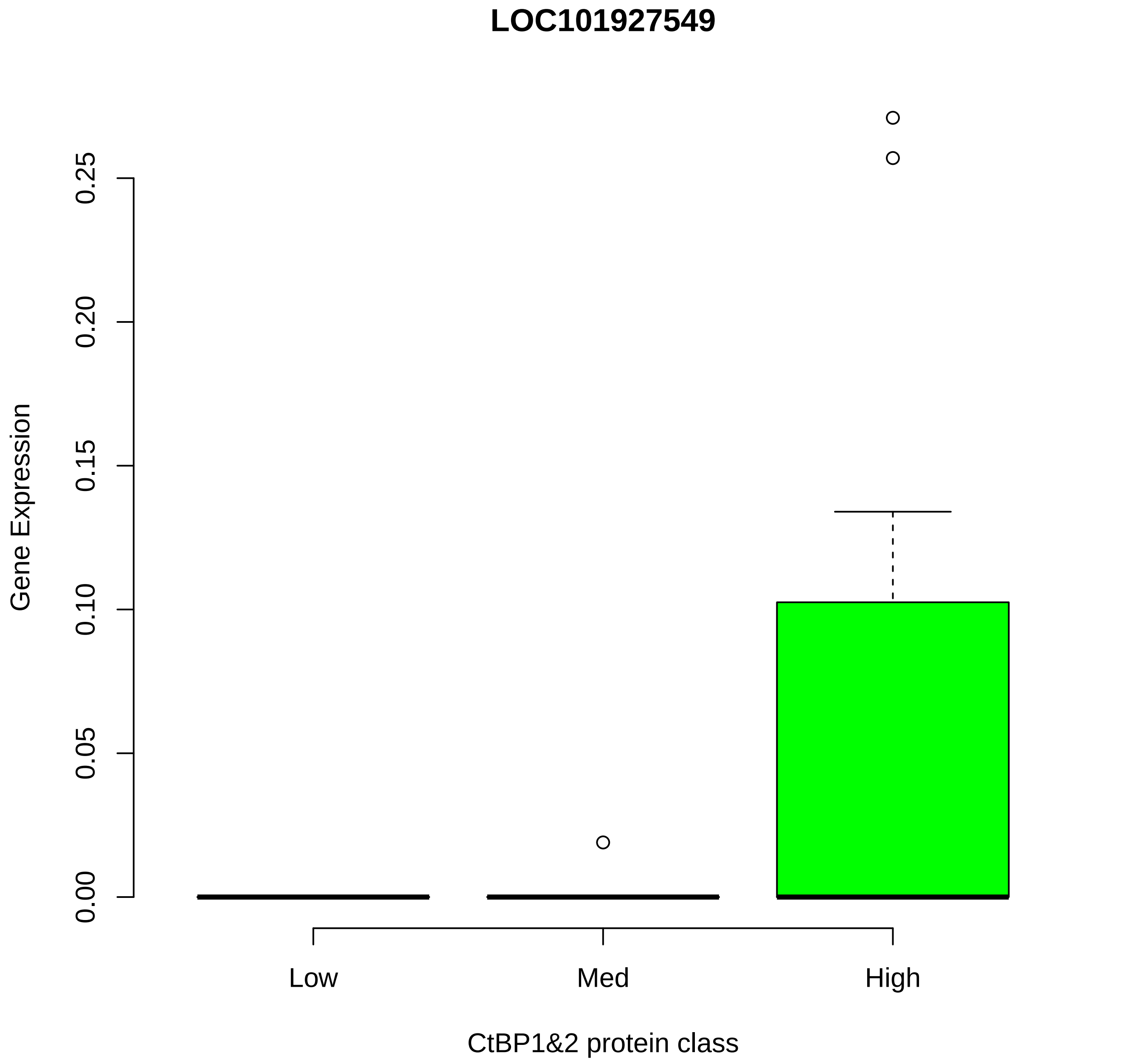

LOC101927549
Gene Expression
0.00
0.05
0.10
0.15
0.20
0.25
Low
Med
High
CtBP1&2 protein class

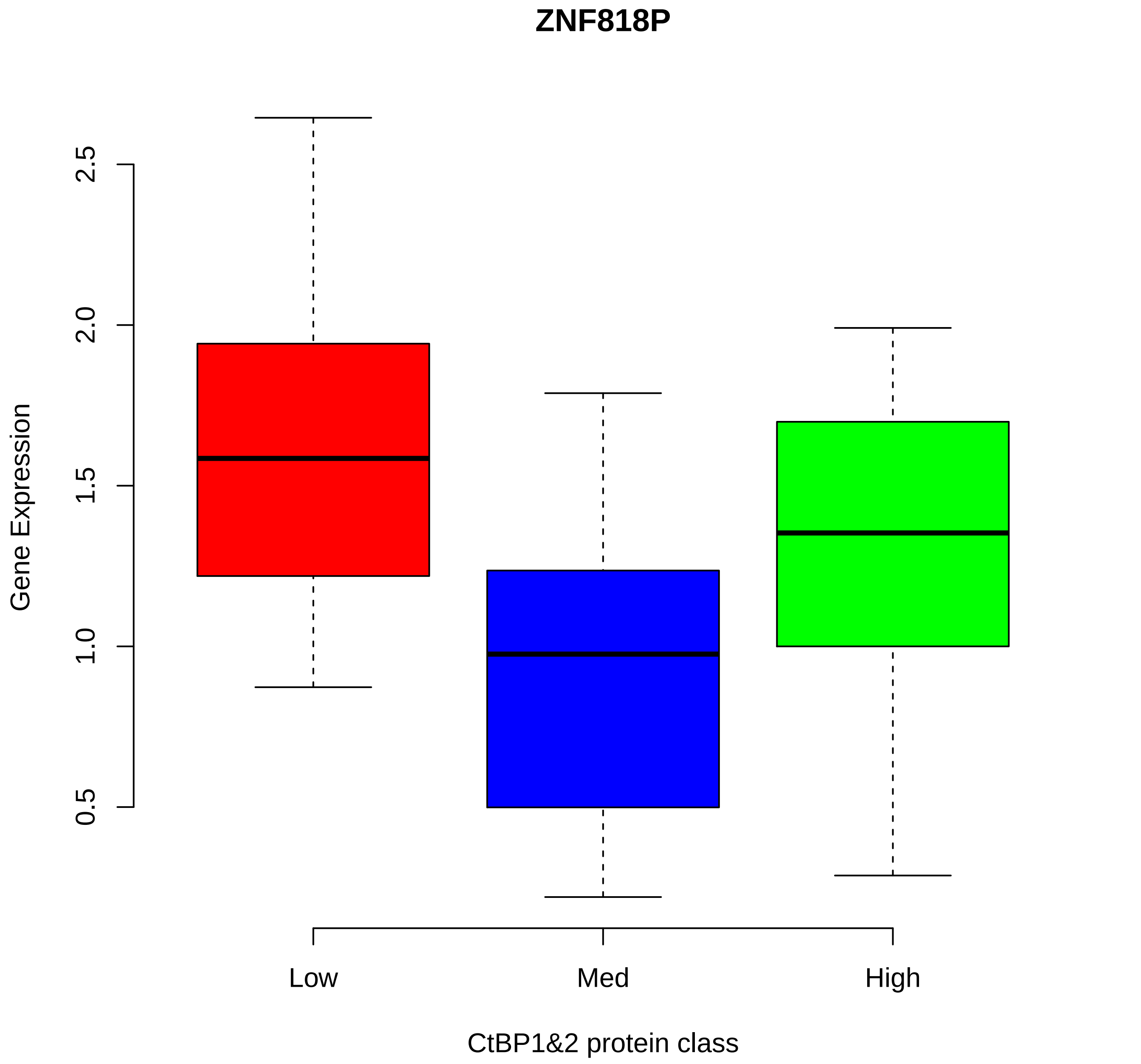

ZNF818P
Gene Expression
0.5
1.0
1.5
2.0
2.5
Low
Med
High
CtBP1&2 protein class

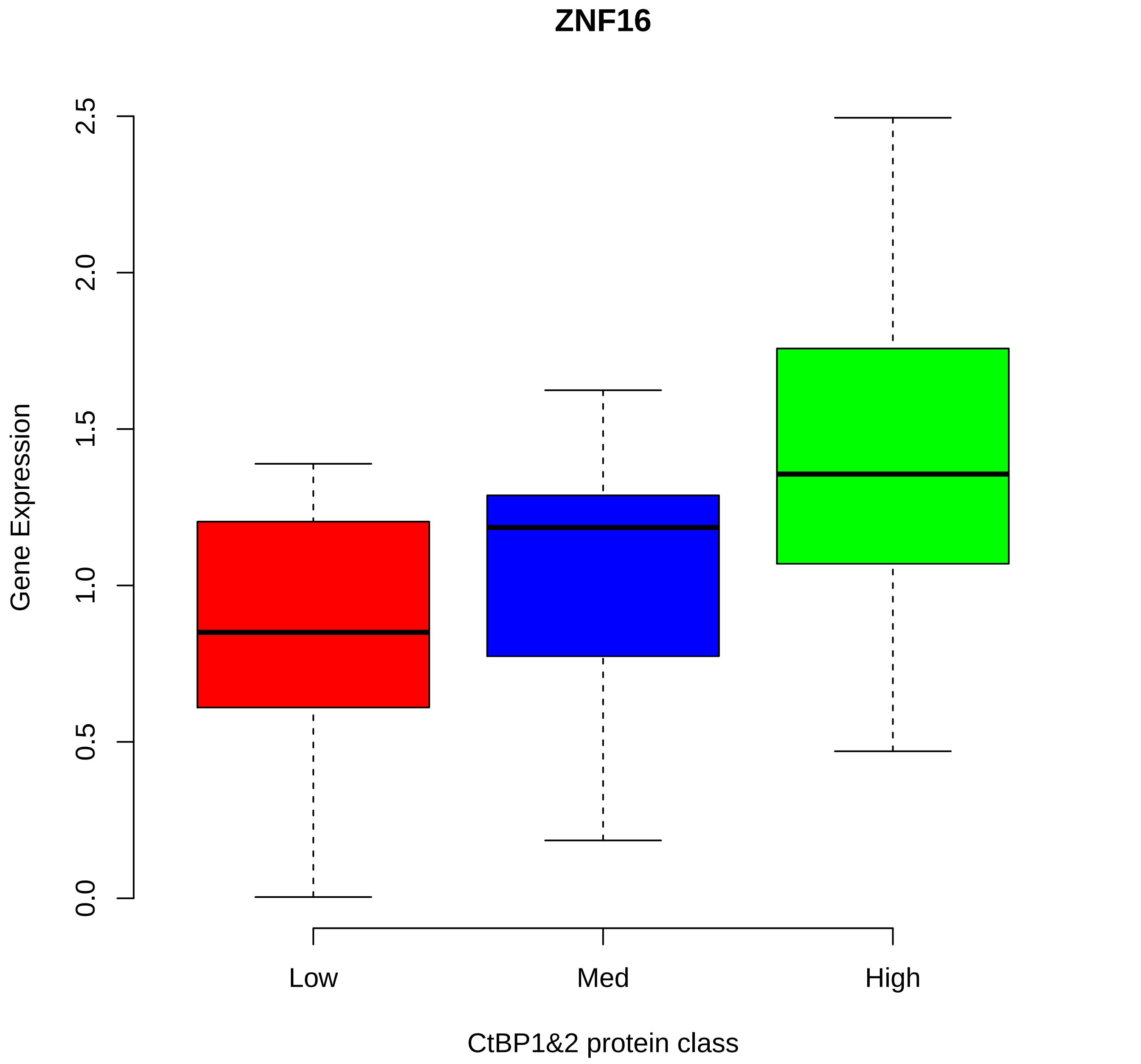
ZNF16
Gene Expression
0.0
0.5
1.0
1.5
2.0
2.5
Low
Med
High
CtBP1&2 protein class

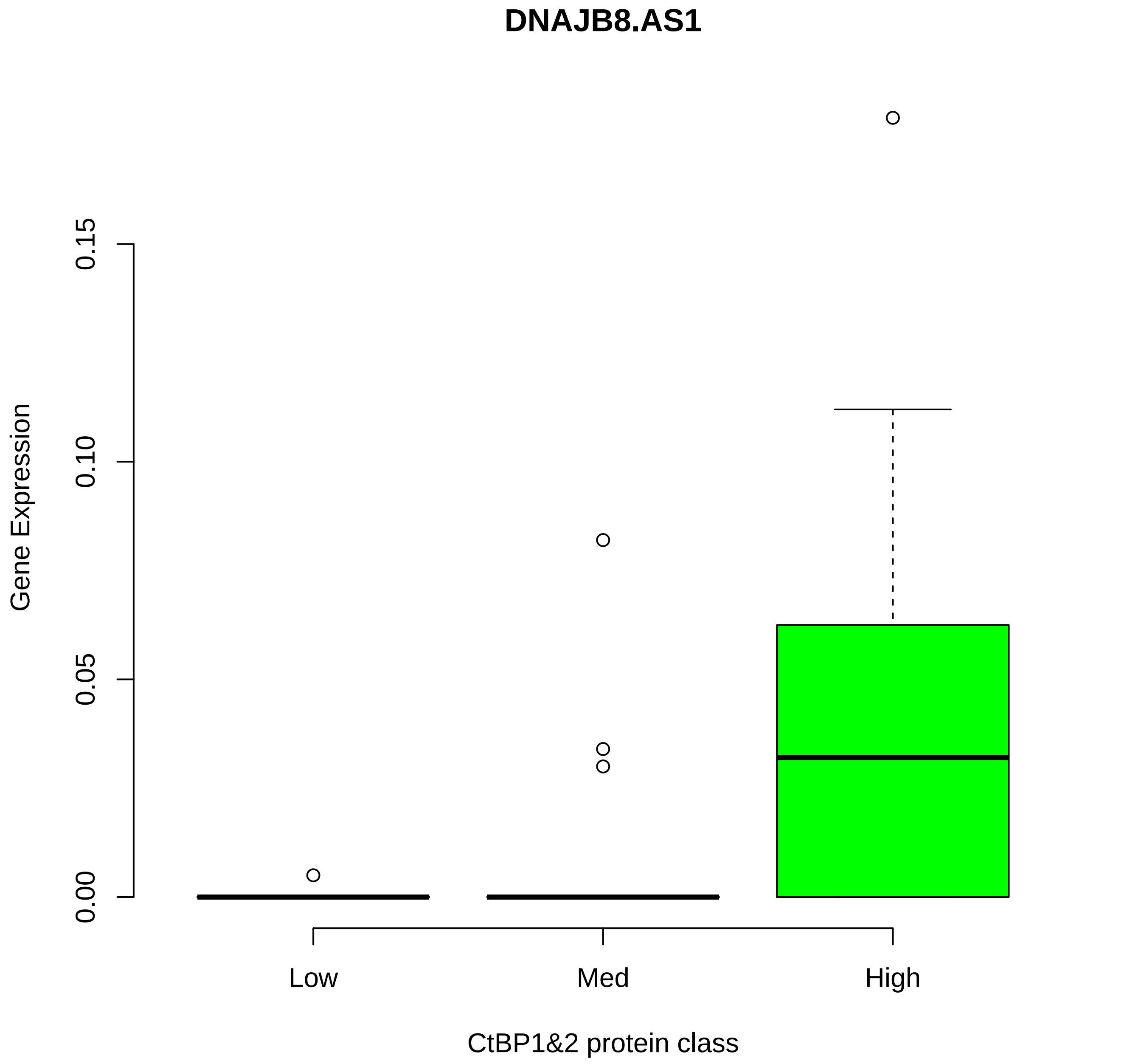

DNAJB8.AS1
Gene Expression
0.00
0.05
0.10
0.15
Low
Med
High
CtBP1&2 protein class

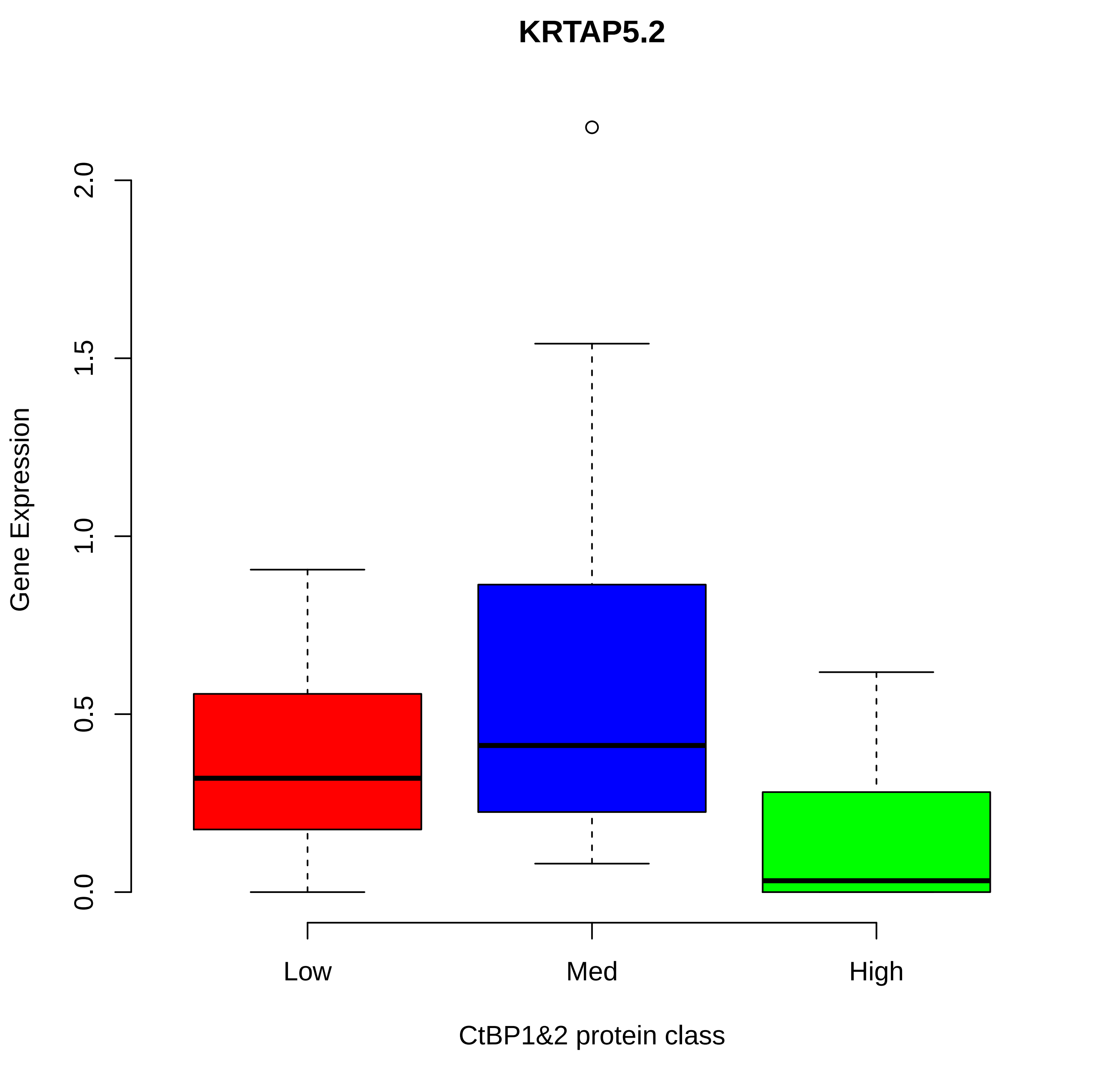
KRTAP5.2
Gene Expression
0.0
0.5
1.0
1.5
2.0
Low
Med
High
CtBP1&2 protein class

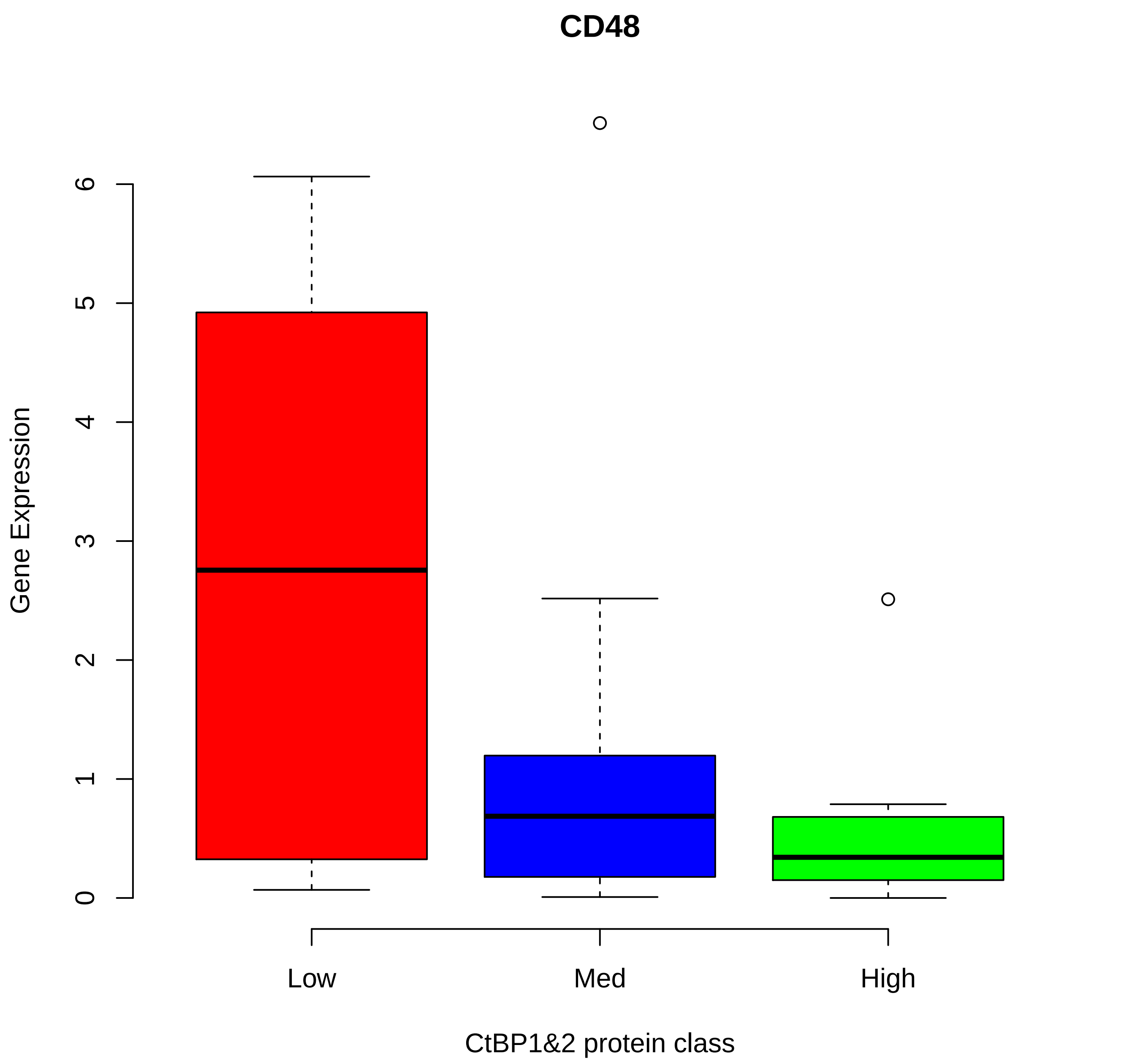
CD48
Gene Expression
0
1
2
3
4
5
6
Low
Med
High
CtBP1&2 protein class

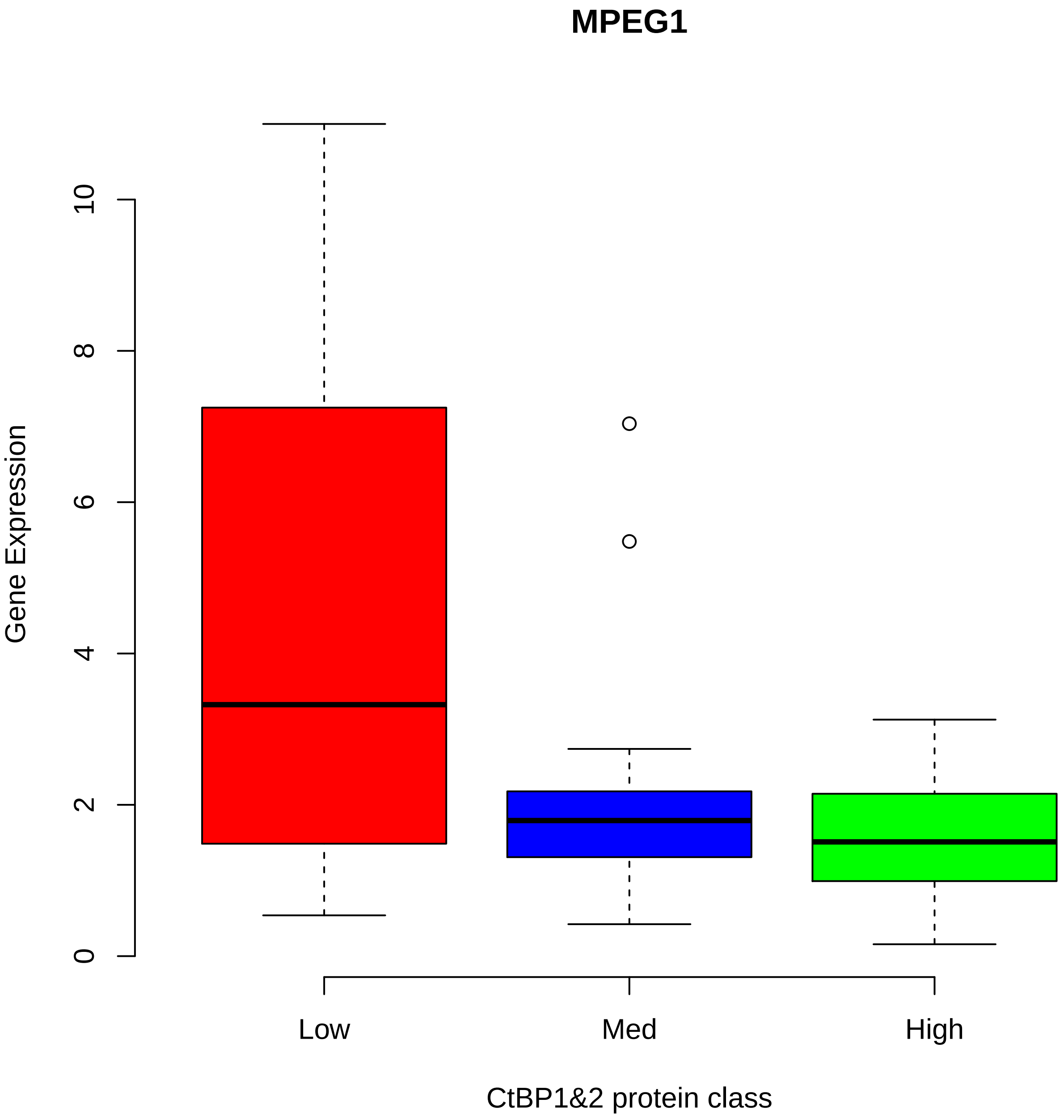

MPEG1
Gene Expression
0
2
4
6
8
10
Low
Med
High
CtBP1&2 protein class

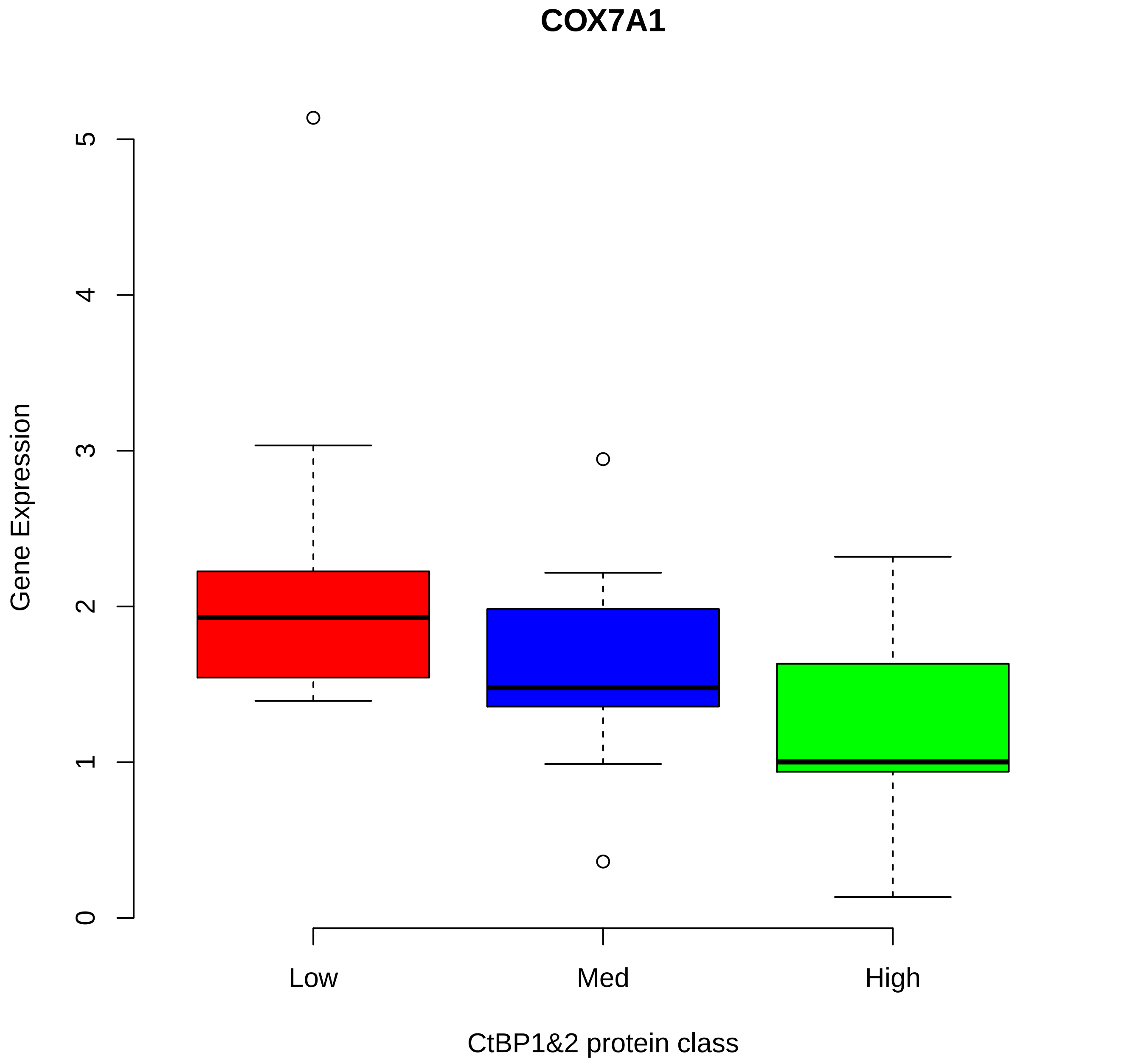

COX7A1
Gene Expression
0
1
2
3
4
5
Low
Med
High
CtBP1&2 protein class

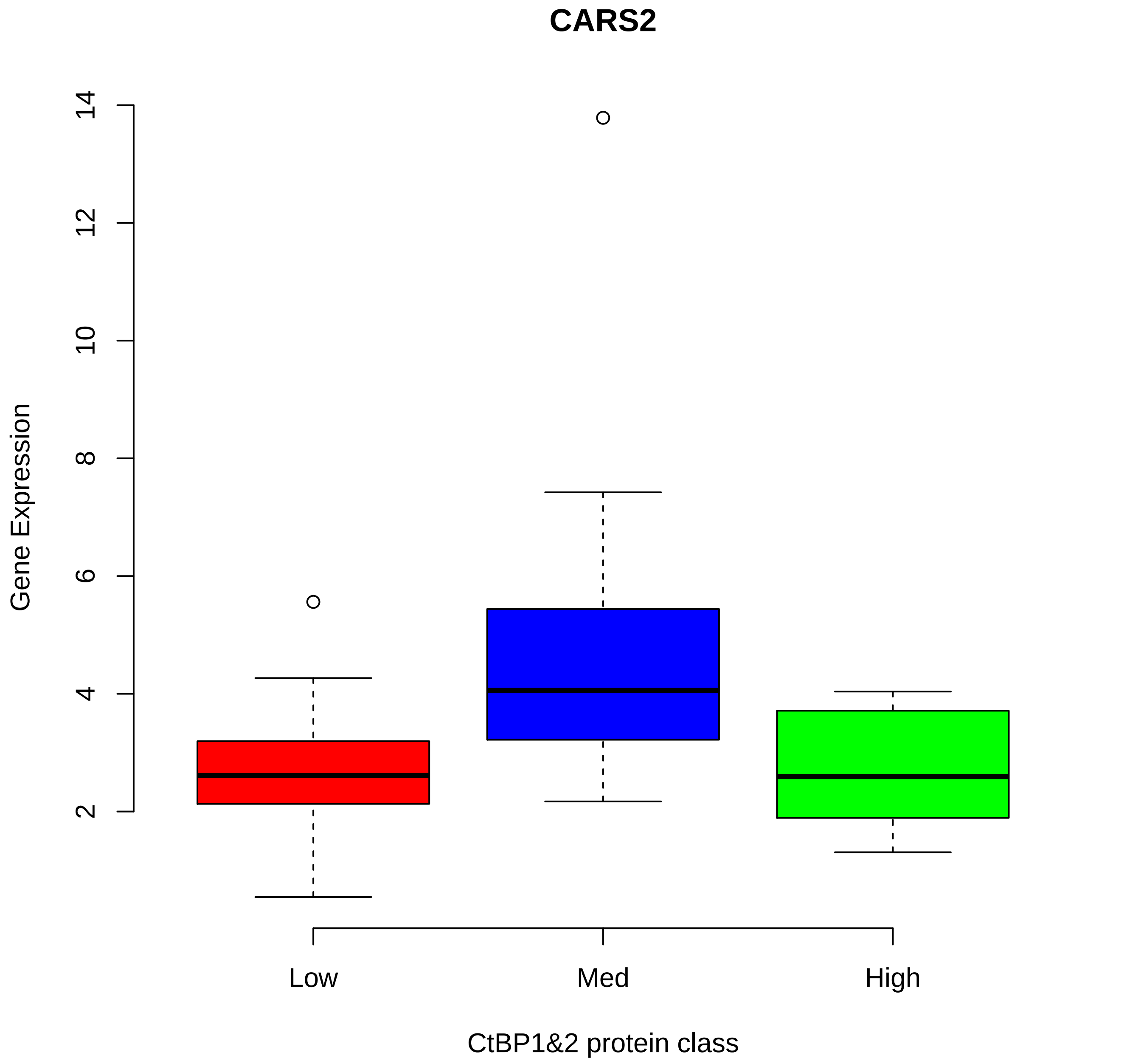

CARS2
Gene Expression
2
4
6
8
10
12
14
Low
Med
High
CtBP1&2 protein class

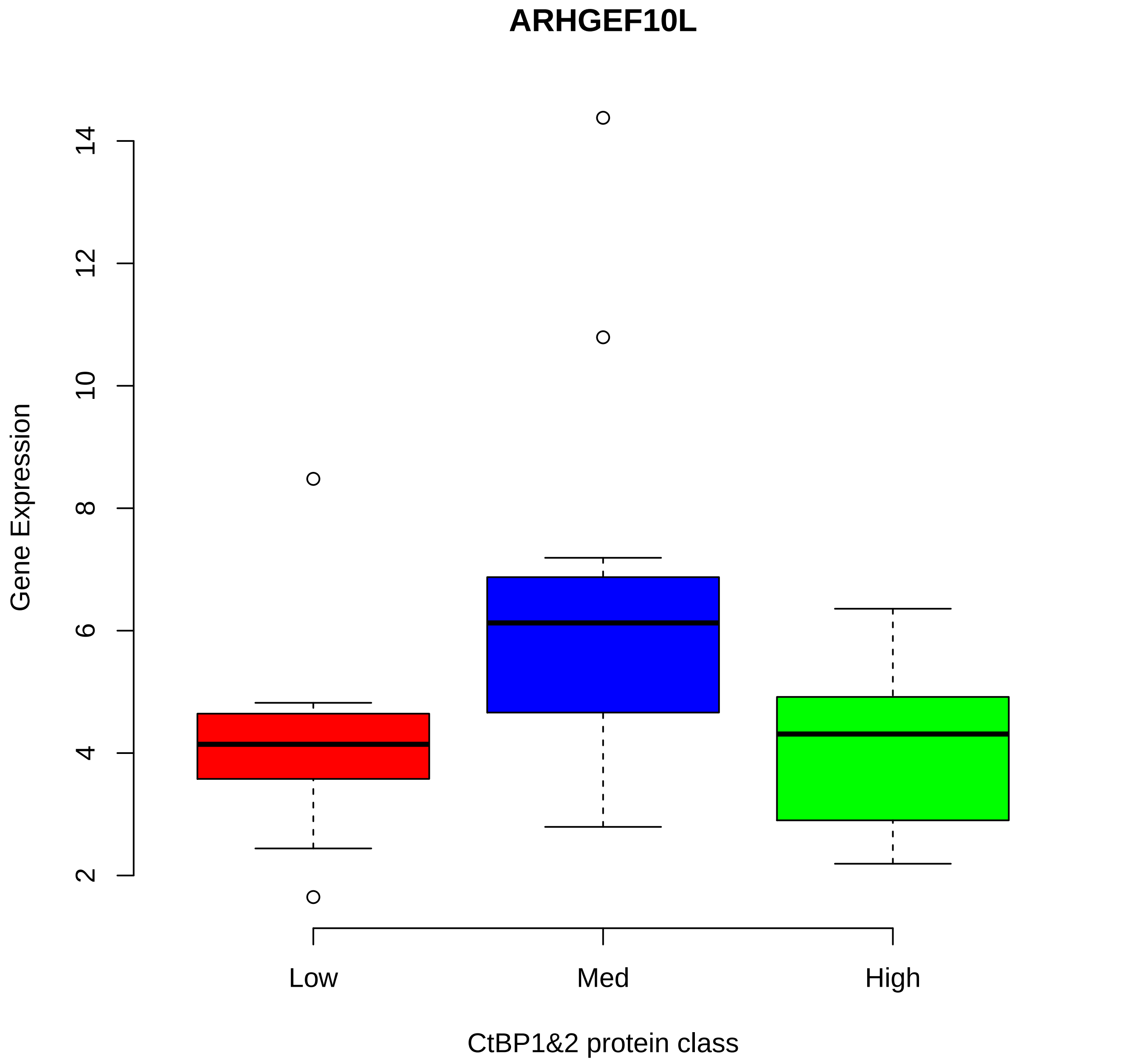

ARHGEF10L
Gene Expression
2
4
6
8
10
12
14
Low
Med
High
CtBP1&2 protein class

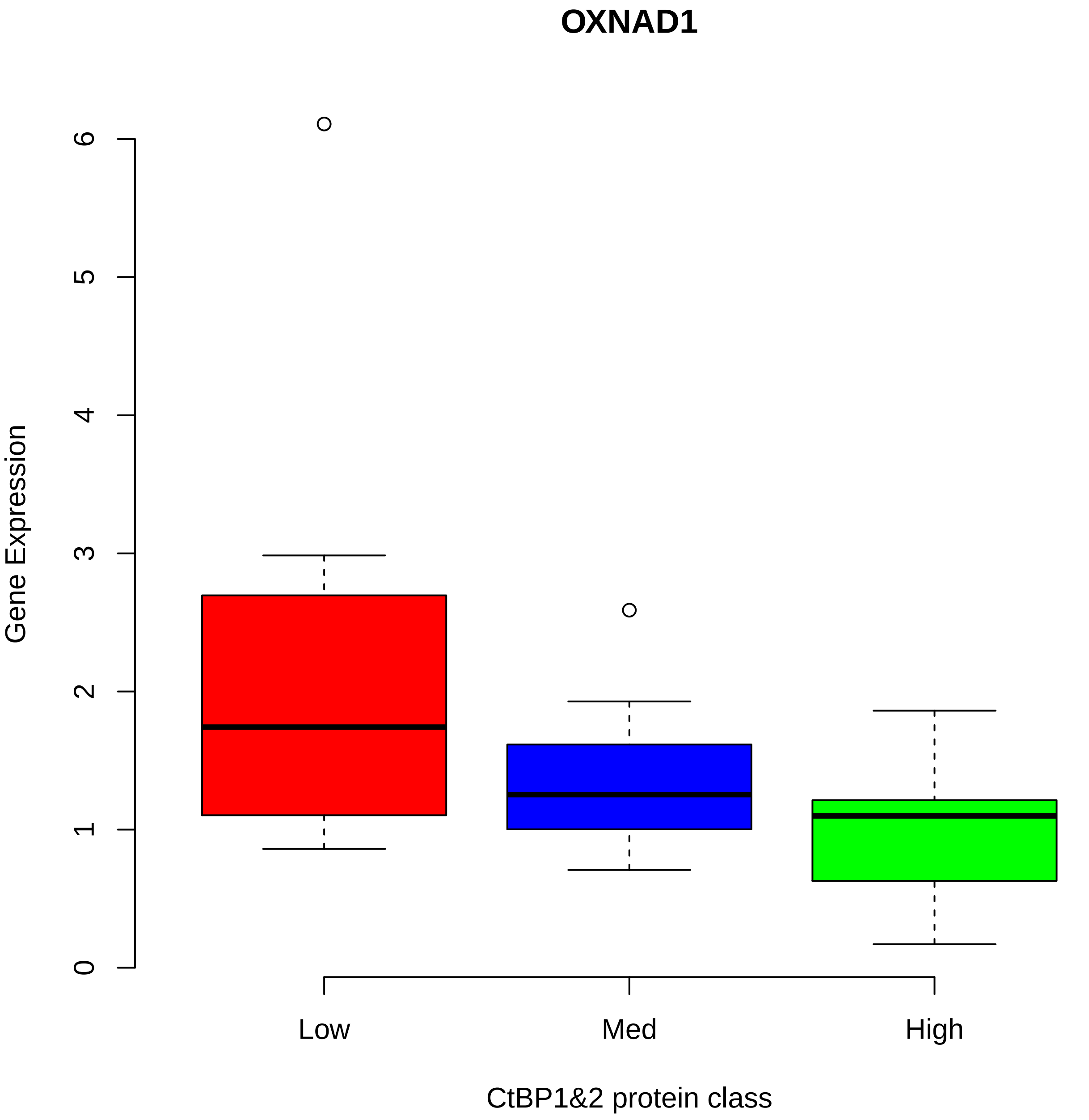
OXNAD1
Gene Expression
0
1
2
3
4
5
6
Low
Med
High
CtBP1&2 protein class

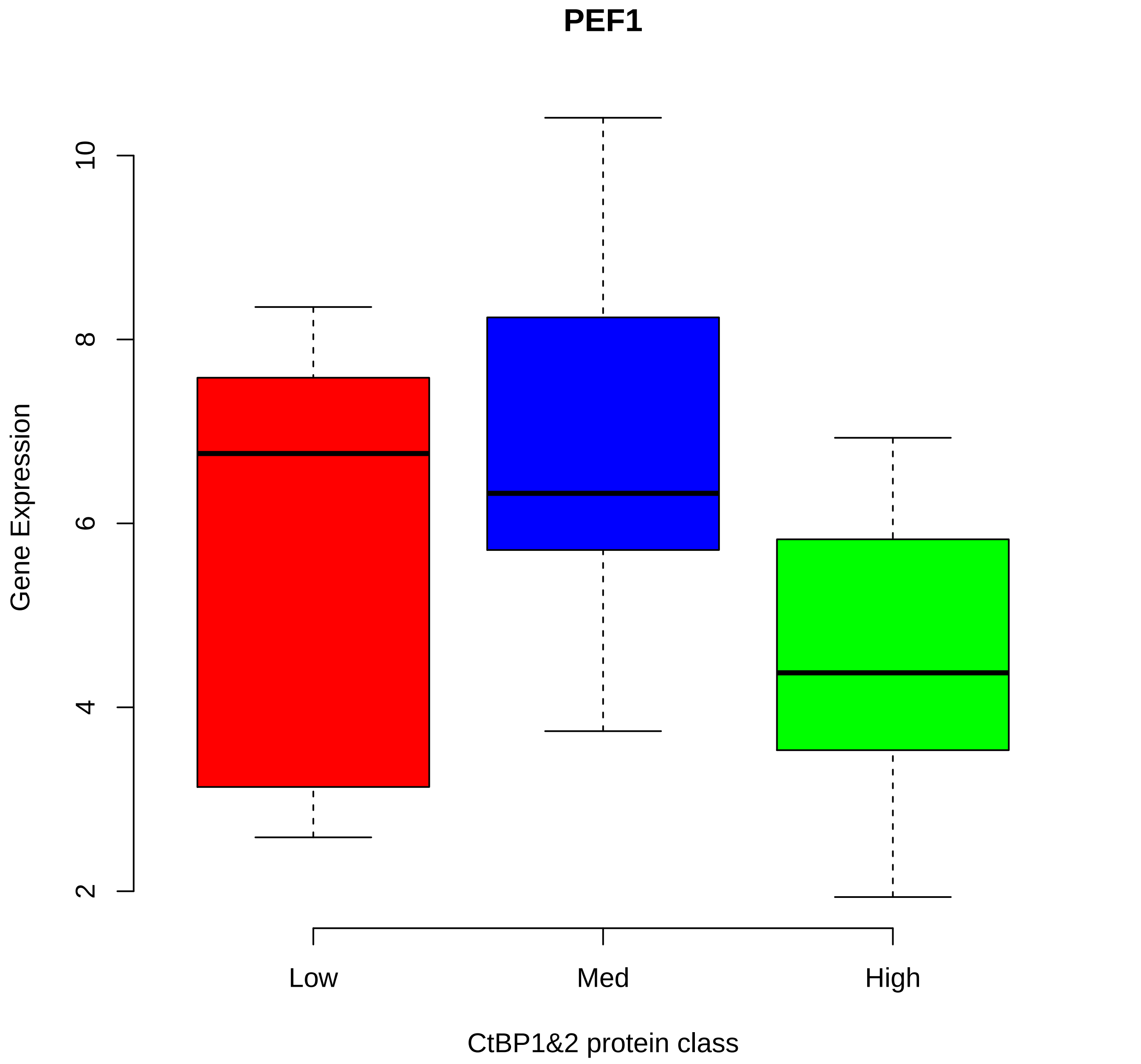

PEF1
Gene Expression
10
8
6
4
2
Low
Med
High
CtBP1&2 protein class

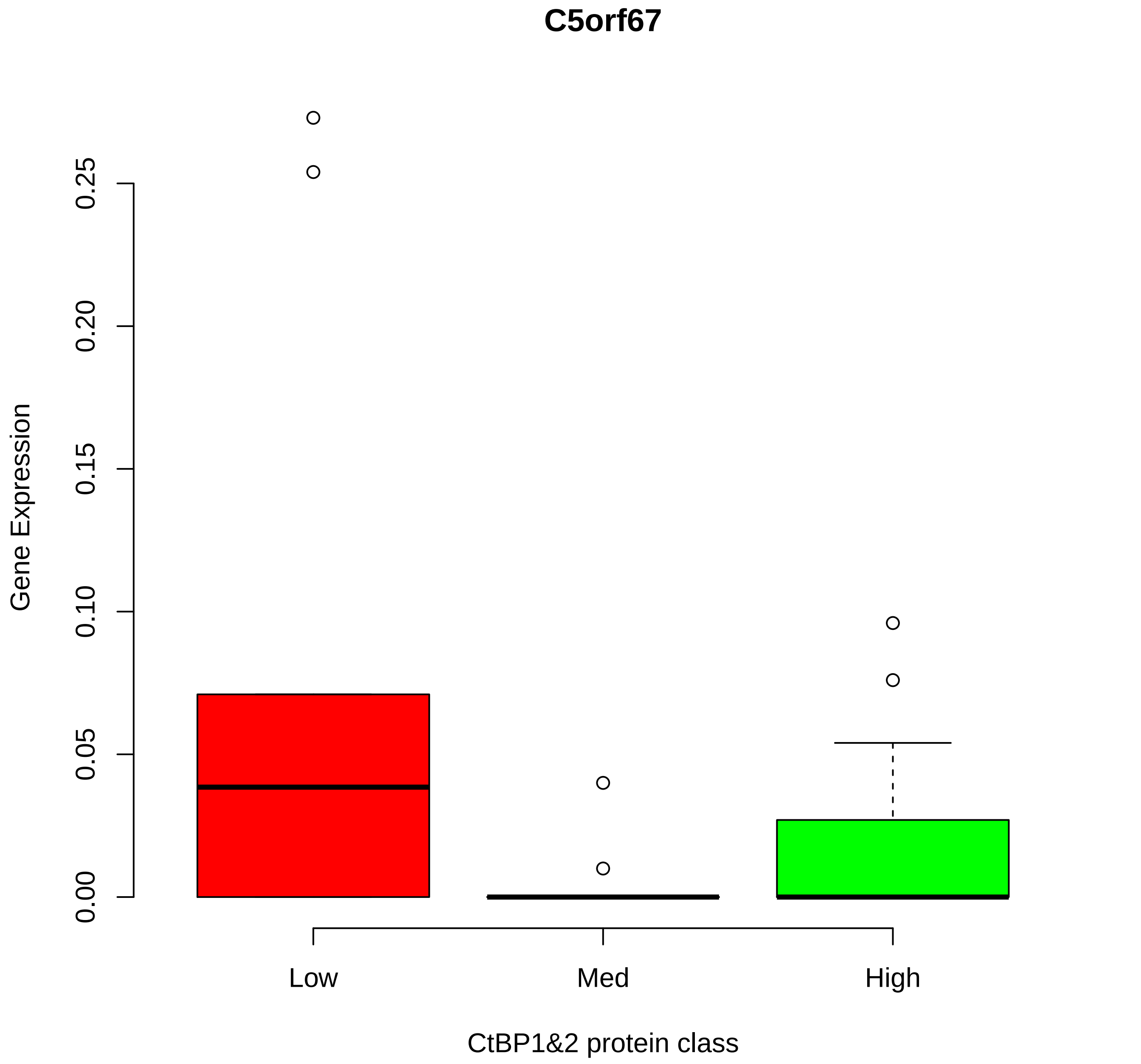

C5orf67
Gene Expression
0.00
0.05
0.10
0.15
0.20
0.25
Low
Med
High
CtBP1&2 protein class

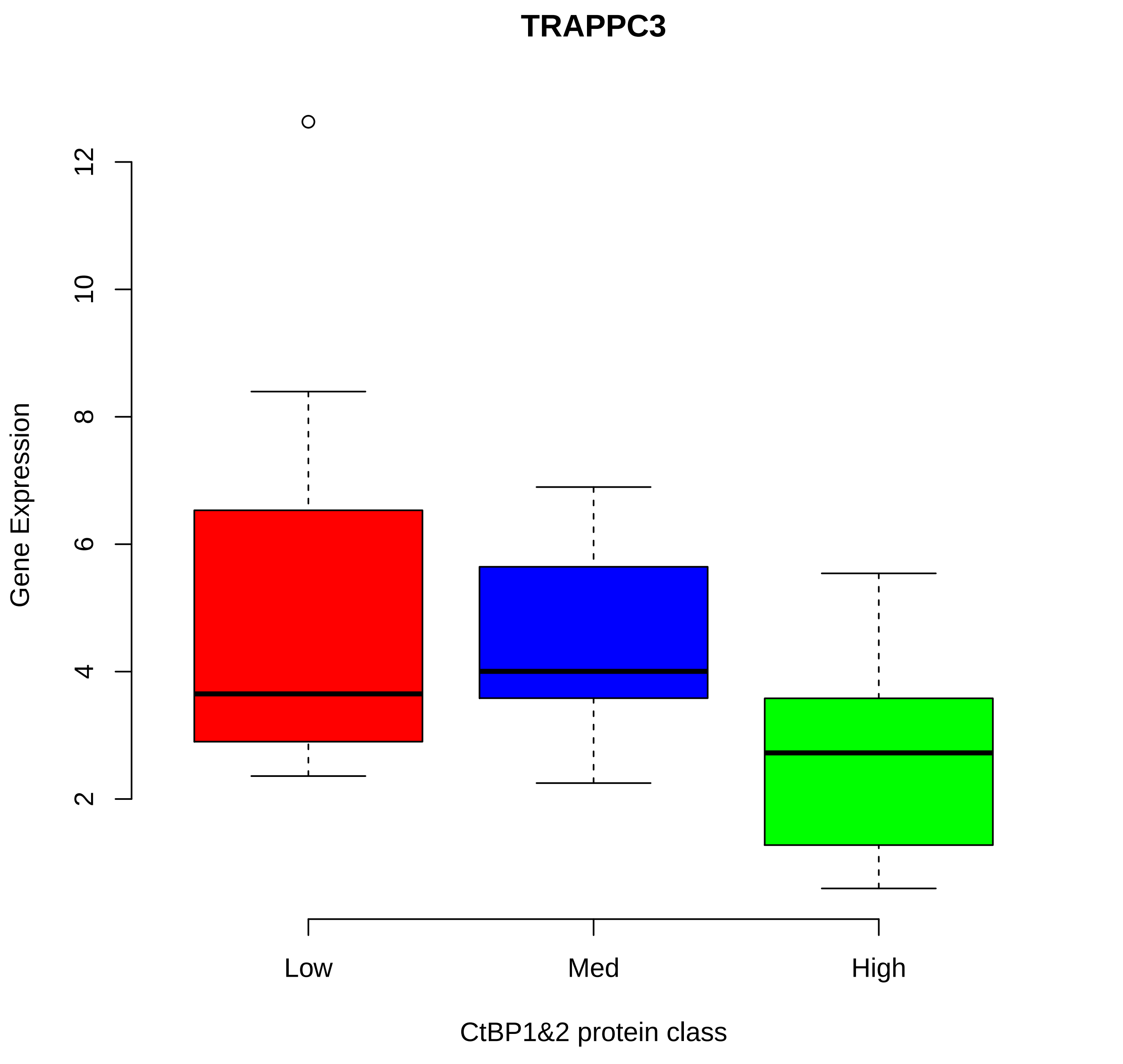

TRAPPC3
Gene Expression
12
10
8
6
4
2
Low
Med
High
CtBP1&2 protein class

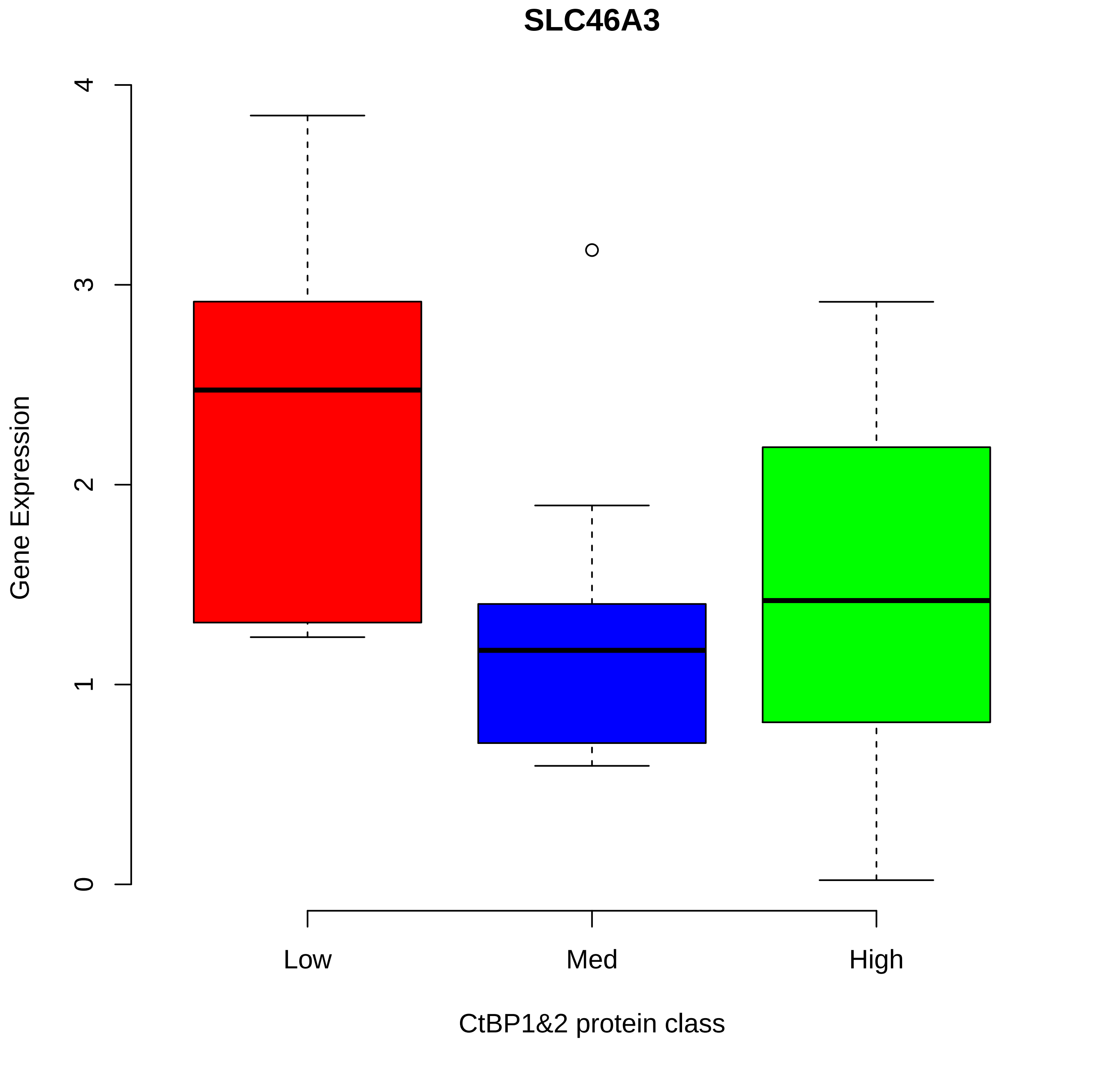

SLC46A3
Gene Expression
0
1
2
3
4
Low
Med
High
CtBP1&2 protein class

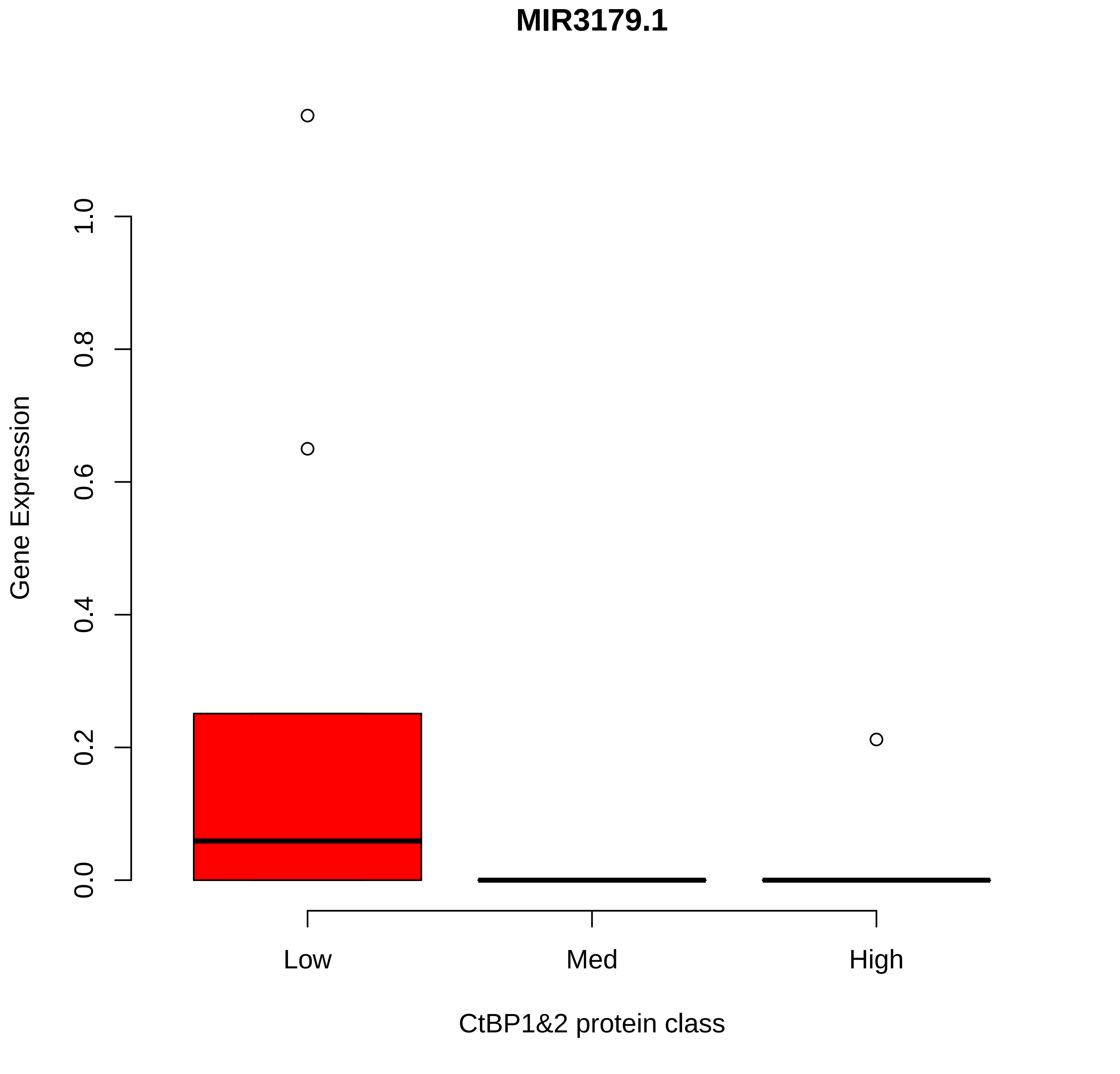
MIR3179.1
Gene Expression
0.0
0.2
0.4
0.6
0.8
1.0
Low
Med
High
CtBP1&2 protein class

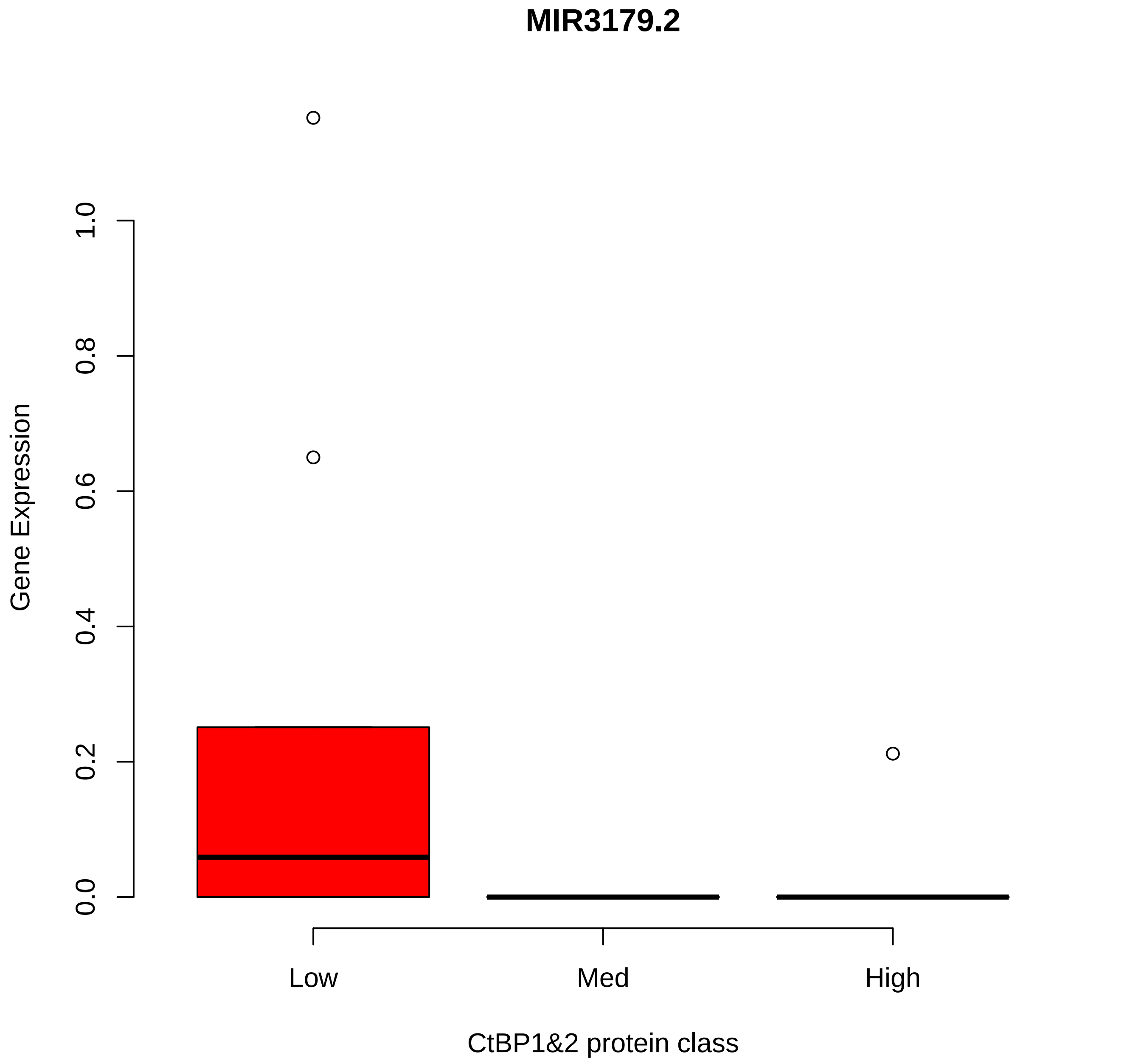

MIR3179.2
Gene Expression
0.0
0.2
0.4
0.6
0.8
1.0
Low
Med
High
CtBP1&2 protein class

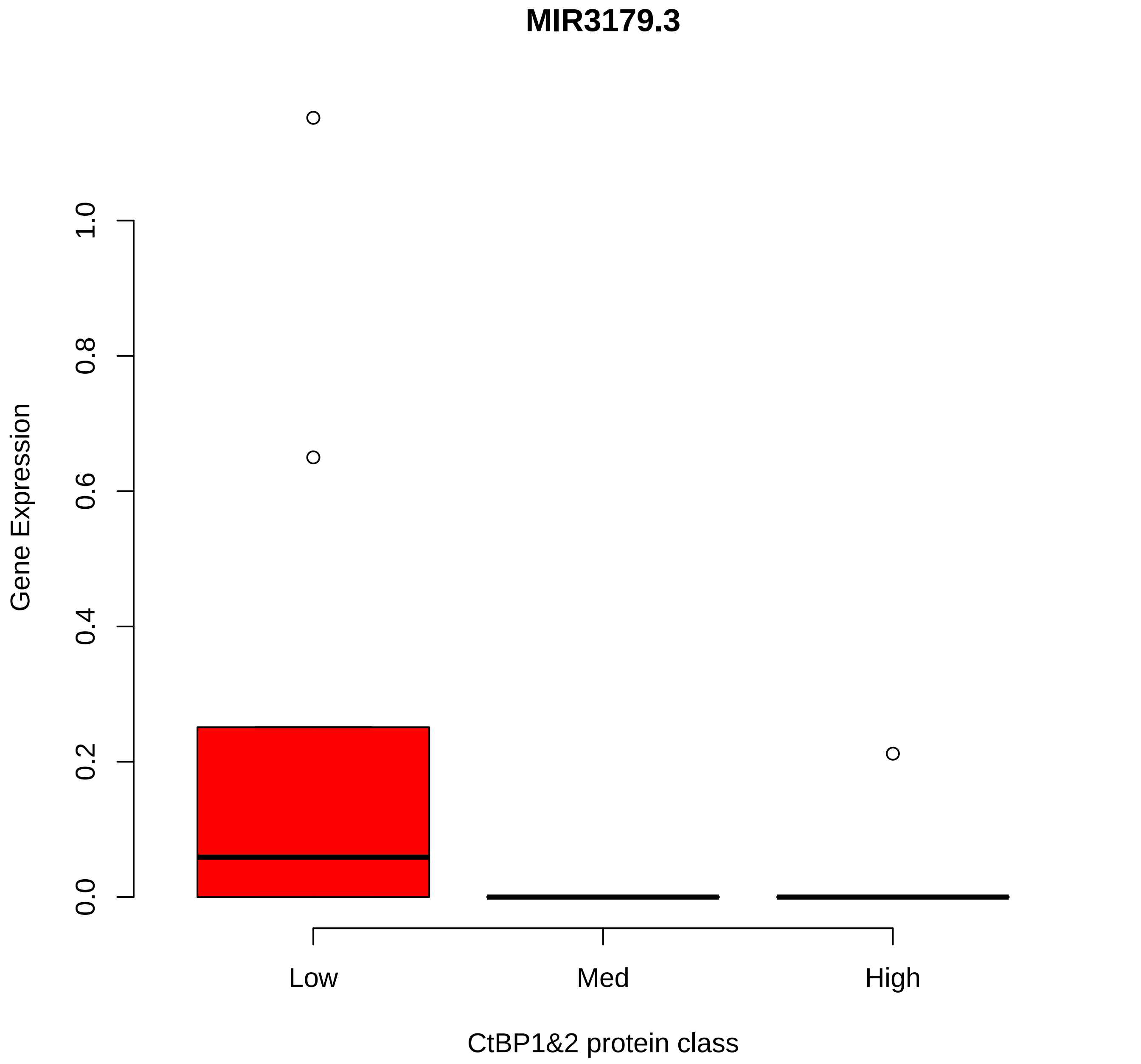

MIR3179.3
Gene Expression
0.0
0.2
0.4
0.6
0.8
1.0
Low
Med
High
CtBP1&2 protein class

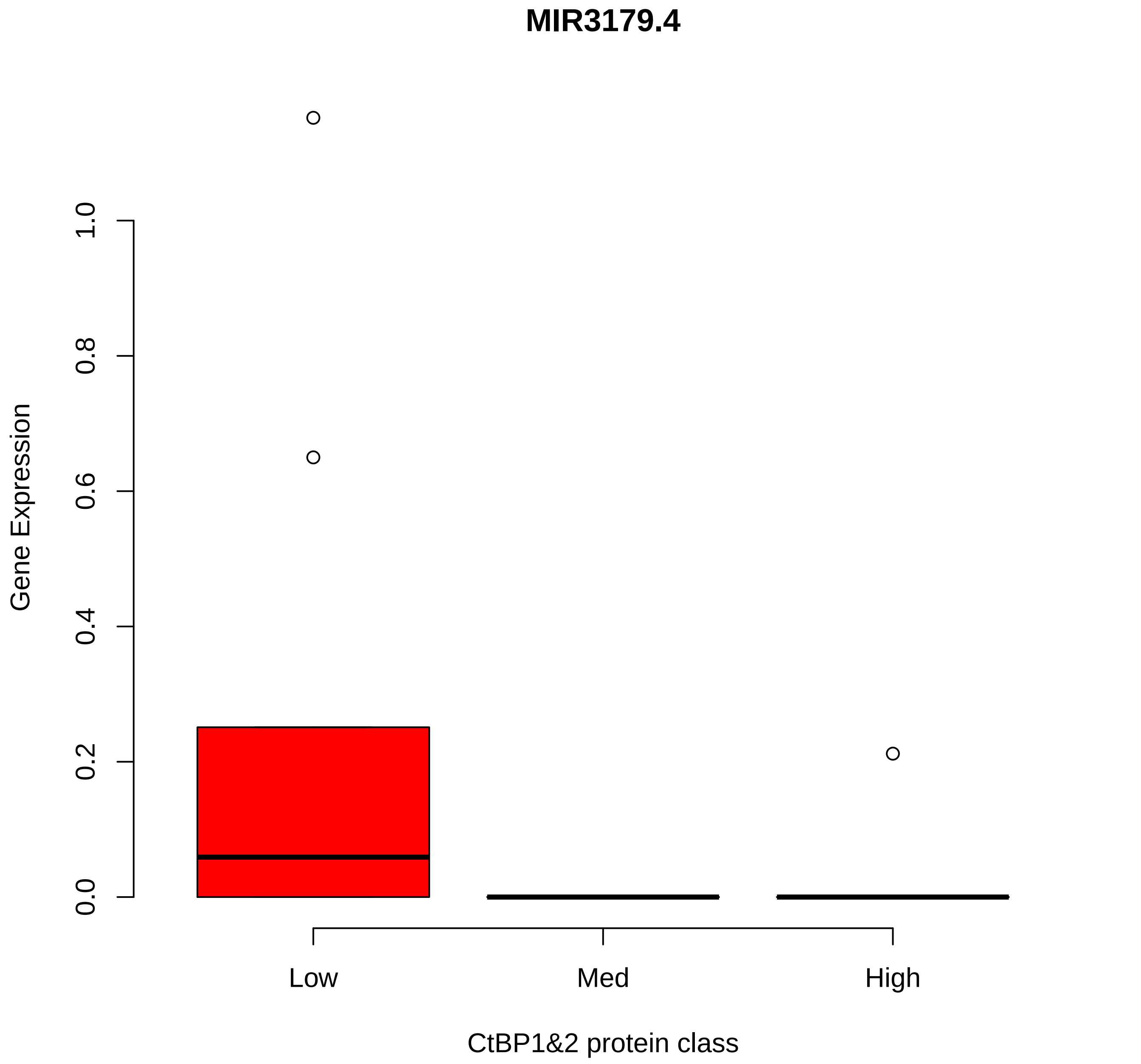

MIR3179.4
Gene Expression
0.0
0.2
0.4
0.6
0.8
1.0
Low
Med
High
CtBP1&2 protein class

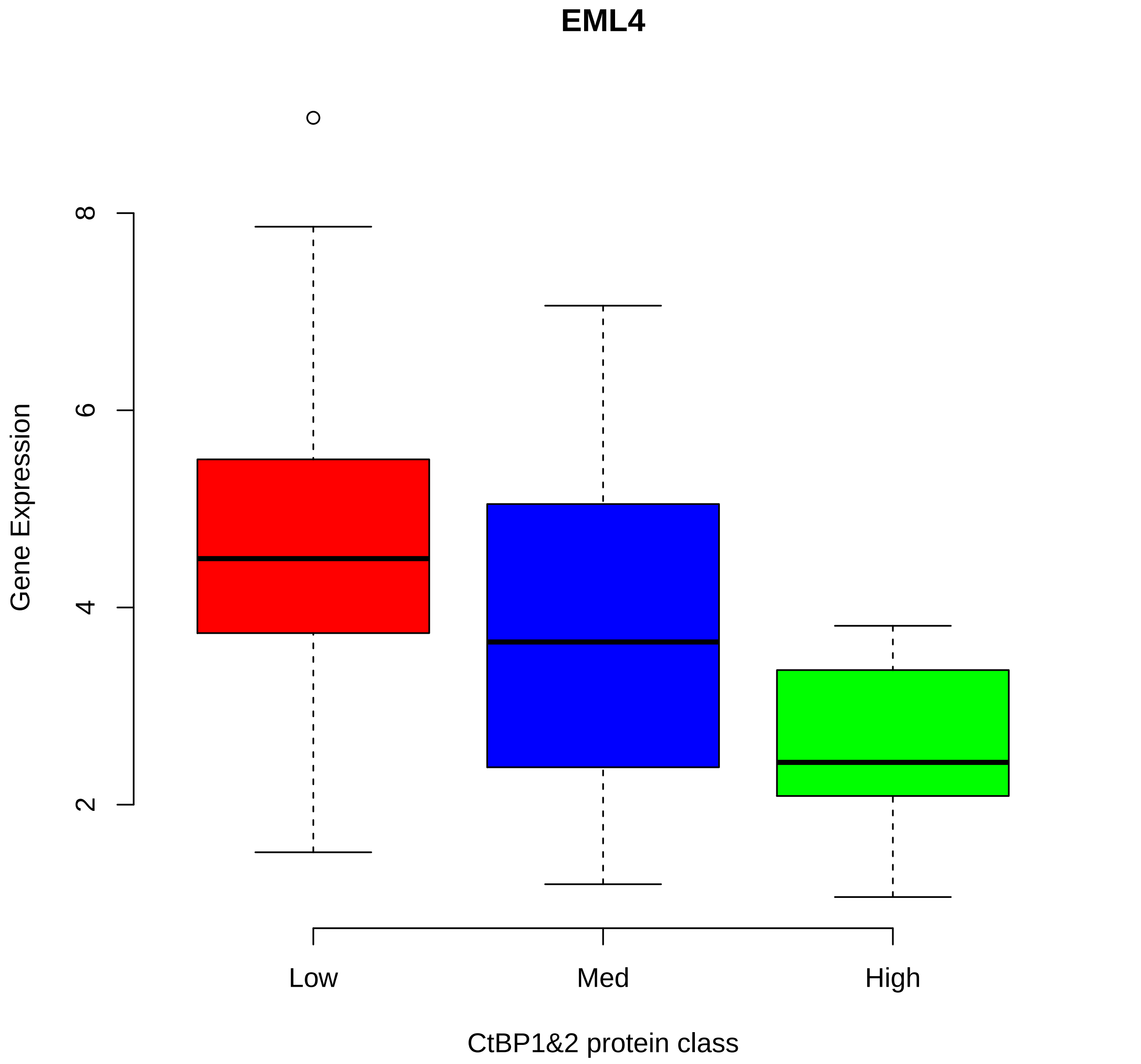
EML4
Gene Expression
2
4
6
8
Low
Med
High
CtBP1&2 protein class

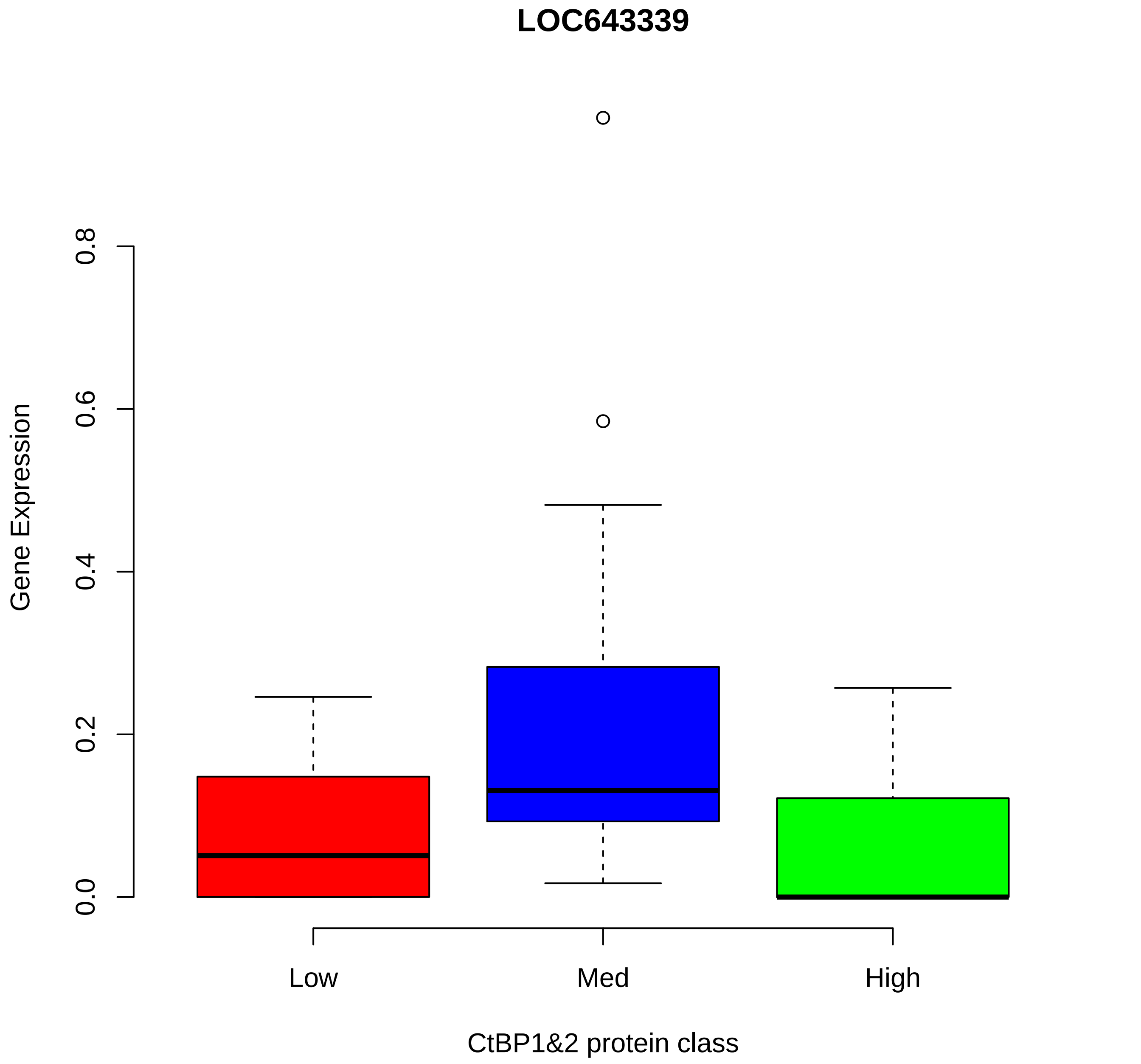
LOC643339
Gene Expression
0.0
0.2
0.4
0.6
0.8
Low
Med
High
CtBP1&2 protein class

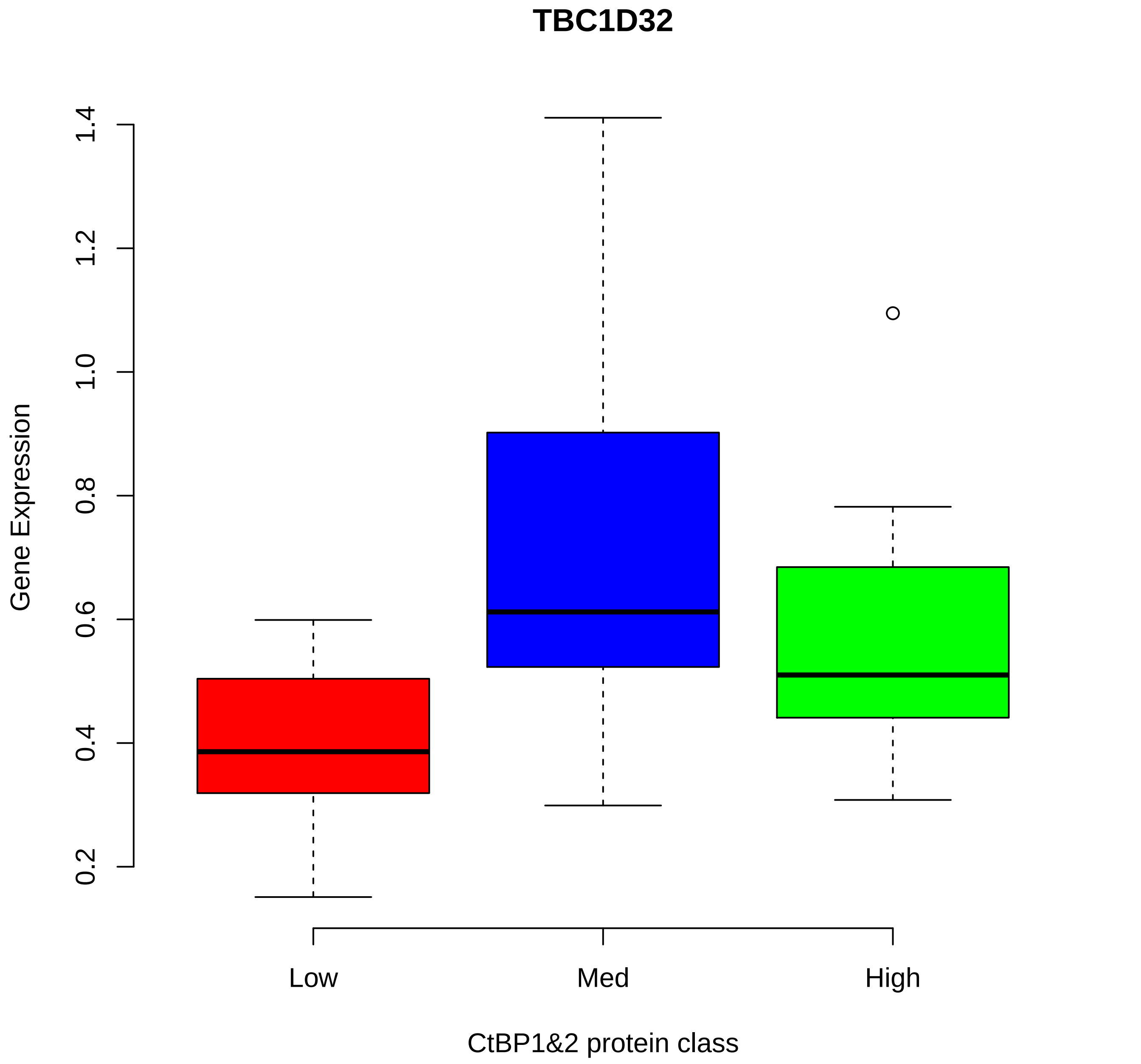
TBC1D32
Gene Expression
1.4
1.2
1.0
0.8
0.6
0.4
0.2
Low
Med
High
CtBP1&2 protein class

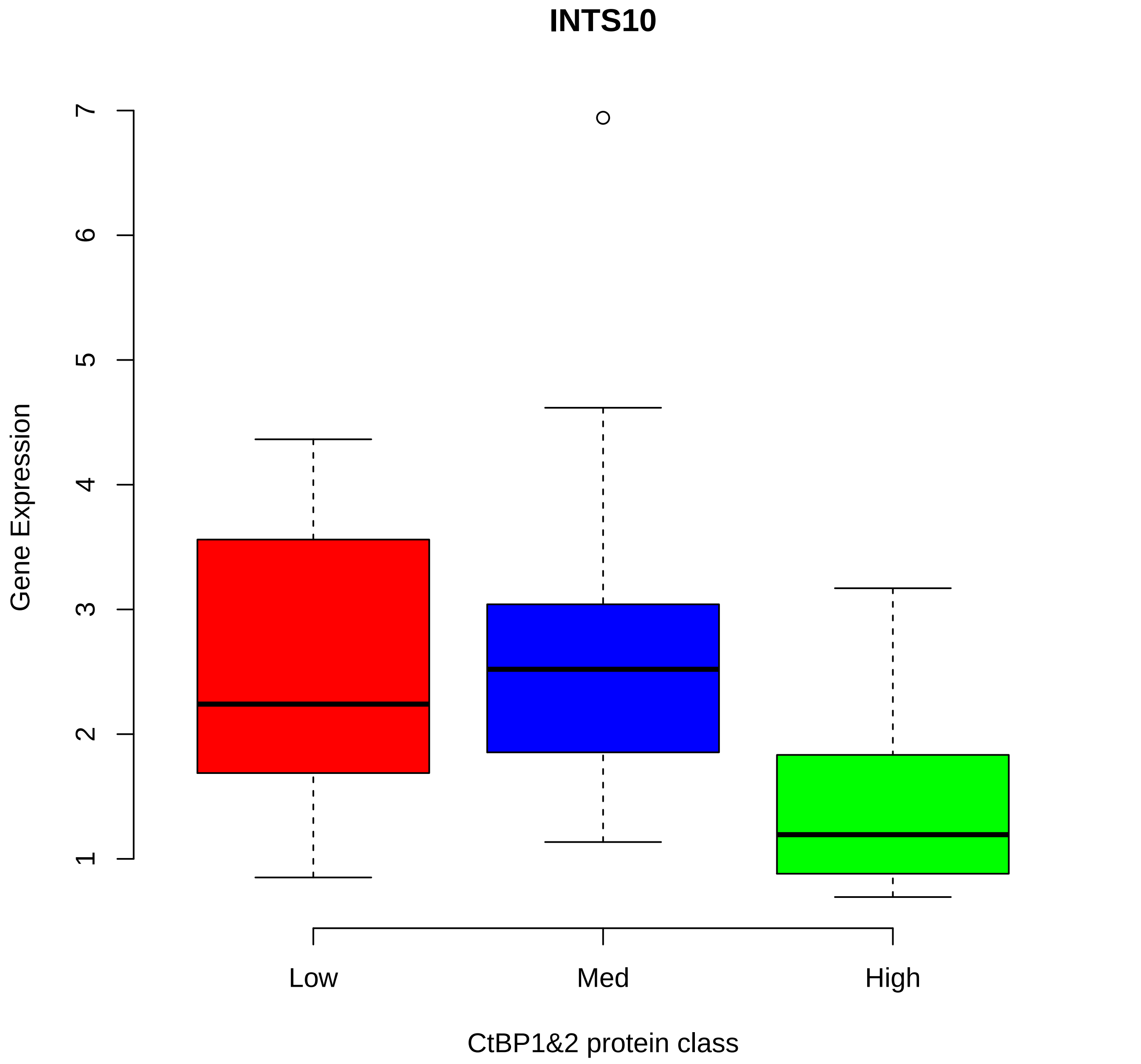
INTS10
Gene Expression
1
2
3
4
5
6
7
Low
Med
High
CtBP1&2 protein class

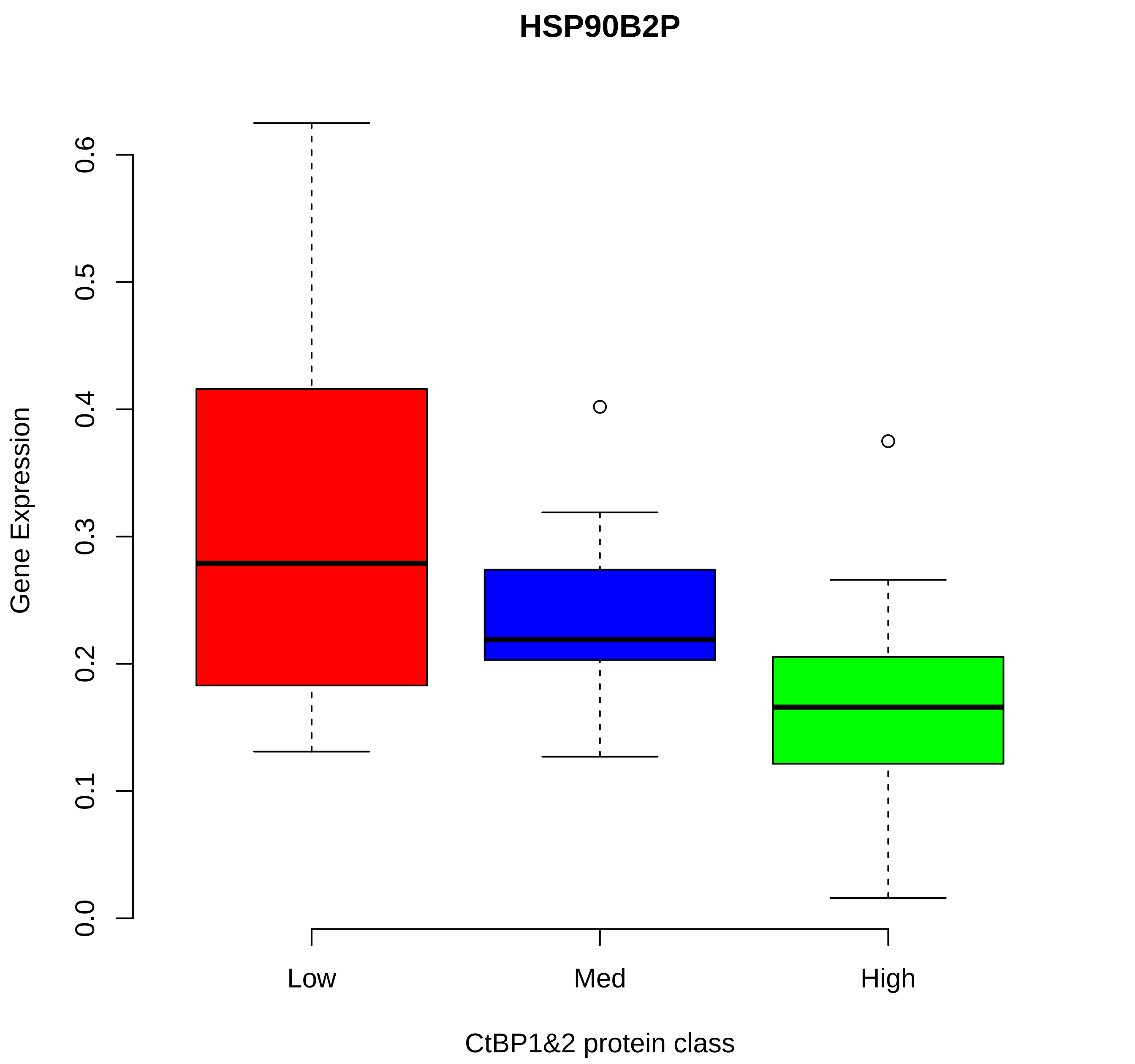

HSP90B2P
Gene Expression
0.0
0.1
0.2
0.3
0.4
0.5
0.6
Low
Med
High
CtBP1&2 protein class

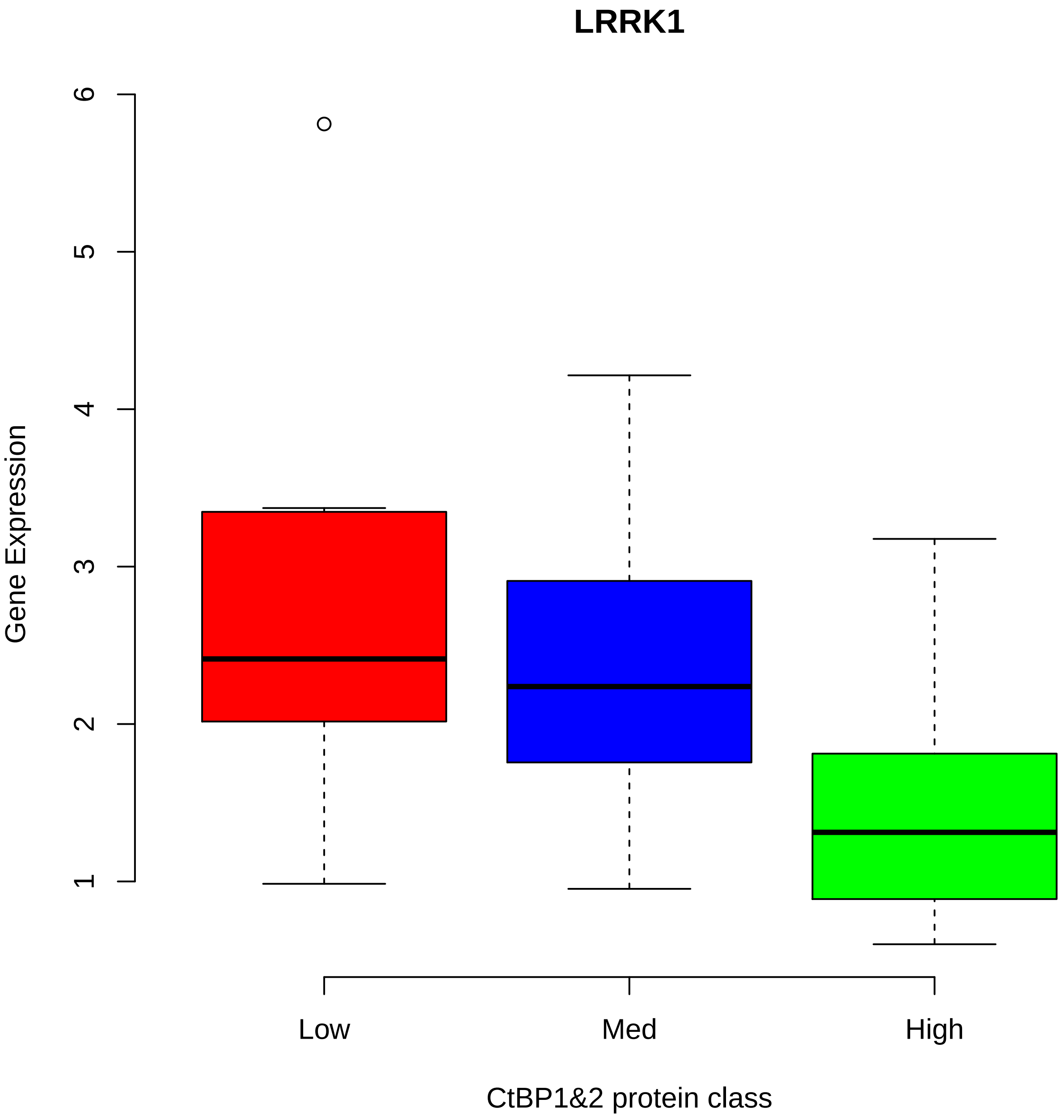

LRRK1
Gene Expression
1
2
3
4
5
6
Low
Med
High
CtBP1&2 protein class

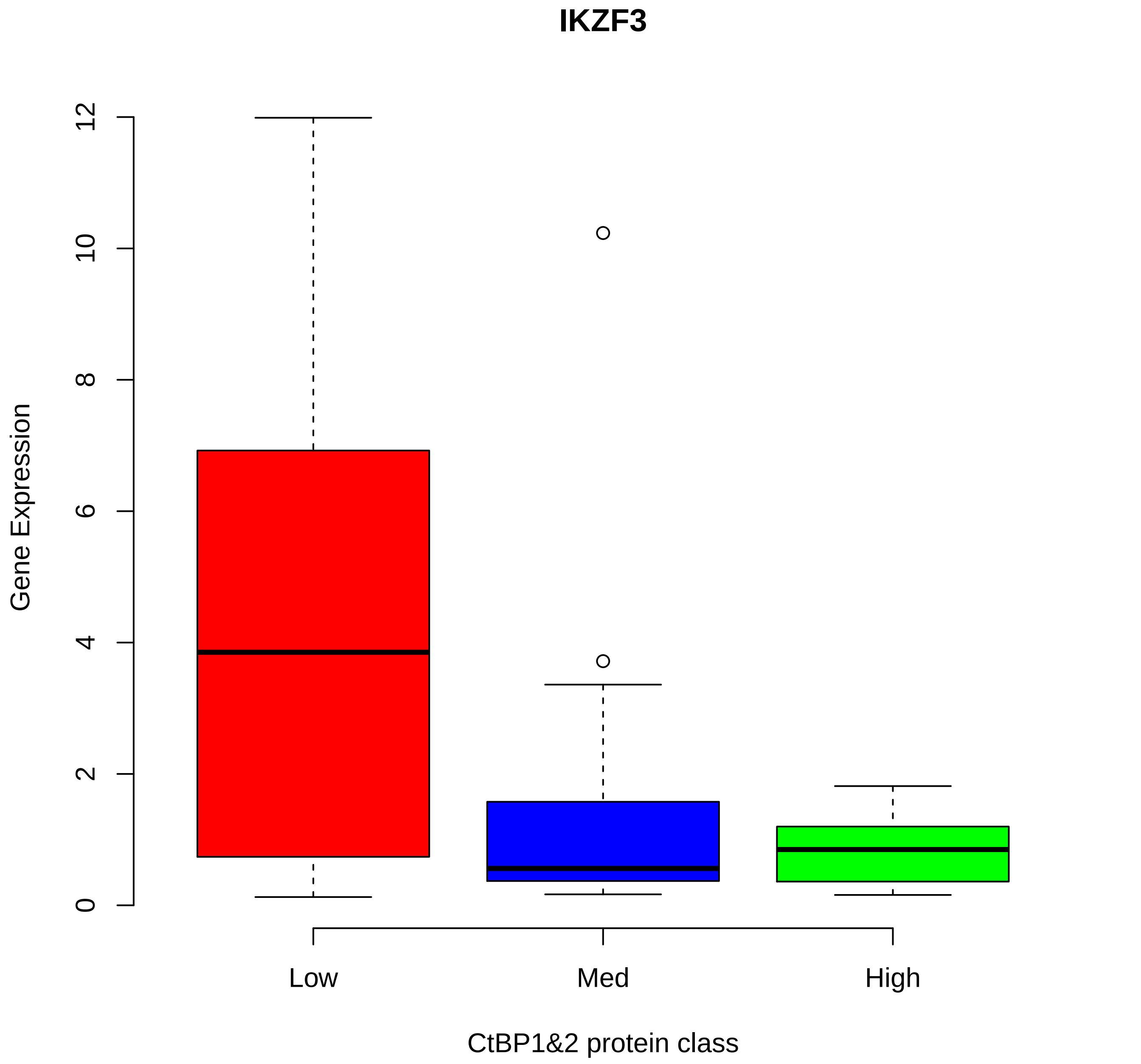
IKZF3
Gene Expression
0
2
4
6
8
10
12
Low
Med
High
CtBP1&2 protein class

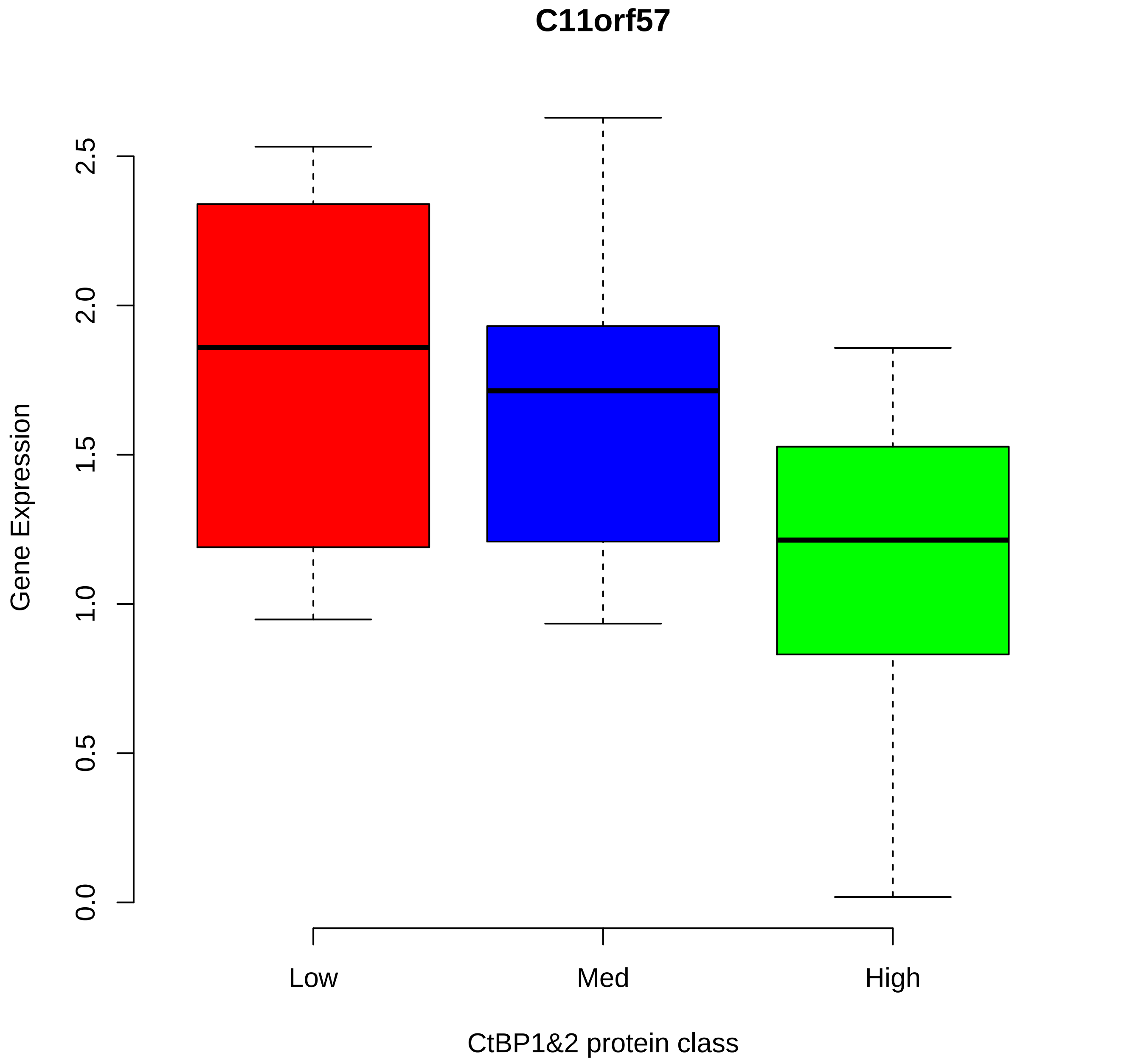

C11orf57
Gene Expression
0.0
0.5
1.0
1.5
2.0
2.5
Low
Med
High
CtBP1&2 protein class

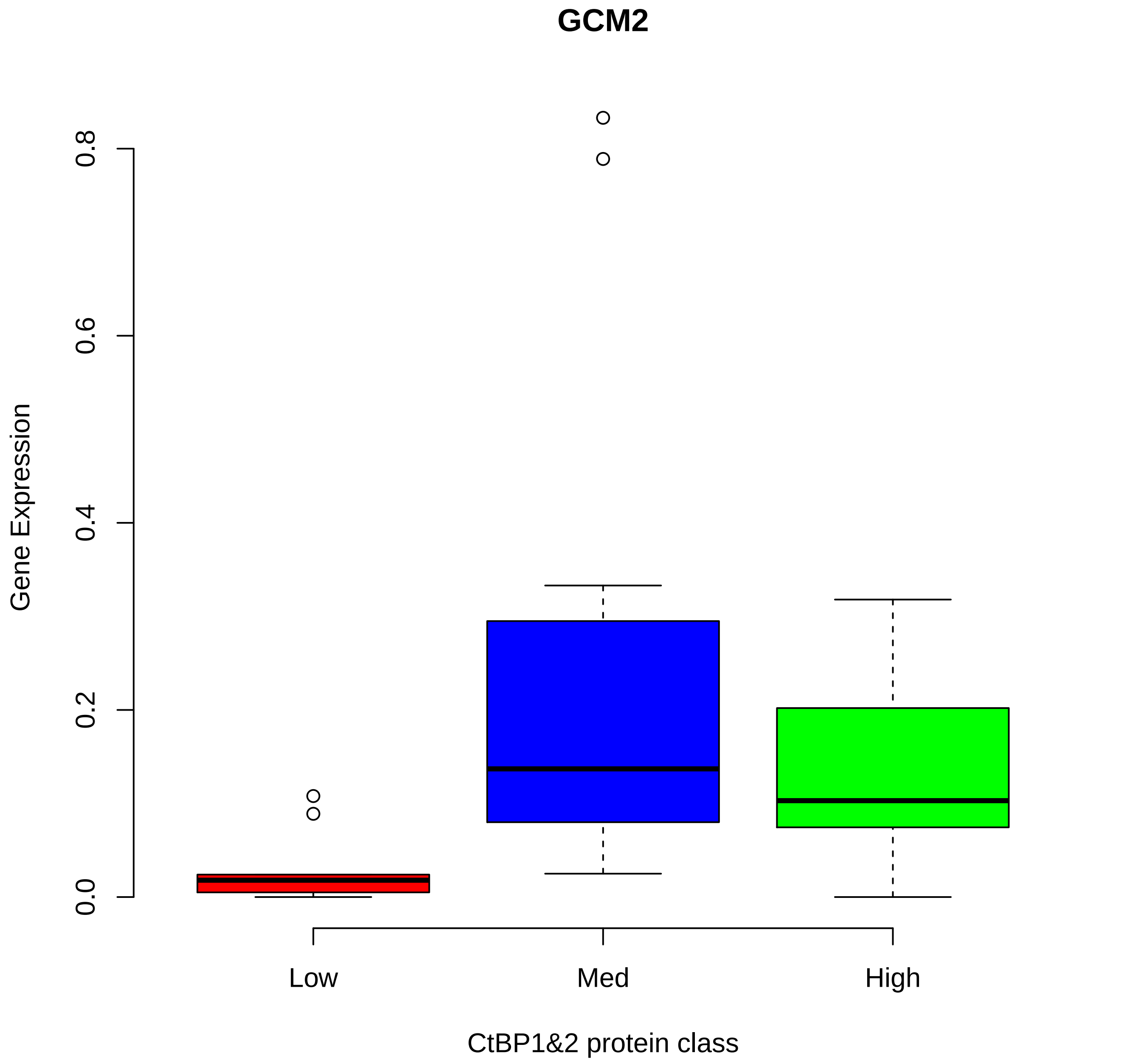
GCM2
Gene Expression
0.0
0.2
0.4
0.6
0.8
Low
Med
High
CtBP1&2 protein class

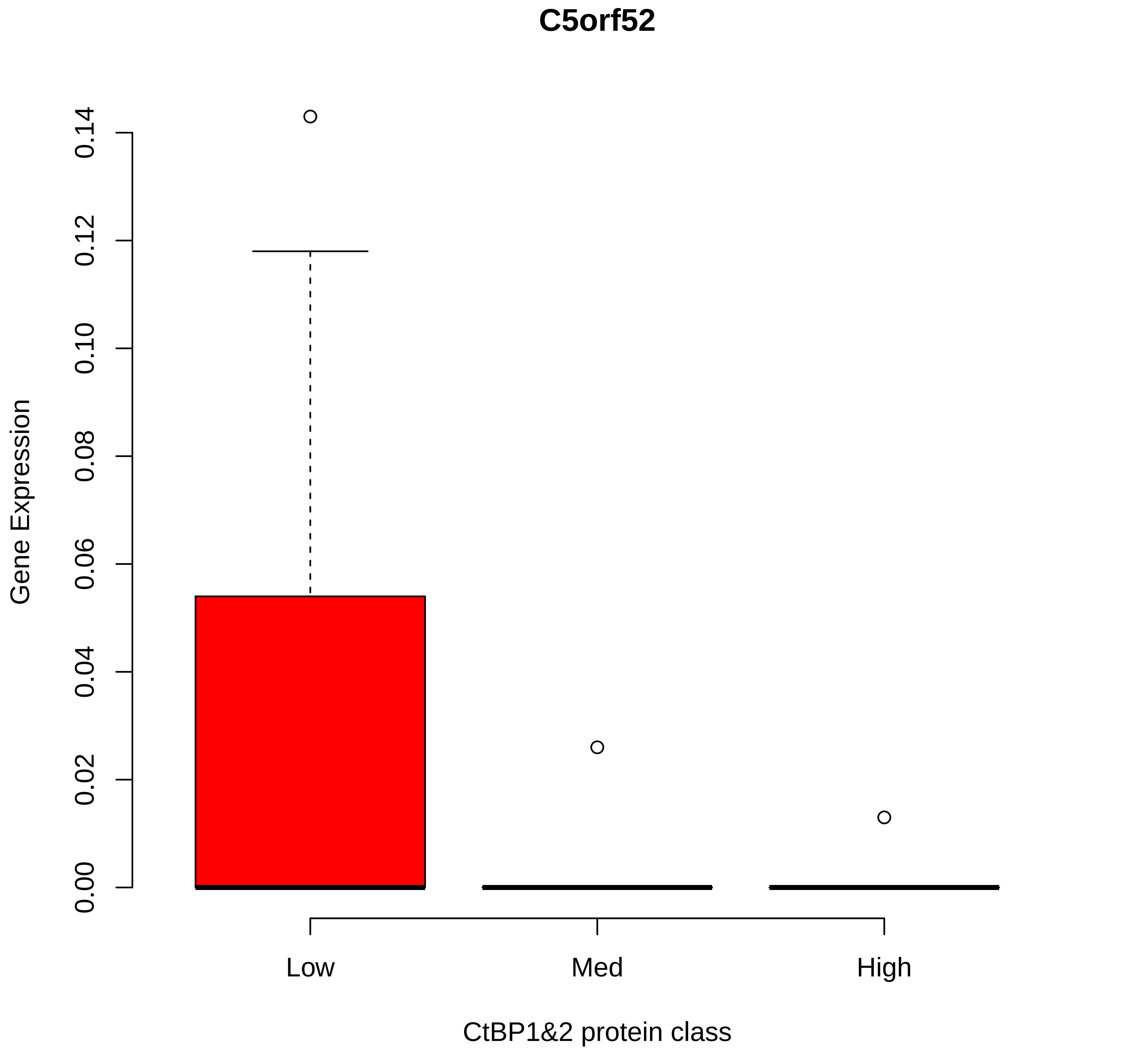
C5orf52
Gene Expression
0.00
0.02
0.04
0.06
0.08
0.10
0.12
0.14
Low
Med
High
CtBP1&2 protein class

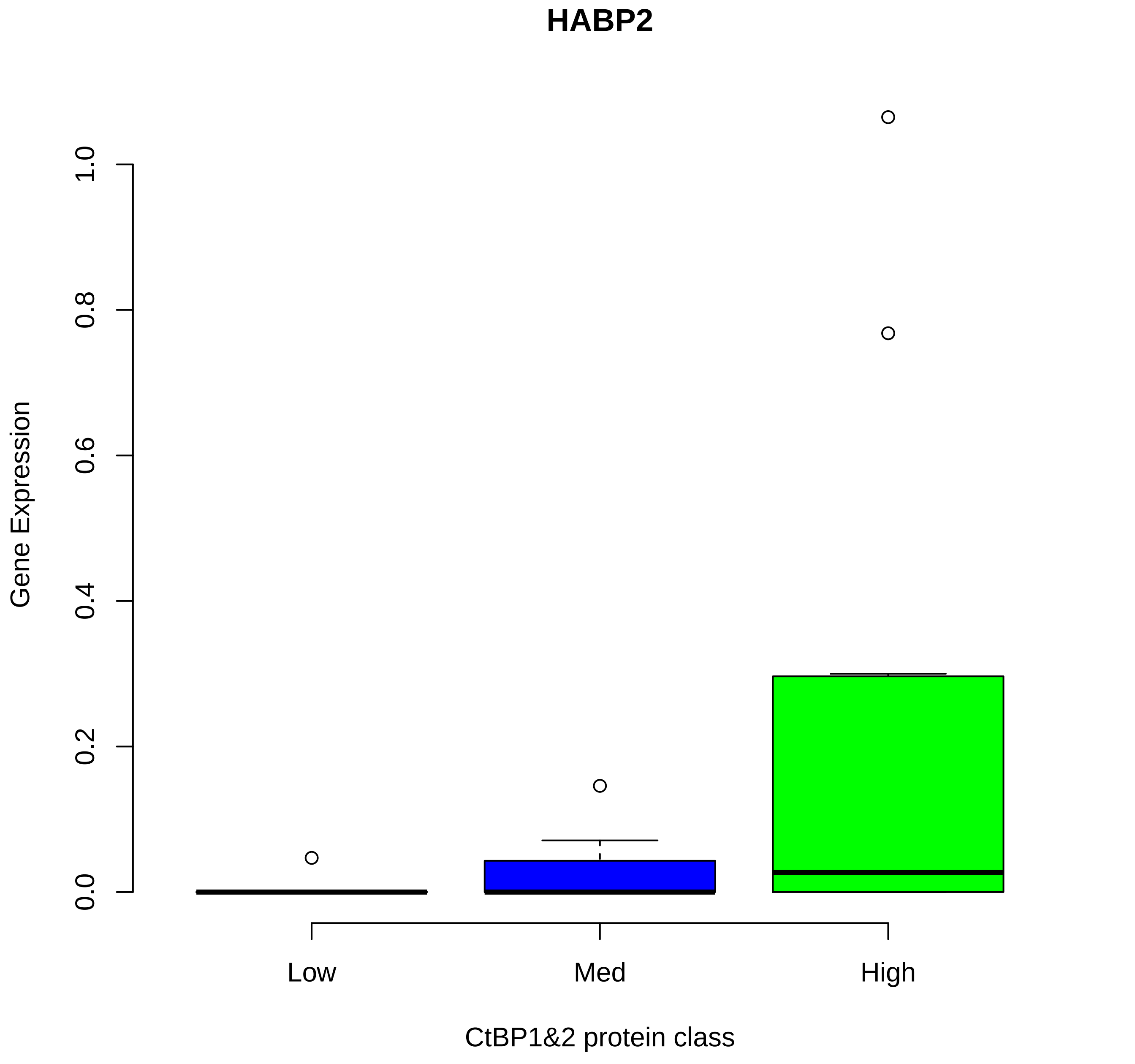
HABP2
Gene Expression
0.0
0.2
0.4
0.6
0.8
1.0
Low
Med
High
CtBP1&2 protein class

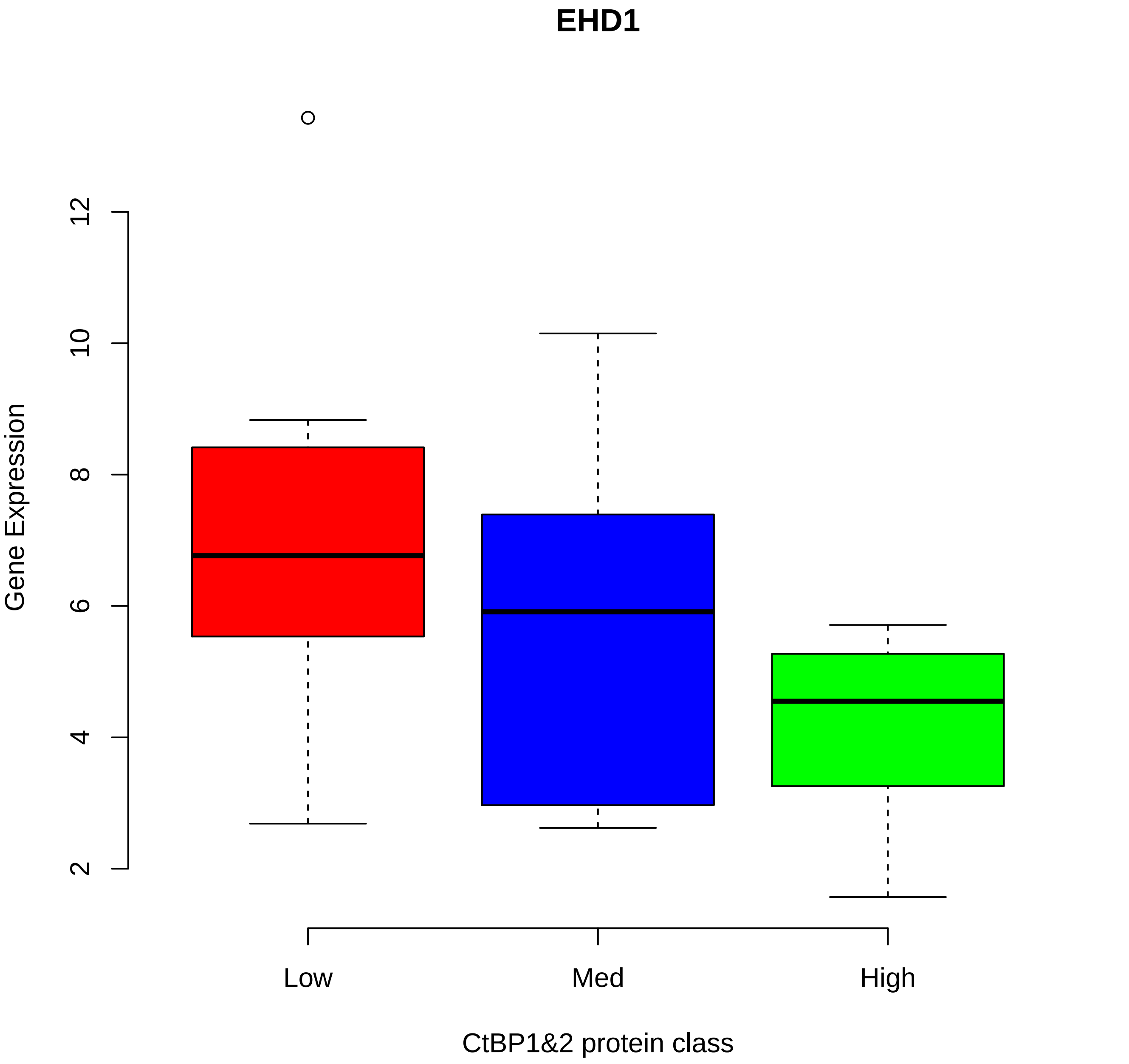
EHD1
Gene Expression
2
4
6
8
10
12
Low
Med
High
CtBP1&2 protein class

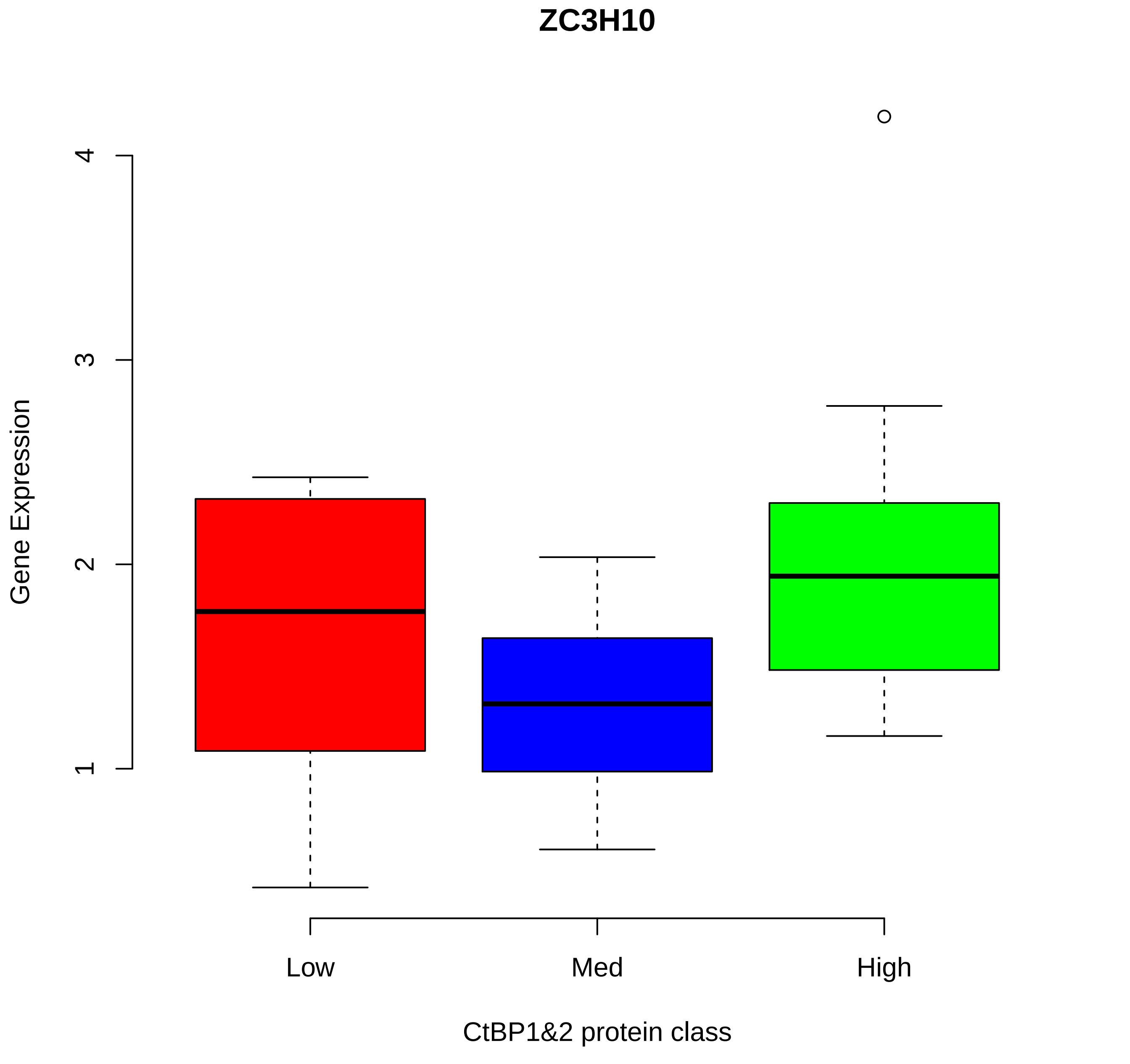
ZC3H10
Gene Expression
4
3
2
1
Low
Med
High
CtBP1&2 protein class

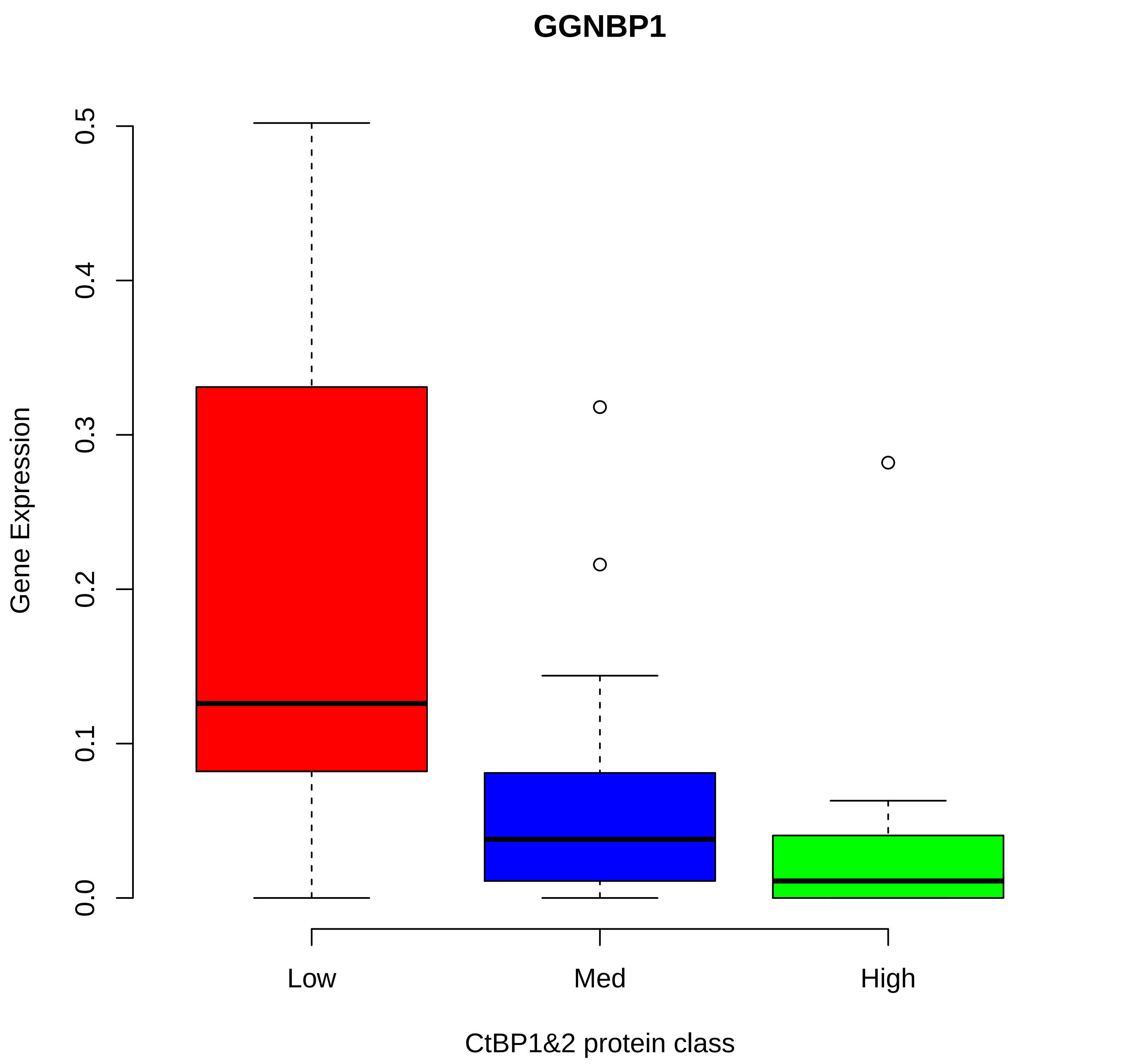
GGNBP1
Gene Expression
0.0
0.1
0.2
0.3
0.4
0.5
Low
Med
High
CtBP1&2 protein class

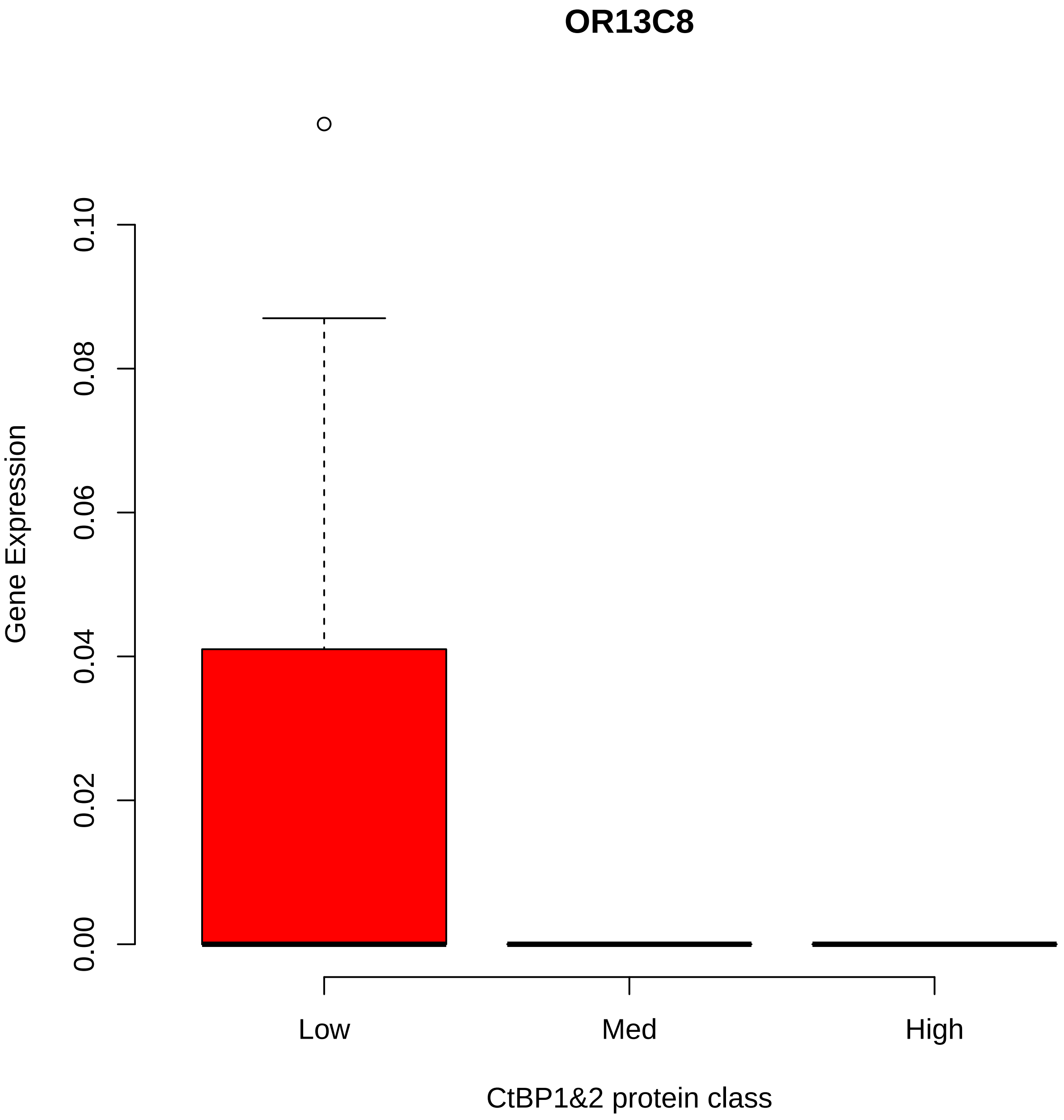

OR13C8
Gene Expression
0.00
0.02
0.04
0.06
0.08
0.10
Low
Med
High
CtBP1&2 protein class

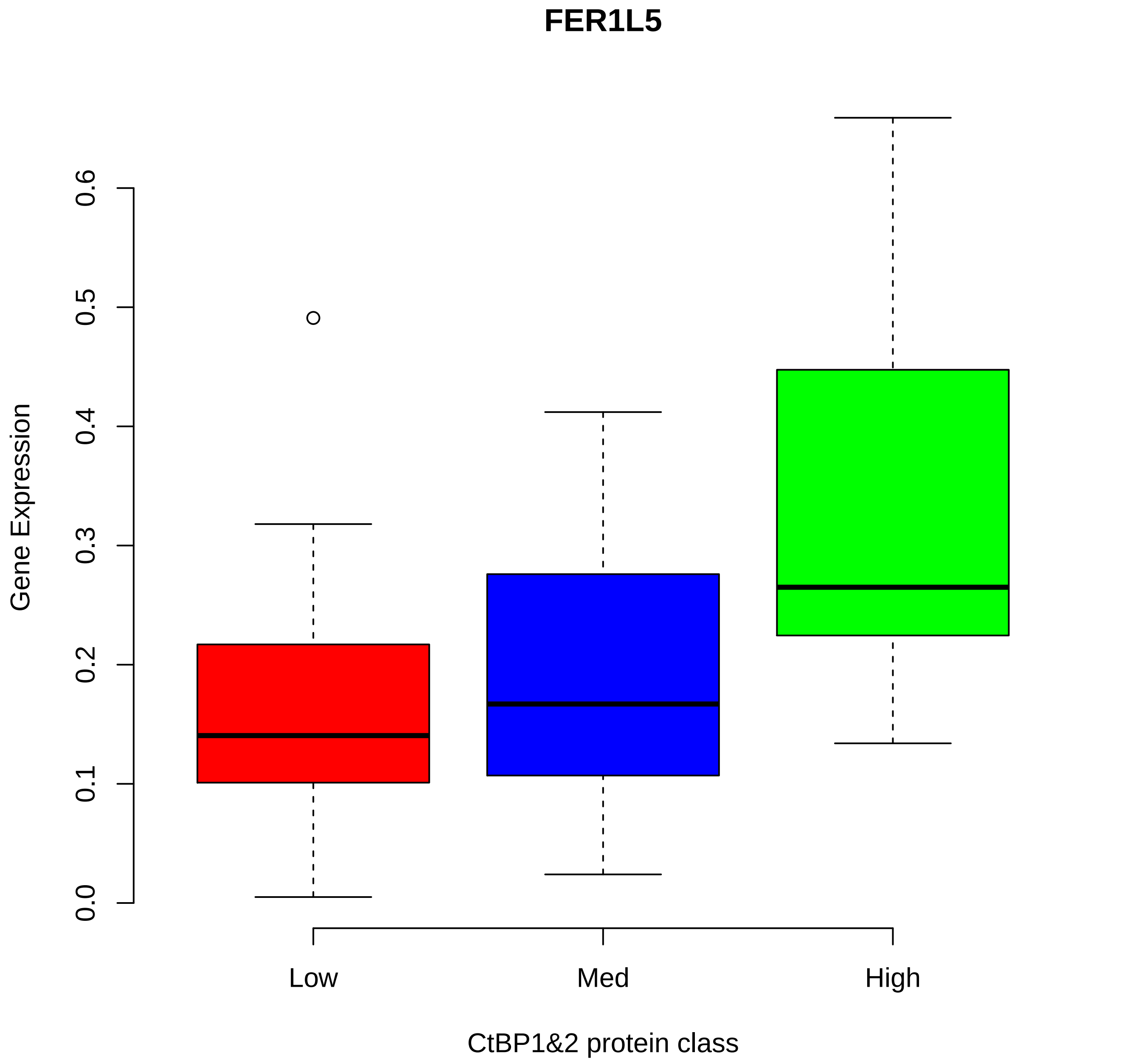

FER1L5
Gene Expression
0.0
0.1
0.2
0.3
0.4
0.5
0.6
Low
Med
High
CtBP1&2 protein class

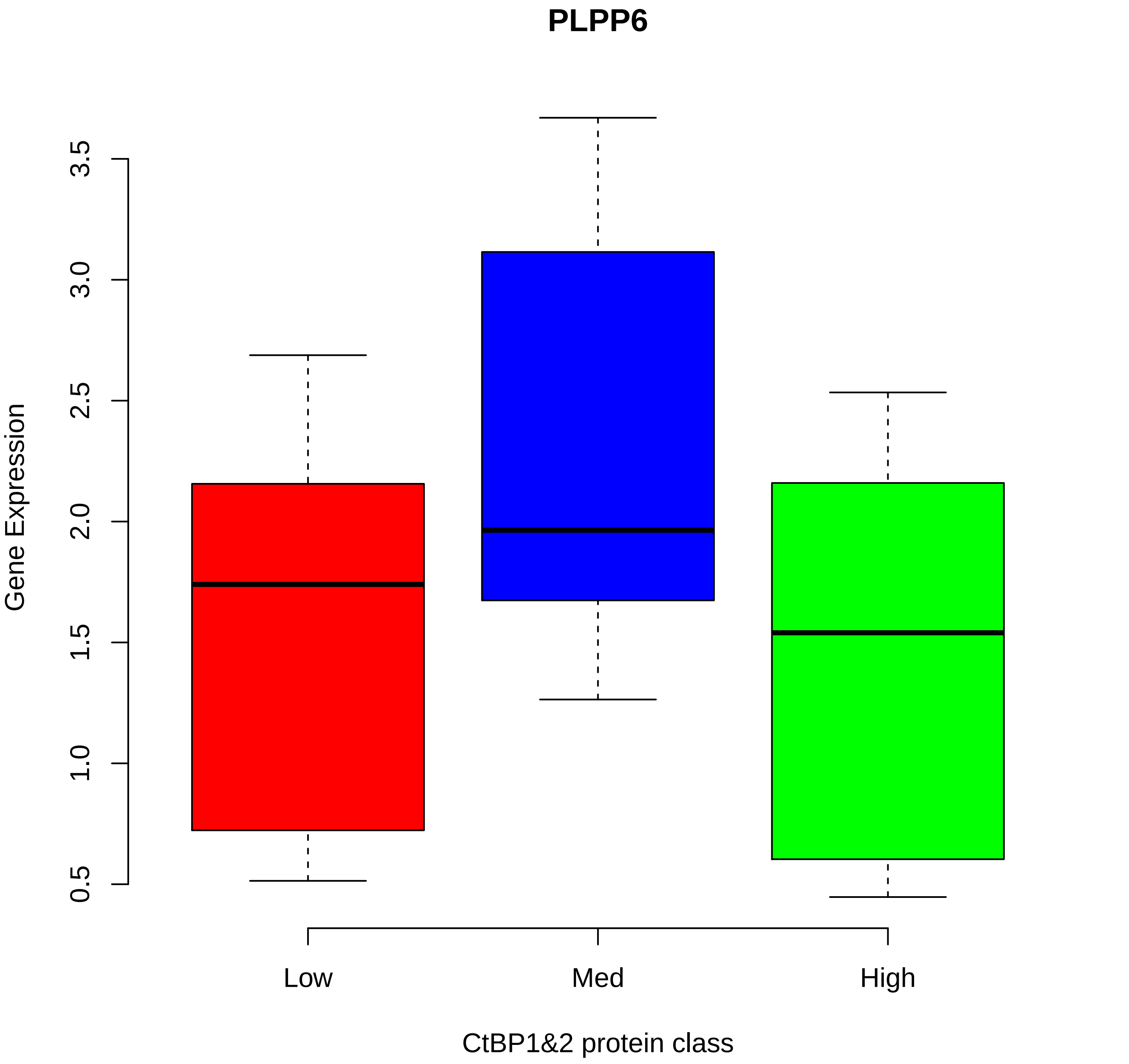
PLPP6
Gene Expression
0.5
1.0
1.5
2.0
2.5
3.0
3.5
Low
Med
High
CtBP1&2 protein class

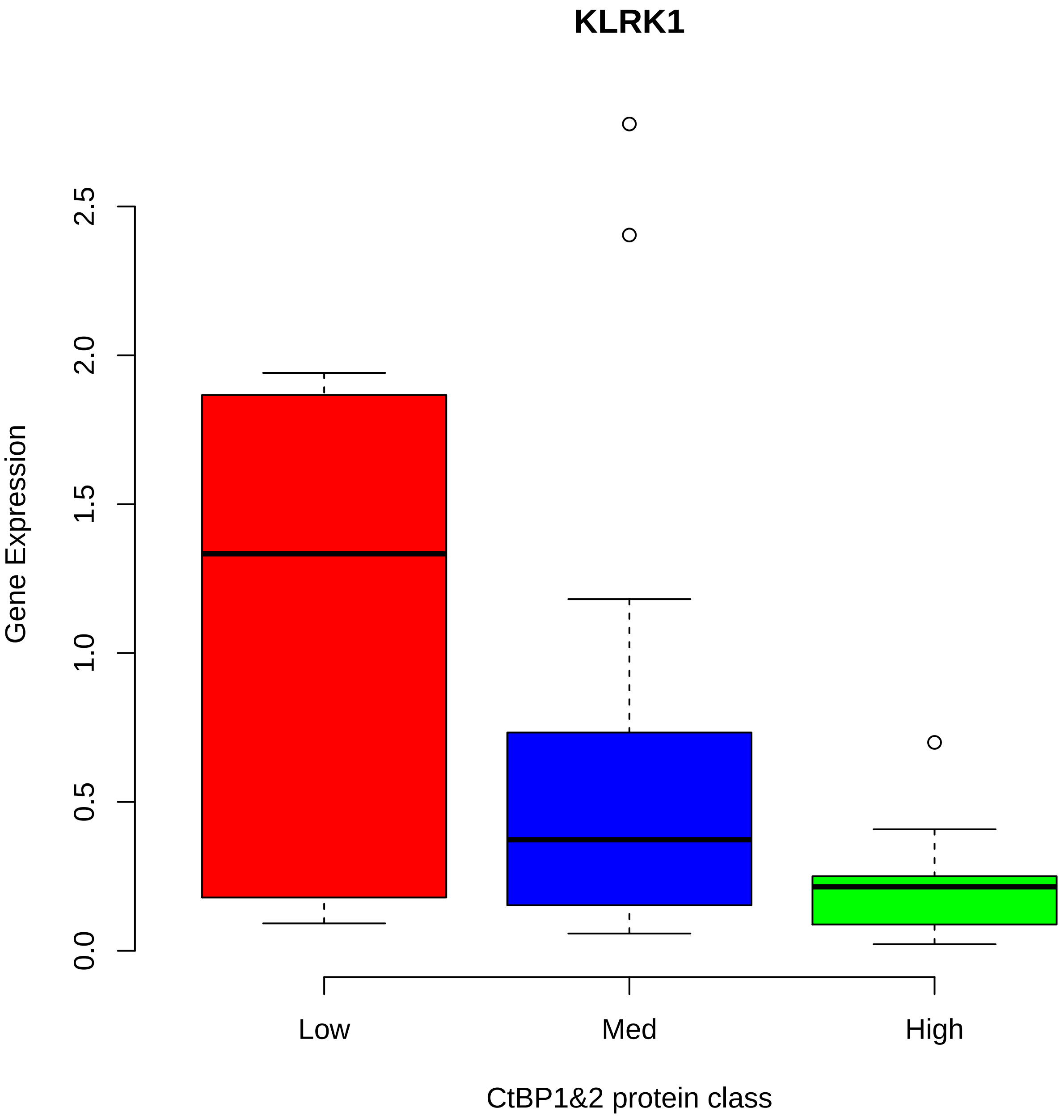

KLRK1
Gene Expression
0.0
0.5
1.0
1.5
2.0
2.5
Low
Med
High
CtBP1&2 protein class

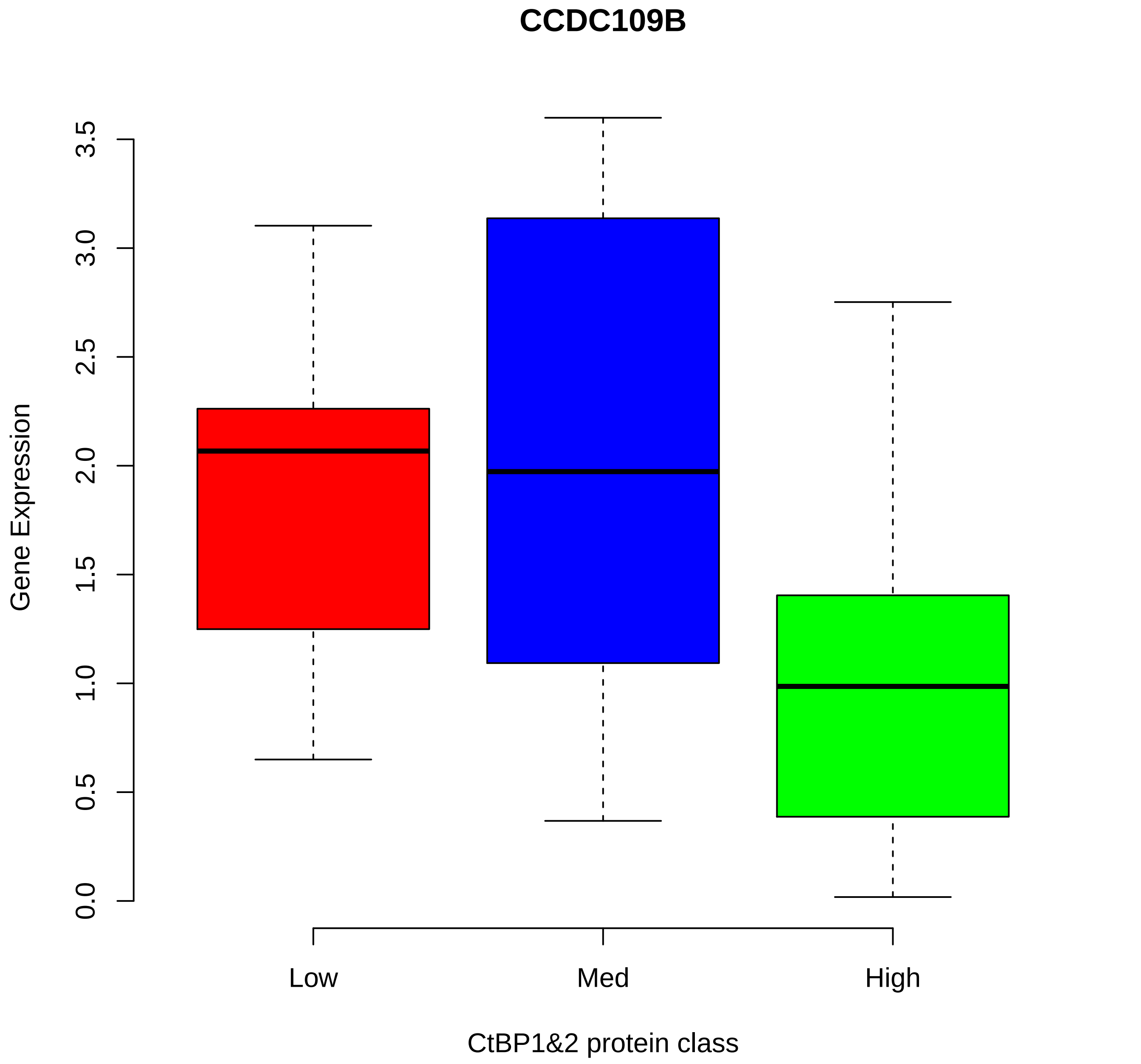

CCDC109B
Gene Expression
0.0
0.5
1.0
1.5
2.0
2.5
3.0
3.5
Low
Med
High
CtBP1&2 protein class

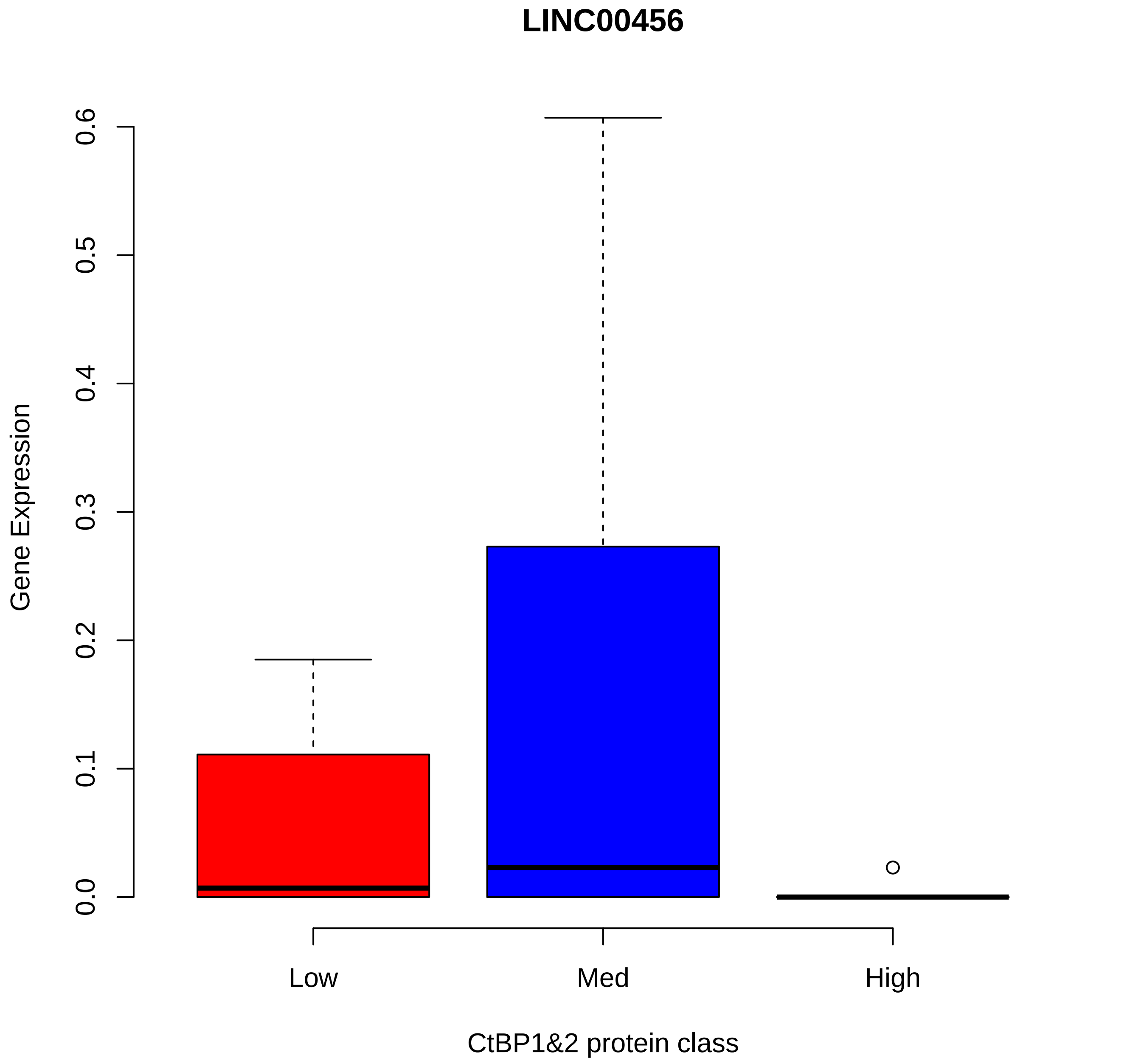
LINC00456
Gene Expression
0.0
0.1
0.2
0.3
0.4
0.5
0.6
Low
Med
High
CtBP1&2 protein class

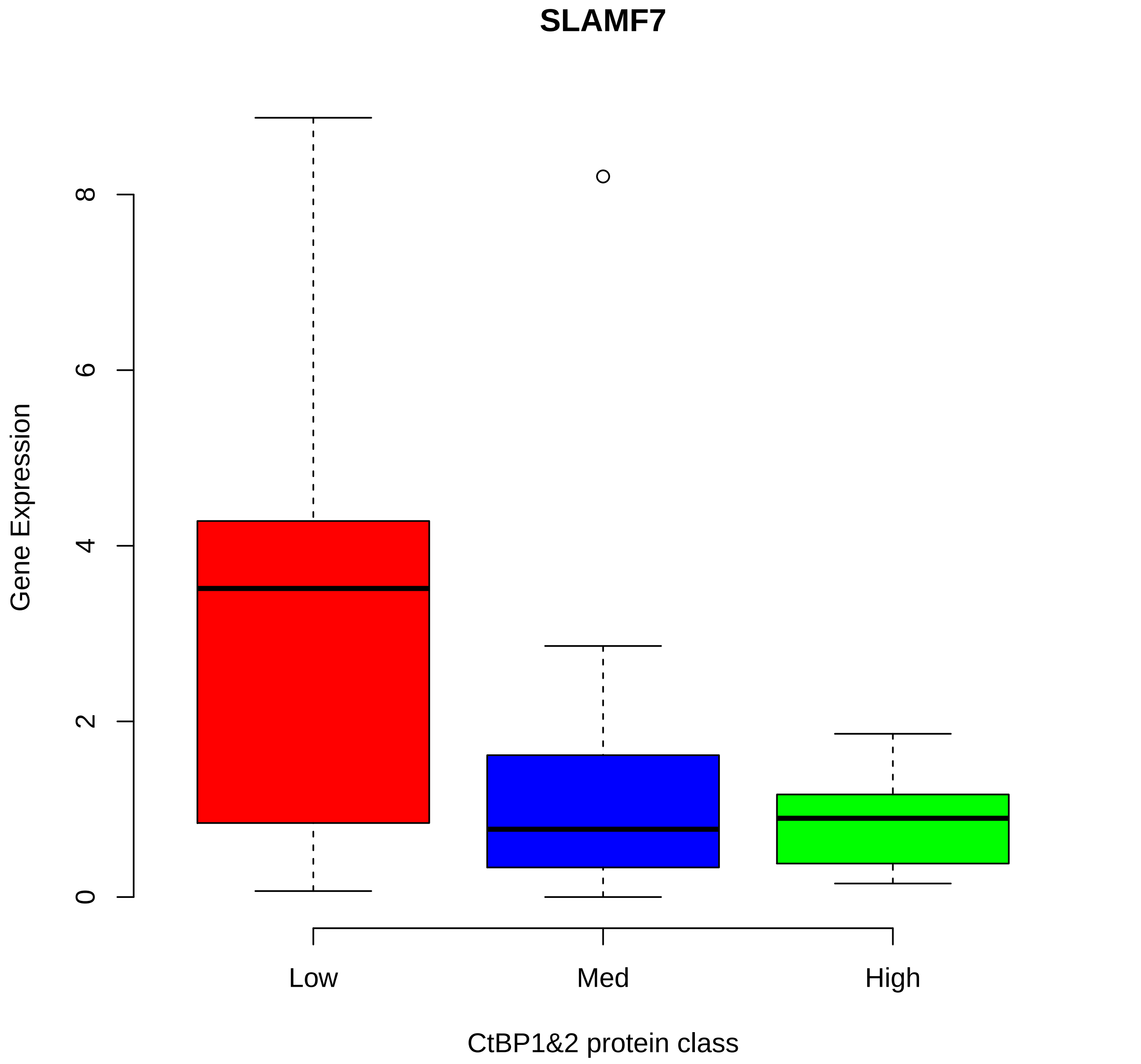
SLAMF7
Gene Expression
0
2
4
6
8
Low
Med
High
CtBP1&2 protein class

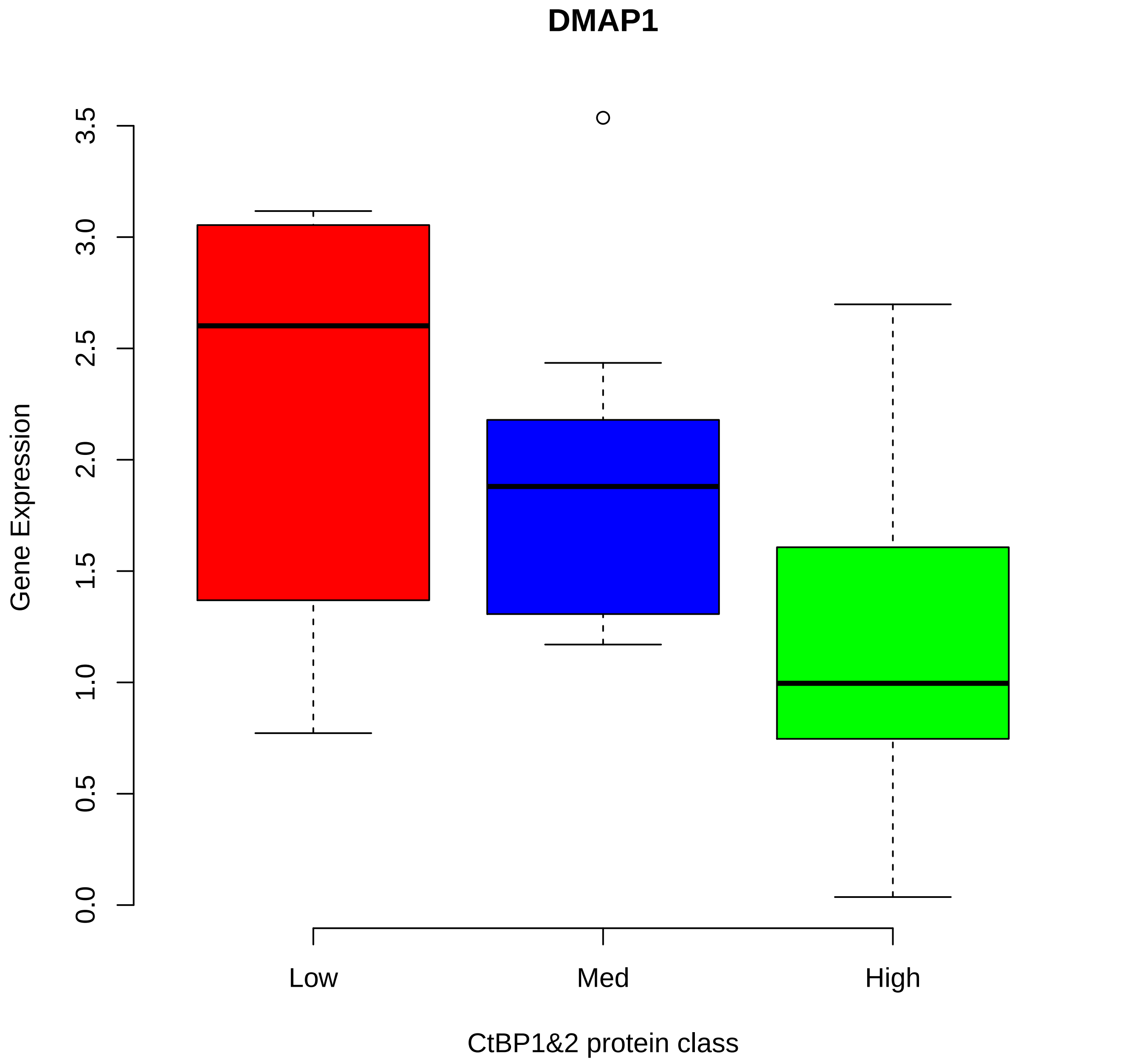
DMAP1
Gene Expression
0.0
0.5
1.0
1.5
2.0
2.5
3.0
3.5
Low
Med
High
CtBP1&2 protein class

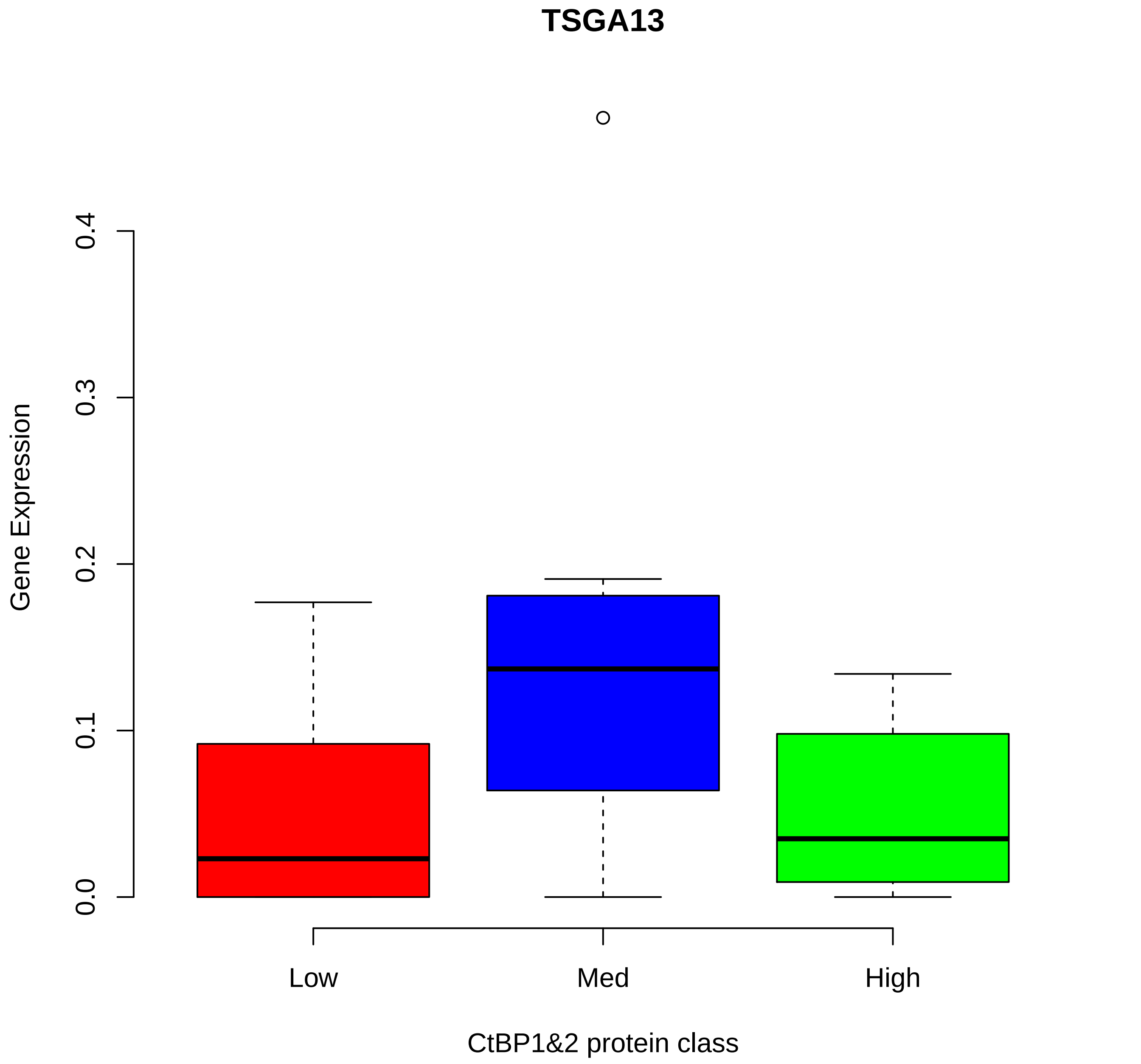
TSGA13
Gene Expression
0.0
0.1
0.2
0.3
0.4
Low
Med
High
CtBP1&2 protein class

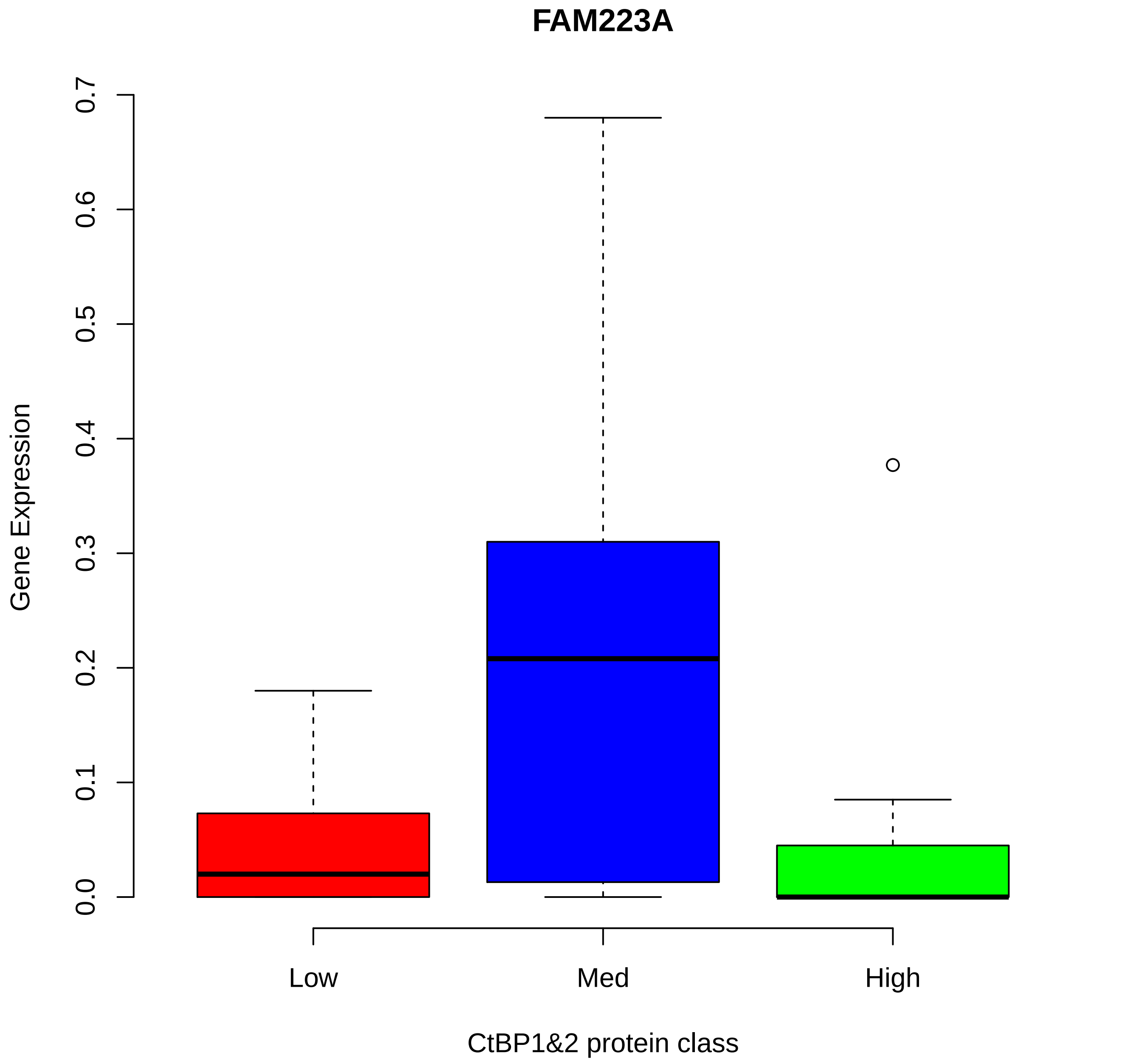

FAM223A
Gene Expression
0.0
0.1
0.2
0.3
0.4
0.5
0.6
0.7
Low
Med
High
CtBP1&2 protein class

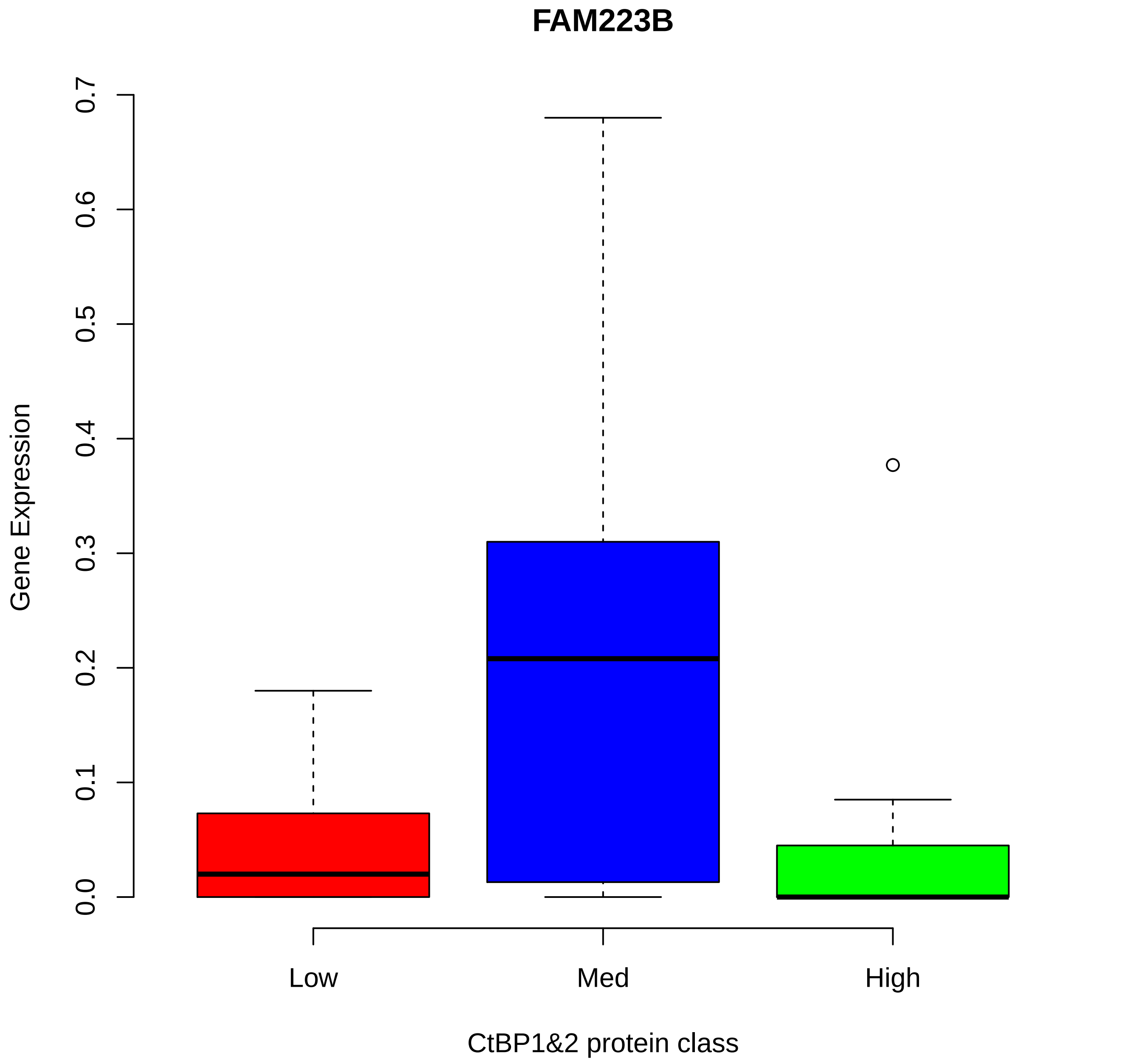

FAM223B
Gene Expression
0.0
0.1
0.2
0.3
0.4
0.5
0.6
0.7
Low
Med
High
CtBP1&2 protein class

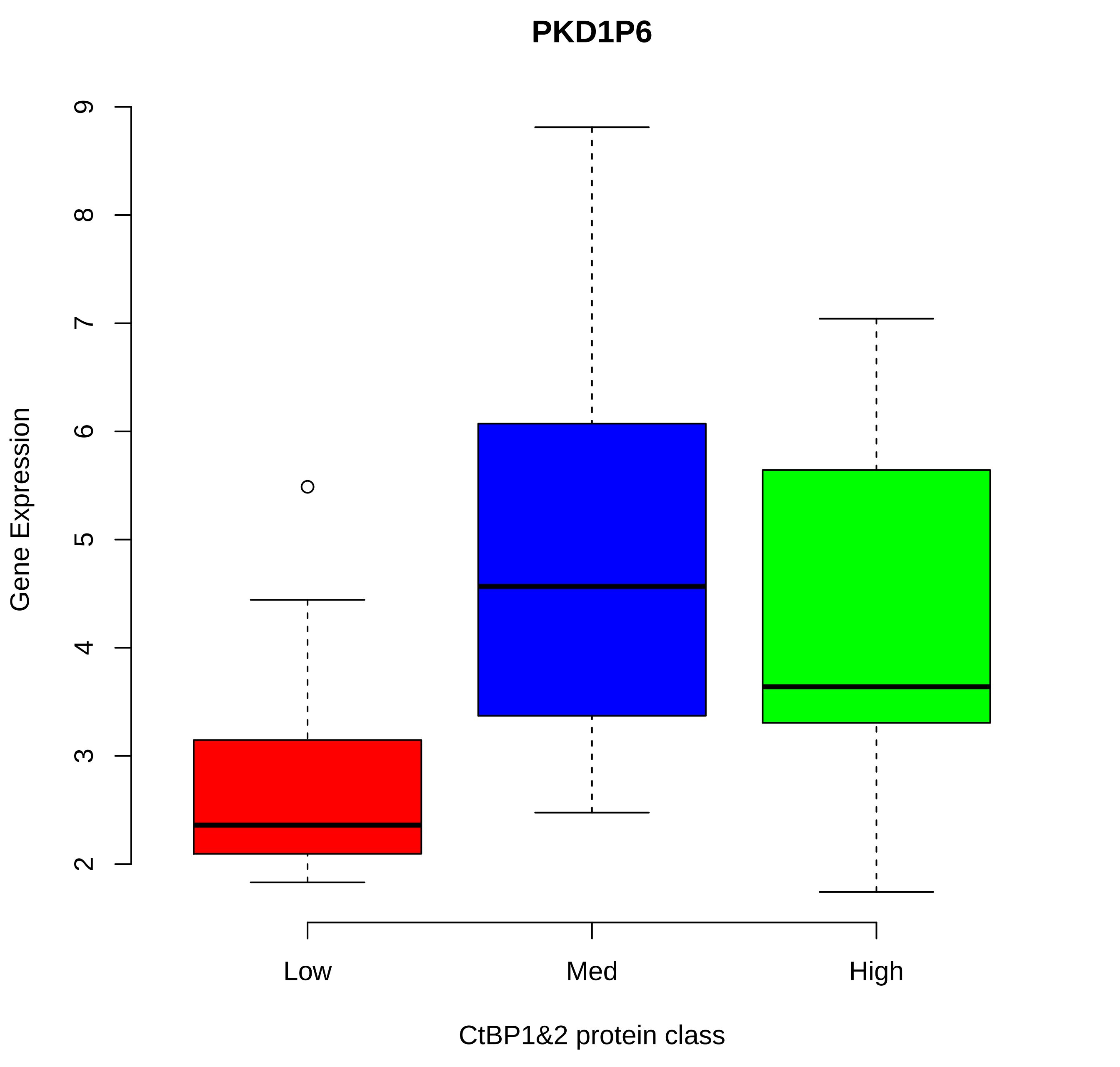

PKD1P6
Gene Expression
2
3
4
5
6
7
8
9
Low
Med
High
CtBP1&2 protein class

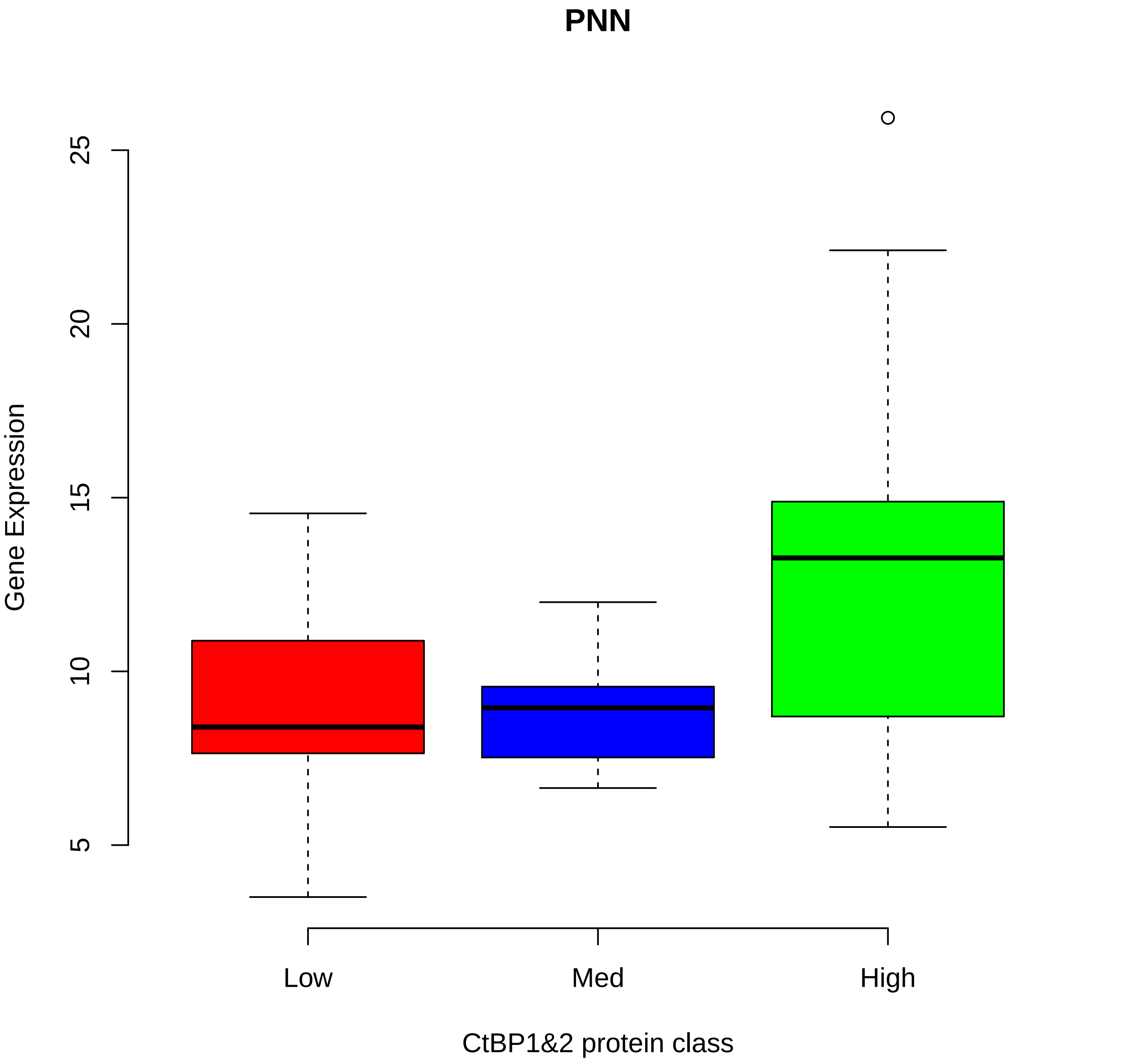
PNN
Gene Expression
25
20
15
10
5
Low
Med
High
CtBP1&2 protein class

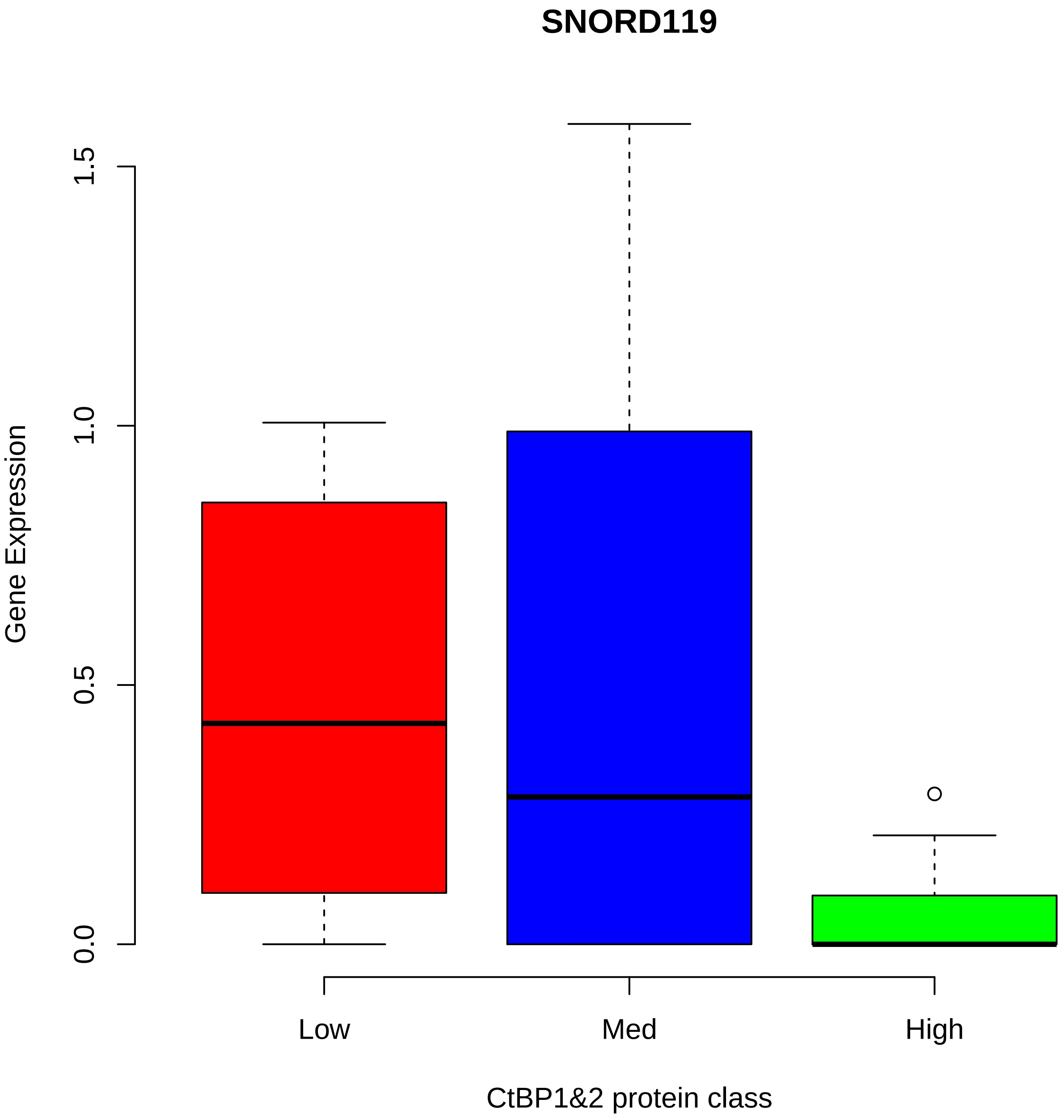

SNORD119
Gene Expression
0.0
0.5
1.0
1.5
Low
Med
High
CtBP1&2 protein class

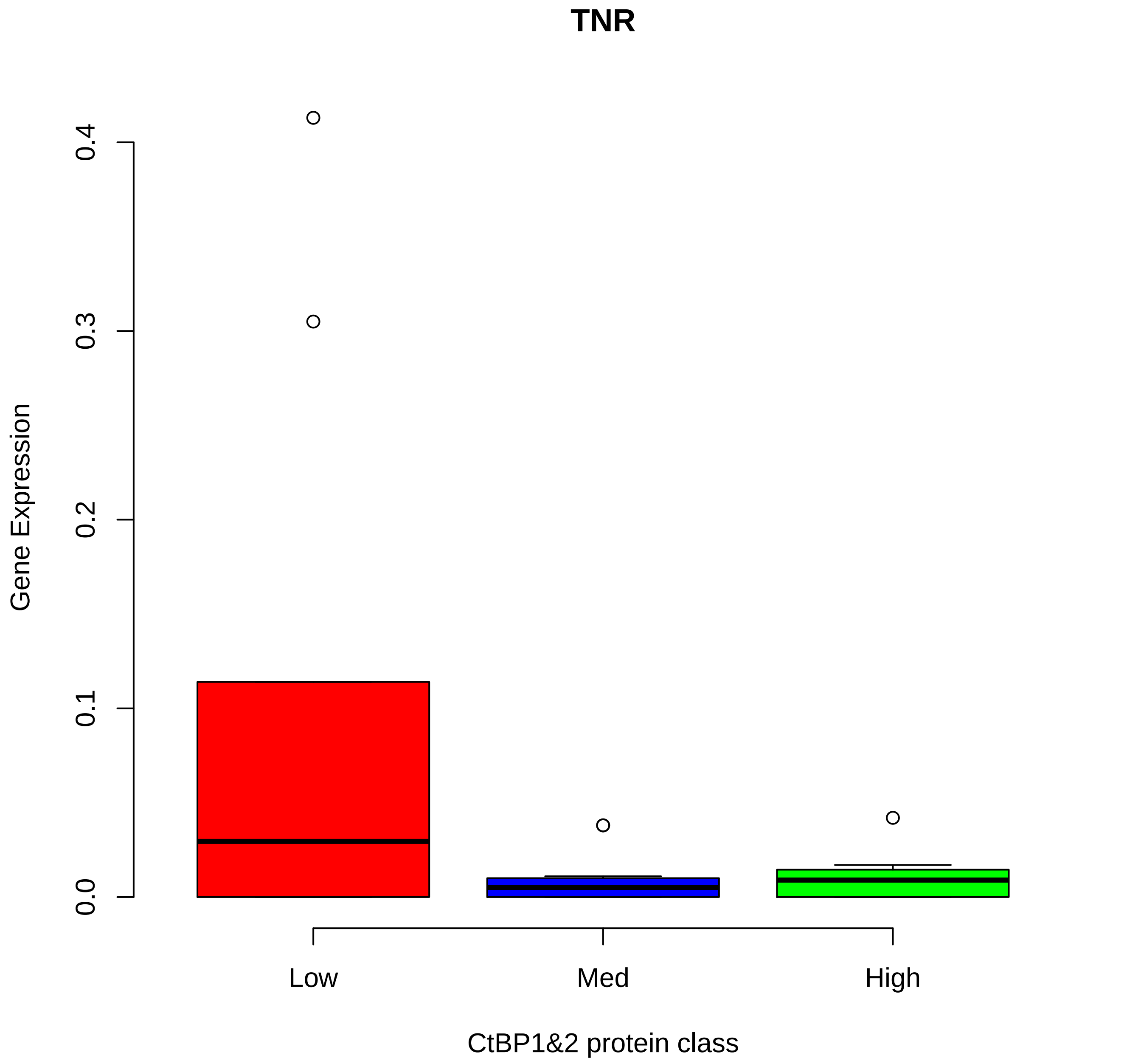
TNR
Gene Expression
0.0
0.1
0.2
0.3
0.4
Low
Med
High
CtBP1&2 protein class

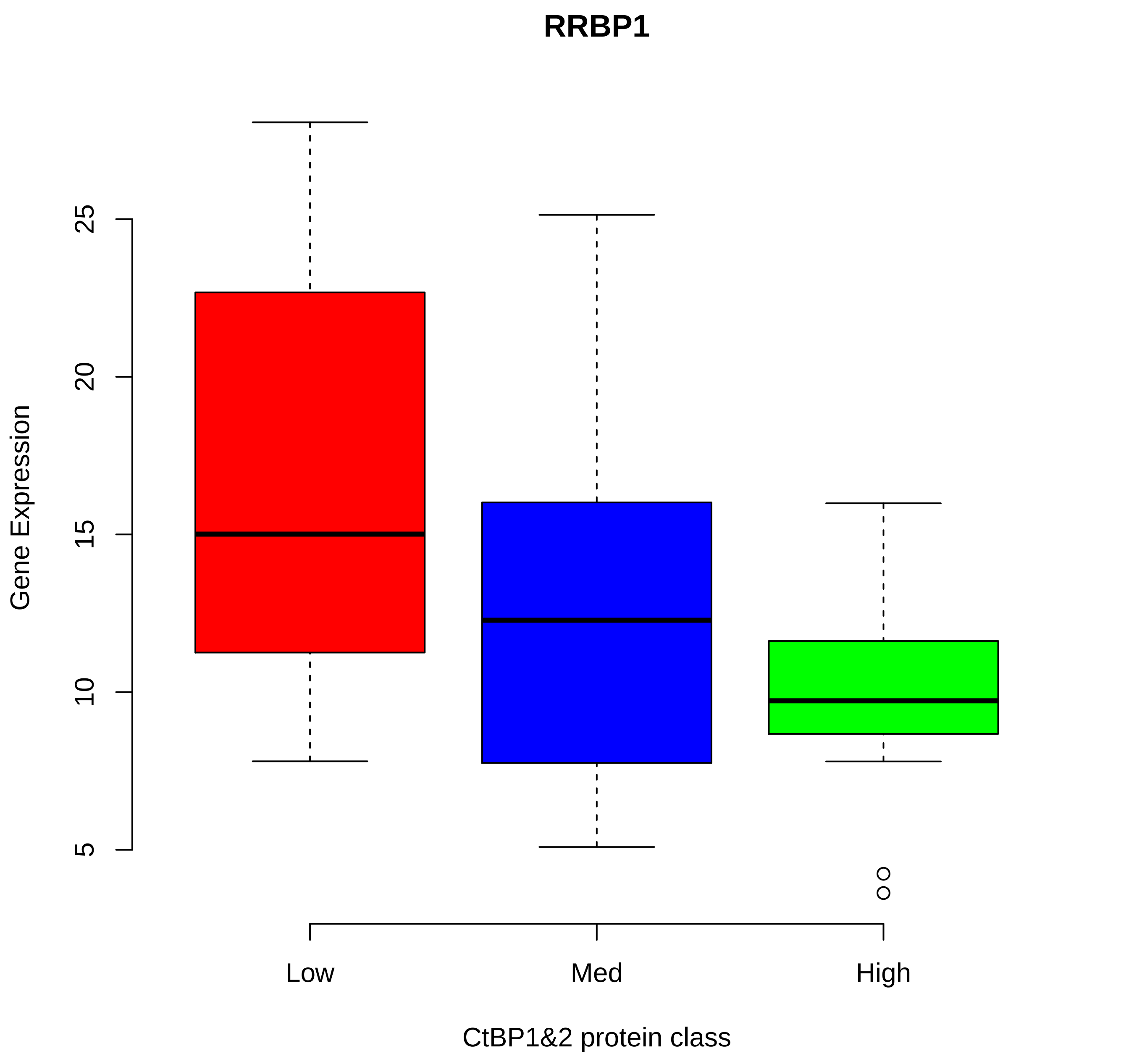
RRBP1
Gene Expression
25
20
15
10
5
Low
Med
High
CtBP1&2 protein class

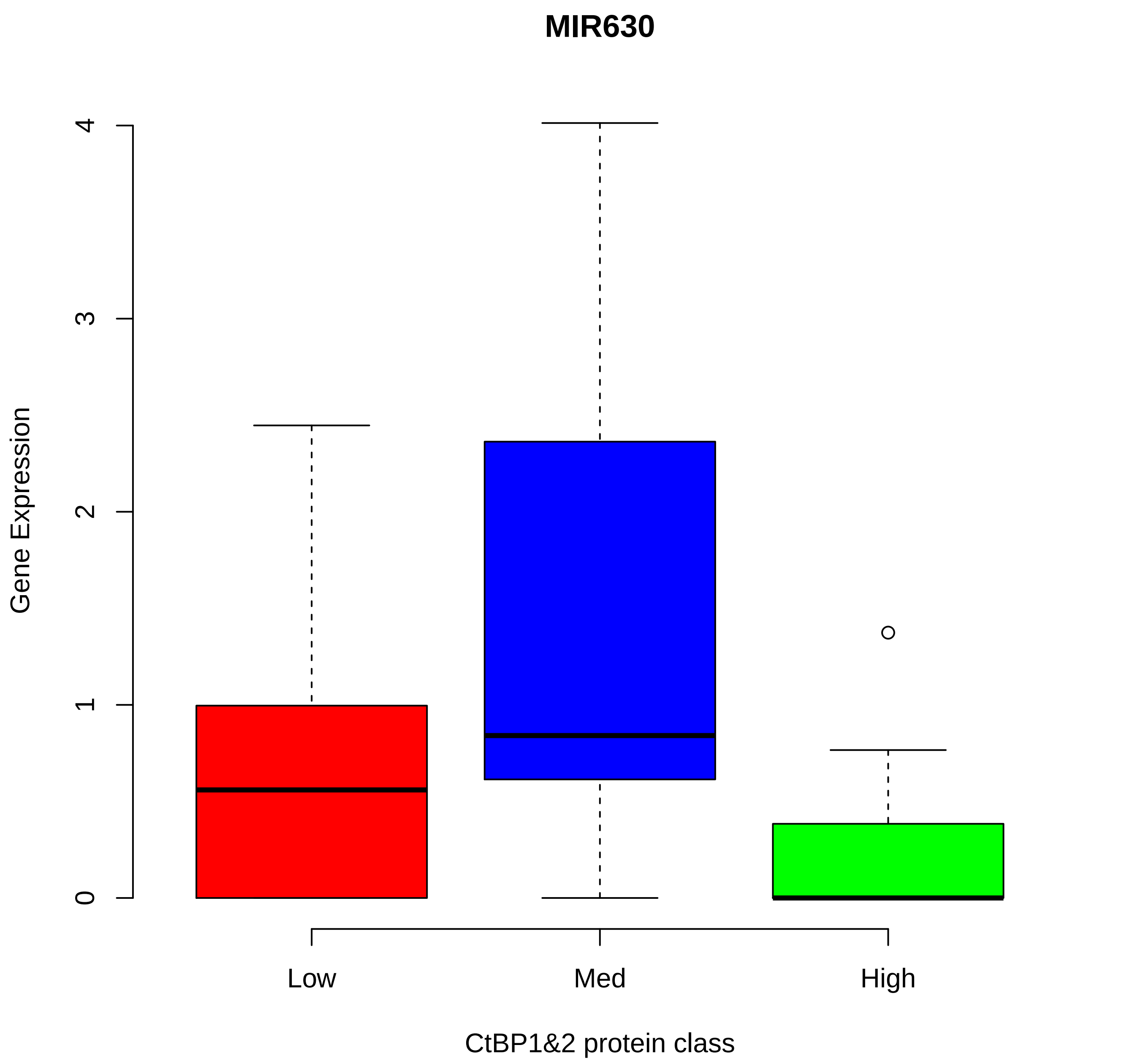

MIR630
Gene Expression
0
1
2
3
4
Low
Med
High
CtBP1&2 protein class

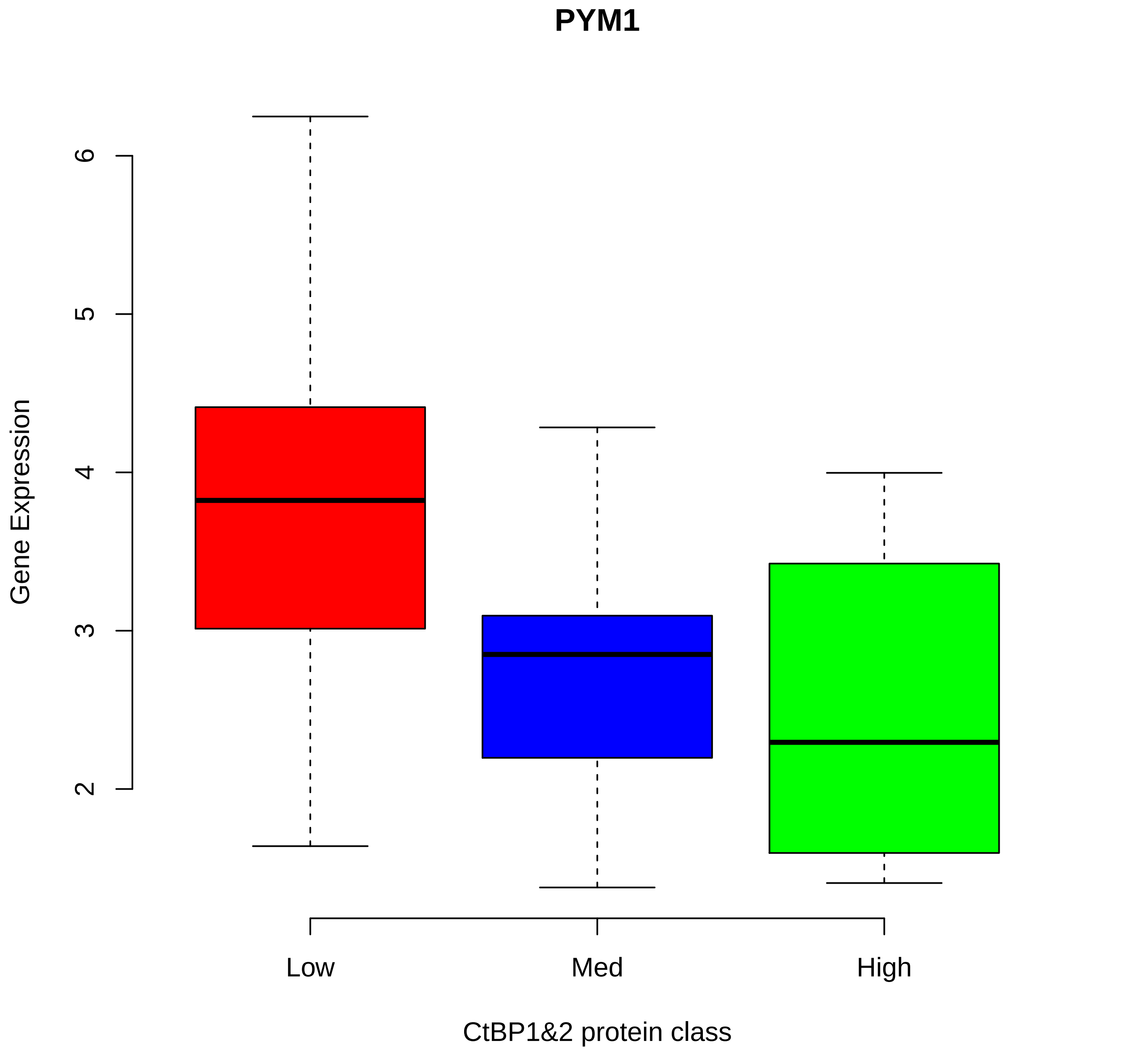

PYM1
Gene Expression
6
5
4
3
2
Low
Med
High
CtBP1&2 protein class

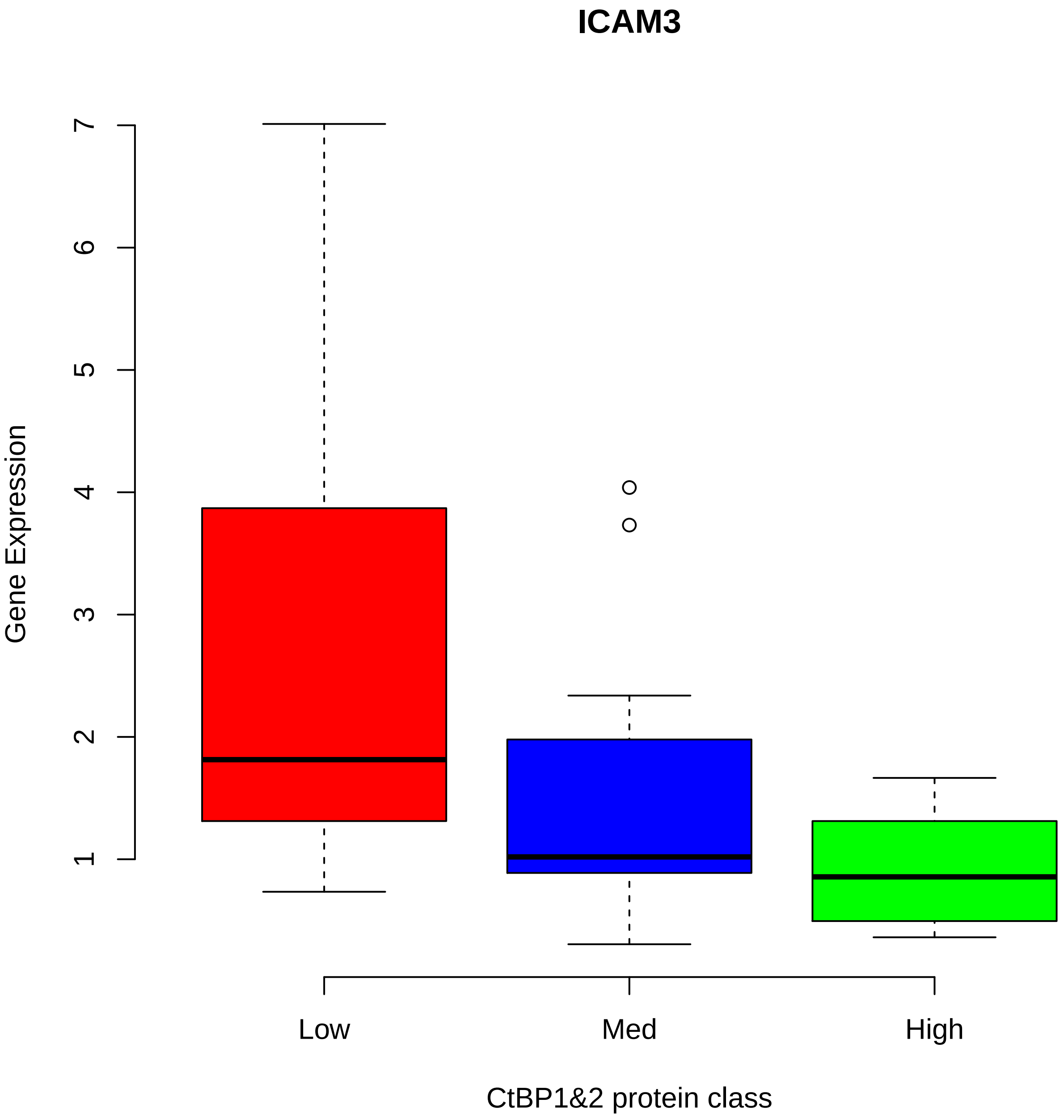
ICAM3
Gene Expression
1
2
3
4
5
6
7
Low
Med
High
CtBP1&2 protein class

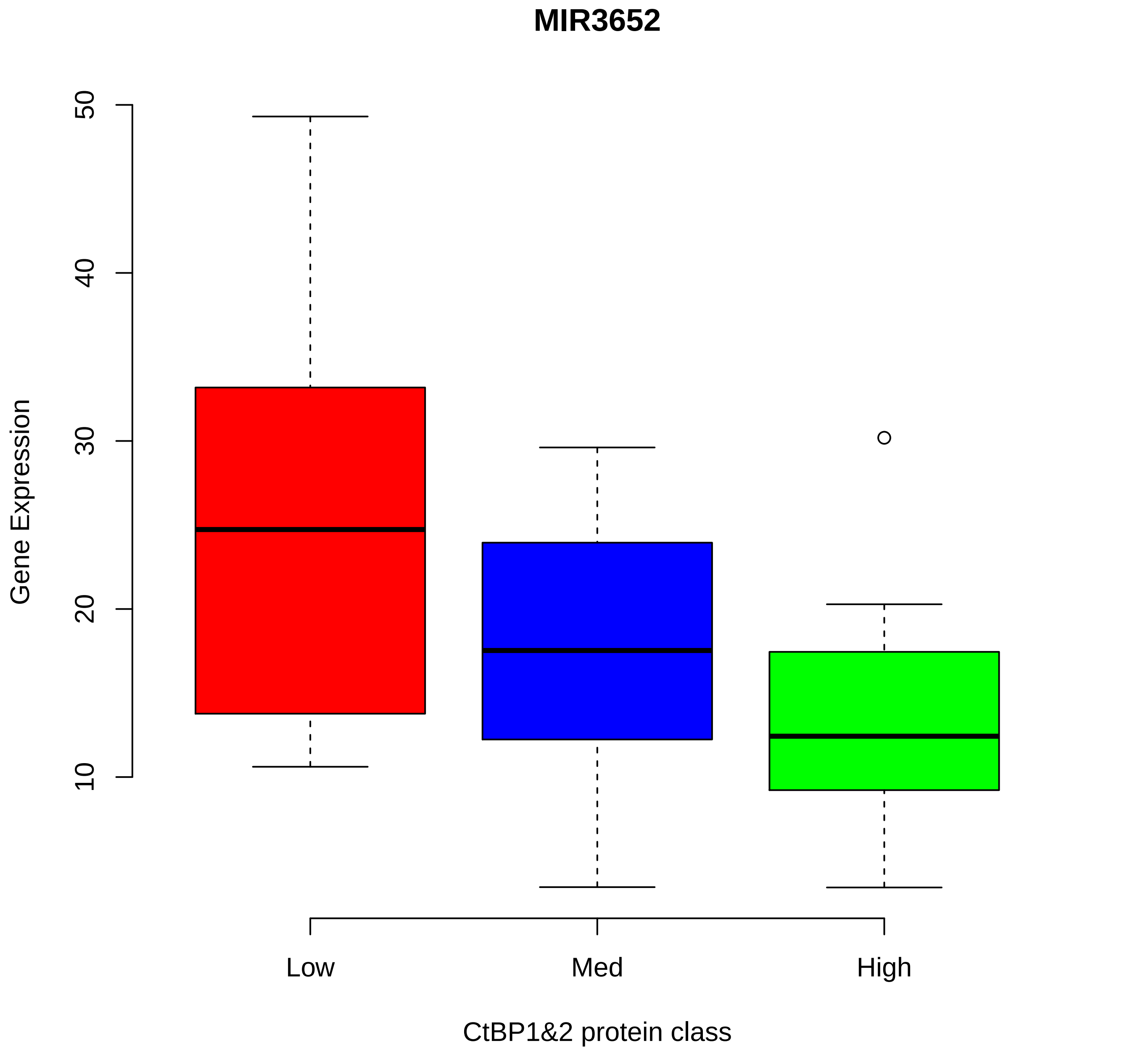

MIR3652
Gene Expression
10
20
30
40
50
Low
Med
High
CtBP1&2 protein class

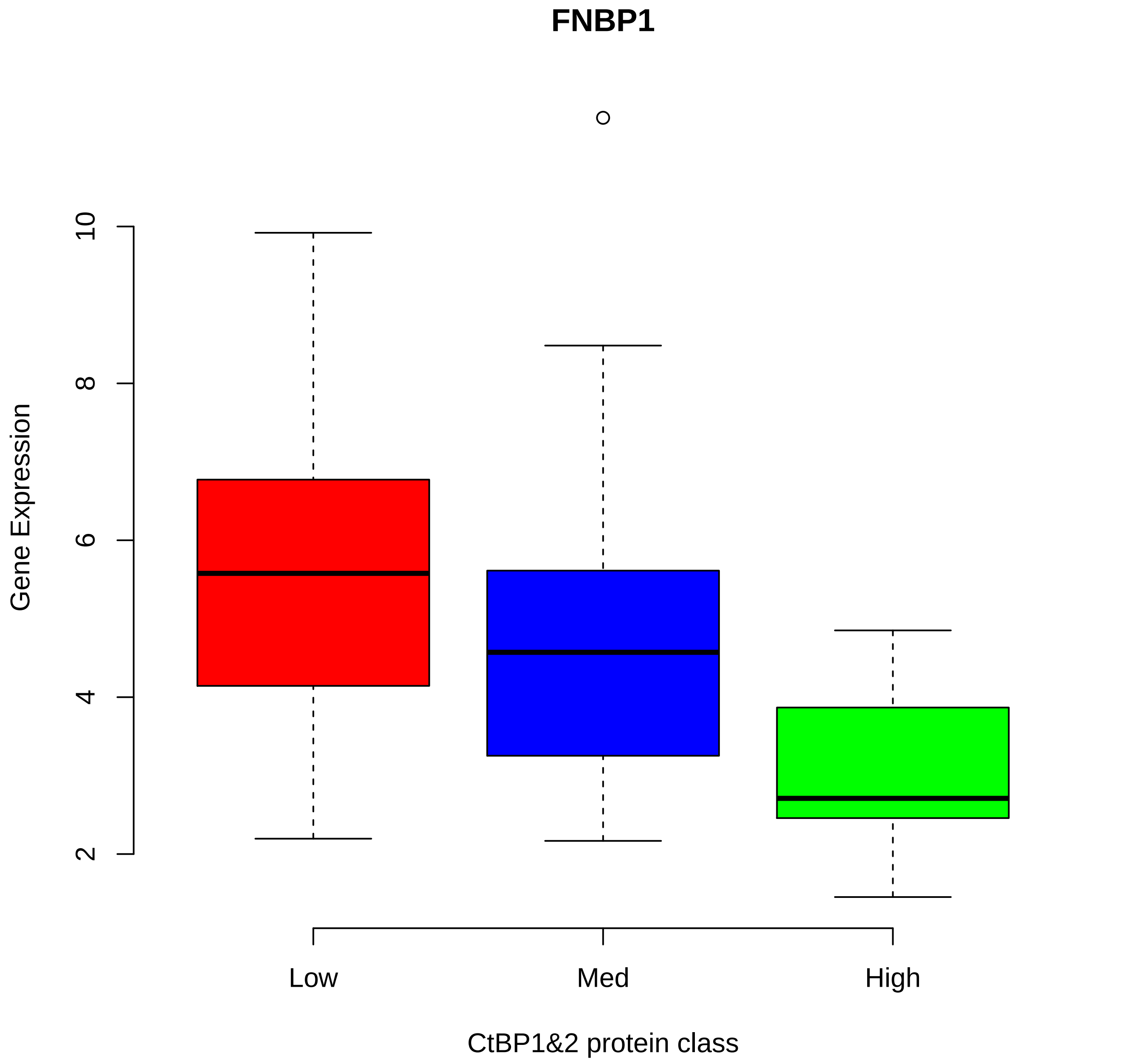
FNBP1
Gene Expression
2
4
6
8
10
Low
Med
High
CtBP1&2 protein class

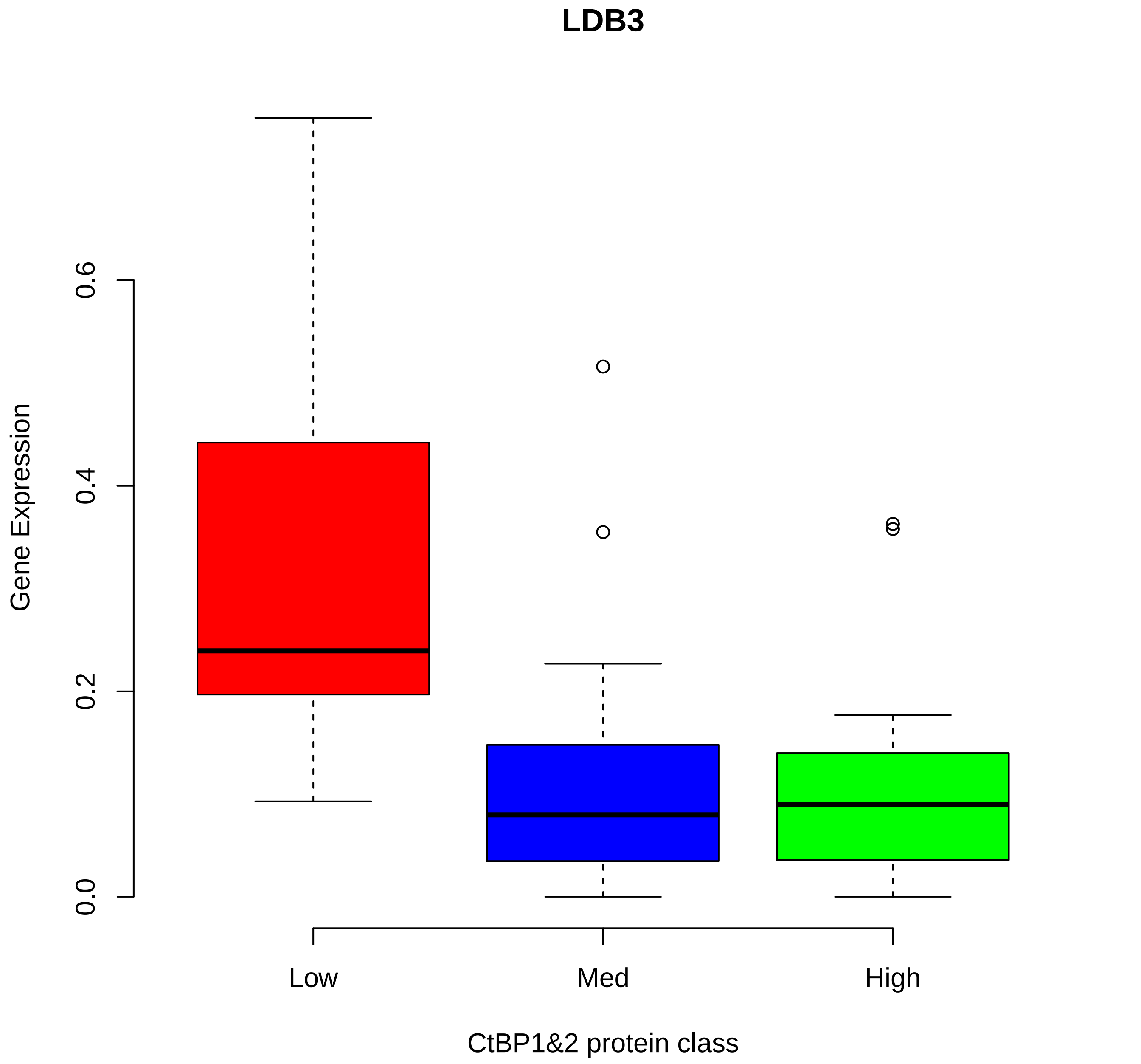
LDB3
Gene Expression
0.0
0.2
0.4
0.6
Low
Med
High
CtBP1&2 protein class

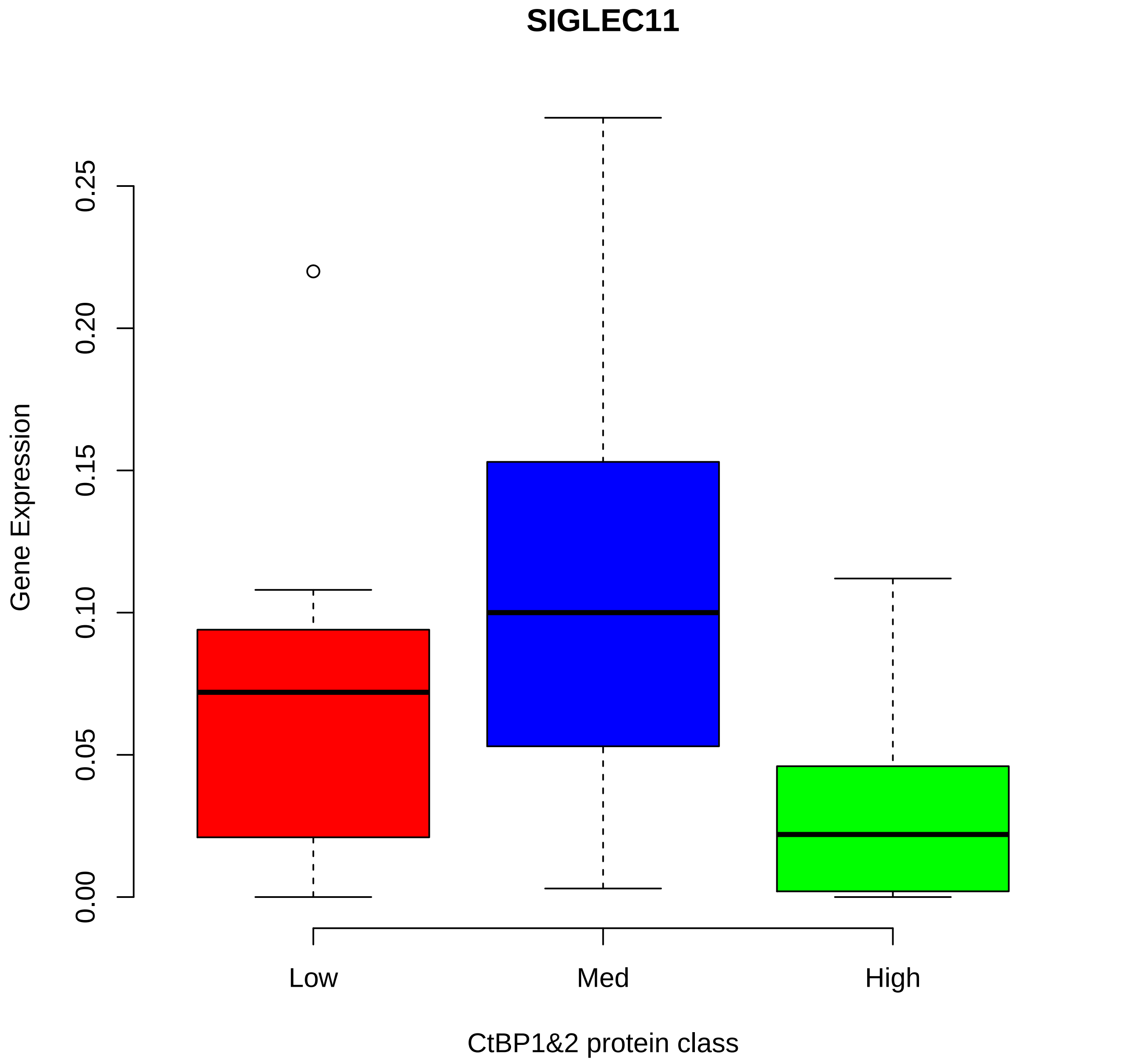

SIGLEC11
Gene Expression
0.00
0.05
0.10
0.15
0.20
0.25
Low
Med
High
CtBP1&2 protein class

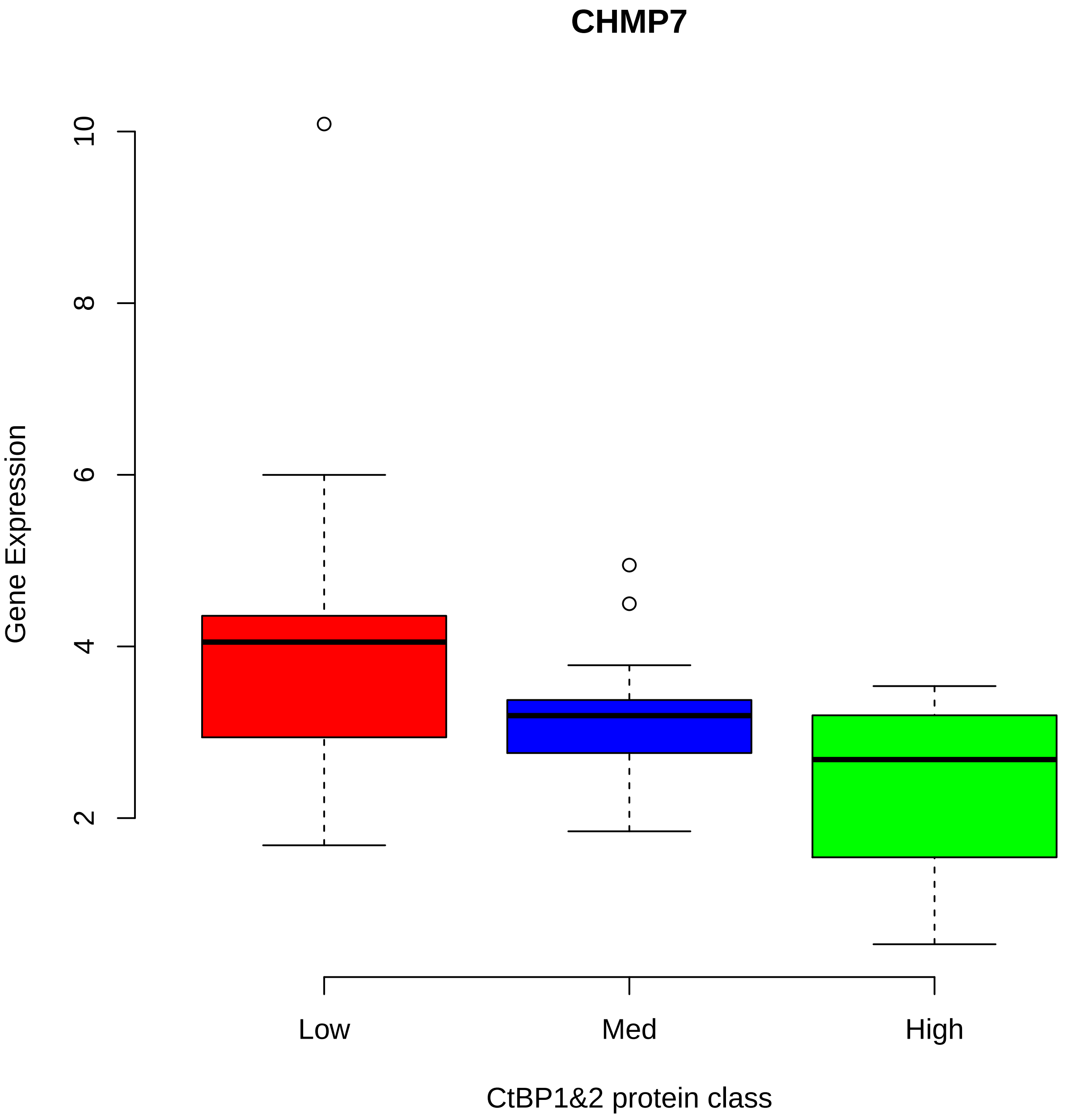

CHMP7
Gene Expression
2
4
6
8
10
Low
Med
High
CtBP1&2 protein class

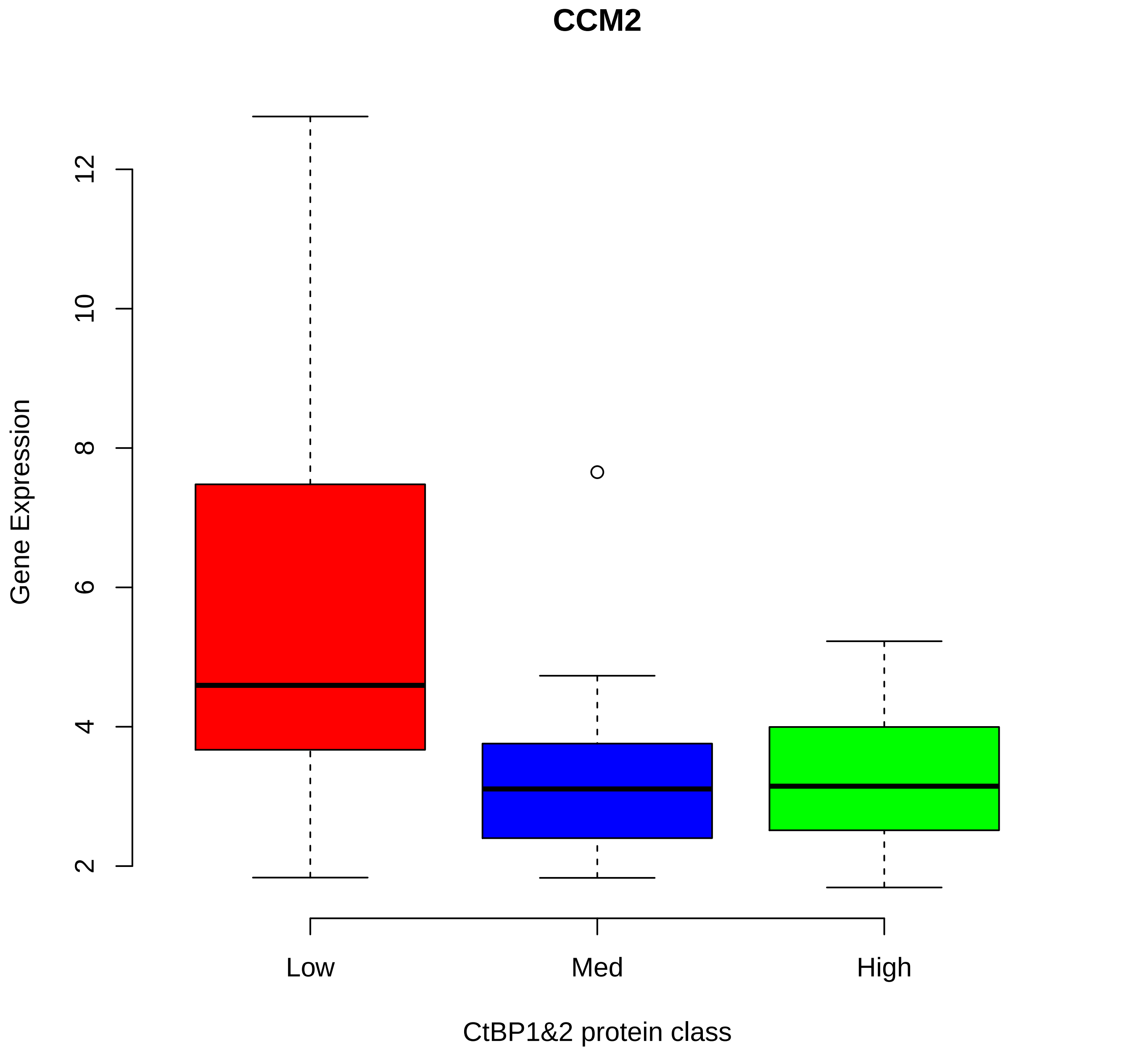
CCM2
Gene Expression
2
4
6
8
10
12
Low
Med
High
CtBP1&2 protein class

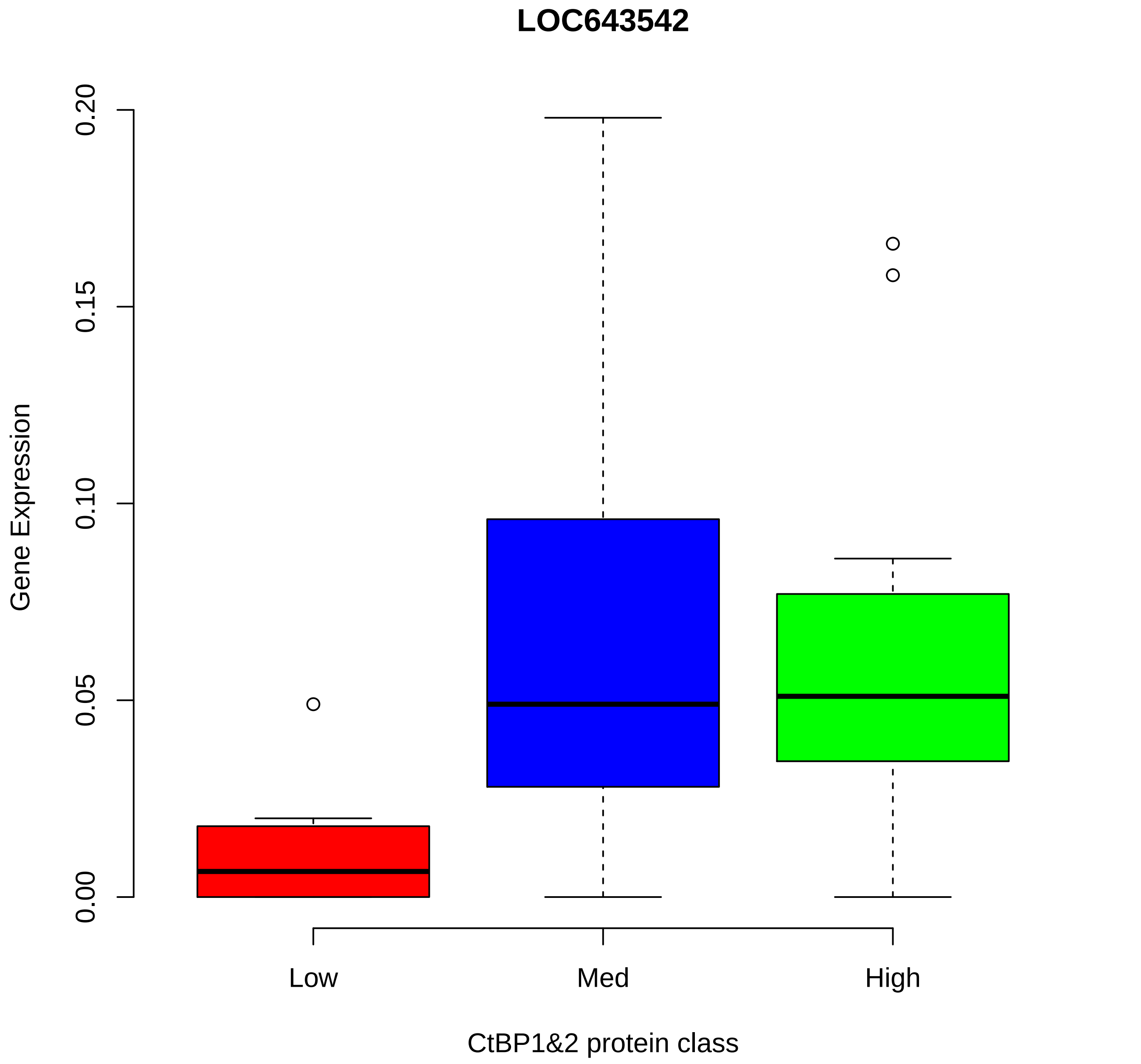
LOC643542
Gene Expression
0.00
0.05
0.10
0.15
0.20
Low
Med
High
CtBP1&2 protein class

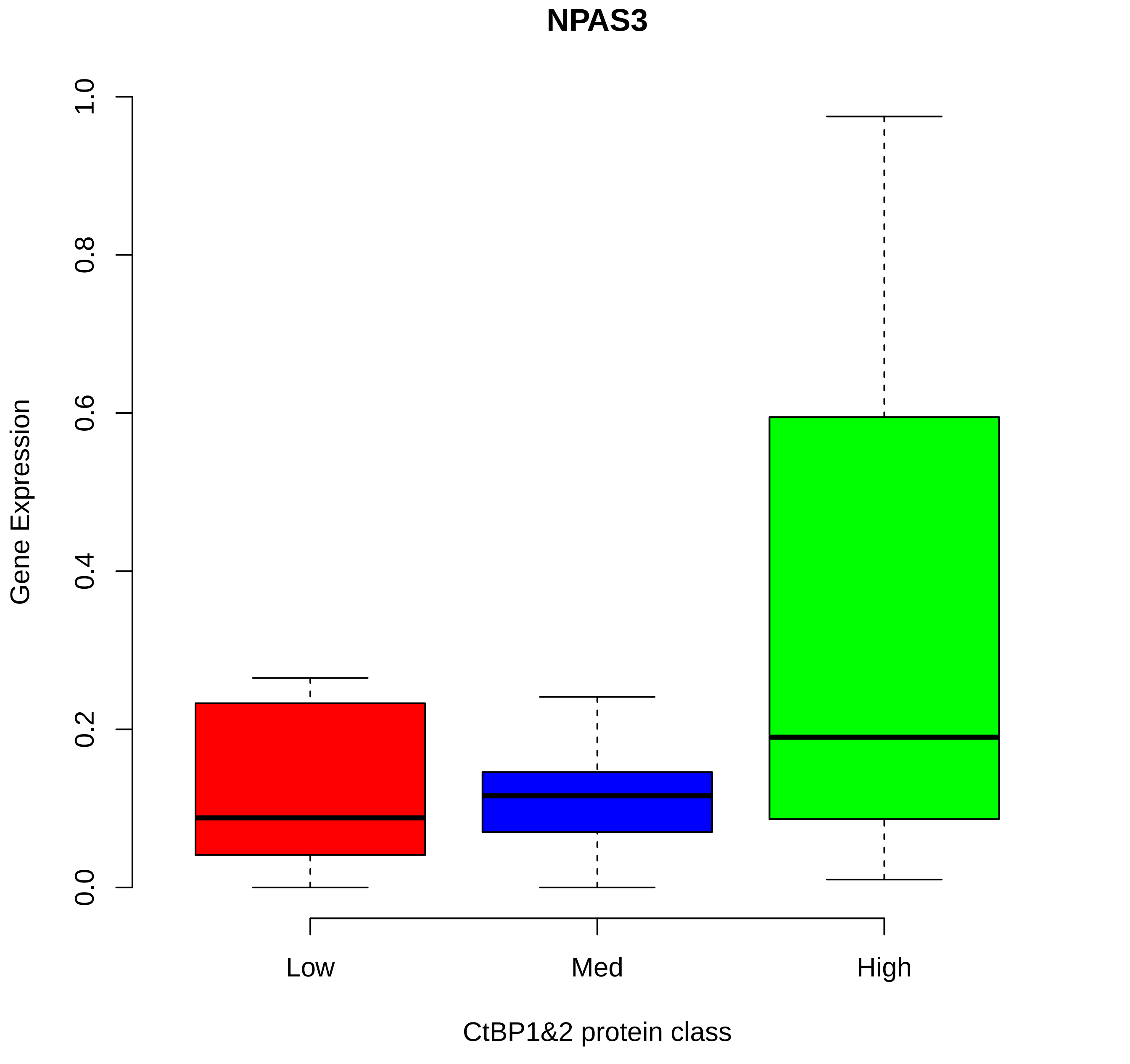

NPAS3
Gene Expression
0.0
0.2
0.4
0.6
0.8
1.0
Low
Med
High
CtBP1&2 protein class

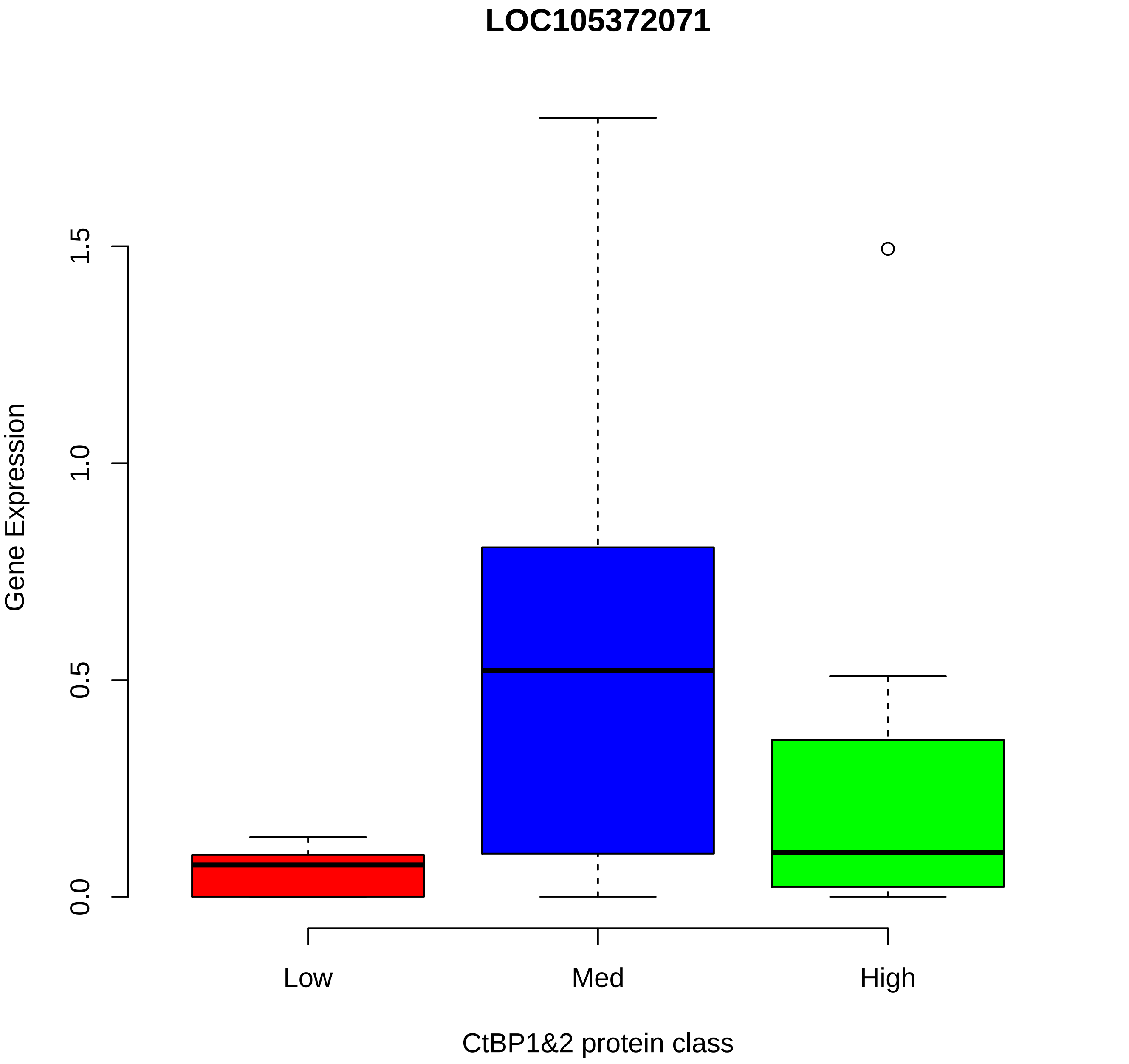
LOC105372071
Gene Expression
0.0
0.5
1.0
1.5
Low
Med
High
CtBP1&2 protein class

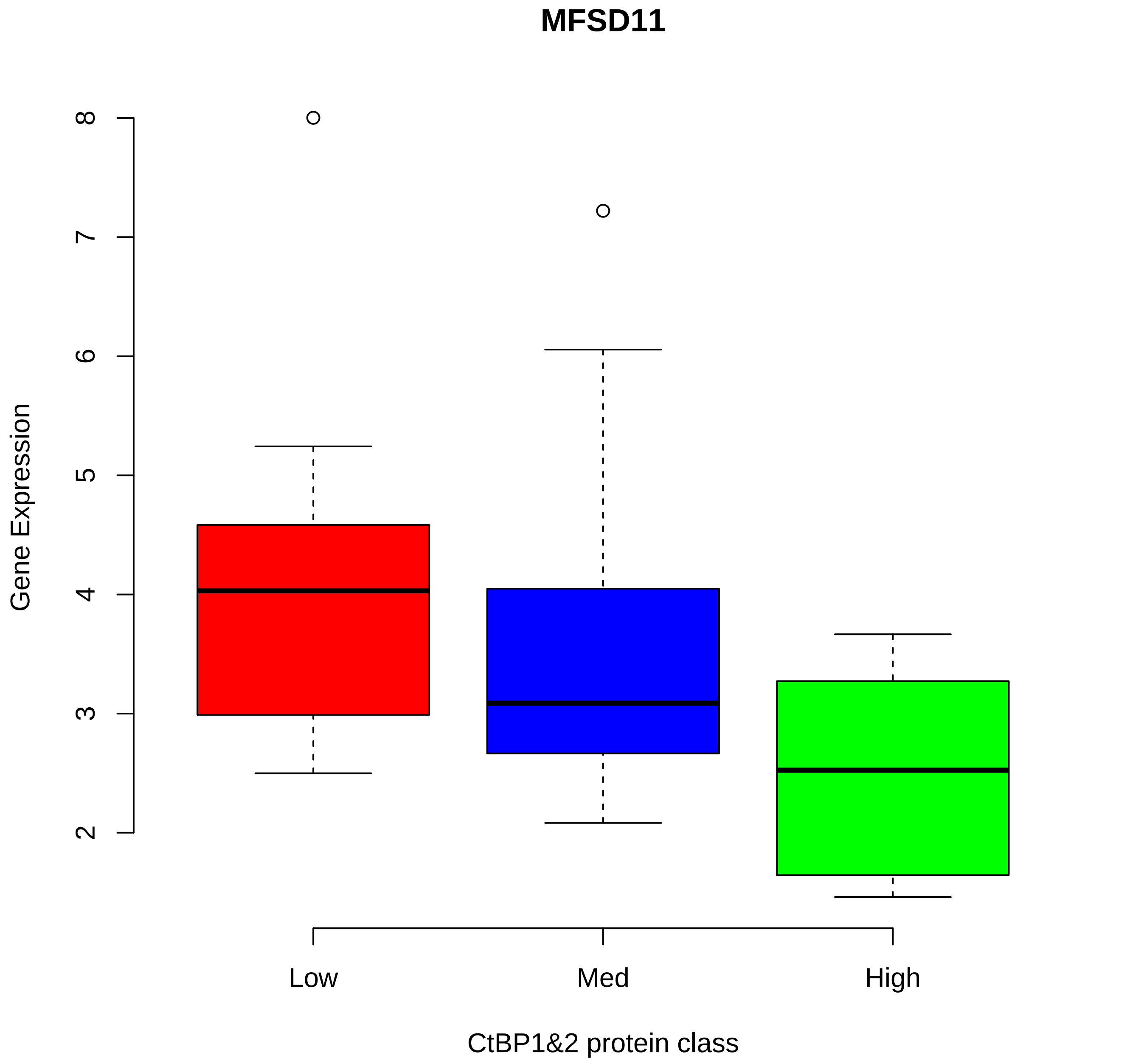
MFSD11
Gene Expression
2
3
4
5
6
7
8
Low
Med
High
CtBP1&2 protein class

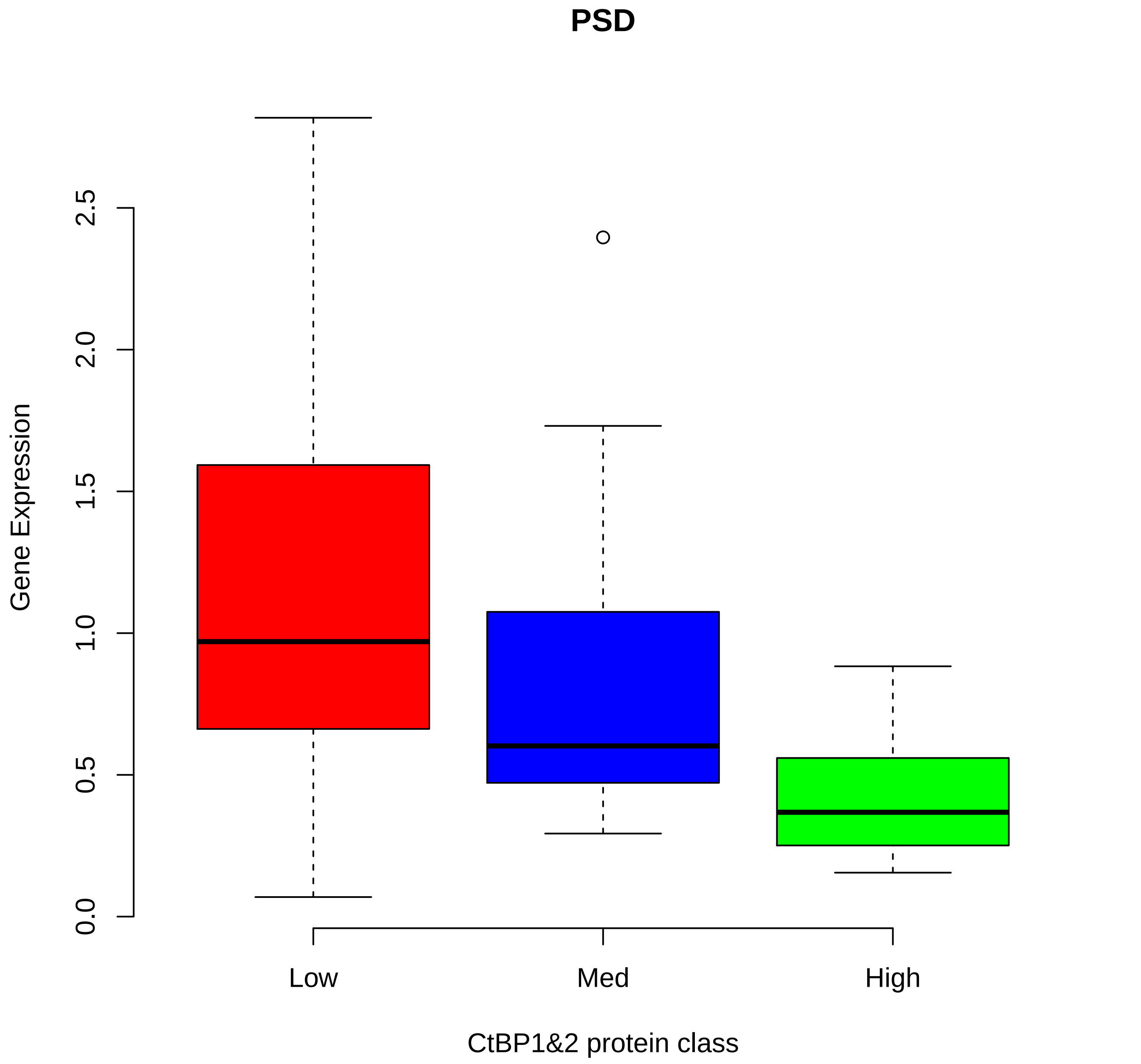
PSD
Gene Expression
0.0
0.5
1.0
1.5
2.0
2.5
Low
Med
High
CtBP1&2 protein class

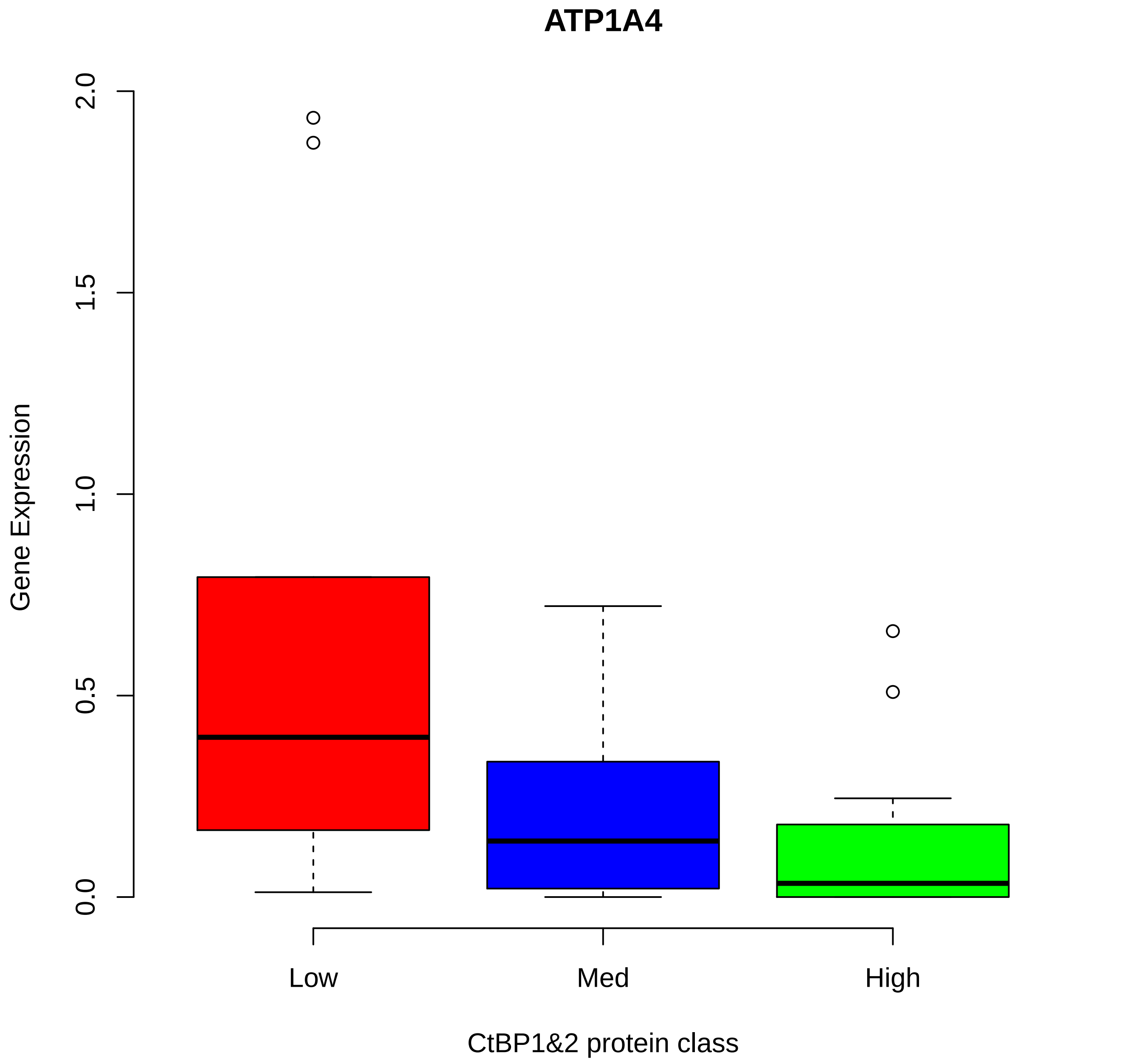

ATP1A4
Gene Expression
2.0
1.5
1.0
0.5
0.0
Low
Med
High
CtBP1&2 protein class

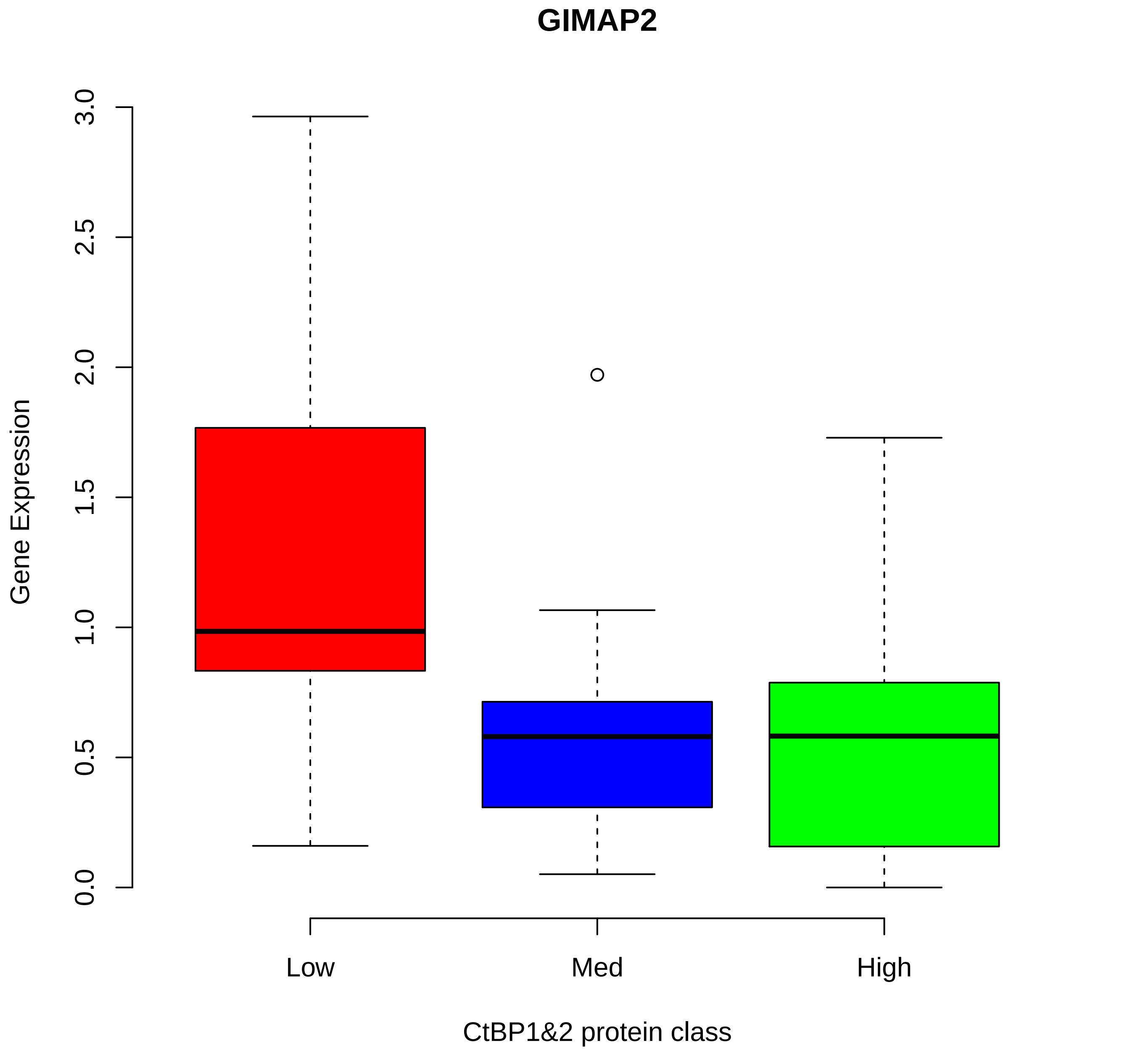
GIMAP2
Gene Expression
0.0
0.5
1.0
1.5
2.0
2.5
3.0
Low
Med
High
CtBP1&2 protein class

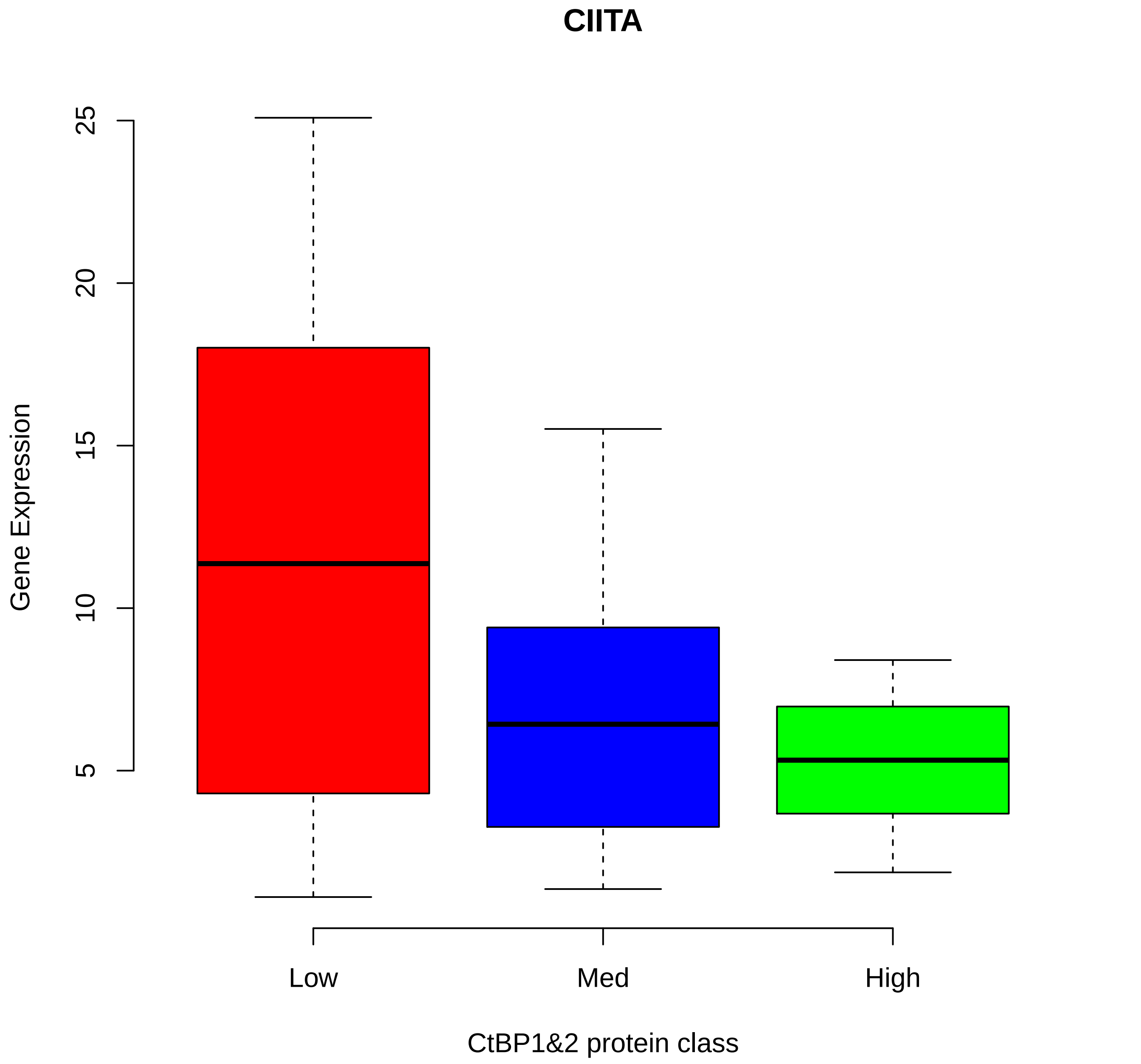

CIITA
Gene Expression
25
20
15
10
5
Low
Med
High
CtBP1&2 protein class

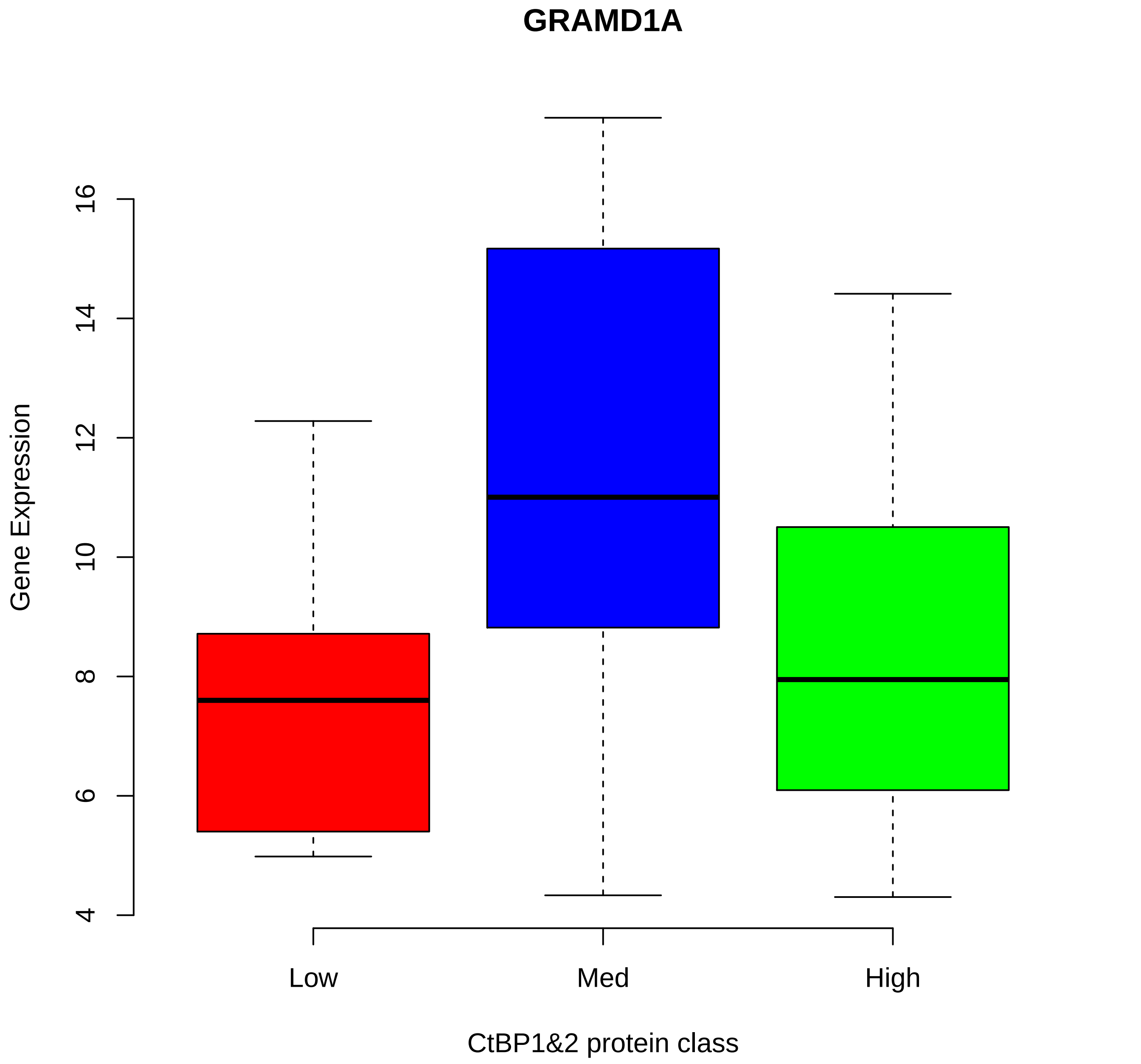
GRAMD1A
Gene Expression
4
6
8
10
12
14
16
Low
Med
High
CtBP1&2 protein class

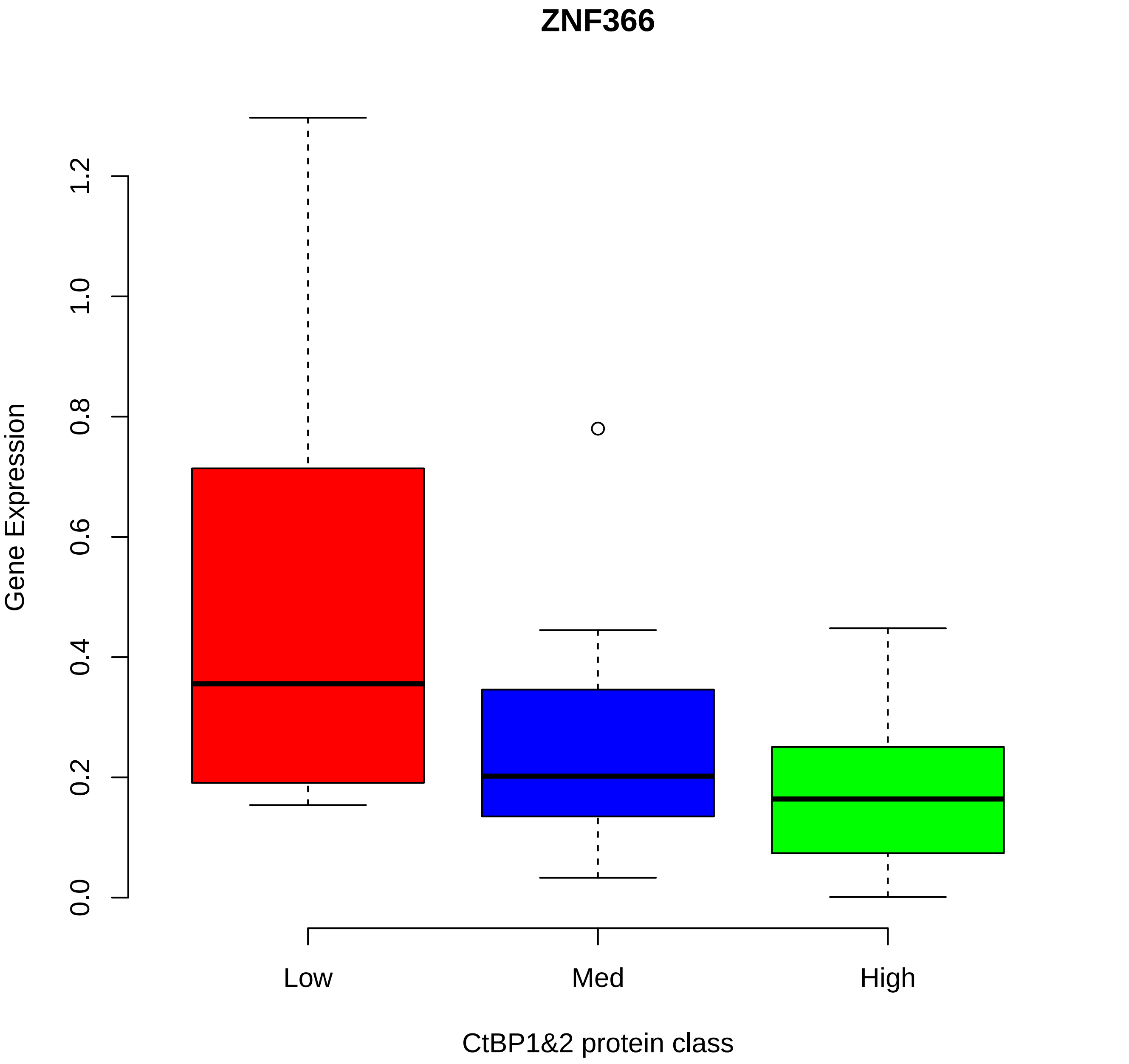
ZNF366
Gene Expression
0.0
0.2
0.4
0.6
0.8
1.0
1.2
Low
Med
High
CtBP1&2 protein class

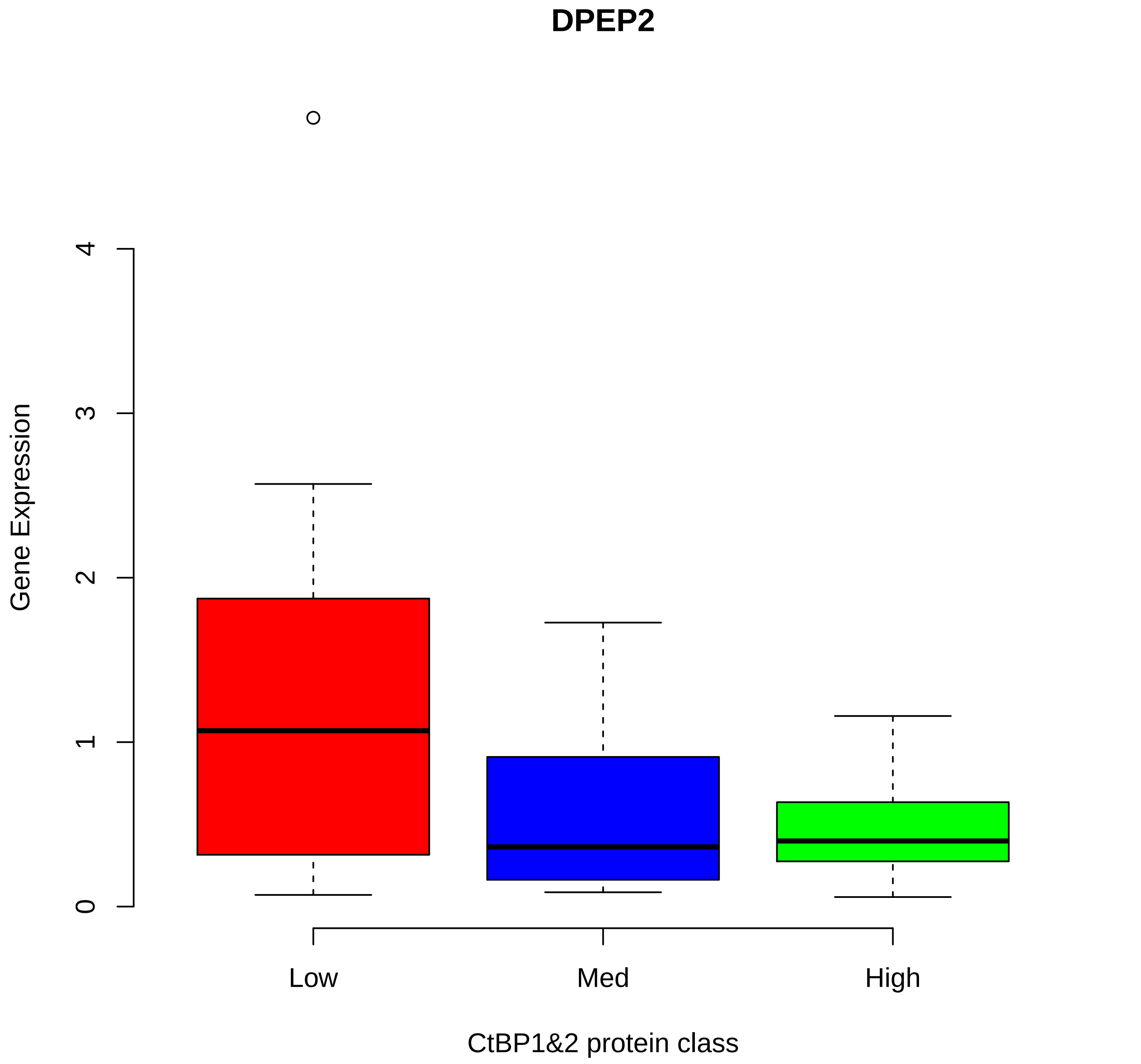

DPEP2
Gene Expression
0
1
2
3
4
Low
Med
High
CtBP1&2 protein class

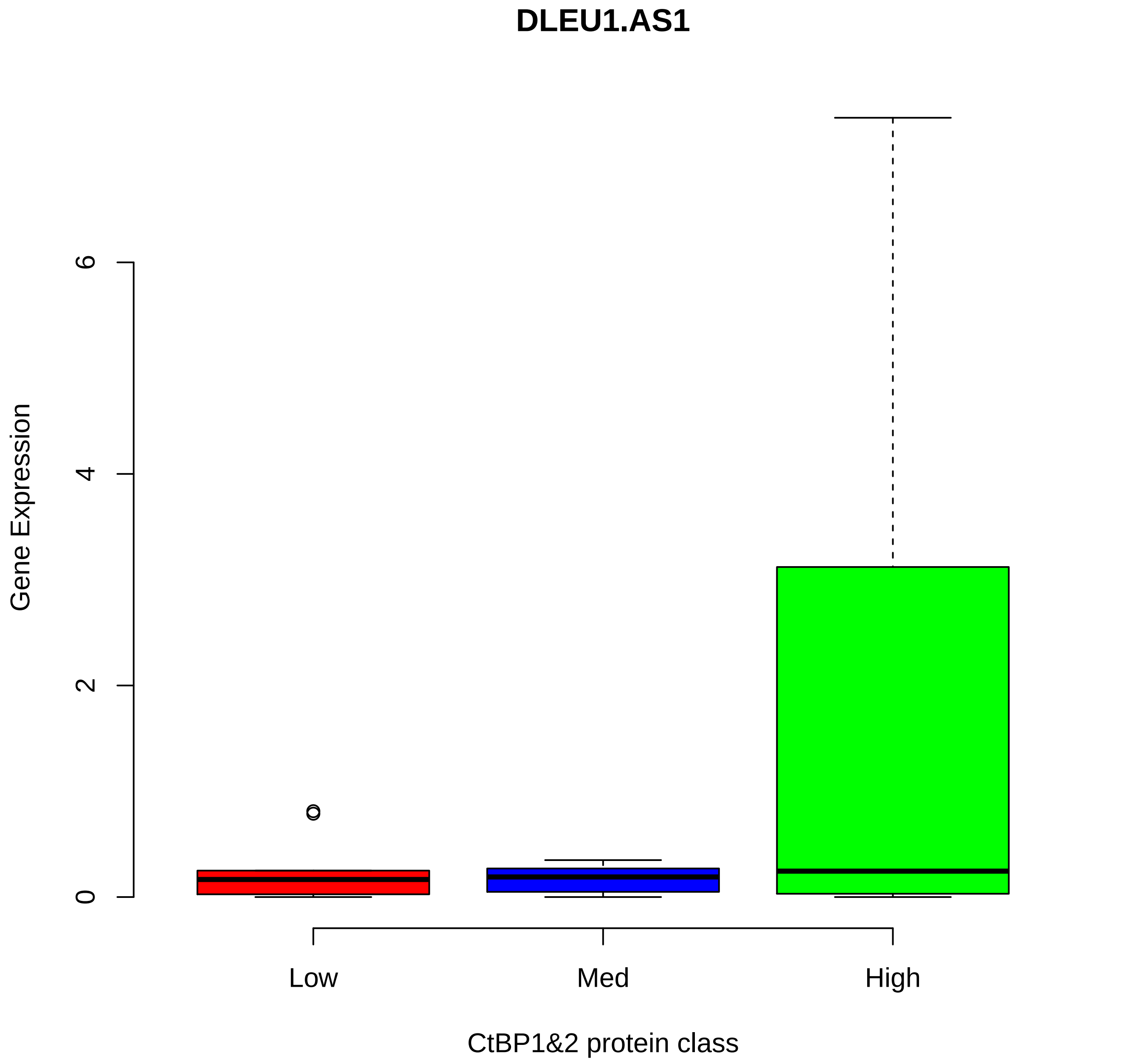

DLEU1.AS1
Gene Expression
0
2
4
6
Low
Med
High
CtBP1&2 protein class

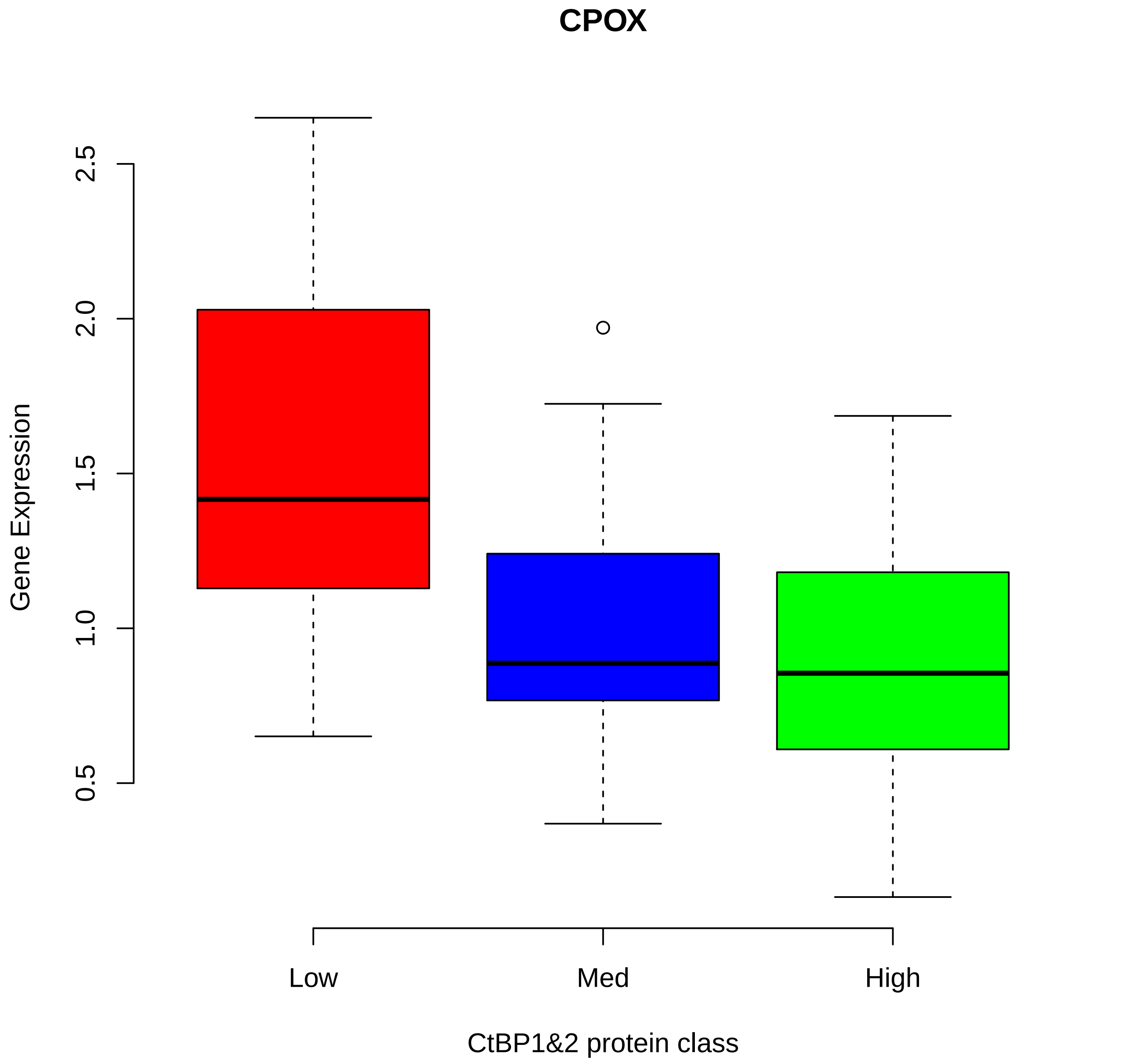

CPOX
Gene Expression
0.5
1.0
1.5
2.0
2.5
Low
Med
High
CtBP1&2 protein class

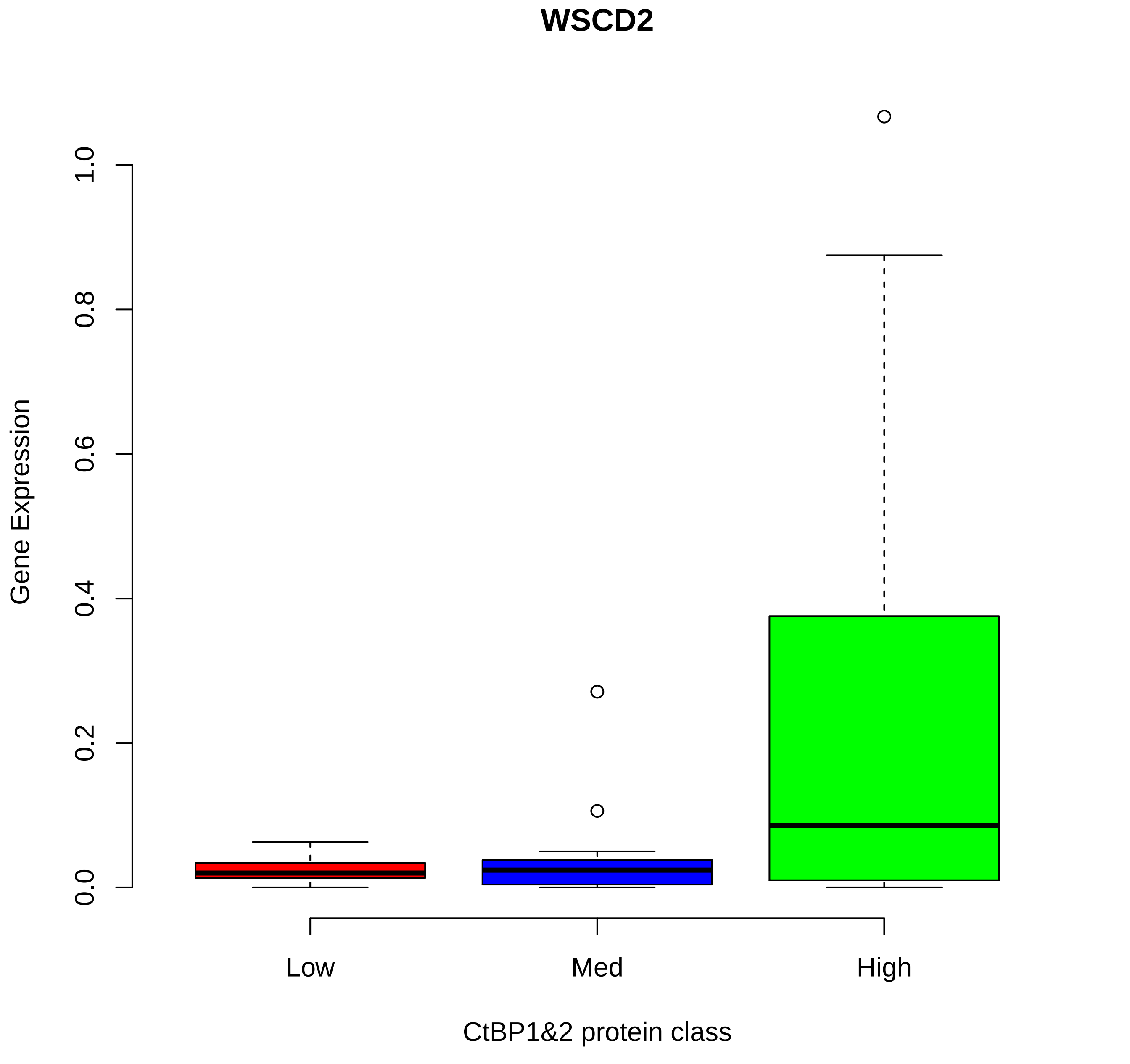
WSCD2
Gene Expression
0.0
0.2
0.4
0.6
0.8
1.0
Low
Med
High
CtBP1&2 protein class

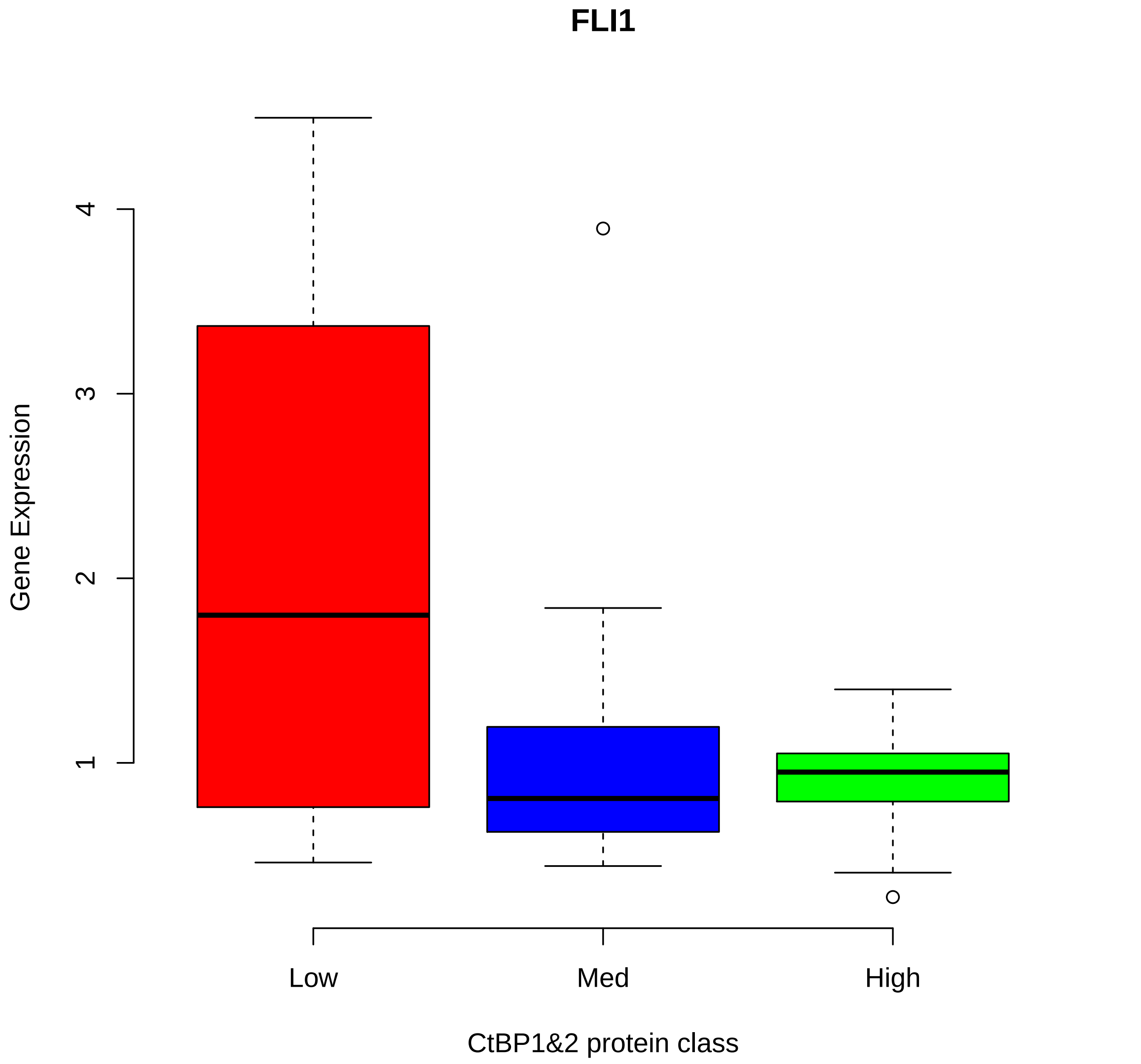

FLI1
Gene Expression
1
2
3
4
Low
Med
High
CtBP1&2 protein class

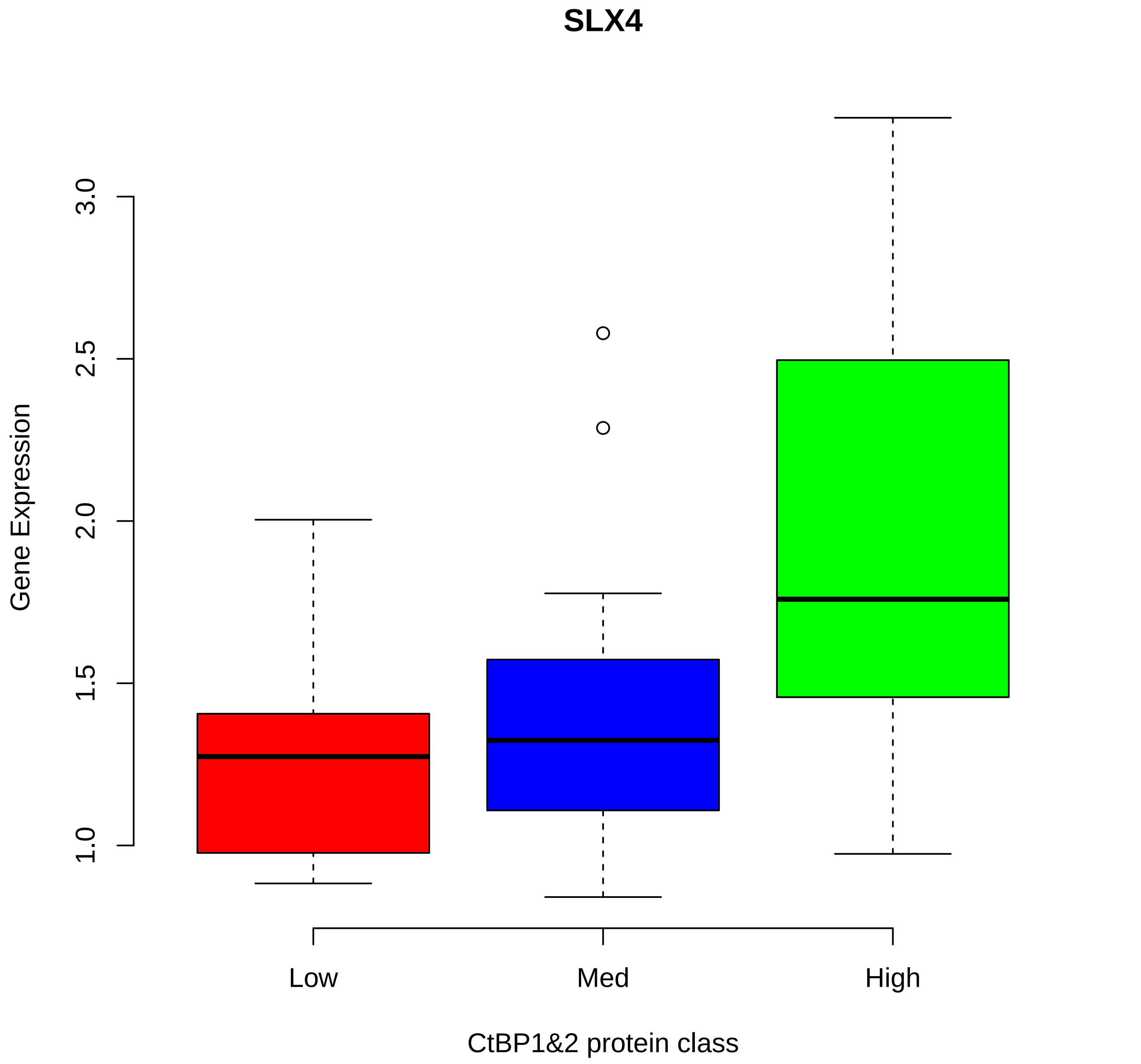

SLX4
Gene Expression
3.0
2.5
2.0
1.5
1.0
Low
Med
High
CtBP1&2 protein class

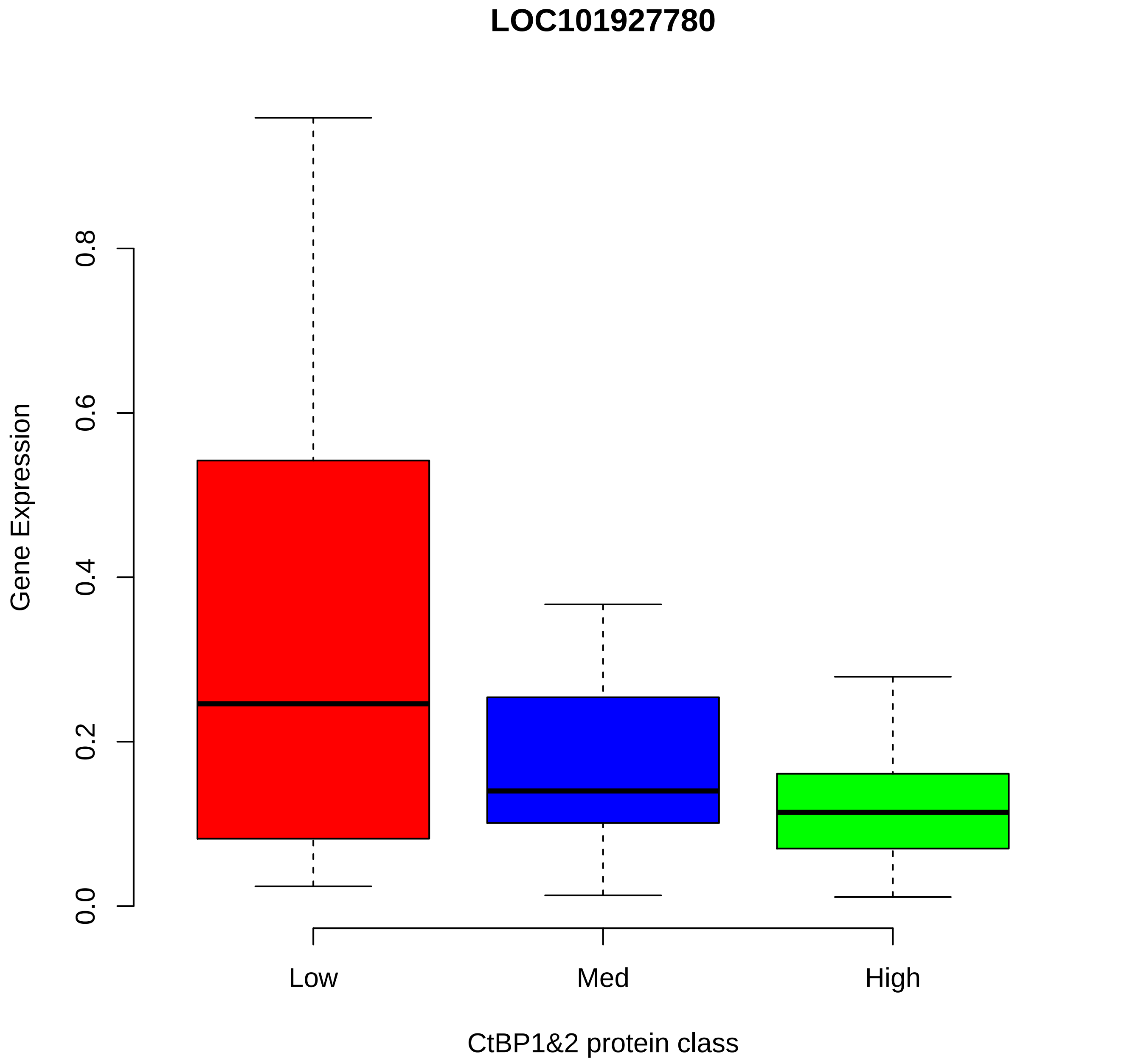
LOC101927780
Gene Expression
0.0
0.2
0.4
0.6
0.8
Low
Med
High
CtBP1&2 protein class

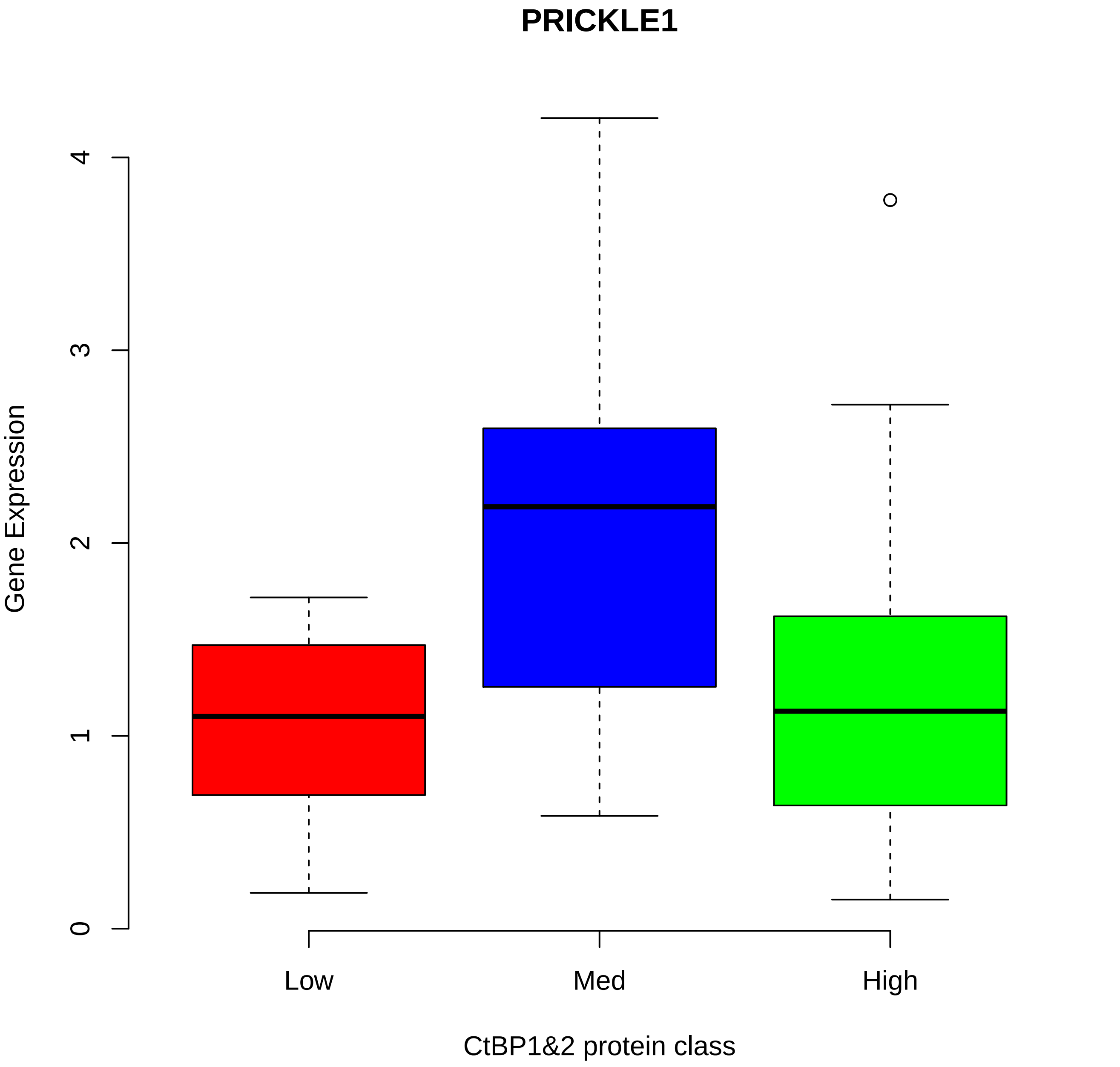
PRICKLE1
Gene Expression
0
1
2
3
4
Low
Med
High
CtBP1&2 protein class

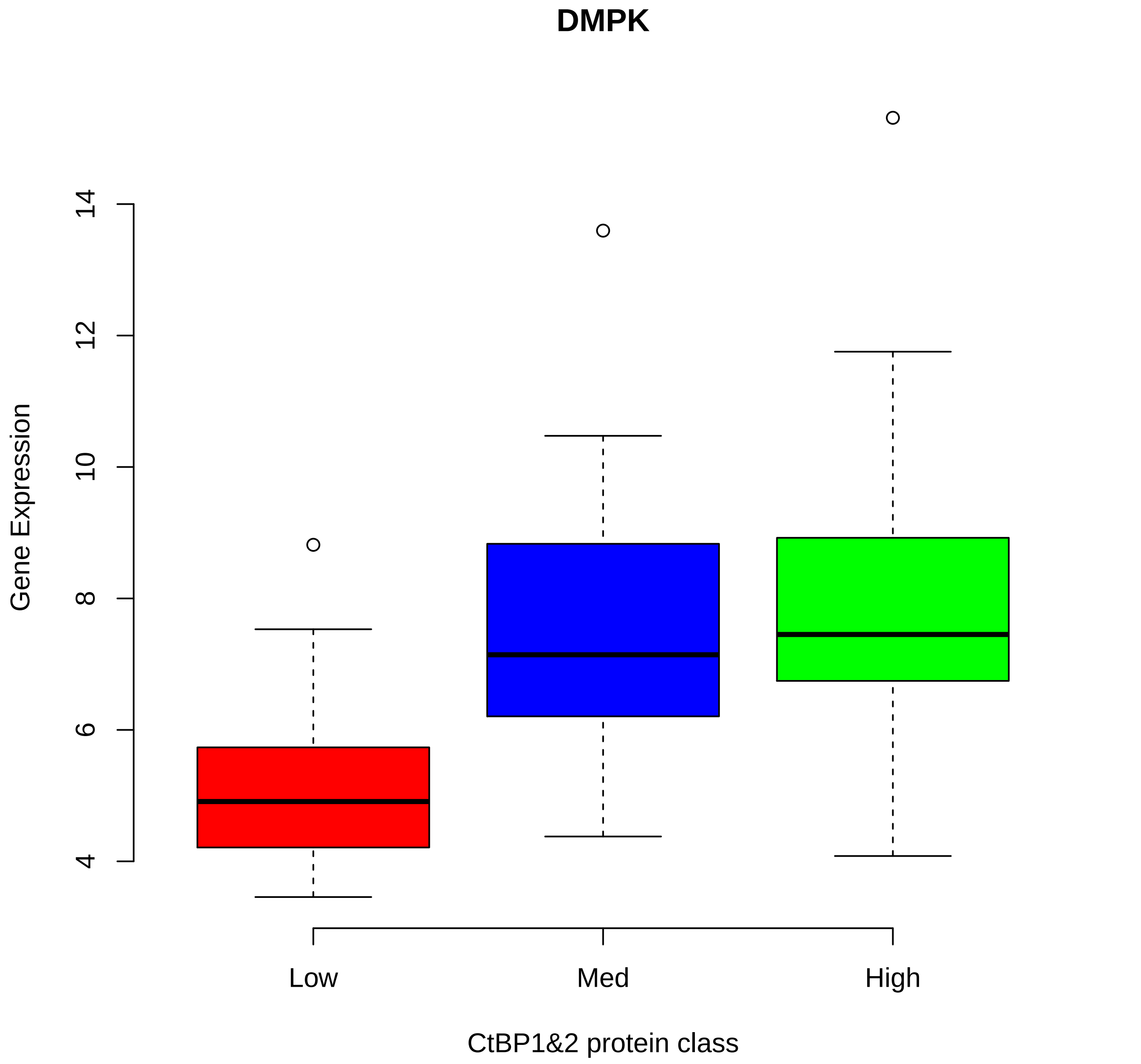
DMPK
Gene Expression
4
6
8
10
12
14
Low
Med
High
CtBP1&2 protein class

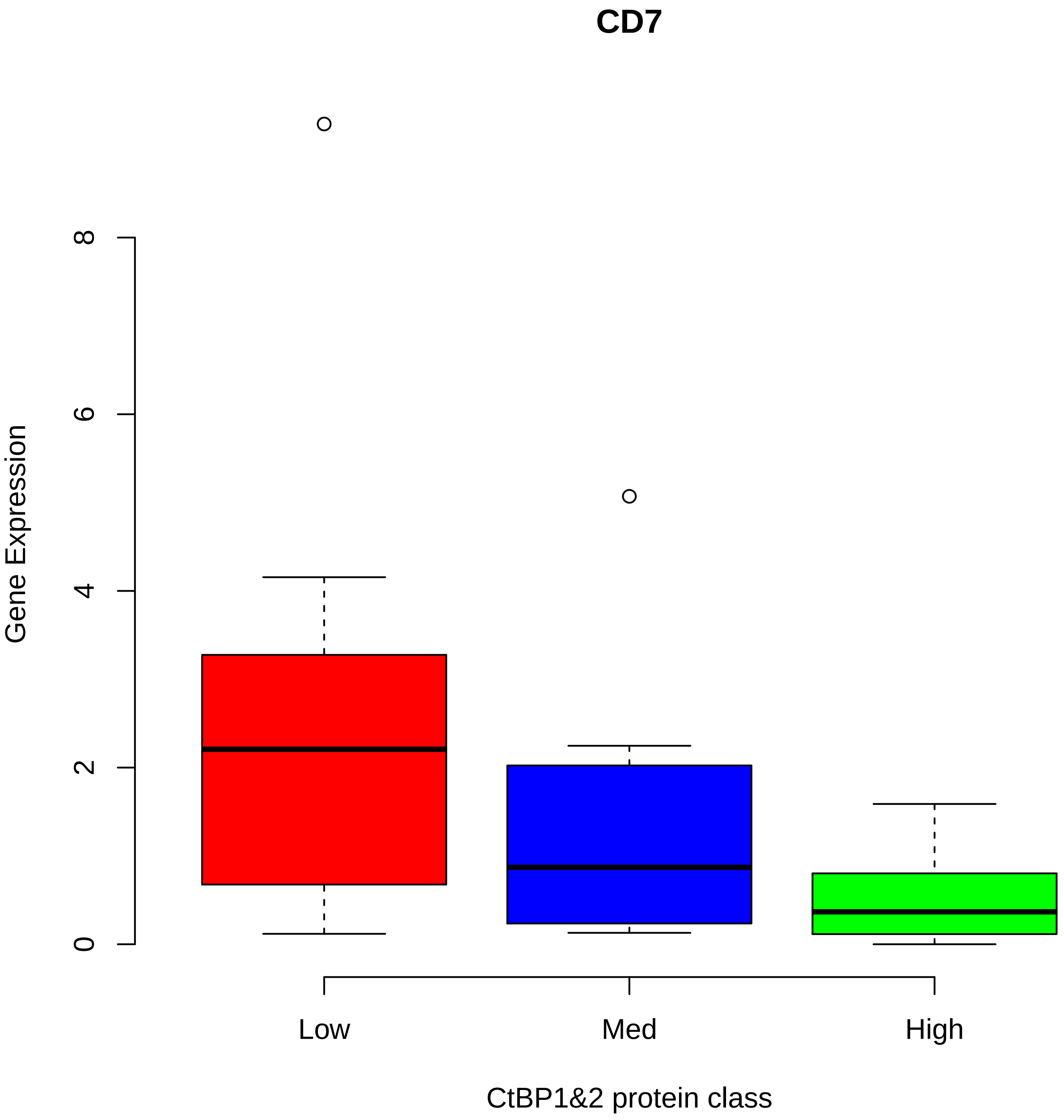

CD7
Gene Expression
0
2
4
6
8
Low
Med
High
CtBP1&2 protein class

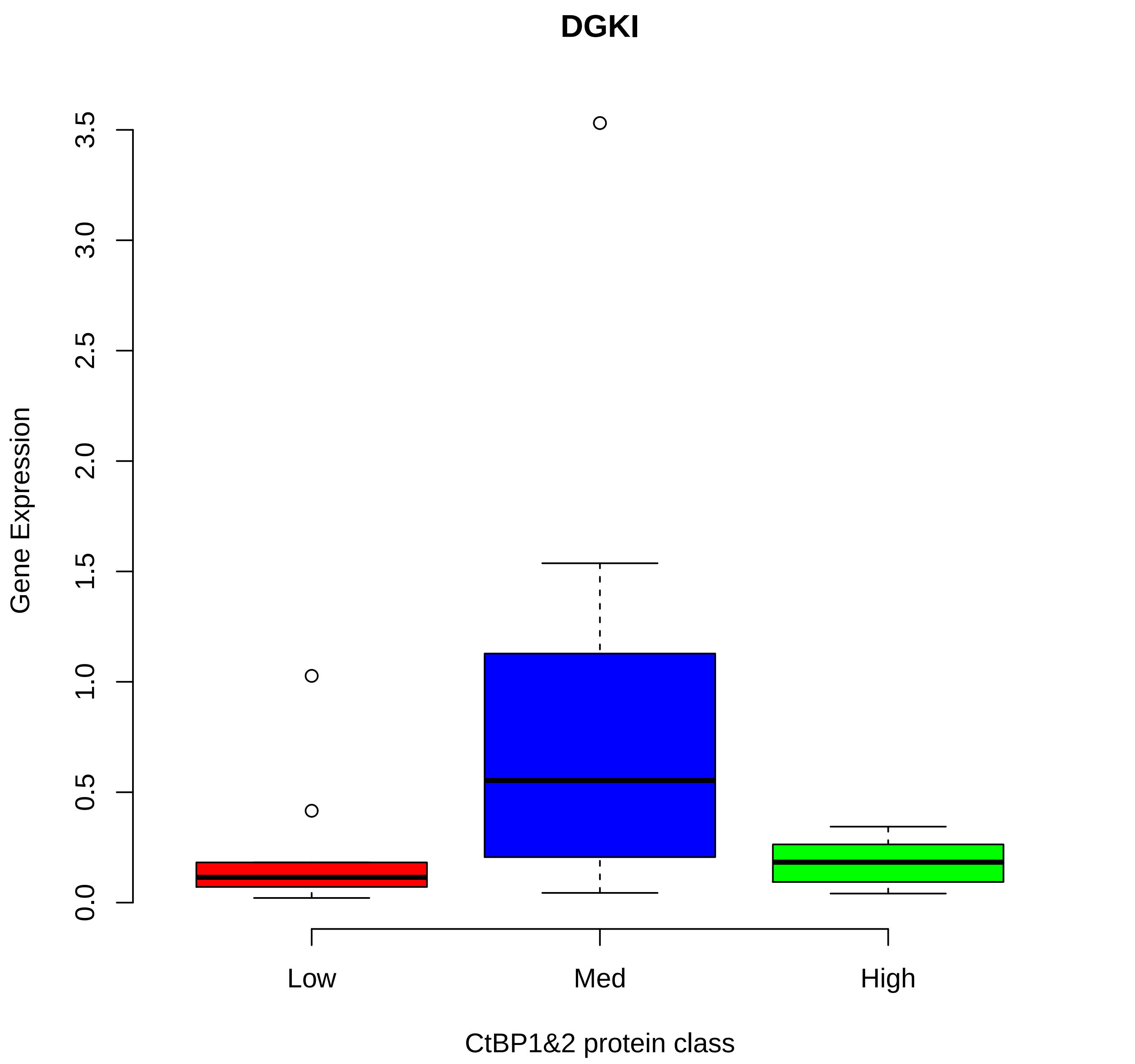

DGKI
Gene Expression
0.0
0.5
1.0
1.5
2.0
2.5
3.0
3.5
Low
Med
High
CtBP1&2 protein class

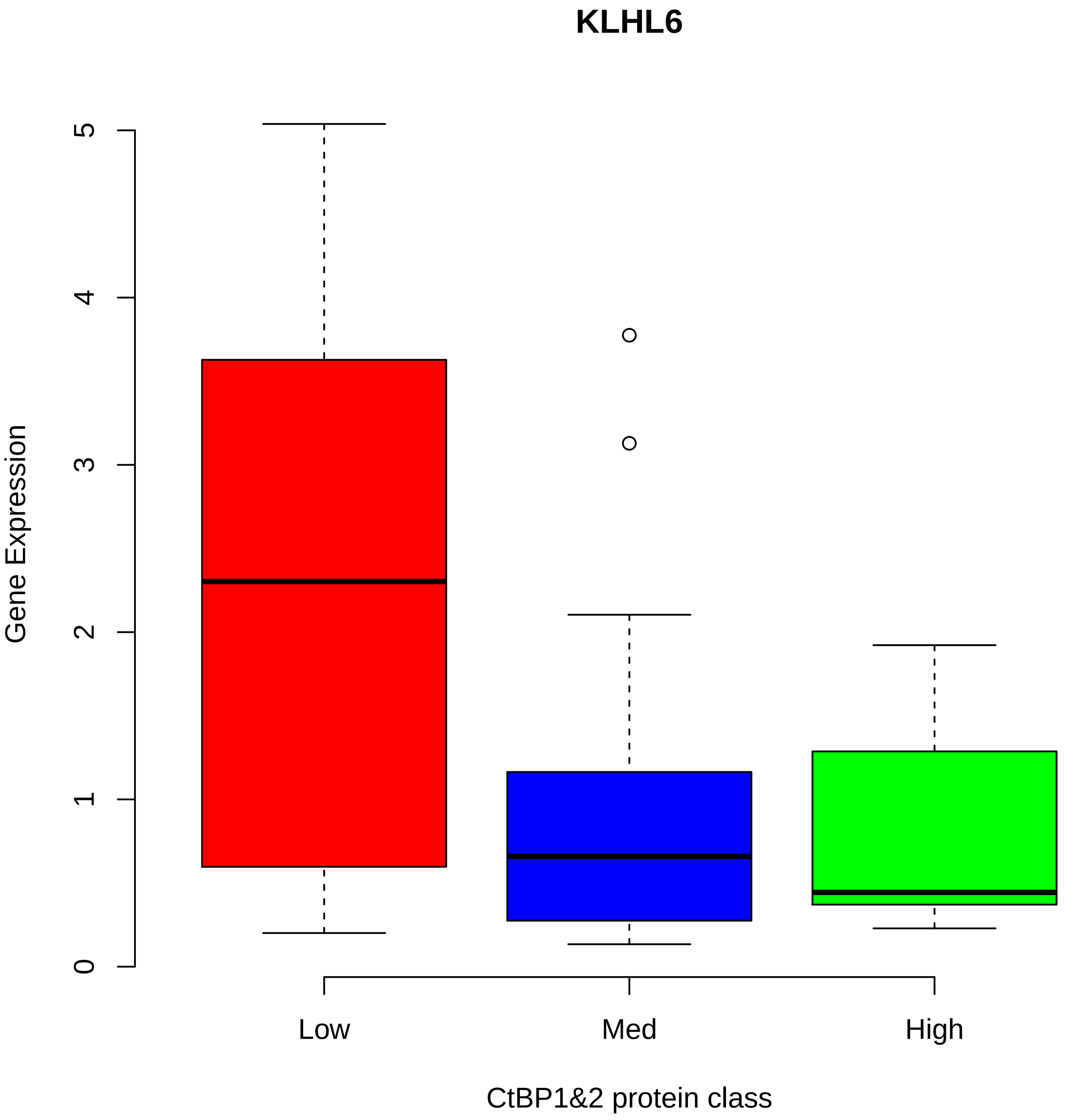
KLHL6
Gene Expression
0
1
2
3
4
5
Low
Med
High
CtBP1&2 protein class

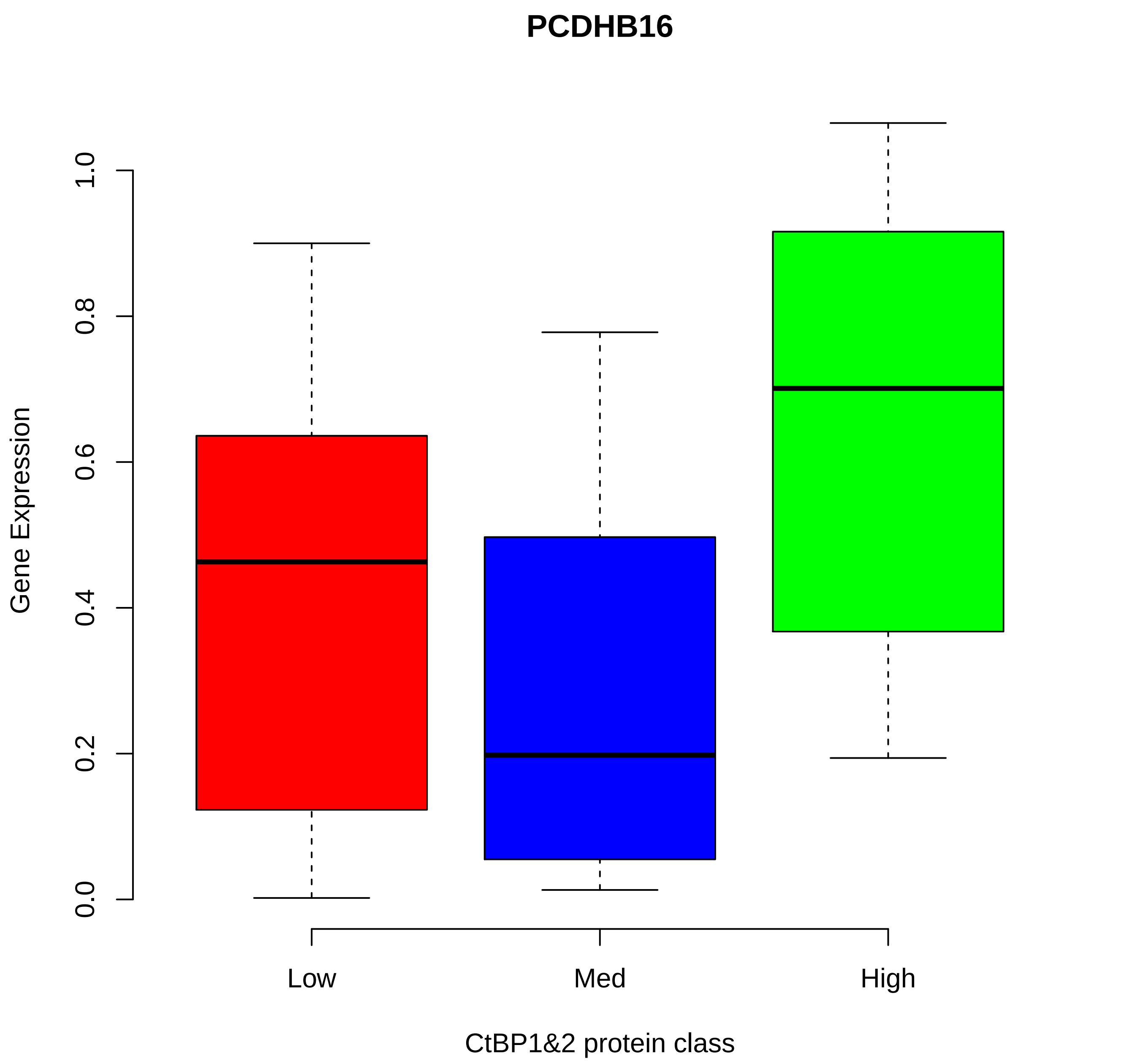

PCDHB16
Gene Expression
0.0
0.2
0.4
0.6
0.8
1.0
Low
Med
High
CtBP1&2 protein class

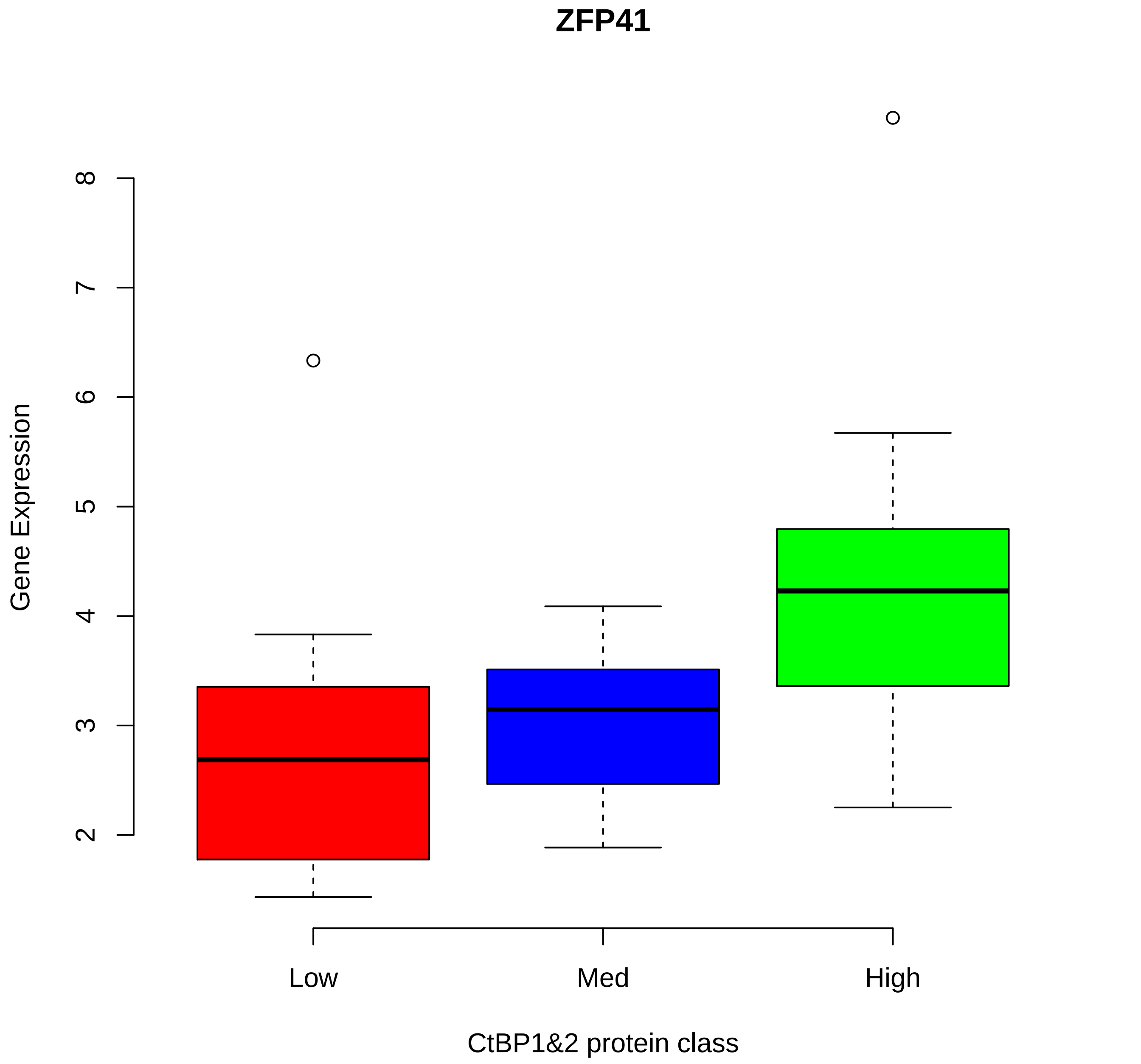
ZFP41
Gene Expression
2
3
4
5
6
7
8
Low
Med
High
CtBP1&2 protein class

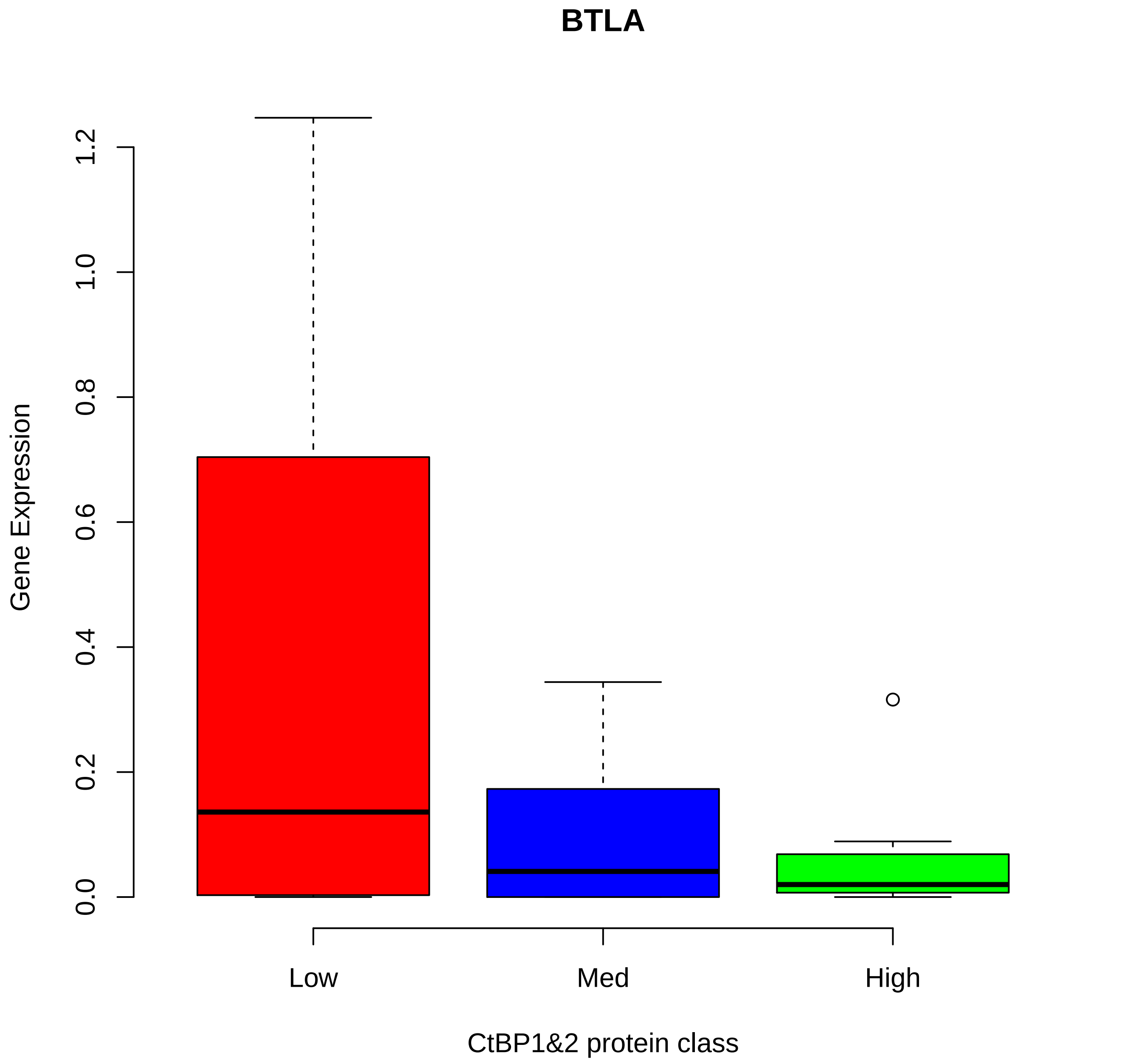
BTLA
Gene Expression
0.0
0.2
0.4
0.6
0.8
1.0
1.2
Low
Med
High
CtBP1&2 protein class

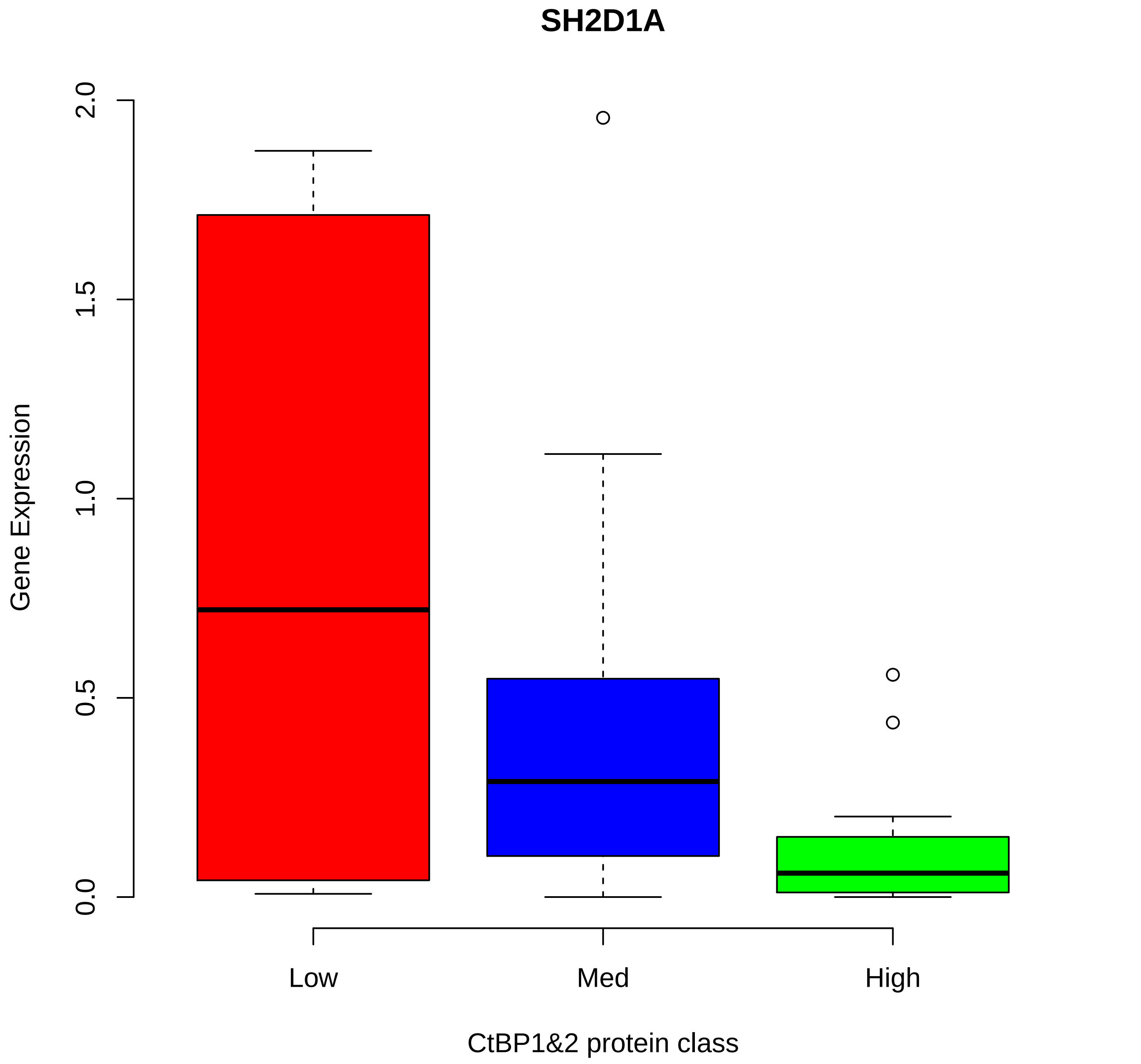

SH2D1A
Gene Expression
0.0
0.5
1.0
1.5
2.0
Low
Med
High
CtBP1&2 protein class

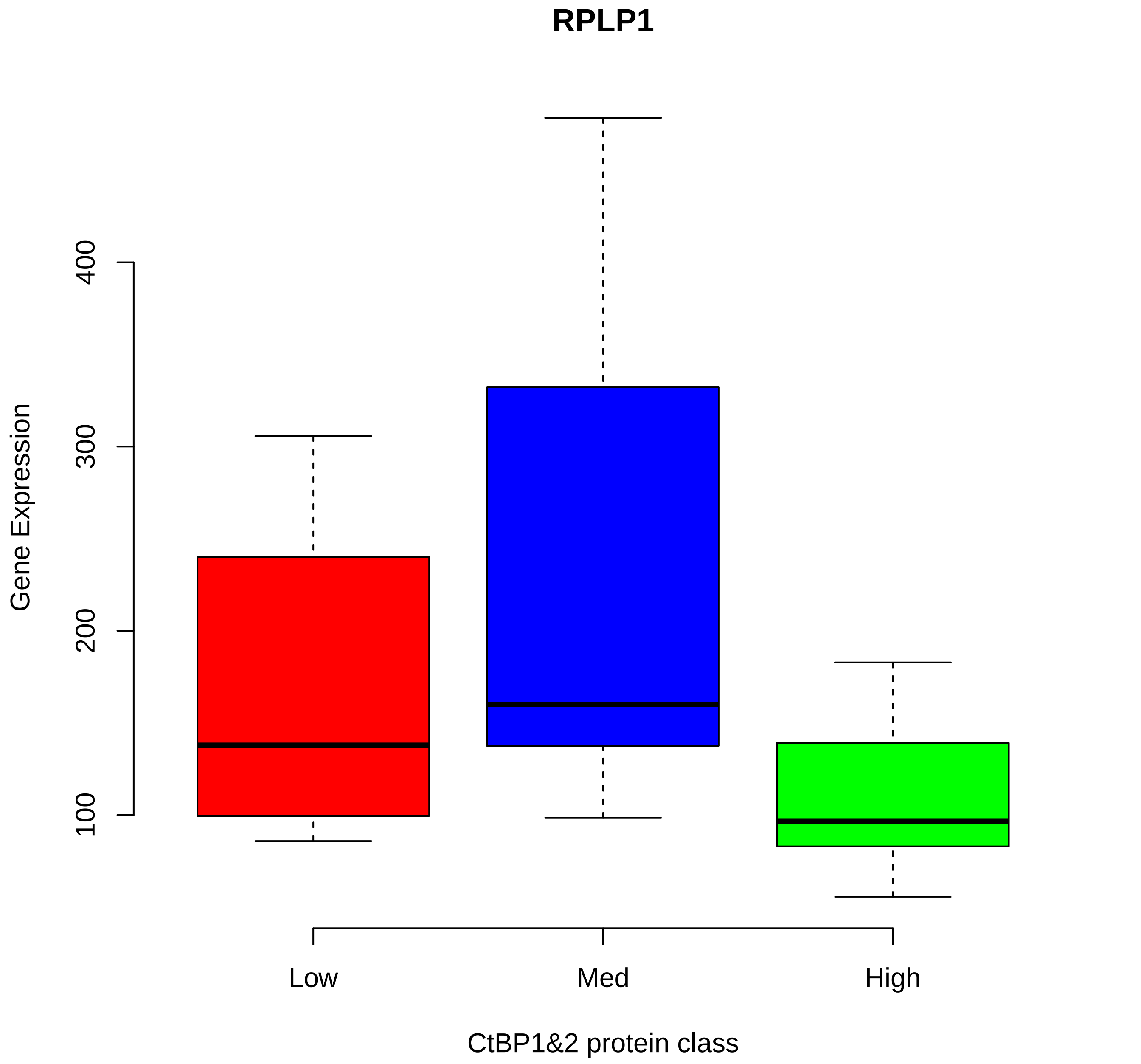
RPLP1
Gene Expression
100
200
300
400
Low
Med
High
CtBP1&2 protein class

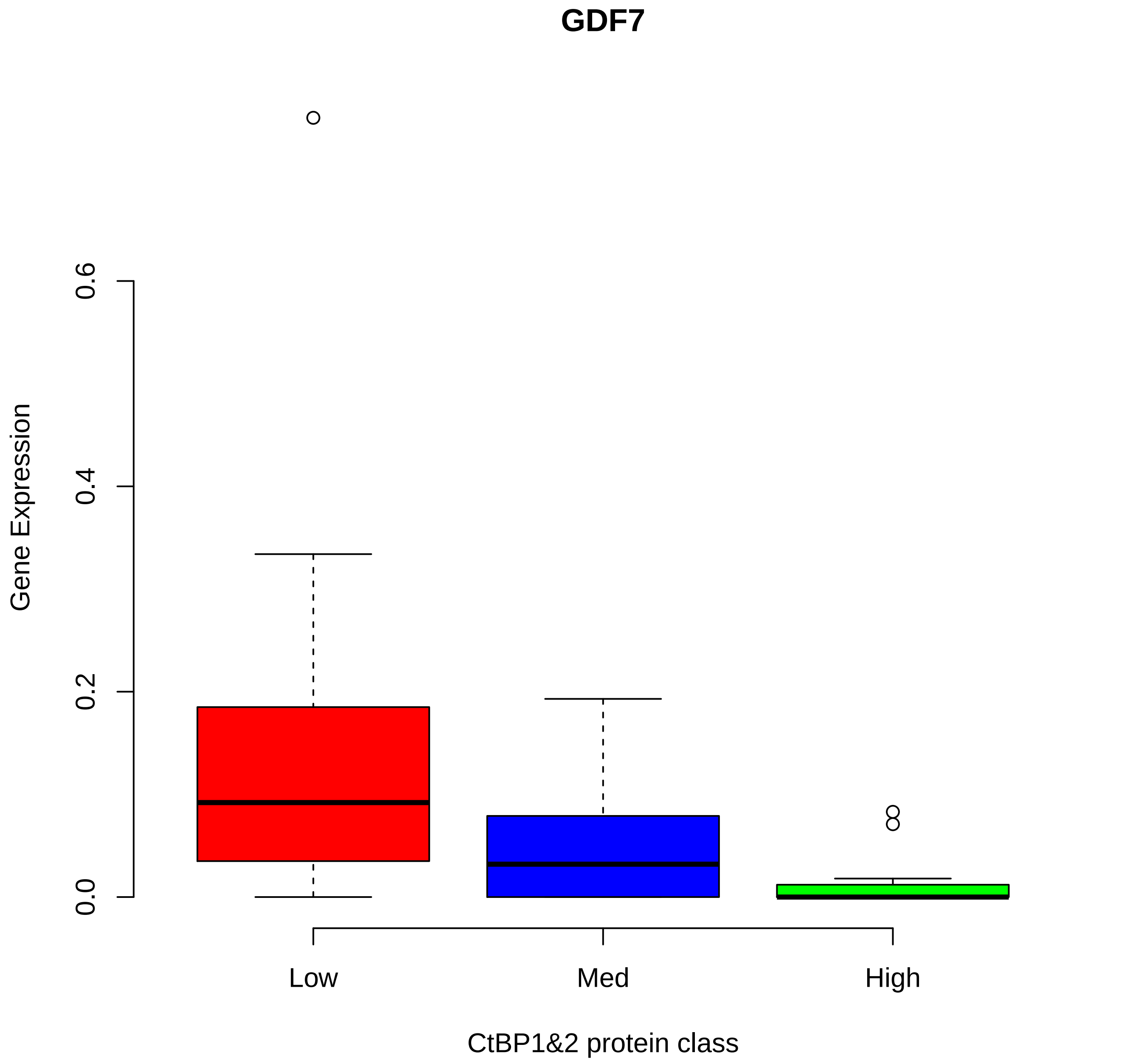
GDF7
Gene Expression
0.0
0.2
0.4
0.6
Low
Med
High
CtBP1&2 protein class

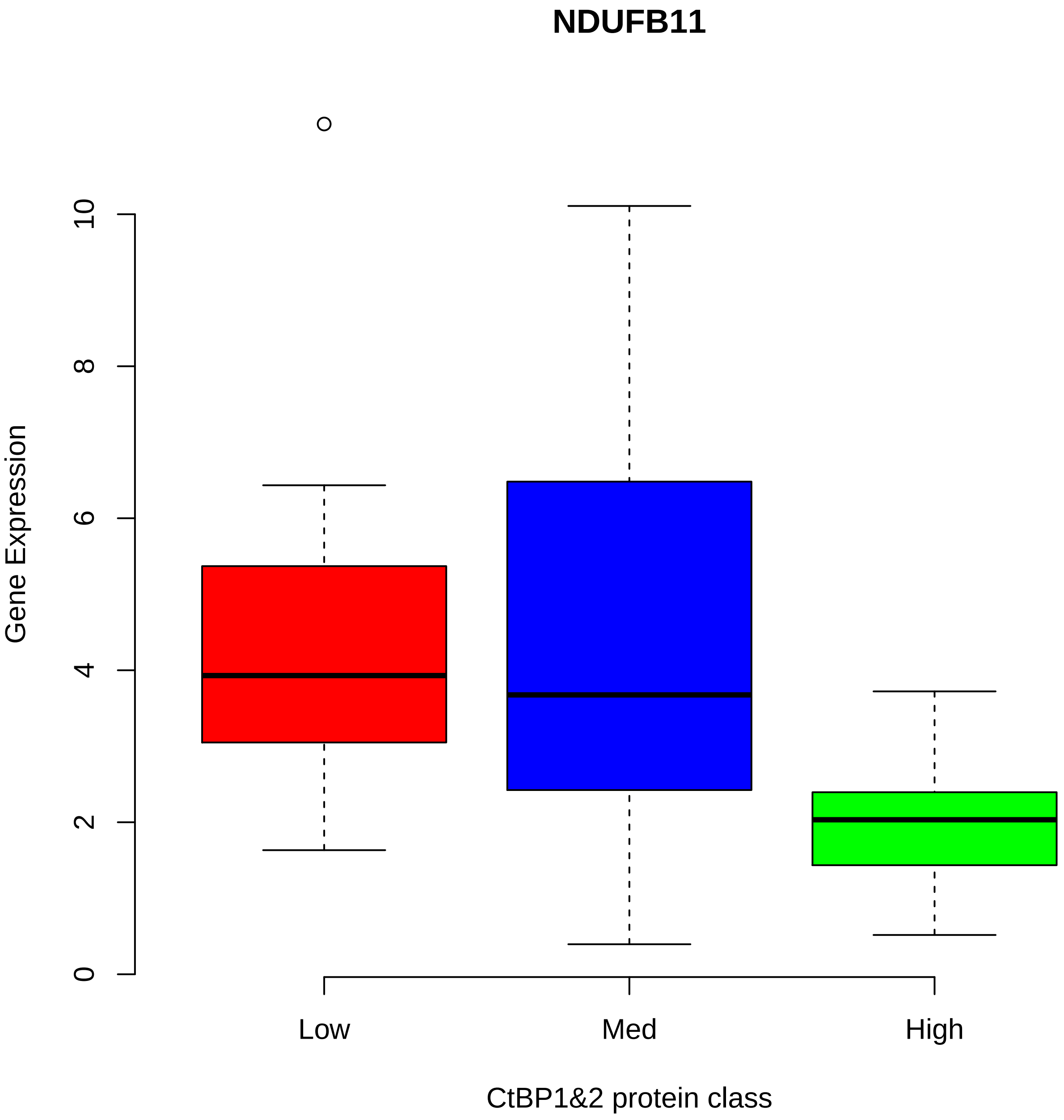
NDUFB11
Gene Expression
0
2
4
6
8
10
Low
Med
High
CtBP1&2 protein class

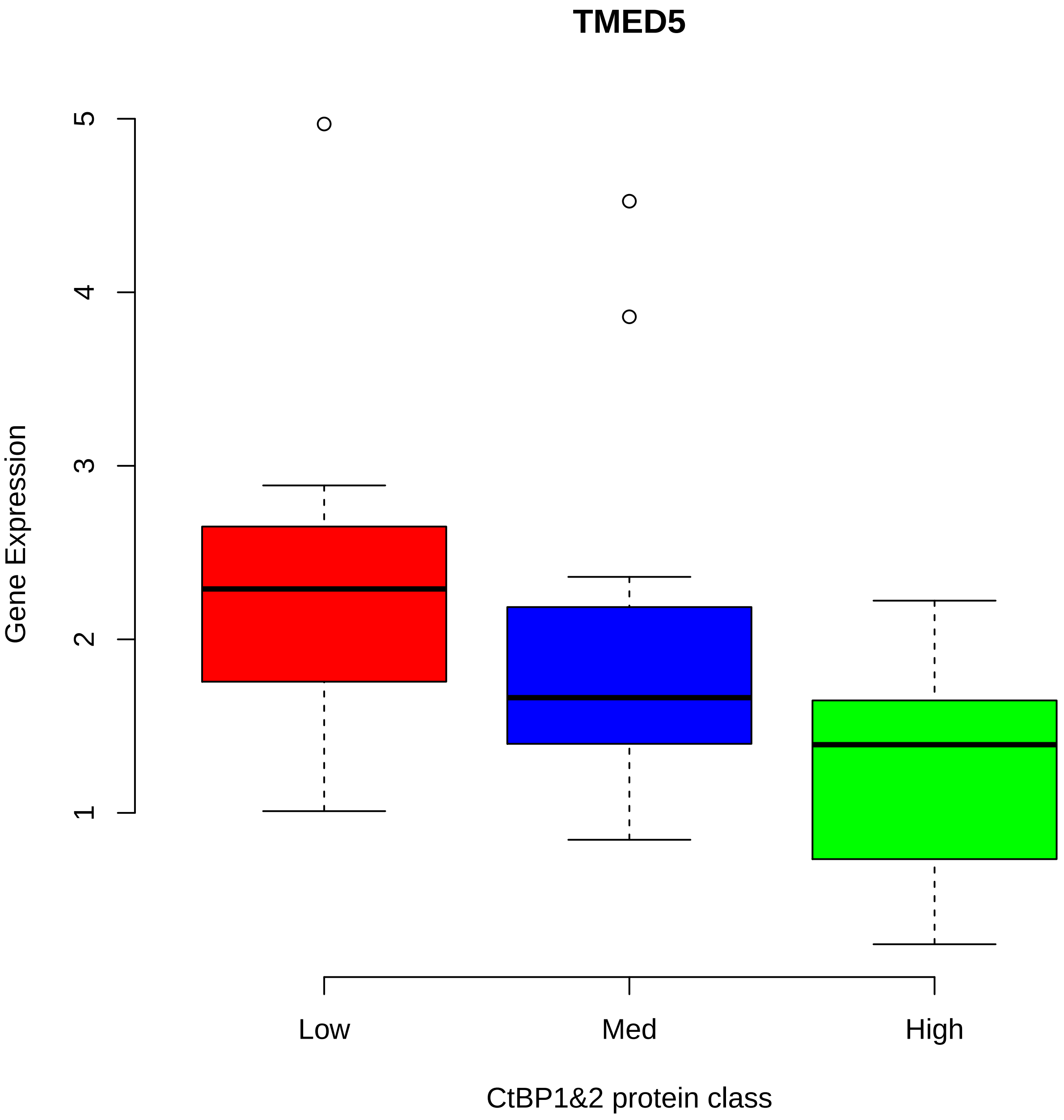

TMED5
Gene Expression
1
2
3
4
5
Low
Med
High
CtBP1&2 protein class

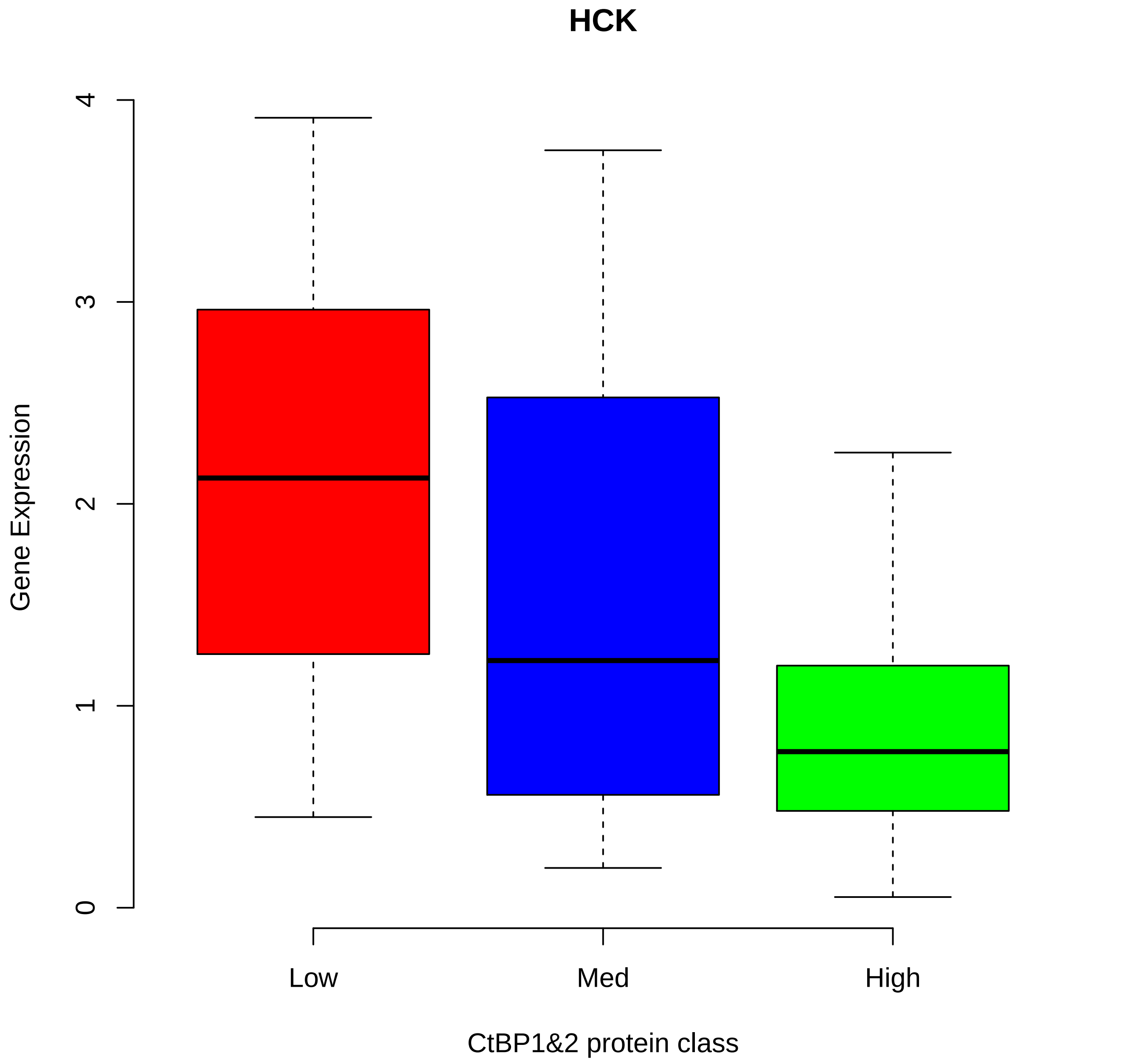
HCK
Gene Expression
0
1
2
3
4
Low
Med
High
CtBP1&2 protein class

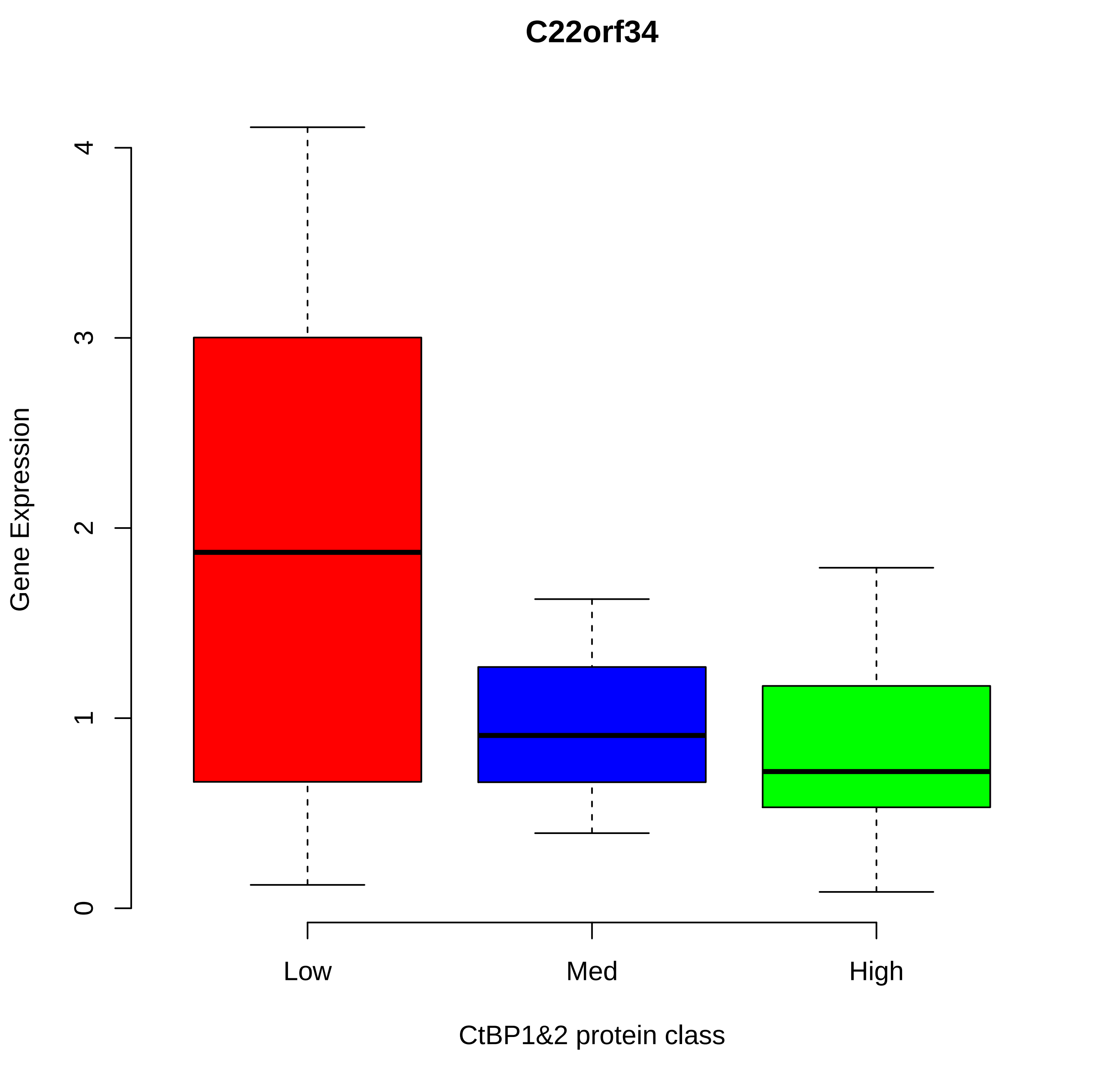
C22orf34
Gene Expression
0
1
2
3
4
Low
Med
High
CtBP1&2 protein class

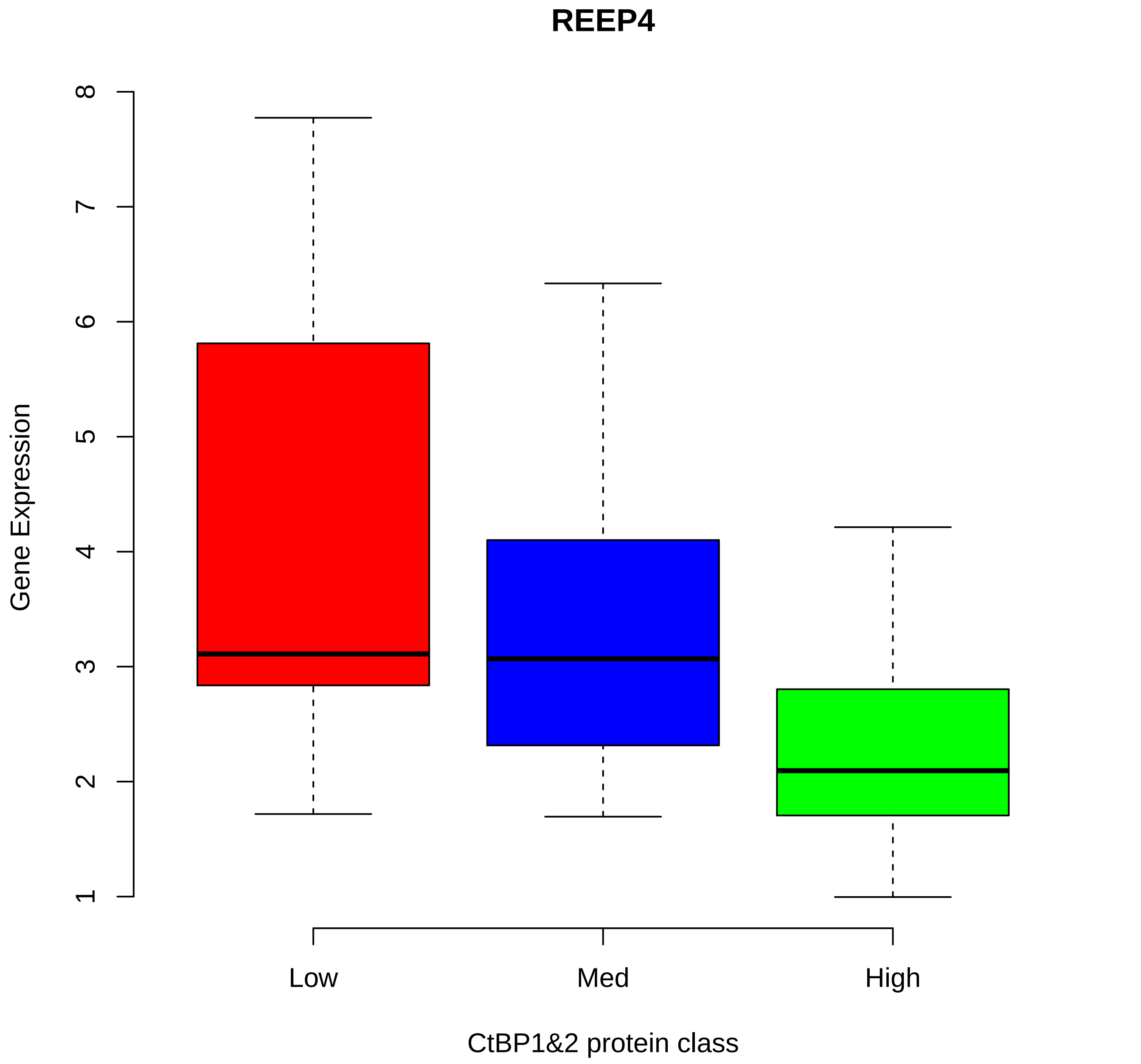

REEP4
Gene Expression
1
2
3
4
5
6
7
8
Low
Med
High
CtBP1&2 protein class

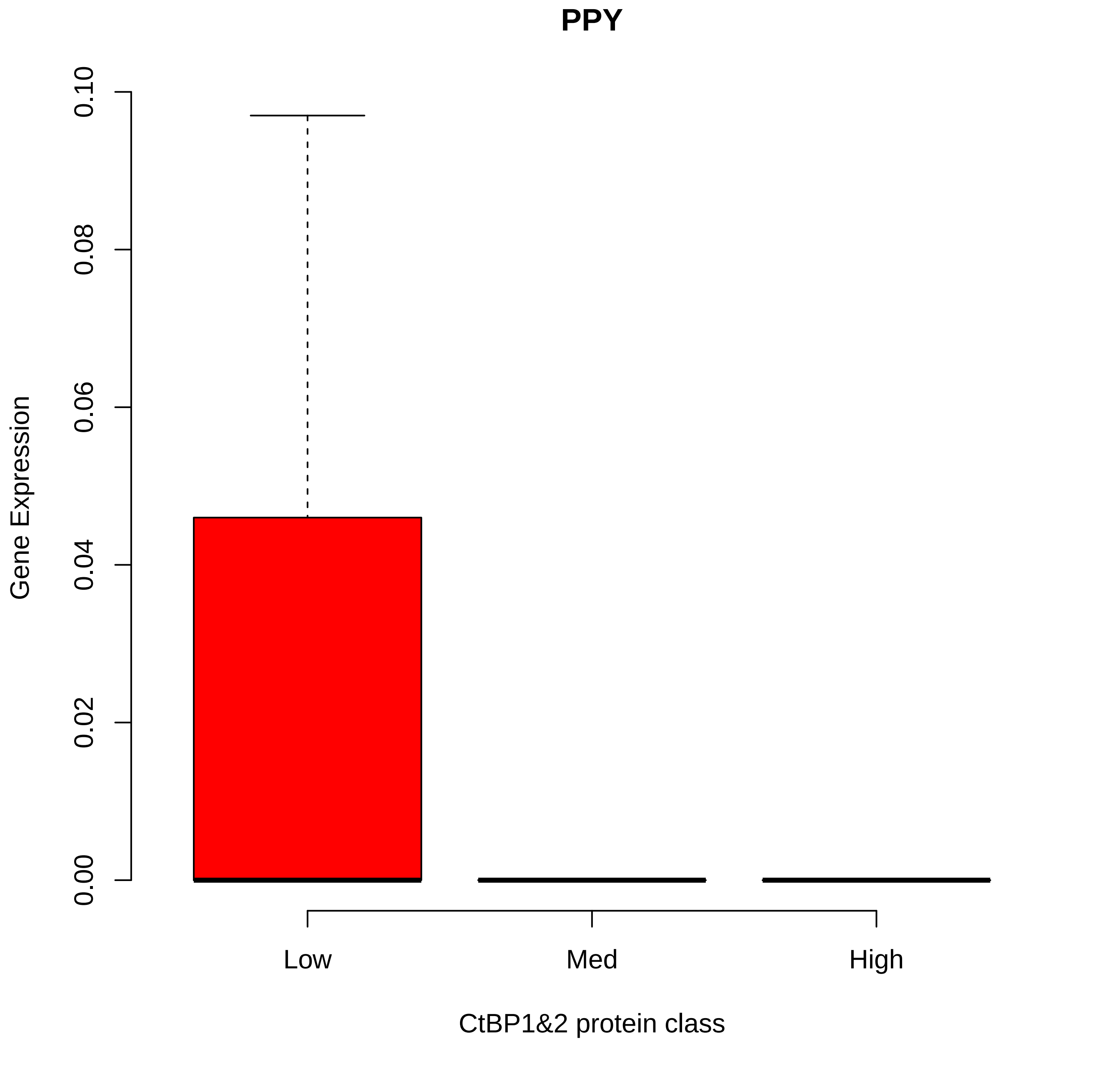

PPY
0.10
0.08
0.06
0.04
0.02
0.00
Gene Expression
Low
Med
High
CtBP1&2 protein class

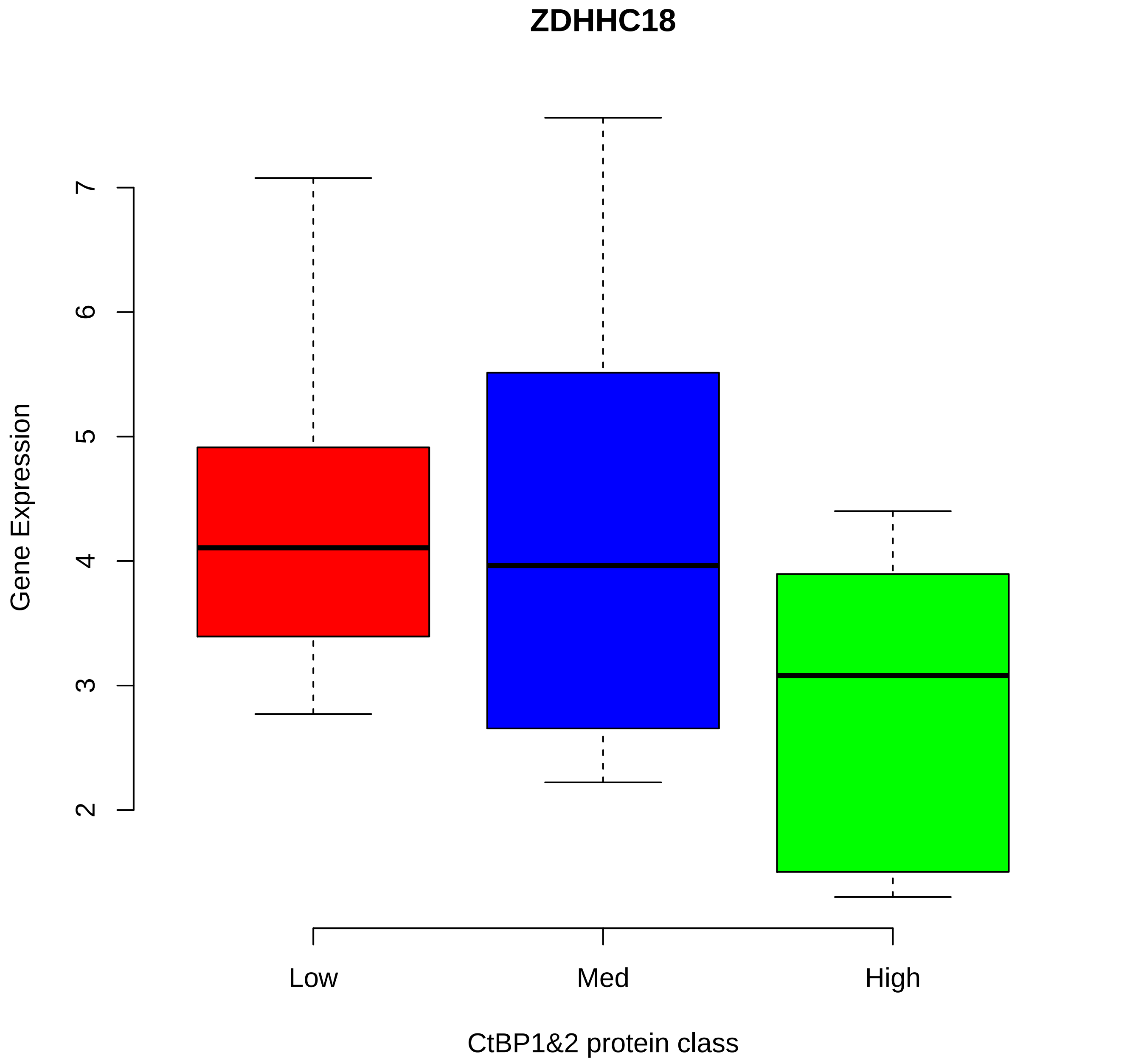

ZDHHC18
Gene Expression
2
3
4
5
6
7
Low
Med
High
CtBP1&2 protein class

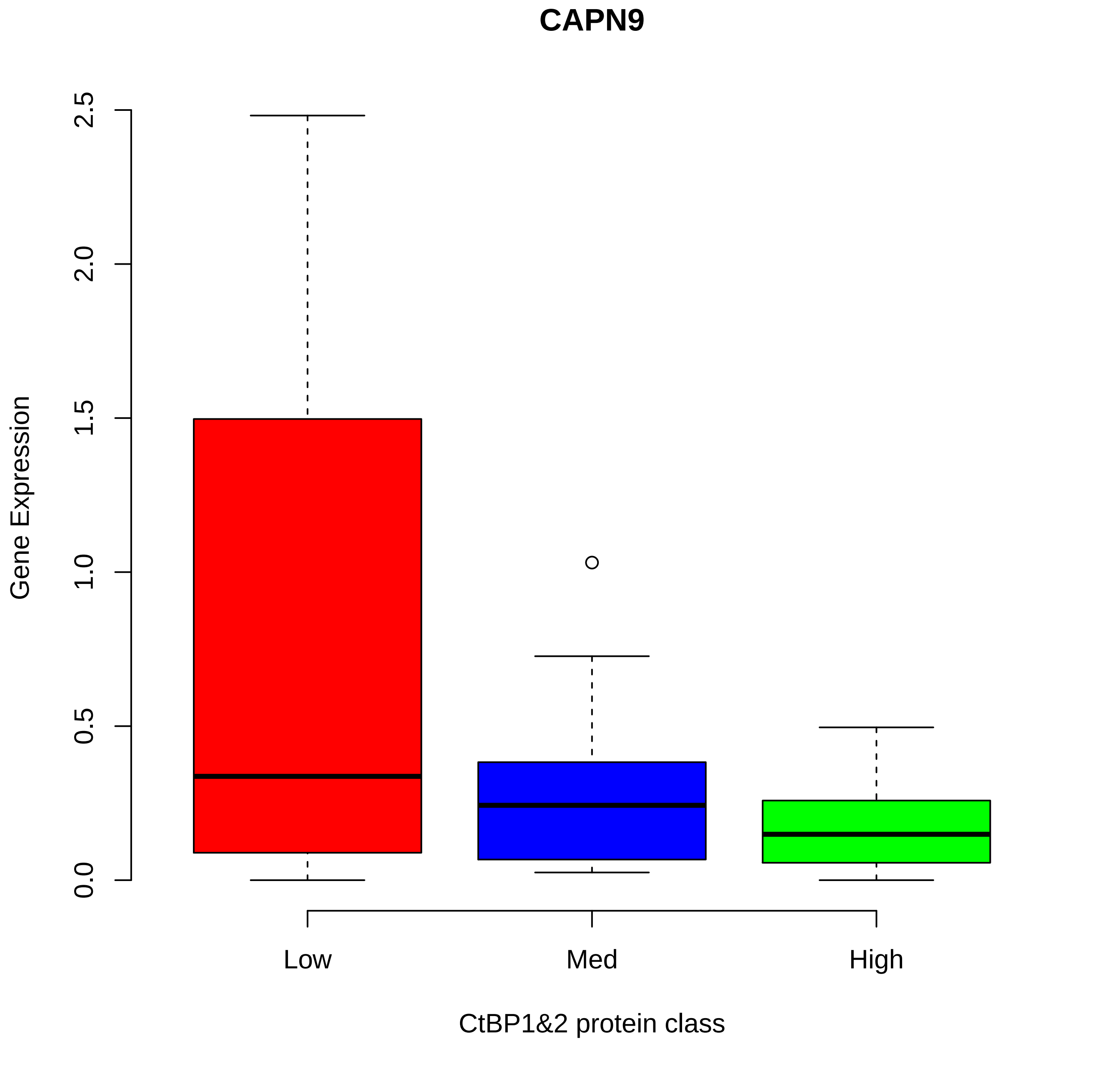

CAPN9
Gene Expression
0.0
0.5
1.0
1.5
2.0
2.5
Low
Med
High
CtBP1&2 protein class

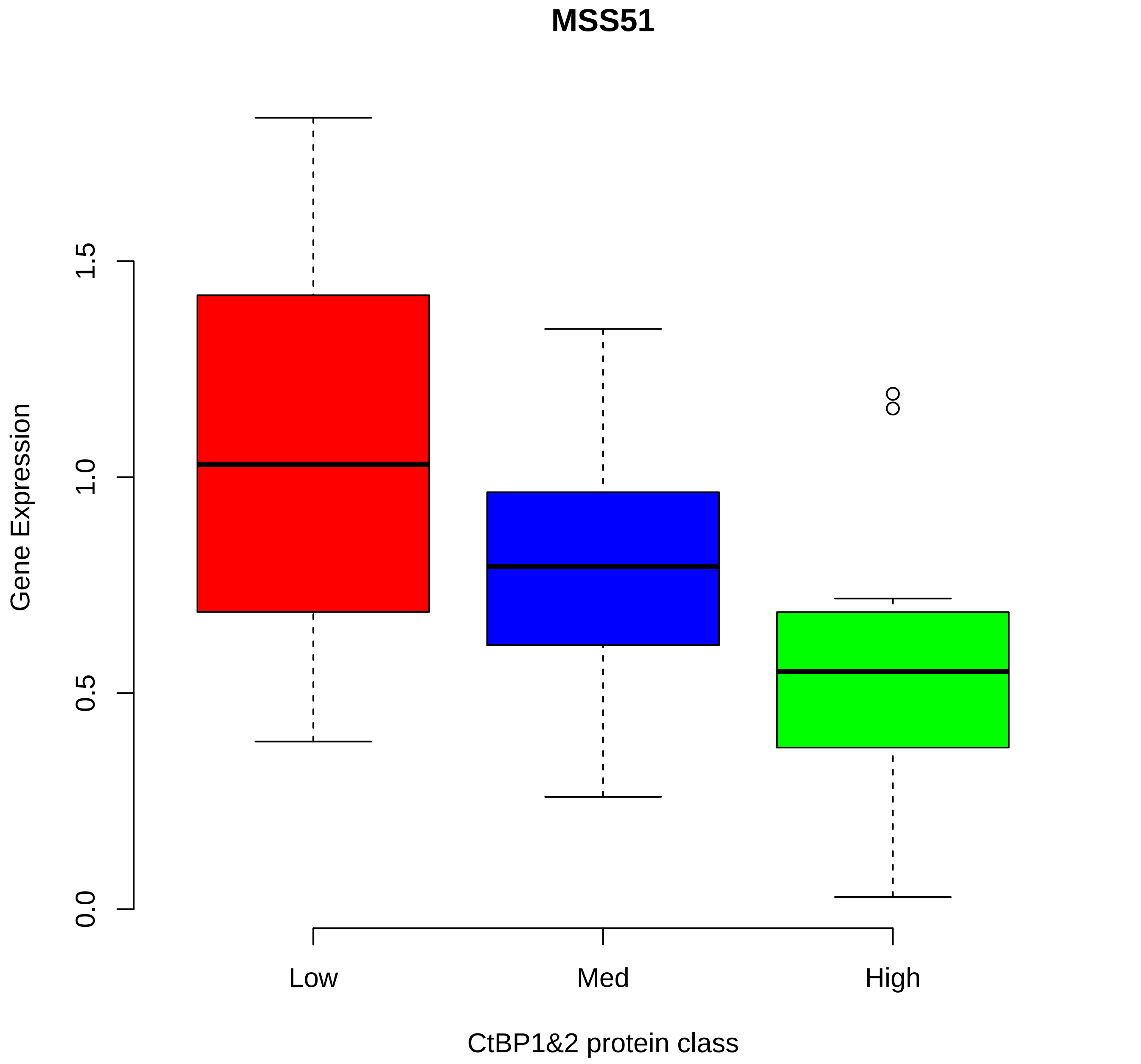

MSS51
Gene Expression
0.0
0.5
1.0
1.5
Low
Med
High
CtBP1&2 protein class

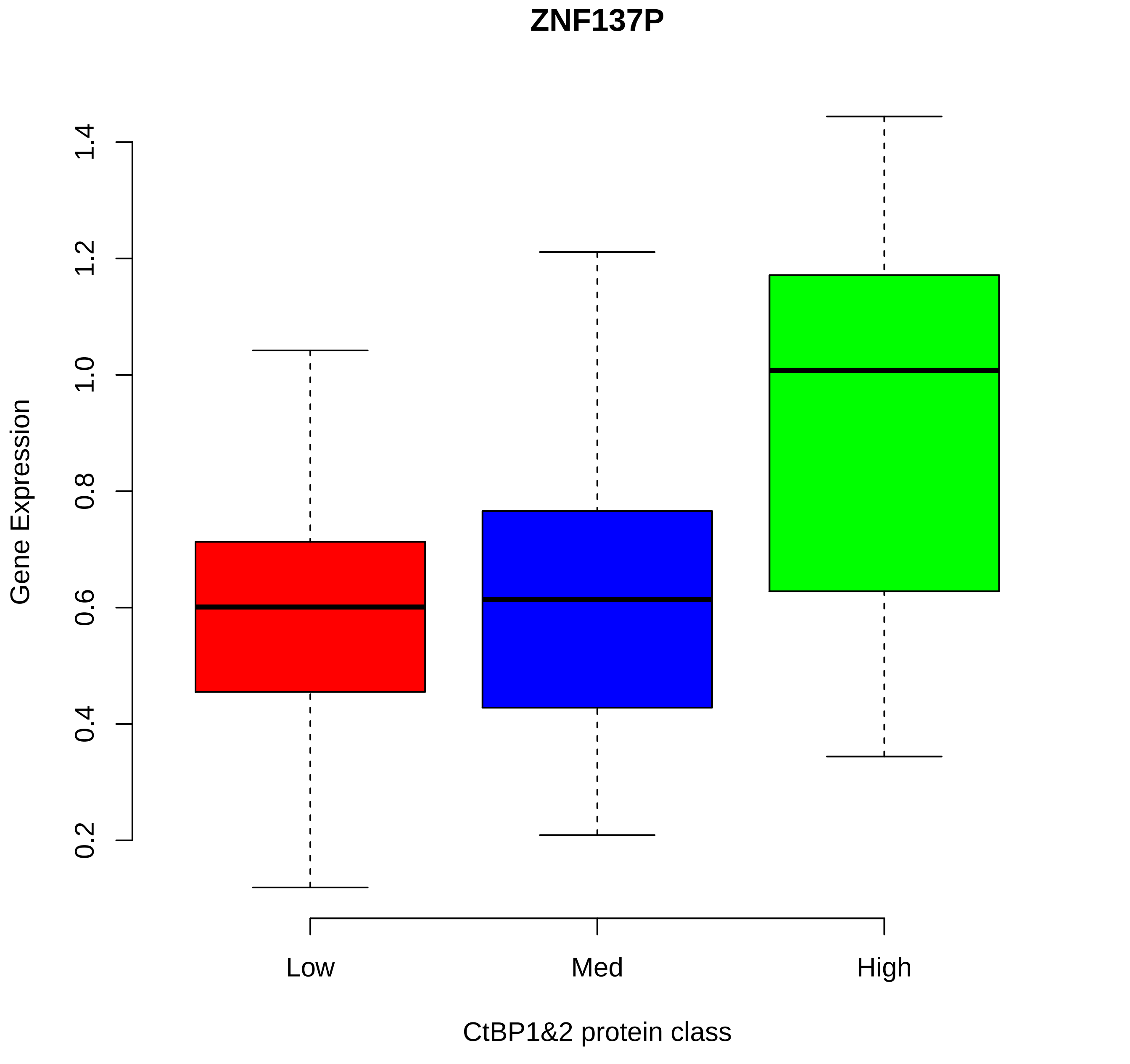

ZNF137P
Gene Expression
0.2
0.4
0.6
0.8
1.0
1.2
1.4
Low
Med
High
CtBP1&2 protein class

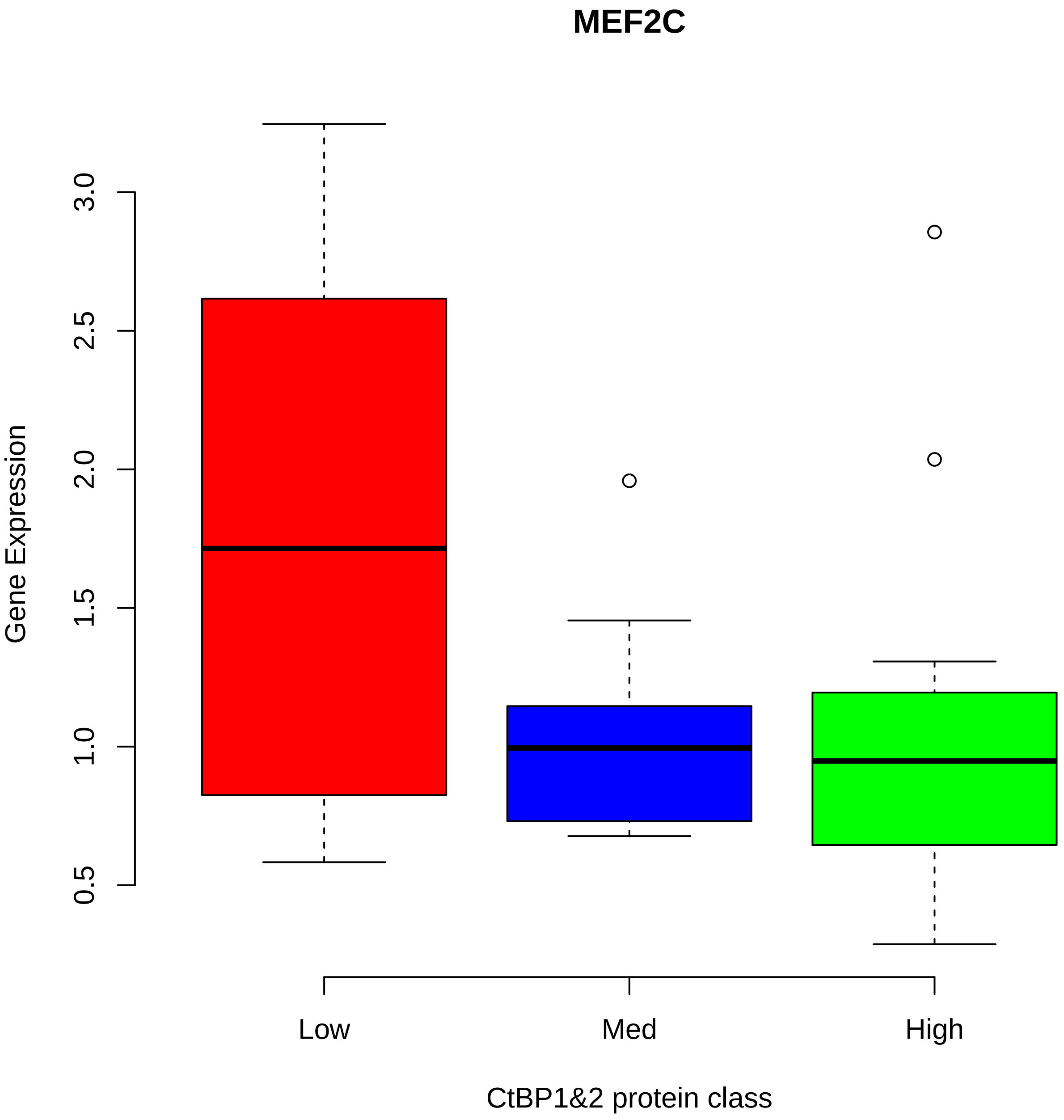
MEF2C
Gene Expression
0.5
1.0
1.5
2.0
2.5
3.0
Low
Med
High
CtBP1&2 protein class

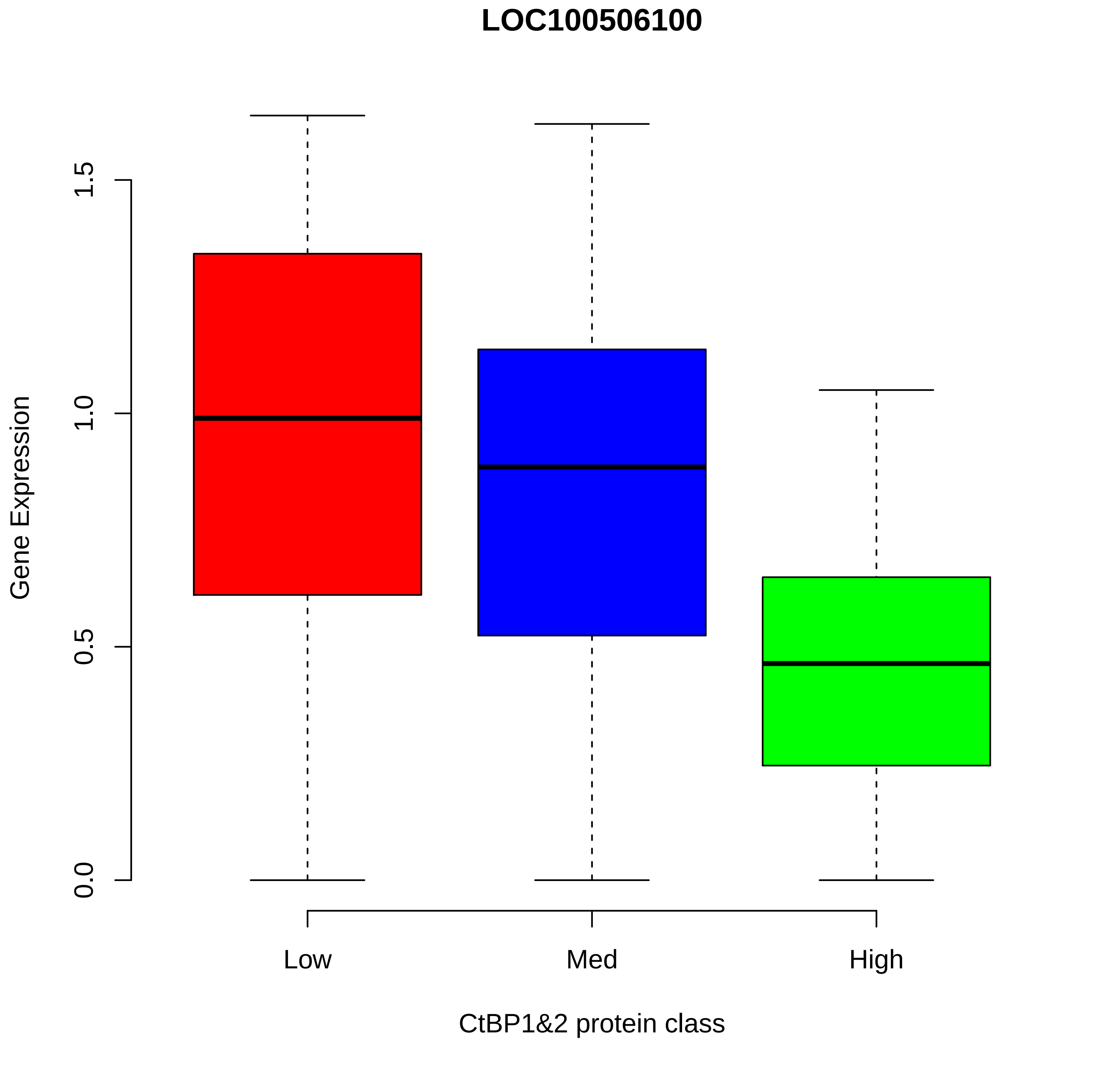

LOC100506100
Gene Expression
0.0
0.5
1.0
1.5
Low
Med
High
CtBP1&2 protein class

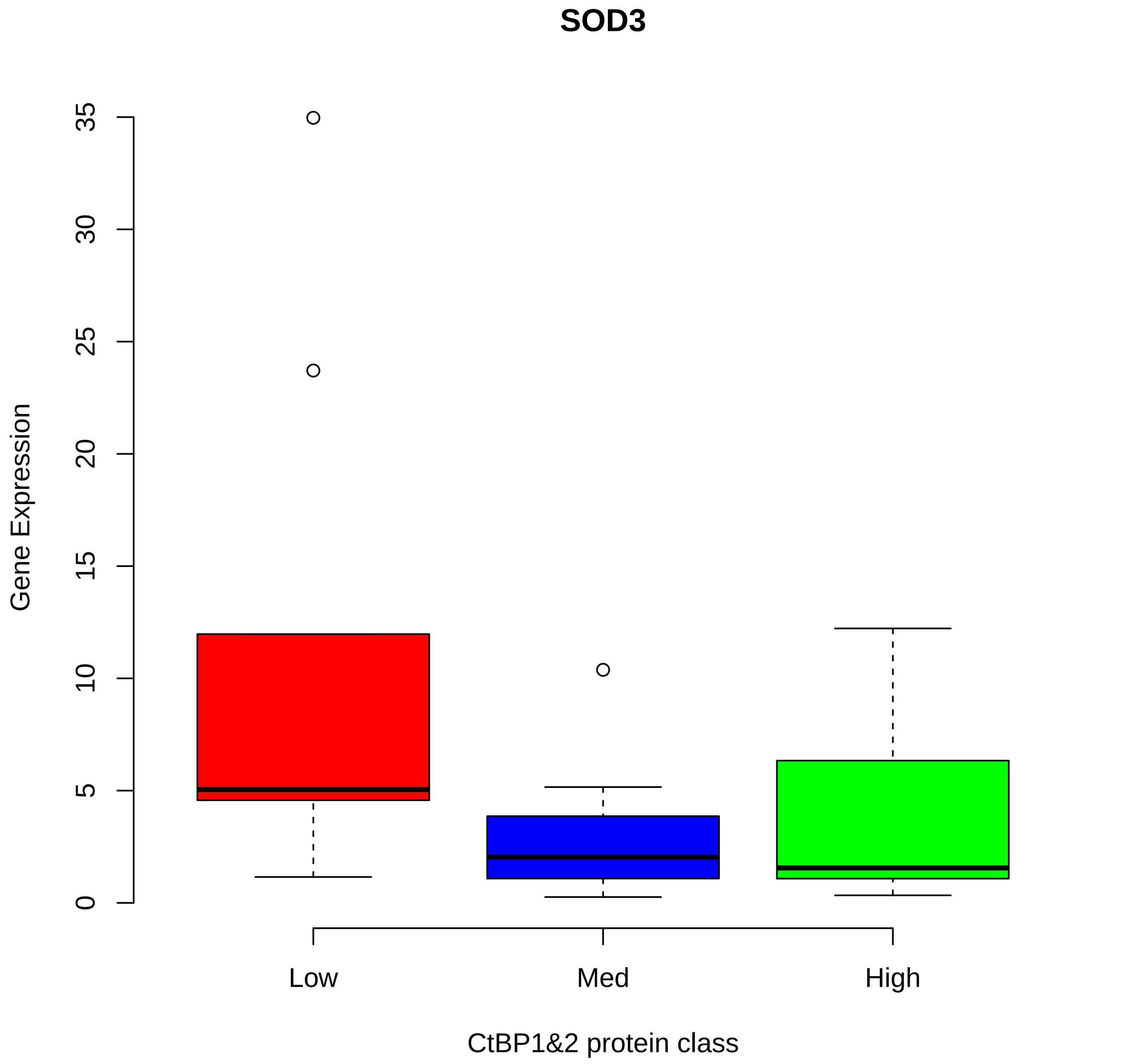
SOD3
Gene Expression
0
5
10
15
20
25
30
35
Low
Med
High
CtBP1&2 protein class

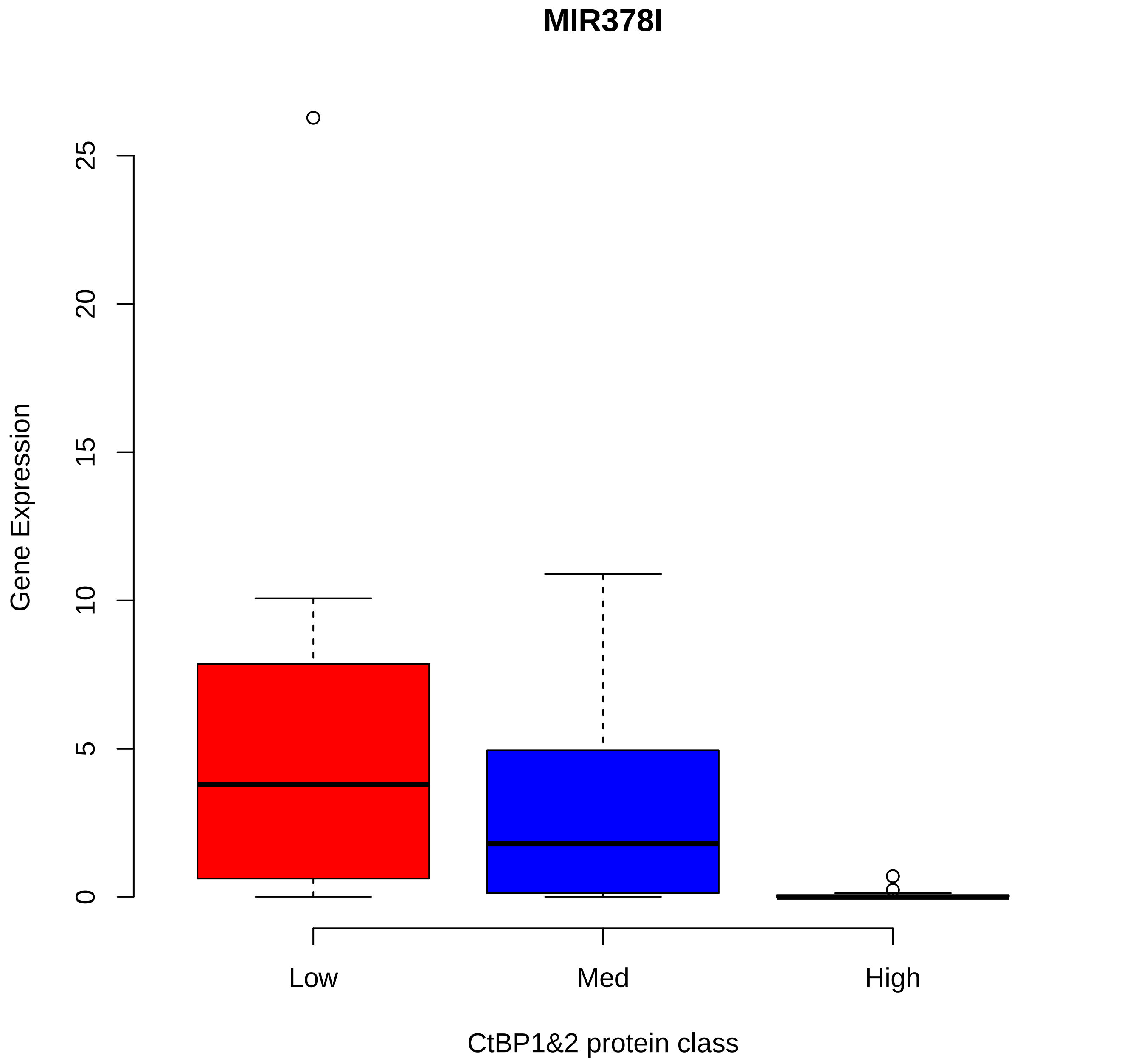

MIR378I
Gene Expression
0
5
10
15
20
25
Low
Med
High
CtBP1&2 protein class

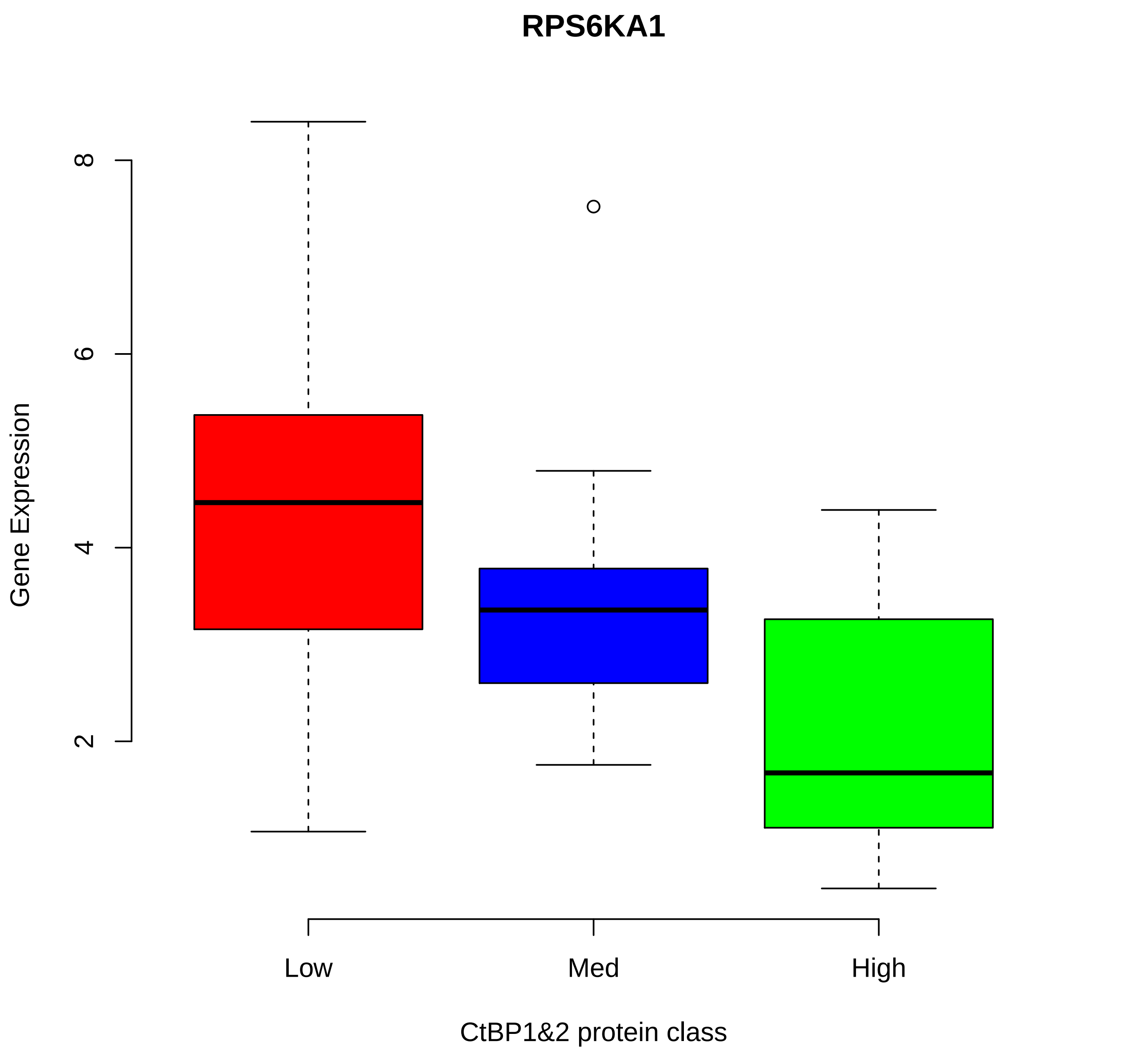

RPS6KA1
Gene Expression
2
4
6
8
Low
Med
High
CtBP1&2 protein class

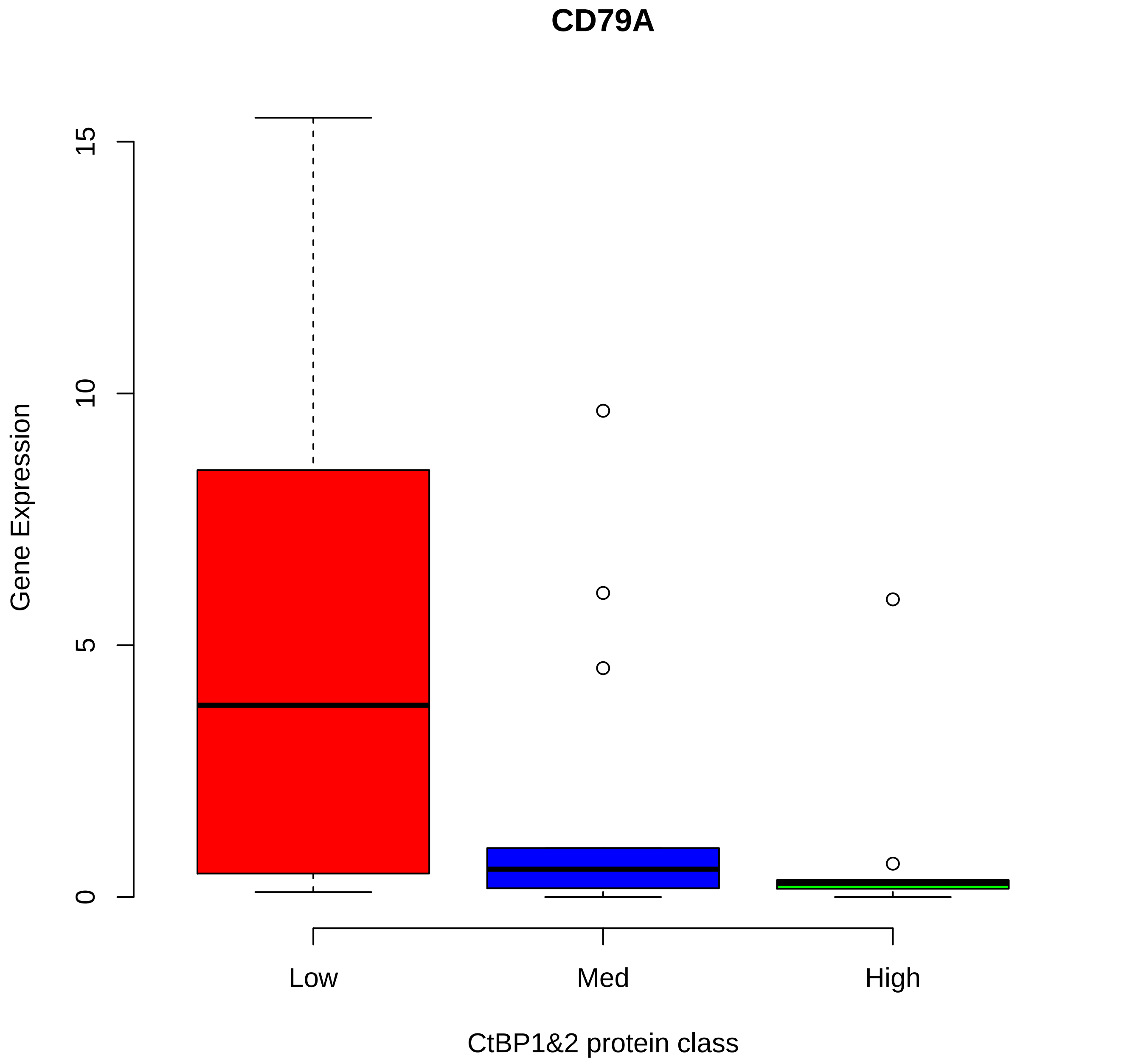
CD79A
Gene Expression
0
5
10
15
Low
Med
High
CtBP1&2 protein class

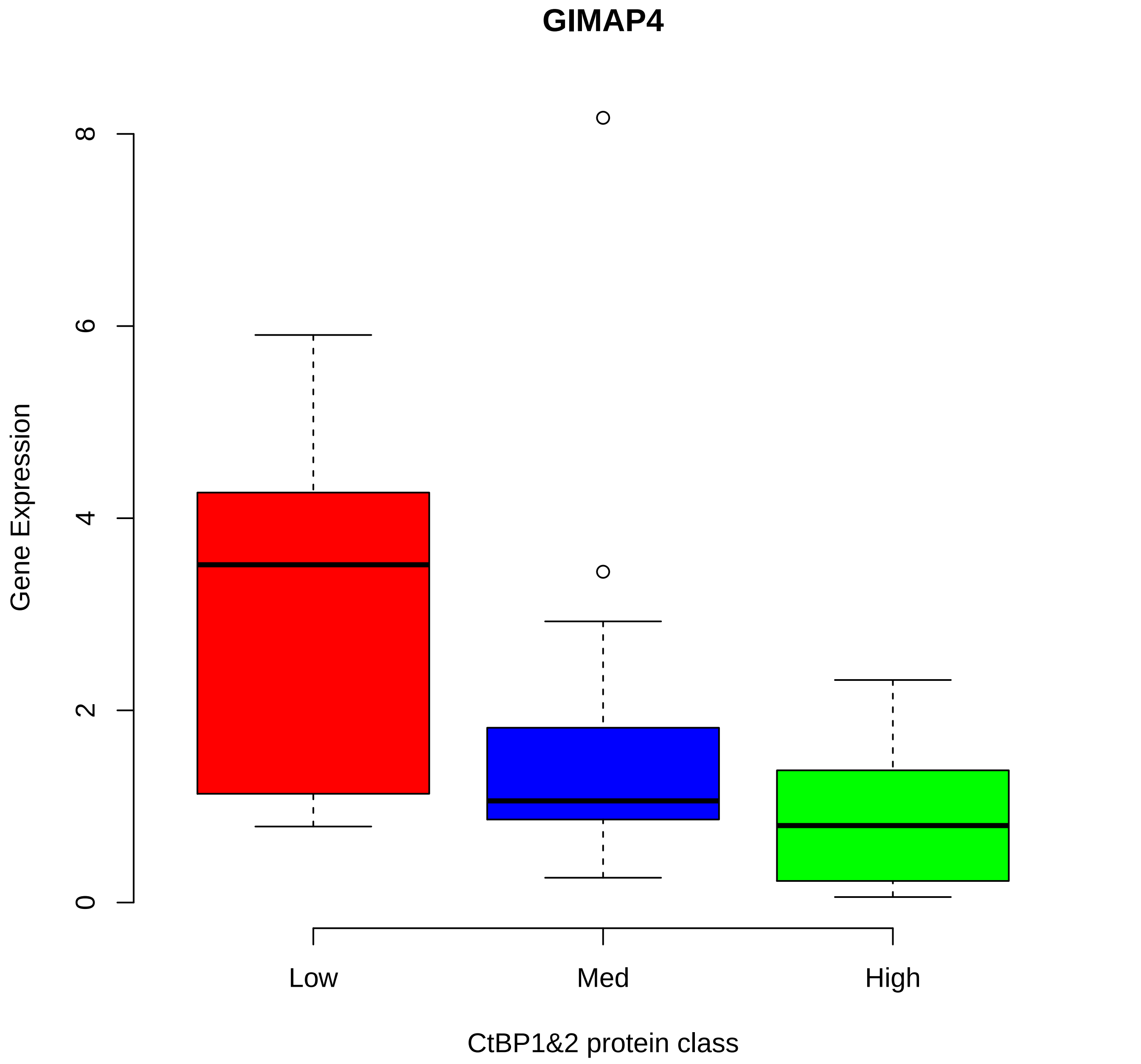
GIMAP4
Gene Expression
0
2
4
6
8
Low
Med
High
CtBP1&2 protein class

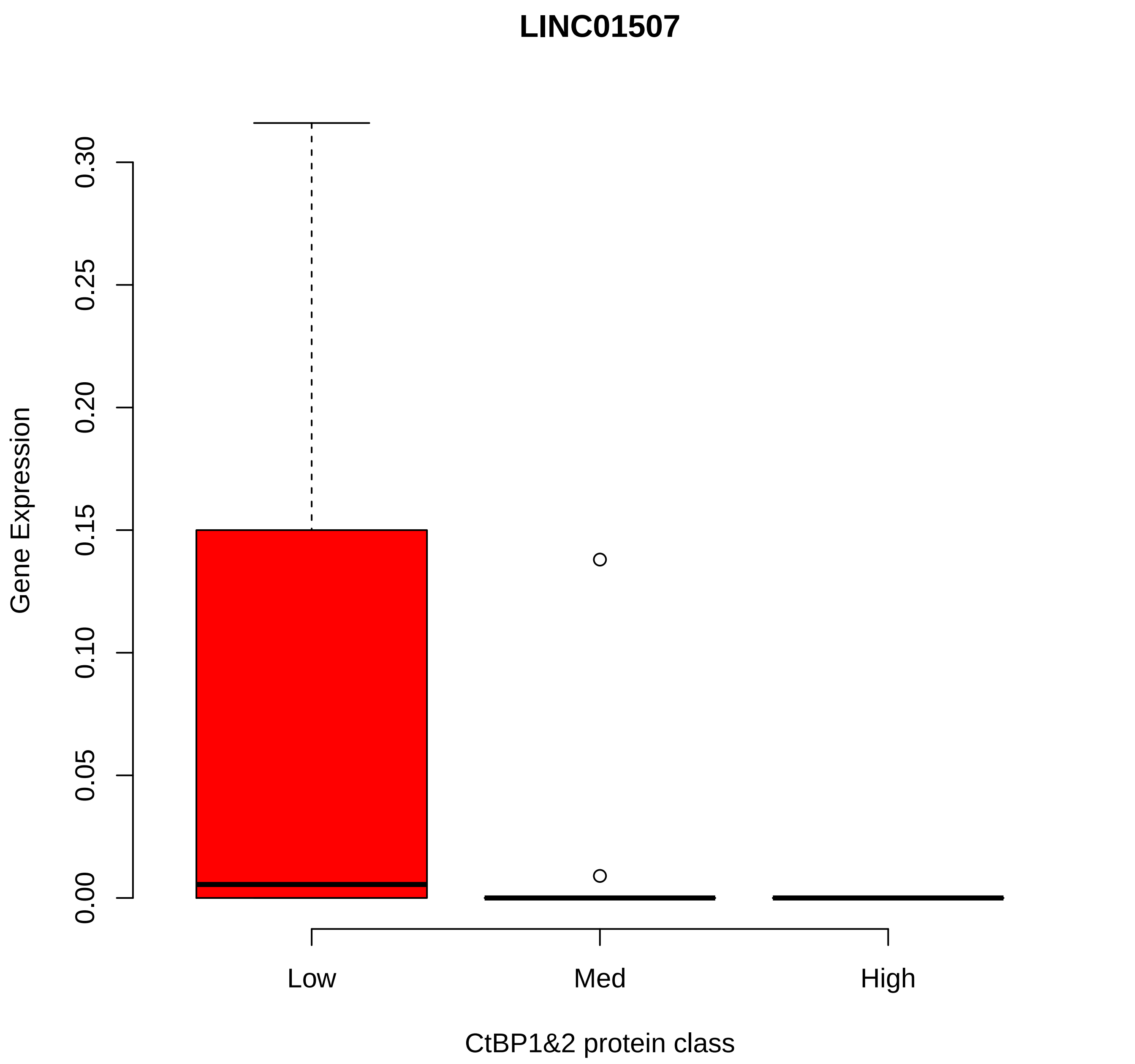

LINC01507
Gene Expression
0.00
0.05
0.10
0.15
0.20
0.25
0.30
Low
Med
High
CtBP1&2 protein class

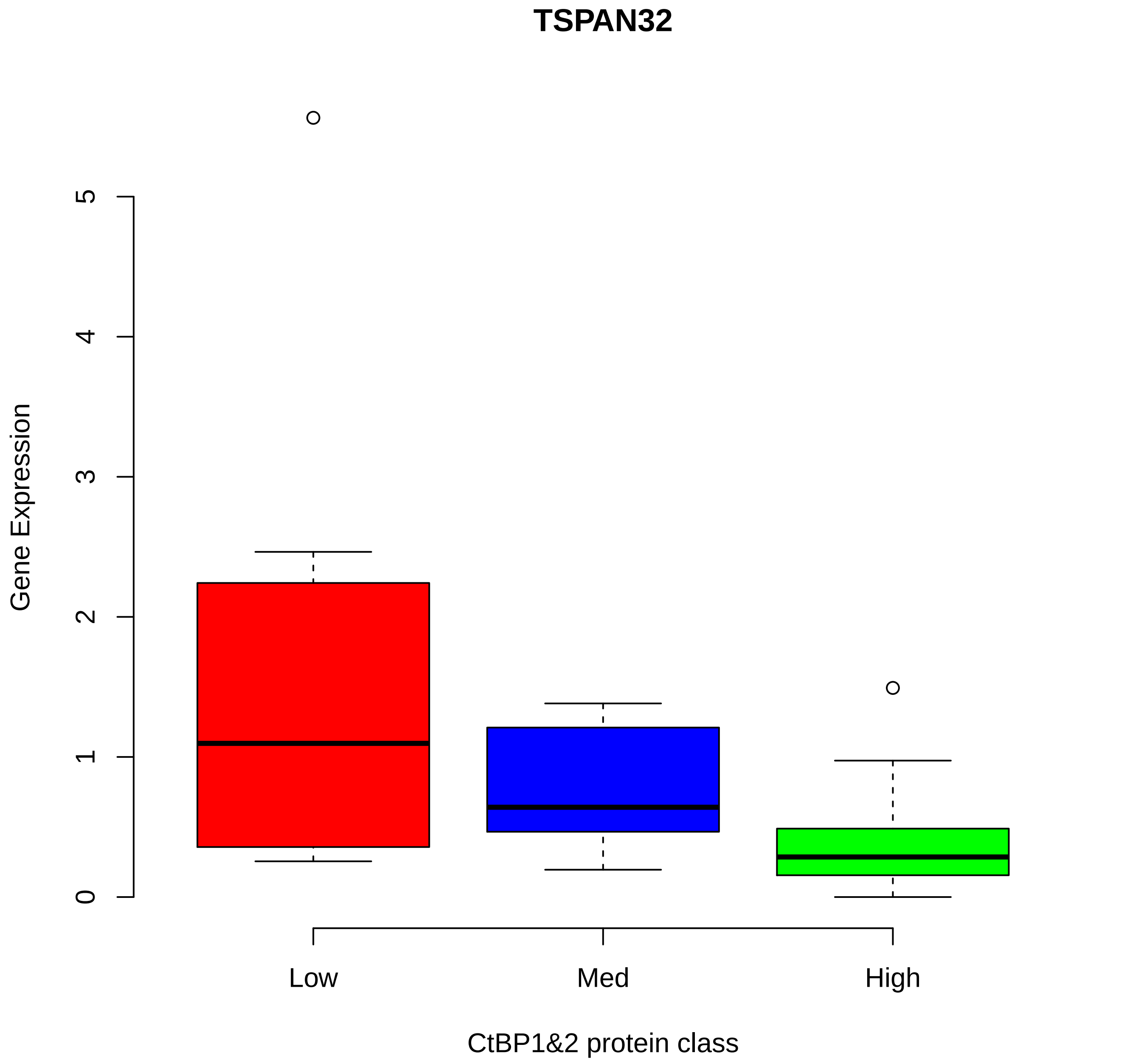

TSPAN32
Gene Expression
0
1
2
3
4
5
Low
Med
High
CtBP1&2 protein class

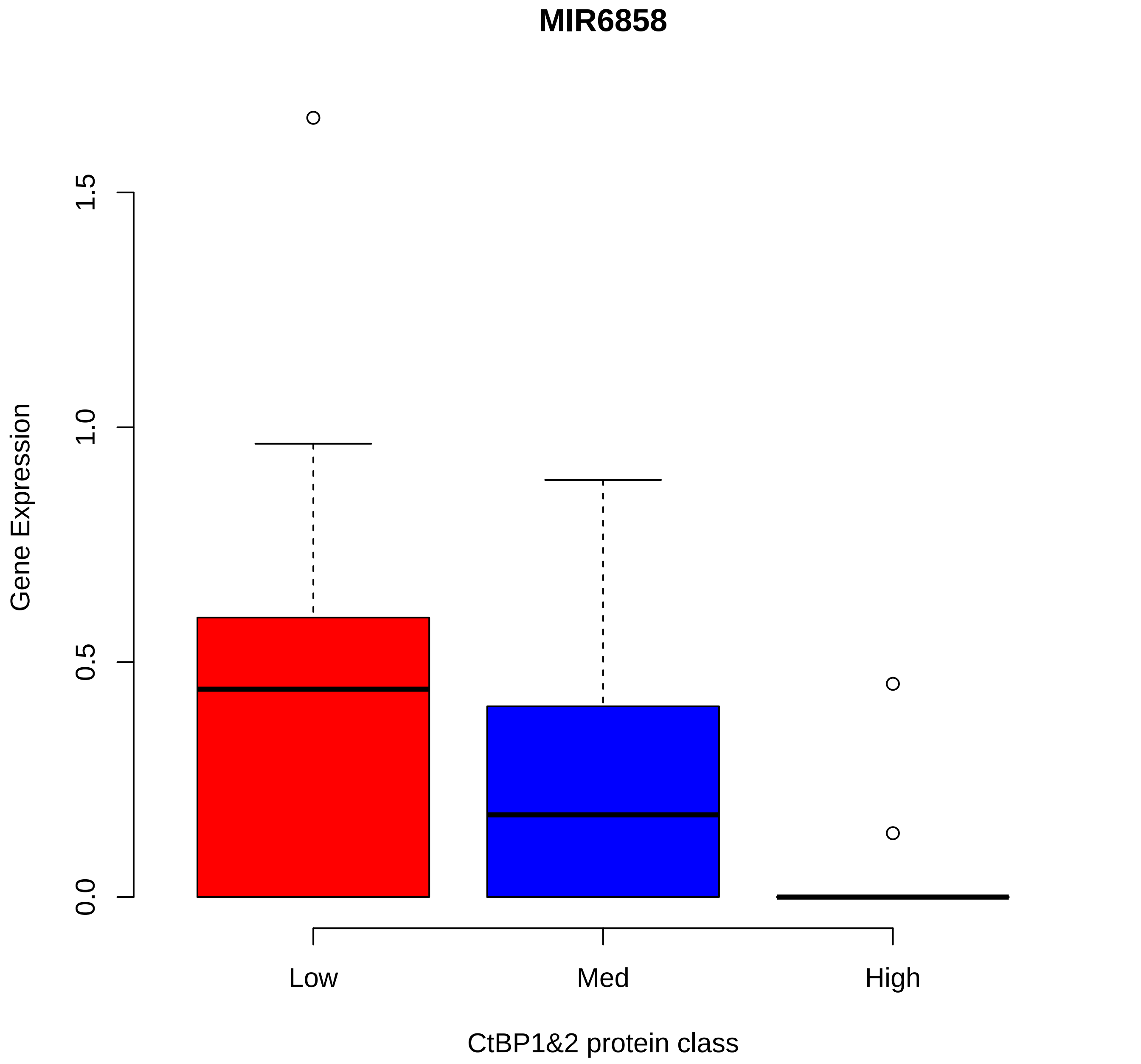

MIR6858
Gene Expression
0.0
0.5
1.0
1.5
Low
Med
High
CtBP1&2 protein class

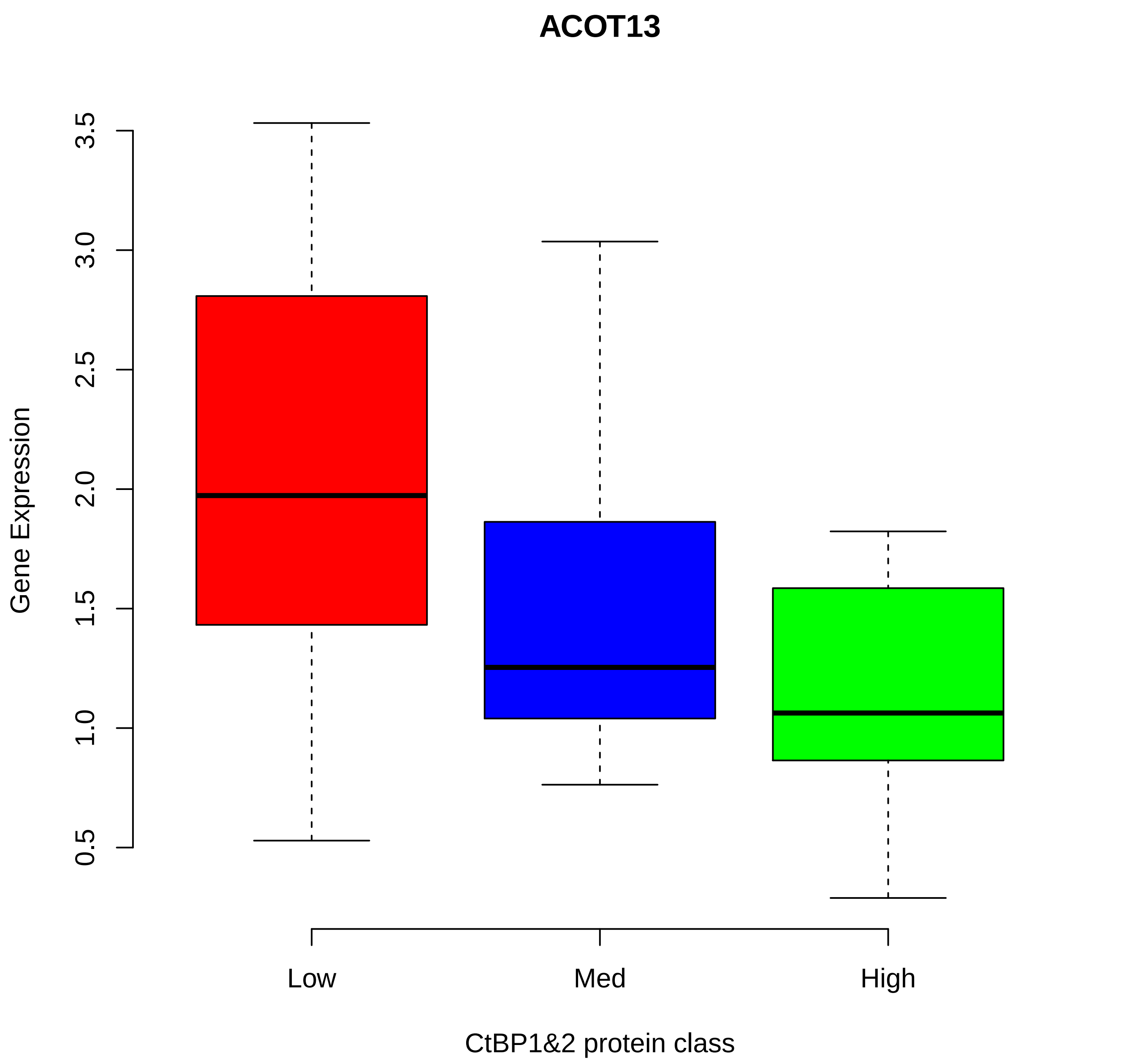

ACOT13
Gene Expression
3.5
3.0
2.5
2.0
1.5
1.0
0.5
Low
Med
High
CtBP1&2 protein class

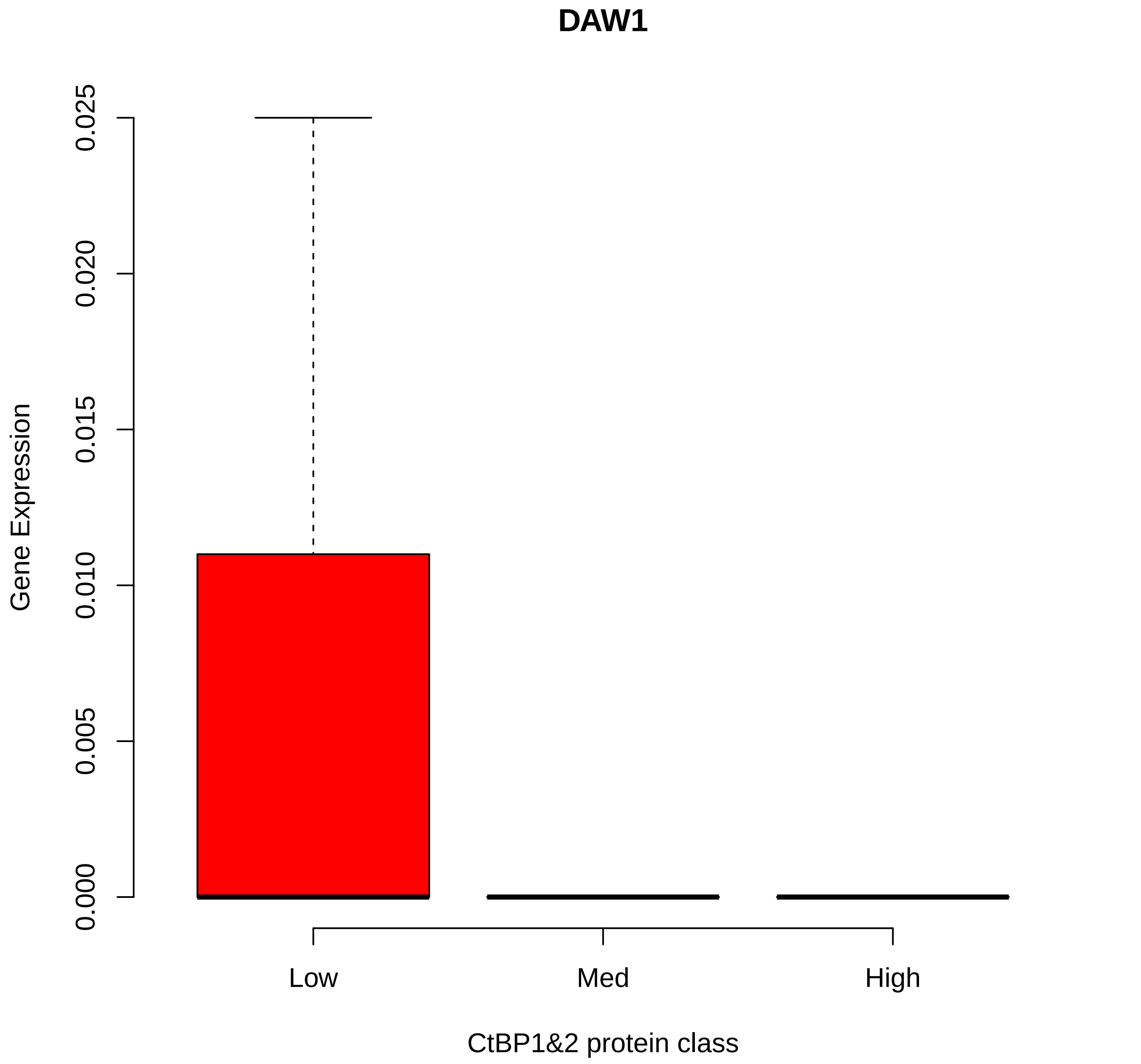

DAW1
Gene Expression
0.000
0.005
0.010
0.015
0.020
0.025
Low
Med
High
CtBP1&2 protein class

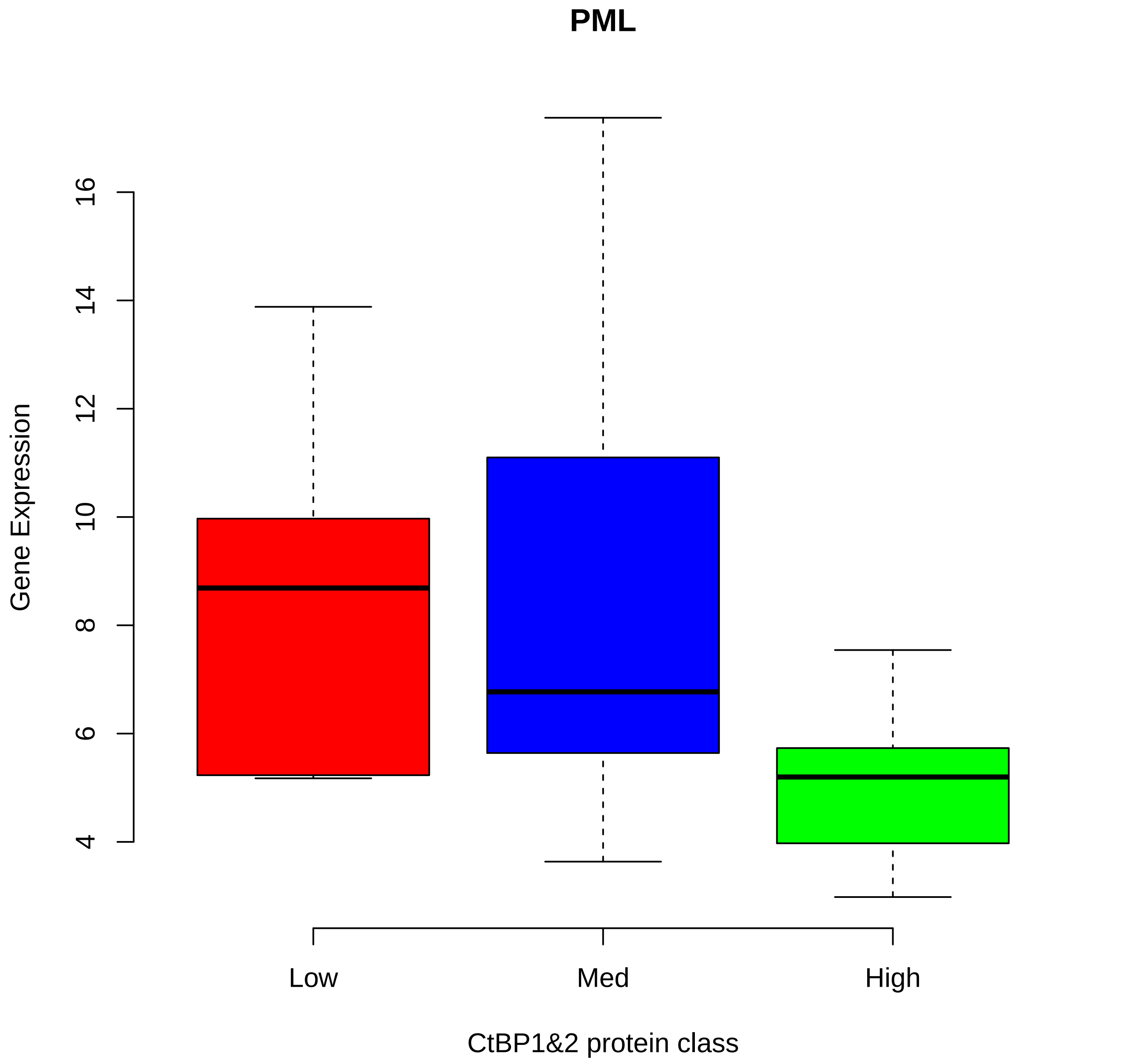
PML
Gene Expression
4
6
8
10
12
14
16
Low
Med
High
CtBP1&2 protein class

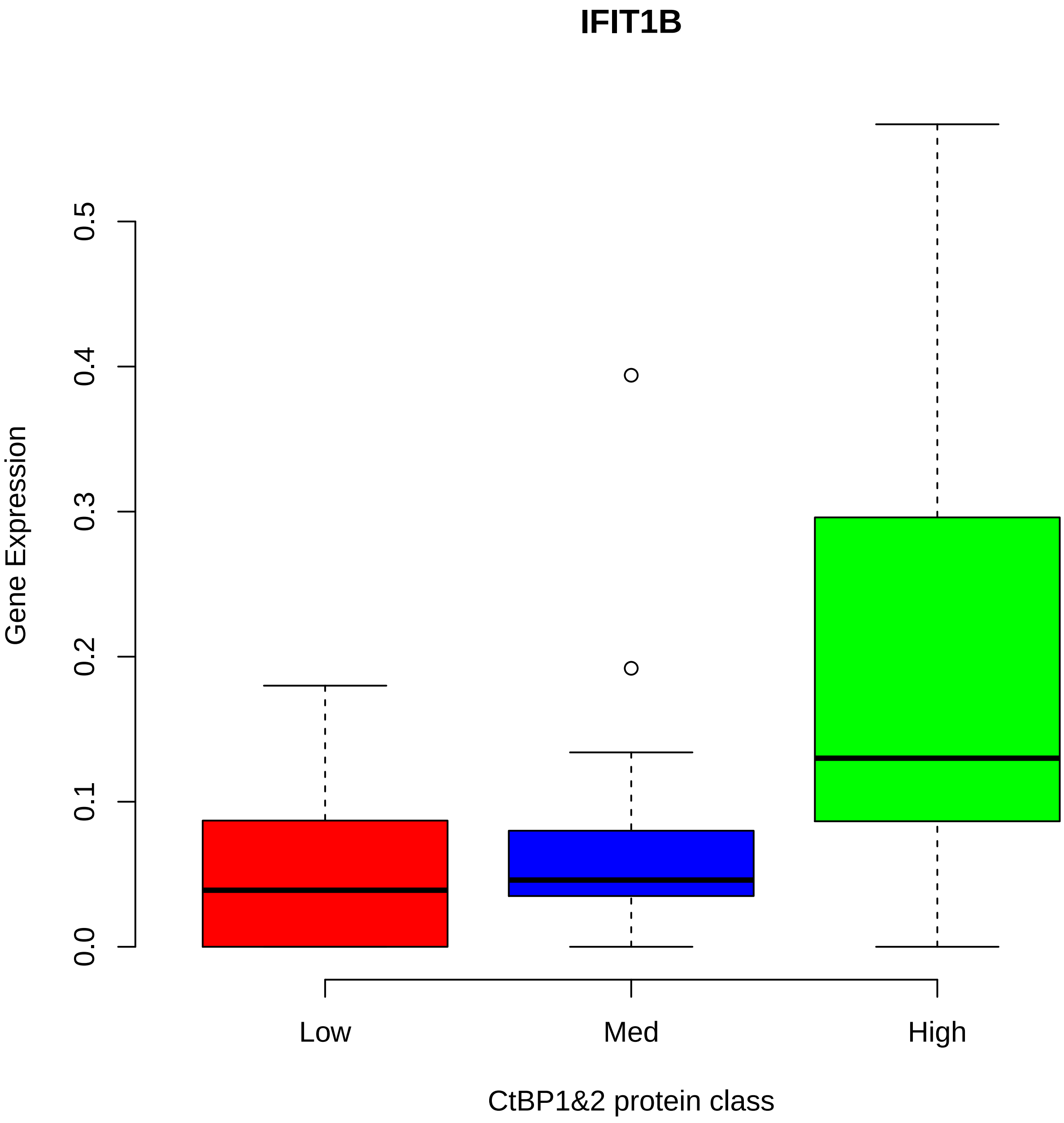
IFIT1B
Gene Expression
0.0
0.1
0.2
0.3
0.4
0.5
Low
Med
High
CtBP1&2 protein class

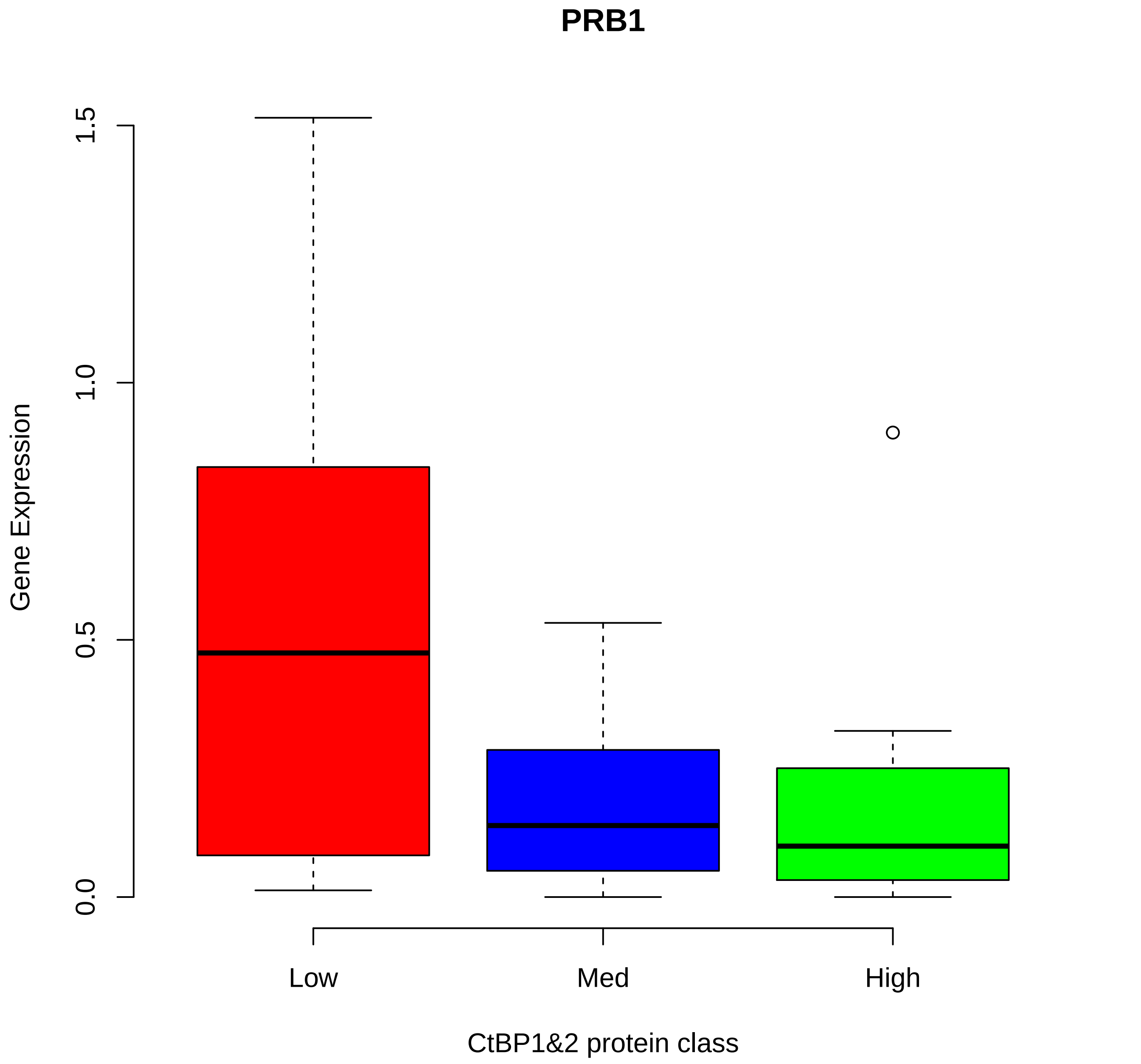
PRB1
Gene Expression
0.0
0.5
1.0
1.5
Low
Med
High
CtBP1&2 protein class

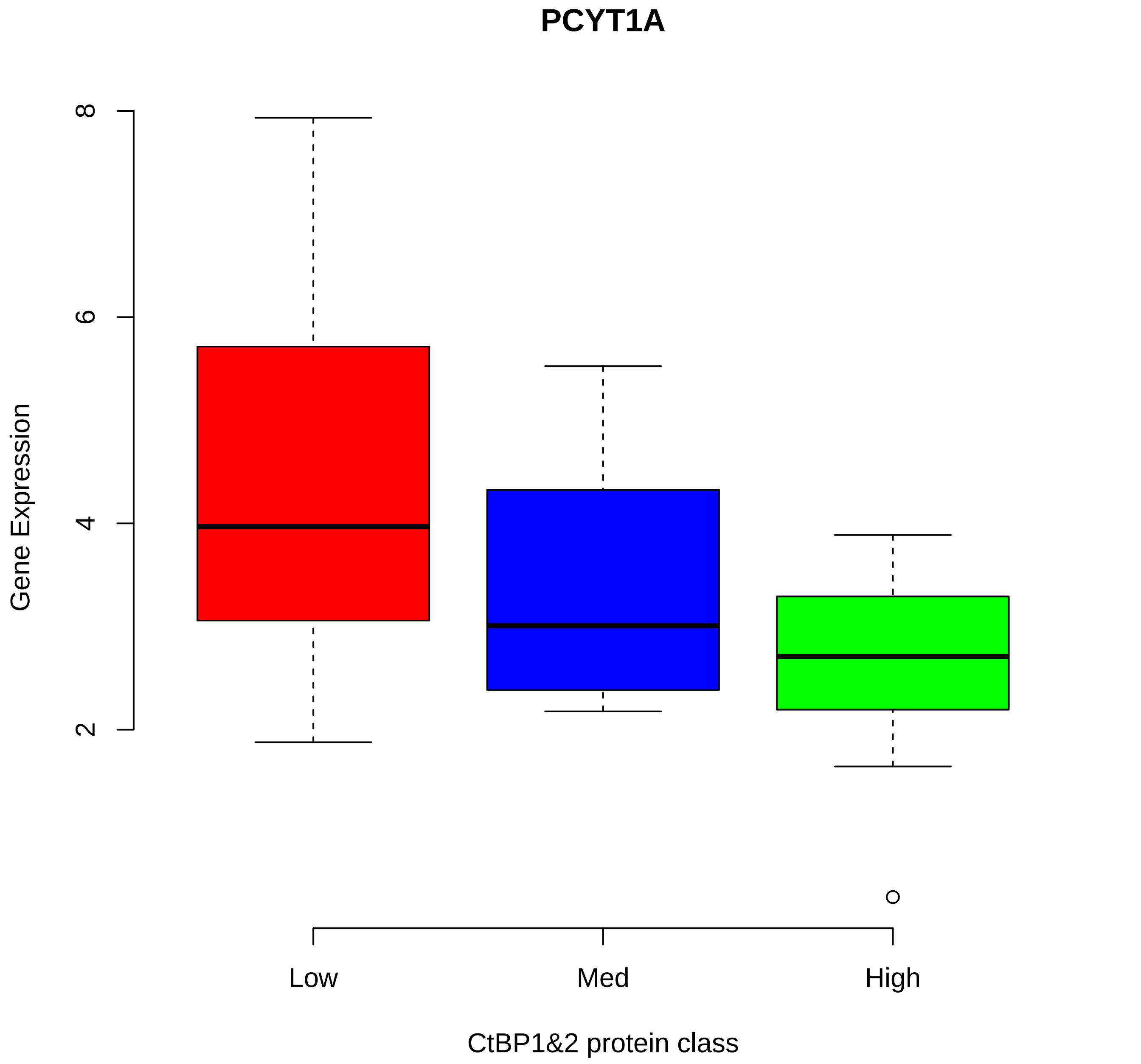
PCYT1A
Gene Expression
8
6
4
2
Low
Med
High
CtBP1&2 protein class

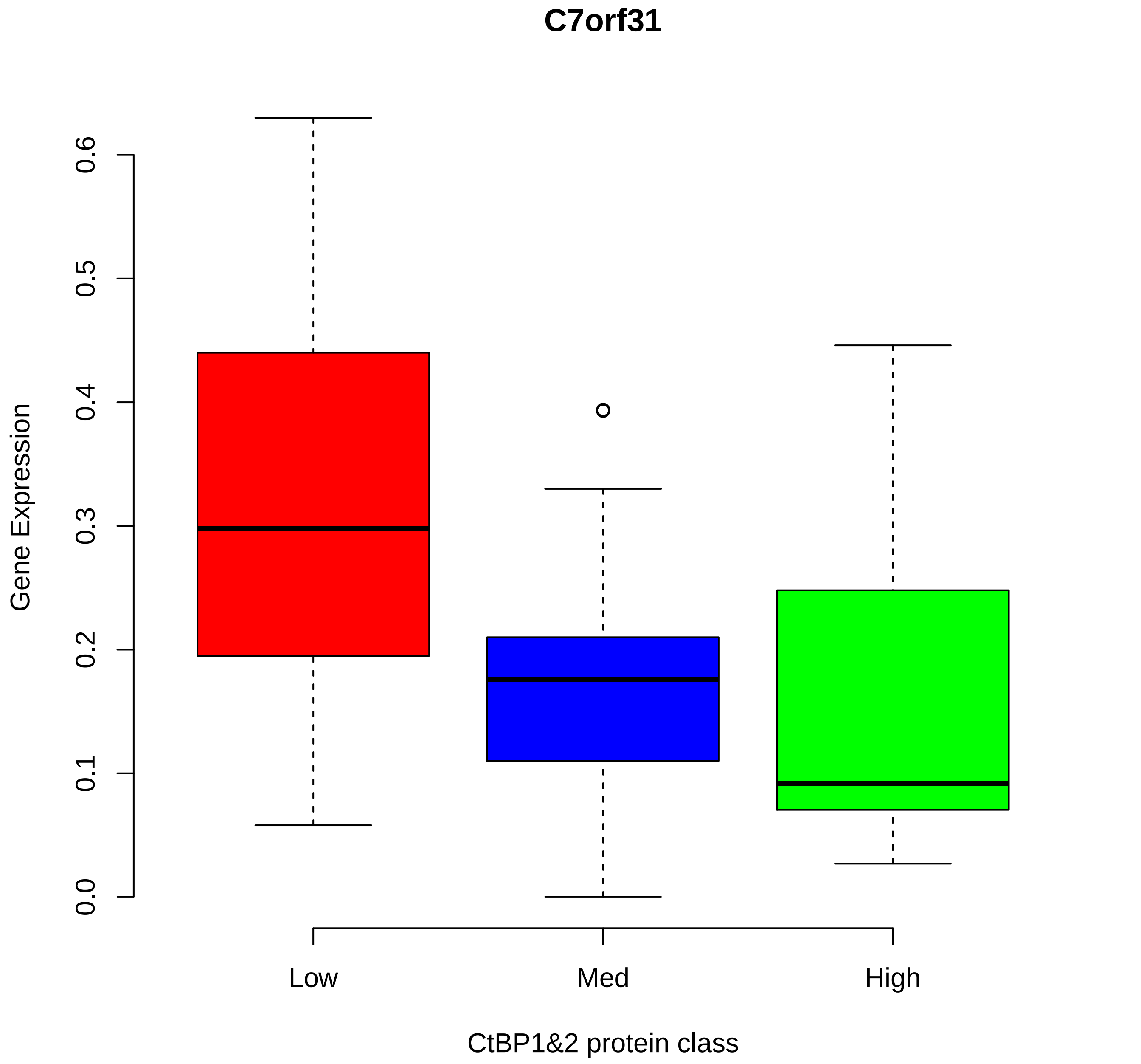

C7orf31
Gene Expression
0.0
0.1
0.2
0.3
0.4
0.5
0.6
Low
Med
High
CtBP1&2 protein class

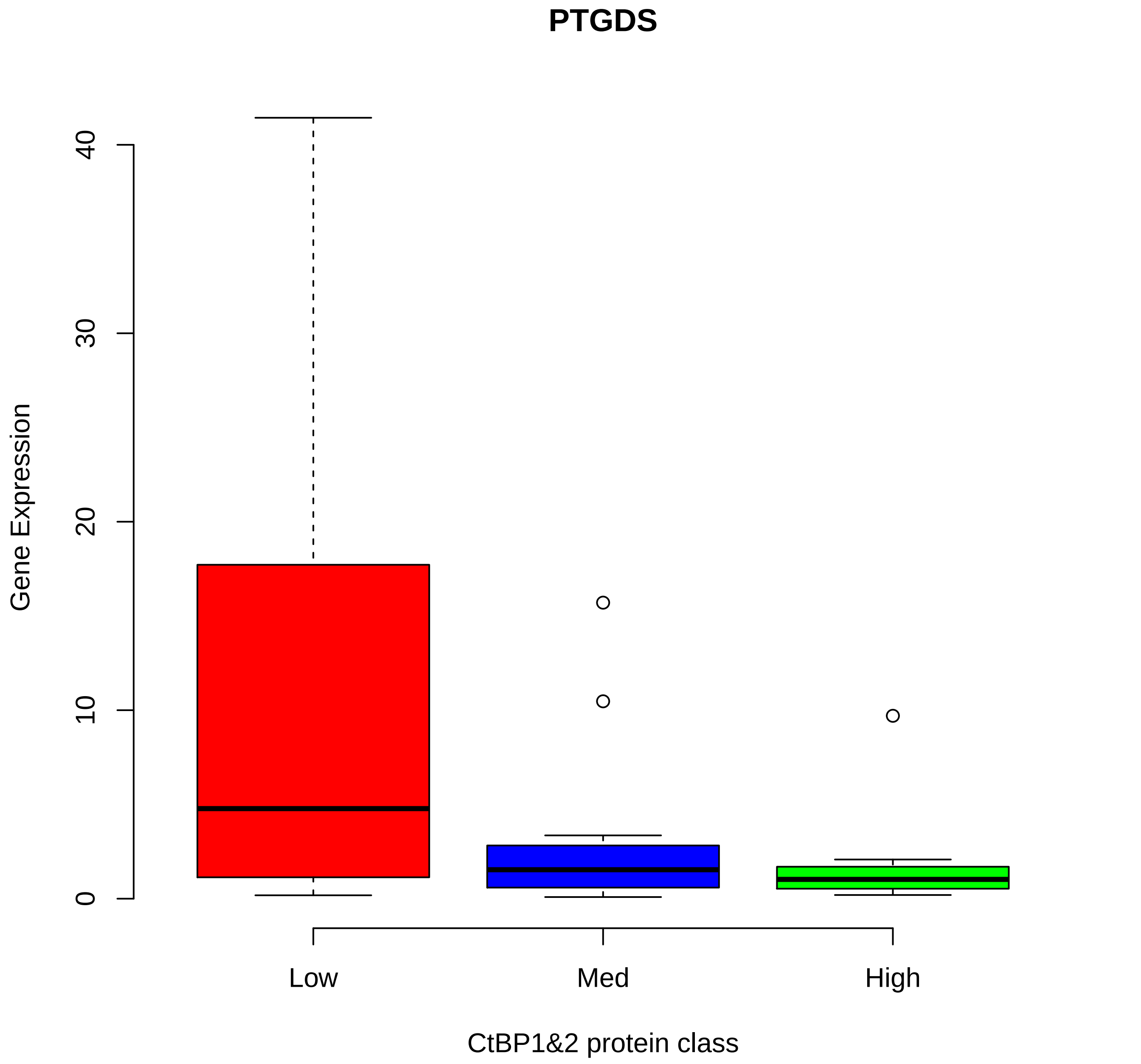
PTGDS
Gene Expression
0
10
20
30
40
Low
Med
High
CtBP1&2 protein class

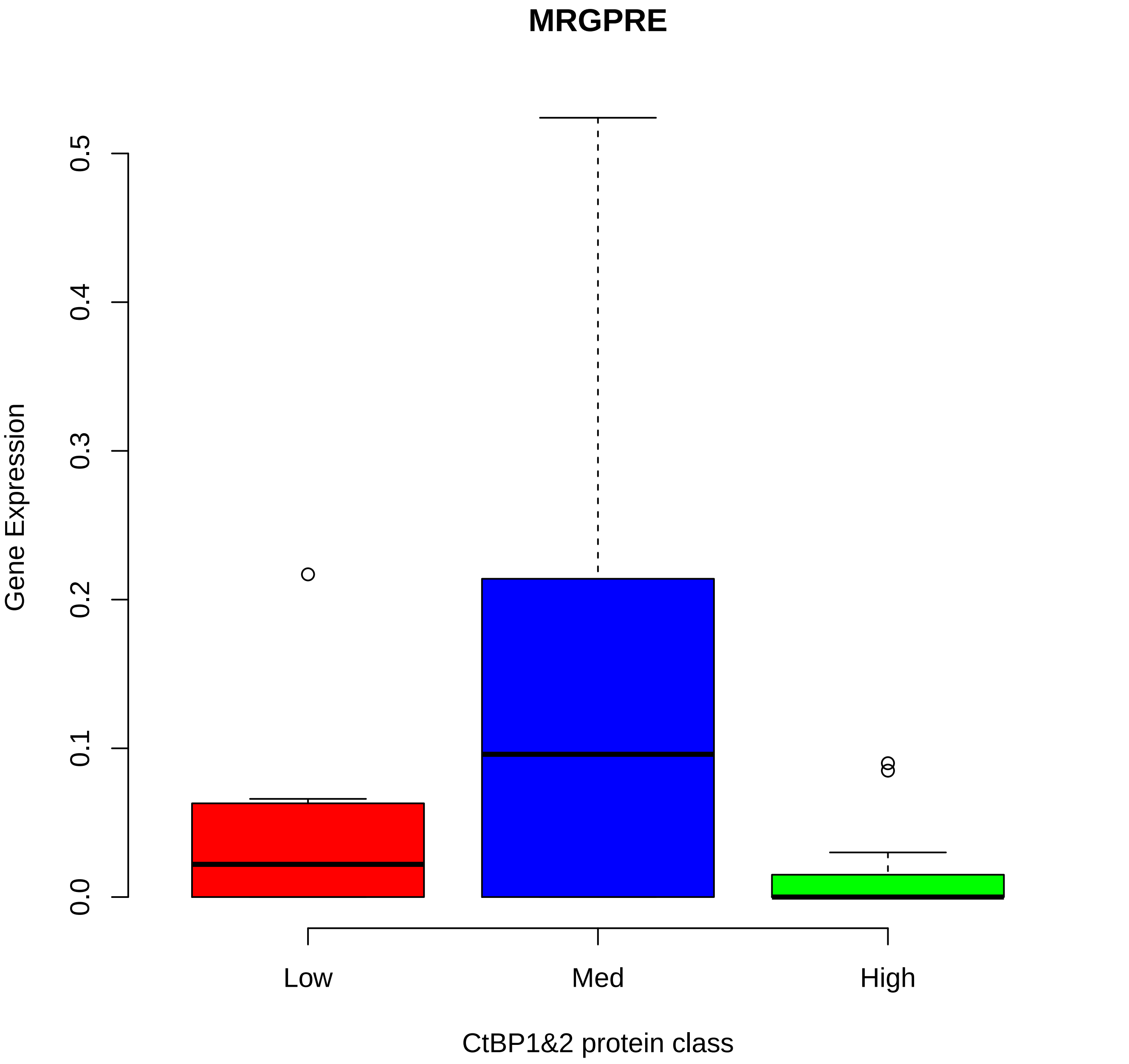
MRGPRE
Gene Expression
0.0
0.1
0.2
0.3
0.4
0.5
Low
Med
High
CtBP1&2 protein class

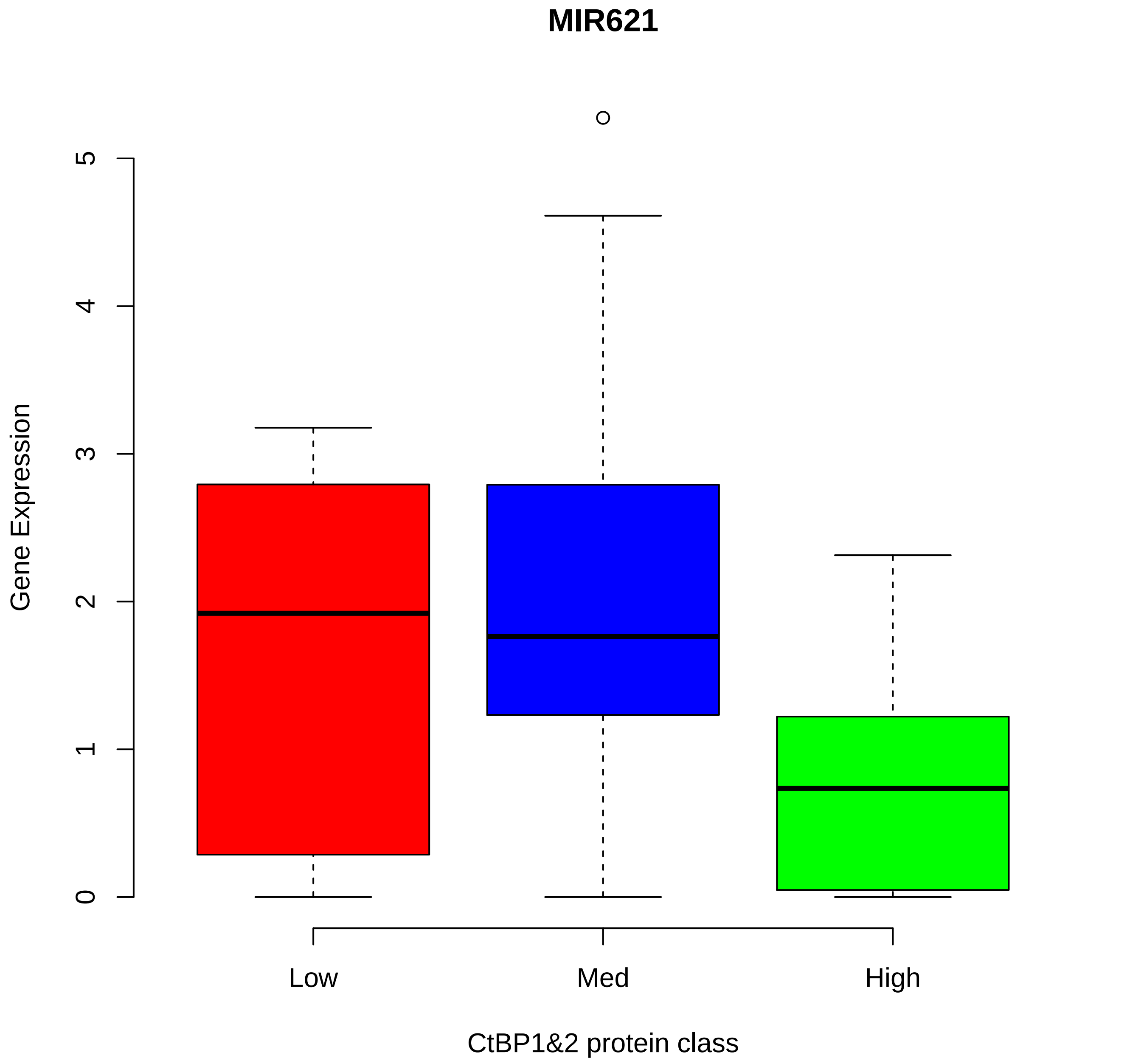

MIR621
Gene Expression
0
1
2
3
4
5
Low
Med
High
CtBP1&2 protein class

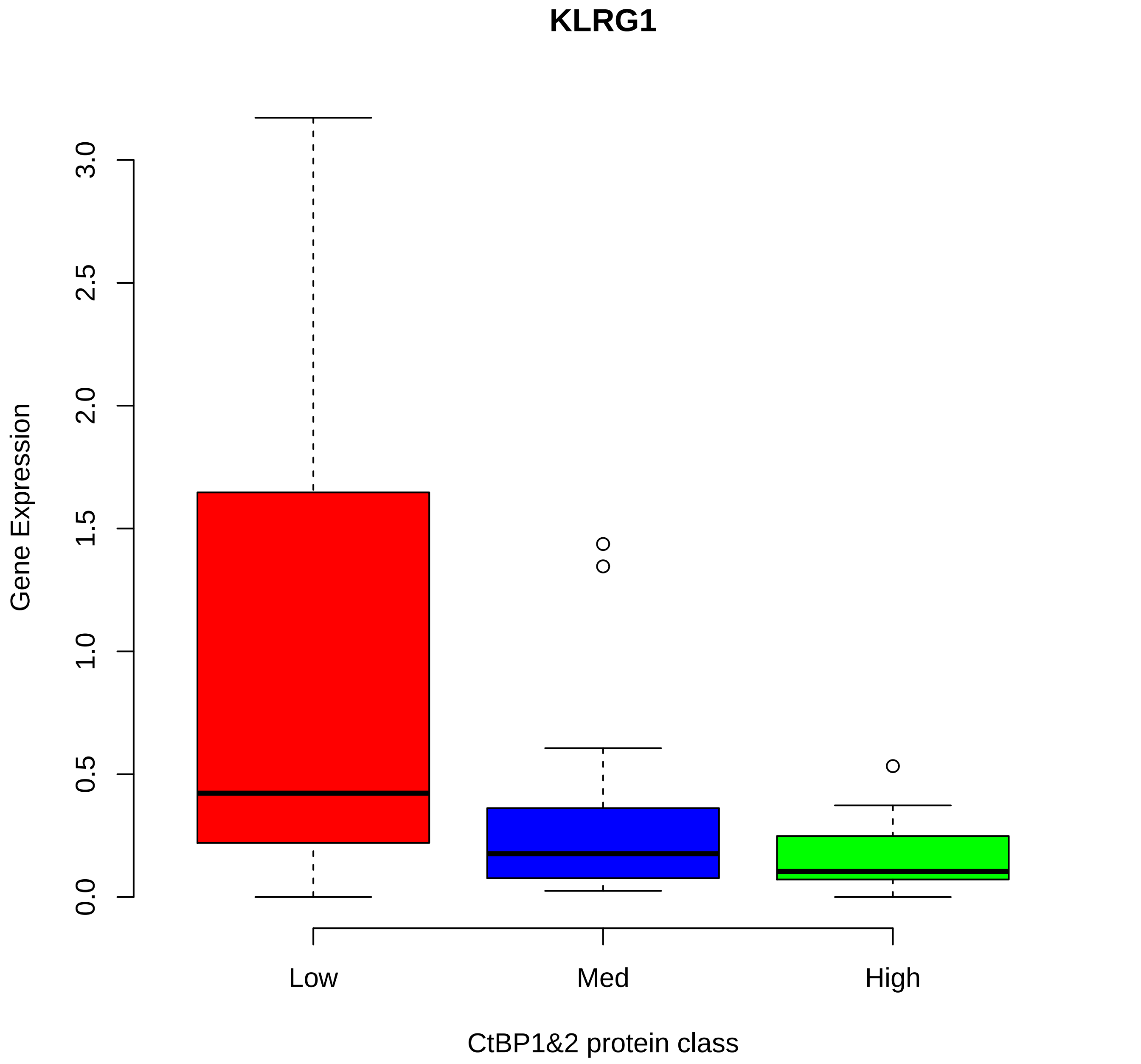

KLRG1
Gene Expression
0.0
0.5
1.0
1.5
2.0
2.5
3.0
Low
Med
High
CtBP1&2 protein class

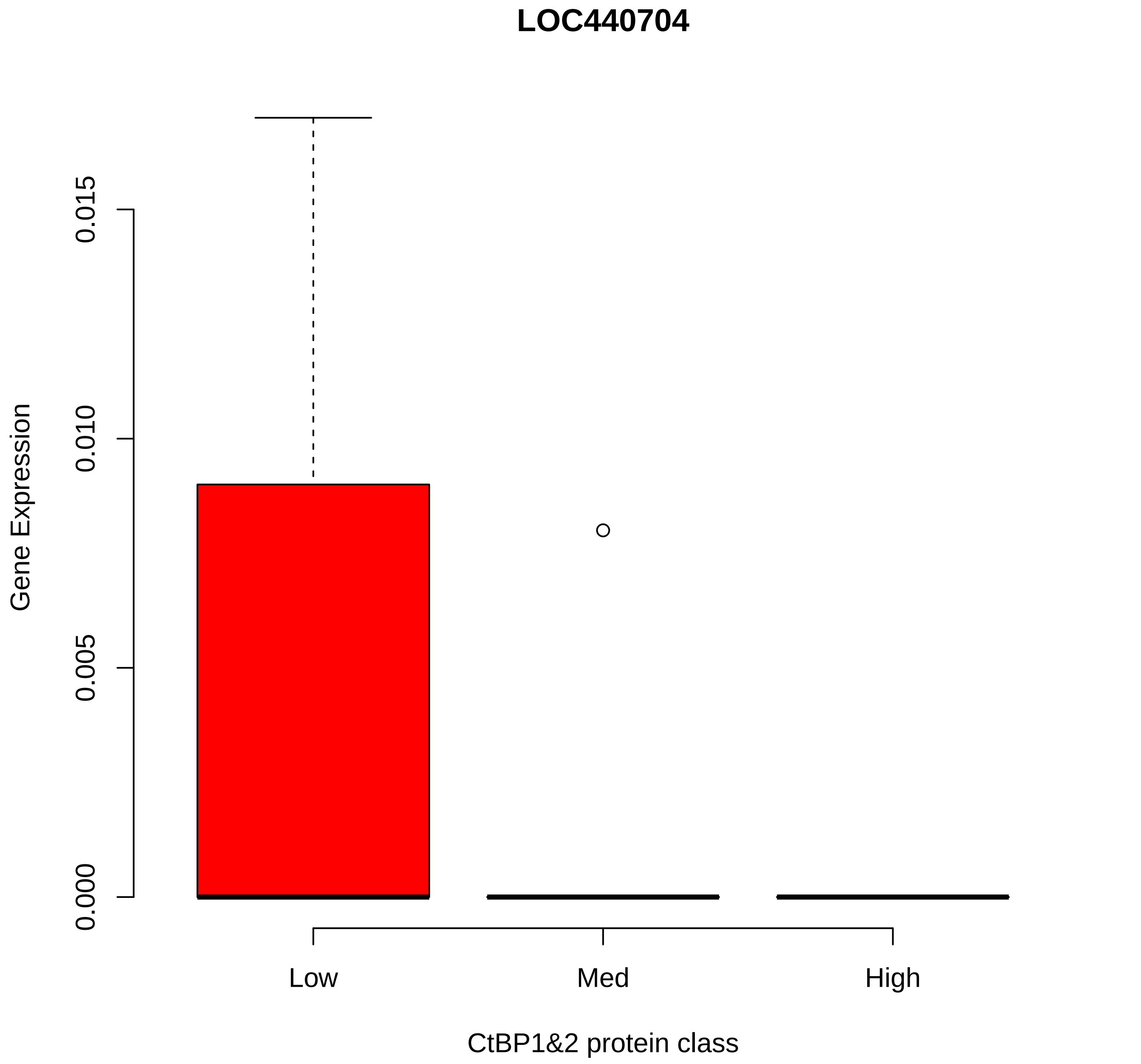
LOC440704
Gene Expression
0.000
0.005
0.010
0.015
Low
Med
High
CtBP1&2 protein class

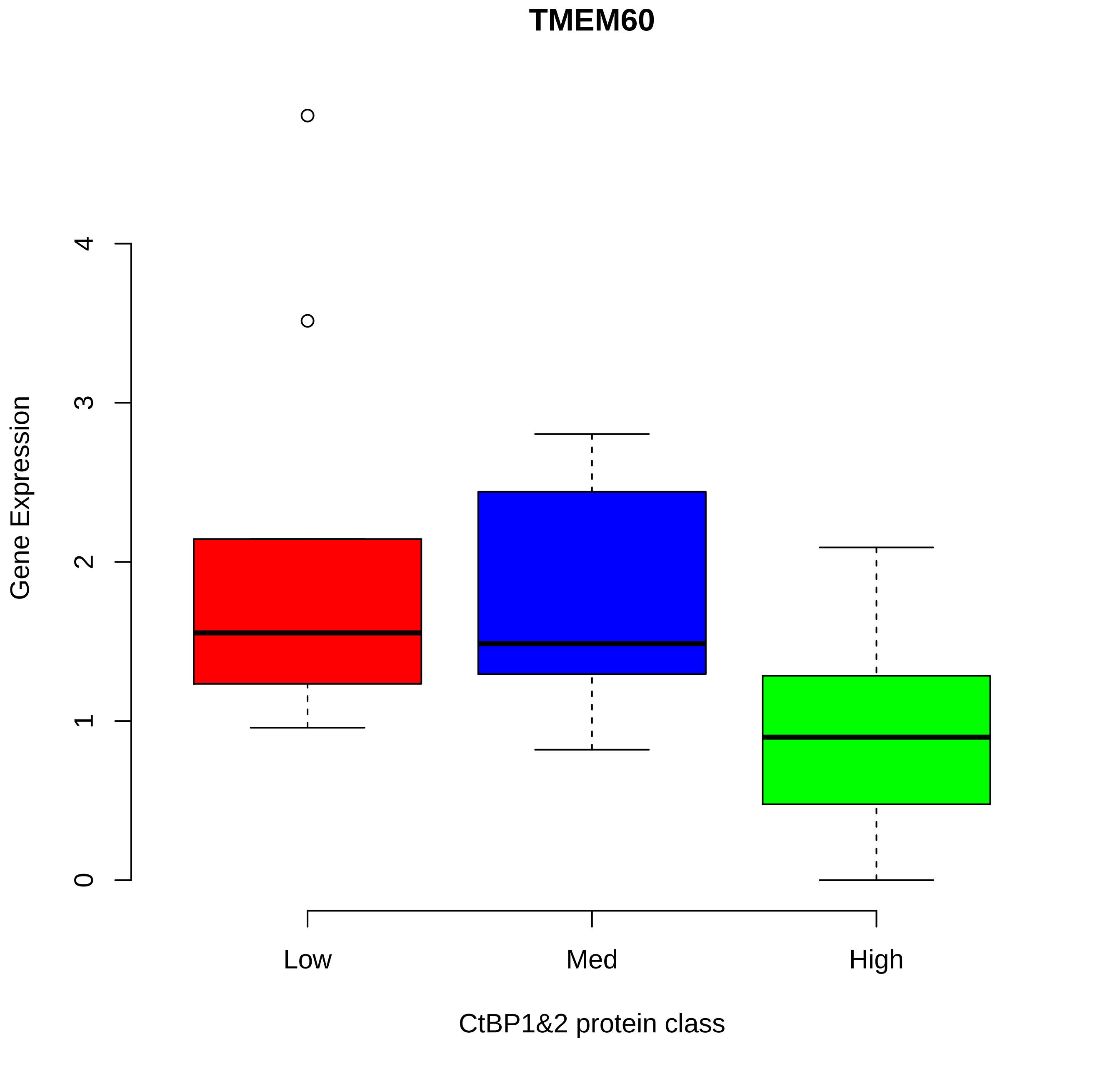
TMEM60
Gene Expression
0
1
2
3
4
Low
Med
High
CtBP1&2 protein class

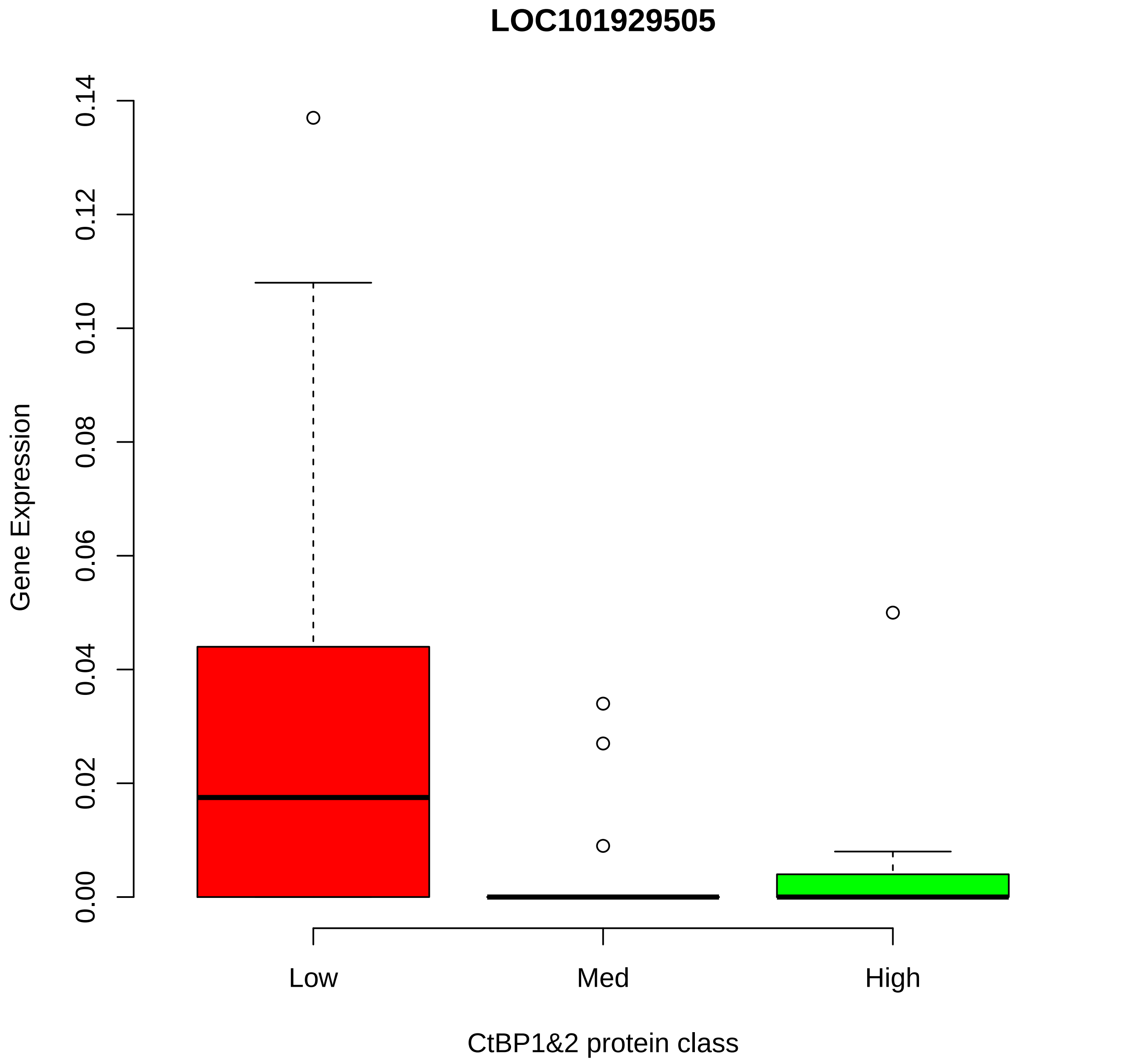

LOC101929505
Gene Expression
0.00
0.02
0.04
0.06
0.08
0.10
0.12
0.14
Low
Med
High
CtBP1&2 protein class

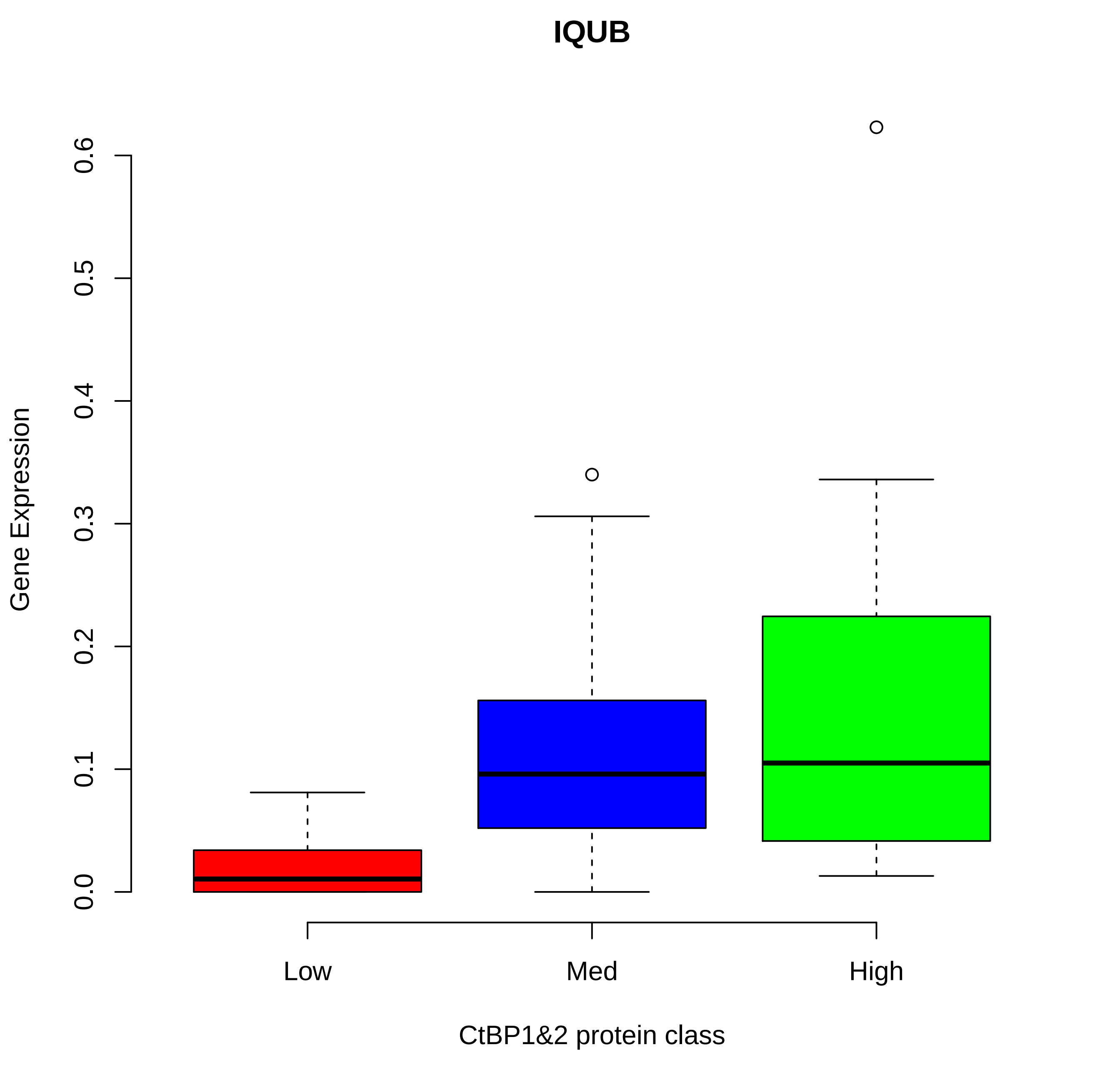

IQUB
Gene Expression
0.0
0.1
0.2
0.3
0.4
0.5
0.6
Low
Med
High
CtBP1&2 protein class

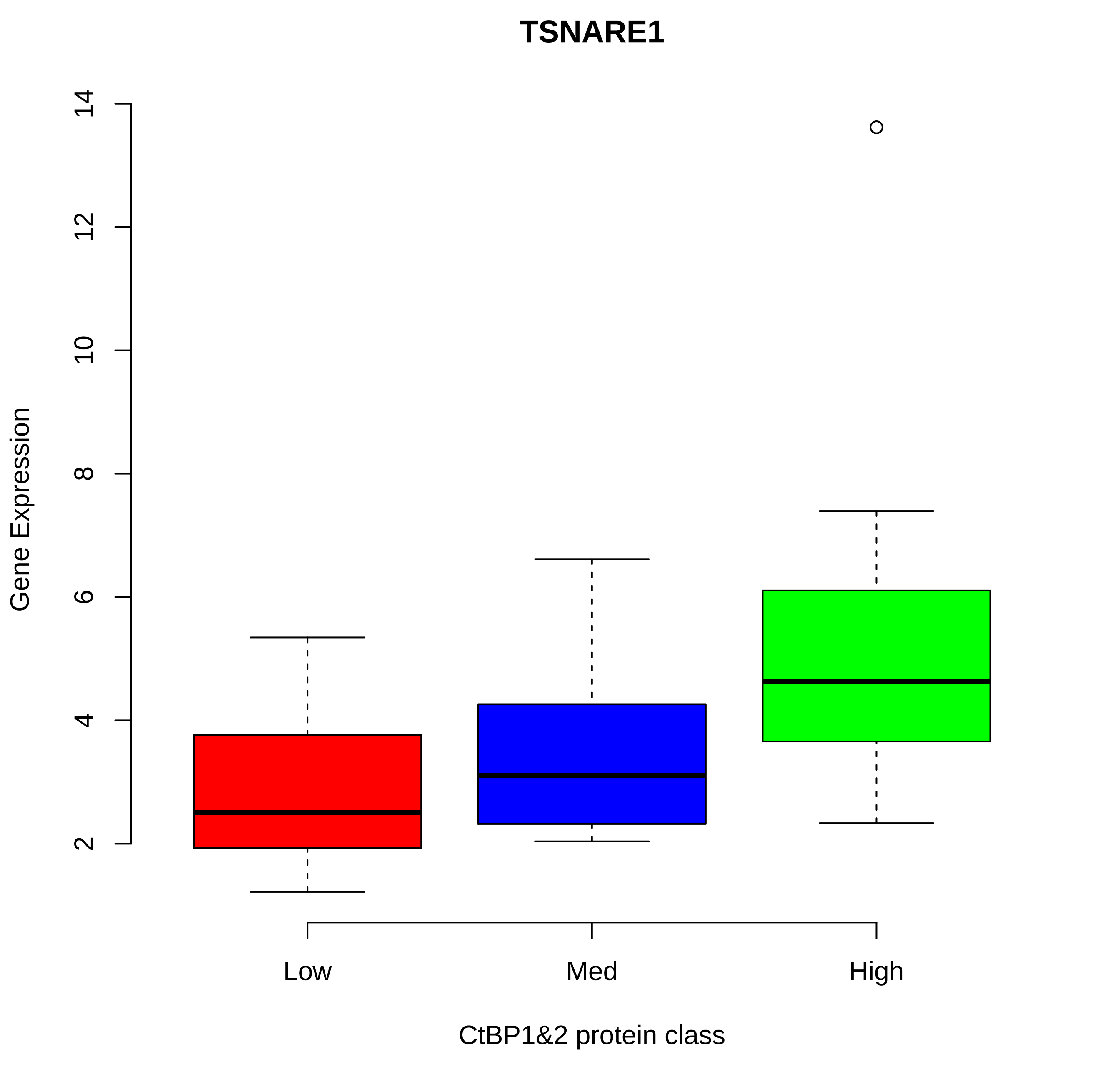

TSNARE1
Gene Expression
2
4
6
8
10
12
14
Low
Med
High
CtBP1&2 protein class

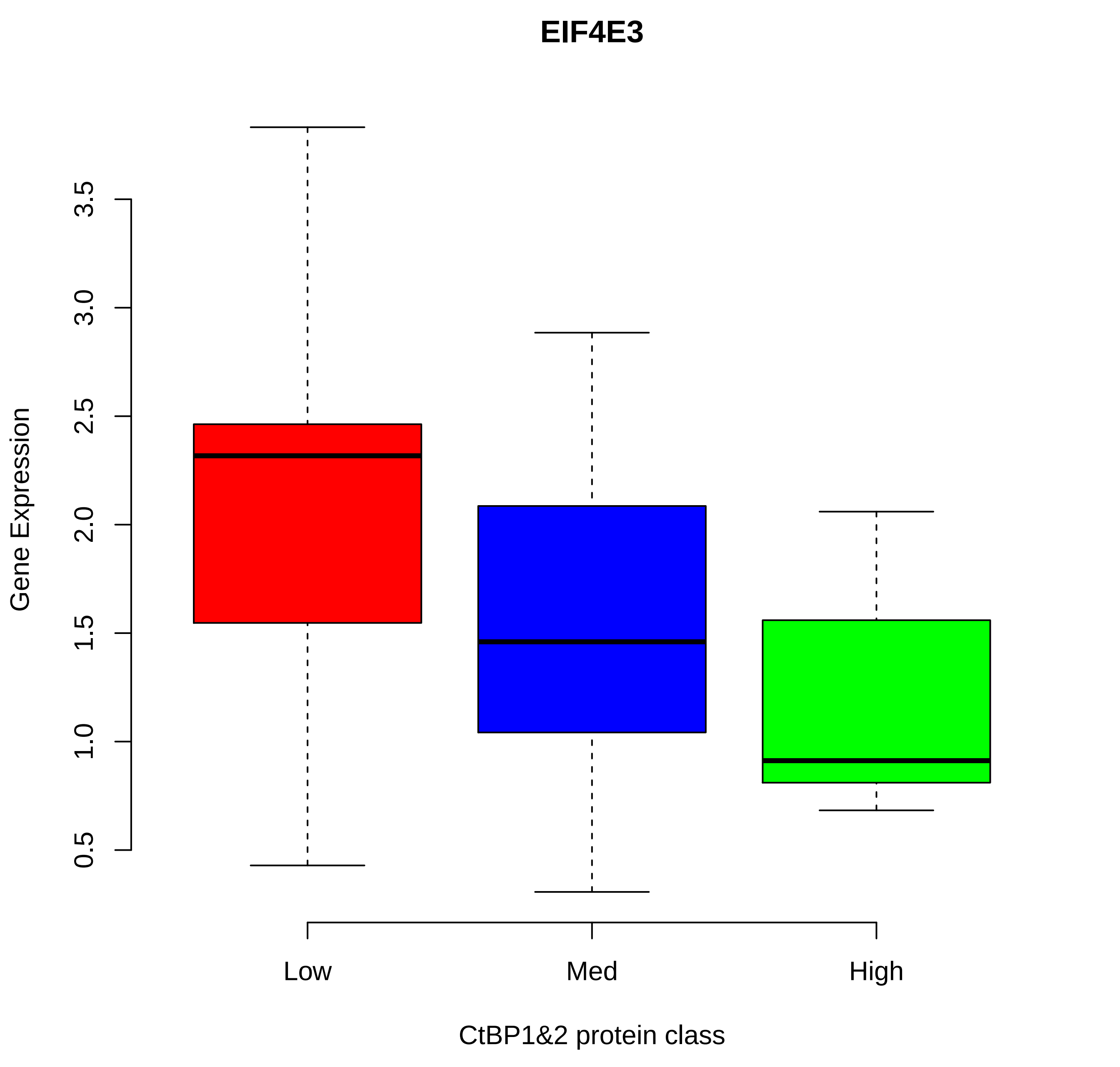

EIF4E3
Gene Expression
0.5
1.0
1.5
2.0
2.5
3.0
3.5
Low
Med
High
CtBP1&2 protein class

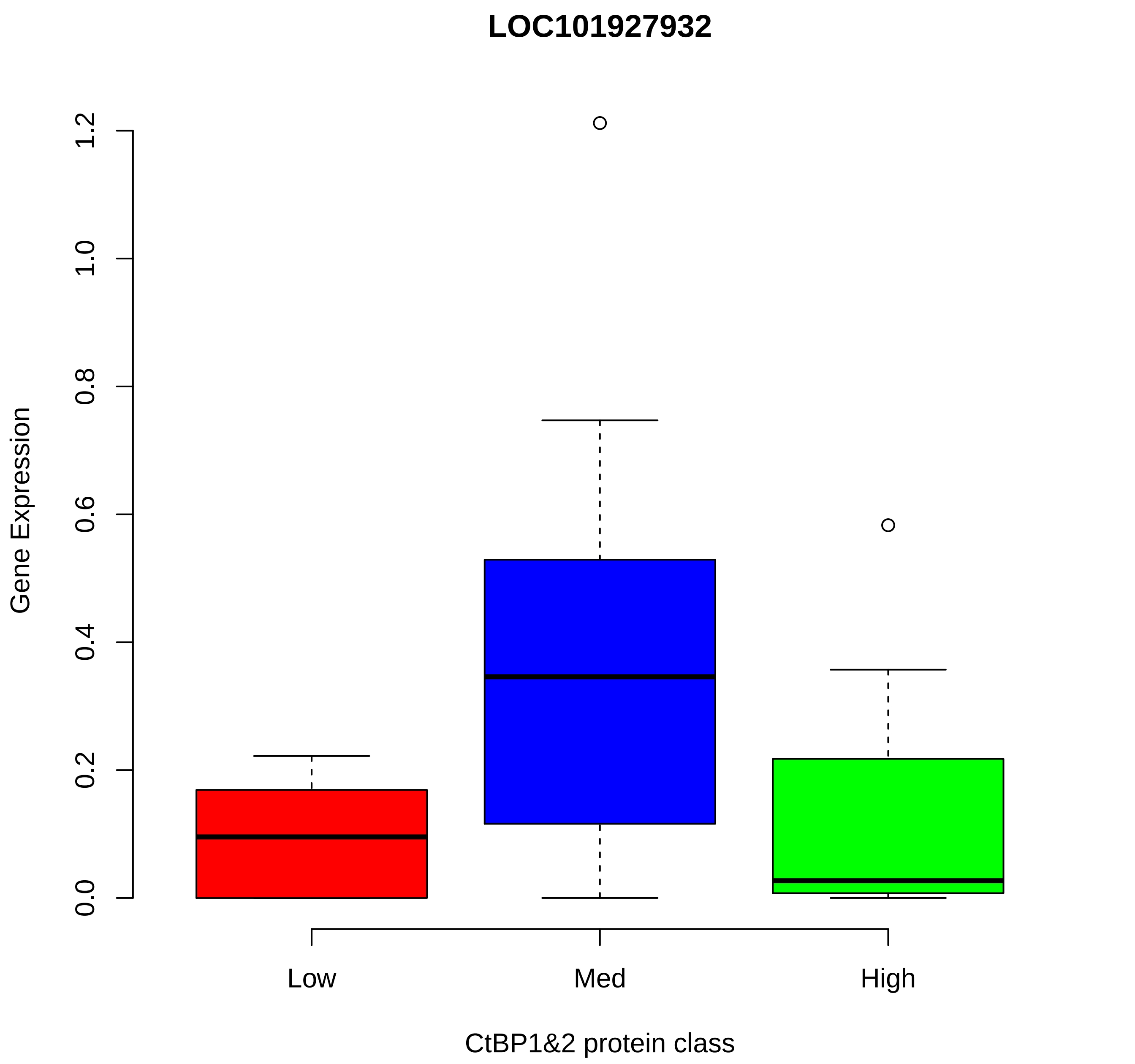

LOC101927932
Gene Expression
0.0
0.2
0.4
0.6
0.8
1.0
1.2
Low
Med
High
CtBP1&2 protein class

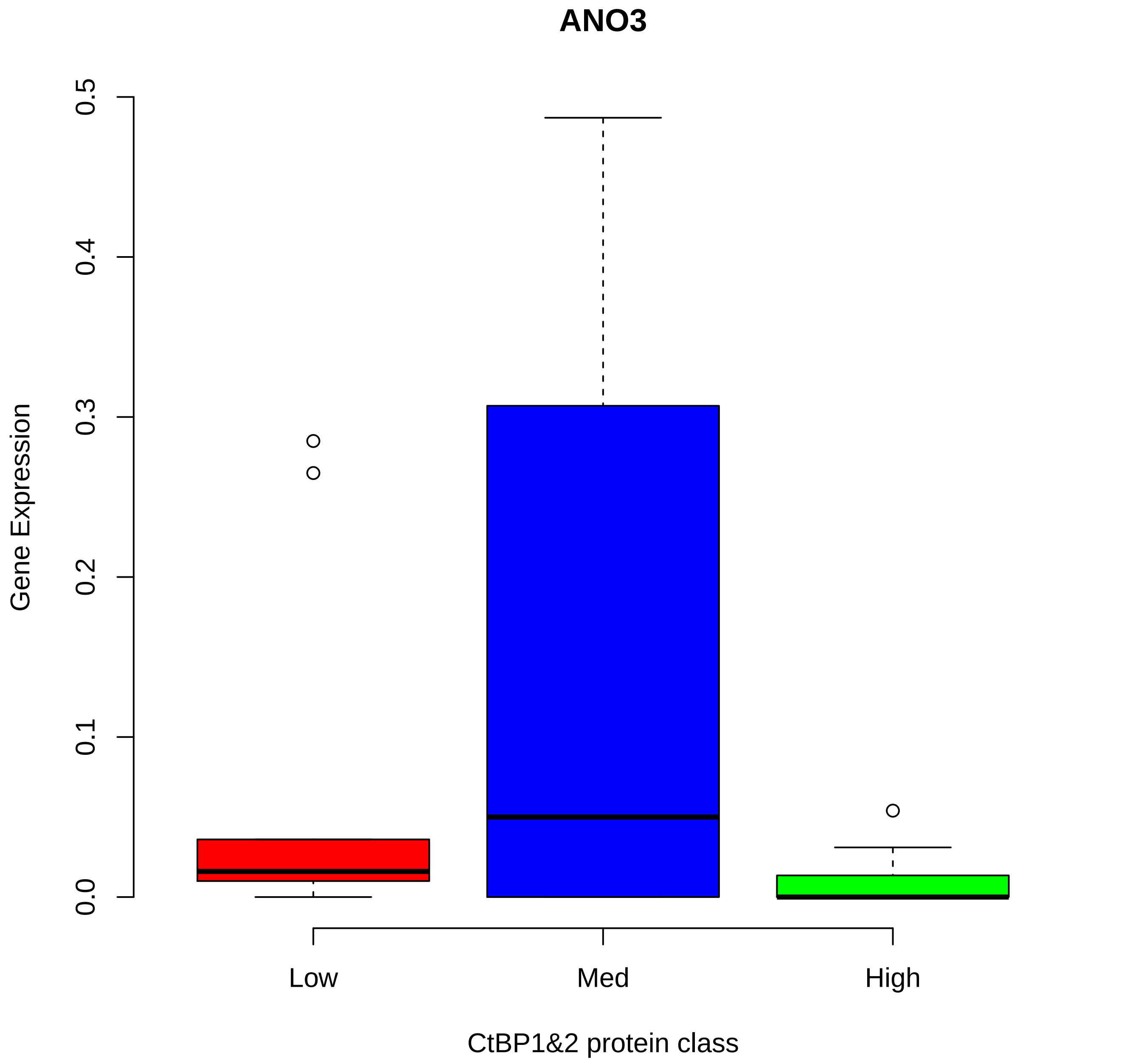
ANO3
Gene Expression
0.0
0.1
0.2
0.3
0.4
0.5
Low
Med
High
CtBP1&2 protein class

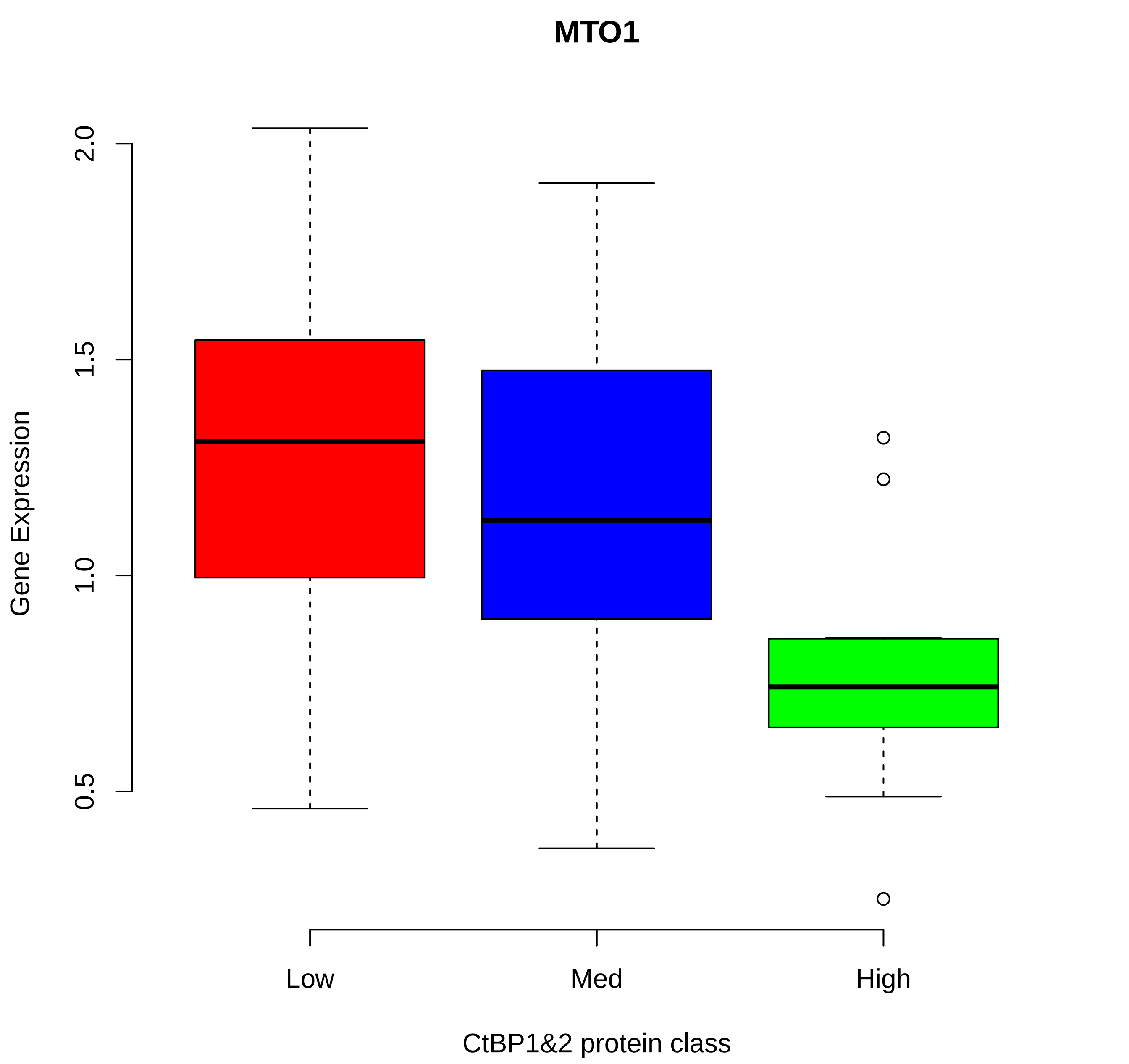

MTO1
Gene Expression
0.5
1.0
1.5
2.0
Low
Med
High
CtBP1&2 protein class

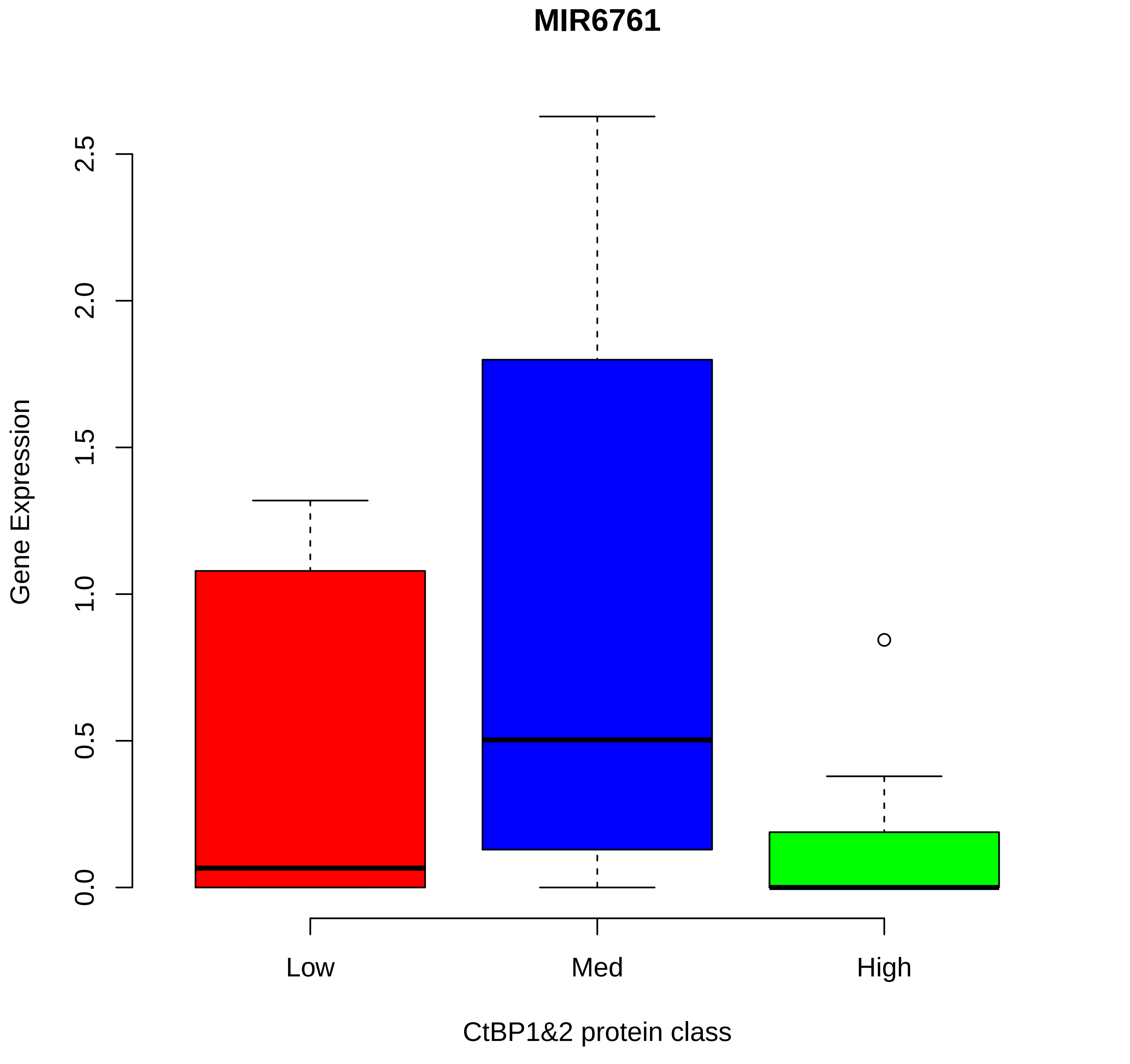

MIR6761
Gene Expression
0.0
0.5
1.0
1.5
2.0
2.5
Low
Med
High
CtBP1&2 protein class

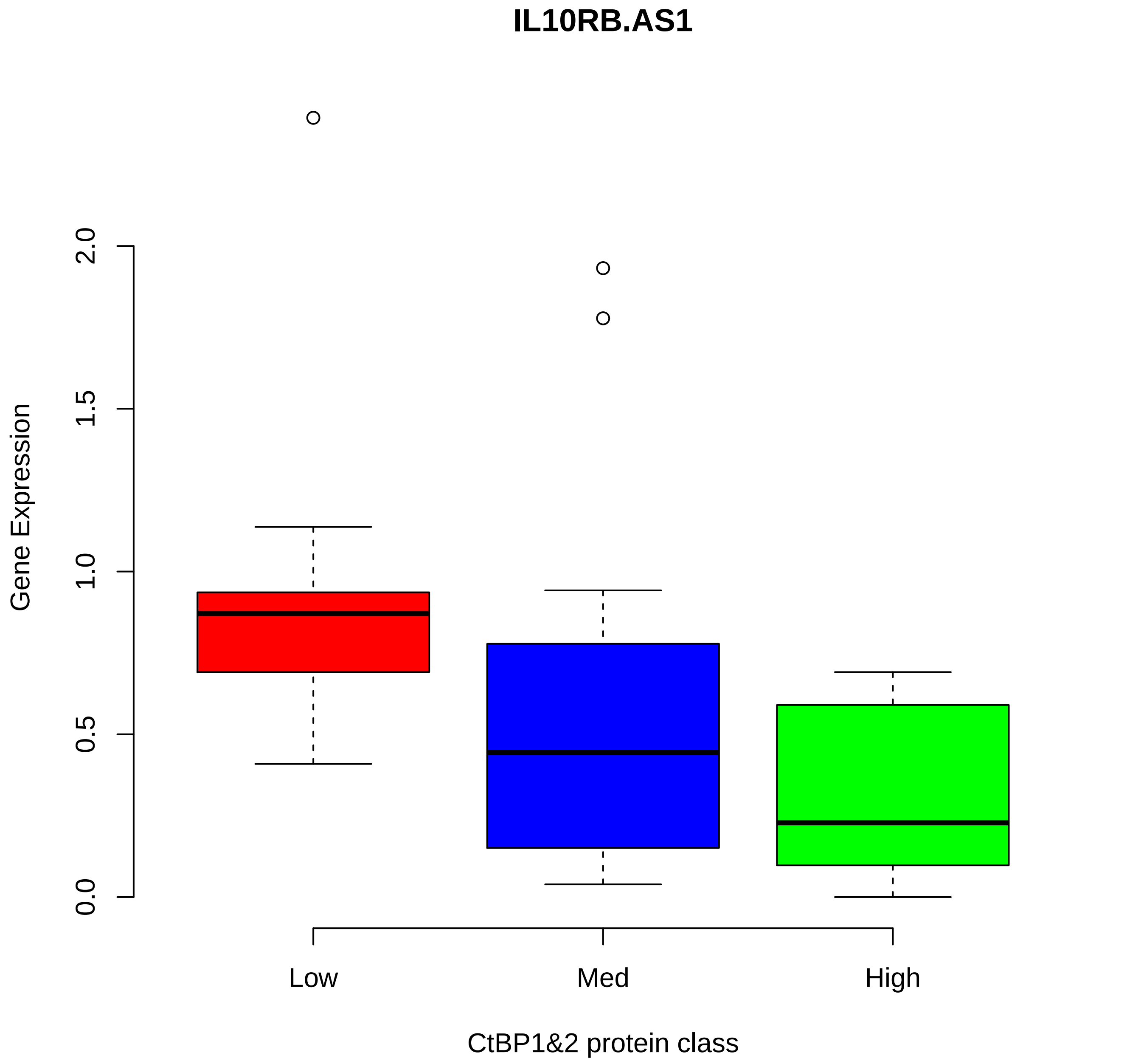
IL10RB.AS1
Gene Expression
0.0
0.5
1.0
1.5
2.0
Low
Med
High
CtBP1&2 protein class

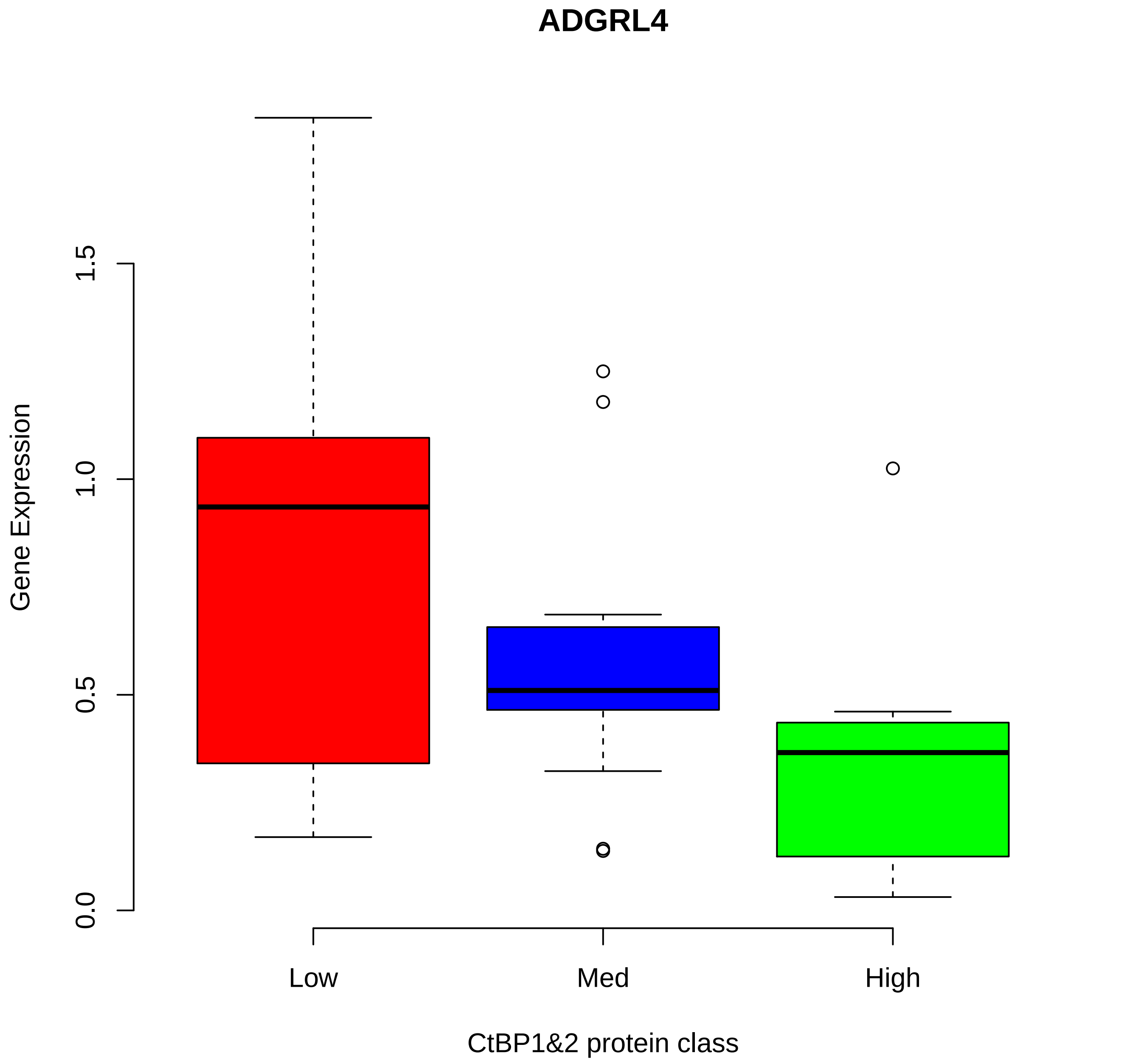
ADGRL4
Gene Expression
0.0
0.5
1.0
1.5
Low
Med
High
CtBP1&2 protein class

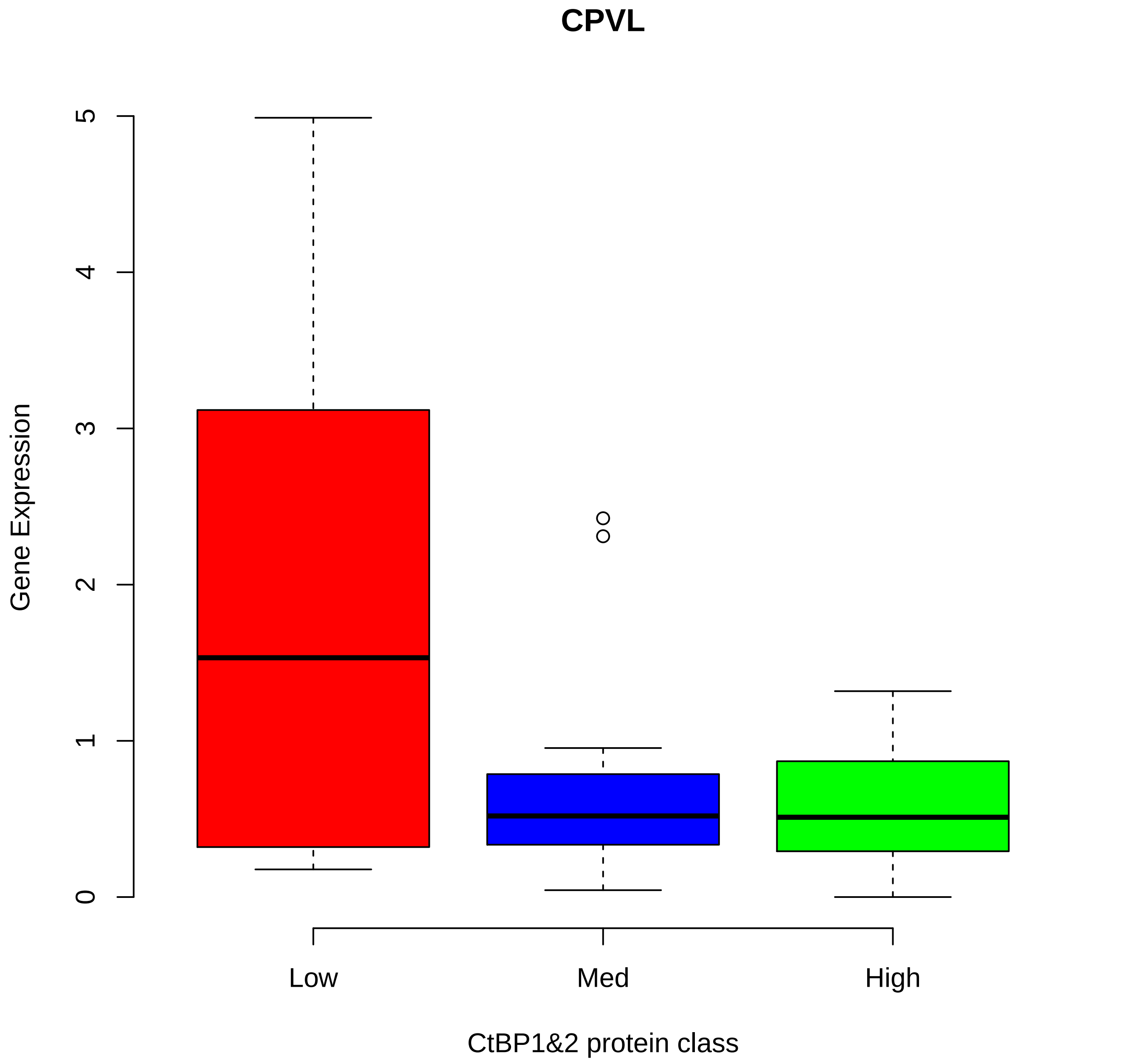
CPVL
Gene Expression
0
1
2
3
4
5
Low
Med
High
CtBP1&2 protein class

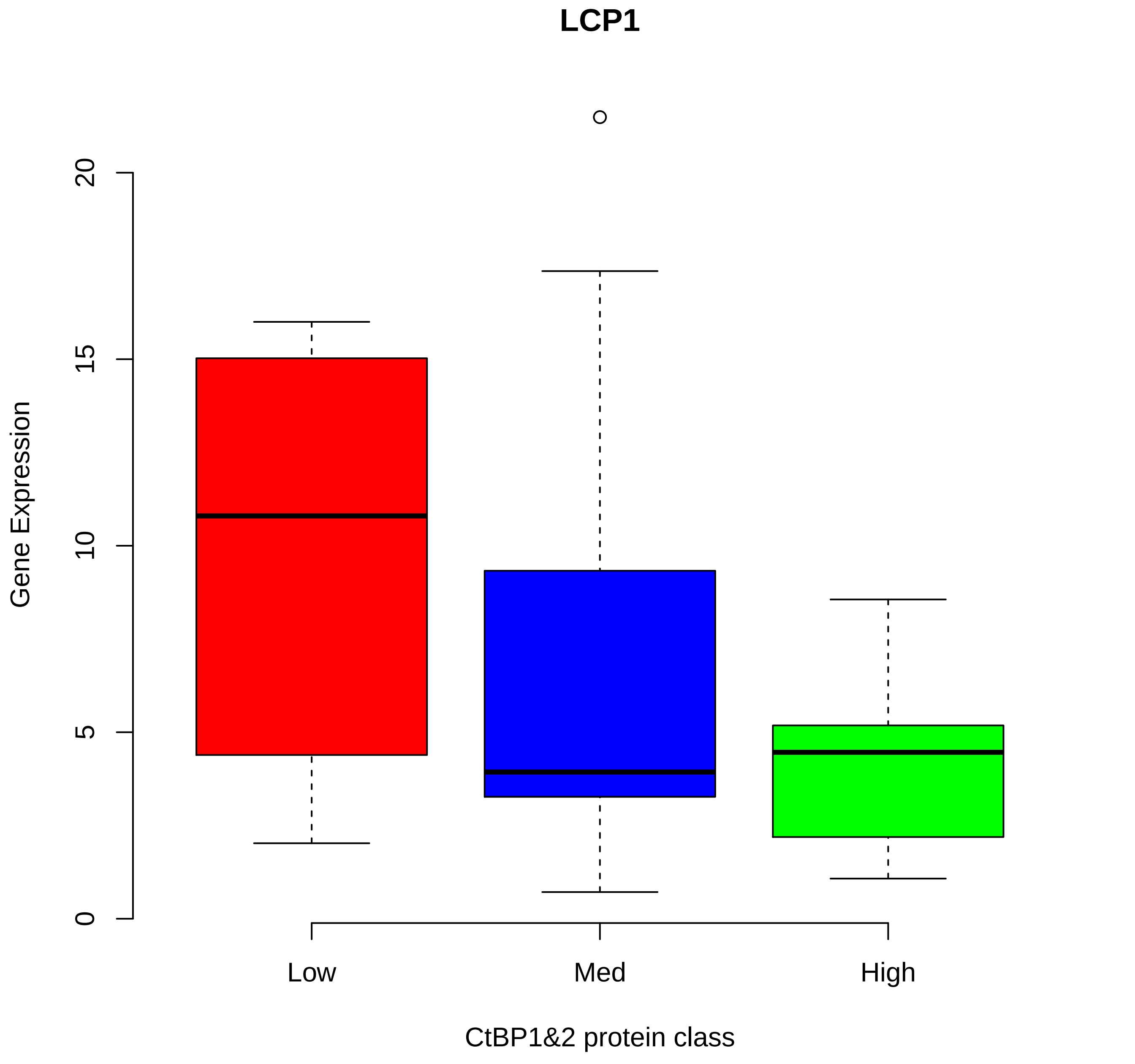
LCP1
Gene Expression
0
5
10
15
20
Low
Med
High
CtBP1&2 protein class

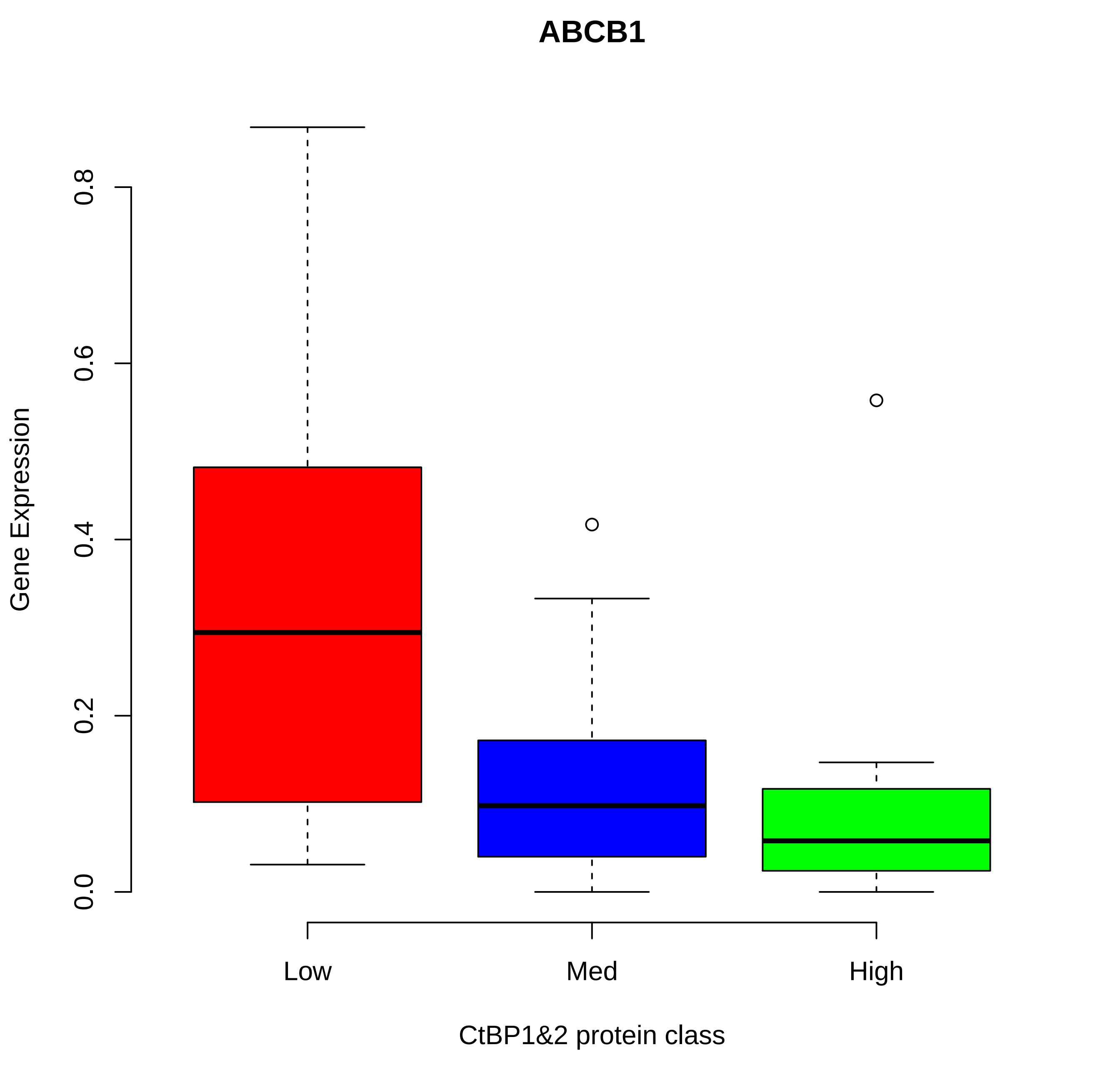
ABCB1
Gene Expression
0.0
0.2
0.4
0.6
0.8
Low
Med
High
CtBP1&2 protein class

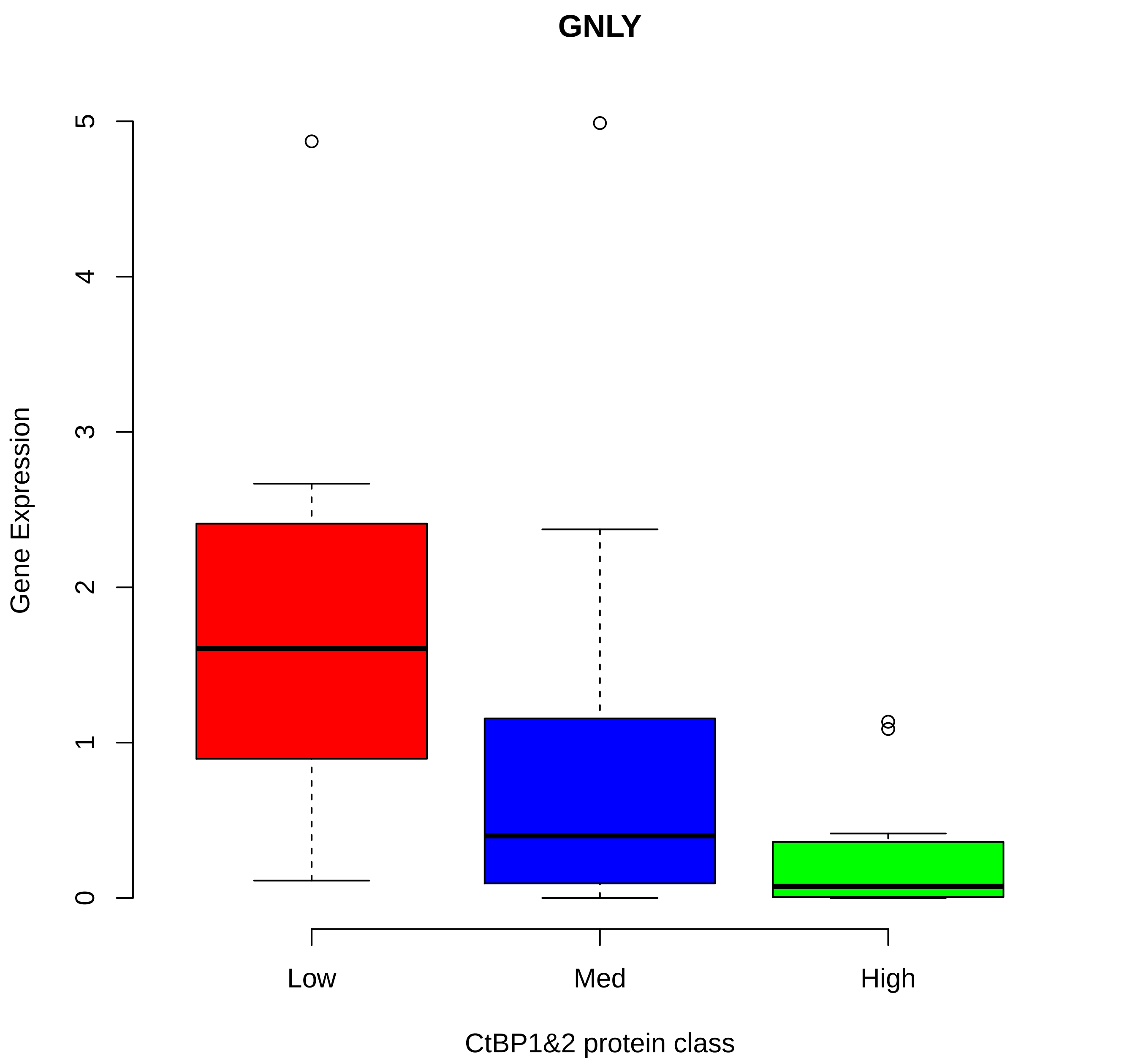
GNLY
Gene Expression
0
1
2
3
4
5
Low
Med
High
CtBP1&2 protein class

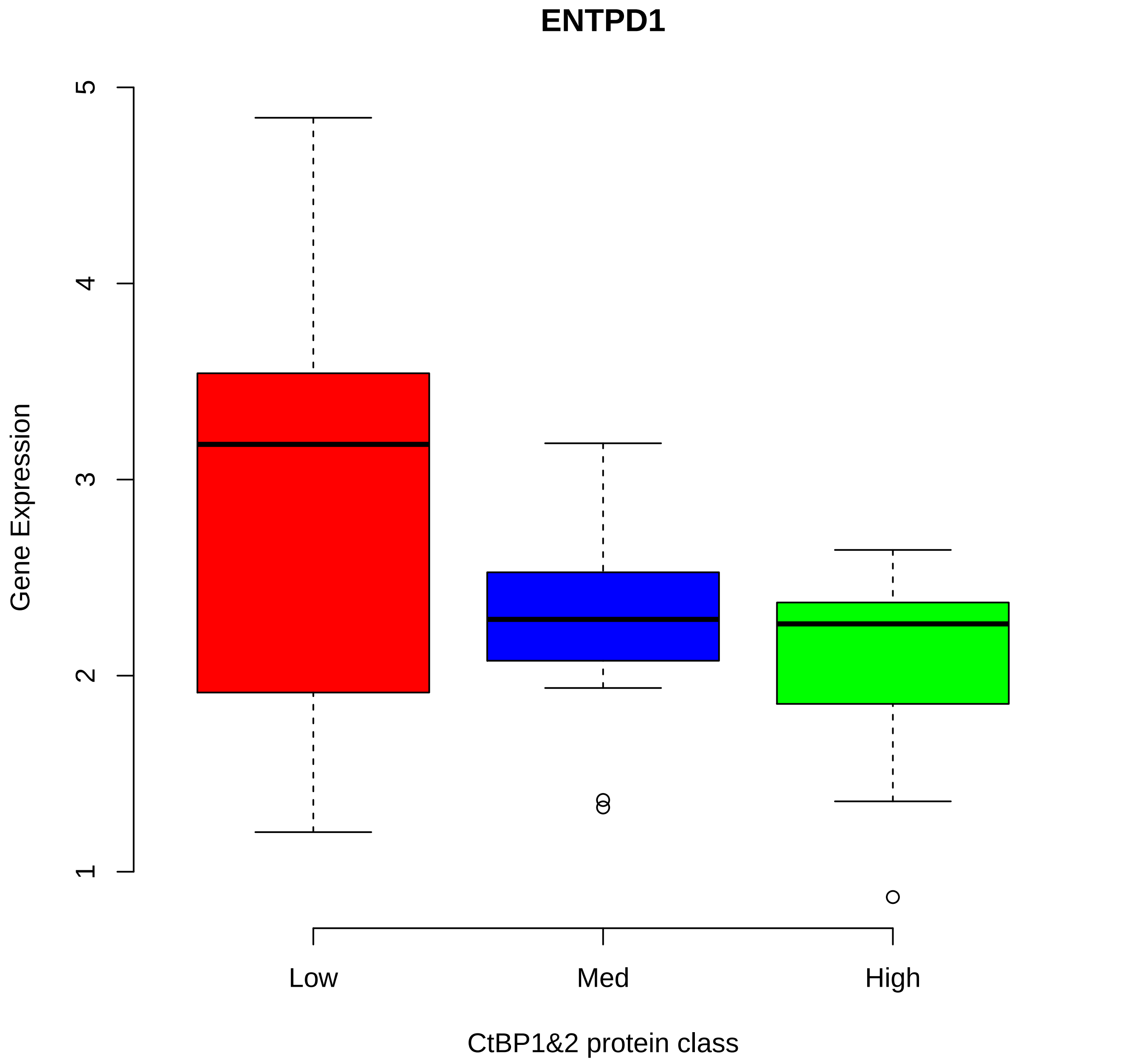

ENTPD1
Gene Expression
1
2
3
4
5
Low
Med
High
CtBP1&2 protein class

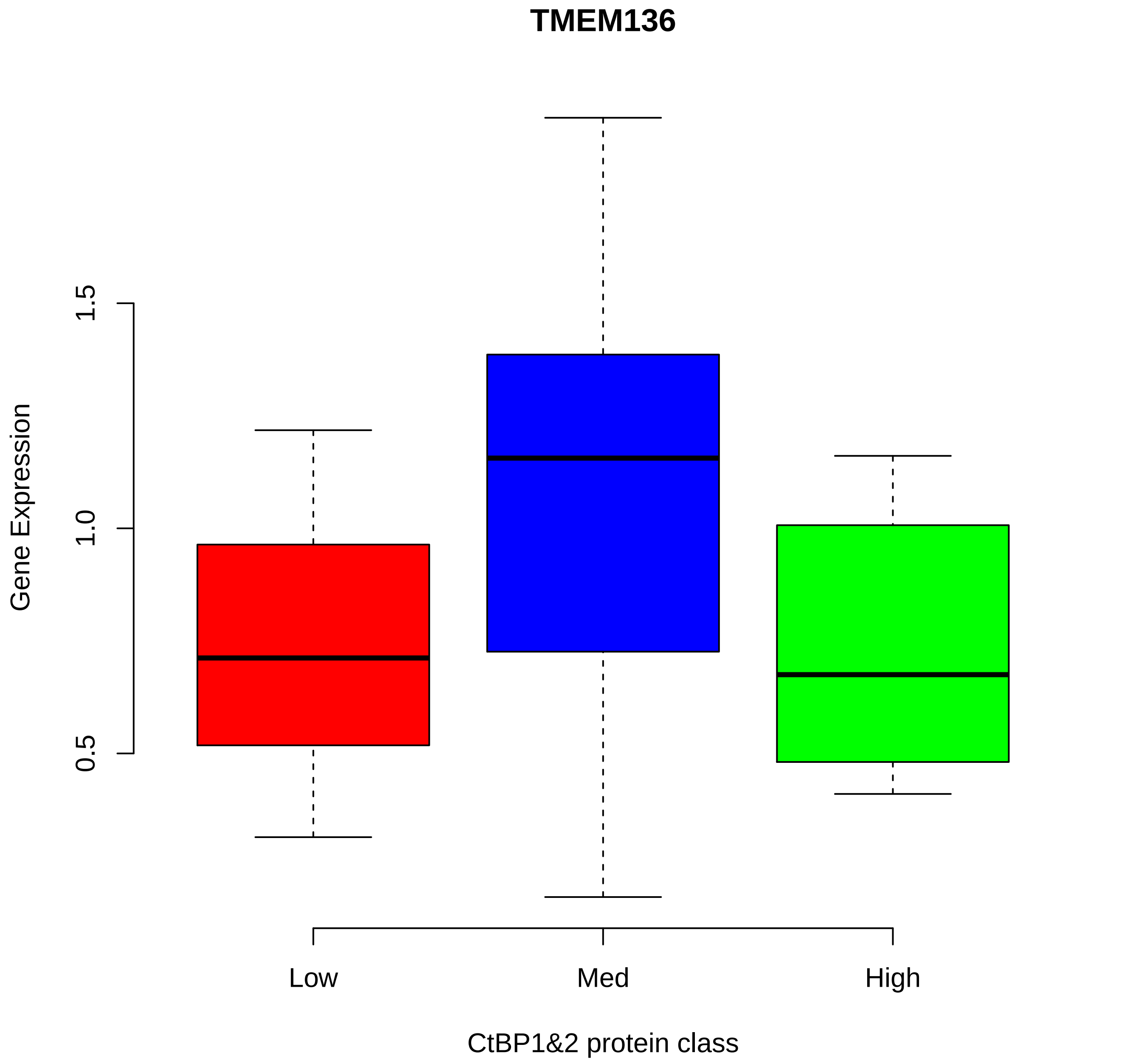

TMEM136
Gene Expression
1.5
1.0
0.5
Low
Med
High
CtBP1&2 protein class

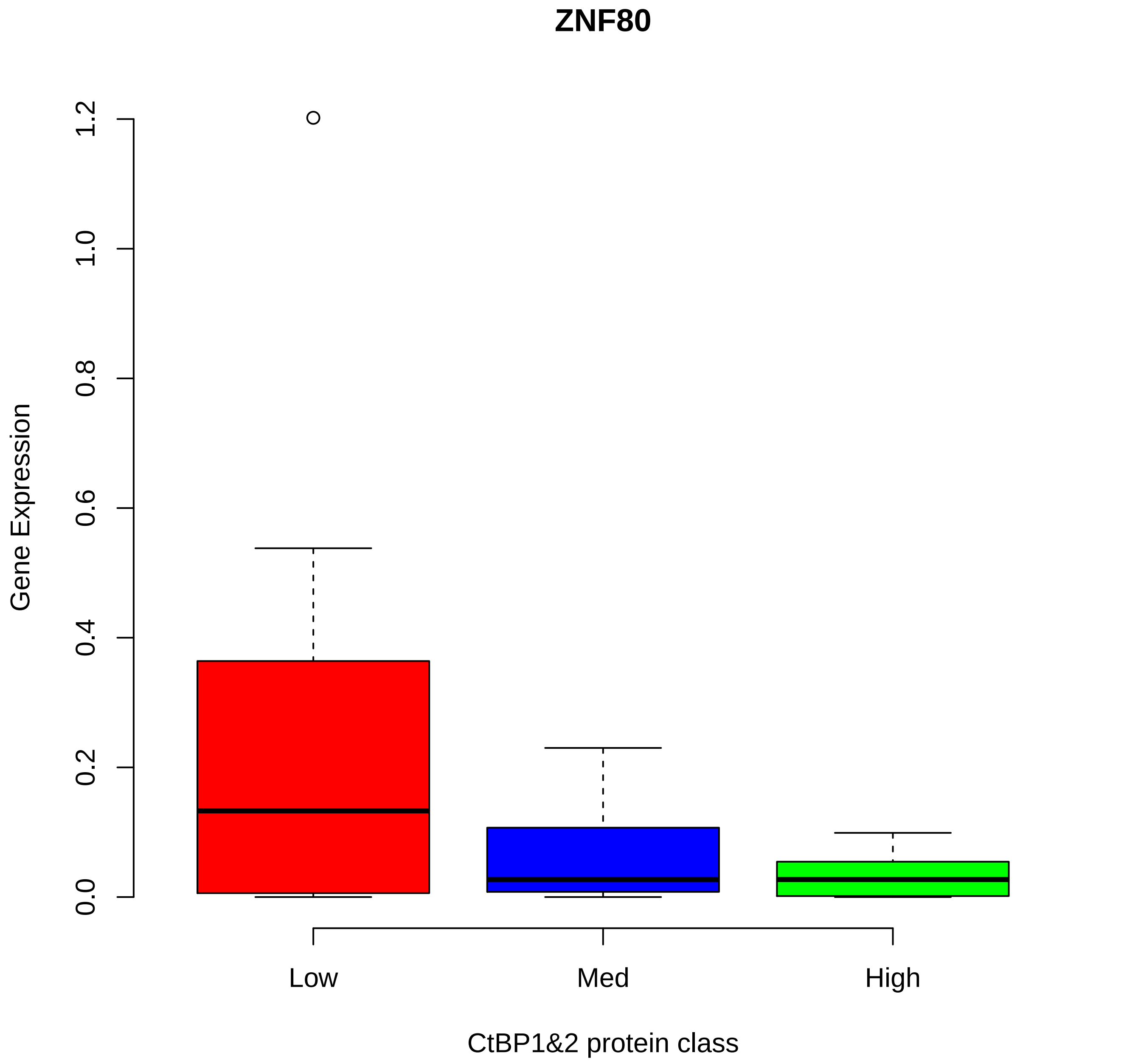
ZNF80
Gene Expression
0.0
0.2
0.4
0.6
0.8
1.0
1.2
Low
Med
High
CtBP1&2 protein class

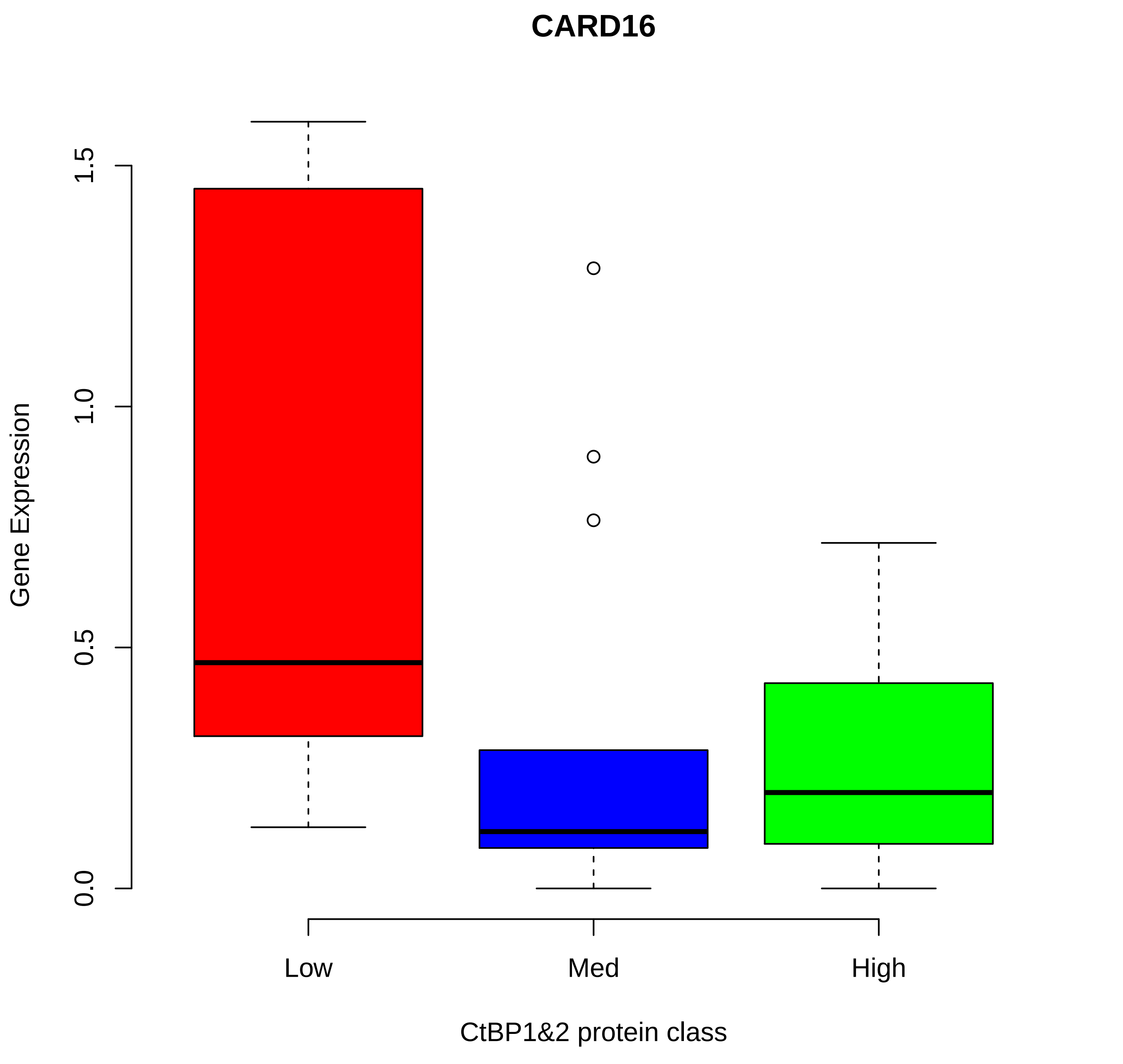
CARD16
Gene Expression
0.0
0.5
1.0
1.5
Low
Med
High
CtBP1&2 protein class

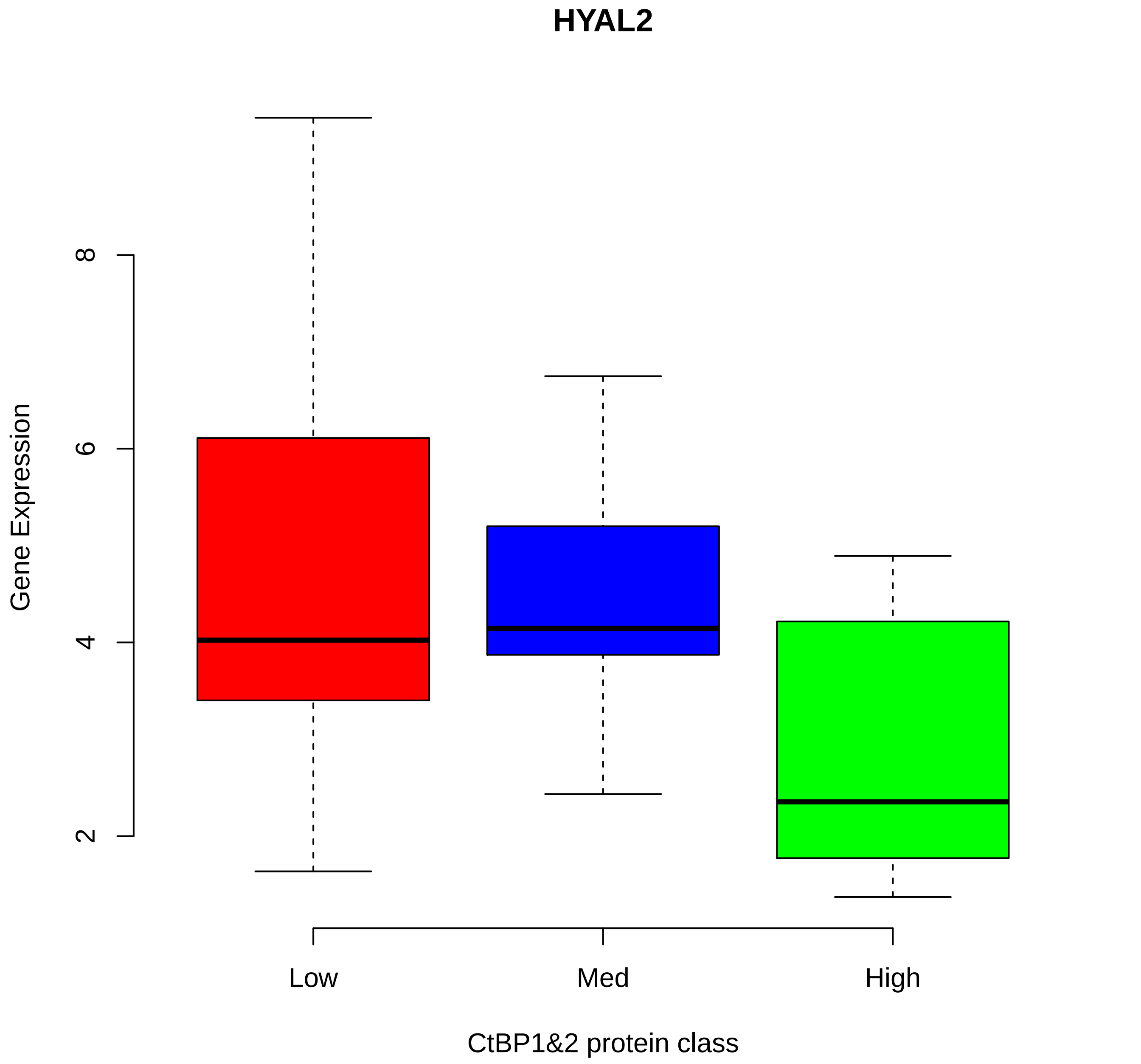
HYAL2
Gene Expression
2
4
6
8
Low
Med
High
CtBP1&2 protein class

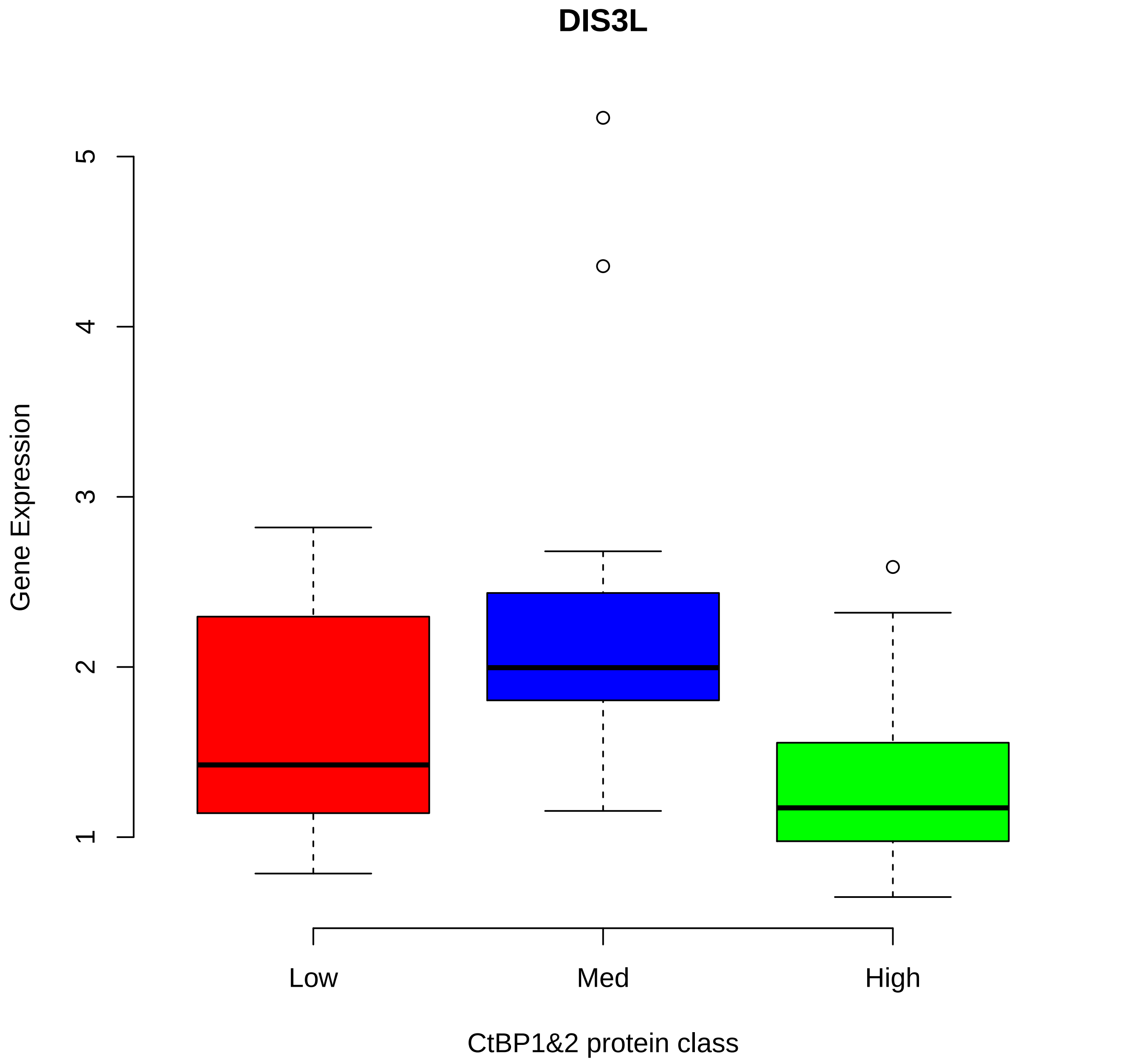
DIS3L
Gene Expression
1
2
3
4
5
Low
Med
High
CtBP1&2 protein class

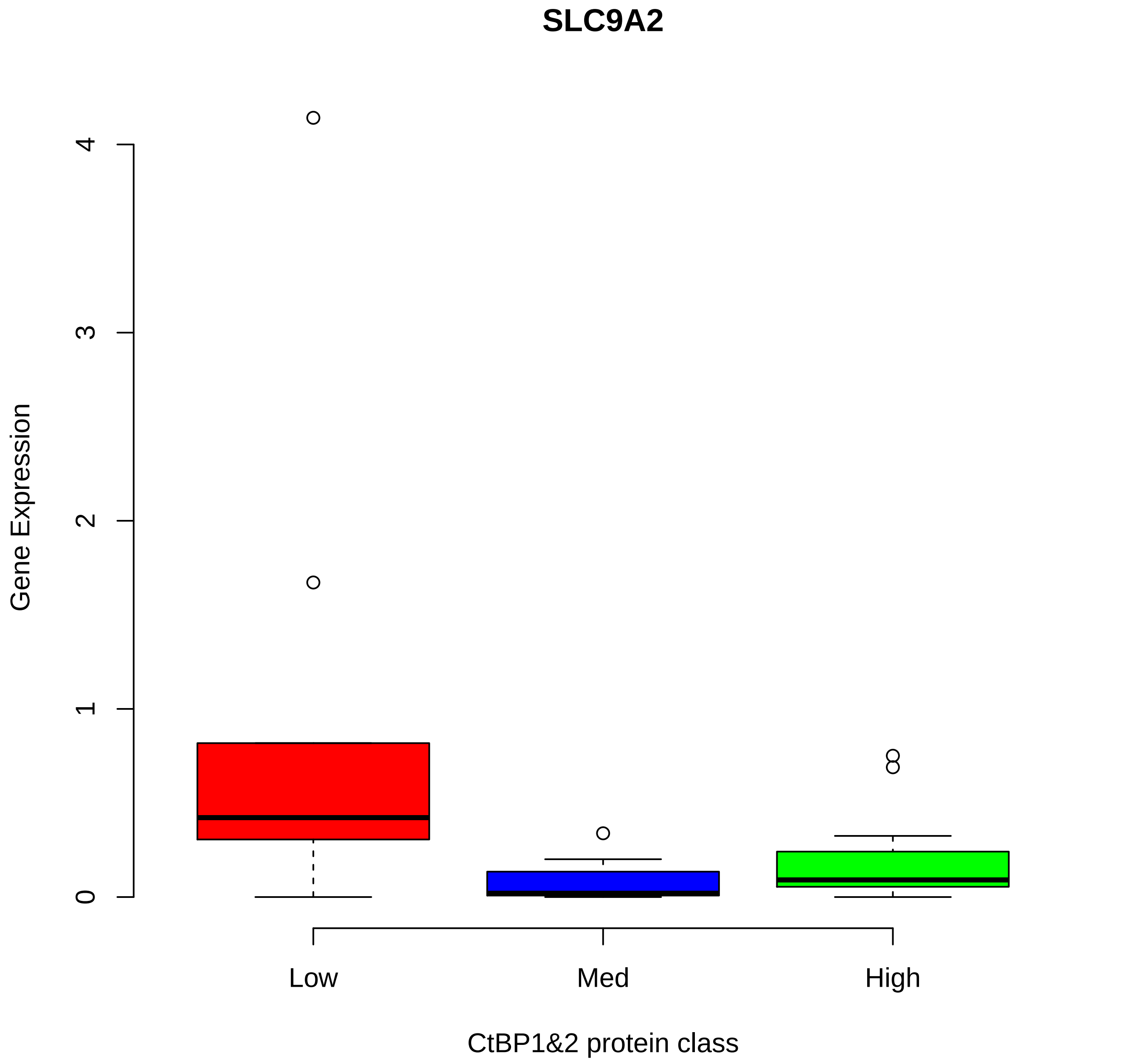

SLC9A2
Gene Expression
0
1
2
3
4
Low
Med
High
CtBP1&2 protein class

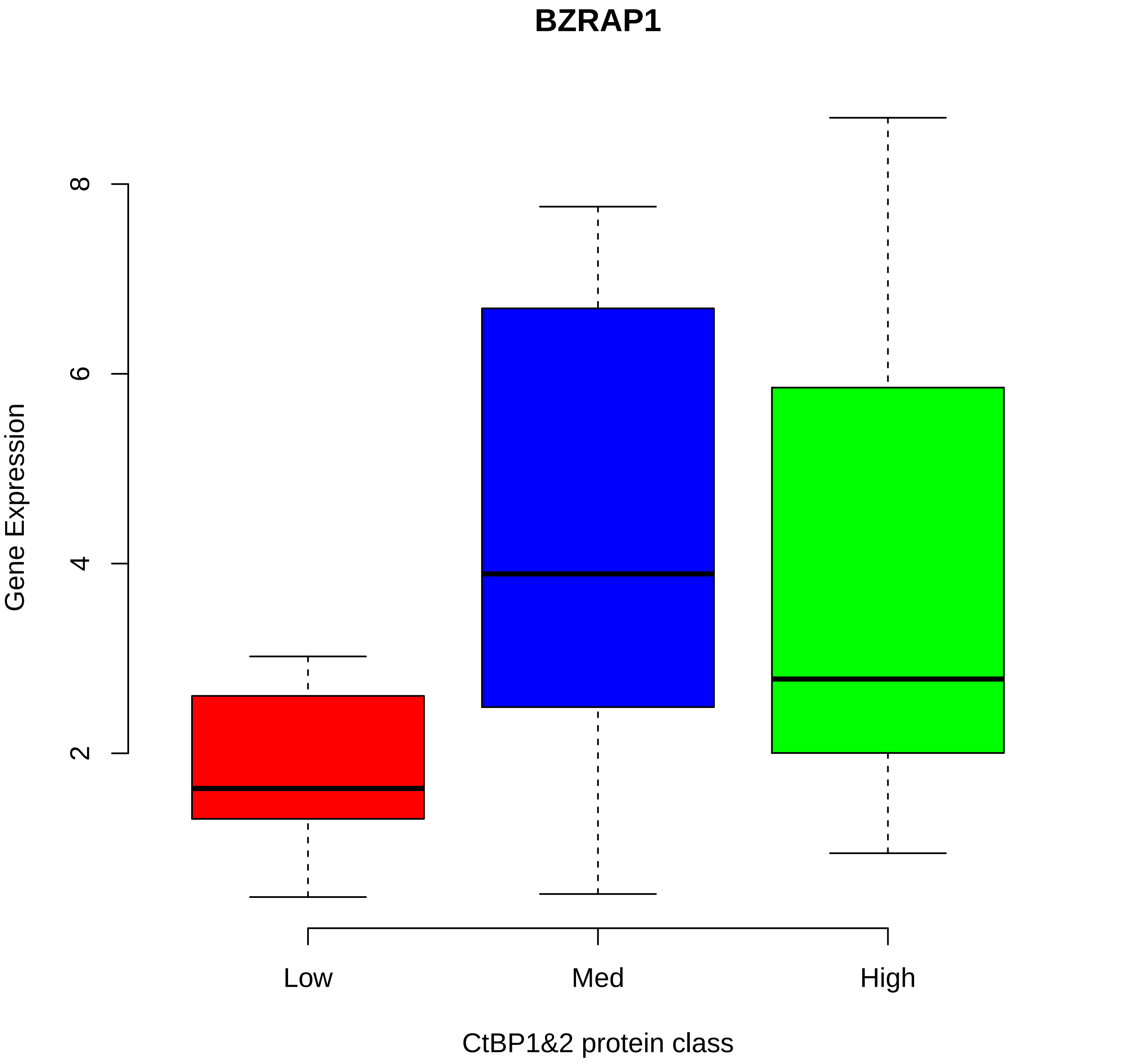

BZRAP1
Gene Expression
2
4
6
8
Low
Med
High
CtBP1&2 protein class

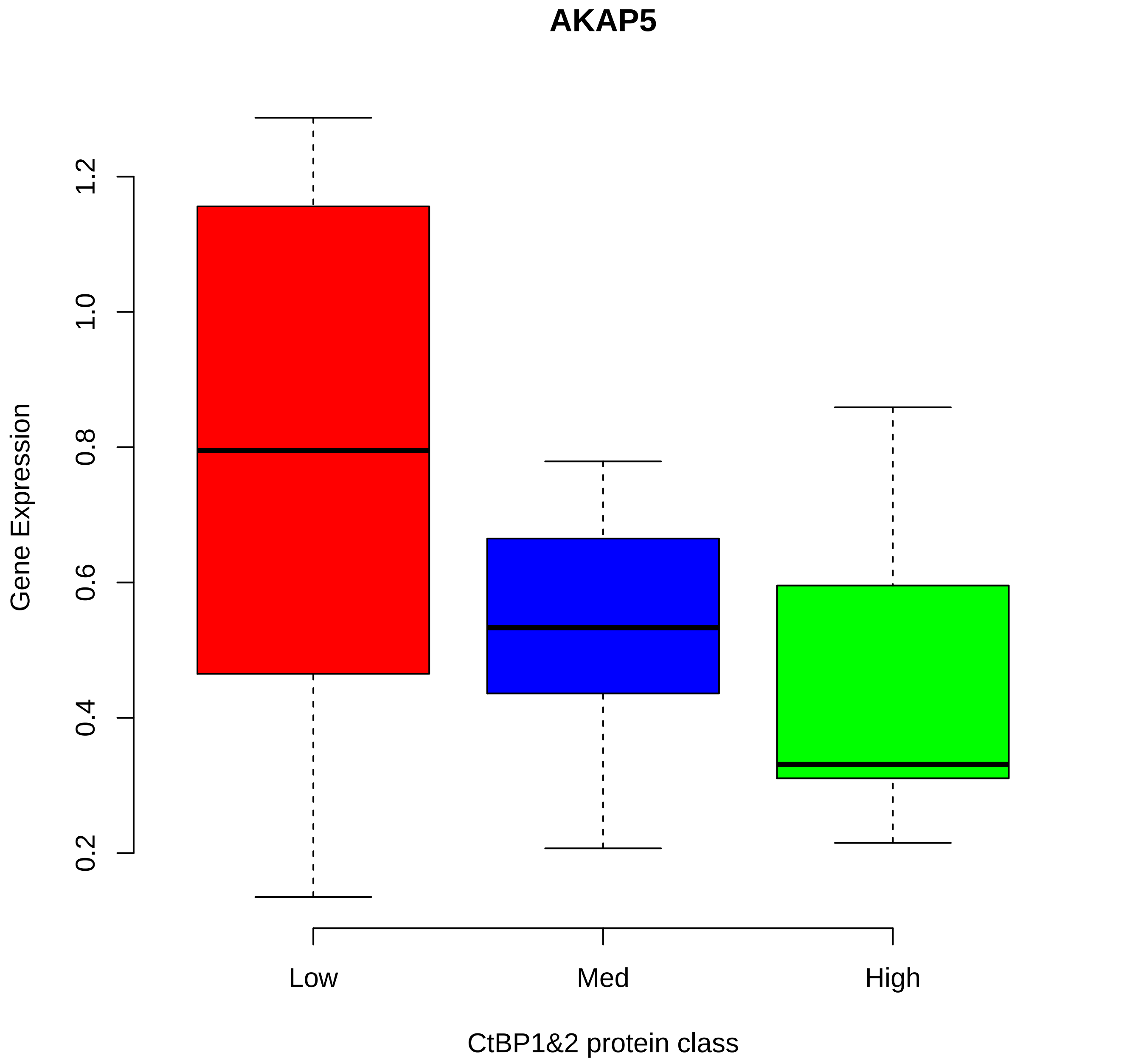

AKAP5
Gene Expression
0.2
0.4
0.6
0.8
1.0
1.2
Low
Med
High
CtBP1&2 protein class

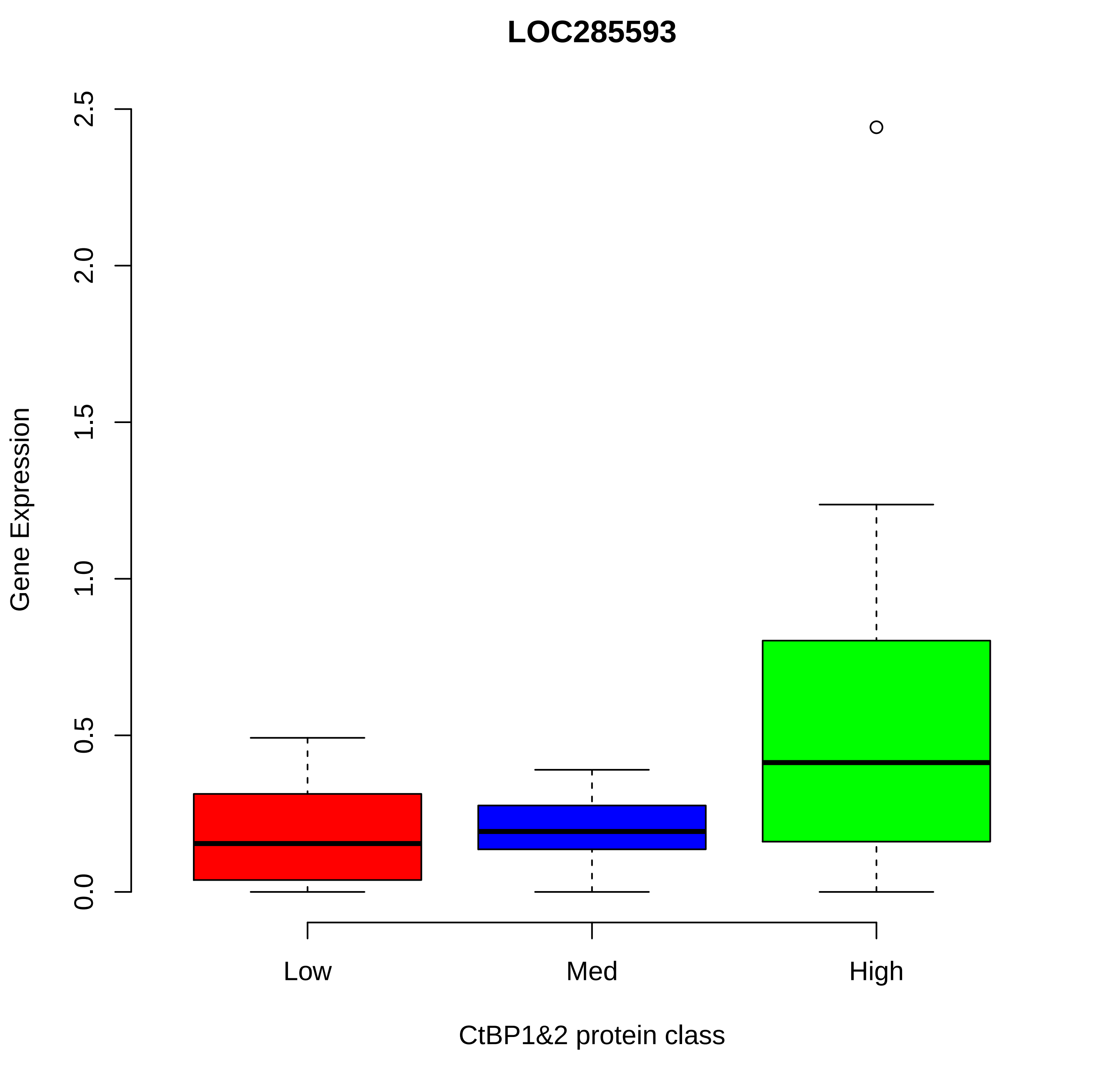

LOC285593
Gene Expression
0.0
0.5
1.0
1.5
2.0
2.5
Low
Med
High
CtBP1&2 protein class

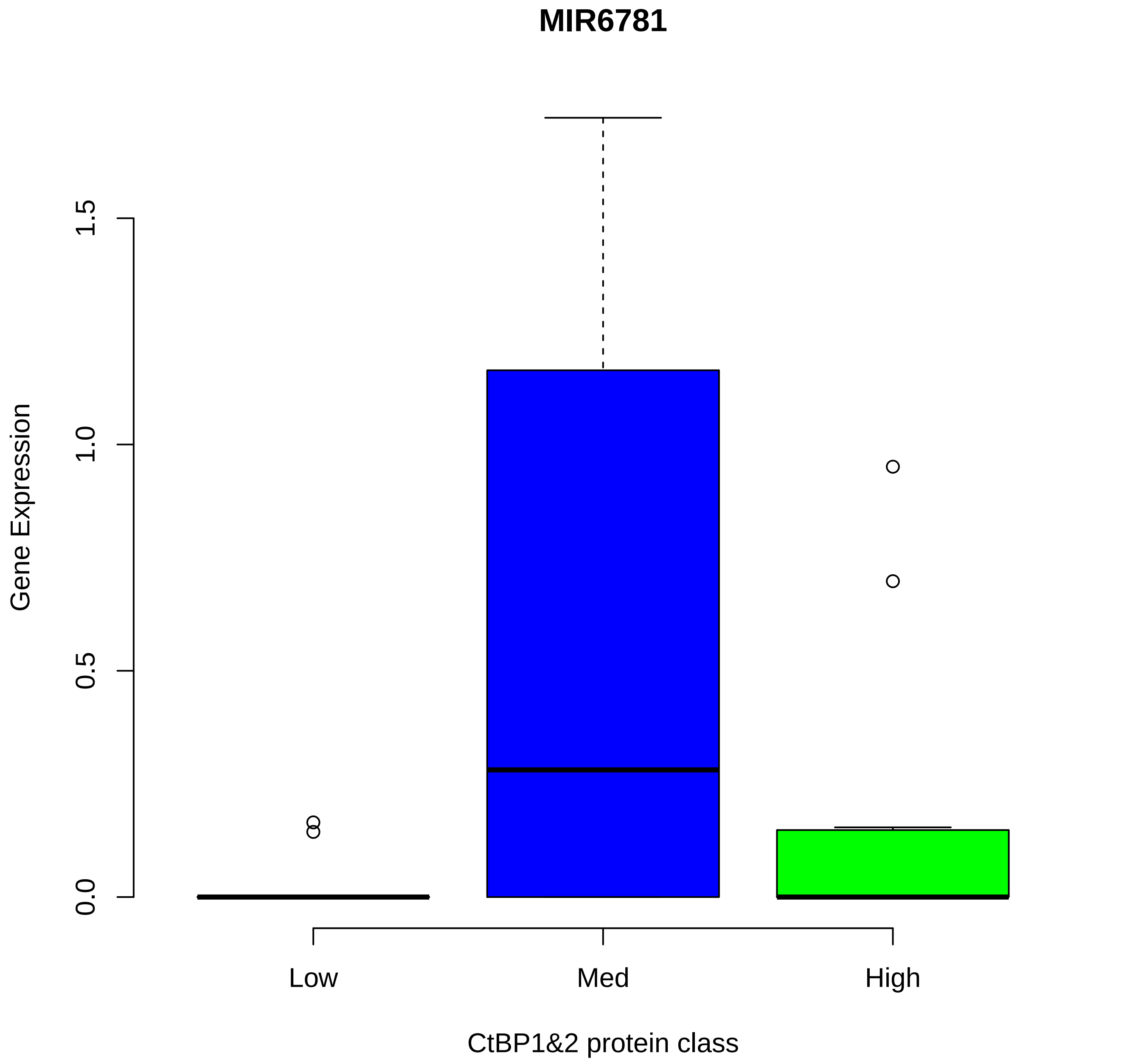

MIR6781
Gene Expression
0.0
0.5
1.0
1.5
Low
Med
High
CtBP1&2 protein class

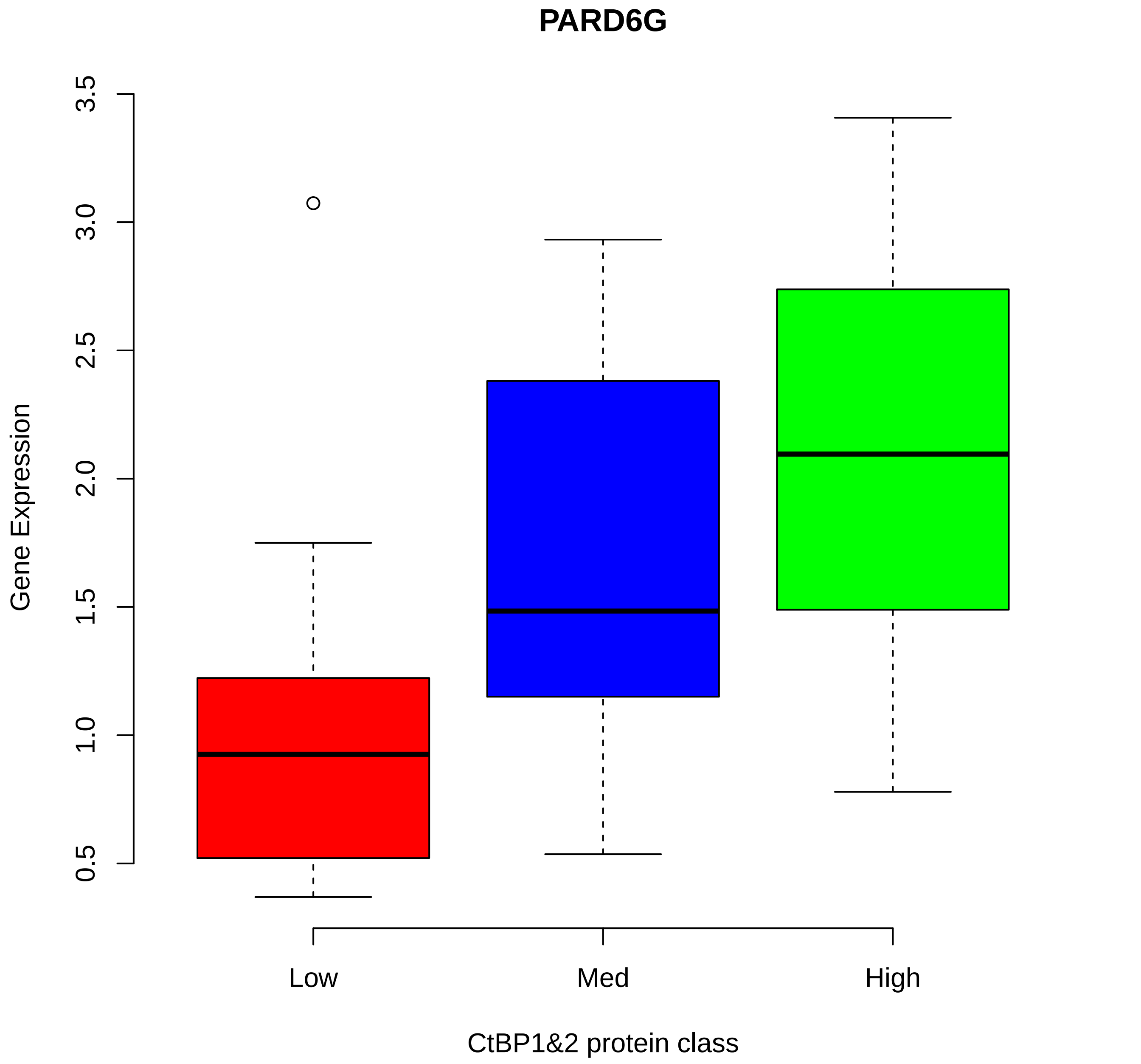
PARD6G
Gene Expression
0.5
1.0
1.5
2.0
2.5
3.0
3.5
Low
Med
High
CtBP1&2 protein class

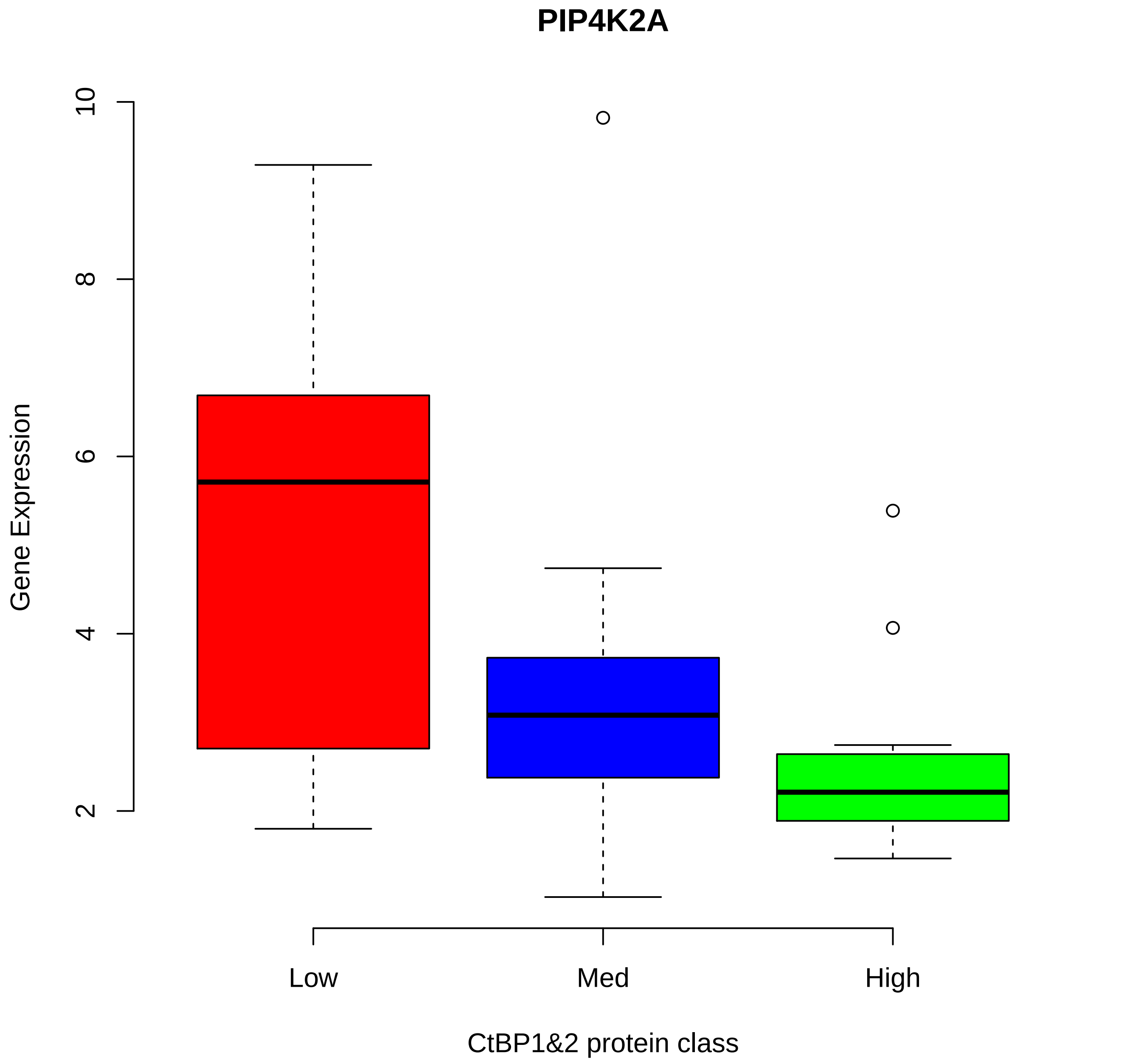
PIP4K2A
Gene Expression
2
4
6
8
10
Low
Med
High
CtBP1&2 protein class

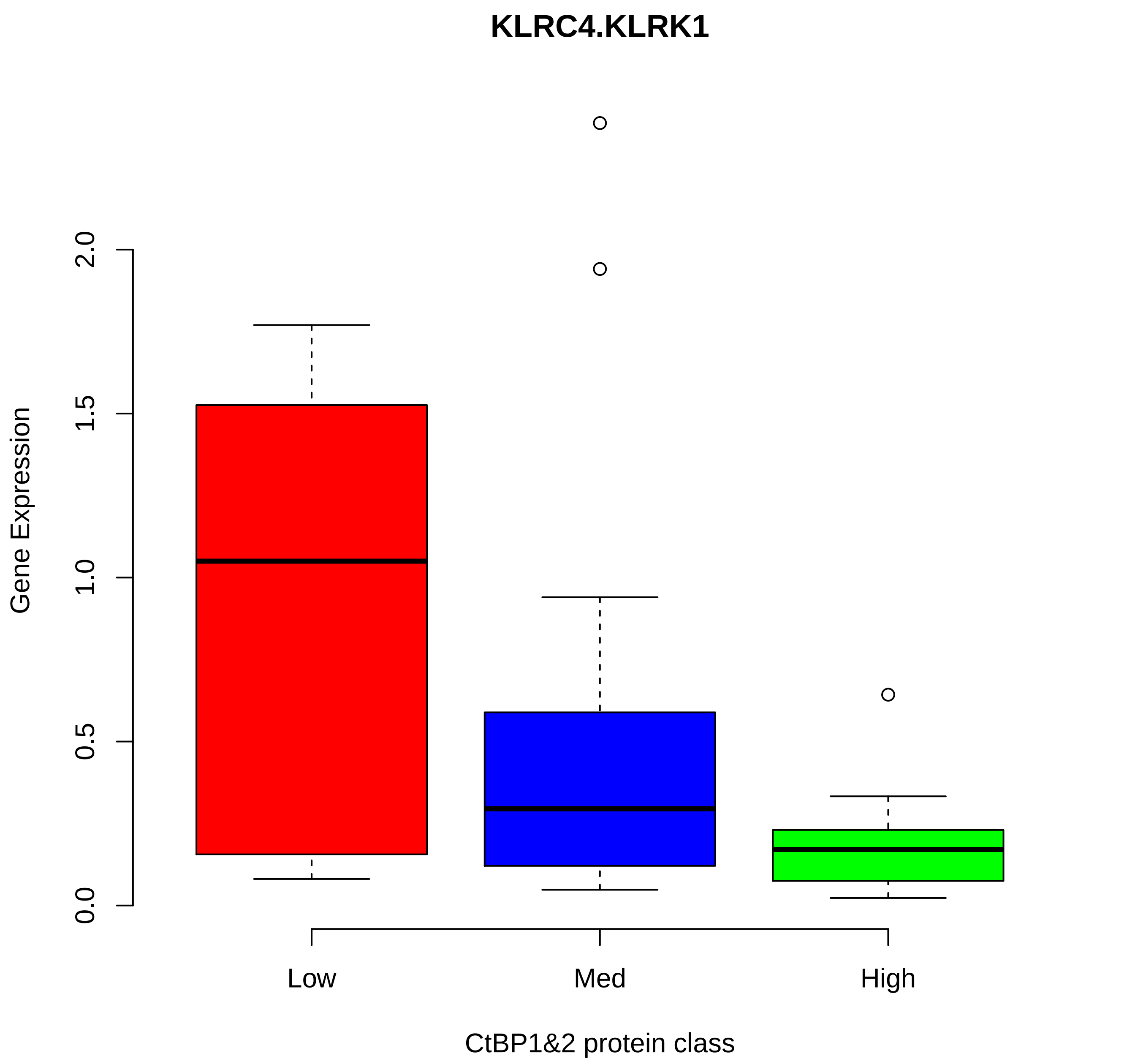

KLRC4.KLRK1
Gene Expression
0.0
0.5
1.0
1.5
2.0
Low
Med
High
CtBP1&2 protein class

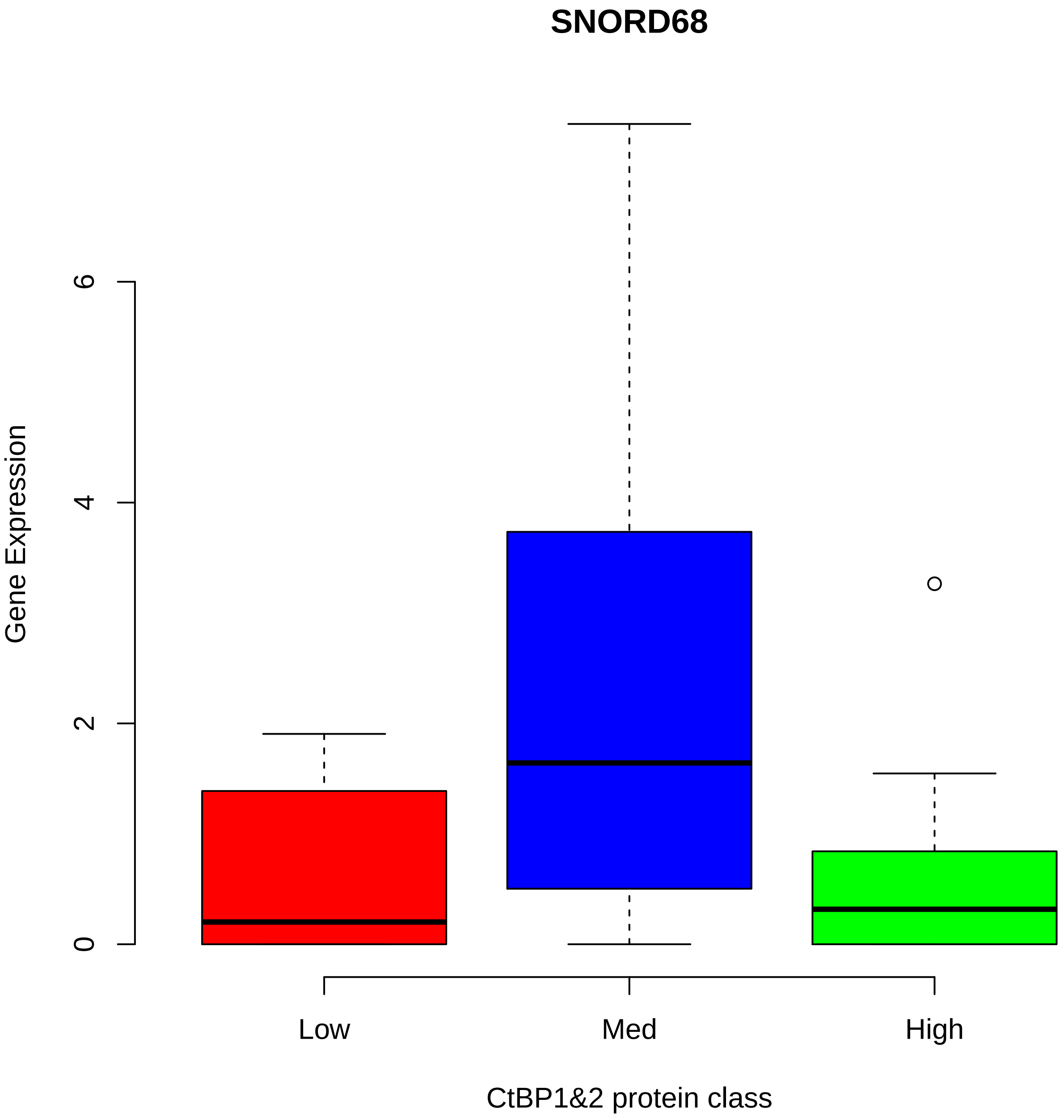
SNORD68
Gene Expression
0
2
4
6
Low
Med
High
CtBP1&2 protein class

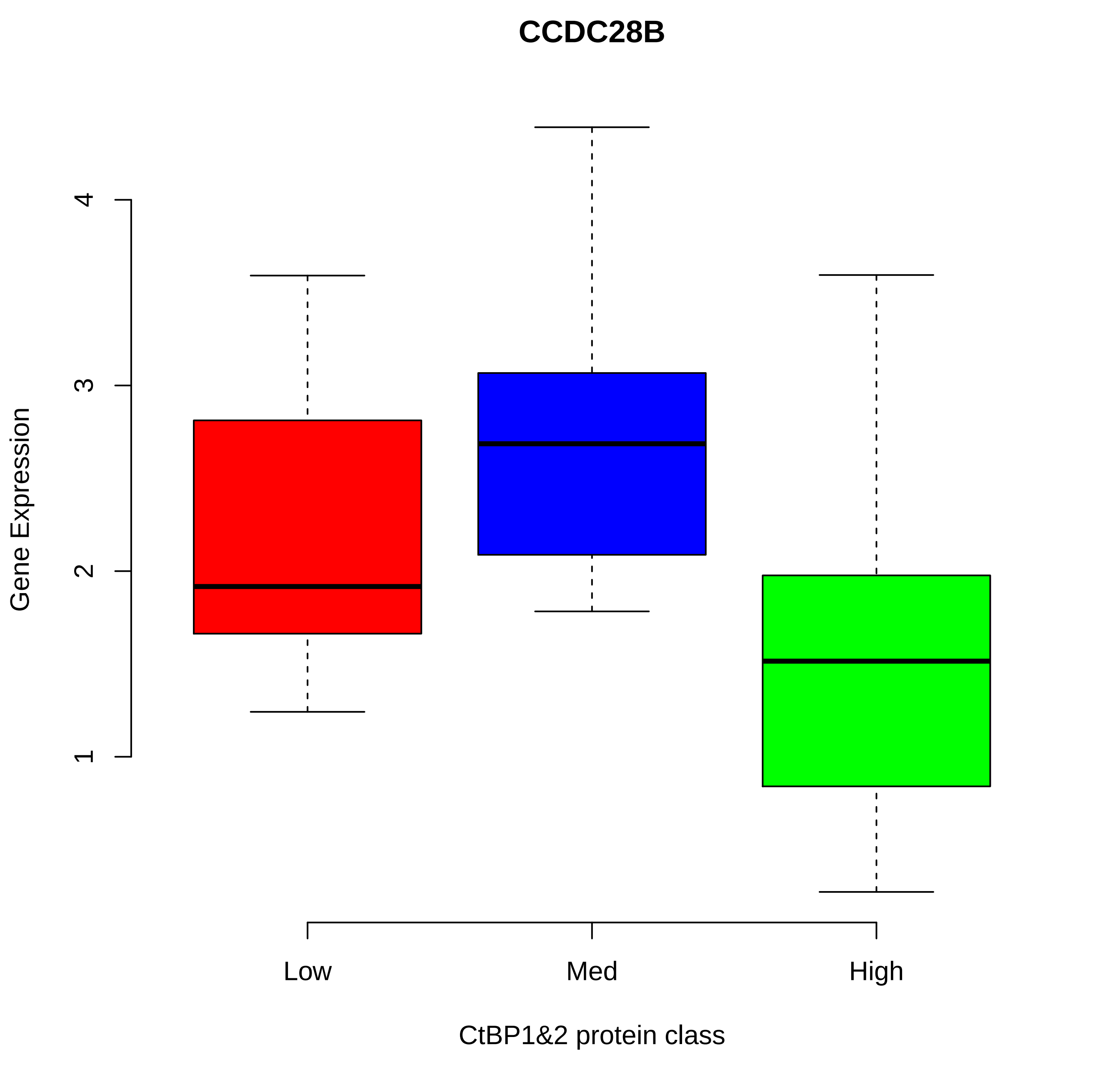
CCDC28B
Gene Expression
1
2
3
4
Low
Med
High
CtBP1&2 protein class

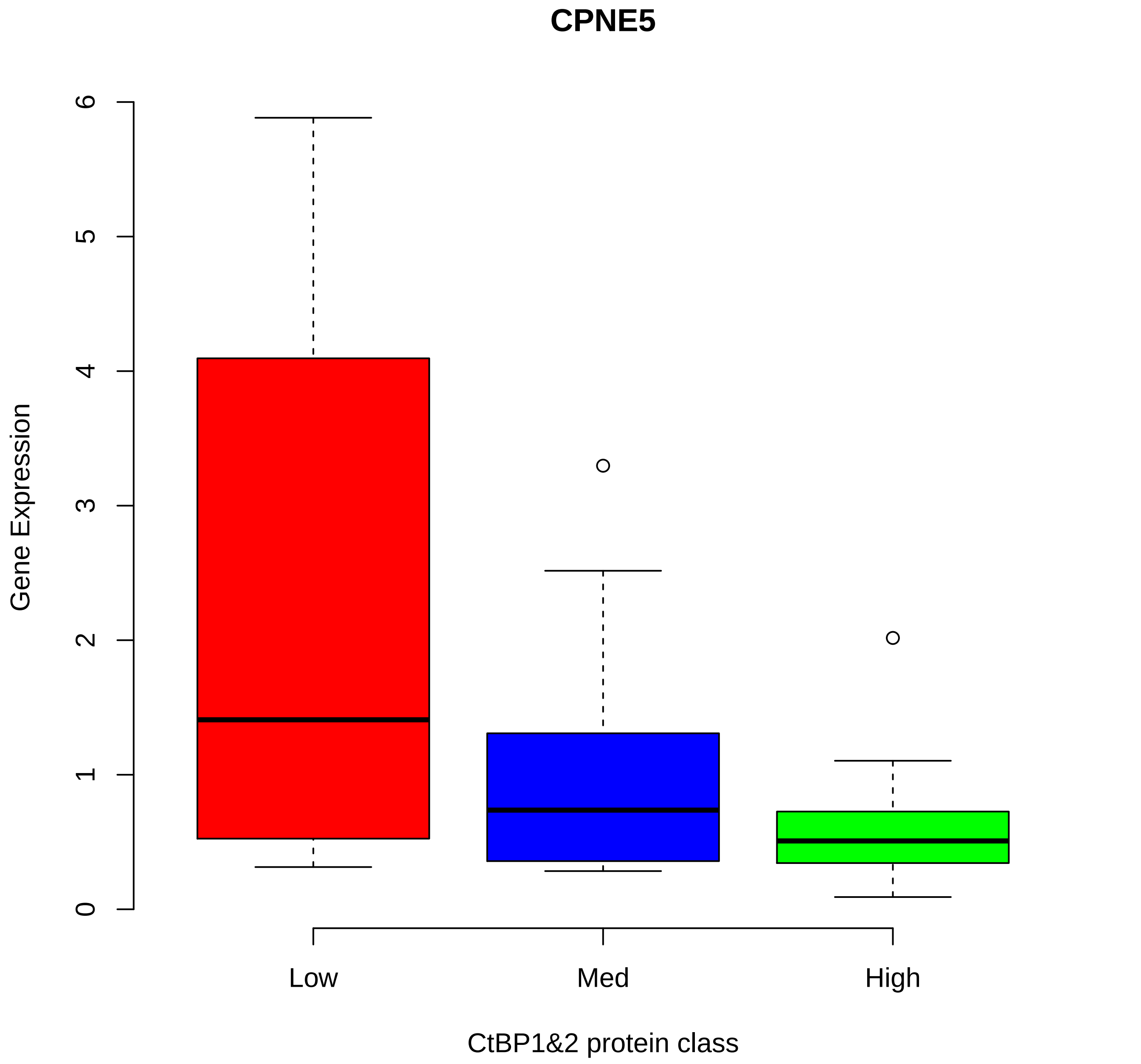
CPNE5
Gene Expression
0
1
2
3
4
5
6
Low
Med
High
CtBP1&2 protein class

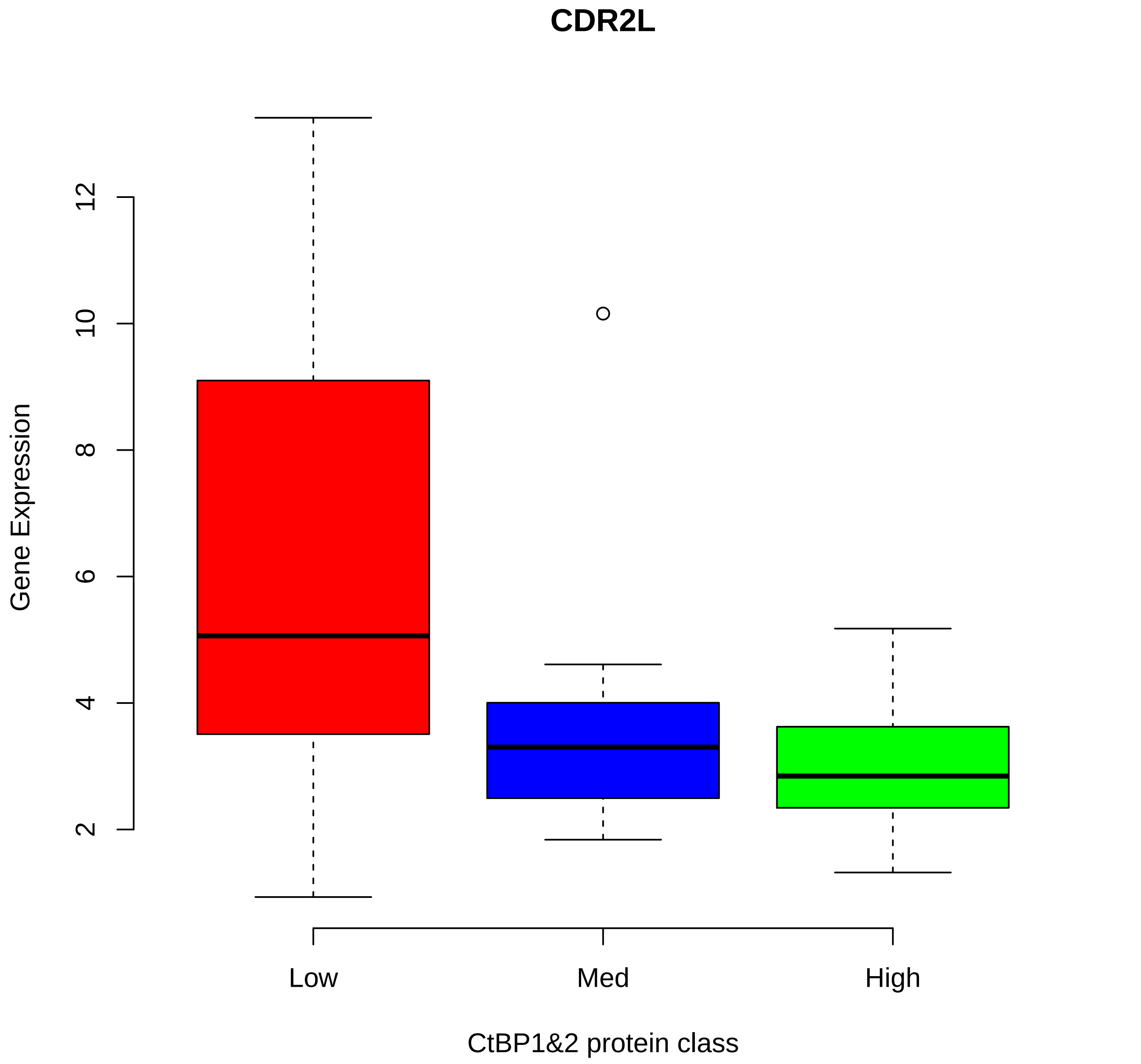

CDR2L
Gene Expression
12
10
8
6
4
2
Low
Med
High
CtBP1&2 protein class

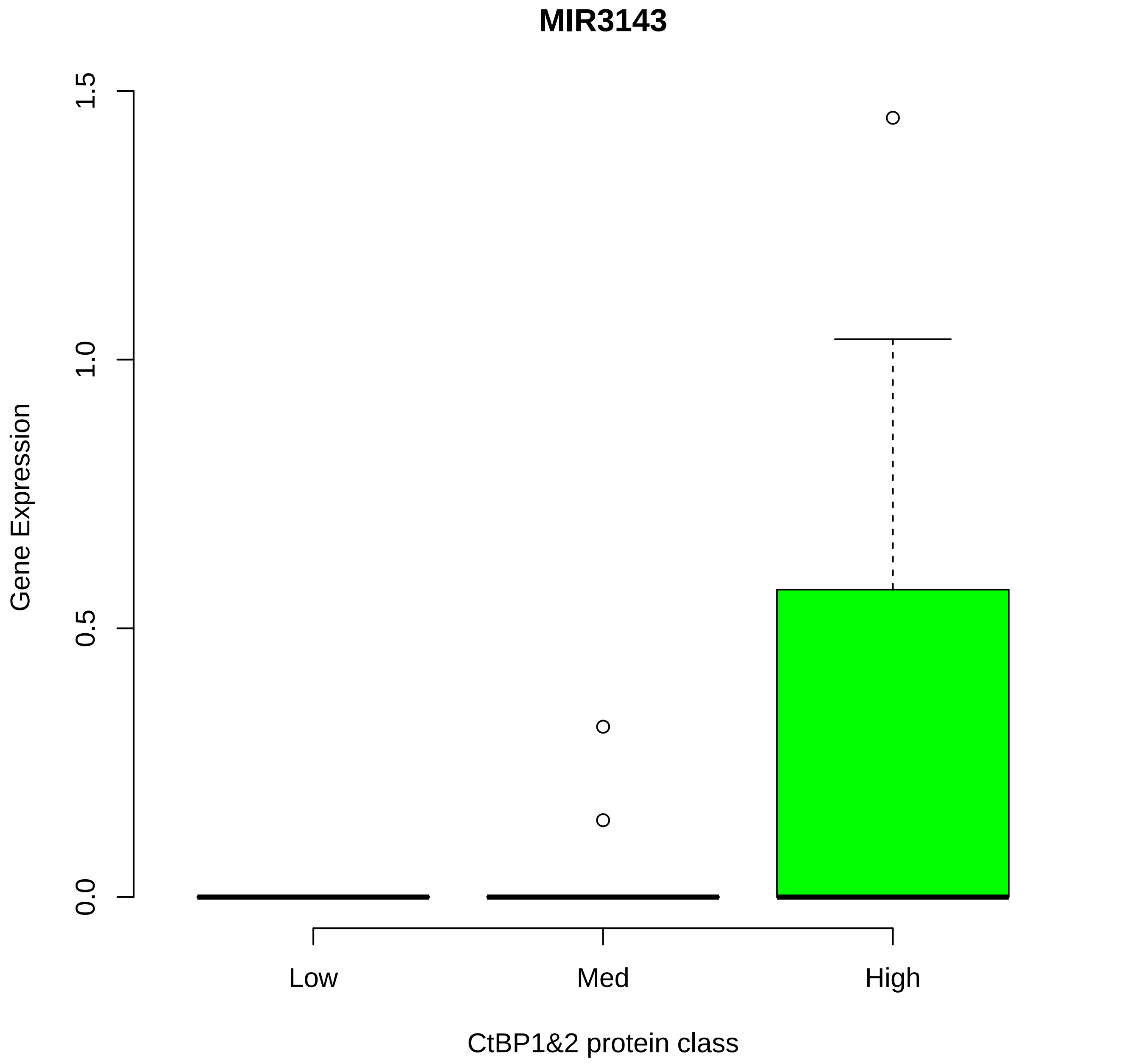
MIR3143
Gene Expression
0.0
0.5
1.0
1.5
Low
Med
High
CtBP1&2 protein class

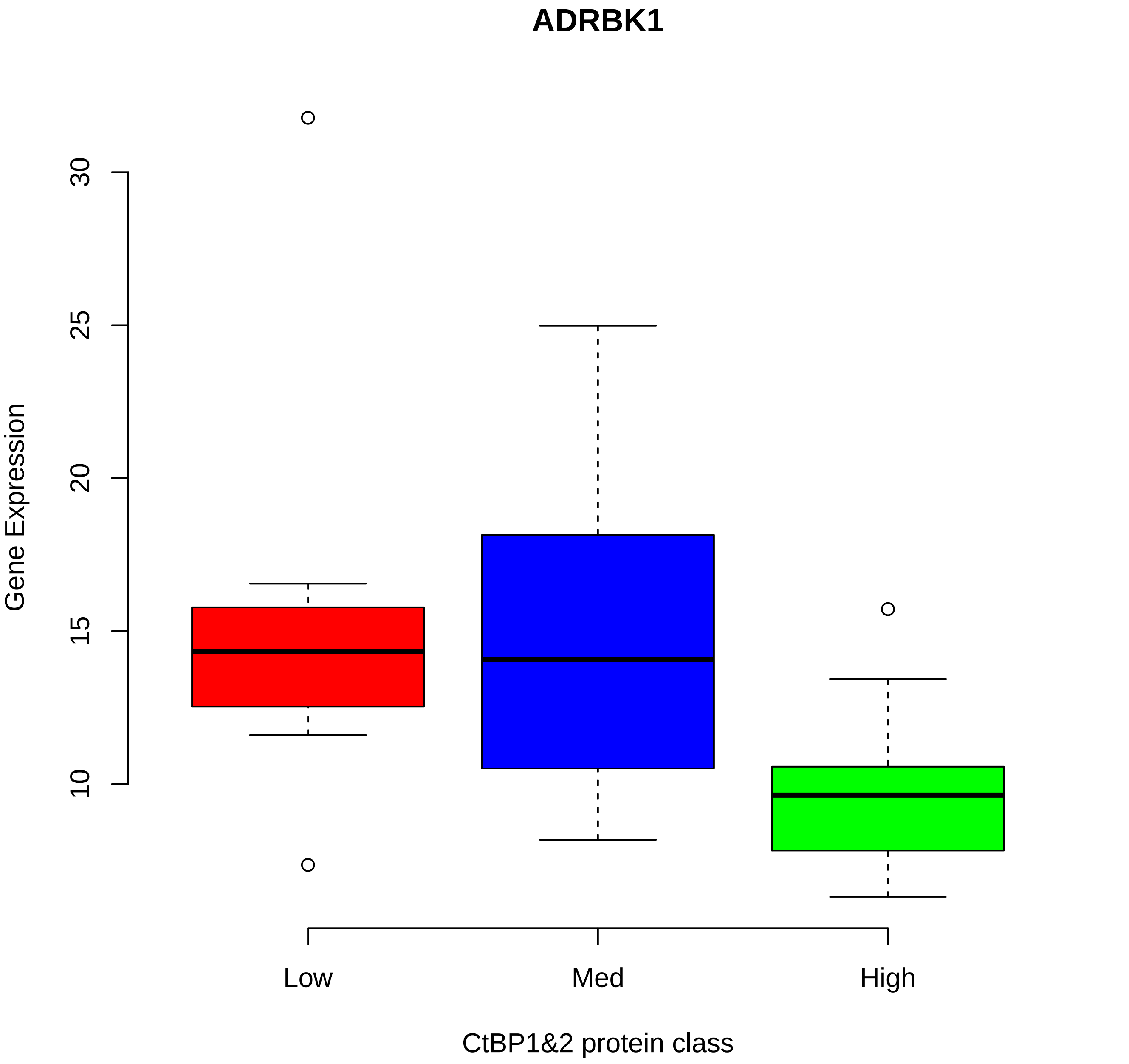

ADRBK1
Gene Expression
30
25
20
15
10
Low
Med
High
CtBP1&2 protein class

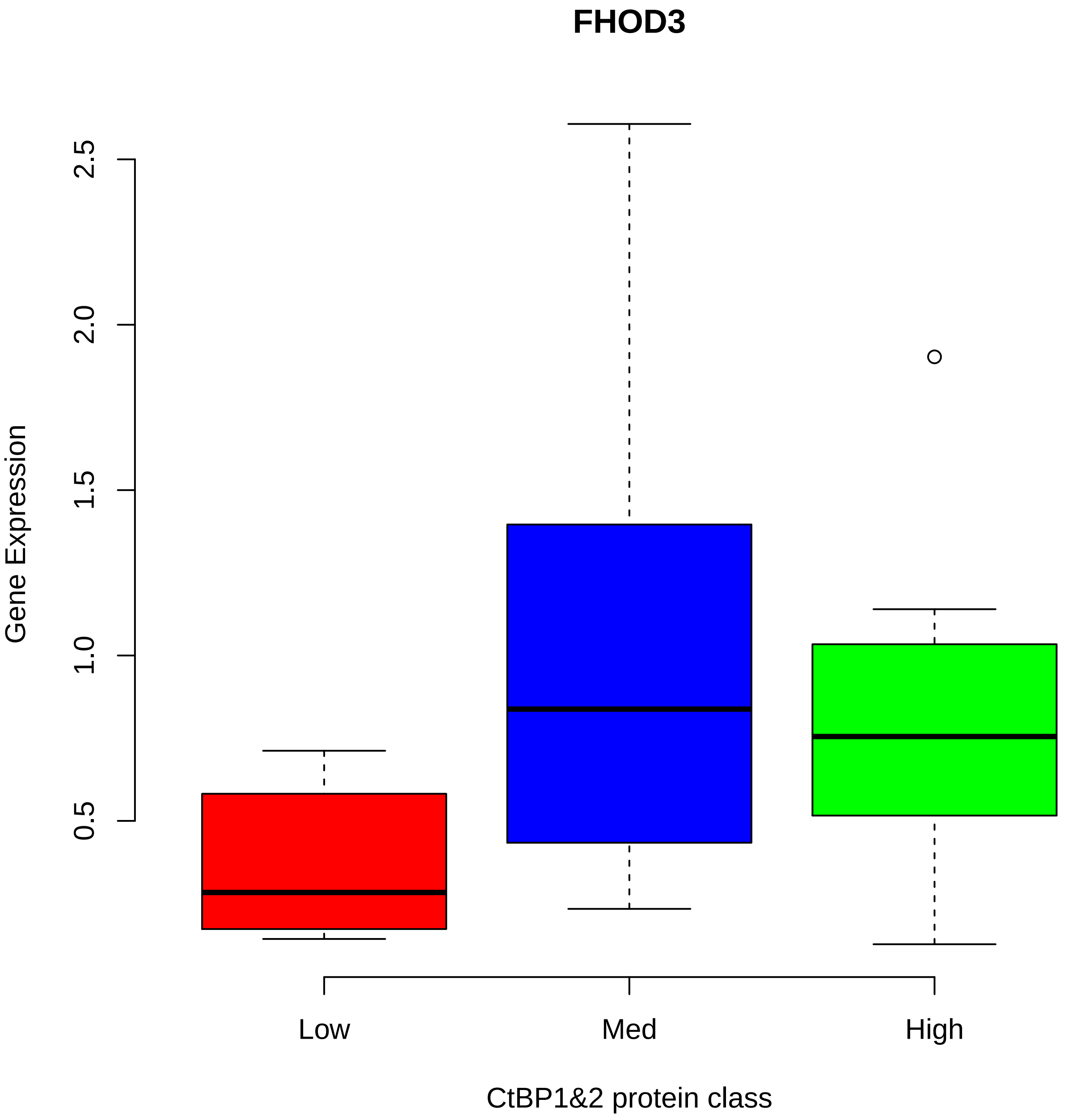
FHOD3
Gene Expression
0.5
1.0
1.5
2.0
2.5
Low
Med
High
CtBP1&2 protein class

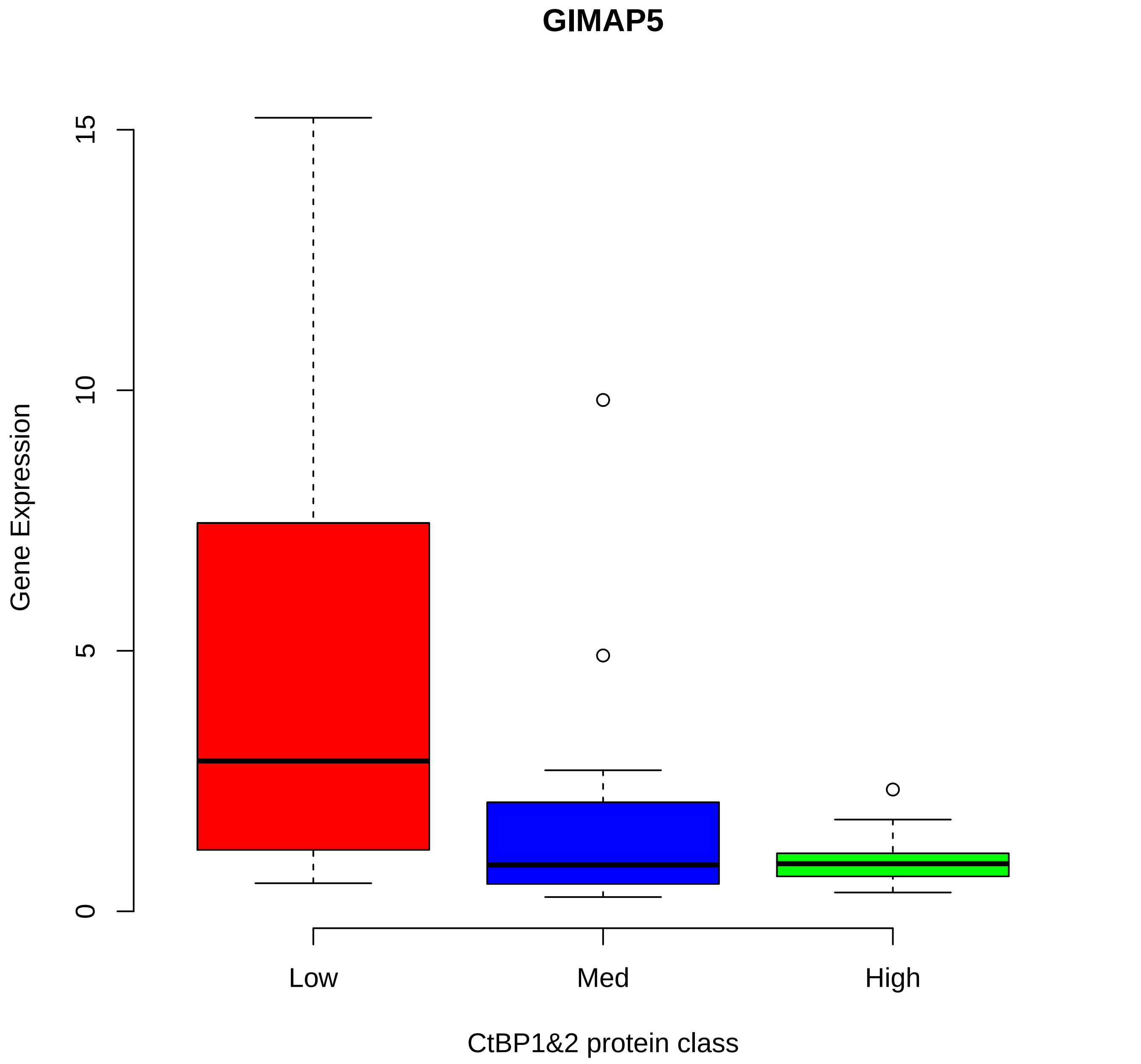
GIMAP5
Gene Expression
0
5
10
15
Low
Med
High
CtBP1&2 protein class

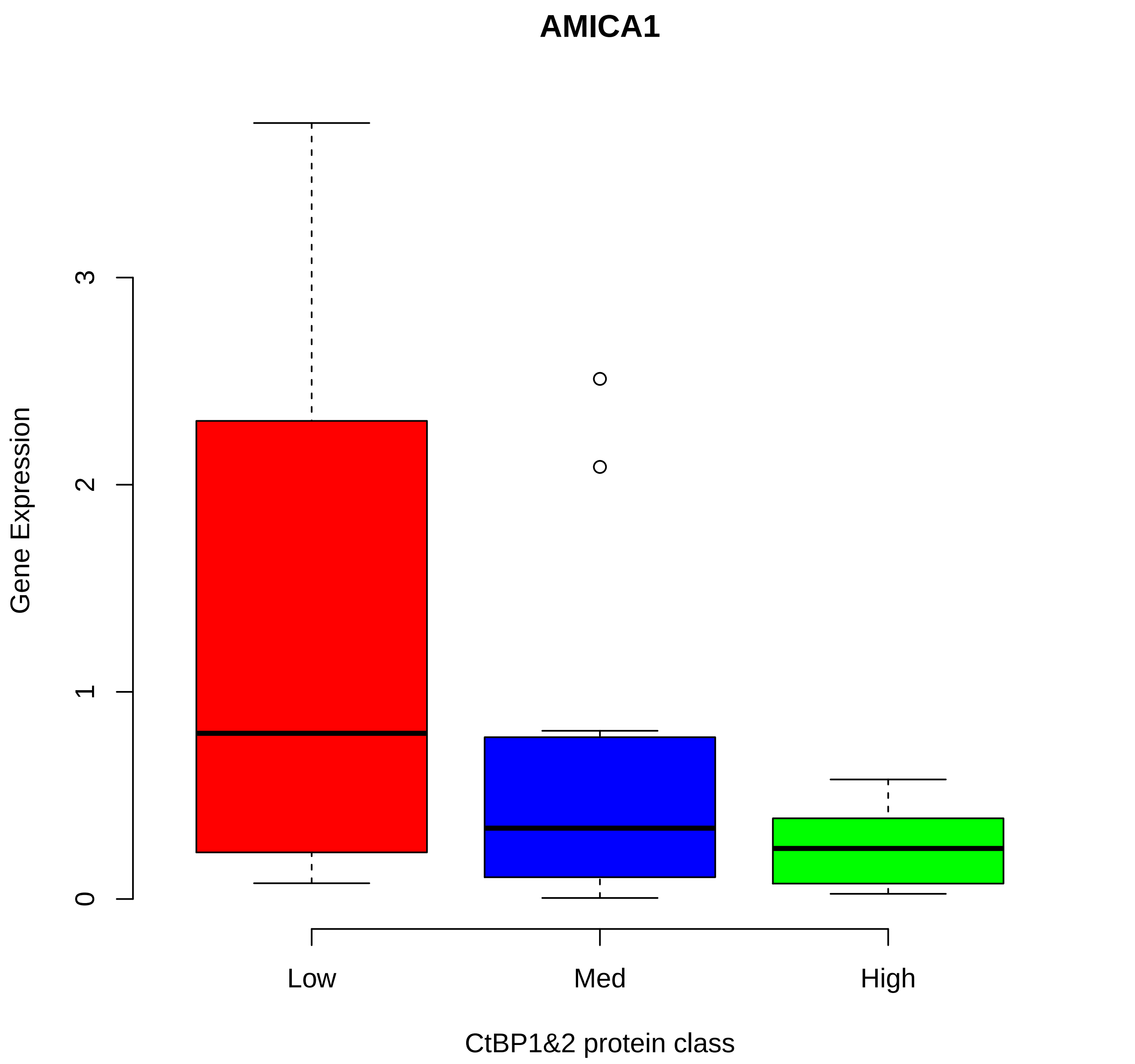

AMICA1
Gene Expression
0
1
2
3
Low
Med
High
CtBP1&2 protein class

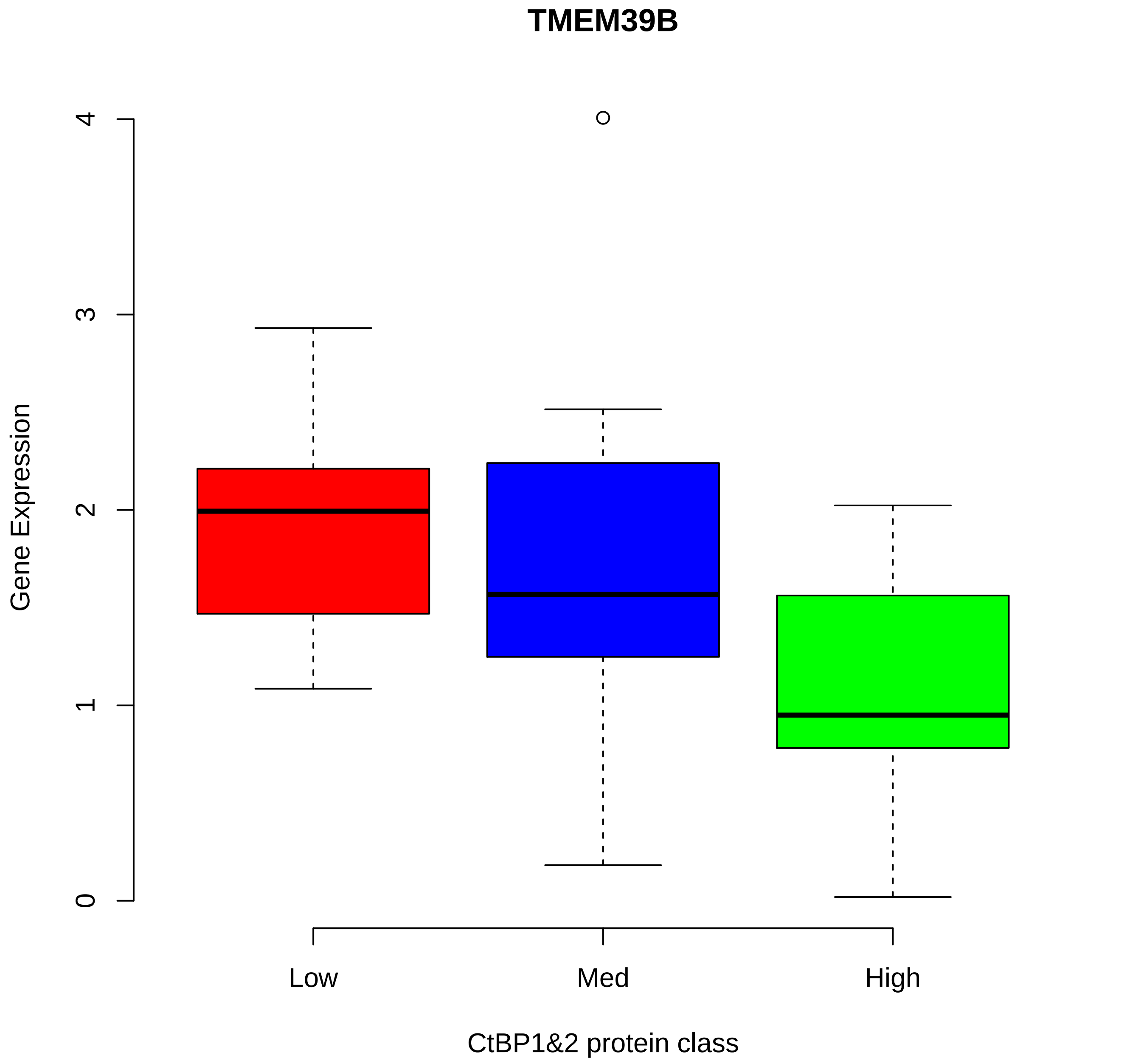
TMEM39B
Gene Expression
0
1
2
3
4
Low
Med
High
CtBP1&2 protein class

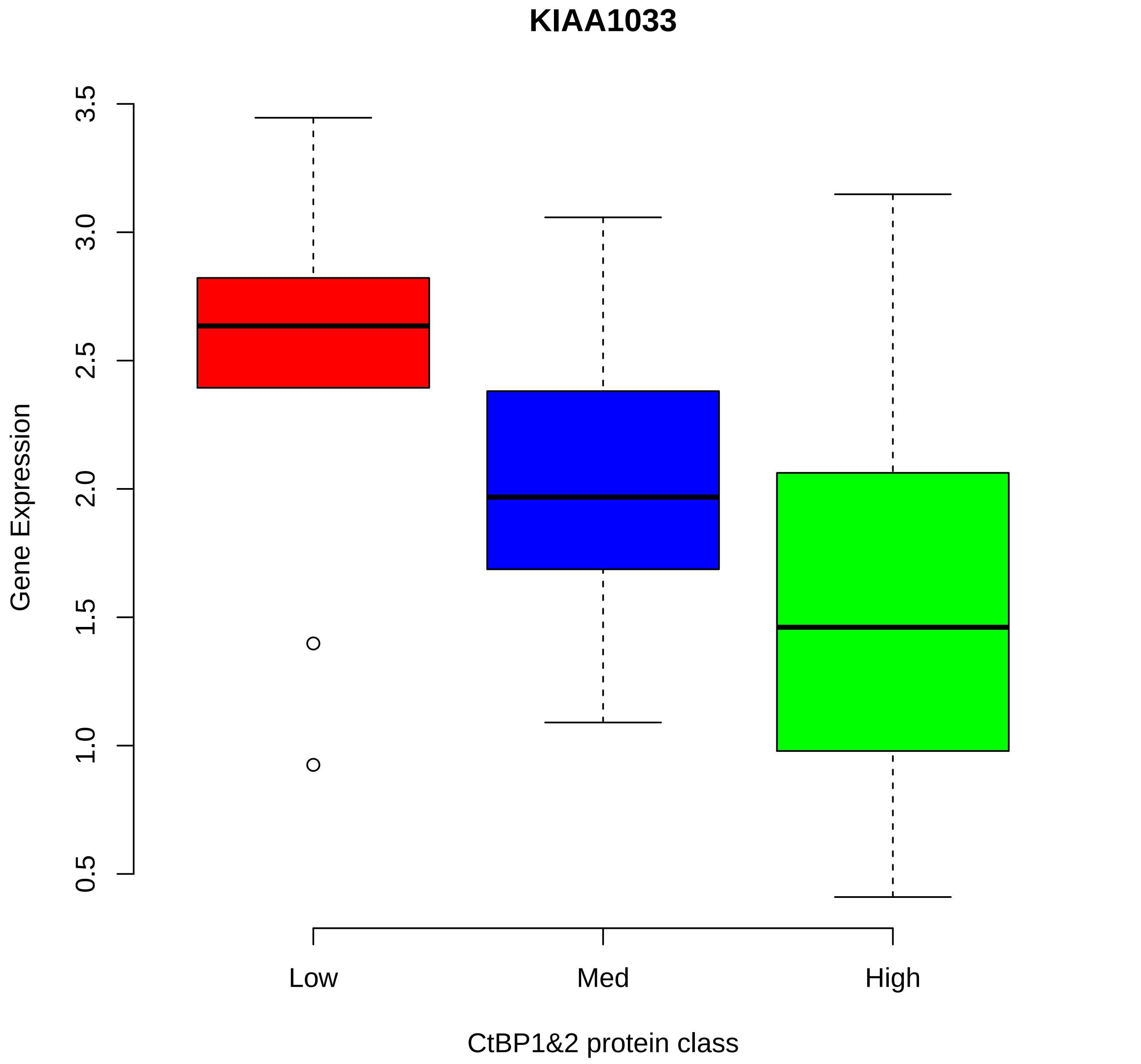
KIAA1033
Gene Expression
0.5
1.0
1.5
2.0
2.5
3.0
3.5
Low
Med
High
CtBP1&2 protein class

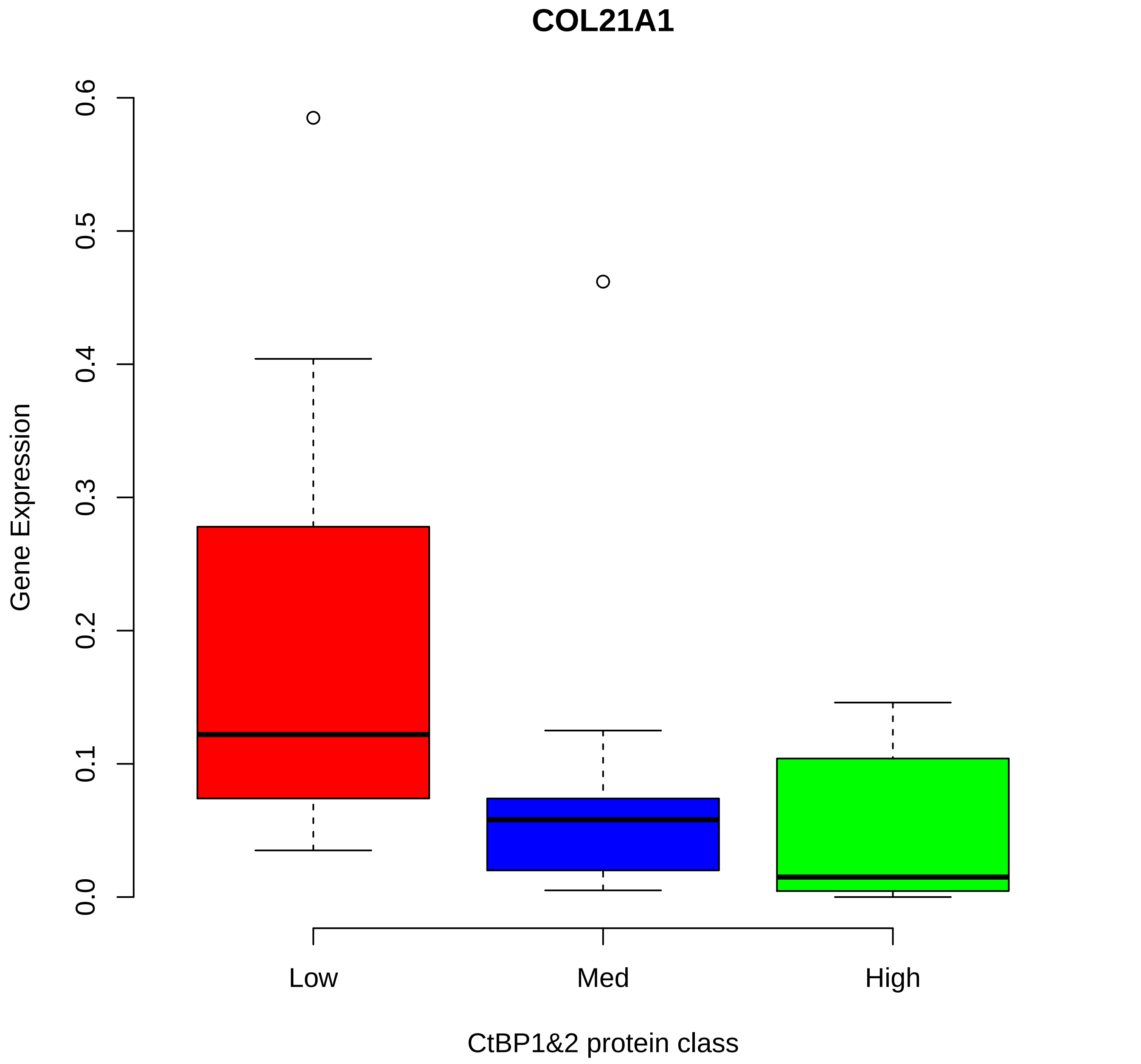
COL21A1
Gene Expression
0.0
0.1
0.2
0.3
0.4
0.5
0.6
Low
Med
High
CtBP1&2 protein class

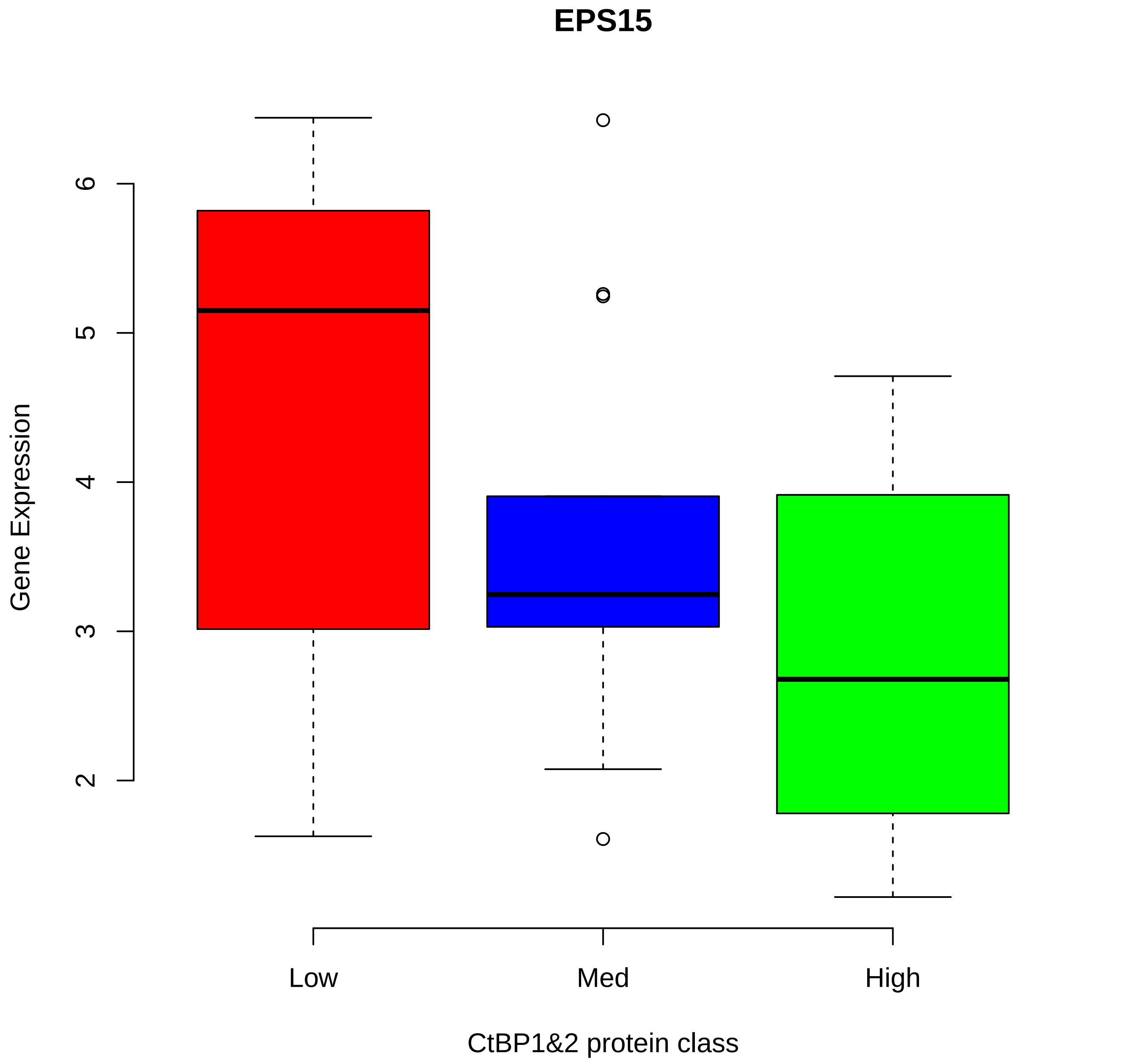

EPS15
Gene Expression
2
3
4
5
6
Low
Med
High
CtBP1&2 protein class

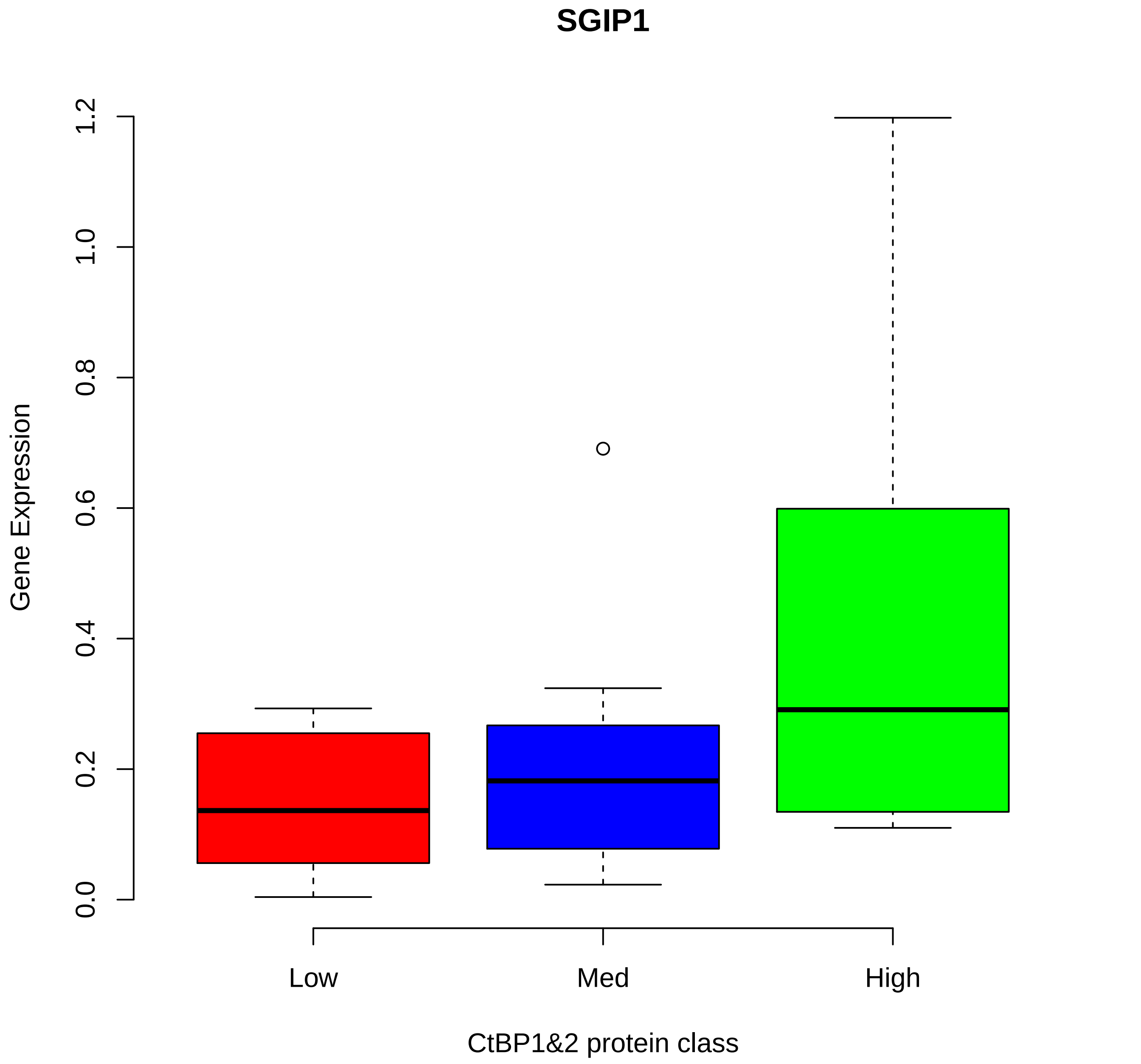
SGIP1
1.2
1.0
0.8
0.6
0.4
0.2
0.0
Gene Expression
Low
Med
High
CtBP1&2 protein class

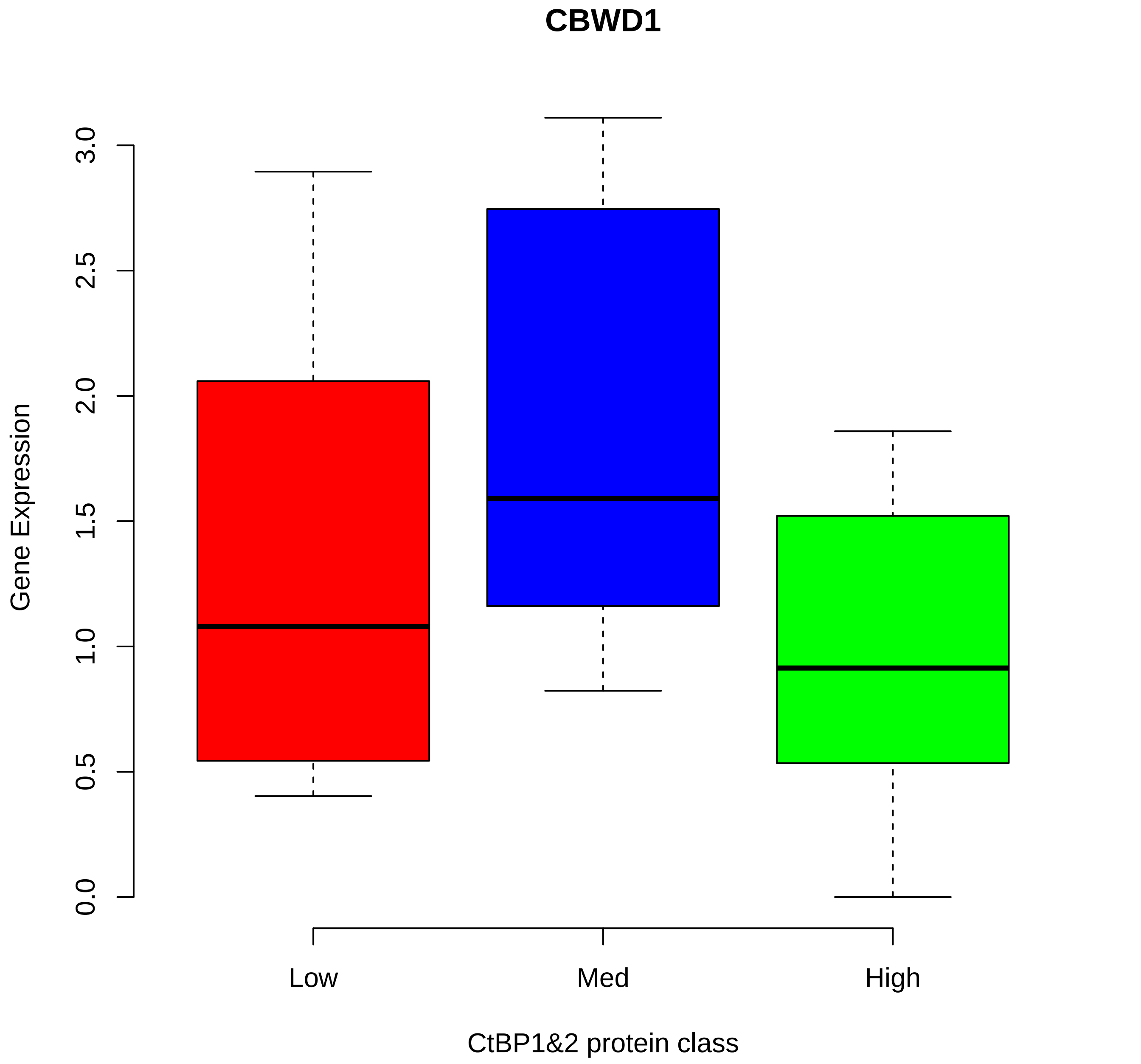

CBWD1
Gene Expression
0.0
0.5
1.0
1.5
2.0
2.5
3.0
Low
Med
High
CtBP1&2 protein class

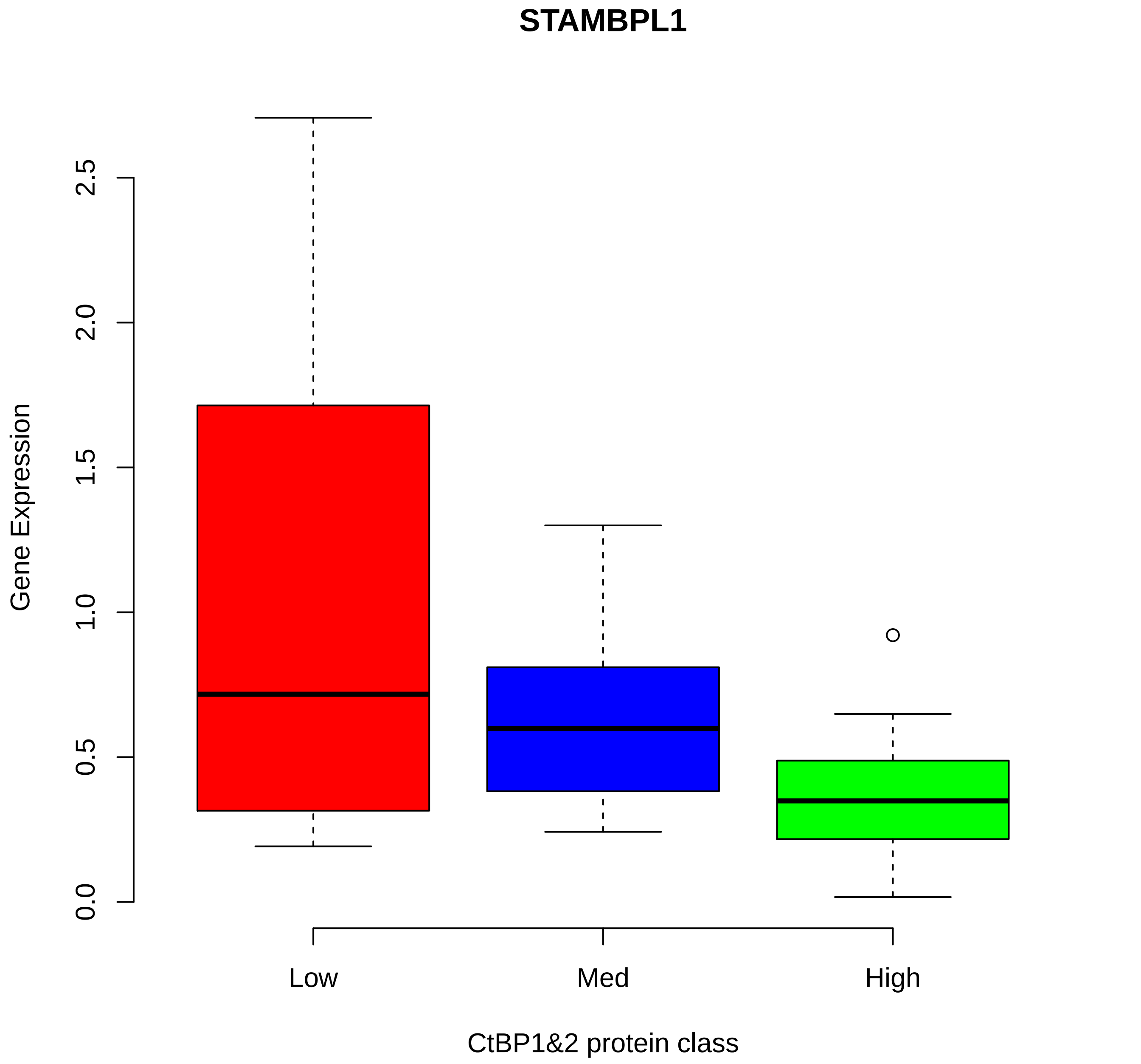
STAMBPL1
Gene Expression
0.0
0.5
1.0
1.5
2.0
2.5
Low
Med
High
CtBP1&2 protein class

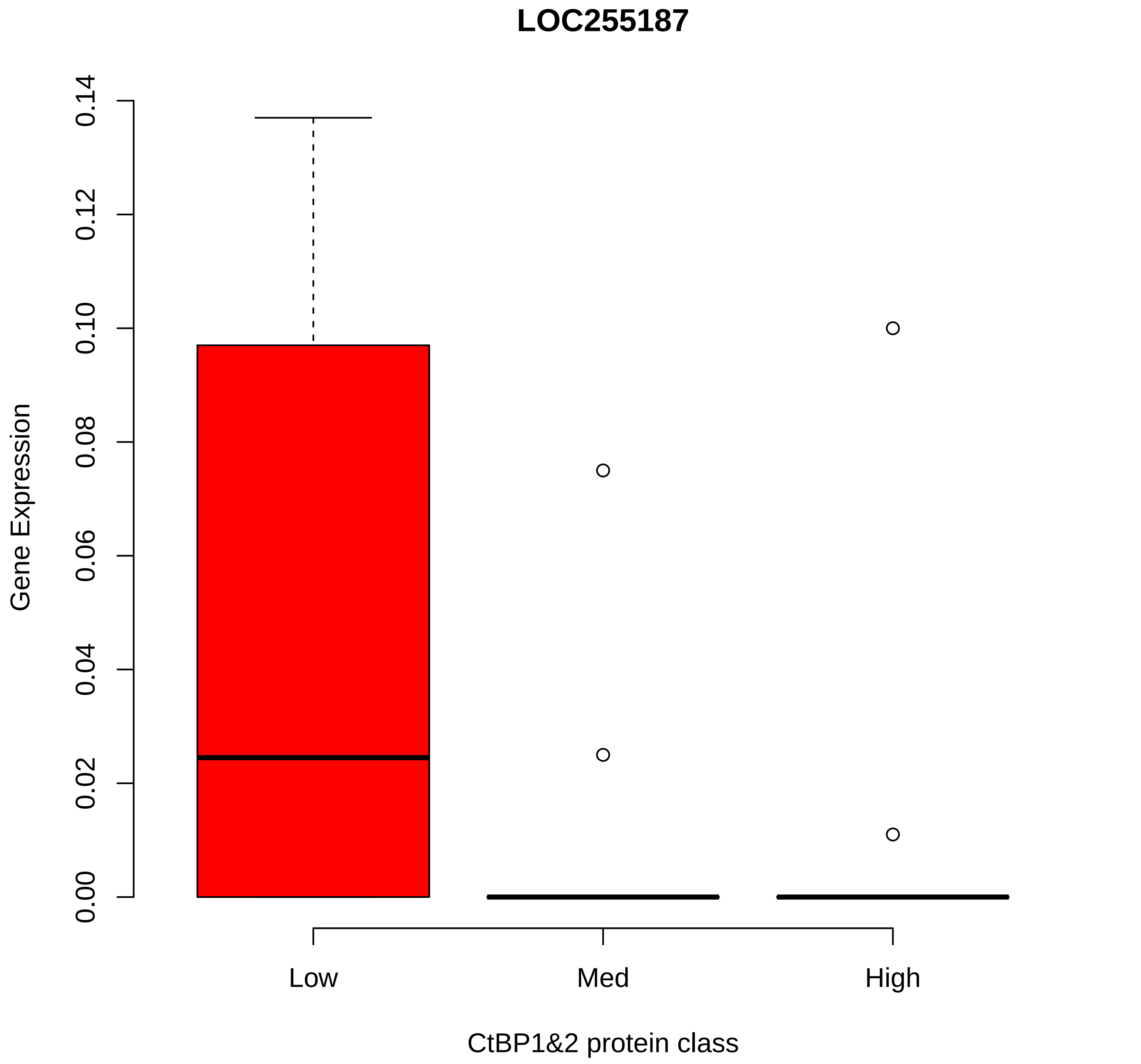
LOC255187
Gene Expression
0.00
0.02
0.04
0.06
0.08
0.10
0.12
0.14
Low
Med
High
CtBP1&2 protein class

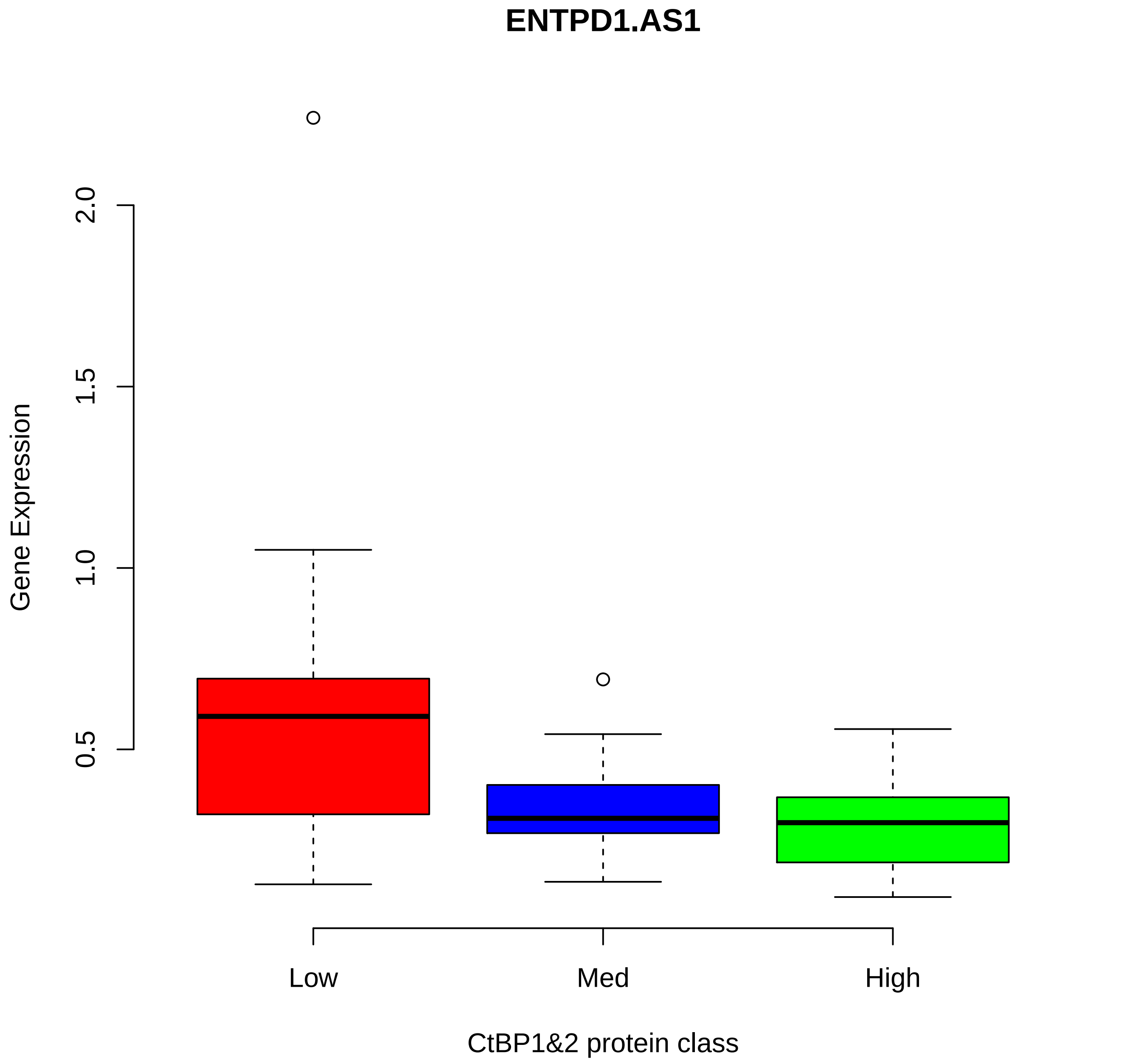

ENTPD1.AS1
Gene Expression
2.0
1.5
1.0
0.5
Low
Med
High
CtBP1&2 protein class

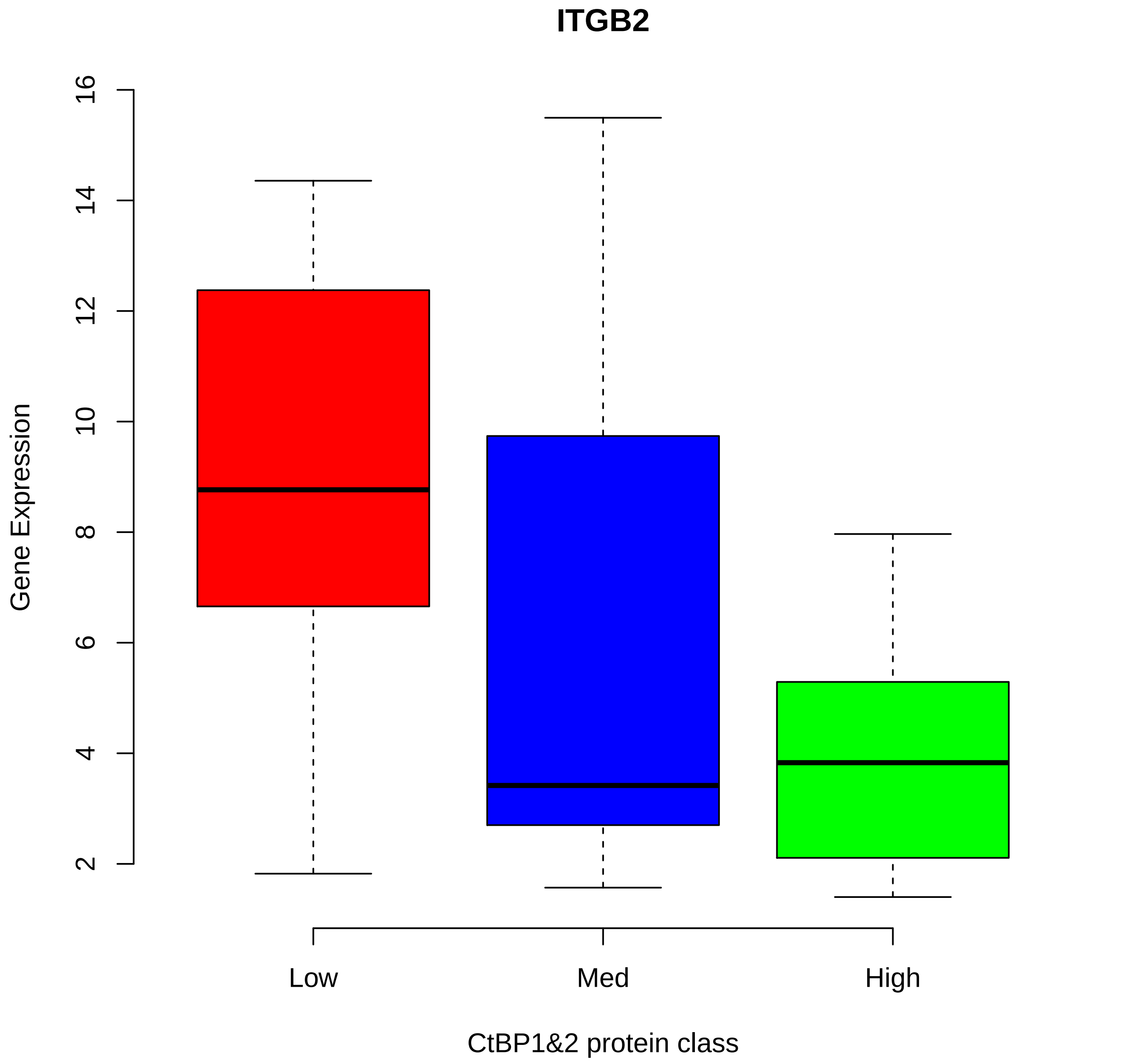

ITGB2
Gene Expression
16
14
12
10
8
6
4
2
Low
Med
High
CtBP1&2 protein class

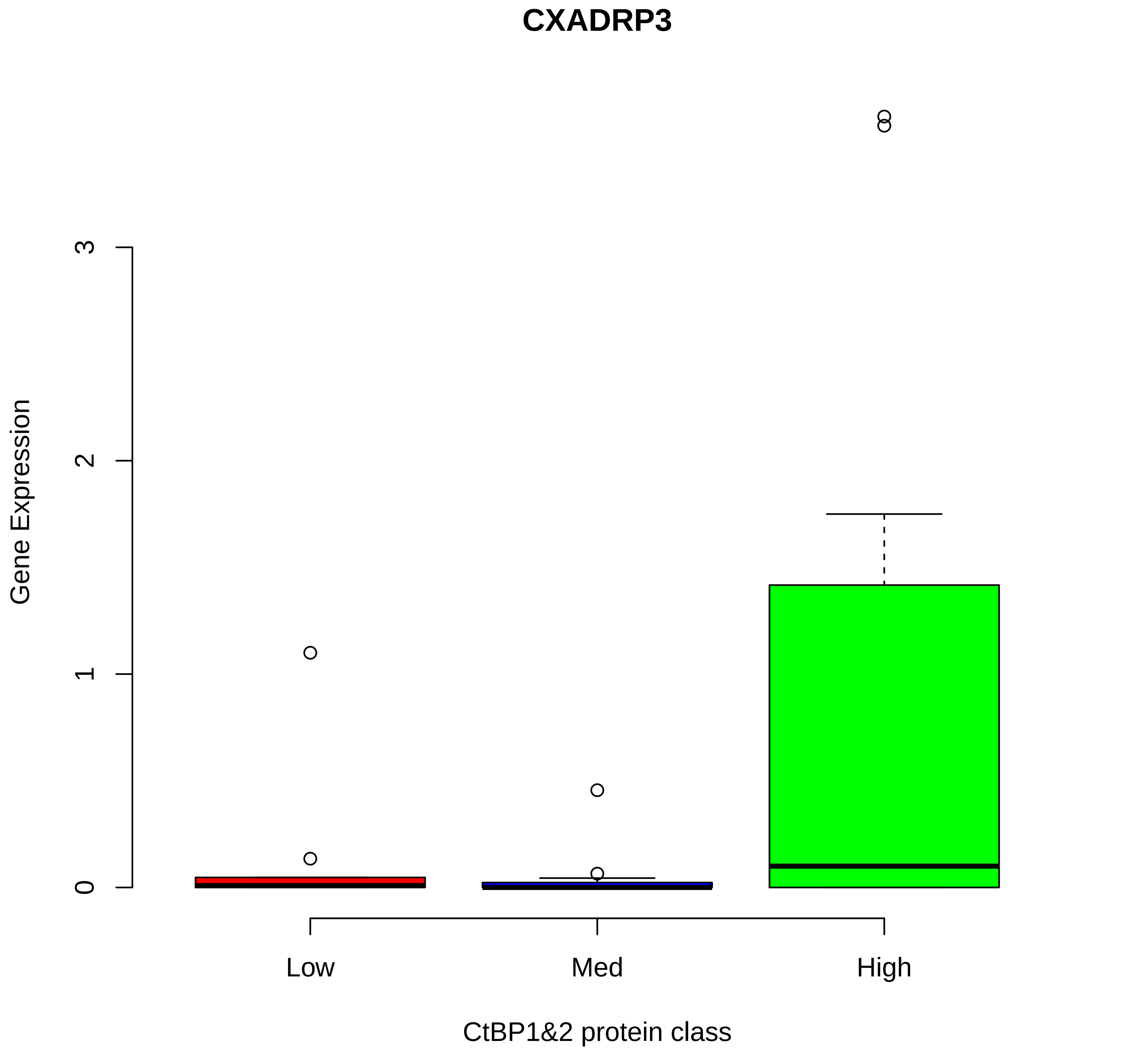
CXADRP3
Gene Expression
0
1
2
3
Low
Med
High
CtBP1&2 protein class

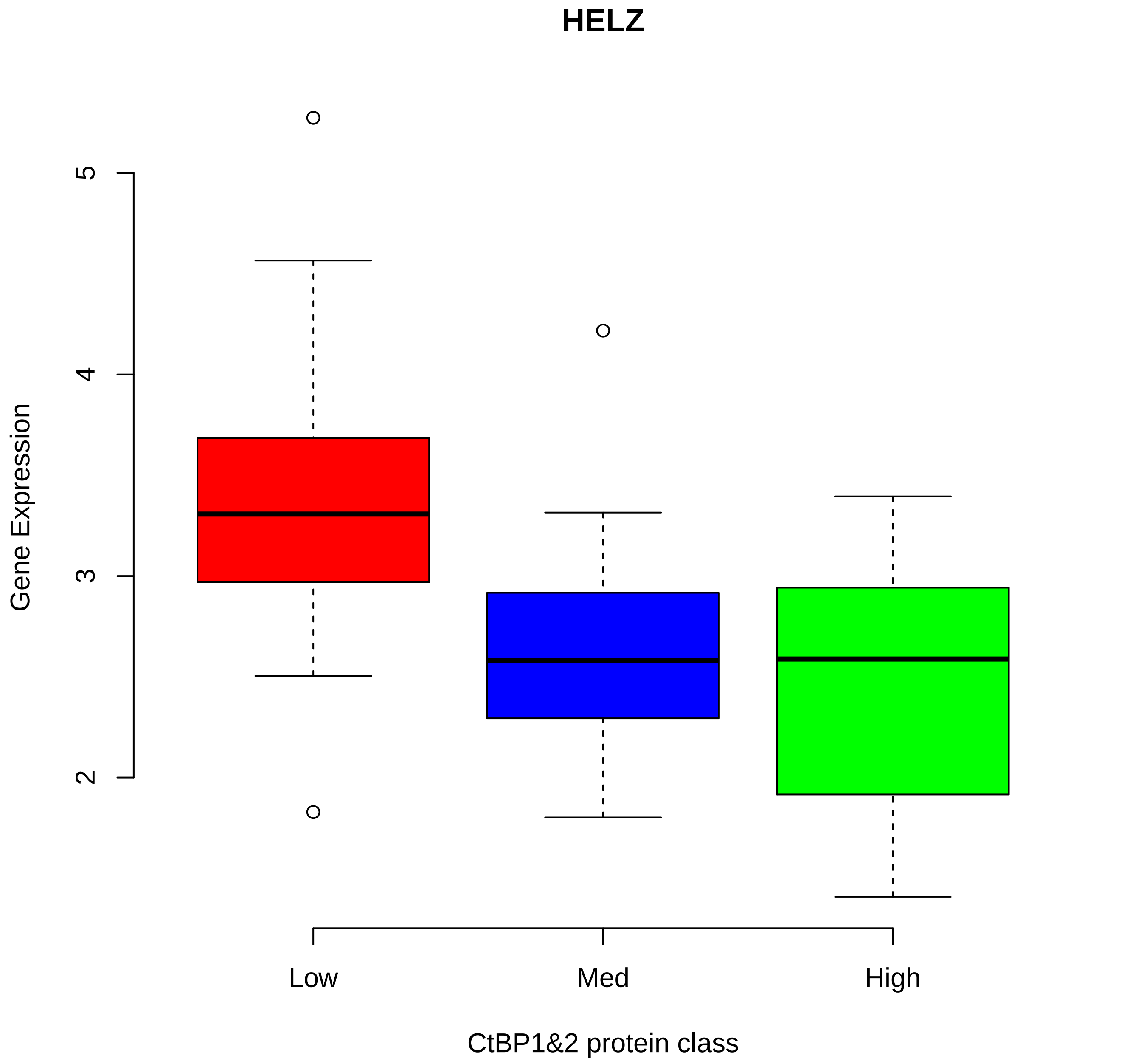
HELZ
Gene Expression
2
3
4
5
Low
Med
High
CtBP1&2 protein class

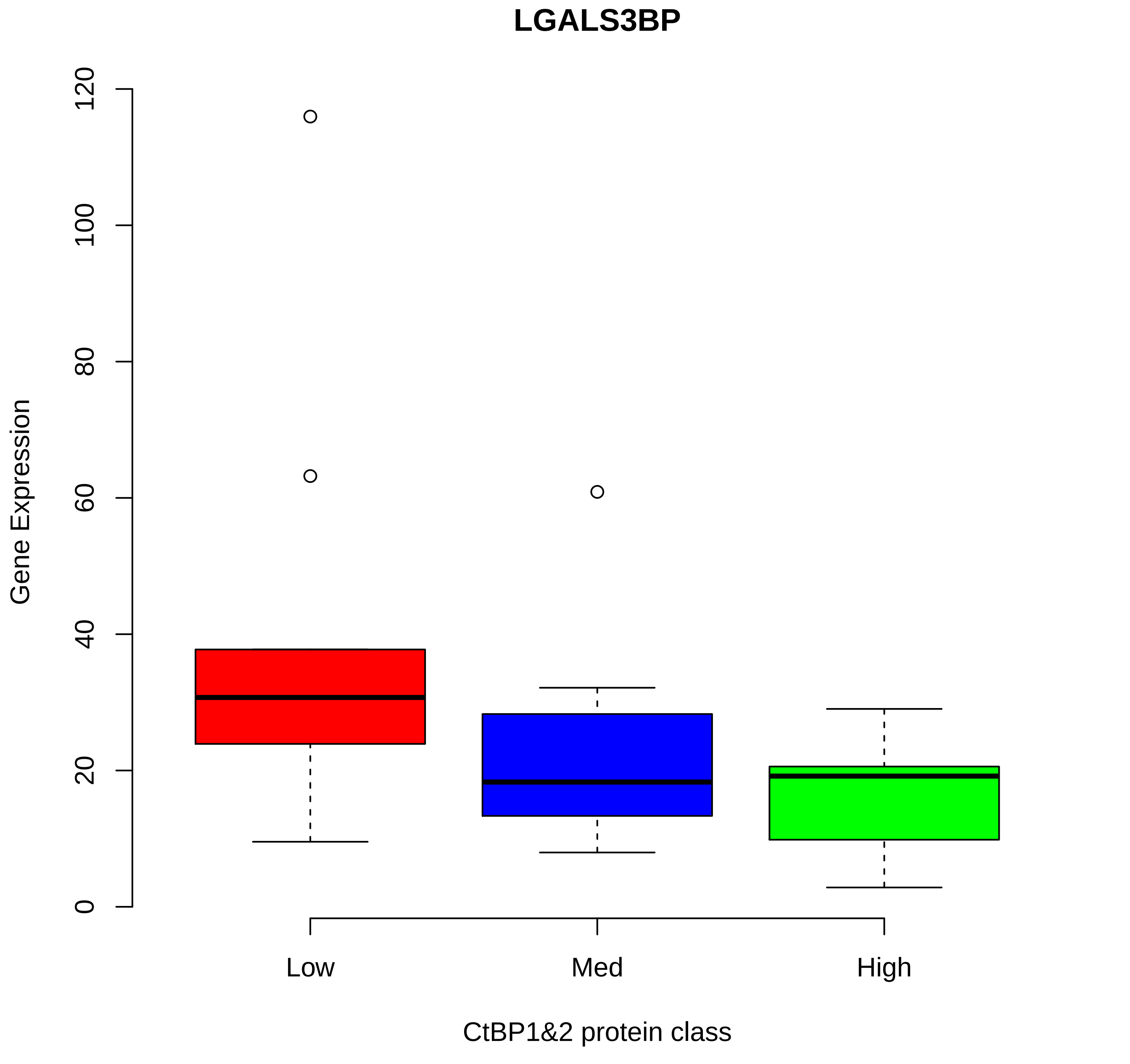

LGALS3BP
Gene Expression
0
20
40
60
80
100
120
Low
Med
High
CtBP1&2 protein class

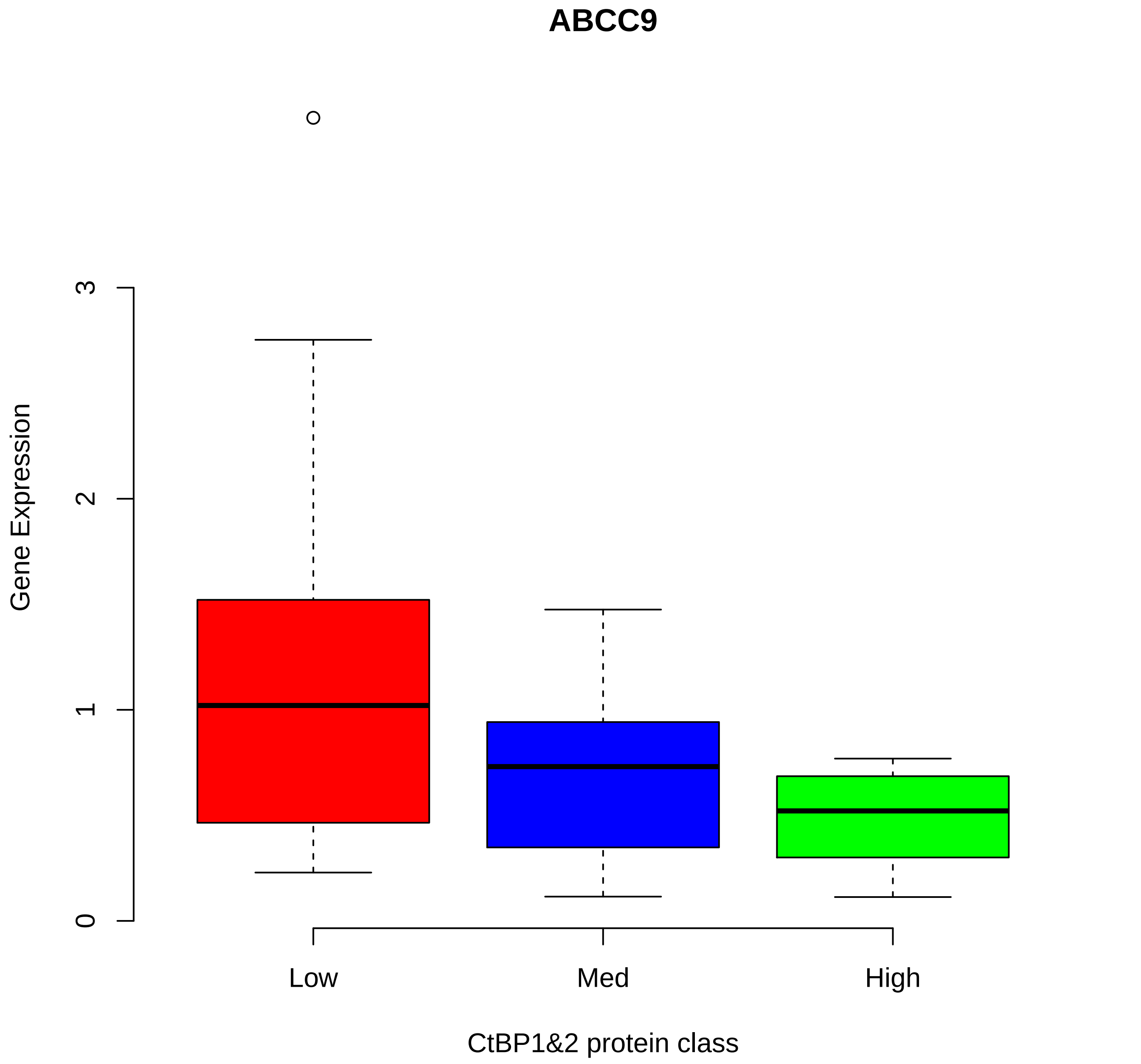

ABCC9
Gene Expression
0
1
2
3
Low
Med
High
CtBP1&2 protein class

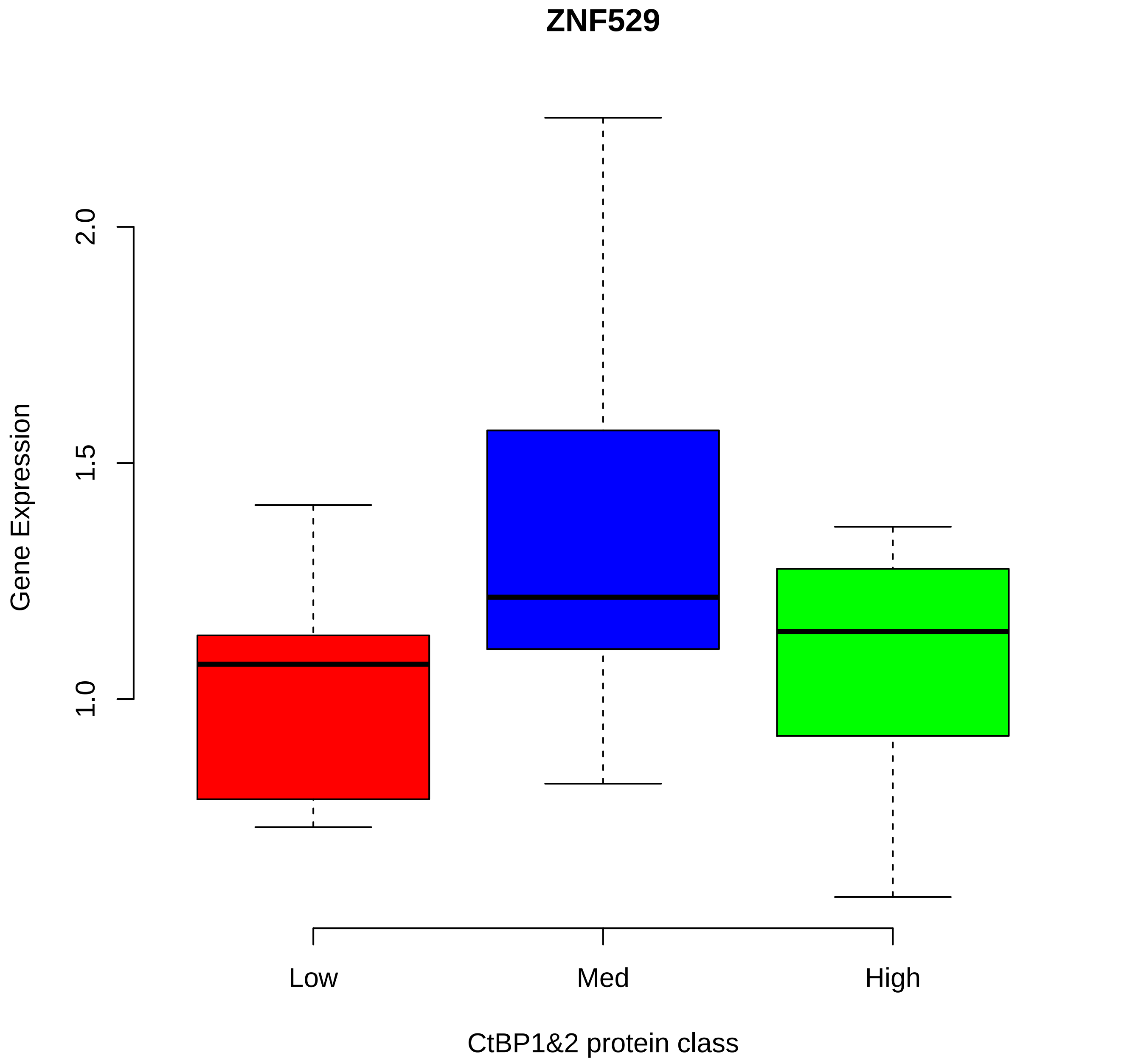

ZNF529
Gene Expression
2.0
1.5
1.0
Low
Med
High
CtBP1&2 protein class

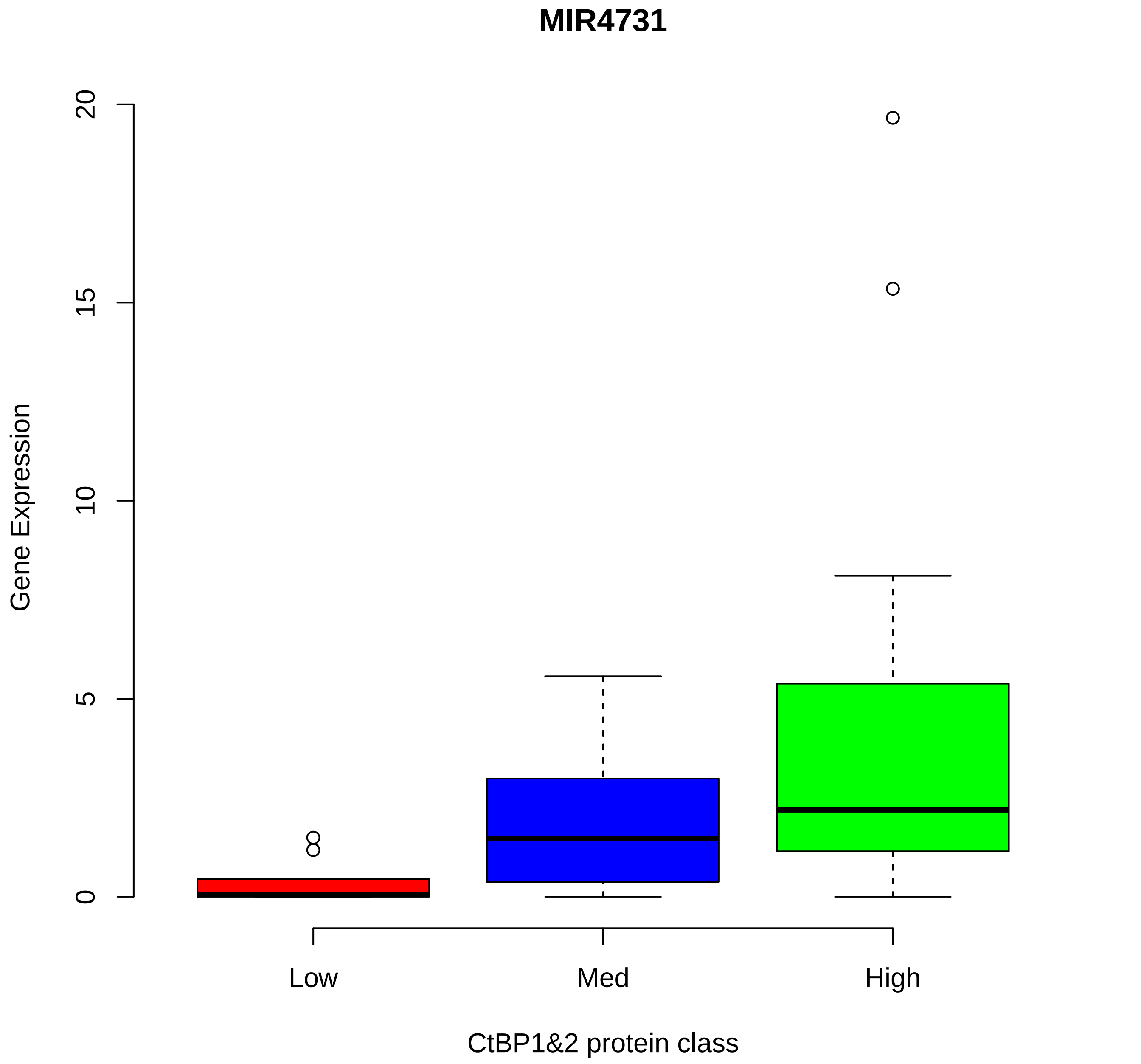
MIR4731
Gene Expression
0
5
10
15
20
Low
Med
High
CtBP1&2 protein class

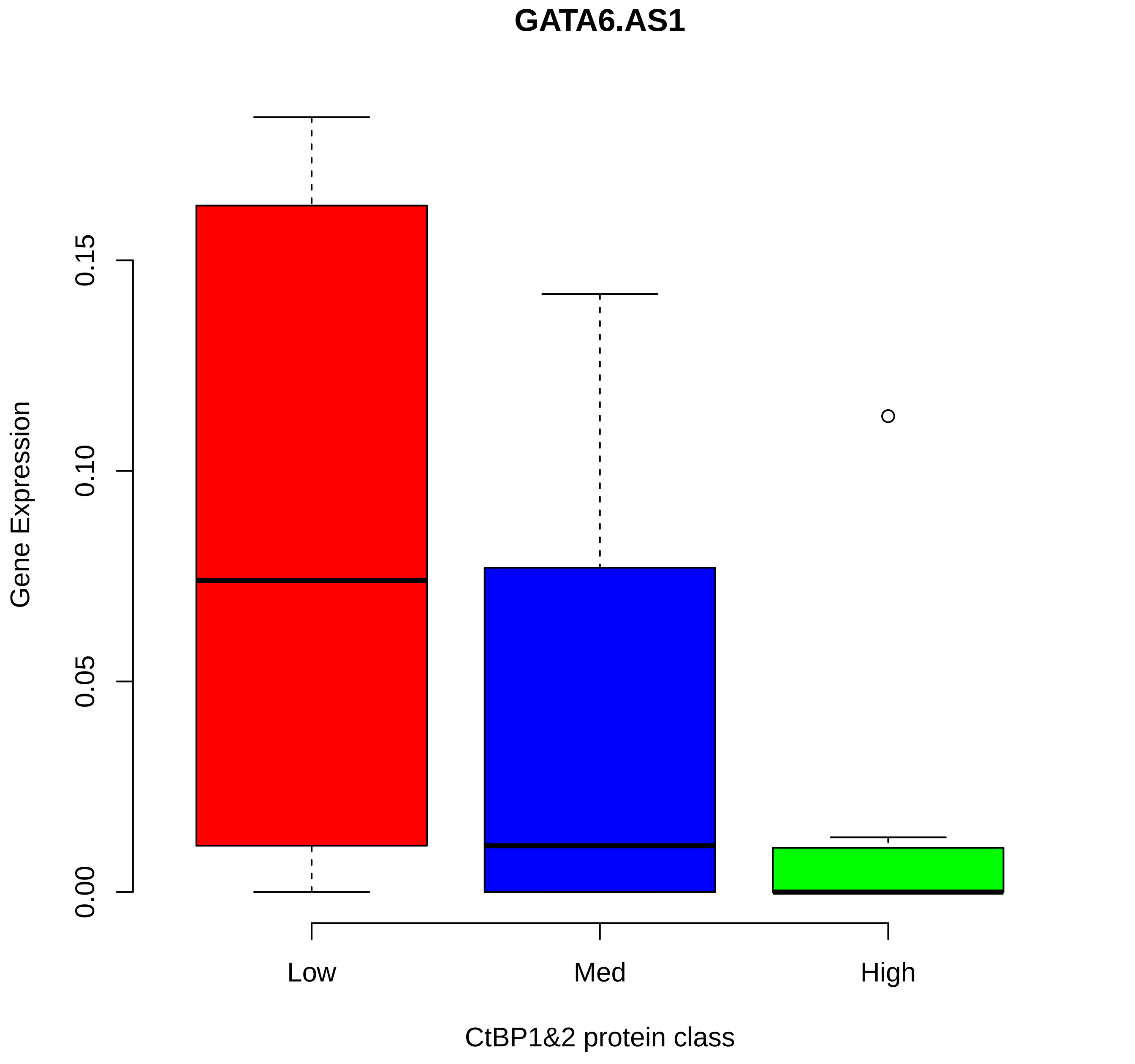
GATA6.AS1
Gene Expression
0.00
0.05
0.10
0.15
Low
Med
High
CtBP1&2 protein class

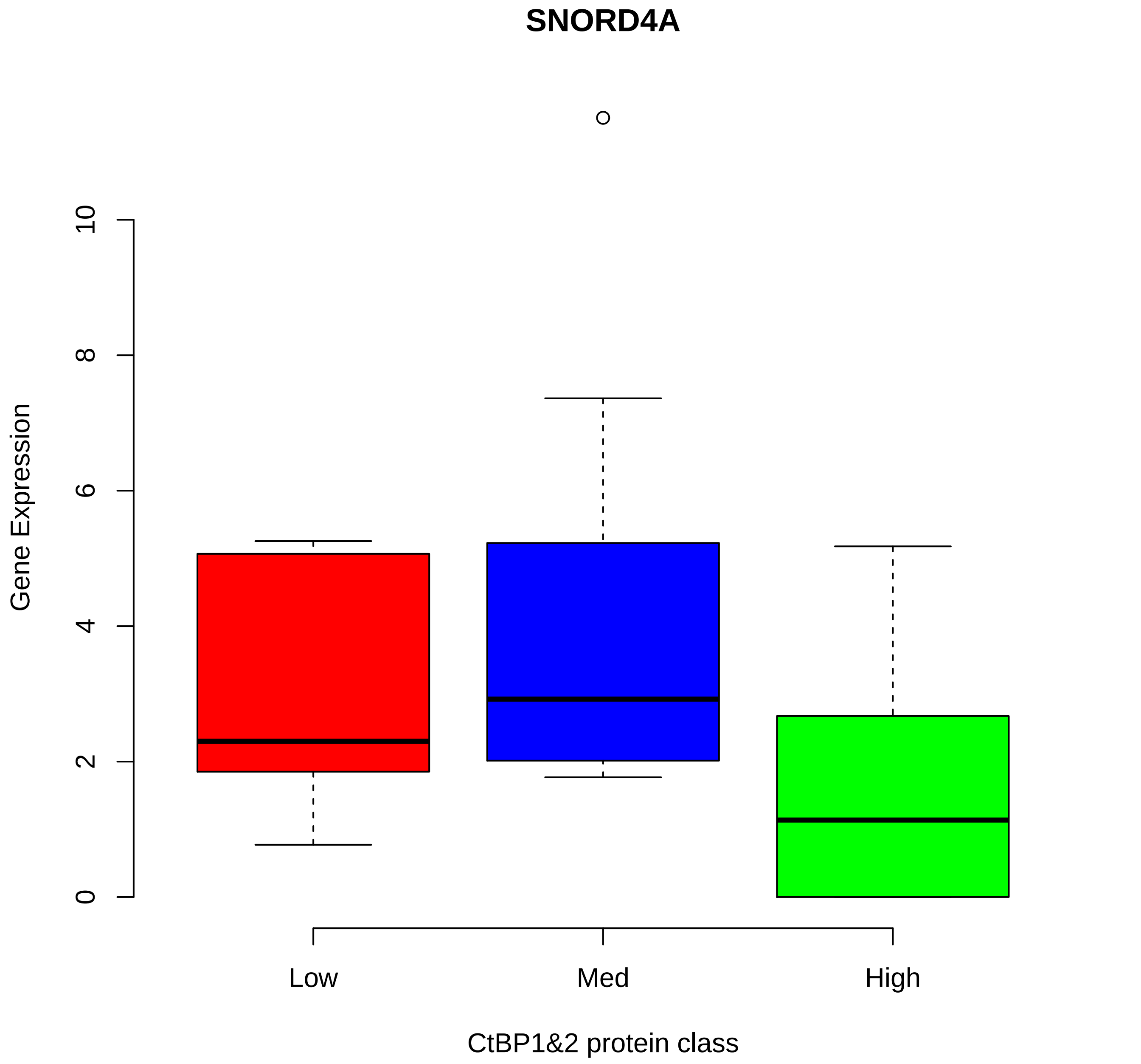

SNOND4A
Gene Expression
0
2
4
6
8
10
Low
Med
High
CtBP1&2 protein class

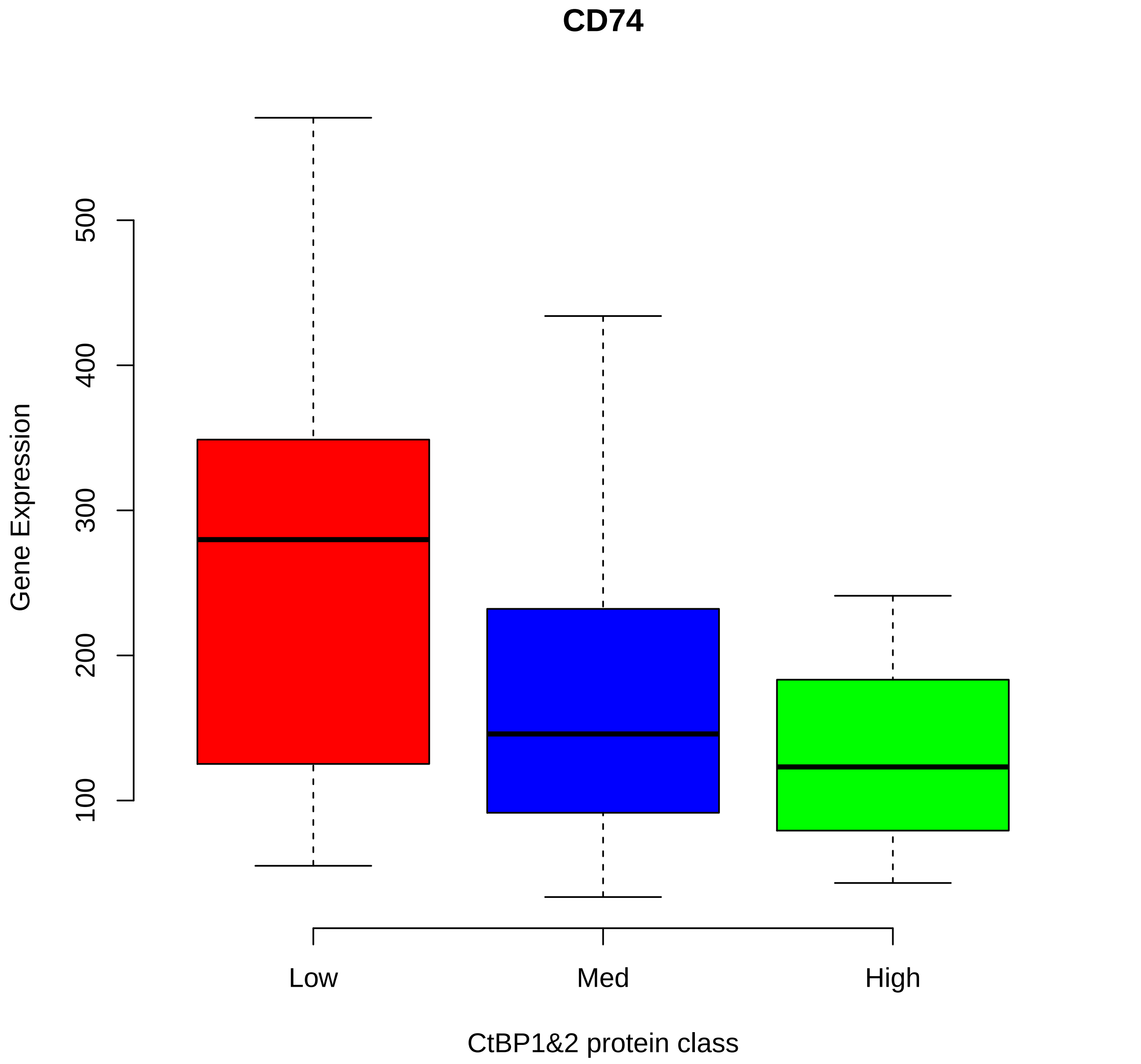

CD74
Gene Expression
100
200
300
400
500
Low
Med
High
CtBP1&2 protein class

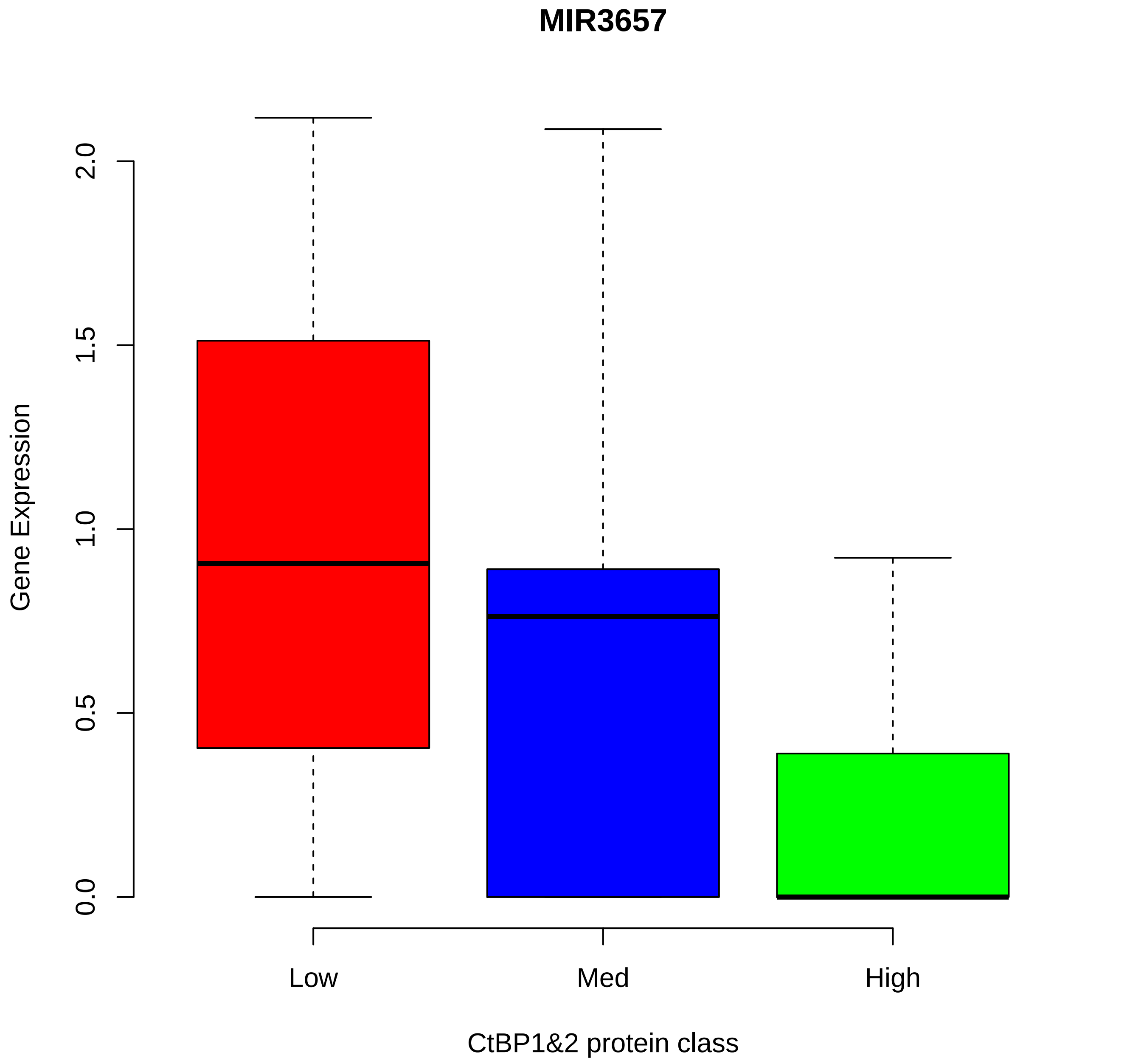

MIR3657
Gene Expression
0.0
0.5
1.0
1.5
2.0
Low
Med
High
CtBP1&2 protein class

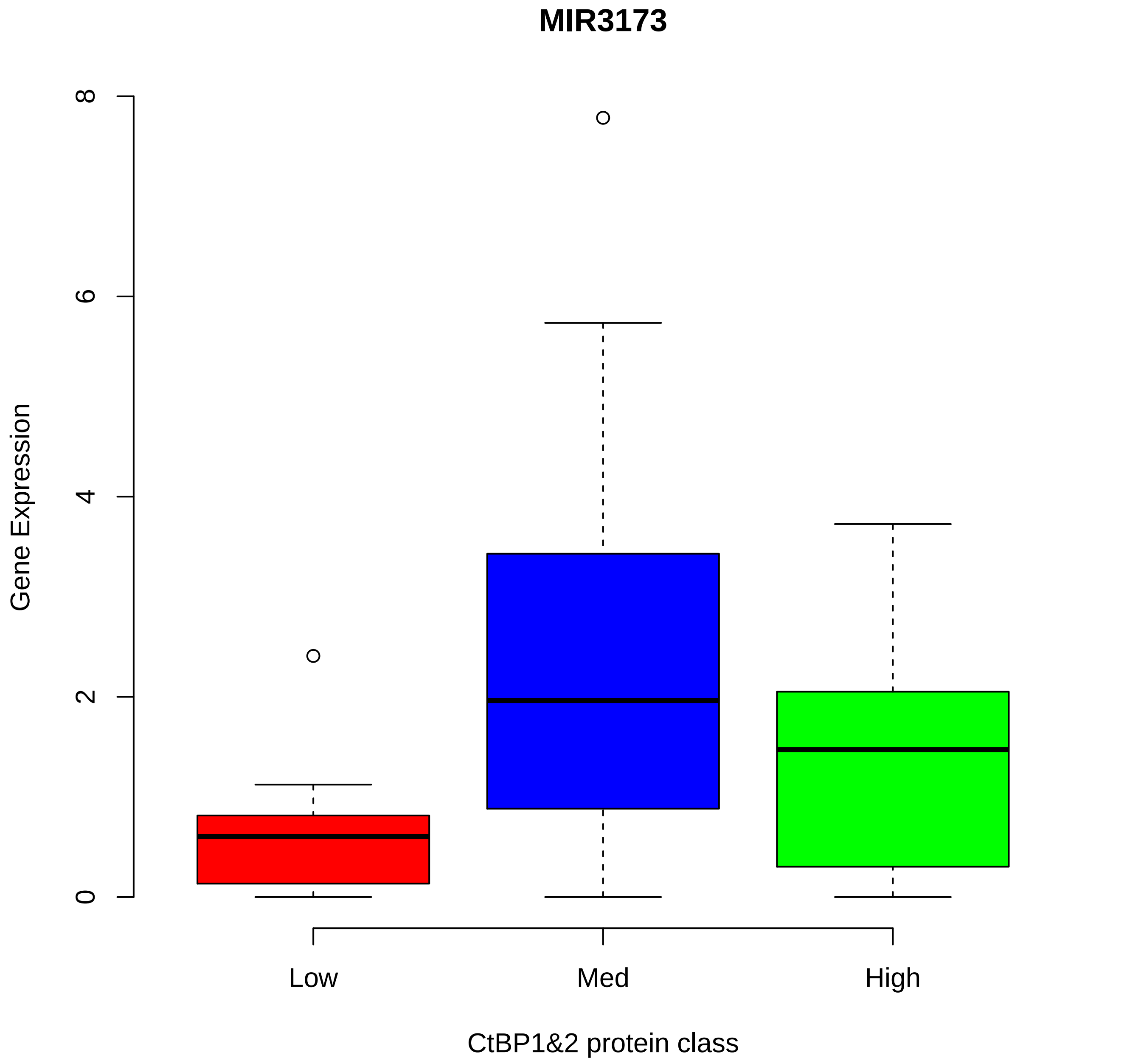

MIR3173
Gene Expression
0
2
4
6
8
Low
Med
High
CtBP1&2 protein class

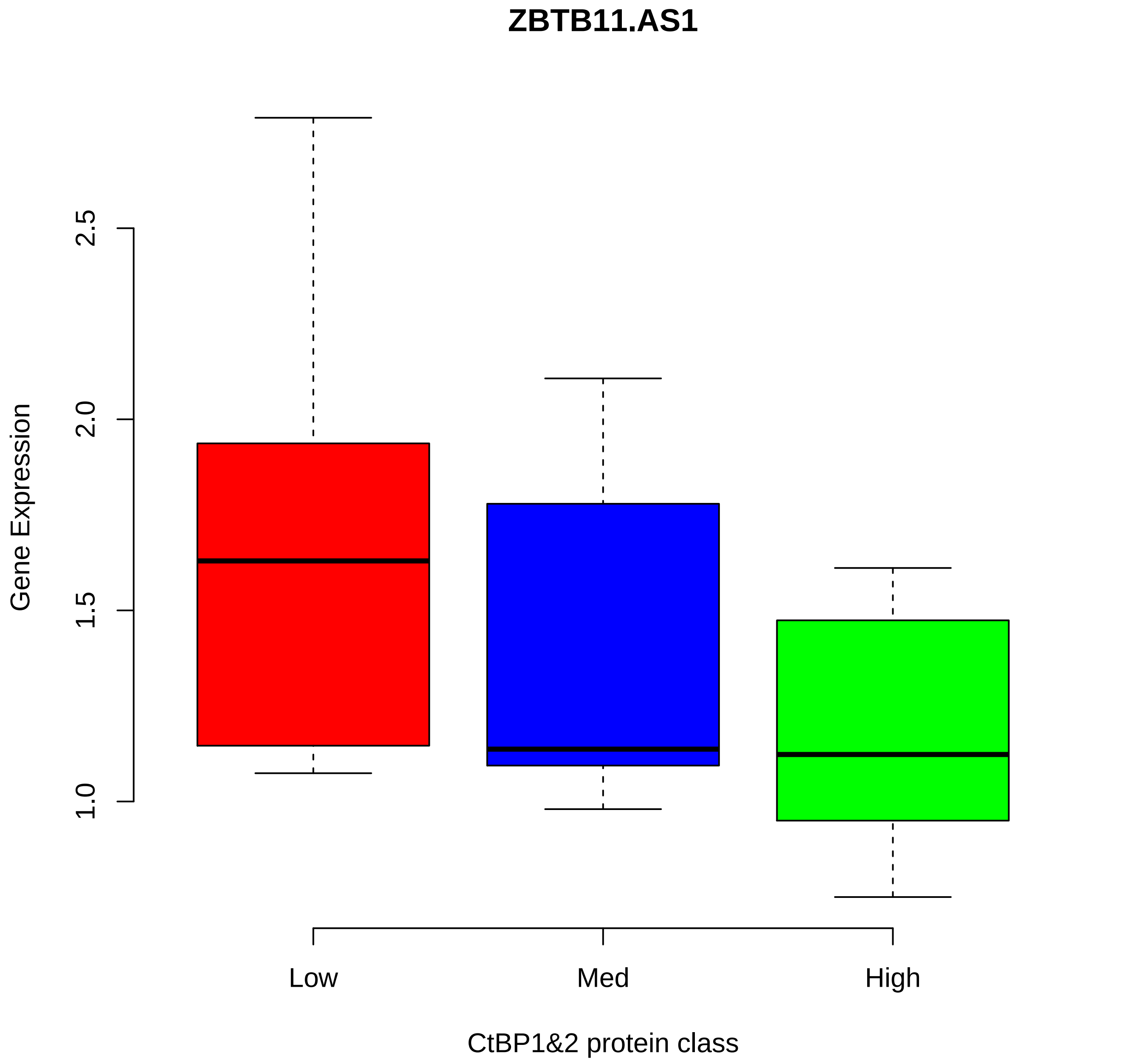
ZBTB11.AS1
Gene Expression
1.0
1.5
2.0
2.5
Low
Med
High
CtBP1&2 protein class

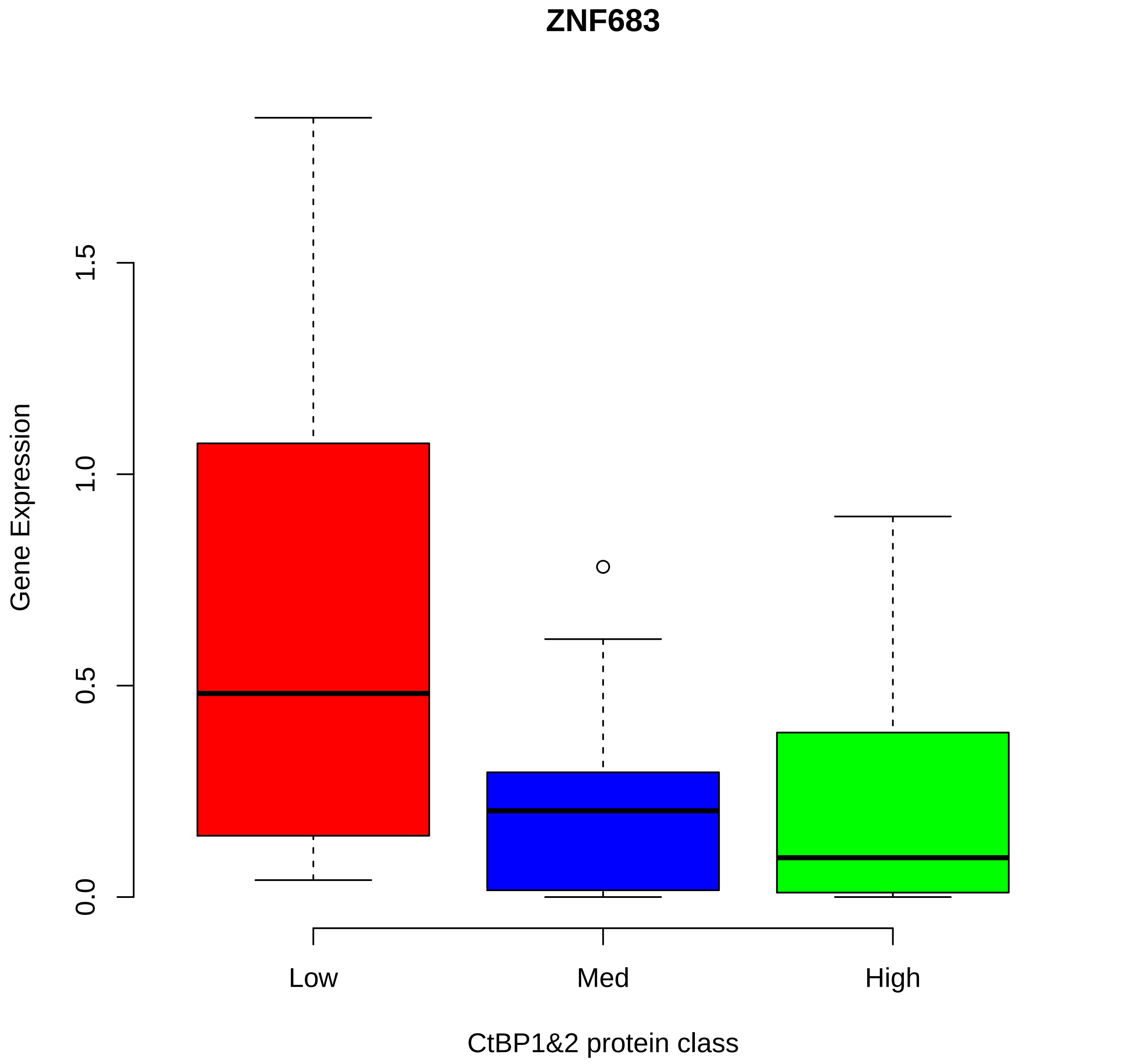

ZNF683
Gene Expression
0.0
0.5
1.0
1.5
Low
Med
High
CtBP1&2 protein class

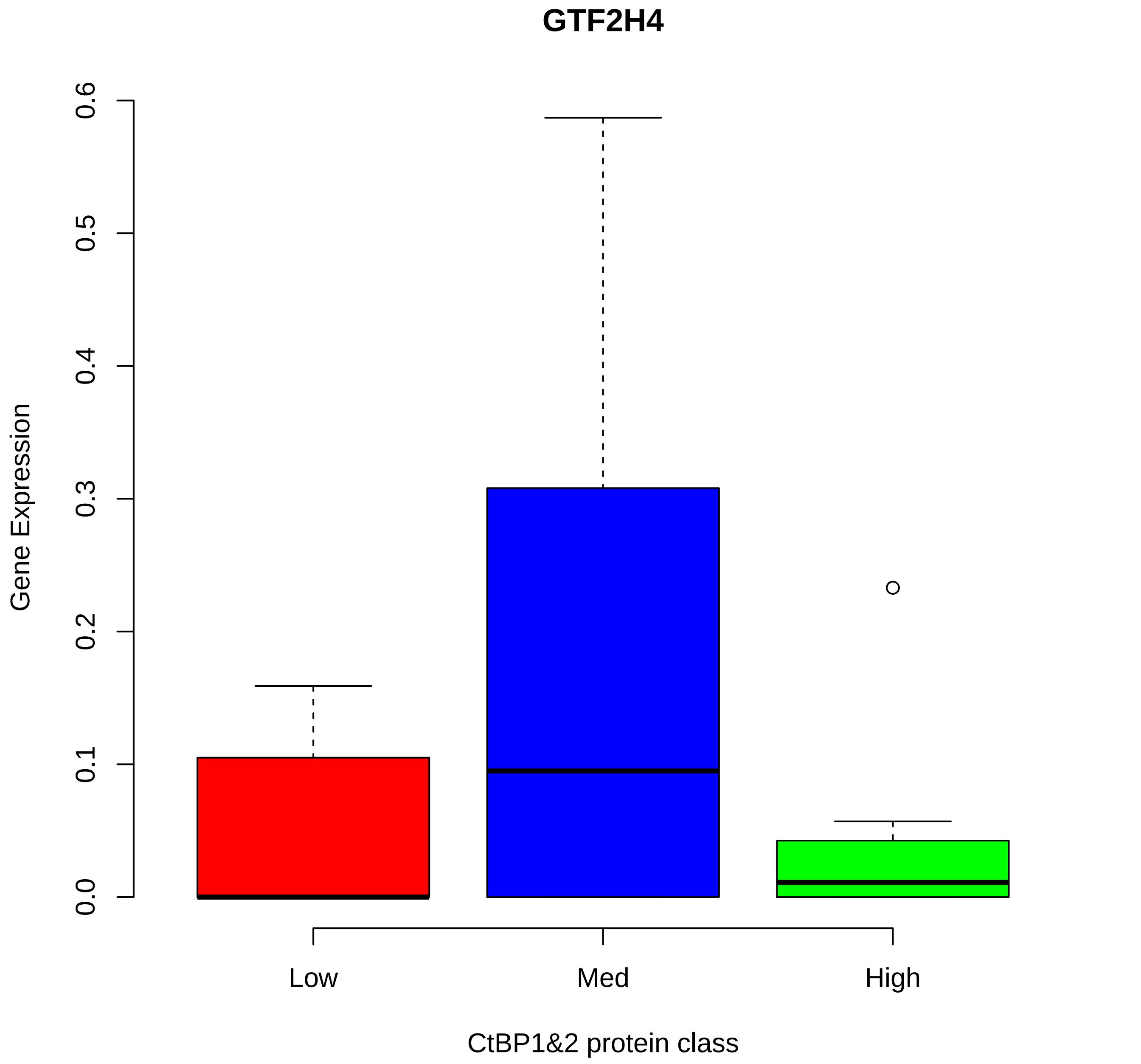
GTF2H4
Gene Expression
0.0
0.1
0.2
0.3
0.4
0.5
0.6
Low
Med
High
CtBP1&2 protein class

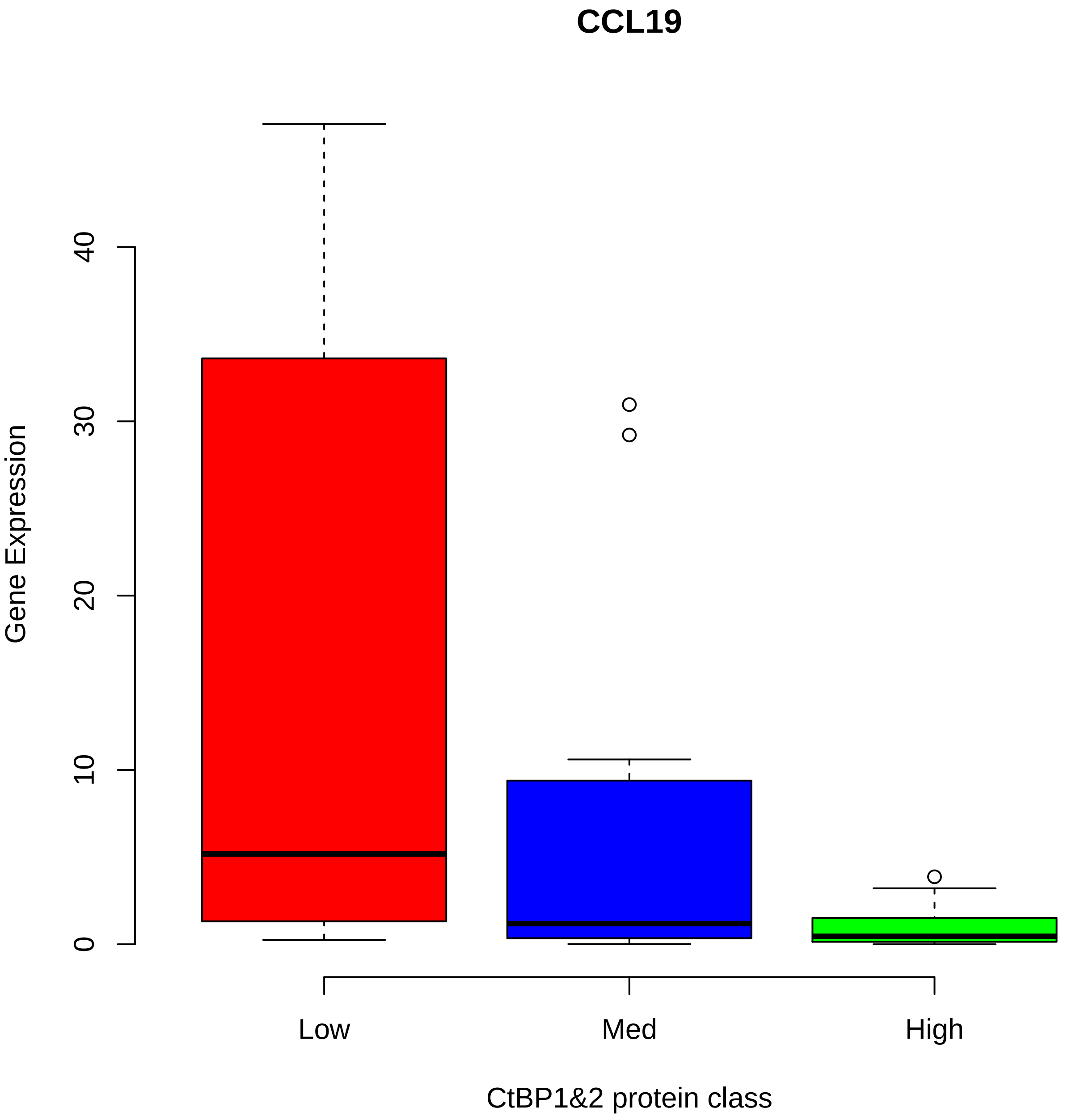
CCL19
Gene Expression
0
10
20
30
40
Low
Med
High
CtBP1&2 protein class

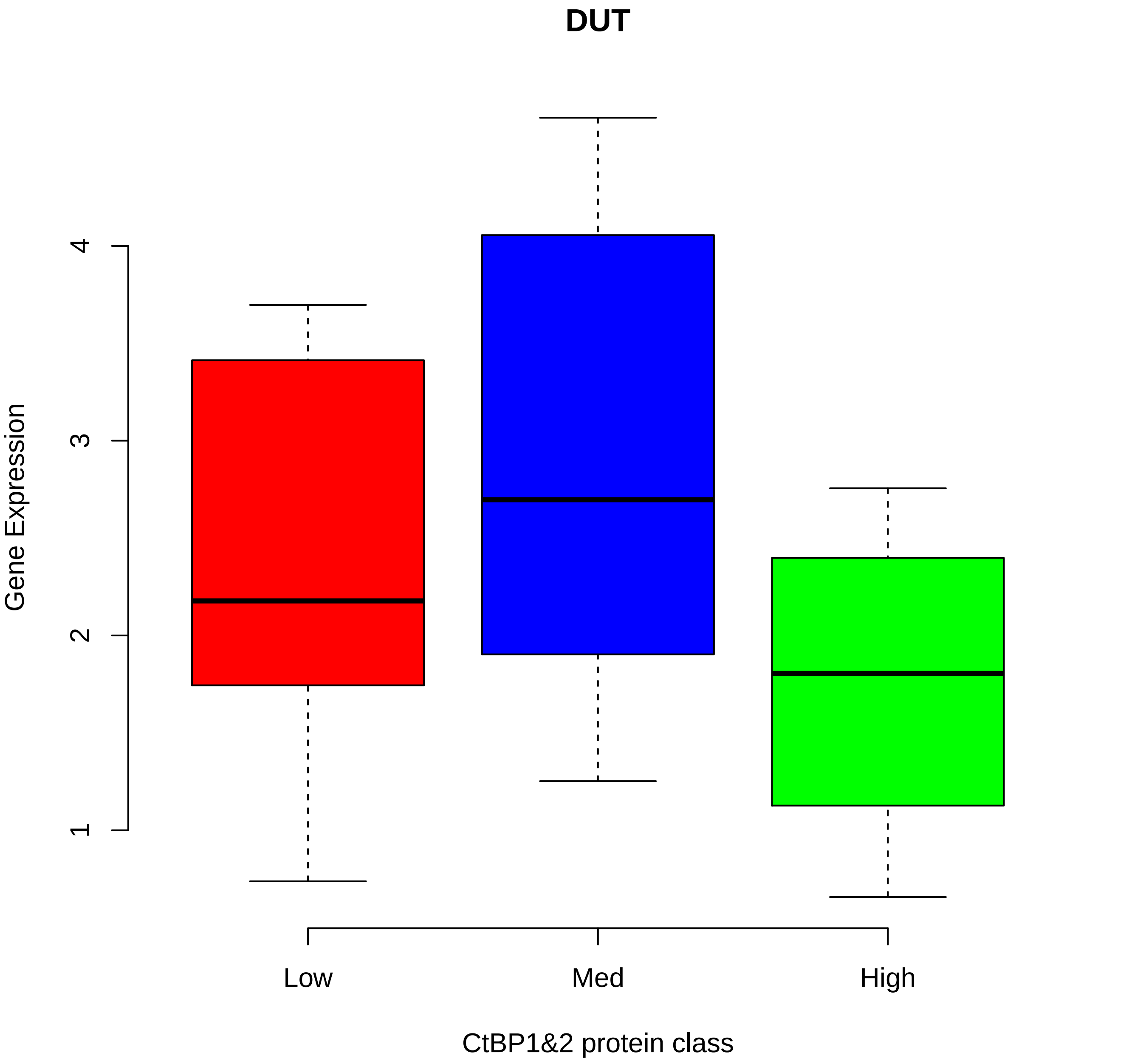
DUT
Gene Expression
1
2
3
4
Low
Med
High
CtBP1&2 protein class

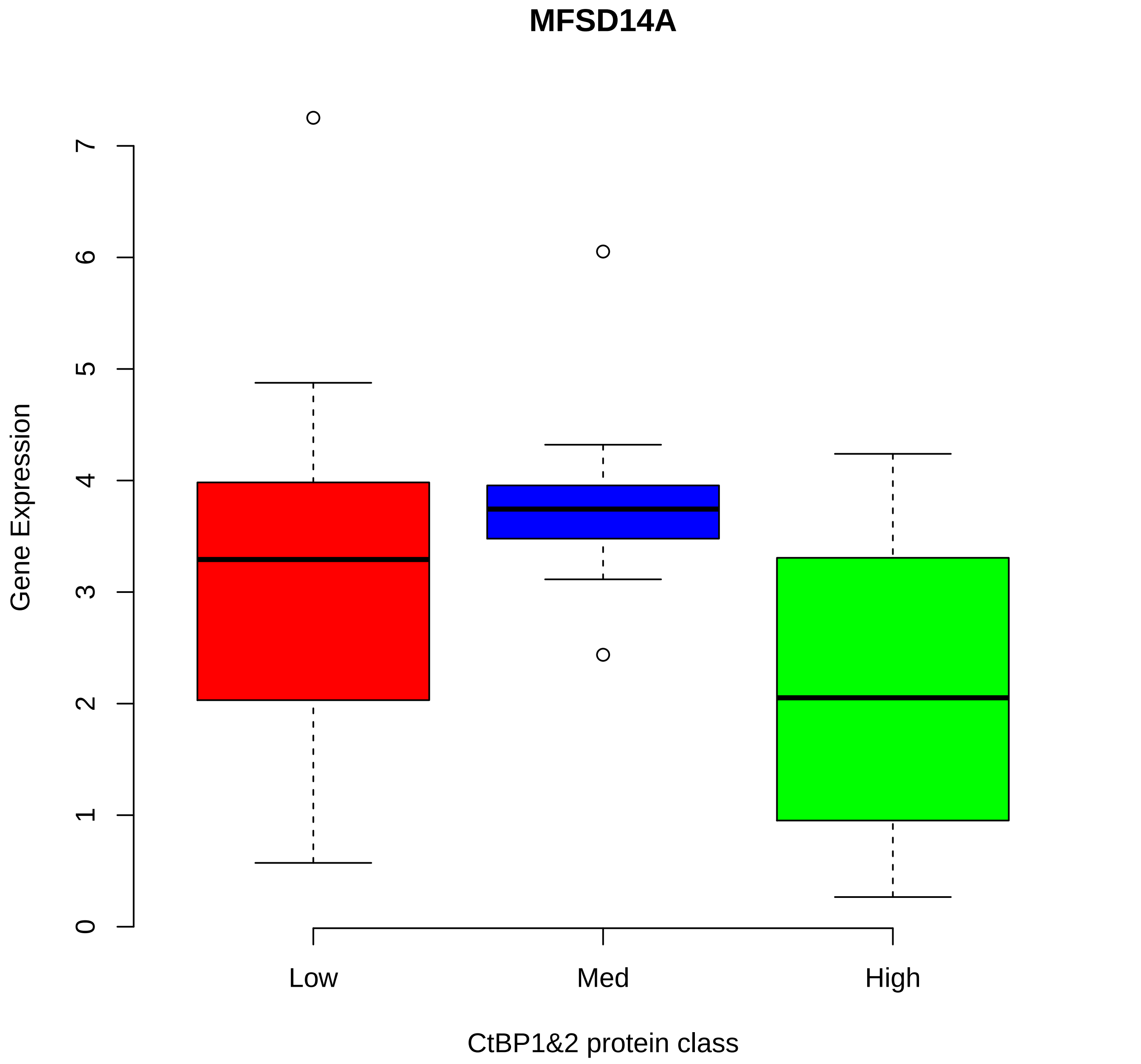
MFSD14A
Gene Expression
0
1
2
3
4
5
6
7
Low
Med
High
CtBP1&2 protein class

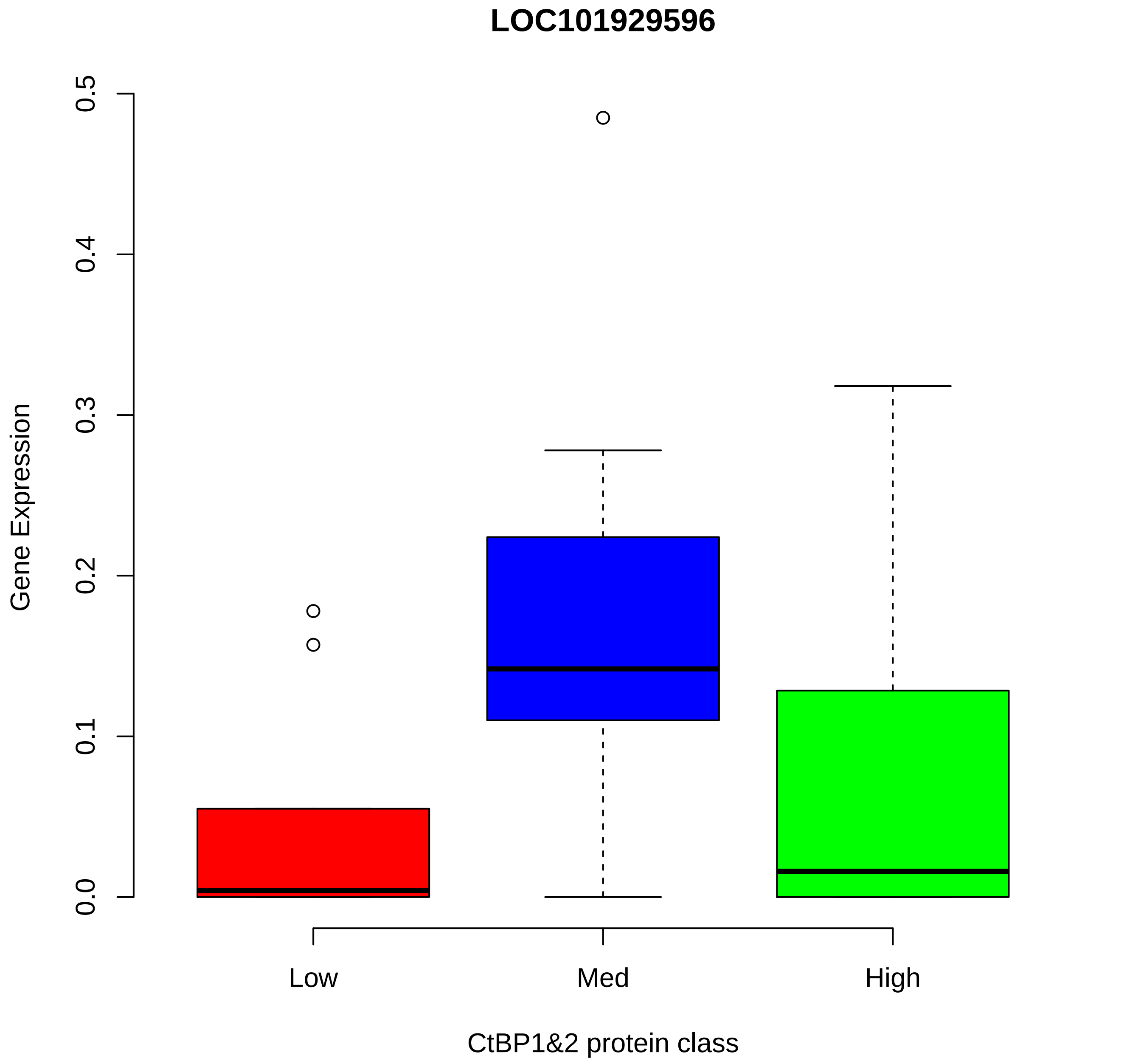
LOC101929596
Gene Expression
0.0
0.1
0.2
0.3
0.4
0.5
Low
Med
High
CtBP1&2 protein class

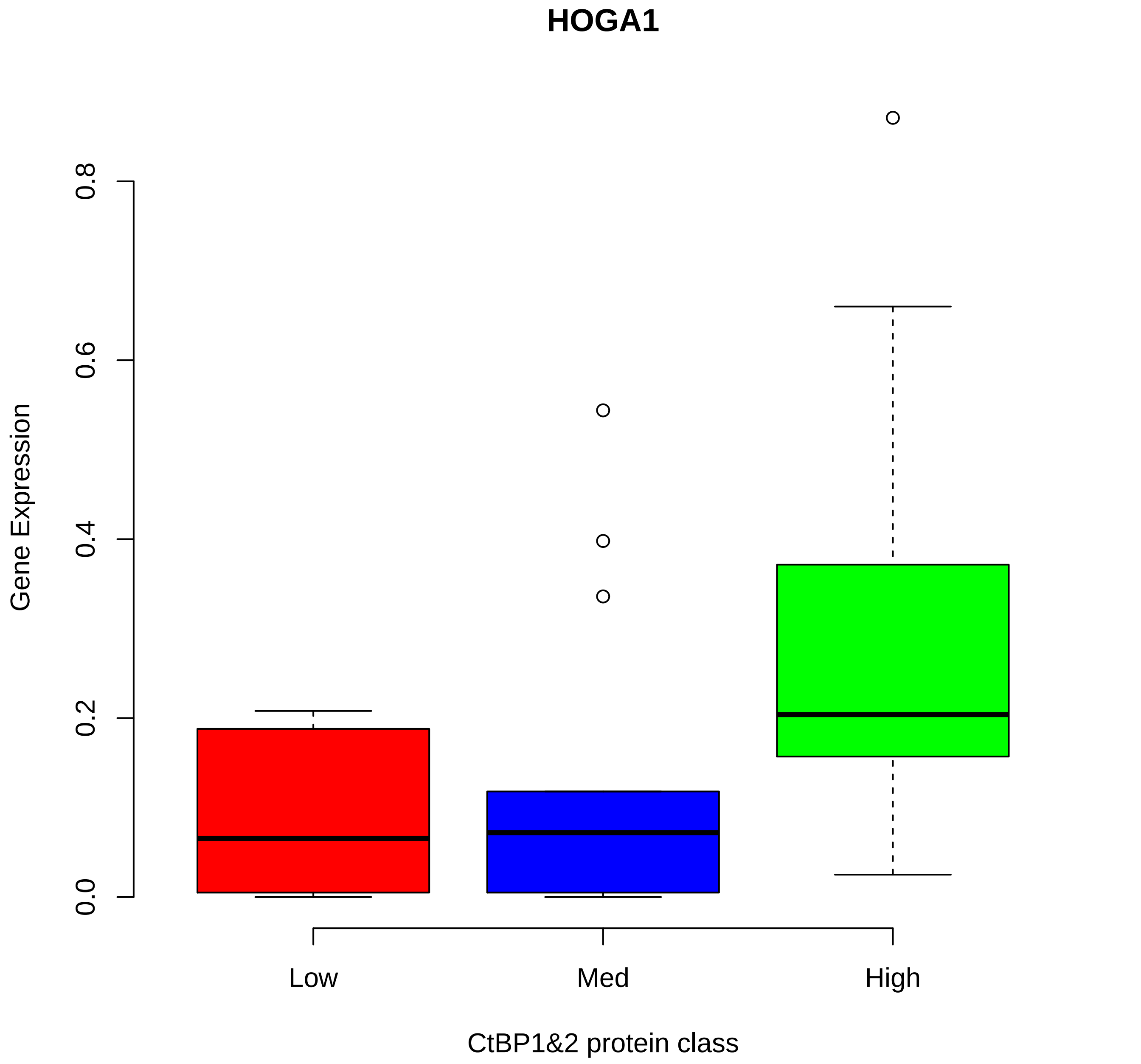
HOGA1
Gene Expression
0.0
0.2
0.4
0.6
0.8
Low
Med
High
CtBP1&2 protein class

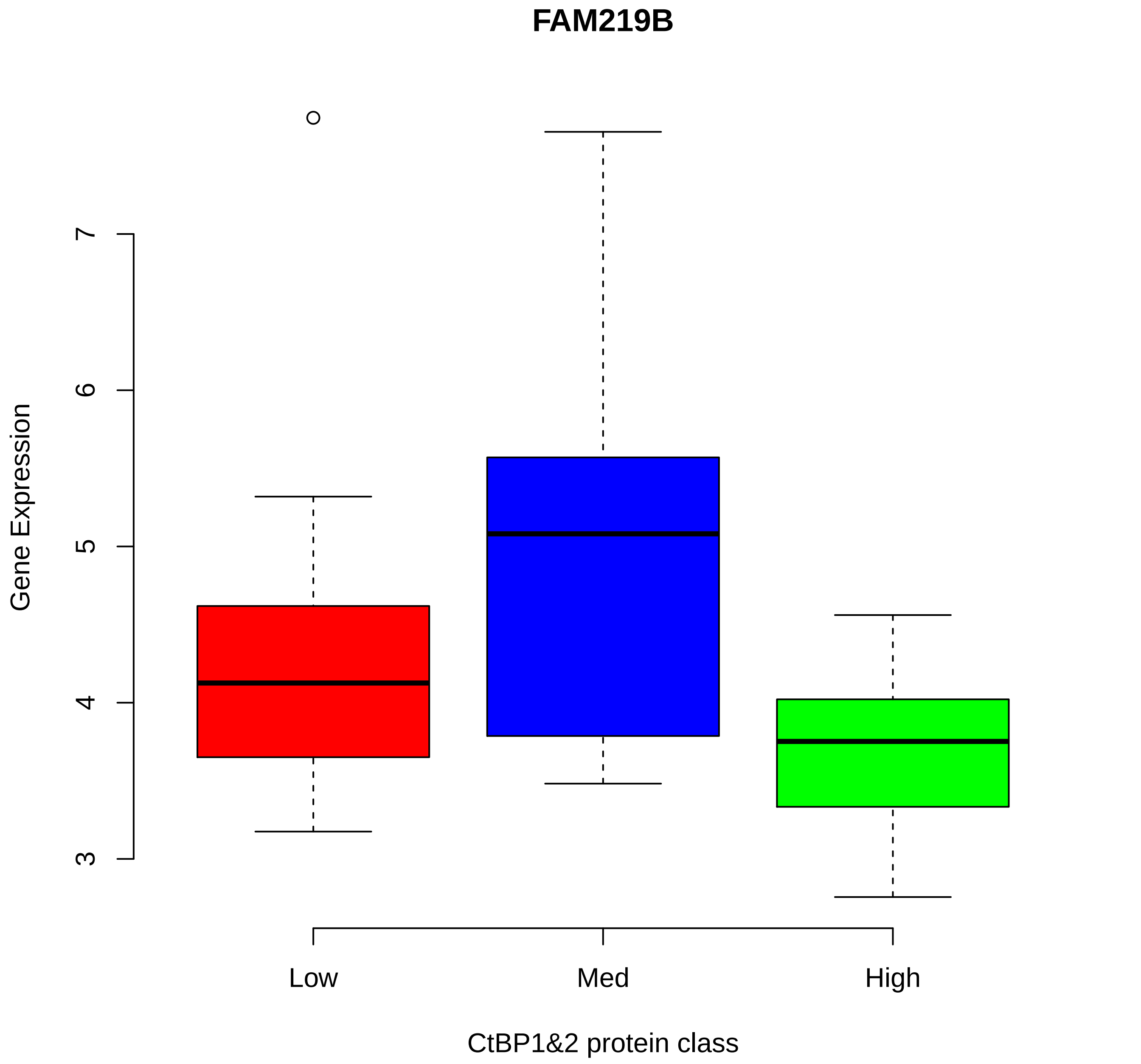
FAM219B
Gene Expression
3
4
5
6
7
Low
Med
High
CtBP1&2 protein class

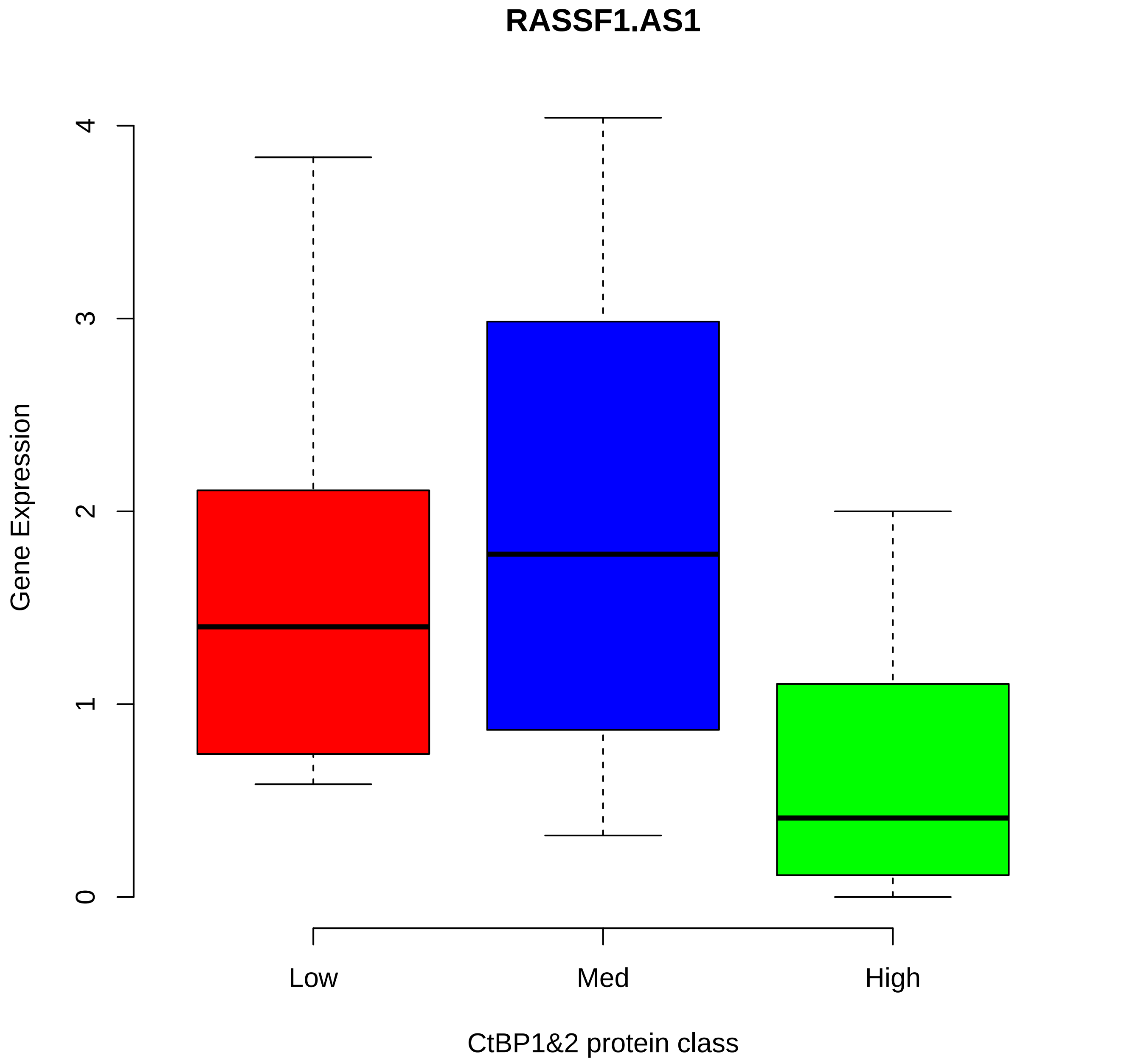
RASSF1.AS1
Gene Expression
0
1
2
3
4
Low
Med
High
CtBP1&2 protein class

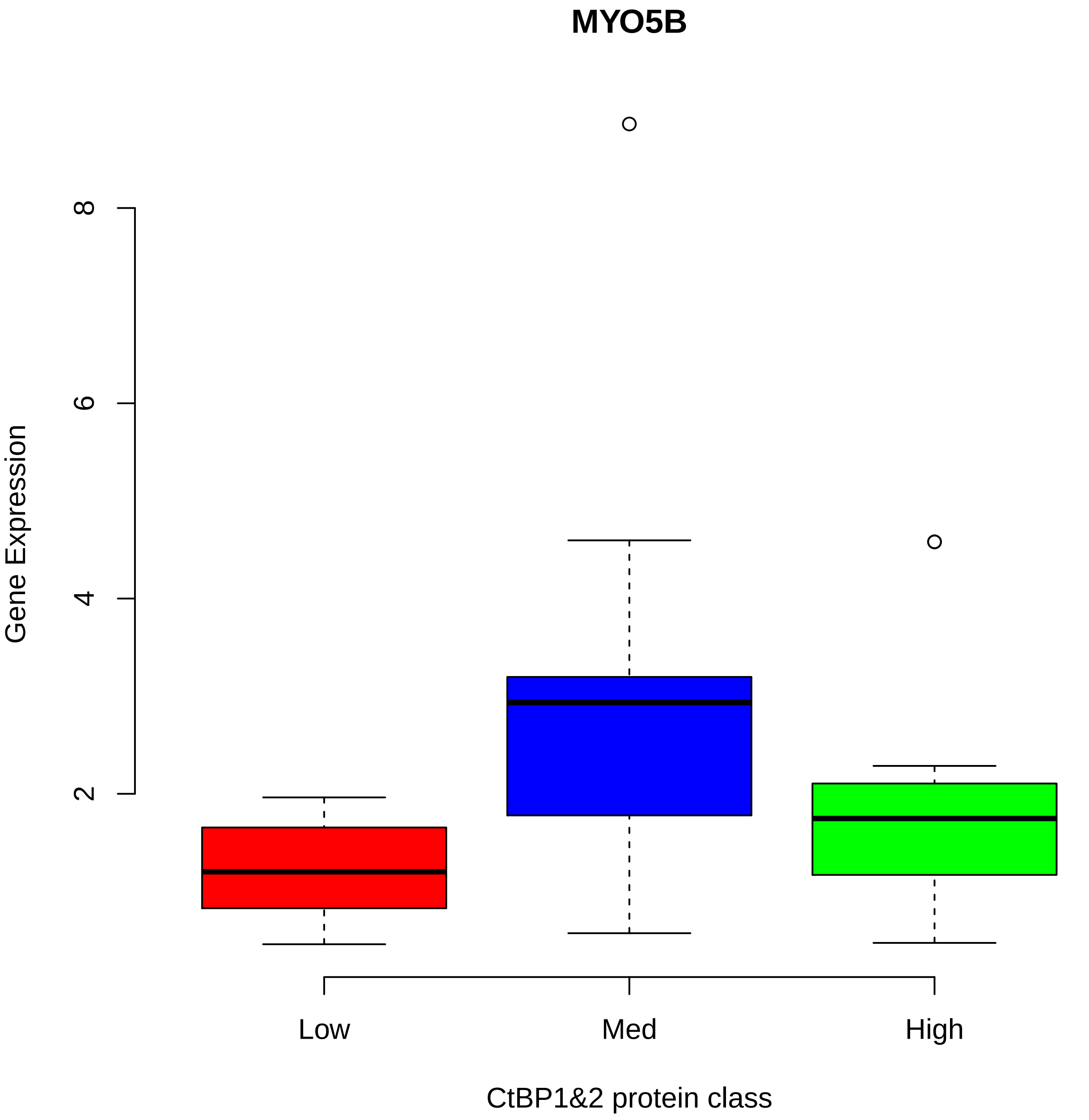

MYO5B
Gene Expression
8
6
4
2
Low
Med
High
CtBP1&2 protein class

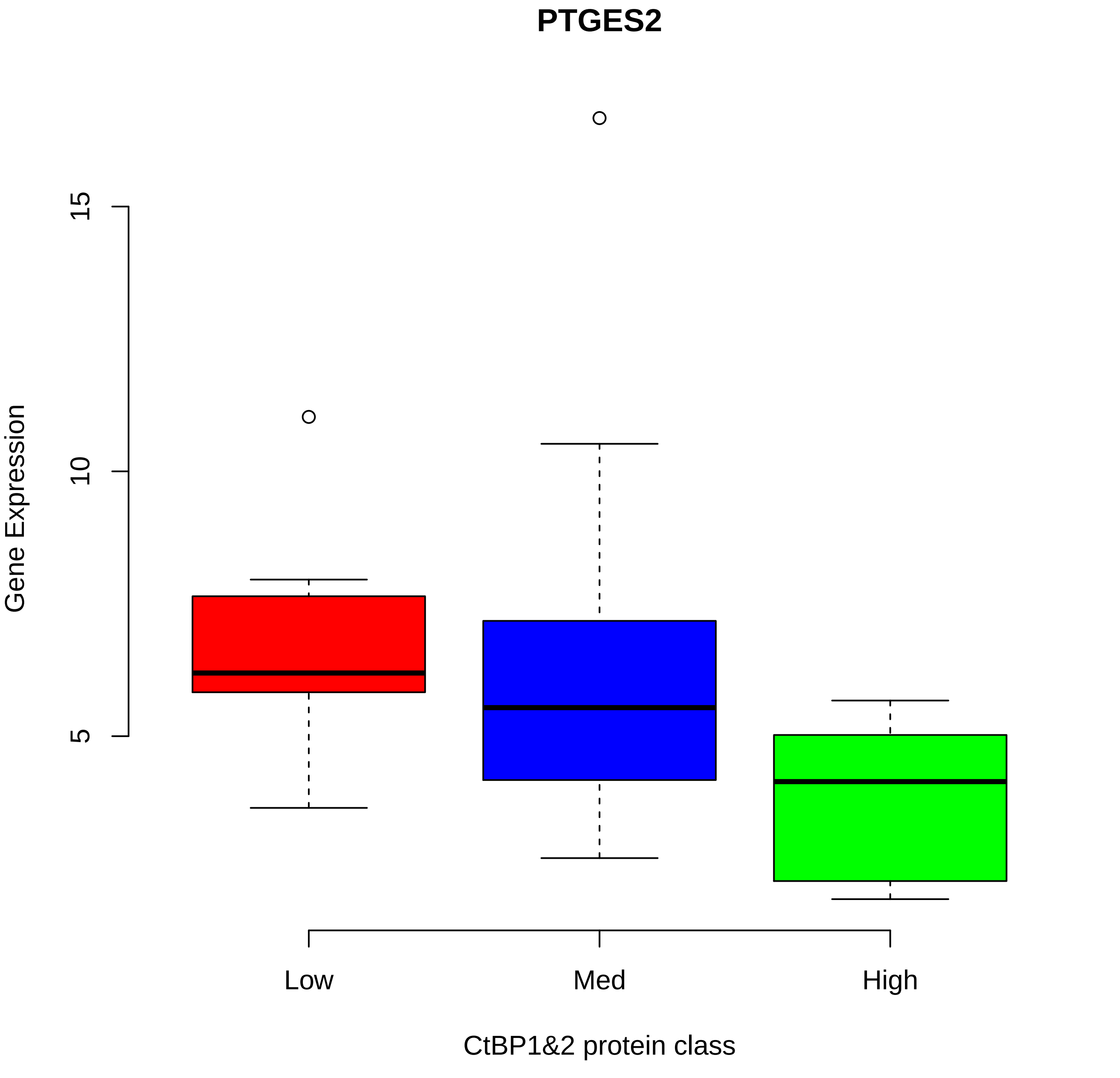
PTGES2
Gene Expression
15
10
5
Low
Med
High
CtBP1&2 protein class

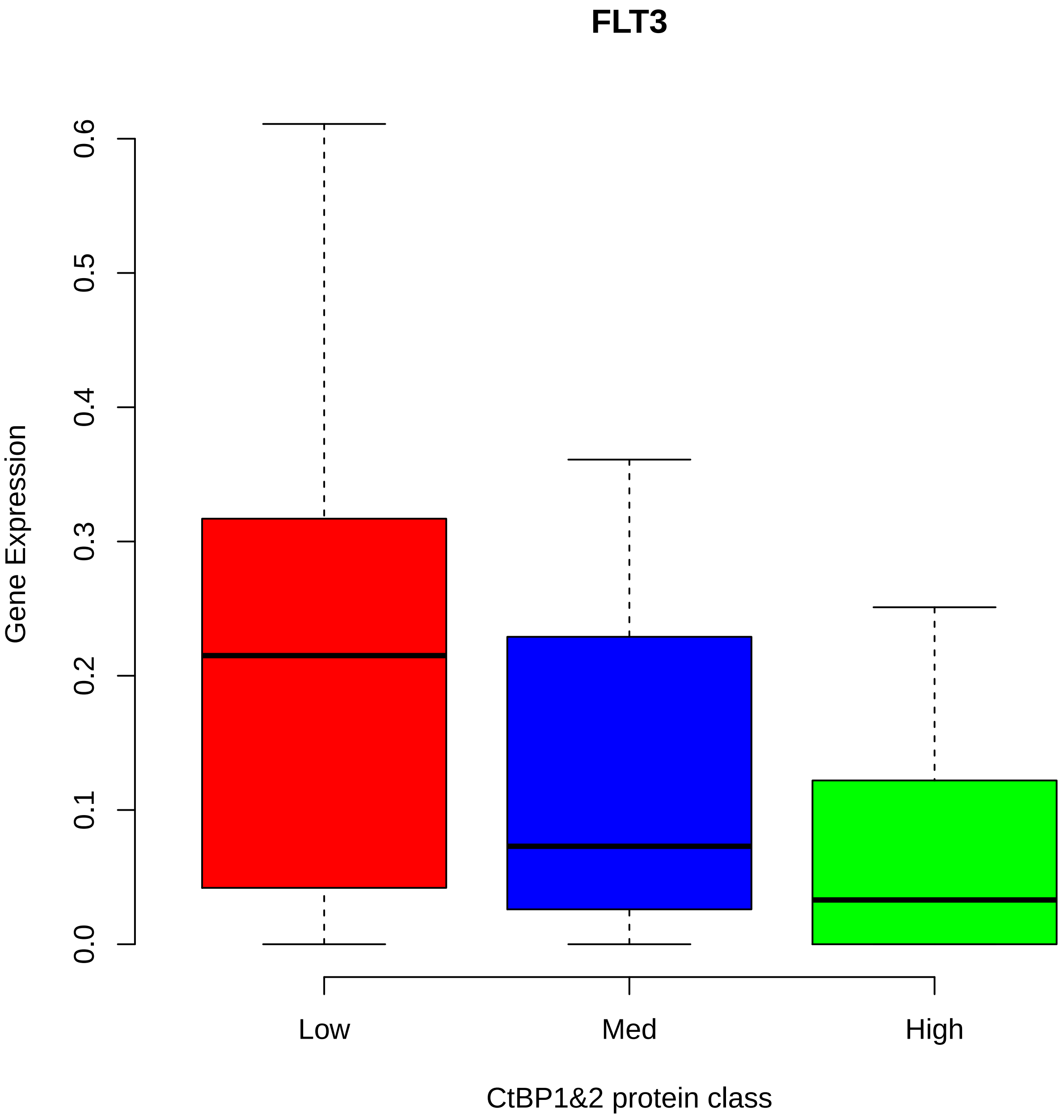

FLT3
Gene Expression
0.0
0.1
0.2
0.3
0.4
0.5
0.6
Low
Med
High
CtBP1&2 protein class

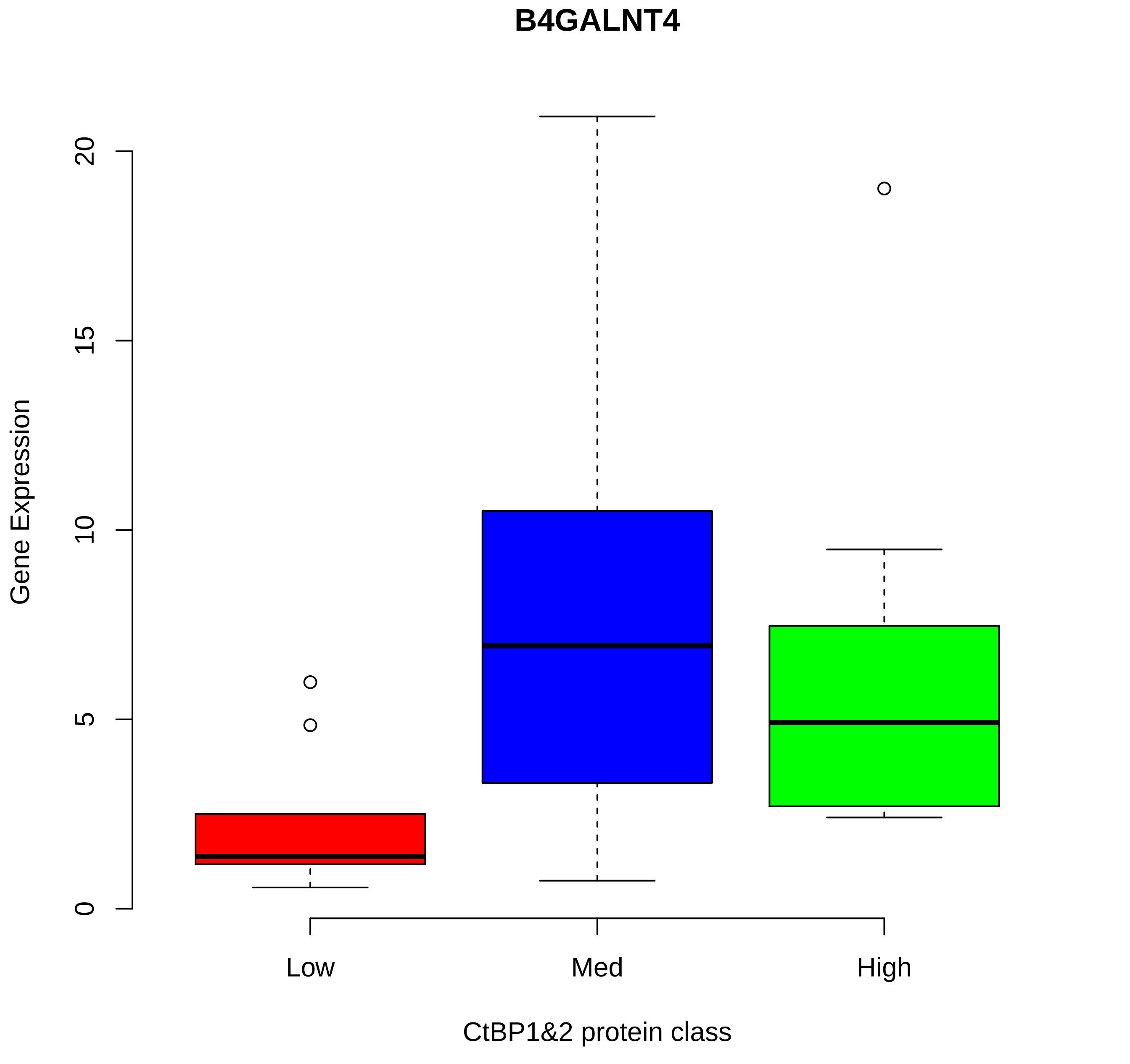

B4GALNT4
Gene Expression
0
5
10
15
20
Low
Med
High
CtBP1&2 protein class

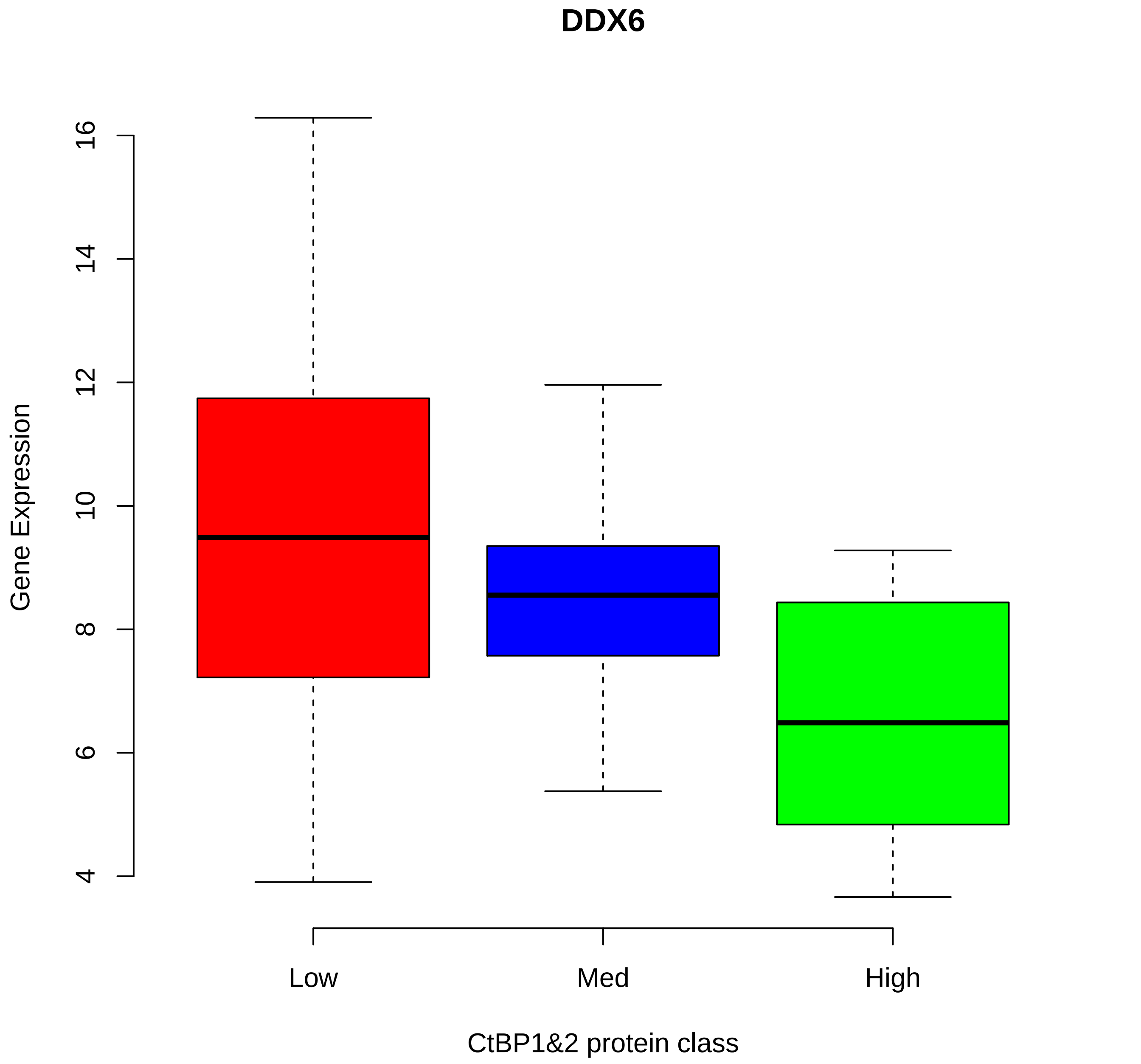

DDX6
Gene Expression
4
6
8
10
12
14
16
Low
Med
High
CtBP1&2 protein class

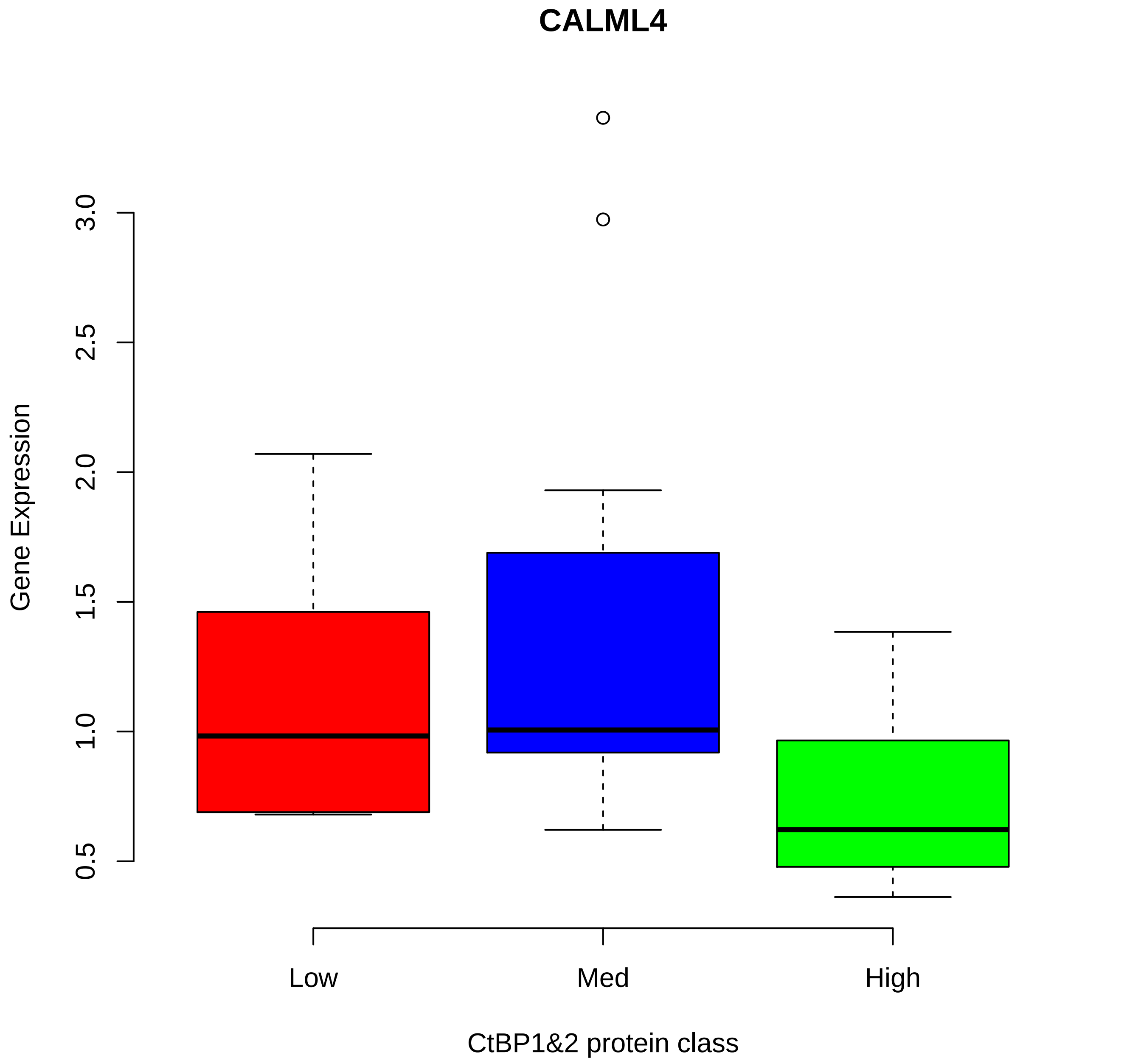

CALML4
Gene Expression
3.0
2.5
2.0
1.5
1.0
0.5
Low
Med
High
CtBP1&2 protein class

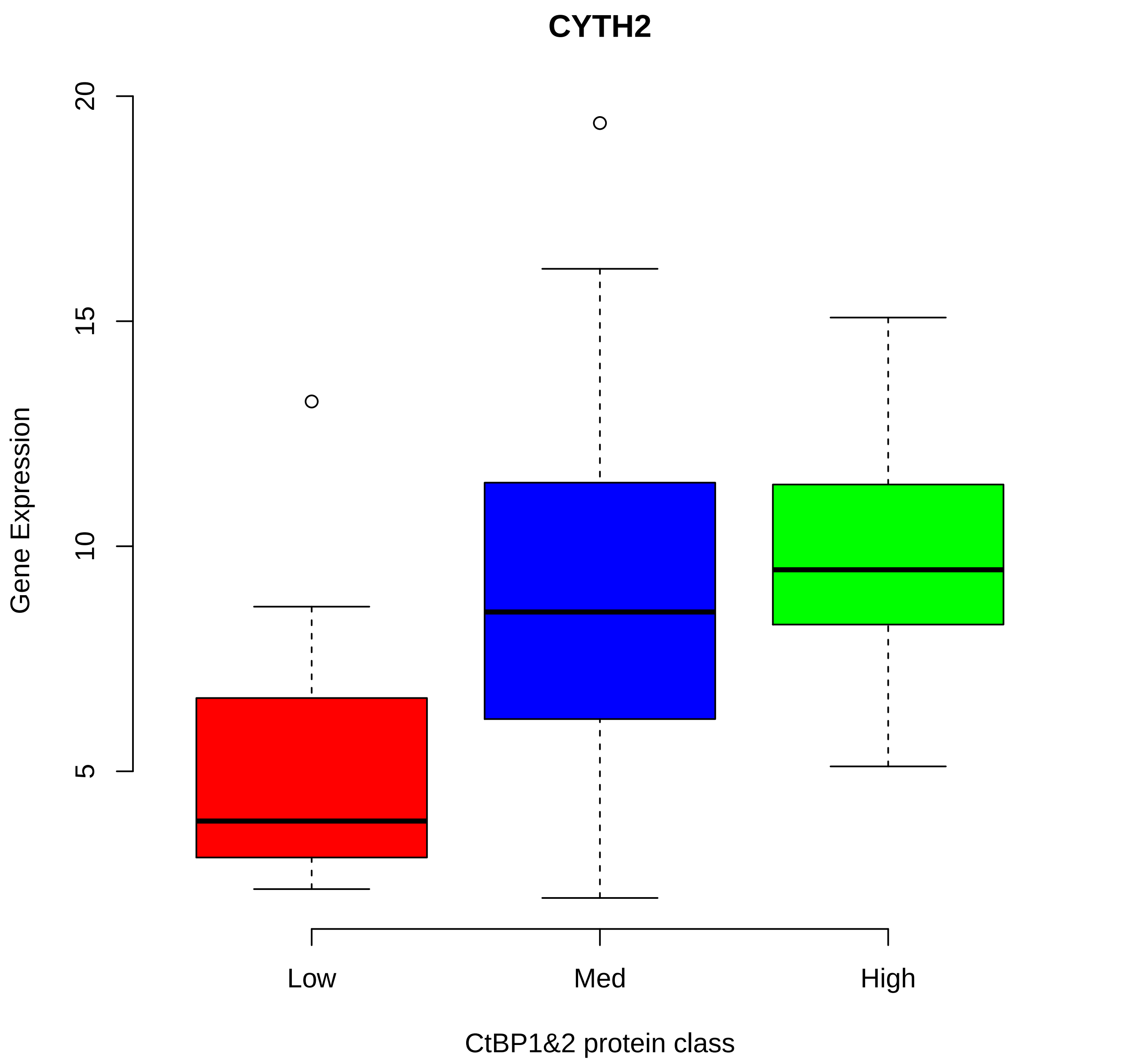
CYTH2
Gene Expression
5
10
15
20
Low
Med
High
CtBP1&2 protein class

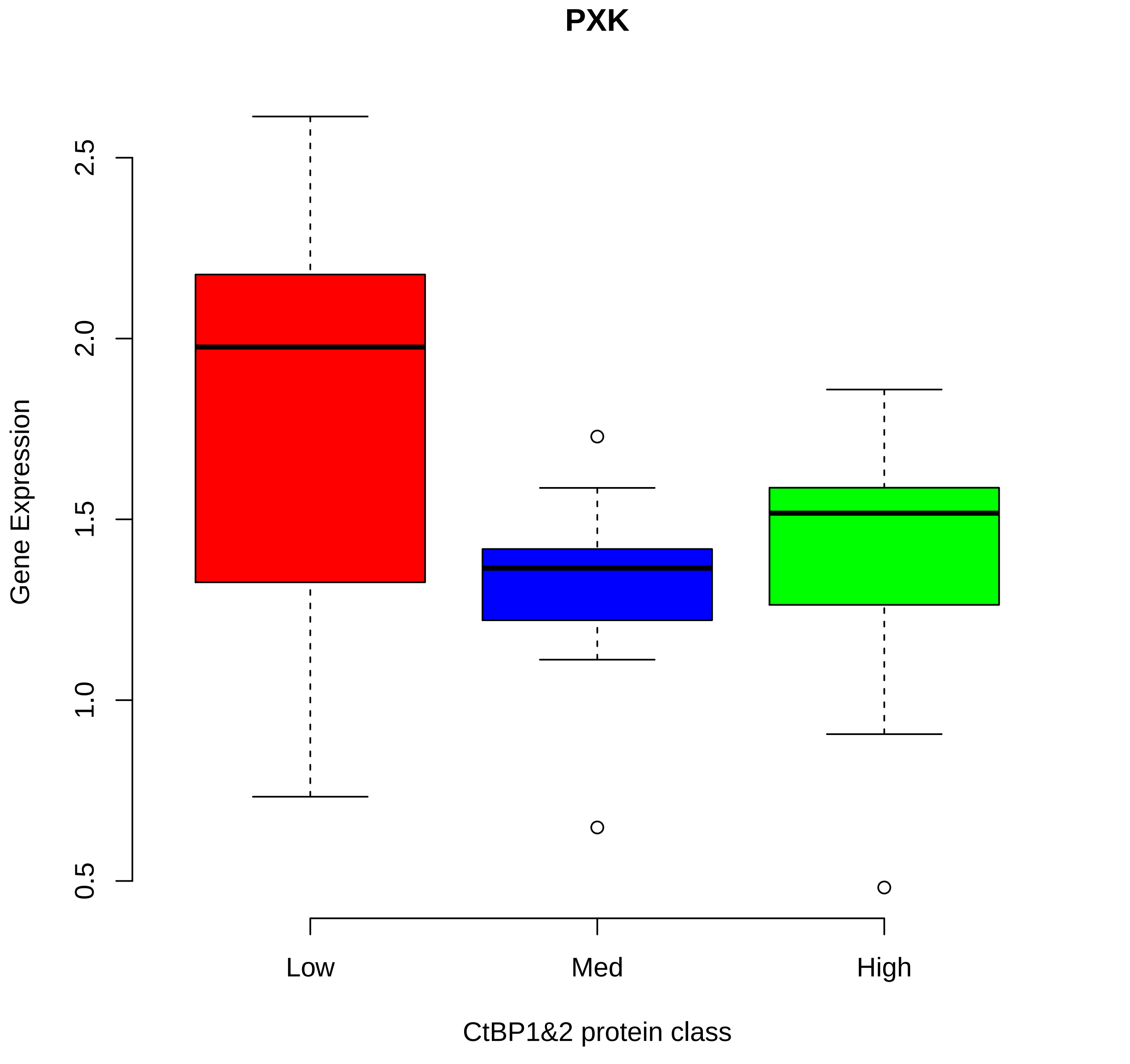

PXK
Gene Expression
0.5
1.0
1.5
2.0
2.5
Low
Med
High
CtBP1&2 protein class

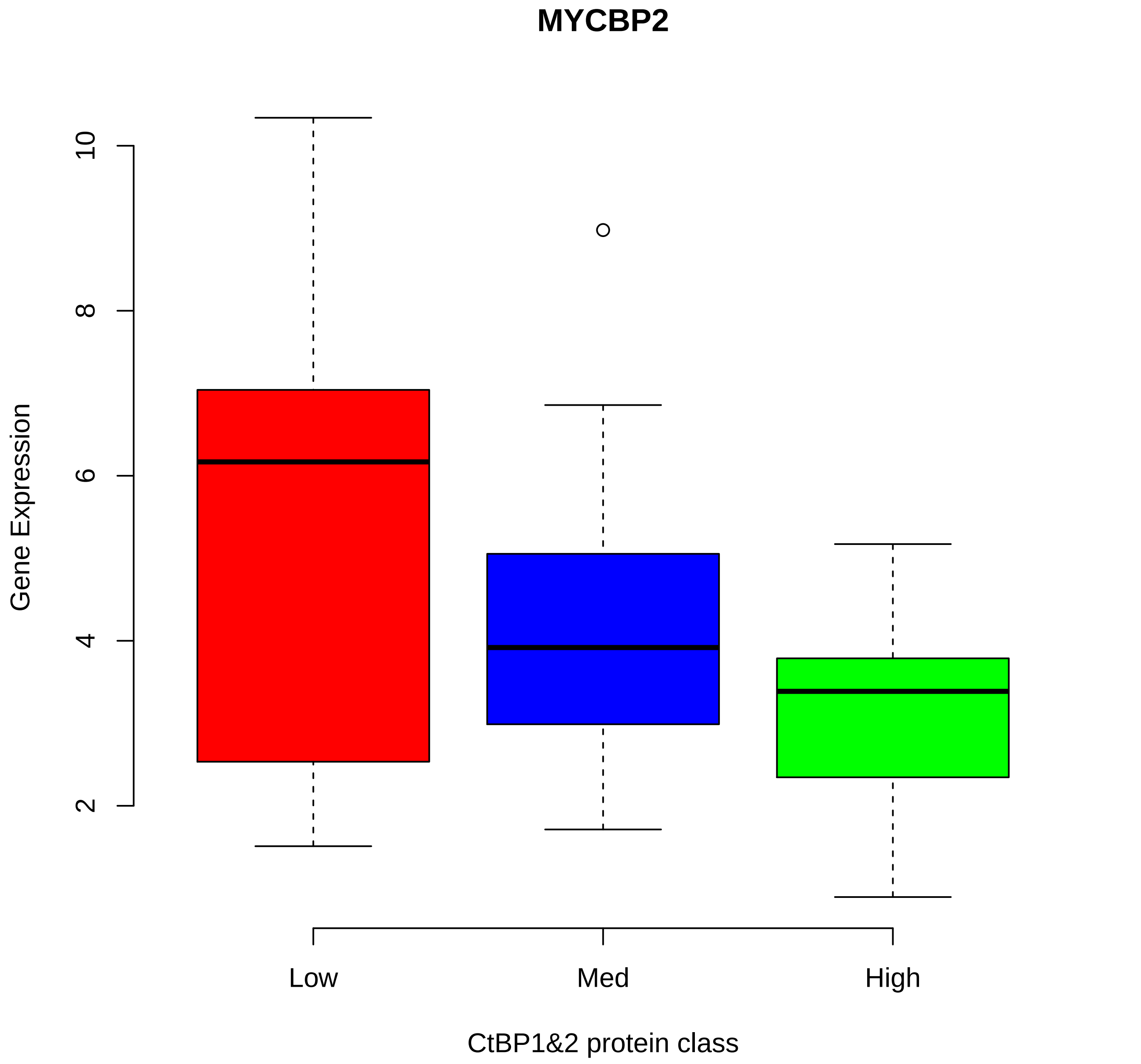
MYCBP2
Gene Expression
10
8
6
4
2
Low
Med
High
CtBP1&2 protein class

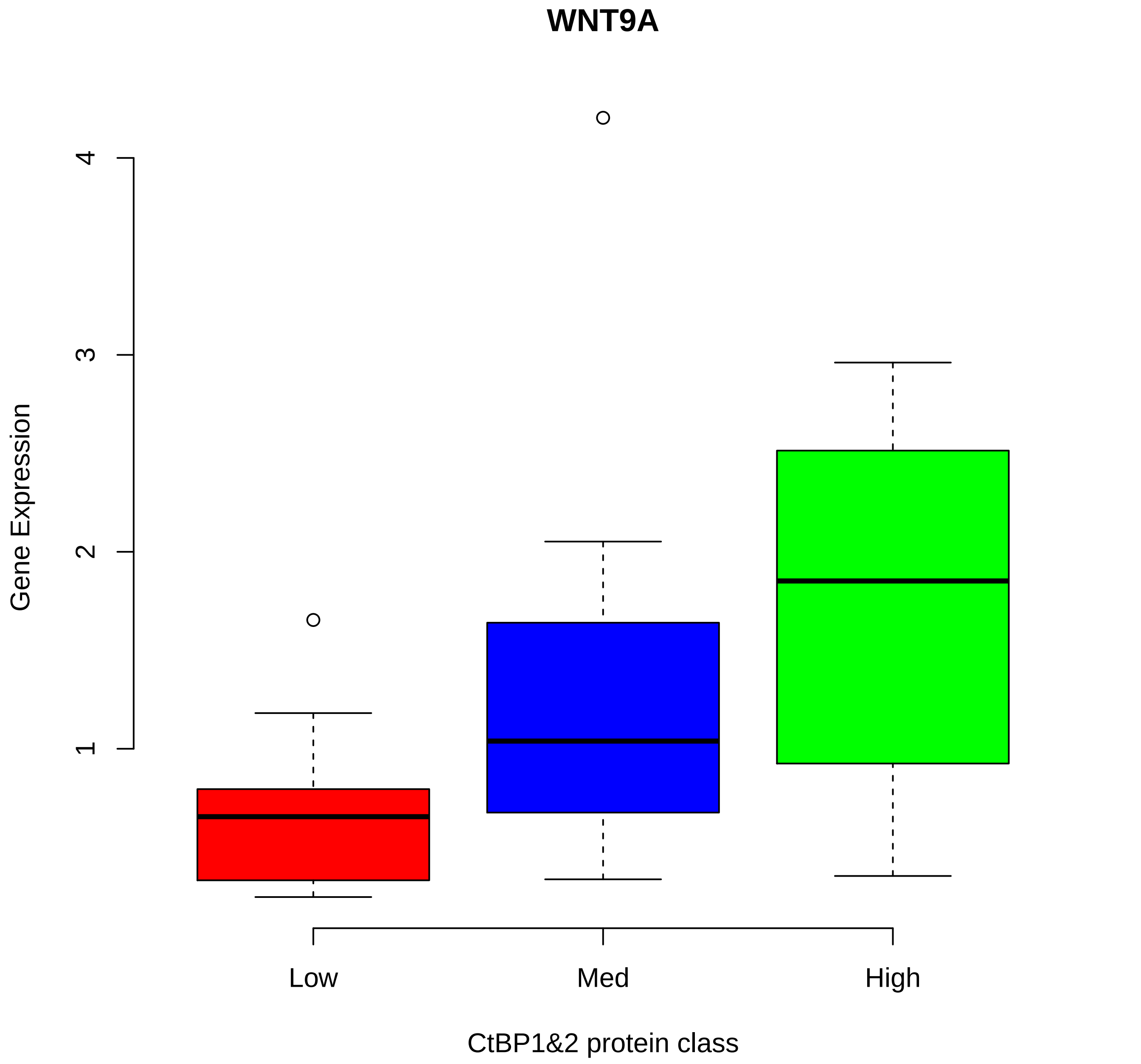
WNT9A
Gene Expression
1
2
3
4
Low
Med
High
CtBP1&2 protein class

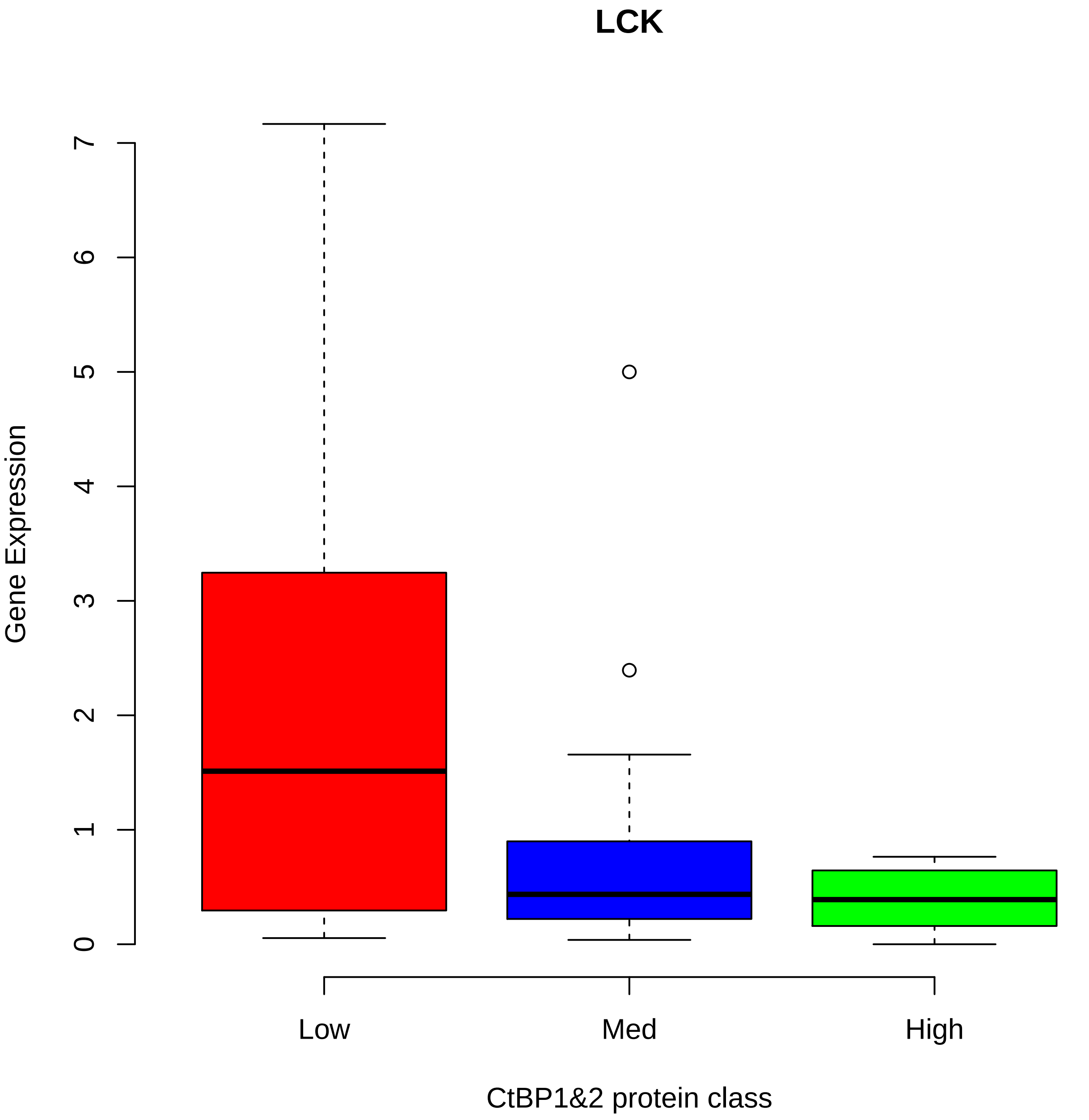
LCK
Gene Expression
0
1
2
3
4
5
6
7
Low
Med
High
CtBP1&2 protein class

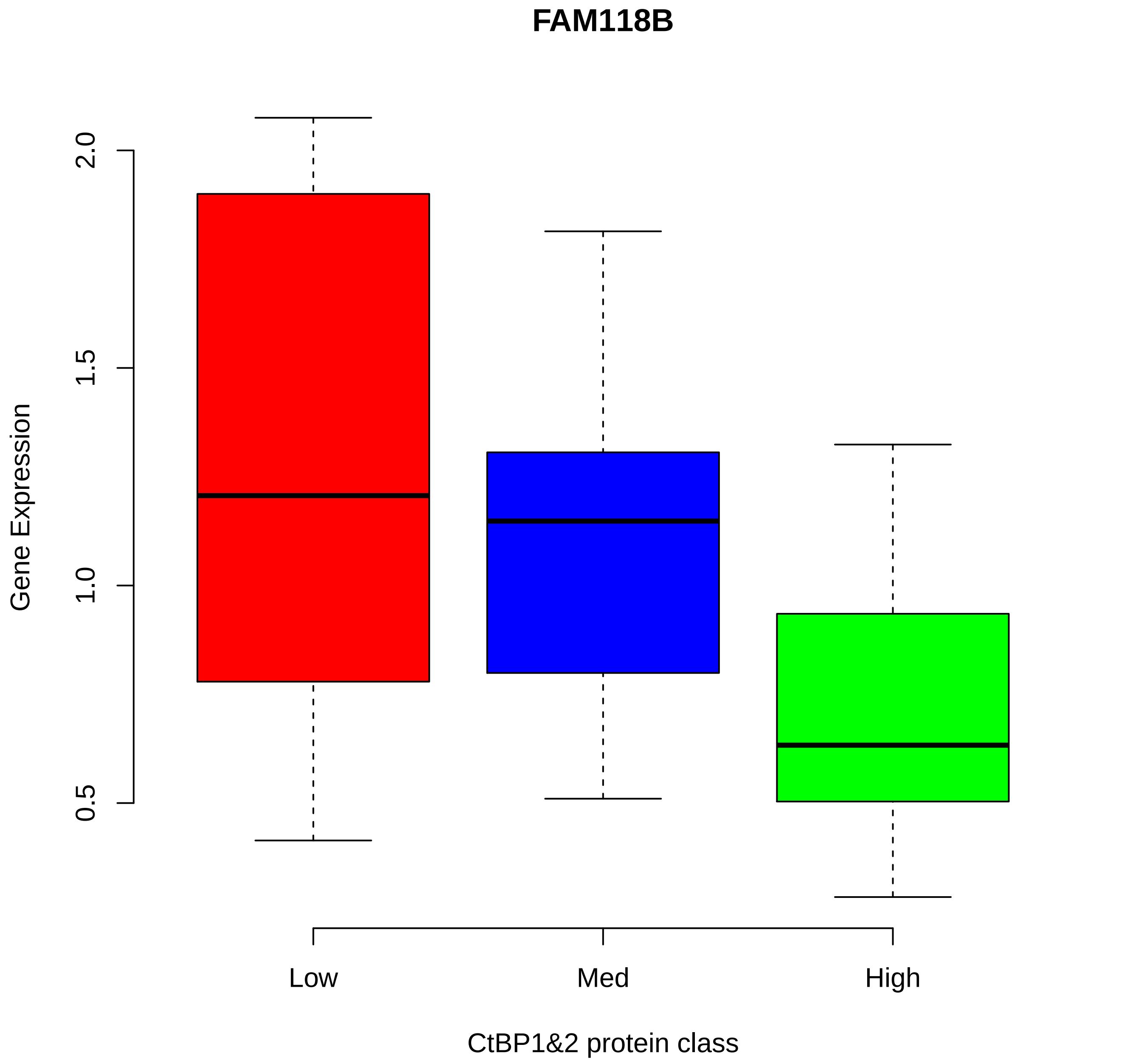

FAM118B
Gene Expression
2.0
1.5
1.0
0.5
Low
Med
High
CtBP1&2 protein class

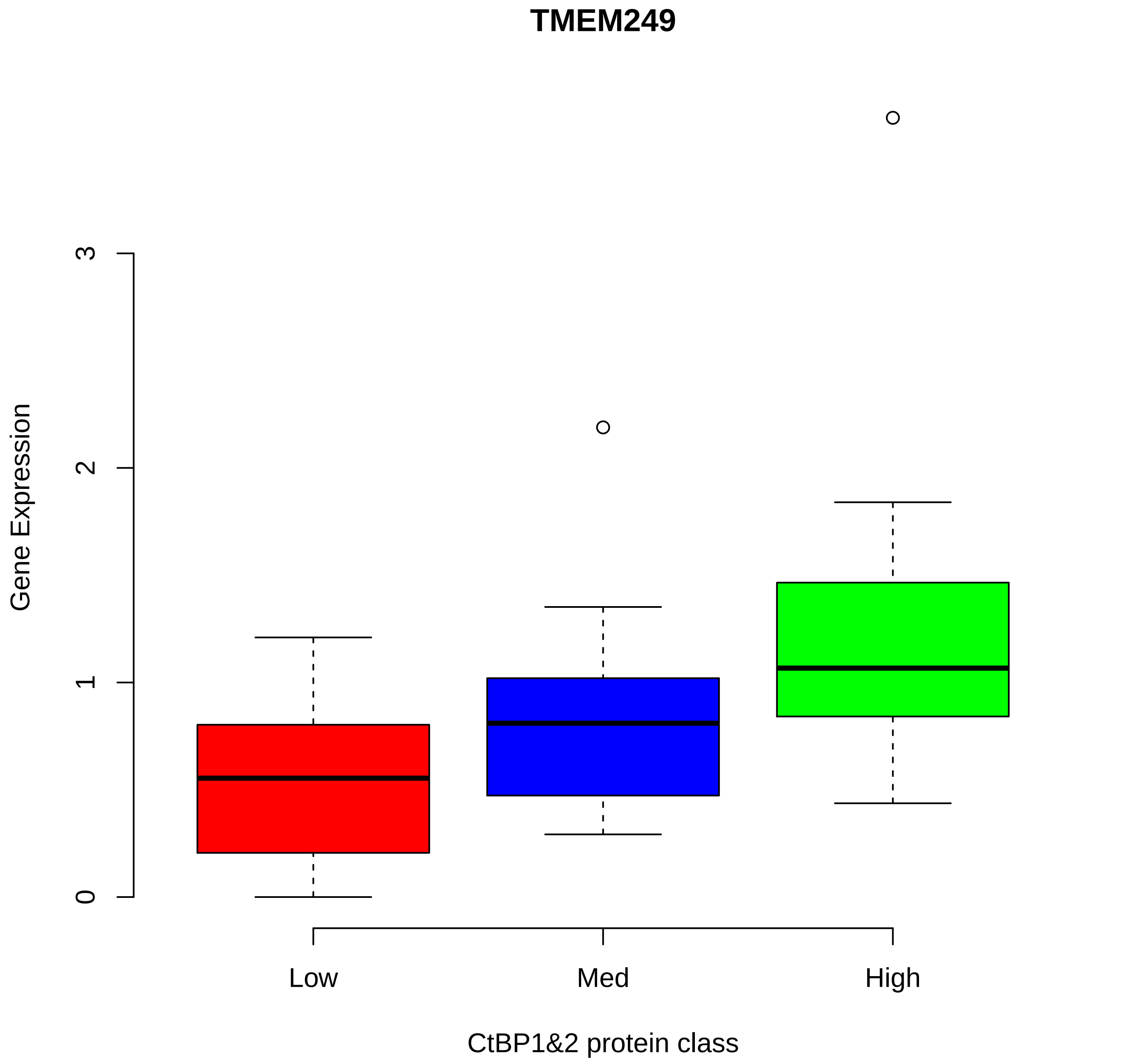
TMEM249
Gene Expression
0
1
2
3
Low
Med
High
CtBP1&2 protein class

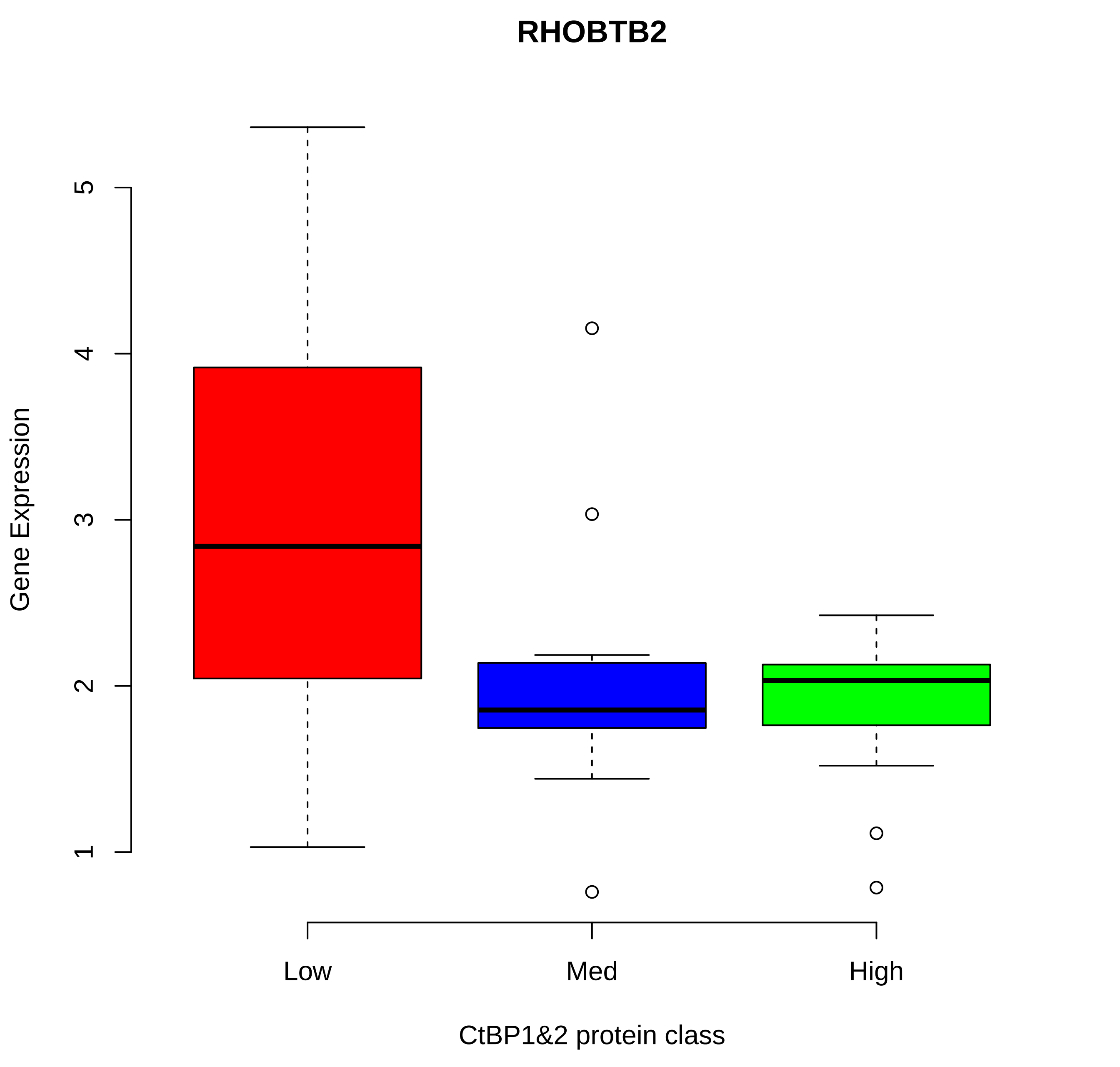
RHOBTB2
Gene Expression
1
2
3
4
5
Low
Med
High
CtBP1&2 protein class

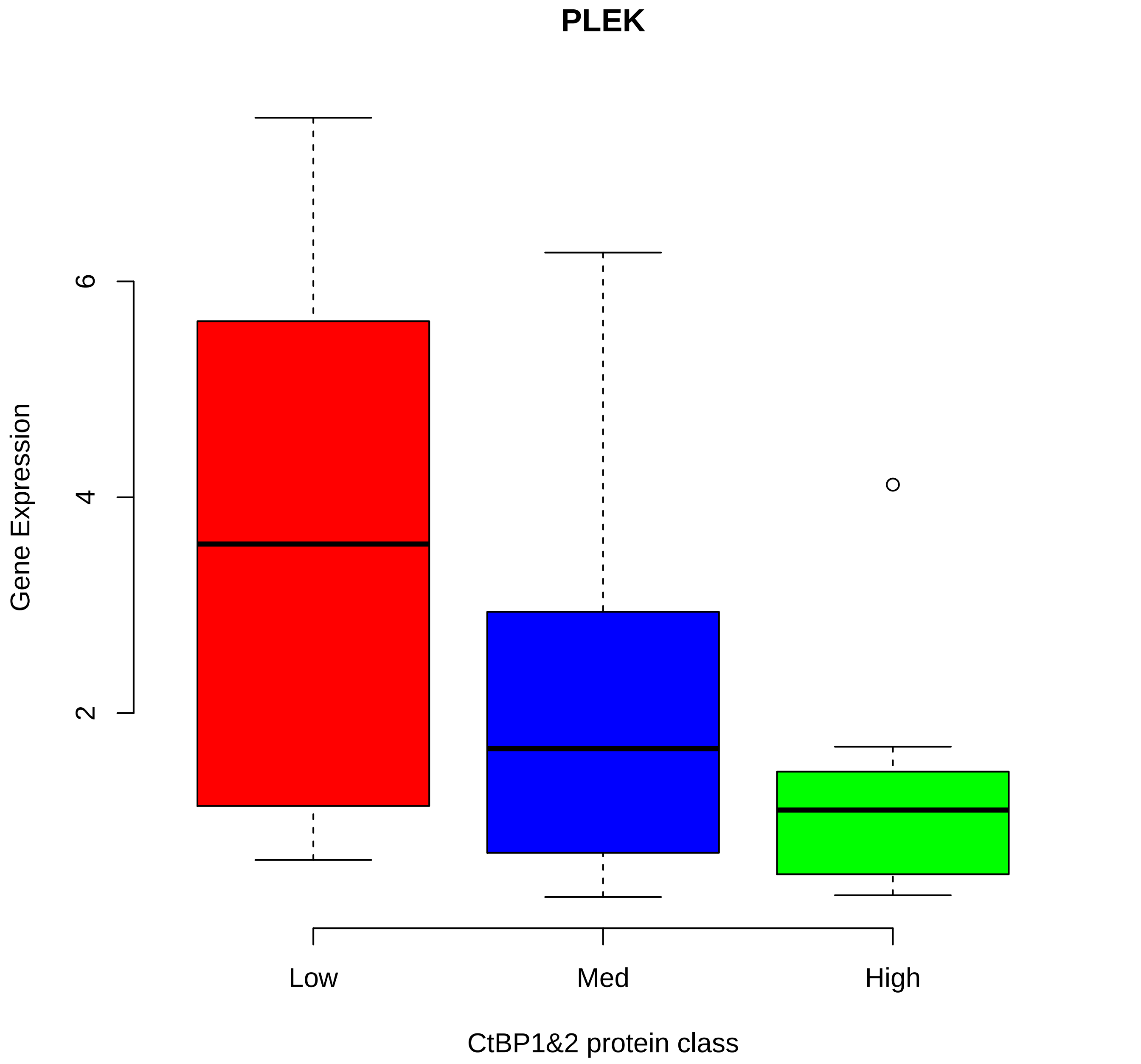
PLEK
Gene Expression
2
4
6
Low
Med
High
CtBP1&2 protein class

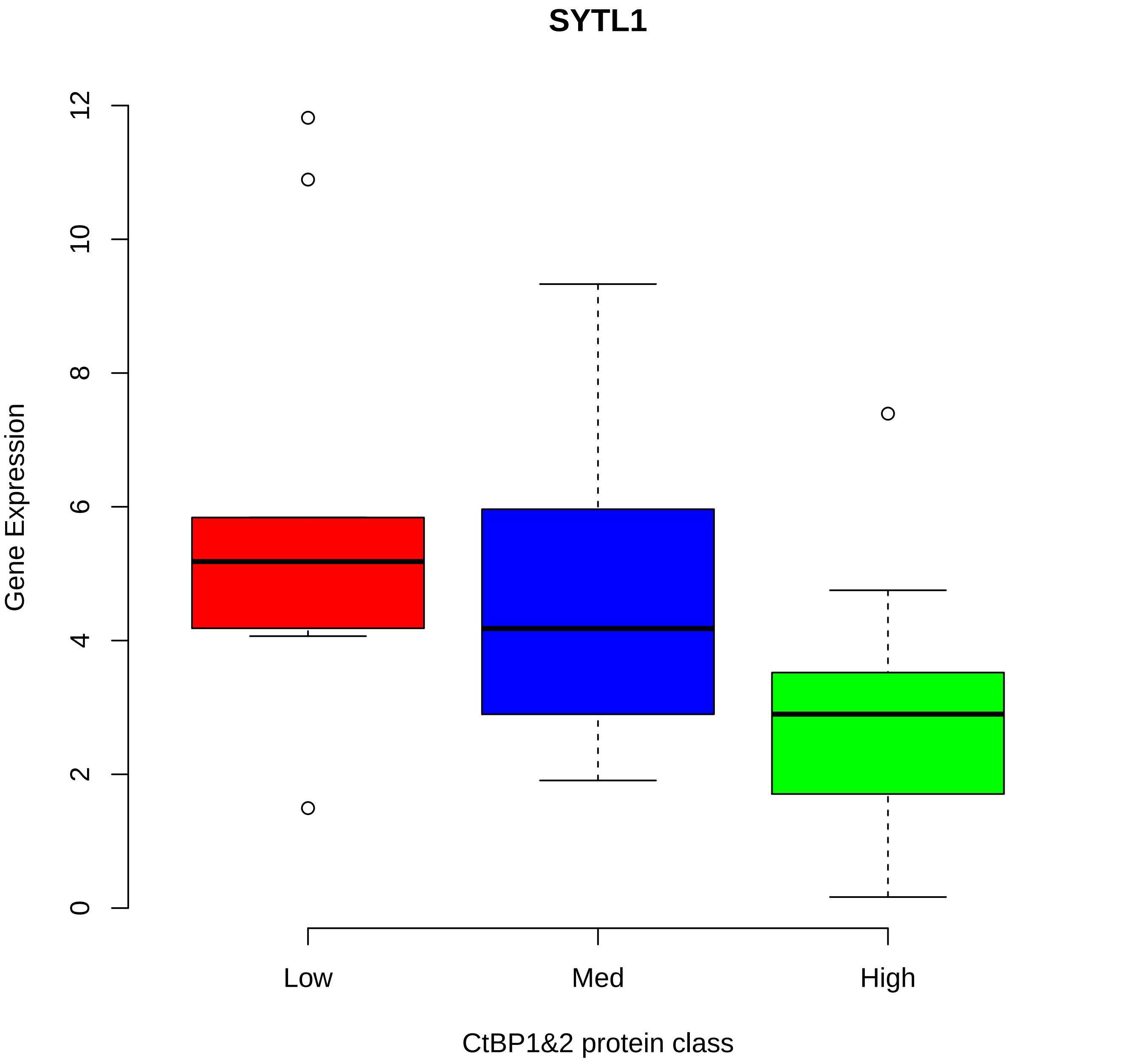
SYTL1
Gene Expression
0
2
4
6
8
10
12
Low
Med
High
CtBP1&2 protein class

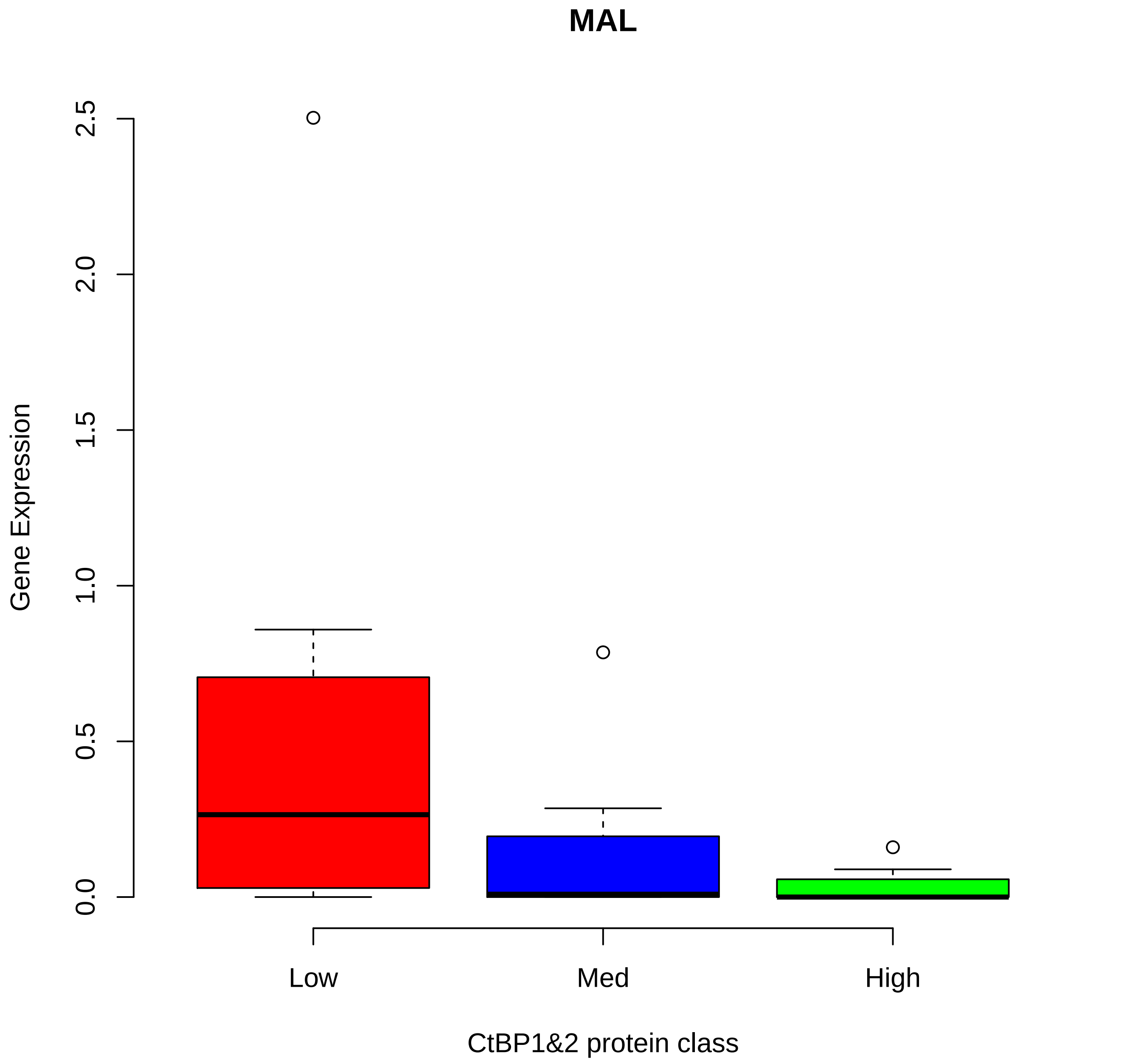
MAL
Gene Expression
0.0
0.5
1.0
1.5
2.0
2.5
Low
Med
High
CtBP1&2 protein class

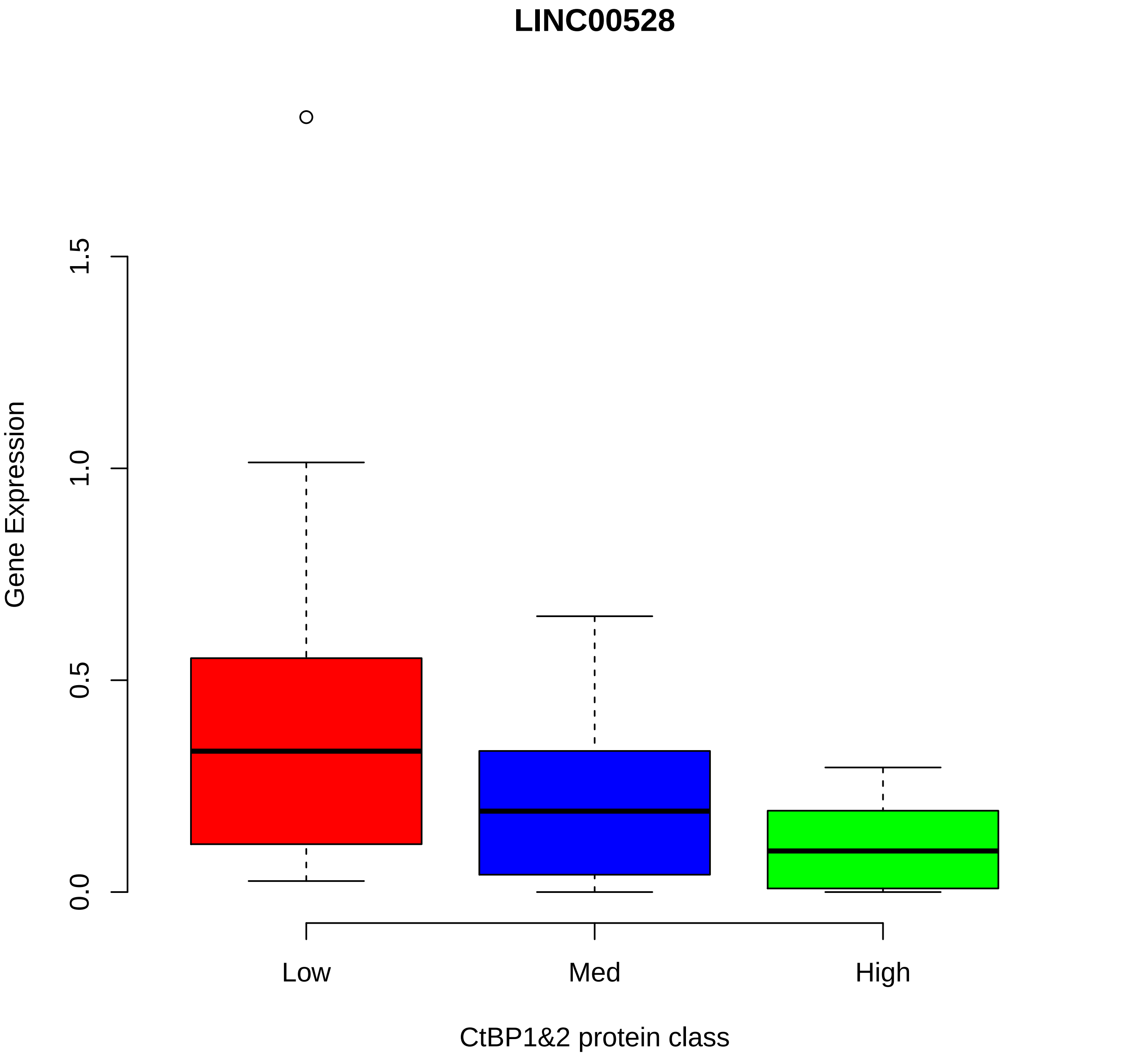
LINC00528
Gene Expression
0.0
0.5
1.0
1.5
Low
Med
High
CtBP1&2 protein class

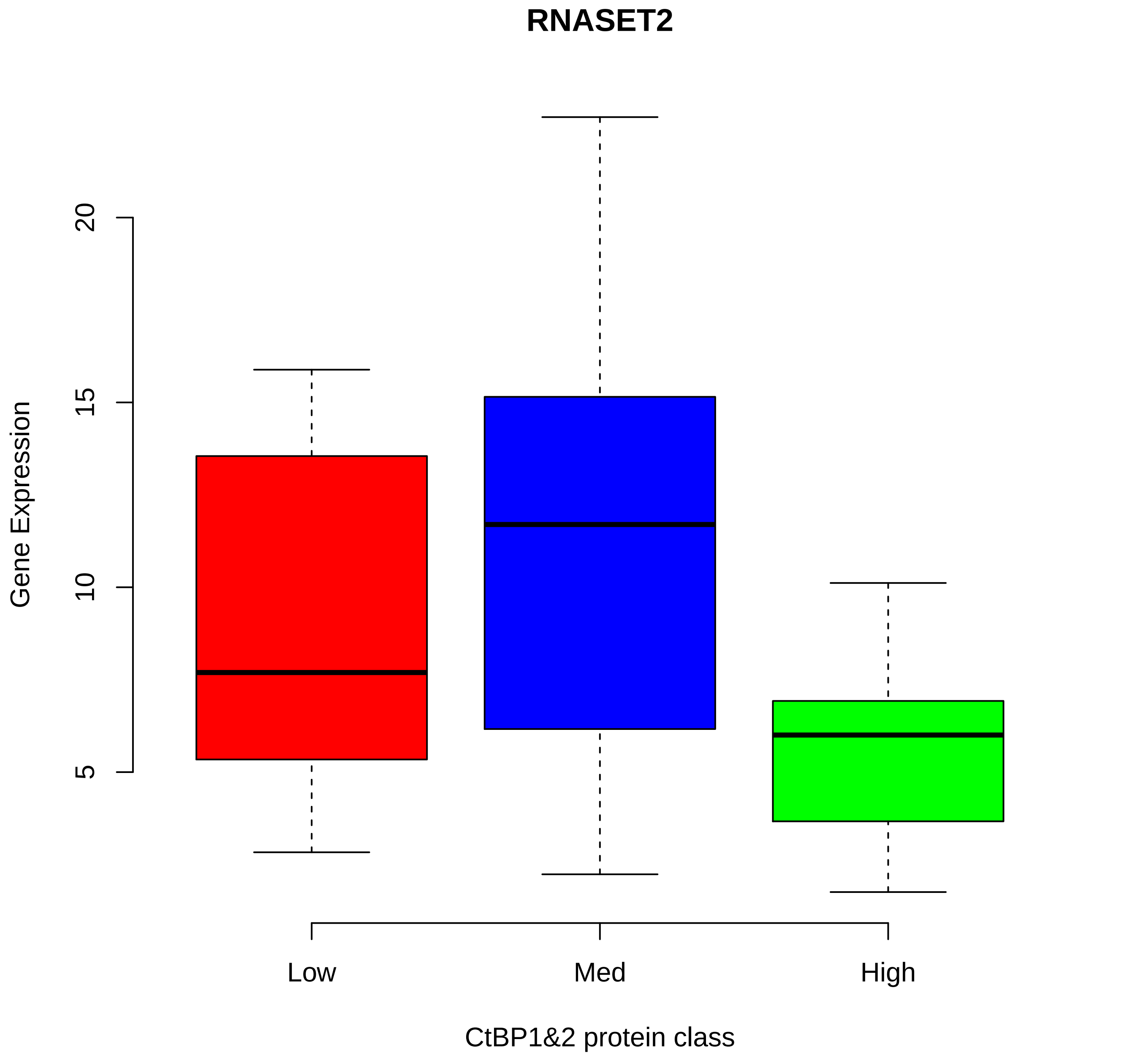

RNASET2
Gene Expression
20
15
10
5
Low
Med
High
CtBP1&2 protein class

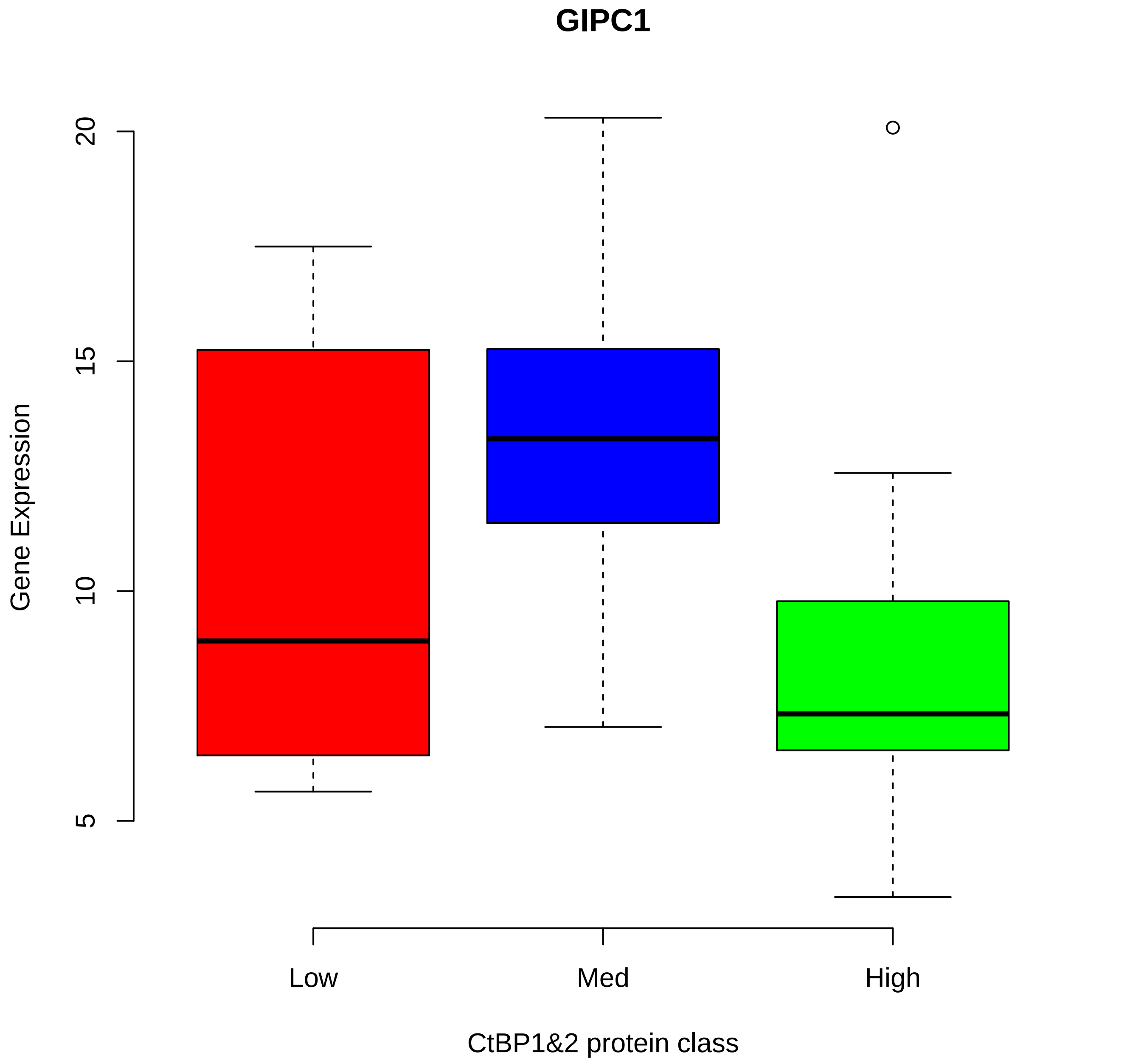
GIPC1
Gene Expression
5
10
15
20
Low
Med
High
CtBP1&2 protein class

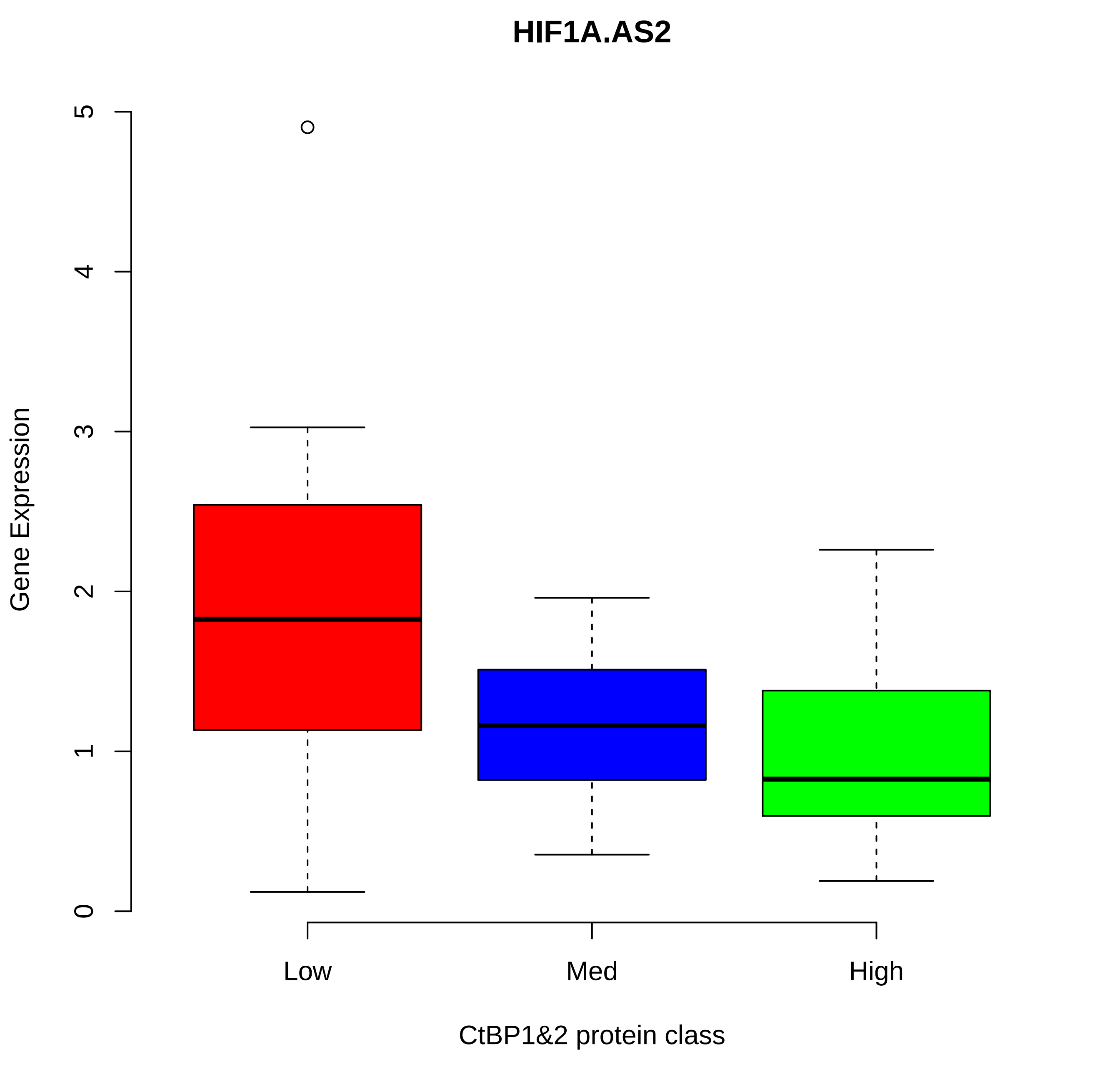

HIF1A.AS2
Gene Expression
0
1
2
3
4
5
Low
Med
High
CtBP1&2 protein class

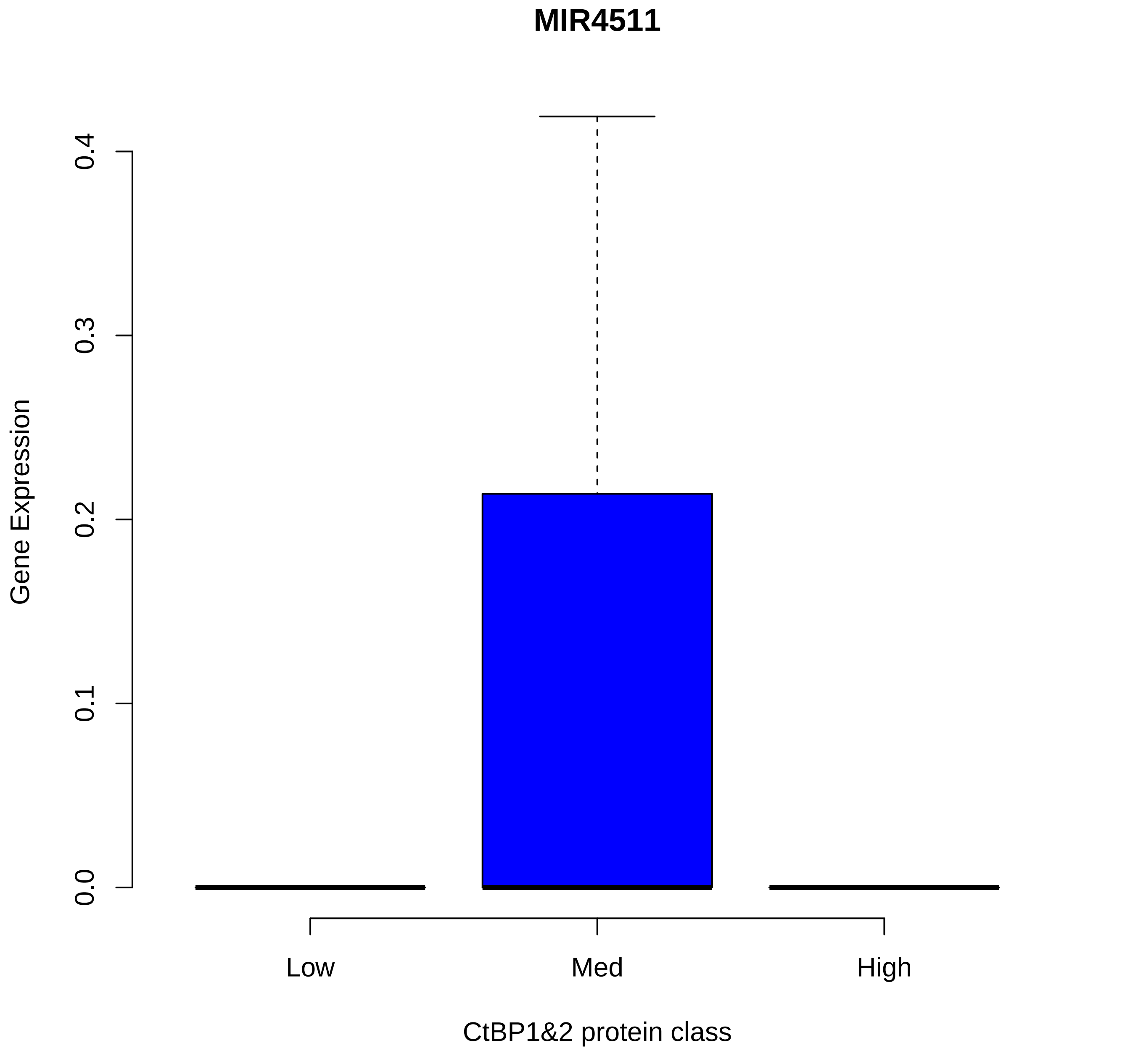
MIR4511
Gene Expression
0.0
0.1
0.2
0.3
0.4
Low
Med
High
CtBP1&2 protein class

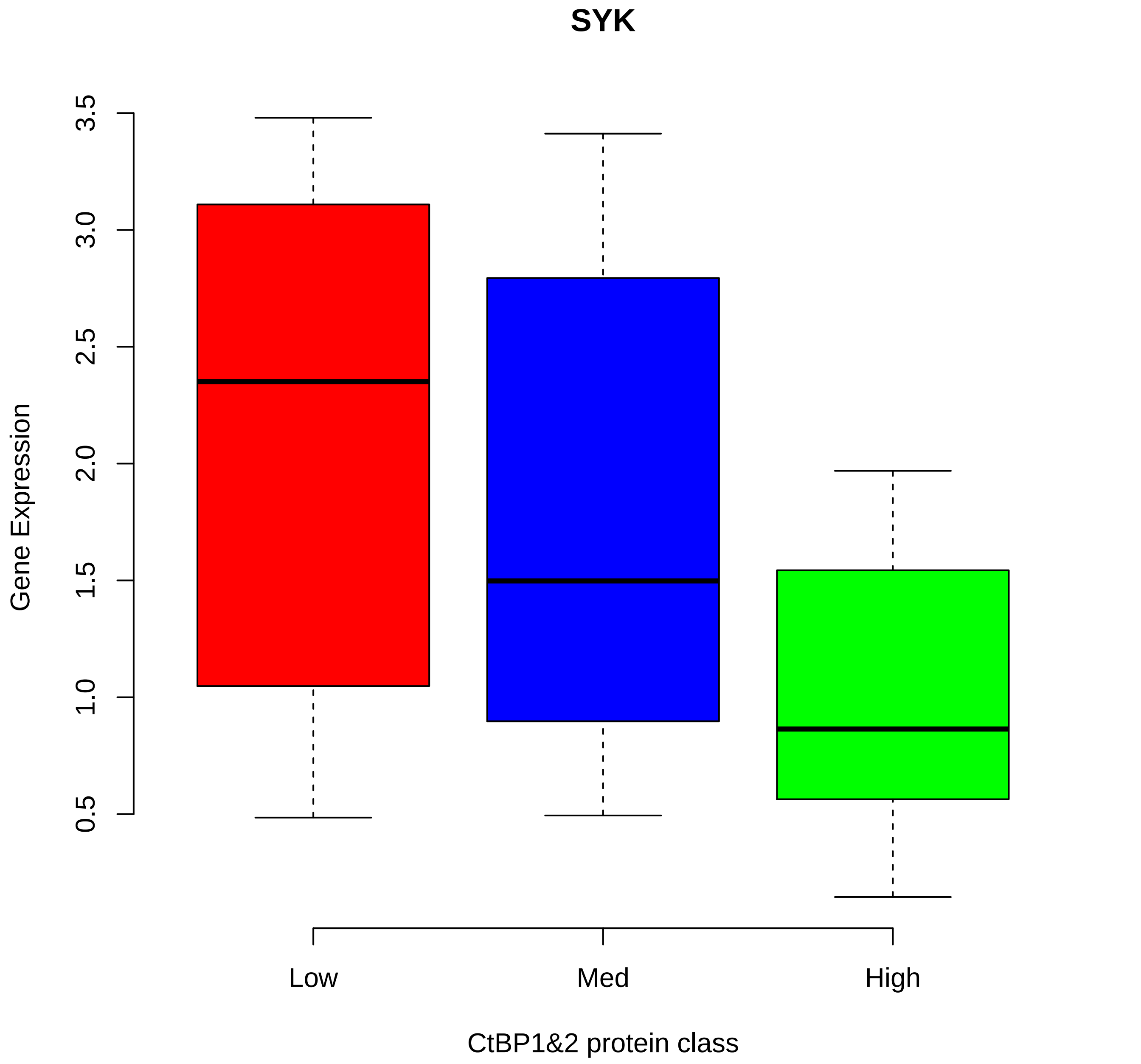
SYK
Gene Expression
0.5
1.0
1.5
2.0
2.5
3.0
3.5
Low
Med
High
CtBP1&2 protein class

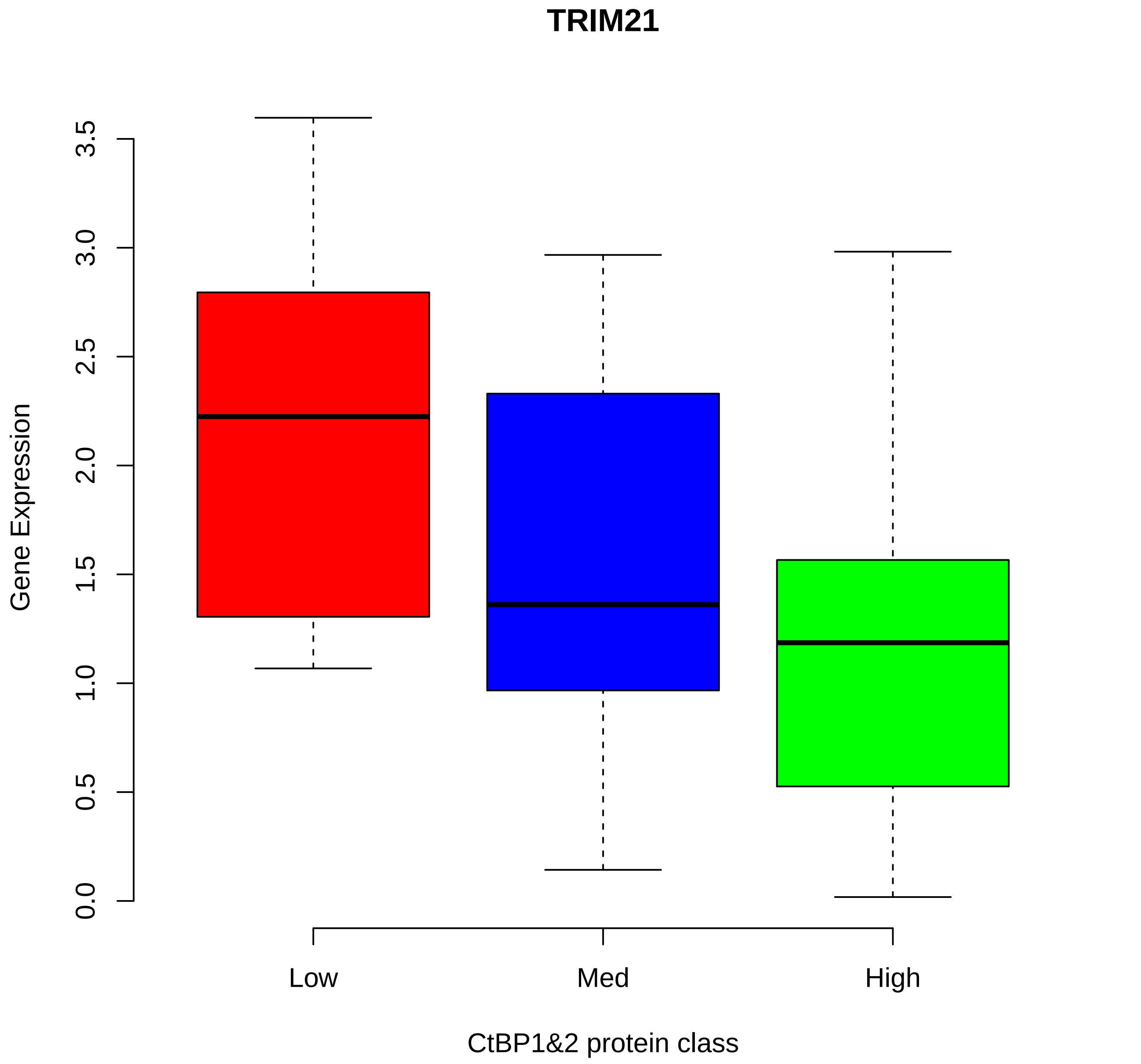

TRIM21
Gene Expression
0.0
0.5
1.0
1.5
2.0
2.5
3.0
3.5
Low
Med
High
CtBP1&2 protein class

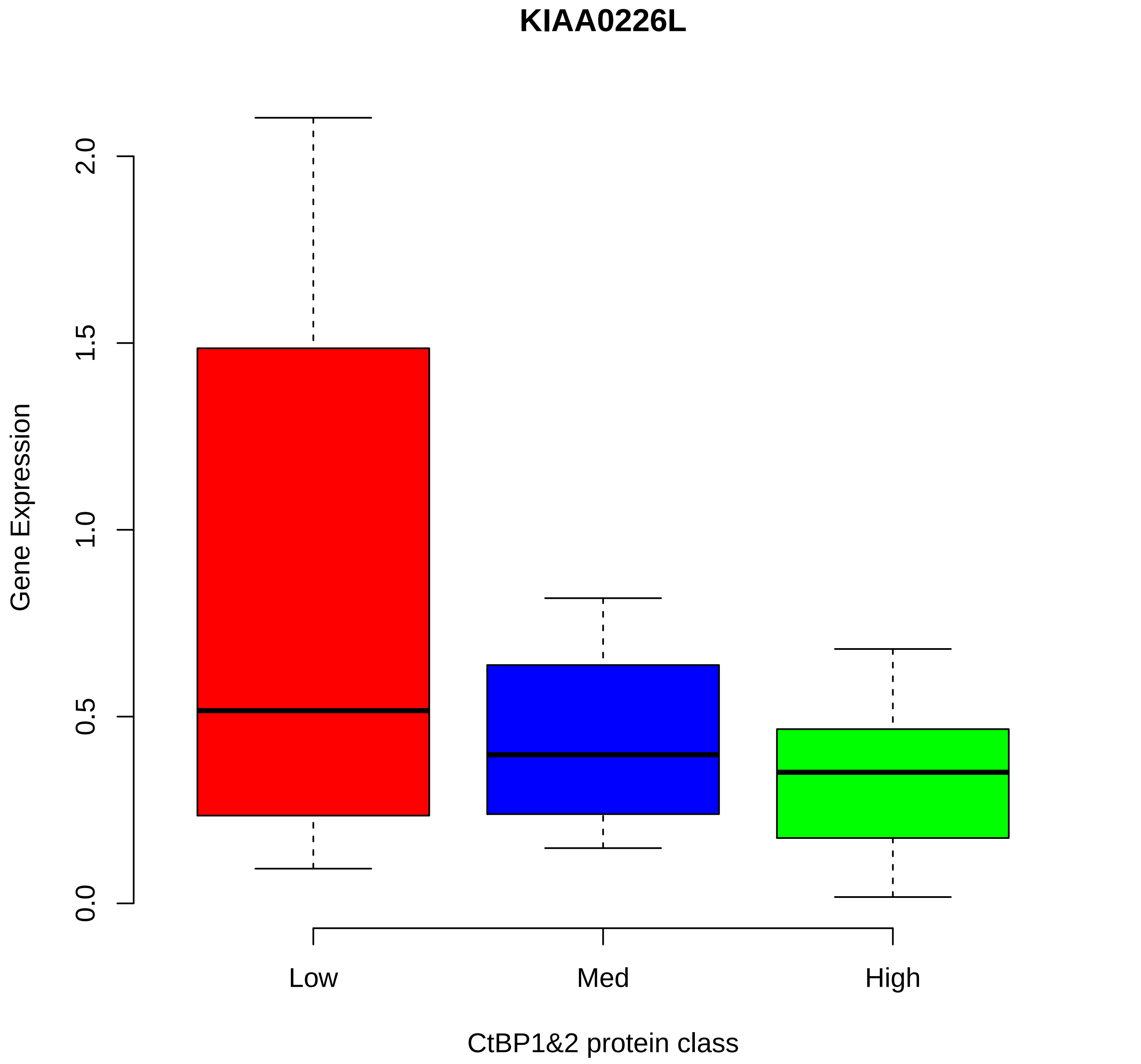

KIAA0226L
Gene Expression
0.0
0.5
1.0
1.5
2.0
Low
Med
High
CtBP1&2 protein class

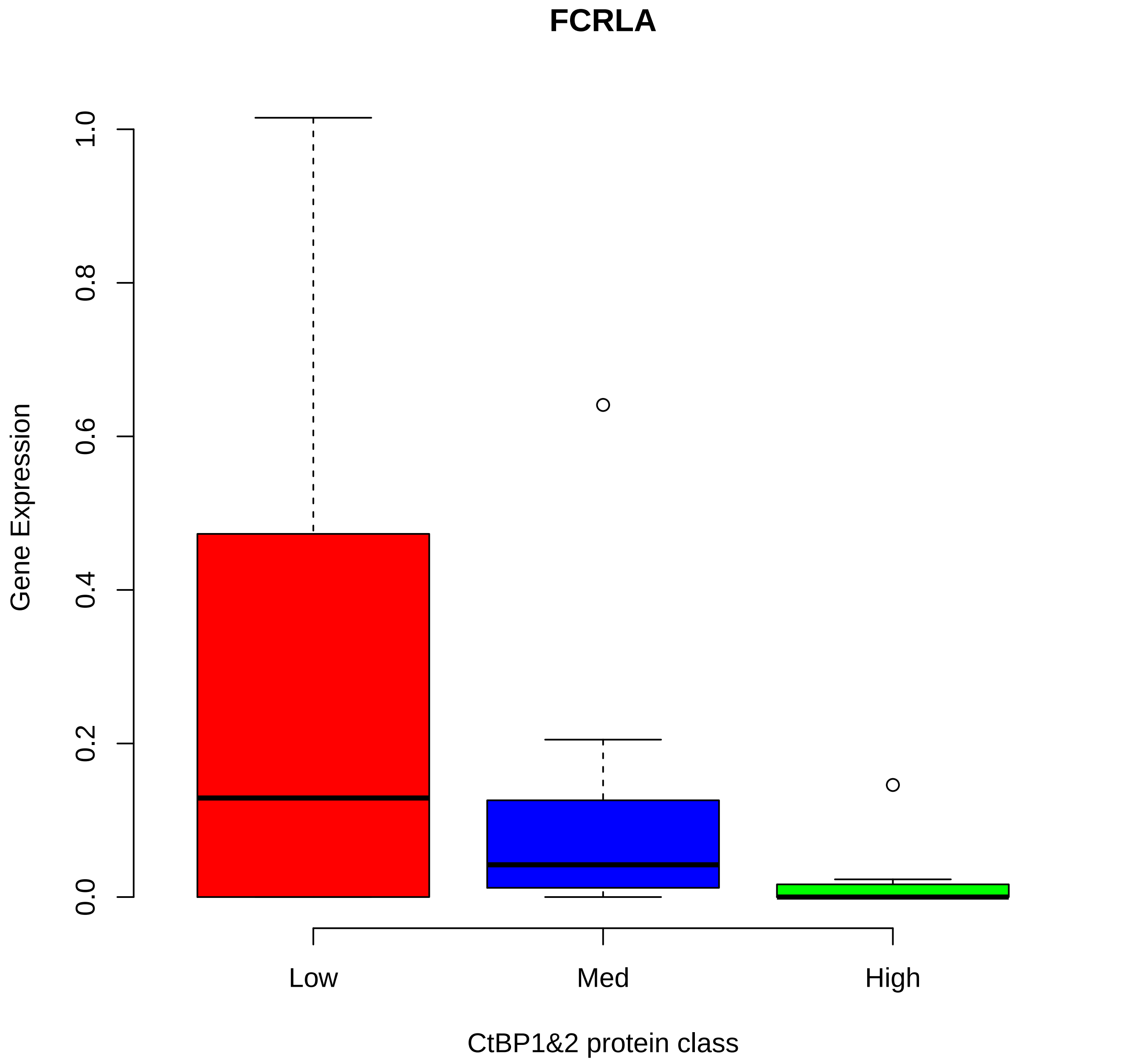
FCRLA
Gene Expression
0.0
0.2
0.4
0.6
0.8
1.0
Low
Med
High
CtBP1&2 protein class

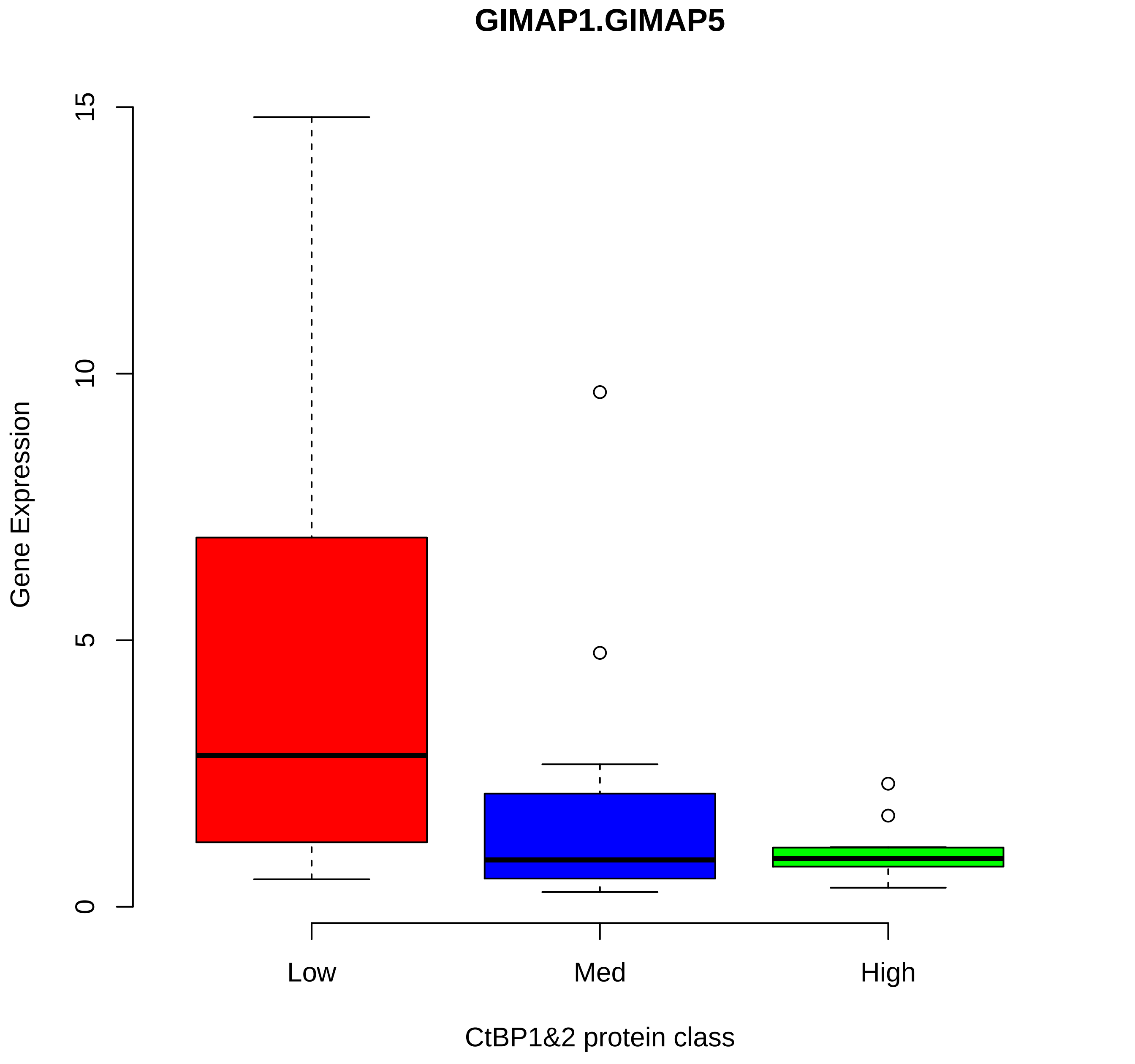
GIMAP1.GIMAP5
Gene Expression
0
5
10
15
Low
Med
High
CtBP1&2 protein class

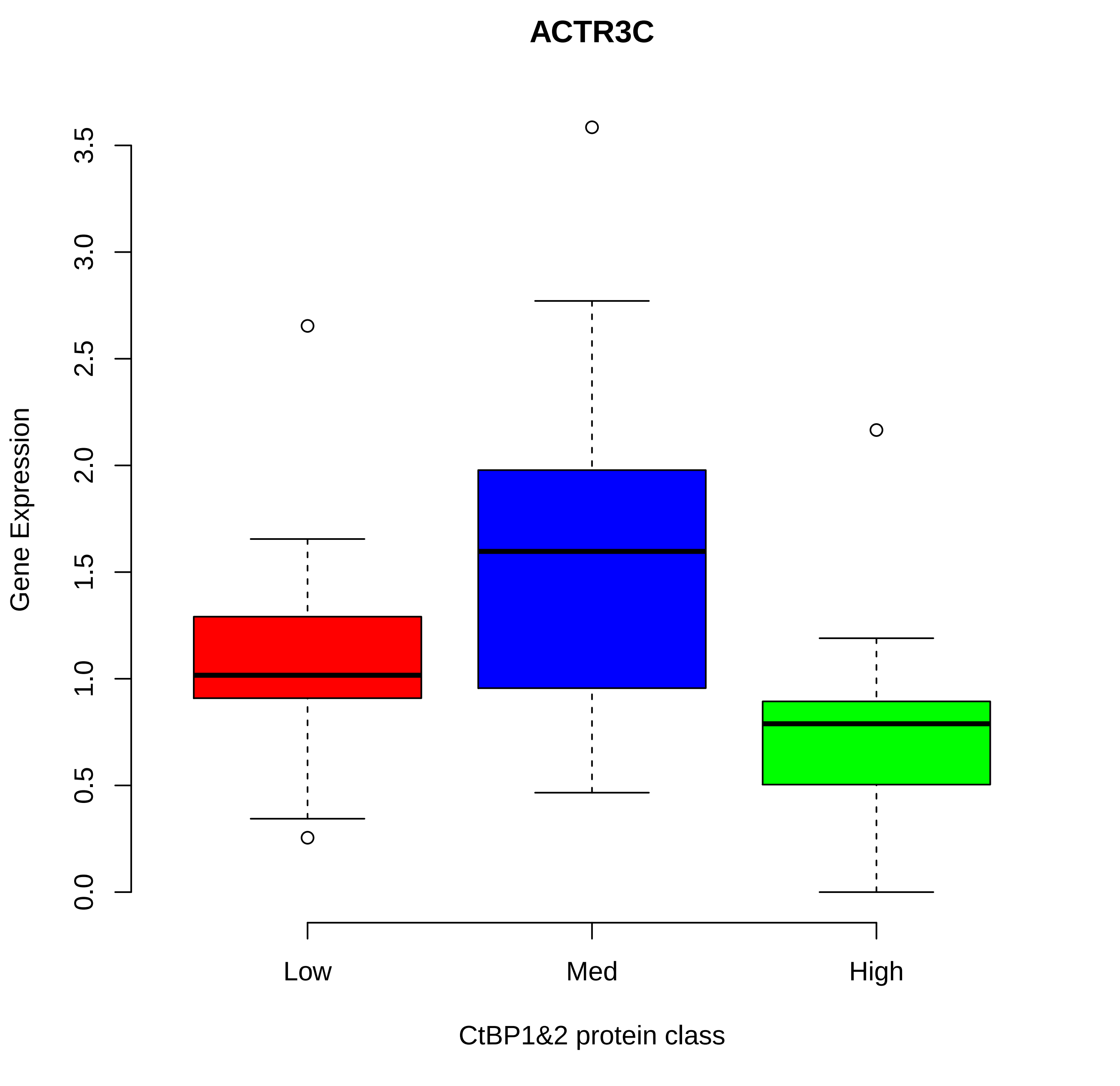
ACTR3C
Gene Expression
0.0
0.5
1.0
1.5
2.0
2.5
3.0
3.5
Low
Med
High
CtBP1&2 protein class

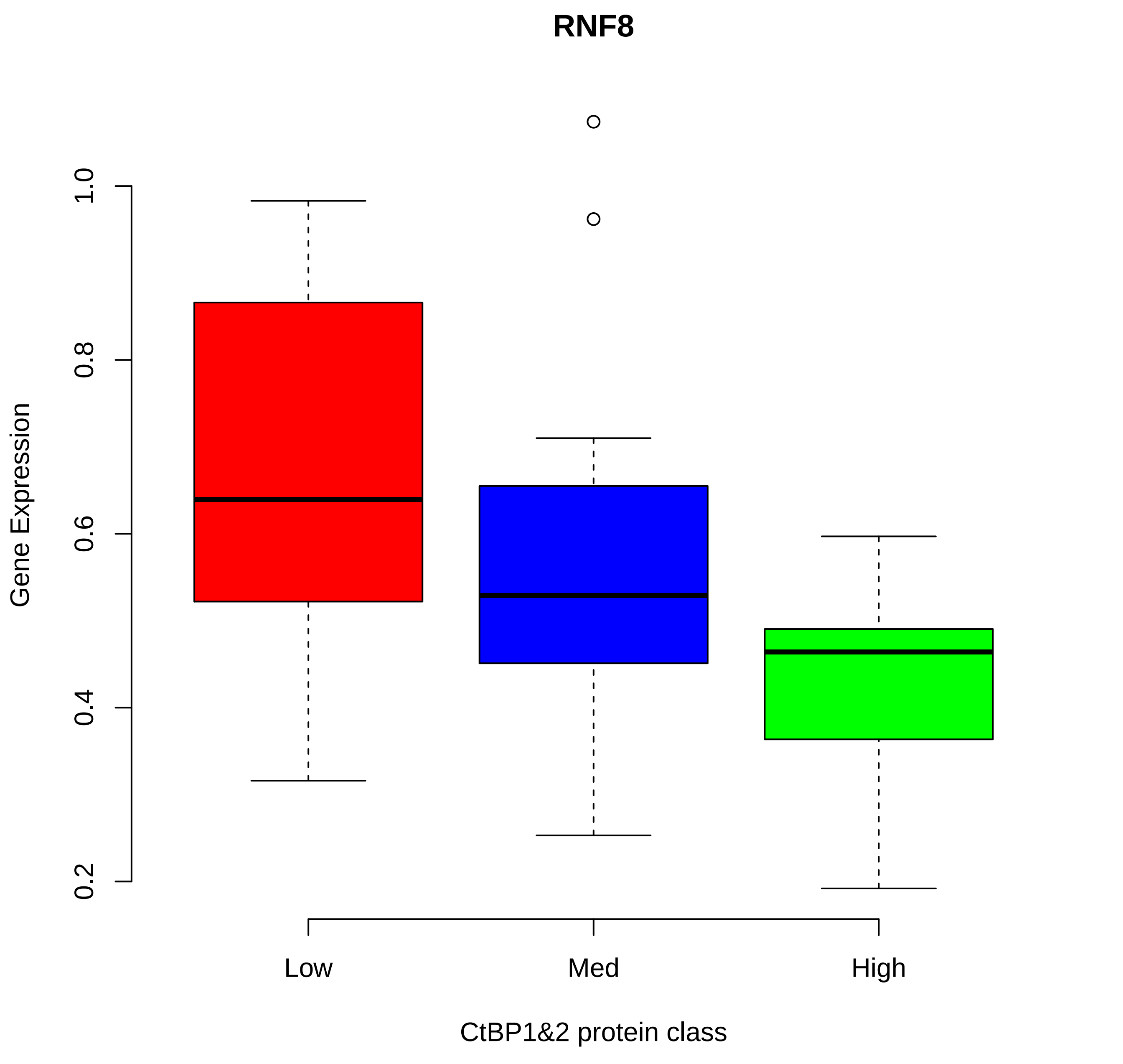
RNF8
Gene Expression
0.2
0.4
0.6
0.8
1.0
Low
Med
High
CtBP1&2 protein class

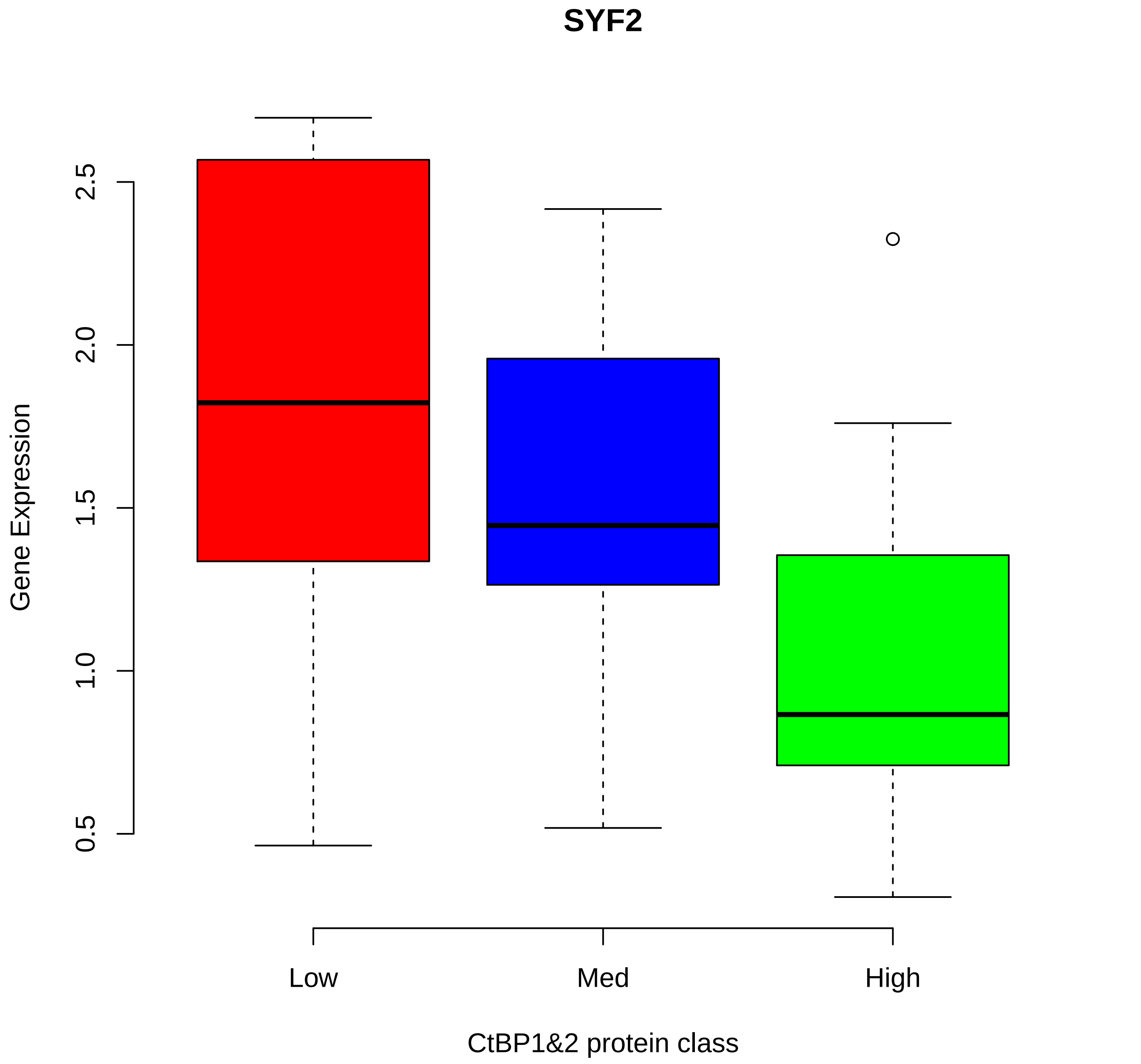
SYF2
Gene Expression
0.5
1.0
1.5
2.0
2.5
Low
Med
High
CtBP1&2 protein class

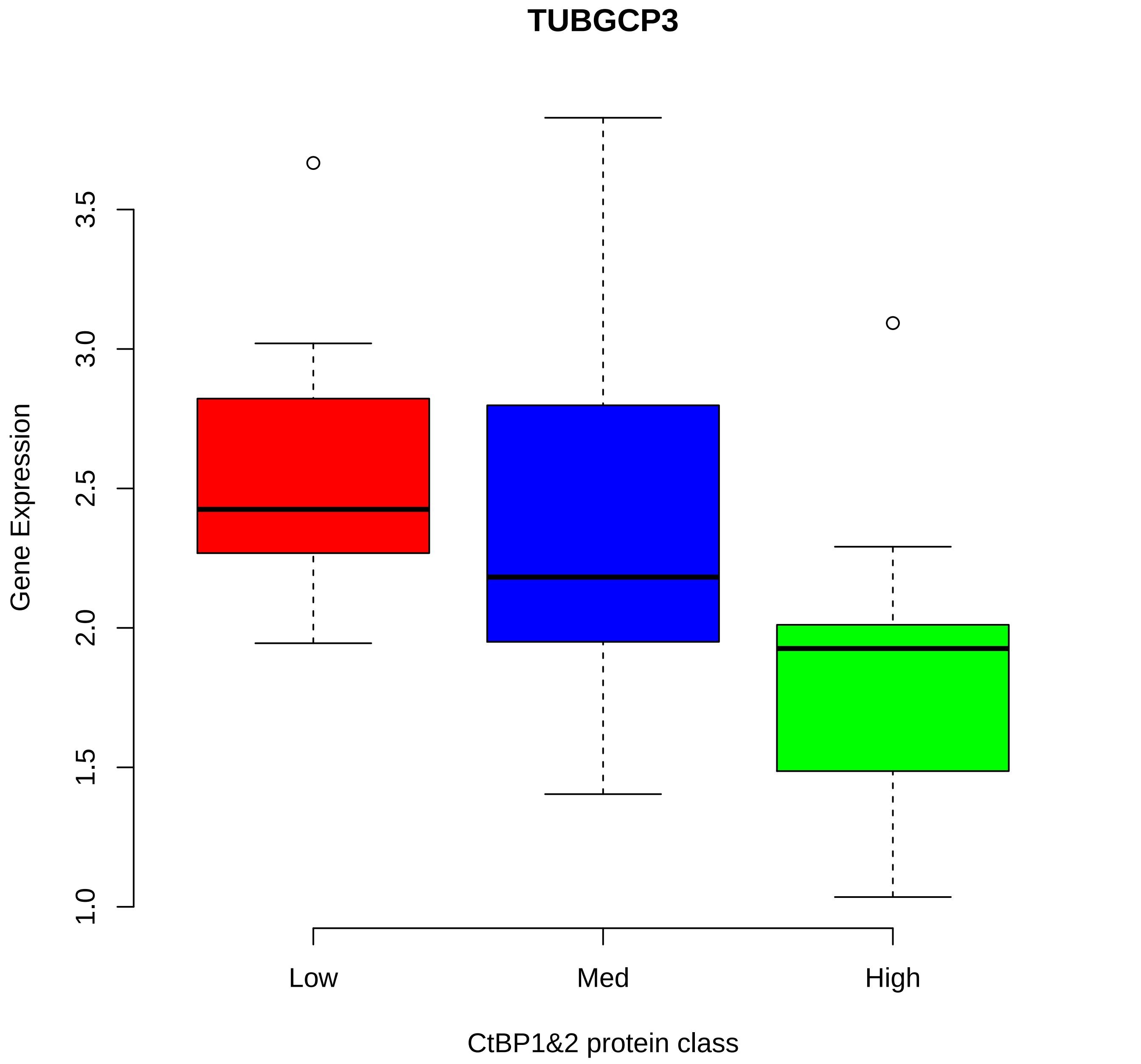
TUBGCP3
Gene Expression
1.0
1.5
2.0
2.5
3.0
3.5
Low
Med
High
CtBP1&2 protein class

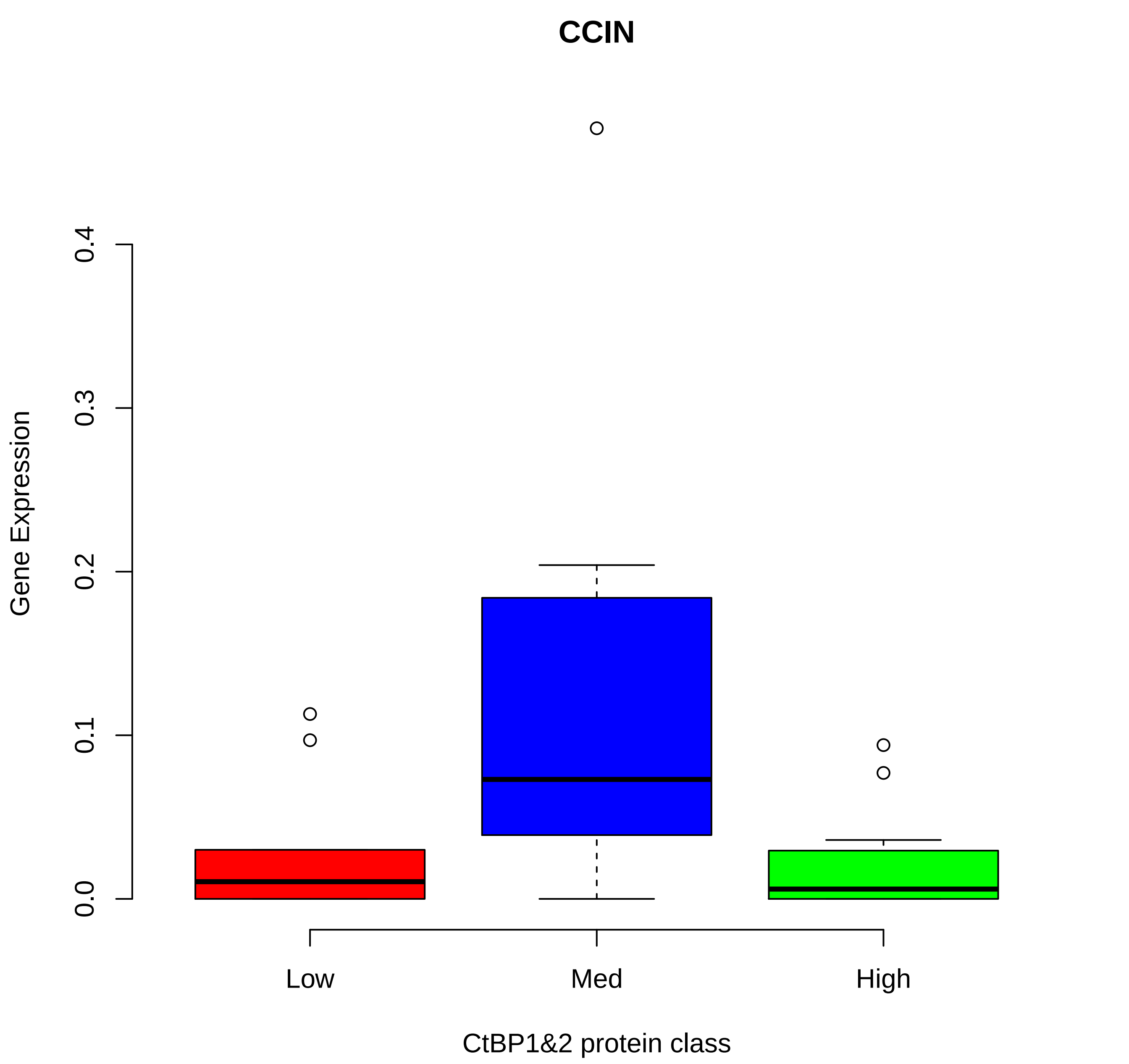
CCIN
Gene Expression
0.0
0.1
0.2
0.3
0.4
Low
Med
High
CtBP1&2 protein class

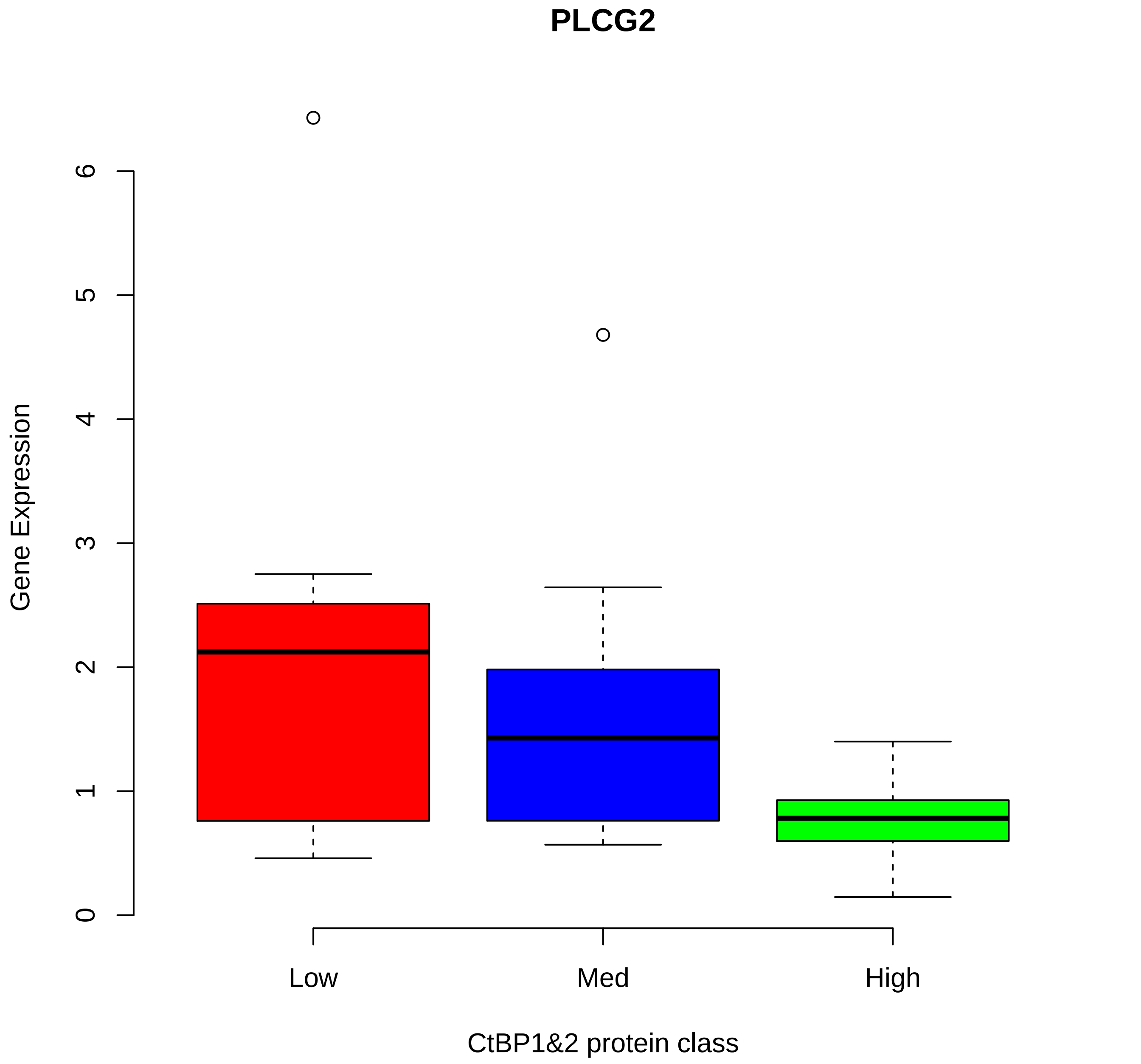

PLCG2
Gene Expression
0
1
2
3
4
5
6
Low
Med
High
CtBP1&2 protein class

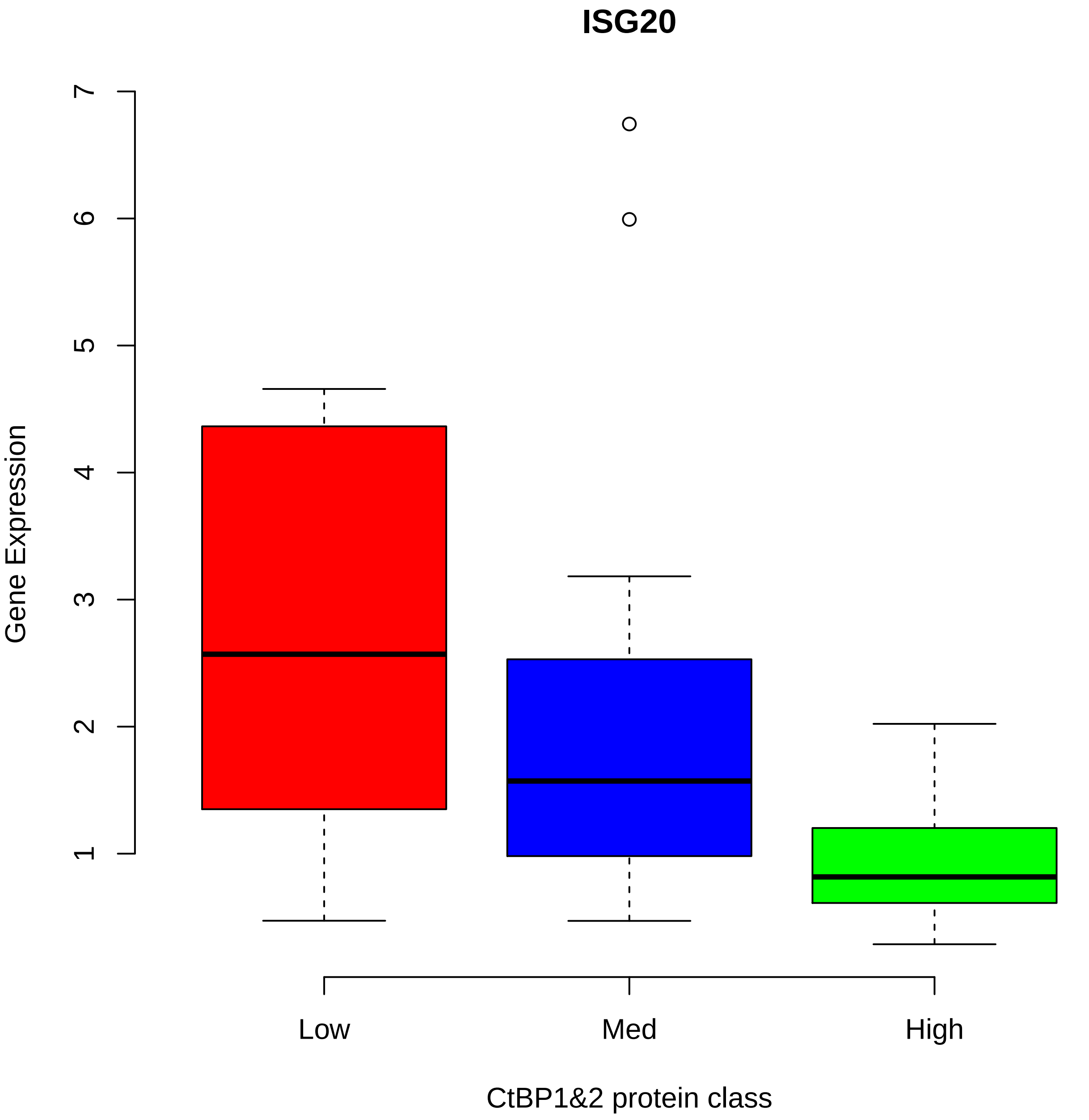
ISG20
Gene Expression
1
2
3
4
5
6
7
Low
Med
High
CtBP1&2 protein class

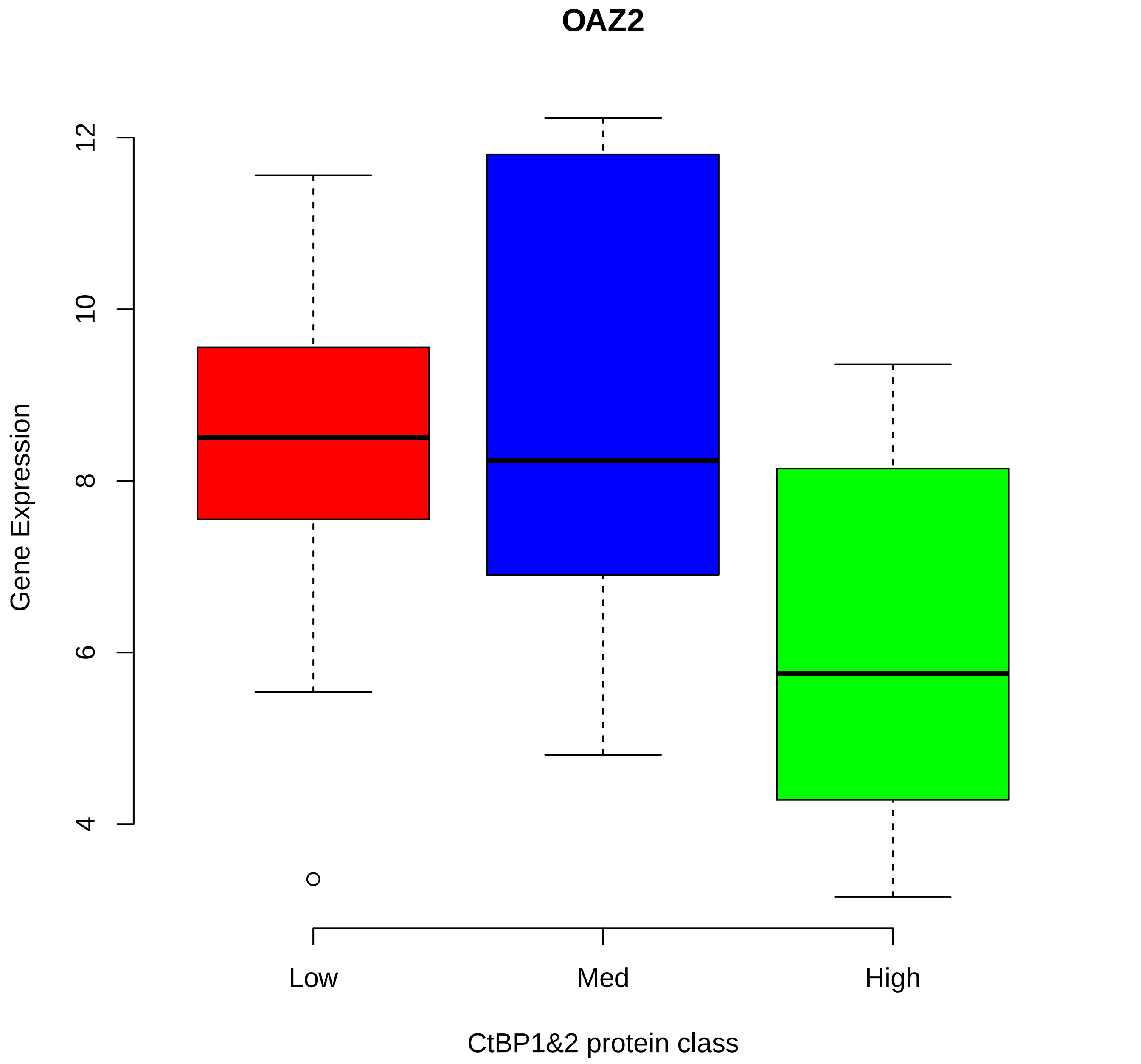

OAZ2
Gene Expression
12
10
8
6
4
Low
Med
High
CtBP1&2 protein class

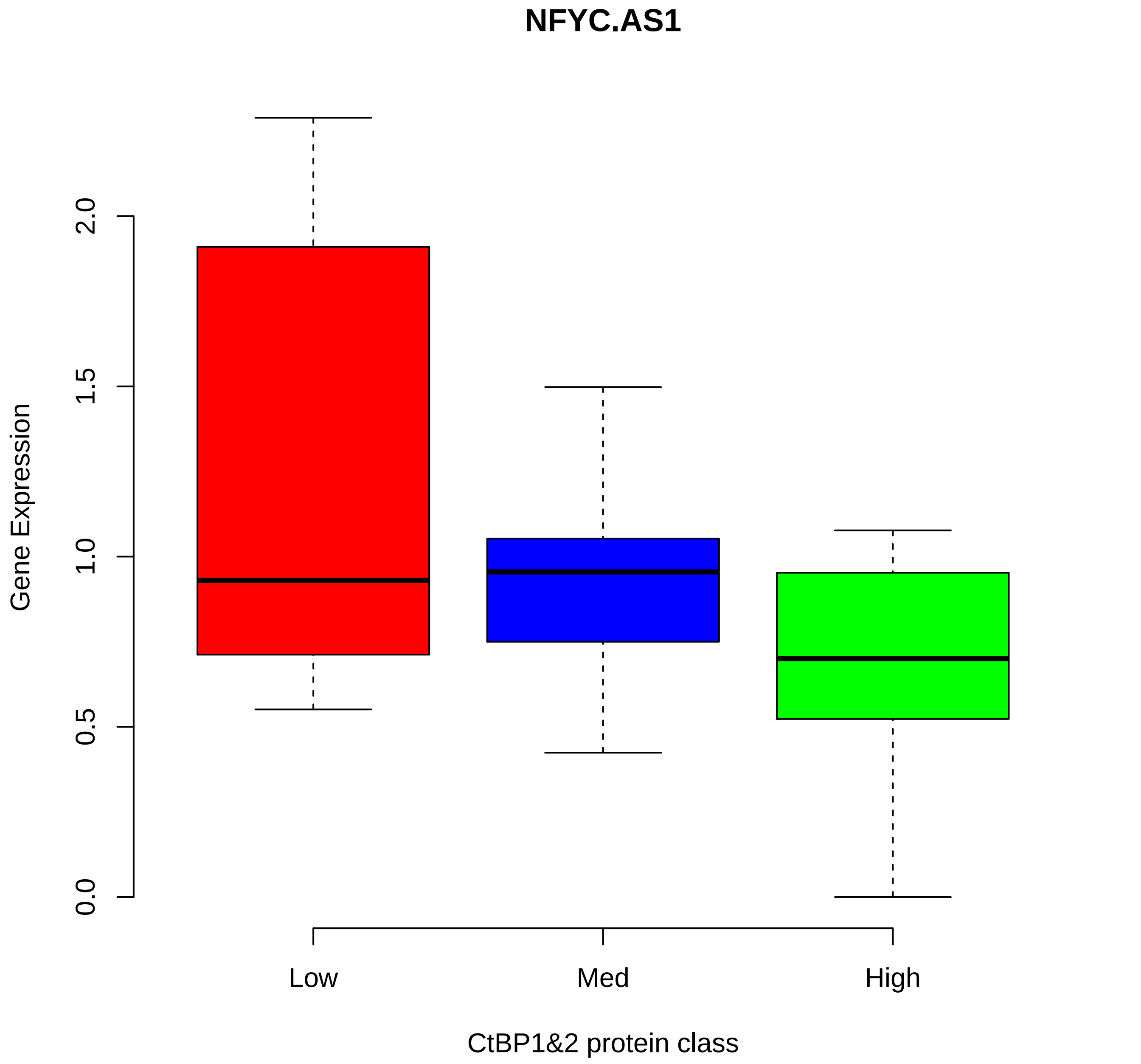

NFYC.AS1
Gene Expression
0.0
0.5
1.0
1.5
2.0
Low
Med
High
CtBP1&2 protein class

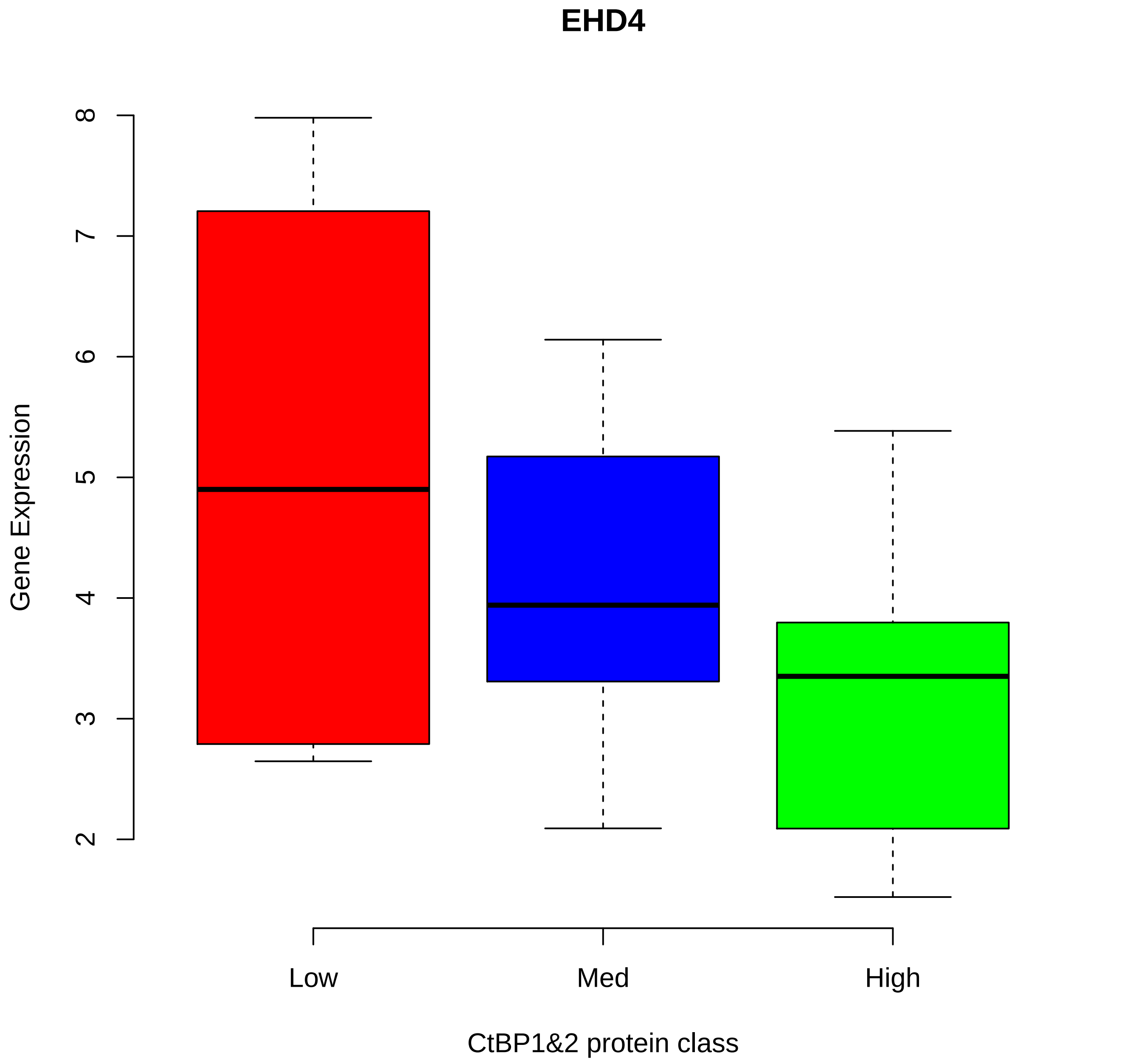
EHD4
Gene Expression
2
3
4
5
6
7
8
Low
Med
High
CtBP1&2 protein class

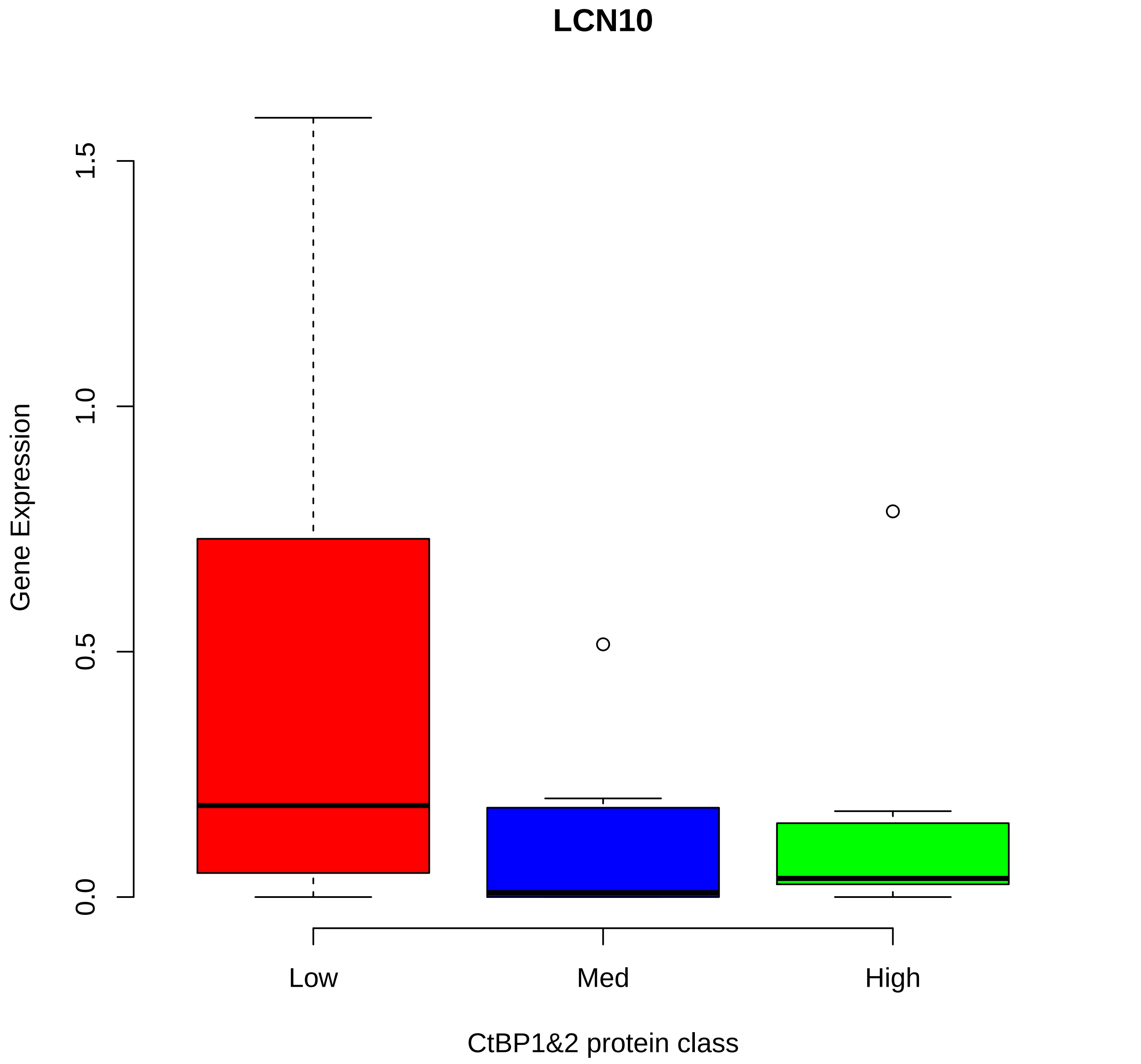
LCN10
Gene Expression
0.0
0.5
1.0
1.5
Low
Med
High
CtBP1&2 protein class

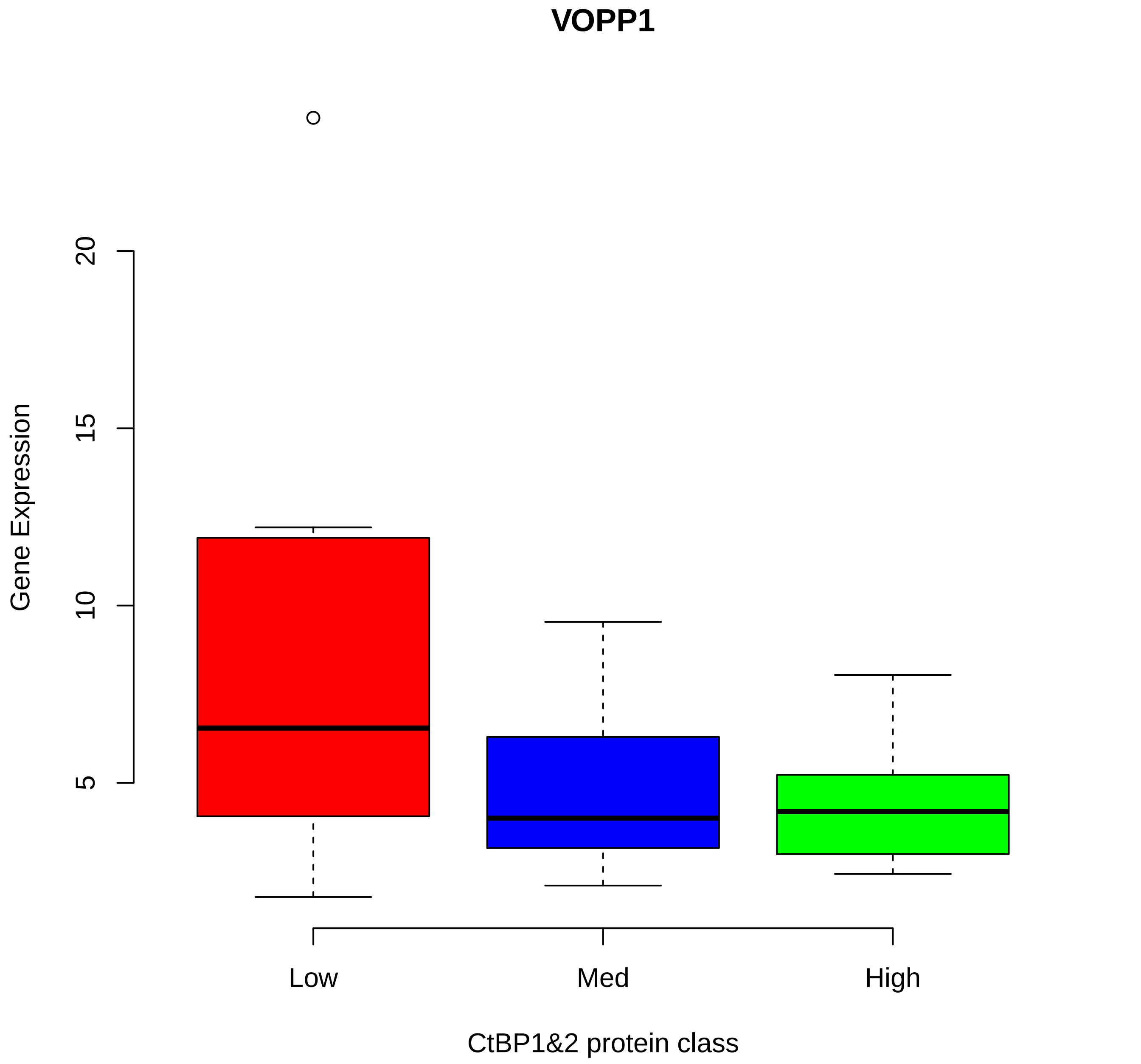

VOPP1
Gene Expression
20
15
10
5
Low
Med
High
CtBP1&2 protein class

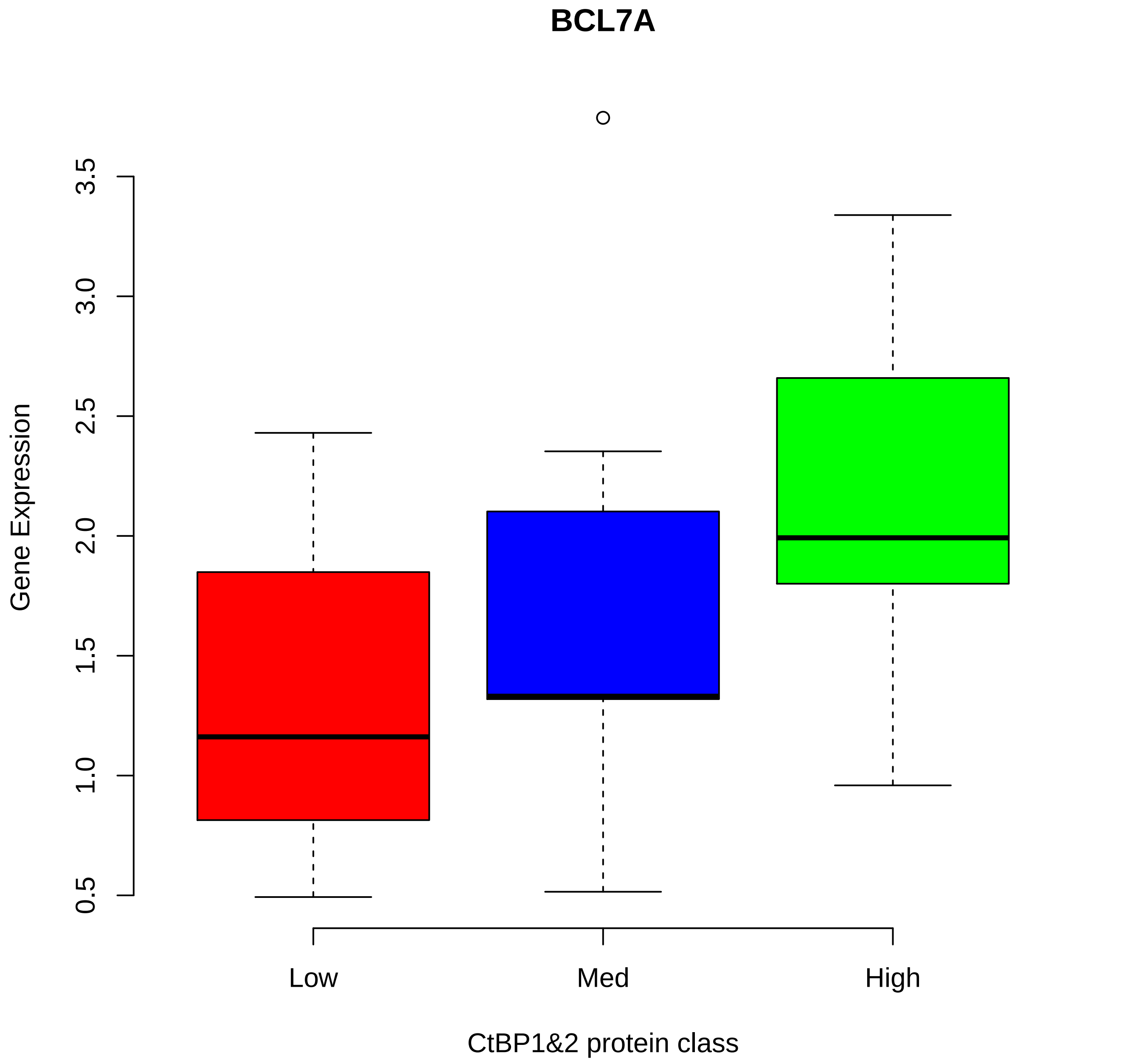
BCL7A
Gene Expression
0.5
1.0
1.5
2.0
2.5
3.0
3.5
Low
Med
High
CtBP1&2 protein class

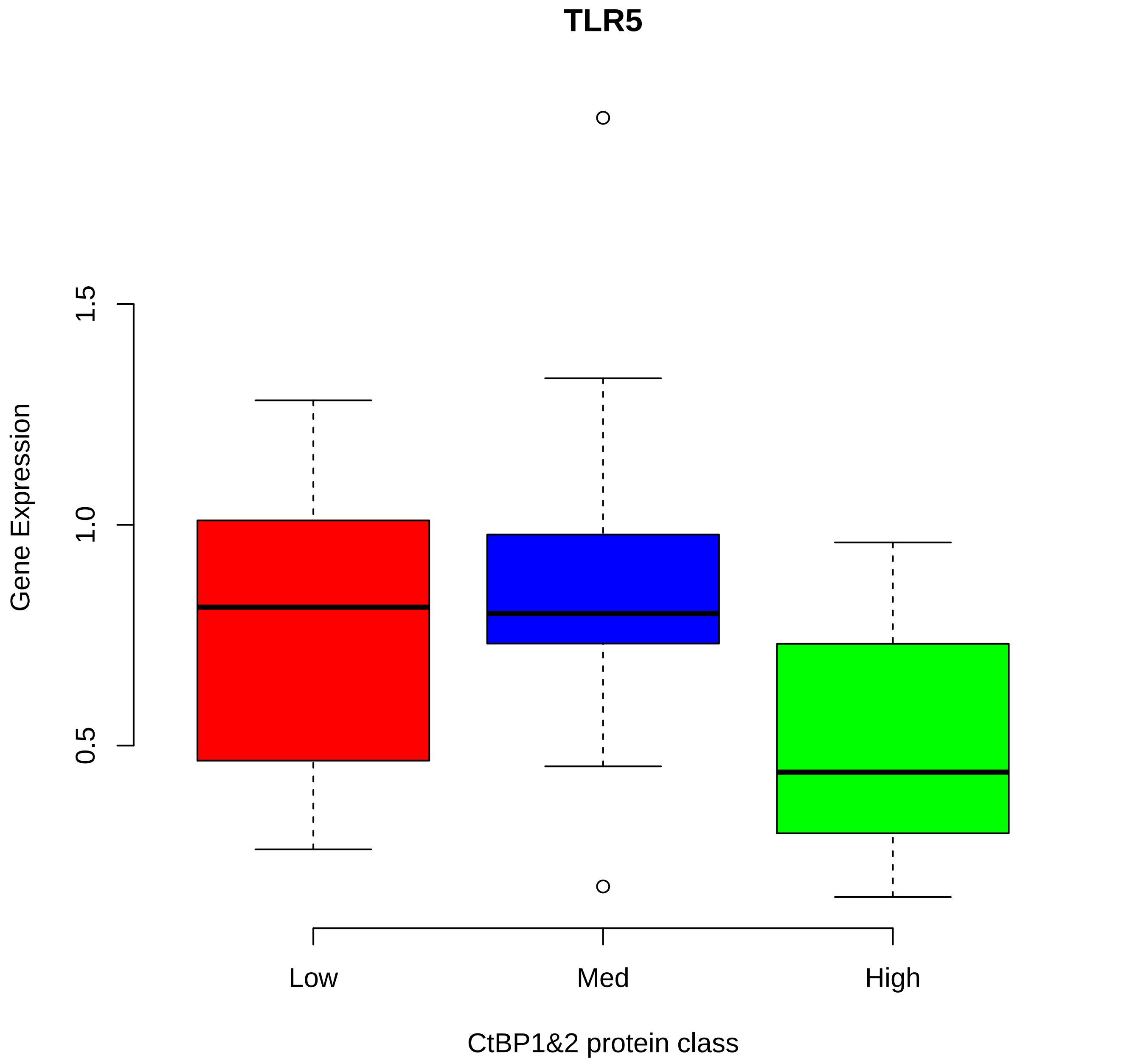

TLR5
Gene Expression
0.5
1.0
1.5
Low
Med
High
CtBP1&2 protein class

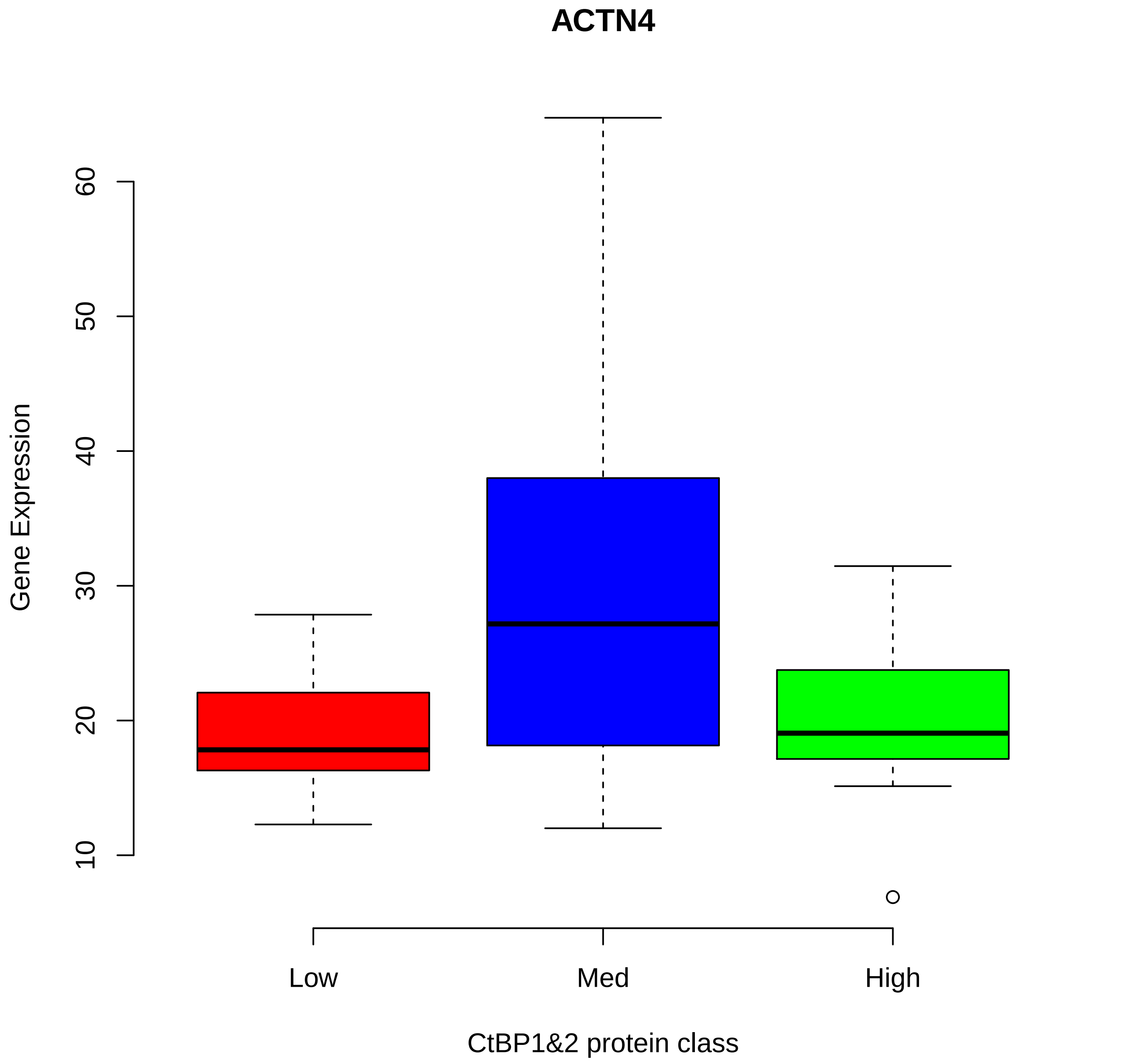

ACTN4
Gene Expression
10
20
30
40
50
60
Low
Med
High
CtBP1&2 protein class

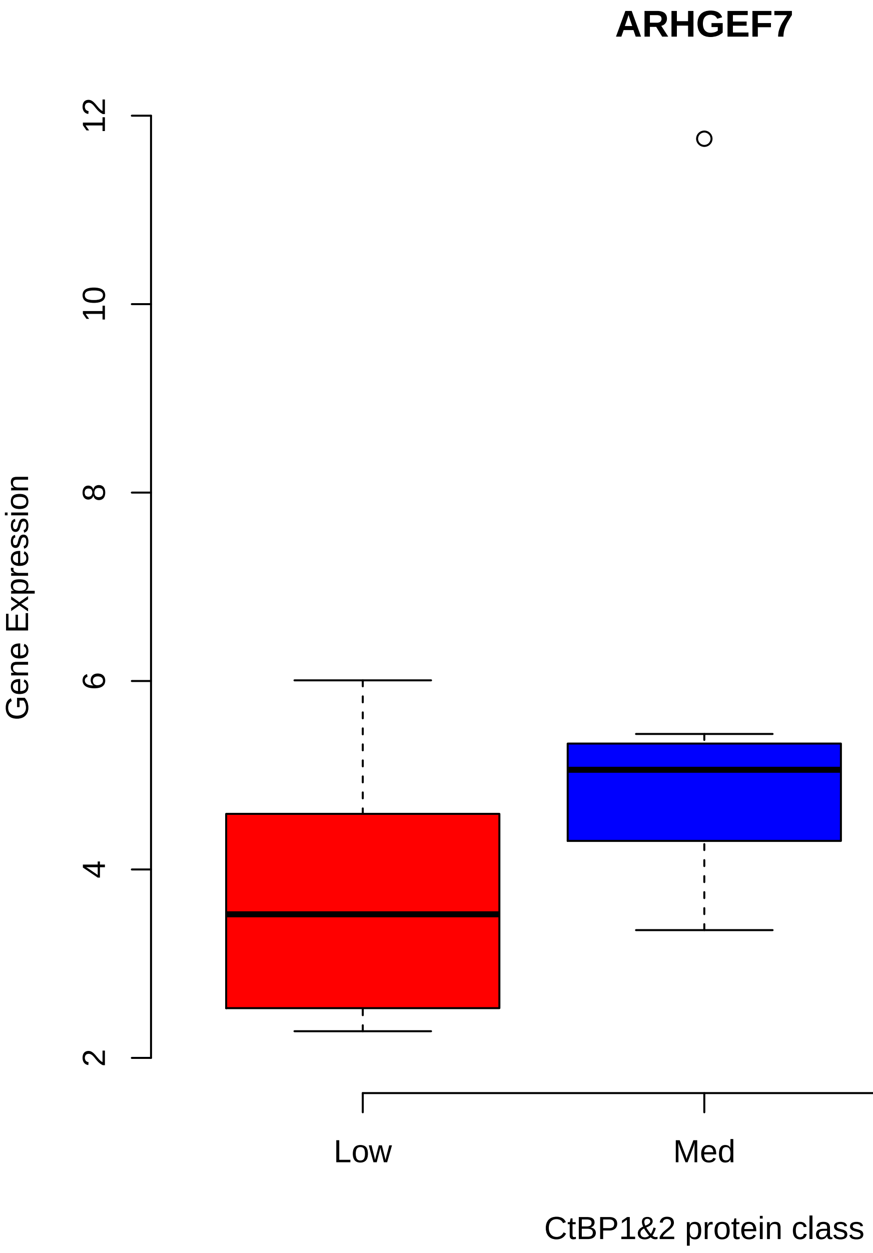
ARHGEF7
Gene Expression
2
4
6
8
10
12
Low
Med
High
CtBP1&2 protein class

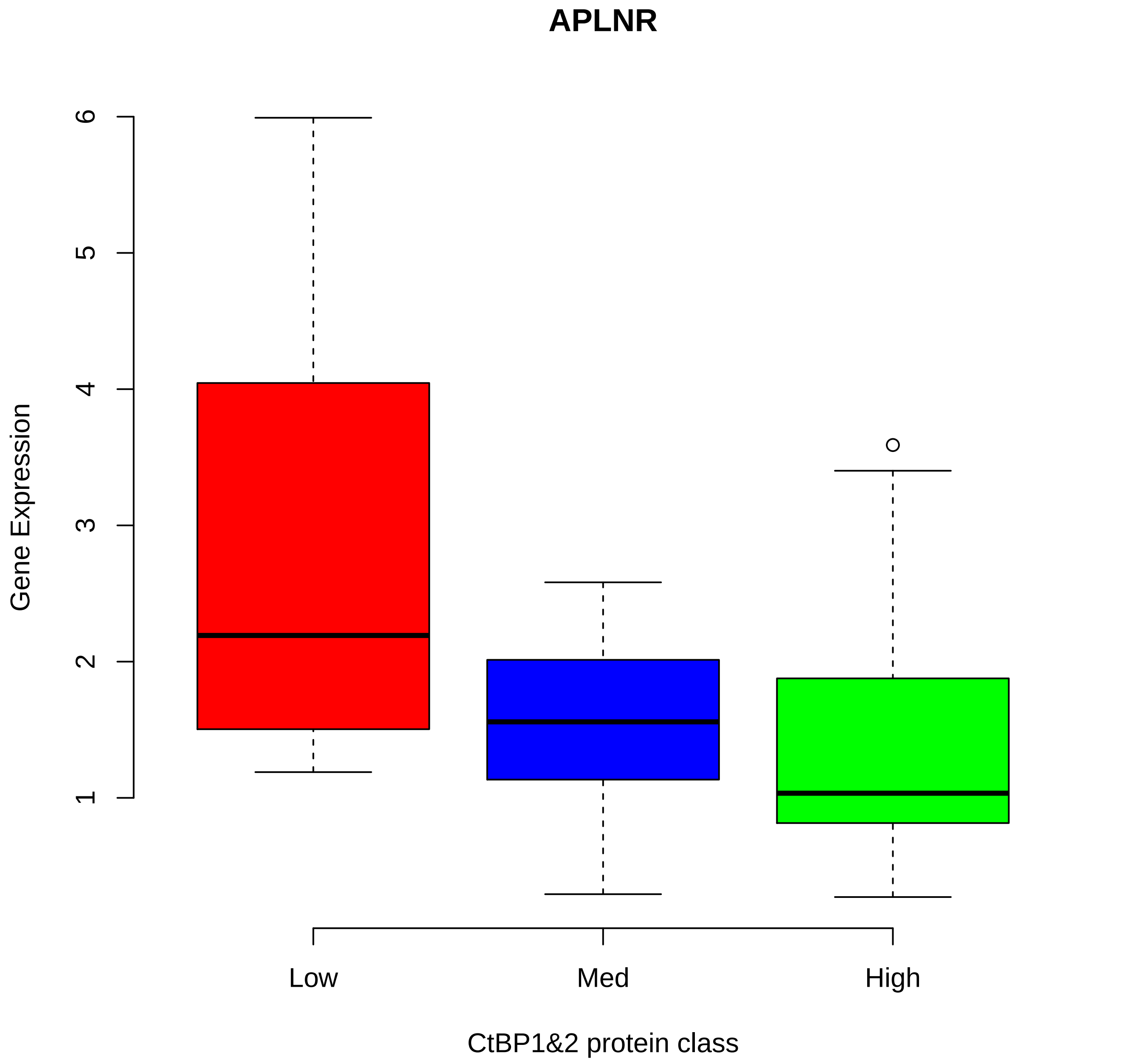
APLNR
Gene Expression
1
2
3
4
5
6
Low
Med
High
CtBP1&2 protein class

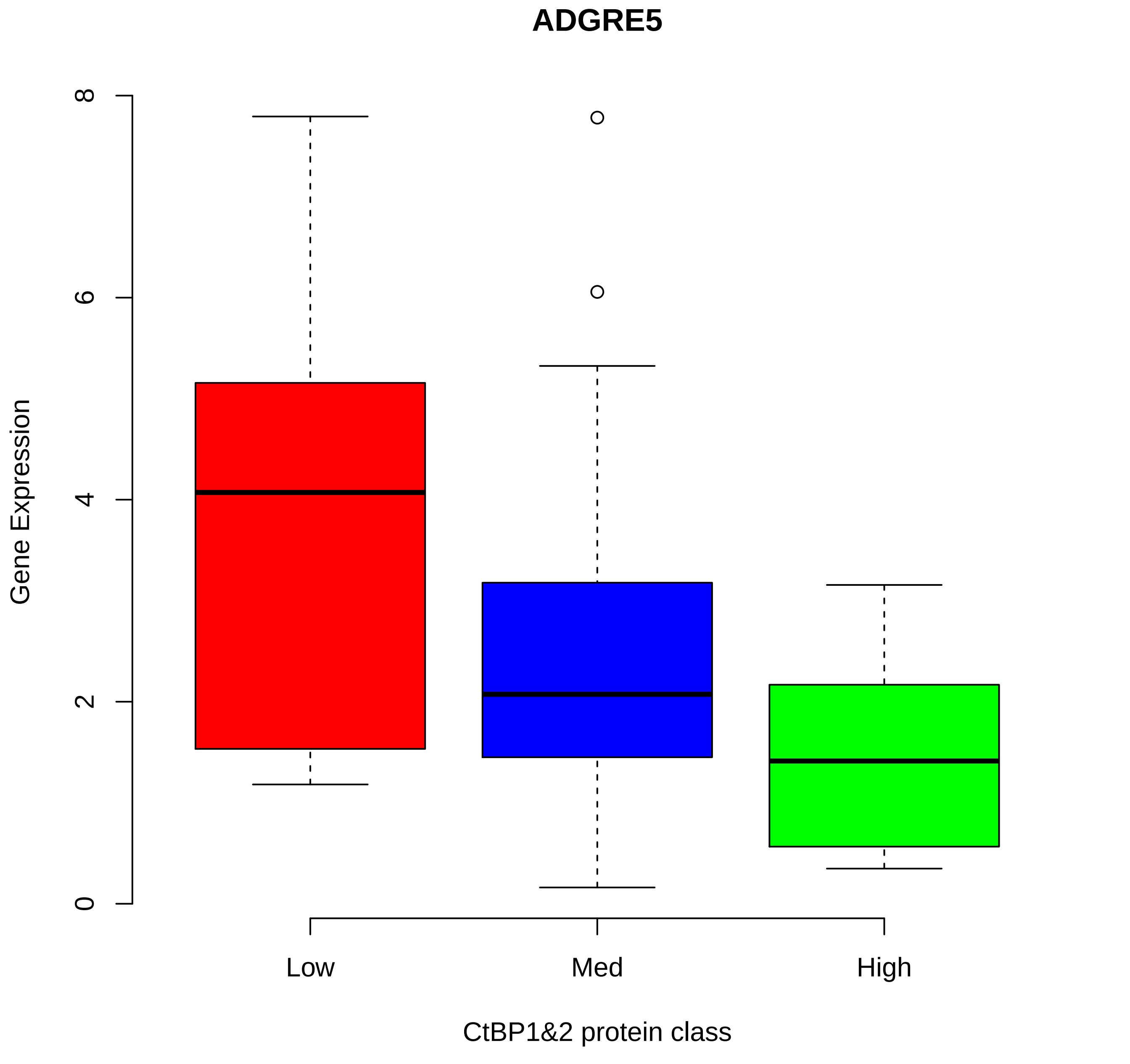

ADGRE5
Gene Expression
0
2
4
6
8
Low
Med
High
CtBP1&2 protein class

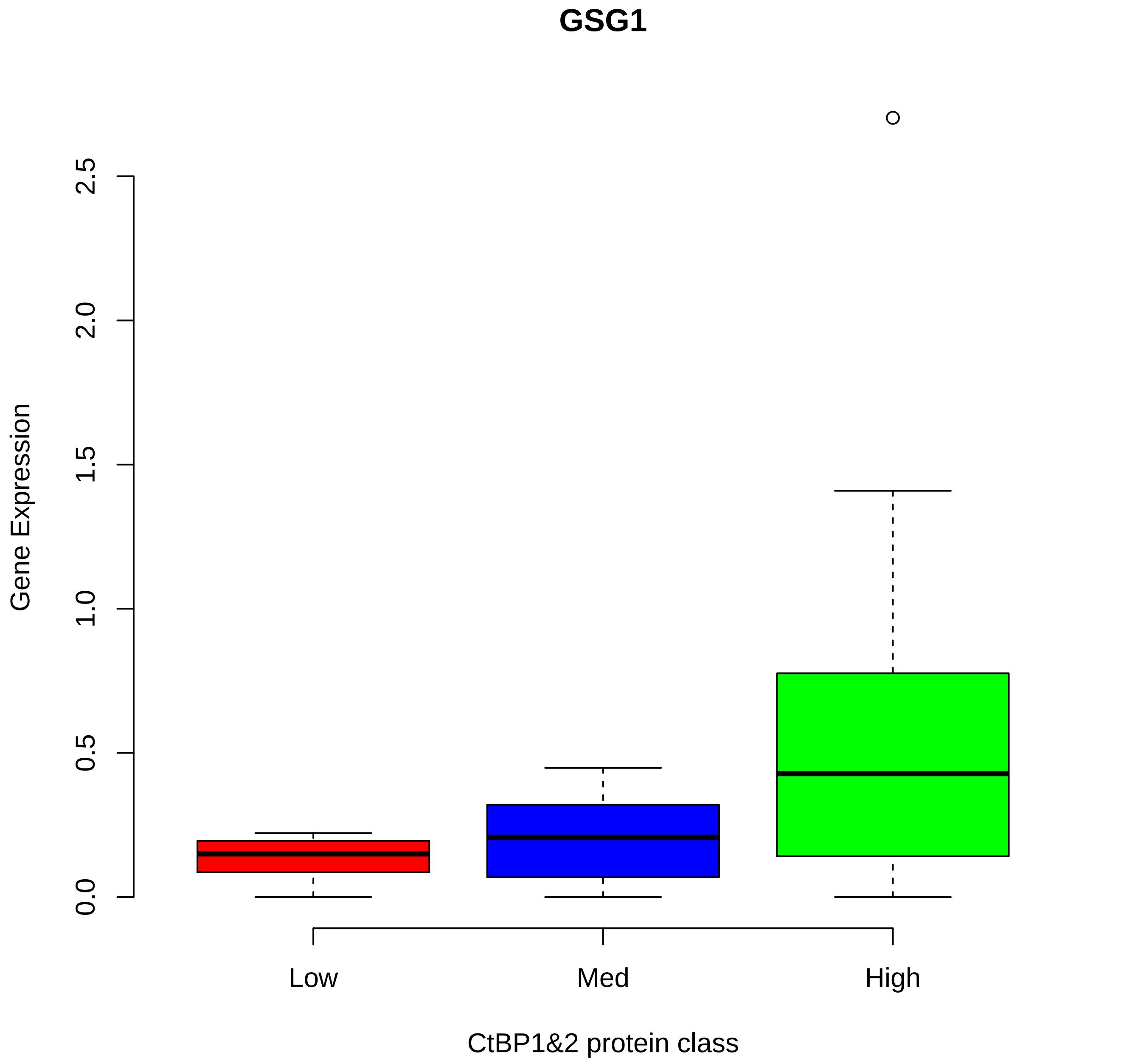
GSG1
Gene Expression
0.0
0.5
1.0
1.5
2.0
2.5
Low
Med
High
CtBP1&2 protein class

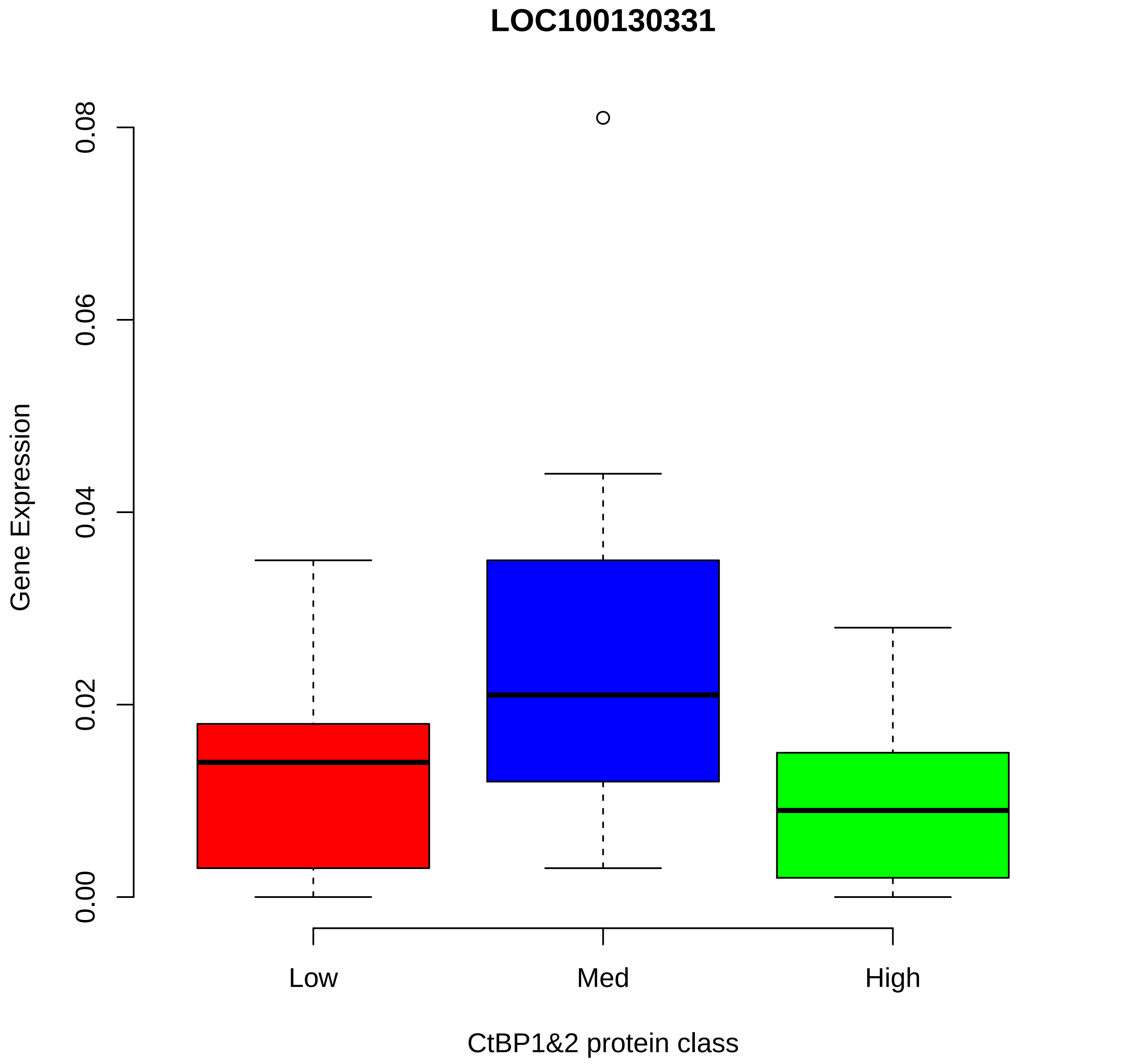

LOC100130331
Gene Expression
0.00
0.02
0.04
0.06
0.08
Low
Med
High
CtBP1&2 protein class

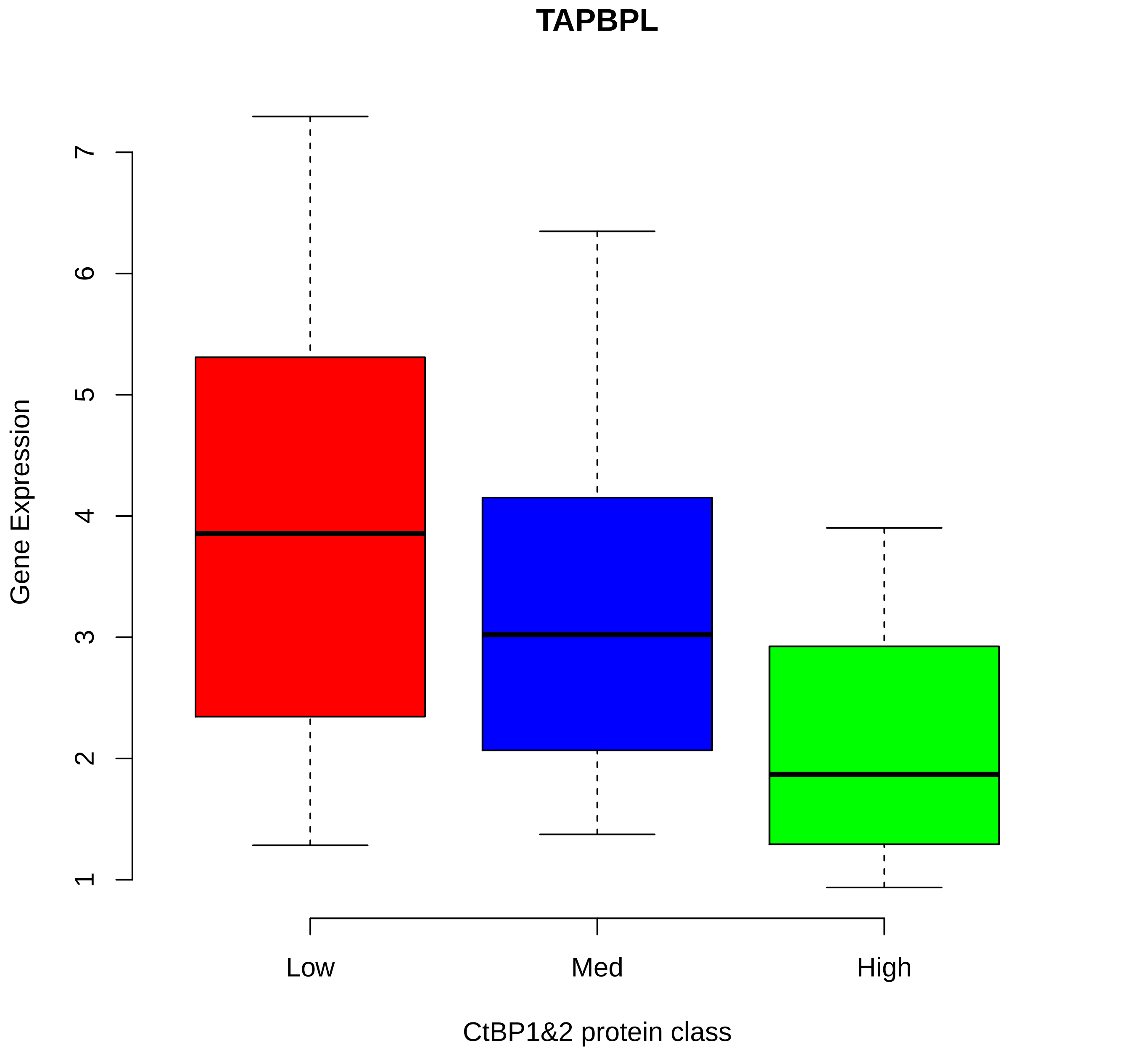

TAPBPL
Gene Expression
1
2
3
4
5
6
7
Low
Med
High
CtBP1&2 protein class

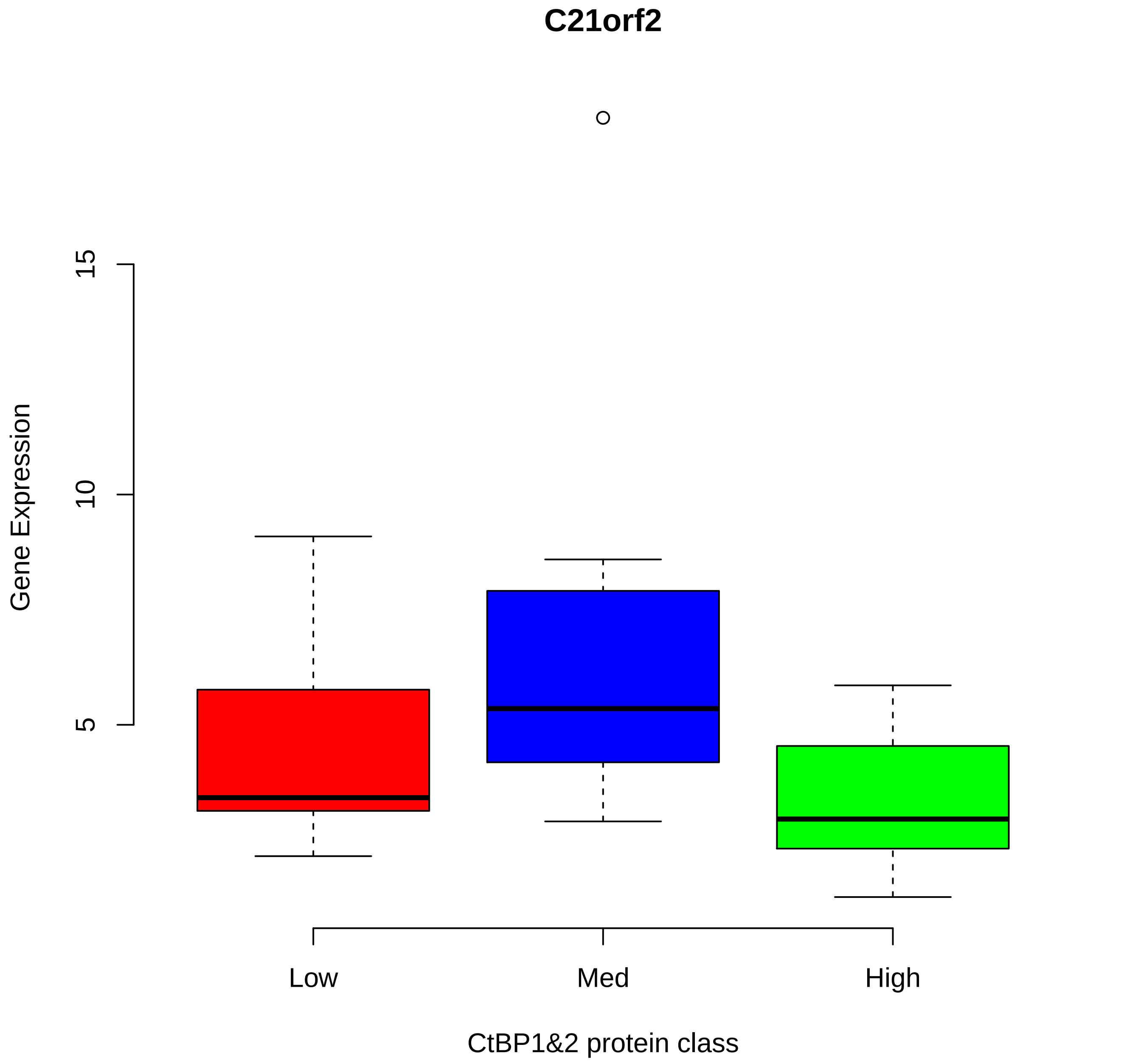
C21orf2
Gene Expression
15
10
5
Low
Med
High
CtBP1&2 protein class

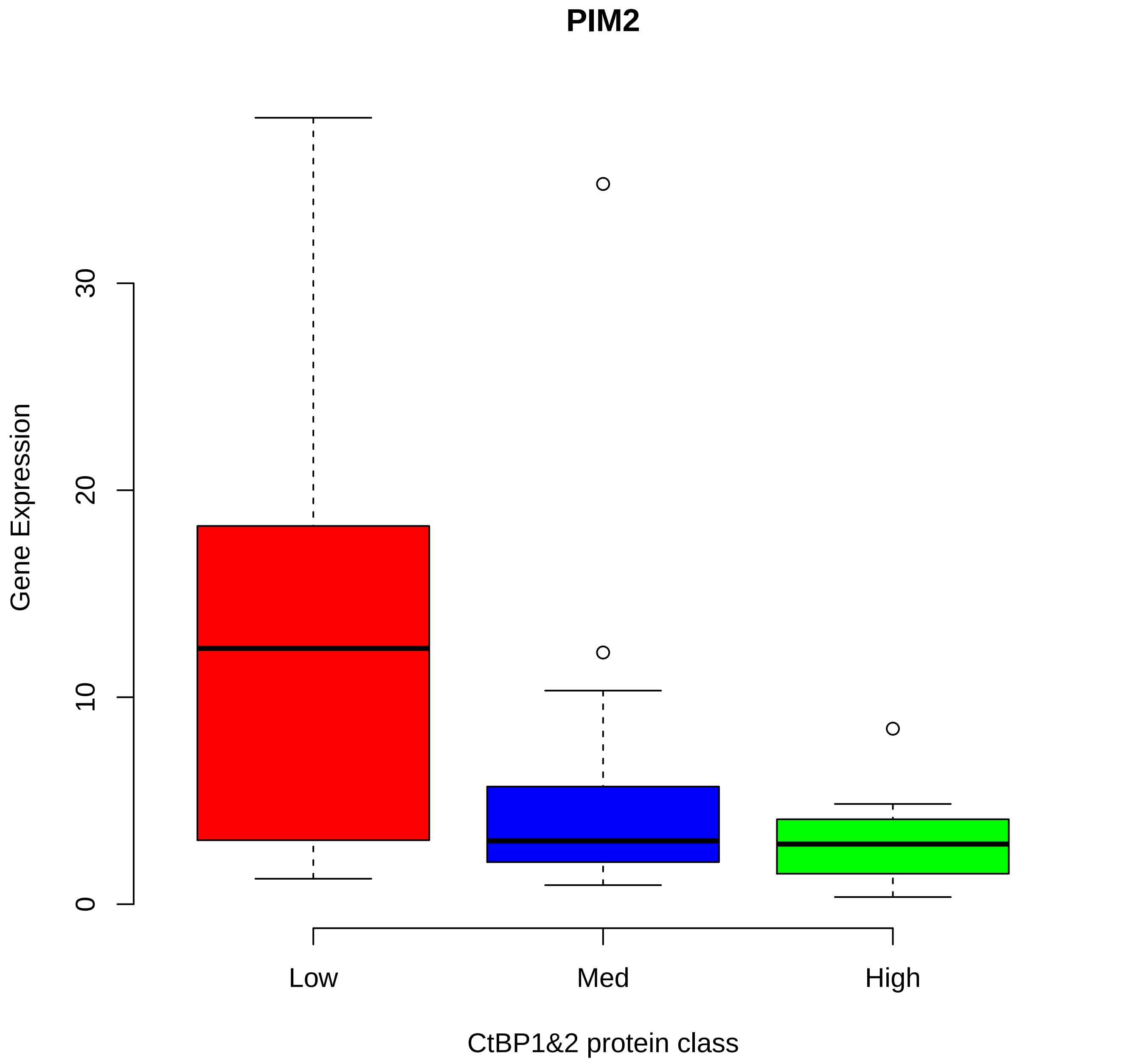
PIM2
Gene Expression
0
10
20
30
Low
Med
High
CtBP1&2 protein class

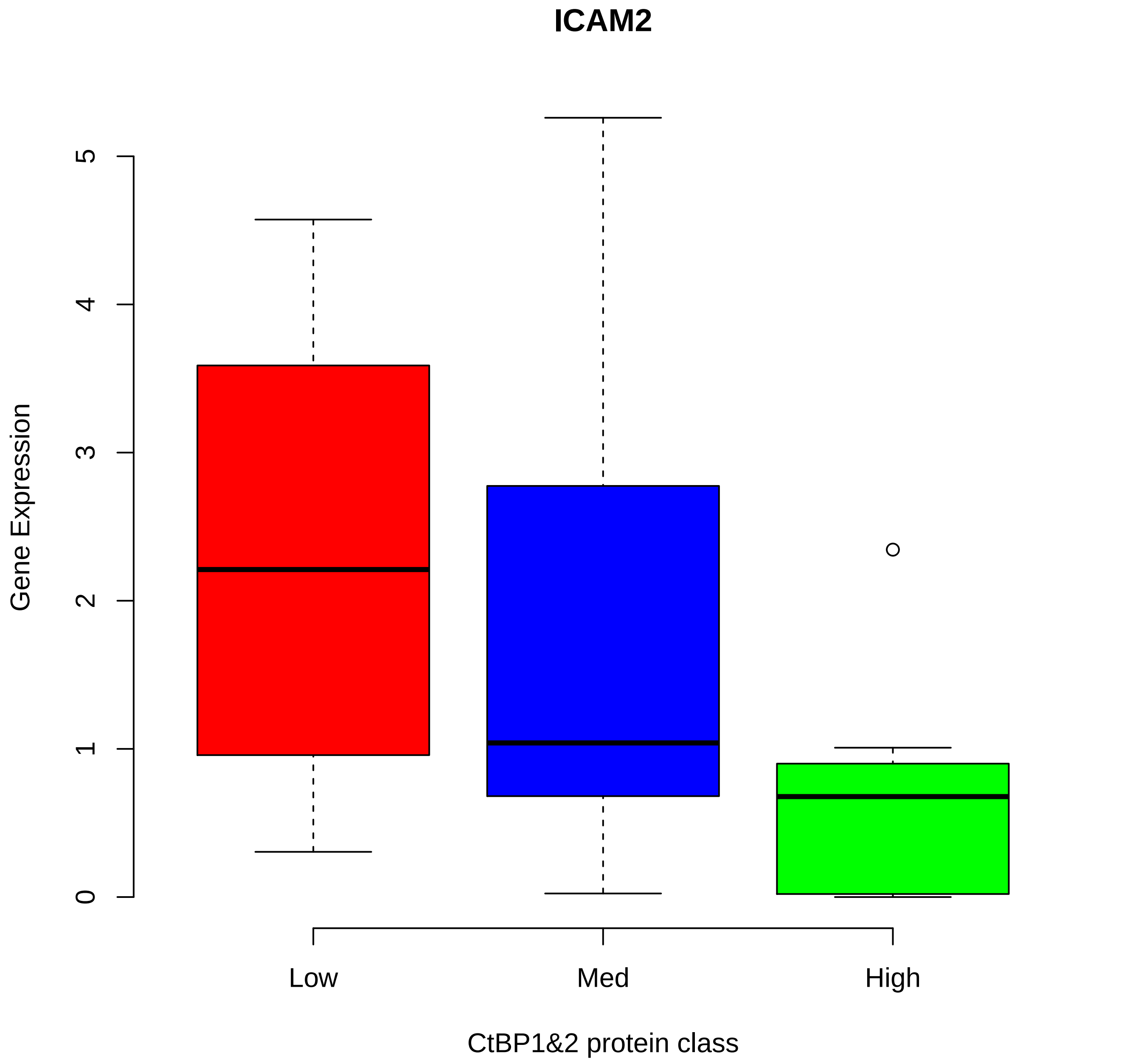
ICAM2
Gene Expression
0
1
2
3
4
5
Low
Med
High
CtBP1&2 protein class

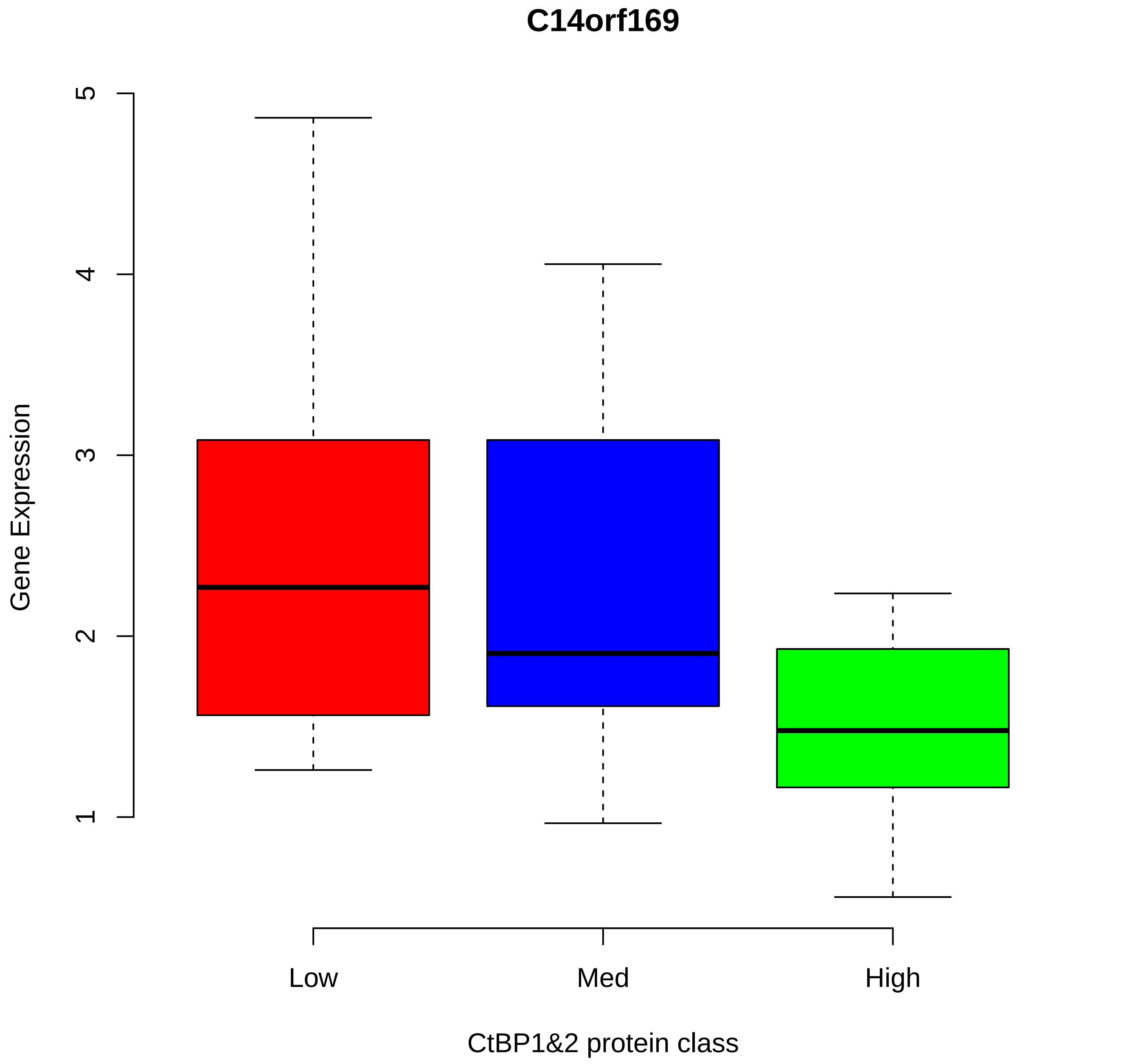
C14orf169
Gene Expression
5
4
3
2
1
Low
Med
High
CtBP1&2 protein class

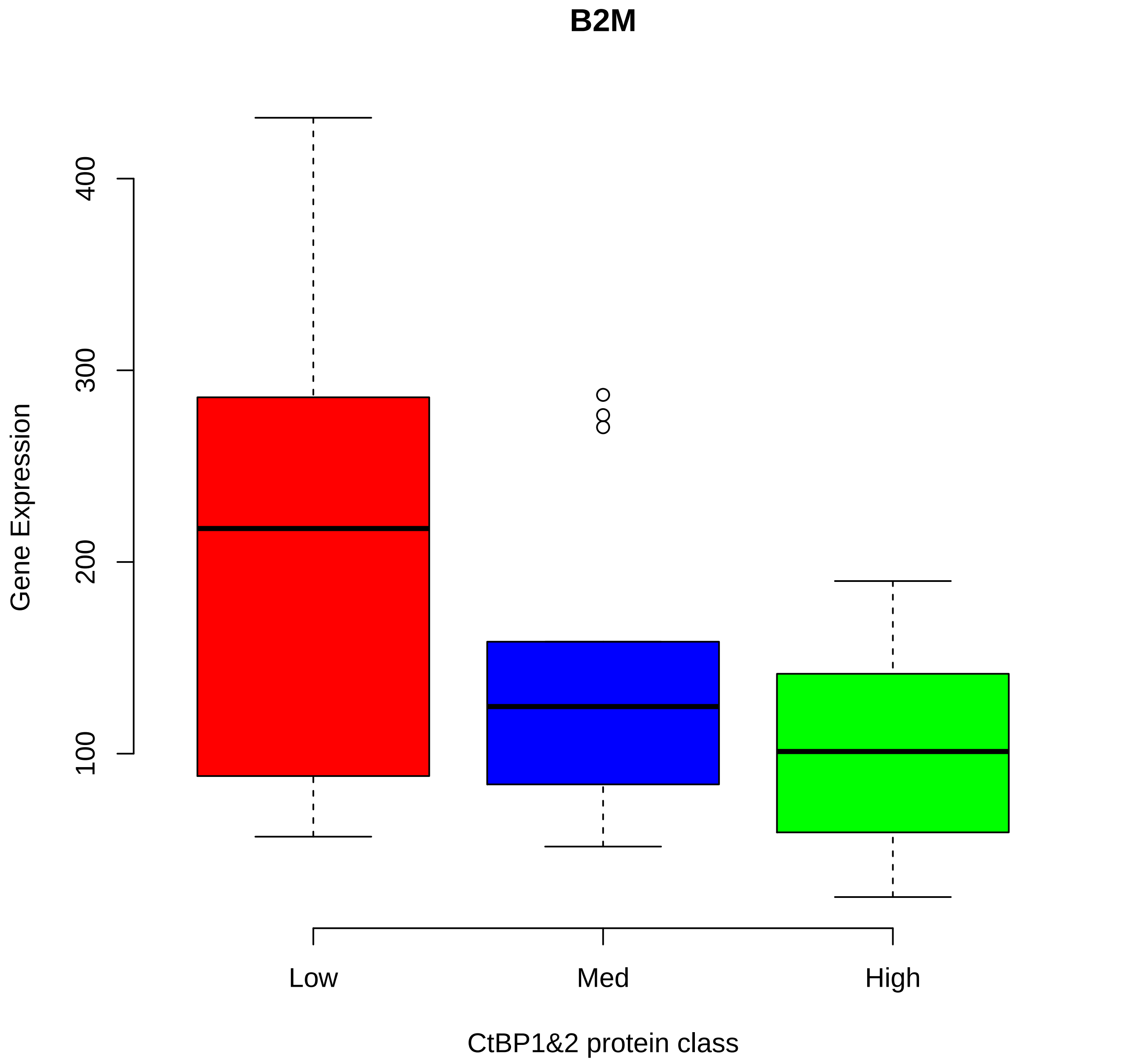
B2M
Gene Expression
100
200
300
400
Low
Med
High
CtBP1&2 protein class

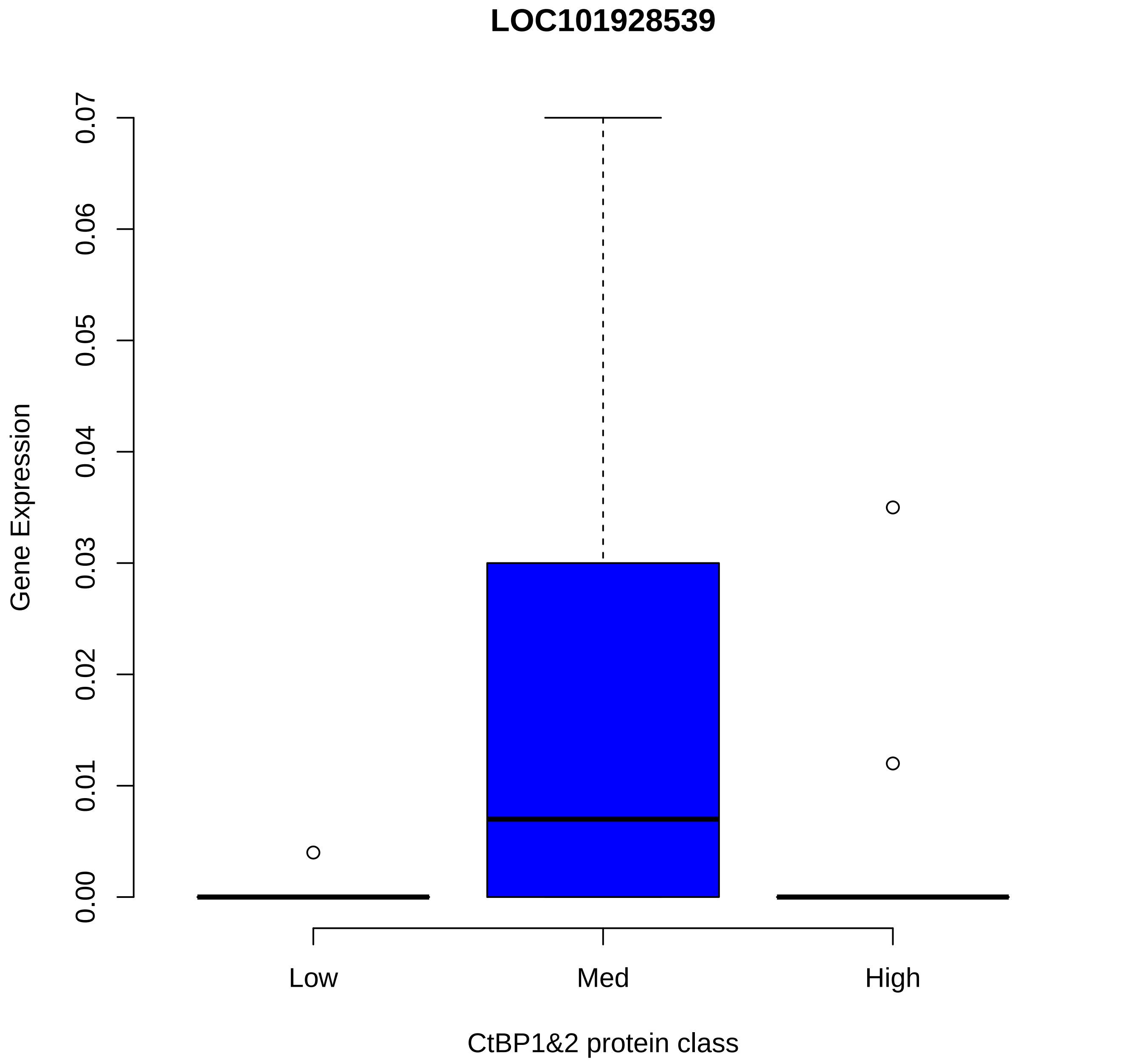

LOC101928539
Gene Expression
0.00
0.01
0.02
0.03
0.04
0.05
0.06
0.07
Low
Med
High
CtBP1&2 protein class

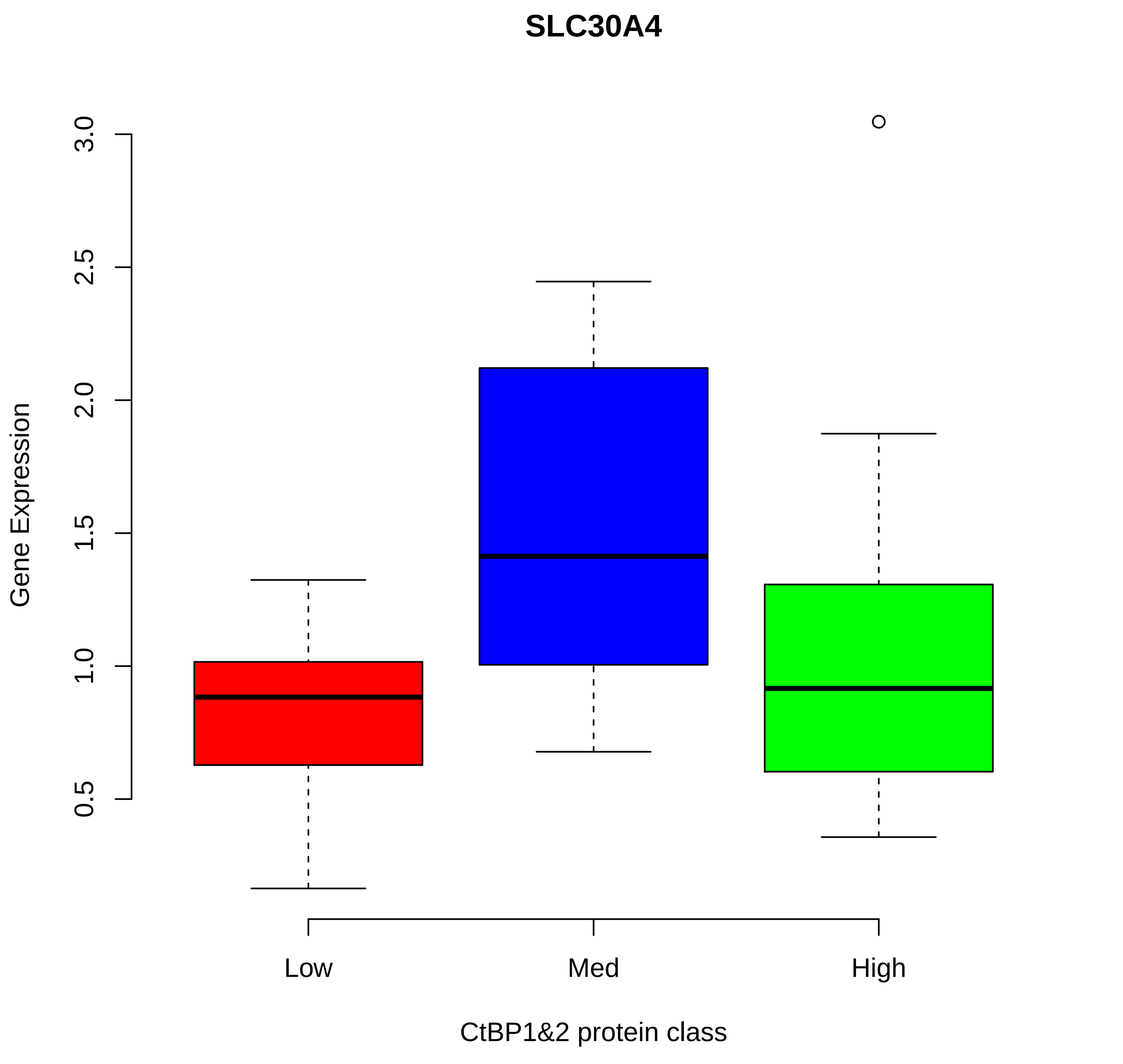
SLC30A4
Gene Expression
3.0
2.5
2.0
1.5
1.0
0.5
Low
Med
High
CtBP1&2 protein class

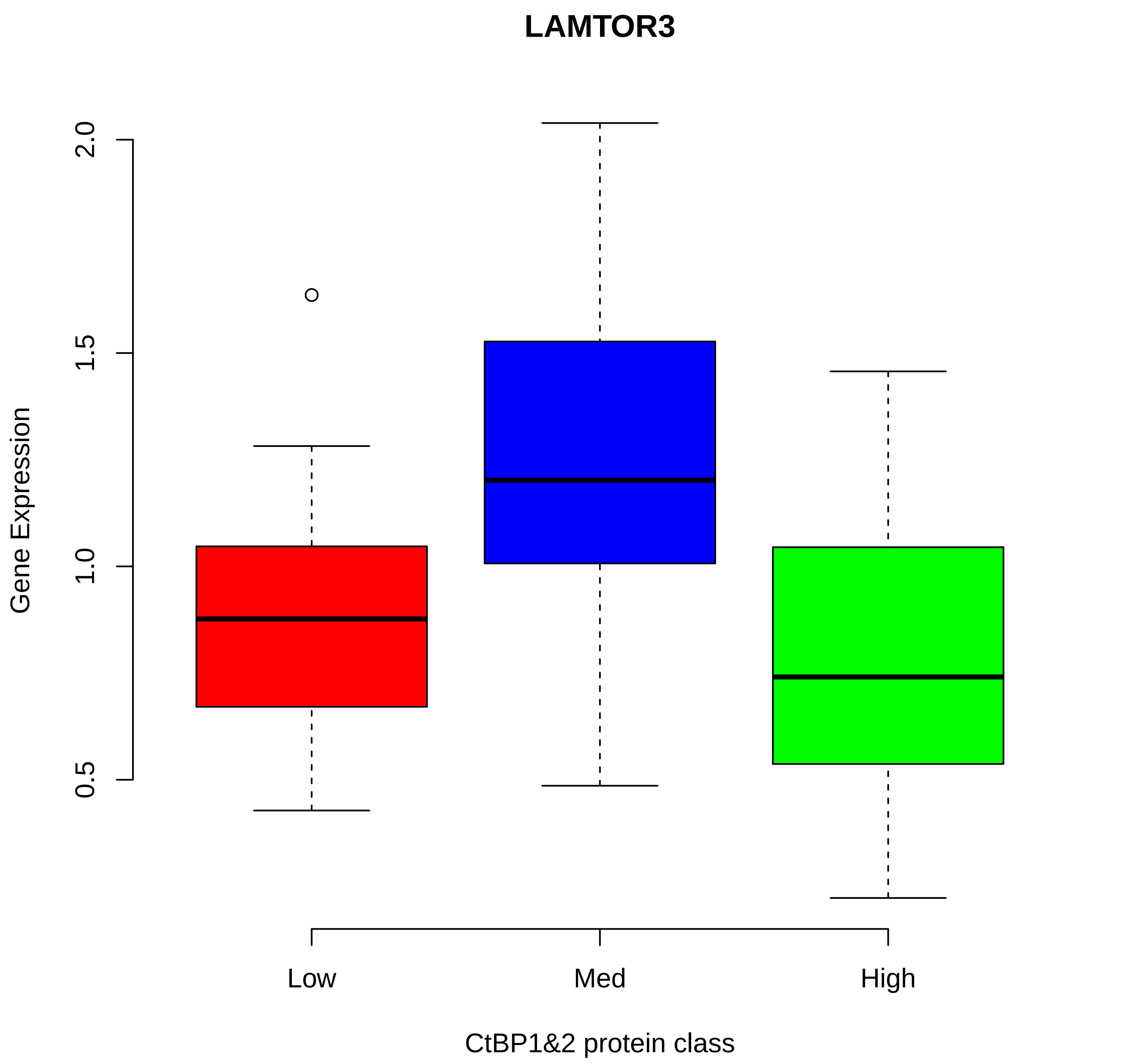
LAMTOR3
Gene Expression
2.0
1.5
1.0
0.5
Low
Med
High
CtBP1&2 protein class

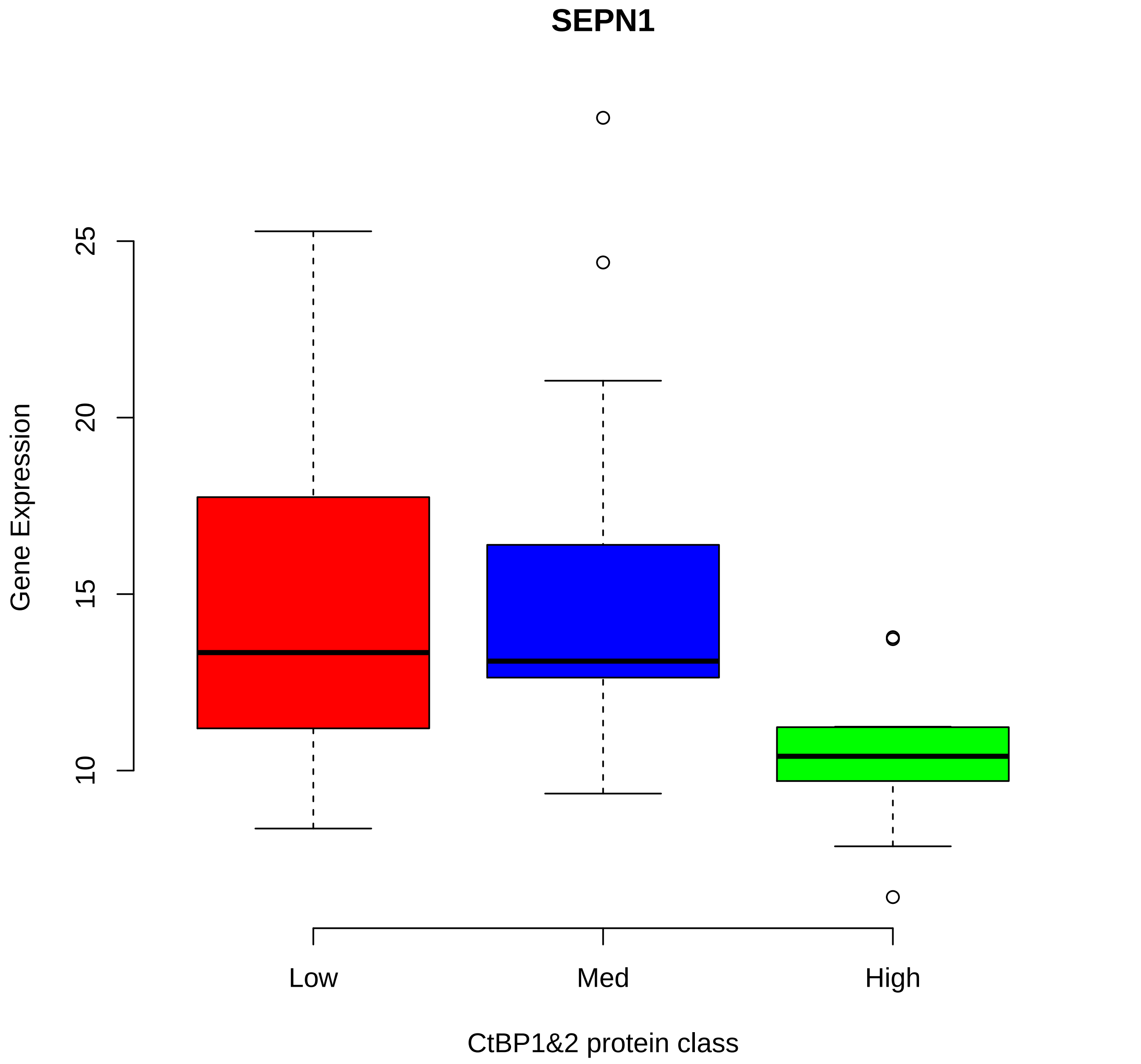

SEPN1
Gene Expression
10
15
20
25
Low
Med
High
CtBP1&2 protein class

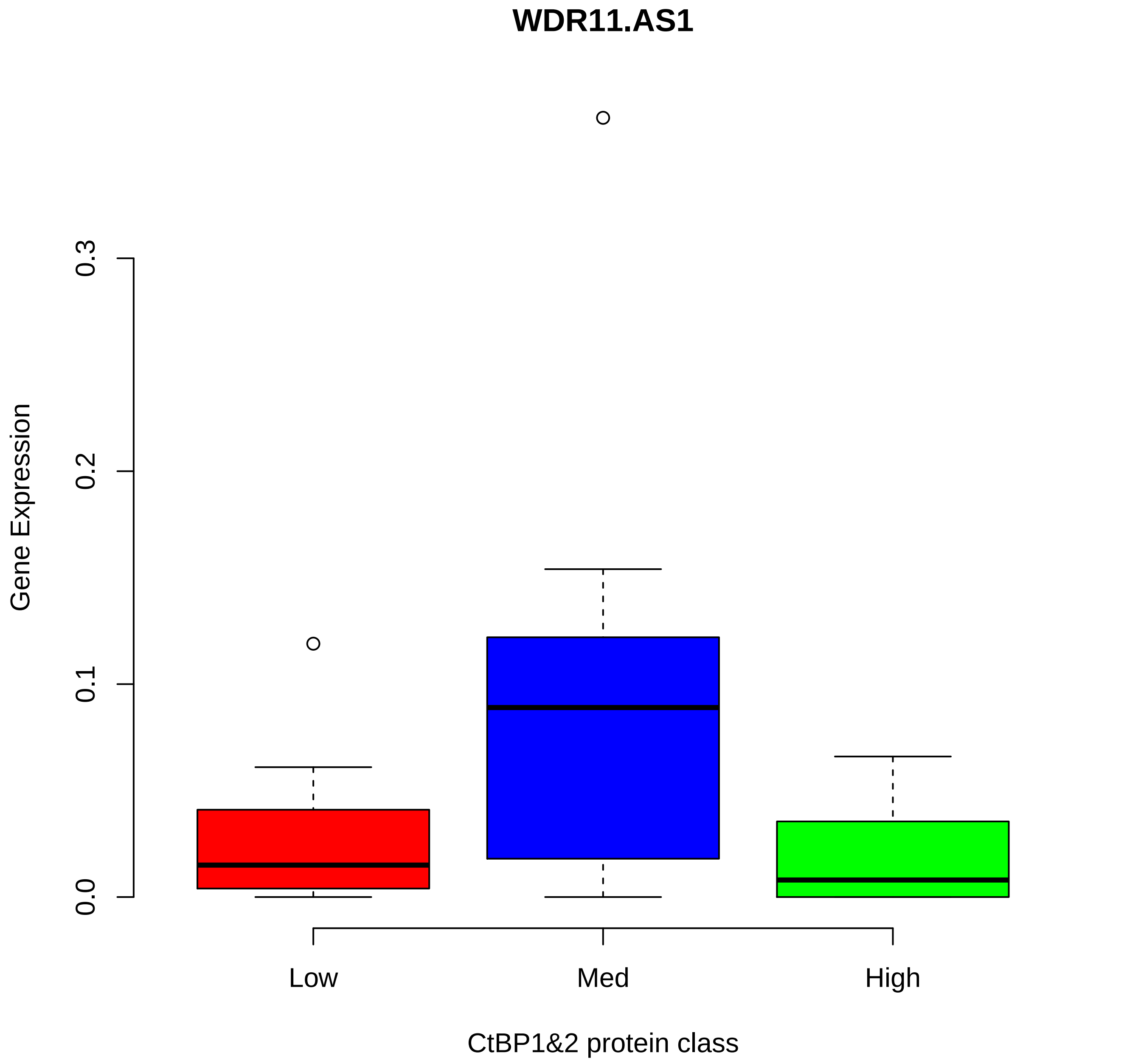
WDR11.AS1
Gene Expression
0.0
0.1
0.2
0.3
Low
Med
High
CtBP1&2 protein class

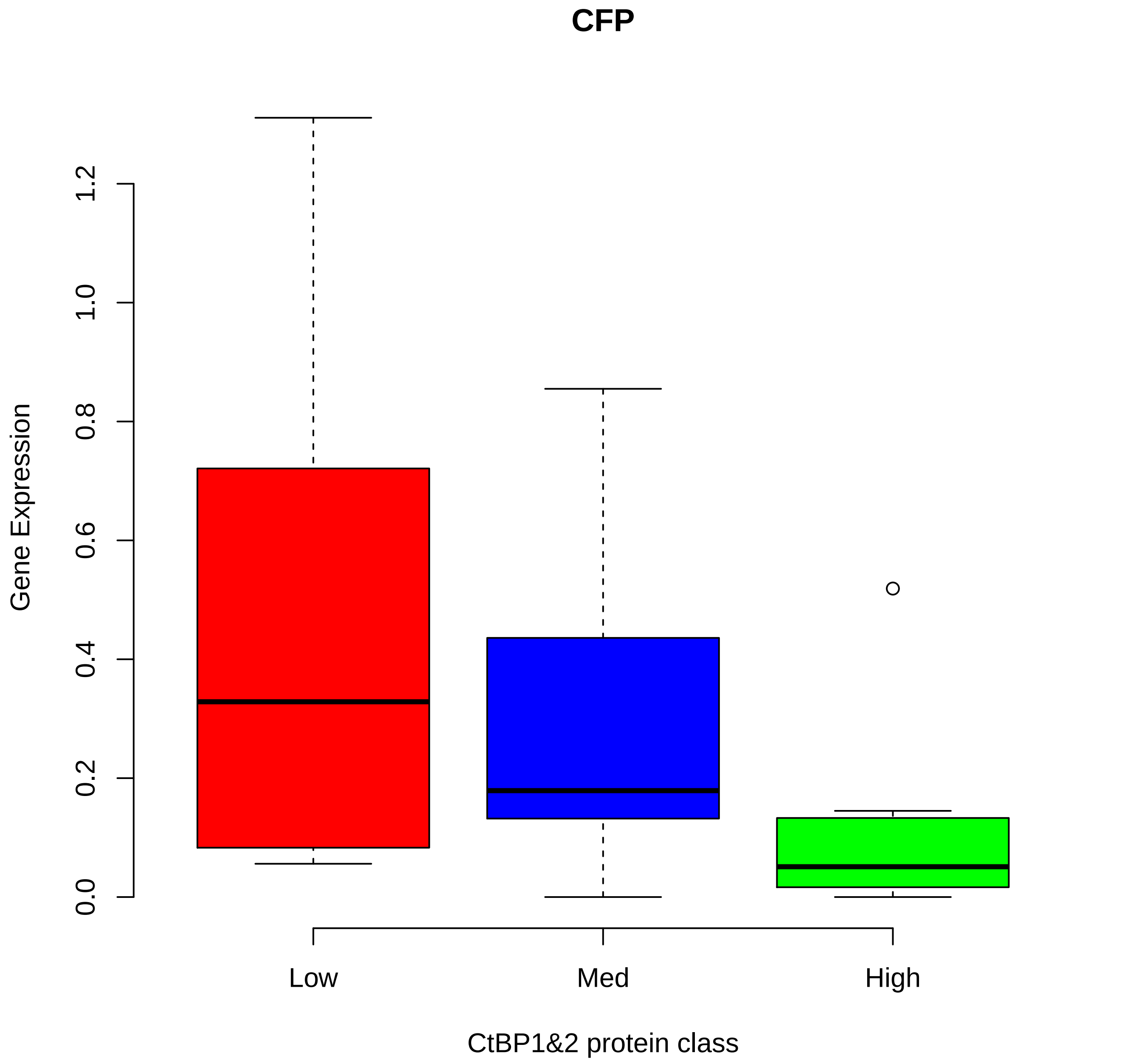

CFP
Gene Expression
0.0
0.2
0.4
0.6
0.8
1.0
1.2
Low
Med
High
CtBP1&2 protein class

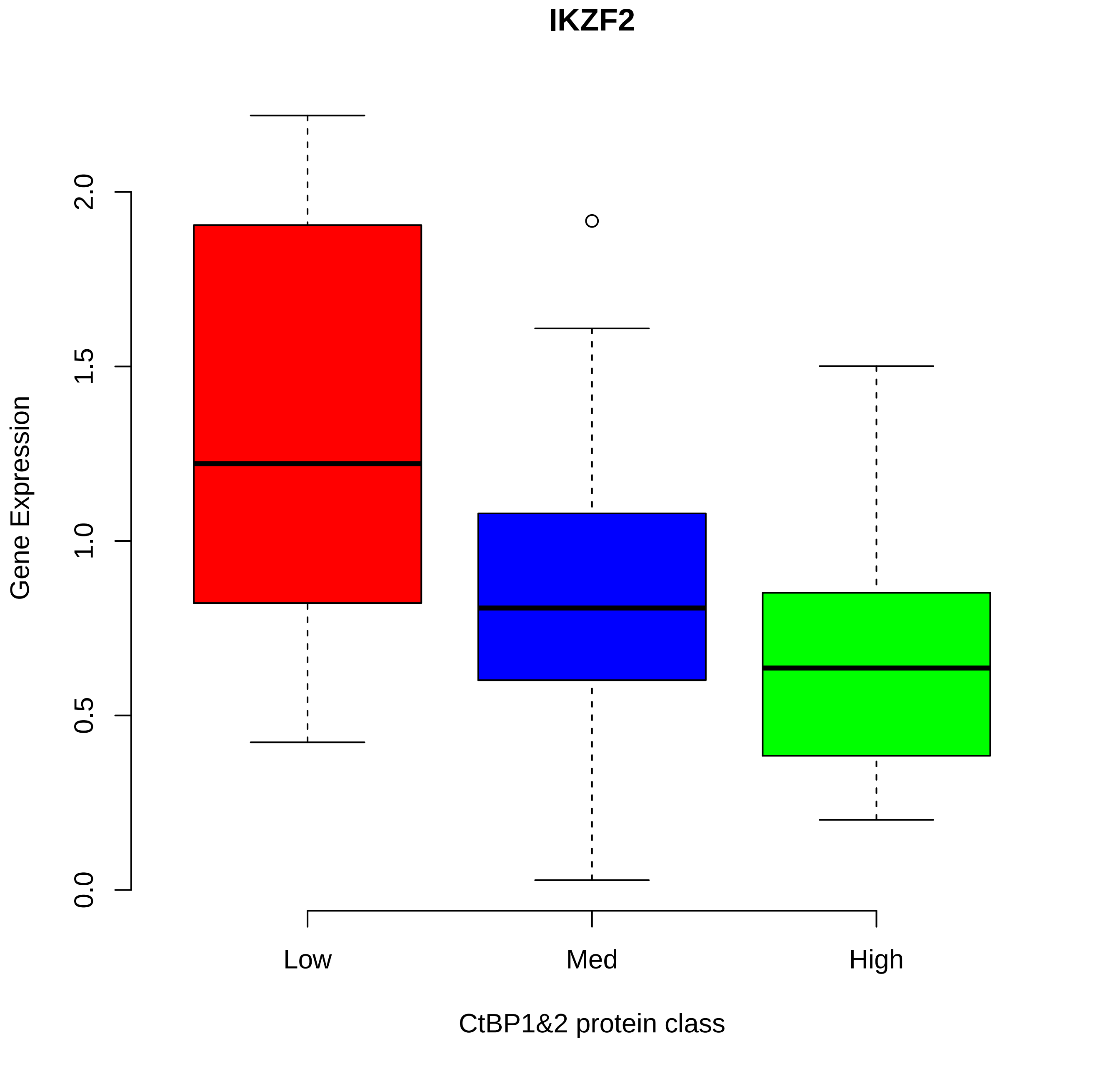
IKZF2
Gene Expression
0.0
0.5
1.0
1.5
2.0
Low
Med
High
CtBP1&2 protein class

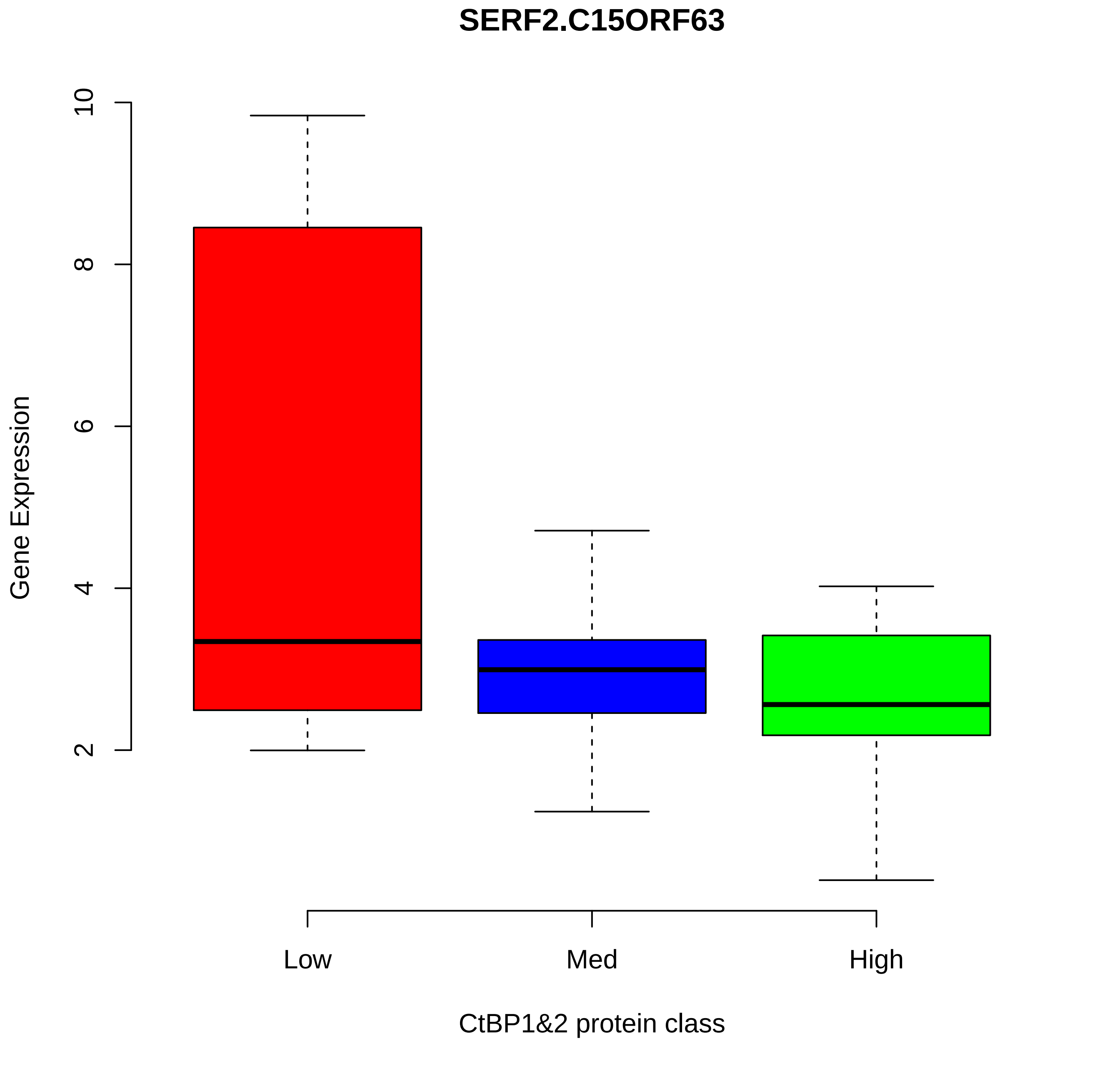

SERF2.C15ORF63
Gene Expression
2
4
6
8
10
Low
Med
High
CtBP1&2 protein class

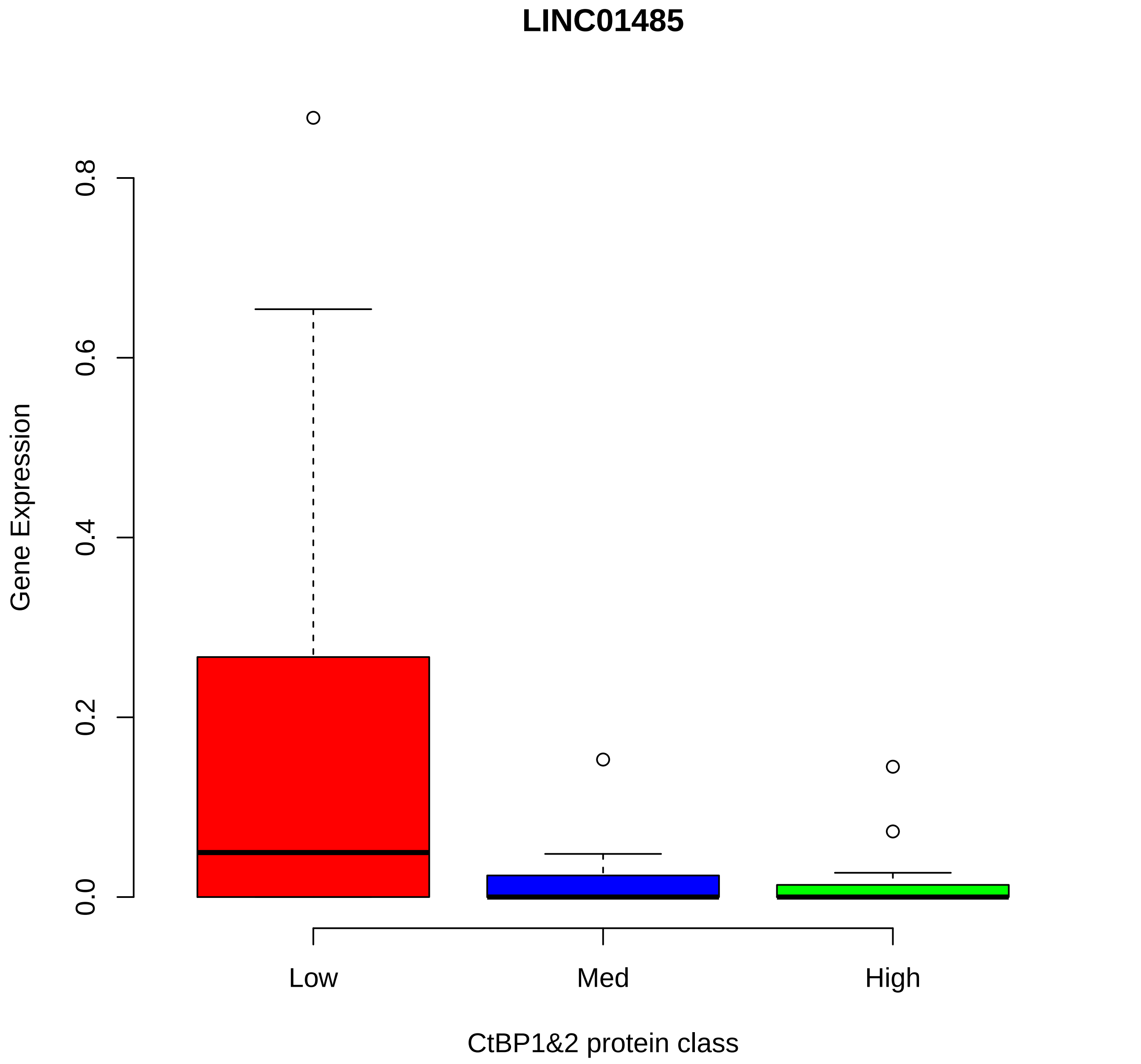
LINC01485
Gene Expression
0.0
0.2
0.4
0.6
0.8
Low
Med
High
CtBP1&2 protein class

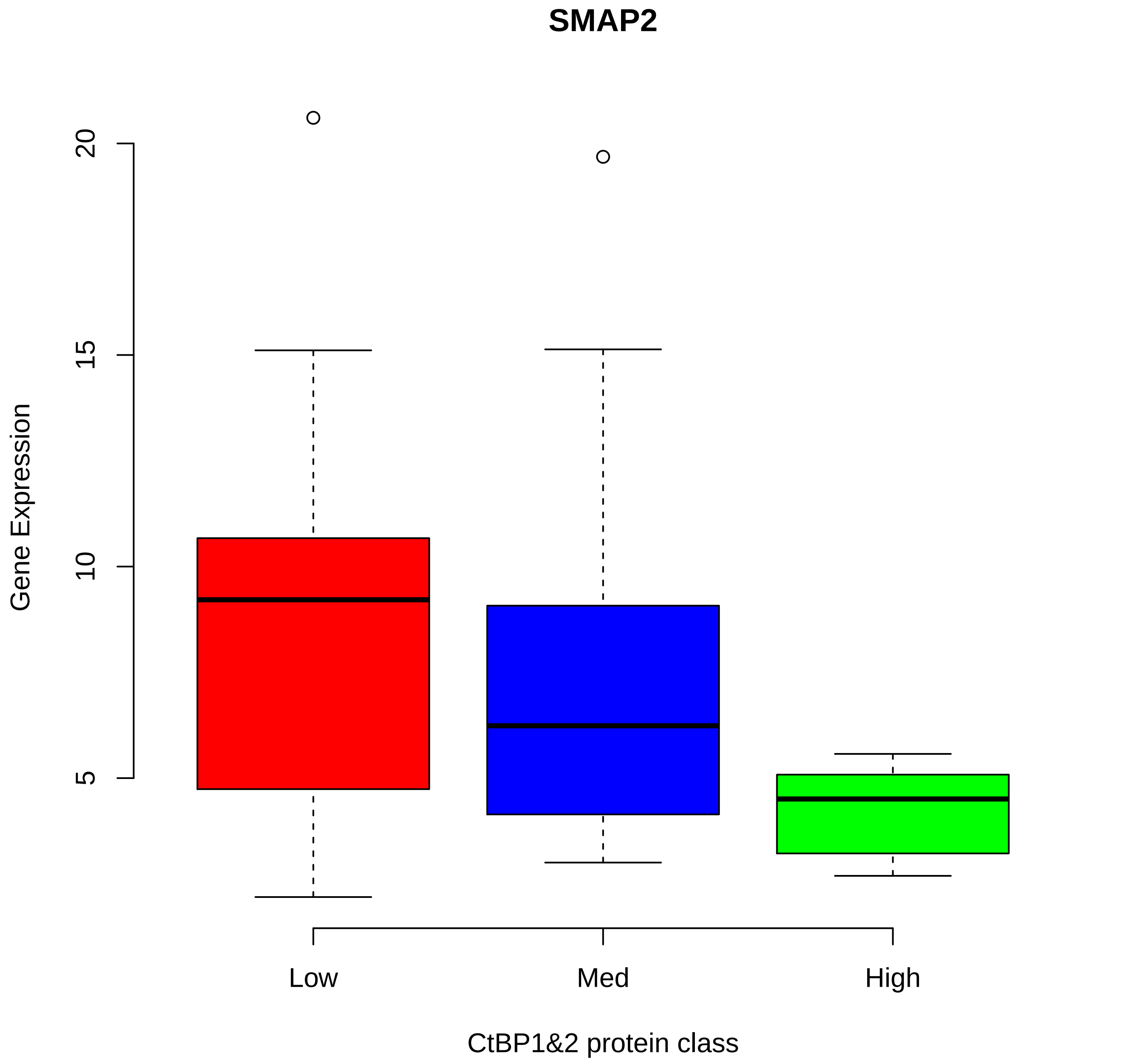
SMAP2
Gene Expression
20
15
10
5
Low
Med
High
CtBP1&2 protein class

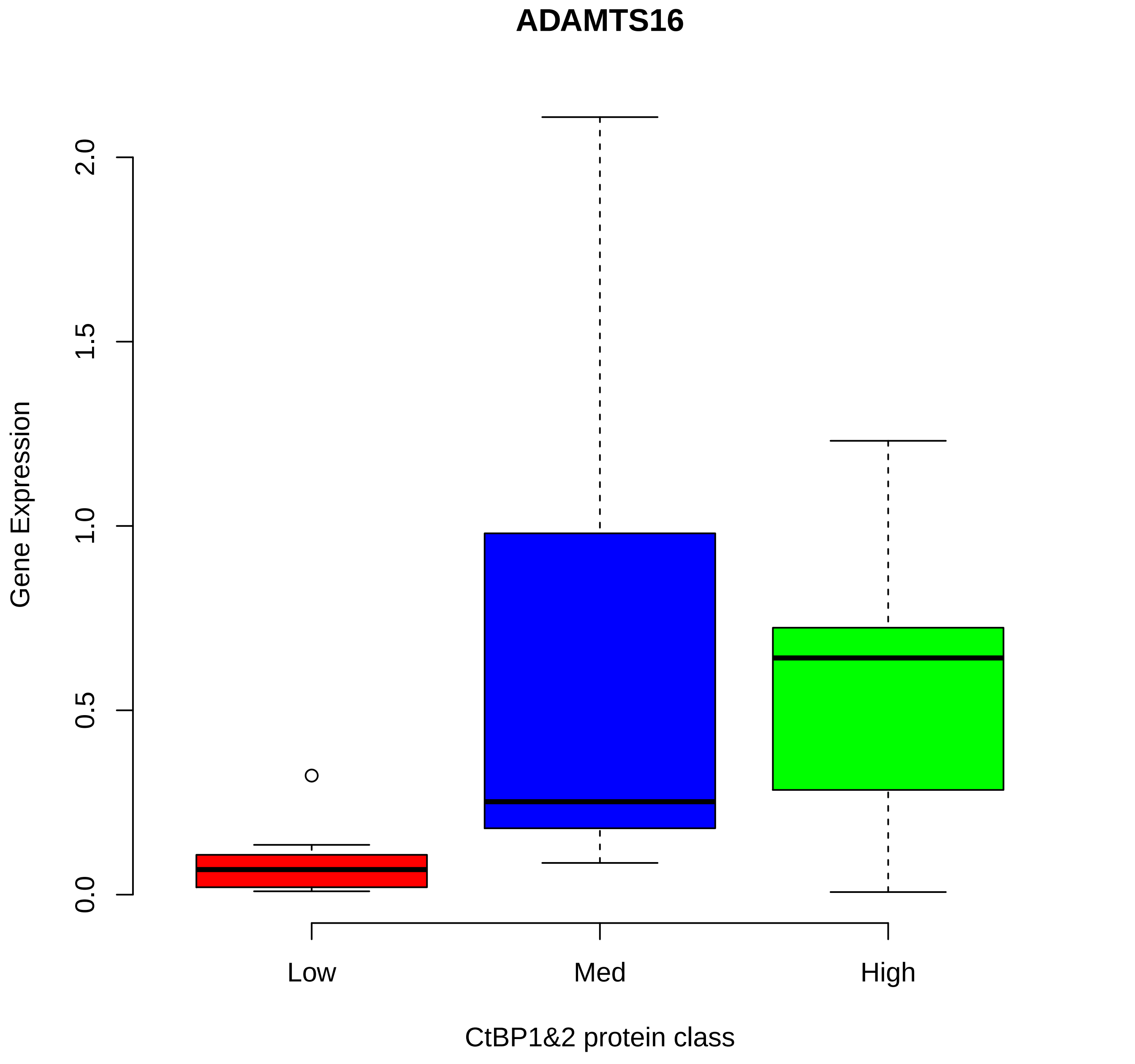

ADAMTS16
Gene Expression
0.0
0.5
1.0
1.5
2.0
Low
Med
High
CtBP1&2 protein class

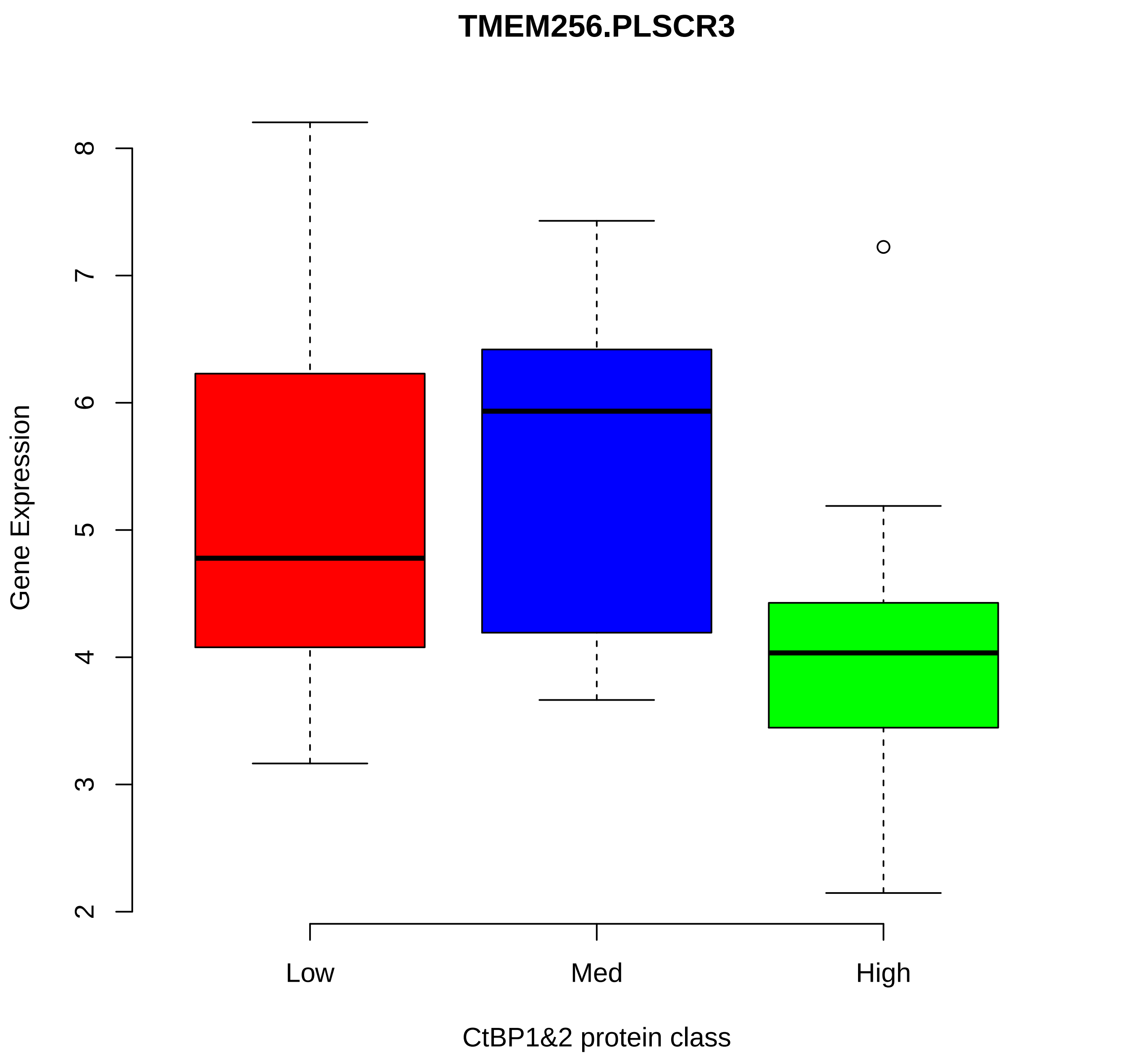

TMEM256.PLSCR3
Gene Expression
2
3
4
5
6
7
8
Low
Med
High
CtBP1&2 protein class

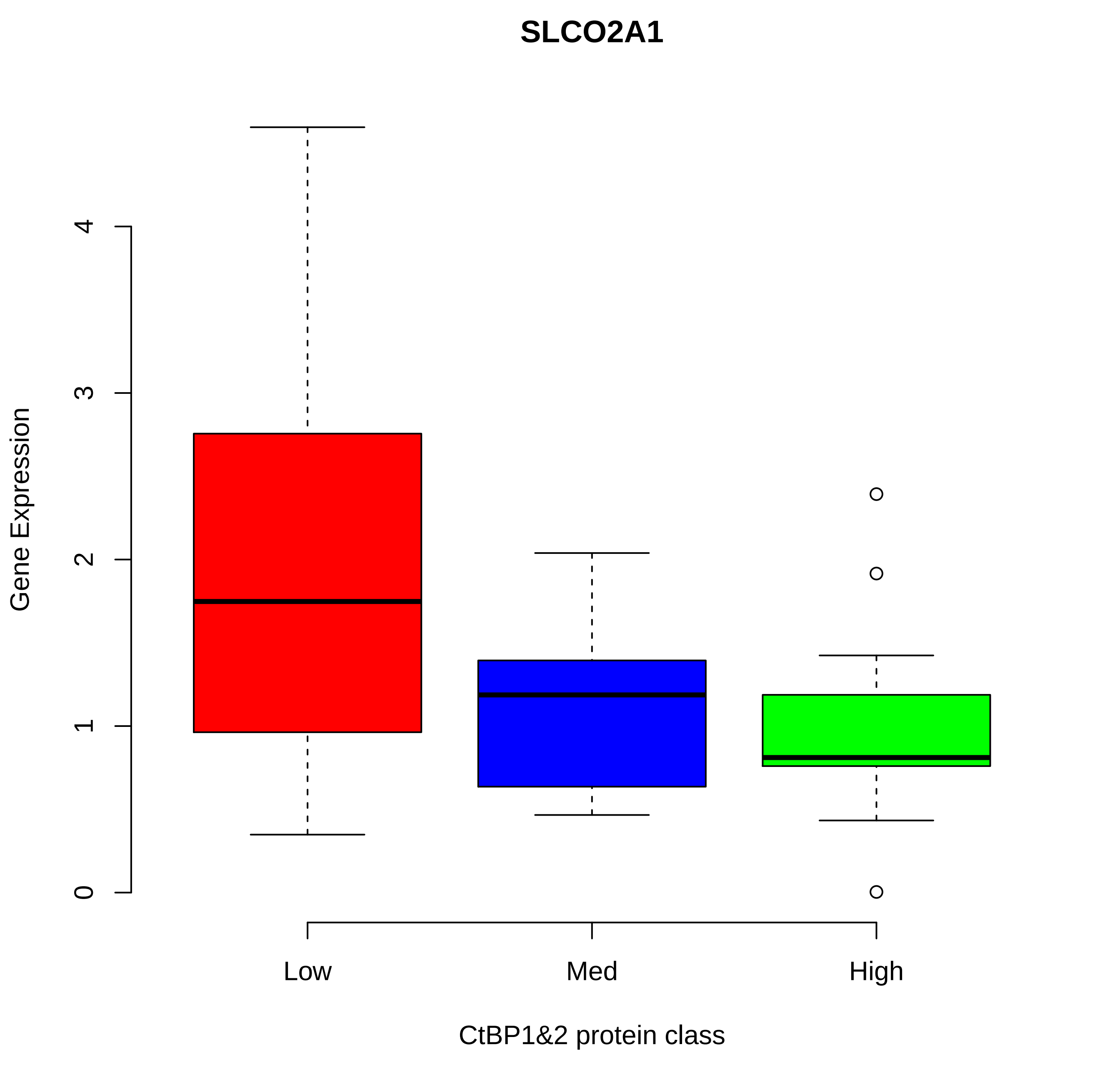

SLCO2A1
Gene Expression
0
1
2
3
4
Low
Med
High
CtBP1&2 protein class

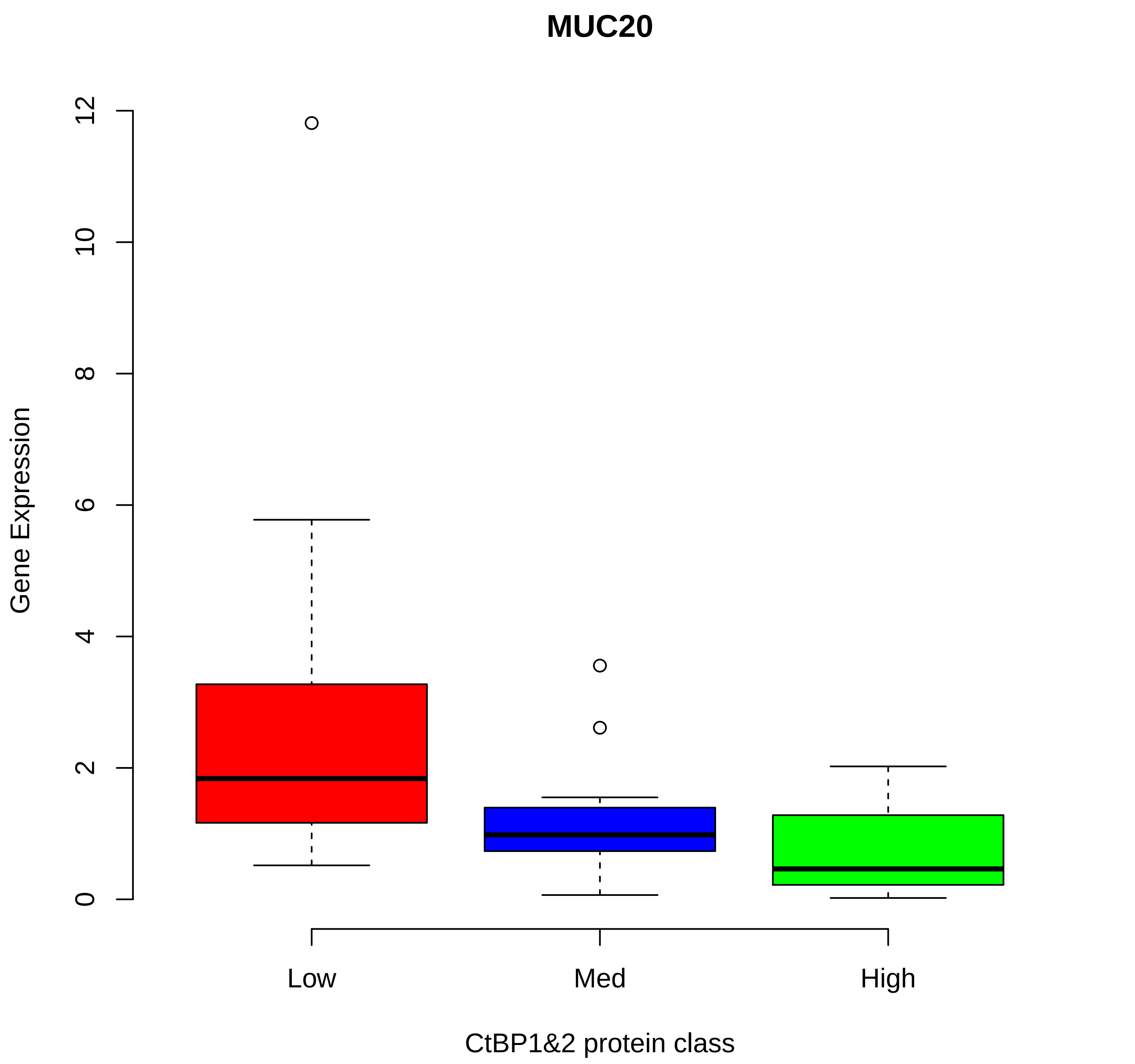
MUC20
Gene Expression
0
2
4
6
8
10
12
Low
Med
High
CtBP1&2 protein class

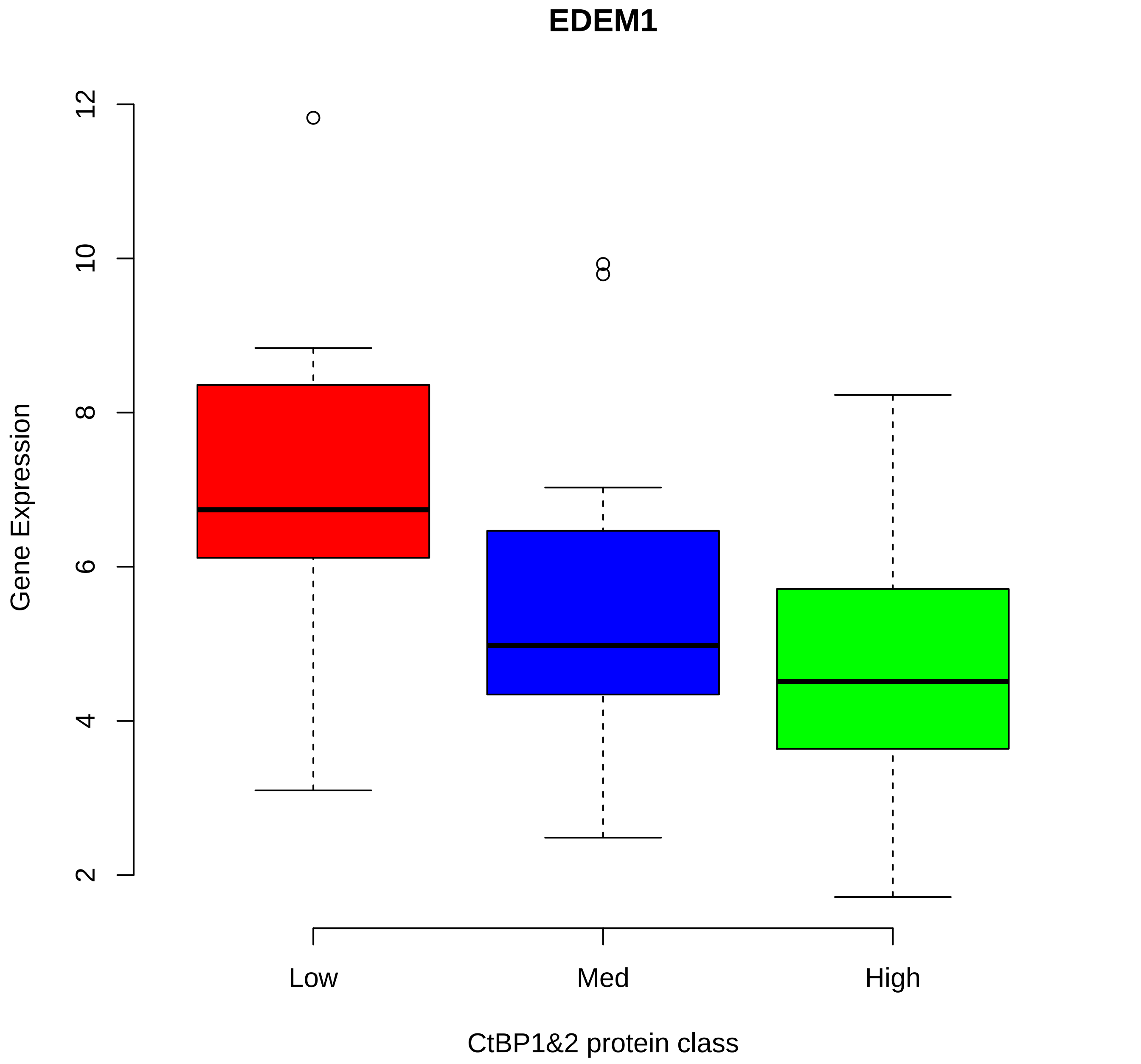

EDEM1
Gene Expression
12
10
8
6
4
2
Low
Med
High
CtBP1&2 protein class

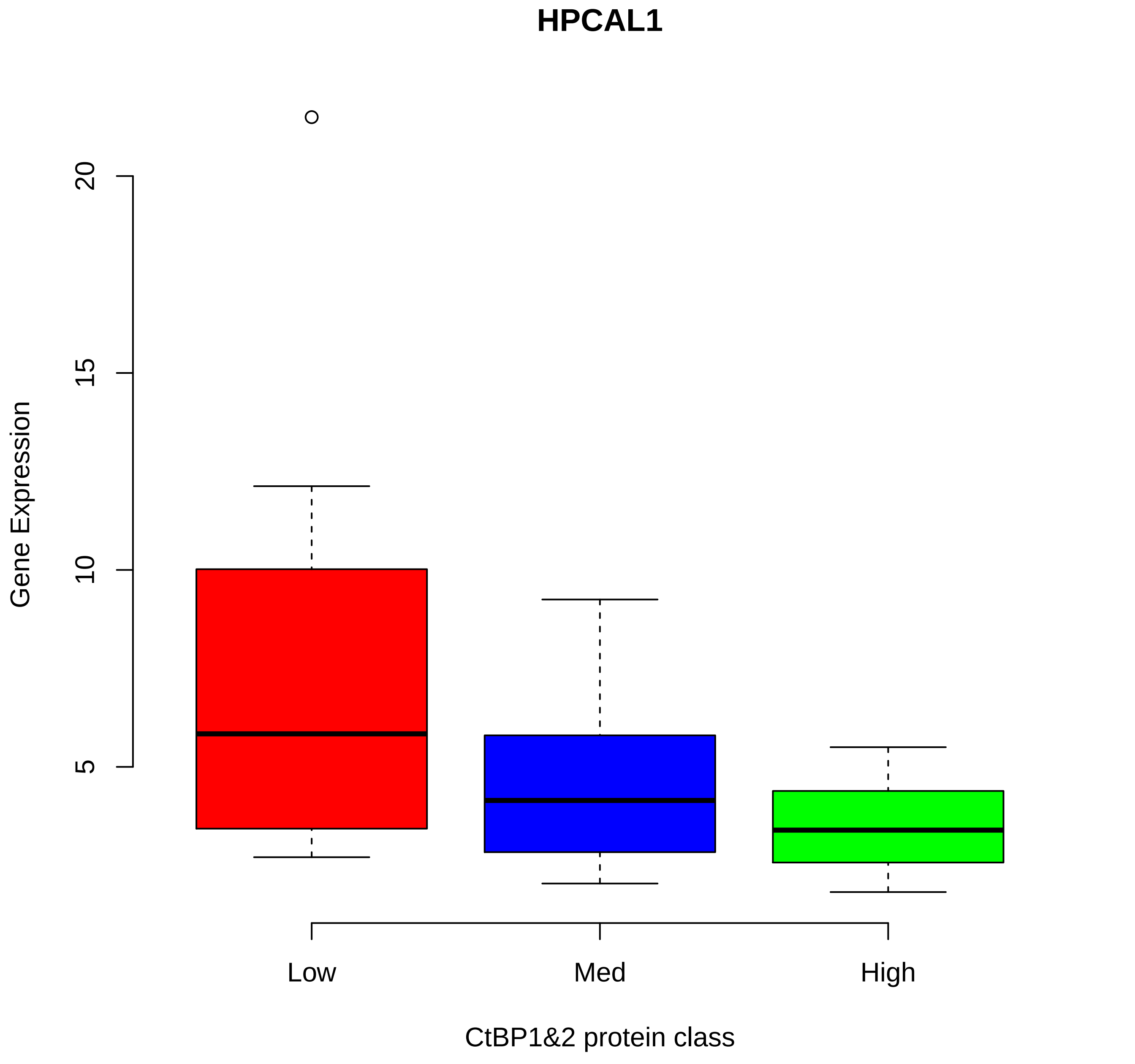

HPCAL1
20
15
10
5
Gene Expression
Low
Med
High
CtBP1&2 protein class

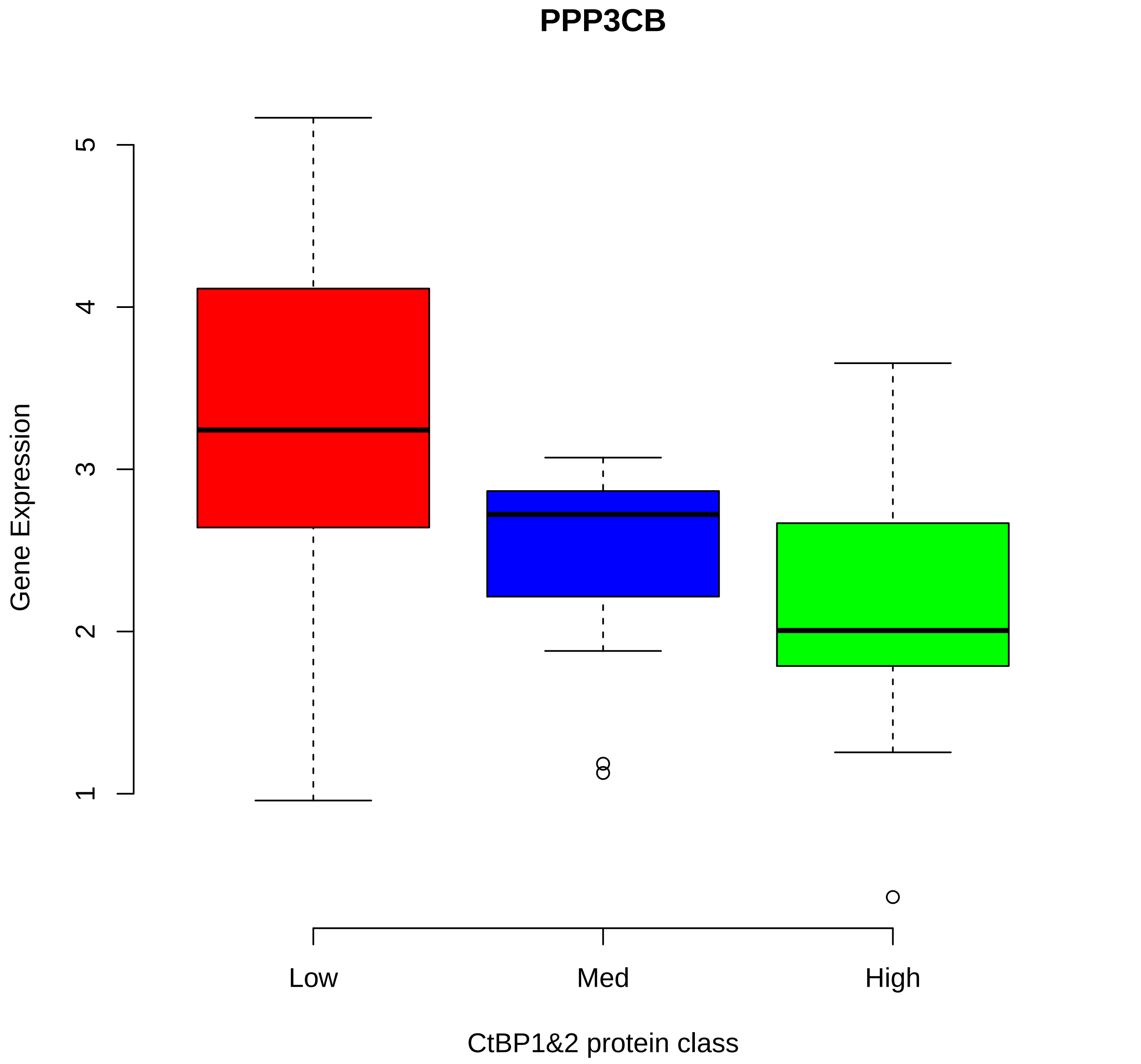
PPP3CB
Gene Expression
1
2
3
4
5
Low
Med
High
CtBP1&2 protein class

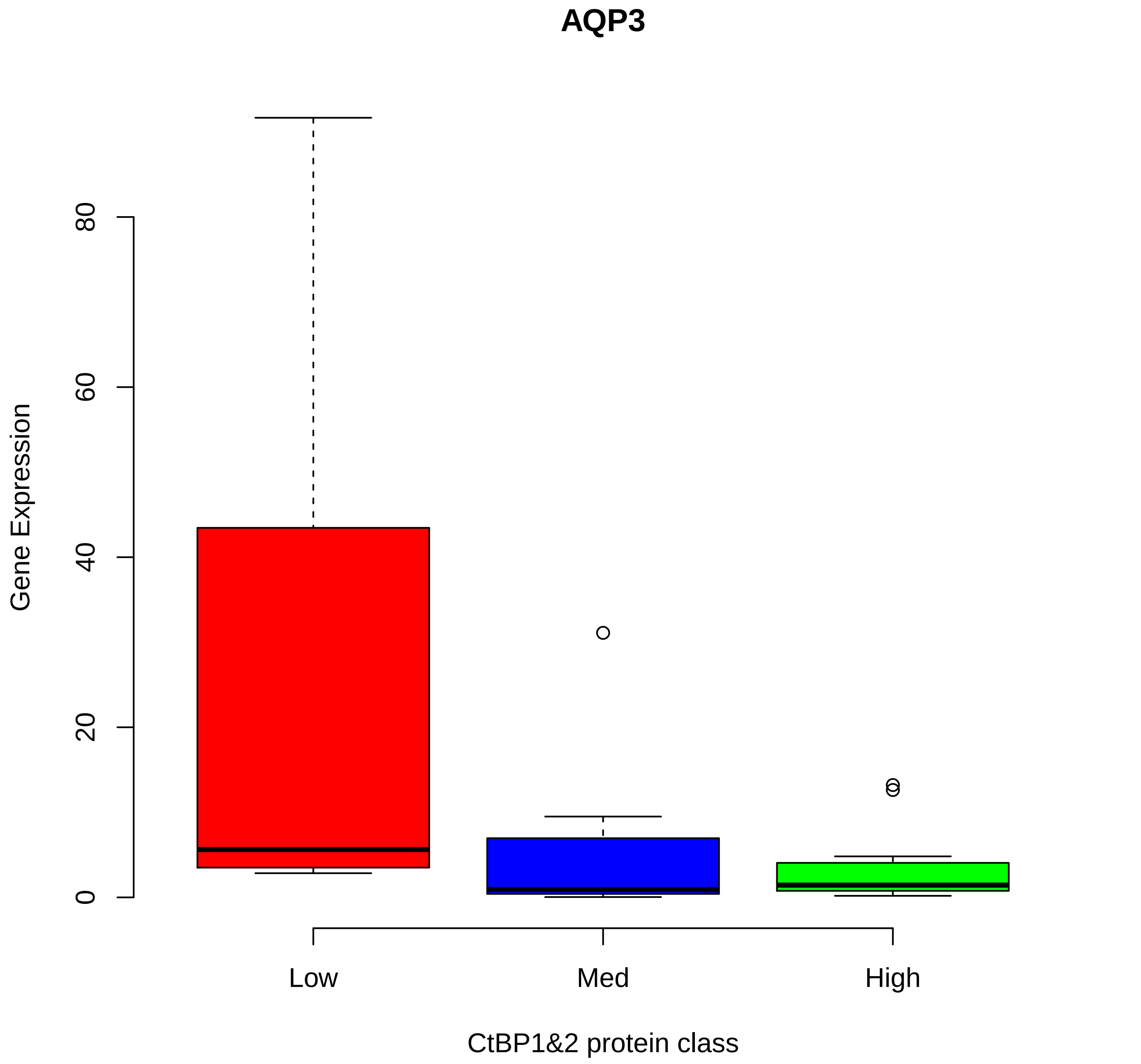

AQP3
Gene Expression
0
20
40
60
80
Low
Med
High
CtBP1&2 protein class

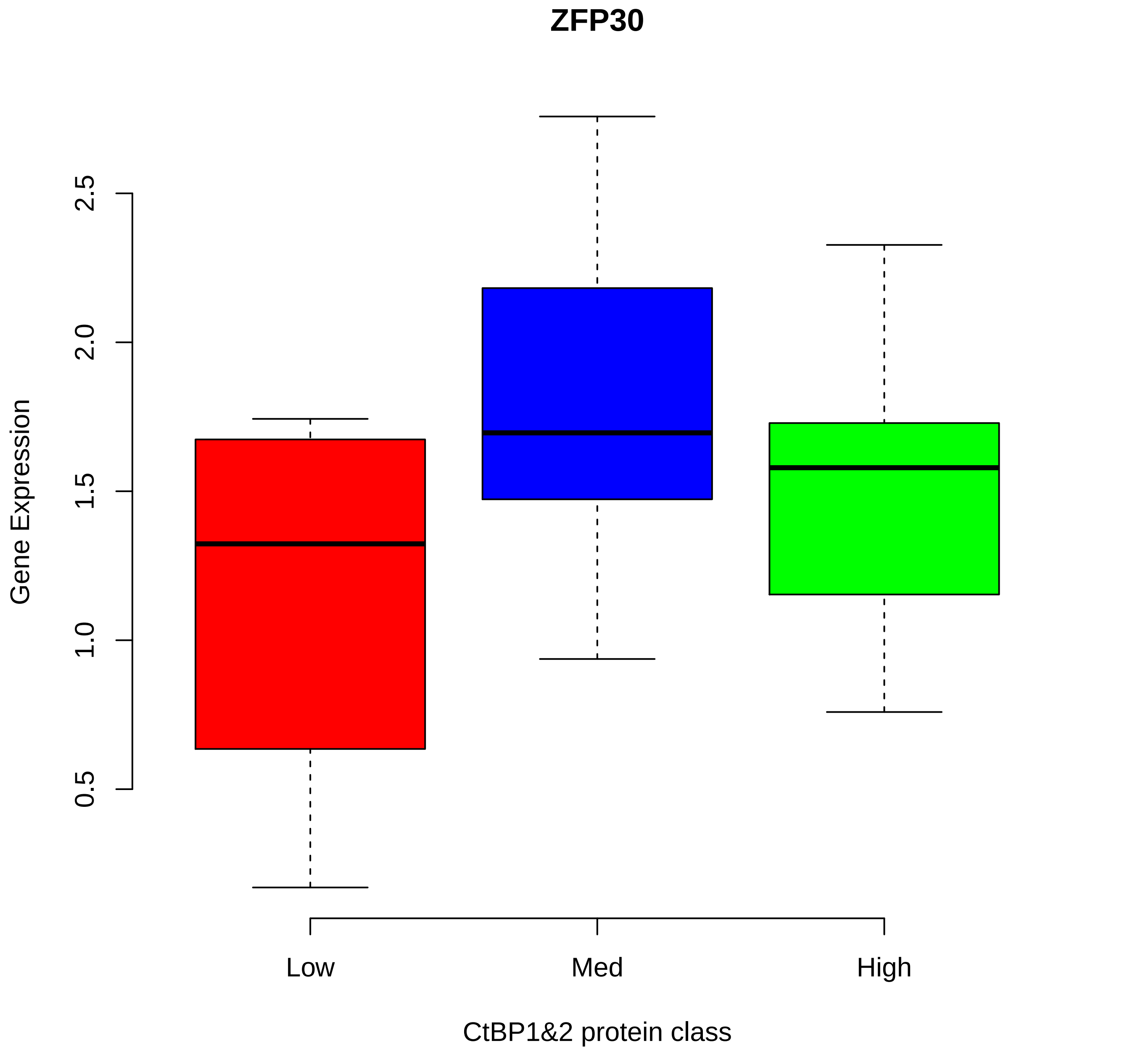
ZFP30
Gene Expression
0.5
1.0
1.5
2.0
2.5
Low
Med
High
CtBP1&2 protein class

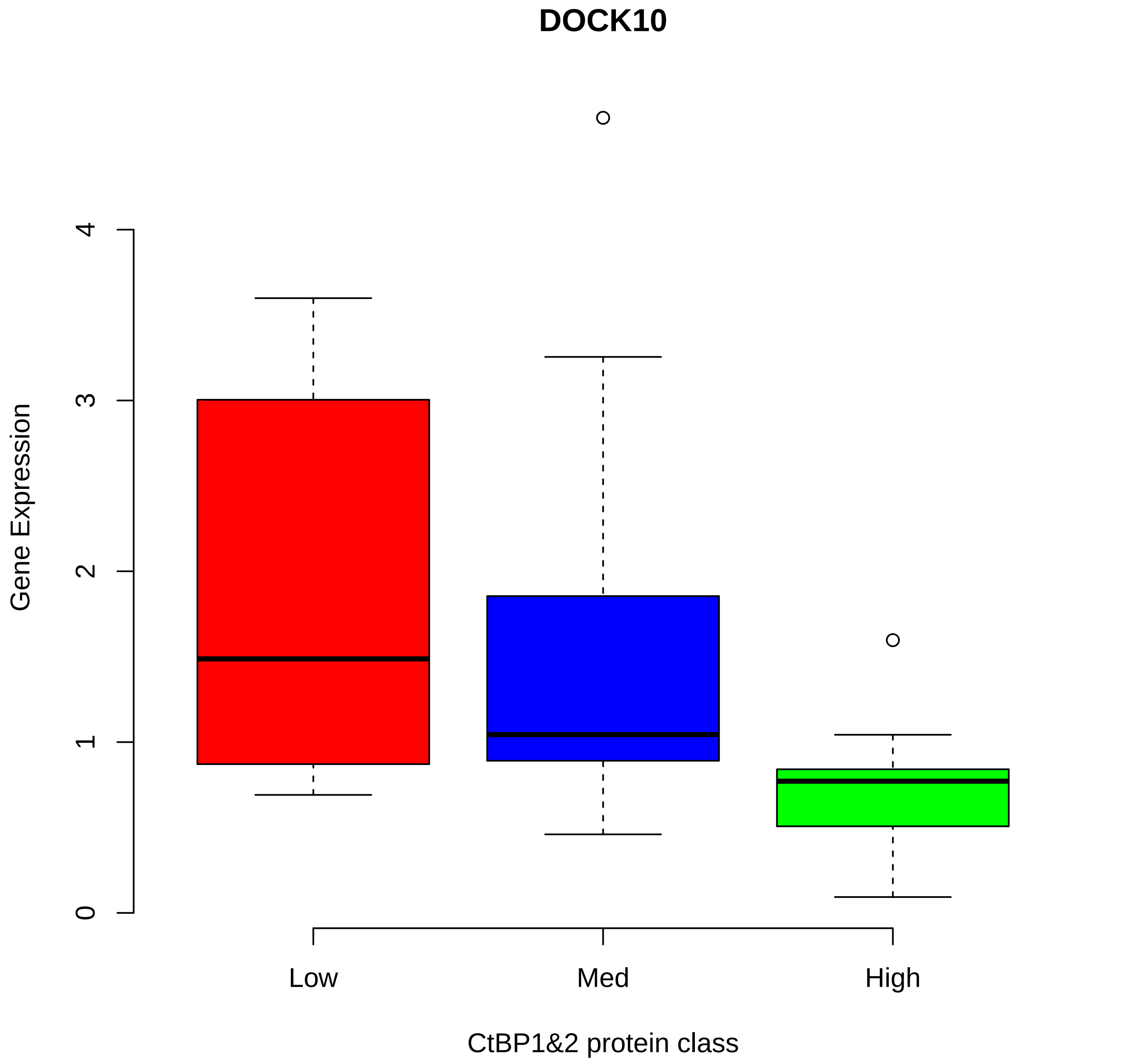
DOCK10
Gene Expression
0
1
2
3
4
Low
Med
High
CtBP1&2 protein class

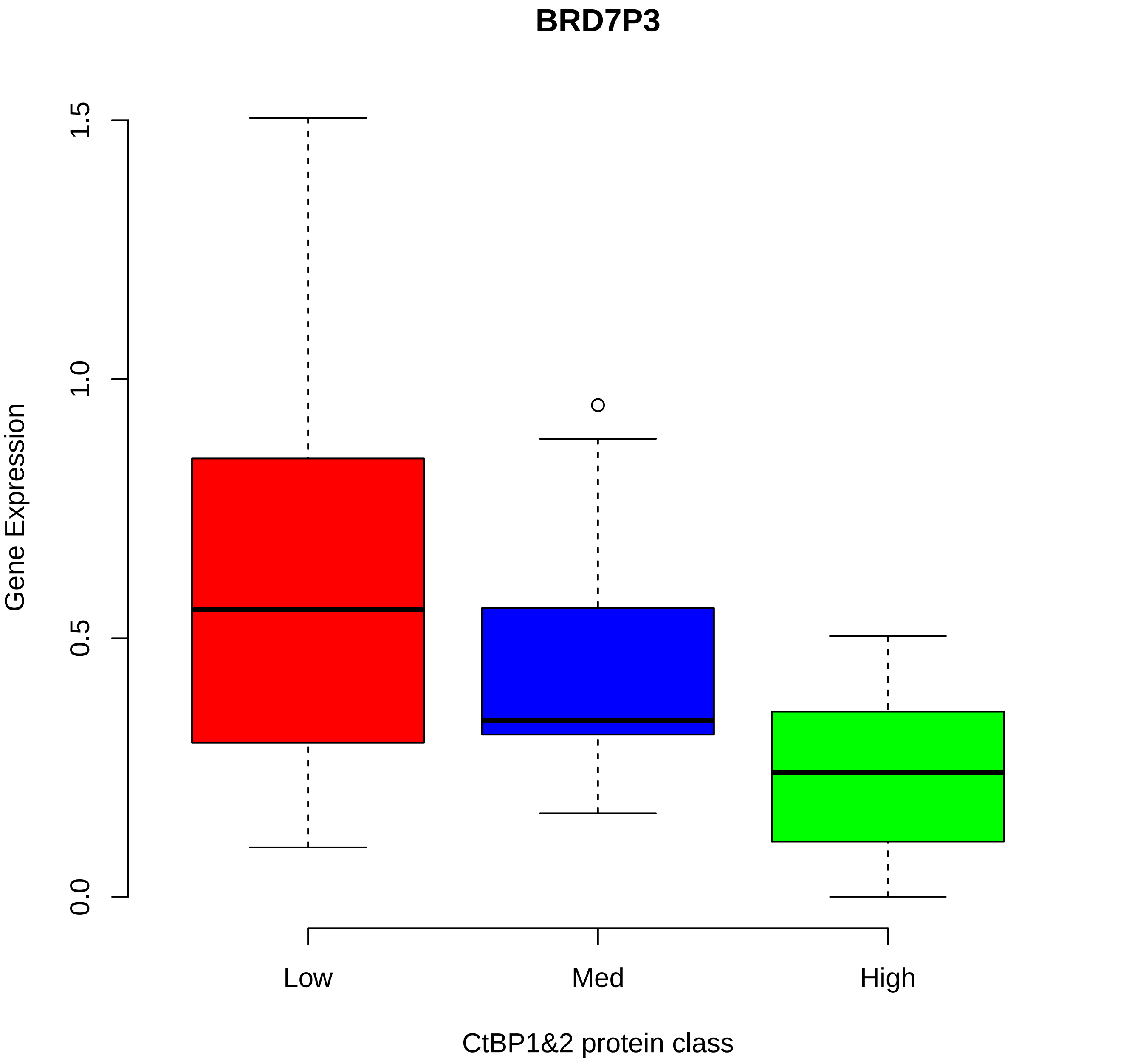

BRD7P3
Gene Expression
1.5
1.0
0.5
0.0
Low
Med
High
CtBP1&2 protein class

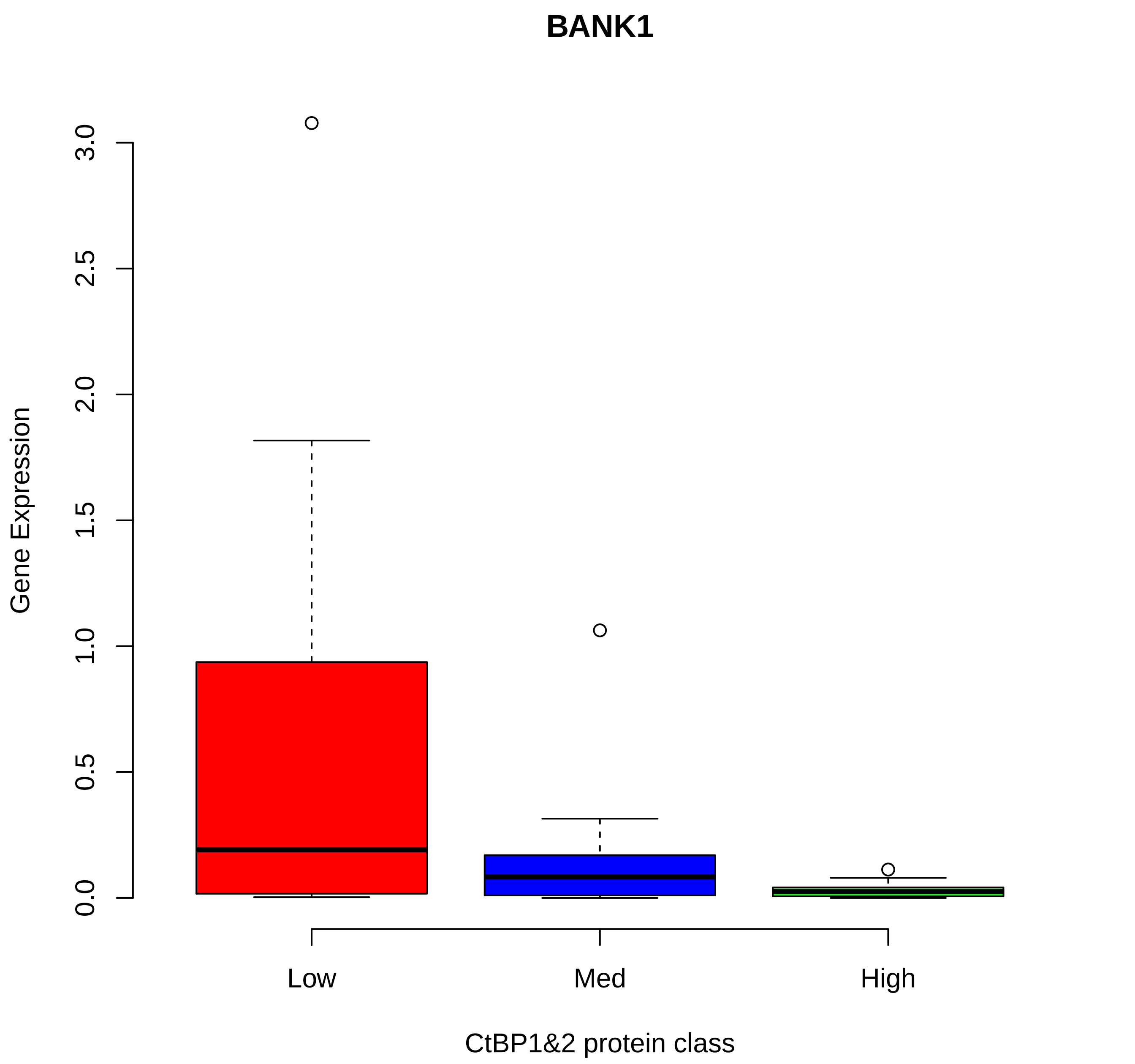
BANK1
Gene Expression
0.0
0.5
1.0
1.5
2.0
2.5
3.0
Low
Med
High
CtBP1&2 protein class

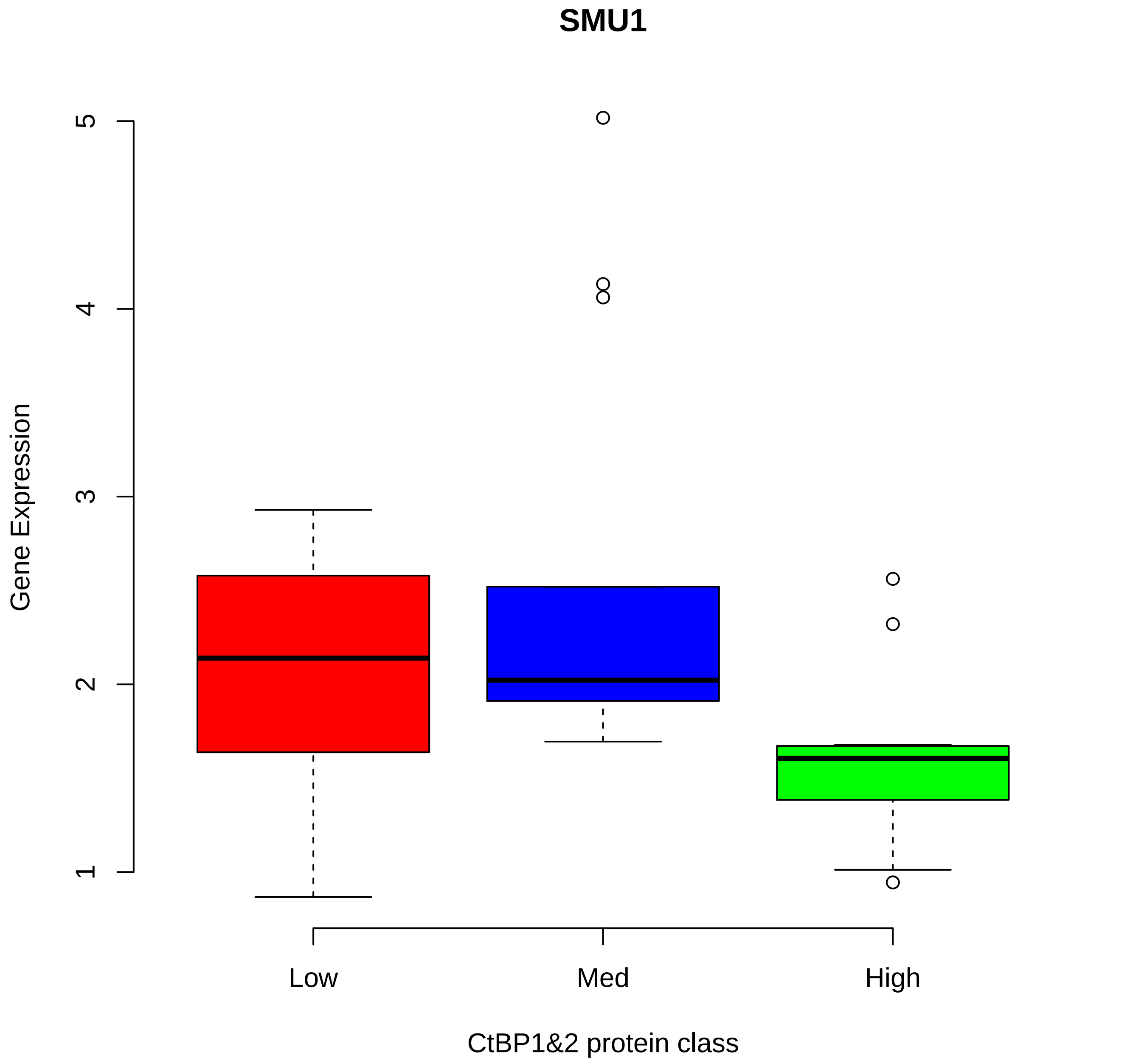

SMU1
Gene Expression
1
2
3
4
5
Low
Med
High
CtBP1&2 protein class

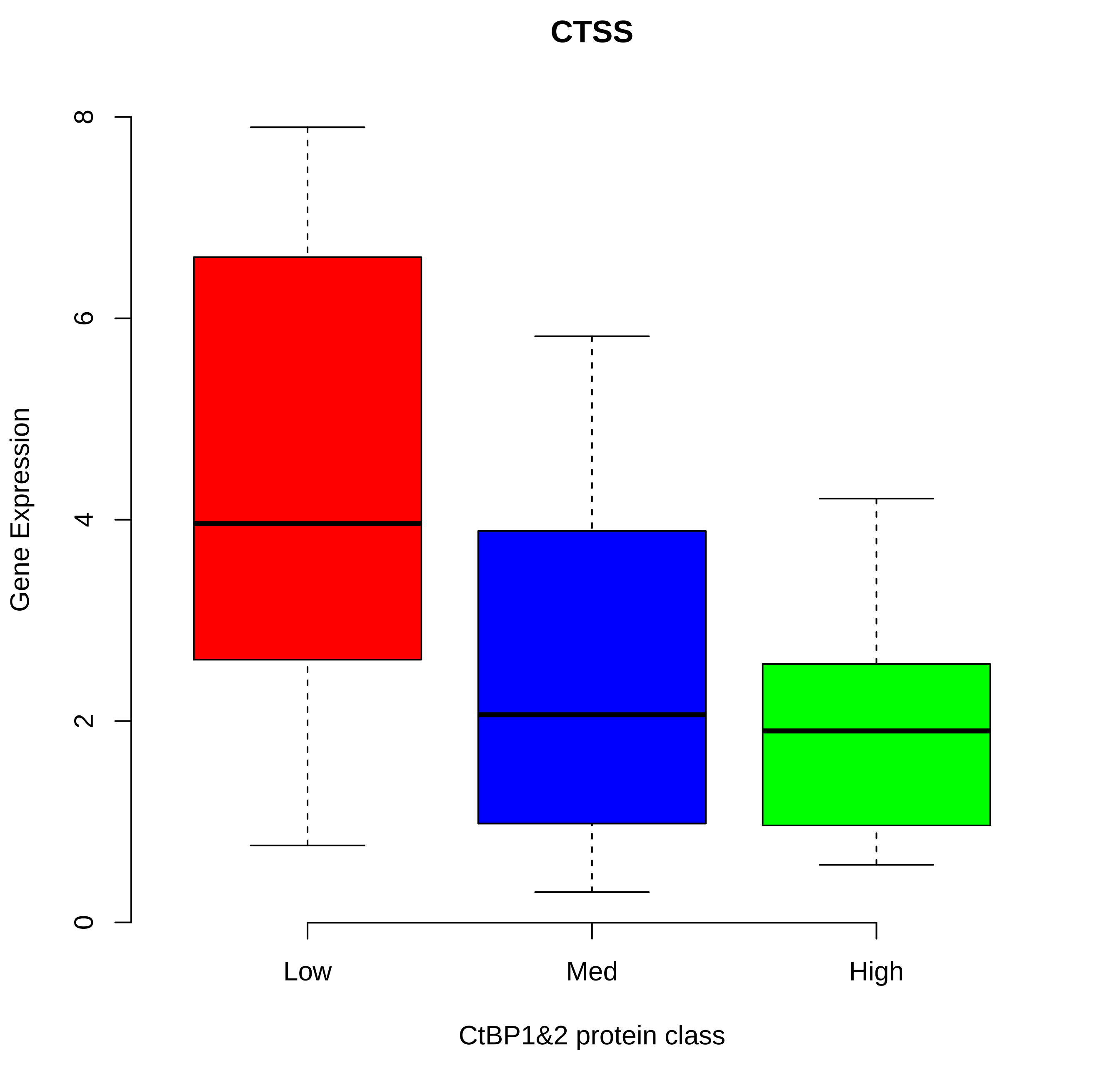

CTSS
Gene Expression
0
2
4
6
8
Low
Med
High
CtBP1&2 protein class

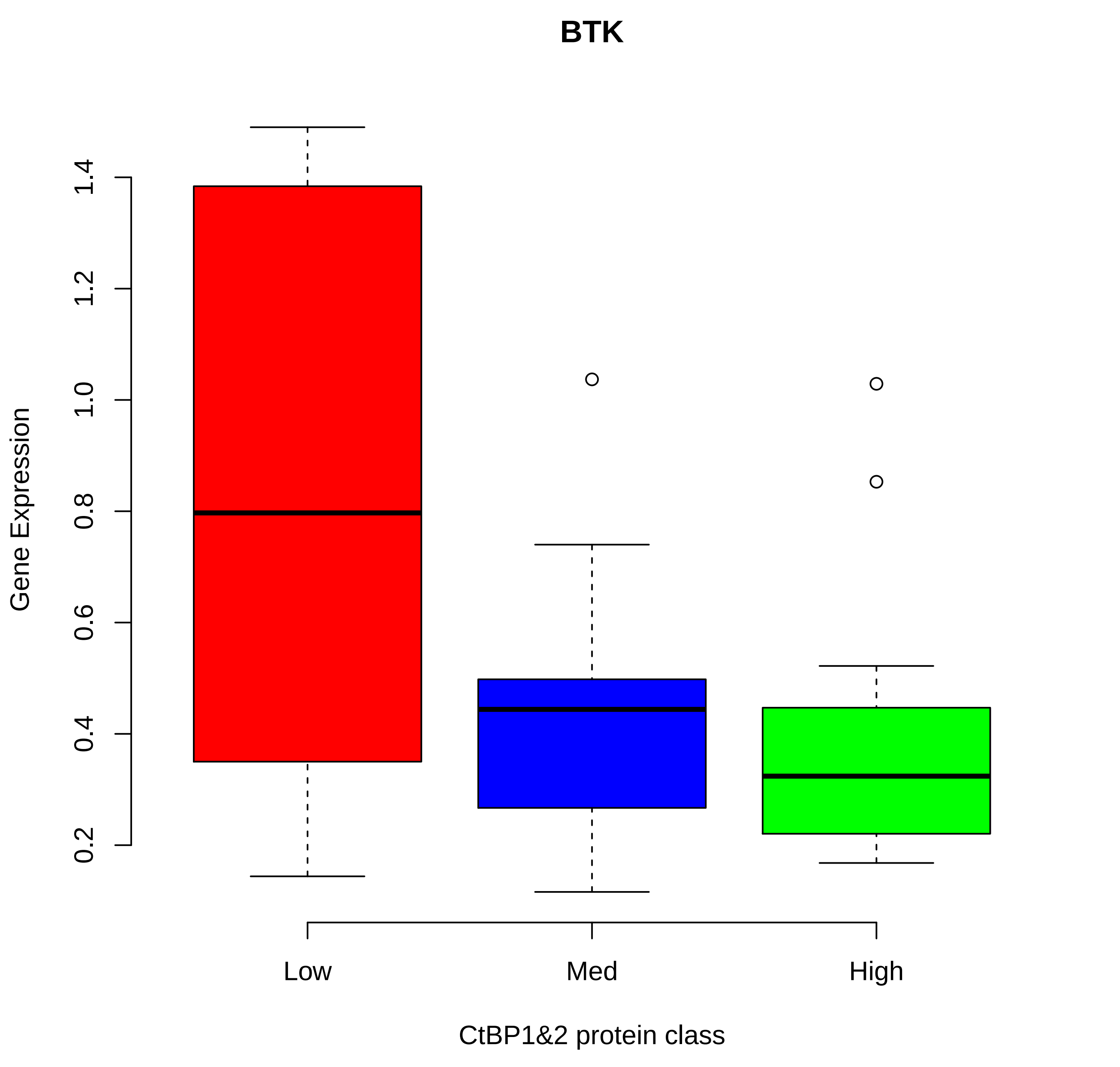
BTK
Gene Expression
0.2
0.4
0.6
0.8
1.0
1.2
1.4
Low
Med
High
CtBP1&2 protein class

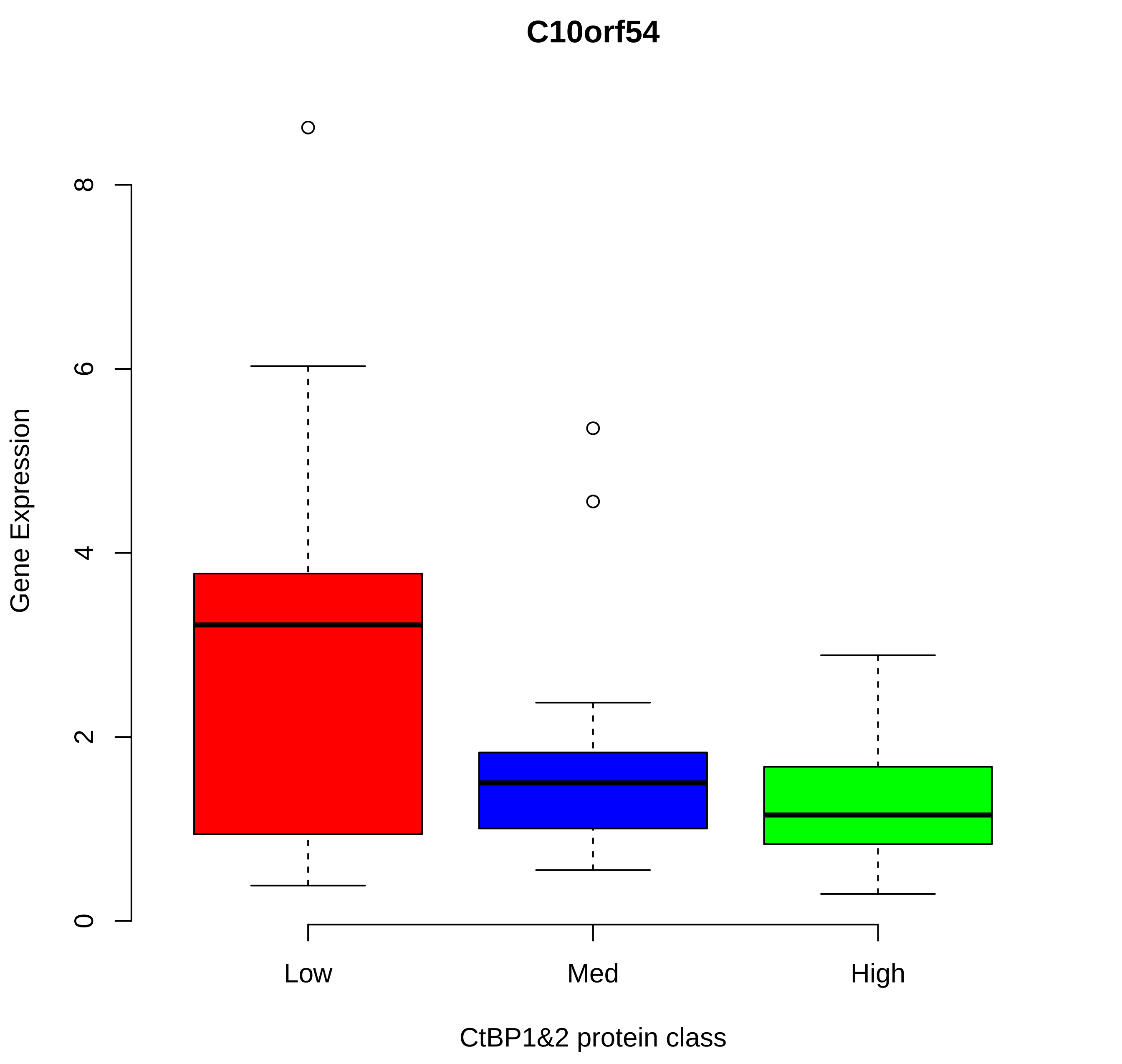

C10orf54
Gene Expression
0
2
4
6
8
Low
Med
High
CtBP1&2 protein class

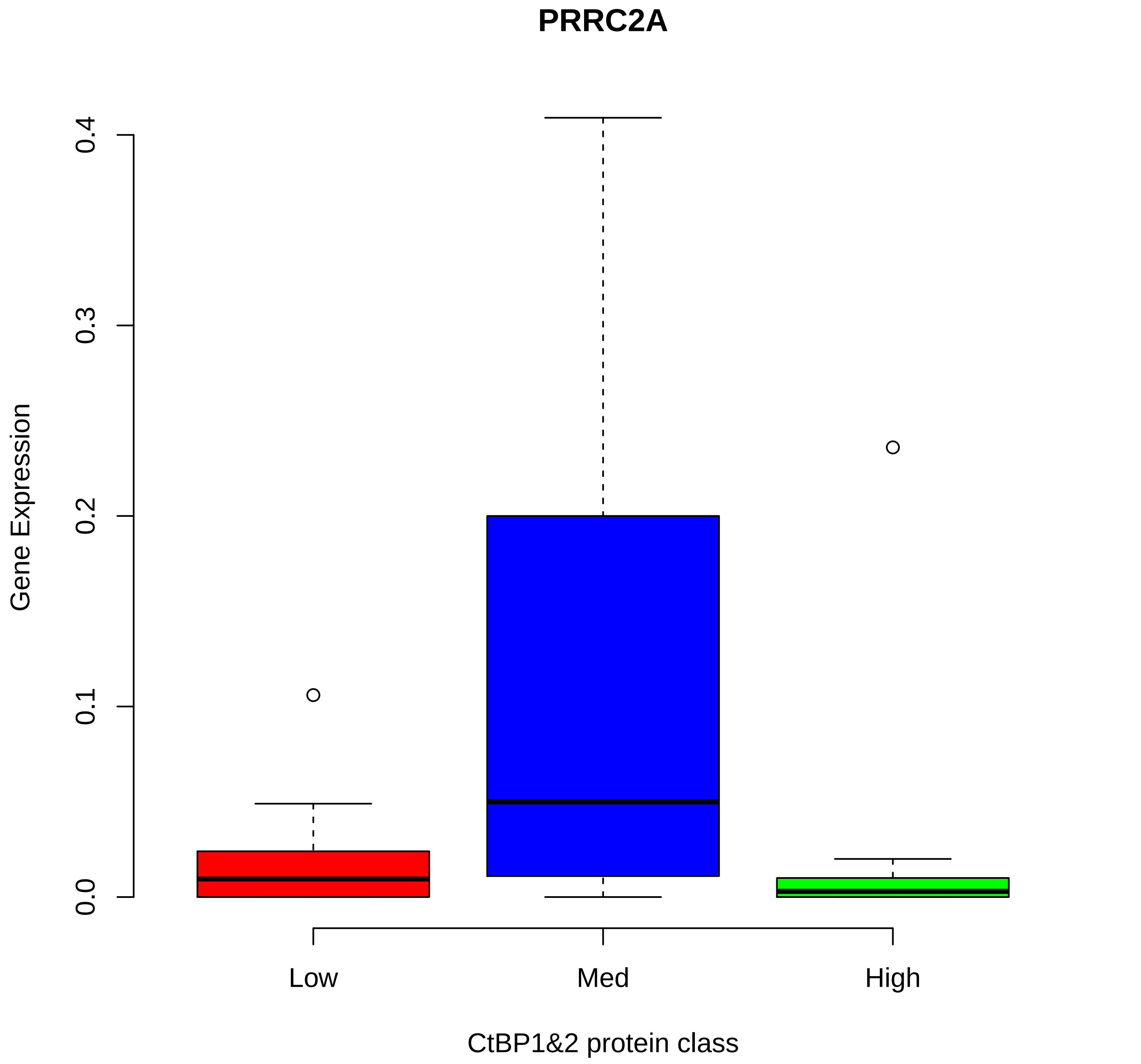

PRRC2A
Gene Expression
0.0
0.1
0.2
0.3
0.4
Low
Med
High
CtBP1&2 protein class

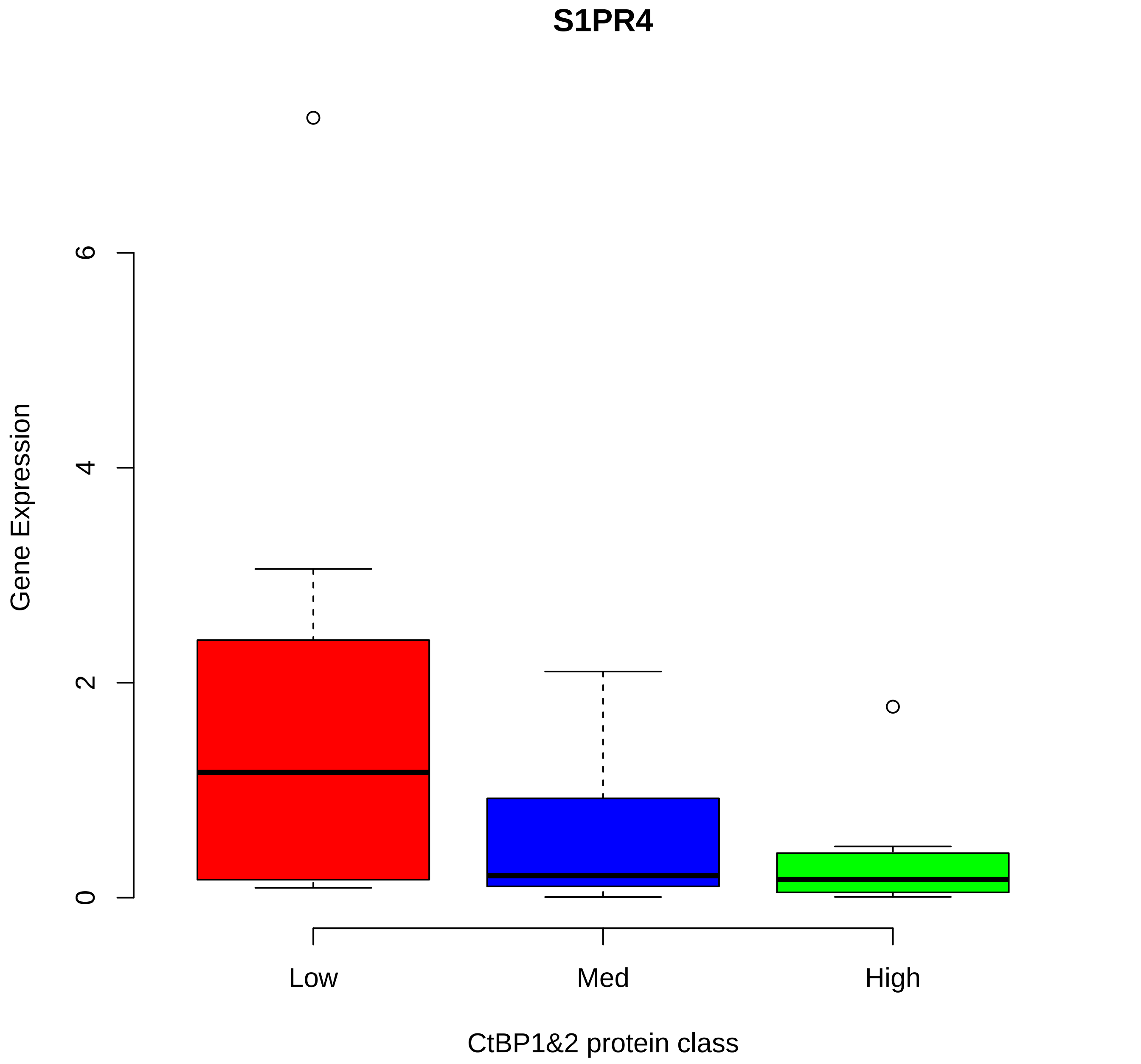

S1PR4
Gene Expression
0
2
4
6
Low
Med
High
CtBP1&2 protein class

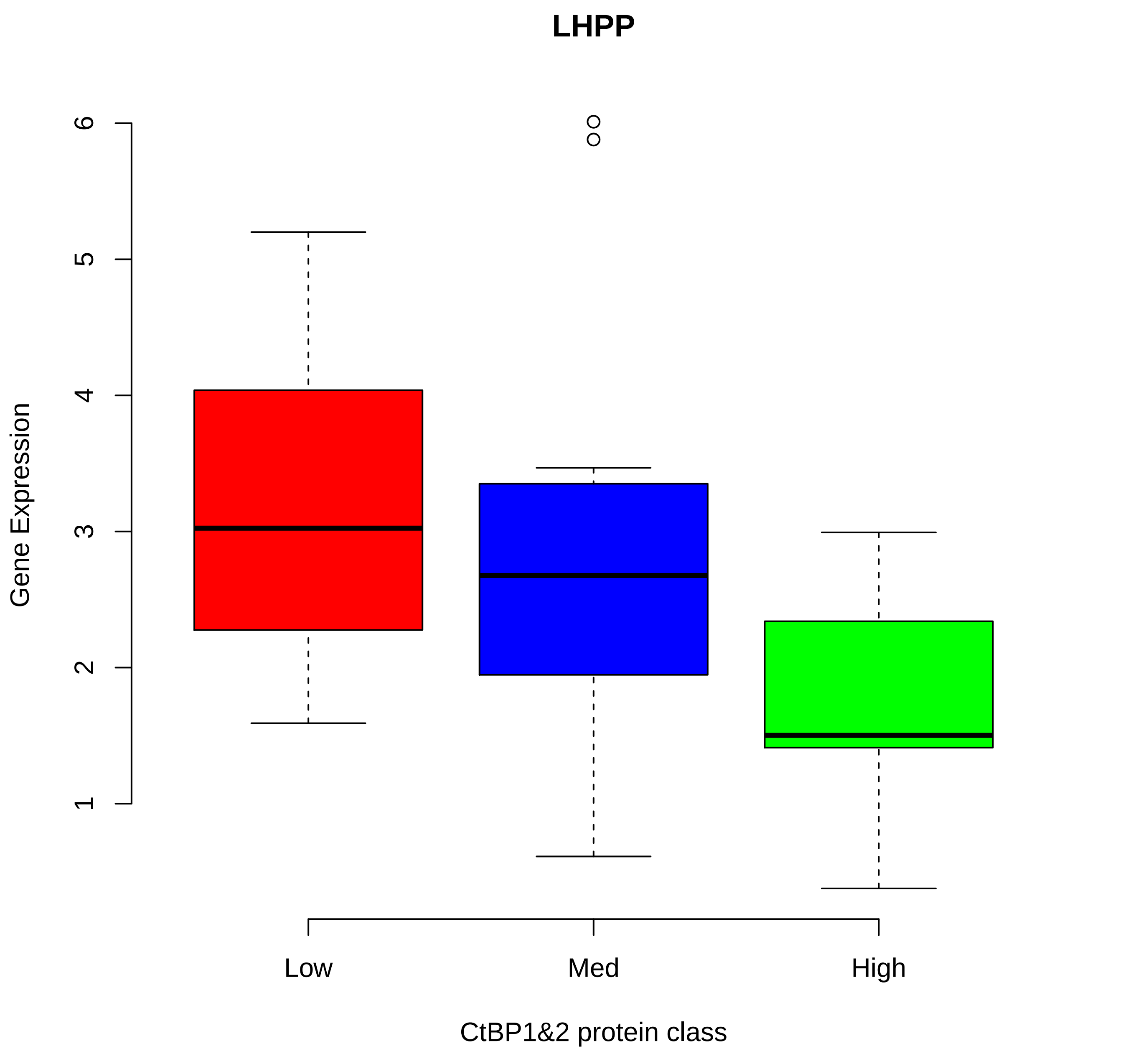

LHPP
Gene Expression
1
2
3
4
5
6
Low
Med
High
CtBP1&2 protein class

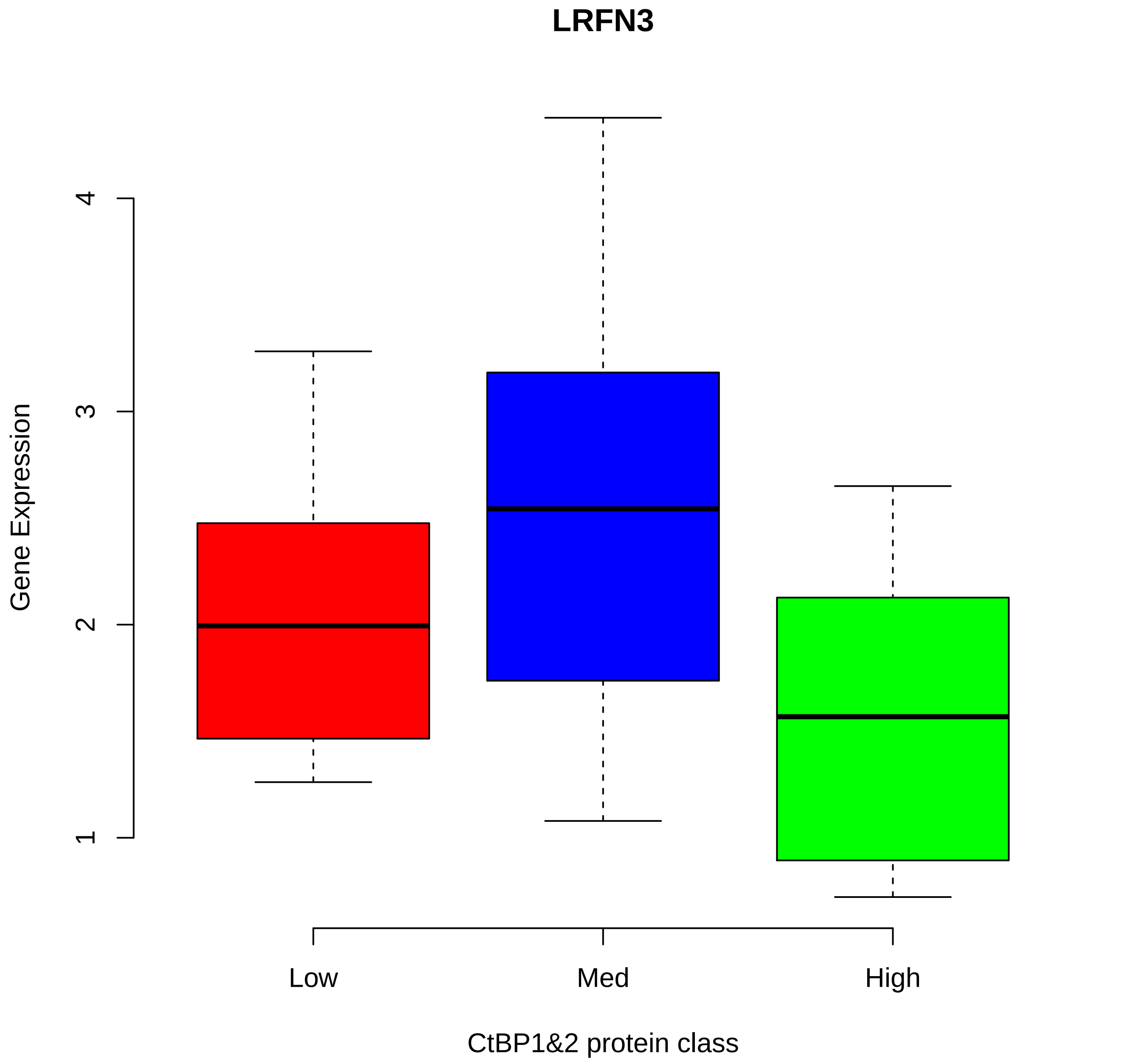
LRFN3
Gene Expression
1
2
3
4
Low
Med
High
CtBP1&2 protein class

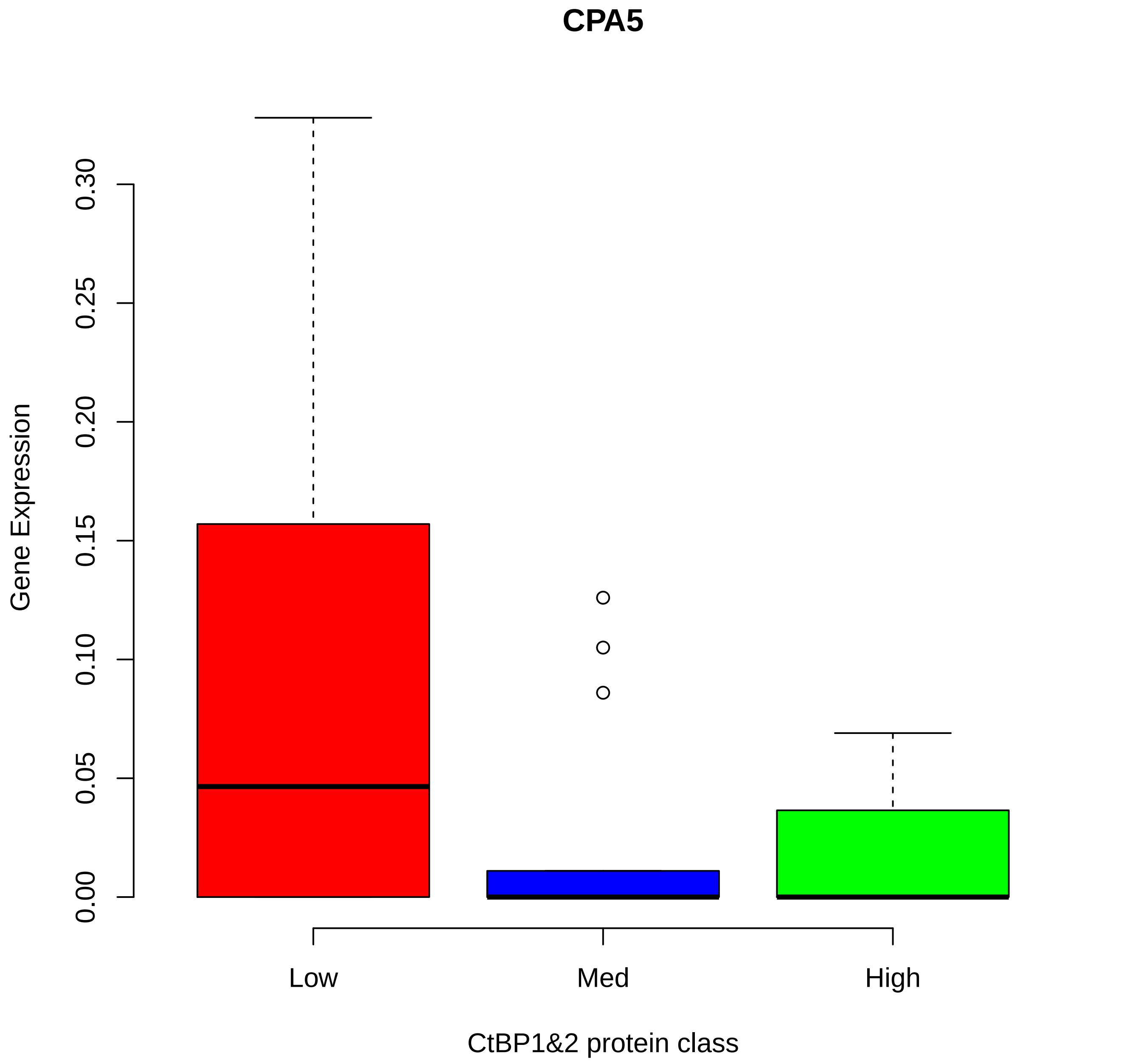

CPA5
Gene Expression
0.00
0.05
0.10
0.15
0.20
0.25
0.30
Low
Med
High
CtBP1&2 protein class

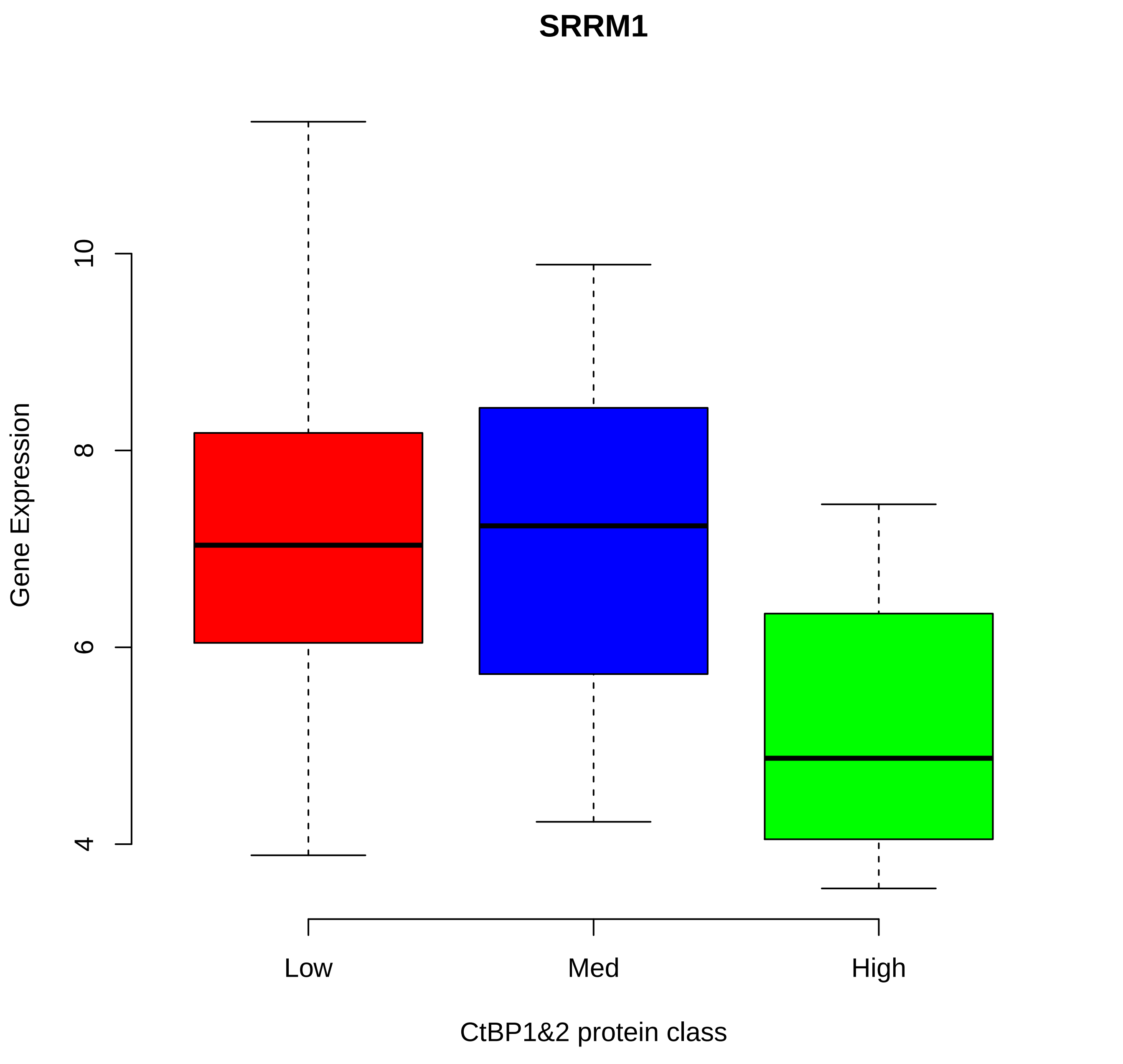

SRRM1
Gene Expression
10
8
6
4
Low
Med
High
CtBP1&2 protein class

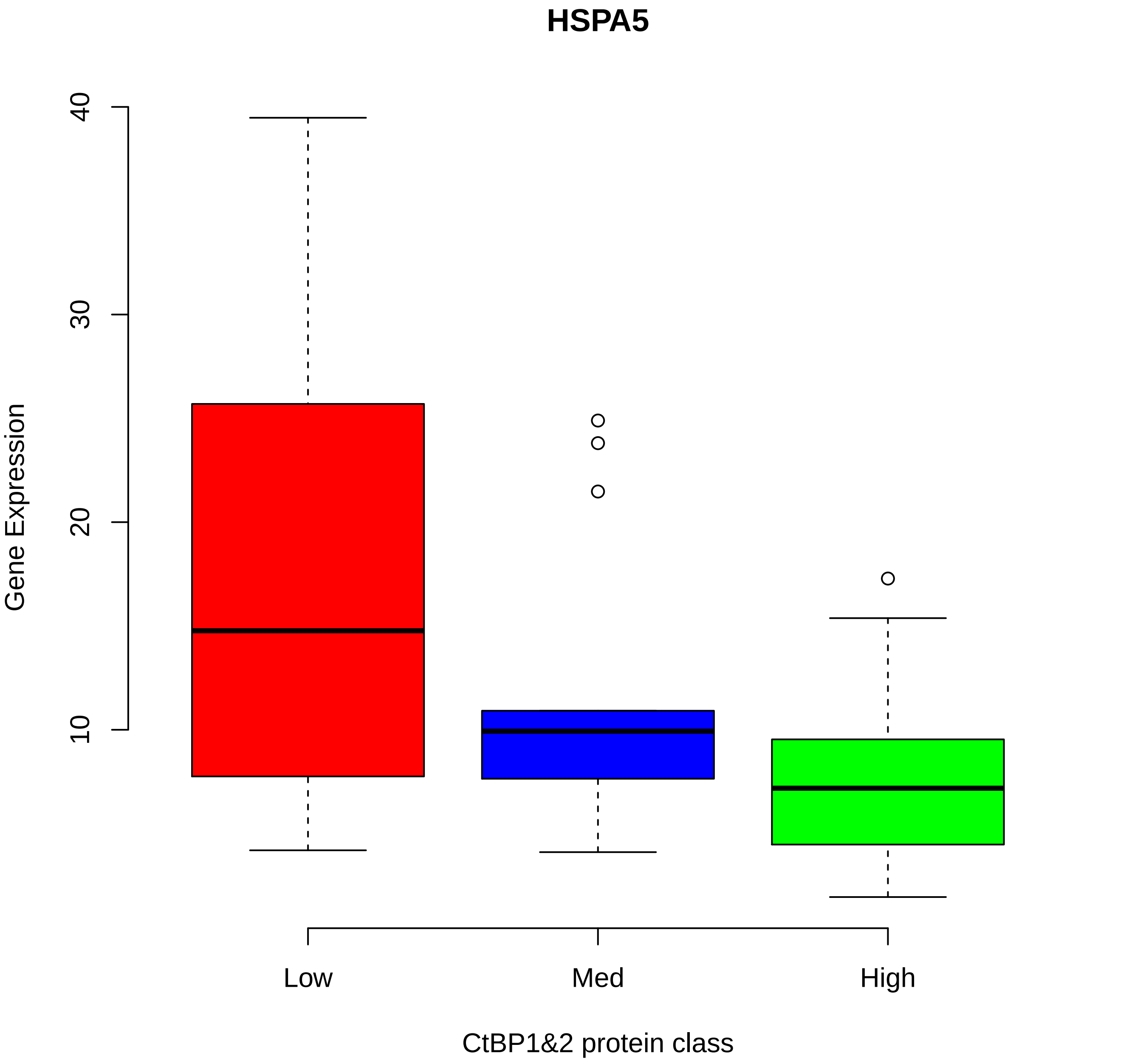
HSPA5
Gene Expression
40
30
20
10
Low
Med
High
CtBP1&2 protein class

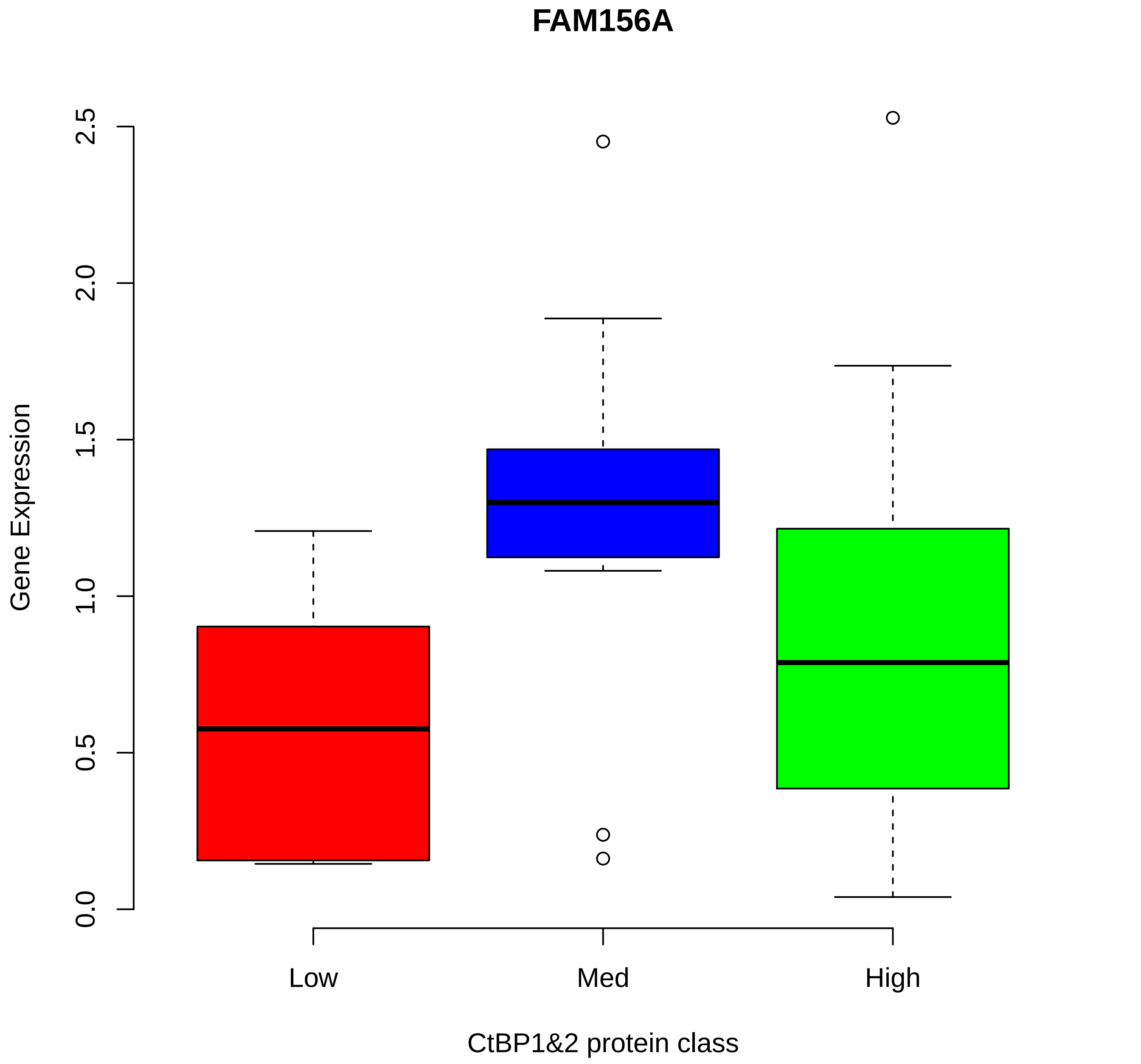
FAM156A
Gene Expression
0.0
0.5
1.0
1.5
2.0
2.5
Low
Med
High
CtBP1&2 protein class

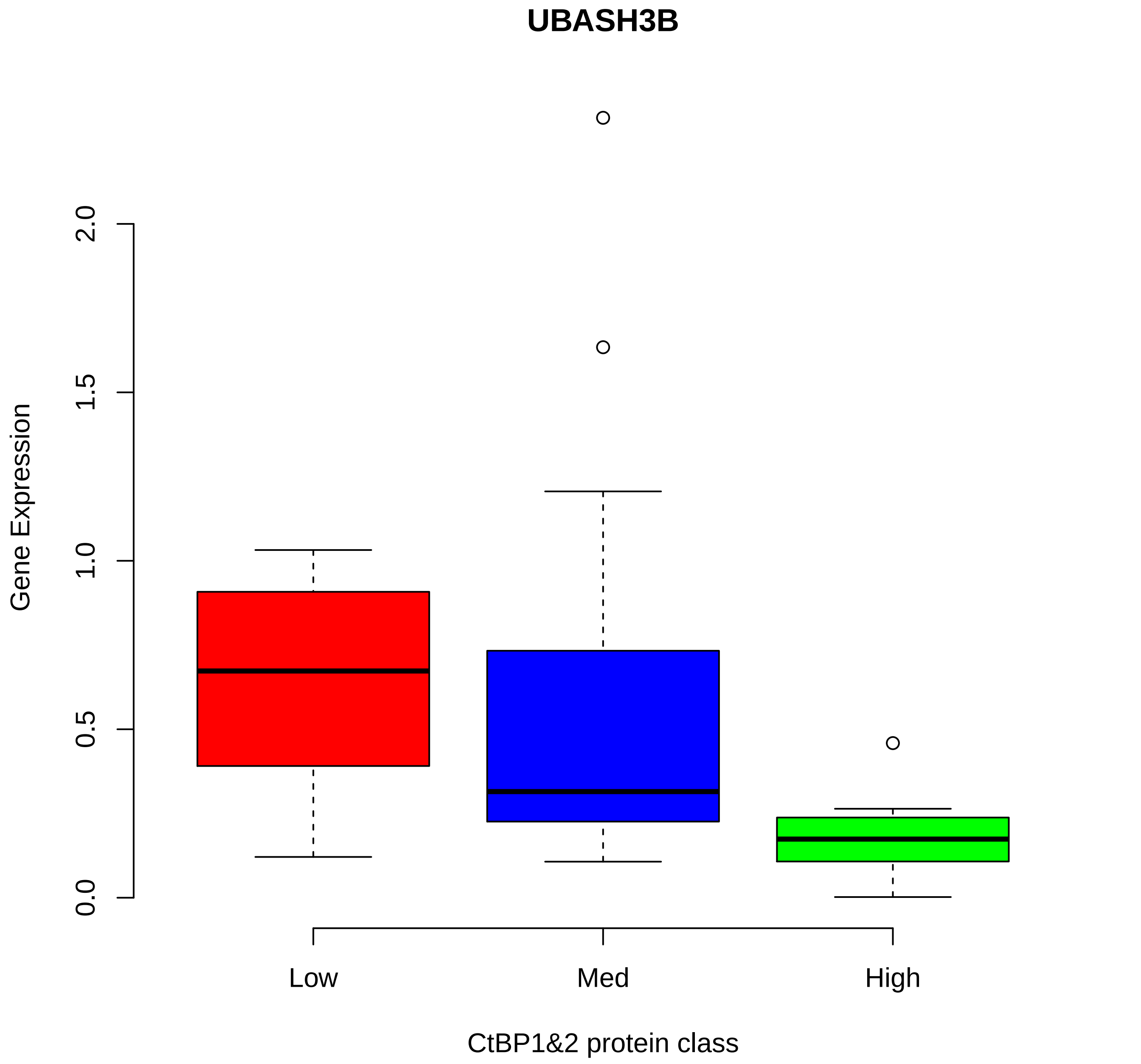
UBASH3B
Gene Expression
0.0
0.5
1.0
1.5
2.0
Low
Med
High
CtBP1&2 protein class

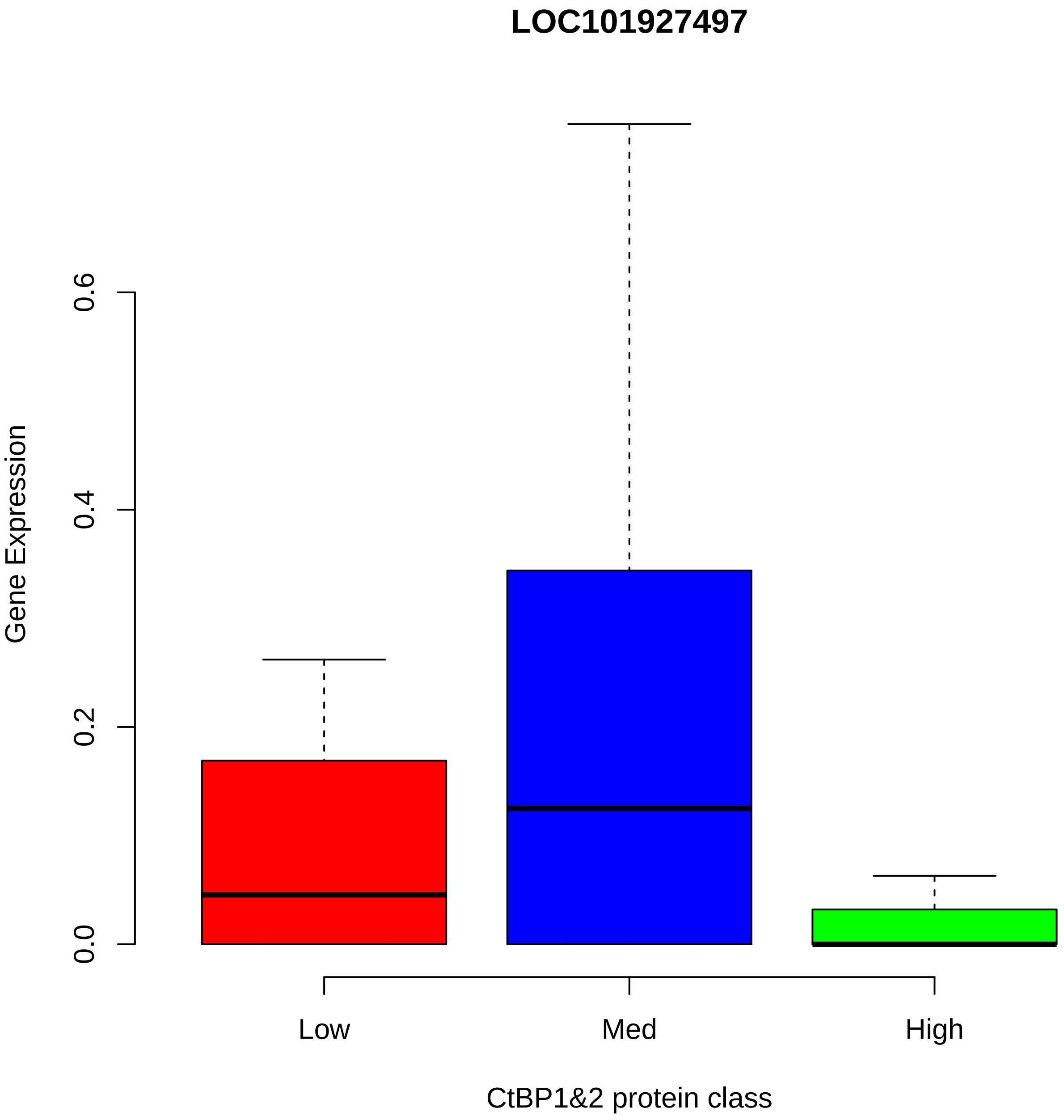

LOC101927497
Gene Expression
0.0
0.2
0.4
0.6
Low
Med
High
CtBP1&2 protein class

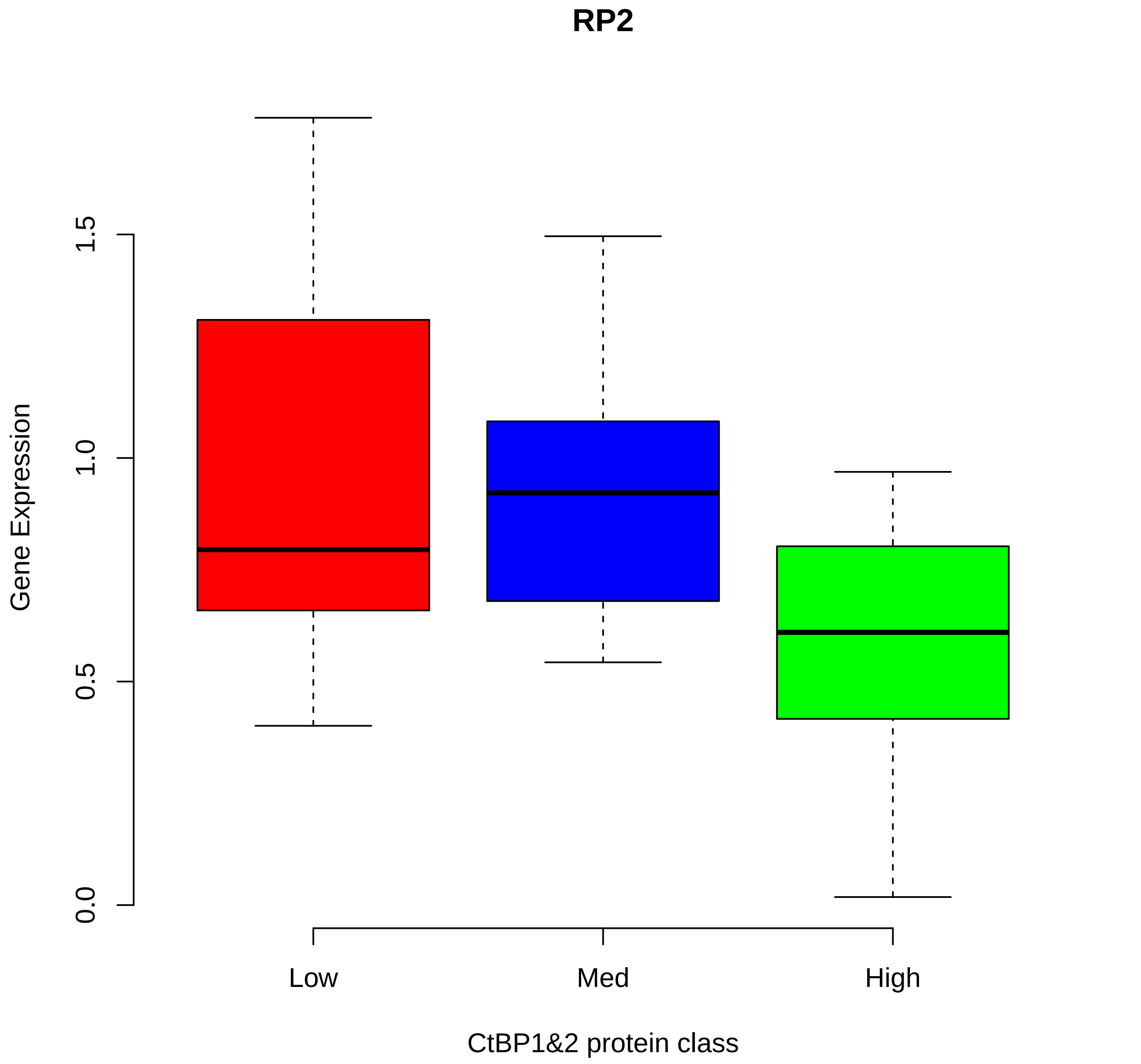
RP2
Gene Expression
0.0
0.5
1.0
1.5
Low
Med
High
CtBP1&2 protein class

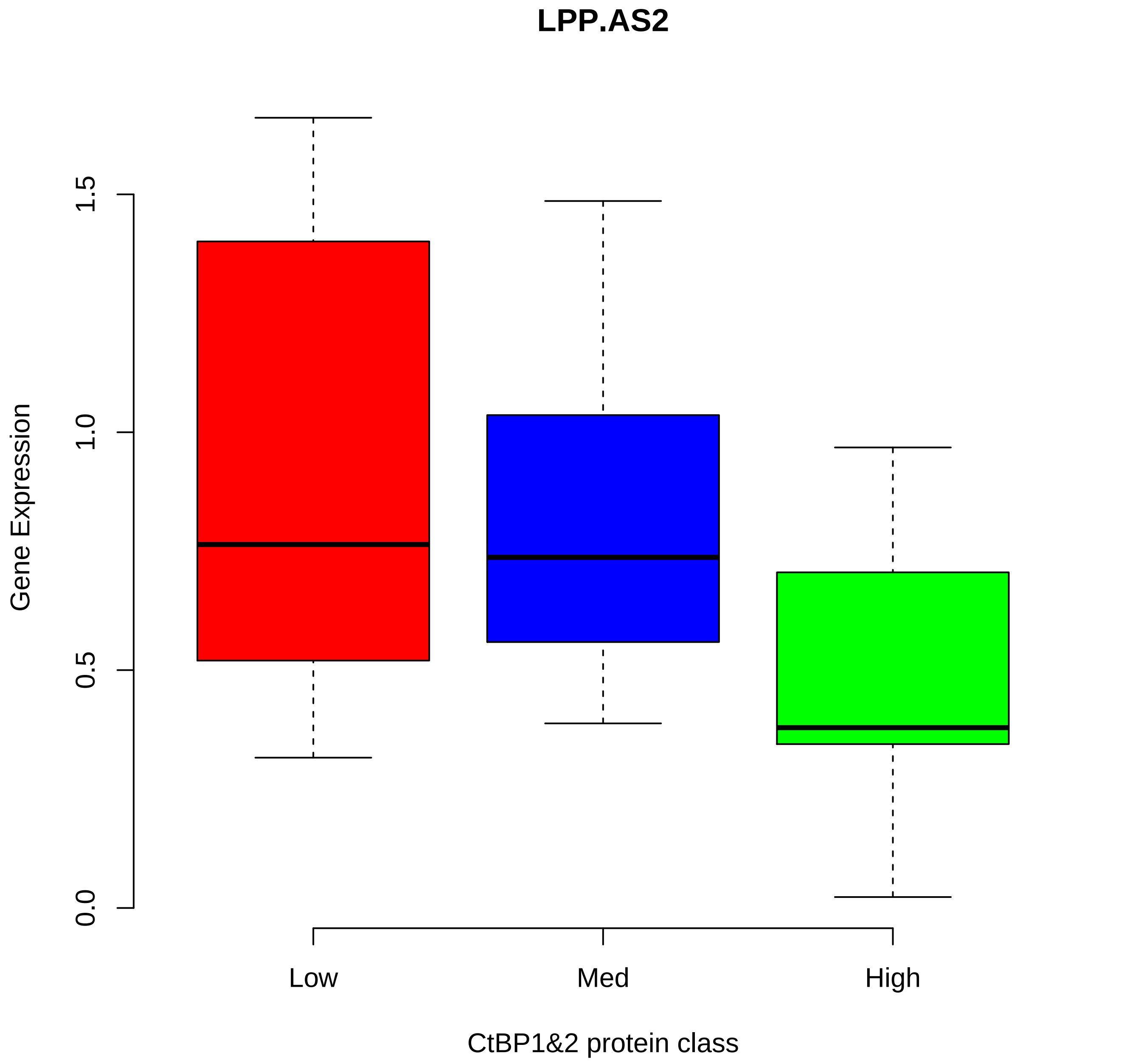
LPP.AS2
Gene Expression
0.0
0.5
1.0
1.5
Low
Med
High
CtBP1&2 protein class

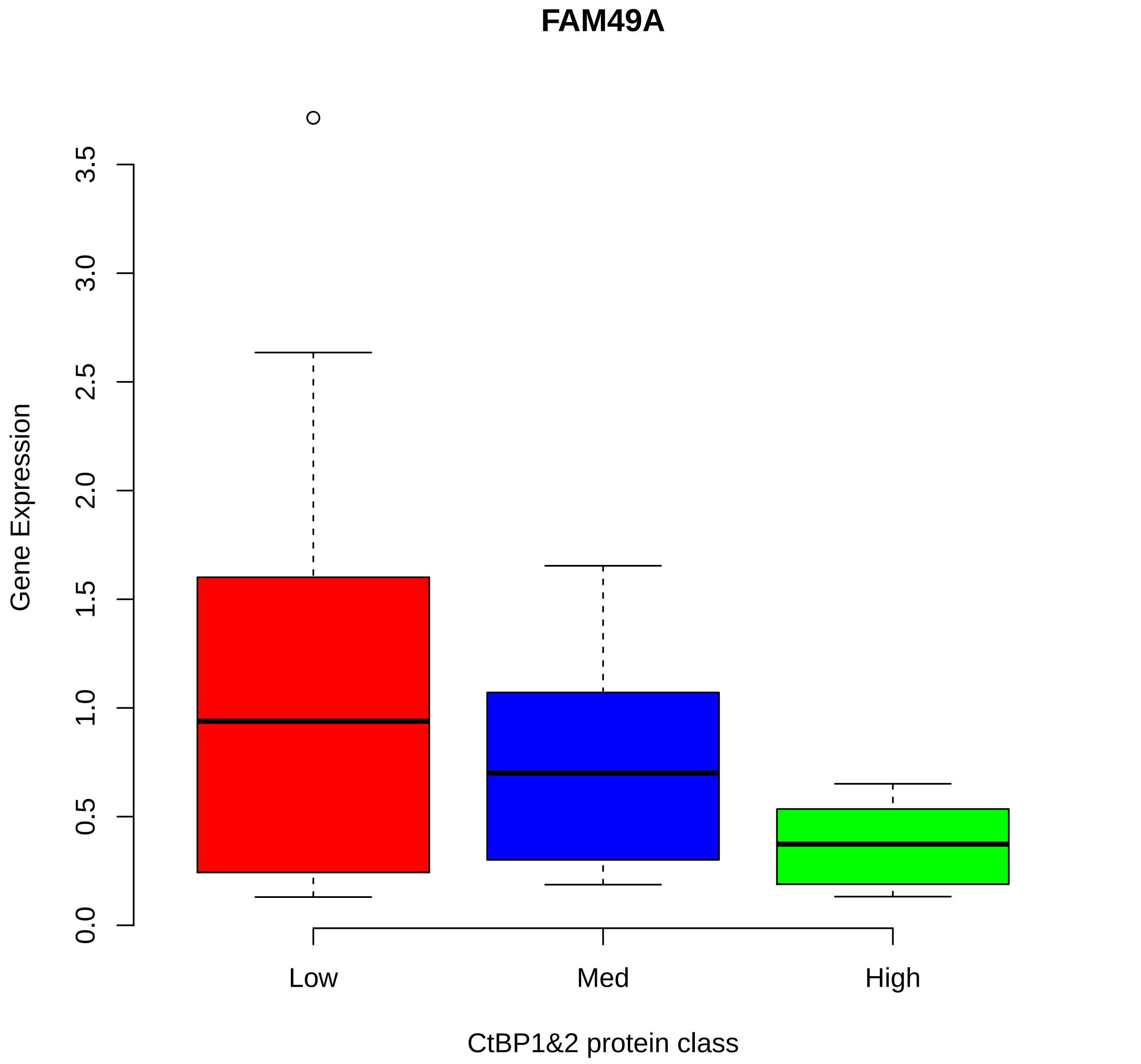

FAM49A
Gene Expression
0.0
0.5
1.0
1.5
2.0
2.5
3.0
3.5
Low
Med
High
CtBP1&2 protein class

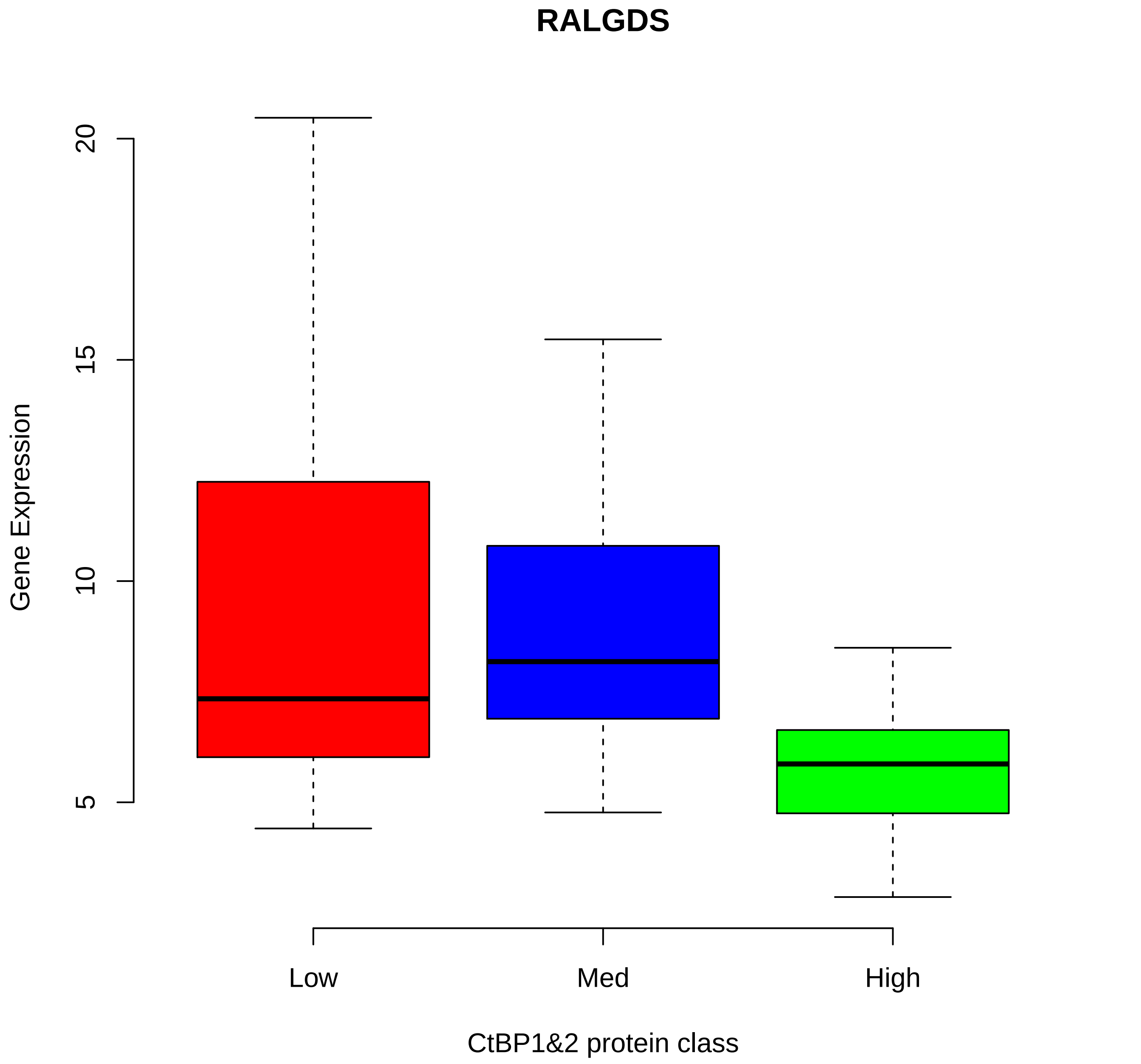

RALGDS
Gene Expression
20
15
10
5
Low
Med
High
CtBP1&2 protein class

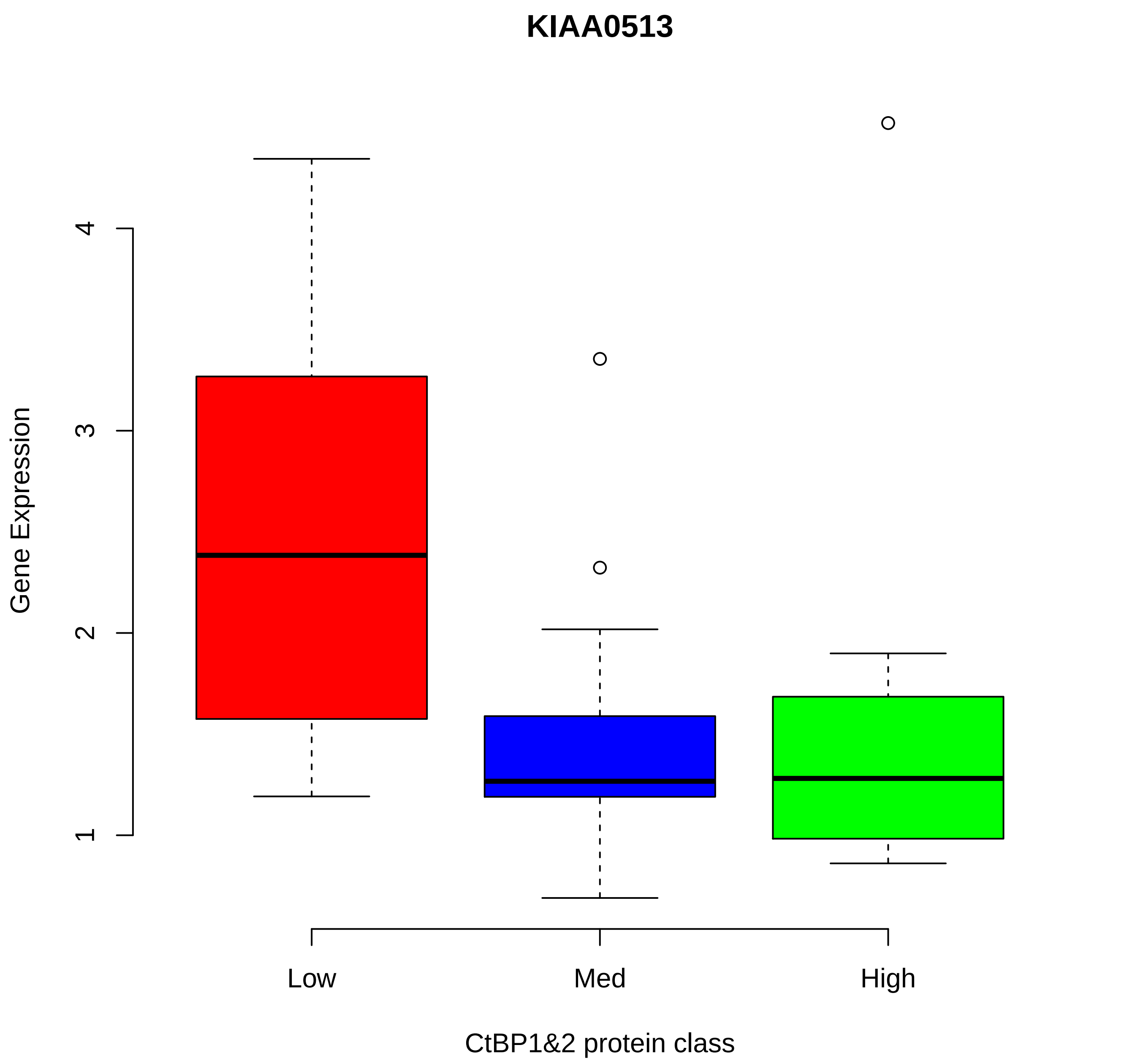
KIAA0513
Gene Expression
1
2
3
4
Low
Med
High
CtBP1&2 protein class

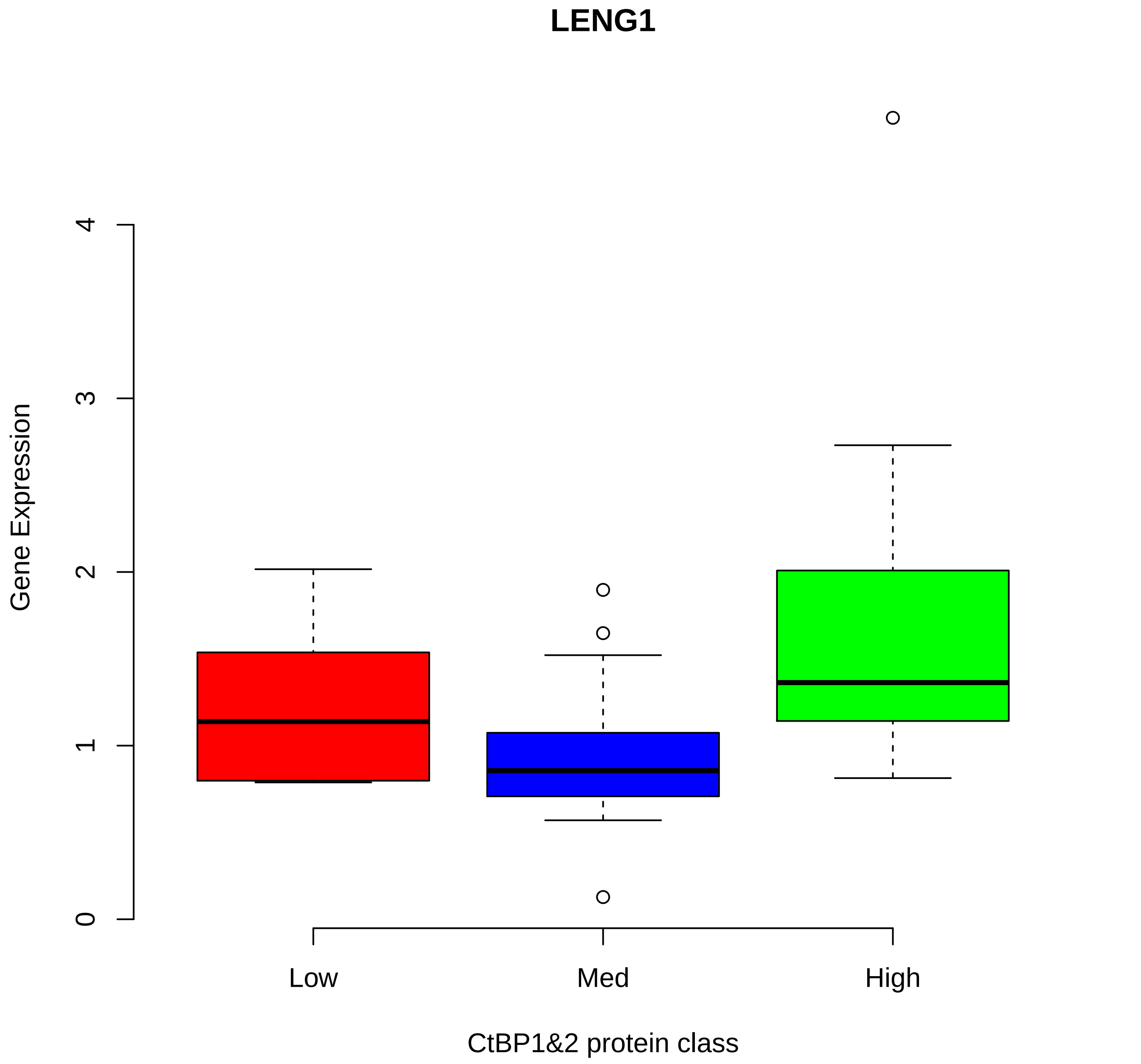
LENG1
Gene Expression
0
1
2
3
4
Low
Med
High
CtBP1&2 protein class

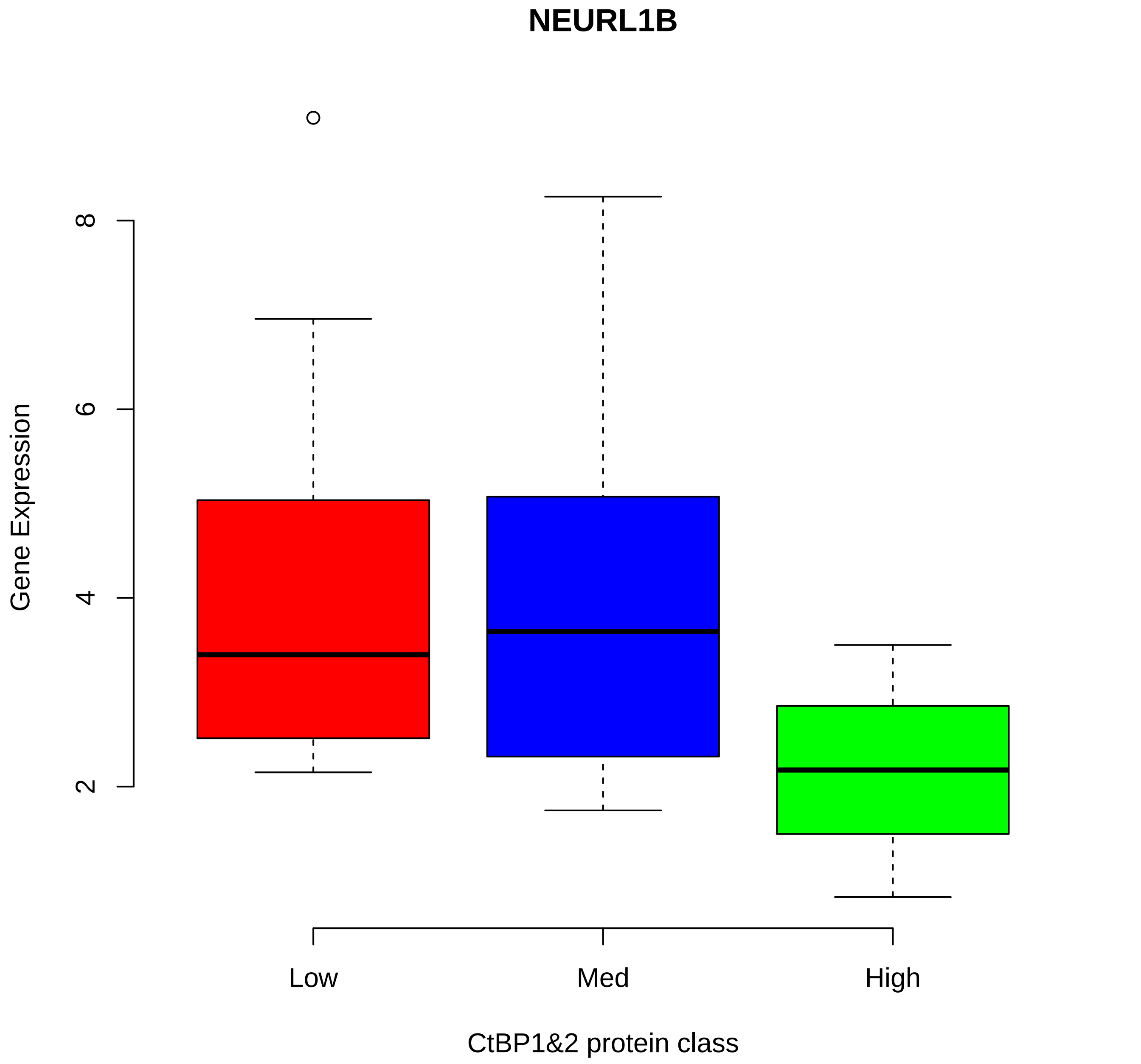
NEURL1B
Gene Expression
2
4
6
8
Low
Med
High
CtBP1&2 protein class

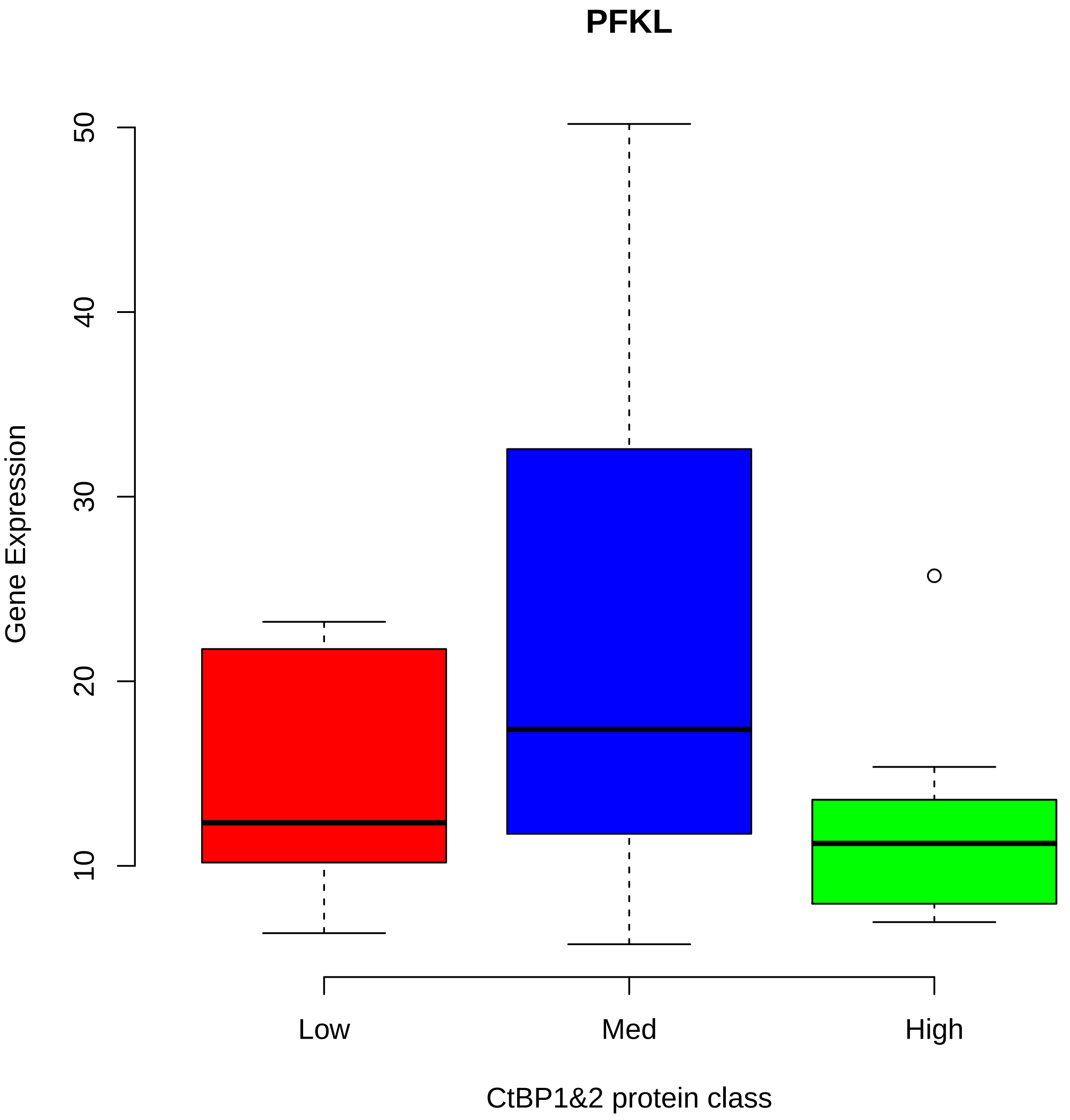

PFKL
Gene Expression
50
40
30
20
10
Low
Med
High
CtBP1&2 protein class

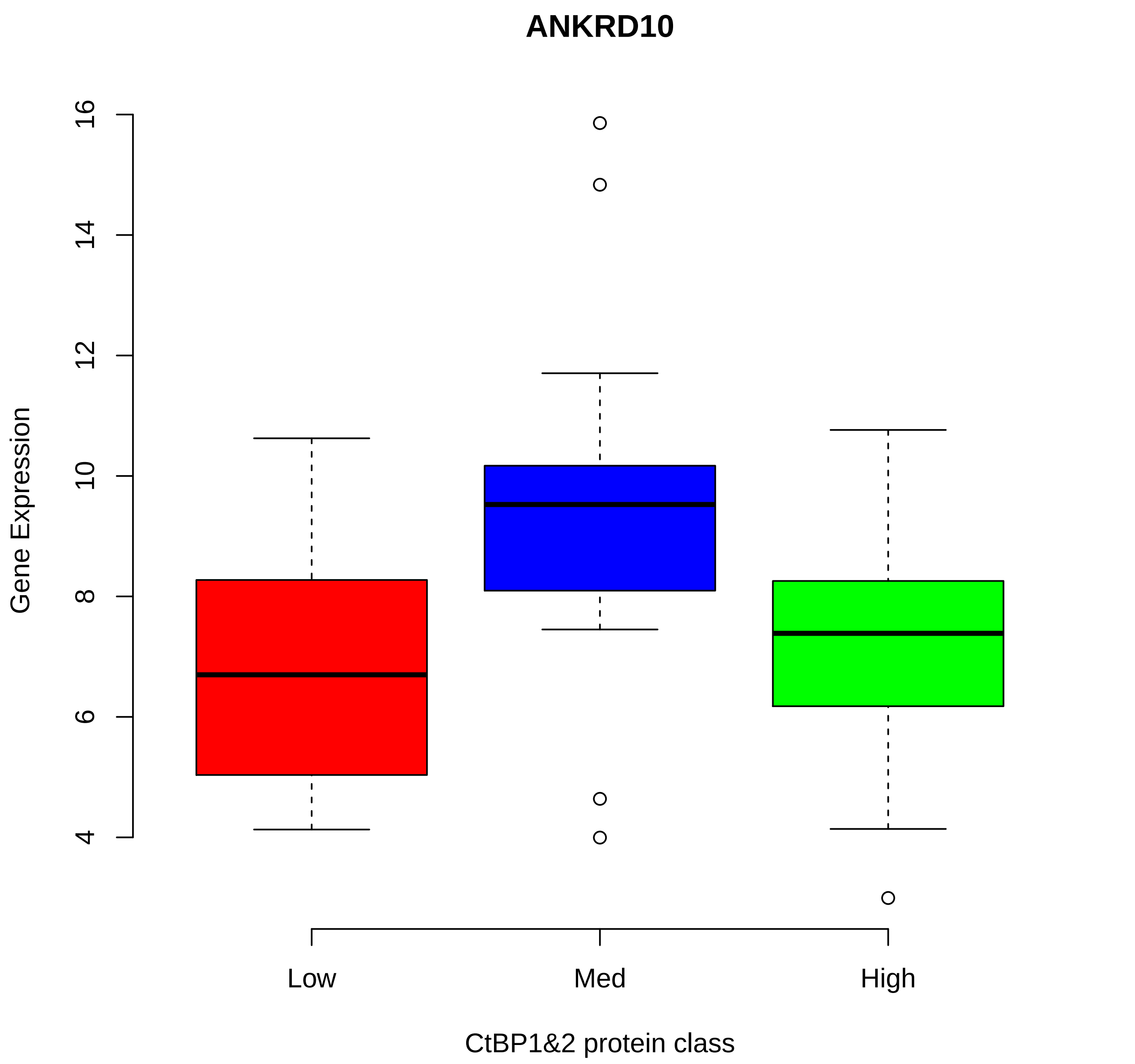

ANKRD10
Gene Expression
4
6
8
10
12
14
16
Low
Med
High
CtBP1&2 protein class

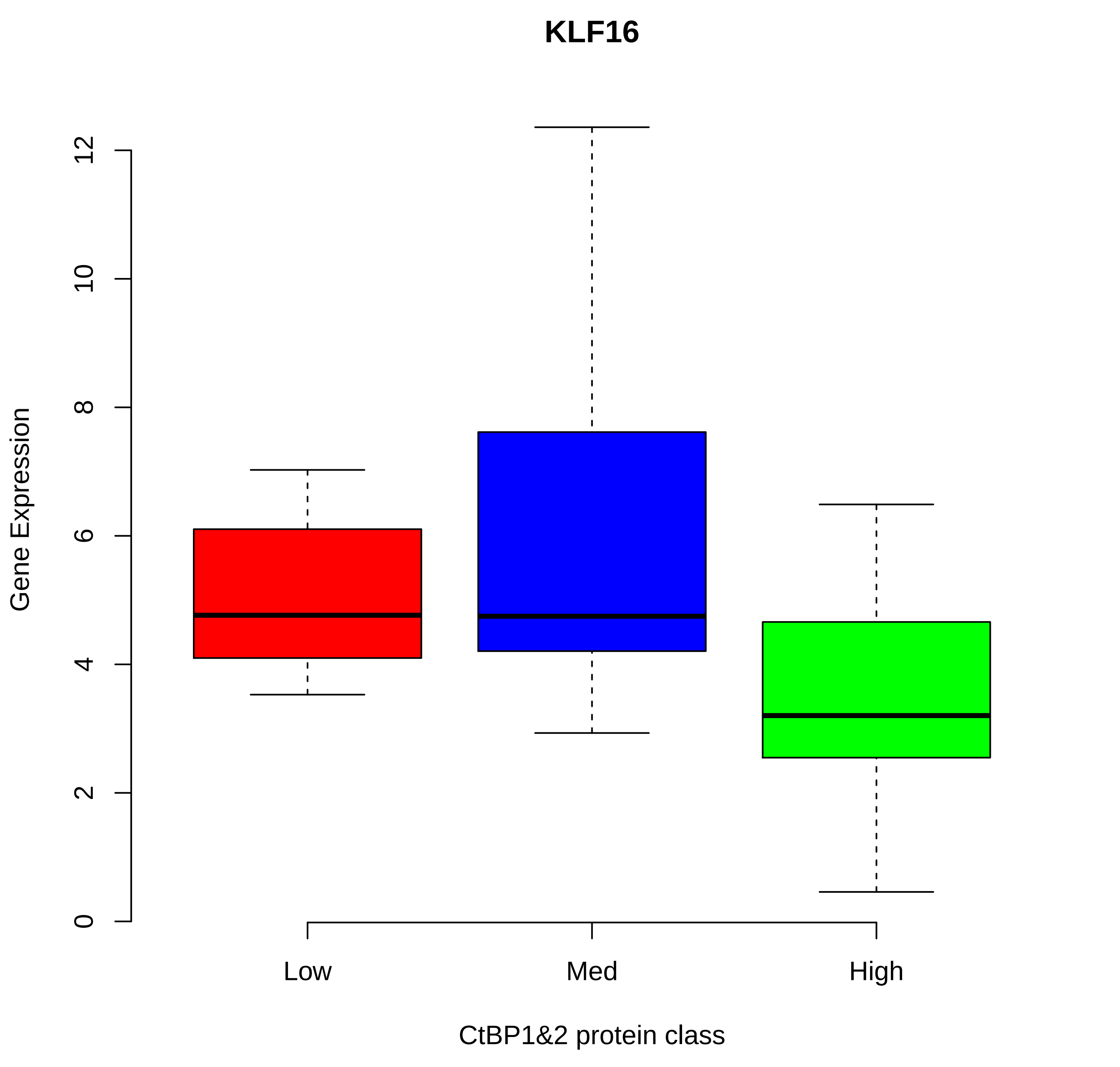

KLF16
Gene Expression
0
2
4
6
8
10
12
Low
Med
High
CtBP1&2 protein class

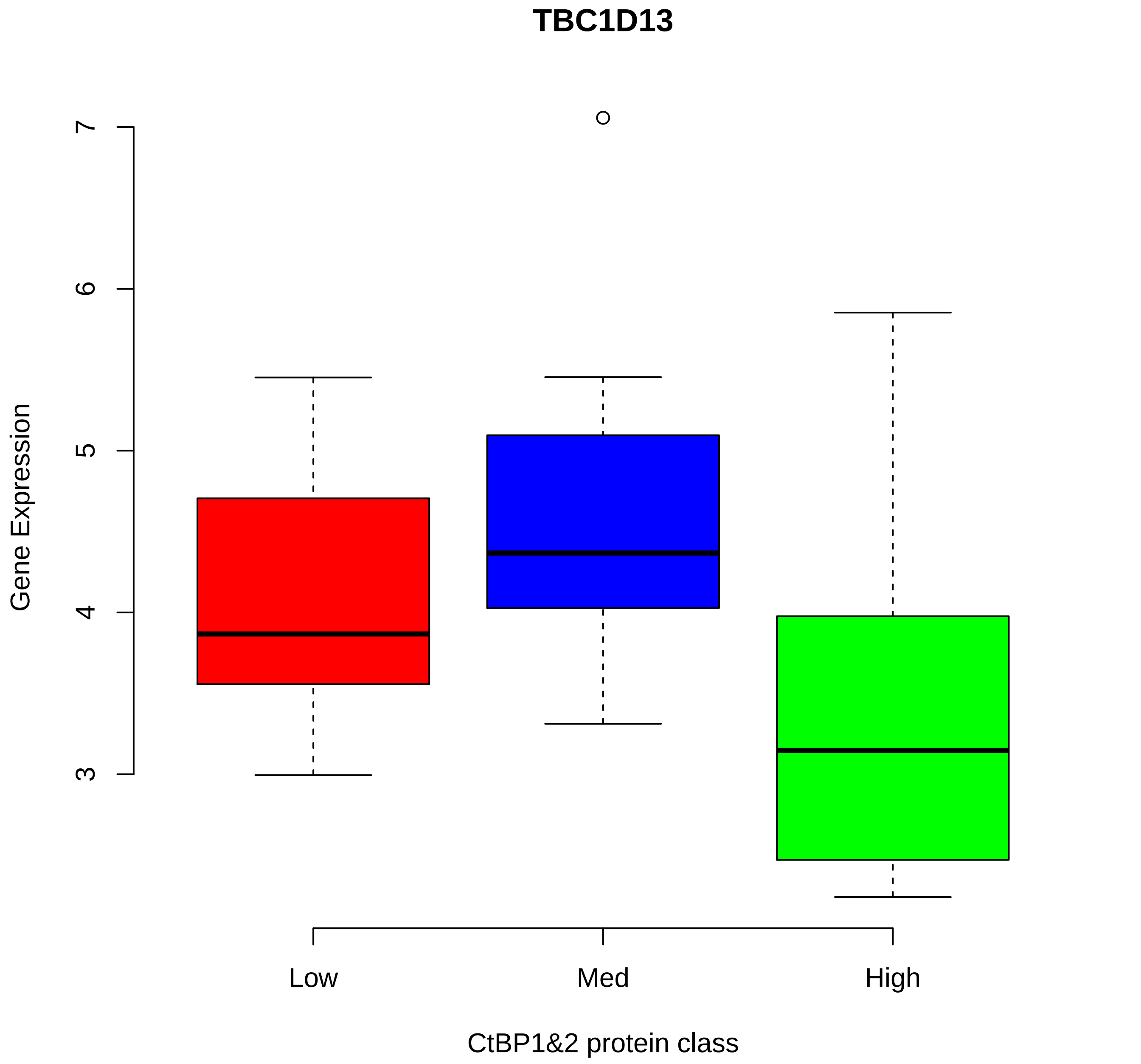
TBC1D13
Gene Expression
7
6
5
4
3
Low
Med
High
CtBP1&2 protein class

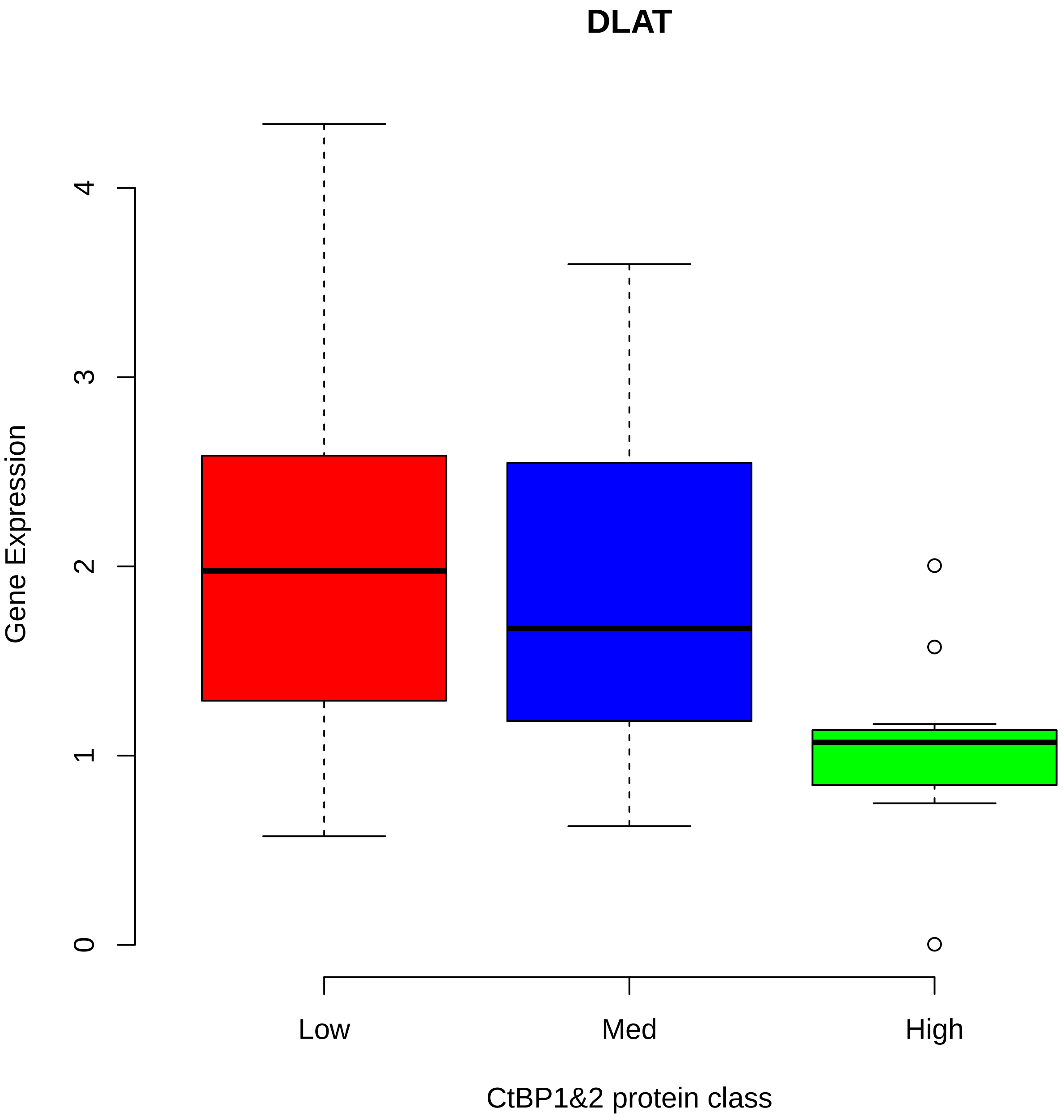
DLAT
Gene Expression
0
1
2
3
4
Low
Med
High
CtBP1&2 protein class

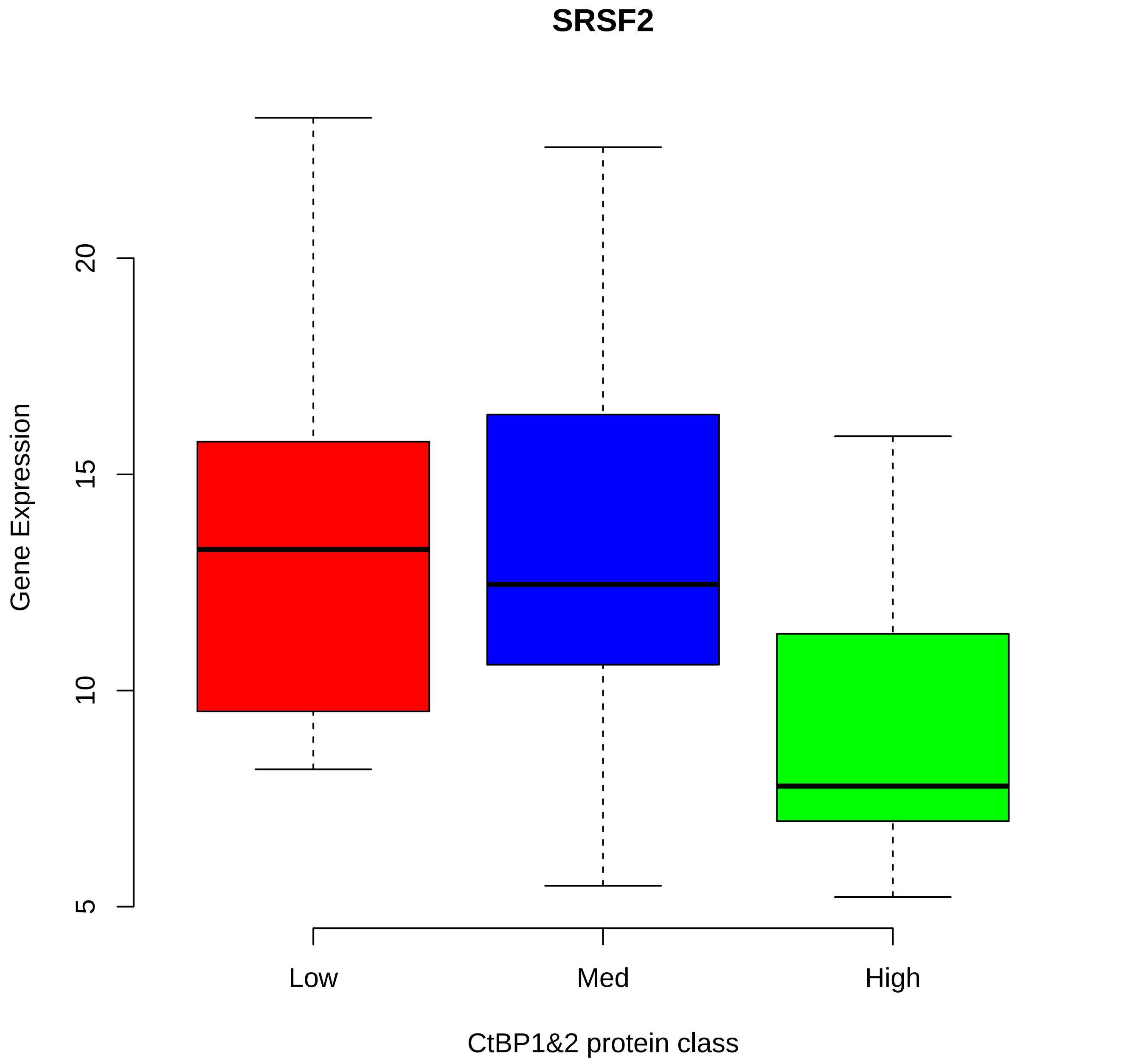

SRSF2
Gene Expression
20
15
10
5
Low
Med
High
CtBP1&2 protein class

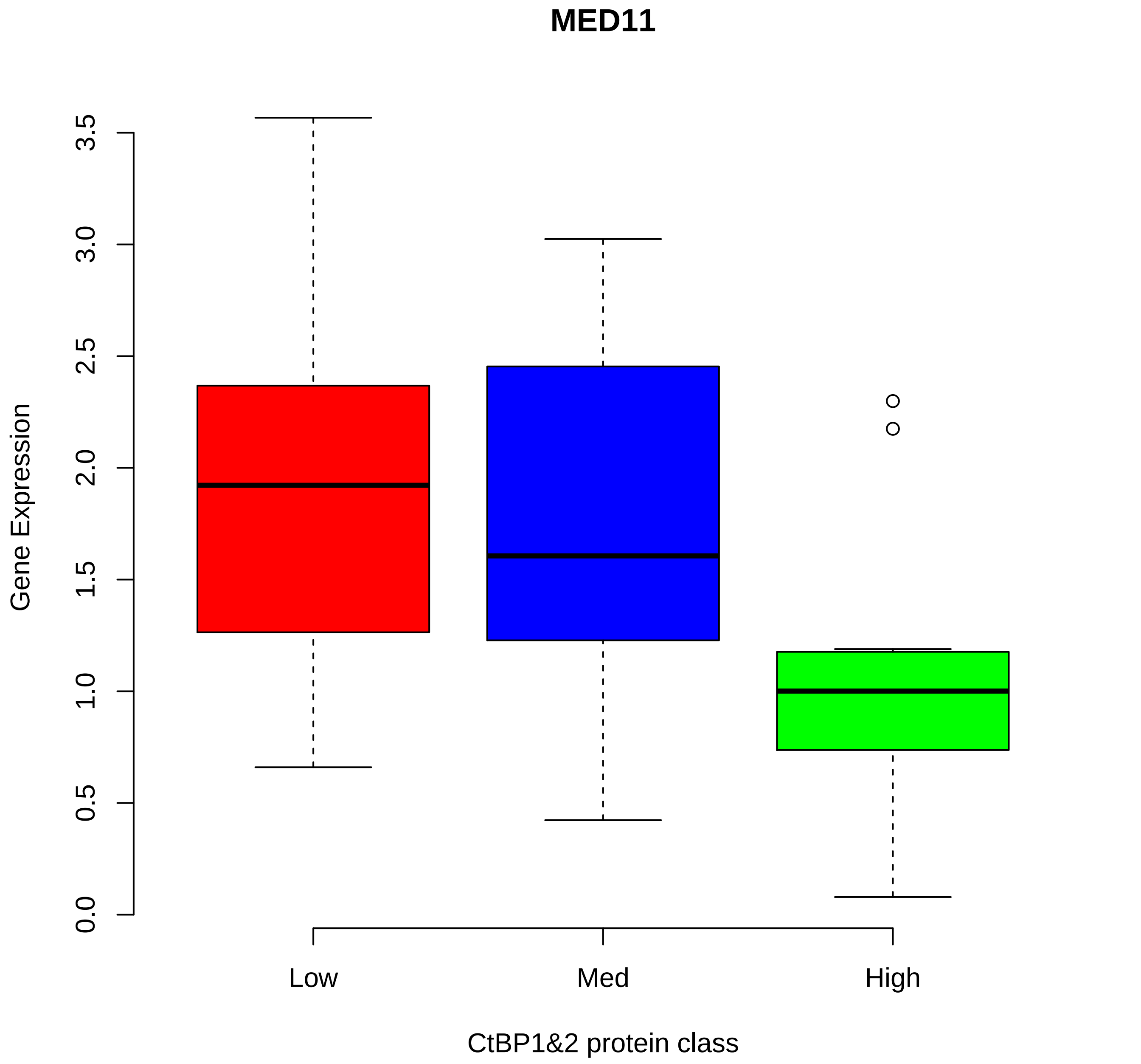

MED11
Gene Expression
0.0
0.5
1.0
1.5
2.0
2.5
3.0
3.5
Low
Med
High
CtBP1&2 protein class

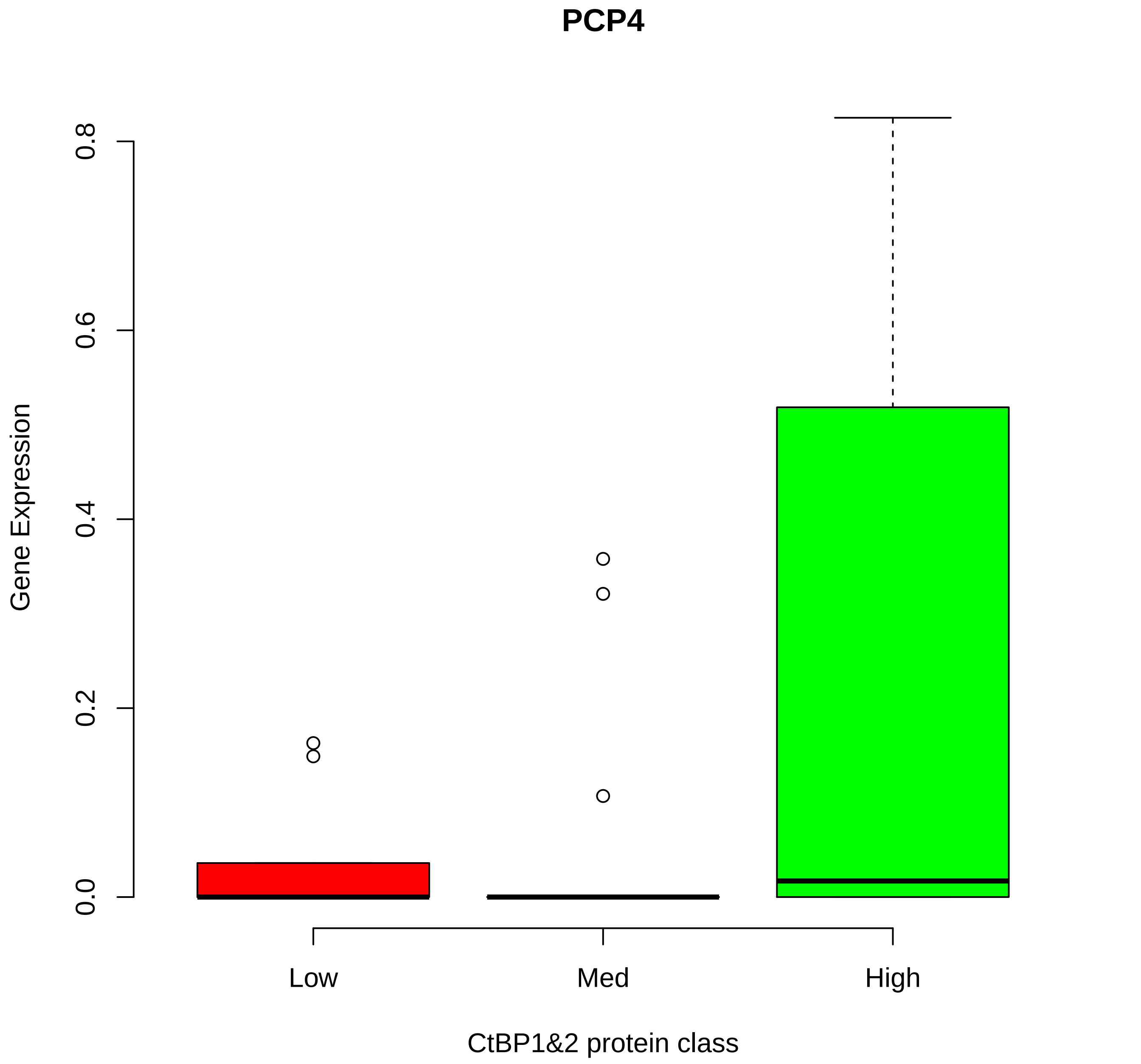

PCP4
Gene Expression
0.0
0.2
0.4
0.6
0.8
Low
Med
High
CtBP1&2 protein class

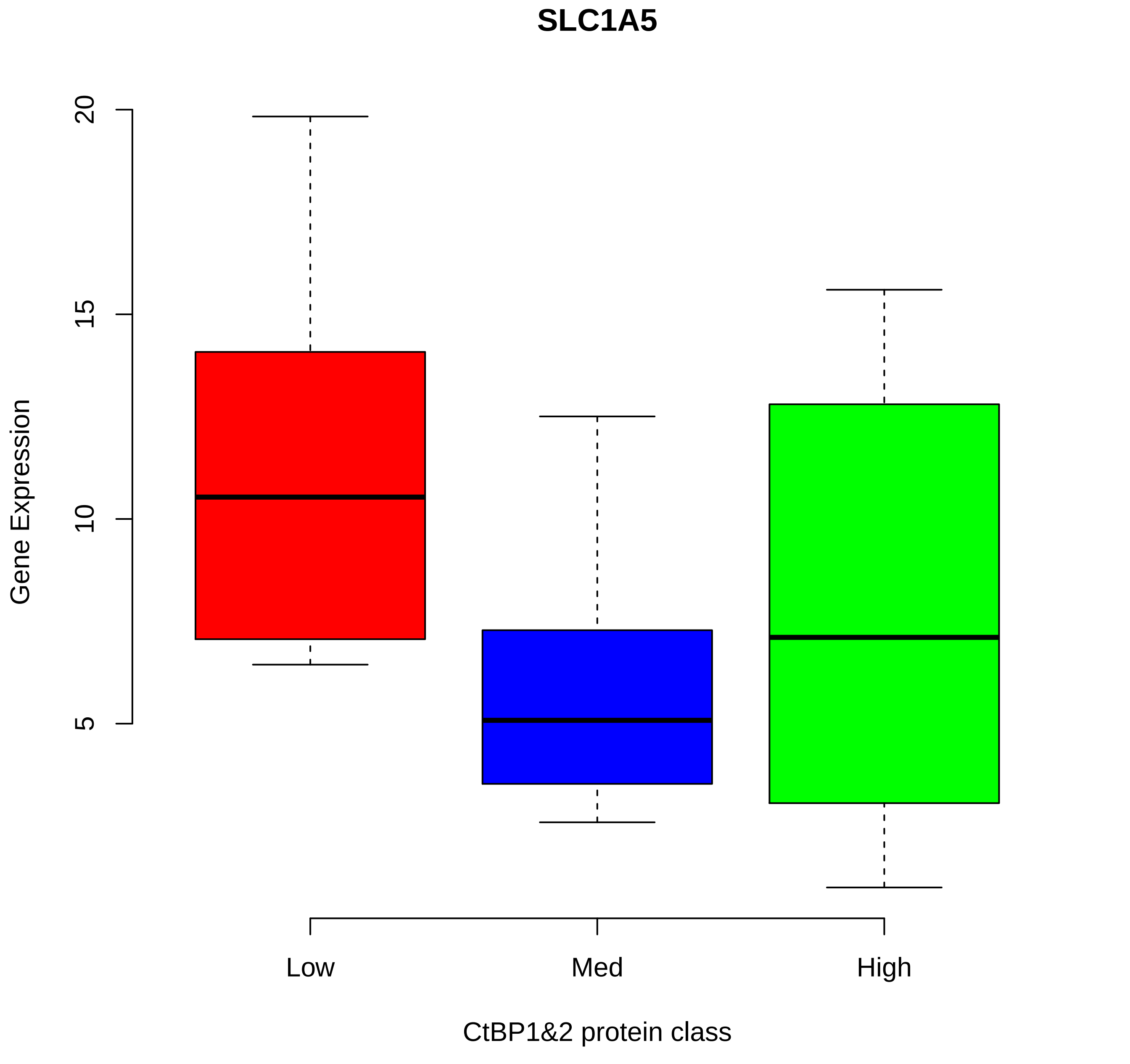
SLC1A5
Gene Expression
20
15
10
5
Low
Med
High
CtBP1&2 protein class

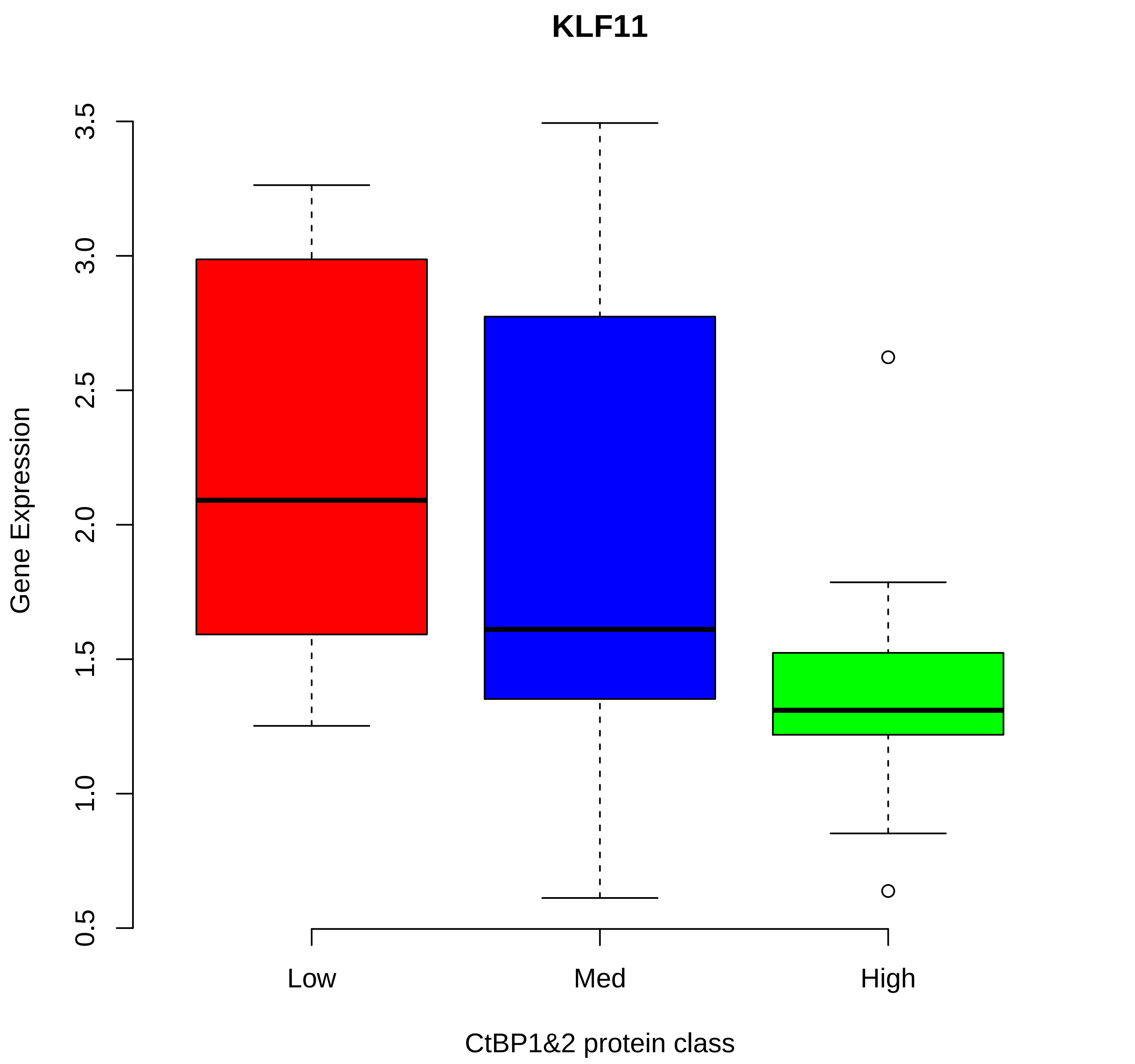
KLF11
Gene Expression
0.5
1.0
1.5
2.0
2.5
3.0
3.5
Low
Med
High
CtBP1&2 protein class

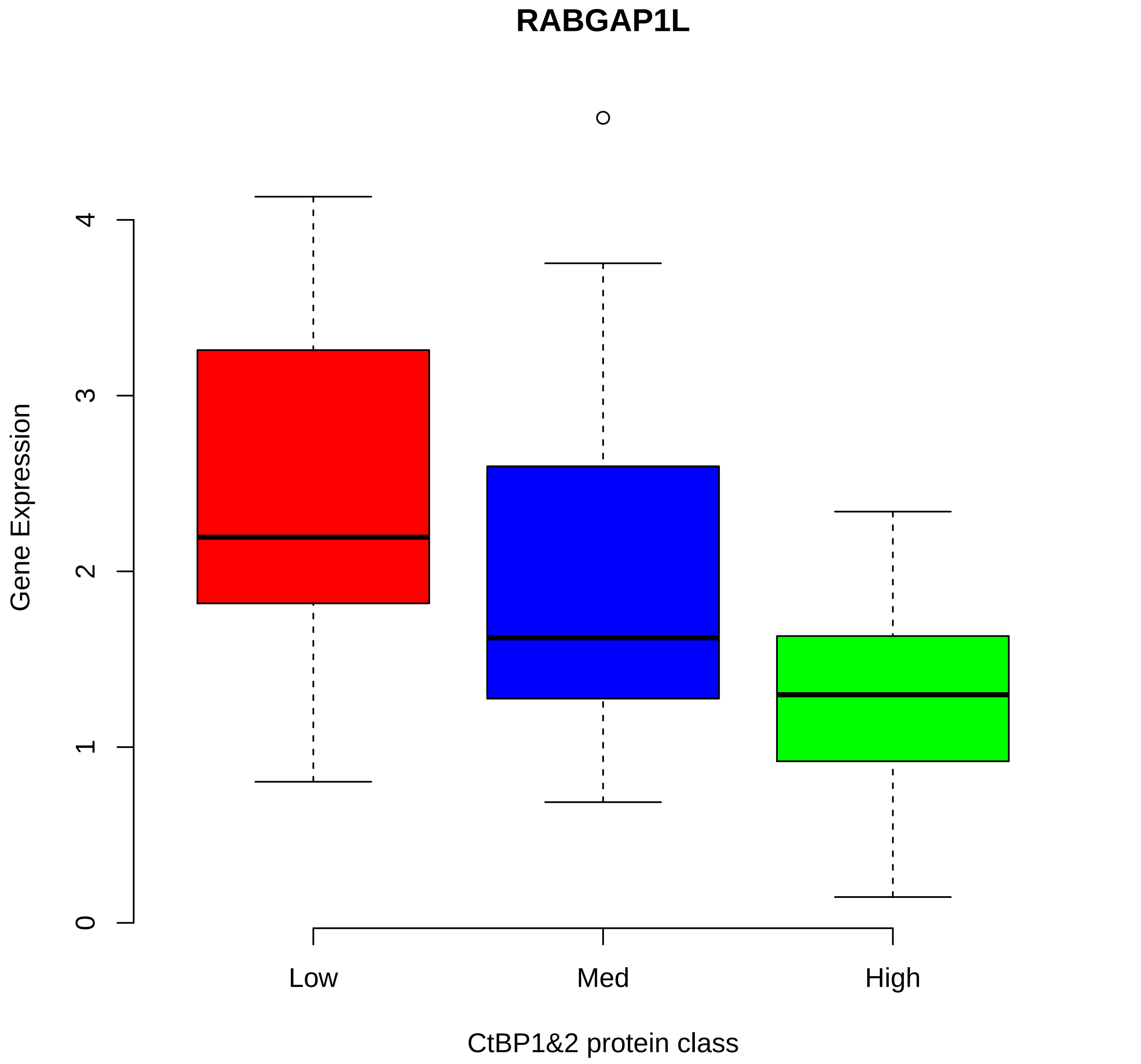
RABGAP1L
Gene Expression
0
1
2
3
4
Low
Med
High
CtBP1&2 protein class

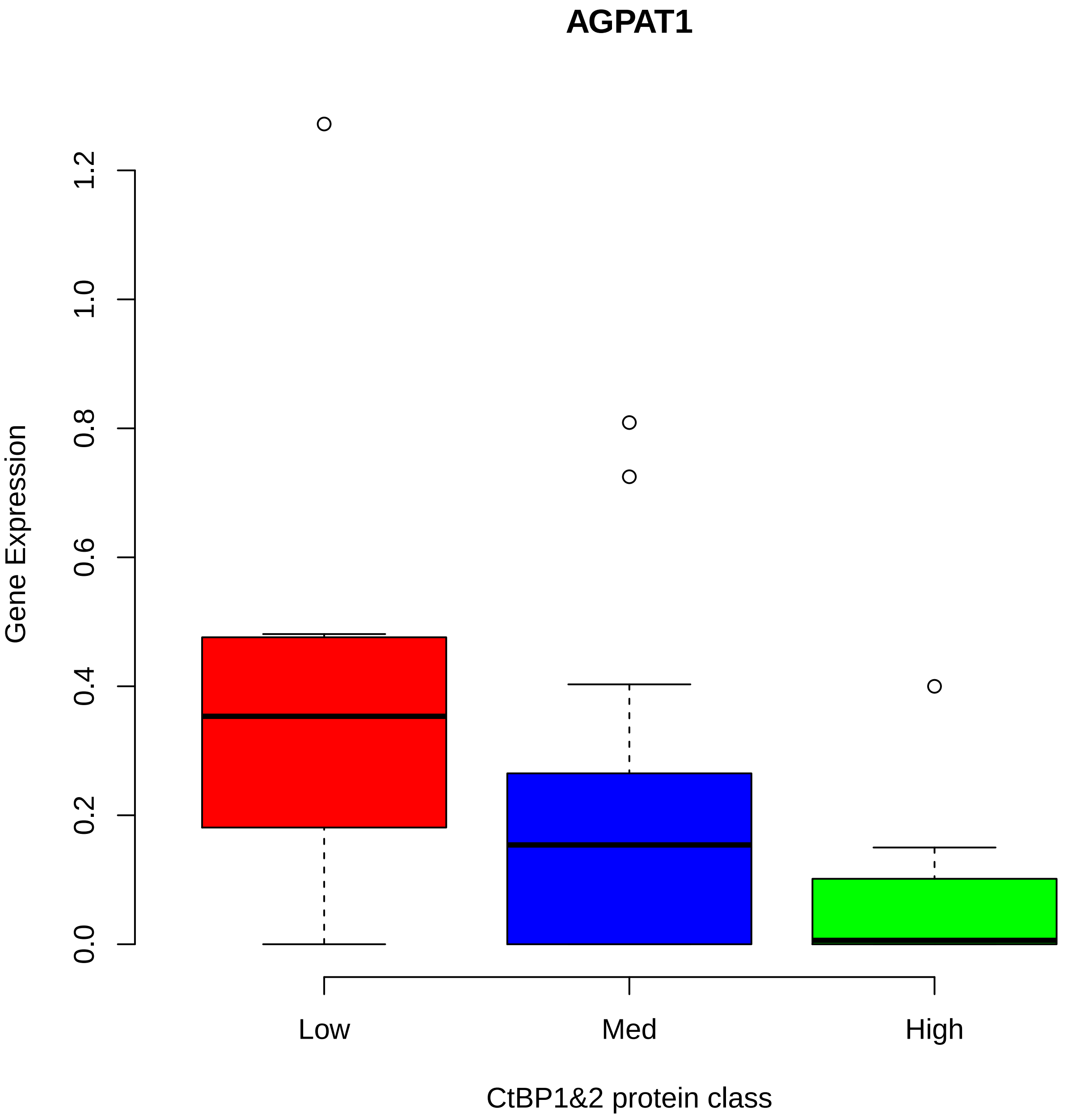
AGPAT1
Gene Expression
0.0
0.2
0.4
0.6
0.8
1.0
1.2
Low
Med
High
CtBP1&2 protein class

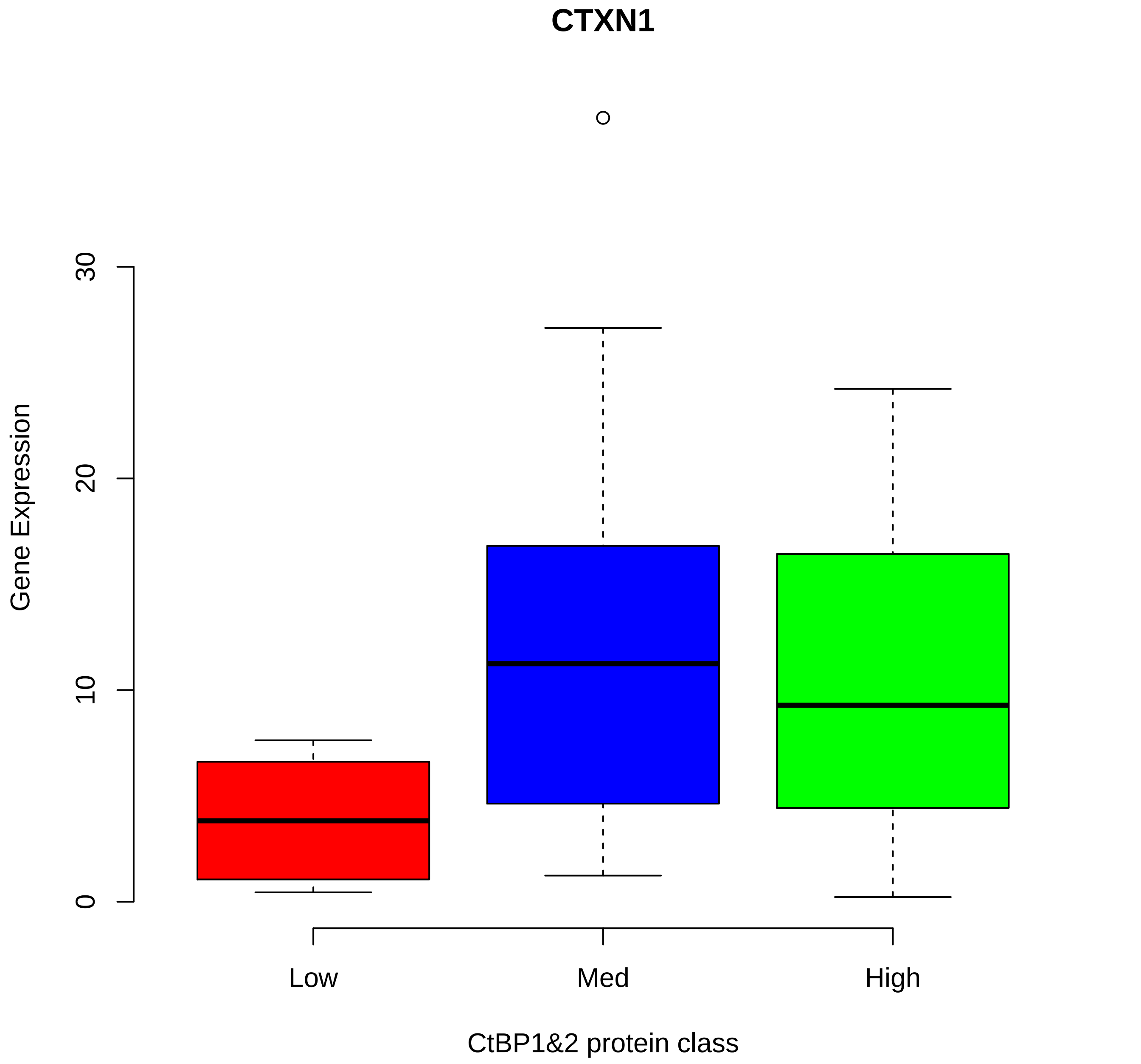

CTXN1
Gene Expression
0
10
20
30
Low
Med
High
CtBP1&2 protein class

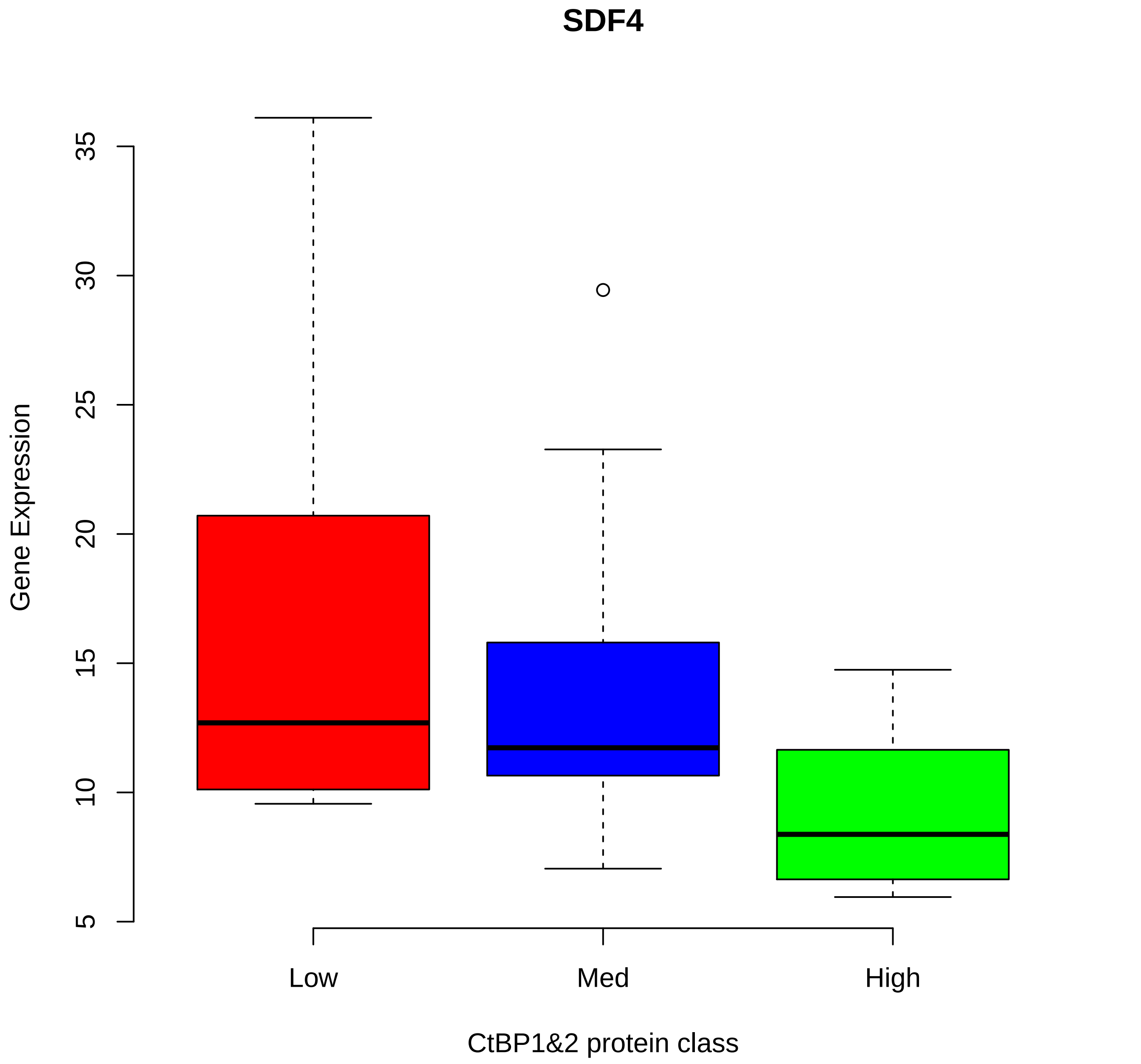
SDF4
Gene Expression
35
30
25
20
15
10
5
Low
Med
High
CtBP1&2 protein class

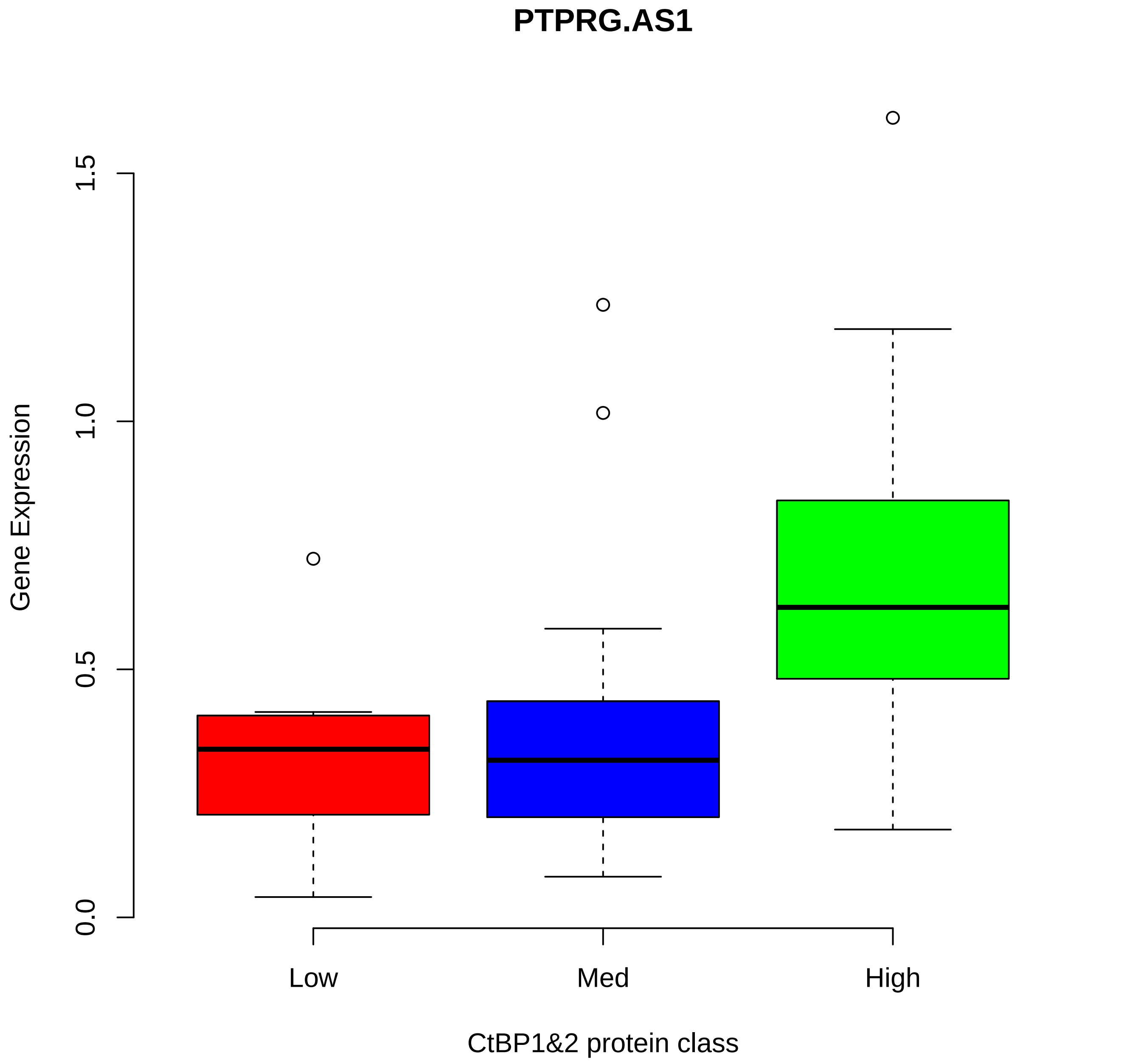
PTPRG.AS1
Gene Expression
0.0
0.5
1.0
1.5
Low
Med
High
CtBP1&2 protein class

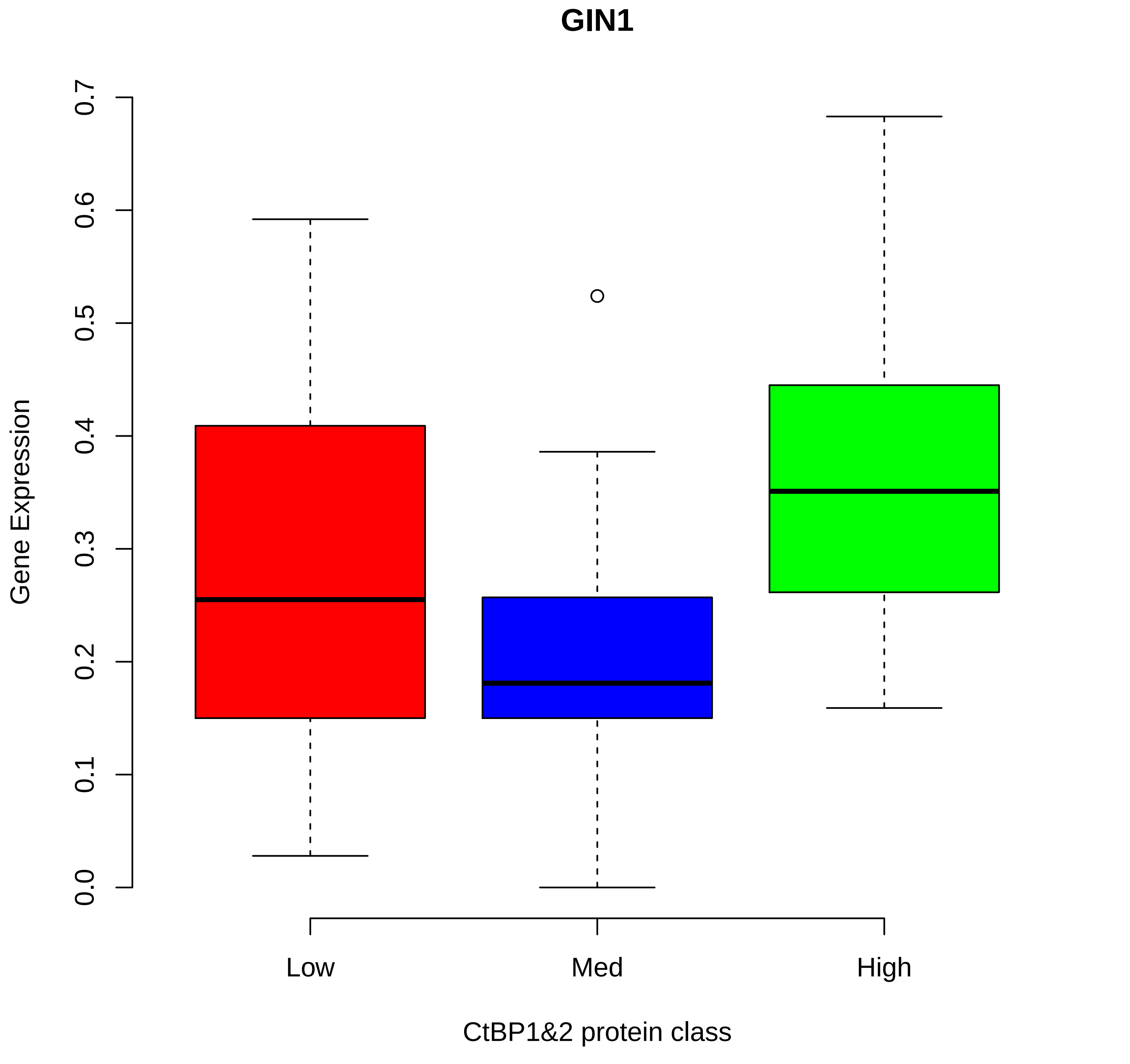
GIN1
Gene Expression
0.0
0.1
0.2
0.3
0.4
0.5
0.6
0.7
Low
Med
High
CtBP1&2 protein class

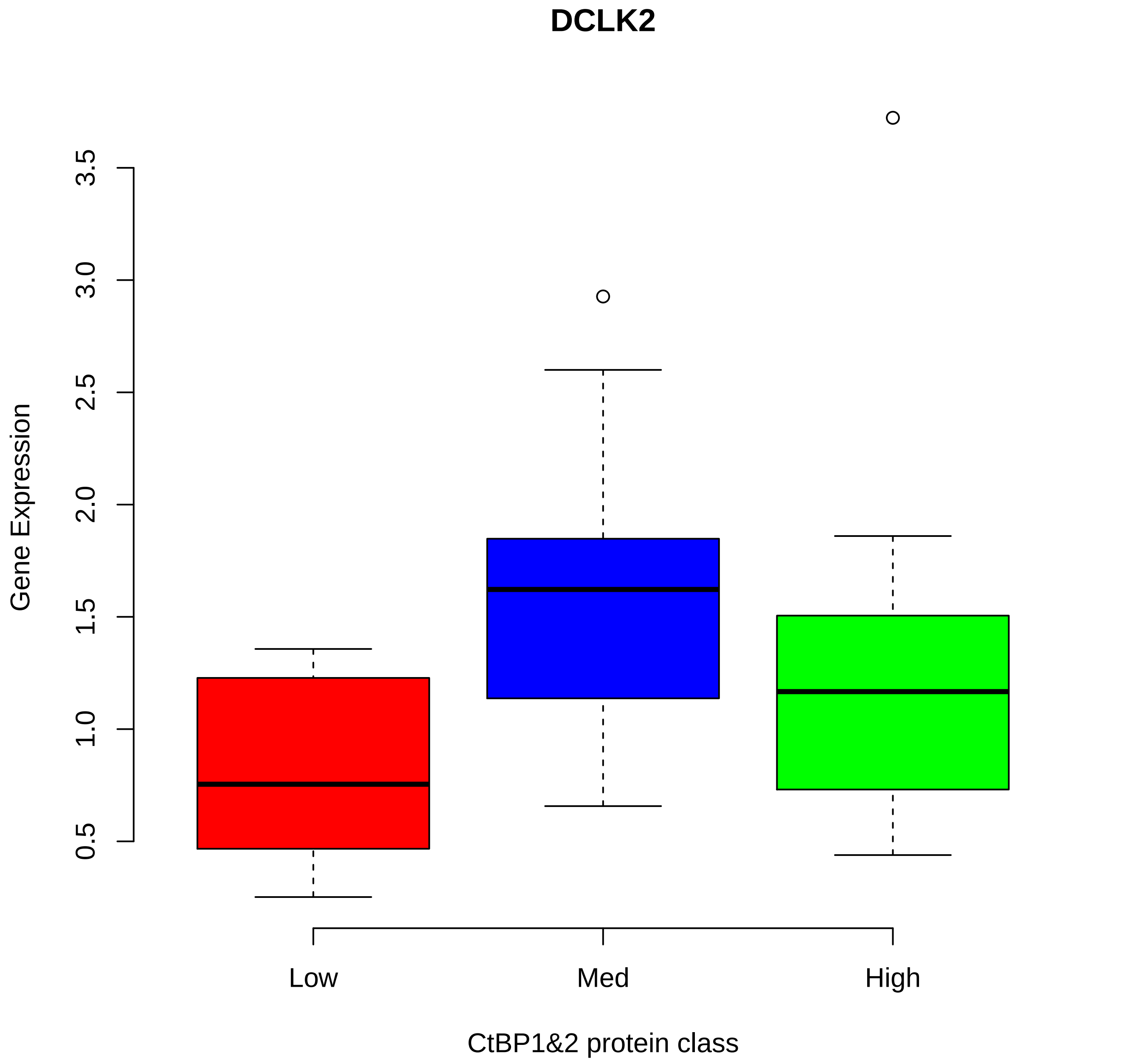

DCLK2
Gene Expression
0.5
1.0
1.5
2.0
2.5
3.0
3.5
Low
Med
High
CtBP1&2 protein class

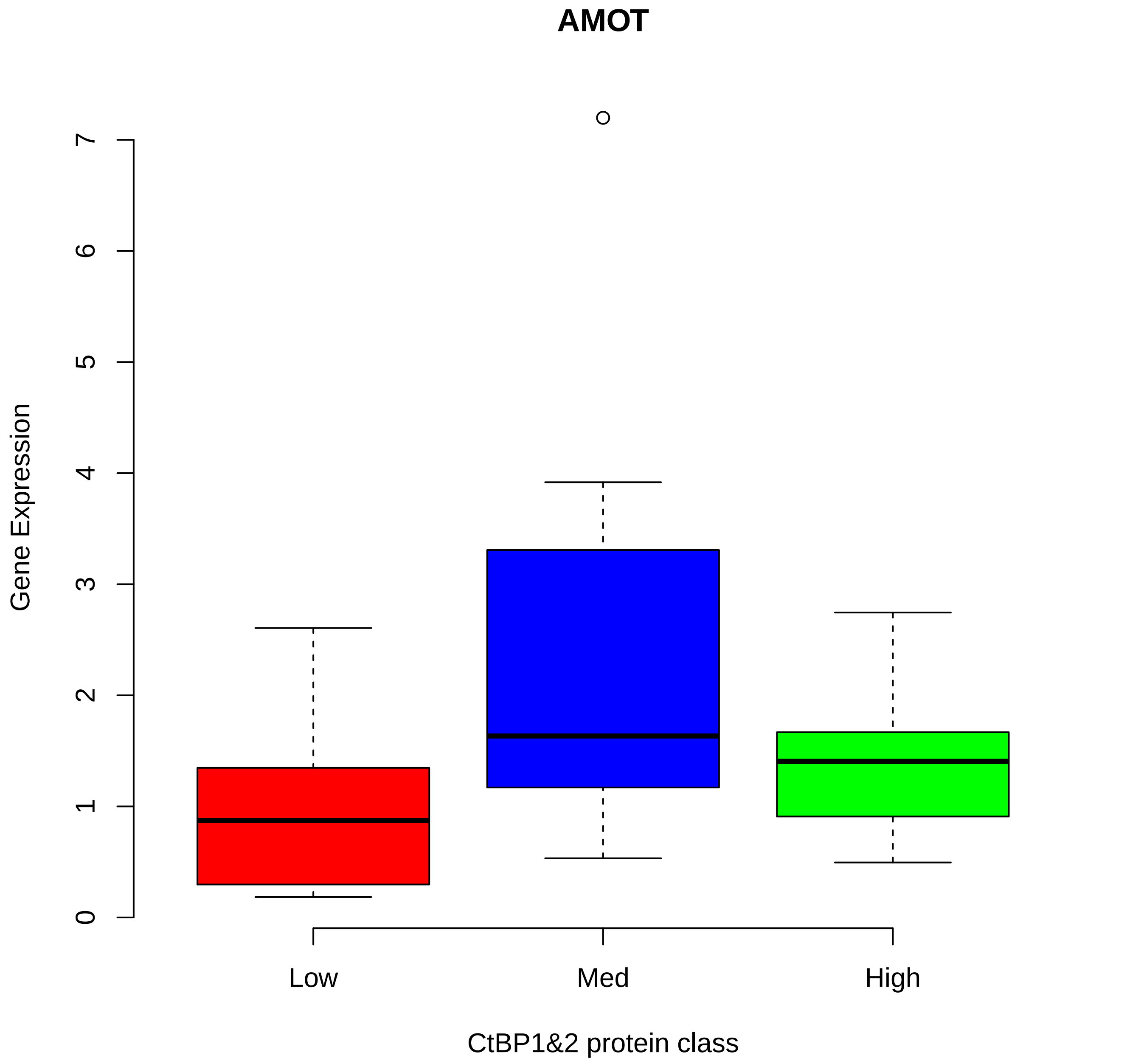

AMOT
Gene Expression
0
1
2
3
4
5
6
7
Low
Med
High
CtBP1&2 protein class

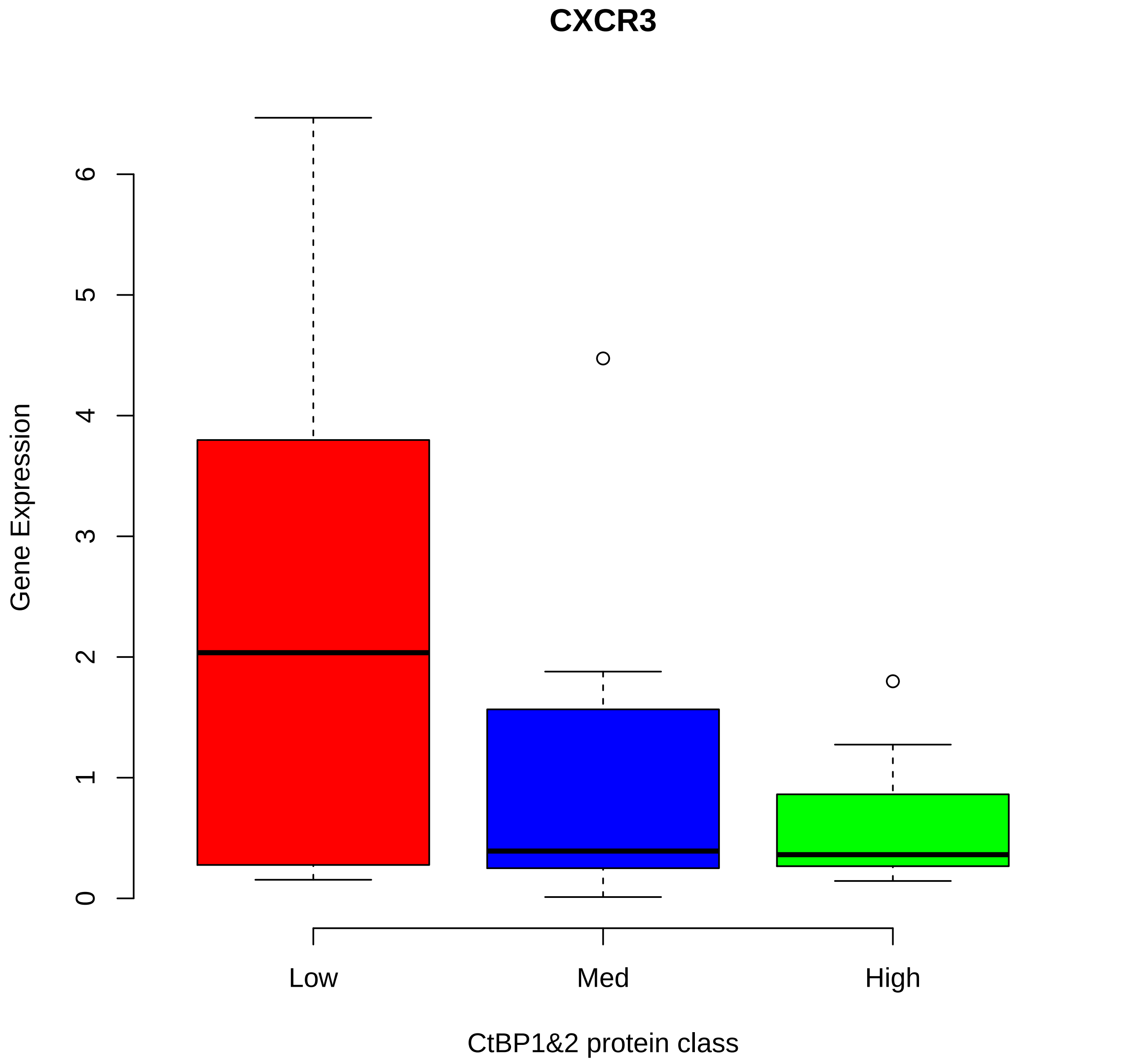

CXCR3
Gene Expression
0
1
2
3
4
5
6
Low
Med
High
CtBP1&2 protein class

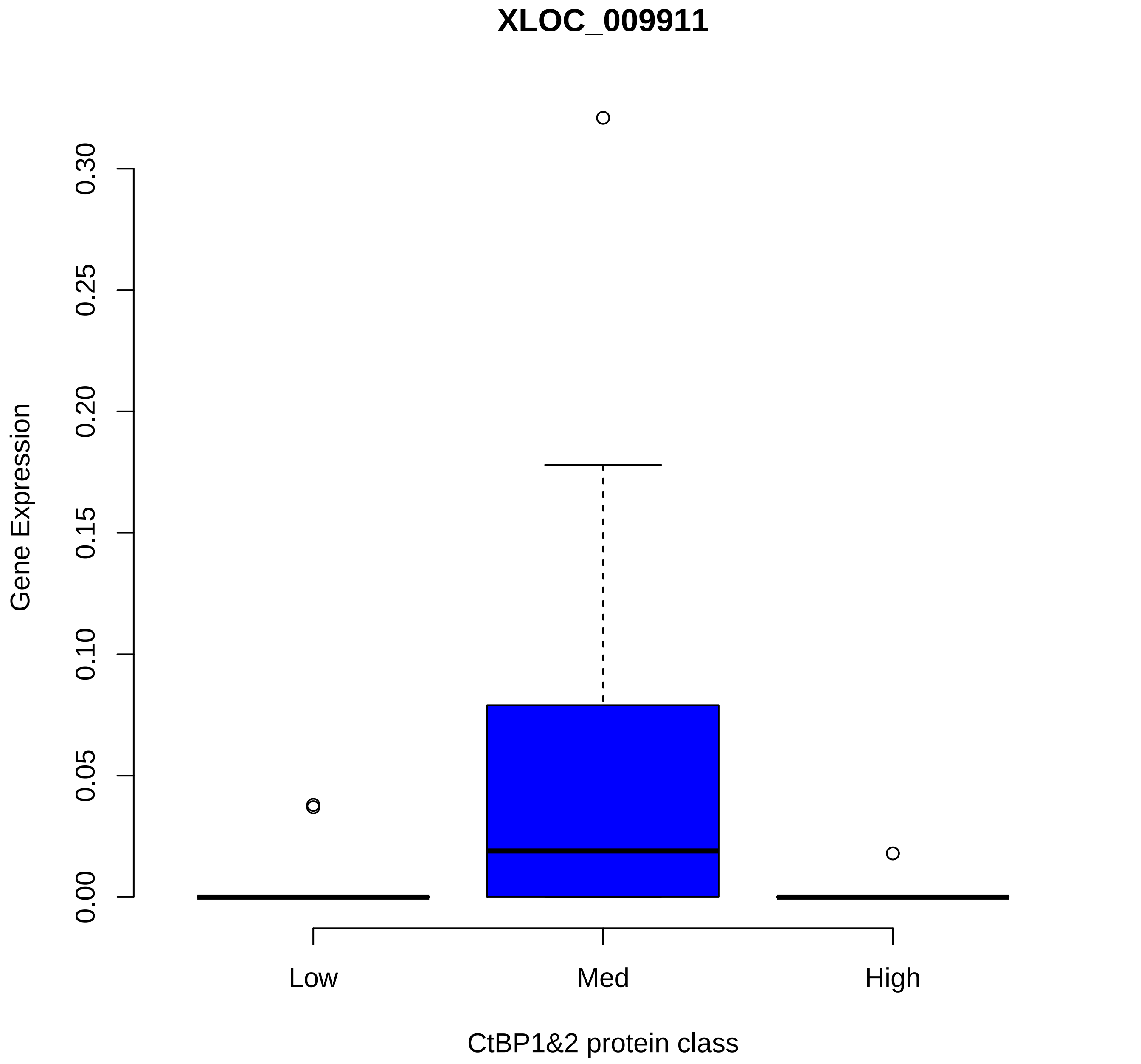
XLOC_009911
Gene Expression
0.00
0.05
0.10
0.15
0.20
0.25
0.30
Low
Med
High
CtBP1&2 protein class

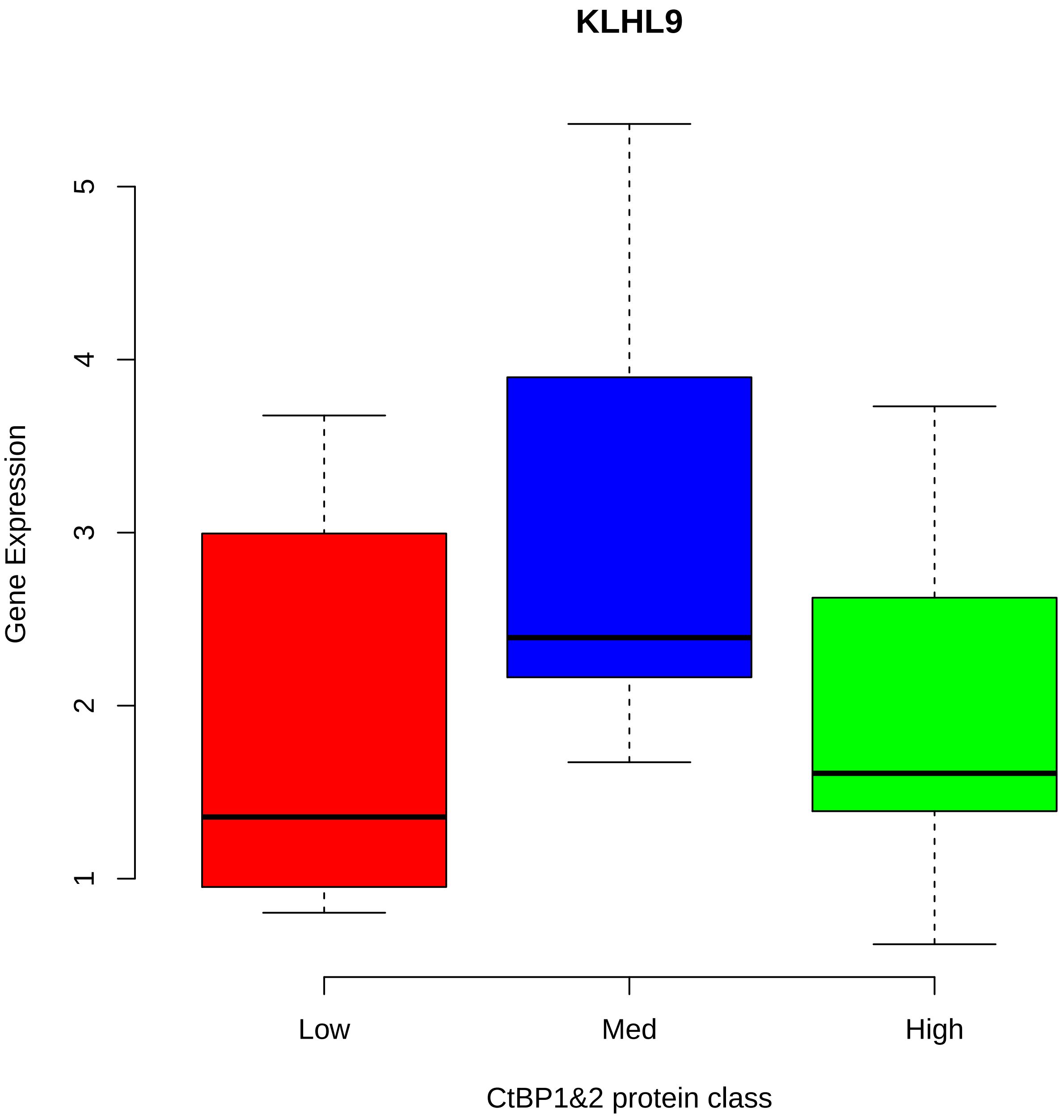

KLHL9
Gene Expression
1
2
3
4
5
Low
Med
High
CtBP1&2 protein class

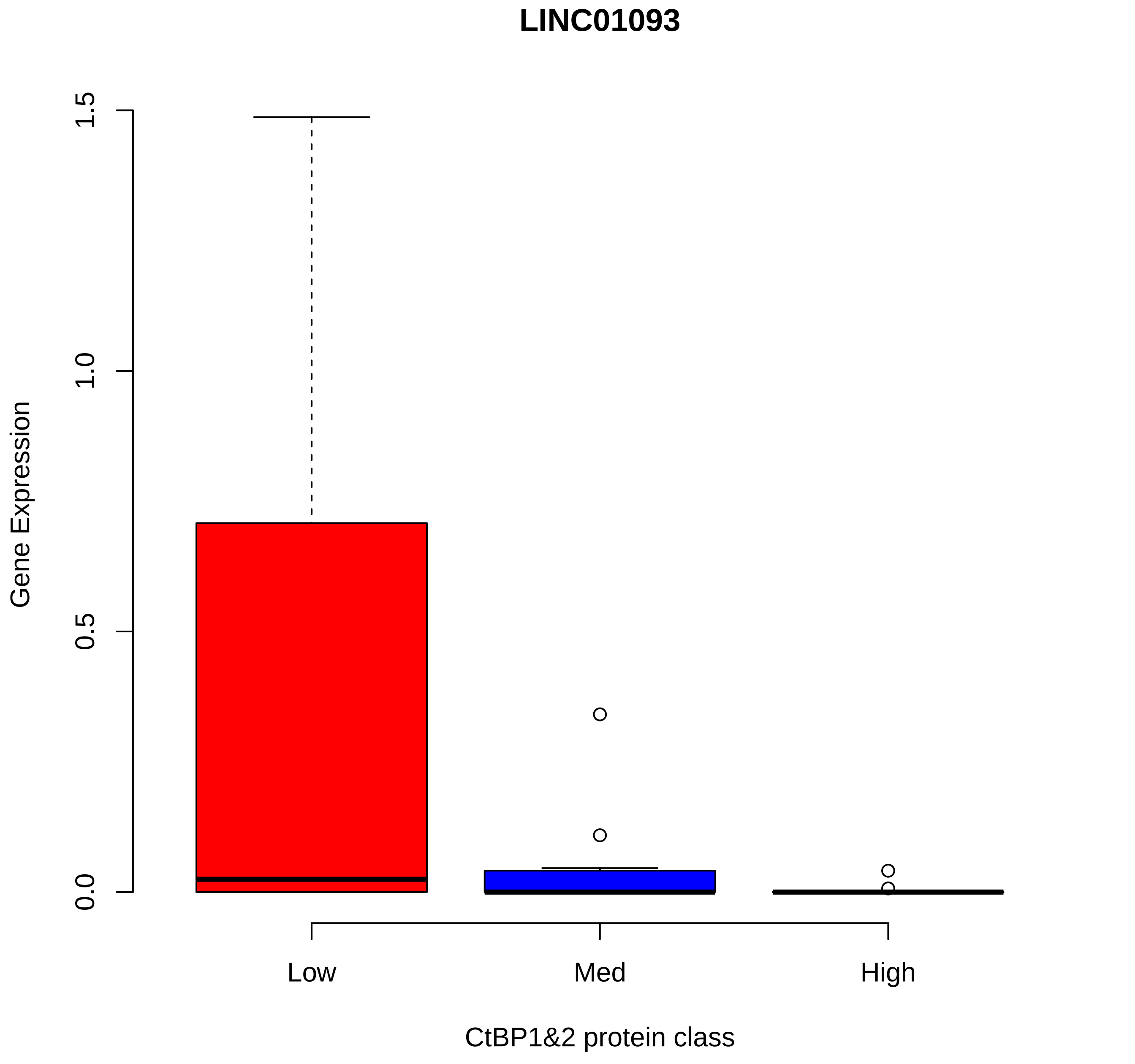
LINC01093
Gene Expression
0.0
0.5
1.0
1.5
Low
Med
High
CtBP1&2 protein class

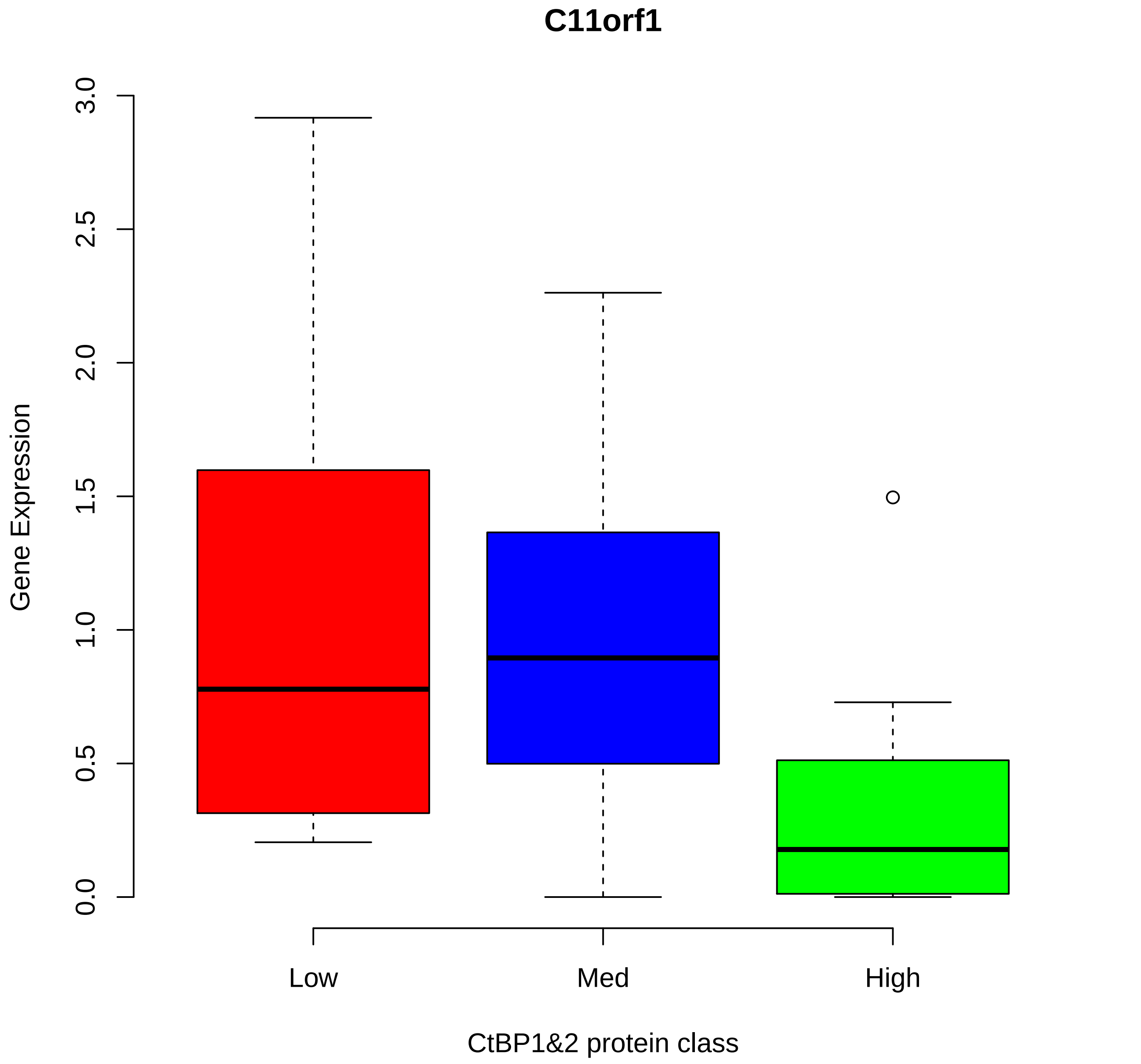

C11orf1
Gene Expression
0.0
0.5
1.0
1.5
2.0
2.5
3.0
Low
Med
High
CtBP1&2 protein class

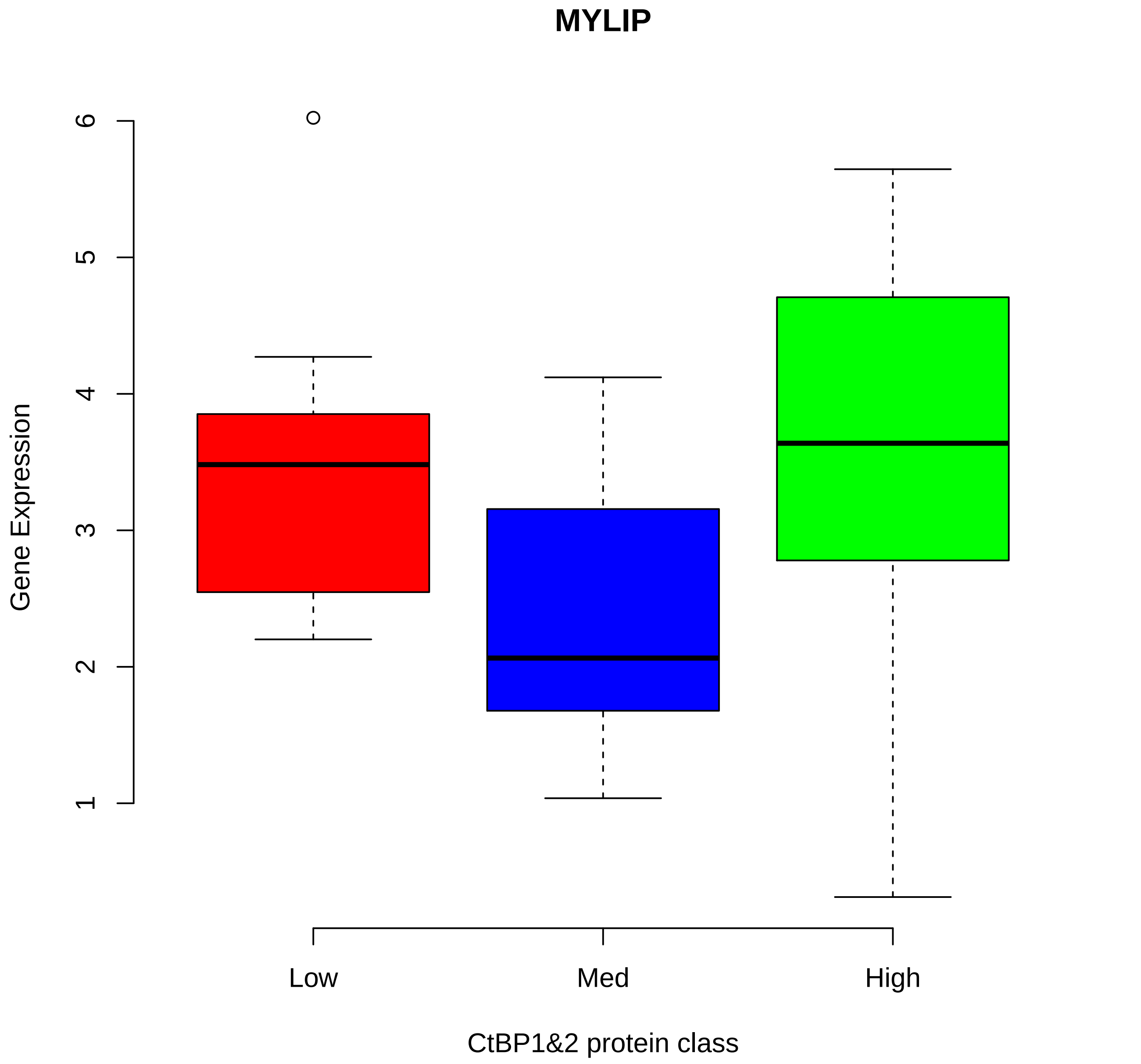

MYLIP
Gene Expression
1
2
3
4
5
6
Low
Med
High
CtBP1&2 protein class

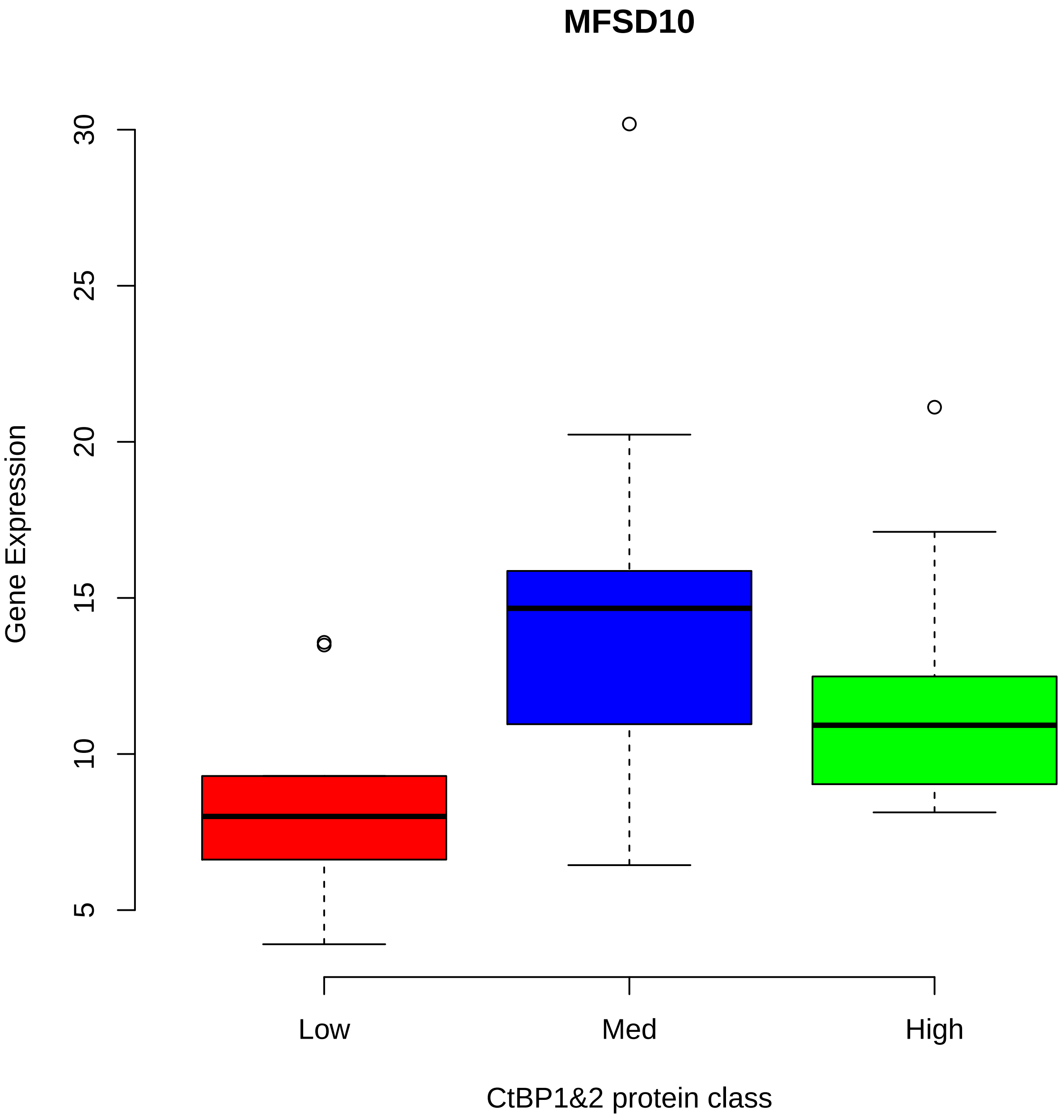
MFSD10
Gene Expression
30
25
20
15
10
5
Low
Med
High
CtBP1&2 protein class

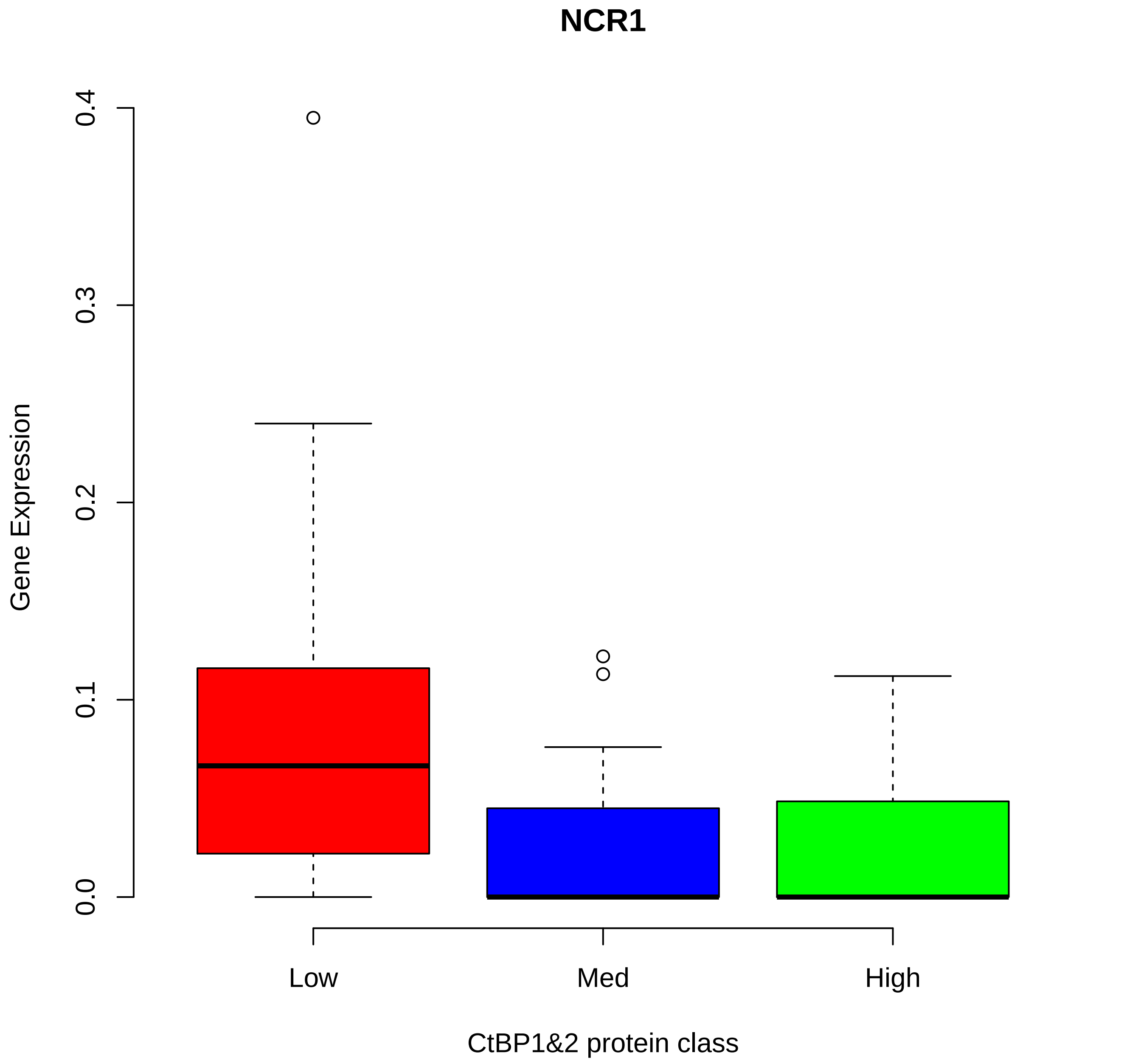
NCR1
Gene Expression
0.0
0.1
0.2
0.3
0.4
Low
Med
High
CtBP1&2 protein class

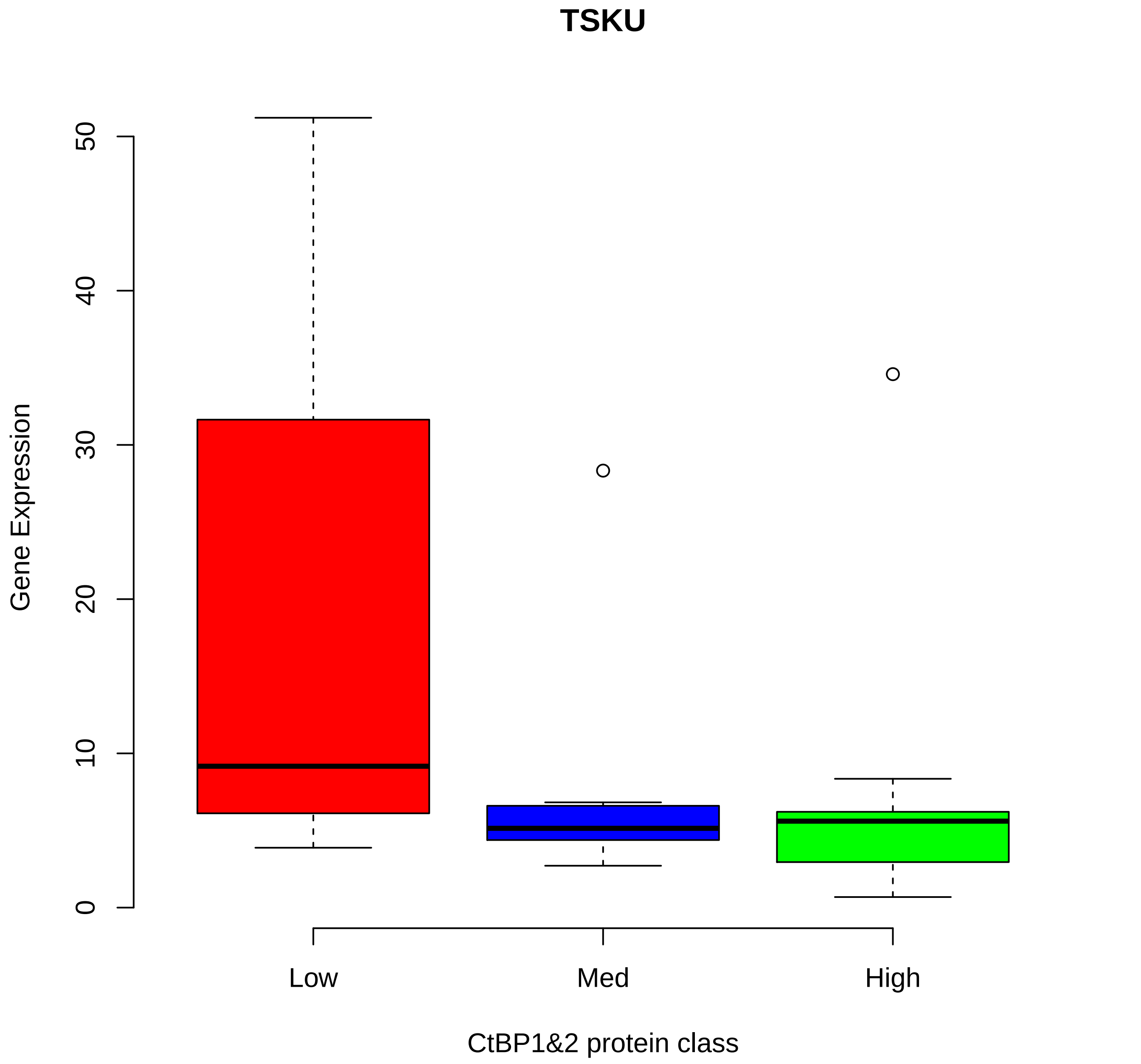

TSKU
Gene Expression
0
10
20
30
40
50
Low
Med
High
CtBP1&2 protein class

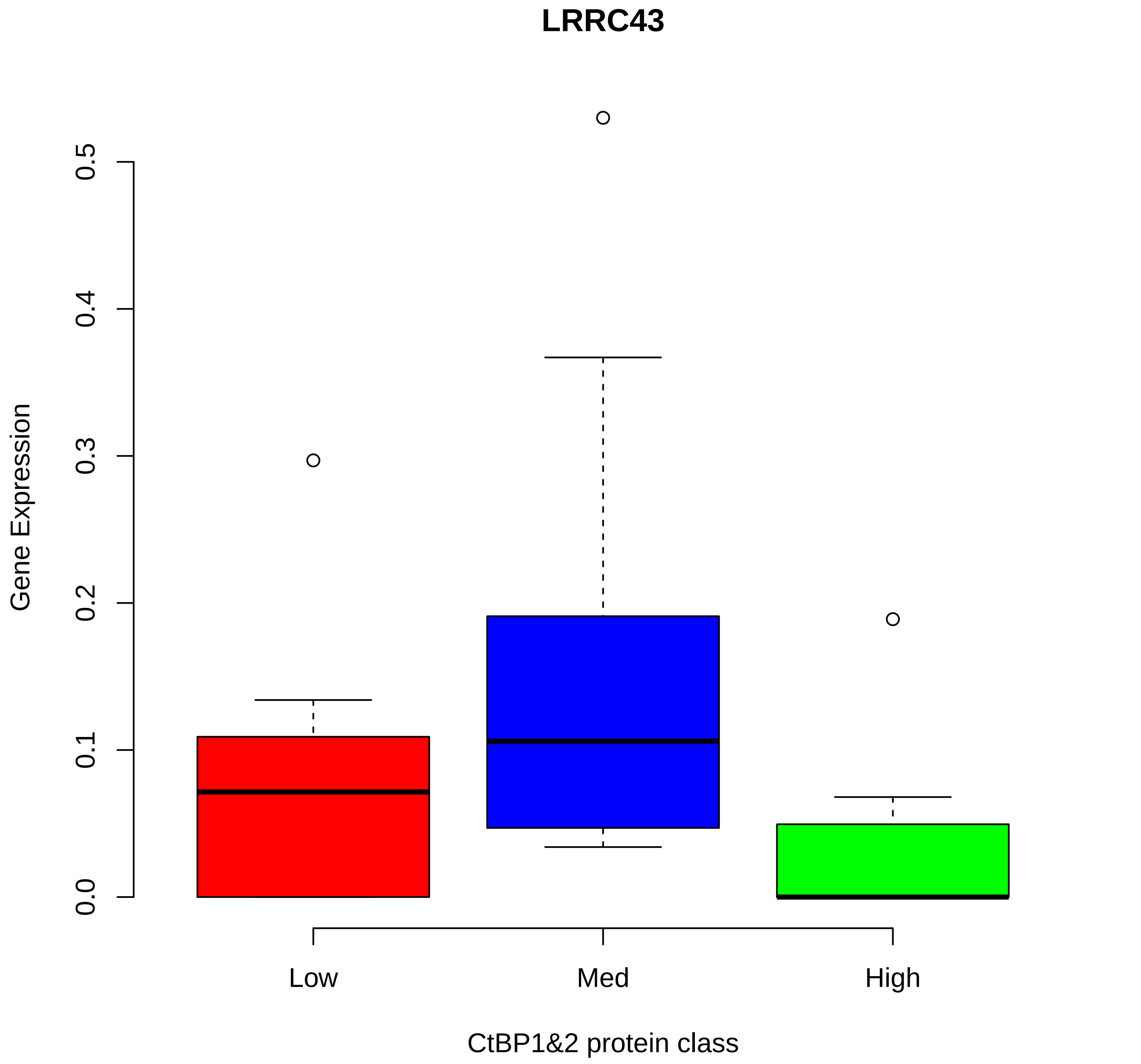
LRRC43
Gene Expression
0.0
0.1
0.2
0.3
0.4
0.5
Low
Med
High
CtBP1&2 protein class

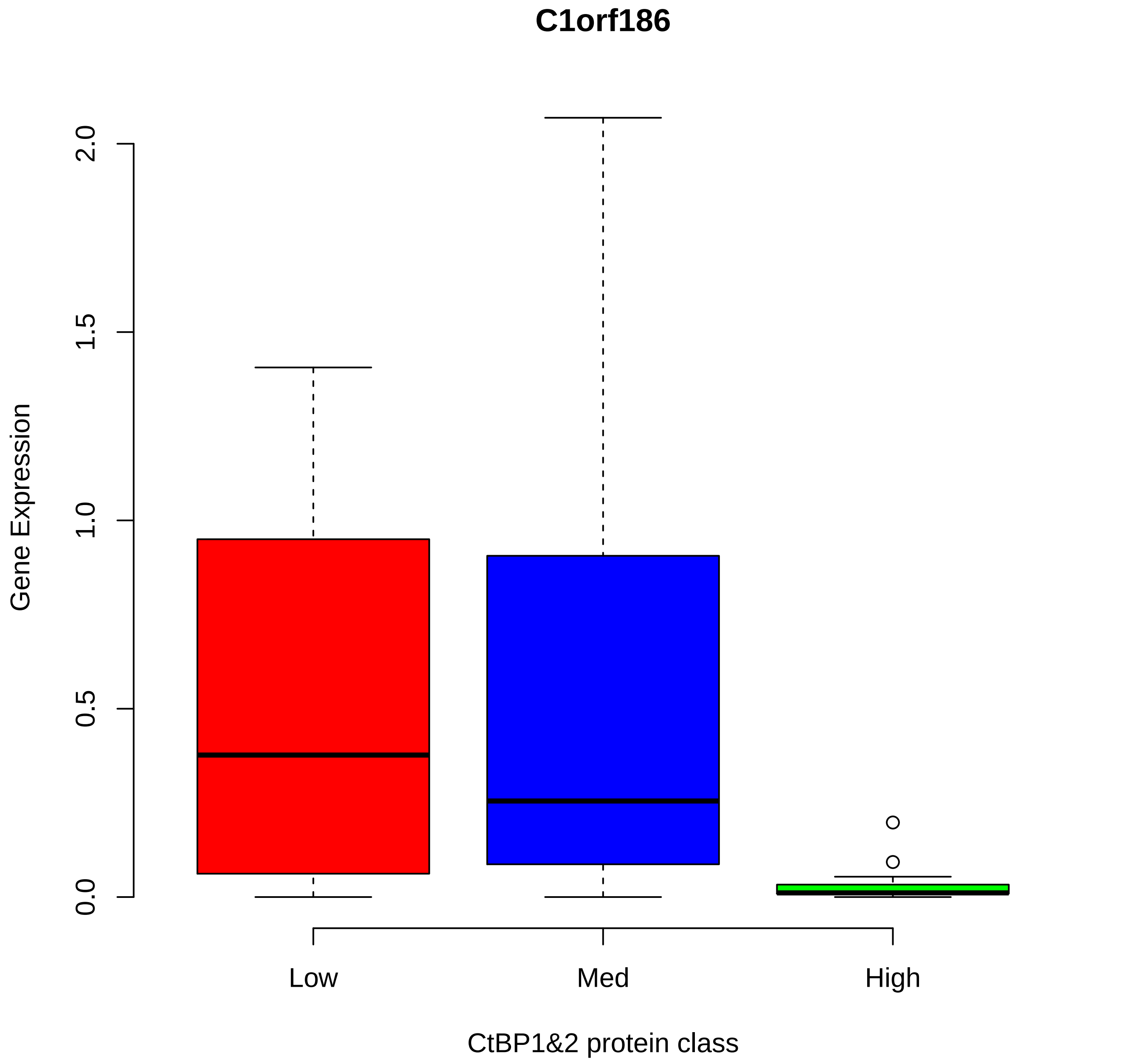
C1orf186
Gene Expression
0.0
0.5
1.0
1.5
2.0
Low
Med
High
CtBP1&2 protein class

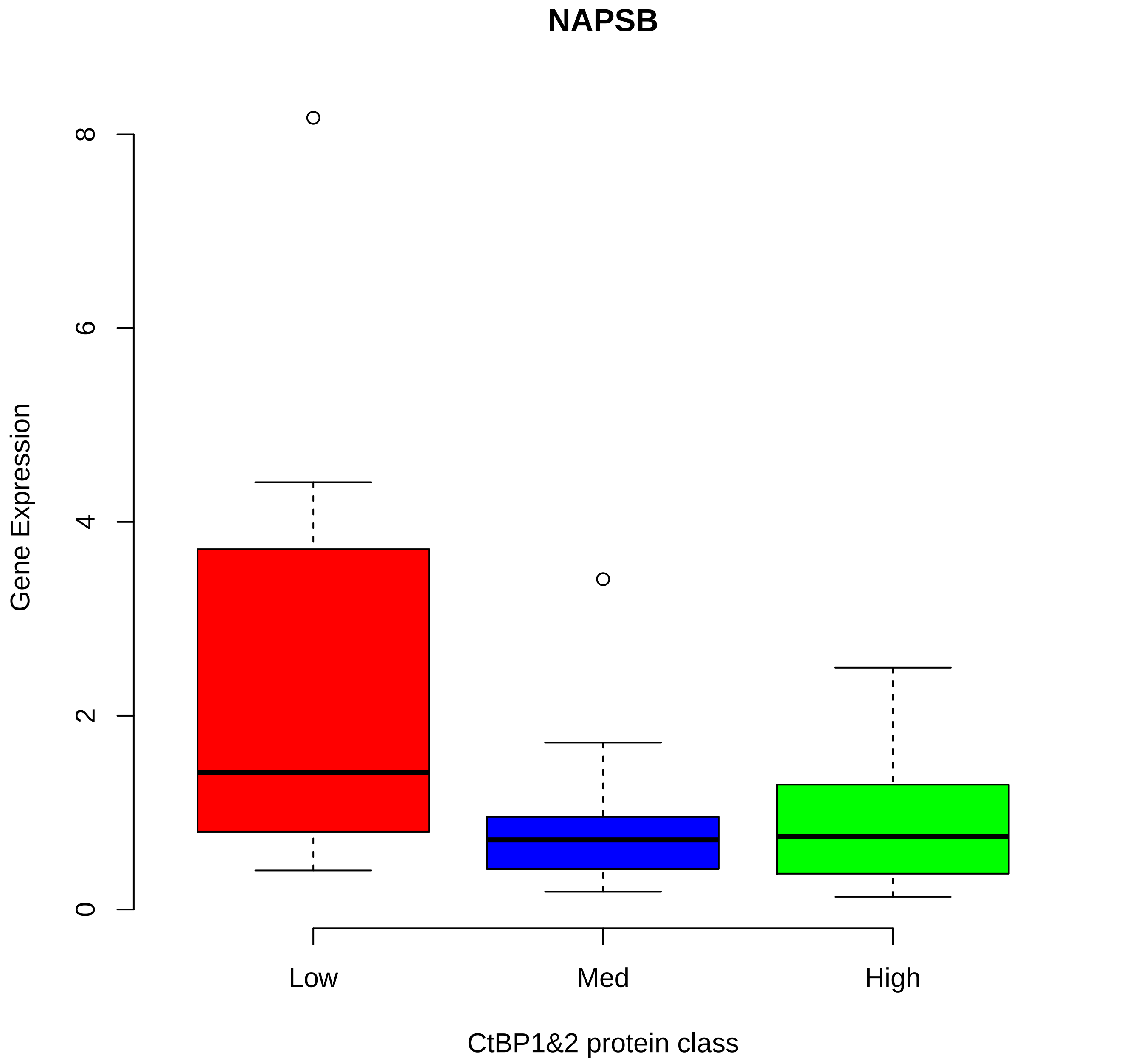

NAPSB
Gene Expression
0
2
4
6
8
Low
Med
High
CtBP1&2 protein class

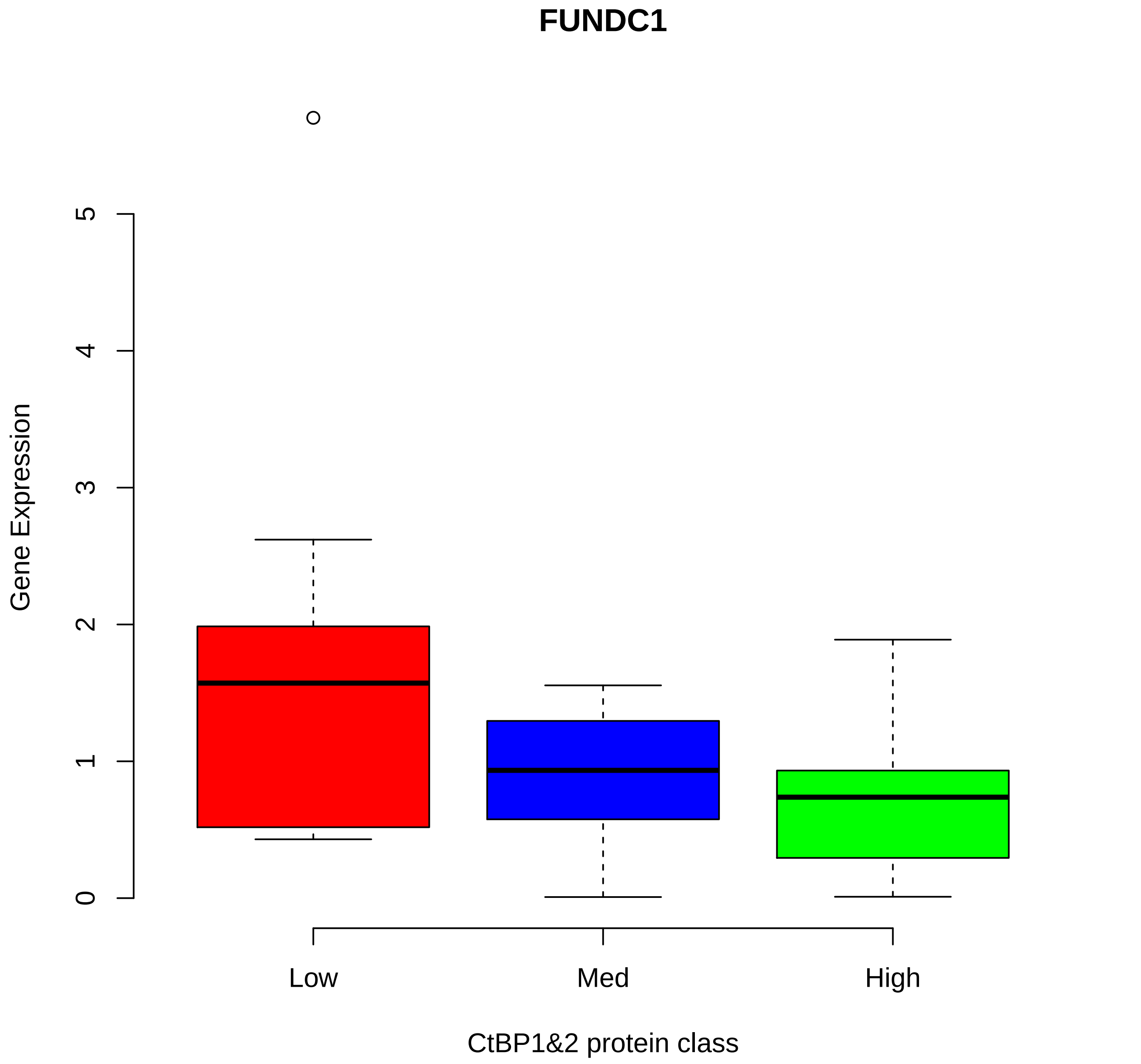

FUNDC1
Gene Expression
0
1
2
3
4
5
Low
Med
High
CtBP1&2 protein class

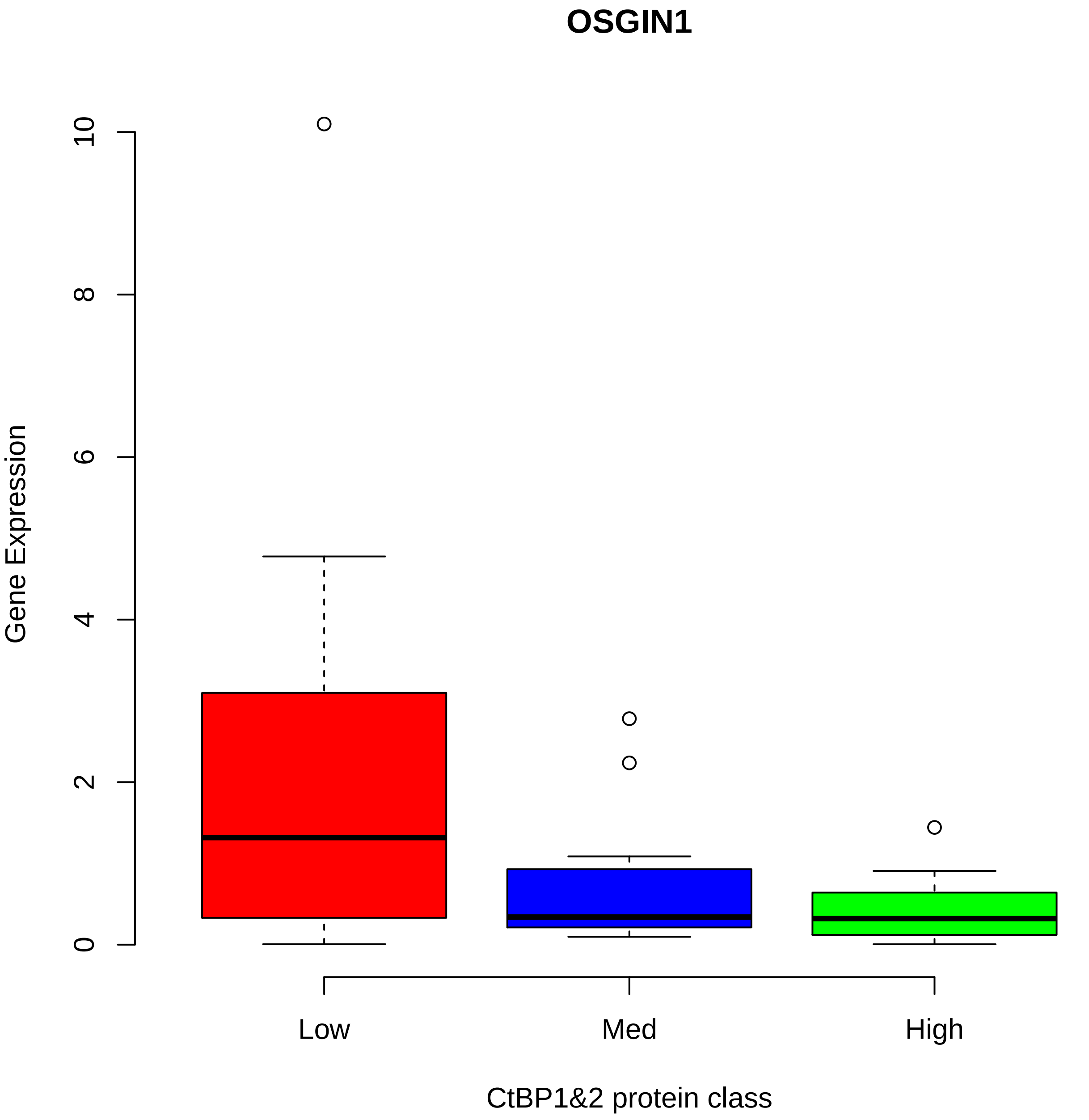
OSGIN1
Gene Expression
0
2
4
6
8
10
Low
Med
High
CtBP1&2 protein class

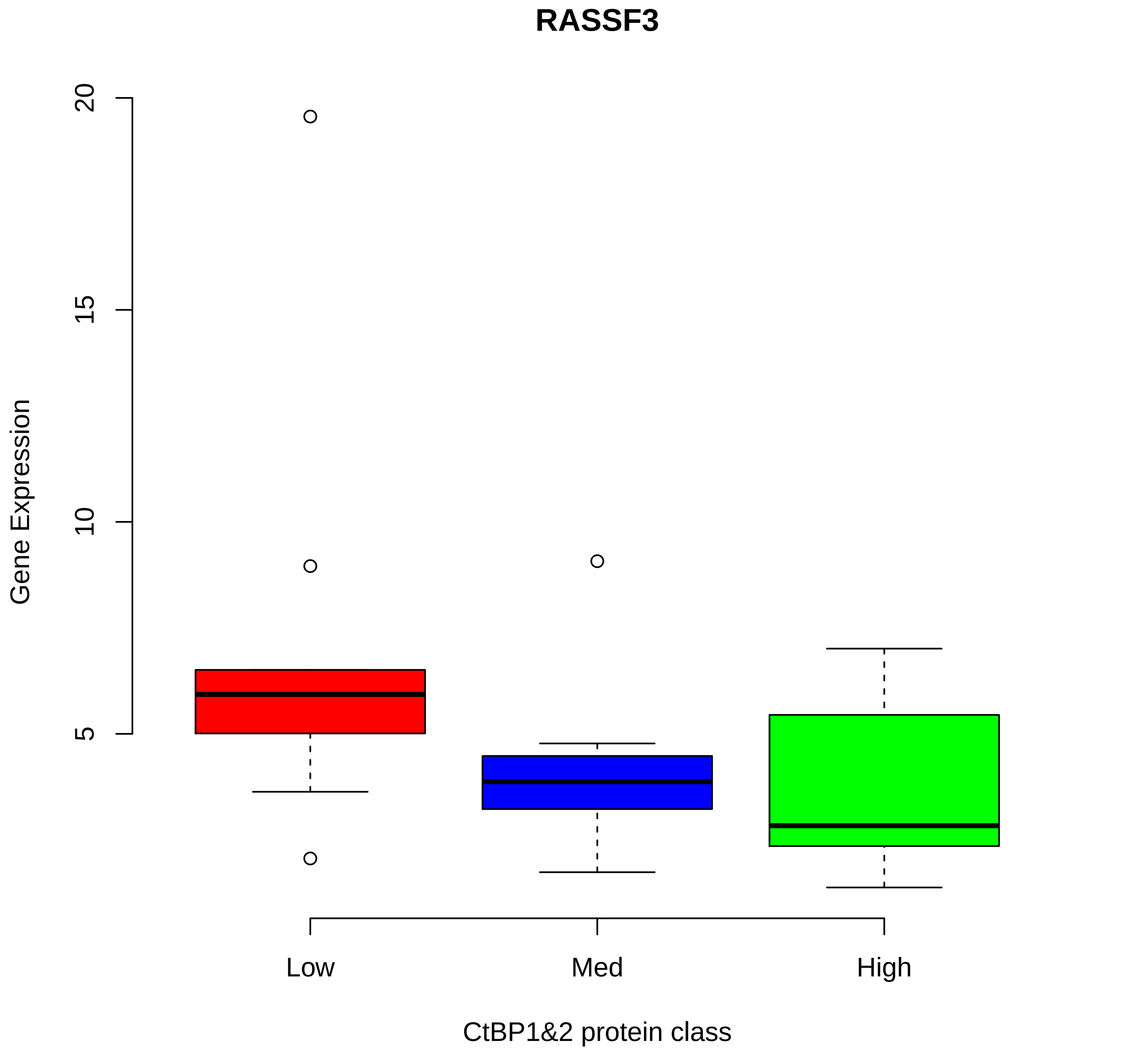
RASSF3
Gene Expression
20
15
10
5
Low
Med
High
CtBP1&2 protein class

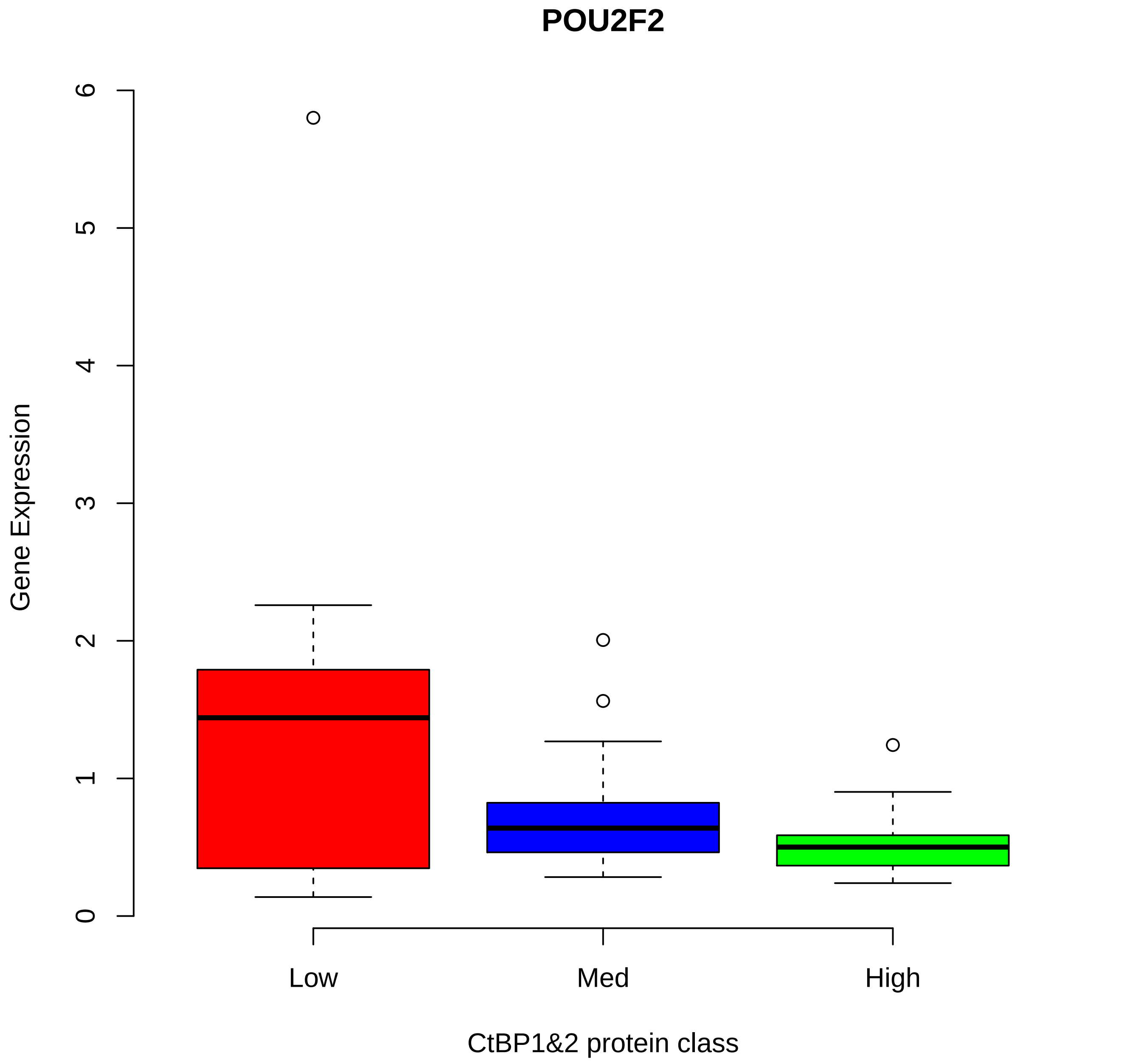

POU2F2
Gene Expression
0
1
2
3
4
5
6
Low
Med
High
CtBP1&2 protein class

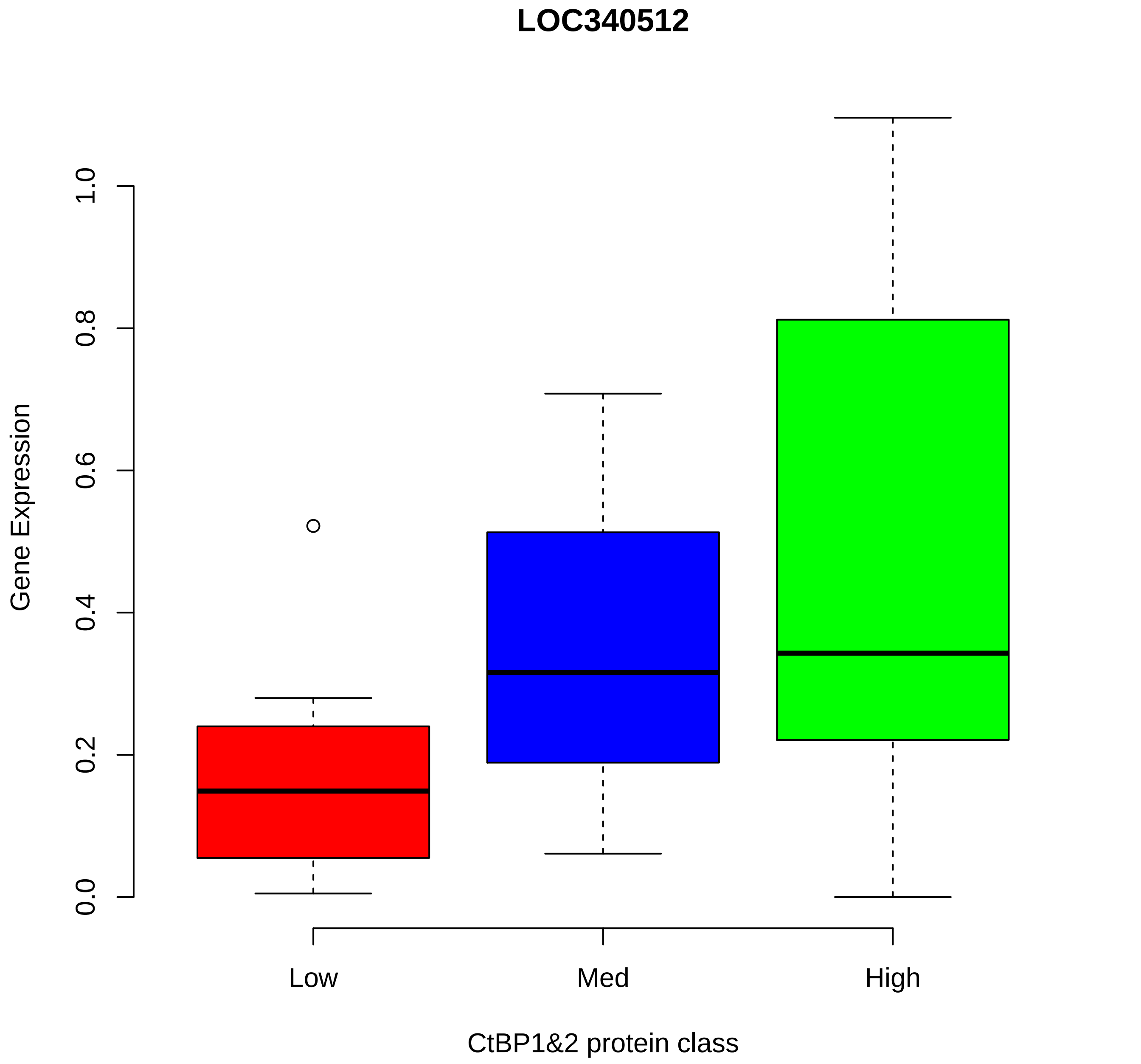

LOC340512
Gene Expression
0.0
0.2
0.4
0.6
0.8
1.0
Low
Med
High
CtBP1&2 protein class

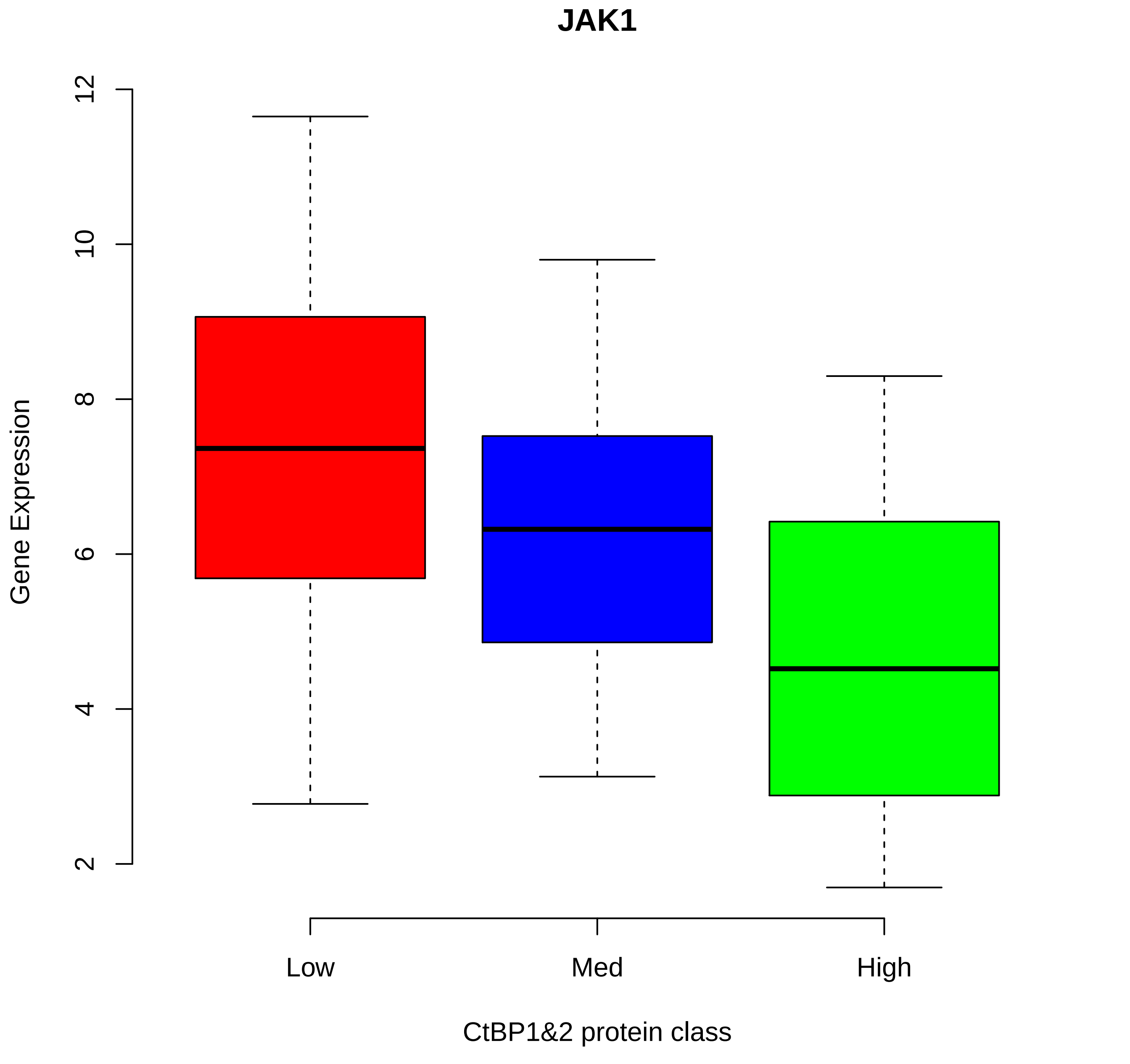

JAK1
Gene Expression
12
10
8
6
4
2
Low
Med
High
CtBP1&2 protein class

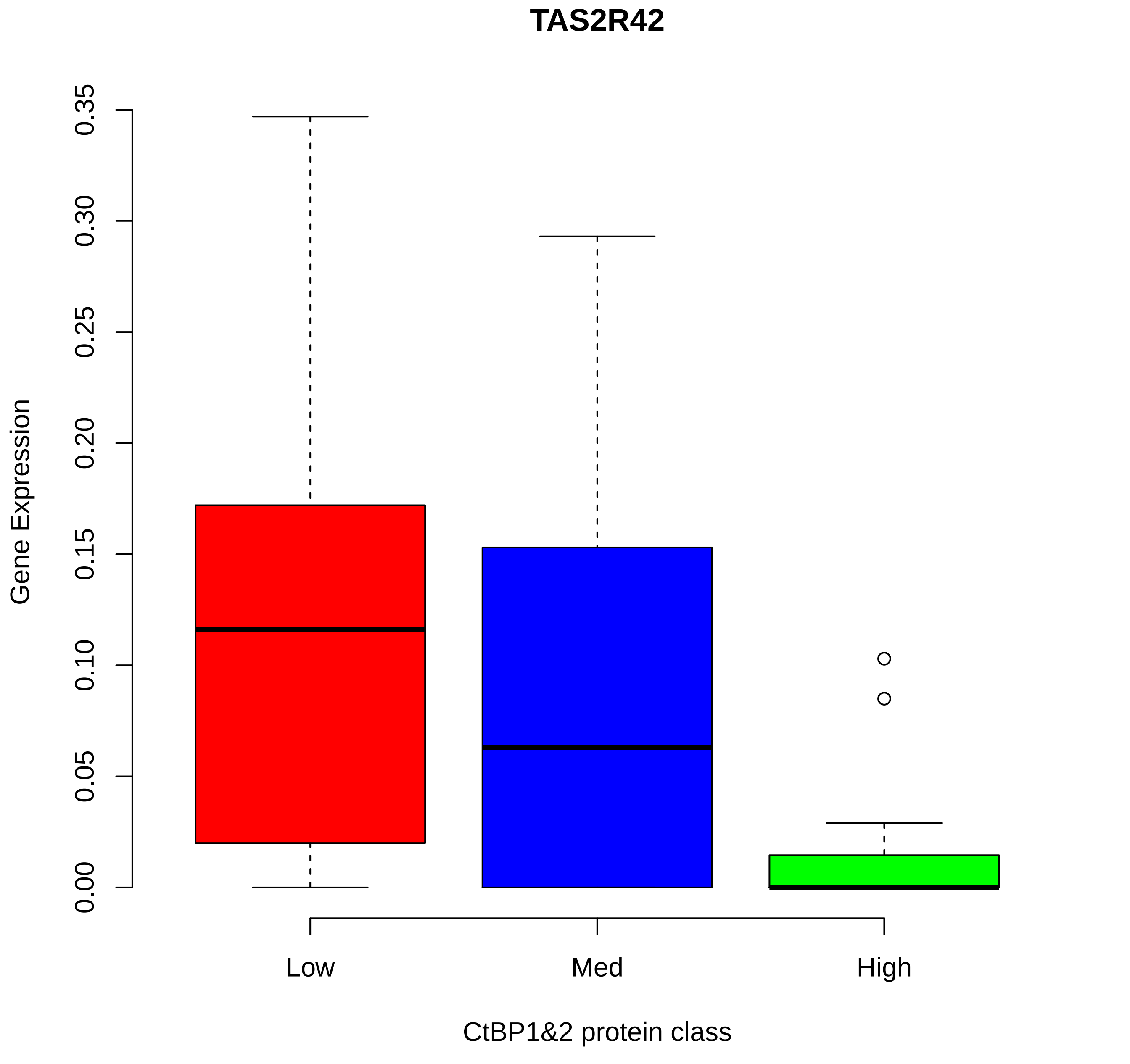

TAS2R42
Gene Expression
0.00
0.05
0.10
0.15
0.20
0.25
0.30
0.35
Low
Med
High
CtBP1&2 protein class

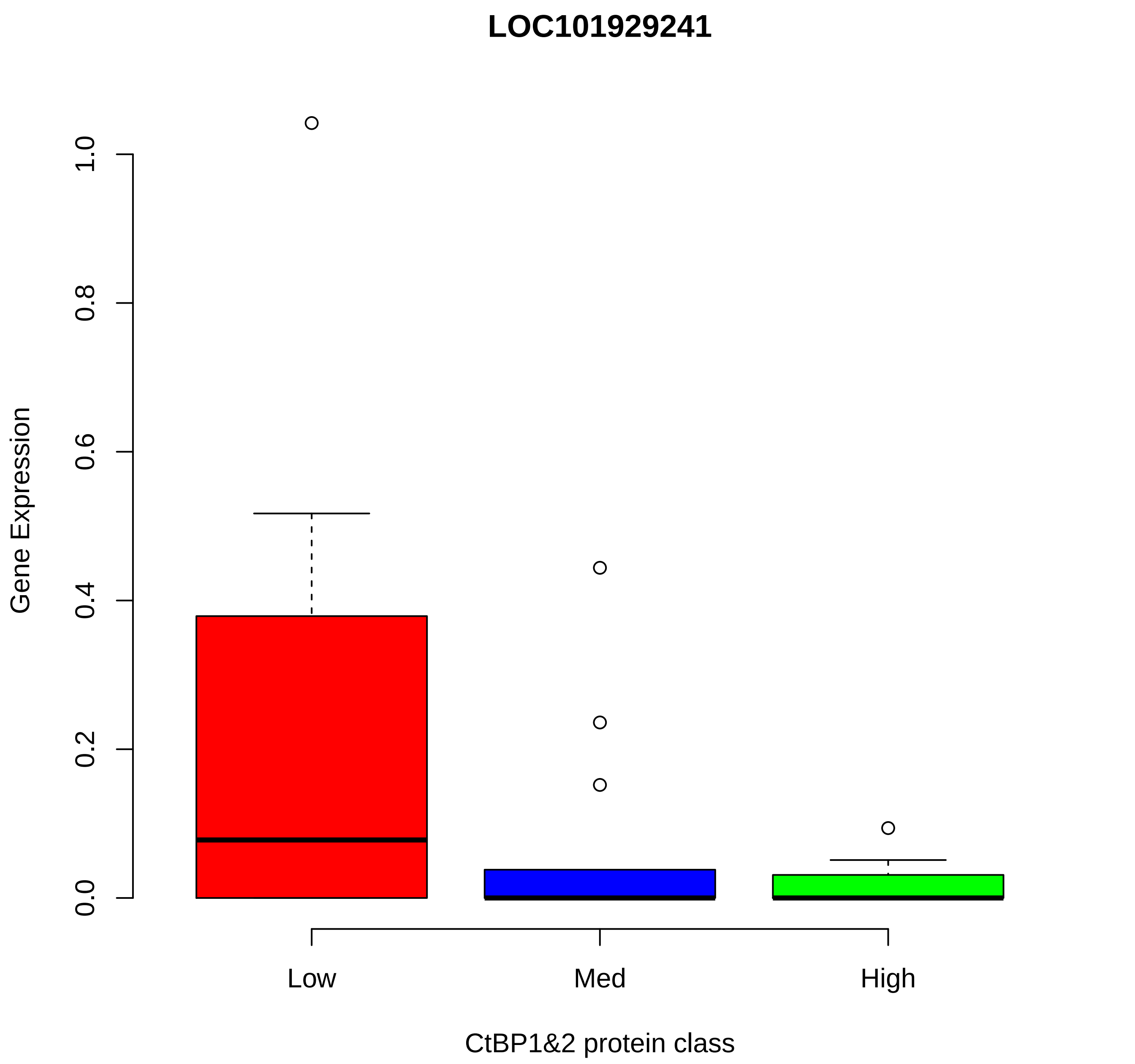

LOC101929241
Gene Expression
0.0
0.2
0.4
0.6
0.8
1.0
Low
Med
High
CtBP1&2 protein class

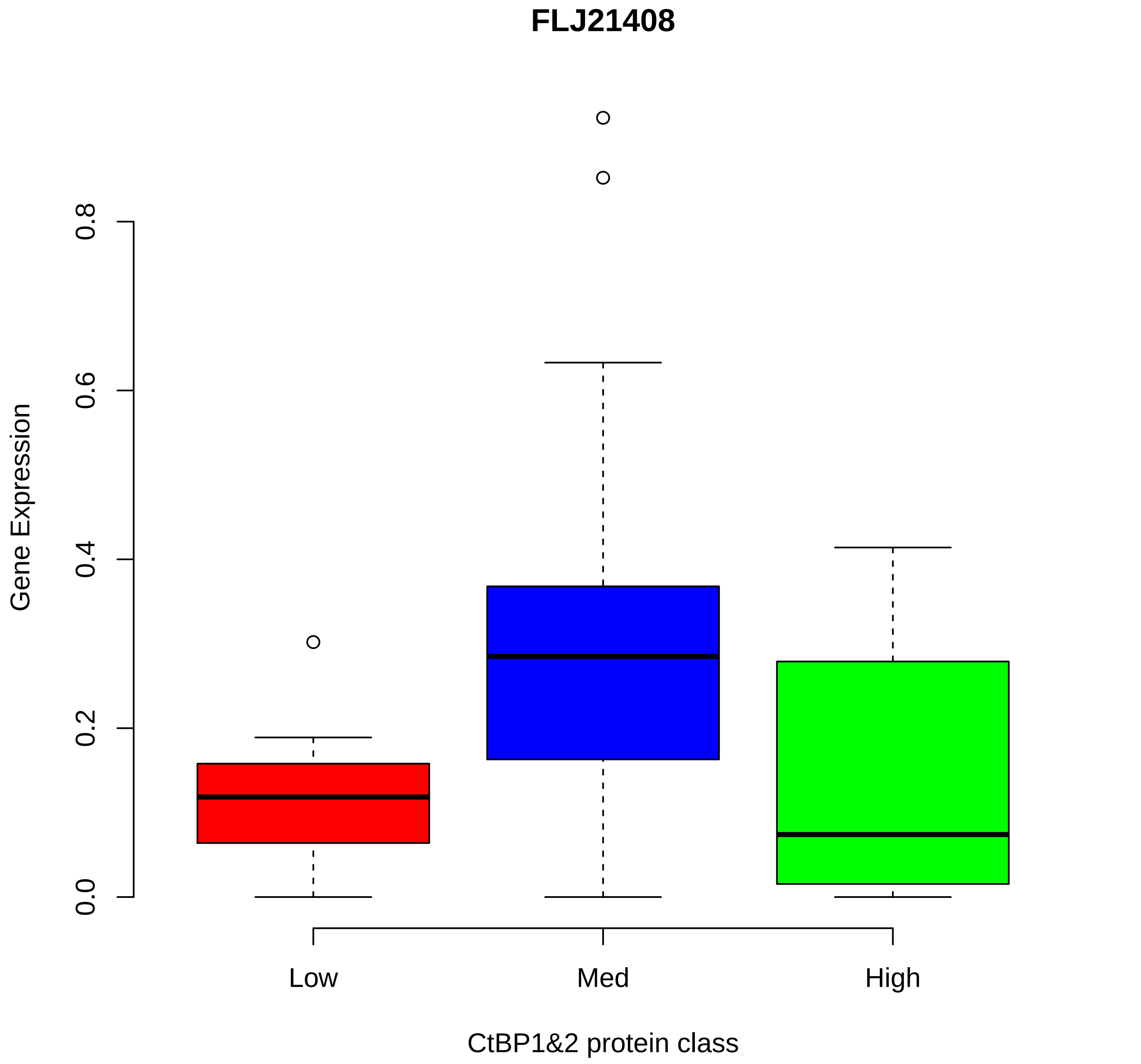

FLJ21408
Gene Expression
0.0
0.2
0.4
0.6
0.8
Low
Med
High
CtBP1&2 protein class

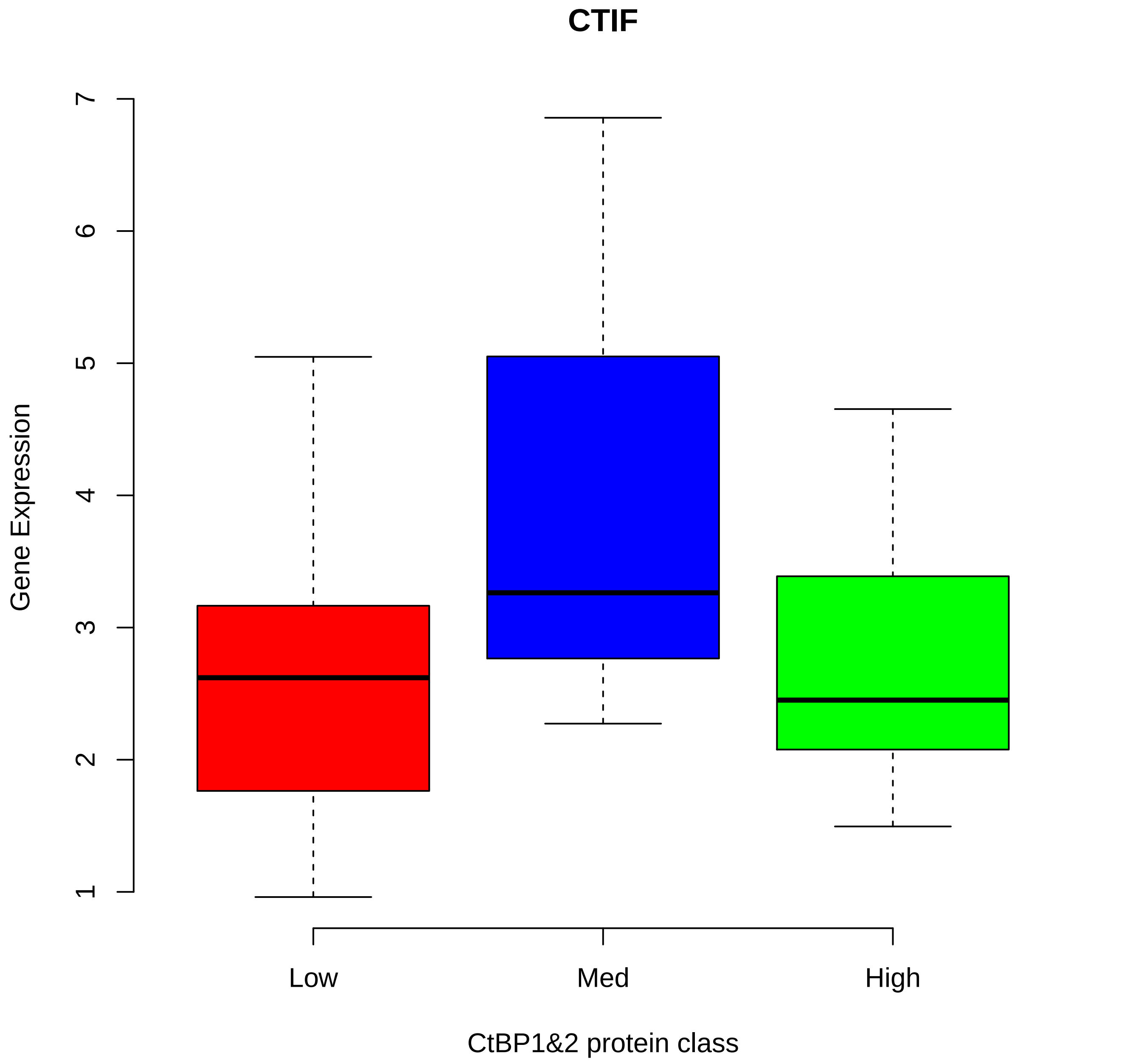
CTIF
Gene Expression
1
2
3
4
5
6
7
Low
Med
High
CtBP1&2 protein class

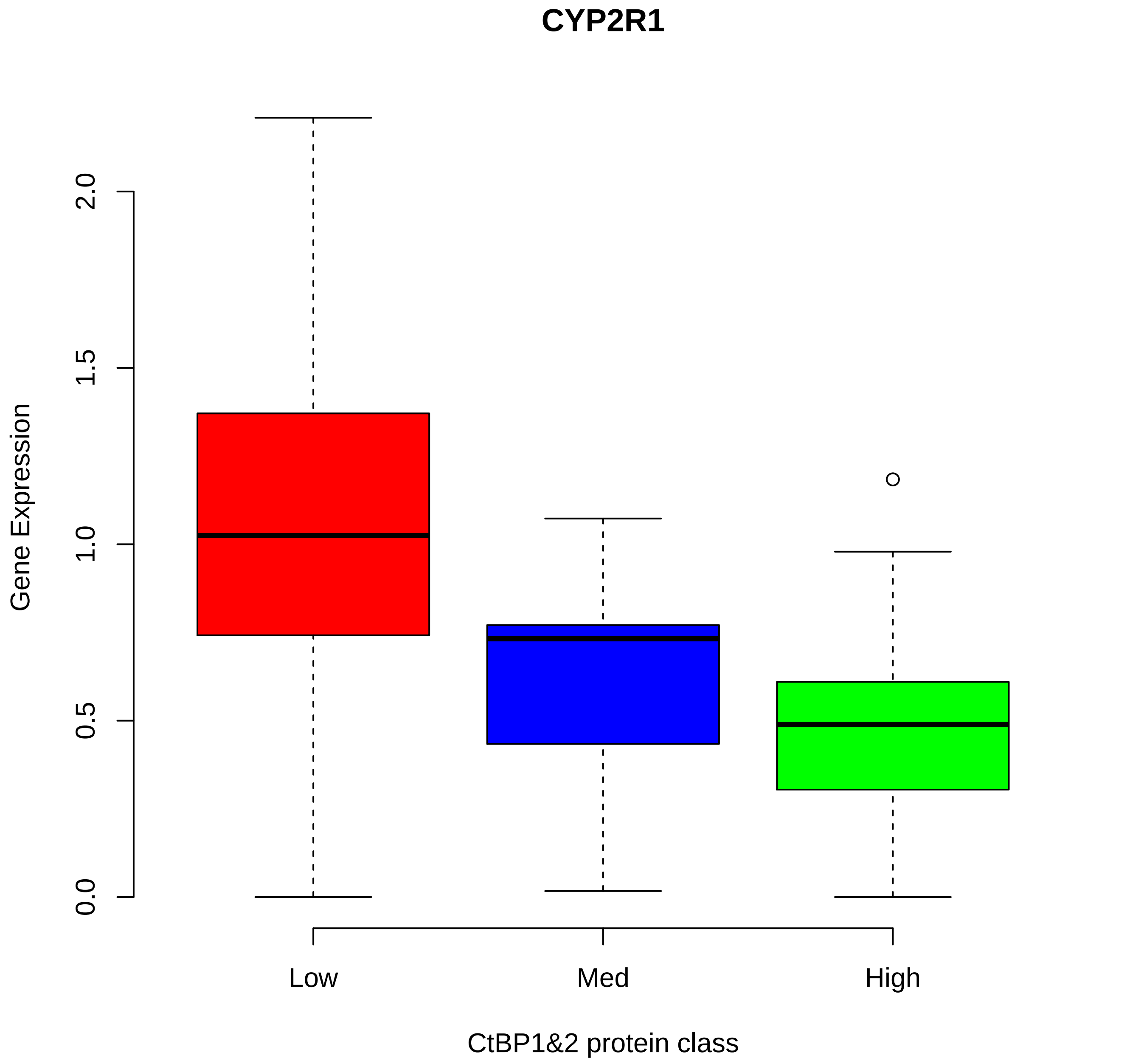

CYP2R1
Gene Expression
0.0
0.5
1.0
1.5
2.0
Low
Med
High
CtBP1&2 protein class

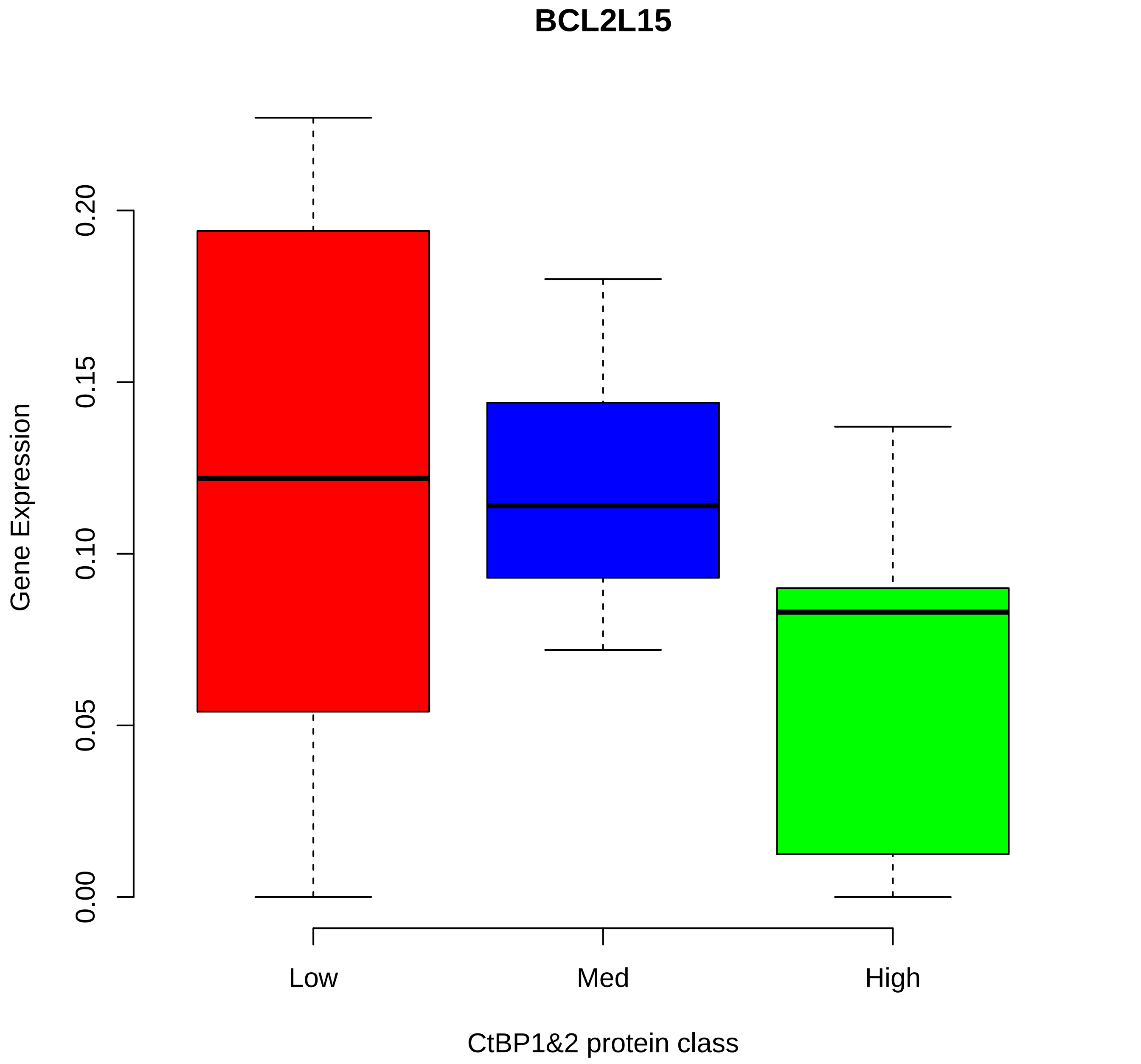

BCL2L15
Gene Expression
0.00
0.05
0.10
0.15
0.20
Low
Med
High
CtBP1&2 protein class

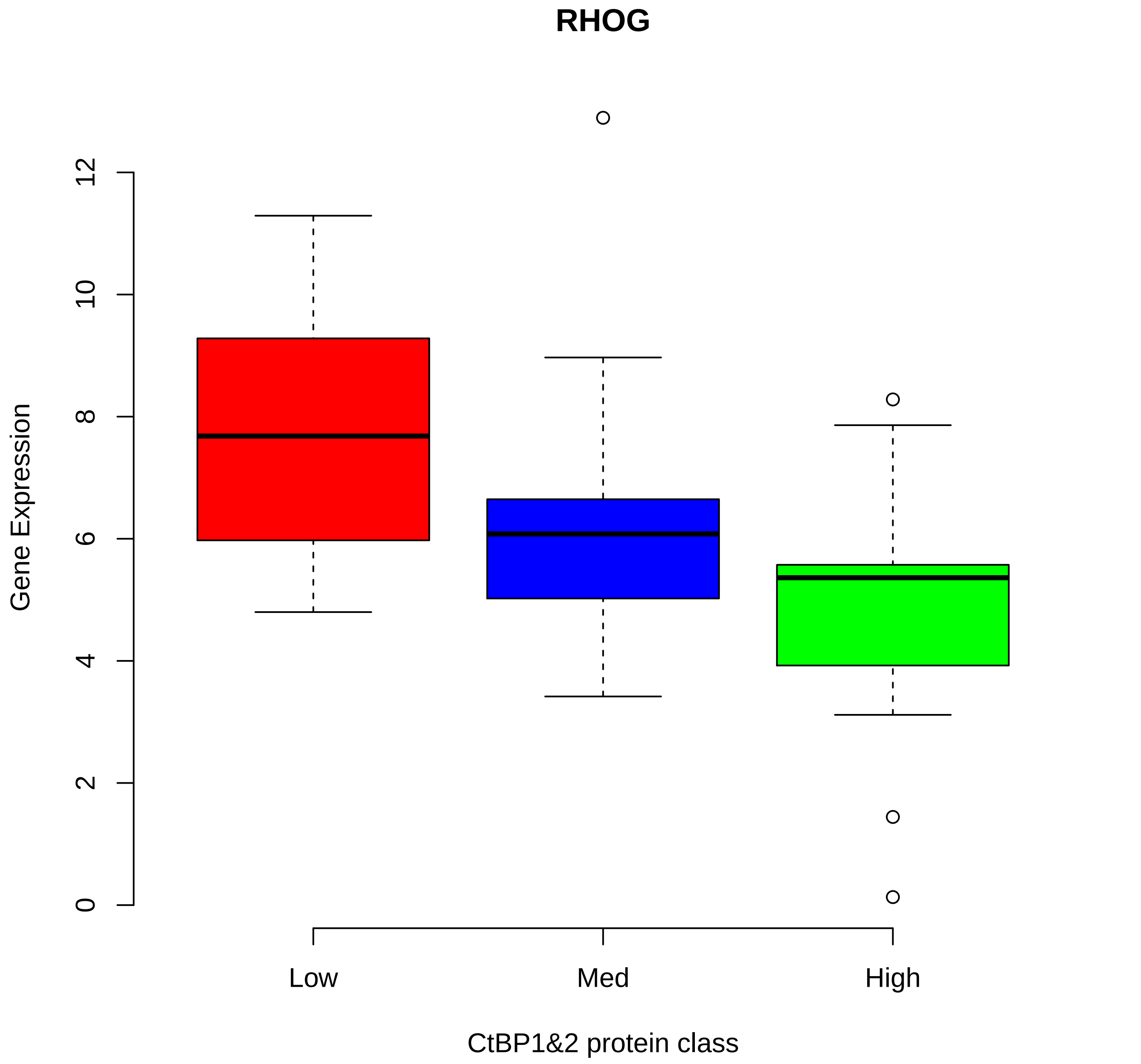

RHOG
Gene Expression
0
2
4
6
8
10
12
Low
Med
High
CtBP1&2 protein class

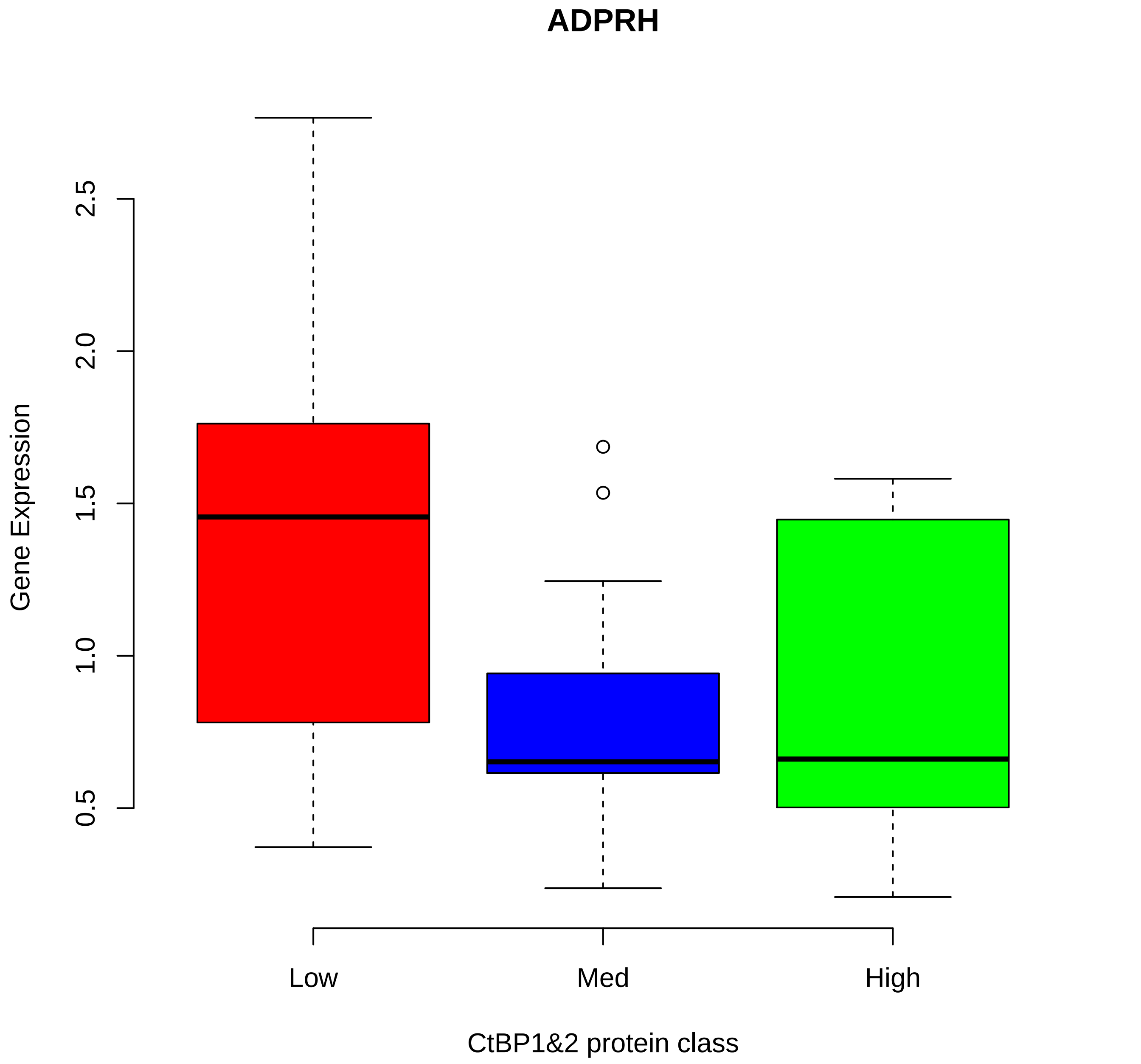
ADPRH
Gene Expression
0.5
1.0
1.5
2.0
2.5
Low
Med
High
CtBP1&2 protein class

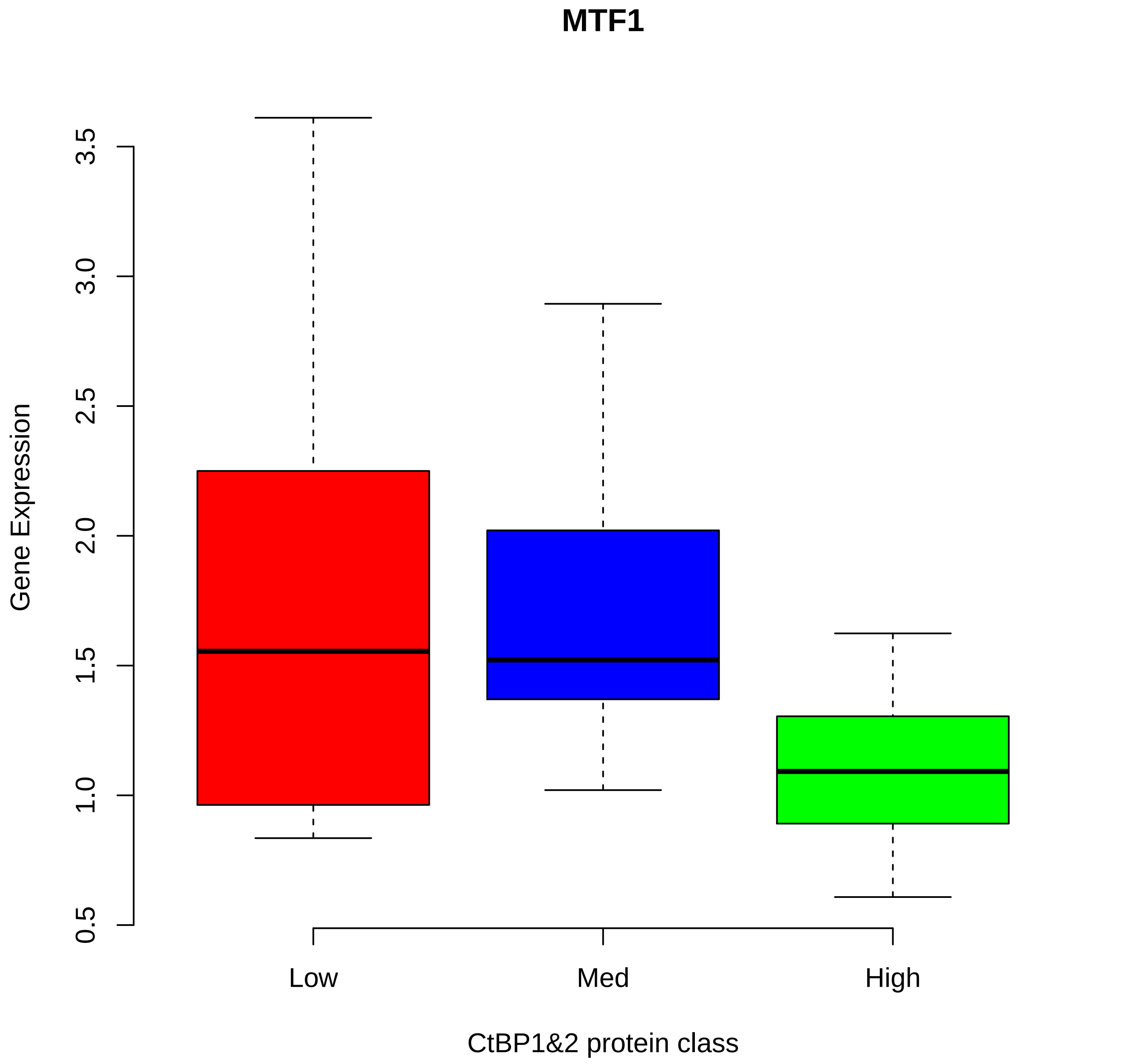

MTF1
Gene Expression
3.5
3.0
2.5
2.0
1.5
1.0
0.5
Low
Med
High
CtBP1&2 protein class

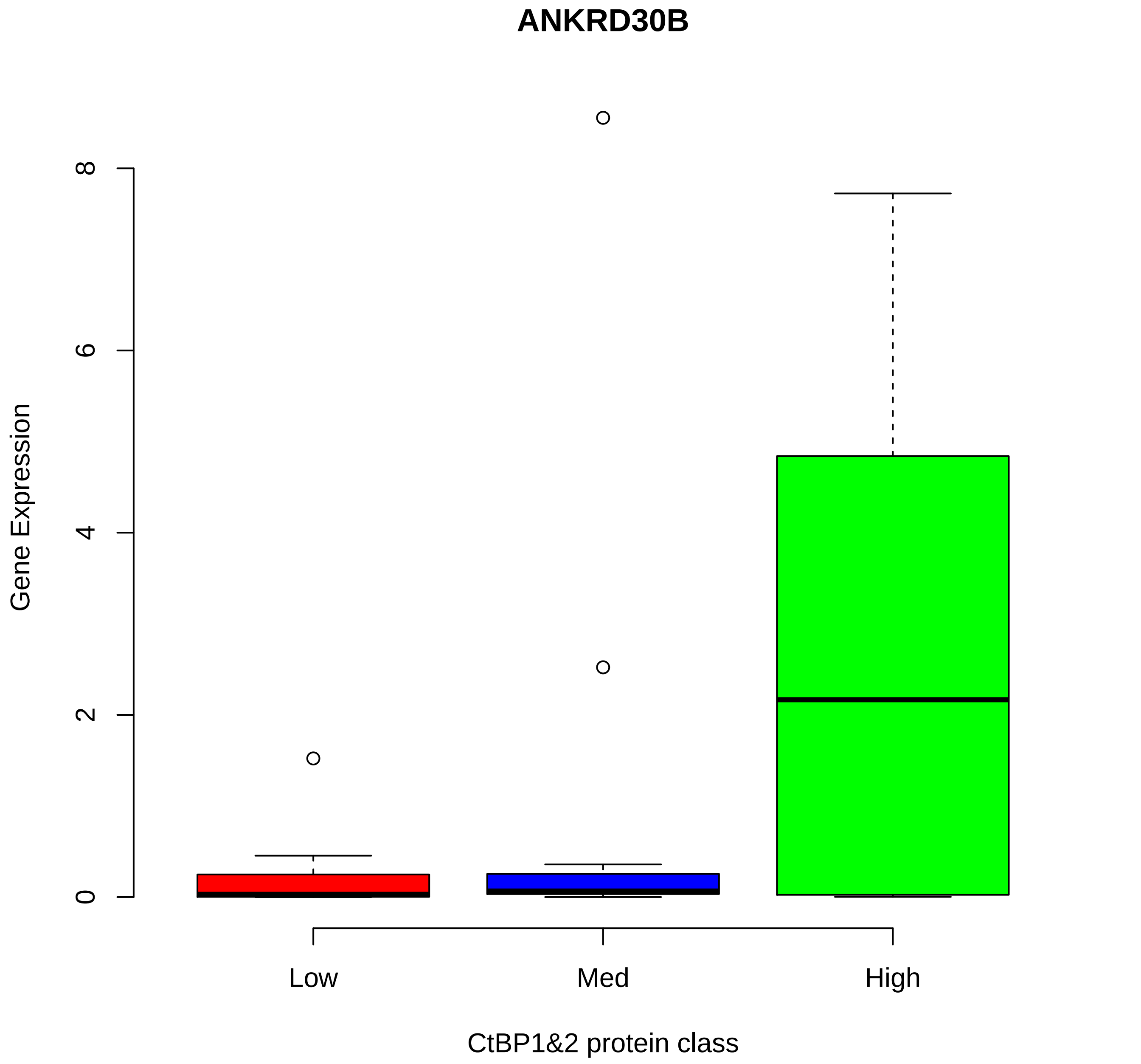
ANKRD30B
Gene Expression
0
2
4
6
8
Low
Med
High
CtBP1&2 protein class

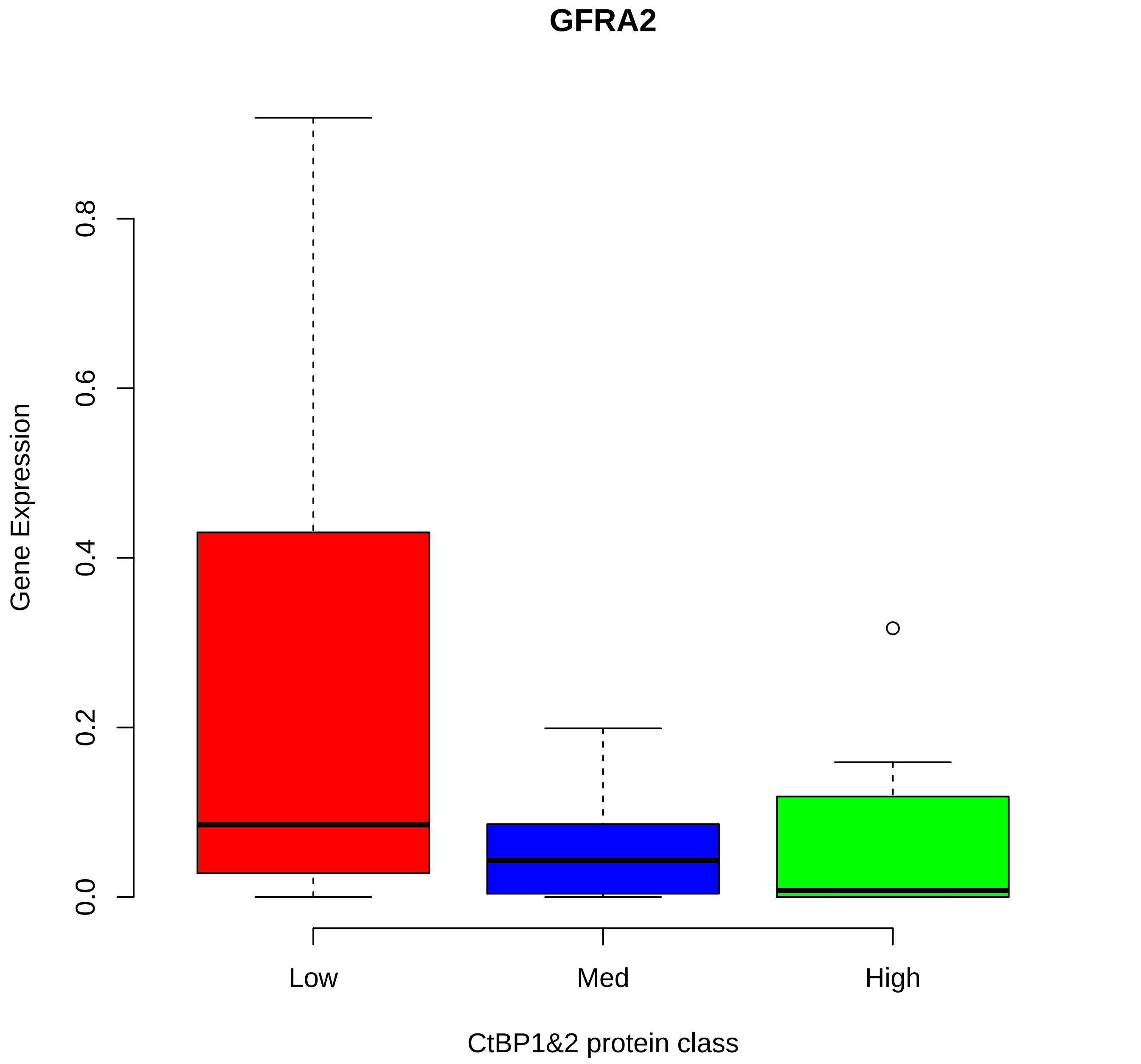

GFRA2
Gene Expression
0.0
0.2
0.4
0.6
0.8
Low
Med
High
CtBP1&2 protein class

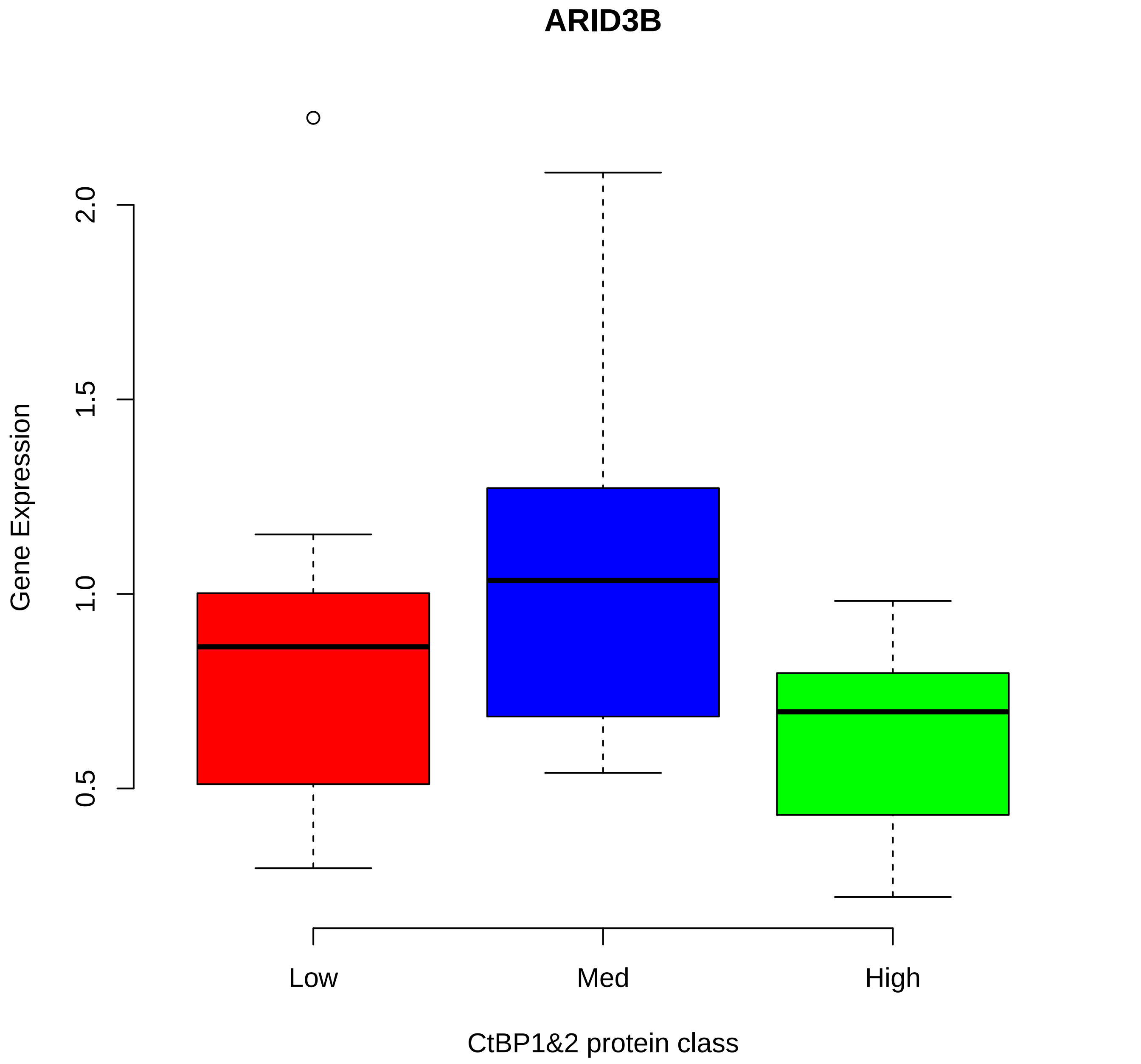
ARID3B
Gene Expression
0.5
1.0
1.5
2.0
Low
Med
High
CtBP1&2 protein class

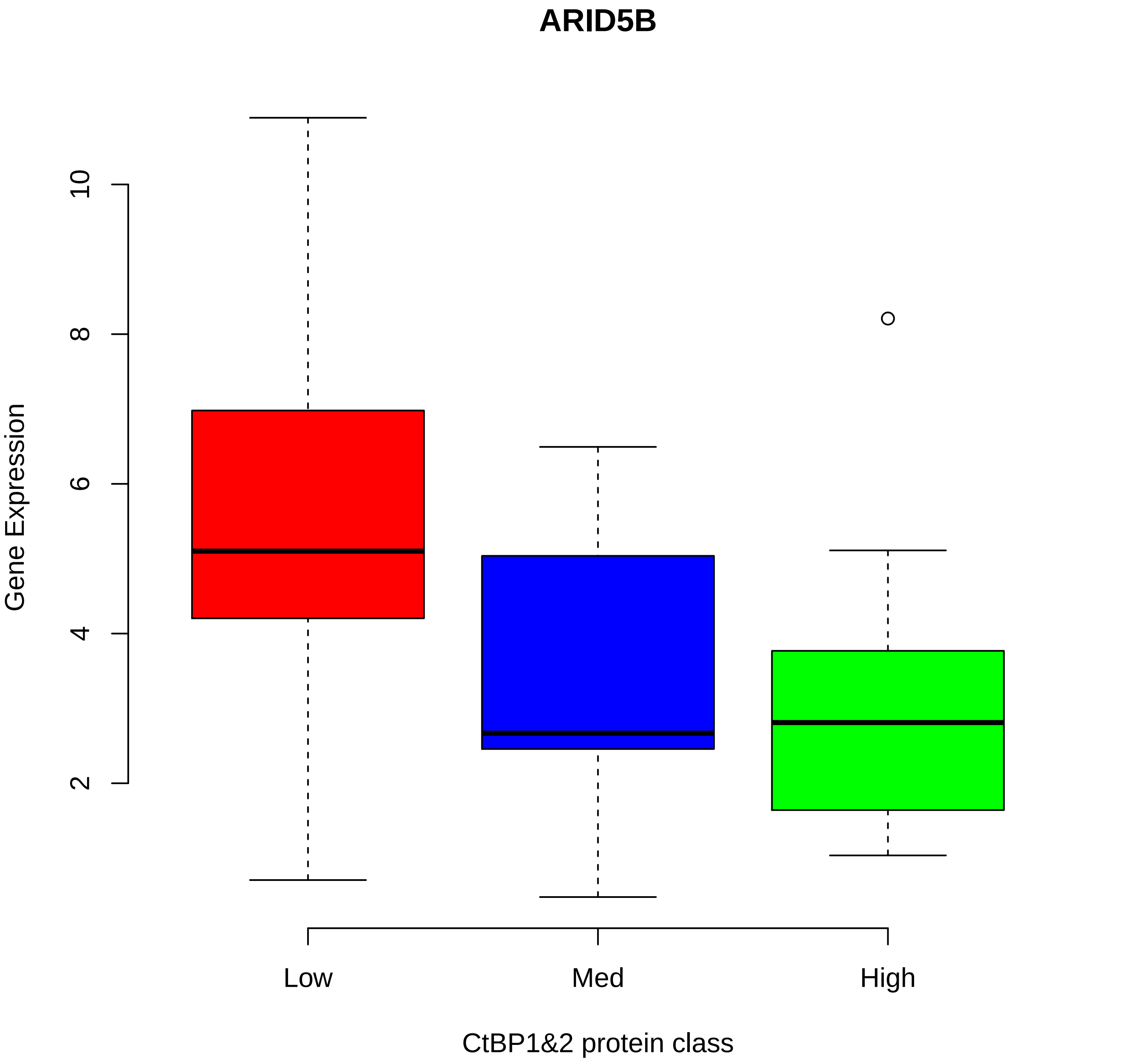
ARID5B
Gene Expression
10
8
6
4
2
Low
Med
High
CtBP1&2 protein class

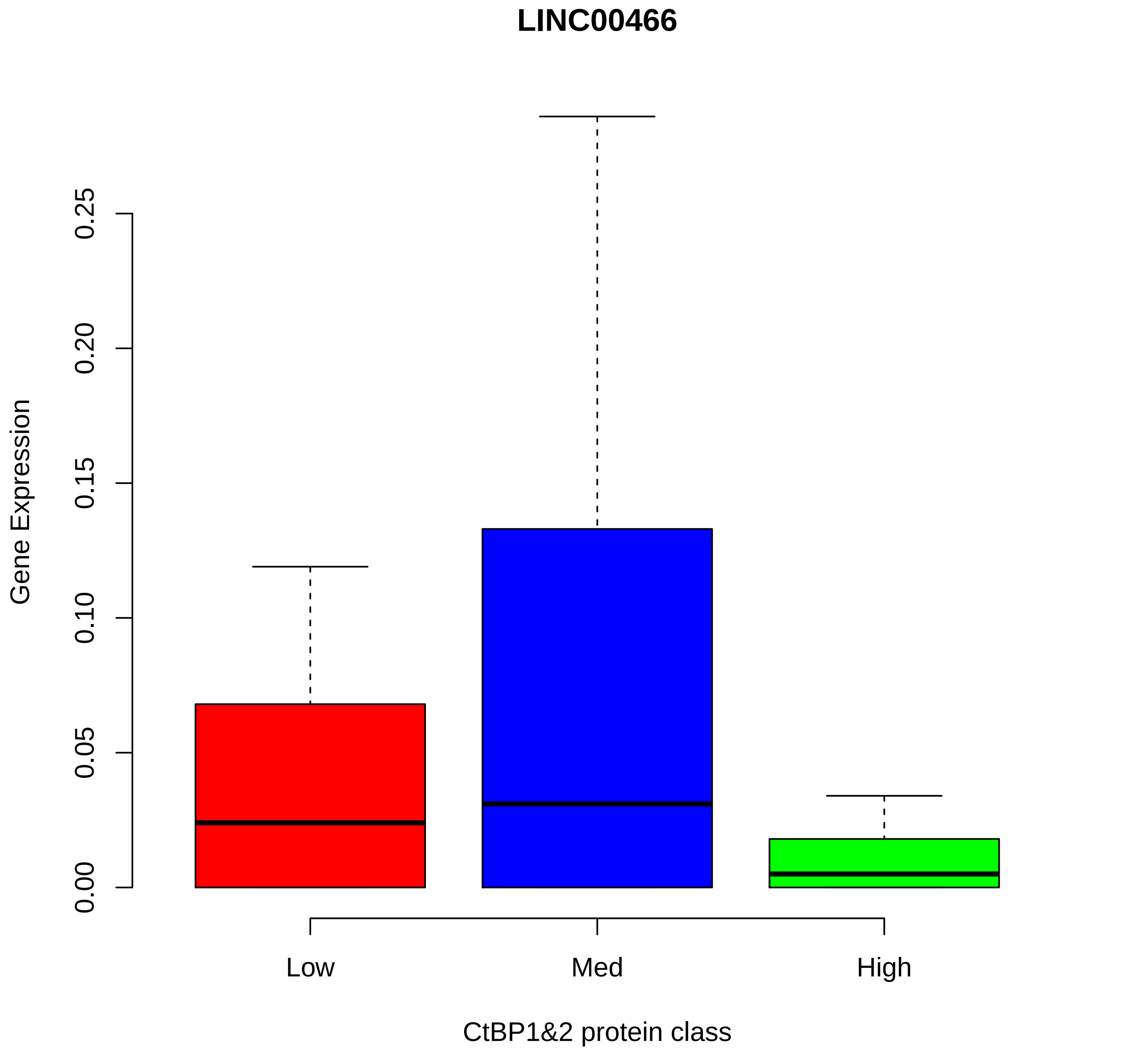
LINC00466
Gene Expression
0.00
0.05
0.10
0.15
0.20
0.25
Low
Med
High
CtBP1&2 protein class

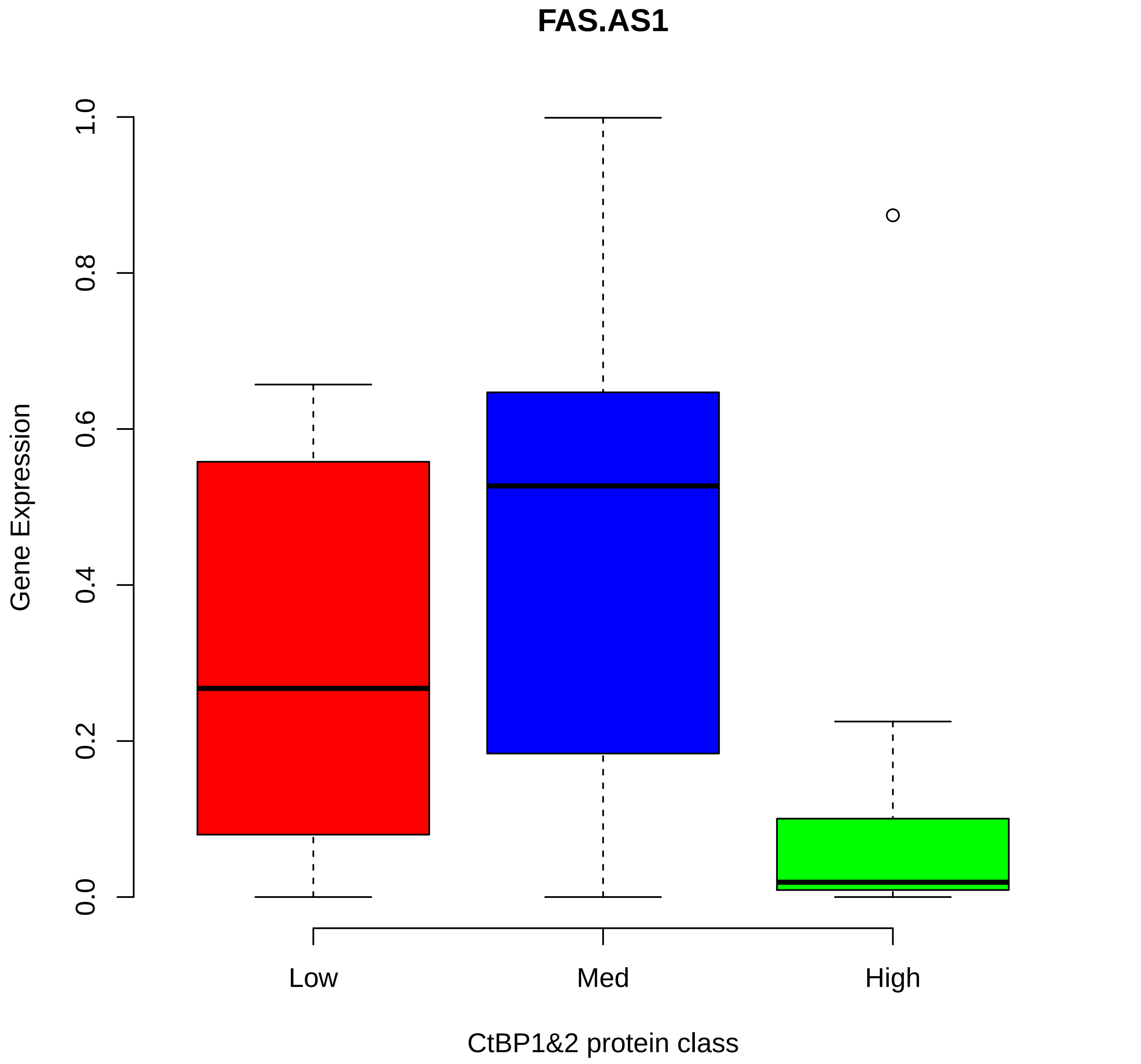

FAS.AS1
Gene Expression
0.0
0.2
0.4
0.6
0.8
1.0
Low
Med
High
CtBP1&2 protein class

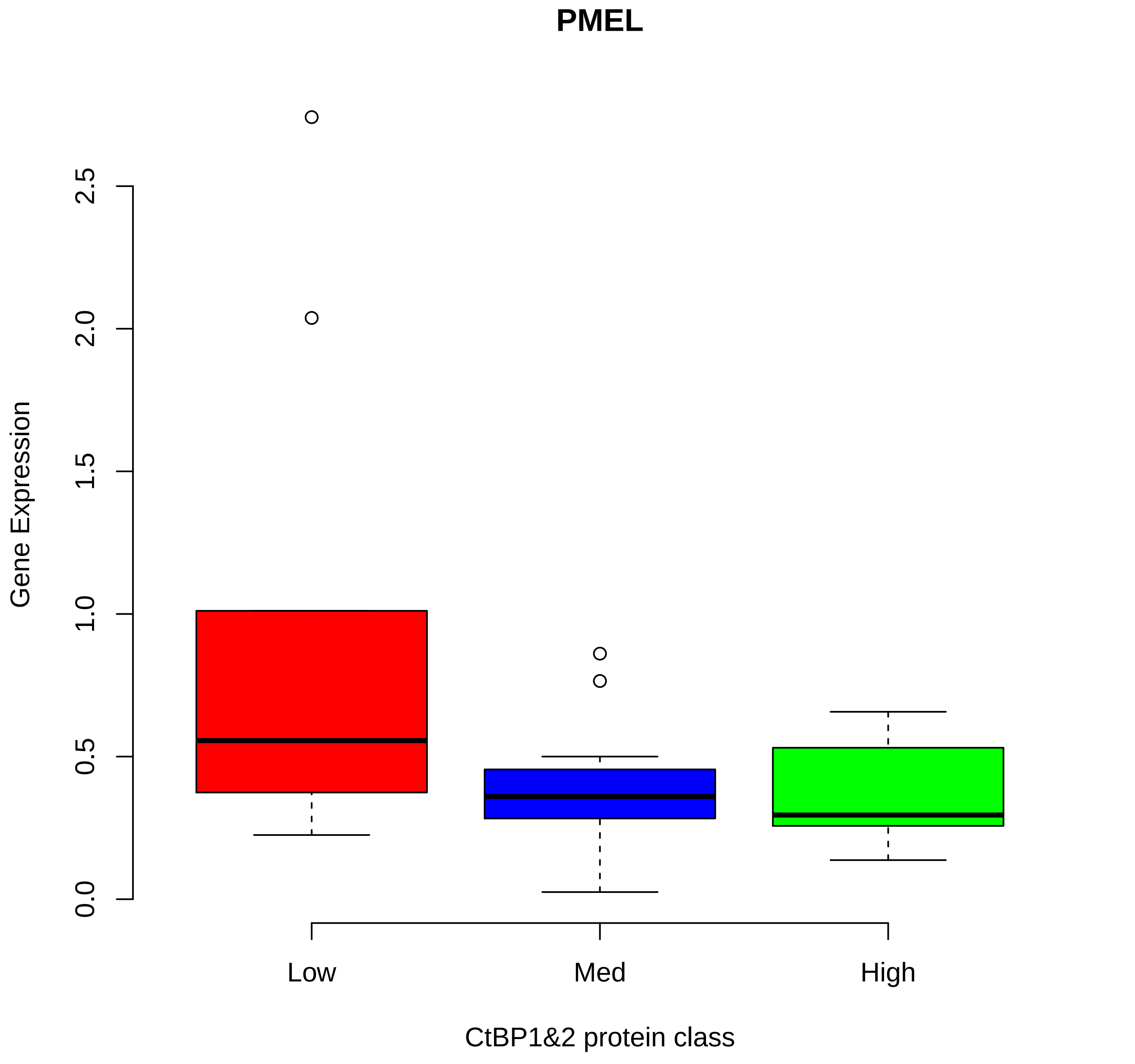

PMEL
Gene Expression
0.0
0.5
1.0
1.5
2.0
2.5
Low
Med
High
CtBP1&2 protein class

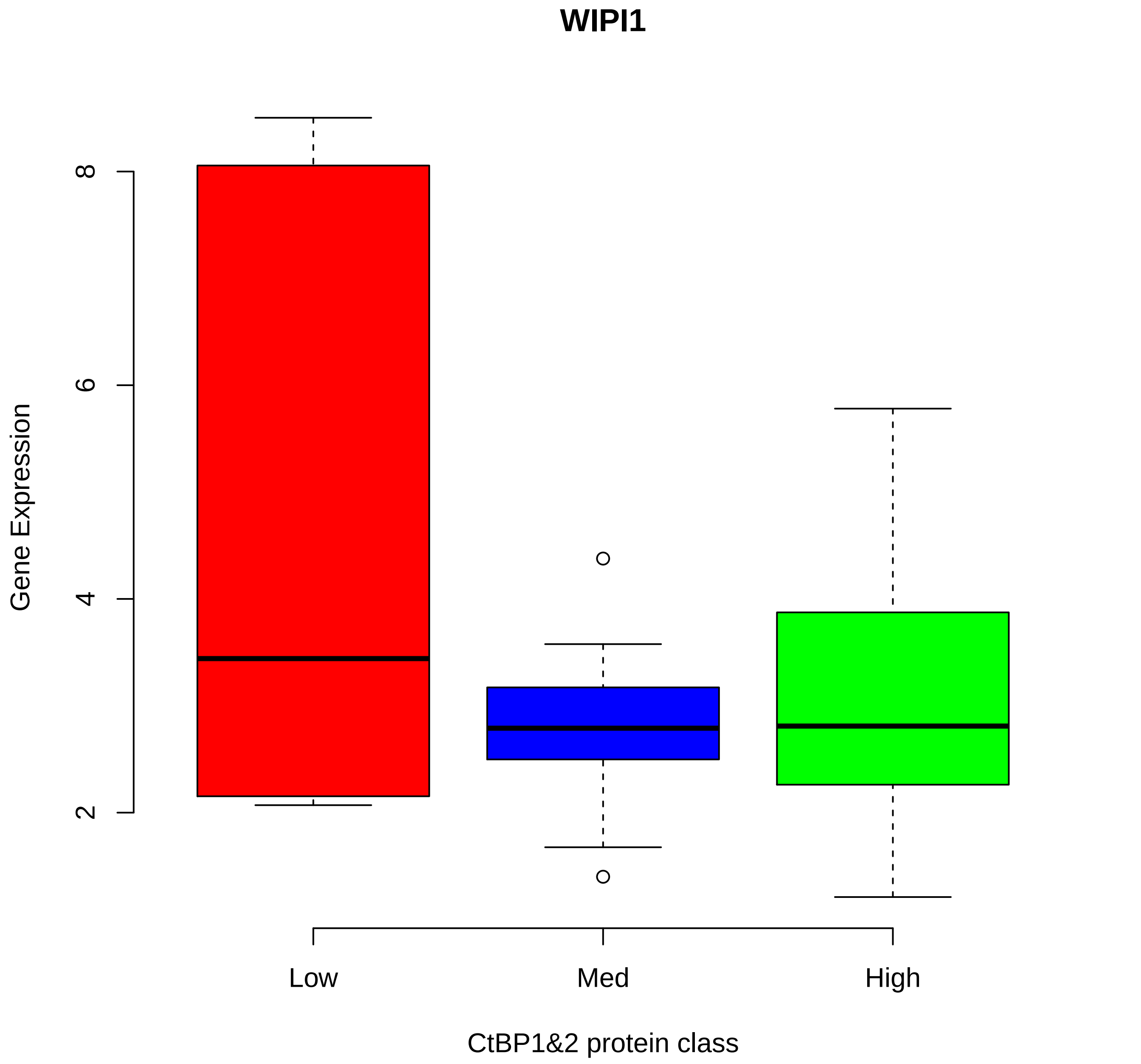
WIPI1
Gene Expression
8
6
4
2
Low
Med
High
CtBP1&2 protein class

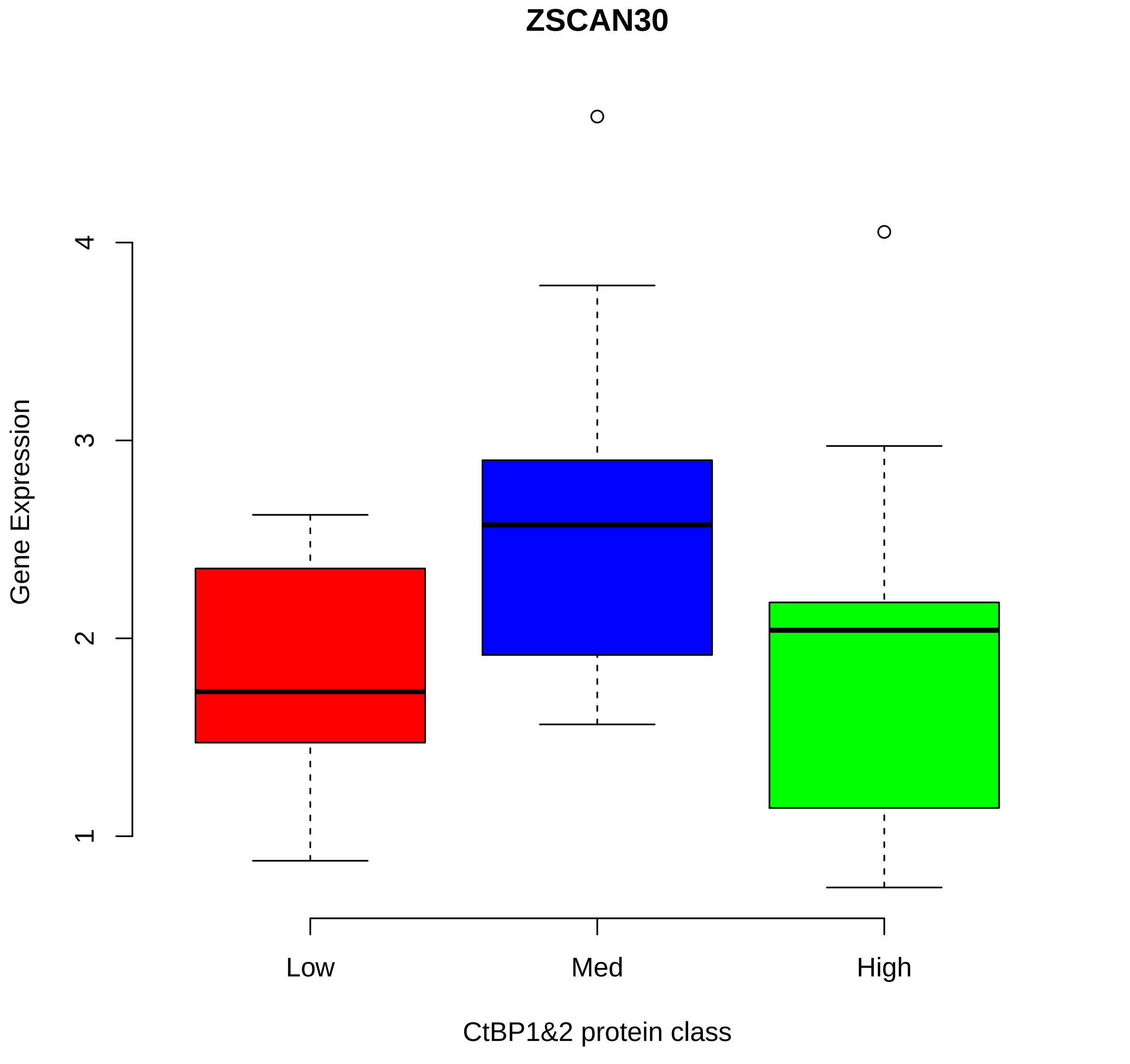
ZSCAN30
Gene Expression
1
2
3
4
Low
Med
High
CtBP1&2 protein class

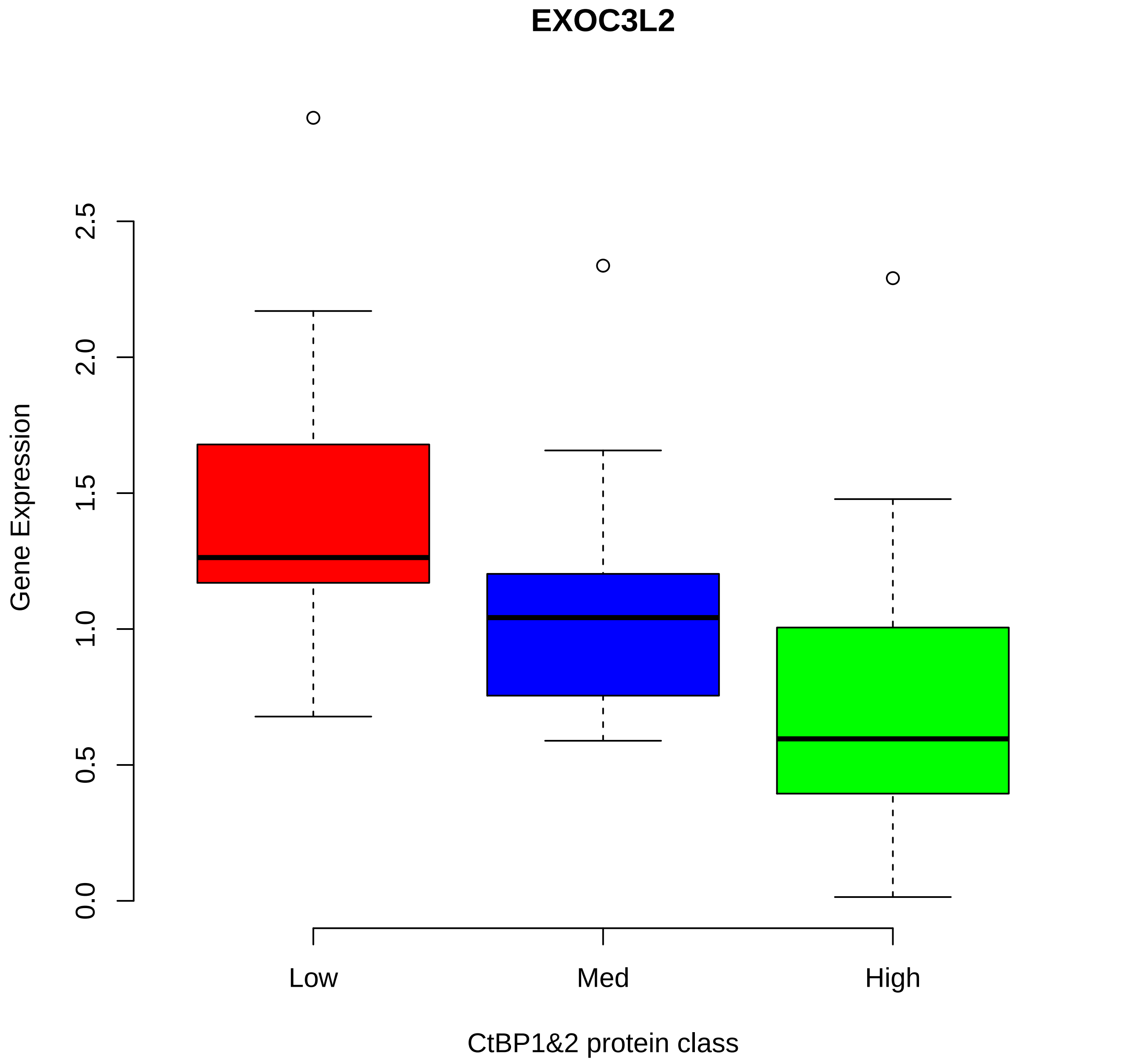

EXOC3L2
Gene Expression
0.0
0.5
1.0
1.5
2.0
2.5
Low
Med
High
CtBP1&2 protein class

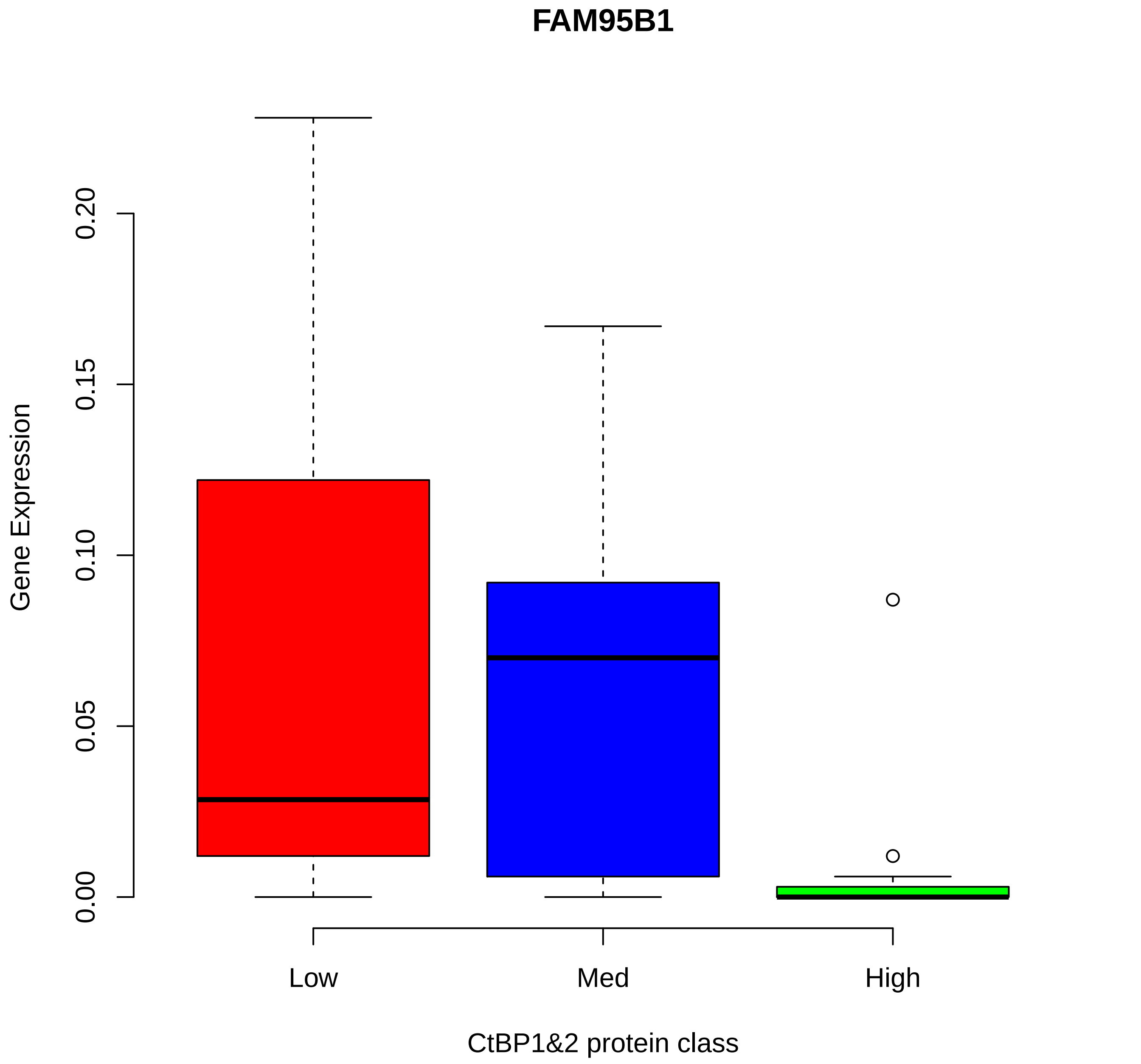
FAM95B1
Gene Expression
0.00
0.05
0.10
0.15
0.20
Low
Med
High
CtBP1&2 protein class

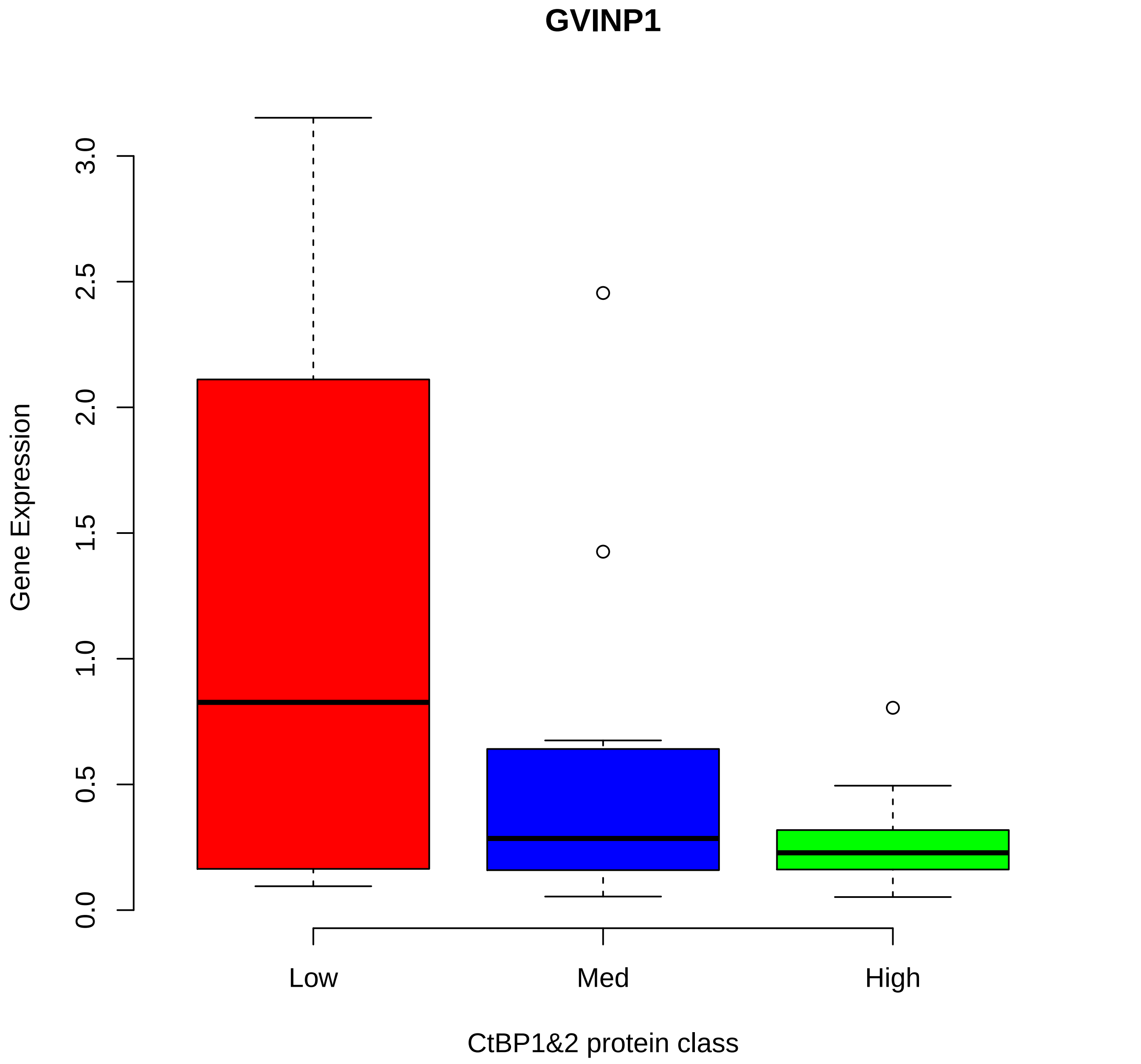
GVINP1
Gene Expression
0.0
0.5
1.0
1.5
2.0
2.5
3.0
Low
Med
High
CtBP1&2 protein class

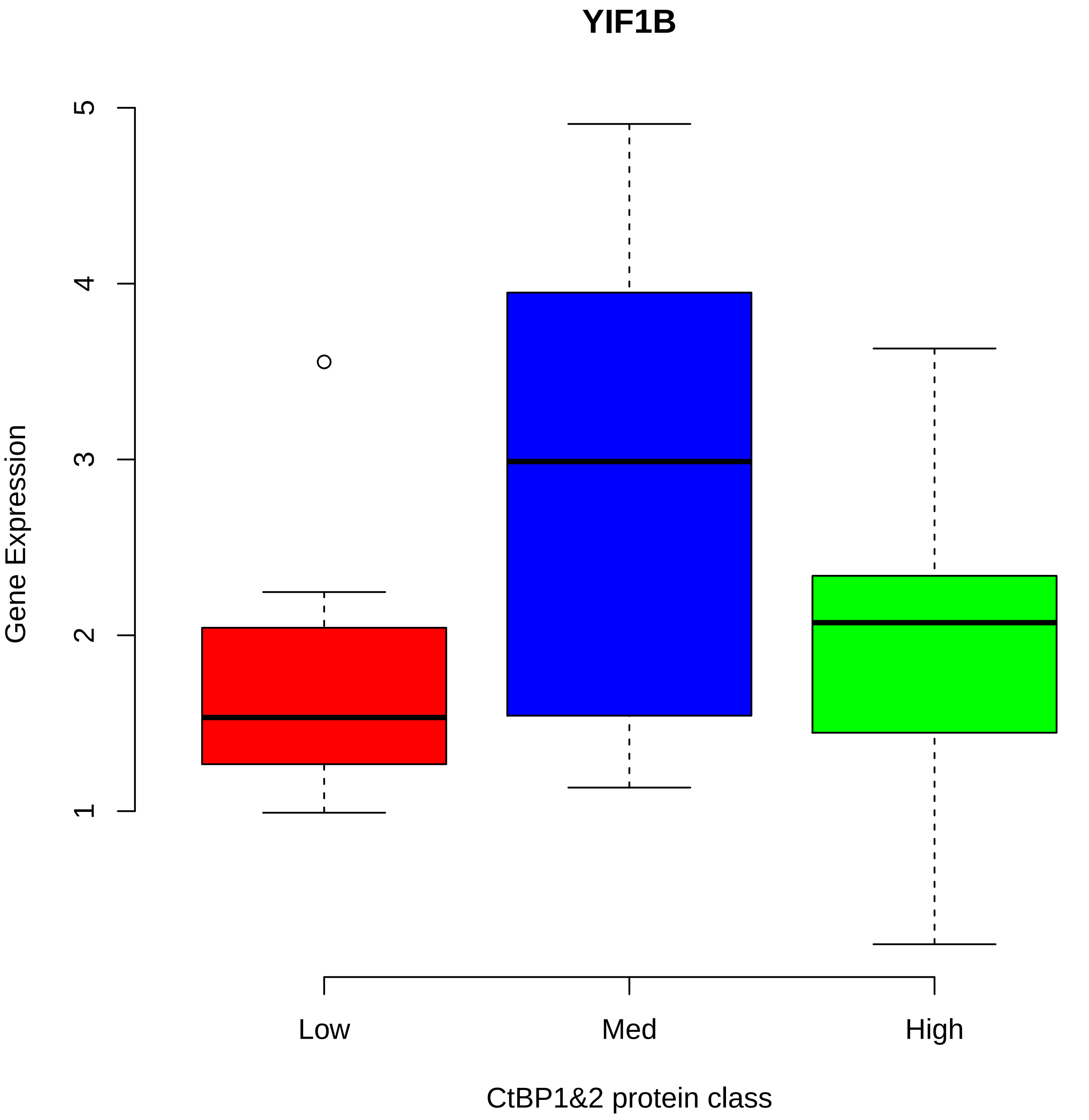
YIF1B
Gene Expression
5
4
3
2
1
Low
Med
High
CtBP1&2 protein class

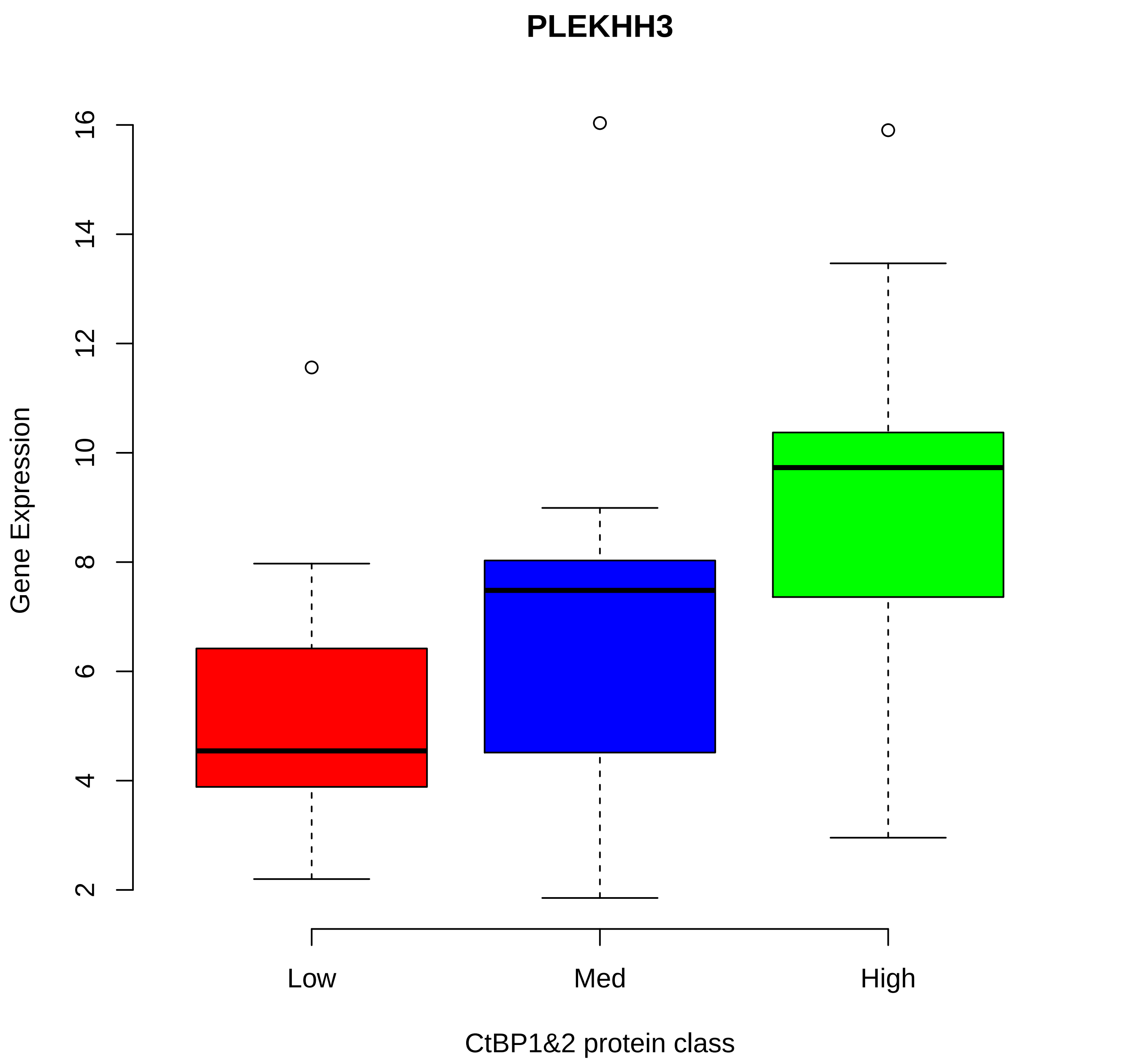
PLEKHH3
Gene Expression
16
14
12
10
8
6
4
2
Low
Med
High
CtBP1&2 protein class

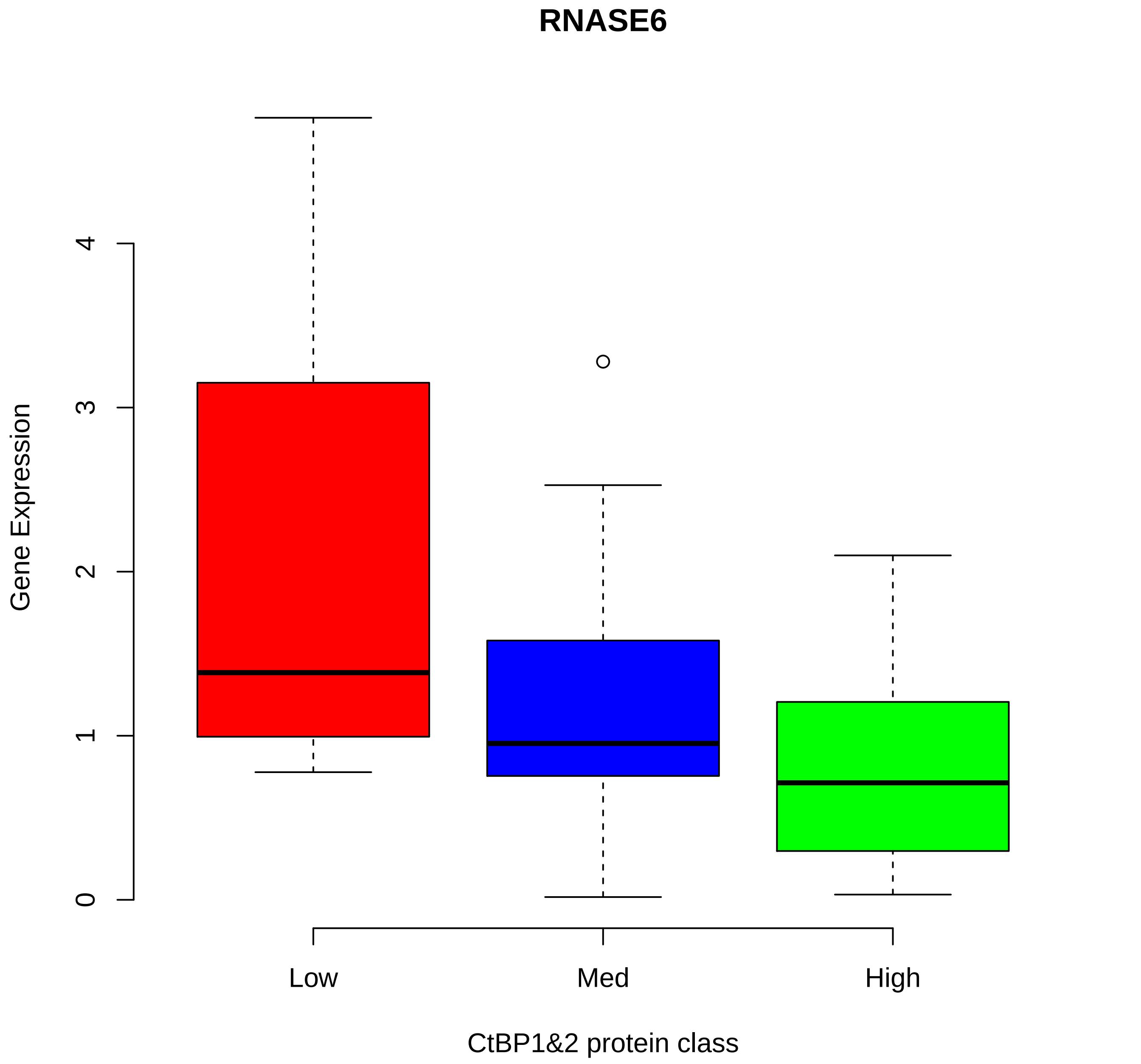
RNASE6
Gene Expression
0
1
2
3
4
Low
Med
High
CtBP1&2 protein class

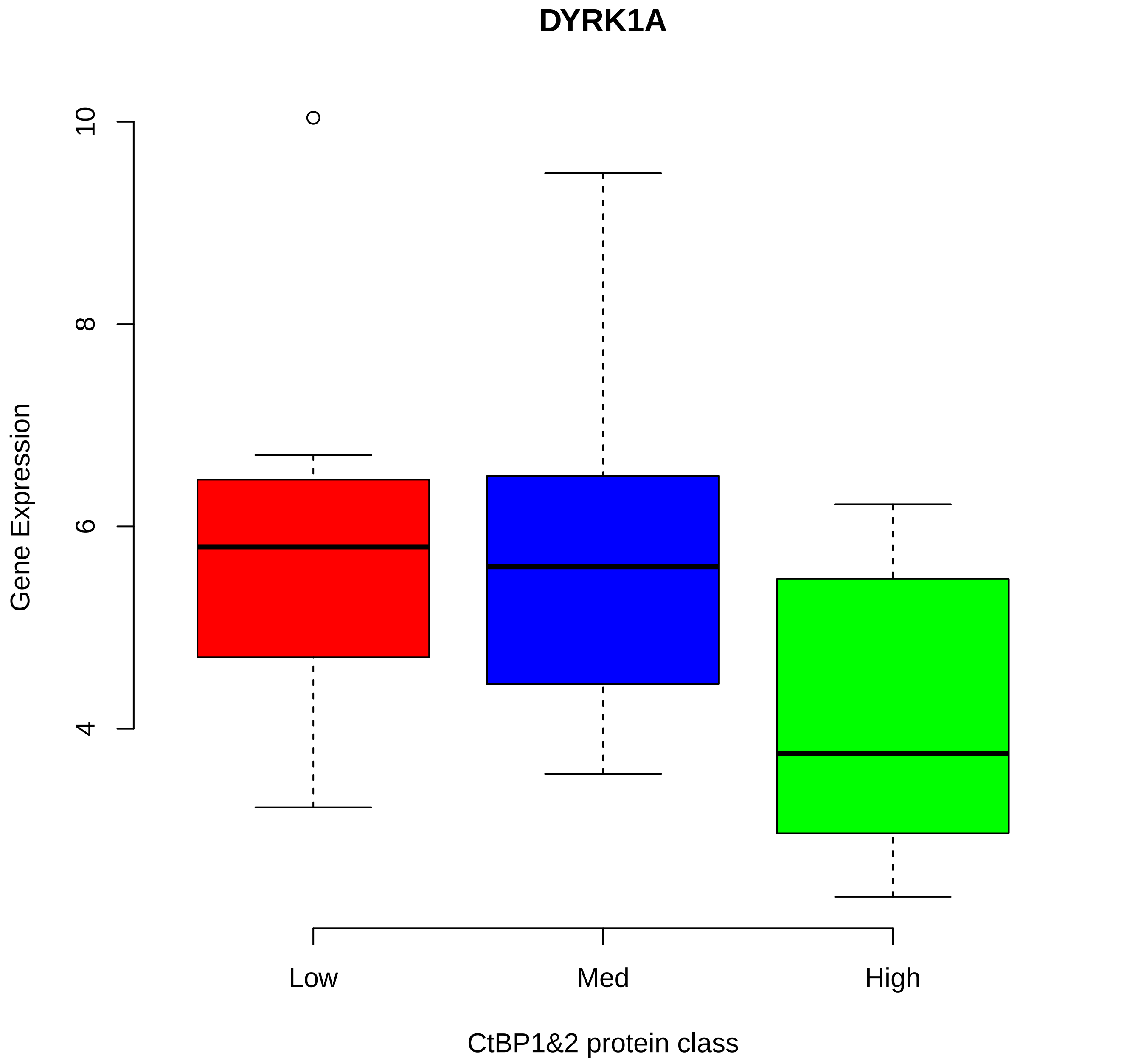

DYRK1A
Gene Expression
10
8
6
4
Low
Med
High
CtBP1&2 protein class

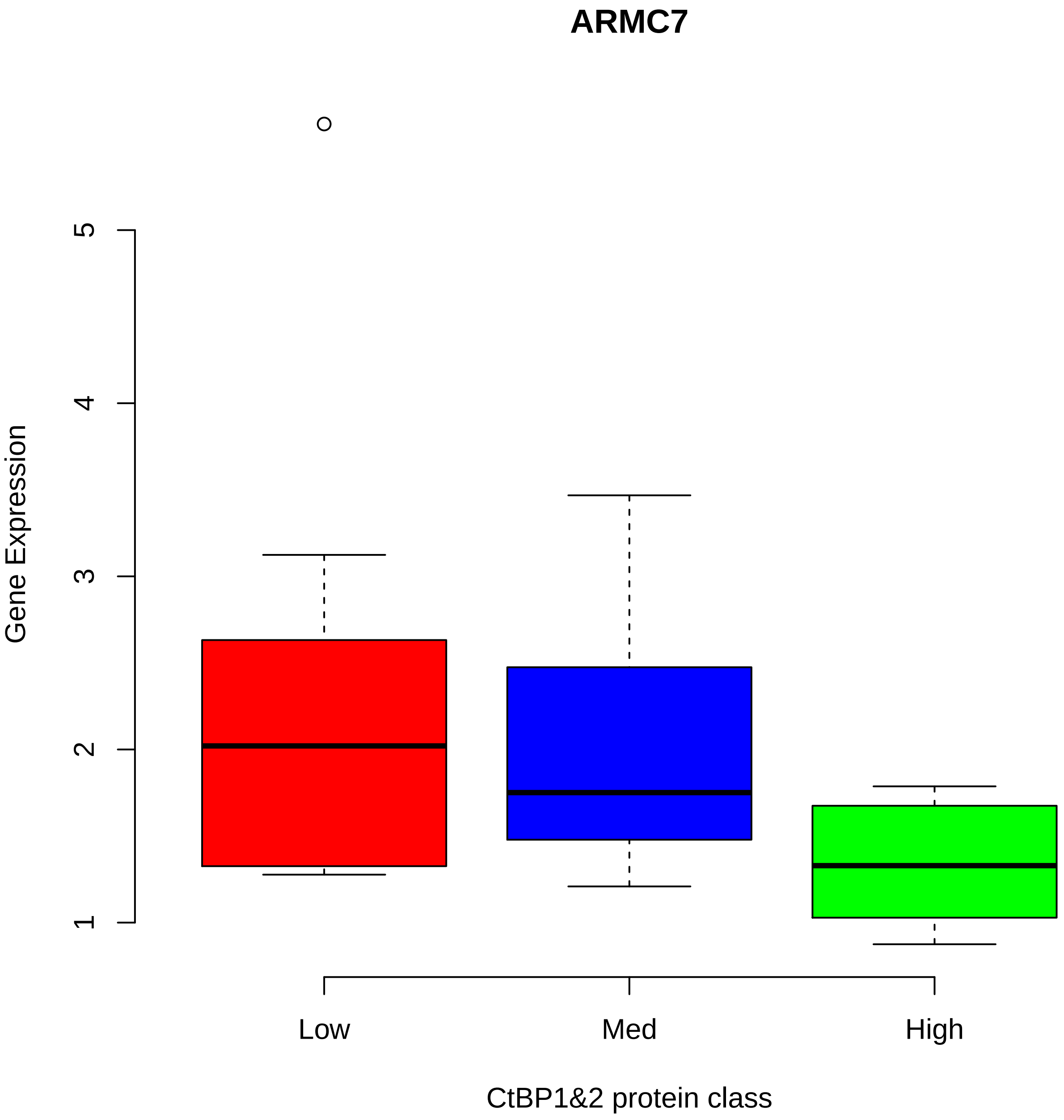
ARMC7
Gene Expression
1
2
3
4
5
Low
Med
High
CtBP1&2 protein class

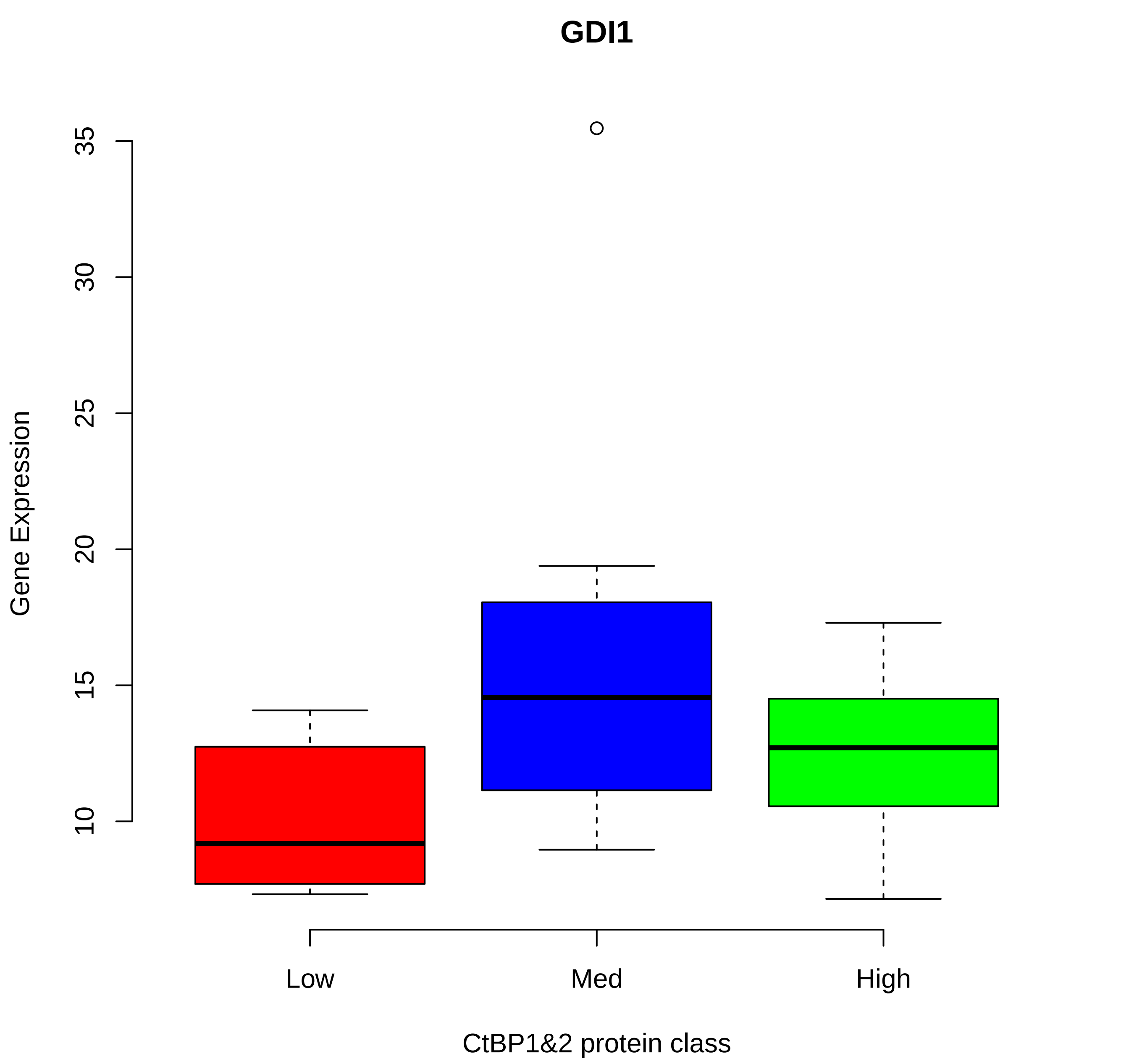

GDI1
Gene Expression
35
30
25
20
15
10
Low
Med
High
CtBP1&2 protein class

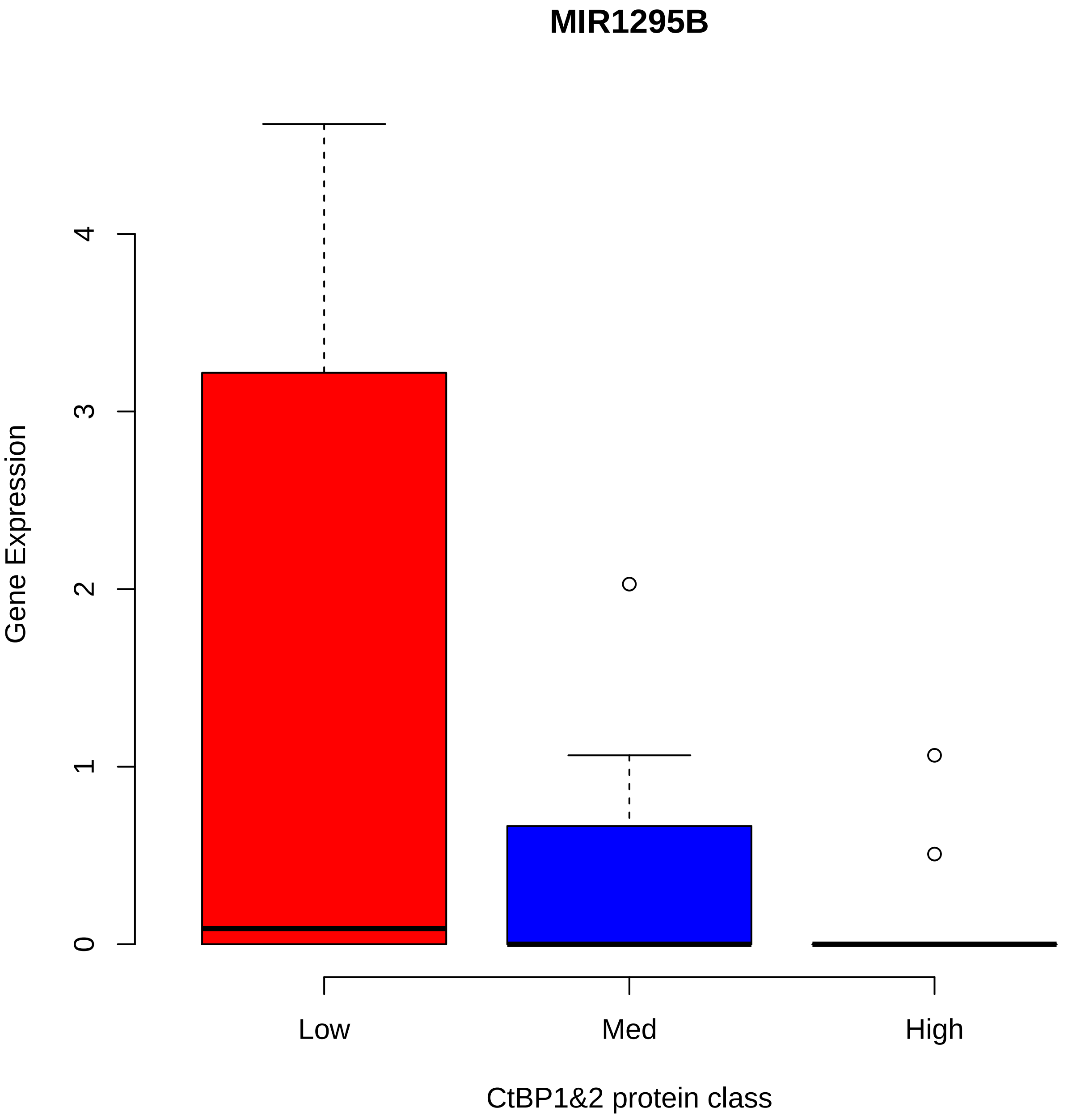

MIR1295B
Gene Expression
0
1
2
3
4
Low
Med
High
CtBP1&2 protein class

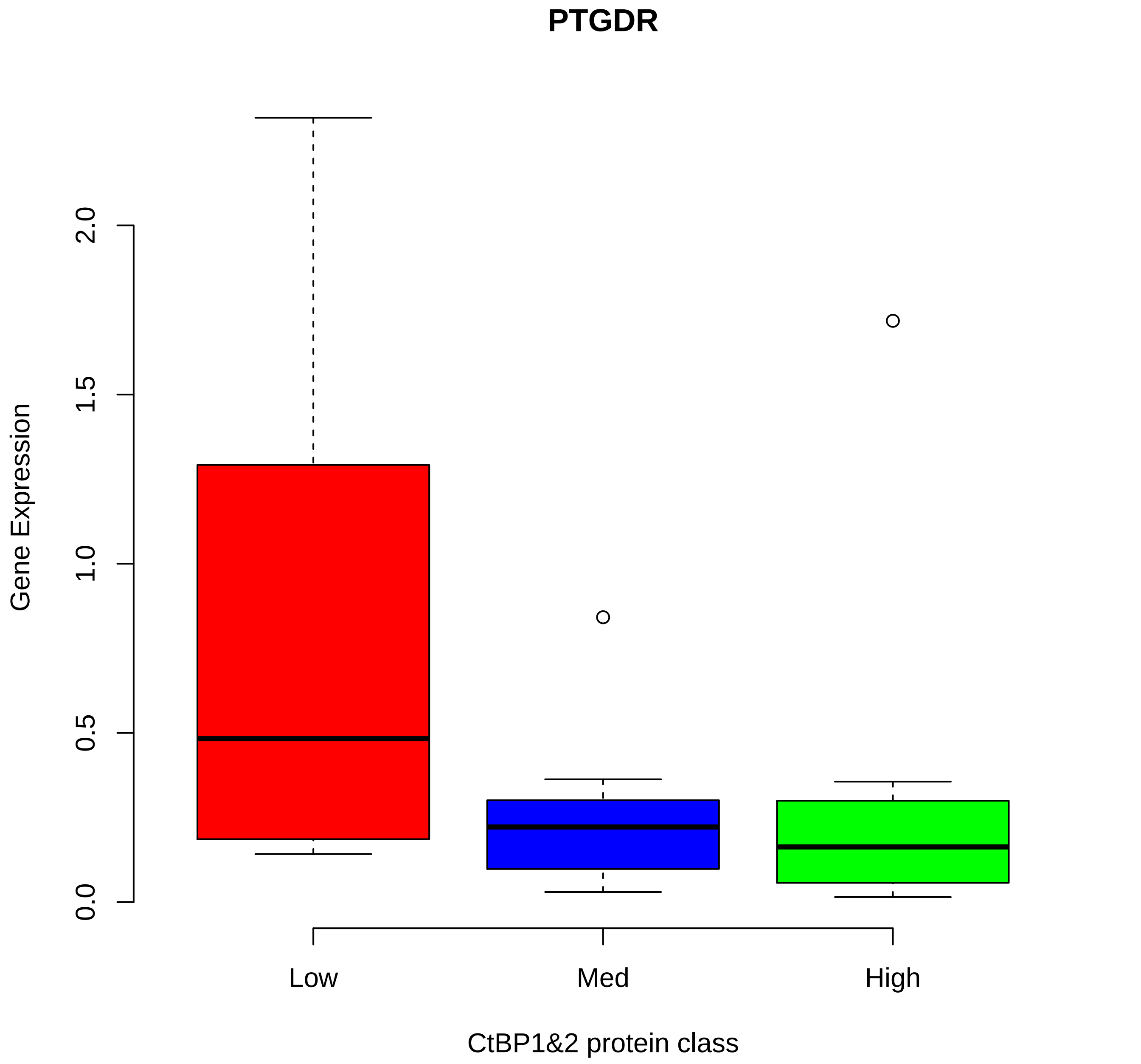
PTGDR
Gene Expression
0.0
0.5
1.0
1.5
2.0
Low
Med
High
CtBP1&2 protein class

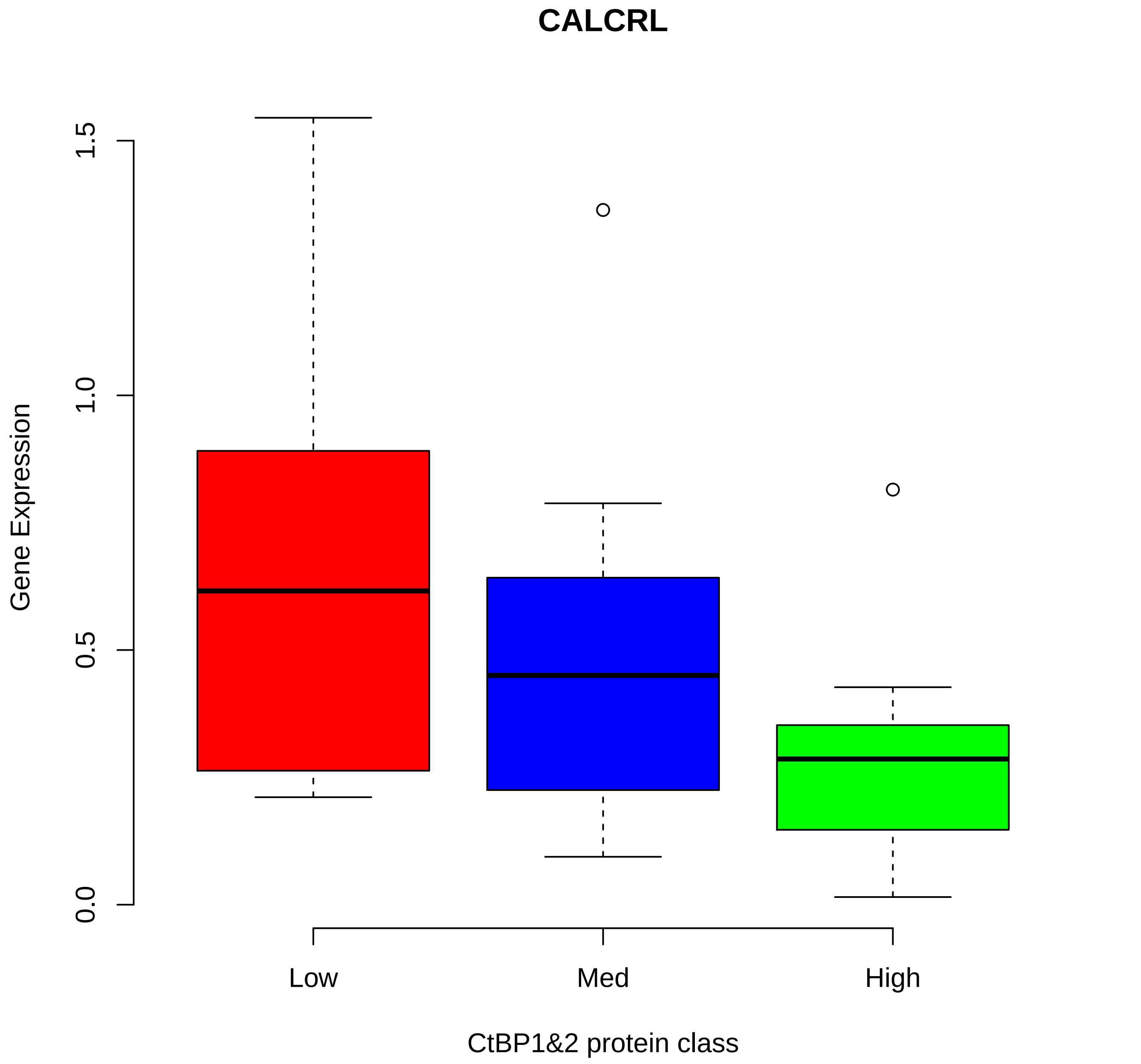
CALCRL
Gene Expression
0.0
0.5
1.0
1.5
Low
Med
High
CtBP1&2 protein class

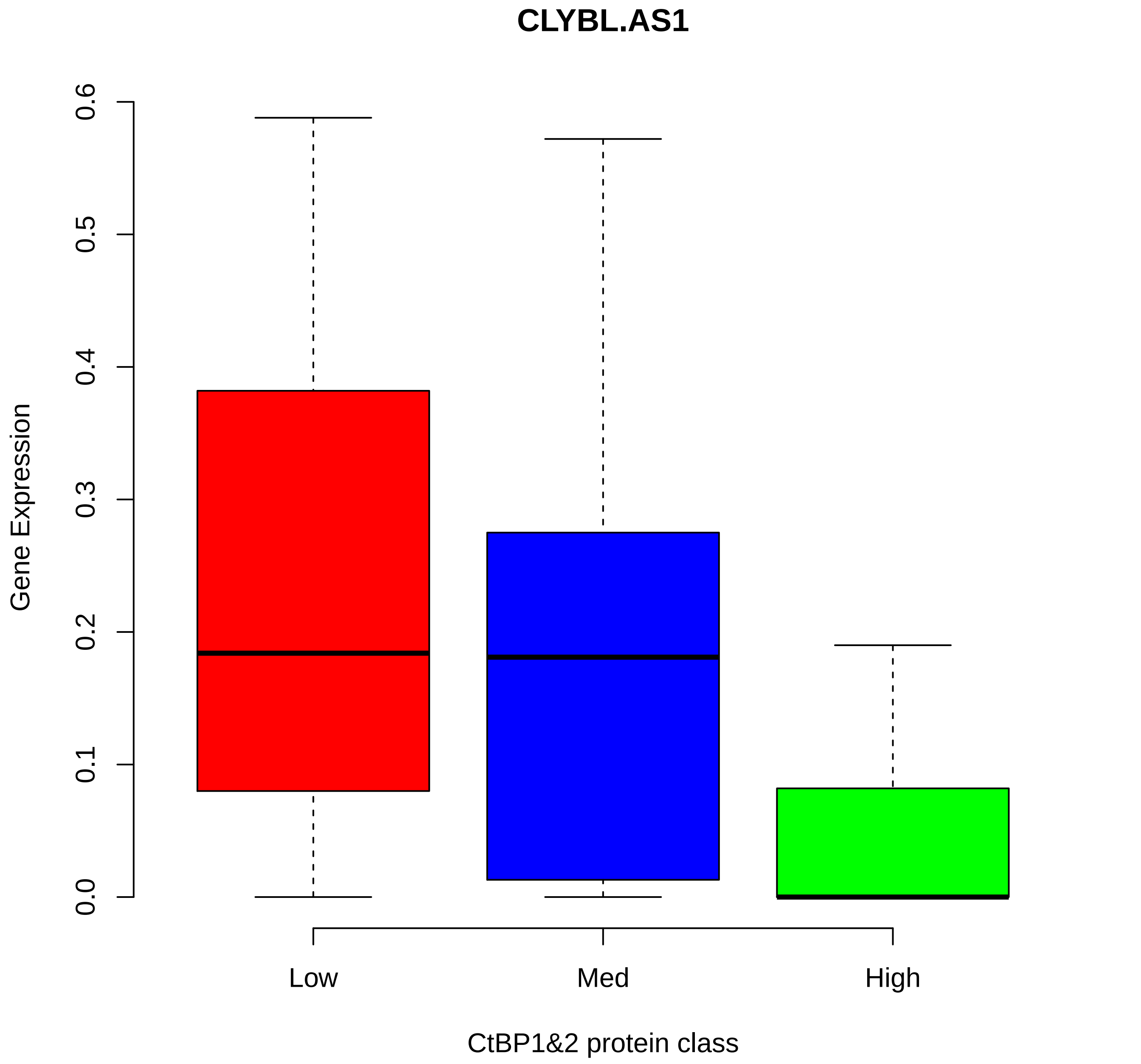

CLYBL.AS1
Gene Expression
0.0
0.1
0.2
0.3
0.4
0.5
0.6
Low
Med
High
CtBP1&2 protein class

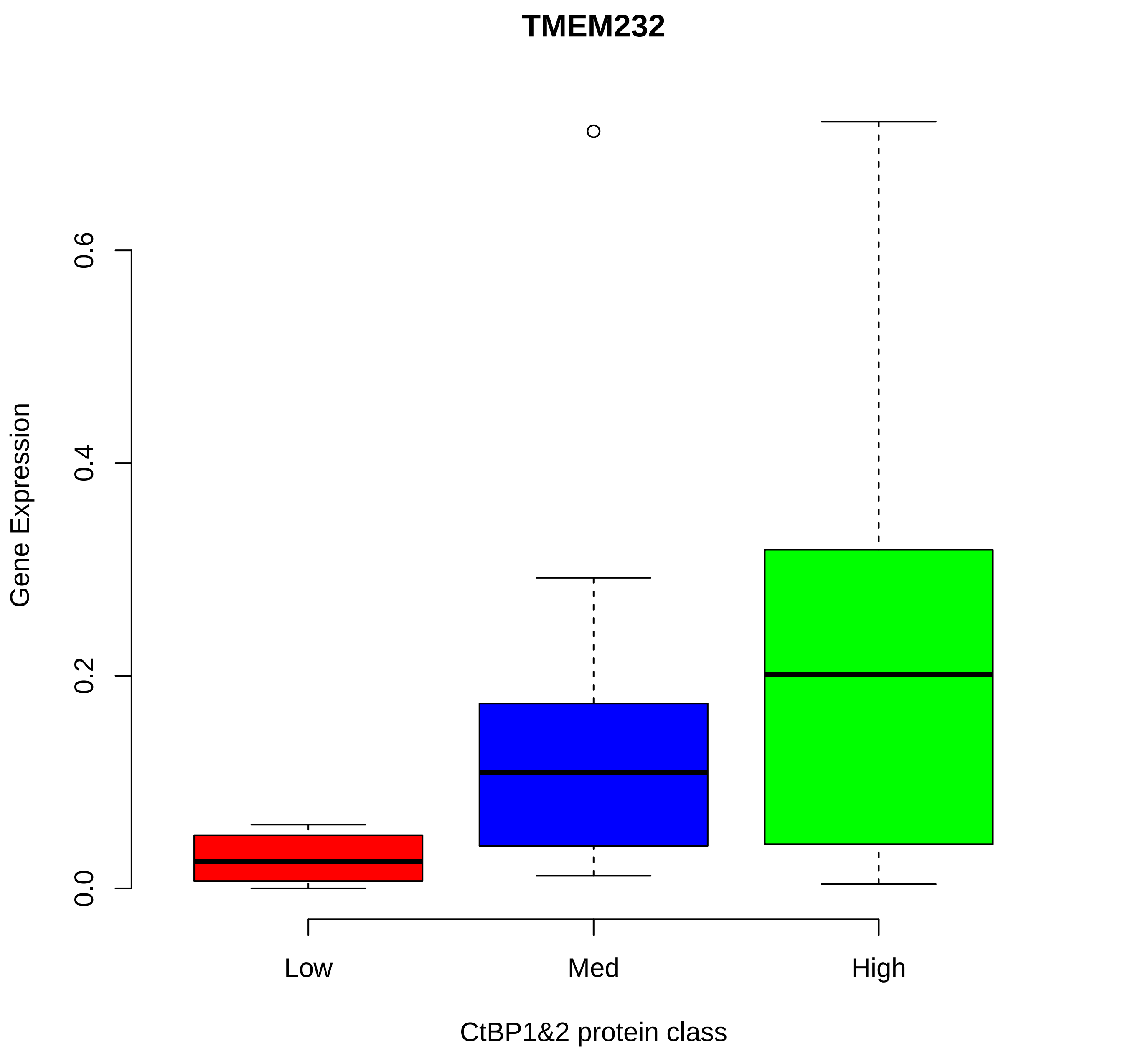

TMEM232
Gene Expression
0.0
0.2
0.4
0.6
Low
Med
High
CtBP1&2 protein class

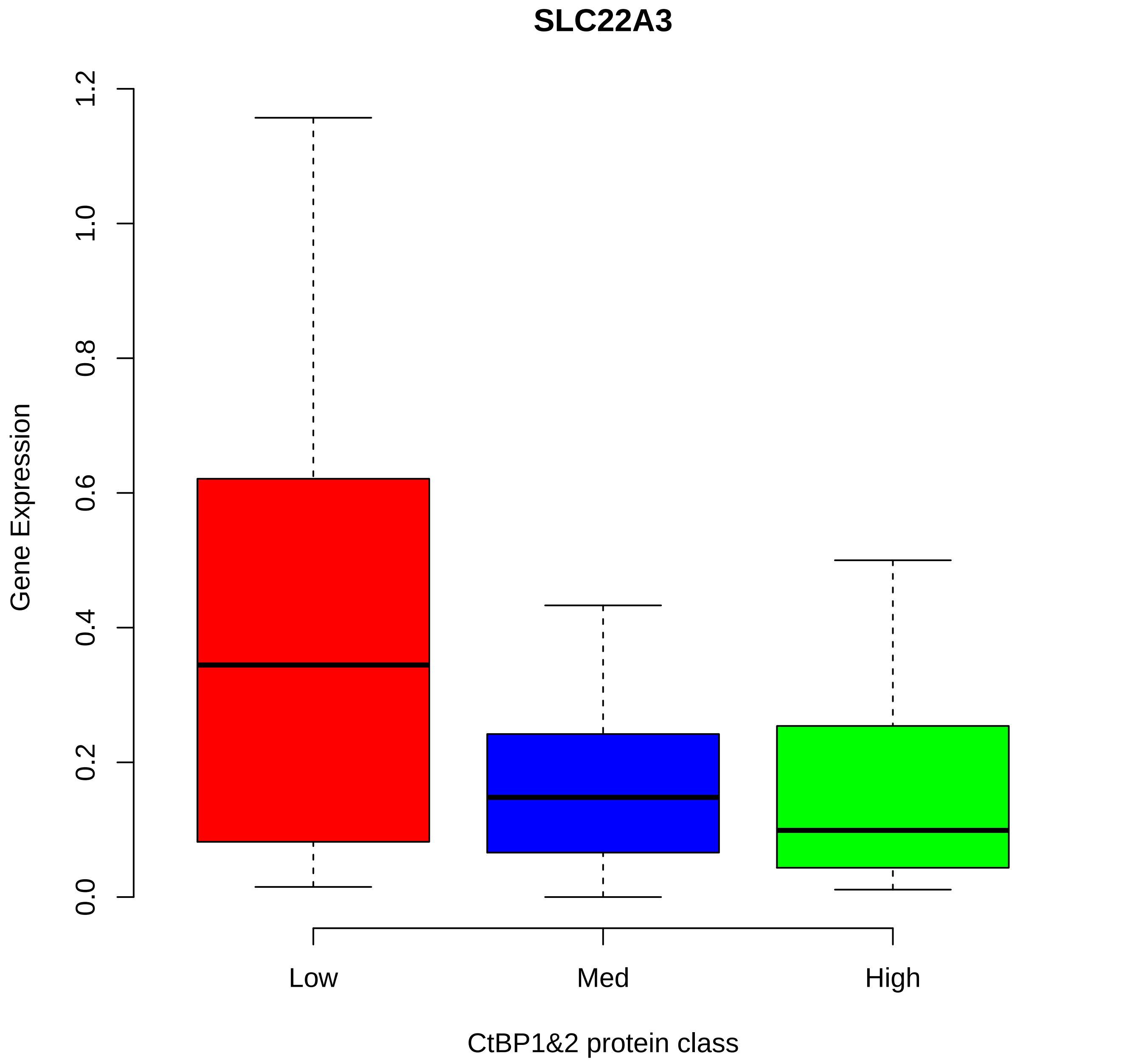

SLC22A3
Gene Expression
0.0
0.2
0.4
0.6
0.8
1.0
1.2
Low
Med
High
CtBP1&2 protein class

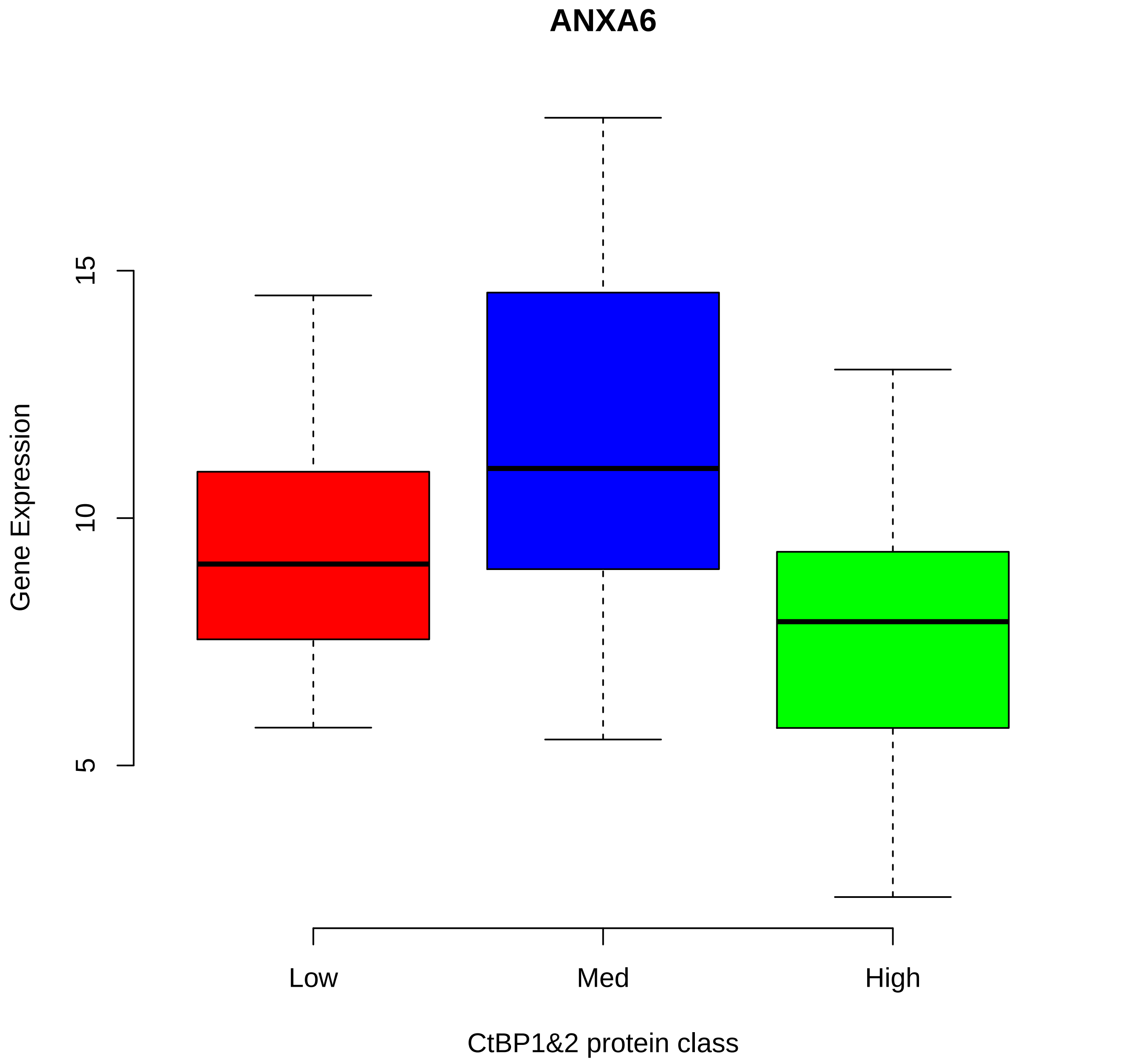
ANXA6
Gene Expression
15
10
5
Low
Med
High
CtBP1&2 protein class

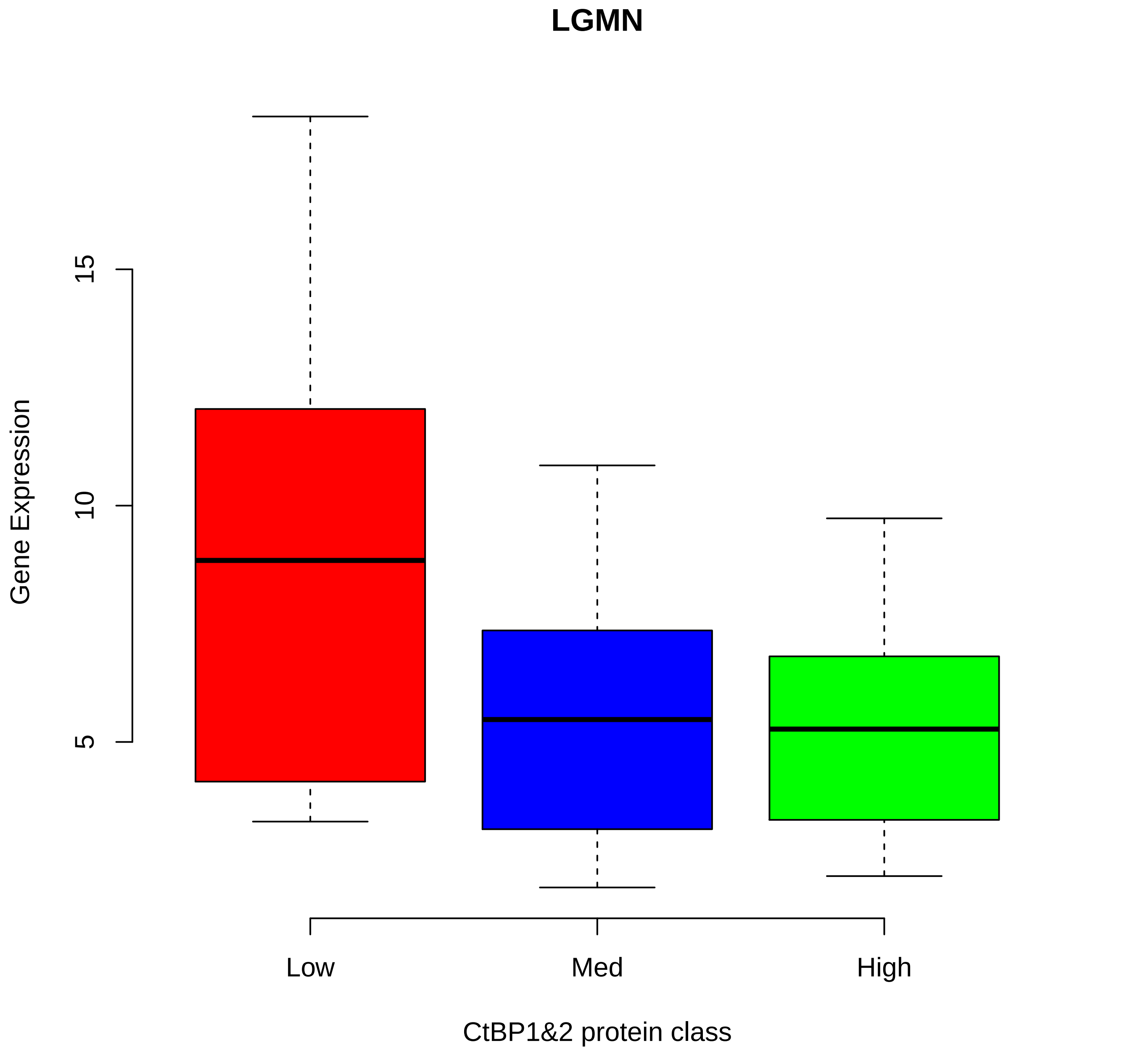
LGMN
Gene Expression
15
10
5
Low
Med
High
CtBP1&2 protein class

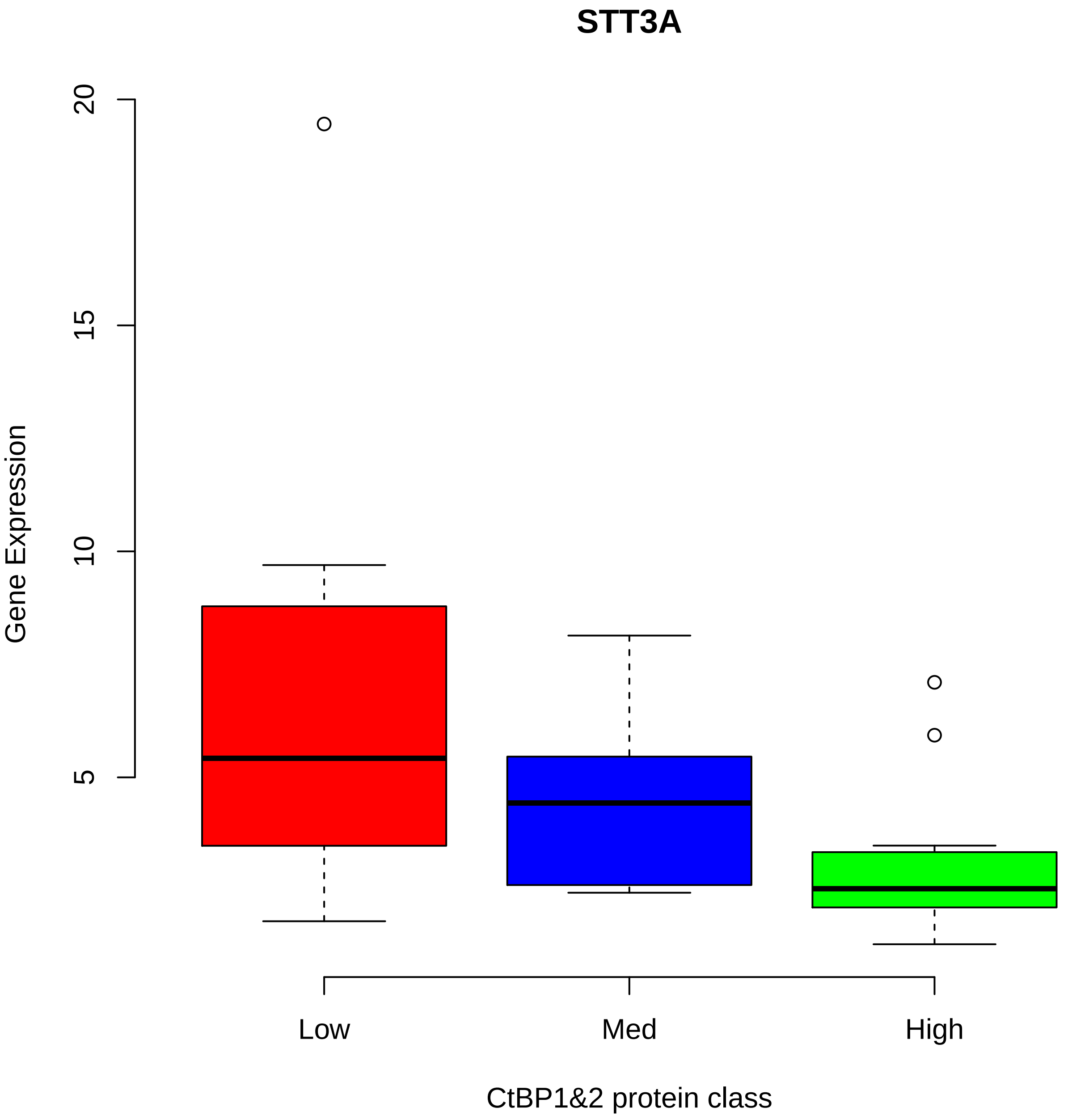

STT3A
Gene Expression
20
15
10
5
Low
Med
High
CtBP1&2 protein class

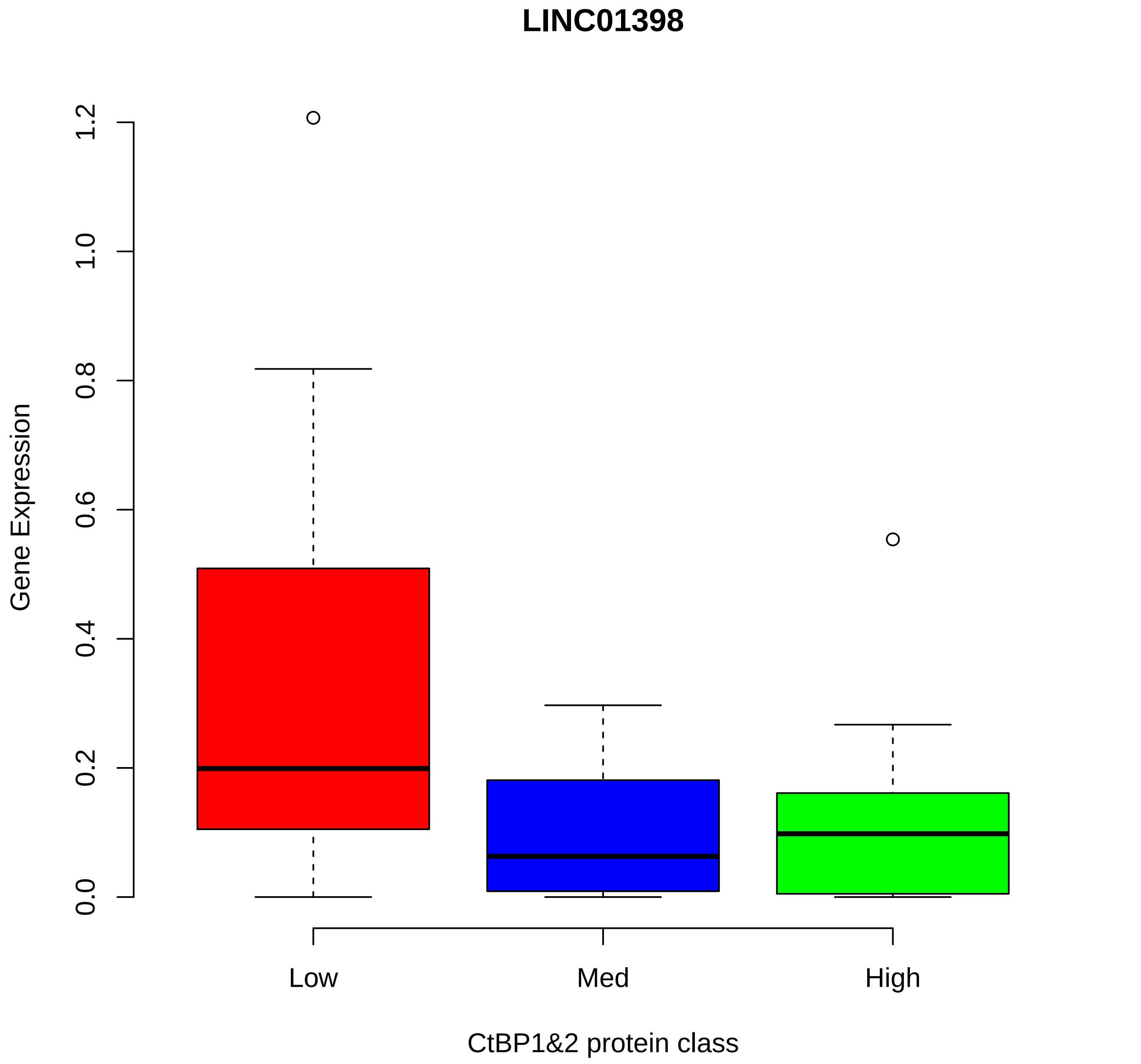
LINC01398
Gene Expression
0.0
0.2
0.4
0.6
0.8
1.0
1.2
Low
Med
High
CtBP1&2 protein class

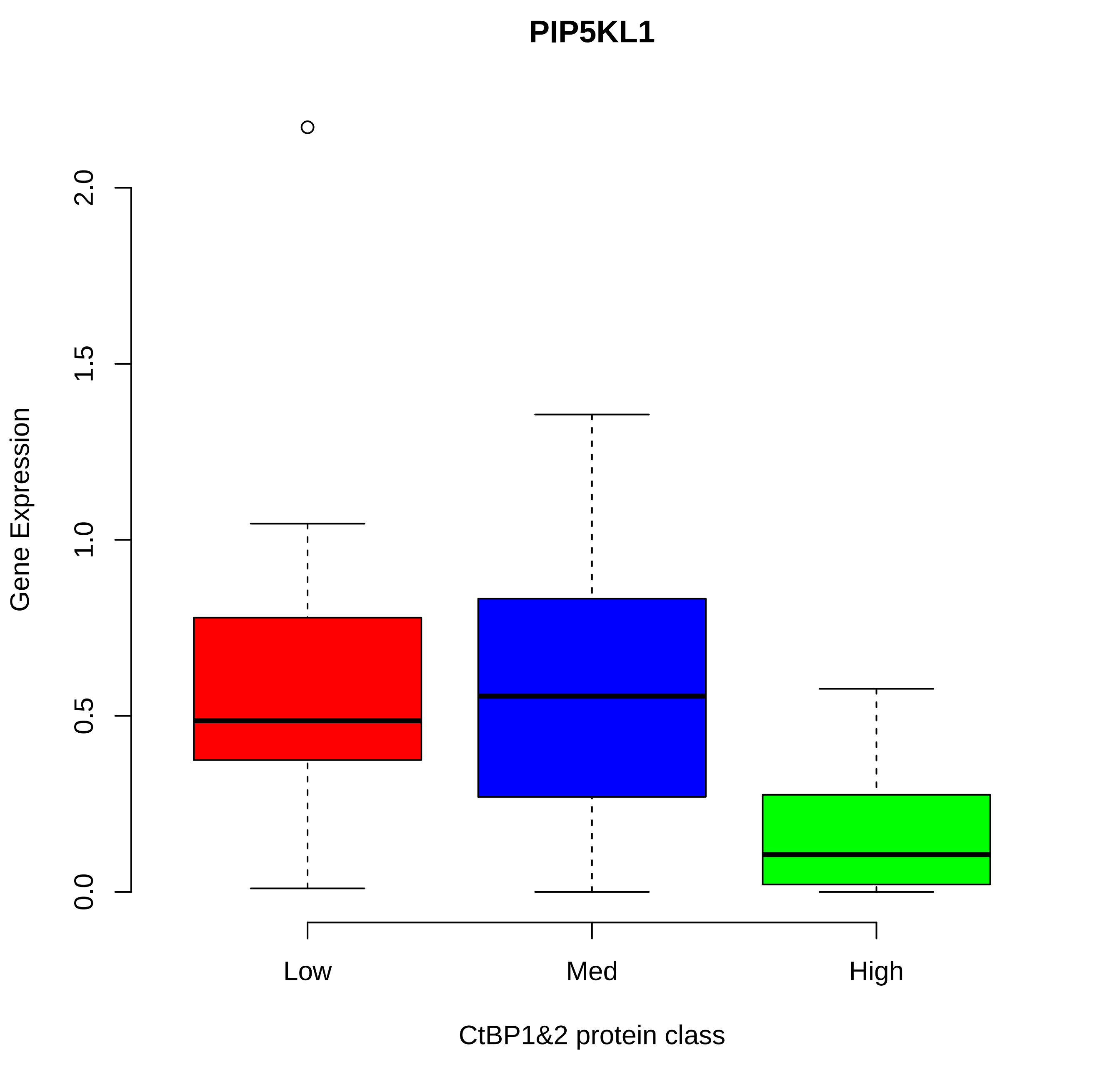

PIP5KL1
Gene Expression
0.0
0.5
1.0
1.5
2.0
Low
Med
High
CtBP1&2 protein class

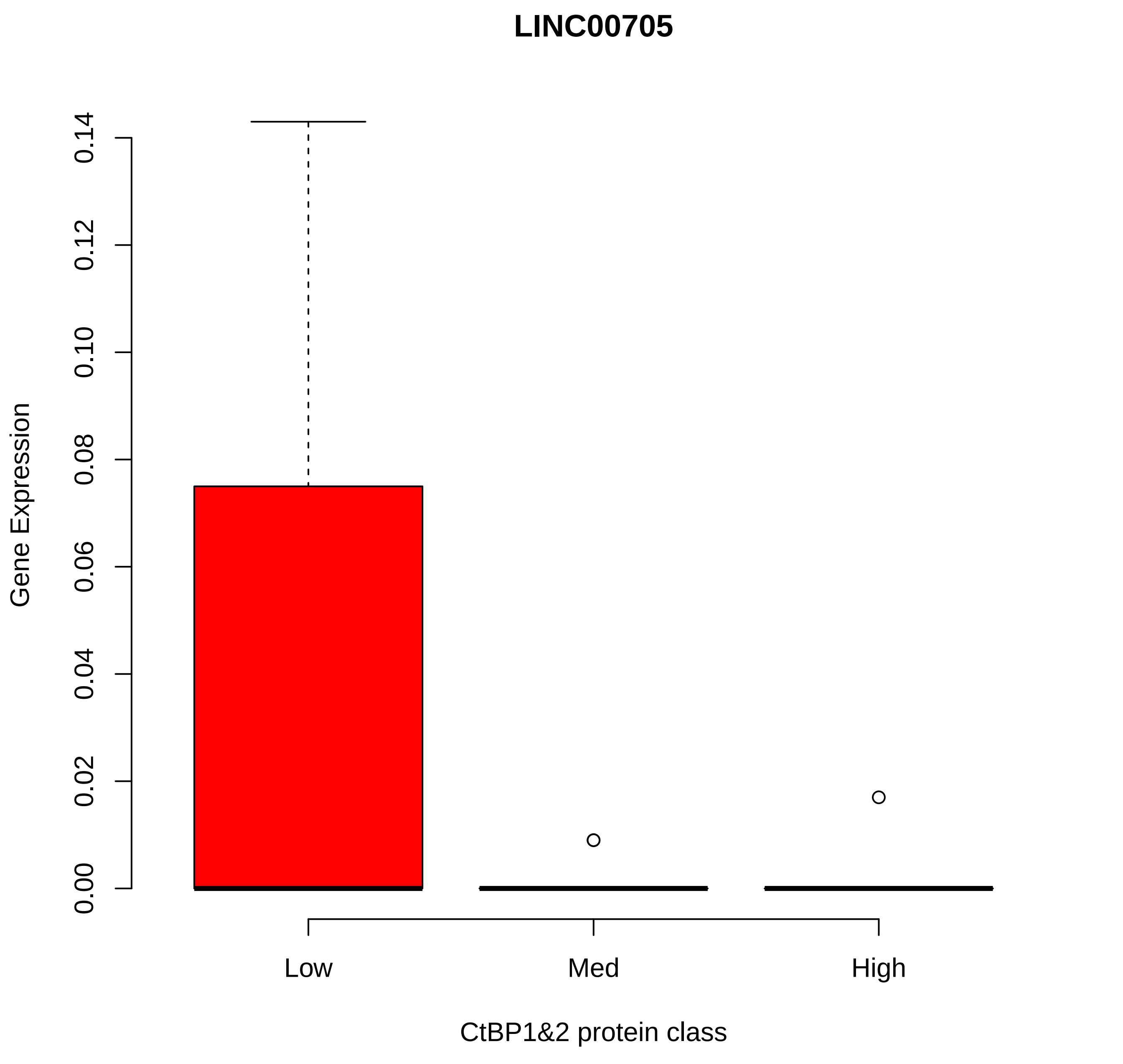
LINC00705
Gene Expression
0.00
0.02
0.04
0.06
0.08
0.10
0.12
0.14
Low
Med
High
CtBP1&2 protein class

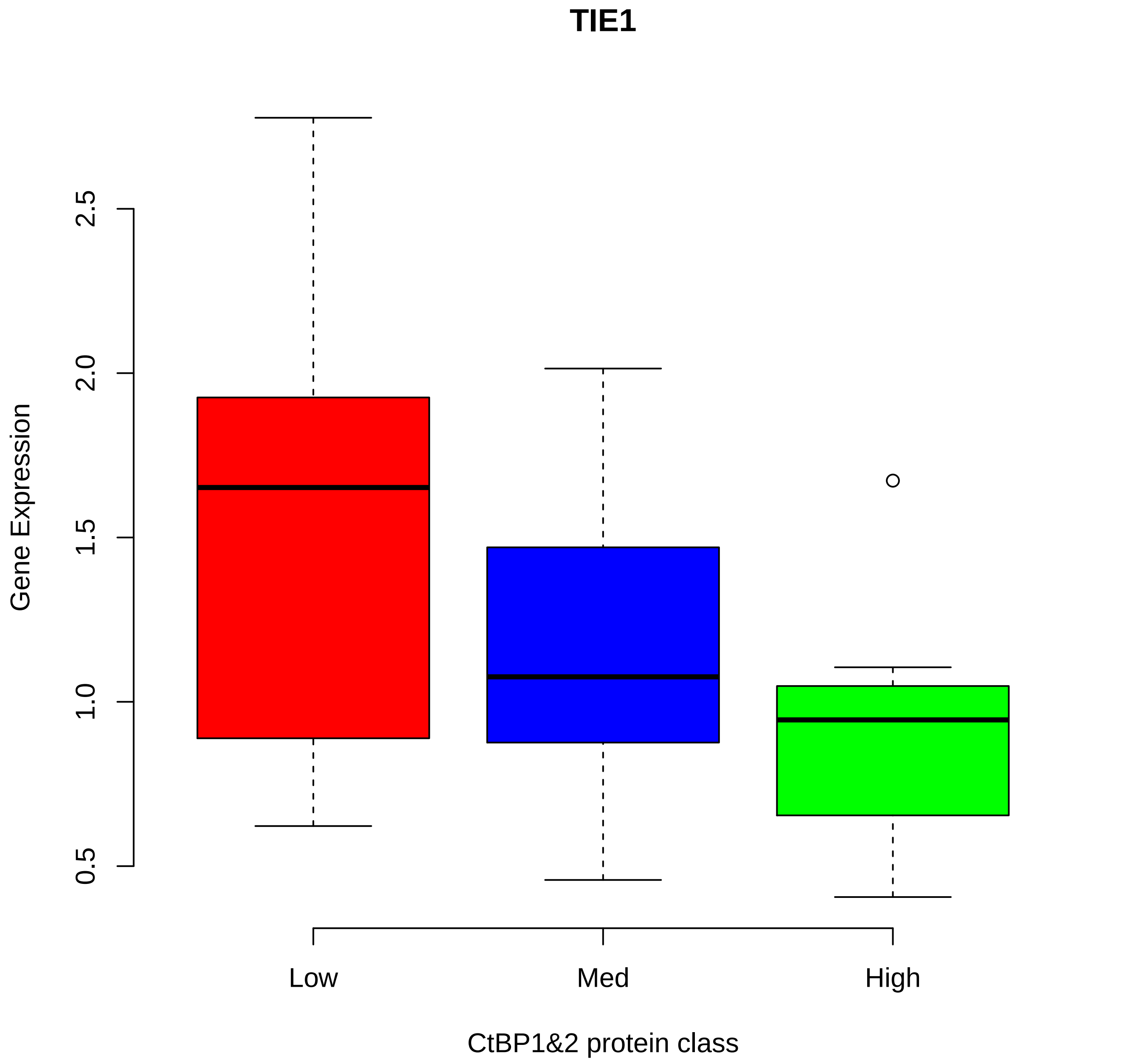

TIE1
Gene Expression
2.5
2.0
1.5
1.0
0.5
Low
Med
High
CtBP1&2 protein class

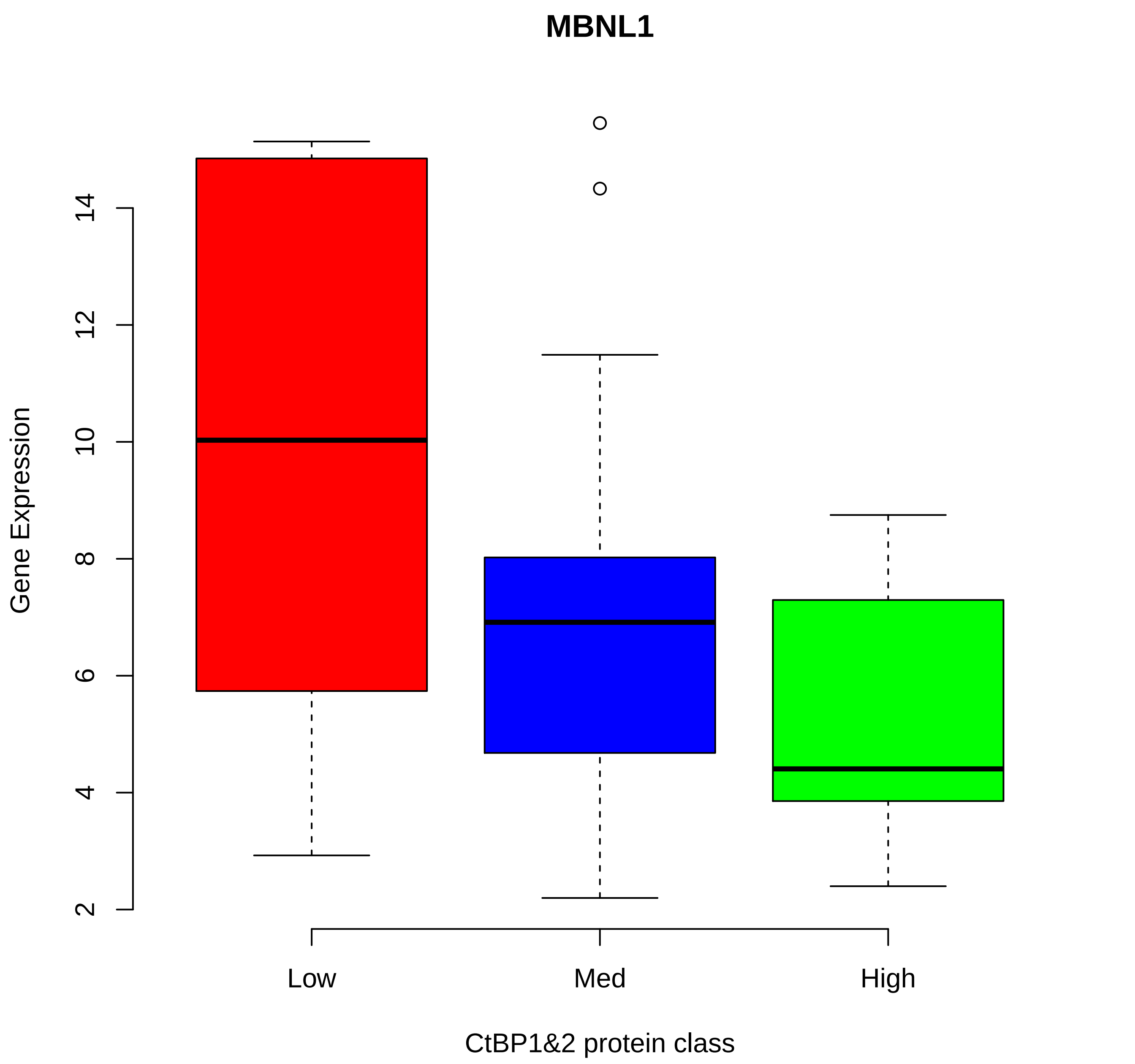

MBNL1
Gene Expression
2
4
6
8
10
12
14
Low
Med
High
CtBP1&2 protein class

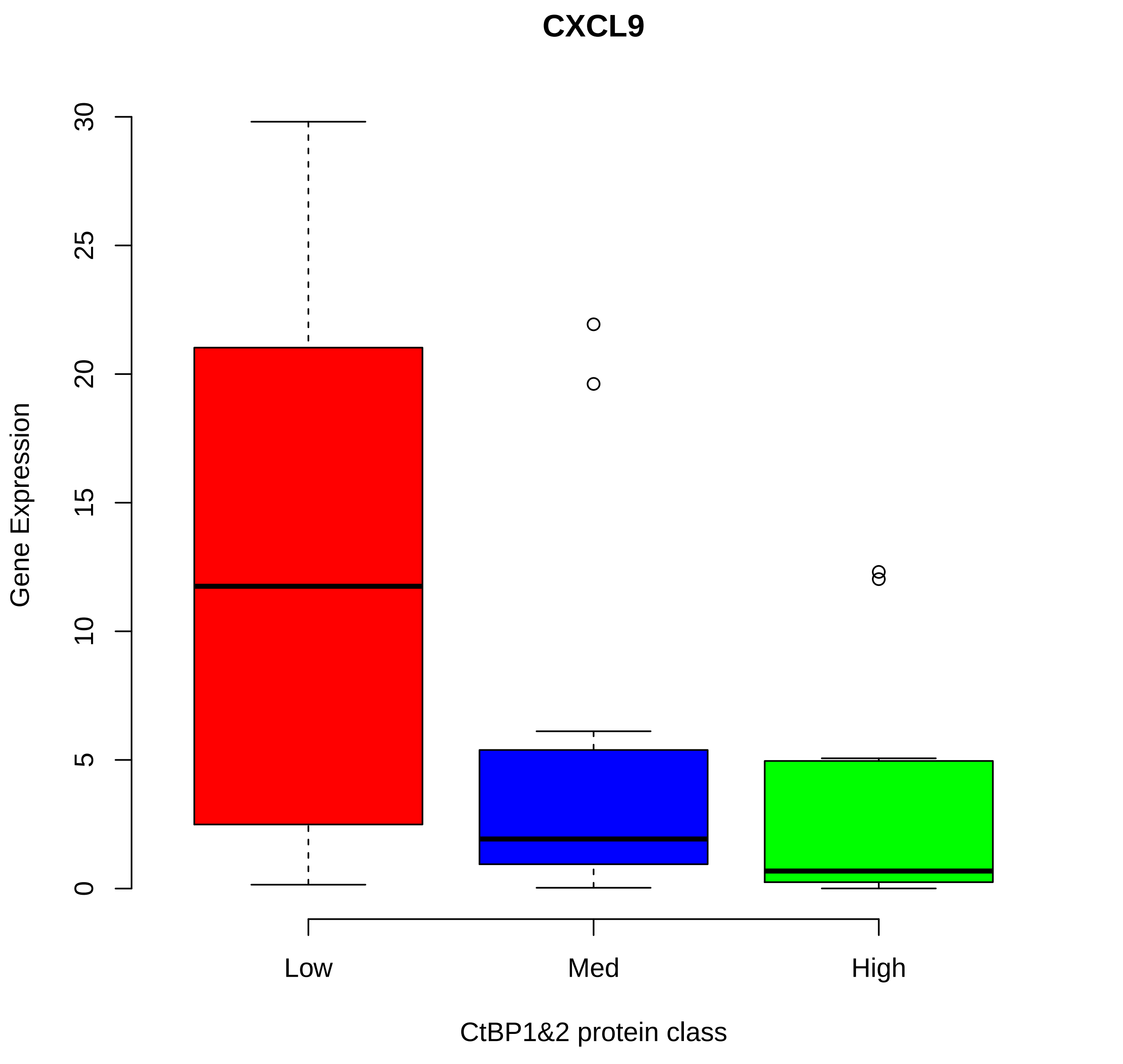
CXCL9
Gene Expression
0
5
10
15
20
25
30
Low
Med
High
CtBP1&2 protein class

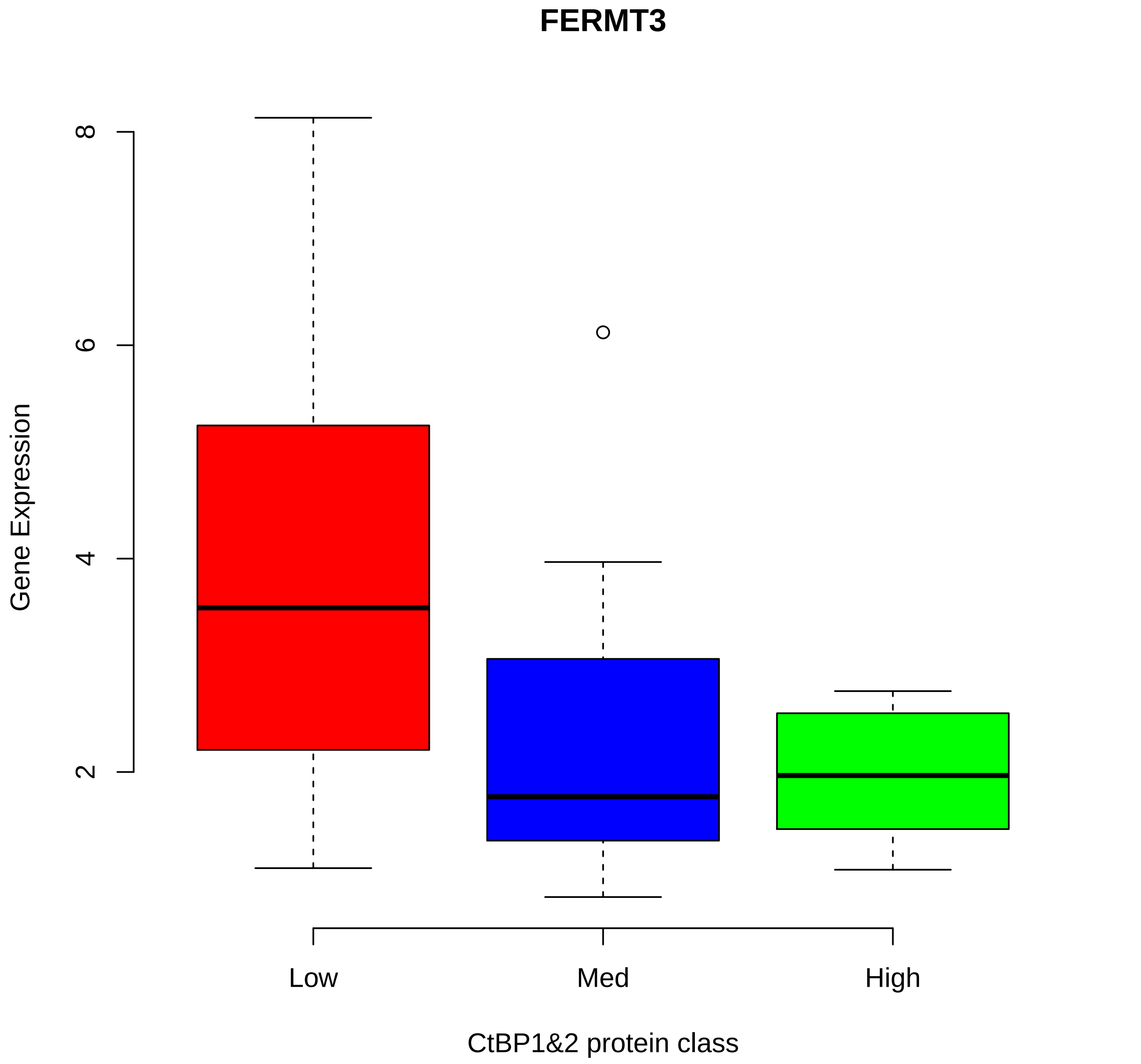

FERMT3
Gene Expression
2
4
6
8
Low
Med
High
CtBP1&2 protein class

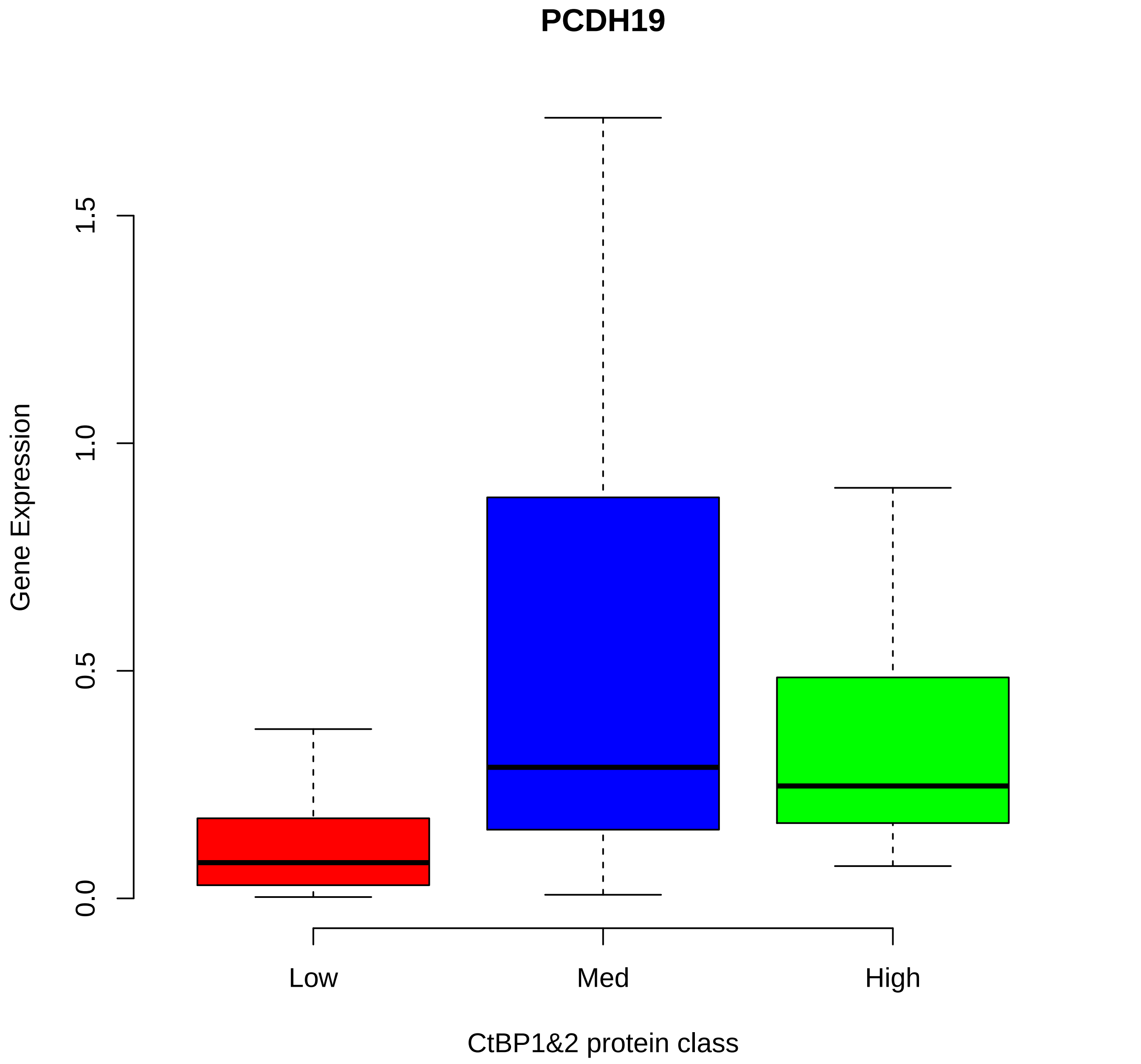
PCDH19
Gene Expression
0.0
0.5
1.0
1.5
Low
Med
High
CtBP1&2 protein class

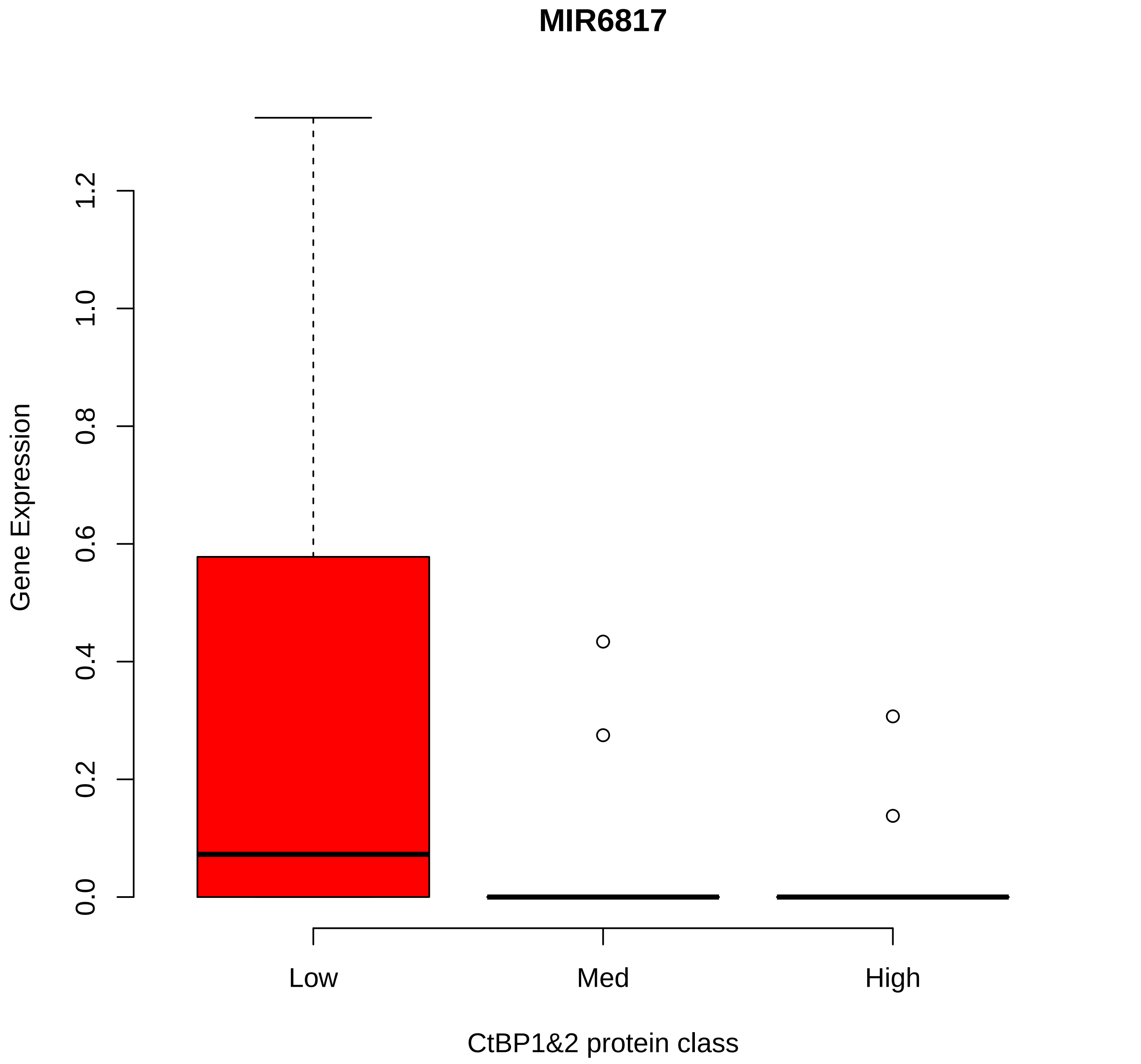

MIR6817
Gene Expression
0.0
0.2
0.4
0.6
0.8
1.0
1.2
Low
Med
High
CtBP1&2 protein class

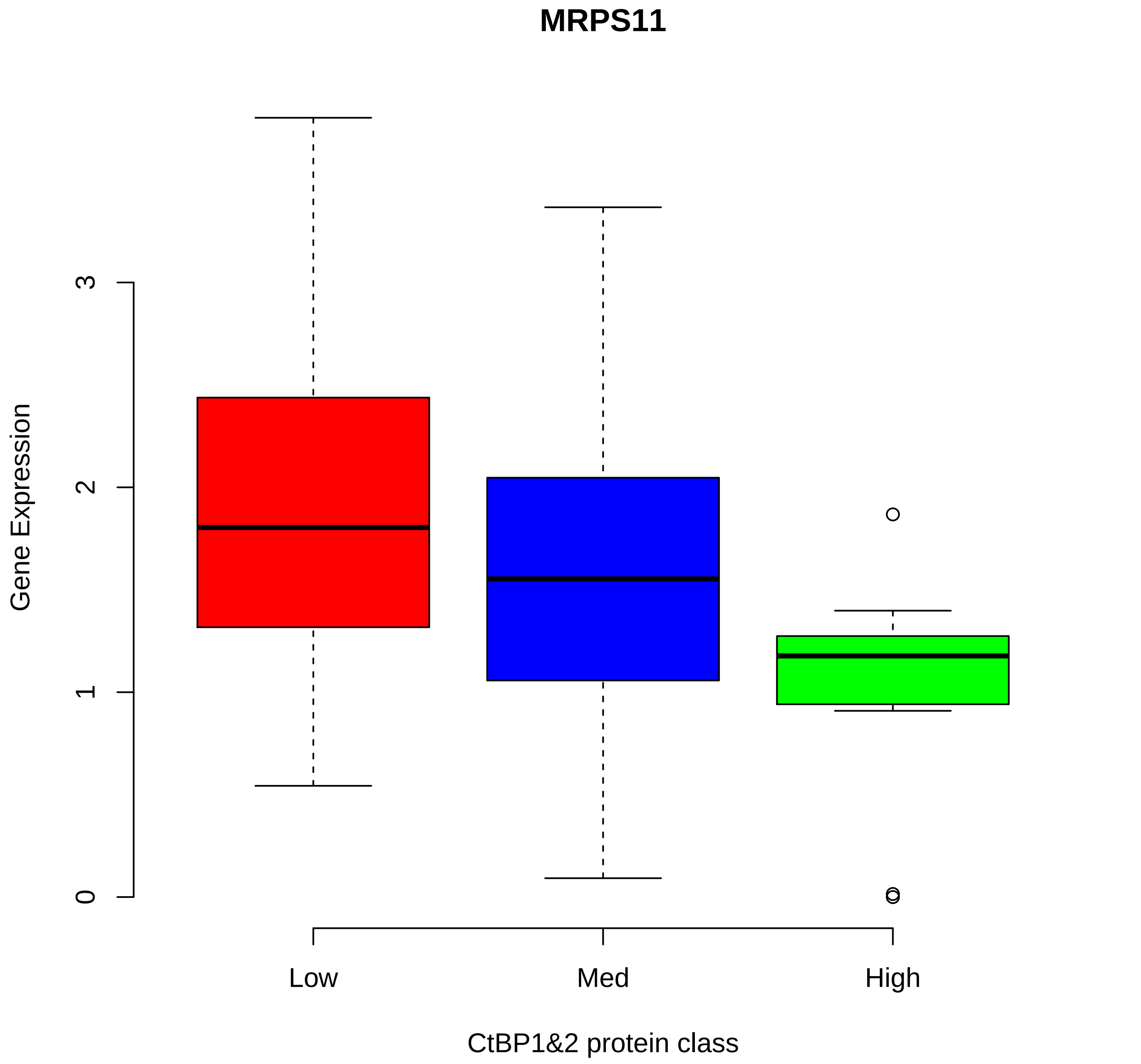

MRPS11
Gene Expression
0
1
2
3
Low
Med
High
CtBP1&2 protein class

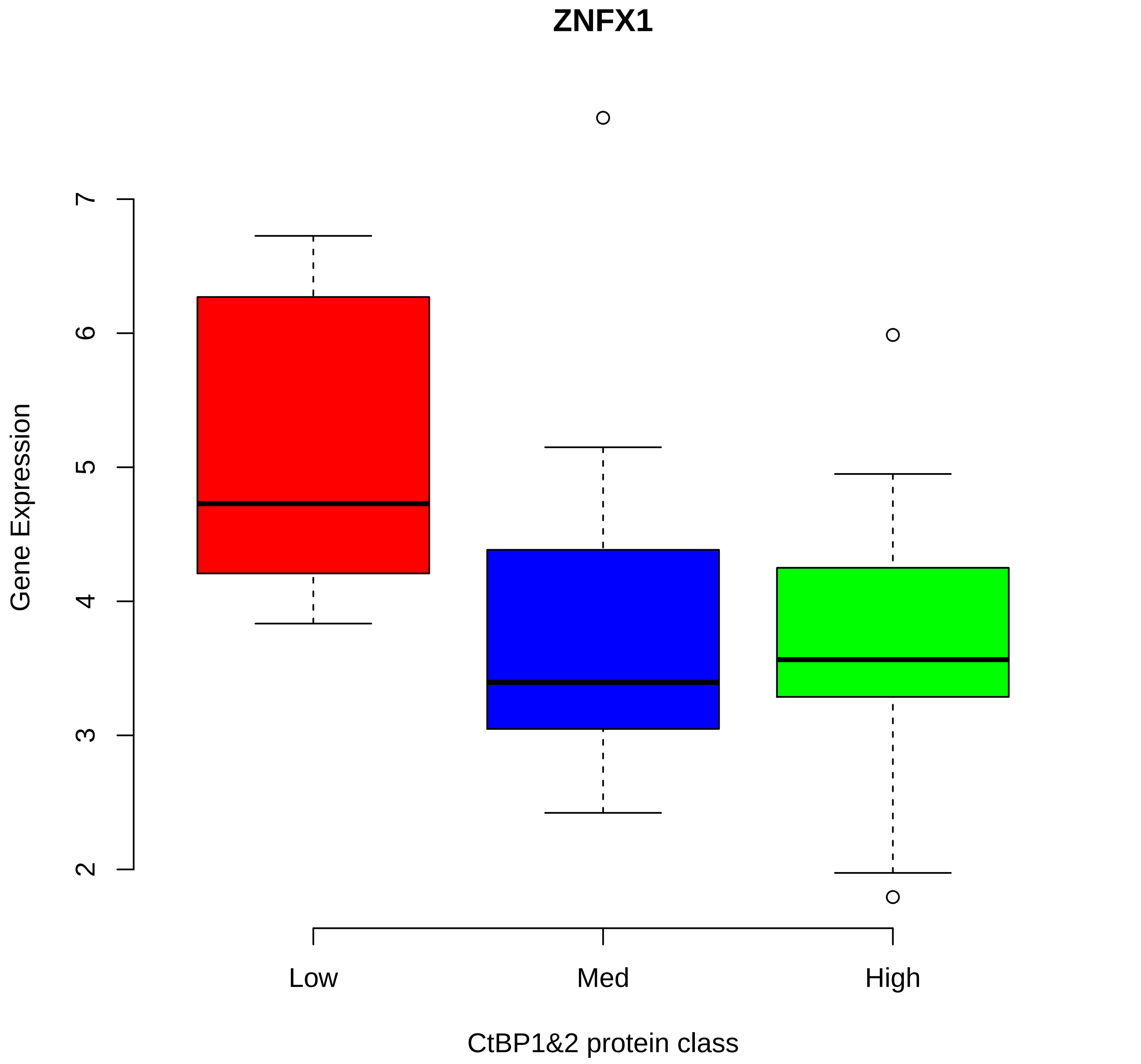
ZNFX1
Gene Expression
2
3
4
5
6
7
Low
Med
High
CtBP1&2 protein class

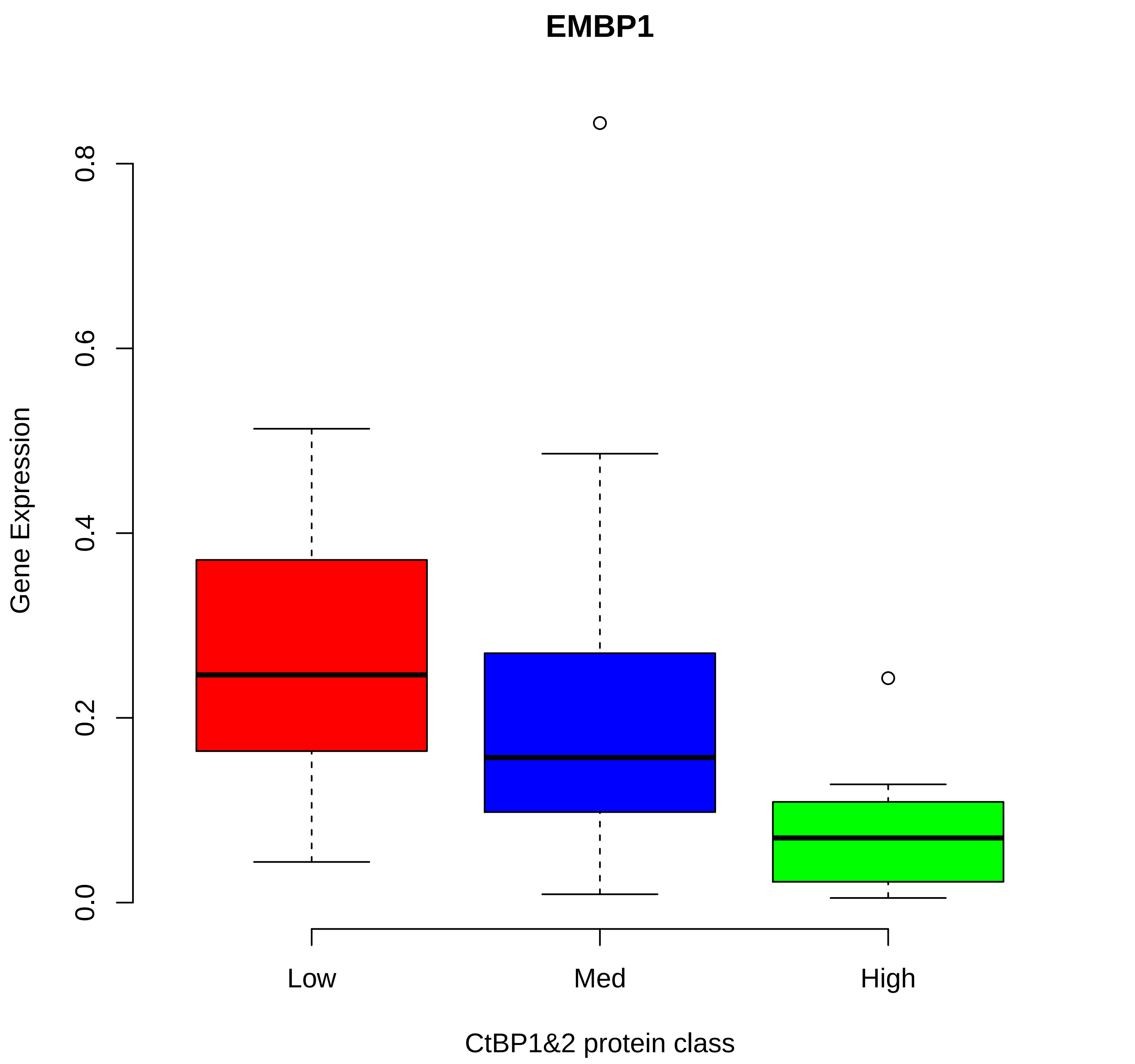

EMBP1
Gene Expression
0.0
0.2
0.4
0.6
0.8
Low
Med
High
CtBP1&2 protein class

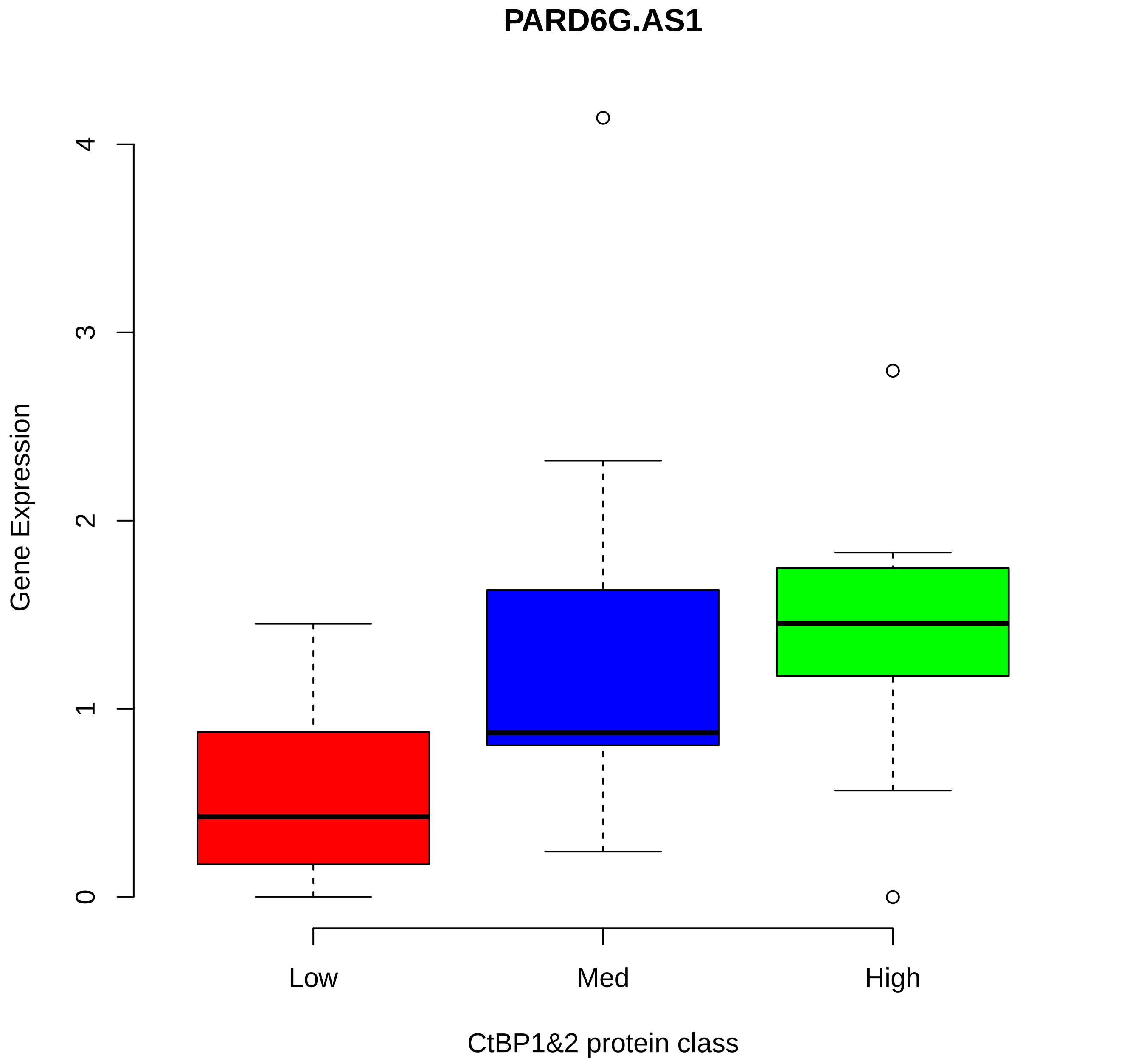

PARD6G.AS1
Gene Expression
0
1
2
3
4
Low
Med
High
CtBP1&2 protein class

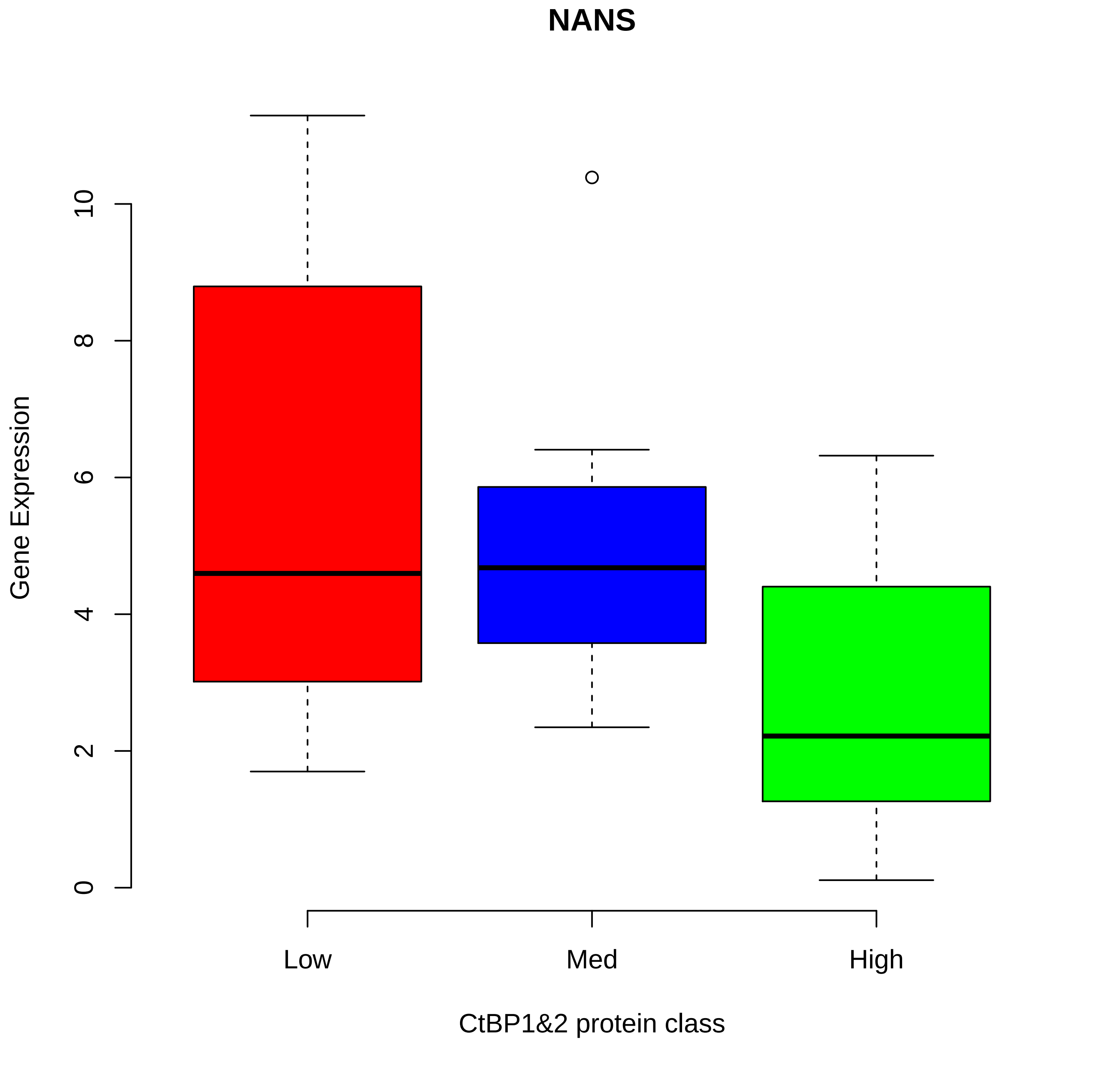
NANS
Gene Expression
0
2
4
6
8
10
Low
Med
High
CtBP1&2 protein class

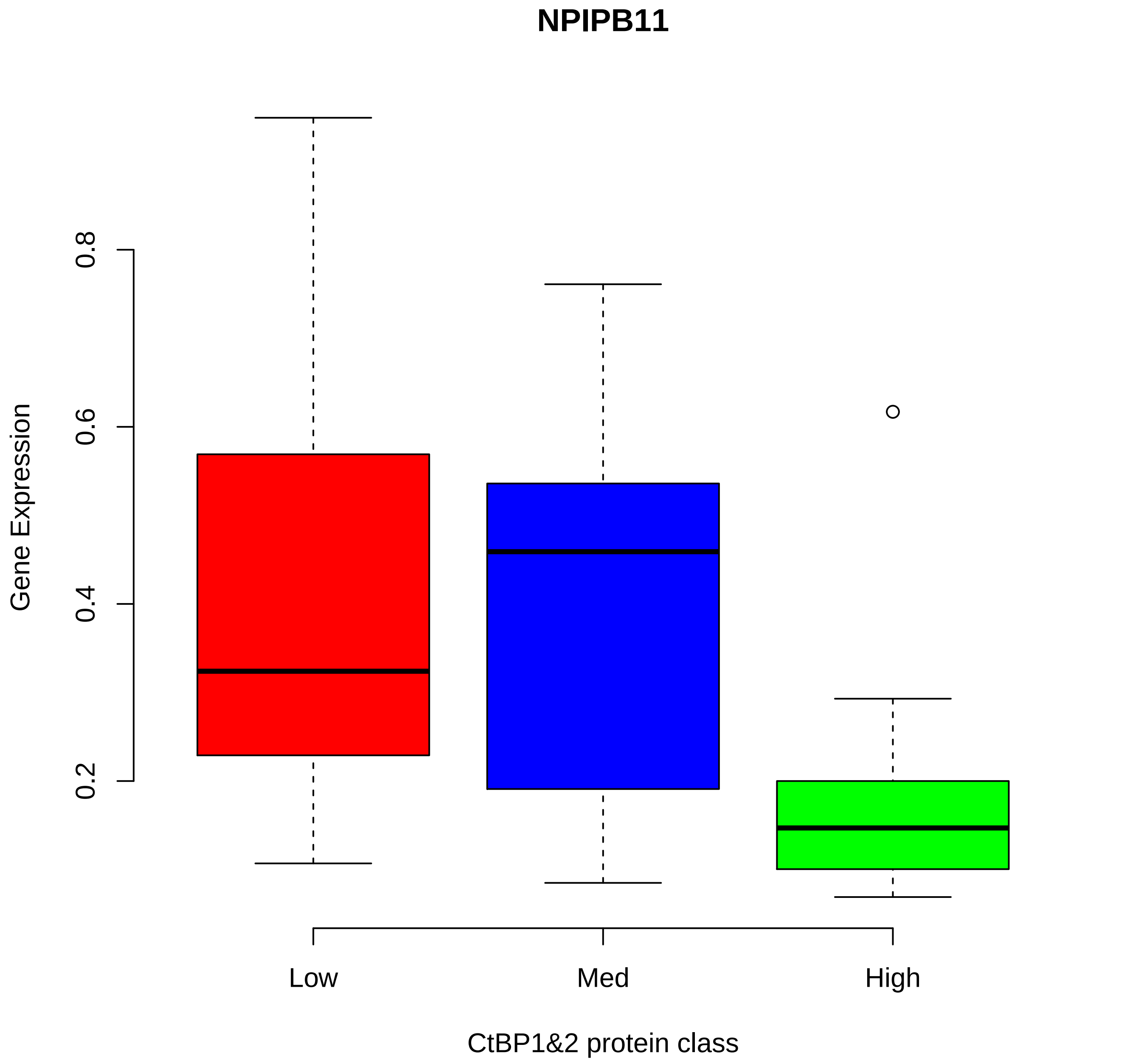
NPIPB11
Gene Expression
0.2
0.4
0.6
0.8
Low
Med
High
CtBP1&2 protein class

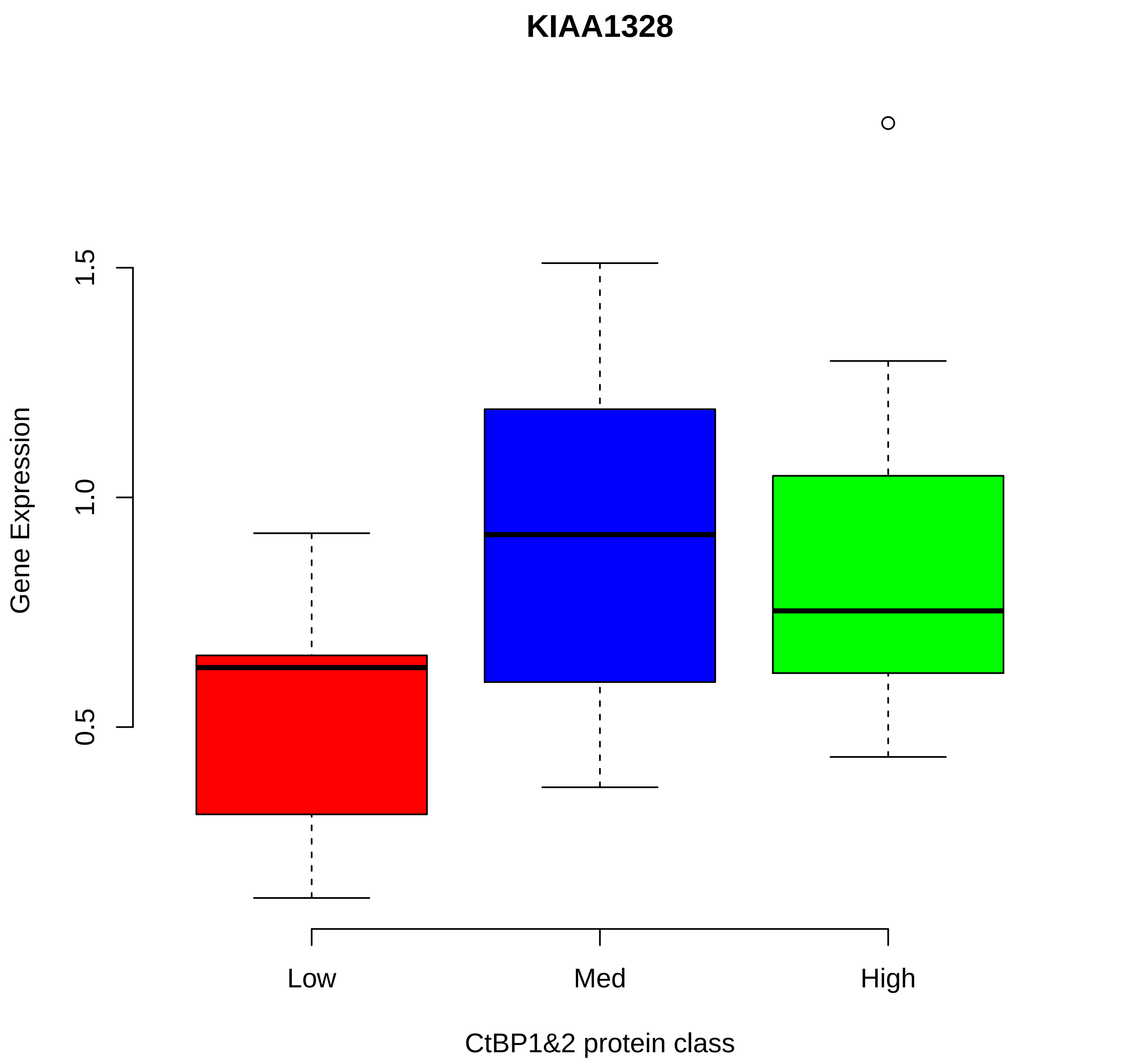
KIAA1328
Gene Expression
1.5
1.0
0.5
Low
Med
High
CtBP1&2 protein class

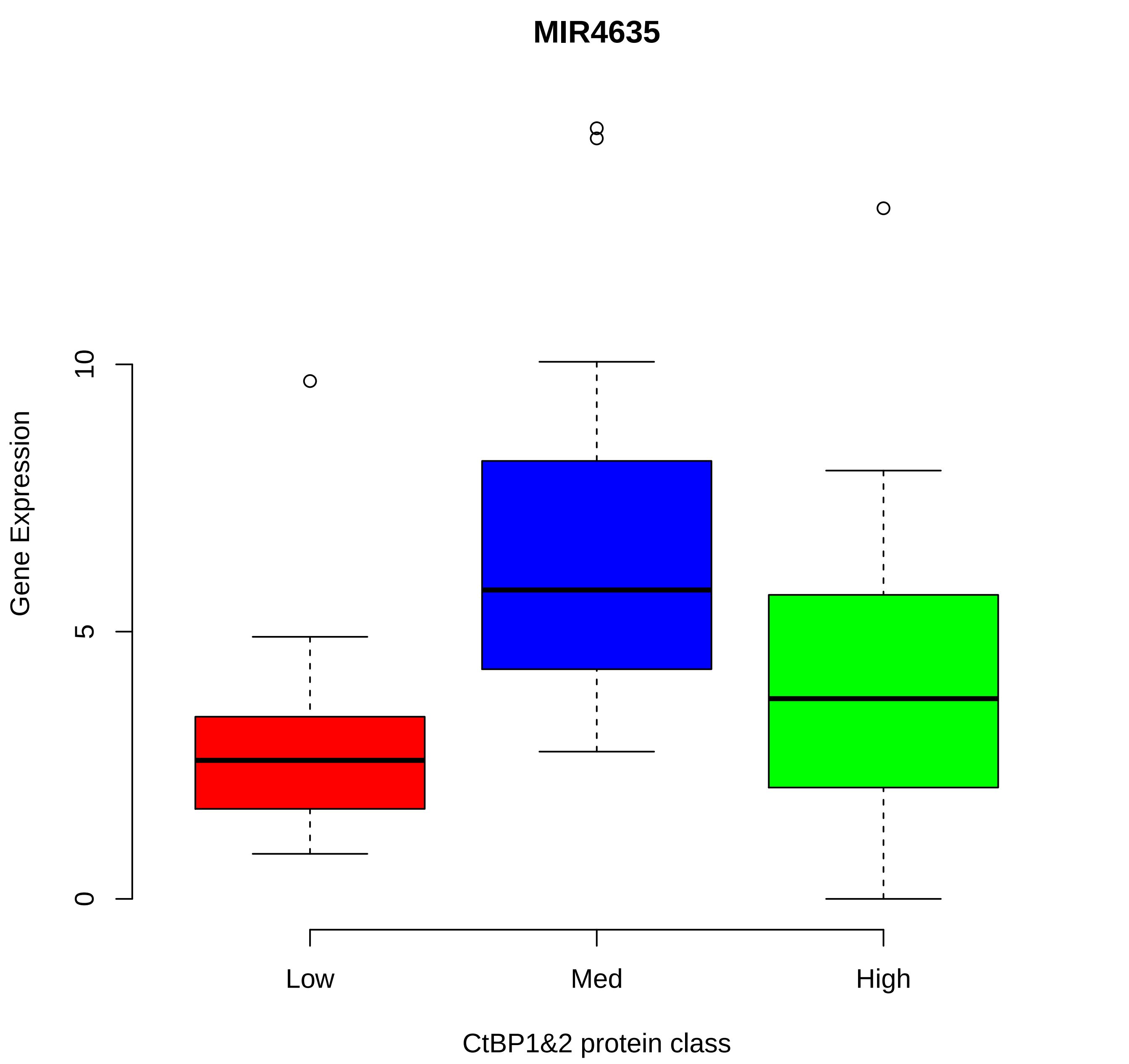
MIR4635
Gene Expression
0
5
10
Low
Med
High
CtBP1&2 protein class

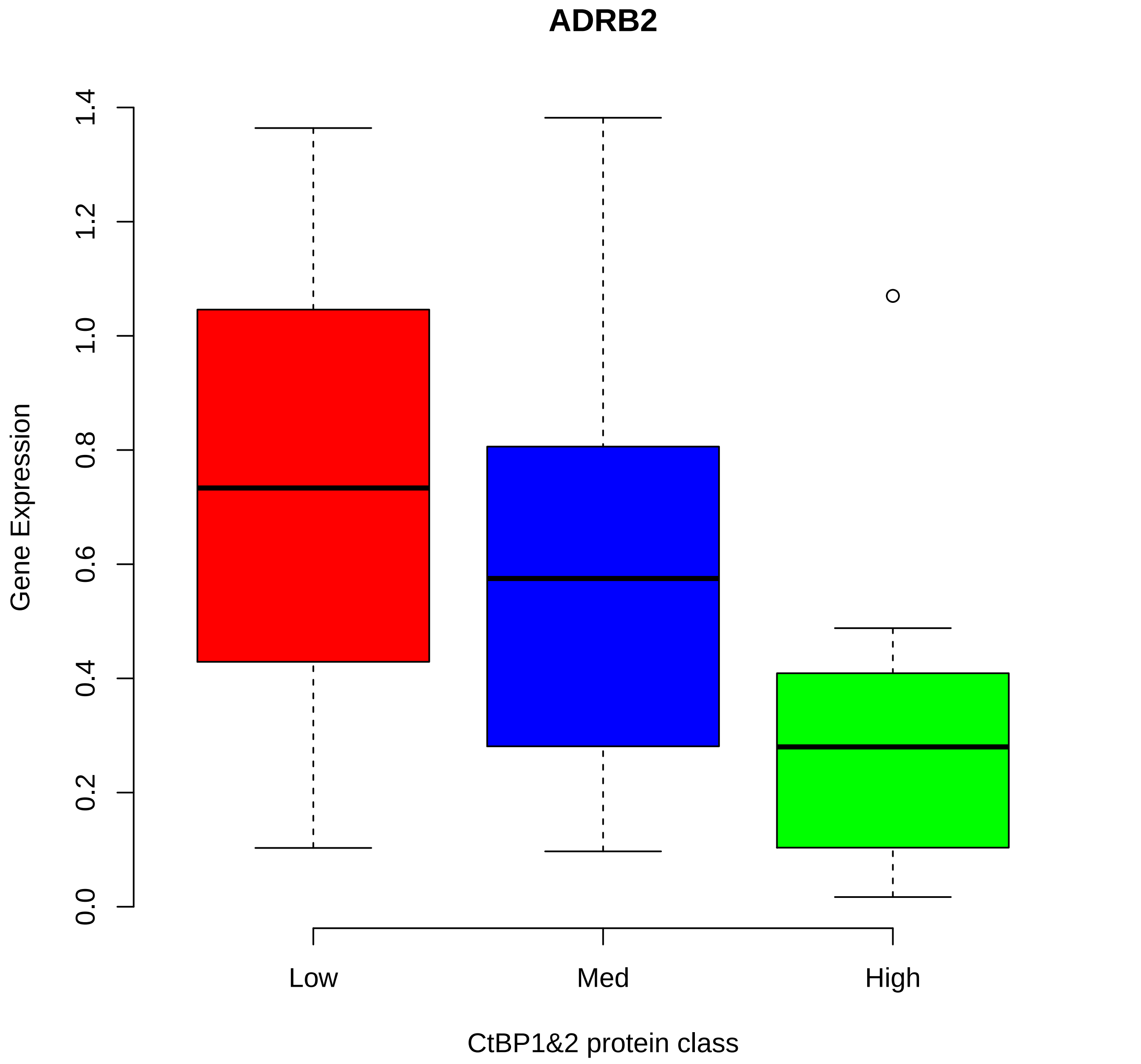
ADRB2
Gene Expression
0.0
0.2
0.4
0.6
0.8
1.0
1.2
1.4
Low
Med
High
CtBP1&2 protein class

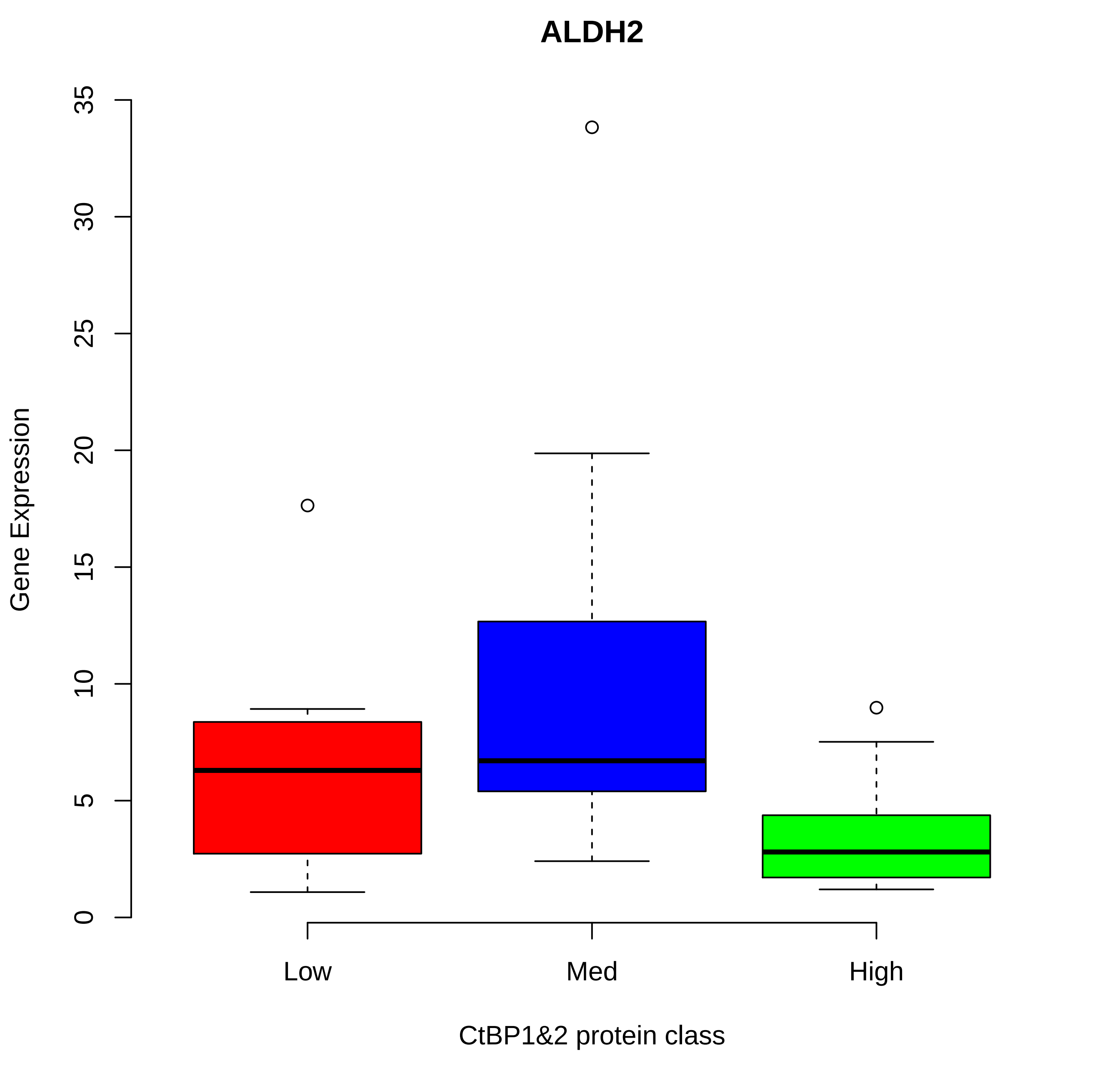

ALDH2
Gene Expression
0
5
10
15
20
25
30
35
Low
Med
High
CtBP1&2 protein class

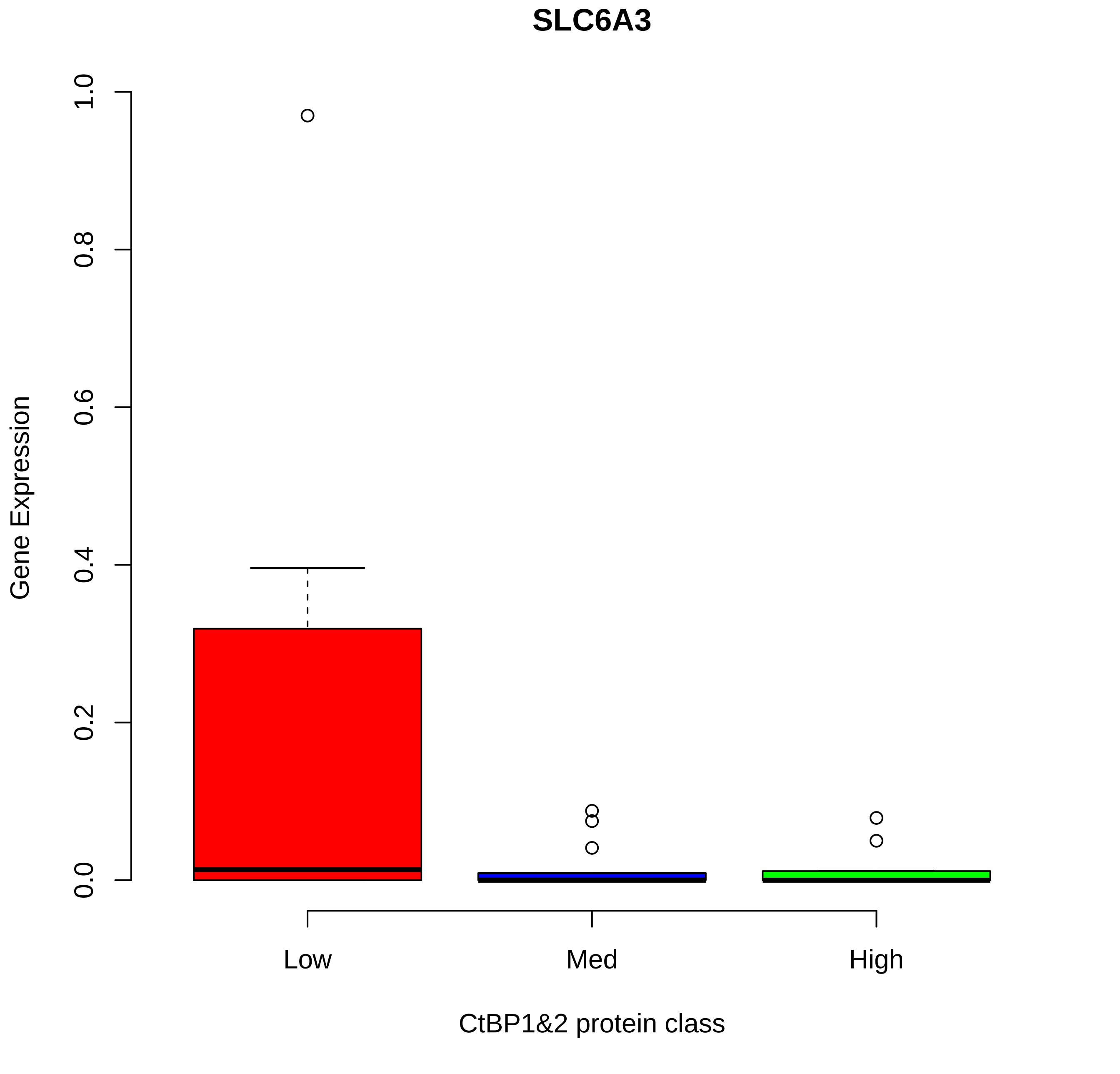

SLC6A3
Gene Expression
0.0
0.2
0.4
0.6
0.8
1.0
Low
Med
High
CtBP1&2 protein class

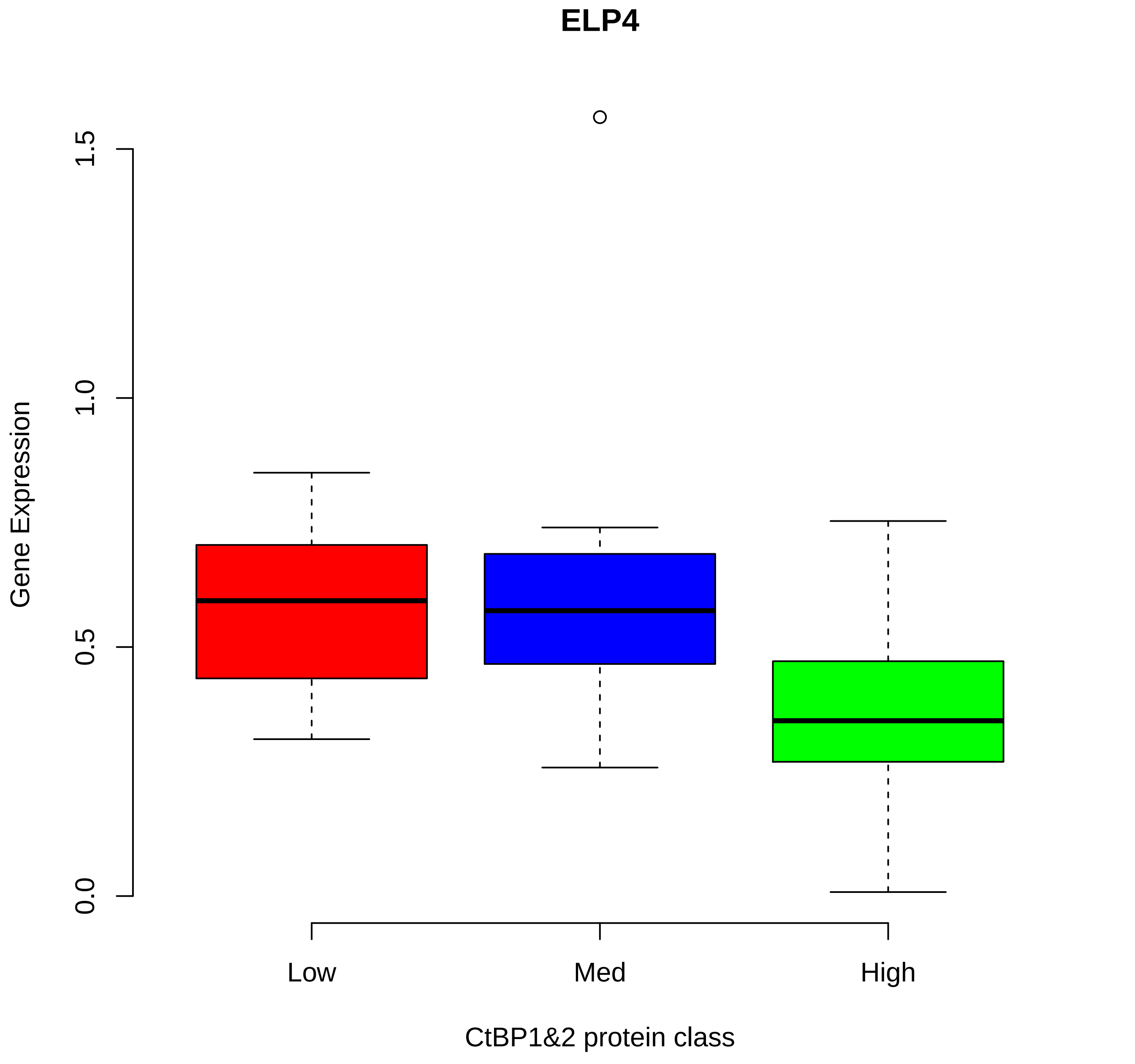

ELP4
Gene Expression
0.0
0.5
1.0
1.5
Low
Med
High
CtBP1&2 protein class

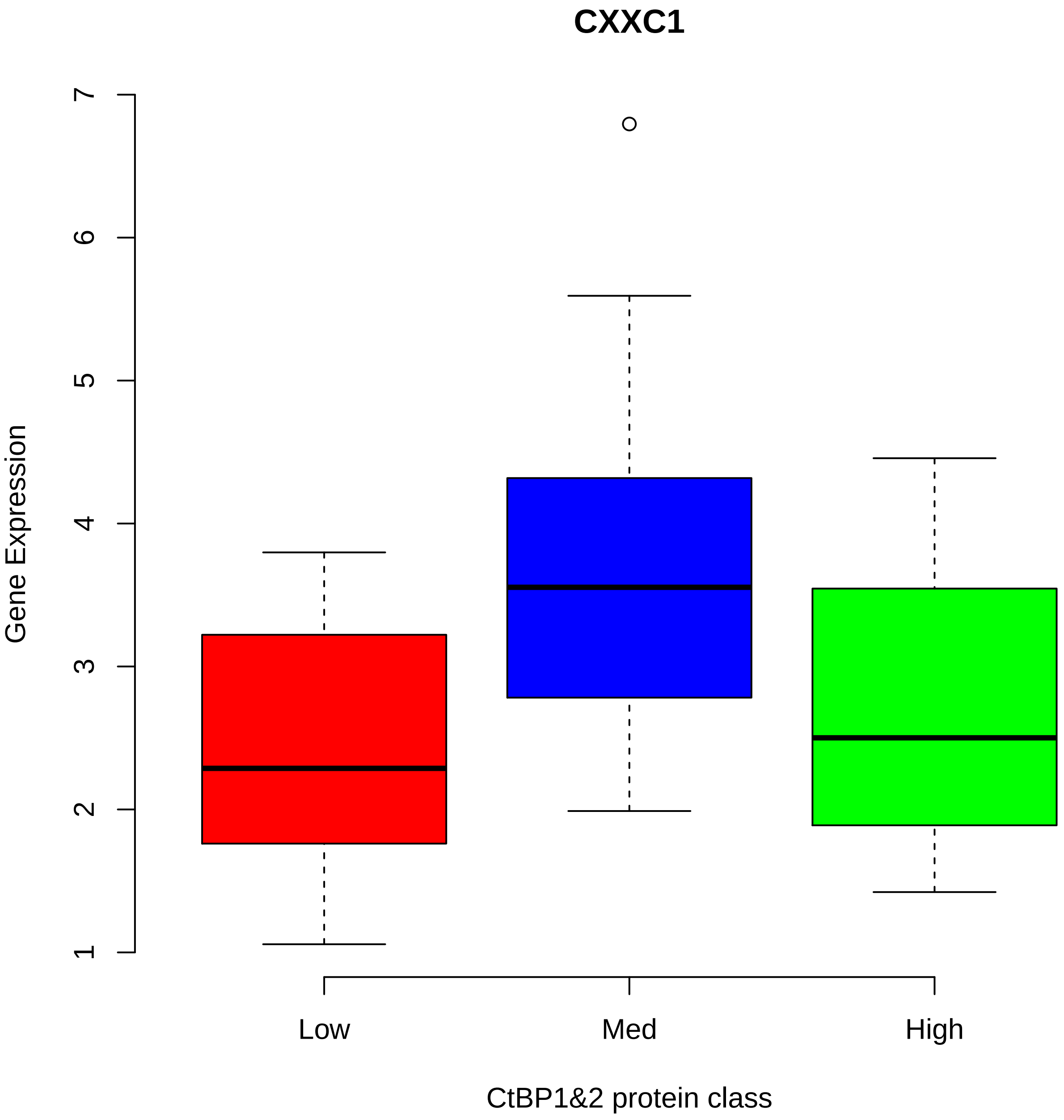
CXXC1
Gene Expression
1
2
3
4
5
6
7
Low
Med
High
CtBP1&2 protein class

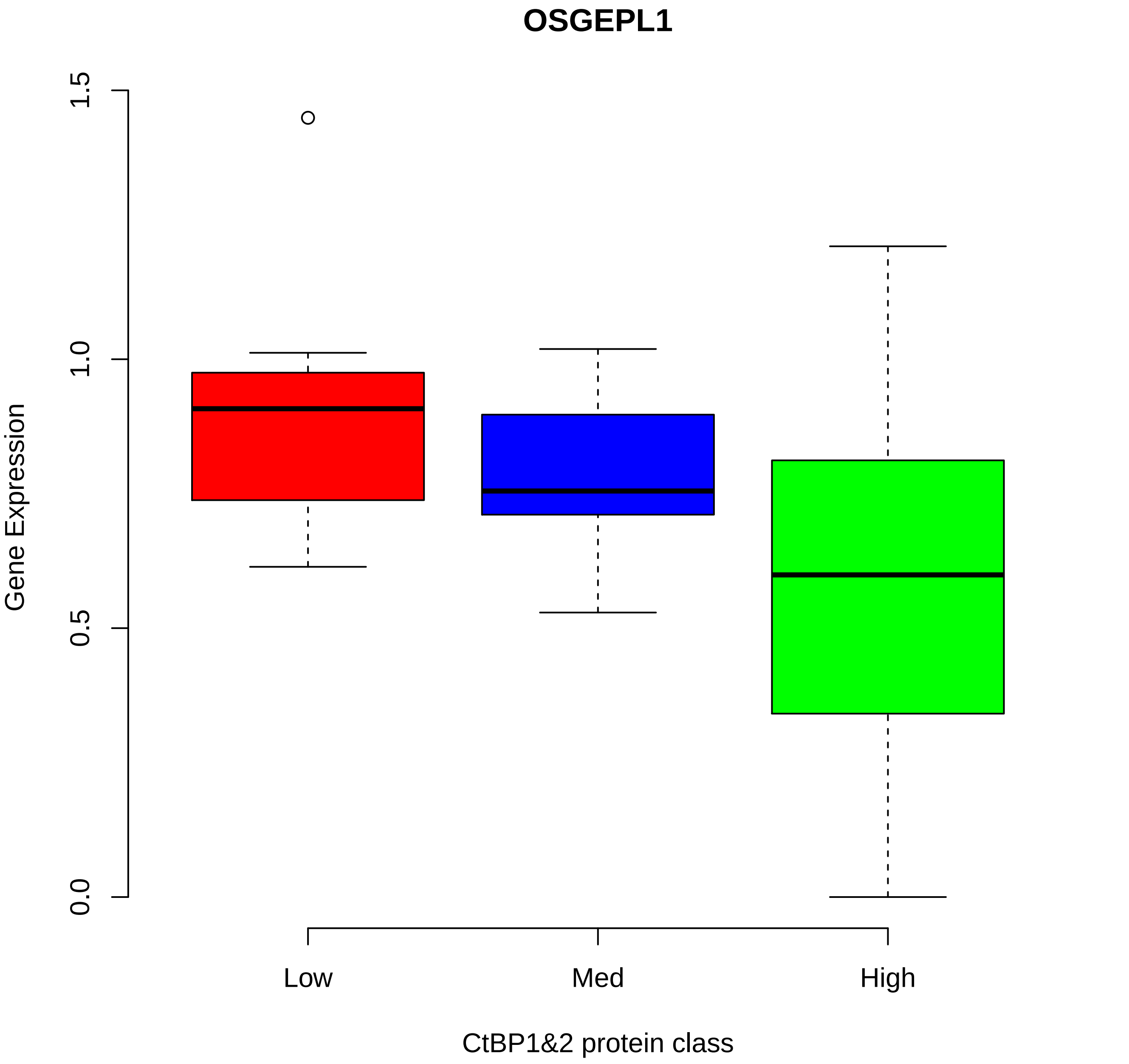

OSGEPL1
Gene Expression
0.0
0.5
1.0
1.5
Low
Med
High
CtBP1&2 protein class

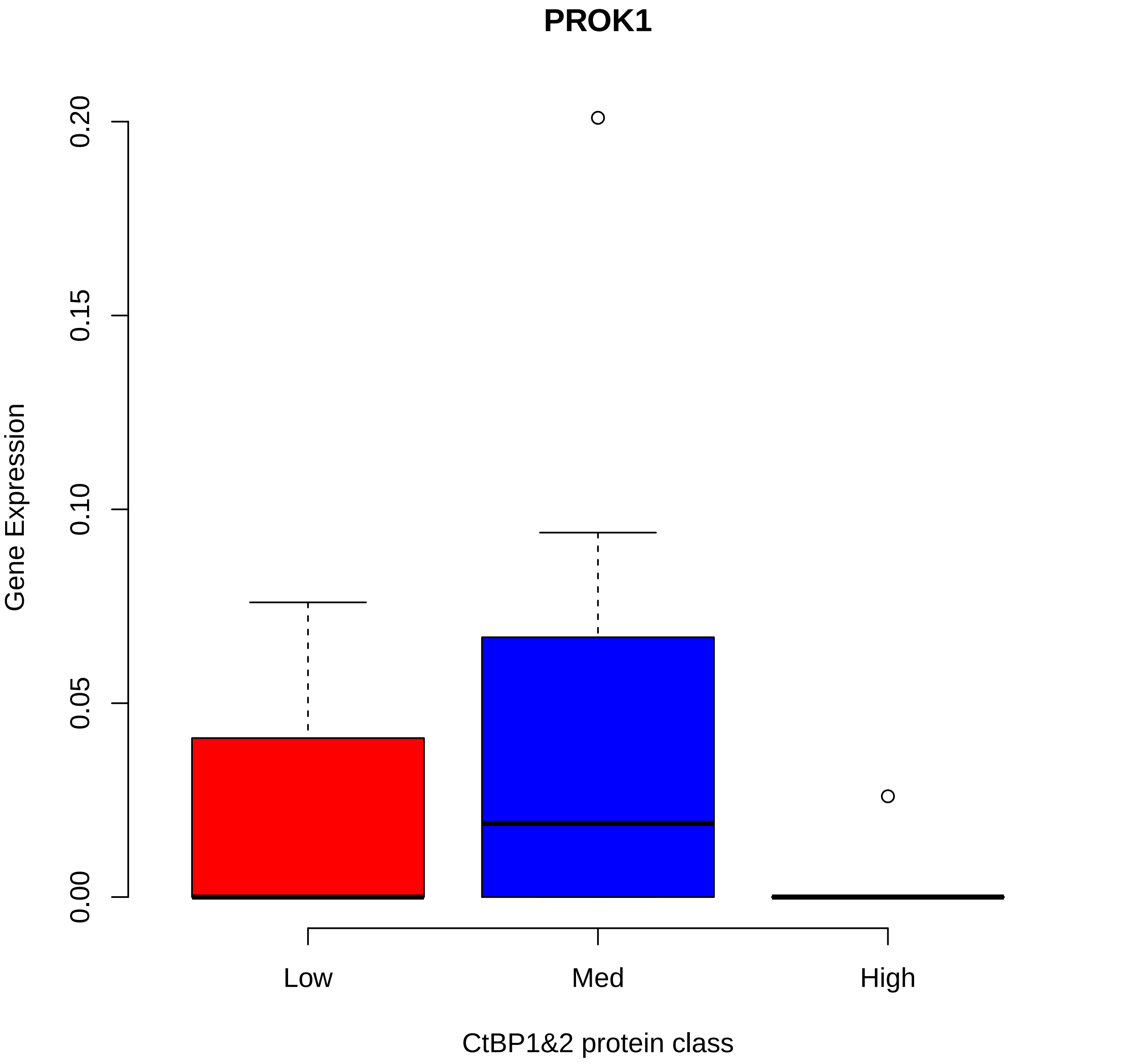

PROK1
Gene Expression
0.00
0.05
0.10
0.15
0.20
Low
Med
High
CtBP1&2 protein class

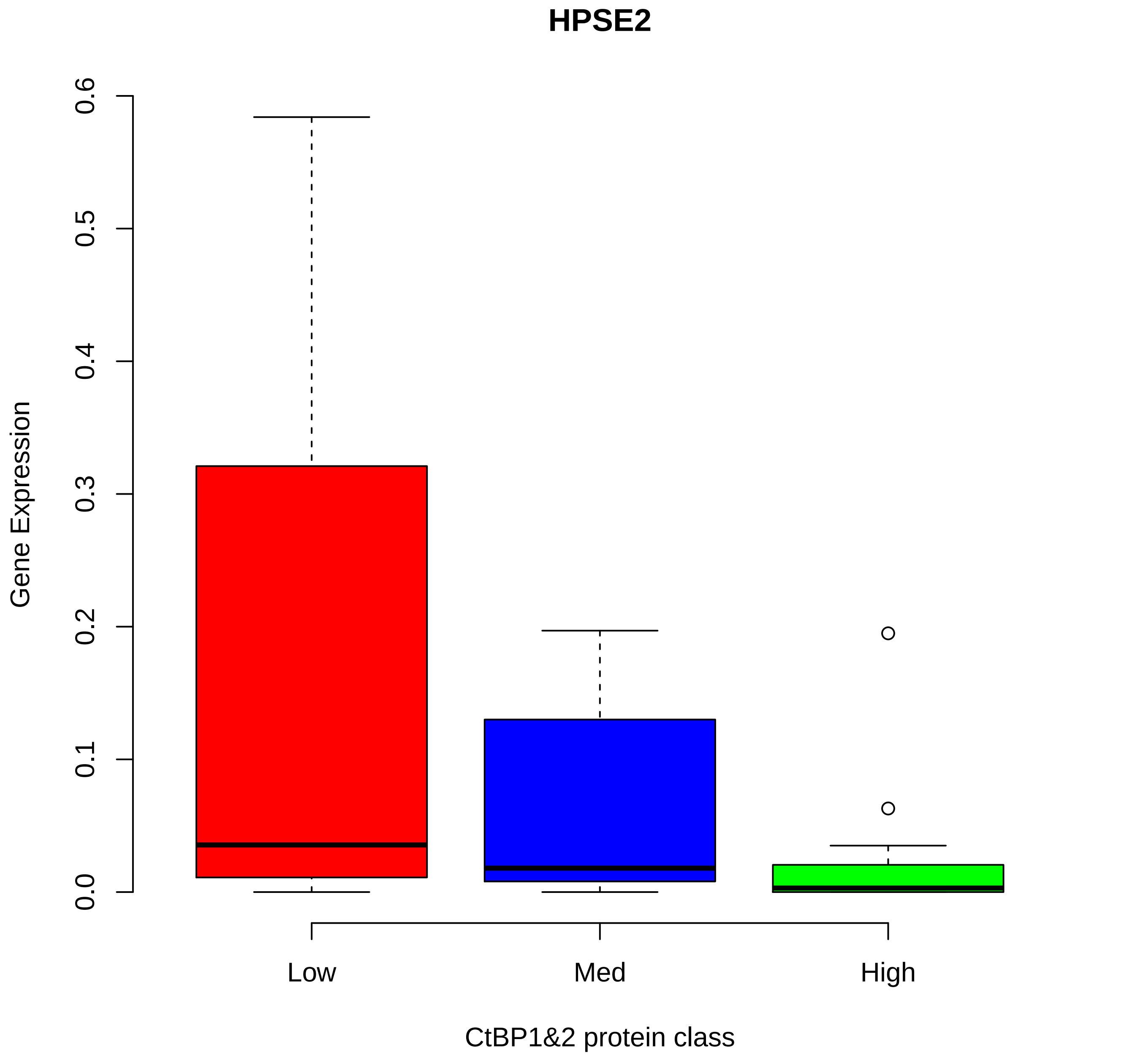

HPSE2
Gene Expression
0.0
0.1
0.2
0.3
0.4
0.5
0.6
Low
Med
High
CtBP1&2 protein class

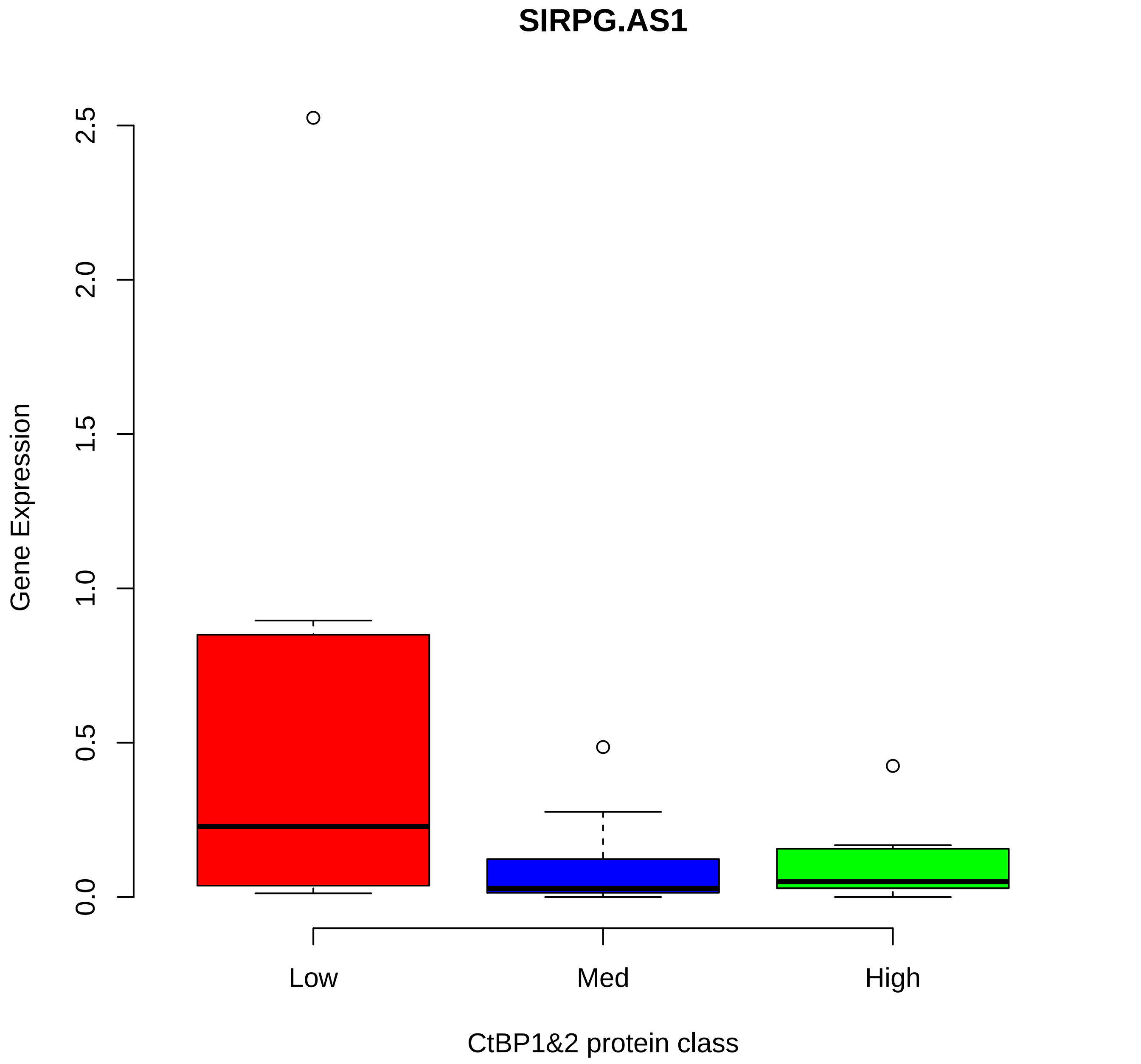

SIRPG.AS1
Gene Expression
0.0
0.5
1.0
1.5
2.0
2.5
Low
Med
High
CtBP1&2 protein class

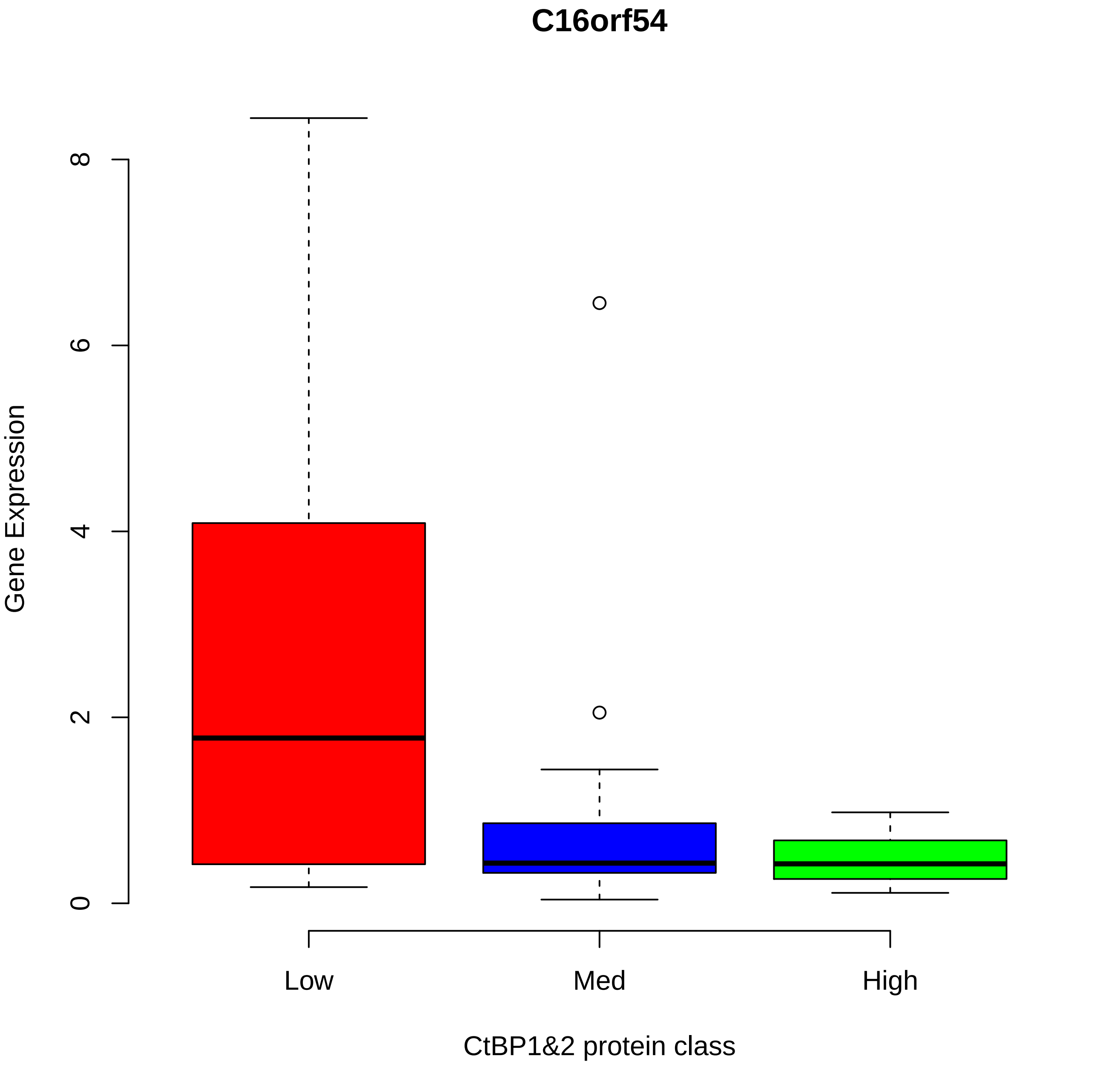

C16orf54
Gene Expression
0
2
4
6
8
Low
Med
High
CtBP1&2 protein class

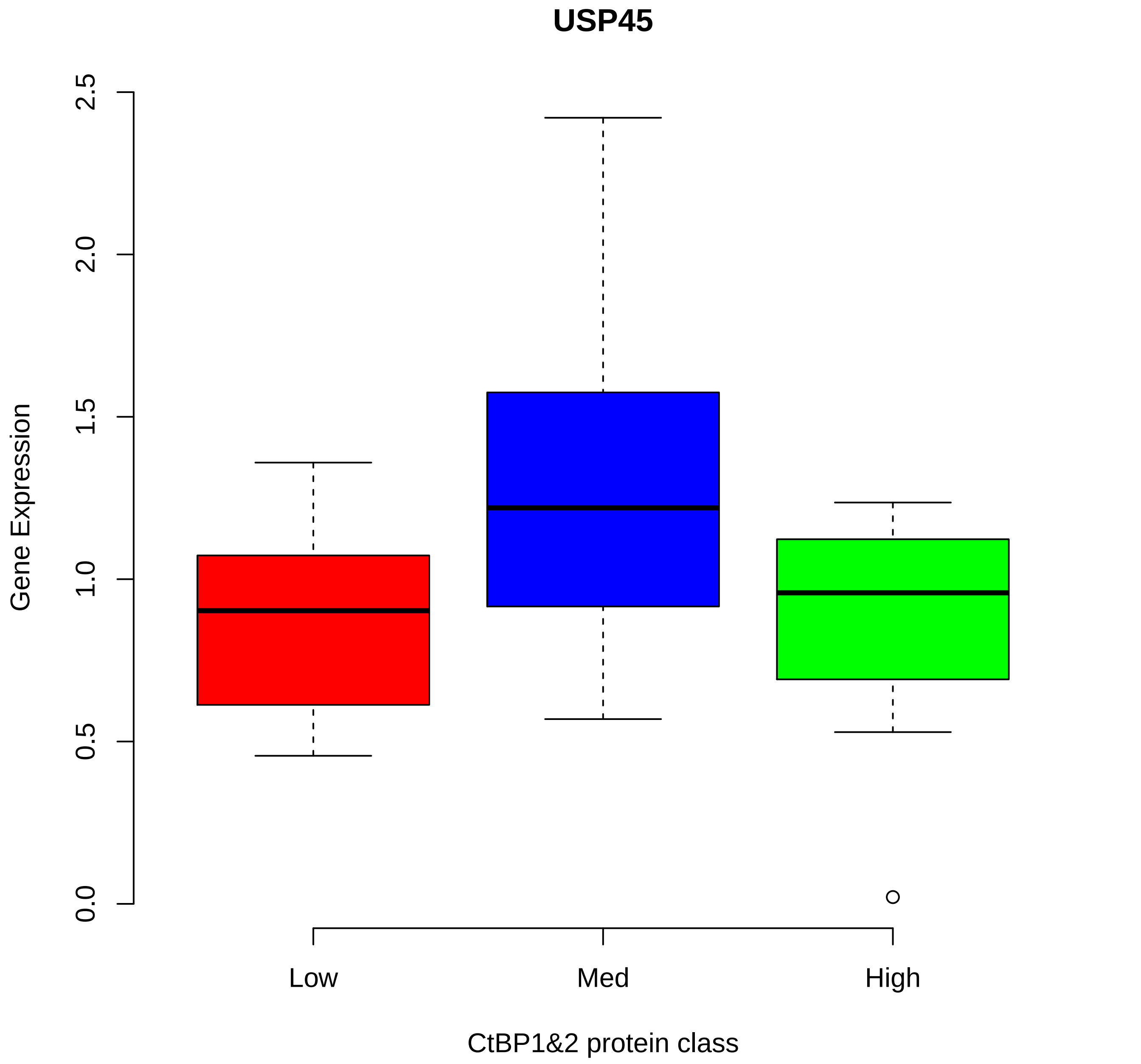

USP45
Gene Expression
0.0
0.5
1.0
1.5
2.0
2.5
Low
Med
High
CtBP1&2 protein class

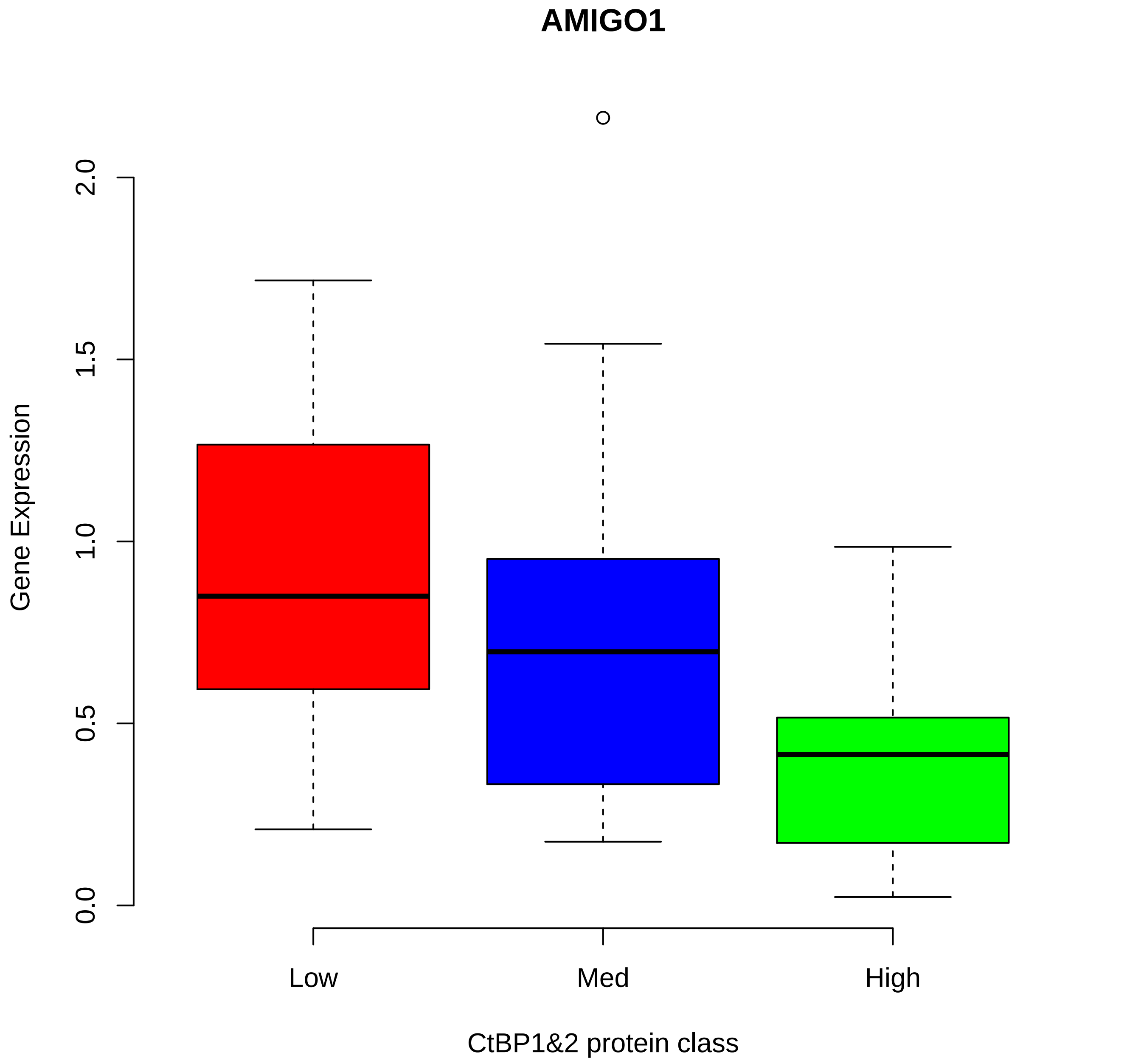
AMIGO1
Gene Expression
0.0
0.5
1.0
1.5
2.0
Low
Med
High
CtBP1&2 protein class

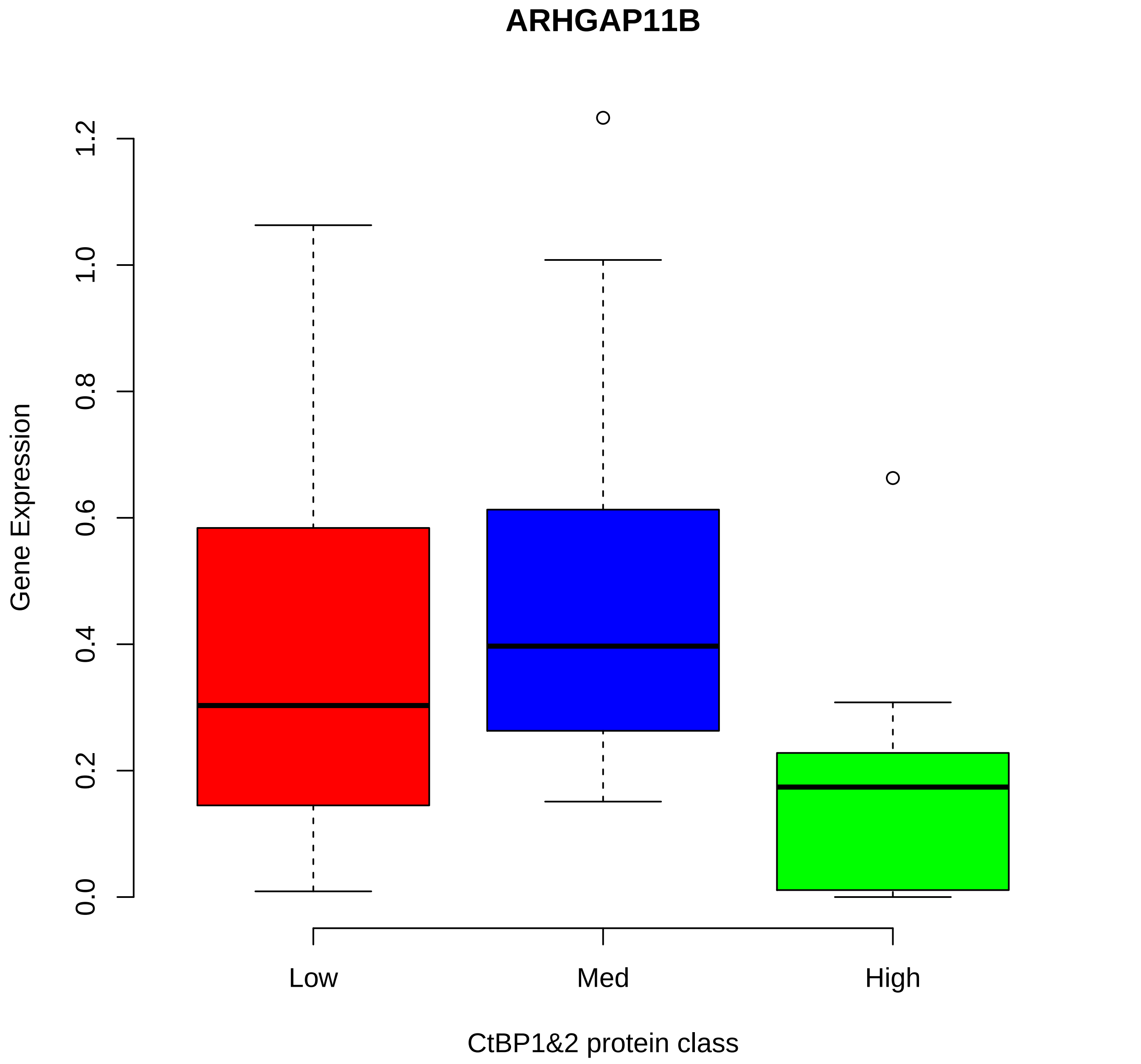

ARHGAP11B
Gene Expression
0.0
0.2
0.4
0.6
0.8
1.0
1.2
Low
Med
High
CtBP1&2 protein class

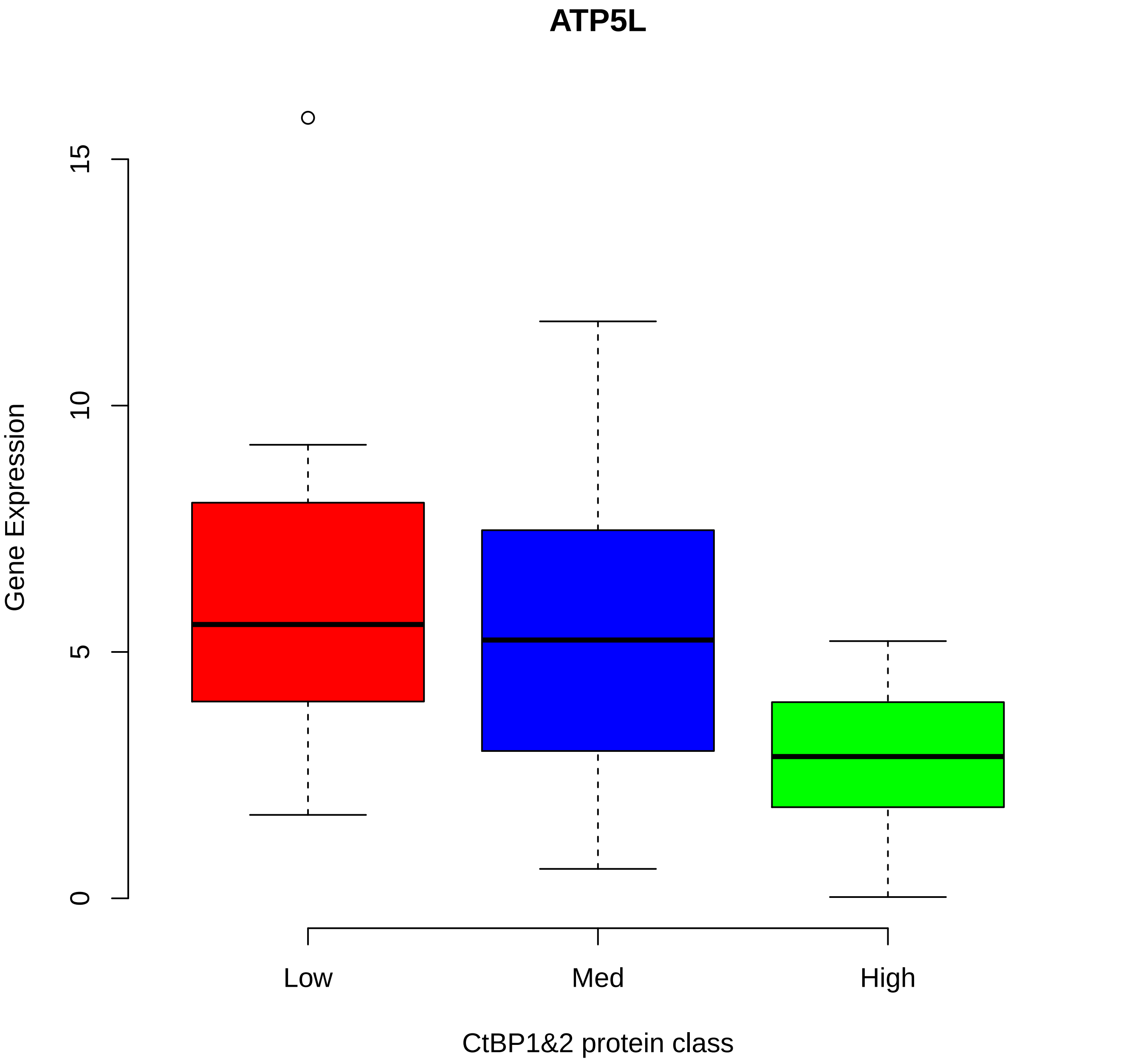
ATP5L
Gene Expression
0
5
10
15
Low
Med
High
CtBP1&2 protein class

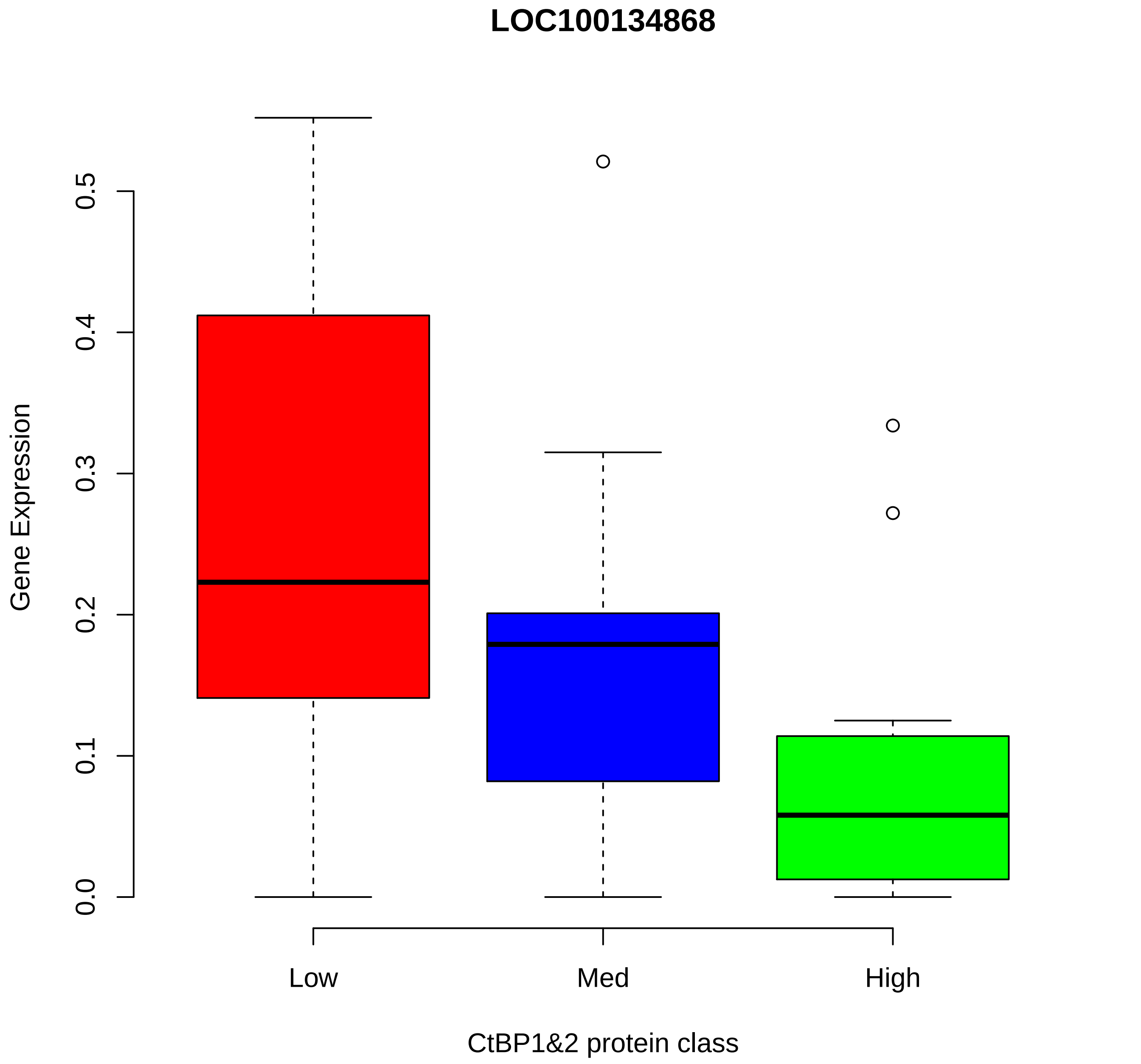
LOC100134868
Gene Expression
0.0
0.1
0.2
0.3
0.4
0.5
Low
Med
High
CtBP1&2 protein class

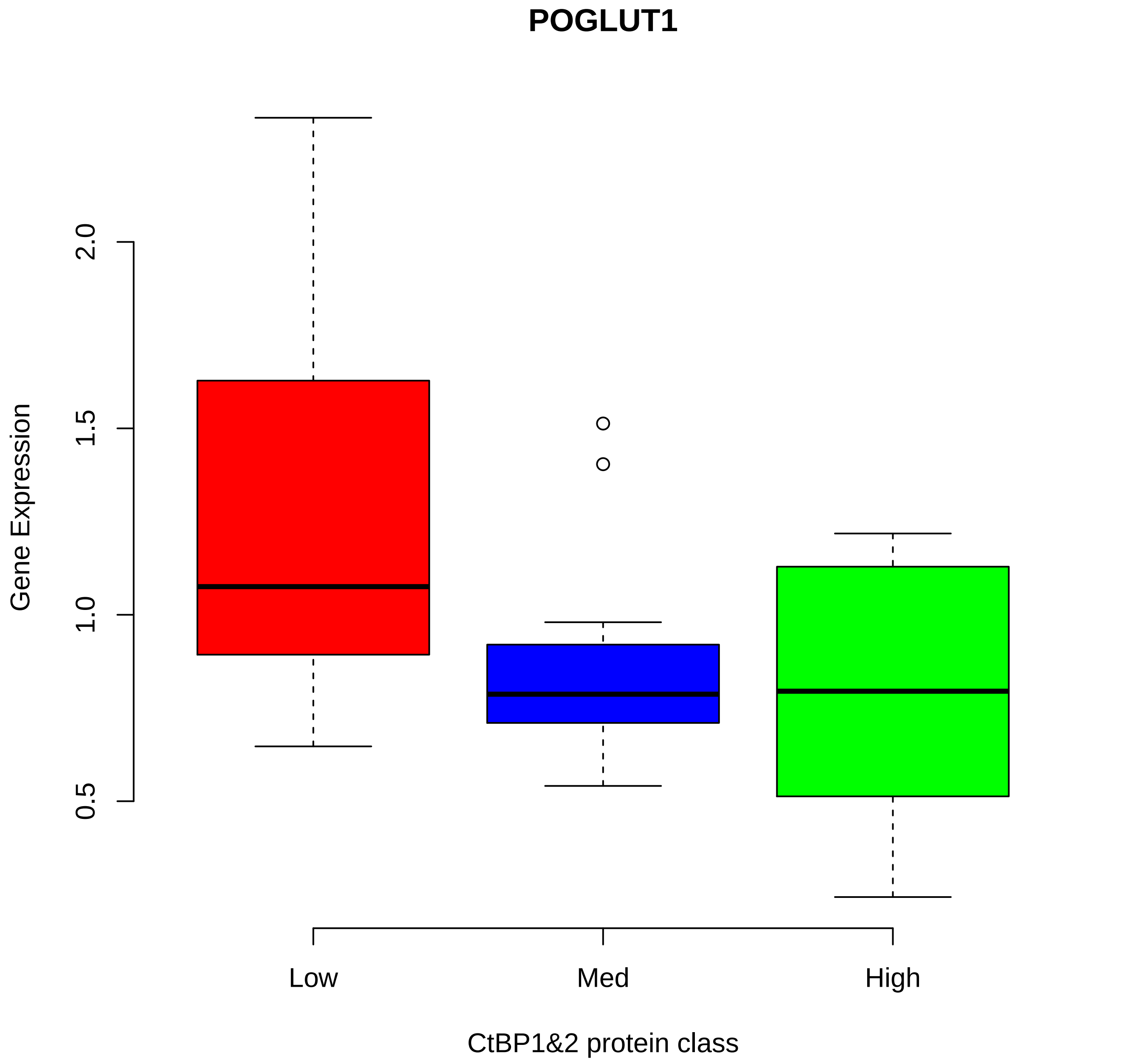

POGLUT1
Gene Expression
0.5
1.0
1.5
2.0
Low
Med
High
CtBP1&2 protein class

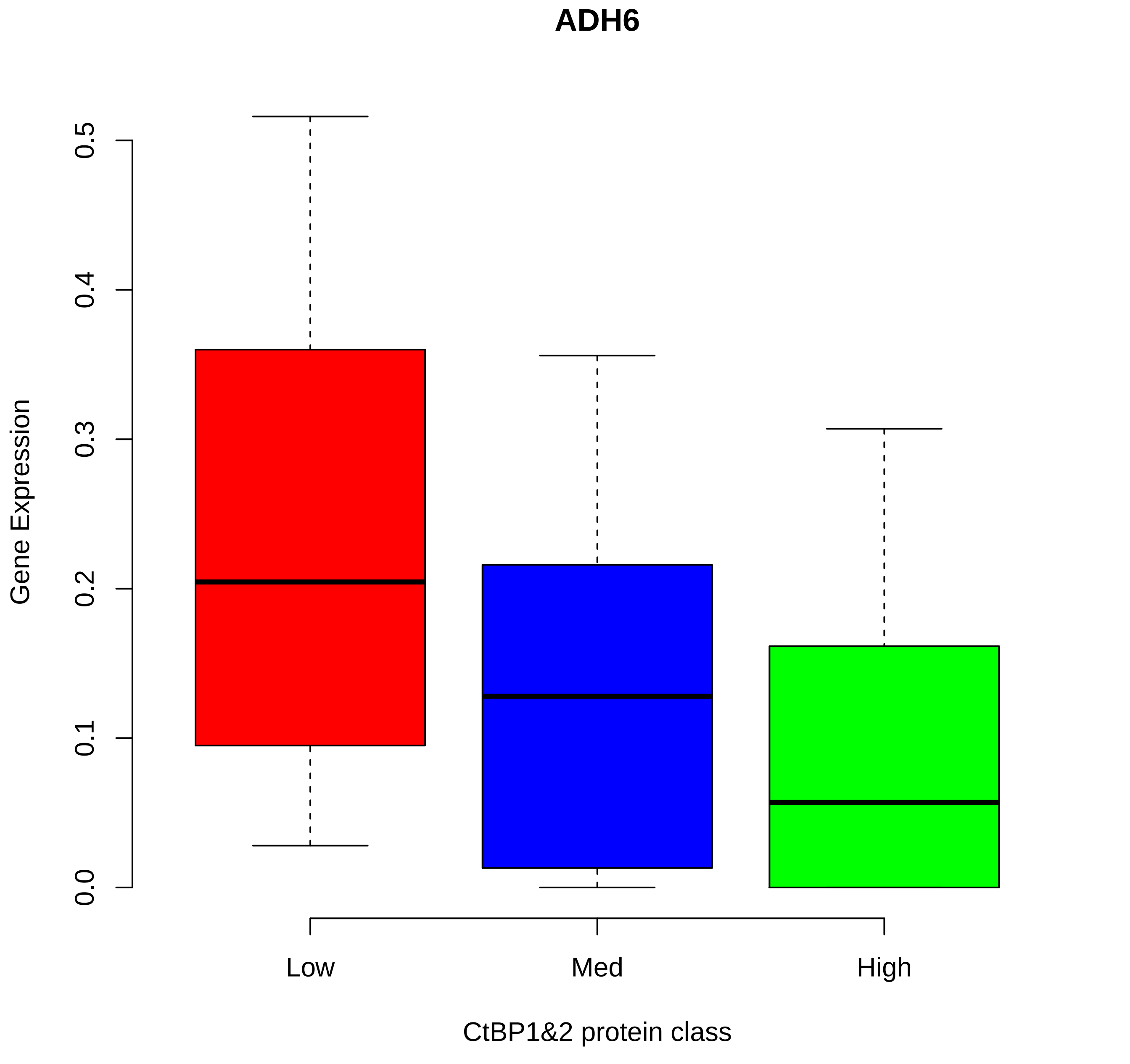
ADH6
Gene Expression
0.0
0.1
0.2
0.3
0.4
0.5
Low
Med
High
CtBP1&2 protein class

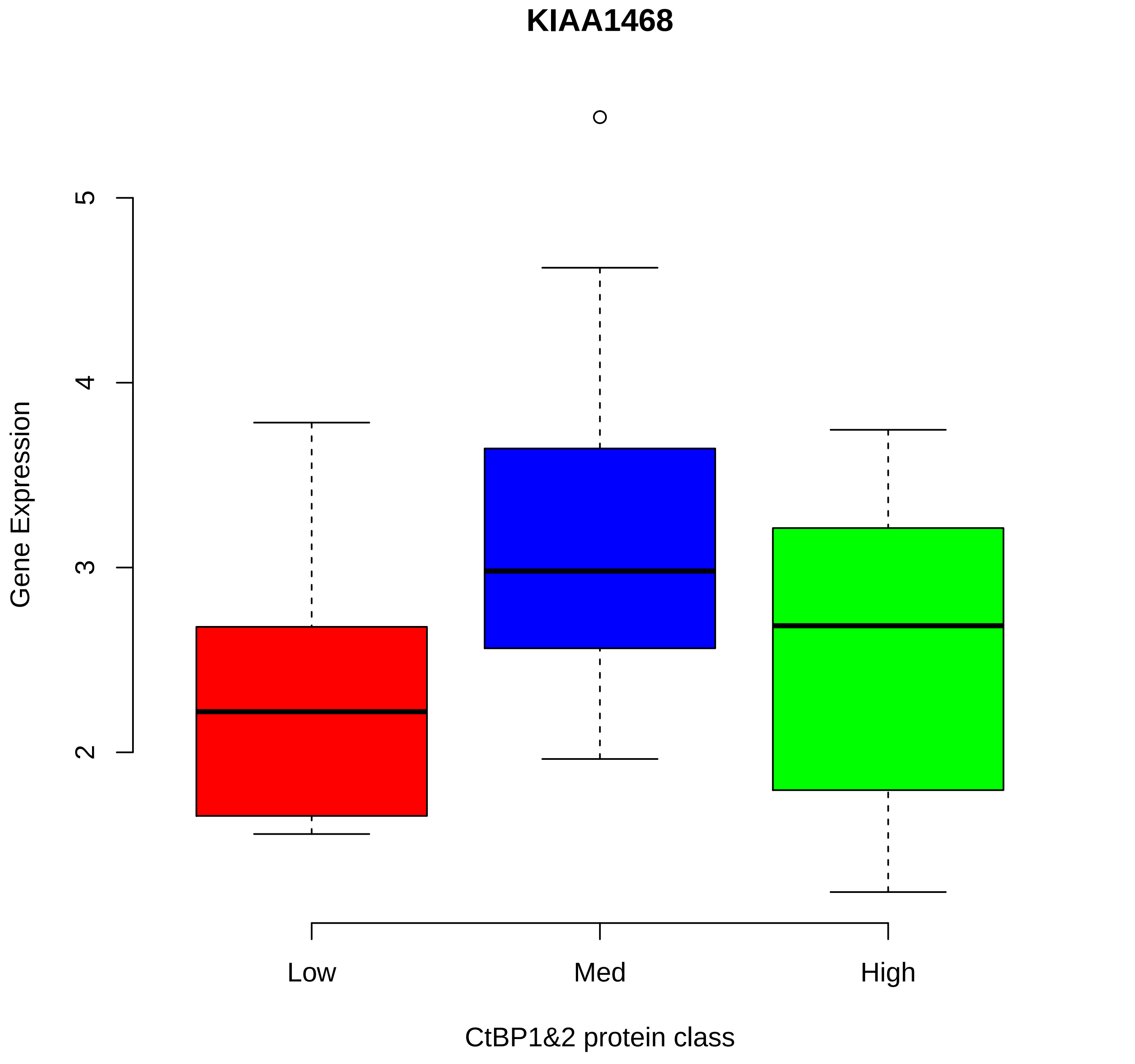
KIAA1468
Gene Expression
2
3
4
5
Low
Med
High
CtBP1&2 protein class

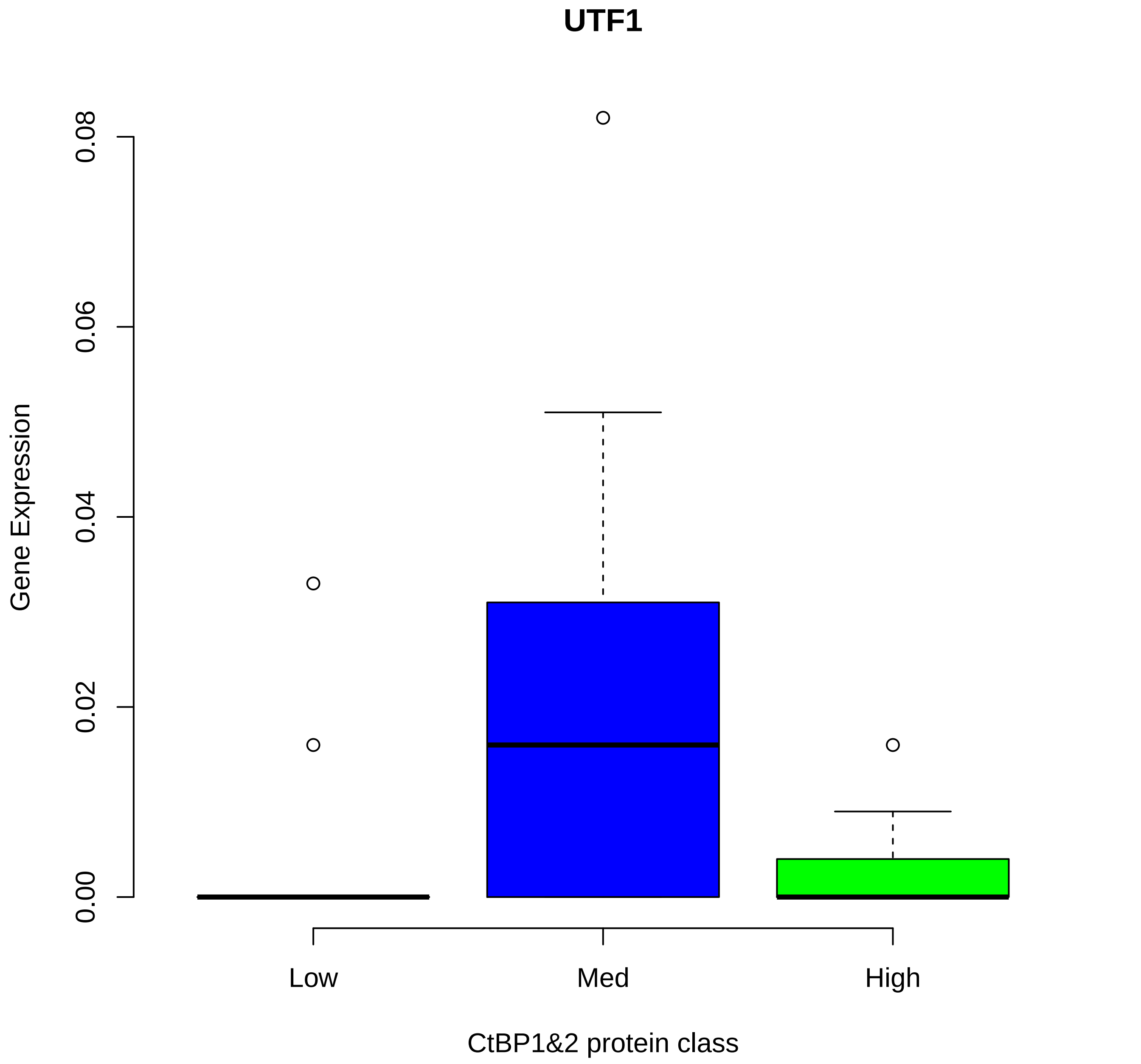
UTF1
Gene Expression
0.00
0.02
0.04
0.06
0.08
Low
Med
High
CtBP1&2 protein class

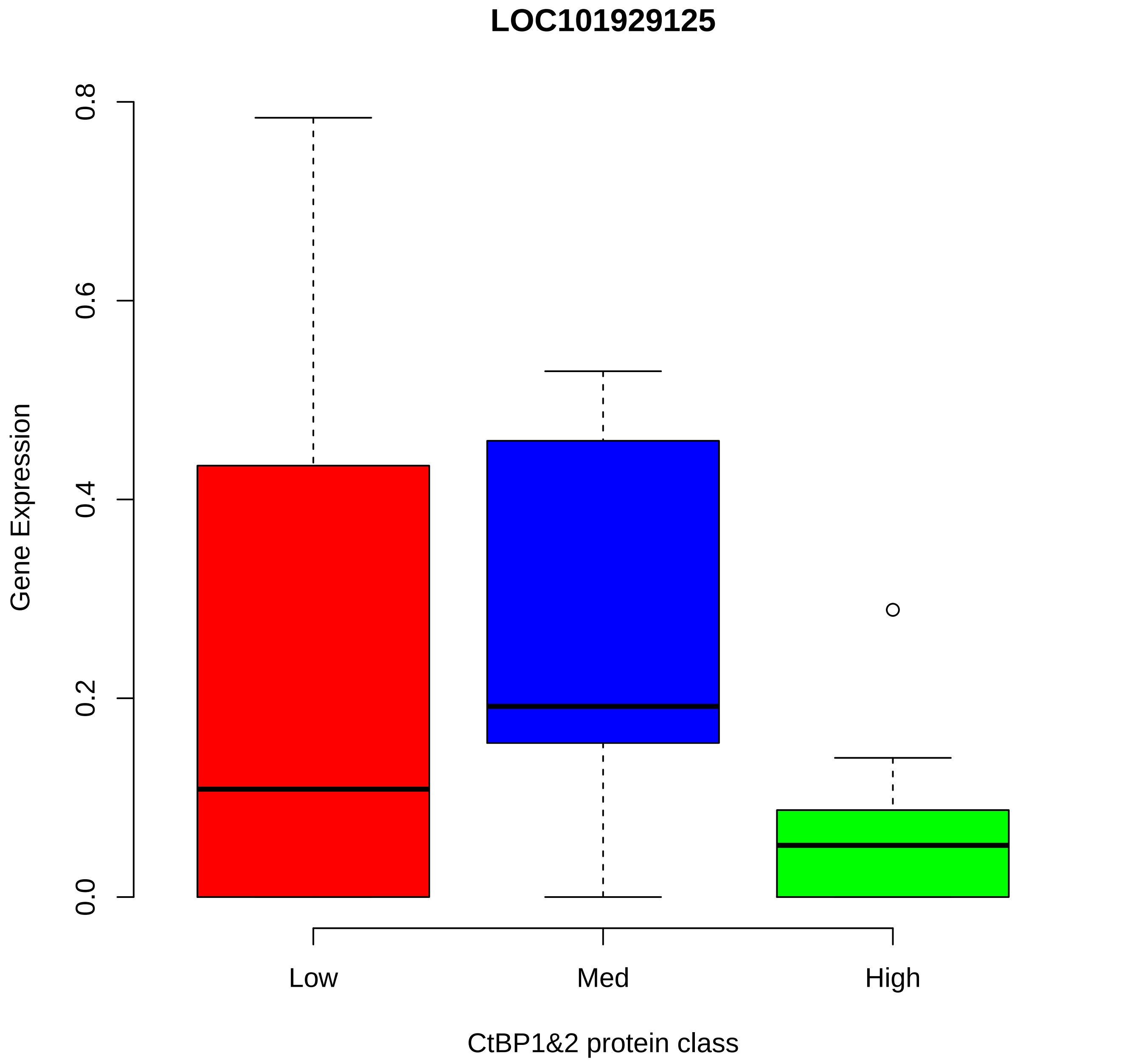
LOC101929125
Gene Expression
0.0
0.2
0.4
0.6
0.8
Low
Med
High
CtBP1&2 protein class

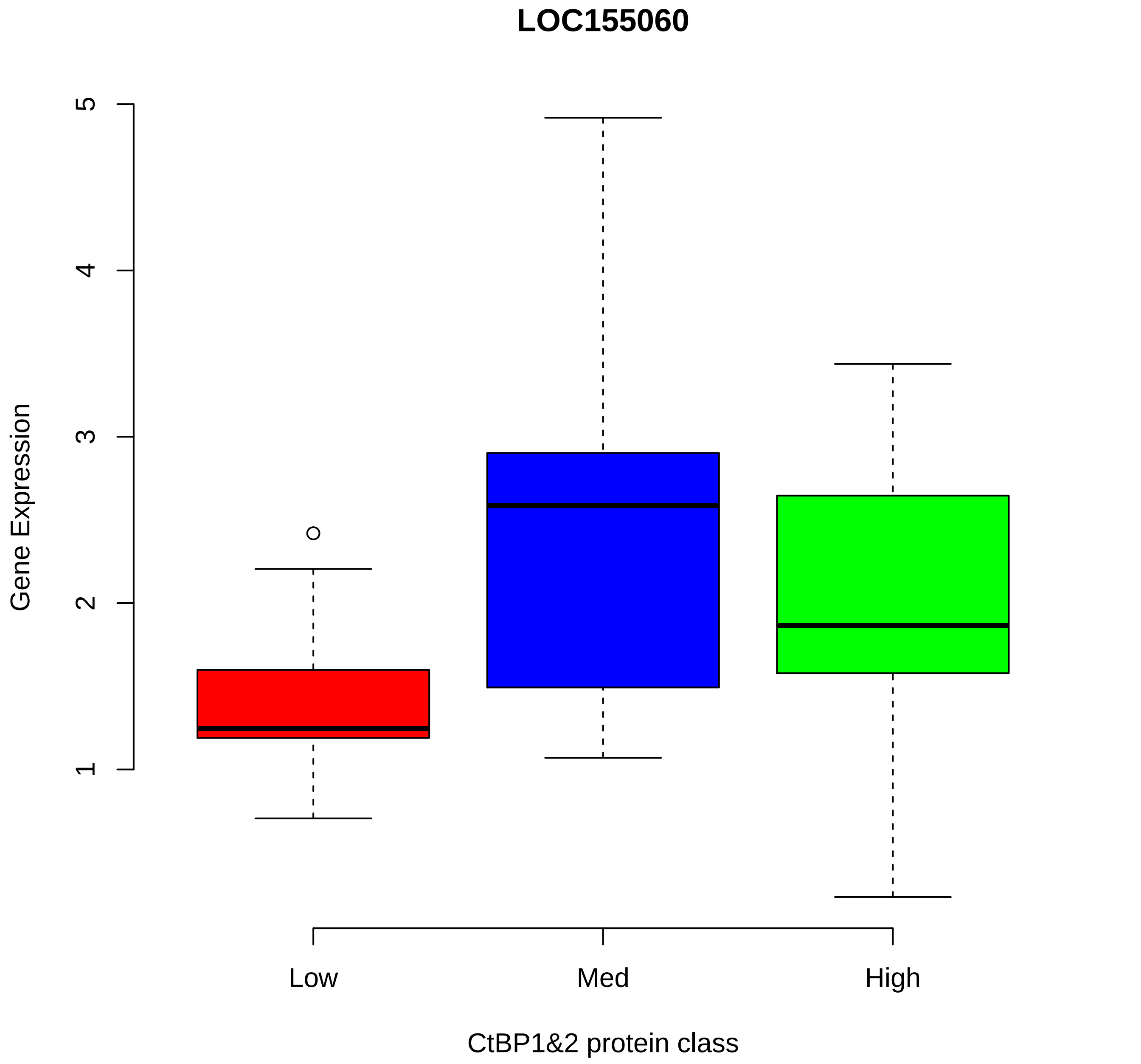
LOC155060
Gene Expression
1
2
3
4
5
Low
Med
High
CtBP1&2 protein class

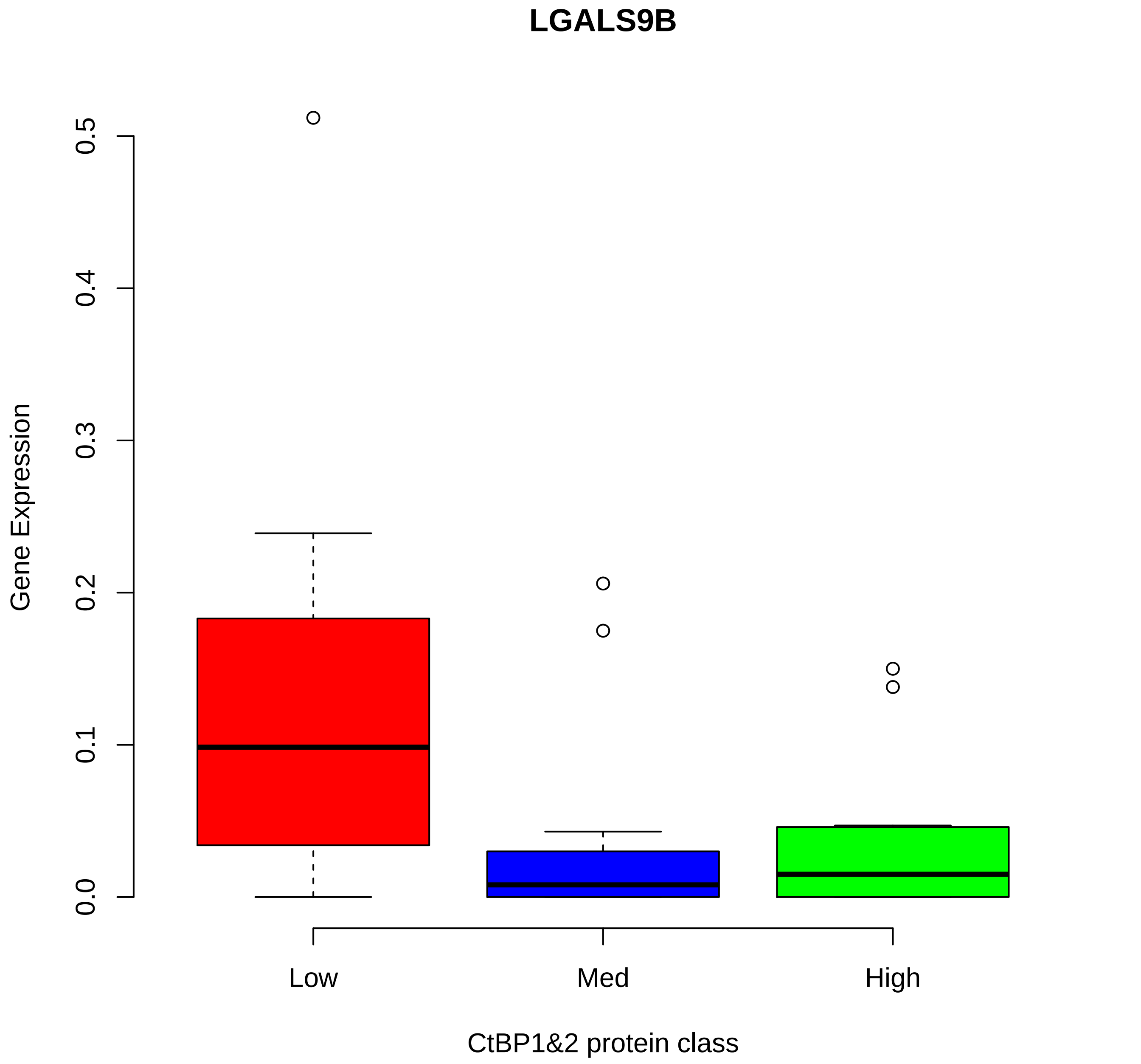

LGALS9B
Gene Expression
0.0
0.1
0.2
0.3
0.4
0.5
Low
Med
High
CtBP1&2 protein class

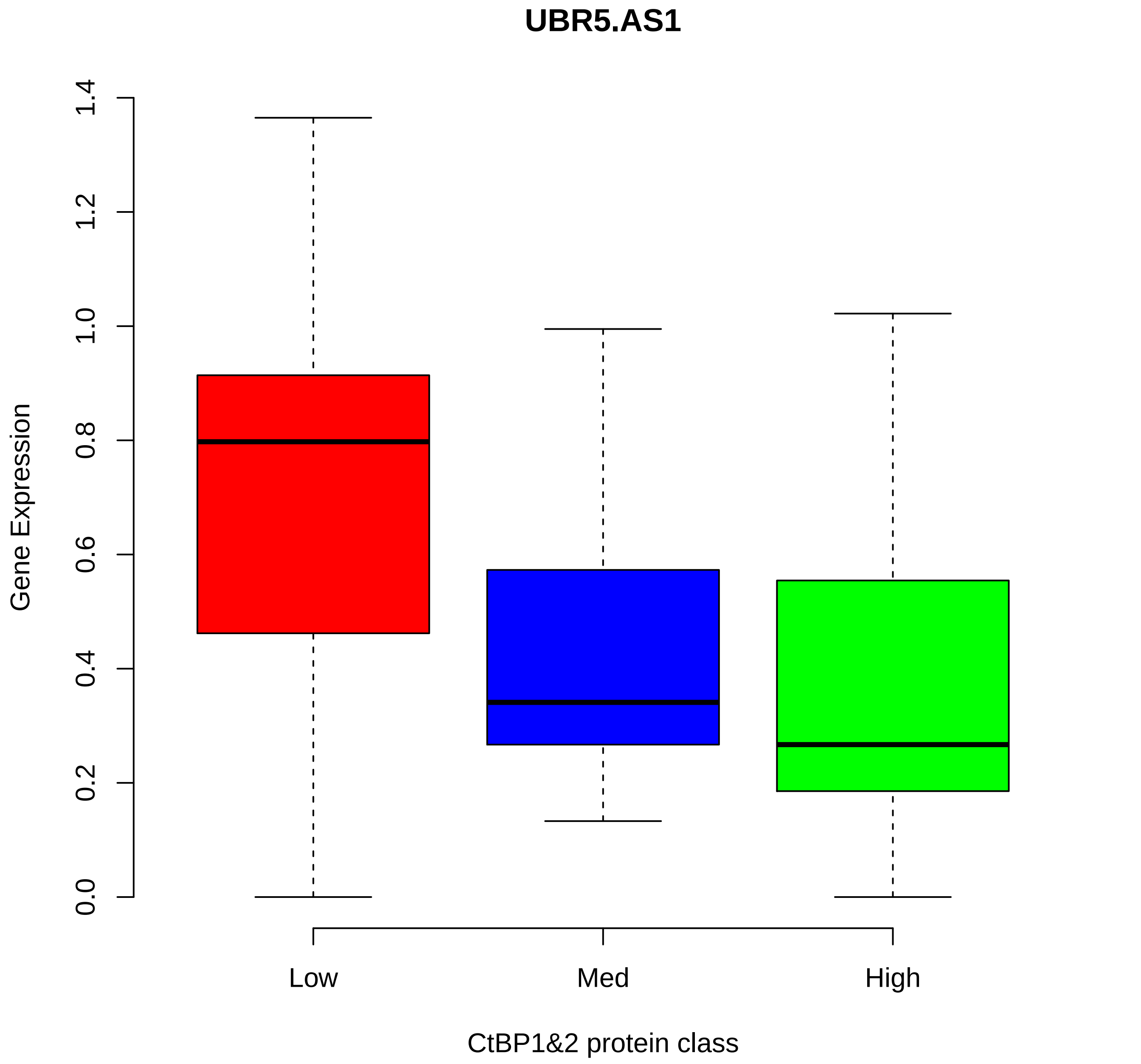
UBR5.AS1
Gene Expression
0.0
0.2
0.4
0.6
0.8
1.0
1.2
1.4
Low
Med
High
CtBP1&2 protein class

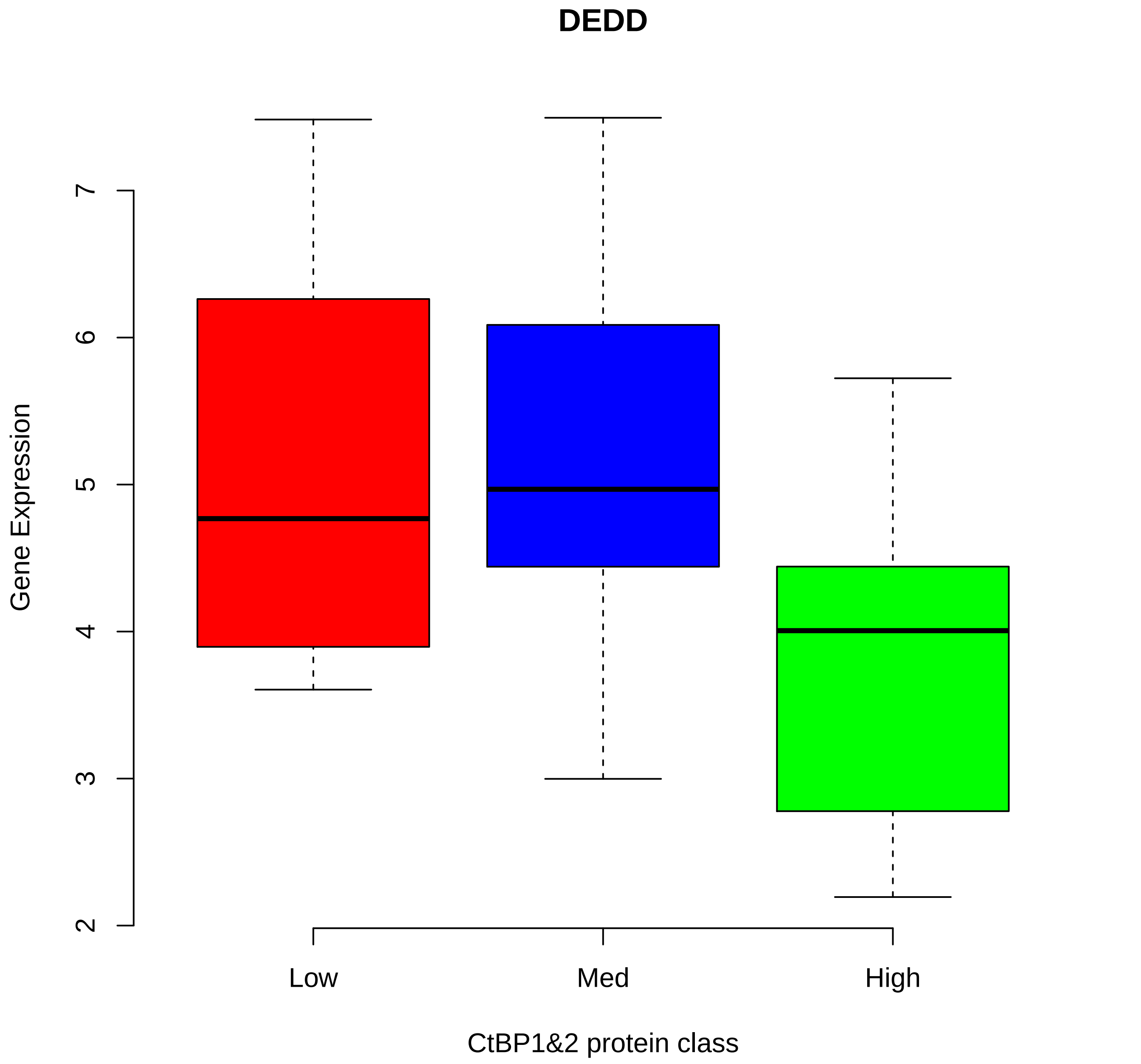

DEDD
Gene Expression
2
3
4
5
6
7
Low
Med
High
CtBP1&2 protein class

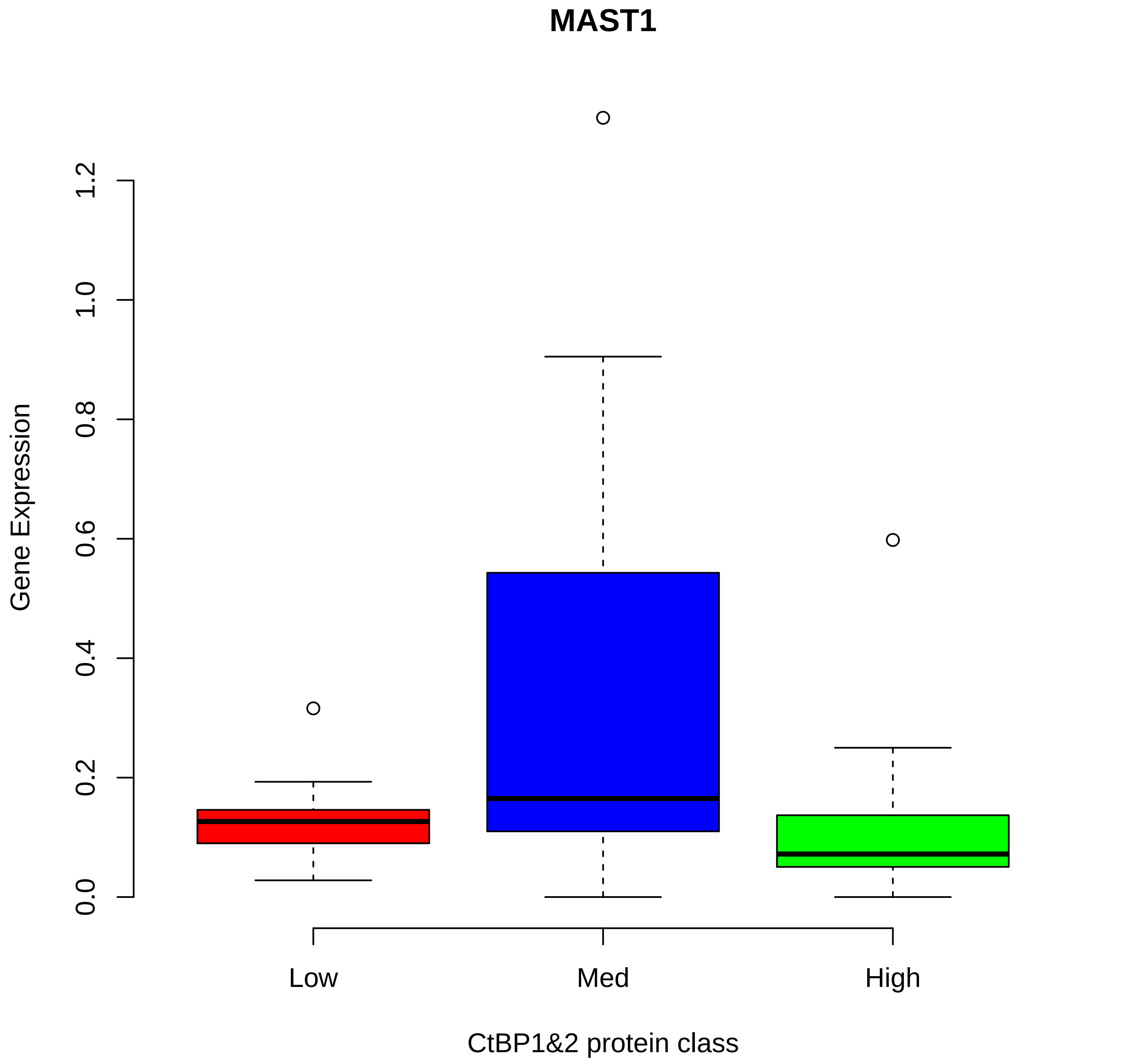

MAST1
Gene Expression
0.0
0.2
0.4
0.6
0.8
1.0
1.2
Low
Med
High
CtBP1&2 protein class

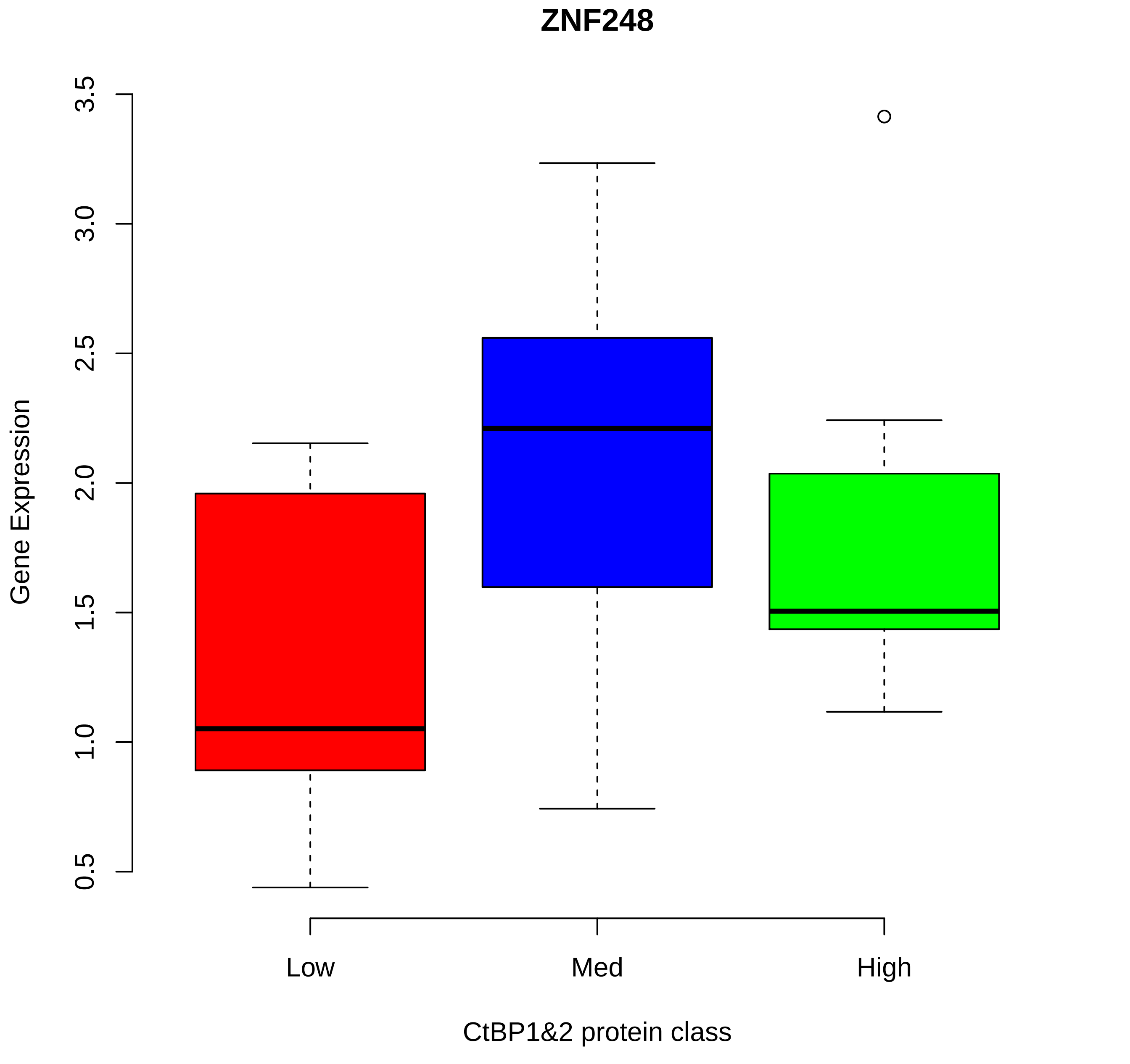
ZNF248
Gene Expression
0.5
1.0
1.5
2.0
2.5
3.0
3.5
Low
Med
High
CtBP1&2 protein class

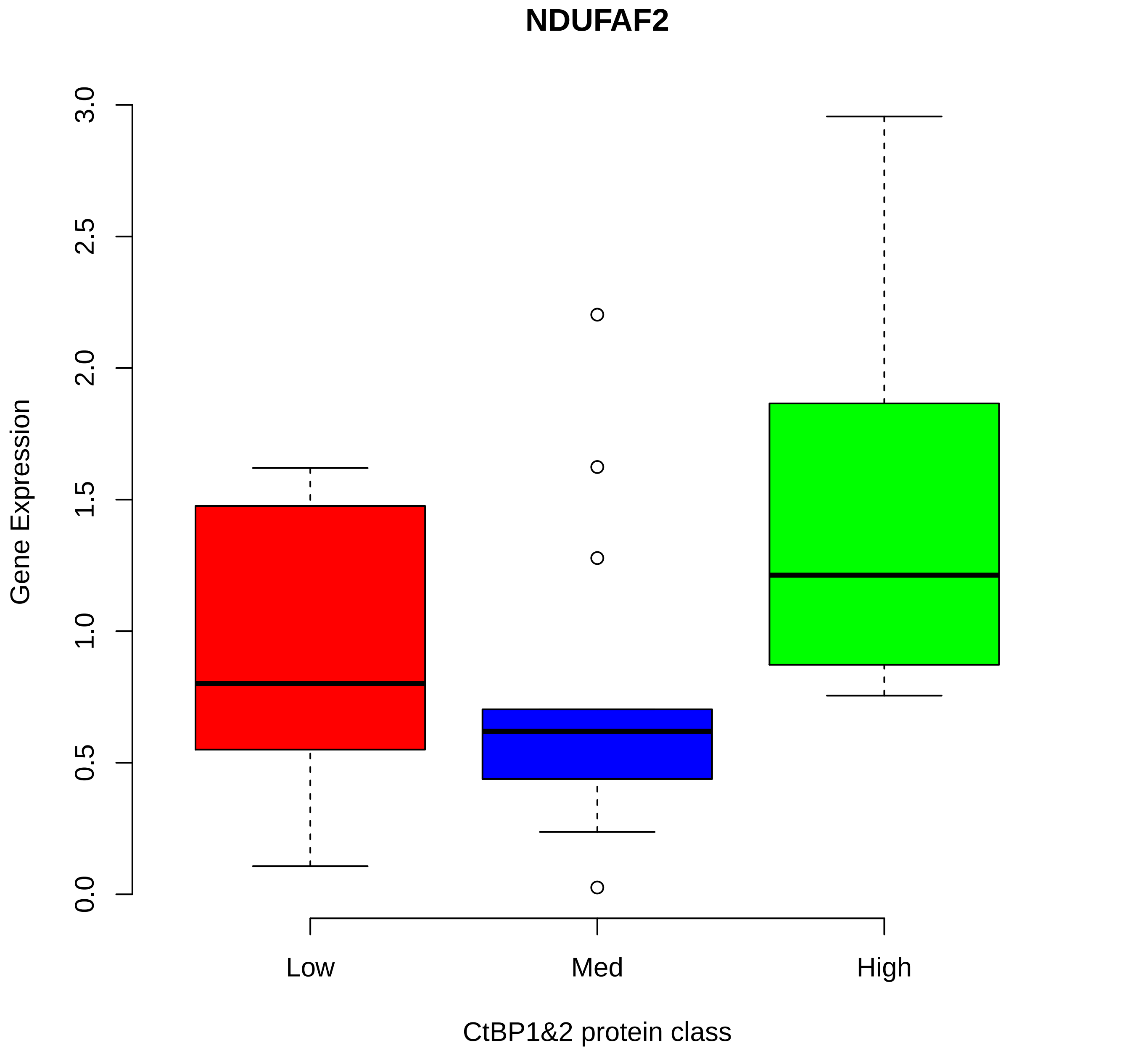

NDUFAF2
Gene Expression
3.0
2.5
2.0
1.5
1.0
0.5
0.0
Low
Med
High
CtBP1&2 protein class

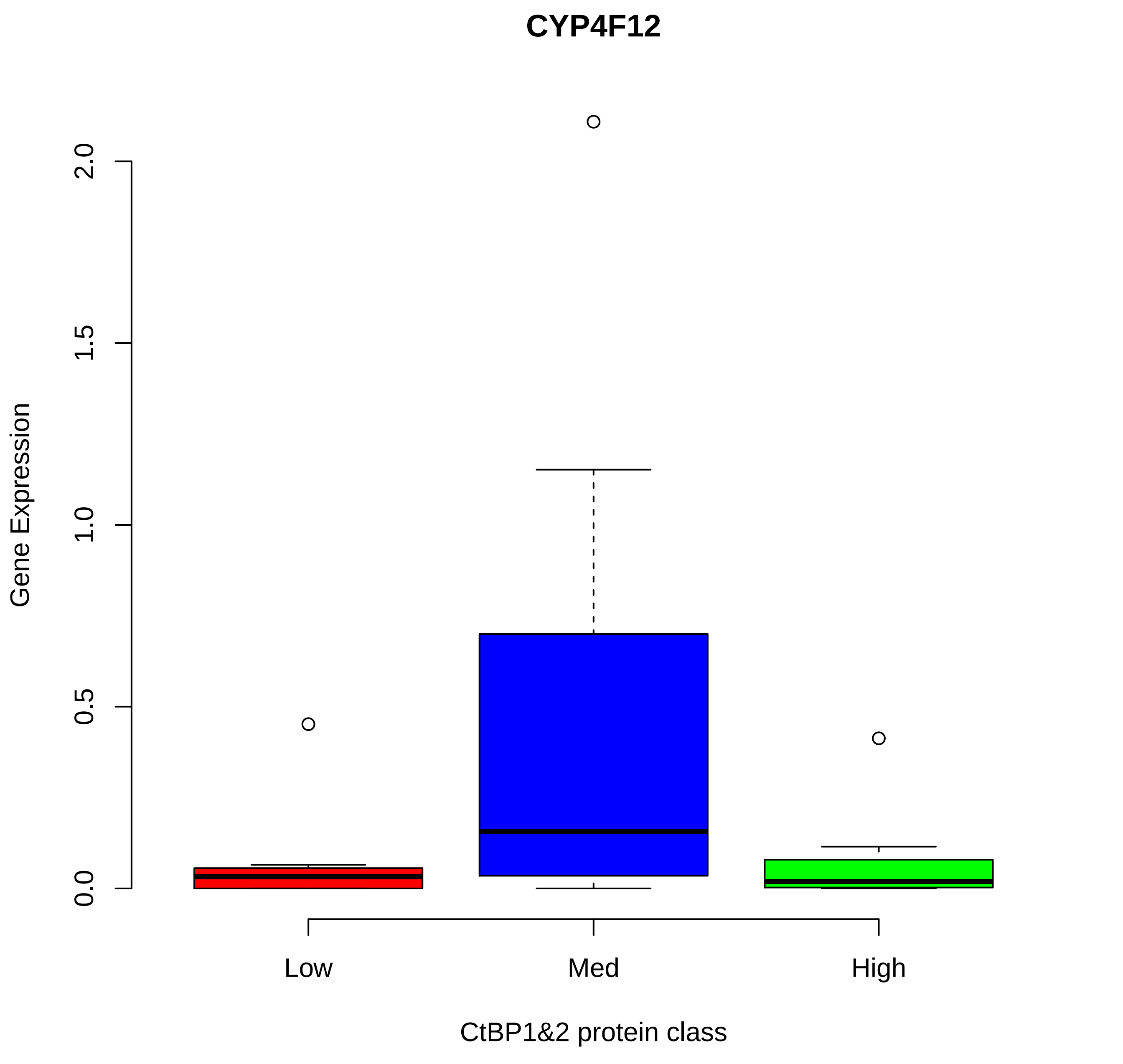
CYP4F12
Gene Expression
0.0
0.5
1.0
1.5
2.0
Low
Med
High
CtBP1&2 protein class

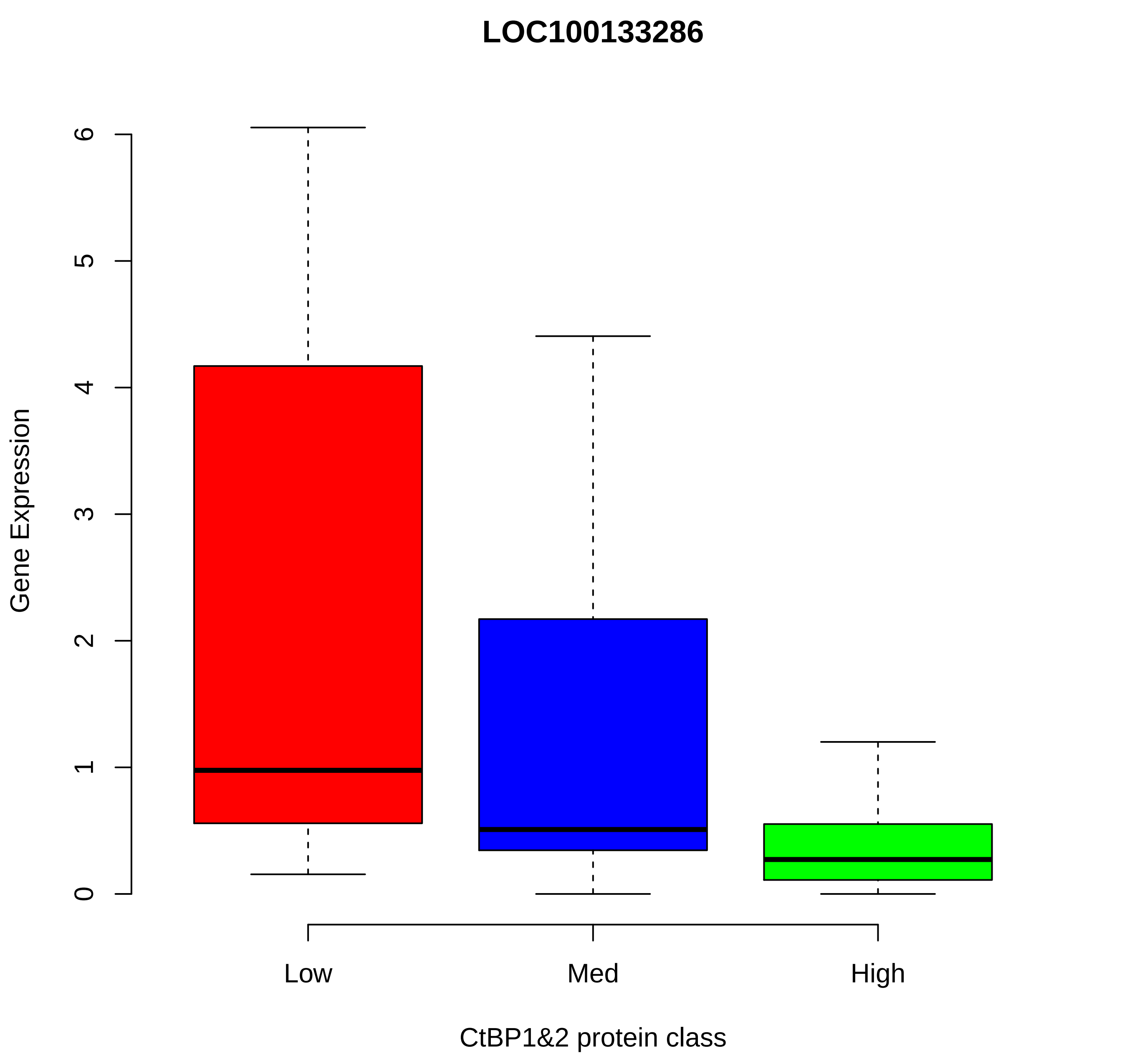
LOC100133286
Gene Expression
0
1
2
3
4
5
6
Low
Med
High
CtBP1&2 protein class

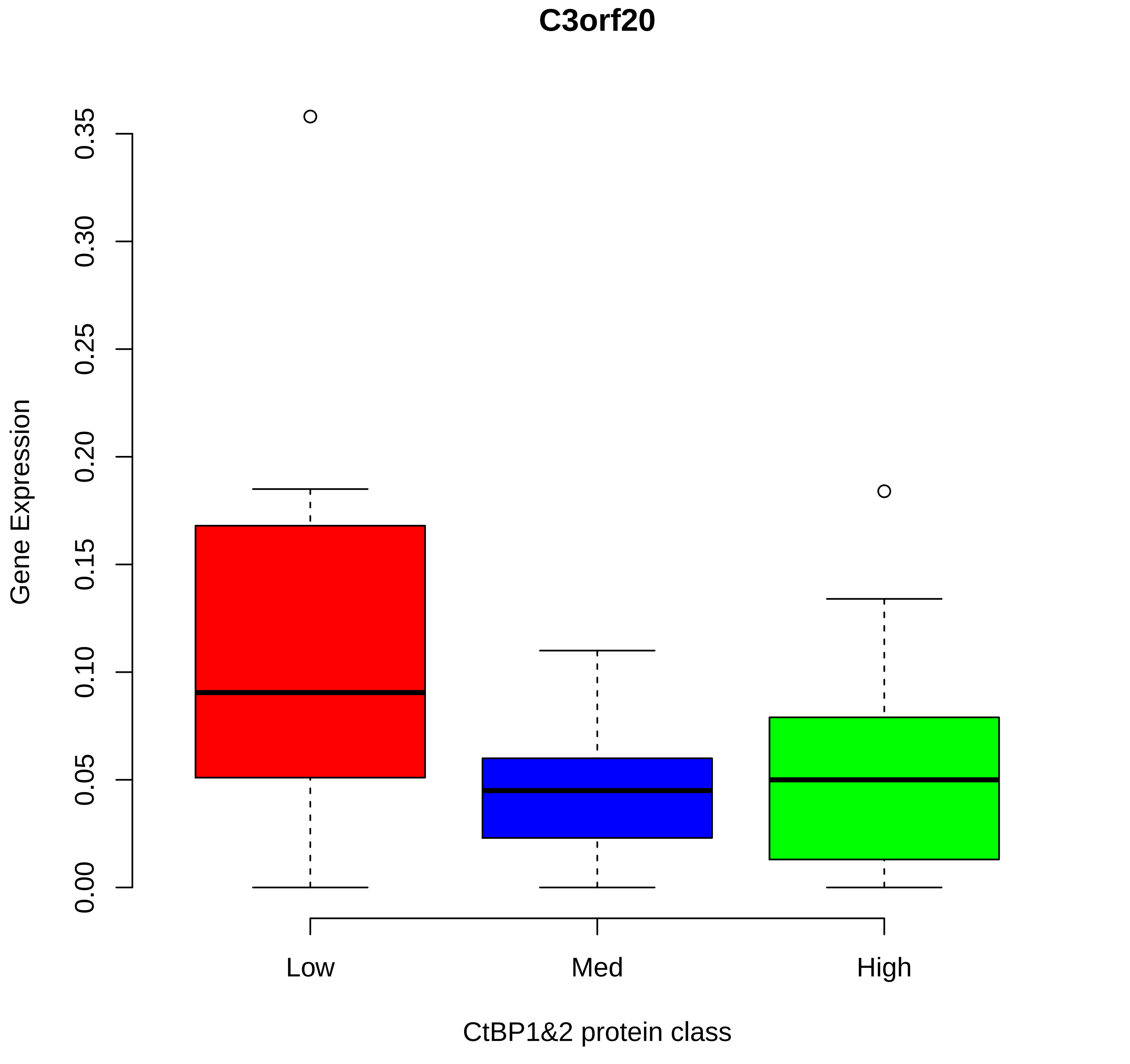
C3orf20
Gene Expression
0.00
0.05
0.10
0.15
0.20
0.25
0.30
0.35
Low
Med
High
CtBP1&2 protein class

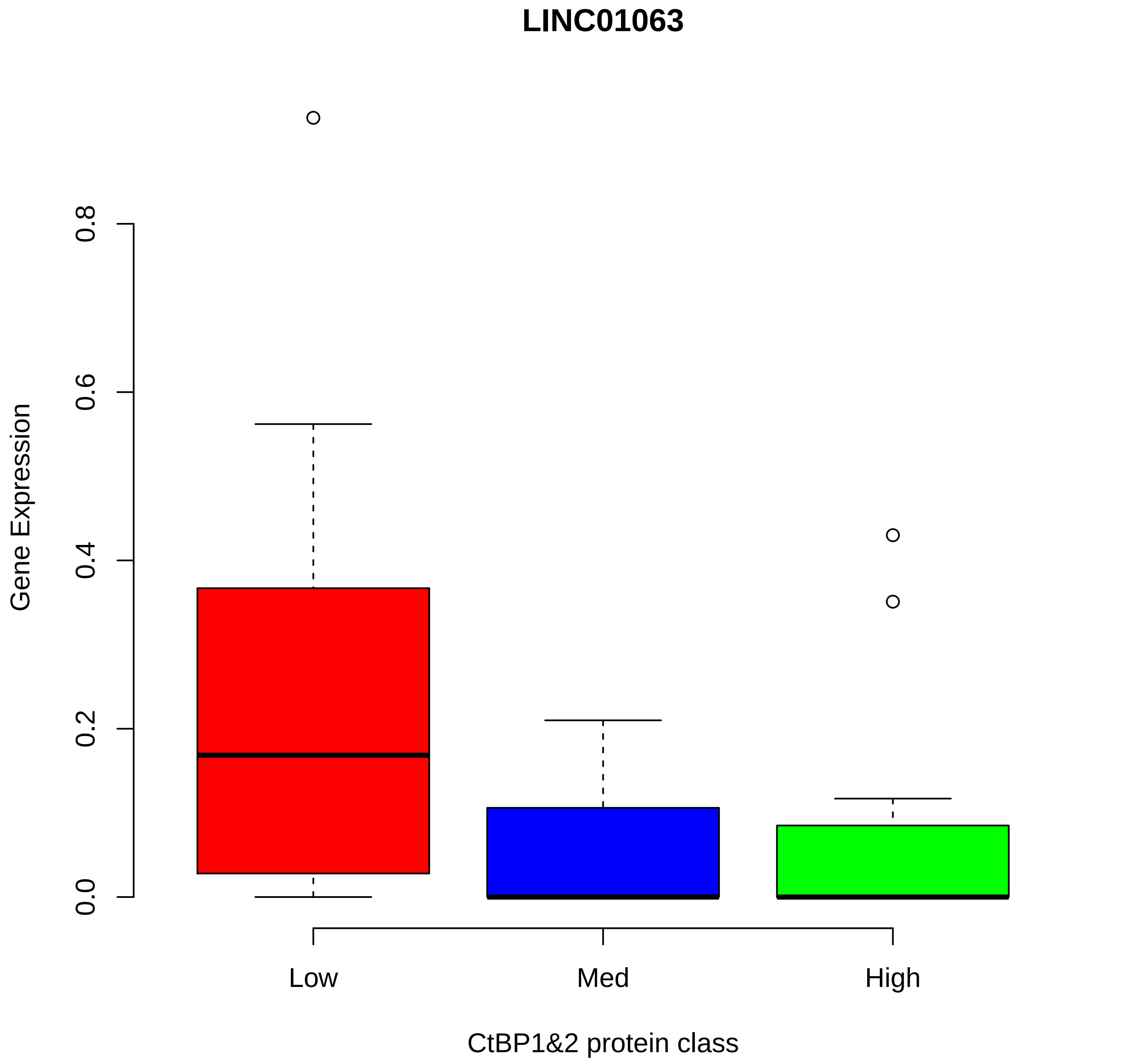
LINC01063
Gene Expression
0.0
0.2
0.4
0.6
0.8
Low
Med
High
CtBP1&2 protein class

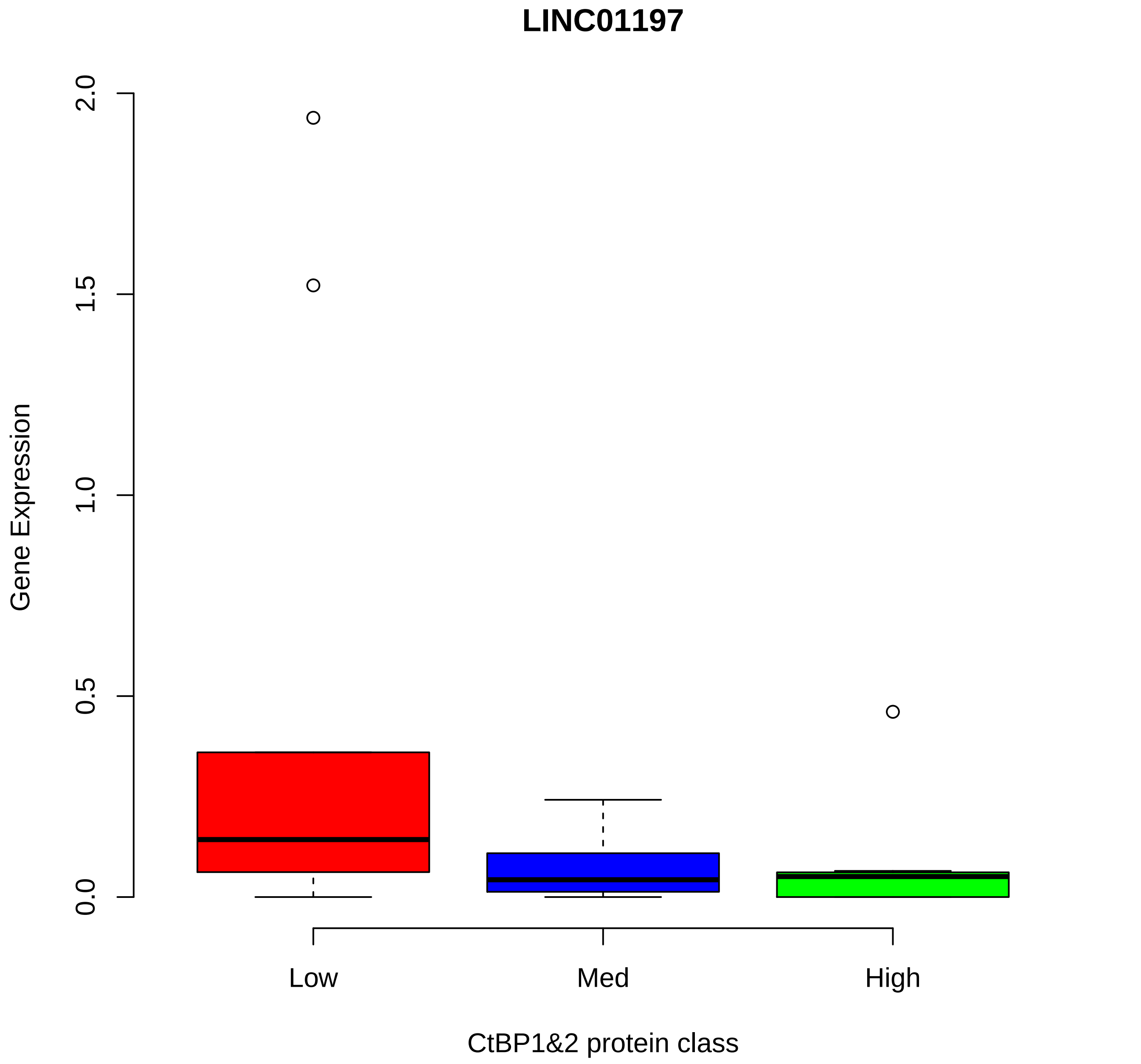

LINC01197
Gene Expression
0.0
0.5
1.0
1.5
2.0
Low
Med
High
CtBP1&2 protein class

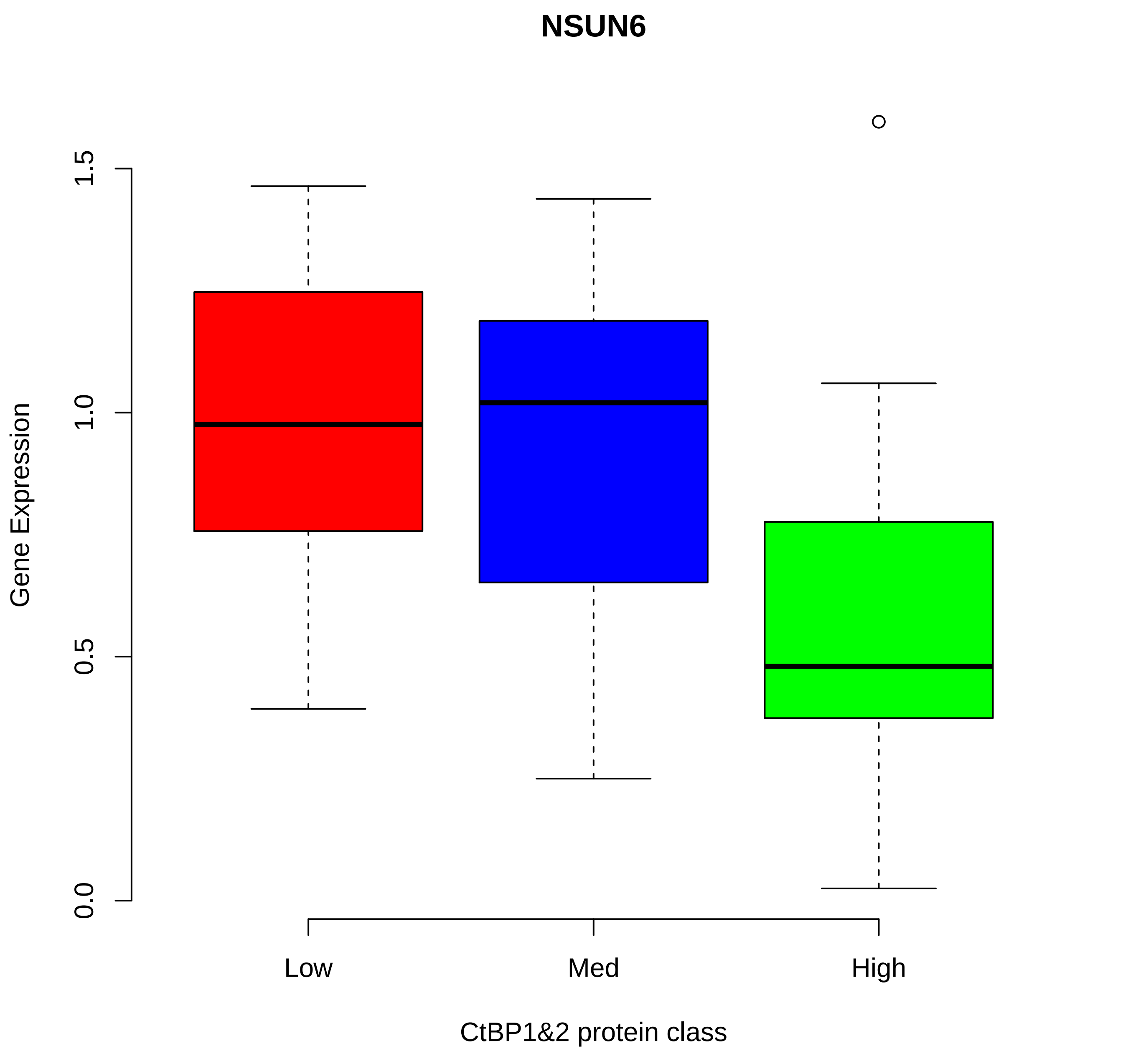
NSUN6
Gene Expression
0.0
0.5
1.0
1.5
Low
Med
High
CtBP1&2 protein class

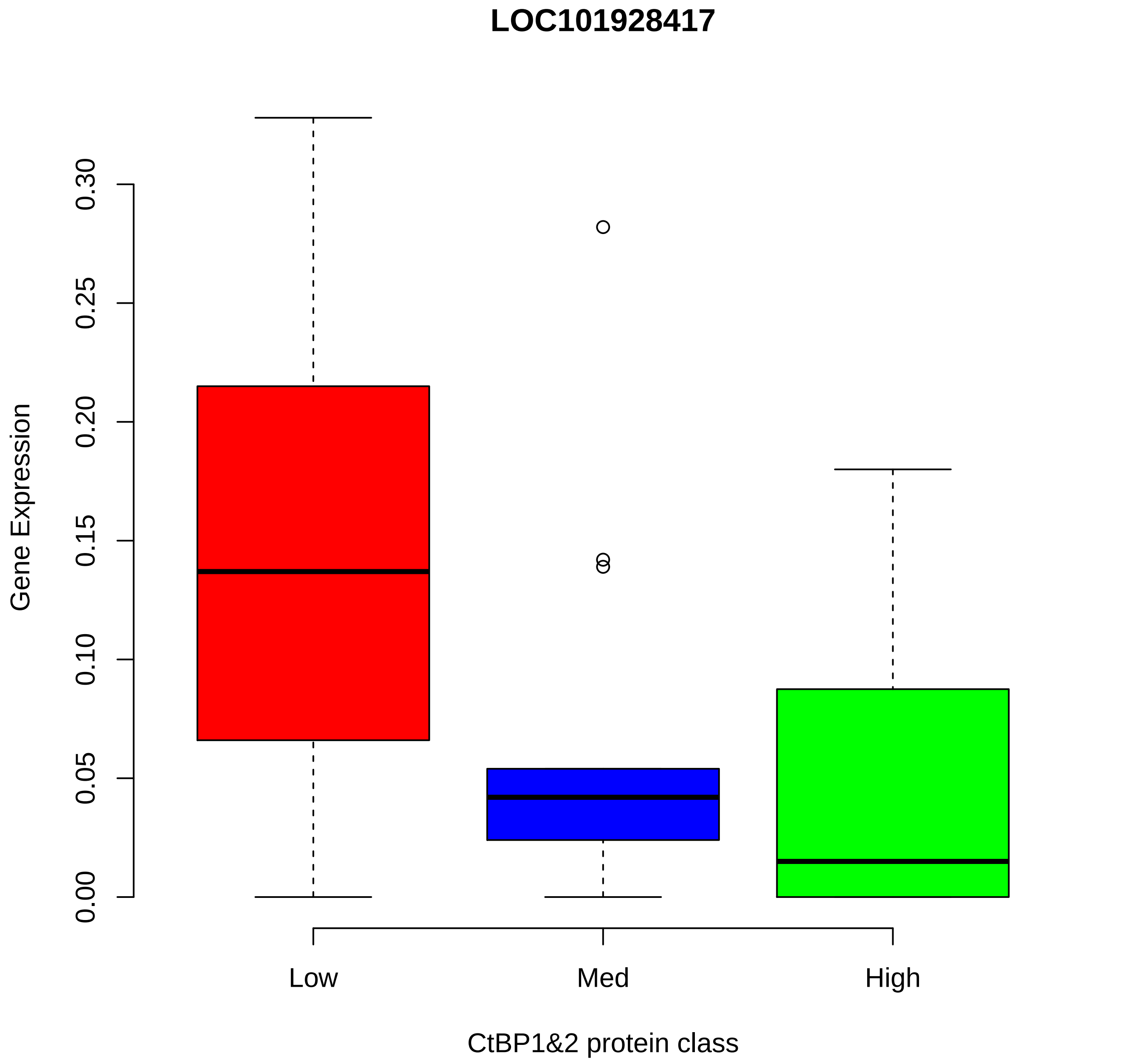

LOC101928417
Gene Expression
0.00
0.05
0.10
0.15
0.20
0.25
0.30
Low
Med
High
CtBP1&2 protein class

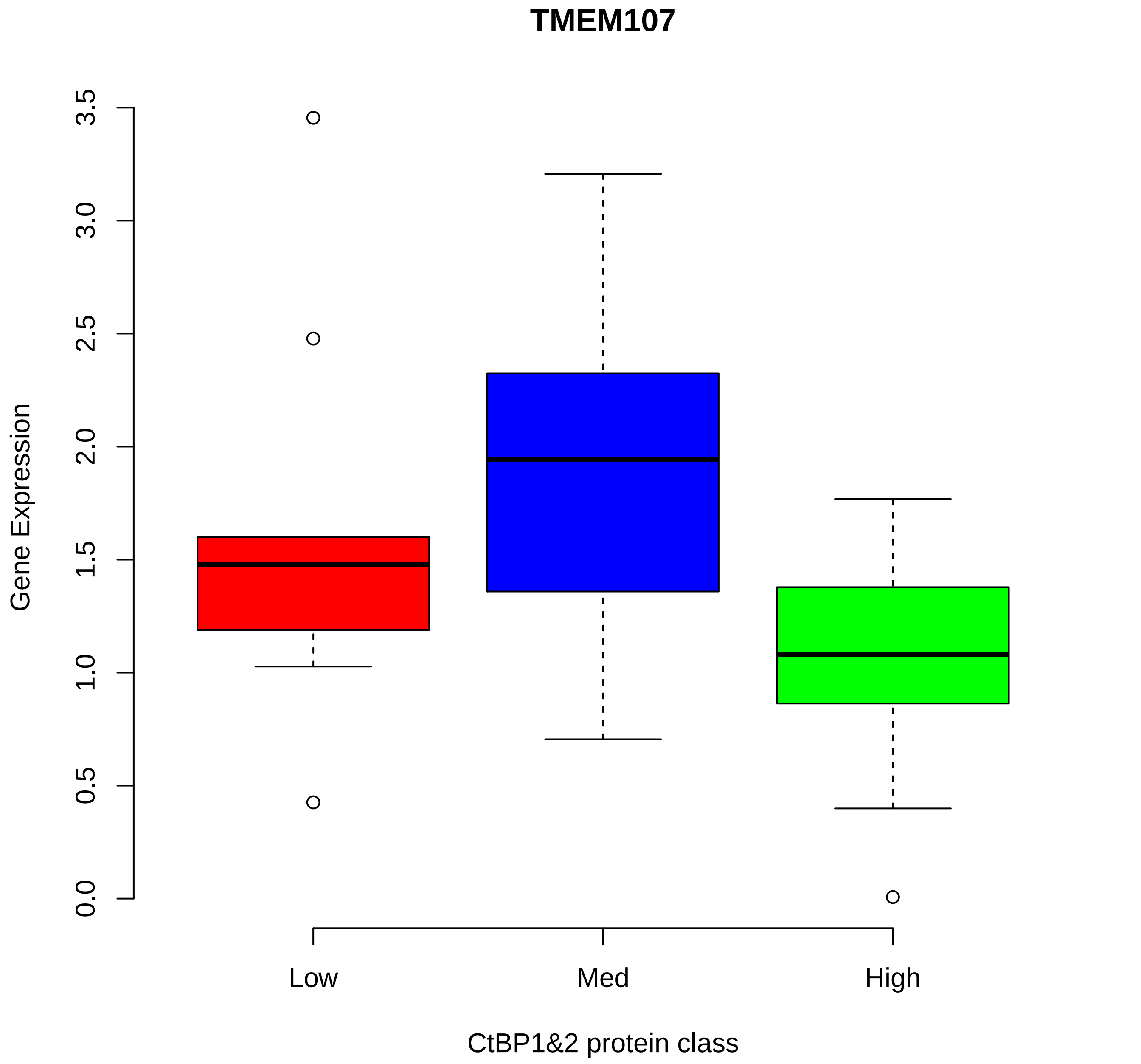
TMEM107
Gene Expression
0.0
0.5
1.0
1.5
2.0
2.5
3.0
3.5
Low
Med
High
CtBP1&2 protein class

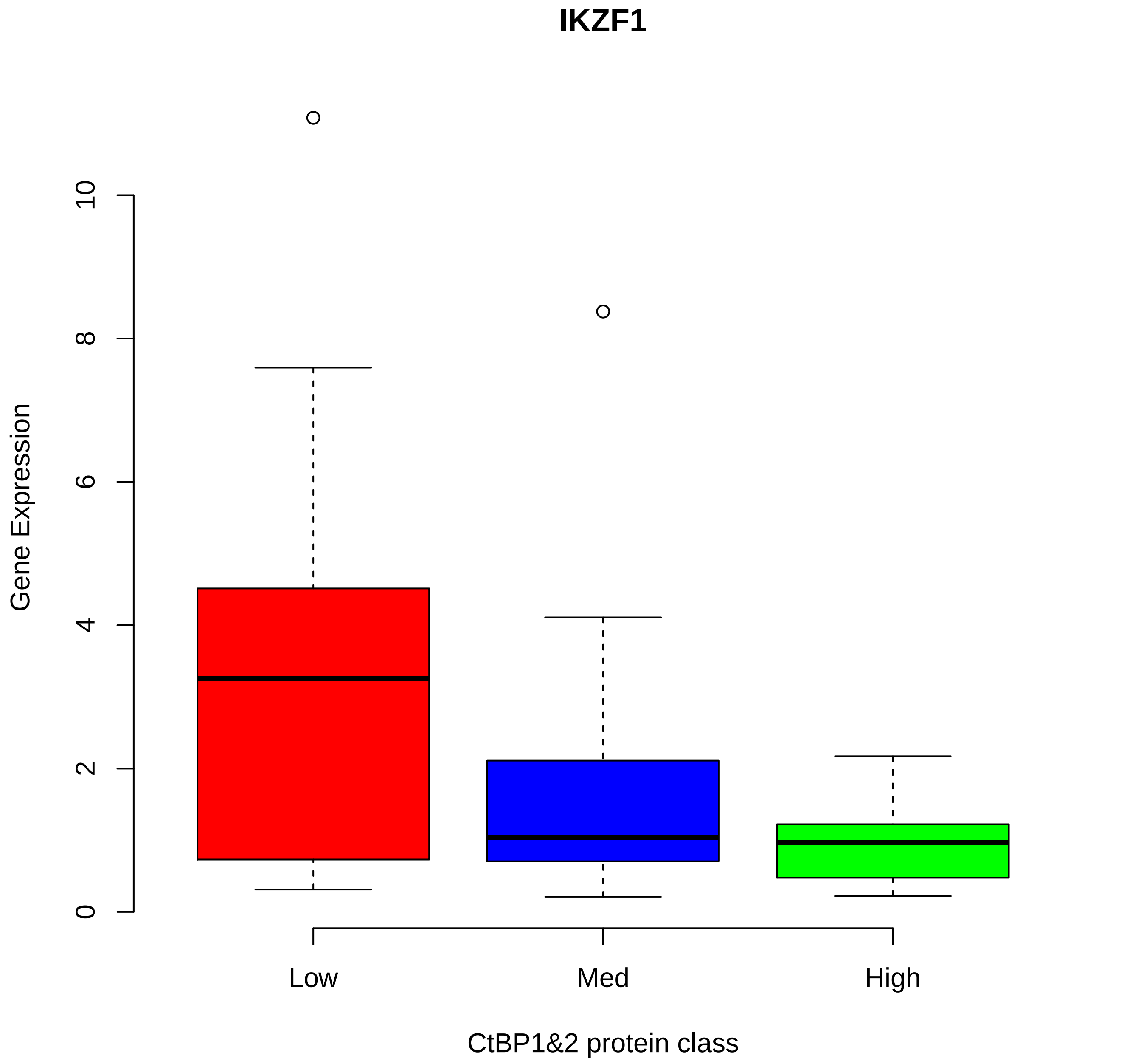
IKZF1
Gene Expression
0
2
4
6
8
10
Low
Med
High
CtBP1&2 protein class

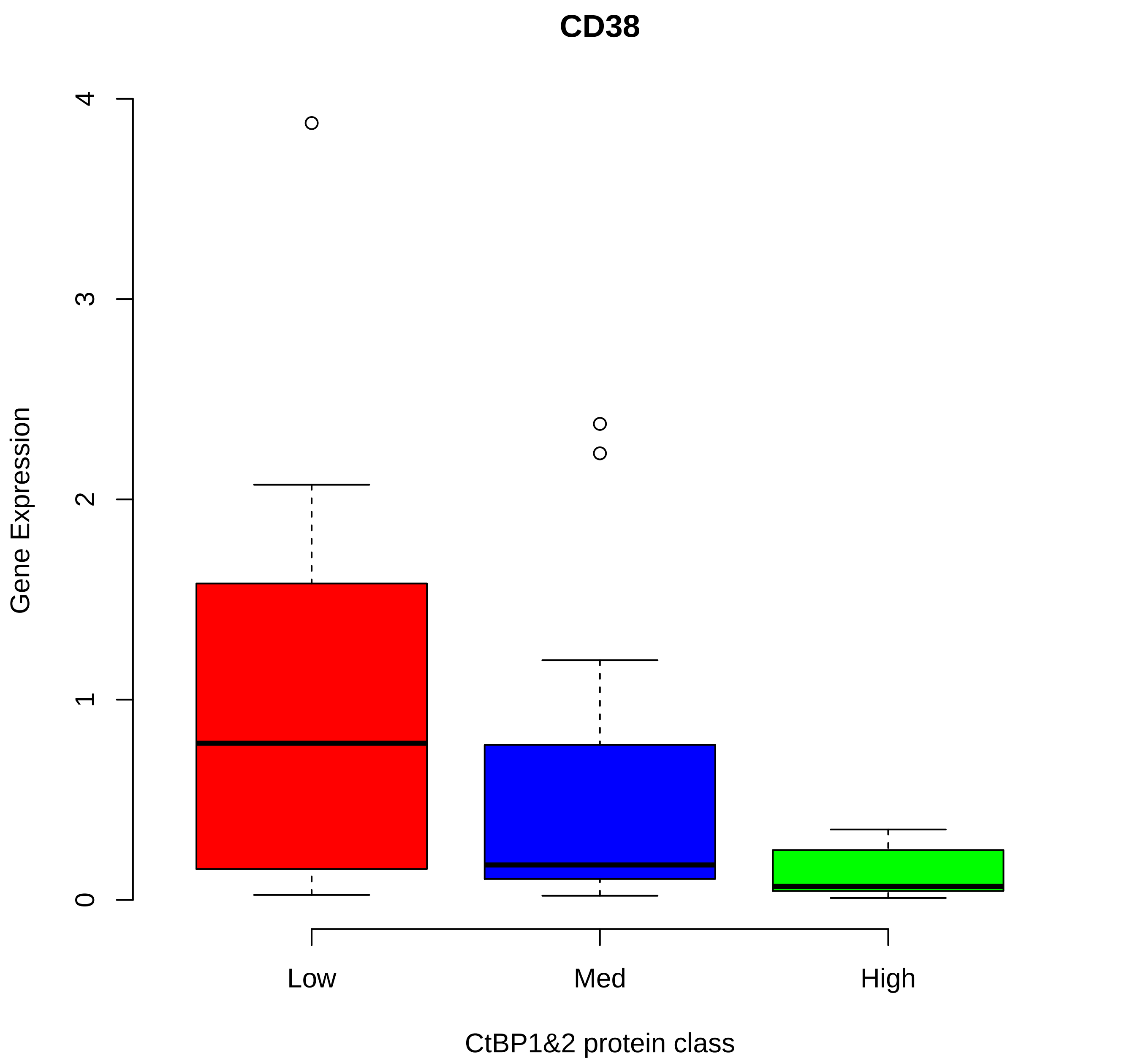
CD38
Gene Expression
0
1
2
3
4
Low
Med
High
CtBP1&2 protein class

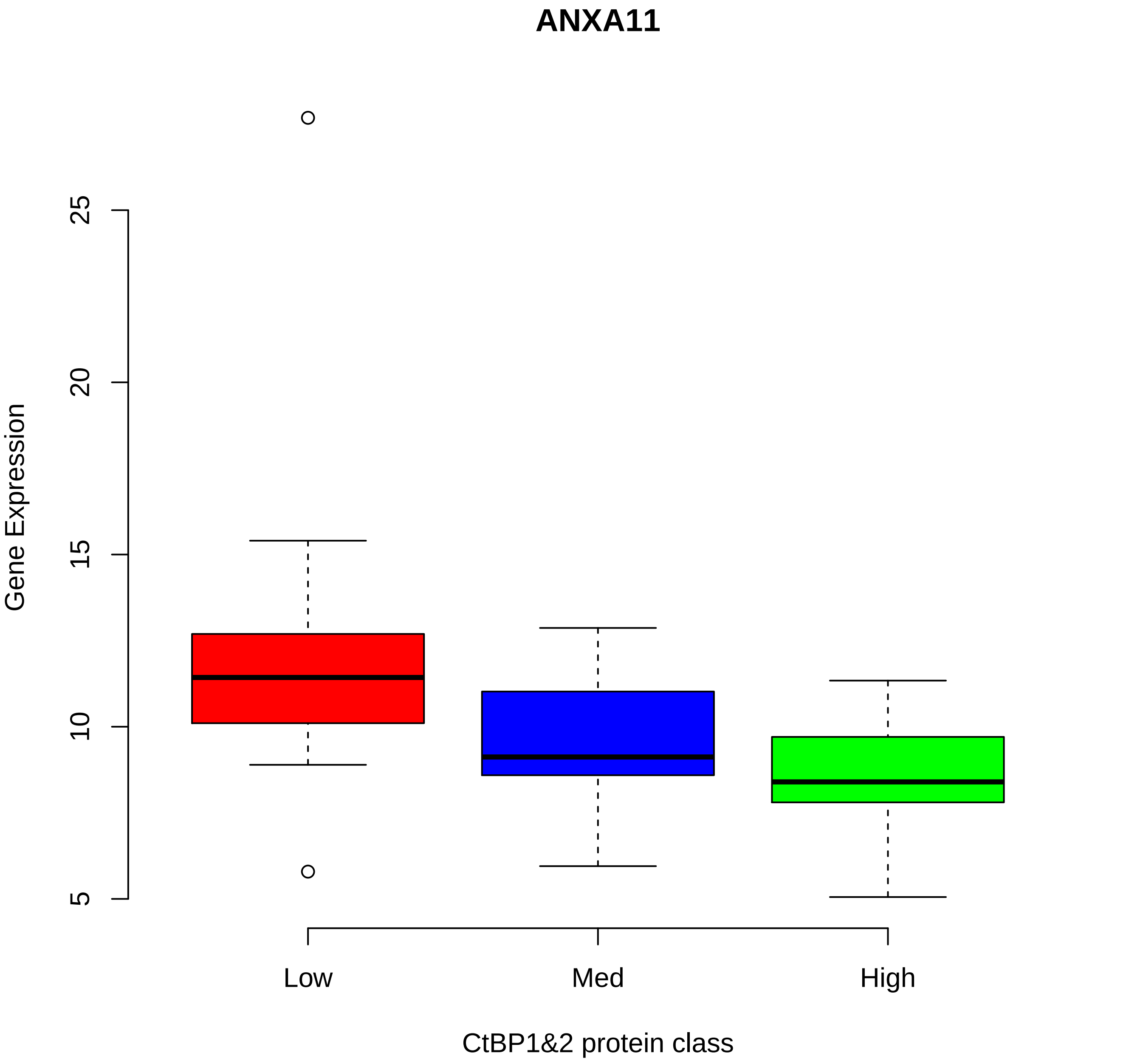
ANXA11
Gene Expression
25
20
15
10
5
Low
Med
High
CtBP1&2 protein class

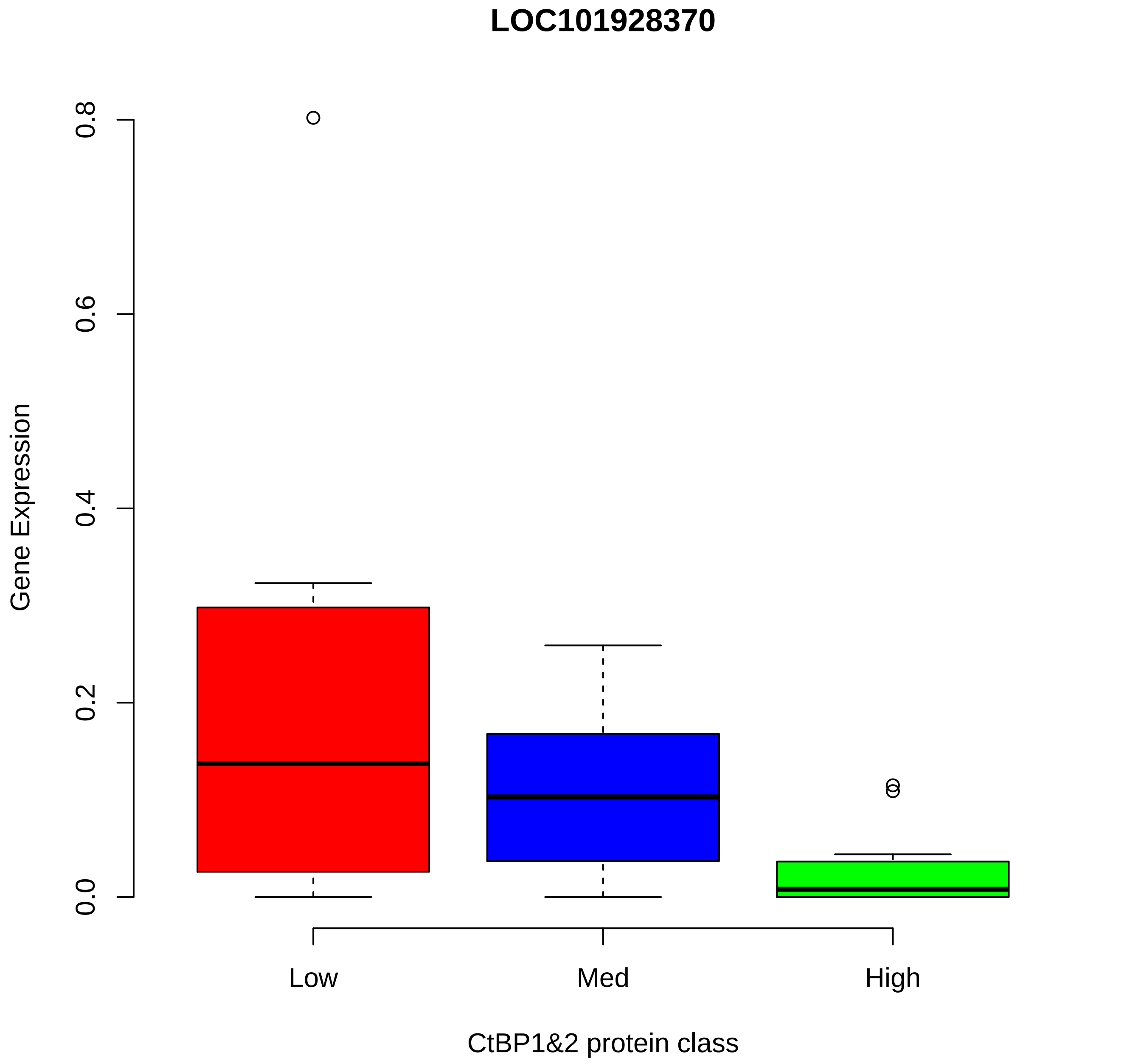
LOC101928370
Gene Expression
0.0
0.2
0.4
0.6
0.8
Low
Med
High
CtBP1&2 protein class

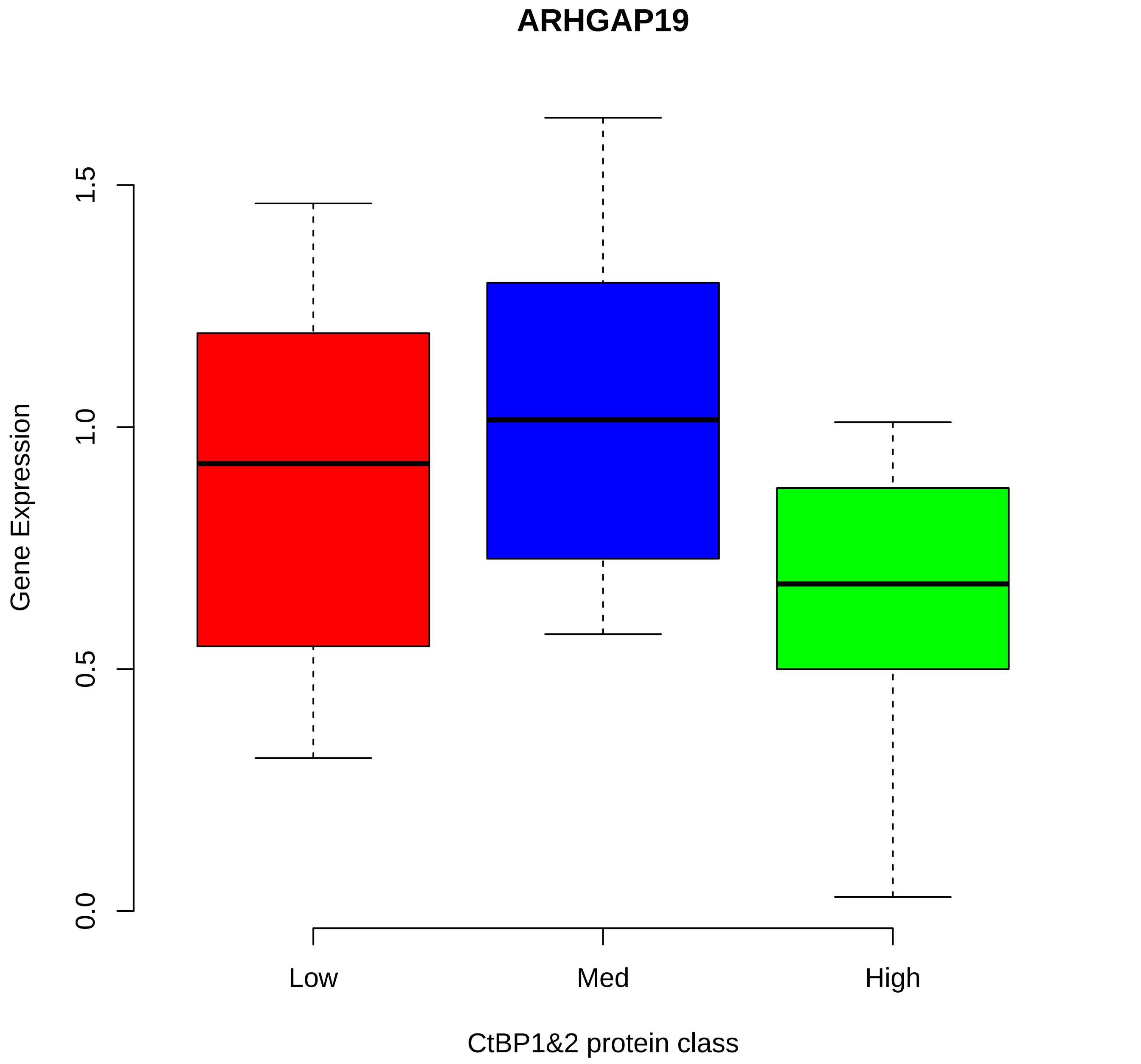

ARHGAP19
Gene Expression
0.0
0.5
1.0
1.5
Low
Med
High
CtBP1&2 protein class

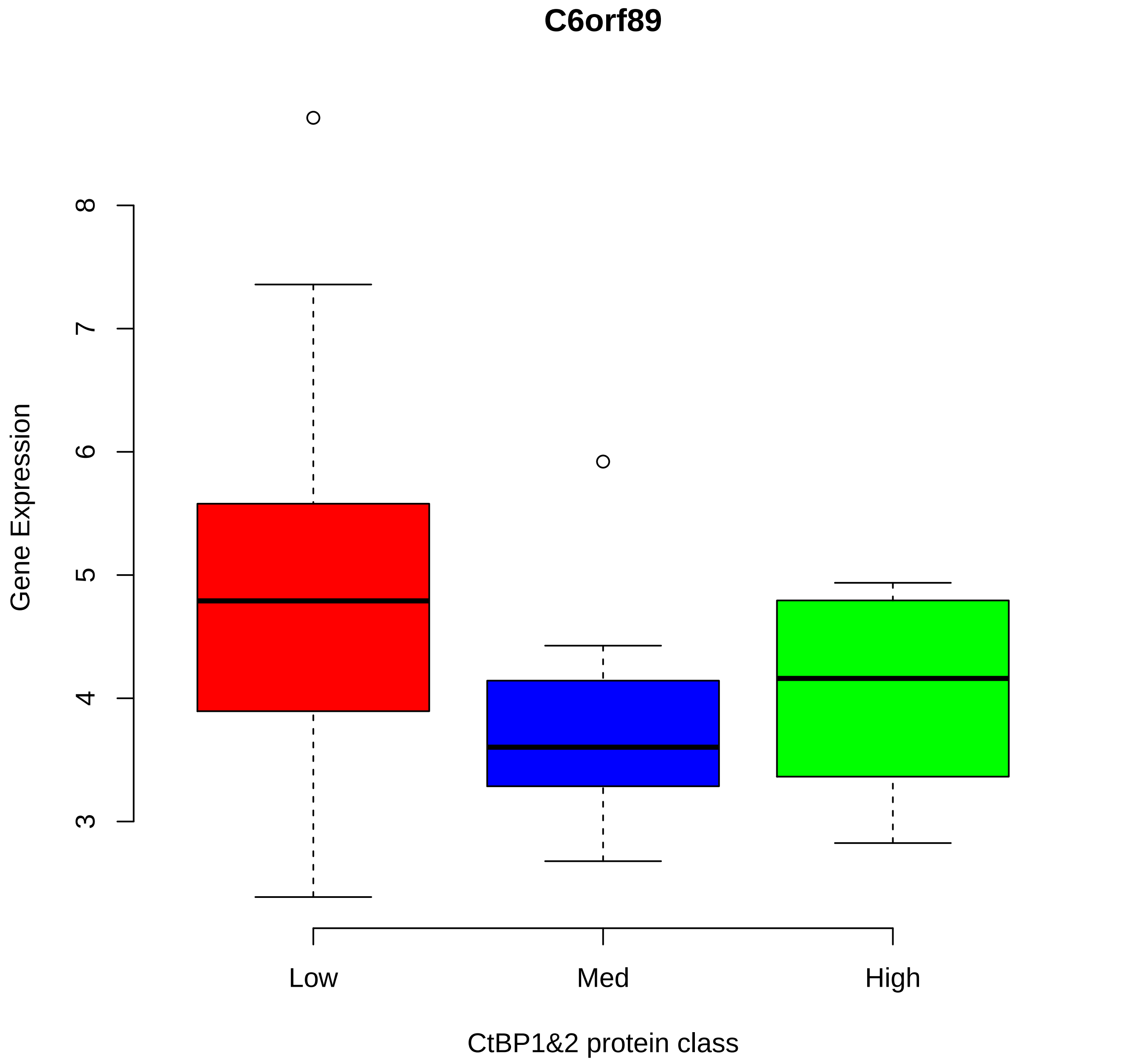

C6orf89
Gene Expression
3
4
5
6
7
8
Low
Med
High
CtBP1&2 protein class

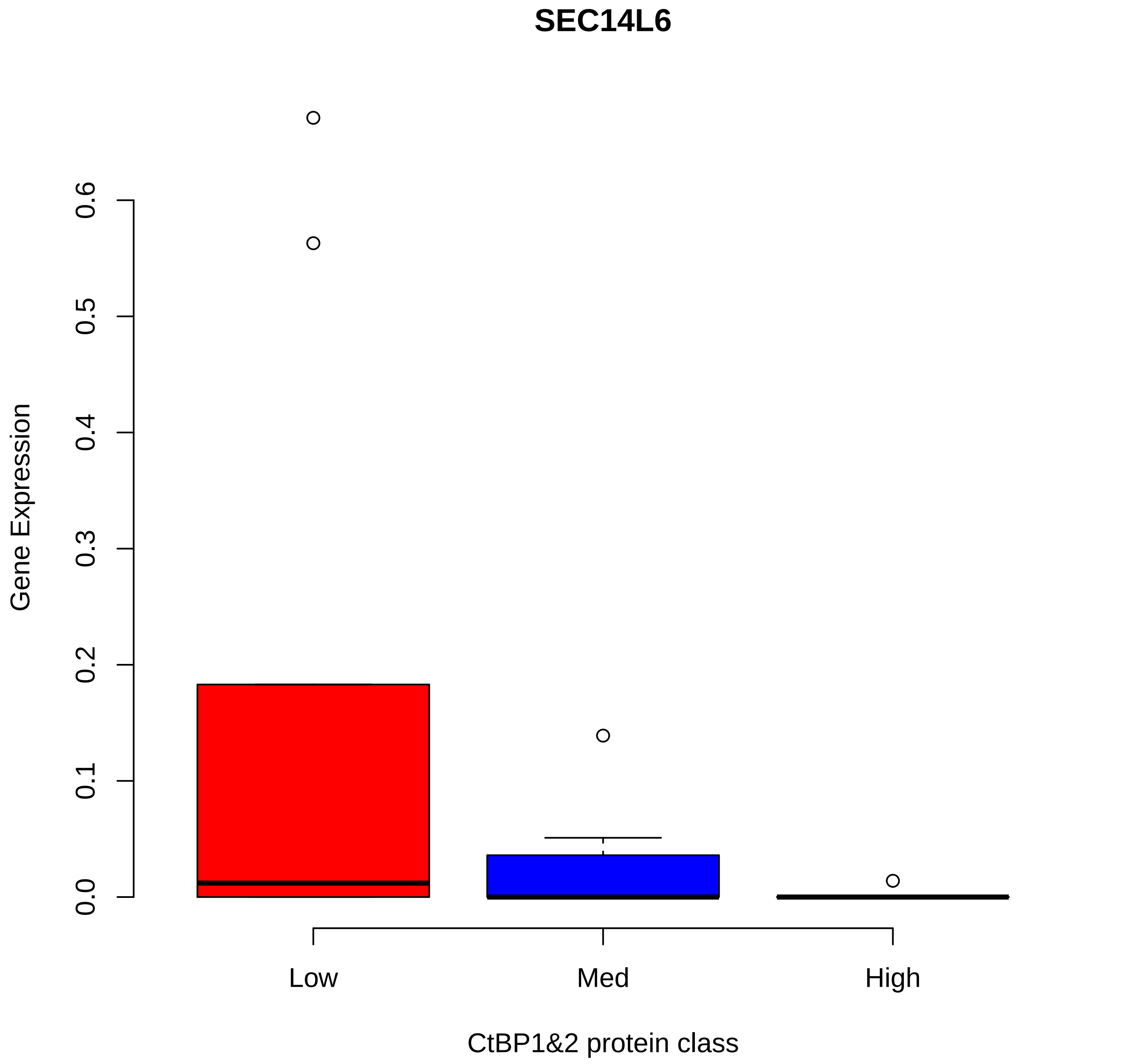
SEC14L6
Gene Expression
0.0
0.1
0.2
0.3
0.4
0.5
0.6
Low
Med
High
CtBP1&2 protein class

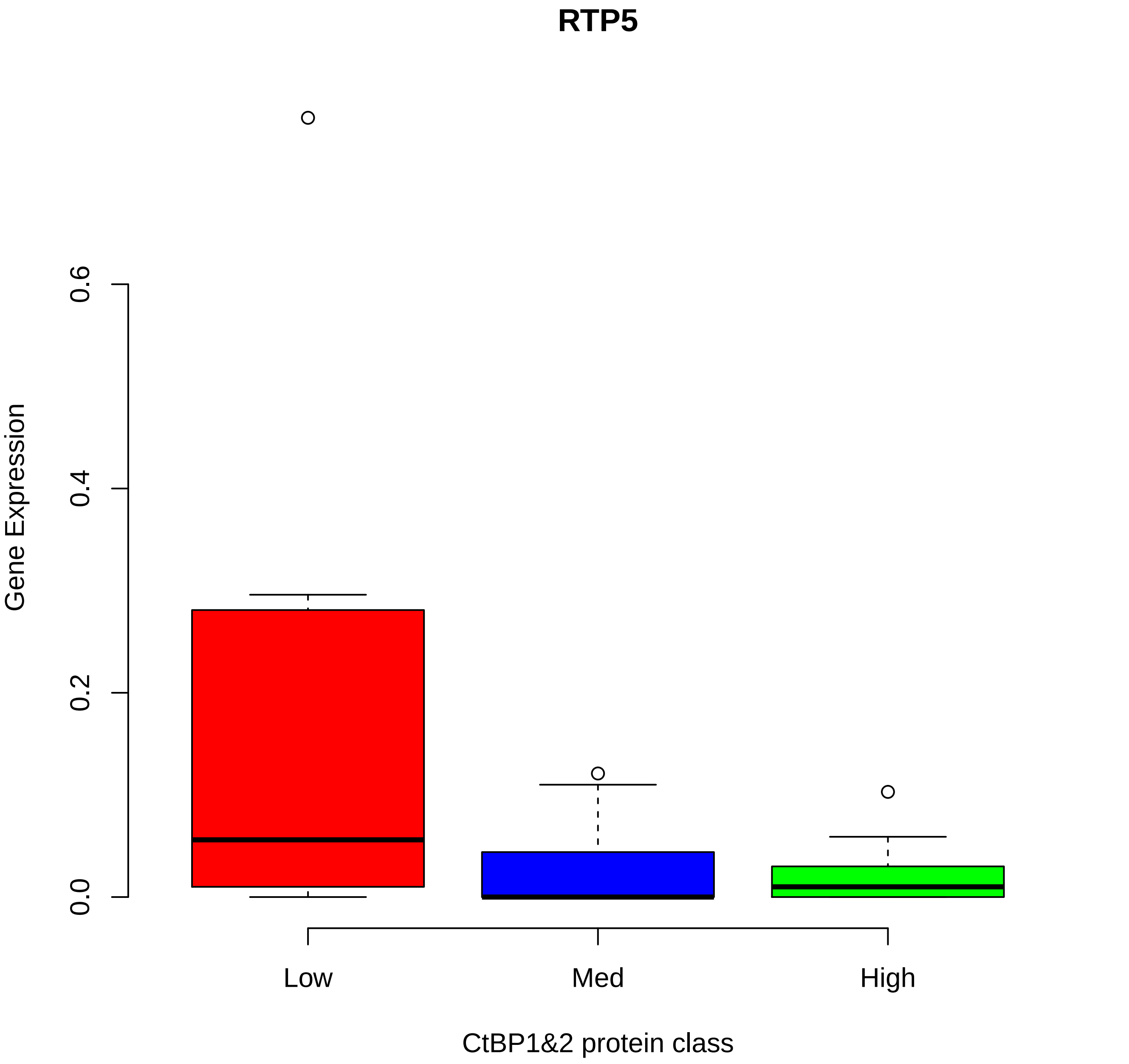
RTP5
Gene Expression
0.0
0.2
0.4
0.6
Low
Med
High
CtBP1&2 protein class

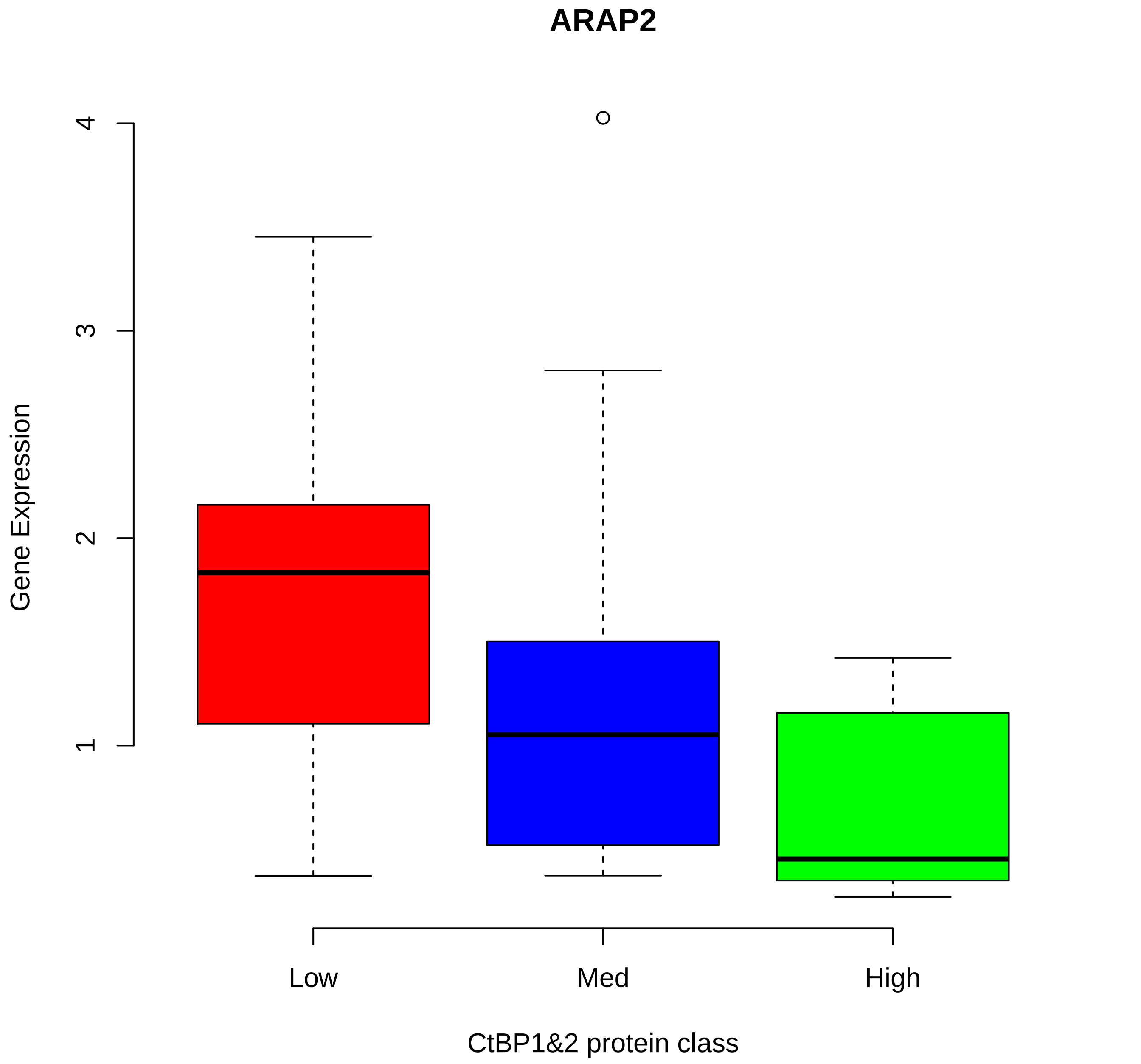
ARAP2
Gene Expression
4
3
2
1
Low
Med
High
CtBP1&2 protein class

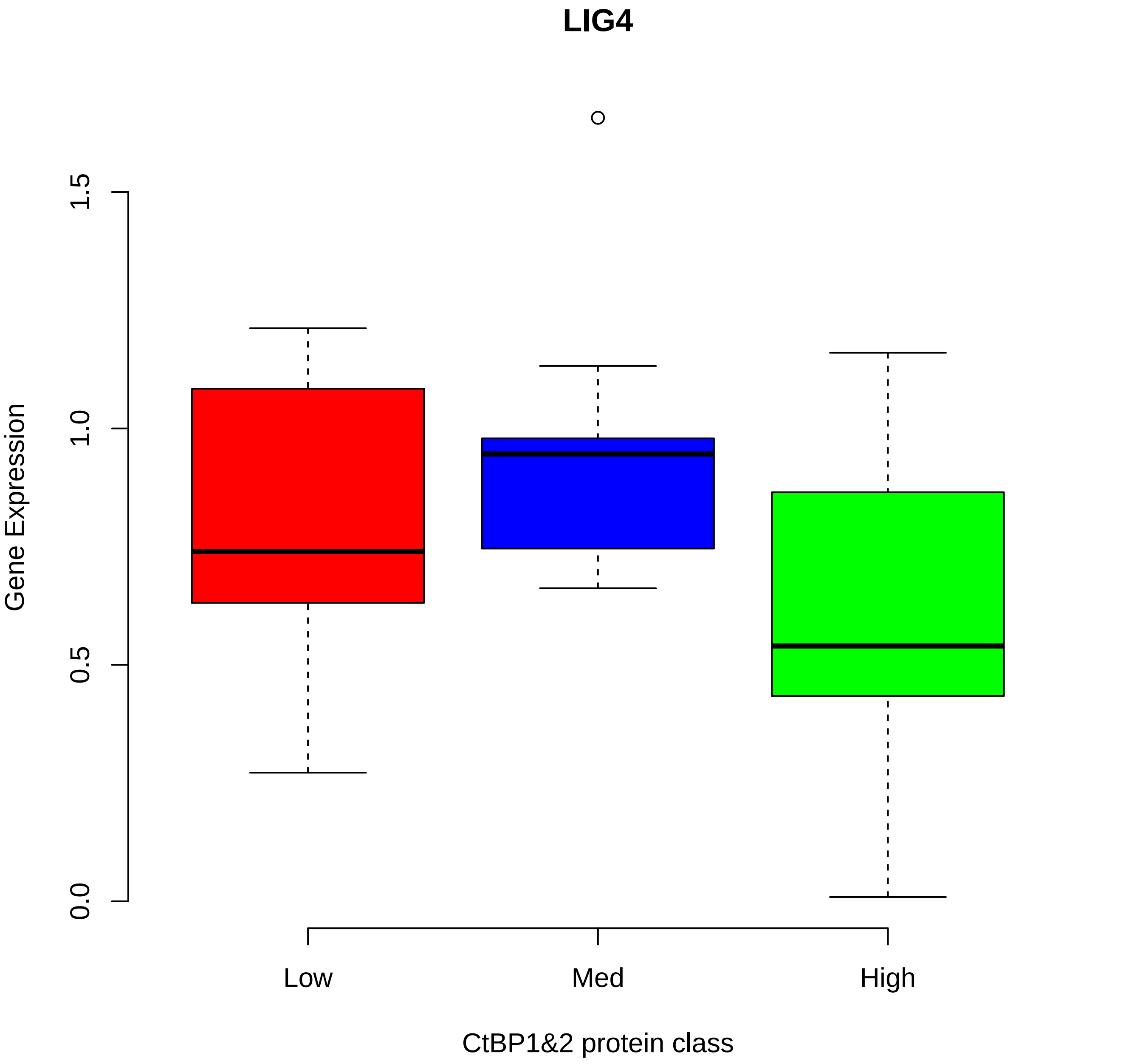
LIG4
Gene Expression
0.0
0.5
1.0
1.5
Low
Med
High
CtBP1&2 protein class

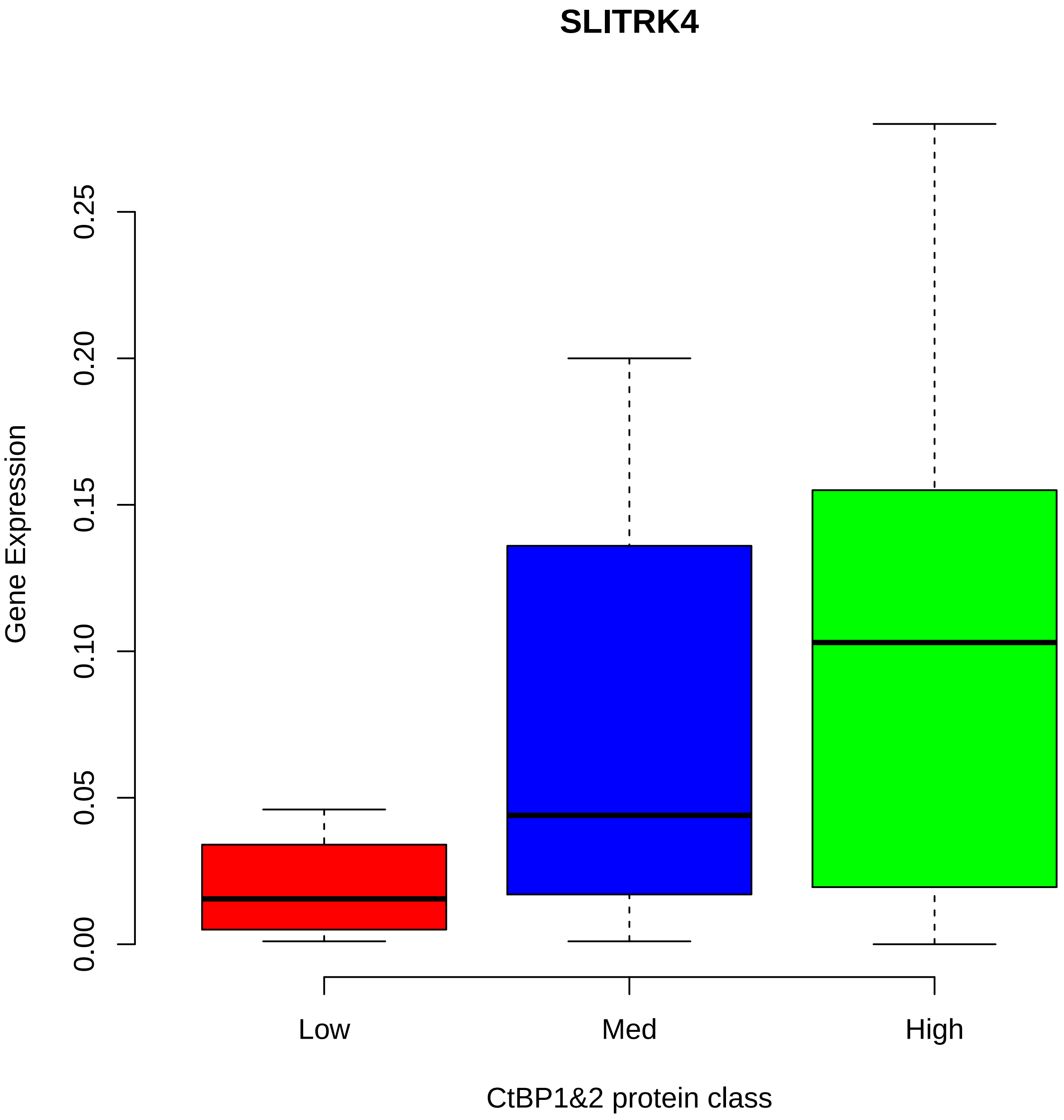

SLITRK4
Gene Expression
0.00
0.05
0.10
0.15
0.20
0.25
Low
Med
High
CtBP1&2 protein class

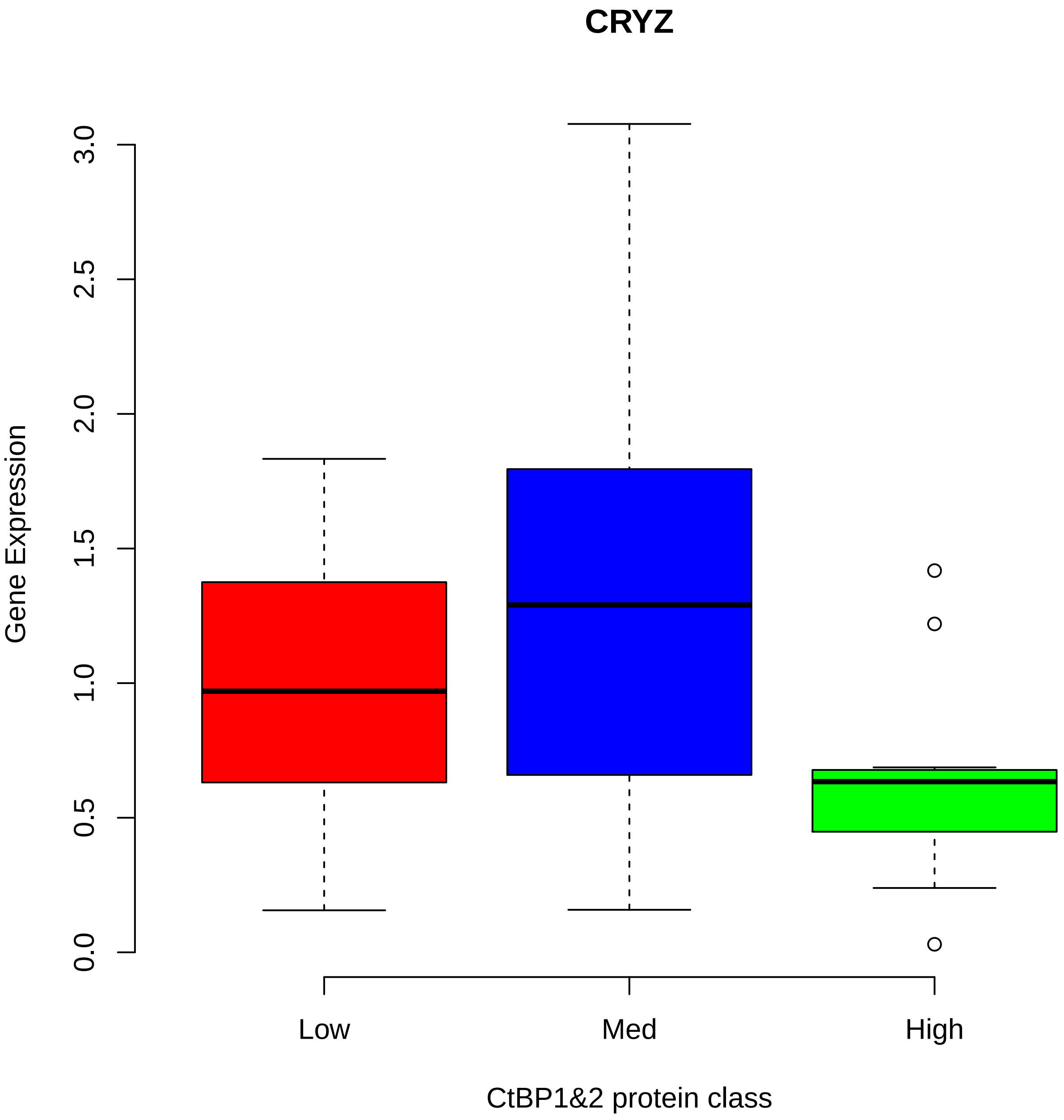

CRYZ
Gene Expression
0.0
0.5
1.0
1.5
2.0
2.5
3.0
Low
Med
High
CtBP1&2 protein class

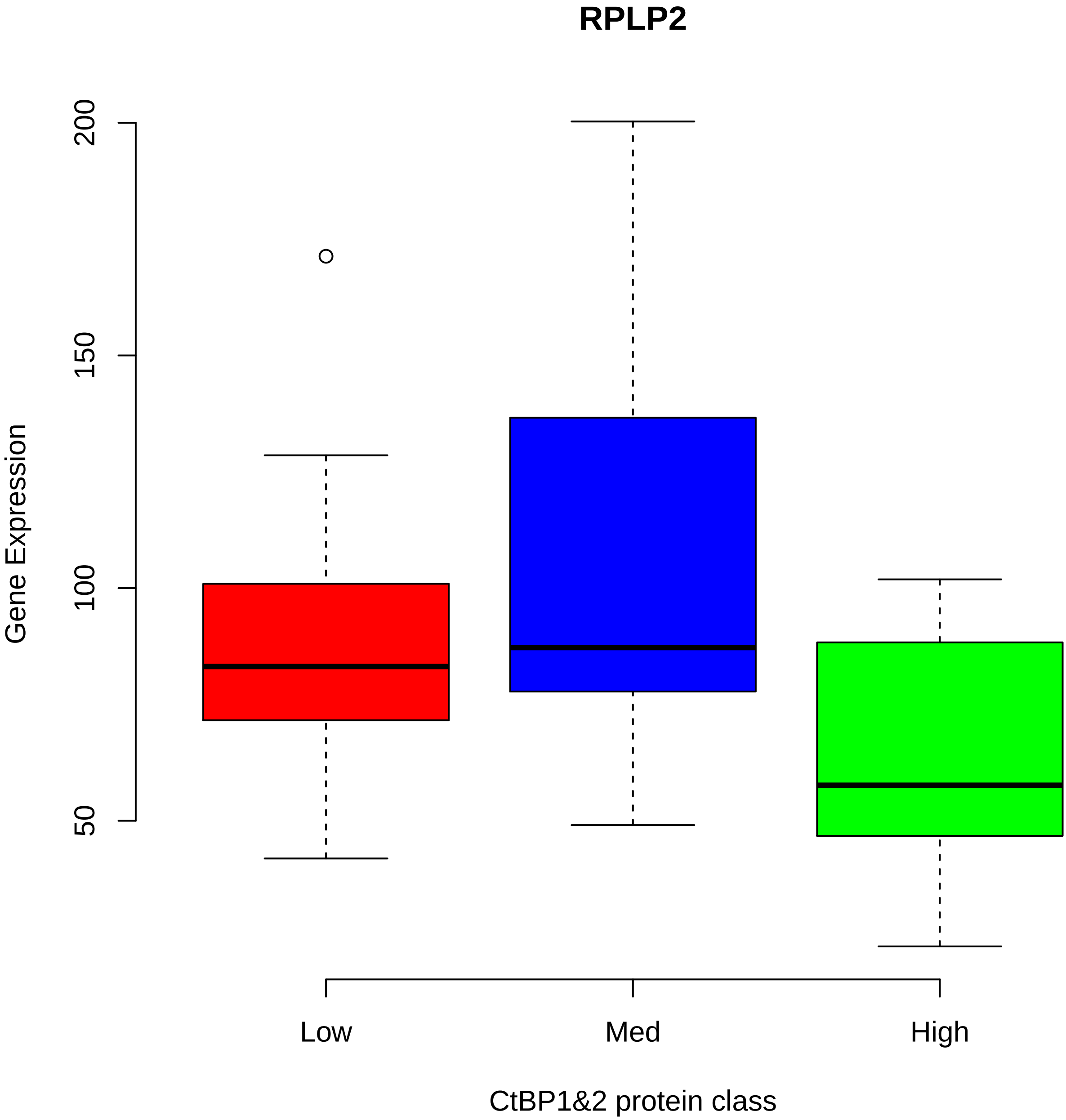
RPLP2
Gene Expression
200
150
100
50
Low
Med
High
CtBP1&2 protein class

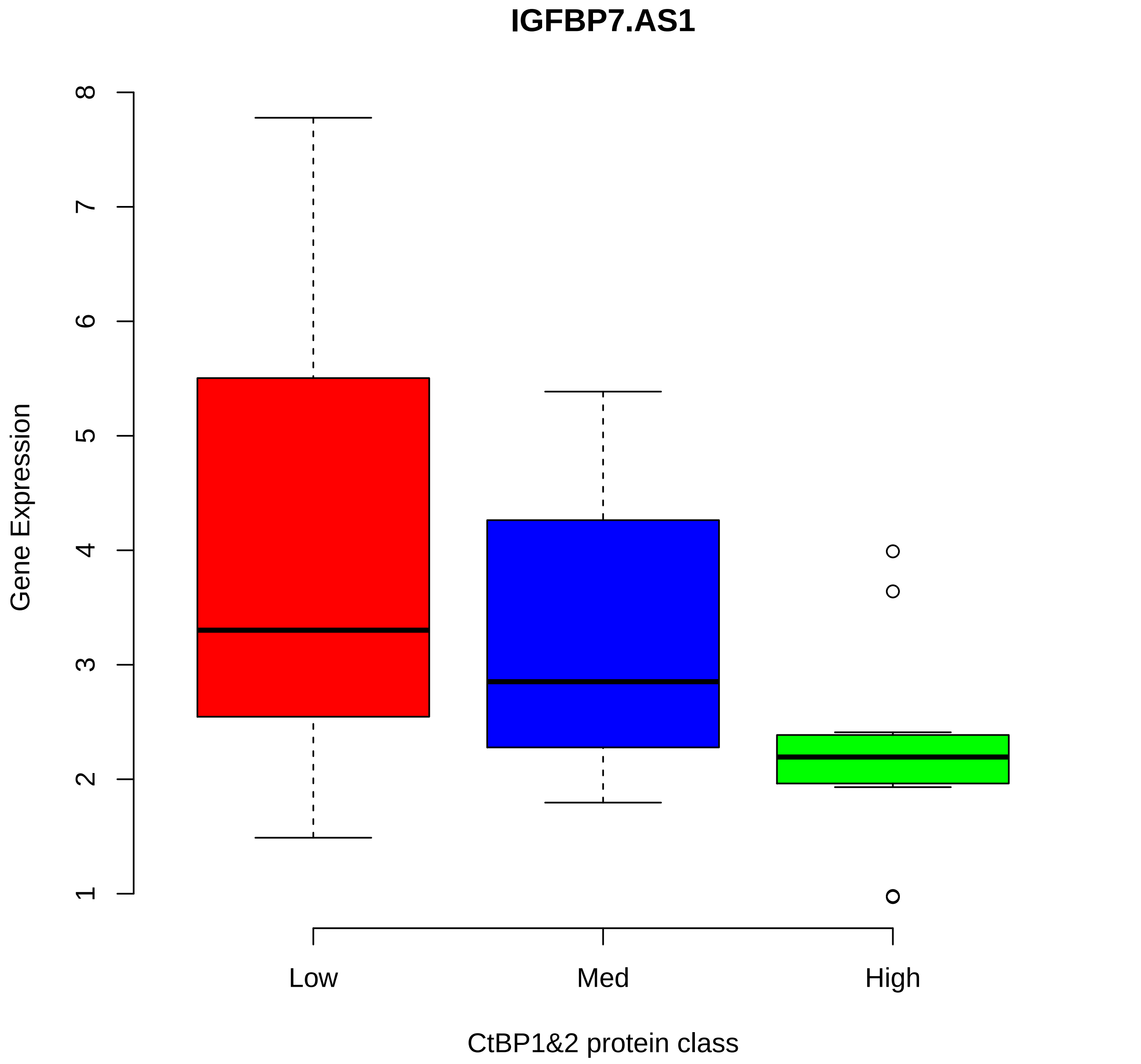
IGFBP7.AS1
Gene Expression
1
2
3
4
5
6
7
8
Low
Med
High
CtBP1&2 protein class

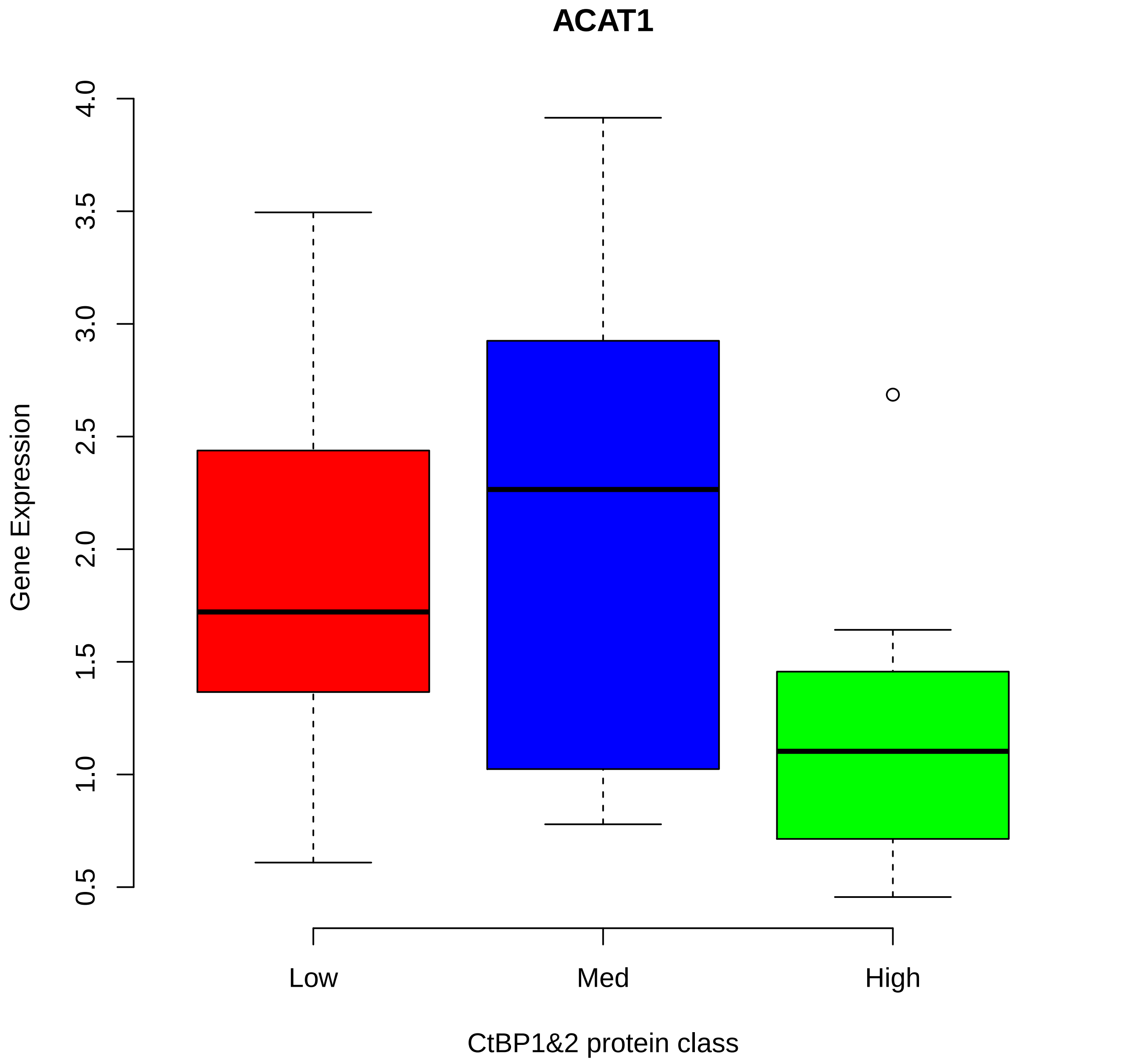

ACAT1
Gene Expression
4.0
3.5
3.0
2.5
2.0
1.5
1.0
0.5
Low
Med
High
CtBP1&2 protein class

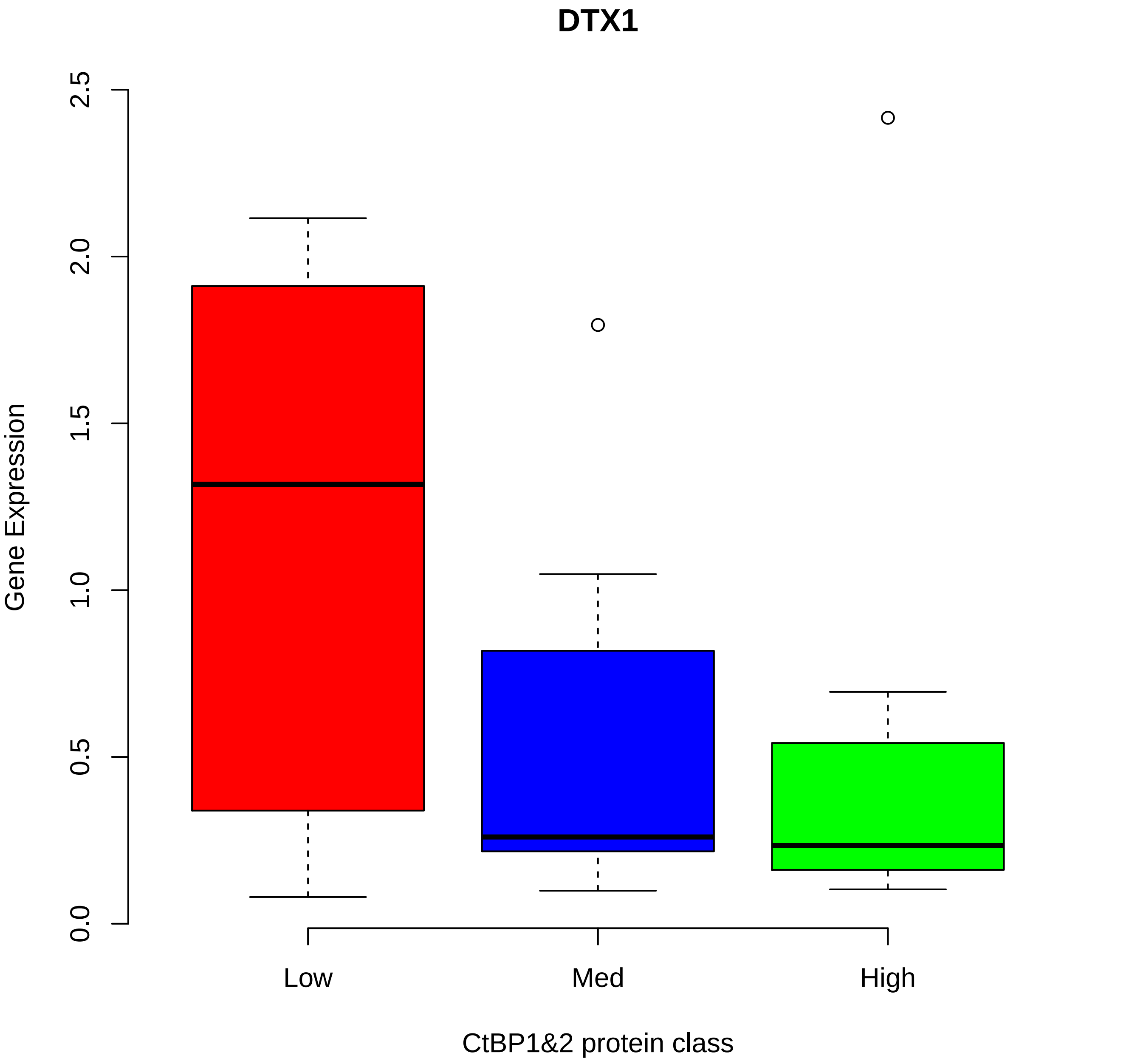
DTX1
Gene Expression
0.0
0.5
1.0
1.5
2.0
2.5
Low
Med
High
CtBP1&2 protein class

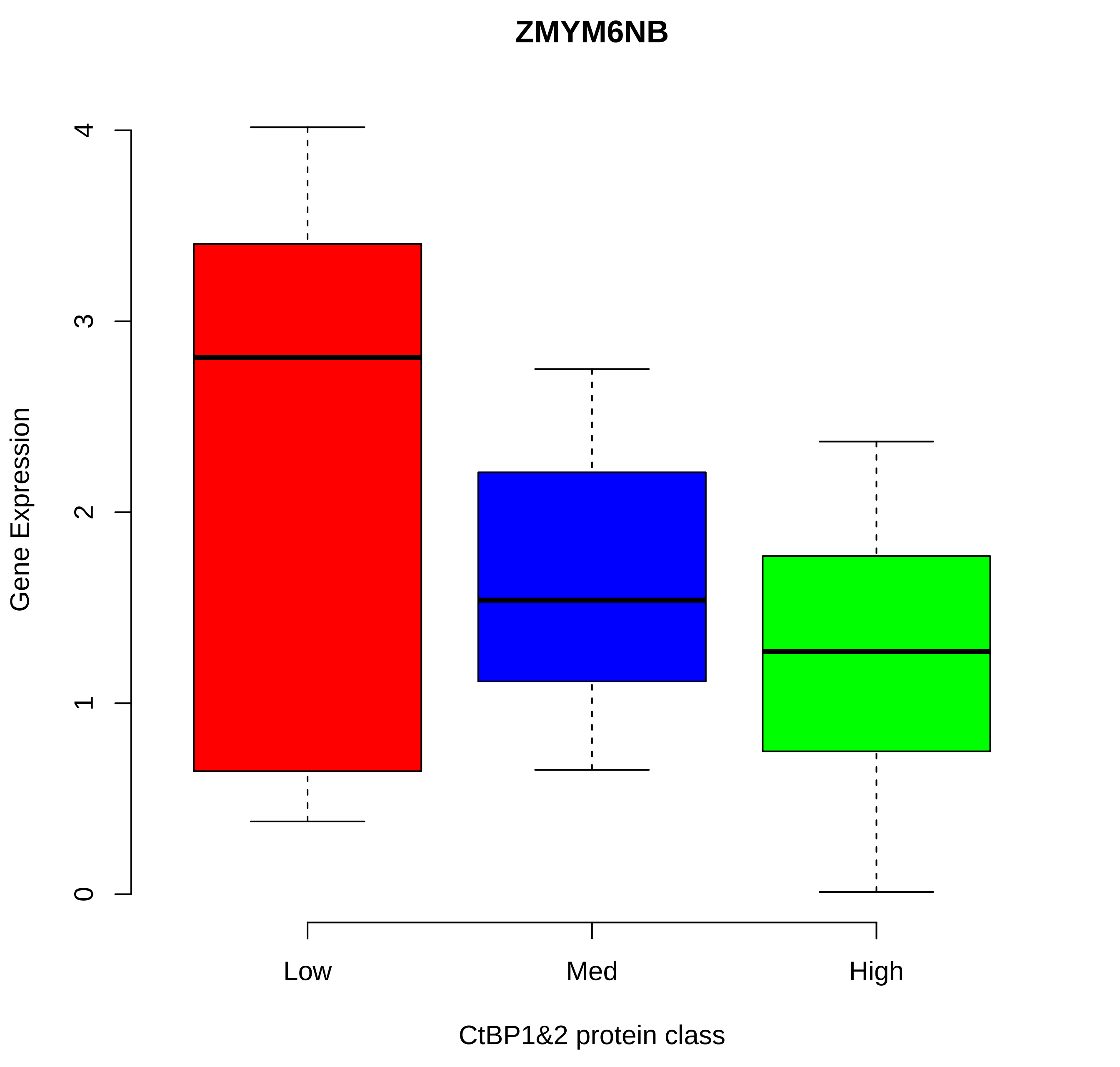
ZMYM6NB
Gene Expression
0
1
2
3
4
Low
Med
High
CtBP1&2 protein class

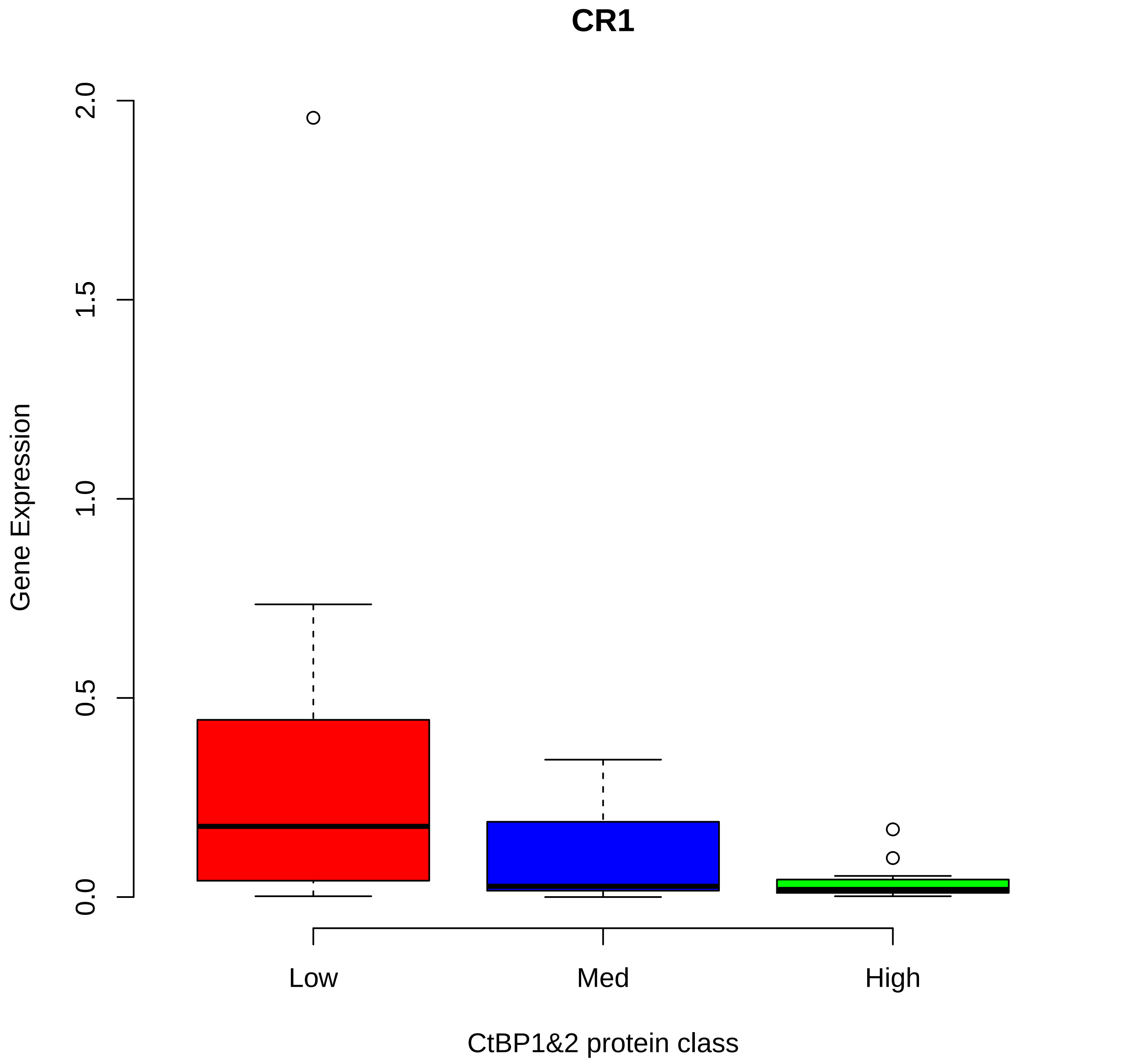
CR1
Gene Expression
0.0
0.5
1.0
1.5
2.0
Low
Med
High
CtBP1&2 protein class

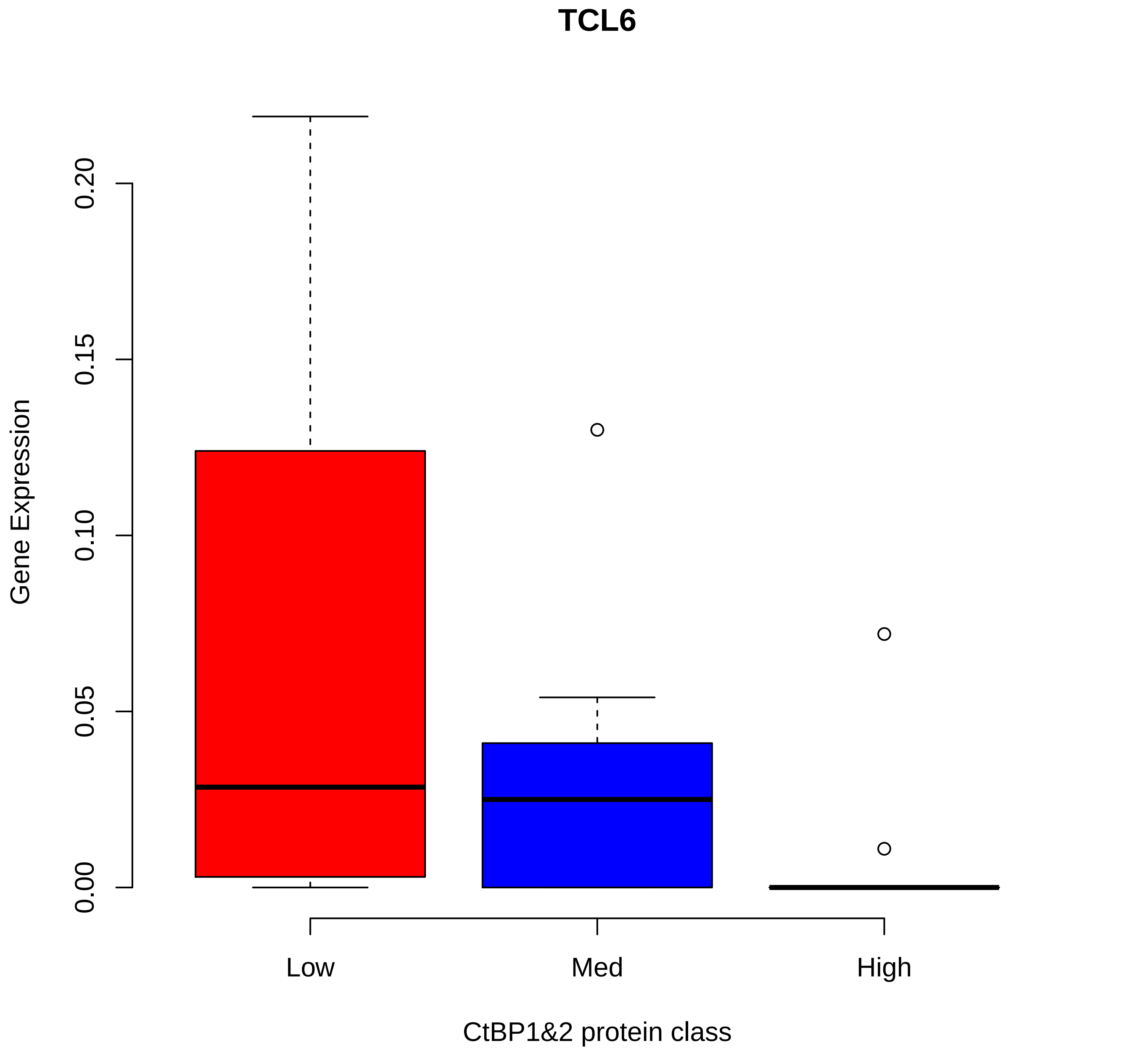
TCL6
Gene Expression
0.00
0.05
0.10
0.15
0.20
Low
Med
High
CtBP1&2 protein class

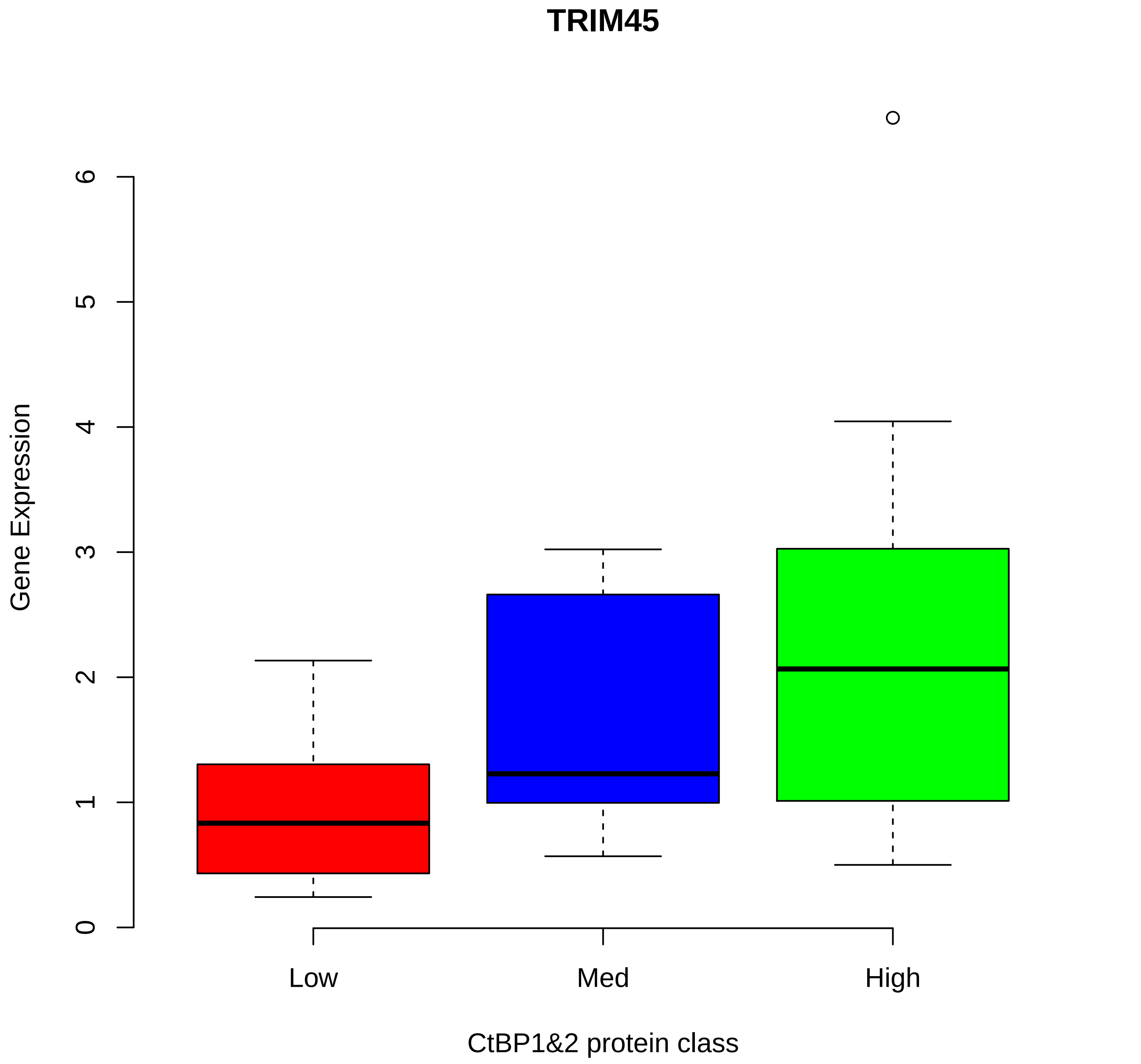

TRIM45
Gene Expression
0
1
2
3
4
5
6
Low
Med
High
CtBP1&2 protein class

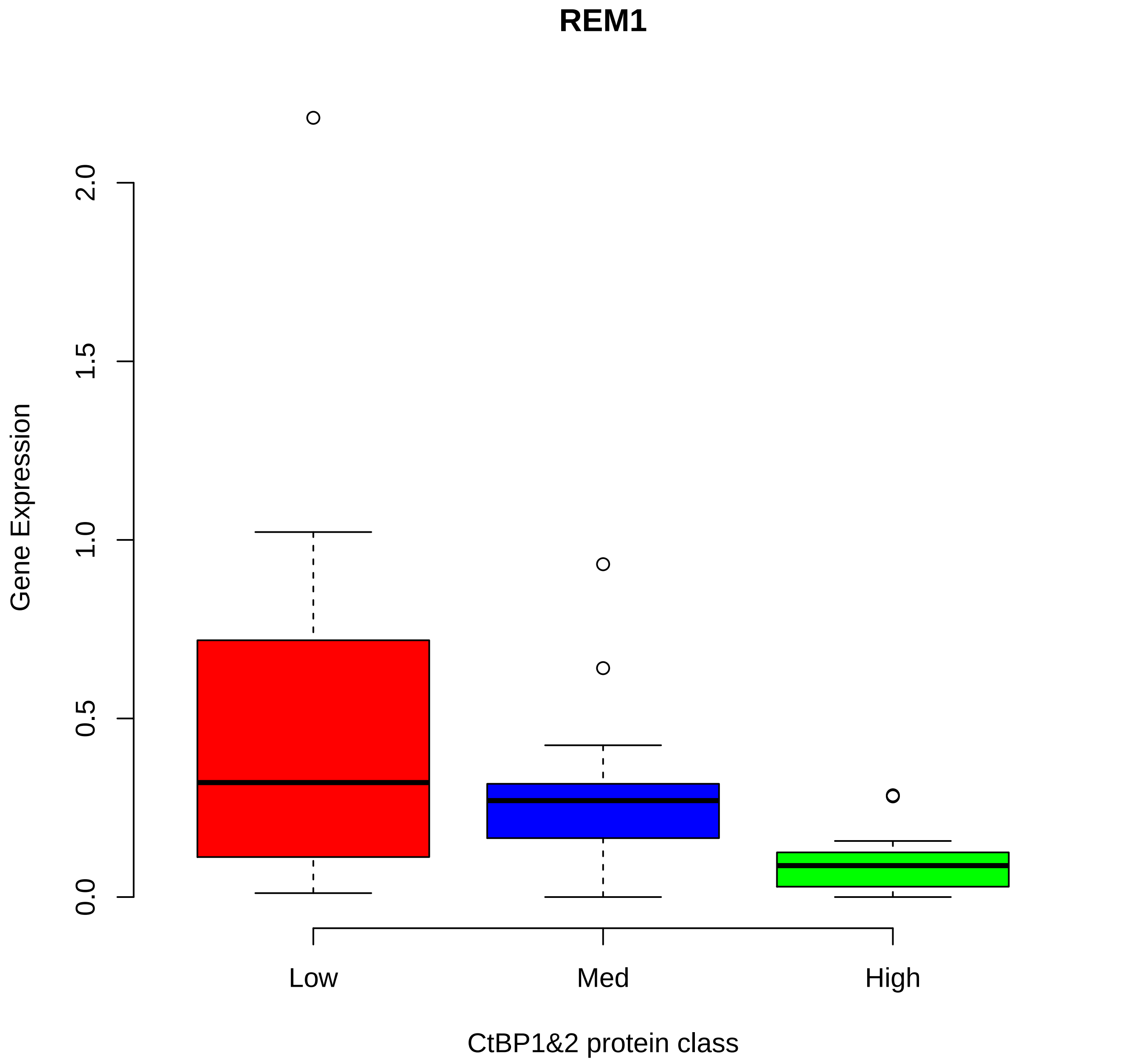
REM1
Gene Expression
0.0
0.5
1.0
1.5
2.0
Low
Med
High
CtBP1&2 protein class

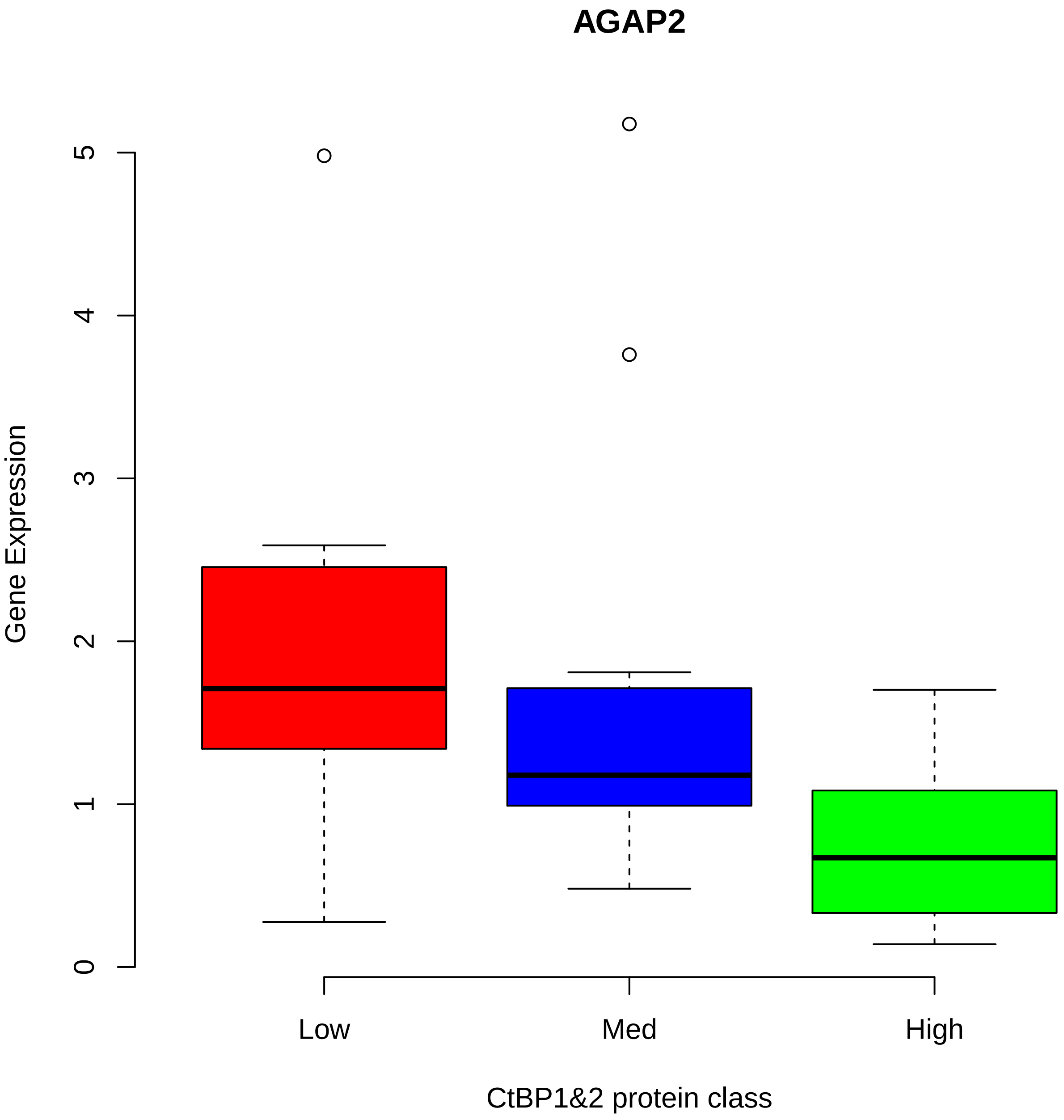
AGAP2
Gene Expression
0
1
2
3
4
5
Low
Med
High
CtBP1&2 protein class

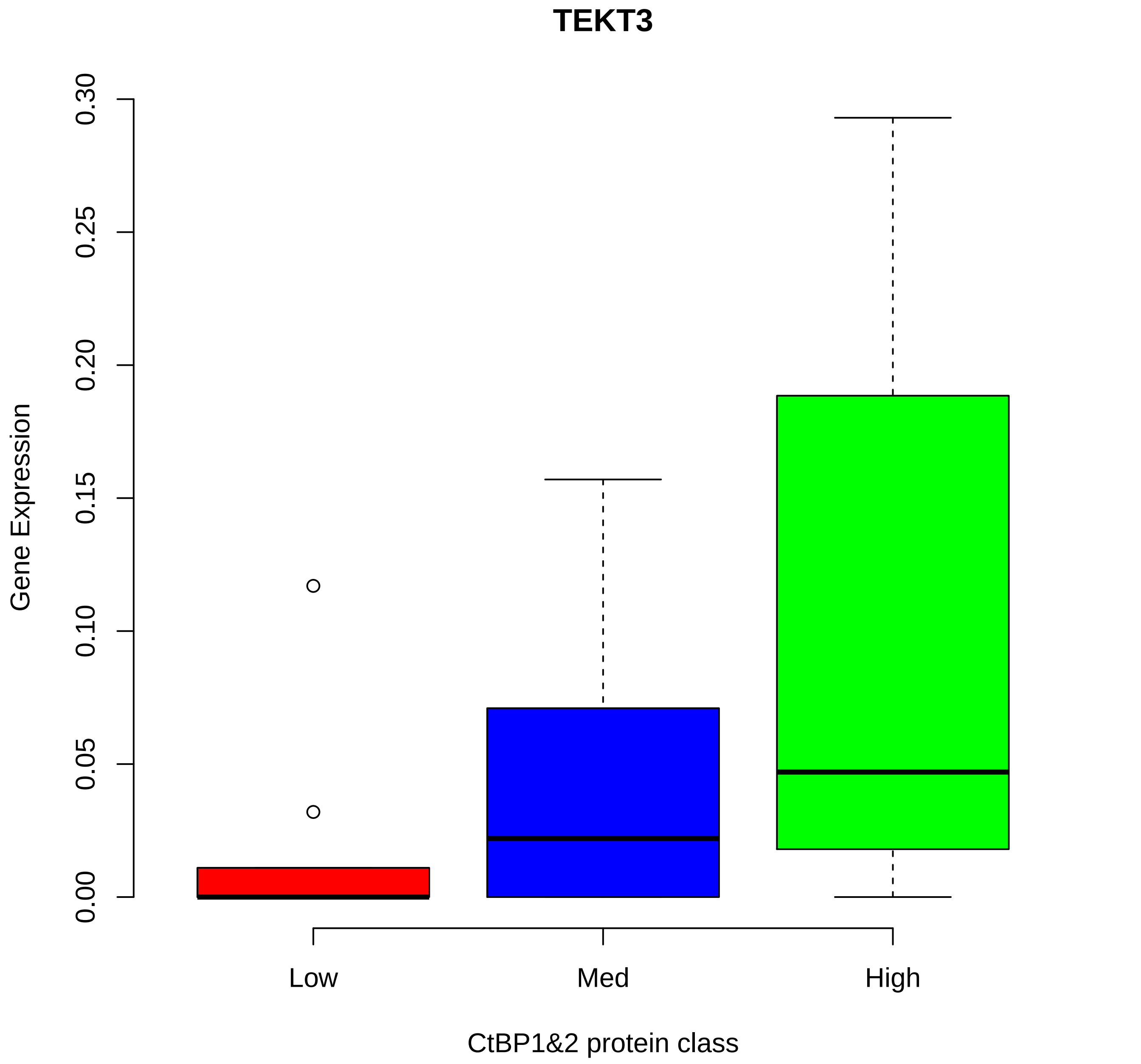
TEKT3
Gene Expression
0.00
0.05
0.10
0.15
0.20
0.25
0.30
Low
Med
High
CtBP1&2 protein class

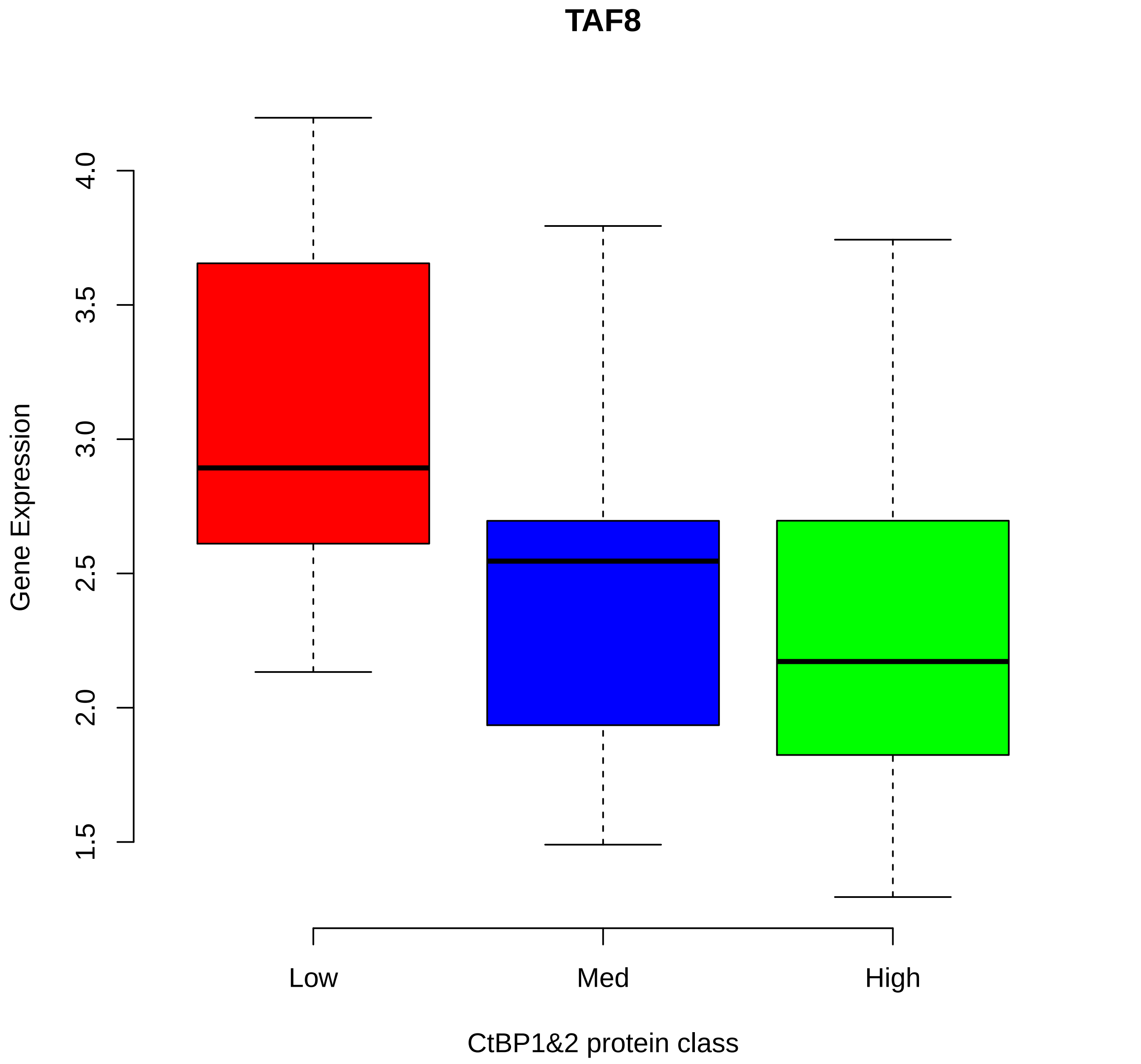

TAF8
4.0
3.5
3.0
2.5
2.0
1.5
Gene Expression
Low
Med
High
CtBP1&2 protein class

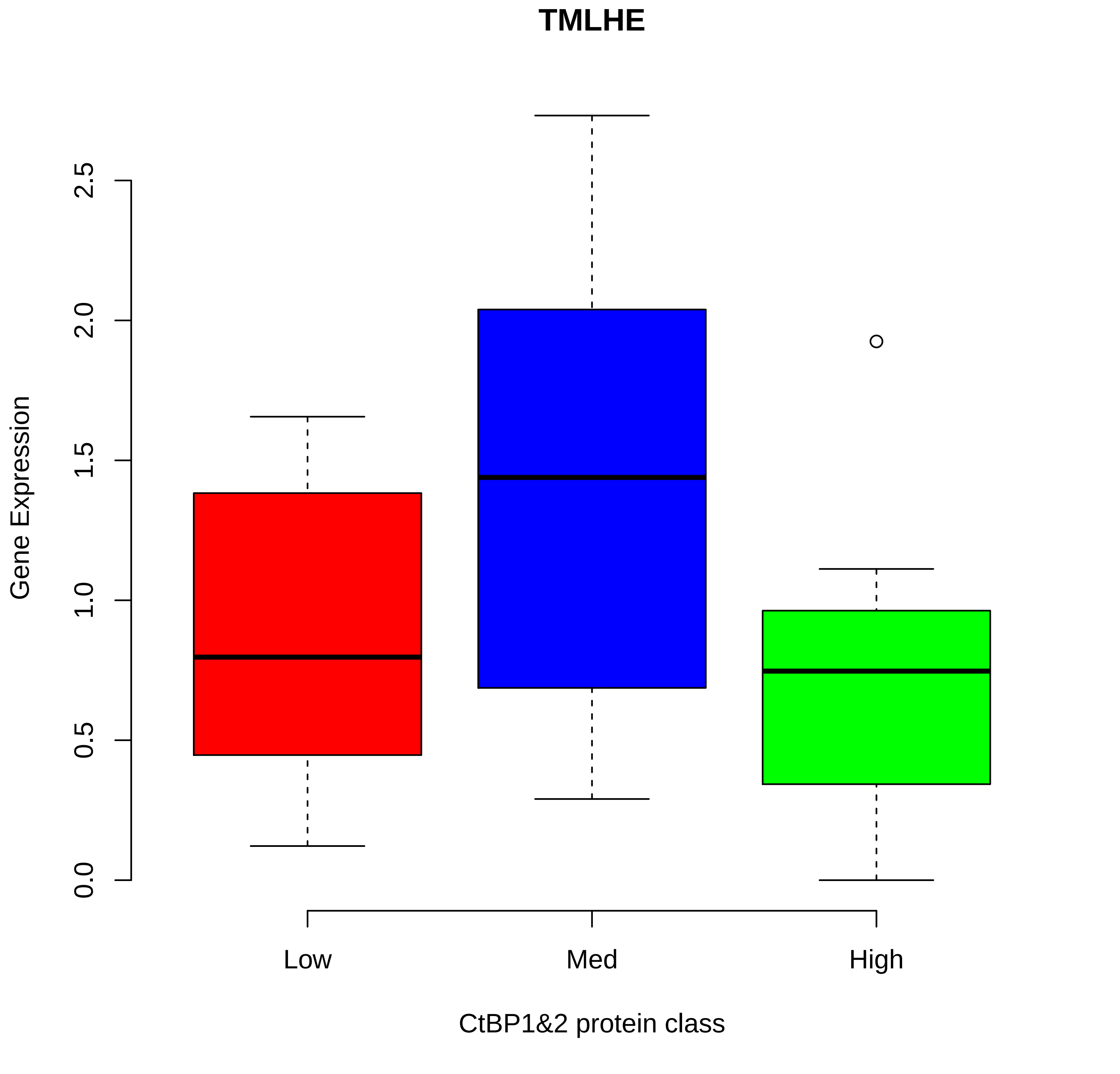
TMLHE
Gene Expression
0.0
0.5
1.0
1.5
2.0
2.5
Low
Med
High
CtBP1&2 protein class

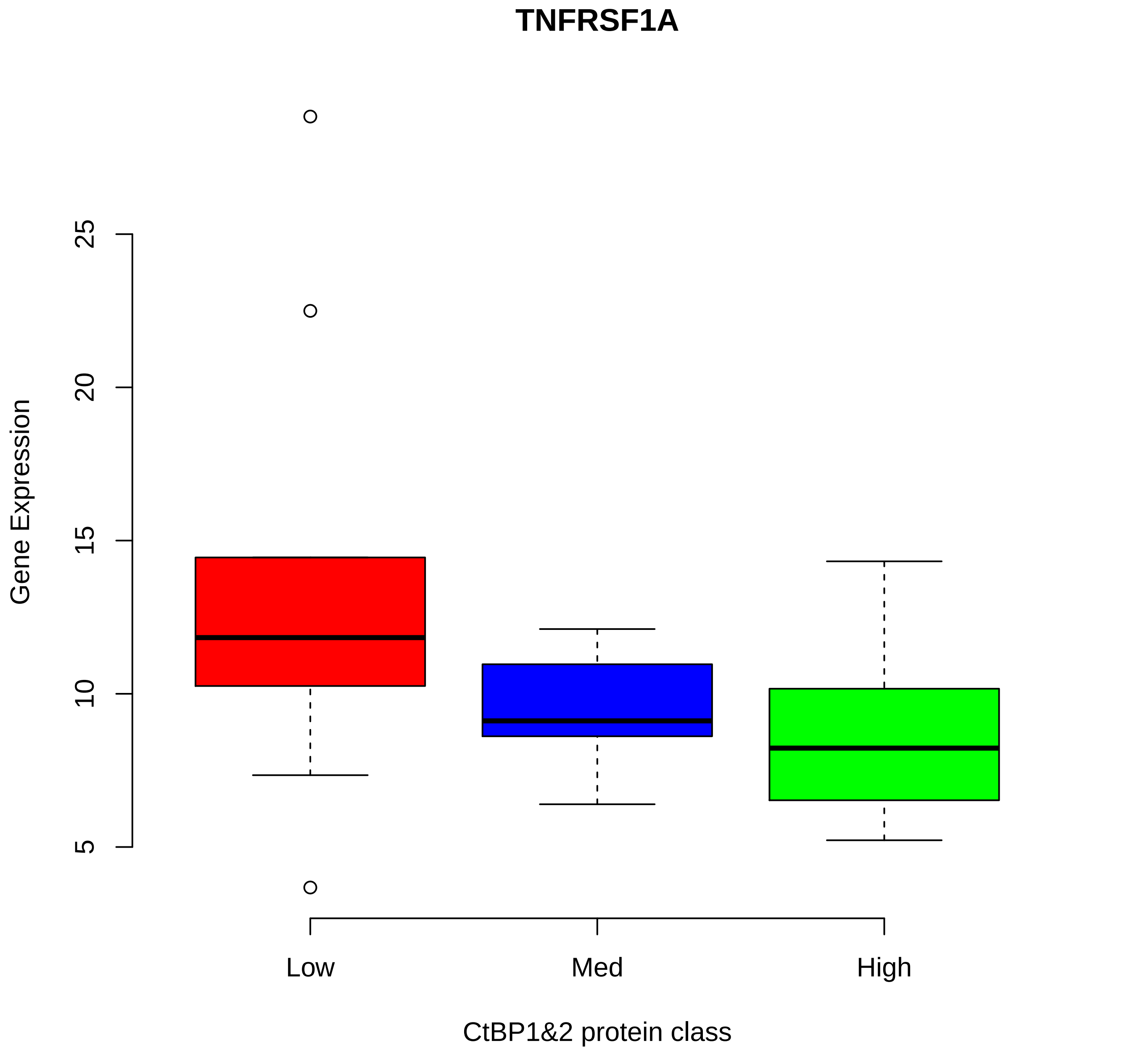
TNFRSF1A
Gene Expression
5
10
15
20
25
Low
Med
High
CtBP1&2 protein class

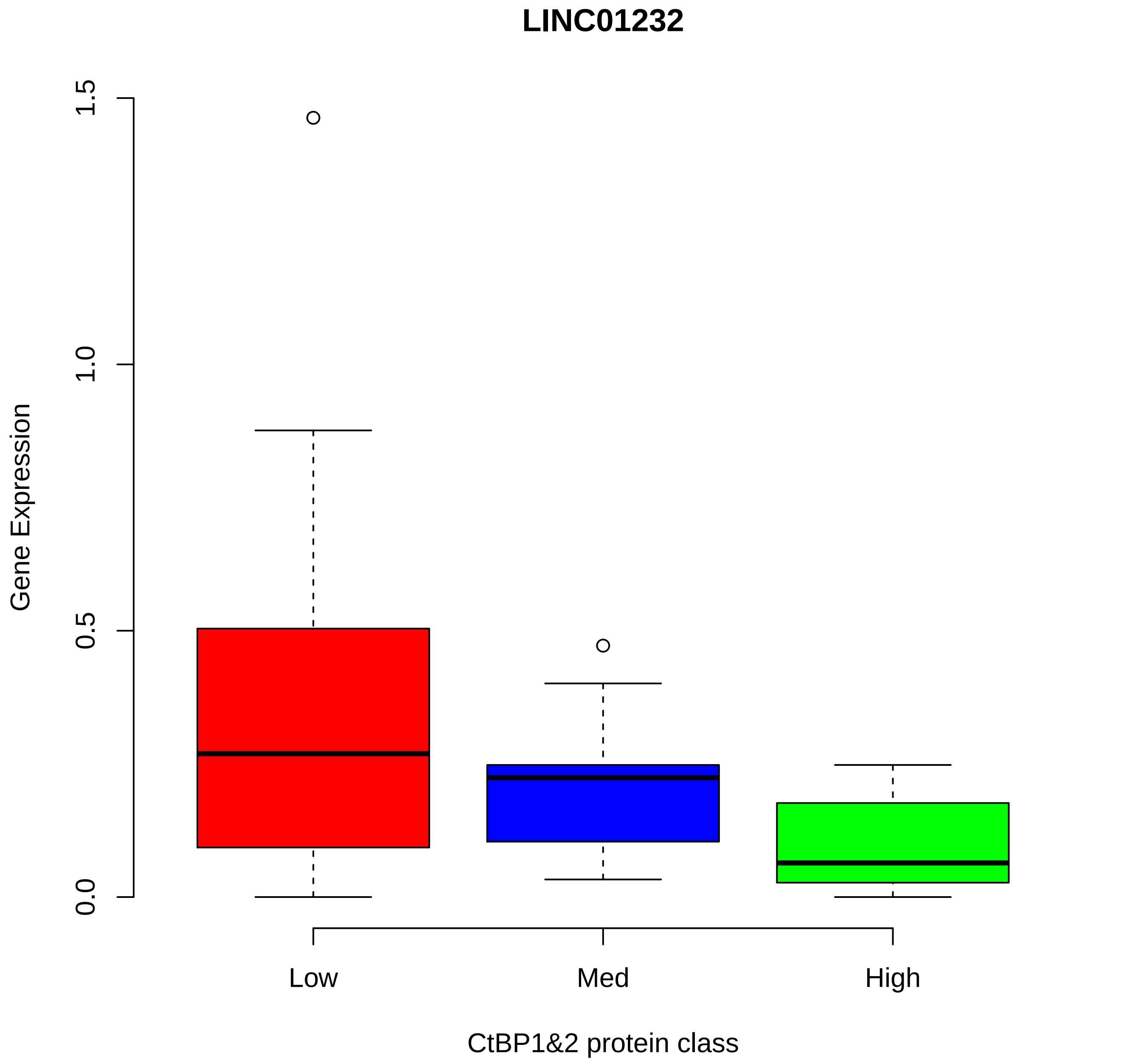
LINC01232
Gene Expression
0.0
0.5
1.0
1.5
Low
Med
High
CtBP1&2 protein class

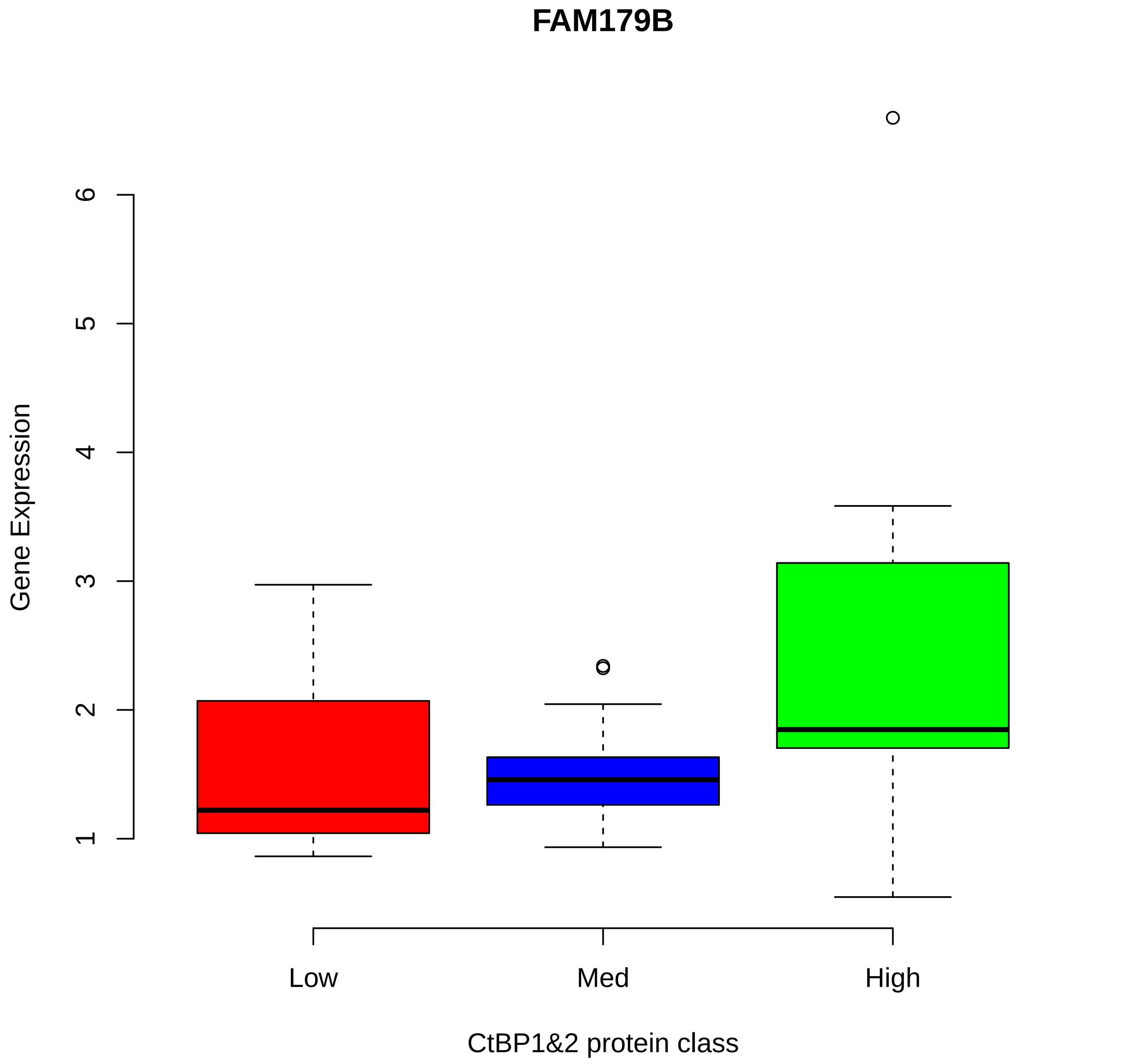

FAM179B
Gene Expression
1
2
3
4
5
6
Low
Med
High
CtBP1&2 protein class

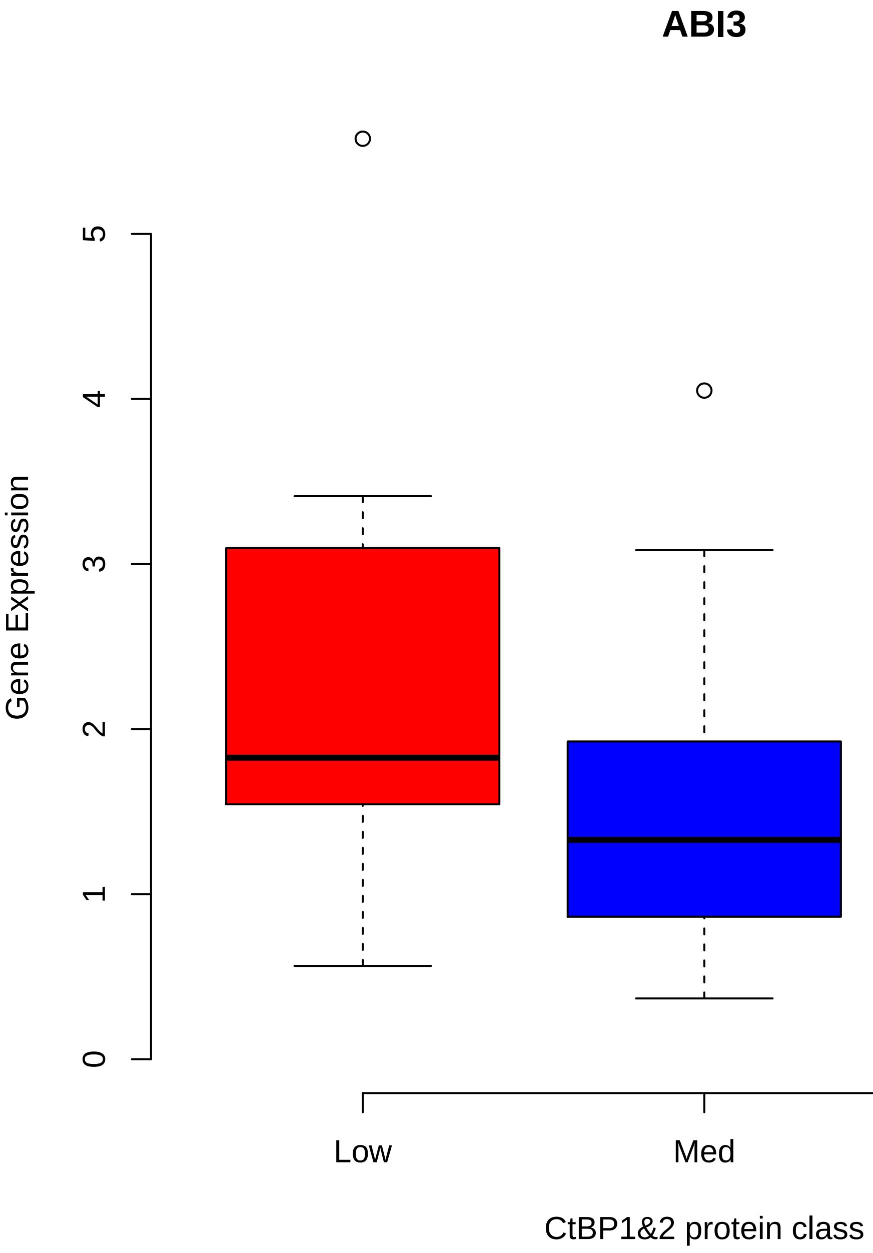
ABI3
Gene Expression
0
1
2
3
4
5
Low
Med
High
CtBP1&2 protein class

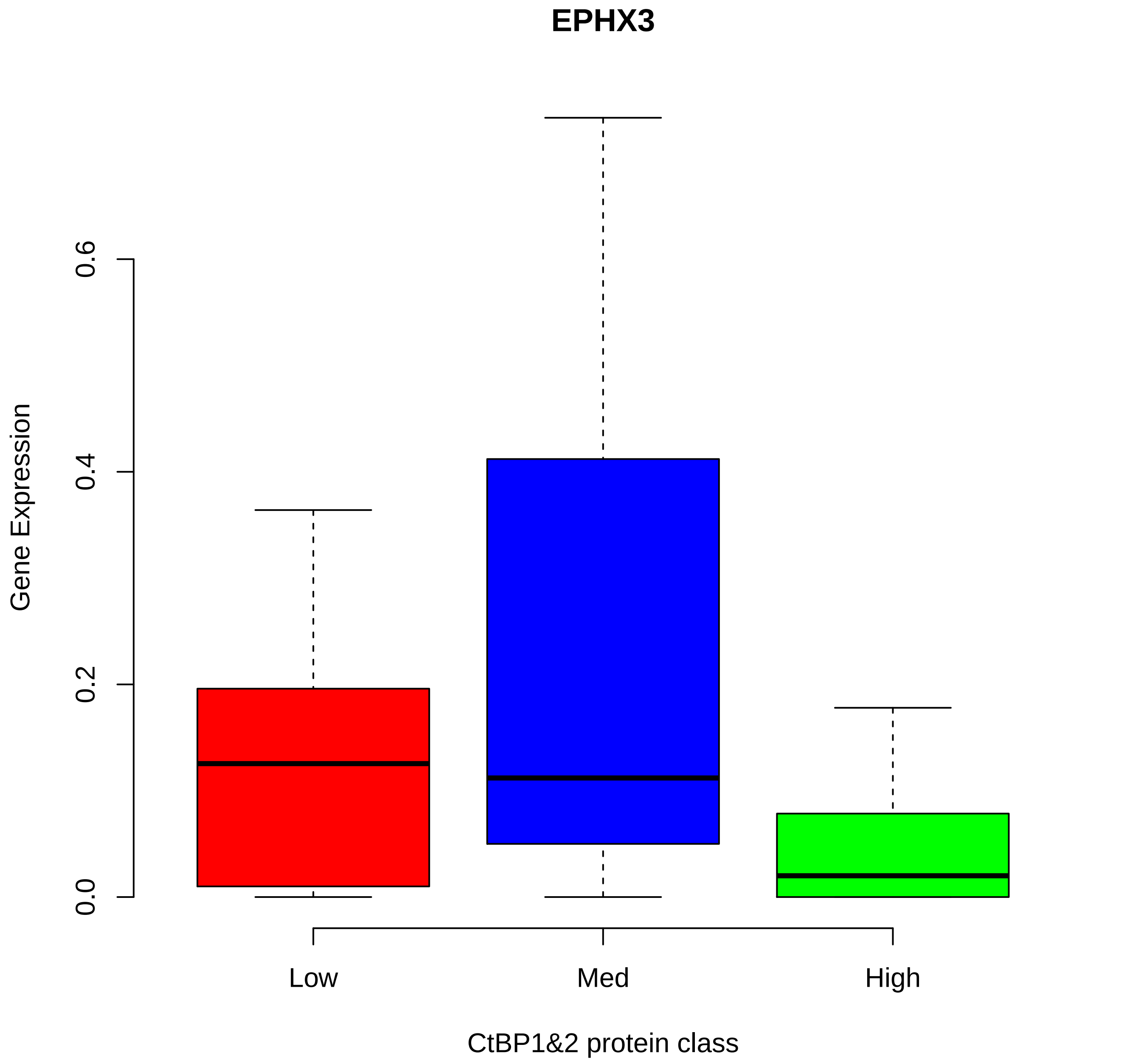

EPHX3
Gene Expression
0.0
0.2
0.4
0.6
Low
Med
High
CtBP1&2 protein class

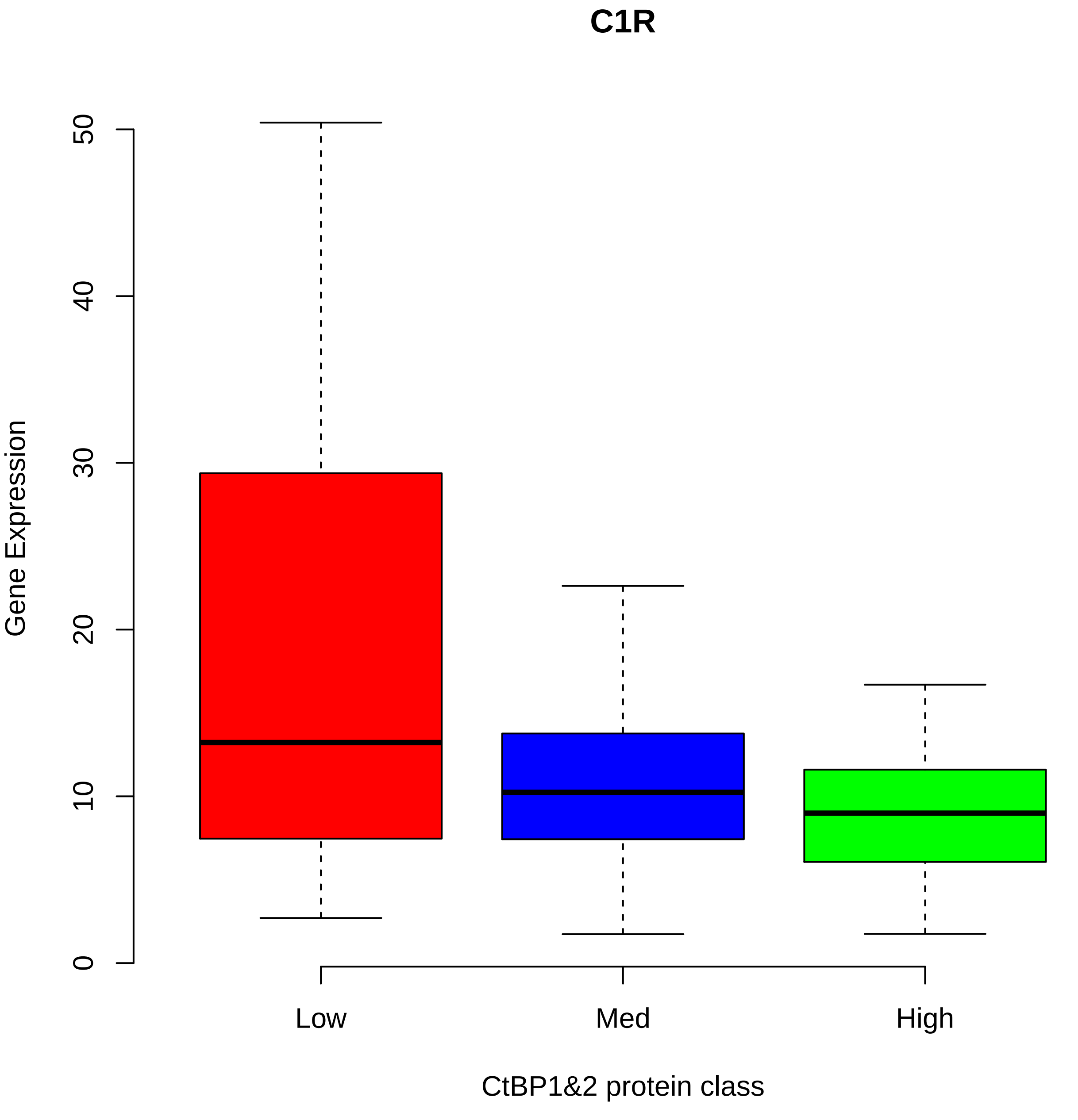

C1R
Gene Expression
0
10
20
30
40
50
Low
Med
High
CtBP1&2 protein class

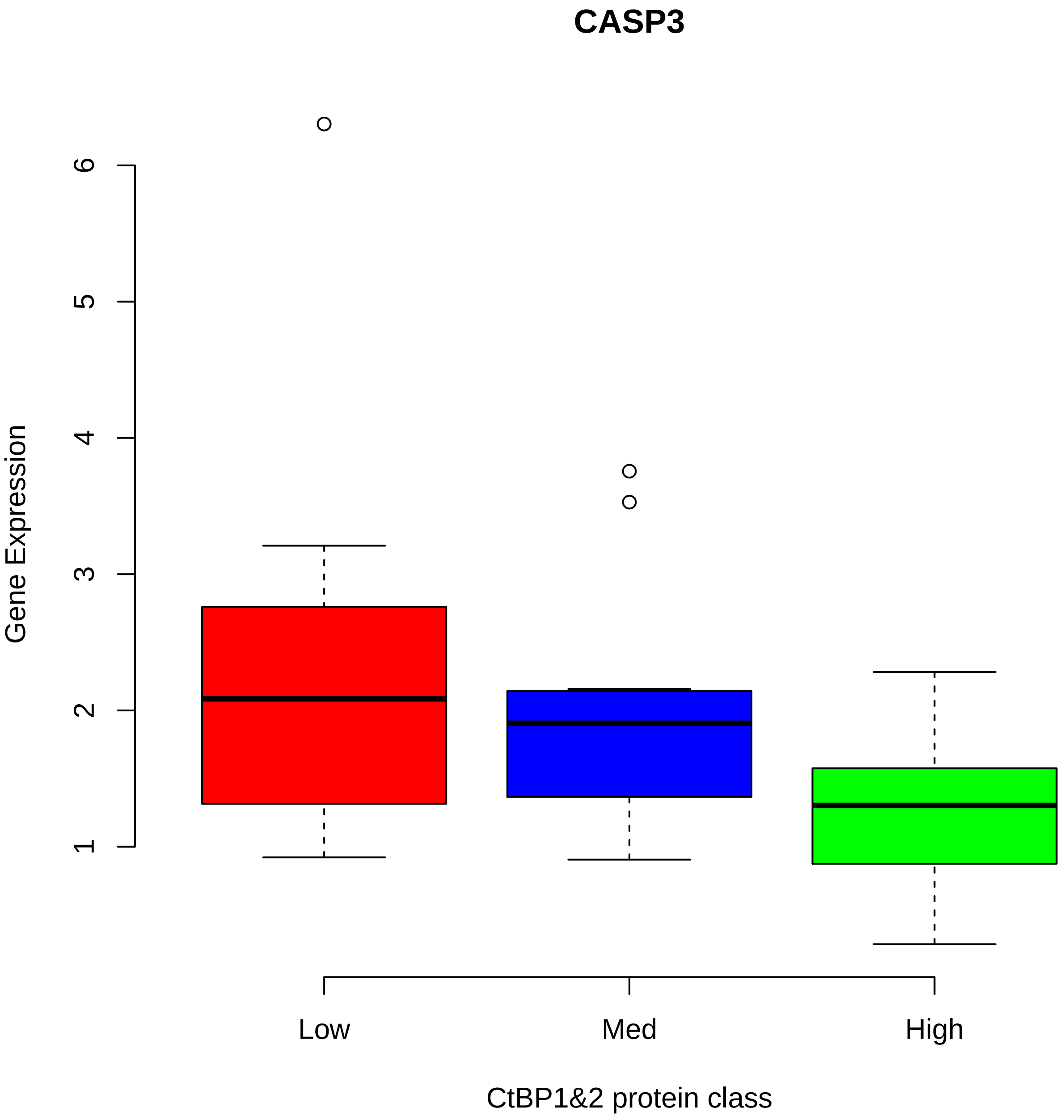
CASP3
Gene Expression
6
5
4
3
2
1
Low
Med
High
CtBP1&2 protein class

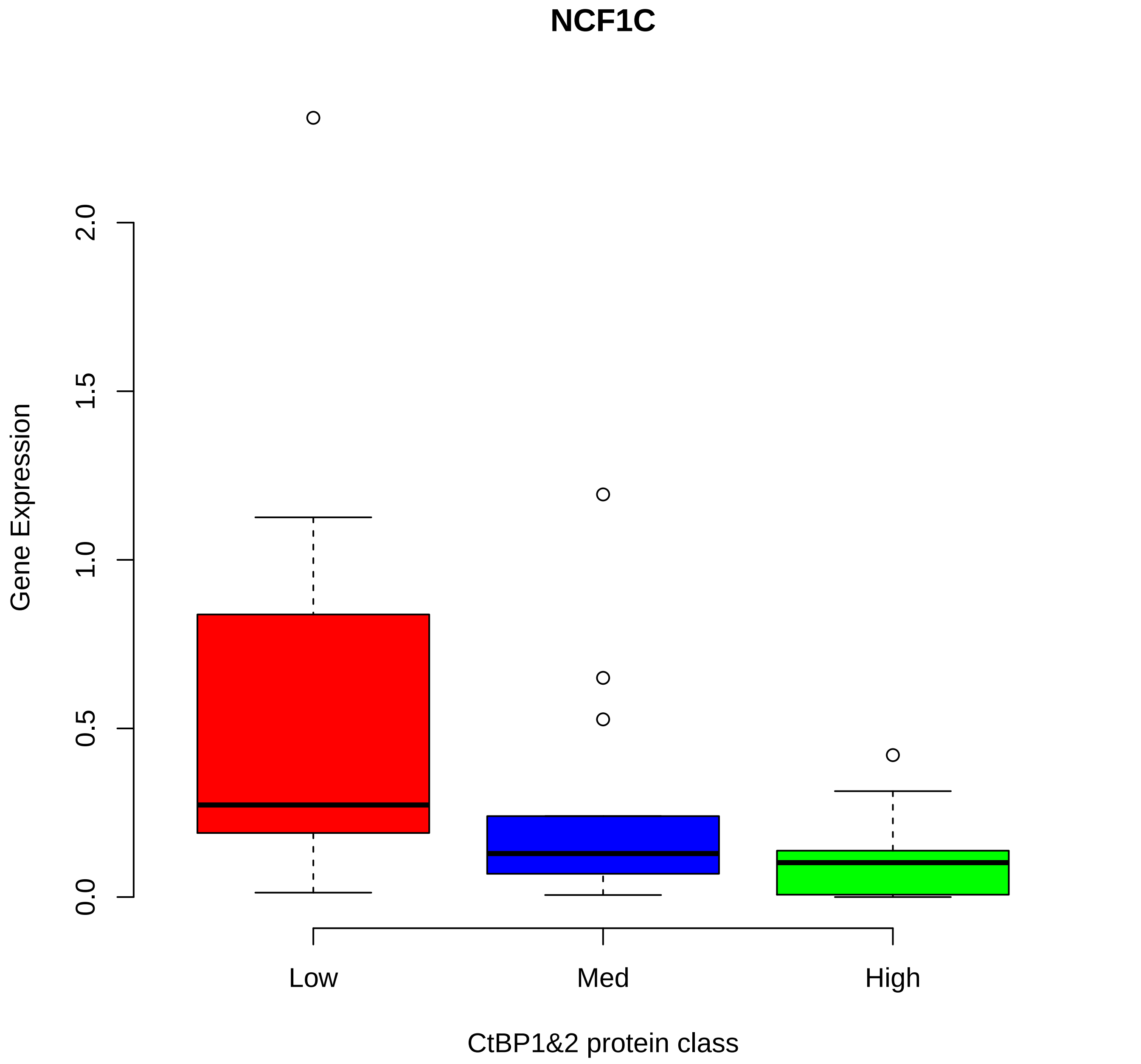

NCF1C
Gene Expression
0.0
0.5
1.0
1.5
2.0
Low
Med
High
CtBP1&2 protein class

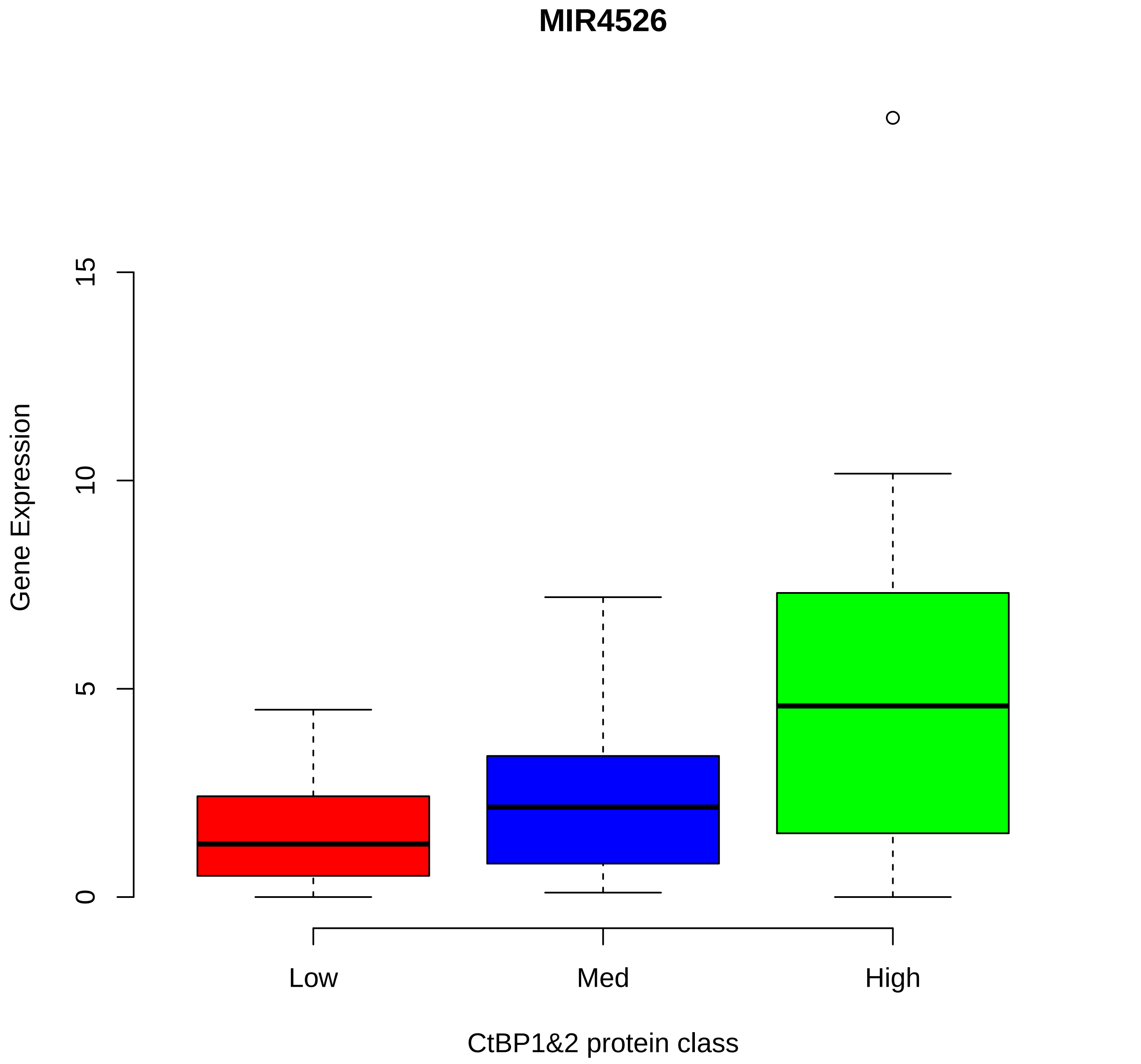

MIR4526
Gene Expression
0
5
10
15
Low
Med
High
CtBP1&2 protein class

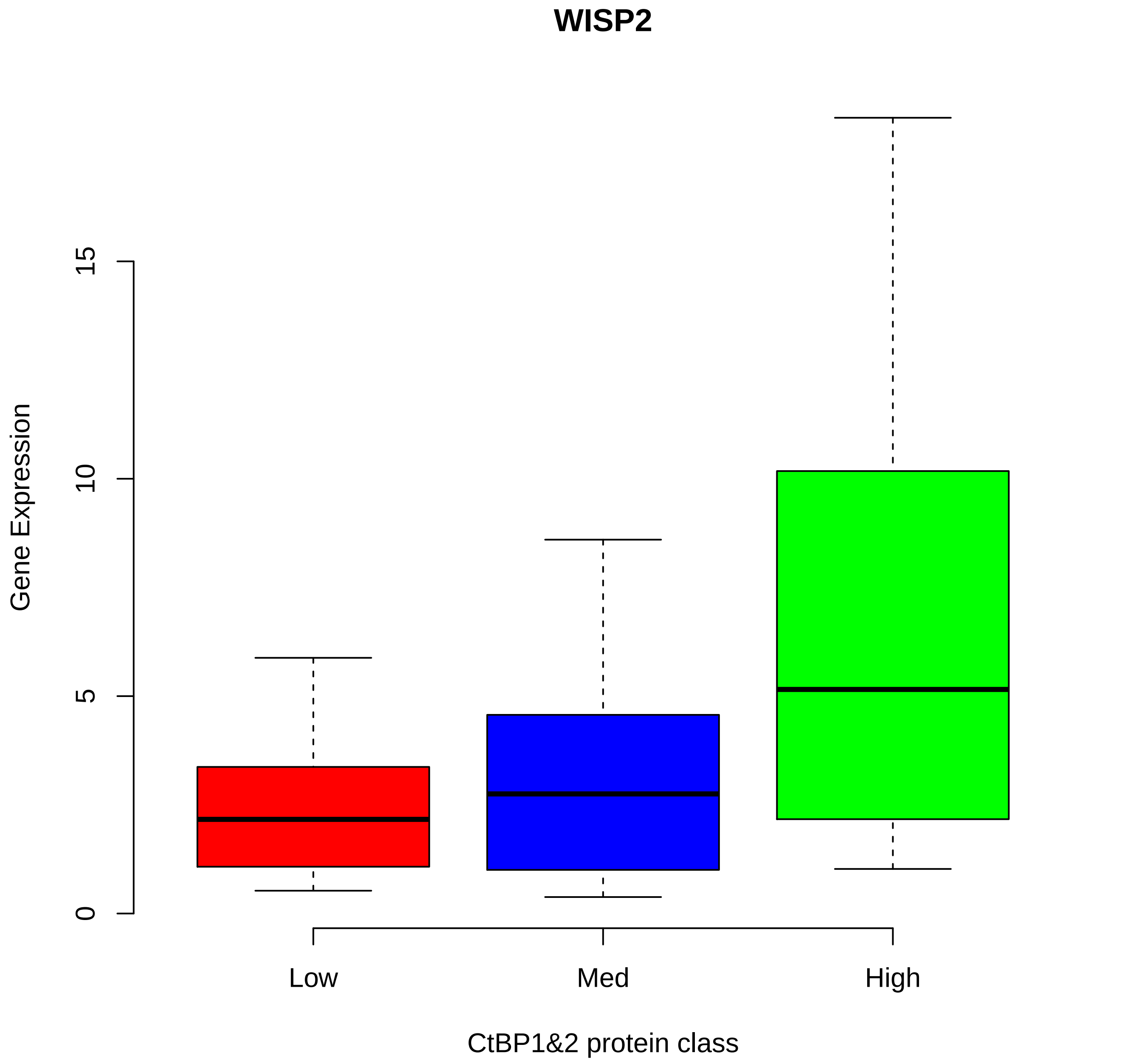

WISP2
Gene Expression
0
5
10
15
Low
Med
High
CtBP1&2 protein class

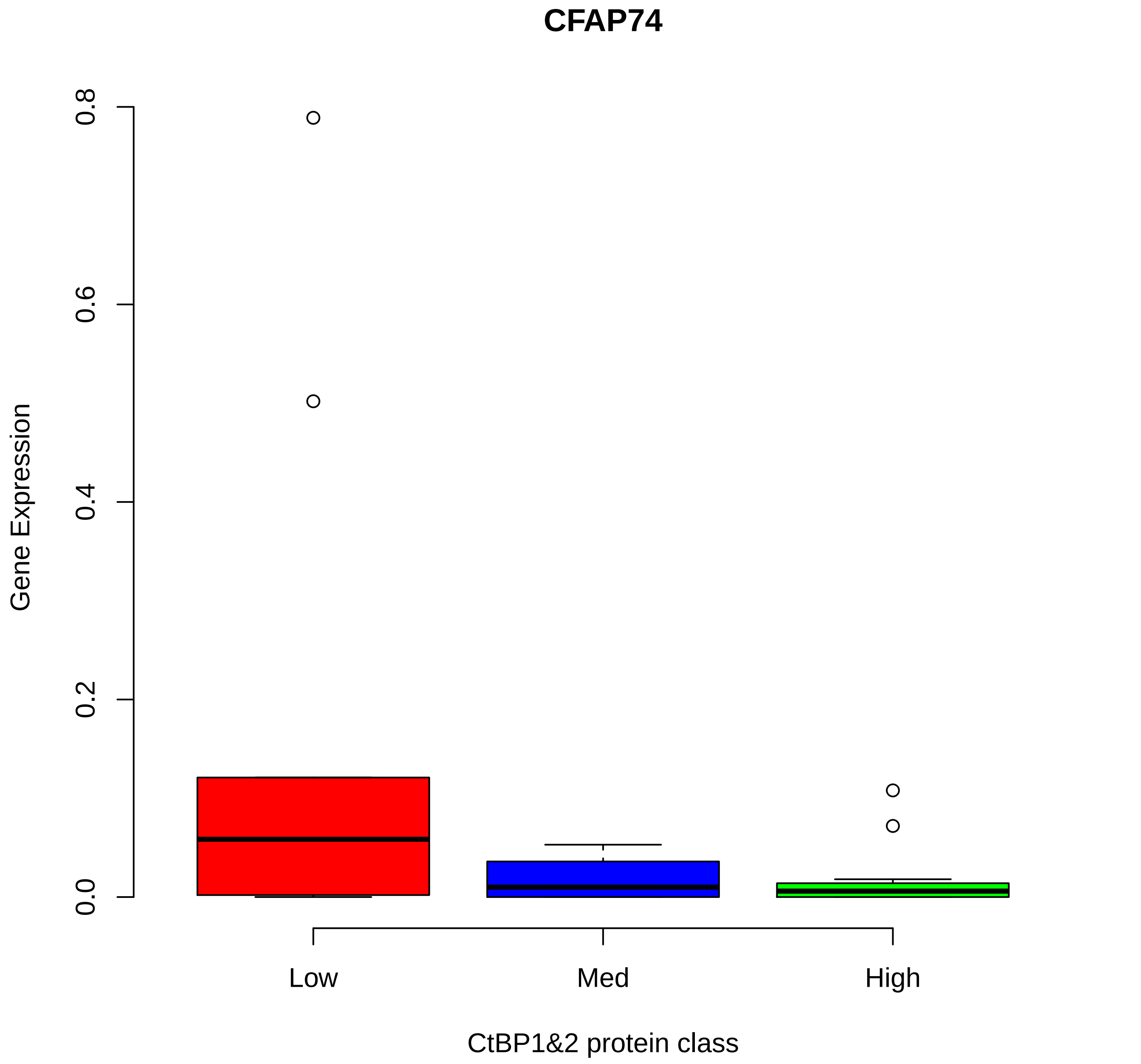
CFAP74
0.8
0.6
0.4
0.2
0.0
Gene Expression
Low
Med
High
CtBP1&2 protein class

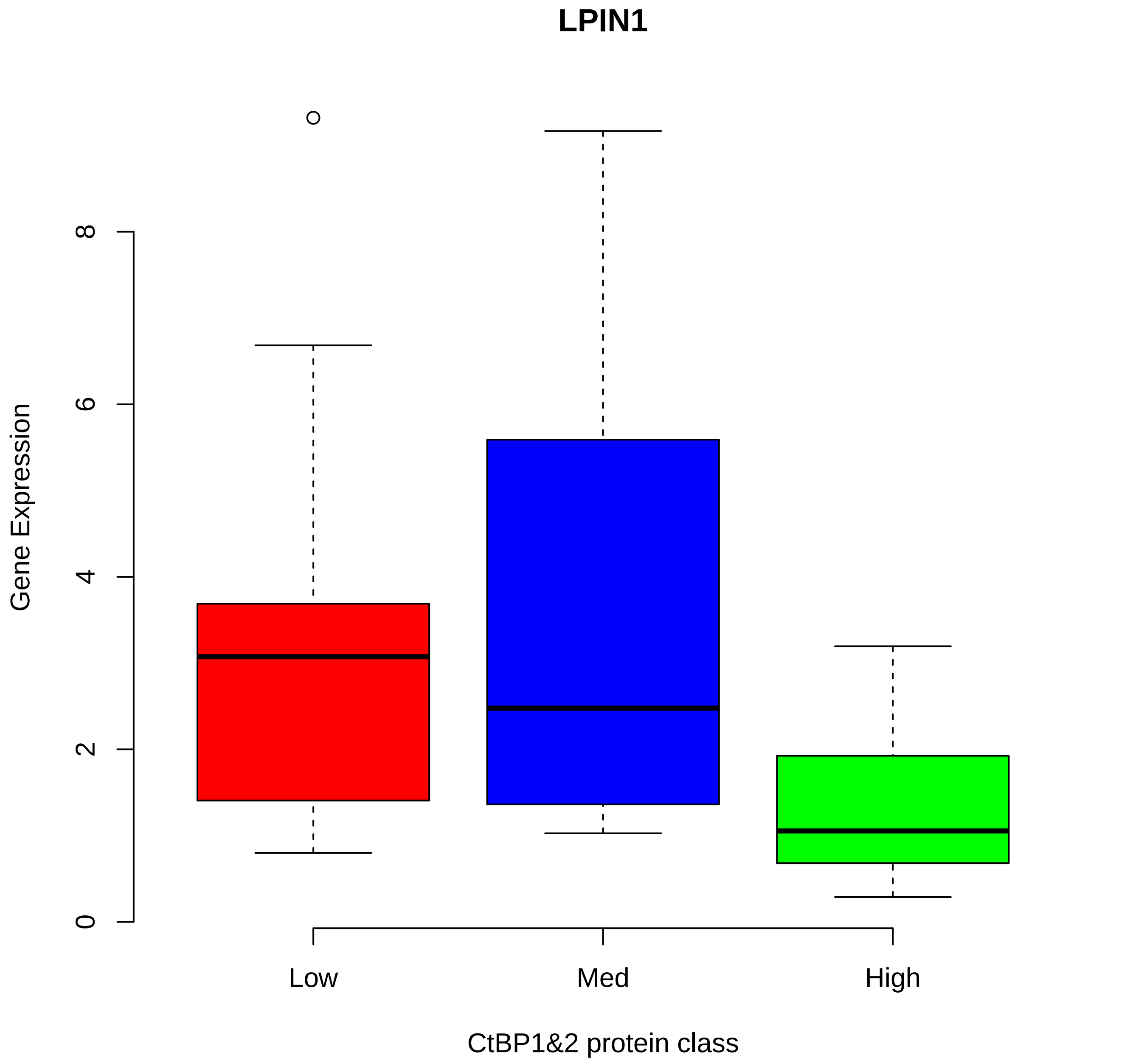

LPIN1
Gene Expression
0
2
4
6
8
Low
Med
High
CtBP1&2 protein class

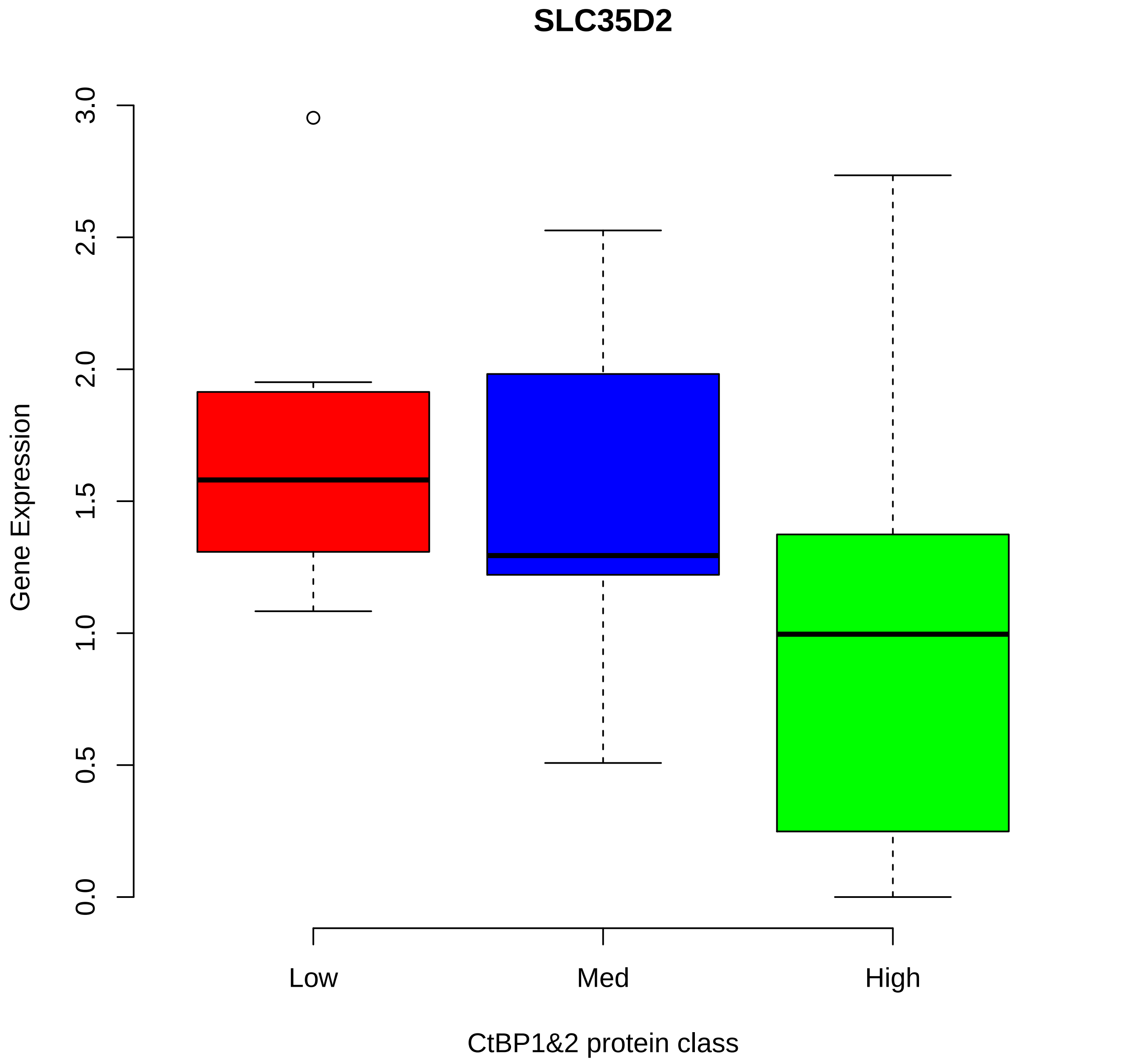

SLC35D2
Gene Expression
0.0
0.5
1.0
1.5
2.0
2.5
3.0
Low
Med
High
CtBP1&2 protein class

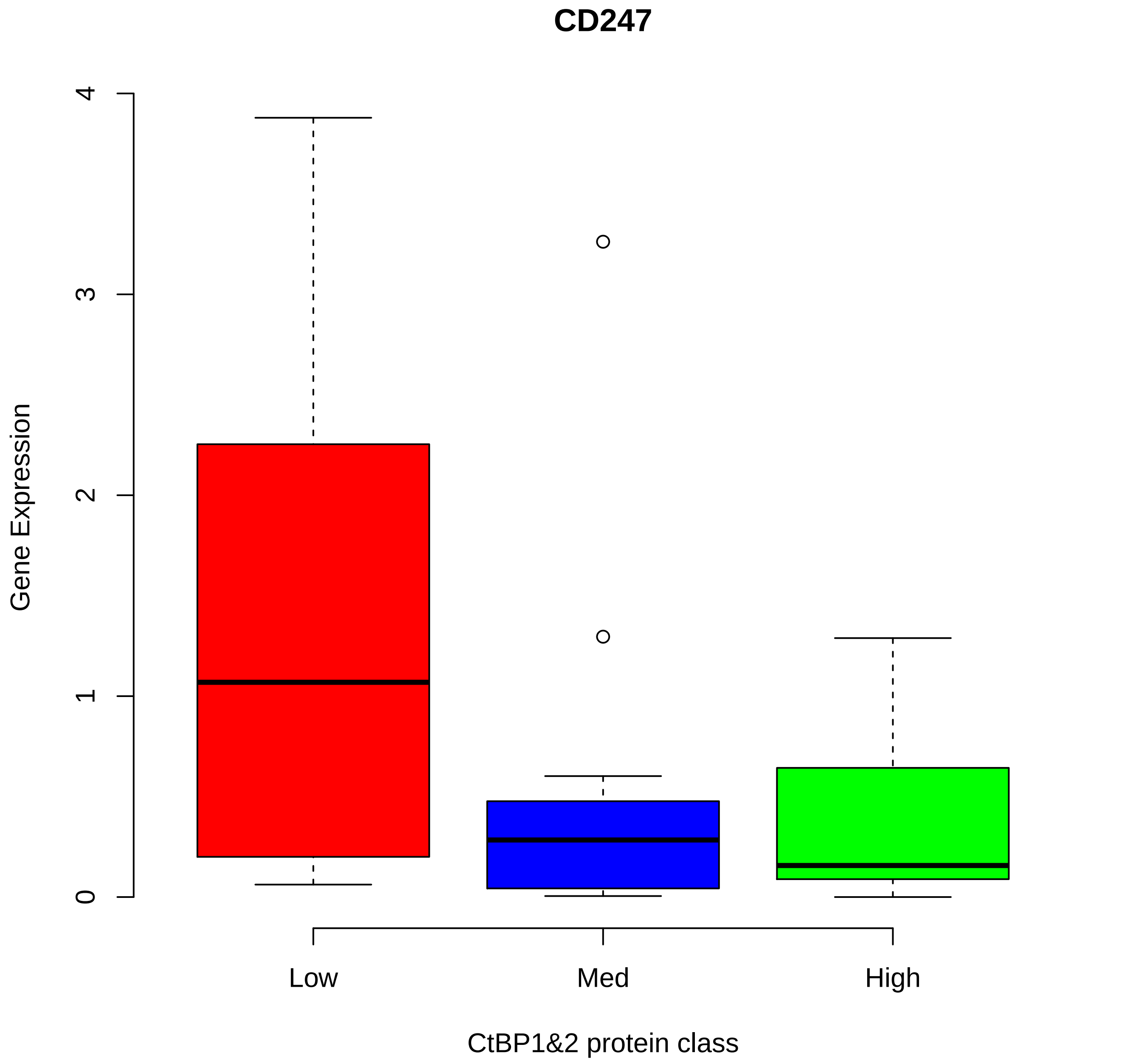
CD247
Gene Expression
0
1
2
3
4
Low
Med
High
CtBP1&2 protein class

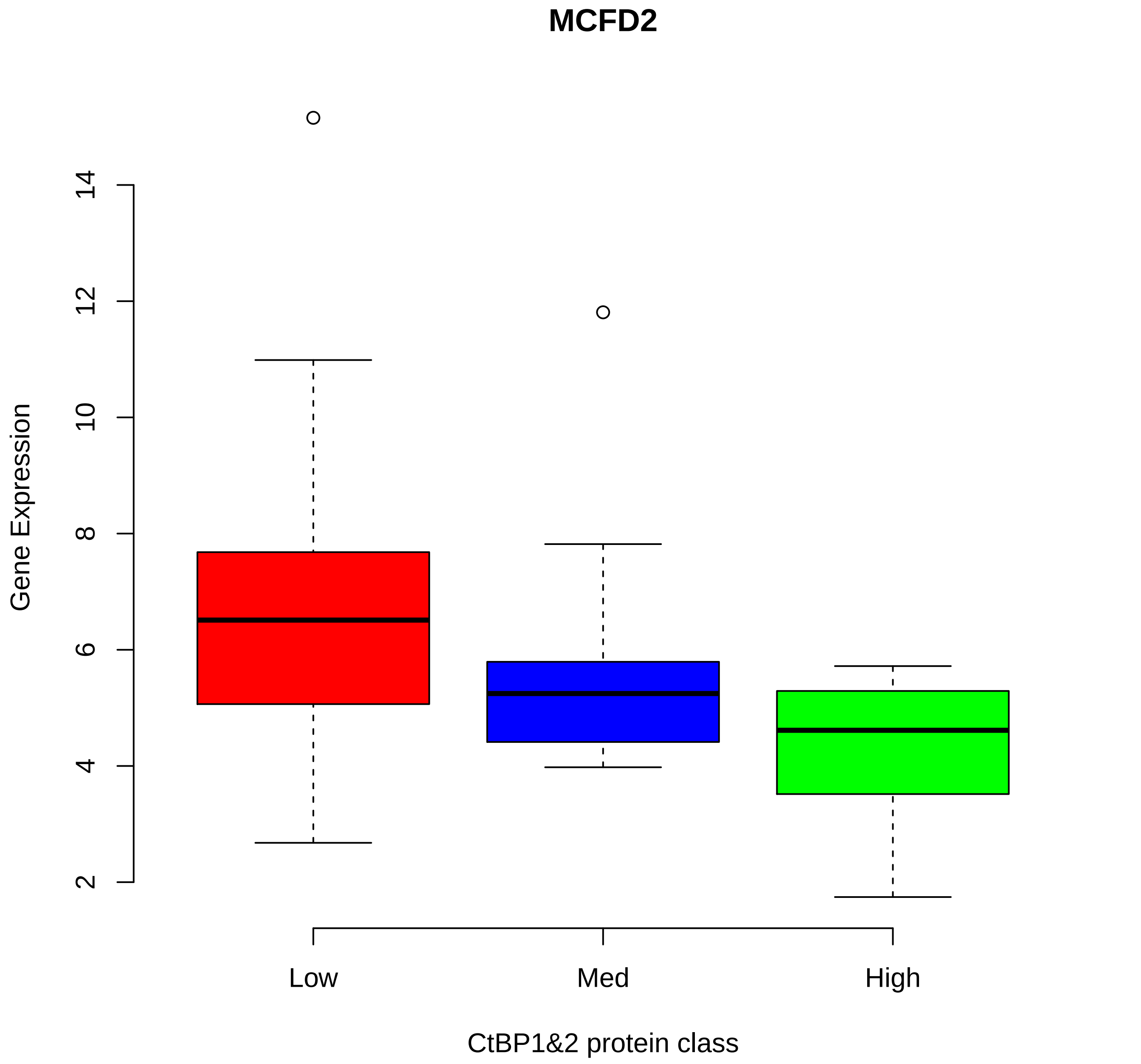

MCFD2
Gene Expression
2
4
6
8
10
12
14
Low
Med
High
CtBP1&2 protein class

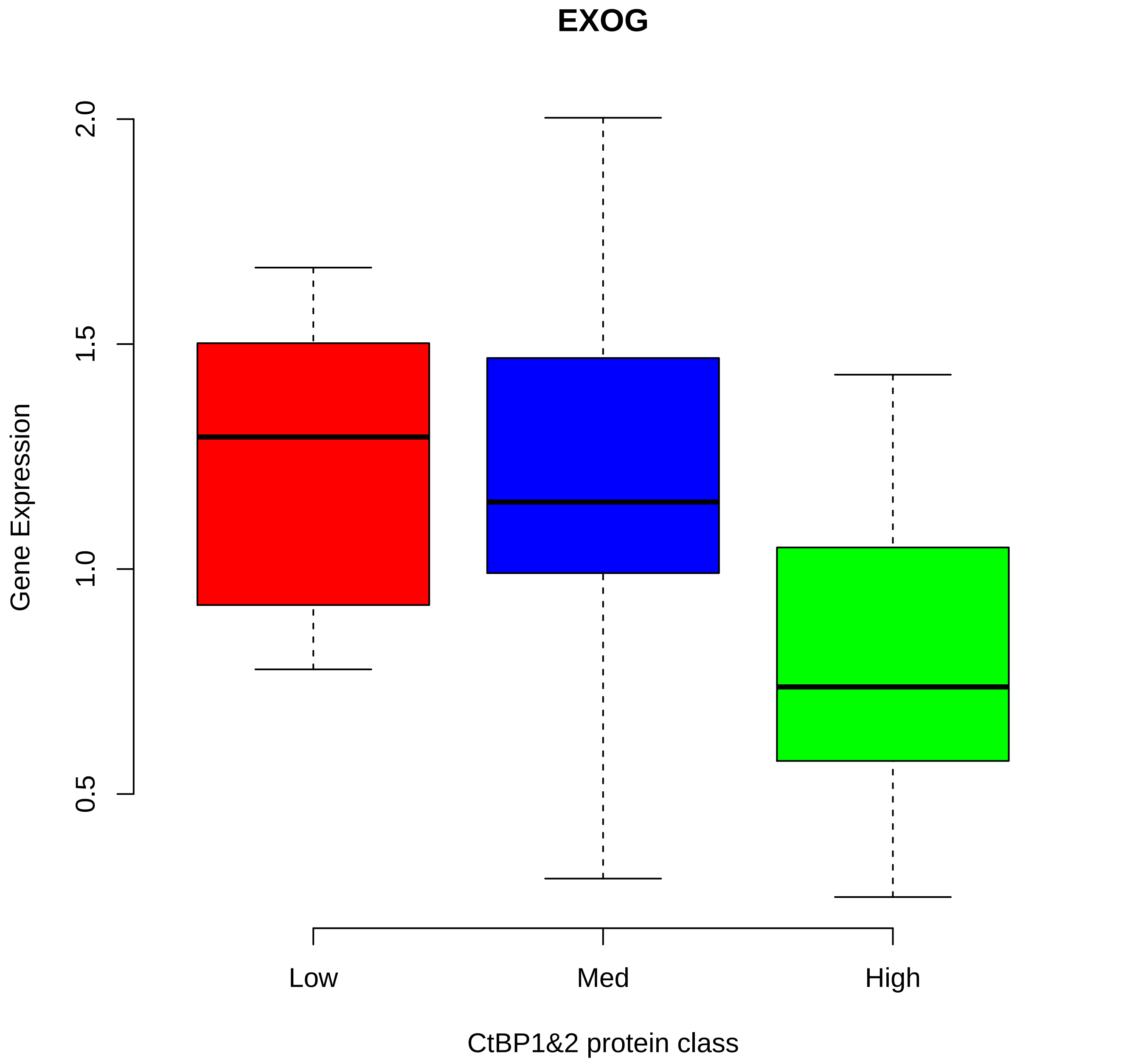
EXOG
Gene Expression
2.0
1.5
1.0
0.5
Low
Med
High
CtBP1&2 protein class

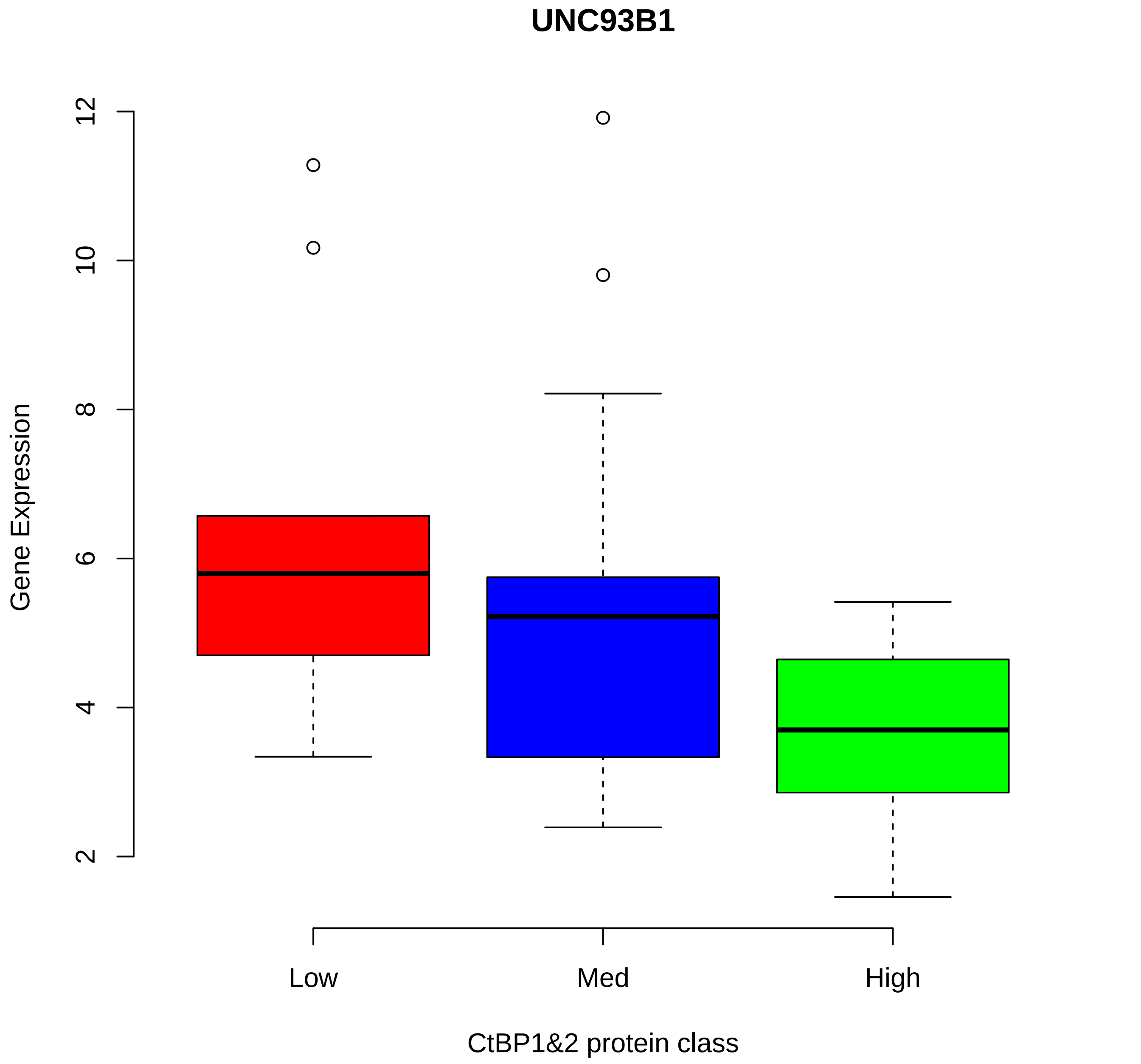
UNC93B1
Gene Expression
12
10
8
6
4
2
Low
Med
High
CtBP1&2 protein class

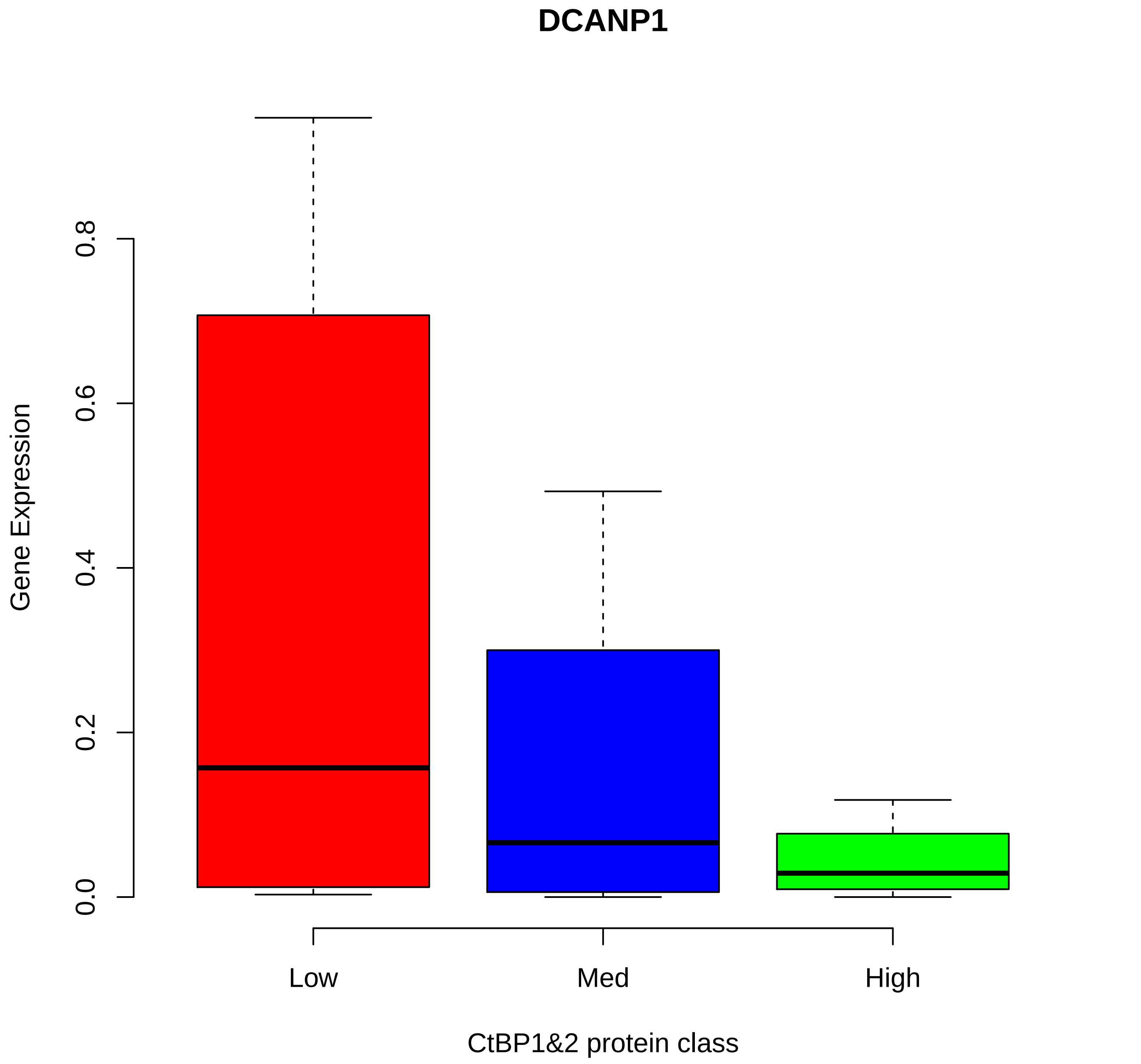
DCANP1
Gene Expression
0.0
0.2
0.4
0.6
0.8
Low
Med
High
CtBP1&2 protein class

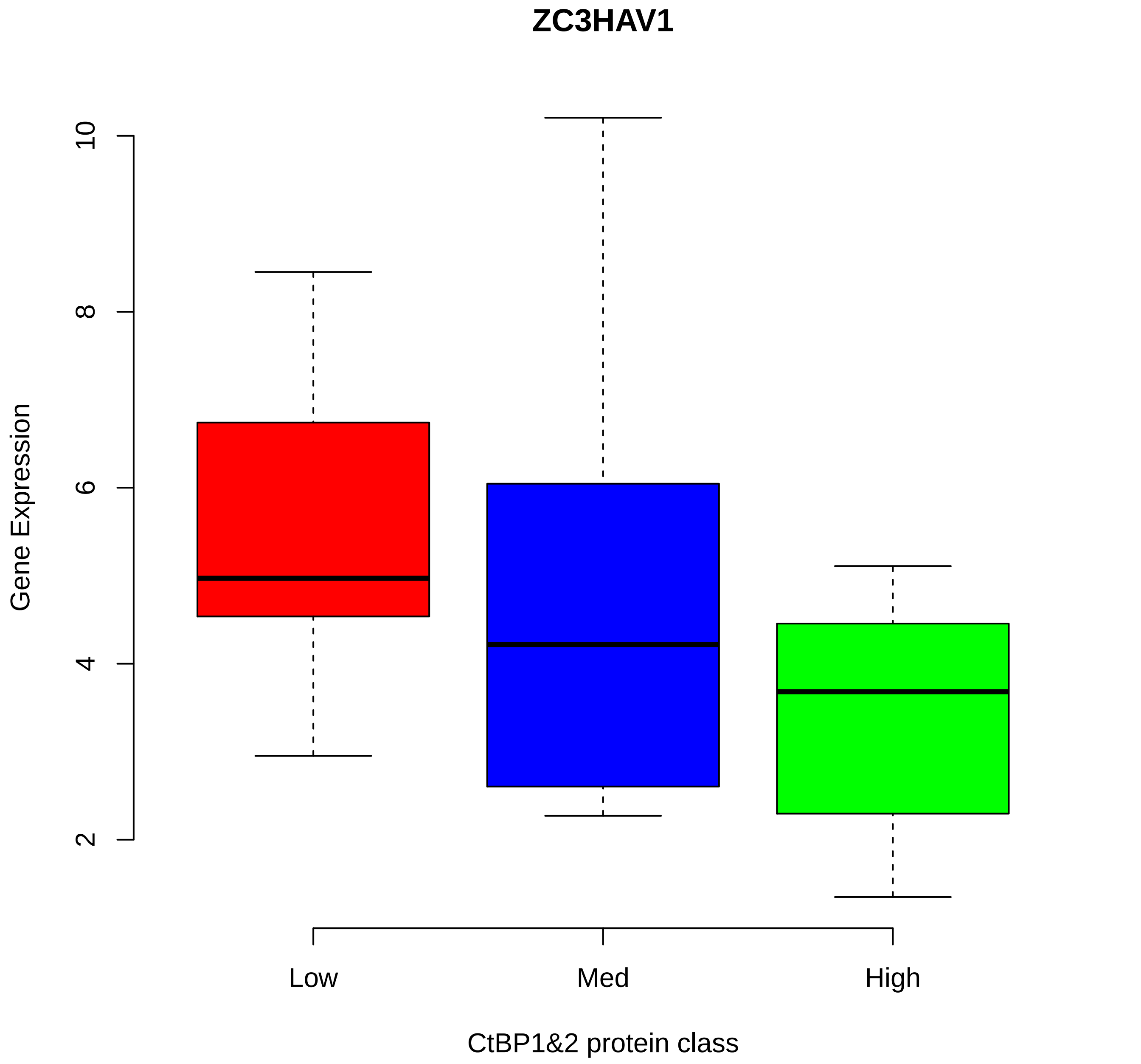
ZC3HAV1
Gene Expression
2
4
6
8
10
Low
Med
High
CtBP1&2 protein class

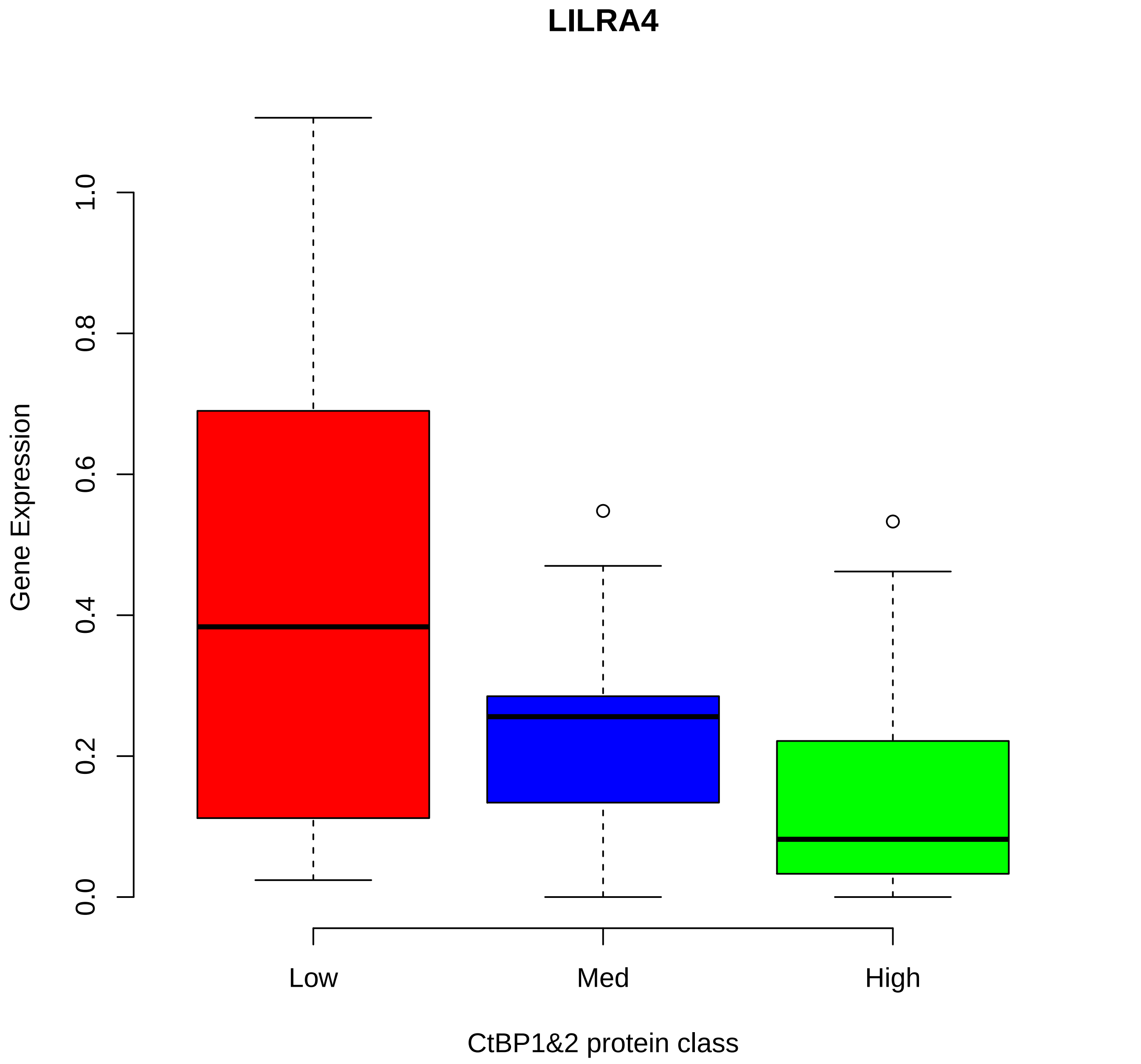
LILRA4
Gene Expression
0.0
0.2
0.4
0.6
0.8
1.0
Low
Med
High
CtBP1&2 protein class

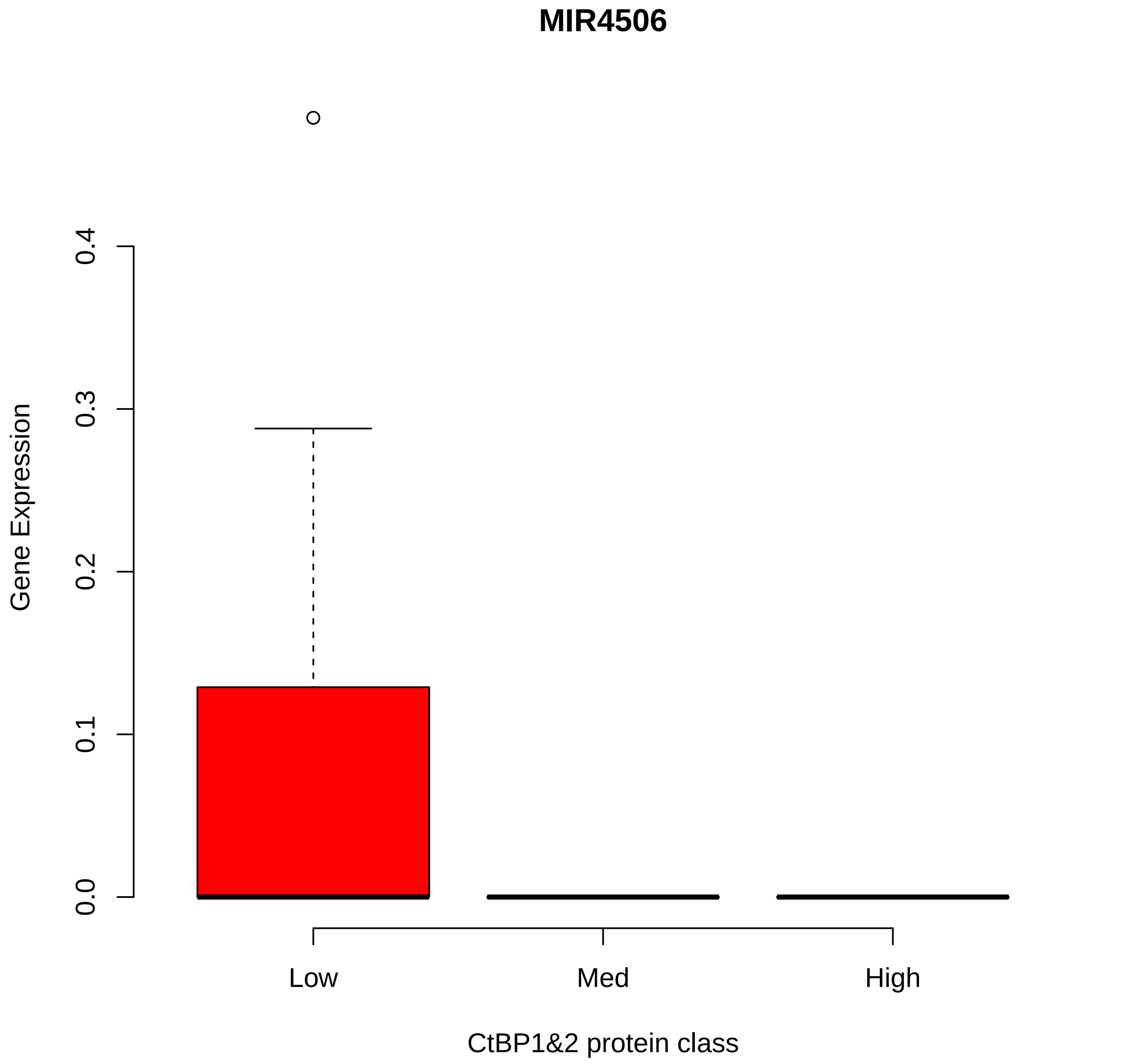
MIR4506
Gene Expression
0.0
0.1
0.2
0.3
0.4
Low
Med
High
CtBP1&2 protein class

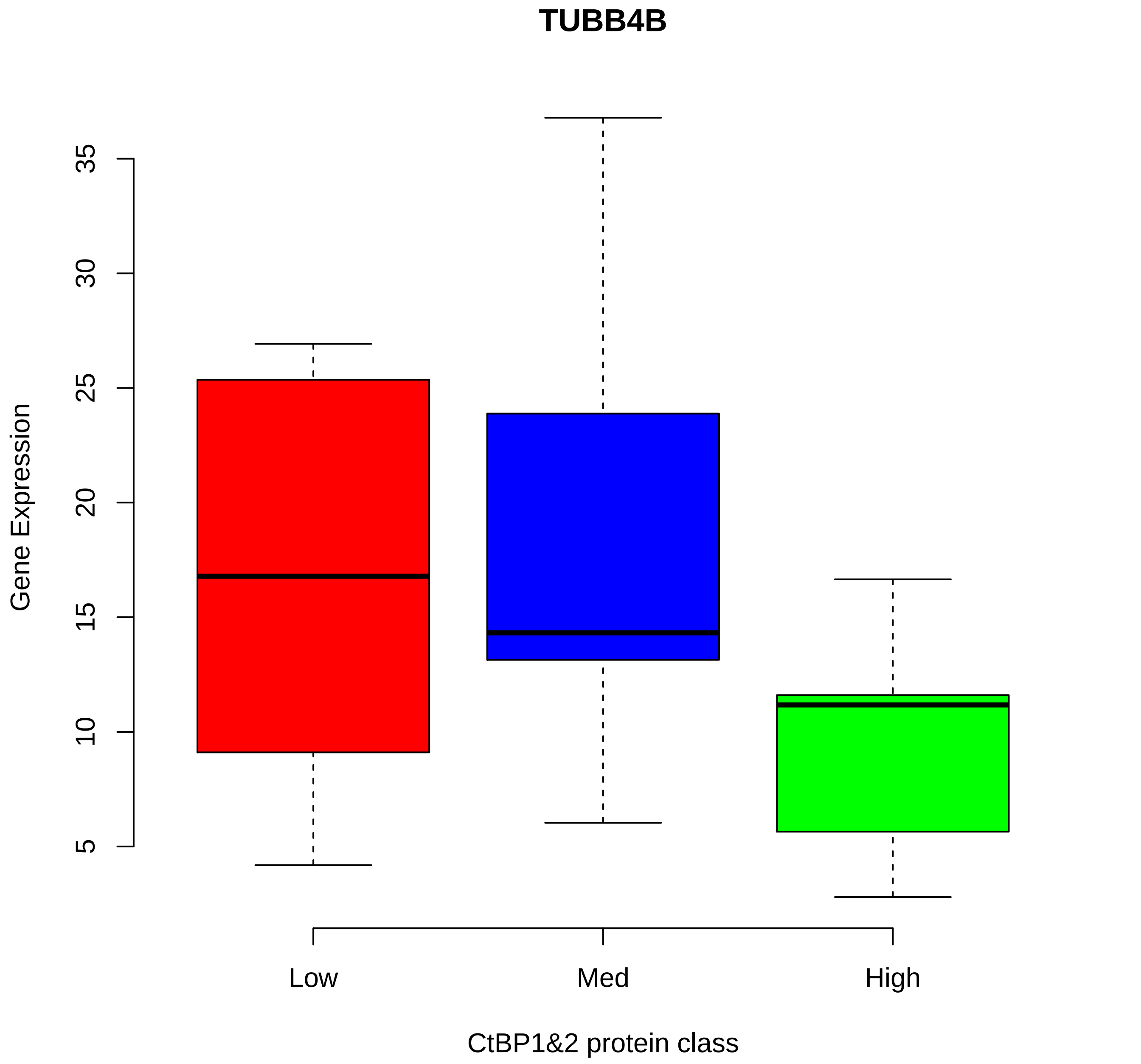

TUBB4B
Gene Expression
35
30
25
20
15
10
5
Low
Med
High
CtBP1&2 protein class

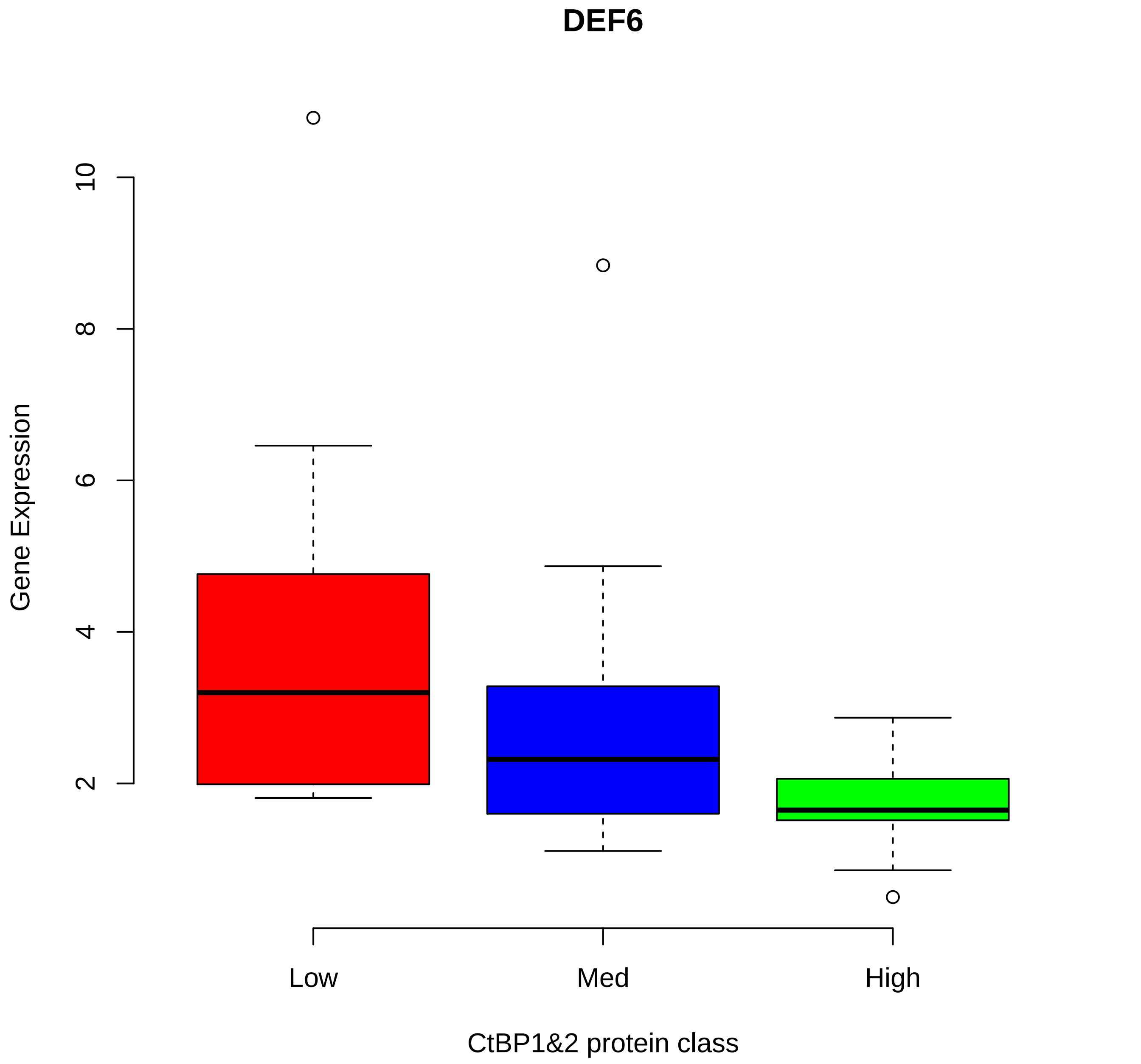
DEF6
Gene Expression
2
4
6
8
10
Low
Med
High
CtBP1&2 protein class

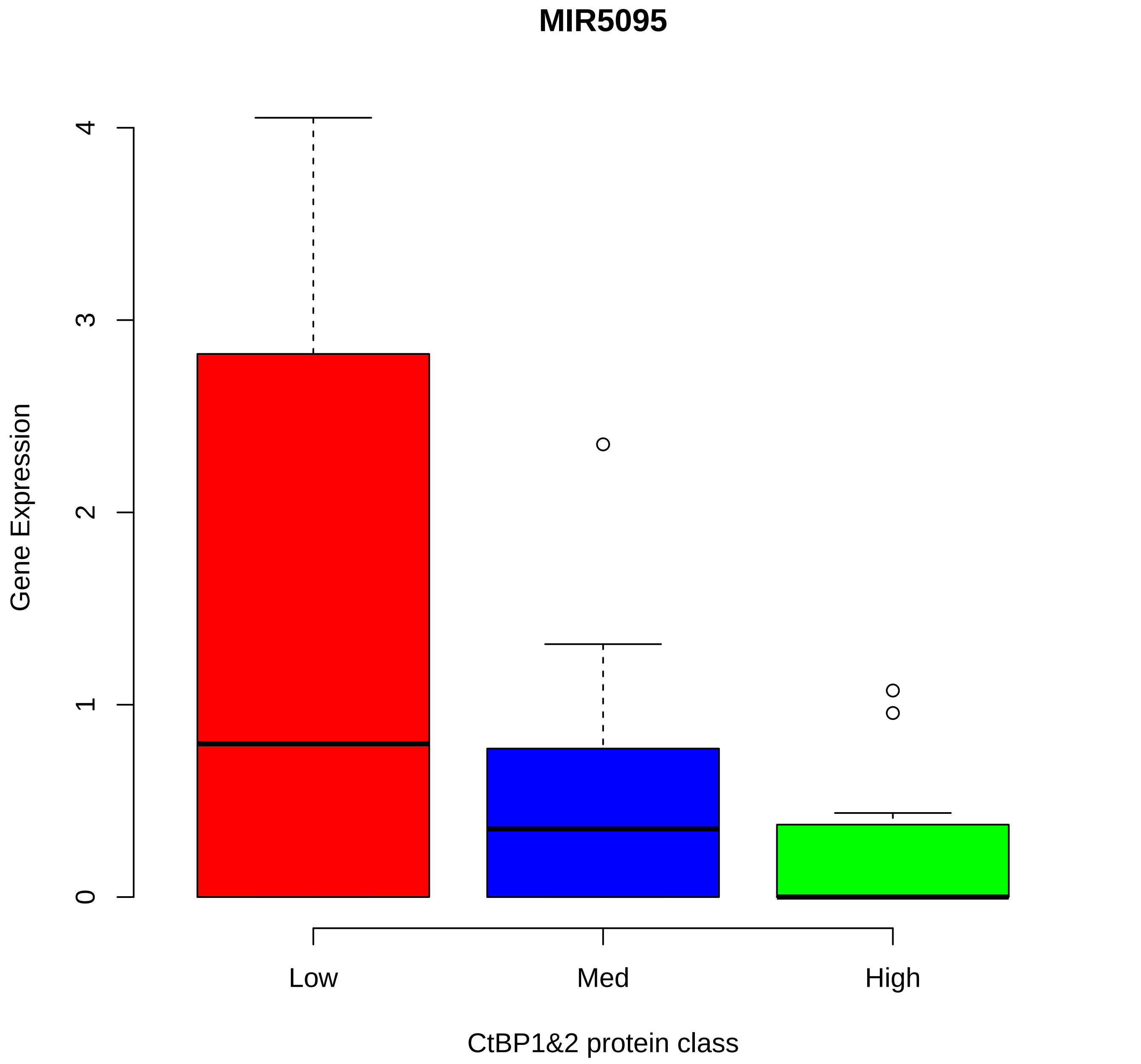
MIR5095
Gene Expression
0
1
2
3
4
Low
Med
High
CtBP1&2 protein class

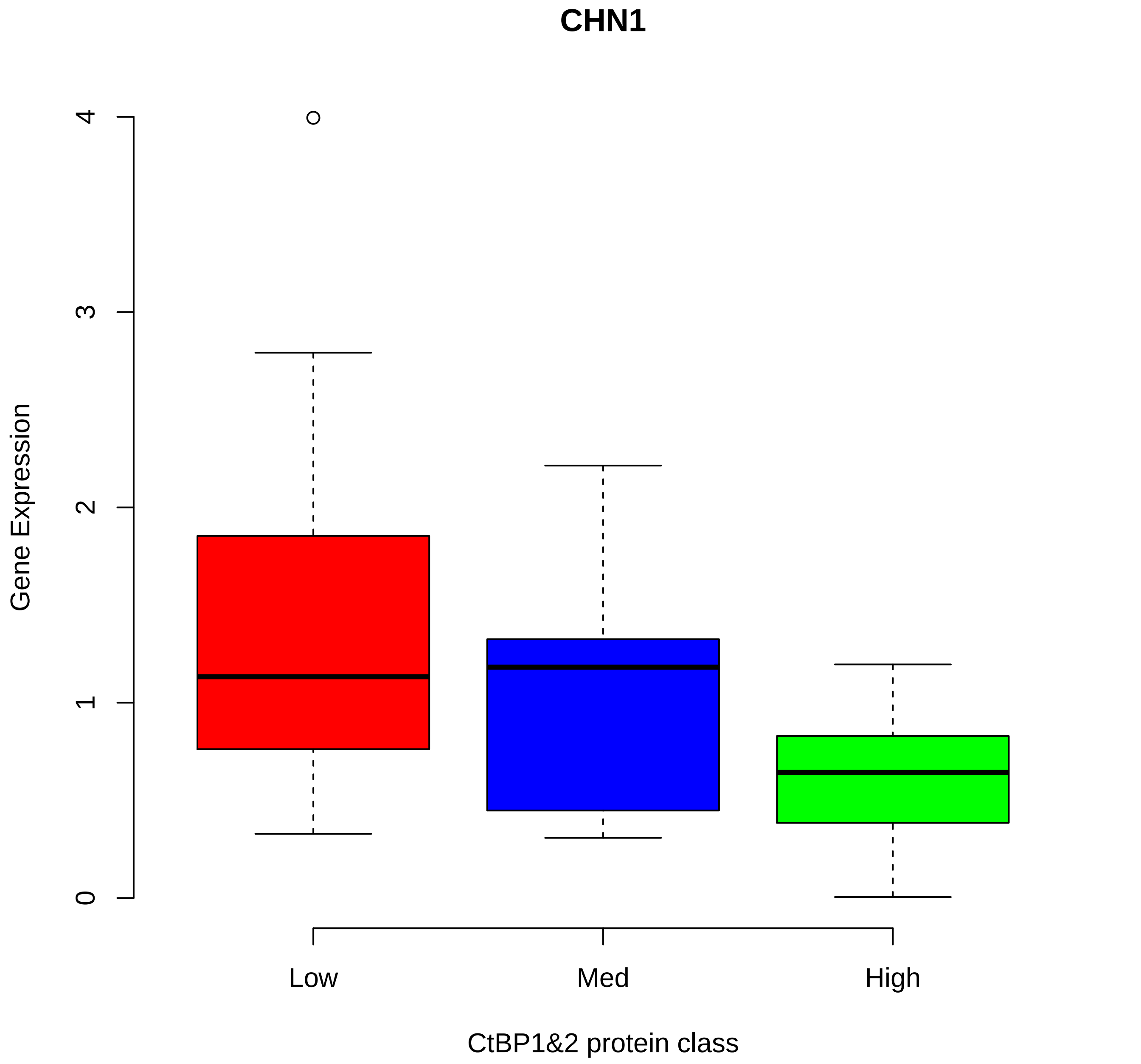

CHN1
Gene Expression
0
1
2
3
4
Low
Med
High
CtBP1&2 protein class

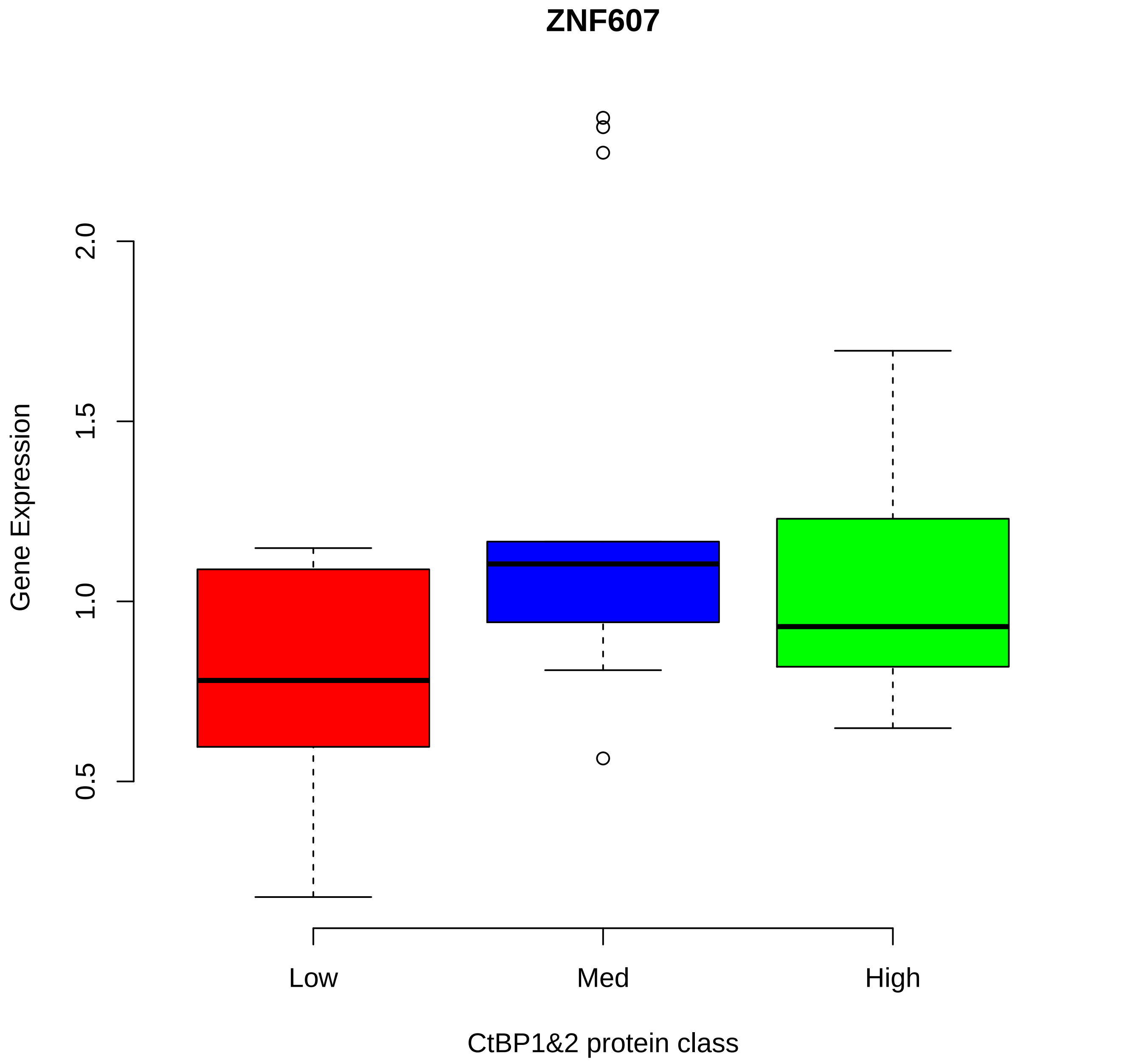
ZNF607
Gene Expression
2.0
1.5
1.0
0.5
Low
Med
High
CtBP1&2 protein class

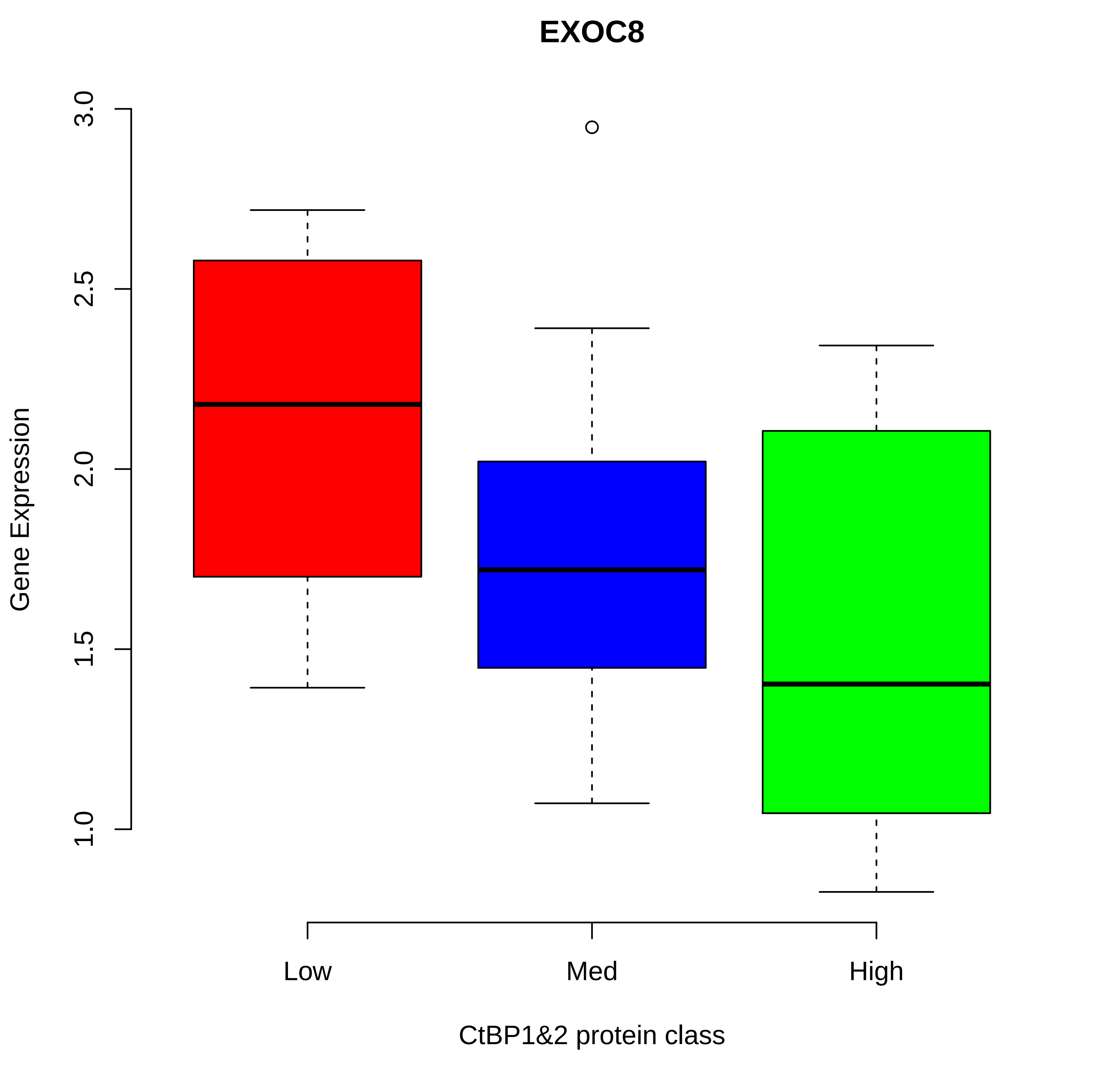
EXOC8
Gene Expression
3.0
2.5
2.0
1.5
1.0
Low
Med
High
CtBP1&2 protein class

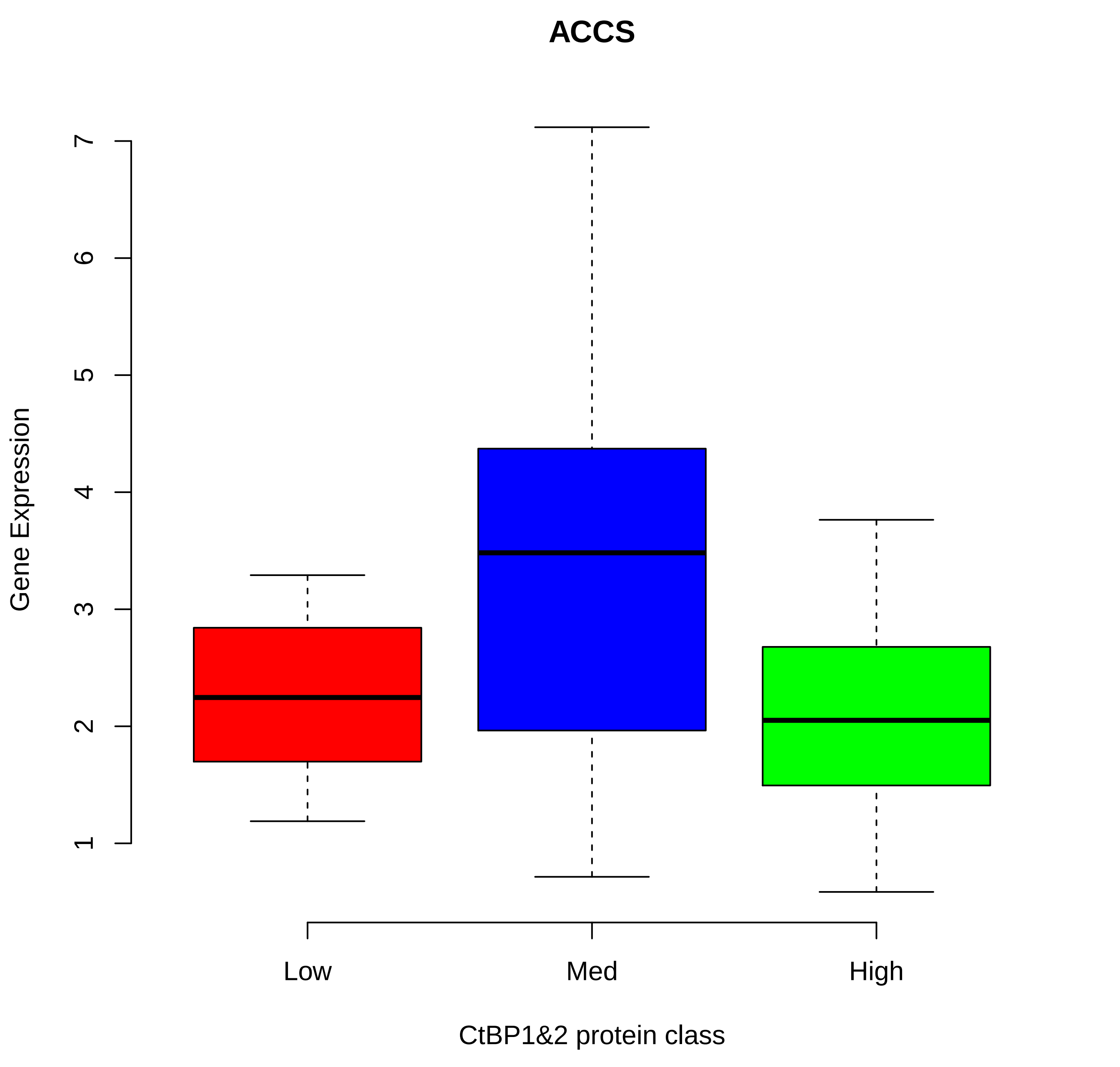
ACCS
Gene Expression
1
2
3
4
5
6
7
Low
Med
High
CtBP1&2 protein class

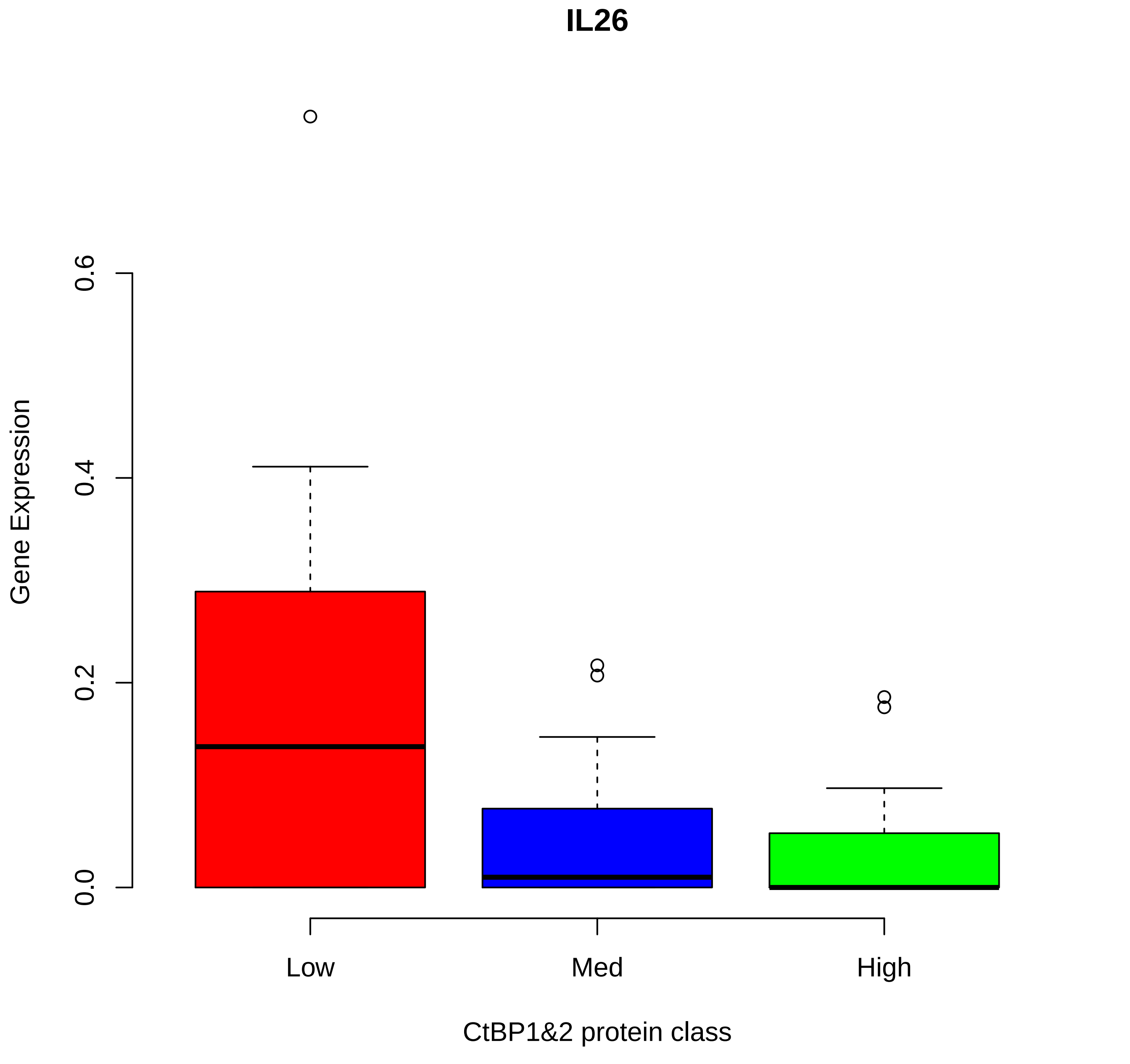
IL26
Gene Expression
0.0
0.2
0.4
0.6
Low
Med
High
CtBP1&2 protein class

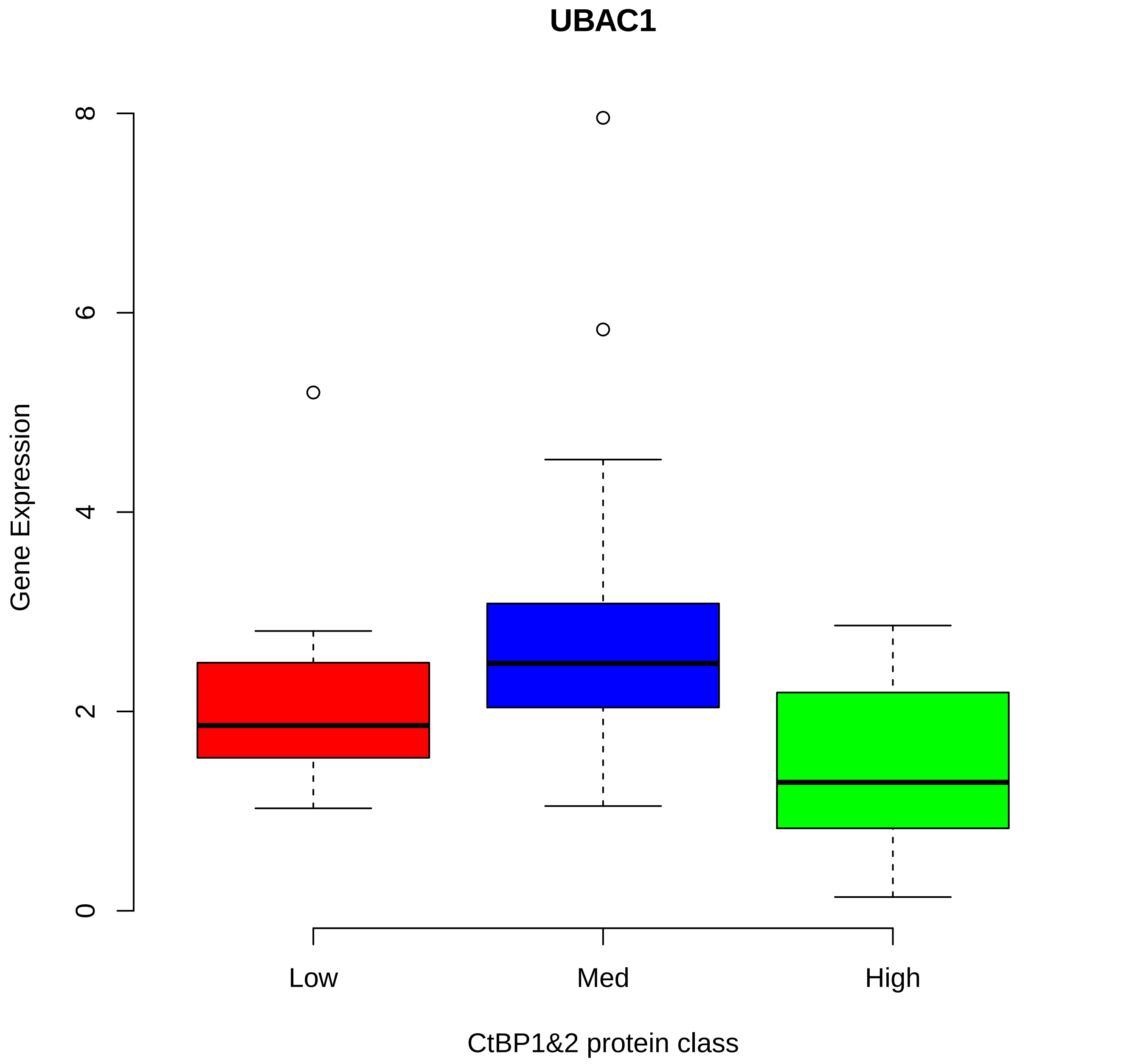
UBAC1
Gene Expression
0
2
4
6
8
Low
Med
High
CtBP1&2 protein class

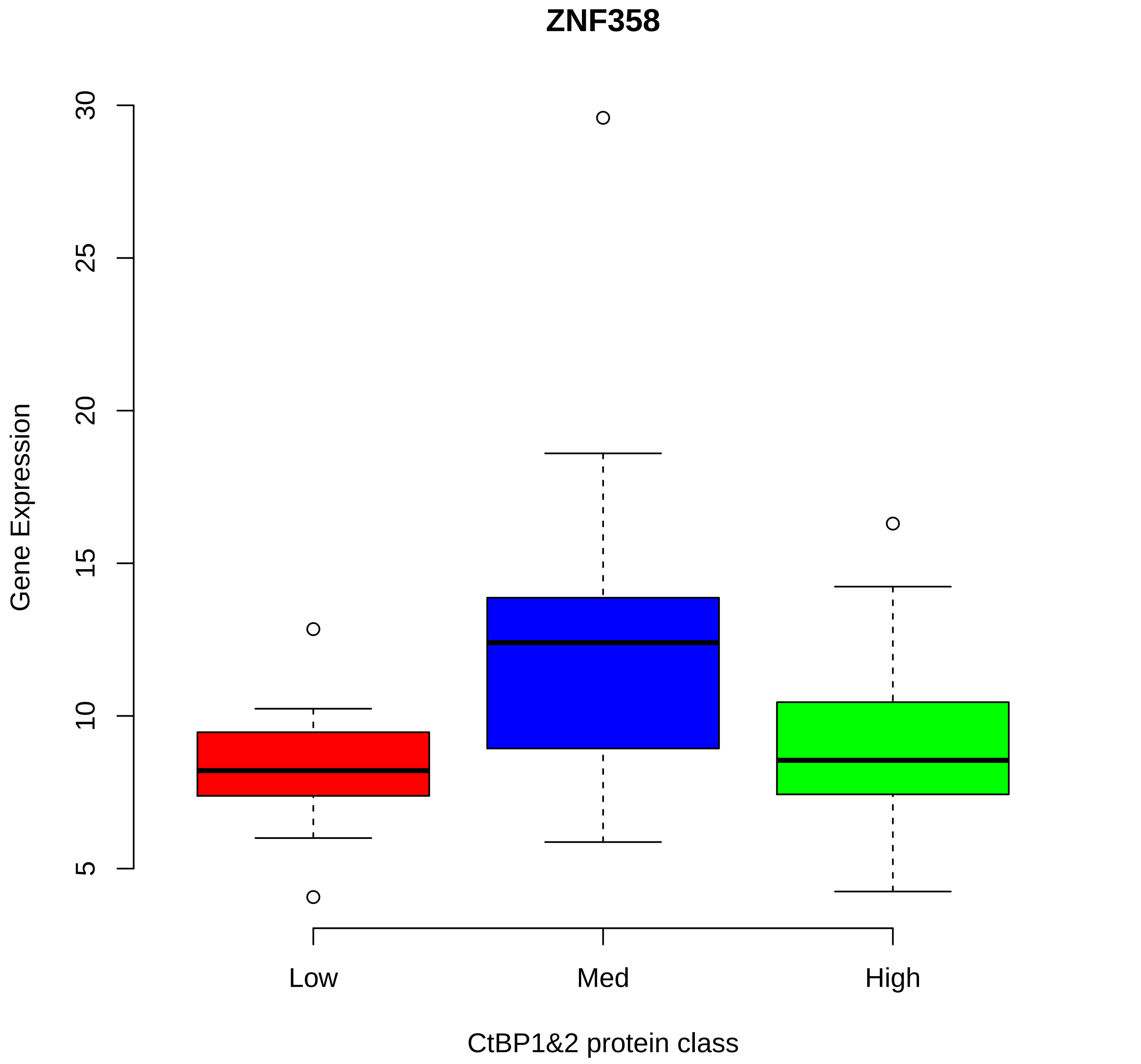
ZNF358
Gene Expression
30
25
20
15
10
5
Low
Med
High
CtBP1&2 protein class

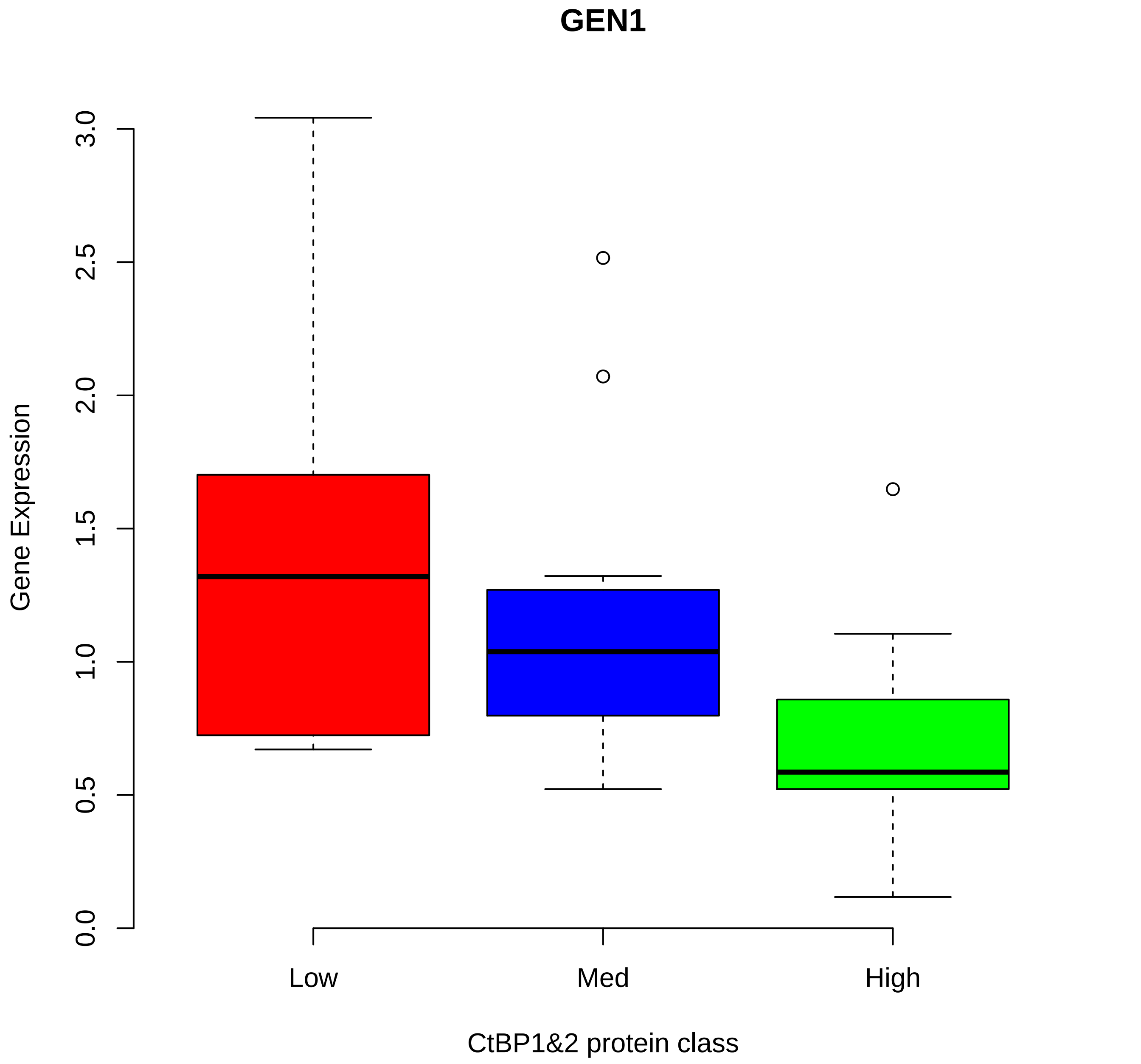
GEN1
Gene Expression
0.0
0.5
1.0
1.5
2.0
2.5
3.0
Low
Med
High
CtBP1&2 protein class

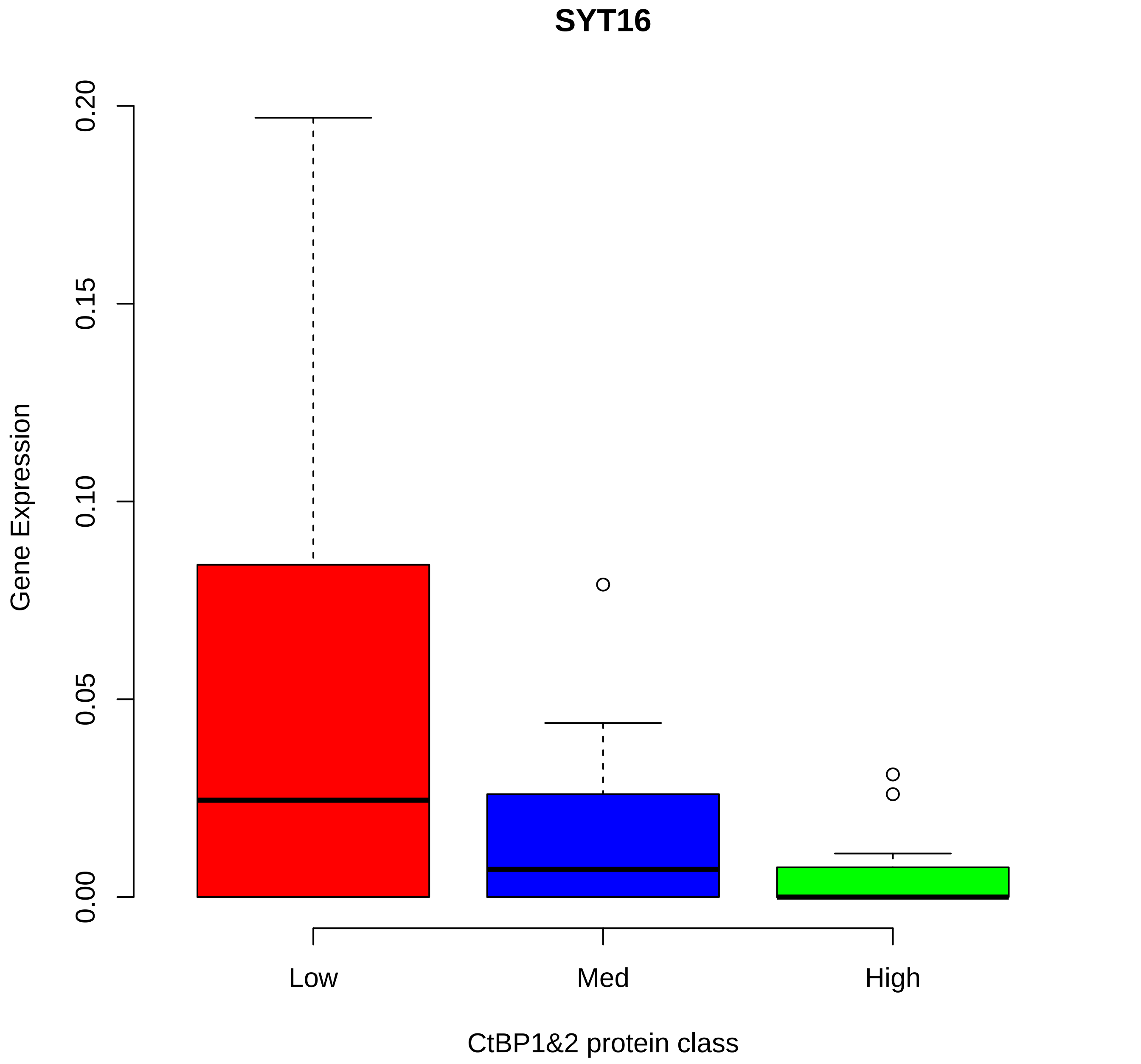
SYT16
Gene Expression
0.00
0.05
0.10
0.15
0.20
Low
Med
High
CtBP1&2 protein class

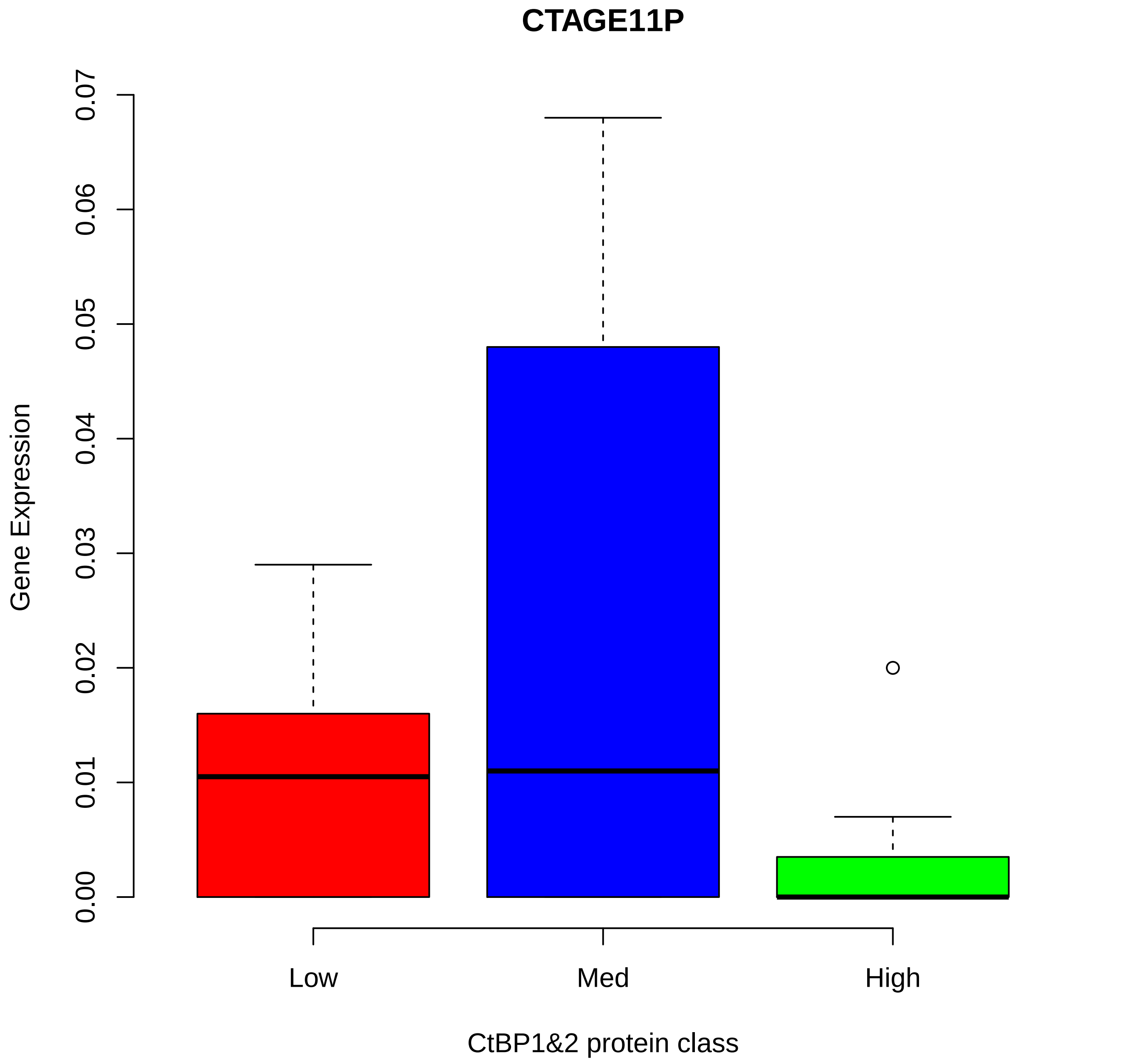

CTAGE11P
Gene Expression
0.00
0.01
0.02
0.03
0.04
0.05
0.06
0.07
Low
Med
High
CtBP1&2 protein class

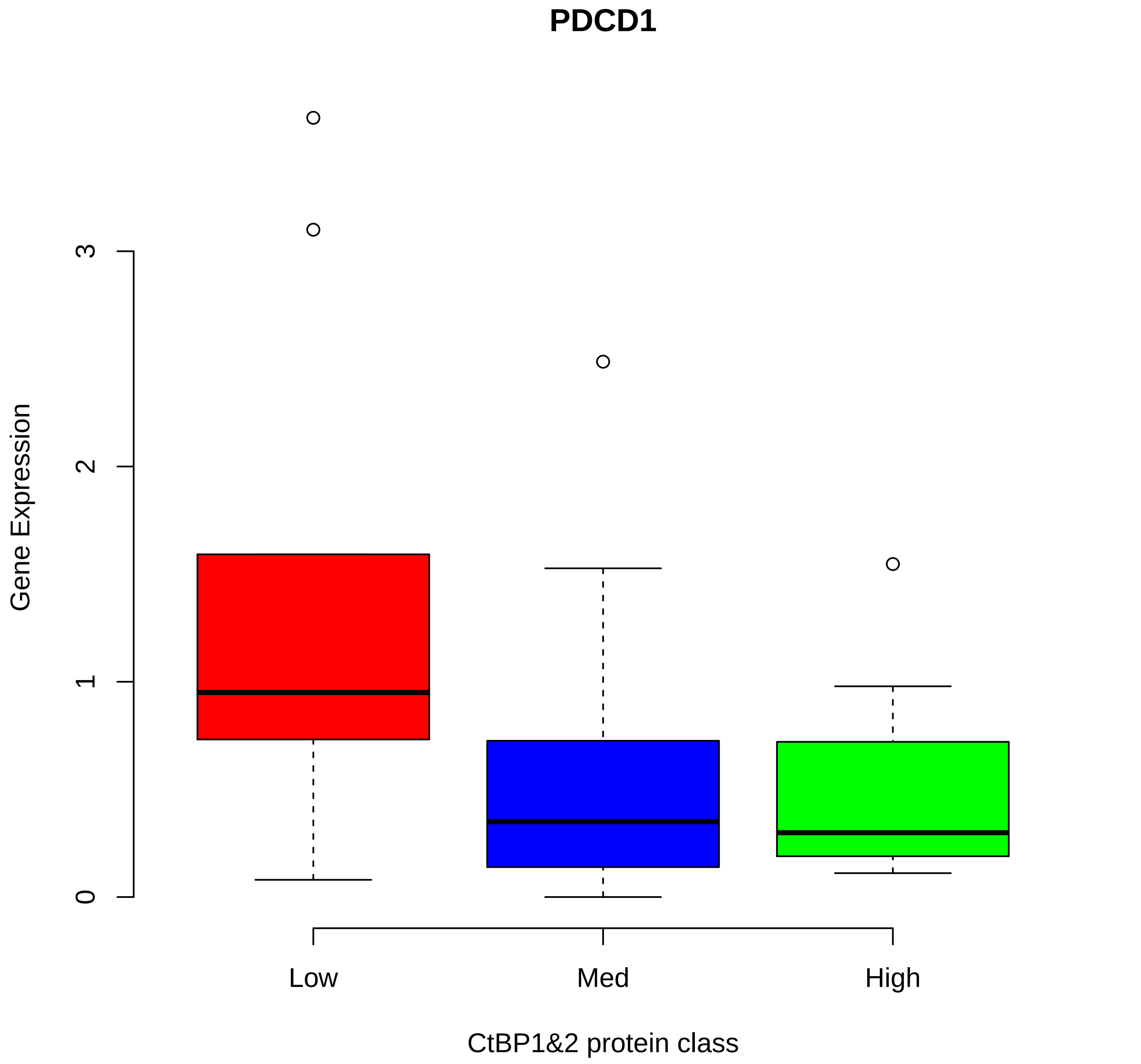
PDCD1
Gene Expression
0
1
2
3
Low
Med
High
CtBP1&2 protein class

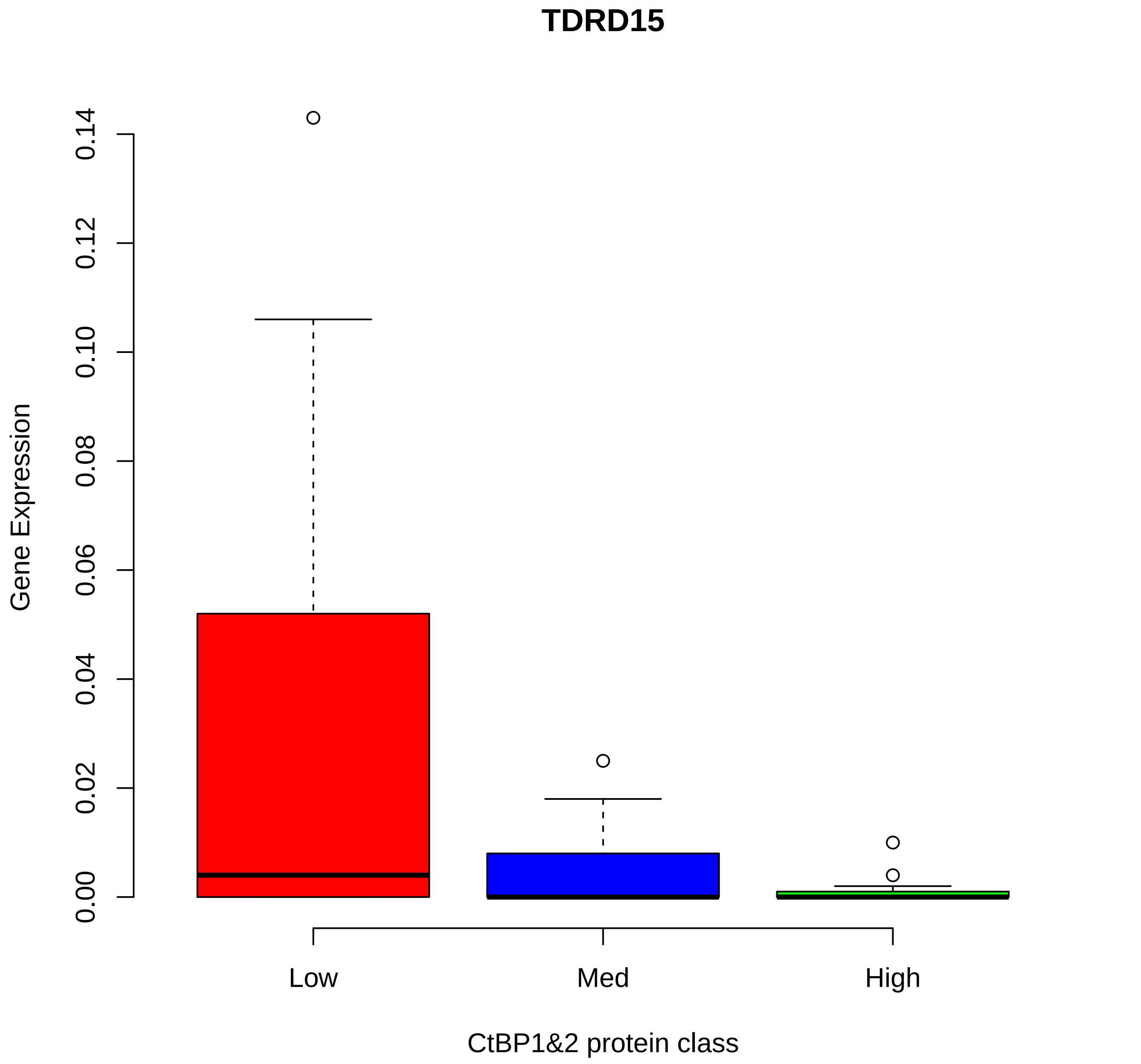

TDRD15
Gene Expression
0.00
0.02
0.04
0.06
0.08
0.10
0.12
0.14
Low
Med
High
CtBP1&2 protein class

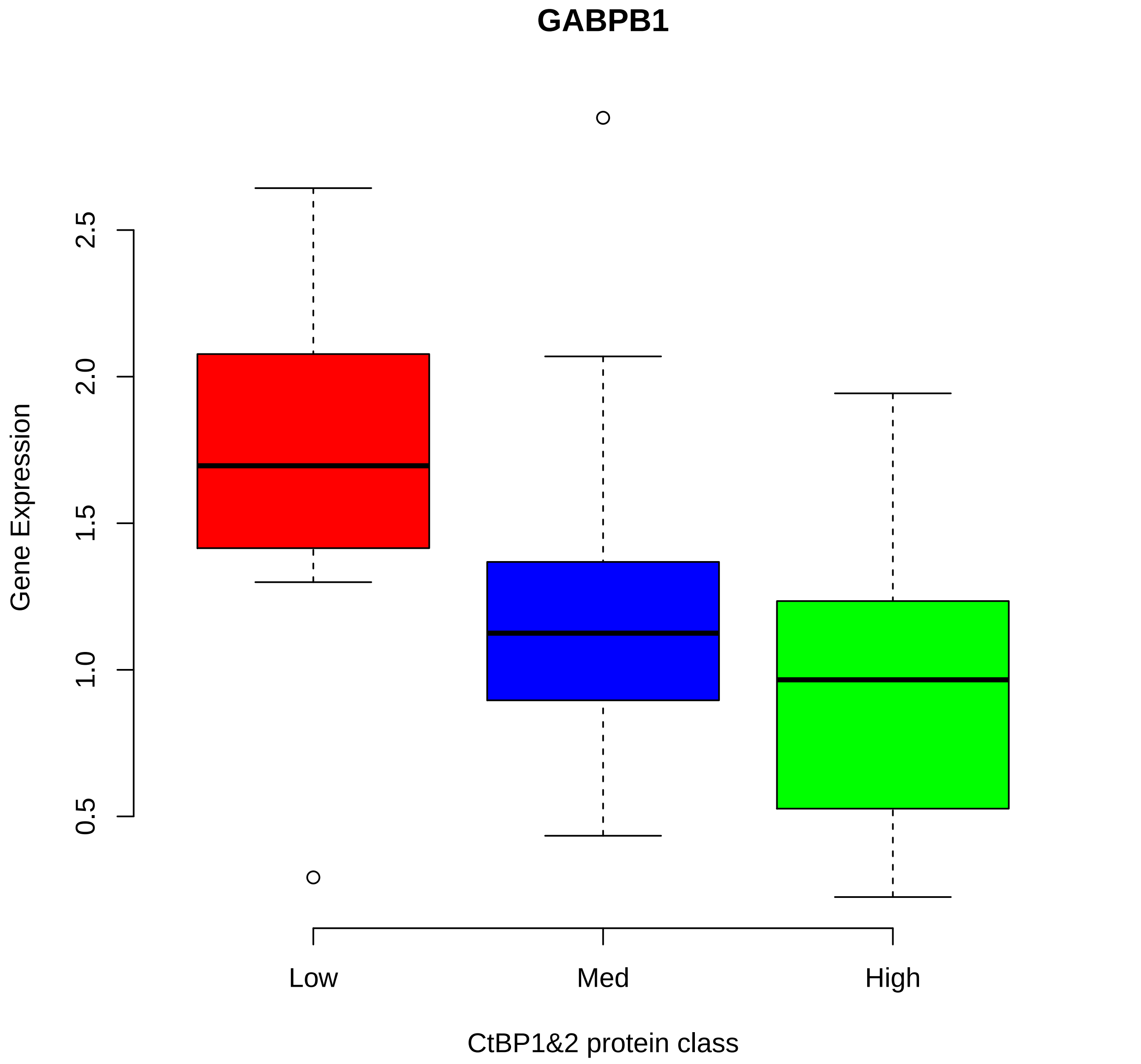
GABPB1
Gene Expression
0.5
1.0
1.5
2.0
2.5
Low
Med
High
CtBP1&2 protein class

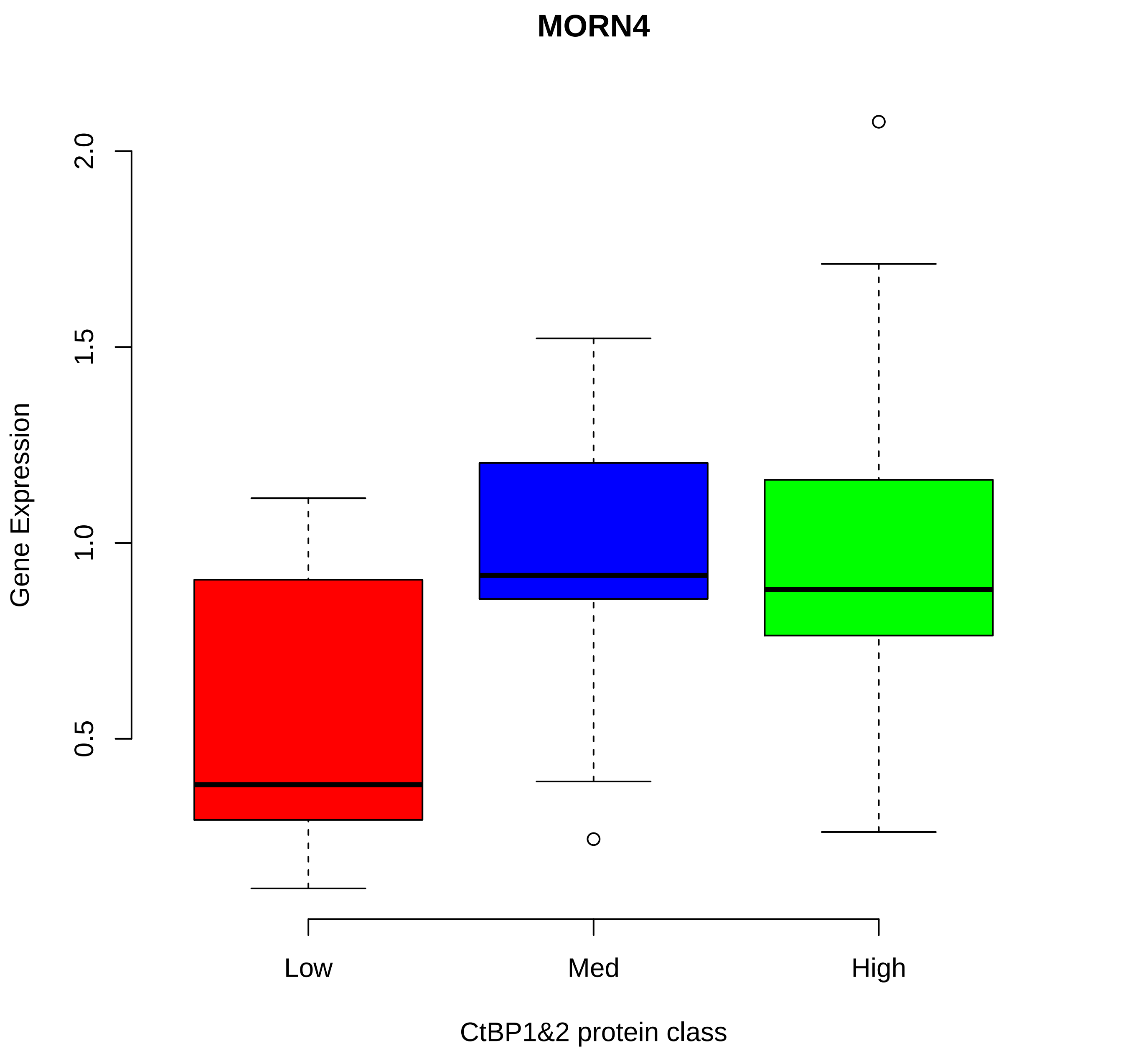
MORN4
Gene Expression
0.5
1.0
1.5
2.0
Low
Med
High
CtBP1&2 protein class

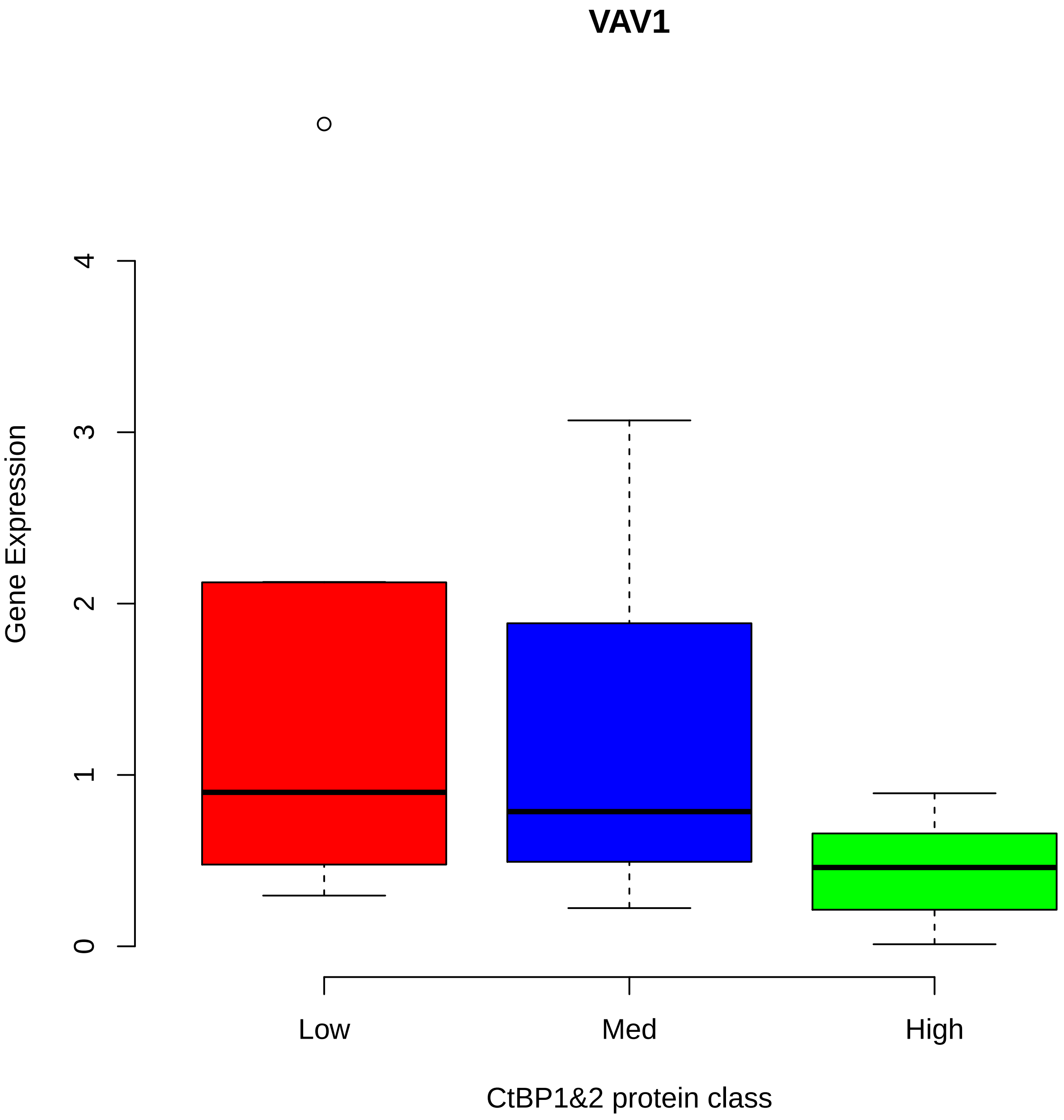
VAV1
Gene Expression
0
1
2
3
4
Low
Med
High
CtBP1&2 protein class

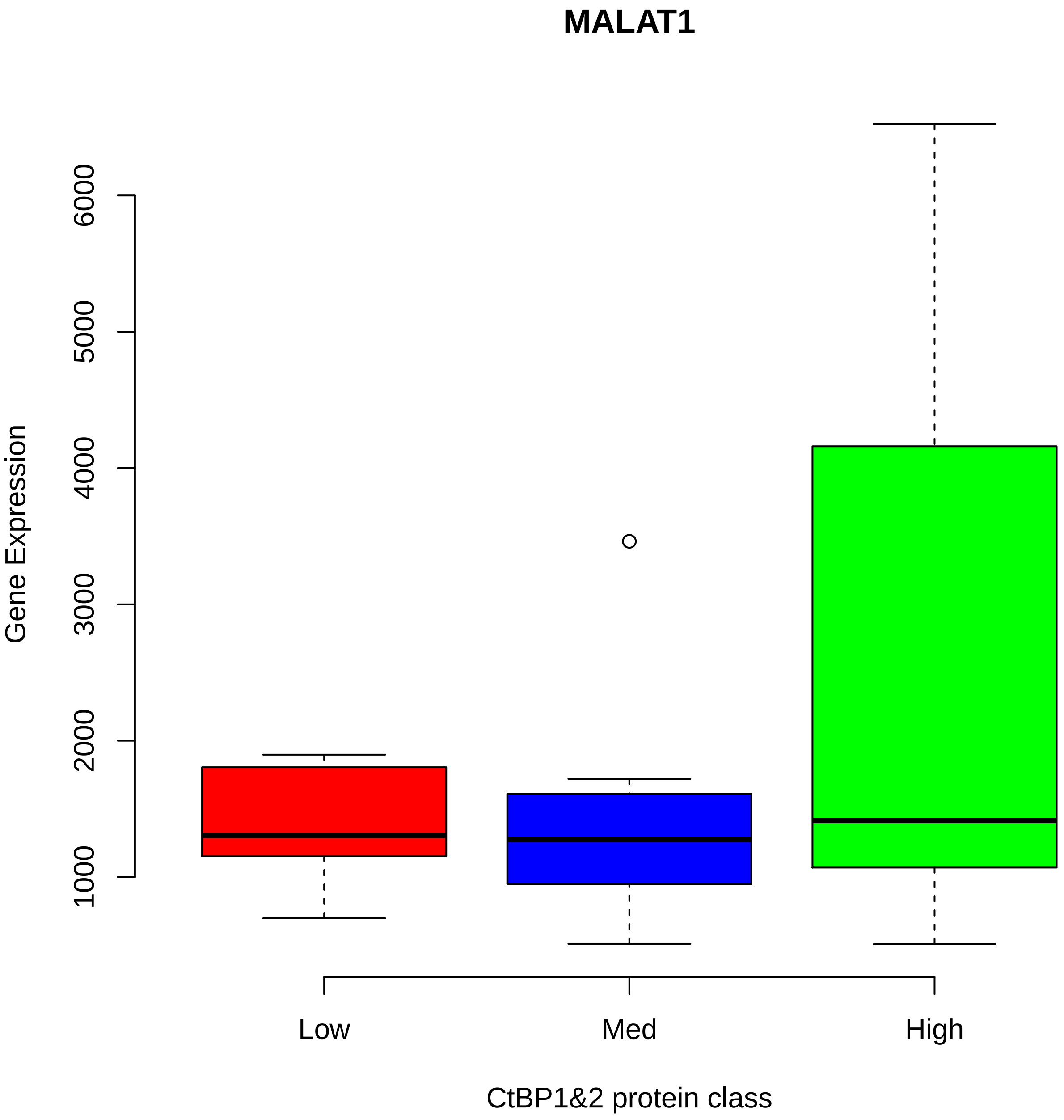

MALAT1
Gene Expression
1000
2000
3000
4000
5000
6000
Low
Med
High
CtBP1&2 protein class

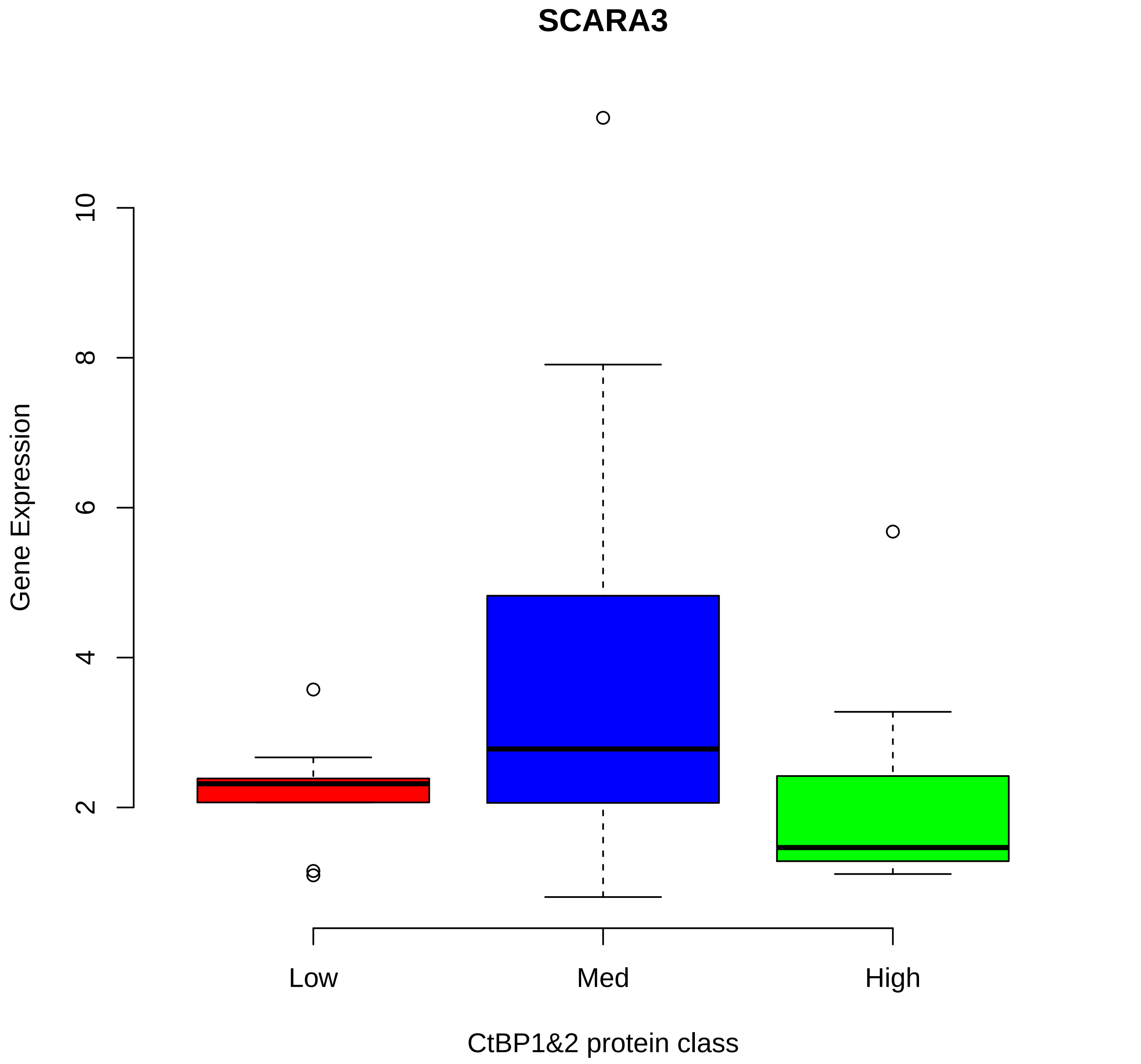

SCARA3
Gene Expression
2
4
6
8
10
Low
Med
High
CtBP1&2 protein class

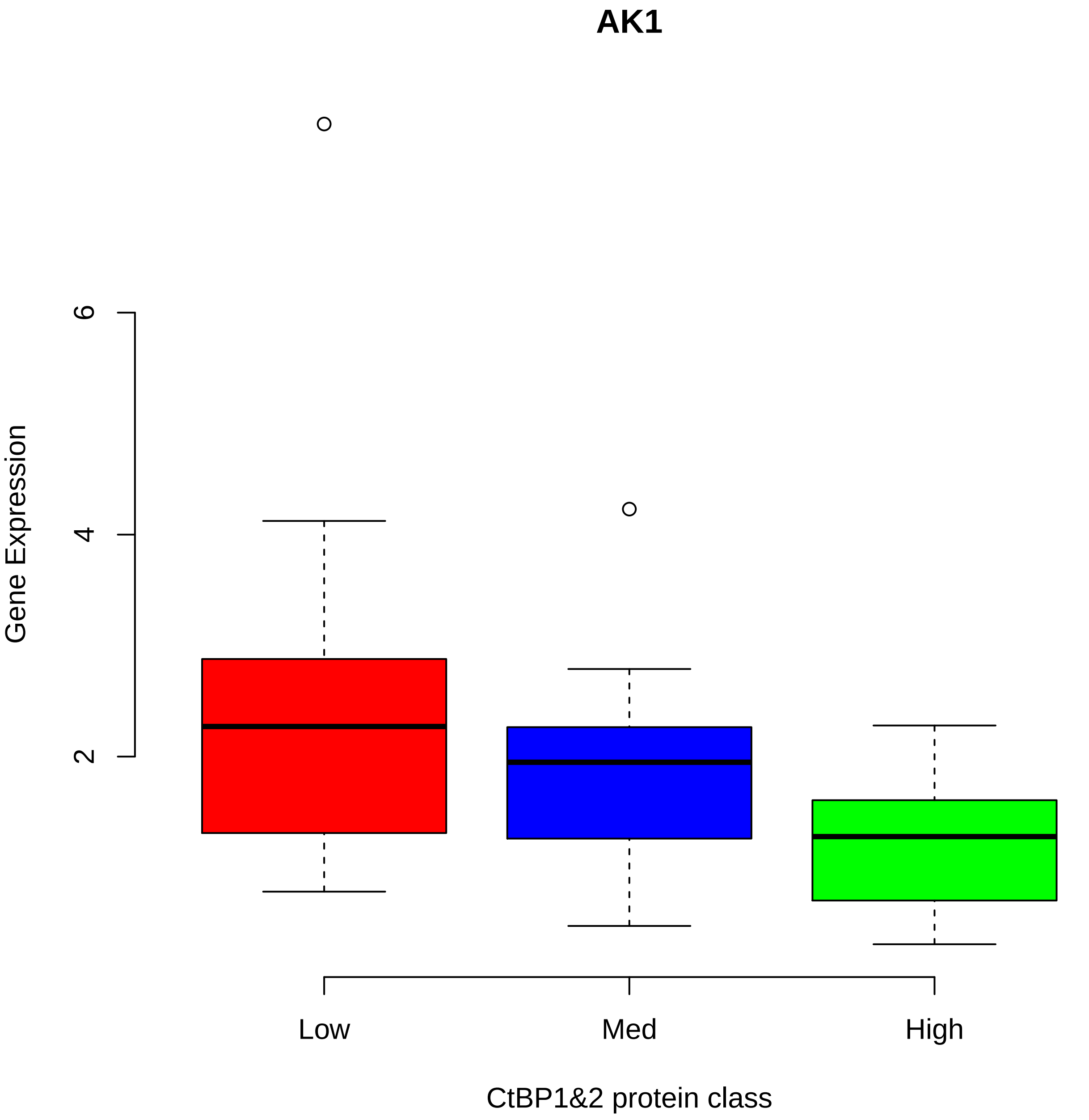
AK1
Gene Expression
6
4
2
Low
Med
High
CtBP1&2 protein class

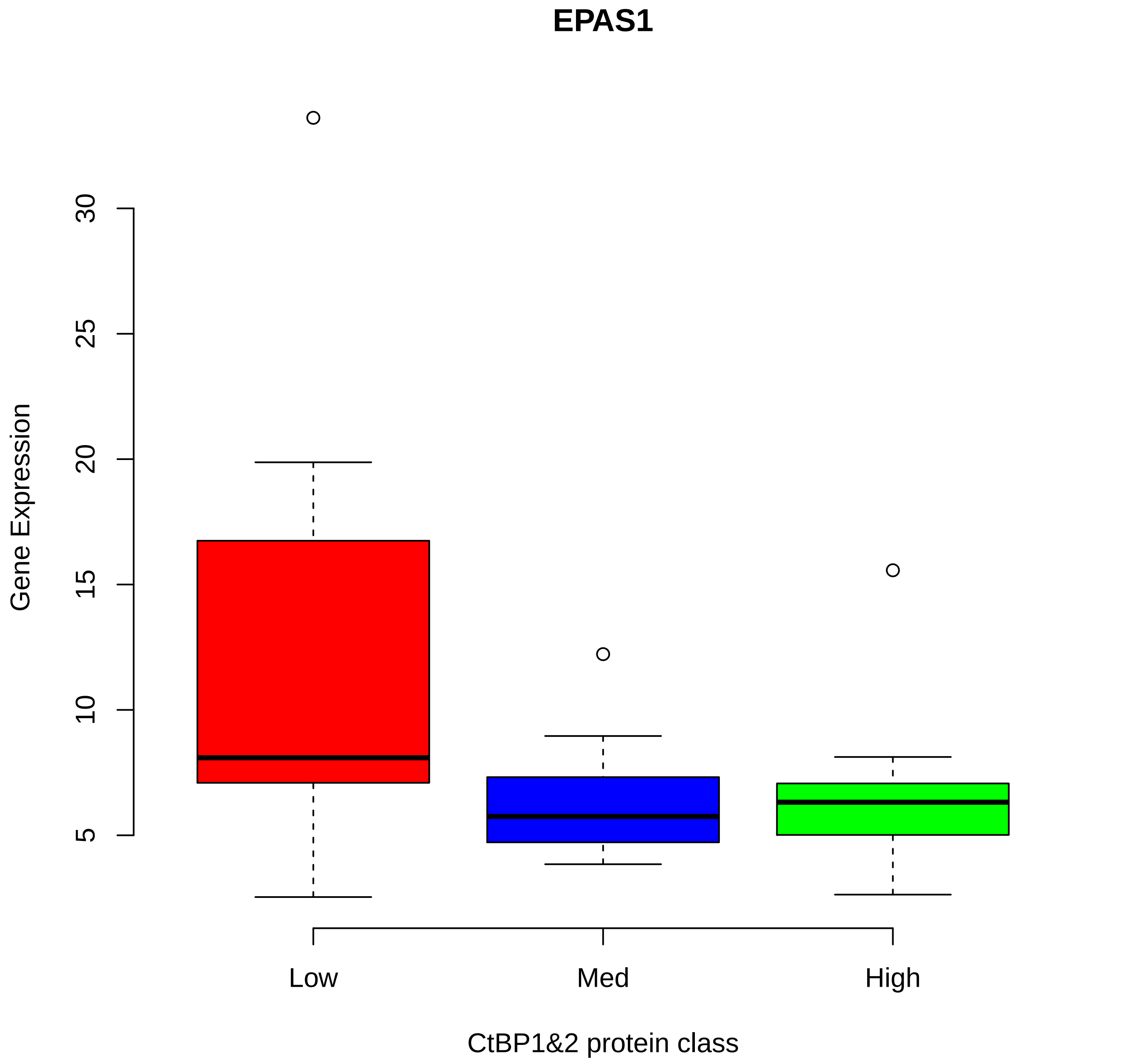
EPAS1
Gene Expression
30
25
20
15
10
5
Low
Med
High
CtBP1&2 protein class

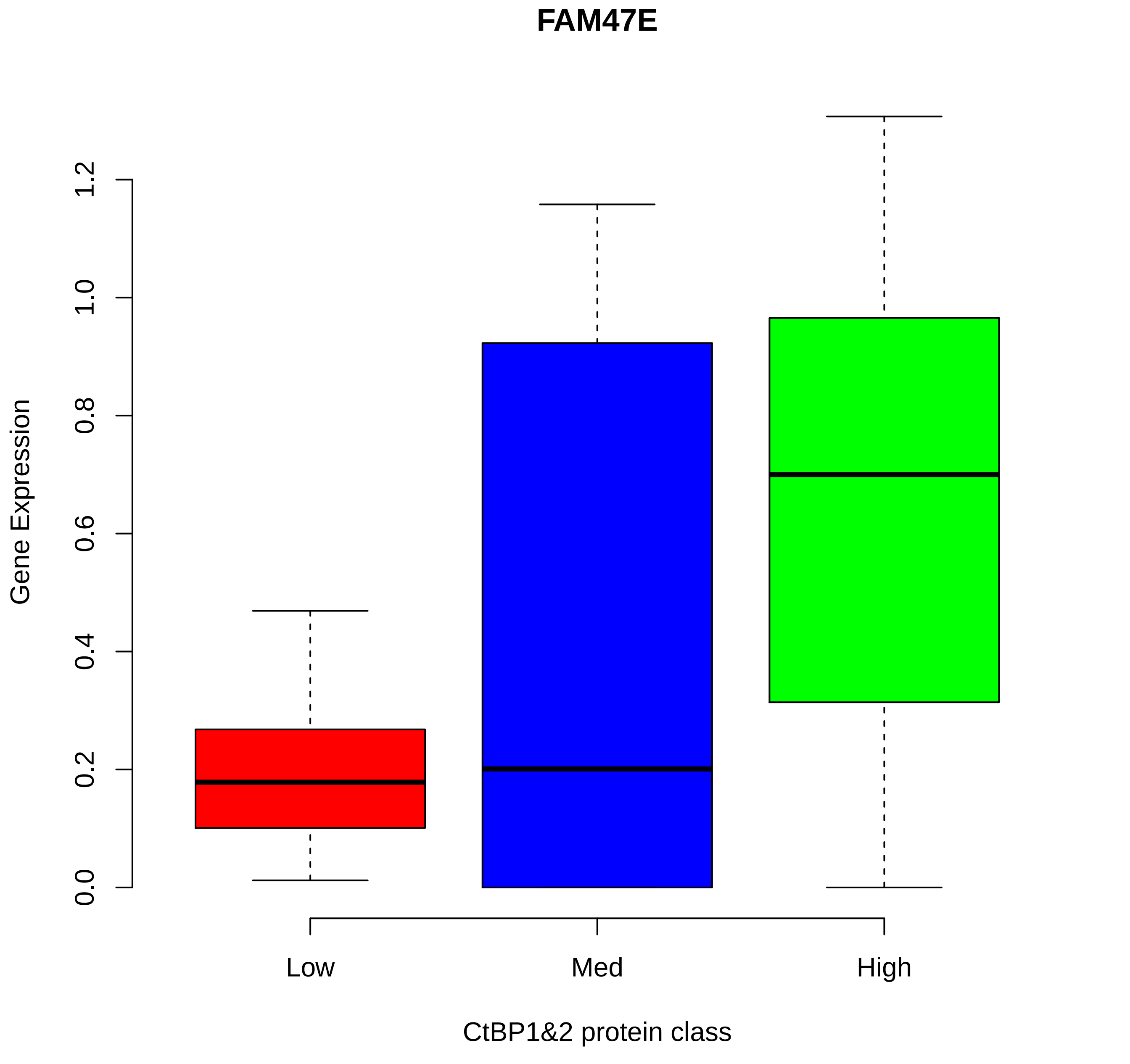
FAM47E
Gene Expression
0.0
0.2
0.4
0.6
0.8
1.0
1.2
Low
Med
High
CtBP1&2 protein class

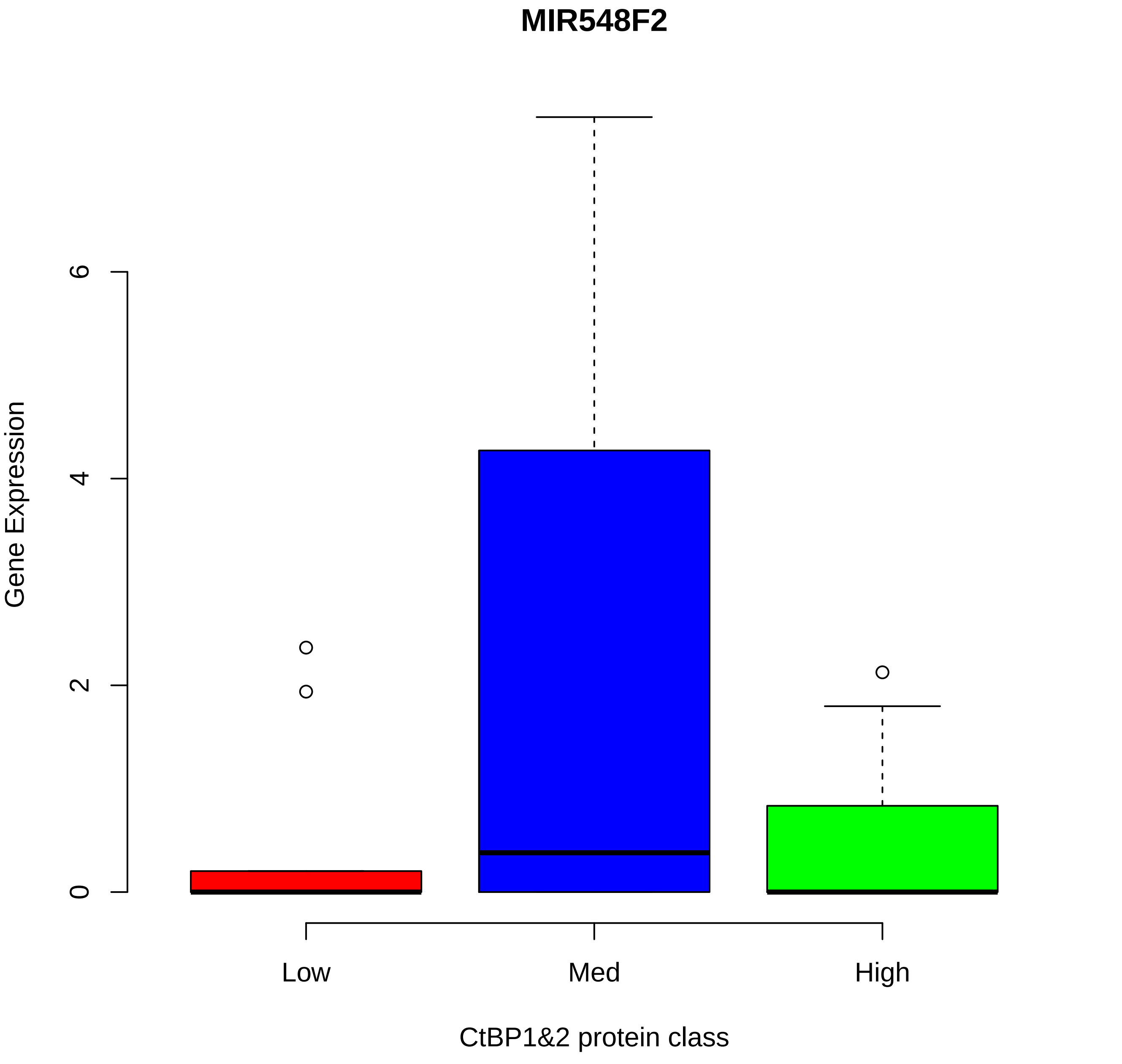

MIR548F2
Gene Expression
0
2
4
6
Low
Med
High
CtBP1&2 protein class

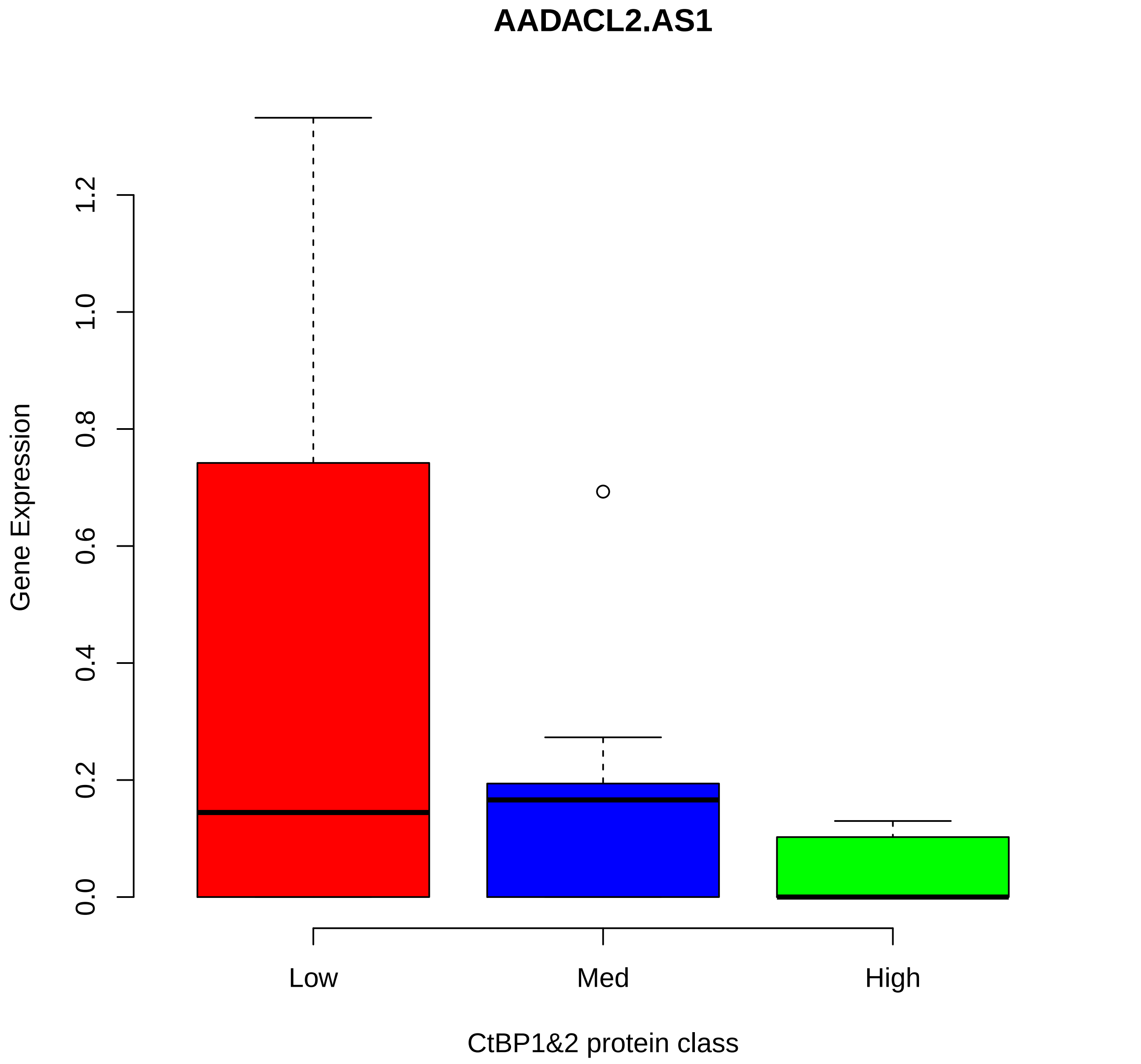
AADACL2.AS1
Gene Expression
0.0
0.2
0.4
0.6
0.8
1.0
1.2
Low
Med
High
CtBP1&2 protein class

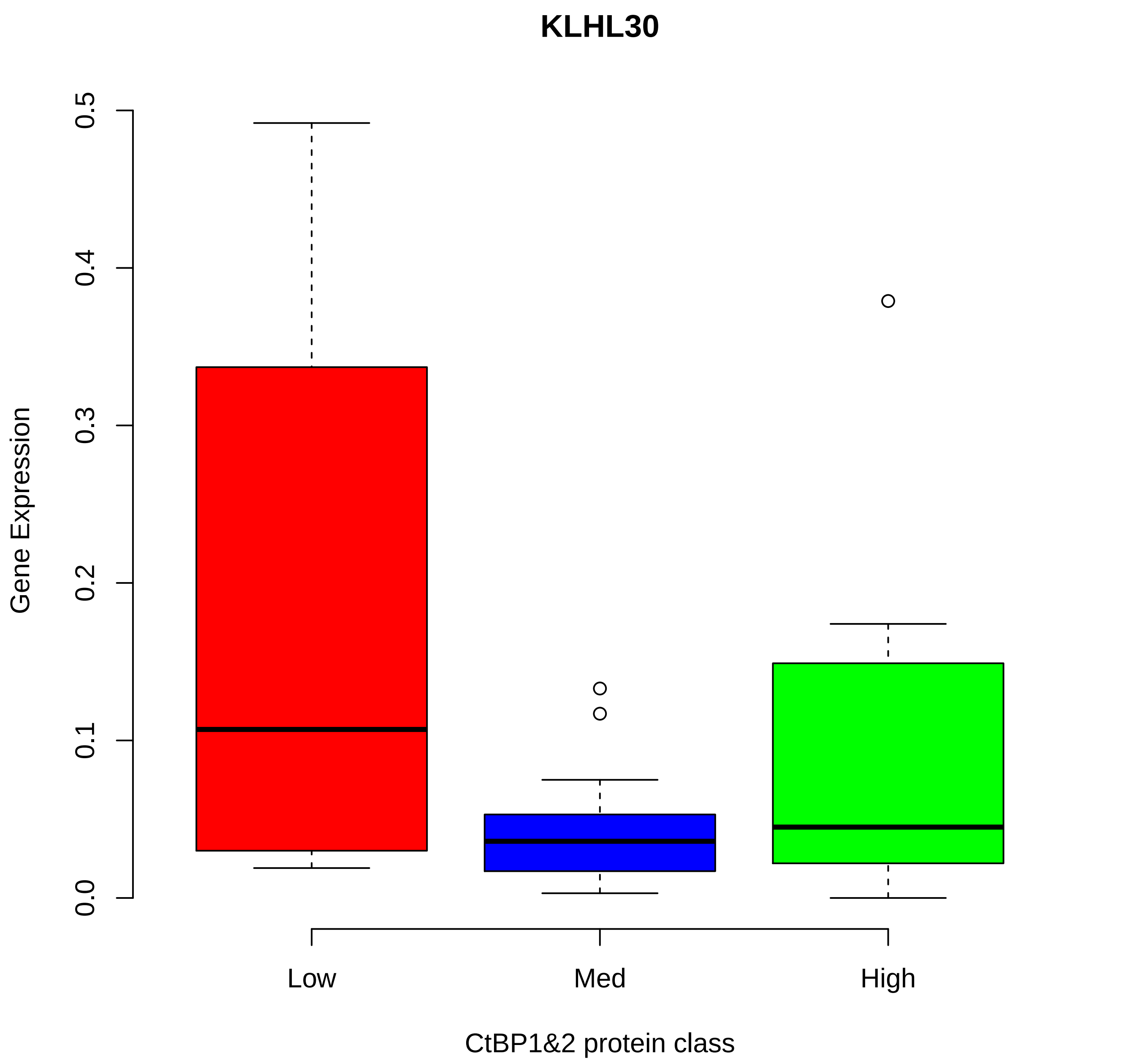
KLHL30
Gene Expression
0.0
0.1
0.2
0.3
0.4
0.5
Low
Med
High
CtBP1&2 protein class

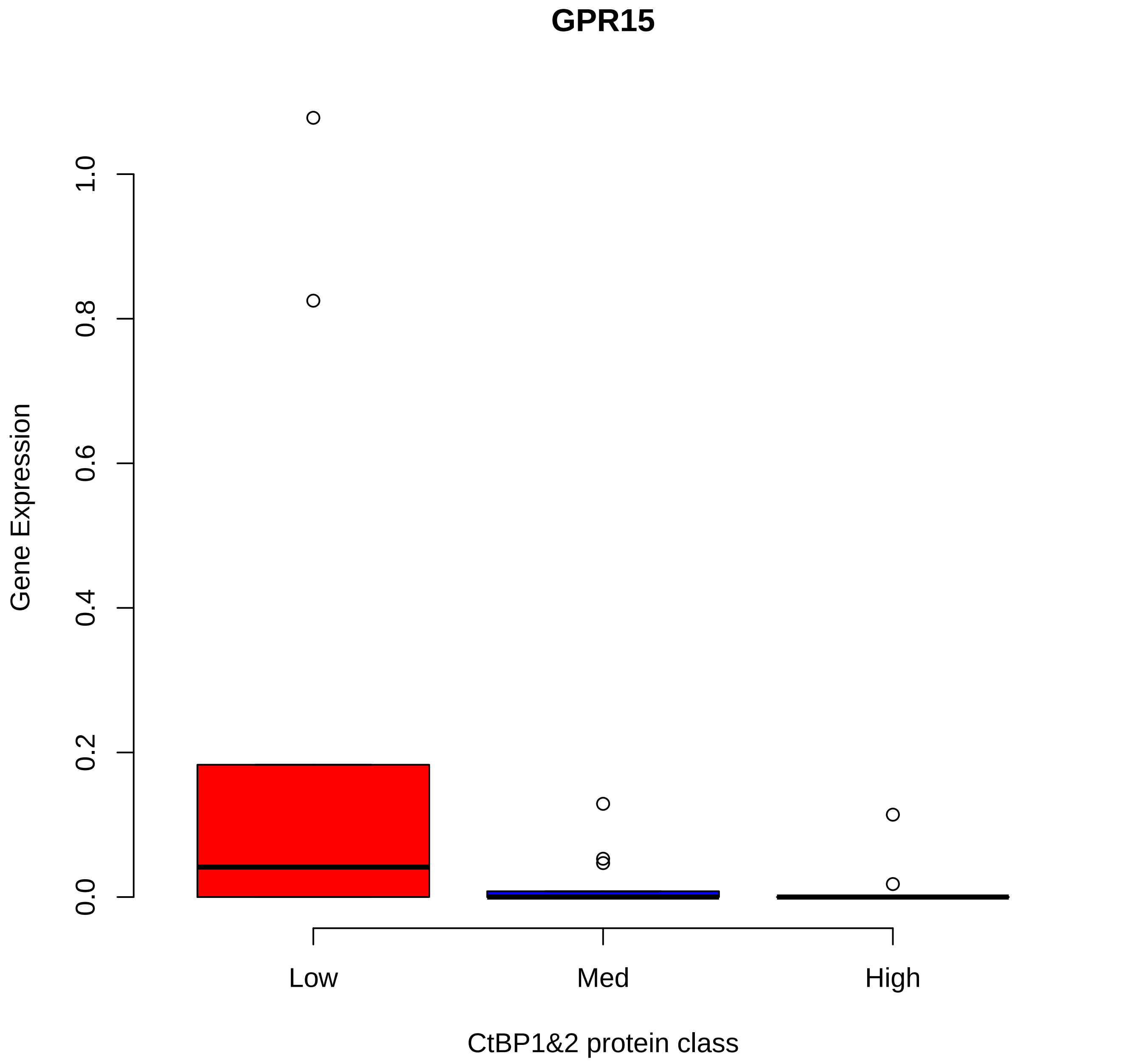
GPR15
Gene Expression
0.0
0.2
0.4
0.6
0.8
1.0
Low
Med
High
CtBP1&2 protein class

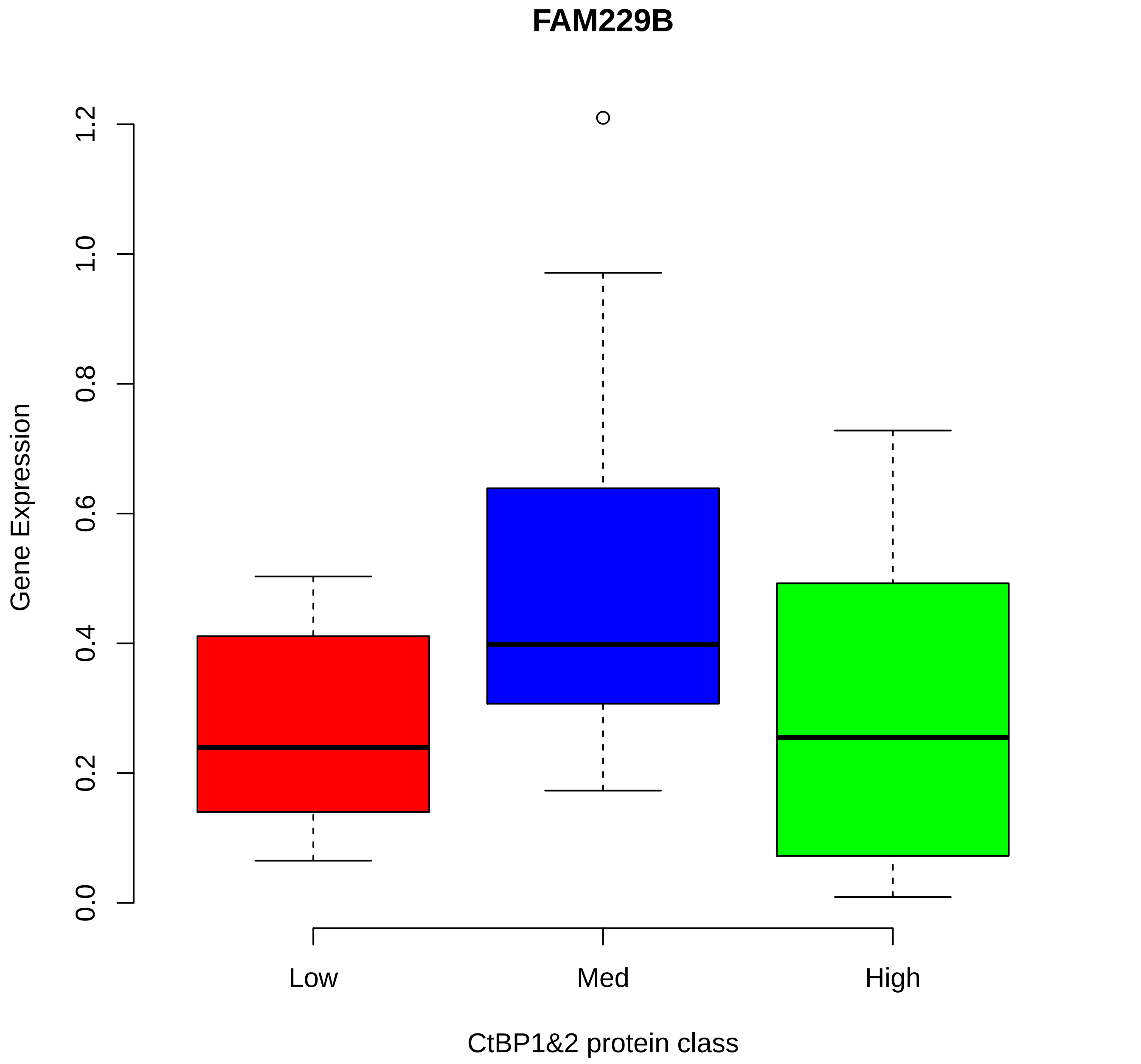
FAM229B
Gene Expression
0.0
0.2
0.4
0.6
0.8
1.0
1.2
Low
Med
High
CtBP1&2 protein class

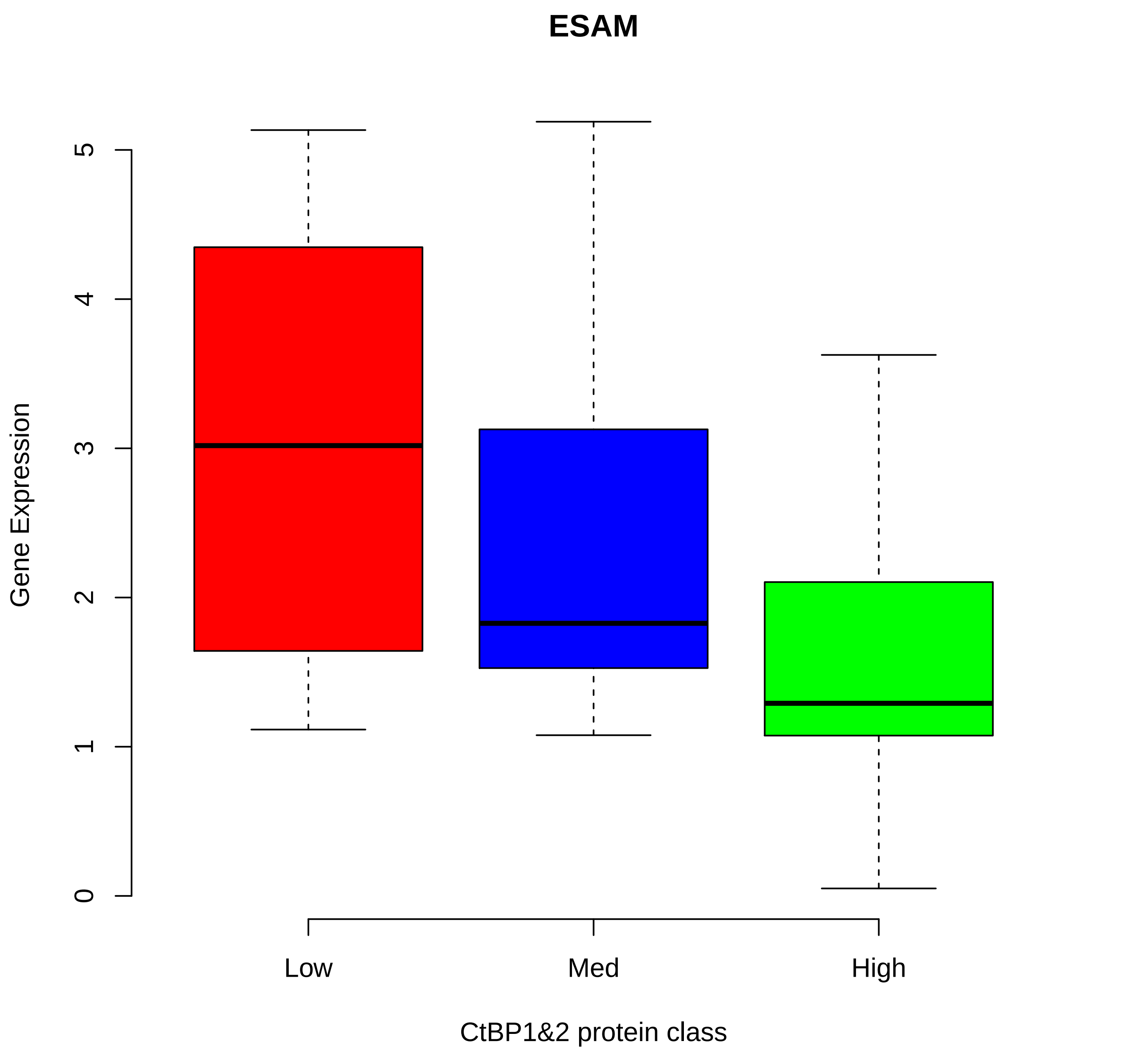
ESAM
Gene Expression
0
1
2
3
4
5
Low
Med
High
CtBP1&2 protein class

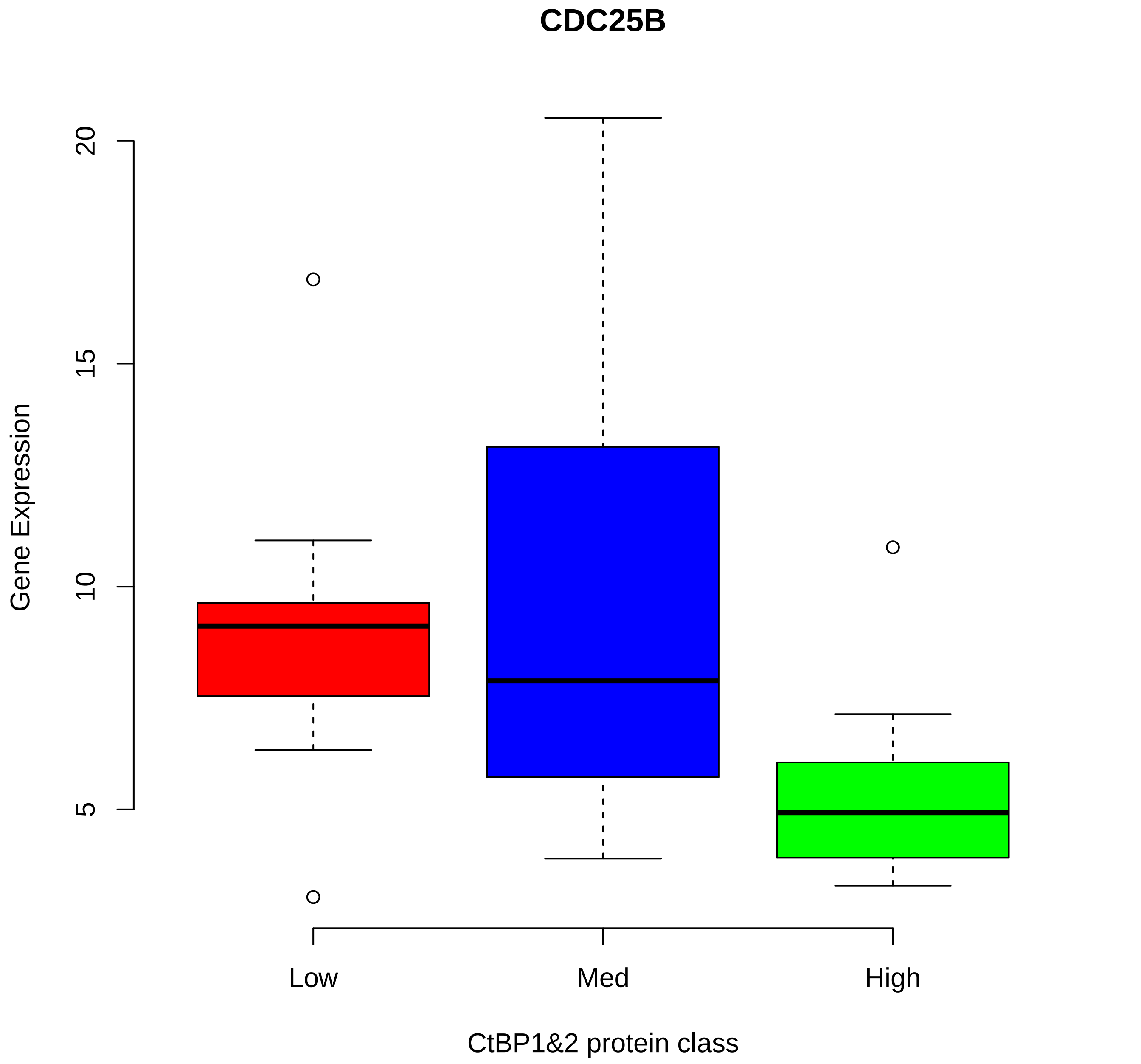
CDC25B
Gene Expression
20
15
10
5
Low
Med
High
CtBP1&2 protein class

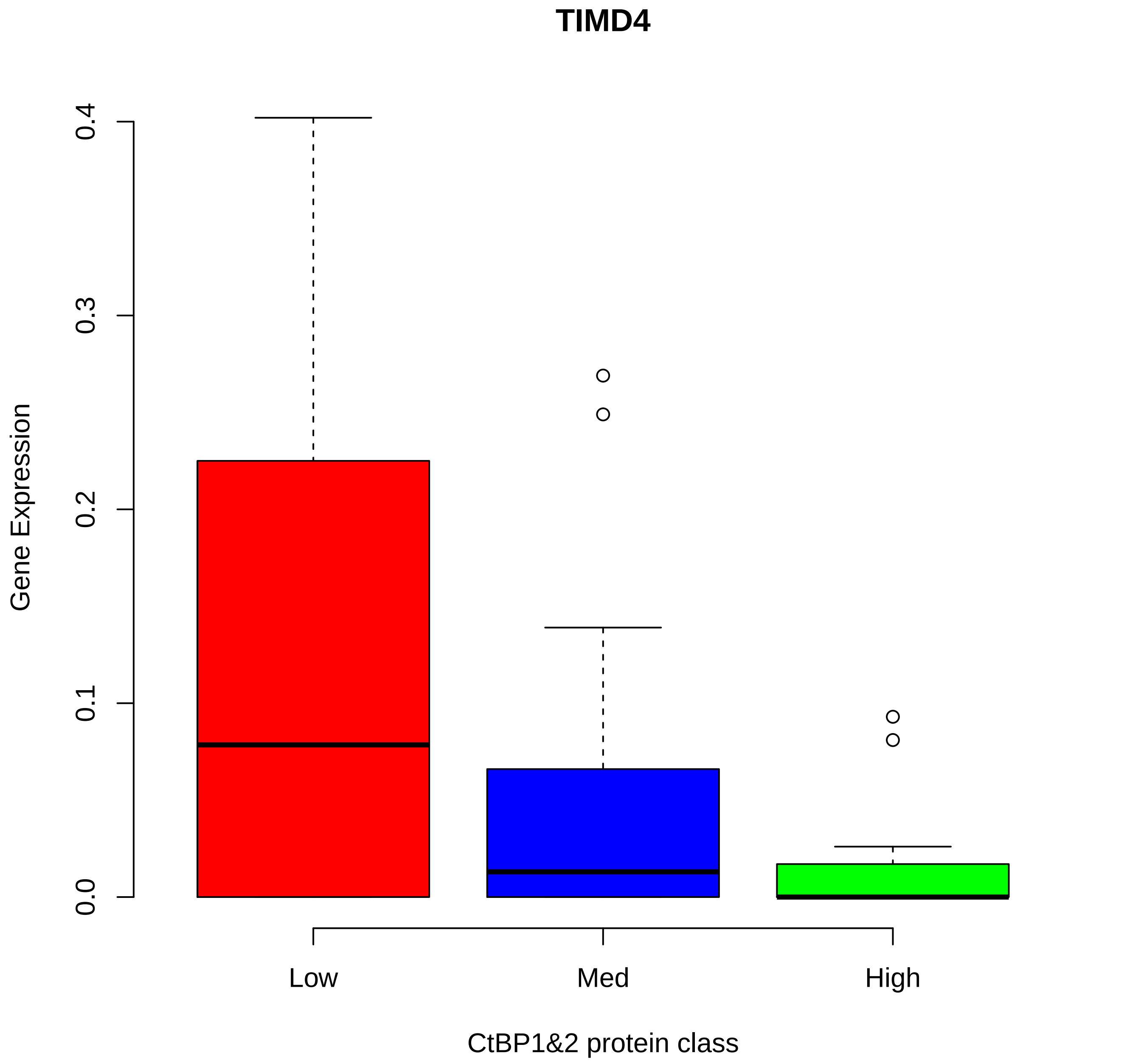
TIMD4
Gene Expression
0.0
0.1
0.2
0.3
0.4
Low
Med
High
CtBP1&2 protein class

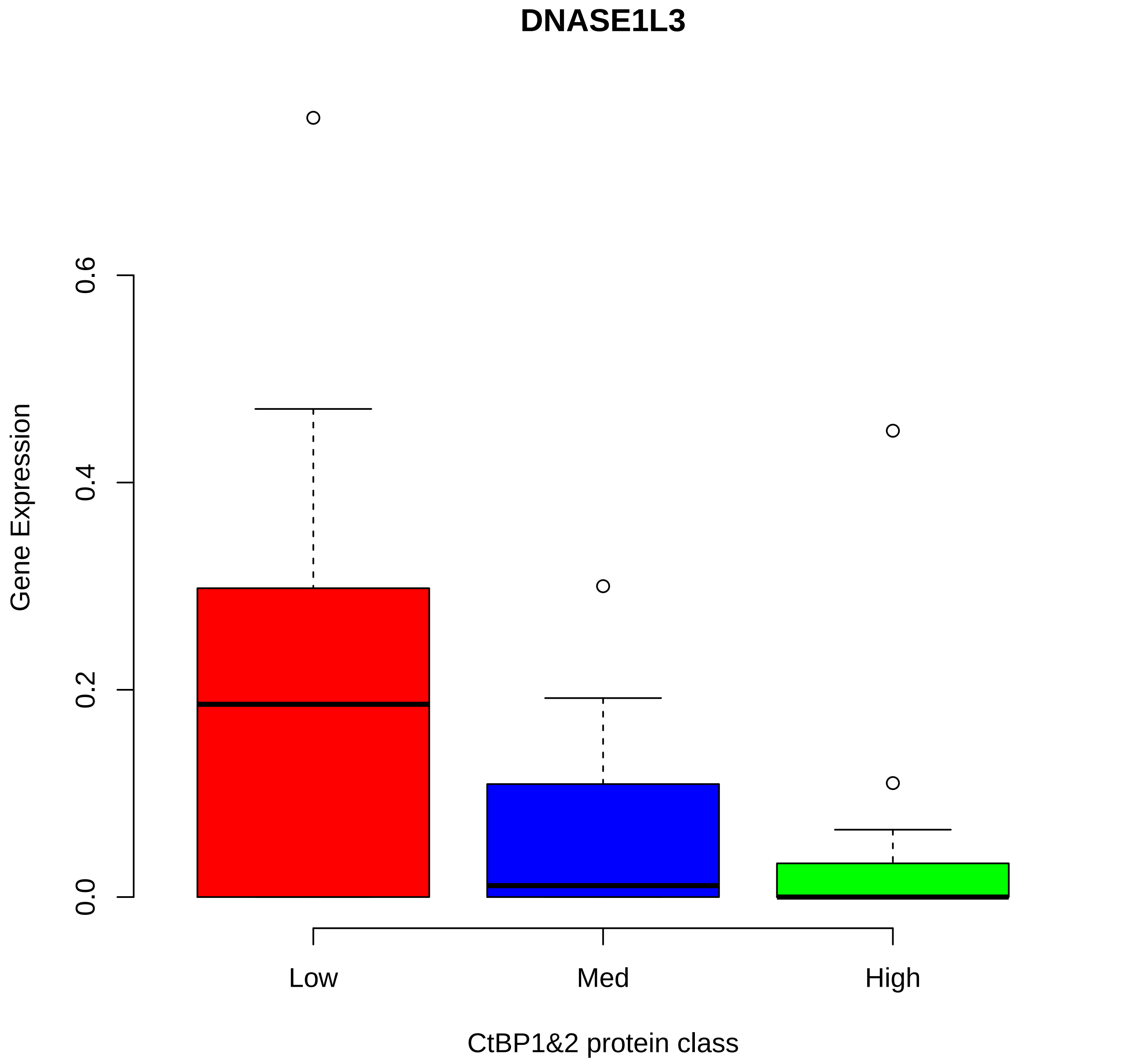
DNASE1L3
Gene Expression
0.0
0.2
0.4
0.6
Low
Med
High
CtBP1&2 protein class

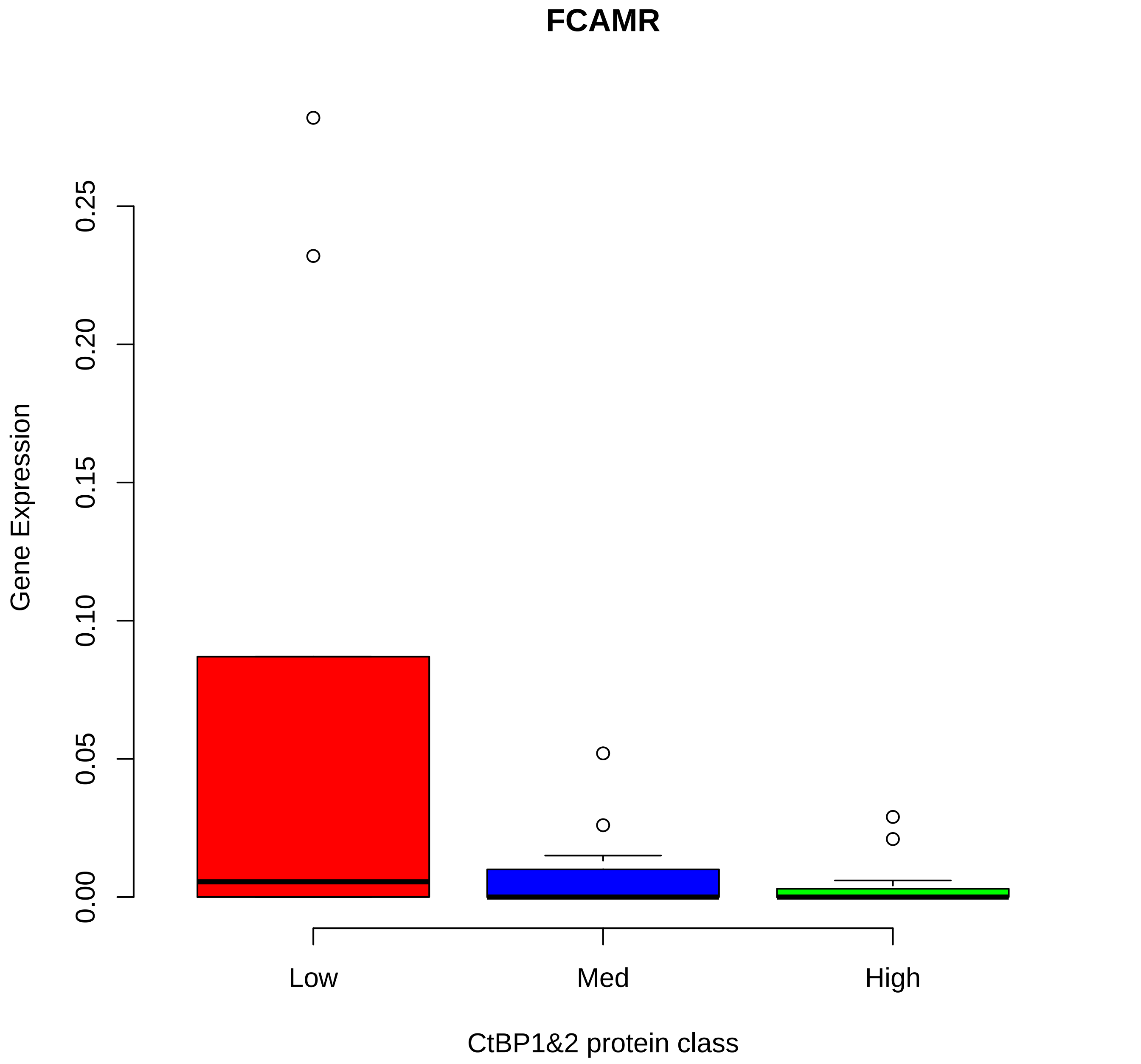
FCAMR
Gene Expression
0.00
0.05
0.10
0.15
0.20
0.25
Low
Med
High
CtBP1&2 protein class

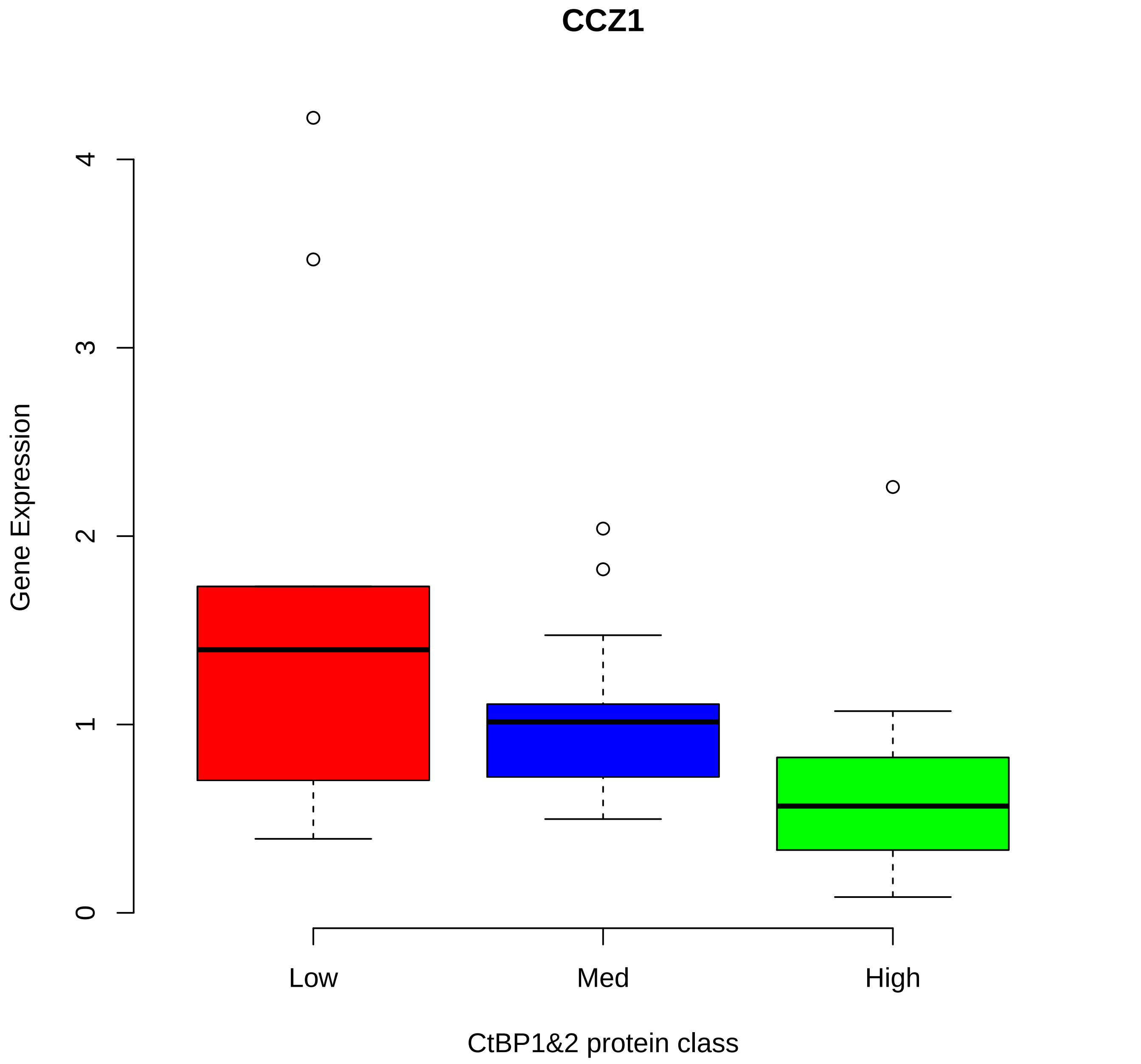

CCZ1
Gene Expression
0
1
2
3
4
Low
Med
High
CtBP1&2 protein class

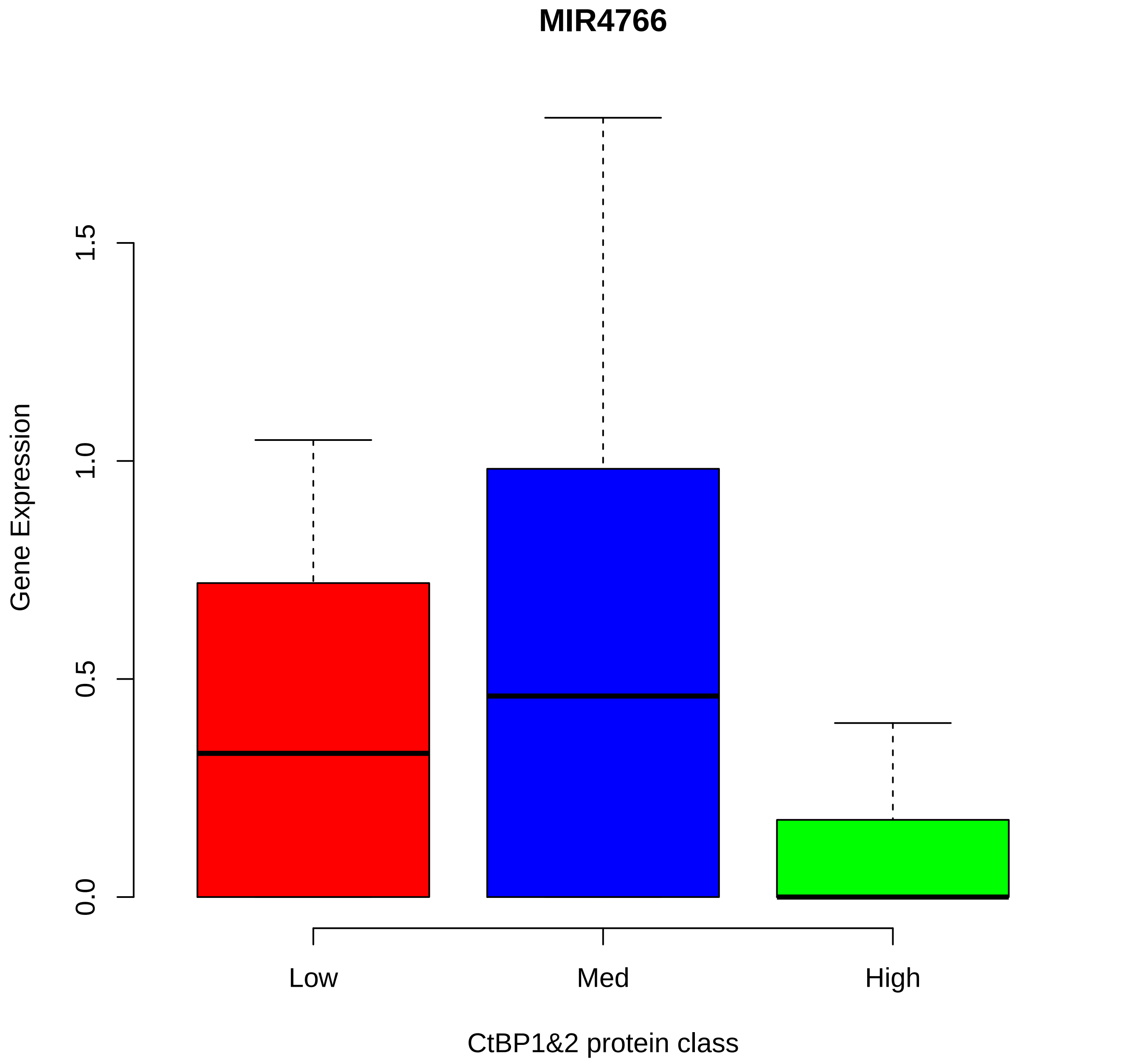

MIR4766
Gene Expression
0.0
0.5
1.0
1.5
Low
Med
High
CtBP1&2 protein class

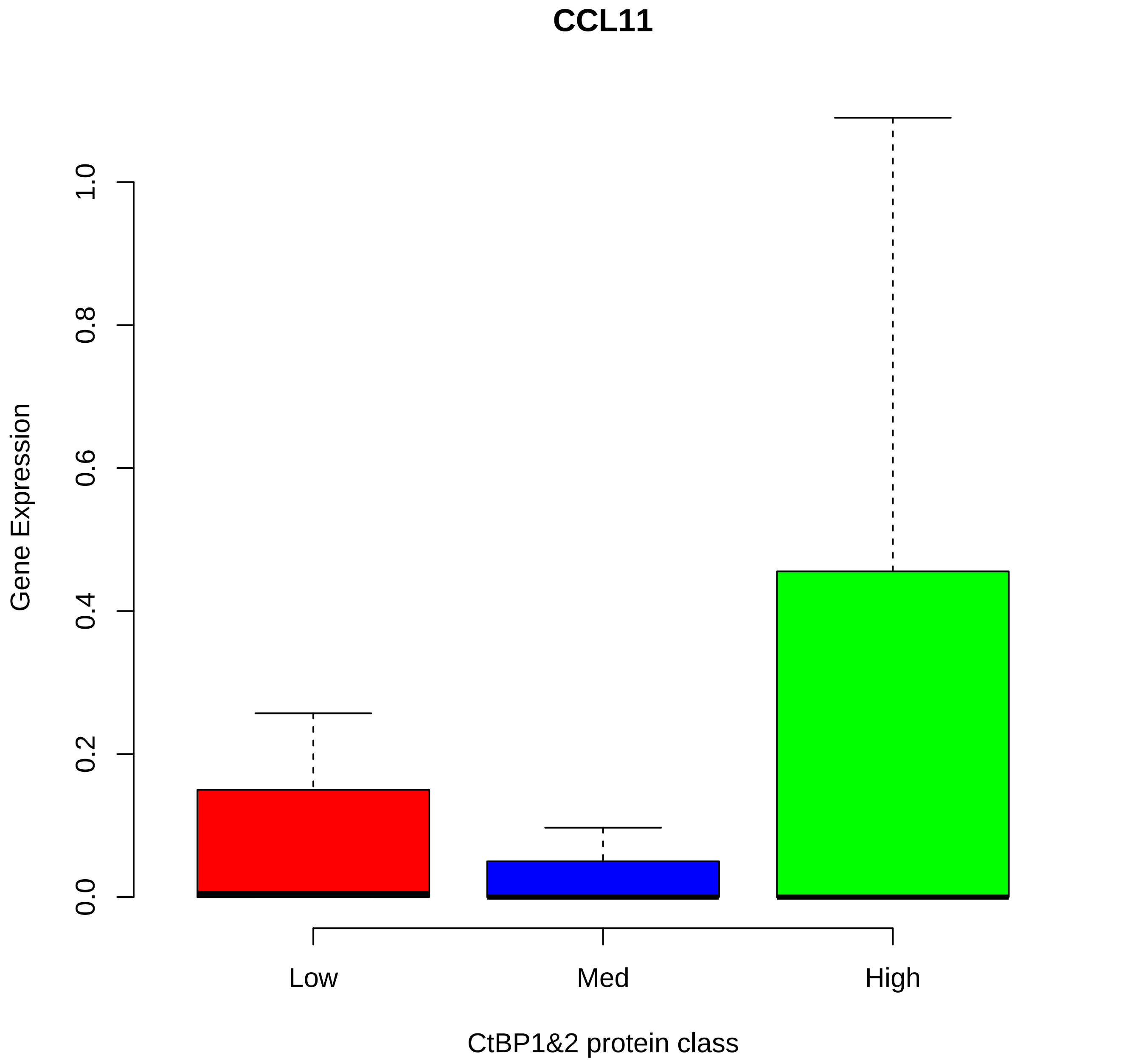

CCL11
Gene Expression
0.0
0.2
0.4
0.6
0.8
1.0
Low
Med
High
CtBP1&2 protein class

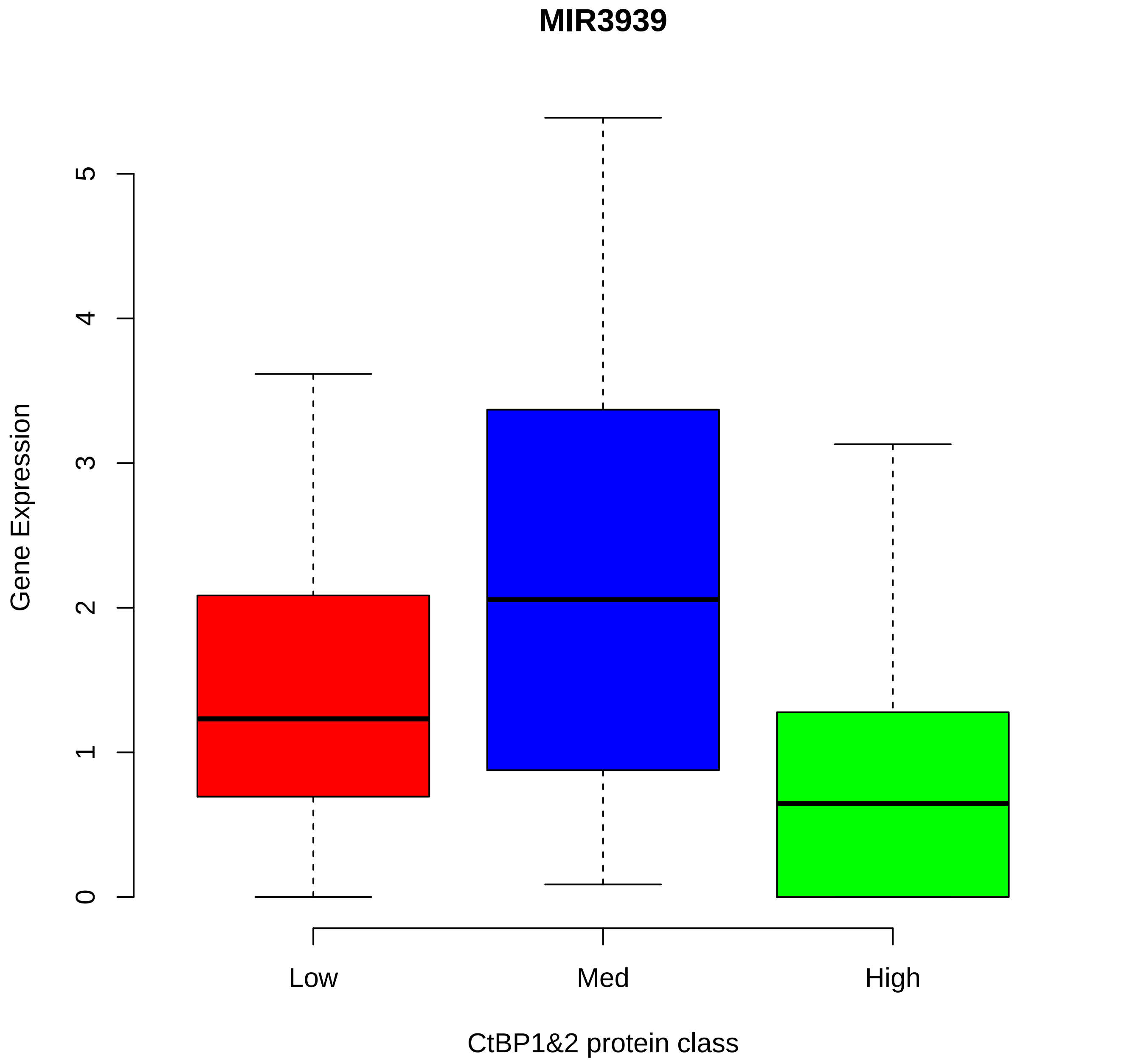

MIR3939
Gene Expression
0
1
2
3
4
5
Low
Med
High
CtBP1&2 protein class

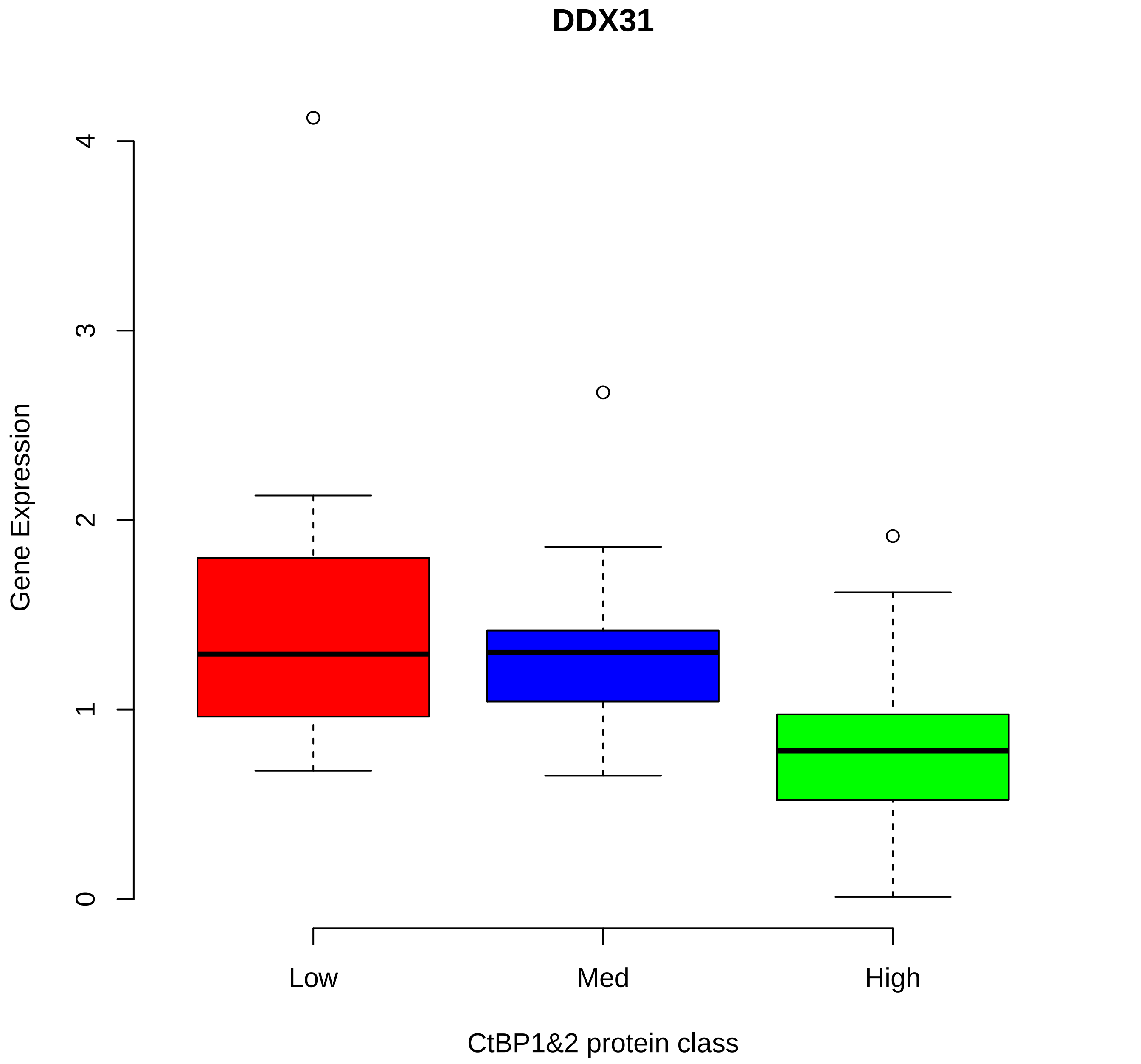
DDX31
Gene Expression
0
1
2
3
4
Low
Med
High
CtBP1&2 protein class

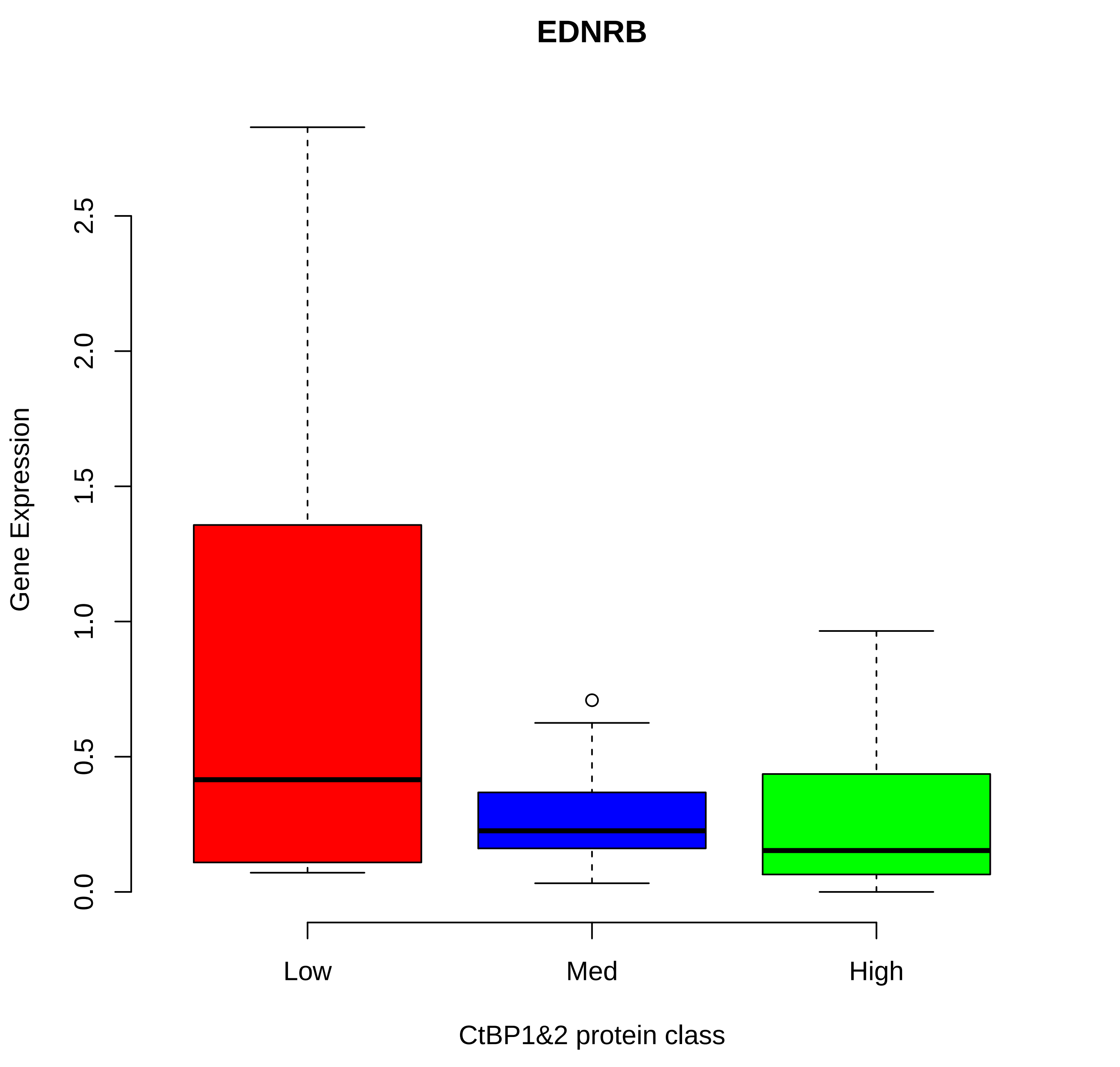

EDNRB
Gene Expression
0.0
0.5
1.0
1.5
2.0
2.5
Low
Med
High
CtBP1&2 protein class

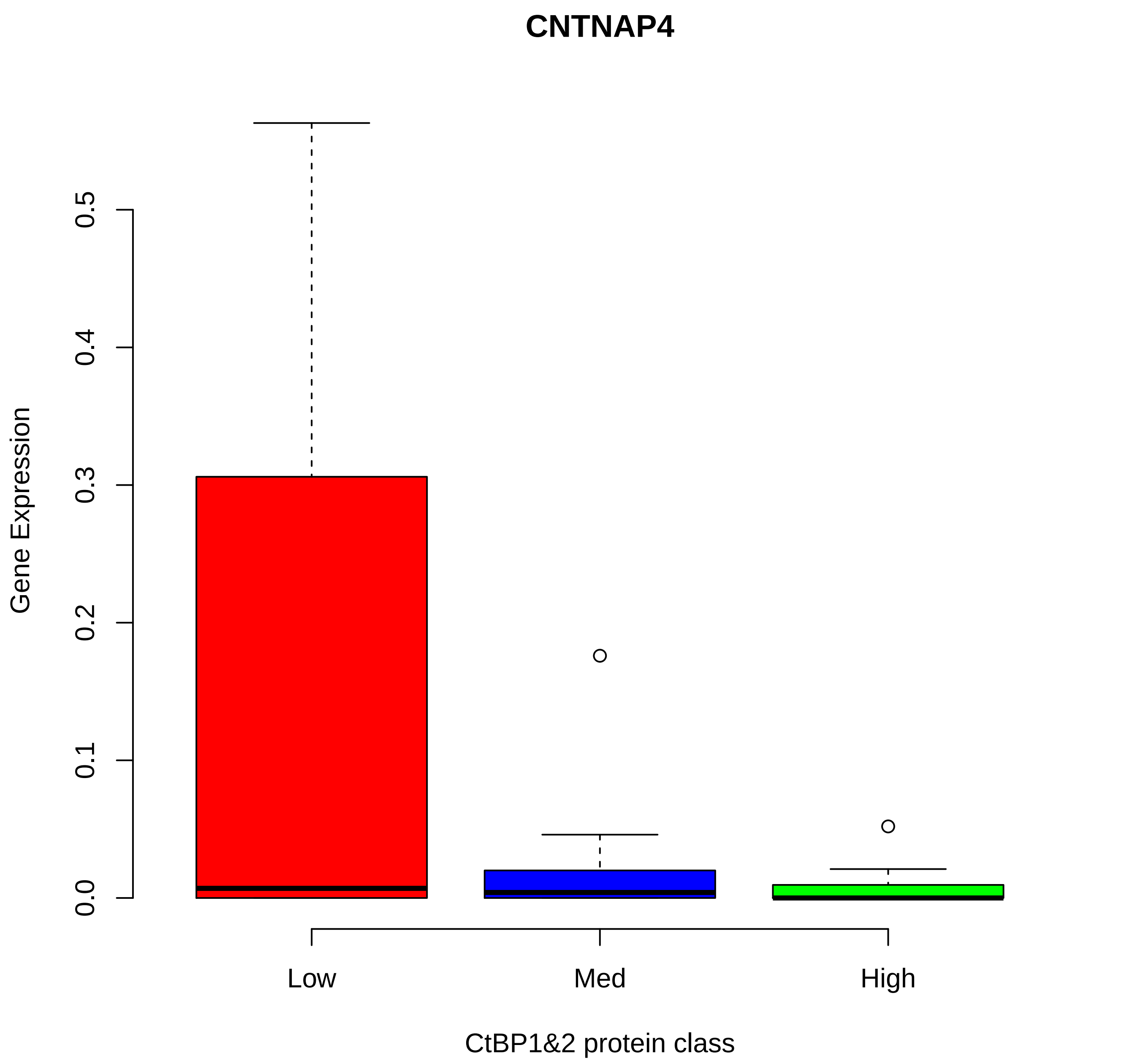

CNTNAP4
Gene Expression
0.0
0.1
0.2
0.3
0.4
0.5
Low
Med
High
CtBP1&2 protein class

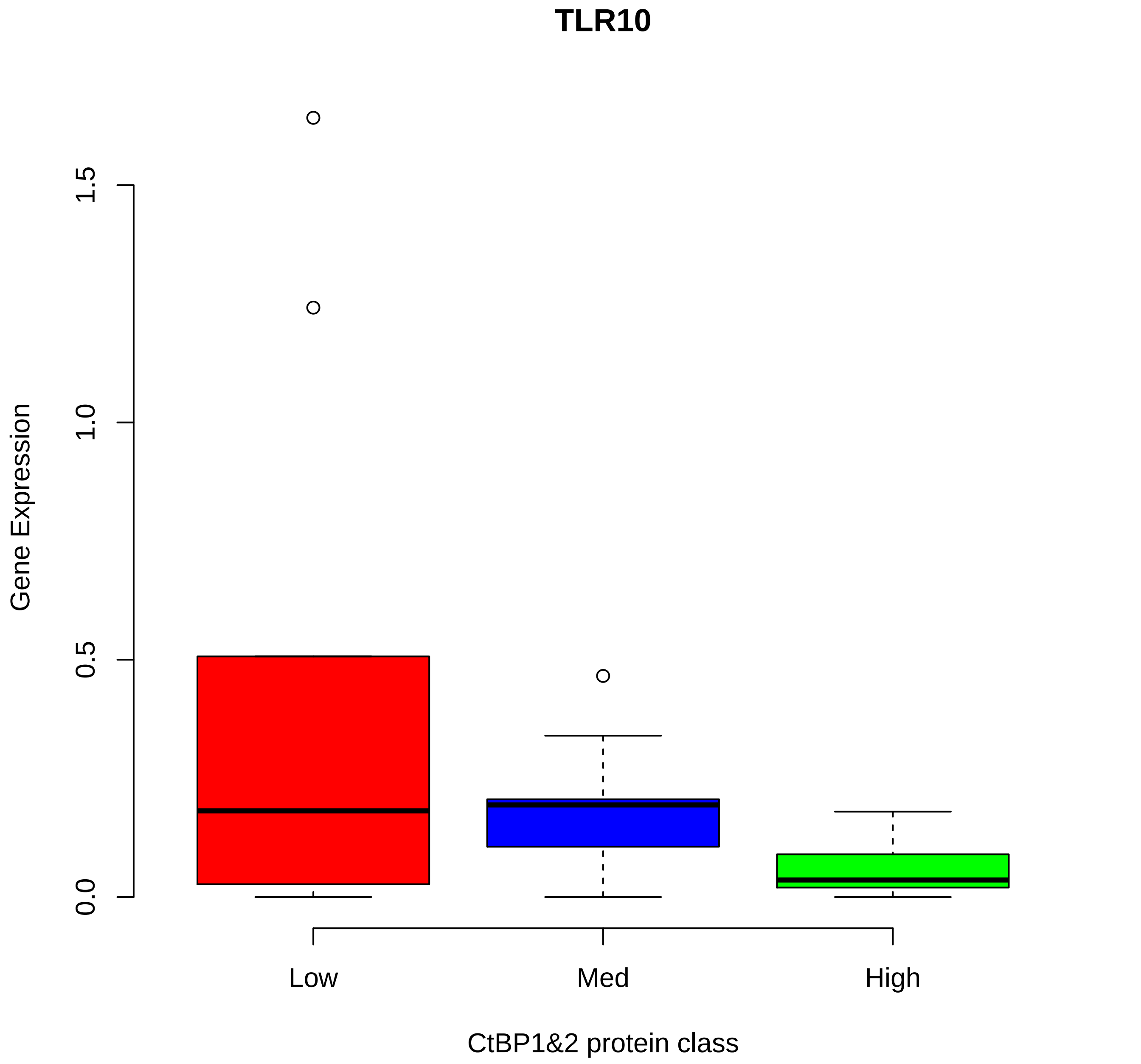
TLR10
Gene Expression
0.0
0.5
1.0
1.5
Low
Med
High
CtBP1&2 protein class

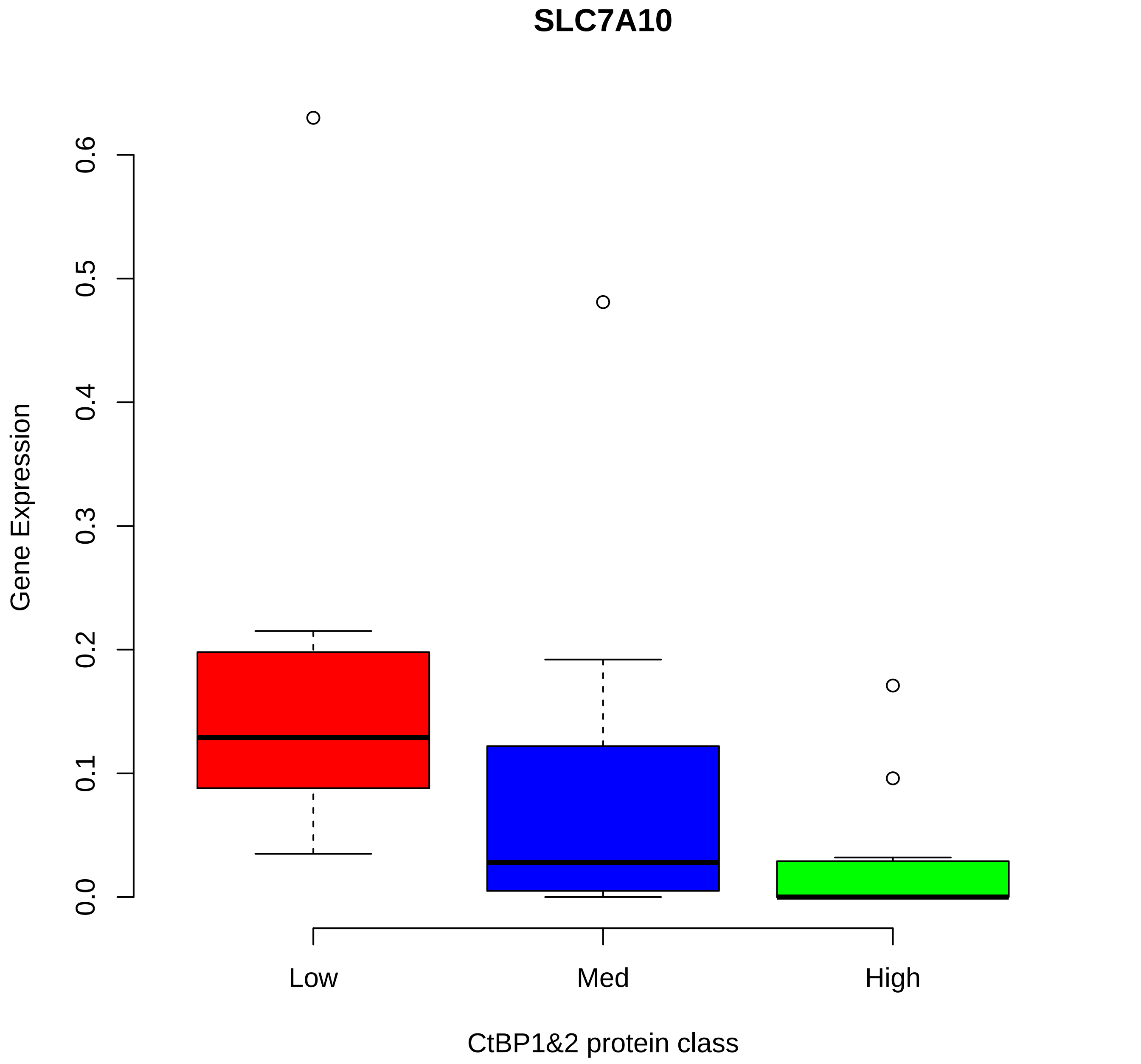

SLC7A10
Gene Expression
0.0
0.1
0.2
0.3
0.4
0.5
0.6
Low
Med
High
CtBP1&2 protein class

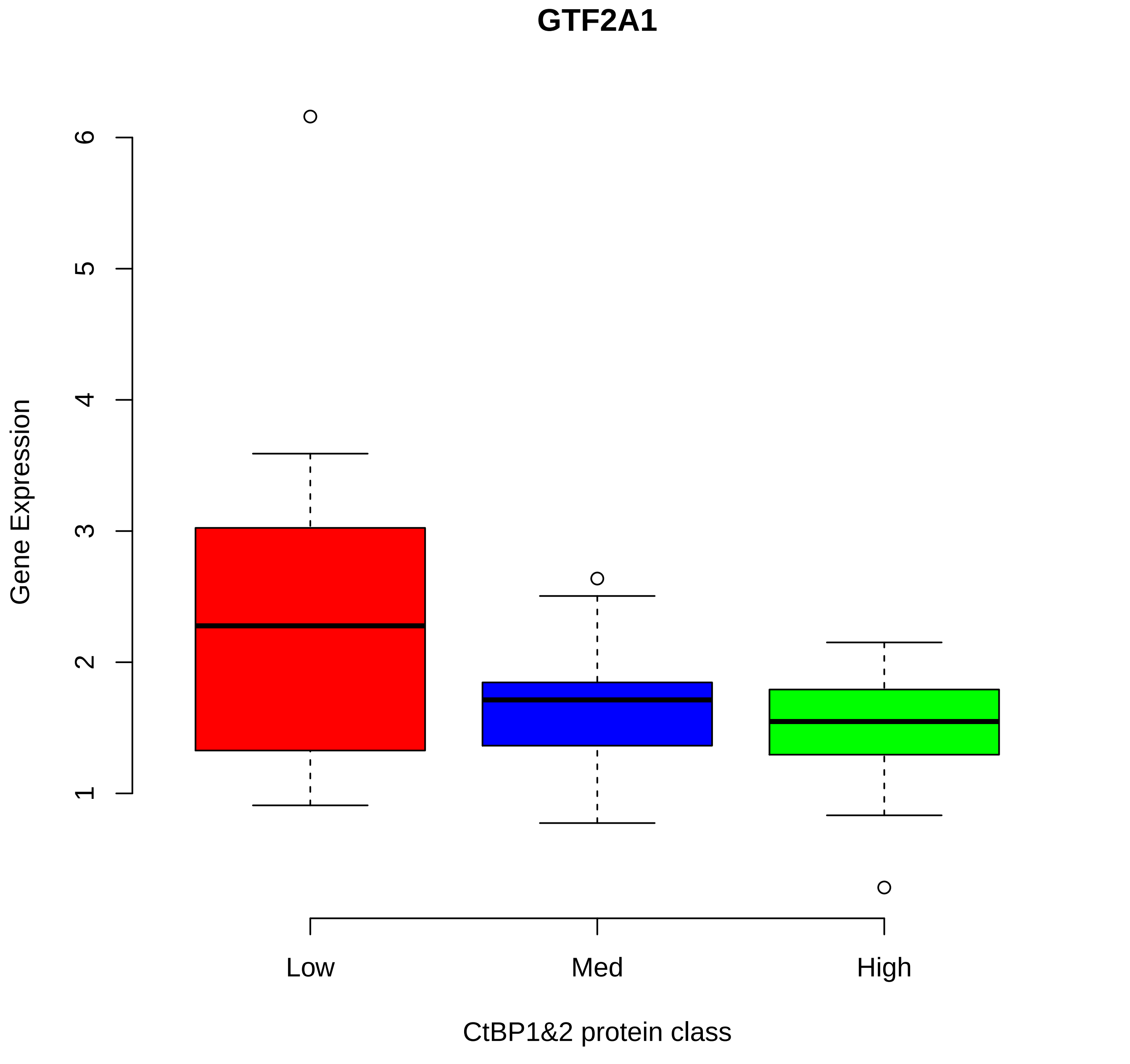
GTF2A1
Gene Expression
1
2
3
4
5
6
Low
Med
High
CtBP1&2 protein class

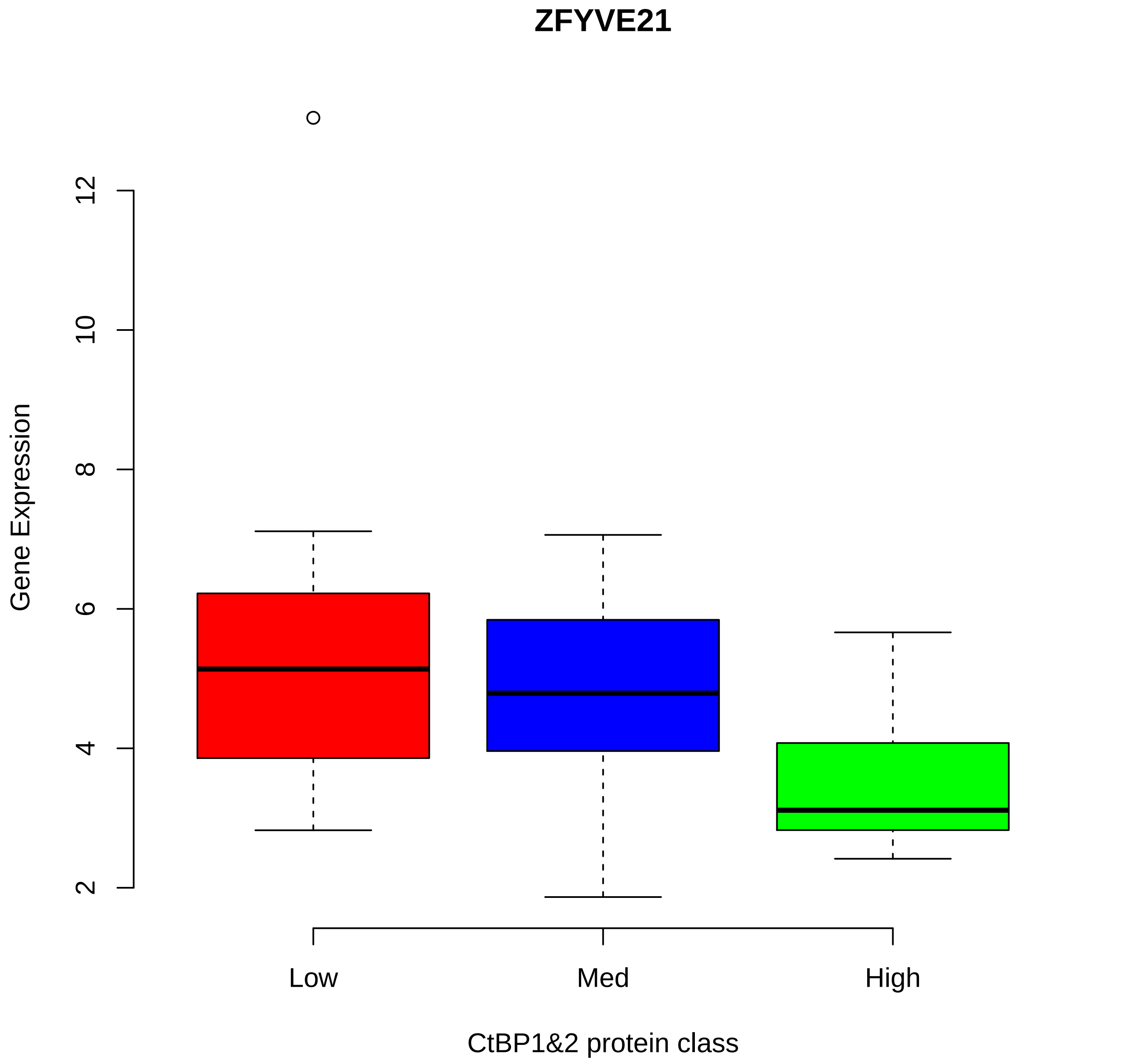
ZFYVE21
Gene Expression
2
4
6
8
10
12
Low
Med
High
CtBP1&2 protein class

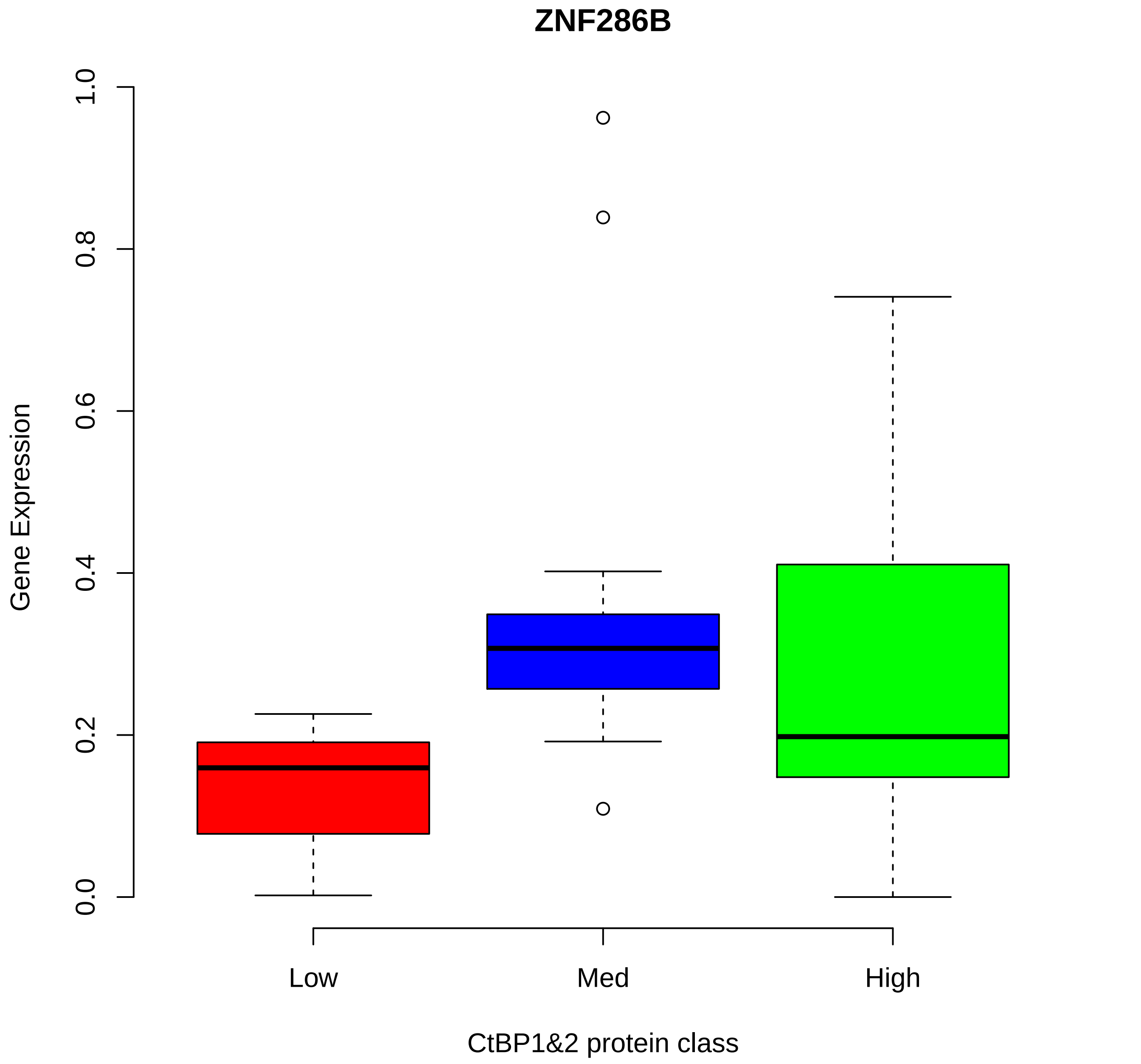

ZNF286B
Gene Expression
0.0
0.2
0.4
0.6
0.8
1.0
Low
Med
High
CtBP1&2 protein class

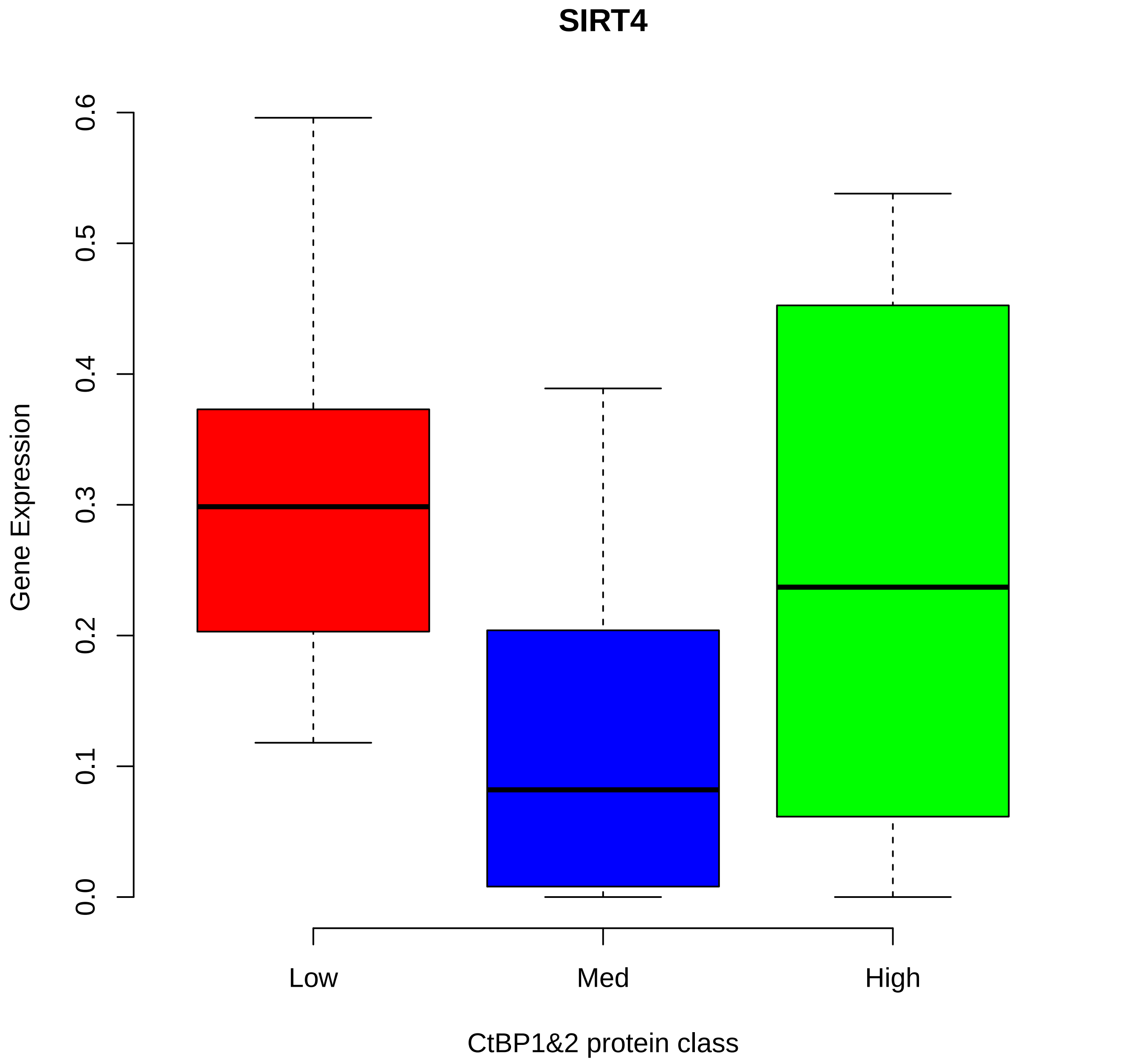
SIRT4
Gene Expression
0.0
0.1
0.2
0.3
0.4
0.5
0.6
Low
Med
High
CtBP1&2 protein class

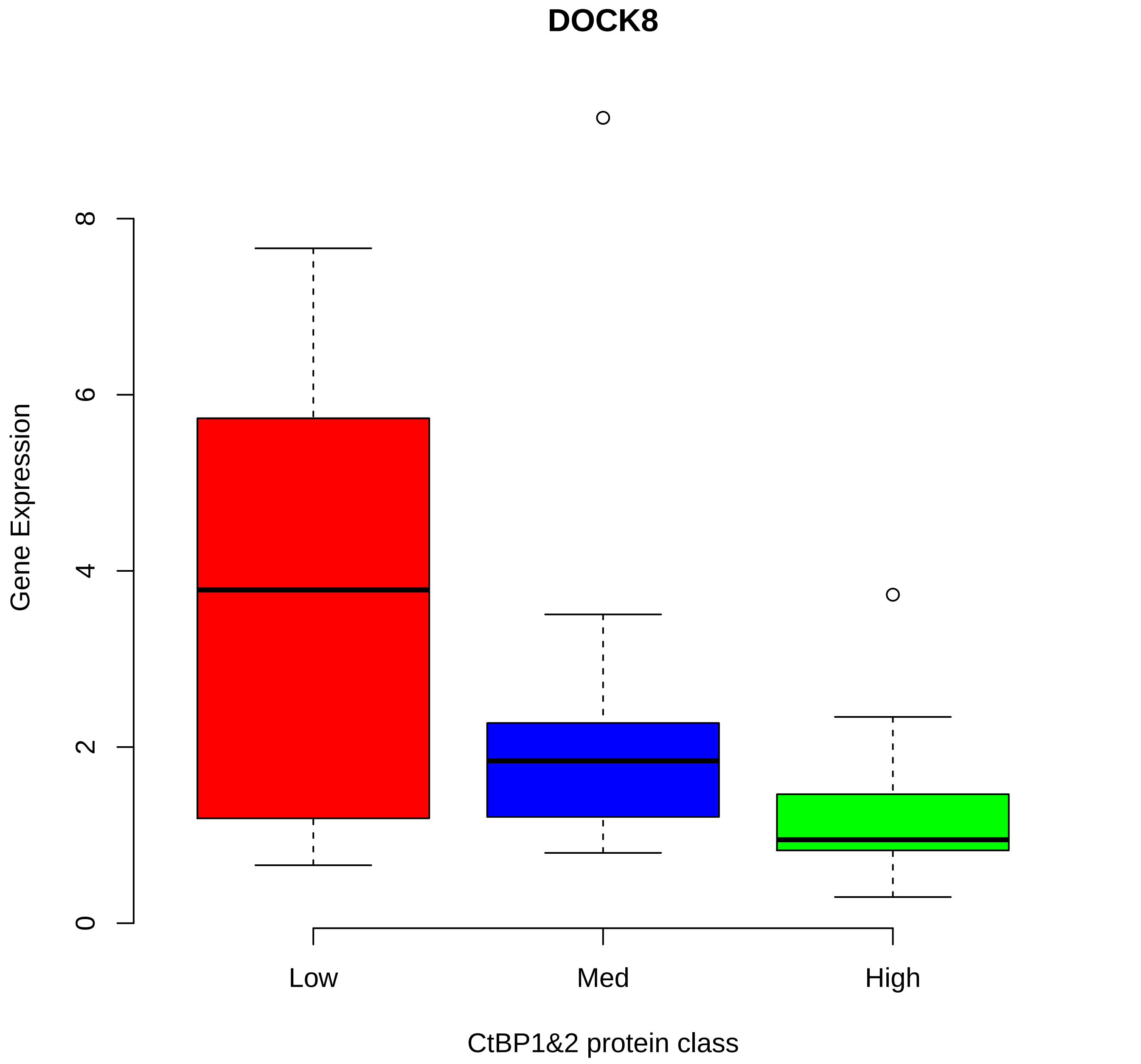
DOCK8
Gene Expression
0
2
4
6
8
Low
Med
High
CtBP1&2 protein class

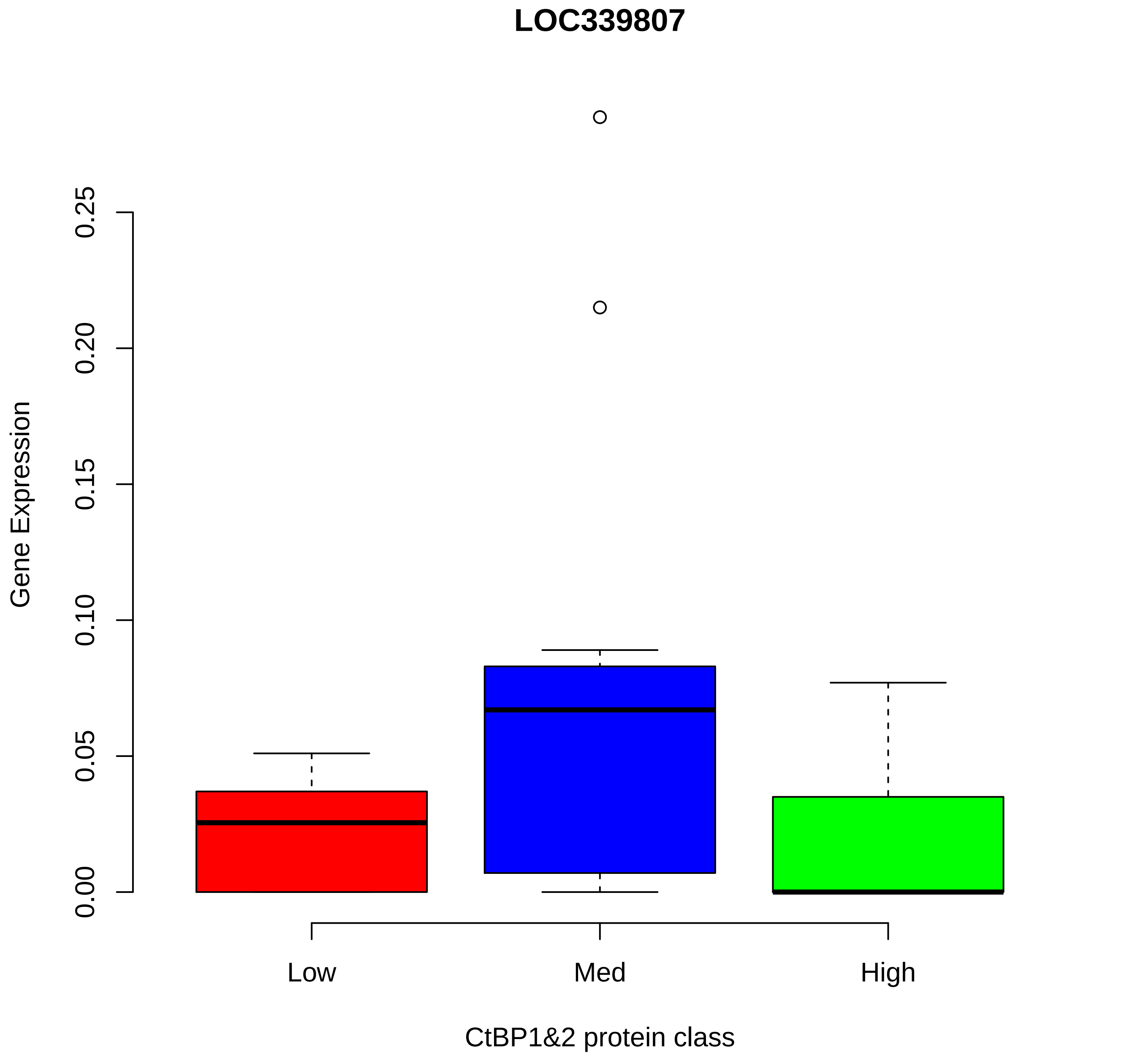

LOC339807
Gene Expression
0.00
0.05
0.10
0.15
0.20
0.25
Low
Med
High
CtBP1&2 protein class

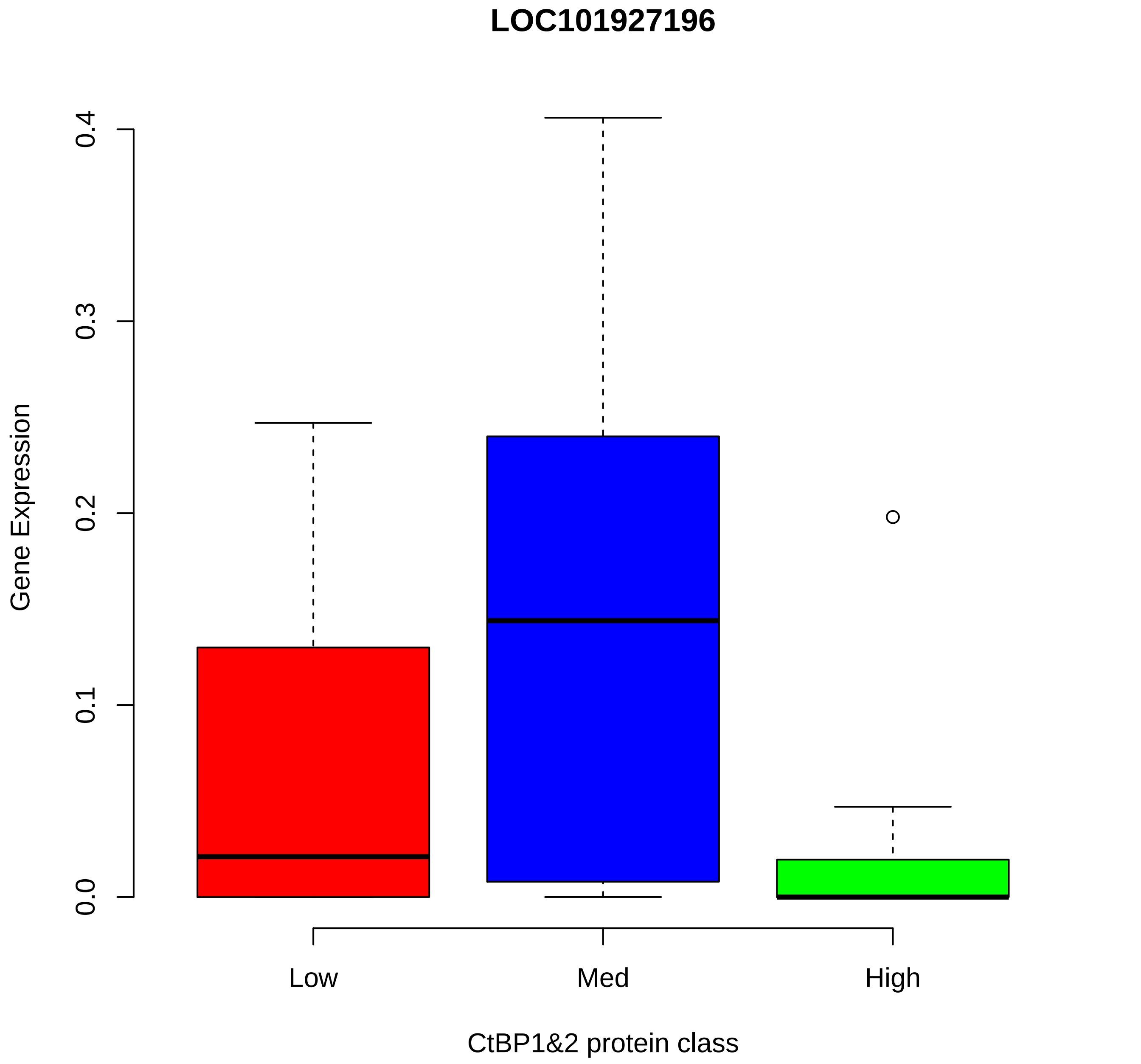

LOC101927196
Gene Expression
0.0
0.1
0.2
0.3
0.4
Low
Med
High
CtBP1&2 protein class

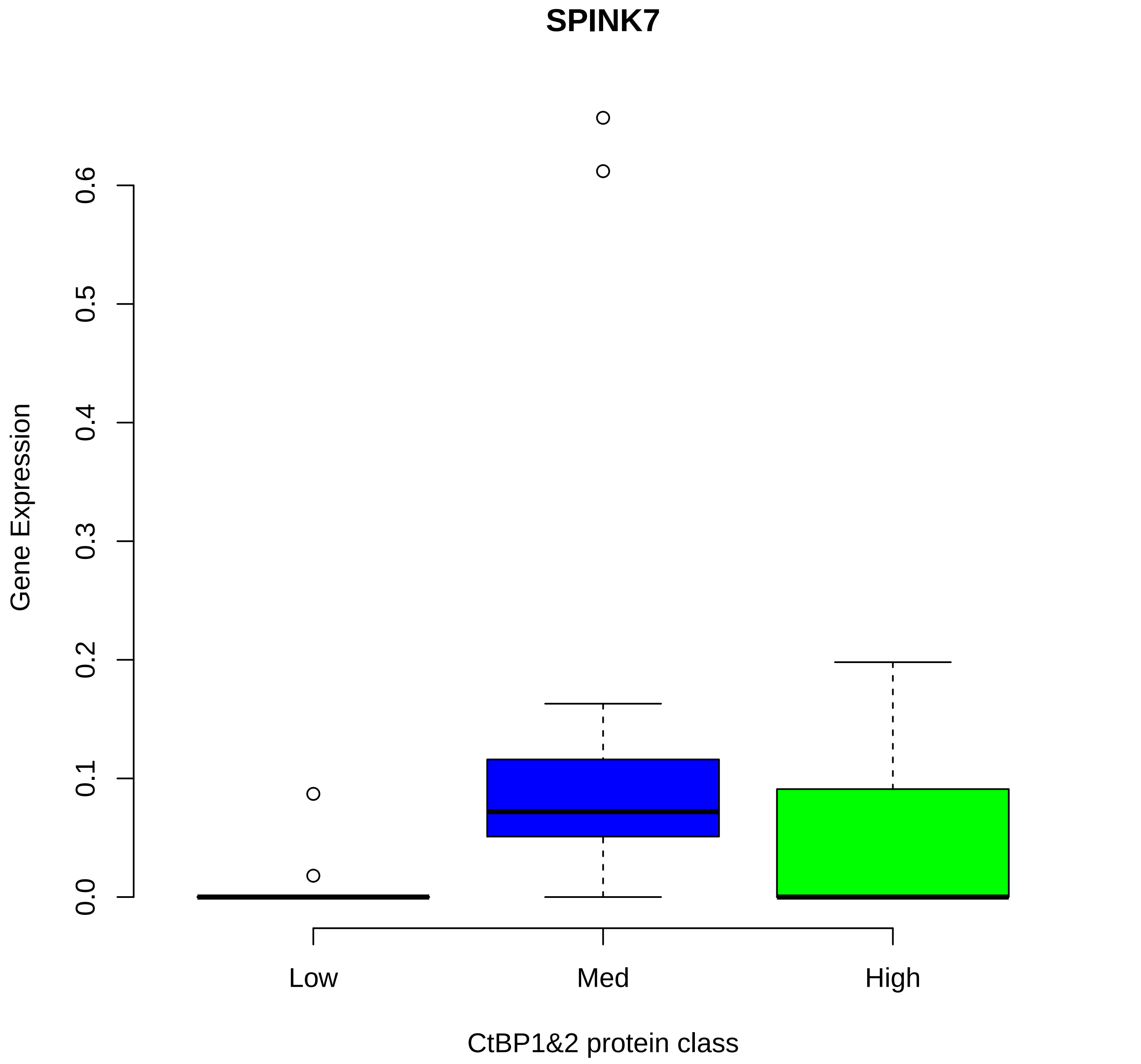
SPINK7
Gene Expression
0.0
0.1
0.2
0.3
0.4
0.5
0.6
Low
Med
High
CtBP1&2 protein class

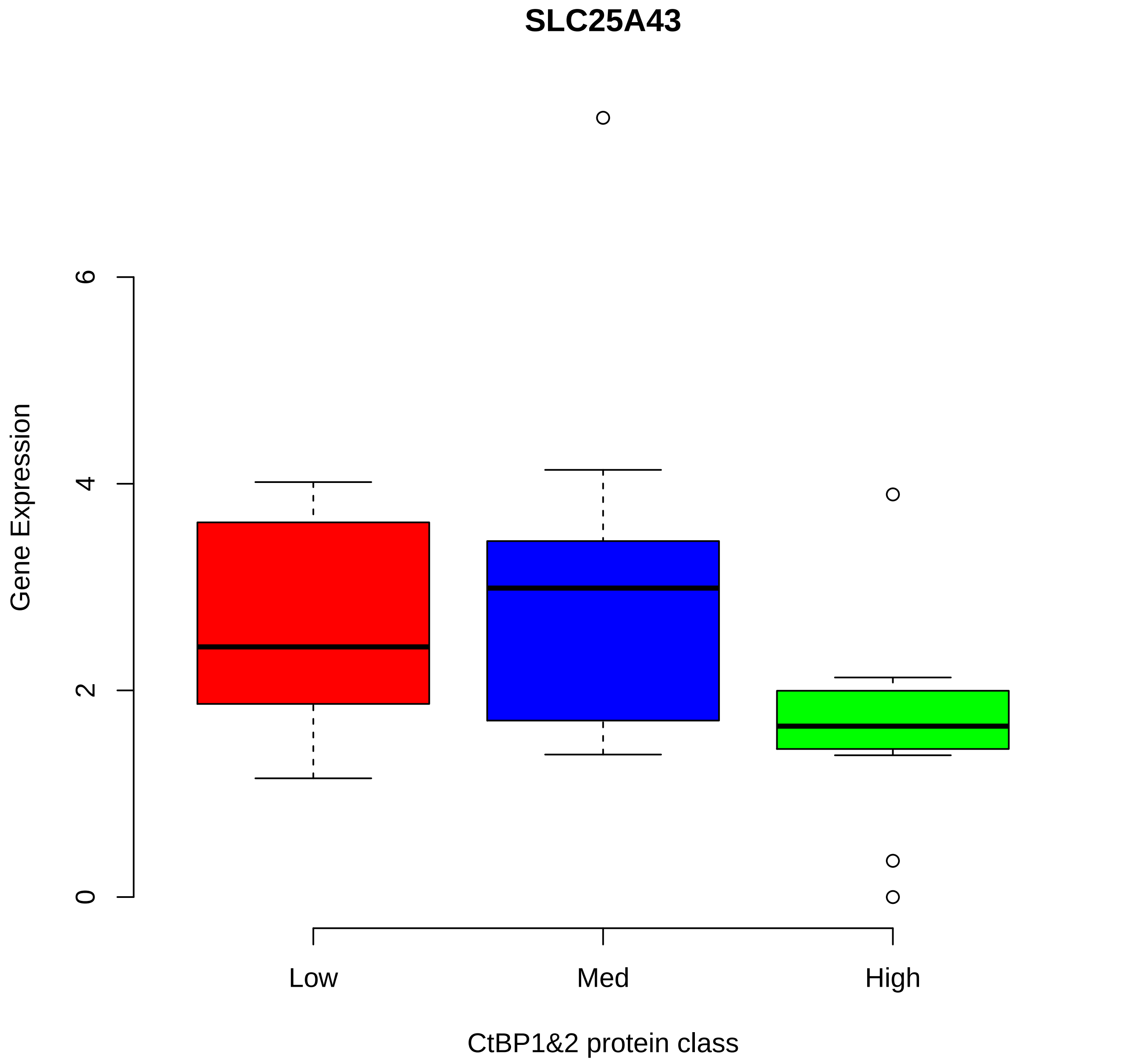
SLC25A43
Gene Expression
0
2
4
6
Low
Med
High
CtBP1&2 protein class

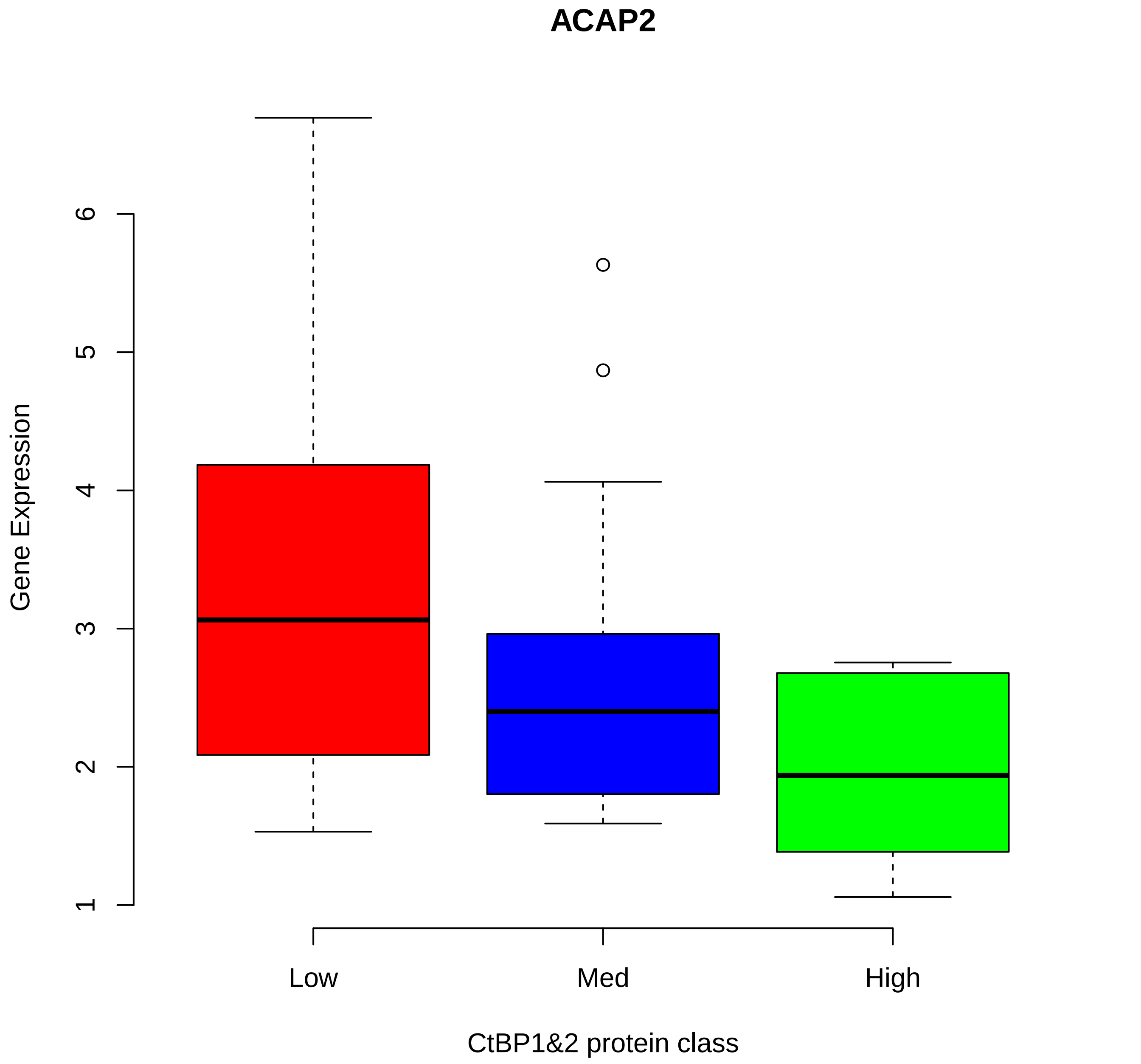

ACAP2
Gene Expression
1
2
3
4
5
6
Low
Med
High
CtBP1&2 protein class

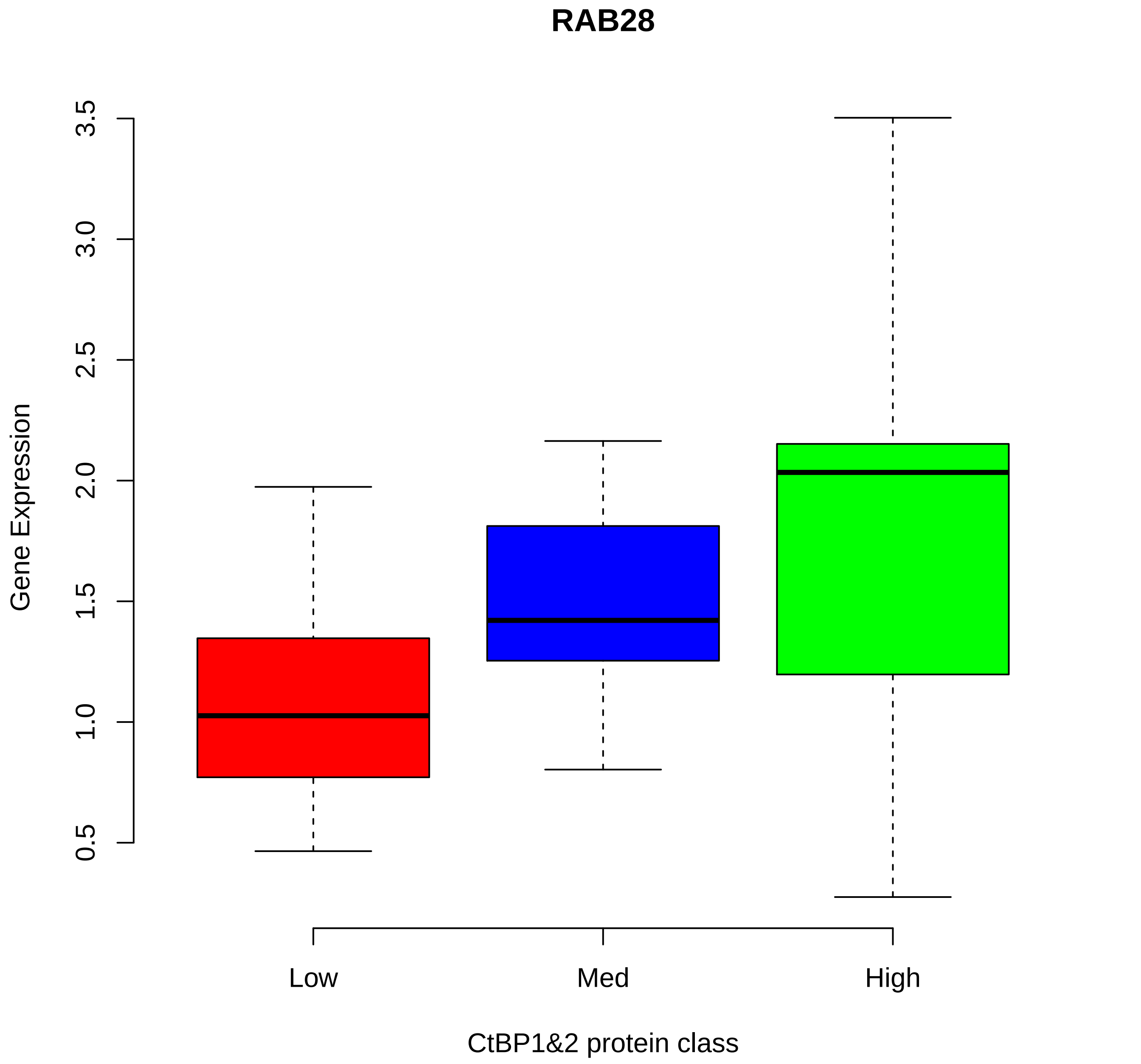

RAB28
Gene Expression
3.5
3.0
2.5
2.0
1.5
1.0
0.5
Low
Med
High
CtBP1&2 protein class

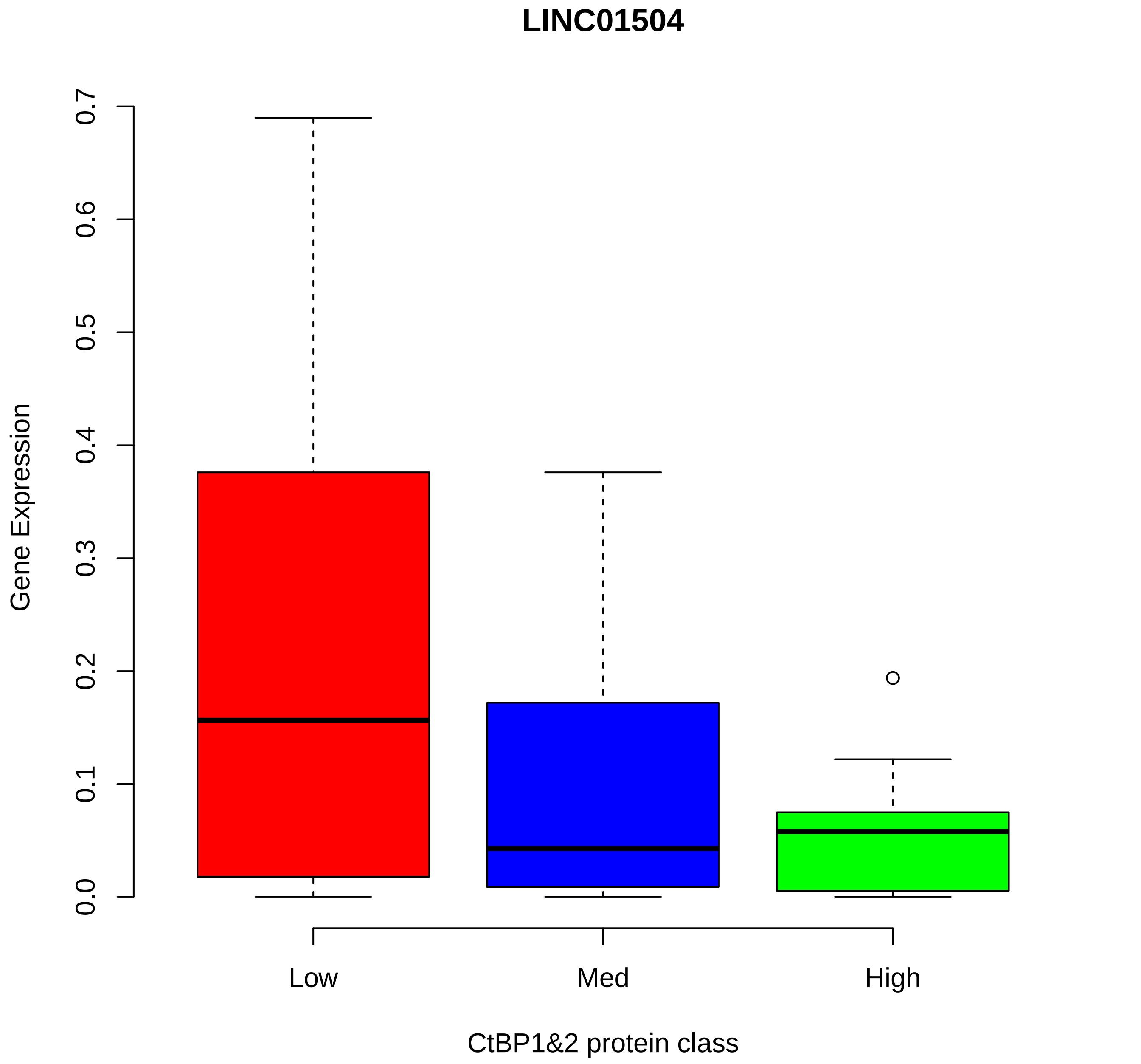

LINC01504
Gene Expression
0.0
0.1
0.2
0.3
0.4
0.5
0.6
0.7
Low
Med
High
CtBP1&2 protein class

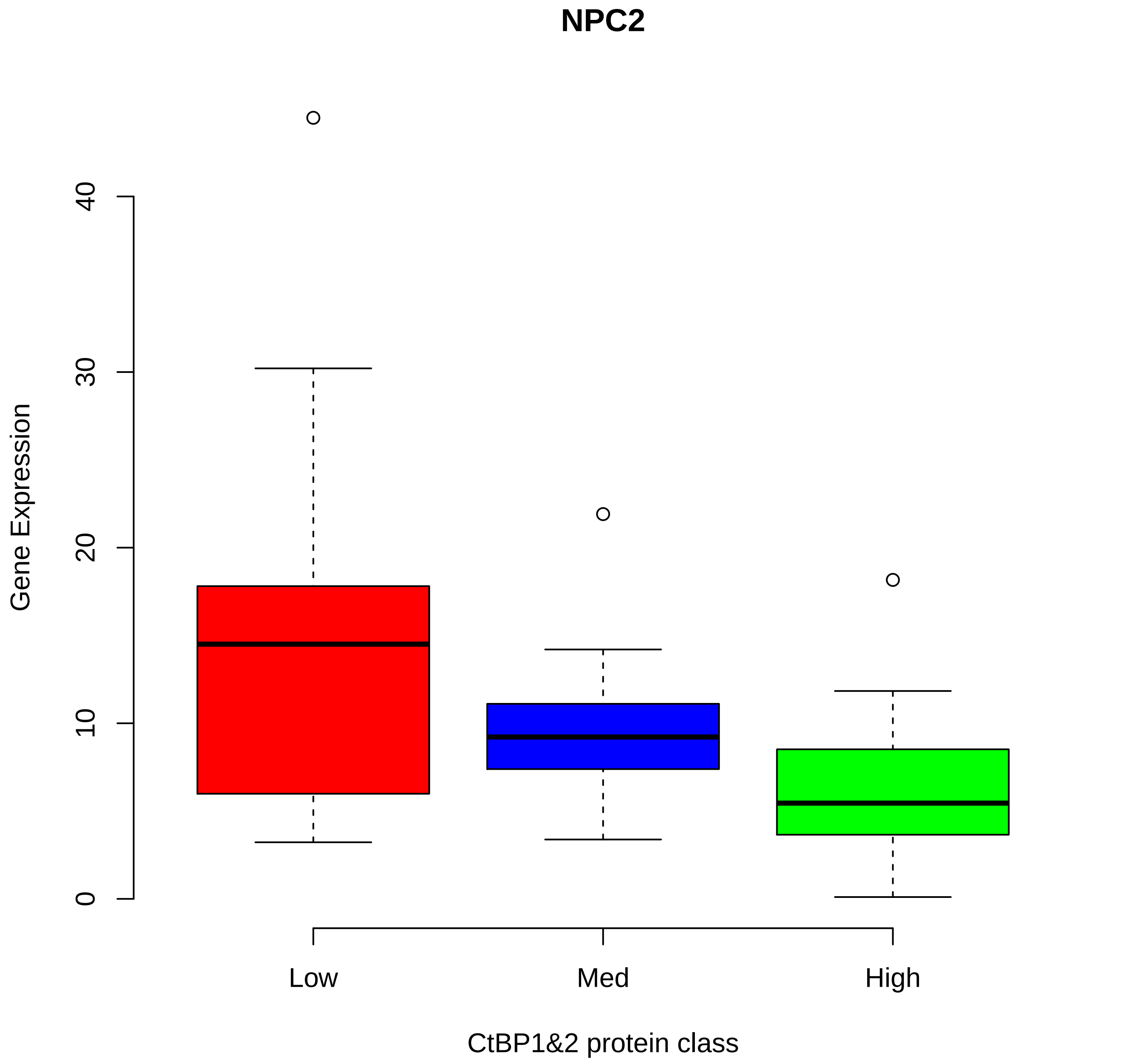
NPC2
Gene Expression
0
10
20
30
40
Low
Med
High
CtBP1&2 protein class

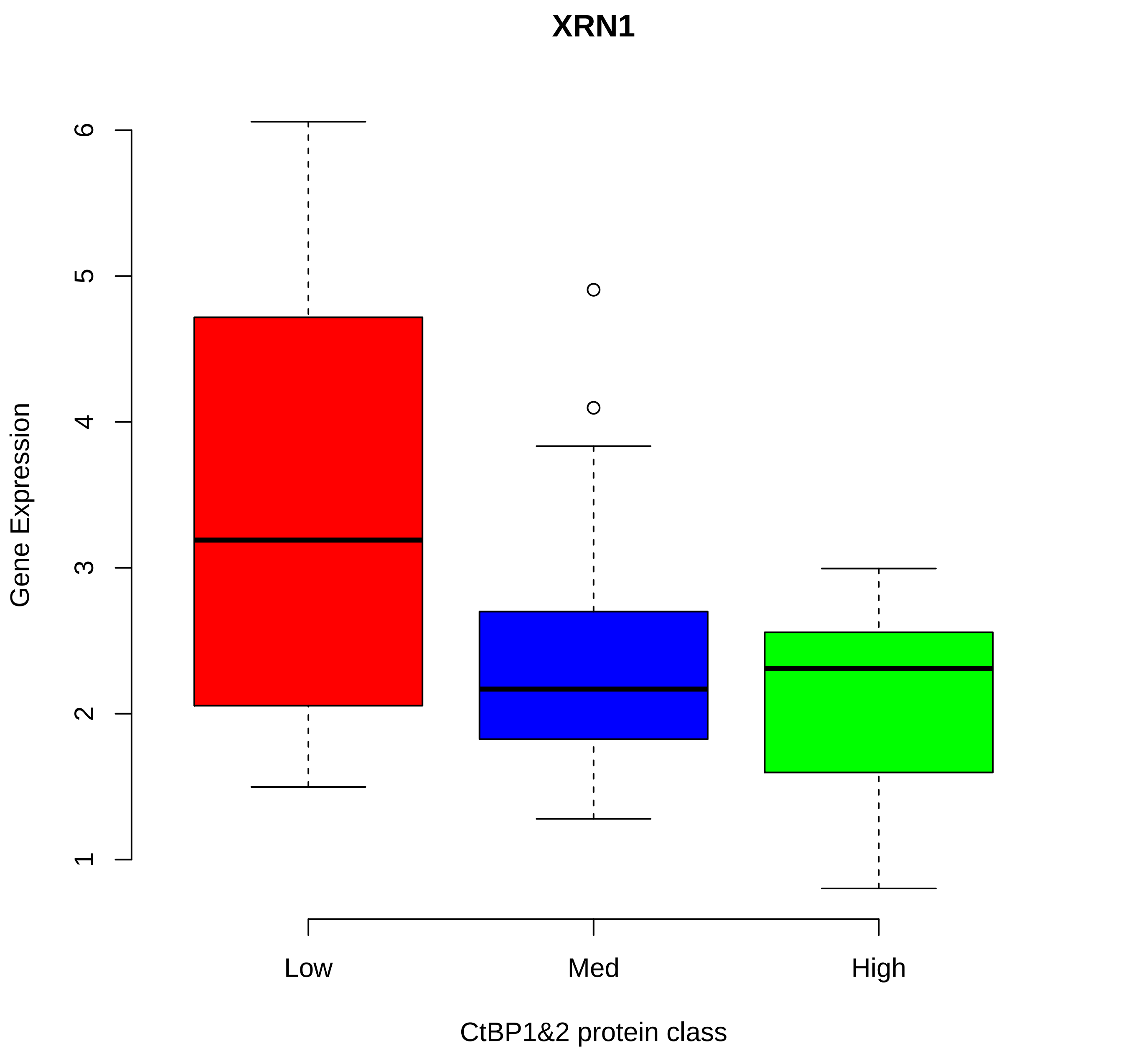

XRN1
Gene Expression
1
2
3
4
5
6
Low
Med
High
CtBP1&2 protein class

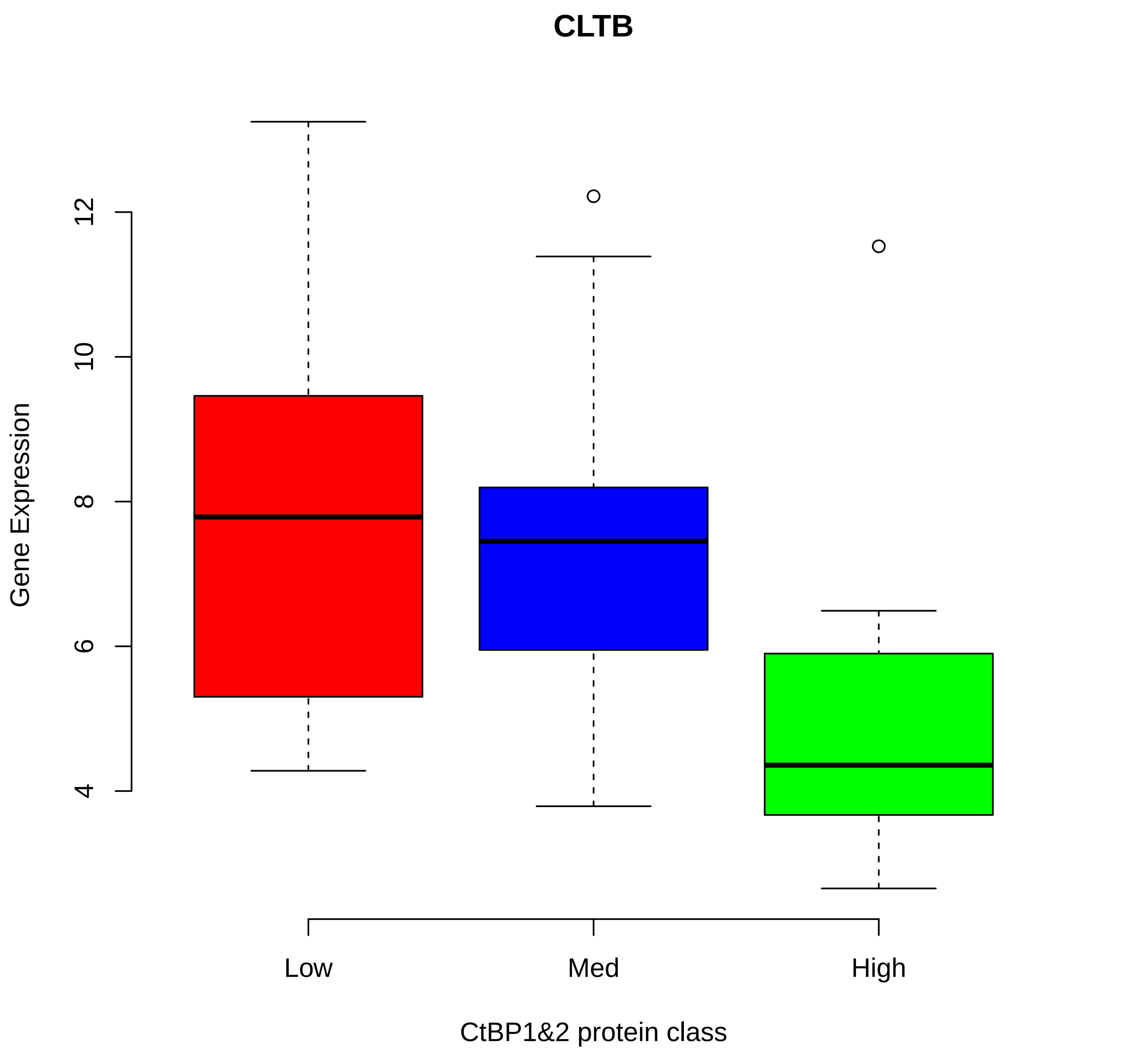
CLTB
Gene Expression
4
6
8
10
12
Low
Med
High
CtBP1&2 protein class

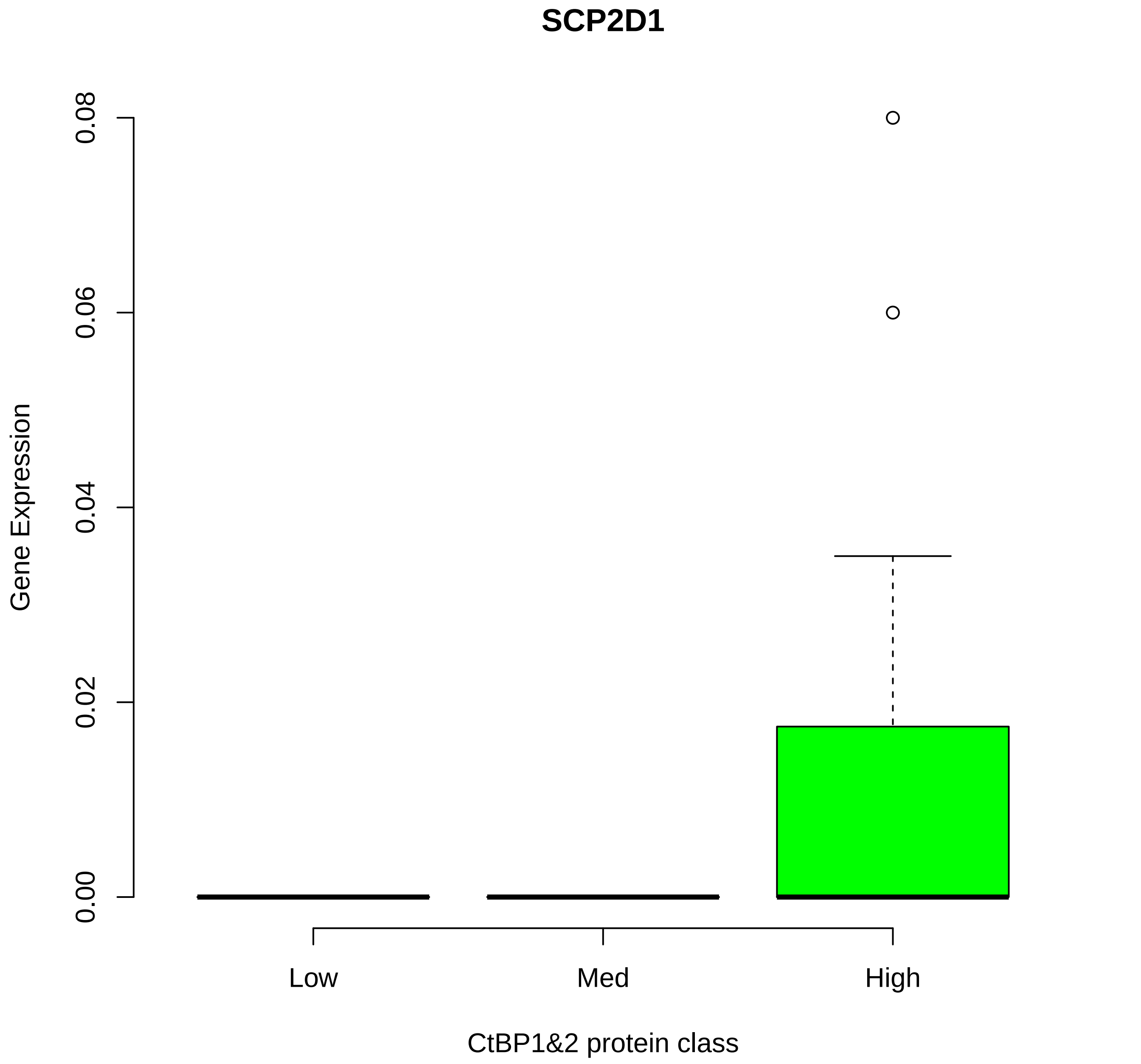

SCP2D1
0.08
0.06
0.04
0.02
0.00
Gene Expression
Low
Med
High
CtBP1&2 protein class

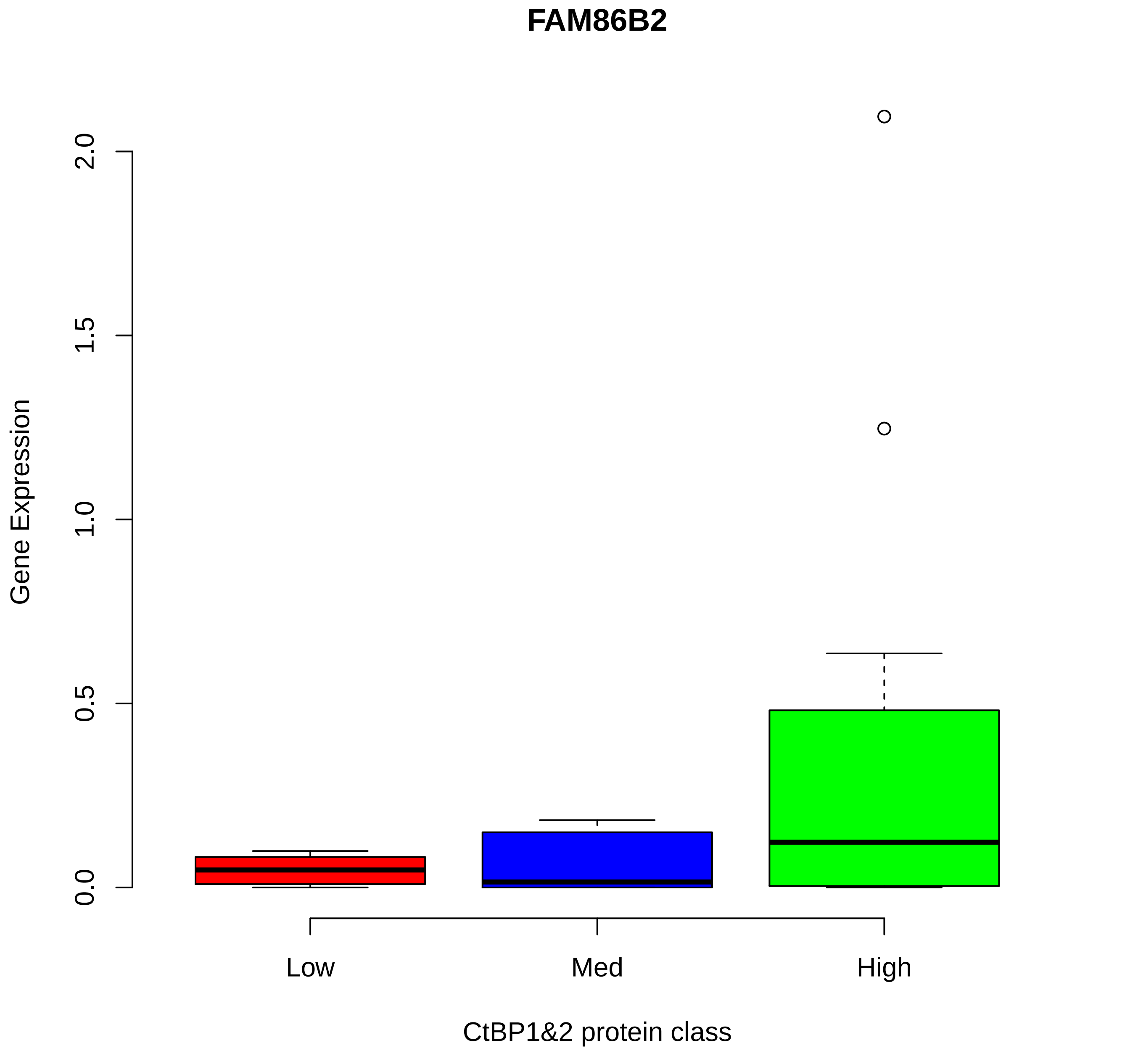

FAM86B2
Gene Expression
0.0
0.5
1.0
1.5
2.0
Low
Med
High
CtBP1&2 protein class

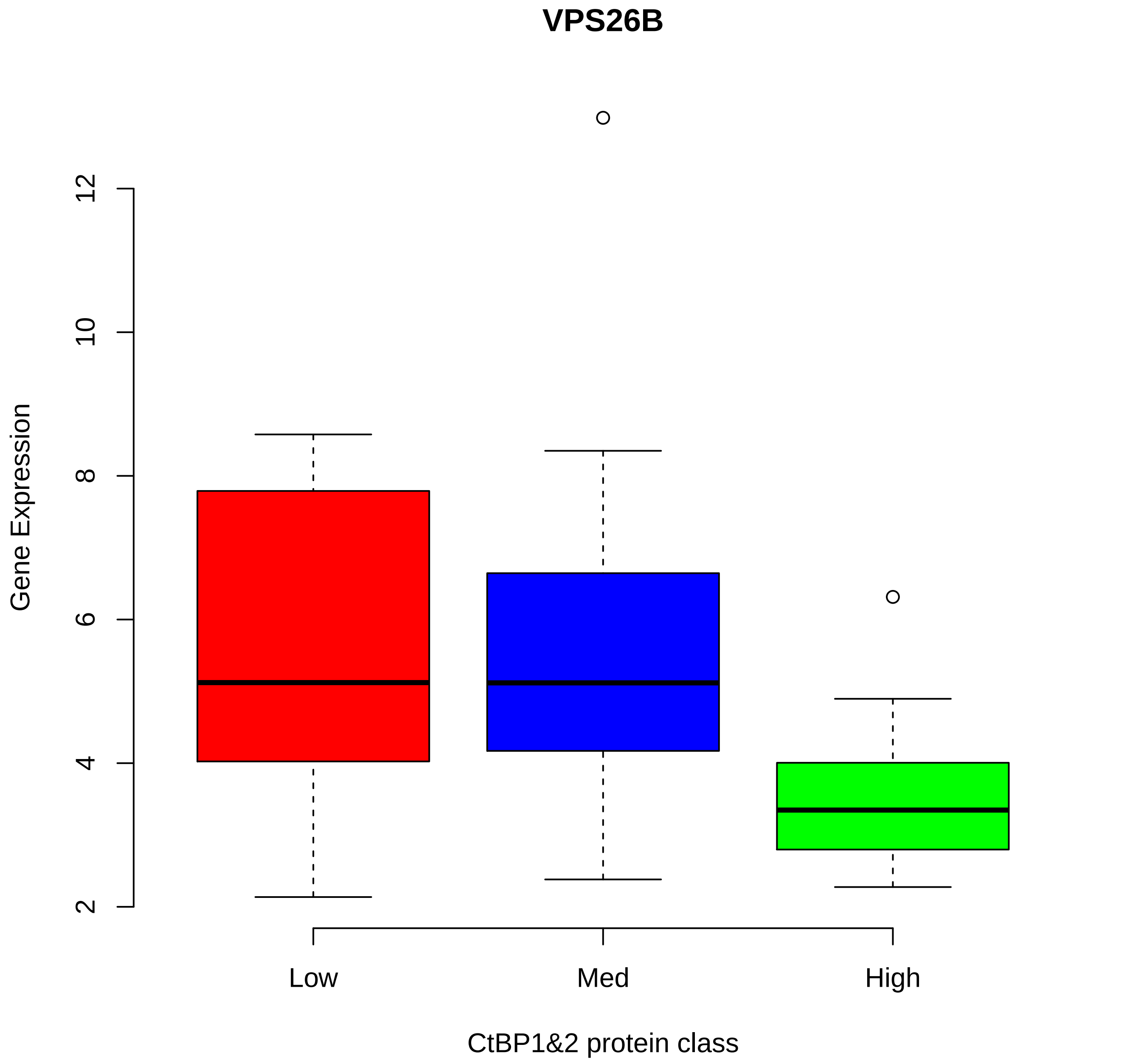
VPS26B
Gene Expression
2
4
6
8
10
12
Low
Med
High
CtBP1&2 protein class

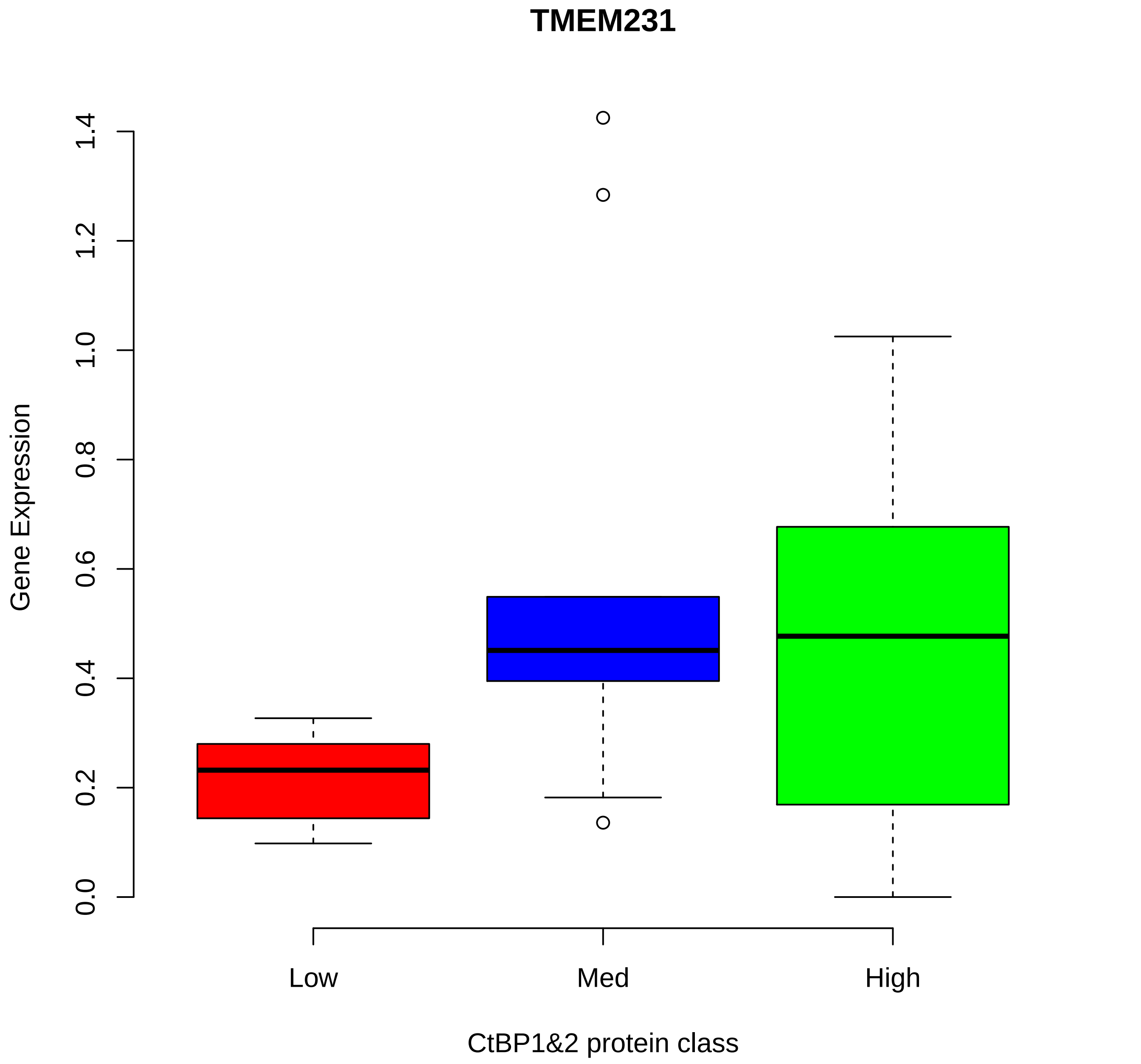
TMEM231
Gene Expression
0.0
0.2
0.4
0.6
0.8
1.0
1.2
1.4
Low
Med
High
CtBP1&2 protein class

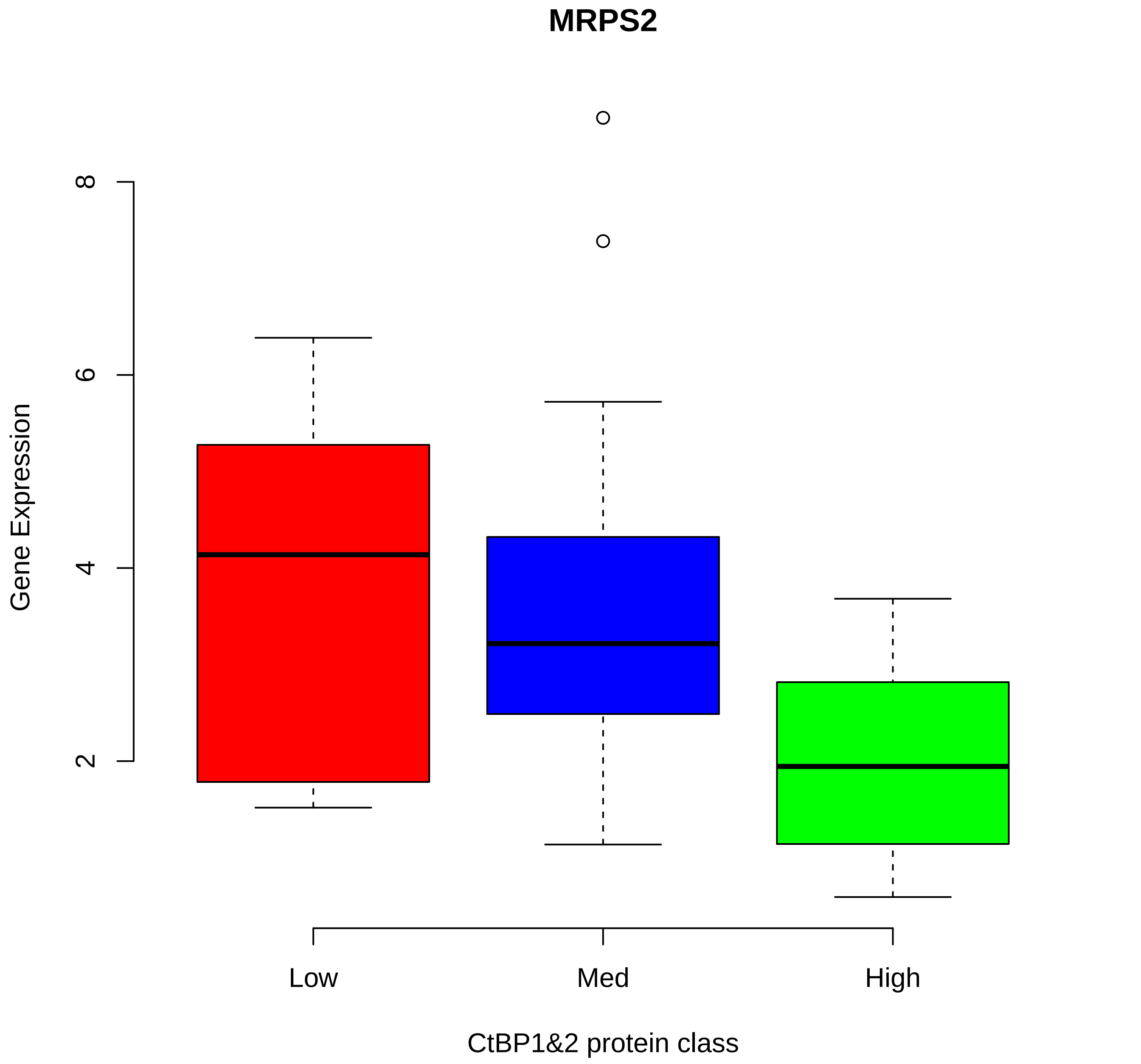
MRPS2
Gene Expression
8
6
4
2
Low
Med
High
CtBP1&2 protein class

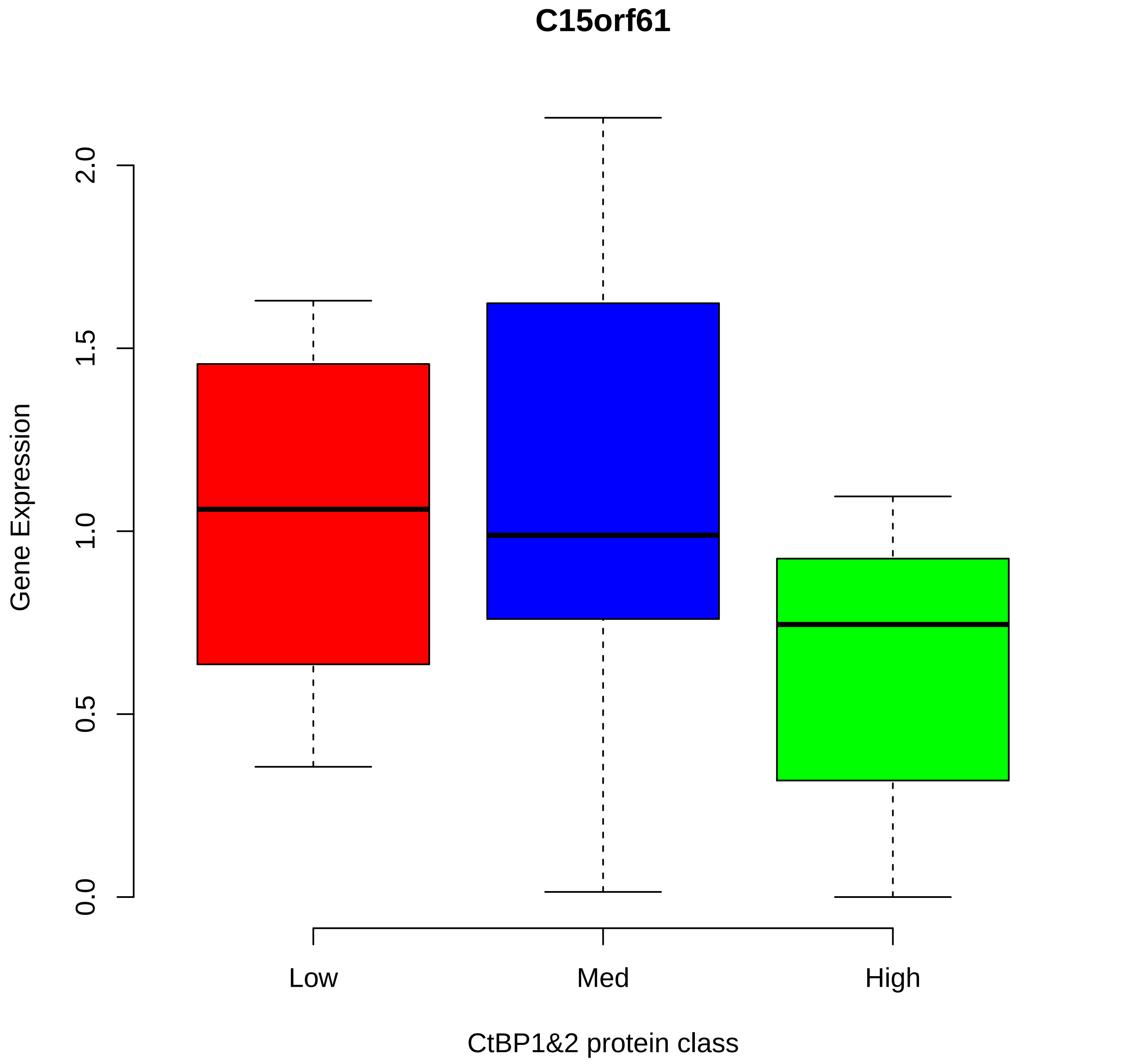

C15orf61
Gene Expression
0.0
0.5
1.0
1.5
2.0
Low
Med
High
CtBP1&2 protein class

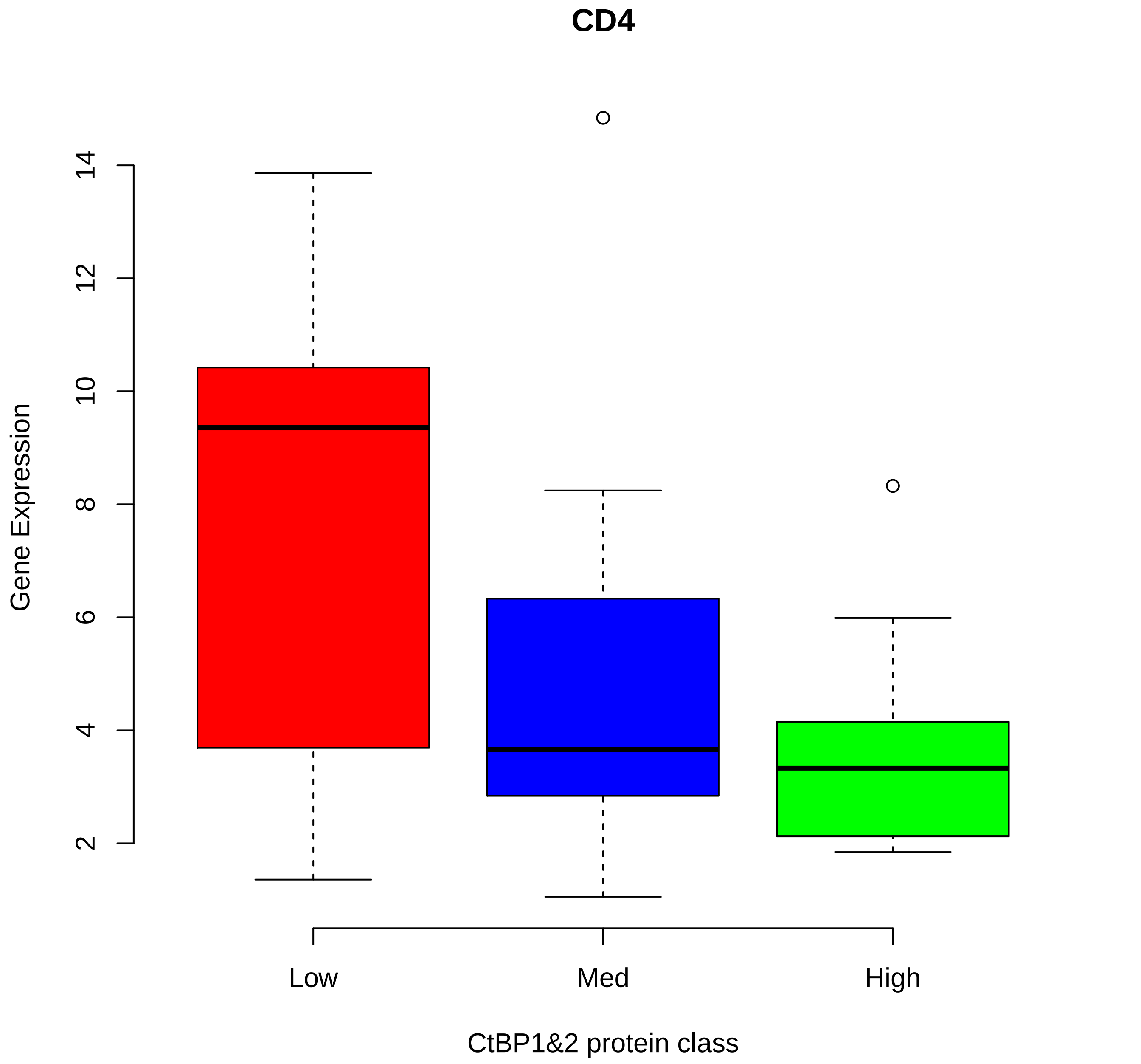
CD4
Gene Expression
2
4
6
8
10
12
14
Low
Med
High
CtBP1&2 protein class

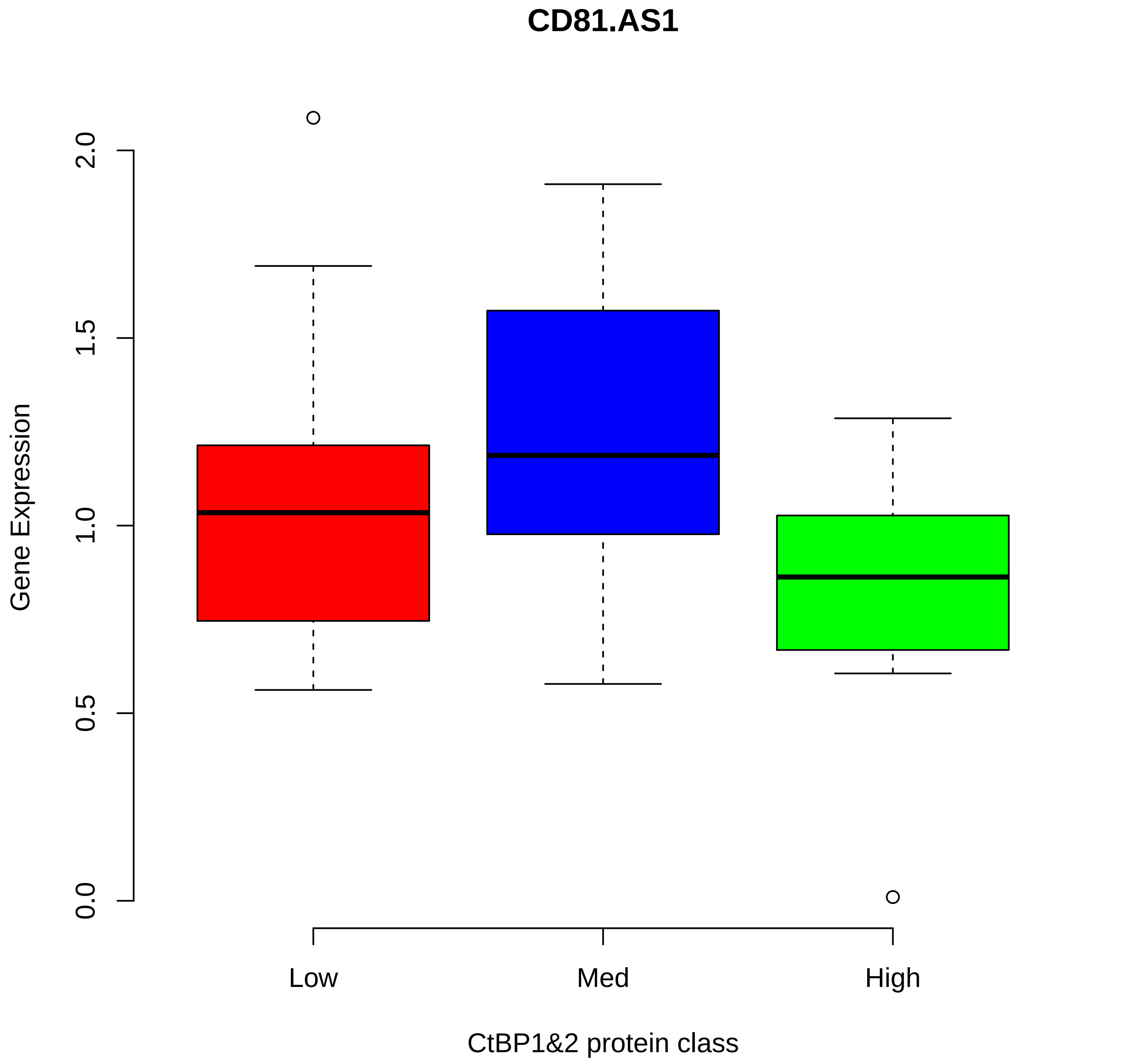

CD81.AS1
Gene Expression
2.0
1.5
1.0
0.5
0.0
Low
Med
High
CtBP1&2 protein class

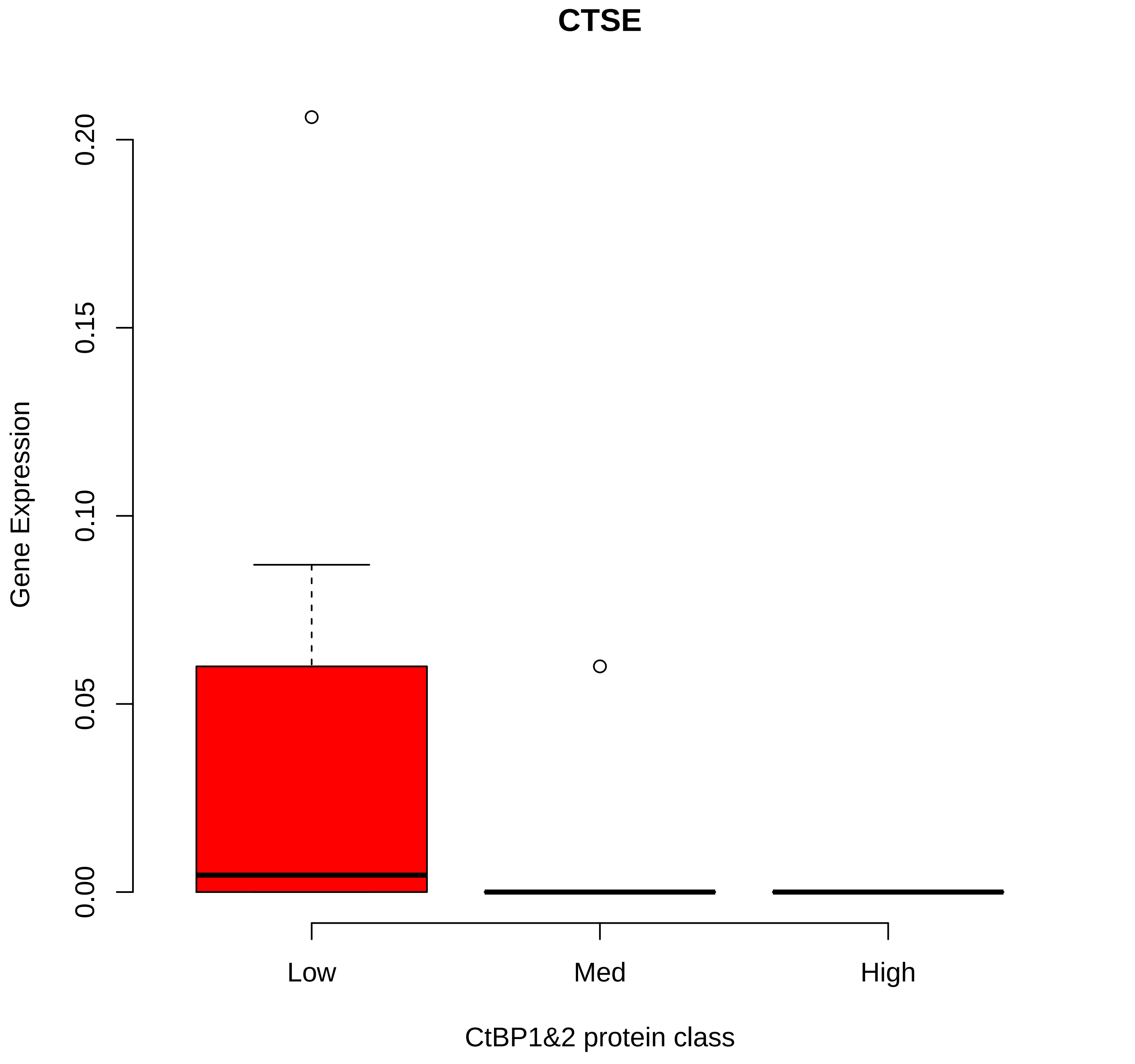

CTSE
Gene Expression
0.20
0.15
0.10
0.05
0.00
Low
Med
High
CtBP1&2 protein class

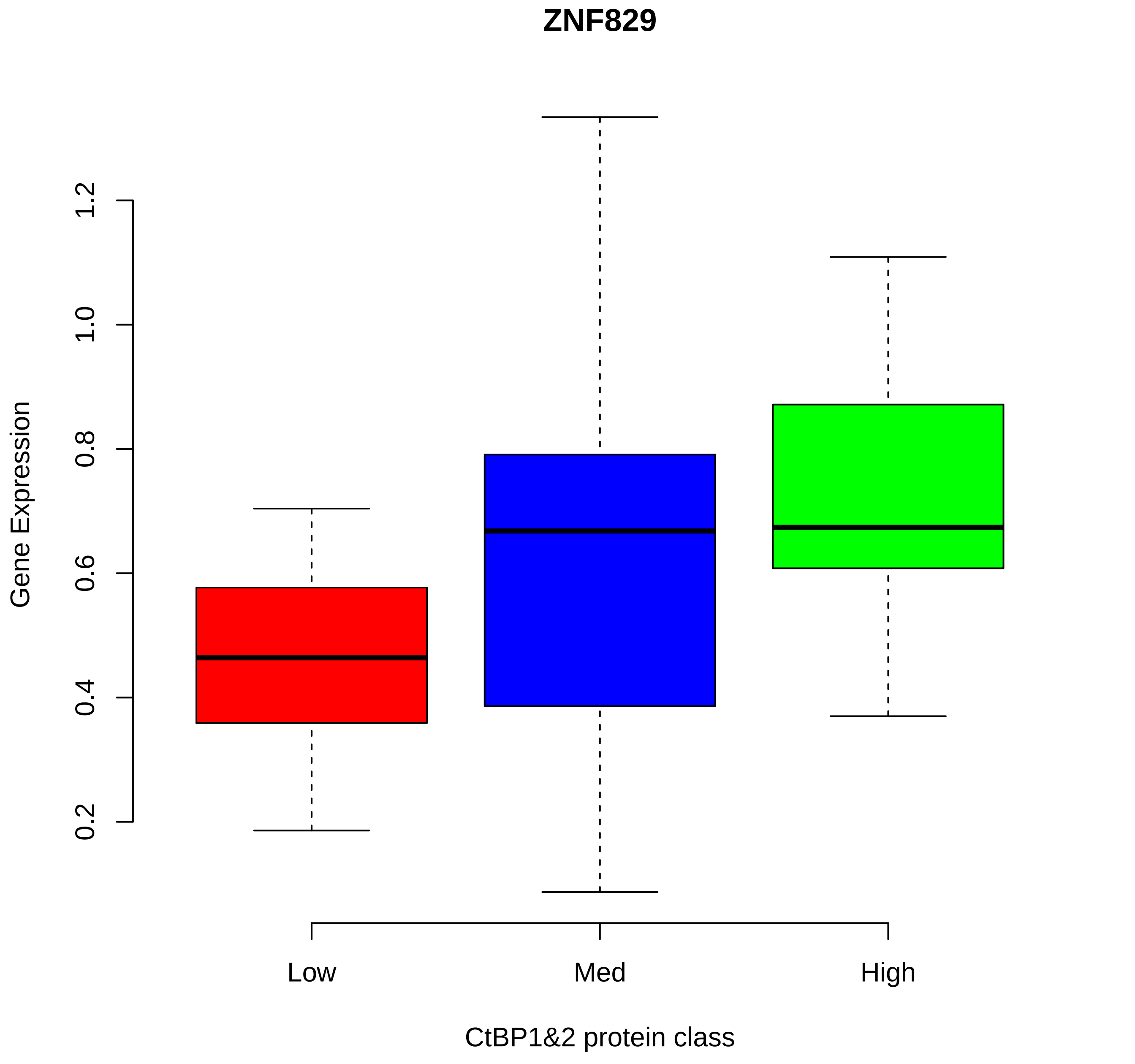

ZNF829
Gene Expression
0.2
0.4
0.6
0.8
1.0
1.2
Low
Med
High
CtBP1&2 protein class

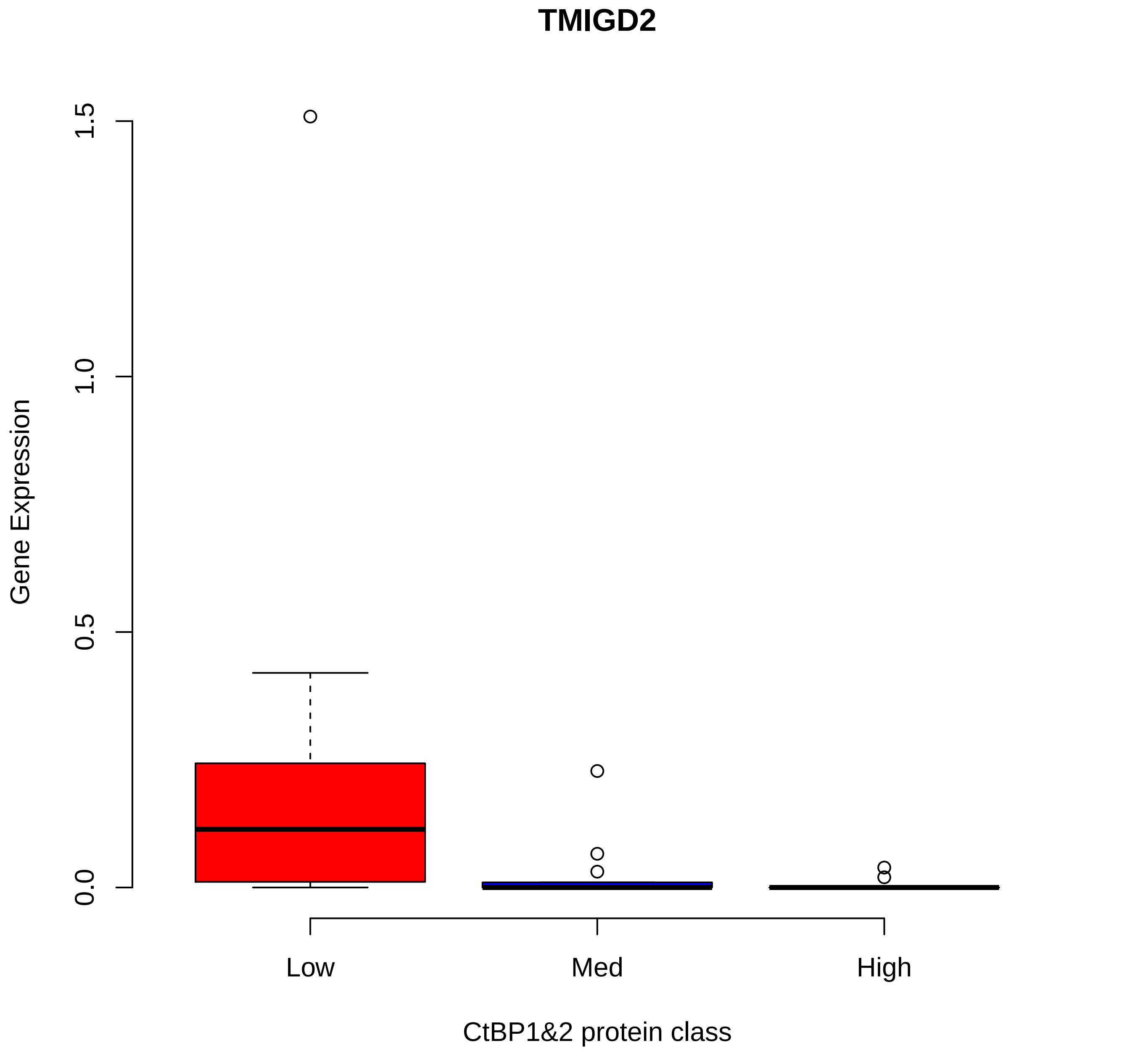
TMIGD2
Gene Expression
0.0
0.5
1.0
1.5
Low
Med
High
CtBP1&2 protein class

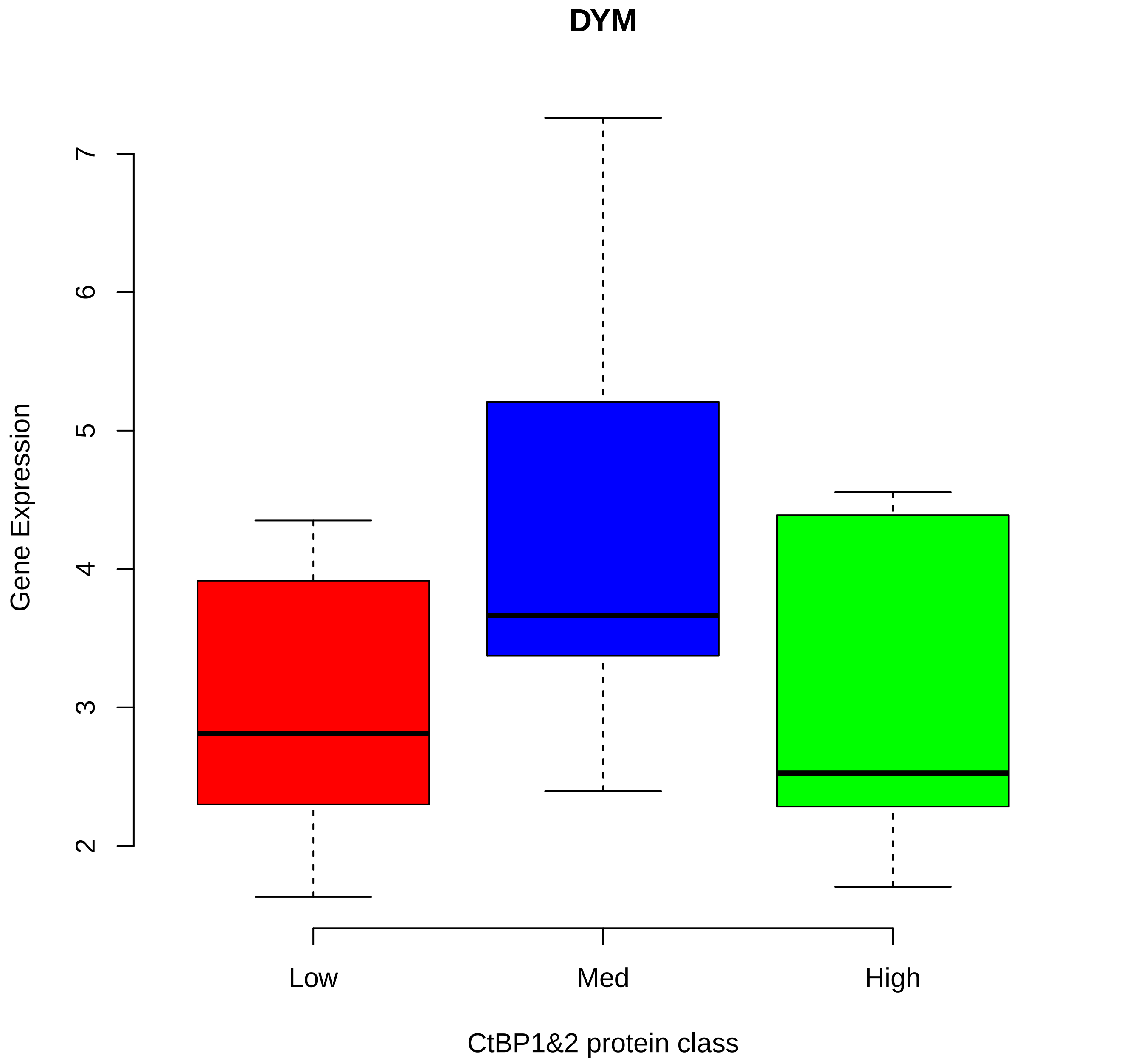

DYM
Gene Expression
2
3
4
5
6
7
Low
Med
High
CtBP1&2 protein class

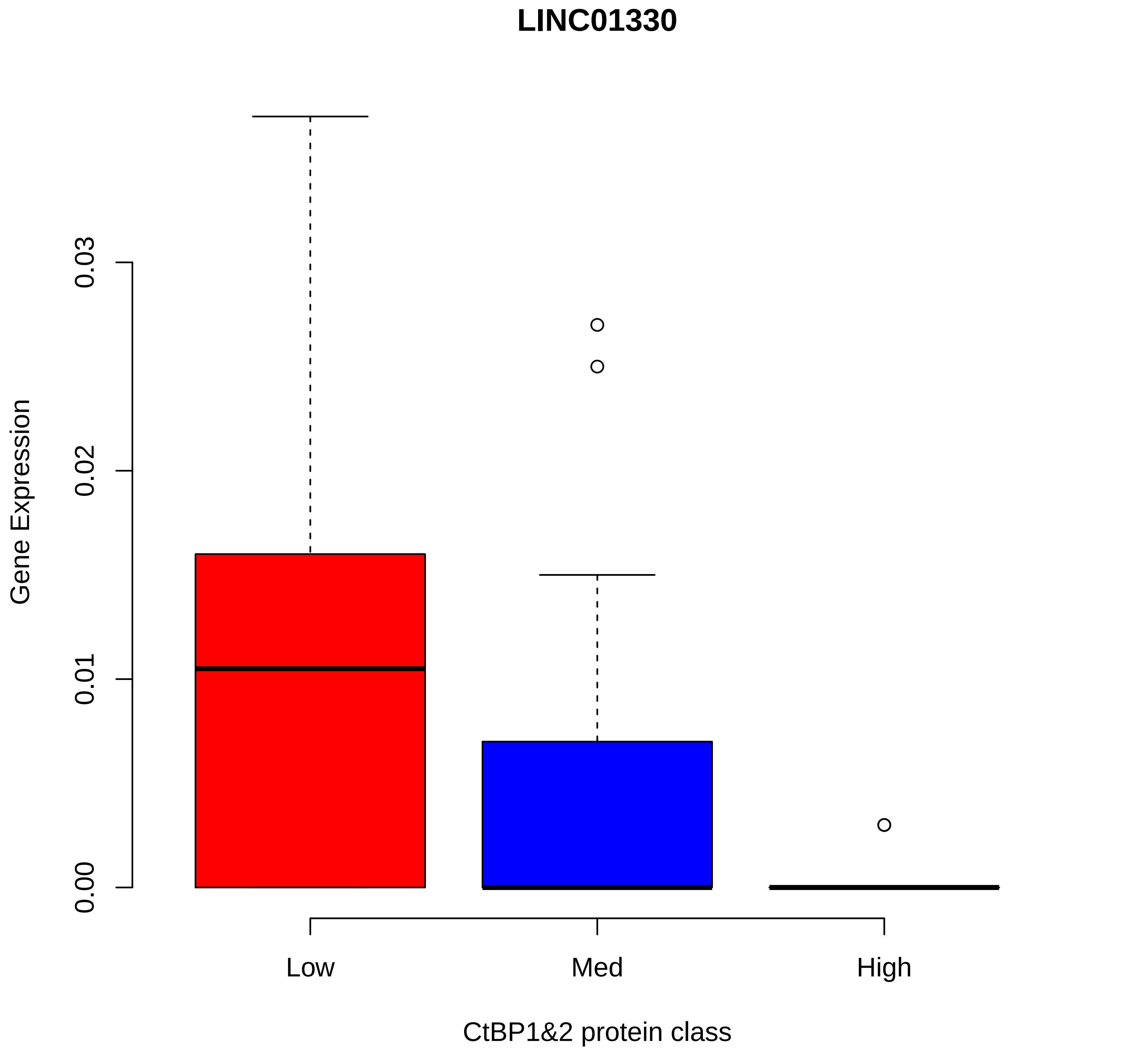

LINC01330
Gene Expression
0.00
0.01
0.02
0.03
Low
Med
High
CtBP1&2 protein class

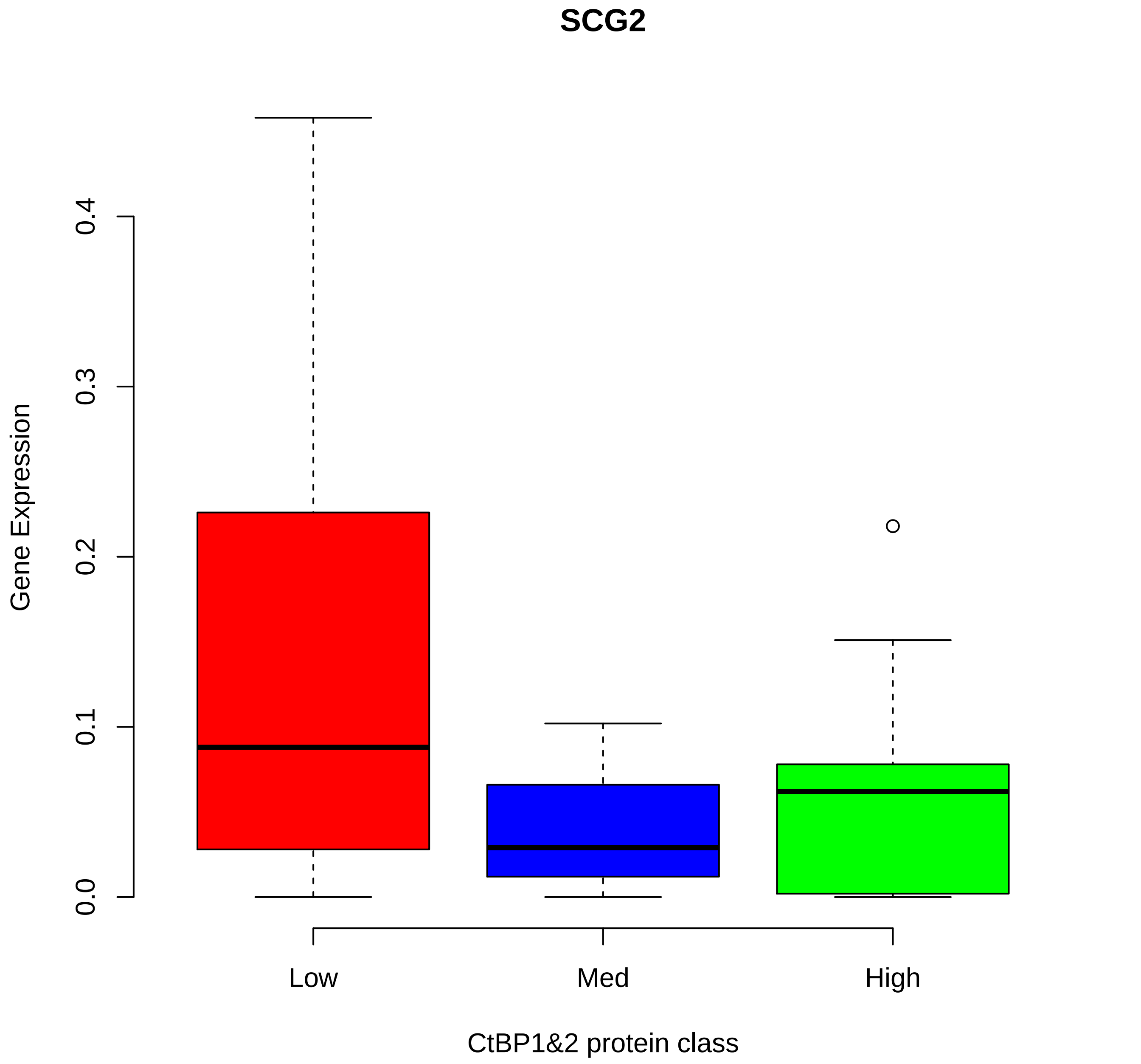

SCG2
Gene Expression
0.0
0.1
0.2
0.3
0.4
Low
Med
High
CtBP1&2 protein class

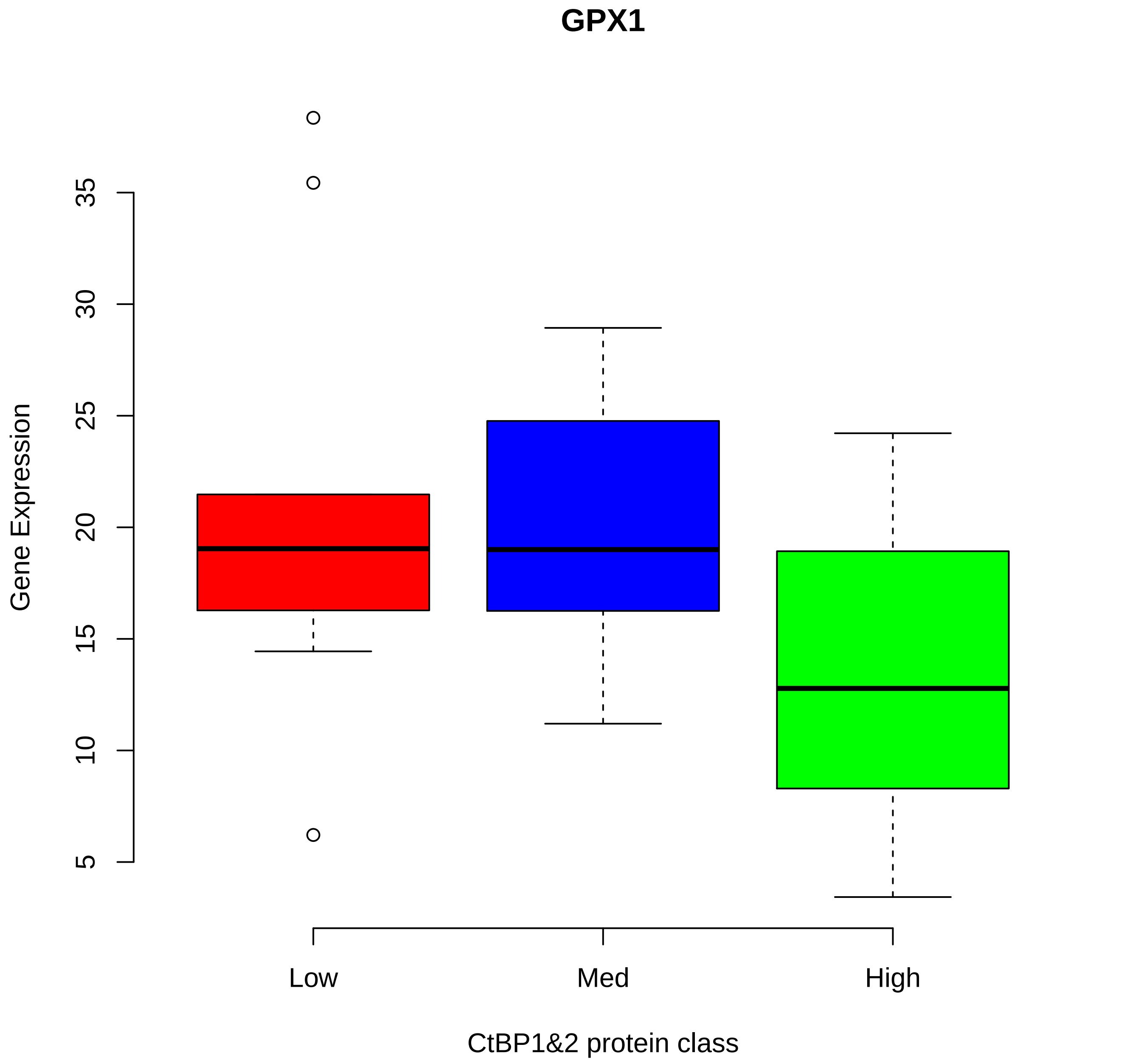
GPX1
Gene Expression
5
10
15
20
25
30
35
Low
Med
High
CtBP1&2 protein class

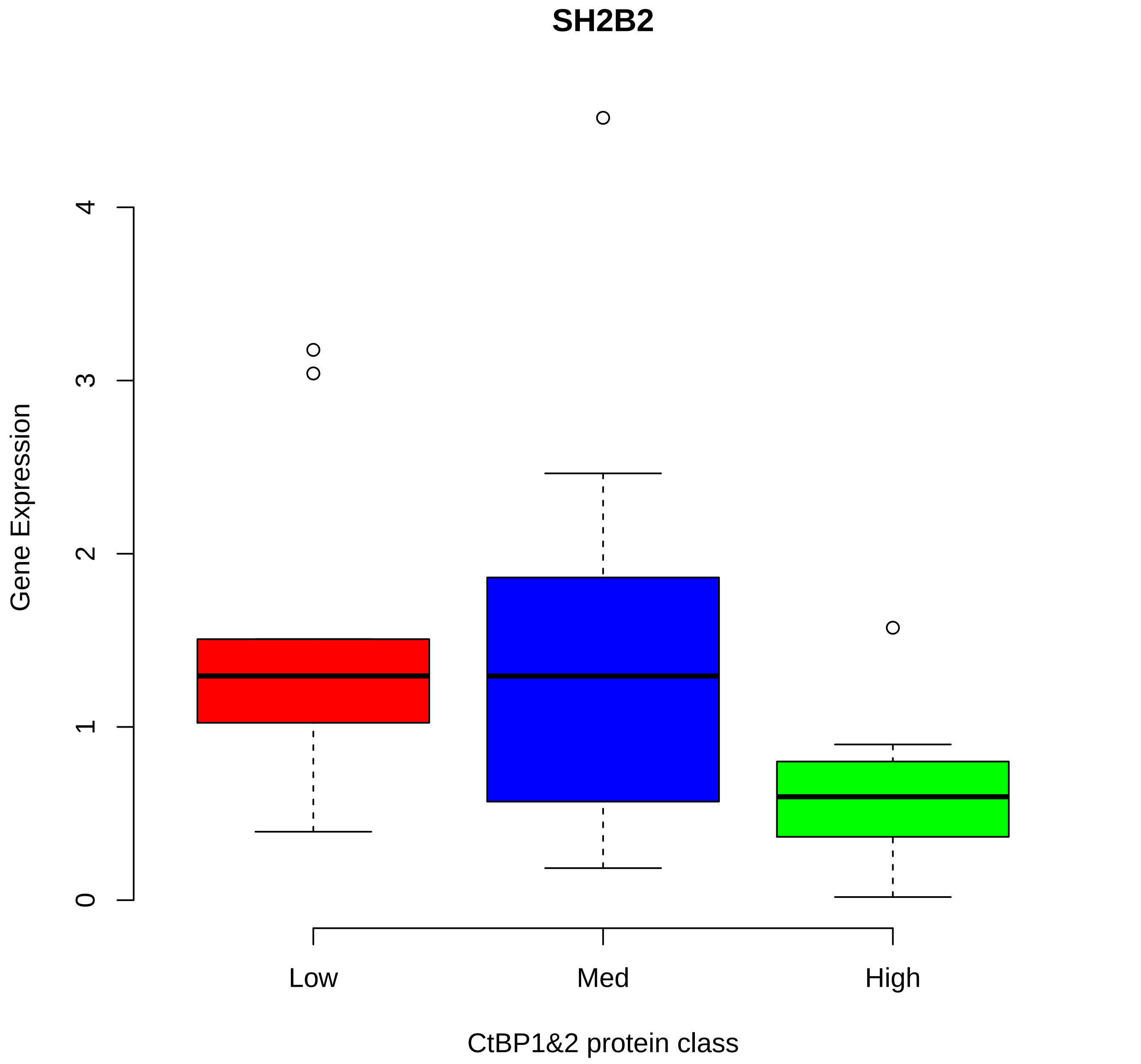
SH2B2
Gene Expression
0
1
2
3
4
Low
Med
High
CtBP1&2 protein class

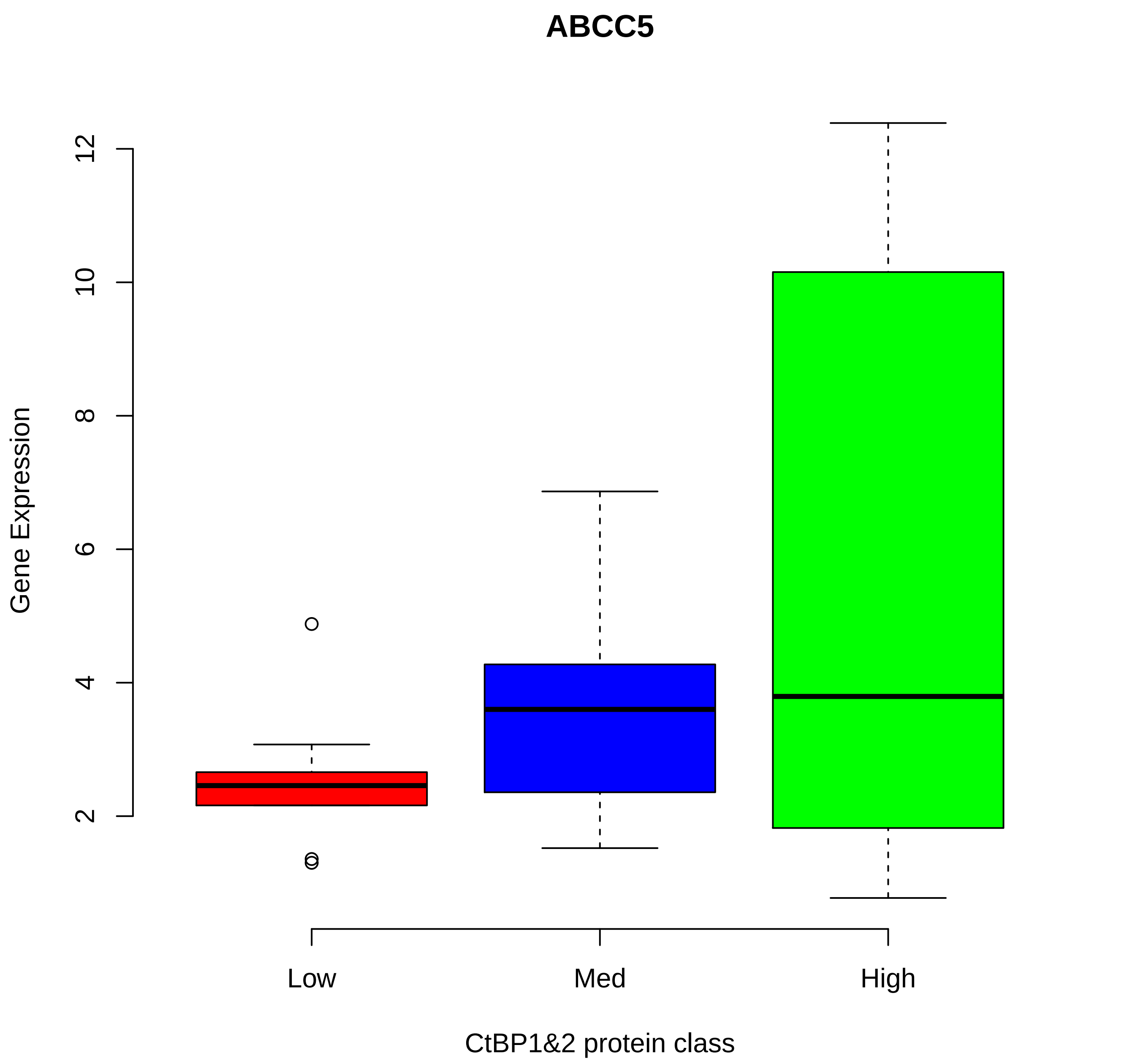

ABCC5
Gene Expression
12
10
8
6
4
2
Low
Med
High
CtBP1&2 protein class

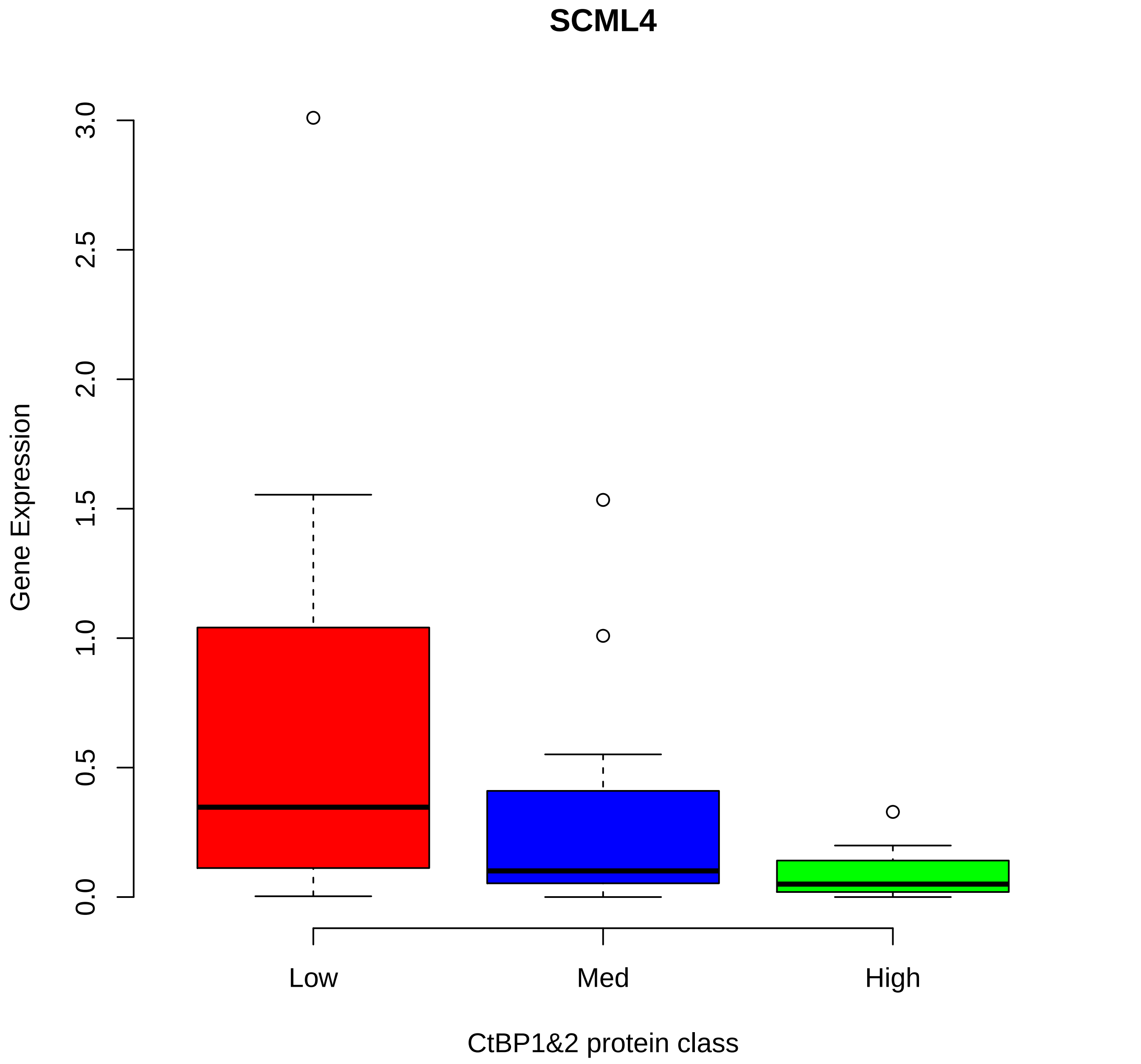
SCML4
Gene Expression
0.0
0.5
1.0
1.5
2.0
2.5
3.0
Low
Med
High
CtBP1&2 protein class

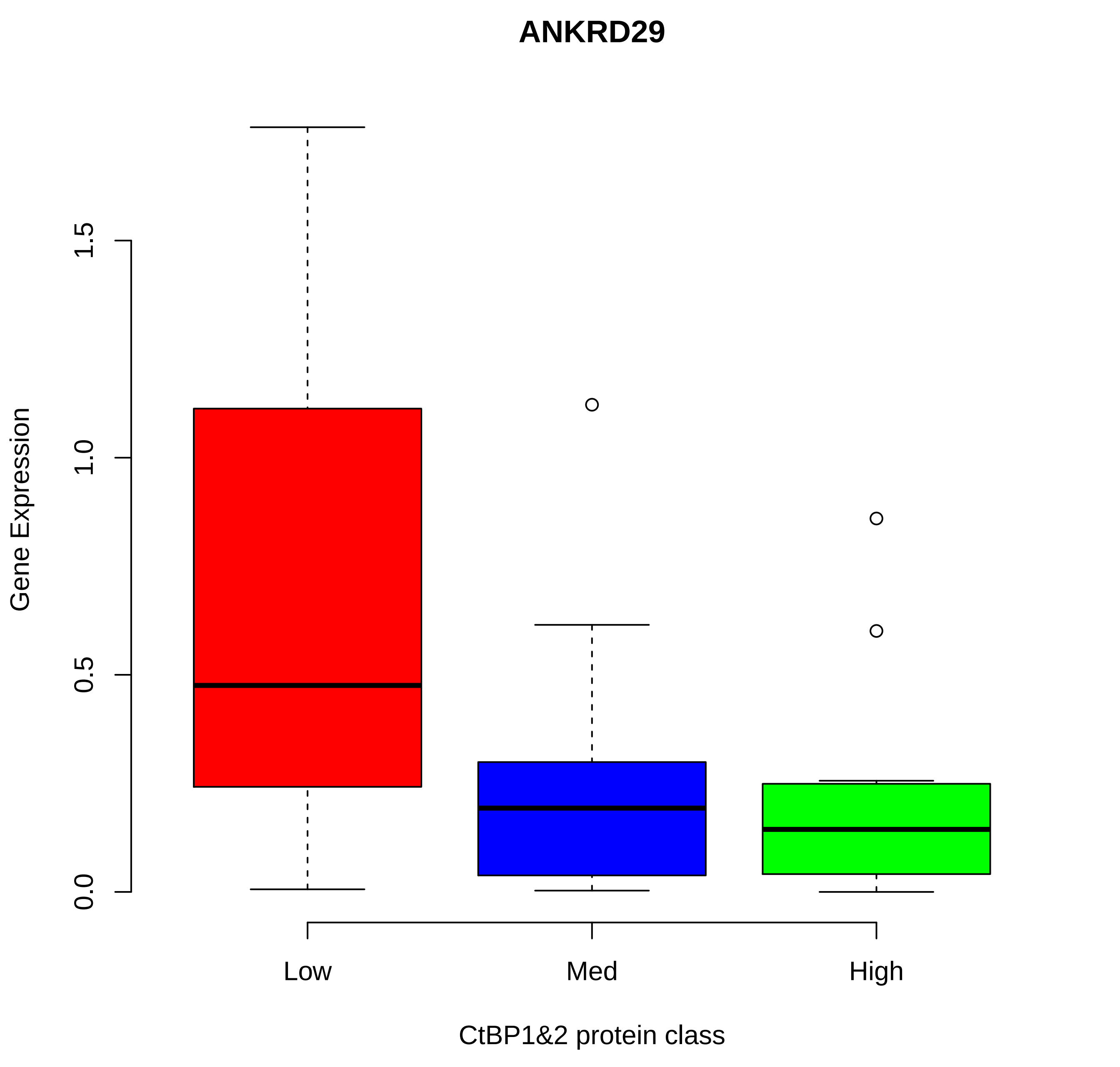

ANKRD29
Gene Expression
0.0
0.5
1.0
1.5
Low
Med
High
CtBP1&2 protein class

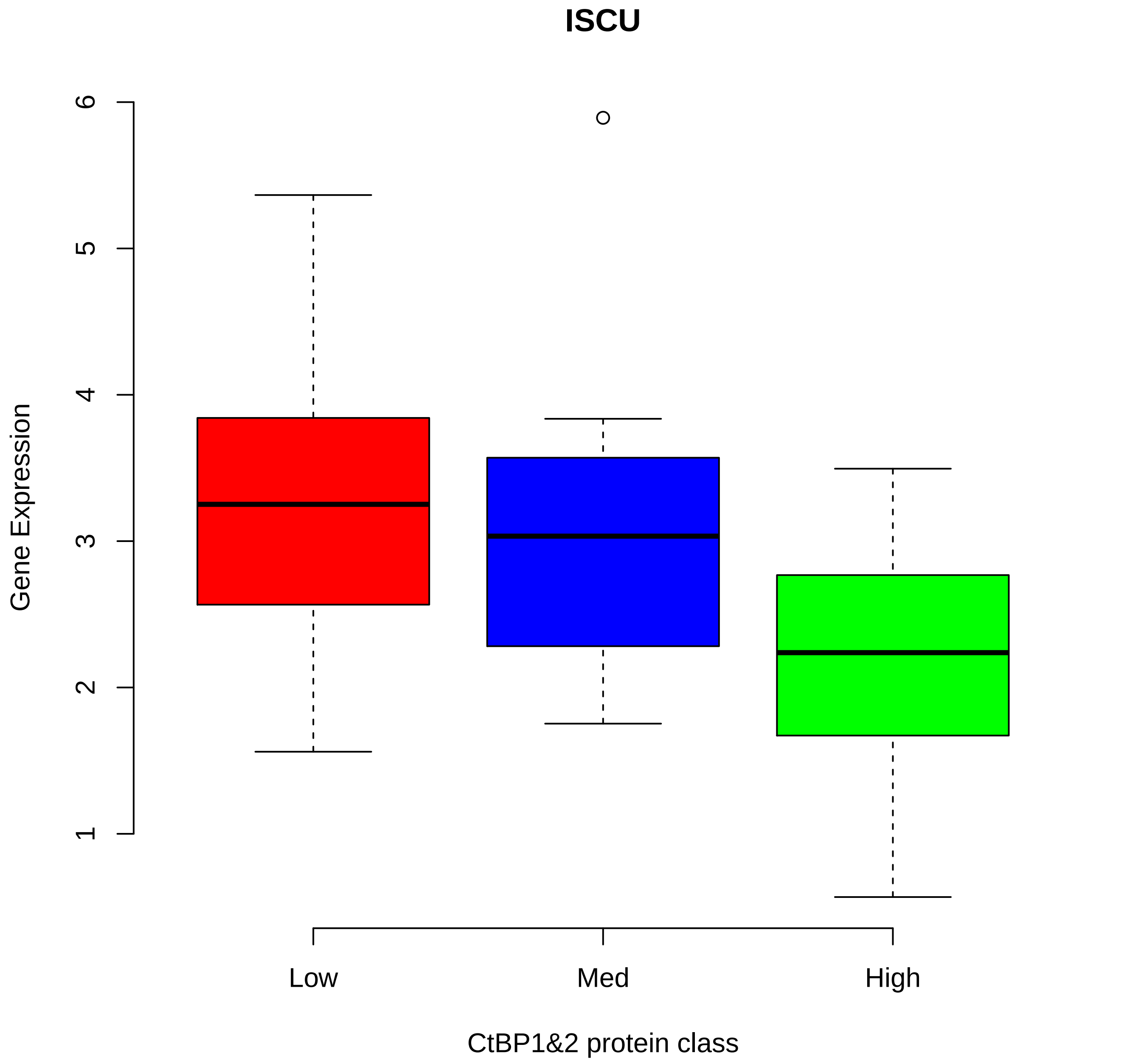
ISCU
Gene Expression
1
2
3
4
5
6
Low
Med
High
CtBP1&2 protein class

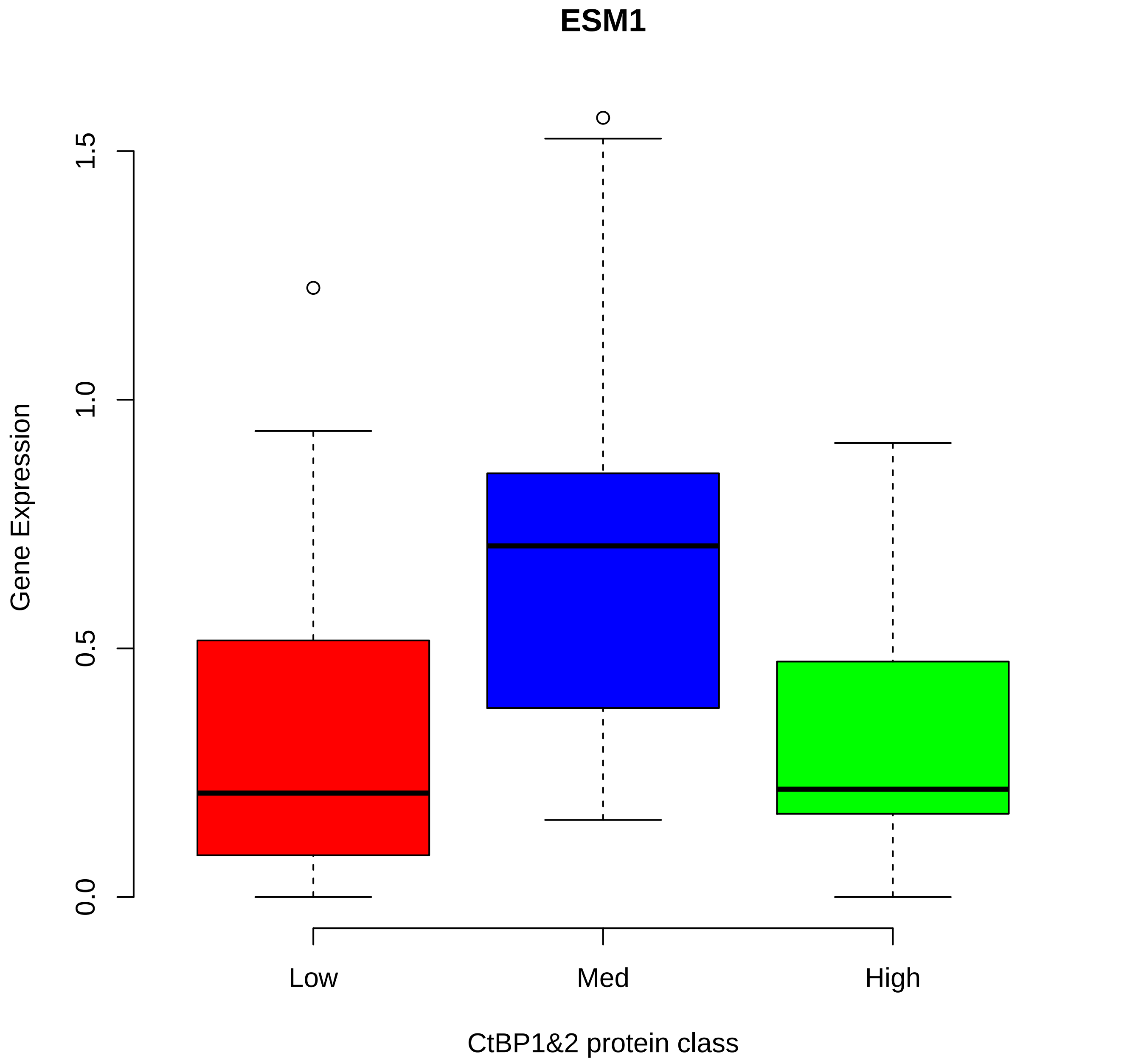

ESM1
Gene Expression
0.0
0.5
1.0
1.5
Low
Med
High
CtBP1&2 protein class

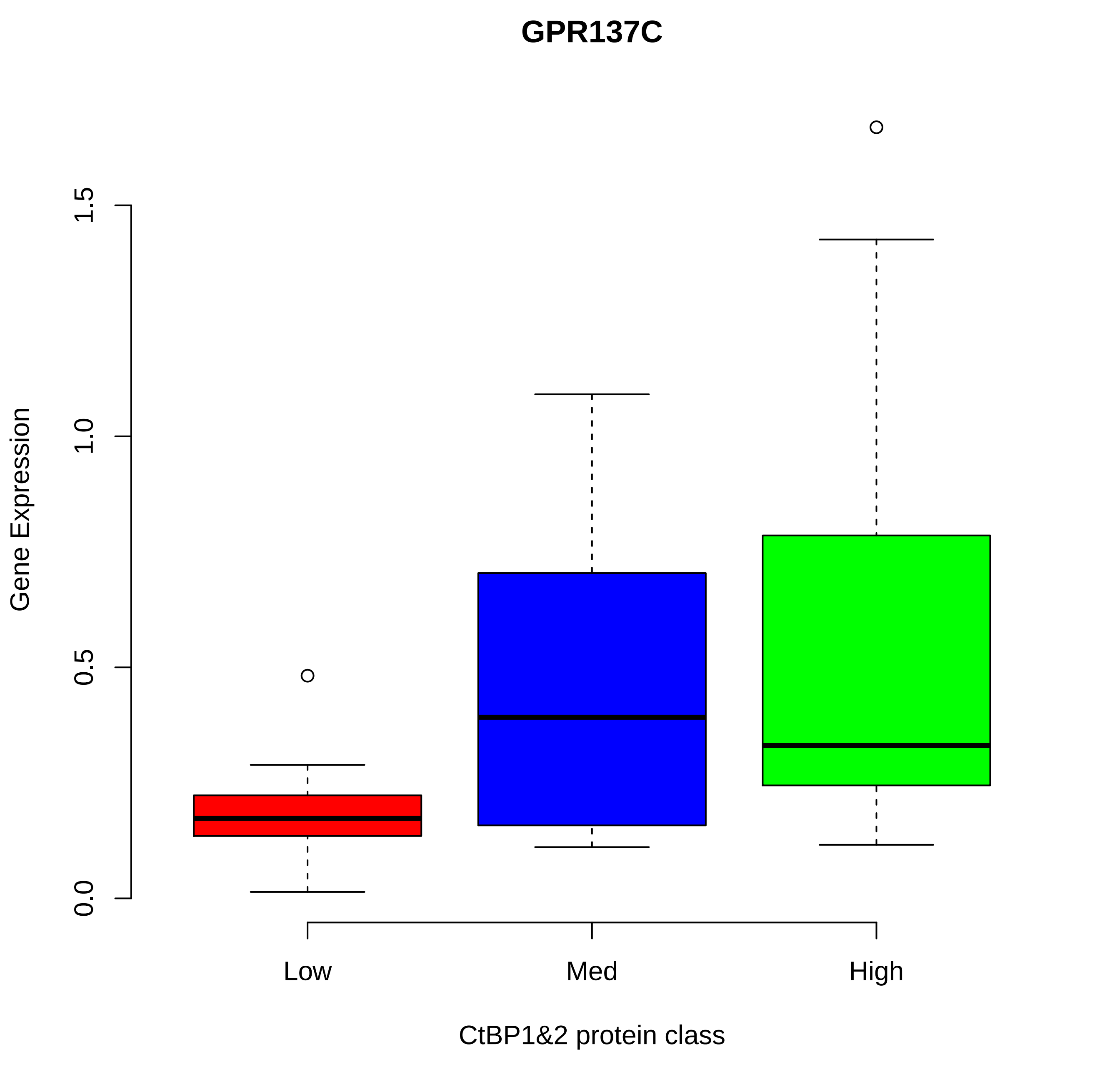
GPR137C
Gene Expression
0.0
0.5
1.0
1.5
Low
Med
High
CtBP1&2 protein class

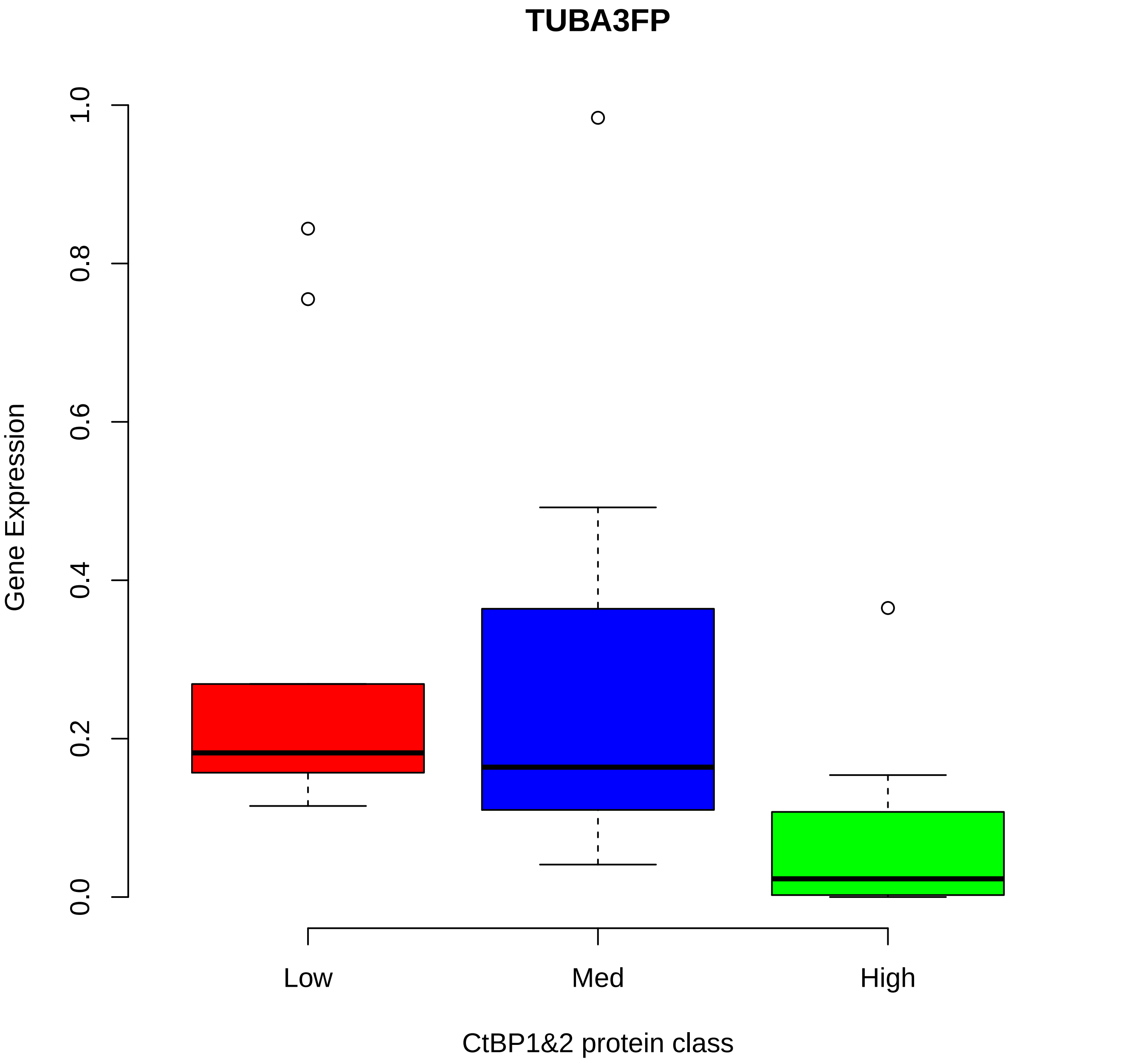

TUBA3FP
Gene Expression
0.0
0.2
0.4
0.6
0.8
1.0
Low
Med
High
CtBP1&2 protein class

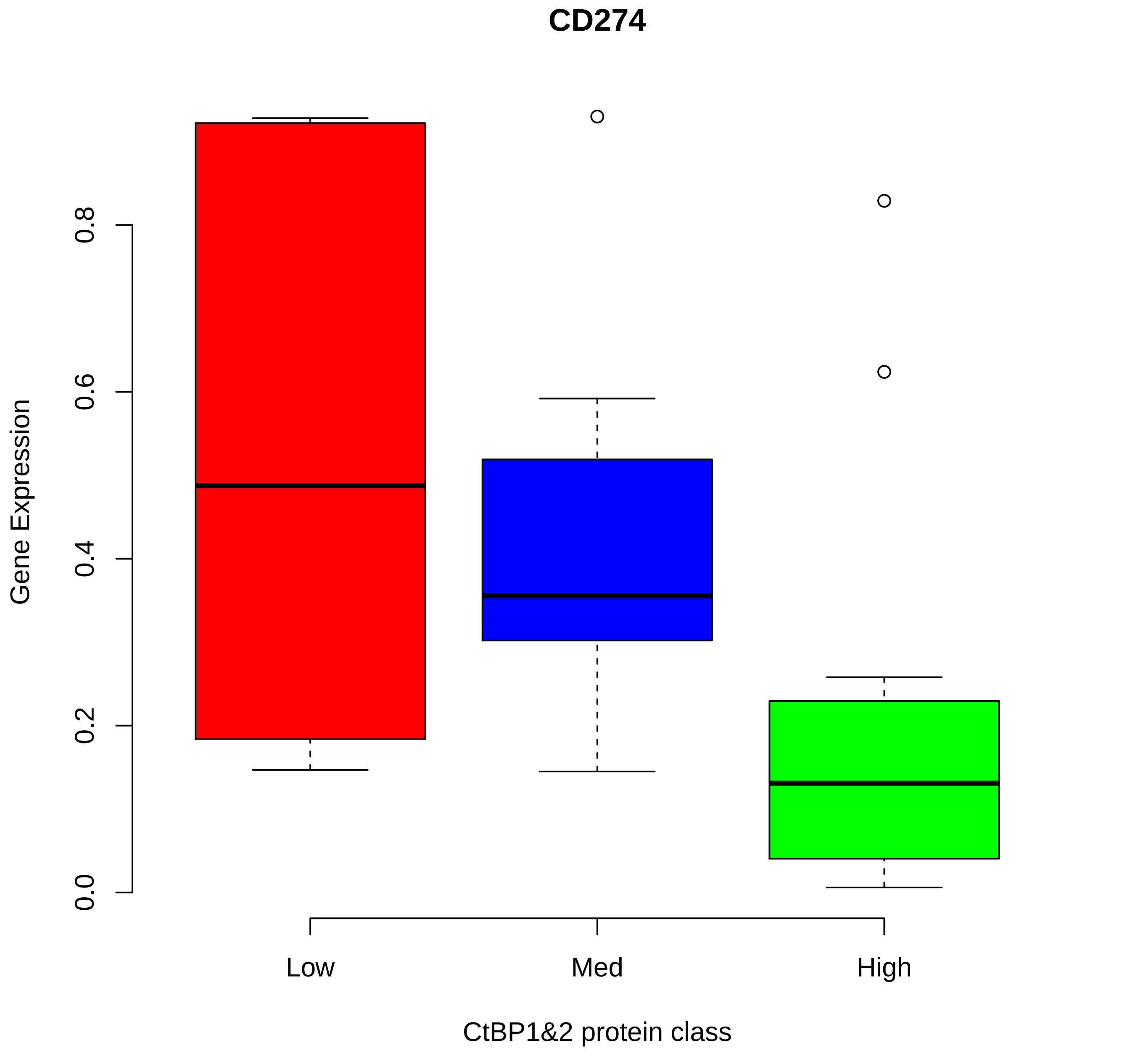
CD274
Gene Expression
0.0
0.2
0.4
0.6
0.8
Low
Med
High
CtBP1&2 protein class

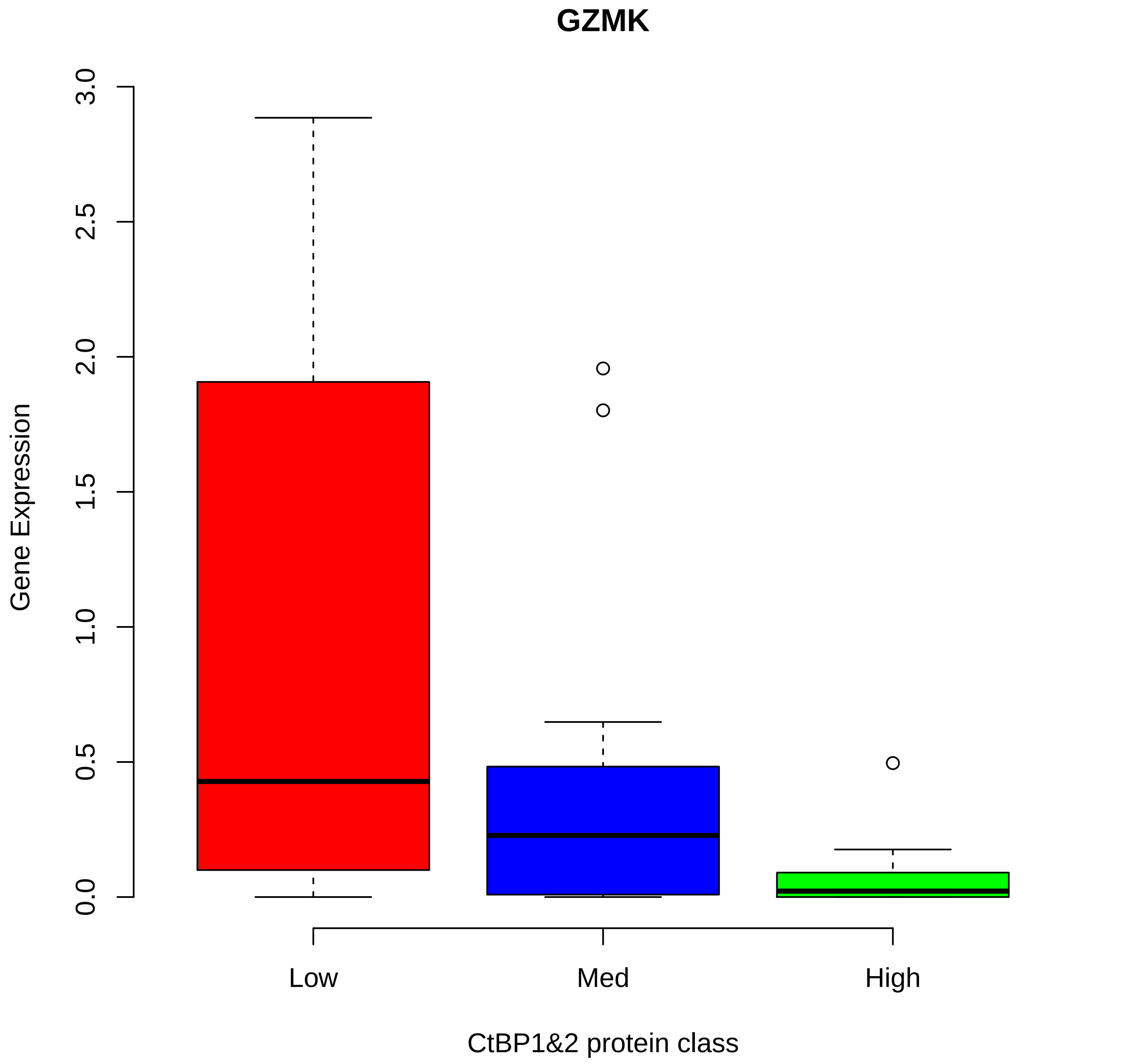

GZMK
3.0
2.5
2.0
1.5
1.0
0.5
0.0
Gene Expression
Low
Med
High
CtBP1&2 protein class

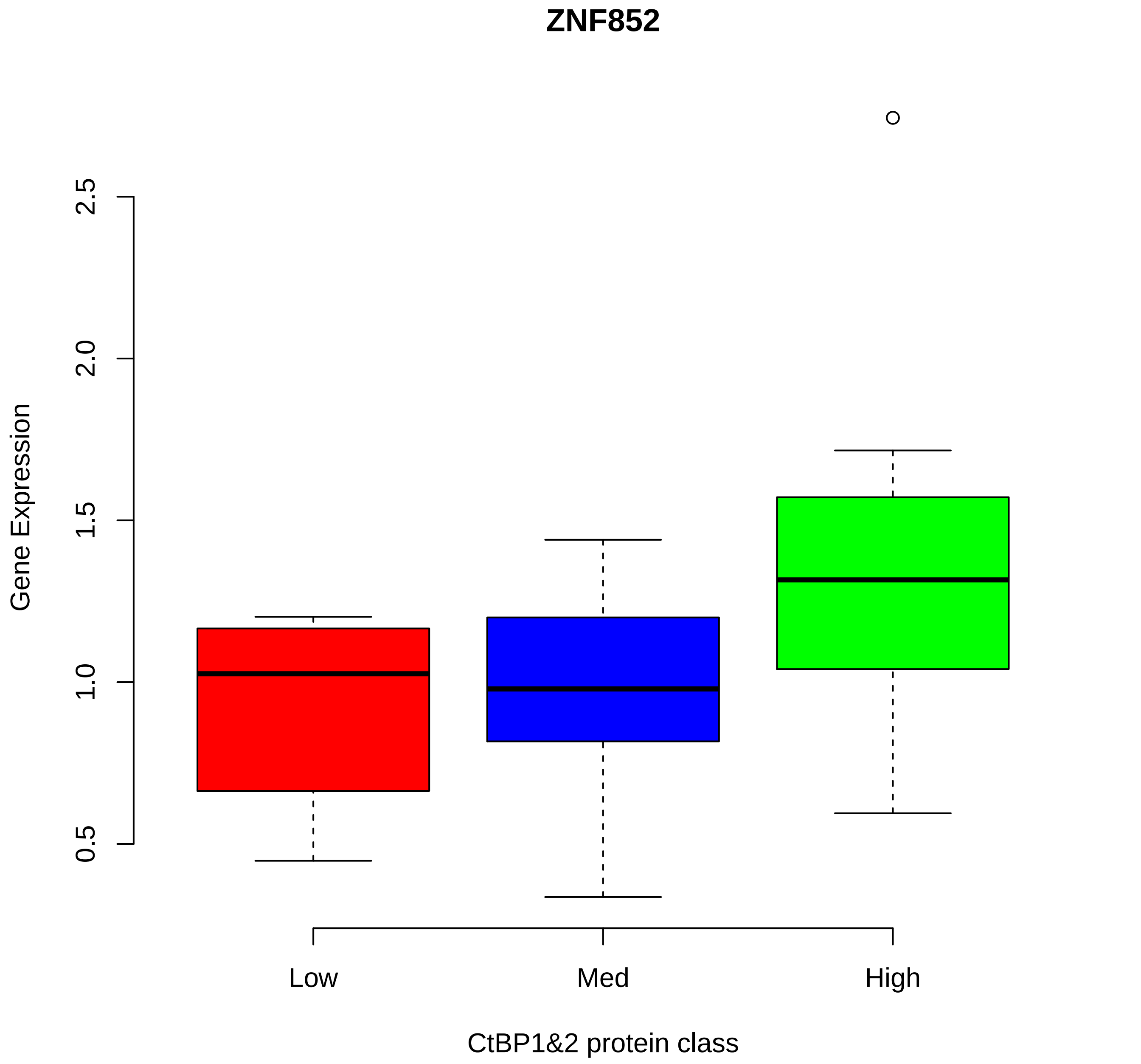

ZNF852
Gene Expression
0.5
1.0
1.5
2.0
2.5
Low
Med
High
CtBP1&2 protein class

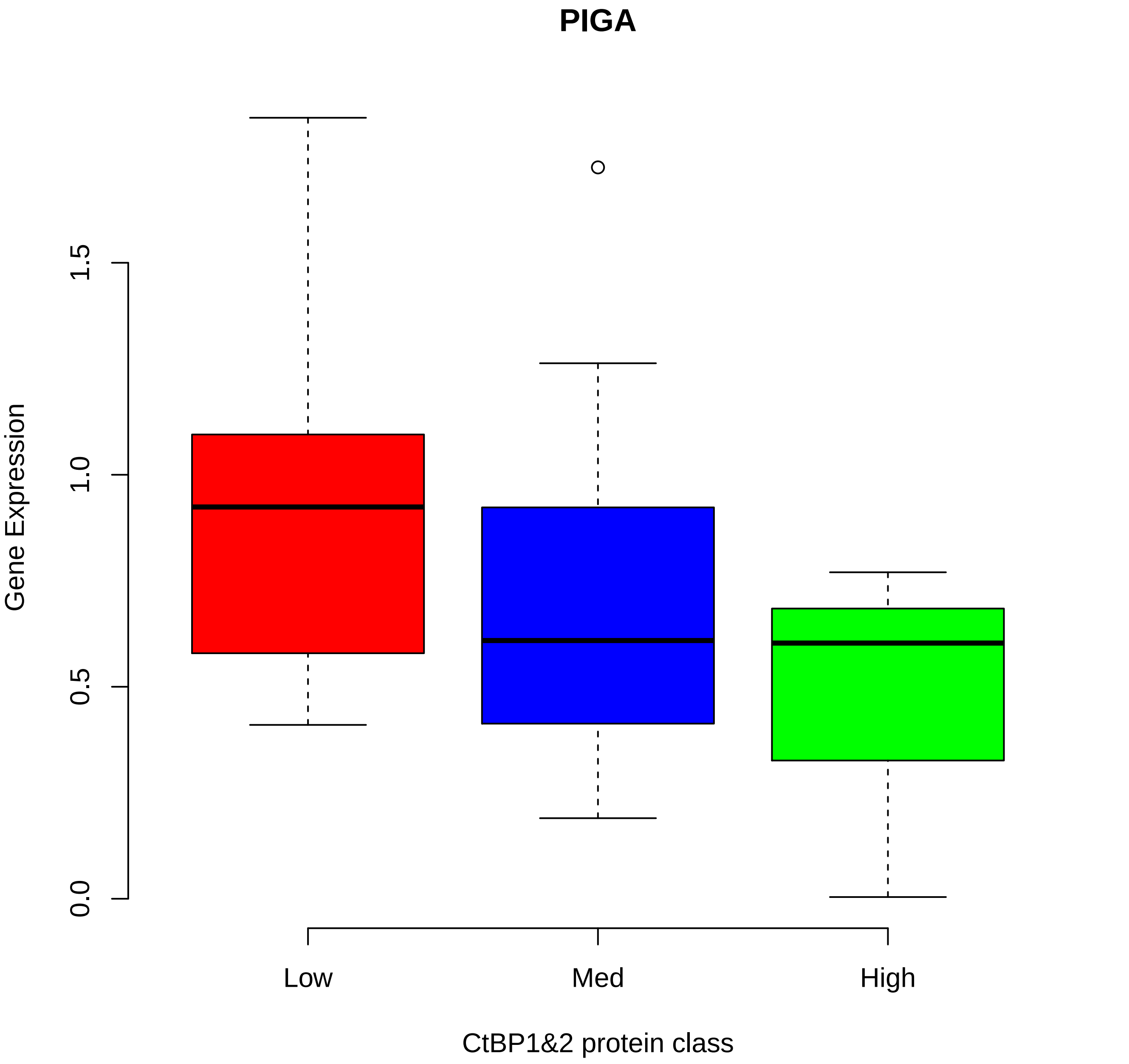

PIGA
Gene Expression
0.0
0.5
1.0
1.5
Low
Med
High
CtBP1&2 protein class

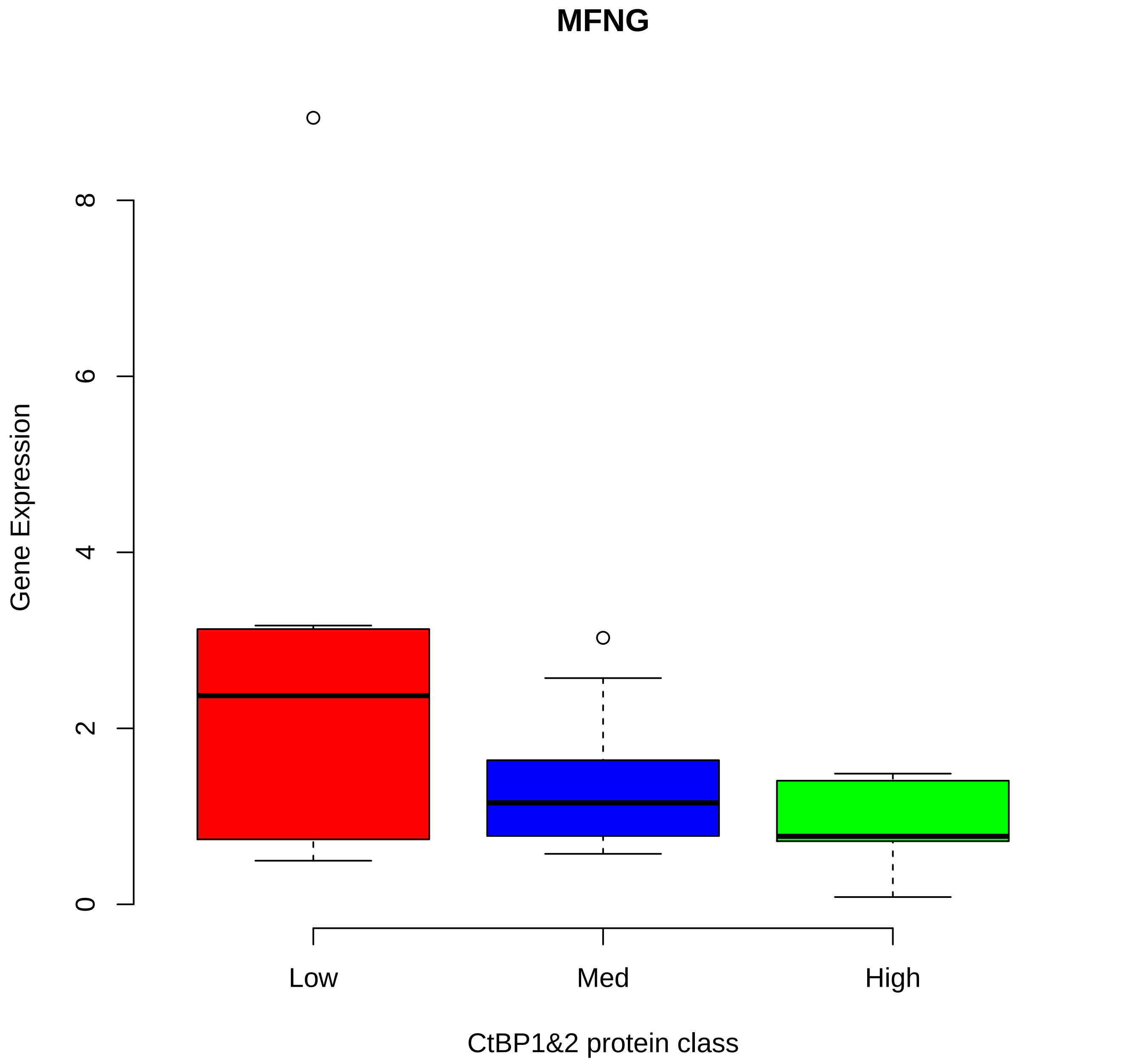
MFNG
Gene Expression
0
2
4
6
8
Low
Med
High
CtBP1&2 protein class

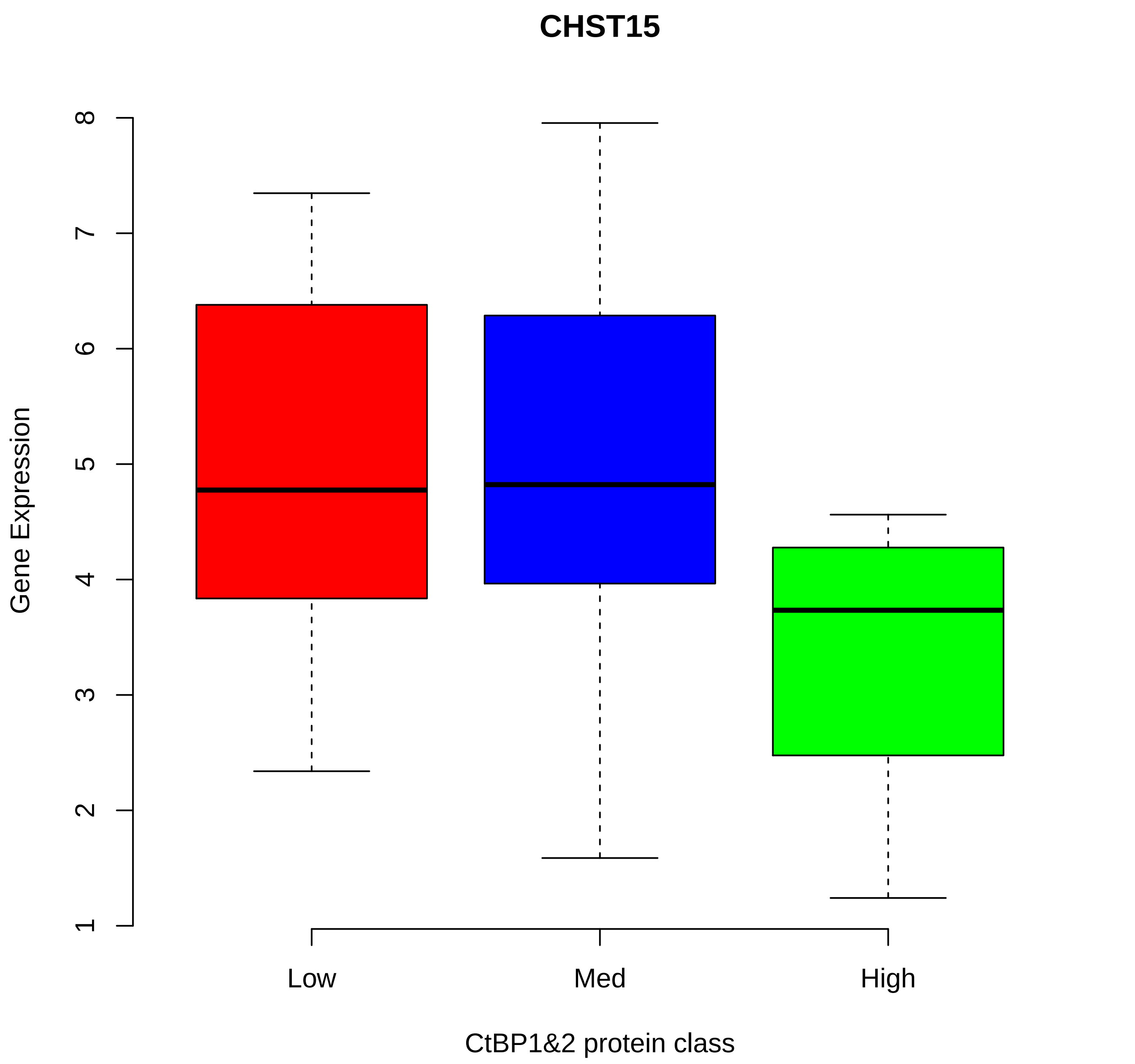
CHST15
Gene Expression
1
2
3
4
5
6
7
8
Low
Med
High
CtBP1&2 protein class

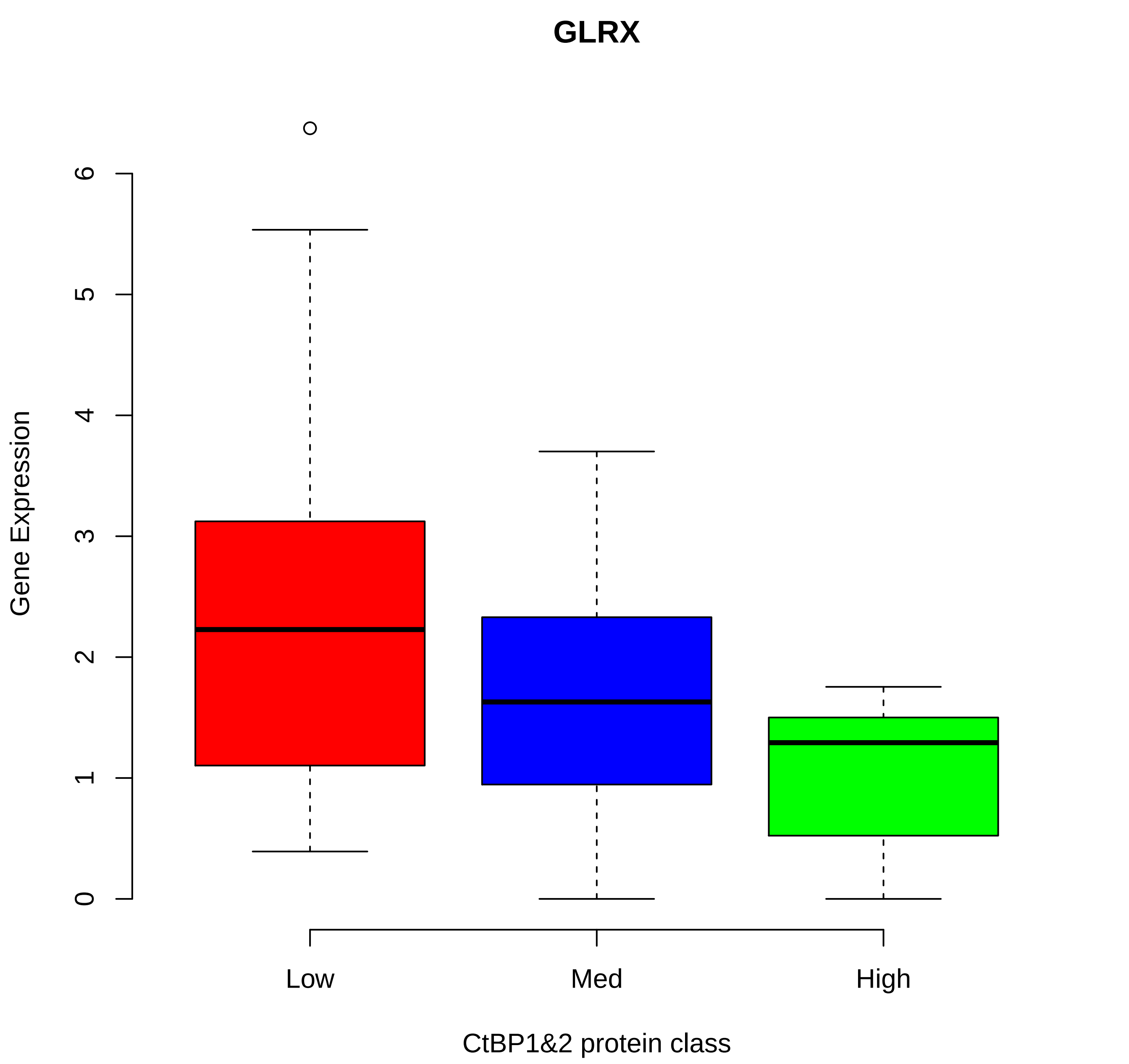

GLRX
Gene Expression
0
1
2
3
4
5
6
Low
Med
High
CtBP1&2 protein class

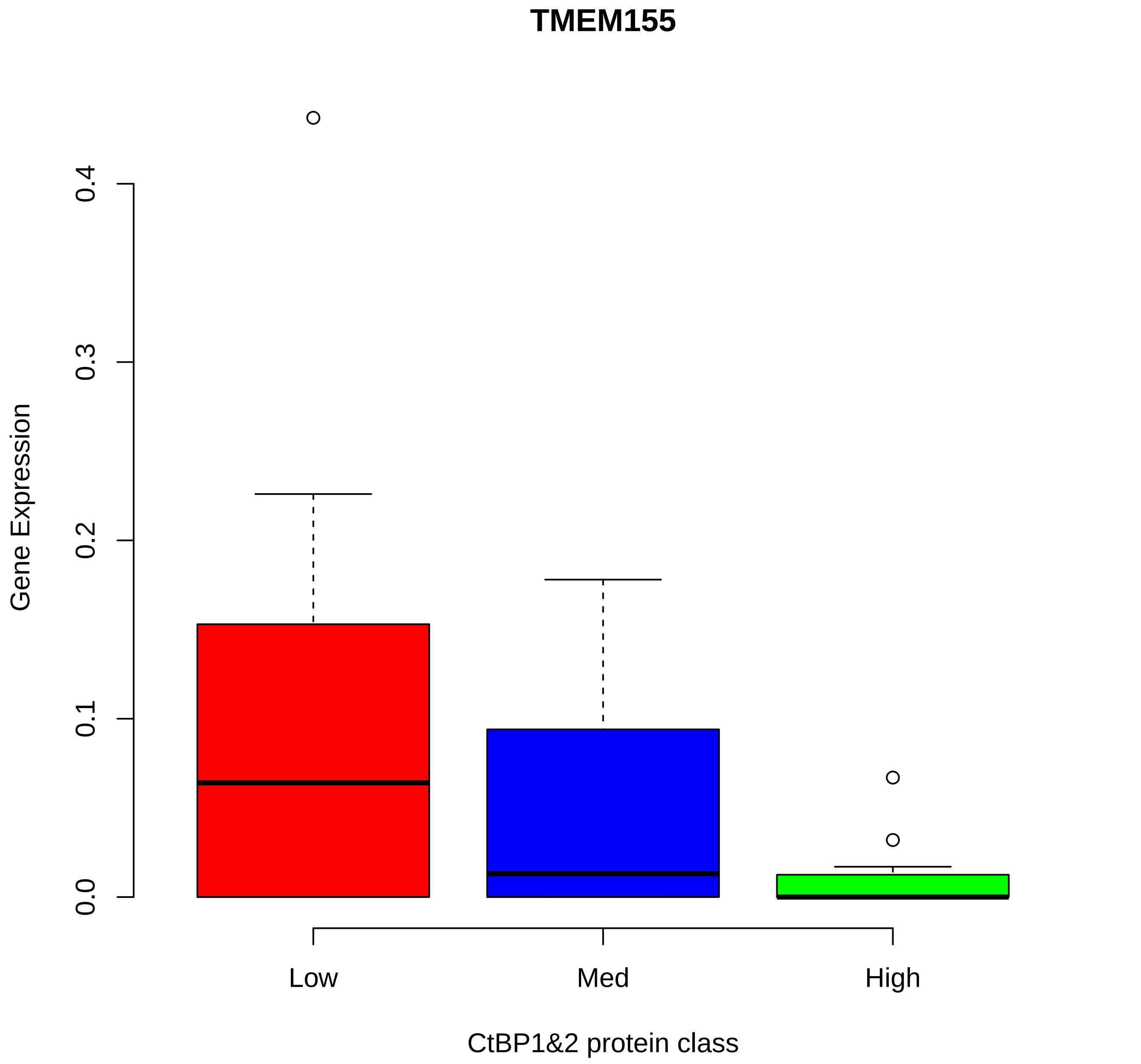

TMEM155
Gene Expression
0.0
0.1
0.2
0.3
0.4
Low
Med
High
CtBP1&2 protein class

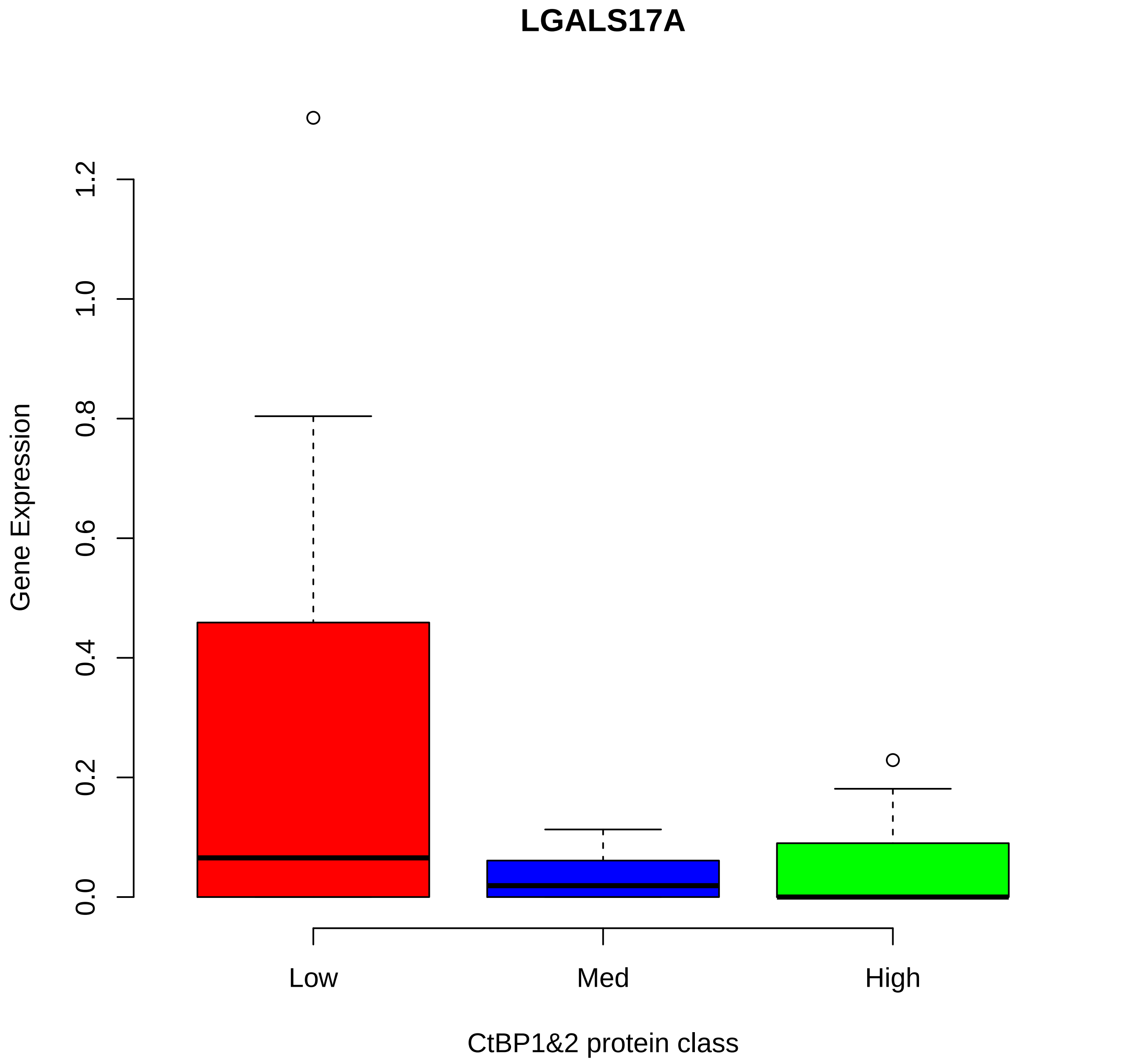
LGALS17A
Gene Expression
0.0
0.2
0.4
0.6
0.8
1.0
1.2
Low
Med
High
CtBP1&2 protein class

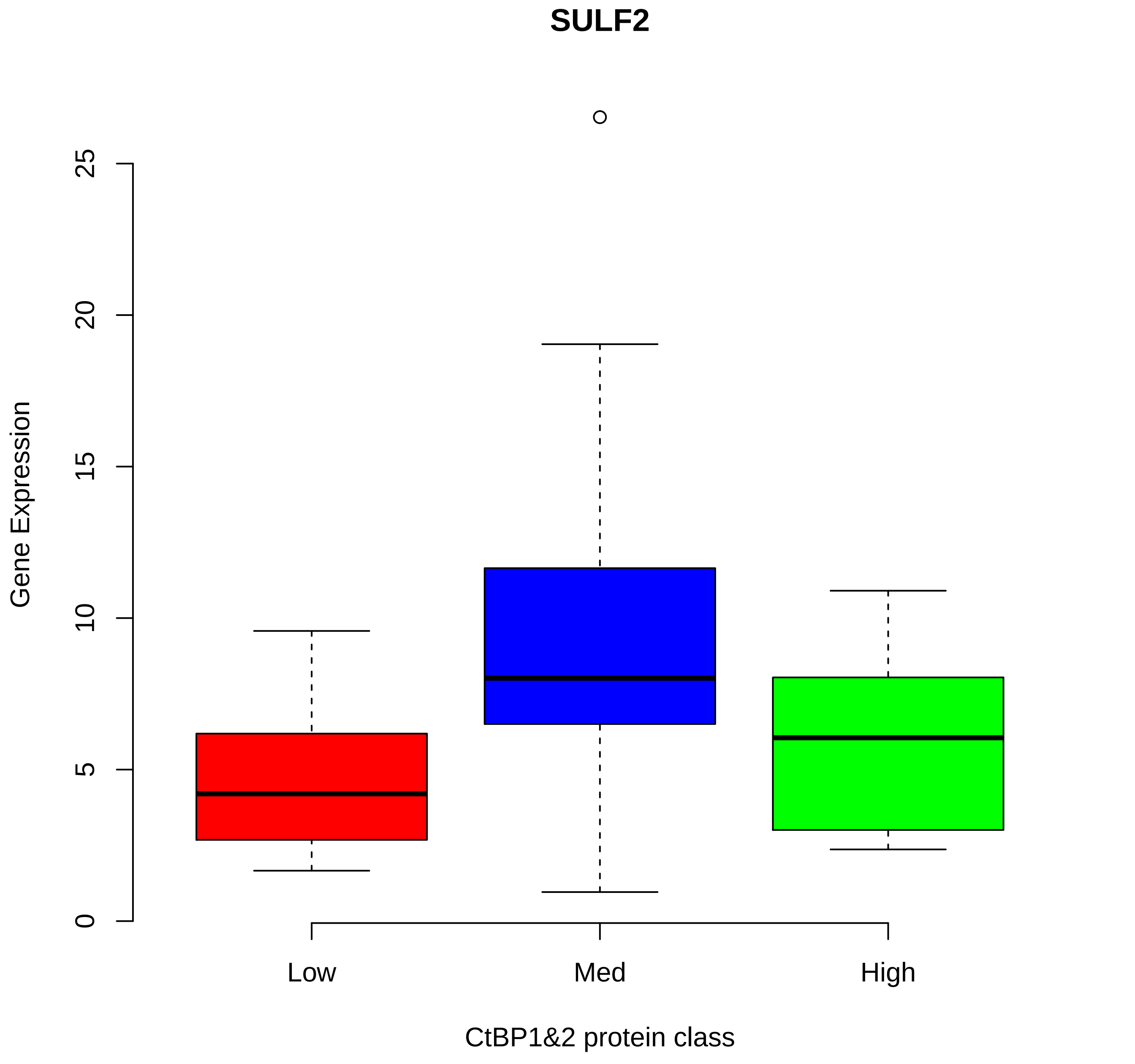

SULF2
Gene Expression
0
5
10
15
20
25
Low
Med
High
CtBP1&2 protein class

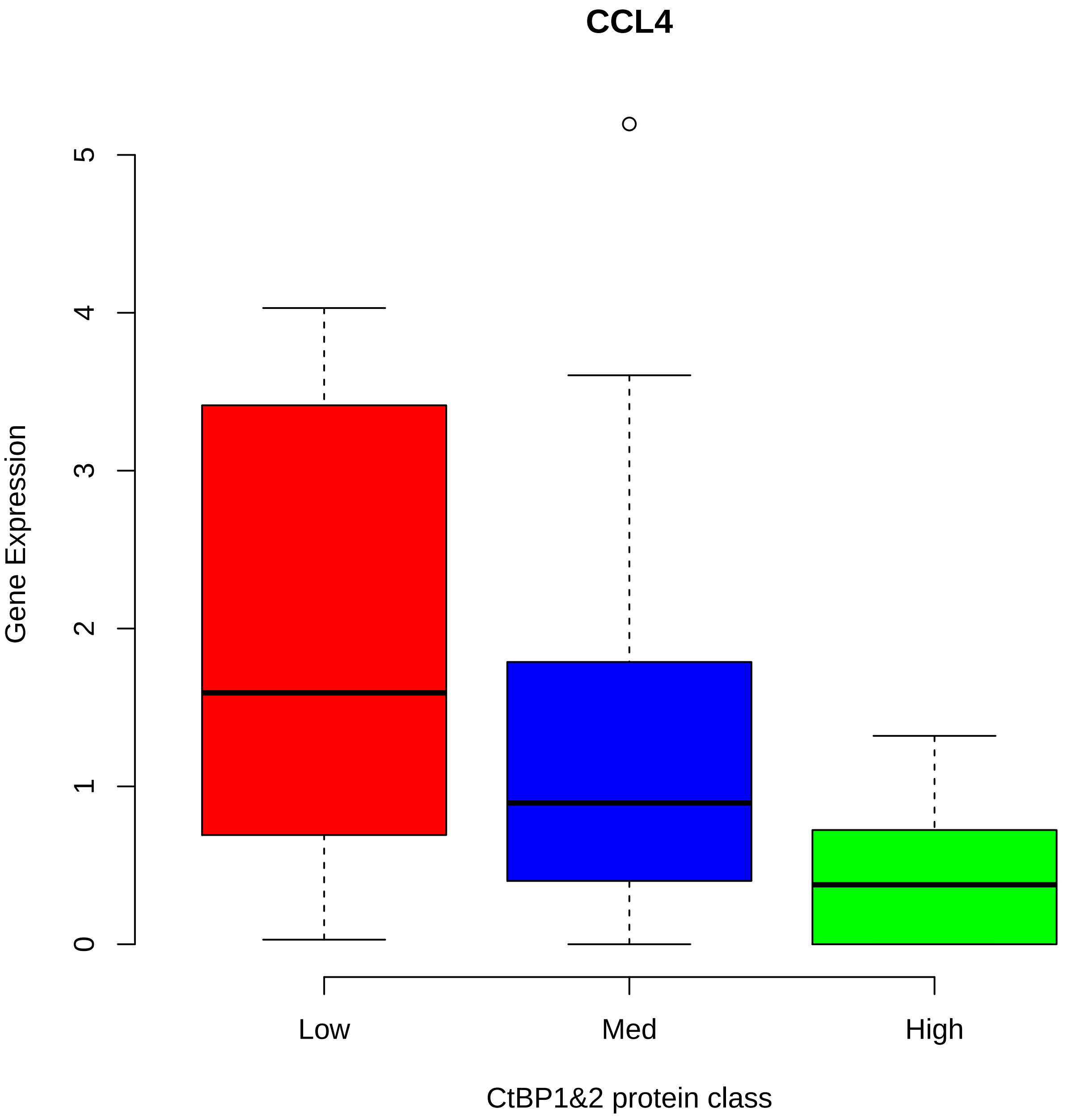
CCL4
Gene Expression
0
1
2
3
4
5
Low
Med
High
CtBP1&2 protein class

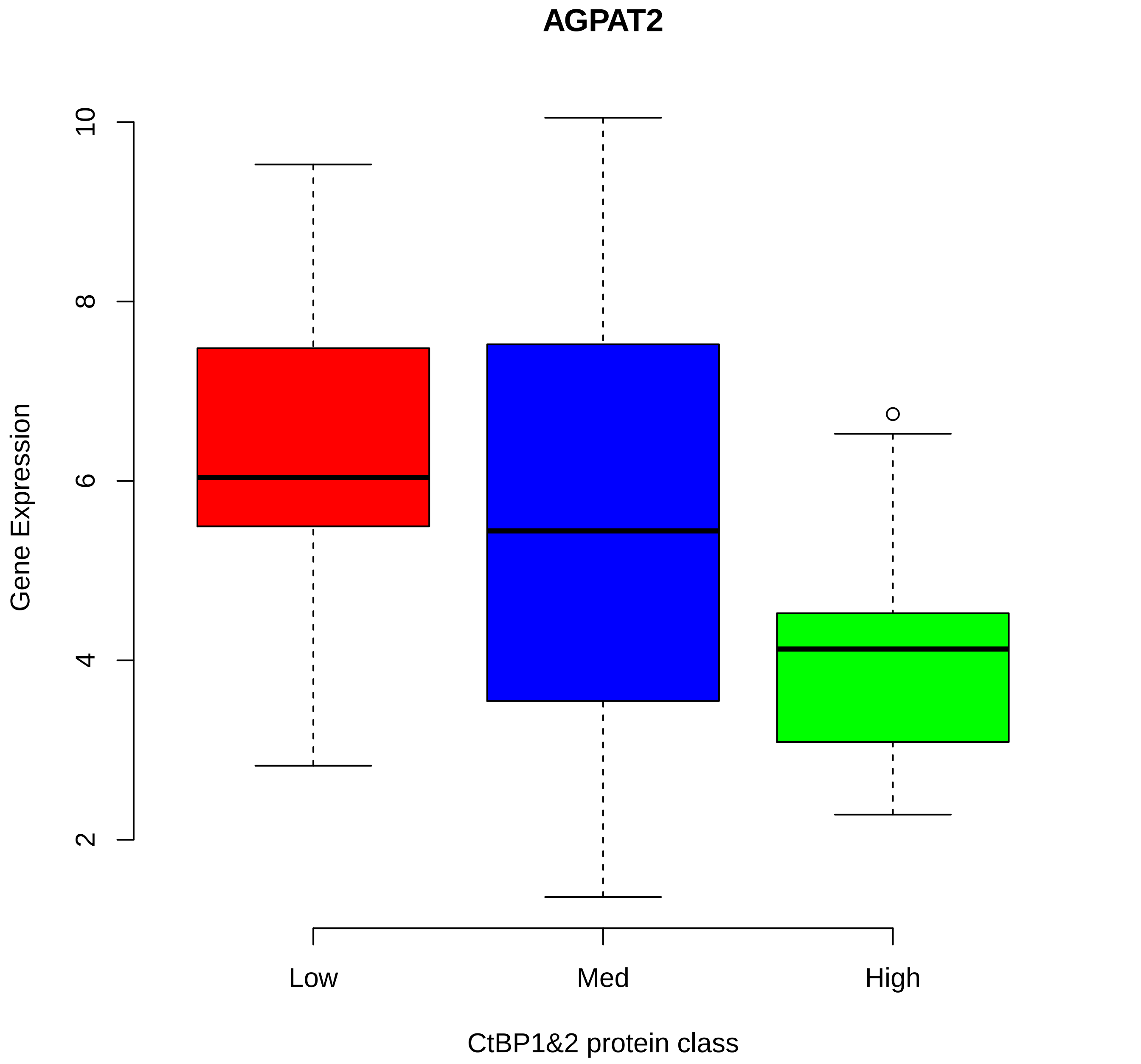
AGPAT2
Gene Expression
10
8
6
4
2
Low
Med
High
CtBP1&2 protein class

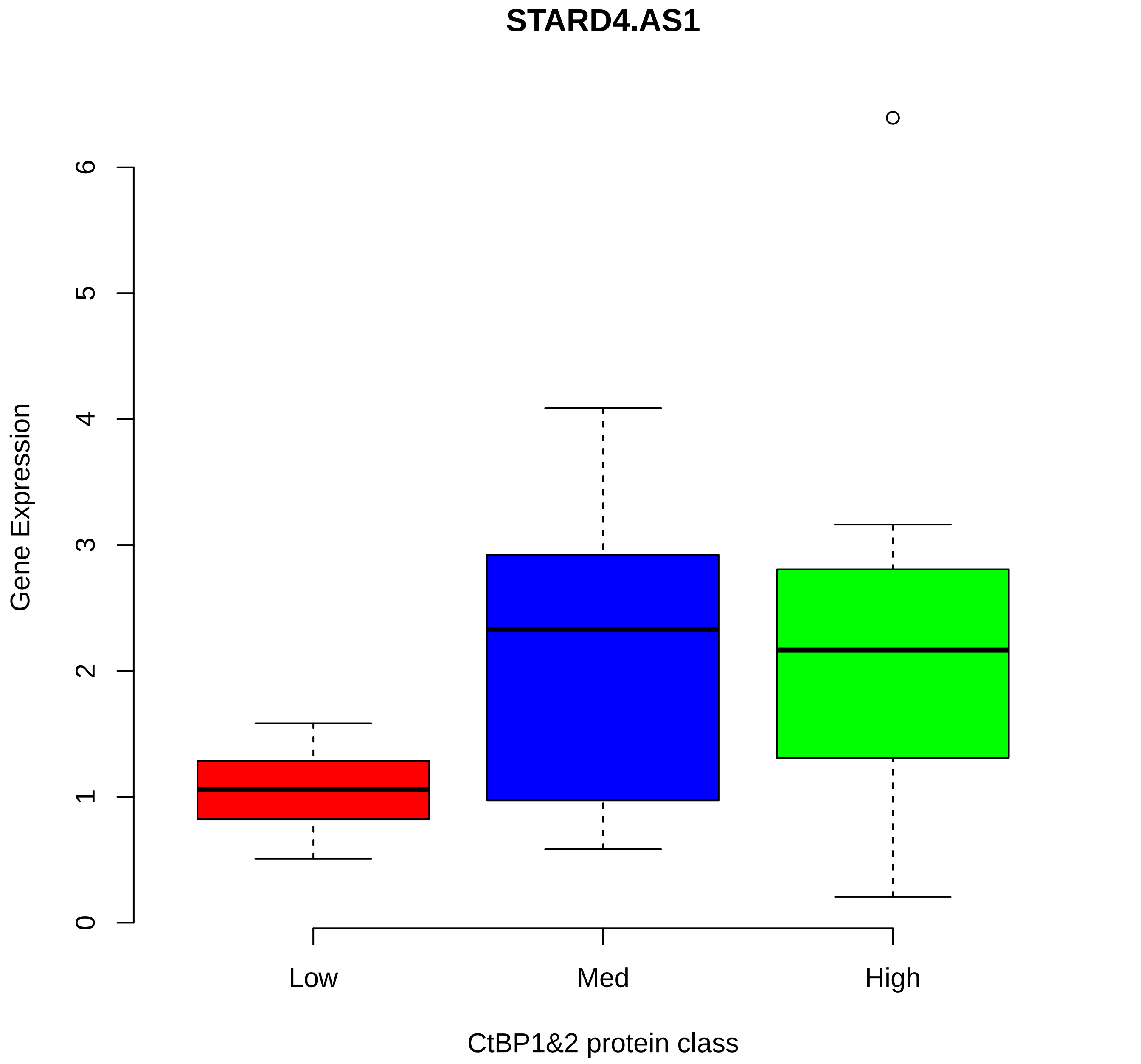
STARD4.AS1
Gene Expression
0
1
2
3
4
5
6
Low
Med
High
CtBP1&2 protein class

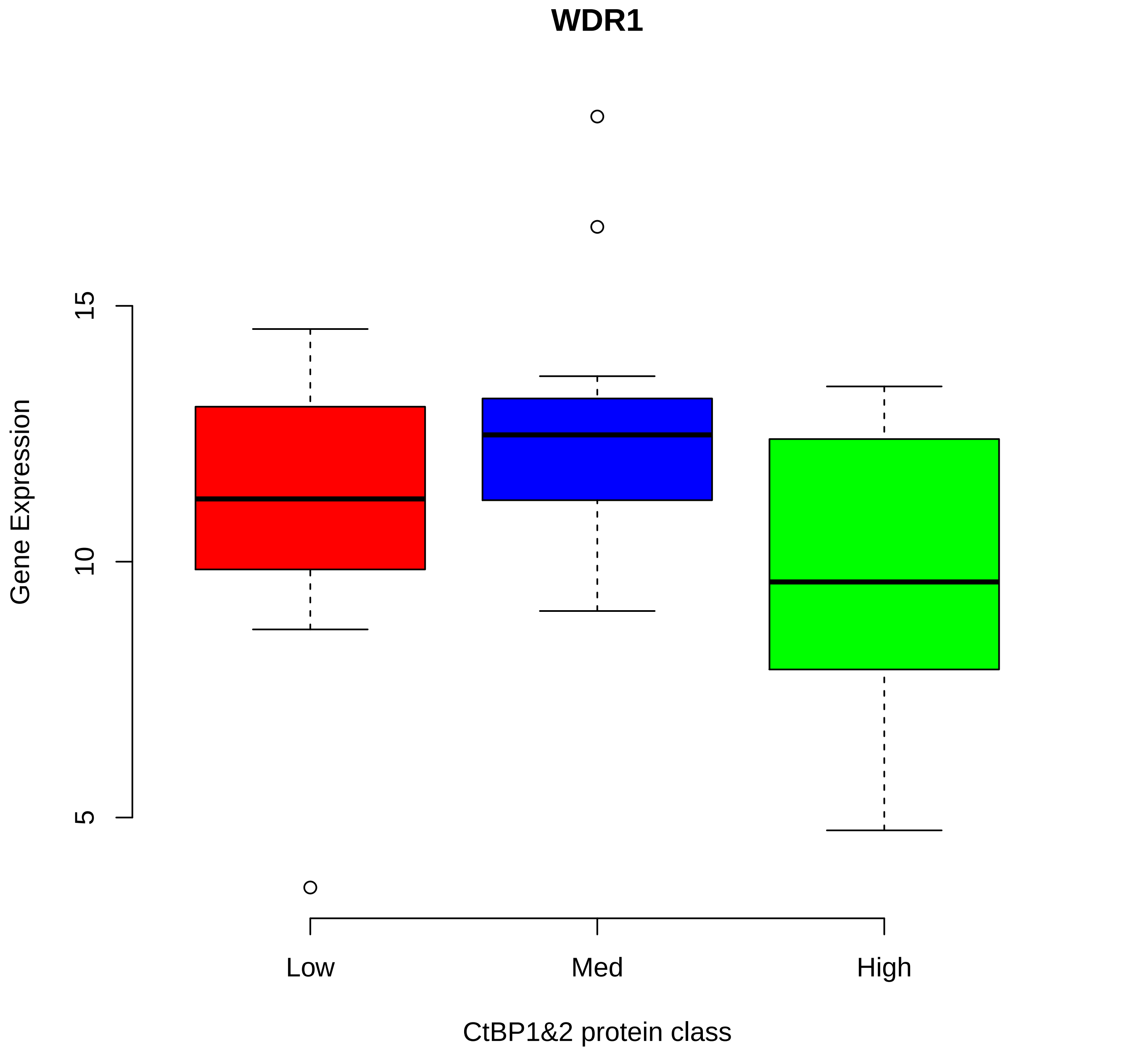

WDR1
Gene Expression
15
10
5
Low
Med
High
CtBP1&2 protein class

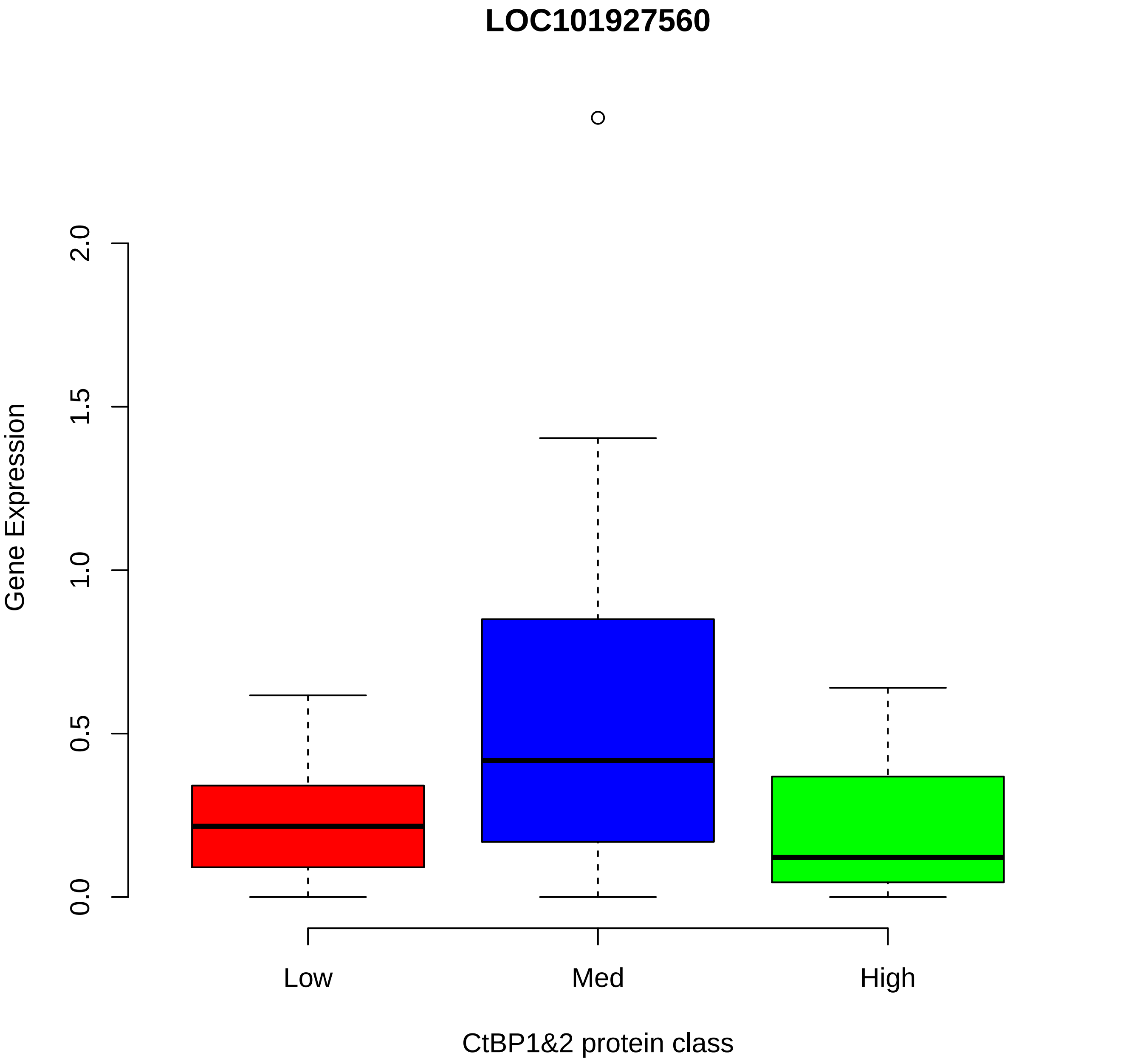
LOC101927560
Gene Expression
0.0
0.5
1.0
1.5
2.0
Low
Med
High
CtBP1&2 protein class

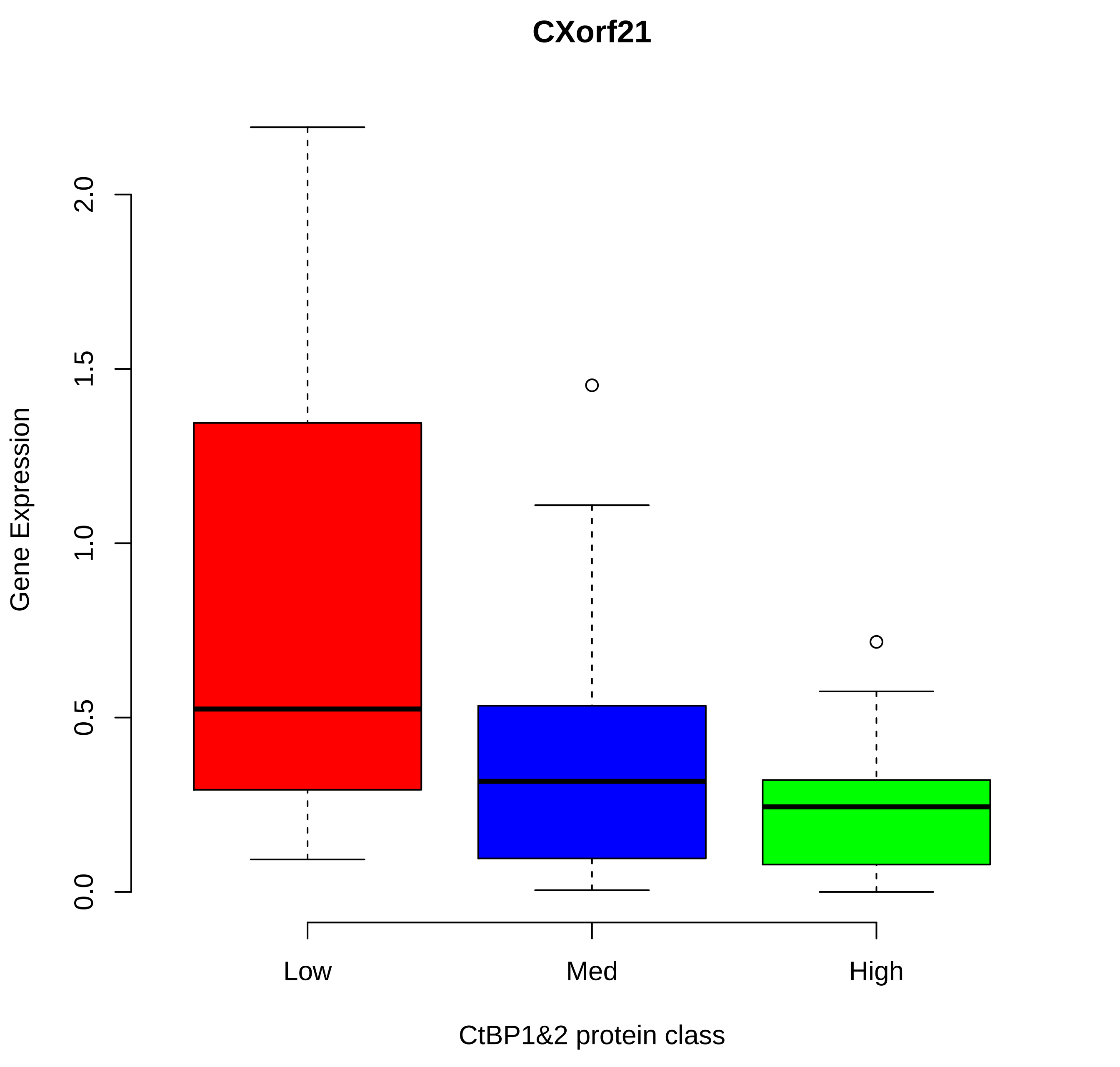
CXorf21
Gene Expression
0.0
0.5
1.0
1.5
2.0
Low
Med
High
CtBP1&2 protein class

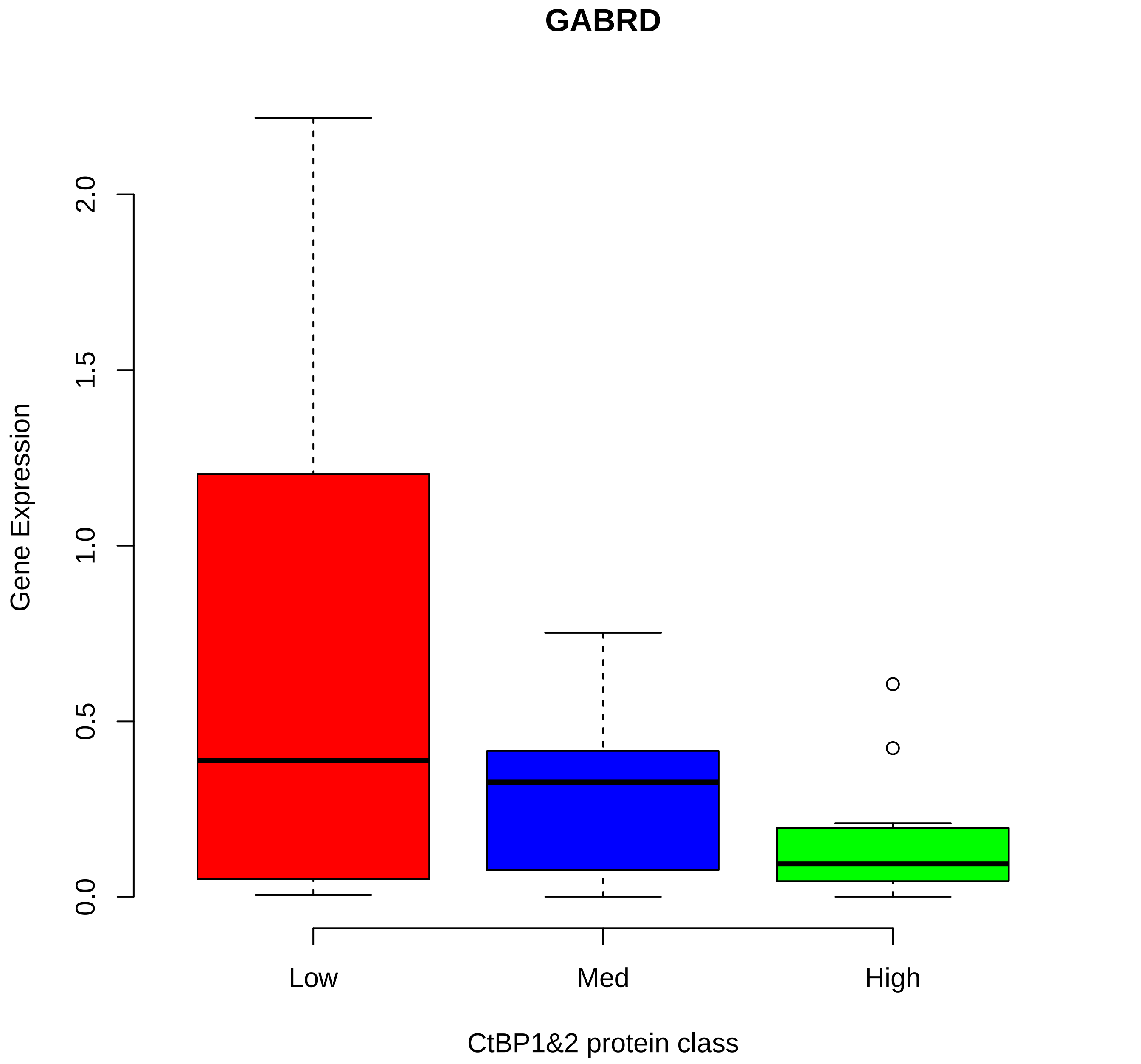

GABRD
Gene Expression
0.0
0.5
1.0
1.5
2.0
Low
Med
High
CtBP1&2 protein class

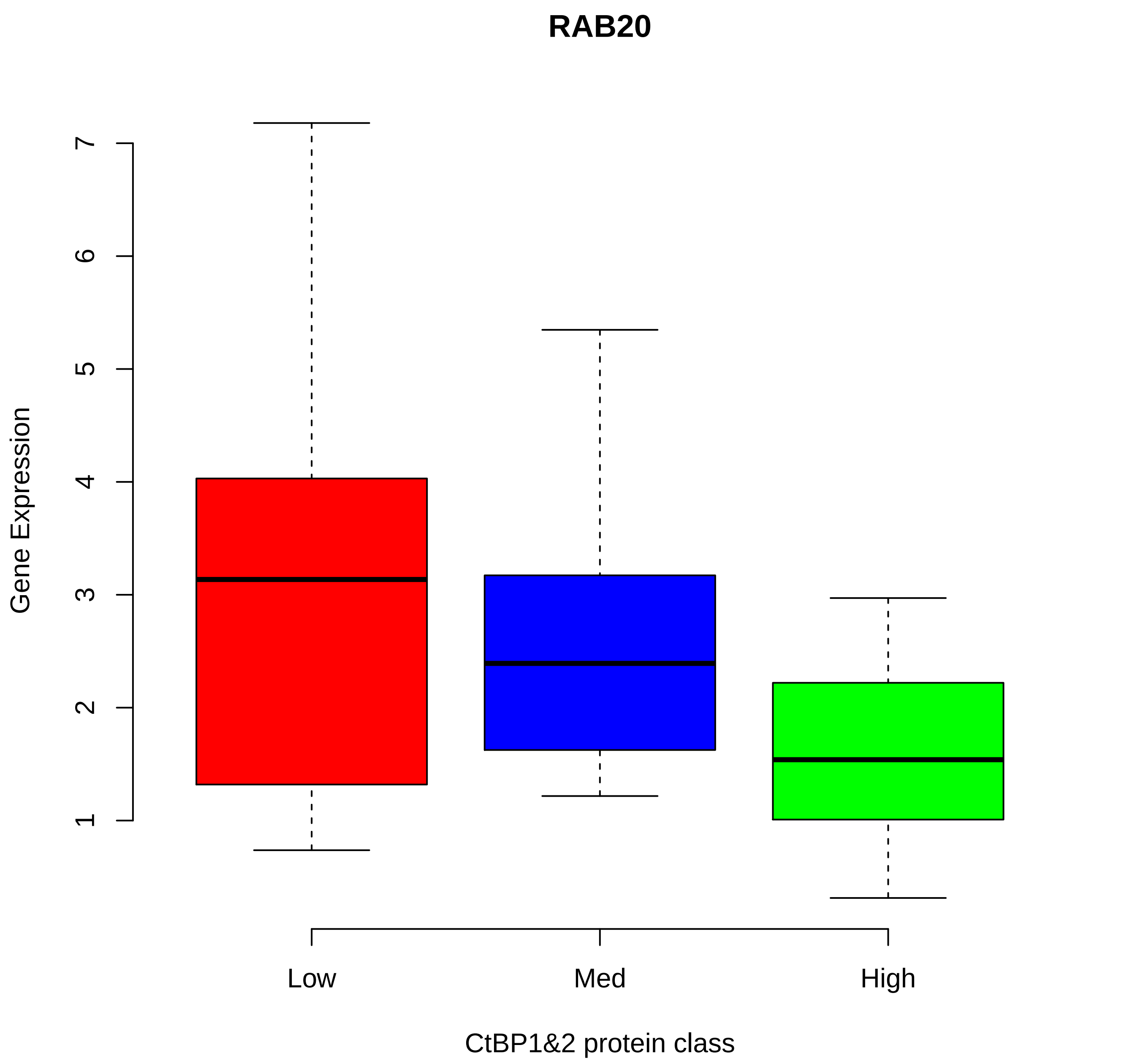

RAB20
Gene Expression
1
2
3
4
5
6
7
Low
Med
High
CtBP1&2 protein class

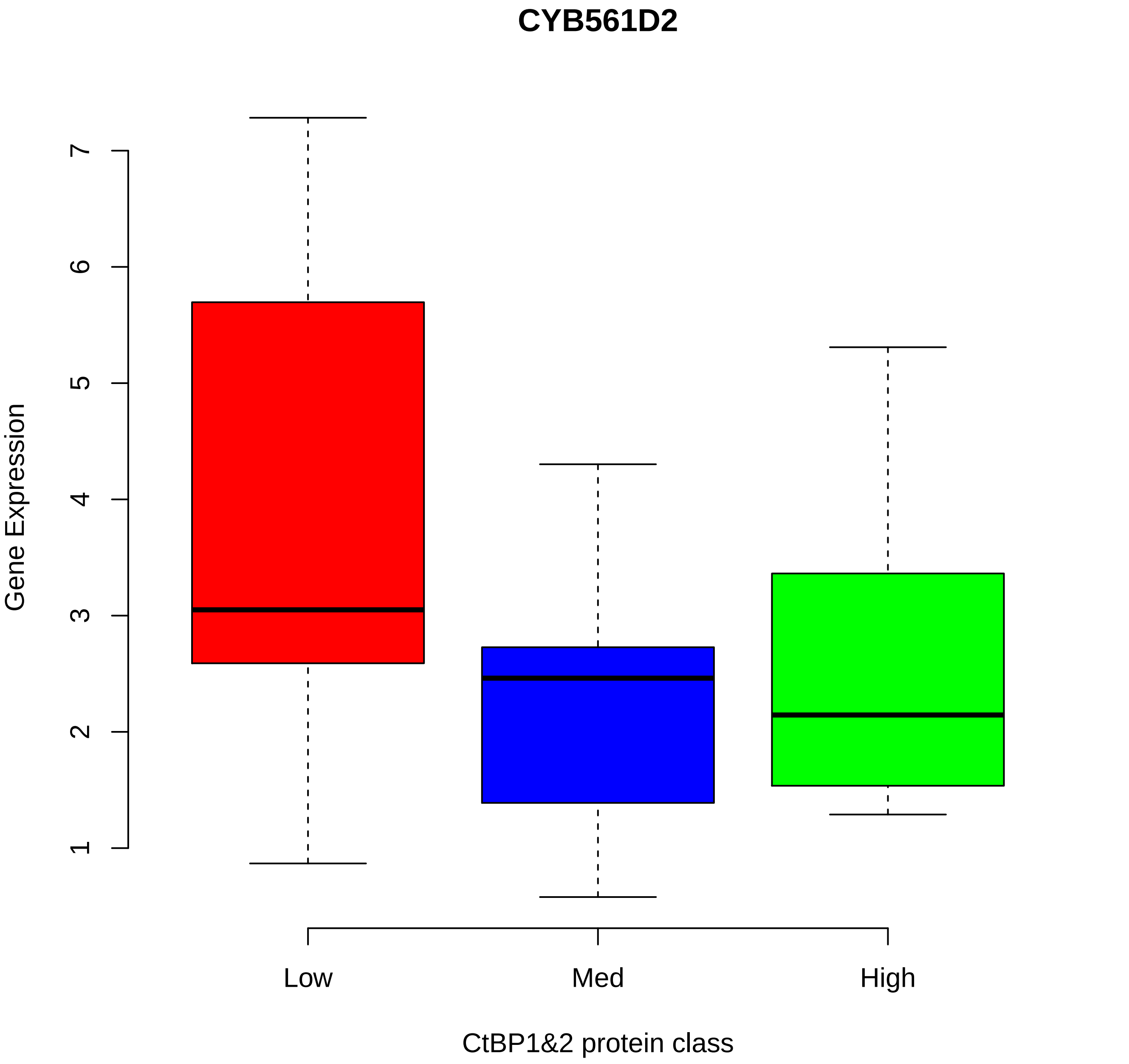
CYB561D2
Gene Expression
1
2
3
4
5
6
7
Low
Med
High
CtBP1&2 protein class

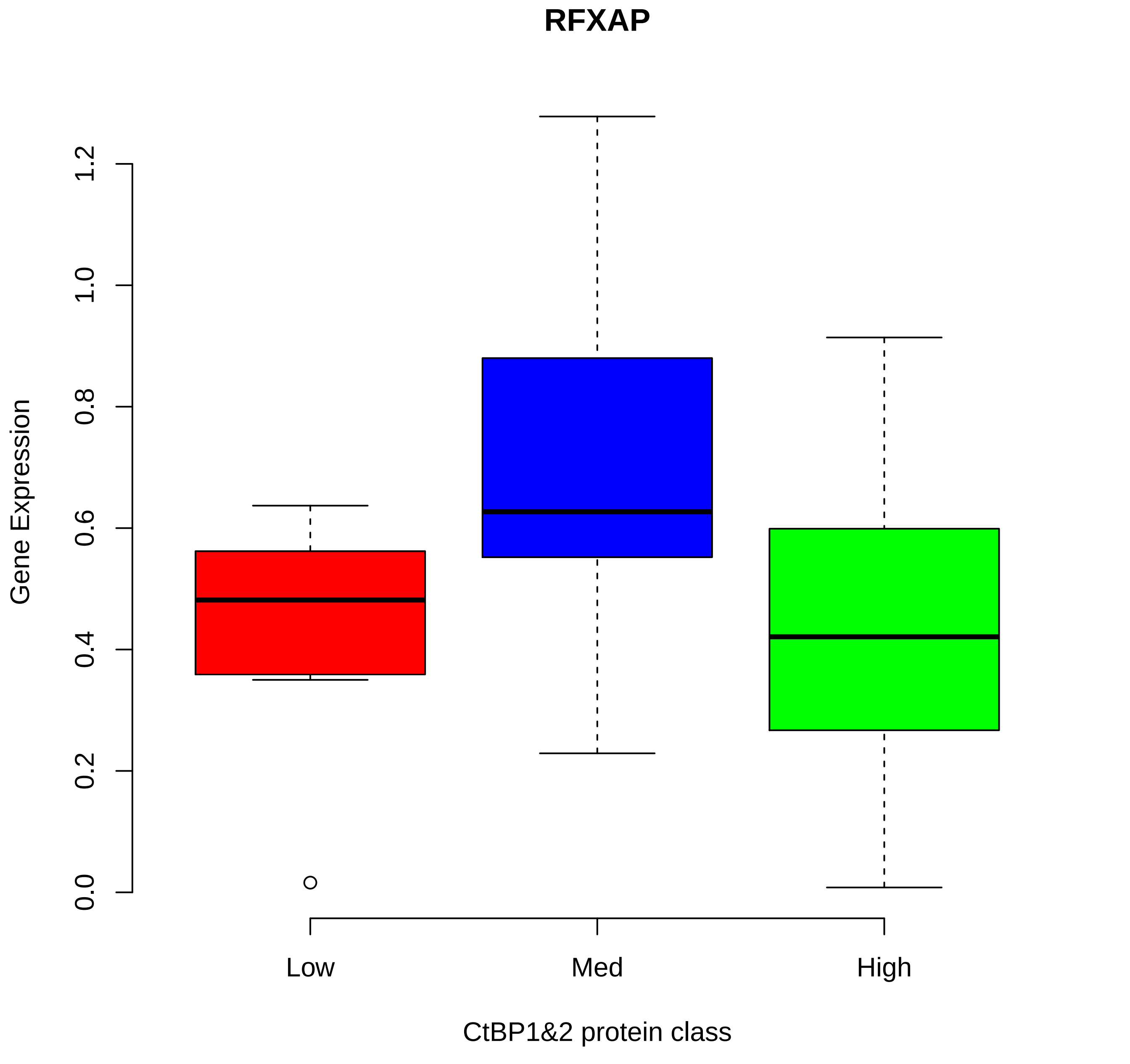
RFXAP
Gene Expression
0.0
0.2
0.4
0.6
0.8
1.0
1.2
Low
Med
High
CtBP1&2 protein class

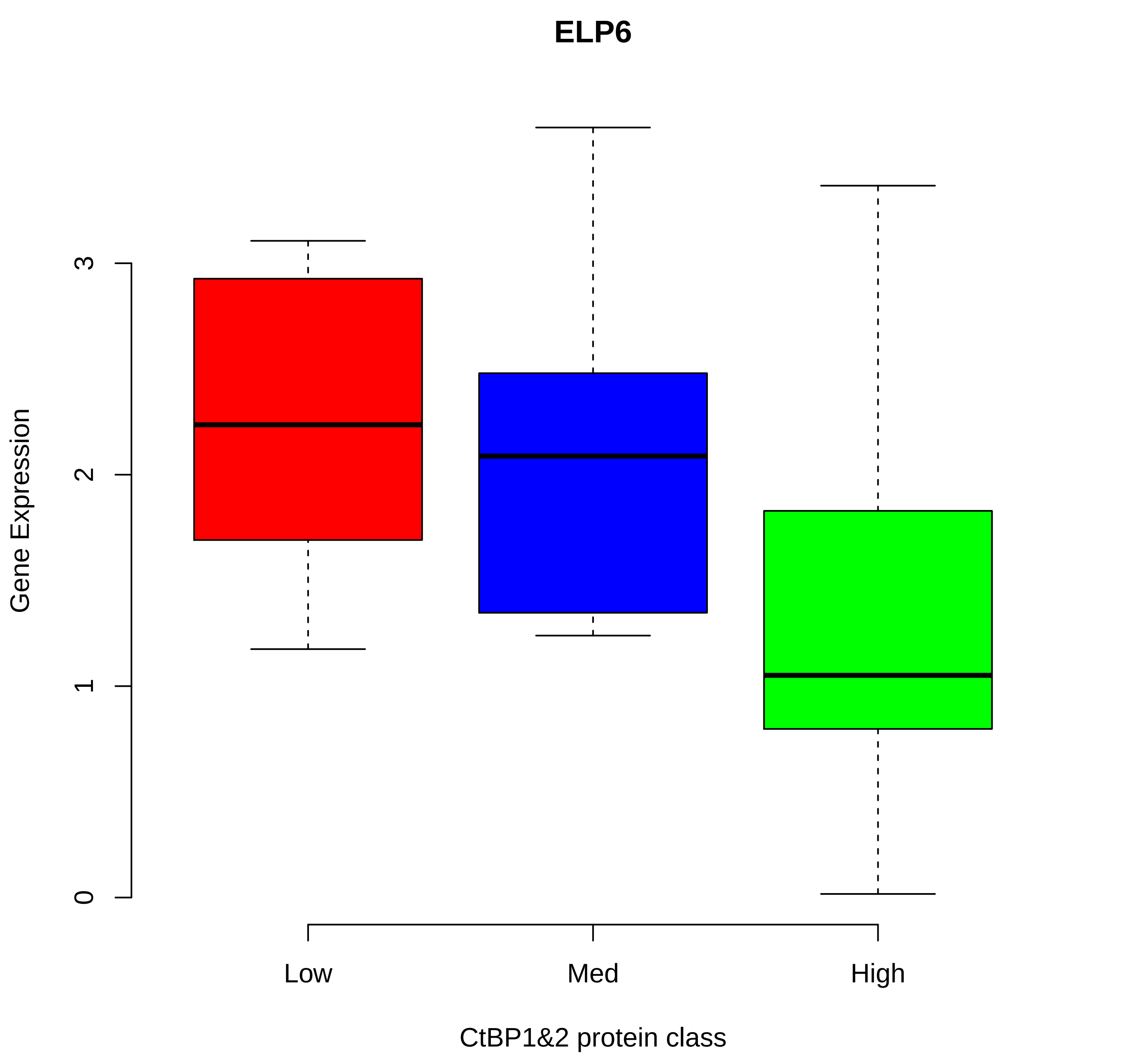
ELP6
Gene Expression
0
1
2
3
Low
Med
High
CtBP1&2 protein class

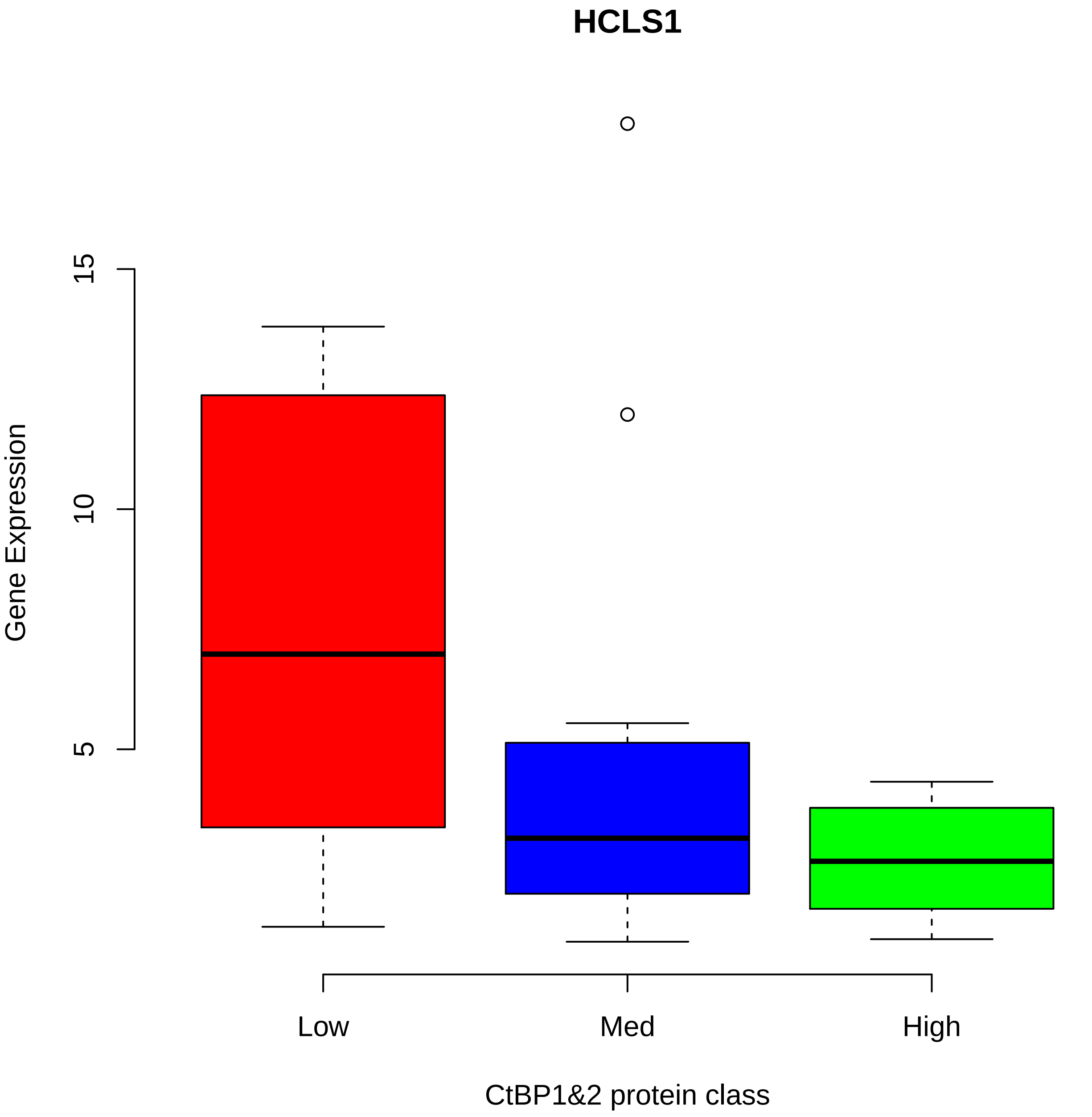

HCLS1
Gene Expression
15
10
5
Low
Med
High
CtBP1&2 protein class

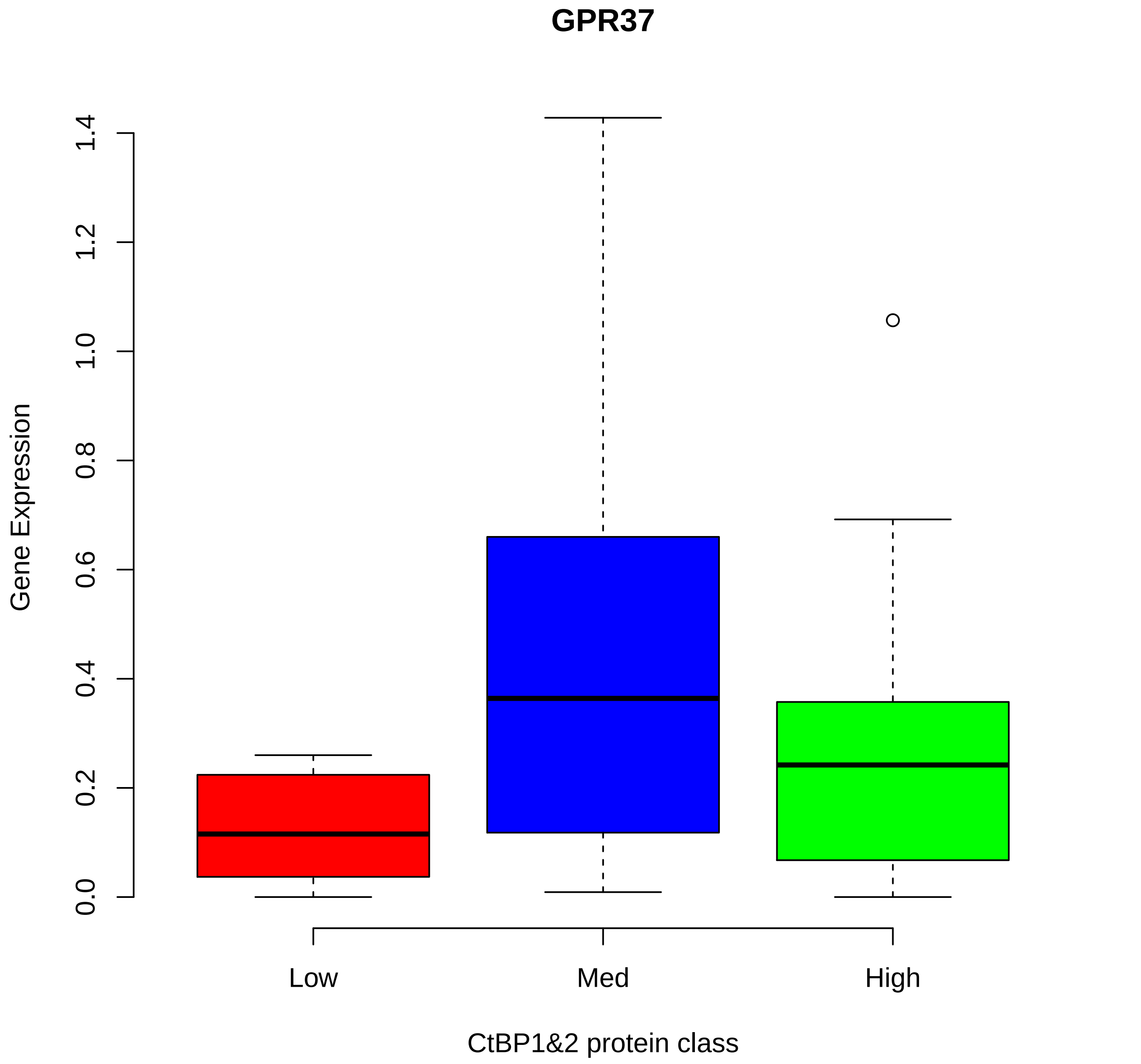
GPR37
Gene Expression
0.0
0.2
0.4
0.6
0.8
1.0
1.2
1.4
Low
Med
High
CtBP1&2 protein class

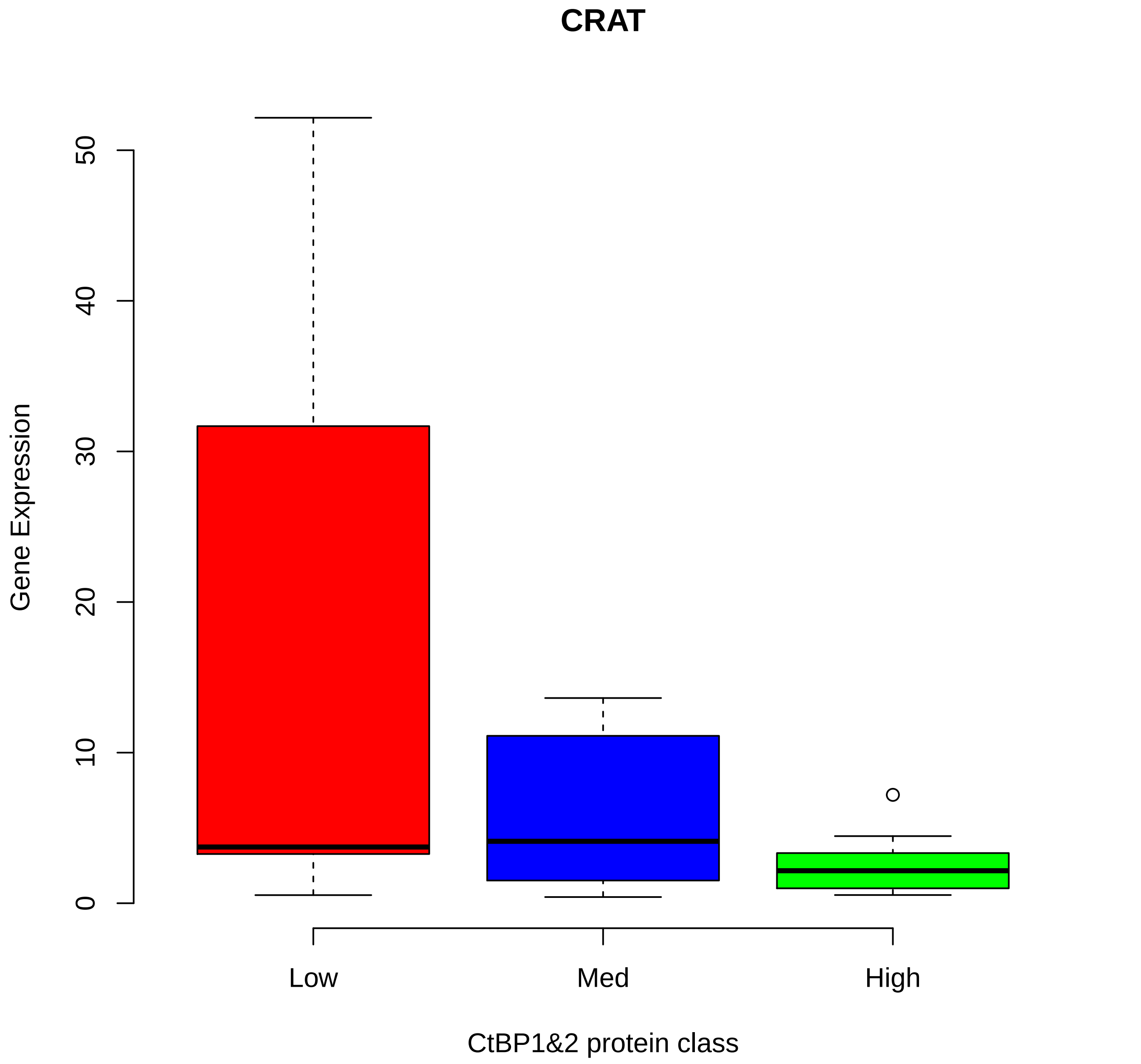
CRAT
Gene Expression
0
10
20
30
40
50
Low
Med
High
CtBP1&2 protein class

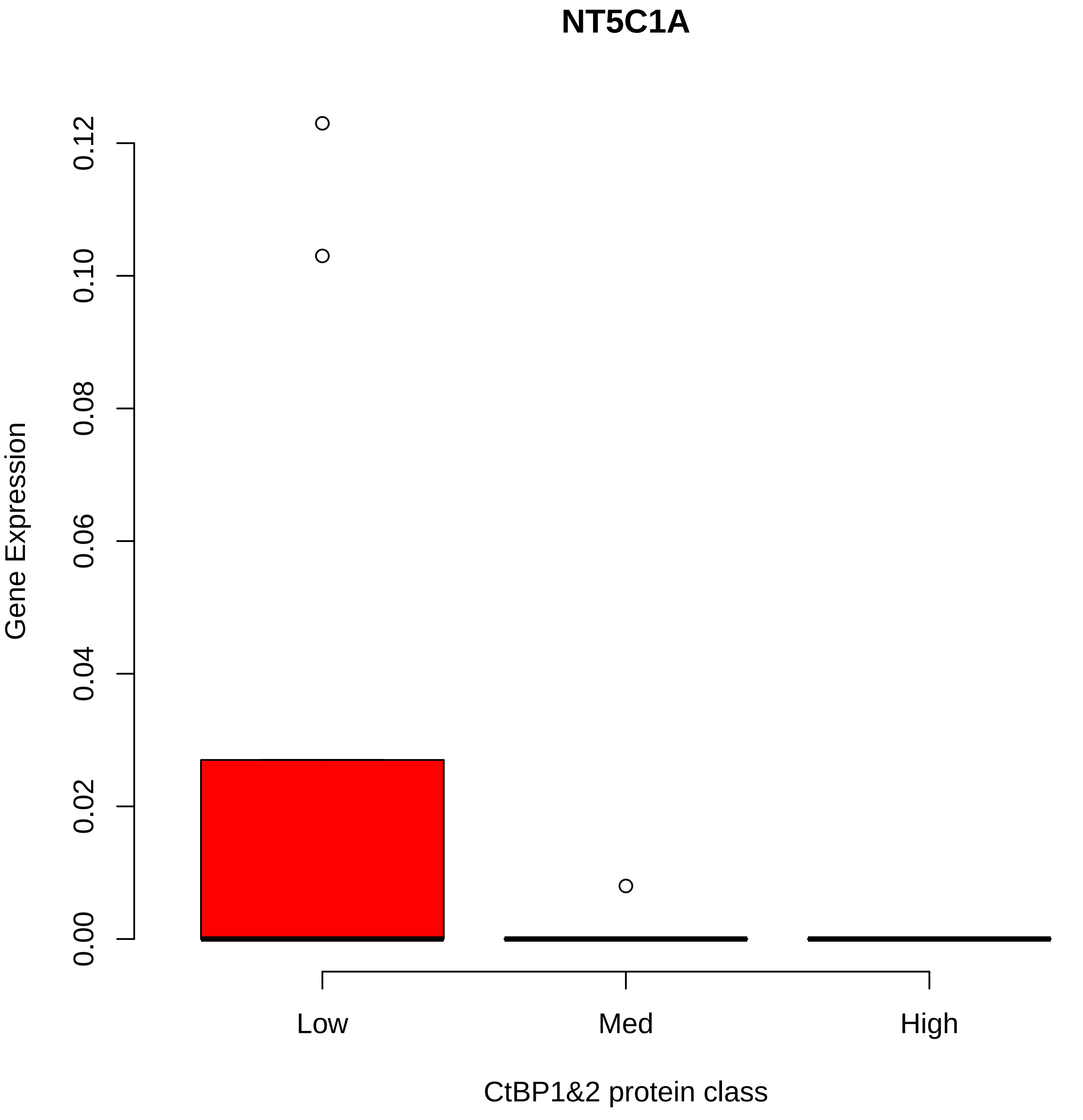
NT5C1A
Gene Expression
0.00
0.02
0.04
0.06
0.08
0.10
0.12
Low
Med
High
CtBP1&2 protein class

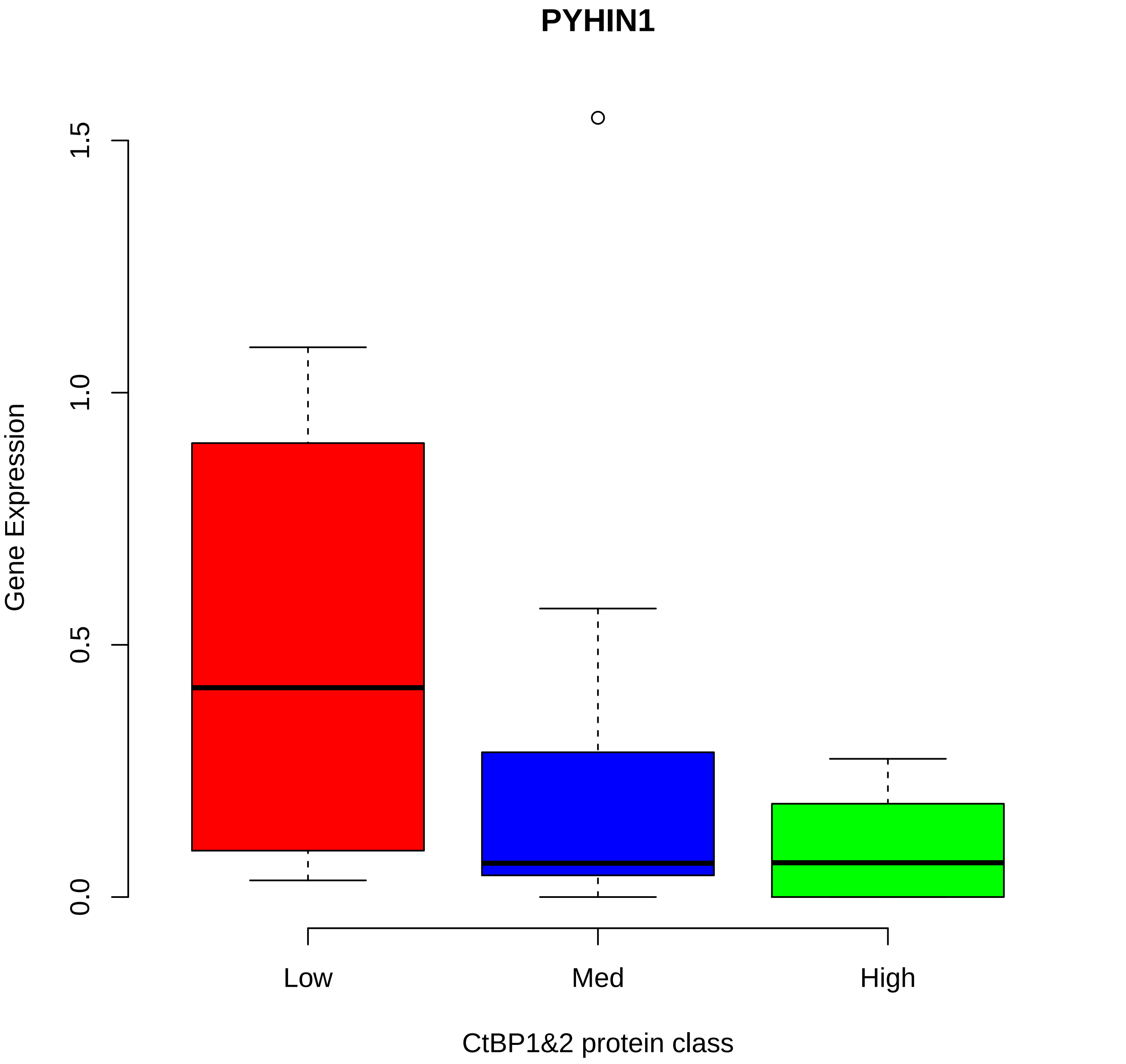
PYHIN1
Gene Expression
0.0
0.5
1.0
1.5
Low
Med
High
CtBP1&2 protein class

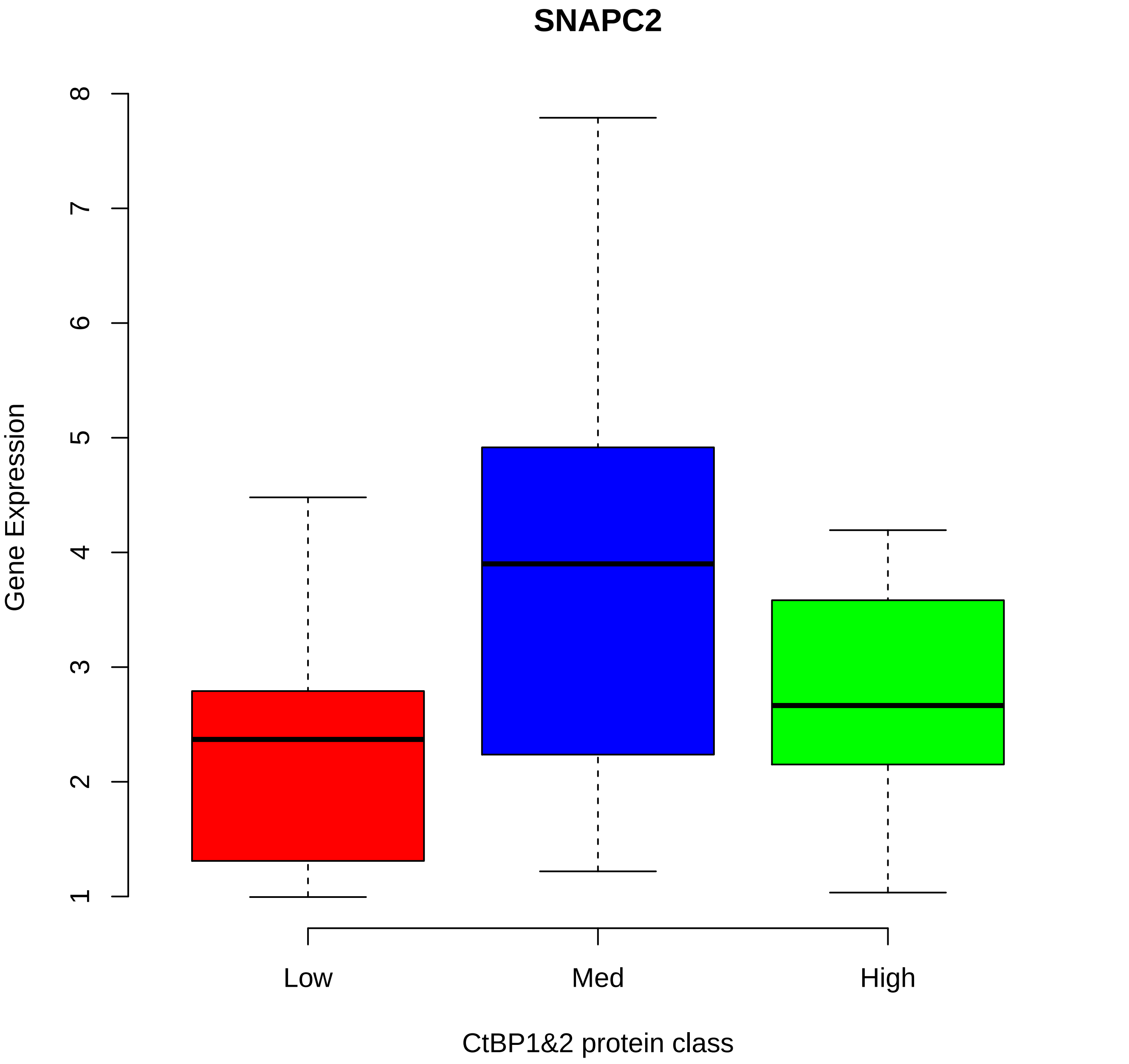
SNAPC2
Gene Expression
1
2
3
4
5
6
7
8
Low
Med
High
CtBP1&2 protein class

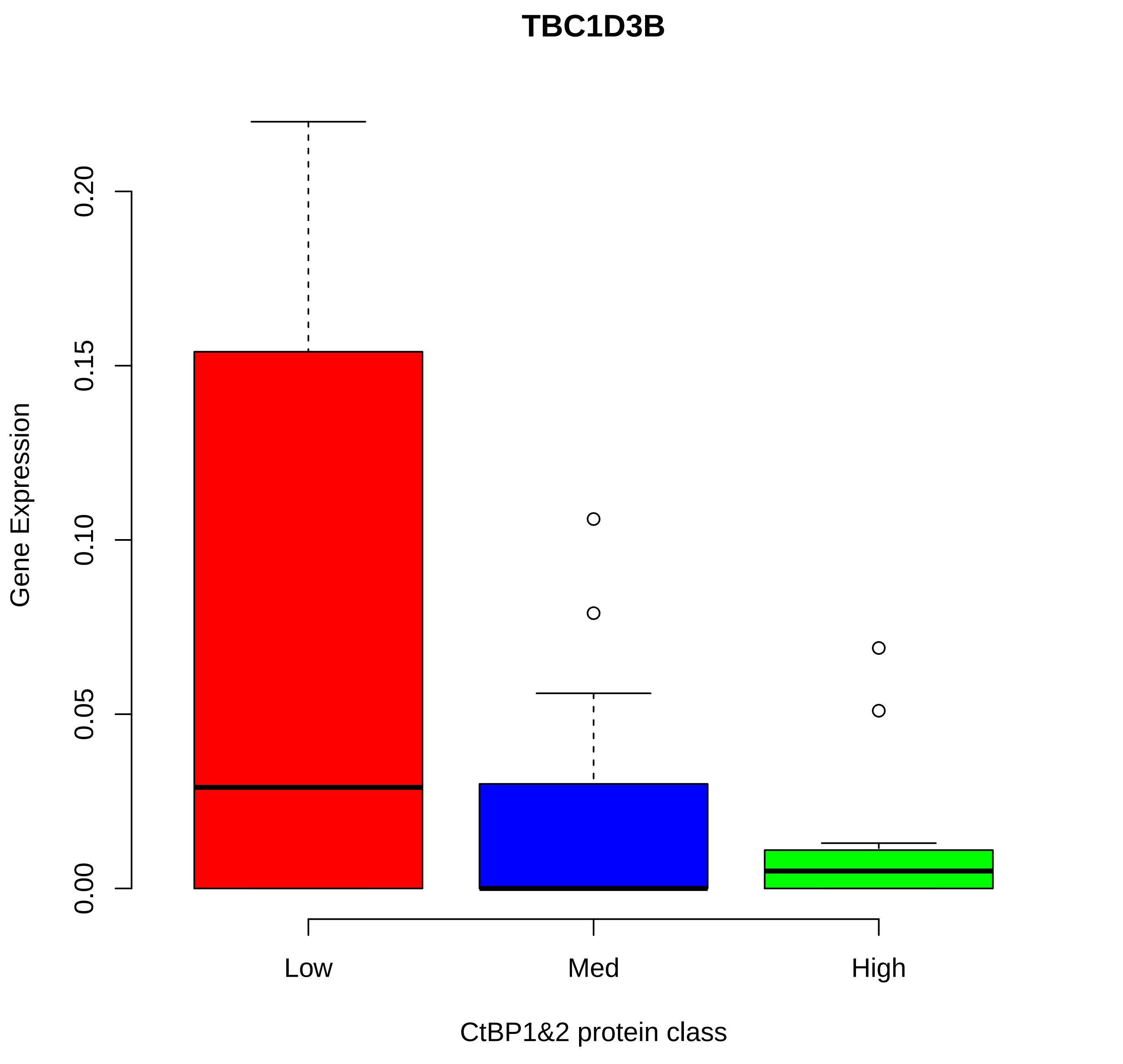

TBC1D3B
Gene Expression
0.00
0.05
0.10
0.15
0.20
Low
Med
High
CtBP1&2 protein class

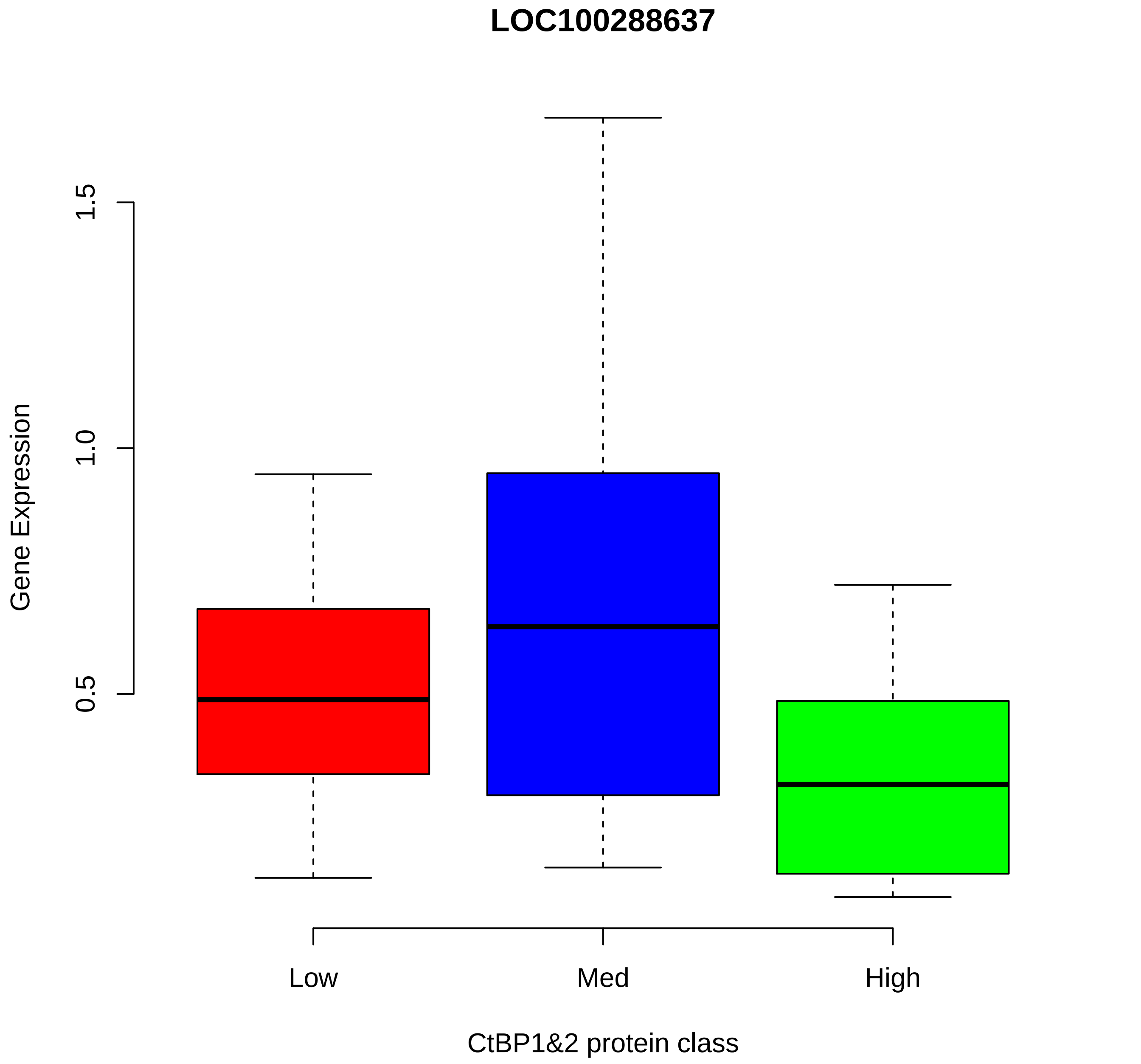
LOC100288637
Gene Expression
1.5
1.0
0.5
Low
Med
High
CtBP1&2 protein class

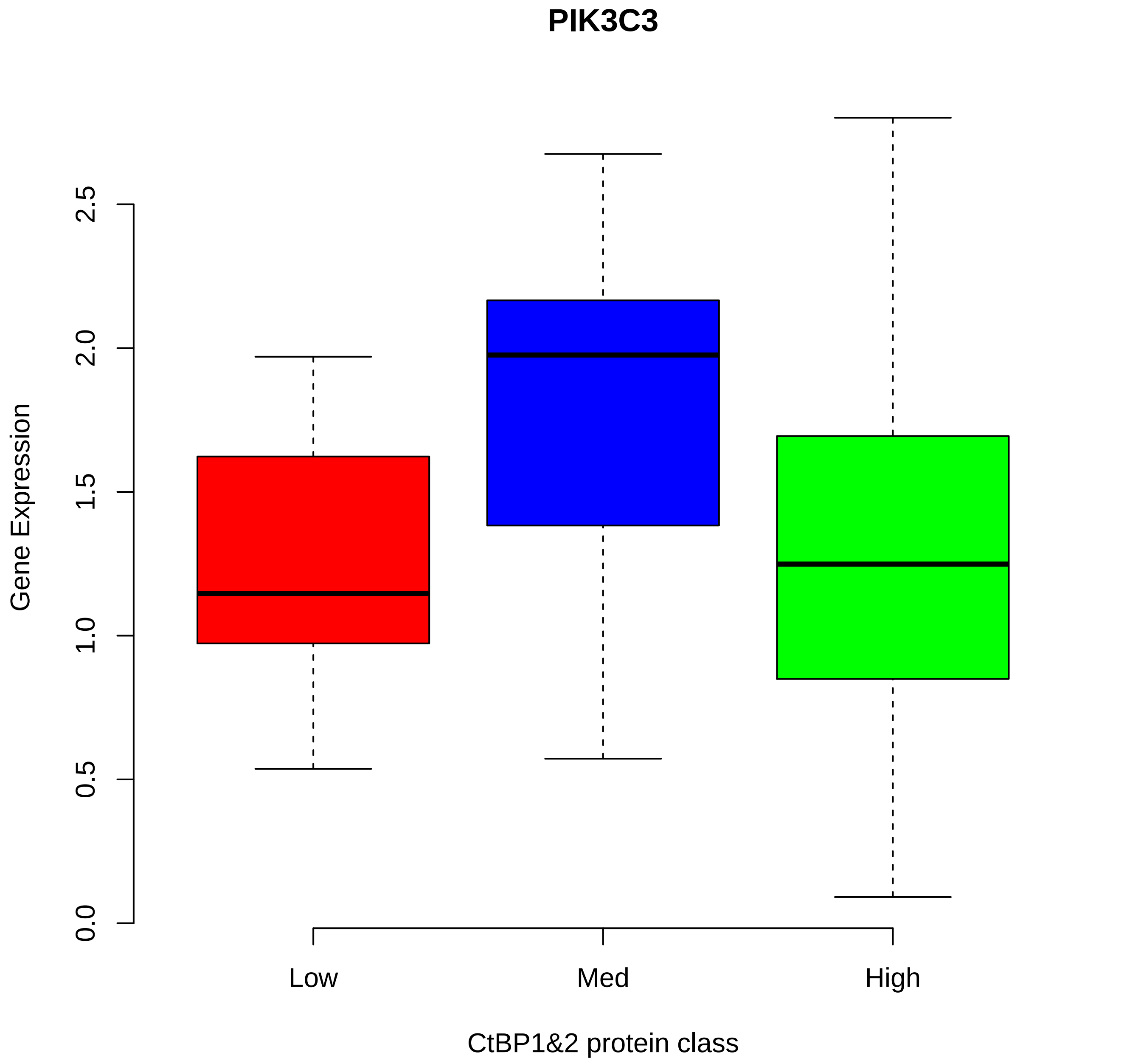

PIK3C3
Gene Expression
0.0
0.5
1.0
1.5
2.0
2.5
Low
Med
High
CtBP1&2 protein class

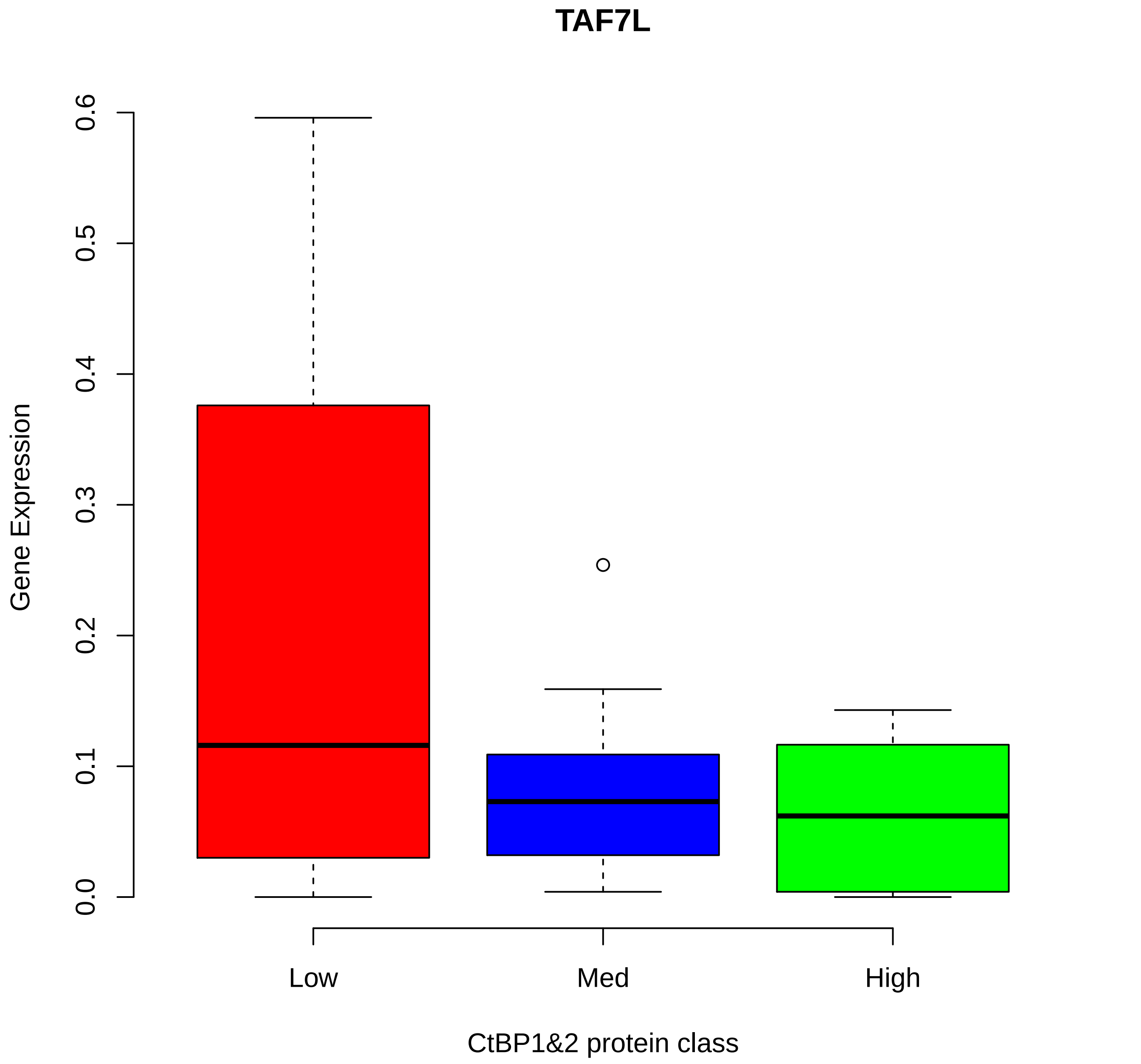

TAF7L
Gene Expression
0.0
0.1
0.2
0.3
0.4
0.5
0.6
Low
Med
High
CtBP1&2 protein class

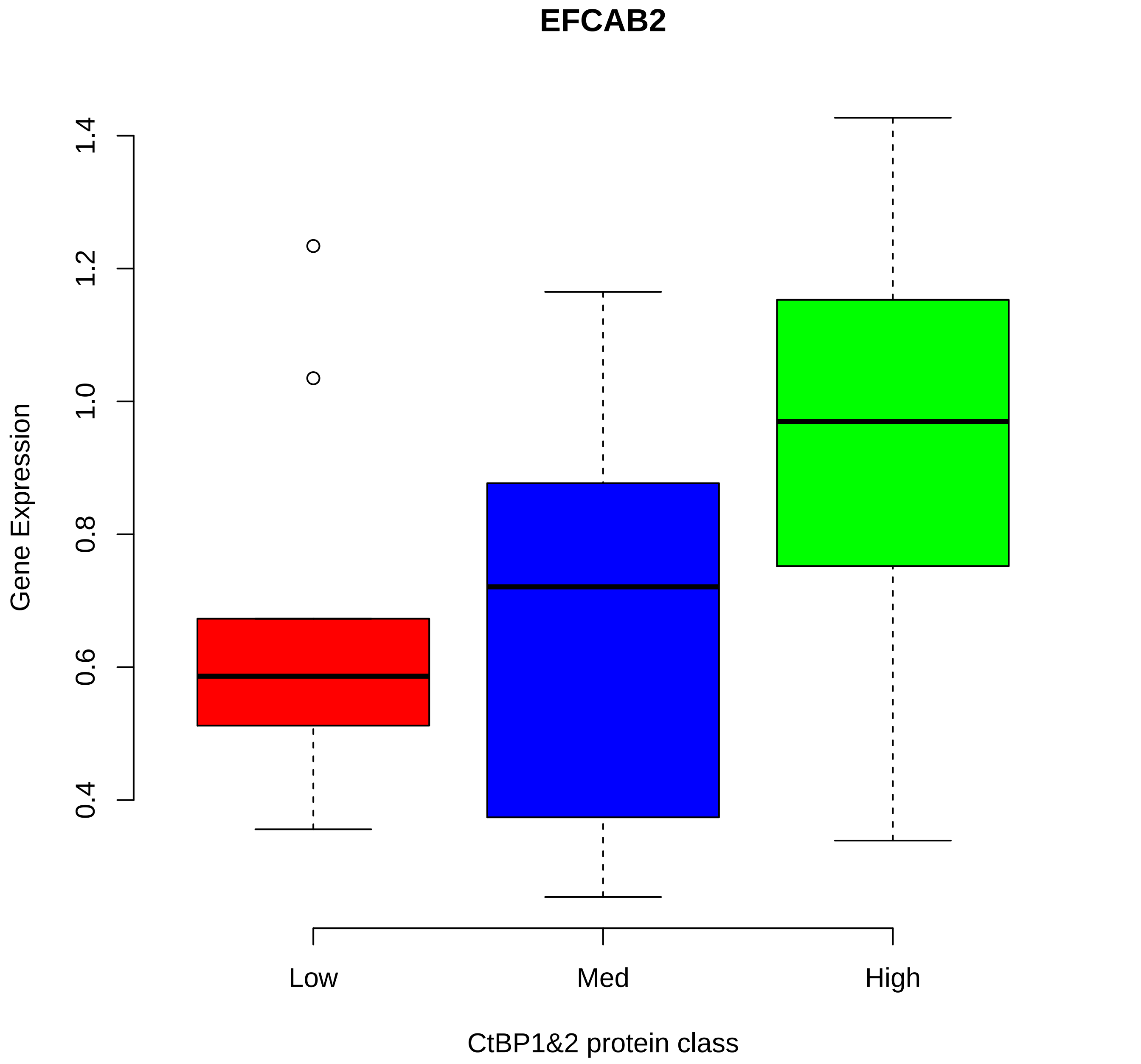
EFCAB2
Gene Expression
1.4
1.2
1.0
0.8
0.6
0.4
Low
Med
High
CtBP1&2 protein class

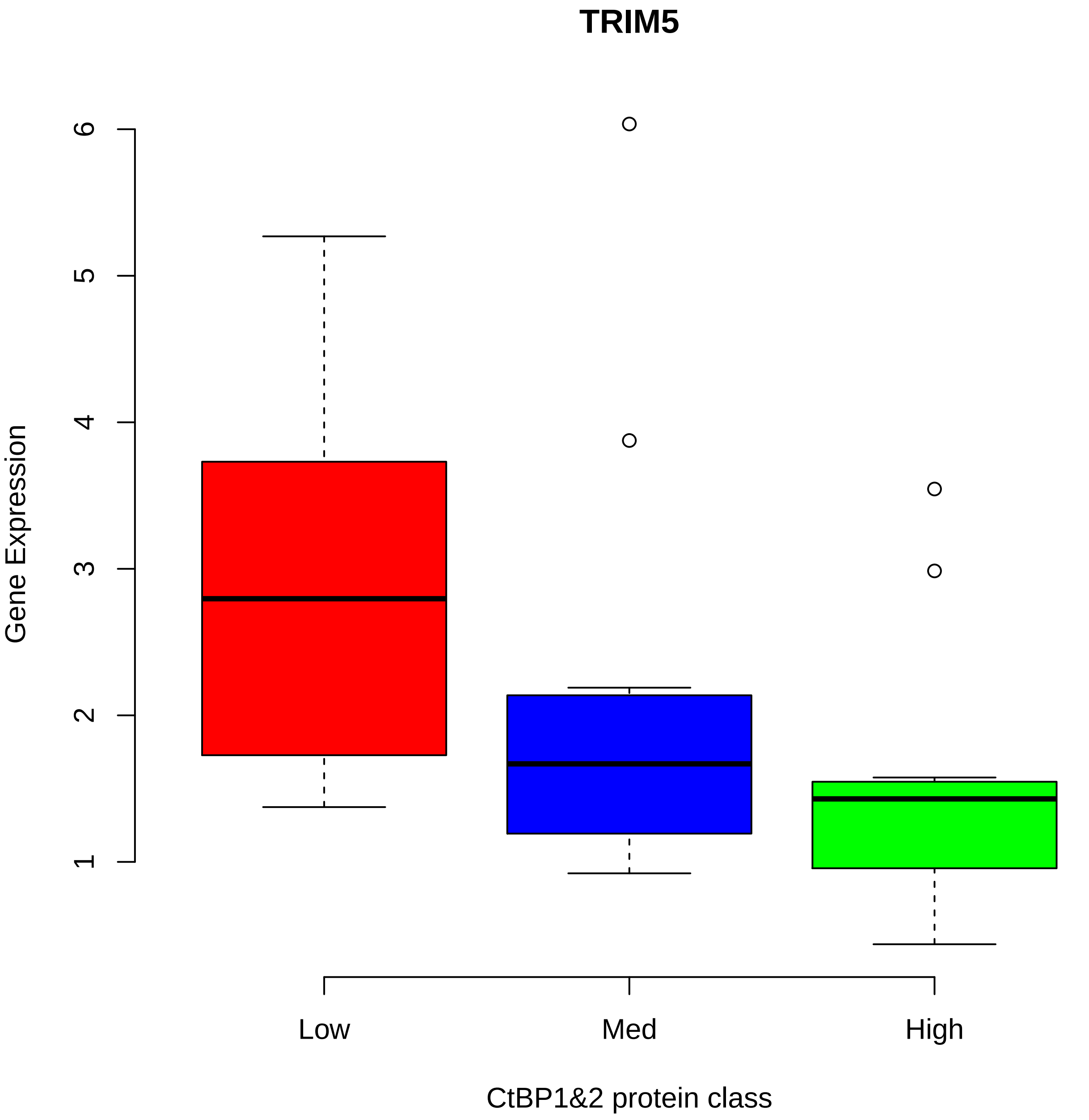
TRIM5
Gene Expression
1
2
3
4
5
6
Low
Med
High
CtBP1&2 protein class

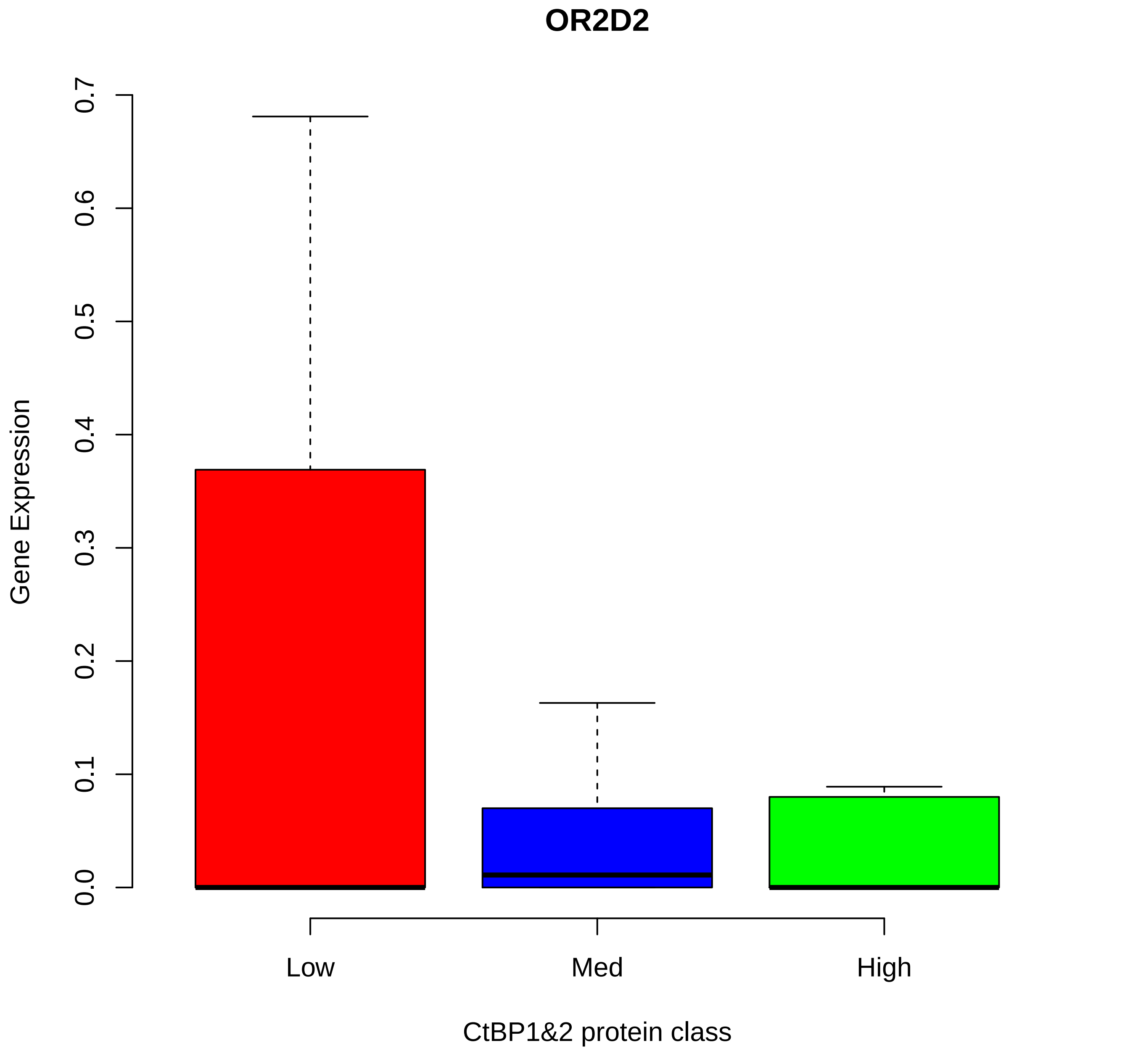

OR2D2
Gene Expression
0.0
0.1
0.2
0.3
0.4
0.5
0.6
0.7
Low
Med
High
CtBP1&2 protein class

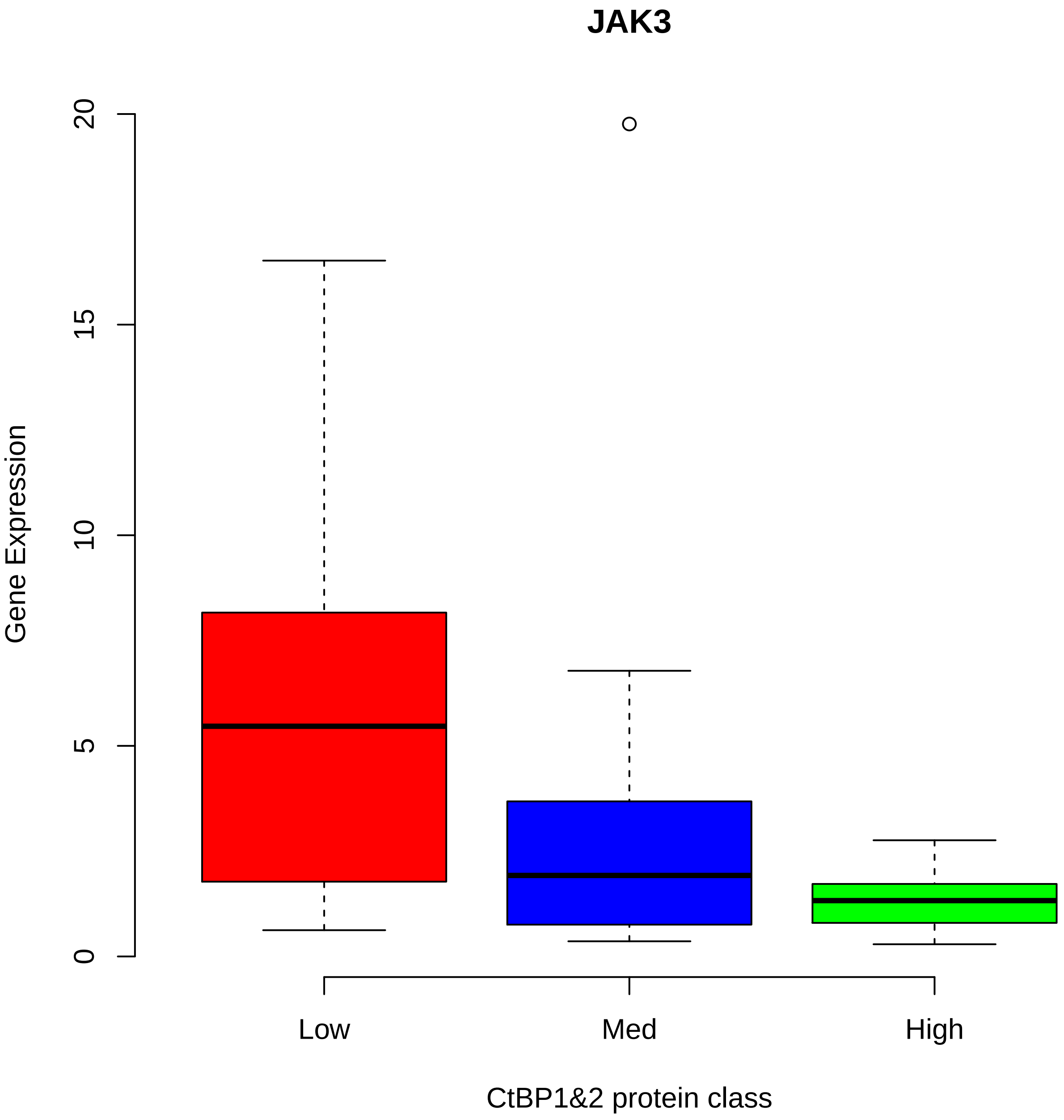
JAK3
Gene Expression
0
5
10
15
20
Low
Med
High
CtBP1&2 protein class

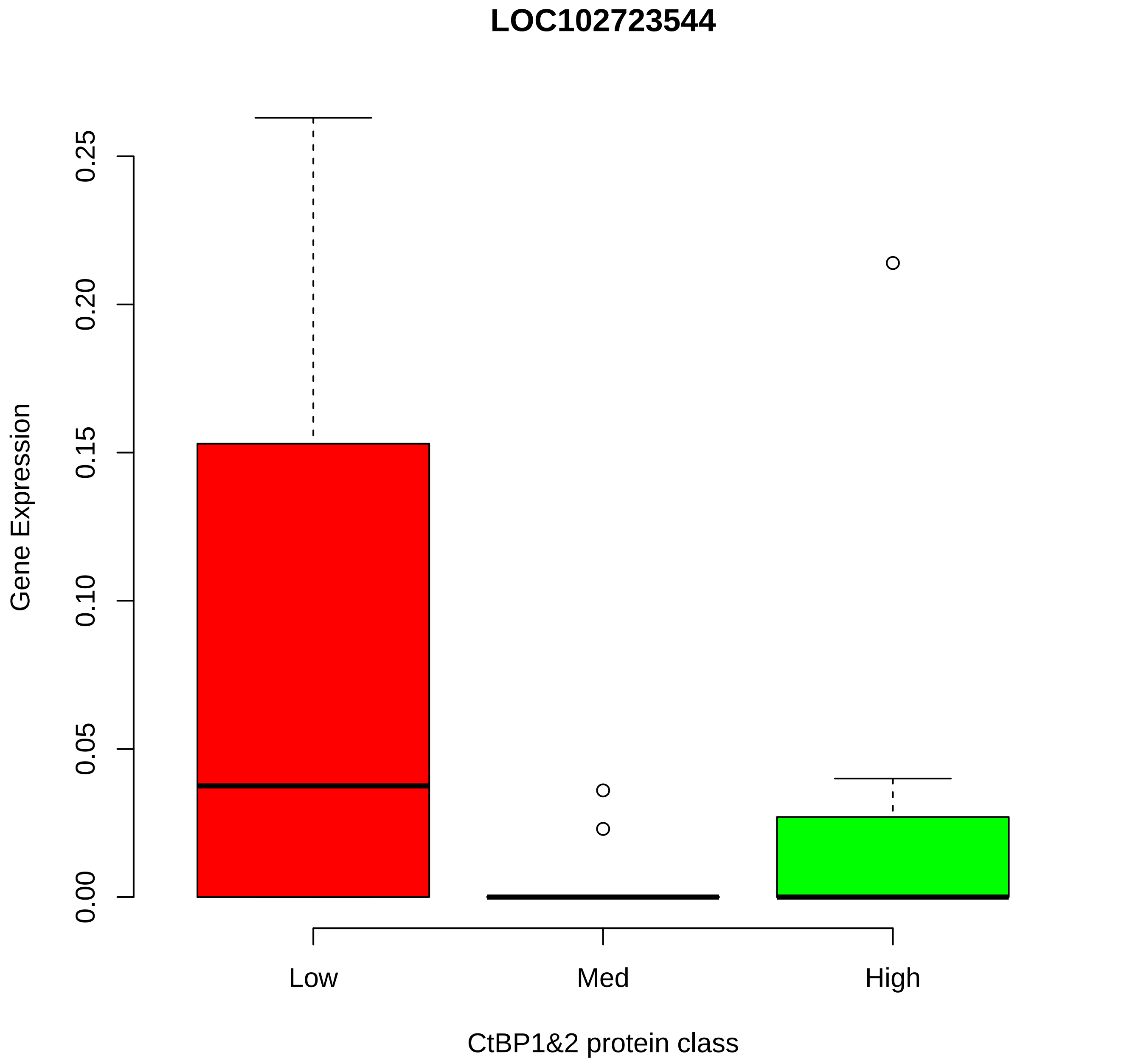

LOC102723544
Gene Expression
0.00
0.05
0.10
0.15
0.20
0.25
Low
Med
High
CtBP1&2 protein class

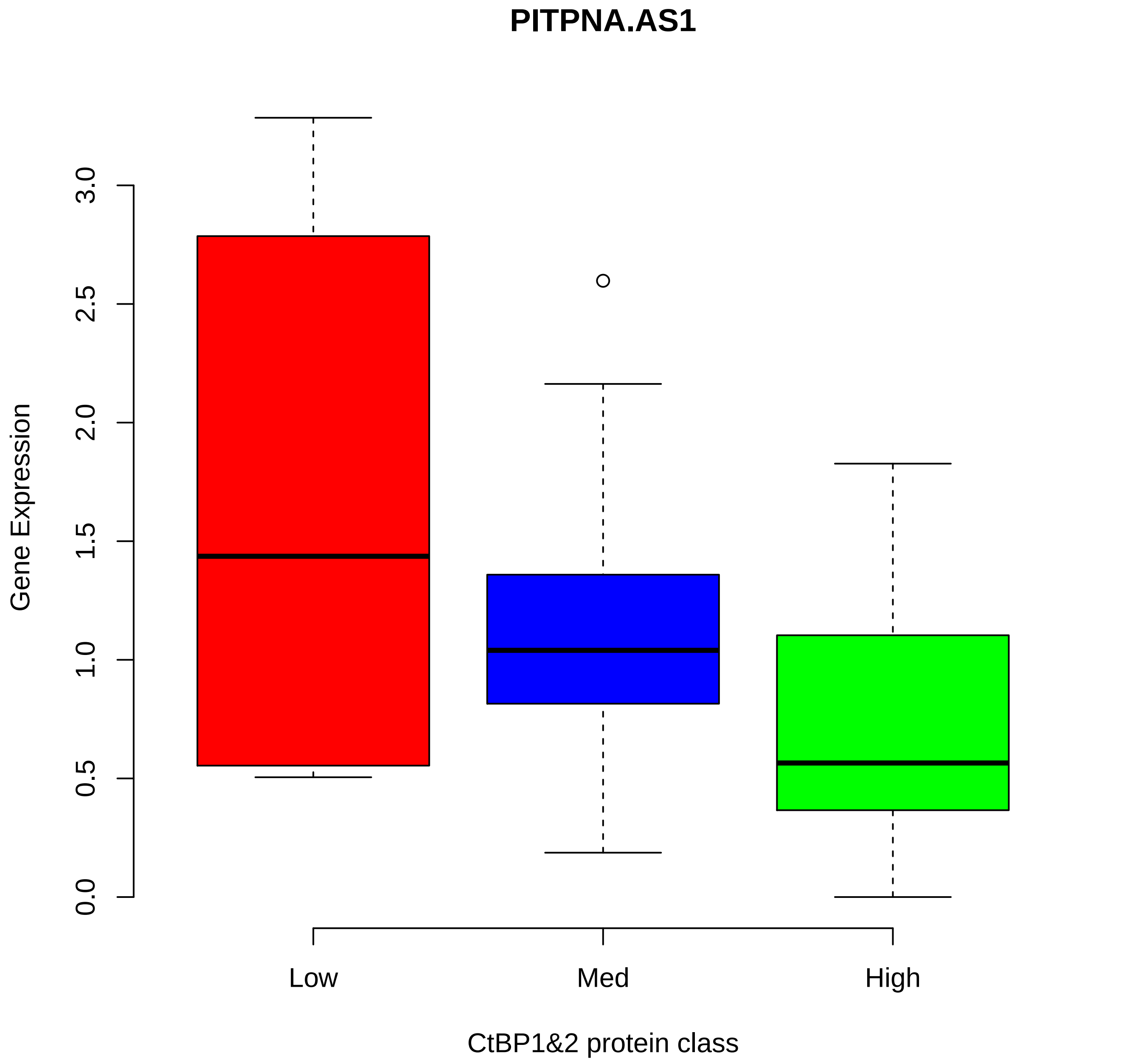

PITPNA.AS1
Gene Expression
0.0
0.5
1.0
1.5
2.0
2.5
3.0
Low
Med
High
CtBP1&2 protein class

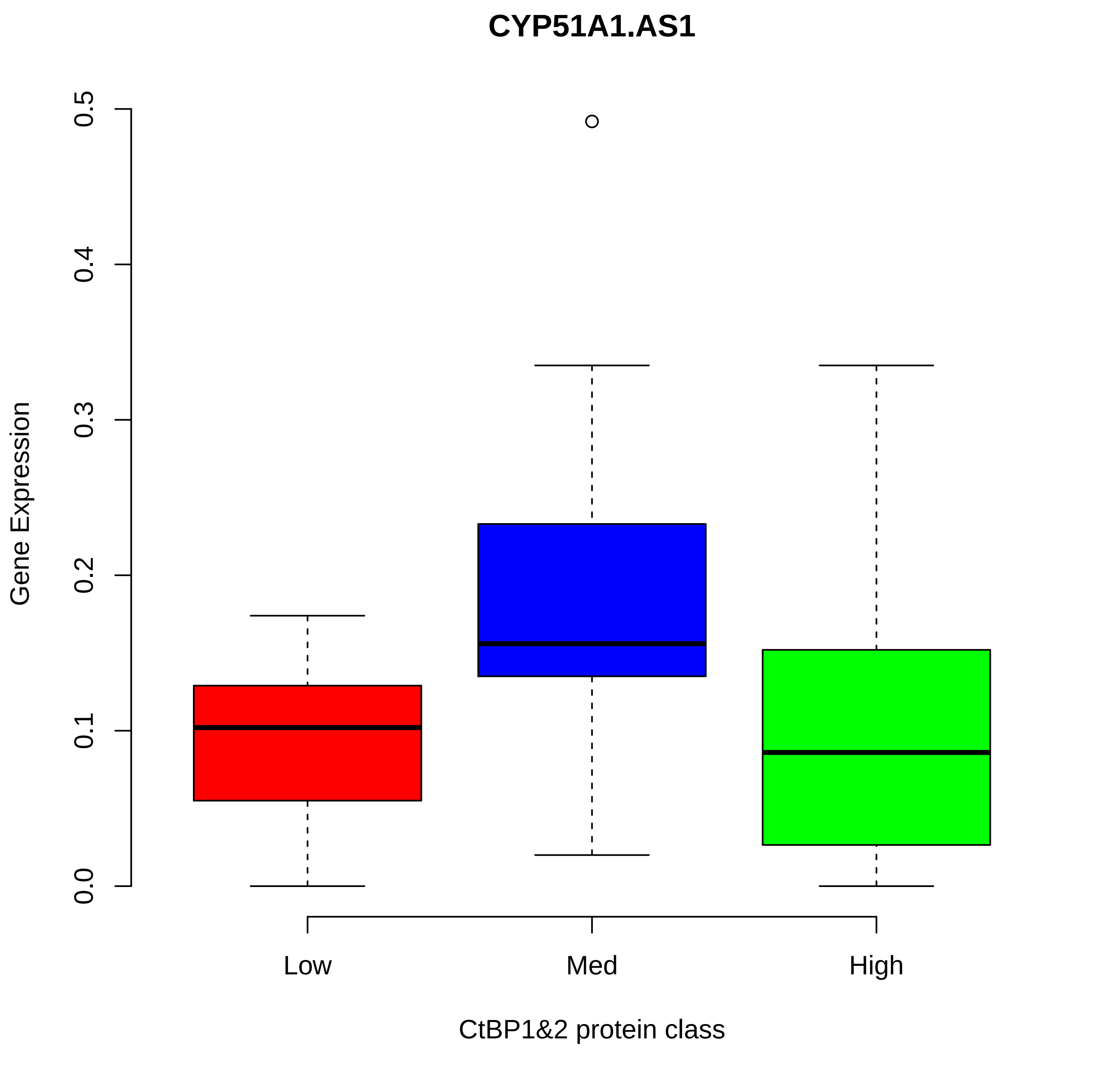
CYP51A1.AS1
Gene Expression
0.0
0.1
0.2
0.3
0.4
0.5
Low
Med
High
CtBP1&2 protein class

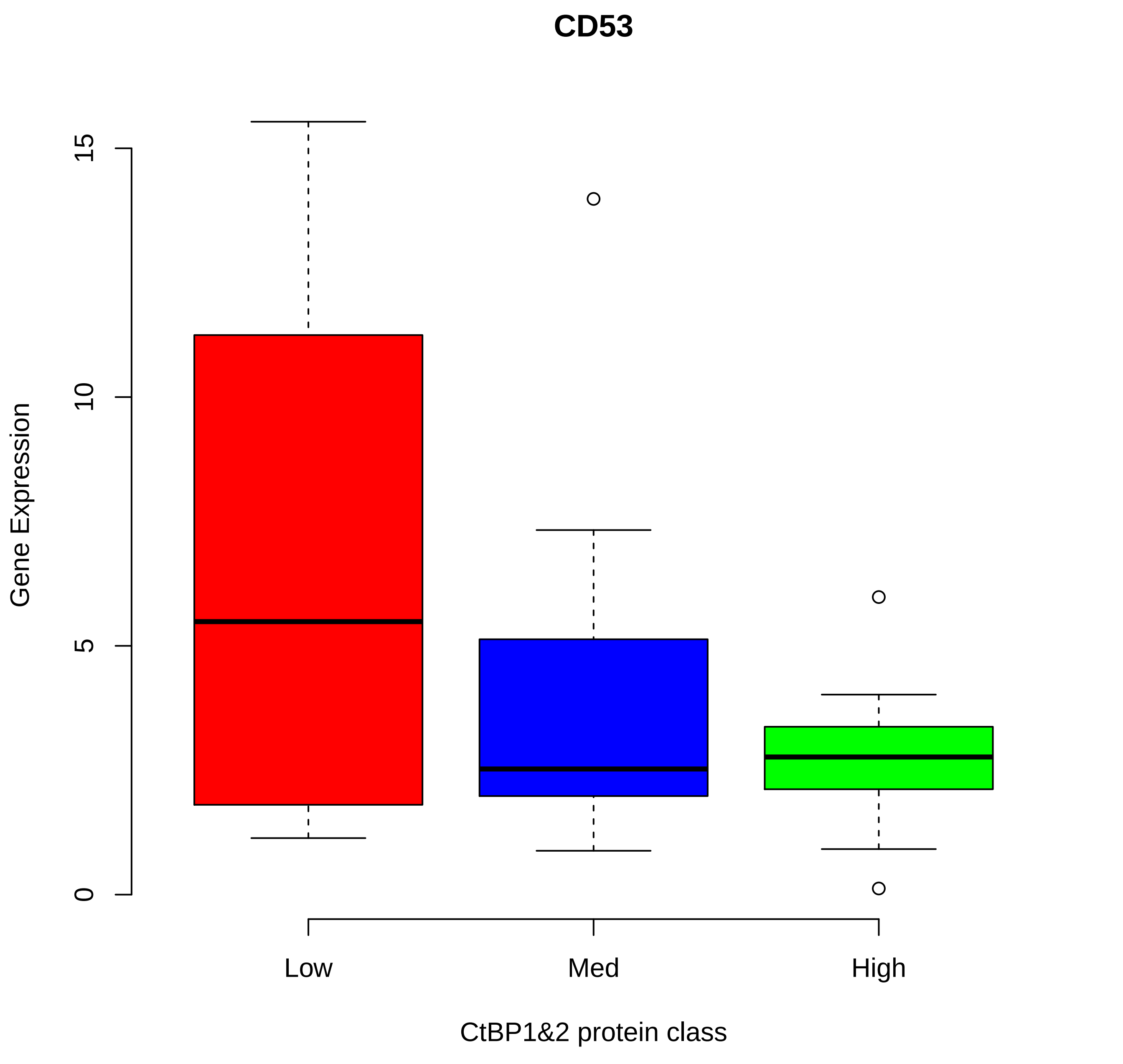

CD53
Gene Expression
15
10
5
0
Low
Med
High
CtBP1&2 protein class

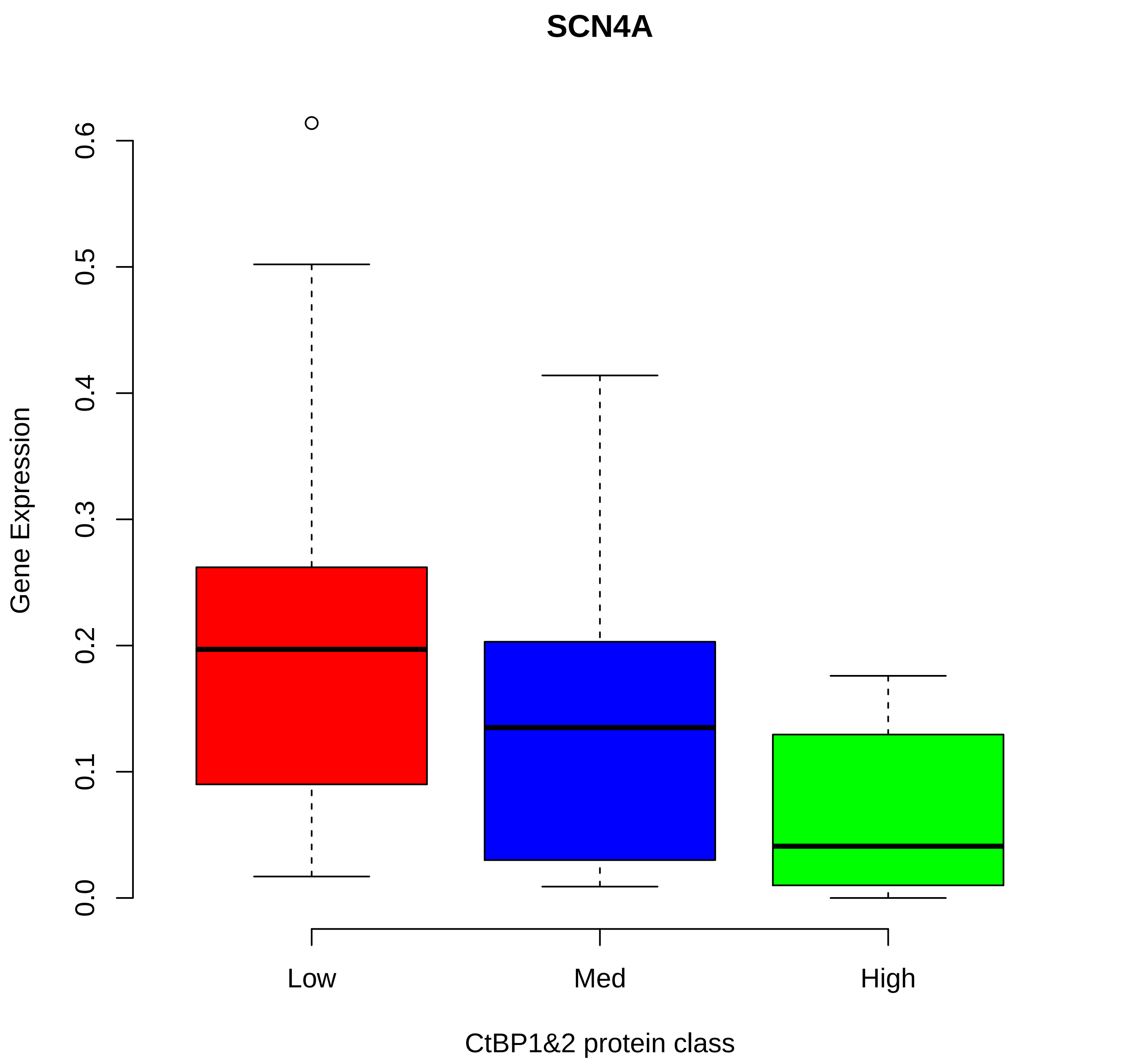

SCN4A
Gene Expression
0.0
0.1
0.2
0.3
0.4
0.5
0.6
Low
Med
High
CtBP1&2 protein class

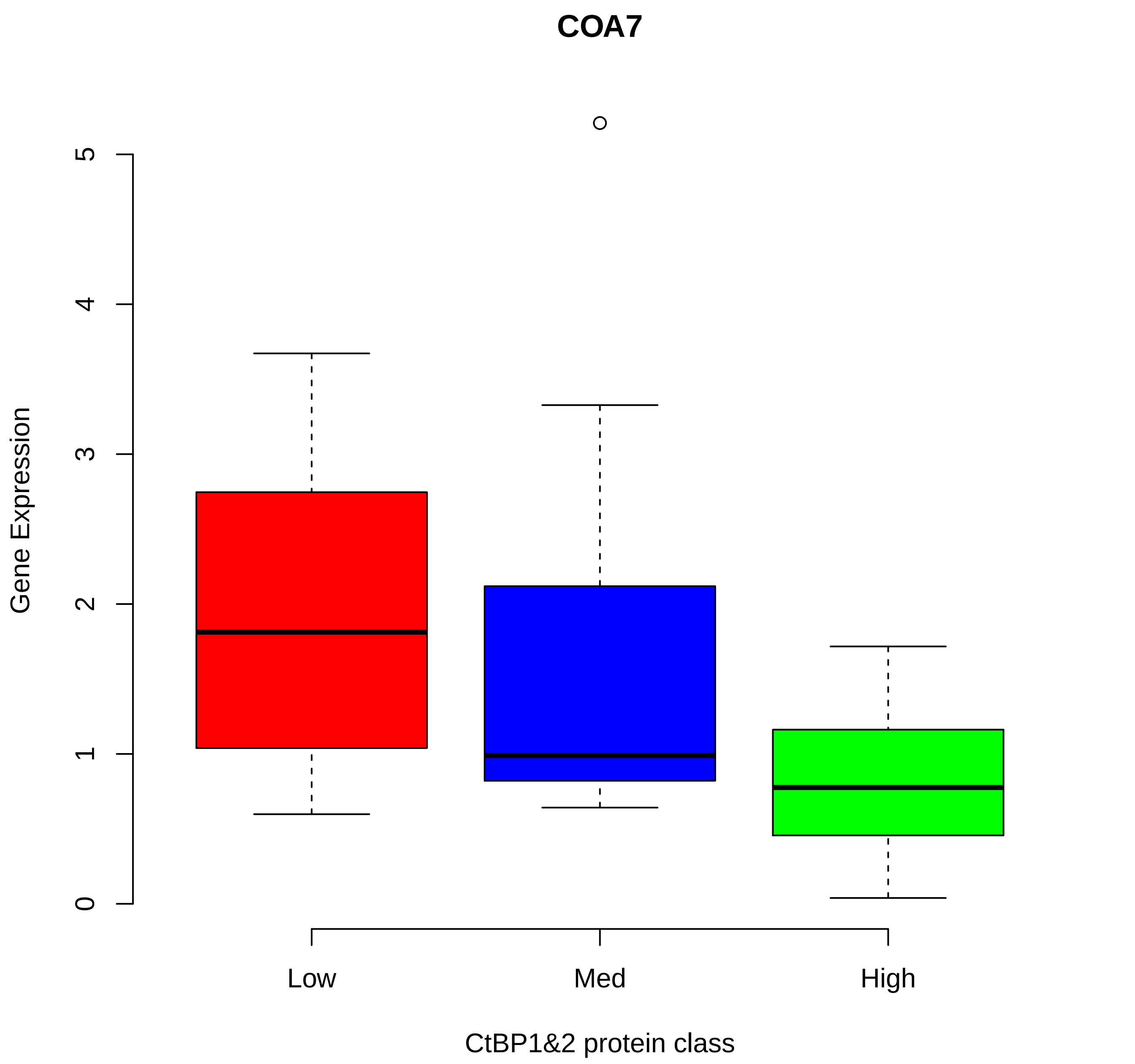
COA7
Gene Expression
0
1
2
3
4
5
Low
Med
High
CtBP1&2 protein class

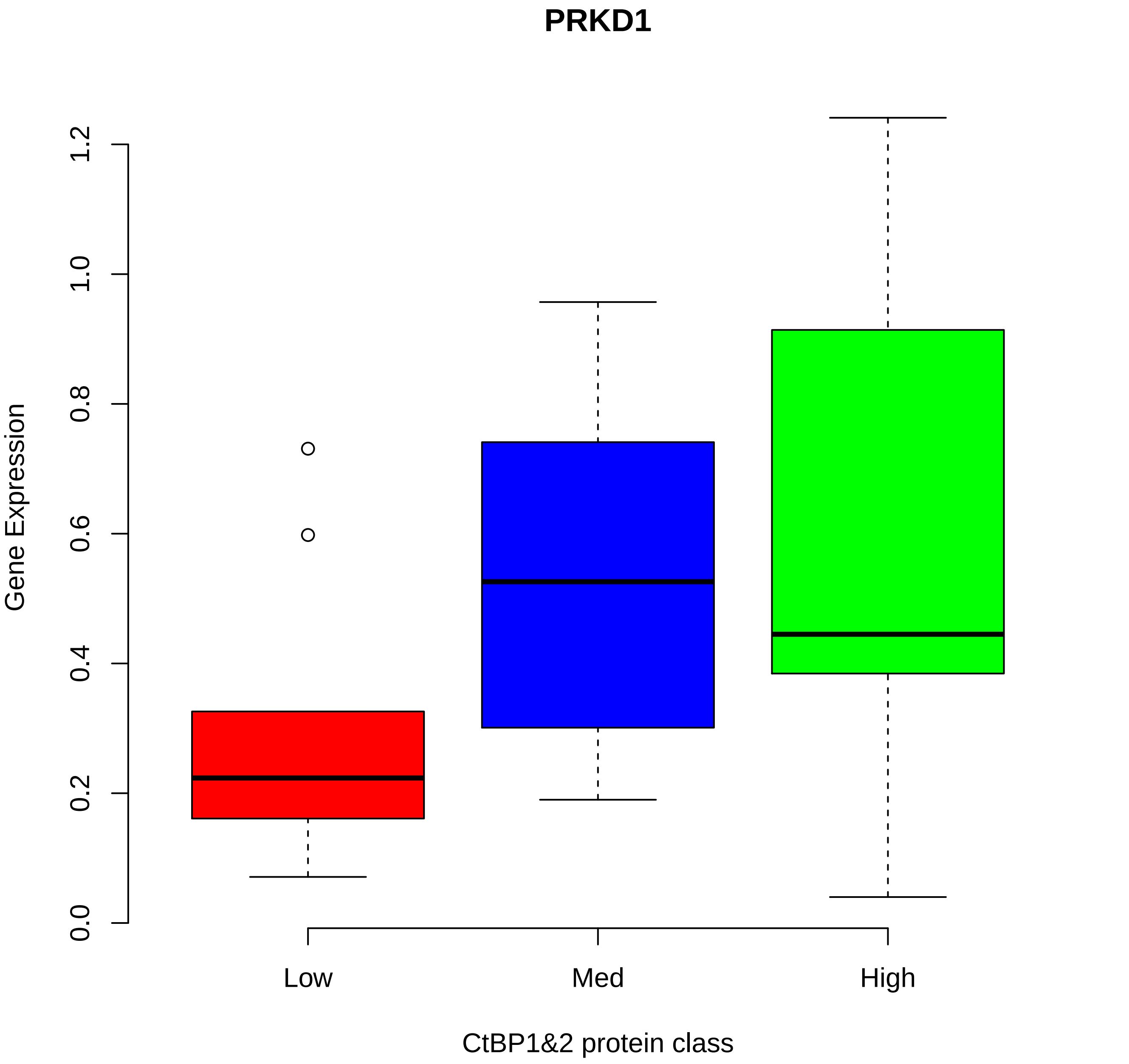
PRKD1
Gene Expression
0.0
0.2
0.4
0.6
0.8
1.0
1.2
Low
Med
High
CtBP1&2 protein class

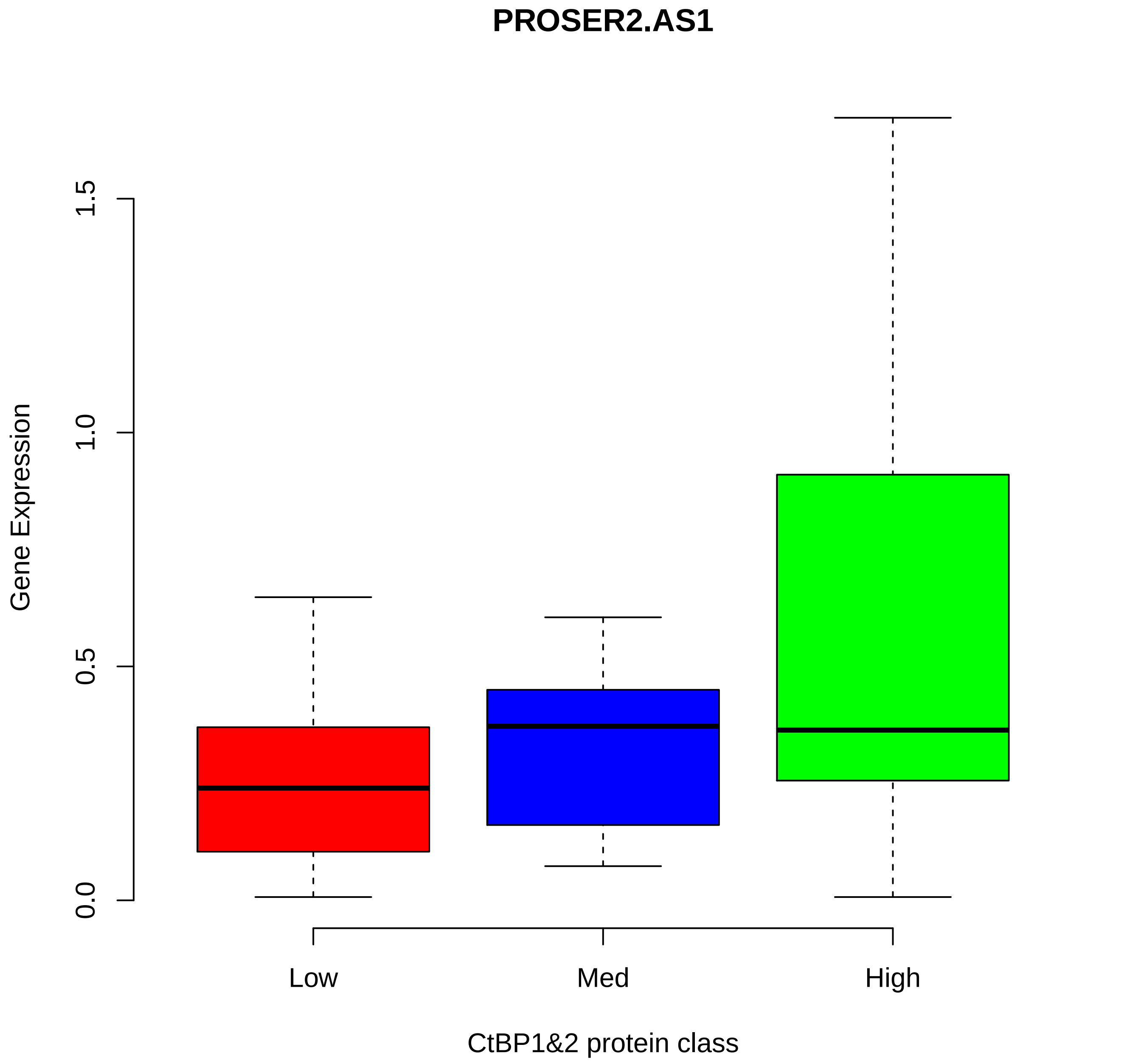

PROSER2.AS1
Gene Expression
0.0
0.5
1.0
1.5
Low
Med
High
CtBP1&2 protein class

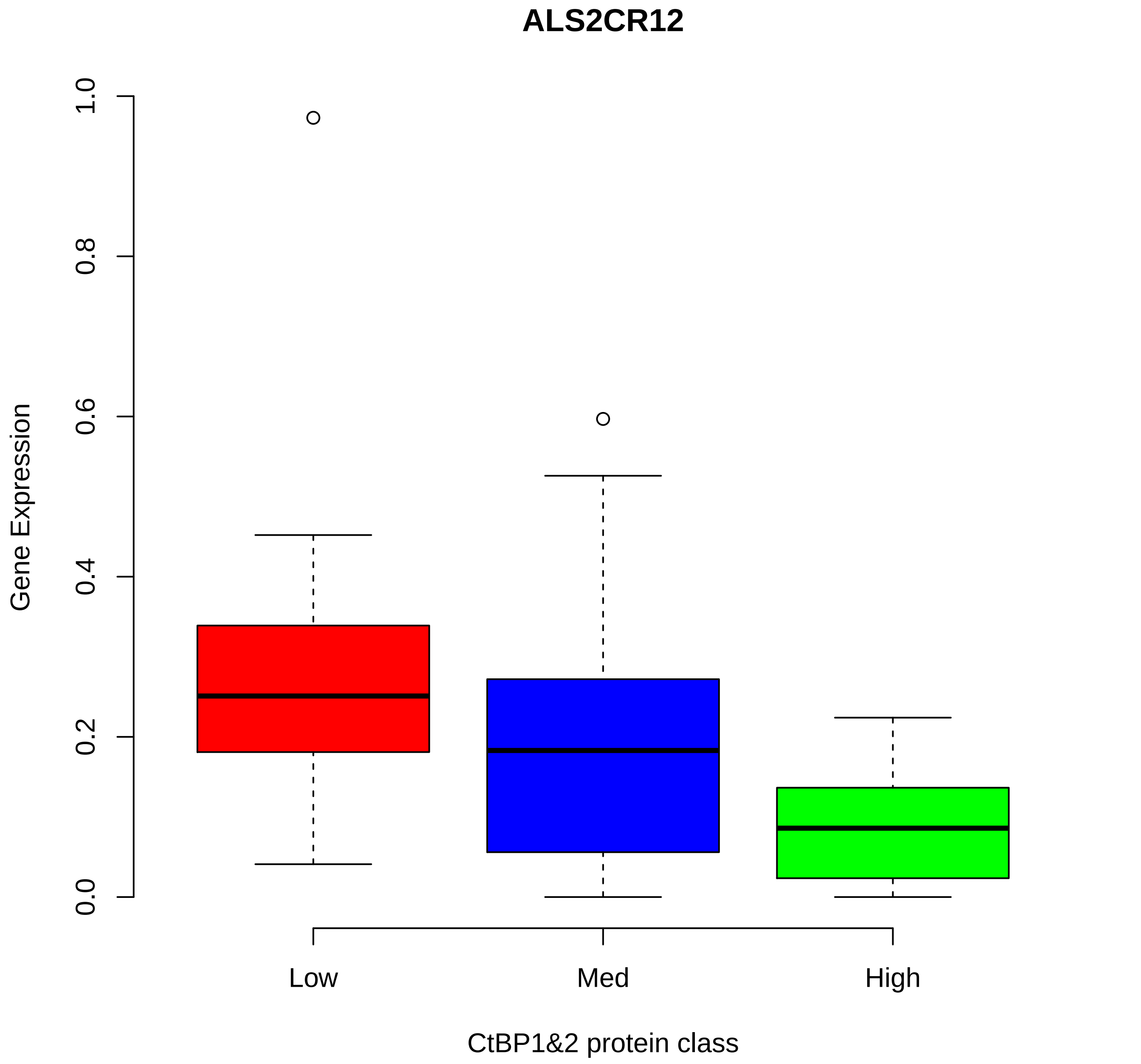
ALS2CR12
Gene Expression
0.0
0.2
0.4
0.6
0.8
1.0
Low
Med
High
CtBP1&2 protein class

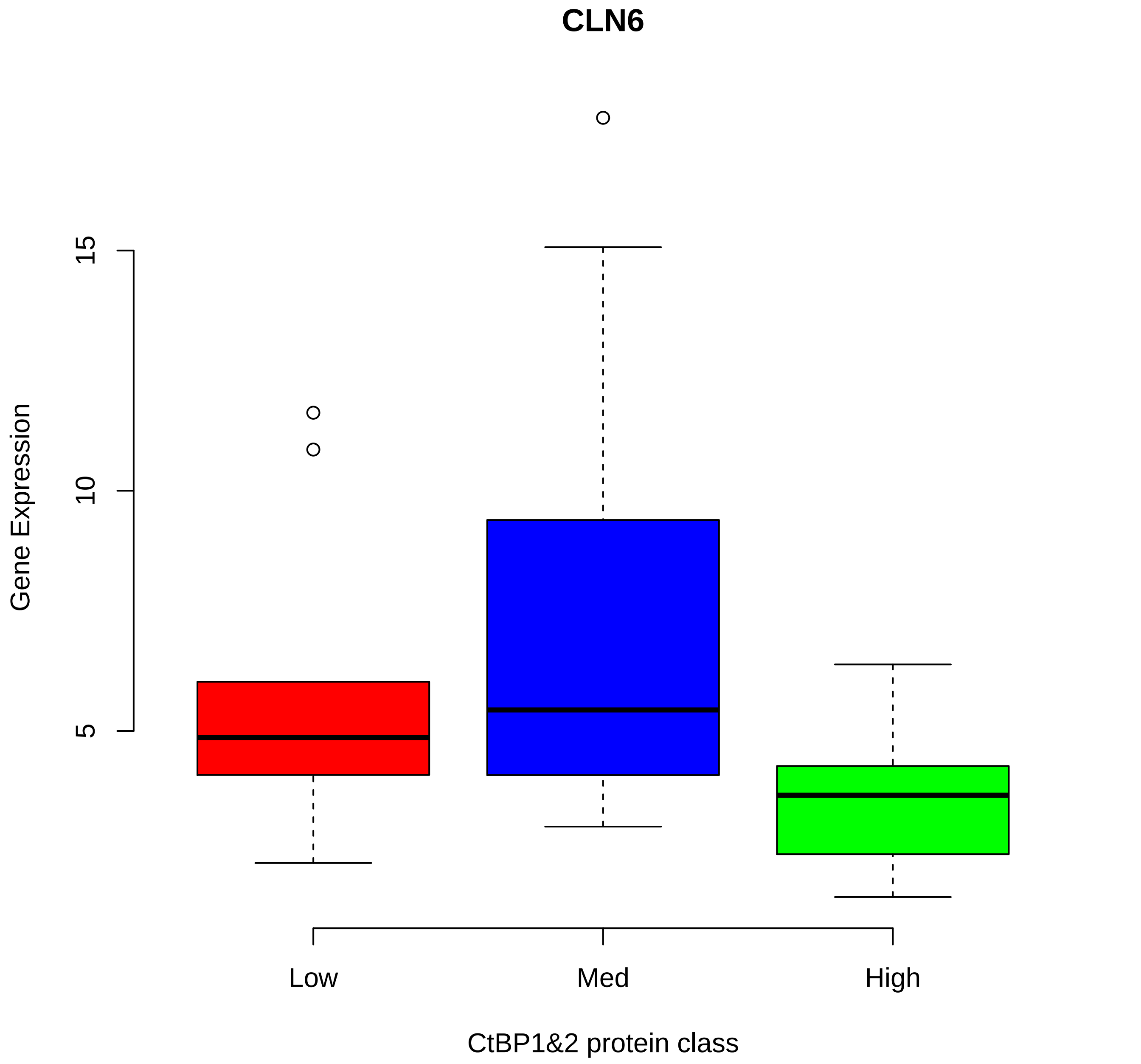
CLN6
Gene Expression
15
10
5
Low
Med
High
CtBP1&2 protein class

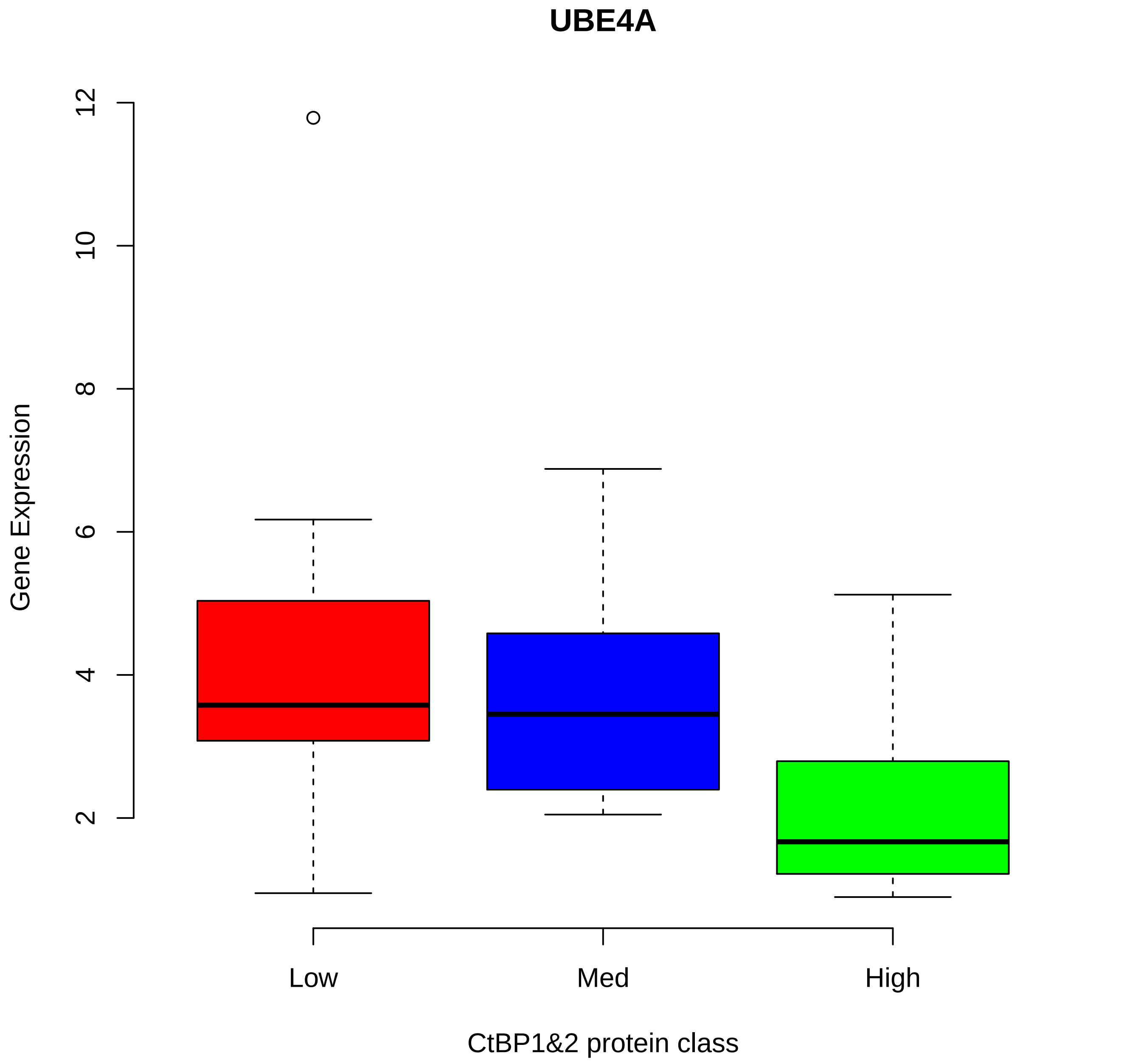
UBE4A
Gene Expression
12
10
8
6
4
2
Low
Med
High
CtBP1&2 protein class

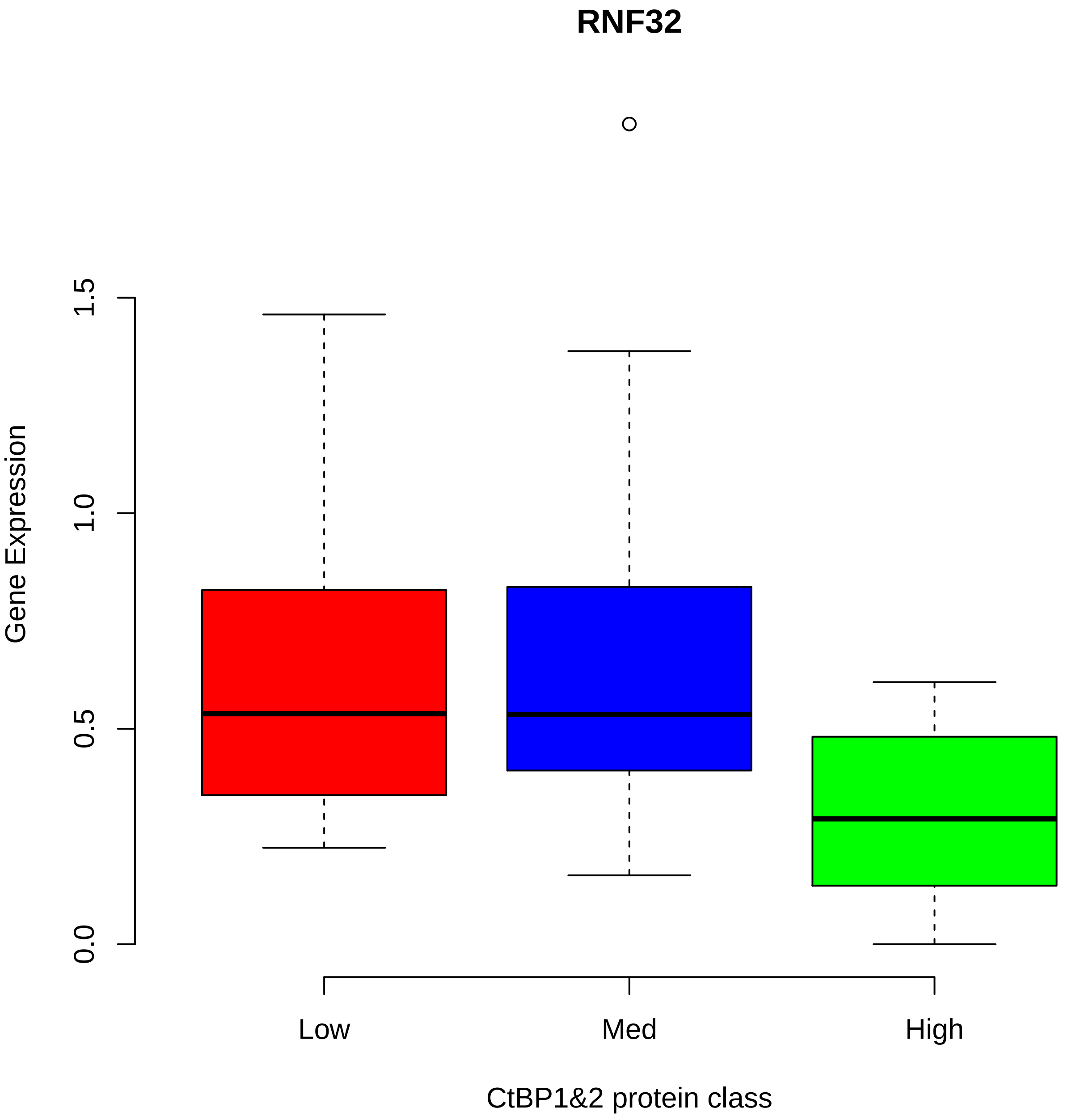

RNF32
Gene Expression
1.5
1.0
0.5
0.0
Low
Med
High
CtBP1&2 protein class

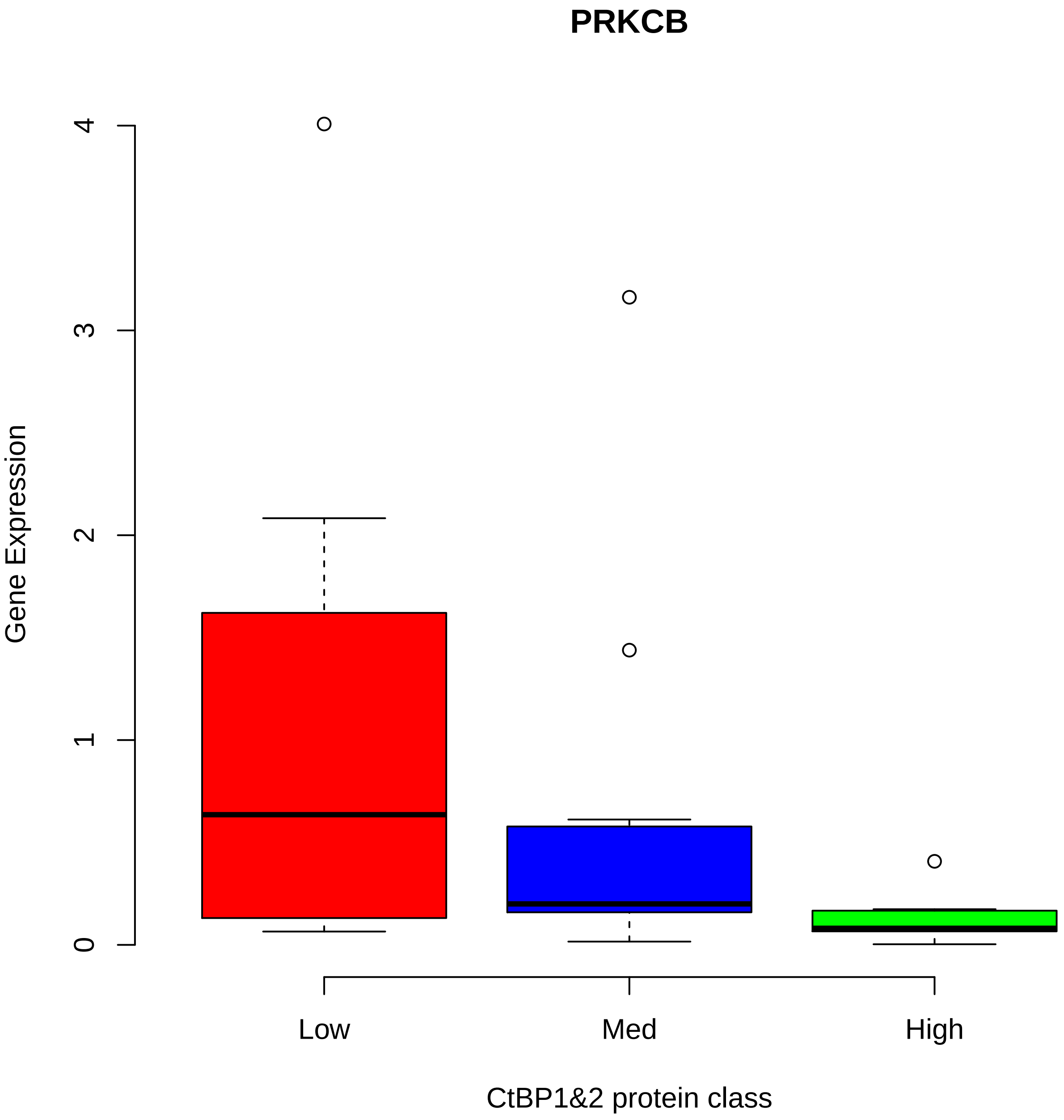
PRKCB
Gene Expression
0
1
2
3
4
Low
Med
High
CtBP1&2 protein class

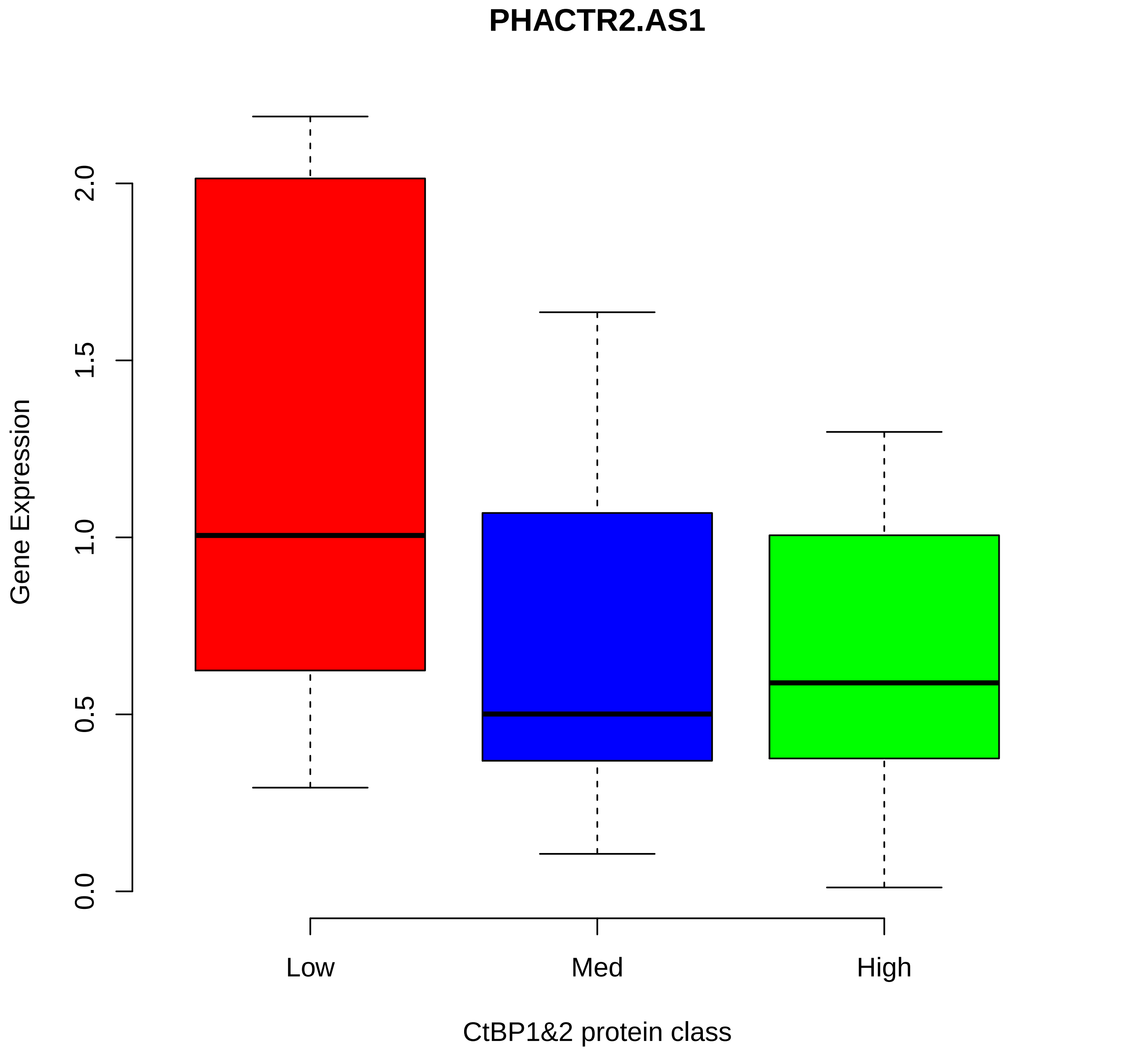
PHACTR2.AS1
Gene Expression
0.0
0.5
1.0
1.5
2.0
Low
Med
High
CtBP1&2 protein class

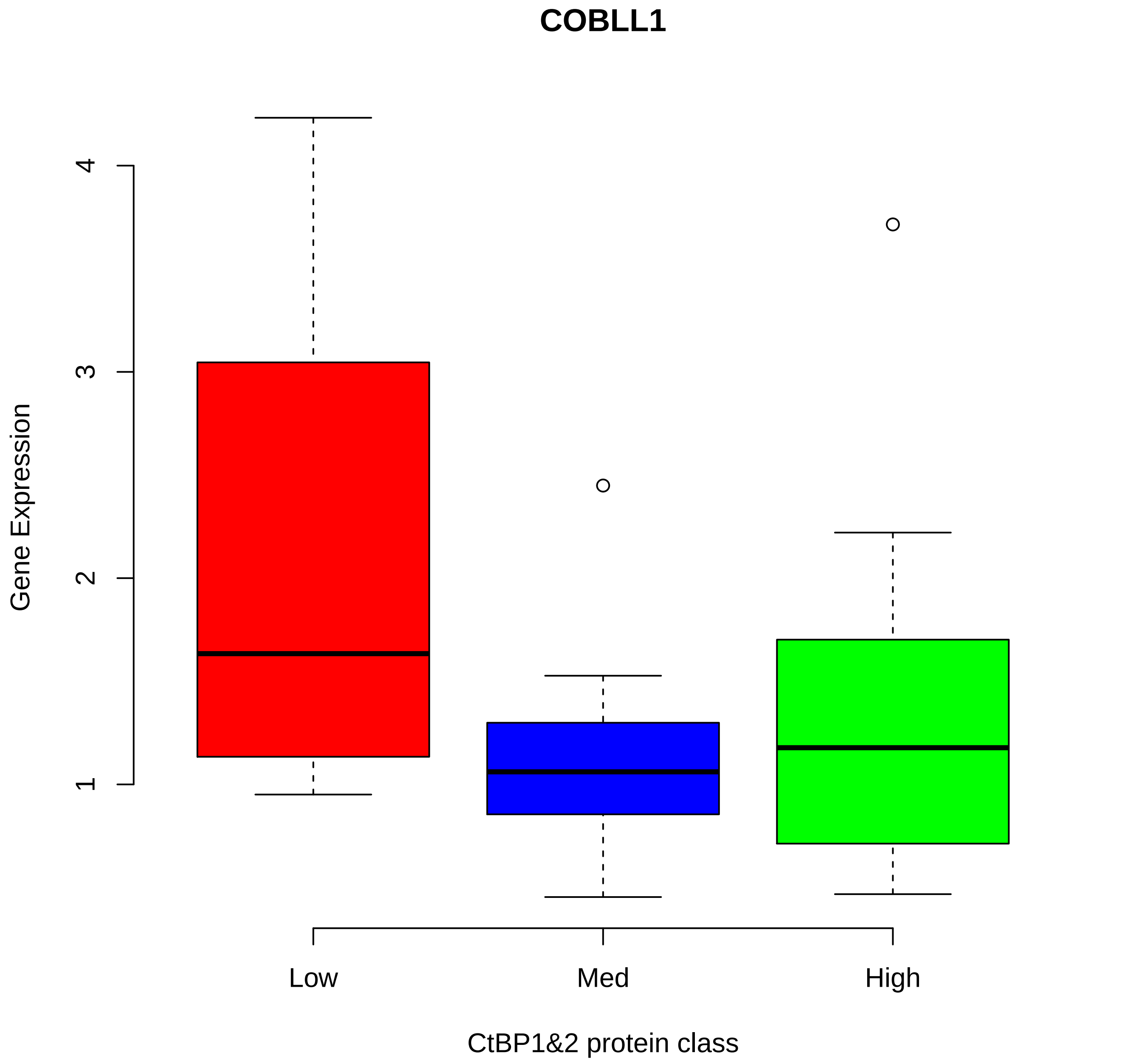
COBLL1
Gene Expression
1
2
3
4
Low
Med
High
CtBP1&2 protein class

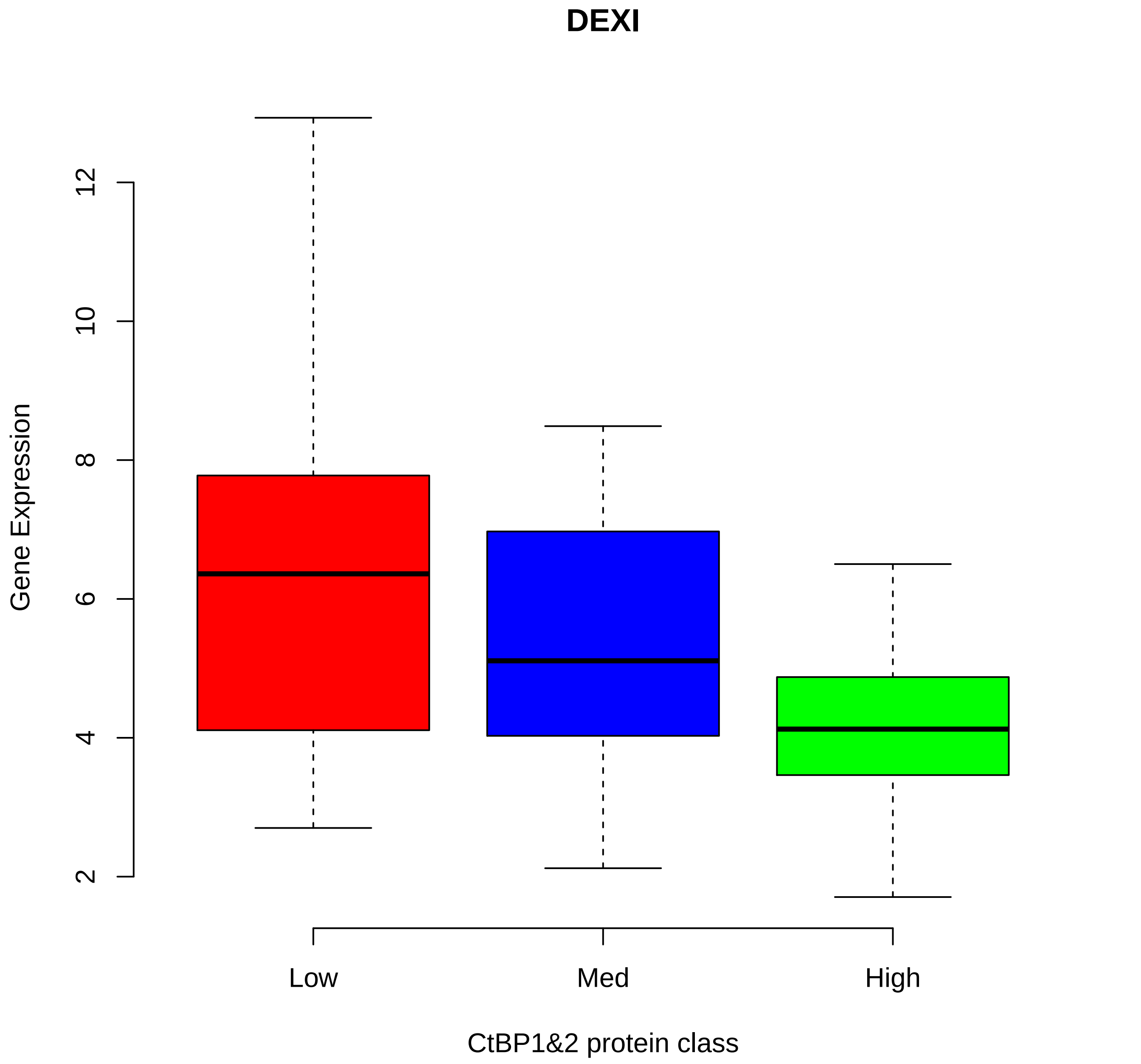

DEXI
Gene Expression
2
4
6
8
10
12
Low
Med
High
CtBP1&2 protein class

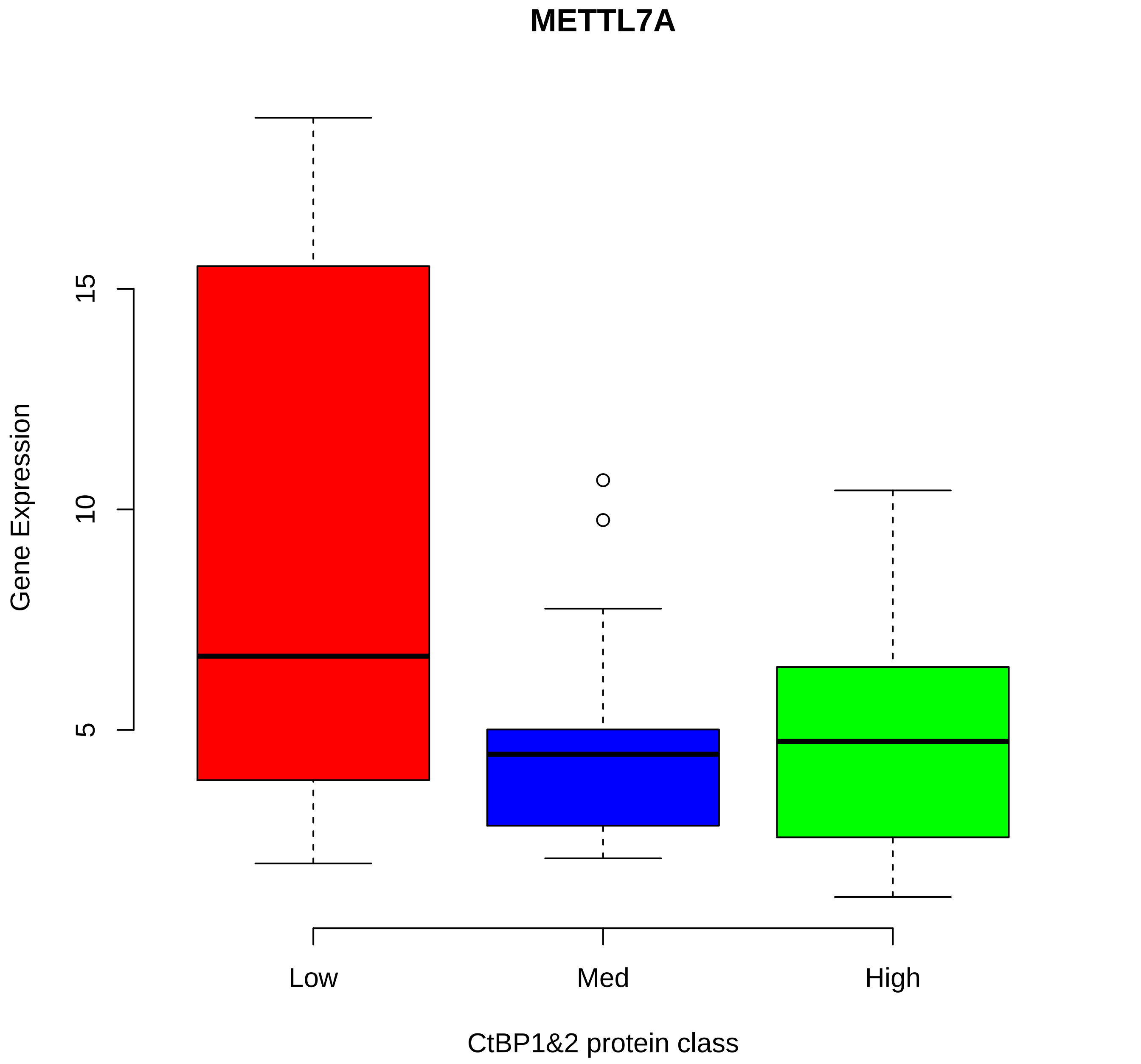

METTL7A
Gene Expression
15
10
5
Low
Med
High
CtBP1&2 protein class

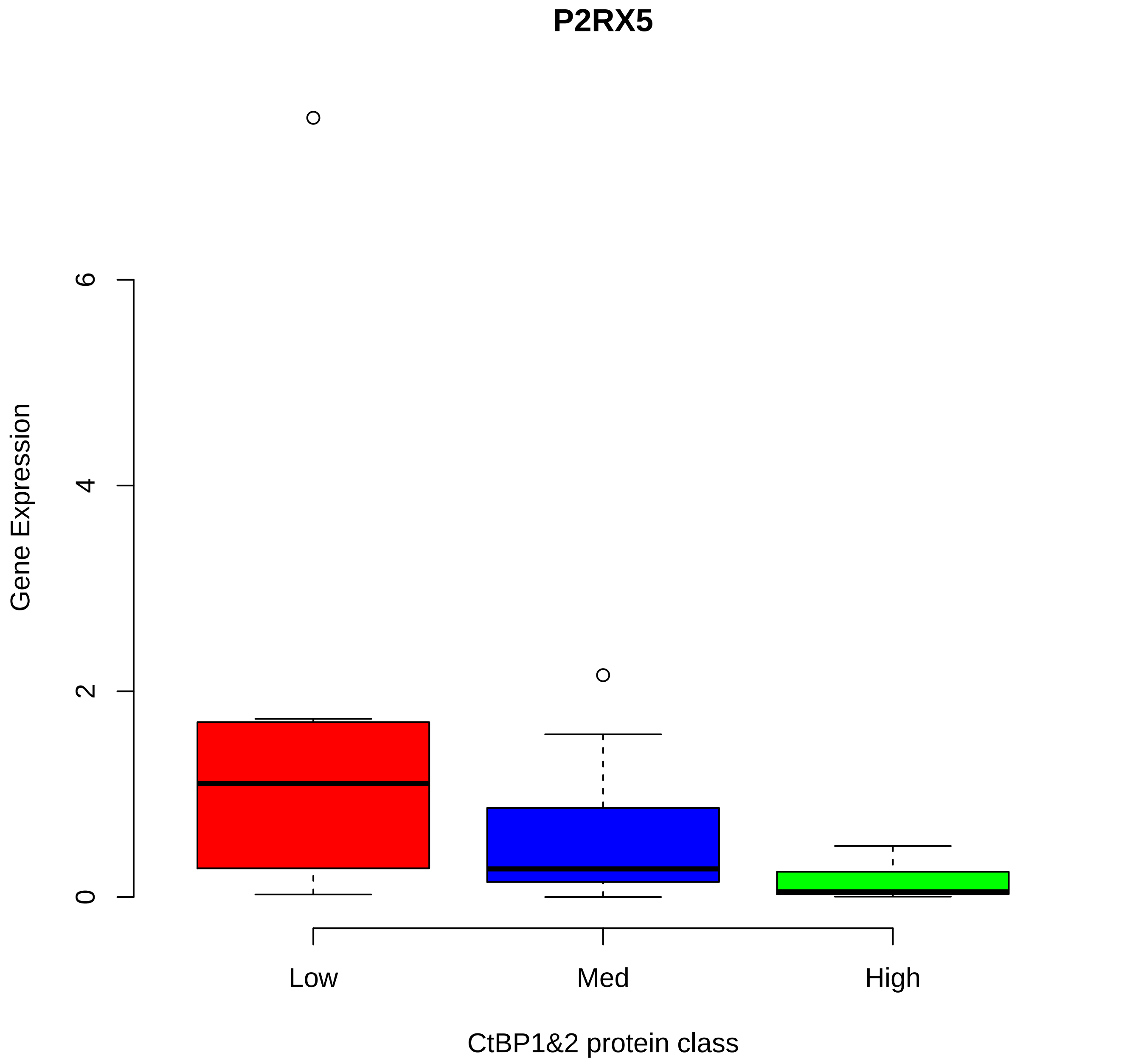

P2RX5
Gene Expression
0
2
4
6
Low
Med
High
CtBP1&2 protein class

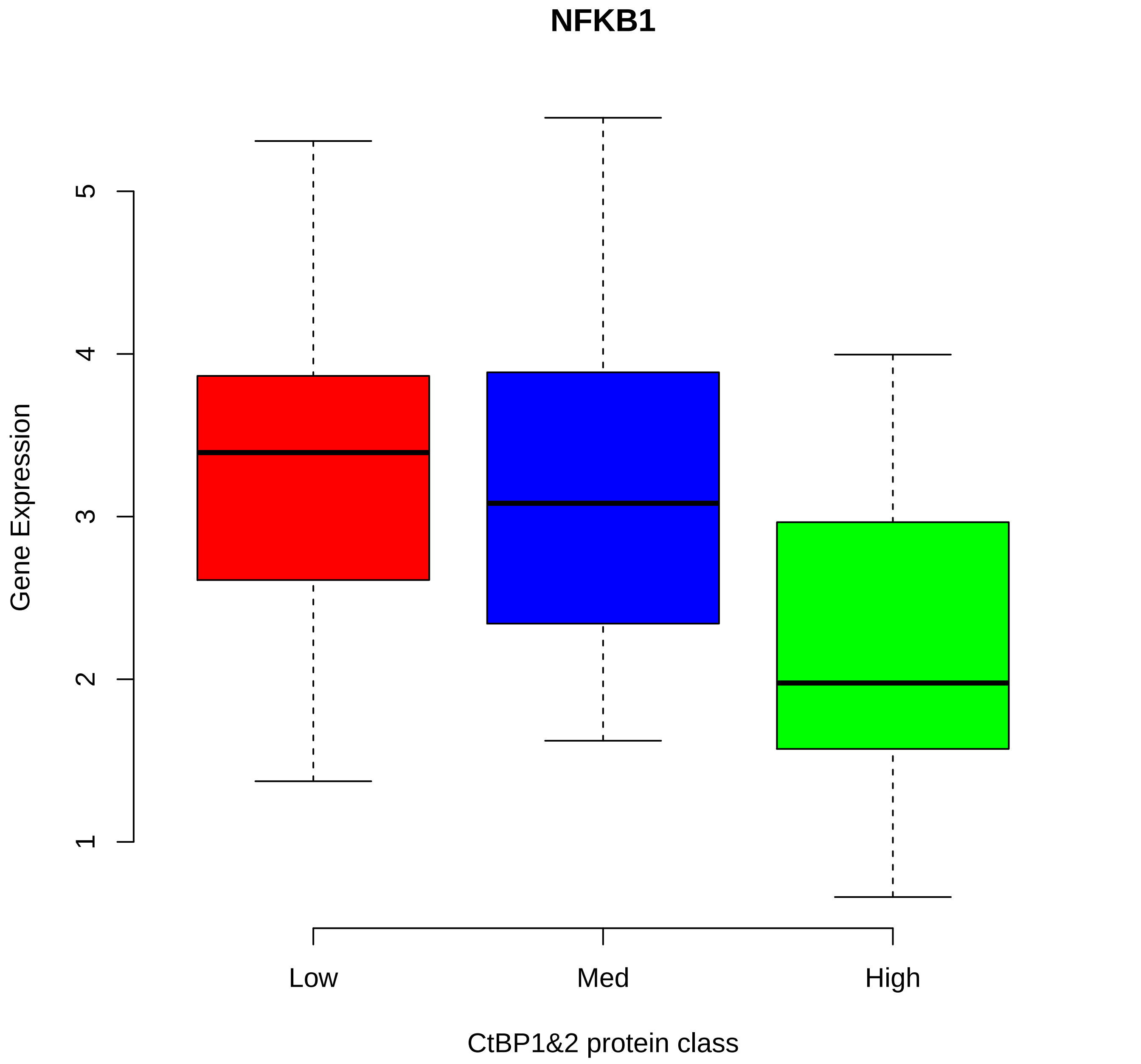
NFKB1
Gene Expression
1
2
3
4
5
Low
Med
High
CtBP1&2 protein class

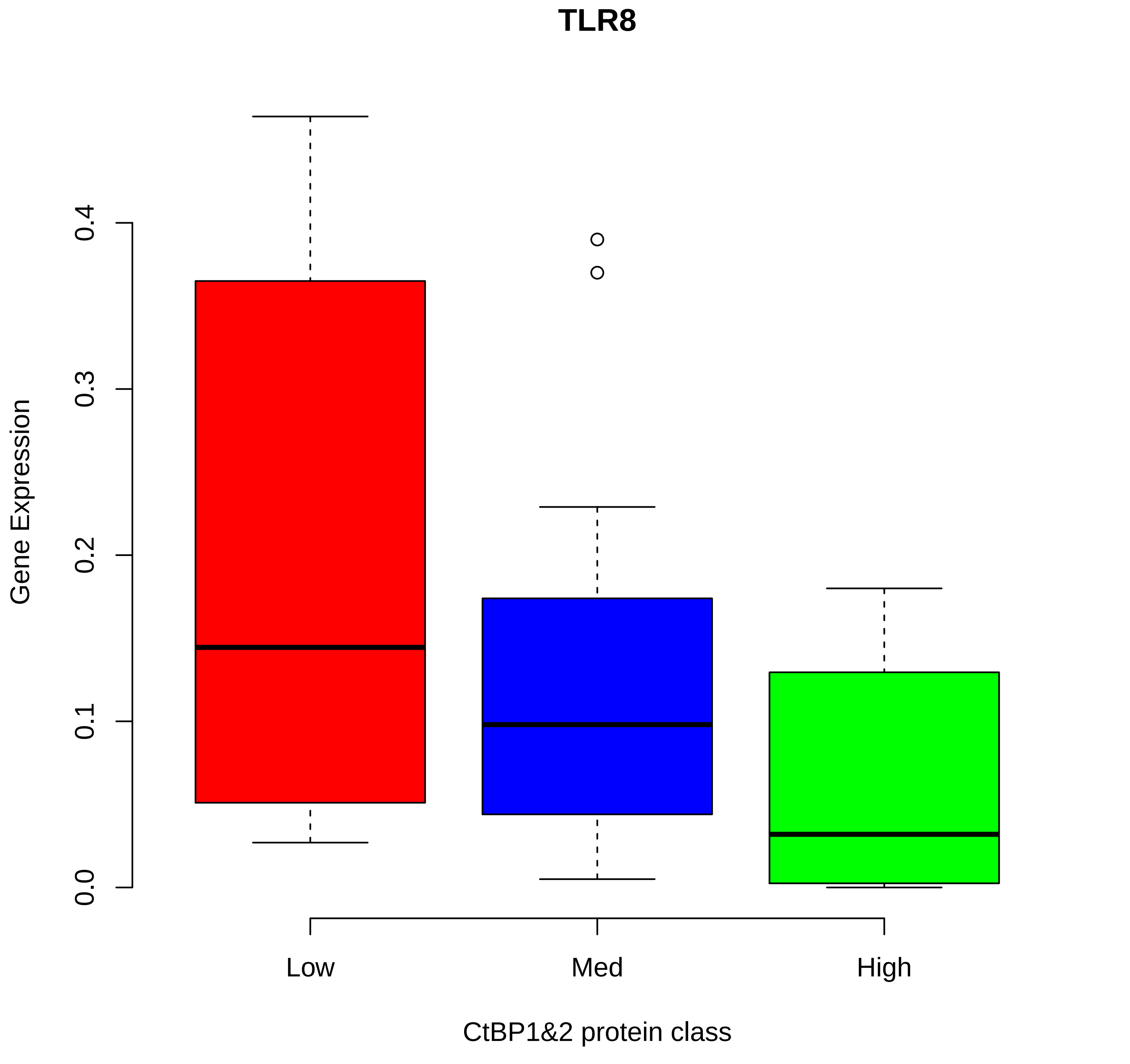
TLR8
Gene Expression
0.0
0.1
0.2
0.3
0.4
Low
Med
High
CtBP1&2 protein class

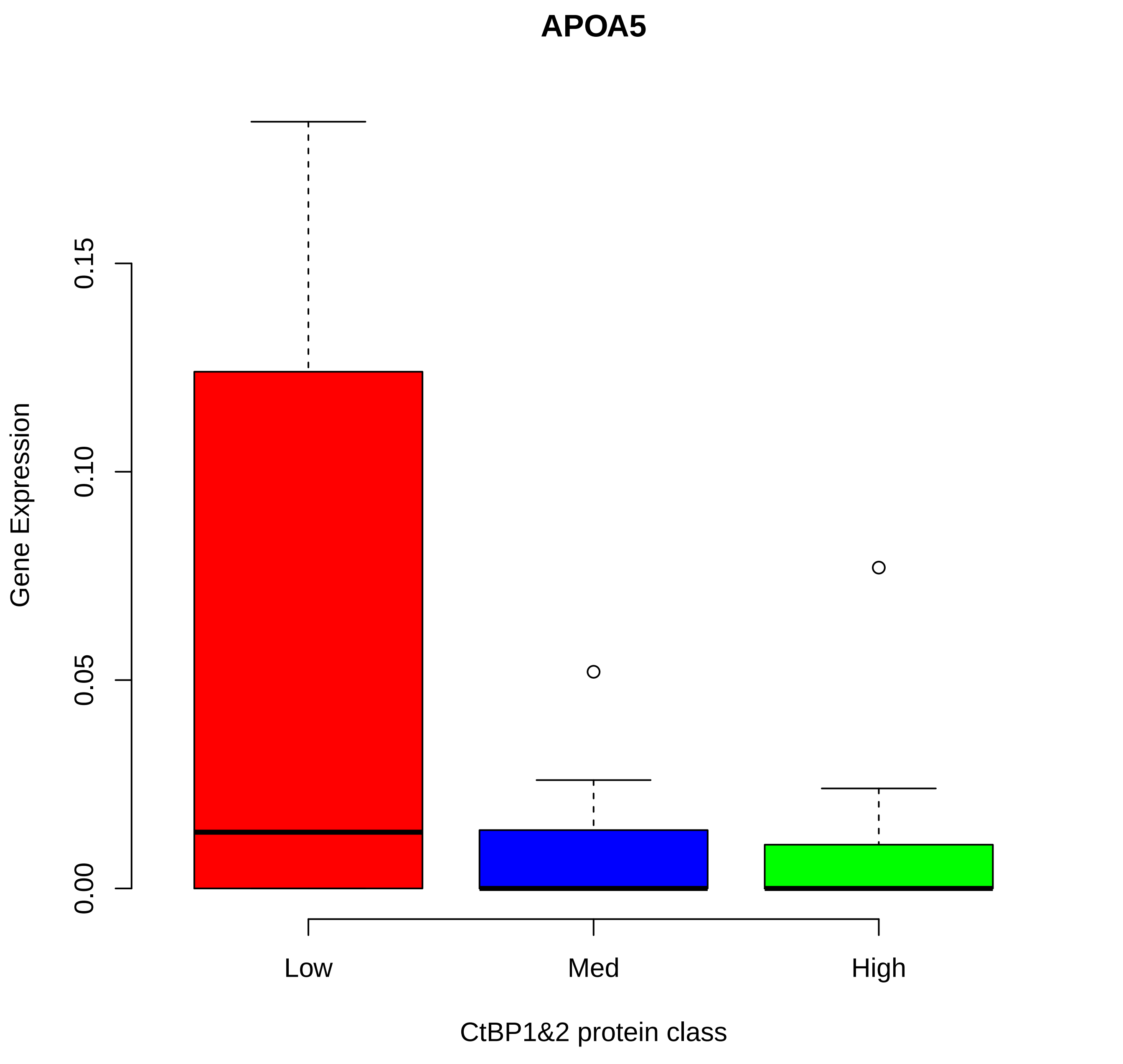
APOA5
Gene Expression
0.00
0.05
0.10
0.15
Low
Med
High
CtBP1&2 protein class

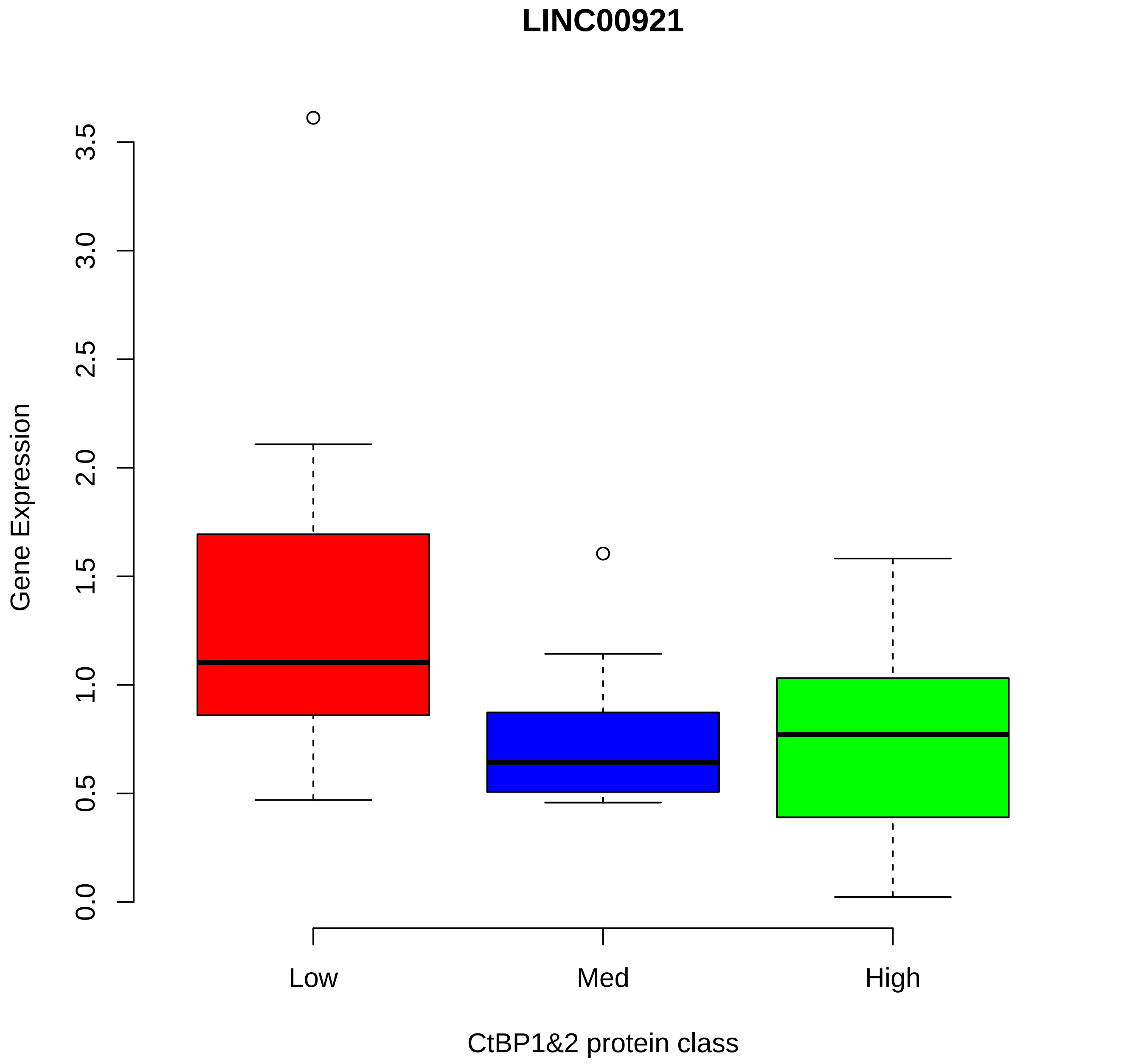

LINC00921
Gene Expression
0.0
0.5
1.0
1.5
2.0
2.5
3.0
3.5
Low
Med
High
CtBP1&2 protein class

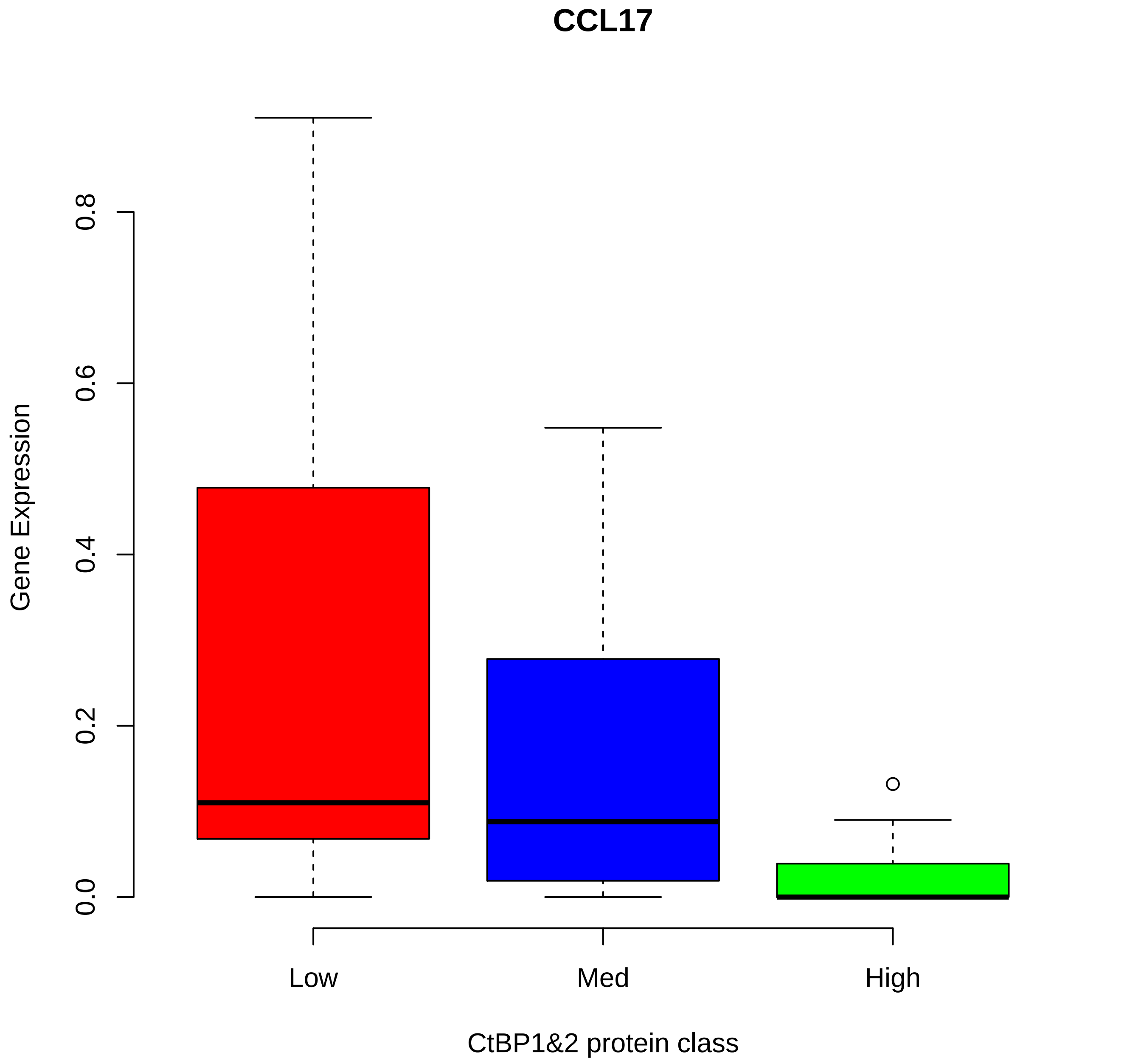
CCL17
Gene Expression
0.0
0.2
0.4
0.6
0.8
Low
Med
High
CtBP1&2 protein class

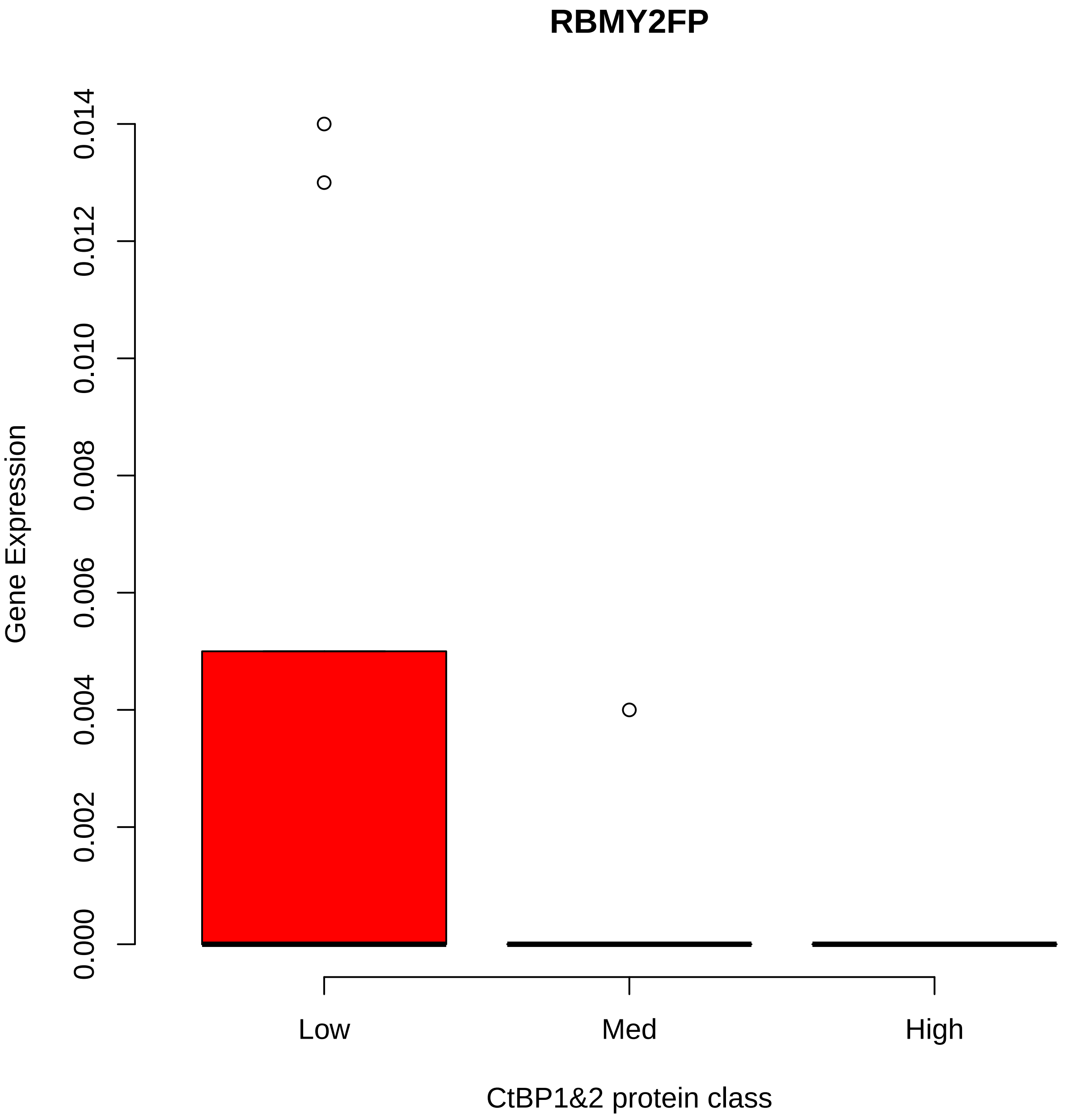
RBMY2FP
Gene Expression
0.000
0.002
0.004
0.006
0.008
0.010
0.012
0.014
Low
Med
High
CtBP1&2 protein class

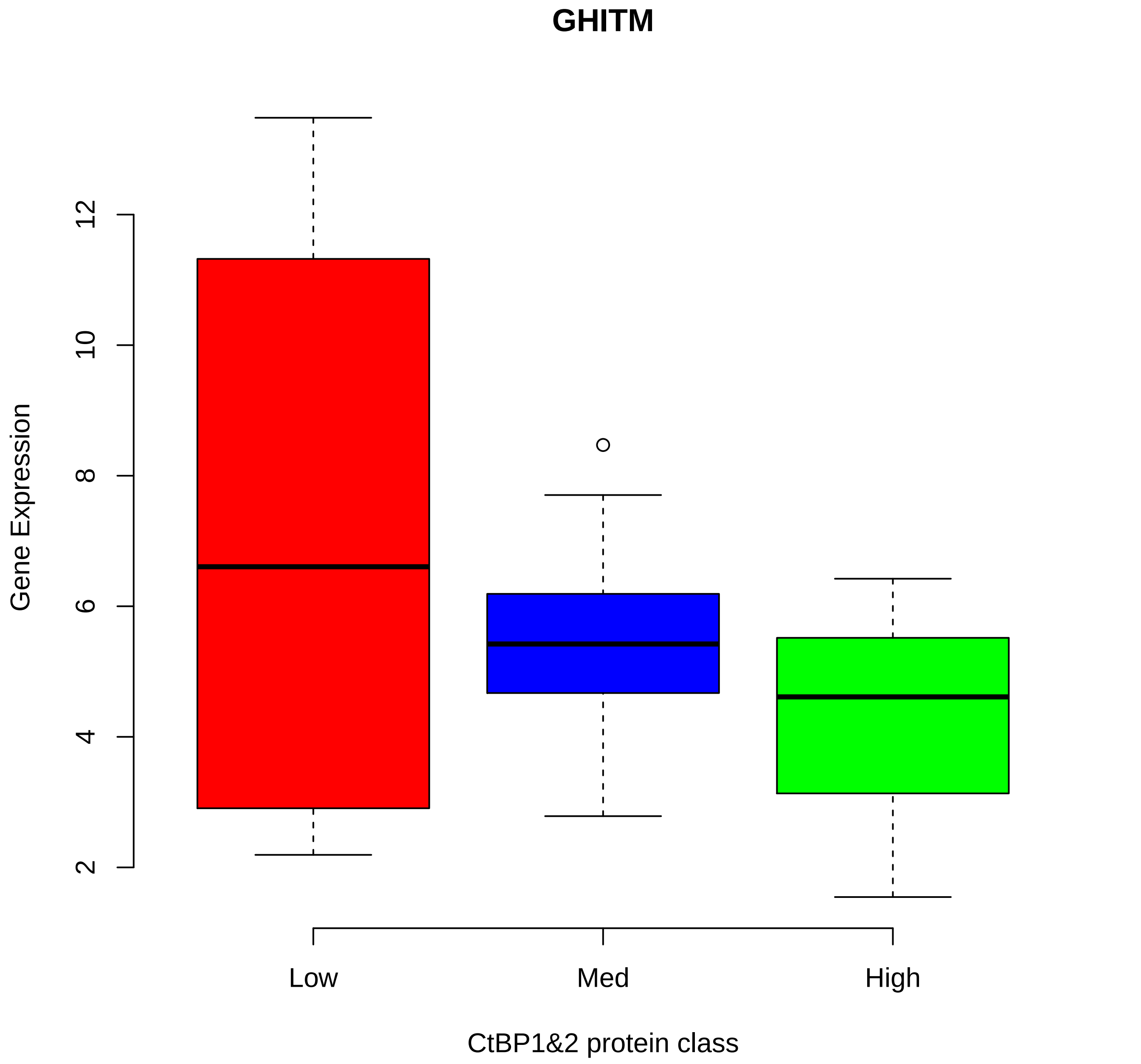
GHITM
Gene Expression
2
4
6
8
10
12
Low
Med
High
CtBP1&2 protein class

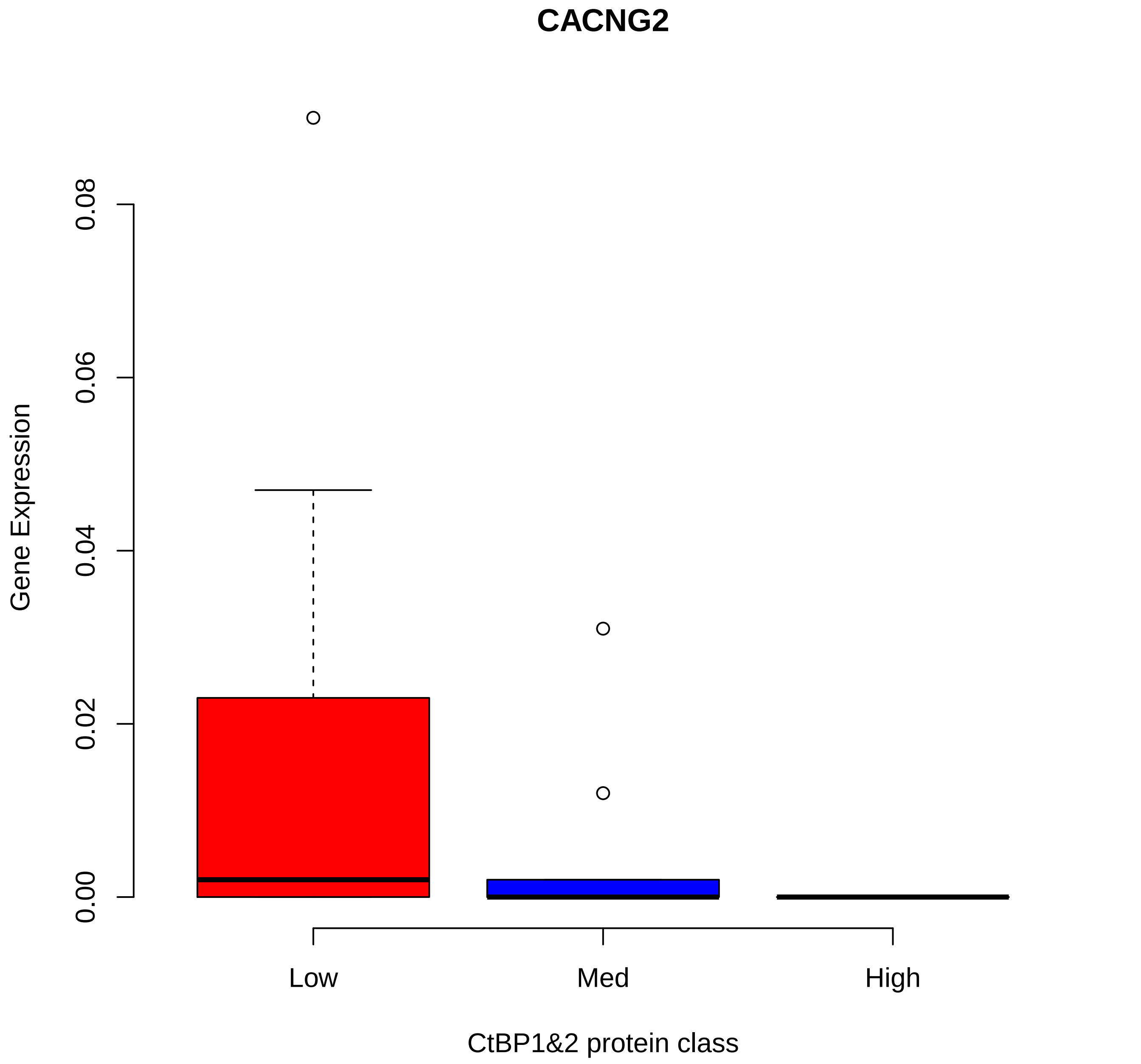

CACNG2
Gene Expression
0.00
0.02
0.04
0.06
0.08
Low
Med
High
CtBP1&2 protein class

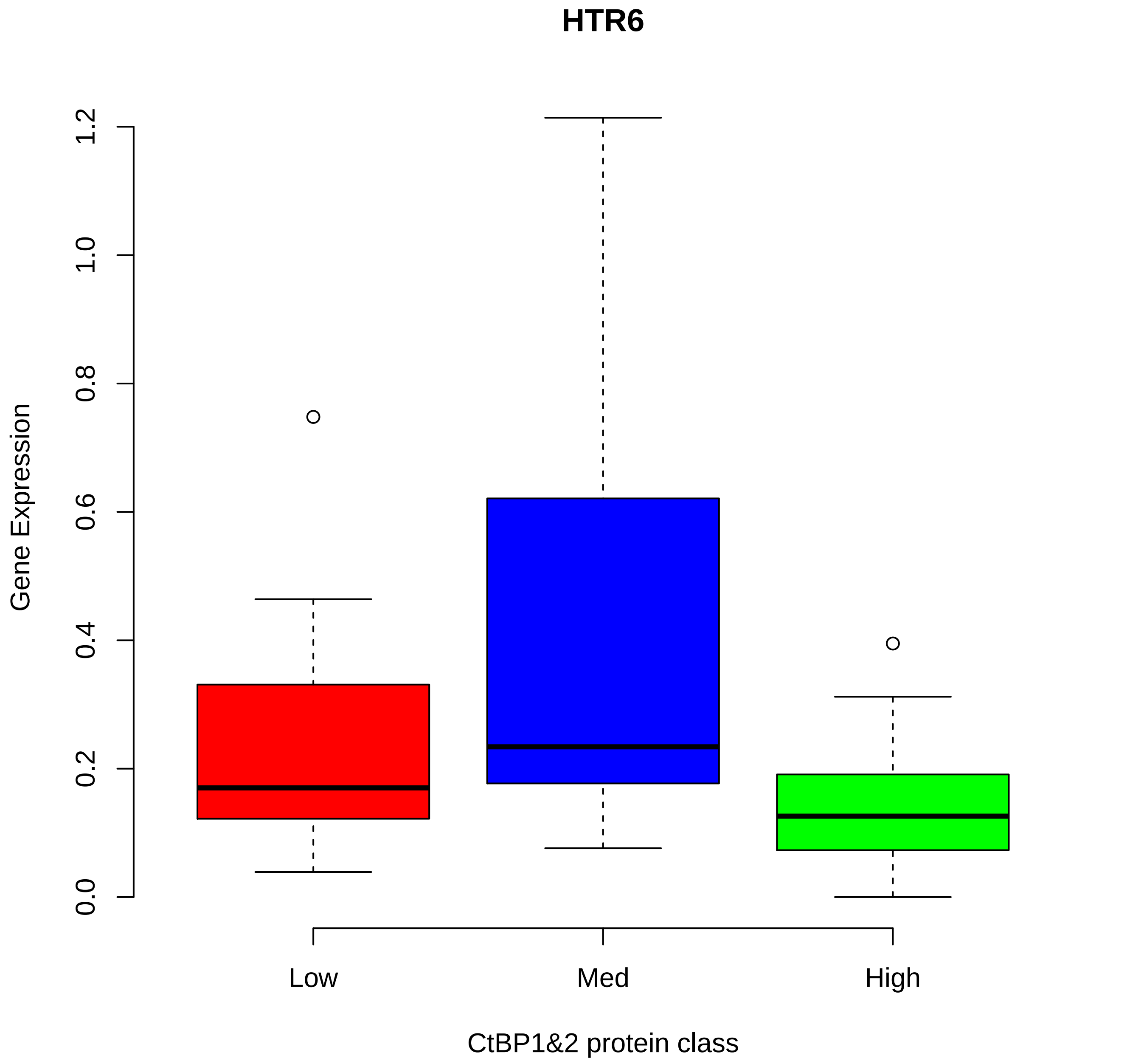

HTR6
Gene Expression
0.0
0.2
0.4
0.6
0.8
1.0
1.2
Low
Med
High
CtBP1&2 protein class

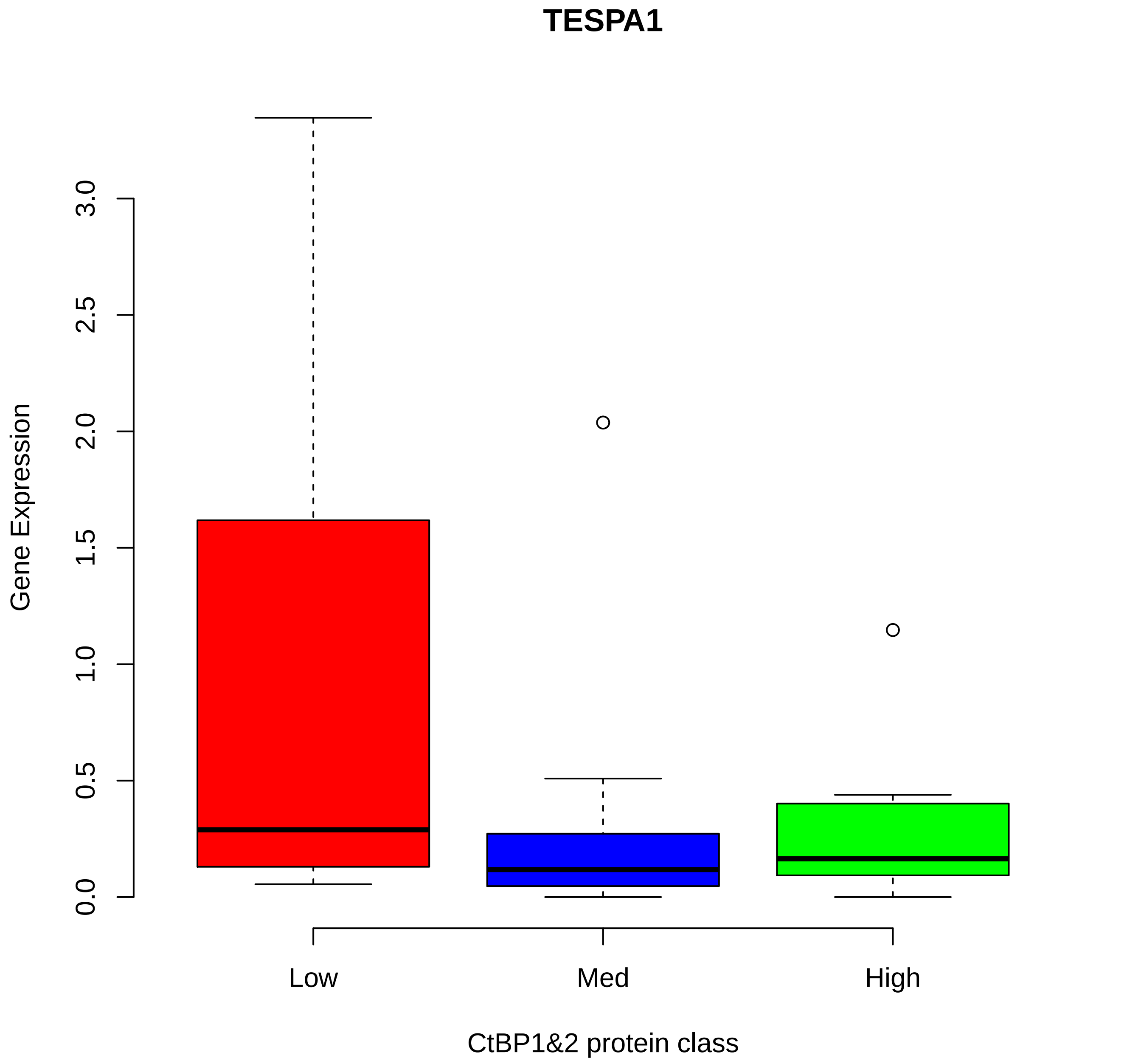

TESPA1
Gene Expression
0.0
0.5
1.0
1.5
2.0
2.5
3.0
Low
Med
High
CtBP1&2 protein class

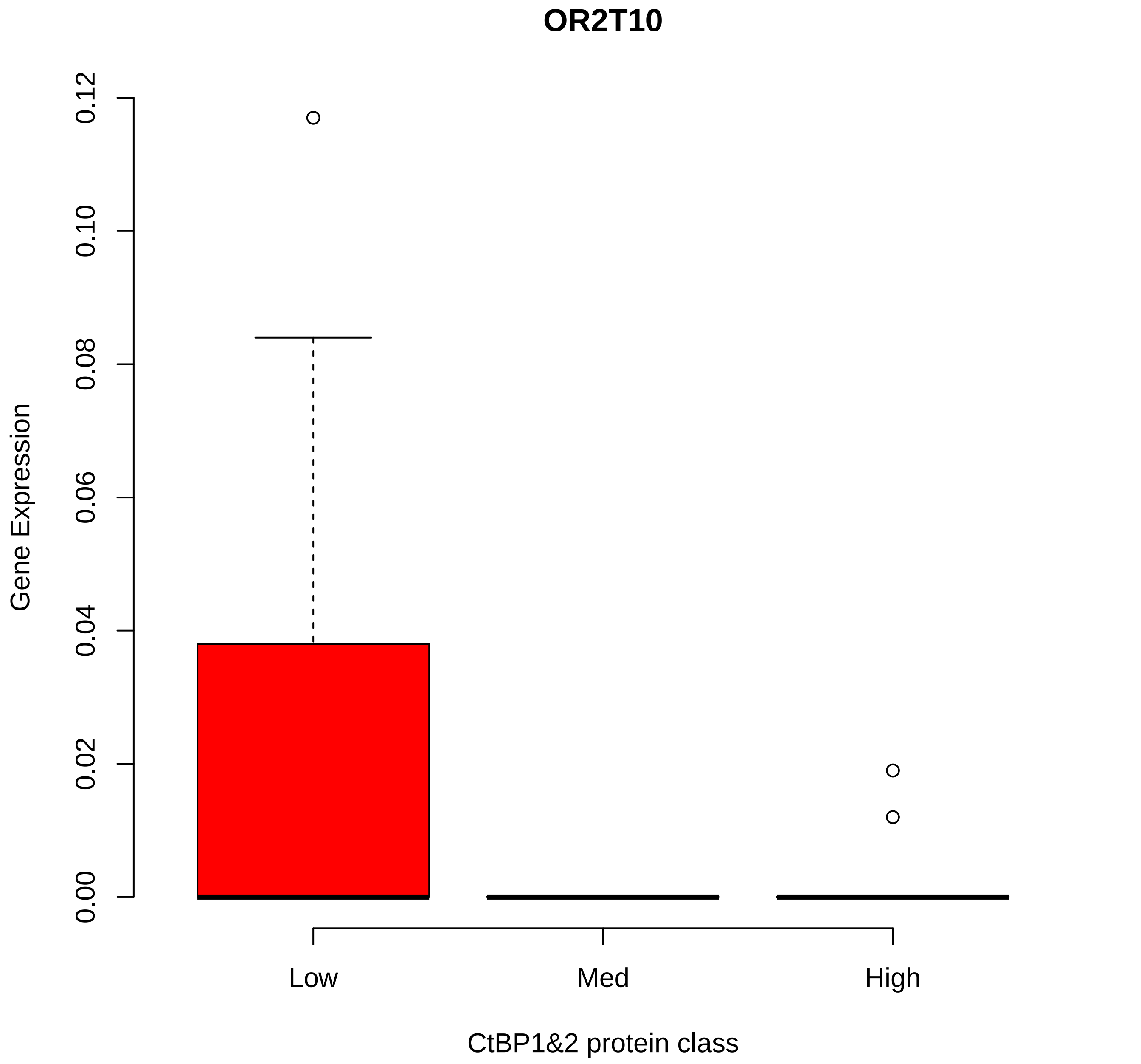
OR2T10
Gene Expression
0.00
0.02
0.04
0.06
0.08
0.10
0.12
Low
Med
High
CtBP1&2 protein class

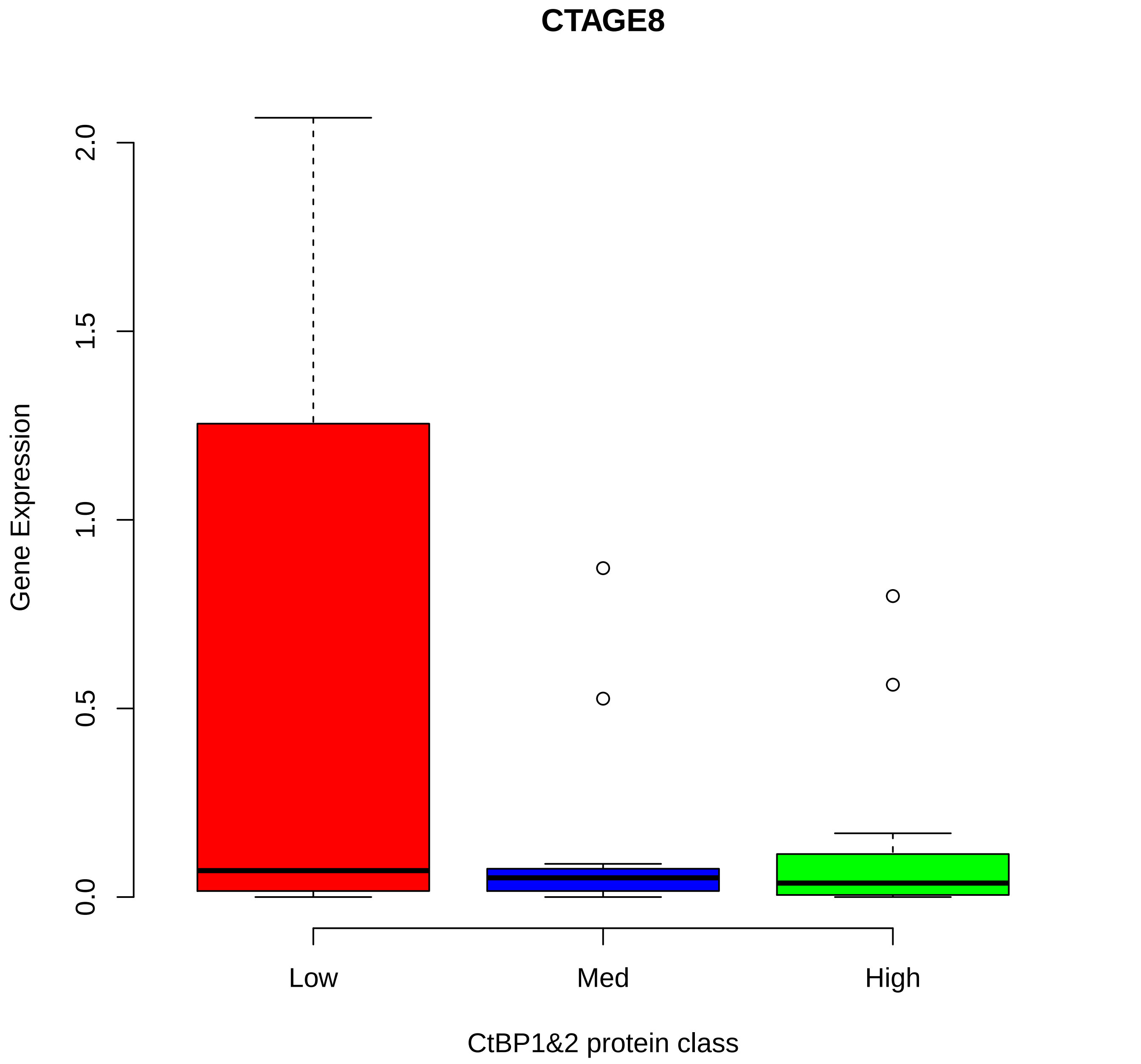

CTAGE8
Gene Expression
0.0
0.5
1.0
1.5
2.0
Low
Med
High
CtBP1&2 protein class

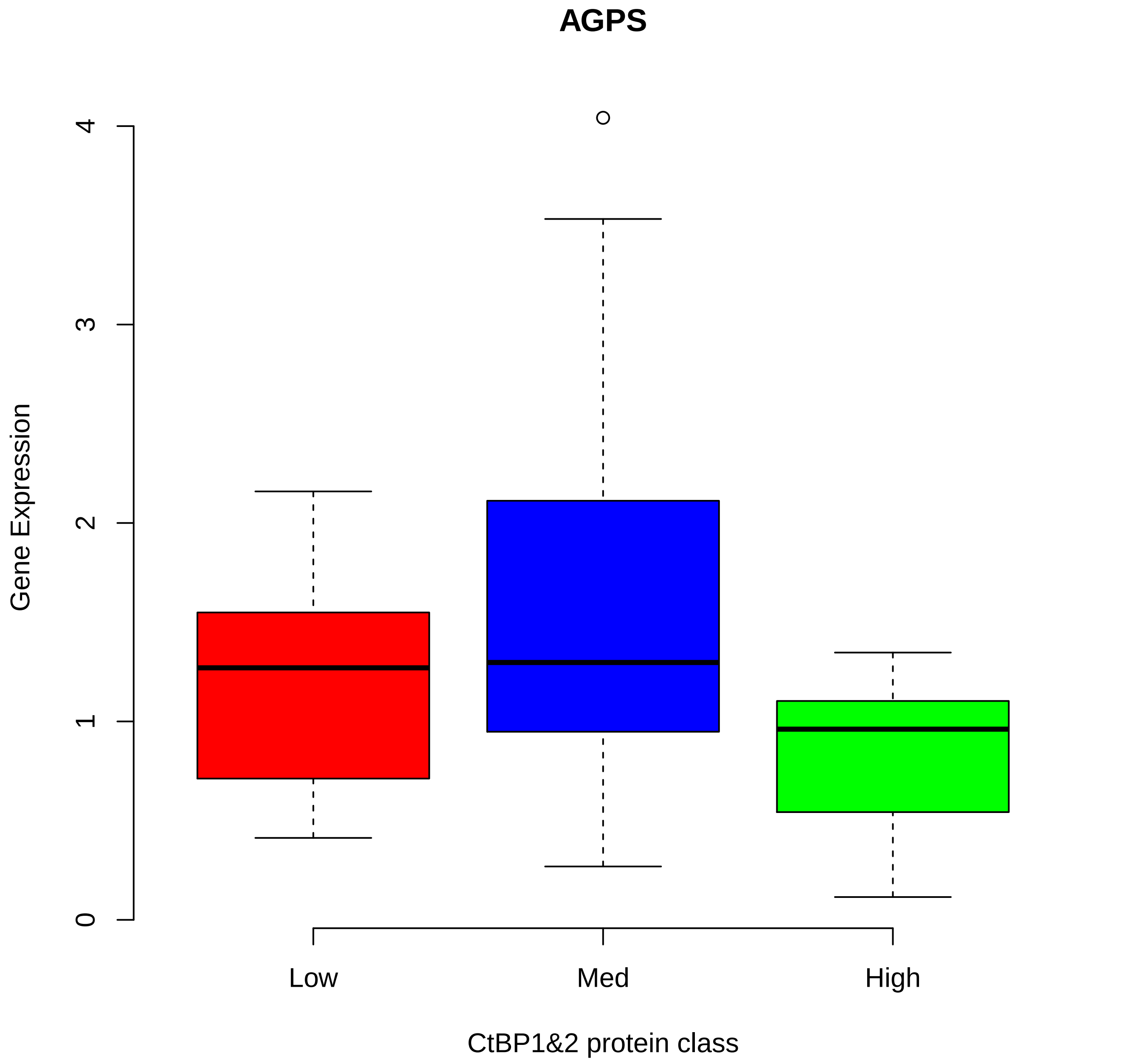
AGPS
Gene Expression
0
1
2
3
4
Low
Med
High
CtBP1&2 protein class

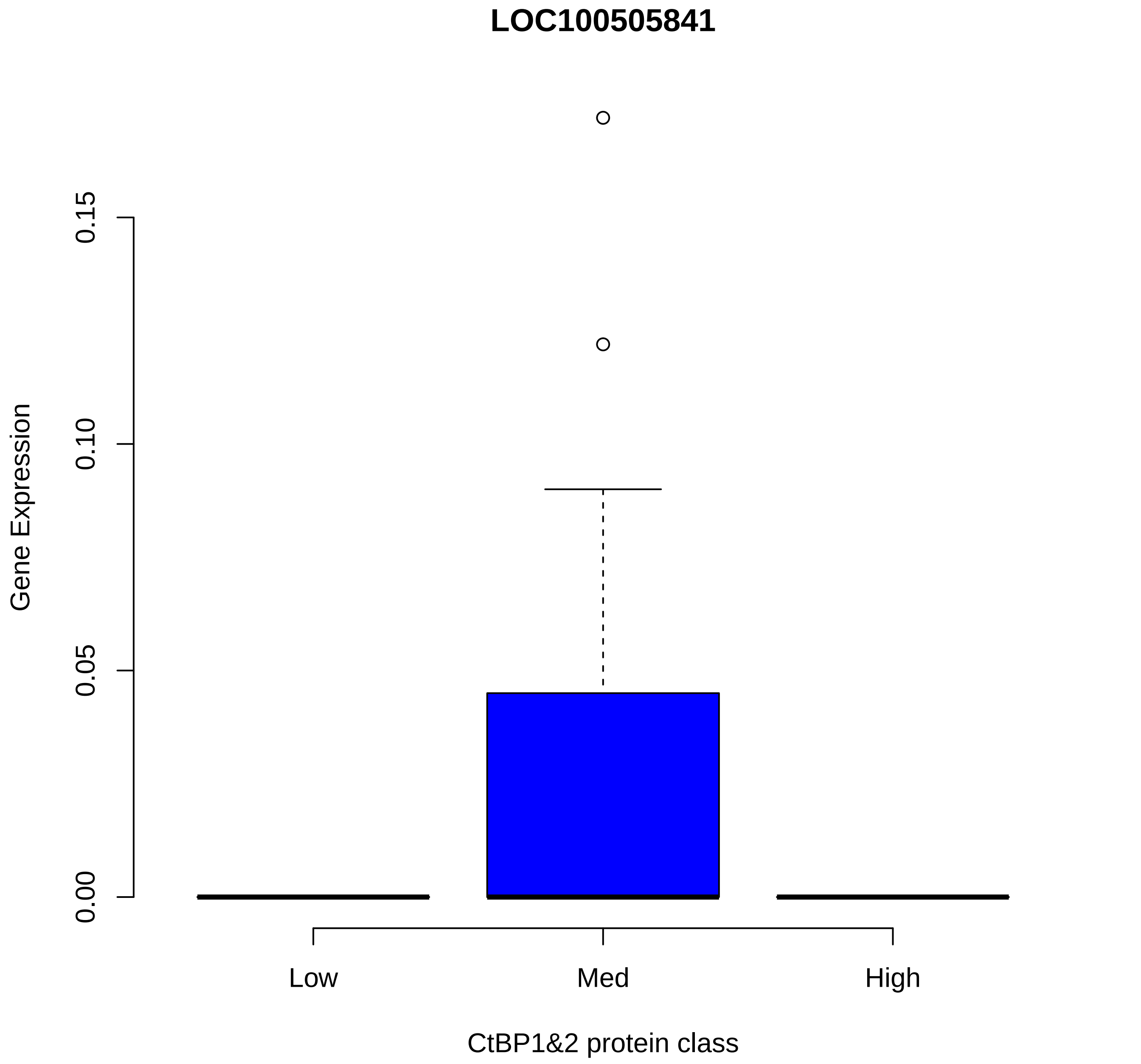
LOC100505841
Gene Expression
0.00
0.05
0.10
0.15
Low
Med
High
CtBP1&2 protein class

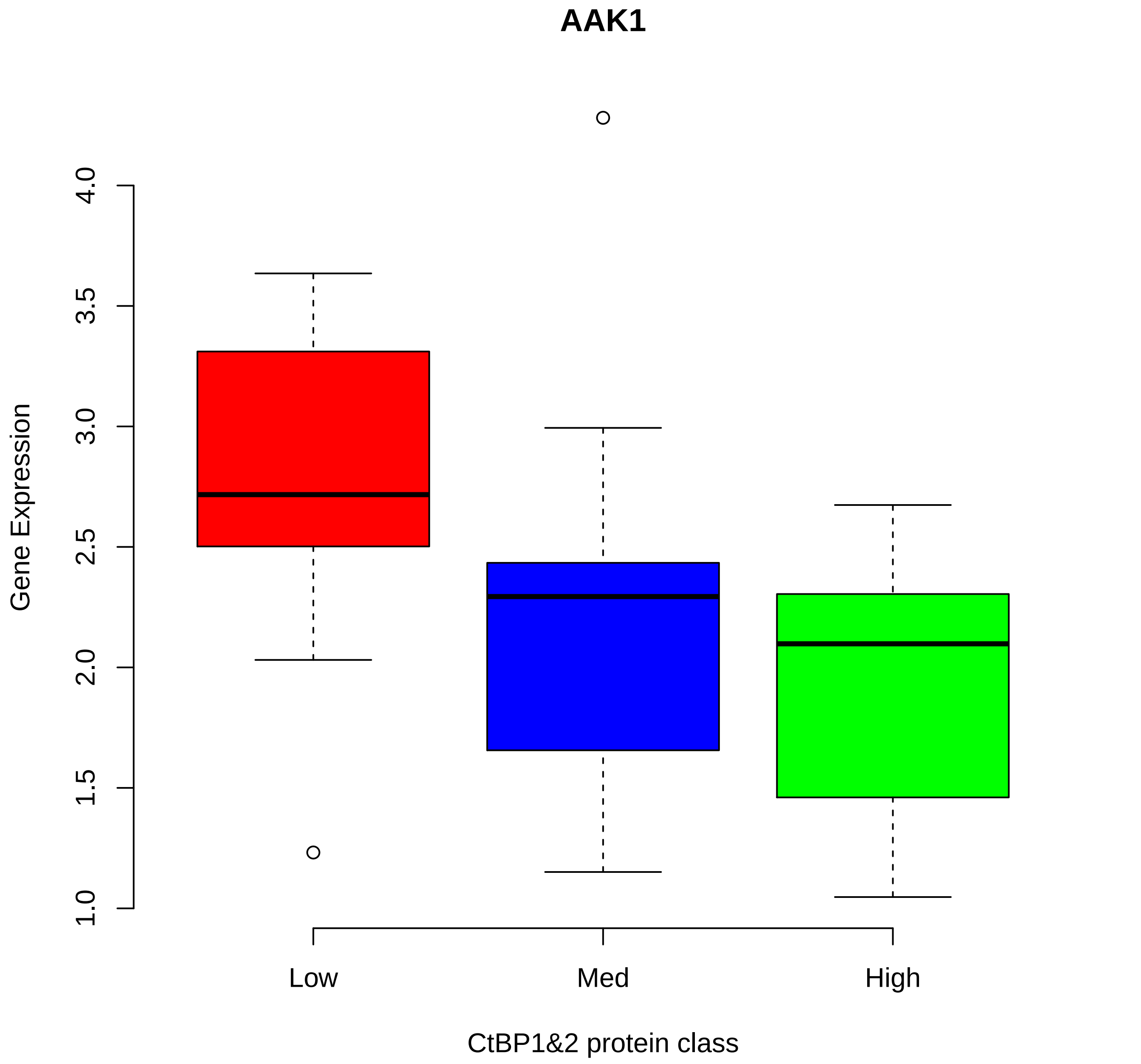
AAK1
Gene Expression
4.0
3.5
3.0
2.5
2.0
1.5
1.0
Low
Med
High
CtBP1&2 protein class

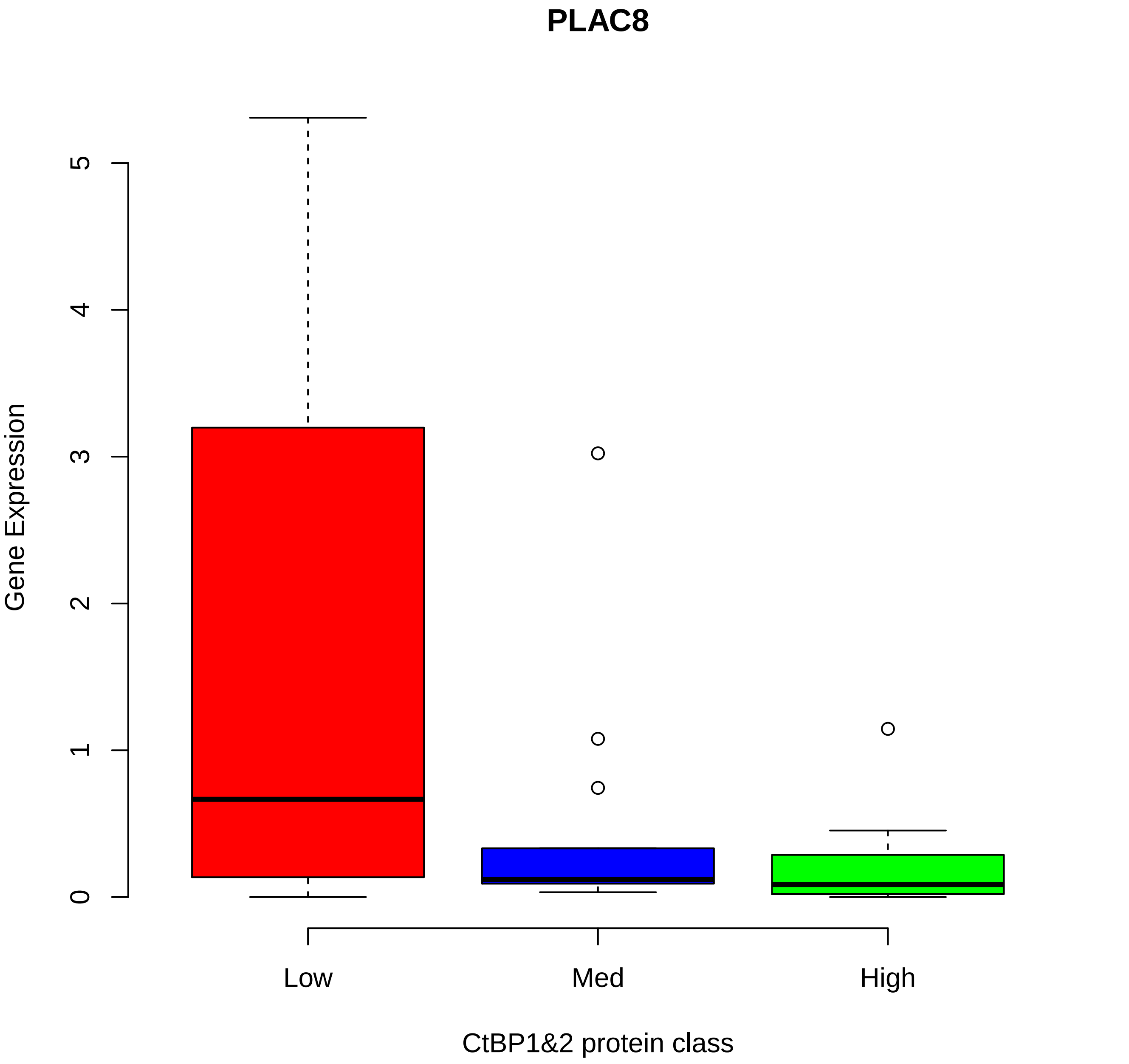

PLAC8
Gene Expression
0
1
2
3
4
5
Low
Med
High
CtBP1&2 protein class

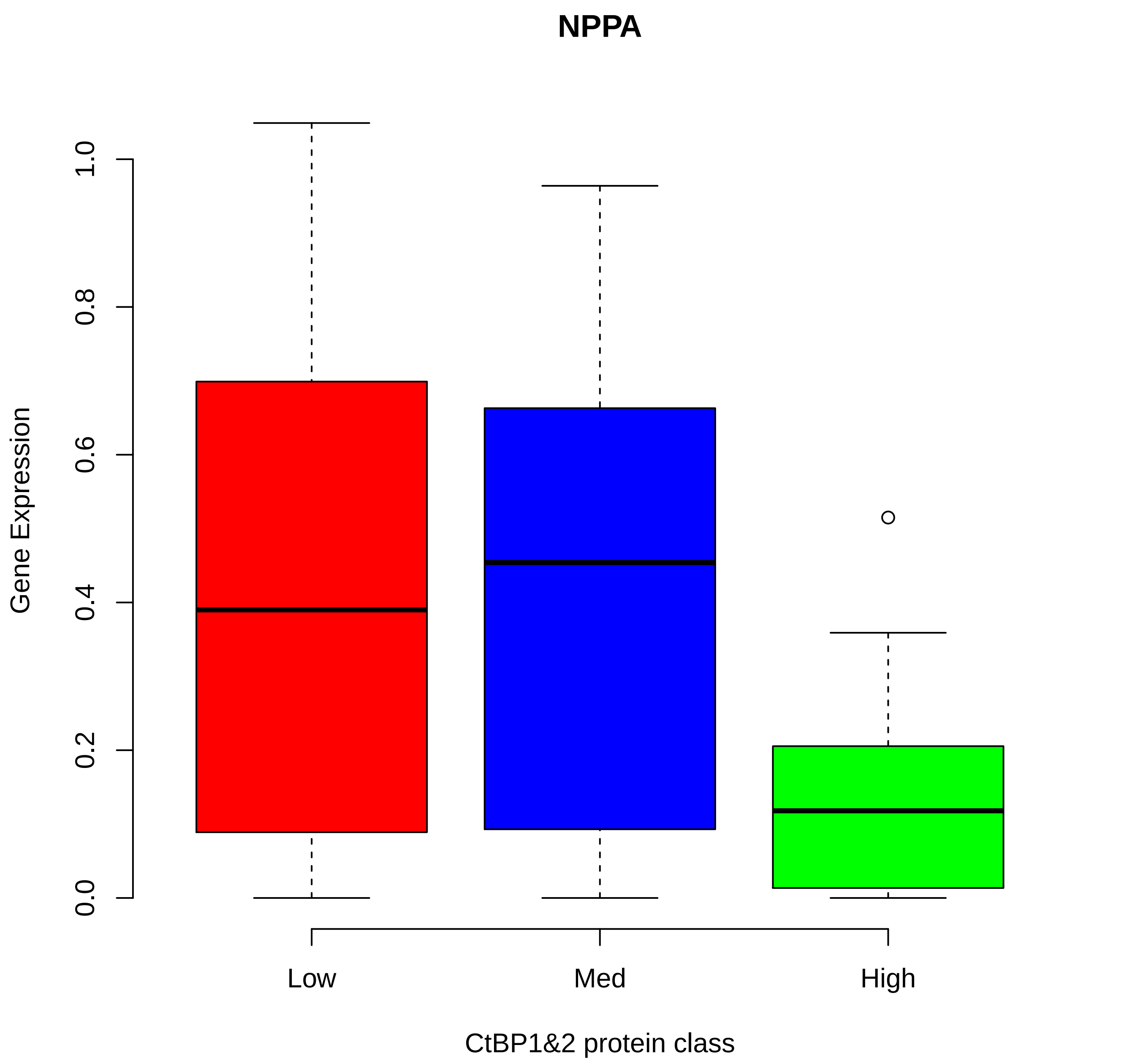

NPPA
Gene Expression
0.0
0.2
0.4
0.6
0.8
1.0
Low
Med
High
CtBP1&2 protein class

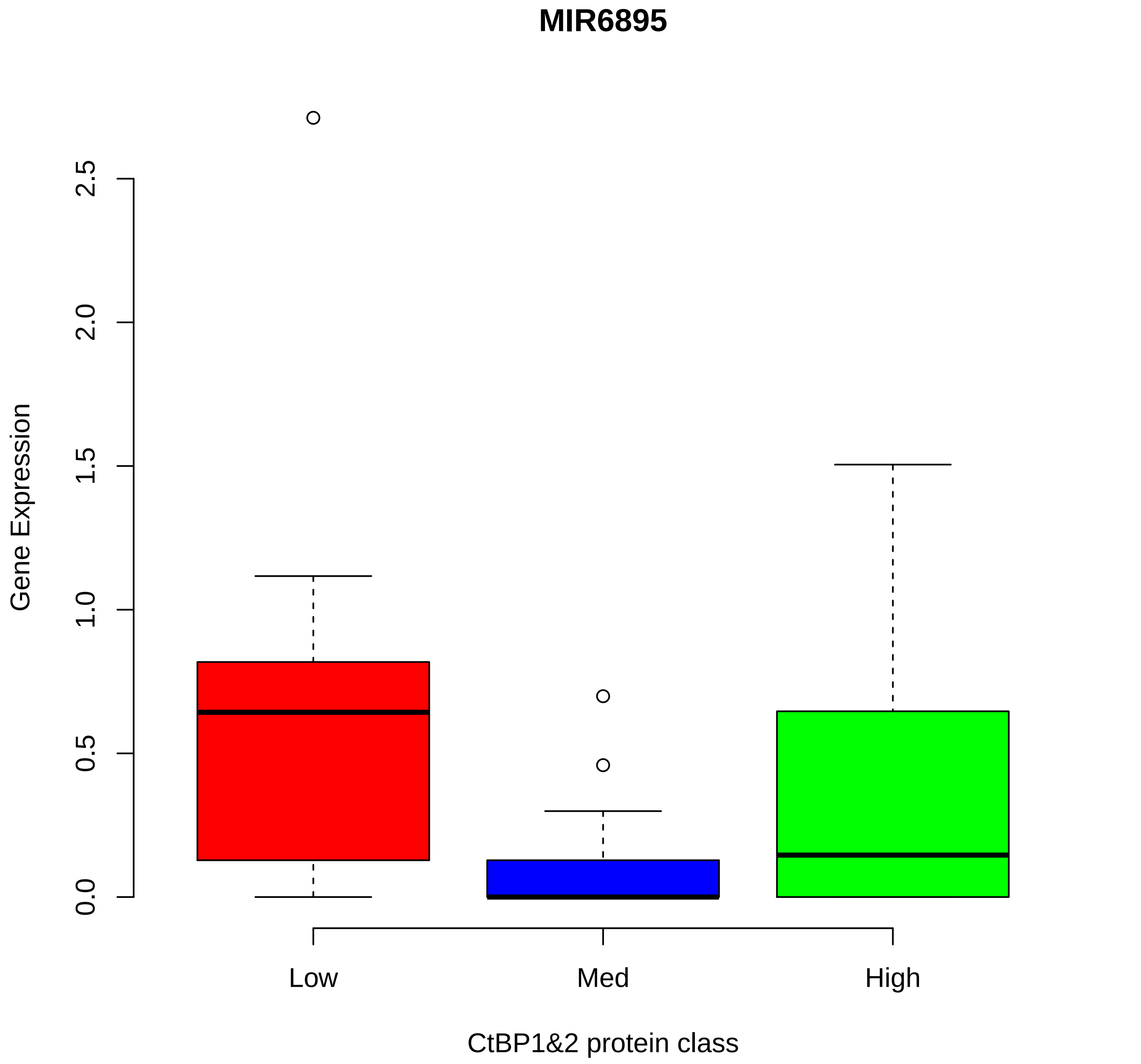
MIR6895
Gene Expression
0.0
0.5
1.0
1.5
2.0
2.5
Low
Med
High
CtBP1&2 protein class

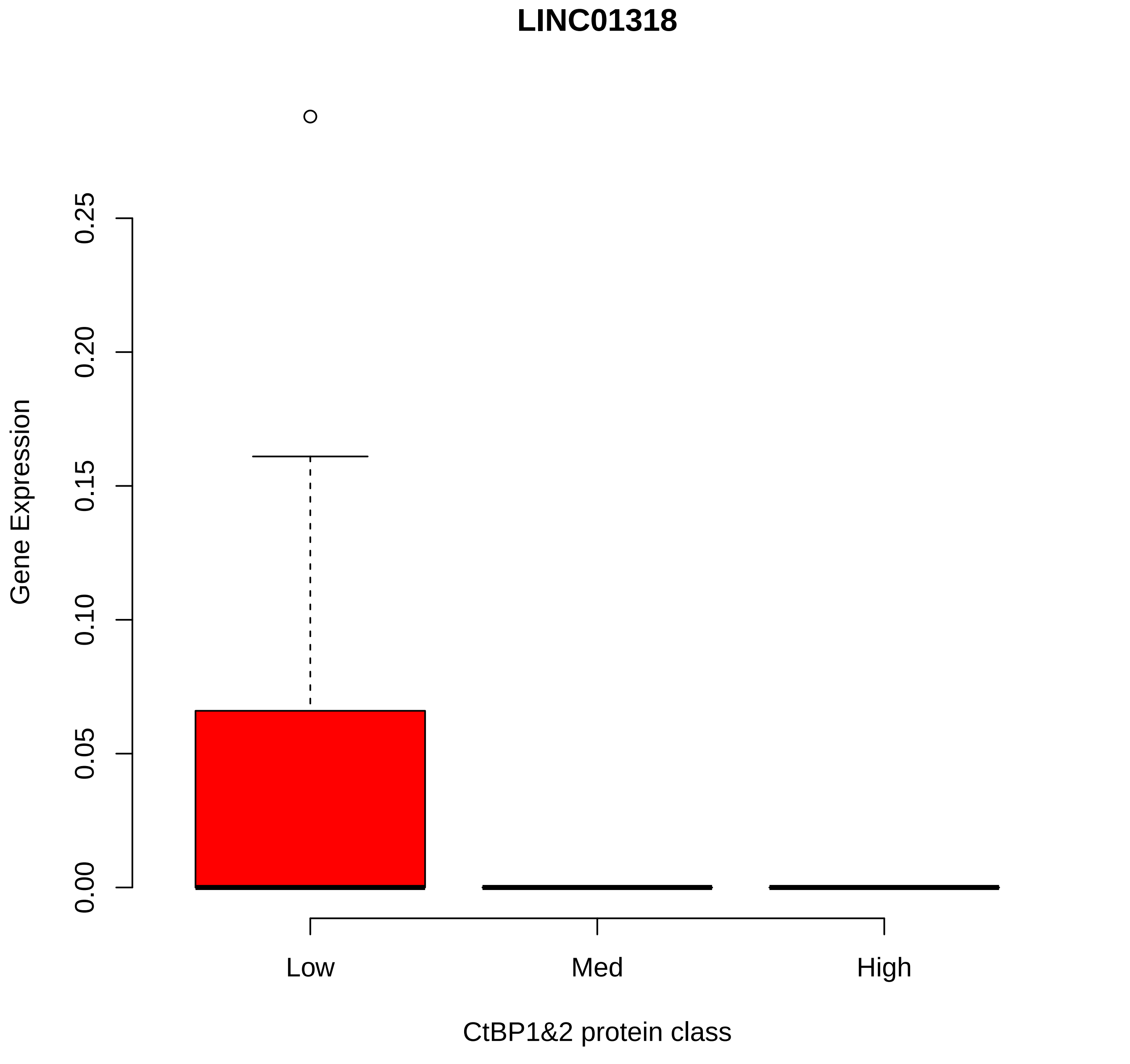
LINC01318
Gene Expression
0.00
0.05
0.10
0.15
0.20
0.25
Low
Med
High
CtBP1&2 protein class

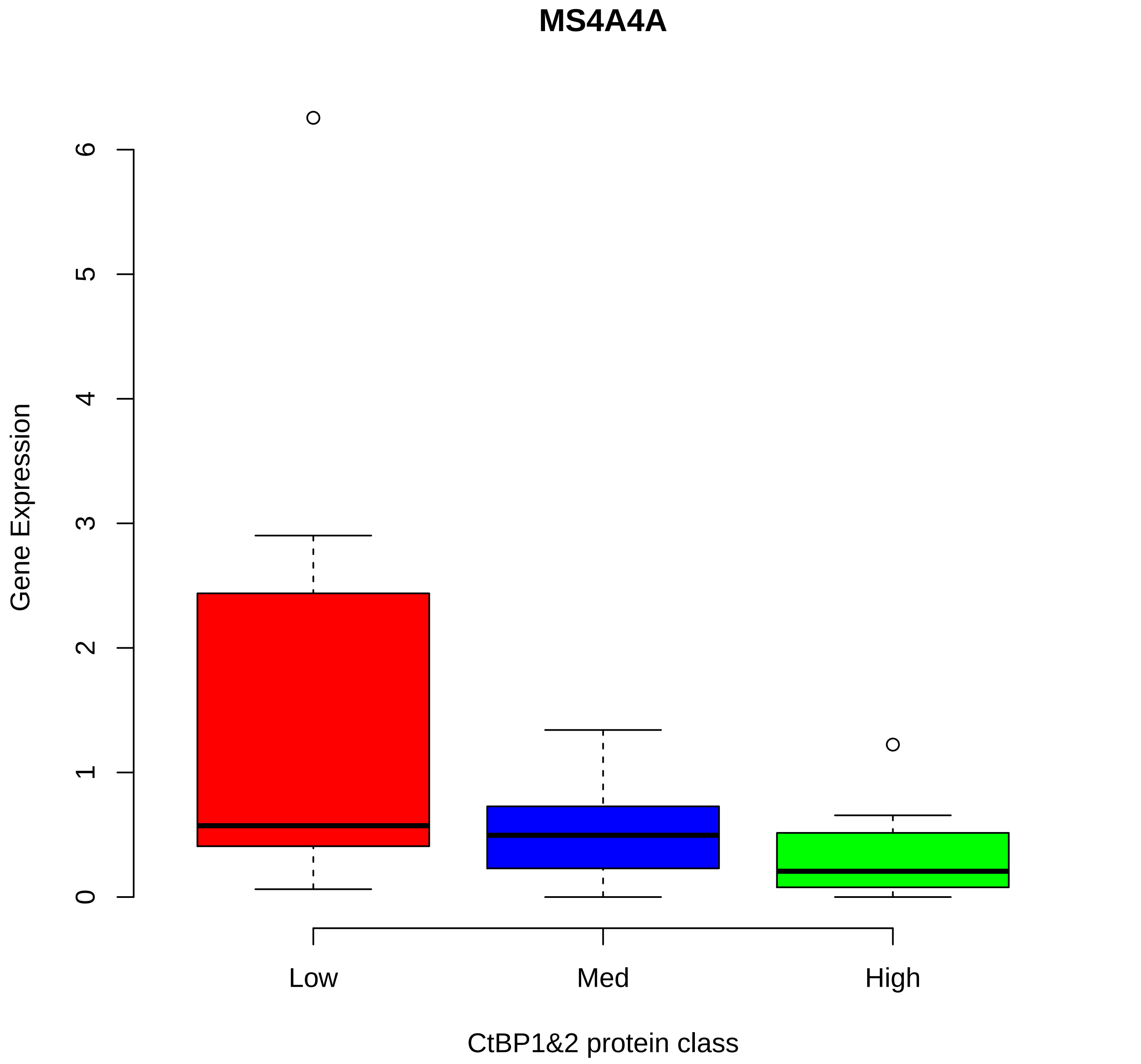

MS4A4A
Gene Expression
0
1
2
3
4
5
6
Low
Med
High
CtBP1&2 protein class

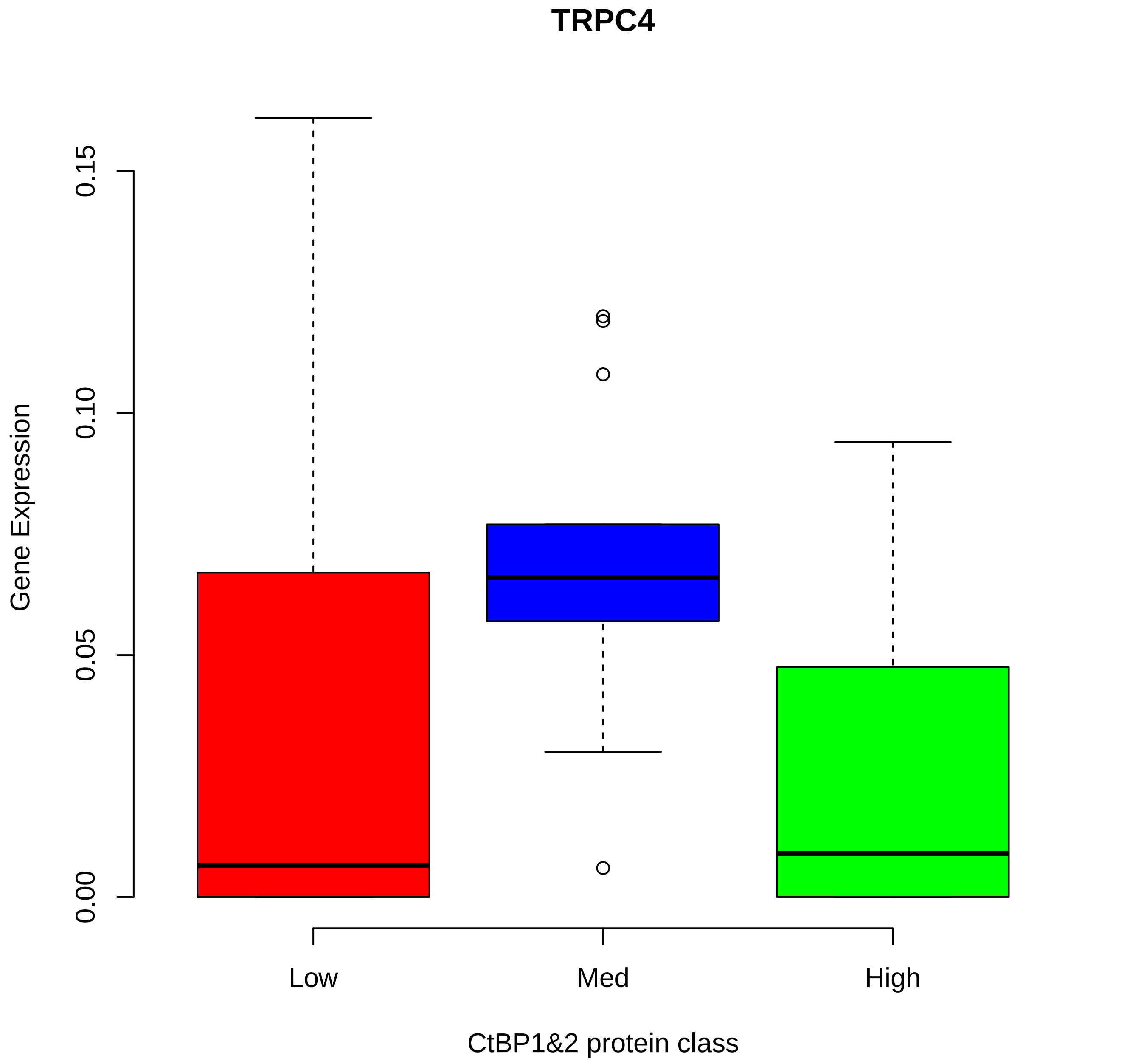

TRPC4
Gene Expression
0.00
0.05
0.10
0.15
Low
Med
High
CtBP1&2 protein class

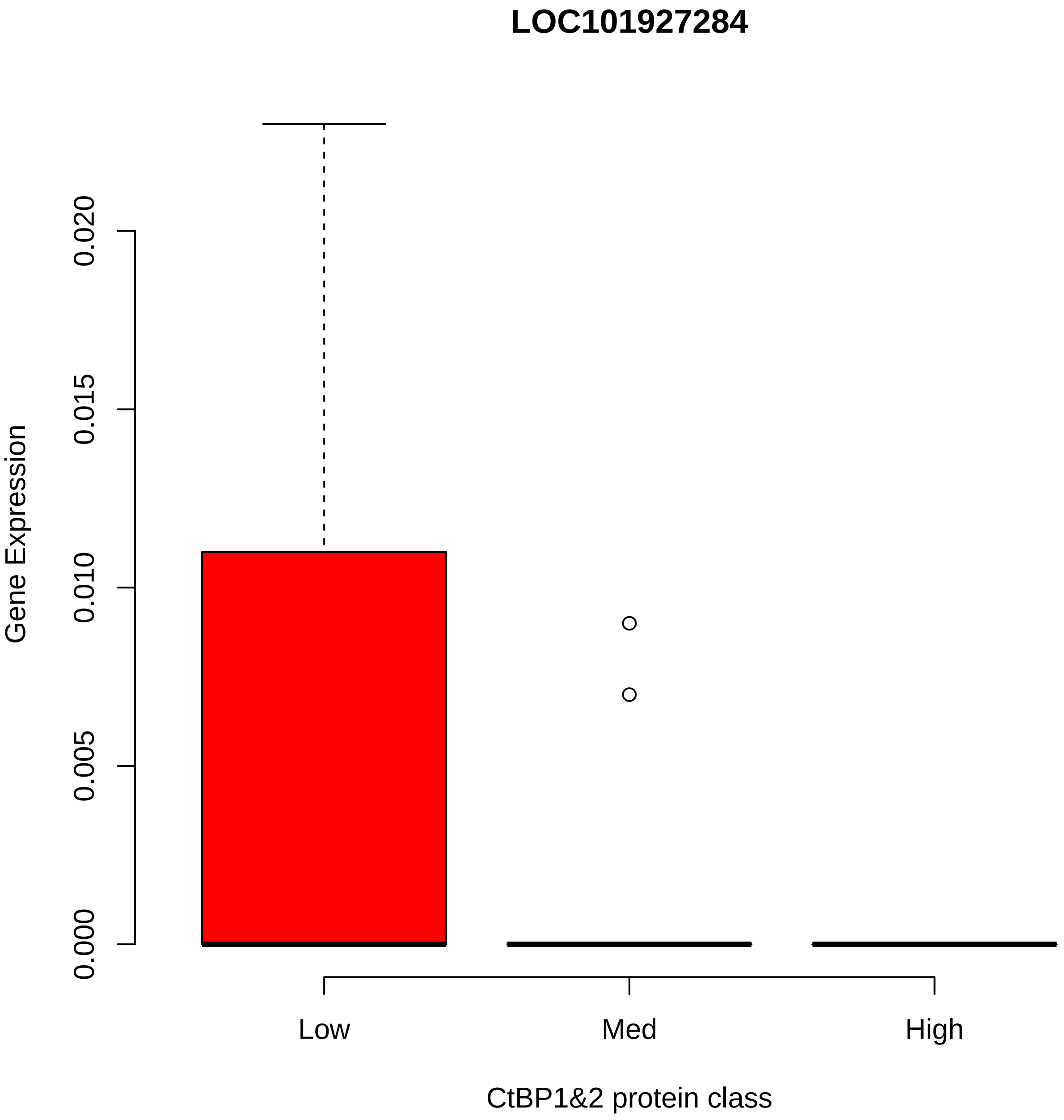

LOC101927284
Gene Expression
0.000
0.005
0.010
0.015
0.020
Low
Med
High
CtBP1&2 protein class

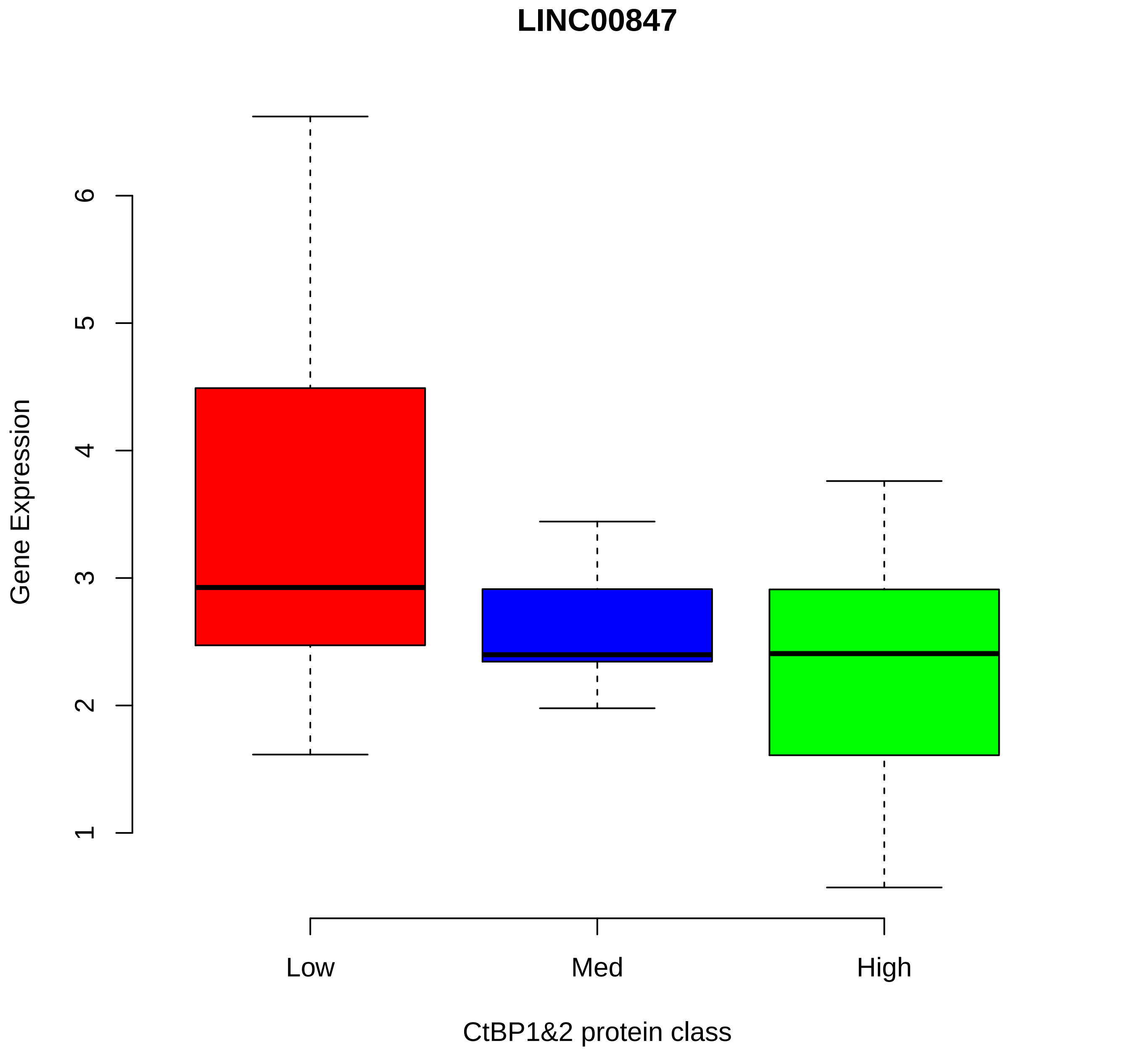

LINC00847
Gene Expression
1
2
3
4
5
6
Low
Med
High
CtBP1&2 protein class

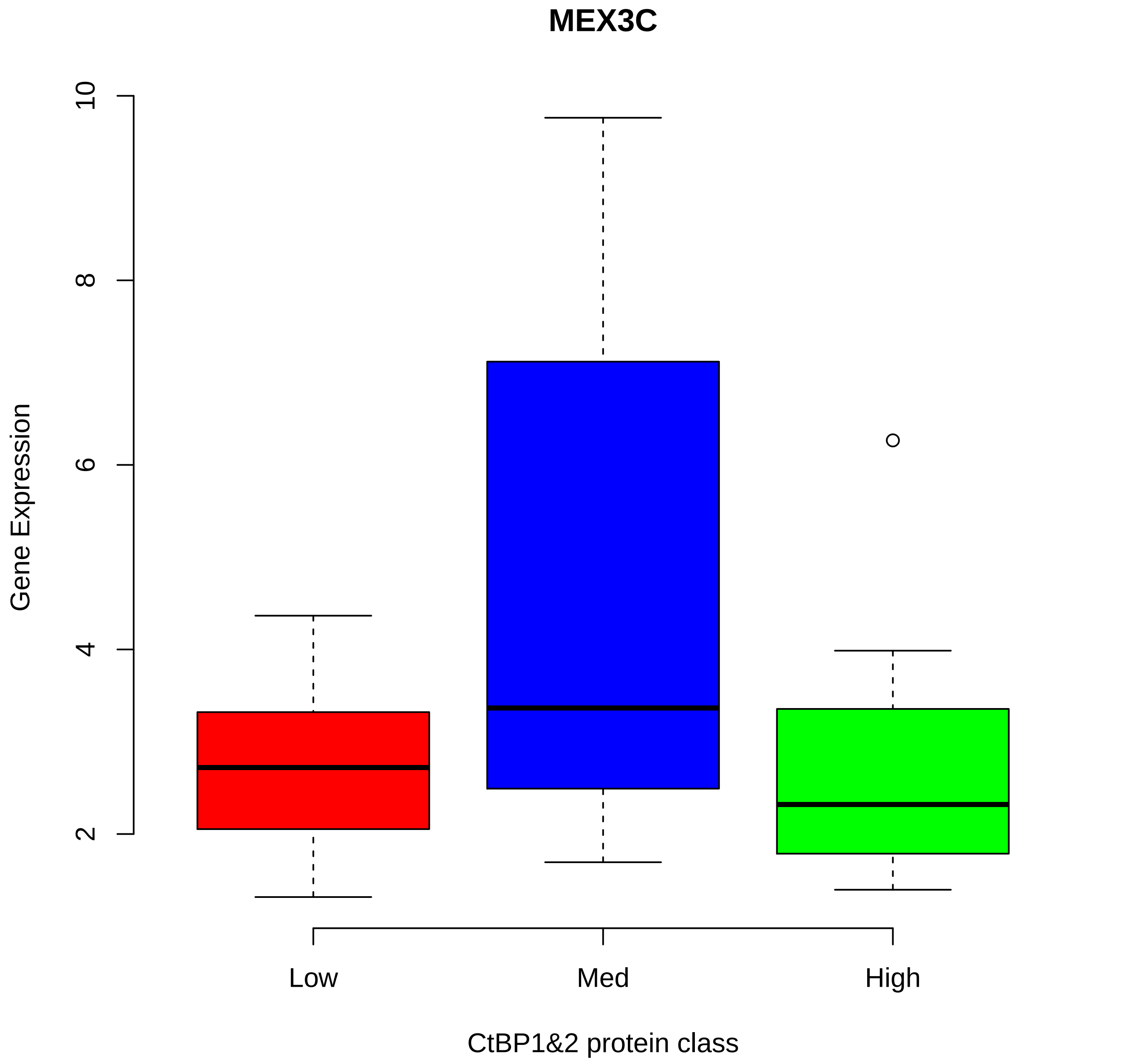
MEX3C
Gene Expression
2
4
6
8
10
Low
Med
High
CtBP1&2 protein class

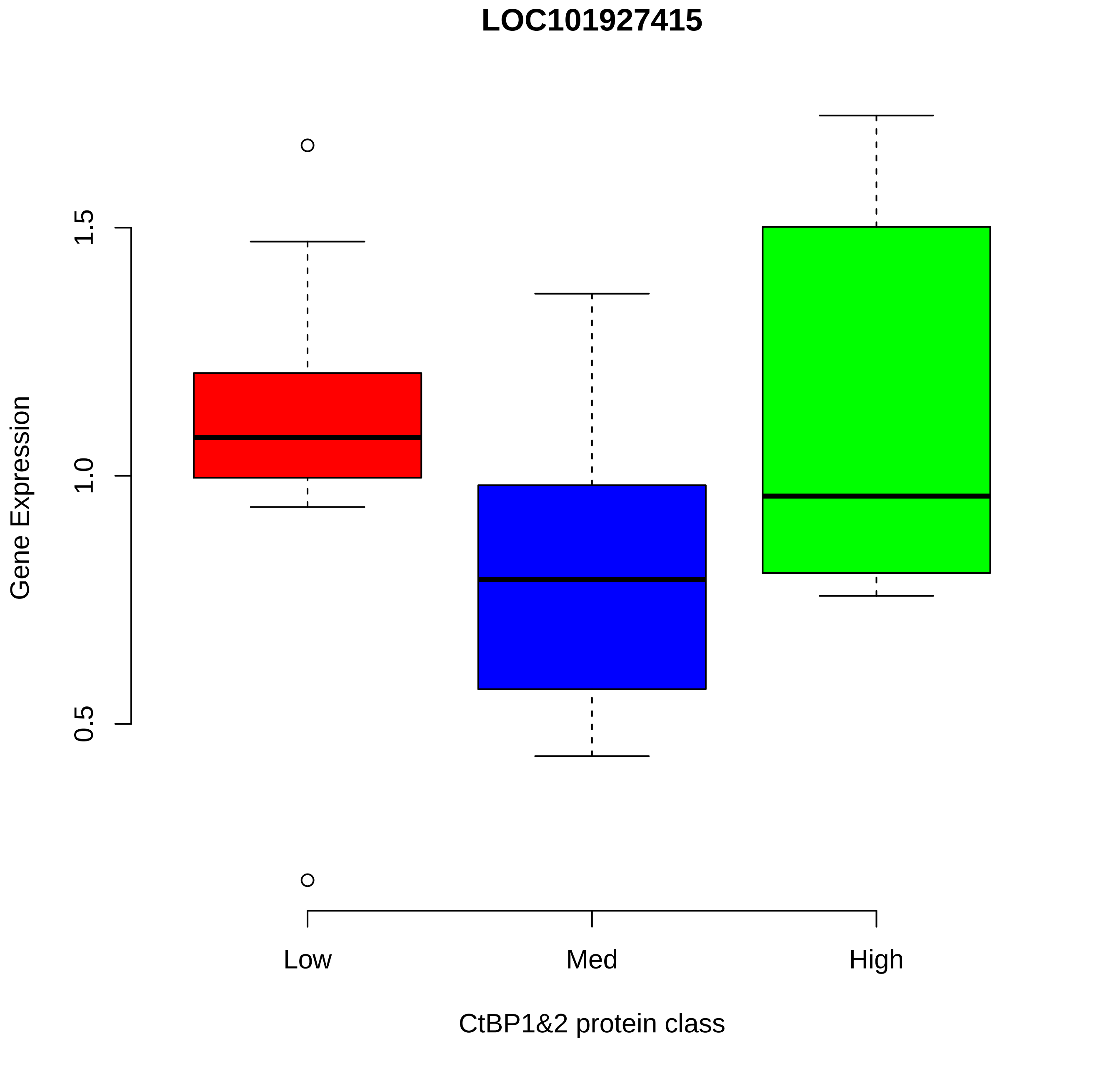

LOC101927415
Gene Expression
1.5
1.0
0.5
Low
Med
High
CtBP1&2 protein class

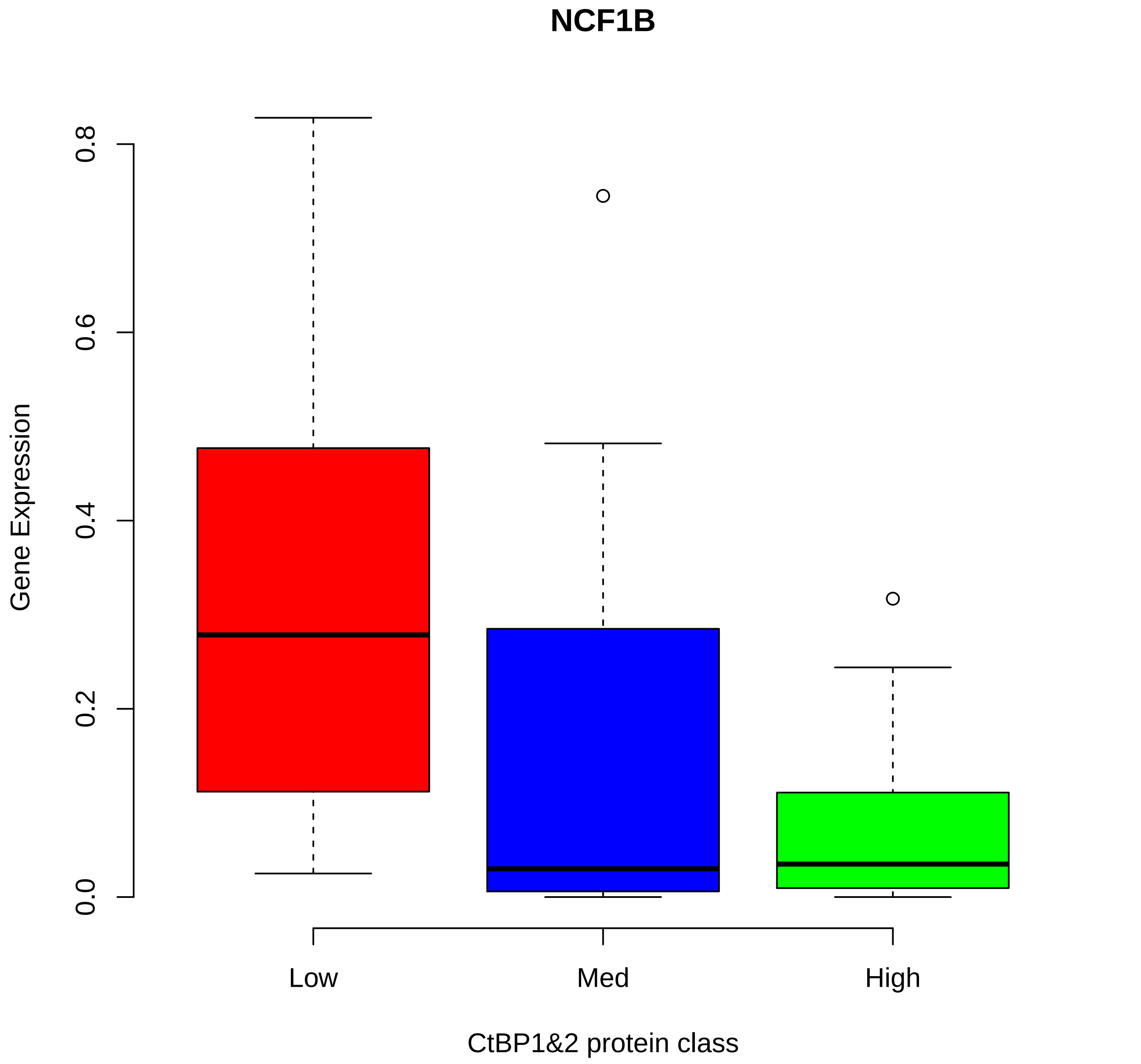

NCF1B
Gene Expression
0.0
0.2
0.4
0.6
0.8
Low
Med
High
CtBP1&2 protein class

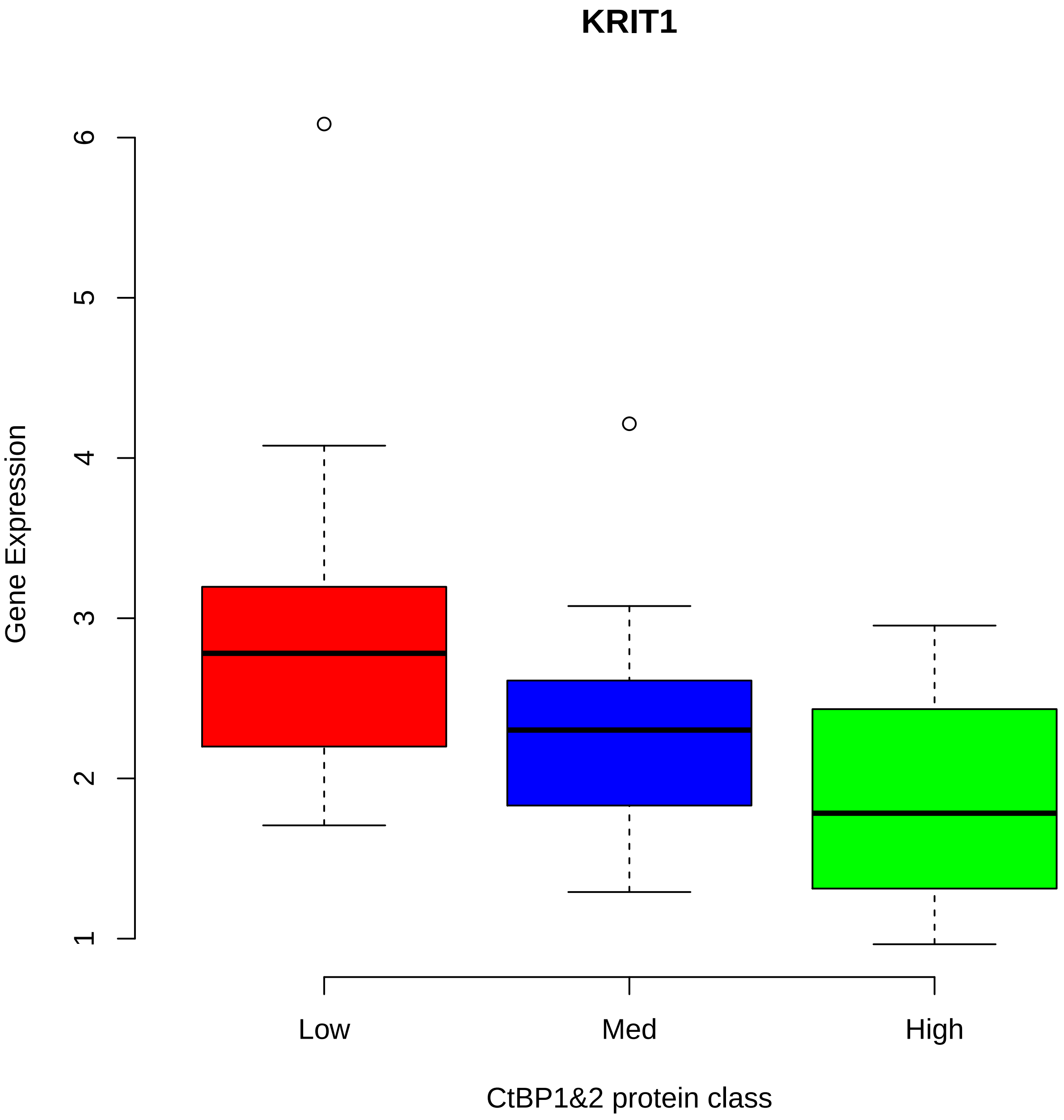

KRIT1
Gene Expression
6
5
4
3
2
1
Low
Med
High
CtBP1&2 protein class

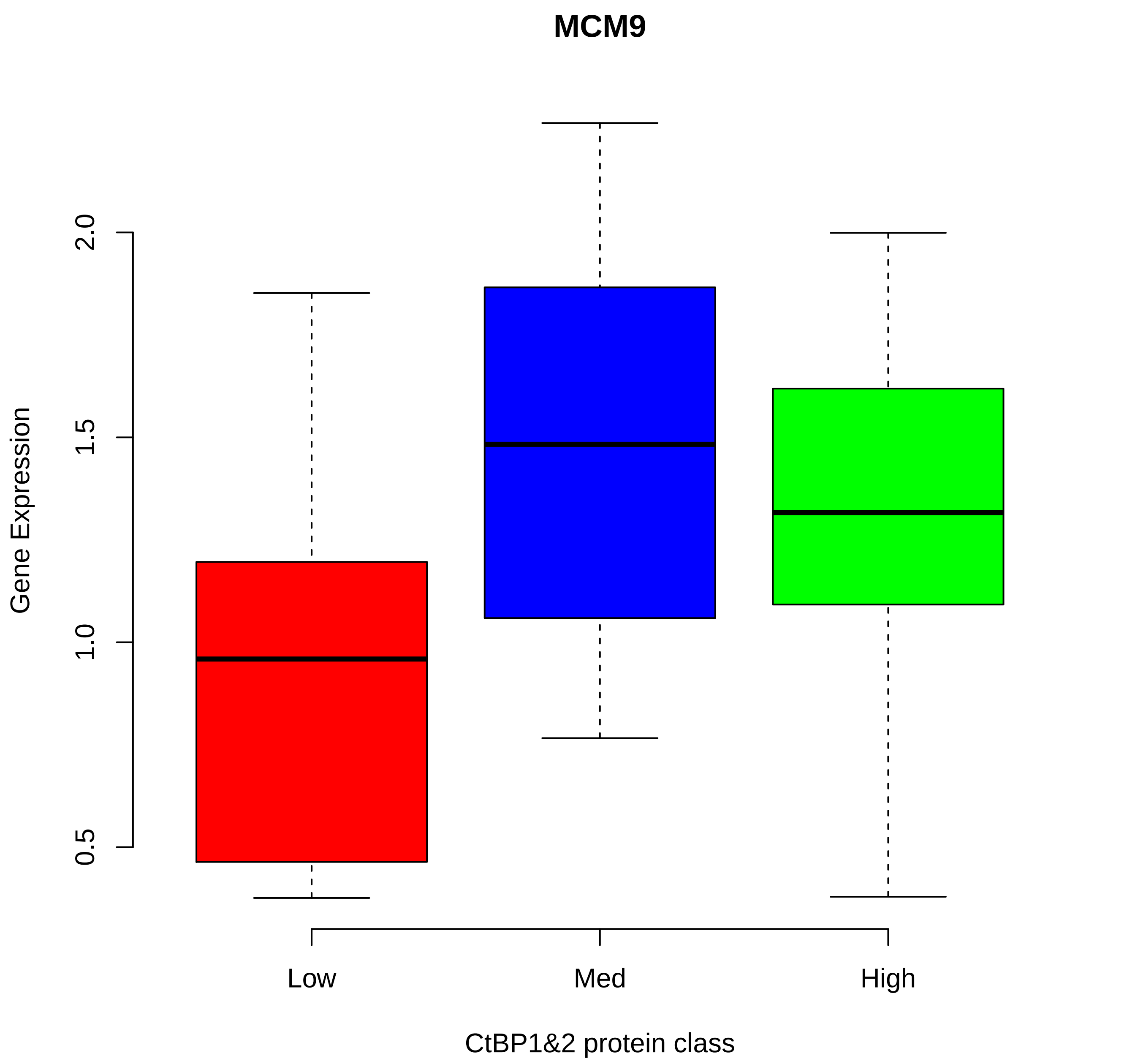

MCM9
Gene Expression
0.5
1.0
1.5
2.0
Low
Med
High
CtBP1&2 protein class

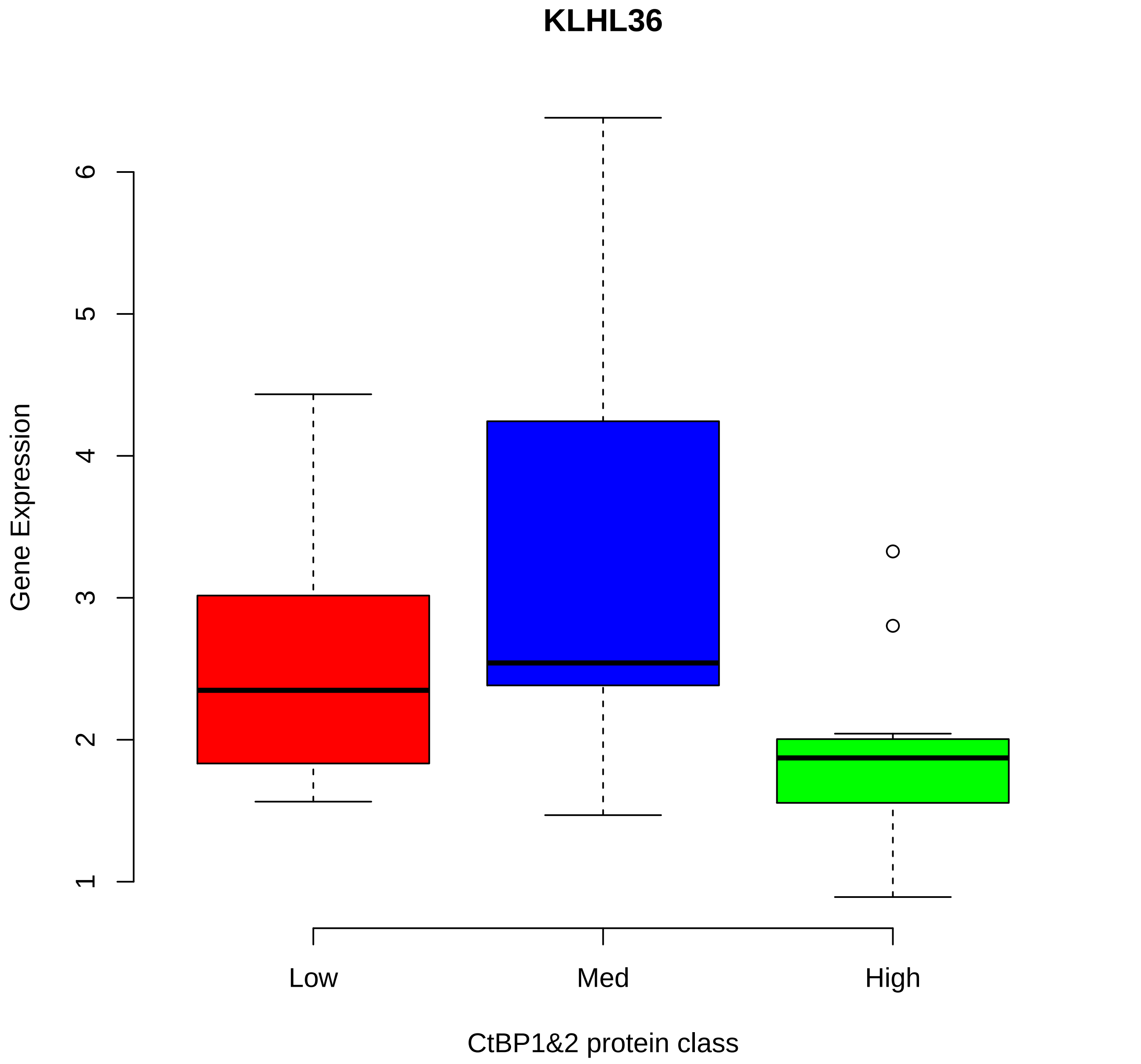

KLHL36
Gene Expression
1
2
3
4
5
6
Low
Med
High
CtBP1&2 protein class

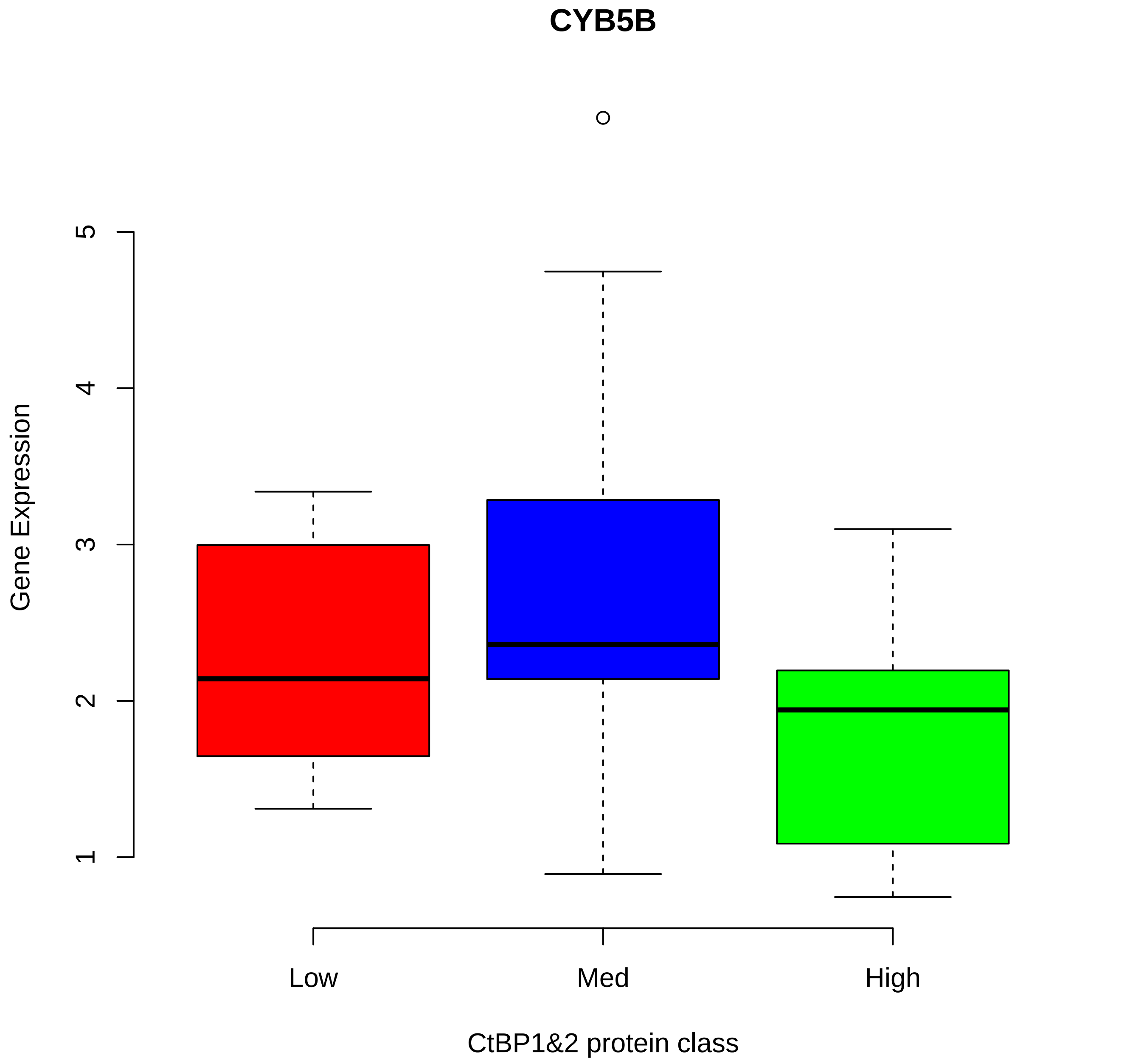
CYB5B
Gene Expression
1
2
3
4
5
Low
Med
High
CtBP1&2 protein class

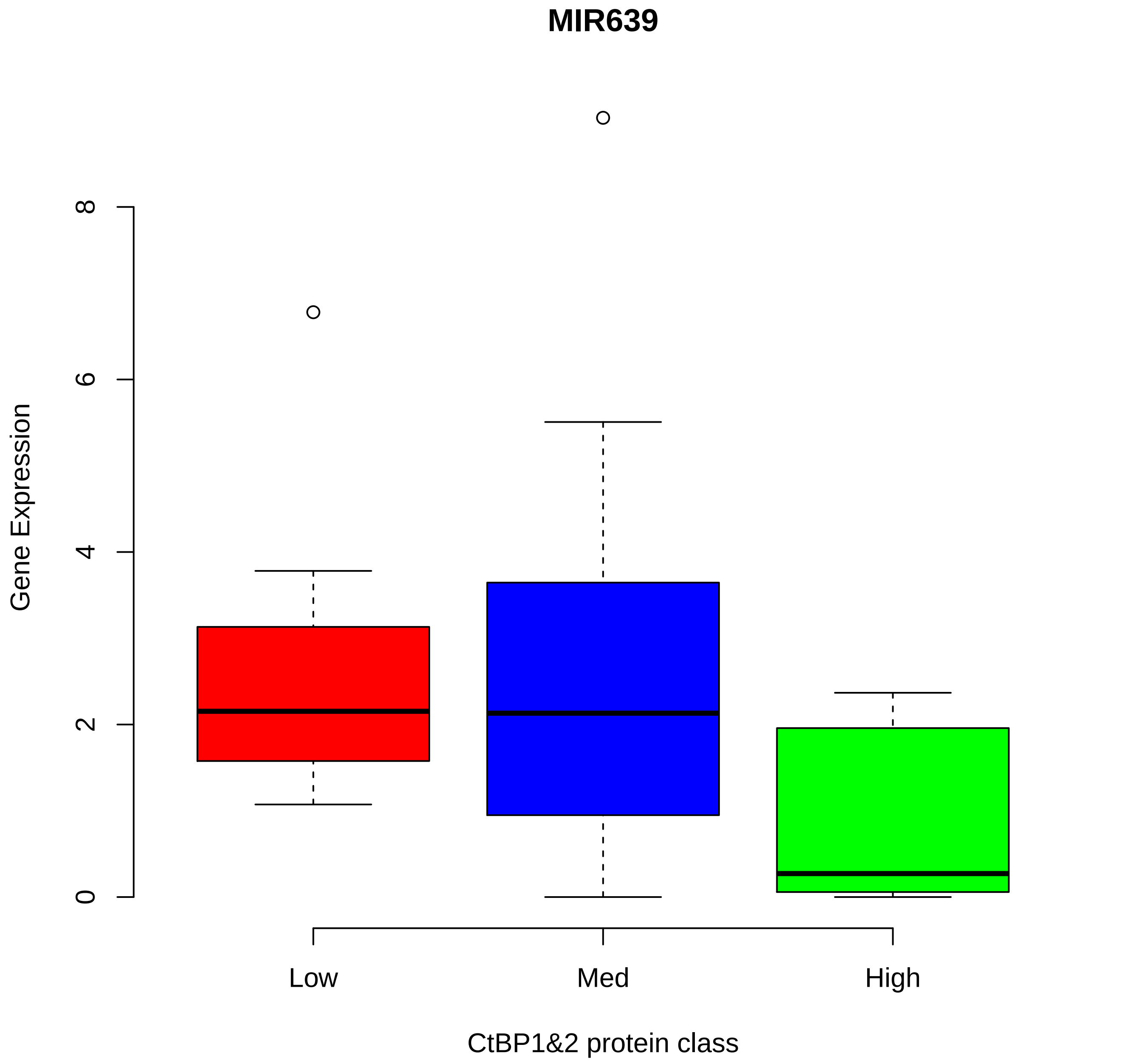

MIR639
Gene Expression
0
2
4
6
8
Low
Med
High
CtBP1&2 protein class

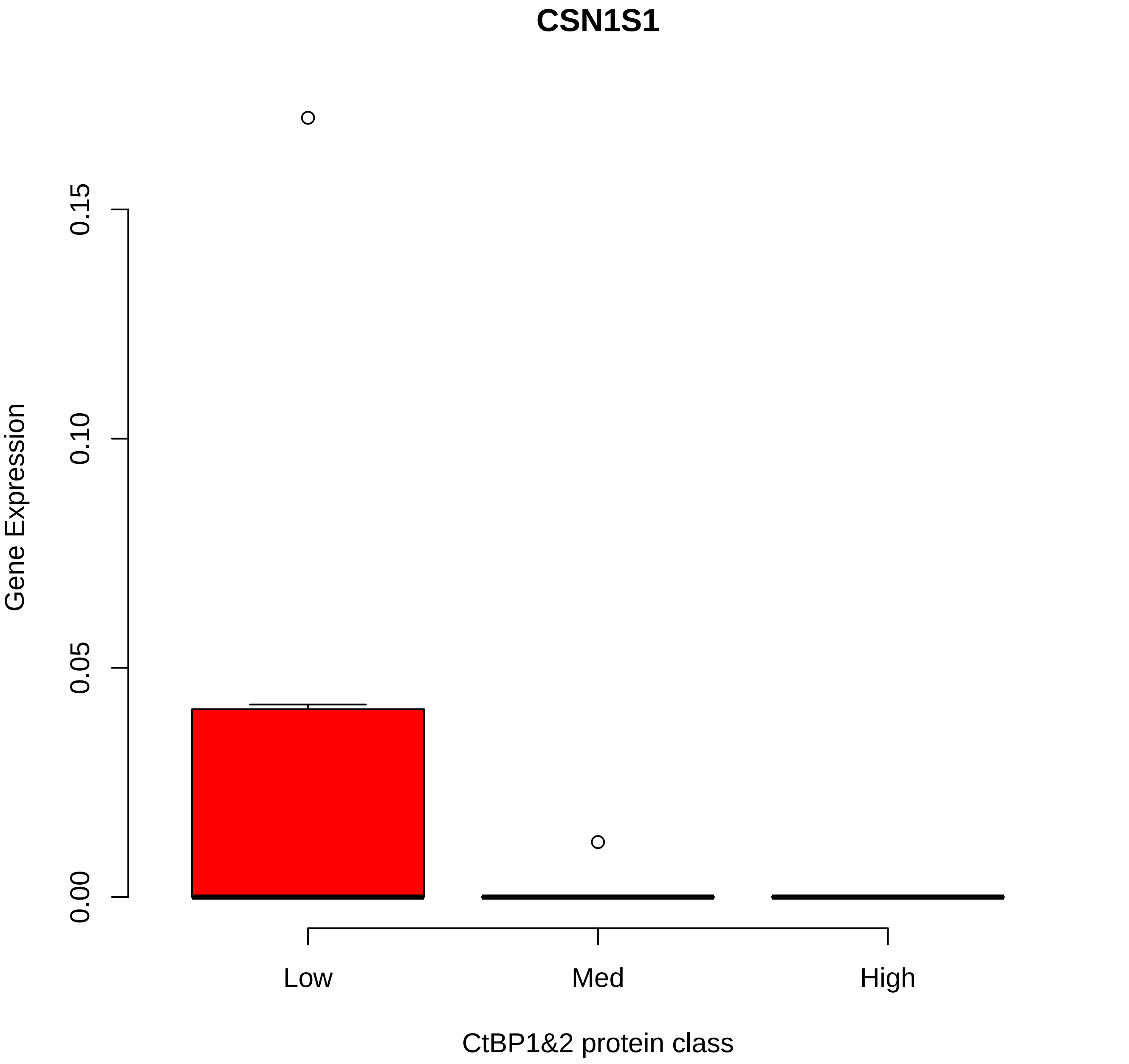
CSN1S1
Gene Expression
0.00
0.05
0.10
0.15
Low
Med
High
CtBP1&2 protein class

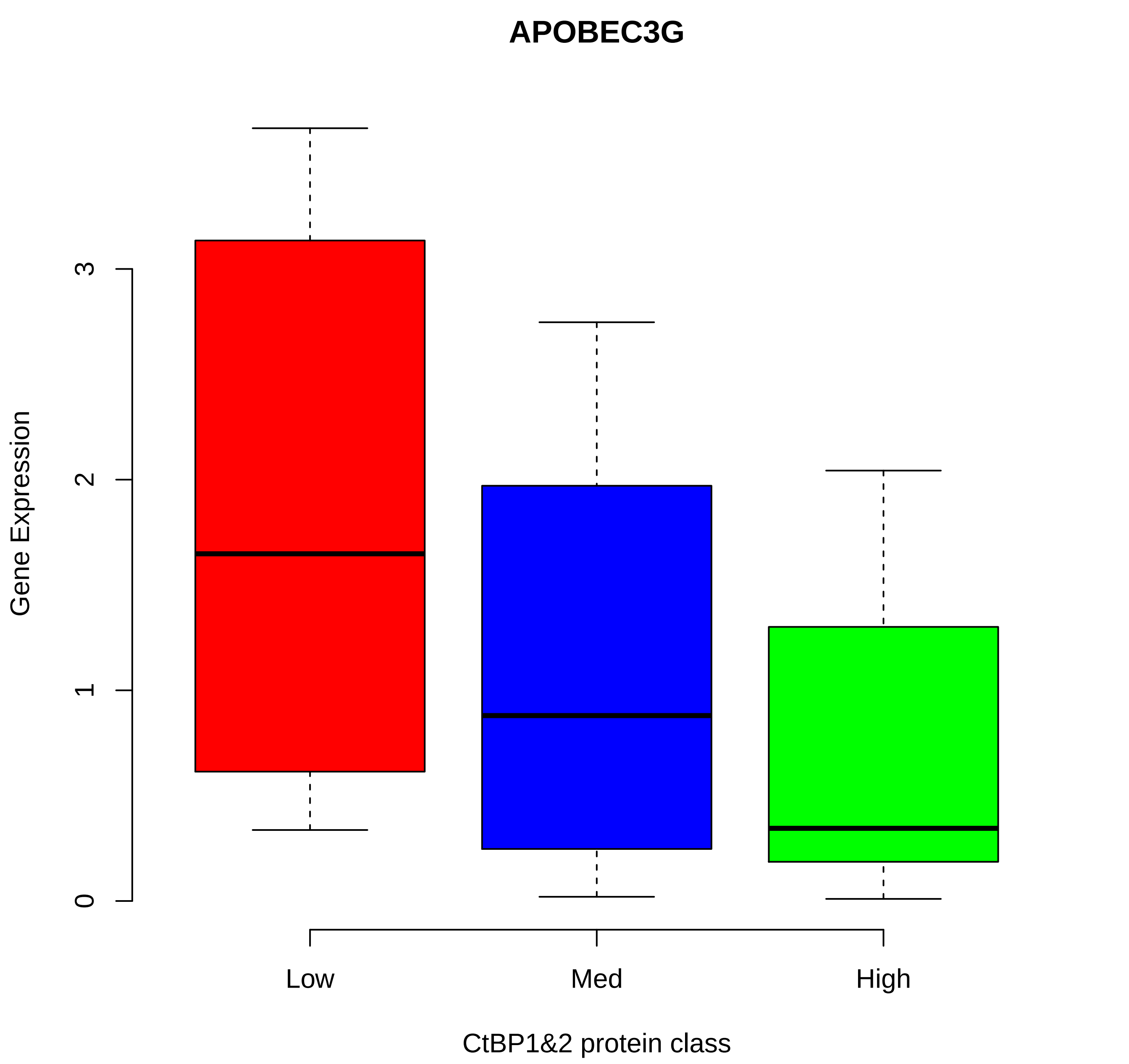

APOBEC3G
Gene Expression
0
1
2
3
Low
Med
High
CtBP1&2 protein class

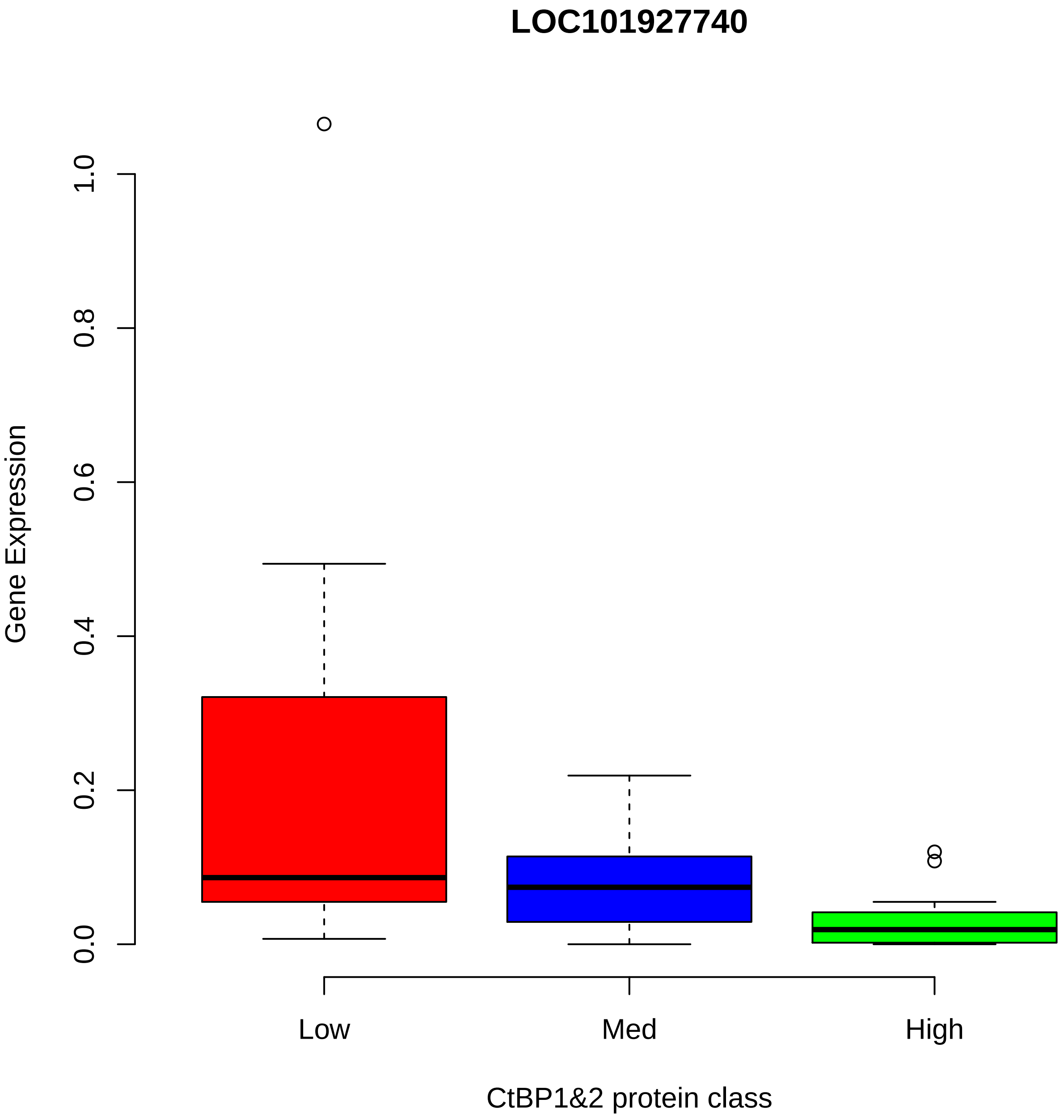

LOC101927740
Gene Expression
0.0
0.2
0.4
0.6
0.8
1.0
Low
Med
High
CtBP1&2 protein class

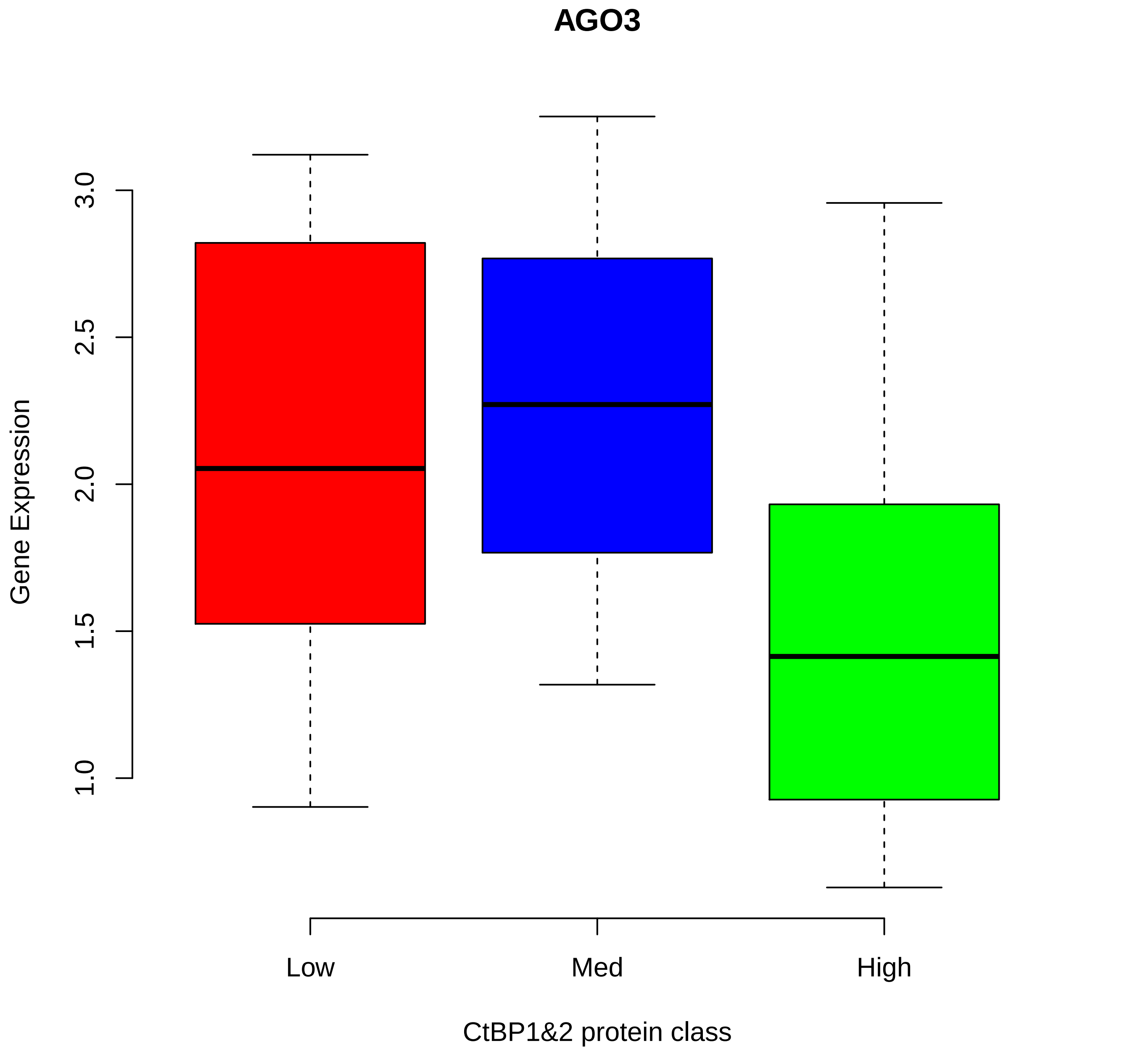
AGO3
Gene Expression
1.0
1.5
2.0
2.5
3.0
Low
Med
High
CtBP1&2 protein class

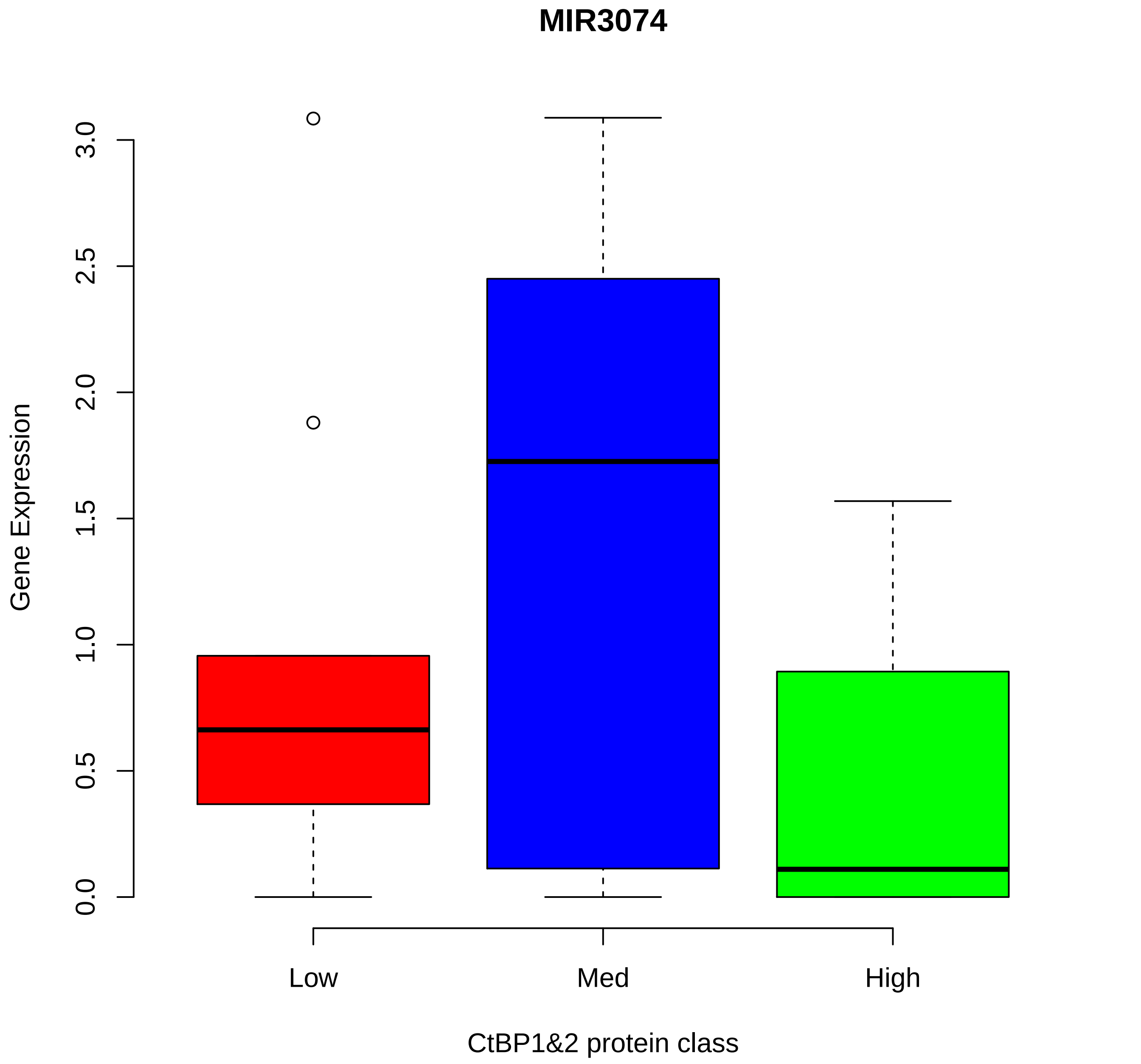
MIR3074
Gene Expression
0.0
0.5
1.0
1.5
2.0
2.5
3.0
Low
Med
High
CtBP1&2 protein class

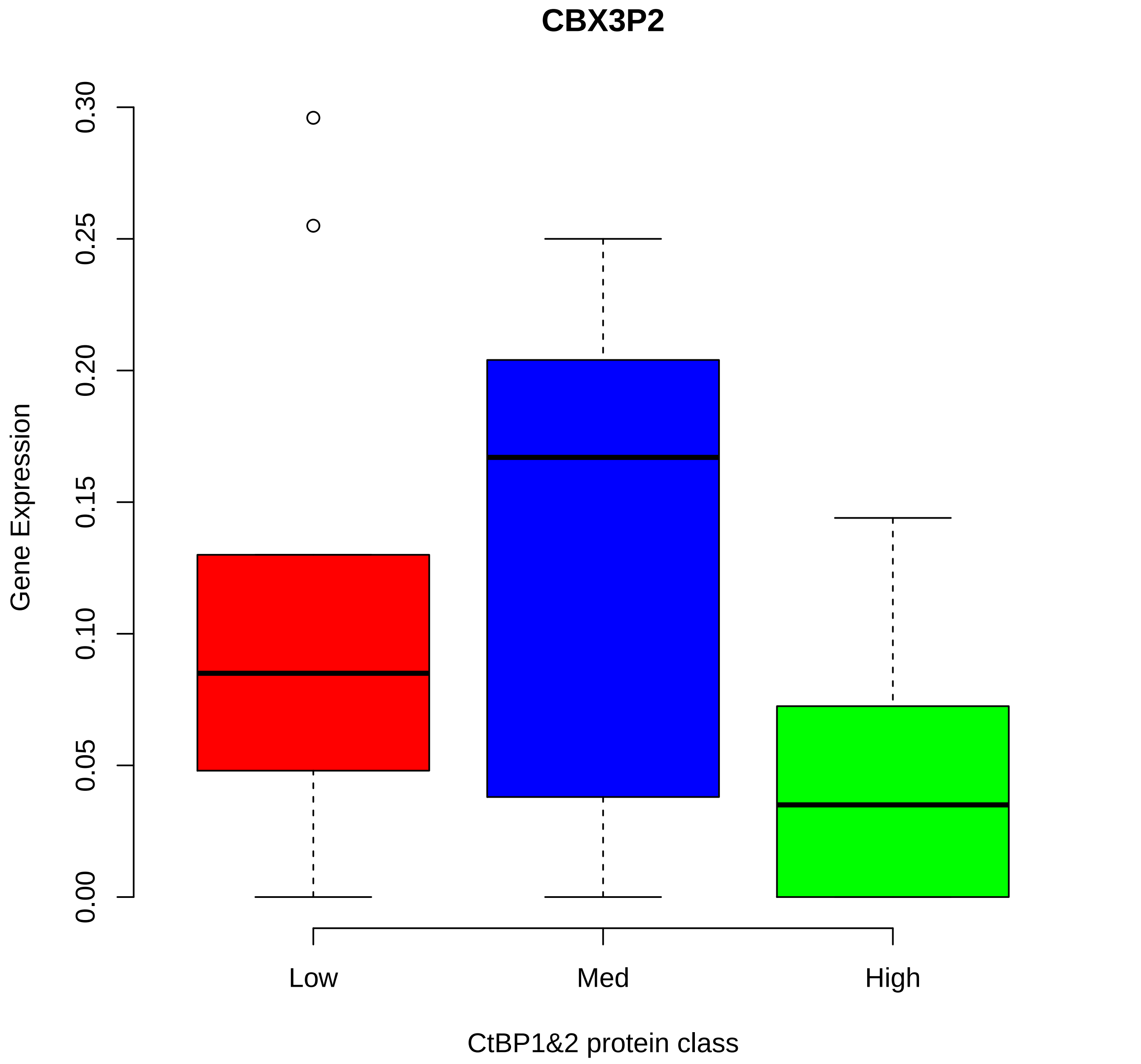

CBX3P2
Gene Expression
0.00
0.05
0.10
0.15
0.20
0.25
0.30
Low
Med
High
CtBP1&2 protein class

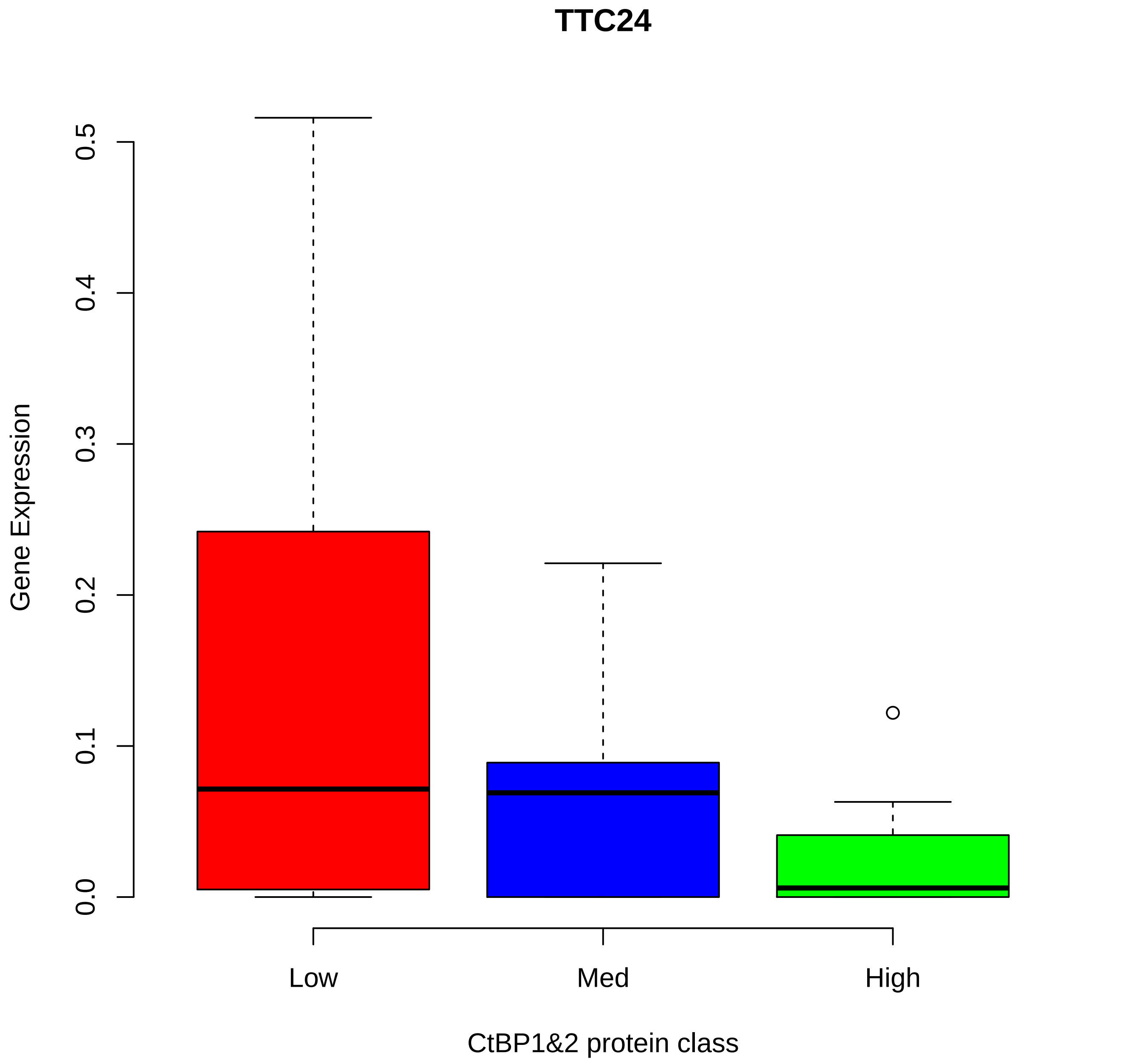
TTC24
Gene Expression
0.0
0.1
0.2
0.3
0.4
0.5
Low
Med
High
CtBP1&2 protein class

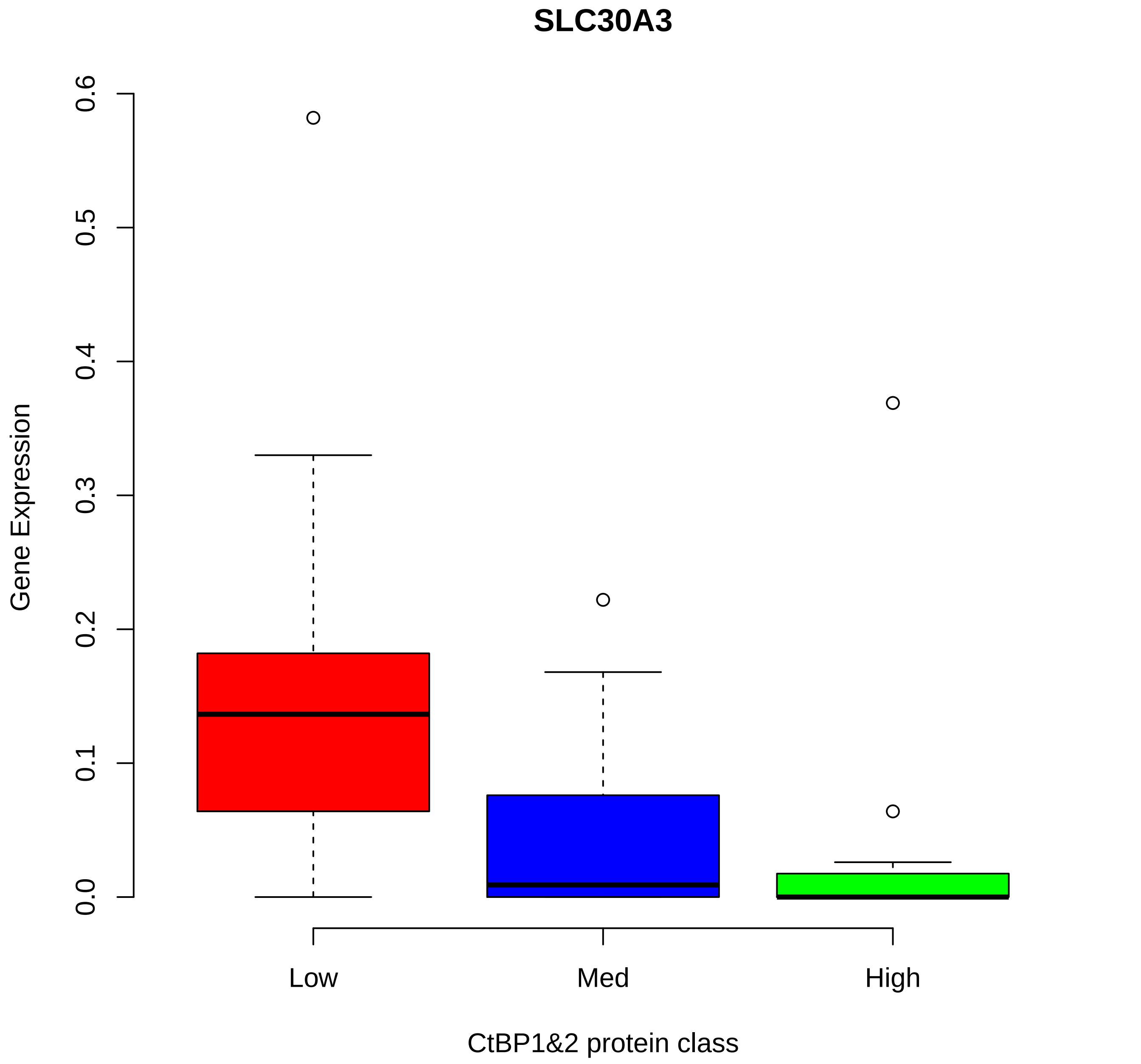

SLC30A3
Gene Expression
0.0
0.1
0.2
0.3
0.4
0.5
0.6
Low
Med
High
CtBP1&2 protein class

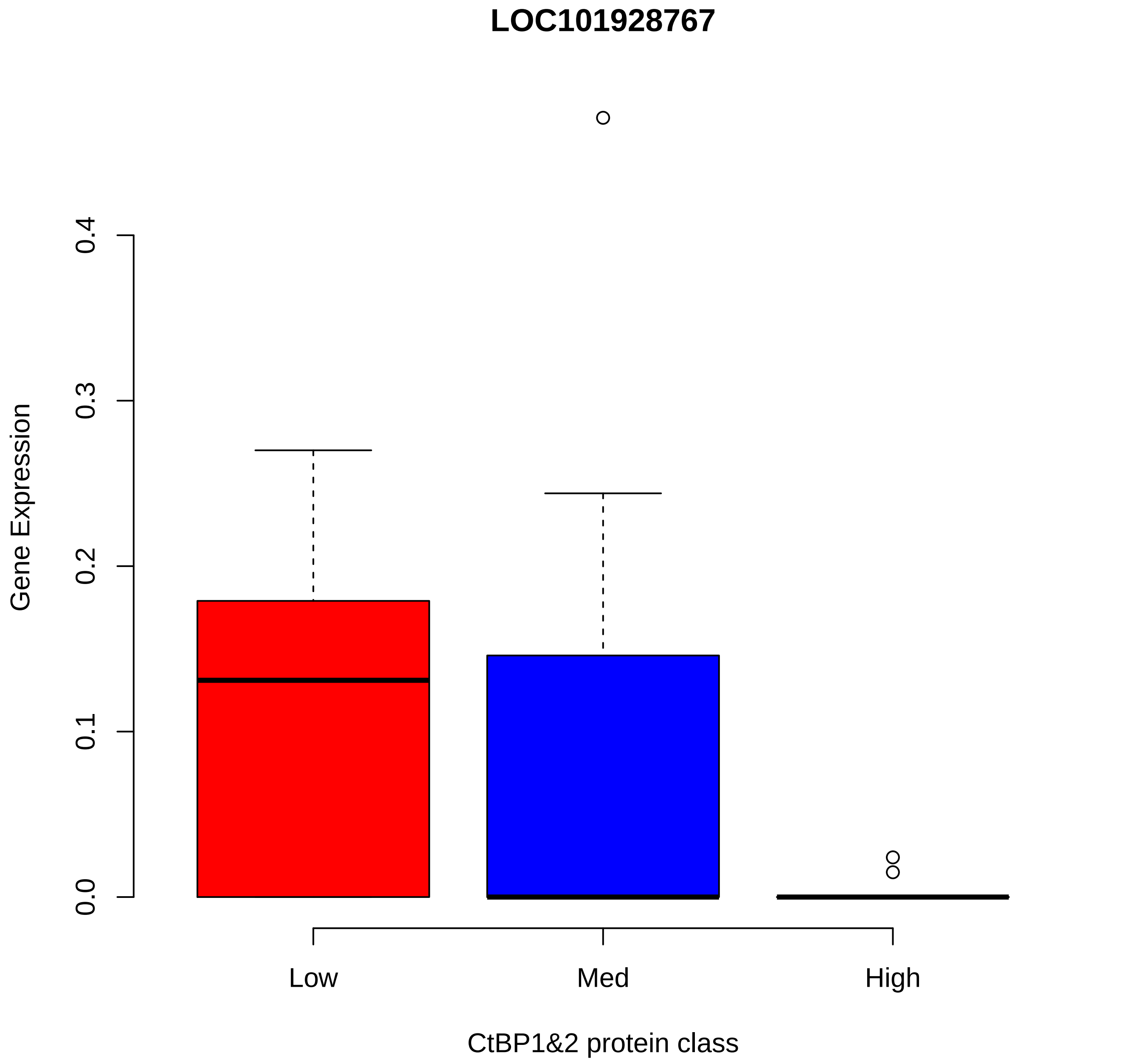

LOC101928767
Gene Expression
0.0
0.1
0.2
0.3
0.4
Low
Med
High
CtBP1&2 protein class

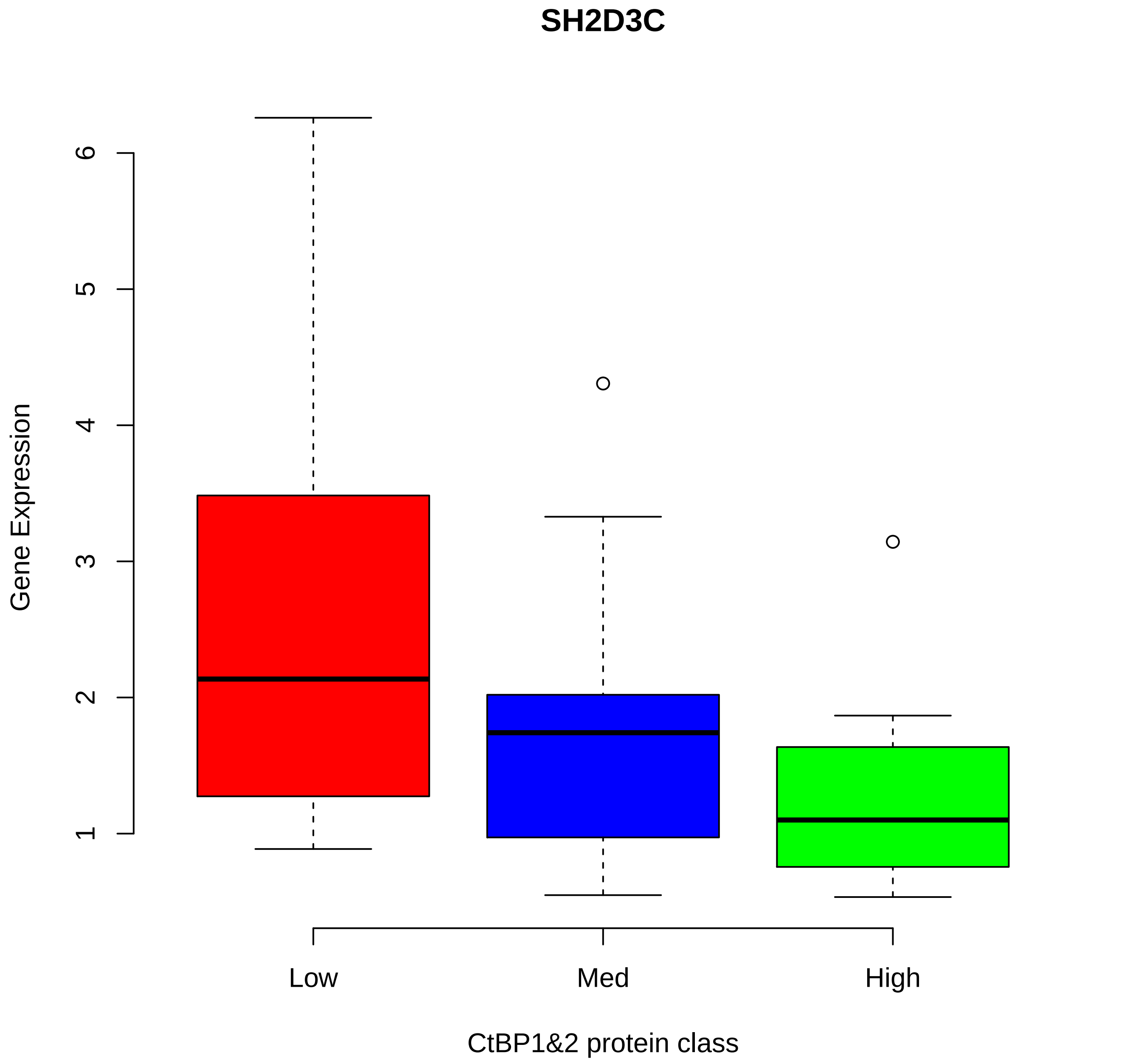
SH2D3C
Gene Expression
1
2
3
4
5
6
Low
Med
High
CtBP1&2 protein class

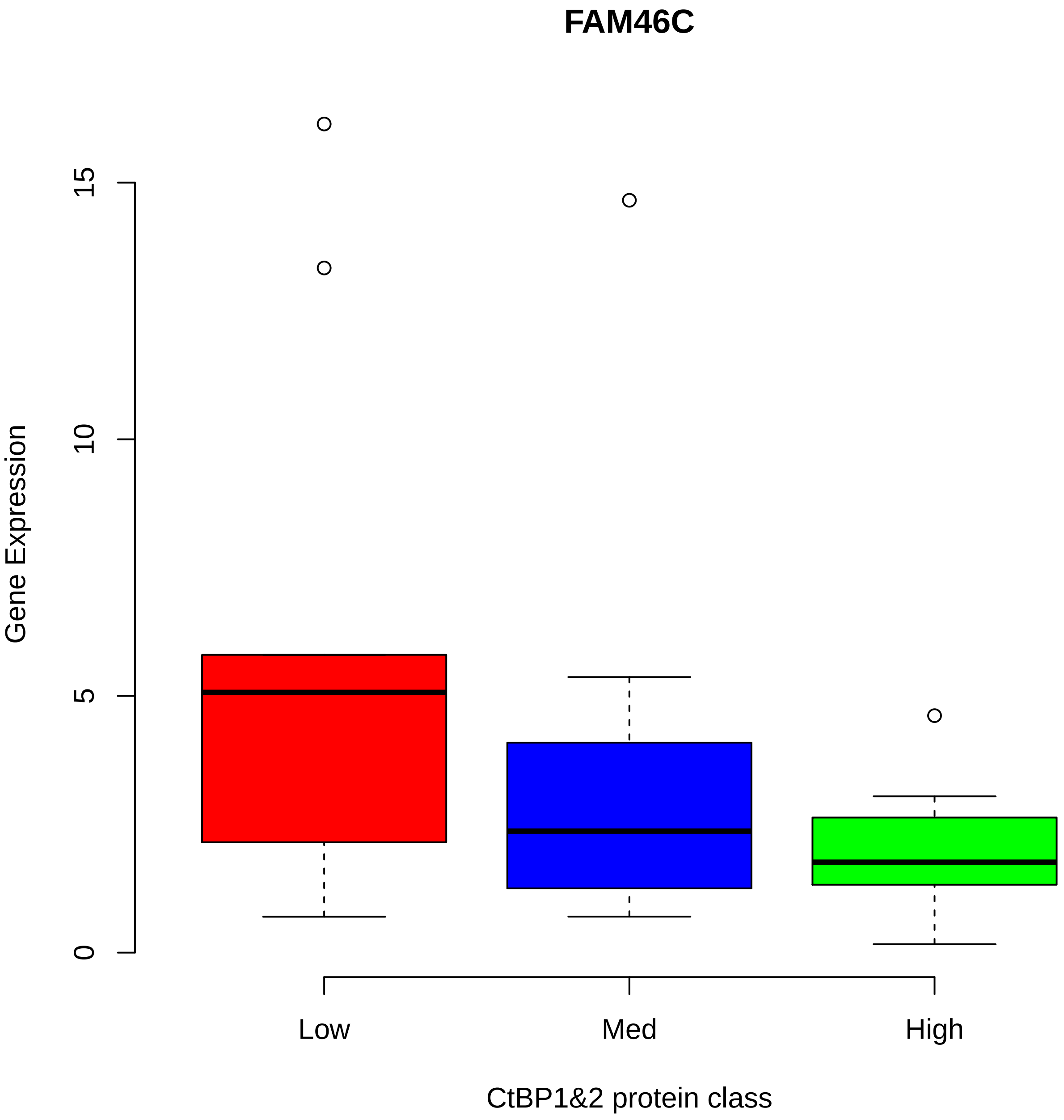

FAM46C
Gene Expression
0
5
10
15
Low
Med
High
CtBP1&2 protein class

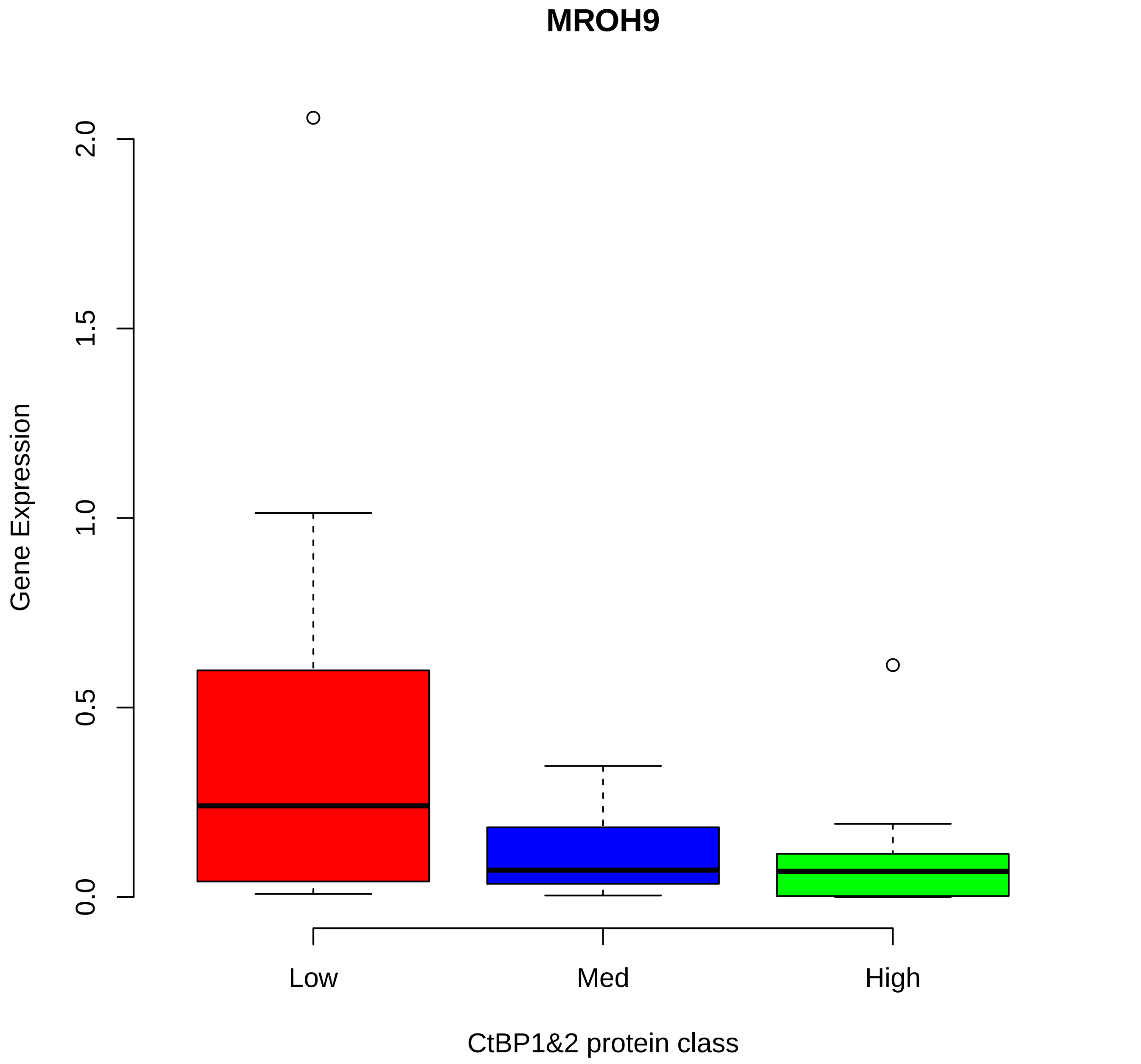
MROH9
Gene Expression
0.0
0.5
1.0
1.5
2.0
Low
Med
High
CtBP1&2 protein class

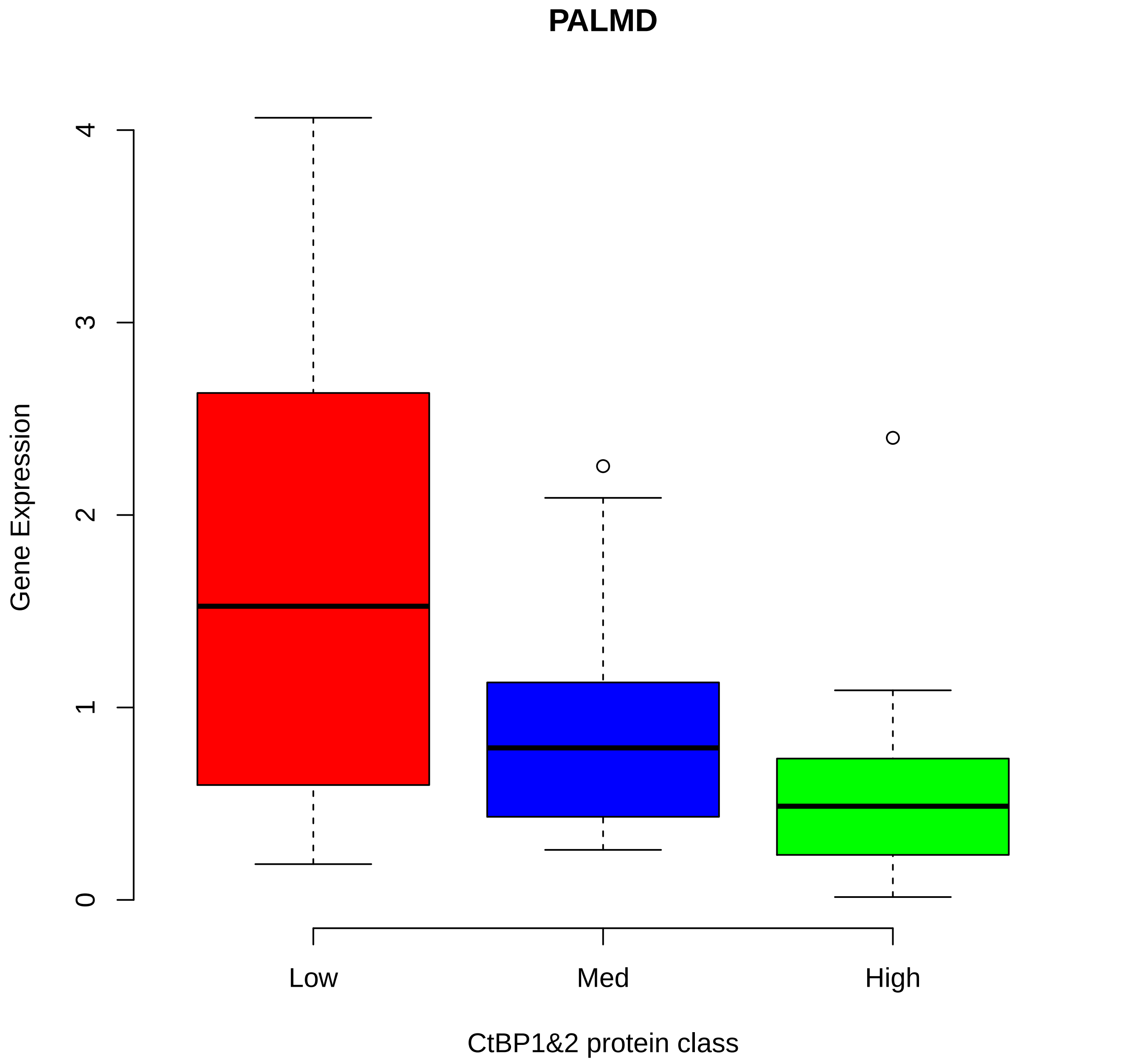
PALMD
Gene Expression
0
1
2
3
4
Low
Med
High
CtBP1&2 protein class

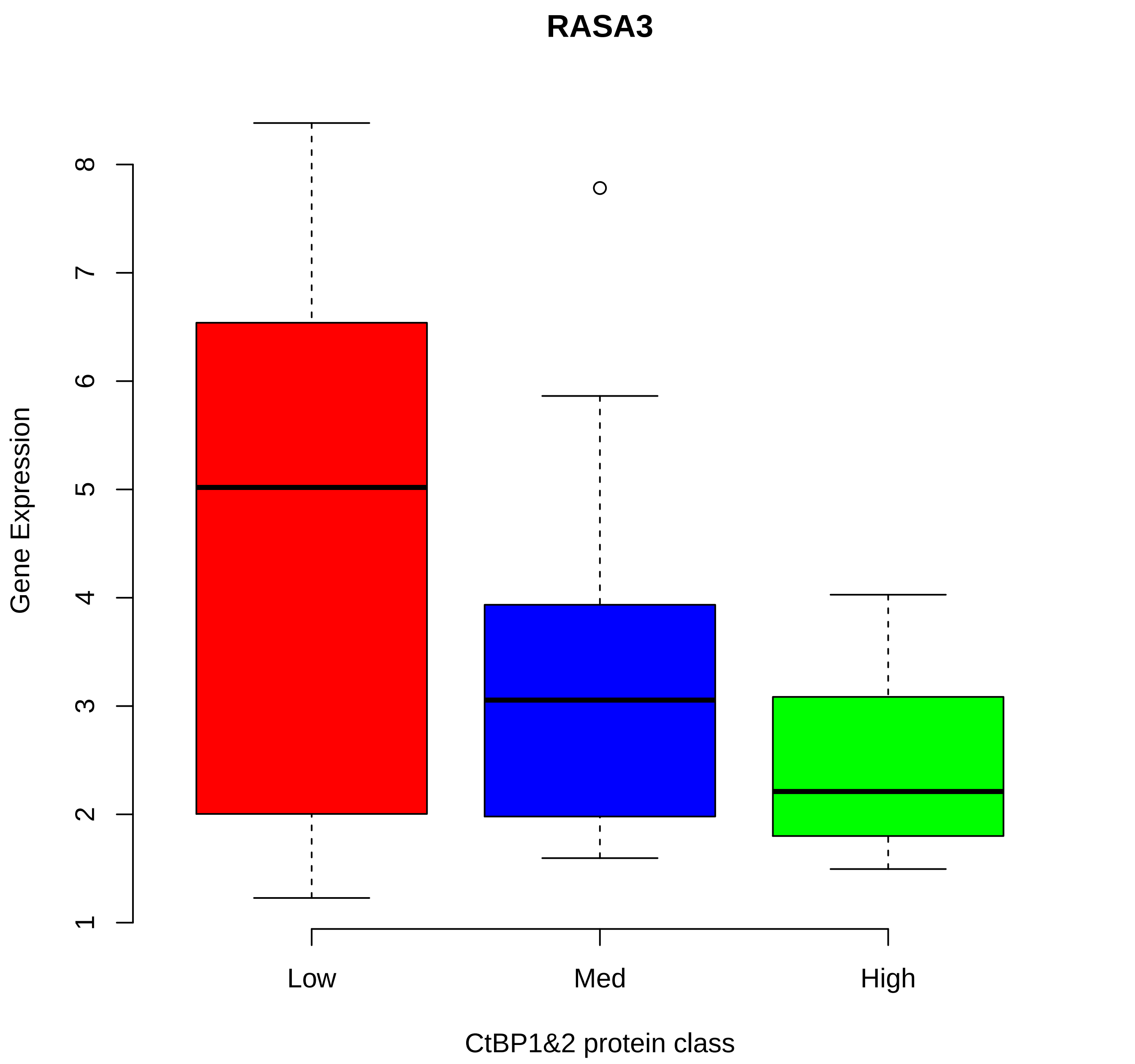
RASA3
Gene Expression
1
2
3
4
5
6
7
8
Low
Med
High
CtBP1&2 protein class

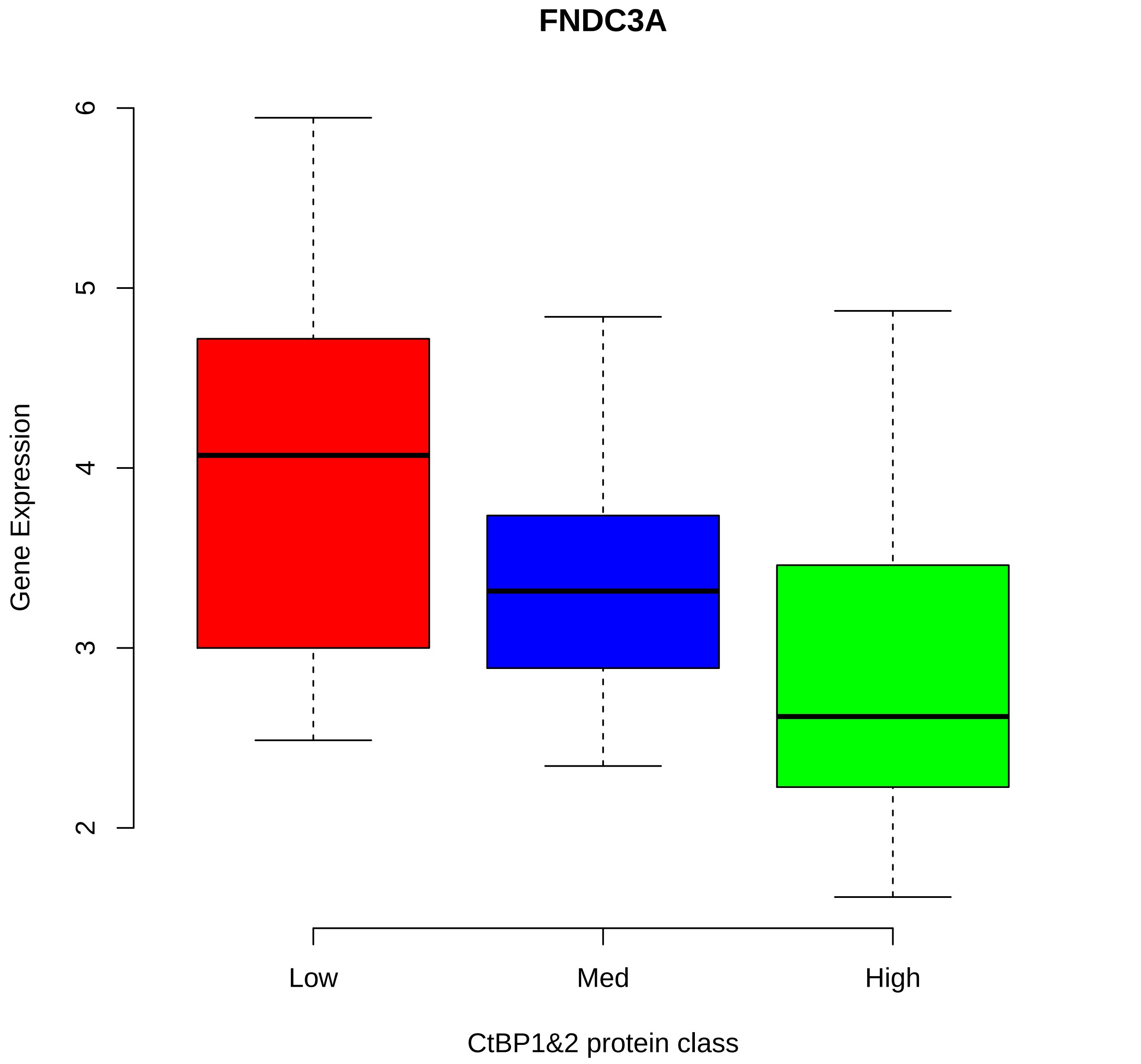

FNDC3A
Gene Expression
2
3
4
5
6
Low
Med
High
CtBP1&2 protein class

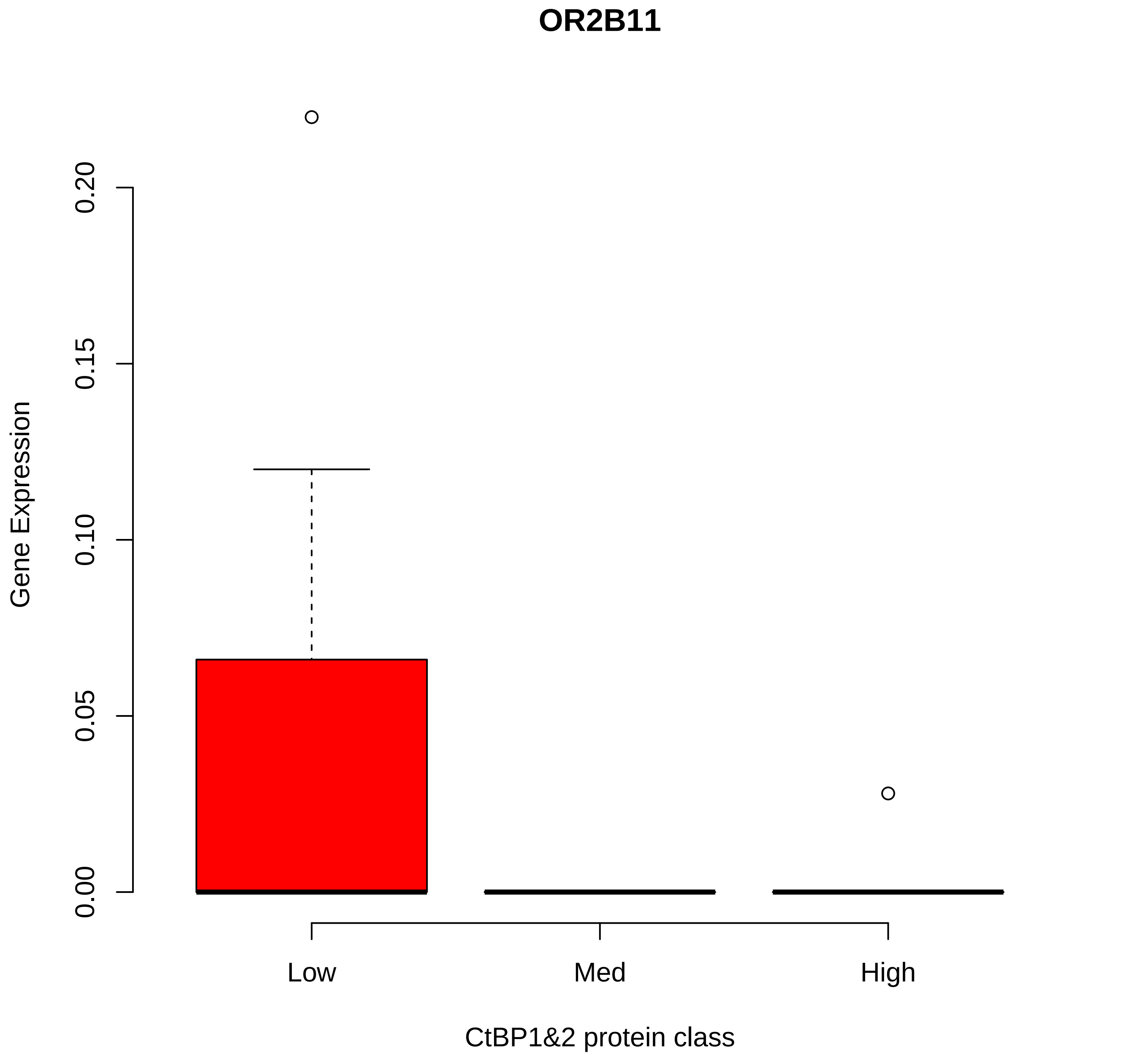

OR2B11
Gene Expression
0.00
0.05
0.10
0.15
0.20
Low
Med
High
CtBP1&2 protein class

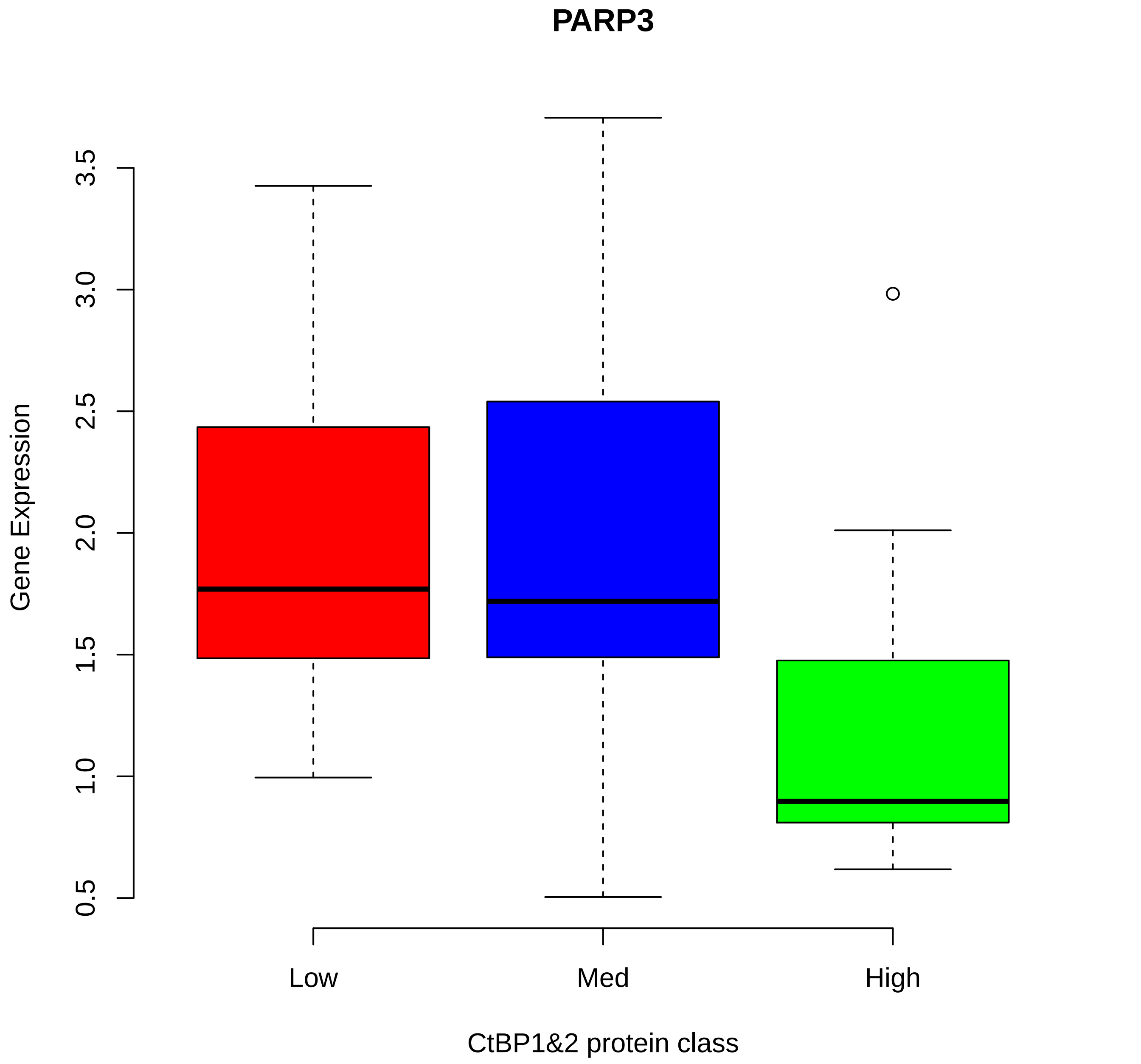

PARP3
Gene Expression
0.5
1.0
1.5
2.0
2.5
3.0
3.5
Low
Med
High
CtBP1&2 protein class

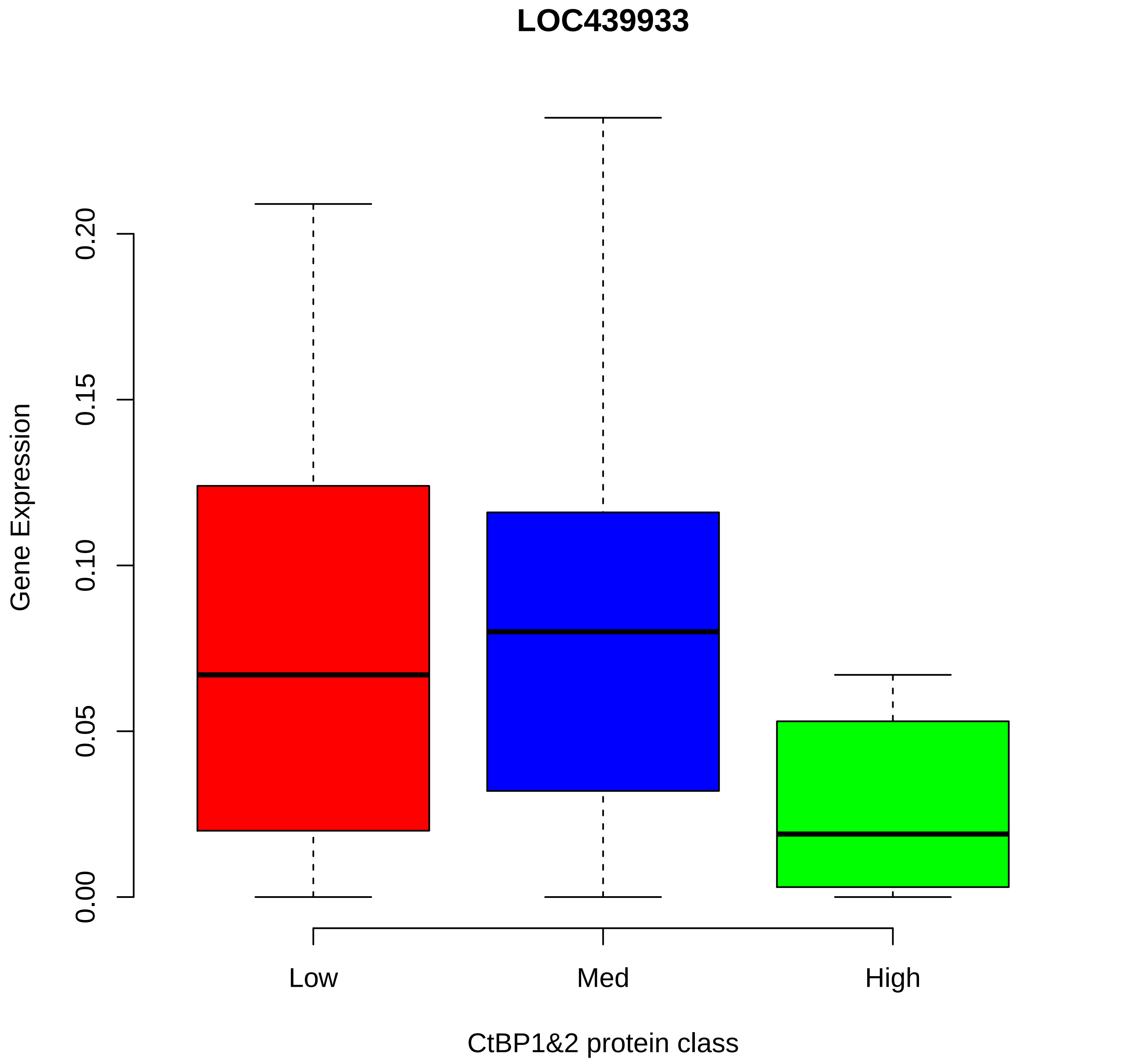
LOC439933
Gene Expression
0.00
0.05
0.10
0.15
0.20
Low
Med
High
CtBP1&2 protein class

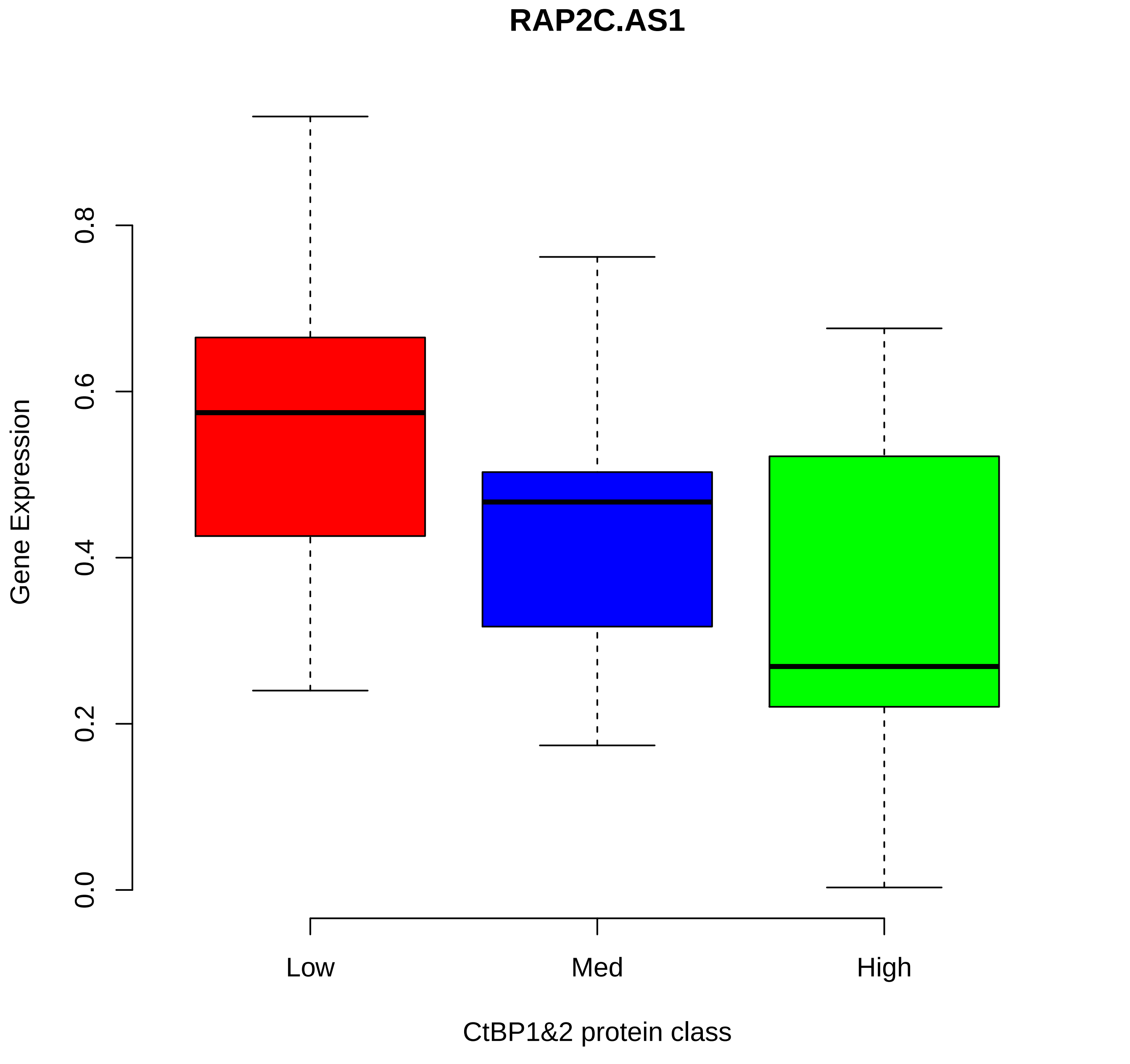
RAP2C.AS1
Gene Expression
0.0
0.2
0.4
0.6
0.8
Low
Med
High
CtBP1&2 protein class

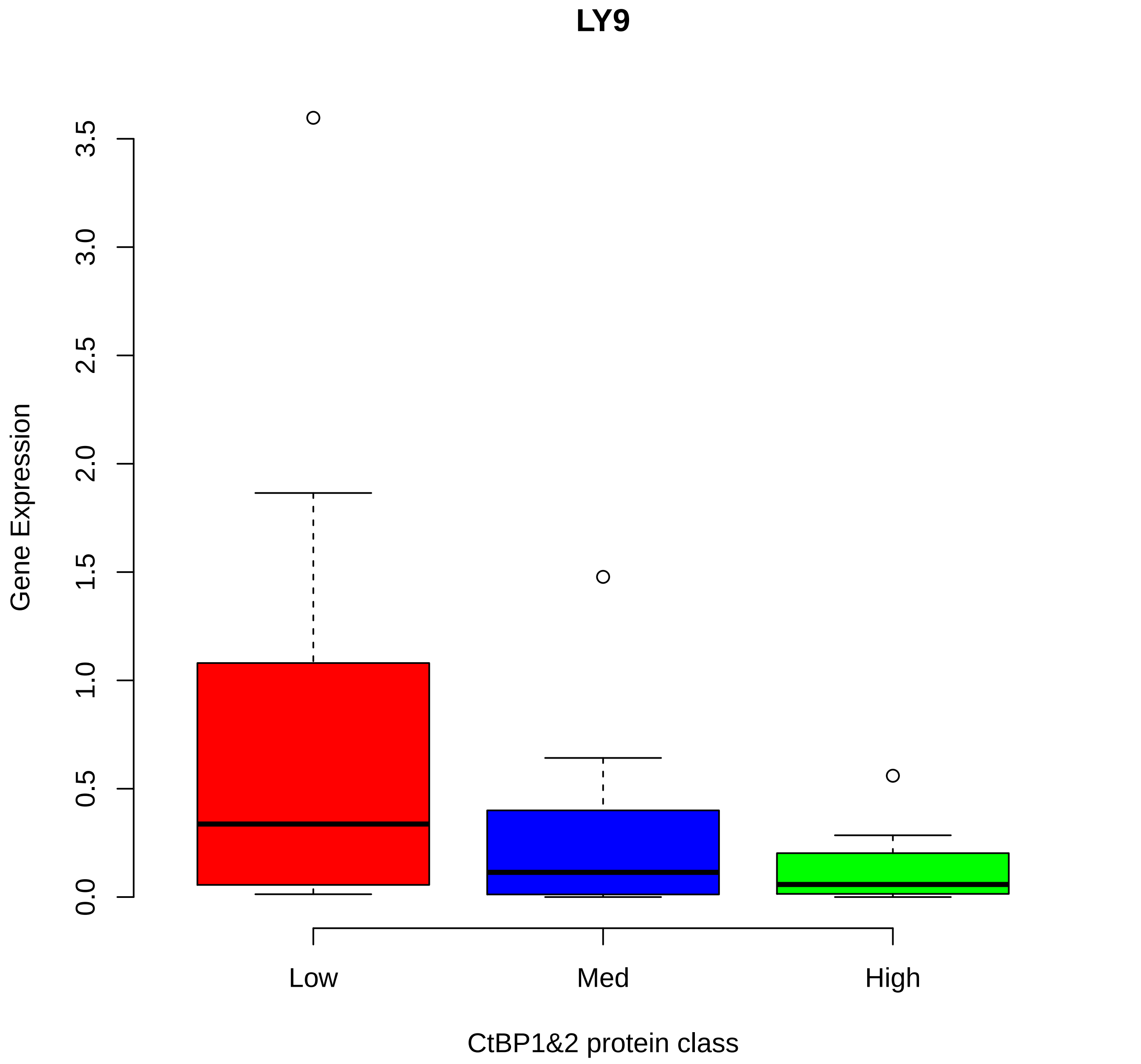
LY9
Gene Expression
0.0
0.5
1.0
1.5
2.0
2.5
3.0
3.5
Low
Med
High
CtBP1&2 protein class

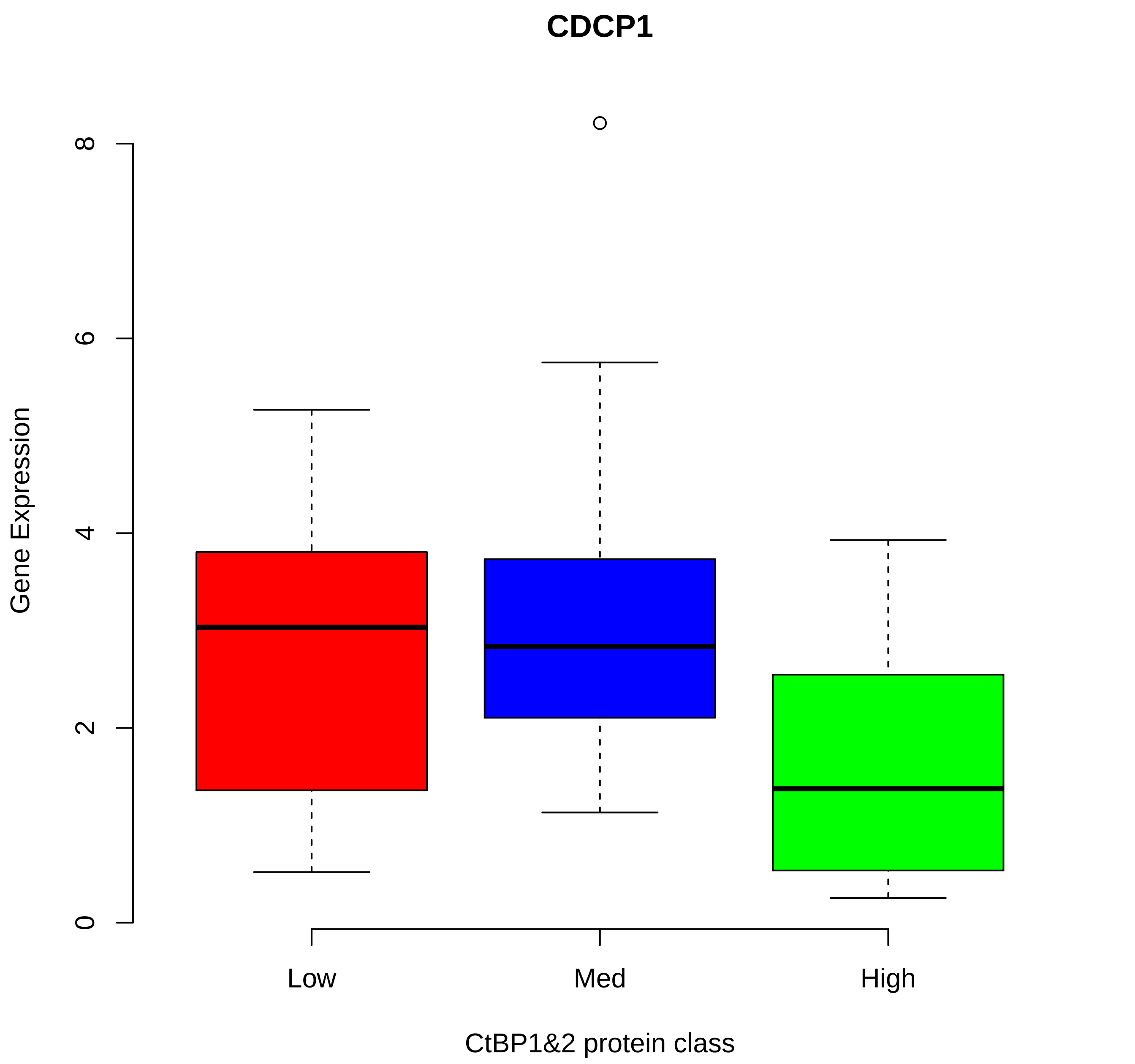

CDCP1
Gene Expression
0
2
4
6
8
Low
Med
High
CtBP1&2 protein class

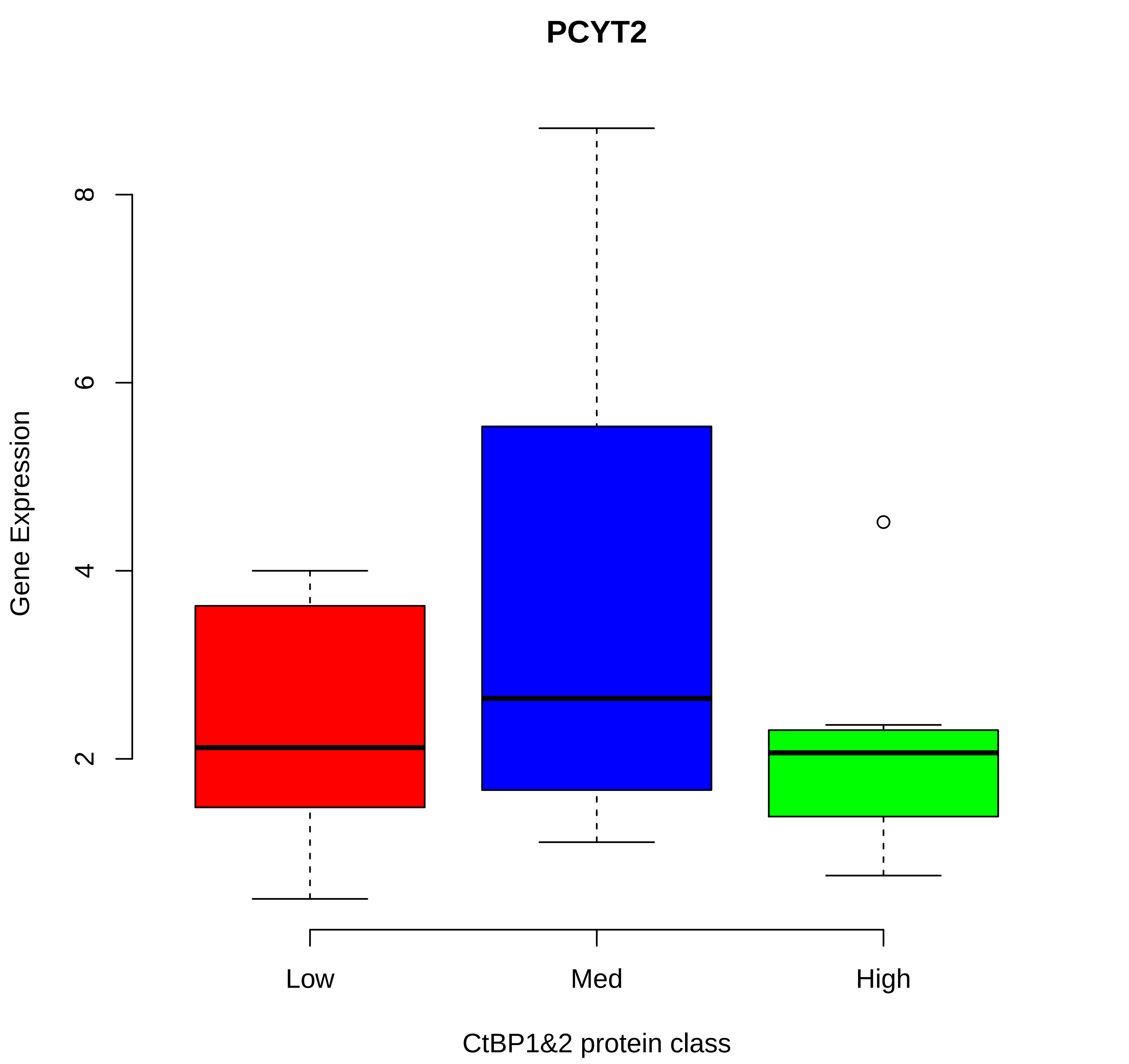
PCYT2
Gene Expression
8
6
4
2
Low
Med
High
CtBP1&2 protein class

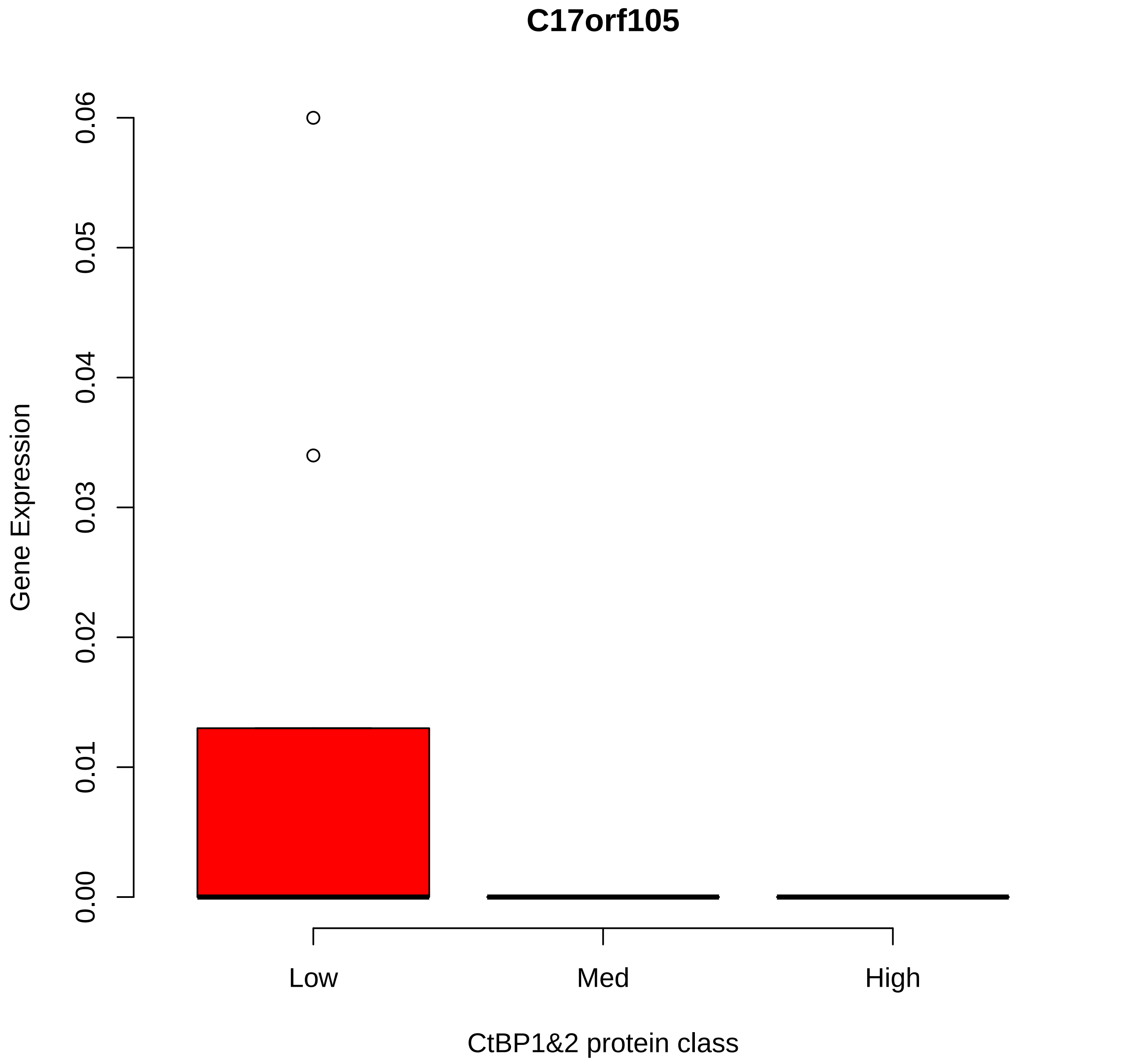
C17orf105
Gene Expression
0.00
0.01
0.02
0.03
0.04
0.05
0.06
Low
Med
High
CtBP1&2 protein class

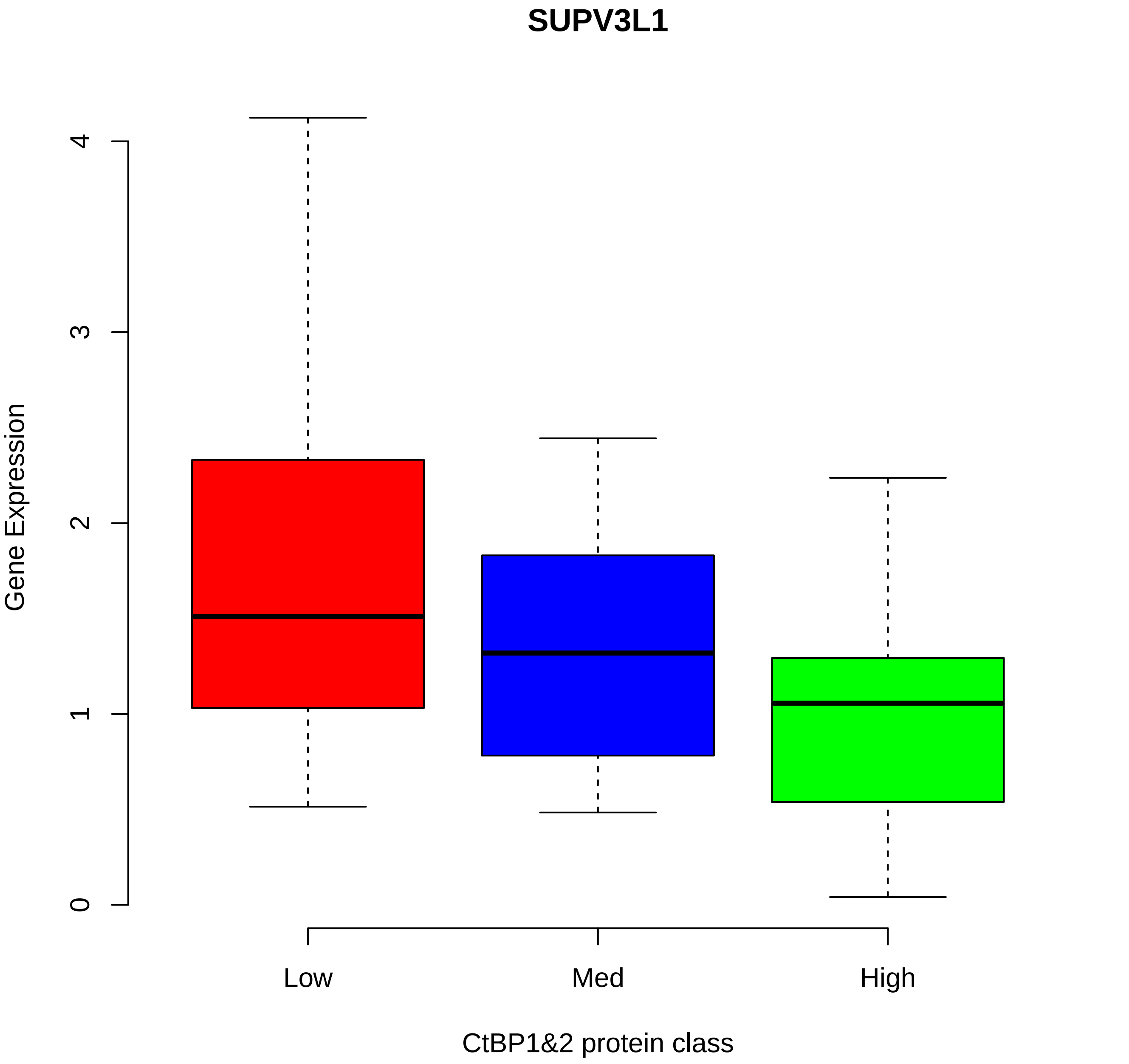
SUPV3L1
Gene Expression
0
1
2
3
4
Low
Med
High
CtBP1&2 protein class

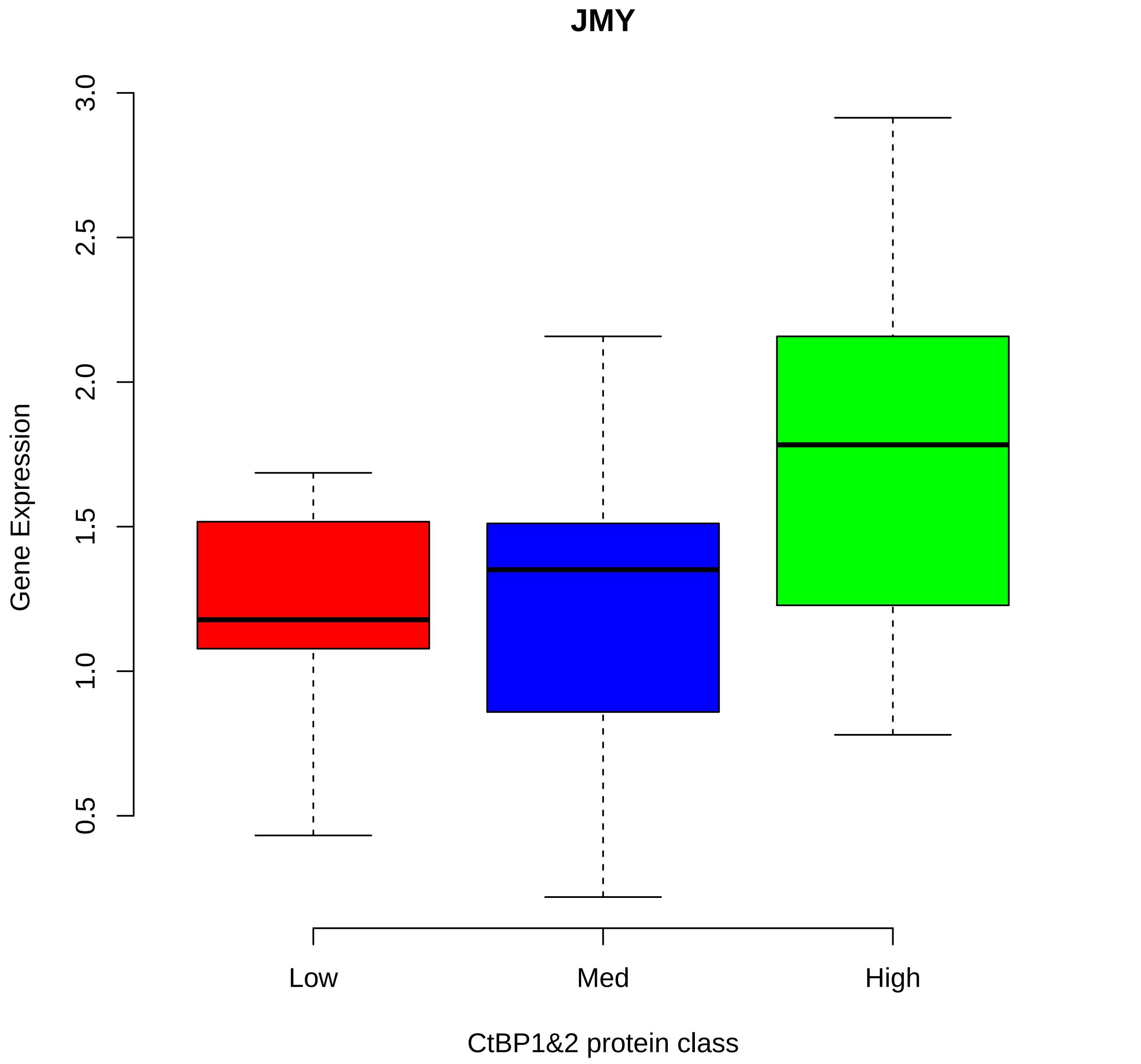
JMY
Gene Expression
3.0
2.5
2.0
1.5
1.0
0.5
Low
Med
High
CtBP1&2 protein class

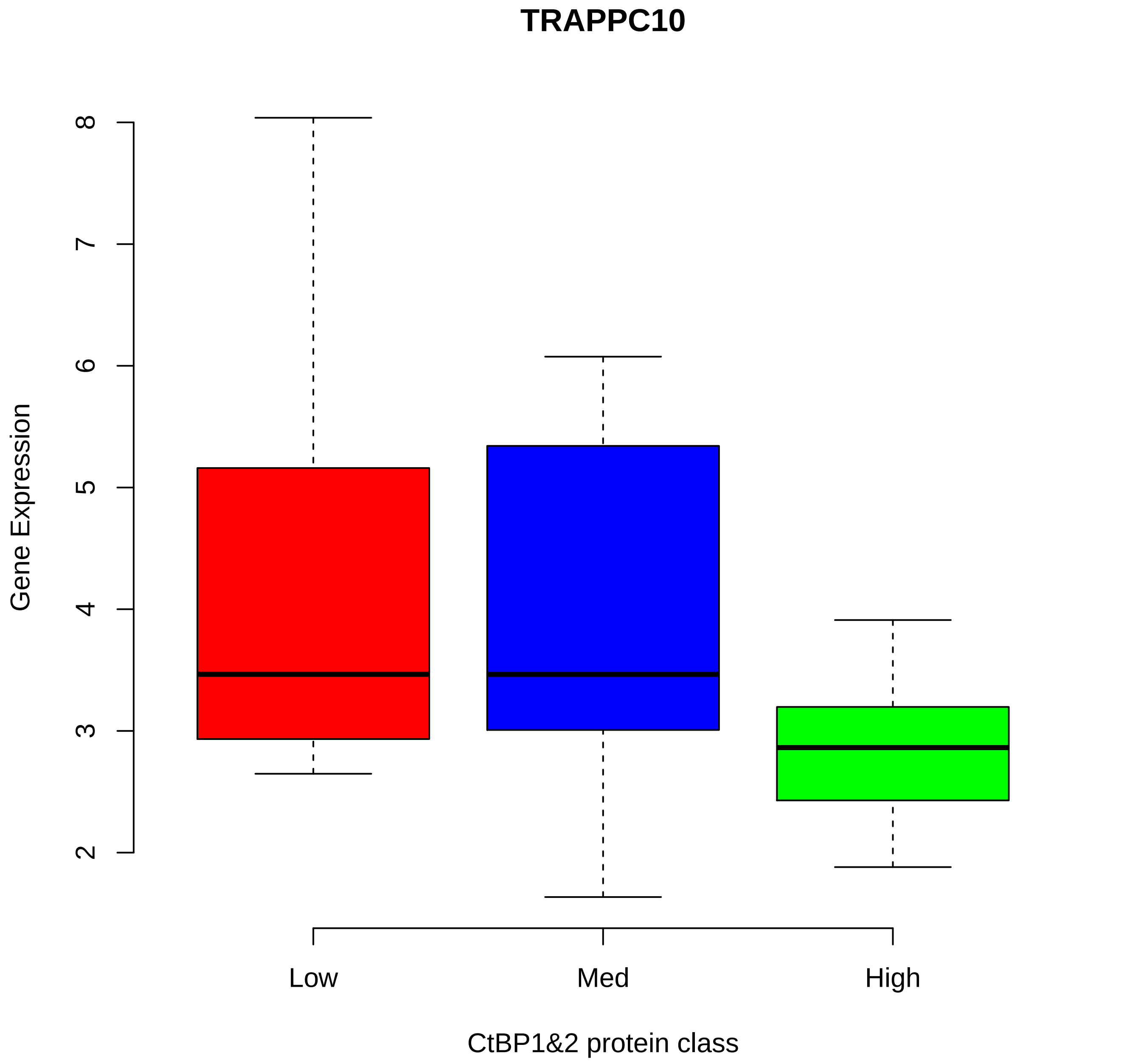
TRAPPC10
Gene Expression
2
3
4
5
6
7
8
Low
Med
High
CtBP1&2 protein class

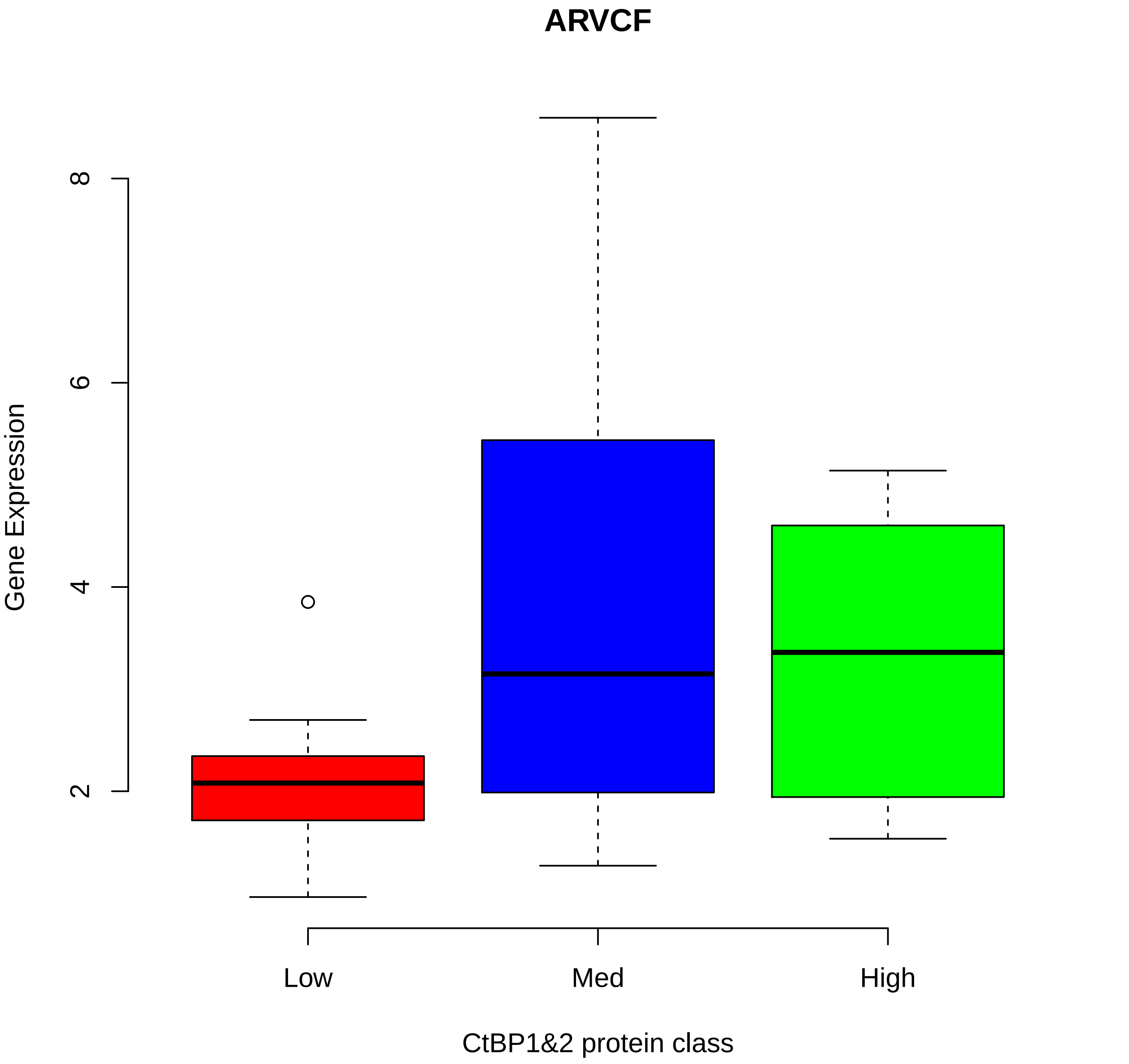
ARVCF
Gene Expression
2
4
6
8
Low
Med
High
CtBP1&2 protein class

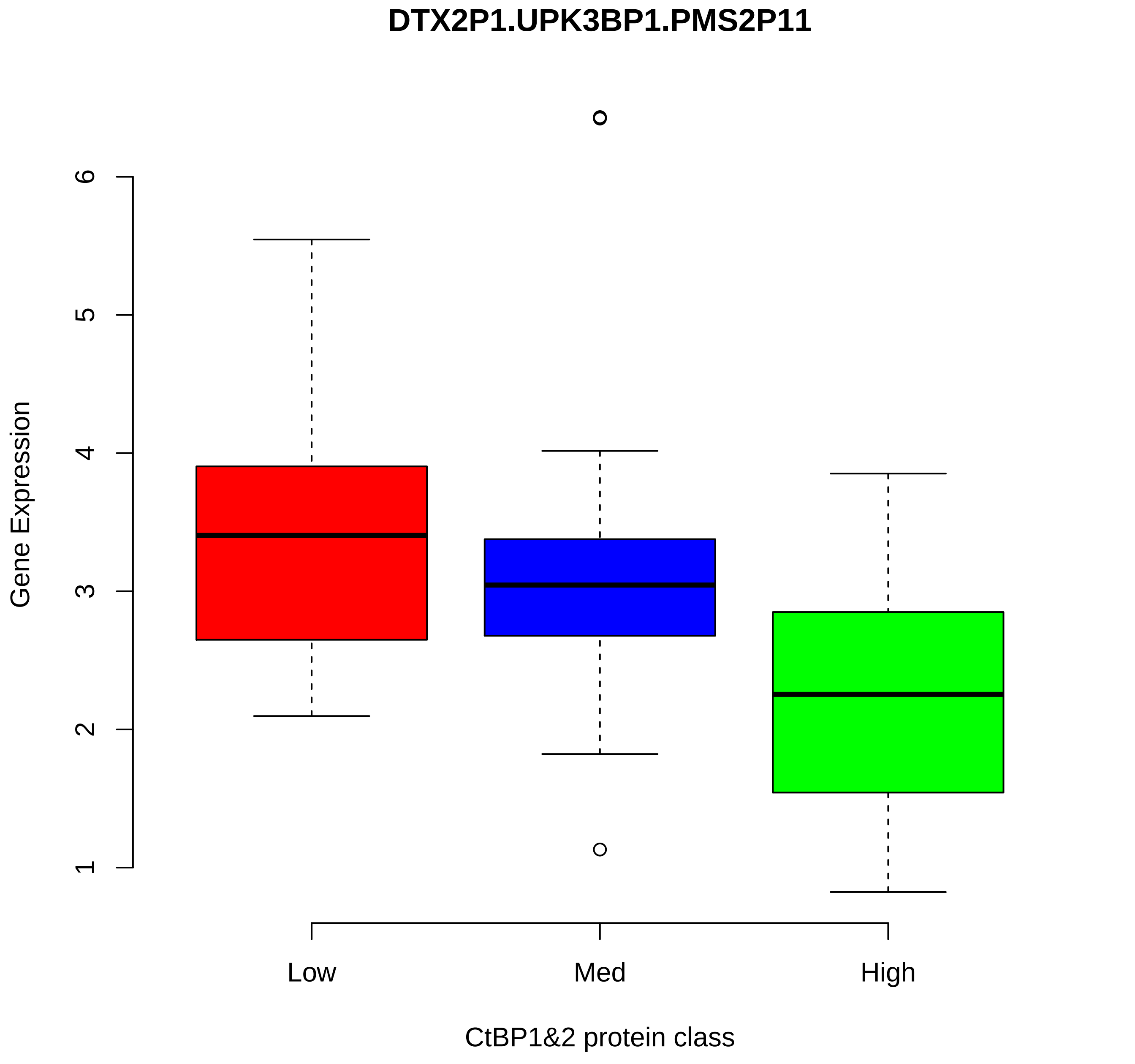
DTX2P1.UPK3BP1.PMS2P11
Gene Expression
1
2
3
4
5
6
Low
Med
High
CtBP1&2 protein class

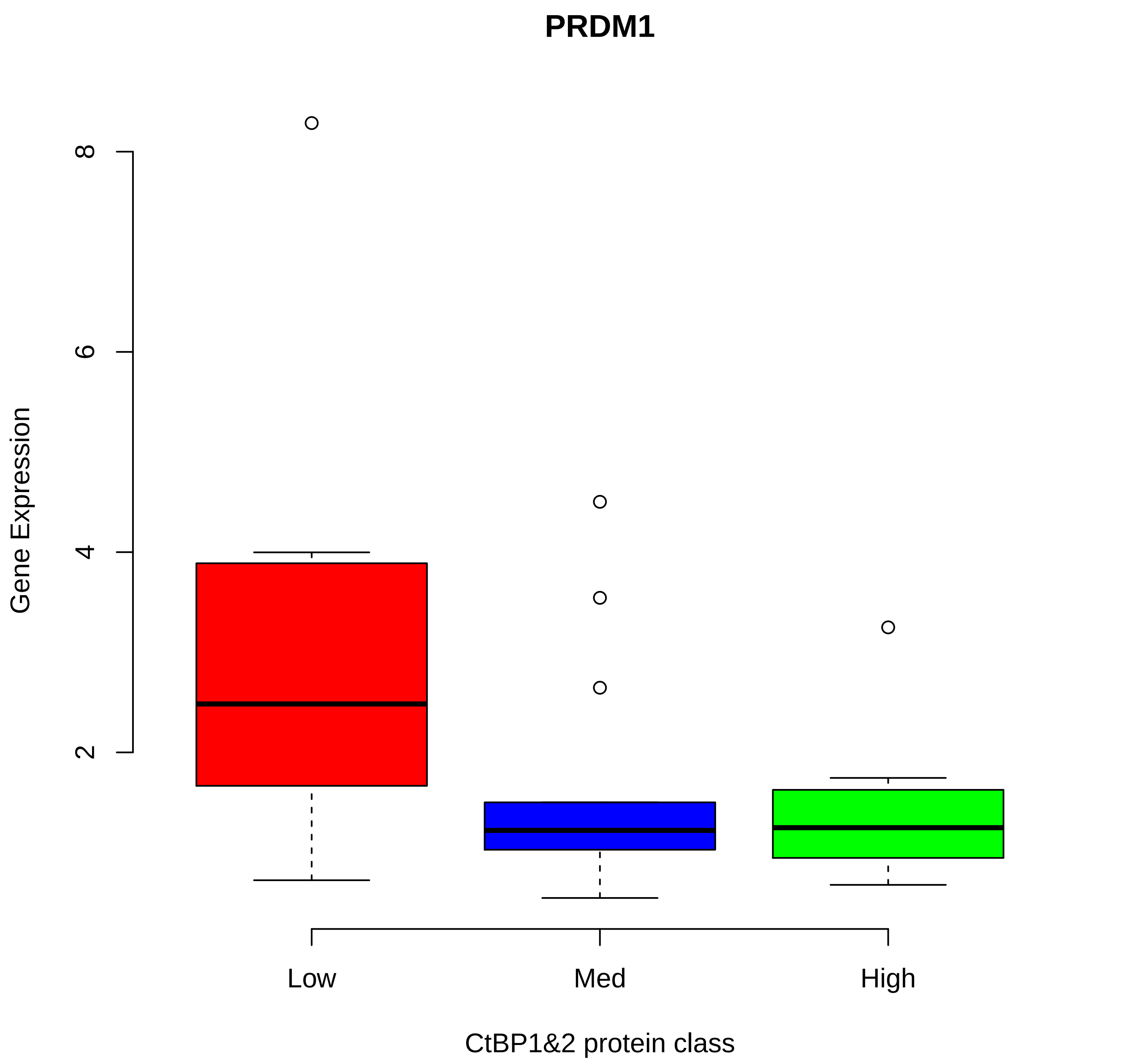
PRDM1
Gene Expression
8
6
4
2
Low
Med
High
CtBP1&2 protein class

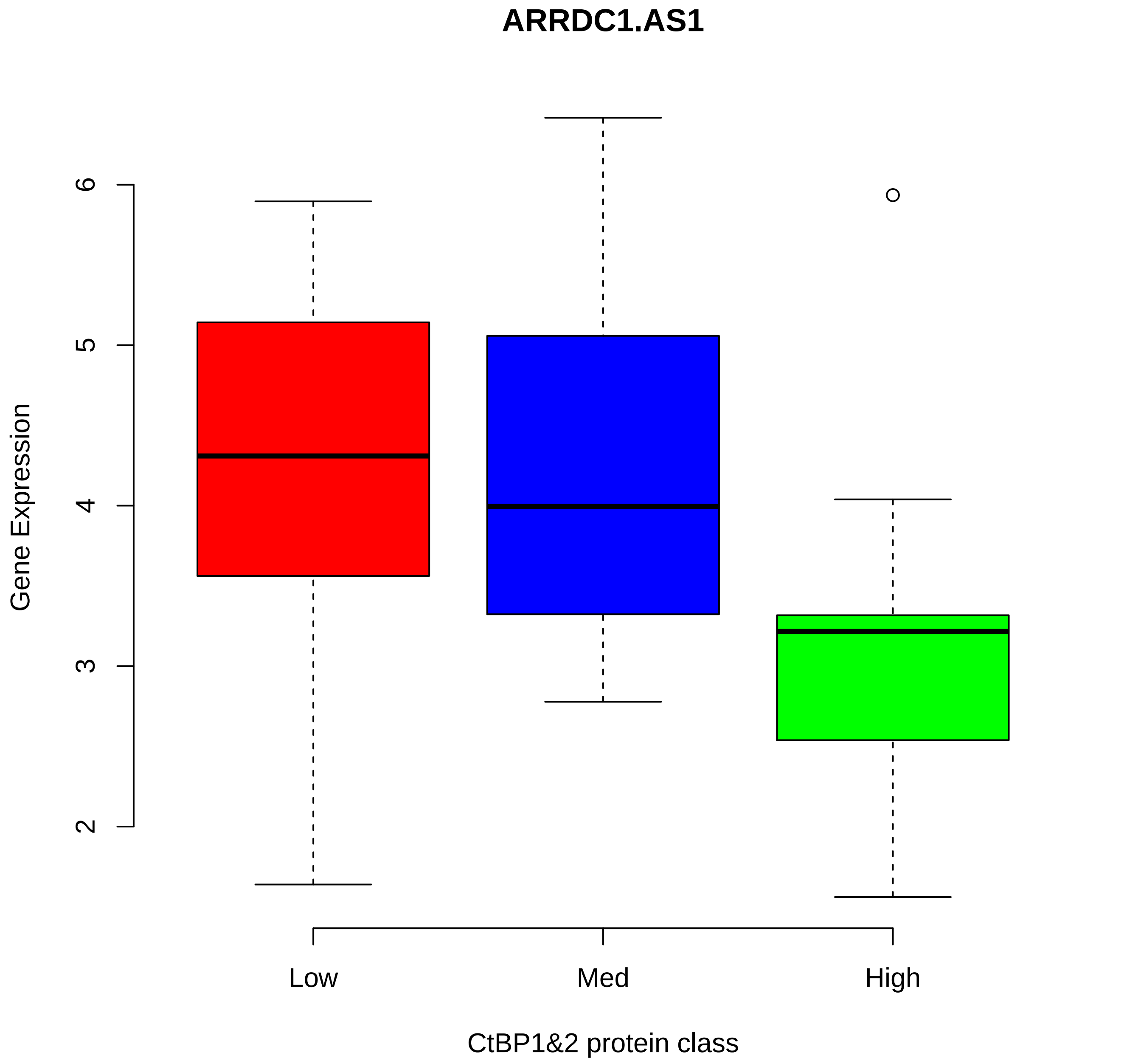
ARRDC1.AS1
Gene Expression
2
3
4
5
6
Low
Med
High
CtBP1&2 protein class

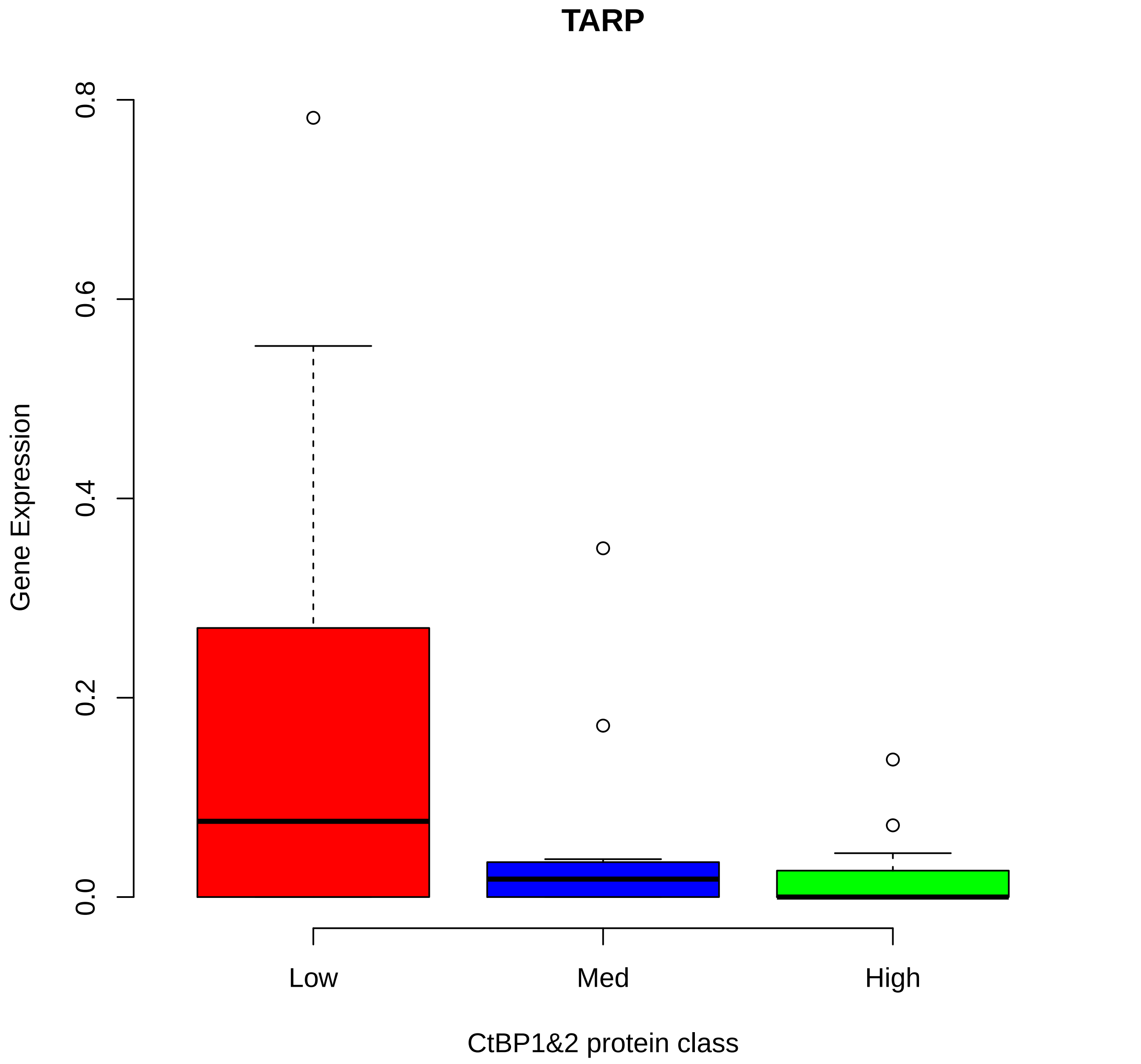

TARP
0.8
0.6
0.4
0.2
0.0
Gene Expression
Low
Med
High
CtBP1&2 protein class

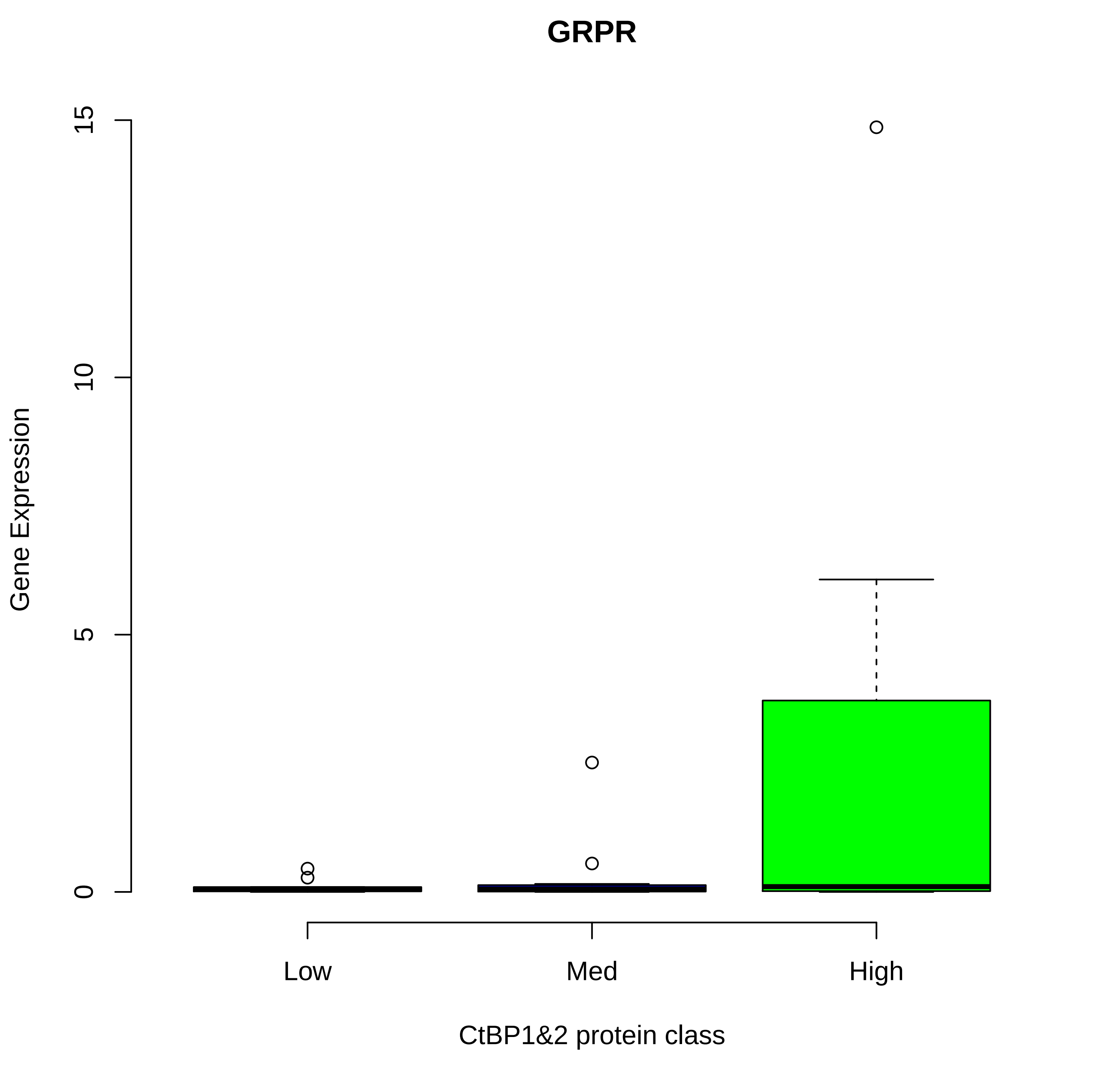
GRPR
Gene Expression
0
5
10
15
Low
Med
High
CtBP1&2 protein class

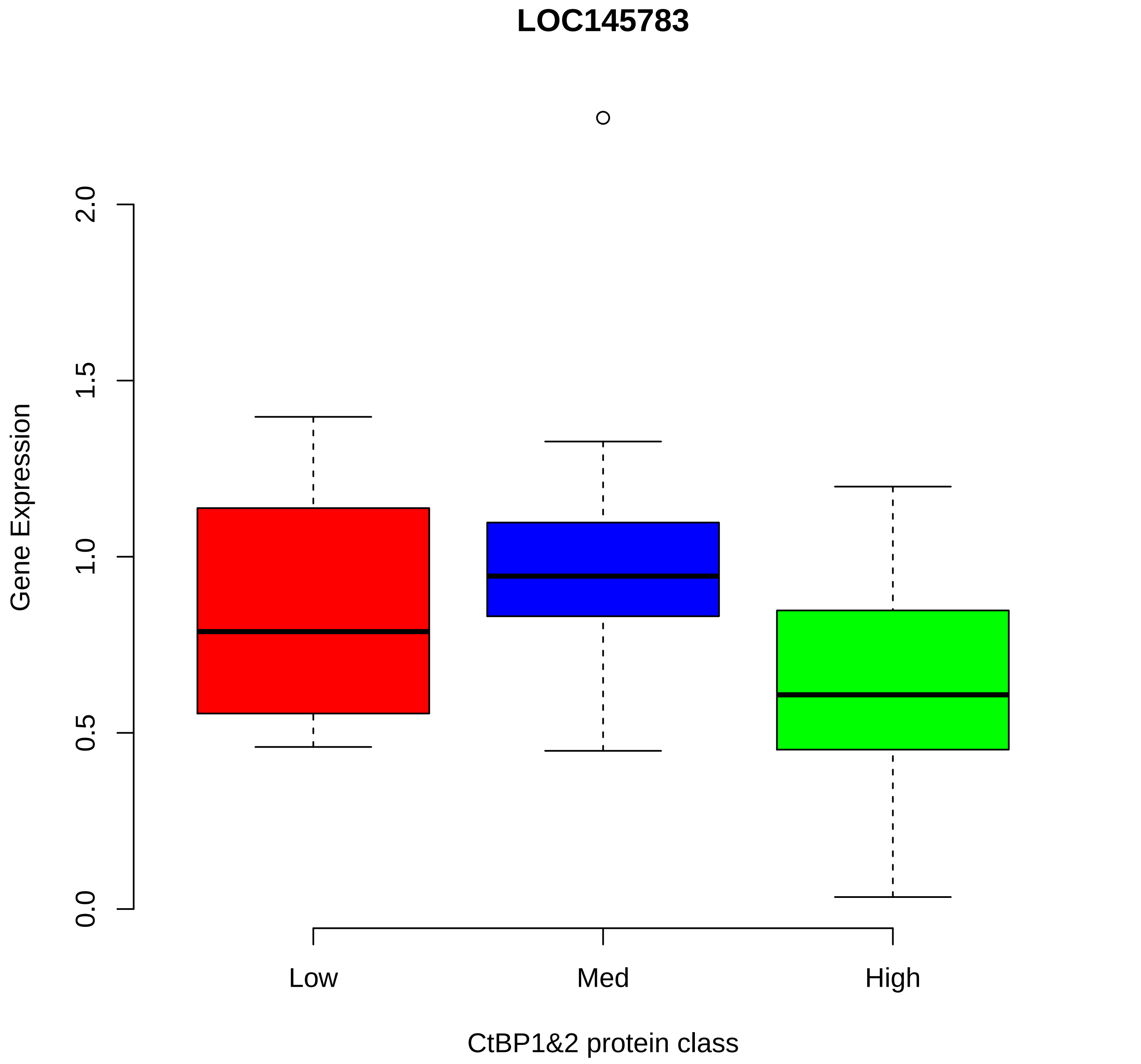
LOC145783
Gene Expression
0.0
0.5
1.0
1.5
2.0
Low
Med
High
CtBP1&2 protein class
