# Supplementary material for: Epigenetic re-wiring of breast cancer by pharmacological targeting of C-terminal binding protein
Source: Cell Death Dis. 2019 Sep 18;10(10):689. doi: 10.1038/s41419-019-1892-7 (PMC6751206; doi:10.1038/s41419-019-1892-7)
Supplement: Supplementary file 6 — Supplementary Table 5. [file 41419_2019_1892_MOESM6_ESM.pdf]

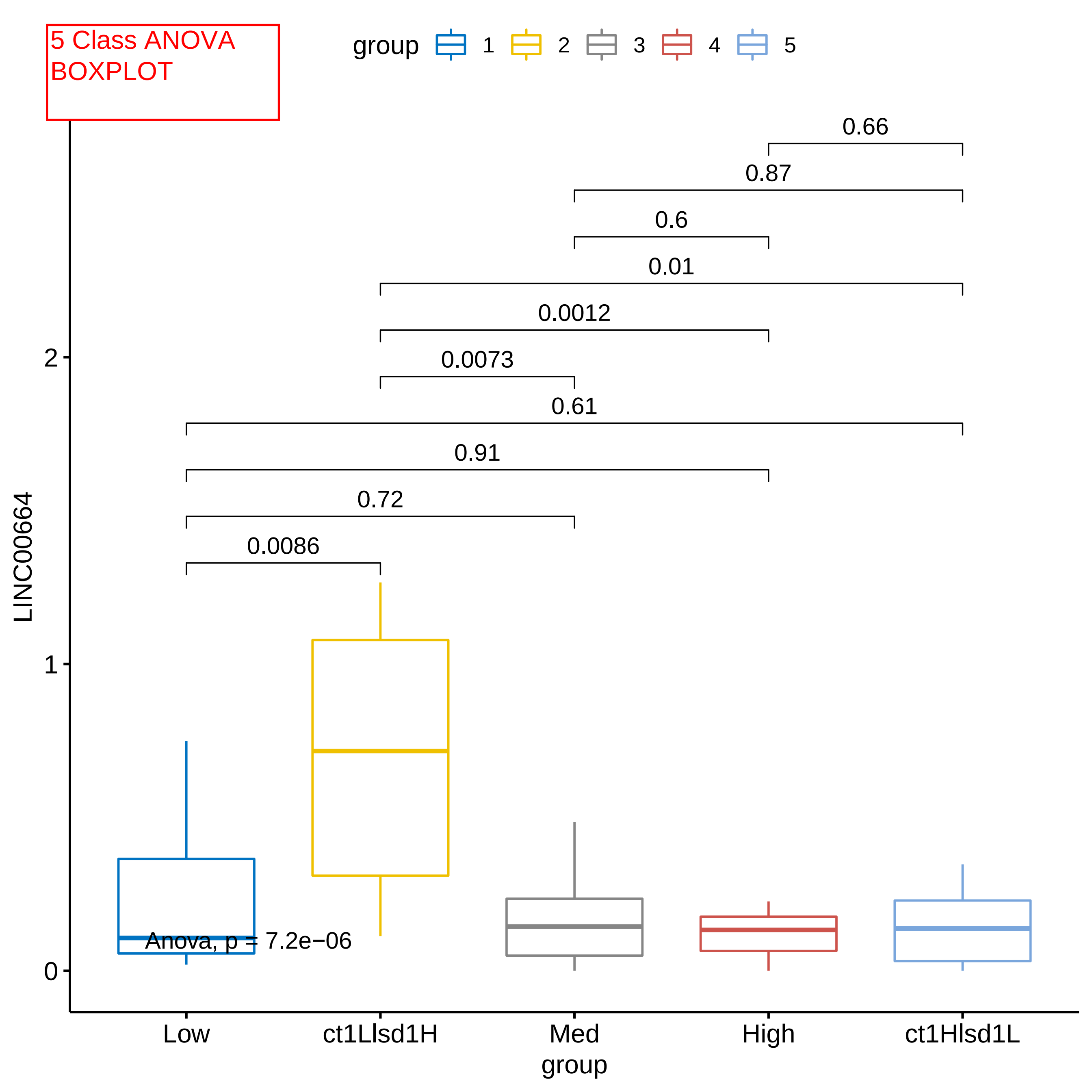

5 Class ANOVA BOXPLOT
group 1 2 3 4 5
0.66
0.87
0.6
0.01
0.0012
0.0073
0.61
0.91
0.72
0.0086
Anova, p = 7.2e−06
LINC00664
0
1
2
Low
ct1Llsd1H
Med
High
ct1Hlsd1L
group

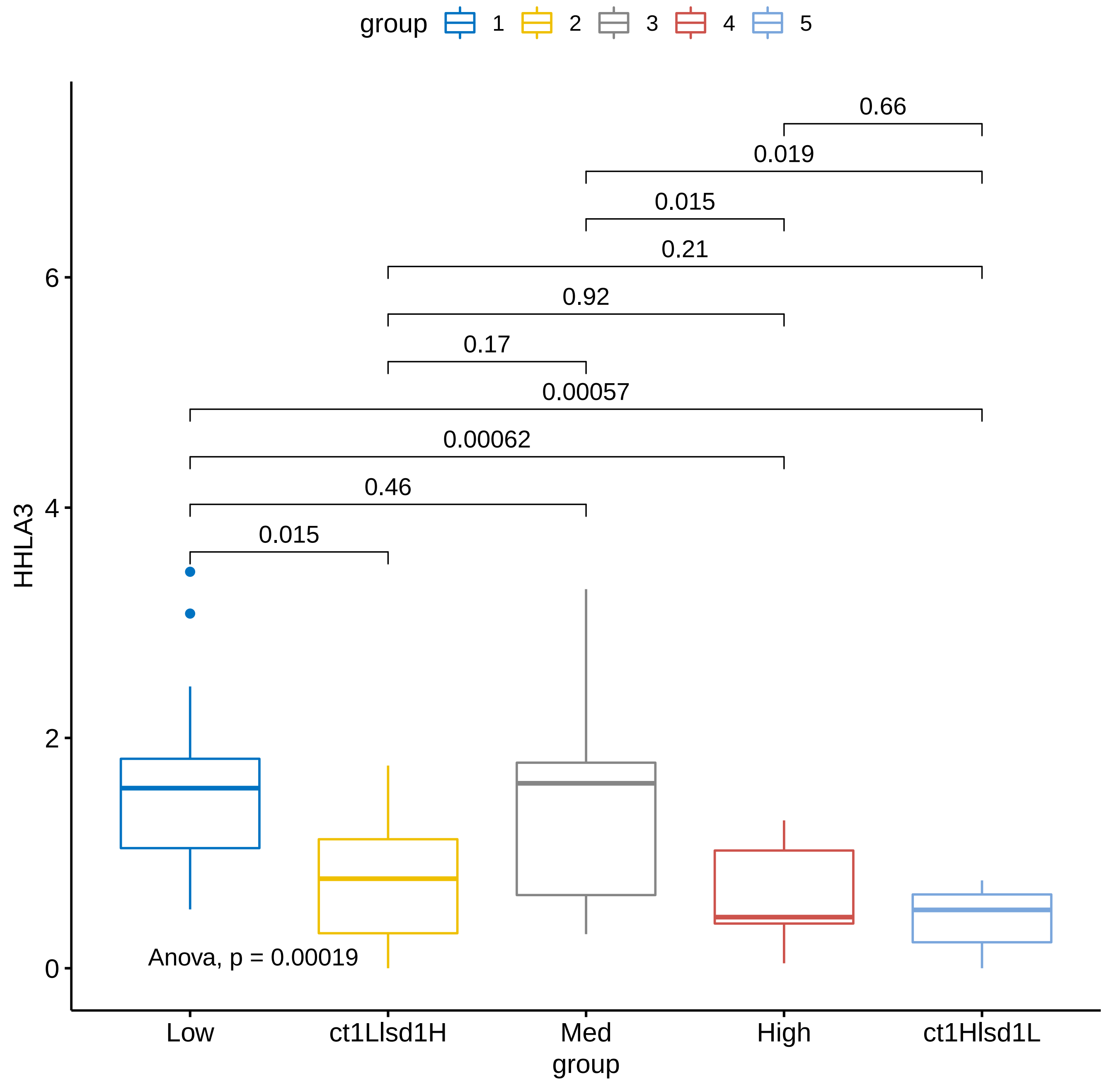
group 1 2 3 4 5
0.66
0.019
0.015
0.21
0.92
0.17
0.00057
0.00062
0.46
0.015
Anova, p = 0.00019
HHLA3
0
2
4
6
Low
ct1Llsd1H
Med
High
ct1Hlsd1L
group

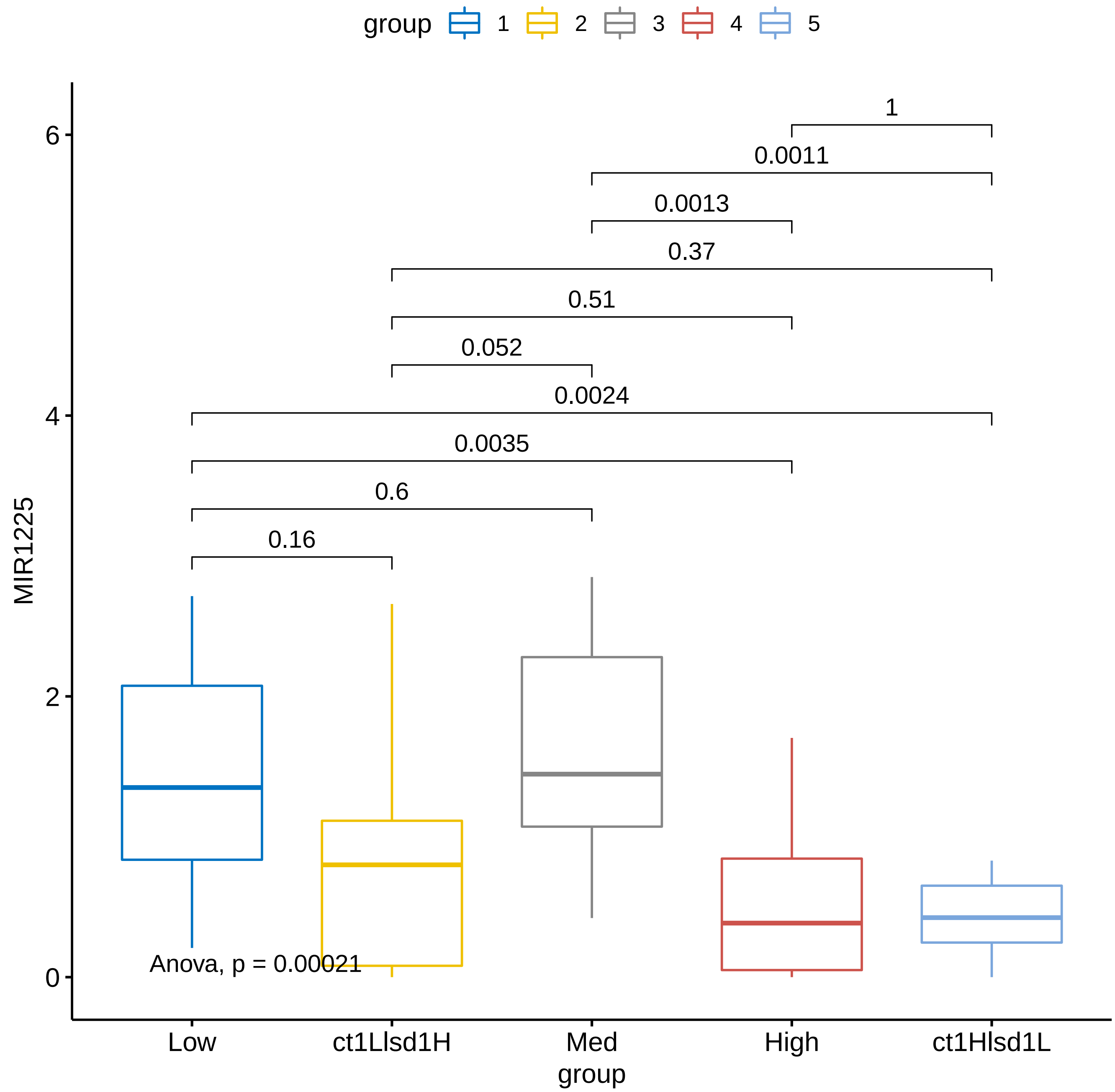

group 1 2 3 4 5
MIR1225
6
4
2
0
1
0.0011
0.0013
0.37
0.51
0.052
0.0024
0.0035
0.6
0.16
Anova, p = 0.00021
Low
ct1Llsd1H
Med
High
ct1Hlsd1L
group

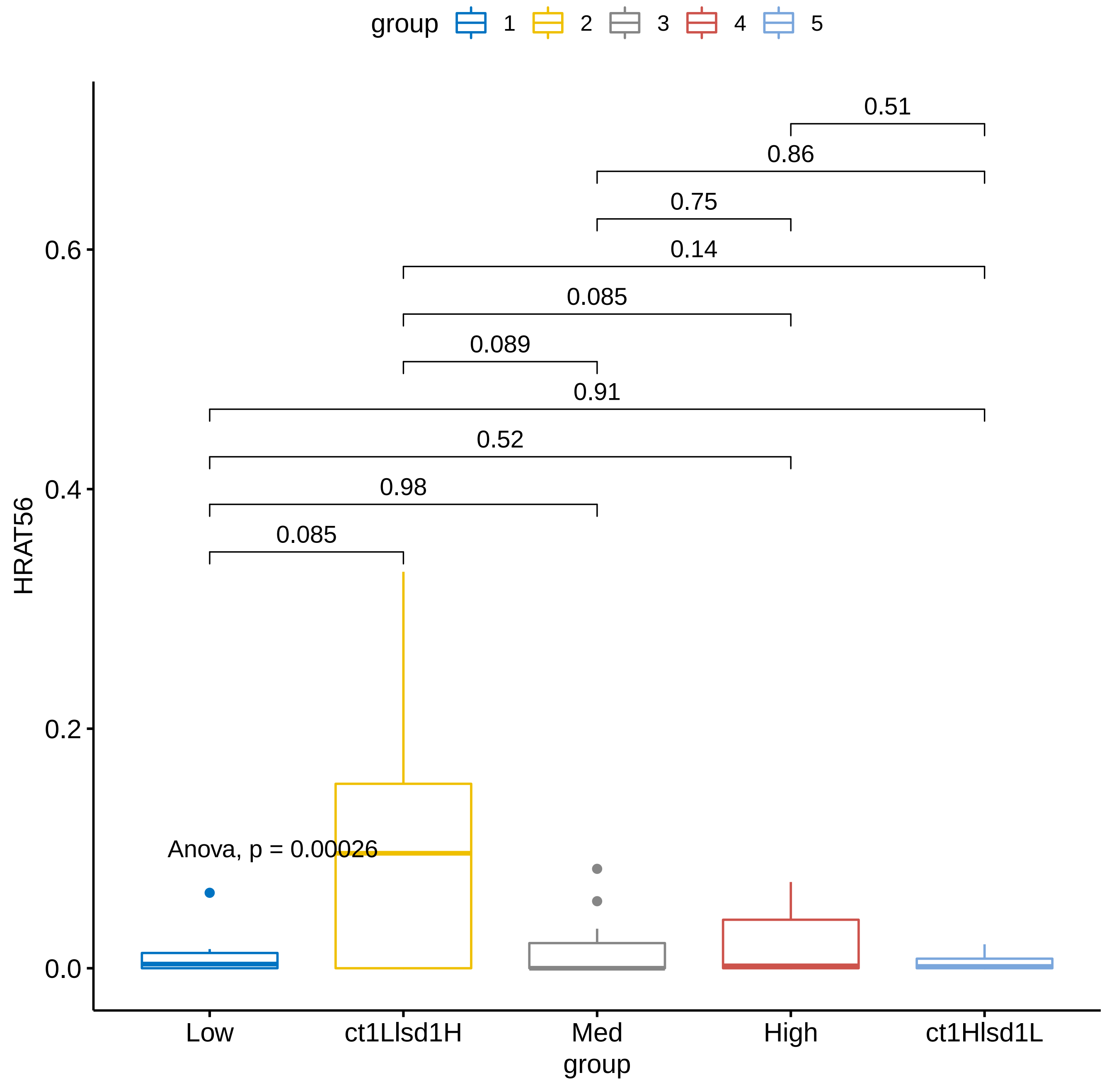
group
1
2
3
4
5
0.51
0.86
0.75
0.14
0.085
0.089
0.91
0.52
0.98
0.085
Anova, p = 0.00026
0.6
0.4
0.2
0.0
HRAT56
Low
ct1Llsd1H
Med
High
ct1Hlsd1L
group

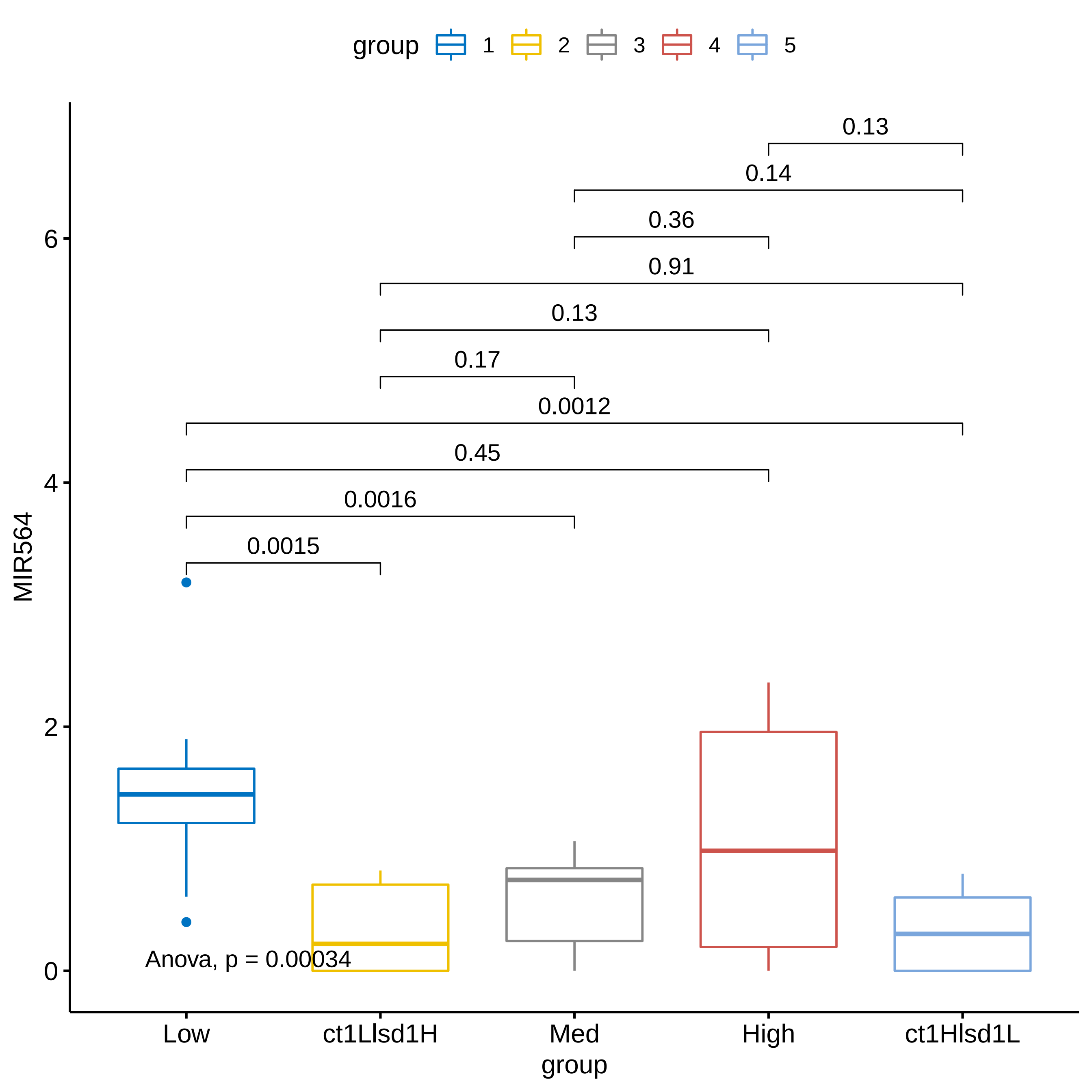

group
1
2
3
4
5
0.13
0.14
0.36
0.91
0.13
0.17
0.0012
0.45
0.0016
0.0015
Anova, p = 0.00034
MIR564
6
4
2
0
Low
ct1Llsd1H
Med
High
ct1Hlsd1L
group

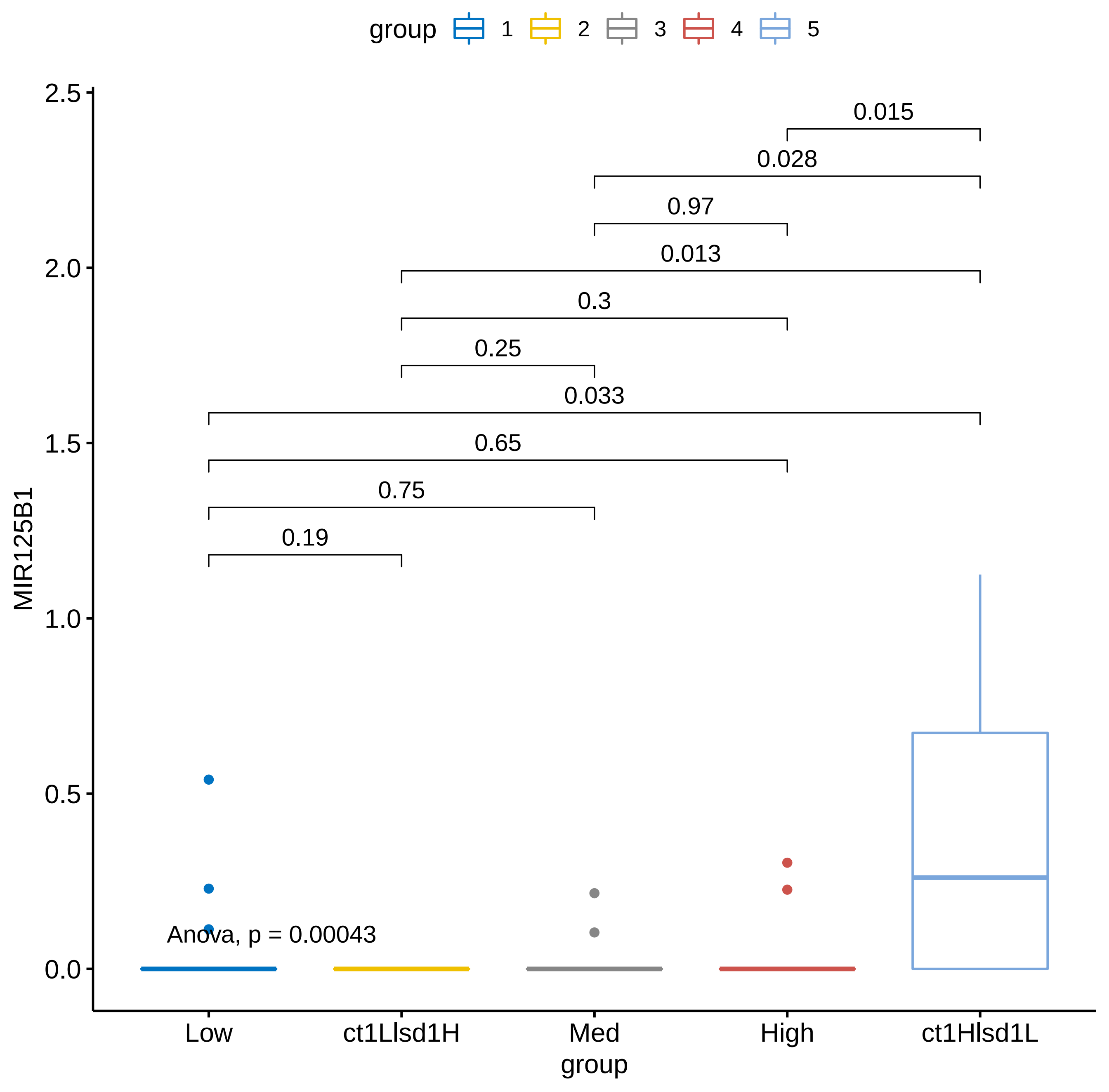

group
1
2
3
4
5
MIR125B1
2.5
2.0
1.5
1.0
0.5
0.0
0.015
0.028
0.97
0.013
0.3
0.25
0.033
0.65
0.75
0.19
Anova, p = 0.00043
Low
ct1Llsd1H
Med
High
ct1Hlsd1L
group

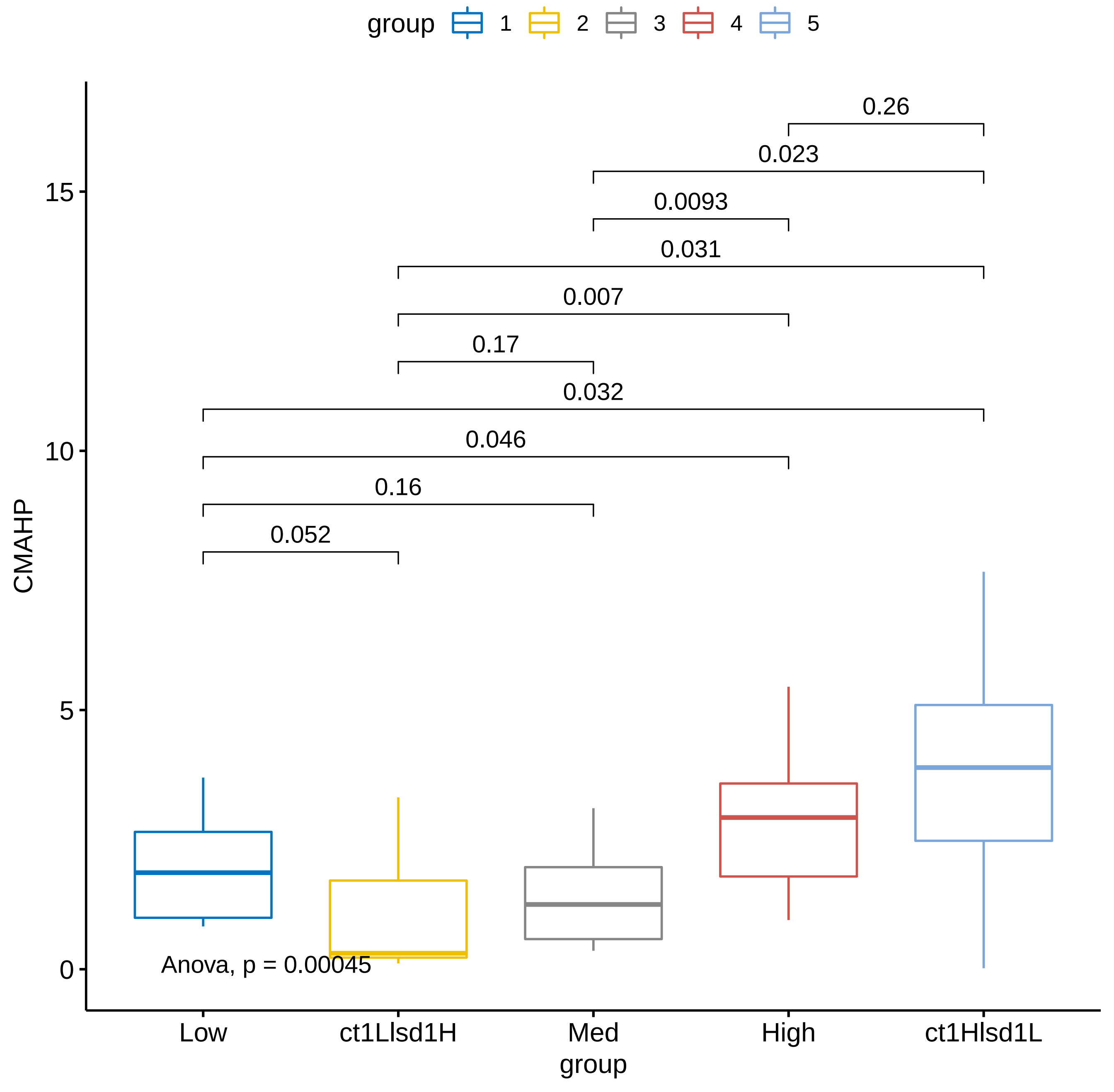

group 1 2 3 4 5
CMAHP
15
10
5
0
0.26
0.023
0.0093
0.031
0.007
0.17
0.032
0.046
0.16
0.052
Anova, p = 0.00045
Low
ct1Llsd1H
Med
High
ct1Hlsd1L
group

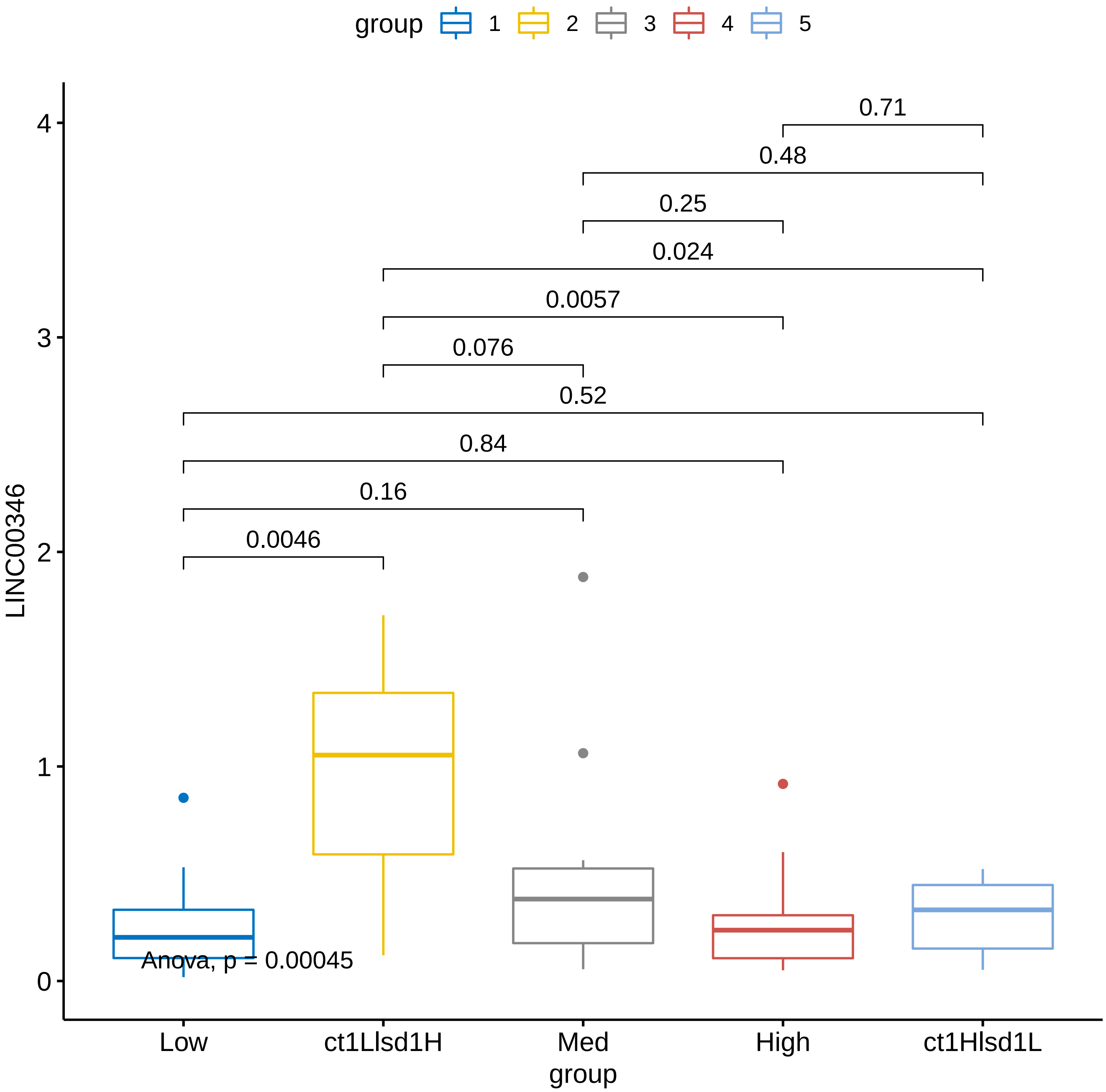
group
1
2
3
4
5
LINC00346
0.71
0.48
0.25
0.024
0.0057
0.076
0.52
0.84
0.16
0.0046
Anova, p = 0.00045
4
3
2
1
0
Low
ct1Llsd1H
Med
High
ct1Hlsd1L
group

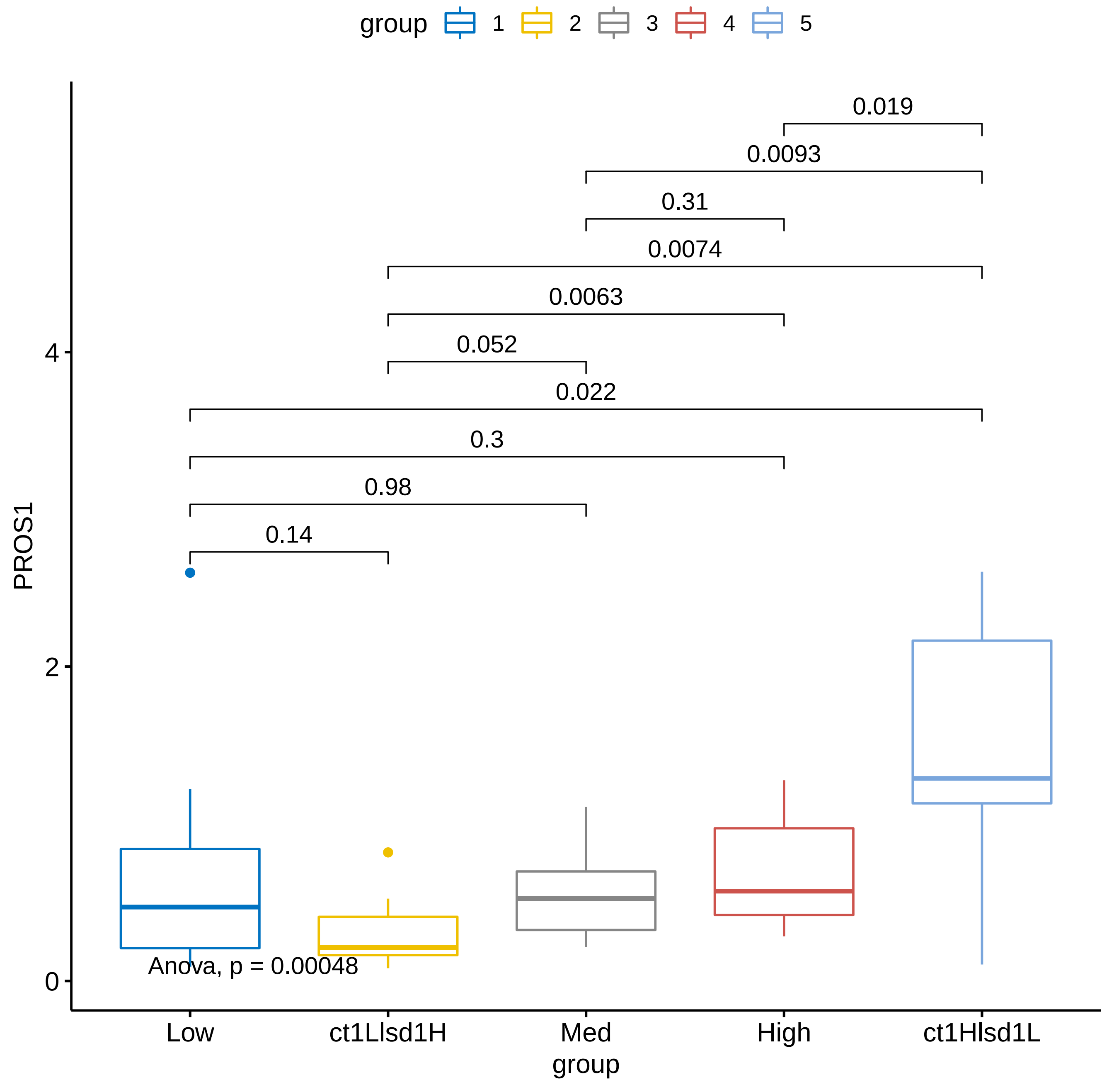
group
1
2
3
4
5
0.019
0.0093
0.31
0.0074
0.0063
0.052
0.022
0.3
0.98
0.14
PROS1
4
2
0
Anova, p = 0.00048
Low
ct1Llsd1H
Med
High
ct1Hlsd1L
group

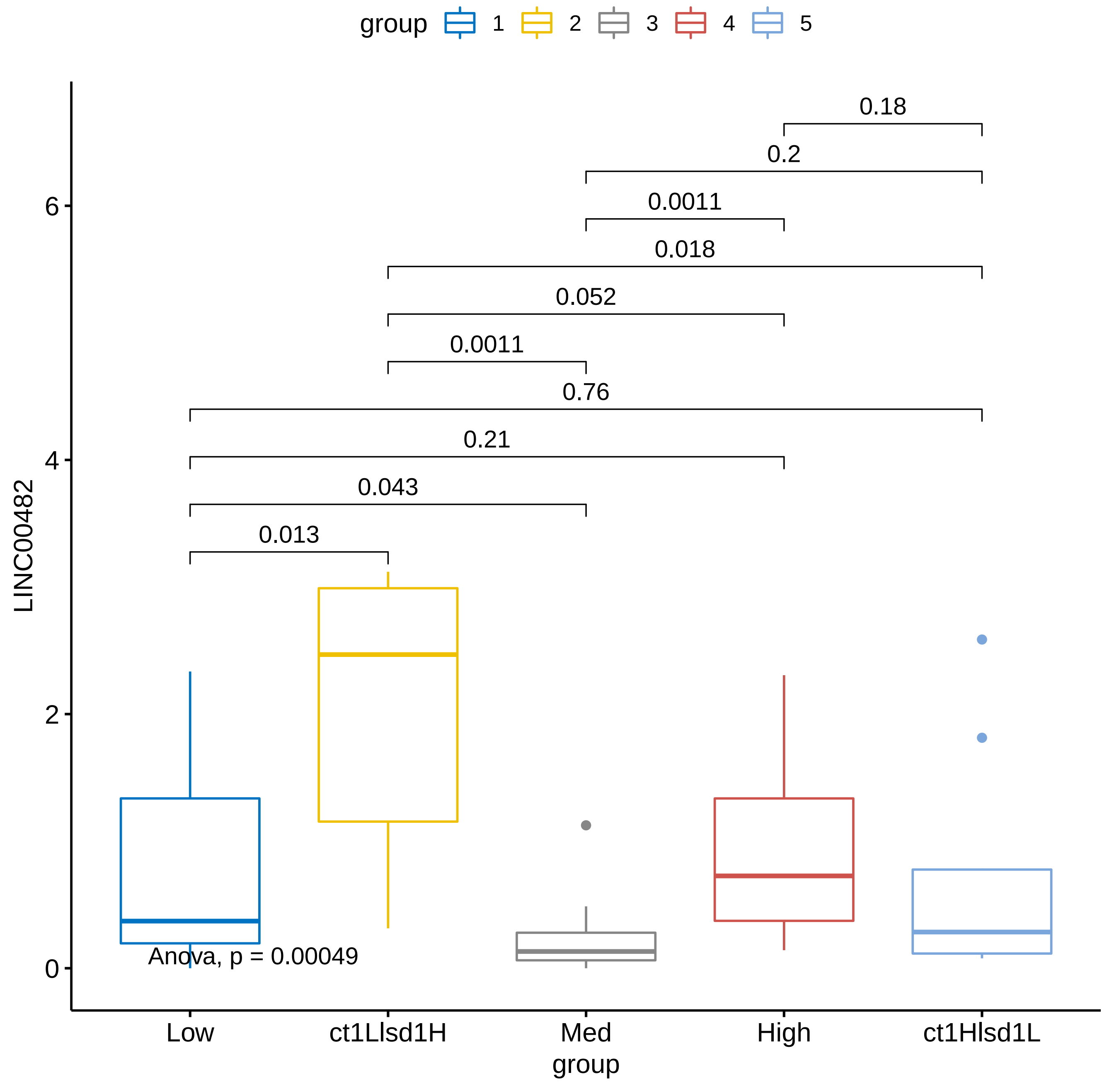
group
1
2
3
4
5
0.18
0.2
0.0011
0.018
0.052
0.0011
0.76
0.21
0.043
0.013
LINC00482
6
4
2
0
Anova, p = 0.00049
Low
ct1Llsd1H
Med
High
ct1Hlsd1L
group

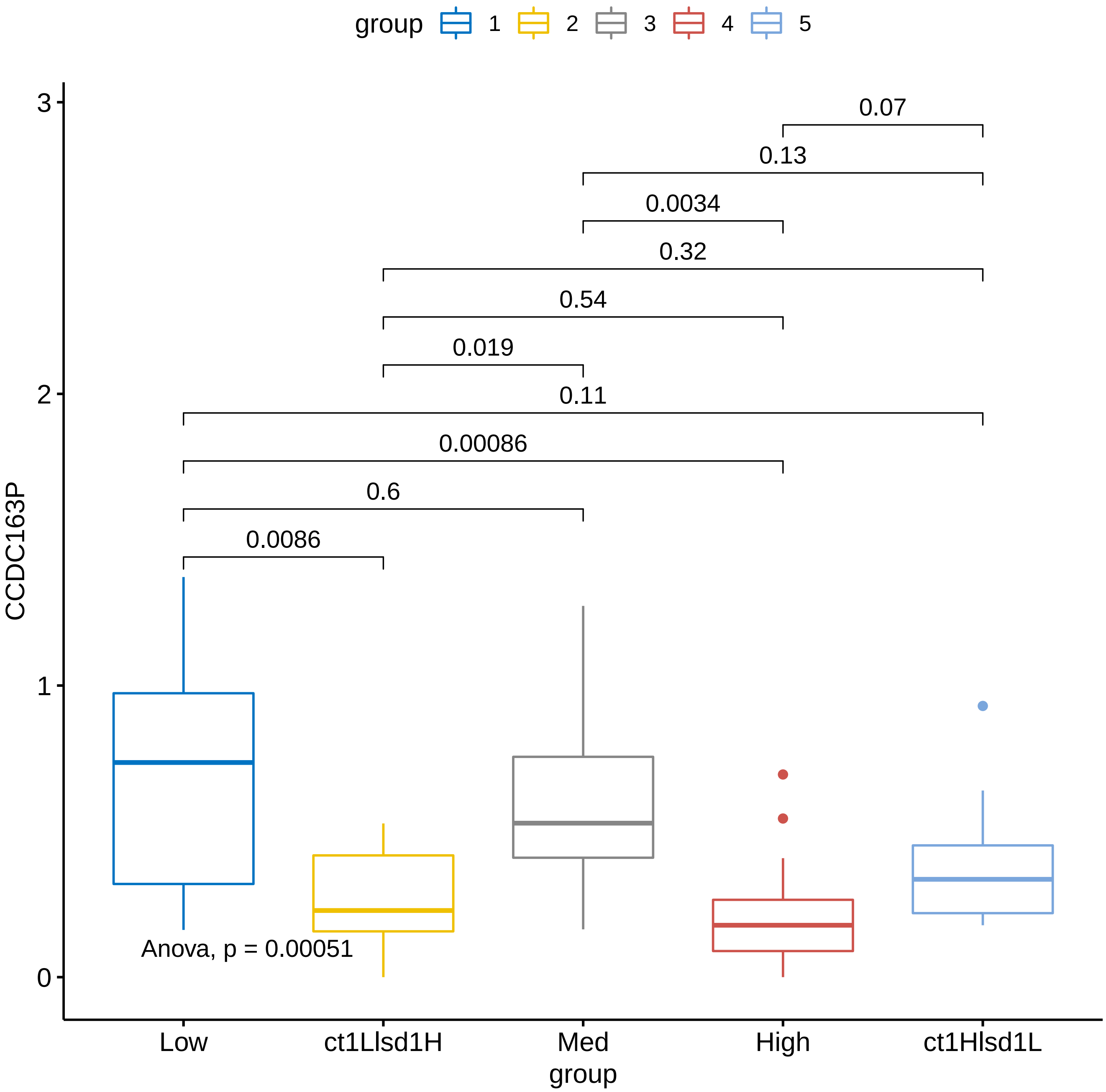

group 1 2 3 4 5
CCDC163P
3
2
1
0
0.07
0.13
0.0034
0.32
0.54
0.019
0.11
0.00086
0.6
0.0086
Anova, p = 0.00051
Low
ct1Llsd1H
Med
High
ct1Hlsd1L
group

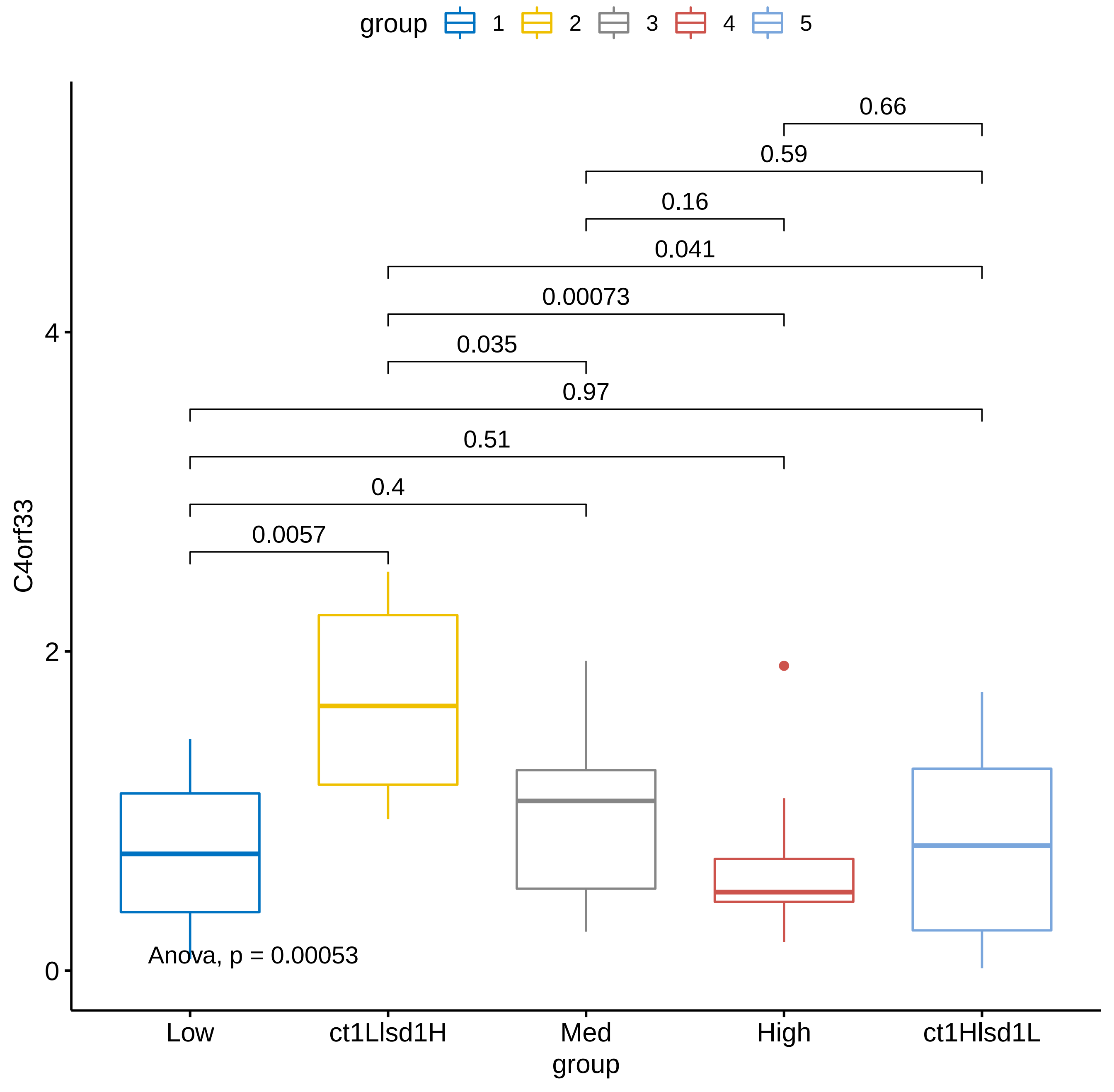
group
1
2
3
4
5
0.66
0.59
0.16
0.041
0.00073
0.035
0.97
0.51
0.4
0.0057
C4orf33
4
2
0
Anova, p = 0.00053
Low
ct1Llsd1H
Med
High
ct1Hlsd1L
group

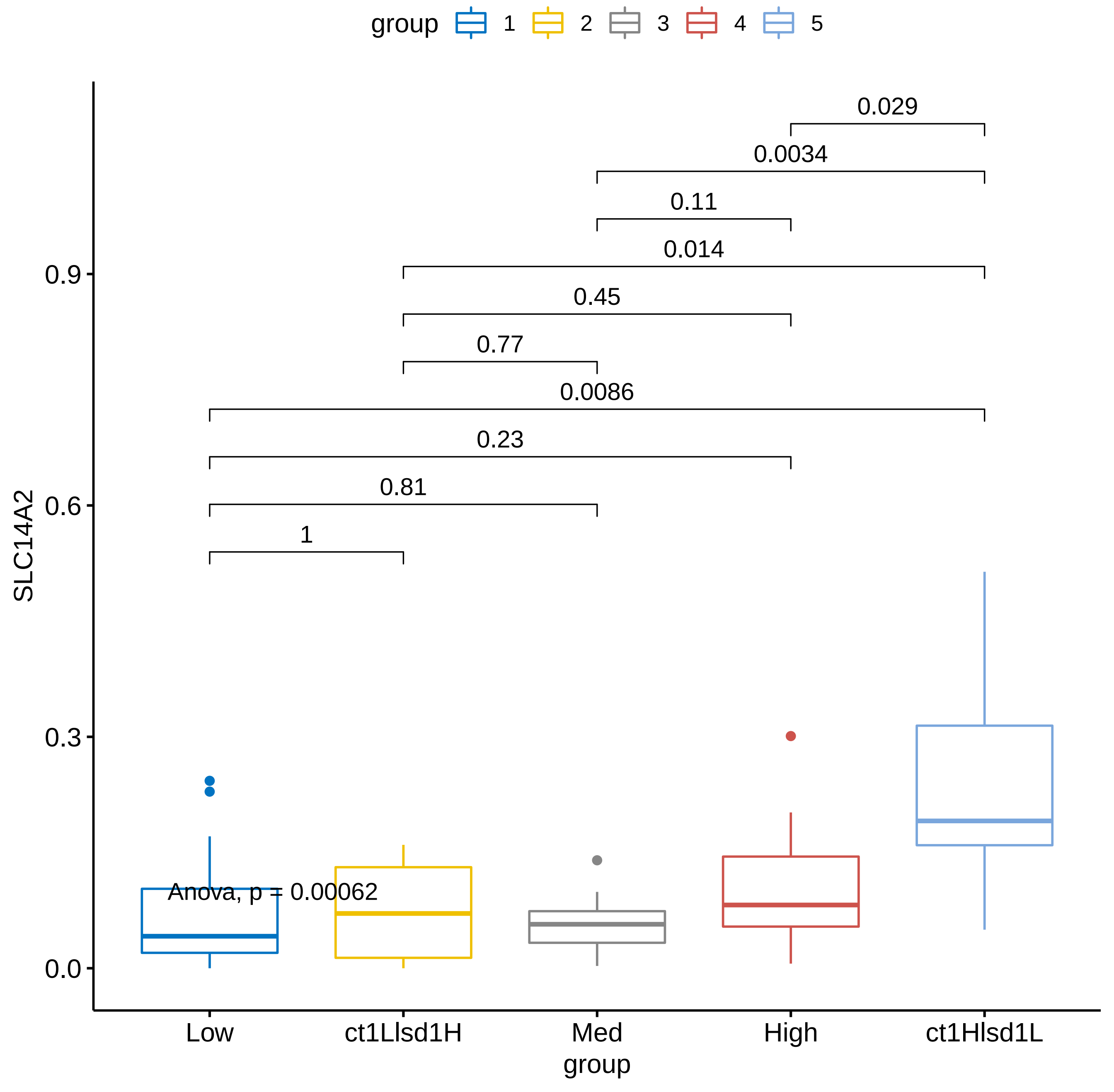
group
1
2
3
4
5
0.029
0.0034
0.11
0.014
0.45
0.77
0.0086
0.23
0.81
1
Anova, p = 0.00062
SLC14A2
0.9
0.6
0.3
0.0
Low
ct1Llsd1H
Med
High
ct1Hlsd1L
group

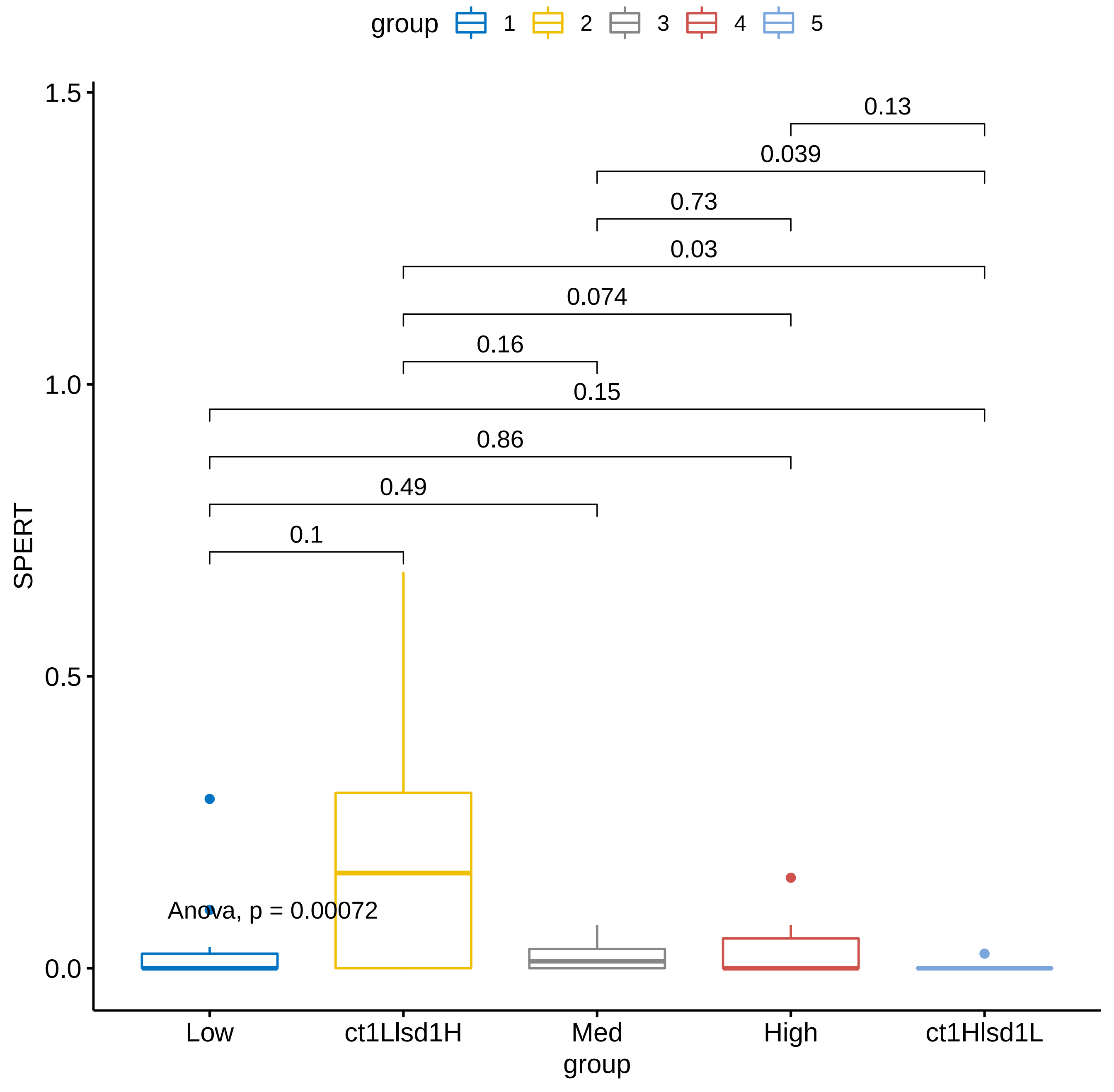

group 1 2 3 4 5
1.5
1.0
0.5
0.0
SPERT
0.13
0.039
0.73
0.03
0.074
0.16
0.15
0.86
0.49
0.1
Anova, p = 0.00072
Low
ct1Llsd1H
Med
High
ct1Hlsd1L
group

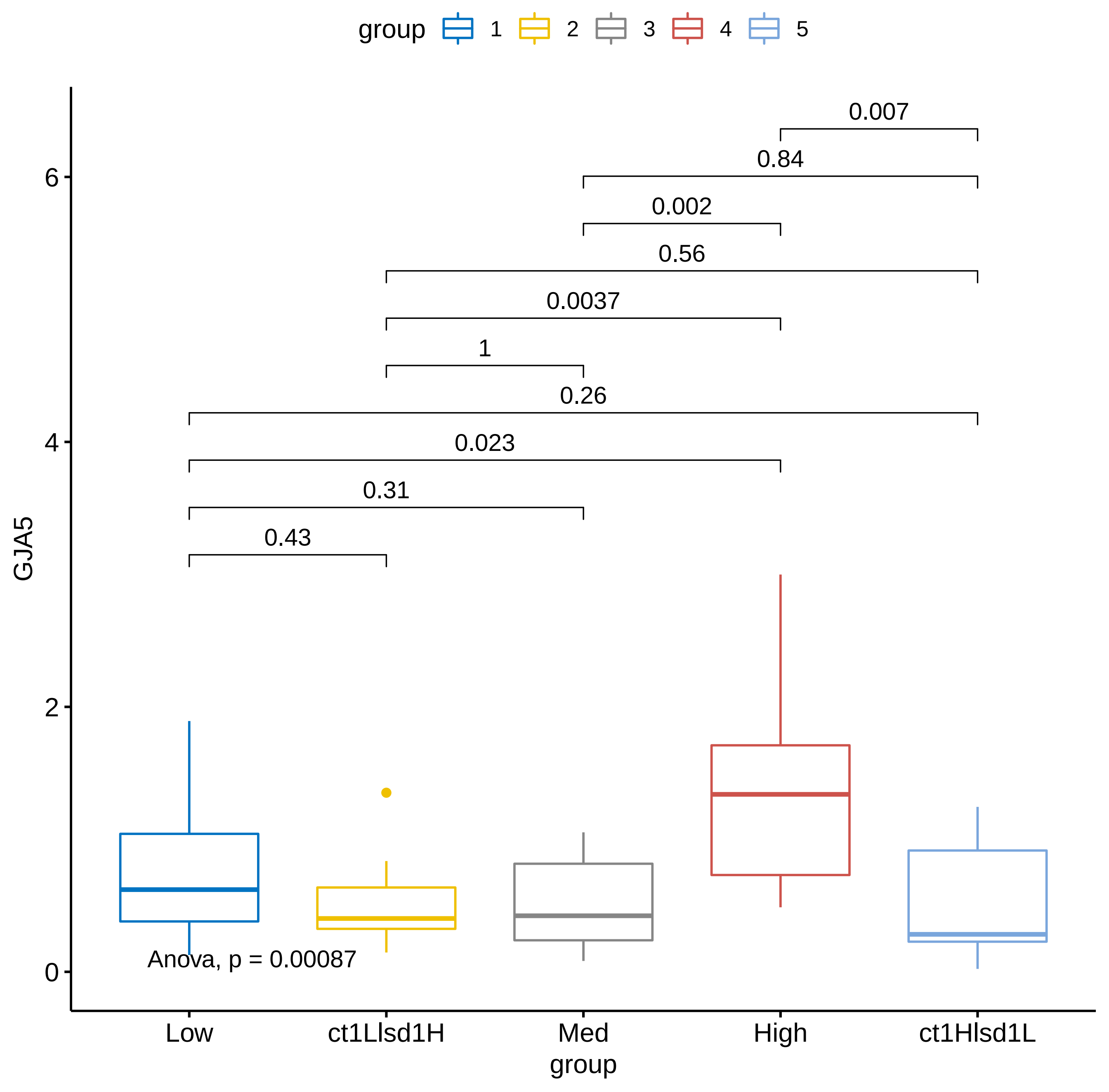
group
1
2
3
4
5
0.007
0.84
0.002
0.56
0.0037
1
0.26
0.023
0.31
0.43
Anova, p = 0.00087
GJA5
6
4
2
0
Low
ct1Llsd1H
Med
High
ct1Hlsd1L
group

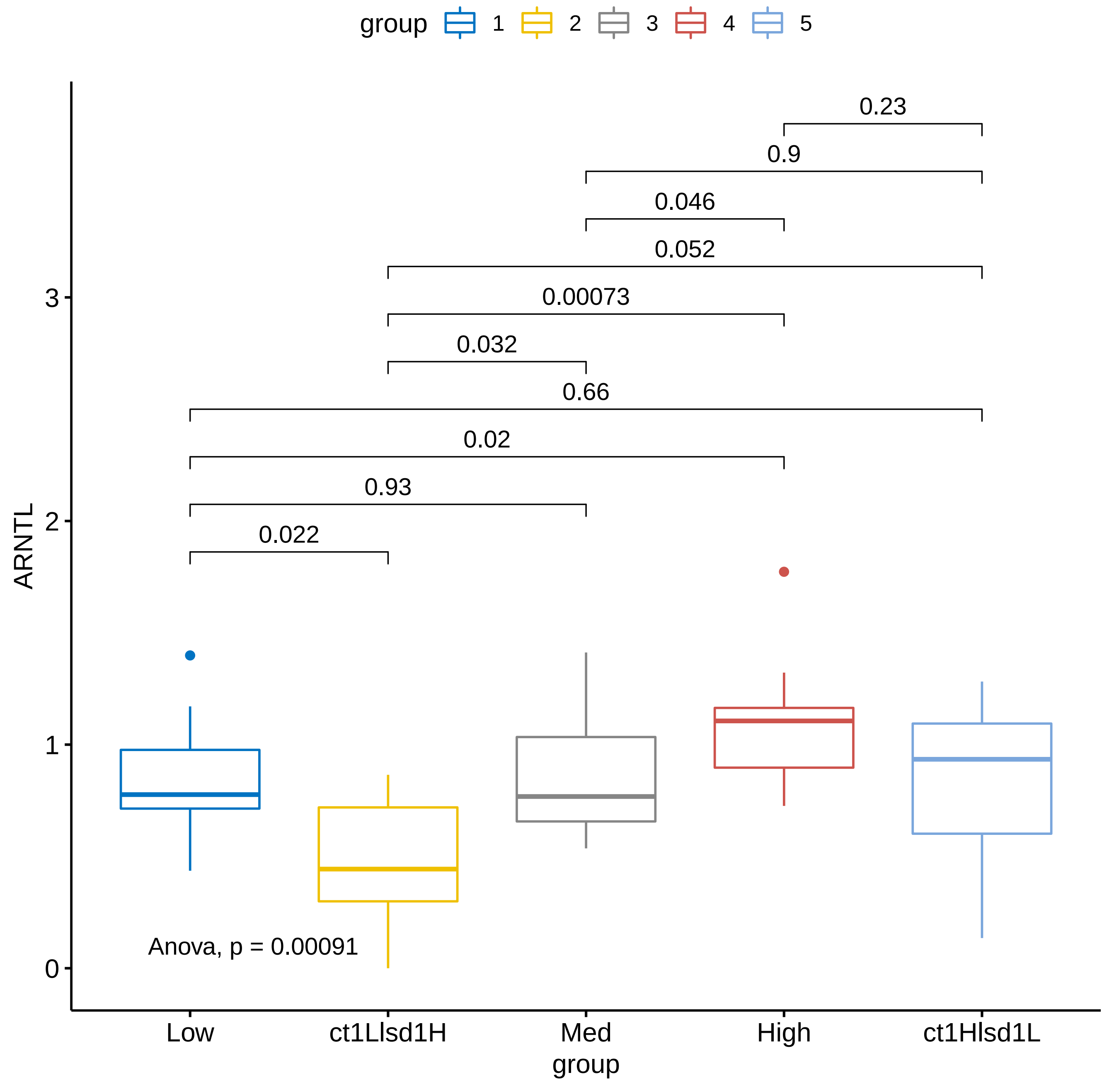

group
1
2
3
4
5
ARNTL
0
1
2
3
0.23
0.9
0.046
0.052
0.00073
0.032
0.66
0.02
0.93
0.022
Anova, p = 0.00091
Low
ct1Llsd1H
Med
High
ct1Hlsd1L
group

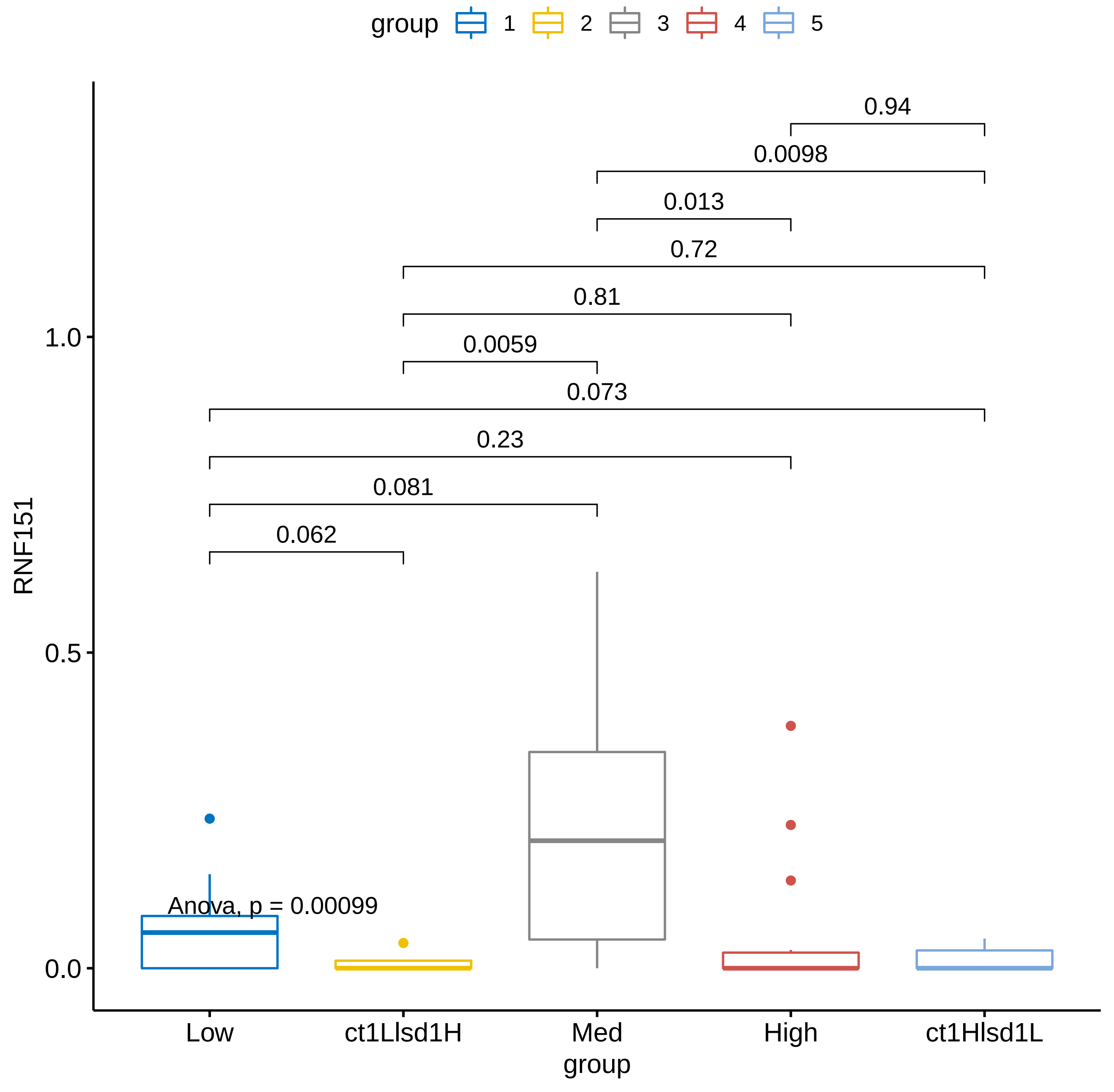
group
1
2
3
4
5
0.94
0.0098
0.013
0.72
0.81
0.0059
0.073
0.23
0.081
0.062
Anova, p = 0.00099
RNF151
1.0
0.5
0.0
Low
ct1Llsd1H
Med
High
ct1Hlsd1L
group

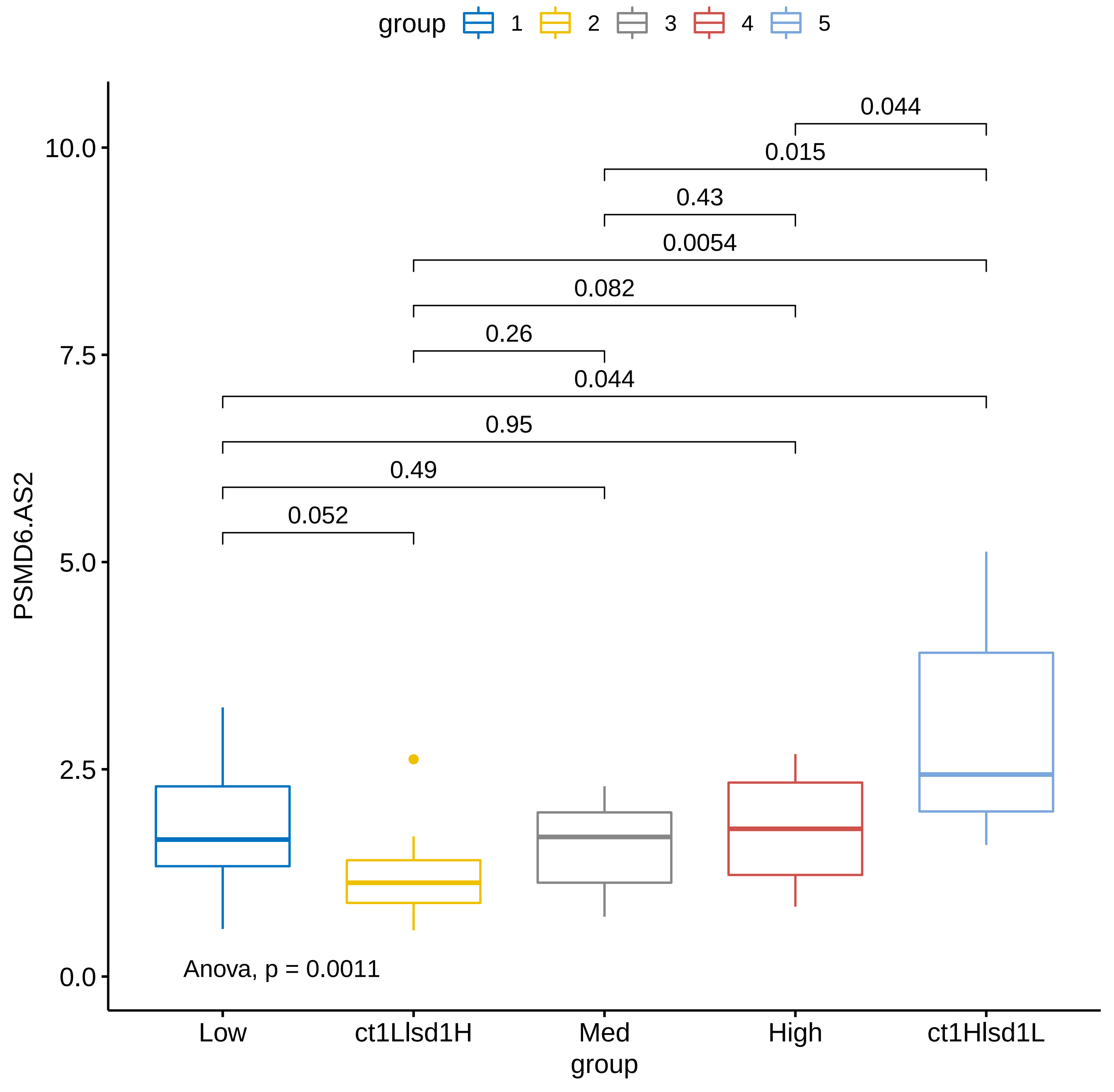

group 1 2 3 4 5
PSMD6.AS2
10.0
7.5
5.0
2.5
0.0
0.044
0.015
0.43
0.0054
0.082
0.26
0.044
0.95
0.49
0.052
Anova, p = 0.0011
Low
ct1Llsd1H
Med
High
ct1Hlsd1L
group

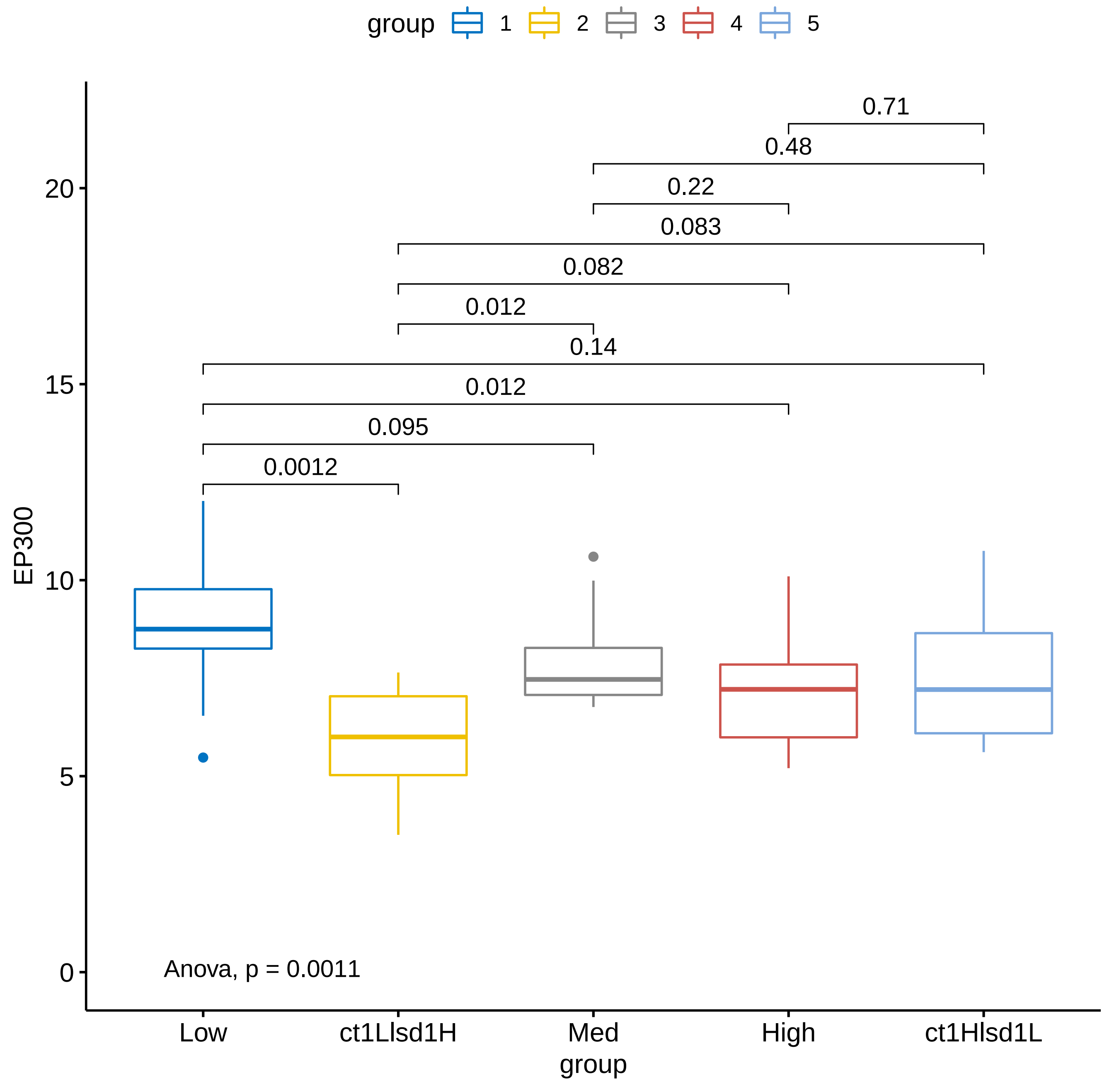

group 1 2 3 4 5
0.71
0.48
0.22
0.083
0.082
0.012
0.14
0.012
0.095
0.0012
EP300
20
15
10
5
0
Anova, p = 0.0011
Low
ct1Llsd1H
Med
High
ct1Hlsd1L
group

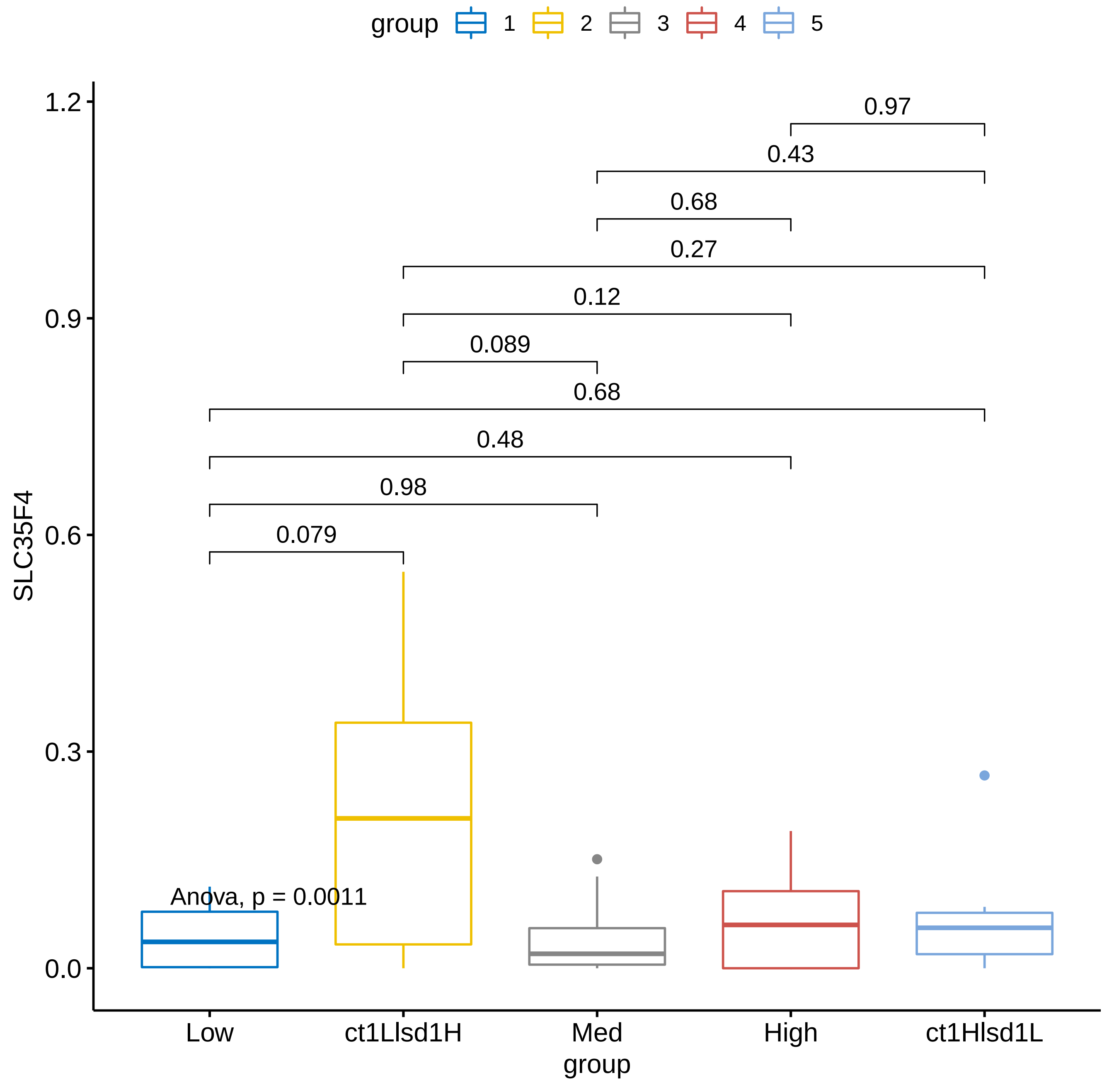
group
1
2
3
4
5
SLC35F4
1.2
0.9
0.6
0.3
0.0
0.97
0.43
0.68
0.27
0.12
0.089
0.68
0.48
0.98
0.079
Anova, p = 0.0011
Low
ct1Llsd1H
Med
High
ct1Hlsd1L
group

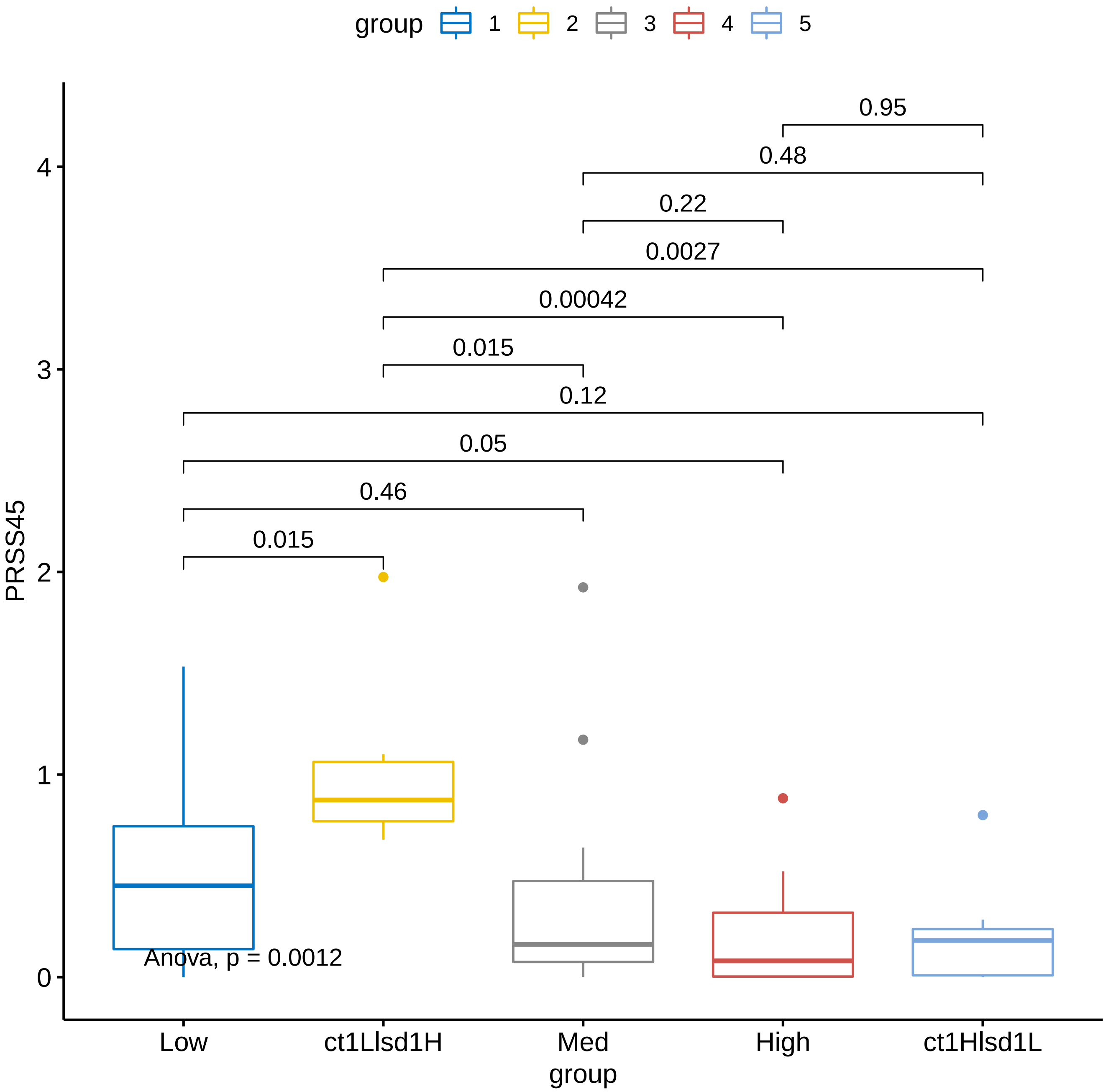

group
1
2
3
4
5
0.95
0.48
0.22
0.0027
0.00042
0.015
0.12
0.05
0.46
0.015
Anova, p = 0.0012
PRSS45
0
1
2
3
4
Low
ct1Llsd1H
Med
High
ct1Hlsd1L
group

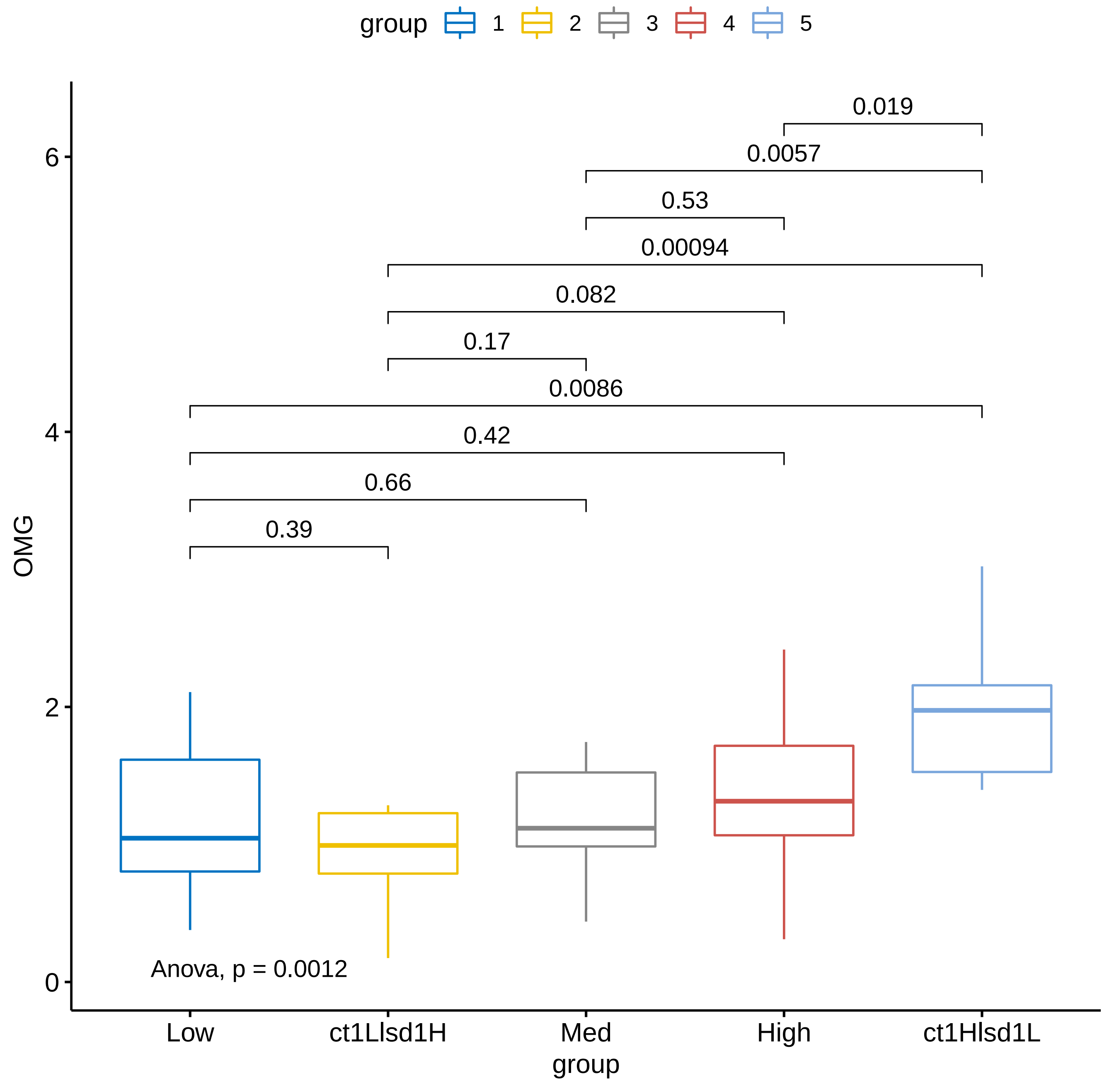
group
1
2
3
4
5
0.019
0.0057
0.53
0.00094
0.082
0.17
0.0086
0.42
0.66
0.39
6
4
2
0
OMG
Anova, p = 0.0012
Low
ct1Llsd1H
Med
High
ct1Hlsd1L
group

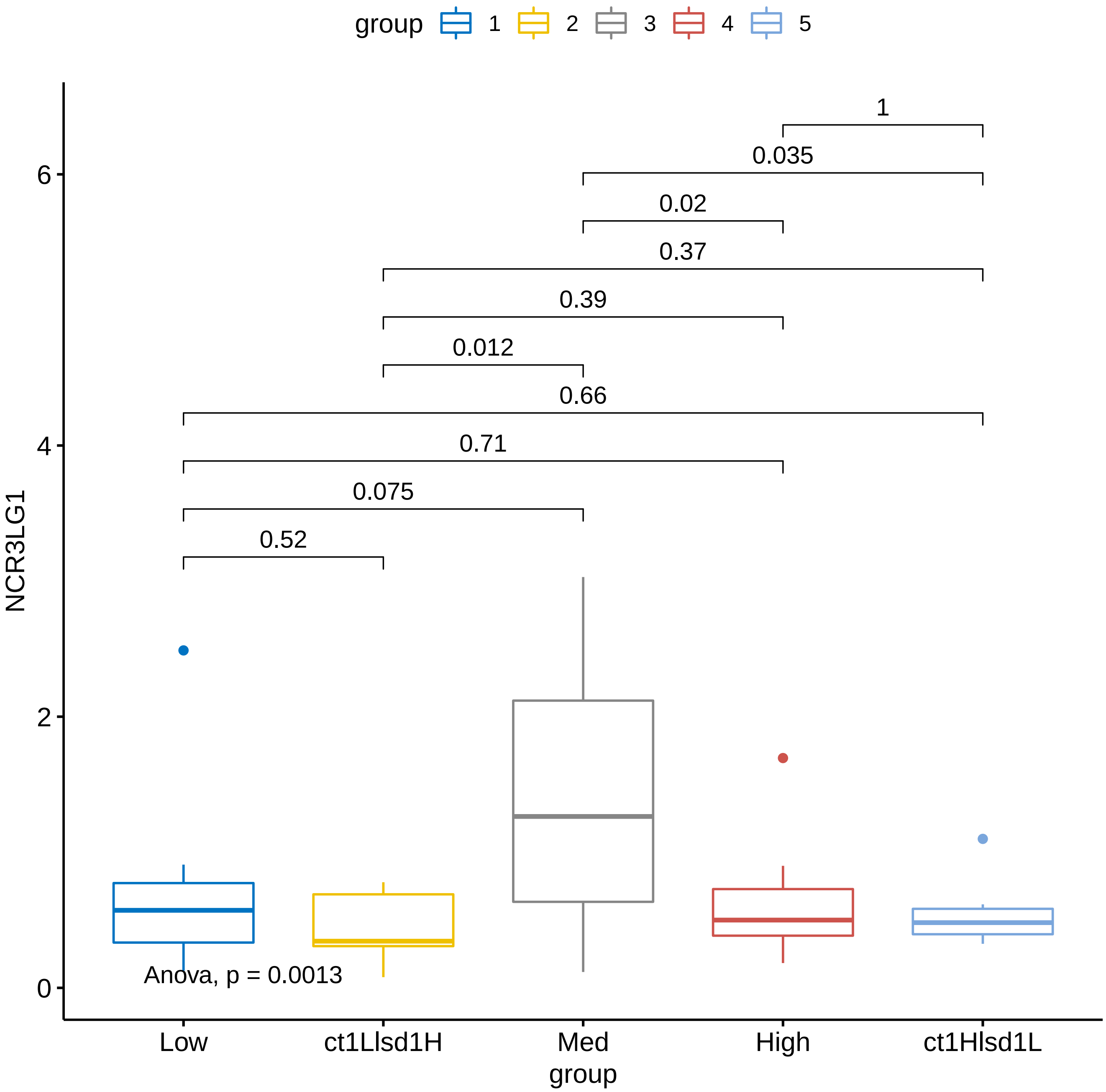

group
1
2
3
4
5
NCR3LG1
6
4
2
0
1
0.035
0.02
0.37
0.39
0.012
0.66
0.71
0.075
0.52
Anova, p = 0.0013
Low
ct1Llsd1H
Med
High
ct1Hlsd1L
group

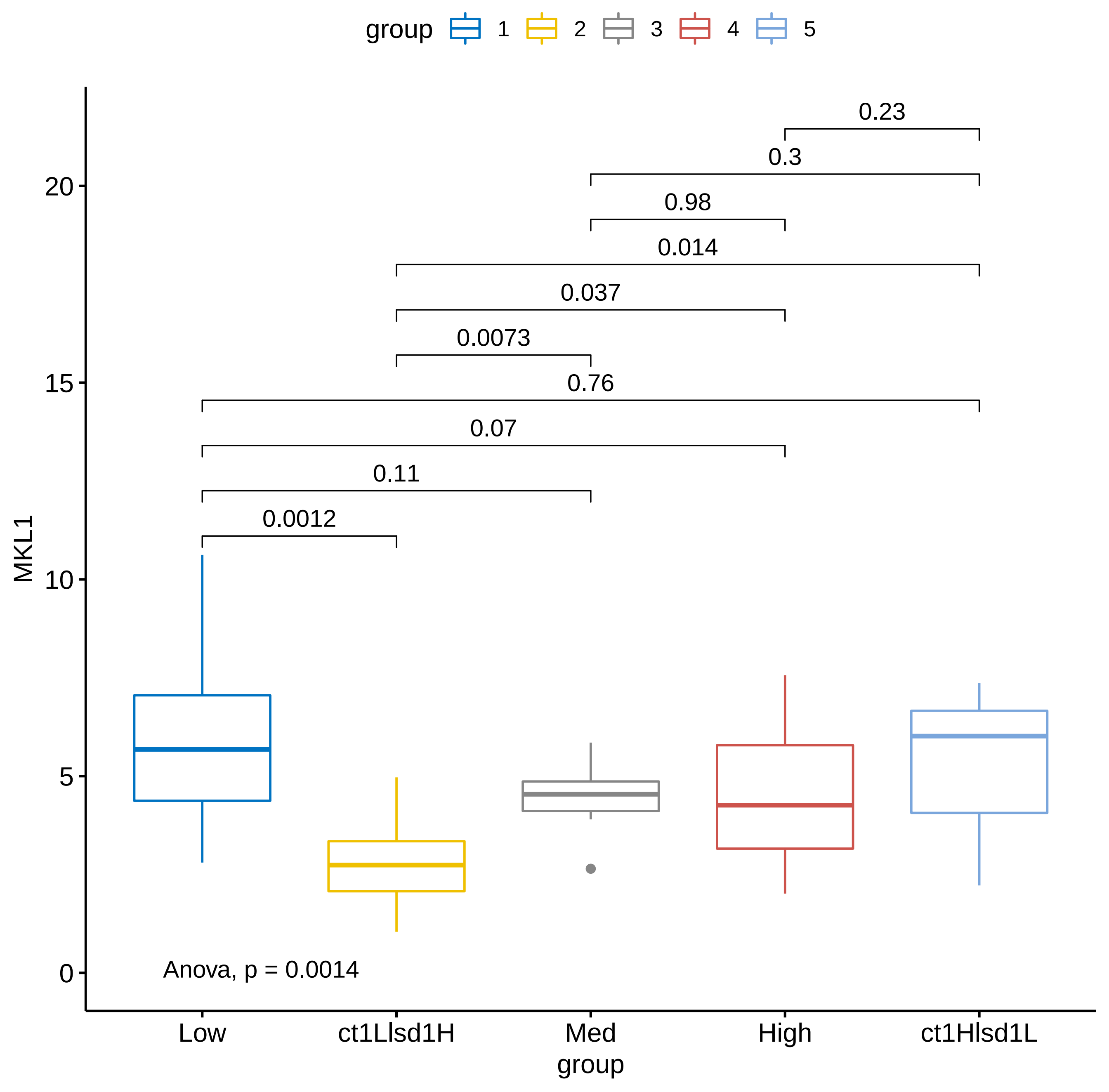

group
1
2
3
4
5
0.23
0.3
0.98
0.014
0.037
0.0073
0.76
0.07
0.11
0.0012
Anova, p = 0.0014
MKL1
20
15
10
5
0
Low
ct1Llsd1H
Med
High
ct1Hlsd1L
group

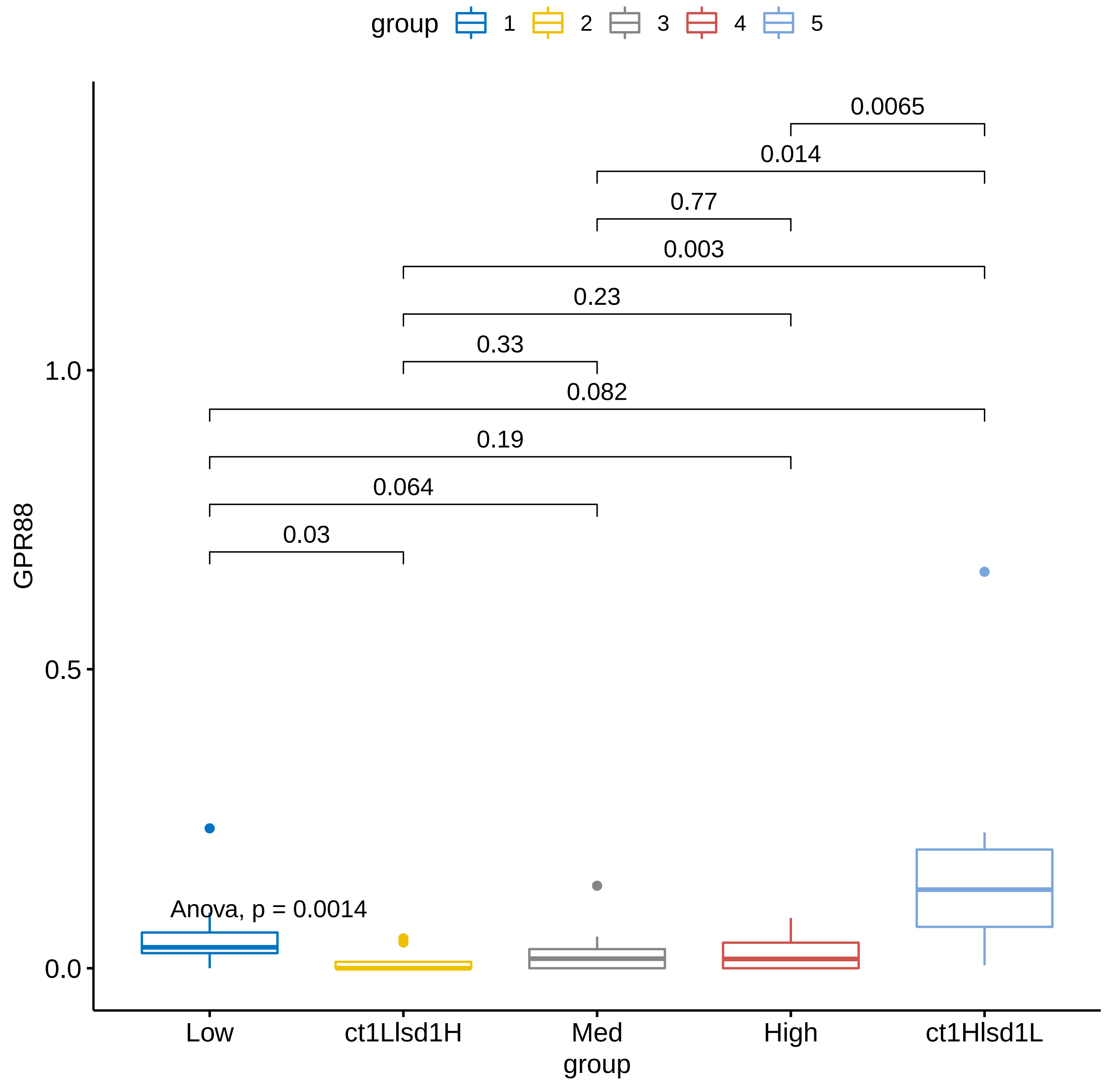

group
1
2
3
4
5
0.0065
0.014
0.77
0.003
0.23
0.33
0.082
0.19
0.064
0.03
Anova, p = 0.0014
GPR88
1.0
0.5
0.0
Low
ct1Llsd1H
Med
High
ct1Hlsd1L
group

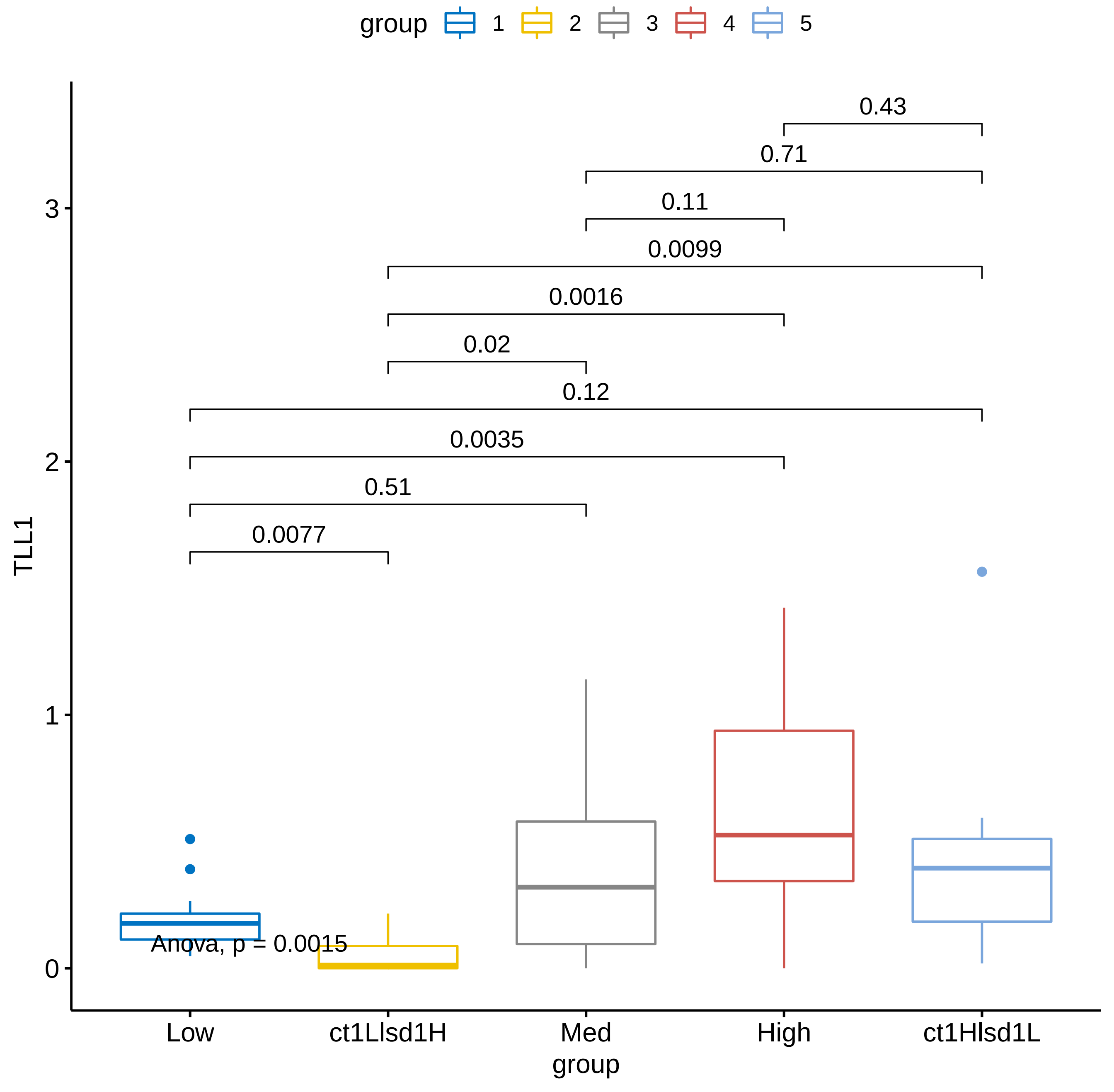

group 1 2 3 4 5
0.43
0.71
0.11
0.0099
0.0016
0.02
0.12
0.0035
0.51
0.0077
TLL1
3
2
1
0
Anova, p = 0.0015
Low
ct1Llsd1H
Med
High
ct1Hlsd1L
group

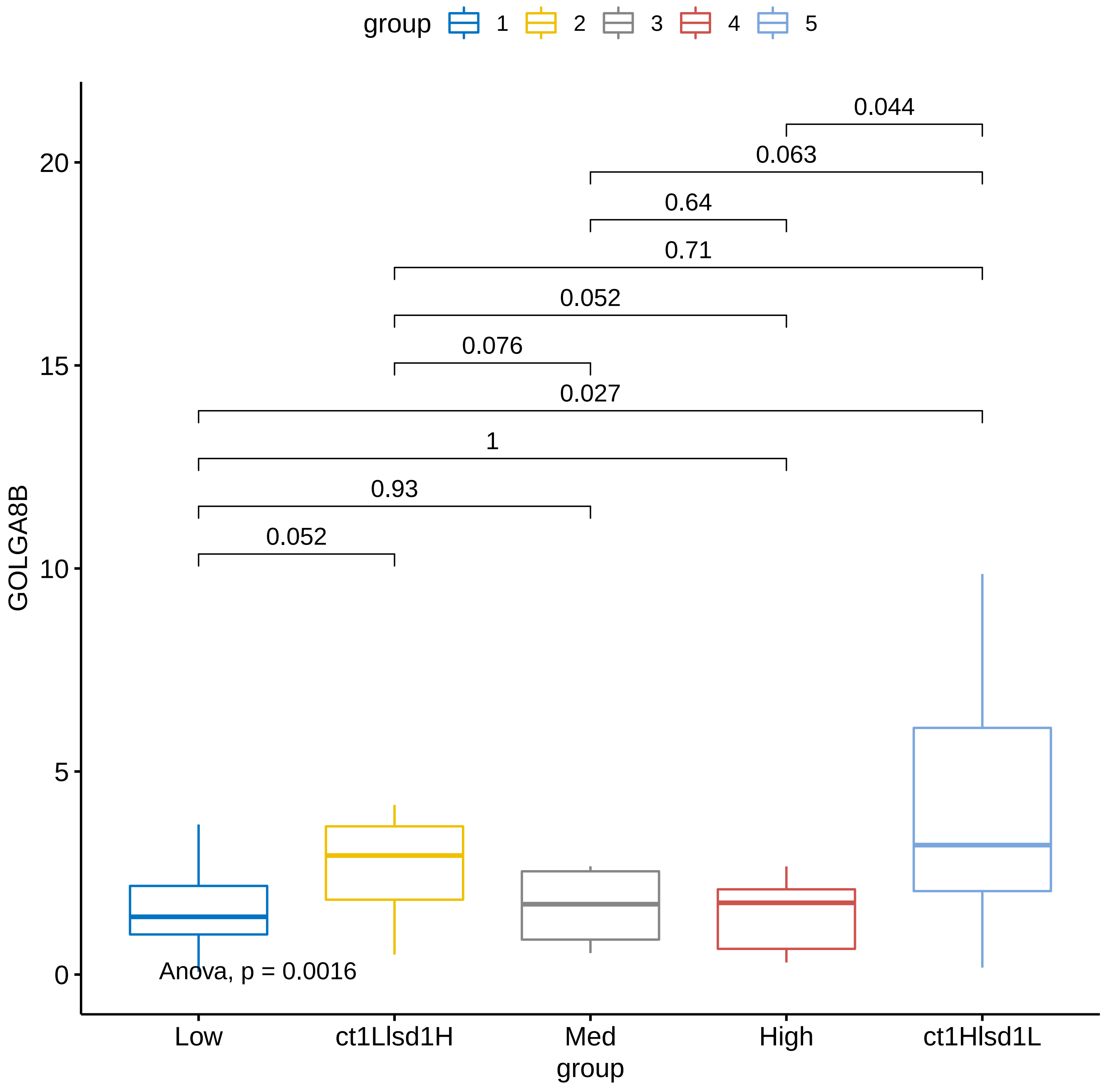

group
1
2
3
4
5
0.044
0.063
0.64
0.71
0.052
0.076
0.027
1
0.93
0.052
20
15
10
5
0
GOLGA8B
Anova, p = 0.0016
Low
ct1Llsd1H
Med
High
ct1Hlsd1L
group

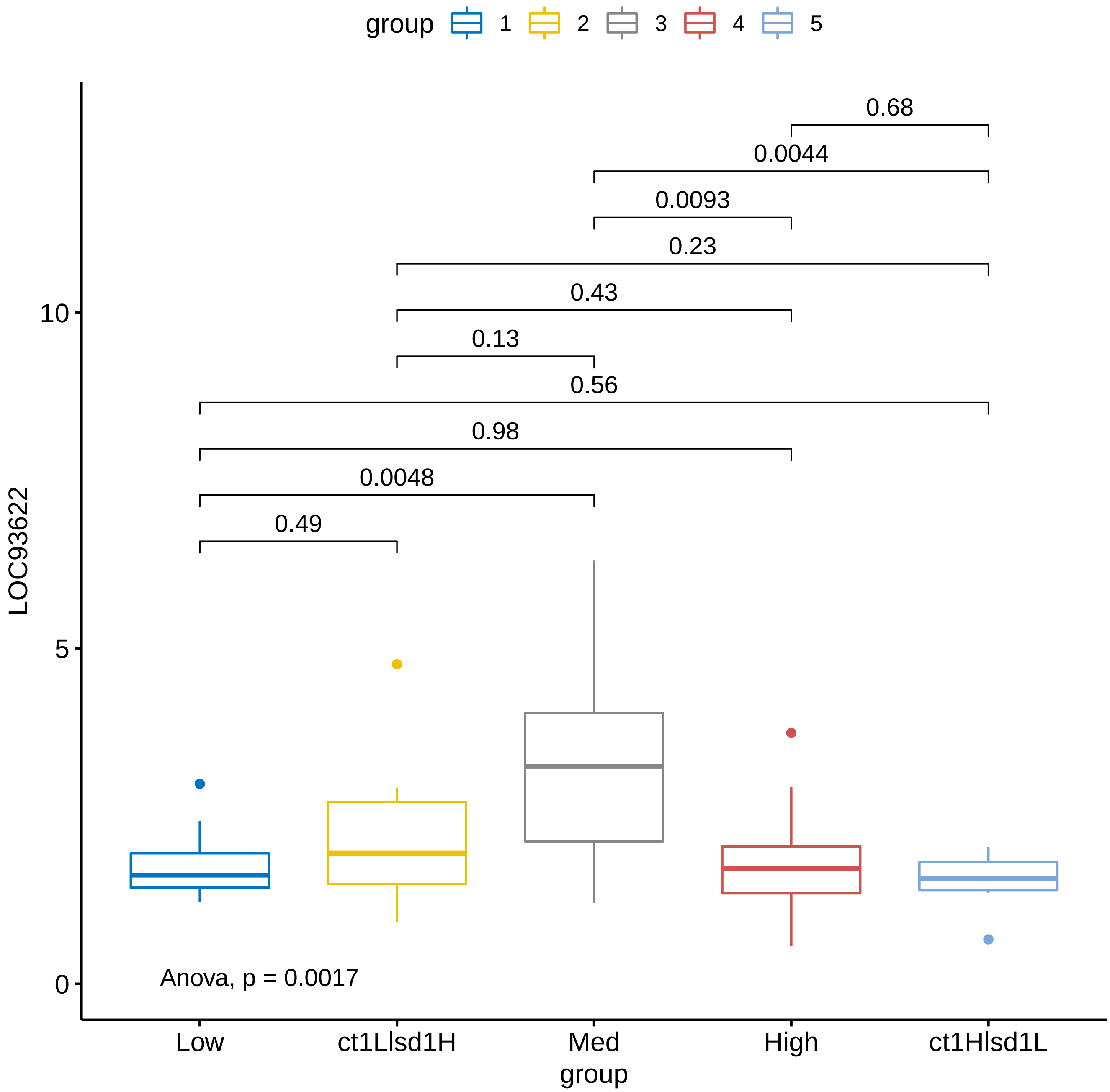
group
1
2
3
4
5
0.68
0.0044
0.0093
0.23
0.43
0.13
0.56
0.98
0.0048
0.49
10
5
0
LOC93622
Anova, p = 0.0017
Low
ct1Llsd1H
Med
High
ct1Hlsd1L
group

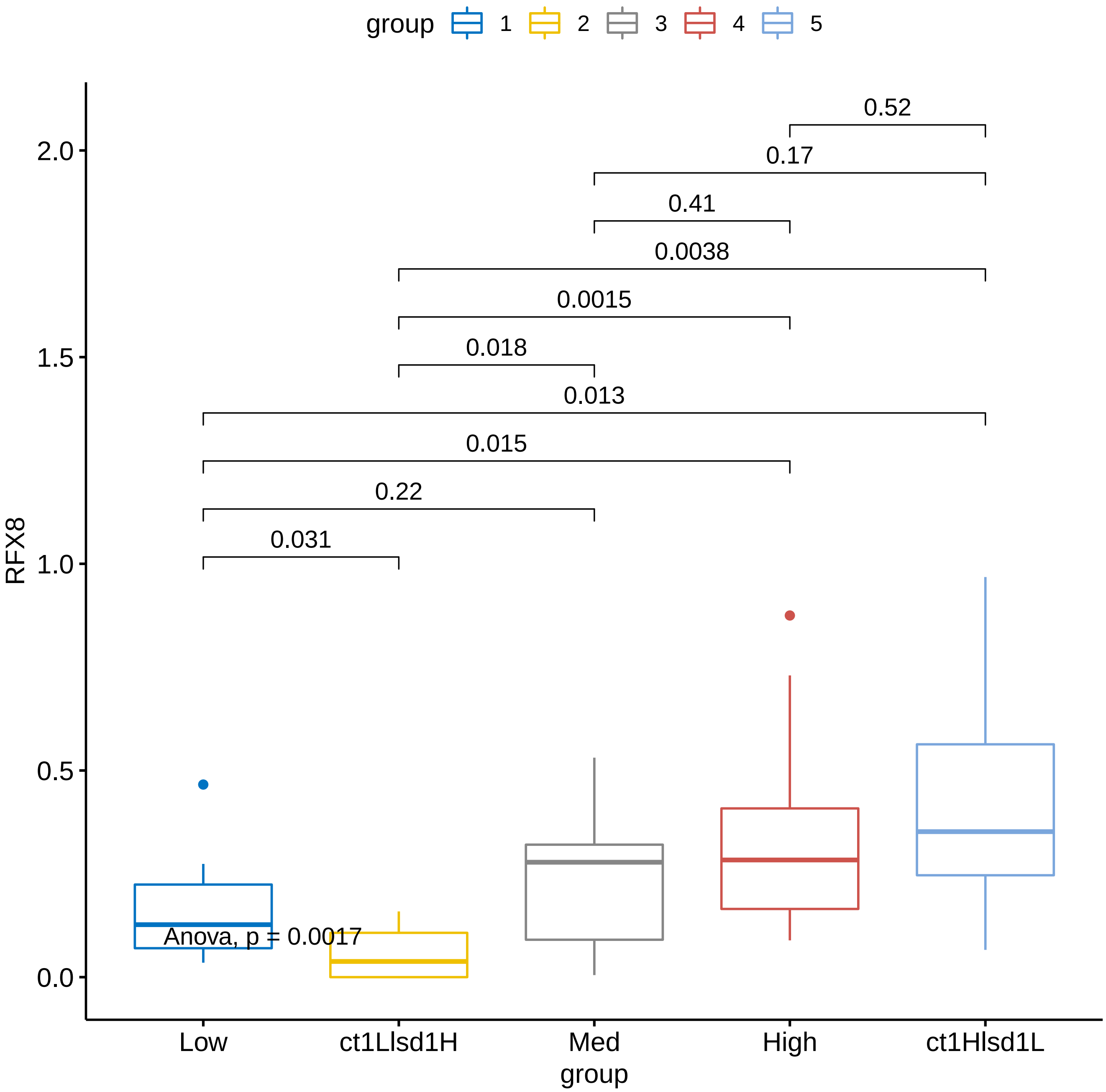
group 1 2 3 4 5
RFX8
2.0
1.5
1.0
0.5
0.0
0.52
0.17
0.41
0.0038
0.0015
0.018
0.013
0.015
0.22
0.031
Anova, p = 0.0017
Low
ct1Llsd1H
Med
High
ct1Hlsd1L
group

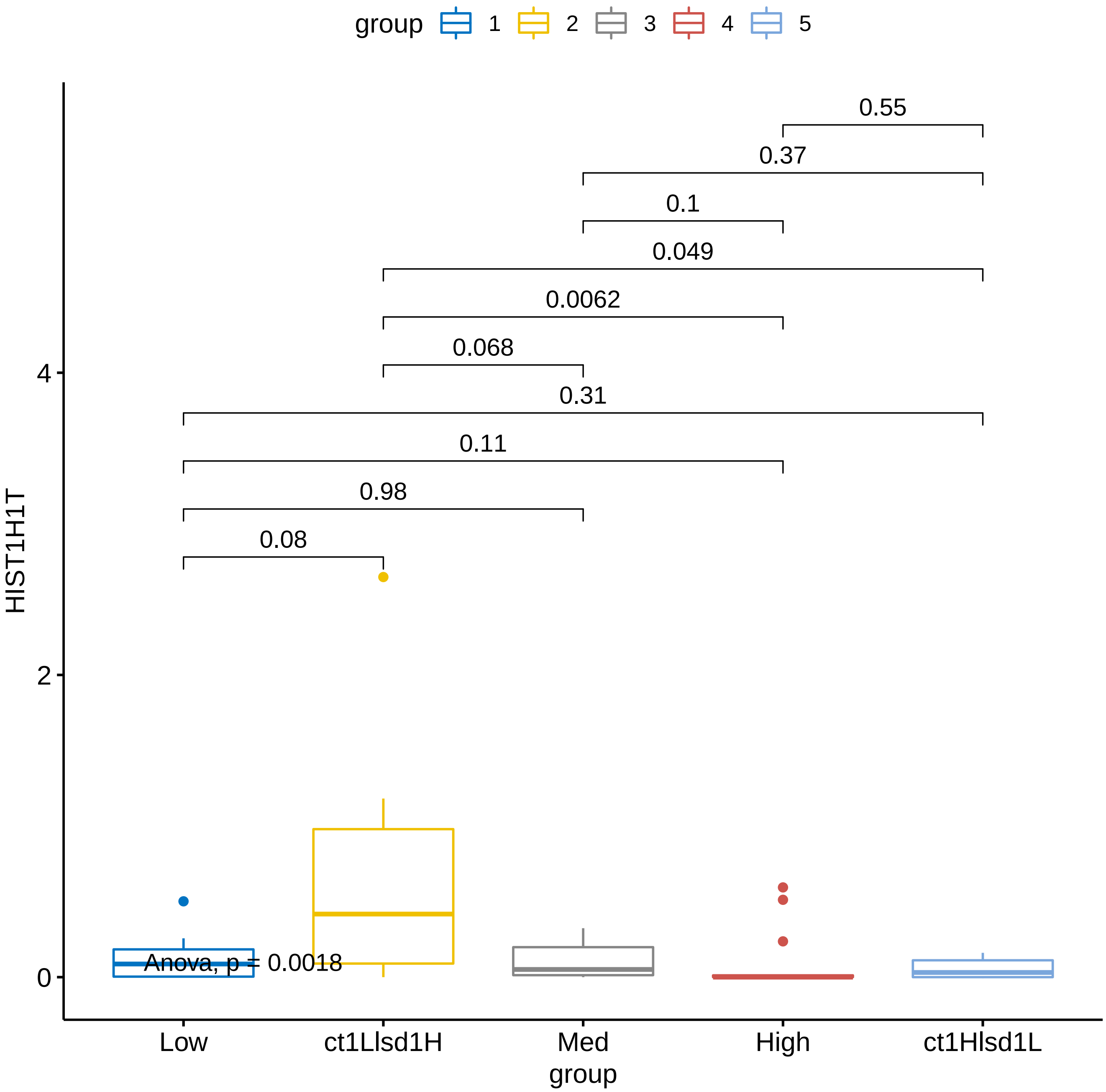
group
1
2
3
4
5
HIST1H1T
0
2
4
0.55
0.37
0.1
0.049
0.0062
0.068
0.31
0.11
0.98
0.08
Anova, p = 0.0018
Low
ct1Llsd1H
Med
High
ct1Hlsd1L
group

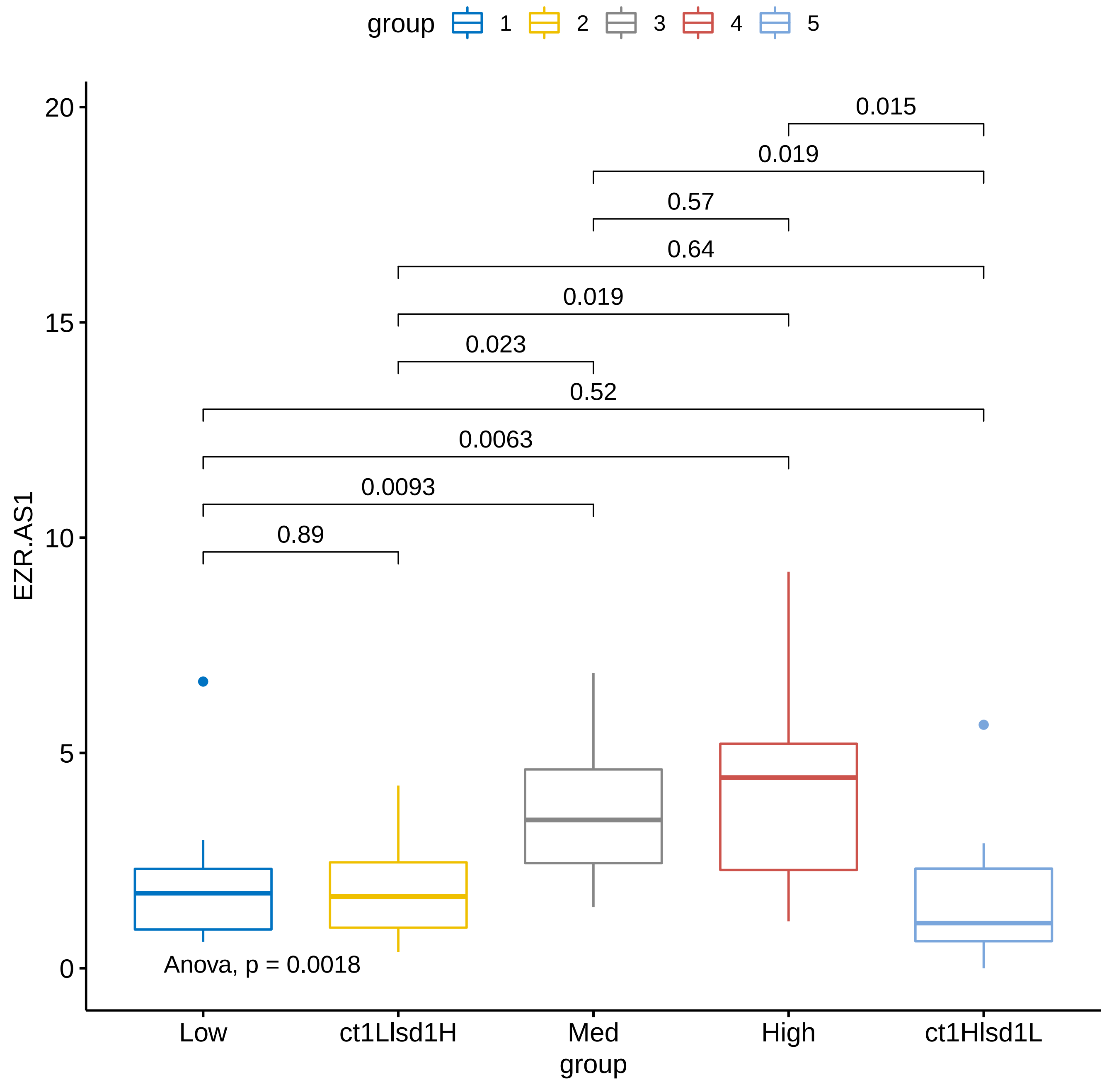
group
1
2
3
4
5
20
15
10
5
0
EZR.AS1
0.015
0.019
0.57
0.64
0.019
0.023
0.52
0.0063
0.0093
0.89
Anova, p = 0.0018
Low
ct1Llsd1H
Med
High
ct1Hlsd1L
group

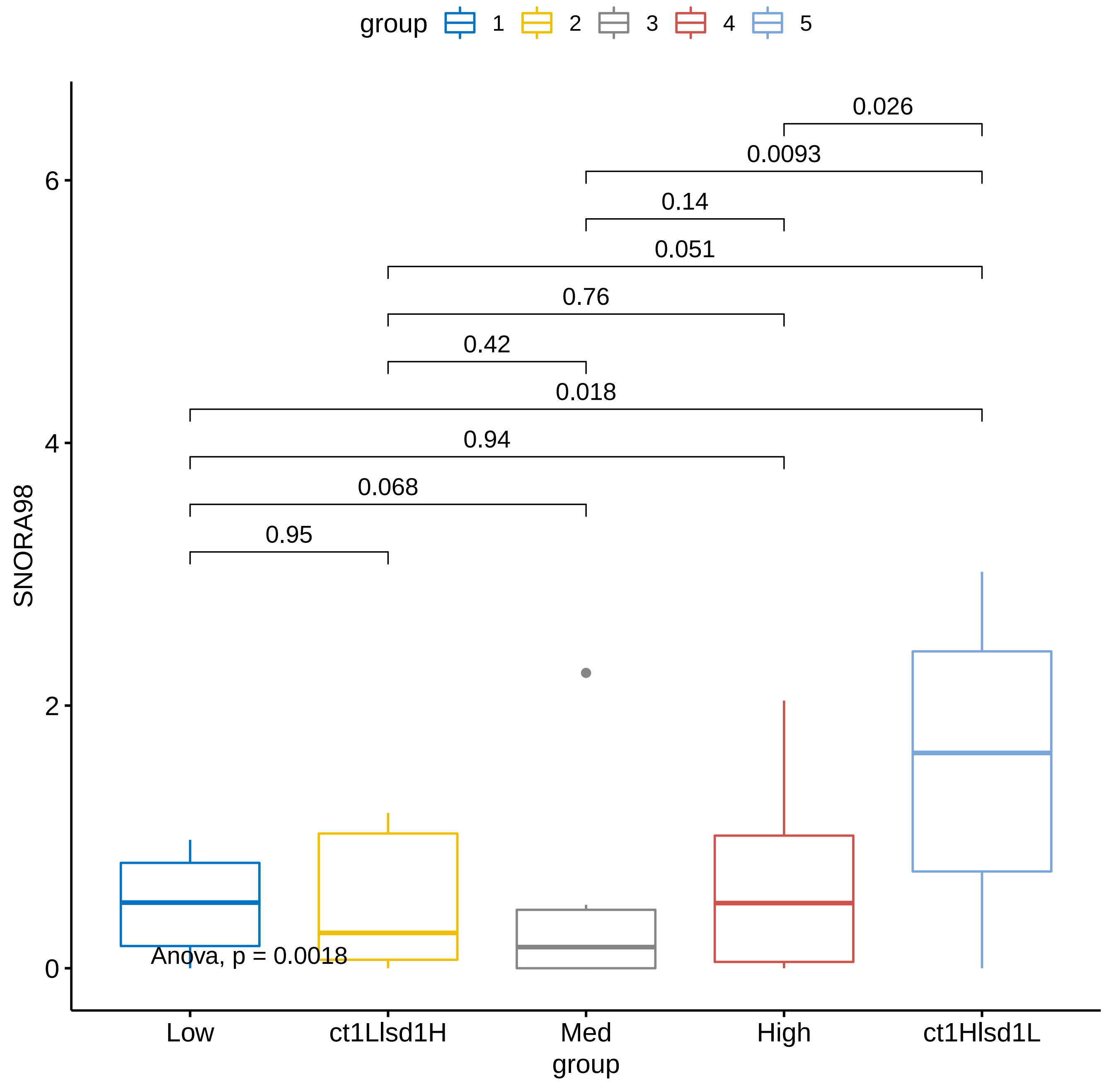
group 1 2 3 4 5
0.026
0.0093
0.14
0.051
0.76
0.42
0.018
0.94
0.068
0.95
SNORA98
6
4
2
0
Anova, p = 0.0018
Low
ct1Llsd1H
Med
High
ct1Hlsd1L
group

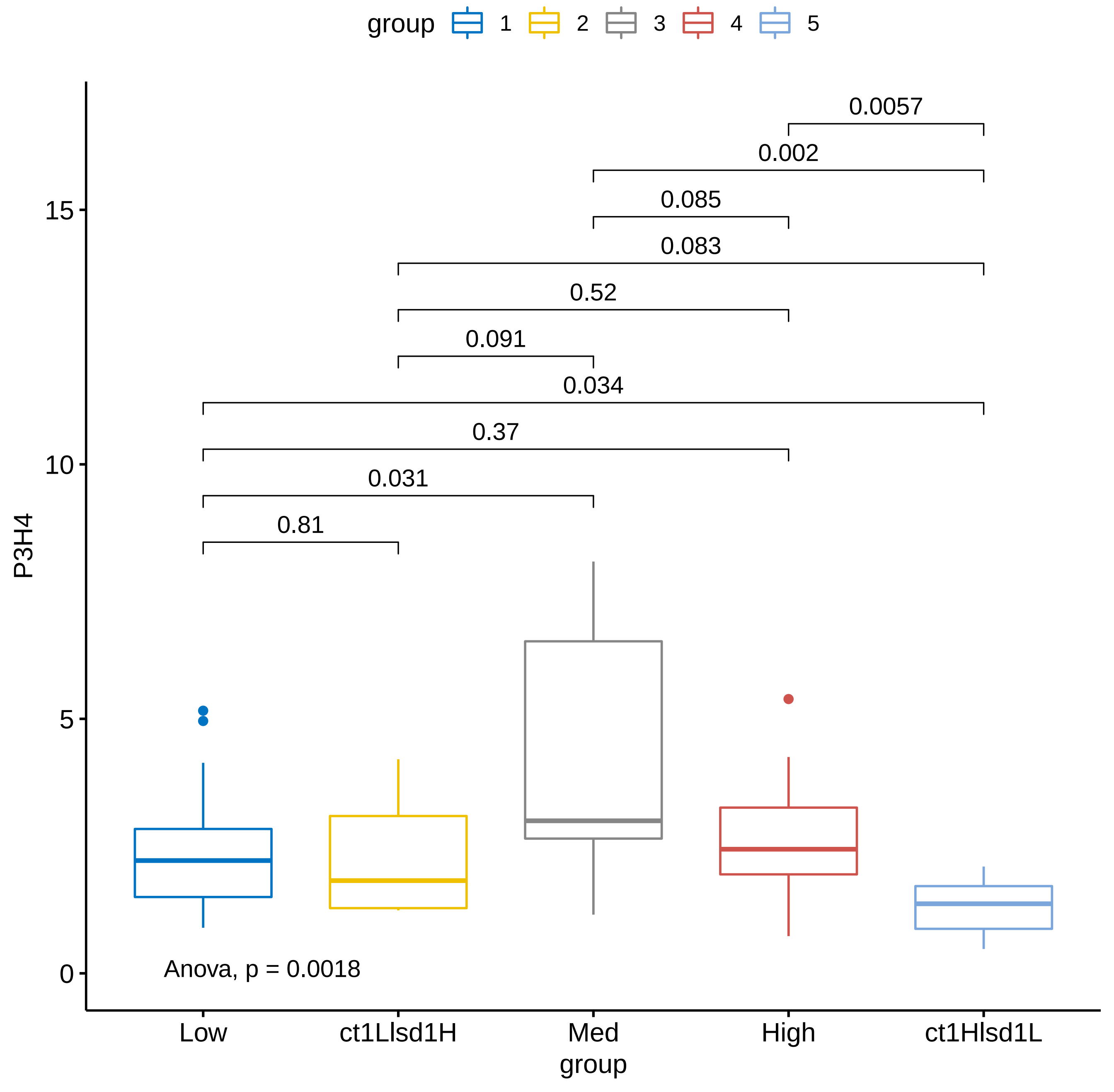
group
1
2
3
4
5
0.0057
0.002
0.085
0.083
0.52
0.091
0.034
0.37
0.031
0.81
15
10
5
0
P3H4
Anova, p = 0.0018
Low
ct1Llsd1H
Med
High
ct1Hlsd1L
group

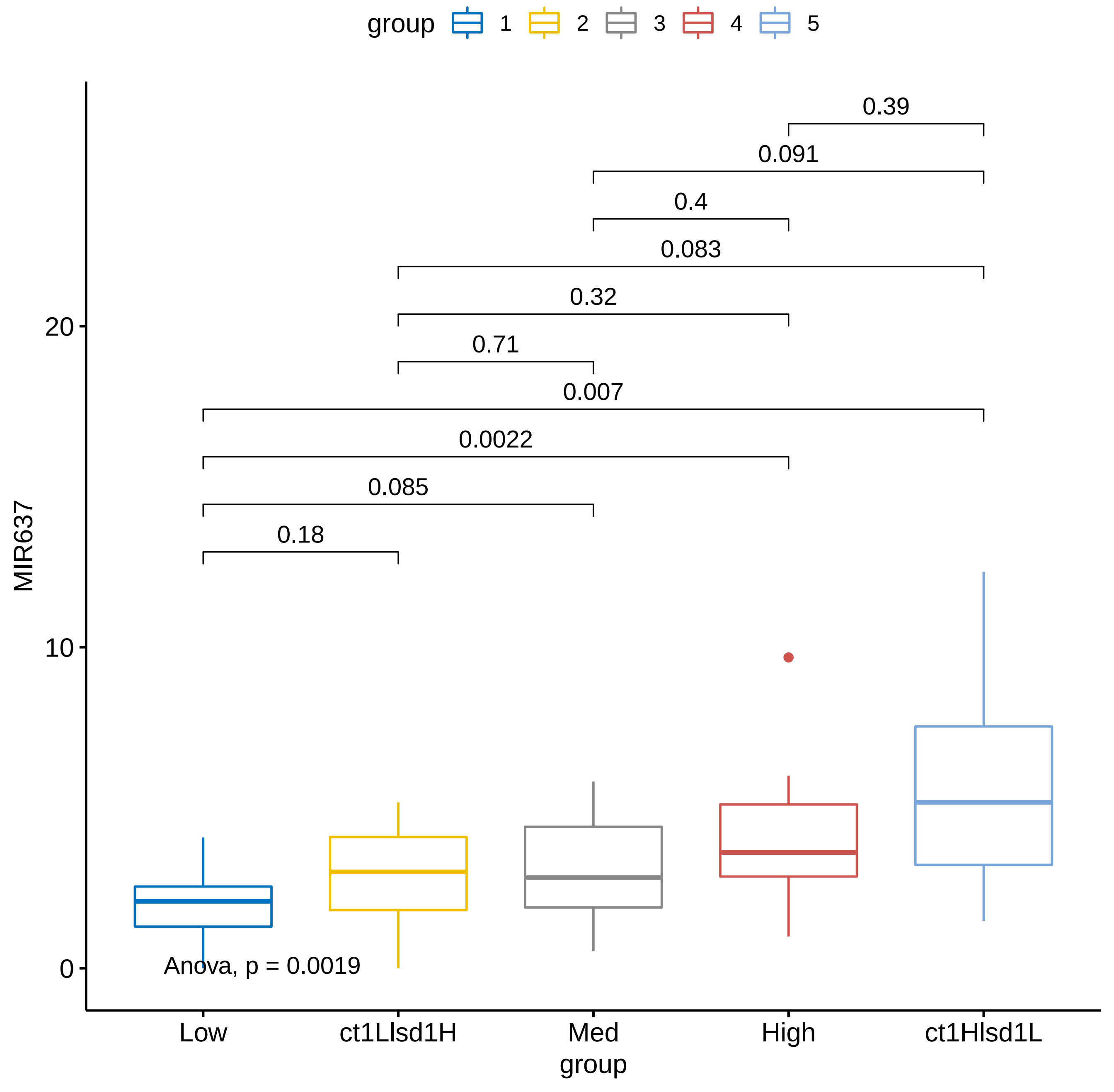

group 1 2 3 4 5
0.39
0.091
0.4
0.083
0.32
0.71
0.007
0.0022
0.085
0.18
Anova, p = 0.0019
MIR637
20
10
0
Low
ct1Llsd1H
Med
High
ct1Hlsd1L
group

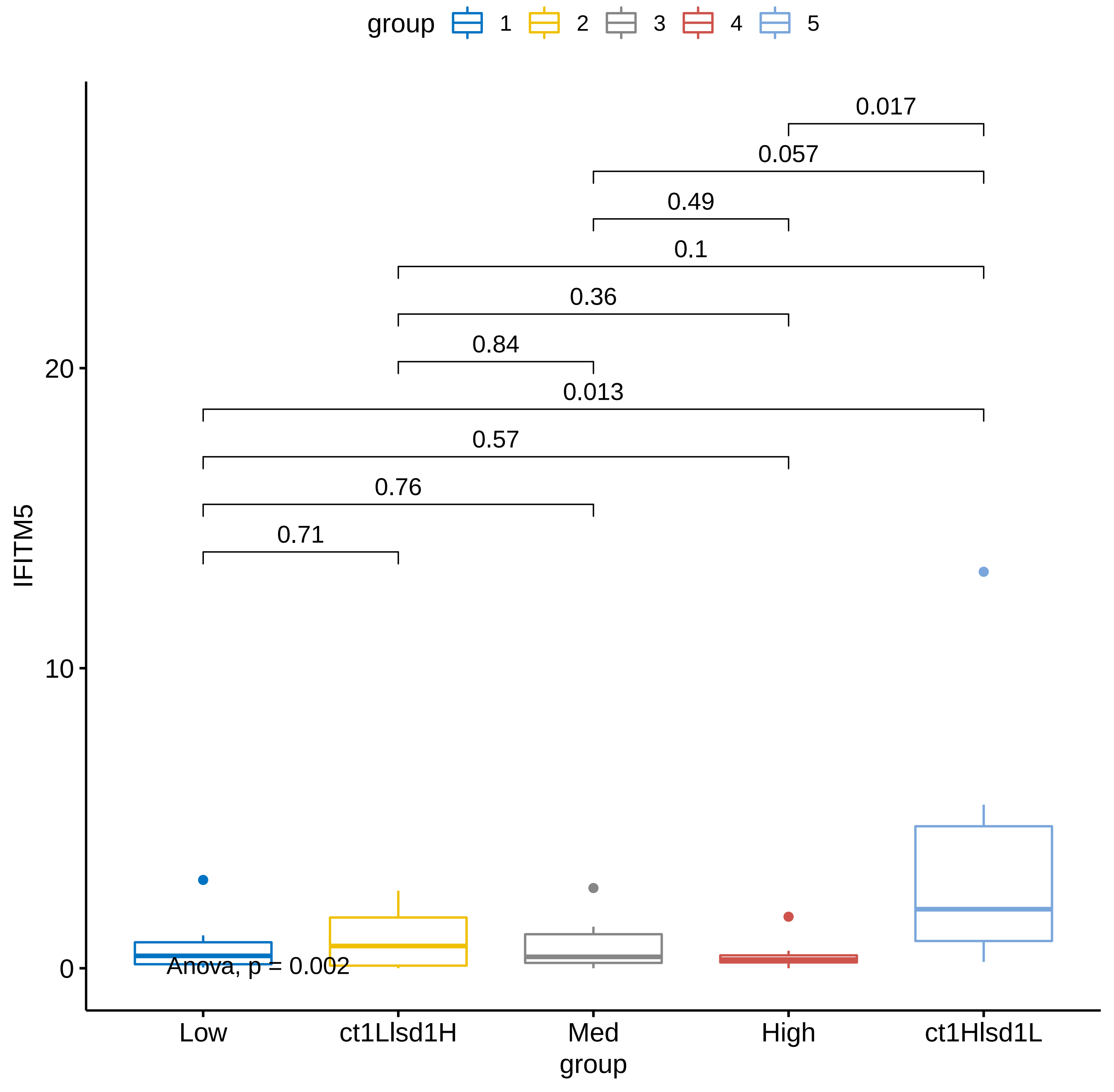

group
1
2
3
4
5
0.017
0.057
0.49
0.1
0.36
0.84
0.013
0.57
0.76
0.71
20
10
0
IFITM5
Anova, p = 0.002
Low
ct1Llsd1H
Med
High
ct1Hlsd1L
group

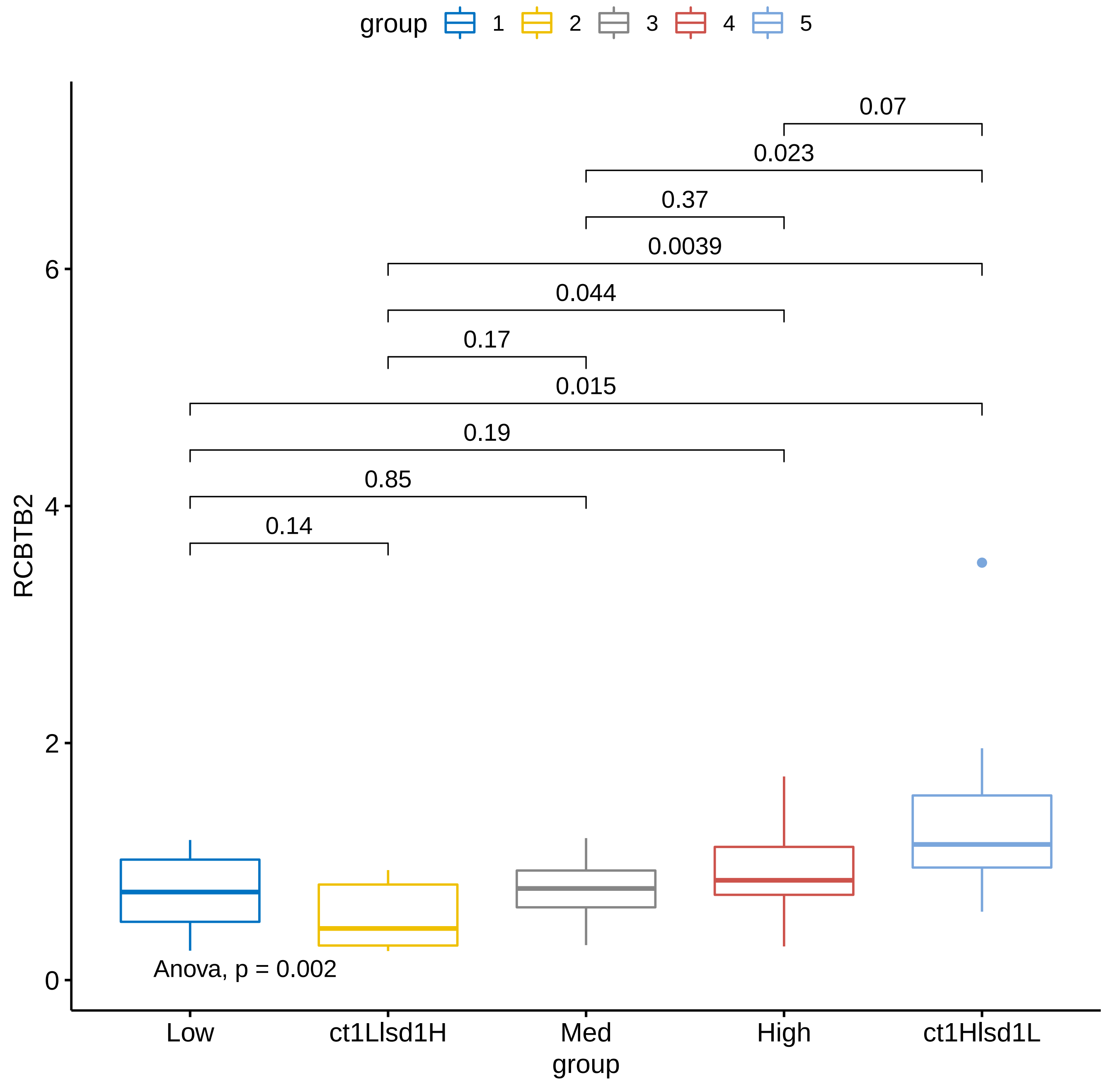
group
1
2
3
4
5
0.07
0.023
0.37
0.0039
0.044
0.17
0.015
0.19
0.85
0.14
6
4
2
0
RCBTB2
Anova, p = 0.002
Low
ct1Llsd1H
Med
High
ct1Hlsd1L
group

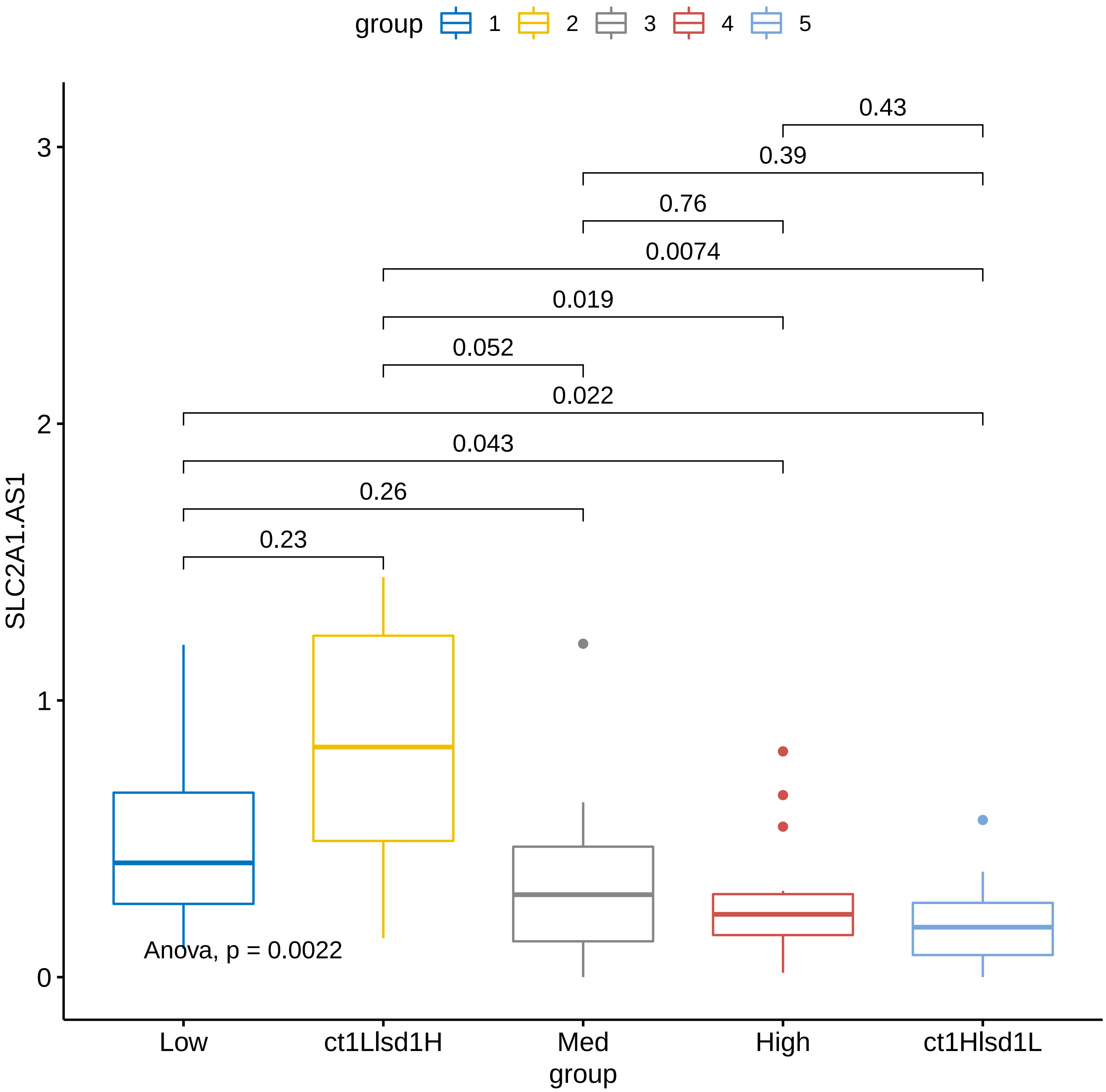

group
1
2
3
4
5
0.43
0.39
0.76
0.0074
0.019
0.052
0.022
0.043
0.26
0.23
Anova, p = 0.0022
SLC2A1.AS1
3
2
1
0
Low
ct1Llsd1H
Med
High
ct1Hlsd1L
group

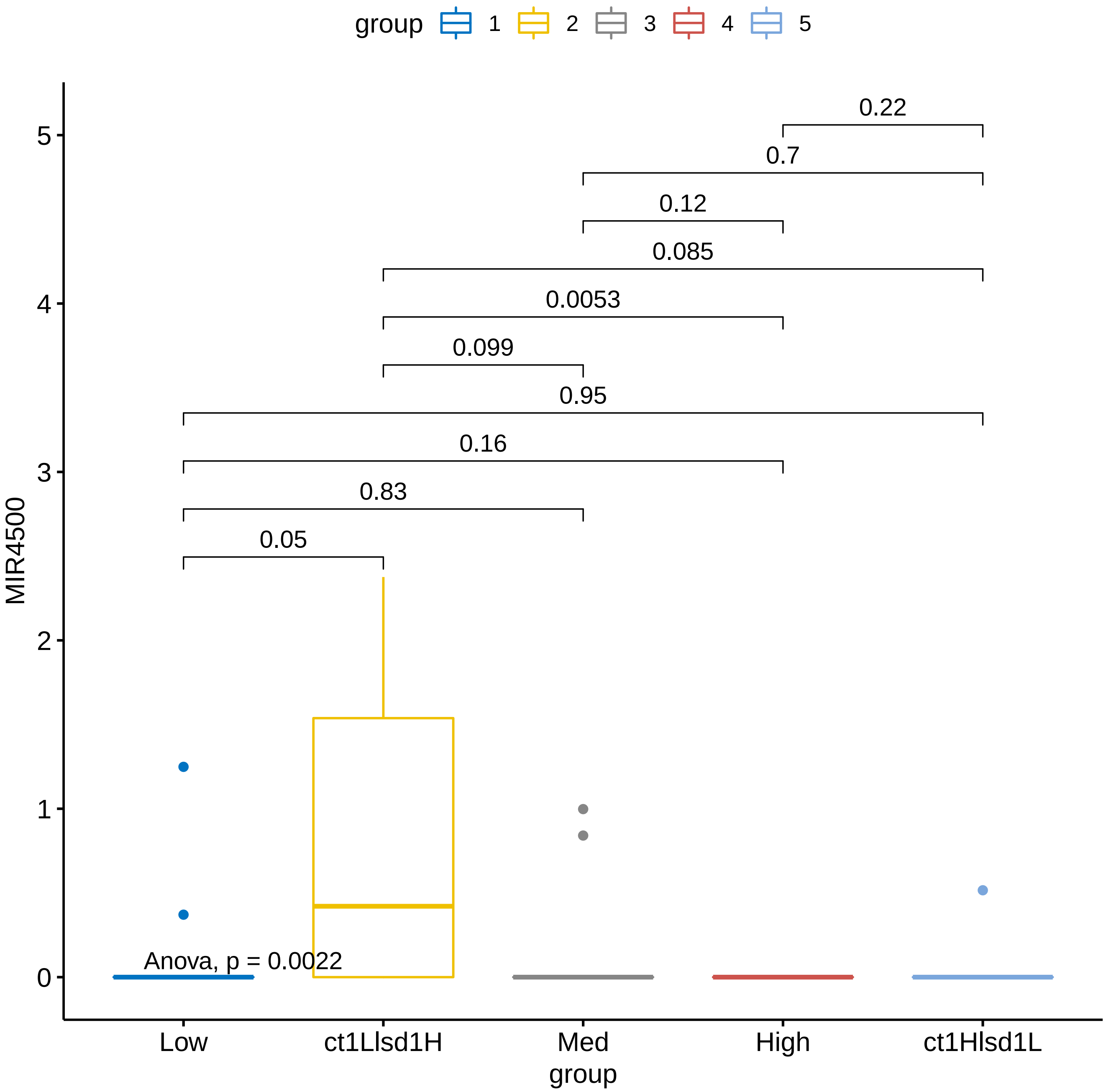
group
1
2
3
4
5
0.22
0.7
0.12
0.085
0.0053
0.099
0.95
0.16
0.83
0.05
Anova, p = 0.0022
MIR4500
0
1
2
3
4
5
Low
ct1Llsd1H
Med
High
ct1Hlsd1L
group

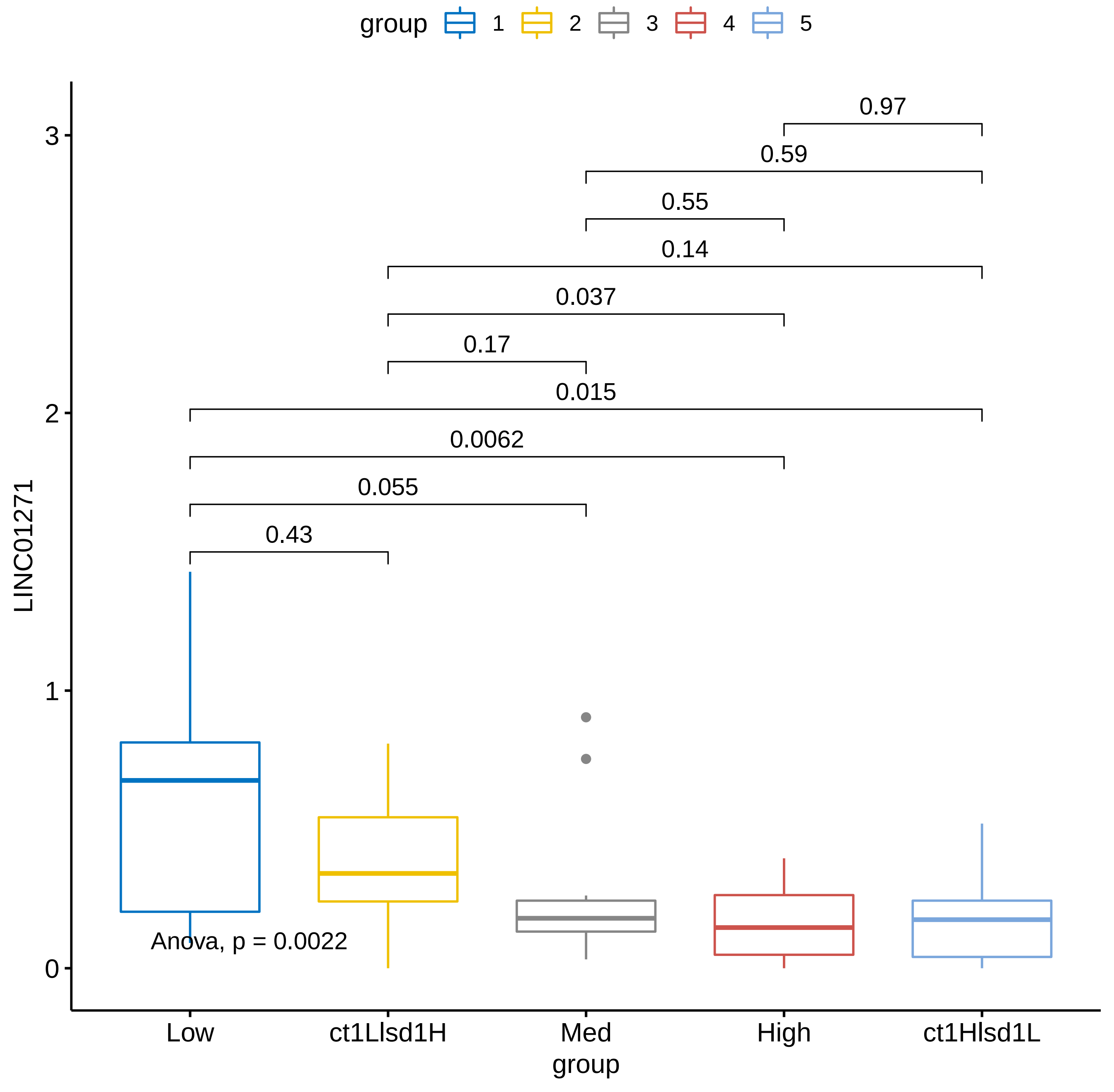
group
1
2
3
4
5
0.97
0.59
0.55
0.14
0.037
0.17
0.015
0.0062
0.055
0.43
Anova, p = 0.0022
3
2
1
0
LINC01271
Low
ct1Llsd1H
Med
High
ct1Hlsd1L
group

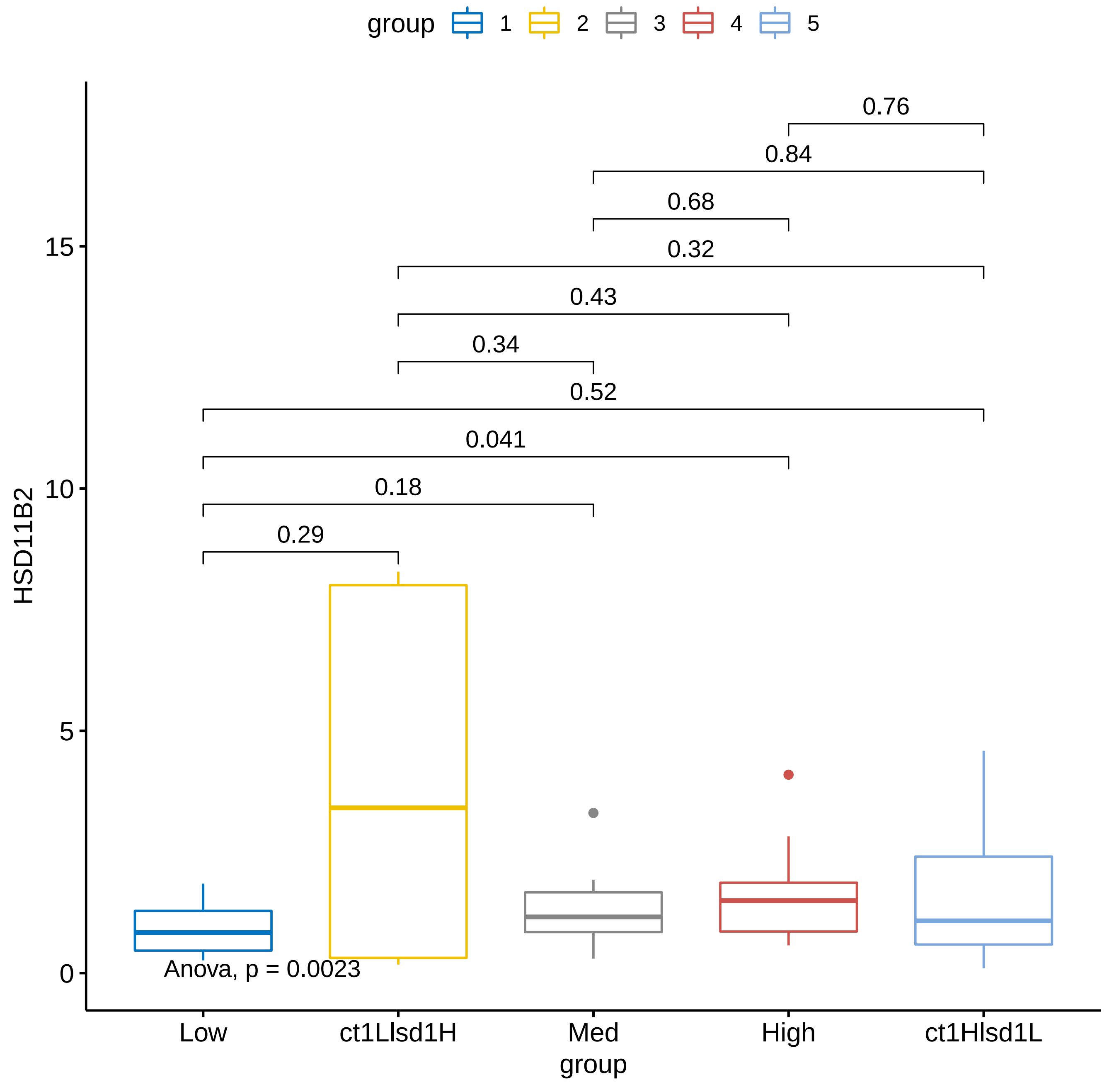

group
1
2
3
4
5
0.76
0.84
0.68
0.32
0.43
0.34
0.52
0.041
0.18
0.29
Anova, p = 0.0023
HSD11B2
15
10
5
0
Low
ct1Llsd1H
Med
High
ct1Hlsd1L
group

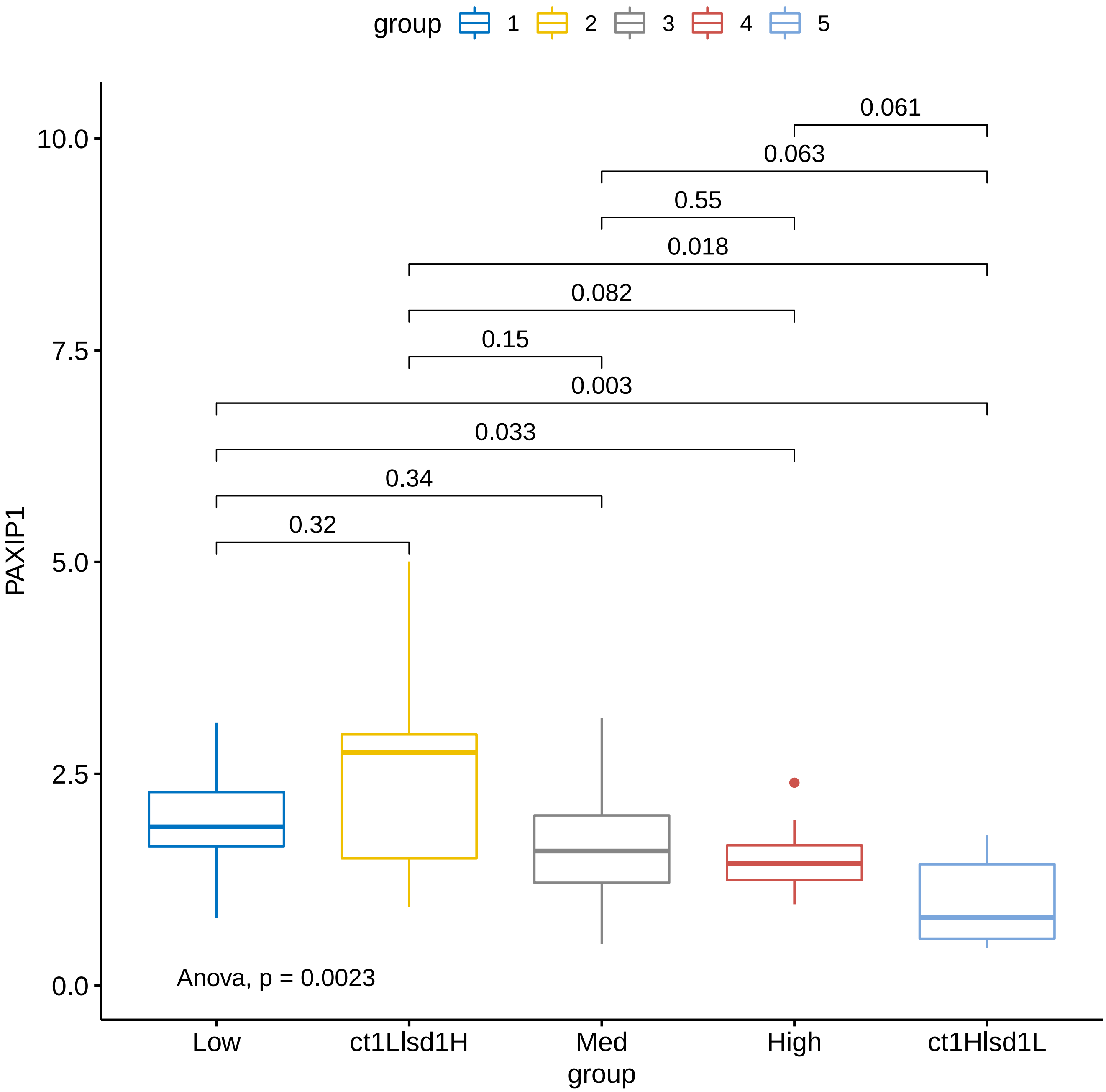
group
1
2
3
4
5
0.061
0.063
0.55
0.018
0.082
0.15
0.003
0.033
0.34
0.32
10.0
7.5
5.0
2.5
0.0
PAXIP1
Anova, p = 0.0023
Low
ct1Llsd1H
Med
High
ct1Hlsd1L
group

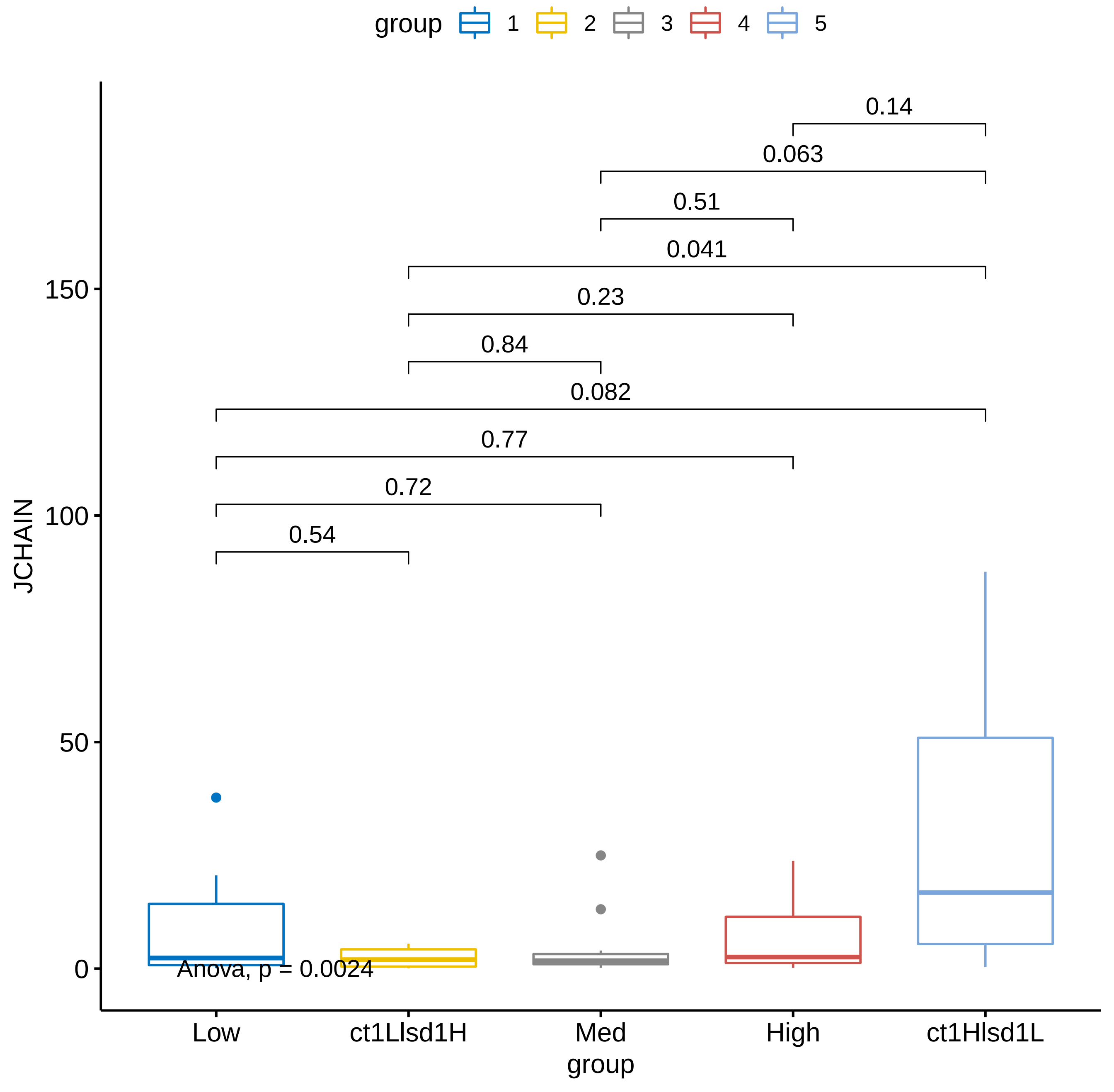
group 1 2 3 4 5
JCHAIN
0
50
100
150
0.14
0.063
0.51
0.041
0.23
0.84
0.082
0.77
0.72
0.54
Anova, p = 0.0024
Low
ct1Llsd1H
Med
High
ct1Hlsd1L
group

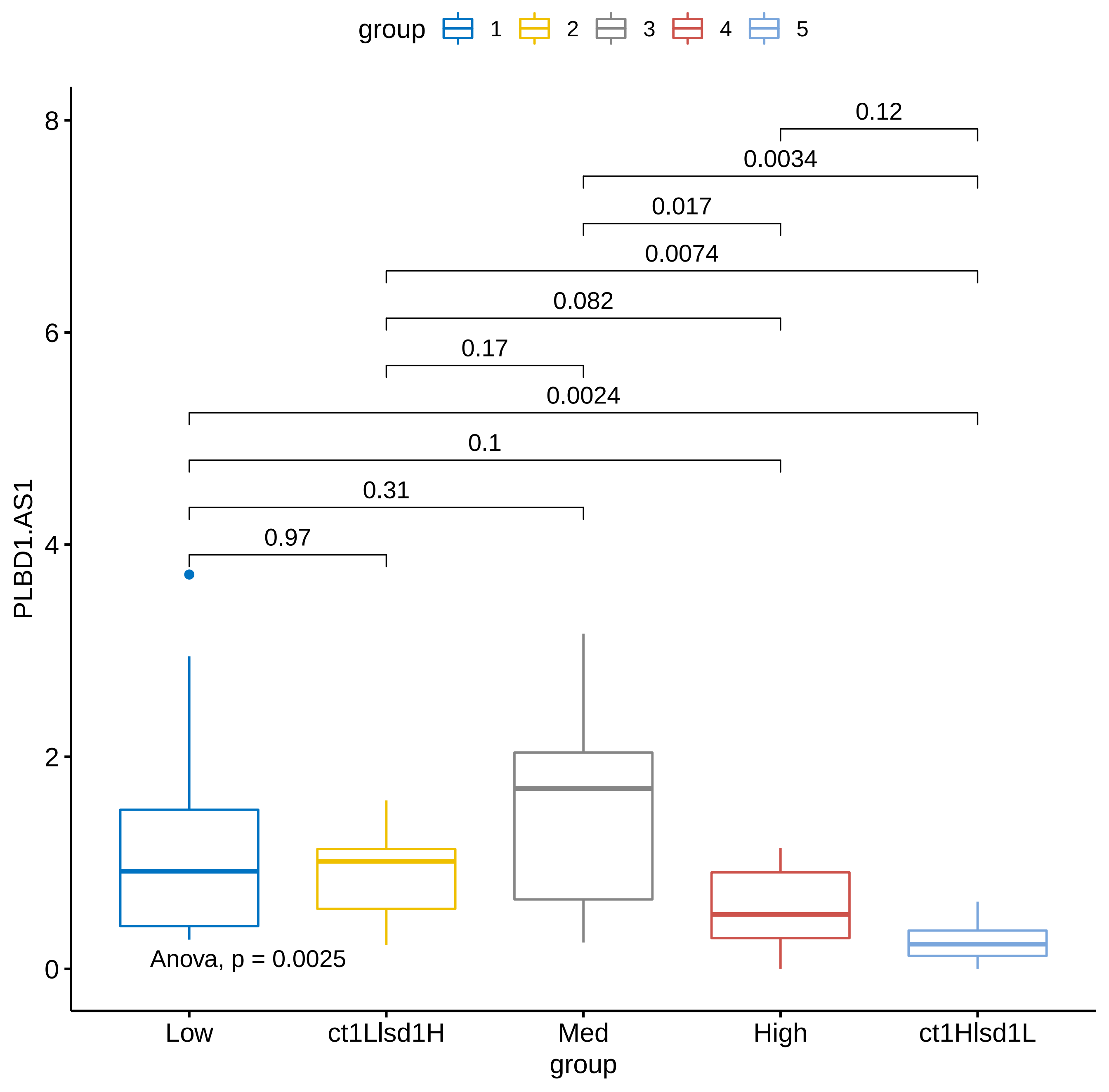
group
1
2
3
4
5
PLBD1.AS1
0
2
4
6
8
0.12
0.0034
0.017
0.0074
0.082
0.17
0.0024
0.1
0.31
0.97
Anova, p = 0.0025
Low
ct1Llsd1H
Med
High
ct1Hlsd1L
group

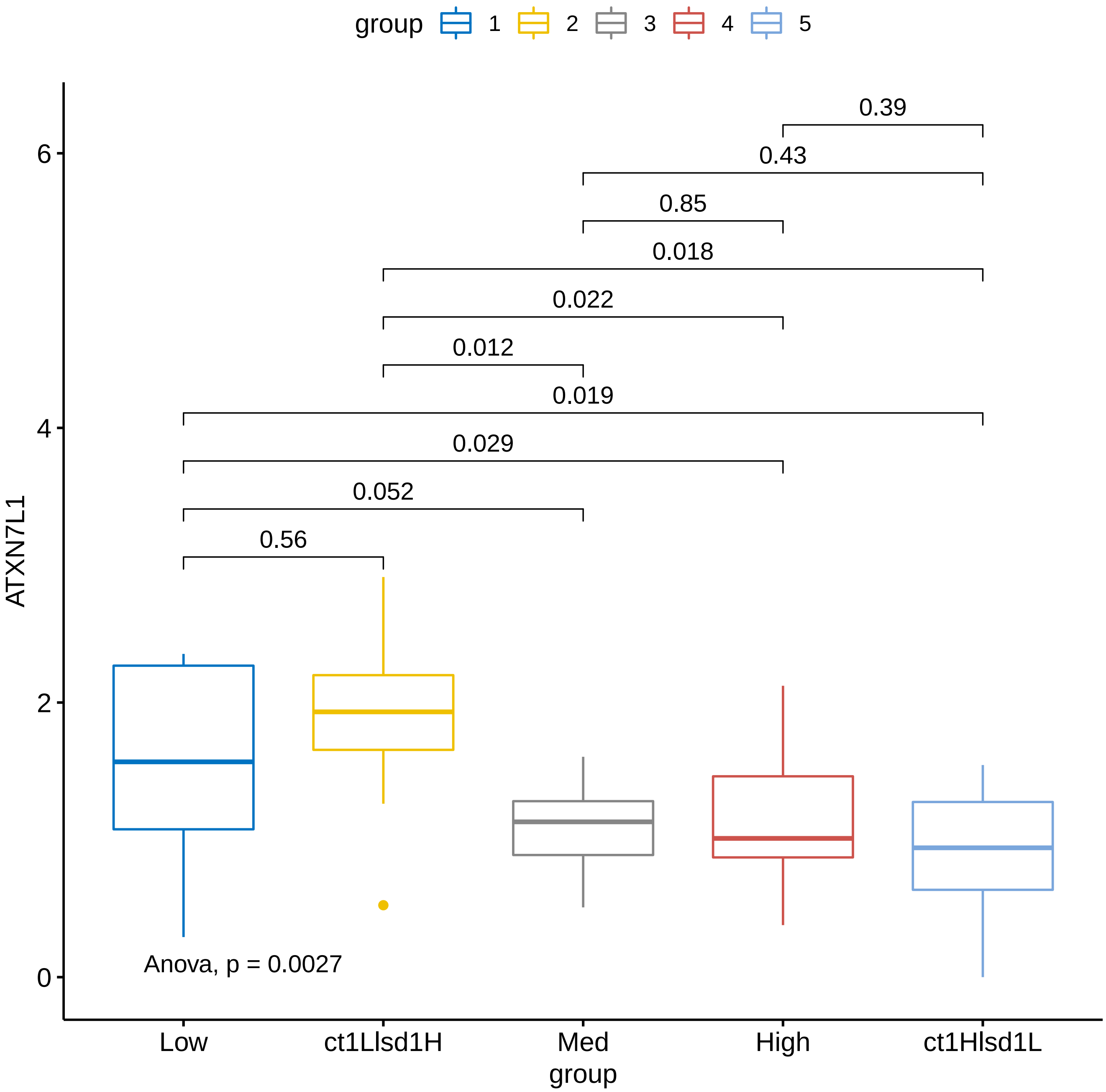
group 1 2 3 4 5
0.39
0.43
0.85
0.018
0.022
0.012
0.019
0.029
0.052
0.56
Anova, p = 0.0027
ATXN7L1
6
4
2
0
Low
ct1Llsd1H
Med
High
ct1Hlsd1L
group

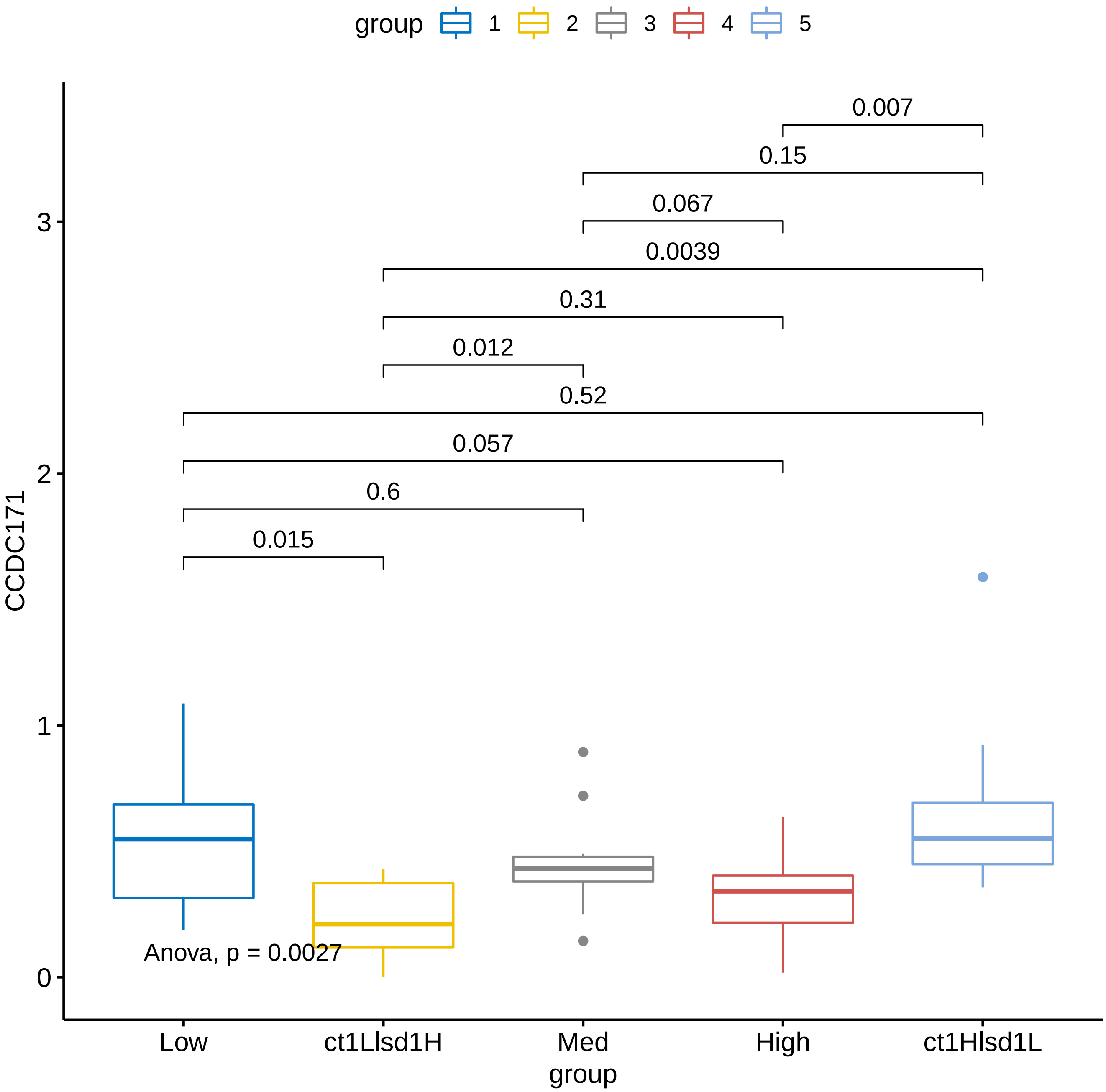
group
1
2
3
4
5
CCDC171
0
1
2
3
0.007
0.15
0.067
0.0039
0.31
0.012
0.52
0.057
0.6
0.015
Anova, p = 0.0027
Low
ct1Llsd1H
Med
High
ct1Hlsd1L
group

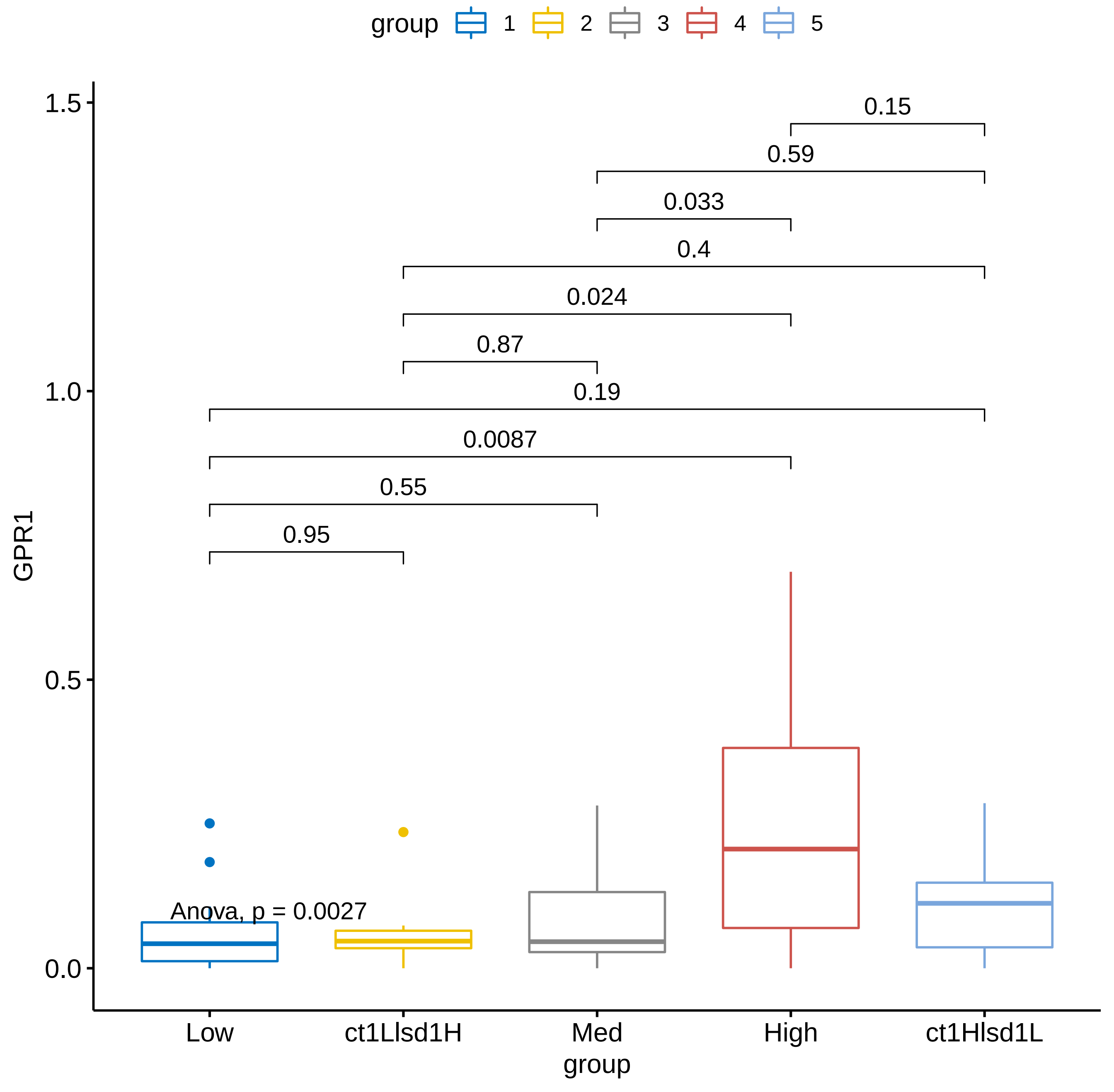
group 1 2 3 4 5
GPR1
1.5
1.0
0.5
0.0
0.15
0.59
0.033
0.4
0.024
0.87
0.19
0.0087
0.55
0.95
Anova, p = 0.0027
Low
ct1Llsd1H
Med
High
ct1Hlsd1L
group

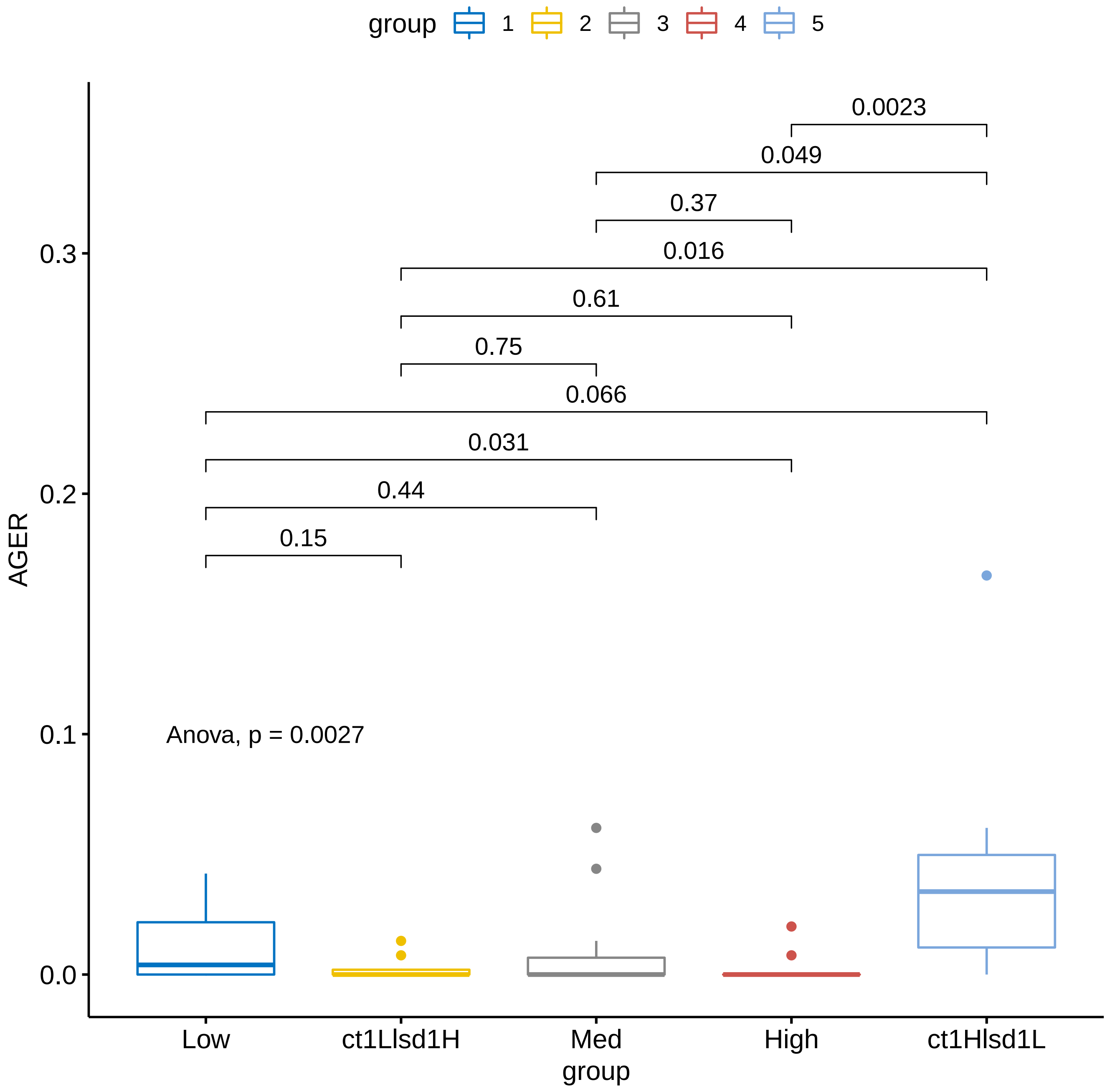
group
1
2
3
4
5
0.0023
0.049
0.37
0.016
0.61
0.75
0.066
0.031
0.44
0.15
Anova, p = 0.0027
0.3
0.2
0.1
0.0
AGER
Low
ct1Llsd1H
Med
High
ct1Hlsd1L
group

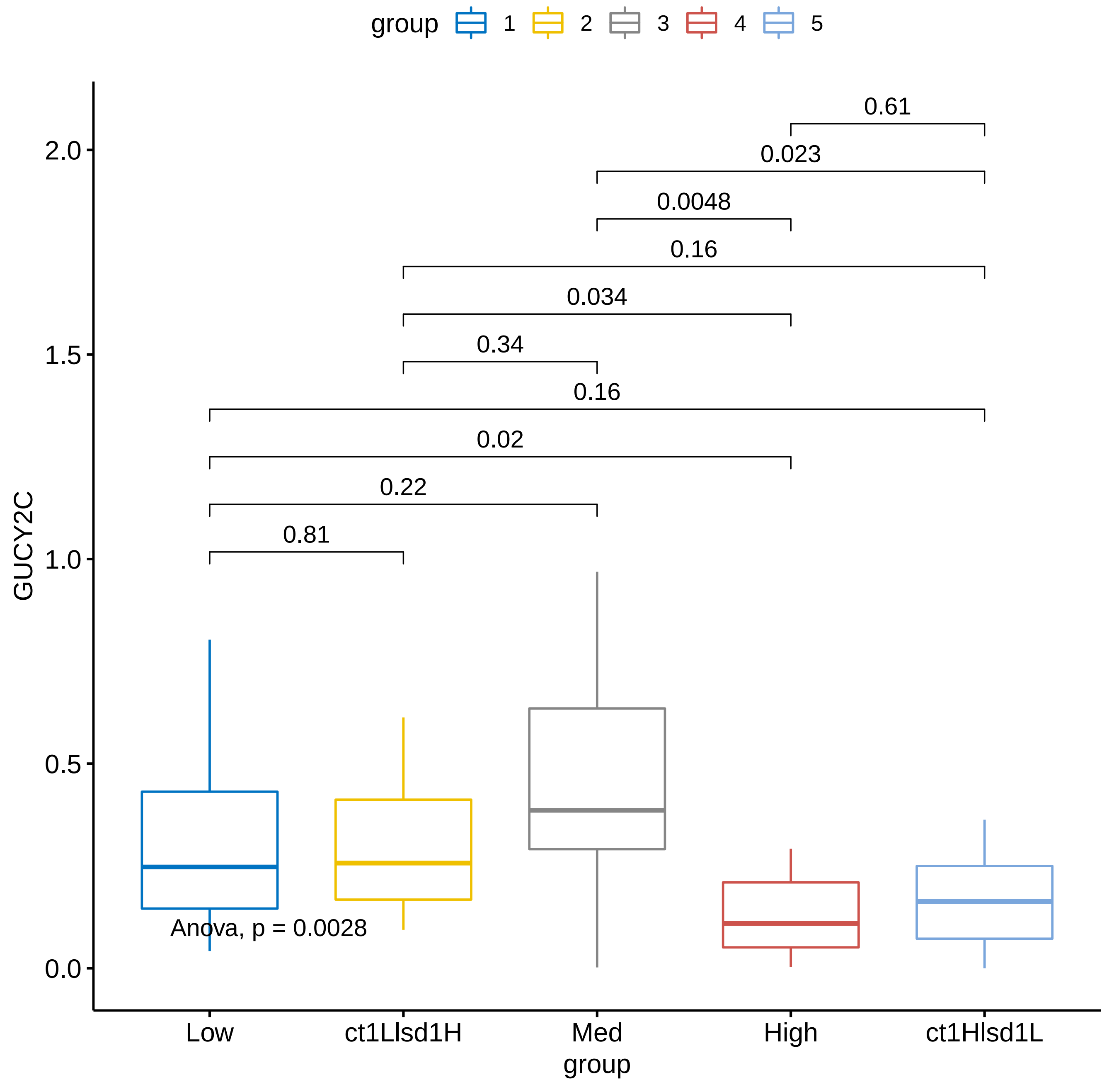
group
1
2
3
4
5
GUCY2C
2.0
1.5
1.0
0.5
0.0
0.61
0.023
0.0048
0.16
0.034
0.34
0.16
0.02
0.22
0.81
Anova, p = 0.0028
Low
ct1Llsd1H
Med
High
ct1Hlsd1L
group

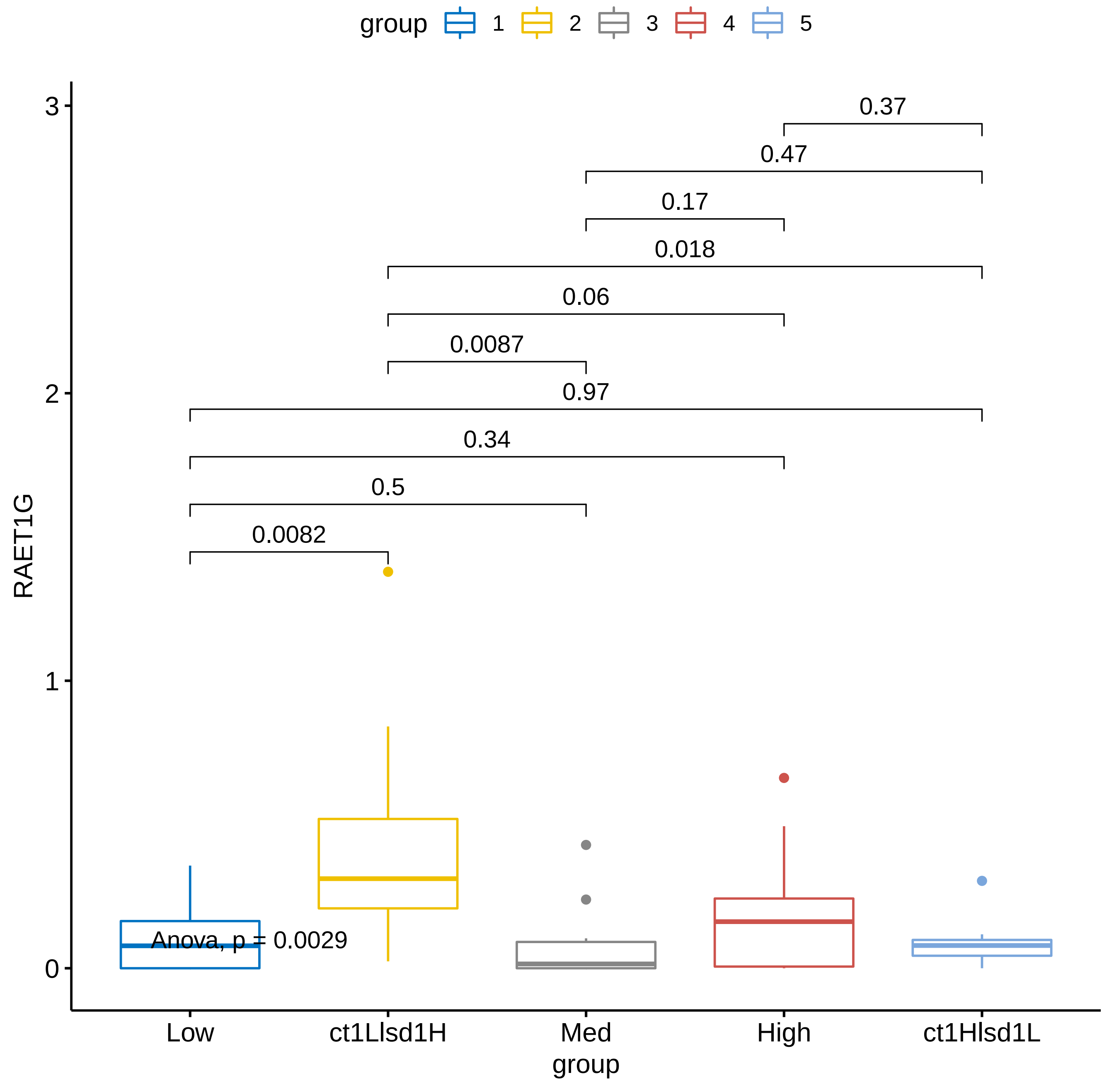

group 1 2 3 4 5
RAET1G
0.37
0.47
0.17
0.018
0.06
0.0087
0.97
0.34
0.5
0.0082
Anova, p = 0.0029
3
2
1
0
Low
ct1Llsd1H
Med
High
ct1Hlsd1L
group

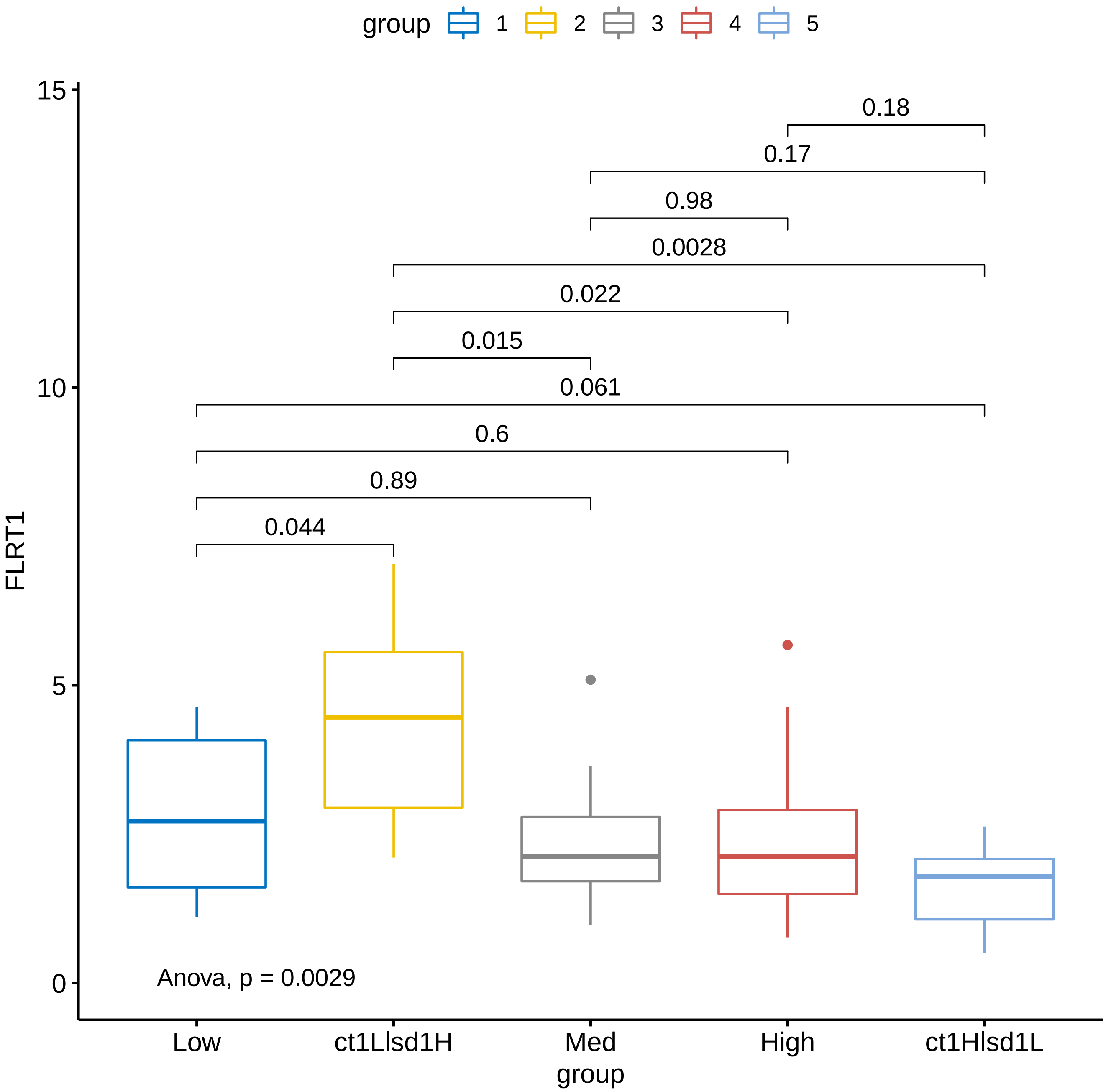
group 1 2 3 4 5
15
10
5
0
FLRT1
0.18
0.17
0.98
0.0028
0.022
0.015
0.061
0.6
0.89
0.044
Anova, p = 0.0029
Low
ct1Llsd1H
Med
High
ct1Hlsd1L
group

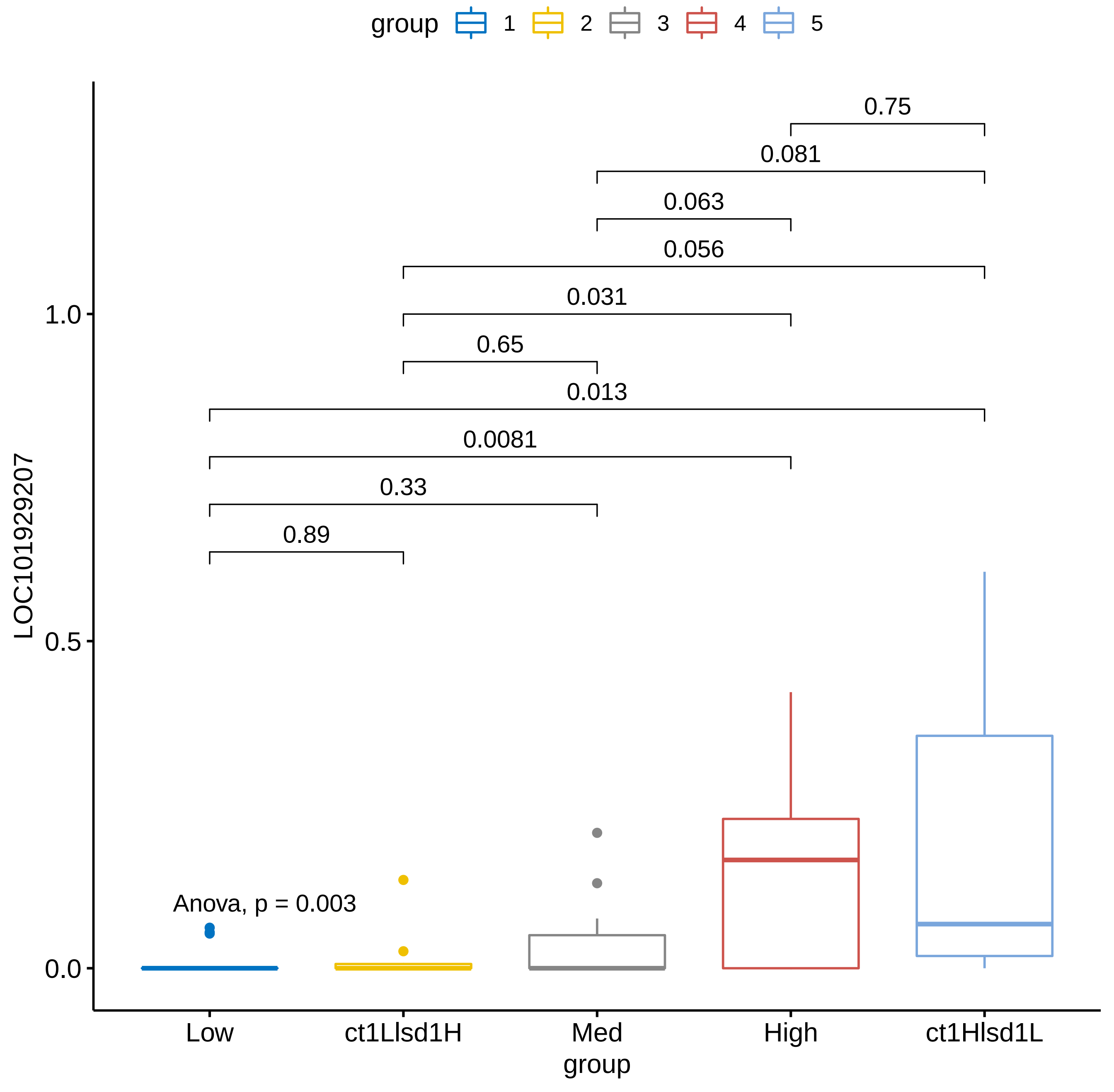

group 1 2 3 4 5
0.75
0.081
0.063
0.056
0.031
0.65
0.013
0.0081
0.33
0.89
Anova, p = 0.003
1.0
0.5
0.0
LOC101929207
Low
ct1Llsd1H
Med
High
ct1Hlsd1L
group

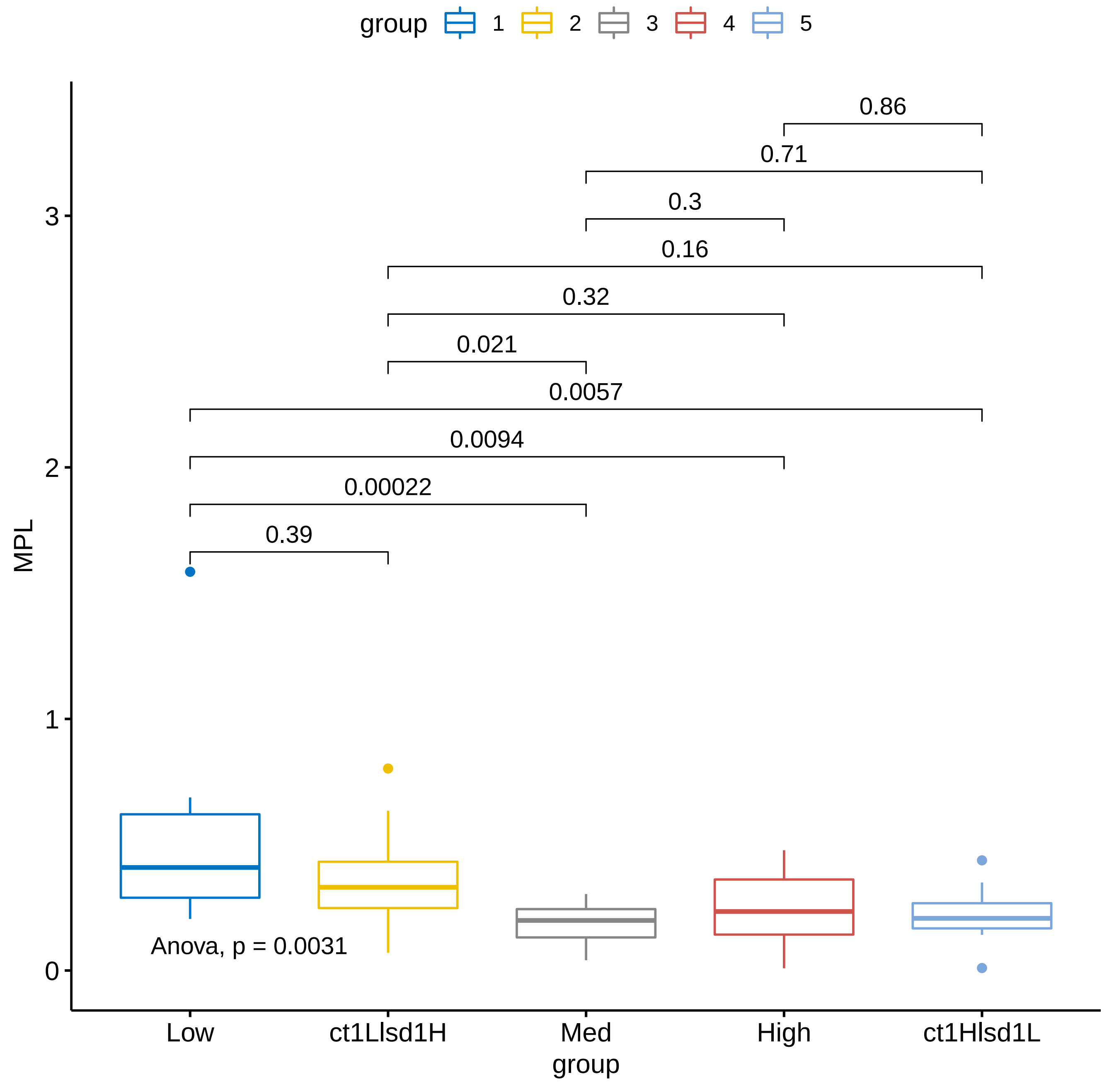
group
1
2
3
4
5
0.86
0.71
0.3
0.16
0.32
0.021
0.0057
0.0094
0.00022
0.39
3
2
1
0
MPL
Anova, p = 0.0031
Low
ct1Llsd1H
Med
High
ct1Hlsd1L
group

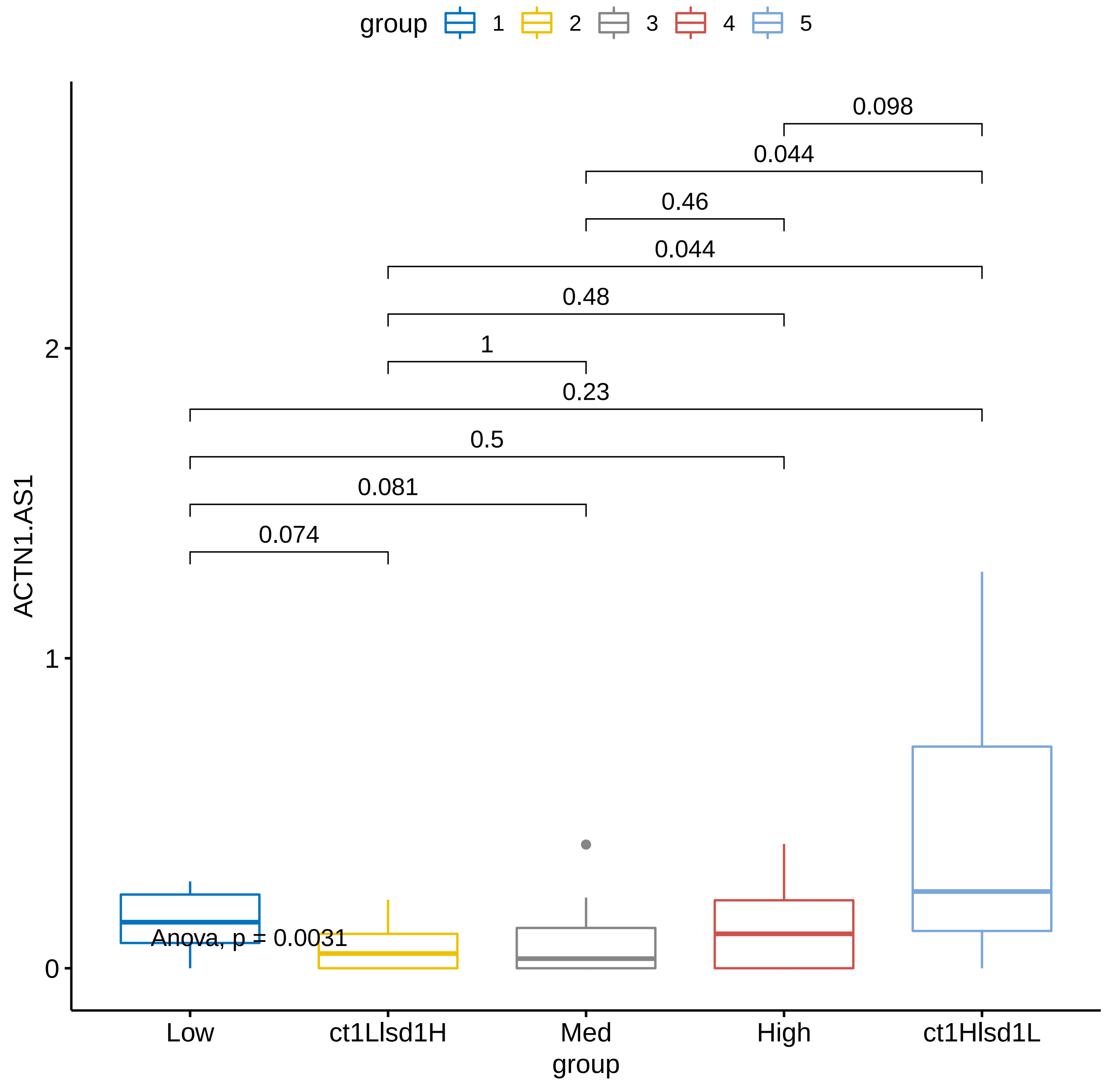
group
1
2
3
4
5
0.098
0.044
0.46
0.044
0.48
1
0.23
0.5
0.081
0.074
ACTN1.AS1
2
1
0
Anova, p = 0.0031
Low
ct1Llsd1H
Med
High
ct1Hlsd1L
group

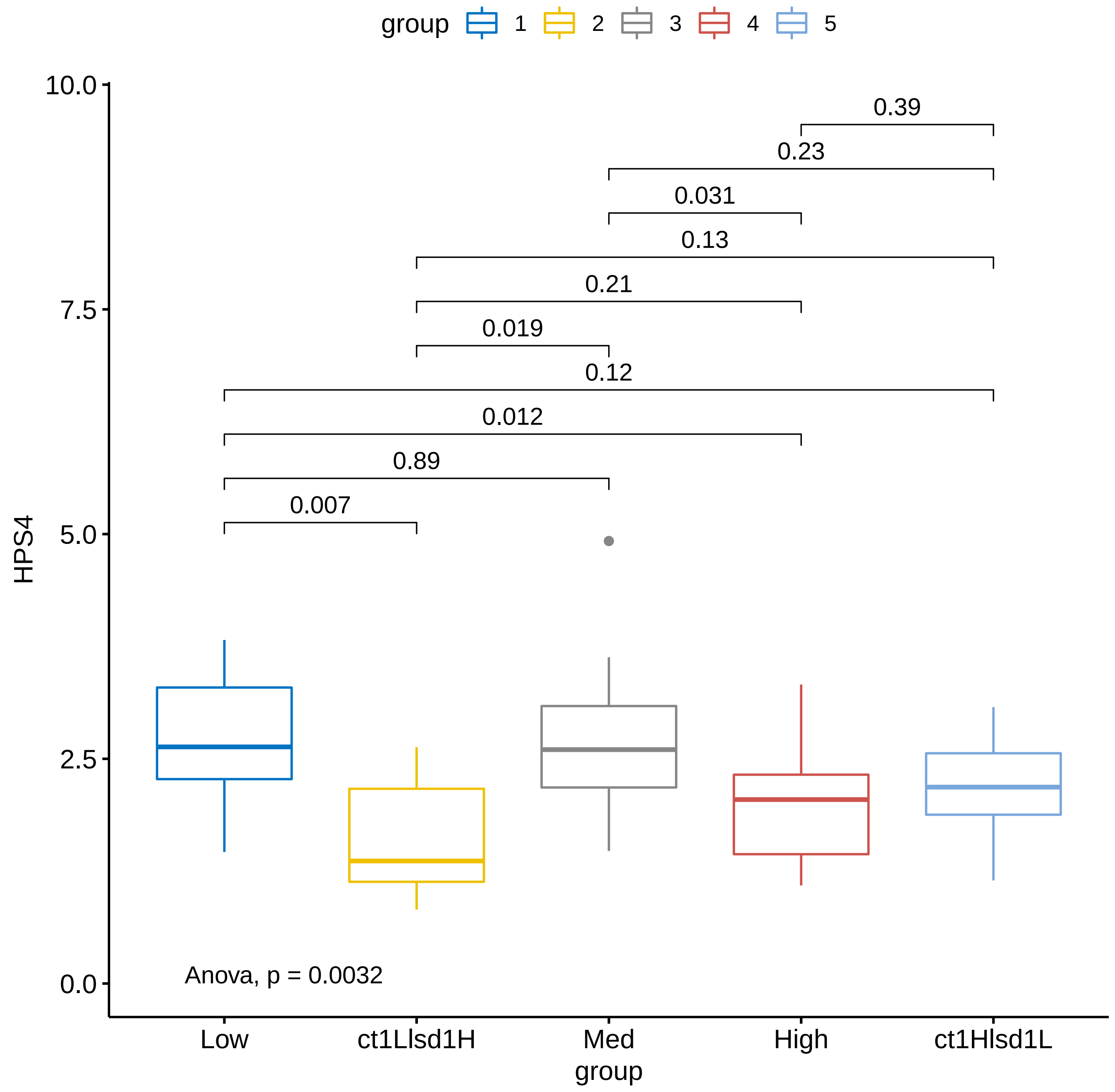

group
1
2
3
4
5
HPS4
10.0
7.5
5.0
2.5
0.0
0.39
0.23
0.031
0.13
0.21
0.019
0.12
0.012
0.89
0.007
Anova, p = 0.0032
Low
ct1Llsd1H
Med
High
ct1Hlsd1L
group

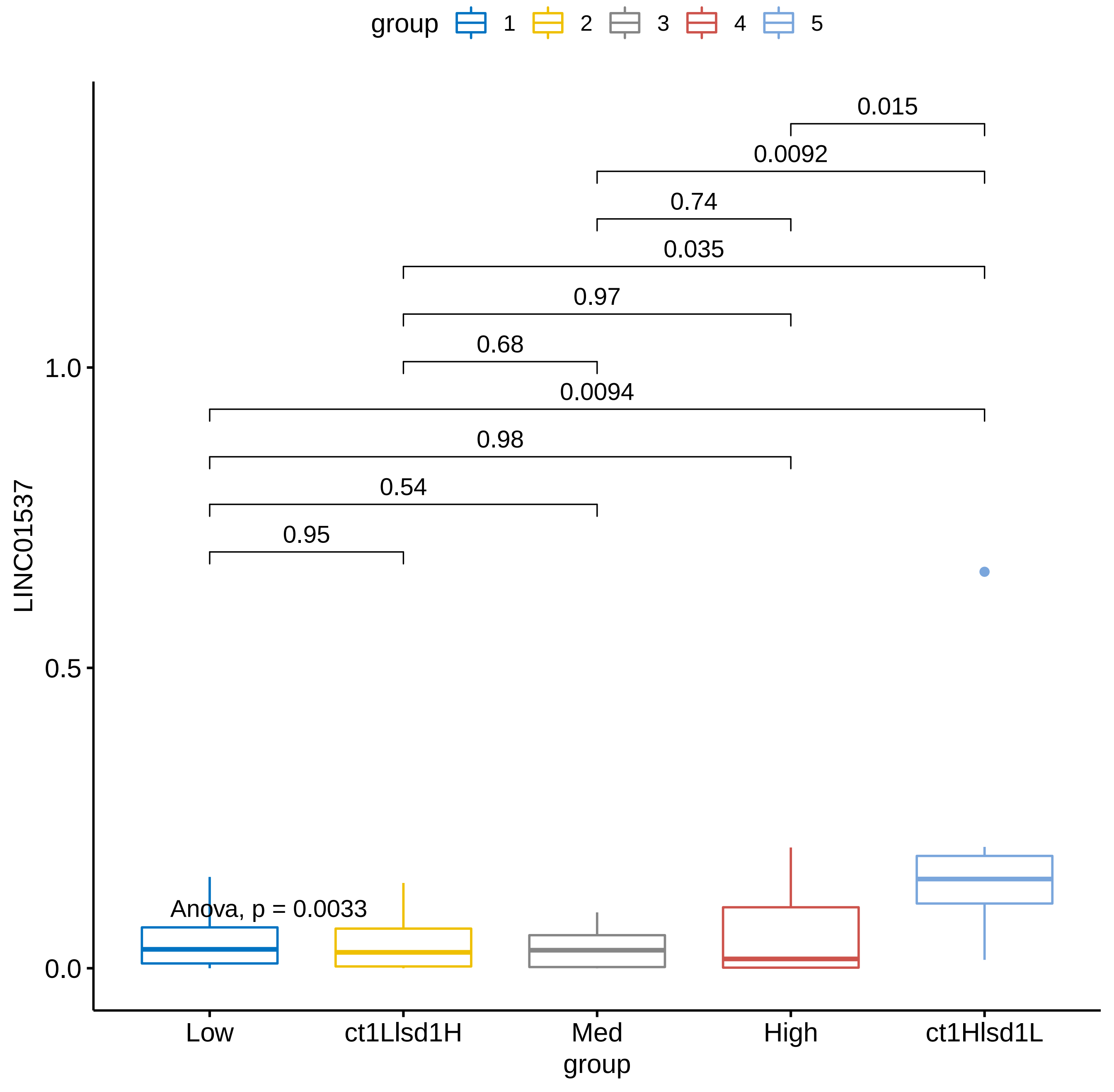

group 1 2 3 4 5
0.015
0.0092
0.74
0.035
0.97
0.68
0.0094
0.98
0.54
0.95
Anova, p = 0.0033
LINC01537
1.0
0.5
0.0
Low
ct1Llsd1H
Med
High
ct1Hlsd1L
group

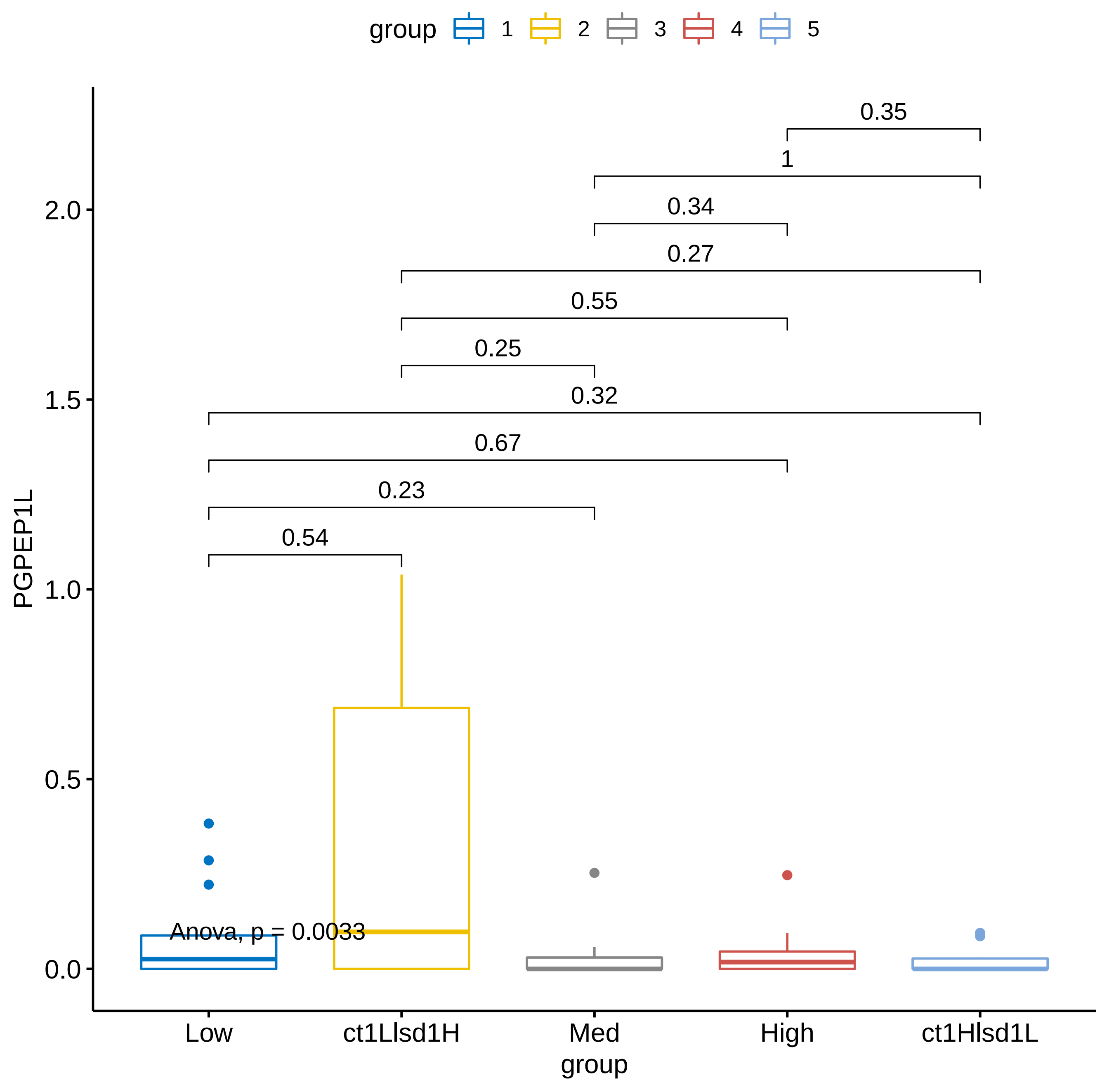

group 1 2 3 4 5
0.35
1
0.34
0.27
0.55
0.25
0.32
0.67
0.23
0.54
Anova, p = 0.0033
PGPEP1L
2.0
1.5
1.0
0.5
0.0
Low
ct1Llsd1H
Med
High
ct1Hlsd1L
group

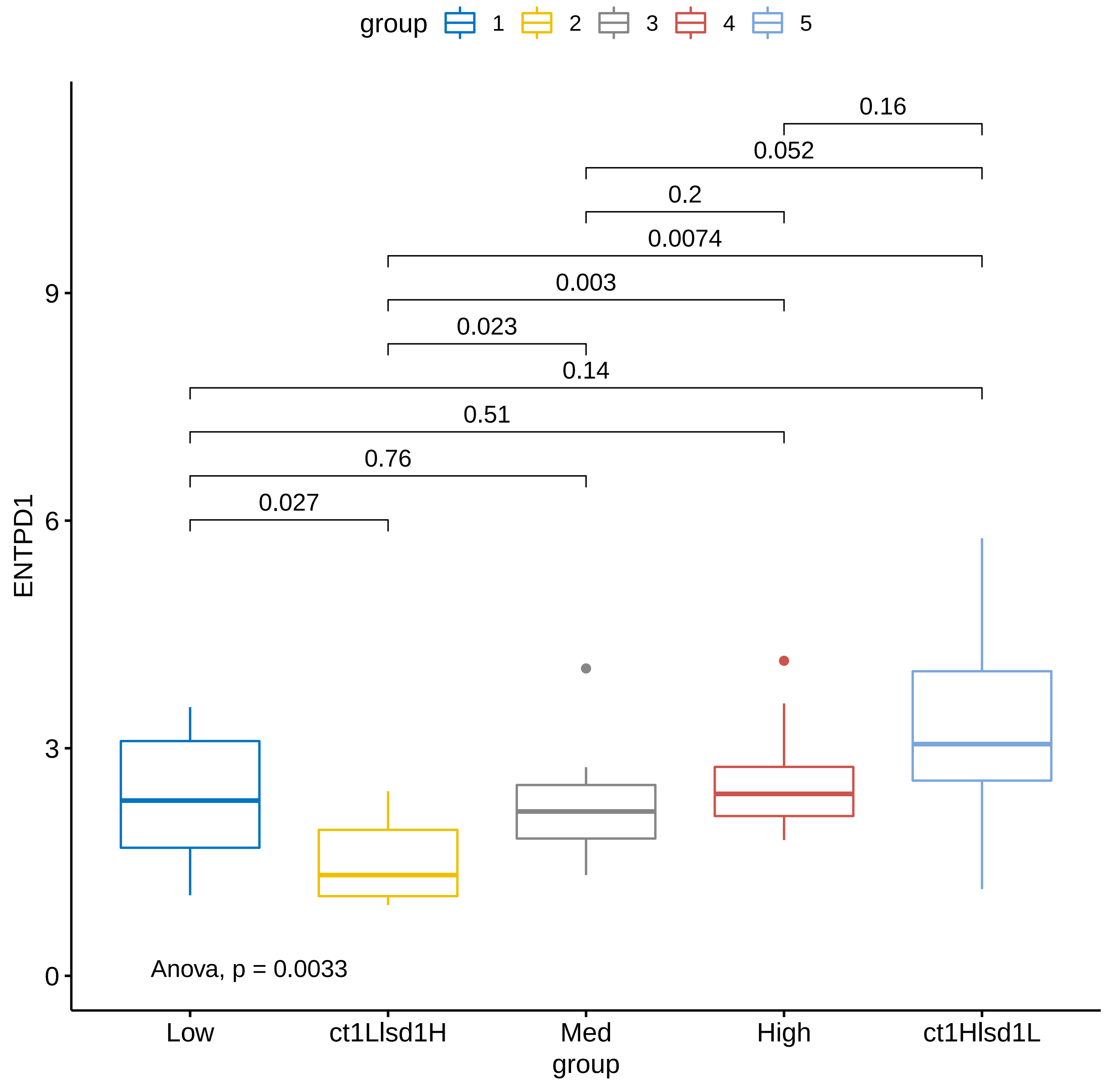

group
1
2
3
4
5
0.16
0.052
0.2
0.0074
0.003
0.023
0.14
0.51
0.76
0.027
ENTPD1
9
6
3
0
Anova, p = 0.0033
Low
ct1Llsd1H
Med
High
ct1Hlsd1L
group

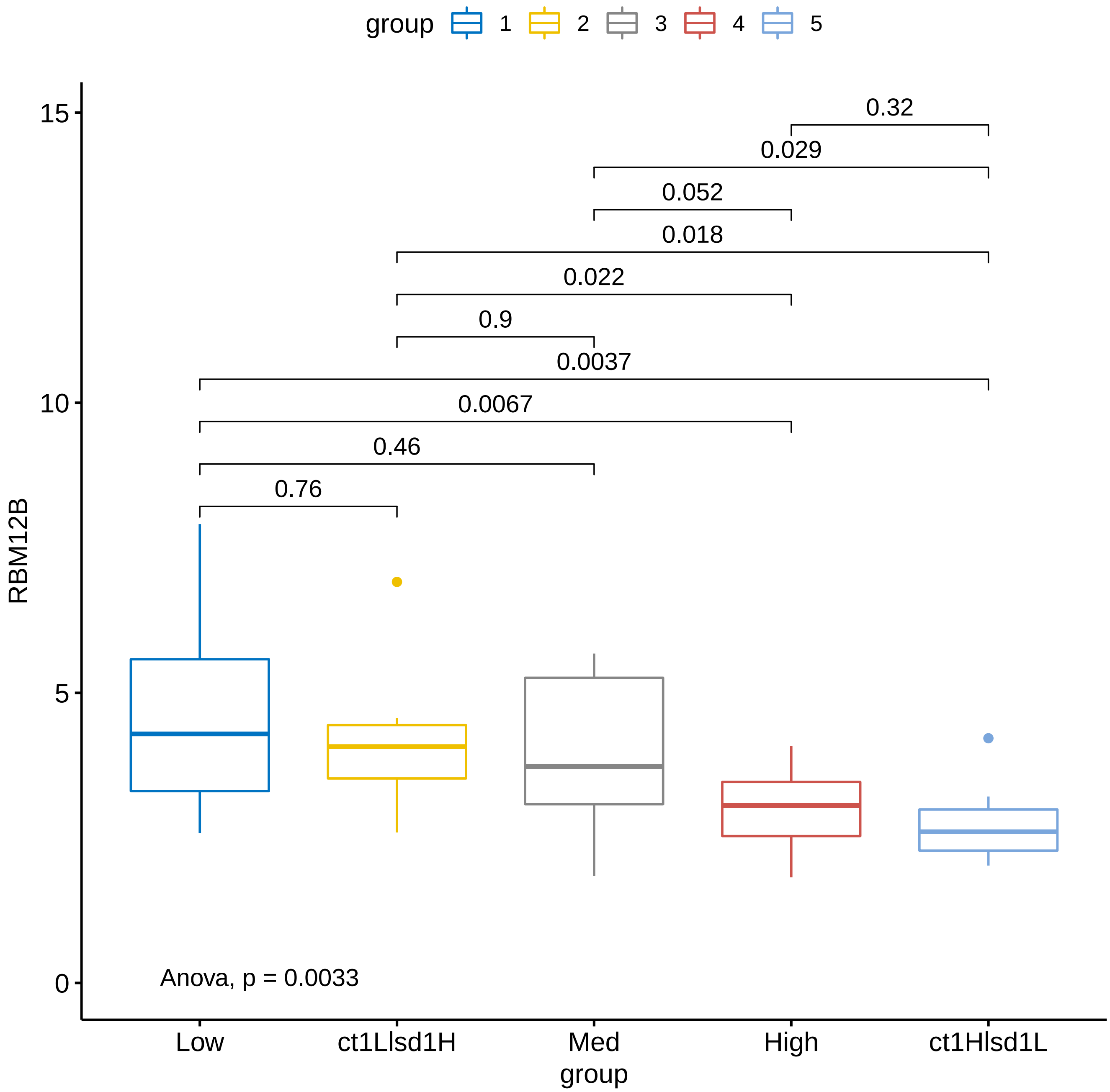
group 1 2 3 4 5
0.32
0.029
0.052
0.018
0.022
0.9
0.0037
0.0067
0.46
0.76
RBM12B
15
10
5
0
Anova, p = 0.0033
Low
ct1Llsd1H
Med
High
ct1Hlsd1L
group

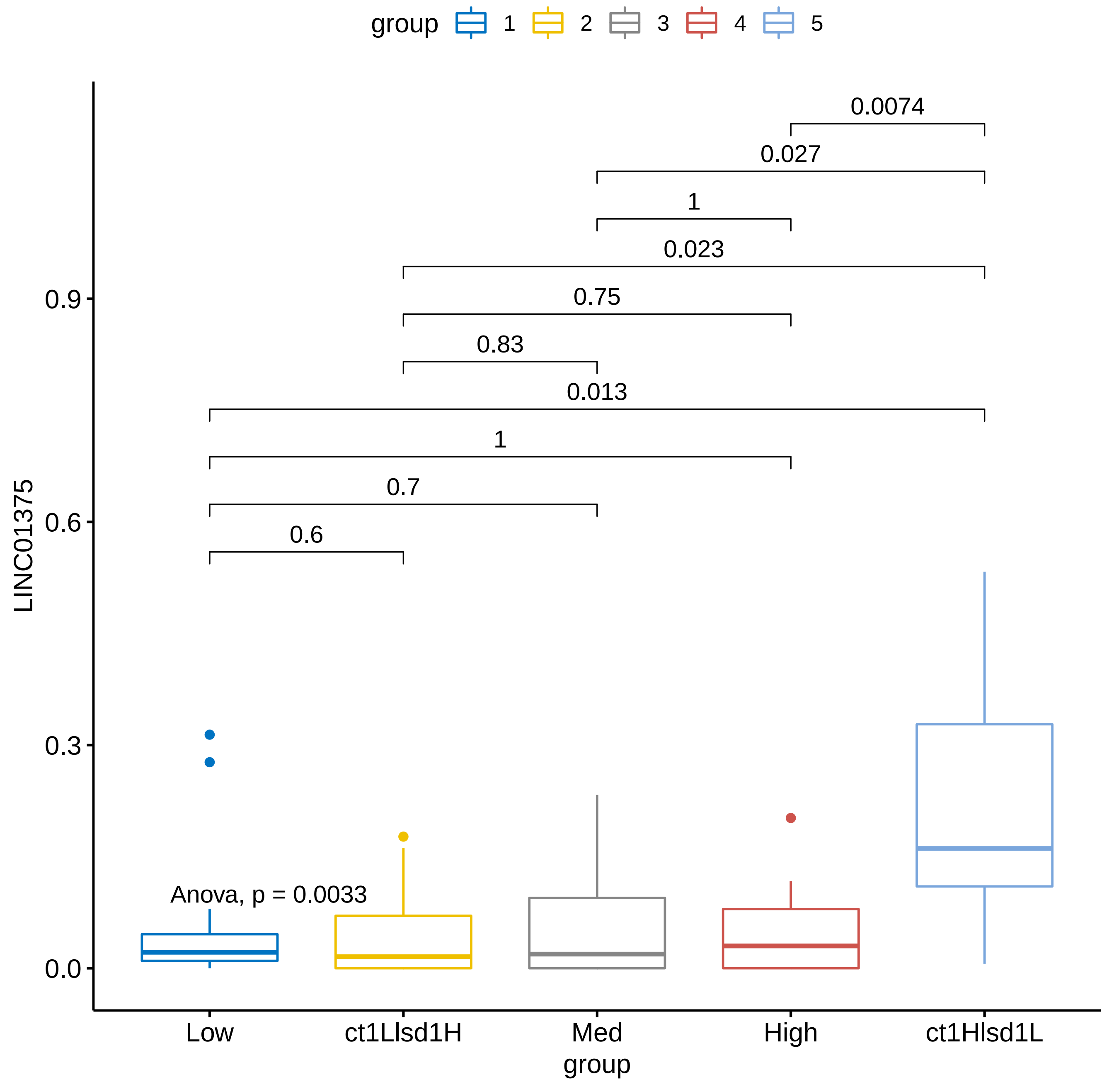
group
1
2
3
4
5
0.0074
0.027
1
0.023
0.75
0.83
0.013
1
0.7
0.6
Anova, p = 0.0033
LINC01375
0.9
0.6
0.3
0.0
Low
ct1Llsd1H
Med
High
ct1Hlsd1L
group

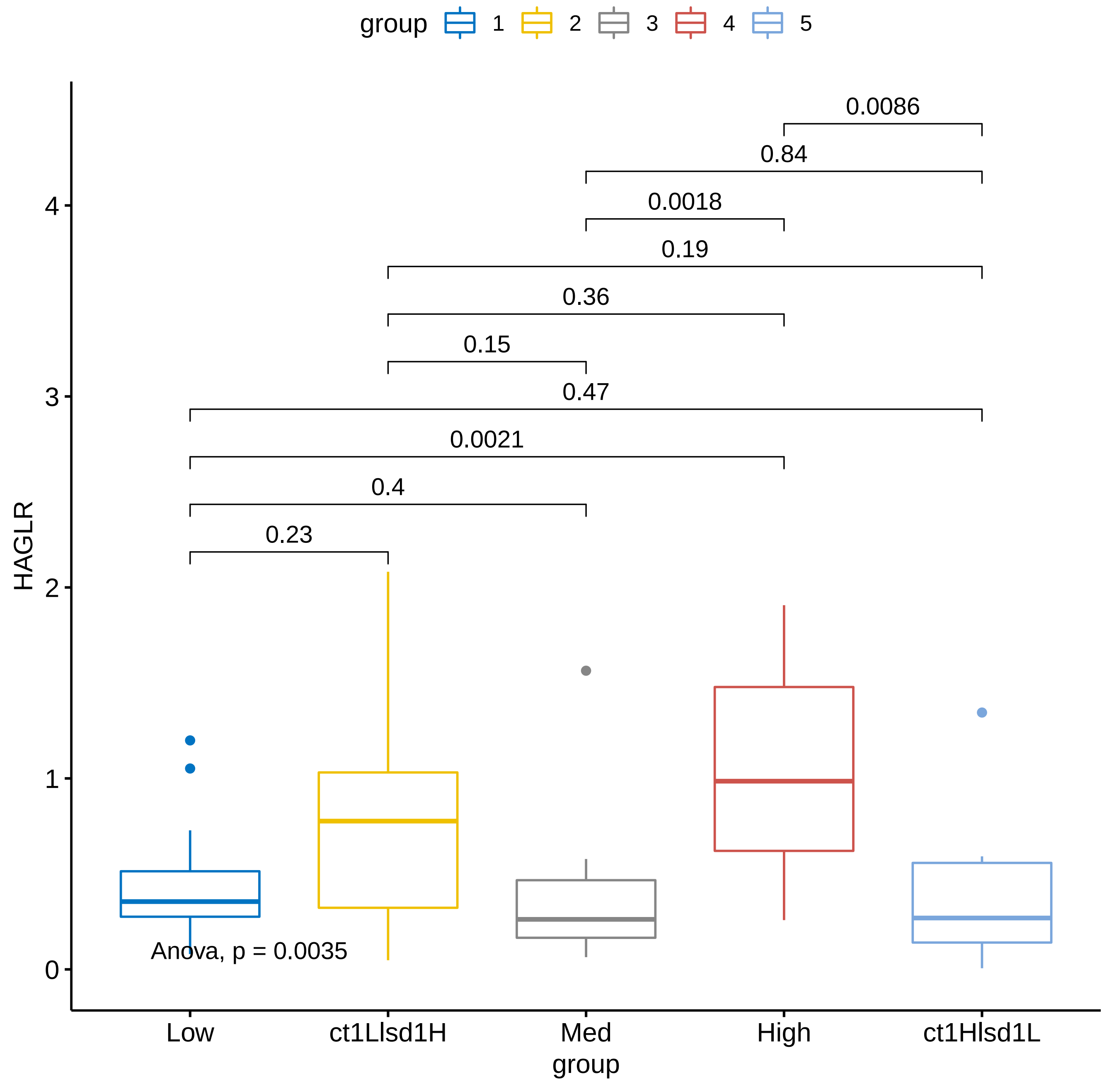

group
1
2
3
4
5
HAGLR
0
1
2
3
4
0.0086
0.84
0.0018
0.19
0.36
0.15
0.47
0.0021
0.4
0.23
Anova, p = 0.0035
Low
ct1Llsd1H
Med
High
ct1Hlsd1L
group

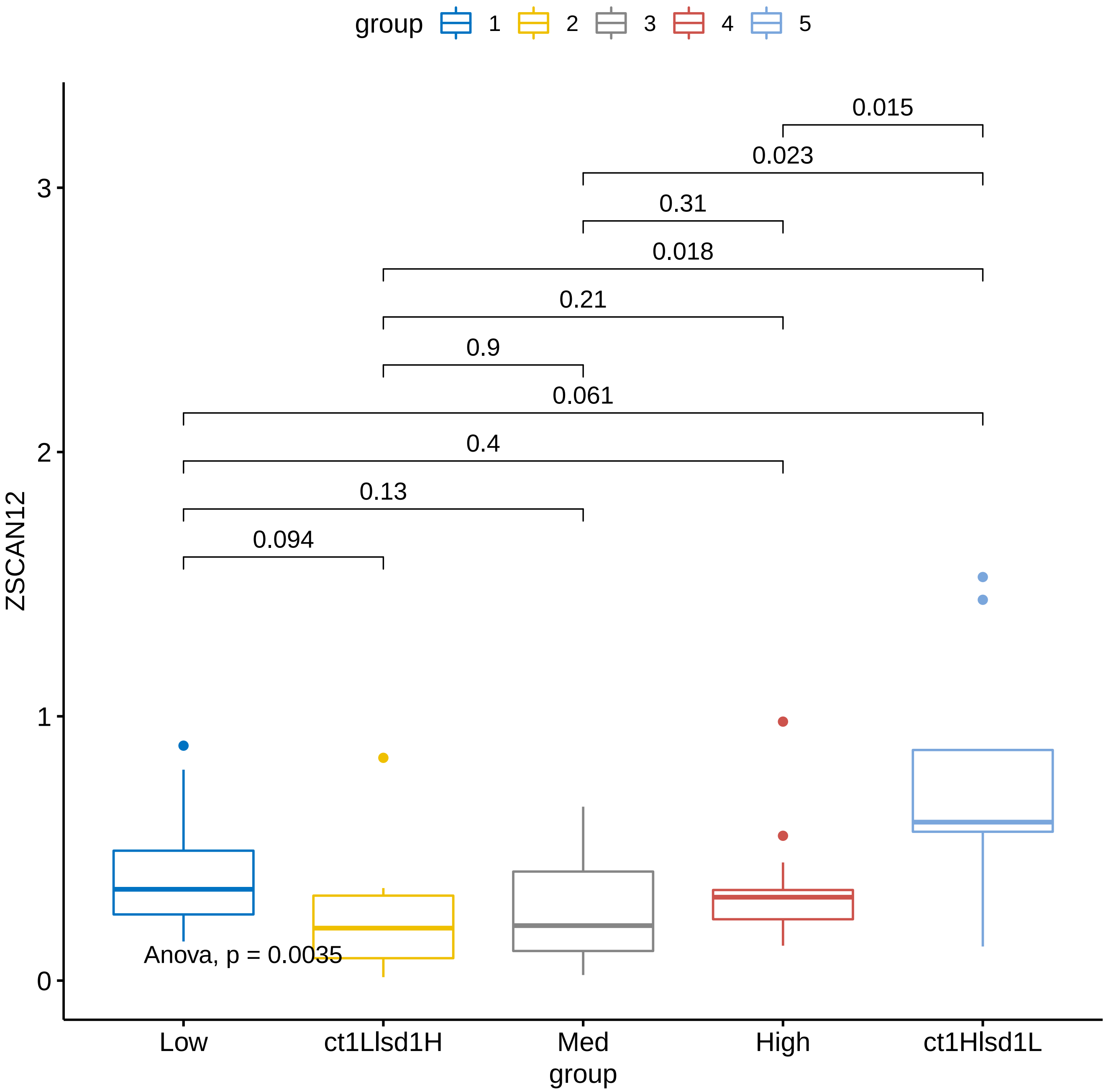

group
1
2
3
4
5
ZSCAN12
3
2
1
0
0.015
0.023
0.31
0.018
0.21
0.9
0.061
0.4
0.13
0.094
Anova, p = 0.0035
Low
ct1Llsd1H
Med
High
ct1Hlsd1L
group

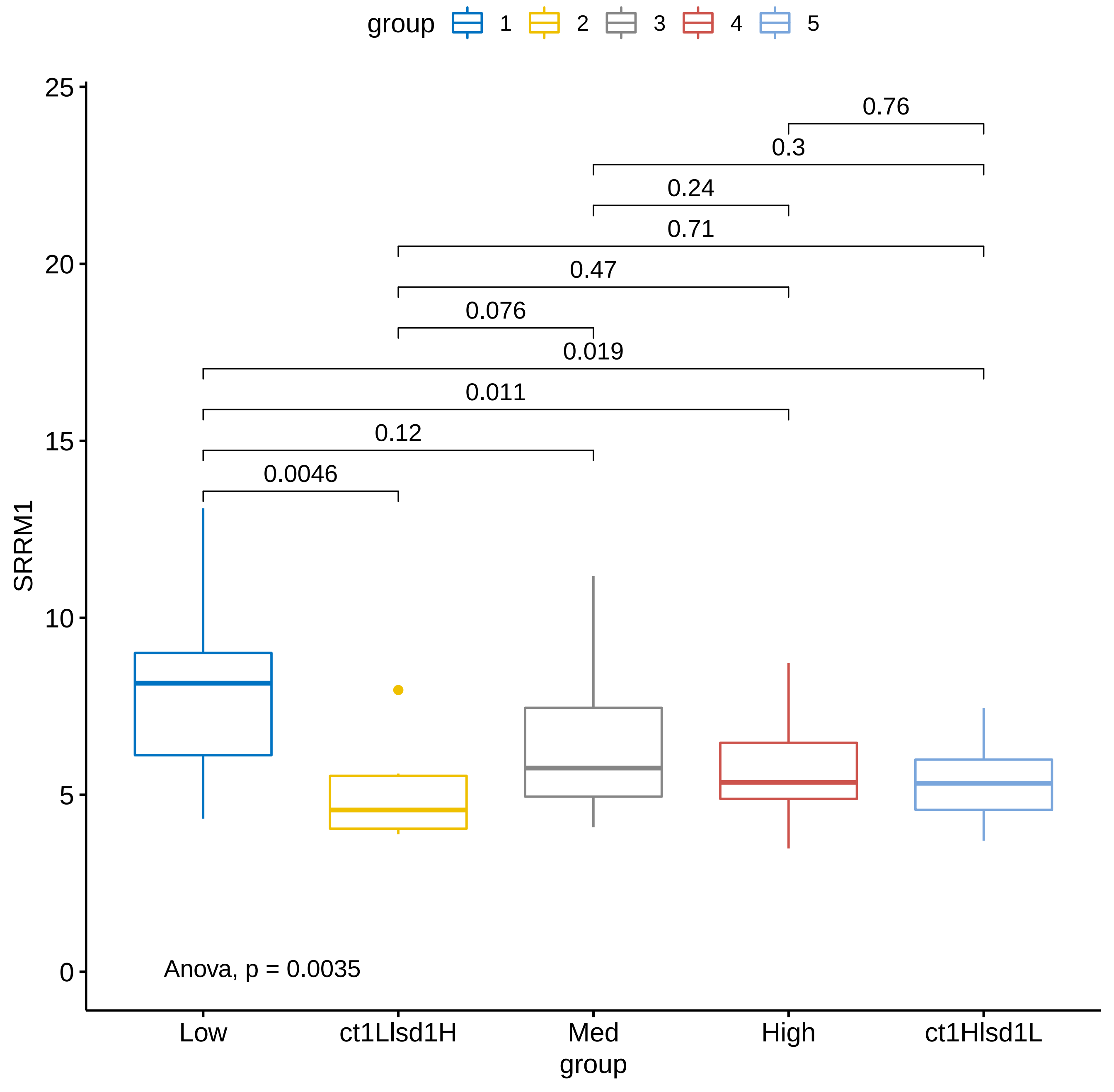

group 1 2 3 4 5
25
20
15
10
5
0
SRRM1
0.76
0.3
0.24
0.71
0.47
0.076
0.019
0.011
0.12
0.0046
Anova, p = 0.0035
Low
ct1Llsd1H
Med
High
ct1Hlsd1L
group

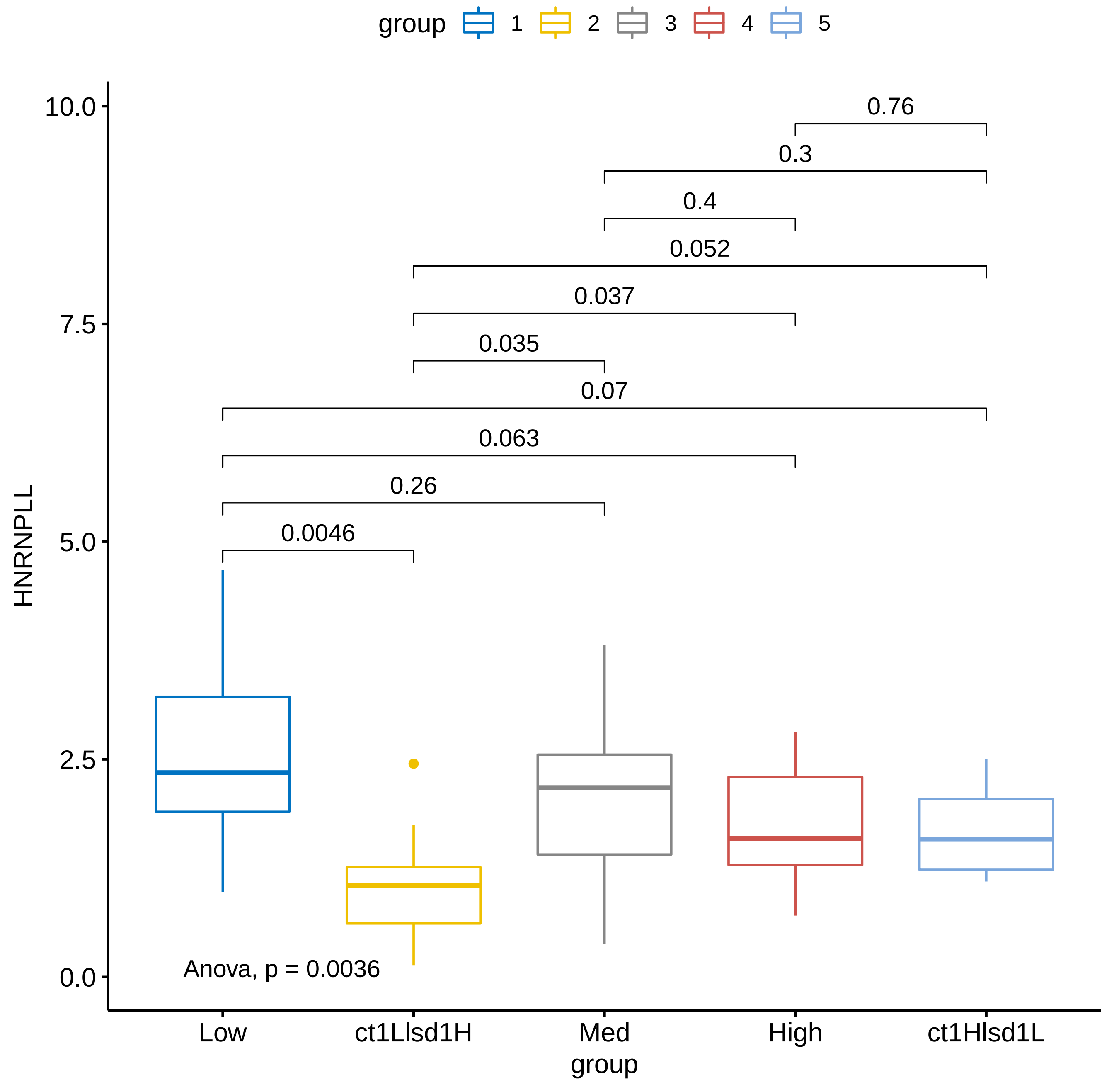
group
1
2
3
4
5
HNRNPLL
10.0
7.5
5.0
2.5
0.0
0.76
0.3
0.4
0.052
0.037
0.035
0.07
0.063
0.26
0.0046
Anova, p = 0.0036
Low
ct1Llsd1H
Med
High
ct1Hlsd1L
group

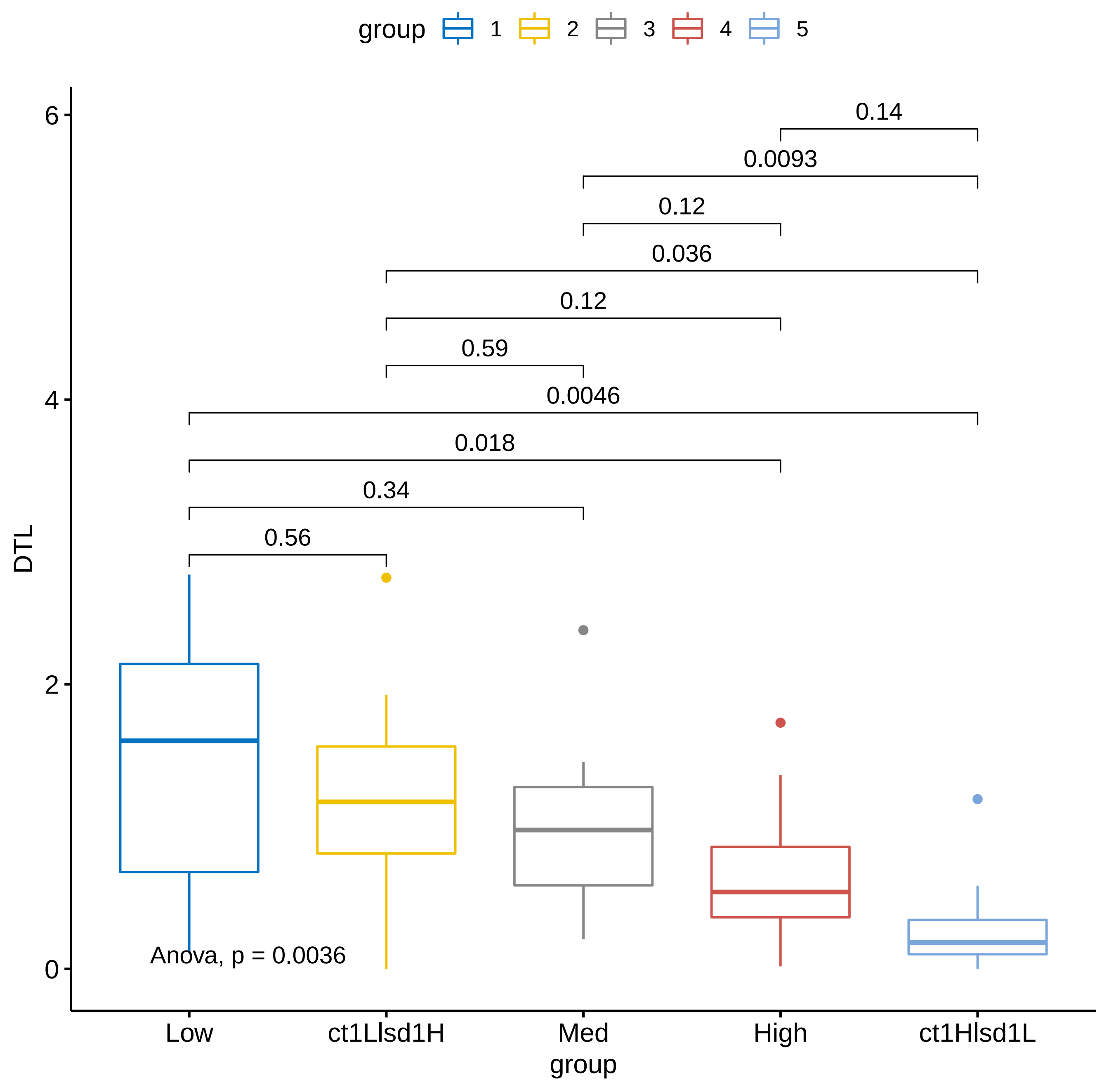

group
1
2
3
4
5
0.14
0.0093
0.12
0.036
0.12
0.59
0.0046
0.018
0.34
0.56
Anova, p = 0.0036
DTL
6
4
2
0
Low
ct1Llsd1H
Med
High
ct1Hlsd1L
group

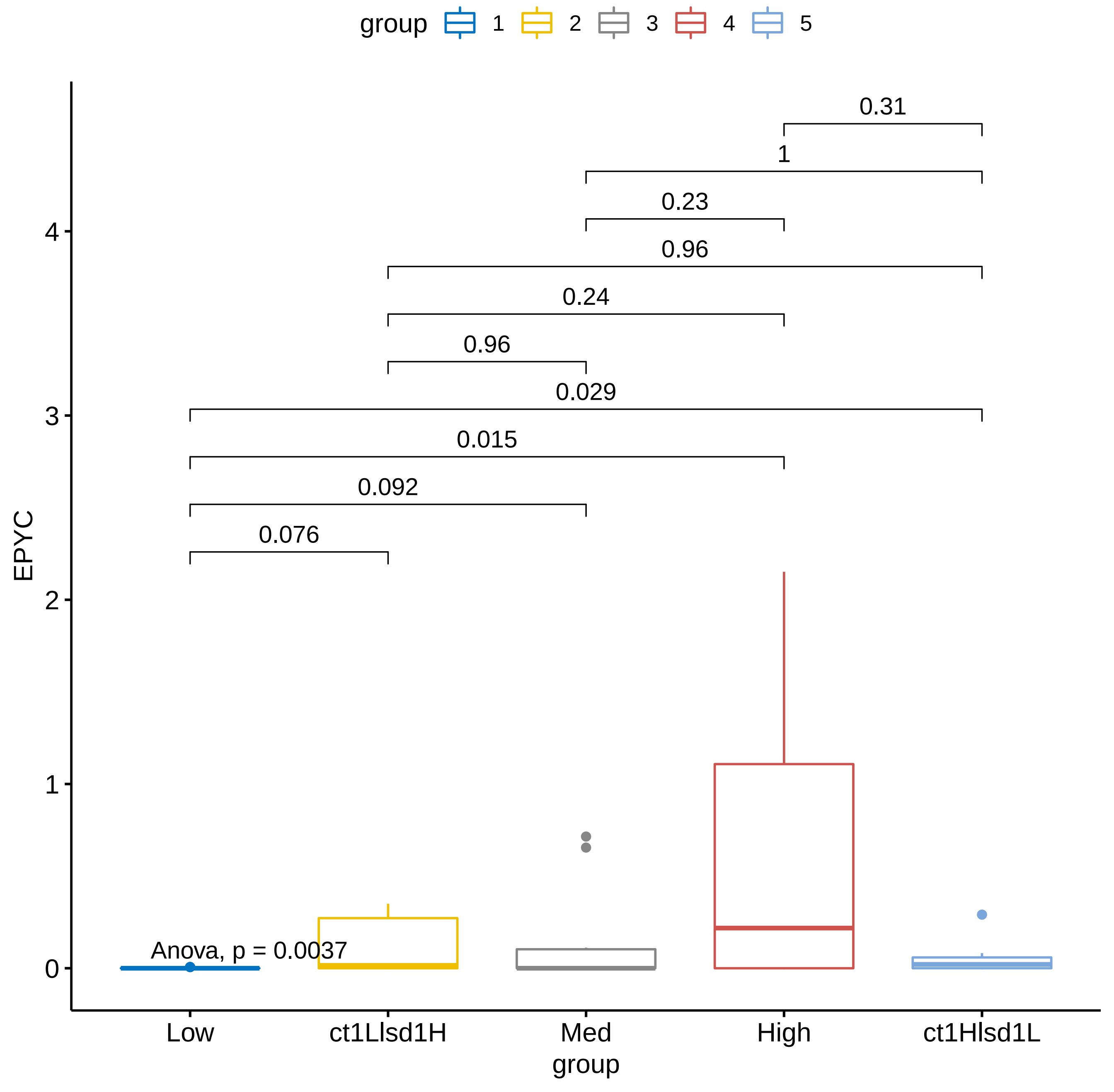

group 1 2 3 4 5
0.31
1
0.23
0.96
0.24
0.96
0.029
0.015
0.092
0.076
Anova, p = 0.0037
EPYC
0
1
2
3
4
Low
ct1Llsd1H
Med
High
ct1Hlsd1L
group

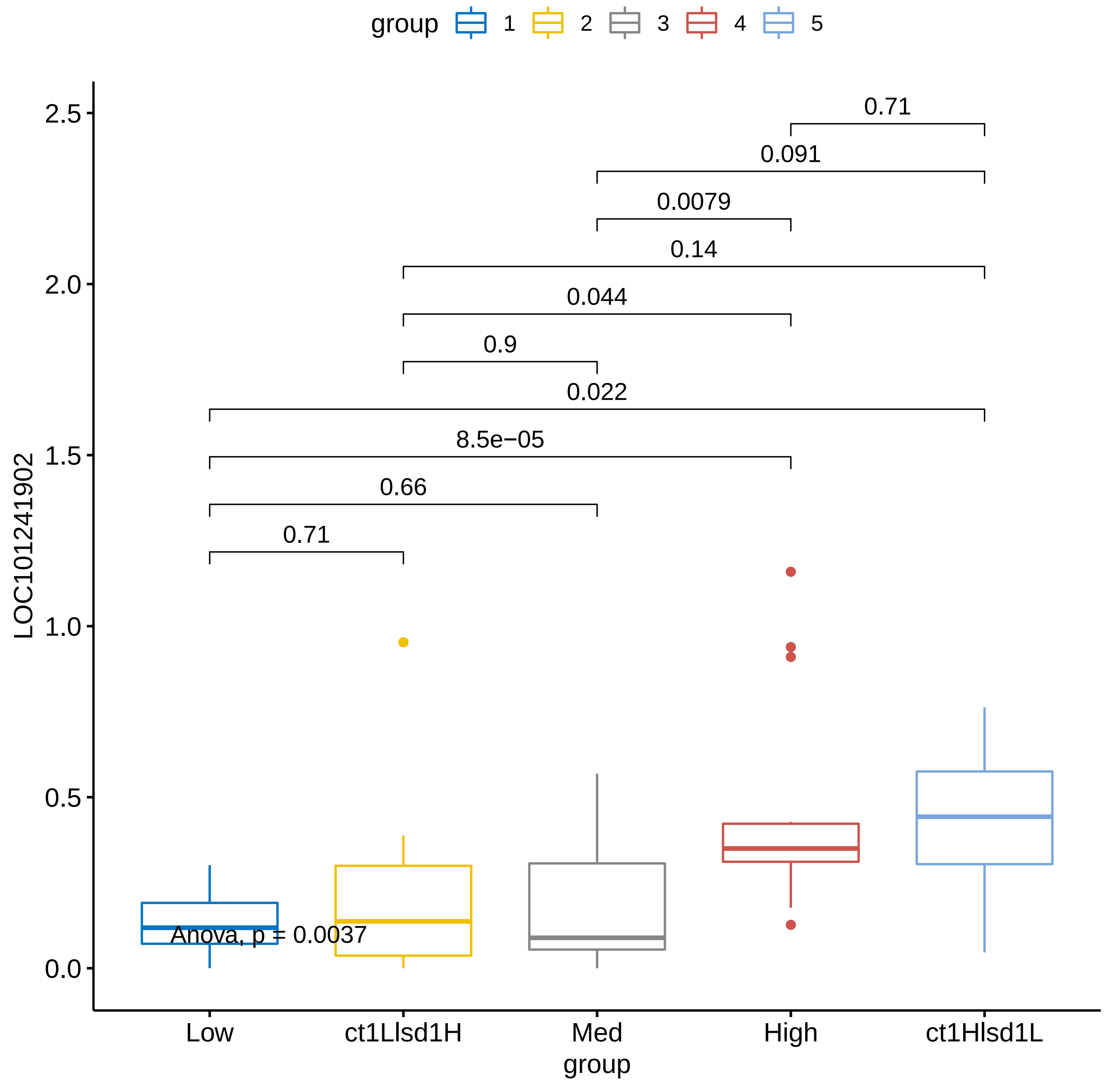

group 1 2 3 4 5
0.71
0.091
0.0079
0.14
0.044
0.9
0.022
8.5e−05
0.66
0.71
Anova, p = 0.0037
LOC101241902
2.5
2.0
1.5
1.0
0.5
0.0
Low
ct1Llsd1H
Med
High
ct1Hlsd1L
group

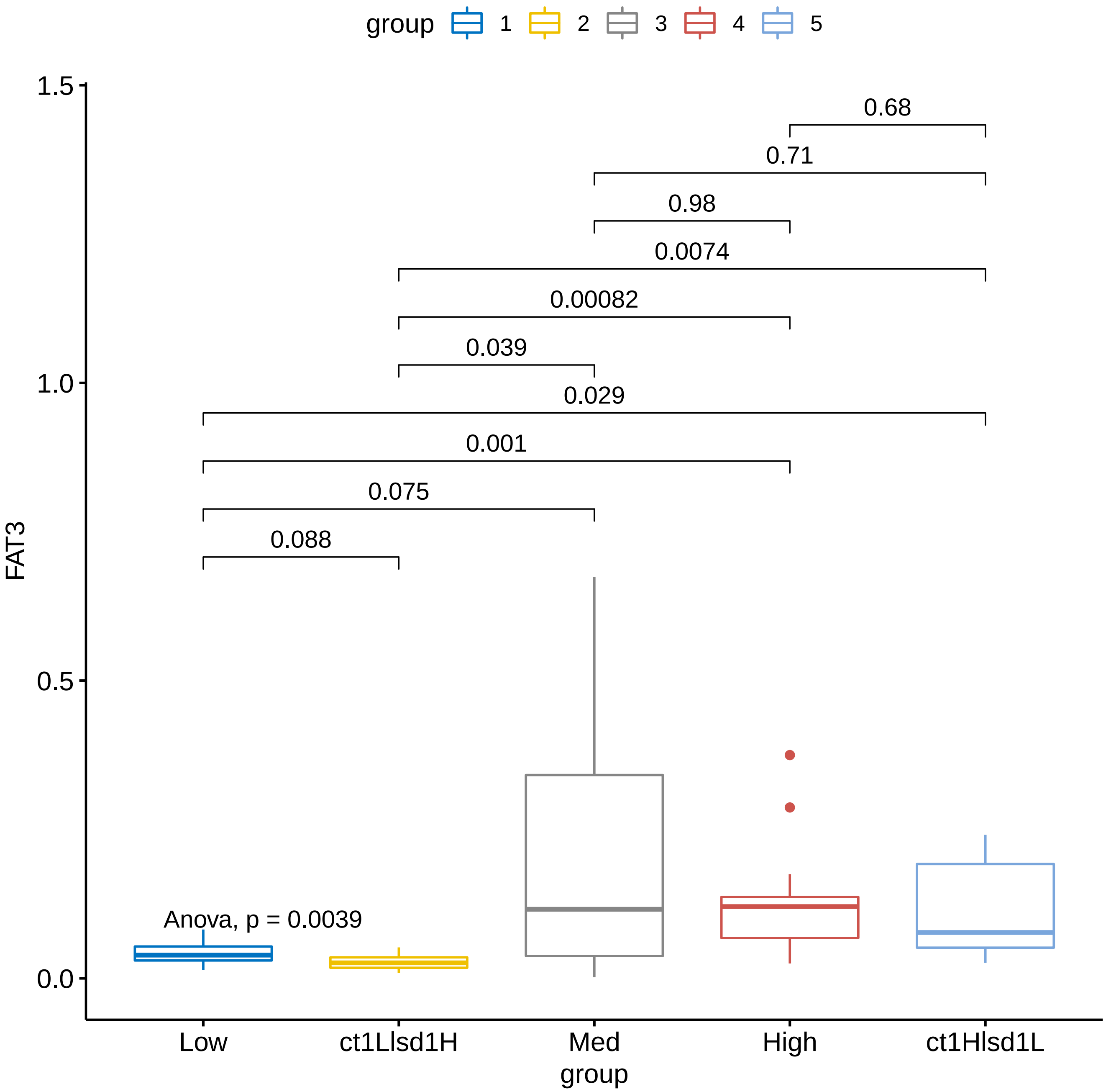
group
1
2
3
4
5
0.68
0.71
0.98
0.0074
0.00082
0.039
0.029
0.001
0.075
0.088
Anova, p = 0.0039
1.5
1.0
0.5
0.0
FAT3
Low
ct1Llsd1H
Med
High
ct1Hlsd1L
group

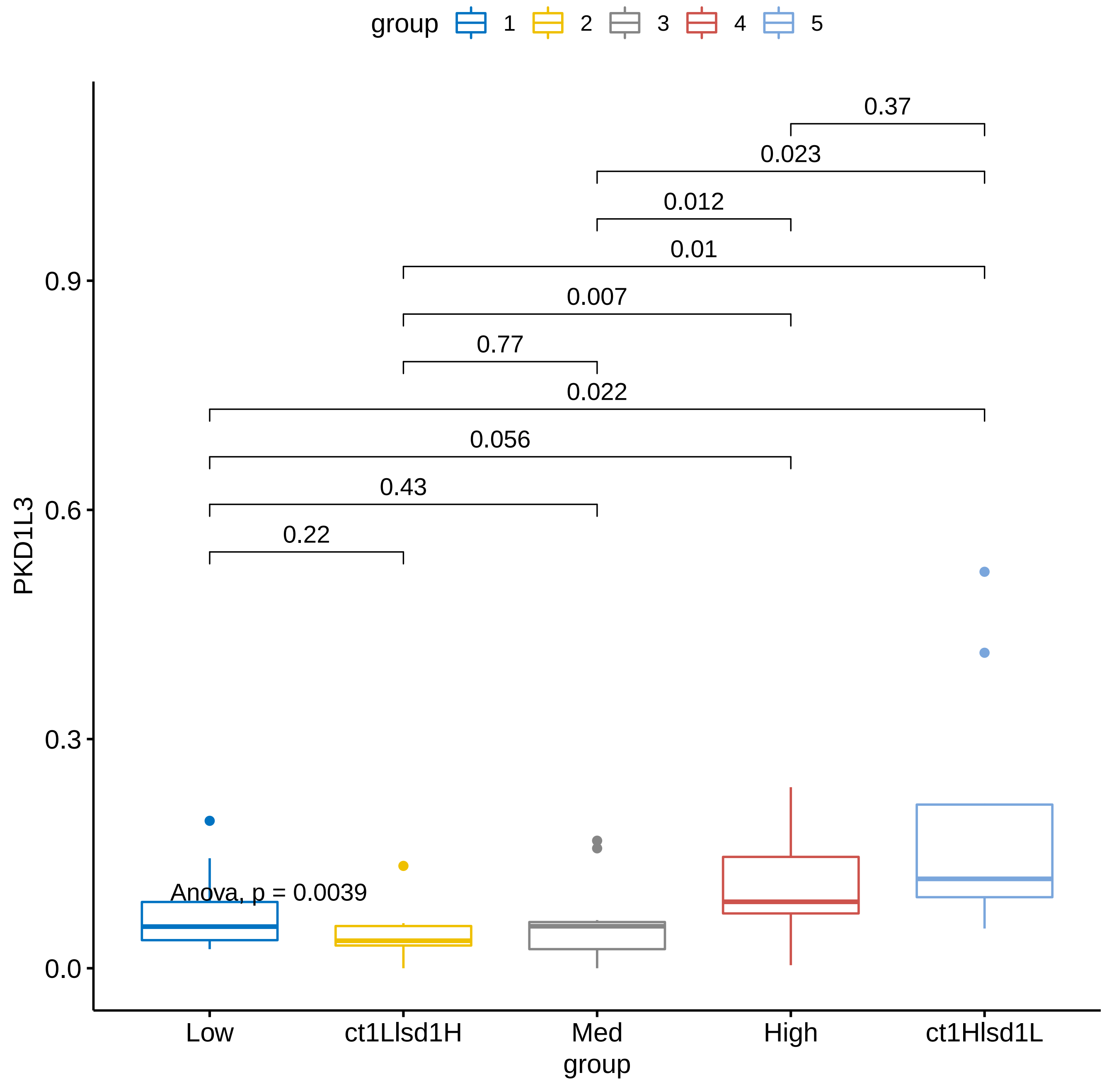

group 1 2 3 4 5
PKD1L3
0.9
0.6
0.3
0.0
0.37
0.023
0.012
0.01
0.007
0.77
0.022
0.056
0.43
0.22
Anova, p = 0.0039
Low
ct1Llsd1H
Med
High
ct1Hlsd1L
group

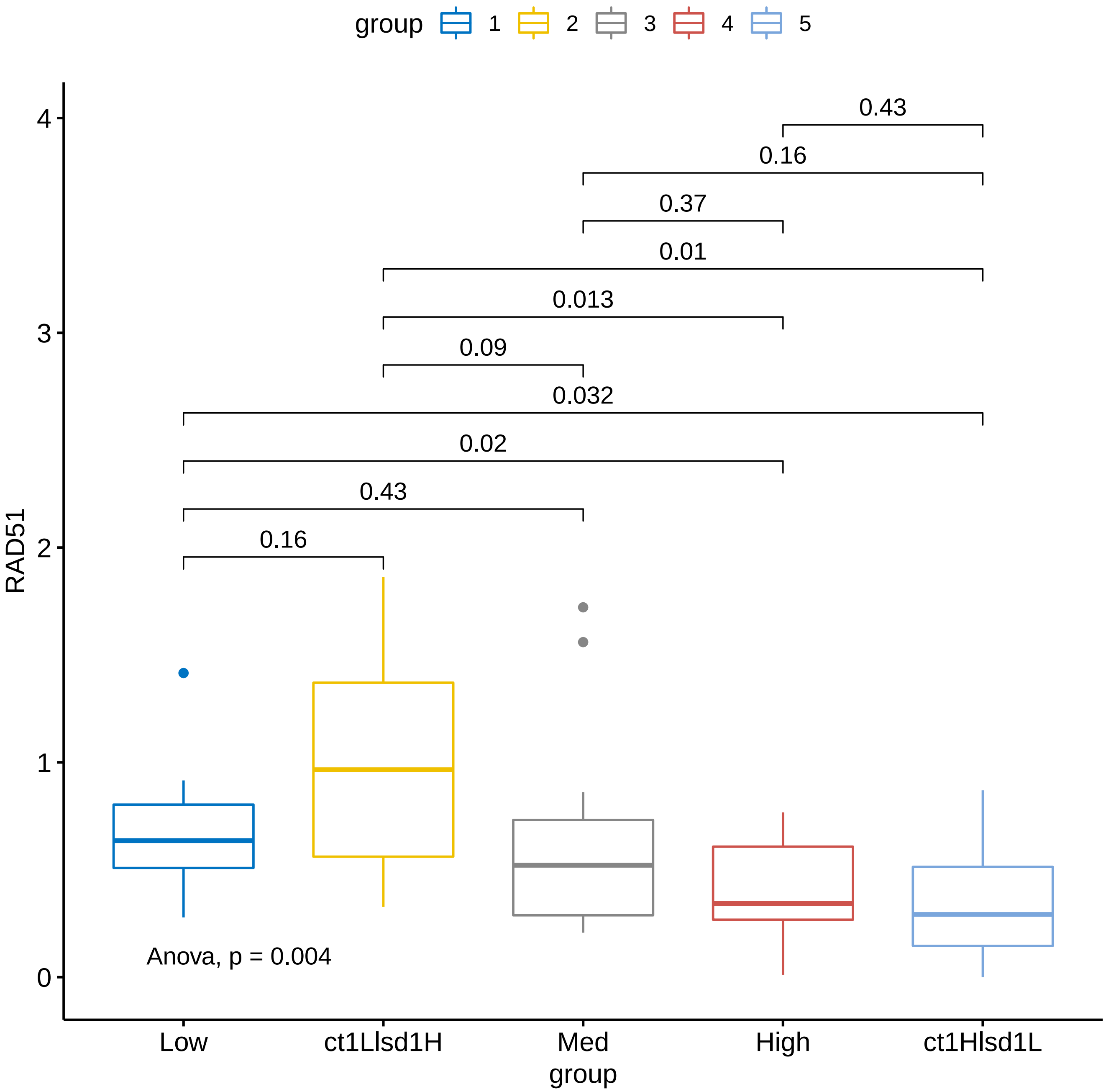
group
1
2
3
4
5
RAD51
4
3
2
1
0
0.43
0.16
0.37
0.01
0.013
0.09
0.032
0.02
0.43
0.16
Anova, p = 0.004
Low
ct1Llsd1H
Med
High
ct1Hlsd1L
group

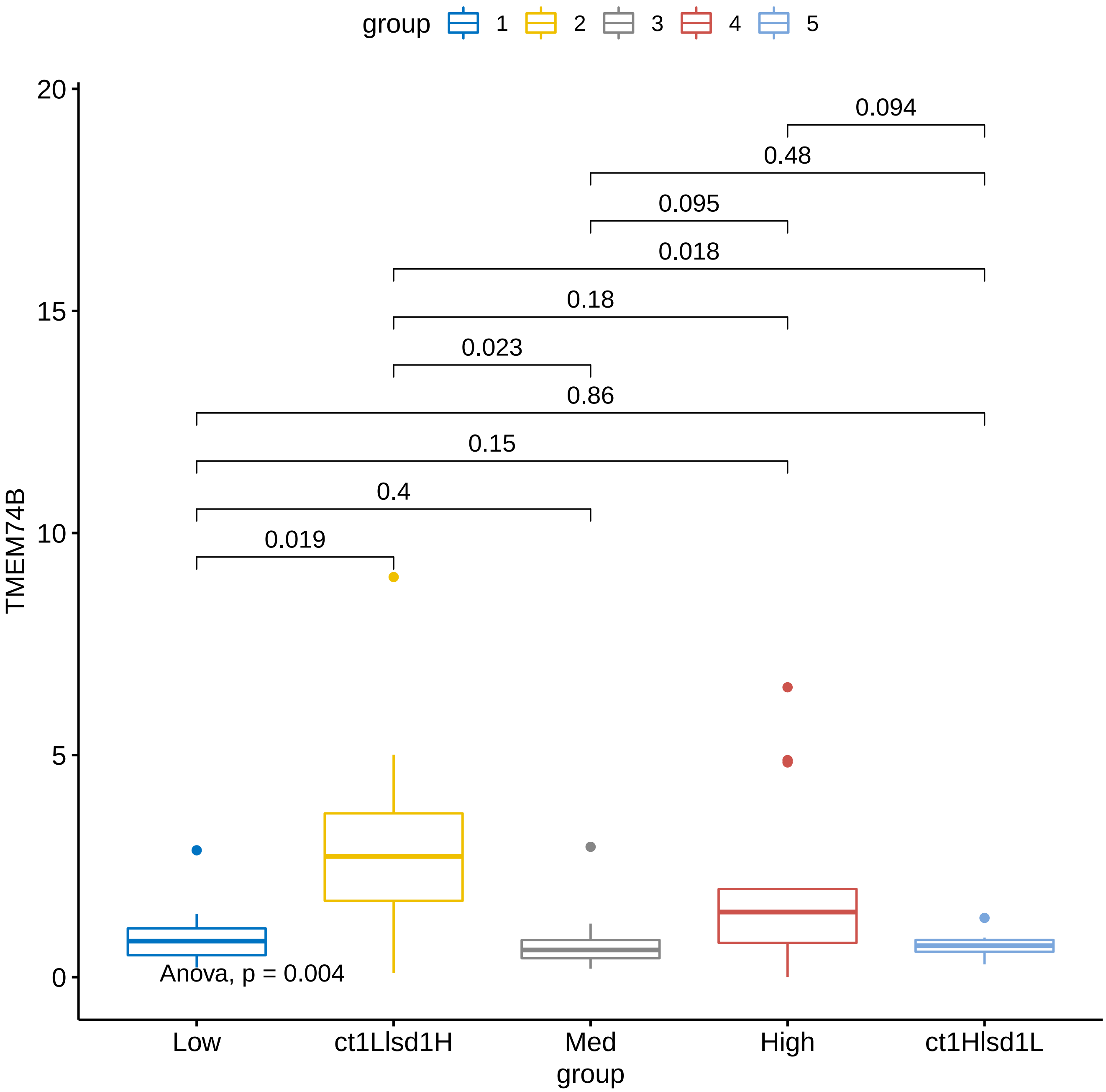

group 1 2 3 4 5
20
15
10
5
0
TMEM74B
0.094
0.48
0.095
0.018
0.18
0.023
0.86
0.15
0.4
0.019
Anova, p = 0.004
Low
ct1Llsd1H
Med
High
ct1Hlsd1L
group

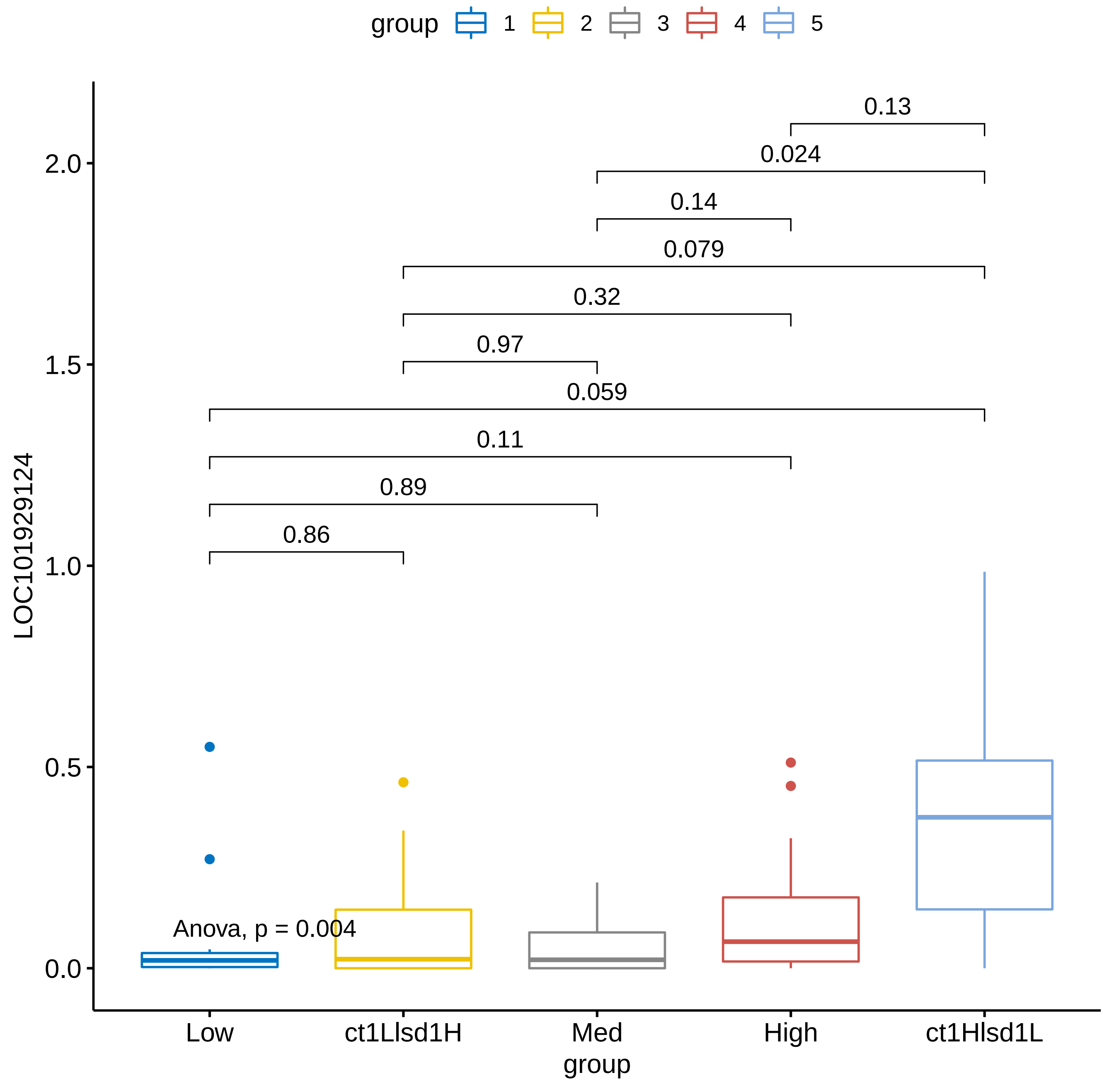

group 1 2 3 4 5
0.13
0.024
0.14
0.079
0.32
0.97
0.059
0.11
0.89
0.86
Anova, p = 0.004
2.0
1.5
1.0
0.5
0.0
LOC101929124
Low
ct1Llsd1H
Med
High
ct1Hlsd1L
group

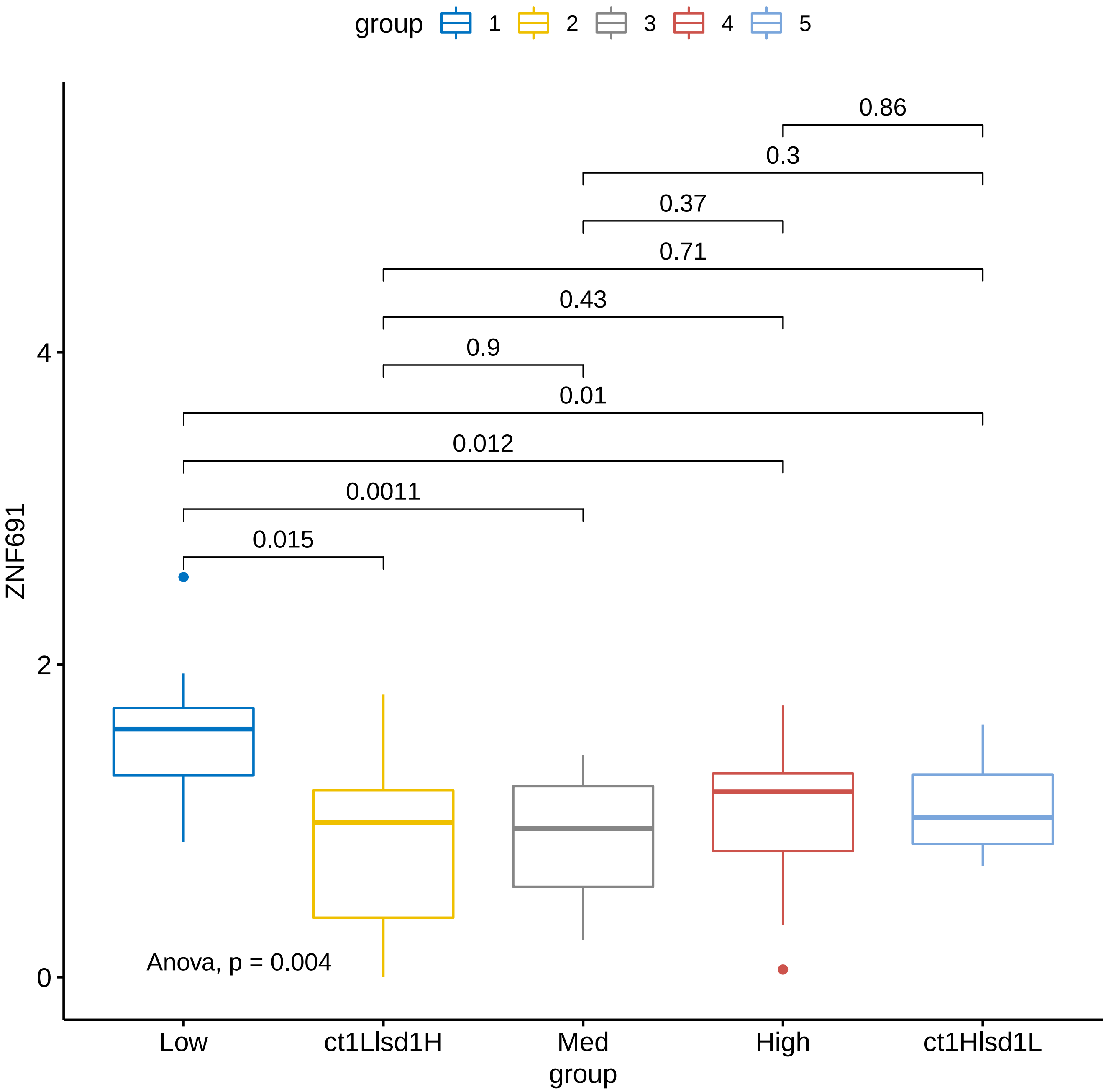

group
1
2
3
4
5
0.86
0.3
0.37
0.71
0.43
0.9
0.01
0.012
0.0011
0.015
ZNF691
4
2
0
Anova, p = 0.004
Low
ct1Llsd1H
Med
High
ct1Hlsd1L
group

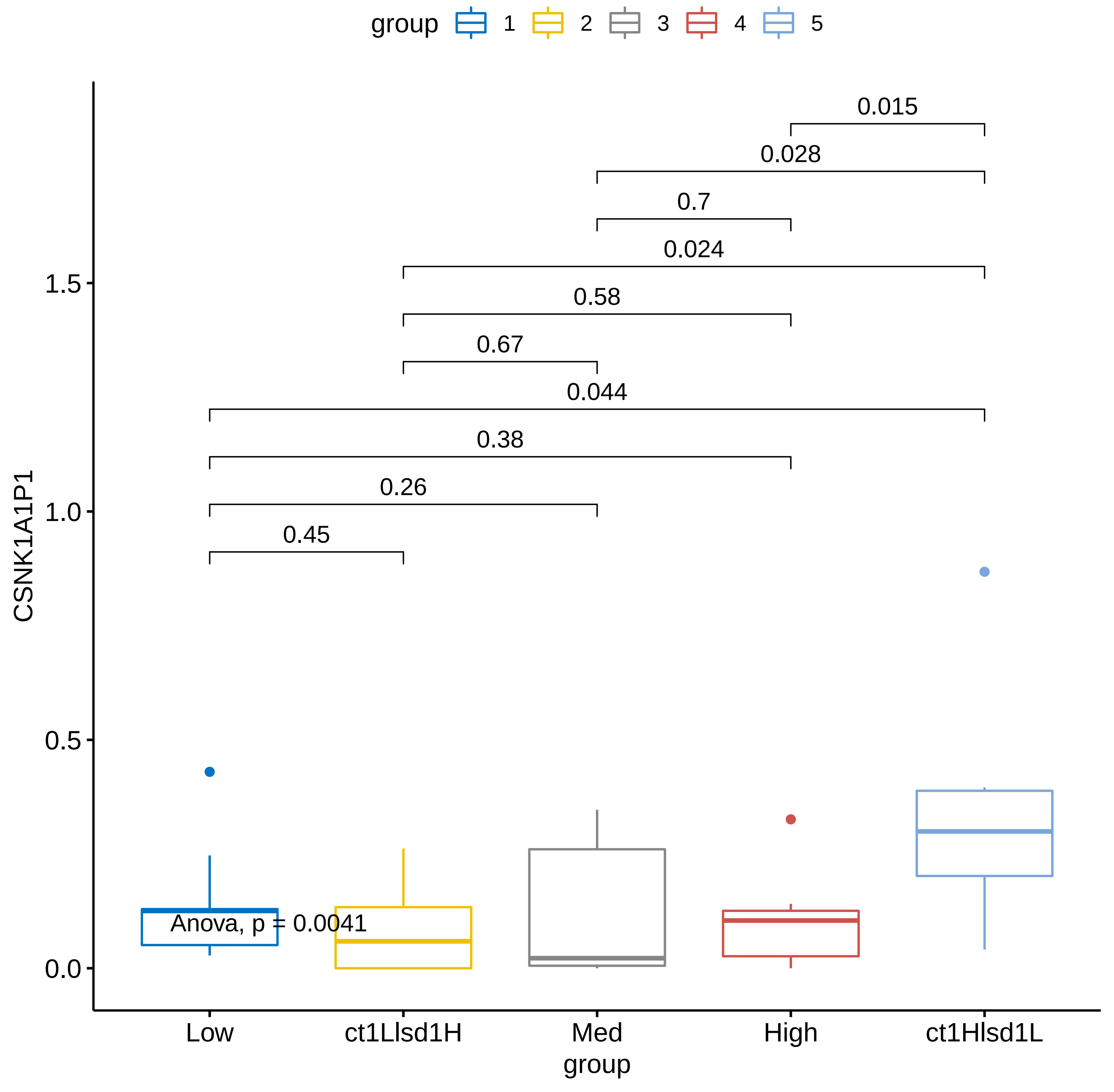
group
1
2
3
4
5
0.015
0.028
0.7
0.024
0.58
0.67
0.044
0.38
0.26
0.45
1.5
1.0
0.5
0.0
CSNK1A1P1
Anova, p = 0.0041
Low
ct1Llsd1H
Med
High
ct1Hlsd1L
group

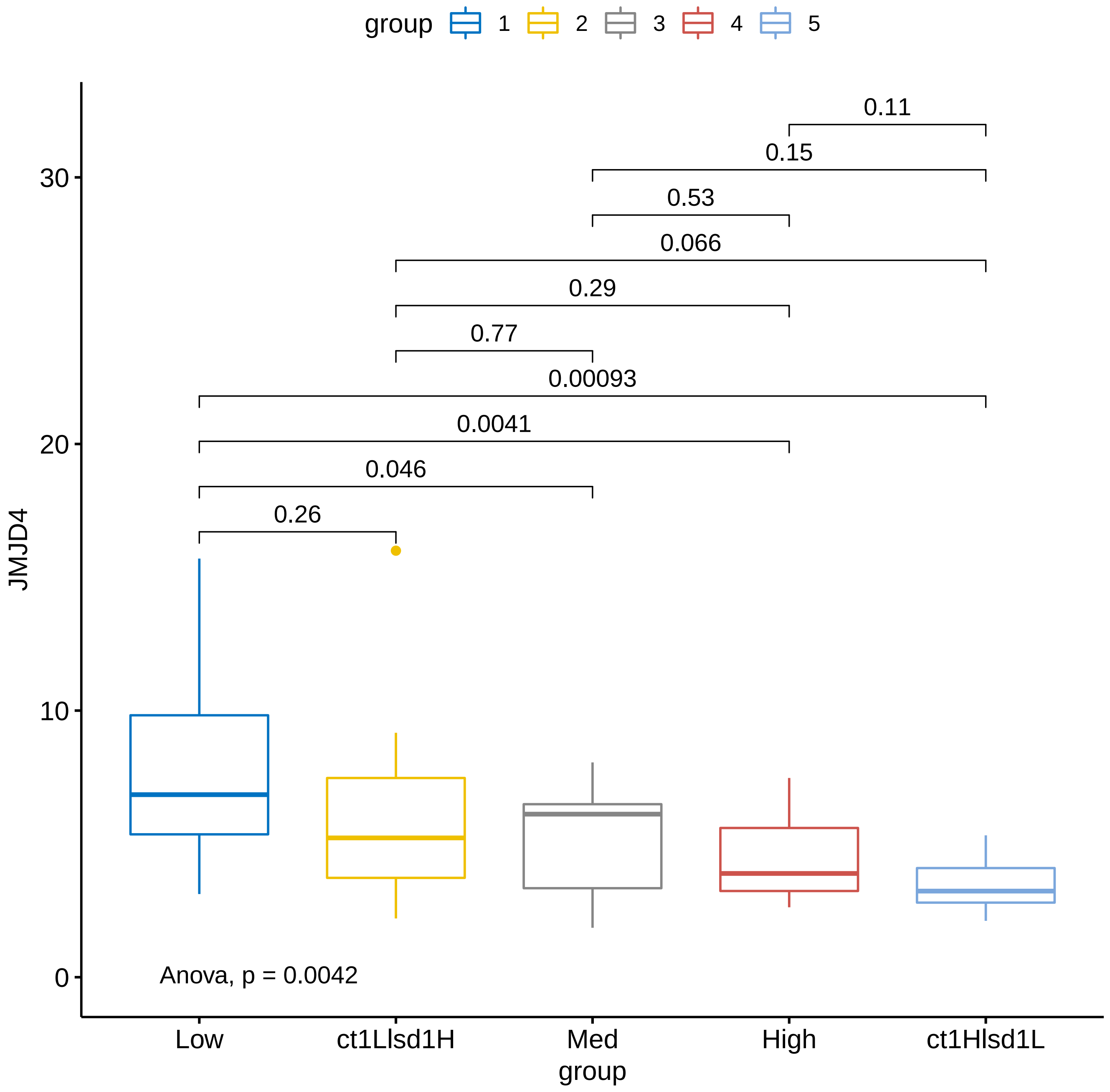
group
1
2
3
4
5
0.11
0.15
0.53
0.066
0.29
0.77
0.00093
0.0041
0.046
0.26
30
20
10
0
JMJD4
Anova, p = 0.0042
Low
ct1Llsd1H
Med
High
ct1Hlsd1L
group

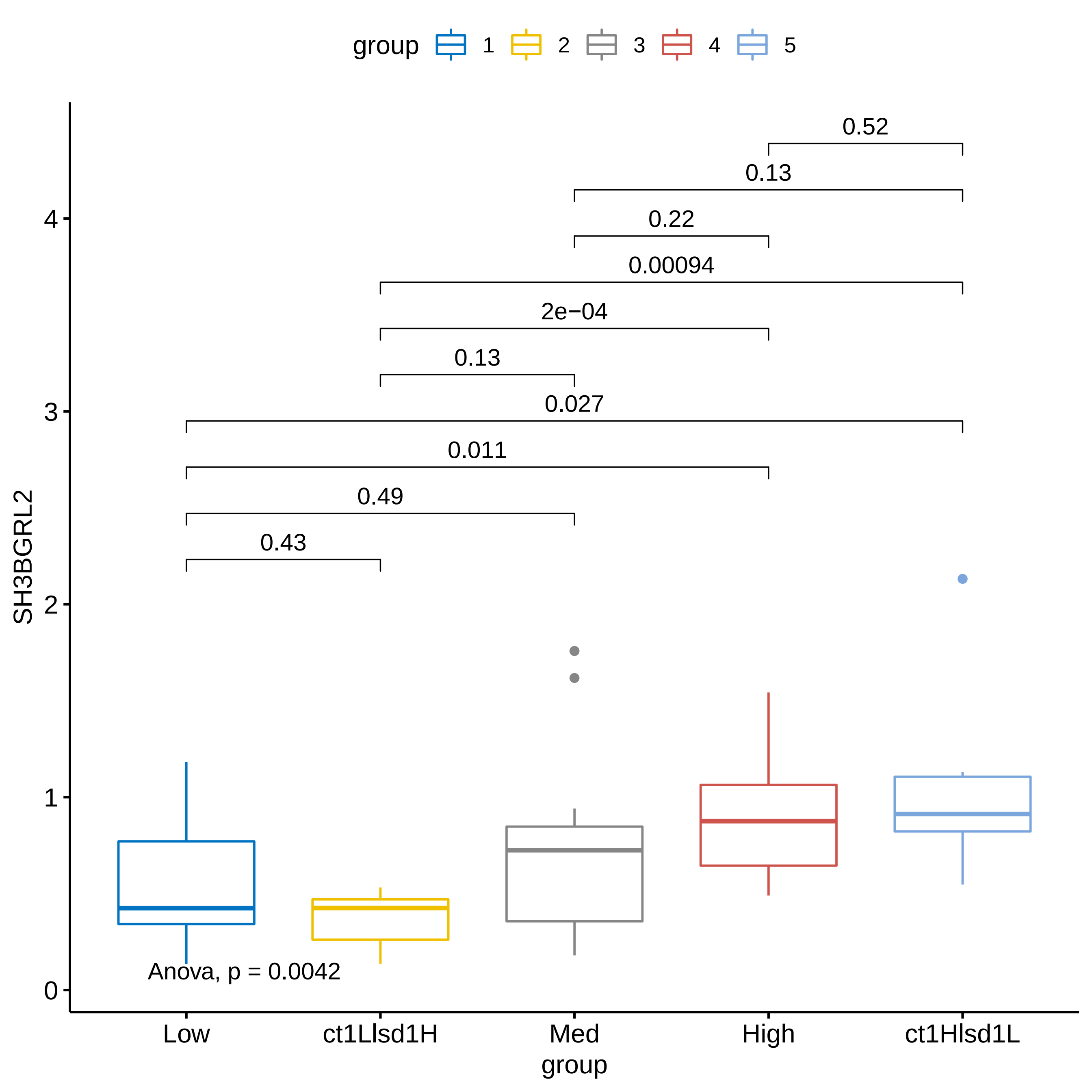

group
1
2
3
4
5
SH3BGRL2
0
1
2
3
4
0.52
0.13
0.22
0.00094
2e−04
0.13
0.027
0.011
0.49
0.43
Anova, p = 0.0042
Low
ct1Llsd1H
Med
High
ct1Hlsd1L
group

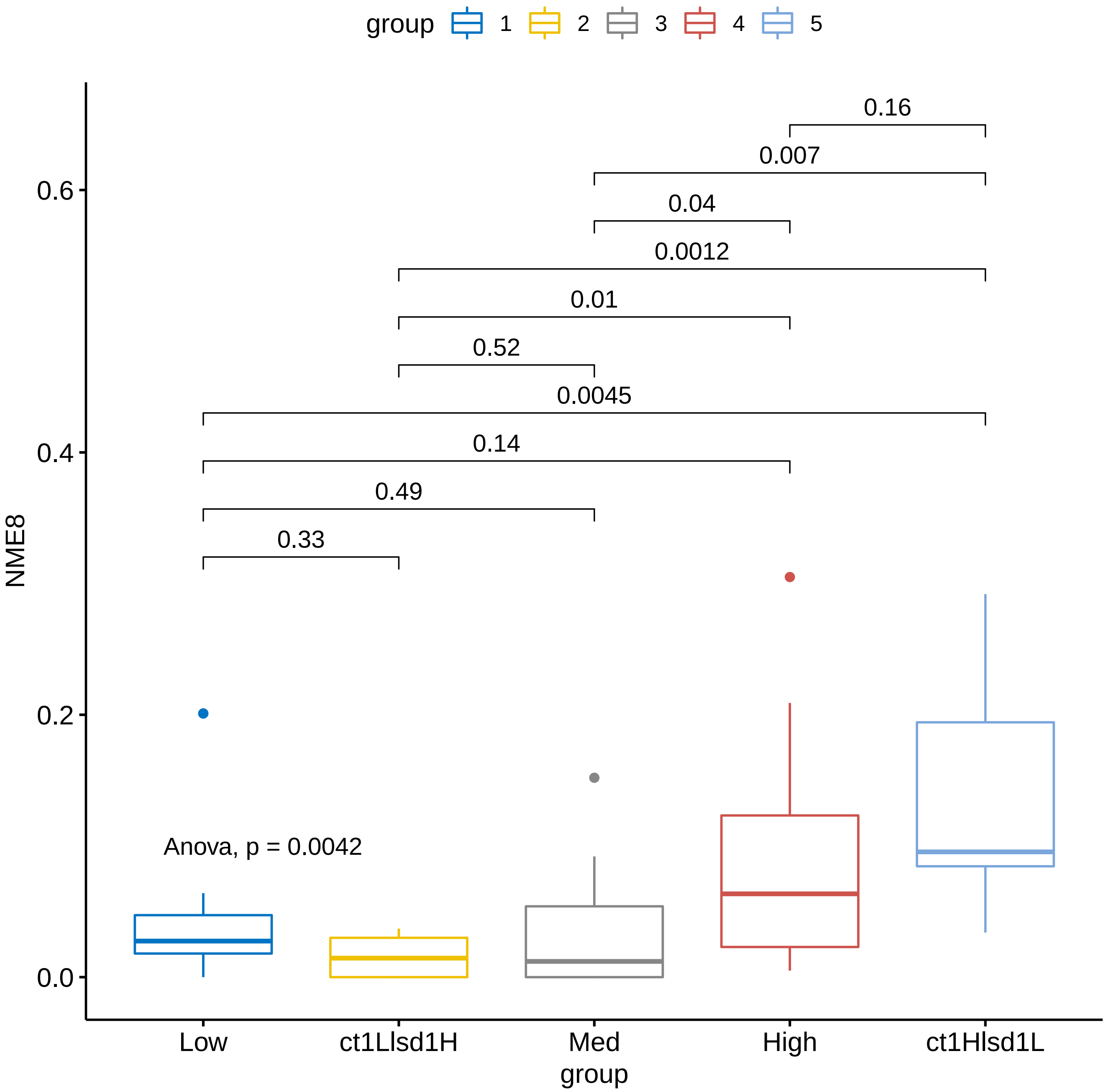

group
1
2
3
4
5
0.16
0.007
0.04
0.0012
0.01
0.52
0.0045
0.14
0.49
0.33
Anova, p = 0.0042
0.6
0.4
0.2
0.0
NME8
Low
ct1Llsd1H
Med
High
ct1Hlsd1L
group

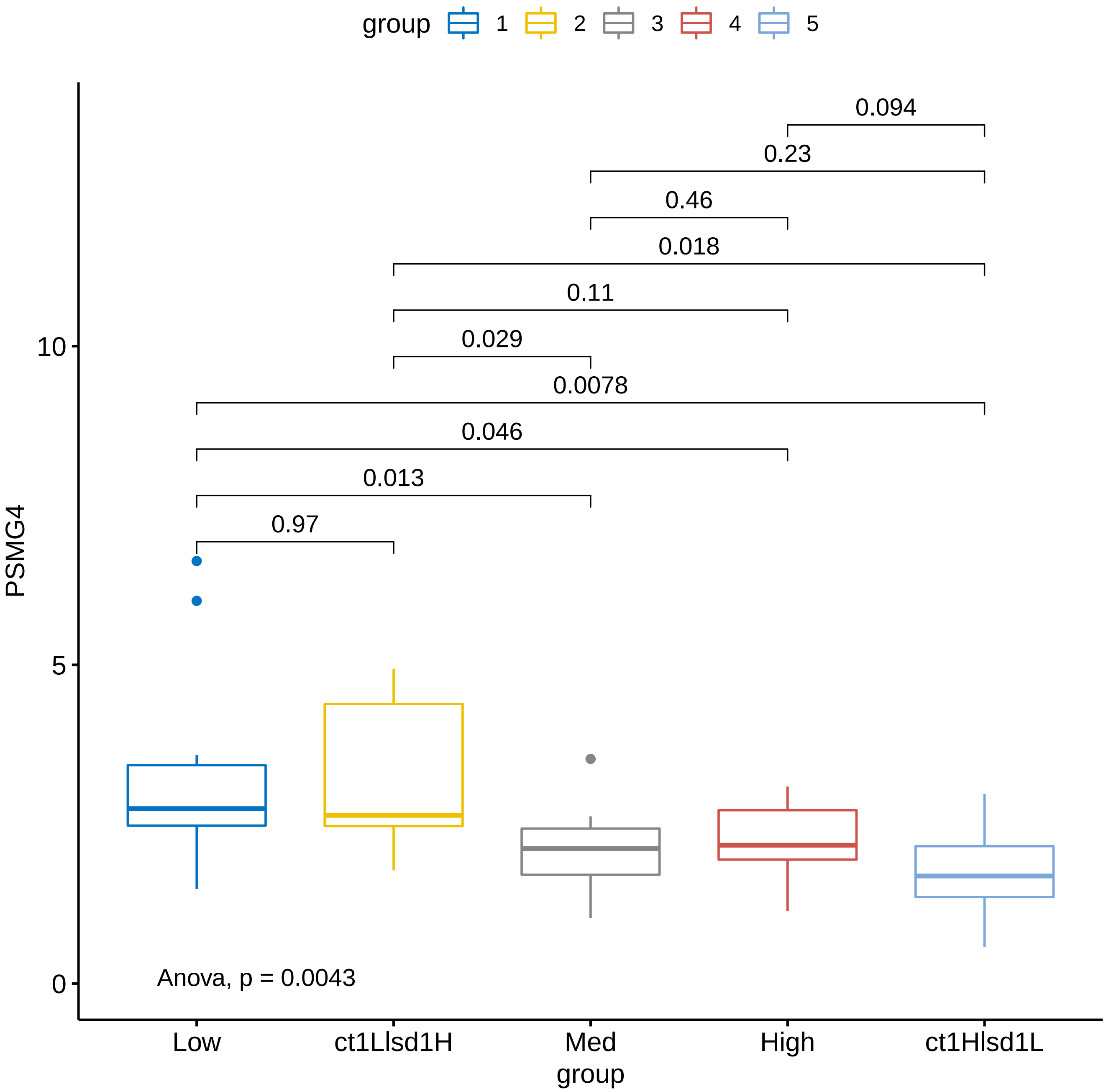
group
1
2
3
4
5
0.094
0.23
0.46
0.018
0.11
0.029
0.0078
0.046
0.013
0.97
10
5
0
PSMG4
Anova, p = 0.0043
Low
ct1Llsd1H
Med
High
ct1Hlsd1L
group

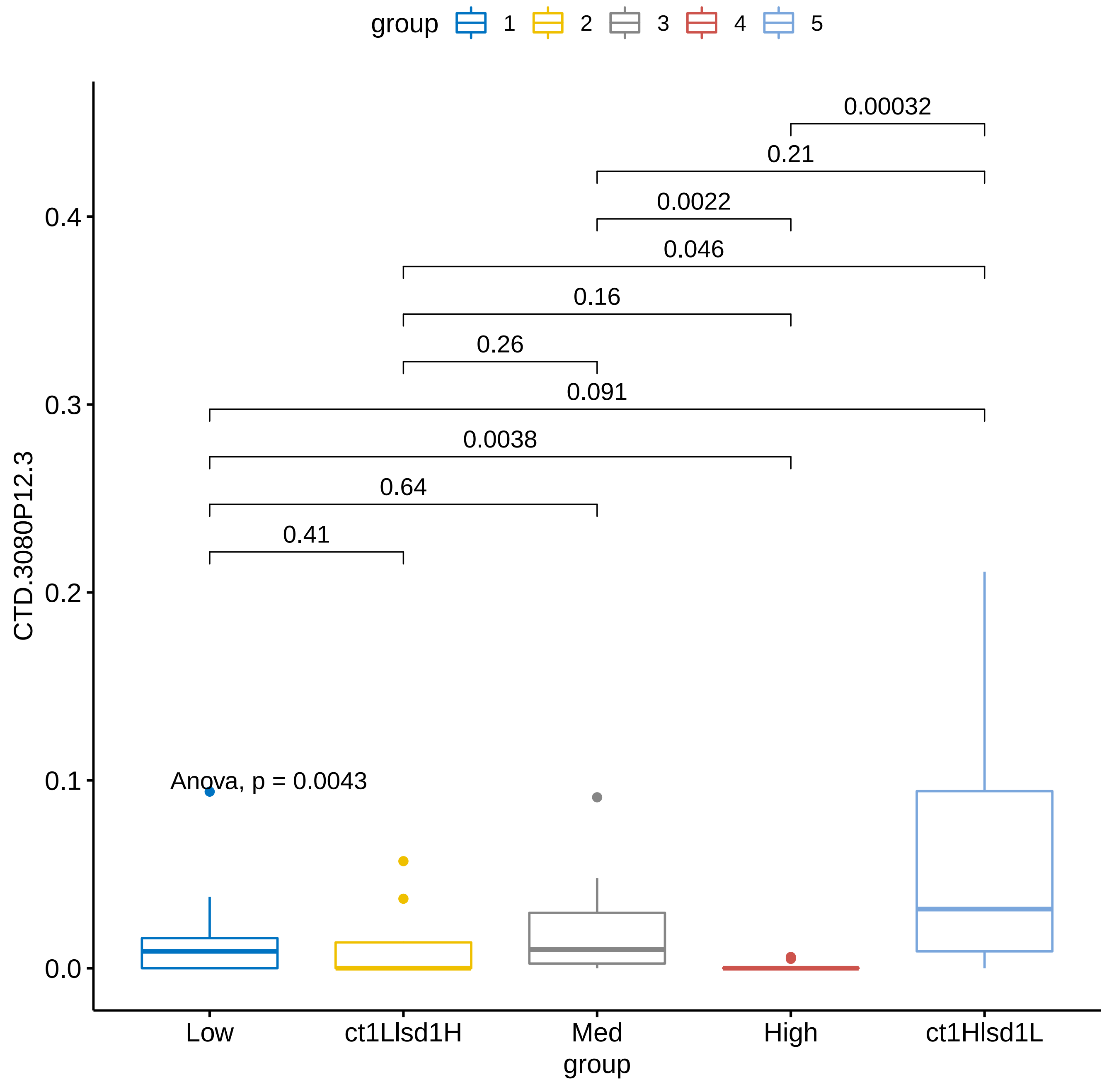

group 1 2 3 4 5
0.00032
0.21
0.0022
0.046
0.16
0.26
0.091
0.0038
0.64
0.41
Anova, p = 0.0043
0.4
0.3
0.2
0.1
0.0
CTD.3080P12.3
Low
ct1Llsd1H
Med
High
ct1Hlsd1L
group

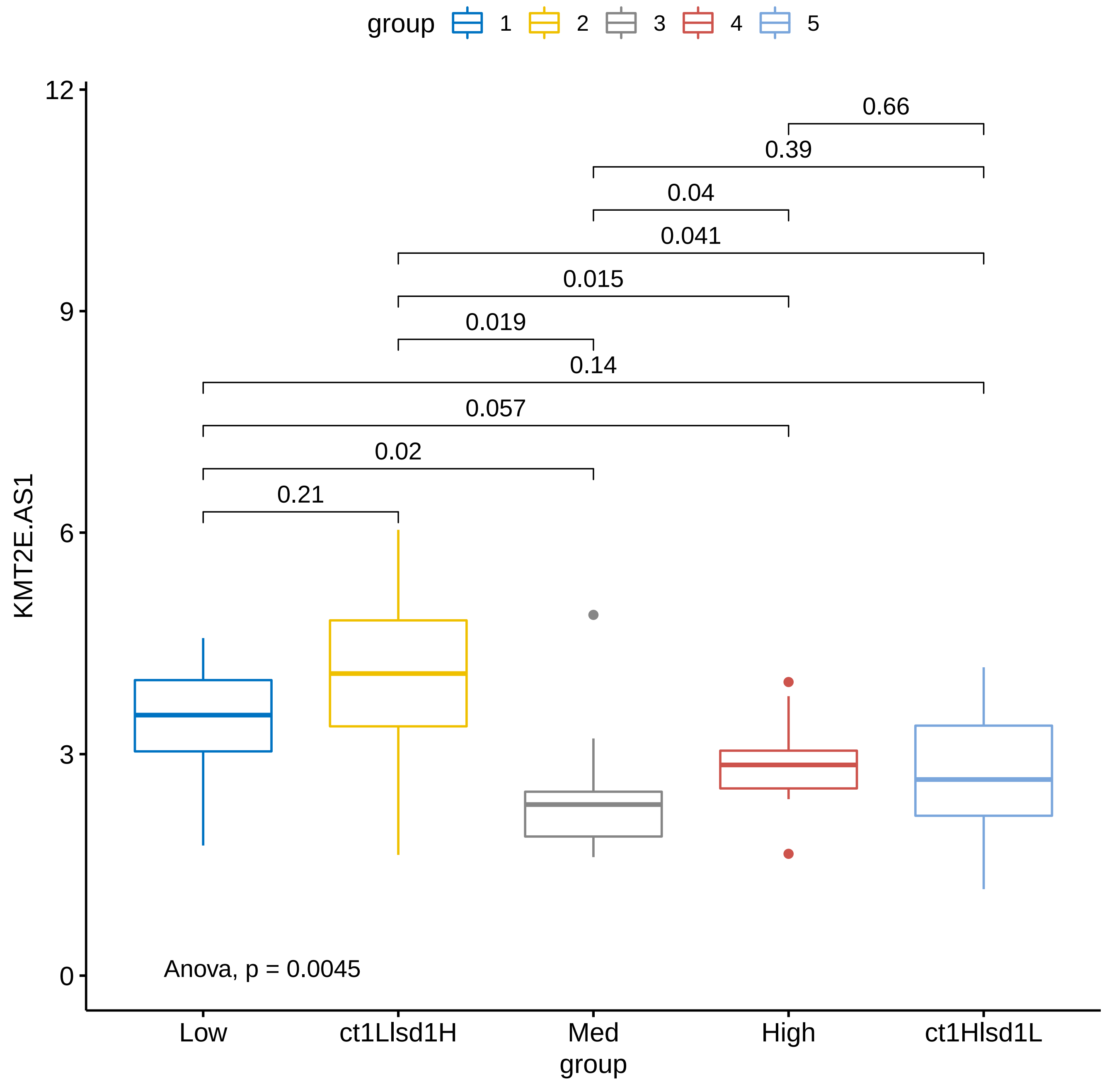

group 1 2 3 4 5
KMT2E.AS1
12
9
6
3
0
0.66
0.39
0.04
0.041
0.015
0.019
0.14
0.057
0.02
0.21
Anova, p = 0.0045
Low
ct1Llsd1H
Med
High
ct1Hlsd1L
group

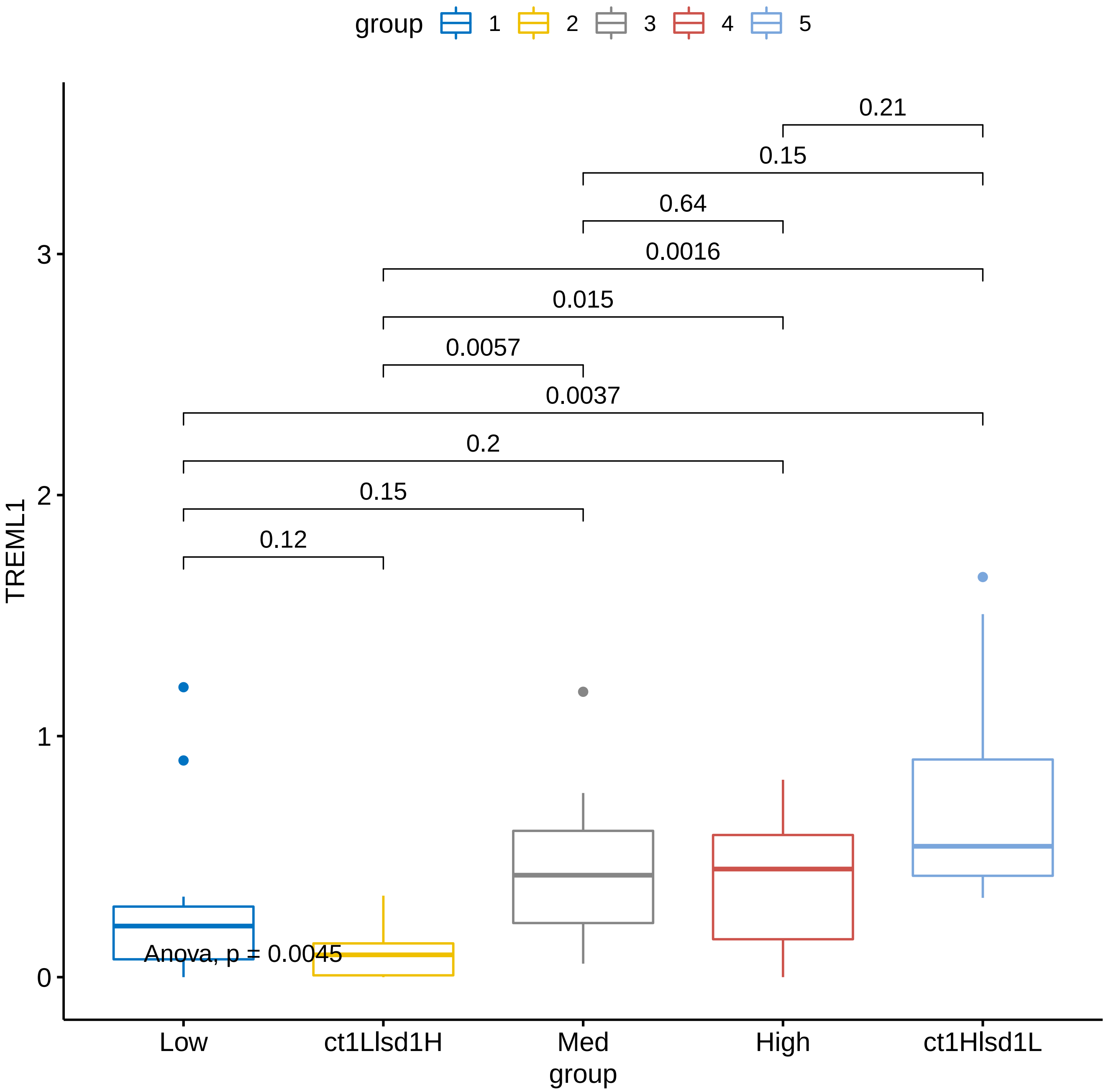

group 1 2 3 4 5
0.21
0.15
0.64
0.0016
0.015
0.0057
0.0037
0.2
0.15
0.12
Anova, p = 0.0045
TREML1
3
2
1
0
Low
ct1Llsd1H
Med
High
ct1Hlsd1L
group

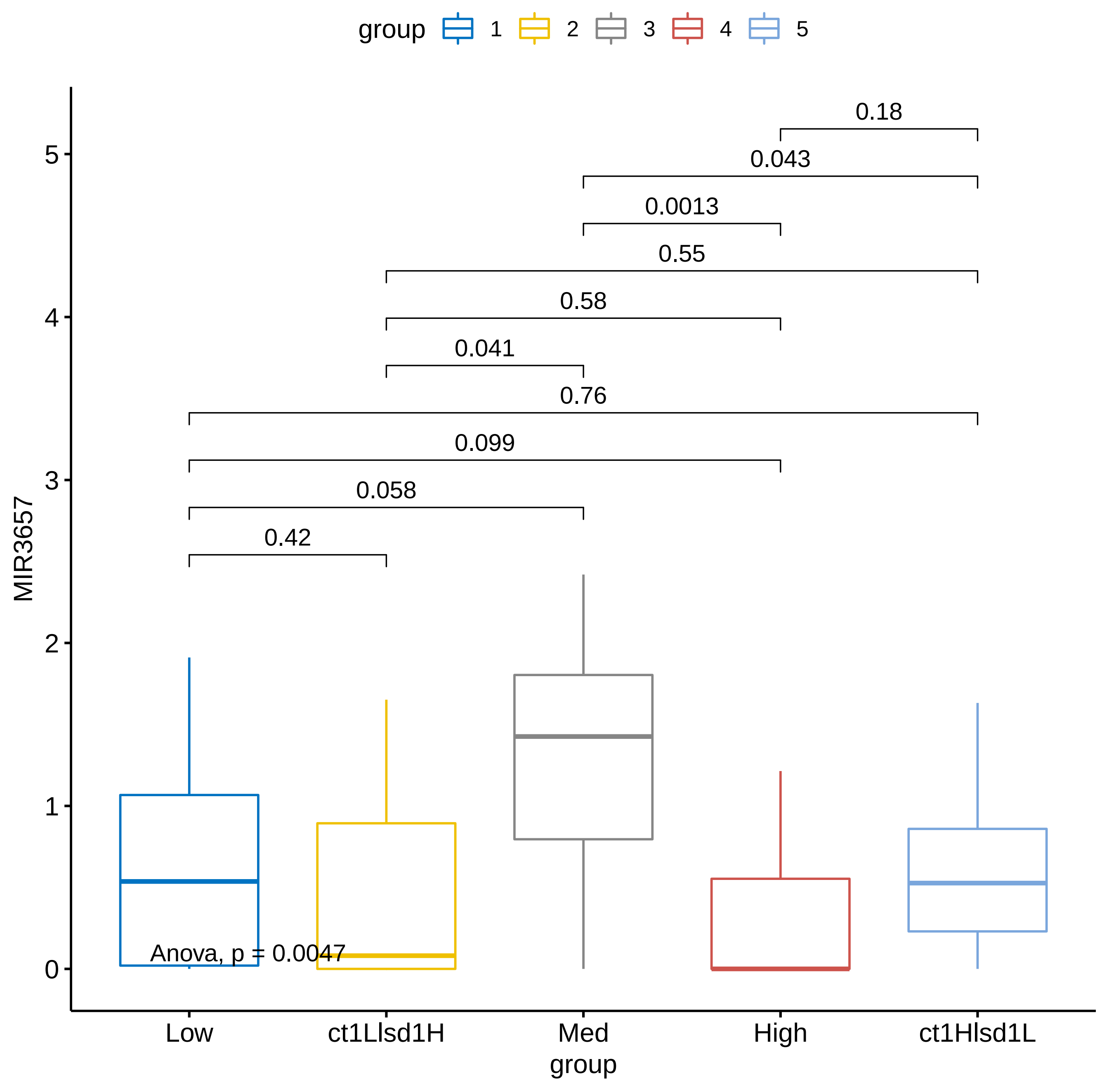
group
1
2
3
4
5
0.18
0.043
0.0013
0.55
0.58
0.041
0.76
0.099
0.058
0.42
Anova, p = 0.0047
MIR3657
0
1
2
3
4
5
Low
ct1Llsd1H
Med
High
ct1Hlsd1L
group

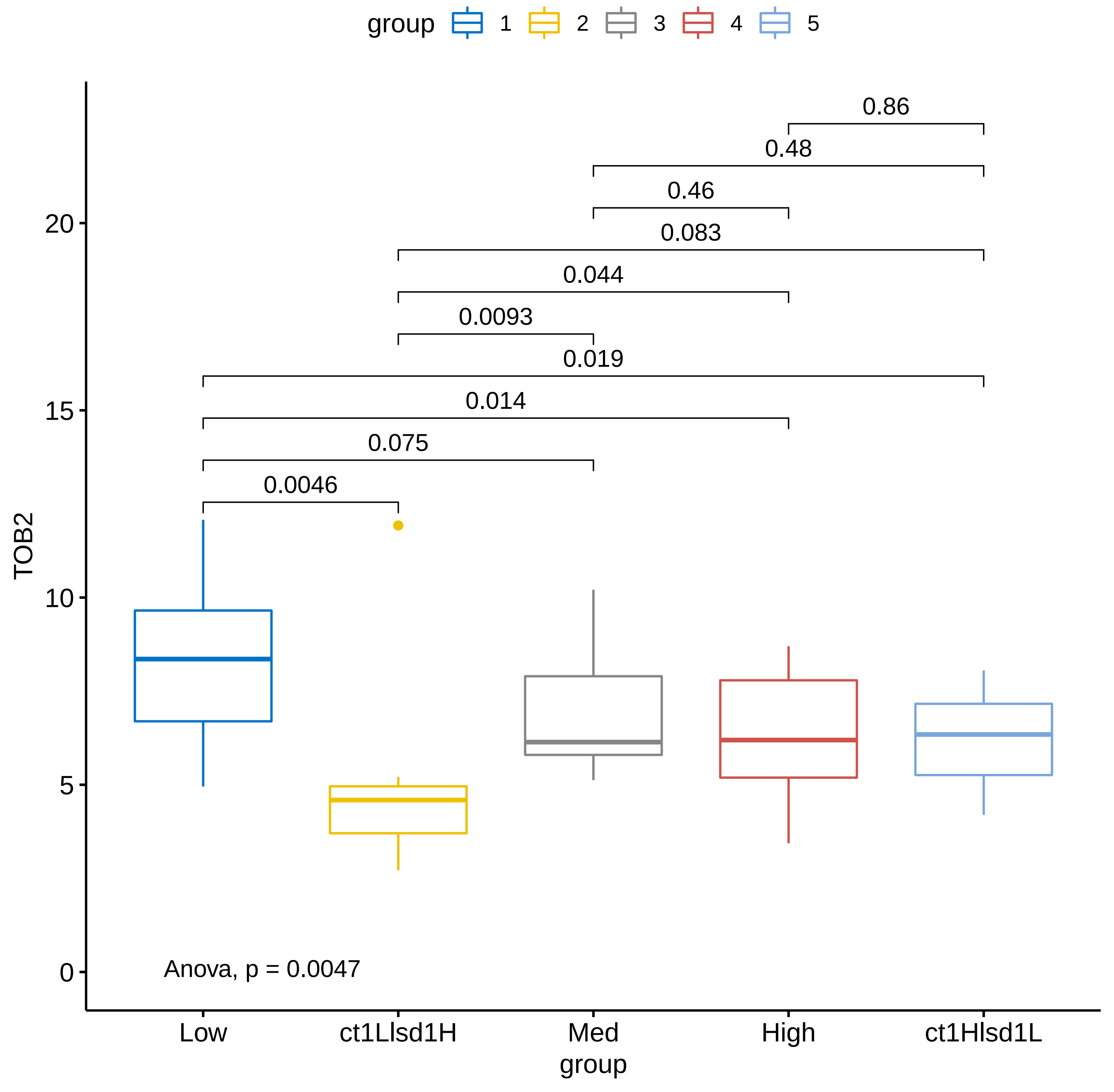
group
1
2
3
4
5
0.86
0.48
0.46
0.083
0.044
0.0093
0.019
0.014
0.075
0.0046
TOB2
20
15
10
5
0
Anova, p = 0.0047
Low
ct1Llsd1H
Med
High
ct1Hlsd1L
group

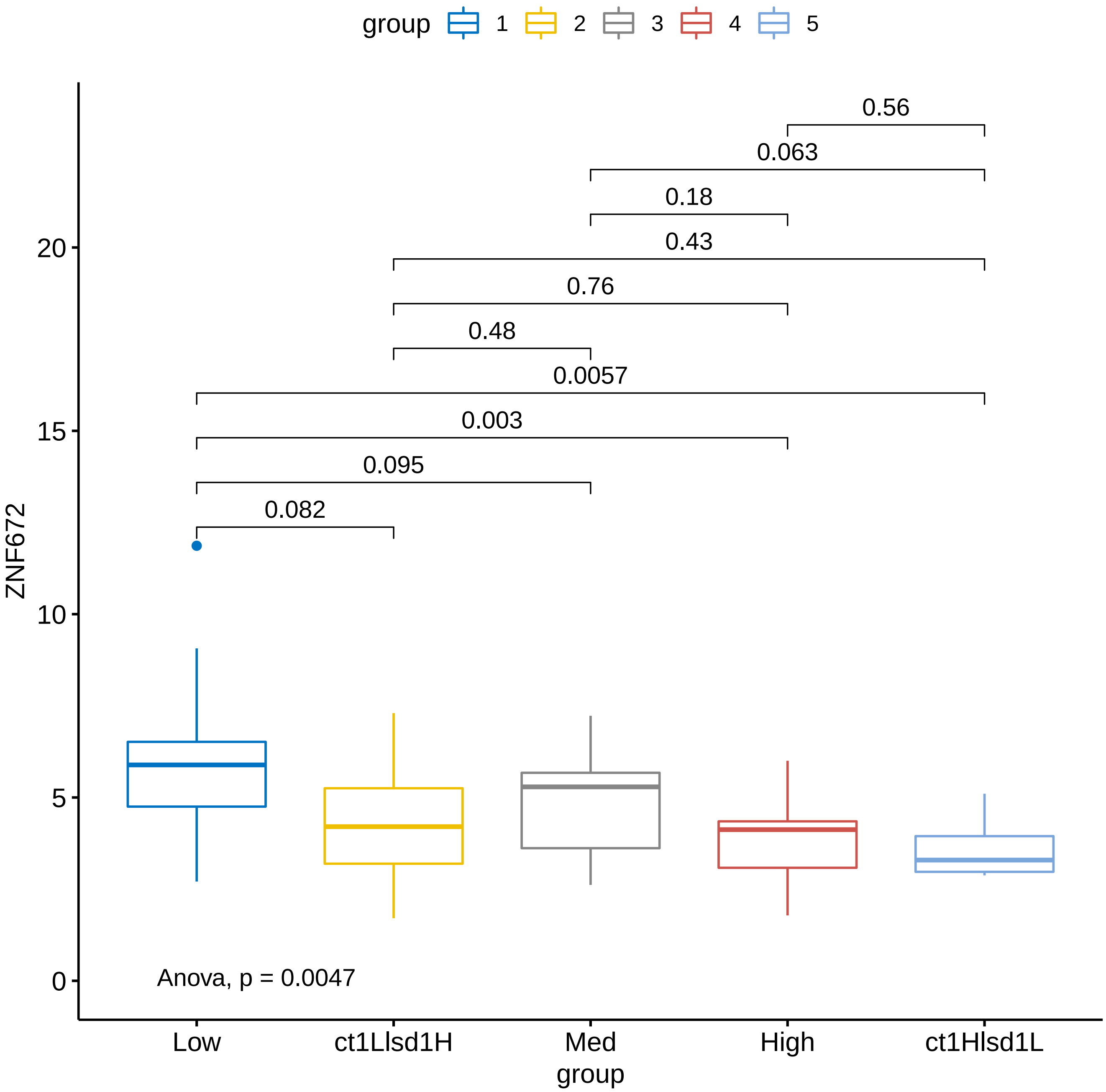

group
1
2
3
4
5
0.56
0.063
0.18
0.43
0.76
0.48
0.0057
0.003
0.095
0.082
20
15
10
5
0
ZNF672
Anova, p = 0.0047
Low
ct1Llsd1H
Med
High
ct1Hlsd1L
group

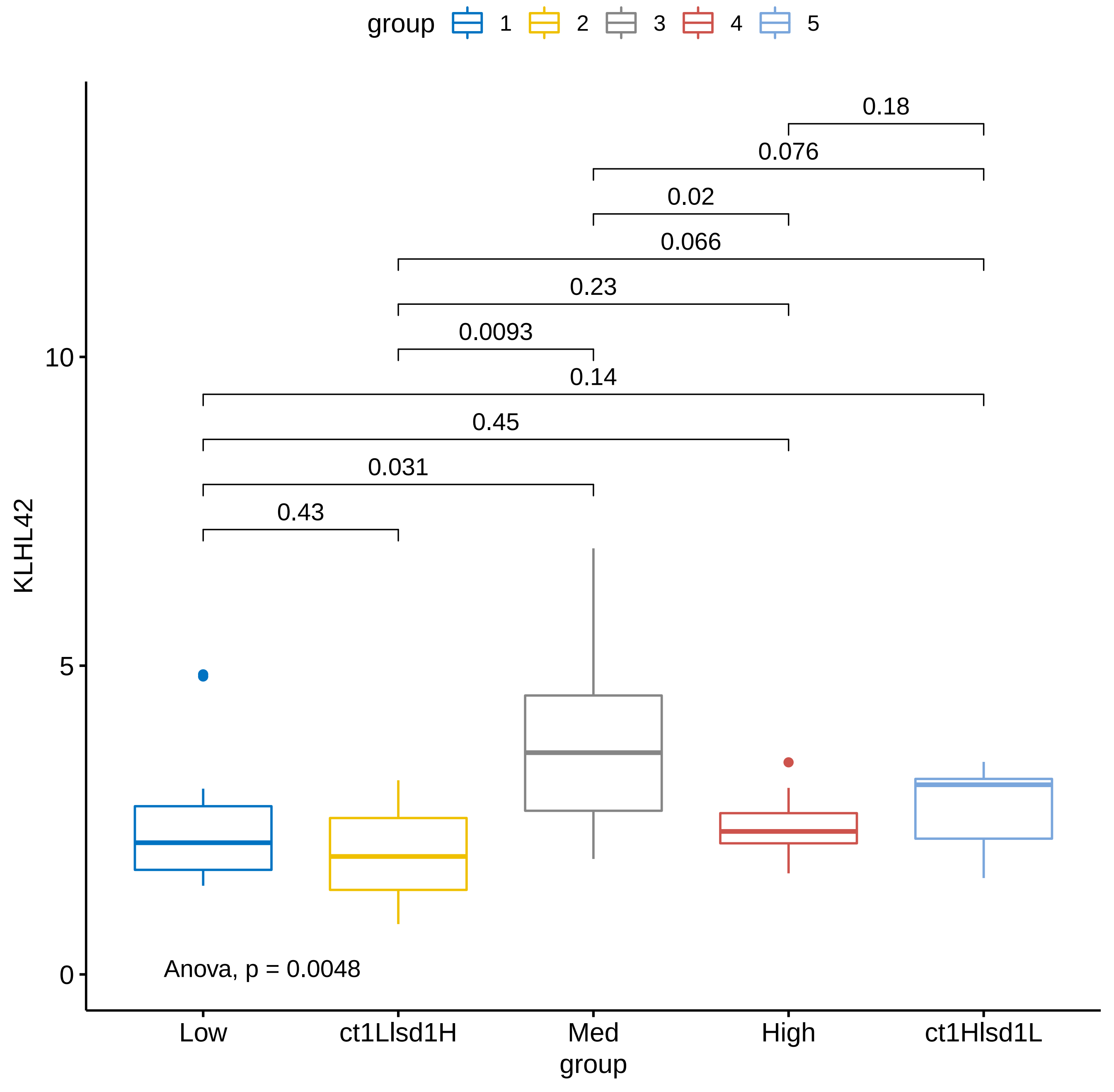

group 1 2 3 4 5
0.18
0.076
0.02
0.066
0.23
0.0093
0.14
0.45
0.031
0.43
KLHL42
10
5
0
Anova, p = 0.0048
Low
ct1Llsd1H
Med
High
ct1Hlsd1L
group

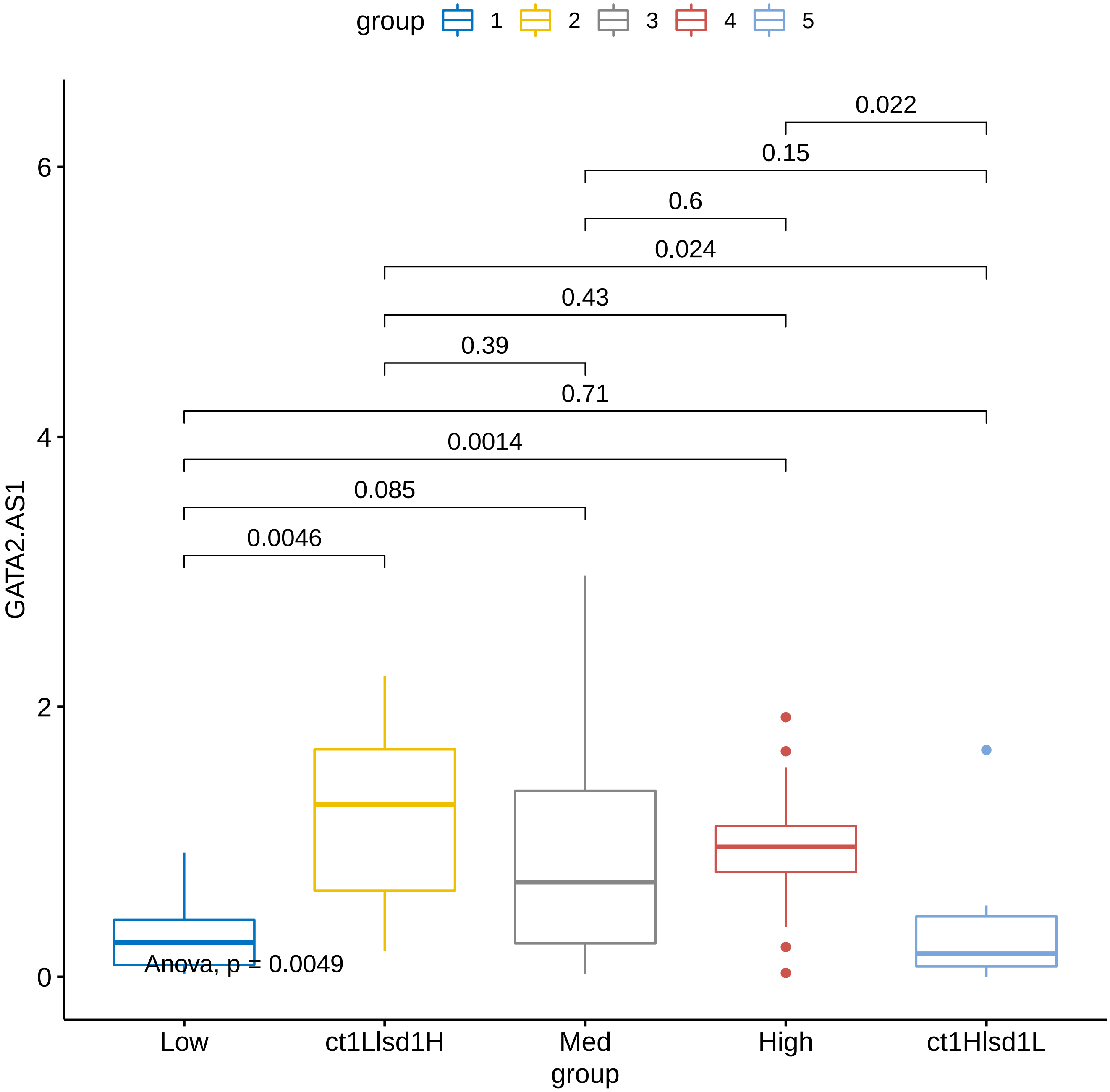

group
1
2
3
4
5
0.022
0.15
0.6
0.024
0.43
0.39
0.71
0.0014
0.085
0.0046
Anova, p = 0.0049
GATA2.AS1
6
4
2
0
Low
ct1Llsd1H
Med
High
ct1Hlsd1L
group

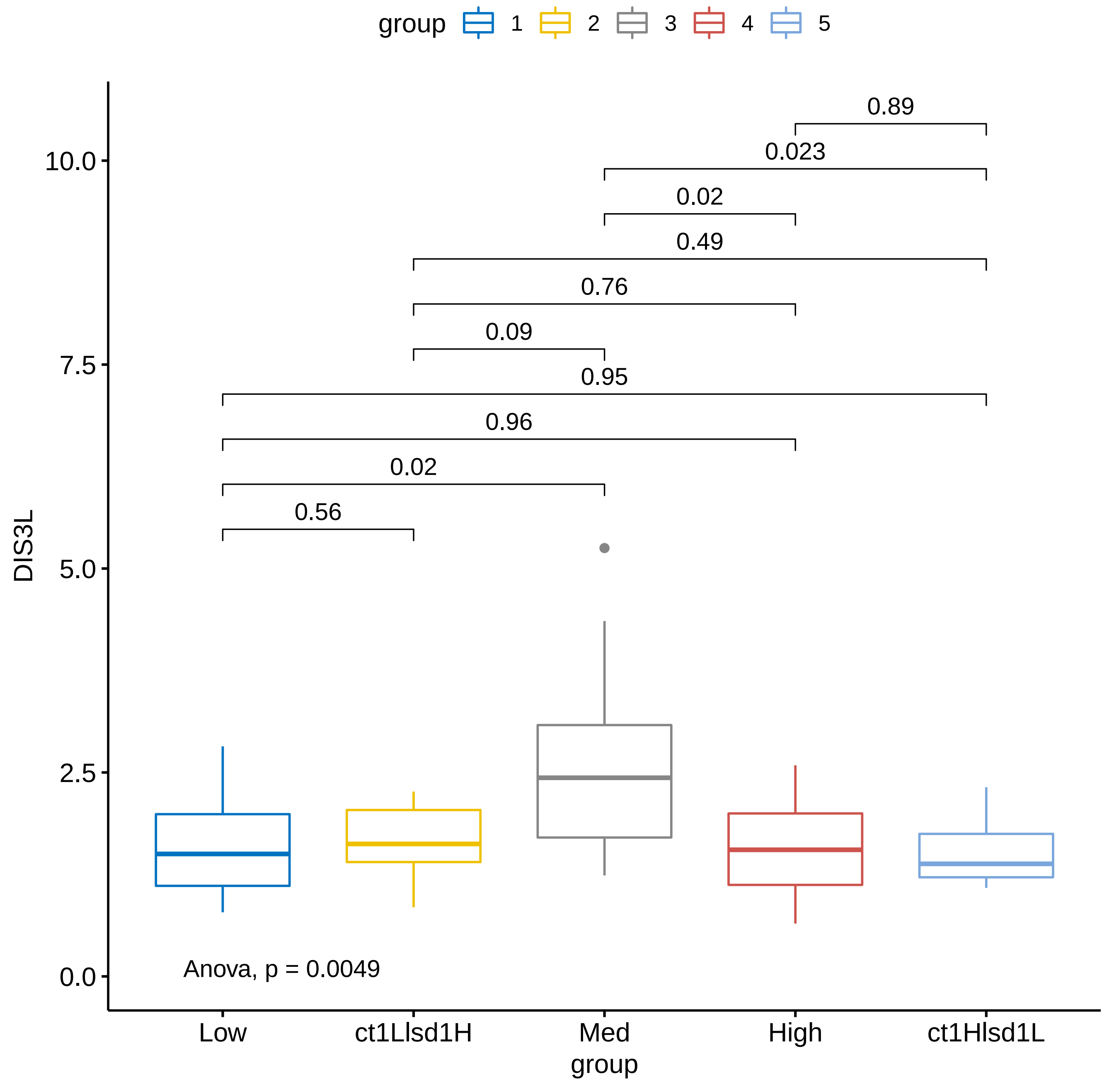
group 1 2 3 4 5
0.89
0.023
0.02
0.49
0.76
0.09
0.95
0.96
0.02
0.56
10.0
7.5
5.0
2.5
0.0
DIS3L
Anova, p = 0.0049
Low
ct1Llsd1H
Med
High
ct1Hlsd1L
group

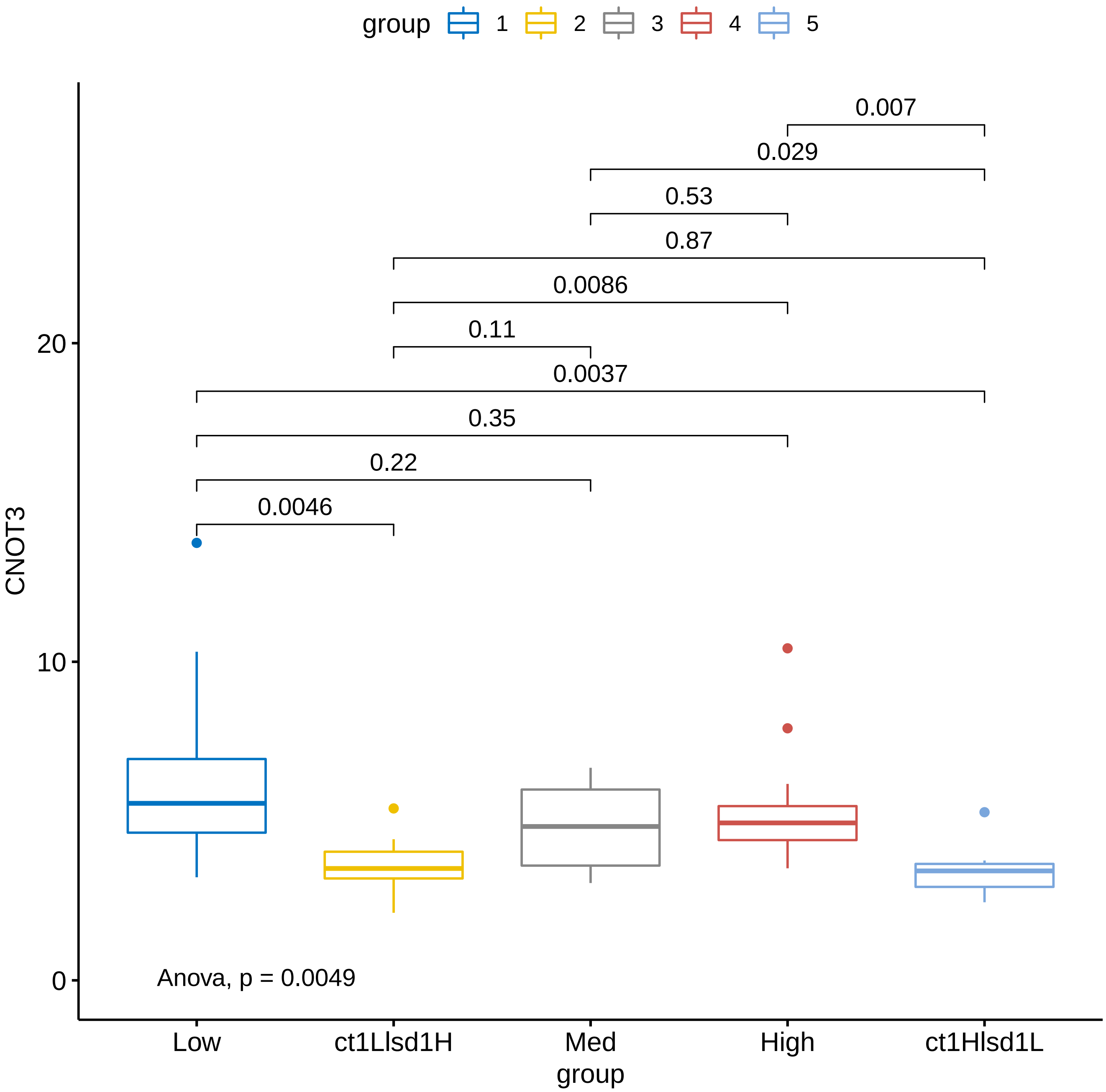
group
1
2
3
4
5
0.007
0.029
0.53
0.87
0.0086
0.11
0.0037
0.35
0.22
0.0046
CNOT3
20
10
0
Anova, p = 0.0049
Low
ct1Llsd1H
Med
High
ct1Hlsd1L
group

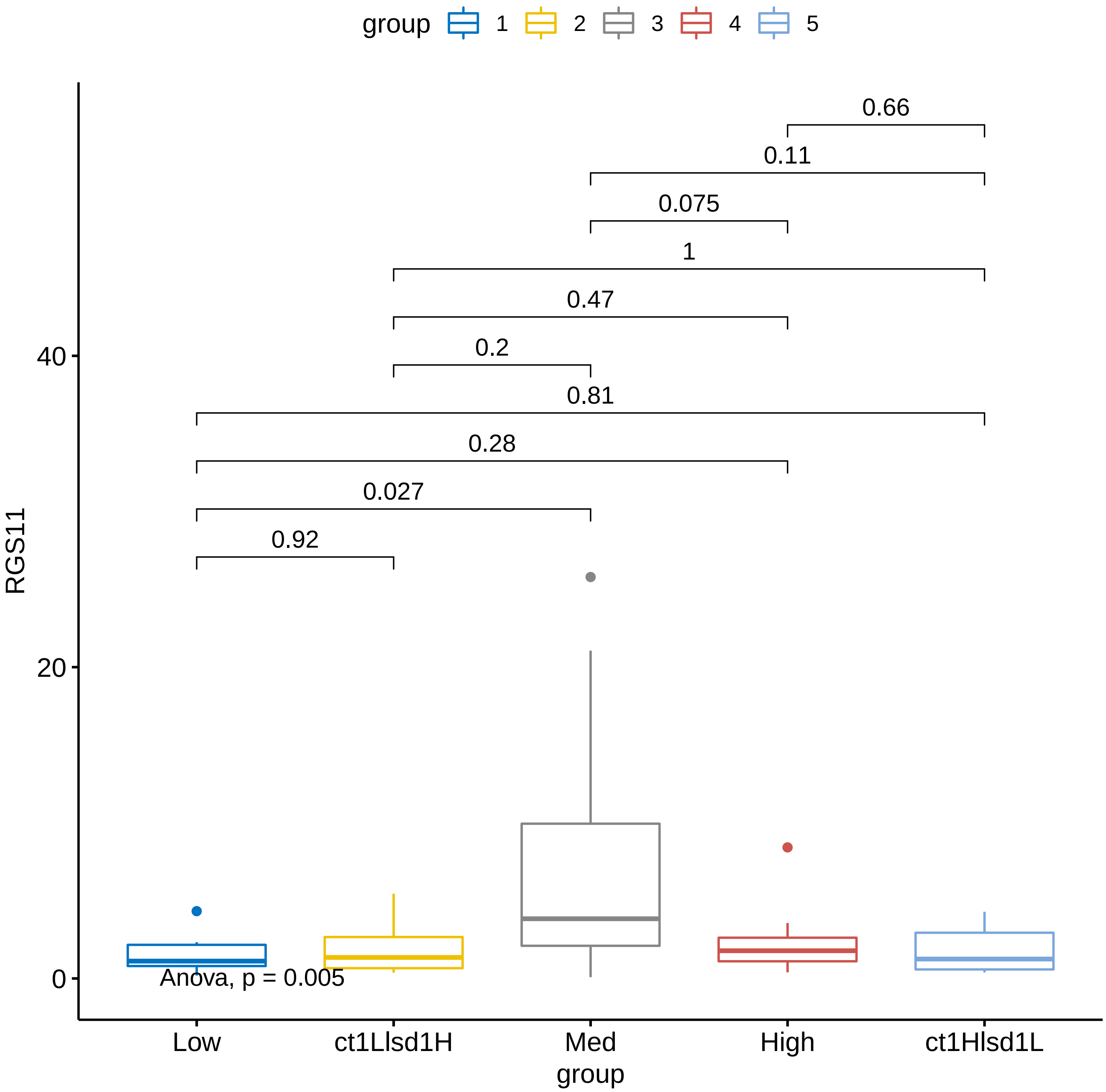
group
1
2
3
4
5
0.66
0.11
0.075
1
0.47
0.2
0.81
0.28
0.027
0.92
RGS11
40
20
0
Anova, p = 0.005
Low
ct1Llsd1H
Med
High
ct1Hlsd1L
group

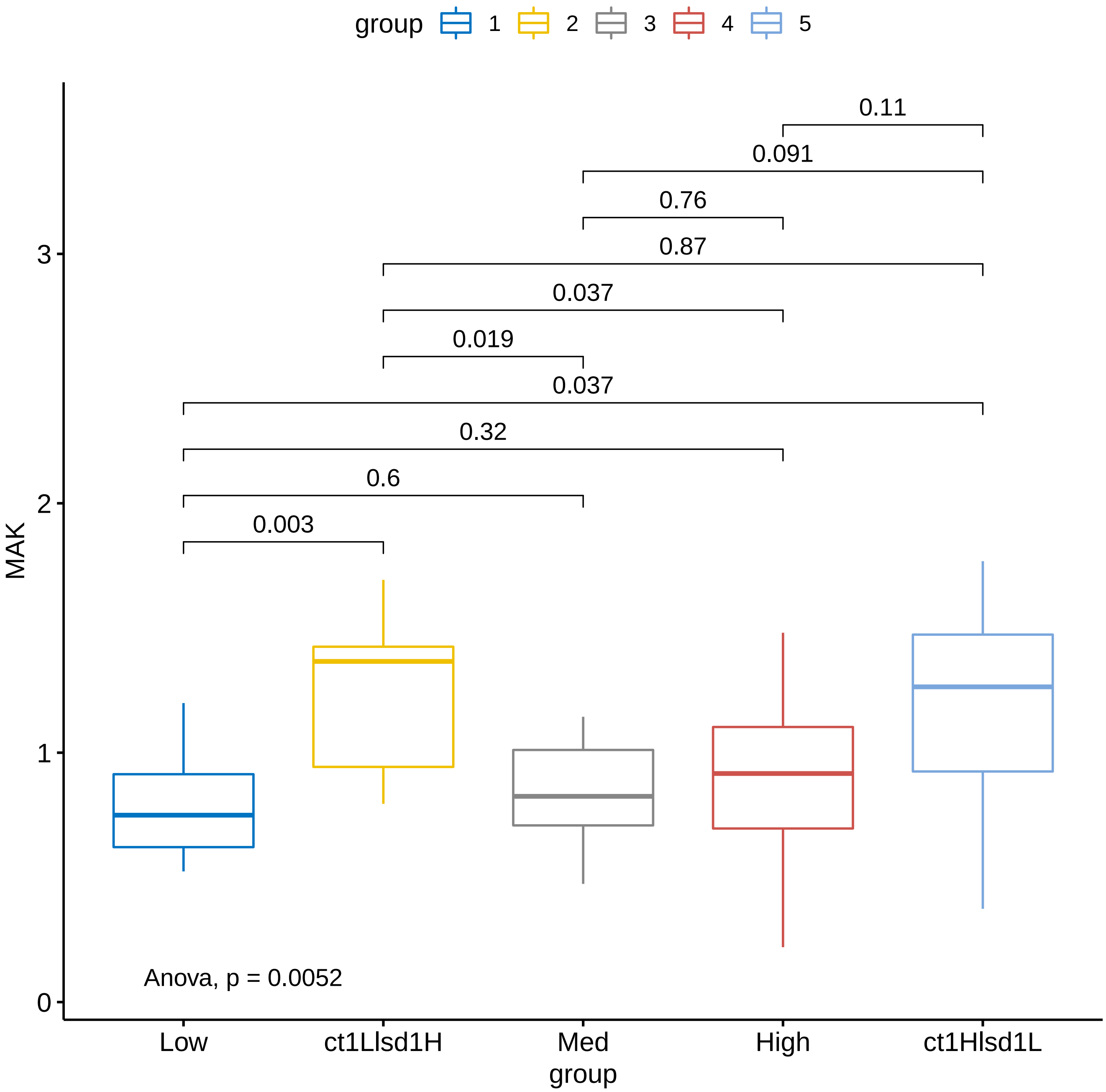
group 1 2 3 4 5
0.11
0.091
0.76
0.87
0.037
0.019
0.037
0.32
0.6
0.003
MAK
3
2
1
0
Anova, p = 0.0052
Low
ct1Llsd1H
Med
High
ct1Hlsd1L
group

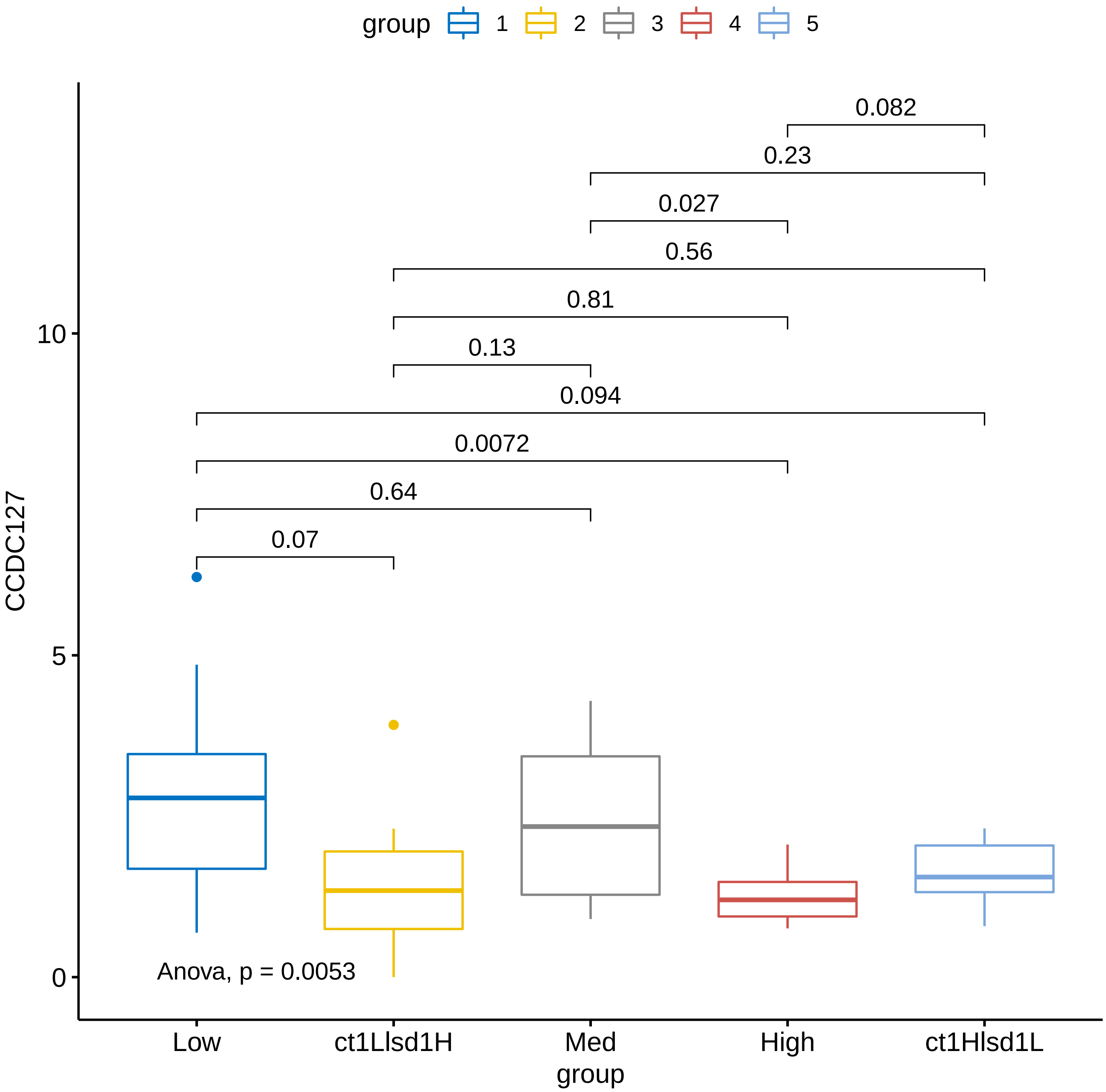

group
1
2
3
4
5
0.082
0.23
0.027
0.56
0.81
0.13
0.094
0.0072
0.64
0.07
Anova, p = 0.0053
CCDC127
10
5
0
Low
ct1Llsd1H
Med
High
ct1Hlsd1L
group

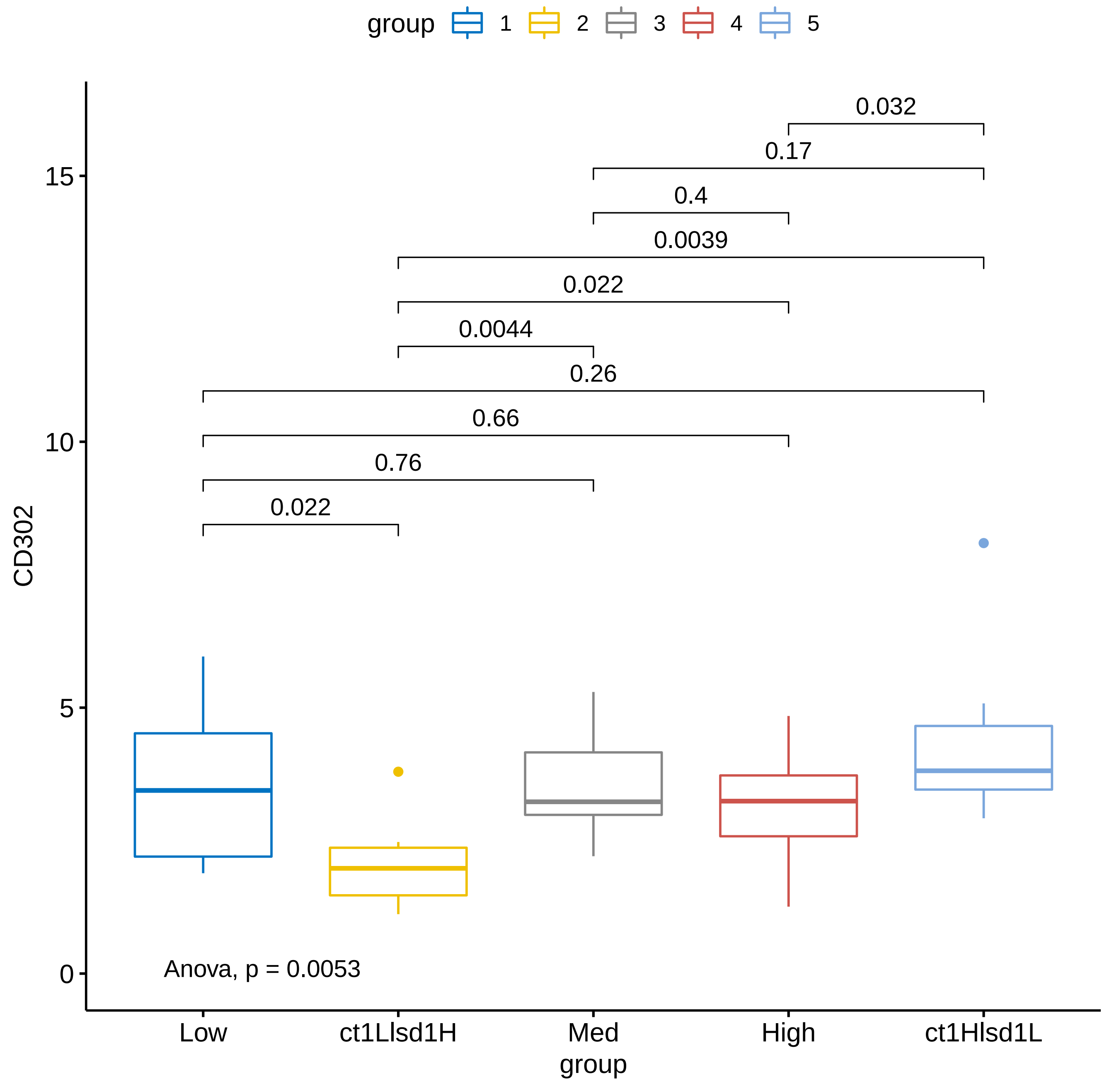
group
1
2
3
4
5
CD302
0
5
10
15
0.032
0.17
0.4
0.0039
0.022
0.0044
0.26
0.66
0.76
0.022
Anova, p = 0.0053
Low
ct1Llsd1H
Med
High
ct1Hlsd1L
group

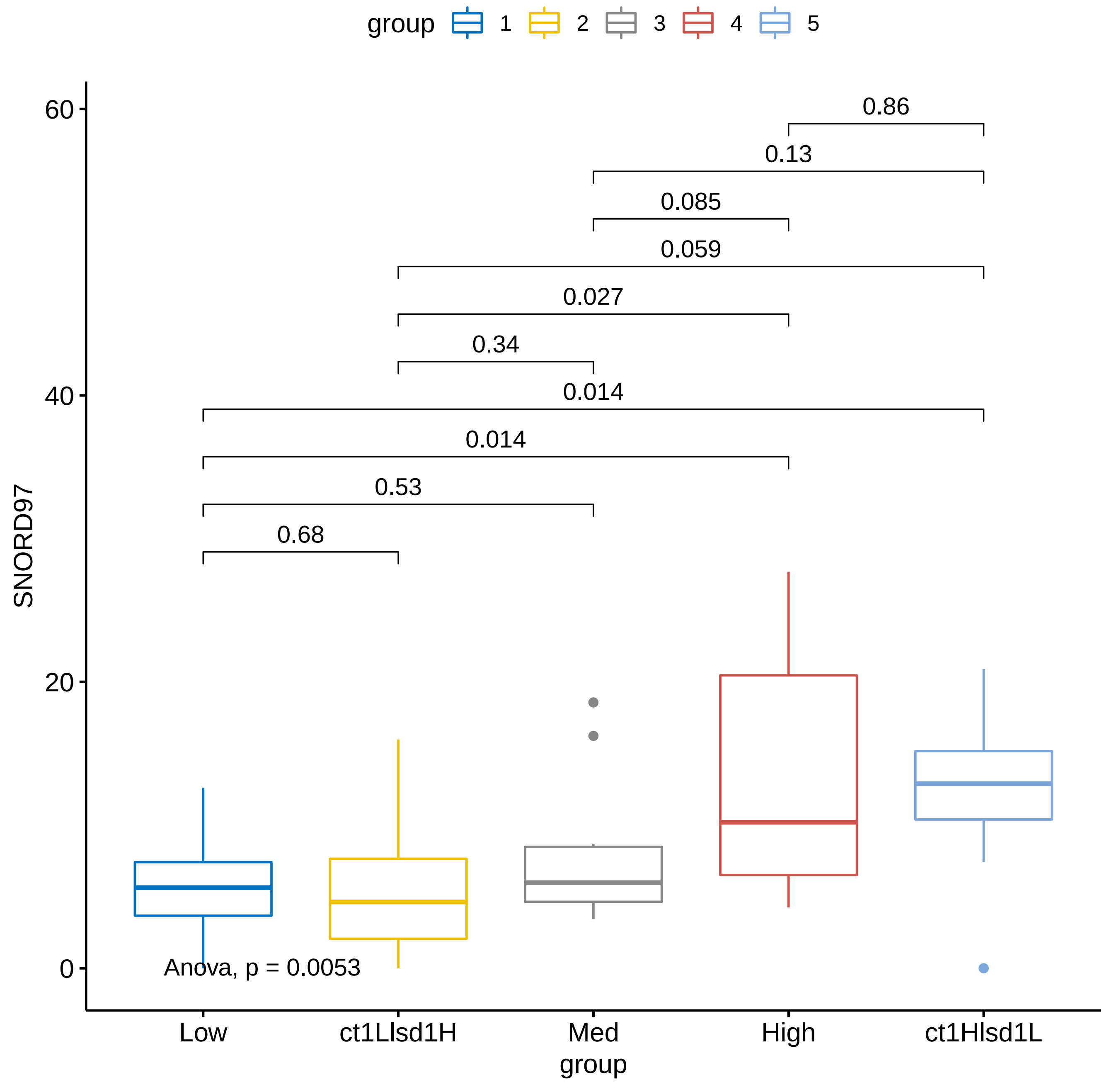

group
1
2
3
4
5
SNORD97
60
40
20
0
0.86
0.13
0.085
0.059
0.027
0.34
0.014
0.014
0.53
0.68
Anova, p = 0.0053
Low
ct1Llsd1H
Med
High
ct1Hlsd1L
group

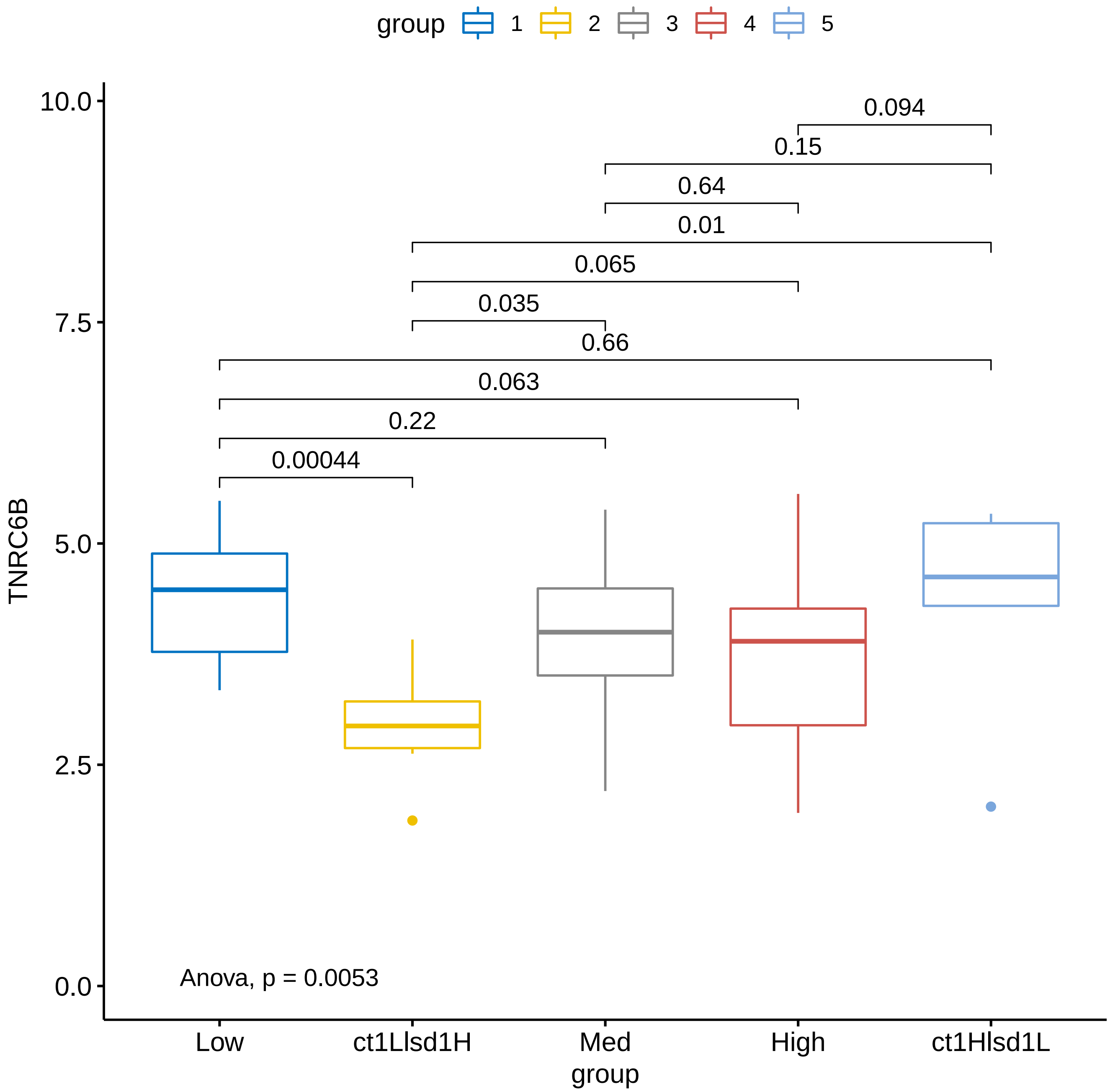

group 1 2 3 4 5
0.094
0.15
0.64
0.01
0.065
0.035
0.66
0.063
0.22
0.00044
10.0
7.5
5.0
2.5
0.0
TNRC6B
Anova, p = 0.0053
Low
ct1Llsd1H
Med
High
ct1Hlsd1L
group

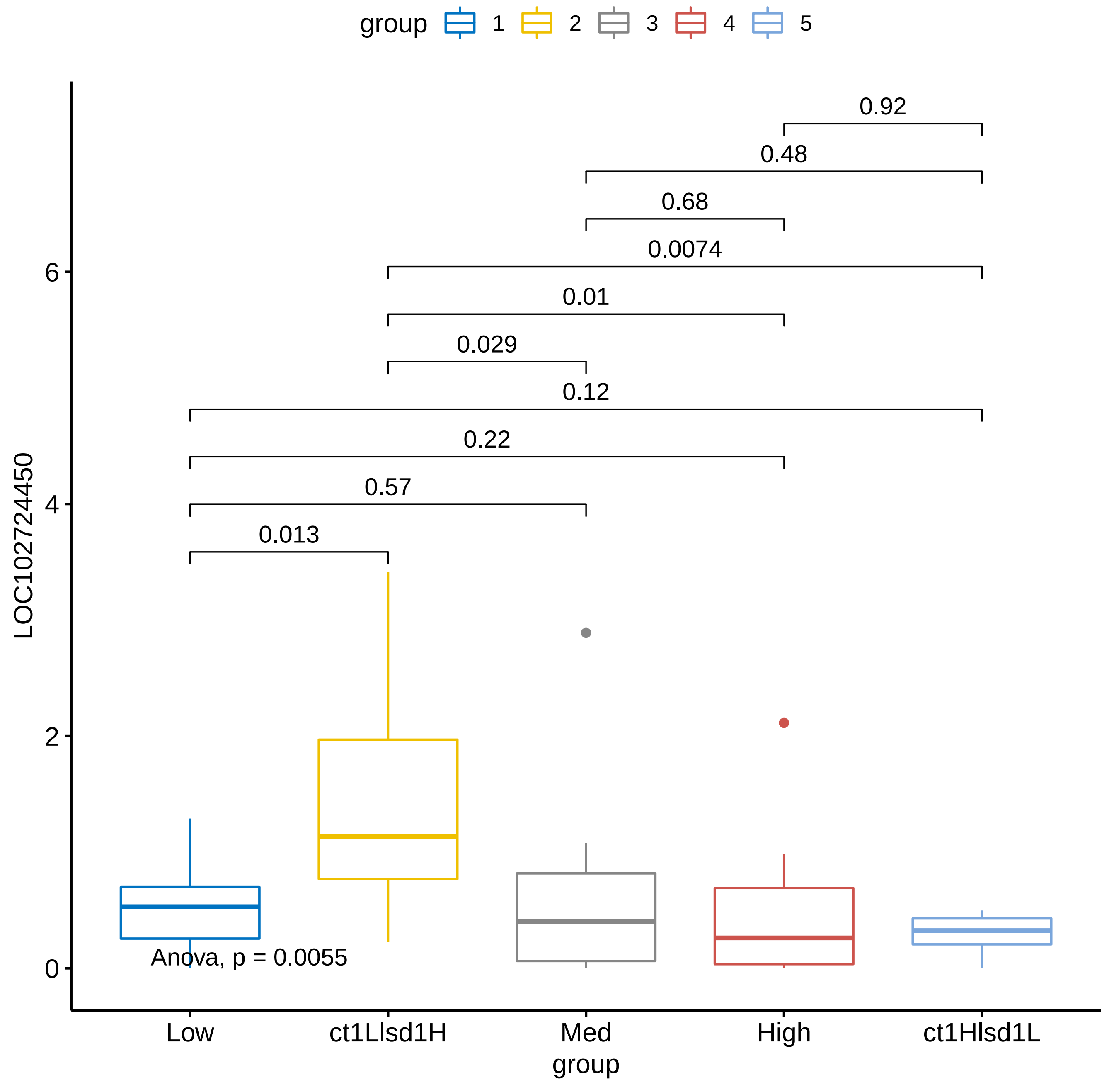

group 1 2 3 4 5
0.92
0.48
0.68
0.0074
0.01
0.029
0.12
0.22
0.57
0.013
Anova, p = 0.0055
6
4
2
0
LOC102724450
Low
ct1Llsd1H
Med
High
ct1Hlsd1L
group

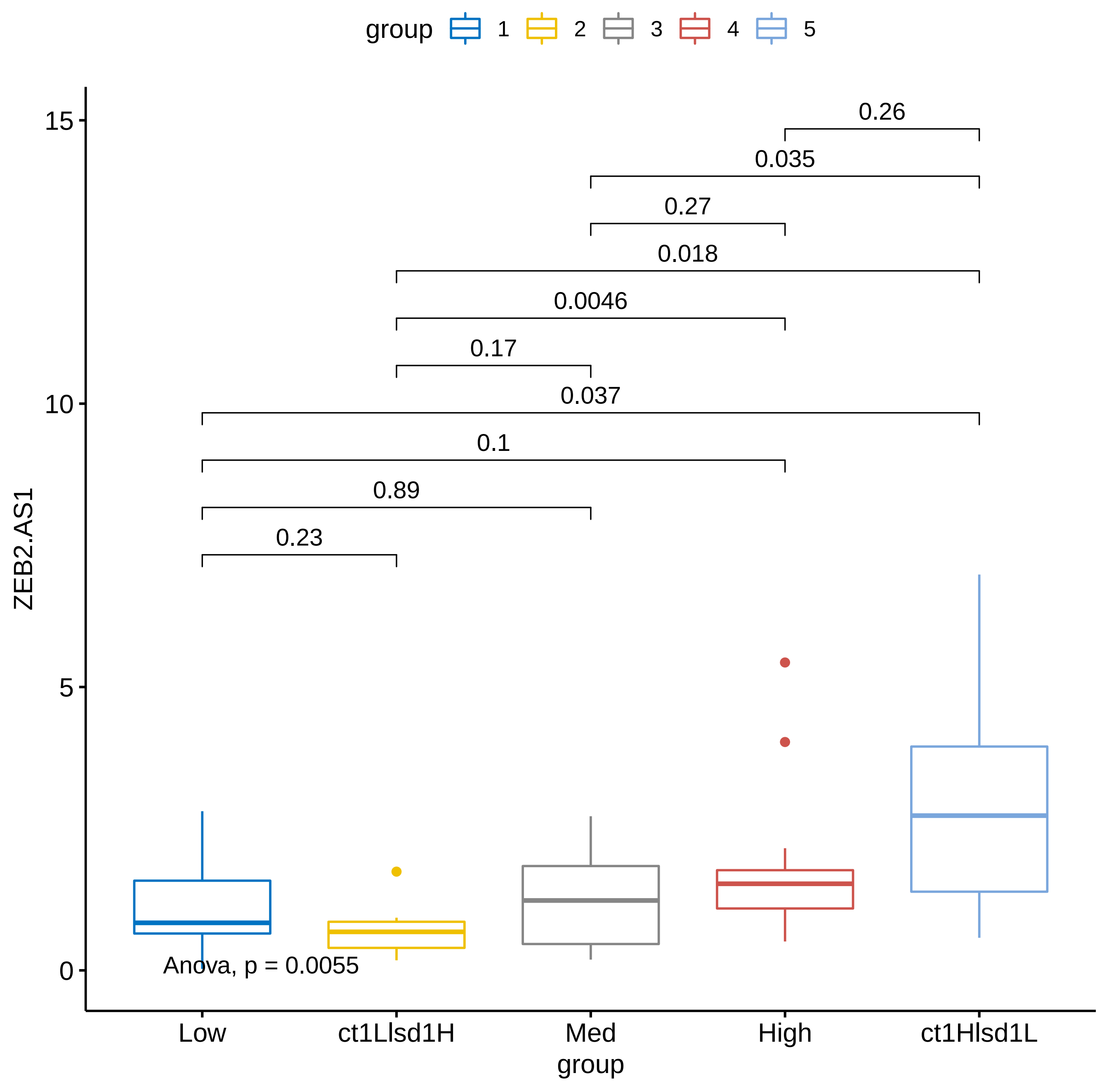

group 1 2 3 4 5
ZEB2.AS1
15
10
5
0
0.26
0.035
0.27
0.018
0.0046
0.17
0.037
0.1
0.89
0.23
Anova, p = 0.0055
Low
ct1Llsd1H
Med
High
ct1Hlsd1L
group

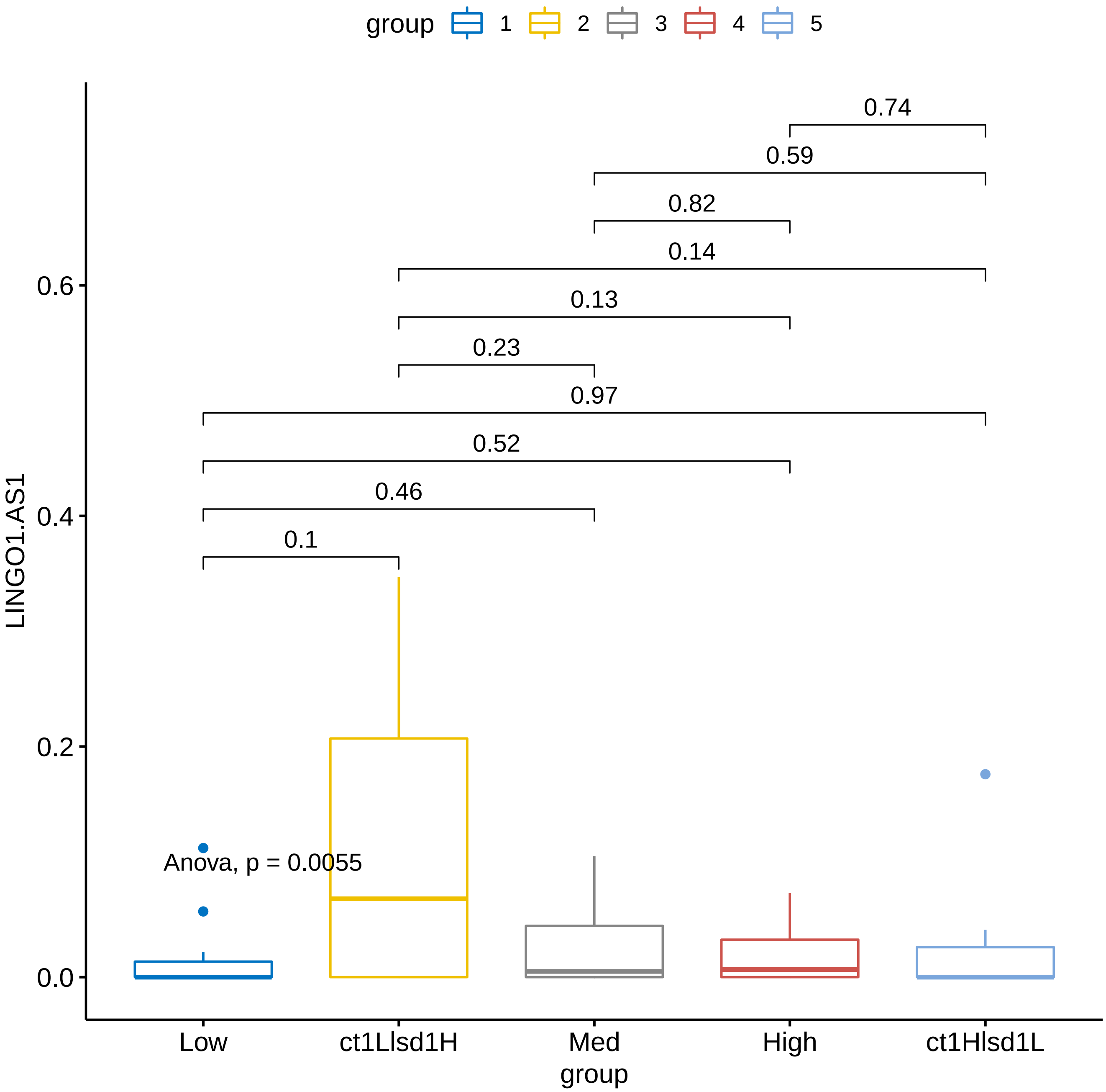

group
1
2
3
4
5
0.74
0.59
0.82
0.14
0.13
0.23
0.97
0.52
0.46
0.1
Anova, p = 0.0055
LINGO1.AS1
0.6
0.4
0.2
0.0
Low
ct1Llsd1H
Med
High
ct1Hlsd1L
group

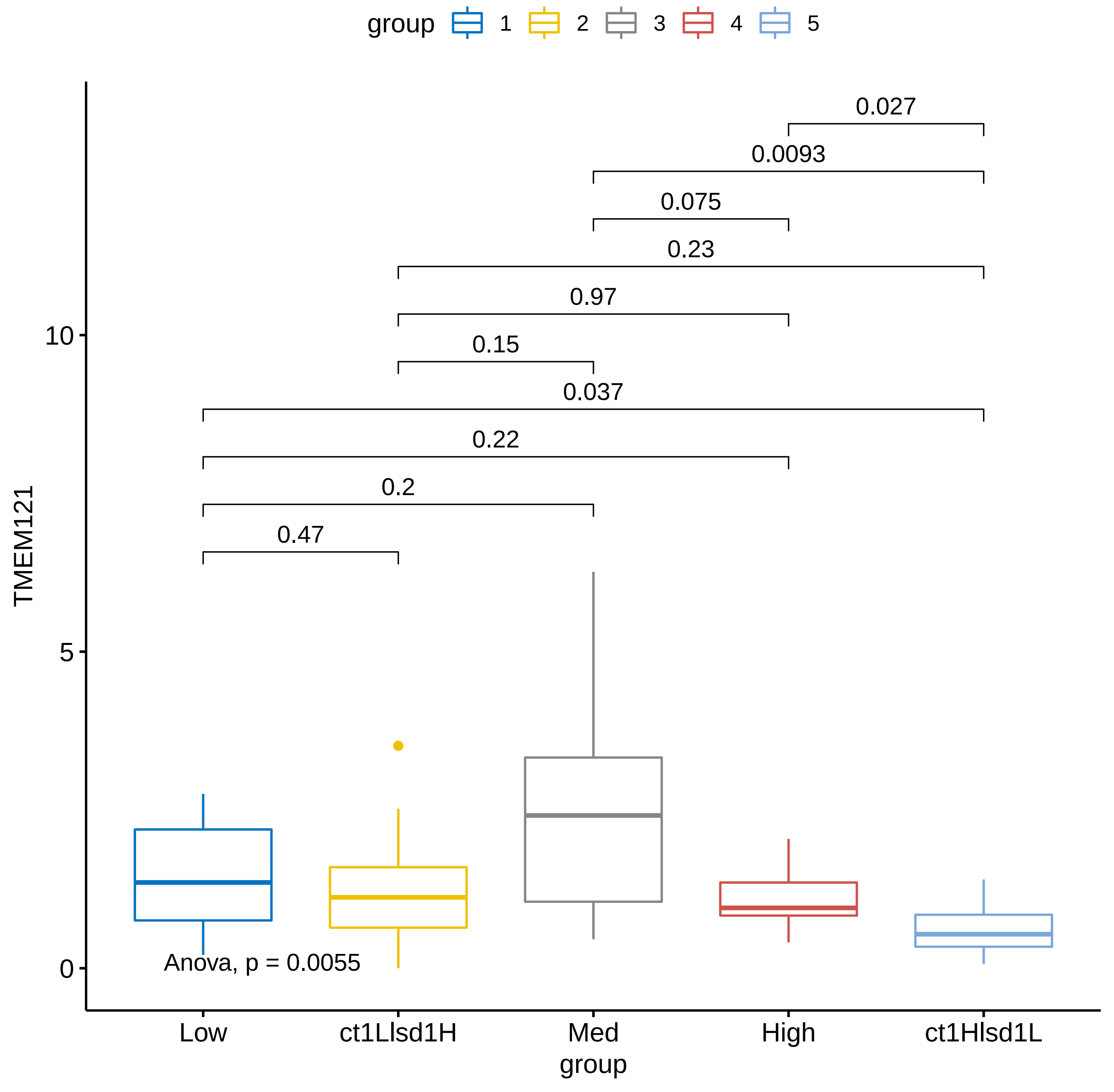
group
1
2
3
4
5
0.027
0.0093
0.075
0.23
0.97
0.15
0.037
0.22
0.2
0.47
Anova, p = 0.0055
TMEM121
10
5
0
Low
ct1Llsd1H
Med
High
ct1Hlsd1L
group

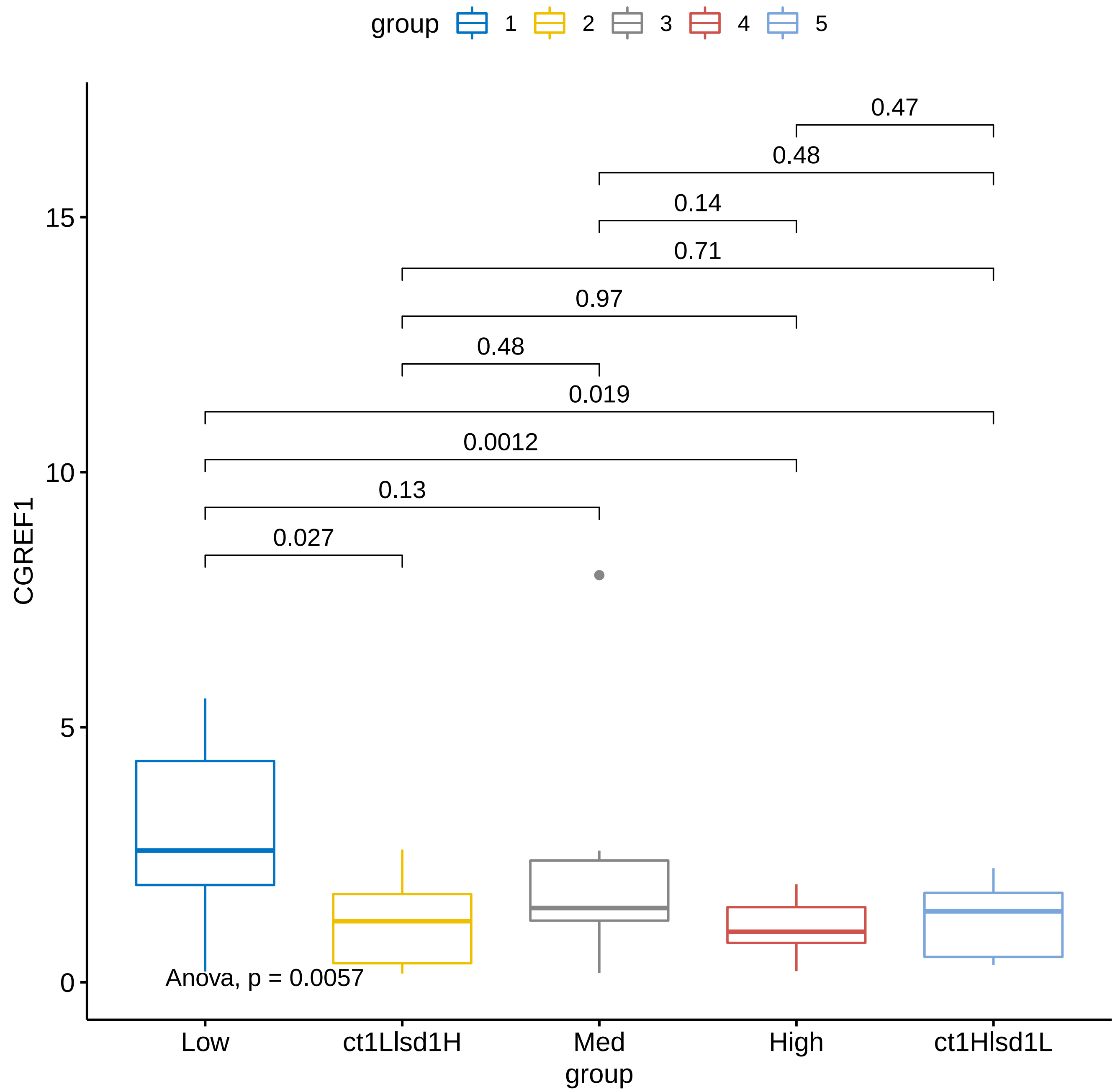

group 1 2 3 4 5
0.47
0.48
0.14
0.71
0.97
0.48
0.019
0.0012
0.13
0.027
CGREF1
15
10
5
0
Anova, p = 0.0057
Low
ct1Llsd1H
Med
High
ct1Hlsd1L
group

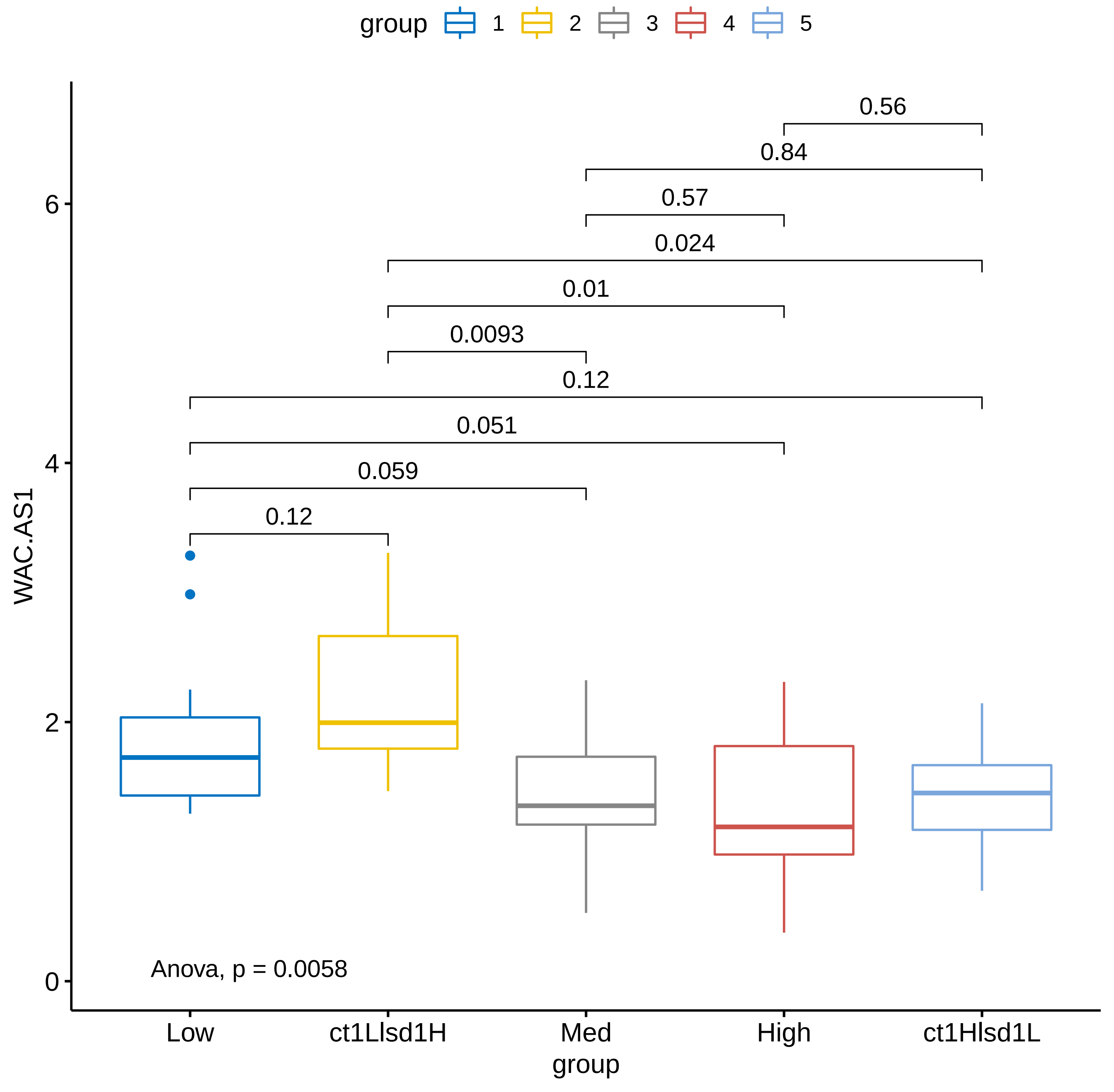

group
1
2
3
4
5
0.56
0.84
0.57
0.024
0.01
0.0093
0.12
0.051
0.059
0.12
6
4
2
0
WAC.AS1
Anova, p = 0.0058
Low
ct1Llsd1H
Med
High
ct1Hlsd1L
group

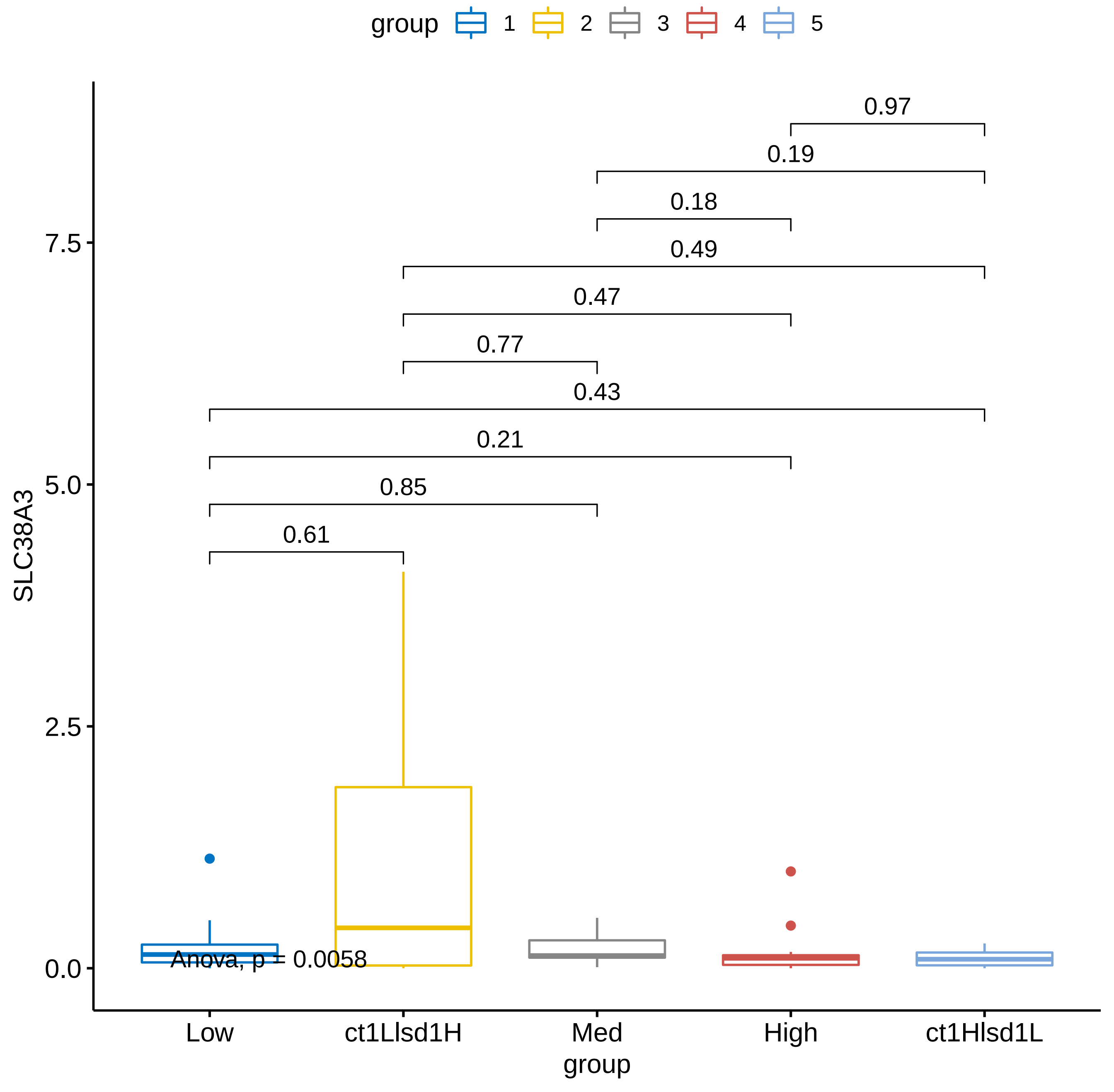

group
1
2
3
4
5
0.97
0.19
0.18
0.49
0.47
0.77
0.43
0.21
0.85
0.61
7.5
5.0
2.5
0.0
SLC38A3
Anova, p = 0.0058
Low
ct1Llsd1H
Med
High
ct1Hlsd1L
group

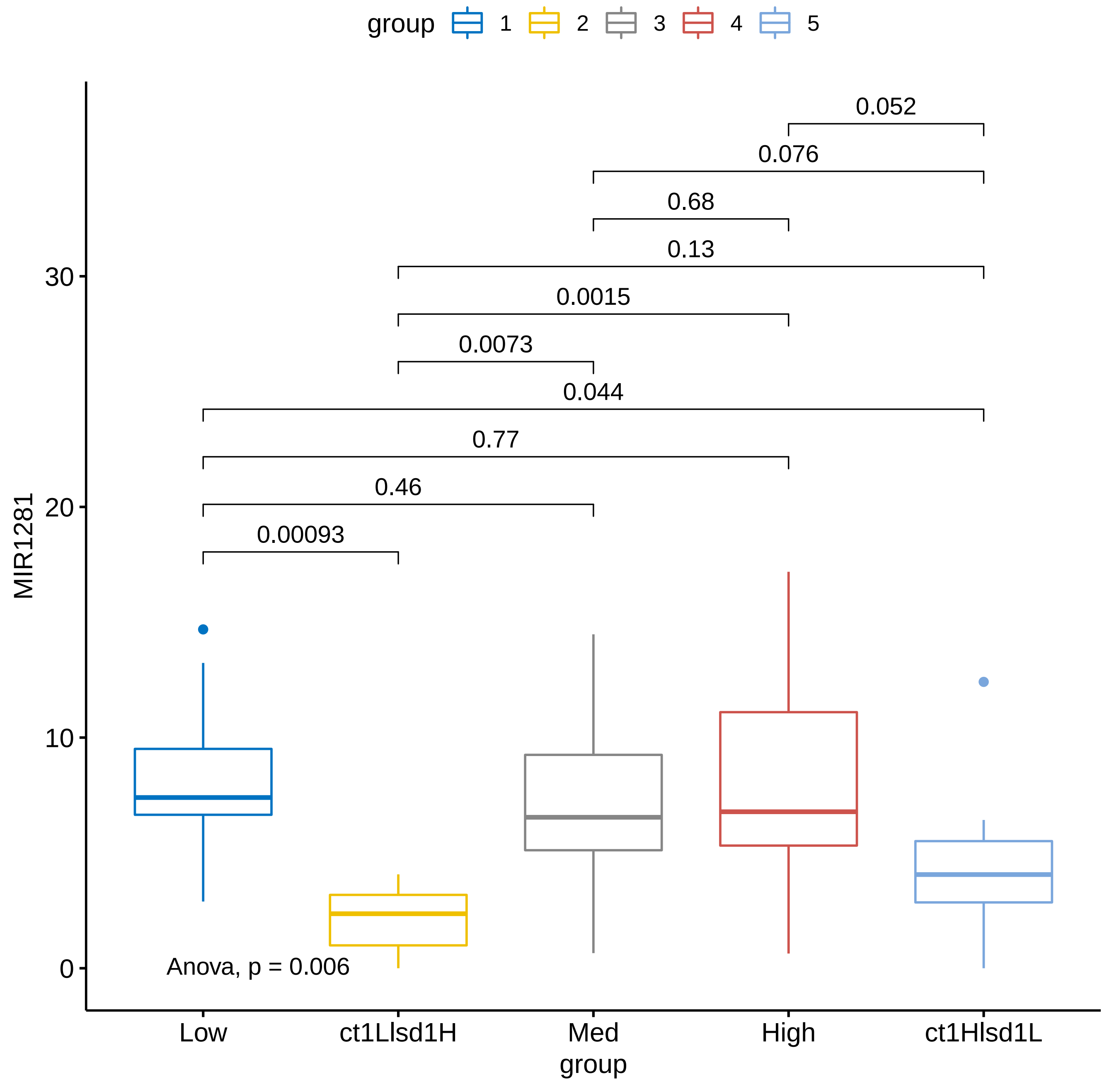

group
1
2
3
4
5
0.052
0.076
0.68
0.13
0.0015
0.0073
0.044
0.77
0.46
0.00093
Anova, p = 0.006
MIR1281
30
20
10
0
Low
ct1Llsd1H
Med
High
ct1Hlsd1L
group

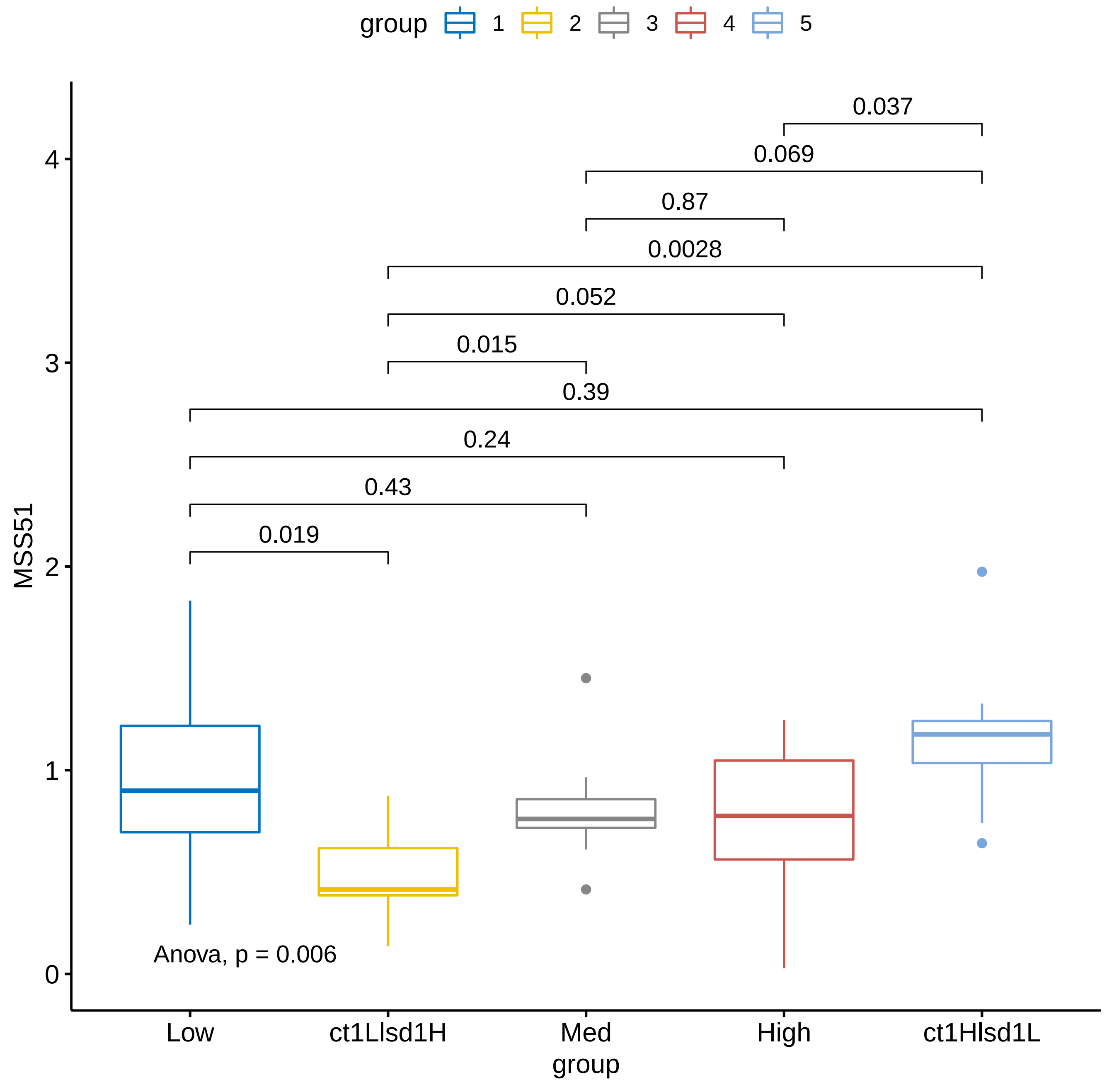

group 1 2 3 4 5
0.037
0.069
0.87
0.0028
0.052
0.015
0.39
0.24
0.43
0.019
MSS51
4
3
2
1
0
Anova, p = 0.006
Low
ct1Llsd1H
Med
High
ct1Hlsd1L
group

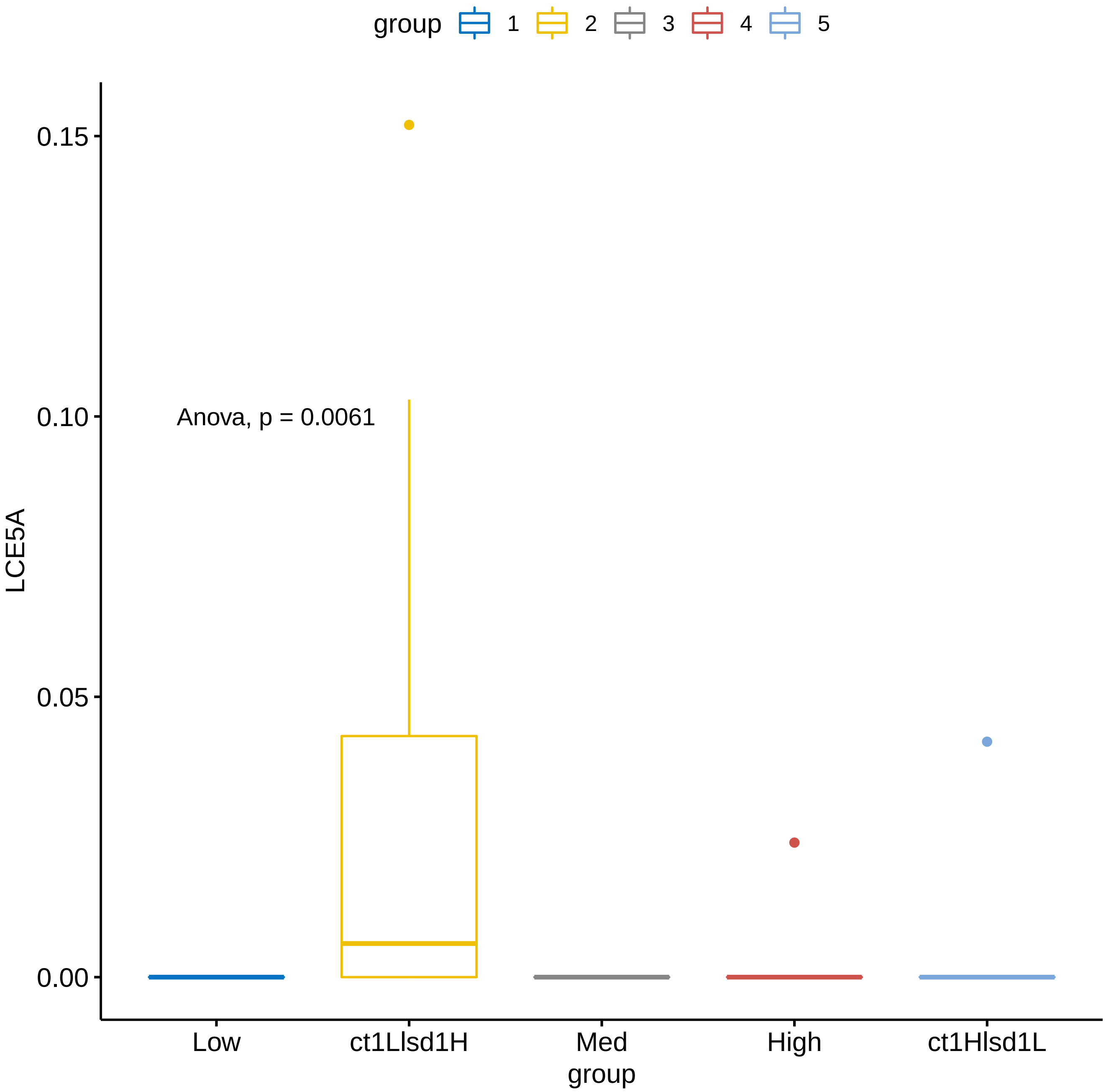

group
1
2
3
4
5
Anova, p = 0.0061
0.15
0.10
0.05
0.00
LCE5A
Low
ct1Llsd1H
Med
High
ct1Hlsd1L
group

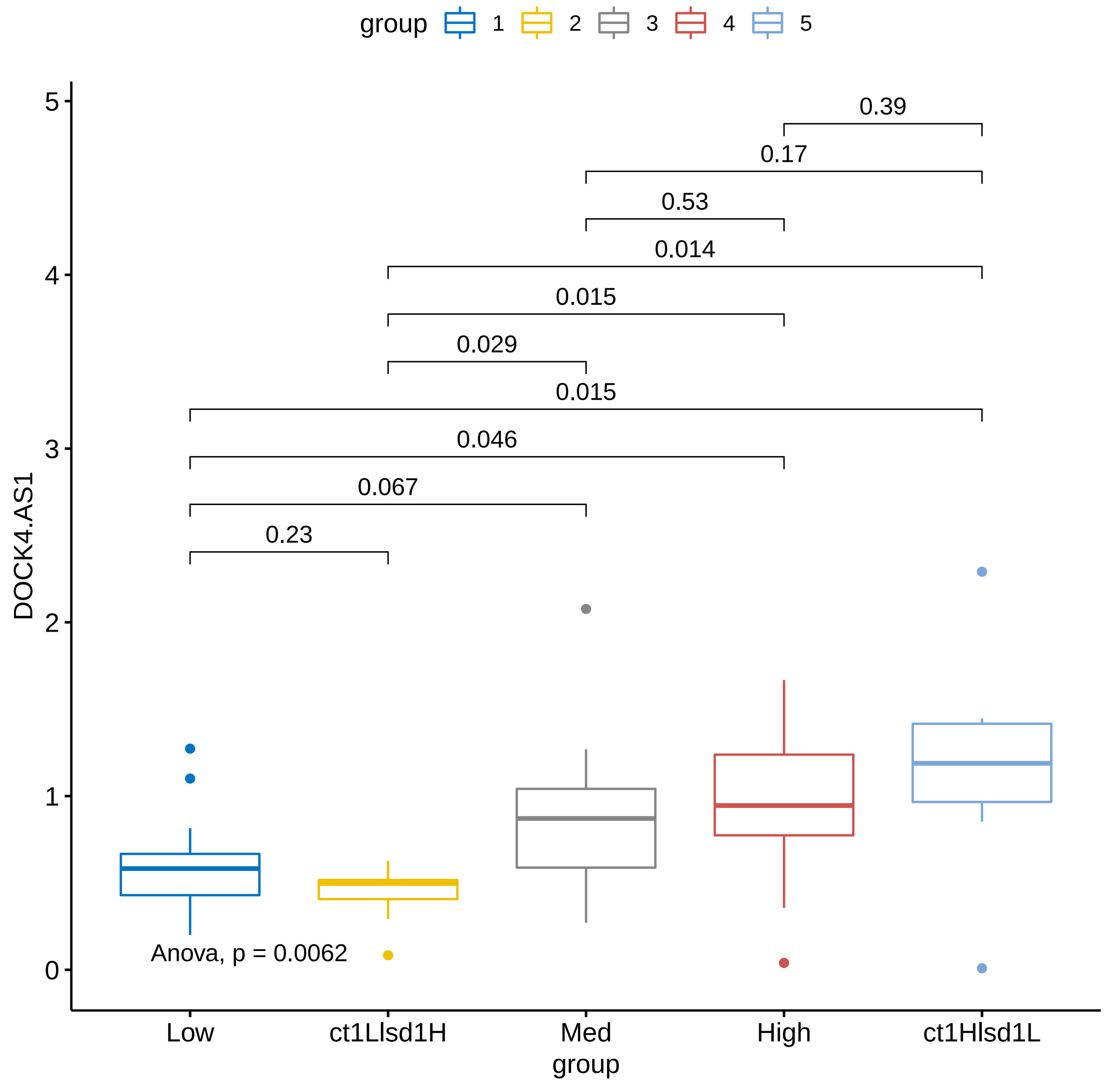

group 1 2 3 4 5
0.39
0.17
0.53
0.014
0.015
0.029
0.015
0.046
0.067
0.23
Anova, p = 0.0062
DOCK4.AS1
0
1
2
3
4
5
Low
ct1Llsd1H
Med
High
ct1Hlsd1L
group

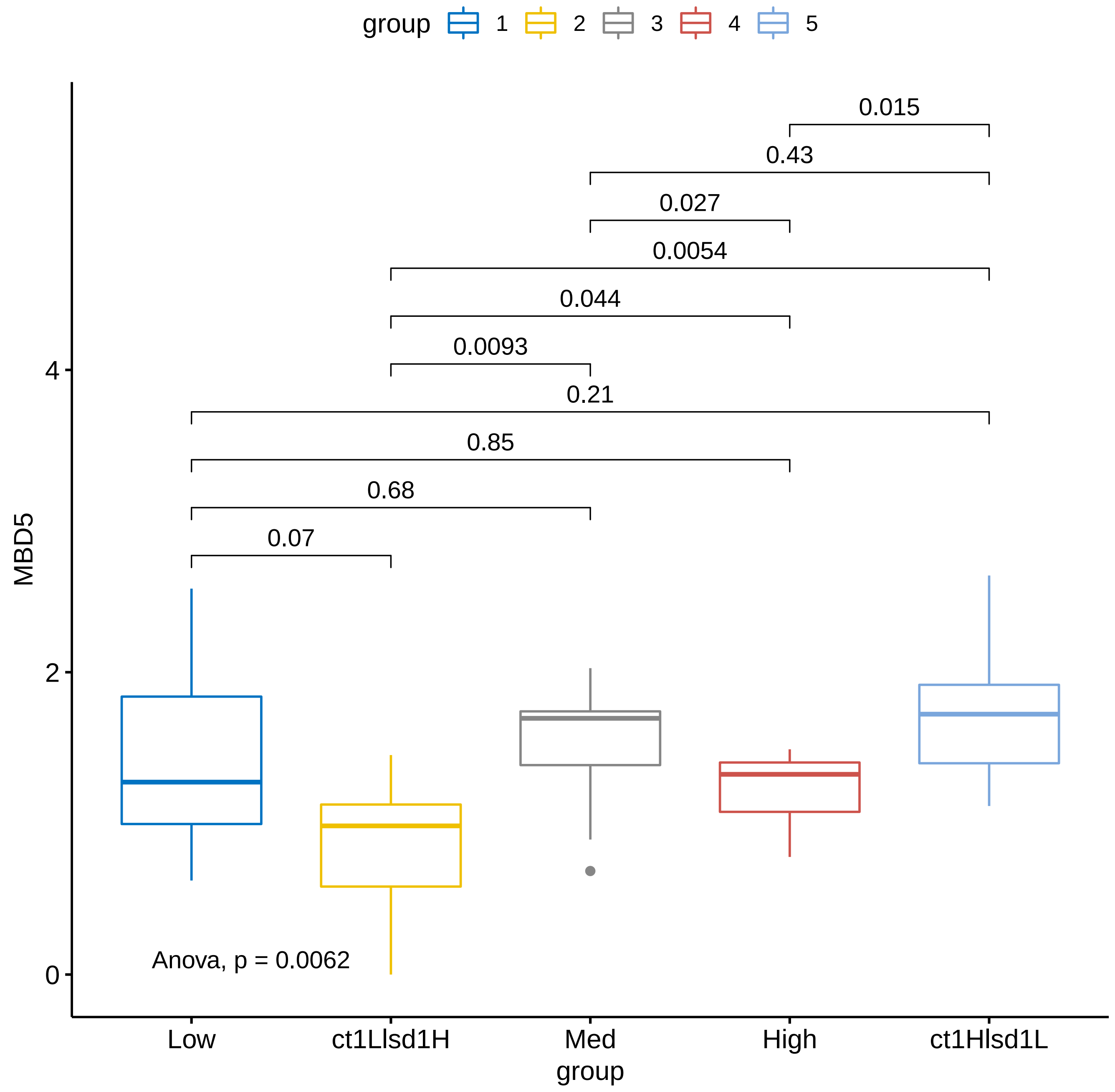
group
1
2
3
4
5
MBD5
0.015
0.43
0.027
0.0054
0.044
0.0093
0.21
0.85
0.68
0.07
4
2
0
Anova, p = 0.0062
Low
ct1Llsd1H
Med
High
ct1Hlsd1L
group

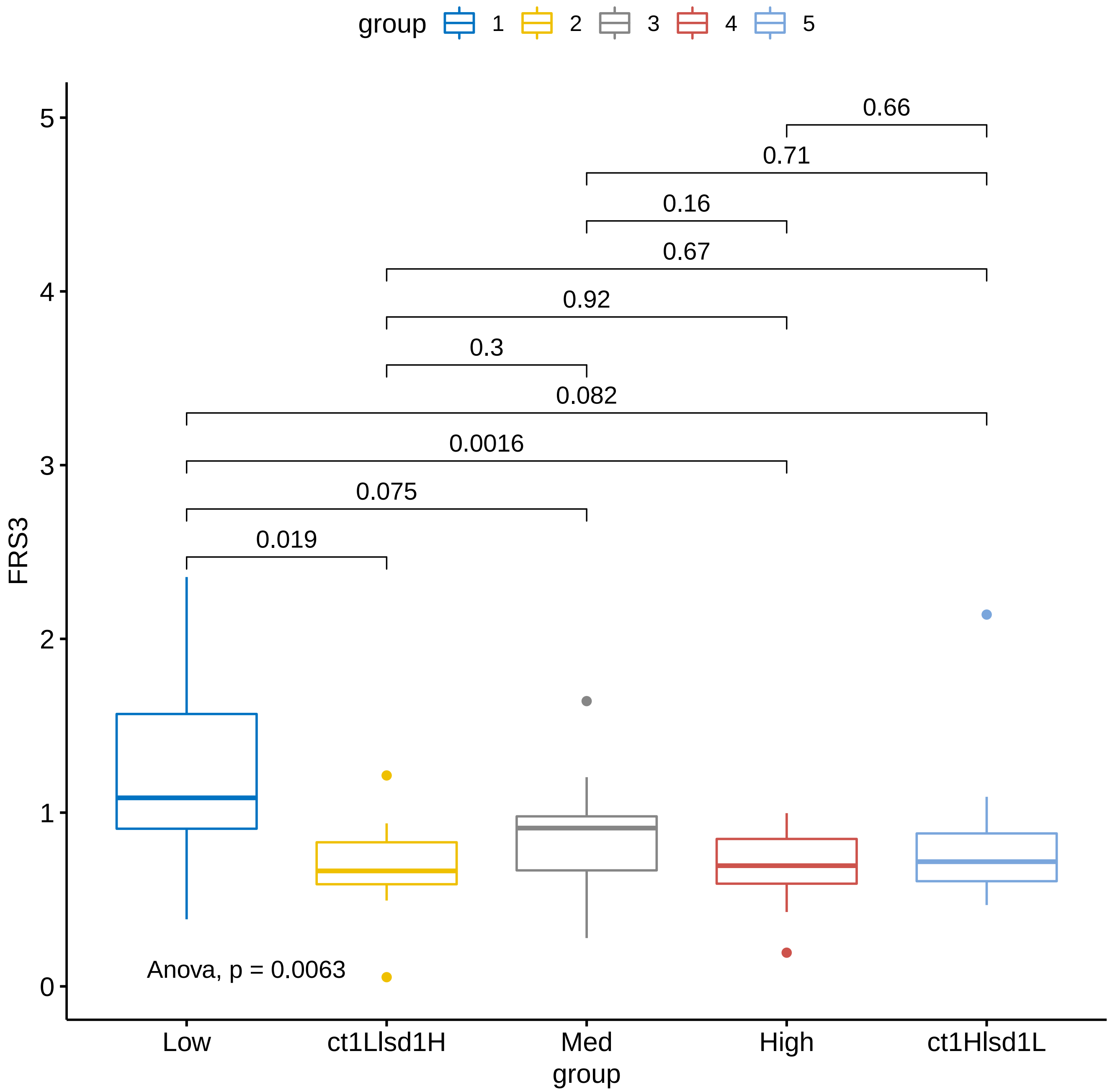

group
1
2
3
4
5
FRS3
0
1
2
3
4
5
0.66
0.71
0.16
0.67
0.92
0.3
0.082
0.0016
0.075
0.019
Anova, p = 0.0063
Low
ct1Llsd1H
Med
High
ct1Hlsd1L
group

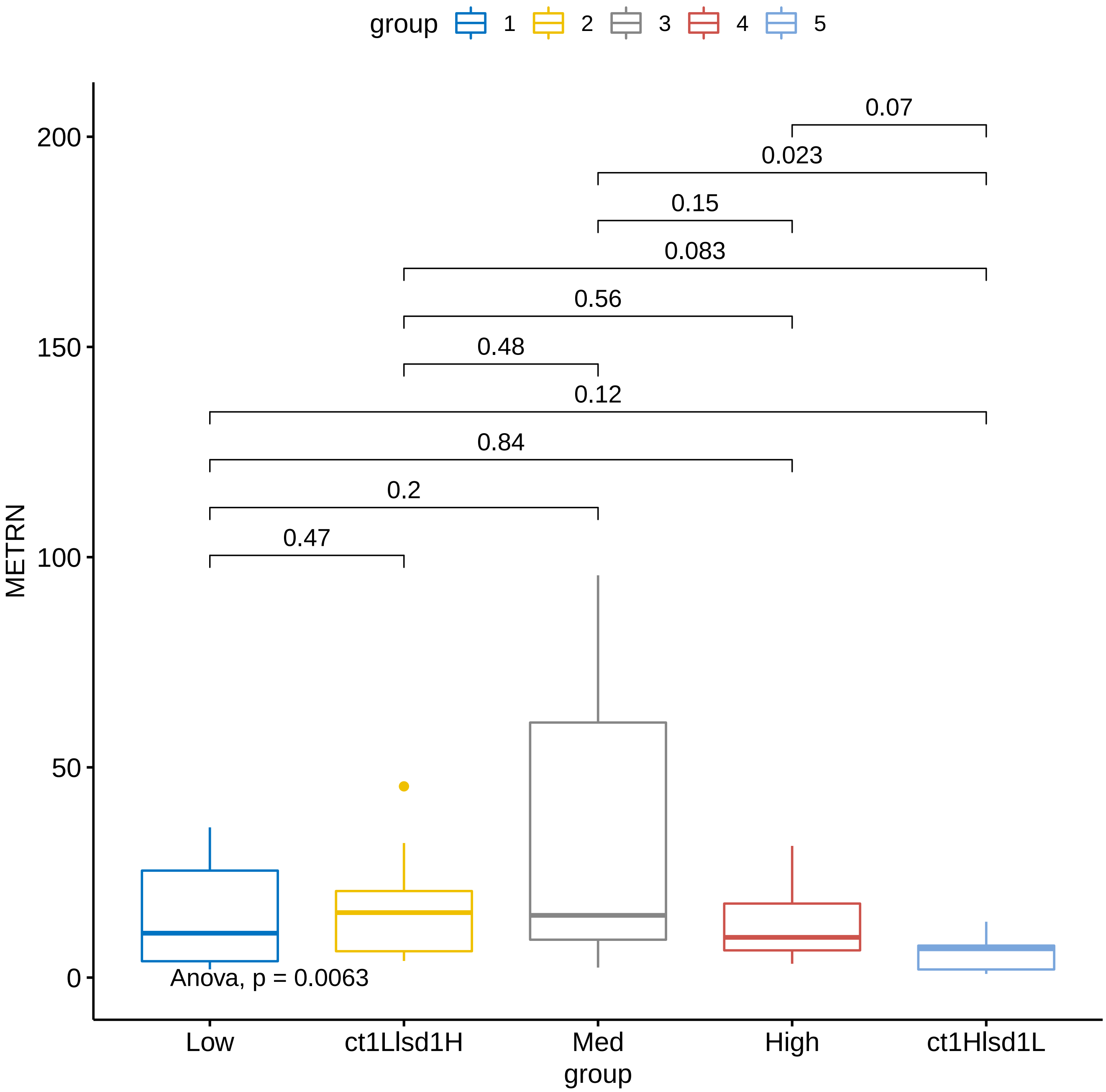
group
1
2
3
4
5
0.07
0.023
0.15
0.083
0.56
0.48
0.12
0.84
0.2
0.47
200
150
100
50
0
METRN
Anova, p = 0.0063
Low
ct1Llsd1H
Med
High
ct1Hlsd1L
group

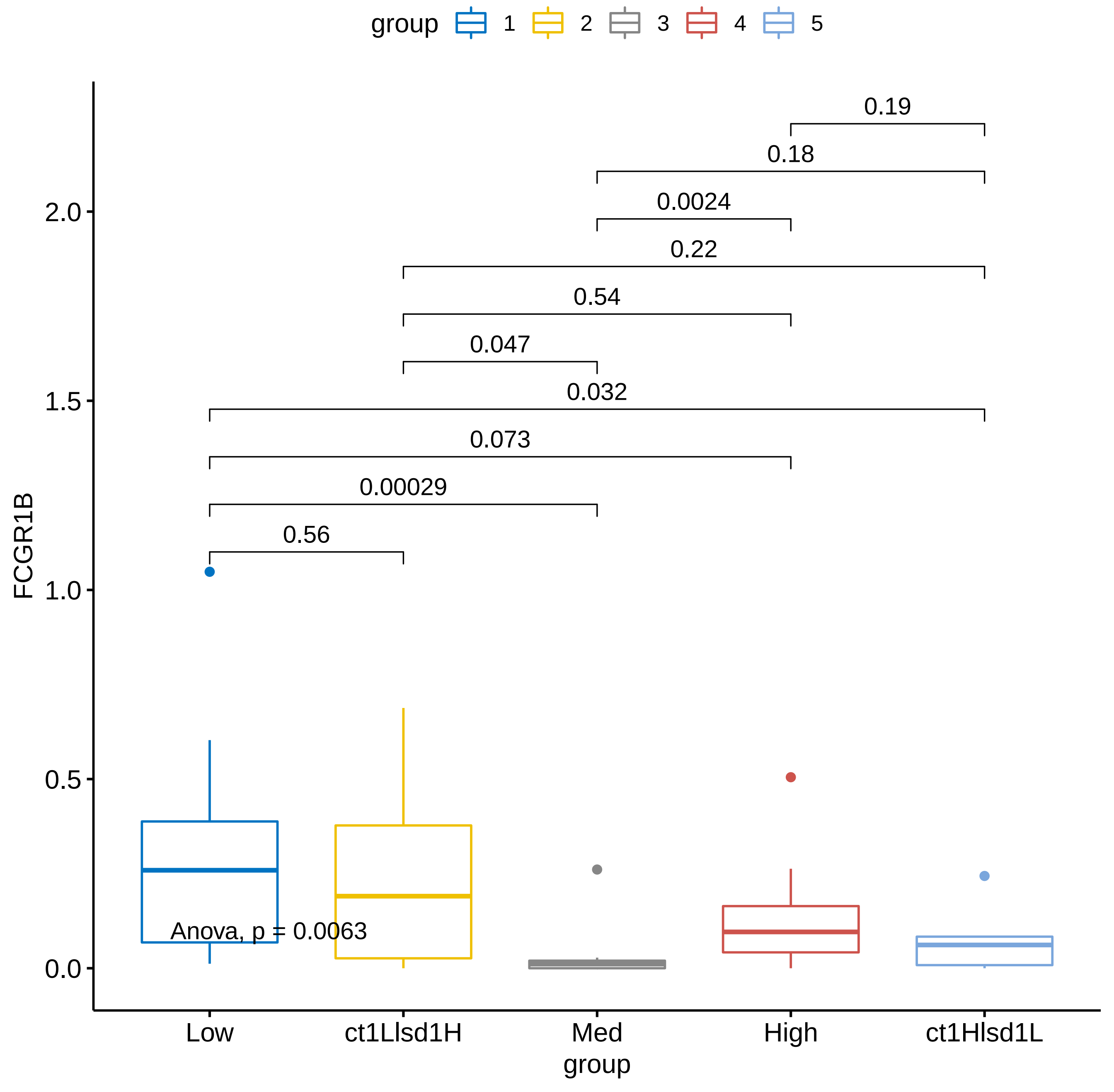

group 1 2 3 4 5
0.19
0.18
0.0024
0.22
0.54
0.047
0.032
0.073
0.00029
0.56
Anova, p = 0.0063
FCGR1B
2.0
1.5
1.0
0.5
0.0
Low
ct1Llsd1H
Med
High
ct1Hlsd1L
group

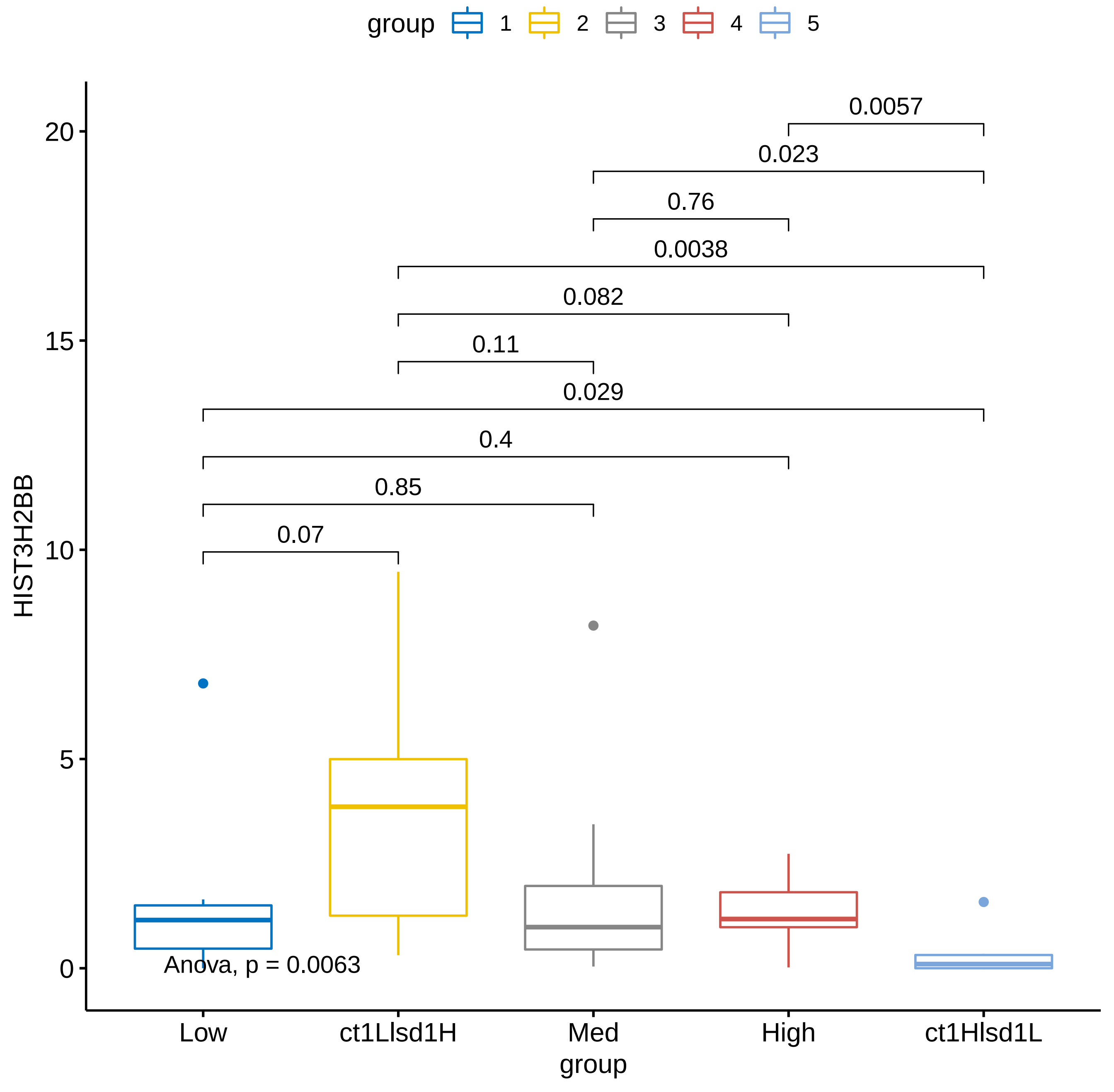

group
1
2
3
4
5
HIST3H2BB
0.0057
0.023
0.76
0.0038
0.082
0.11
0.029
0.4
0.85
0.07
Anova, p = 0.0063
20
15
10
5
0
Low
ct1Llsd1H
Med
High
ct1Hlsd1L
group

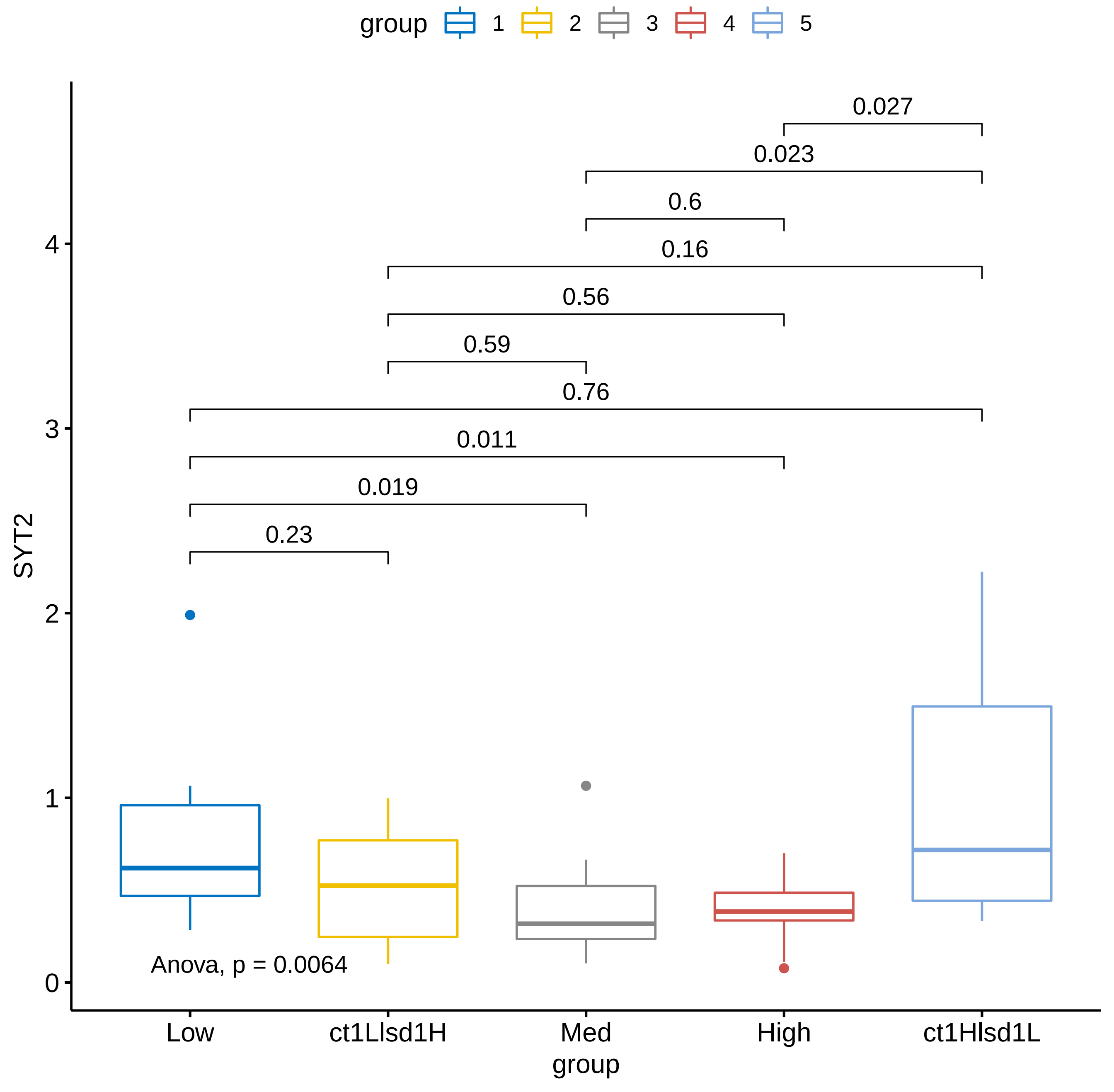

group 1 2 3 4 5
0.027
0.023
0.6
0.16
0.56
0.59
0.76
0.011
0.019
0.23
Anova, p = 0.0064
SYT2
0
1
2
3
4
Low
ct1Llsd1H
Med
High
ct1Hlsd1L
group

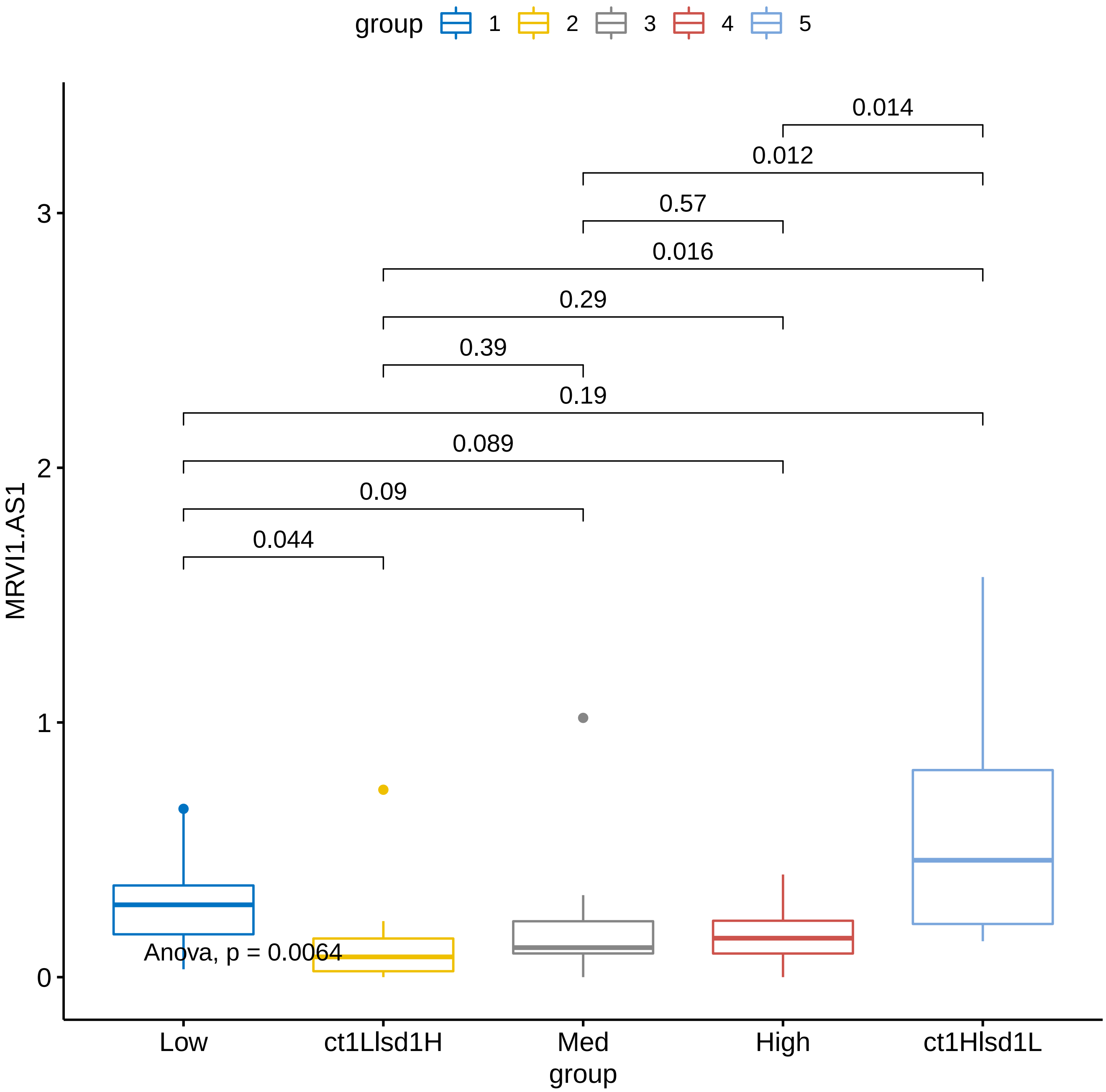

group
1
2
3
4
5
0.014
0.012
0.57
0.016
0.29
0.39
0.19
0.089
0.09
0.044
Anova, p = 0.0064
MRVI1.AS1
3
2
1
0
Low
ct1Llsd1H
Med
High
ct1Hlsd1L
group

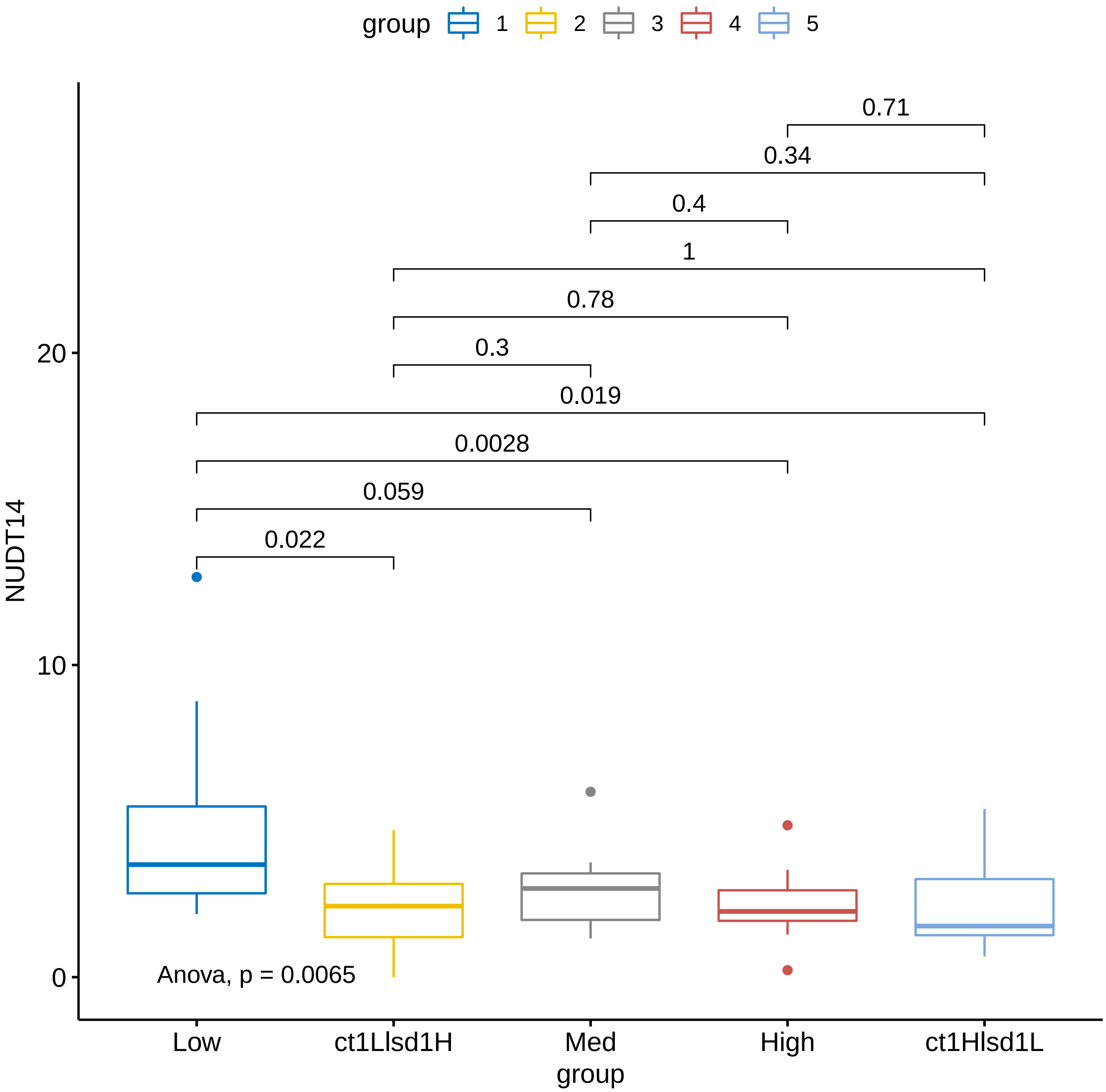
group
1
2
3
4
5
0.71
0.34
0.4
1
0.78
0.3
0.019
0.0028
0.059
0.022
20
10
0
NUDT14
Anova, p = 0.0065
Low
ct1Llsd1H
Med
High
ct1Hlsd1L
group

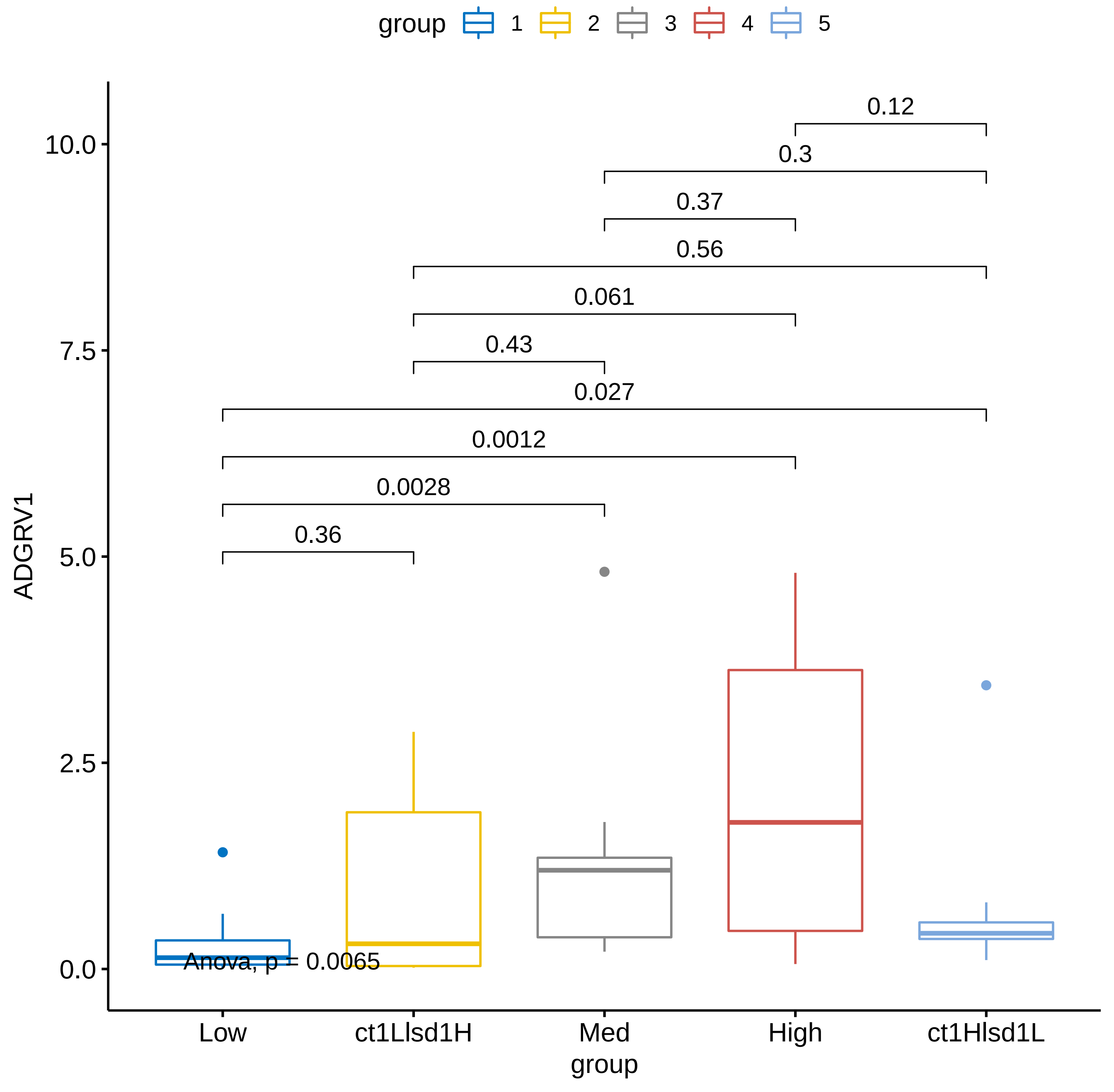

group 1 2 3 4 5
ADGRV1
10.0
7.5
5.0
2.5
0.0
0.12
0.3
0.37
0.56
0.061
0.43
0.027
0.0012
0.0028
0.36
Anova, p = 0.0065
Low
ct1Llsd1H
Med
High
ct1Hlsd1L
group

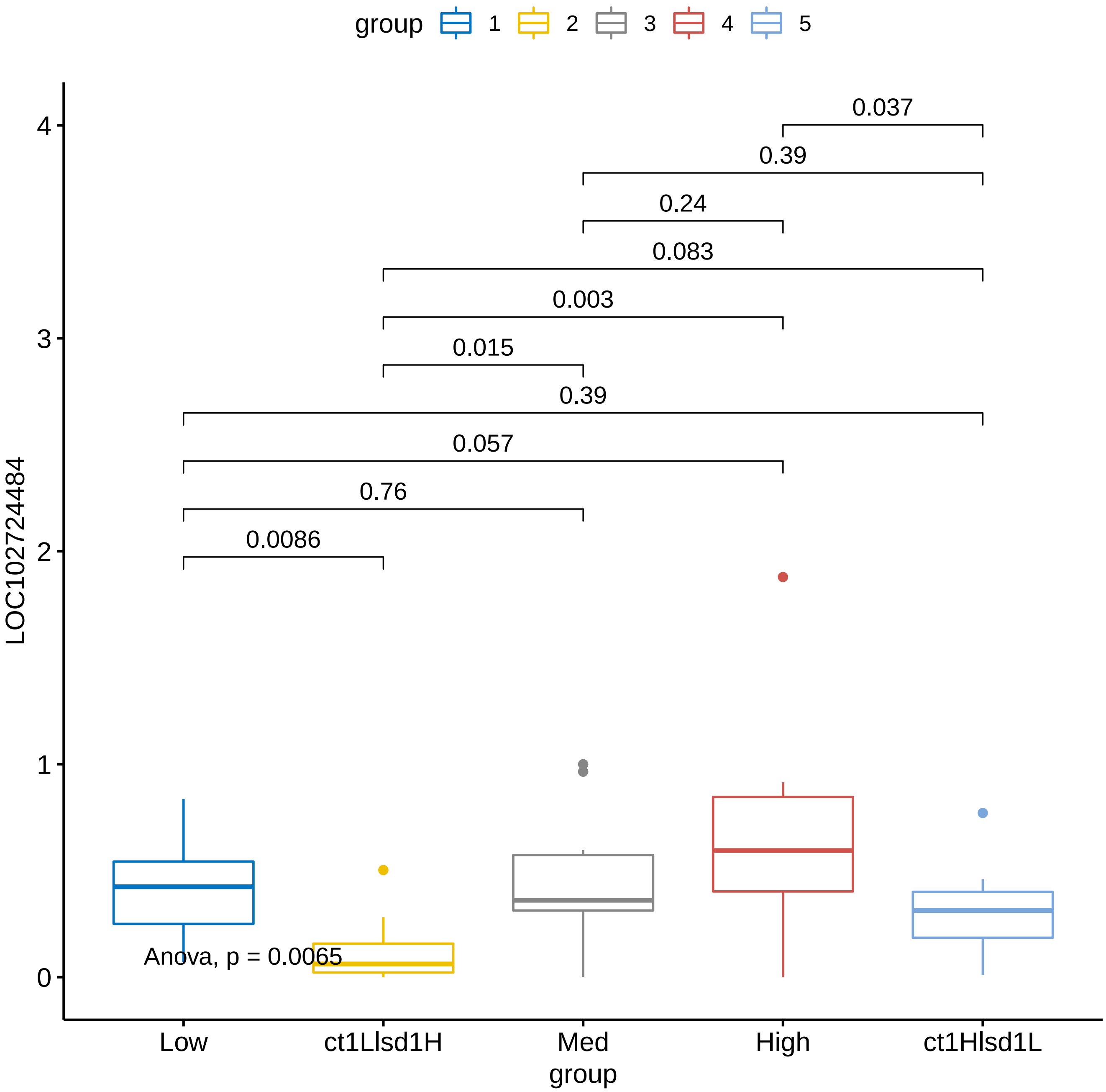
group
1
2
3
4
5
LOC102724484
0
1
2
3
4
0.037
0.39
0.24
0.083
0.003
0.015
0.39
0.057
0.76
0.0086
Anova, p = 0.0065
Low
ct1Llsd1H
Med
High
ct1Hlsd1L
group

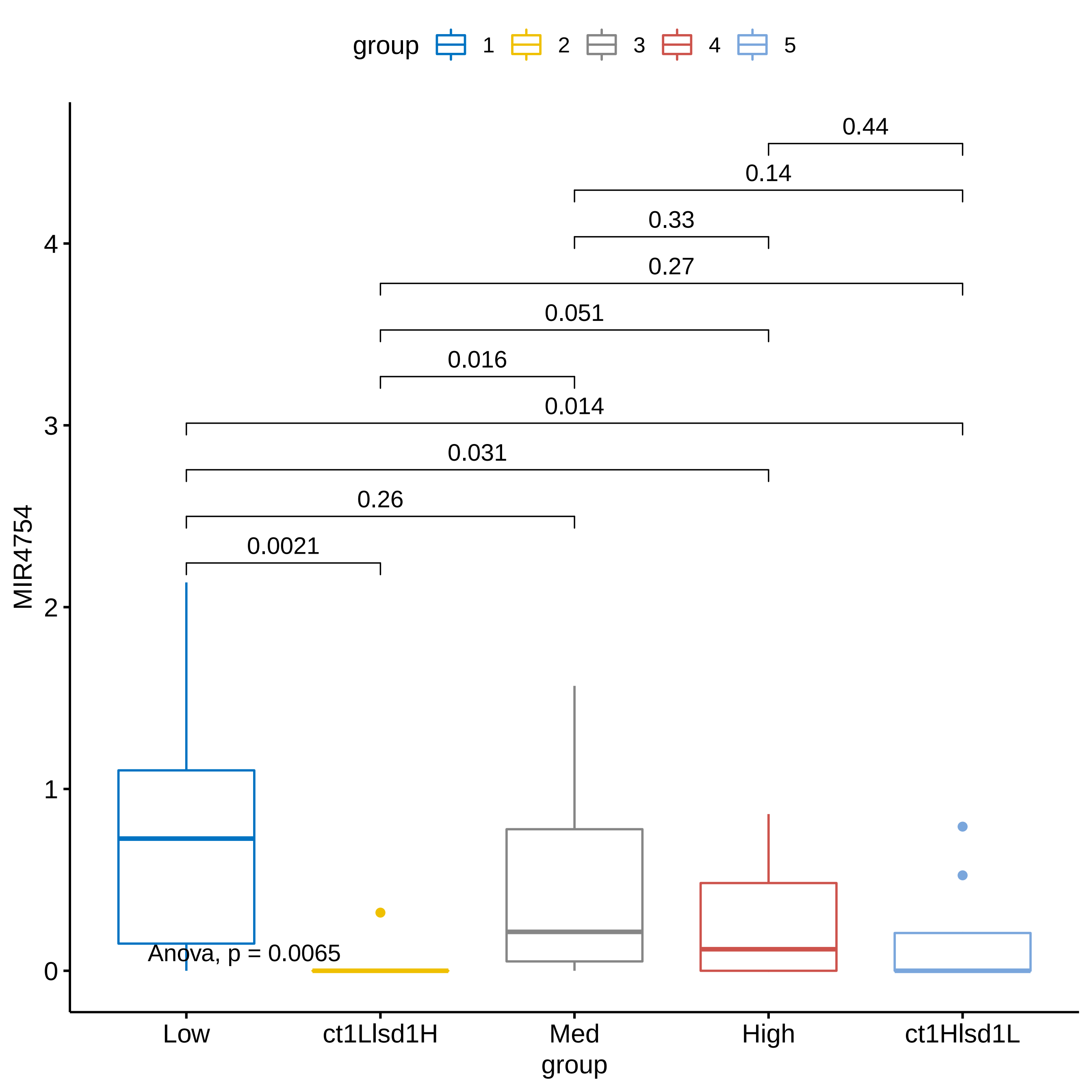
group
1
2
3
4
5
0.44
0.14
0.33
0.27
0.051
0.016
0.014
0.031
0.26
0.0021
Anova, p = 0.0065
MIR4754
0
1
2
3
4
Low
ct1Llsd1H
Med
High
ct1Hlsd1L
group

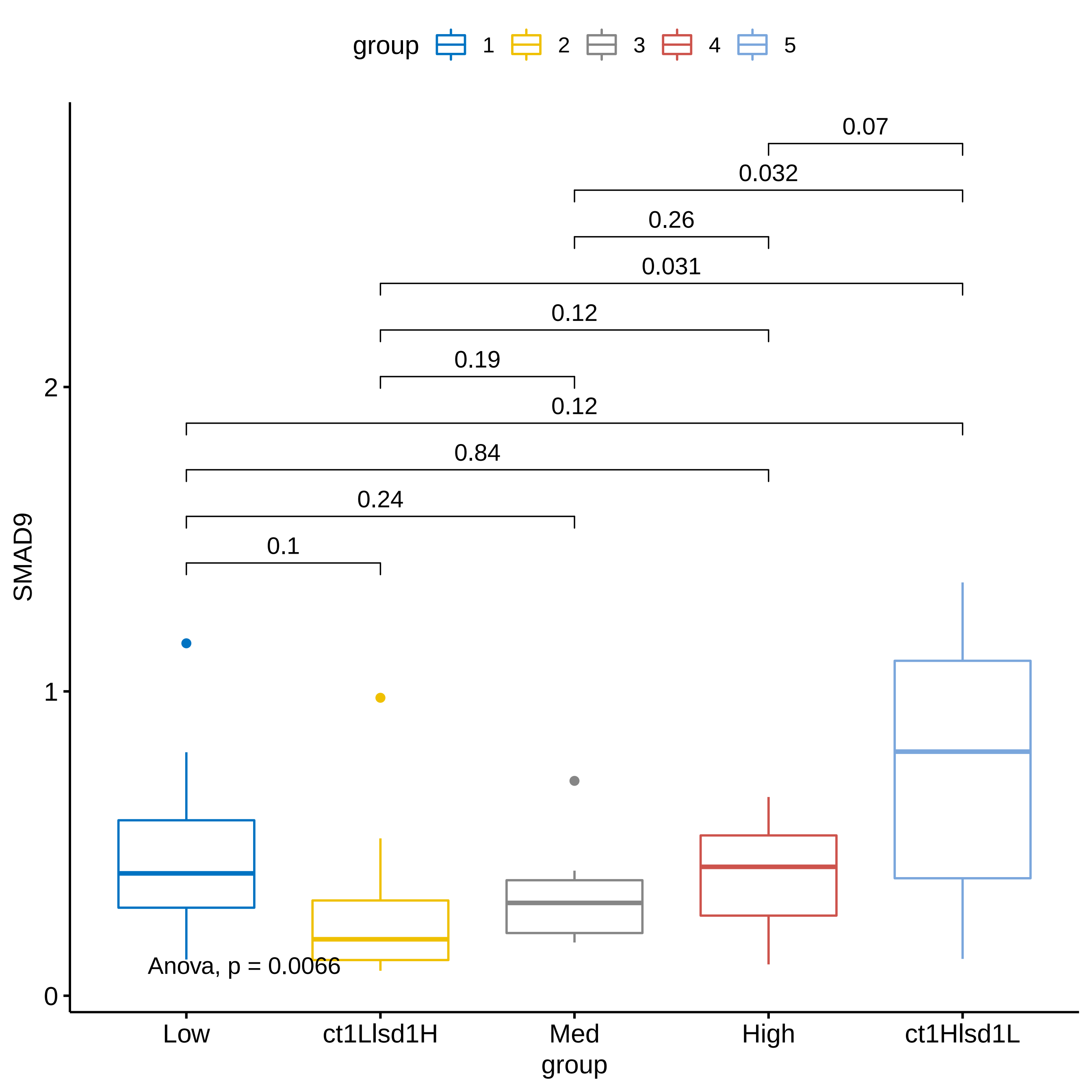

group
1
2
3
4
5
0.07
0.032
0.26
0.031
0.12
0.19
0.12
0.84
0.24
0.1
SMAD9
2
1
0
Anova, p = 0.0066
Low
ct1Llsd1H
Med
High
ct1Hlsd1L
group

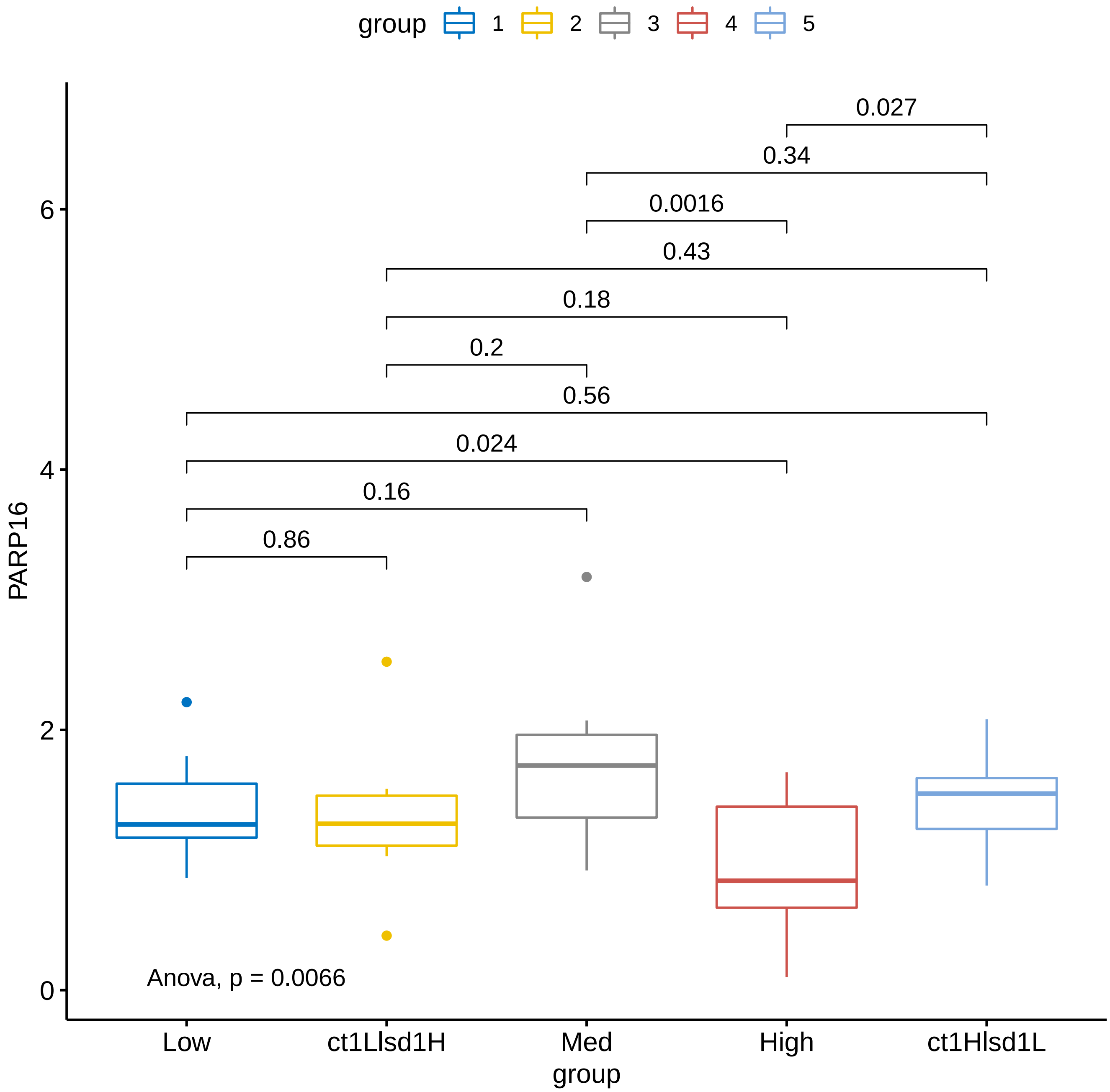

group 1 2 3 4 5
0.027
0.34
0.0016
0.43
0.18
0.2
0.56
0.024
0.16
0.86
PARP16
6
4
2
0
Anova, p = 0.0066
Low
ct1Llsd1H
Med
High
ct1Hlsd1L
group

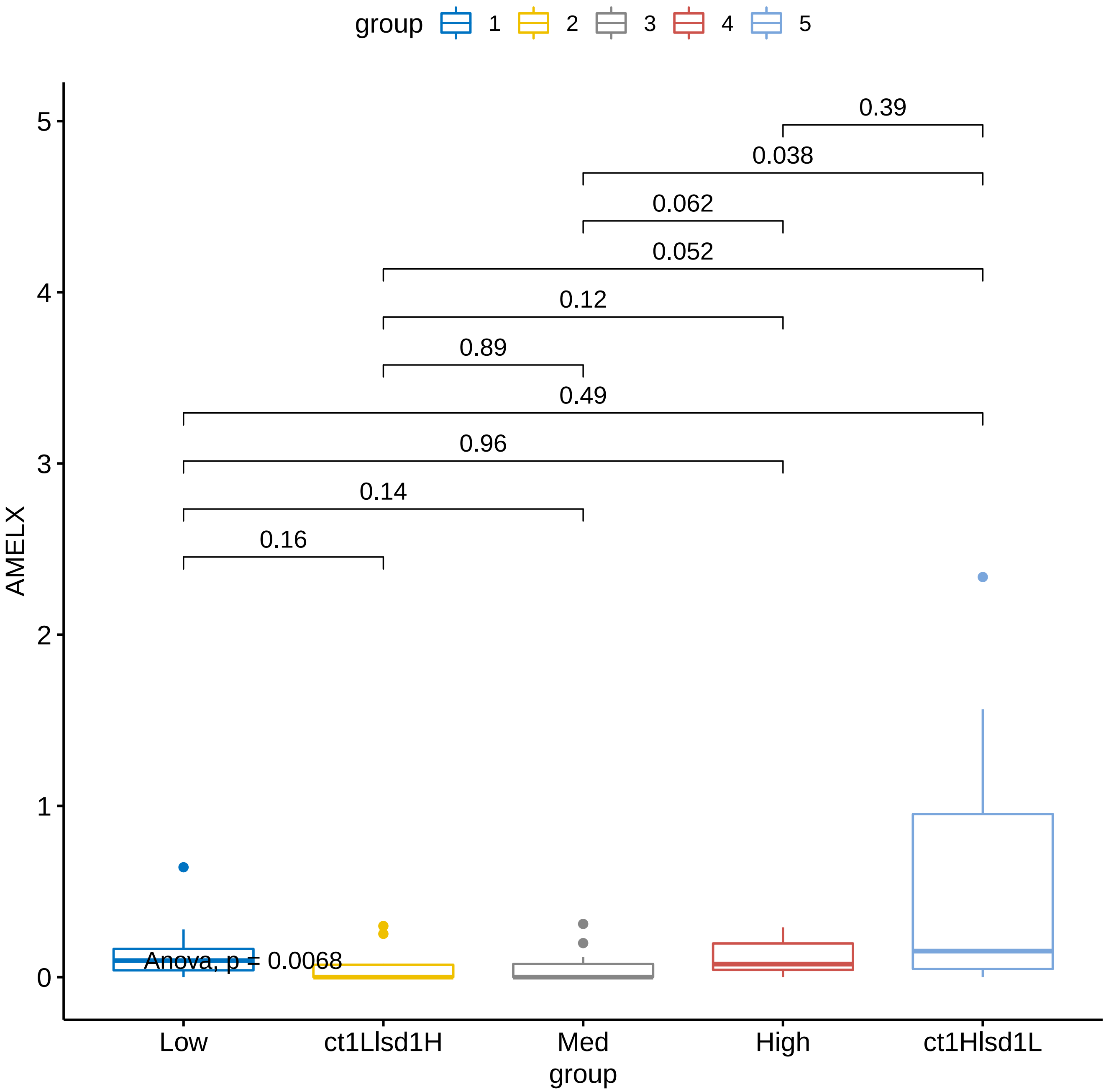

group
1
2
3
4
5
0.39
0.038
0.062
0.052
0.12
0.89
0.49
0.96
0.14
0.16
AMELX
Anova, p = 0.0068
5
4
3
2
1
0
Low
ct1Llsd1H
Med
High
ct1Hlsd1L
group

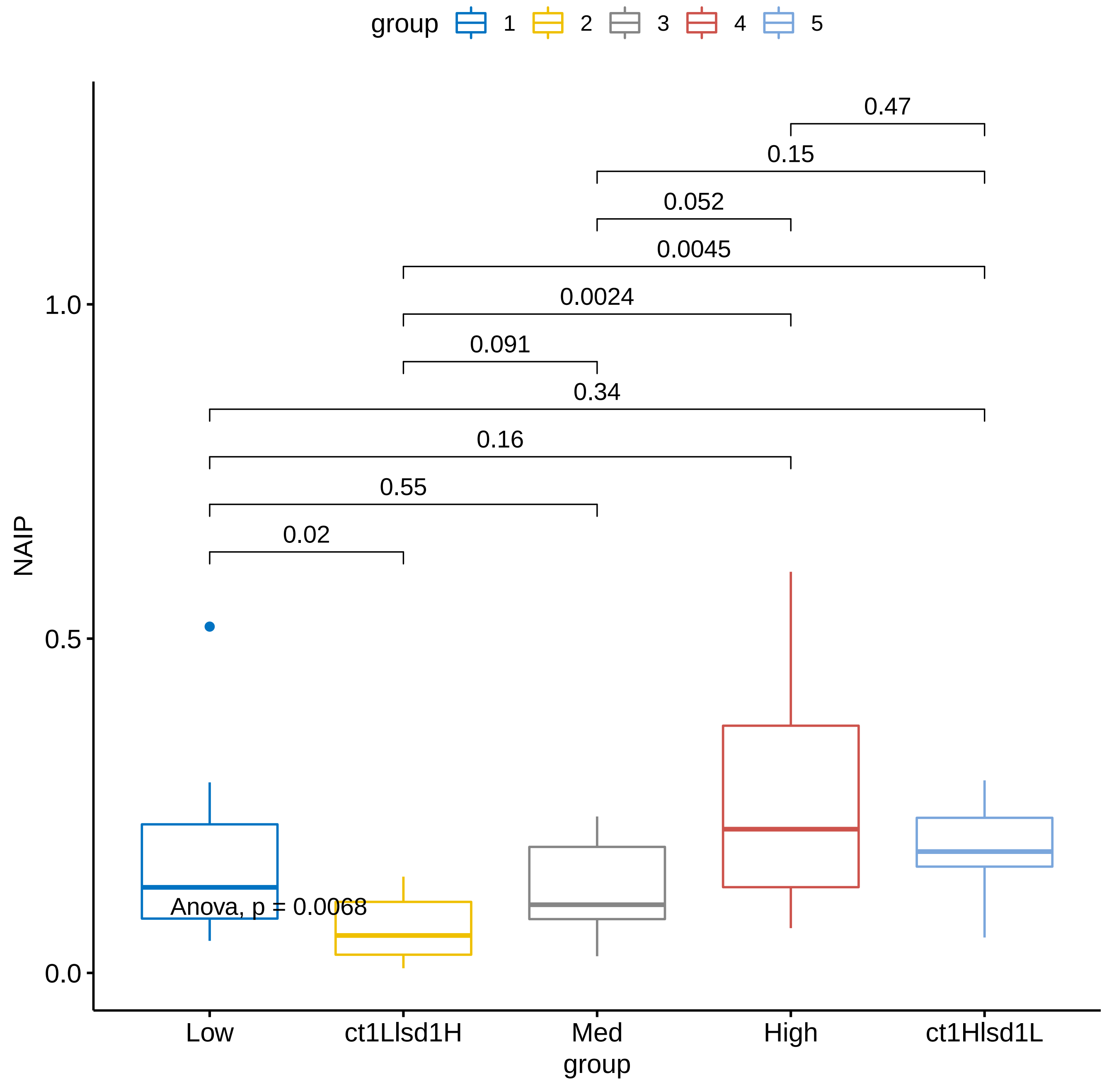

group 1 2 3 4 5
0.47
0.15
0.052
0.0045
0.0024
0.091
0.34
0.16
0.55
0.02
1.0
0.5
0.0
NAIP
Anova, p = 0.0068
Low
ct1Llsd1H
Med
High
ct1Hlsd1L
group

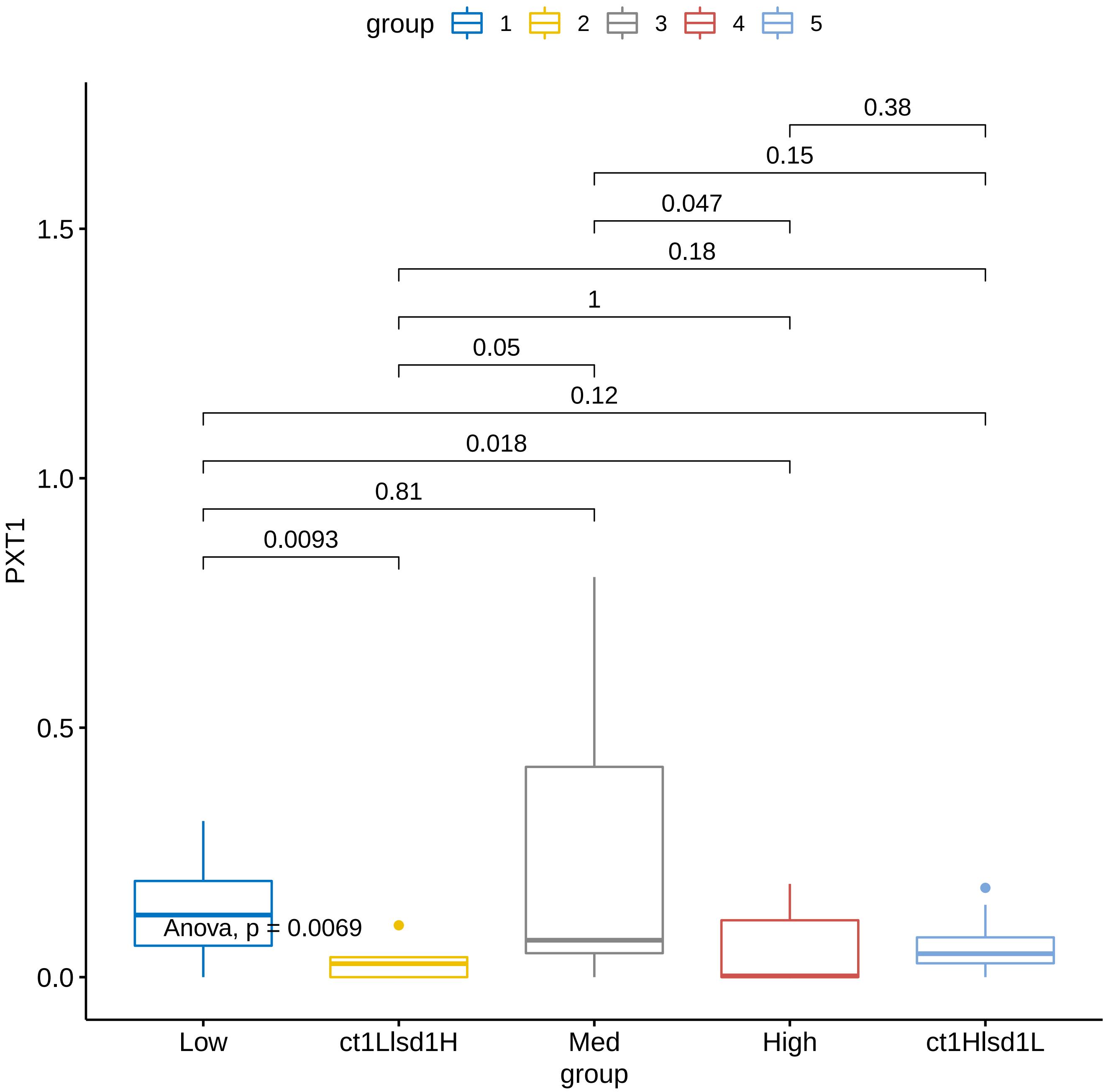

group 1 2 3 4 5
PXT1
1.5
1.0
0.5
0.0
0.38
0.15
0.047
0.18
1
0.05
0.12
0.018
0.81
0.0093
Anova, p = 0.0069
Low
ct1Llsd1H
Med
High
ct1Hlsd1L
group

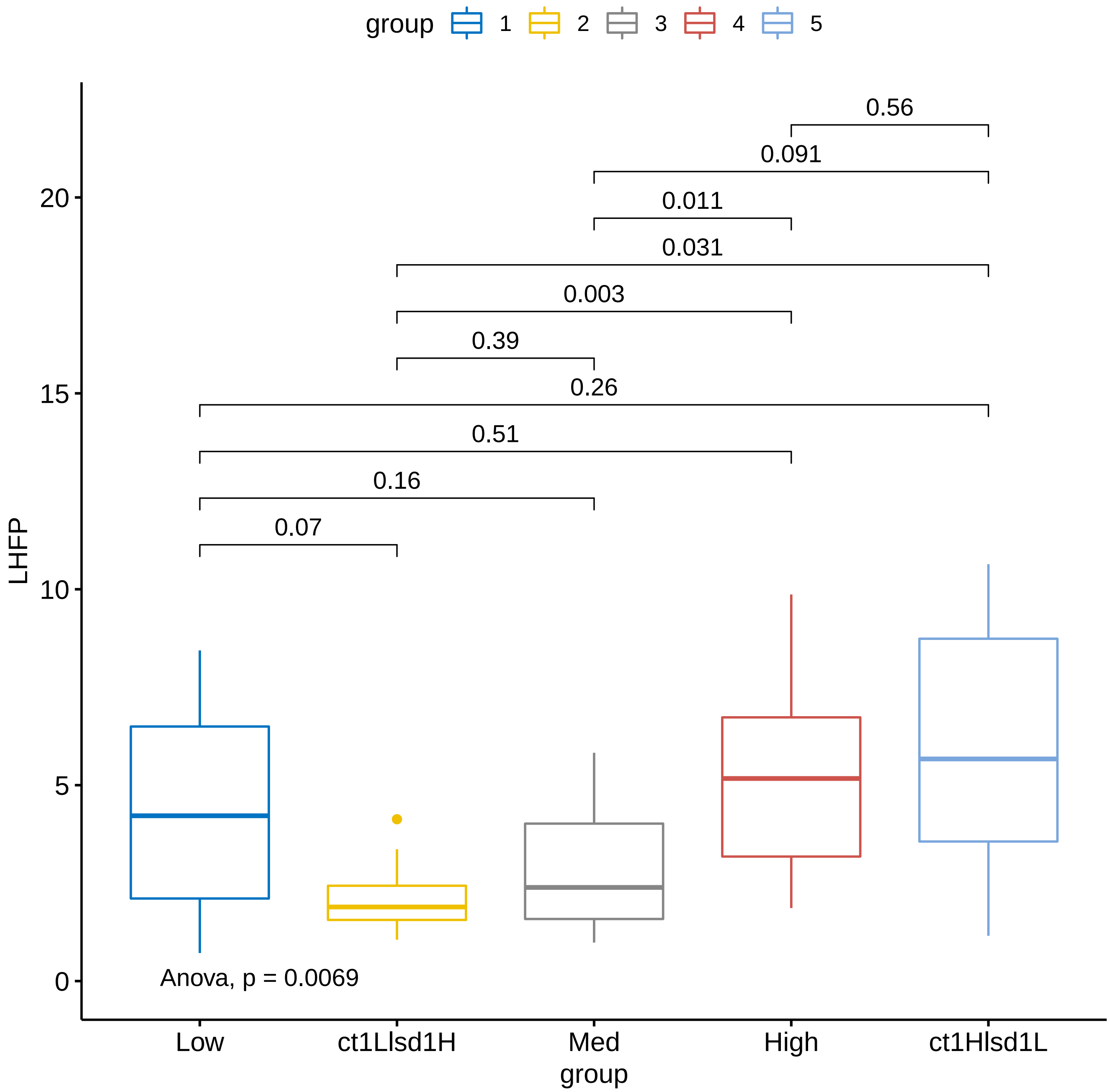
group
1
2
3
4
5
0.56
0.091
0.011
0.031
0.003
0.39
0.26
0.51
0.16
0.07
20
15
10
5
0
LHFP
Anova, p = 0.0069
Low
ct1Llsd1H
Med
High
ct1Hlsd1L
group

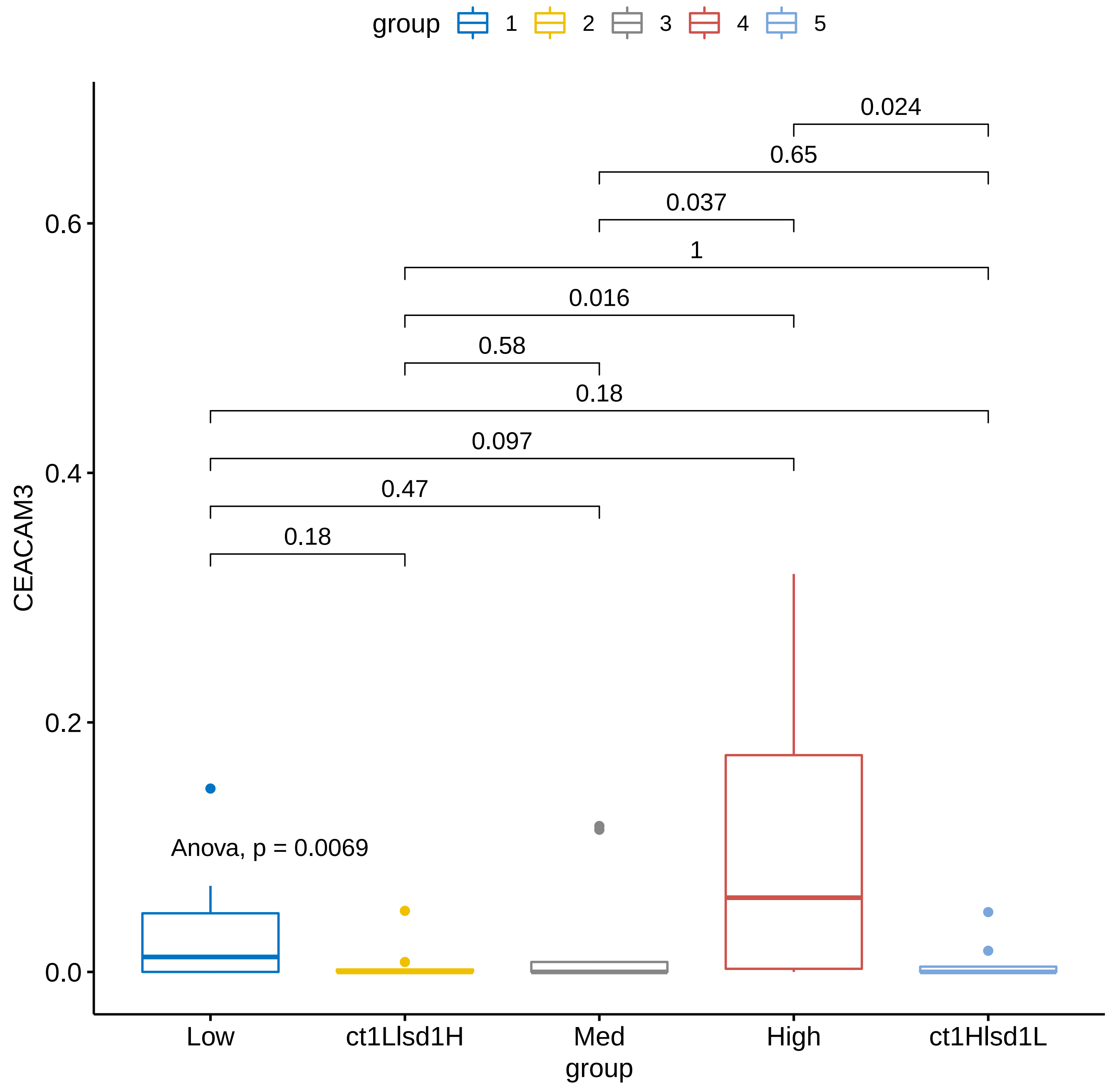
group
1
2
3
4
5
0.024
0.65
0.037
1
0.016
0.58
0.18
0.097
0.47
0.18
Anova, p = 0.0069
CEACAM3
0.6
0.4
0.2
0.0
Low
ct1Llsd1H
Med
High
ct1Hlsd1L
group

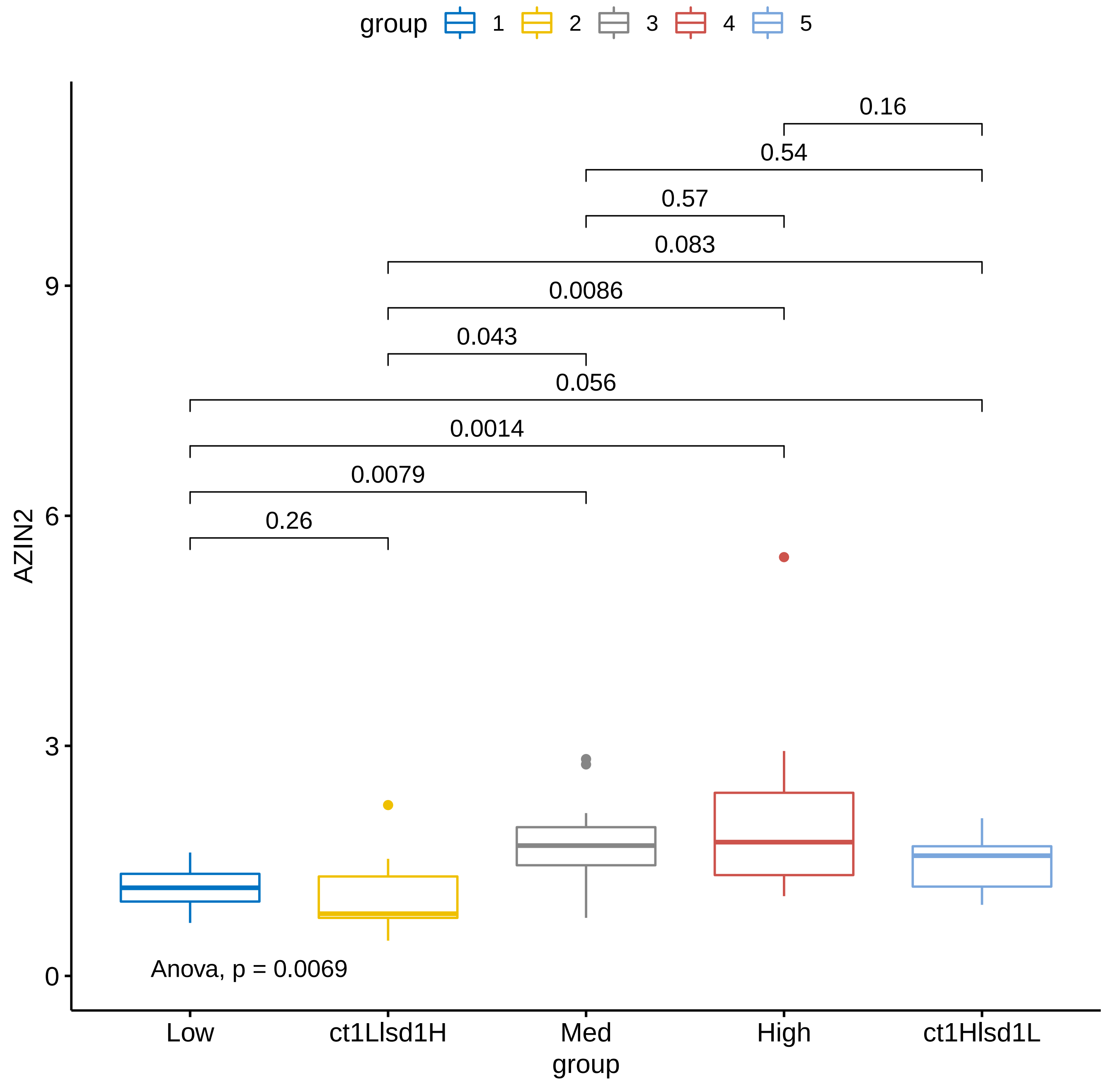

group
1
2
3
4
5
0.16
0.54
0.57
0.083
0.0086
0.043
0.056
0.0014
0.0079
0.26
AZIN2
9
6
3
0
Anova, p = 0.0069
Low
ct1Llsd1H
Med
High
ct1Hlsd1L
group

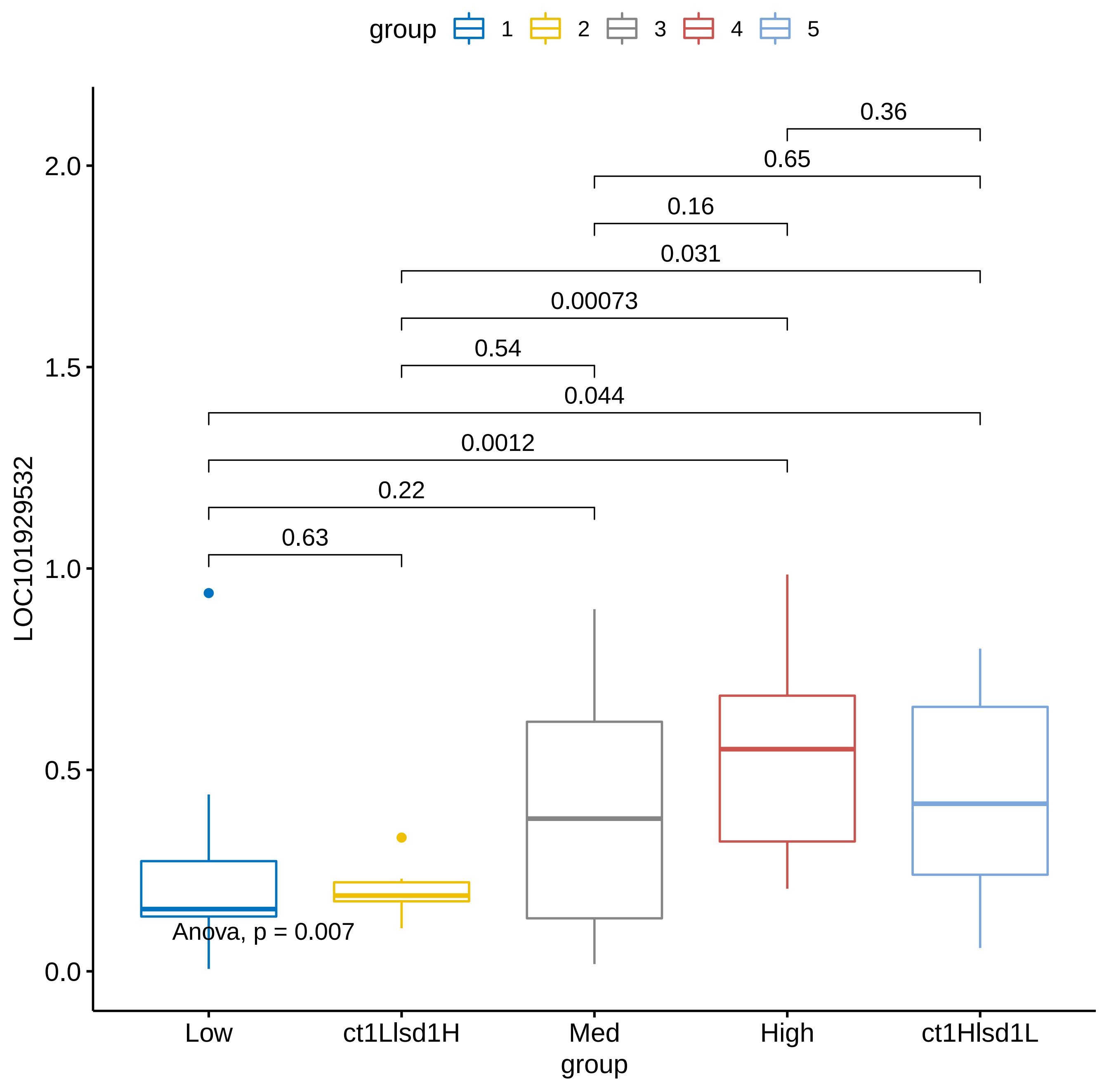
group
1
2
3
4
5
0.36
0.65
0.16
0.031
0.00073
0.54
0.044
0.0012
0.22
0.63
Anova, p = 0.007
2.0
1.5
1.0
0.5
0.0
LOC101929532
Low
ct1Llsd1H
Med
High
ct1Hlsd1L
group

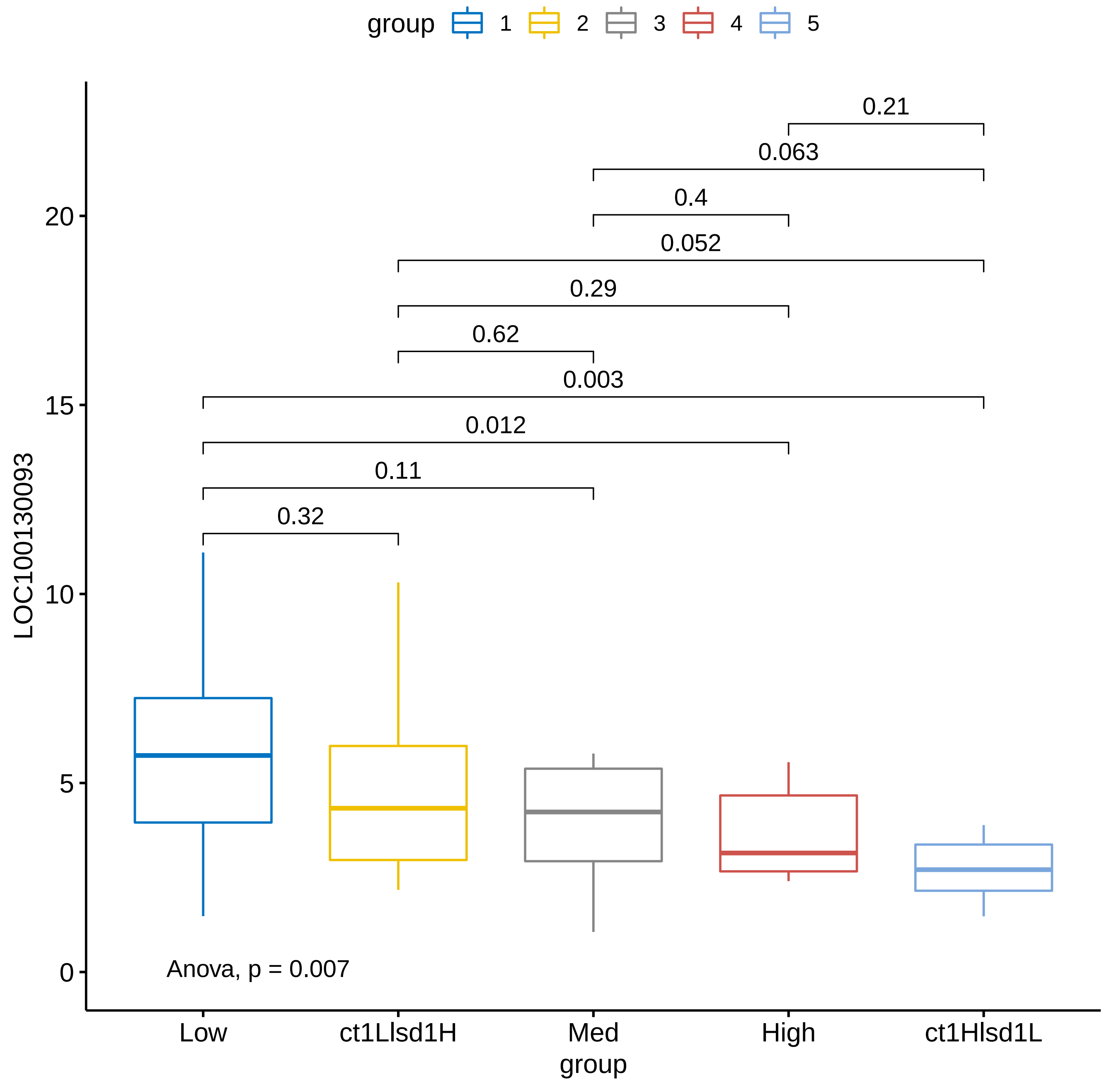

group 1 2 3 4 5
0.21
0.063
0.4
0.052
0.29
0.62
0.003
0.012
0.11
0.32
LOC100130093
20
15
10
5
0
Anova, p = 0.007
Low
ct1Llsd1H
Med
High
ct1Hlsd1L
group

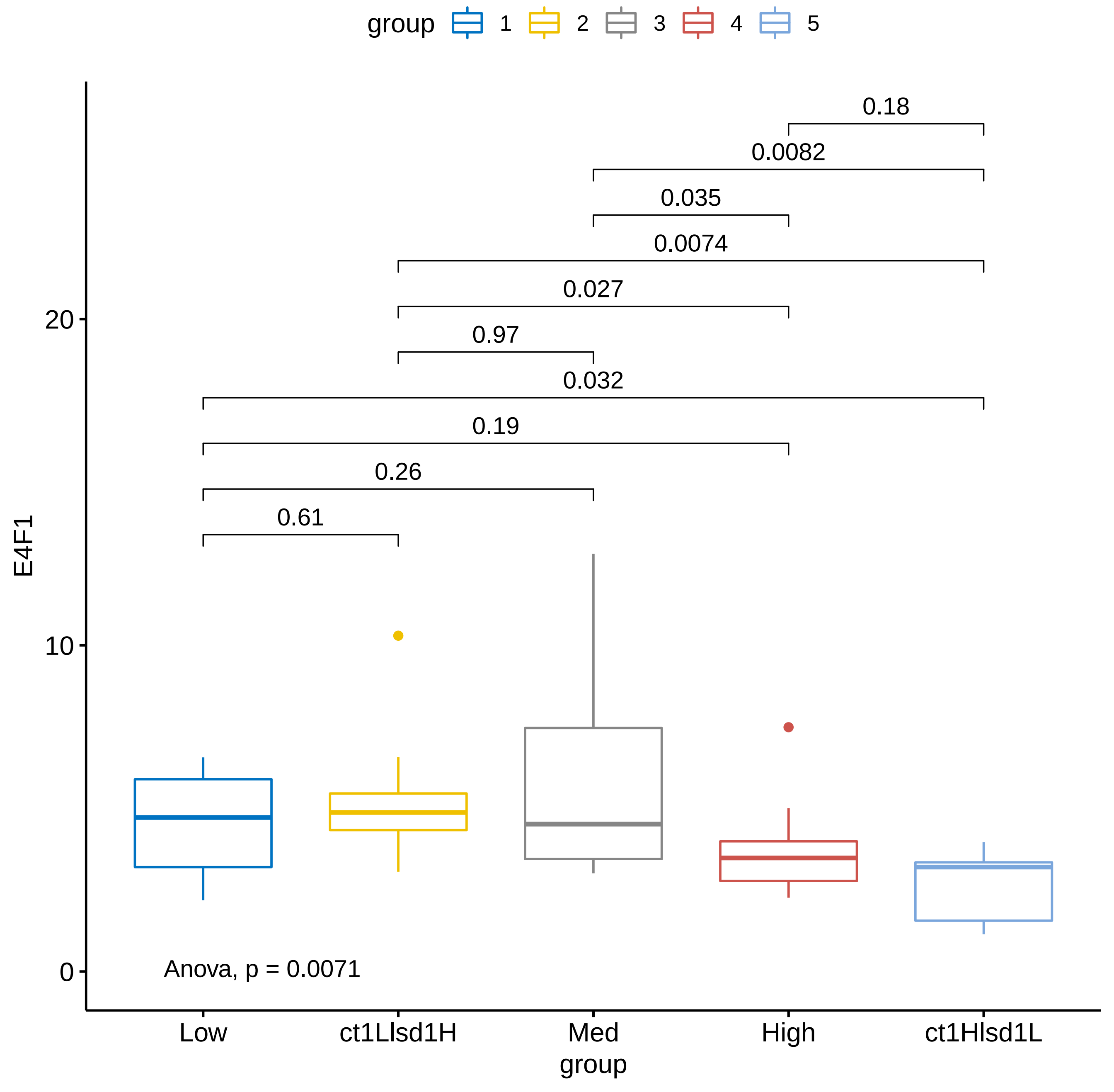
group
1
2
3
4
5
0.18
0.0082
0.035
0.0074
0.027
0.97
0.032
0.19
0.26
0.61
E4F1
20
10
0
Anova, p = 0.0071
Low
ct1Llsd1H
Med
High
ct1Hlsd1L
group

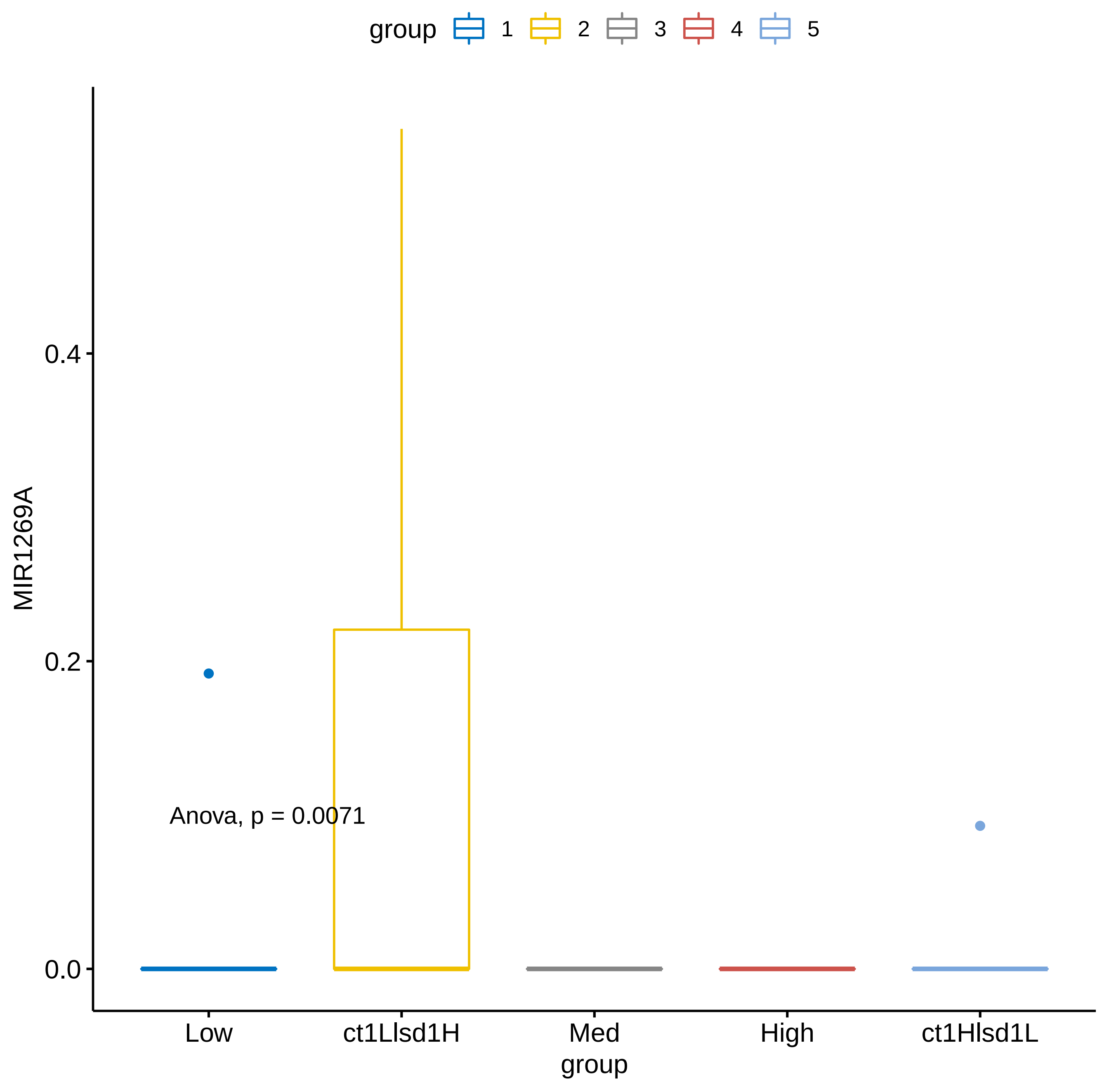
group
1
2
3
4
5
0.4
0.2
0.0
MIR1269A
Anova, p = 0.0071
Low
ct1Llsd1H
Med
High
ct1Hlsd1L
group

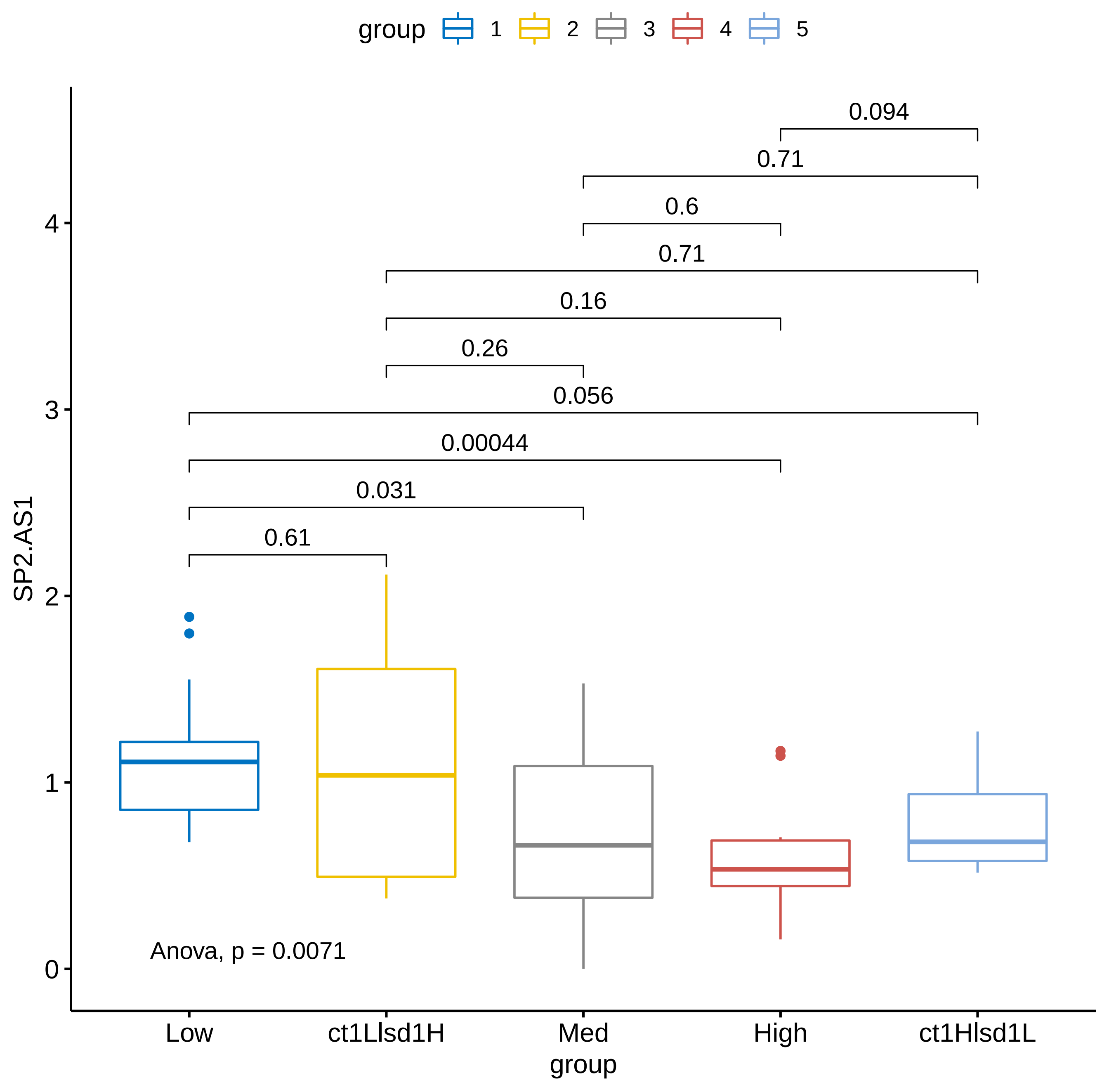
group 1 2 3 4 5
0.094
0.71
0.6
0.71
0.16
0.26
0.056
0.00044
0.031
0.61
SP2.AS1
4
3
2
1
0
Anova, p = 0.0071
Low
ct1Llsd1H
Med
High
ct1Hlsd1L
group

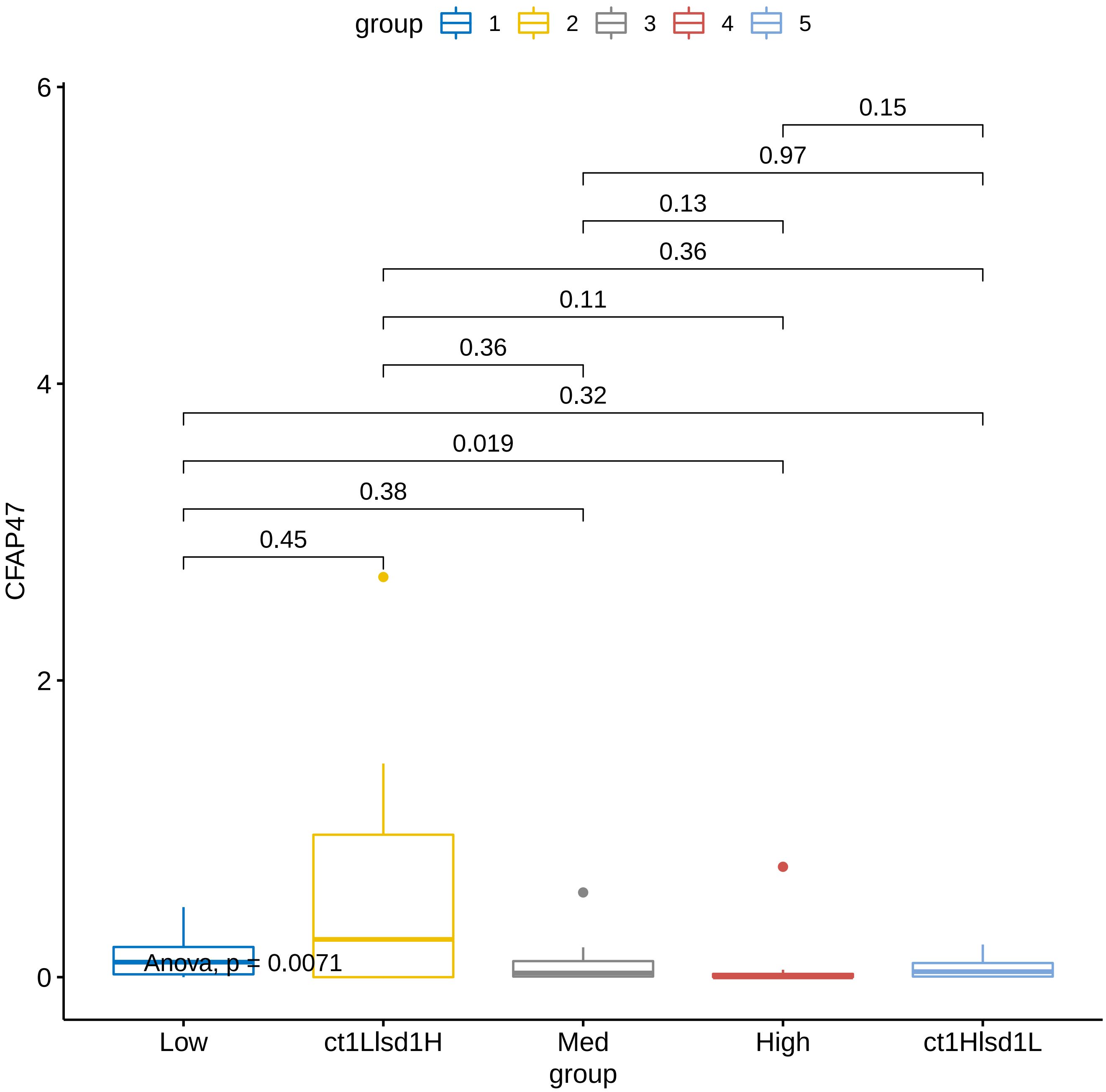
group
1
2
3
4
5
CFAP47
6
4
2
0
0.15
0.97
0.13
0.36
0.11
0.36
0.32
0.019
0.38
0.45
Anova, p = 0.0071
Low
ct1Llsd1H
Med
High
ct1Hlsd1L
group

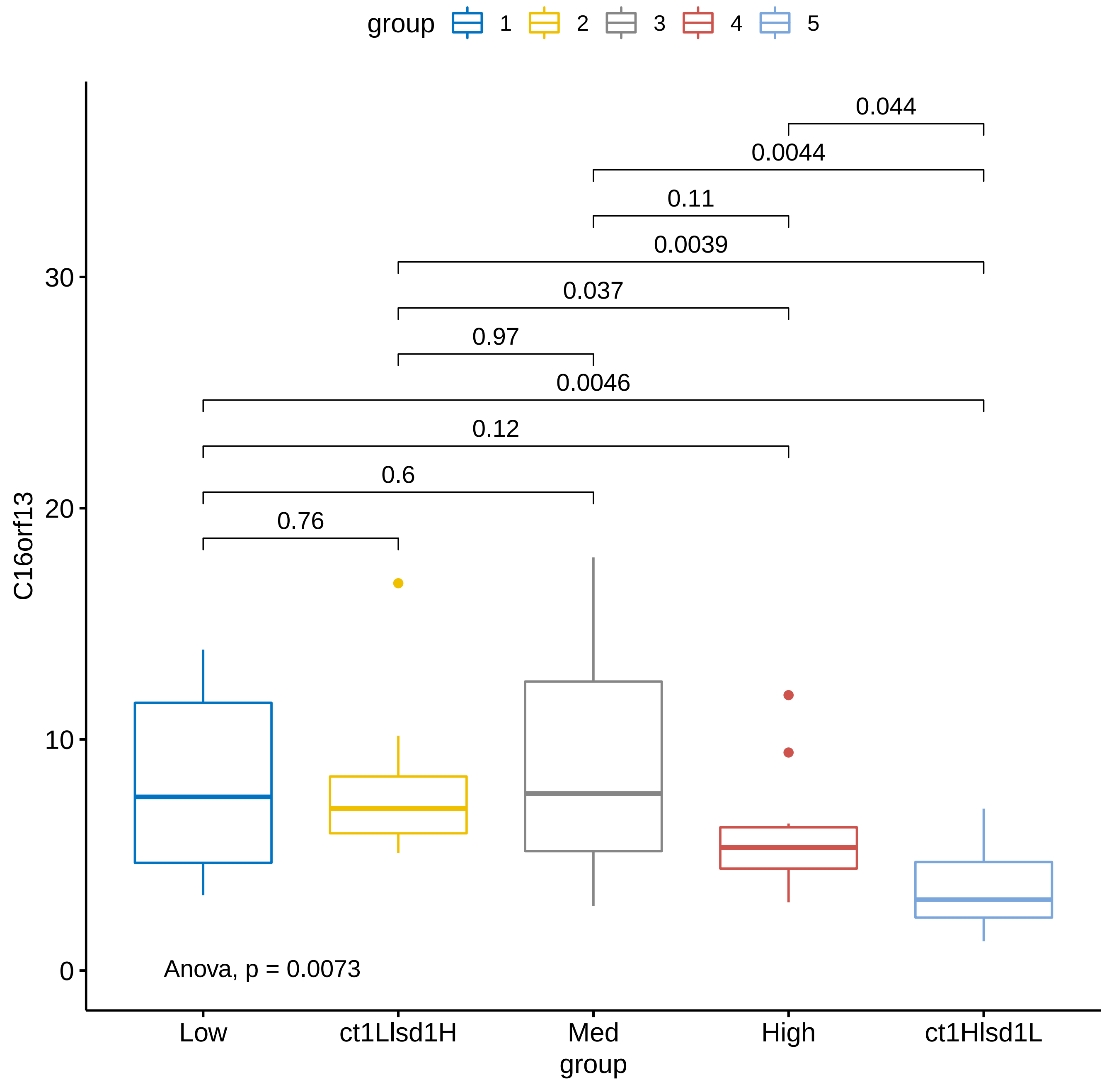

group 1 2 3 4 5
0.044
0.0044
0.11
0.0039
0.037
0.97
0.0046
0.12
0.6
0.76
C16orf13
30
20
10
0
Anova, p = 0.0073
Low
ct1Llsd1H
Med
High
ct1Hlsd1L
group

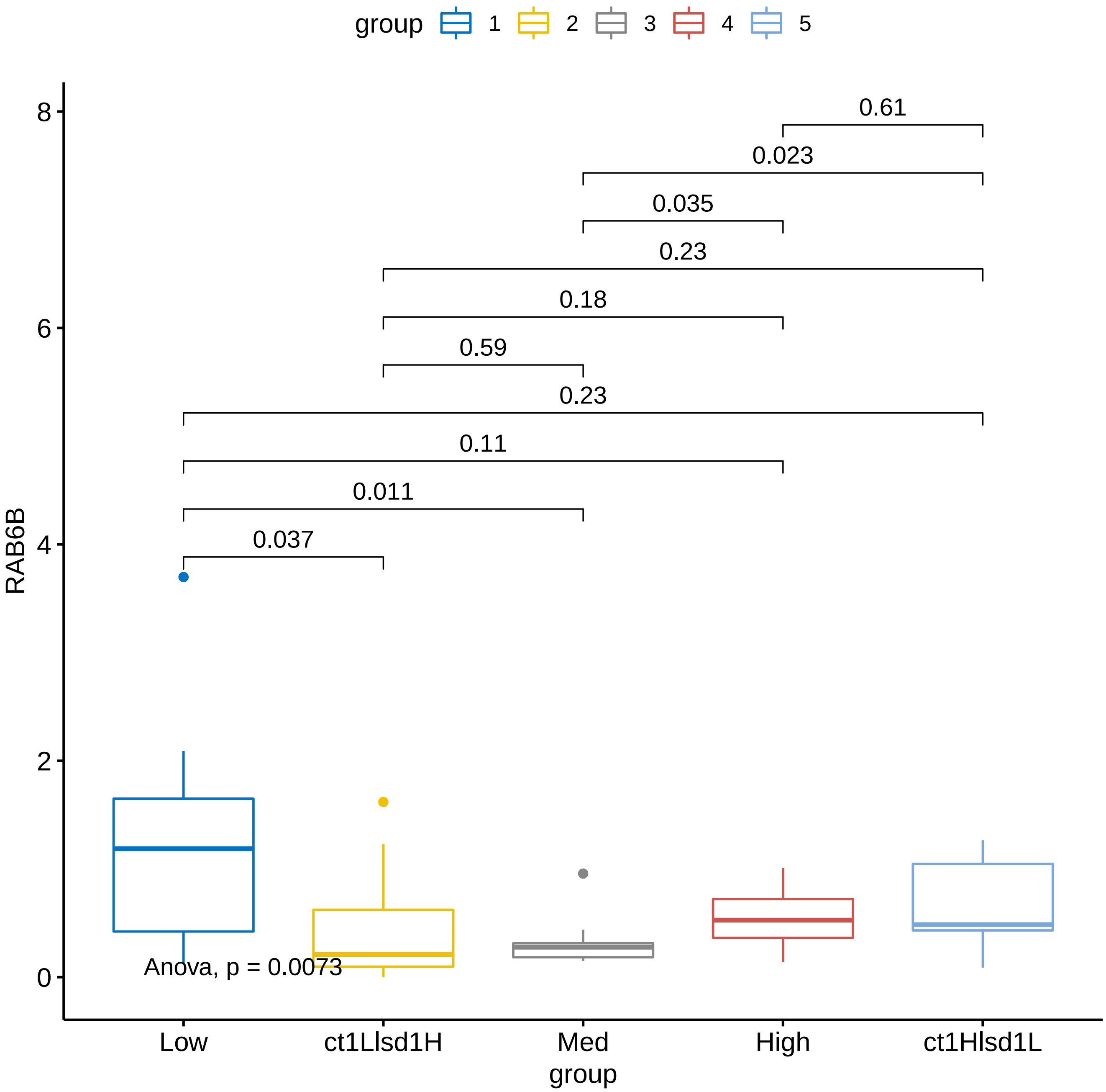

group
1
2
3
4
5
RAB6B
8
6
4
2
0
0.61
0.023
0.035
0.23
0.18
0.59
0.23
0.11
0.011
0.037
Anova, p = 0.0073
Low
ct1Llsd1H
Med
High
ct1Hlsd1L
group

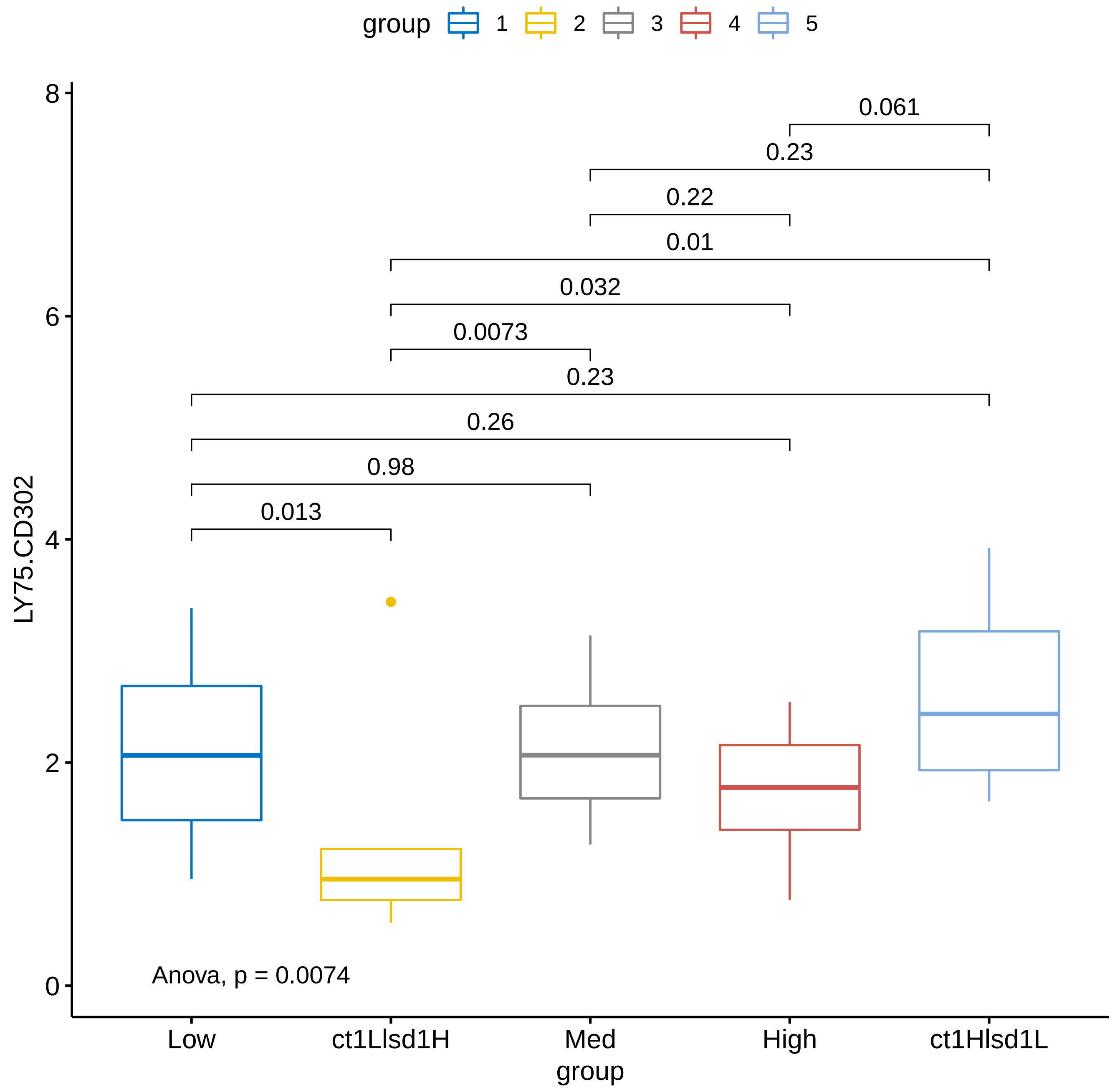

group
1
2
3
4
5
0.061
0.23
0.22
0.01
0.032
0.0073
0.23
0.26
0.98
0.013
LY75.CD302
8
6
4
2
0
Anova, p = 0.0074
Low
ct1Llsd1H
Med
High
ct1Hlsd1L
group

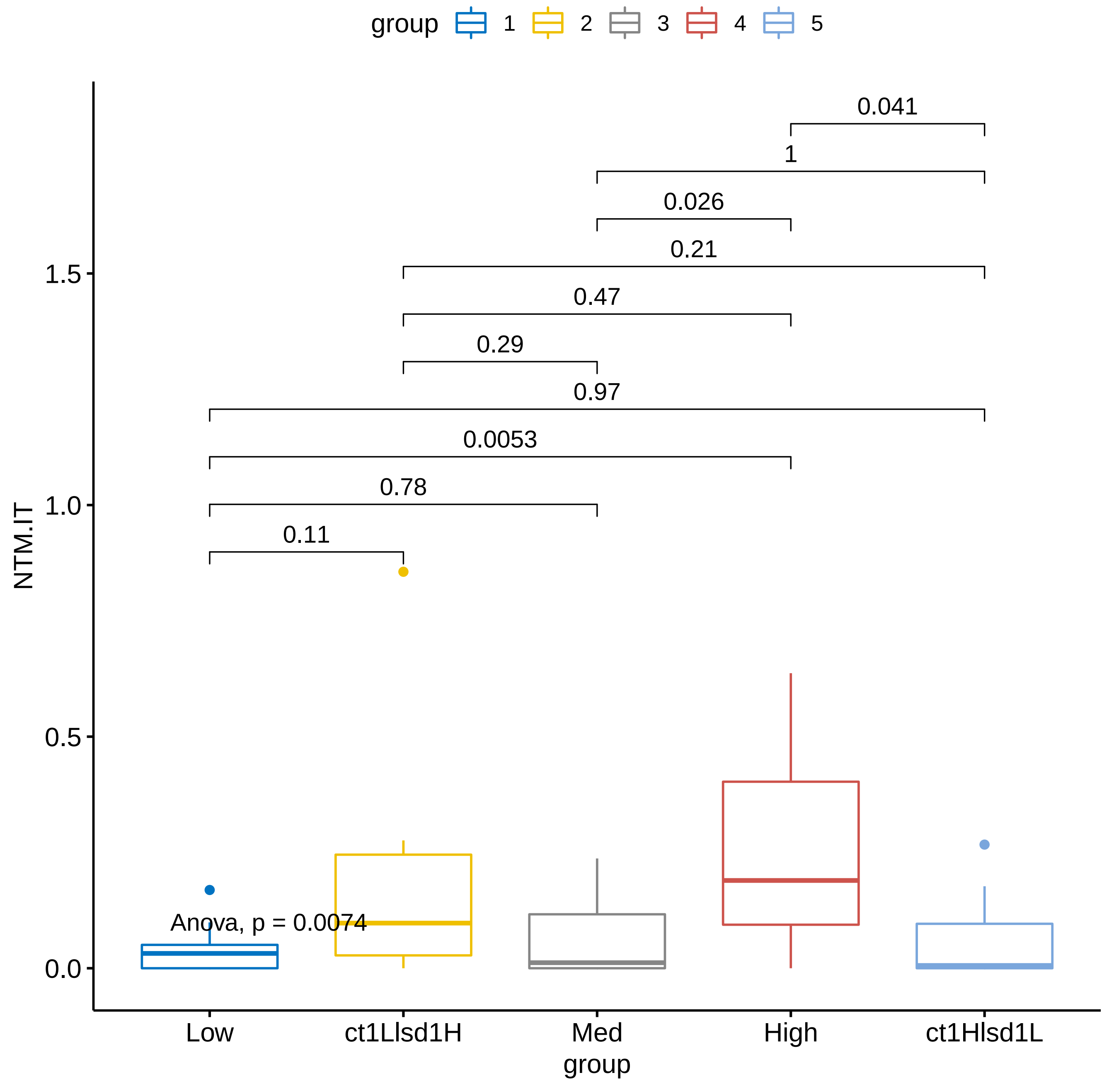

group 1 2 3 4 5
0.041
1
0.026
0.21
0.47
0.29
0.97
0.0053
0.78
0.11
1.5
1.0
0.5
0.0
NTM.IT
Anova, p = 0.0074
Low
ct1Llsd1H
Med
High
ct1Hlsd1L
group

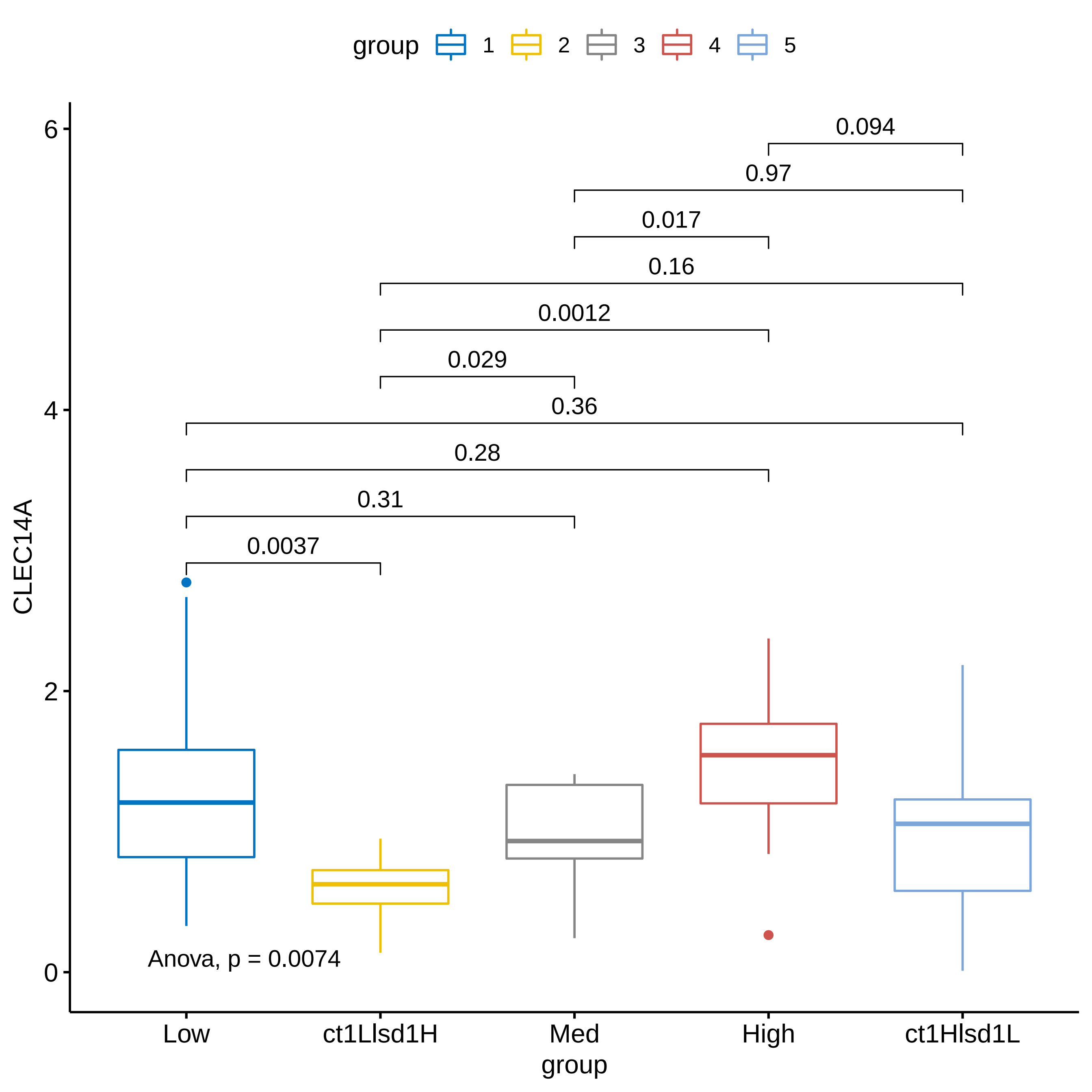
group
1
2
3
4
5
0.094
0.97
0.017
0.16
0.0012
0.029
0.36
0.28
0.31
0.0037
6
4
2
0
CLEC14A
Anova, p = 0.0074
Low
ct1Llsd1H
Med
High
ct1Hlsd1L
group

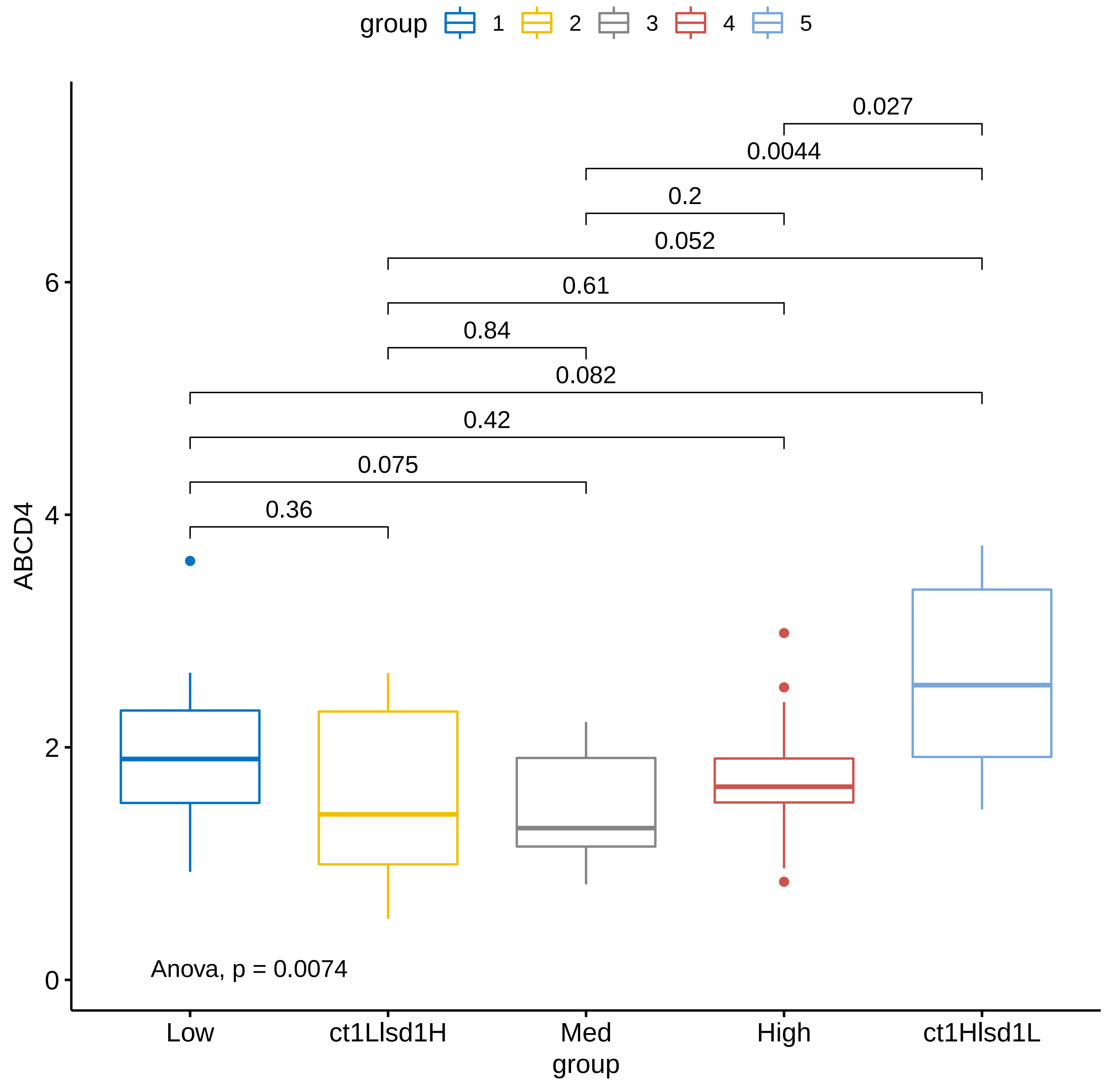

group
1
2
3
4
5
0.027
0.0044
0.2
0.052
0.61
0.84
0.082
0.42
0.075
0.36
Anova, p = 0.0074
ABCD4
6
4
2
0
Low
ct1Llsd1H
Med
High
ct1Hlsd1L
group

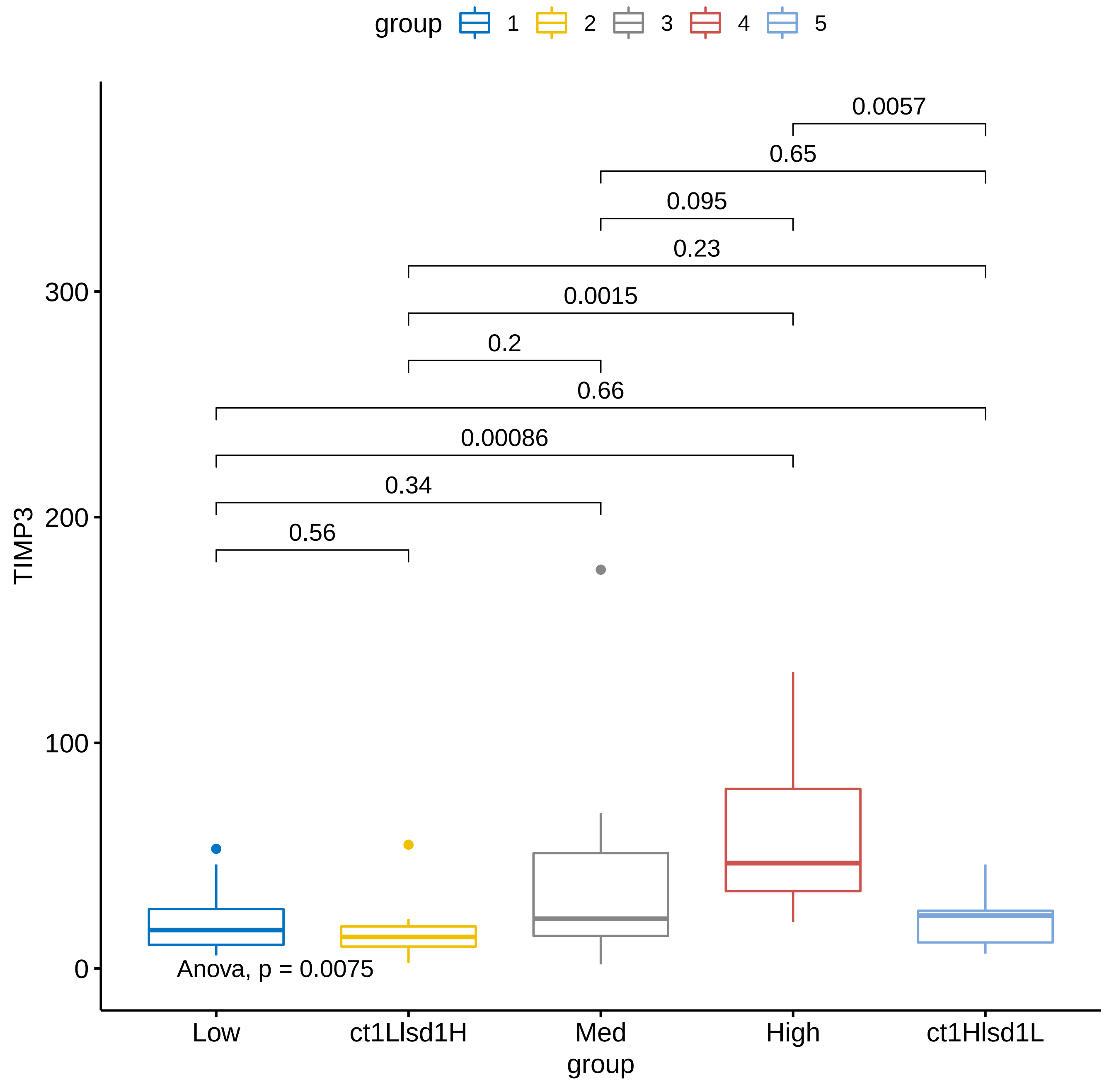

group
1
2
3
4
5
0.0057
0.65
0.095
0.23
0.0015
0.2
0.66
0.00086
0.34
0.56
TIMP3
300
200
100
0
Anova, p = 0.0075
Low
ct1Llsd1H
Med
High
ct1Hlsd1L
group

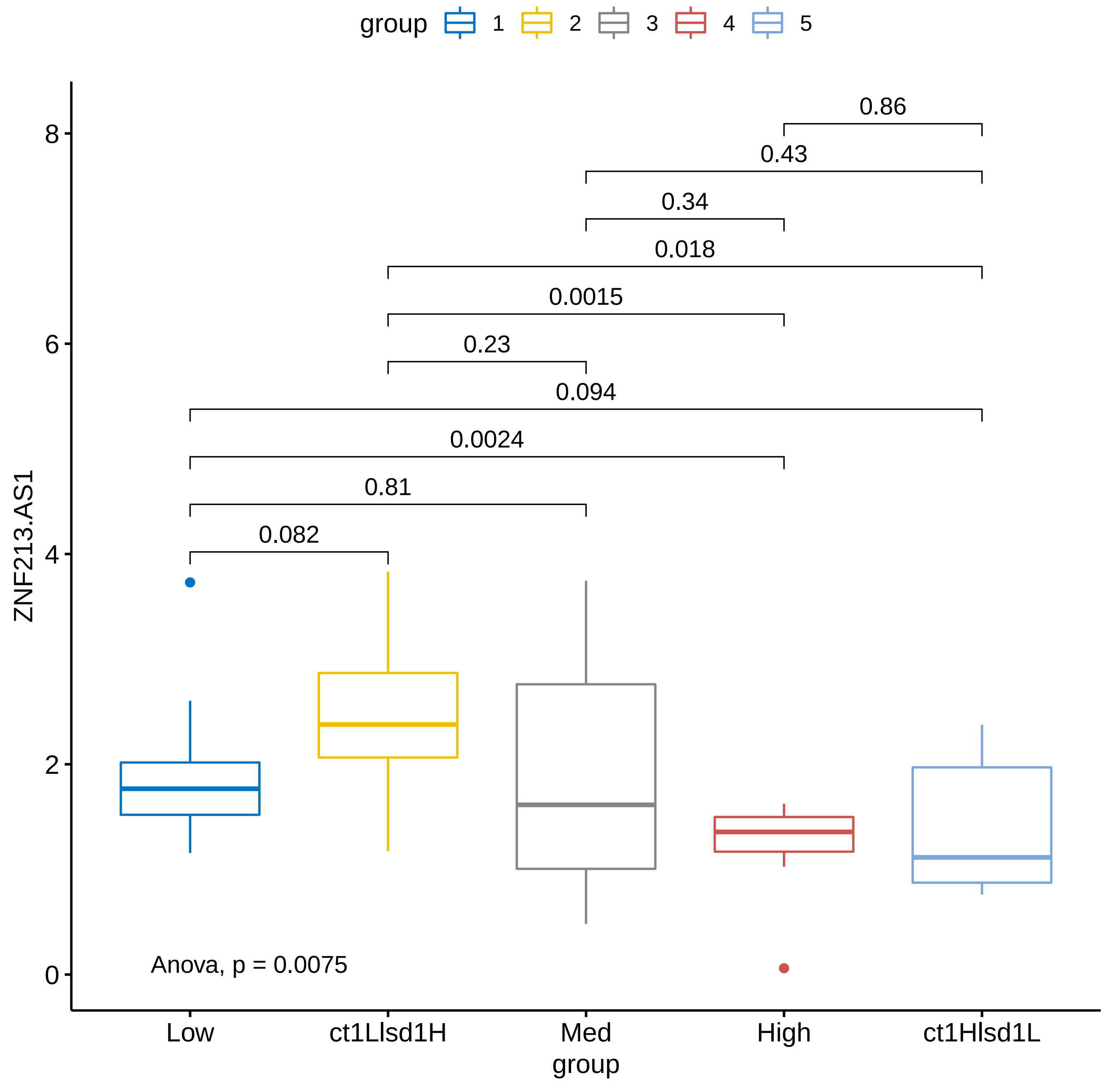

group
1
2
3
4
5
0.86
0.43
0.34
0.018
0.0015
0.23
0.094
0.0024
0.81
0.082
Anova, p = 0.0075
ZNF213.AS1
8
6
4
2
0
Low
ct1Llsd1H
Med
High
ct1Hlsd1L
group

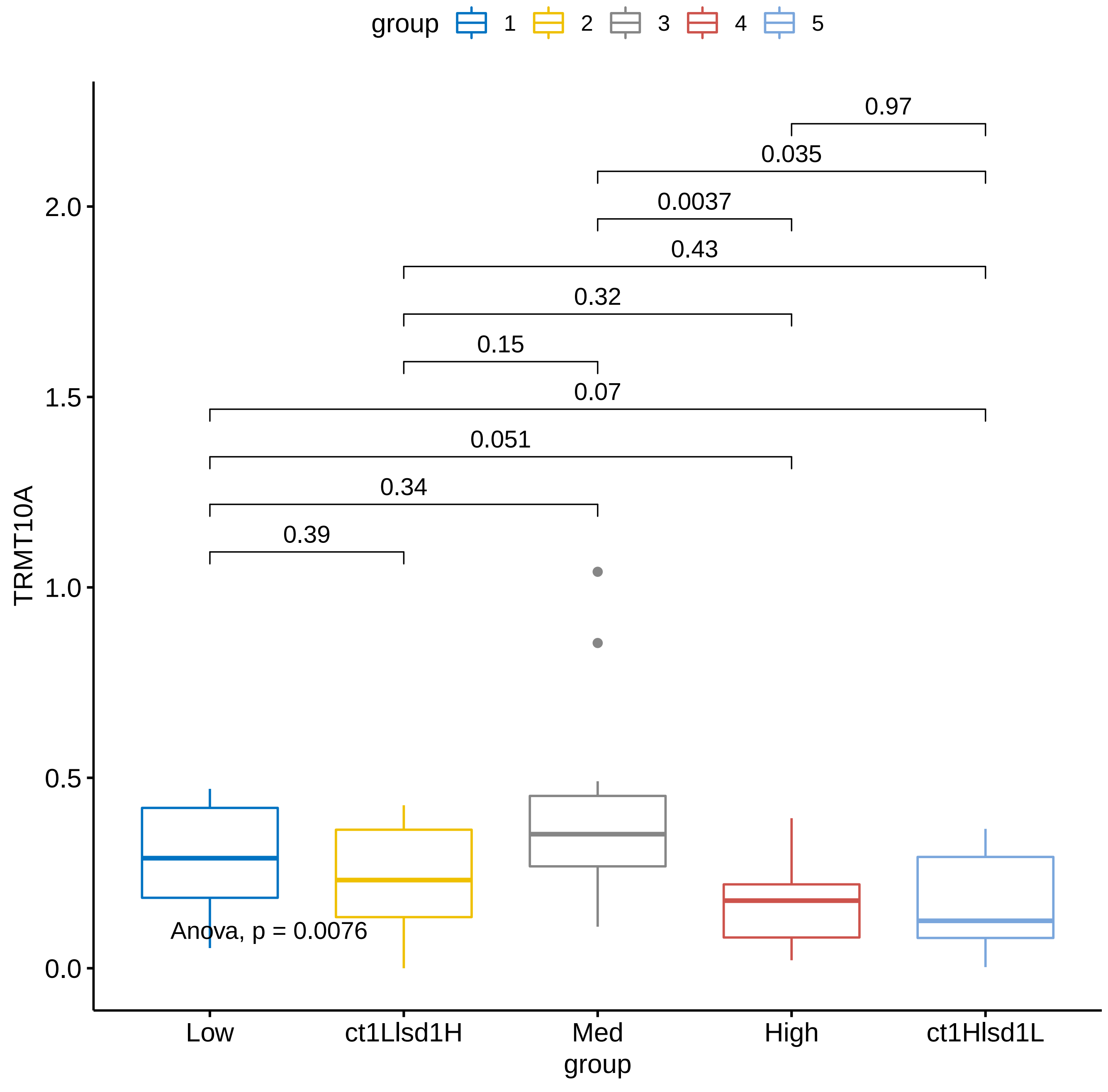

group 1 2 3 4 5
0.97
0.035
0.0037
0.43
0.32
0.15
0.07
0.051
0.34
0.39
Anova, p = 0.0076
TRMT10A
2.0
1.5
1.0
0.5
0.0
Low
ct1Llsd1H
Med
High
ct1Hlsd1L
group

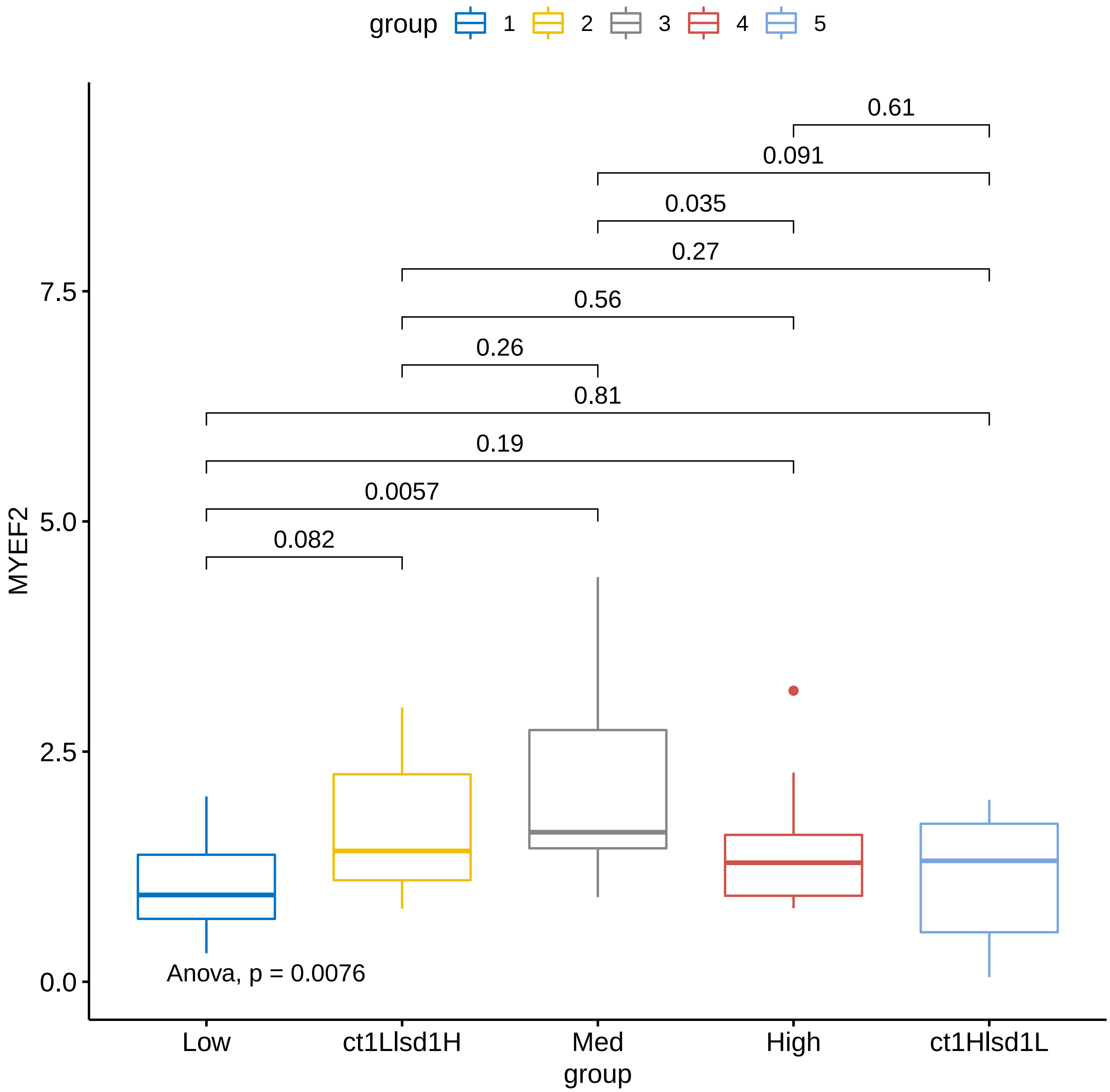

group
1
2
3
4
5
0.61
0.091
0.035
0.27
0.56
0.26
0.81
0.19
0.0057
0.082
7.5
5.0
2.5
0.0
MYEF2
Anova, p = 0.0076
Low
ct1Llsd1H
Med
High
ct1Hlsd1L
group

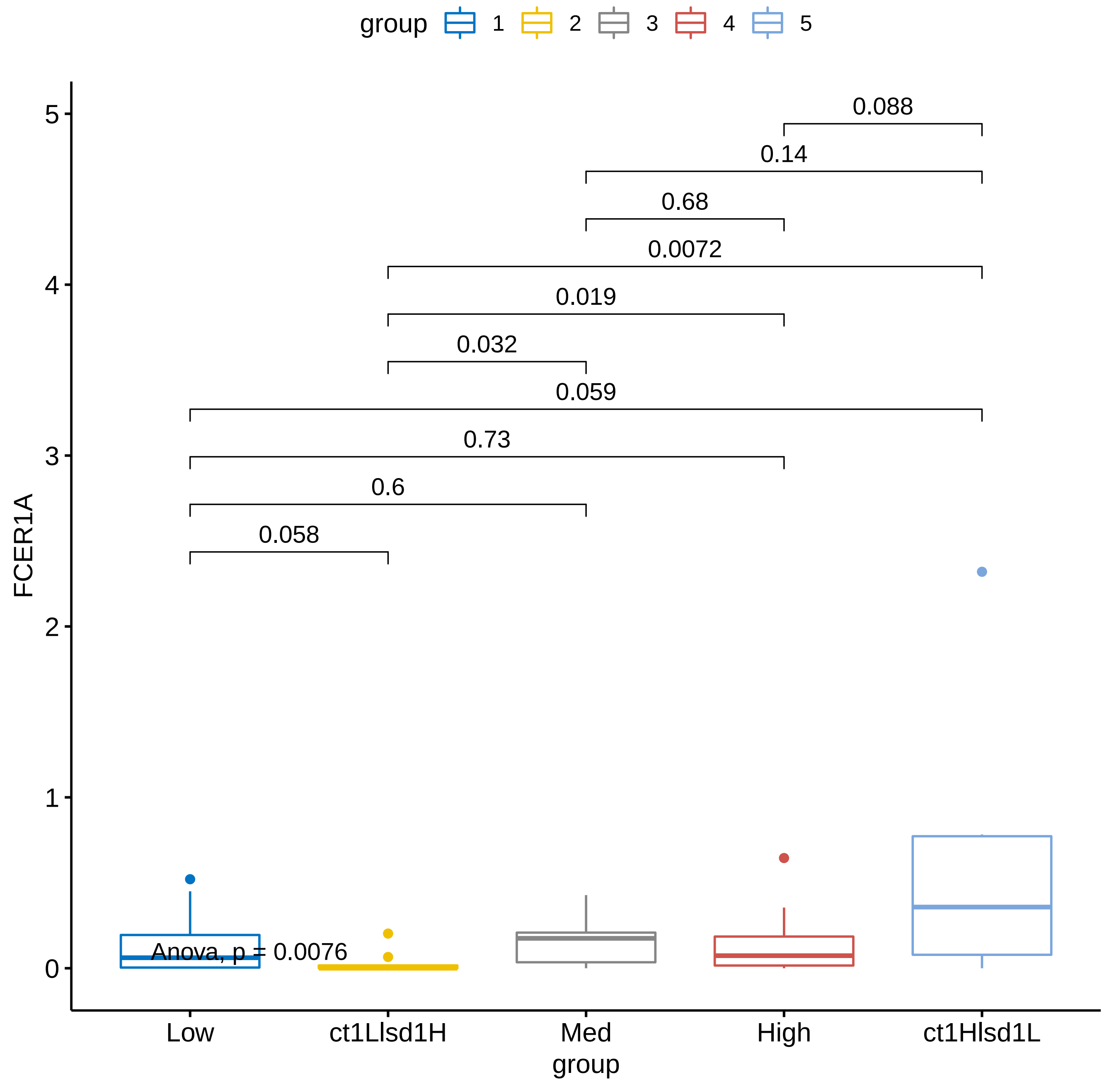
group
1
2
3
4
5
0.088
0.14
0.68
0.0072
0.019
0.032
0.059
0.73
0.6
0.058
Anova, p = 0.0076
FCER1A
5
4
3
2
1
0
Low
ct1Llsd1H
Med
High
ct1Hlsd1L
group

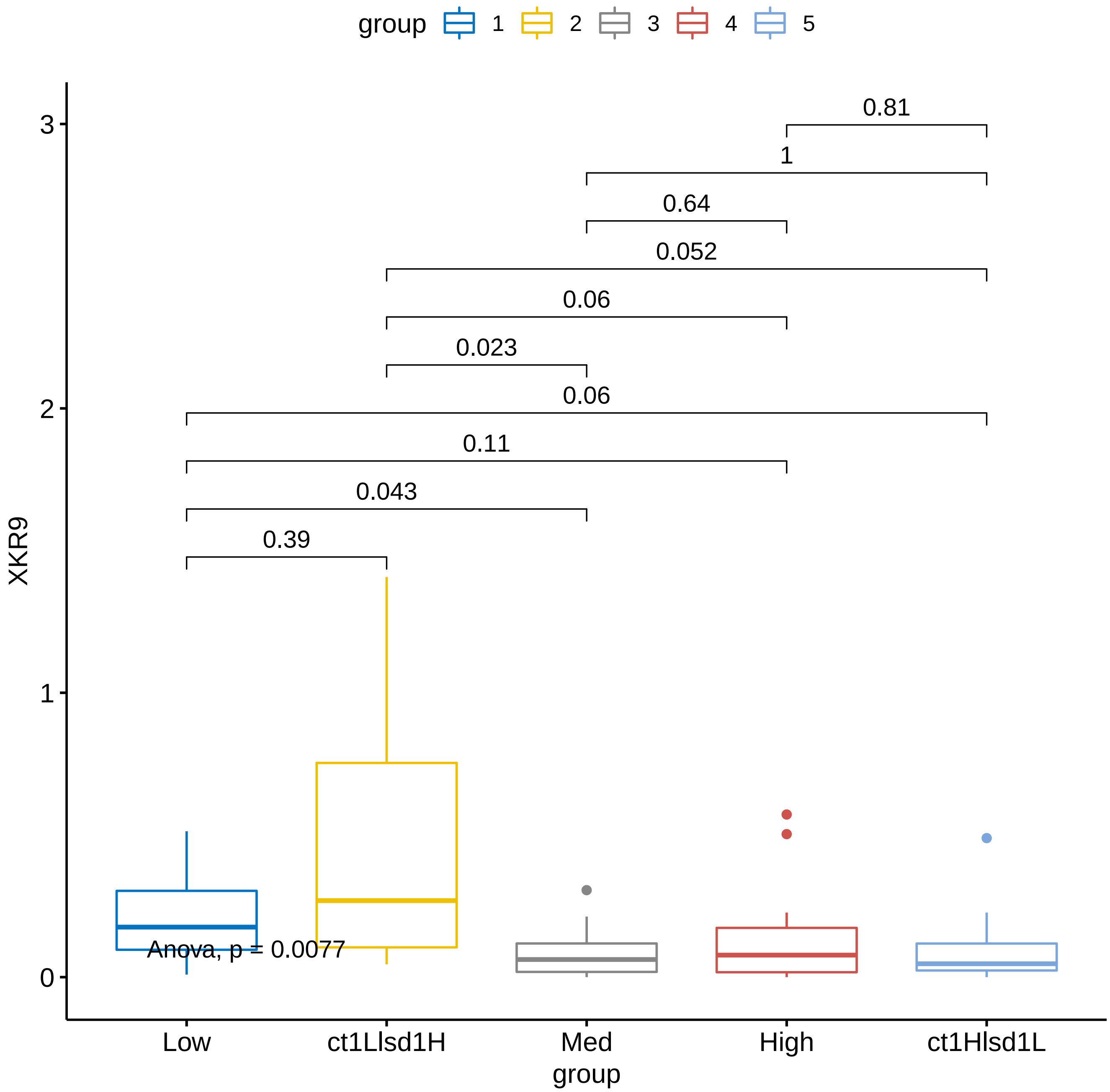
group
1
2
3
4
5
XKR9
0.81
1
0.64
0.052
0.06
0.023
0.06
0.11
0.043
0.39
Anova, p = 0.0077
3
2
1
0
Low
ct1Llsd1H
Med
High
ct1Hlsd1L
group

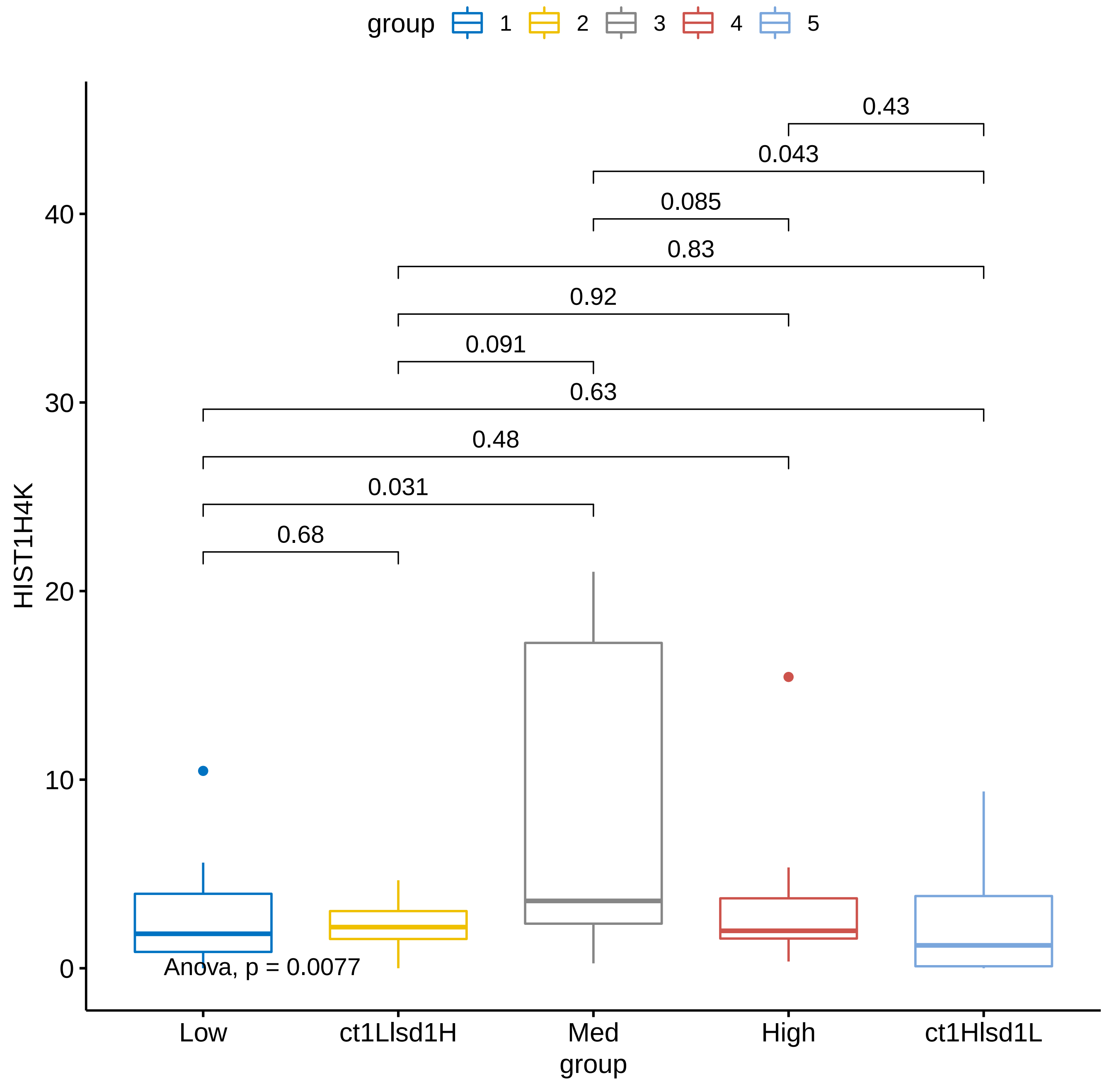

group
1
2
3
4
5
0.43
0.043
0.085
0.83
0.92
0.091
0.63
0.48
0.031
0.68
40
30
20
10
0
HIST1H4K
Anova, p = 0.0077
Low
ct1Llsd1H
Med
High
ct1Hlsd1L
group

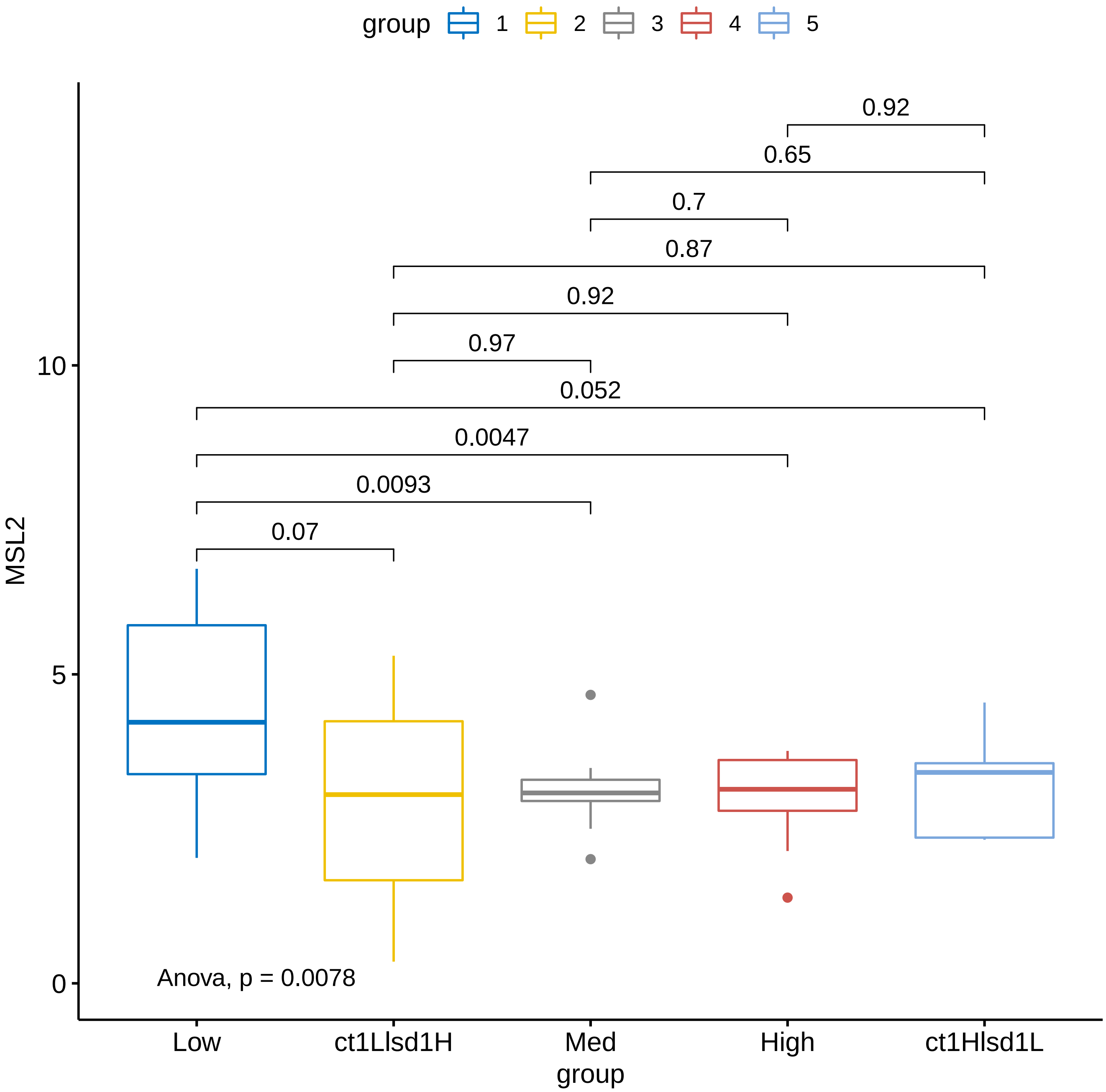
group 1 2 3 4 5
0.92
0.65
0.7
0.87
0.92
0.97
0.052
0.0047
0.0093
0.07
10
5
0
MSL2
Anova, p = 0.0078
Low
ct1Llsd1H
Med
High
ct1Hlsd1L
group

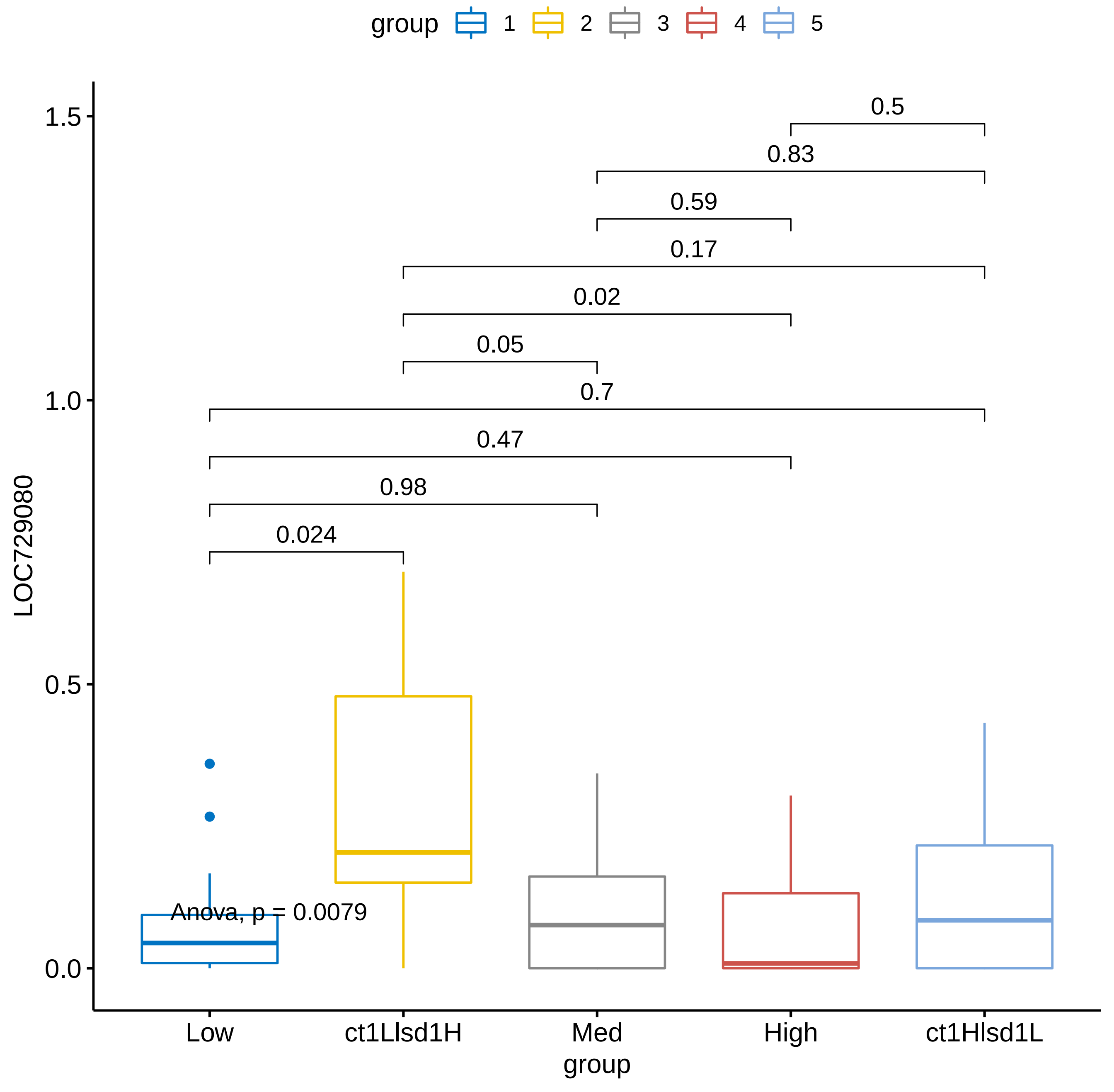

group
1
2
3
4
5
LOC729080
1.5
1.0
0.5
0.0
0.5
0.83
0.59
0.17
0.02
0.05
0.7
0.47
0.98
0.024
Anova, p = 0.0079
Low
ct1Llsd1H
Med
High
ct1Hlsd1L
group

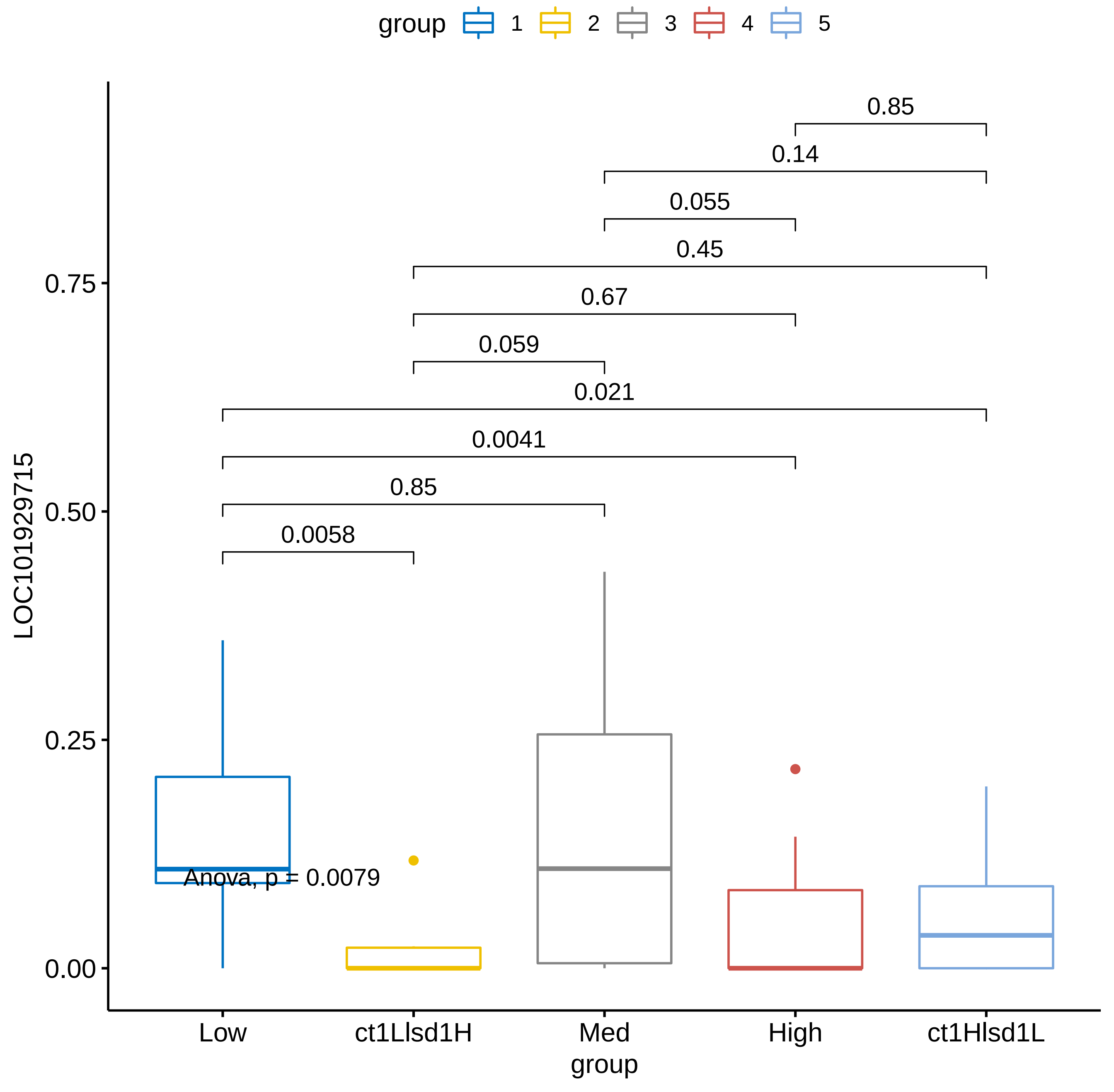

group 1 2 3 4 5
0.85
0.14
0.055
0.45
0.67
0.059
0.021
0.0041
0.85
0.0058
Anova, p = 0.0079
LOC101929715
0.75
0.50
0.25
0.00
Low
ct1Llsd1H
Med
High
ct1Hlsd1L
group

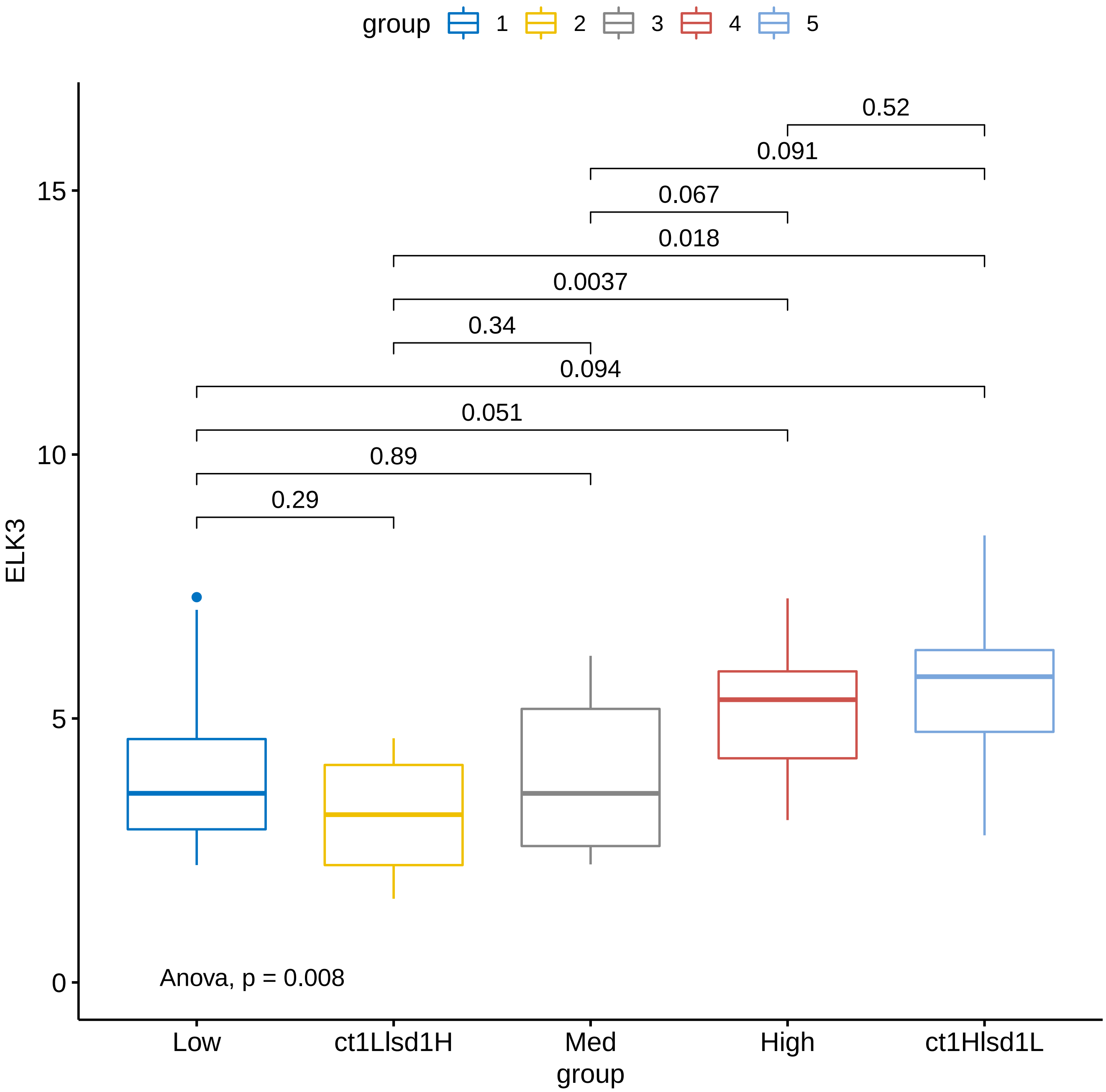
group
1
2
3
4
5
0.52
0.091
0.067
0.018
0.0037
0.34
0.094
0.051
0.89
0.29
15
10
5
0
ELK3
Anova, p = 0.008
Low
ct1Llsd1H
Med
High
ct1Hlsd1L
group

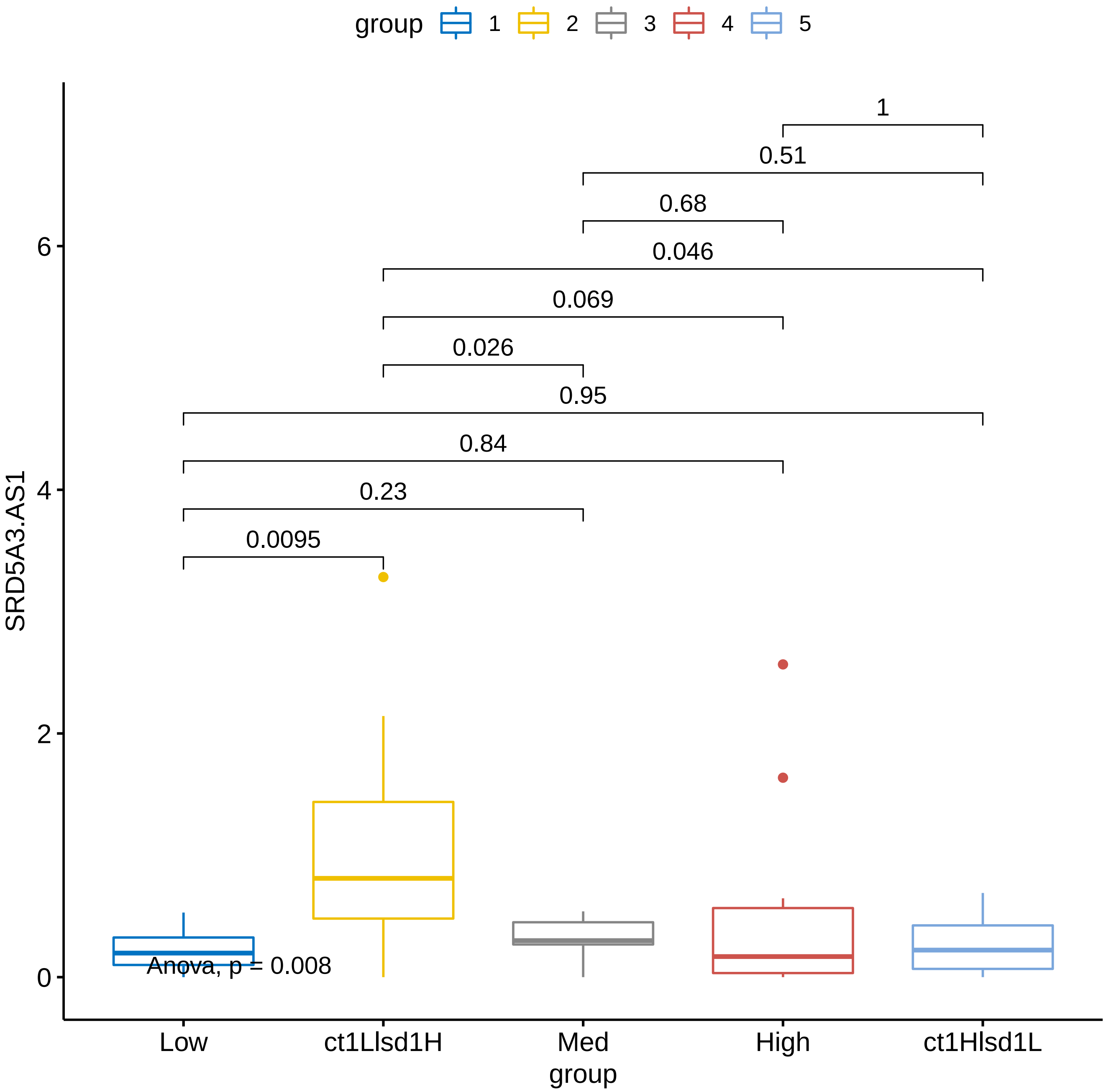

group
1
2
3
4
5
1
0.51
0.68
0.046
0.069
0.026
0.95
0.84
0.23
0.0095
SRD5A3.AS1
6
4
2
0
Anova, p = 0.008
Low
ct1Llsd1H
Med
High
ct1Hlsd1L
group

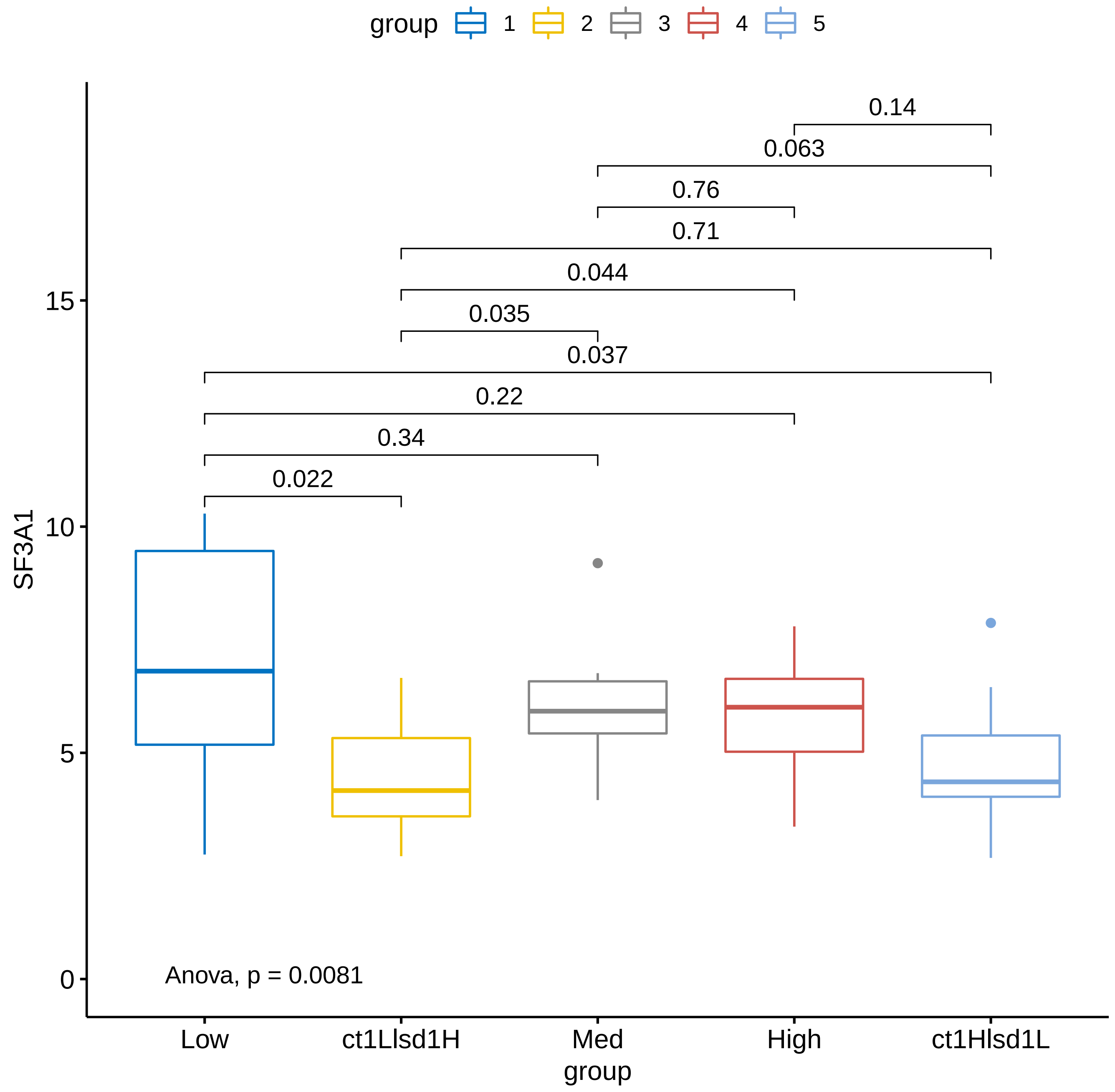
group
1
2
3
4
5
0.14
0.063
0.76
0.71
0.044
0.035
0.037
0.22
0.34
0.022
15
10
5
0
SF3A1
Anova, p = 0.0081
Low
ct1Llsd1H
Med
High
ct1Hlsd1L
group

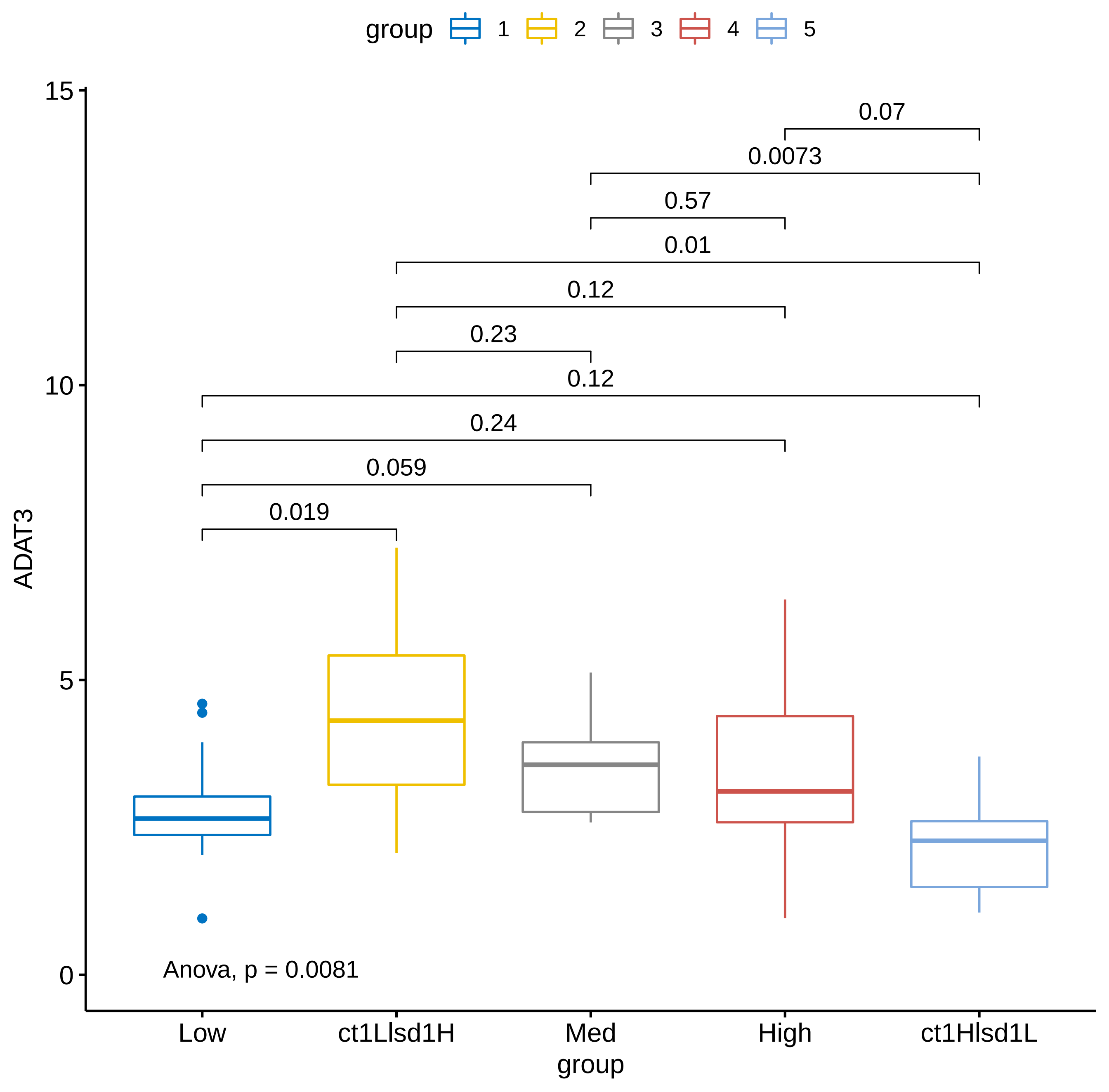

group 1 2 3 4 5
0.07
0.0073
0.57
0.01
0.12
0.23
0.12
0.24
0.059
0.019
15
10
5
0
ADAT3
Anova, p = 0.0081
Low
ct1Llsd1H
Med
High
ct1Hlsd1L
group

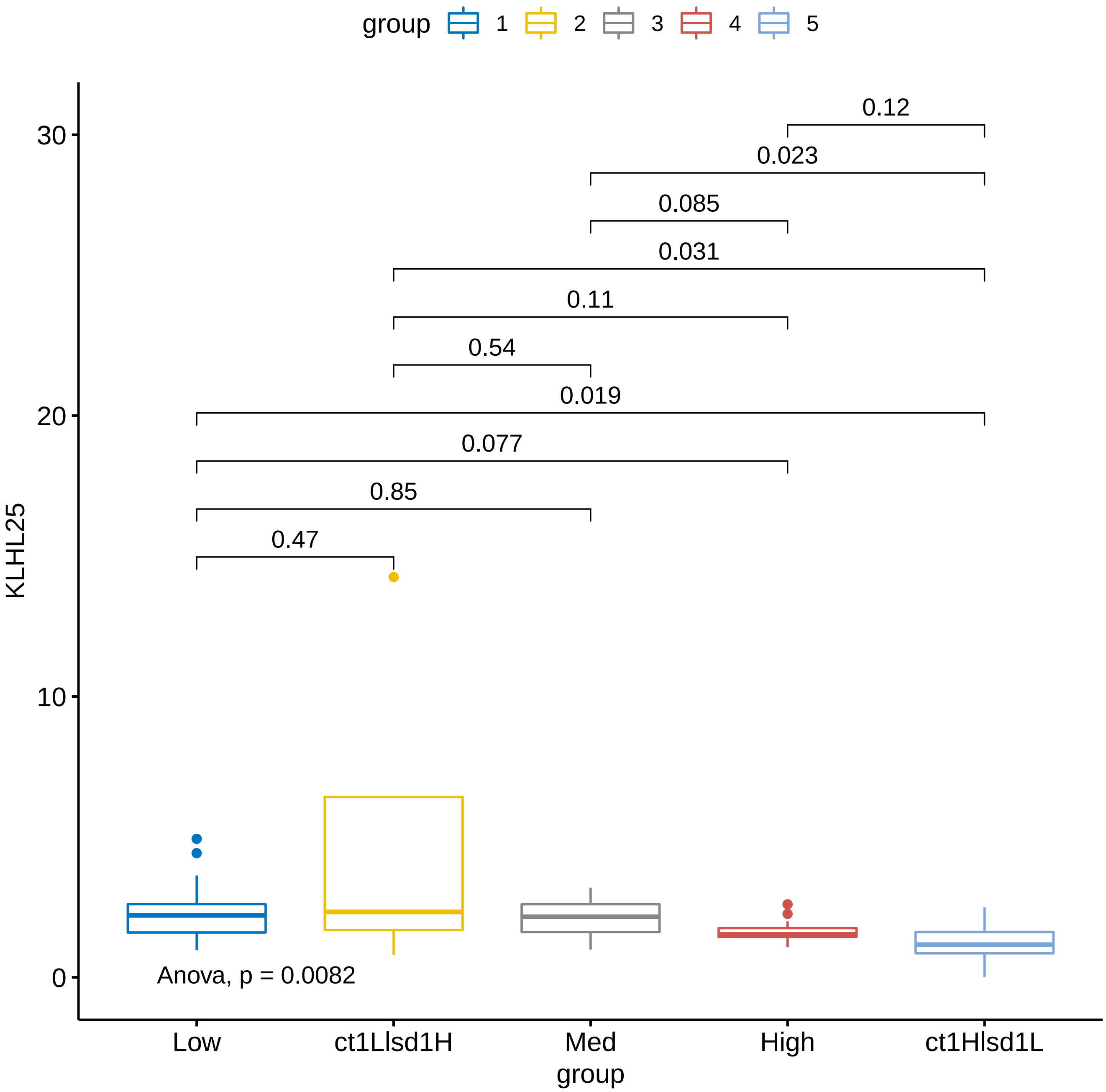
group
1
2
3
4
5
KLHL25
30
20
10
0
0.12
0.023
0.085
0.031
0.11
0.54
0.019
0.077
0.85
0.47
Anova, p = 0.0082
Low
ct1Llsd1H
Med
High
ct1Hlsd1L
group

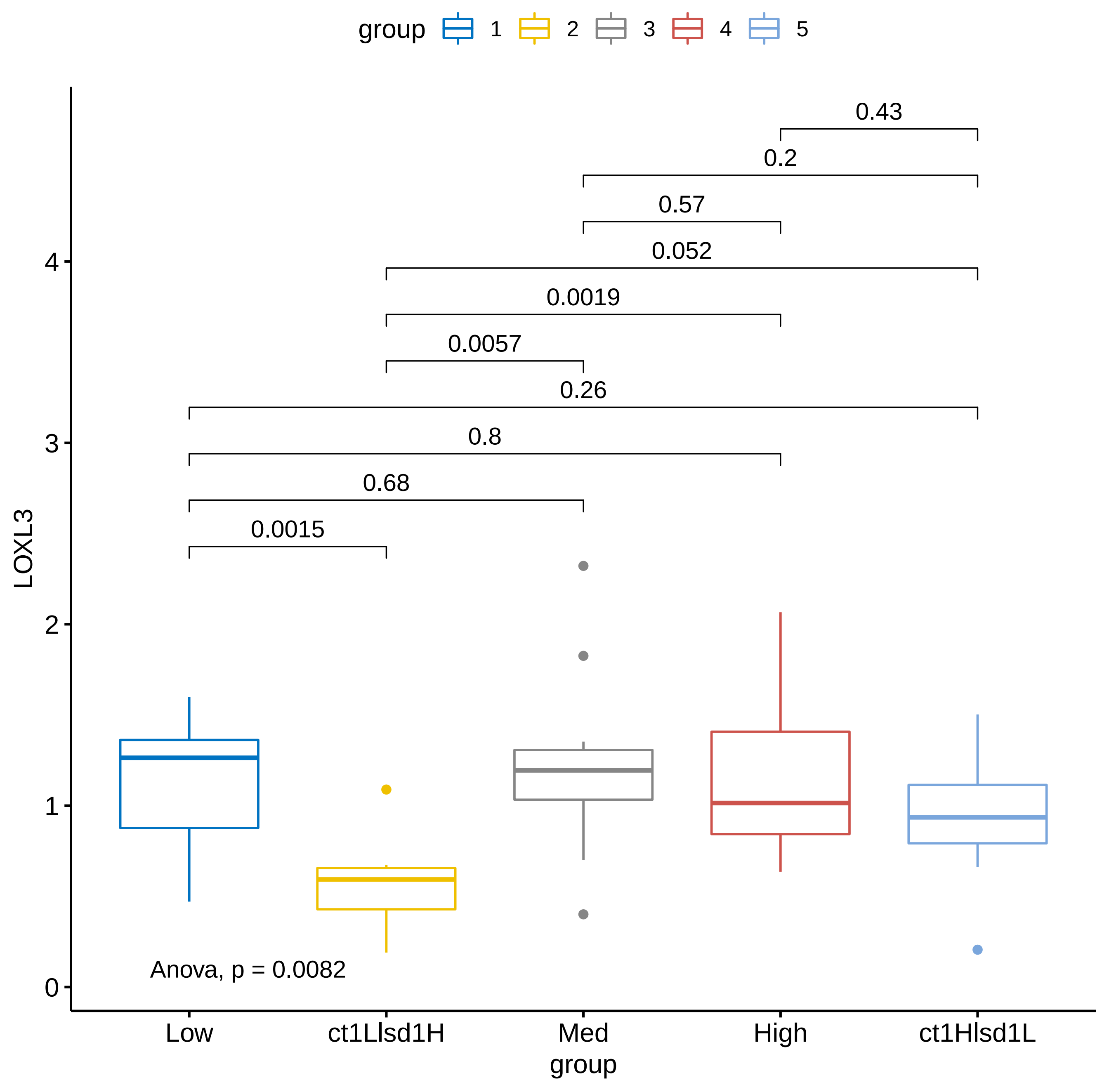
group
1
2
3
4
5
0.43
0.2
0.57
0.052
0.0019
0.0057
0.26
0.8
0.68
0.0015
LOXL3
4
3
2
1
0
Anova, p = 0.0082
Low
ct1Llsd1H
Med
High
ct1Hlsd1L
group

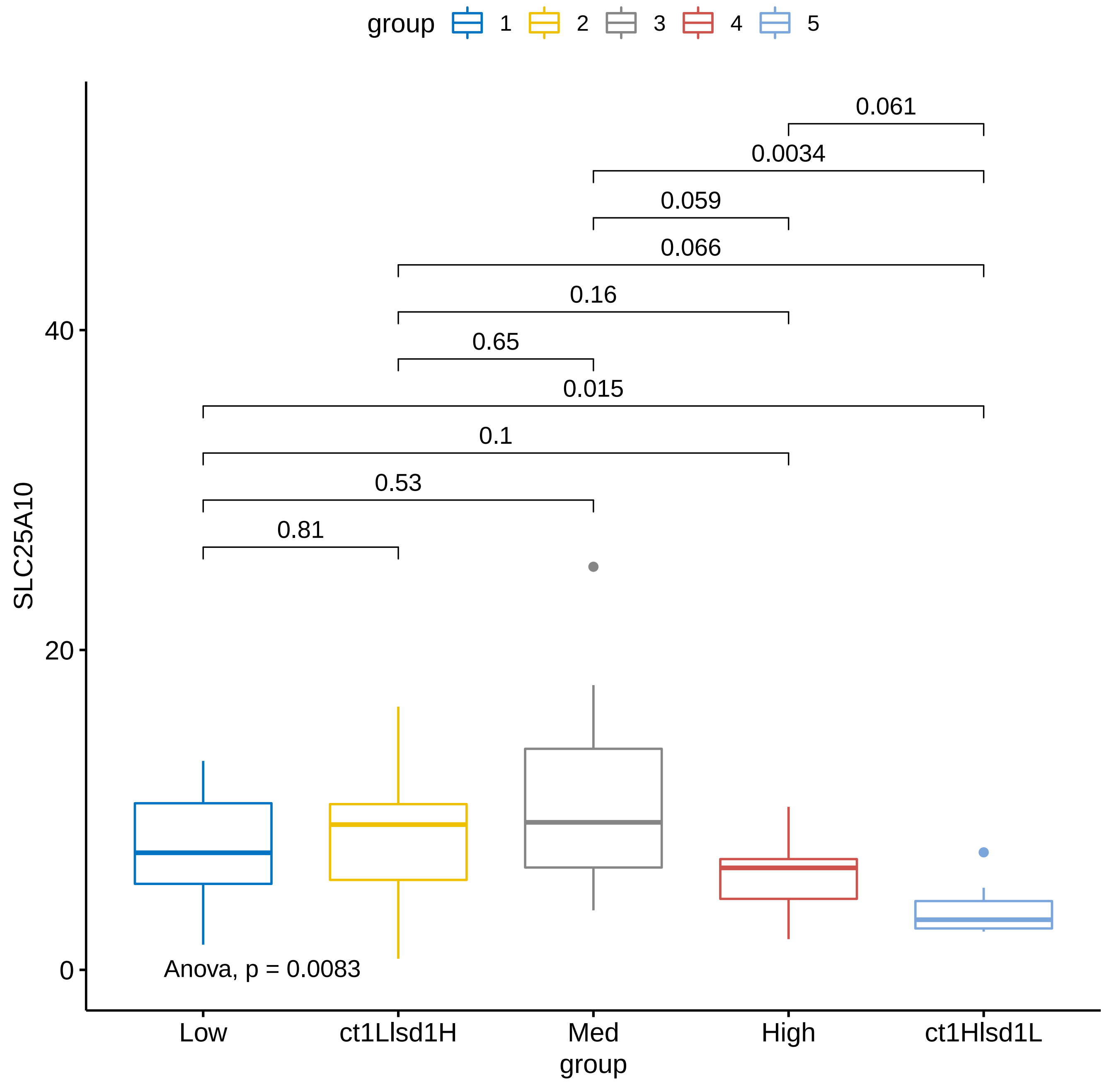

group 1 2 3 4 5
0.061
0.0034
0.059
0.066
0.16
0.65
0.015
0.1
0.53
0.81
SLC25A10
40
20
0
Anova, p = 0.0083
Low
ct1Llsd1H
Med
High
ct1Hlsd1L
group

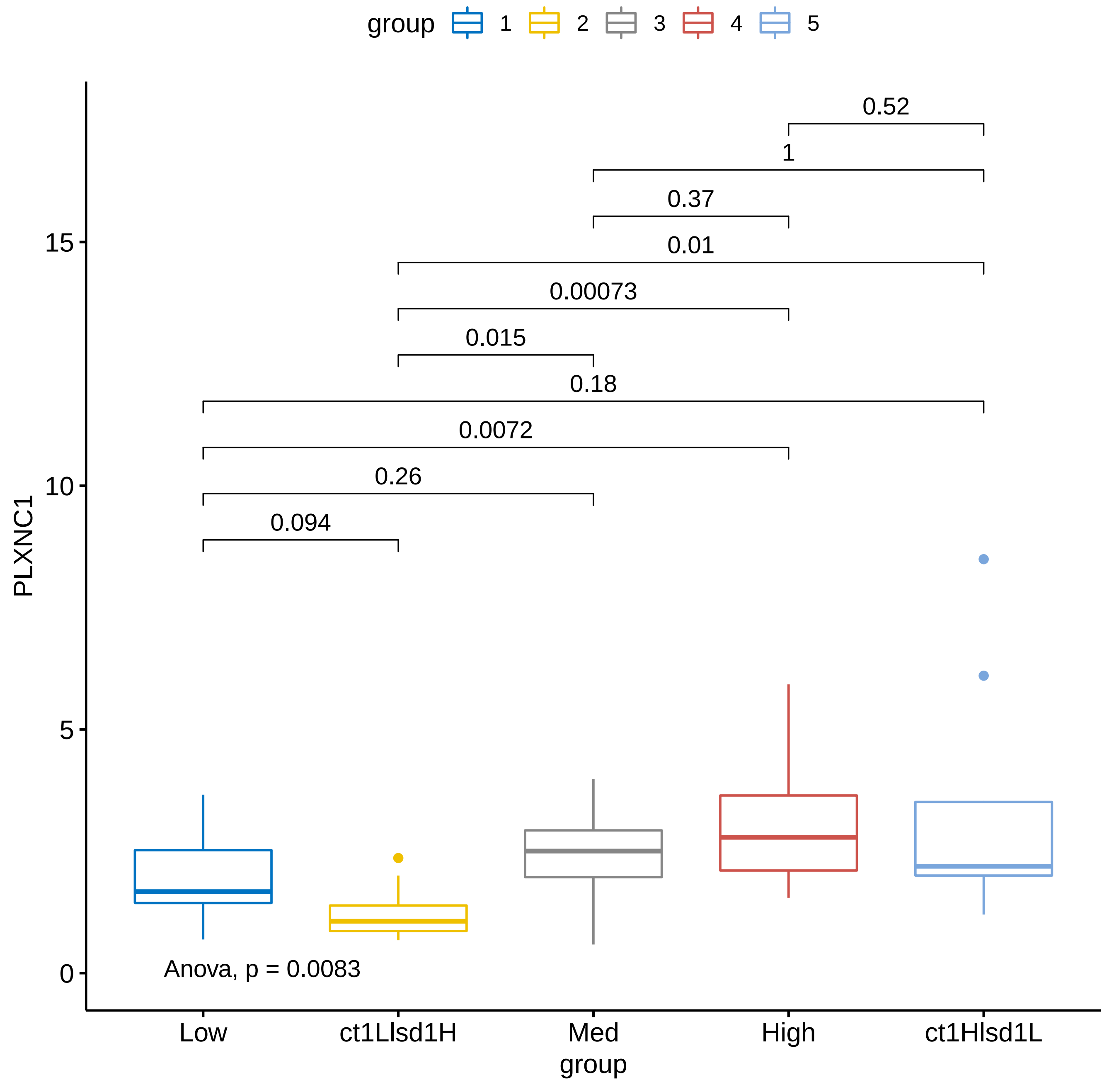

group
1
2
3
4
5
0.52
1
0.37
0.01
0.00073
0.015
0.18
0.0072
0.26
0.094
15
10
5
0
PLXNC1
Anova, p = 0.0083
Low
ct1Llsd1H
Med
High
ct1Hlsd1L
group

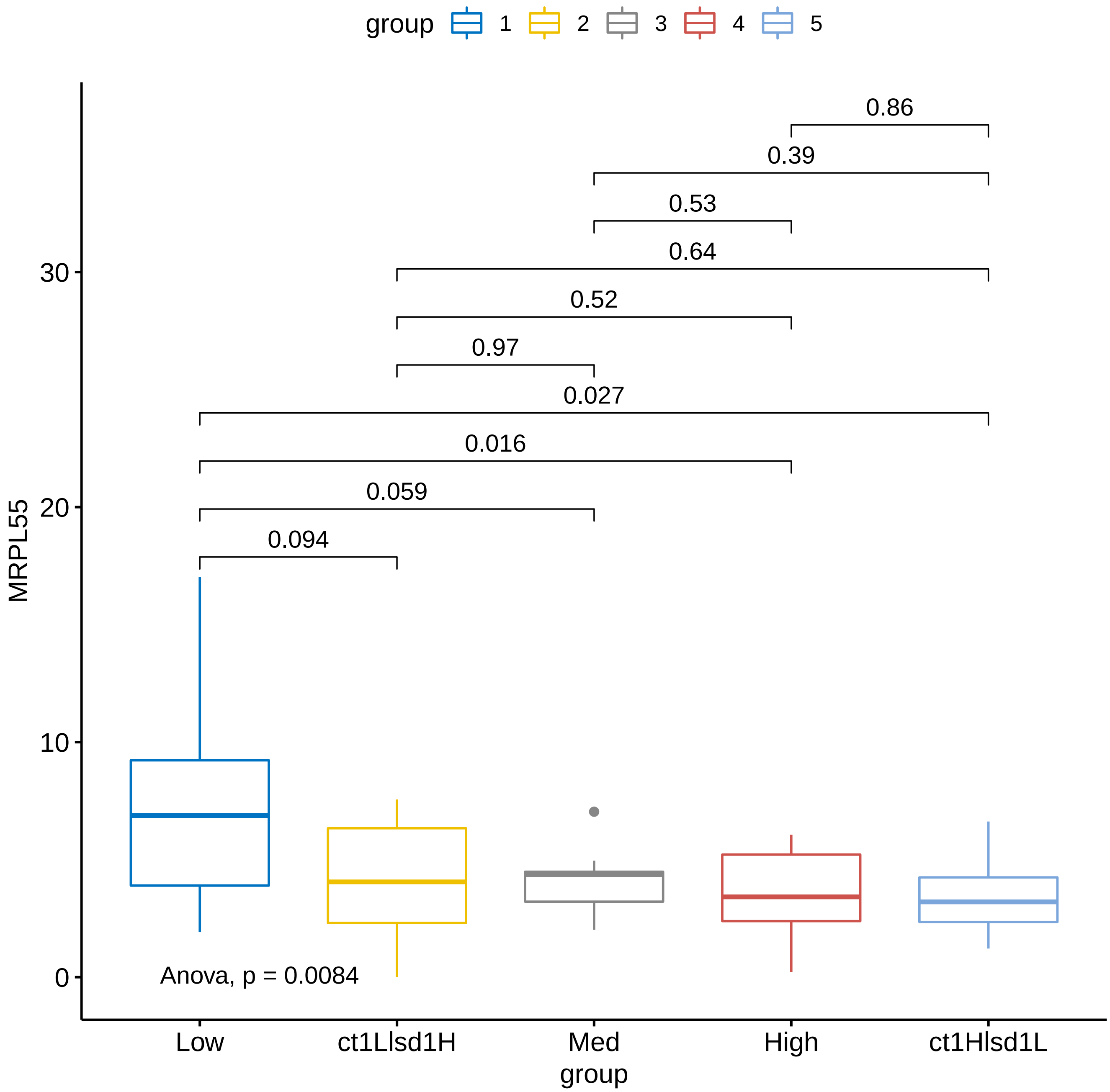
group 1 2 3 4 5
0.86
0.39
0.53
0.64
0.52
0.97
0.027
0.016
0.059
0.094
MRPL55
30
20
10
0
Anova, p = 0.0084
Low
ct1Llsd1H
Med
High
ct1Hlsd1L
group

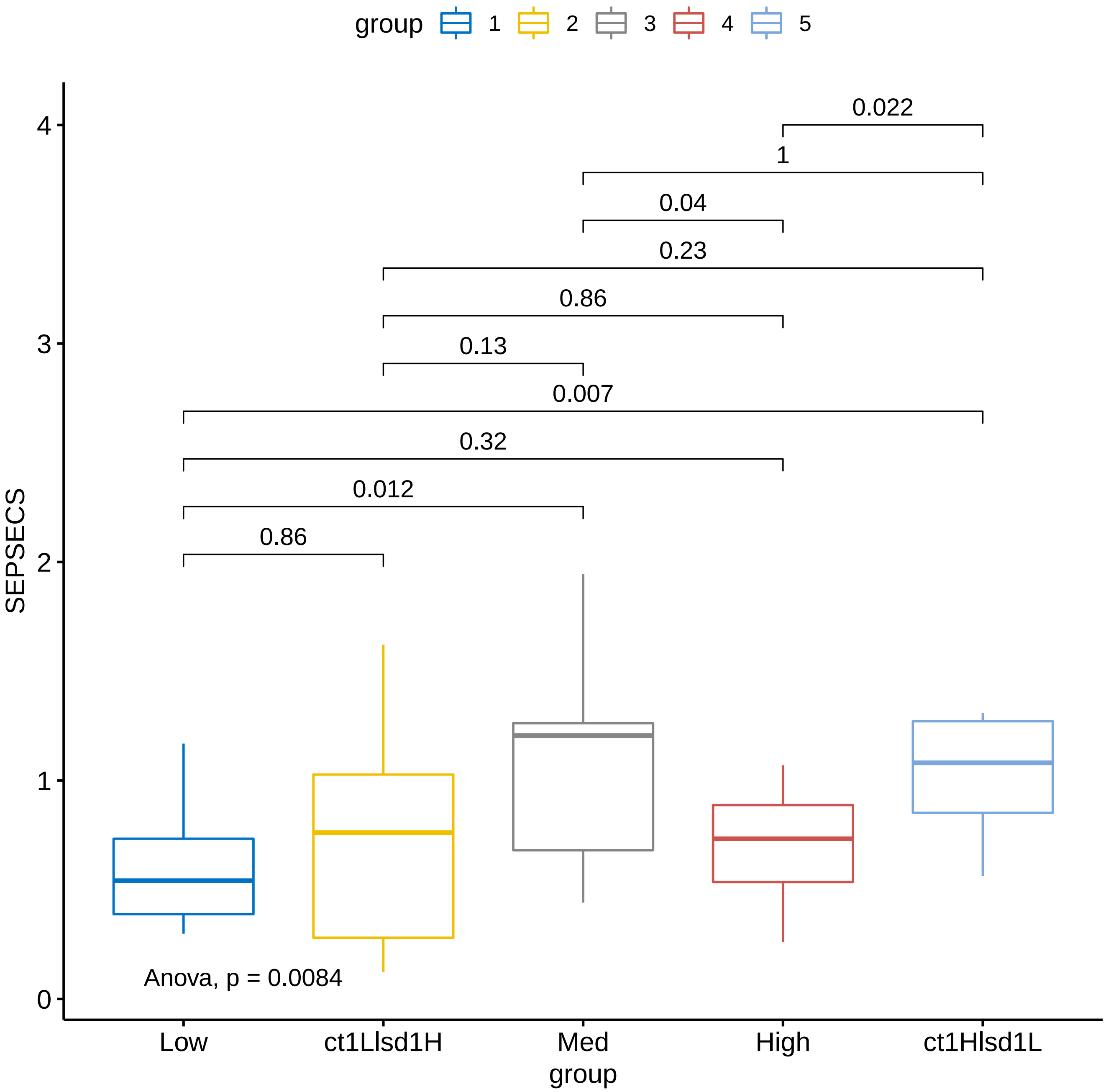
group
1
2
3
4
5
0.022
1
0.04
0.23
0.86
0.13
0.007
0.32
0.012
0.86
Anova, p = 0.0084
SEPSECS
4
3
2
1
0
Low
ct1Llsd1H
Med
High
ct1Hlsd1L
group

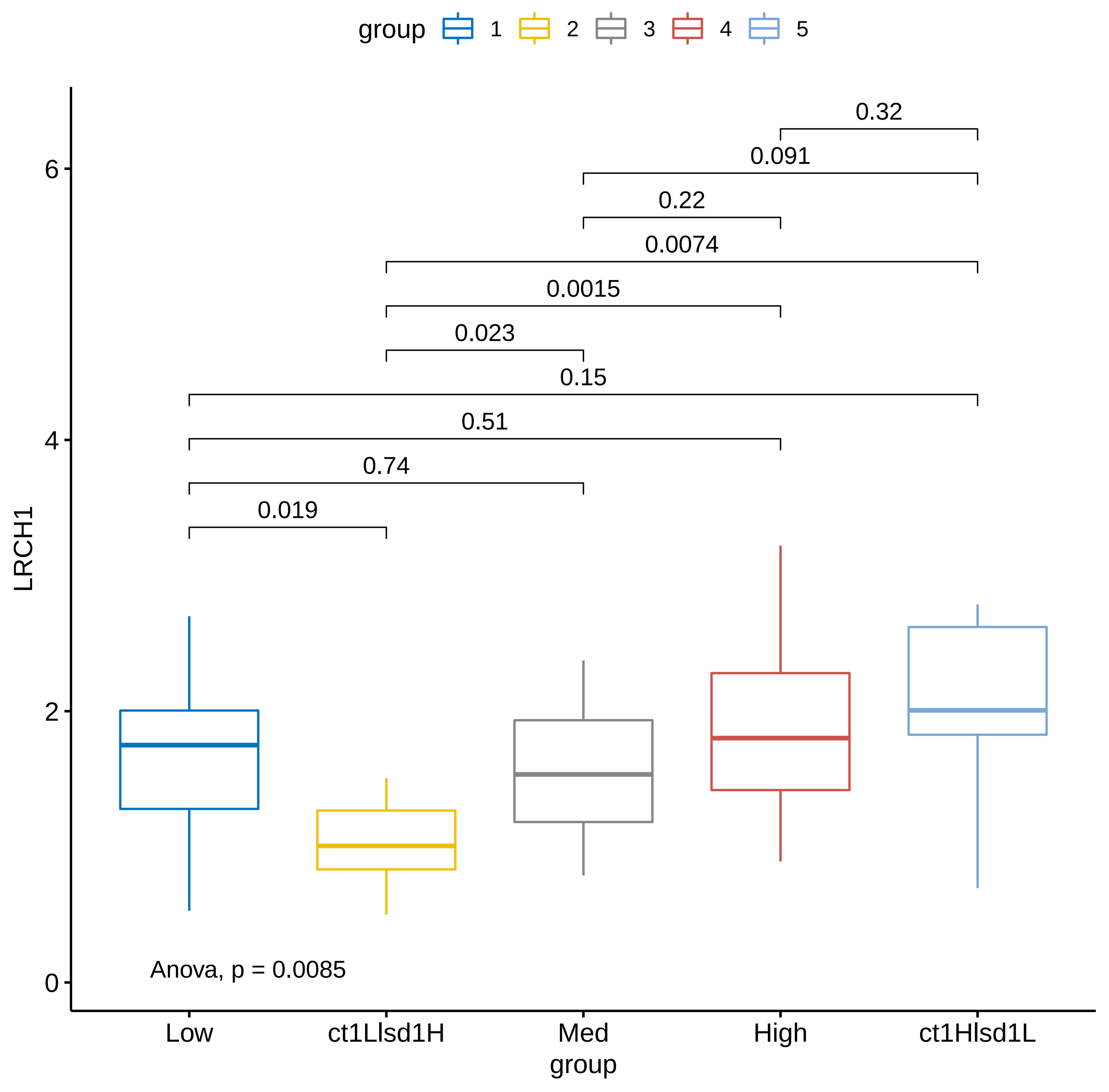
group 1 2 3 4 5
0.32
0.091
0.22
0.0074
0.0015
0.023
0.15
0.51
0.74
0.019
LRCH1
6
4
2
0
Anova, p = 0.0085
Low
ct1Llsd1H
Med
High
ct1Hlsd1L
group

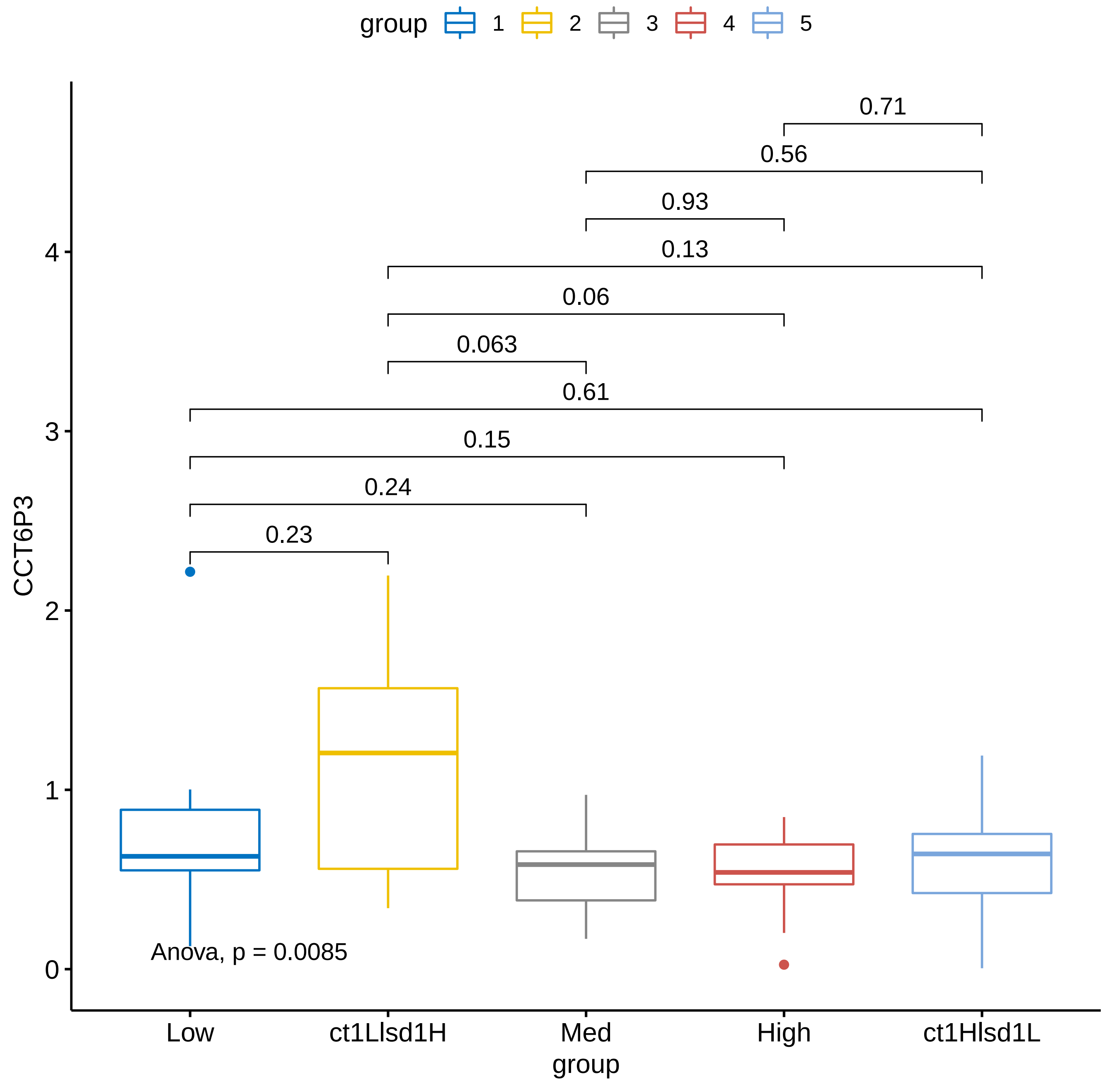

group
1
2
3
4
5
CCT6P3
0.71
0.56
0.93
0.13
0.06
0.063
0.61
0.15
0.24
0.23
Anova, p = 0.0085
0
1
2
3
4
Low
ct1Llsd1H
Med
High
ct1Hlsd1L
group

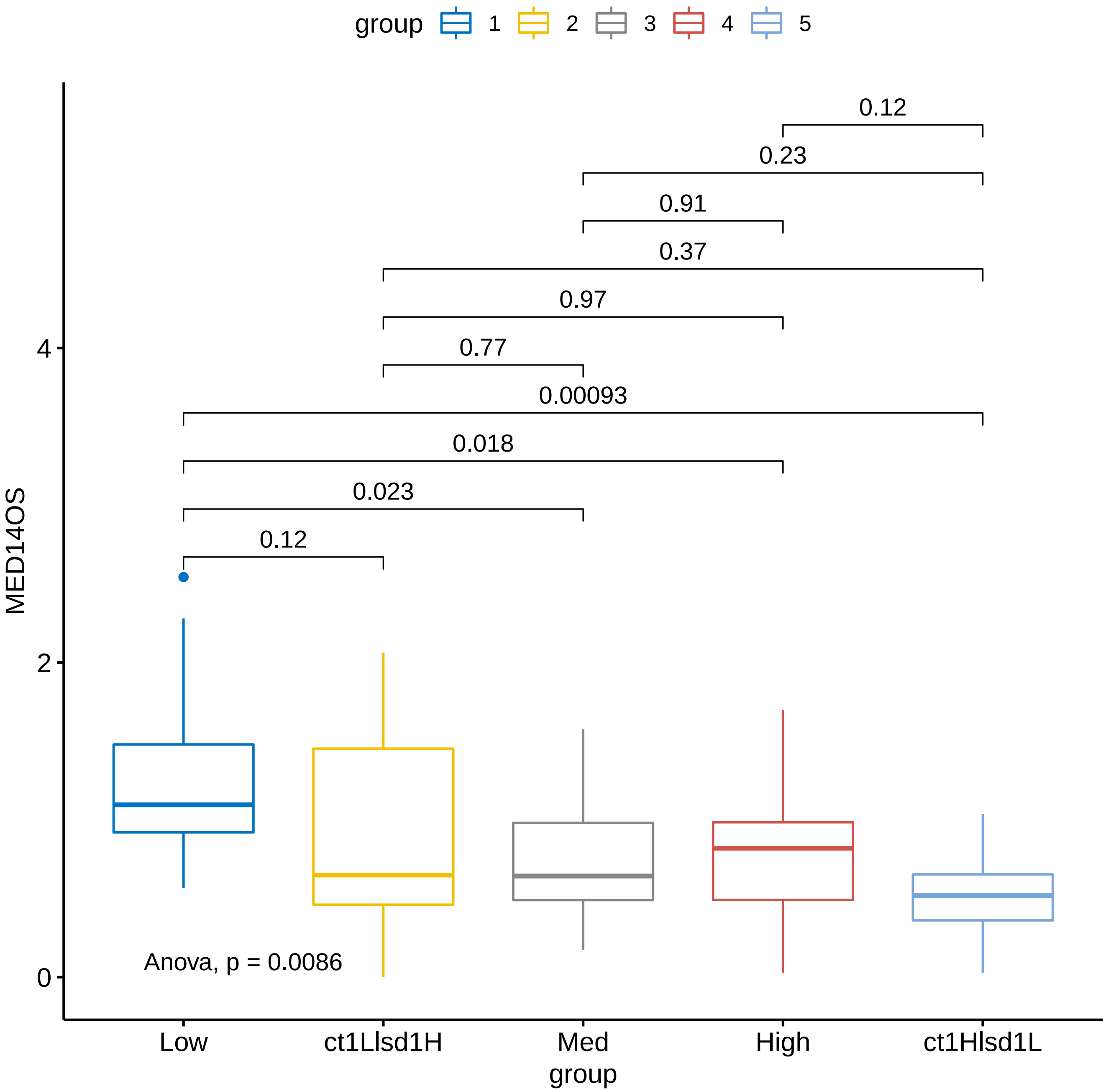

group
1
2
3
4
5
0.12
0.23
0.91
0.37
0.97
0.77
0.00093
0.018
0.023
0.12
MED14OS
4
2
0
Anova, p = 0.0086
Low
ct1Llsd1H
Med
High
ct1Hlsd1L
group

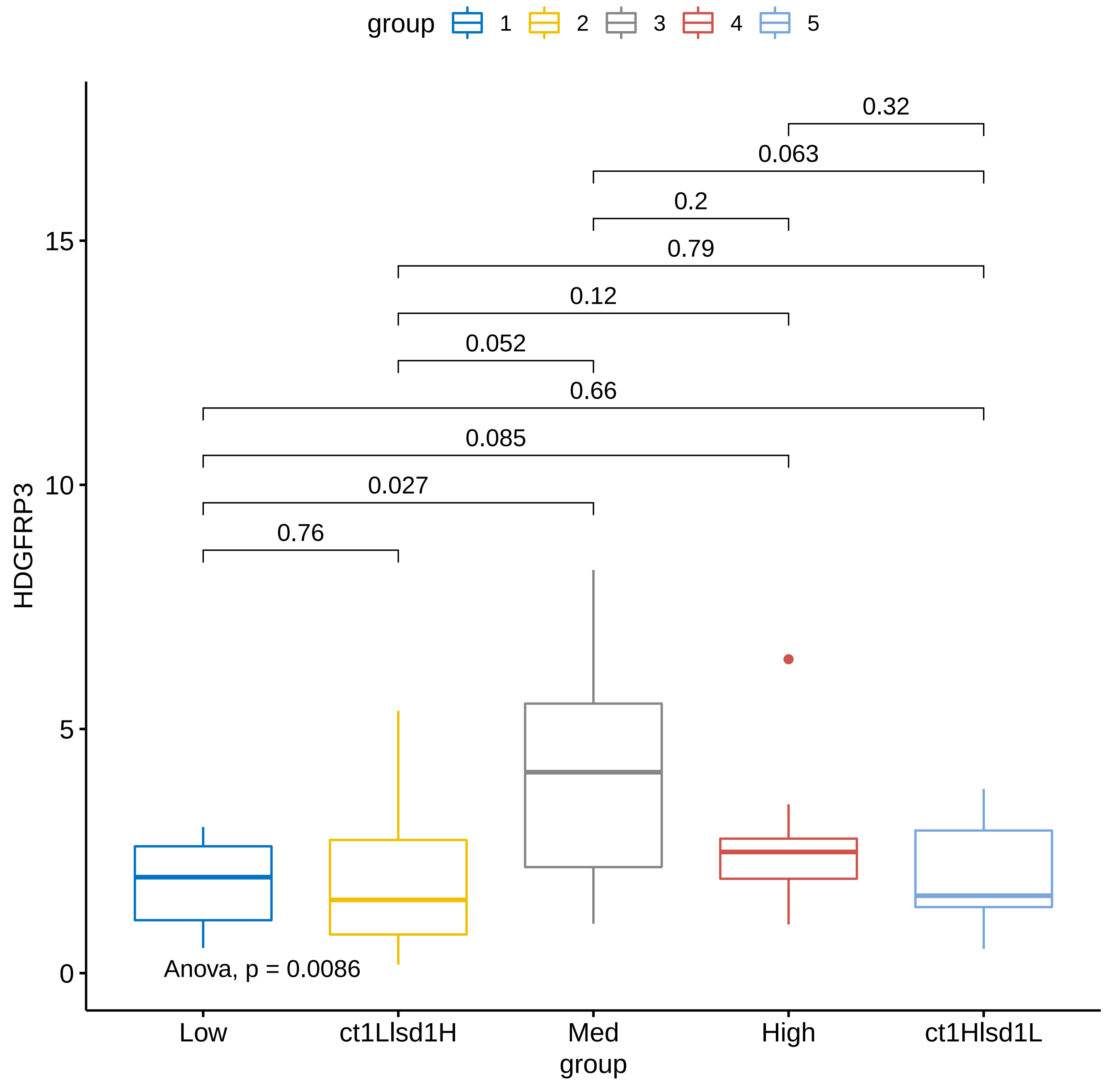
group
1
2
3
4
5
0.32
0.063
0.2
0.79
0.12
0.052
0.66
0.085
0.027
0.76
HDGFRP3
15
10
5
0
Anova, p = 0.0086
Low
ct1Llsd1H
Med
High
ct1Hlsd1L
group

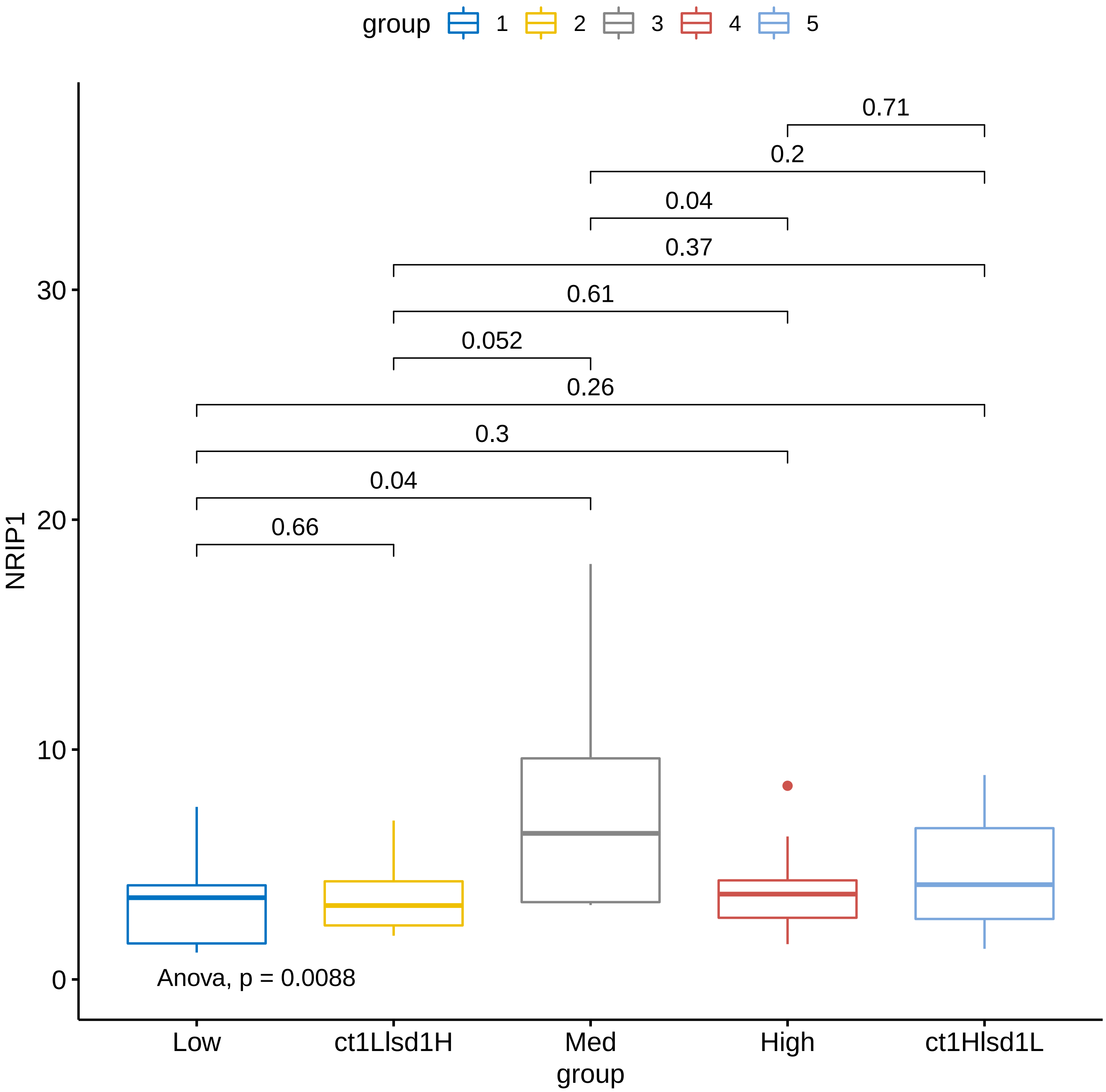
group
1
2
3
4
5
0.71
0.2
0.04
0.37
0.61
0.052
0.26
0.3
0.04
0.66
30
20
10
0
NRIP1
Anova, p = 0.0088
Low
ct1Llsd1H
Med
High
ct1Hlsd1L
group

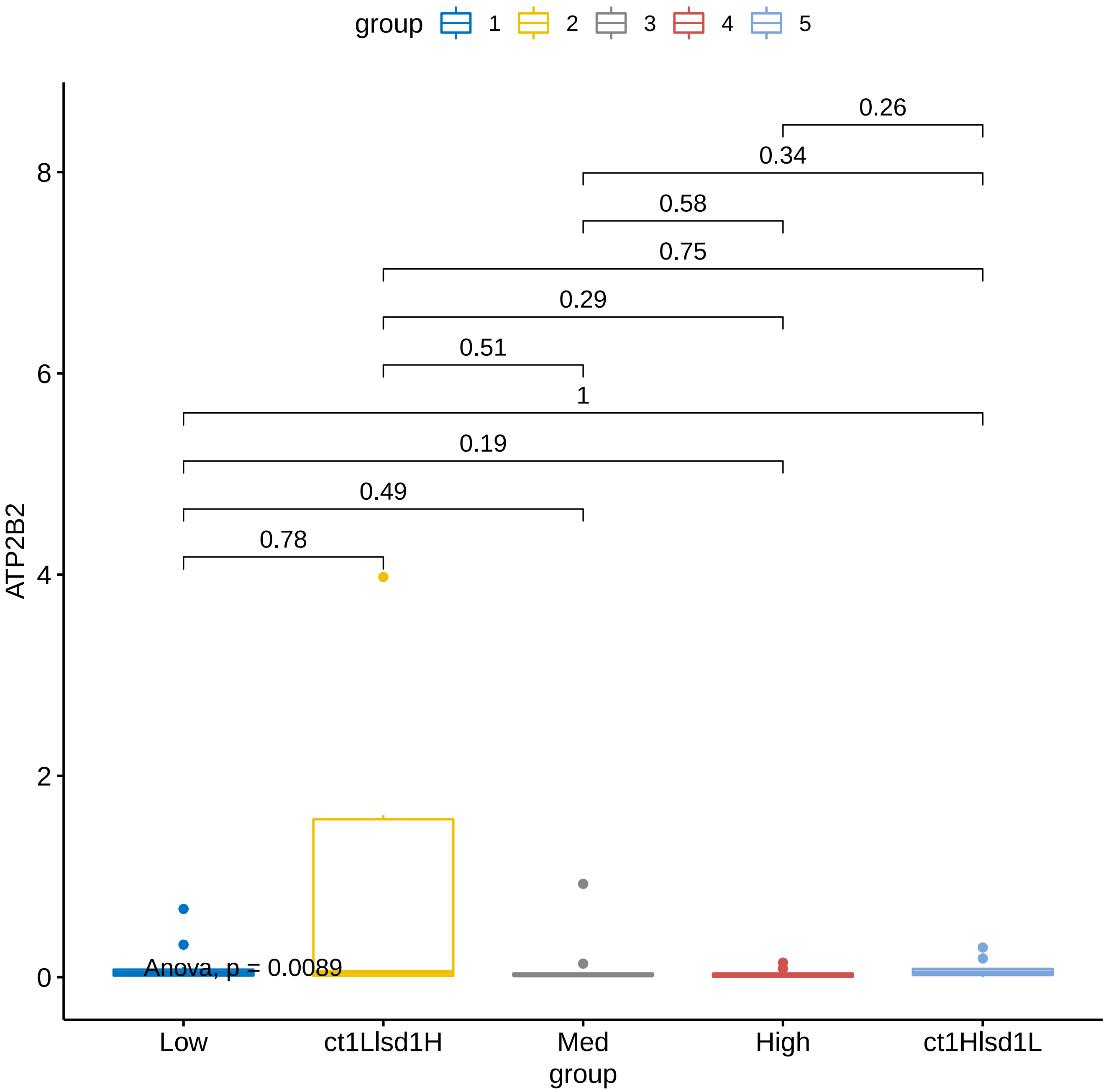

group
1
2
3
4
5
0.26
0.34
0.58
0.75
0.29
0.51
1
0.19
0.49
0.78
ATP2B2
8
6
4
2
0
Anova, p = 0.0089
Low
ct1Llsd1H
Med
High
ct1Hlsd1L
group

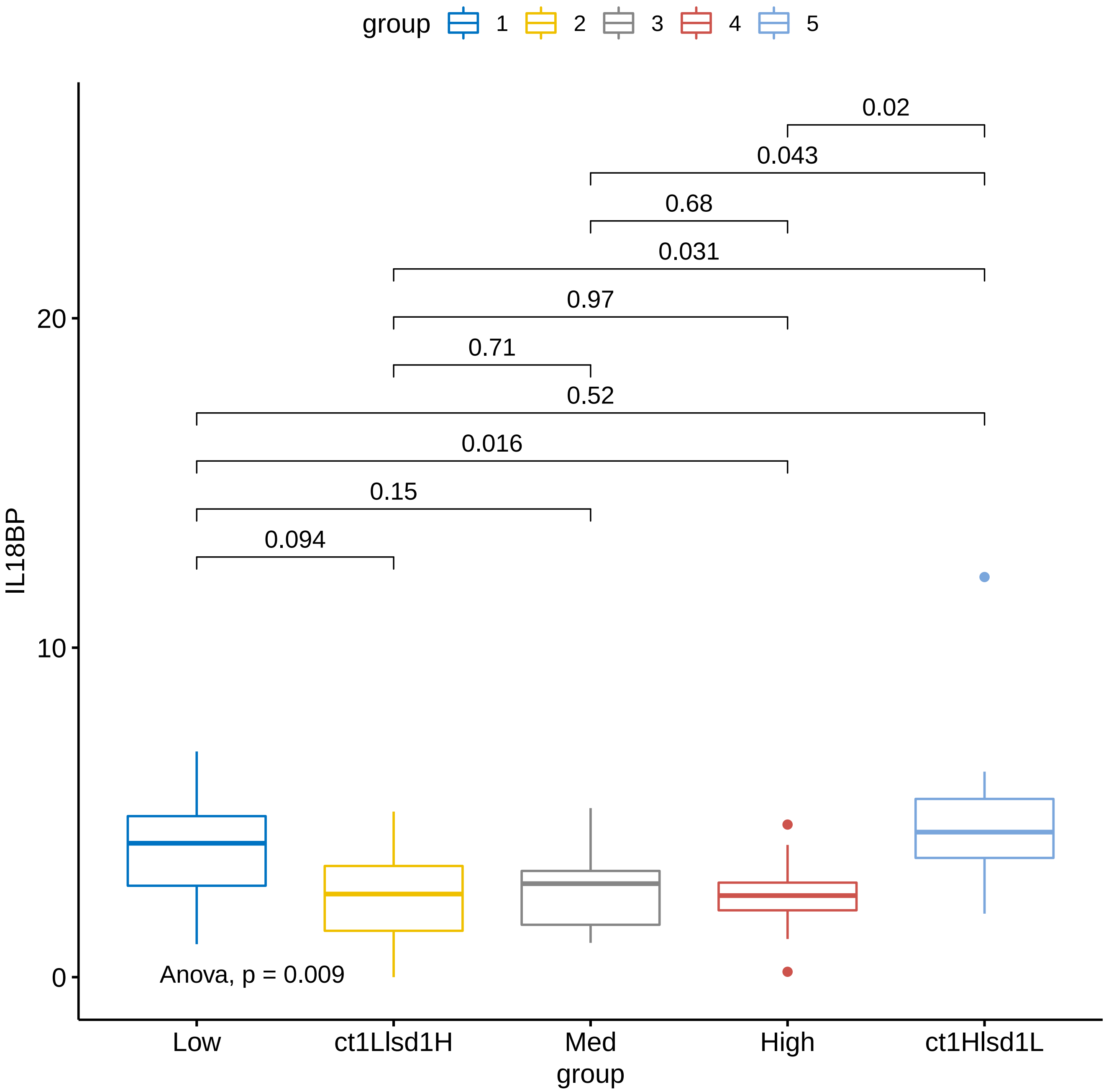

group 1 2 3 4 5
0.02
0.043
0.68
0.031
0.97
0.71
0.52
0.016
0.15
0.094
IL18BP
20
10
0
Anova, p = 0.009
Low
ct1Llsd1H
Med
High
ct1Hlsd1L
group

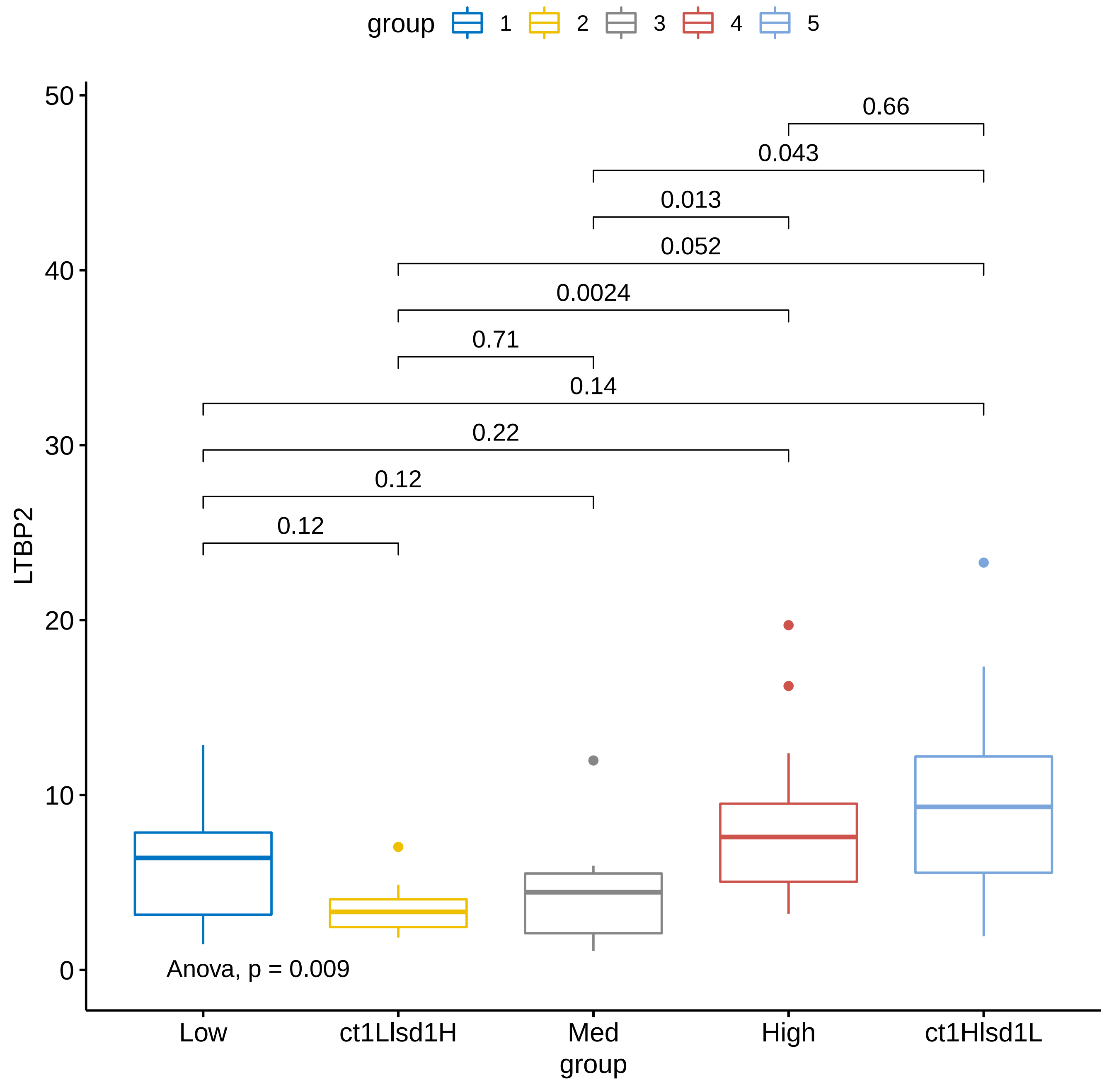

group 1 2 3 4 5
LTBP2
0
10
20
30
40
50
0.66
0.043
0.013
0.052
0.0024
0.71
0.14
0.22
0.12
0.12
Anova, p = 0.009
Low
ct1Llsd1H
Med
High
ct1Hlsd1L
group

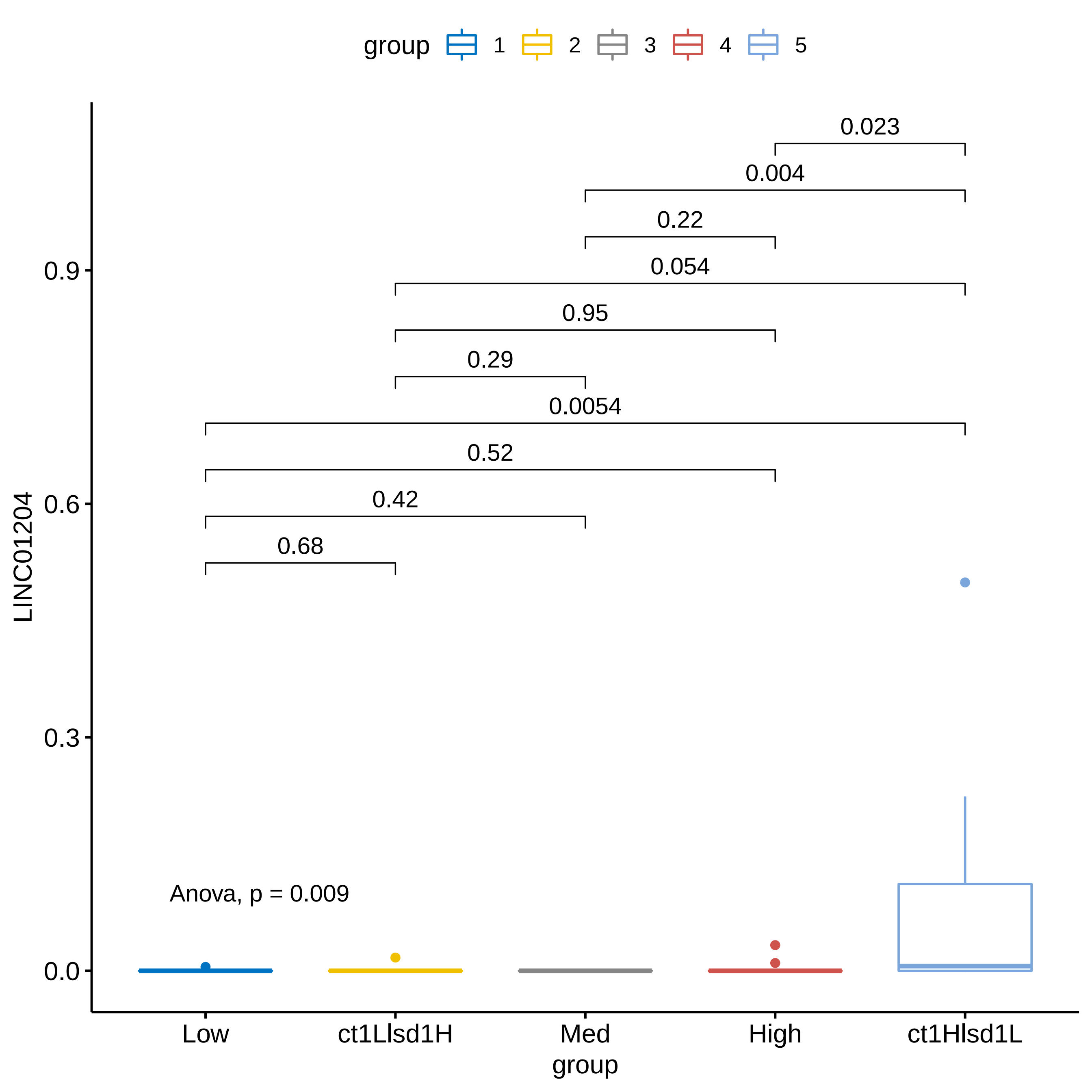

group 1 2 3 4 5
0.023
0.004
0.22
0.054
0.95
0.29
0.0054
0.52
0.42
0.68
0.9
0.6
0.3
0.0
LINC01204
Anova, p = 0.009
Low
ct1Llsd1H
Med
High
ct1Hlsd1L
group

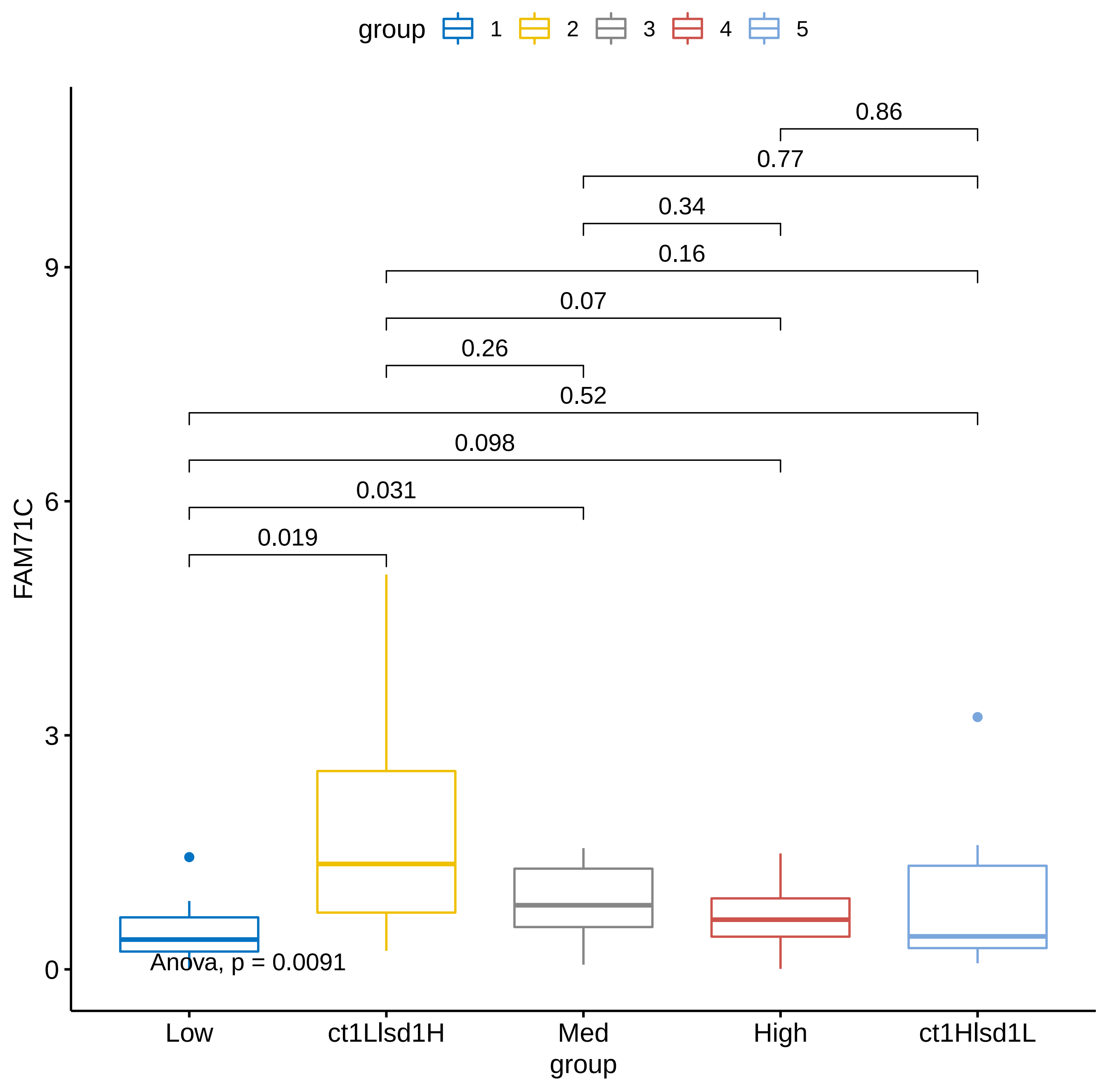

group 1 2 3 4 5
0.86
0.77
0.34
0.16
0.07
0.26
0.52
0.098
0.031
0.019
Anova, p = 0.0091
FAM71C
9
6
3
0
Low
ct1Llsd1H
Med
High
ct1Hlsd1L
group

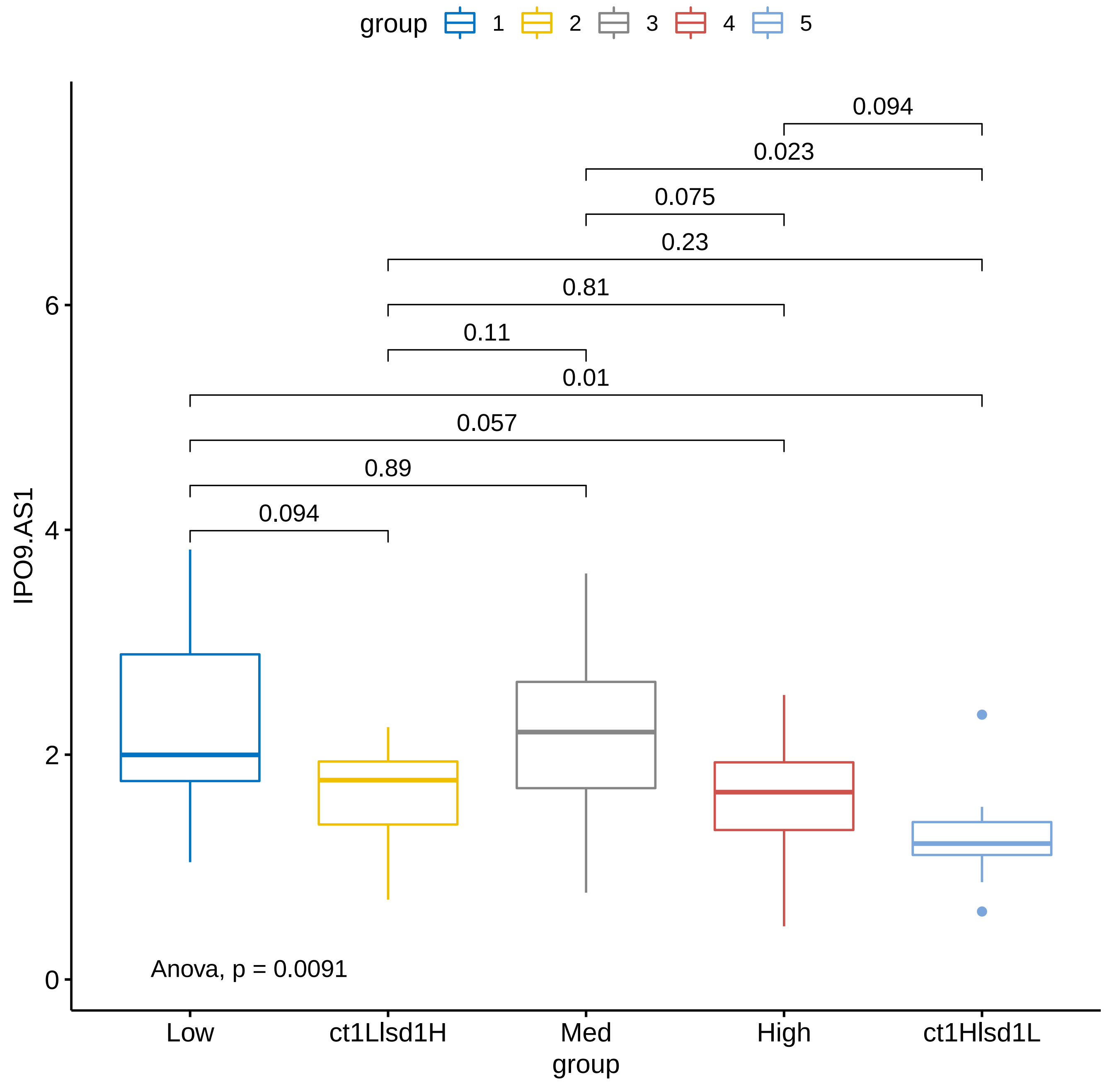

group 1 2 3 4 5
0.094
0.023
0.075
0.23
0.81
0.11
0.01
0.057
0.89
0.094
IPO9.AS1
6
4
2
0
Anova, p = 0.0091
Low
ct1Llsd1H
Med
High
ct1Hlsd1L
group

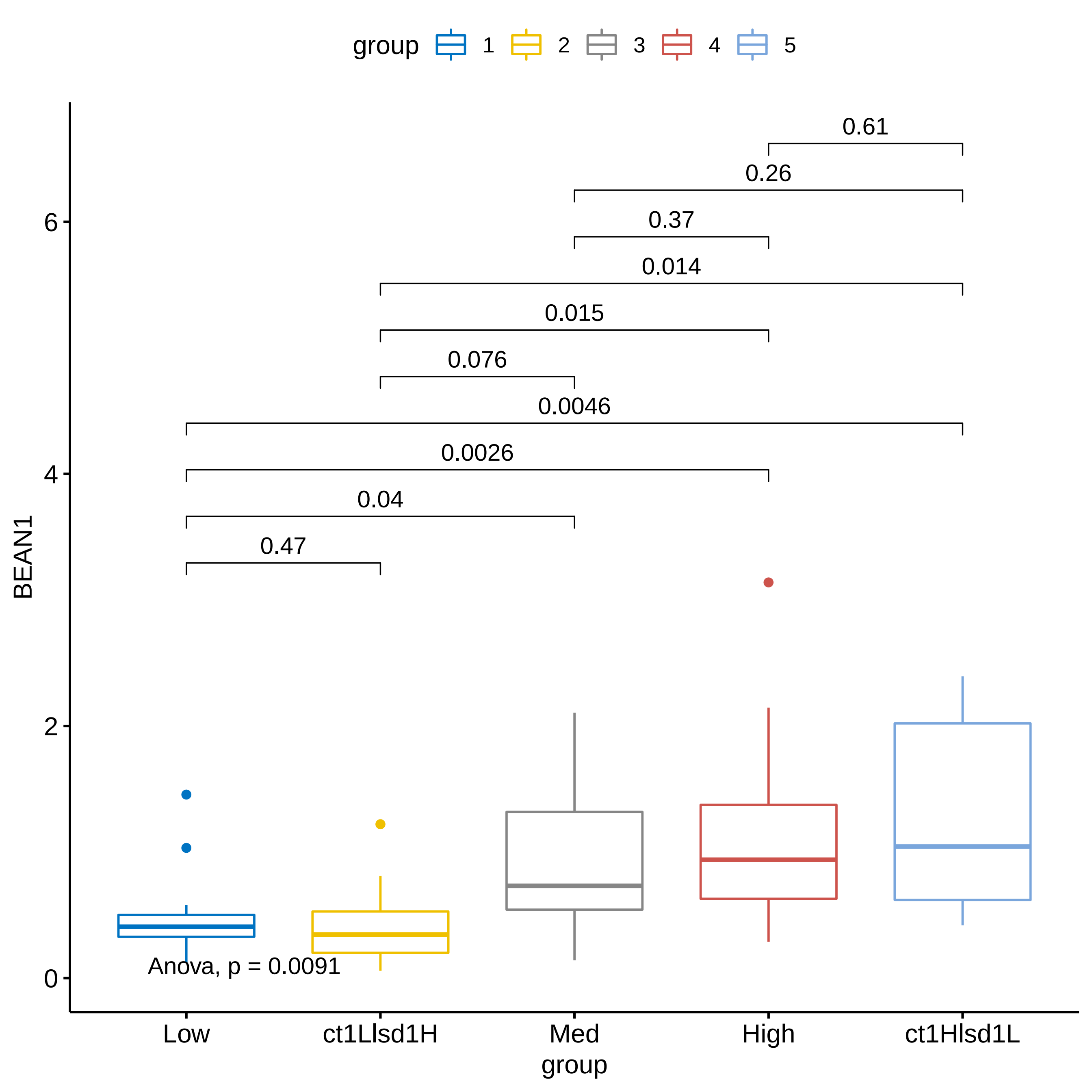
group
1
2
3
4
5
0.61
0.26
0.37
0.014
0.015
0.076
0.0046
0.0026
0.04
0.47
6
4
2
0
BEAN1
Anova, p = 0.0091
Low
ct1Llsd1H
Med
High
ct1Hlsd1L
group

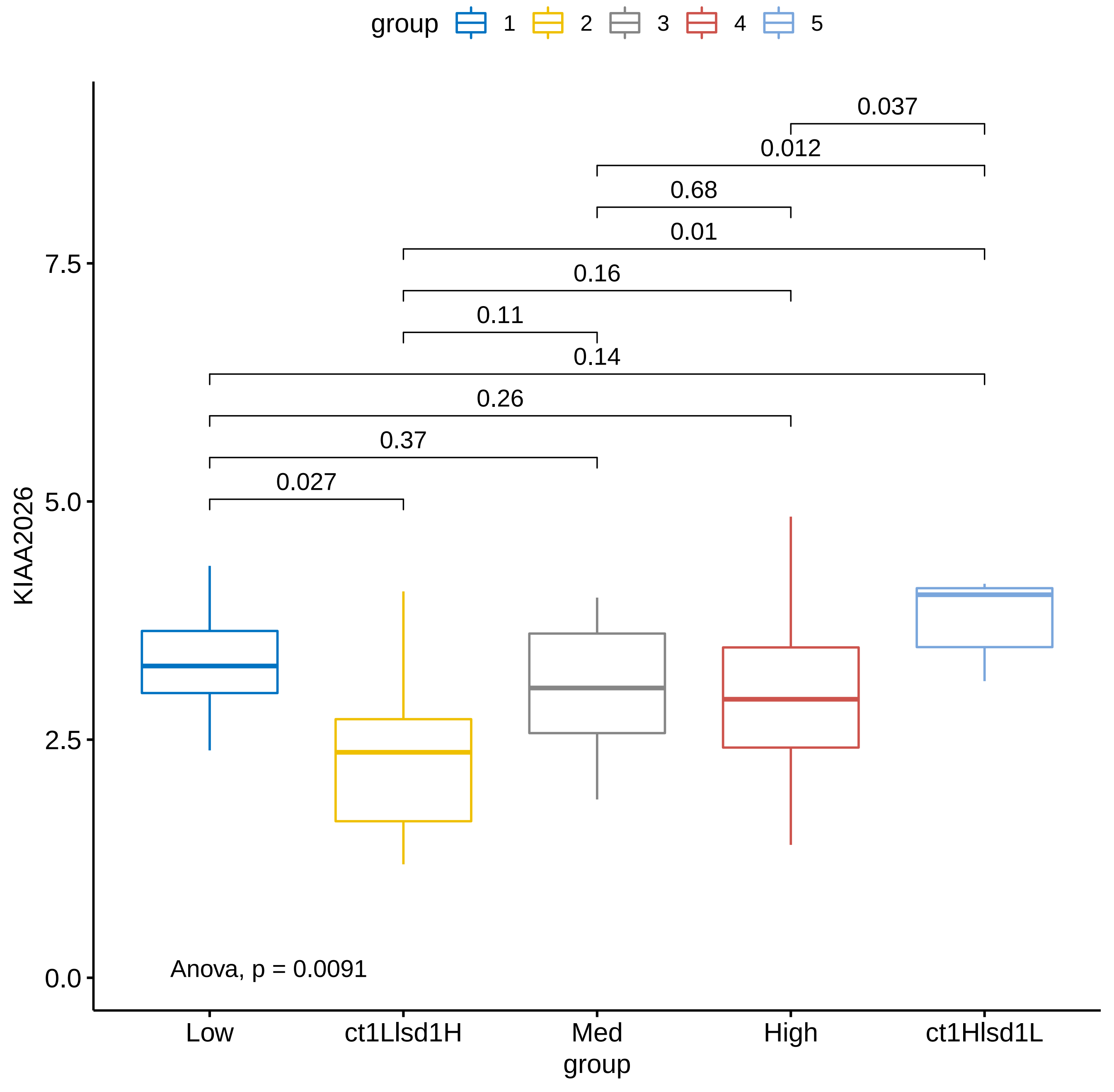
group
1
2
3
4
5
0.037
0.012
0.68
0.01
0.16
0.11
0.14
0.26
0.37
0.027
7.5
5.0
2.5
0.0
KIAA2026
Anova, p = 0.0091
Low
ct1Llsd1H
Med
High
ct1Hlsd1L
group

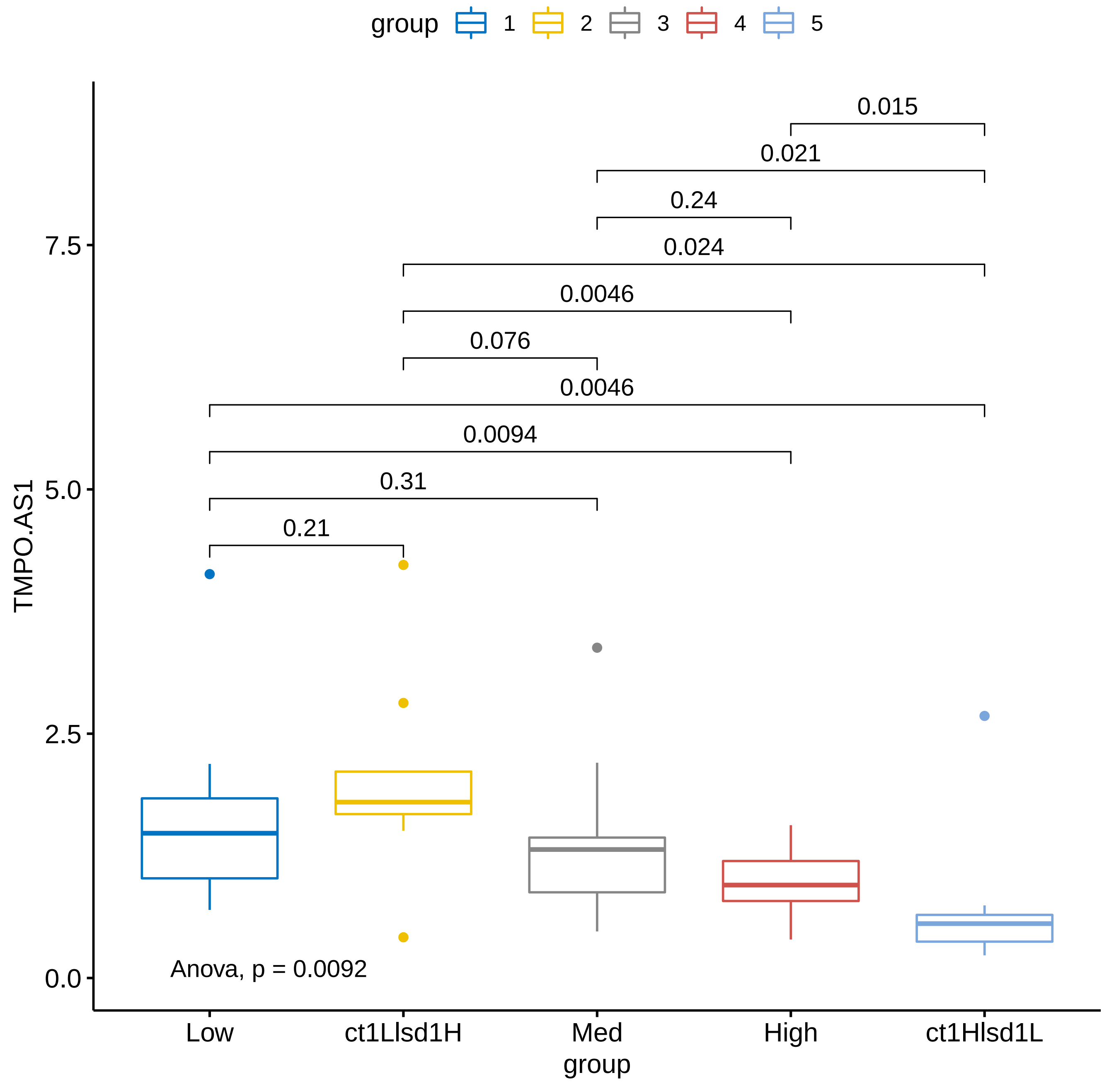

group
1
2
3
4
5
0.015
0.021
0.24
0.024
0.0046
0.076
0.0046
0.0094
0.31
0.21
7.5
5.0
2.5
0.0
TMPO.AS1
Anova, p = 0.0092
Low
ct1Llsd1H
Med
High
ct1Hlsd1L
group

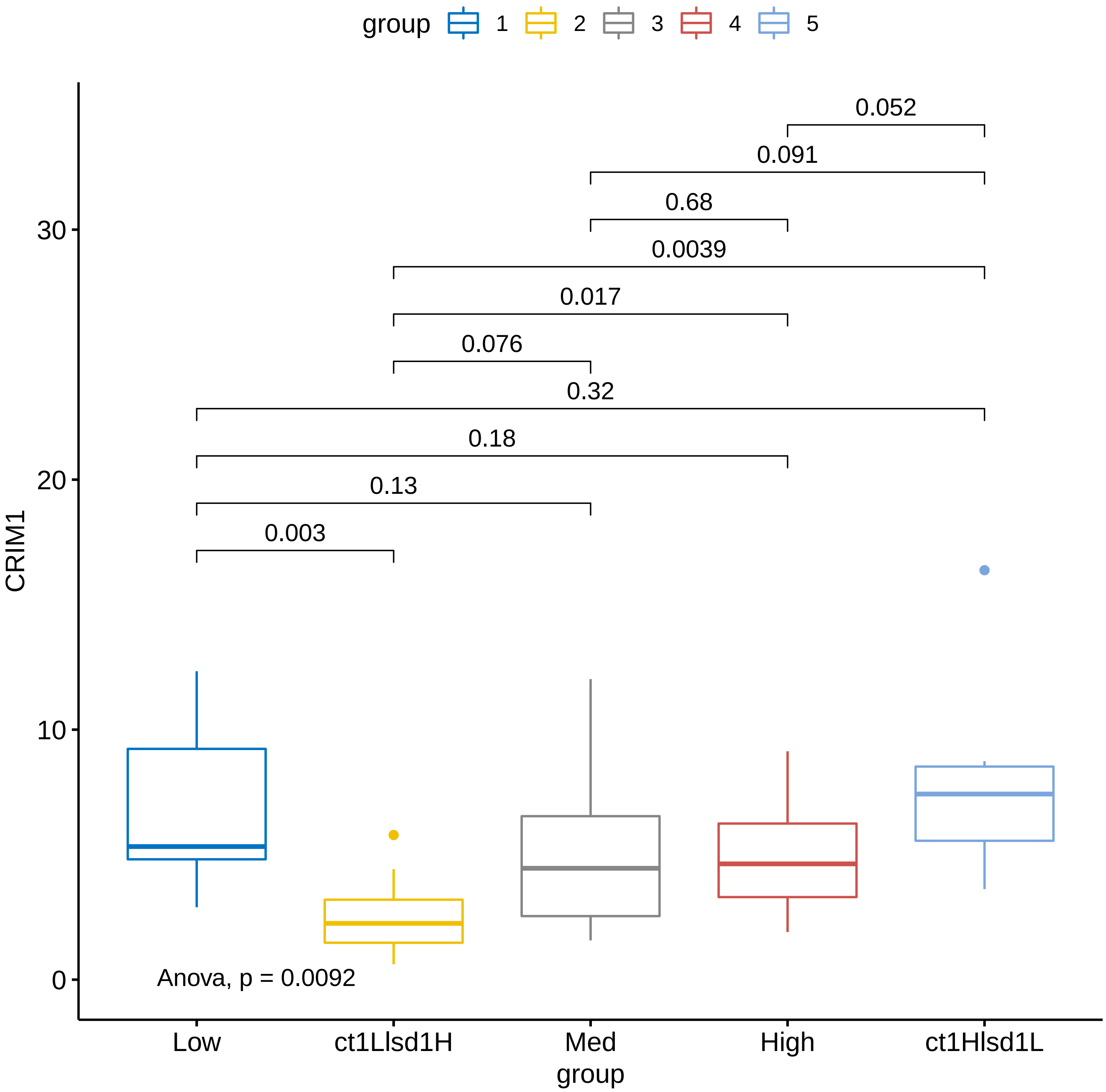
group
1
2
3
4
5
0.052
0.091
0.68
0.0039
0.017
0.076
0.32
0.18
0.13
0.003
30
20
10
0
CRIM1
Anova, p = 0.0092
Low
ct1Llsd1H
Med
High
ct1Hlsd1L
group

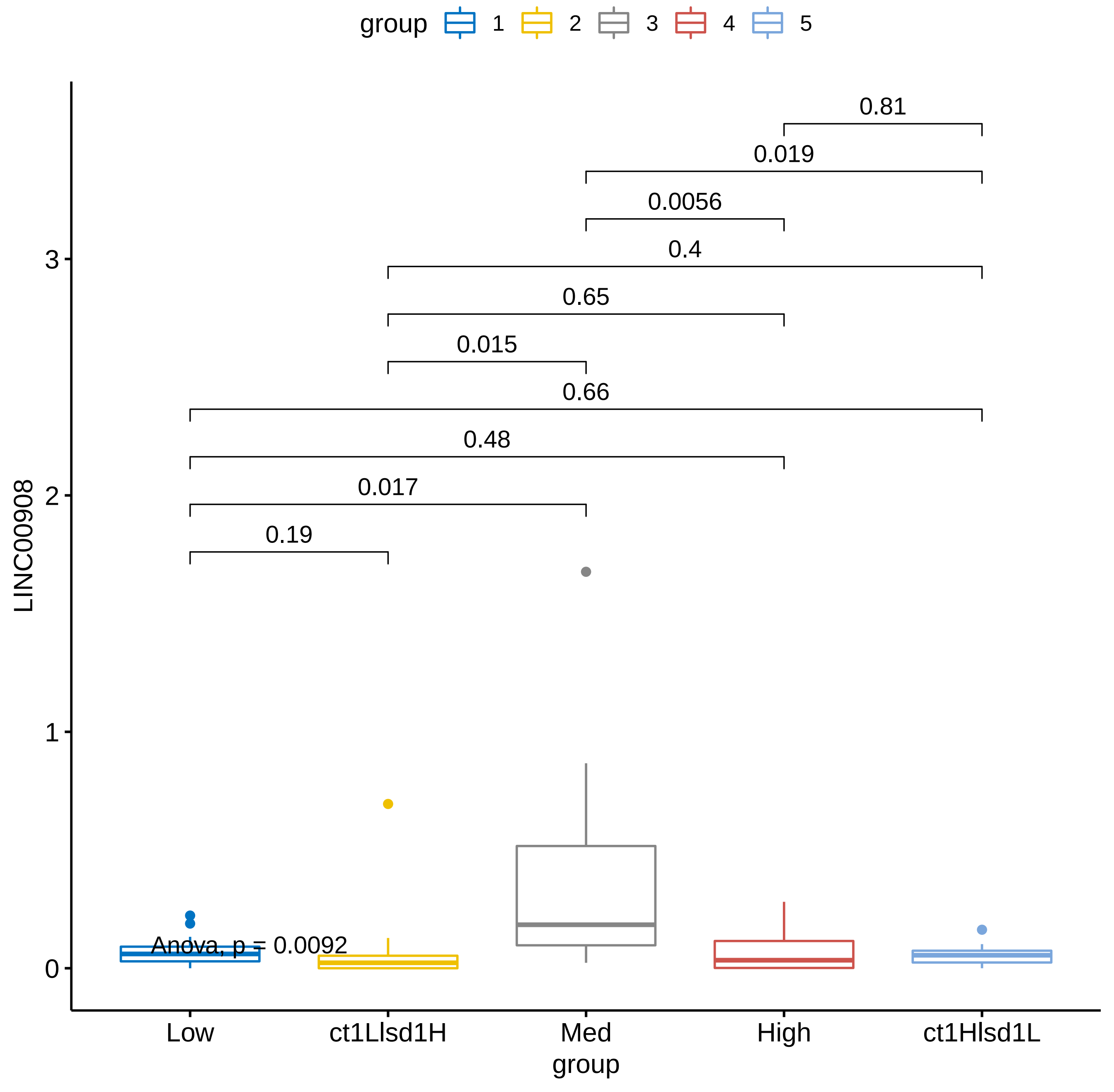

group
1
2
3
4
5
0.81
0.019
0.0056
0.4
0.65
0.015
0.66
0.48
0.017
0.19
Anova, p = 0.0092
LINC00908
3
2
1
0
Low
ct1Llsd1H
Med
High
ct1Hlsd1L
group

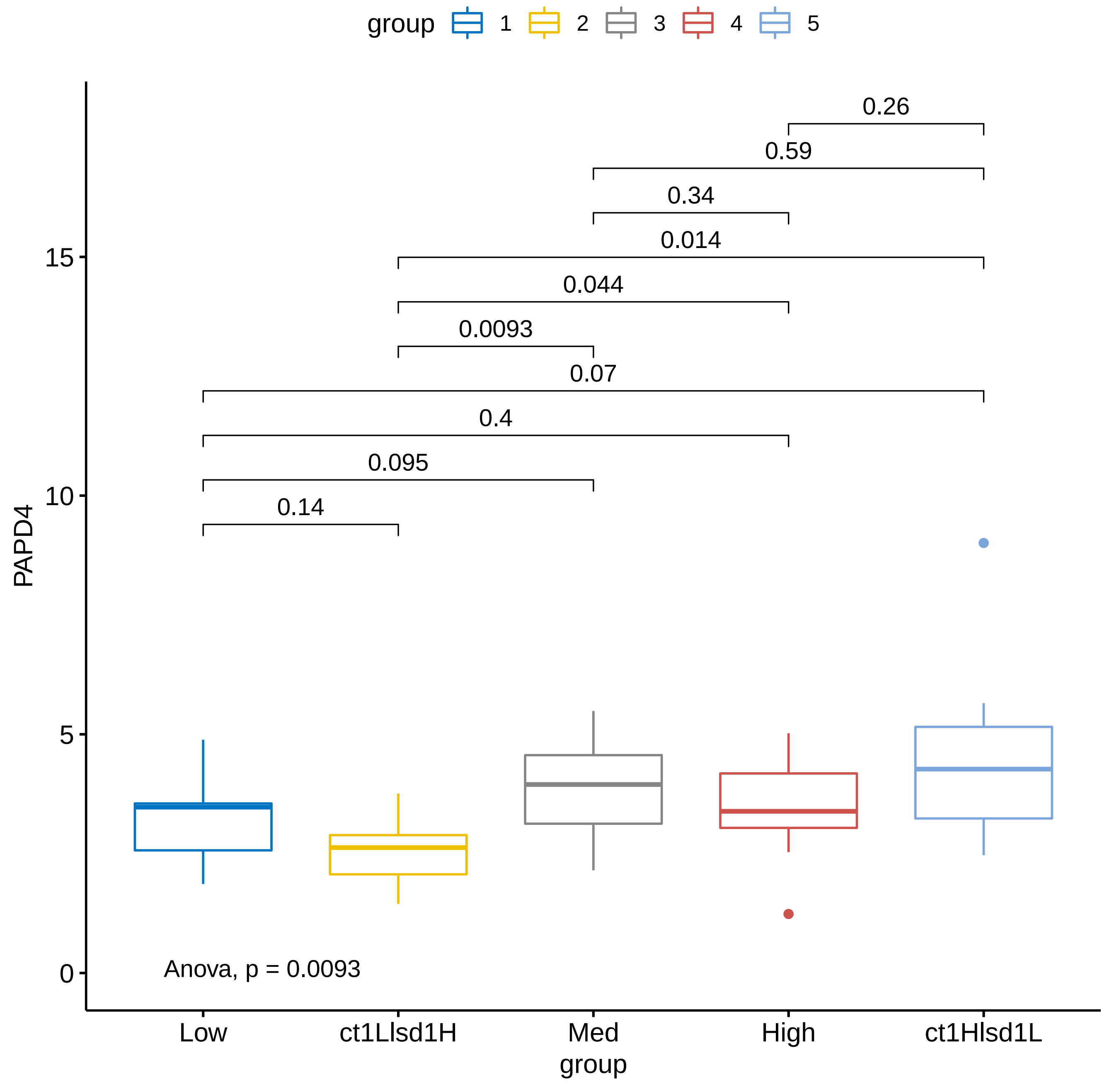

group
1
2
3
4
5
0.26
0.59
0.34
0.014
0.044
0.0093
0.07
0.4
0.095
0.14
15
10
5
0
PAPD4
Anova, p = 0.0093
Low
ct1Llsd1H
Med
High
ct1Hlsd1L
group

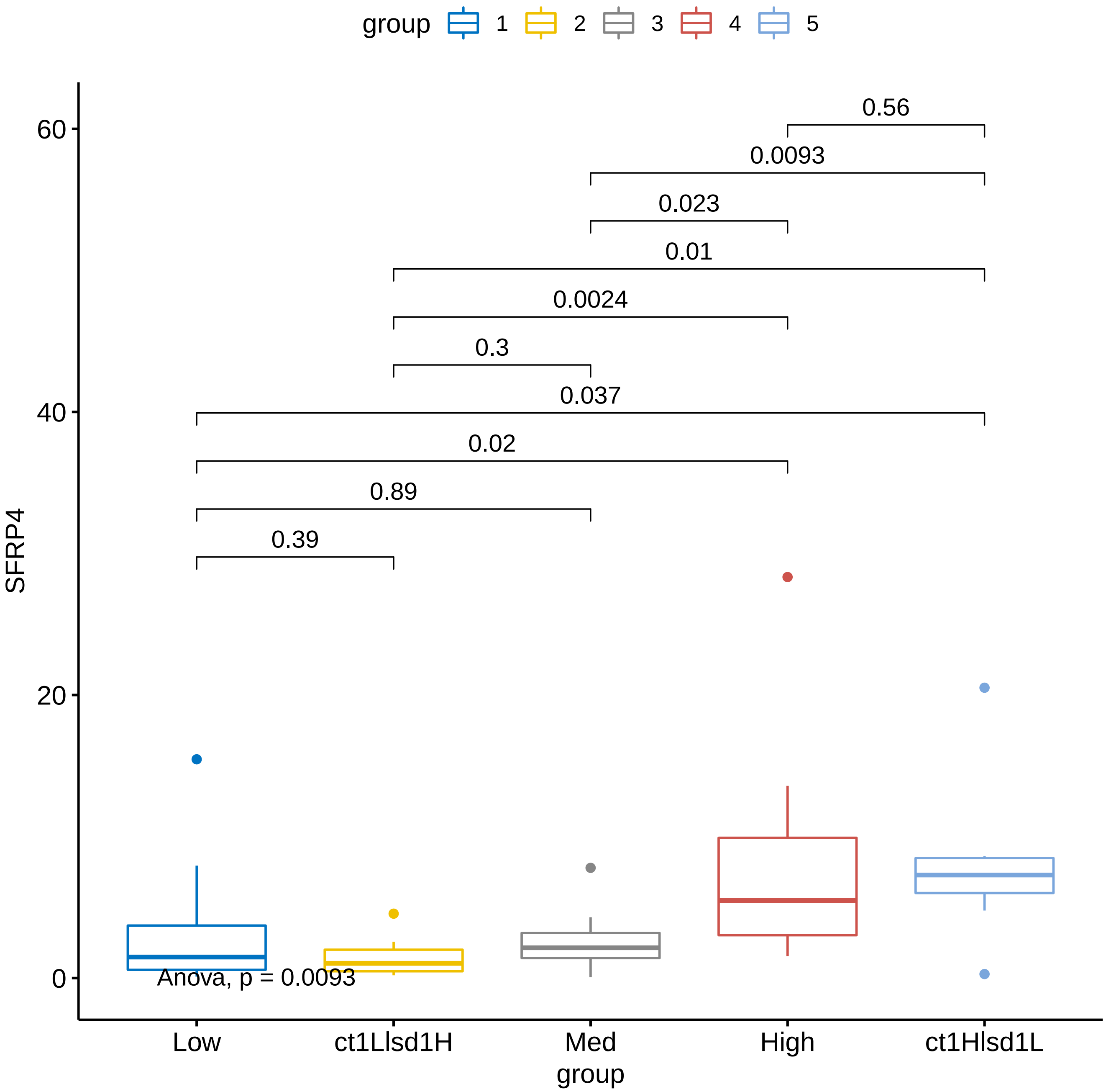

group 1 2 3 4 5
0.56
0.0093
0.023
0.01
0.0024
0.3
0.037
0.02
0.89
0.39
Anova, p = 0.0093
60
40
20
0
SFRP4
Low
ct1Llsd1H
Med
High
ct1Hlsd1L
group

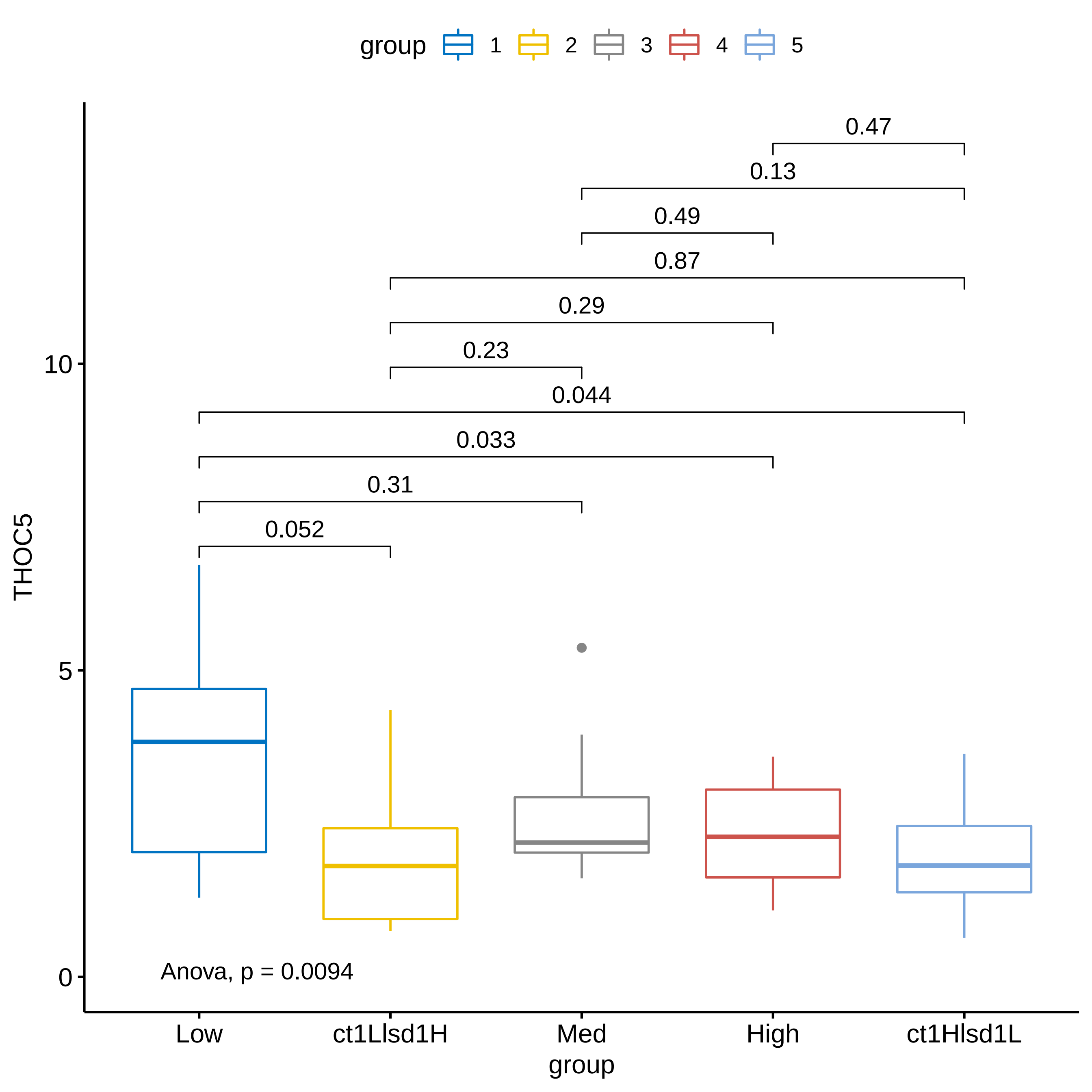
group
1
2
3
4
5
0.47
0.13
0.49
0.87
0.29
0.23
0.044
0.033
0.31
0.052
10
5
0
THOC5
Anova, p = 0.0094
Low
ct1Llsd1H
Med
High
ct1Hlsd1L
group

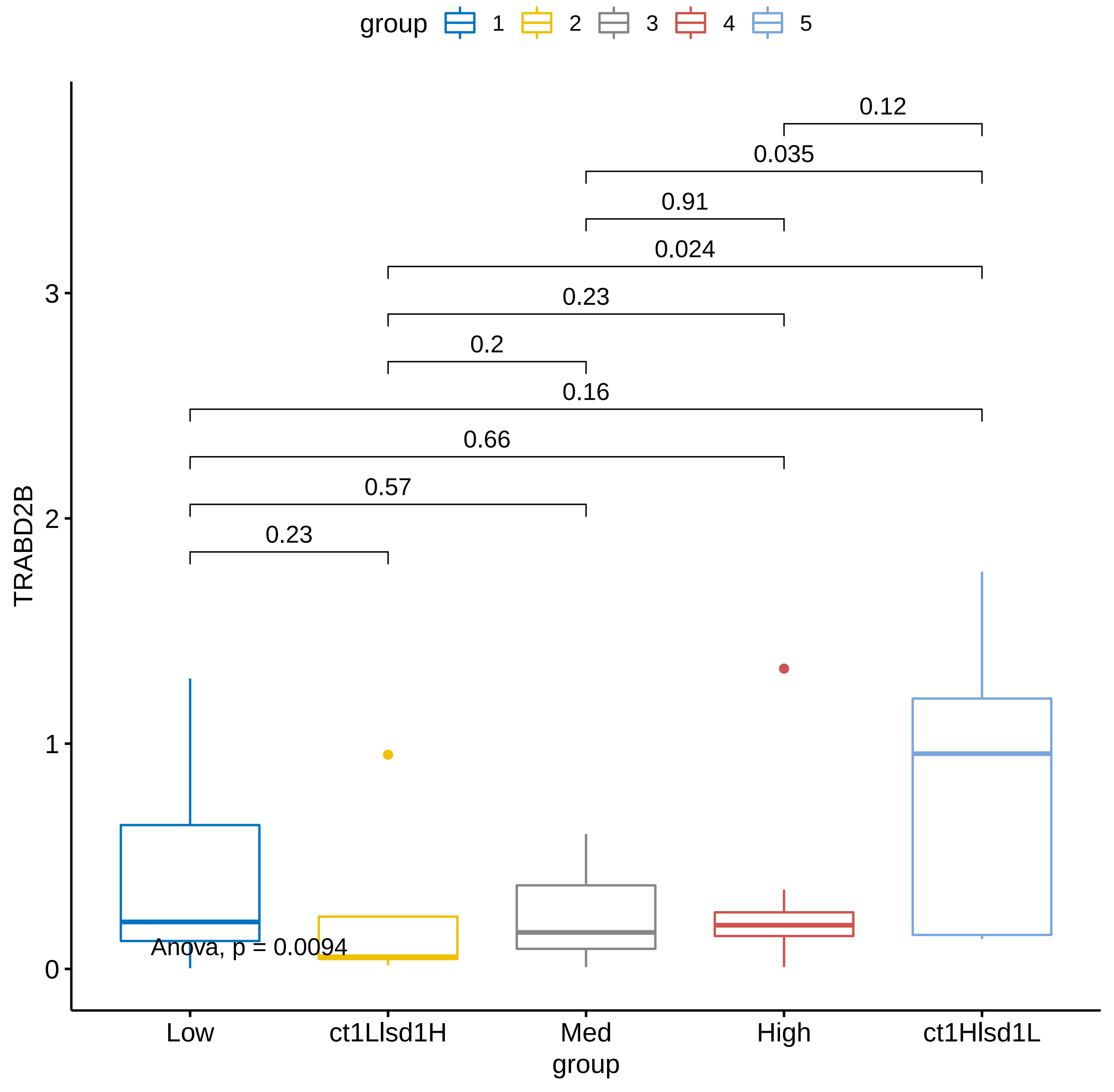

group
1
2
3
4
5
TRABD2B
0
1
2
3
0.12
0.035
0.91
0.024
0.23
0.2
0.16
0.66
0.57
0.23
Anova, p = 0.0094
Low
ct1Llsd1H
Med
High
ct1Hlsd1L
group

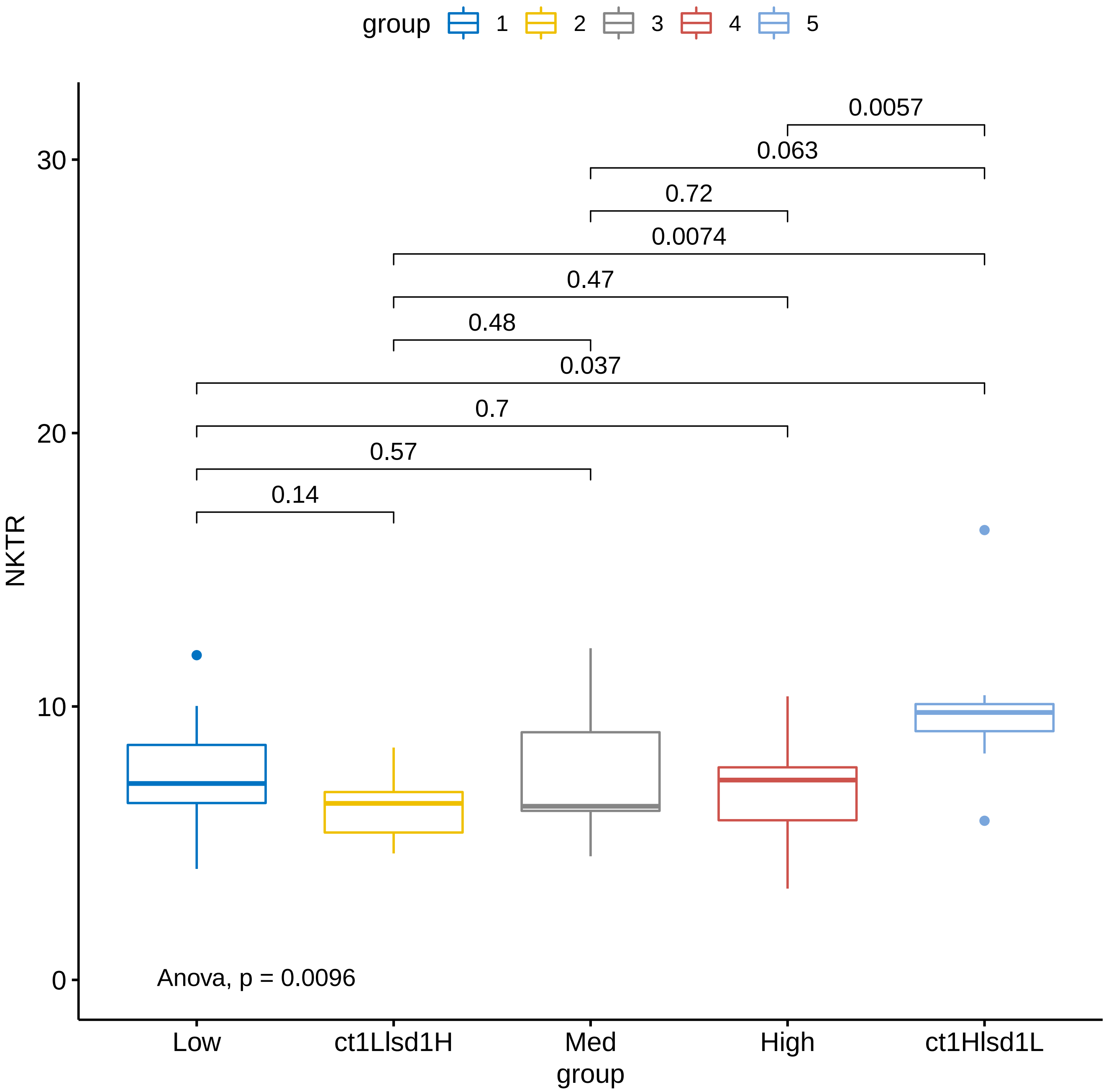
group
1
2
3
4
5
NKTR
30
20
10
0
0.0057
0.063
0.72
0.0074
0.47
0.48
0.037
0.7
0.57
0.14
Anova, p = 0.0096
Low
ct1Llsd1H
Med
High
ct1Hlsd1L
group

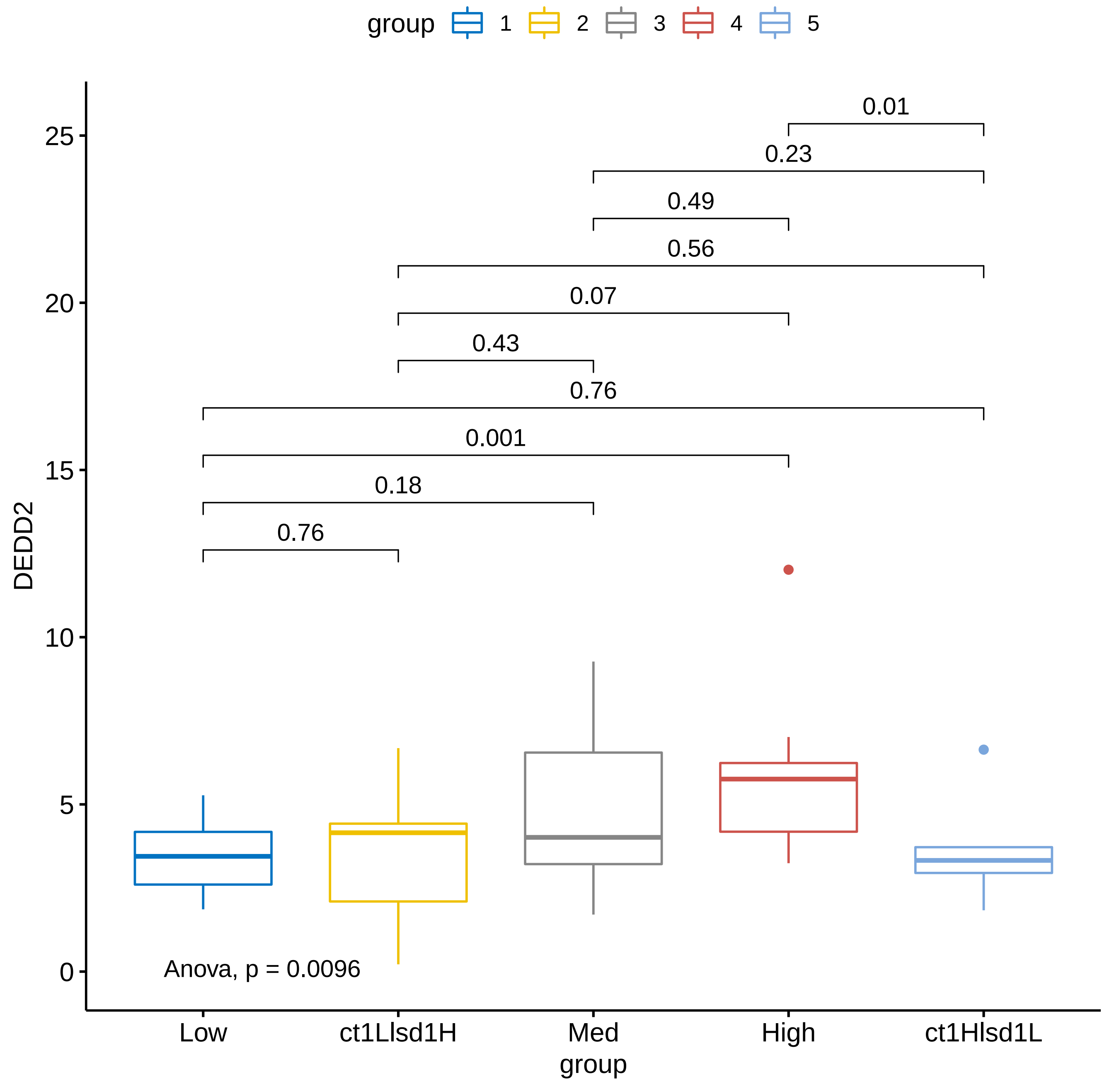

group 1 2 3 4 5
0.01
0.23
0.49
0.56
0.07
0.43
0.76
0.001
0.18
0.76
25
20
15
10
5
0
DEDD2
Anova, p = 0.0096
Low
ct1Llsd1H
Med
High
ct1Hlsd1L
group

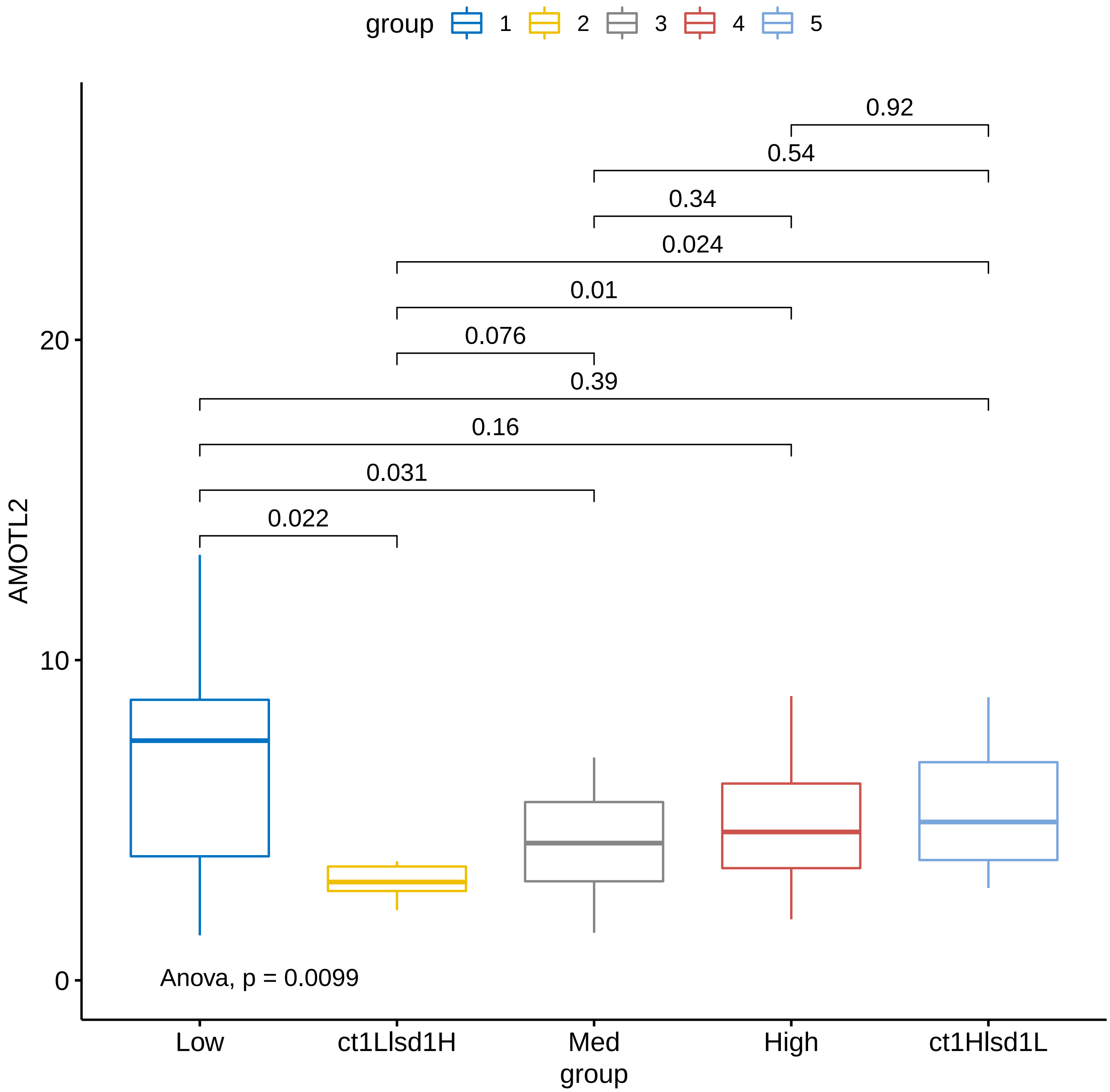

group 1 2 3 4 5
0.92
0.54
0.34
0.024
0.01
0.076
0.39
0.16
0.031
0.022
AMOTL2
20
10
0
Anova, p = 0.0099
Low
ct1Llsd1H
Med
High
ct1Hlsd1L
group

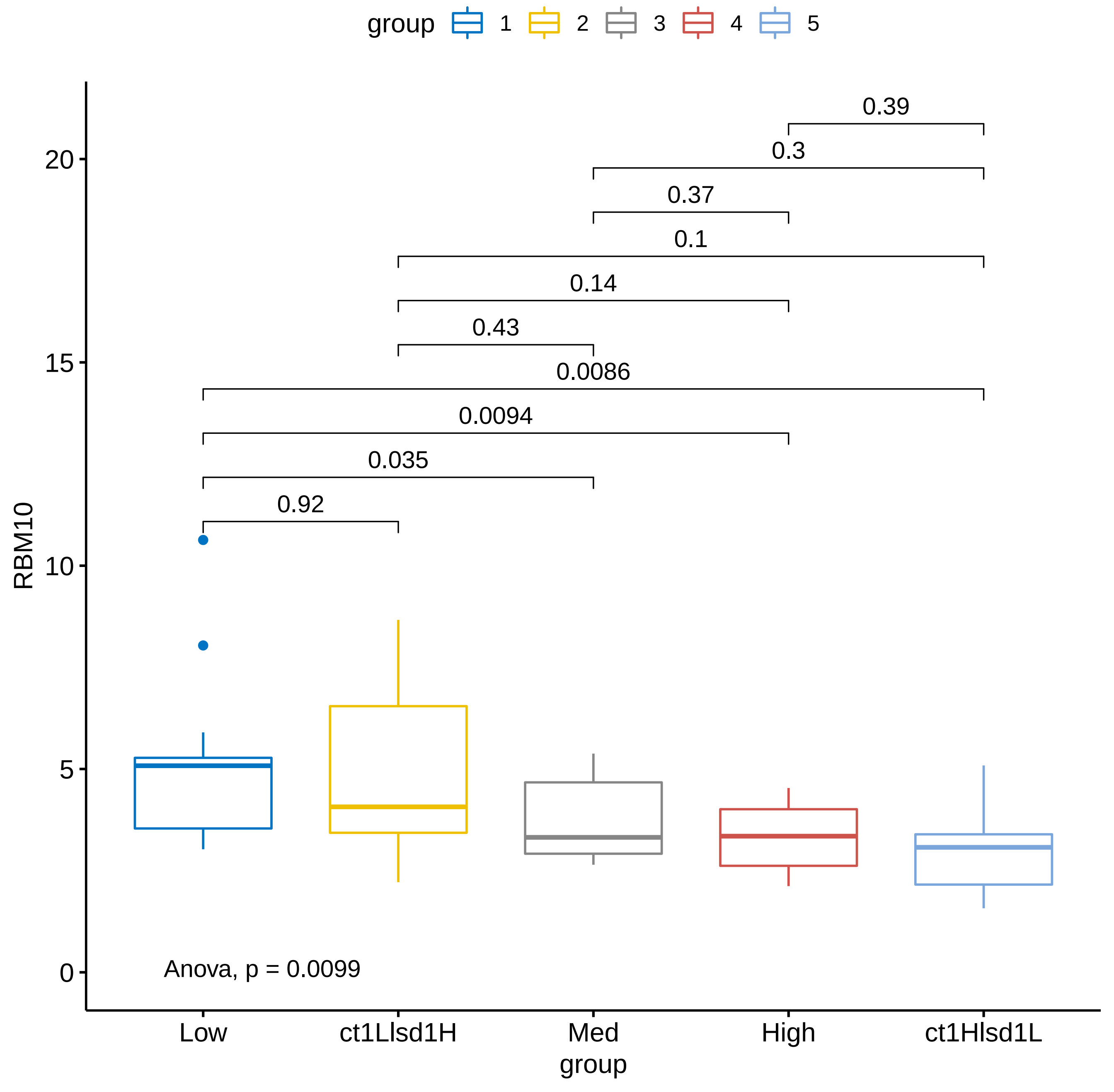

group
1
2
3
4
5
0.39
0.3
0.37
0.1
0.14
0.43
0.0086
0.0094
0.035
0.92
RBM10
20
15
10
5
0
Anova, p = 0.0099
Low
ct1Llsd1H
Med
High
ct1Hlsd1L
group

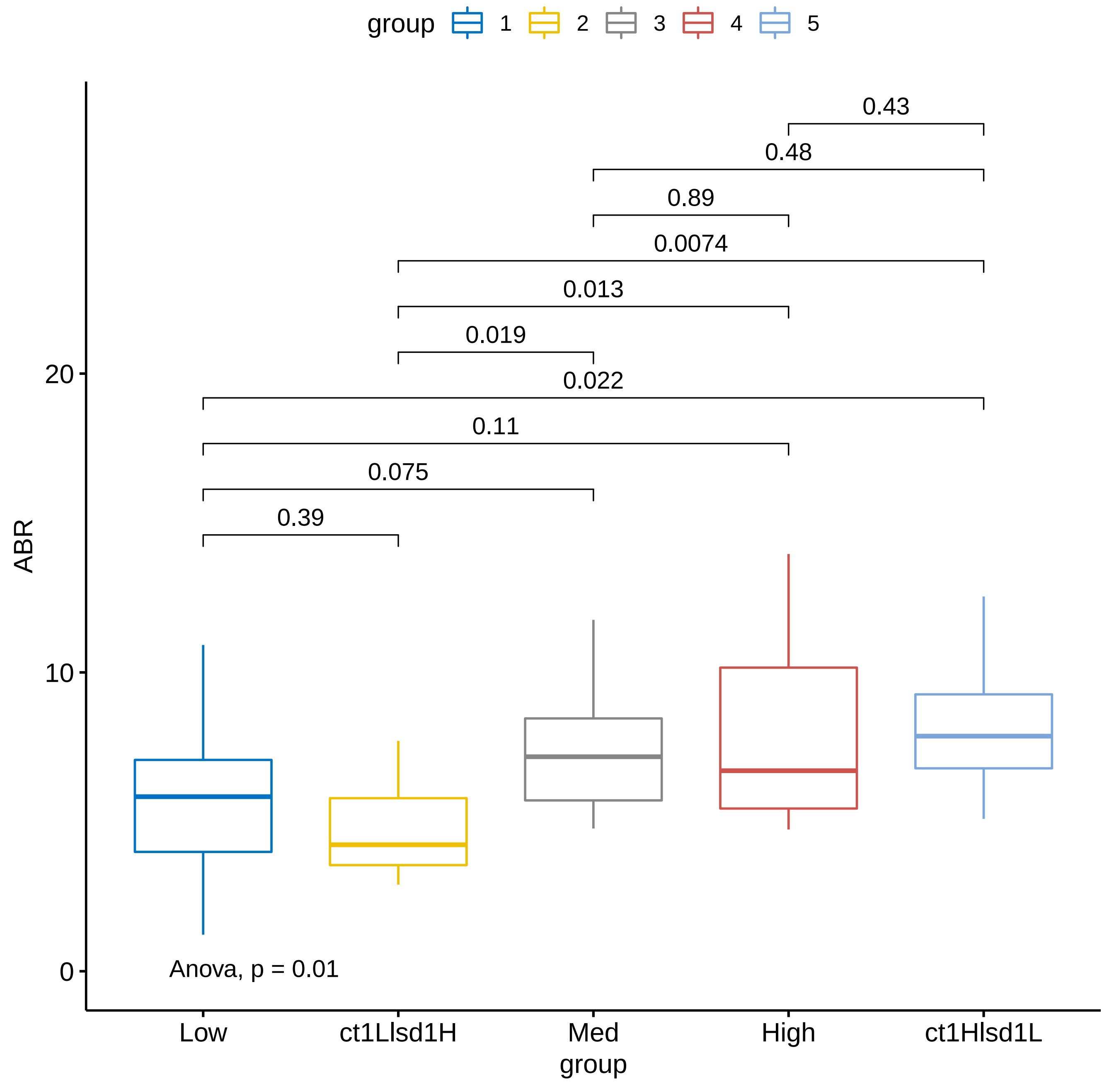

group 1 2 3 4 5
0.43
0.48
0.89
0.0074
0.013
0.019
0.022
0.11
0.075
0.39
ABR
20
10
0
Anova, p = 0.01
Low
ct1Llsd1H
Med
High
ct1Hlsd1L
group

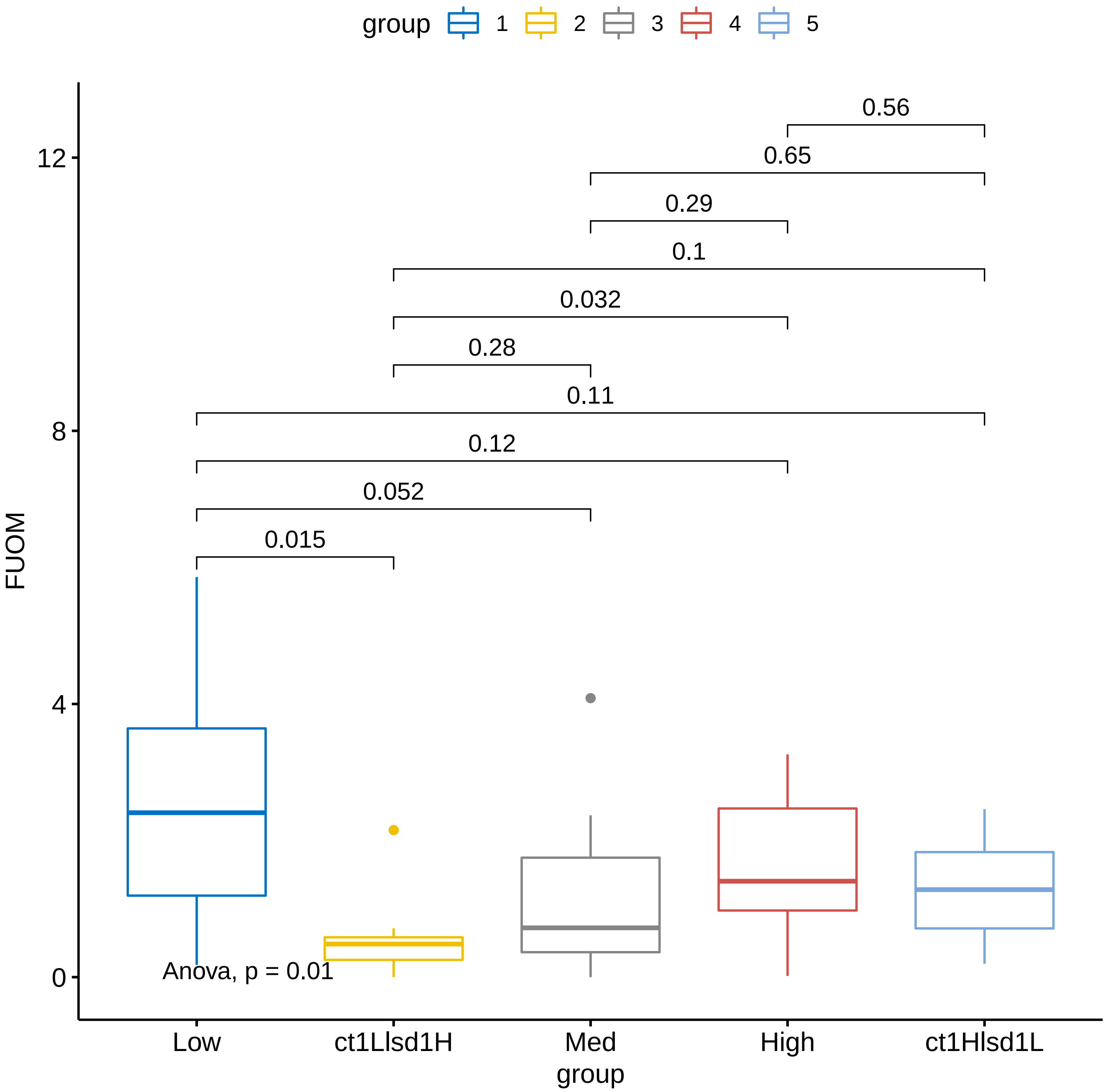
group
1
2
3
4
5
0.56
0.65
0.29
0.1
0.032
0.28
0.11
0.12
0.052
0.015
Anova, p = 0.01
FUOM
12
8
4
0
Low
ct1Llsd1H
Med
High
ct1Hlsd1L
group

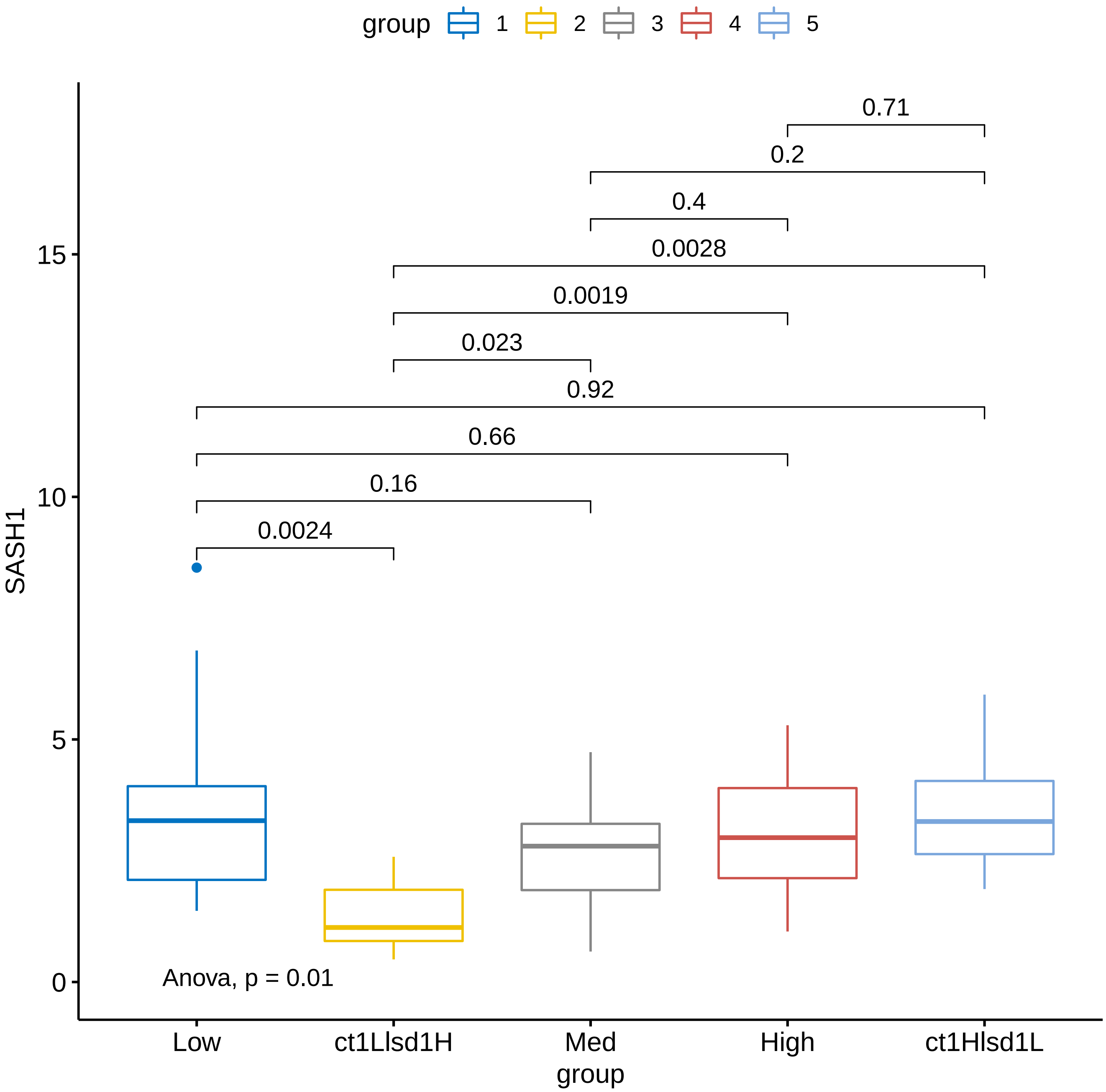

group
1
2
3
4
5
0.71
0.2
0.4
0.0028
0.0019
0.023
0.92
0.66
0.16
0.0024
15
10
5
0
SASH1
Anova, p = 0.01
Low
ct1Llsd1H
Med
High
ct1Hlsd1L
group

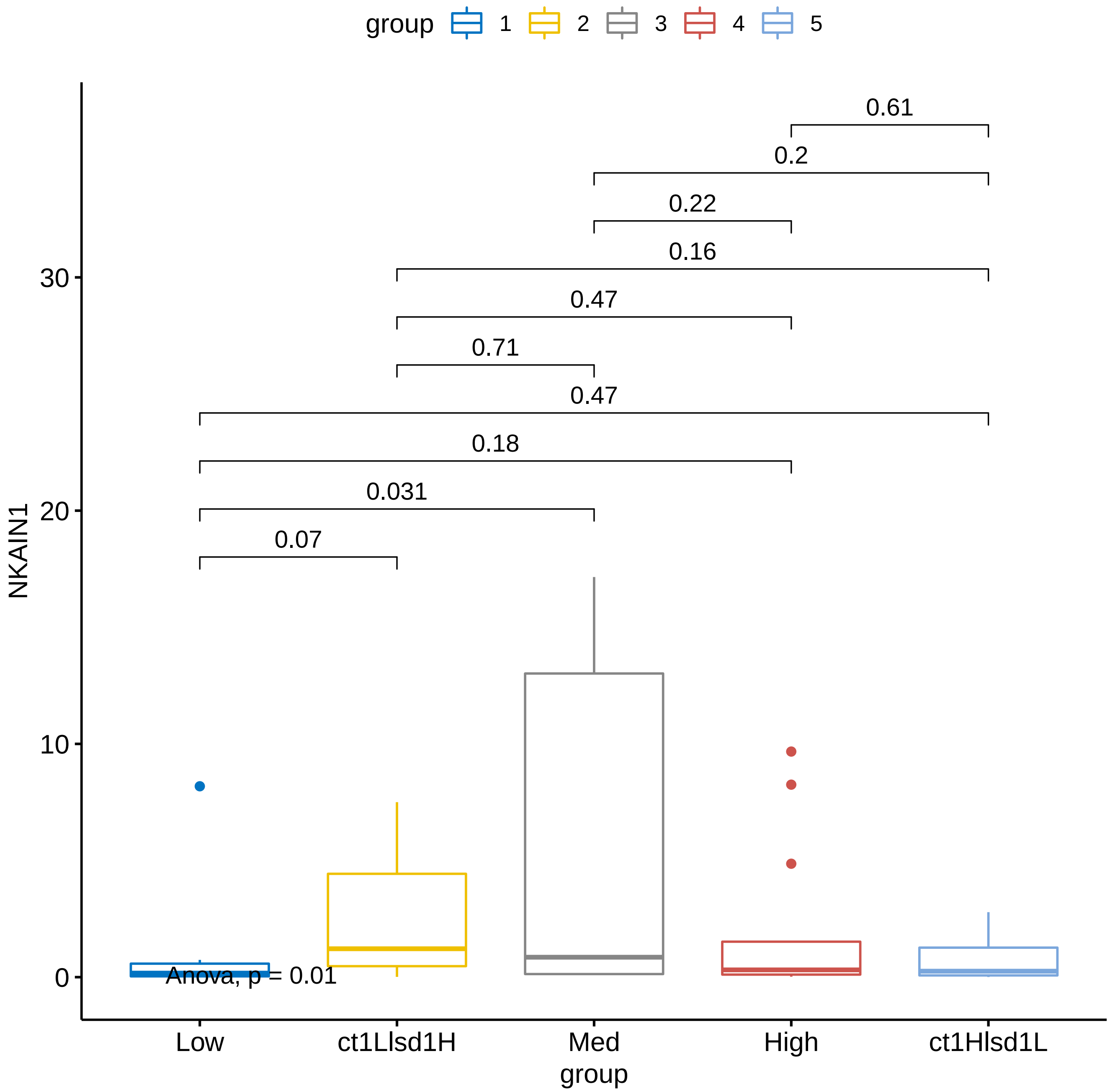
group 1 2 3 4 5
0.61
0.2
0.22
0.16
0.47
0.71
0.47
0.18
0.031
0.07
Anova, p = 0.01
NKAIN1
30
20
10
0
Low
ct1Llsd1H
Med
High
ct1Hlsd1L
group

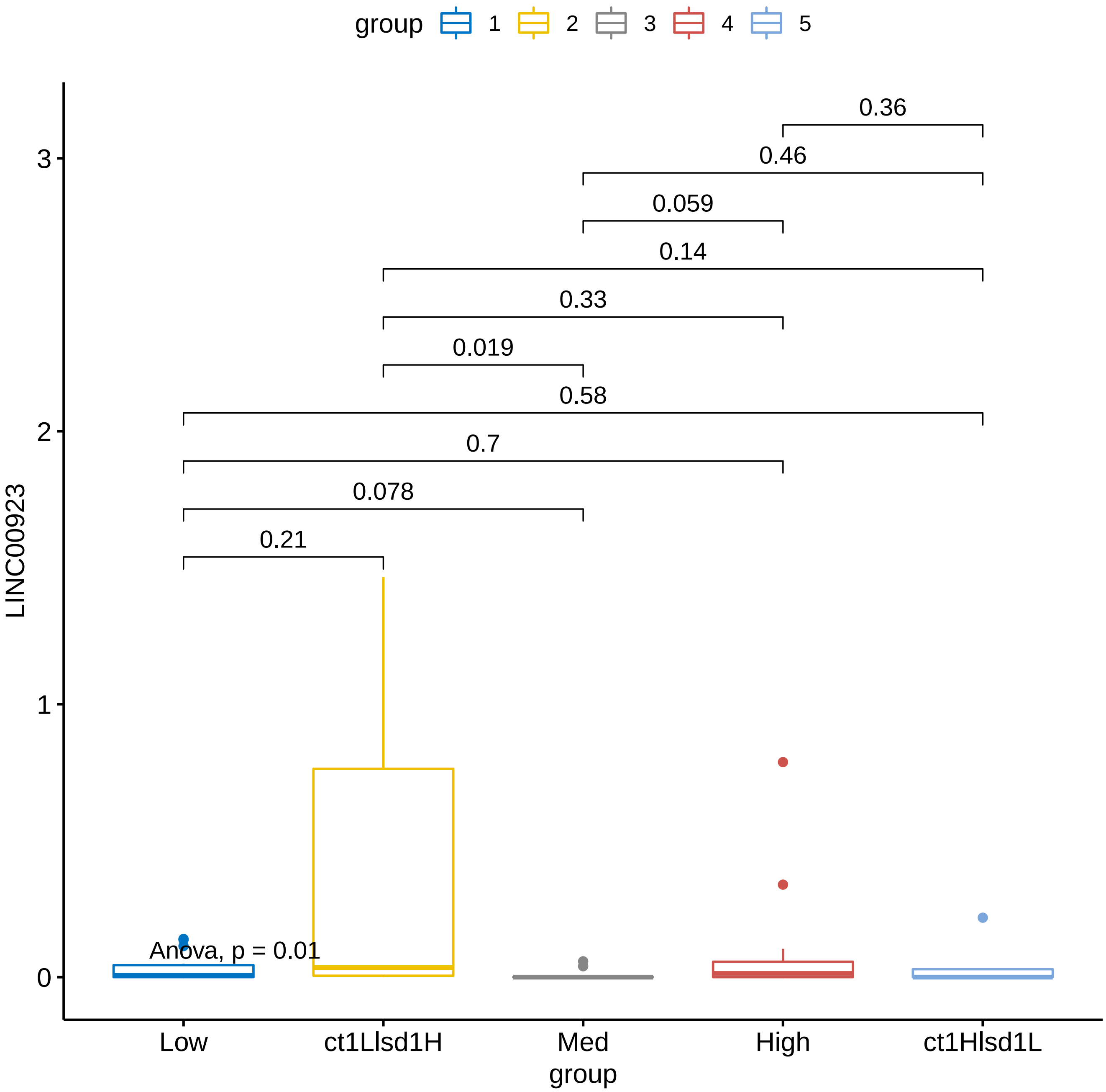

group 1 2 3 4 5
0.36
0.46
0.059
0.14
0.33
0.019
0.58
0.7
0.078
0.21
Anova, p = 0.01
LINC00923
0
1
2
3
Low
ct1Llsd1H
Med
High
ct1Hlsd1L
group

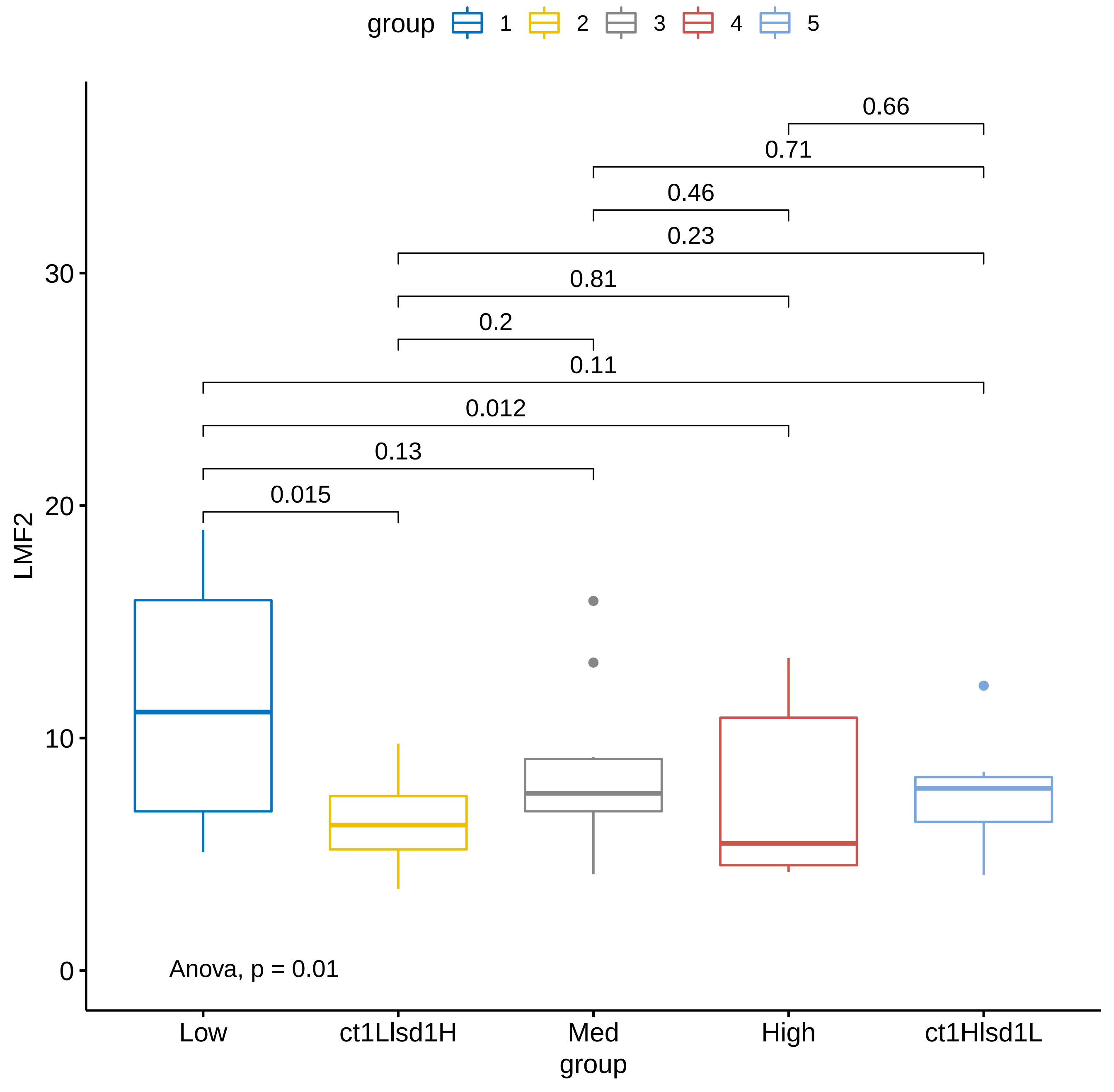

group
1
2
3
4
5
0.66
0.71
0.46
0.23
0.81
0.2
0.11
0.012
0.13
0.015
30
20
10
0
LMF2
Anova, p = 0.01
Low
ct1Llsd1H
Med
High
ct1Hlsd1L
group

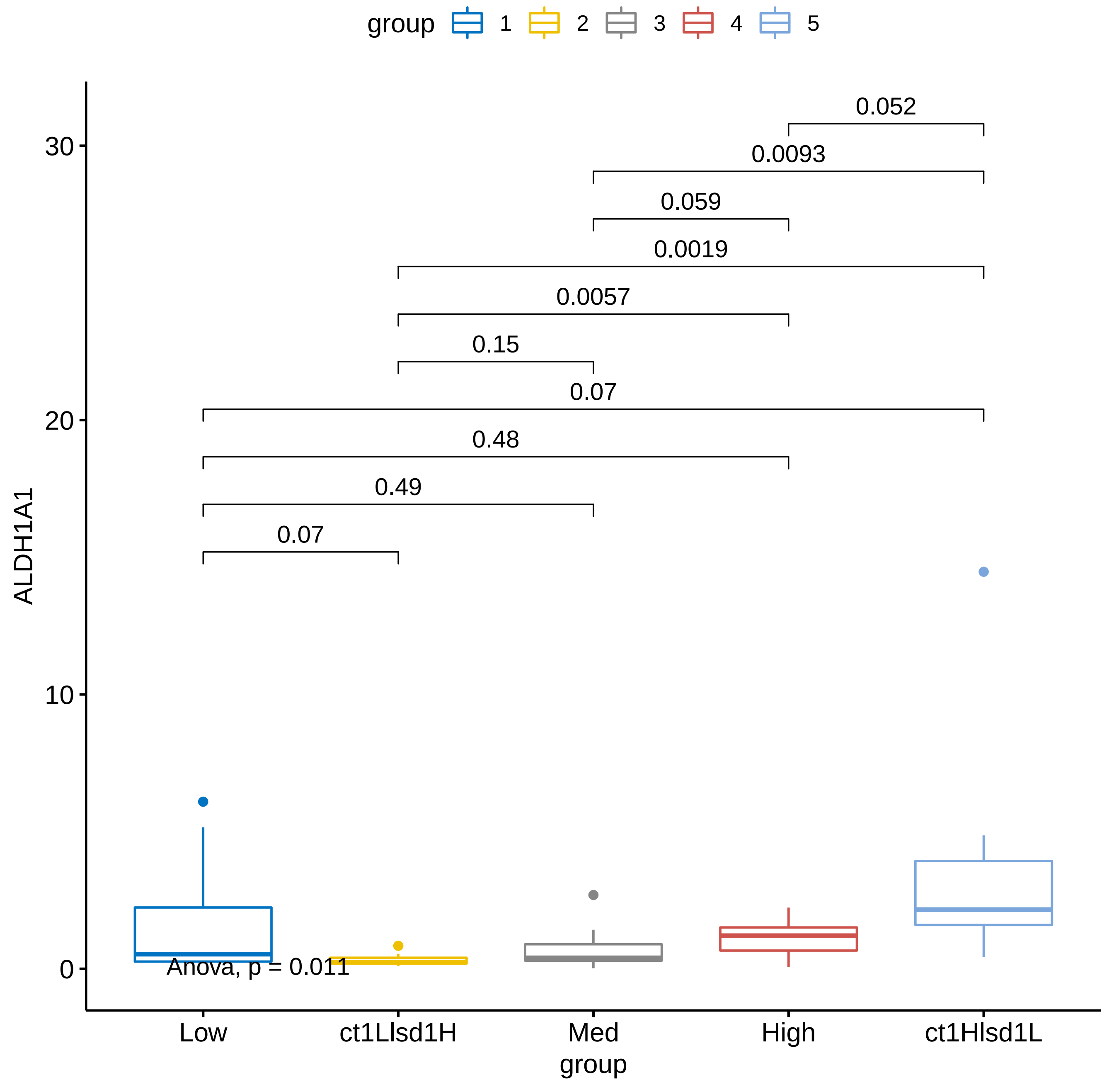
group
1
2
3
4
5
0.052
0.0093
0.059
0.0019
0.0057
0.15
0.07
0.48
0.49
0.07
Anova, p = 0.011
30
20
10
0
ALDH1A1
Low
ct1Llsd1H
Med
High
ct1Hlsd1L
group

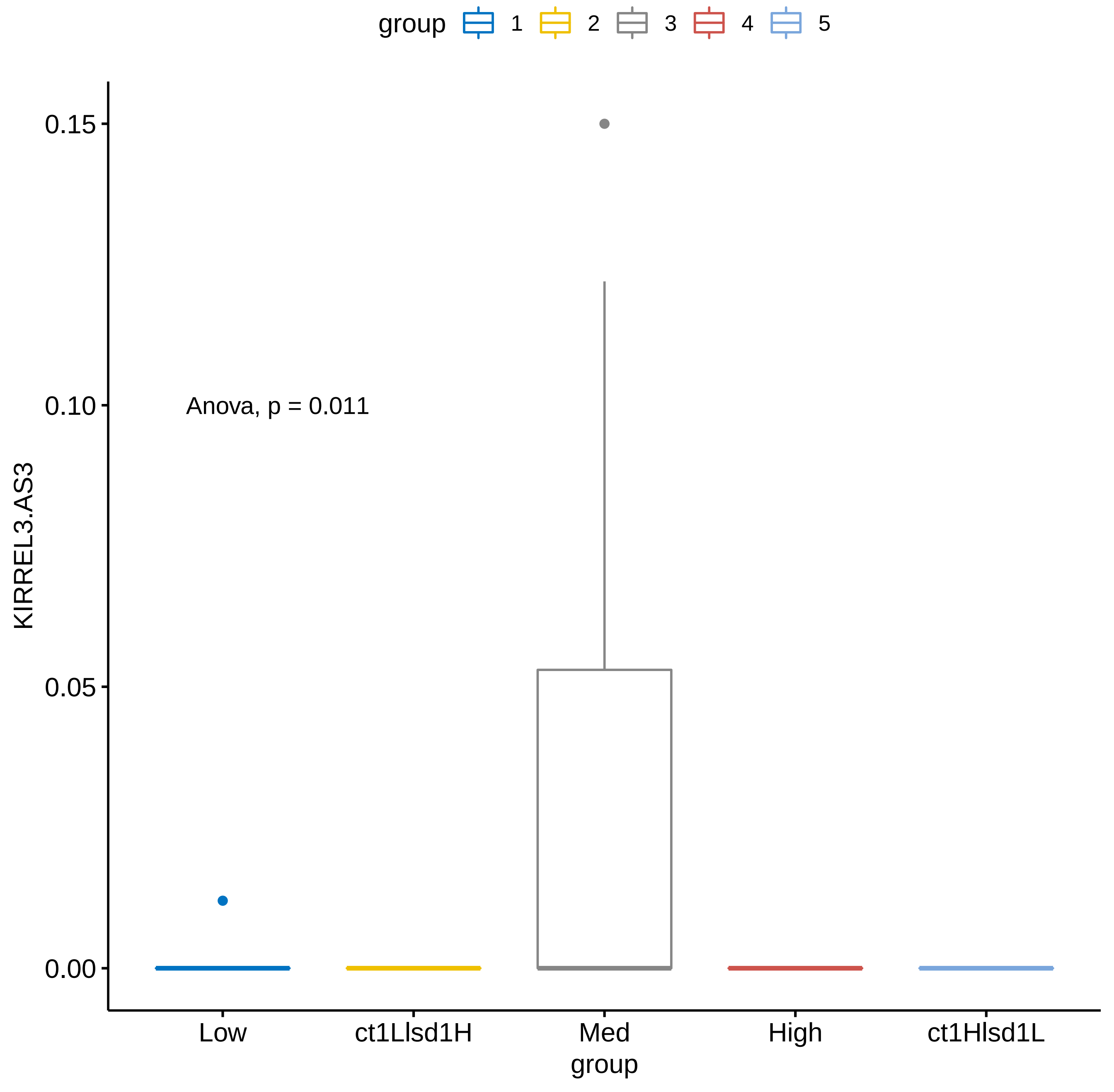
group
1
2
3
4
5
0.15
0.10
0.05
0.00
KIRREL3.AS3
Anova, p = 0.011
Low
ct1Llsd1H
Med
High
ct1Hlsd1L
group

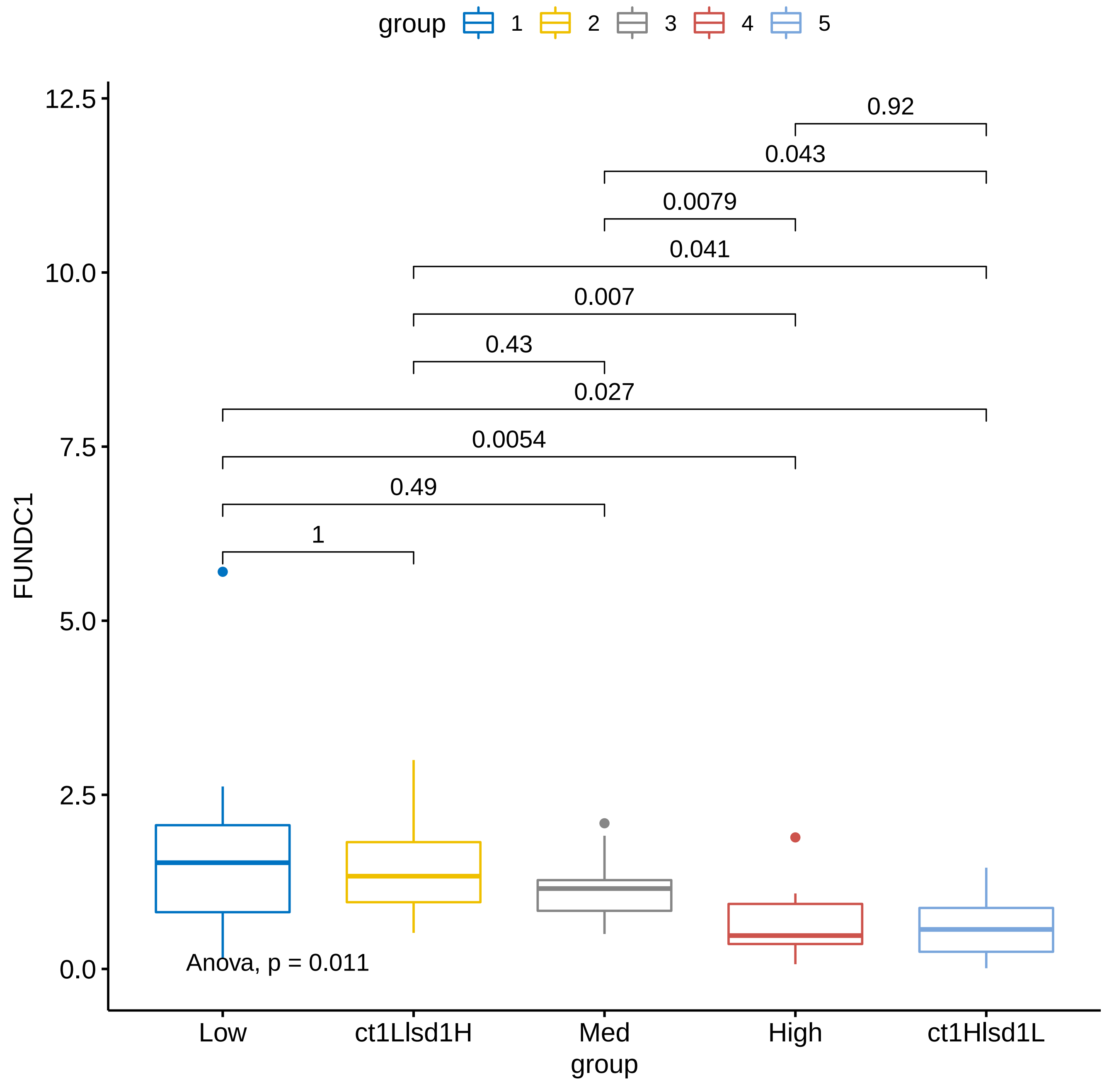

group 1 2 3 4 5
FUNDC1
12.5
10.0
7.5
5.0
2.5
0.0
0.92
0.043
0.0079
0.041
0.007
0.43
0.027
0.0054
0.49
1
Anova, p = 0.011
Low
ct1Llsd1H
Med
High
ct1Hlsd1L
group

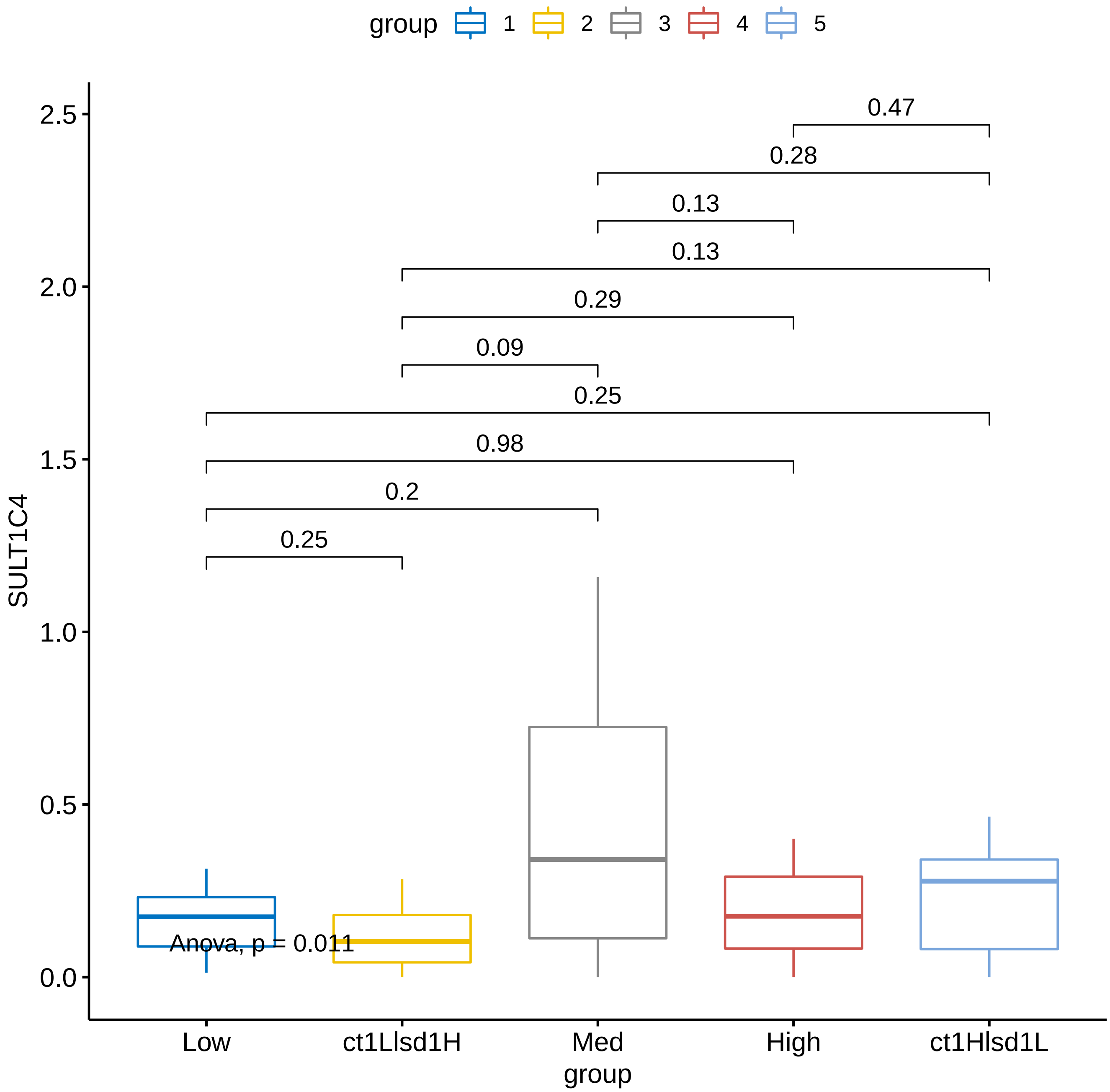

group
1
2
3
4
5
SULT1C4
2.5
2.0
1.5
1.0
0.5
0.0
0.47
0.28
0.13
0.13
0.29
0.09
0.25
0.98
0.2
0.25
Anova, p = 0.011
Low
ct1Llsd1H
Med
High
ct1Hlsd1L
group

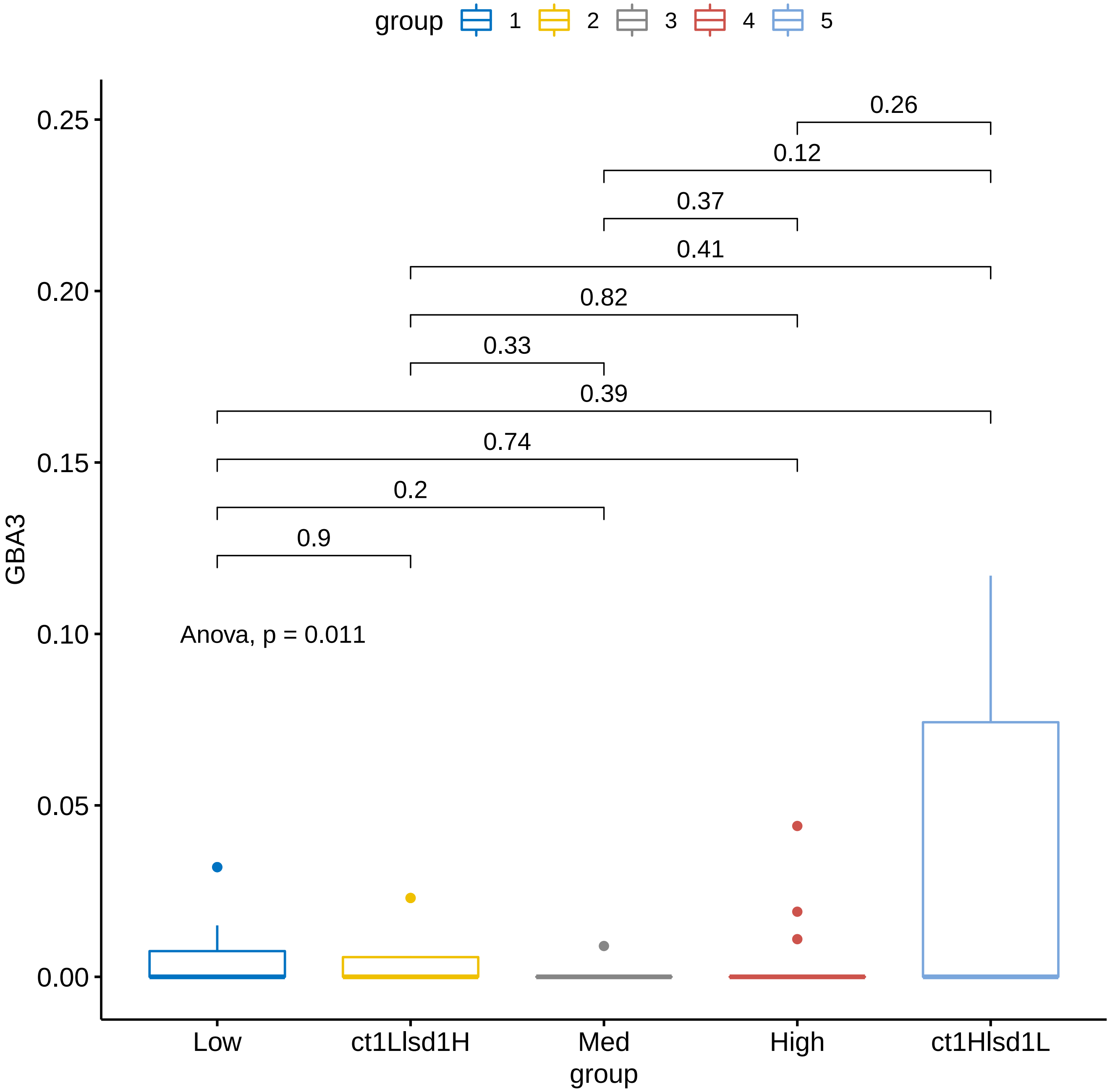

group 1 2 3 4 5
GBA3
0.25
0.20
0.15
0.10
0.05
0.00
0.26
0.12
0.37
0.41
0.82
0.33
0.39
0.74
0.2
0.9
Anova, p = 0.011
Low
ct1Llsd1H
Med
High
ct1Hlsd1L
group

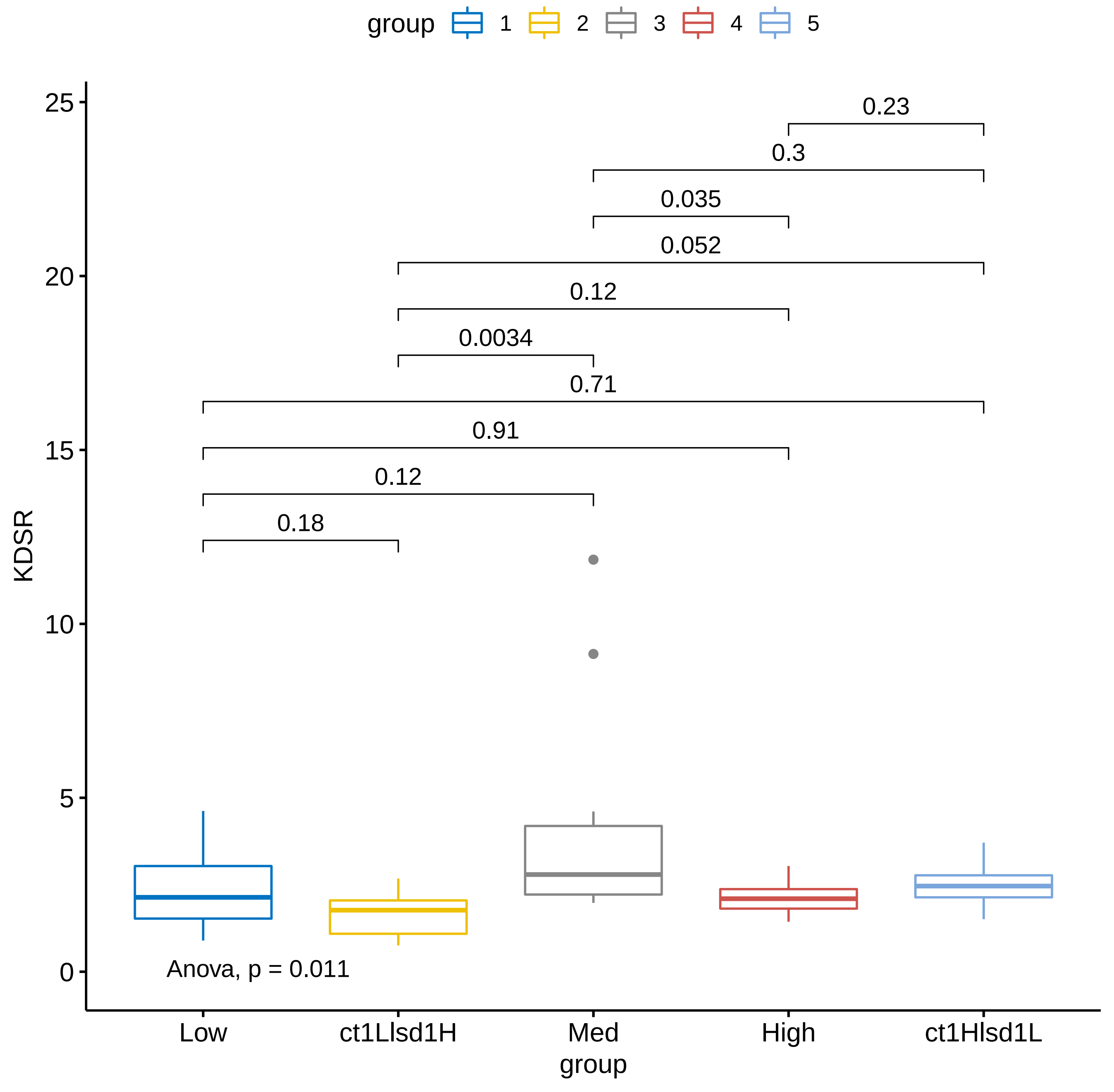

group 1 2 3 4 5
KDSR
25
20
15
10
5
0
0.23
0.3
0.035
0.052
0.12
0.0034
0.71
0.91
0.12
0.18
Anova, p = 0.011
Low
ct1Llsd1H
Med
High
ct1Hlsd1L
group

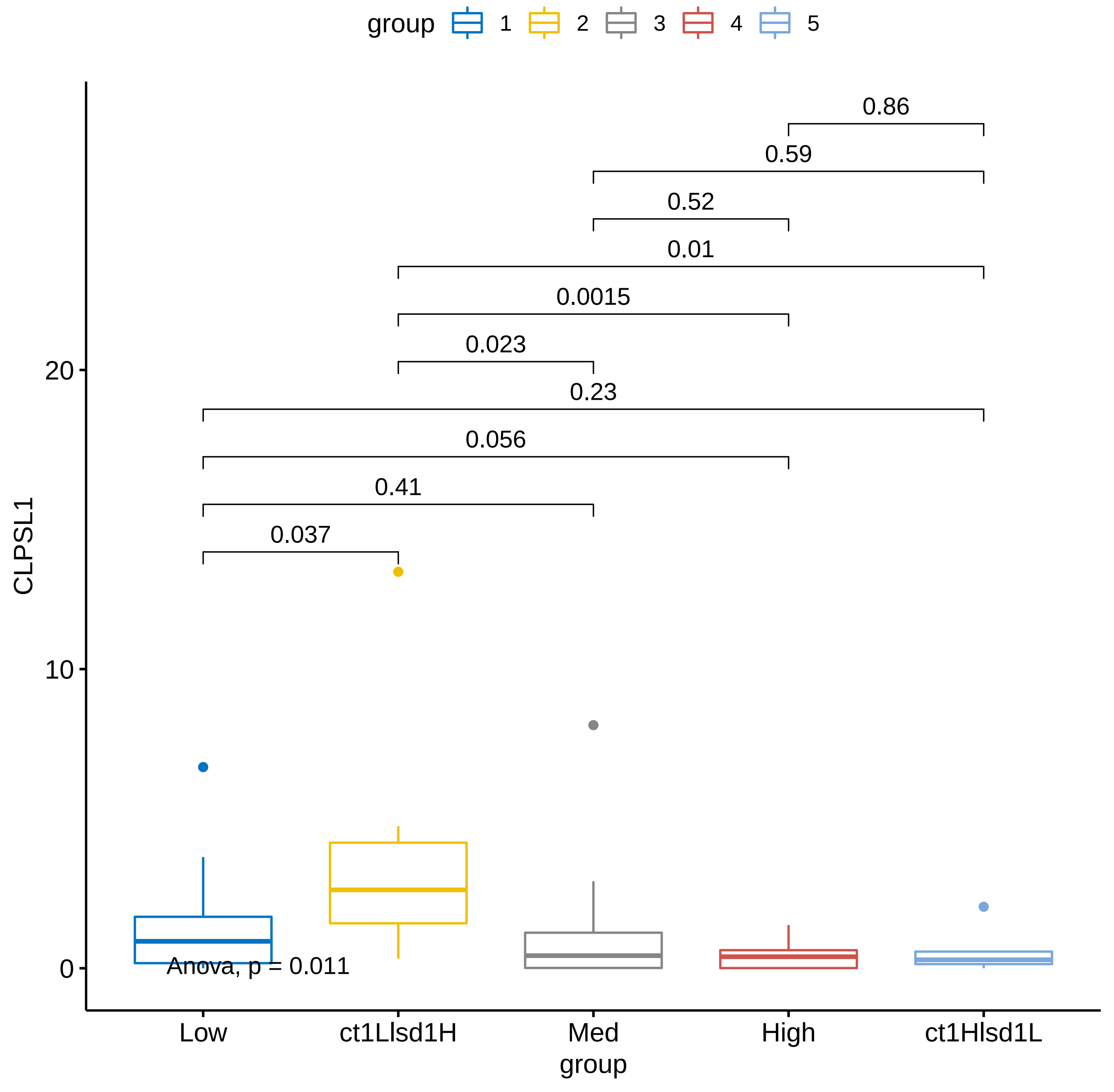

group
1
2
3
4
5
0.86
0.59
0.52
0.01
0.0015
0.023
0.23
0.056
0.41
0.037
20
10
0
CLPSL1
Anova, p = 0.011
Low
ct1Llsd1H
Med
High
ct1Hlsd1L
group

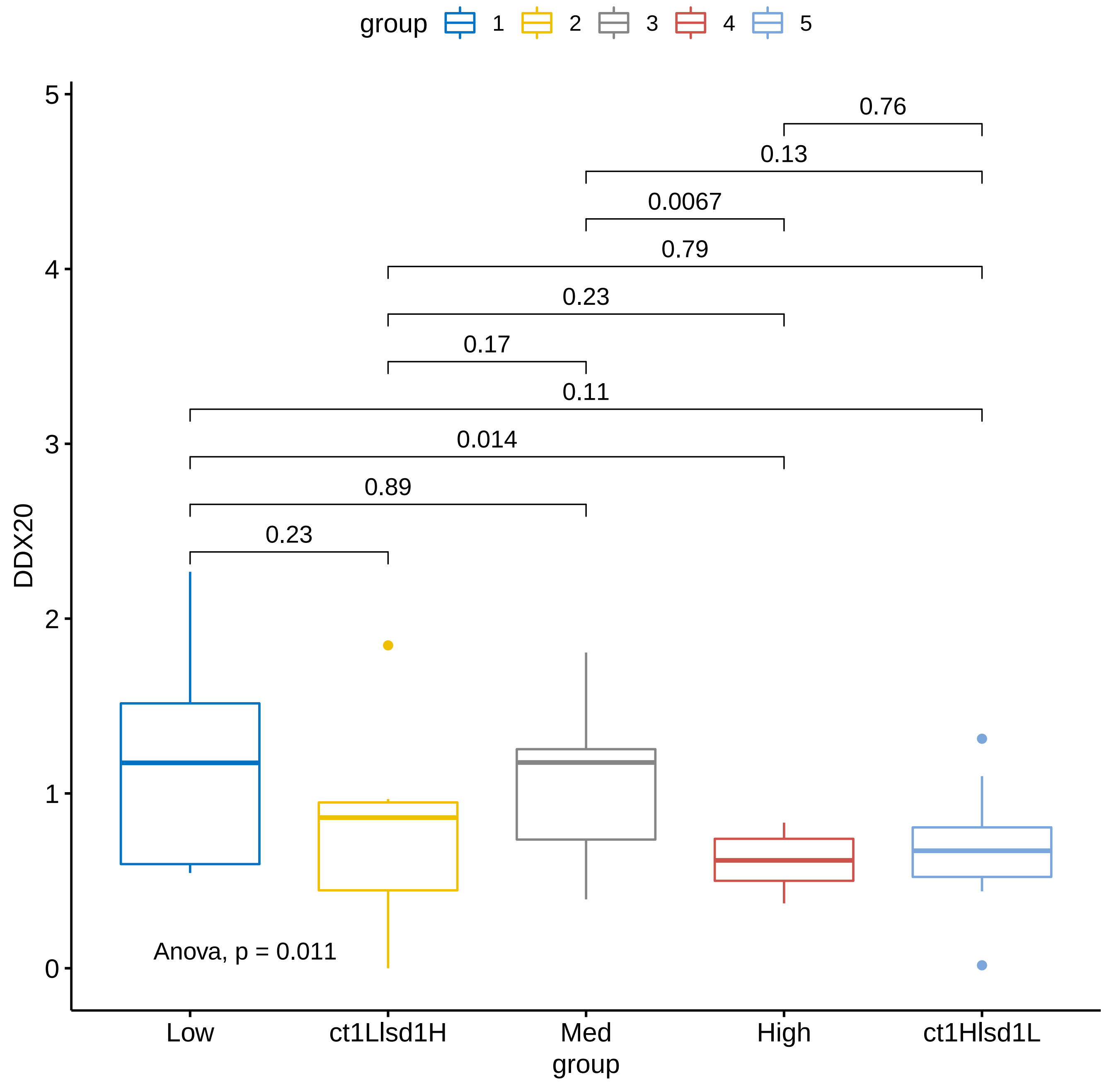

group
1
2
3
4
5
DDX20
0
1
2
3
4
5
0.76
0.13
0.0067
0.79
0.23
0.17
0.11
0.014
0.89
0.23
Anova, p = 0.011
Low
ct1Llsd1H
Med
High
ct1Hlsd1L
group

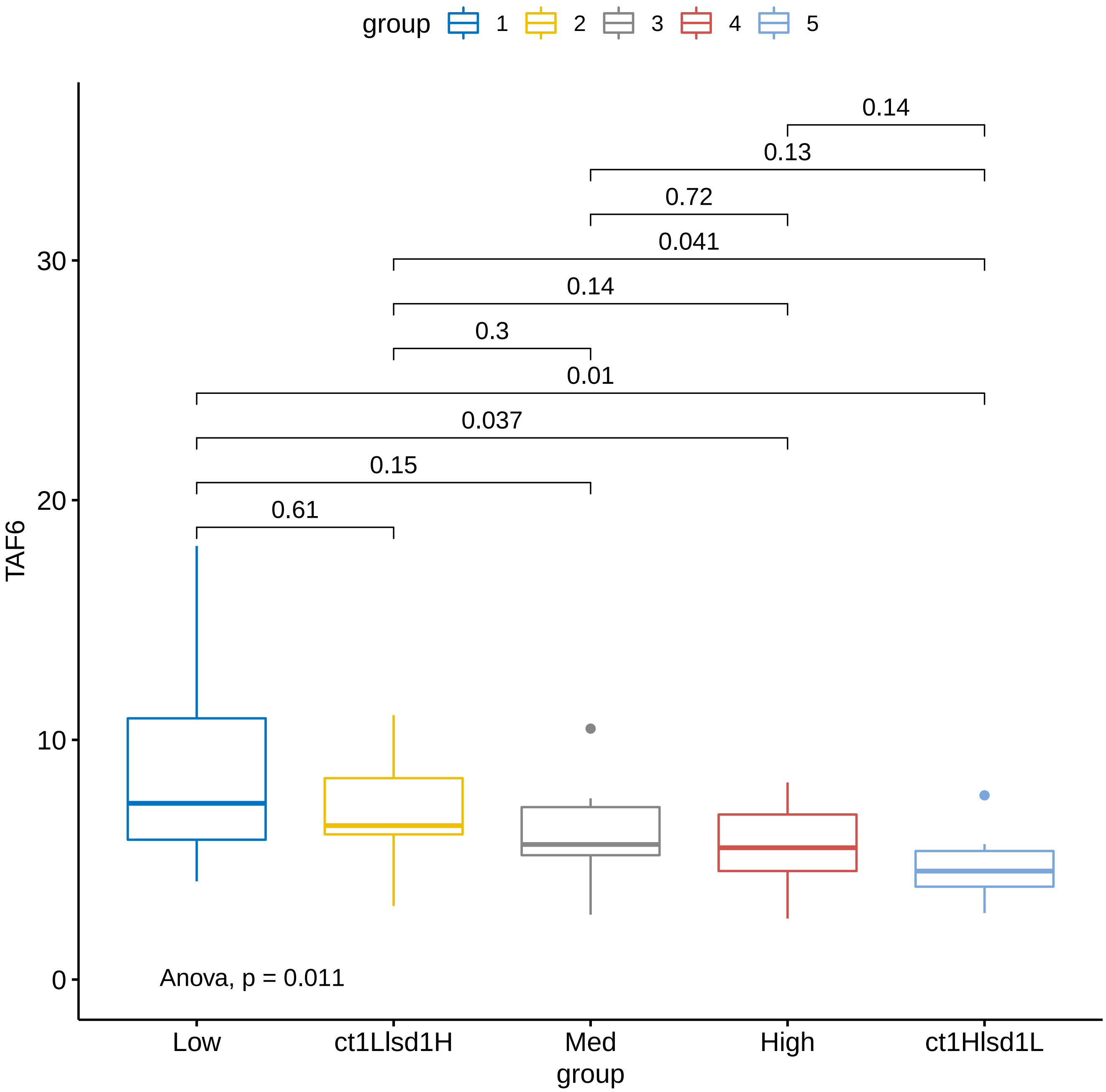
group
1
2
3
4
5
0.14
0.13
0.72
0.041
0.14
0.3
0.01
0.037
0.15
0.61
30
20
10
0
TAF6
Anova, p = 0.011
Low
ct1Llsd1H
Med
High
ct1Hlsd1L
group

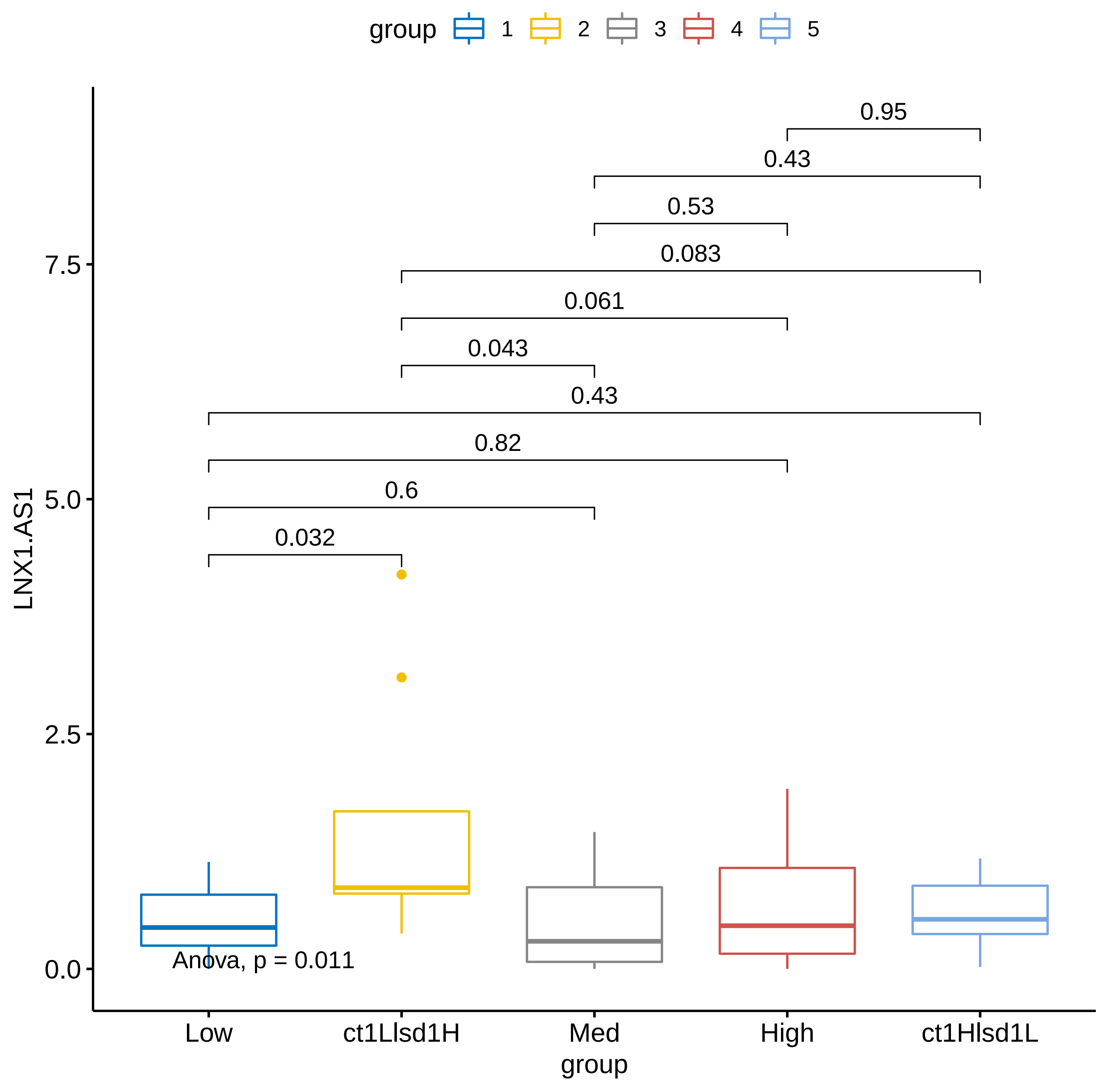

group 1 2 3 4 5
0.95
0.43
0.53
0.083
0.061
0.043
0.43
0.82
0.6
0.032
Anova, p = 0.011
7.5
5.0
2.5
0.0
LNX1.AS1
Low
ct1Llsd1H
Med
High
ct1Hlsd1L
group

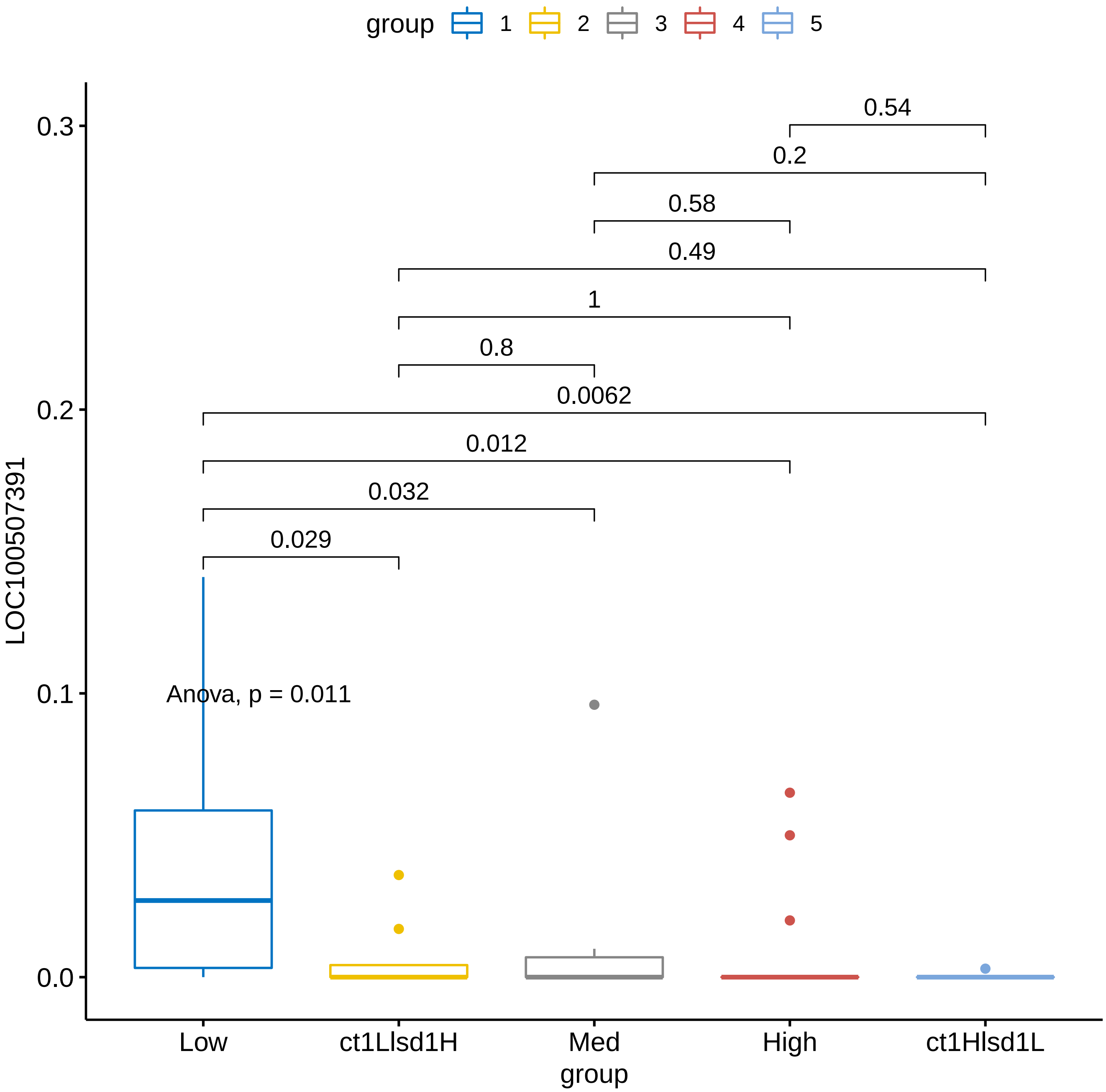

group
1
2
3
4
5
0.54
0.2
0.58
0.49
1
0.8
0.0062
0.012
0.032
0.029
Anova, p = 0.011
0.3
0.2
0.1
0.0
LOC100507391
Low
ct1Llsd1H
Med
High
ct1Hlsd1L
group

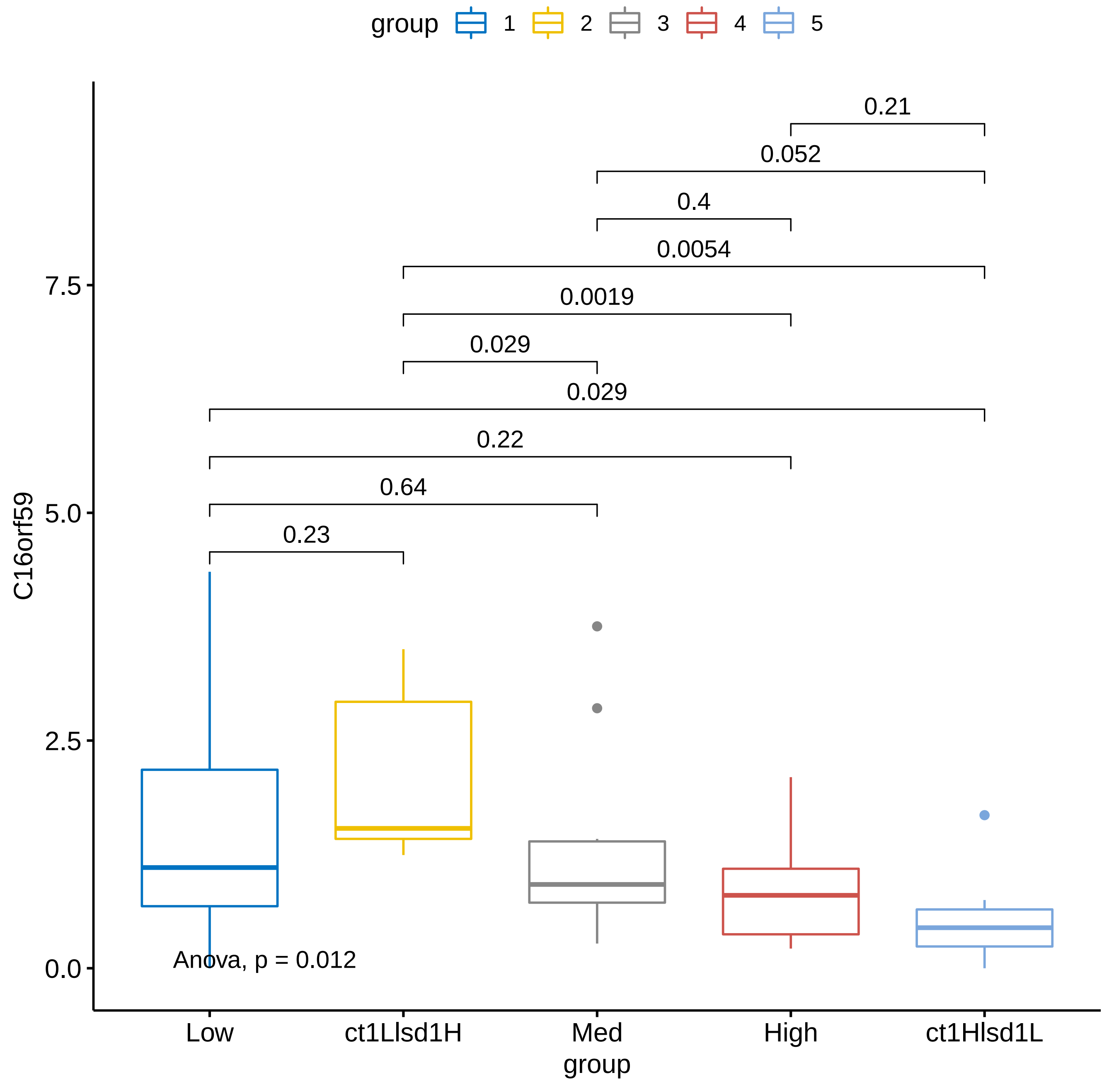

group 1 2 3 4 5
0.21
0.052
0.4
0.0054
0.0019
0.029
0.029
0.22
0.64
0.23
7.5
5.0
2.5
0.0
C16orf59
Anova, p = 0.012
Low
ct1Llsd1H
Med
High
ct1Hlsd1L
group

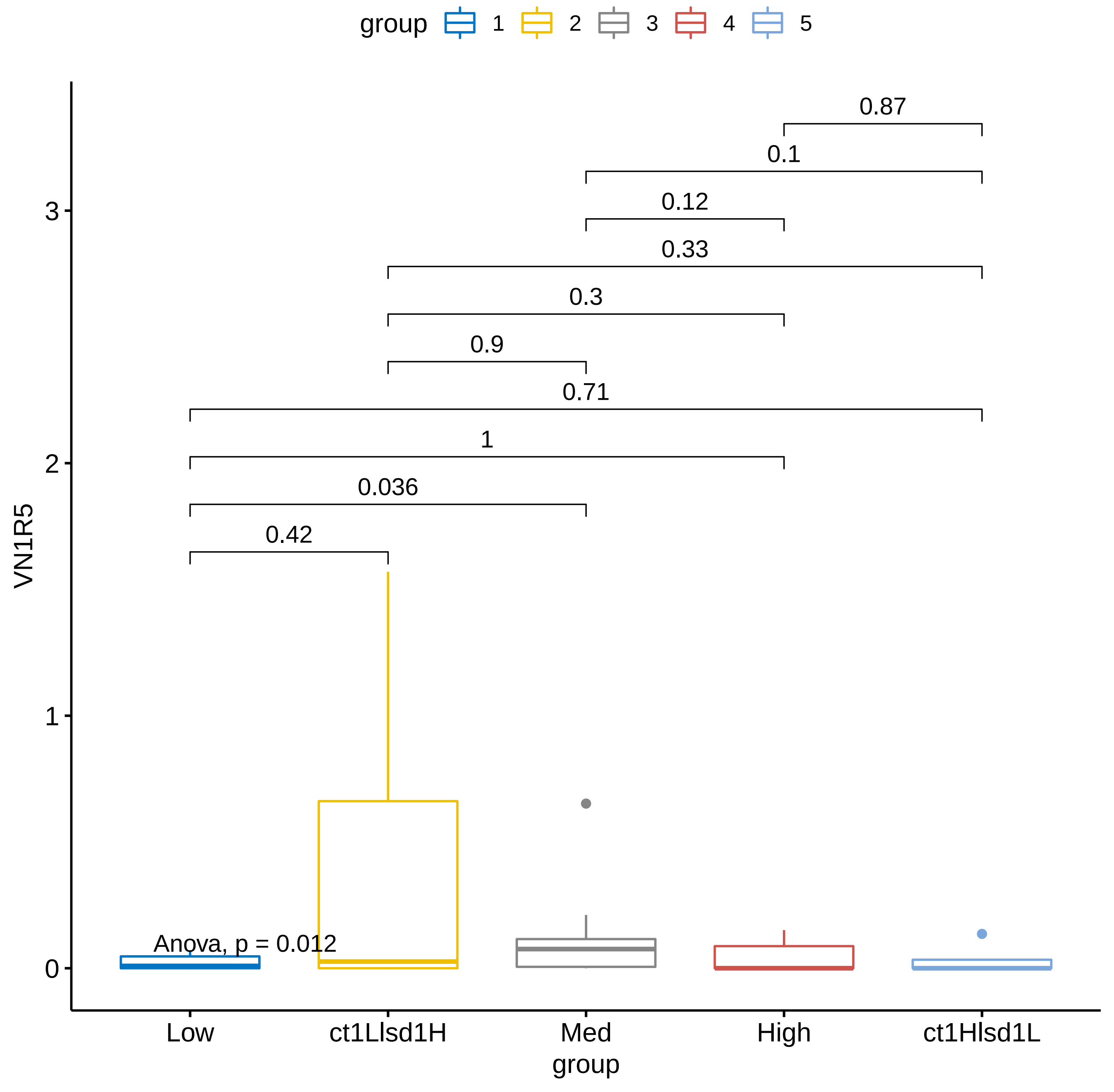

group
1
2
3
4
5
0.87
0.1
0.12
0.33
0.3
0.9
0.71
1
0.036
0.42
Anova, p = 0.012
VN1R5
3
2
1
0
Low
ct1Llsd1H
Med
High
ct1Hlsd1L
group

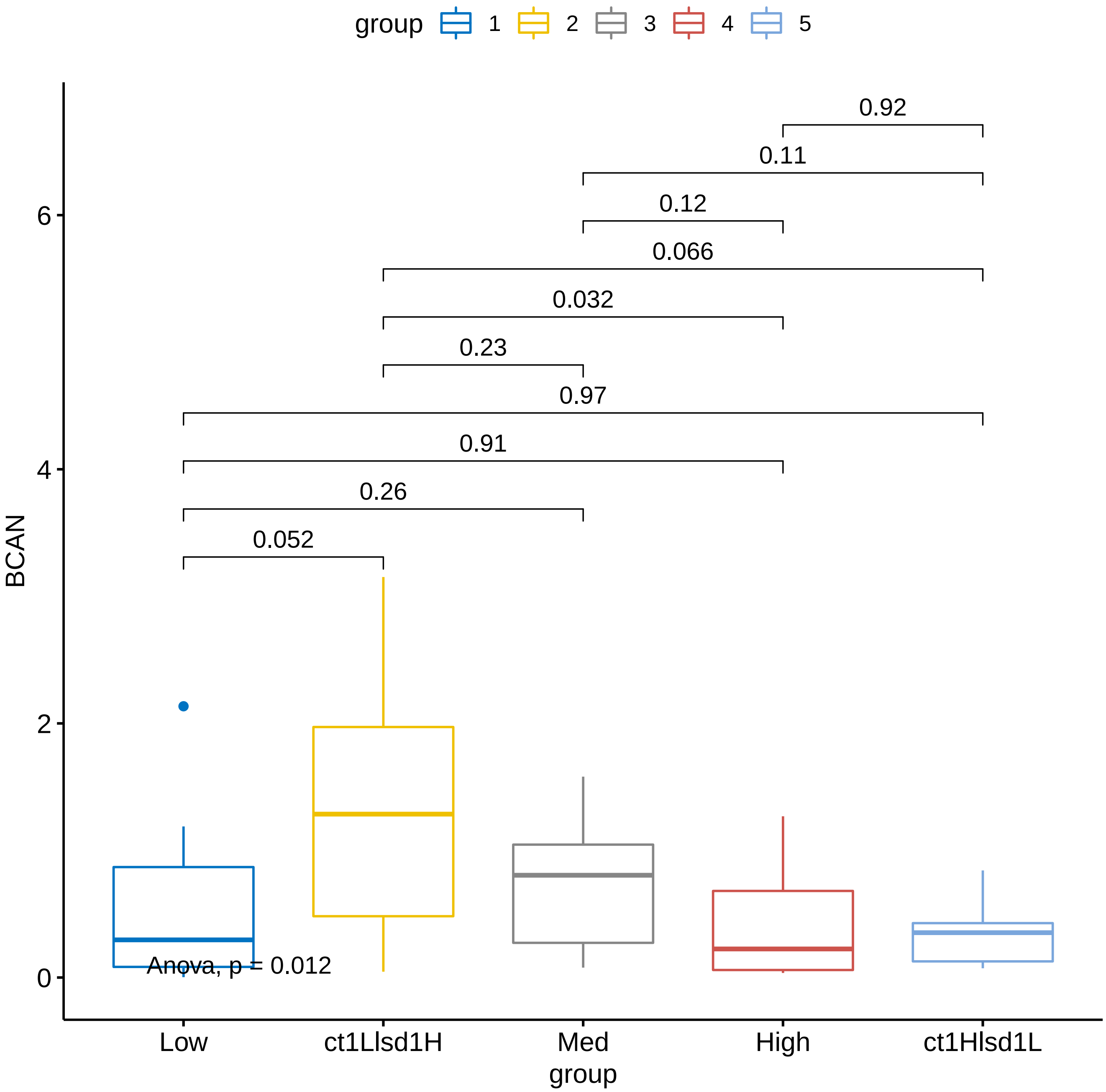

group
1
2
3
4
5
BCAN
6
4
2
0
0.92
0.11
0.12
0.066
0.032
0.23
0.97
0.91
0.26
0.052
Anova, p = 0.012
Low
ct1Llsd1H
Med
High
ct1Hlsd1L
group

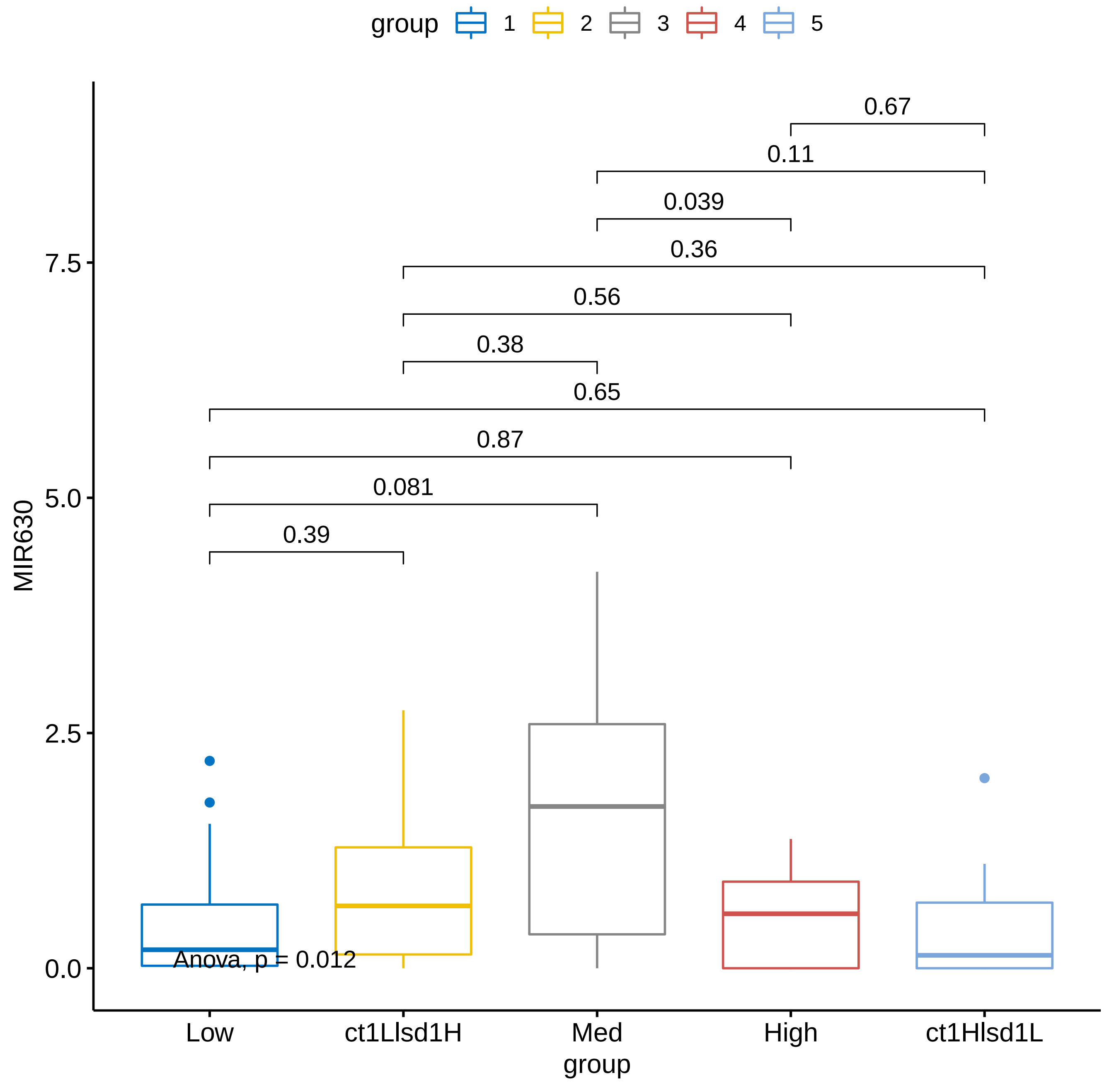

group 1 2 3 4 5
0.67
0.11
0.039
0.36
0.56
0.38
0.65
0.87
0.081
0.39
7.5
5.0
2.5
0.0
MIR630
Anova, p = 0.012
Low
ct1Llsd1H
Med
High
ct1Hlsd1L
group

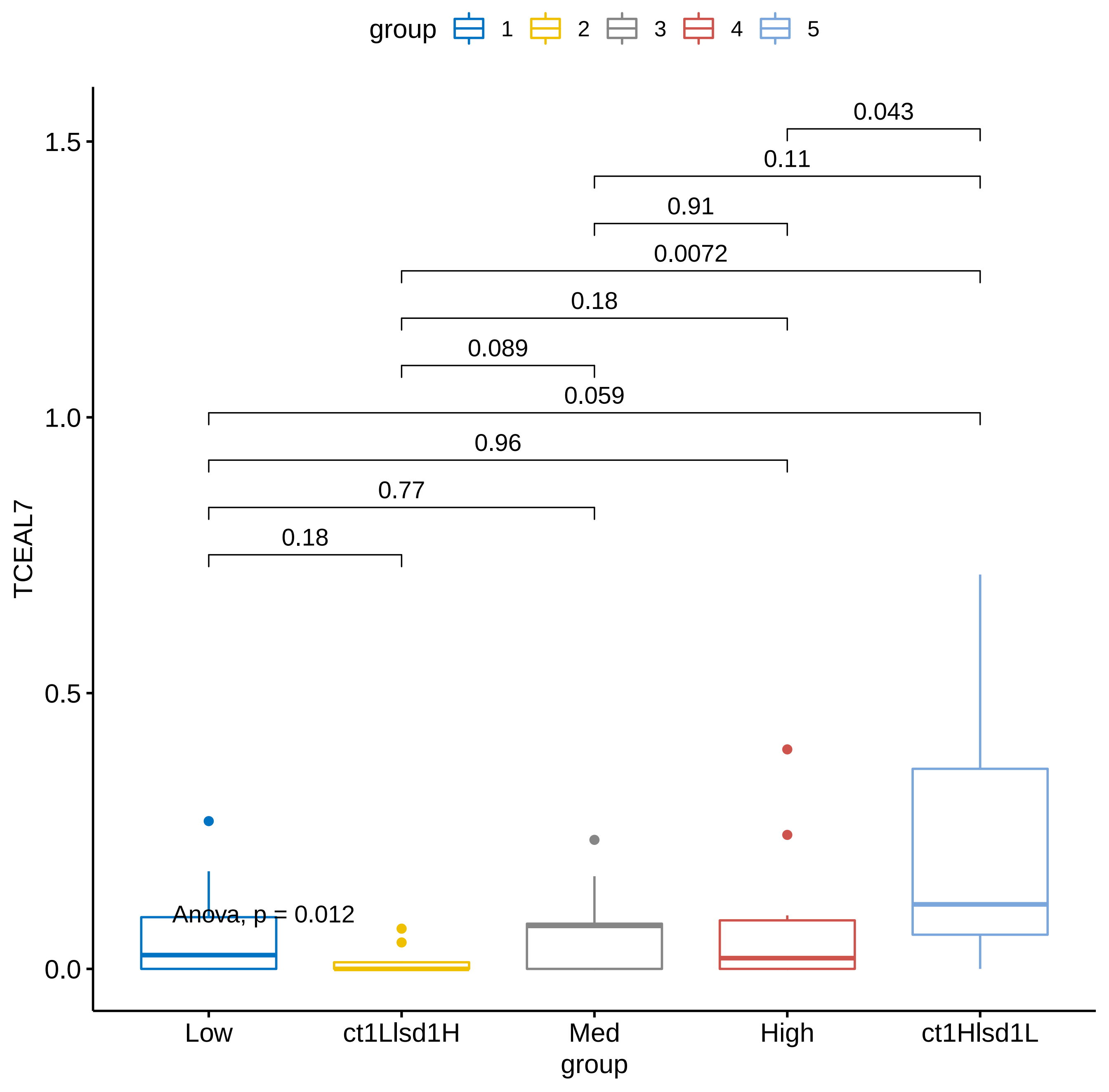
group
1
2
3
4
5
0.043
0.11
0.91
0.0072
0.18
0.089
0.059
0.96
0.77
0.18
1.5
1.0
0.5
0.0
TCEAL7
Anova, p = 0.012
Low
ct1Llsd1H
Med
High
ct1Hlsd1L
group

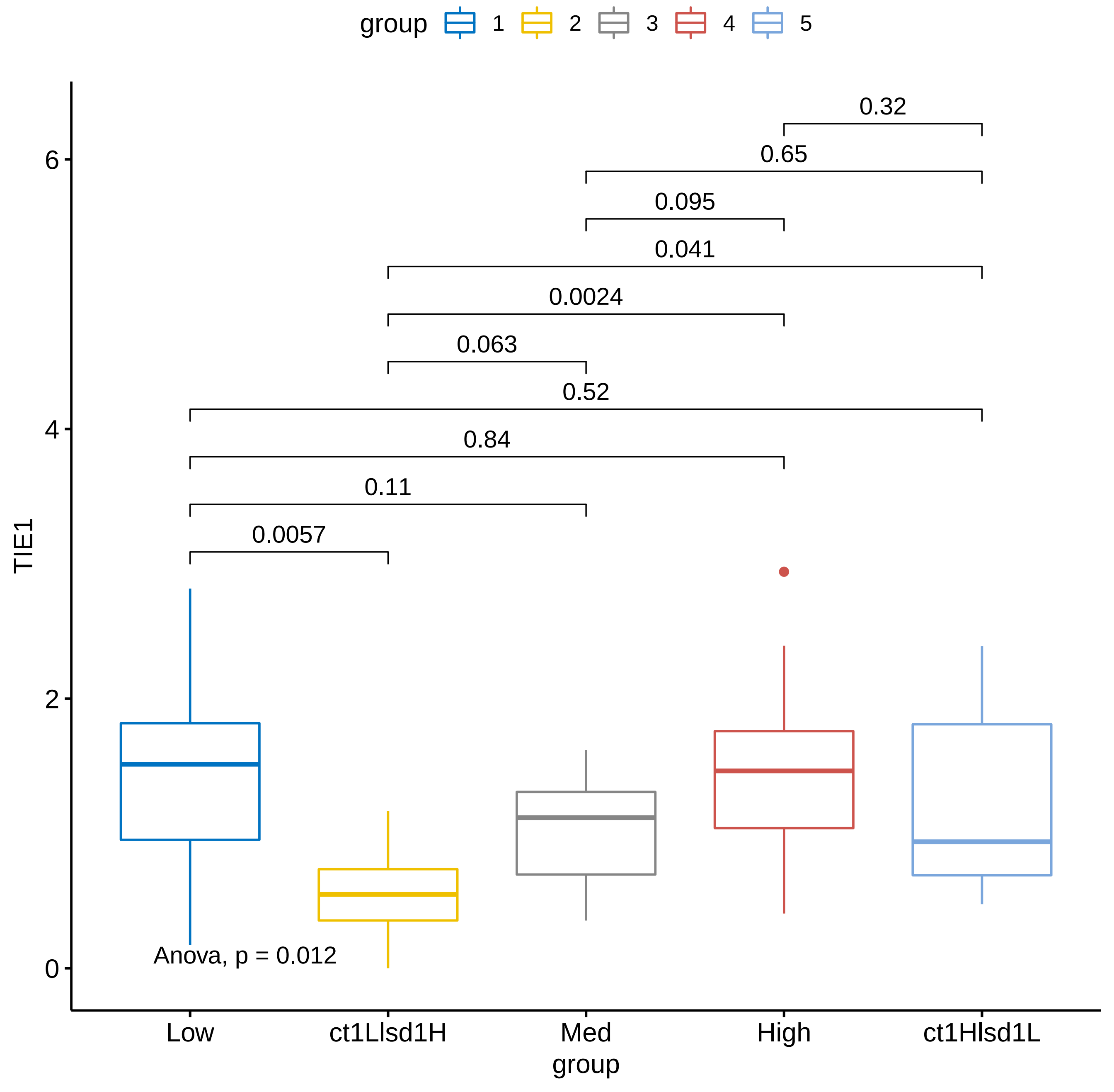
group
1
2
3
4
5
0.32
0.65
0.095
0.041
0.0024
0.063
0.52
0.84
0.11
0.0057
TIE1
6
4
2
0
Anova, p = 0.012
Low
ct1Llsd1H
Med
High
ct1Hlsd1L
group

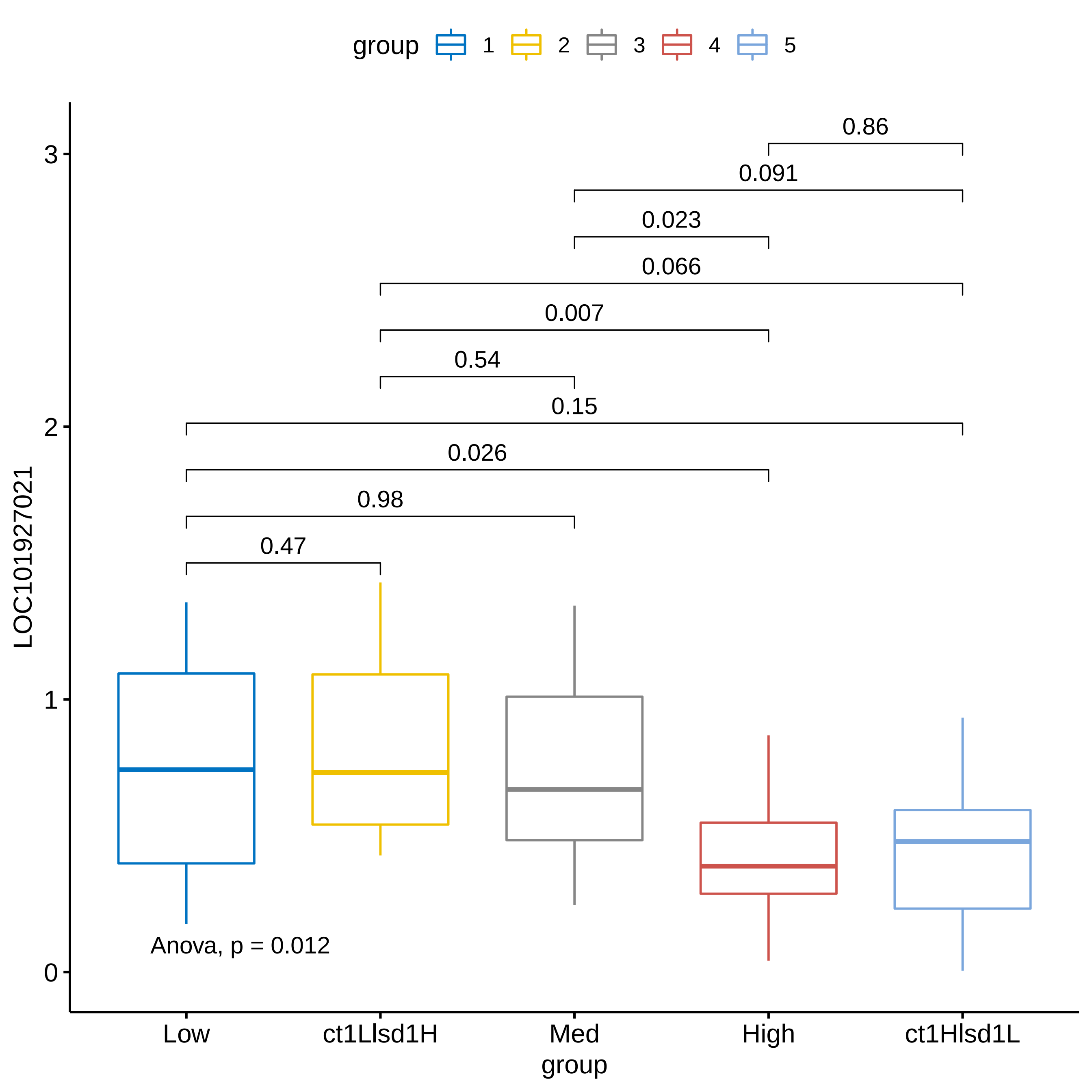
group
1
2
3
4
5
LOC101927021
3
2
1
0
0.86
0.091
0.023
0.066
0.007
0.54
0.15
0.026
0.98
0.47
Anova, p = 0.012
Low
ct1Llsd1H
Med
High
ct1Hlsd1L
group

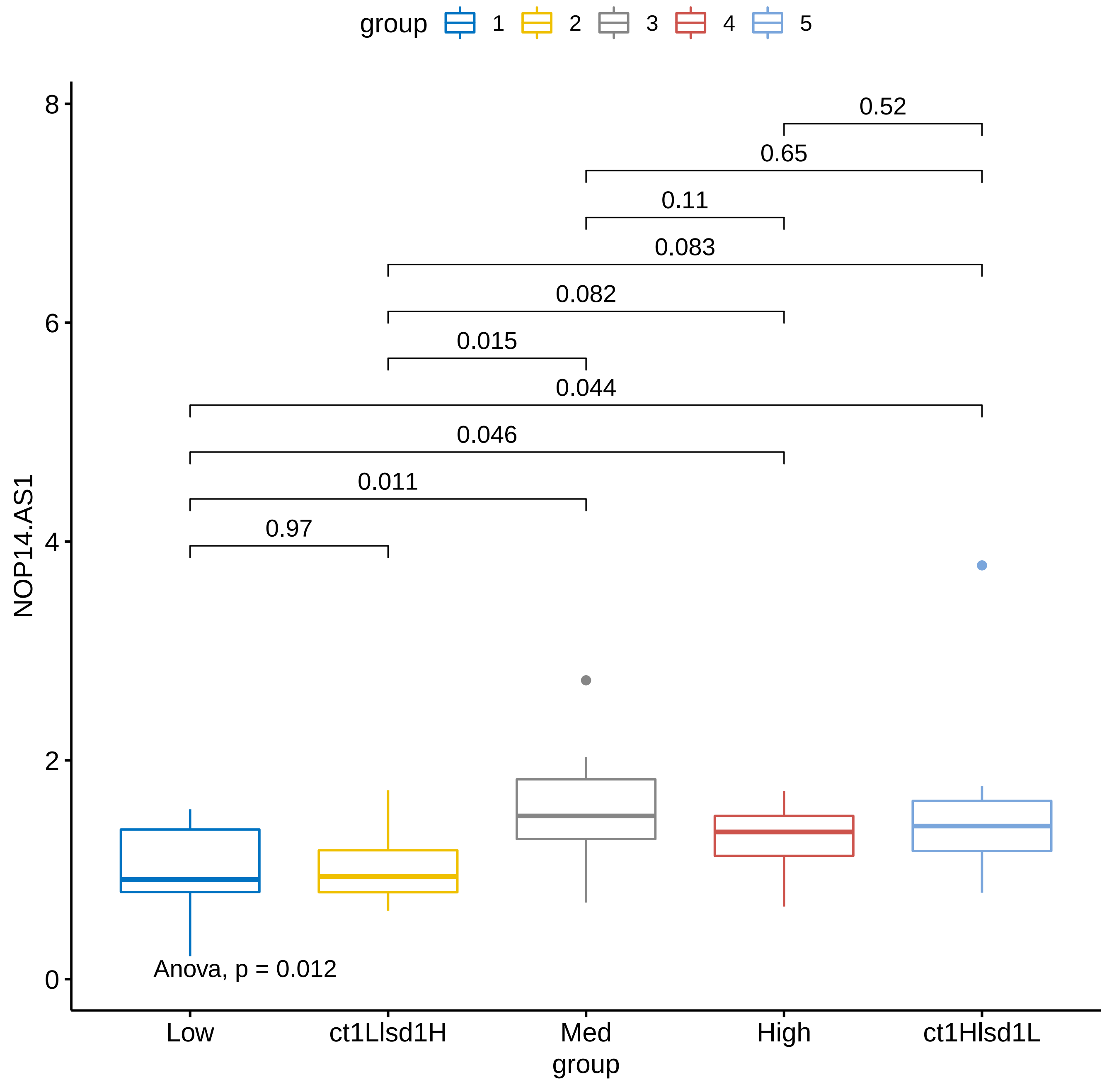
group
1
2
3
4
5
NOP14.AS1
8
6
4
2
0
0.52
0.65
0.11
0.083
0.082
0.015
0.044
0.046
0.011
0.97
Anova, p = 0.012
Low
ct1Llsd1H
Med
High
ct1Hlsd1L
group

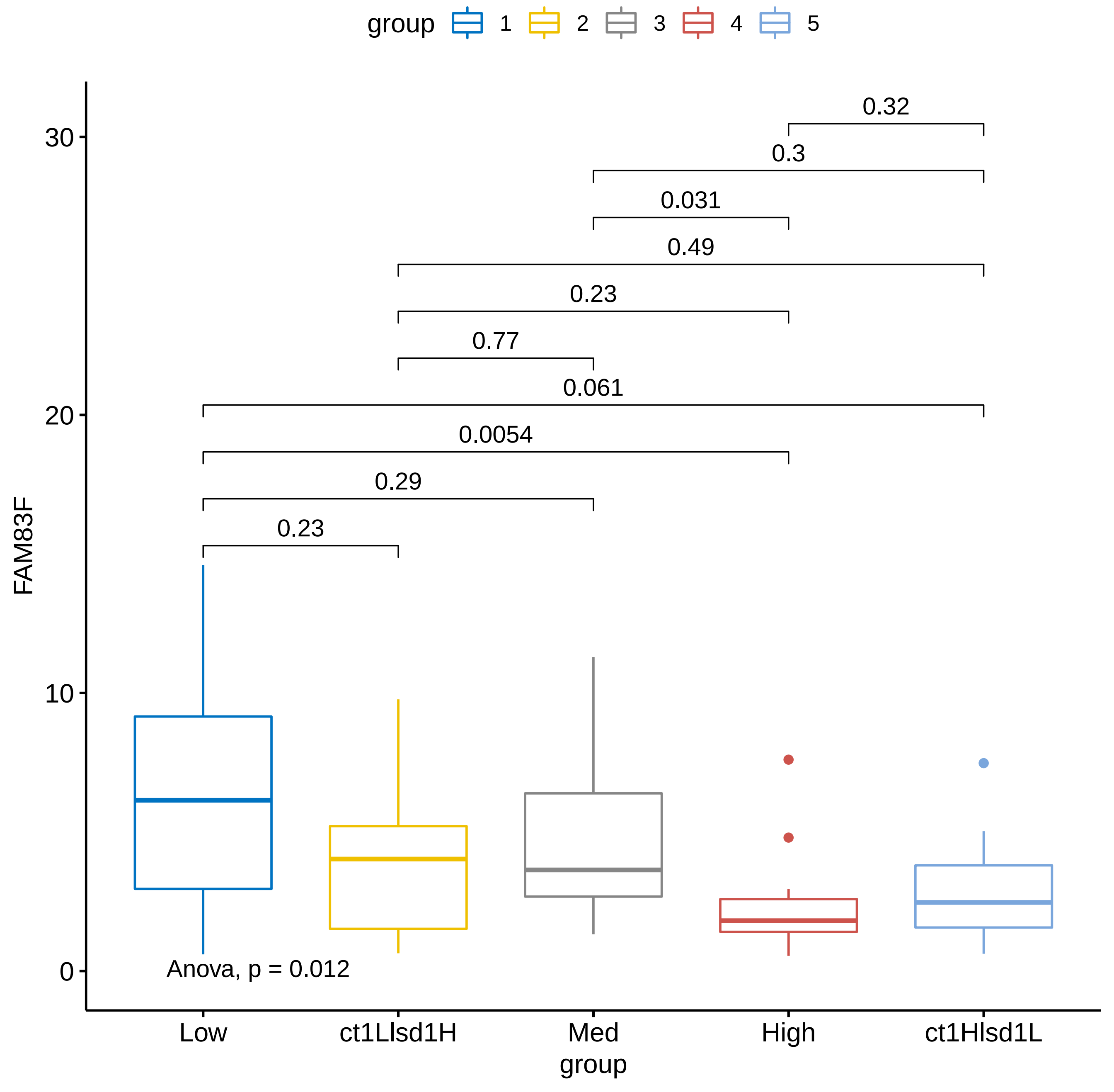
group
1
2
3
4
5
0.32
0.3
0.031
0.49
0.23
0.77
0.061
0.0054
0.29
0.23
30
20
10
0
FAM83F
Anova, p = 0.012
Low
ct1Llsd1H
Med
High
ct1Hlsd1L
group

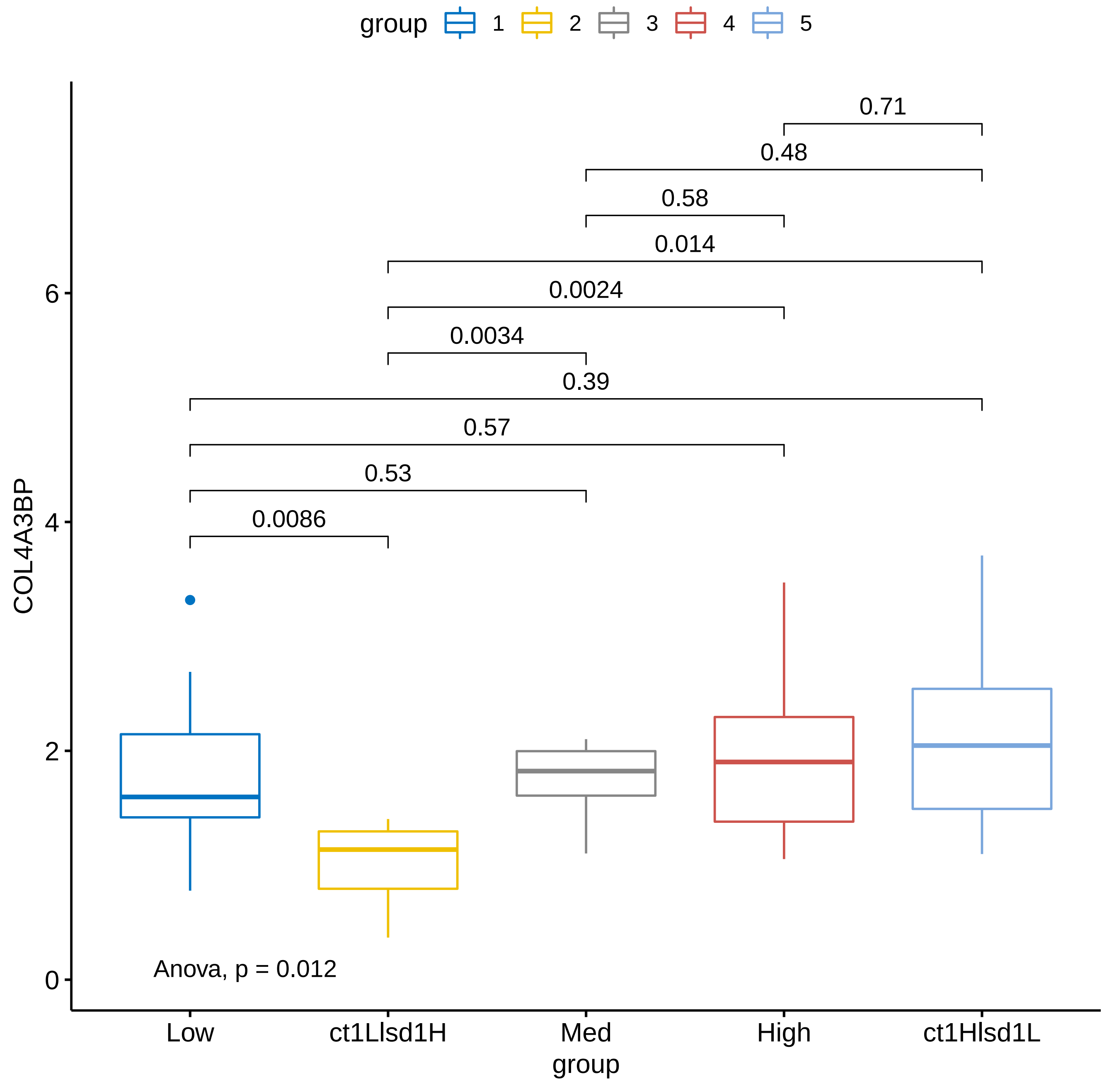

group 1 2 3 4 5
0.71
0.48
0.58
0.014
0.0024
0.0034
0.39
0.57
0.53
0.0086
Anova, p = 0.012
COL4A3BP
0
2
4
6
Low
ct1Llsd1H
Med
High
ct1Hlsd1L
group

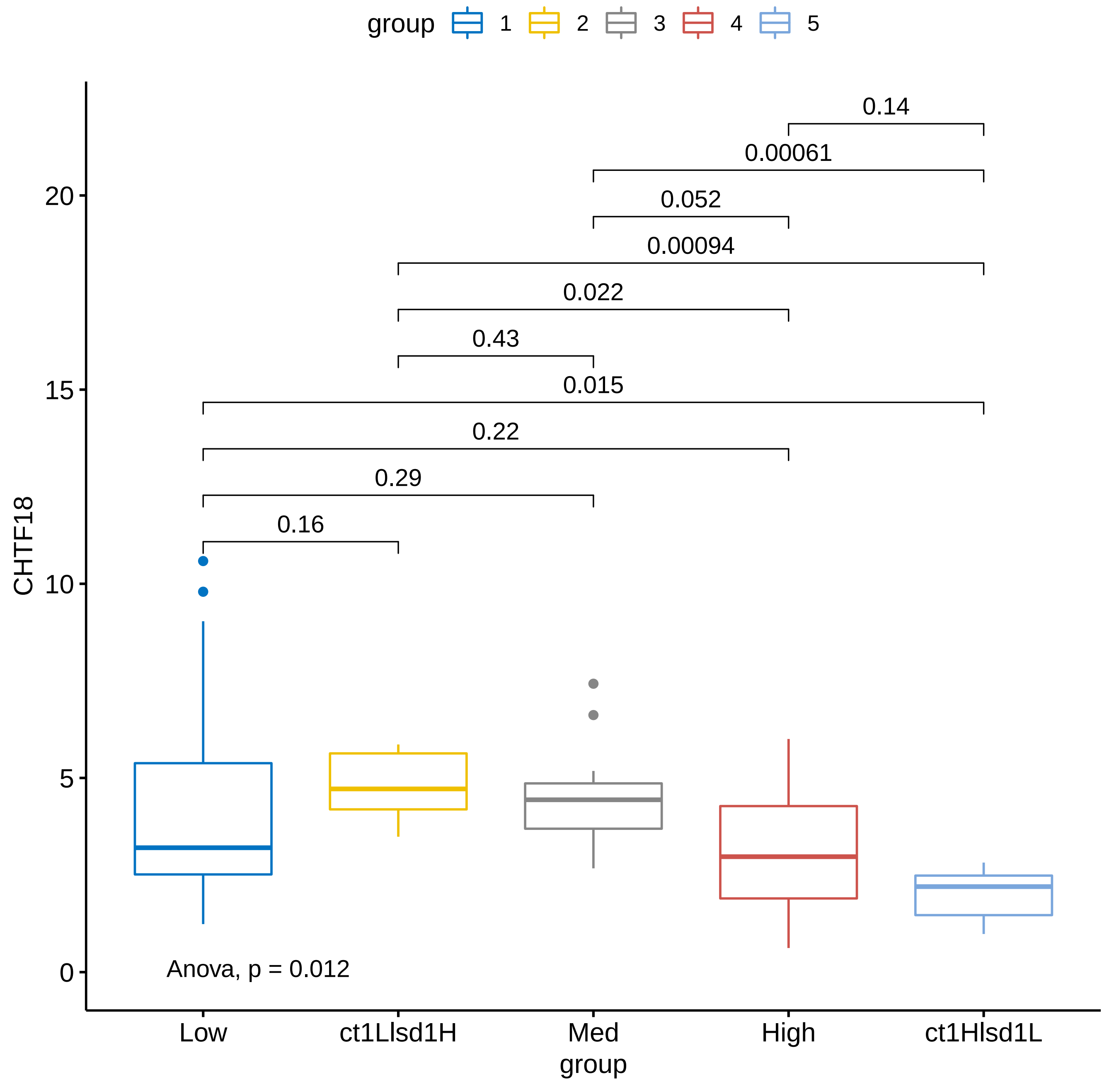
group
1
2
3
4
5
0.14
0.00061
0.052
0.00094
0.022
0.43
0.015
0.22
0.29
0.16
20
15
10
5
0
CHTF18
Anova, p = 0.012
Low
ct1Llsd1H
Med
High
ct1Hlsd1L
group

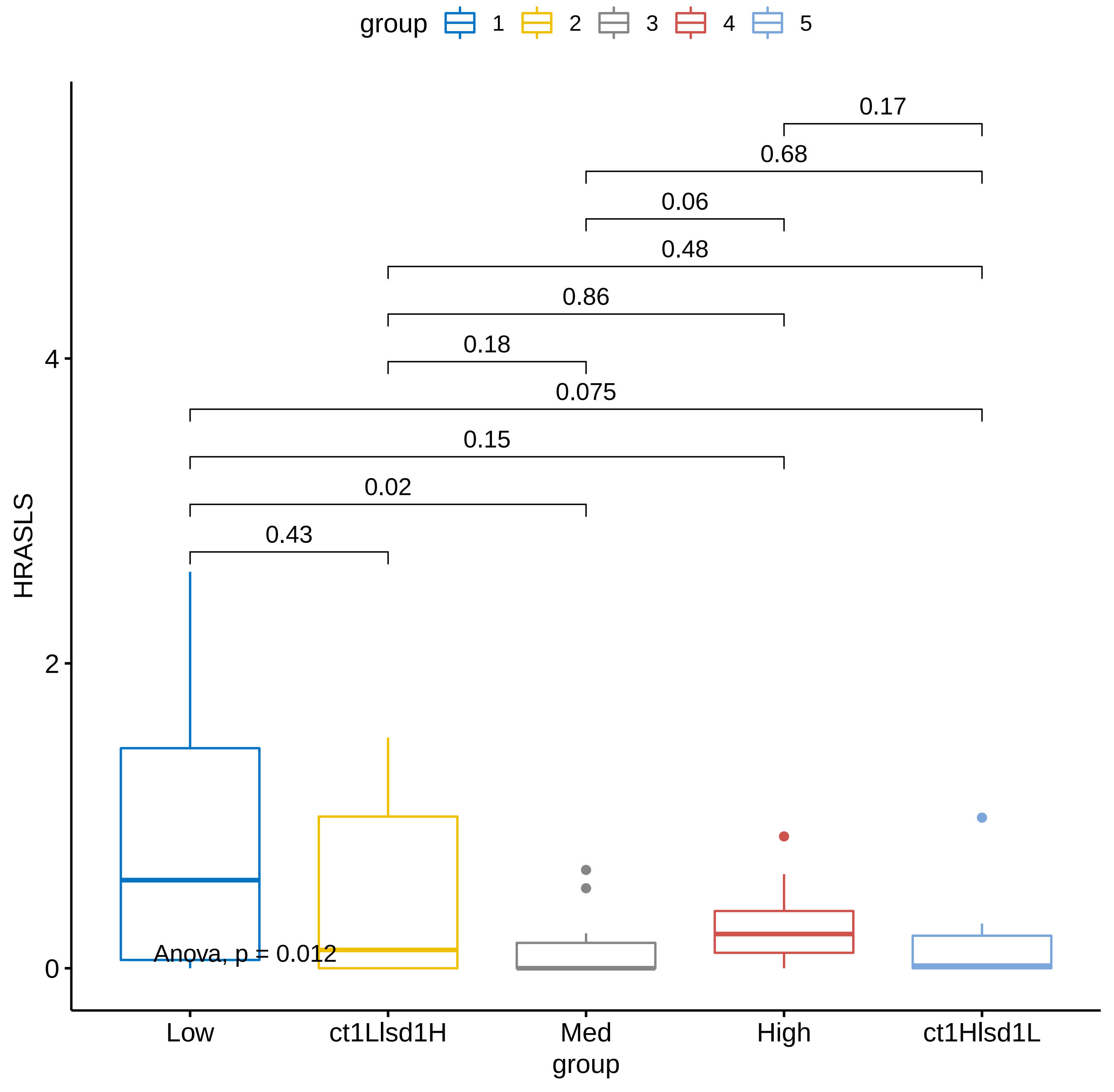

group 1 2 3 4 5
0.17
0.68
0.06
0.48
0.86
0.18
0.075
0.15
0.02
0.43
HRASLS
4
2
0
Anova, p = 0.012
Low
ct1Llsd1H
Med
High
ct1Hlsd1L
group

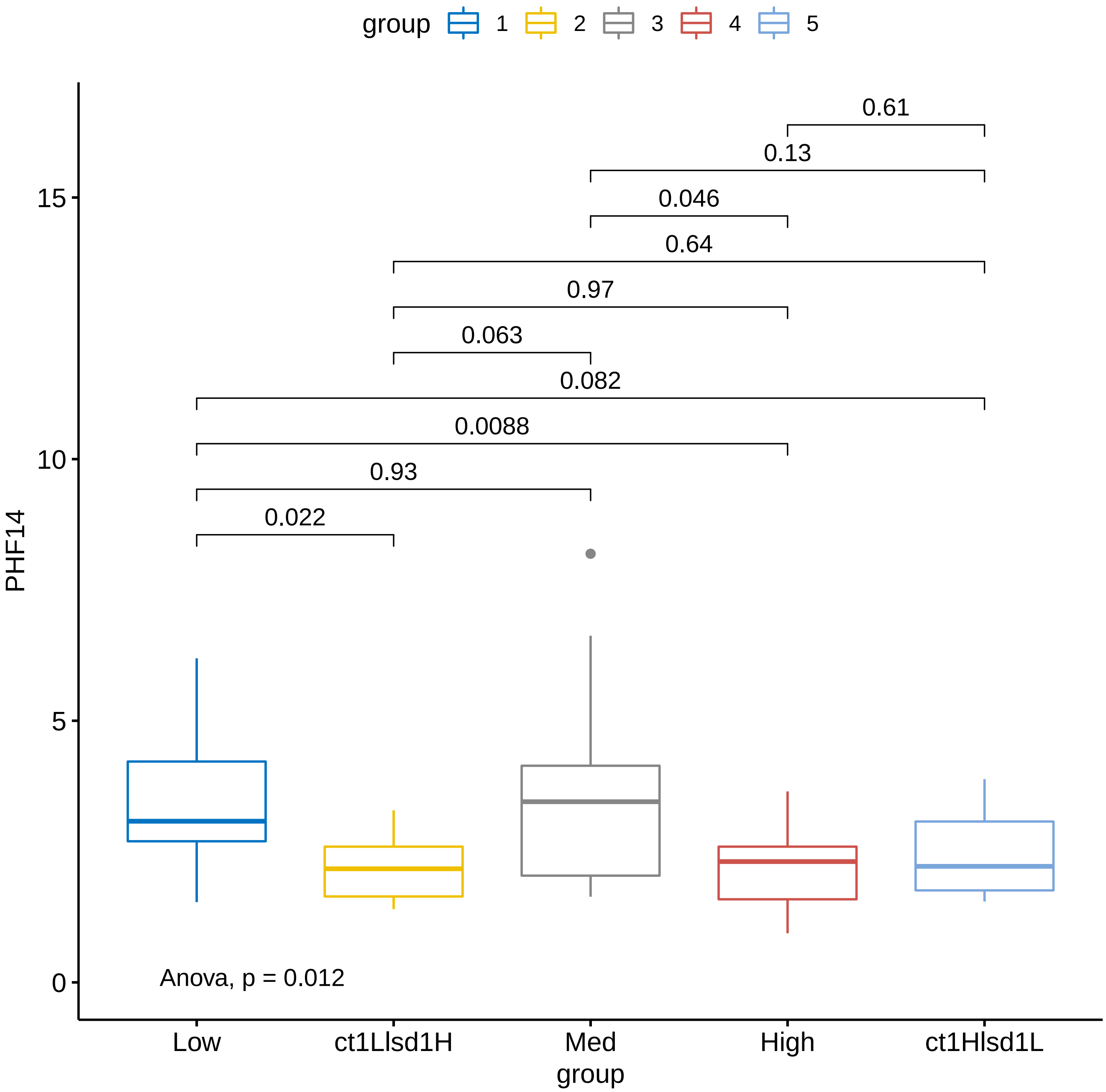

group 1 2 3 4 5
0.61
0.13
0.046
0.64
0.97
0.063
0.082
0.0088
0.93
0.022
15
10
5
0
PHF14
Anova, p = 0.012
Low
ct1Llsd1H
Med
High
ct1Hlsd1L
group

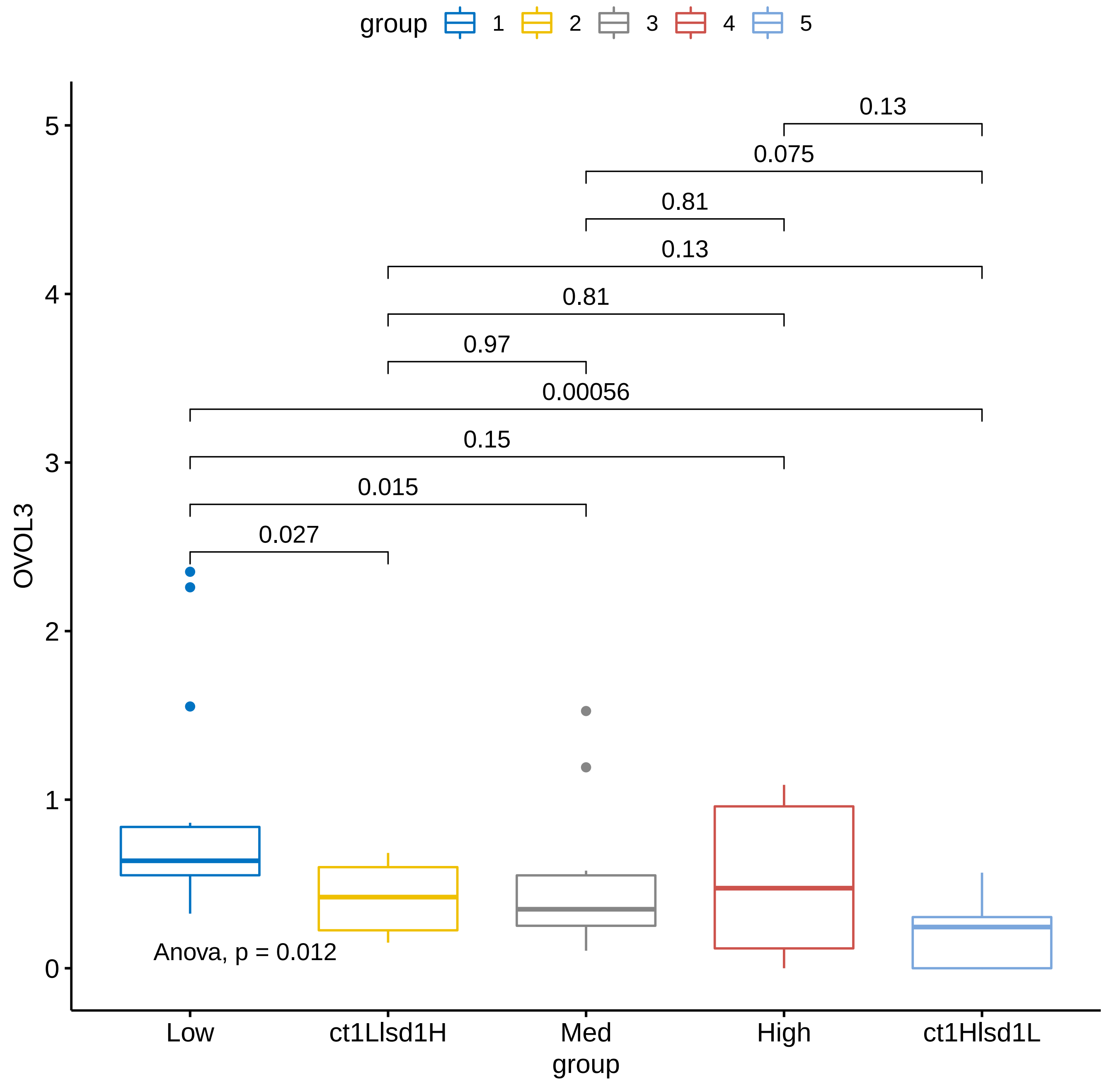
group 1 2 3 4 5
0.13
0.075
0.81
0.13
0.81
0.97
0.00056
0.15
0.015
0.027
Anova, p = 0.012
OVOL3
0
1
2
3
4
5
Low
ct1Llsd1H
Med
High
ct1Hlsd1L
group

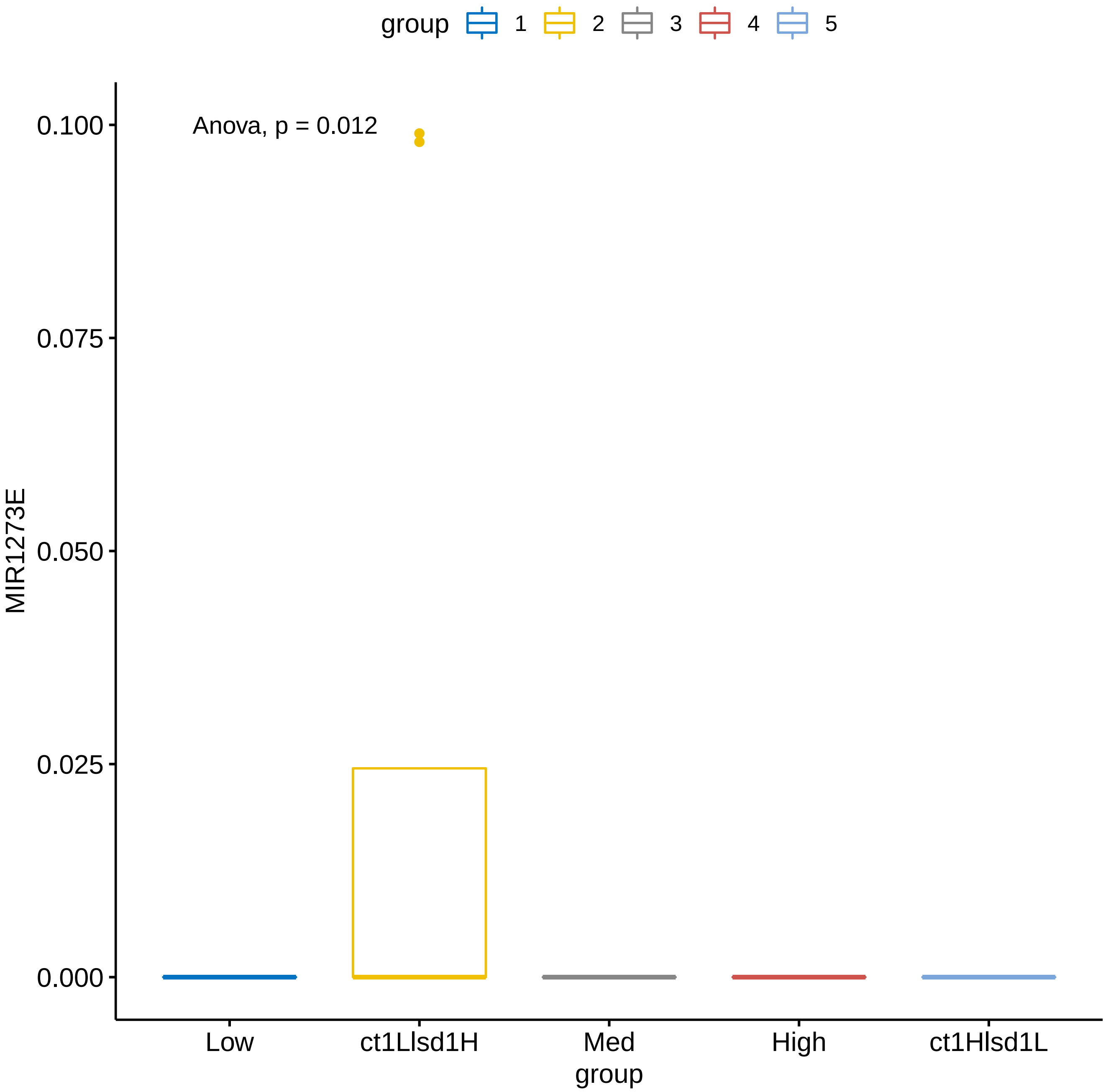

group
1
2
3
4
5
Anova, p = 0.012
0.100
0.075
0.050
0.025
0.000
MIR1273E
Low
ct1Llsd1H
Med
High
ct1Hlsd1L
group

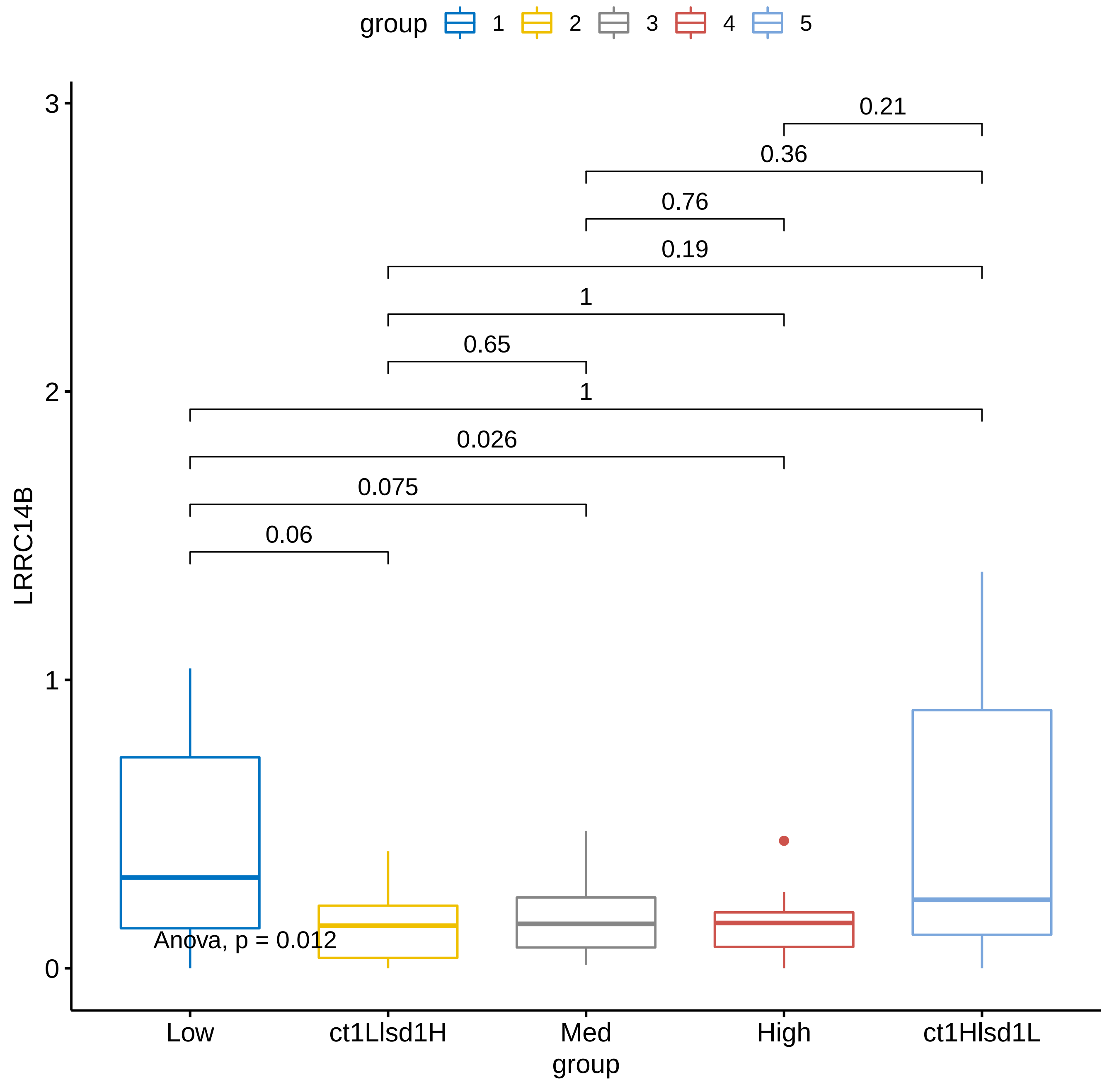

group
1
2
3
4
5
0.21
0.36
0.76
0.19
1
0.65
1
0.026
0.075
0.06
Anova, p = 0.012
LRRC14B
3
2
1
0
Low
ct1Llsd1H
Med
High
ct1Hlsd1L
group

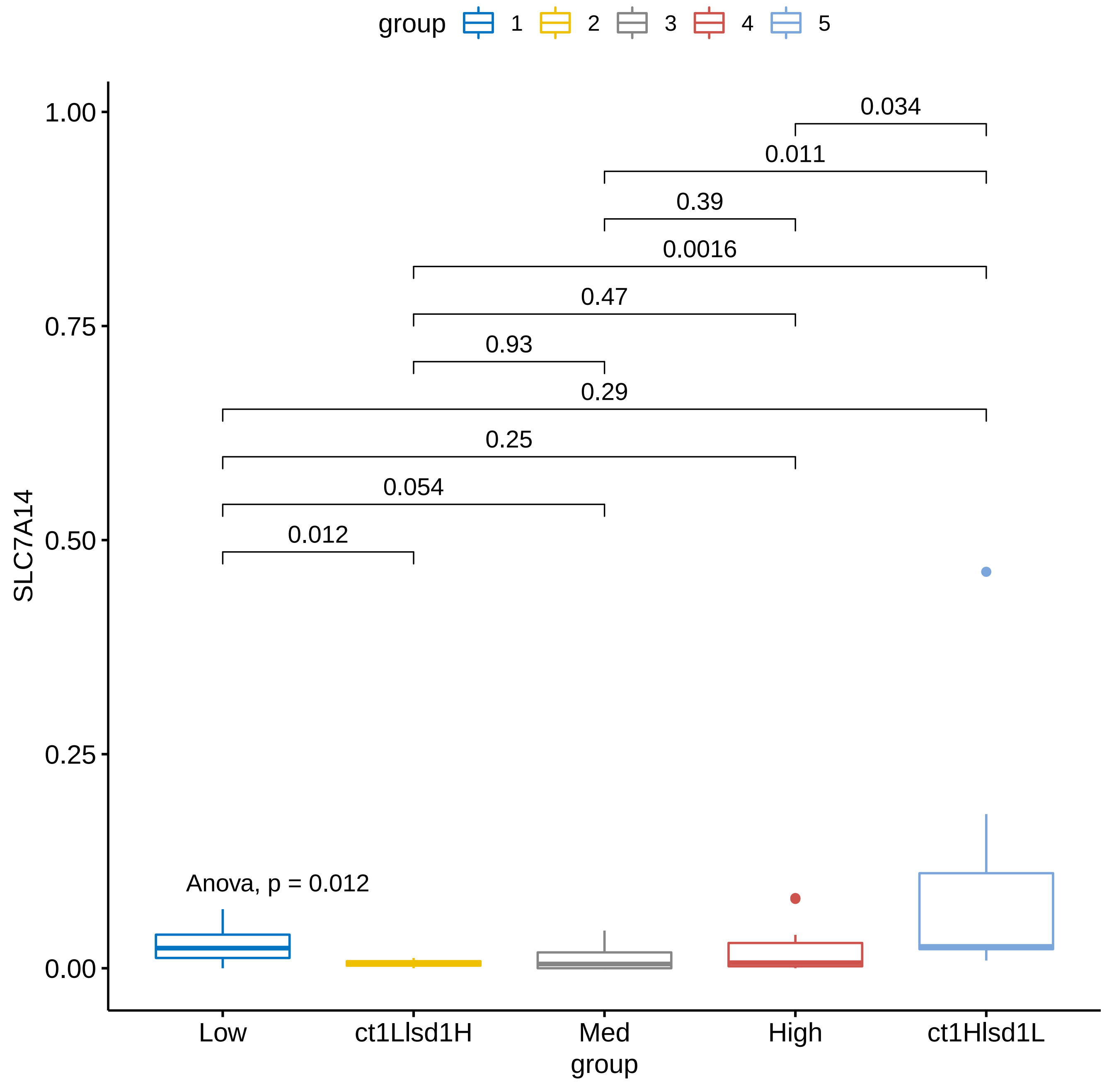

group
1
2
3
4
5
SLC7A14
1.00
0.75
0.50
0.25
0.00
0.034
0.011
0.39
0.0016
0.47
0.93
0.29
0.25
0.054
0.012
Anova, p = 0.012
Low
ct1Llsd1H
Med
High
ct1Hlsd1L
group

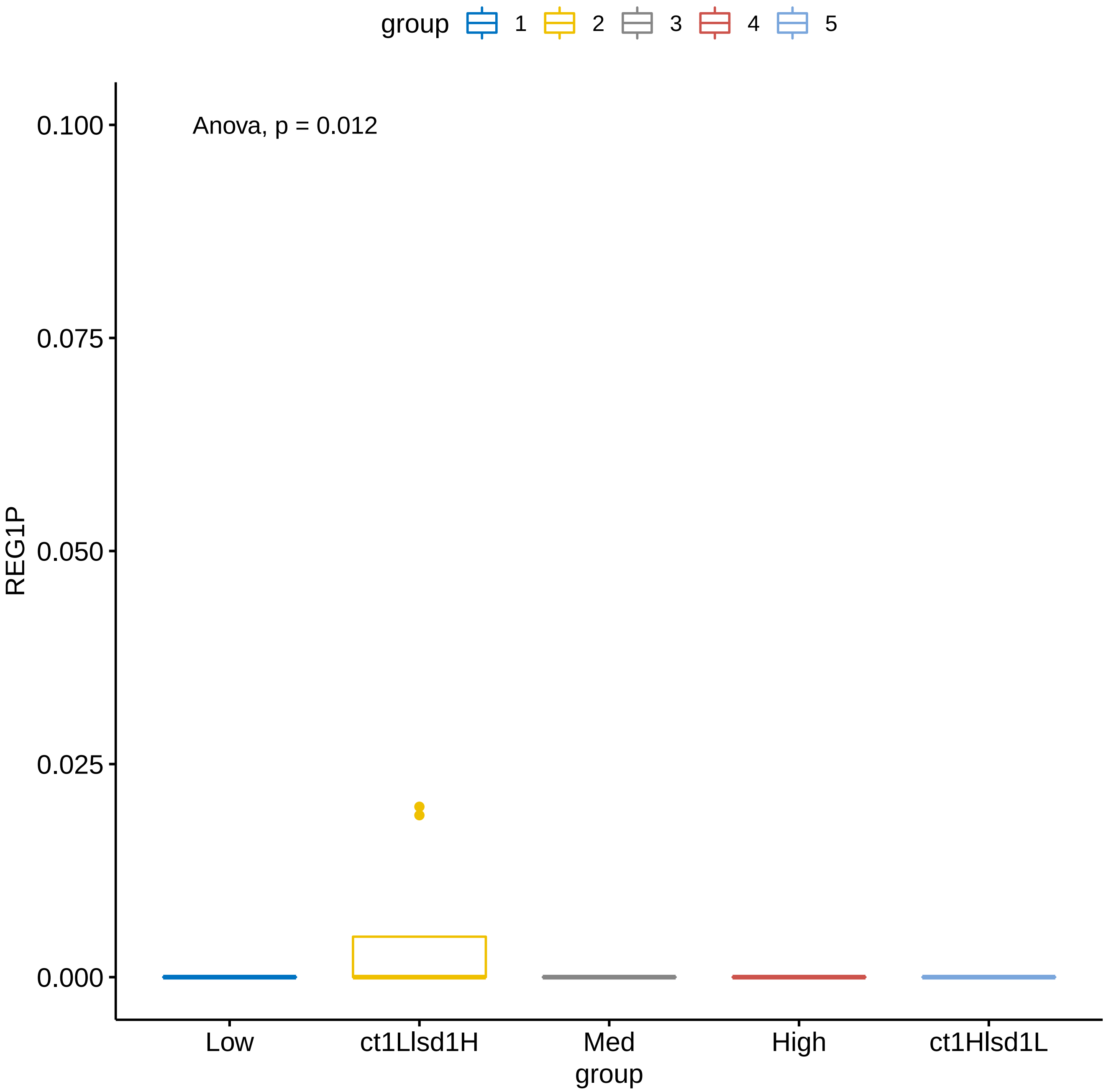

group 1 2 3 4 5
Anova, p = 0.012
0.100
0.075
0.050
0.025
0.000
REG1P
Low
ct1Llsd1H
Med
High
ct1Hlsd1L
group

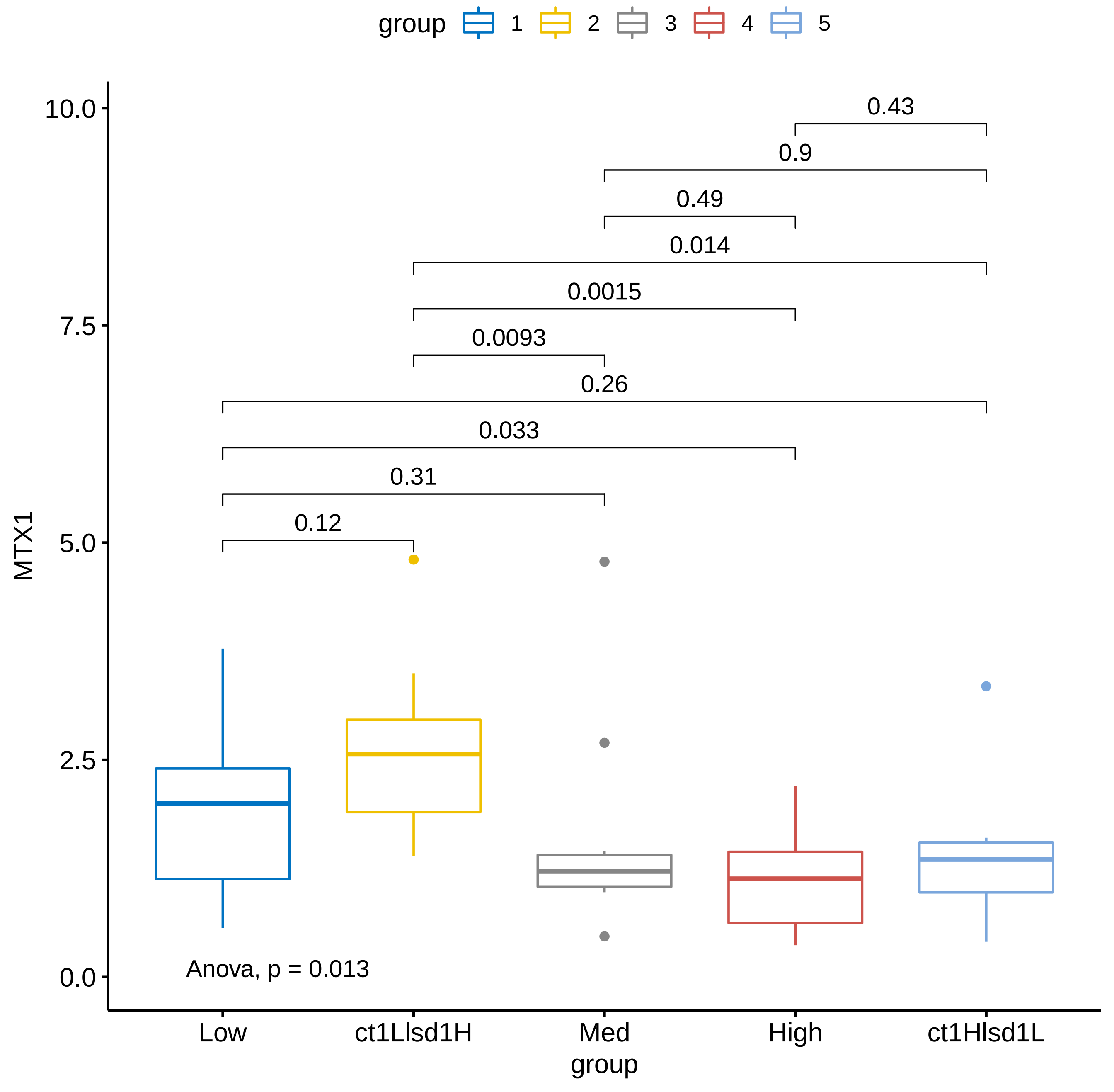

group
1
2
3
4
5
MTX1
10.0
7.5
5.0
2.5
0.0
0.43
0.9
0.49
0.014
0.0015
0.0093
0.26
0.033
0.31
0.12
Anova, p = 0.013
Low
ct1Llsd1H
Med
High
ct1Hlsd1L
group

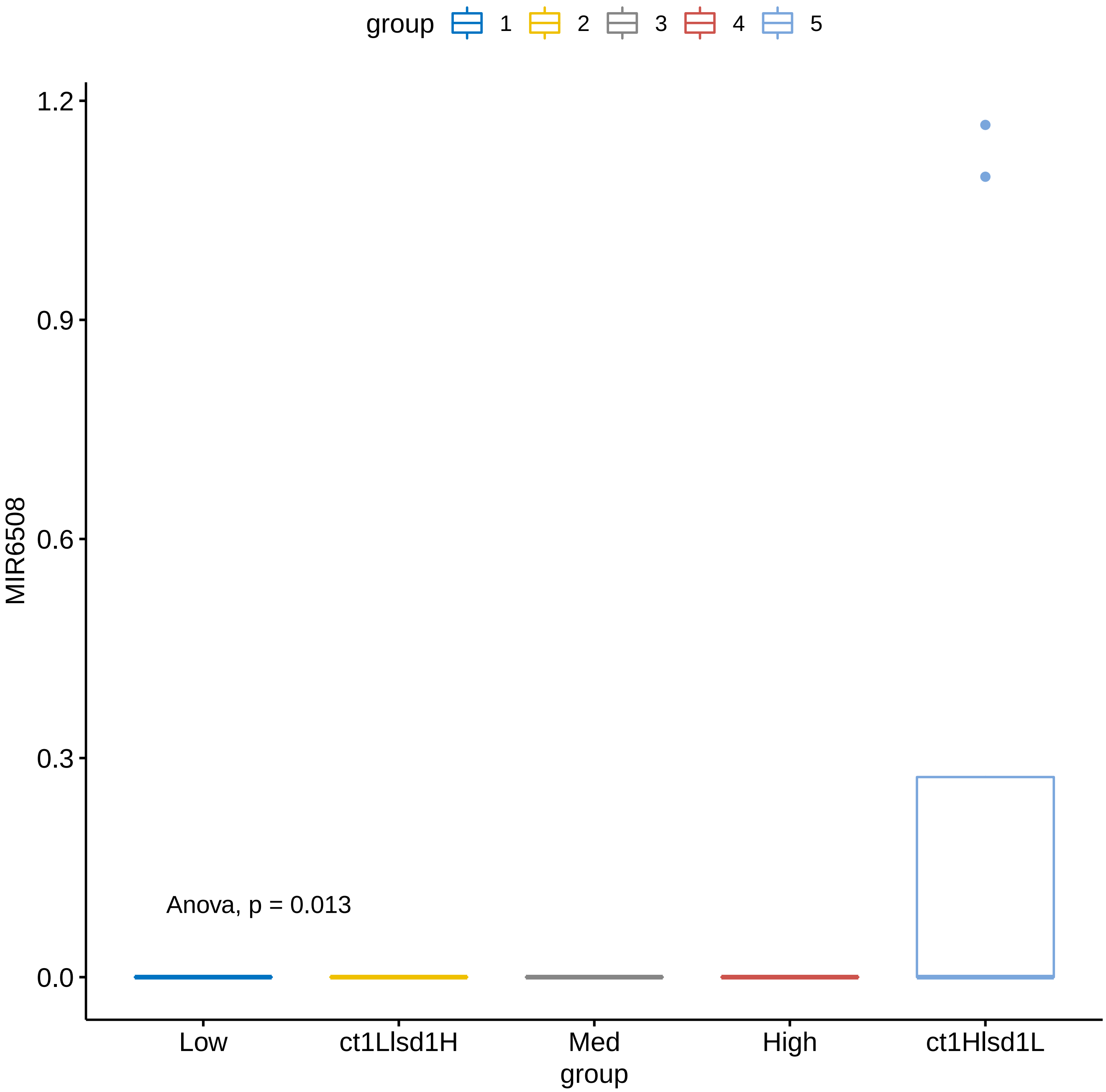

group 1 2 3 4 5
1.2
0.9
0.6
0.3
0.0
MIR6508
Anova, p = 0.013
Low
ct1Llsd1H
Med
High
ct1Hlsd1L
group

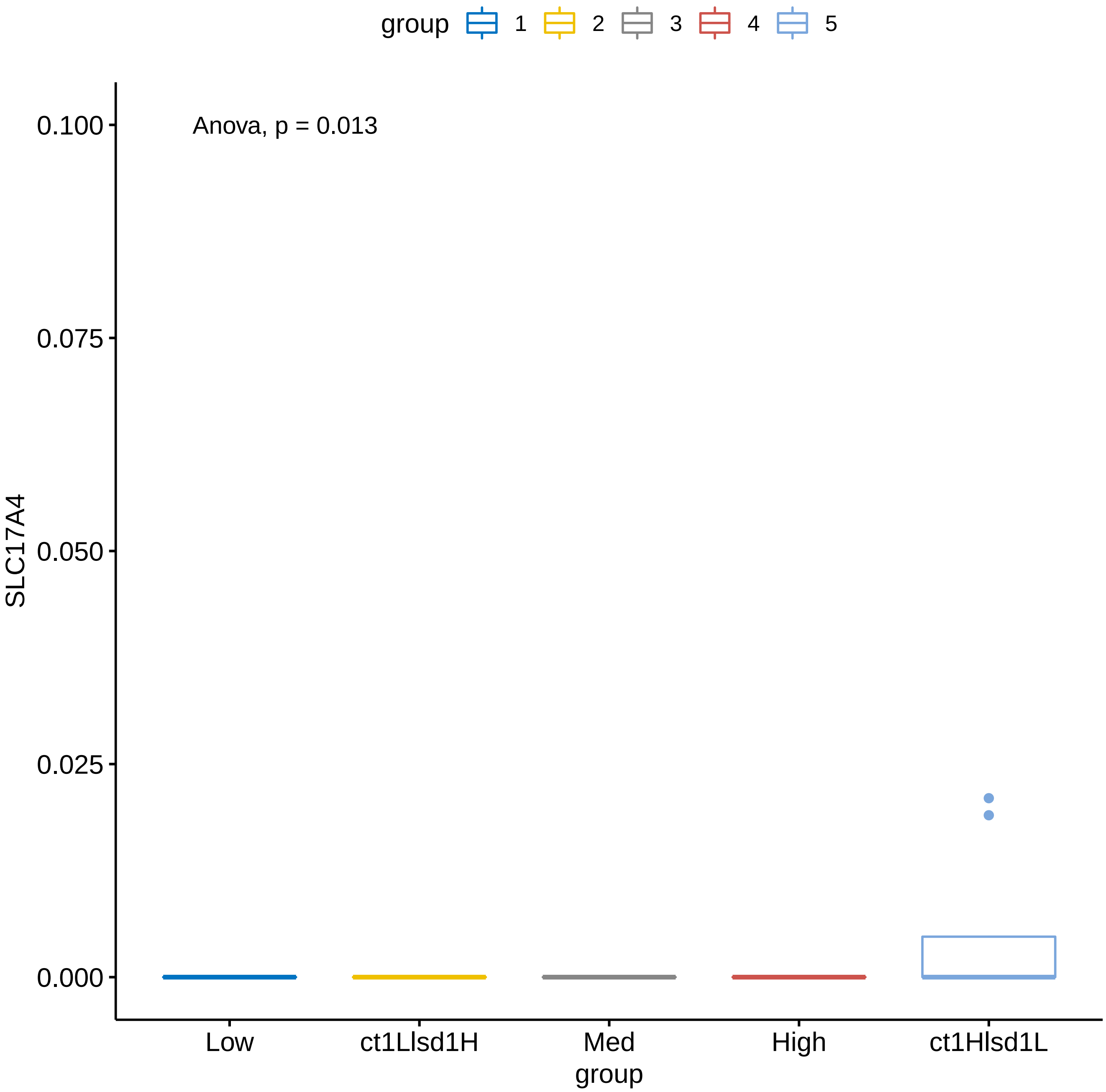

group
1
2
3
4
5
Anova, p = 0.013
0.100
0.075
0.050
0.025
0.000
SLC17A4
Low
ct1Llsd1H
Med
High
ct1Hlsd1L
group

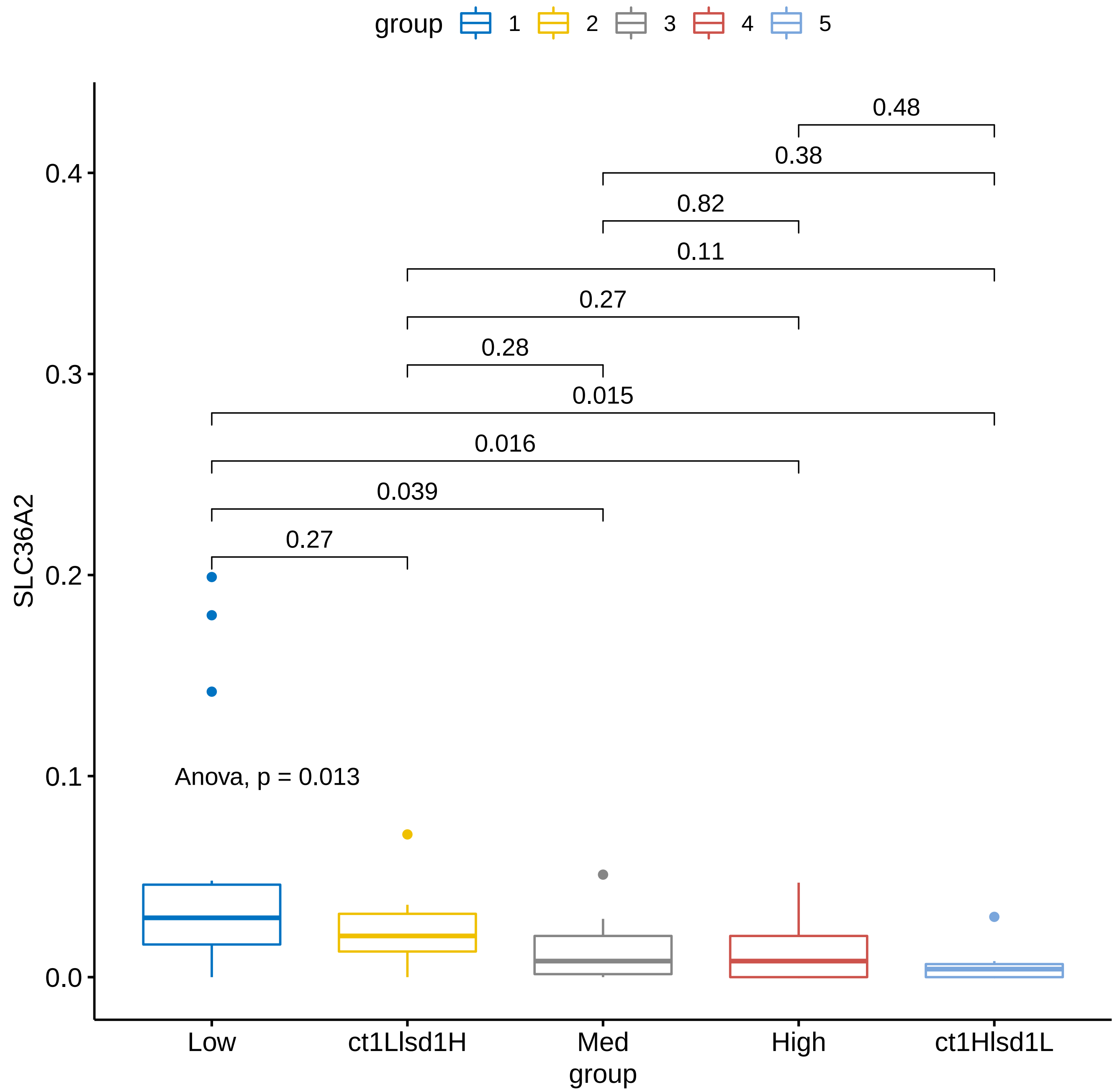

group
1
2
3
4
5
0.48
0.38
0.82
0.11
0.27
0.28
0.015
0.016
0.039
0.27
Anova, p = 0.013
SLC36A2
0.0
0.1
0.2
0.3
0.4
Low
ct1Llsd1H
Med
High
ct1Hlsd1L
group

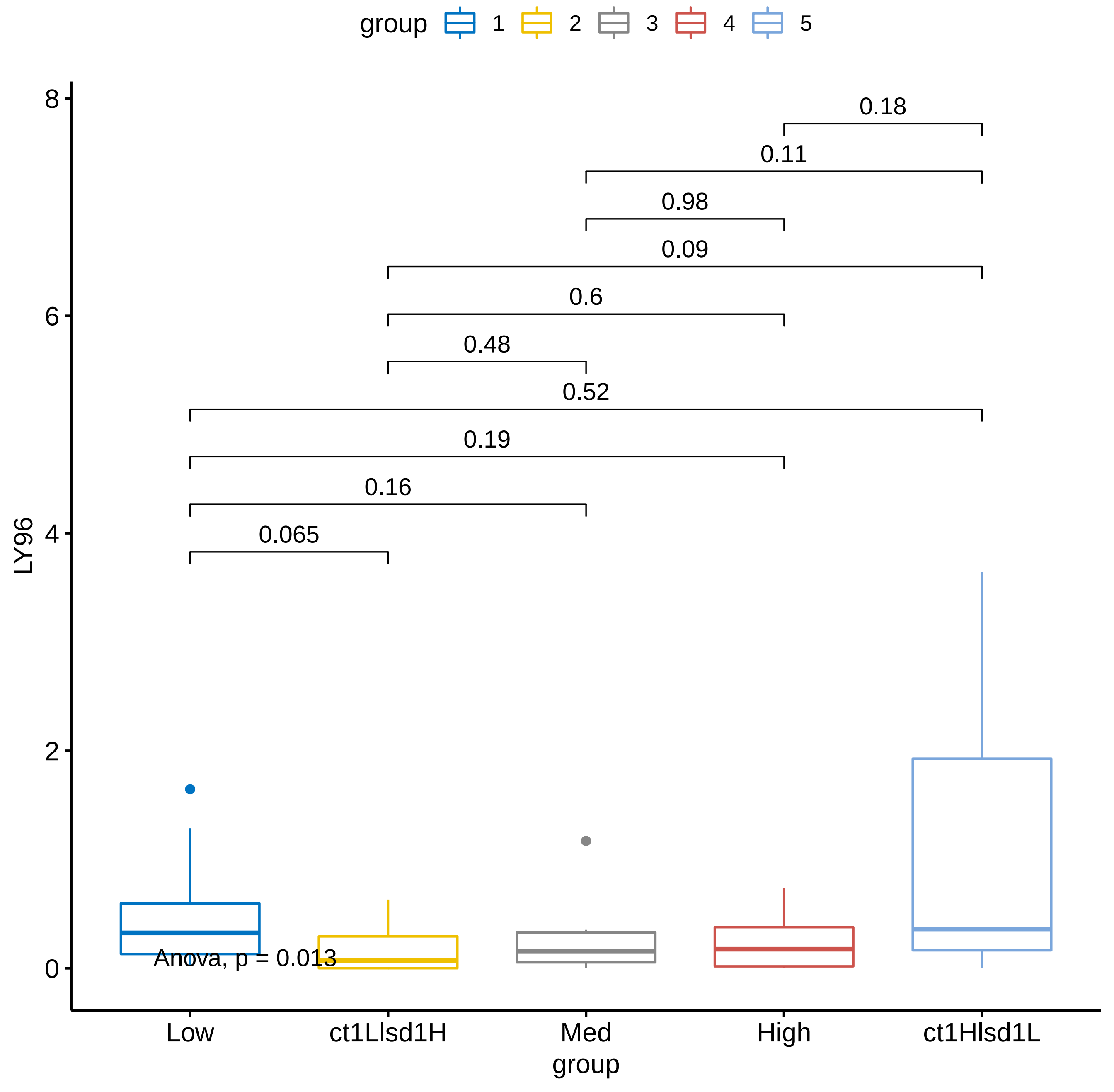

group
1
2
3
4
5
LY96
0
2
4
6
8
0.18
0.11
0.98
0.09
0.6
0.48
0.52
0.19
0.16
0.065
Anova, p = 0.013
Low
ct1Llsd1H
Med
High
ct1Hlsd1L
group

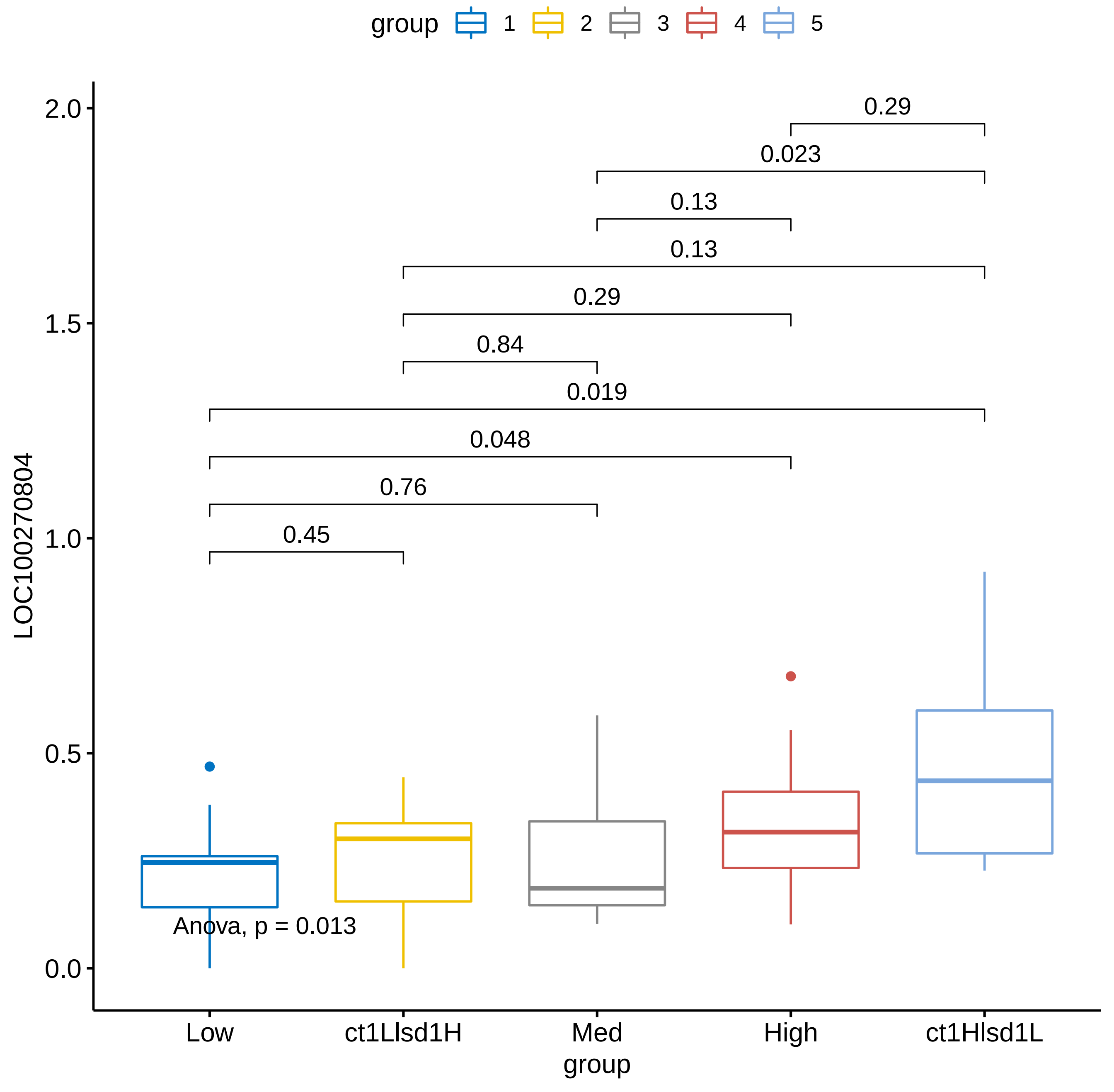
group
1
2
3
4
5
0.29
0.023
0.13
0.13
0.29
0.84
0.019
0.048
0.76
0.45
Anova, p = 0.013
2.0
1.5
1.0
0.5
0.0
LOC100270804
Low
ct1Llsd1H
Med
High
ct1Hlsd1L
group

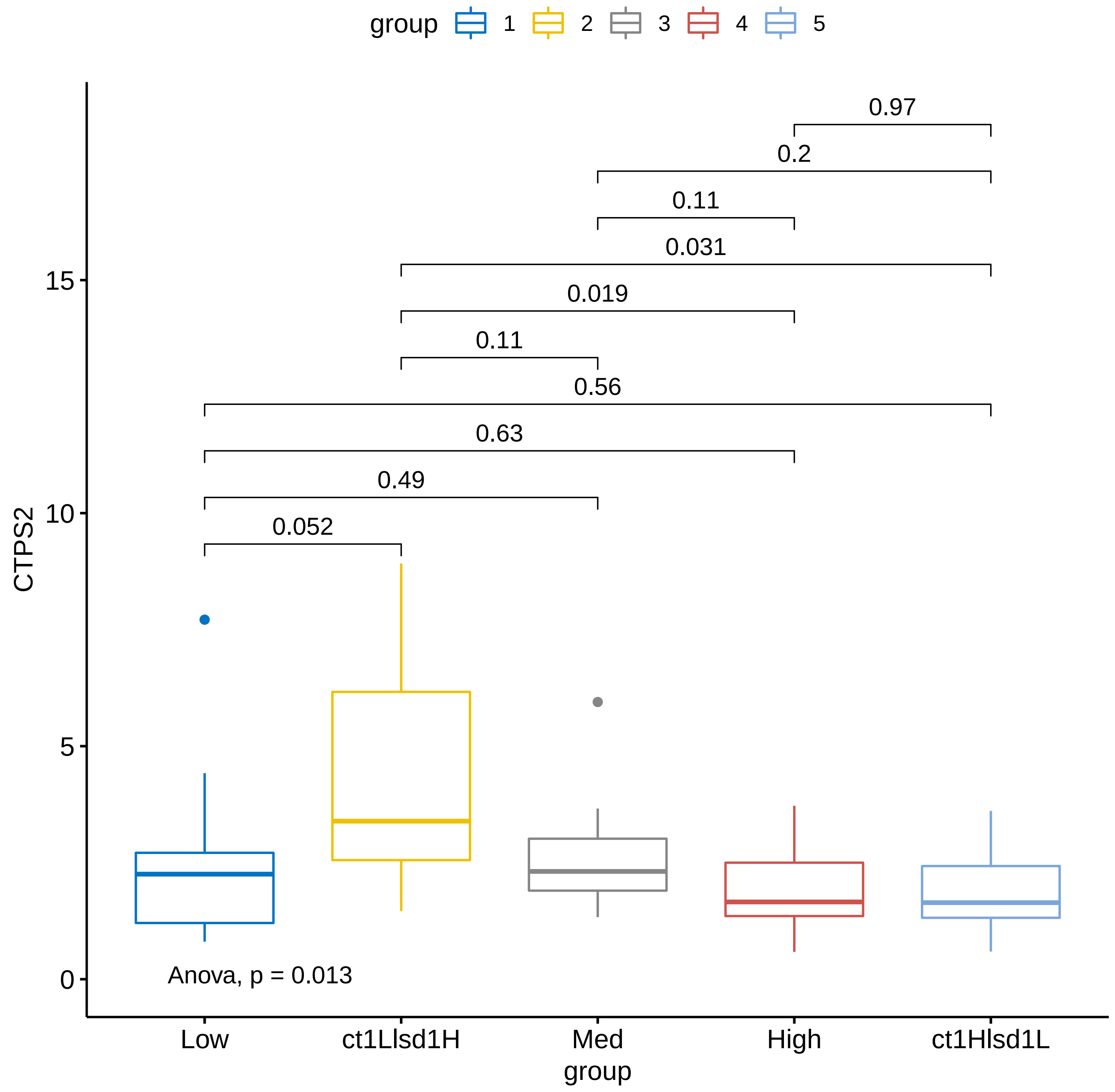
group
1
2
3
4
5
0.97
0.2
0.11
0.031
0.019
0.11
0.56
0.63
0.49
0.052
CTPS2
15
10
5
0
Anova, p = 0.013
Low
ct1Llsd1H
Med
High
ct1Hlsd1L
group

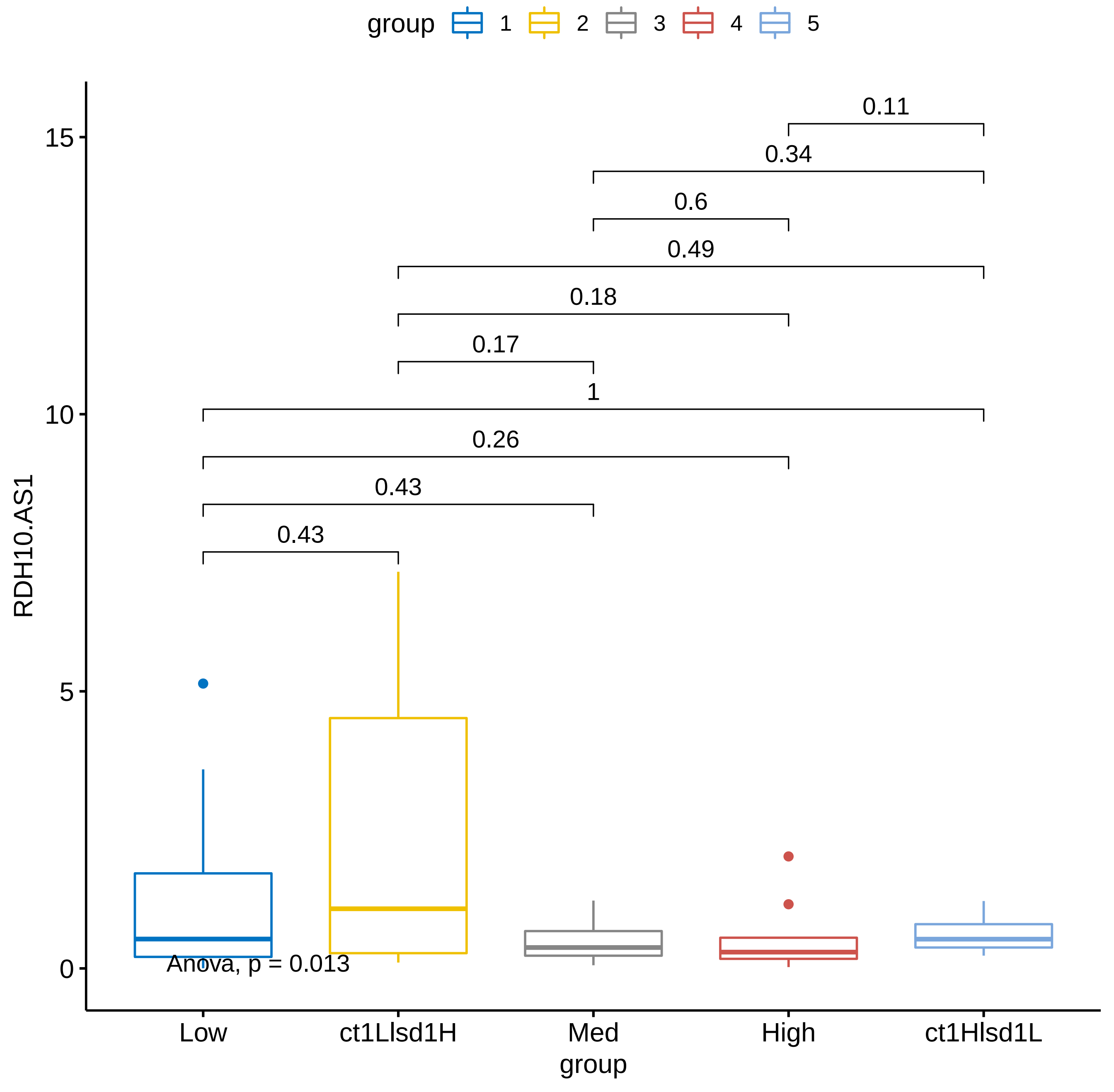
group
1
2
3
4
5
0.11
0.34
0.6
0.49
0.18
0.17
1
0.26
0.43
0.43
Anova, p = 0.013
15
10
5
0
RDH10.AS1
Low
ct1Llsd1H
Med
High
ct1Hlsd1L
group

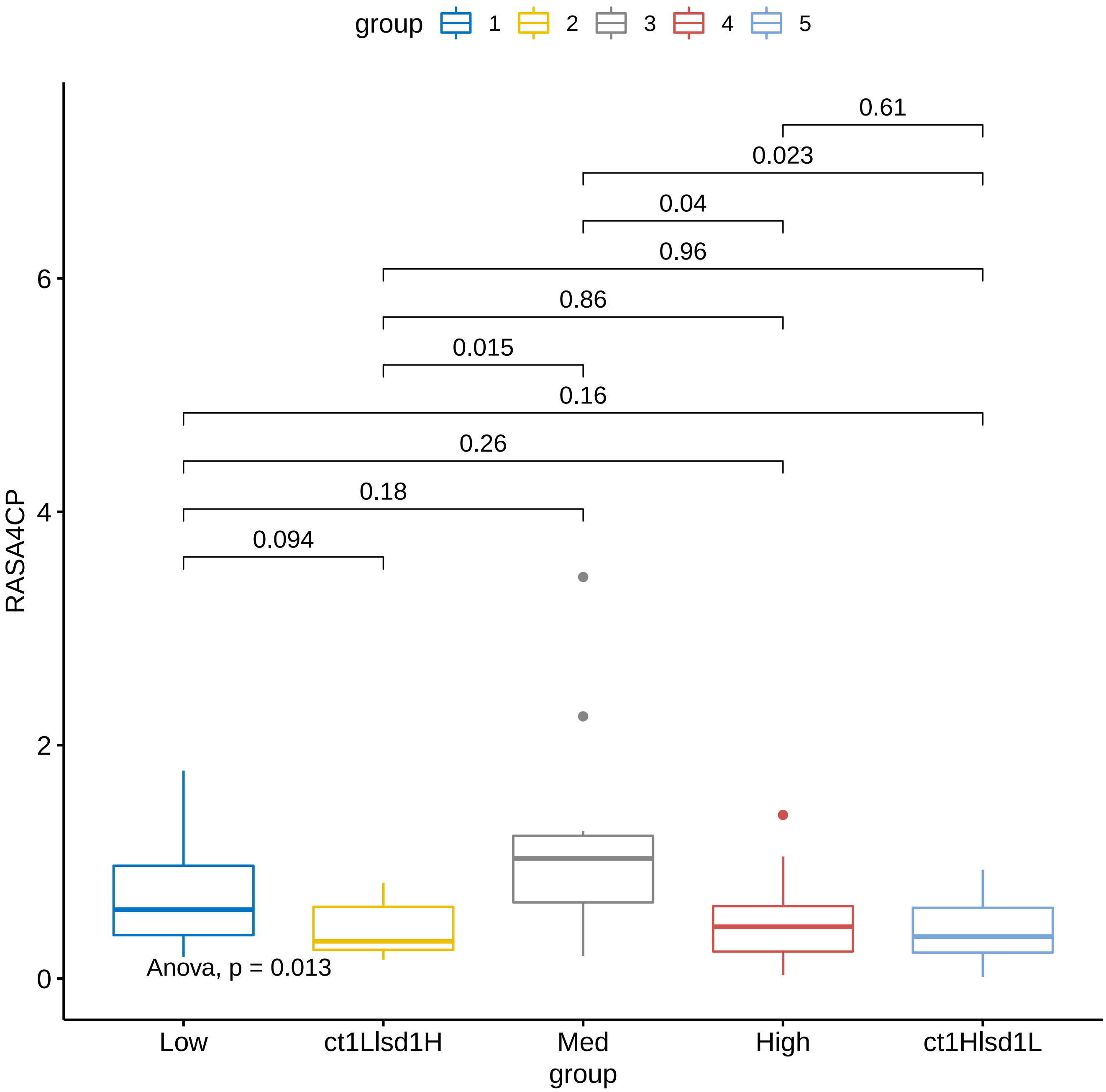
group
1
2
3
4
5
0.61
0.023
0.04
0.96
0.86
0.015
0.16
0.26
0.18
0.094
Anova, p = 0.013
RASA4CP
0
2
4
6
Low
ct1Llsd1H
Med
High
ct1Hlsd1L
group

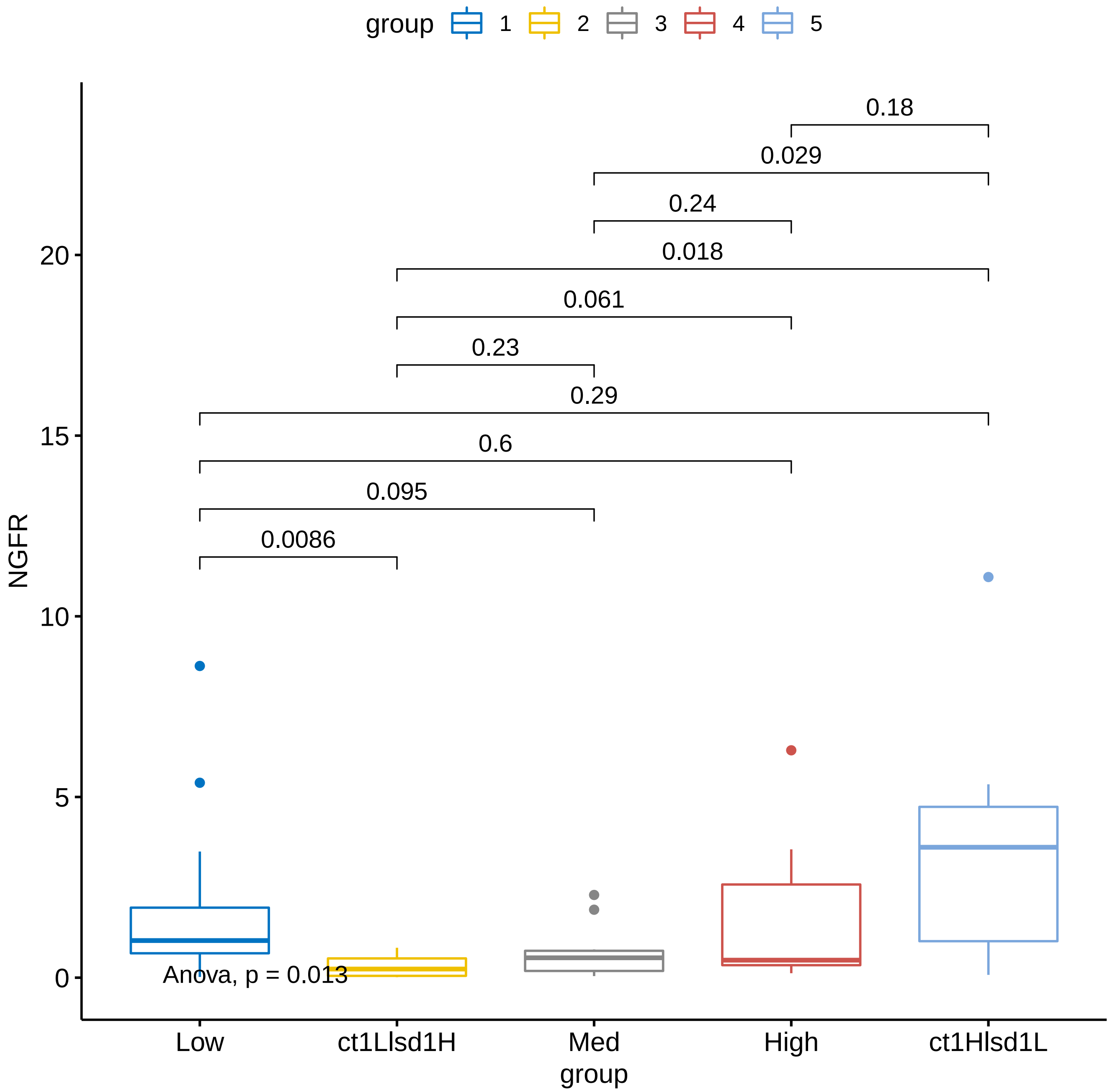

group 1 2 3 4 5
0.18
0.029
0.24
0.018
0.061
0.23
0.29
0.6
0.095
0.0086
Anova, p = 0.013
NGFR
0
5
10
15
20
Low
ct1Llsd1H
Med
High
ct1Hlsd1L
group

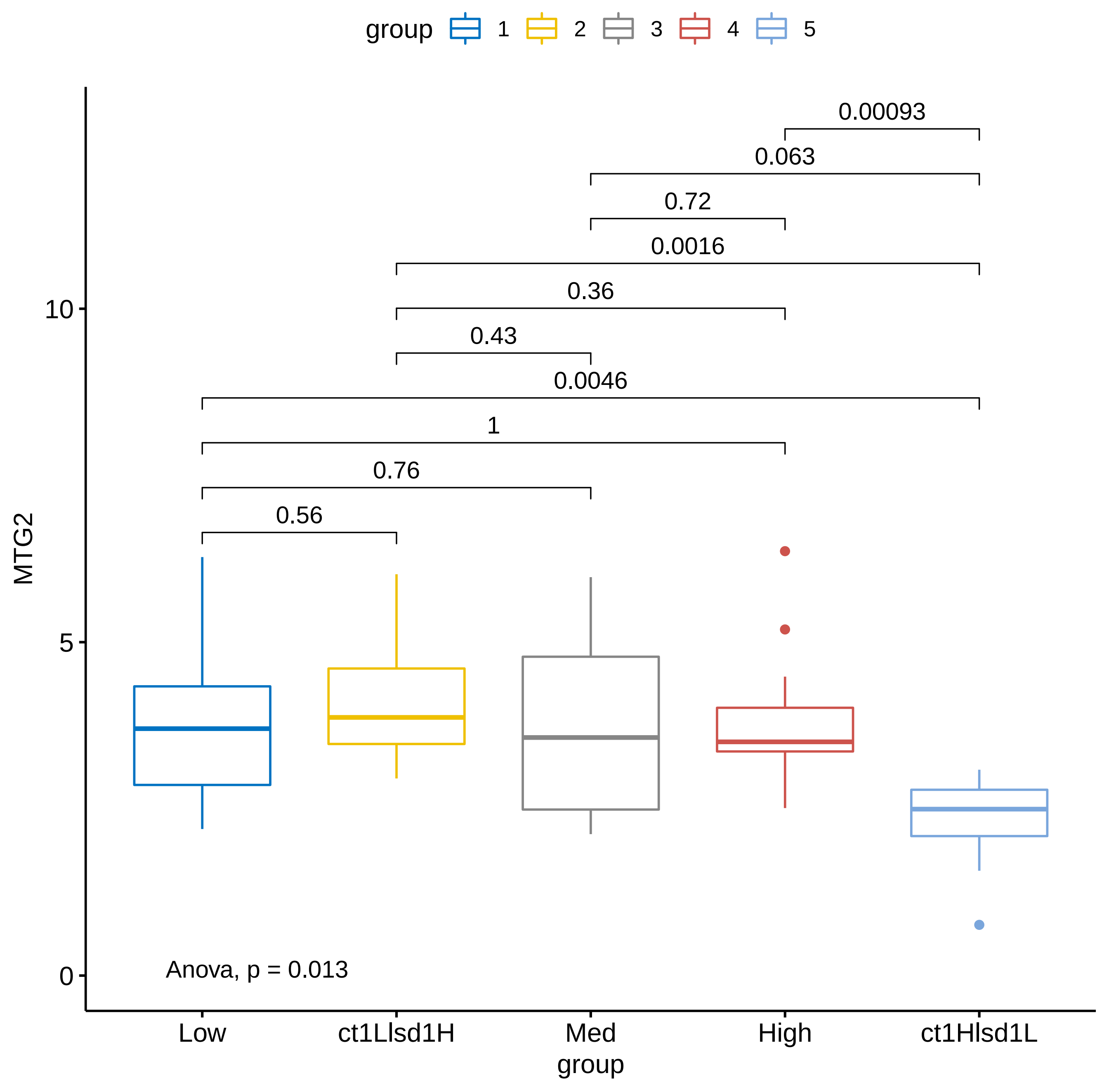

group
1
2
3
4
5
0.00093
0.063
0.72
0.0016
0.36
0.43
0.0046
1
0.76
0.56
10
5
0
MTG2
Anova, p = 0.013
Low
ct1Llsd1H
Med
High
ct1Hlsd1L
group

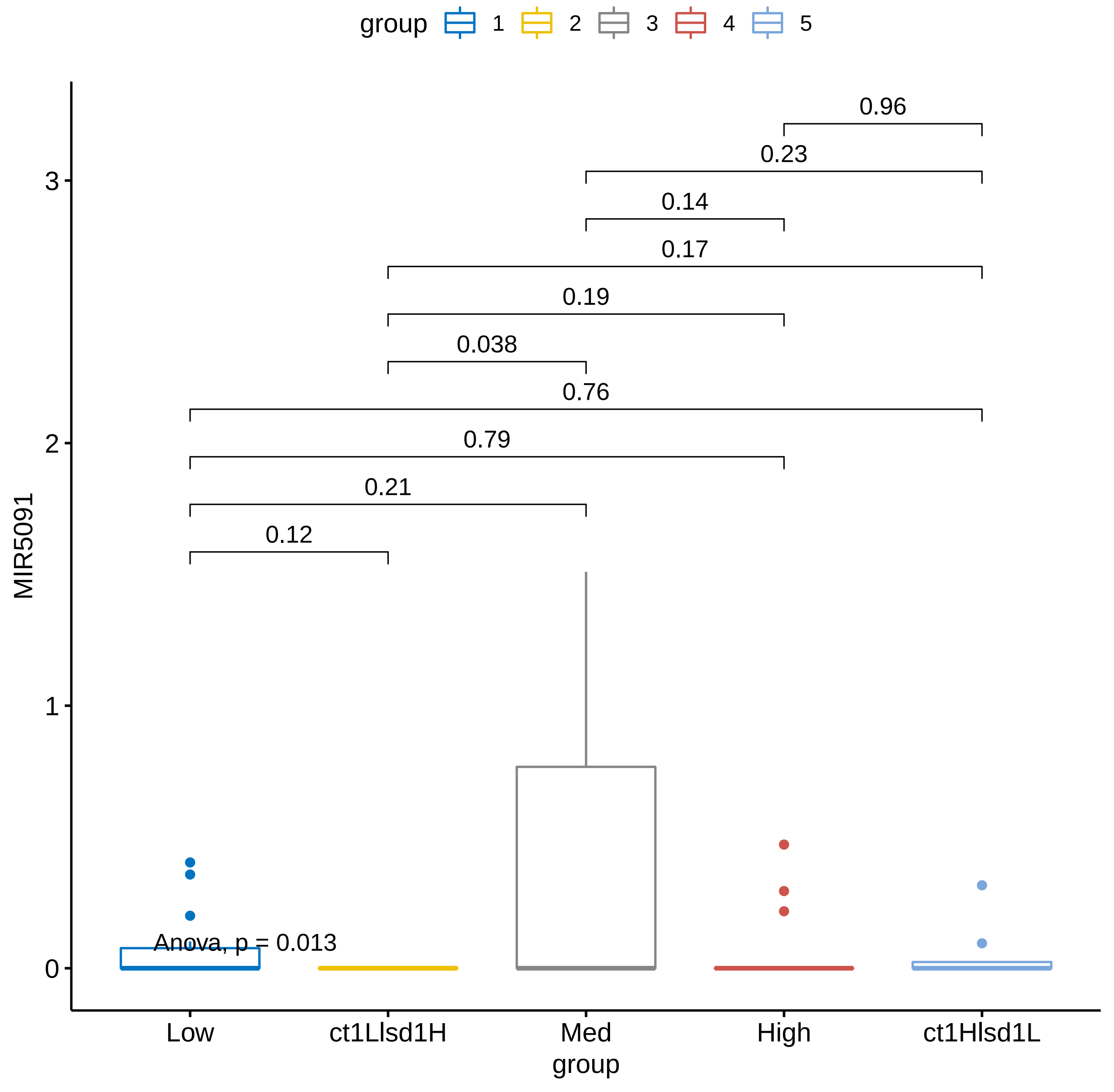

group
1
2
3
4
5
MIR5091
3
2
1
0
0.96
0.23
0.14
0.17
0.19
0.038
0.76
0.79
0.21
0.12
Anova, p = 0.013
Low
ct1Llsd1H
Med
High
ct1Hlsd1L
group

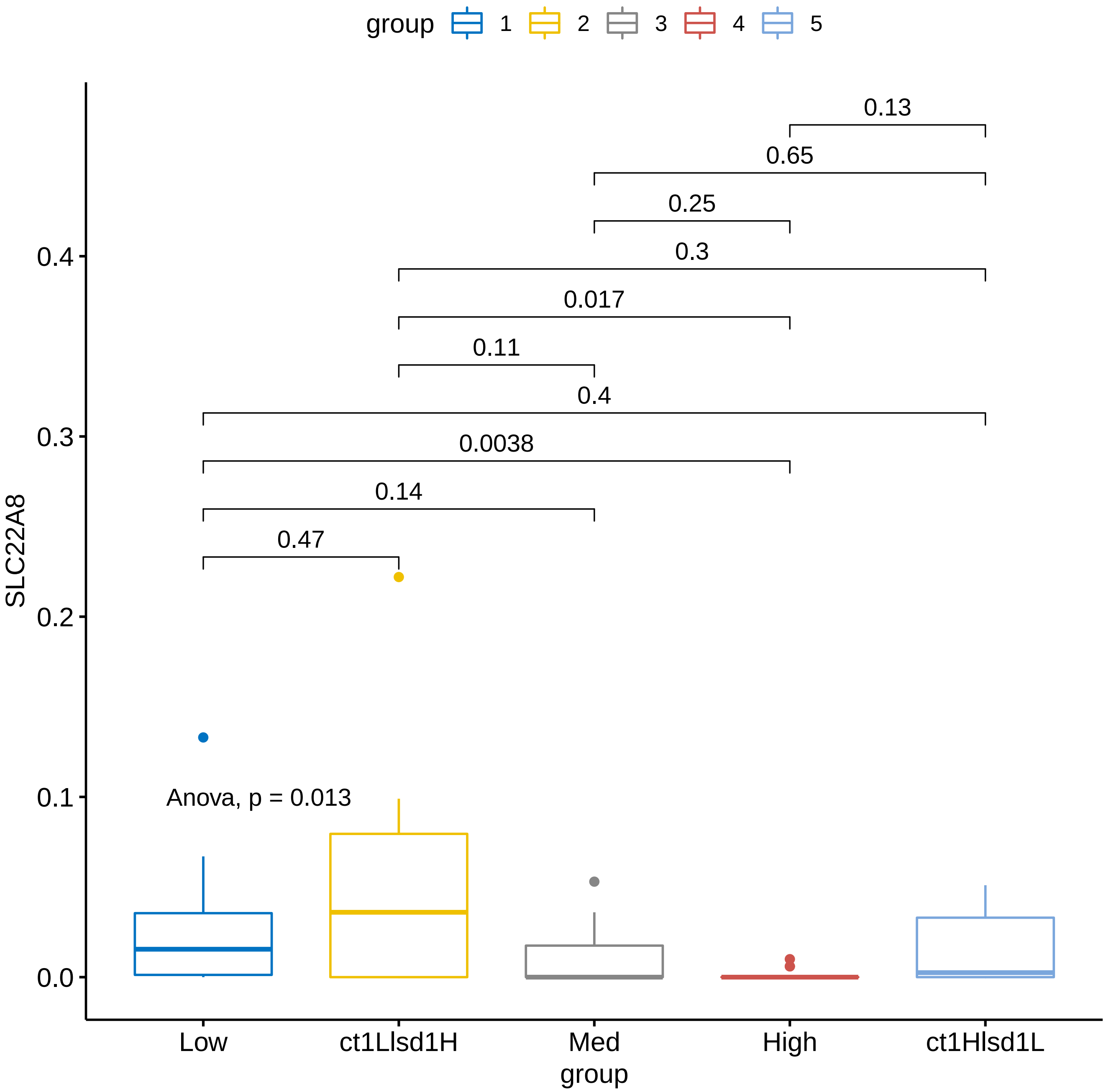

group
1
2
3
4
5
SLC22A8
0.4
0.3
0.2
0.1
0.0
0.13
0.65
0.25
0.3
0.017
0.11
0.4
0.0038
0.14
0.47
Anova, p = 0.013
Low
ct1Llsd1H
Med
High
ct1Hlsd1L
group

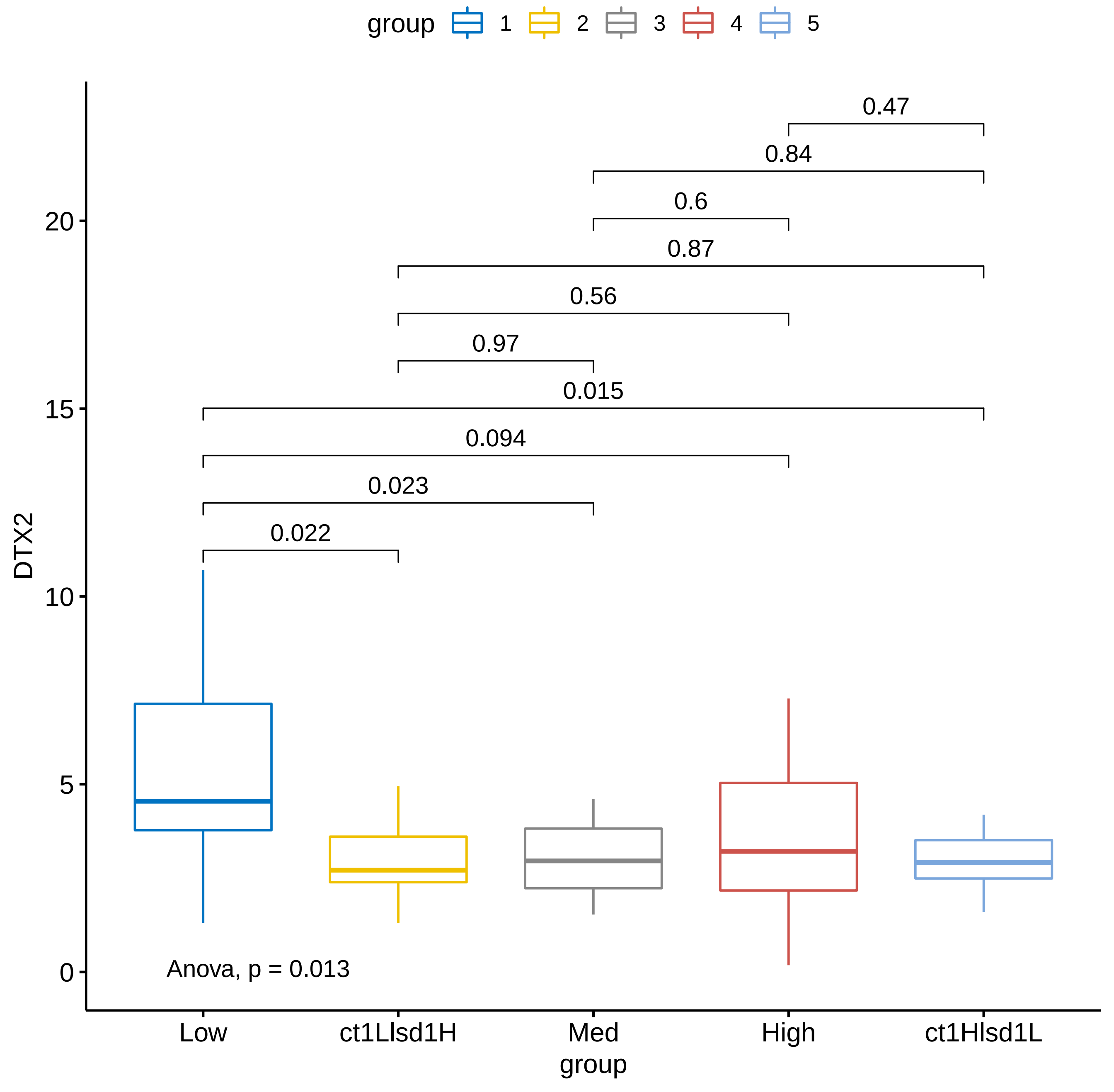

group
1
2
3
4
5
0.47
0.84
0.6
0.87
0.56
0.97
0.015
0.094
0.023
0.022
20
15
10
5
0
DTX2
Anova, p = 0.013
Low
ct1Llsd1H
Med
High
ct1Hlsd1L
group

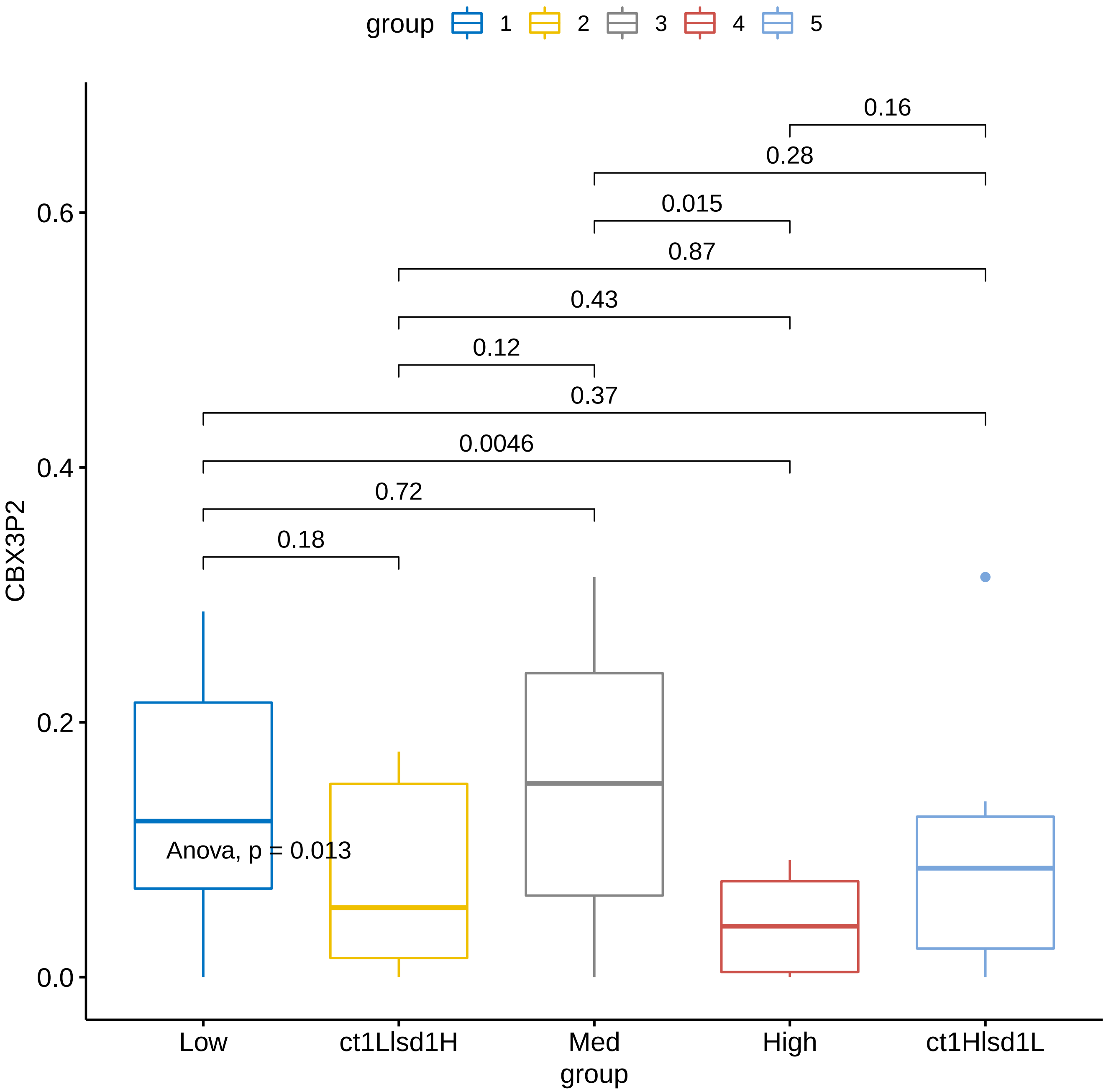
group
1
2
3
4
5
0.16
0.28
0.015
0.87
0.43
0.12
0.37
0.0046
0.72
0.18
Anova, p = 0.013
CBX3P2
0.6
0.4
0.2
0.0
Low
ct1Llsd1H
Med
High
ct1Hlsd1L
group

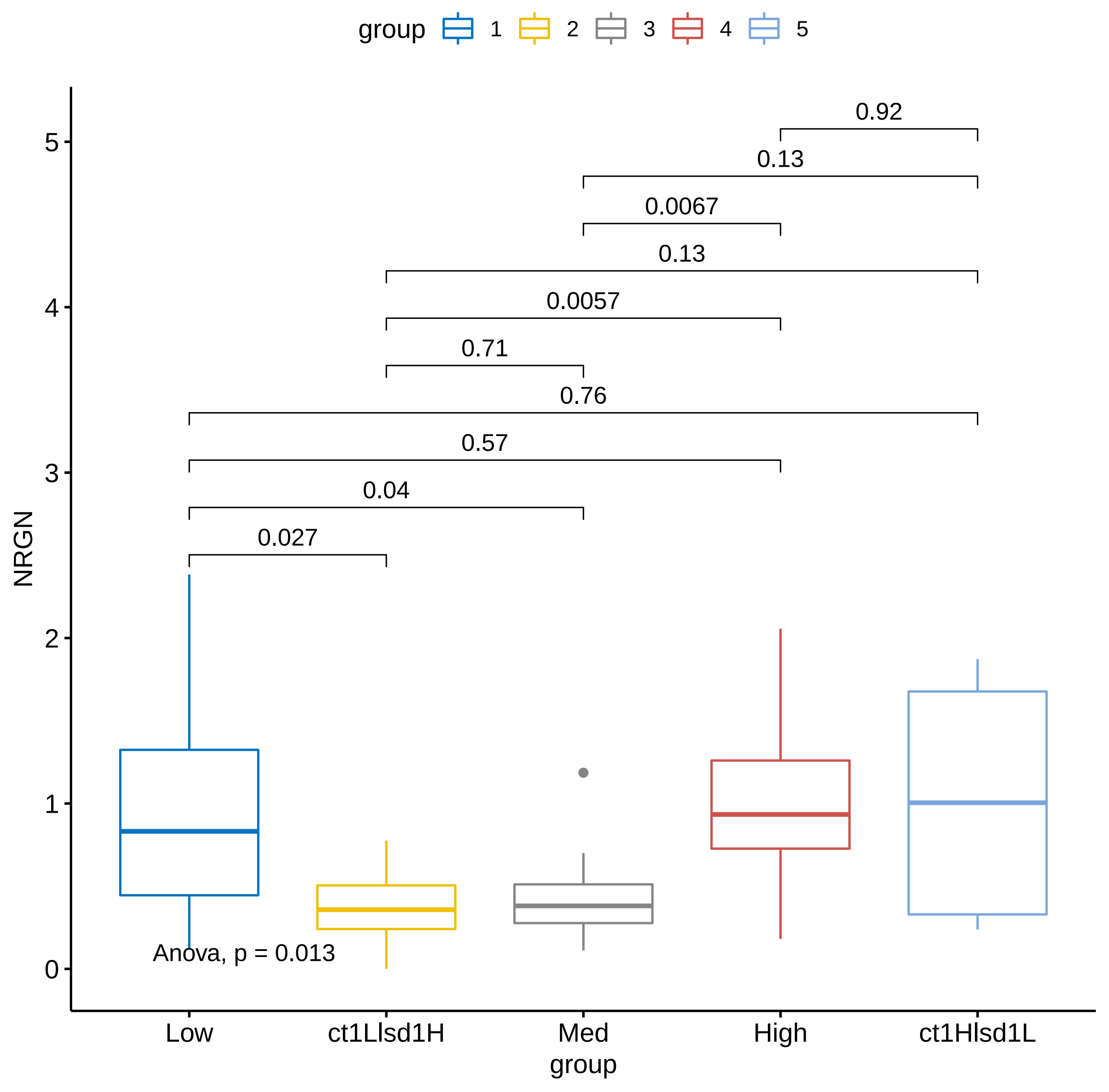

group 1 2 3 4 5
0.92
0.13
0.0067
0.13
0.0057
0.71
0.76
0.57
0.04
0.027
Anova, p = 0.013
NRGN
0
1
2
3
4
5
Low
ct1Llsd1H
Med
High
ct1Hlsd1L
group

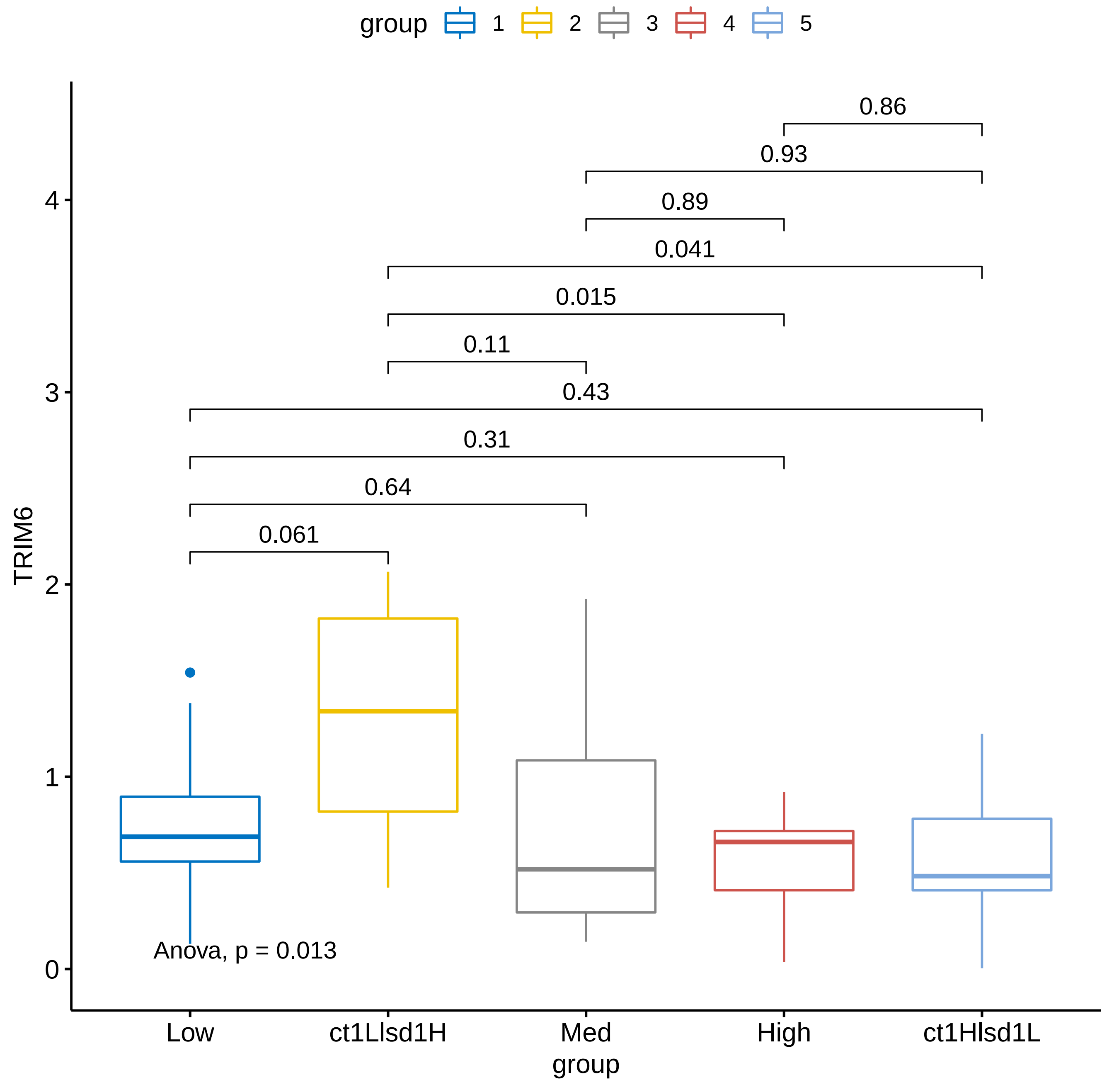

group 1 2 3 4 5
0.86
0.93
0.89
0.041
0.015
0.11
0.43
0.31
0.64
0.061
TRIM6
4
3
2
1
0
Anova, p = 0.013
Low
ct1Llsd1H
Med
High
ct1Hlsd1L
group

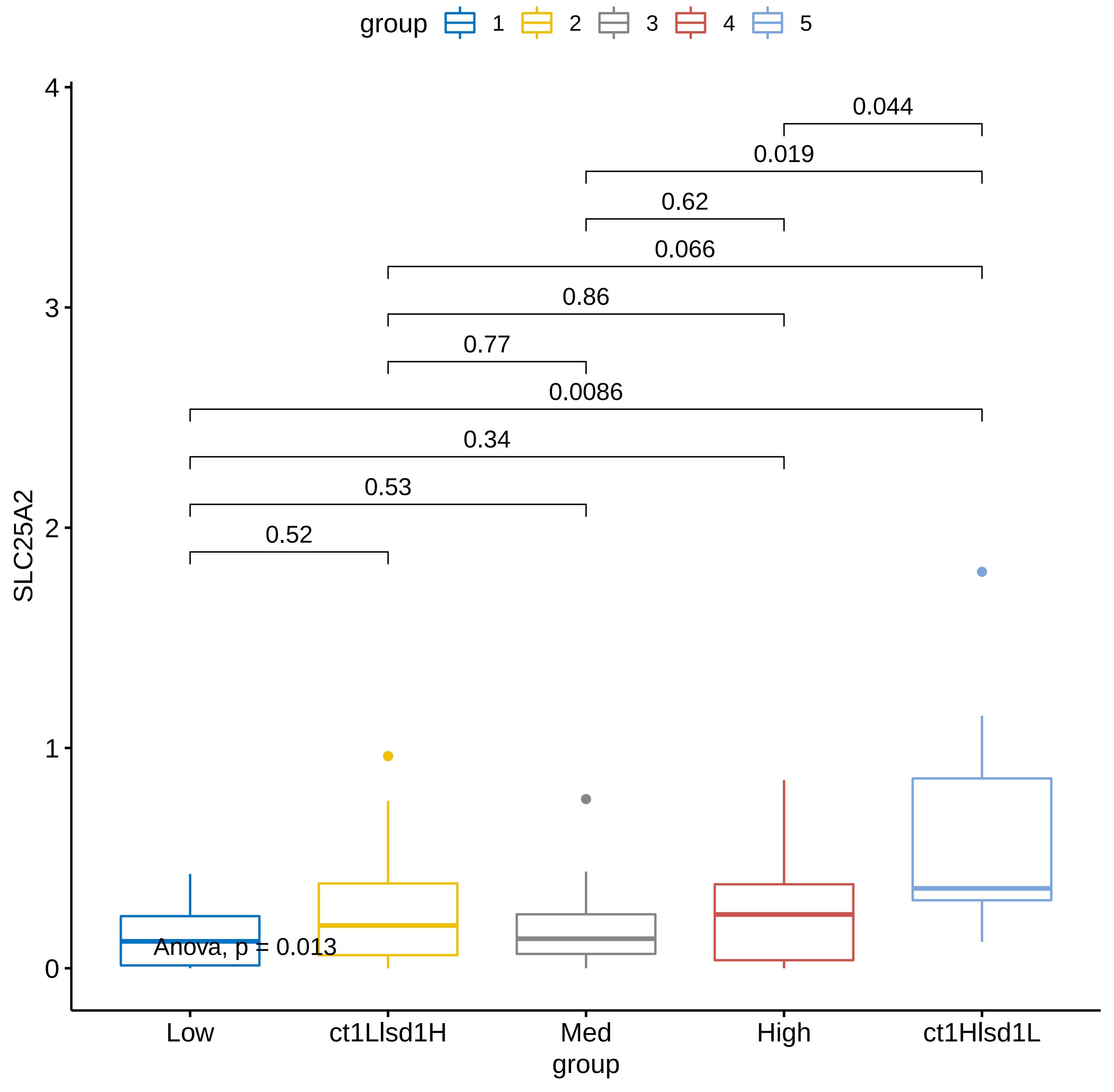

group
1
2
3
4
5
SLC25A2
0
1
2
3
4
0.044
0.019
0.62
0.066
0.86
0.77
0.0086
0.34
0.53
0.52
Anova, p = 0.013
Low
ct1Llsd1H
Med
High
ct1Hlsd1L
group

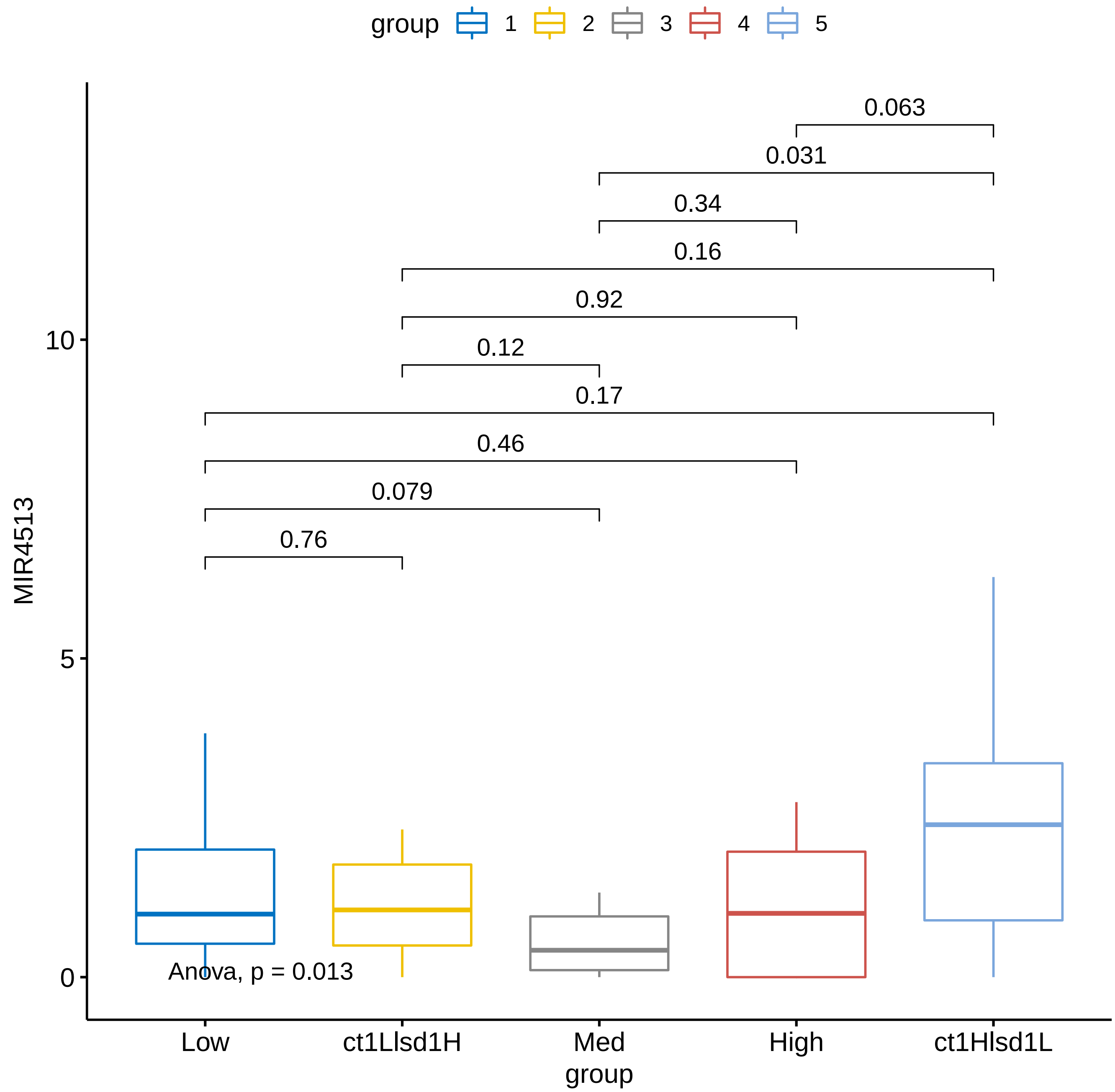

group
1
2
3
4
5
0.063
0.031
0.34
0.16
0.92
0.12
0.17
0.46
0.079
0.76
MIR4513
10
5
0
Anova, p = 0.013
Low
ct1Llsd1H
Med
High
ct1Hlsd1L
group

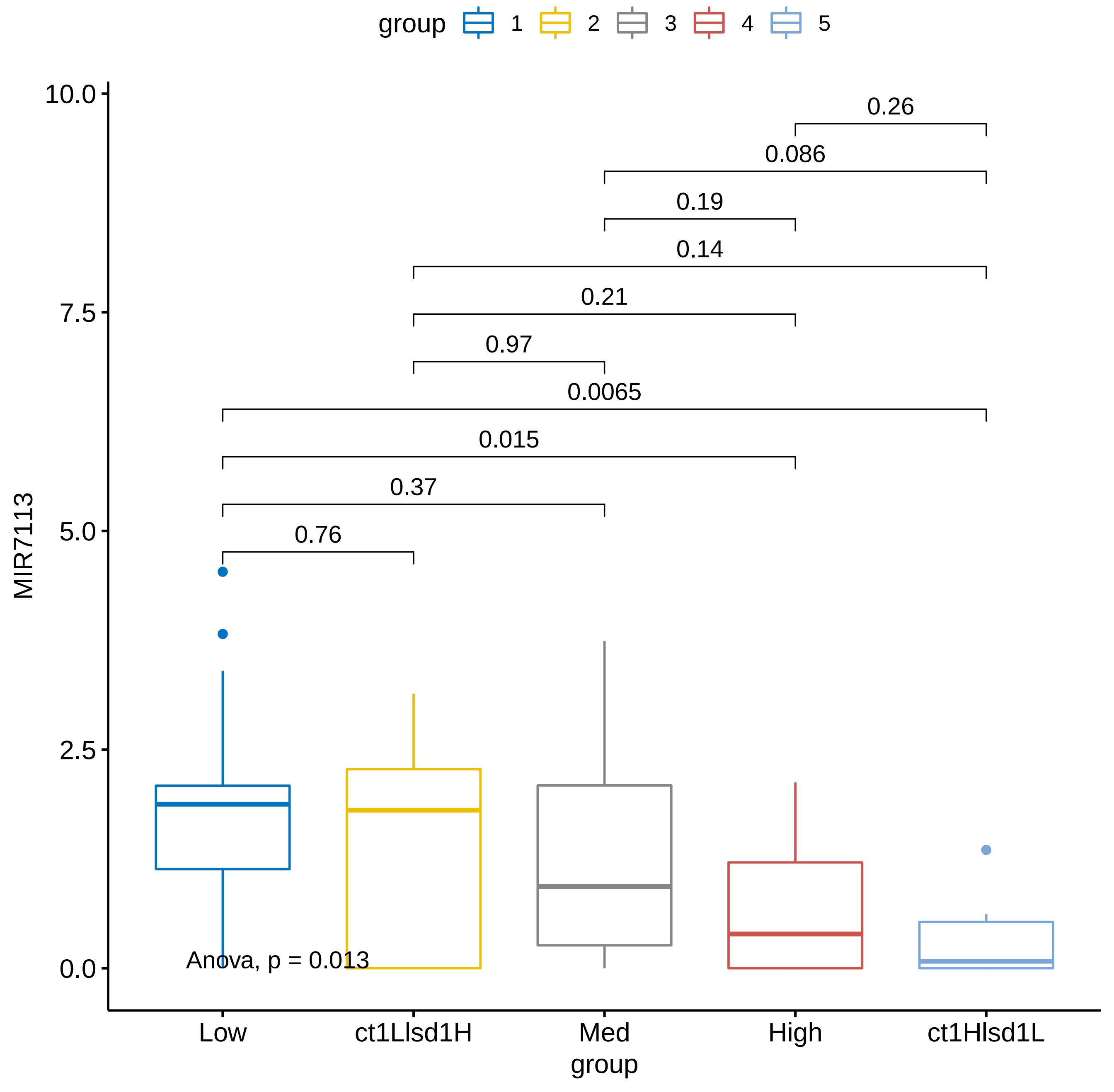

group
1
2
3
4
5
10.0
7.5
5.0
2.5
0.0
MIR7113
0.26
0.086
0.19
0.14
0.21
0.97
0.0065
0.015
0.37
0.76
Anova, p = 0.013
Low
ct1Llsd1H
Med
High
ct1Hlsd1L
group

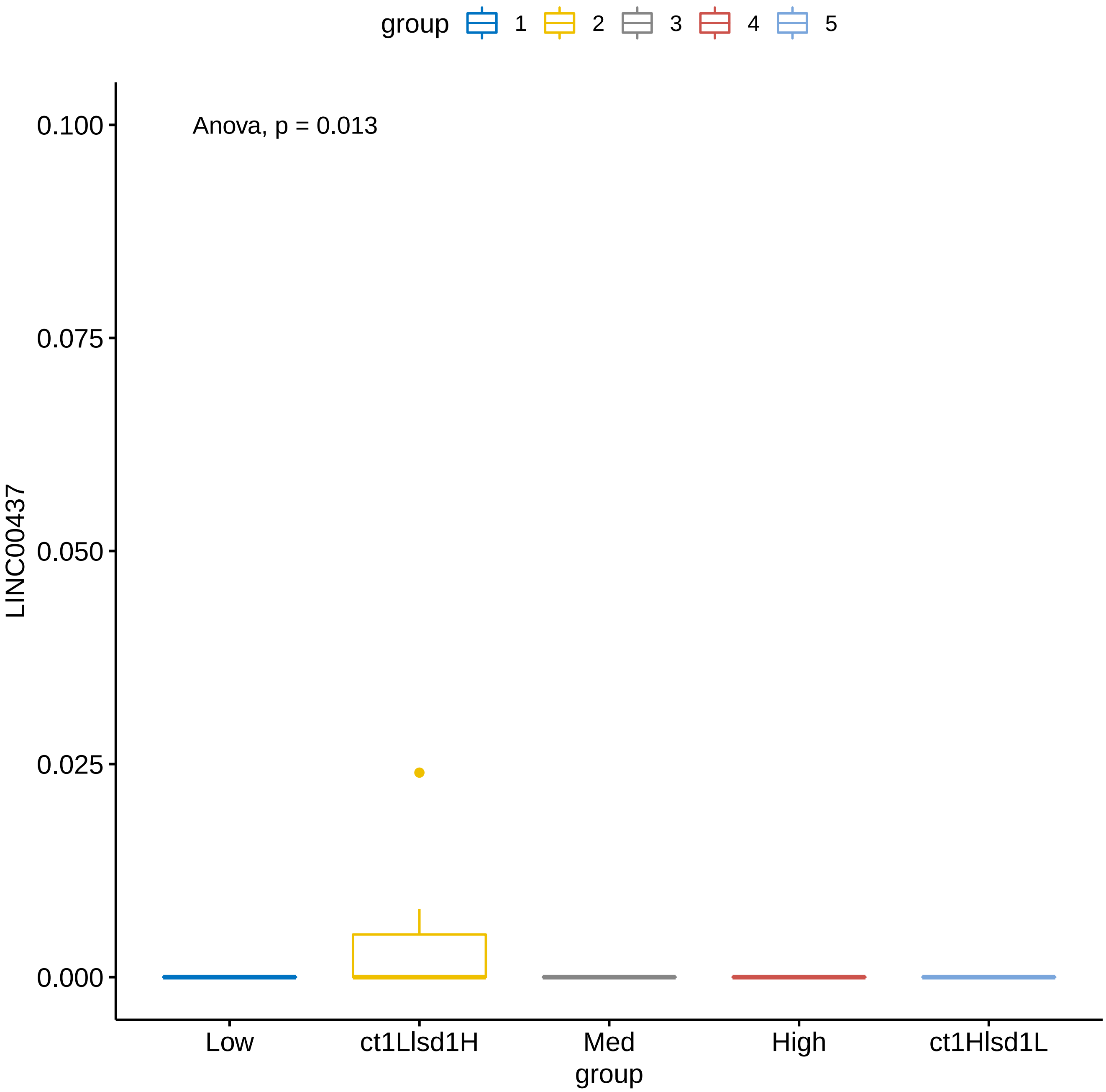
group
1
2
3
4
5
Anova, p = 0.013
LINC00437
0.100
0.075
0.050
0.025
0.000
Low
ct1Llsd1H
Med
High
ct1Hlsd1L
group

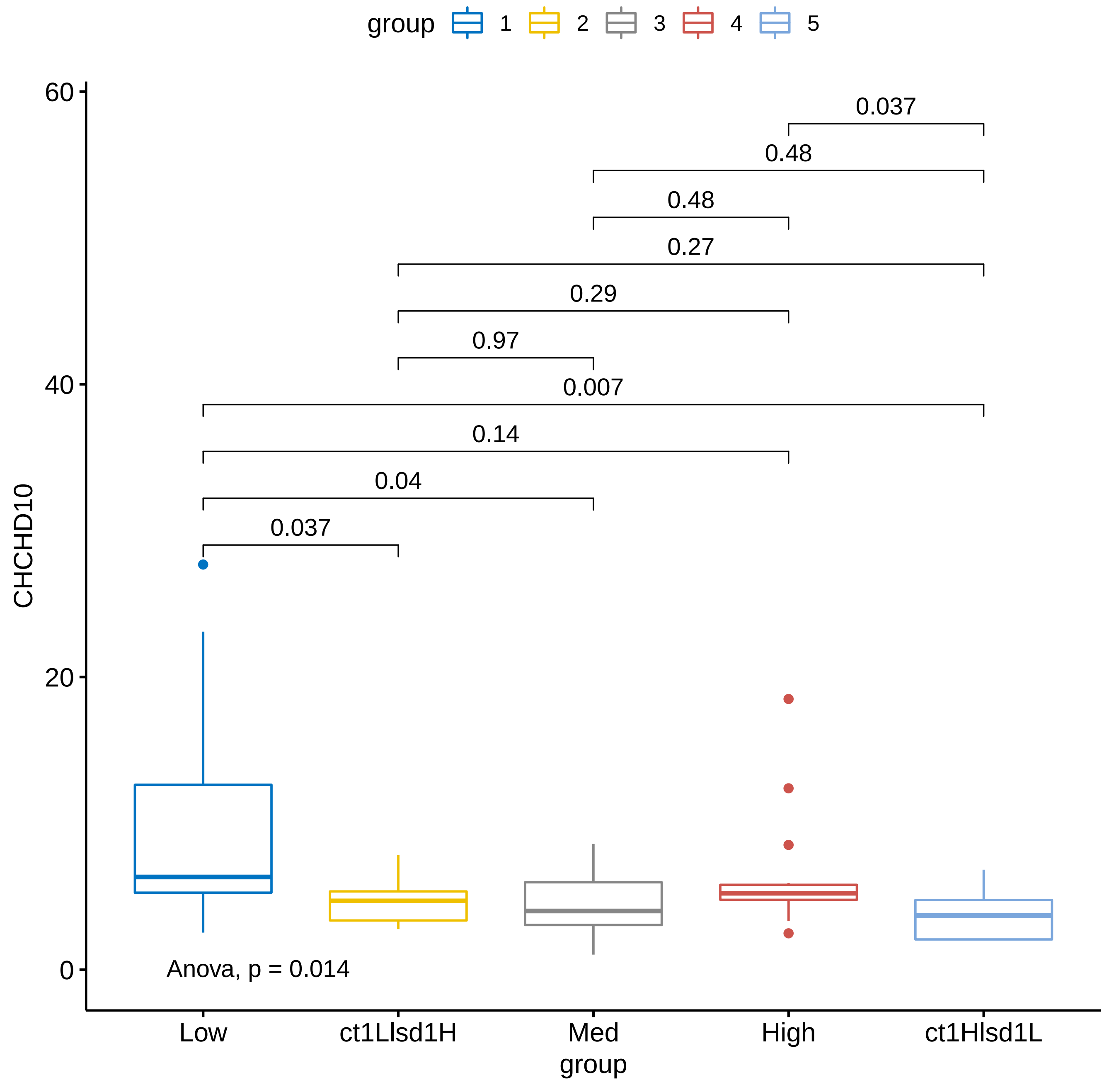

group
1
2
3
4
5
CHCHD10
60
40
20
0
0.037
0.48
0.48
0.27
0.29
0.97
0.007
0.14
0.04
0.037
Anova, p = 0.014
Low
ct1Llsd1H
Med
High
ct1Hlsd1L
group

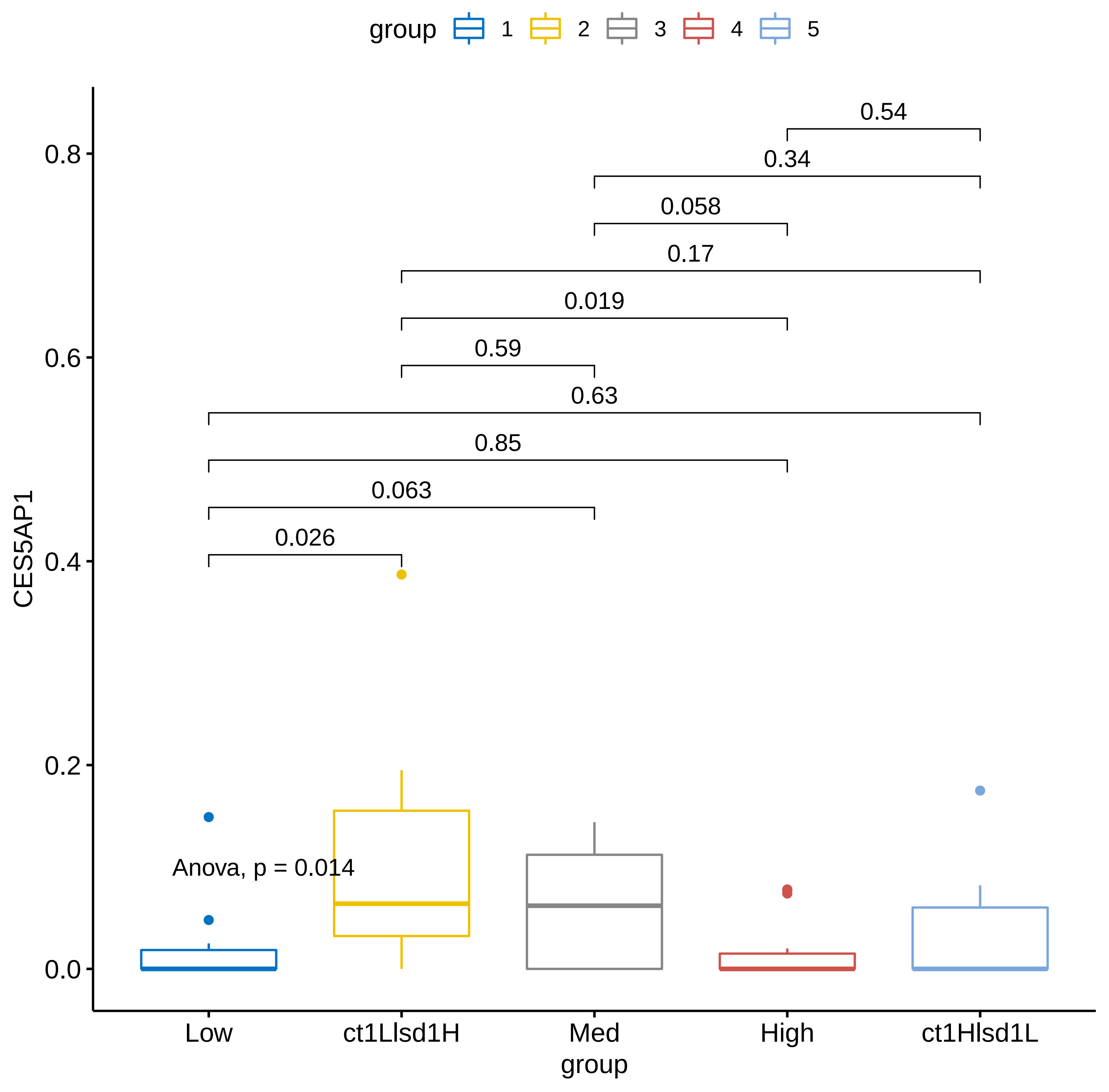

group
1
2
3
4
5
CES5AP1
0.8
0.6
0.4
0.2
0.0
0.54
0.34
0.058
0.17
0.019
0.59
0.63
0.85
0.063
0.026
Anova, p = 0.014
Low
ct1Llsd1H
Med
High
ct1Hlsd1L
group

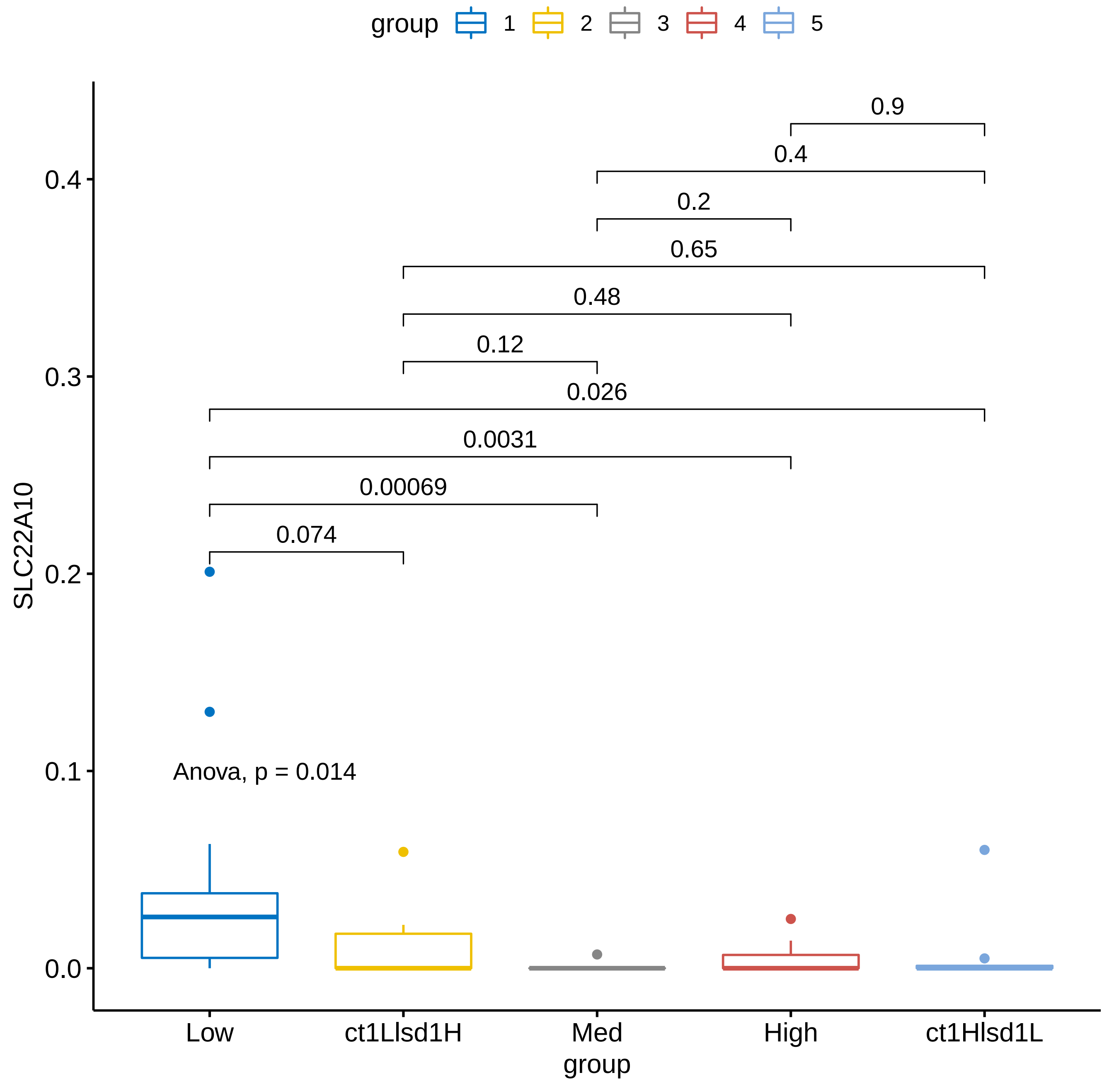
group
1
2
3
4
5
SLC22A10
0.4
0.3
0.2
0.1
0.0
0.9
0.4
0.2
0.65
0.48
0.12
0.026
0.0031
0.00069
0.074
Anova, p = 0.014
Low
ct1Llsd1H
Med
High
ct1Hlsd1L
group

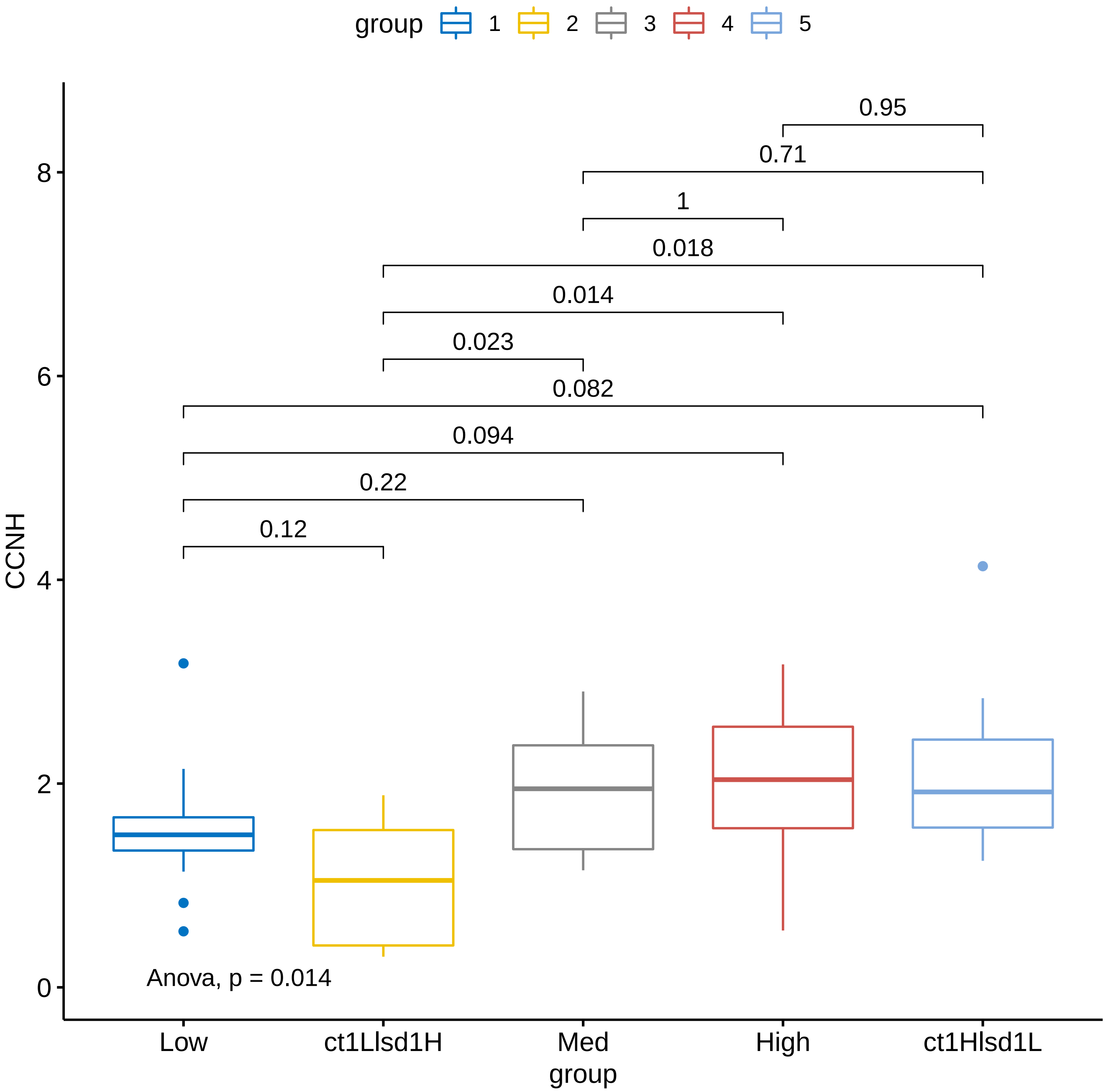
group
1
2
3
4
5
CCNH
8
6
4
2
0
0.95
0.71
1
0.018
0.014
0.023
0.082
0.094
0.22
0.12
Anova, p = 0.014
Low
ct1Llsd1H
Med
High
ct1Hlsd1L
group

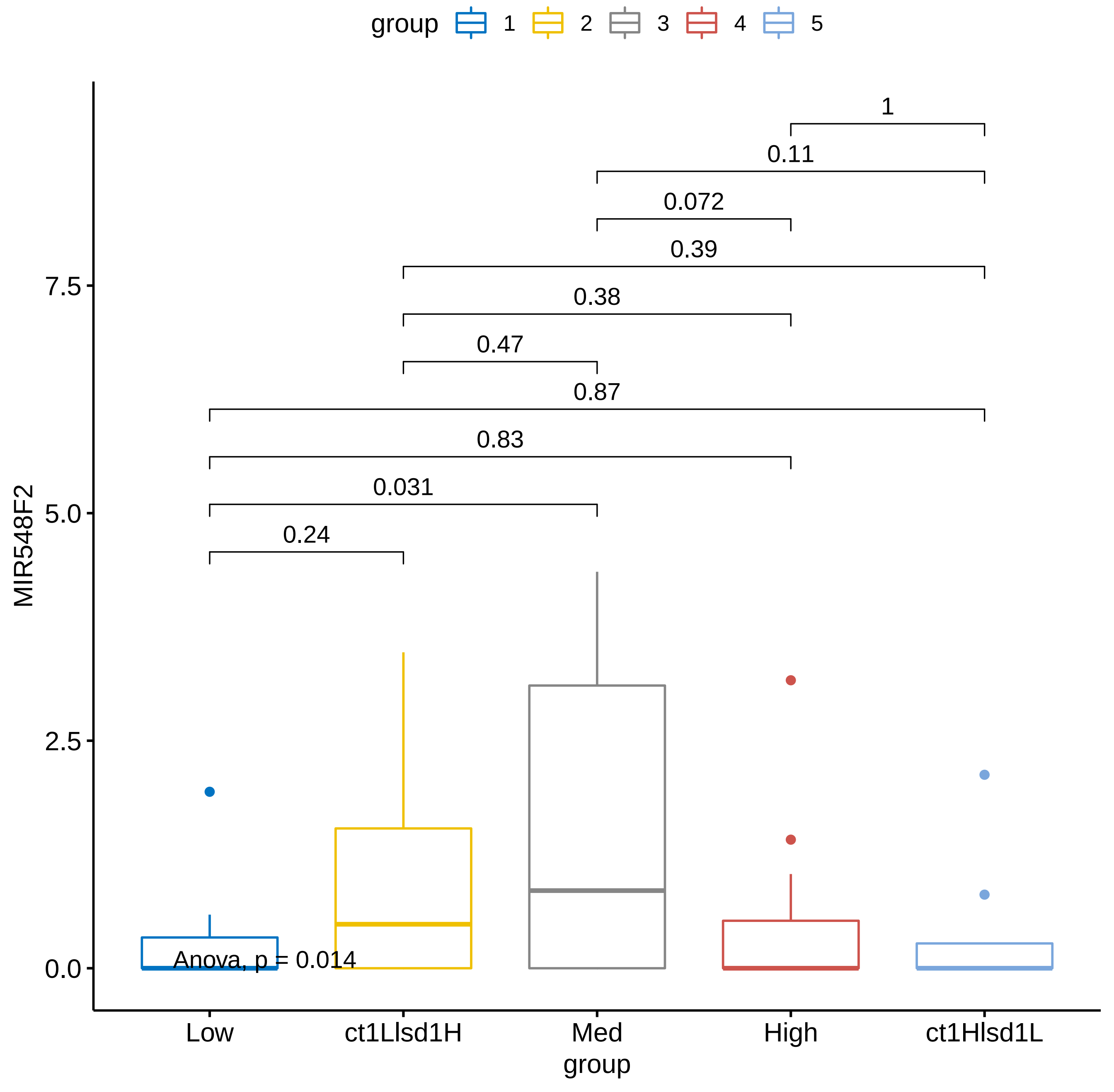
group
1
2
3
4
5
1
0.11
0.072
0.39
0.38
0.47
0.87
0.83
0.031
0.24
7.5
5.0
2.5
0.0
MIR548F2
Anova, p = 0.014
Low
ct1Llsd1H
Med
High
ct1Hlsd1L
group

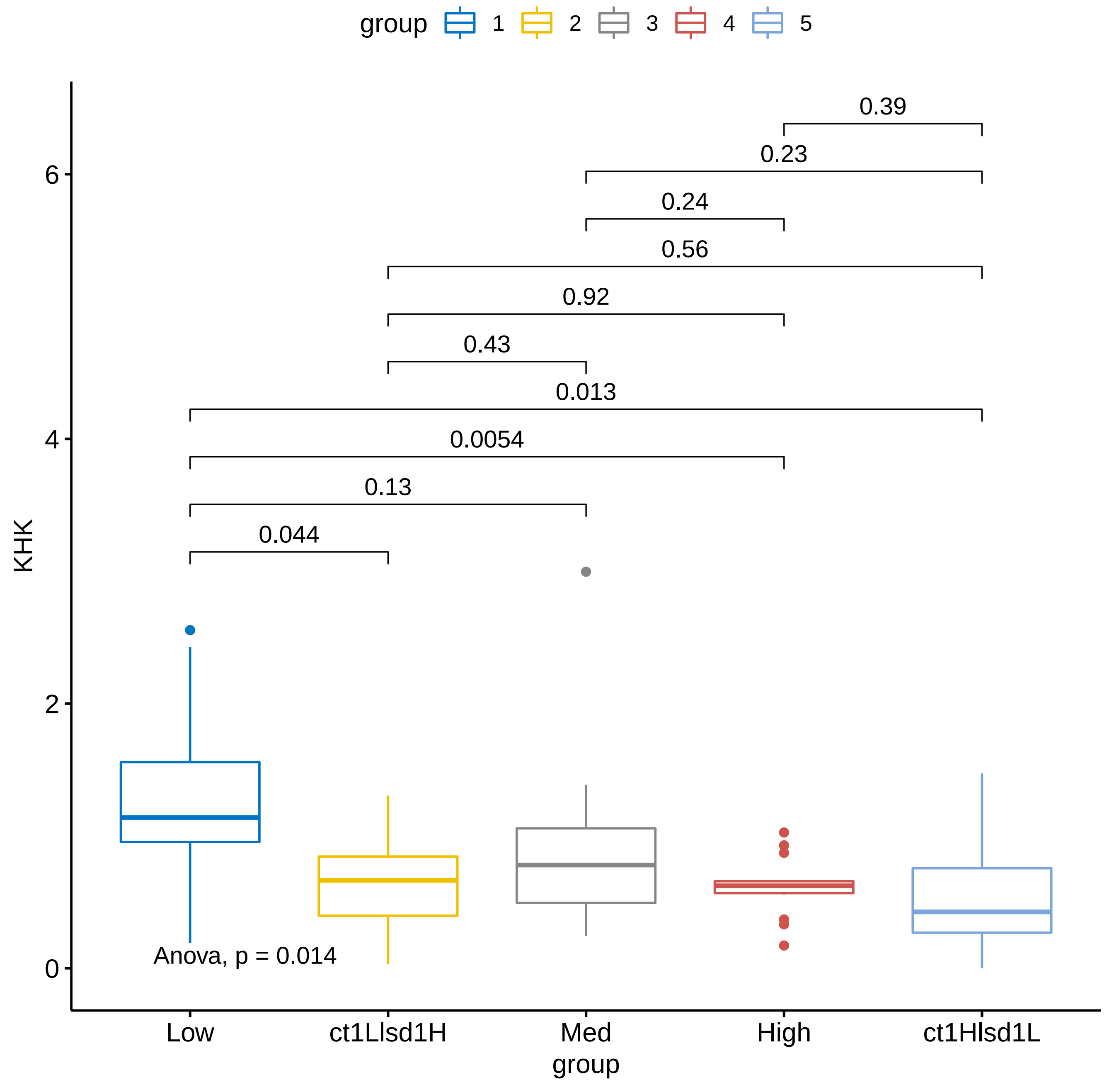
group
1
2
3
4
5
0.39
0.23
0.24
0.56
0.92
0.43
0.013
0.0054
0.13
0.044
6
4
2
0
KHK
Anova, p = 0.014
Low
ct1Llsd1H
Med
High
ct1Hlsd1L
group

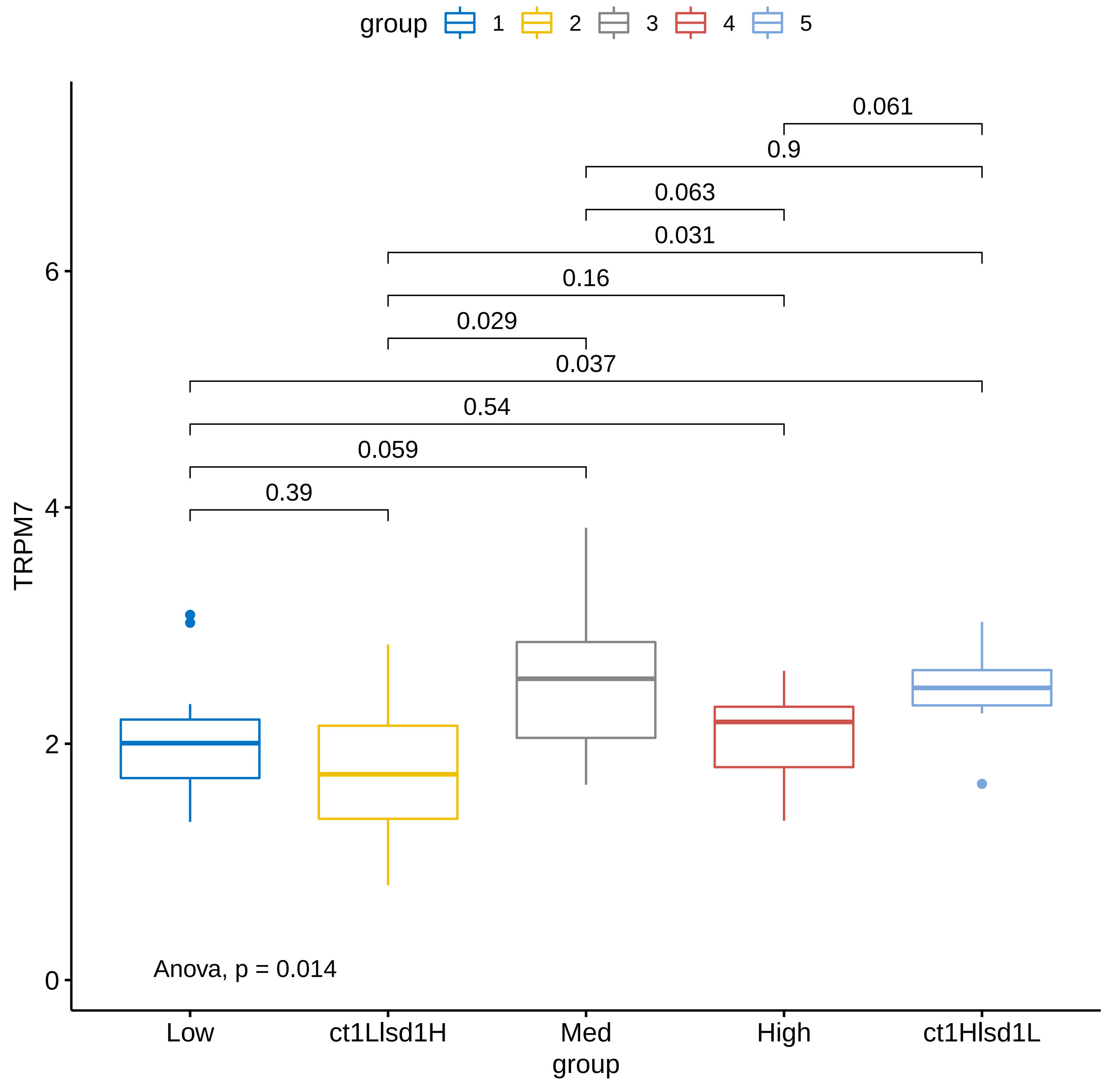

group
1
2
3
4
5
TRPM7
0.061
0.9
0.063
0.031
0.16
0.029
0.037
0.54
0.059
0.39
6
4
2
0
Anova, p = 0.014
Low
ct1Llsd1H
Med
High
ct1Hlsd1L
group

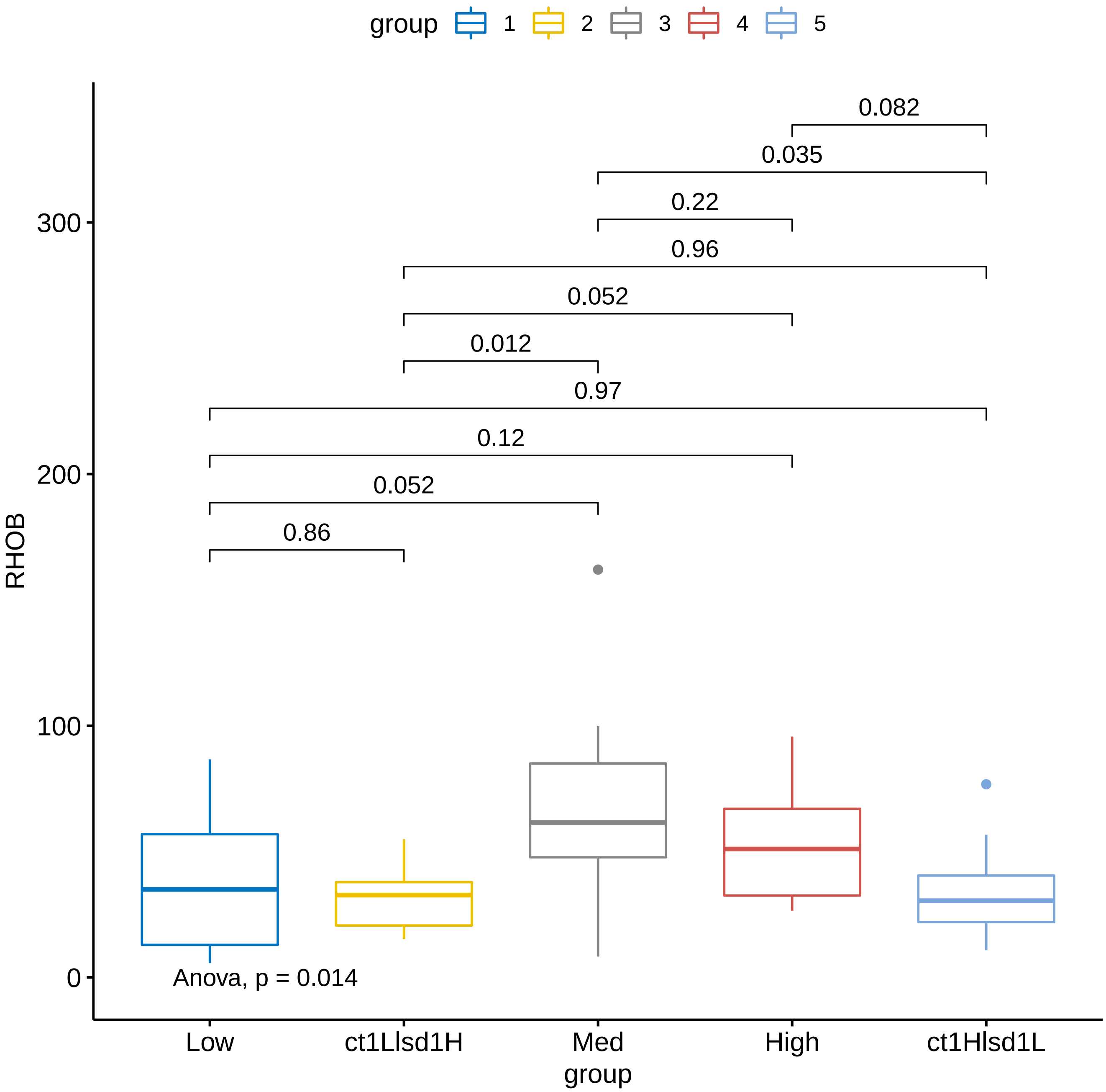

group
1
2
3
4
5
0.082
0.035
0.22
0.96
0.052
0.012
0.97
0.12
0.052
0.86
Anova, p = 0.014
RHOB
300
200
100
0
Low
ct1Llsd1H
Med
High
ct1Hlsd1L
group

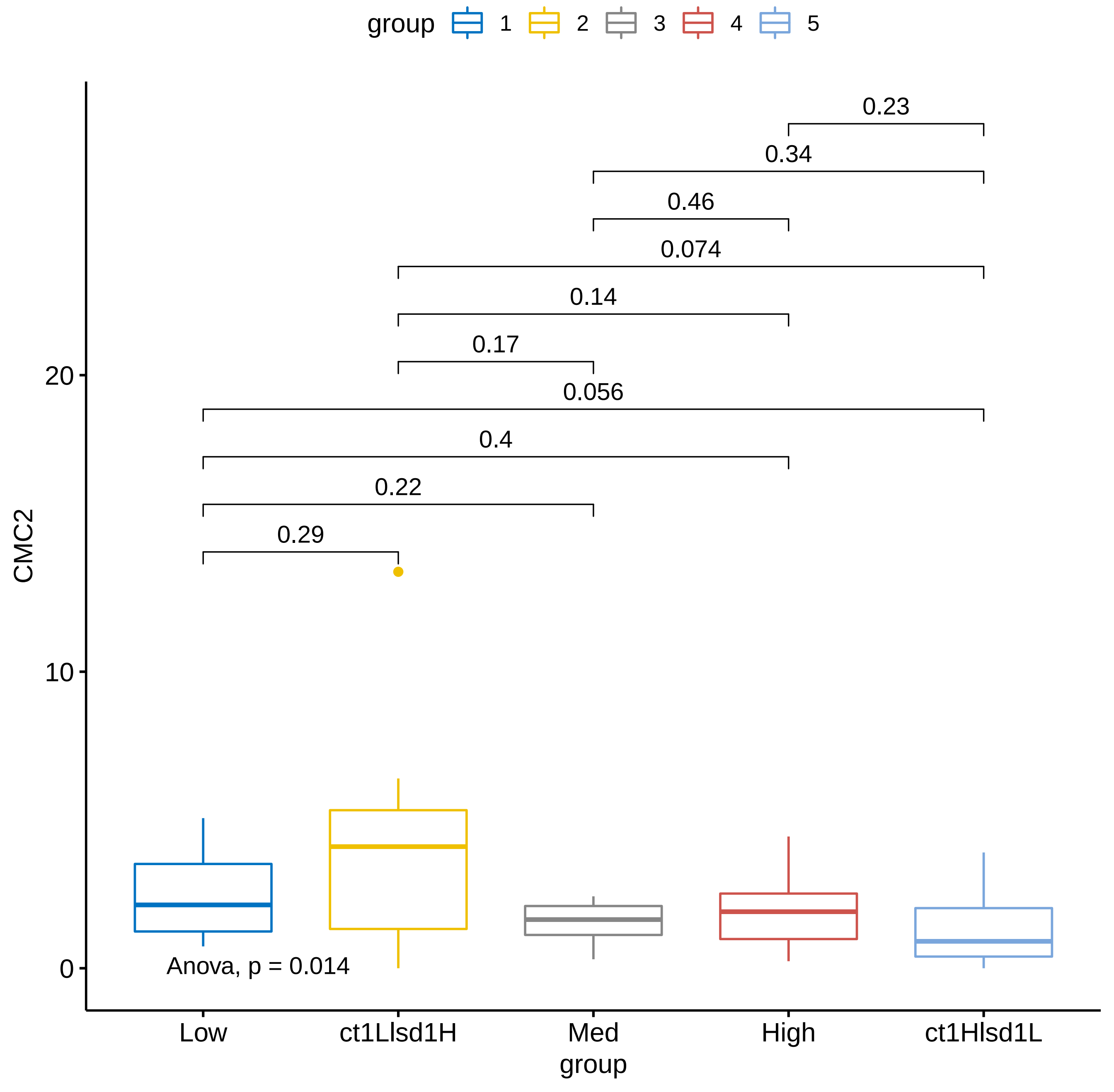
group
1
2
3
4
5
CMC2
0
10
20
0.23
0.34
0.46
0.074
0.14
0.17
0.056
0.4
0.22
0.29
Anova, p = 0.014
Low
ct1Llsd1H
Med
High
ct1Hlsd1L
group

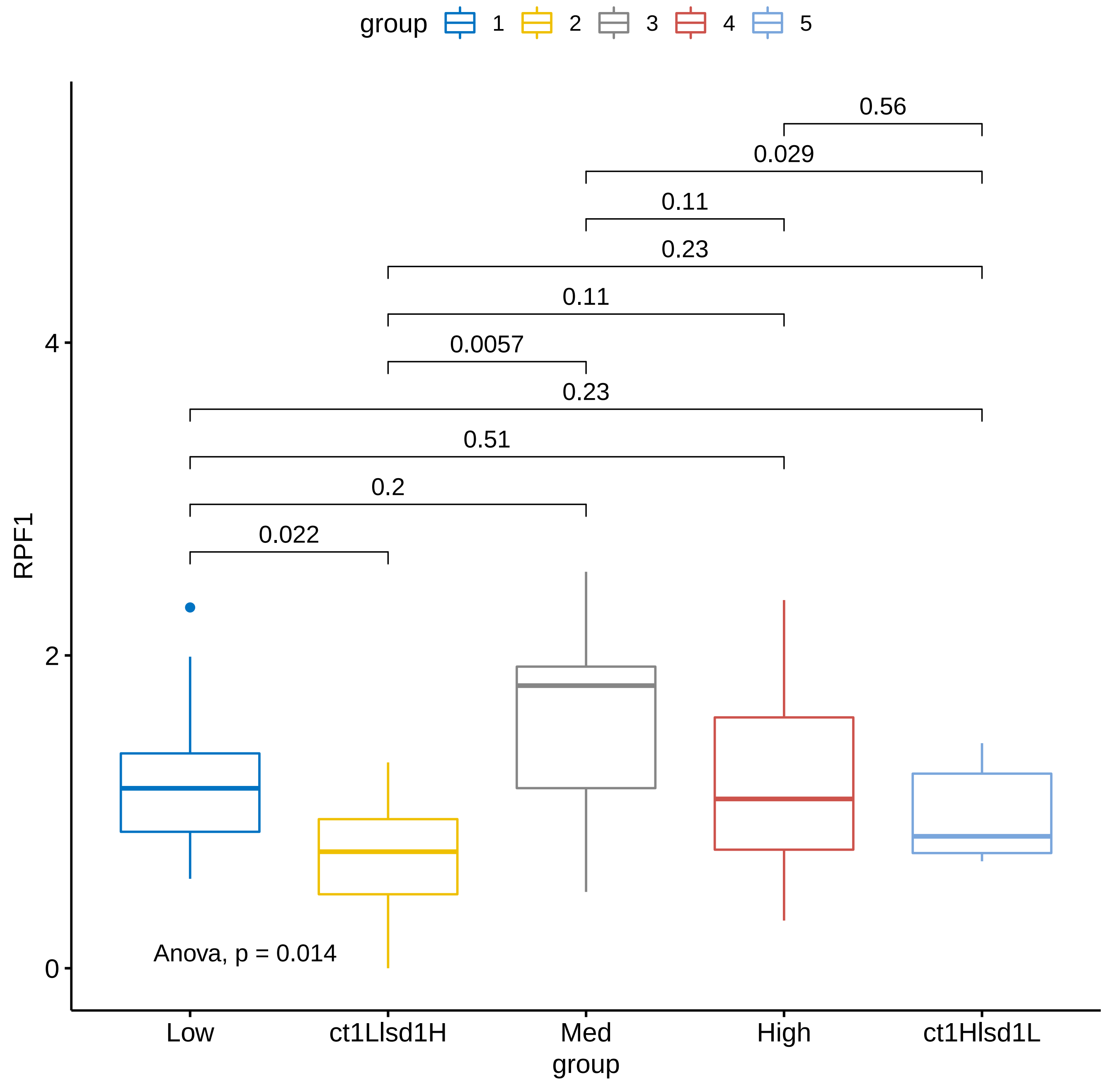
group
1
2
3
4
5
0.56
0.029
0.11
0.23
0.11
0.0057
0.23
0.51
0.2
0.022
RPF1
4
2
0
Anova, p = 0.014
Low
ct1Llsd1H
Med
High
ct1Hlsd1L
group

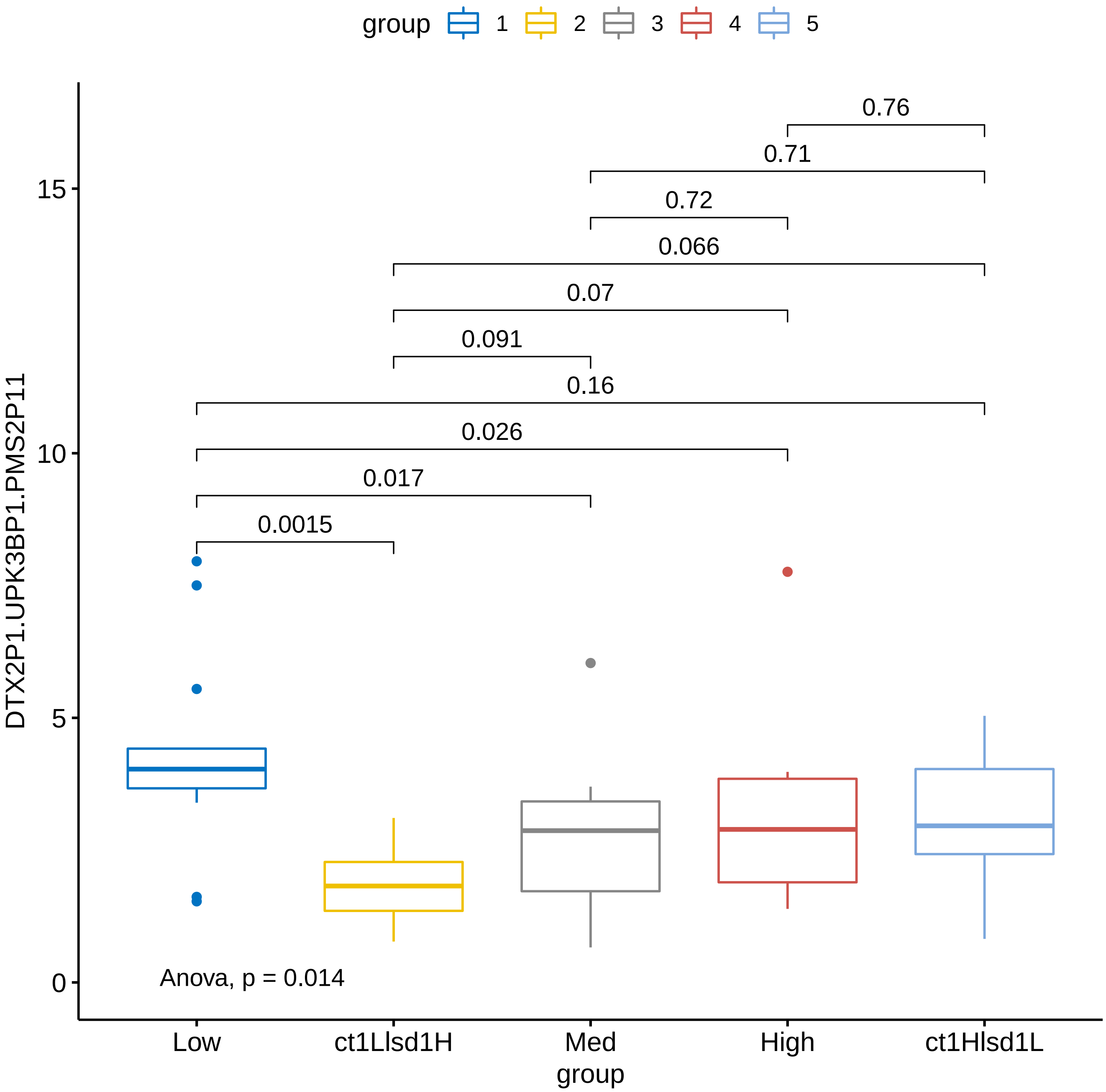

group
1
2
3
4
5
0.76
0.71
0.72
0.066
0.07
0.091
0.16
0.026
0.017
0.0015
DTX2P1.UPK3BP1.PMS2P11
15
10
5
0
Anova, p = 0.014
Low
ct1Llsd1H
Med
High
ct1Hlsd1L
group

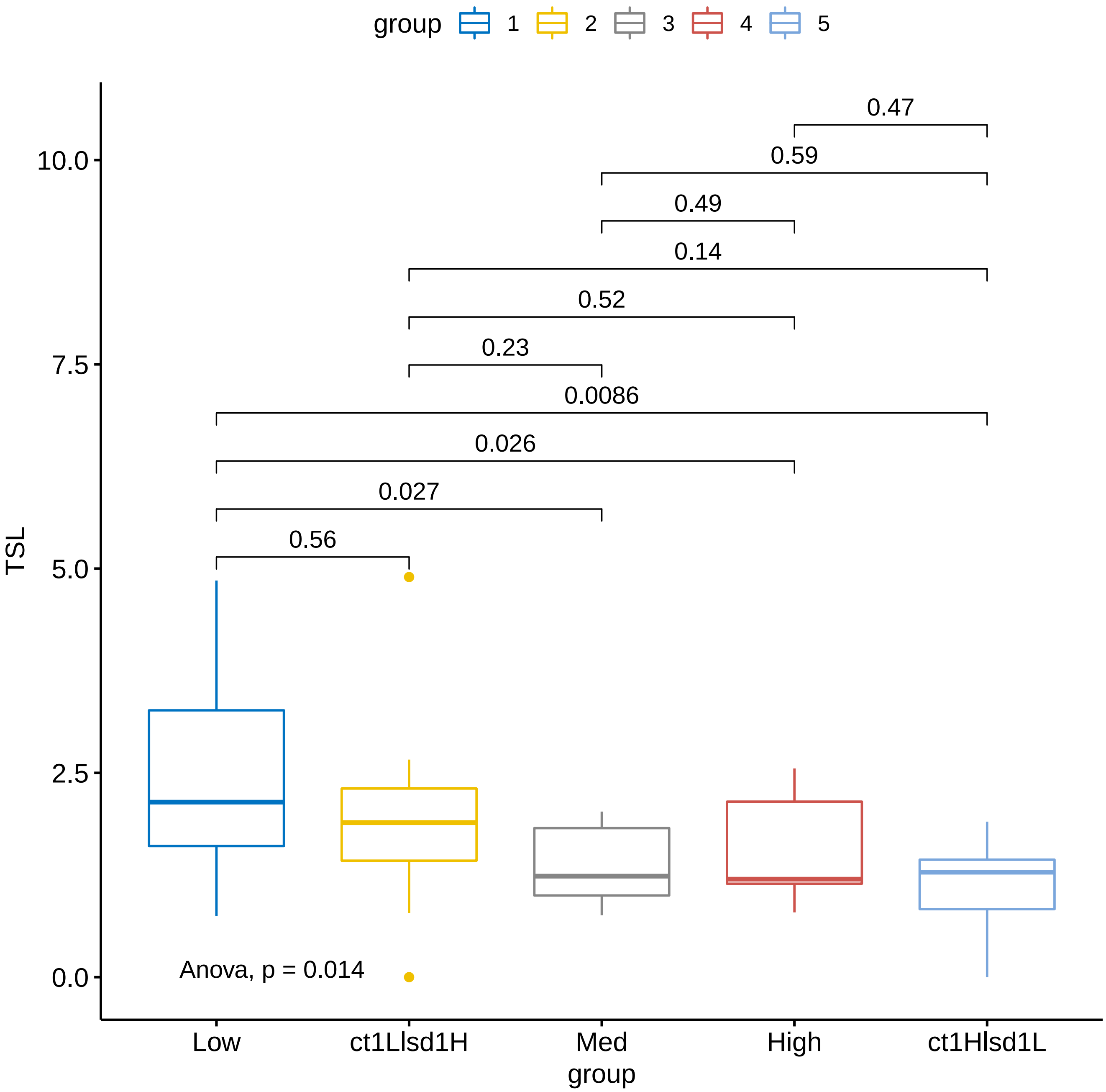

group 1 2 3 4 5
TSL
10.0
7.5
5.0
2.5
0.0
0.47
0.59
0.49
0.14
0.52
0.23
0.0086
0.026
0.027
0.56
Anova, p = 0.014
Low
ct1Llsd1H
Med
High
ct1Hlsd1L
group

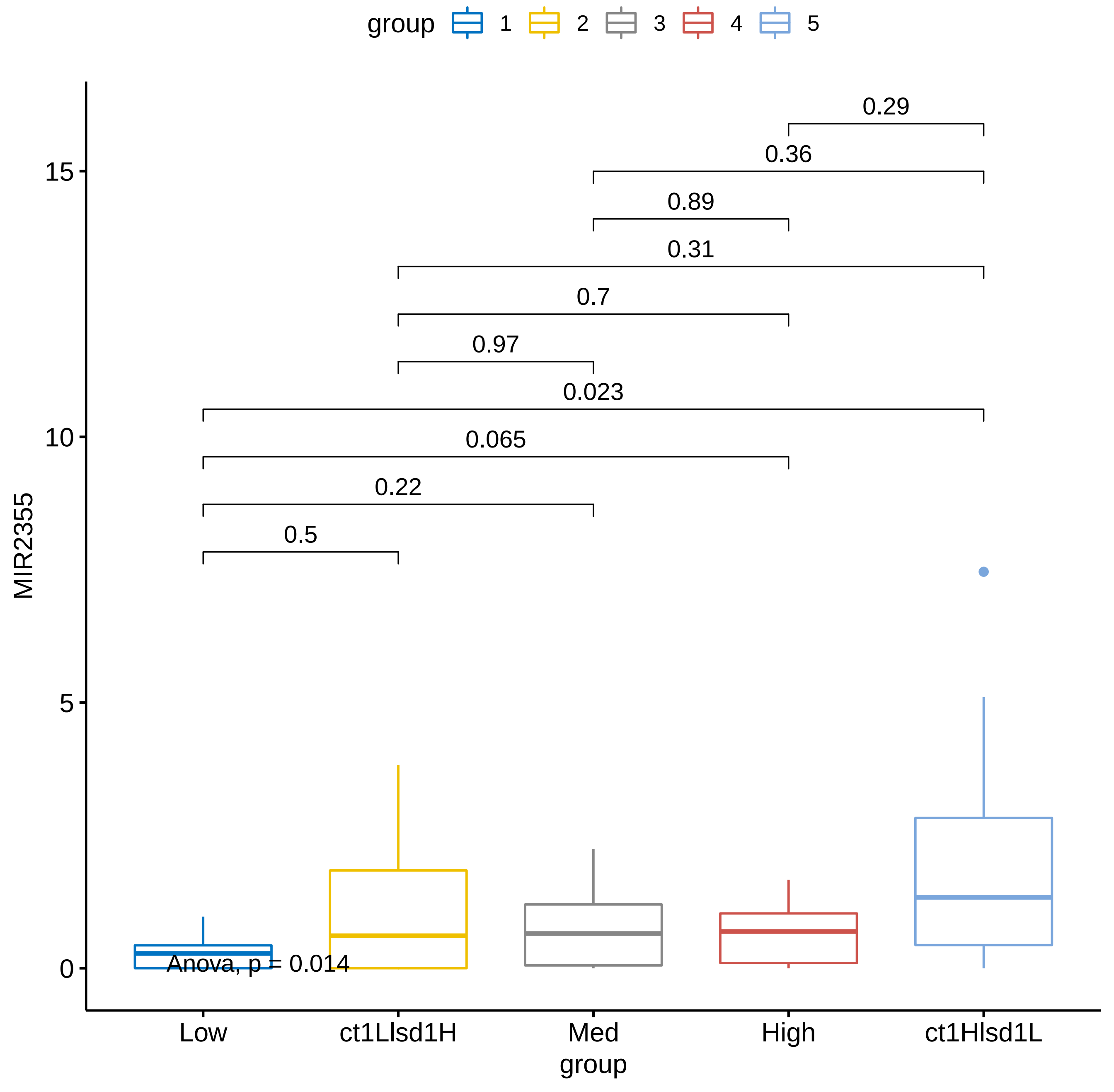

group
1
2
3
4
5
0.29
0.36
0.89
0.31
0.7
0.97
0.023
0.065
0.22
0.5
15
10
5
0
MIR2355
Anova, p = 0.014
Low
ct1Llsd1H
Med
High
ct1Hlsd1L
group

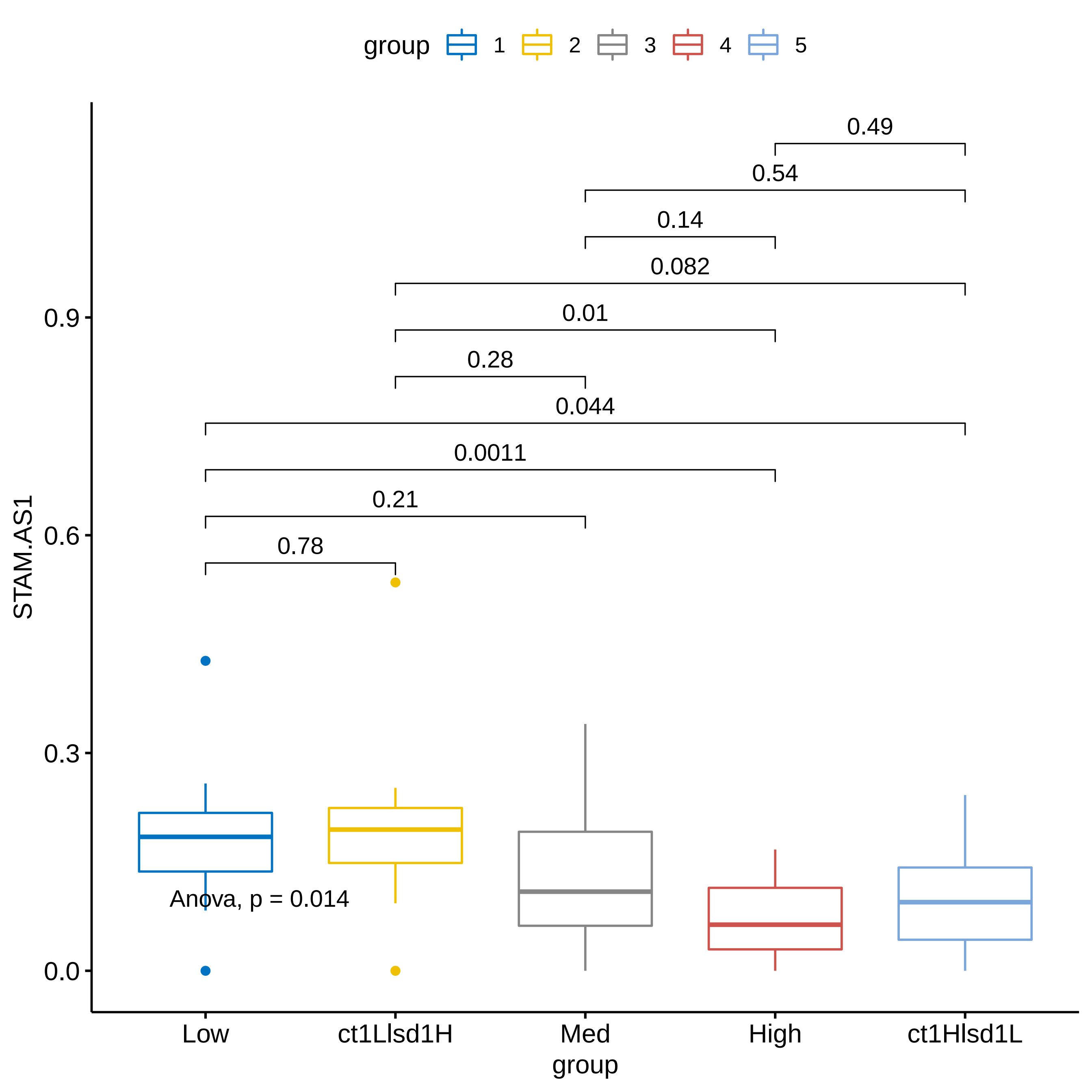
group 1 2 3 4 5
0.49
0.54
0.14
0.082
0.01
0.28
0.044
0.0011
0.21
0.78
Anova, p = 0.014
STAM.AS1
0.9
0.6
0.3
0.0
Low
ct1Llsd1H
Med
High
ct1Hlsd1L
group

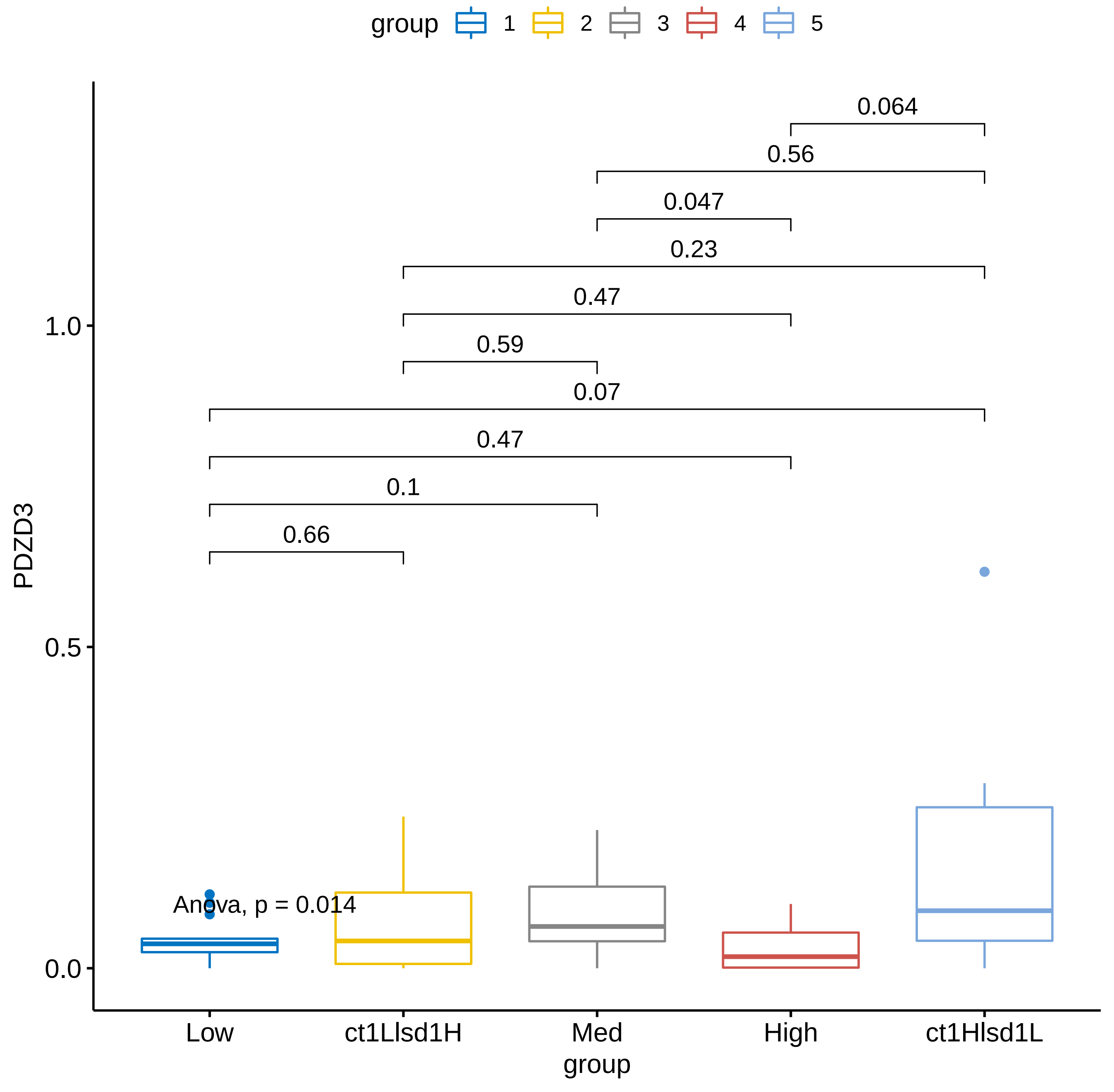

group 1 2 3 4 5
0.064
0.56
0.047
0.23
0.47
0.59
0.07
0.47
0.1
0.66
Anova, p = 0.014
PDZD3
1.0
0.5
0.0
Low
ct1Llsd1H
Med
High
ct1Hlsd1L
group

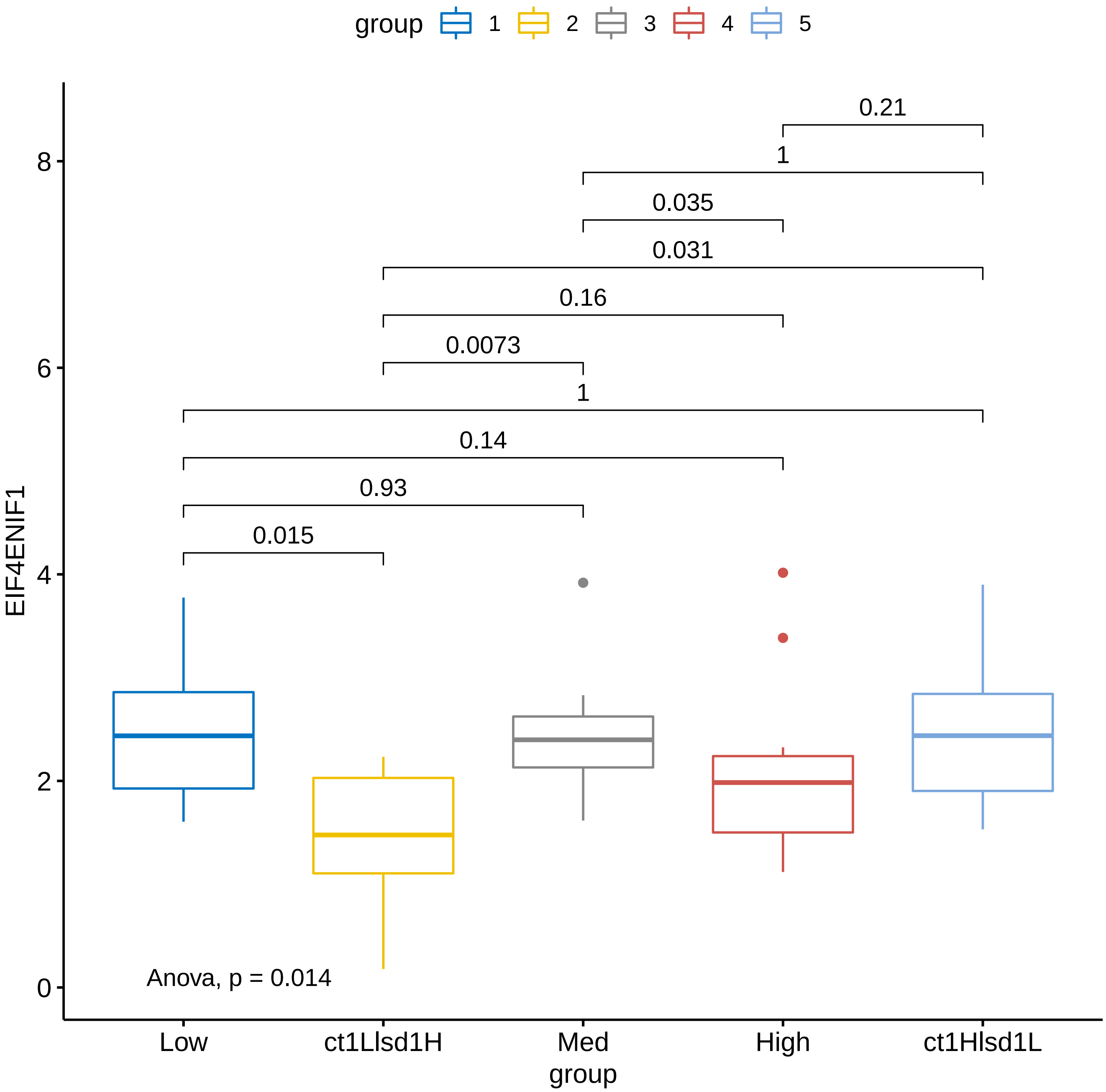
group
1
2
3
4
5
0.21
1
0.035
0.031
0.16
0.0073
1
0.14
0.93
0.015
Anova, p = 0.014
EIF4ENIF1
0
2
4
6
8
Low
ct1Llsd1H
Med
High
ct1Hlsd1L
group

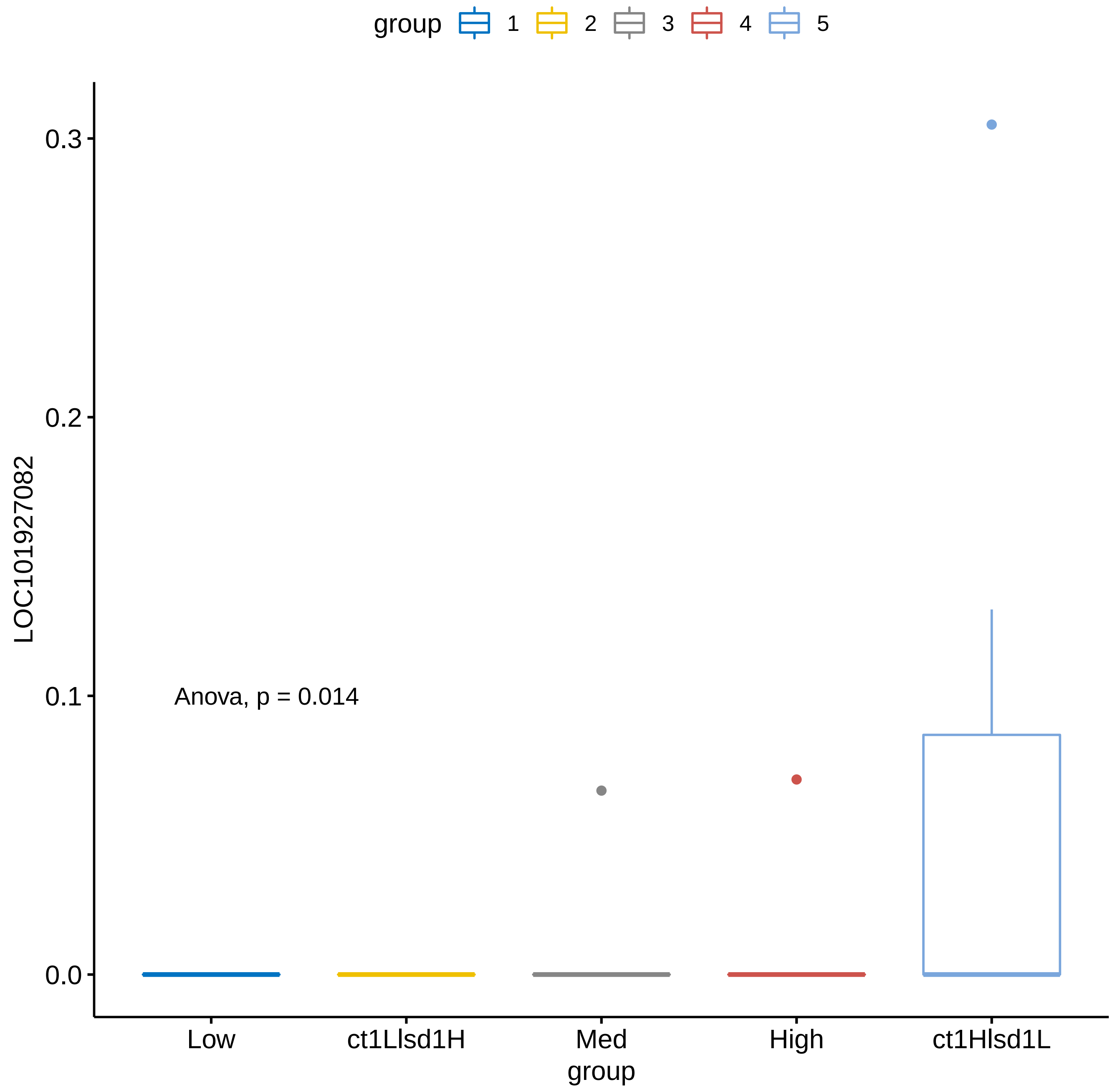
group
1
2
3
4
5
LOC101927082
0.3
0.2
0.1
0.0
Anova, p = 0.014
Low
ct1Llsd1H
Med
High
ct1Hlsd1L
group

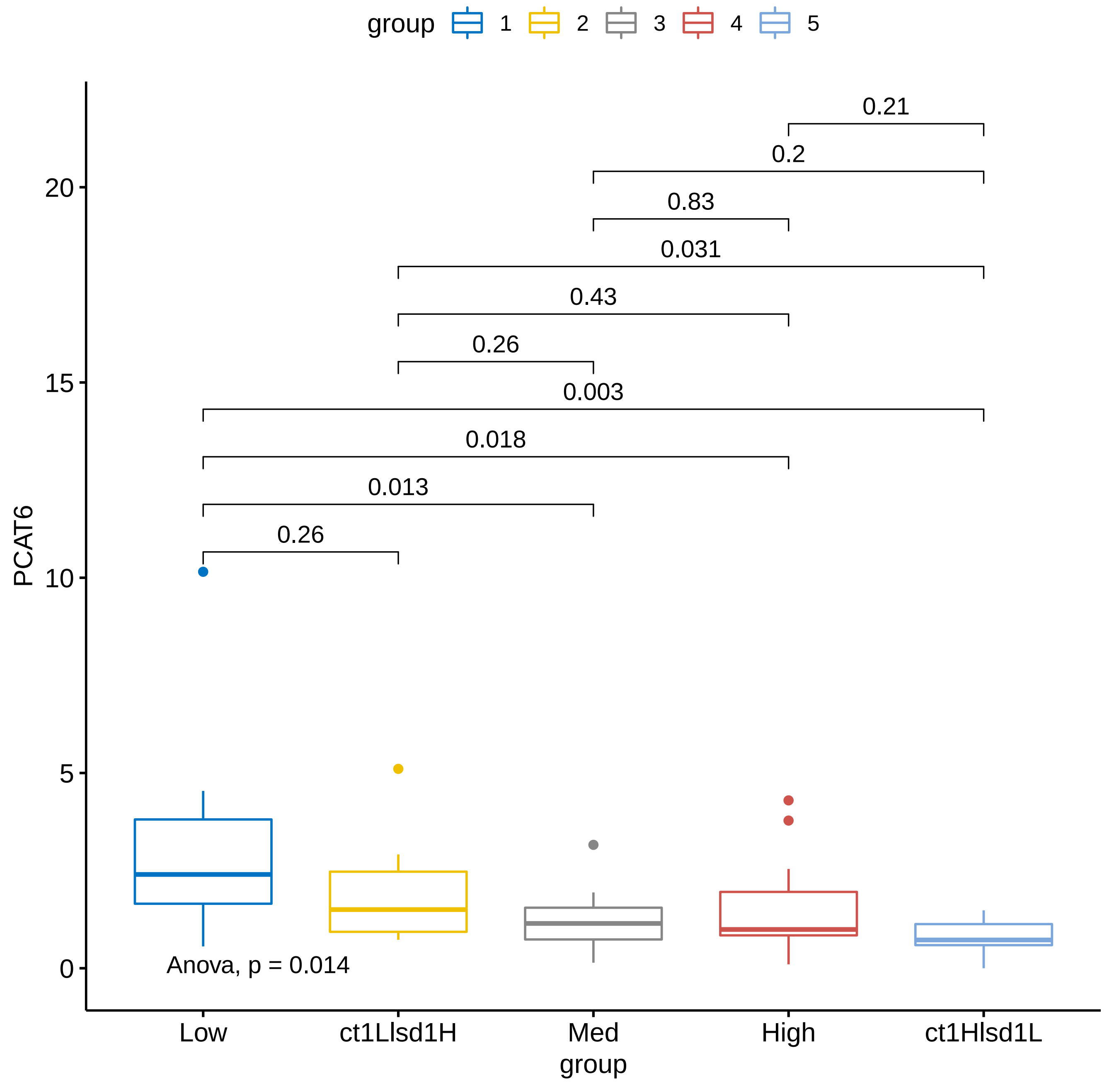

group 1 2 3 4 5
0.21
0.2
0.83
0.031
0.43
0.26
0.003
0.018
0.013
0.26
Anova, p = 0.014
PCAT6
20
15
10
5
0
Low
ct1Llsd1H
Med
High
ct1Hlsd1L
group

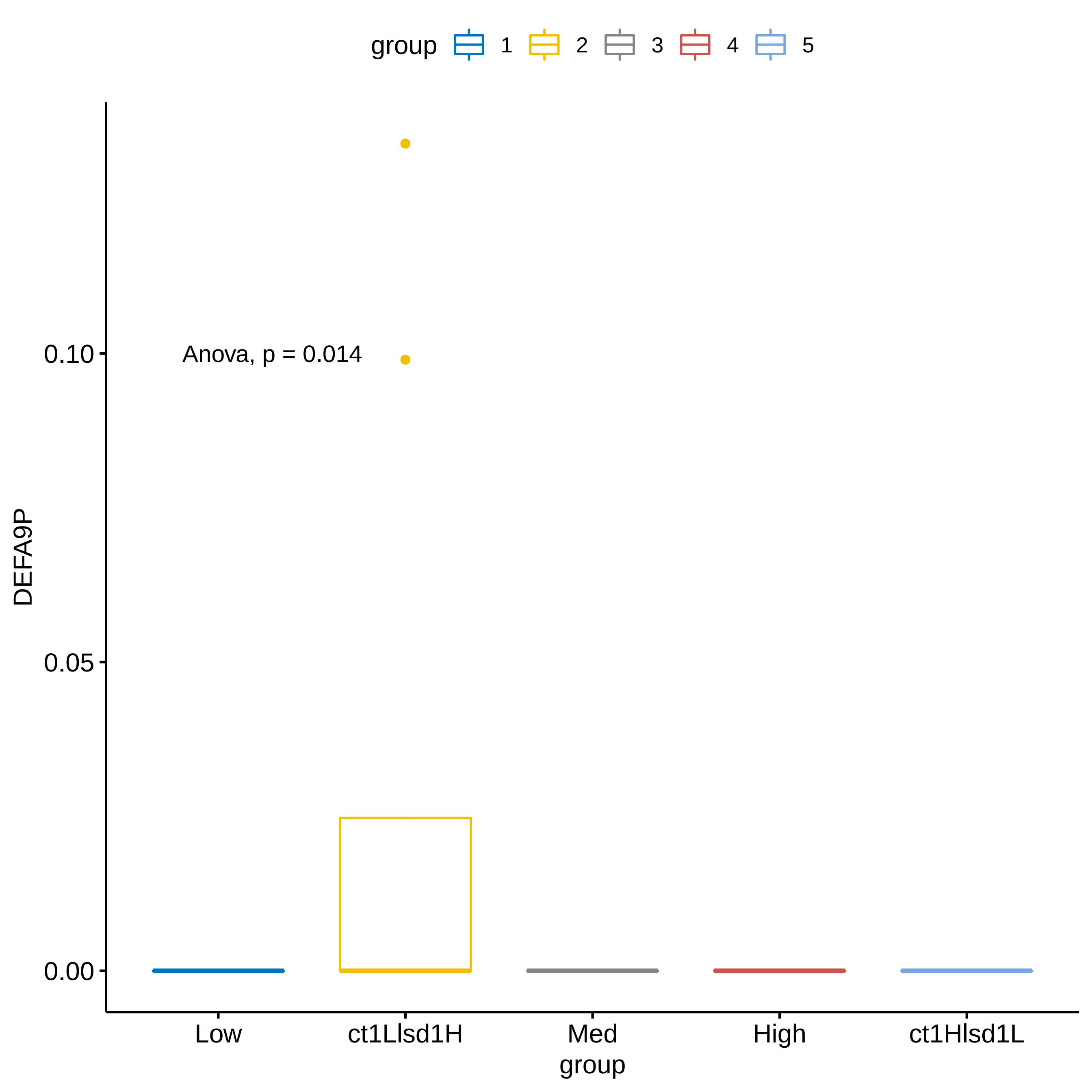

group
1
2
3
4
5
Anova, p = 0.014
DEFA9P
0.10
0.05
0.00
Low
ct1Llsd1H
Med
High
ct1Hlsd1L
group

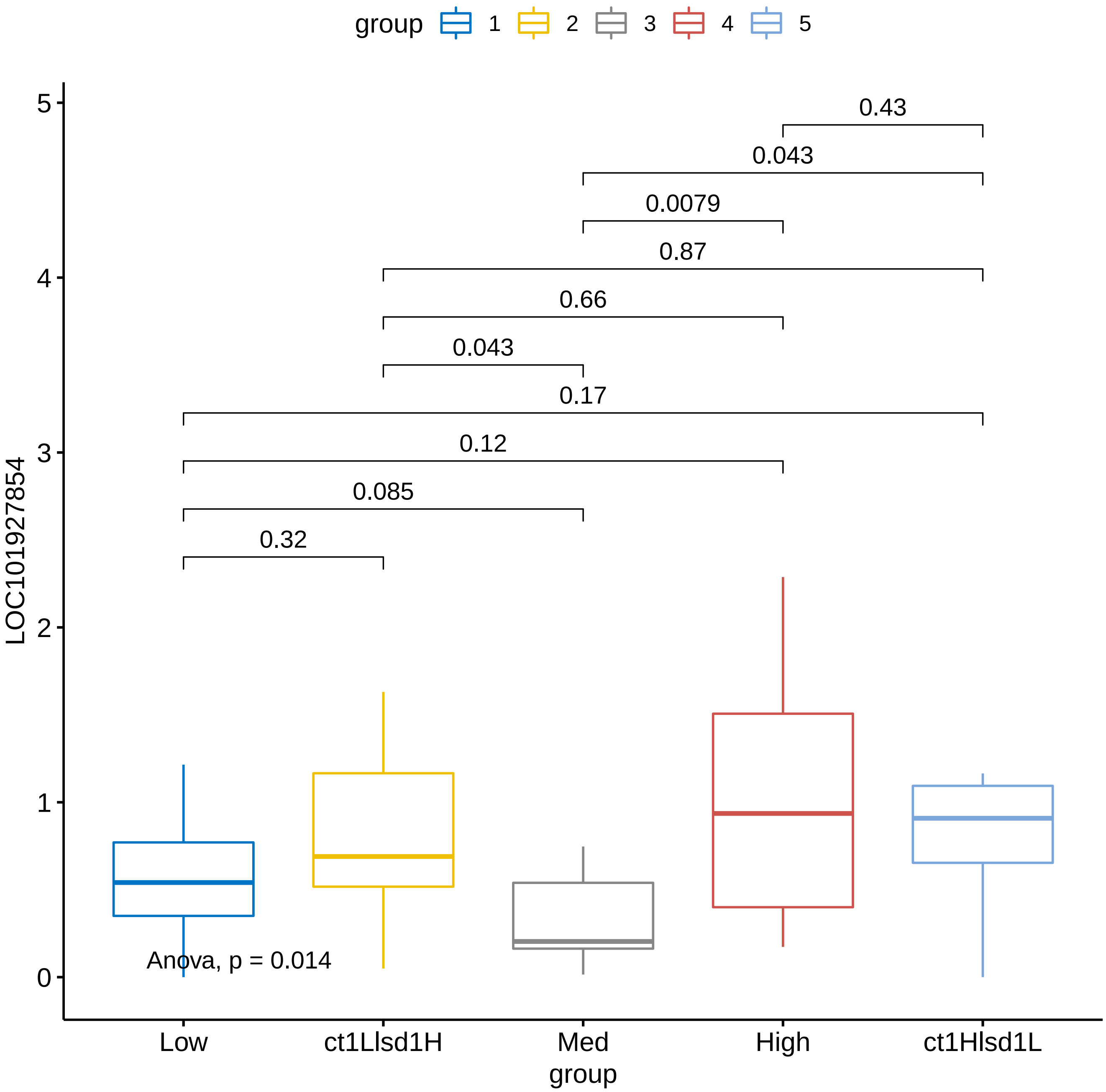
group
1
2
3
4
5
0.43
0.043
0.0079
0.87
0.66
0.043
0.17
0.12
0.085
0.32
Anova, p = 0.014
LOC101927854
0
1
2
3
4
5
Low
ct1Llsd1H
Med
High
ct1Hlsd1L
group

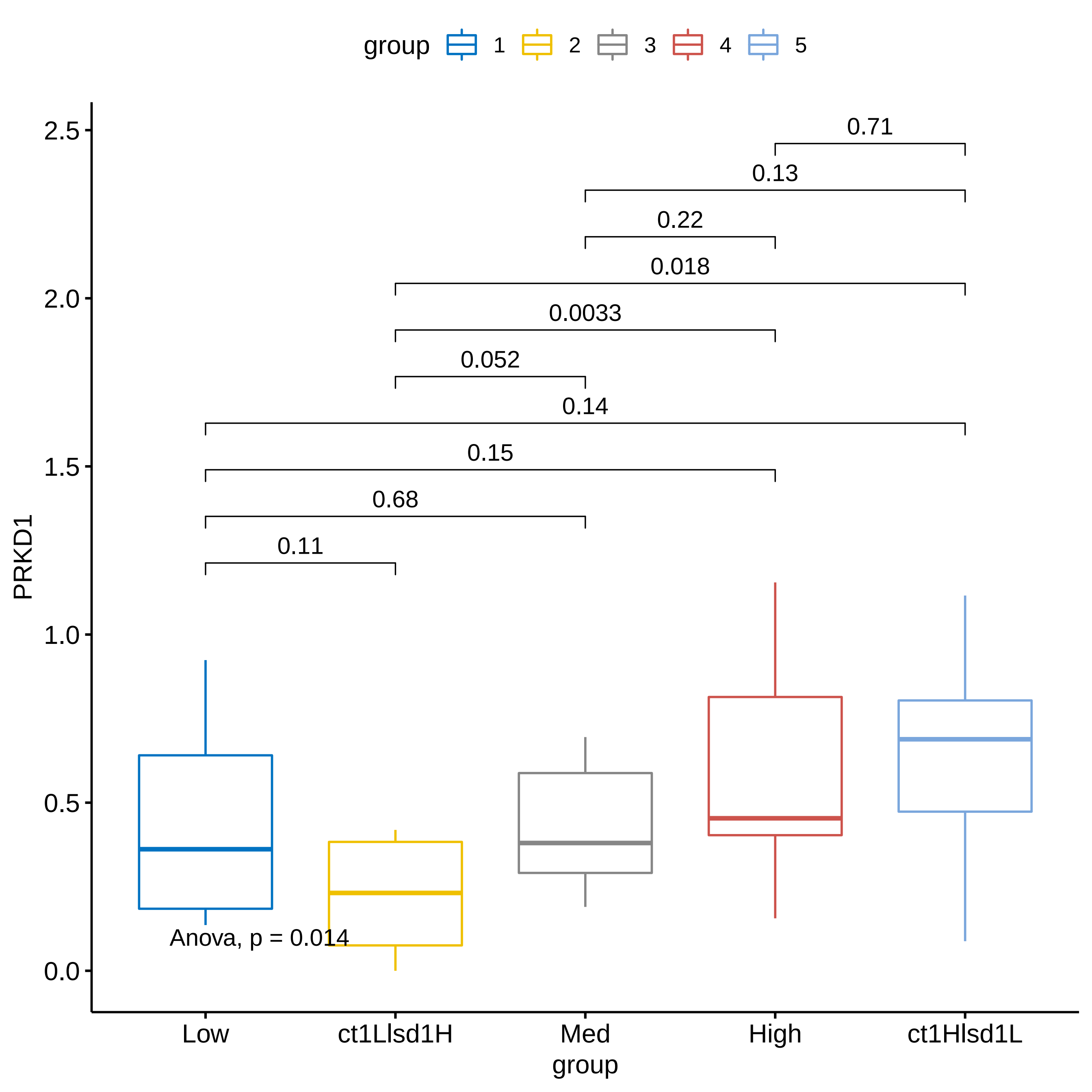

group 1 2 3 4 5
0.71
0.13
0.22
0.018
0.0033
0.052
0.14
0.15
0.68
0.11
Anova, p = 0.014
PRKD1
2.5
2.0
1.5
1.0
0.5
0.0
Low
ct1Llsd1H
Med
High
ct1Hlsd1L
group

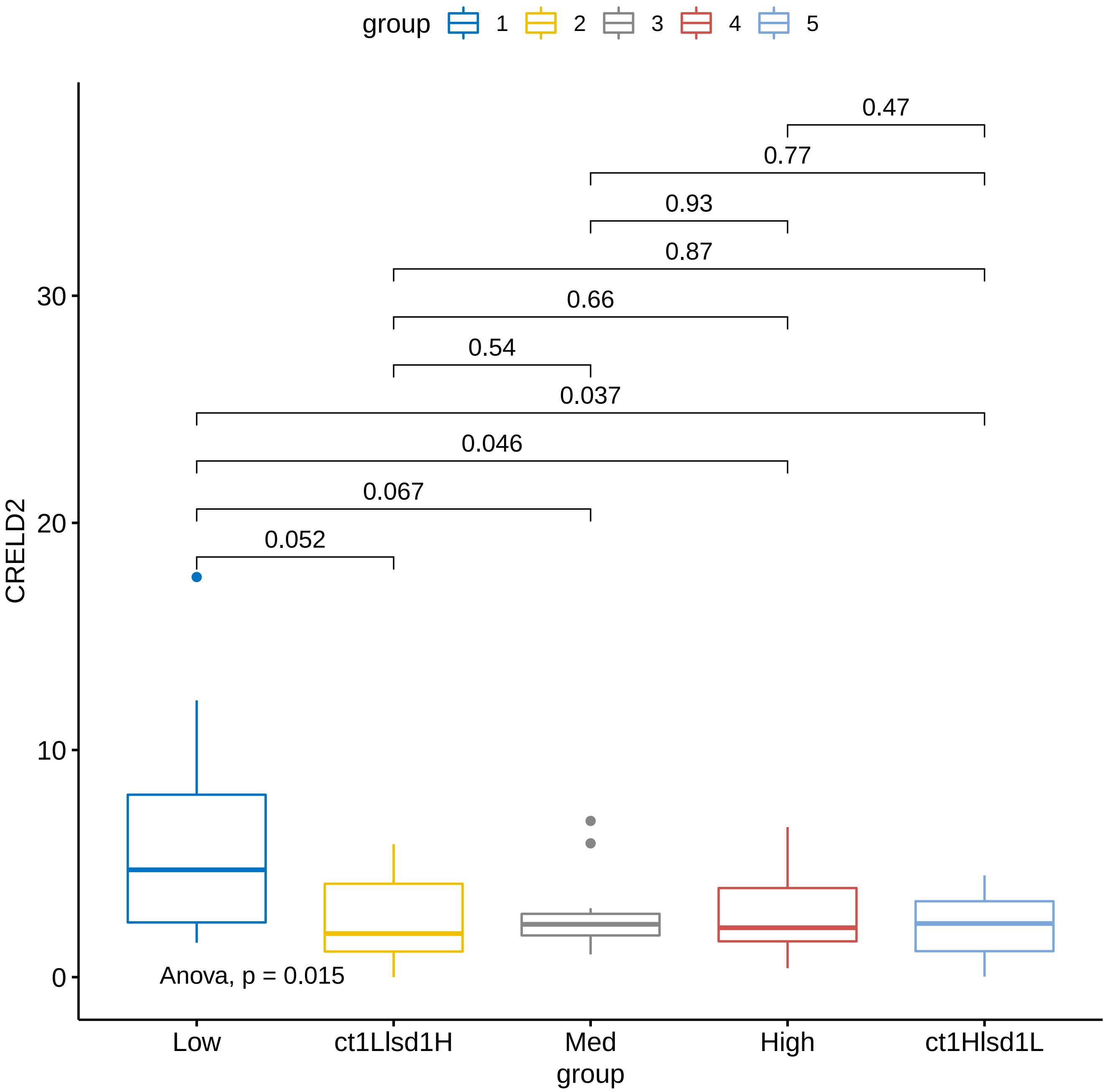
group
1
2
3
4
5
0.47
0.77
0.93
0.87
0.66
0.54
0.037
0.046
0.067
0.052
Anova, p = 0.015
CRELD2
30
20
10
0
Low
ct1Llsd1H
Med
High
ct1Hlsd1L
group

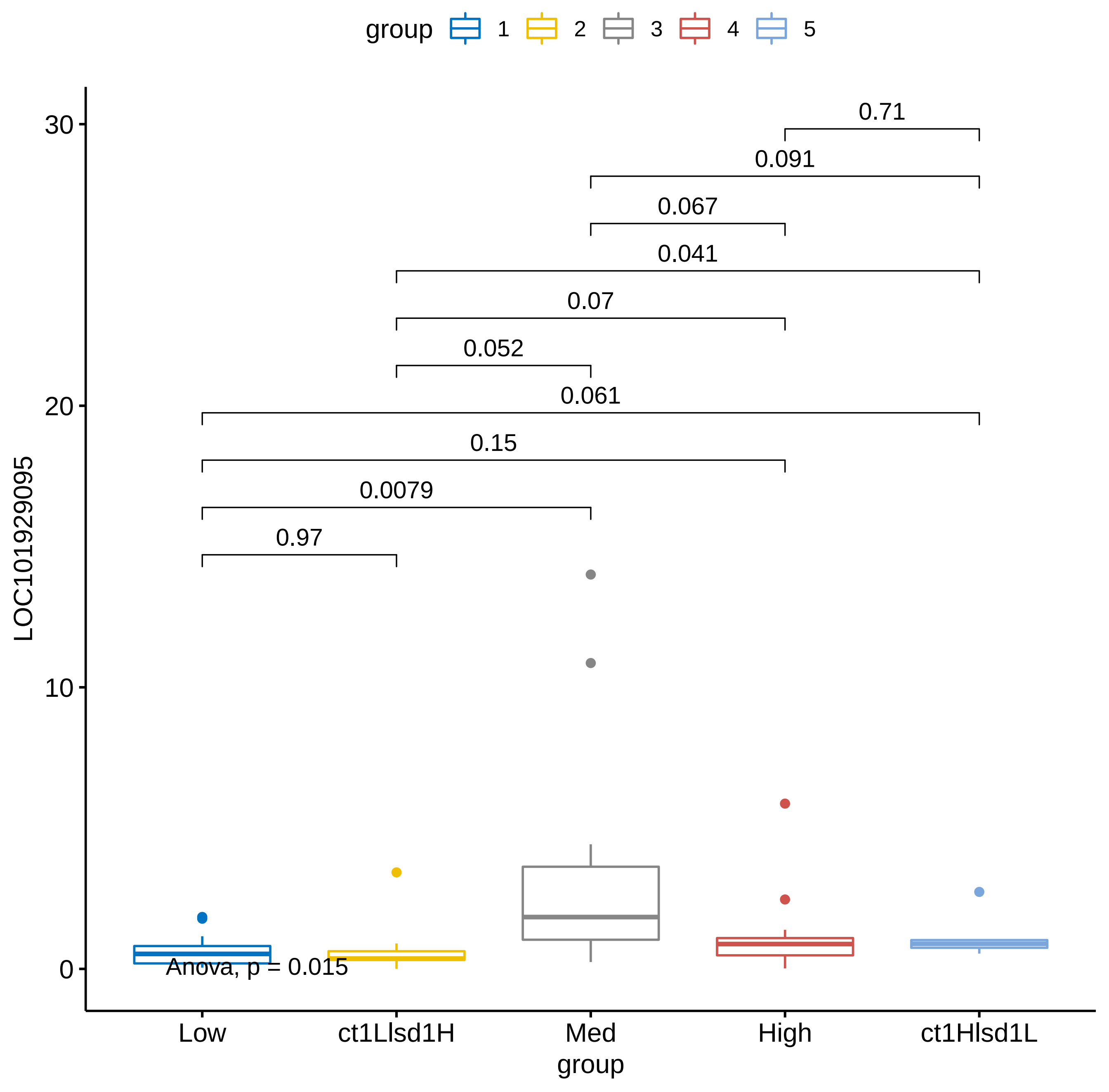

group 1 2 3 4 5
0.71
0.091
0.067
0.041
0.07
0.052
0.061
0.15
0.0079
0.97
30
20
10
0
LOC101929095
Anova, p = 0.015
Low
ct1Llsd1H
Med
High
ct1Hlsd1L
group

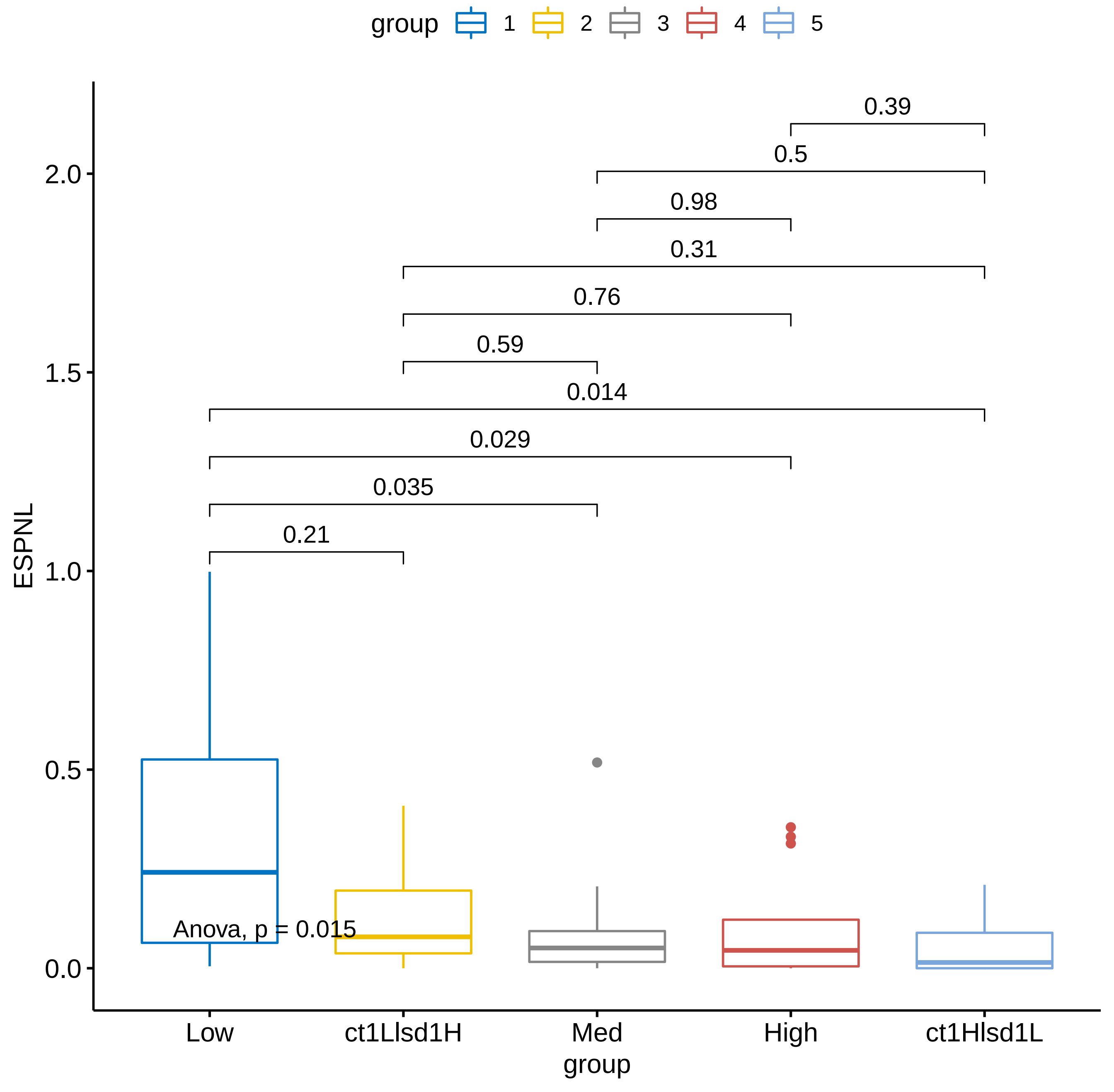

group
1
2
3
4
5
0.39
0.5
0.98
0.31
0.76
0.59
0.014
0.029
0.035
0.21
2.0
1.5
1.0
0.5
0.0
ESPNL
Anova, p = 0.015
Low
ct1Llsd1H
Med
High
ct1Hlsd1L
group

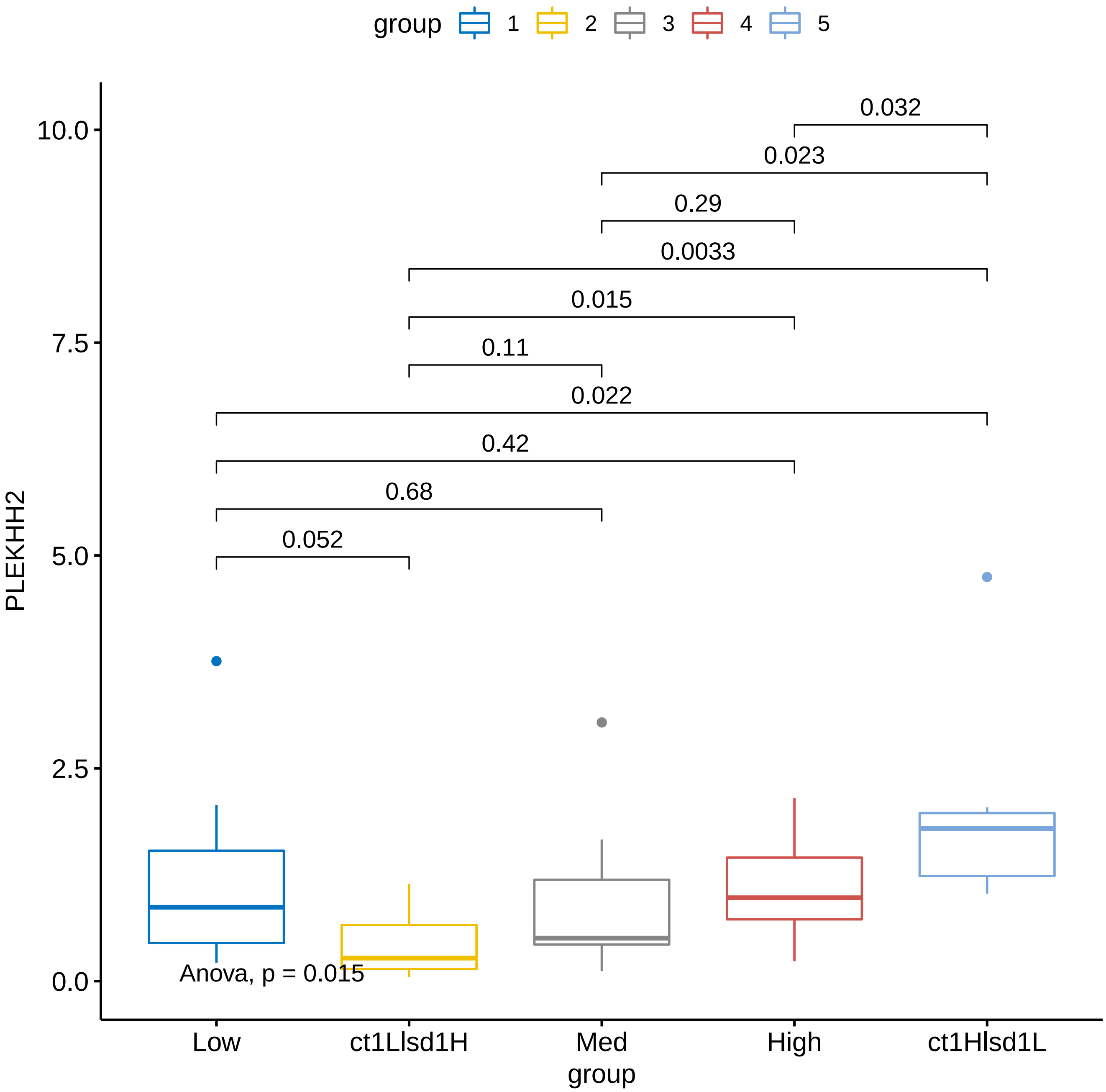

group
1
2
3
4
5
0.032
0.023
0.29
0.0033
0.015
0.11
0.022
0.42
0.68
0.052
10.0
7.5
5.0
2.5
0.0
PLEKHH2
Anova, p = 0.015
Low
ct1Llsd1H
Med
High
ct1Hlsd1L
group

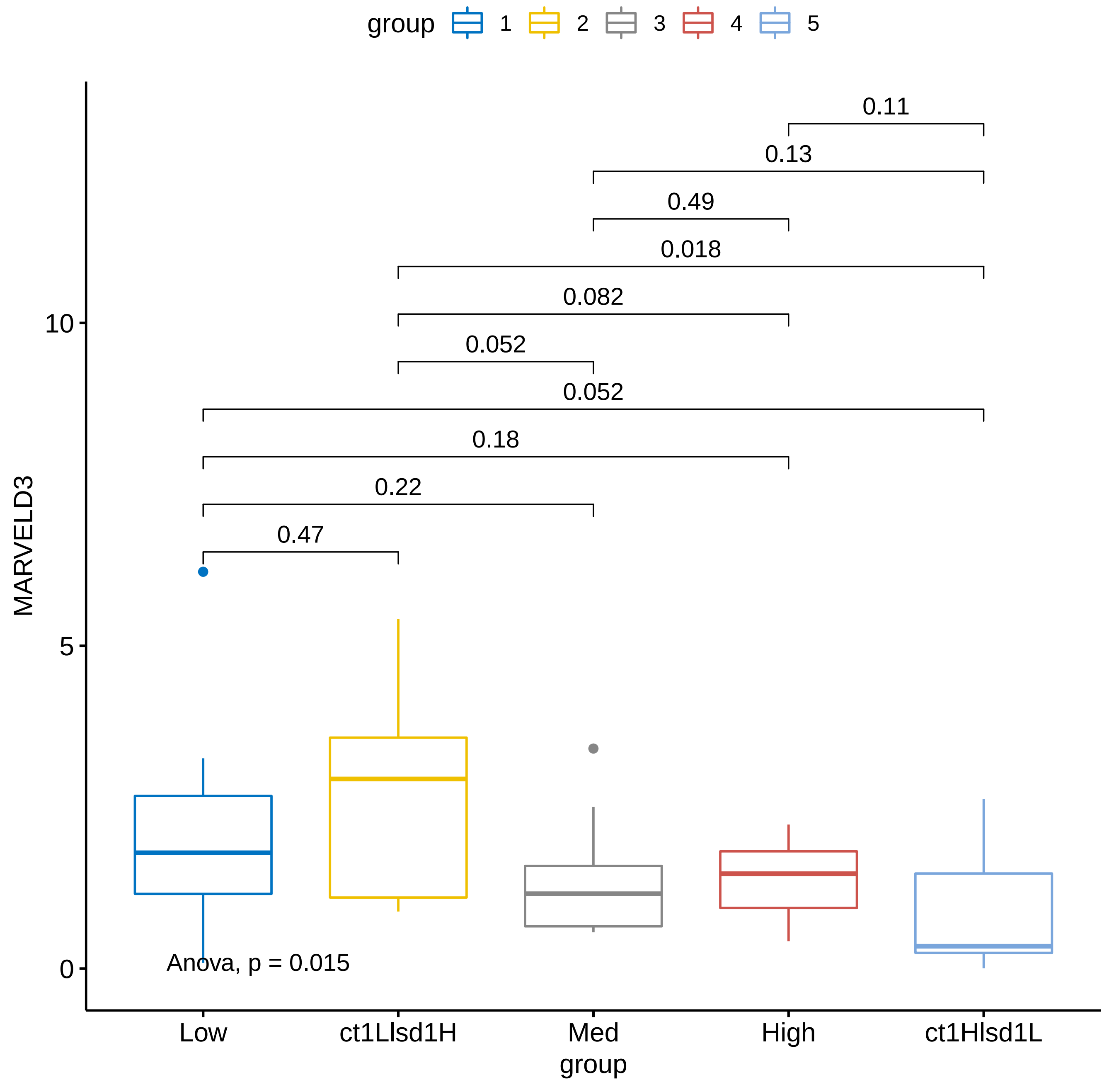

group 1 2 3 4 5
0.11
0.13
0.49
0.018
0.082
0.052
0.052
0.18
0.22
0.47
MARVELD3
10
5
0
Anova, p = 0.015
Low
ct1Llsd1H
Med
High
ct1Hlsd1L
group

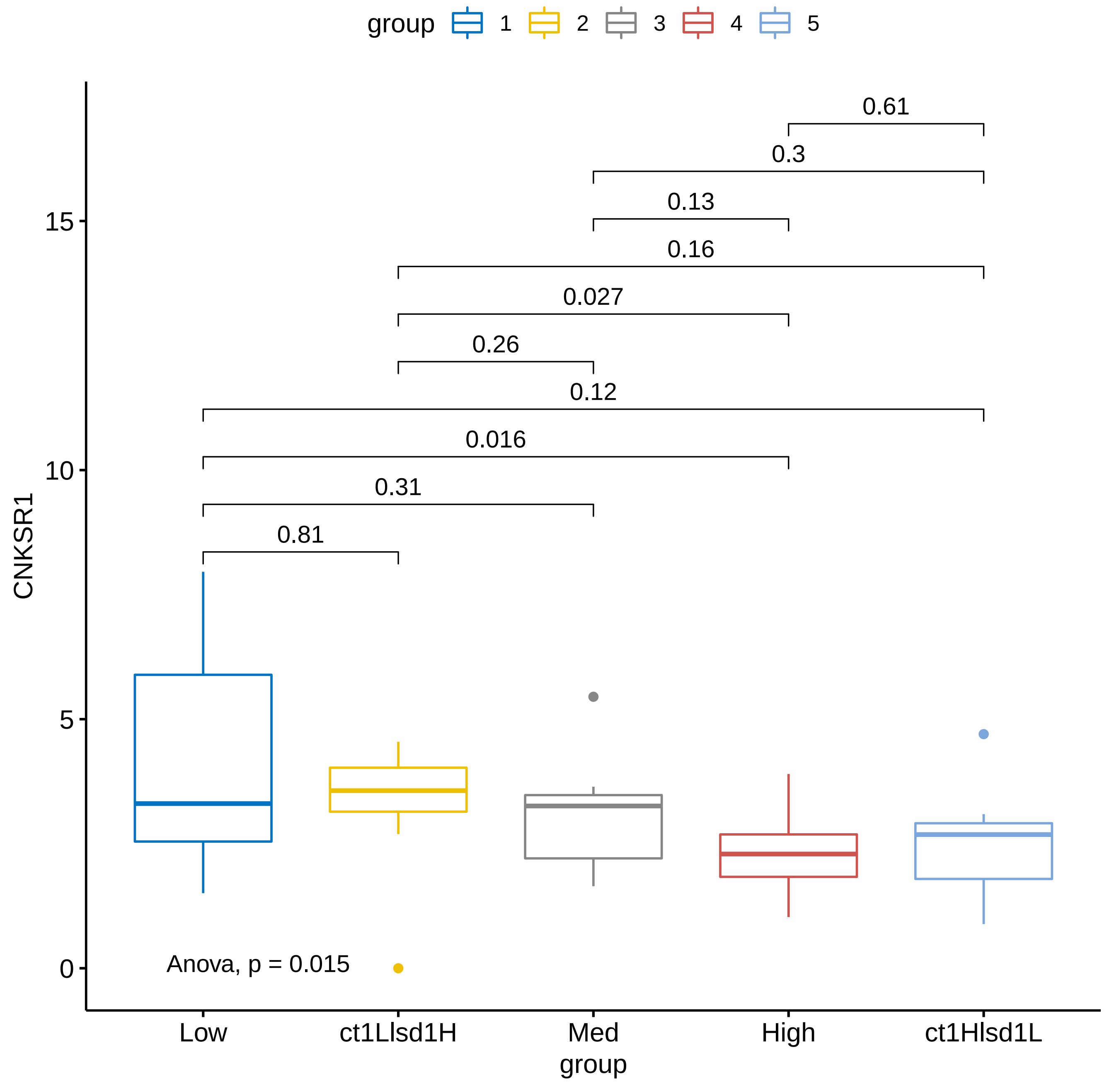

group
1
2
3
4
5
CNKSR1
0.61
0.3
0.13
0.16
0.027
0.26
0.12
0.016
0.31
0.81
15
10
5
0
Anova, p = 0.015
Low
ct1Llsd1H
Med
High
ct1Hlsd1L
group

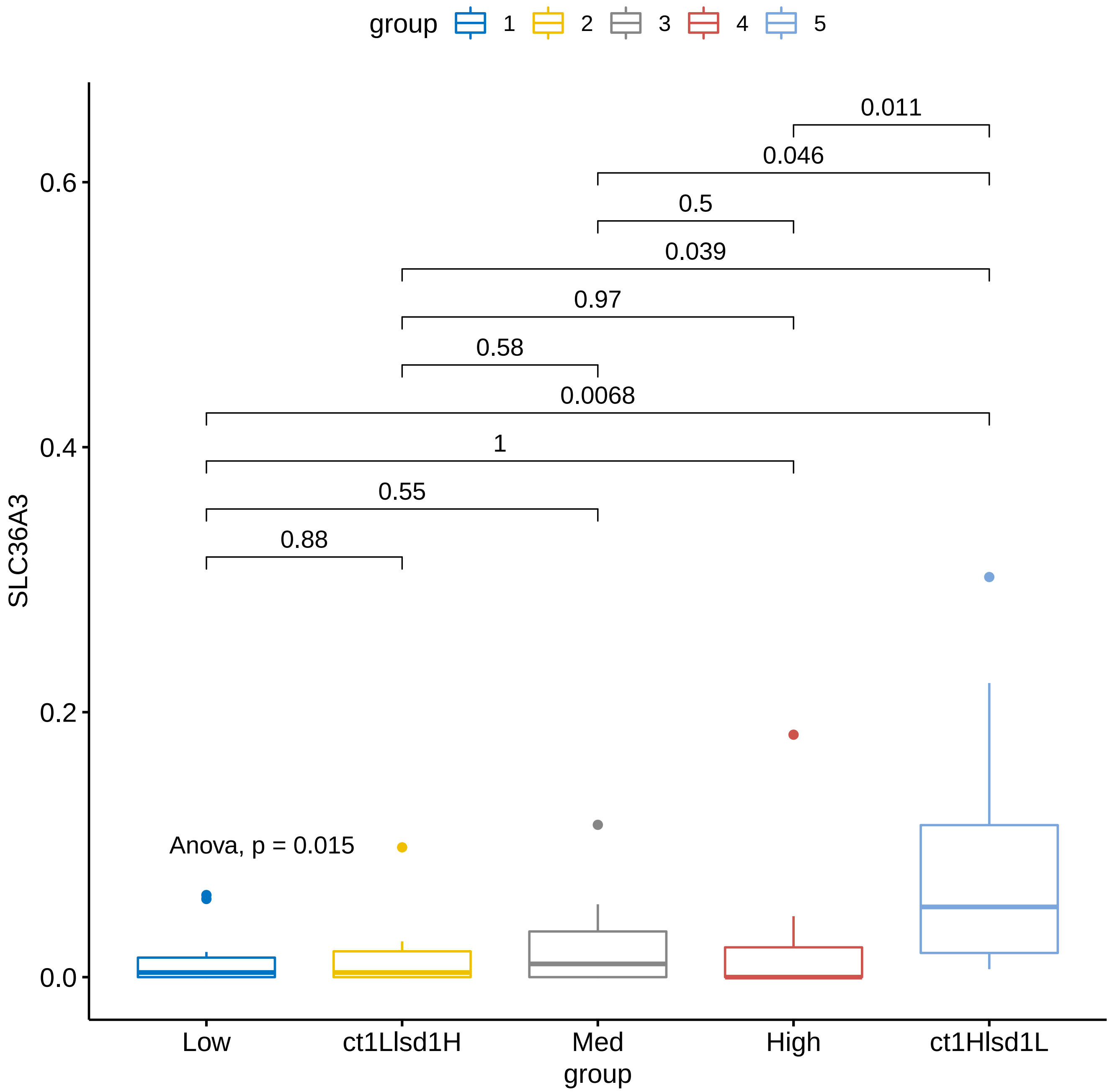

group
1
2
3
4
5
0.011
0.046
0.5
0.039
0.97
0.58
0.0068
1
0.55
0.88
Anova, p = 0.015
SLC36A3
0.6
0.4
0.2
0.0
Low
ct1Llsd1H
Med
High
ct1Hlsd1L
group

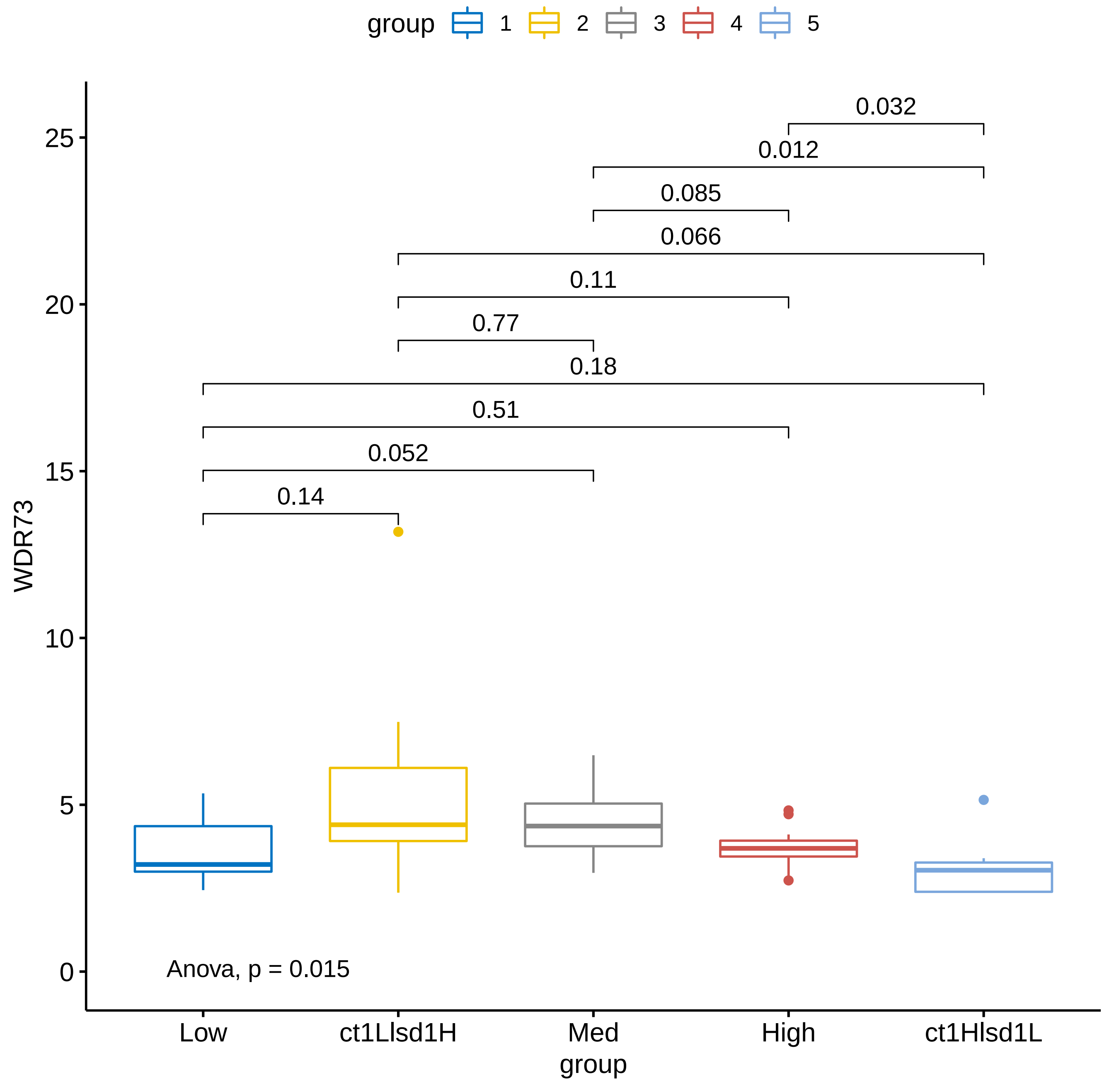
group
1
2
3
4
5
0.032
0.012
0.085
0.066
0.11
0.77
0.18
0.51
0.052
0.14
25
20
15
10
5
0
WDR73
Anova, p = 0.015
Low
ct1Llsd1H
Med
High
ct1Hlsd1L
group

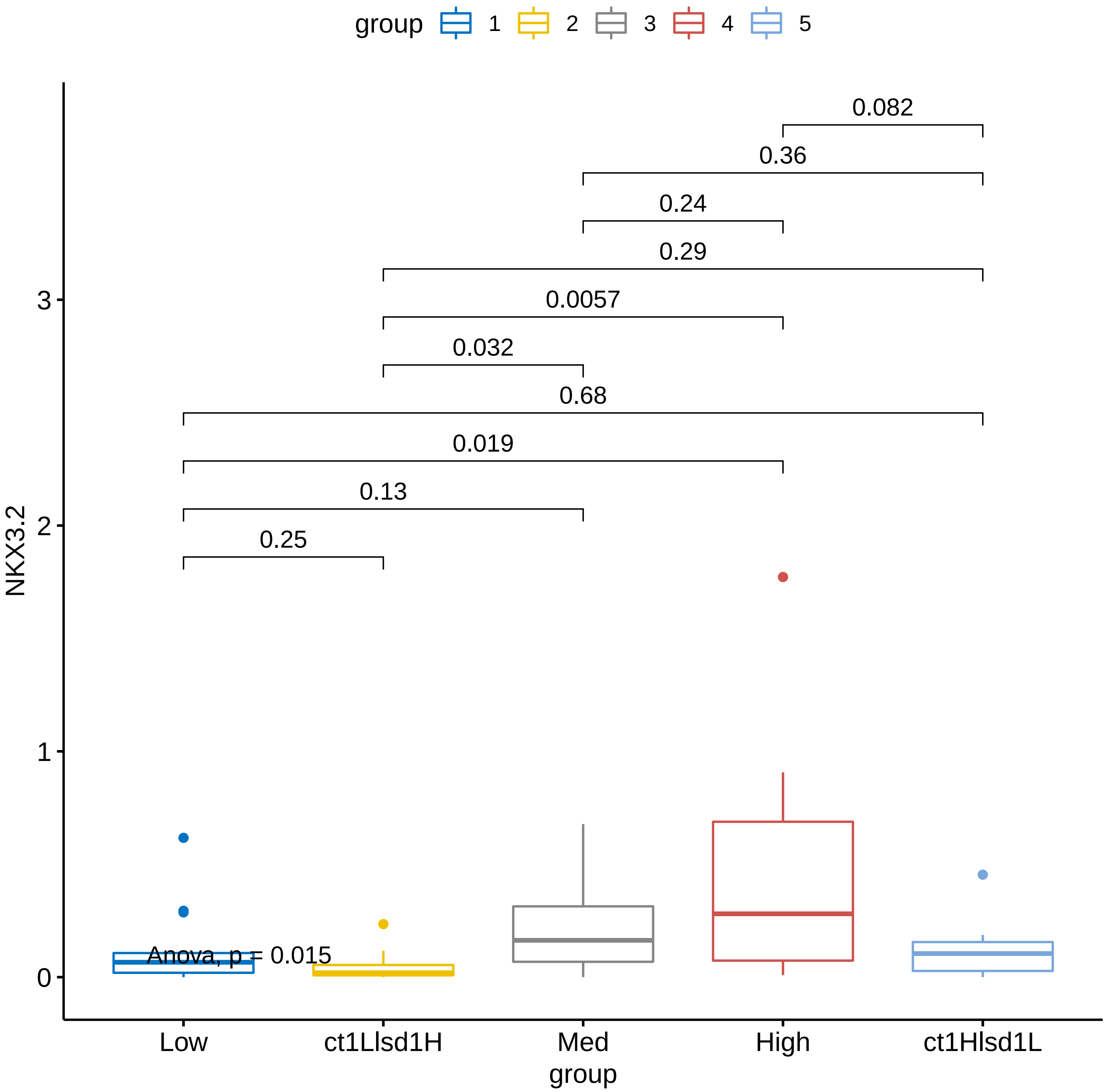

group
1
2
3
4
5
0.082
0.36
0.24
0.29
0.0057
0.032
0.68
0.019
0.13
0.25
Anova, p = 0.015
NKX3.2
3
2
1
0
Low
ct1Llsd1H
Med
High
ct1Hlsd1L
group

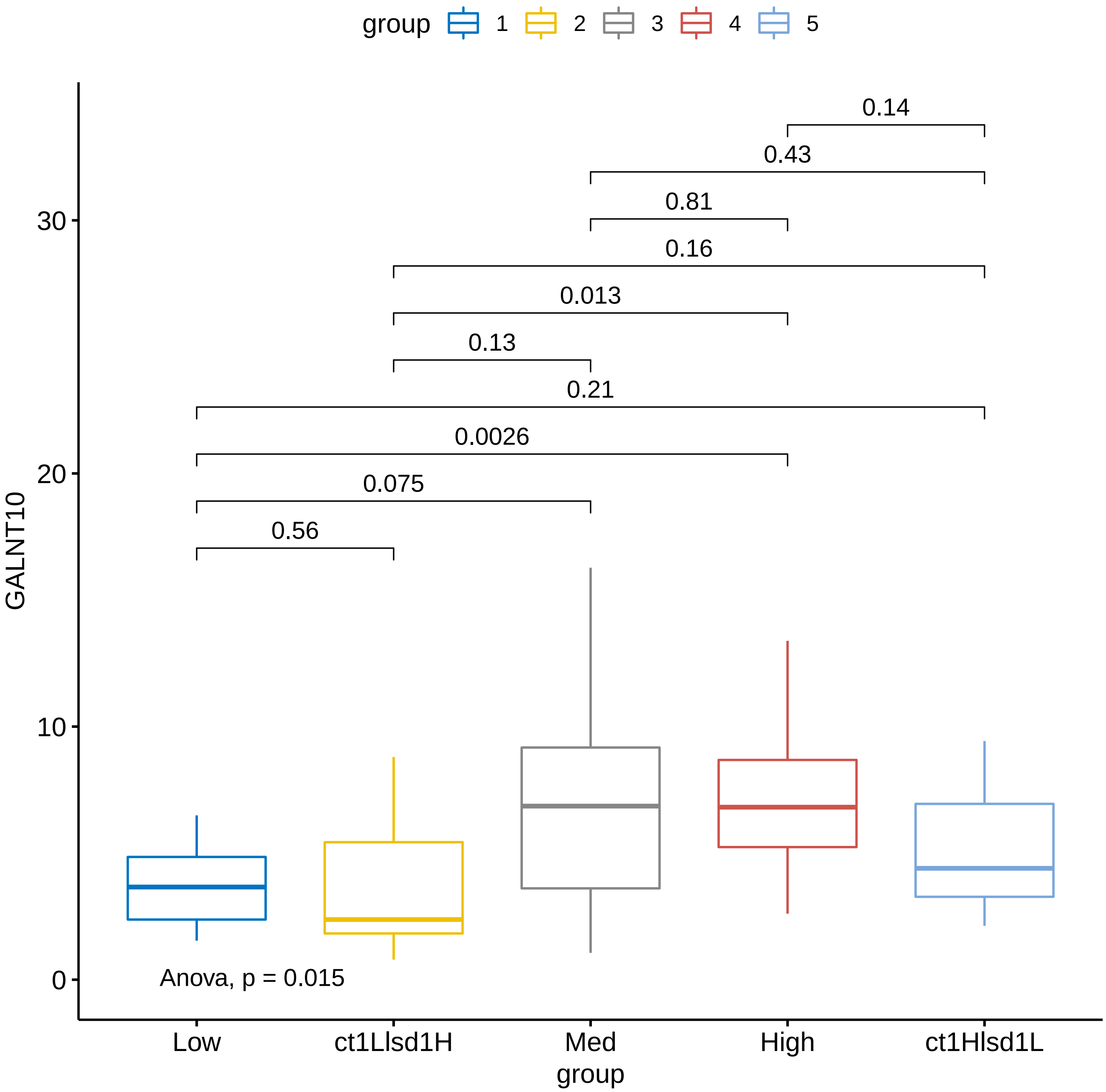
group
1
2
3
4
5
0.14
0.43
0.81
0.16
0.013
0.13
0.21
0.0026
0.075
0.56
30
20
10
0
GALNT10
Anova, p = 0.015
Low
ct1Llsd1H
Med
High
ct1Hlsd1L
group

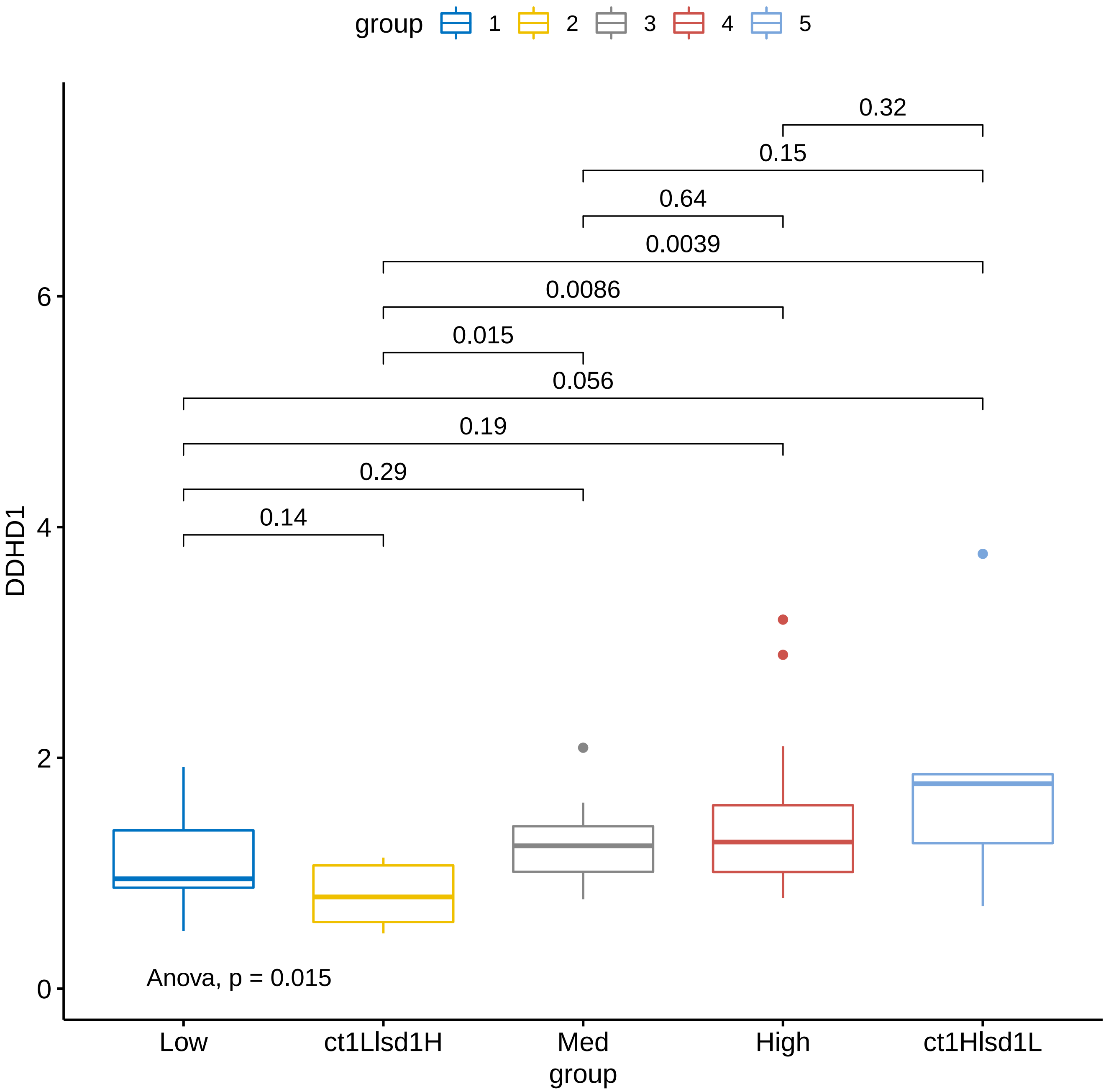

group
1
2
3
4
5
0.32
0.15
0.64
0.0039
0.0086
0.015
0.056
0.19
0.29
0.14
DDHD1
6
4
2
0
Anova, p = 0.015
Low
ct1Llsd1H
Med
High
ct1Hlsd1L
group

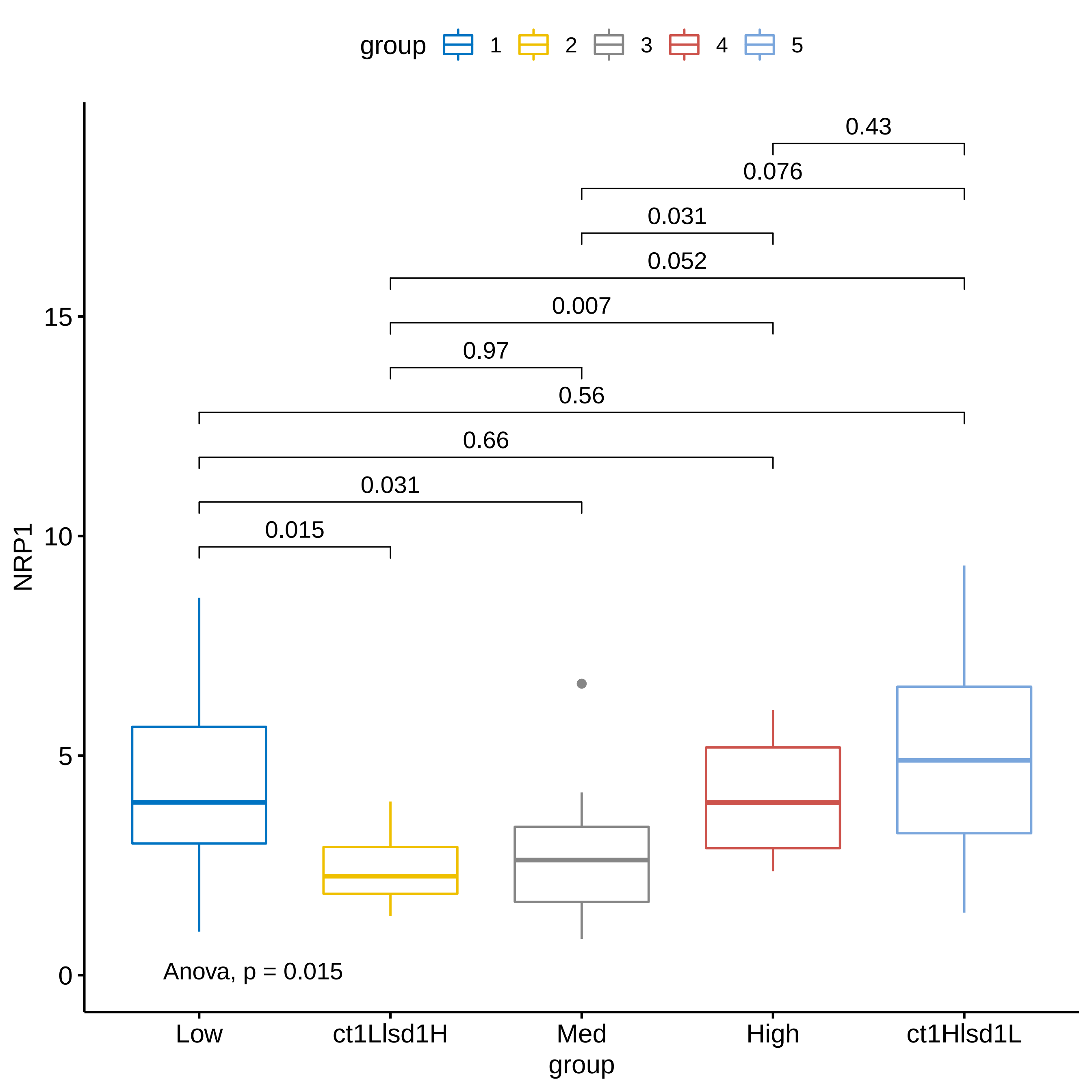
group
1
2
3
4
5
0.43
0.076
0.031
0.052
0.007
0.97
0.56
0.66
0.031
0.015
NRP1
15
10
5
0
Anova, p = 0.015
Low
ct1Llsd1H
Med
High
ct1Hlsd1L
group

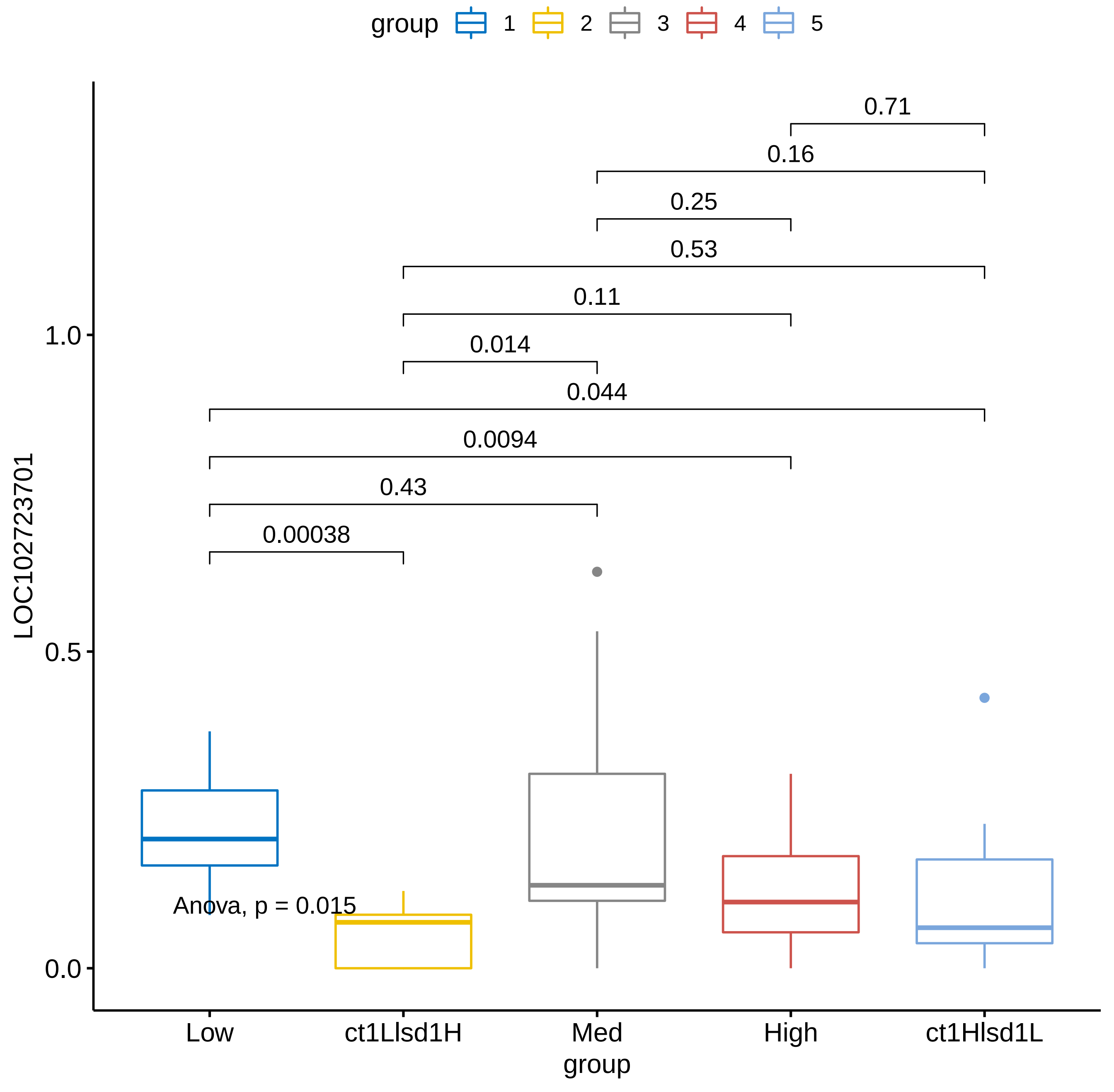

group 1 2 3 4 5
0.71
0.16
0.25
0.53
0.11
1.0
0.014
0.044
0.0094
0.43
0.00038
LOC102723701
0.5
Anova, p = 0.015
0.0
Low
ct1Llsd1H
Med
High
ct1Hlsd1L
group

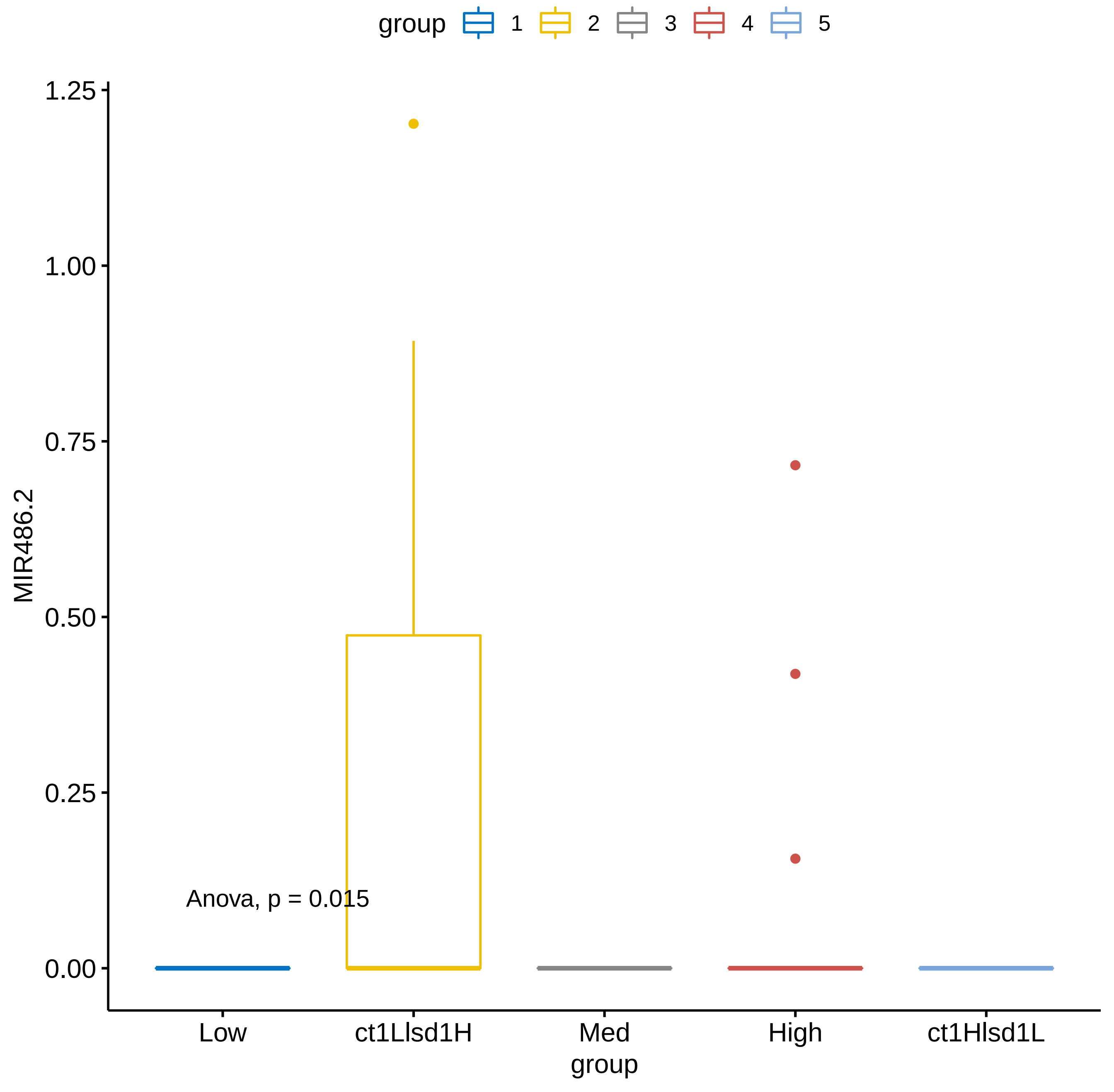

group 1 2 3 4 5
1.25
1.00
0.75
0.50
0.25
0.00
MIR486.2
Anova, p = 0.015
Low
ct1Llsd1H
Med
High
ct1Hlsd1L
group

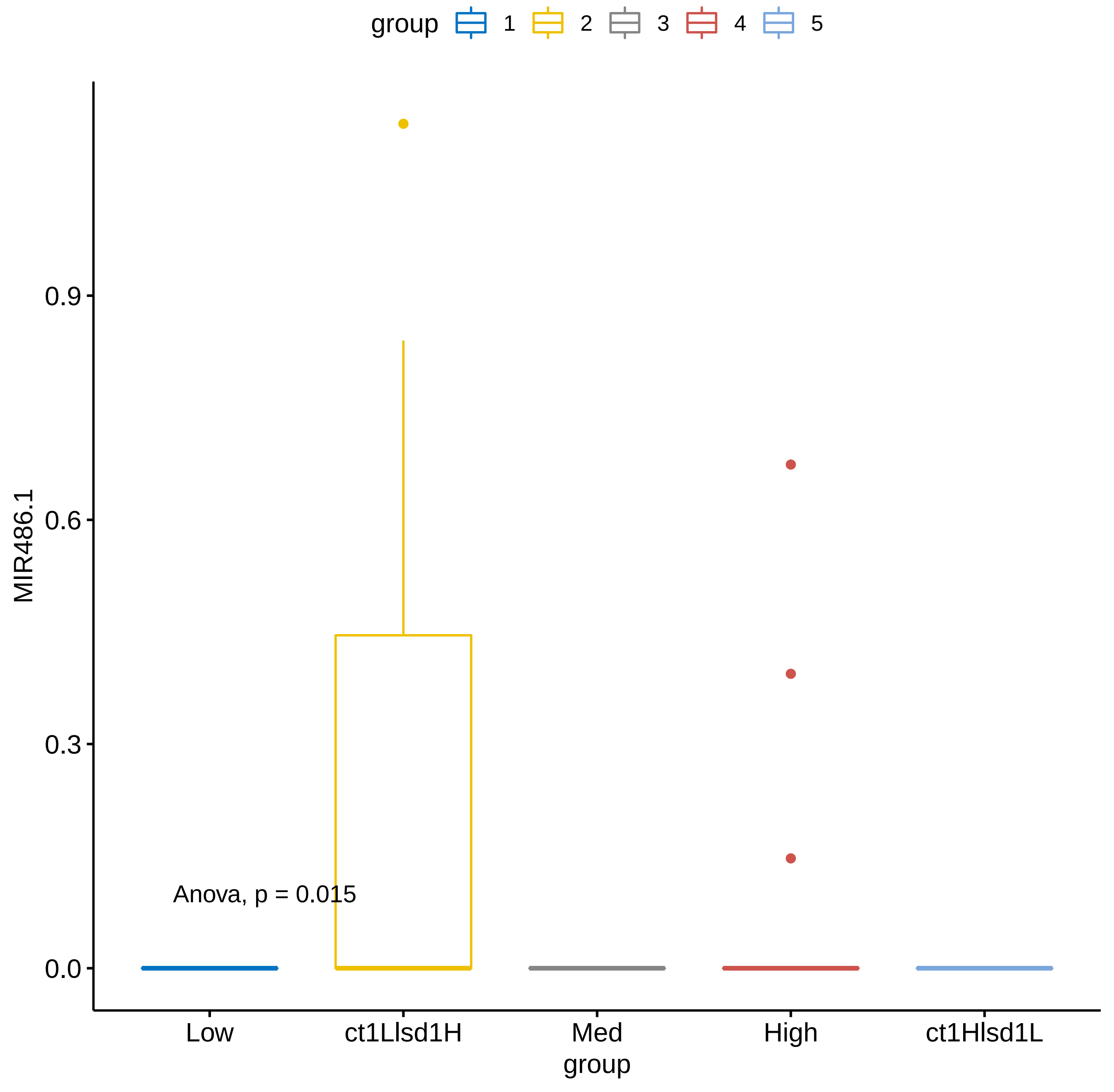

group
1
2
3
4
5
0.9
0.6
0.3
0.0
MIR486.1
Anova, p = 0.015
Low
ct1Llsd1H
Med
High
ct1Hlsd1L
group

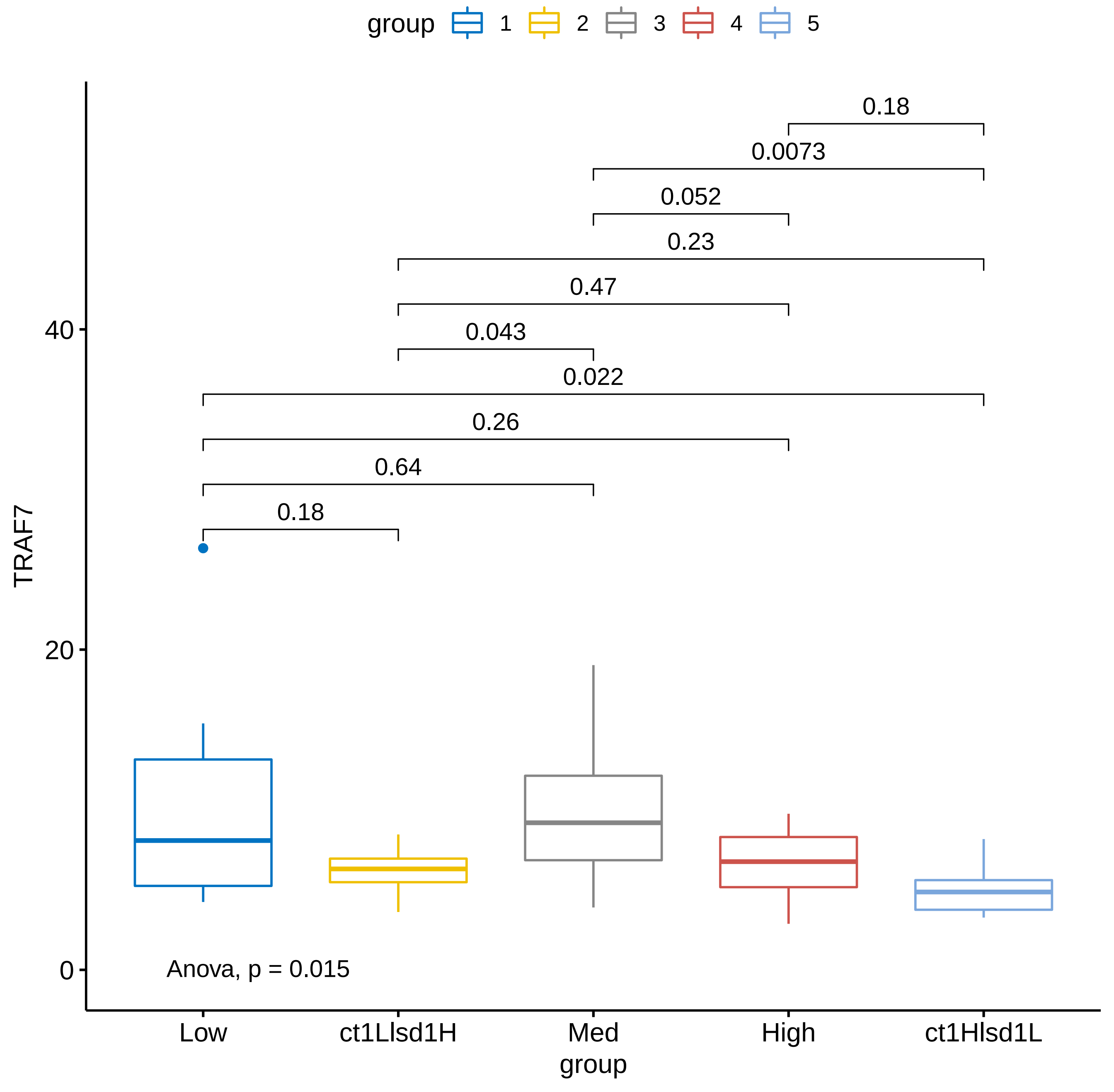
group
1
2
3
4
5
0.18
0.0073
0.052
0.23
0.47
0.043
0.022
0.26
0.64
0.18
40
20
0
TRAF7
Anova, p = 0.015
Low
ct1Llsd1H
Med
High
ct1Hlsd1L
group

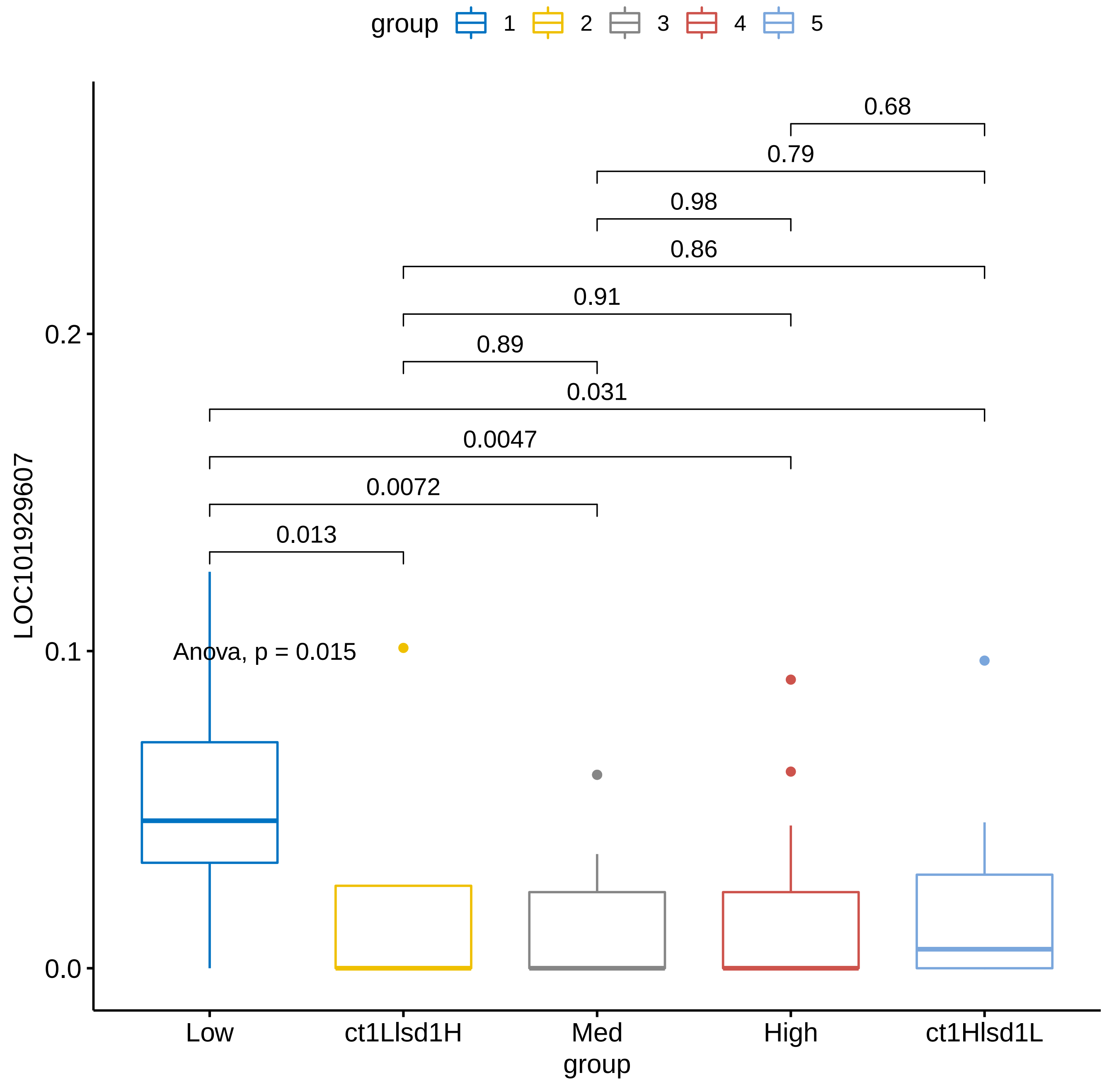

group 1 2 3 4 5
0.68
0.79
0.98
0.86
0.91
0.89
0.031
0.0047
0.0072
0.013
Anova, p = 0.015
LOC101929607
0.2
0.1
0.0
Low
ct1Llsd1H
Med
High
ct1Hlsd1L
group

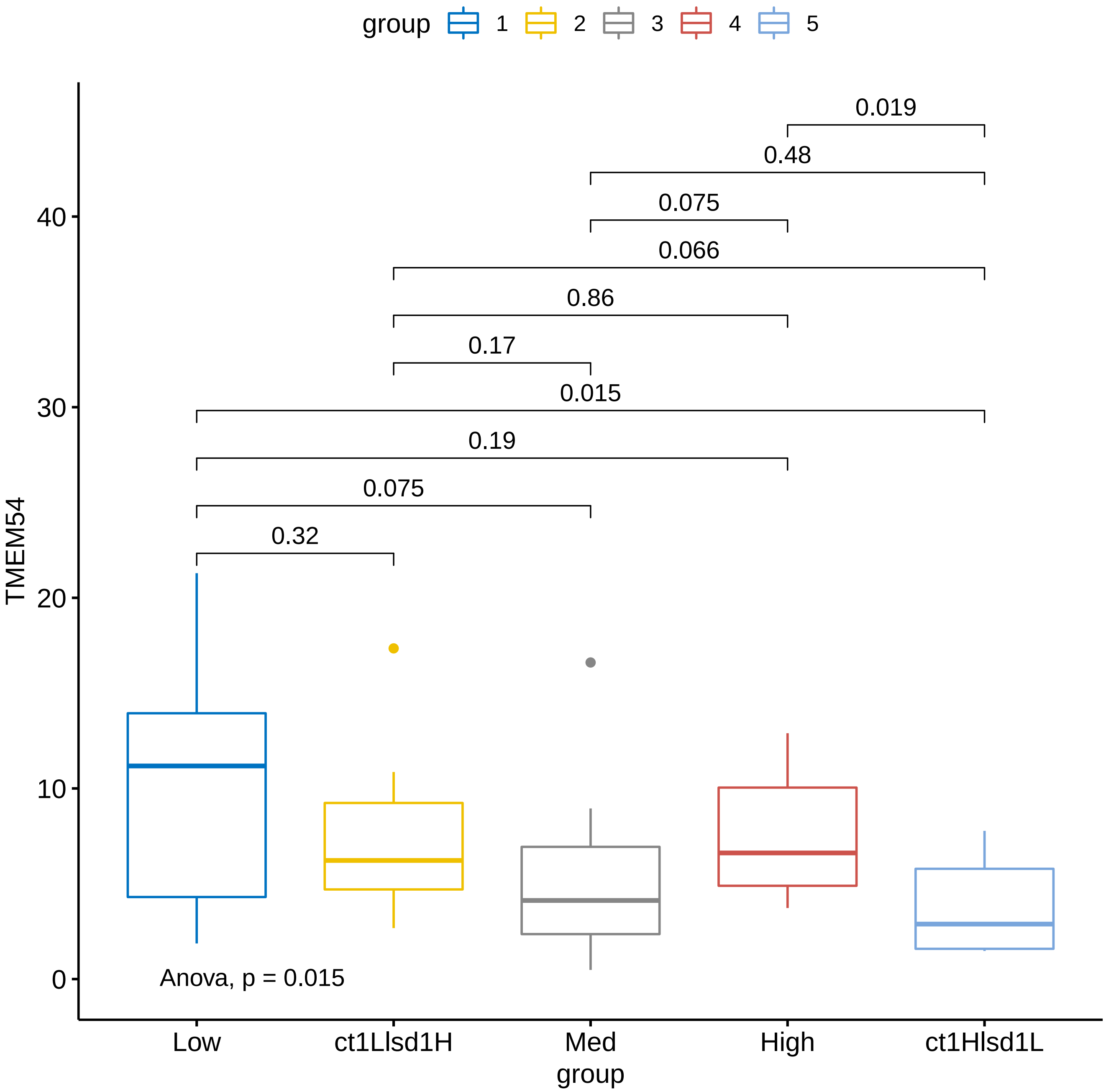
group
1
2
3
4
5
0.019
0.48
0.075
0.066
0.86
0.17
0.015
0.19
0.075
0.32
40
30
20
10
0
TMEM54
Anova, p = 0.015
Low
ct1Llsd1H
Med
High
ct1Hlsd1L
group

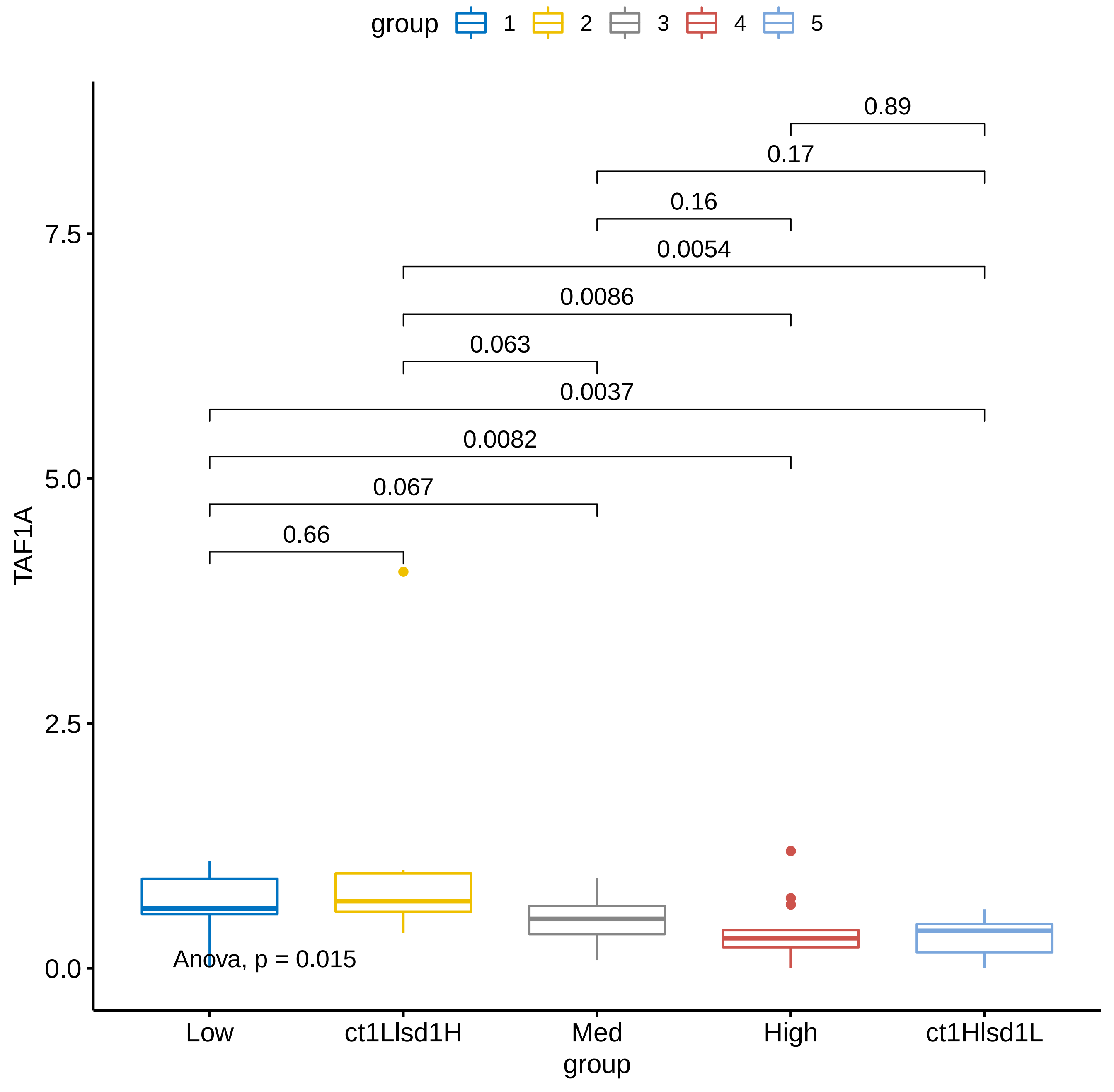

group
1
2
3
4
5
0.89
0.17
0.16
0.0054
0.0086
0.063
0.0037
0.0082
0.067
0.66
7.5
5.0
2.5
0.0
TAF1A
Anova, p = 0.015
Low
ct1Llsd1H
Med
High
ct1Hlsd1L
group

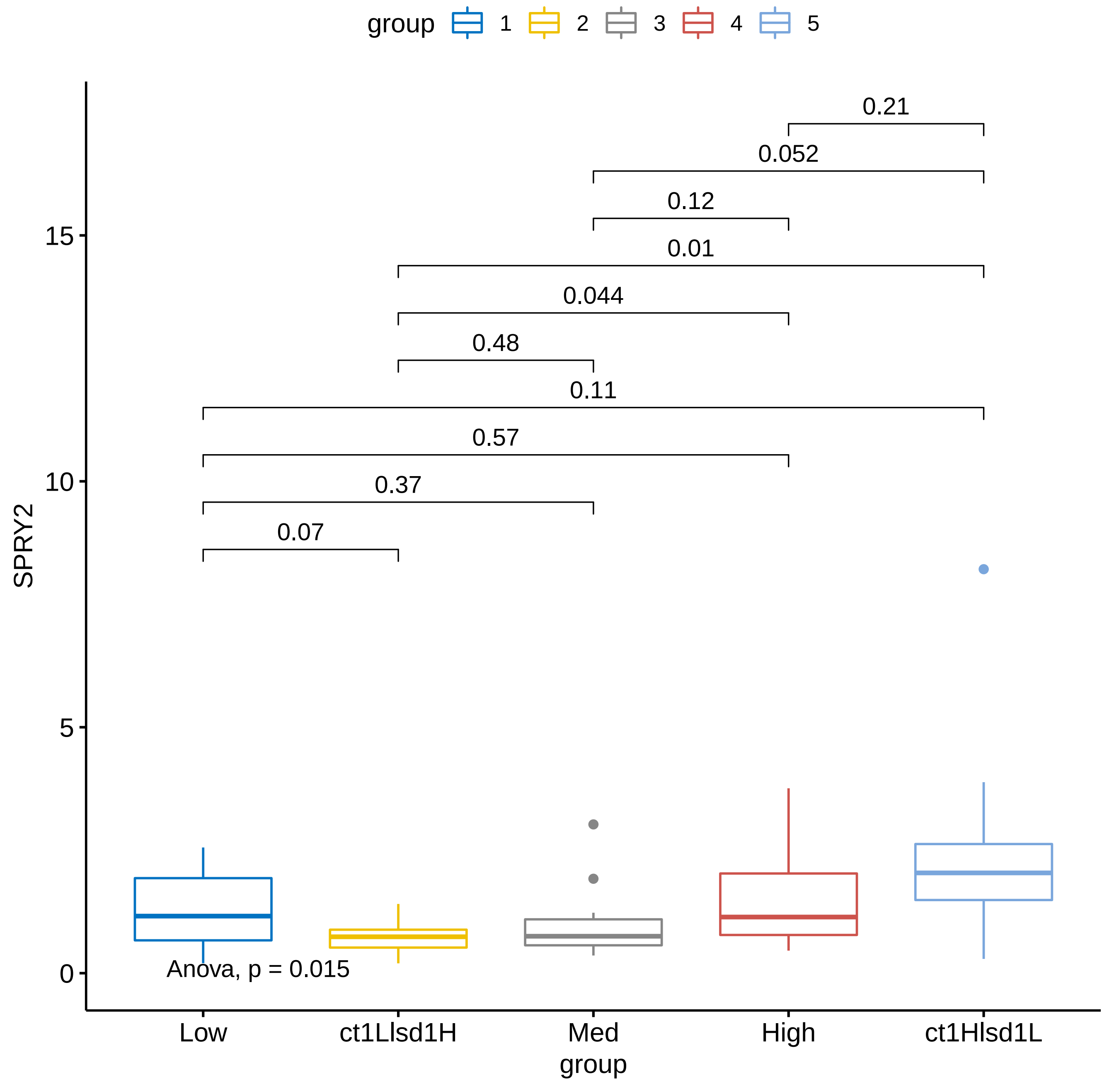

group 1 2 3 4 5
0.21
0.052
0.12
0.01
0.044
0.48
0.11
0.57
0.37
0.07
15
10
5
0
SPRY2
Anova, p = 0.015
Low
ct1Llsd1H
Med
High
ct1Hlsd1L
group

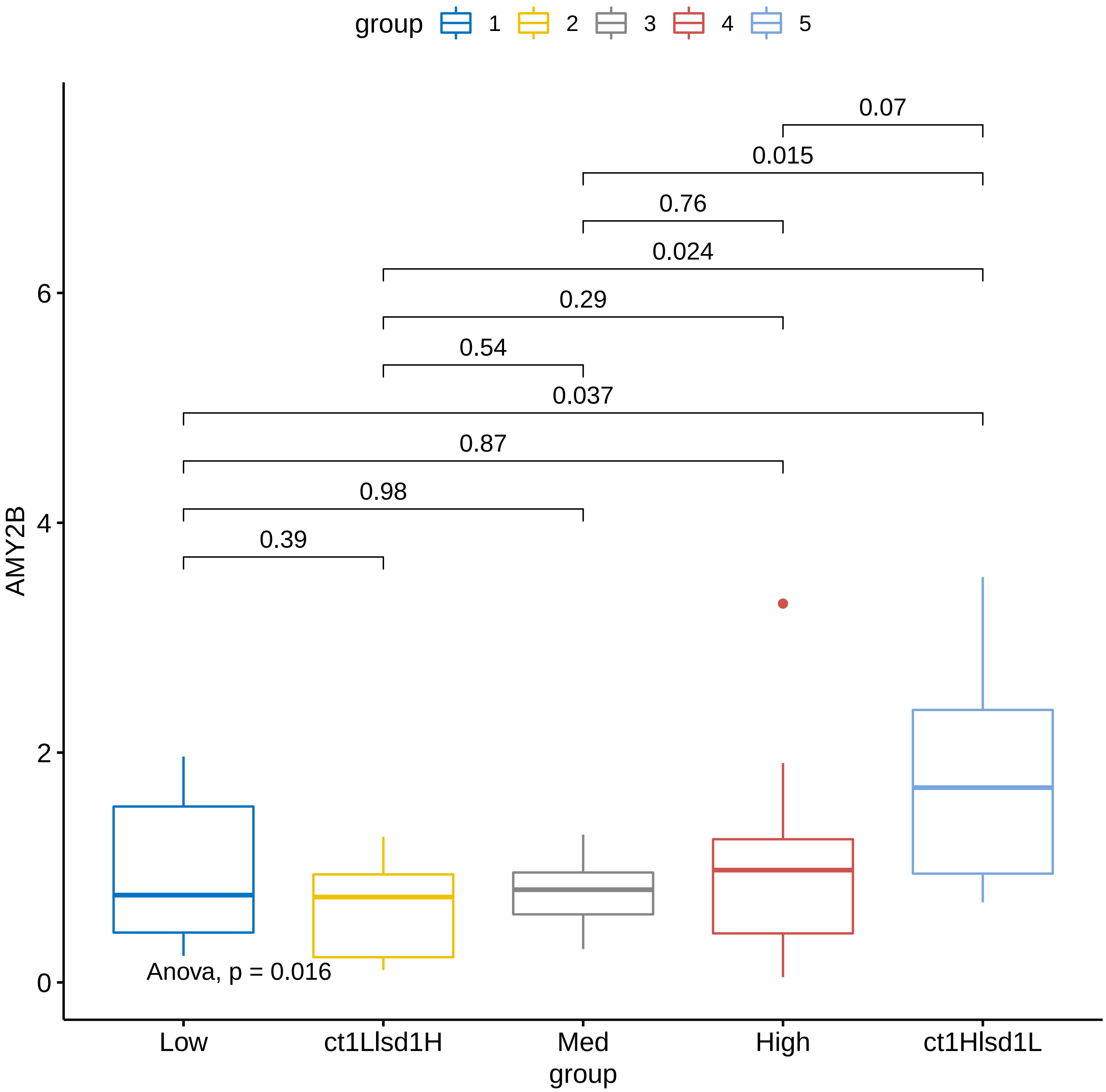

group 1 2 3 4 5
0.07
0.015
0.76
0.024
0.29
0.54
0.037
0.87
0.98
0.39
AMY2B
6
4
2
0
Anova, p = 0.016
Low
ct1Llsd1H
Med
High
ct1Hlsd1L
group

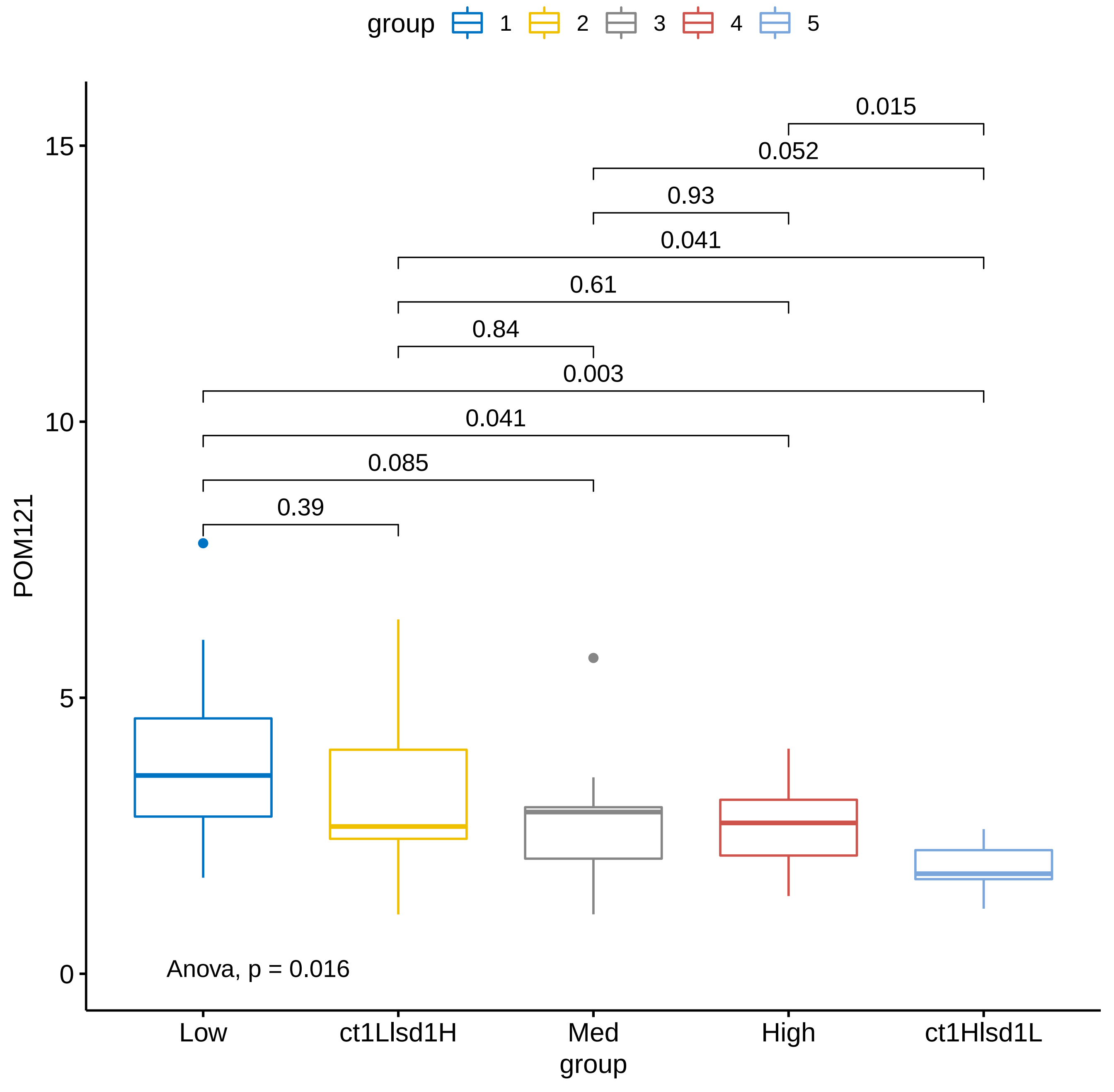
group 1 2 3 4 5
0.015
0.052
0.93
0.041
0.61
0.84
0.003
0.041
0.085
0.39
15
10
5
0
POM121
Anova, p = 0.016
Low
ct1Llsd1H
Med
High
ct1Hlsd1L
group

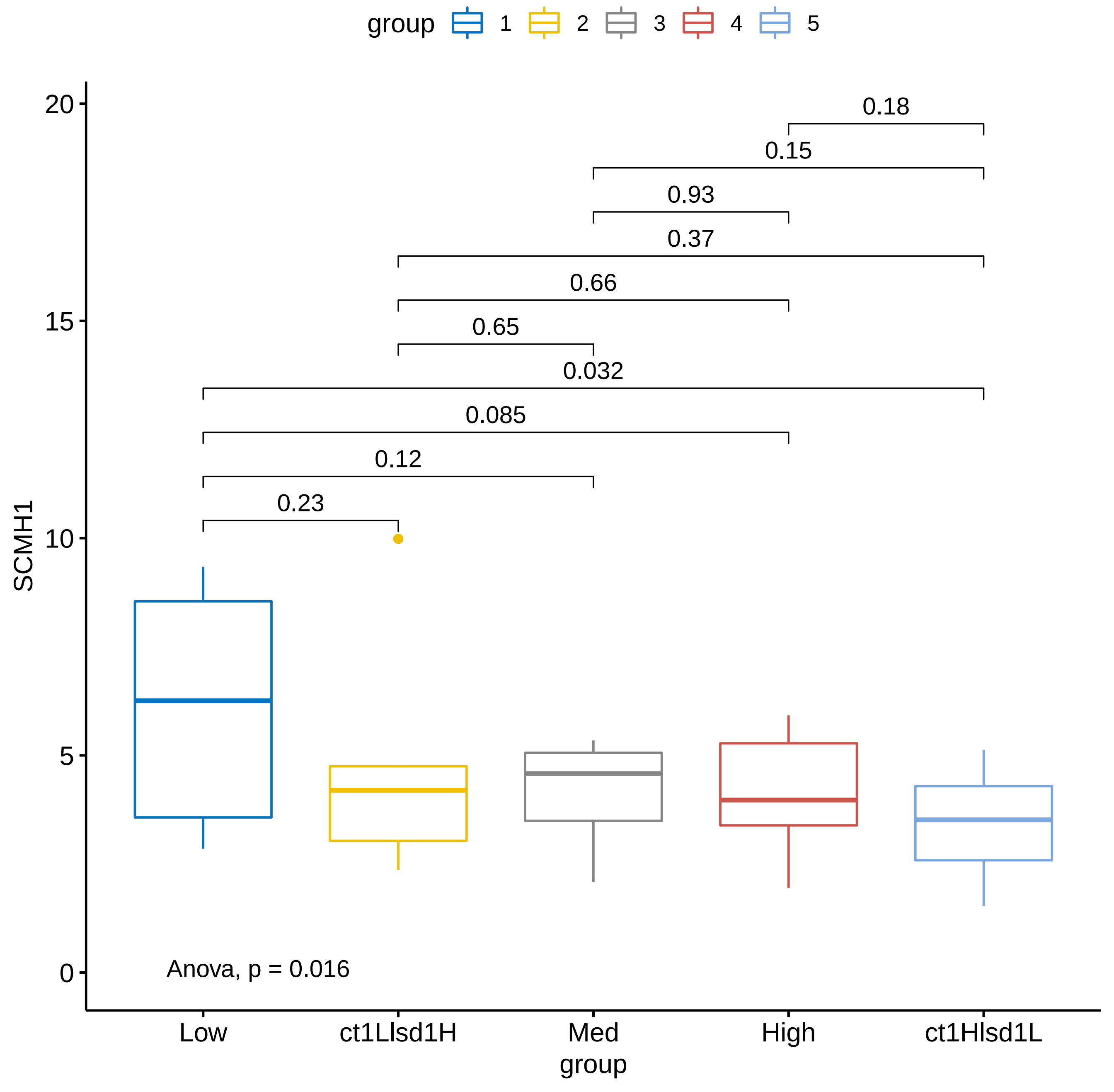

group 1 2 3 4 5
0.18
0.15
0.93
0.37
0.66
0.65
0.032
0.085
0.12
0.23
SCMH1
20
15
10
5
0
Anova, p = 0.016
Low
ct1Llsd1H
Med
High
ct1Hlsd1L
group

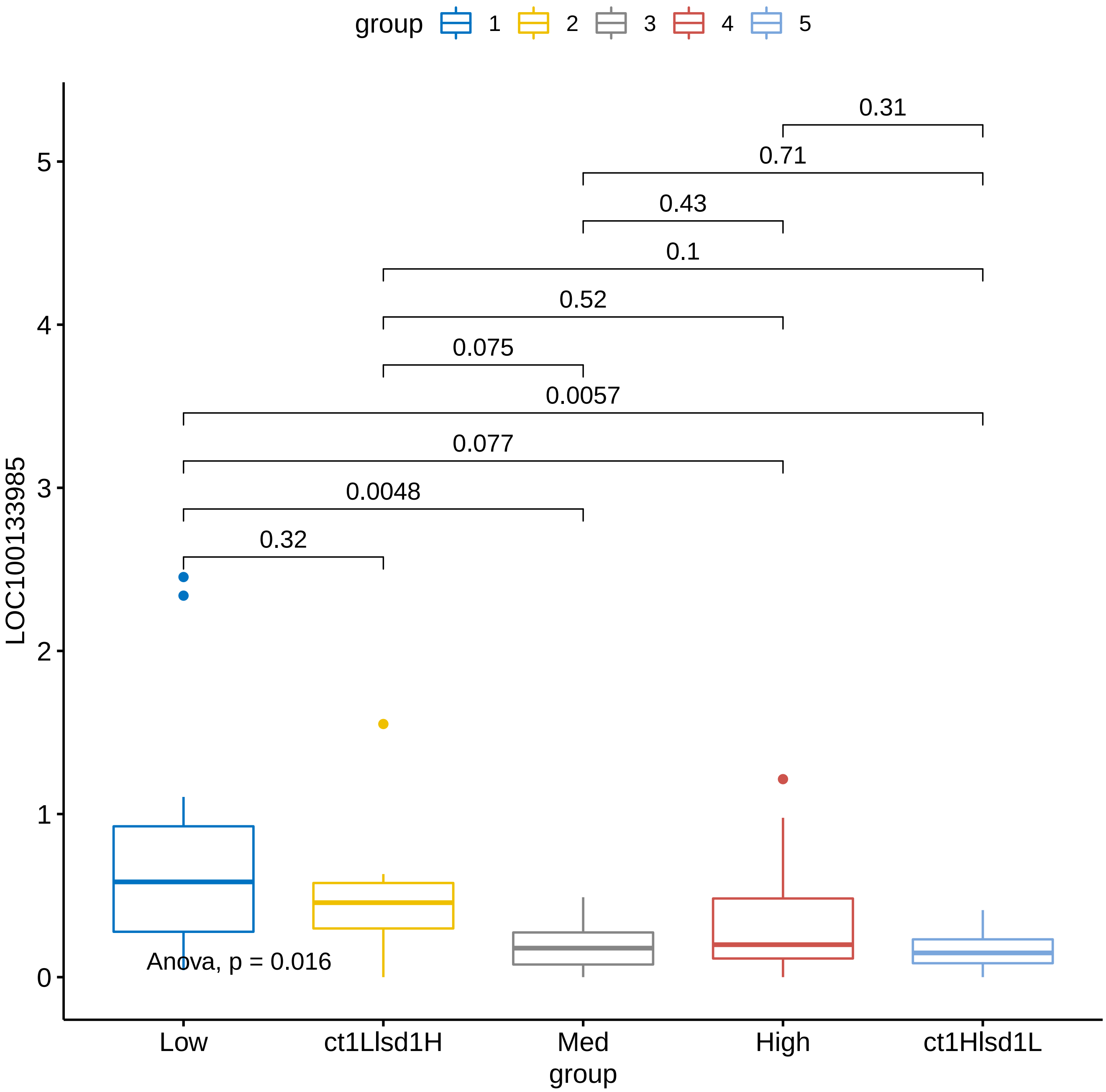
group
1
2
3
4
5
0.31
0.71
0.43
0.1
0.52
0.075
0.0057
0.077
0.0048
0.32
Anova, p = 0.016
LOC100133985
0
1
2
3
4
5
Low
ct1Llsd1H
Med
High
ct1Hlsd1L
group

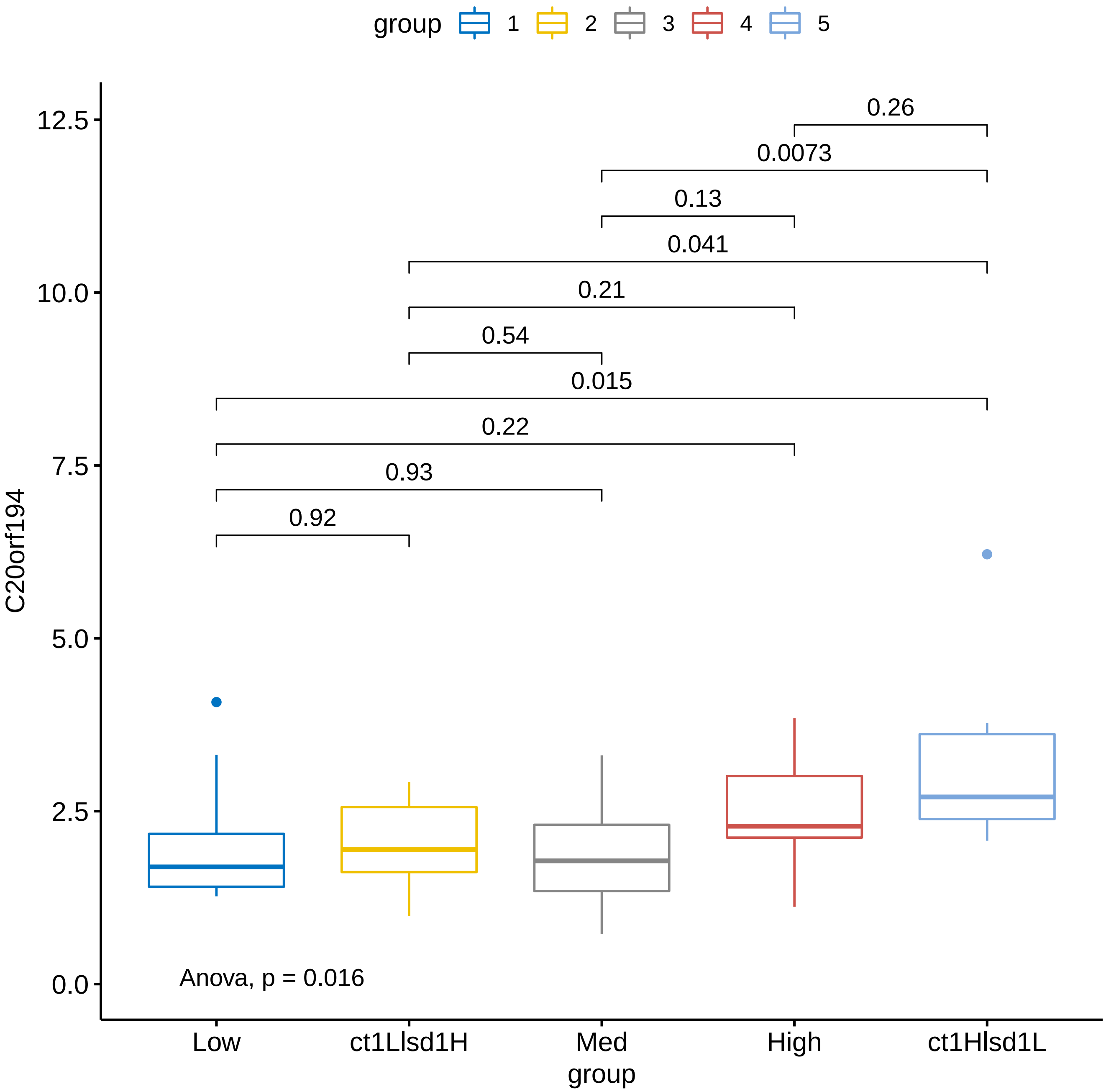

group 1 2 3 4 5
0.26
0.0073
0.13
0.041
0.21
0.54
0.015
0.22
0.93
0.92
Anova, p = 0.016
C20orf194
12.5
10.0
7.5
5.0
2.5
0.0
Low
ct1Llsd1H
Med
High
ct1Hlsd1L
group

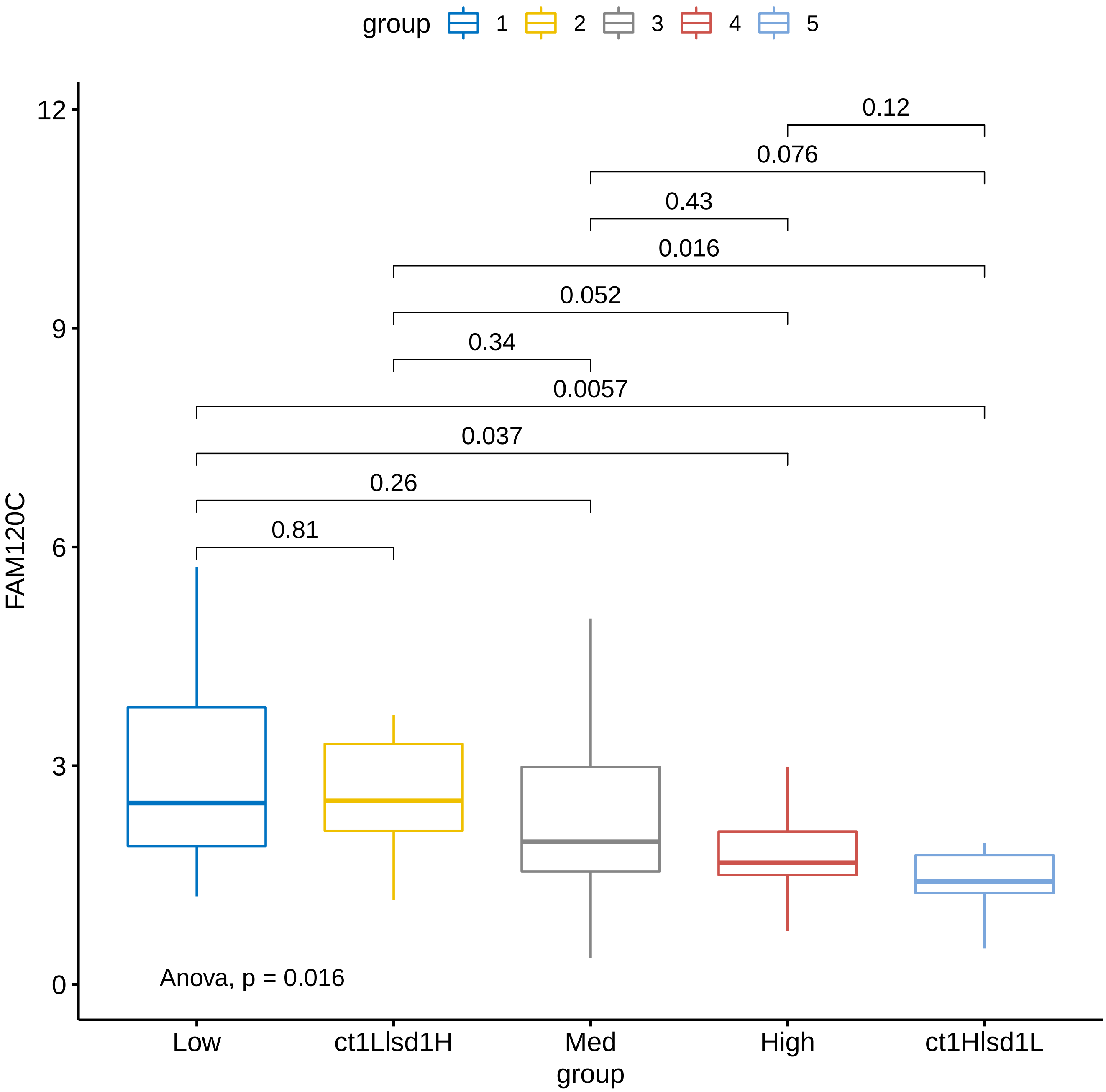
group
1
2
3
4
5
FAM120C
0
3
6
9
12
0.12
0.076
0.43
0.016
0.052
0.34
0.0057
0.037
0.26
0.81
Anova, p = 0.016
Low
ct1Llsd1H
Med
High
ct1Hlsd1L
group

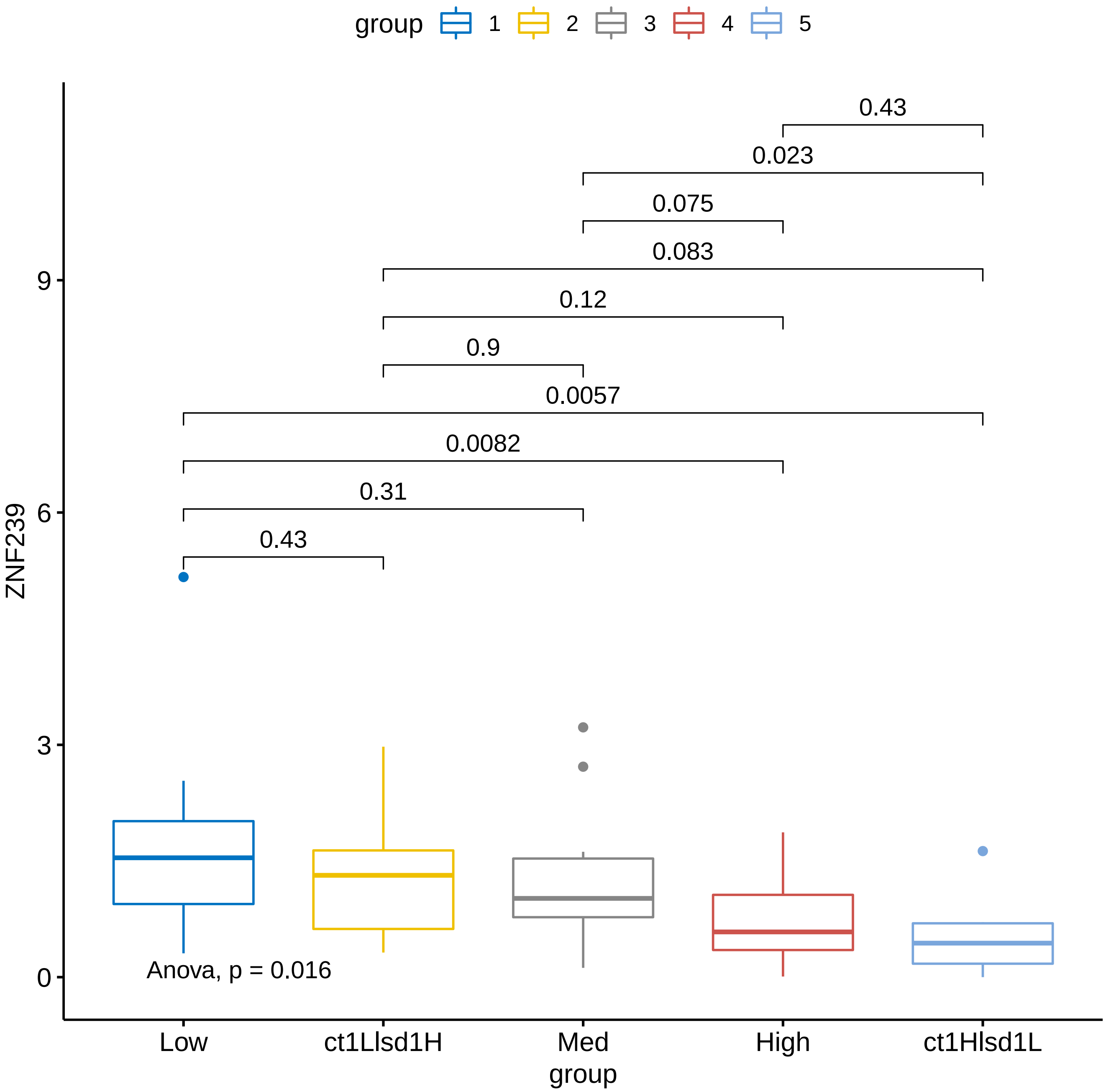
group
1
2
3
4
5
0.43
0.023
0.075
0.083
0.12
0.9
0.0057
0.0082
0.31
0.43
ZNF239
9
6
3
0
Anova, p = 0.016
Low
ct1Llsd1H
Med
High
ct1Hlsd1L
group

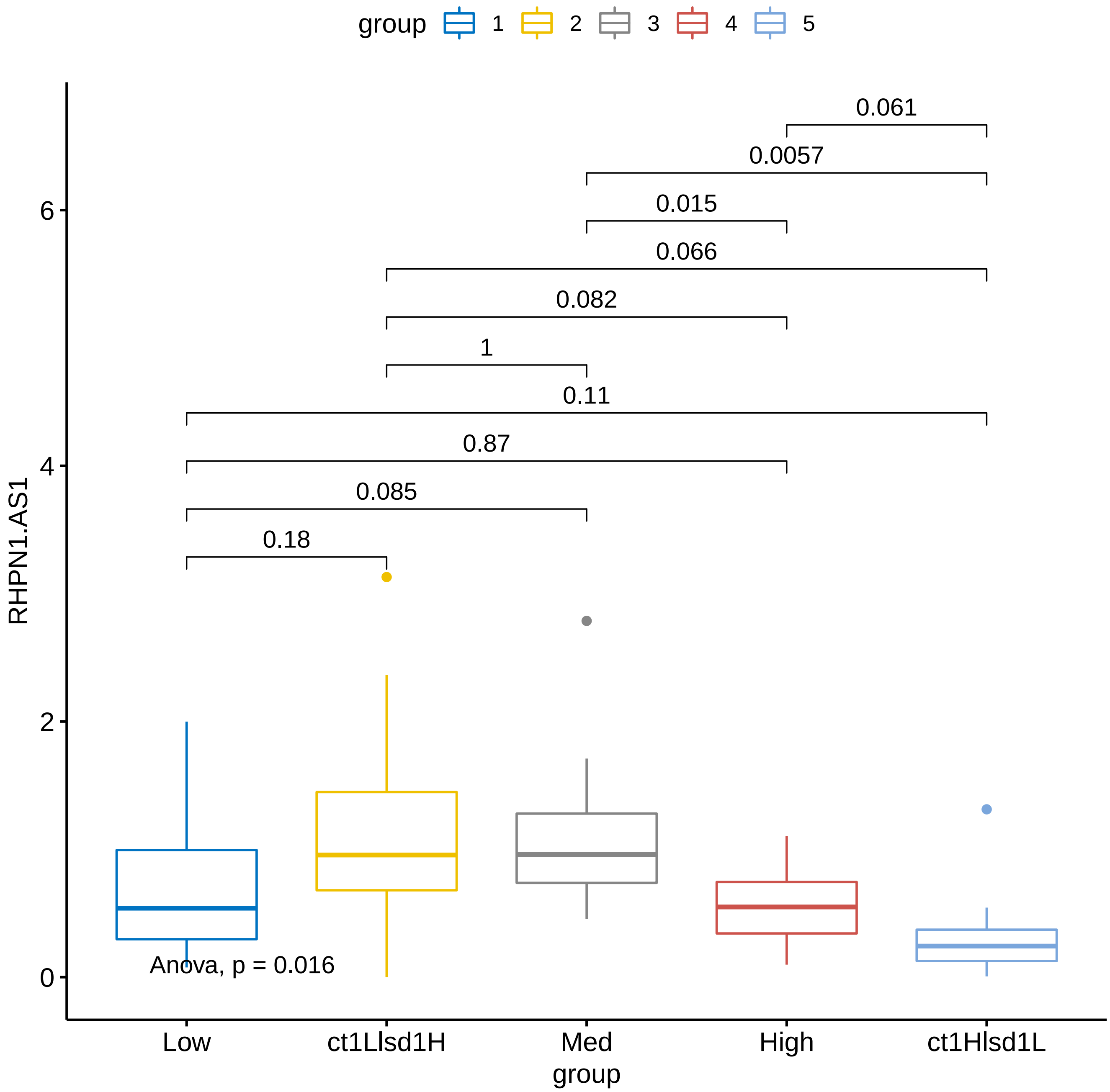

group 1 2 3 4 5
0.061
0.0057
0.015
0.066
0.082
1
0.11
0.87
0.085
0.18
Anova, p = 0.016
RHPN1.AS1
0
2
4
6
Low
ct1Llsd1H
Med
High
ct1Hlsd1L
group

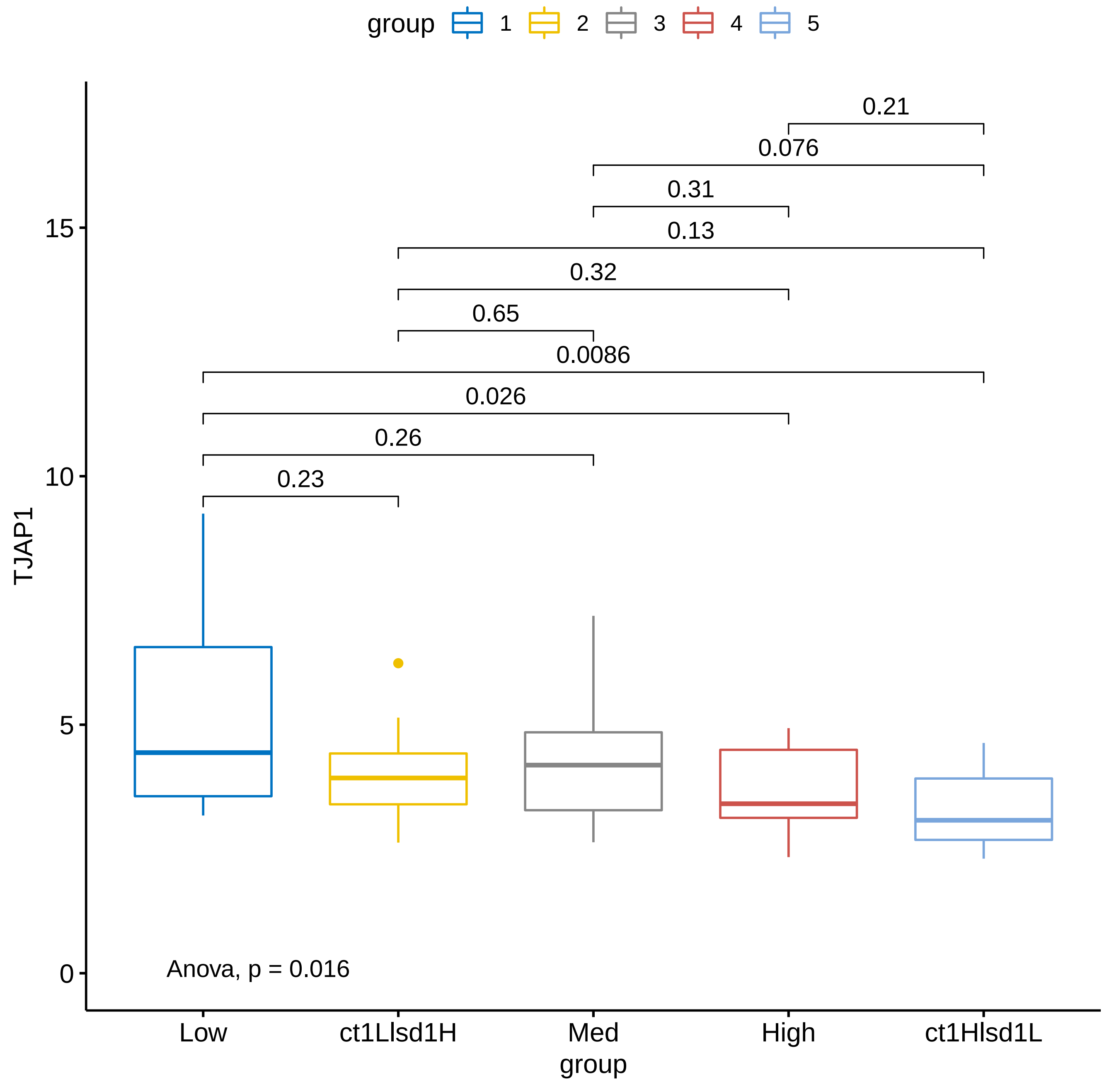

group 1 2 3 4 5
0.21
0.076
0.31
0.13
0.32
0.65
0.0086
0.026
0.26
0.23
TJAP1
15
10
5
0
Anova, p = 0.016
Low
ct1Llsd1H
Med
High
ct1Hlsd1L
group

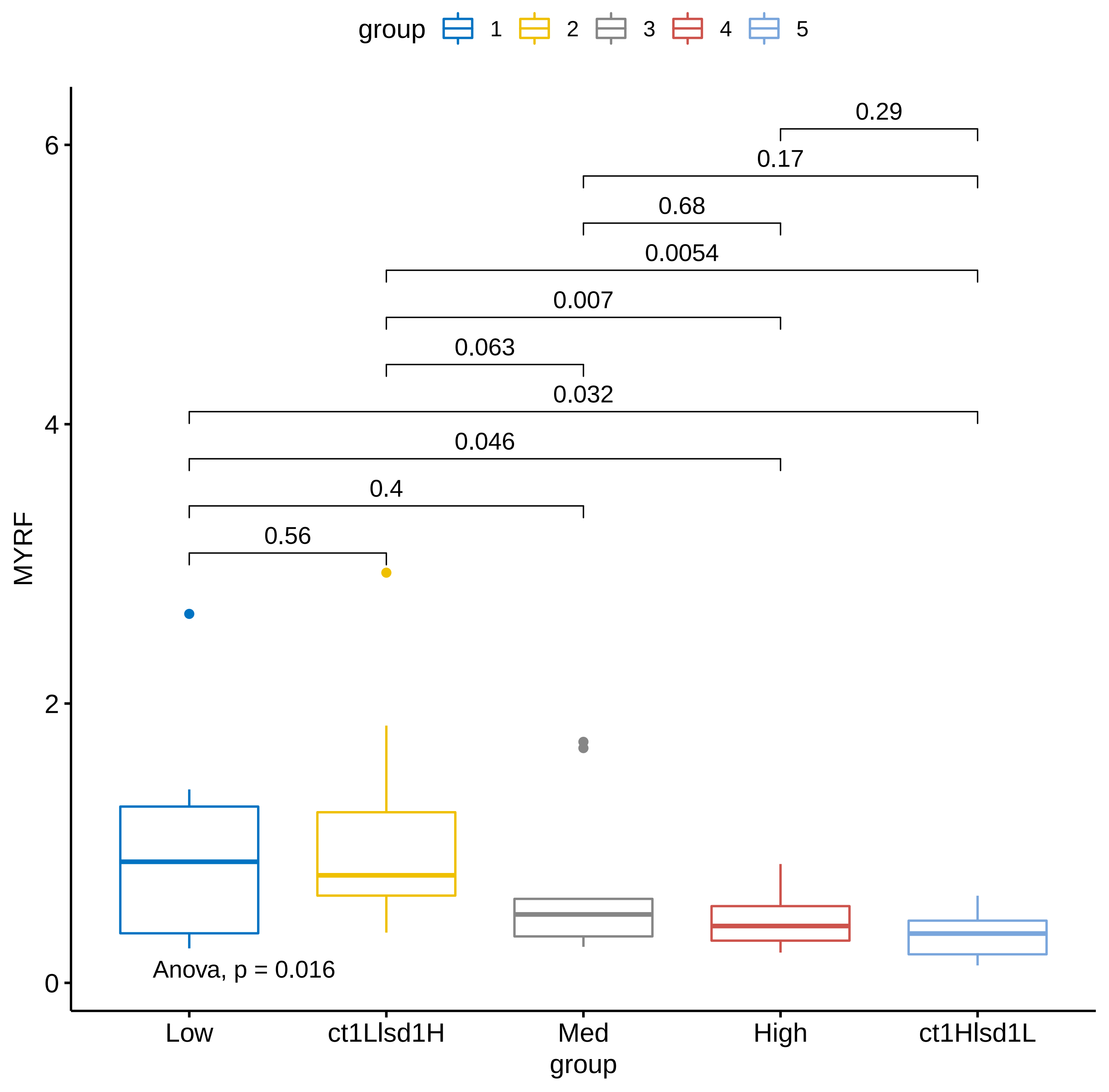

group 1 2 3 4 5
0.29
0.17
0.68
0.0054
0.007
0.063
0.032
0.046
0.4
0.56
Anova, p = 0.016
MYRF
6
4
2
0
Low
ct1Llsd1H
Med
High
ct1Hlsd1L
group

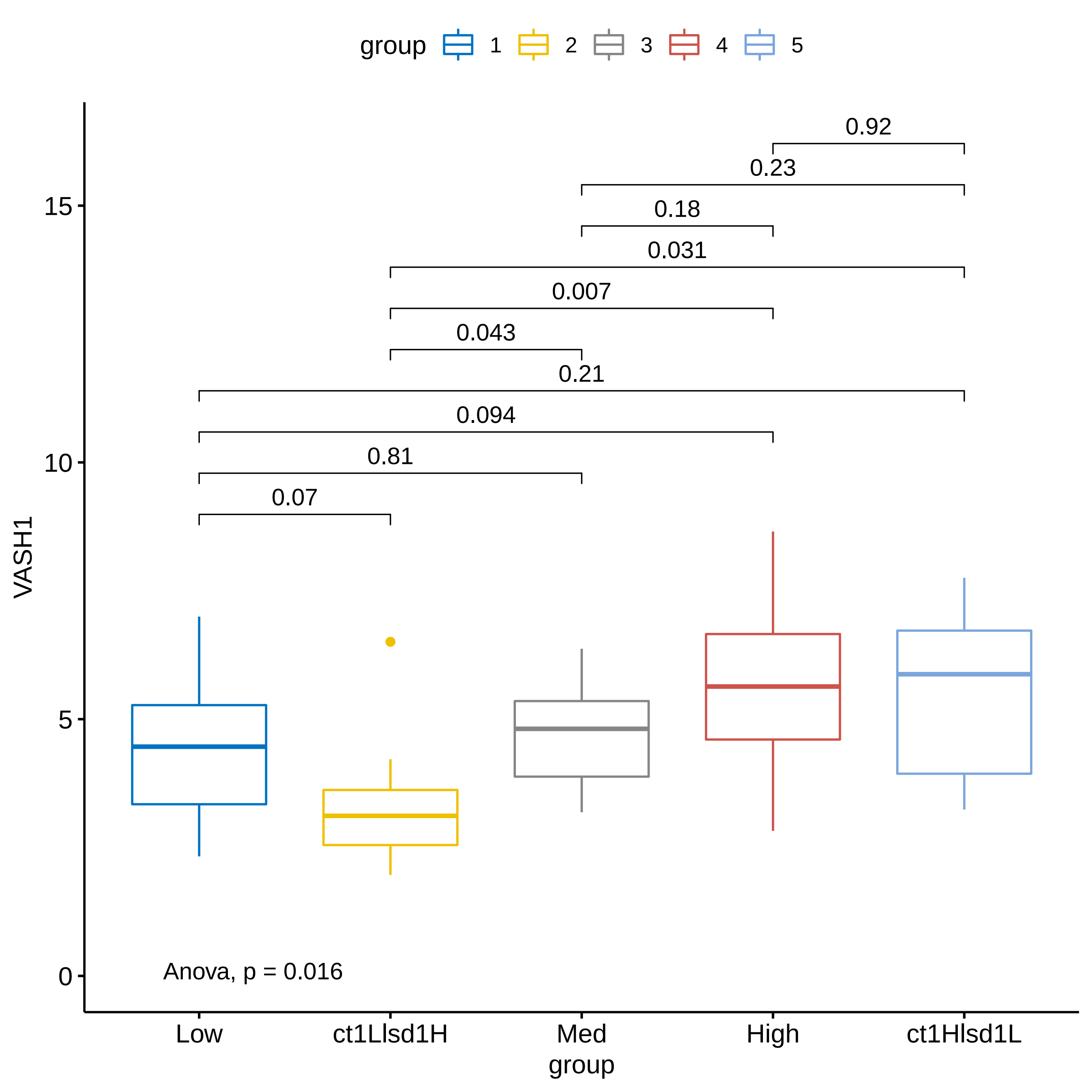

group
1
2
3
4
5
0.92
0.23
0.18
0.031
0.007
0.043
0.21
0.094
0.81
0.07
15
10
5
0
VASH1
Anova, p = 0.016
Low
ct1Llsd1H
Med
High
ct1Hlsd1L
group

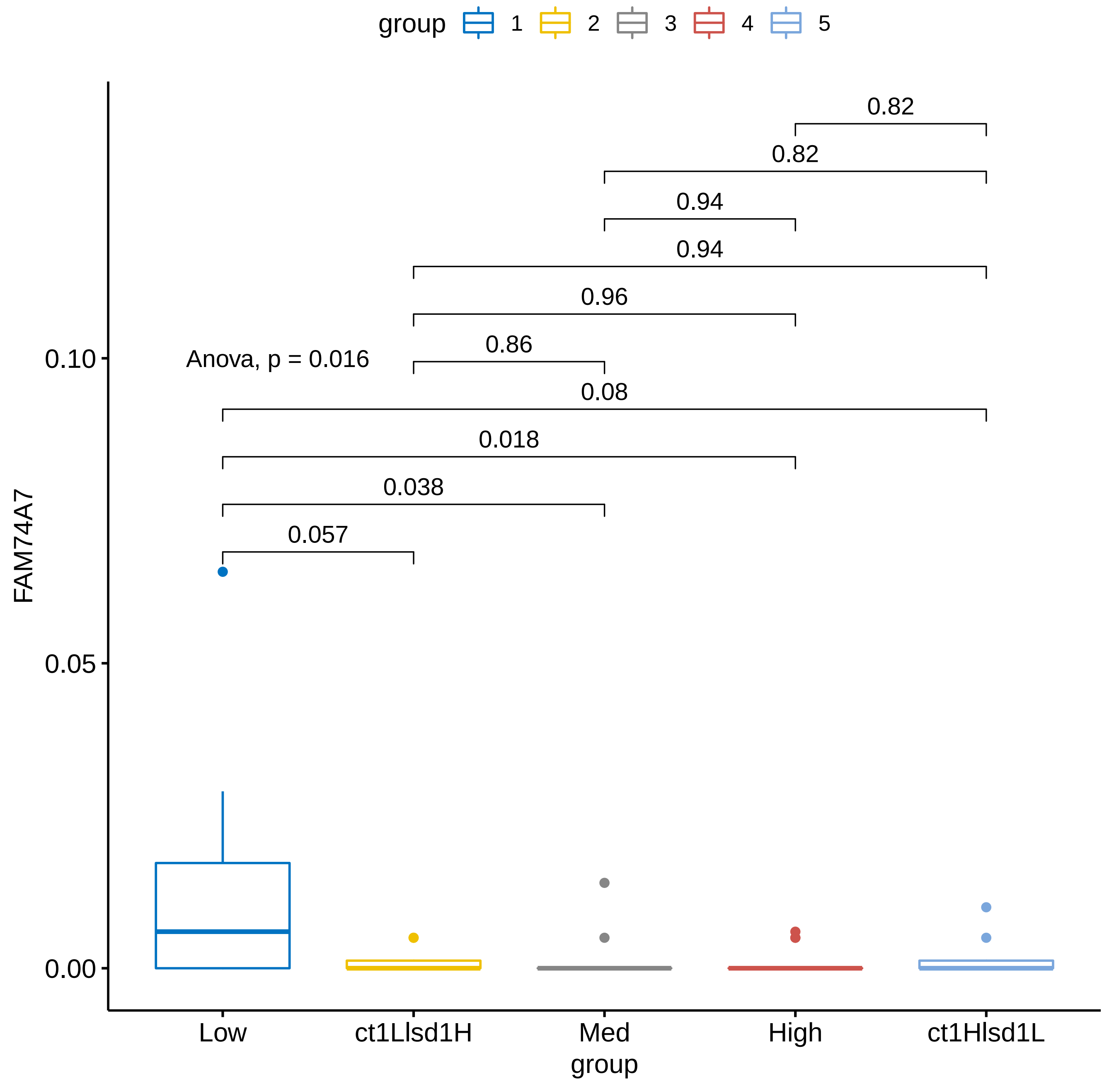
group
1
2
3
4
5
0.82
0.82
0.94
0.94
0.96
0.86
Anova, p = 0.016
0.08
0.018
0.038
0.057
0.10
0.05
0.00
FAM74A7
Low
ct1Llsd1H
Med
High
ct1Hlsd1L
group

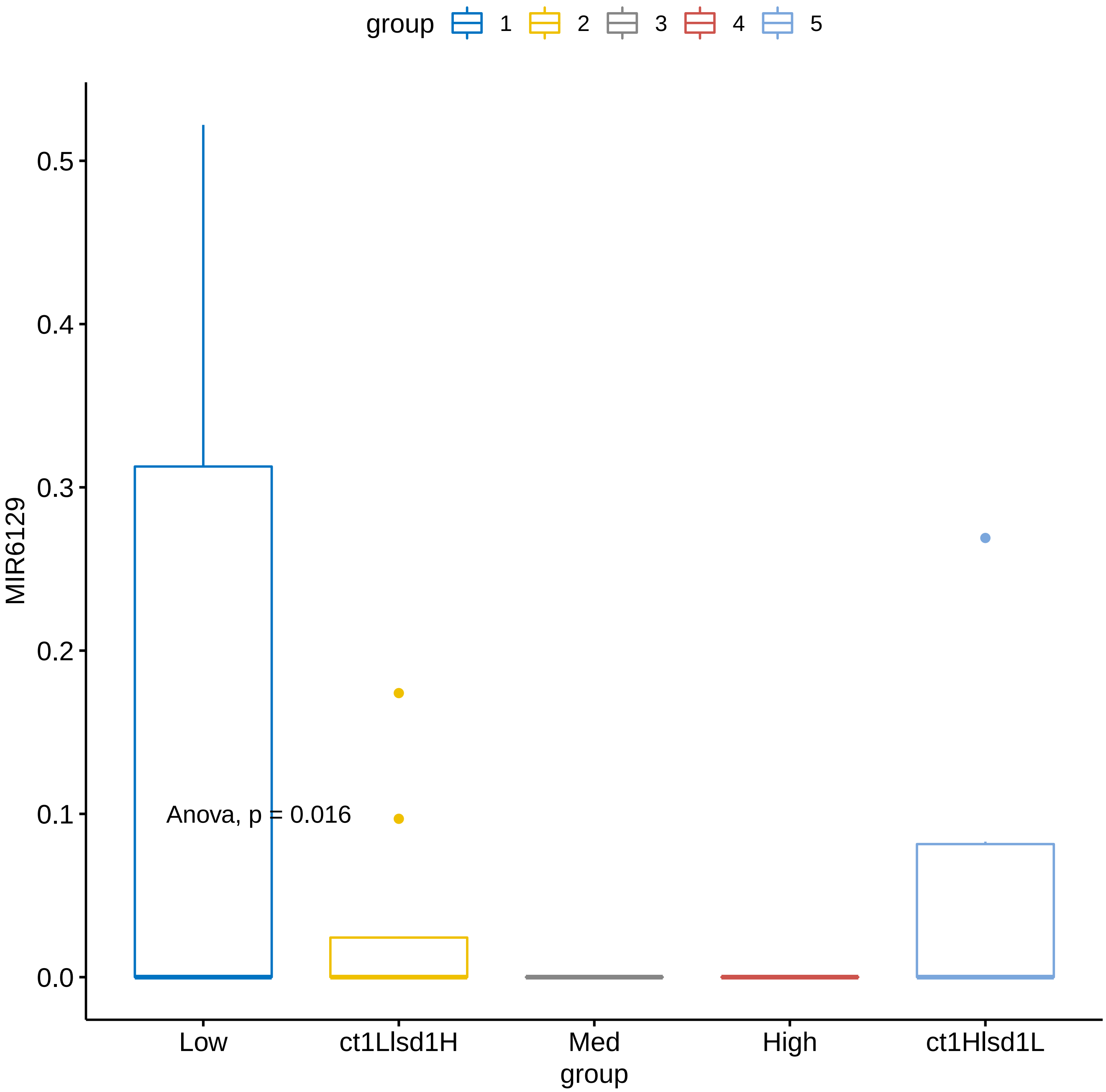
group 1 2 3 4 5
MIR6129
0.5
0.4
0.3
0.2
0.1
0.0
Anova, p = 0.016
Low
ct1Llsd1H
Med
High
ct1Hlsd1L
group

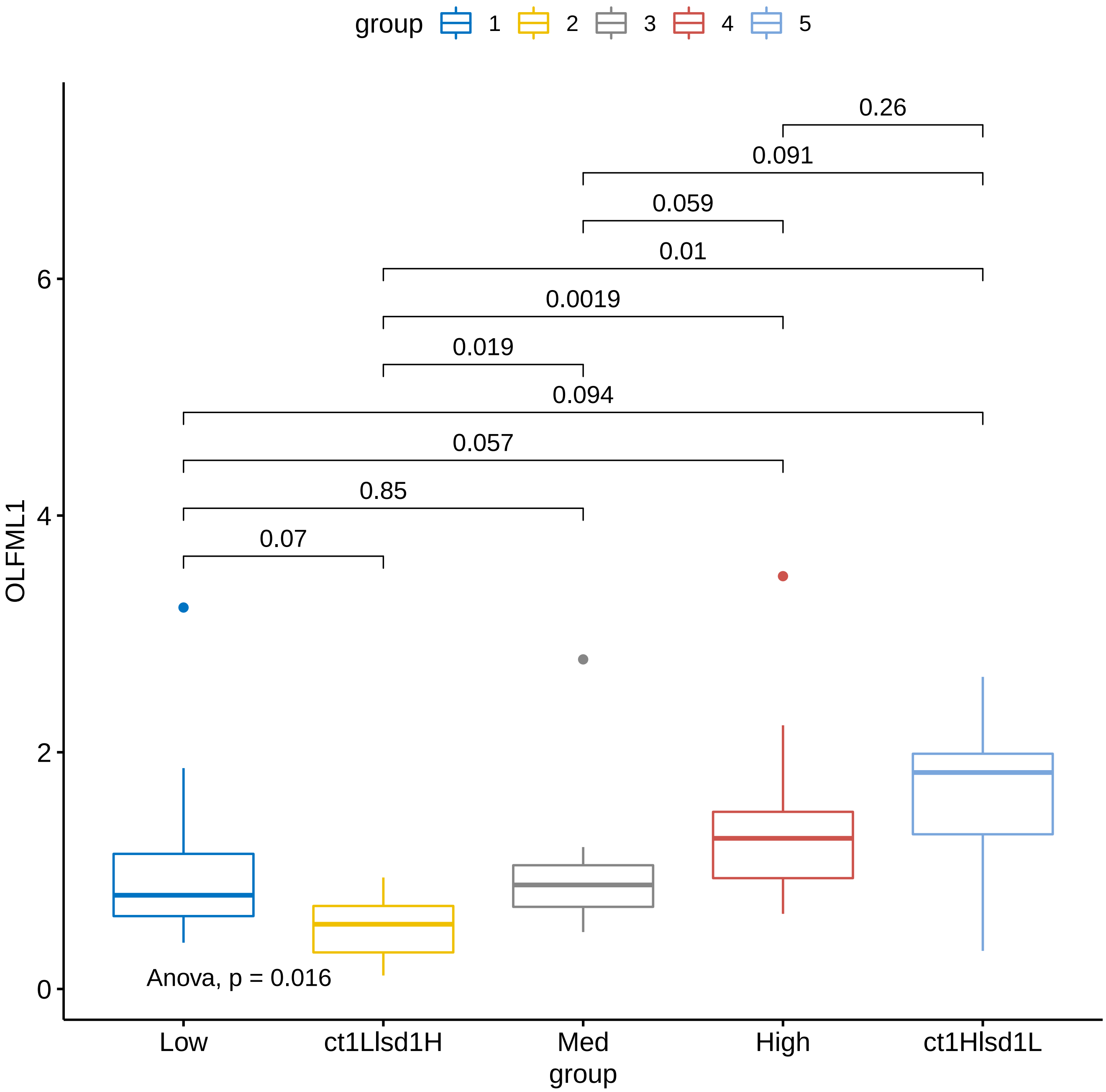

group
1
2
3
4
5
0.26
0.091
0.059
0.01
0.0019
0.019
0.094
0.057
0.85
0.07
Anova, p = 0.016
OLFML1
0
2
4
6
Low
ct1Llsd1H
Med
High
ct1Hlsd1L
group

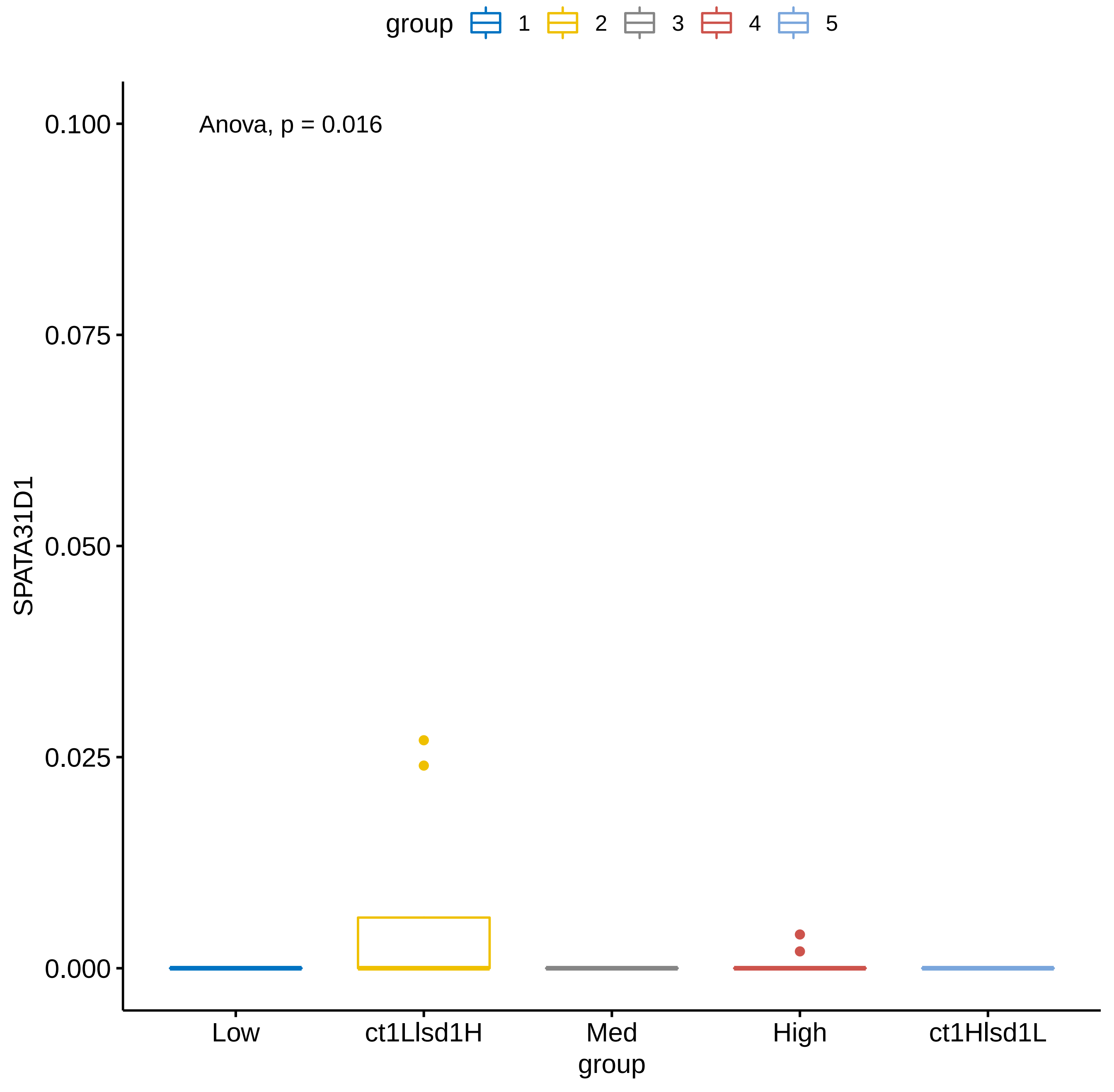

group
1
2
3
4
5
Anova, p = 0.016
SPATA31D1
0.100
0.075
0.050
0.025
0.000
Low
ct1Llsd1H
Med
High
ct1Hlsd1L
group

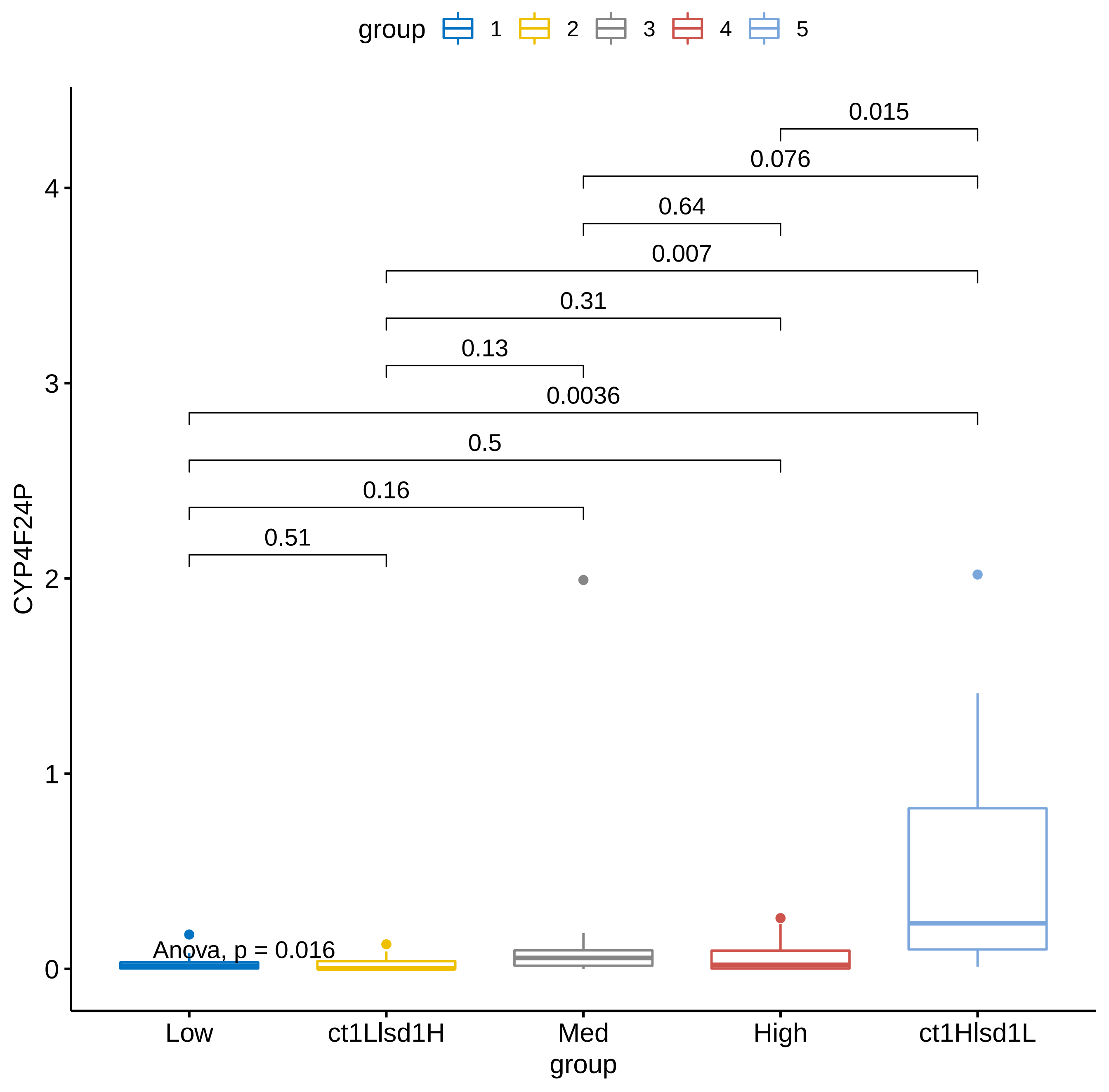

group 1 2 3 4 5
0.015
0.076
0.64
0.007
0.31
0.13
0.0036
0.5
0.16
0.51
Anova, p = 0.016
CYP4F24P
4
3
2
1
0
Low
ct1Llsd1H
Med
High
ct1Hlsd1L
group

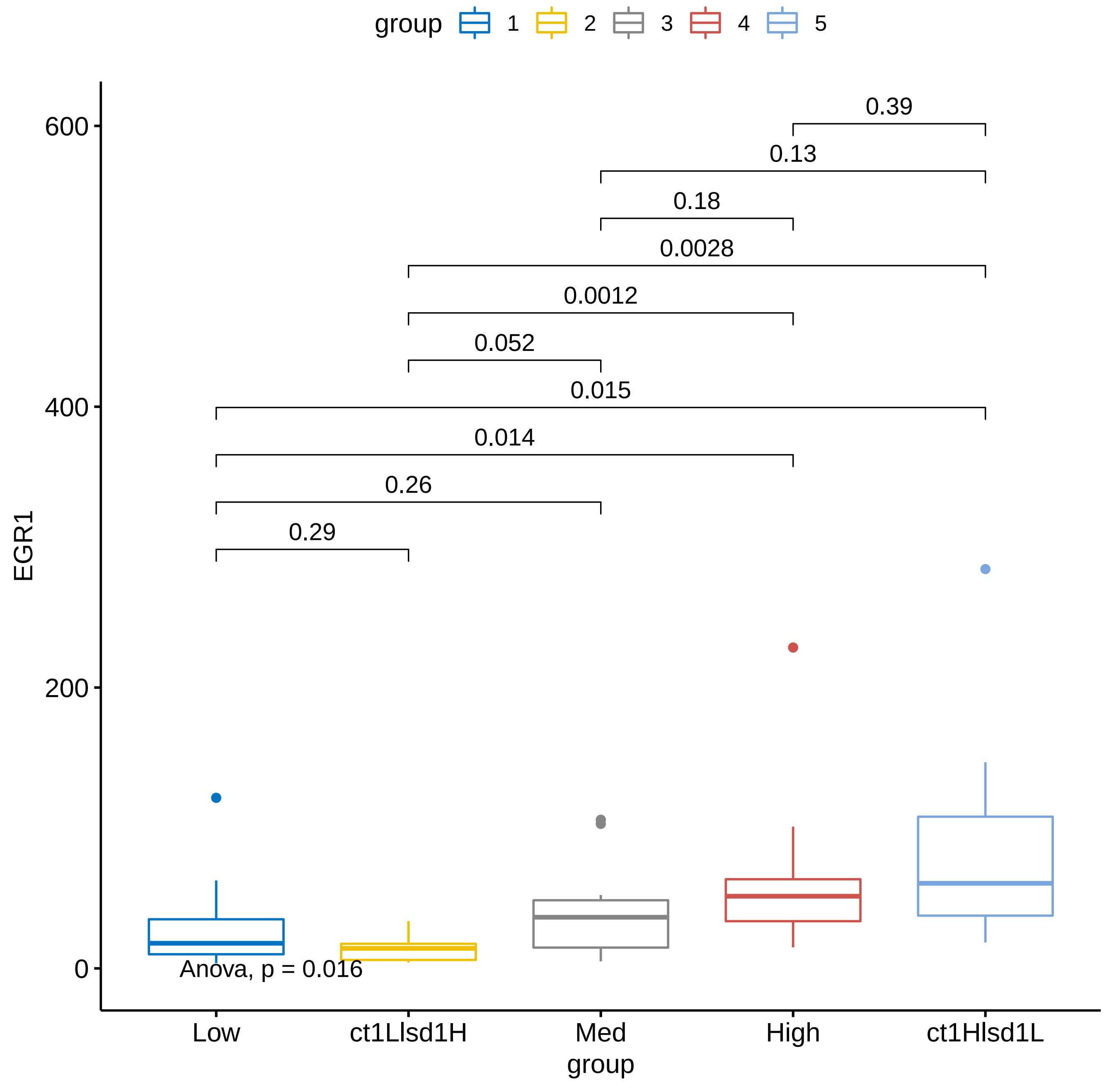
group
1
2
3
4
5
0.39
0.13
0.18
0.0028
0.0012
0.052
0.015
0.014
0.26
0.29
600
400
200
0
EGR1
Anova, p = 0.016
Low
ct1Llsd1H
Med
High
ct1Hlsd1L
group

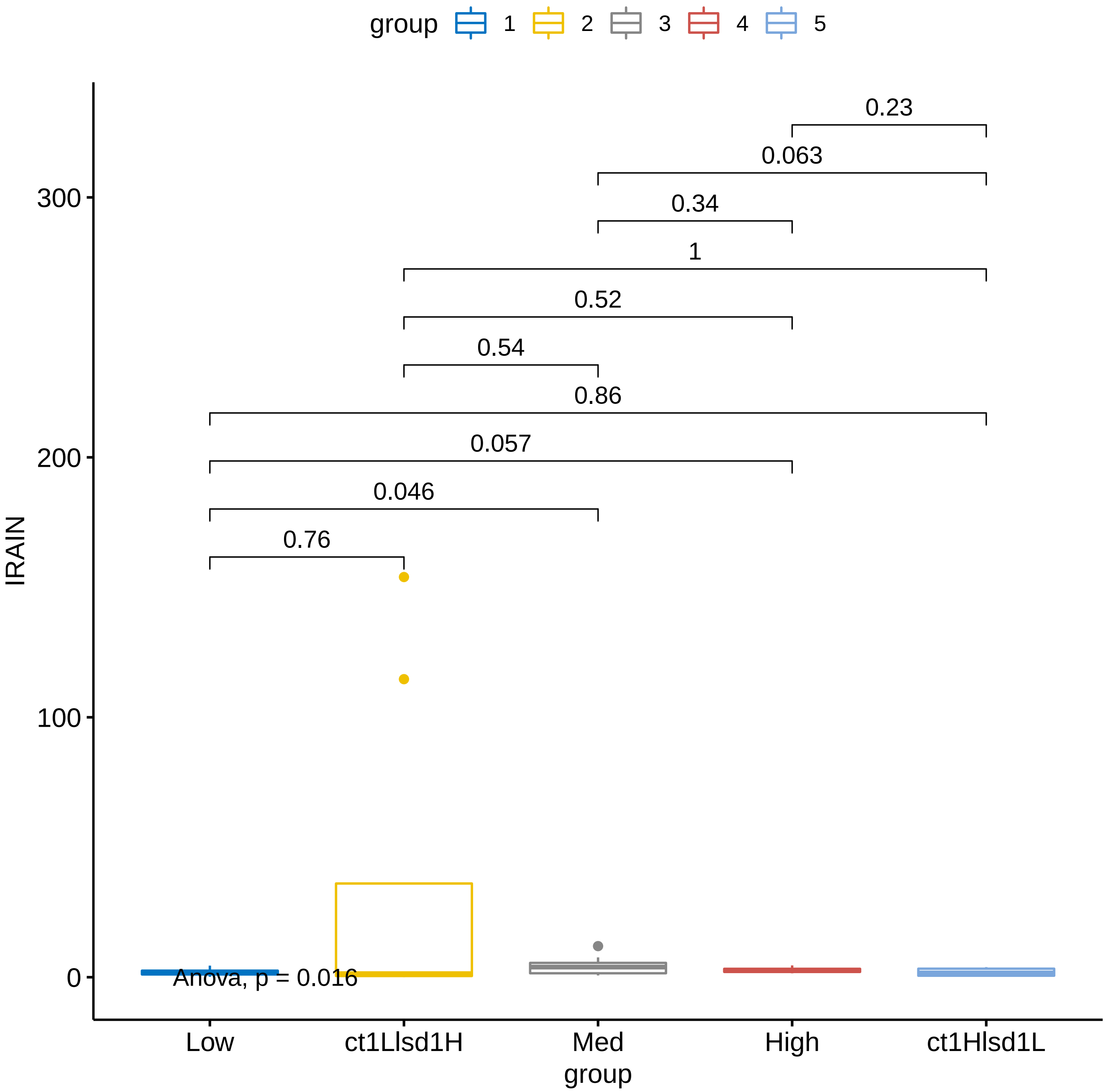

group 1 2 3 4 5
0.23
0.063
0.34
1
0.52
0.54
0.86
0.057
0.046
0.76
300
200
100
0
IRAIN
Anova, p = 0.016
Low
ct1Llsd1H
Med
High
ct1Hlsd1L
group

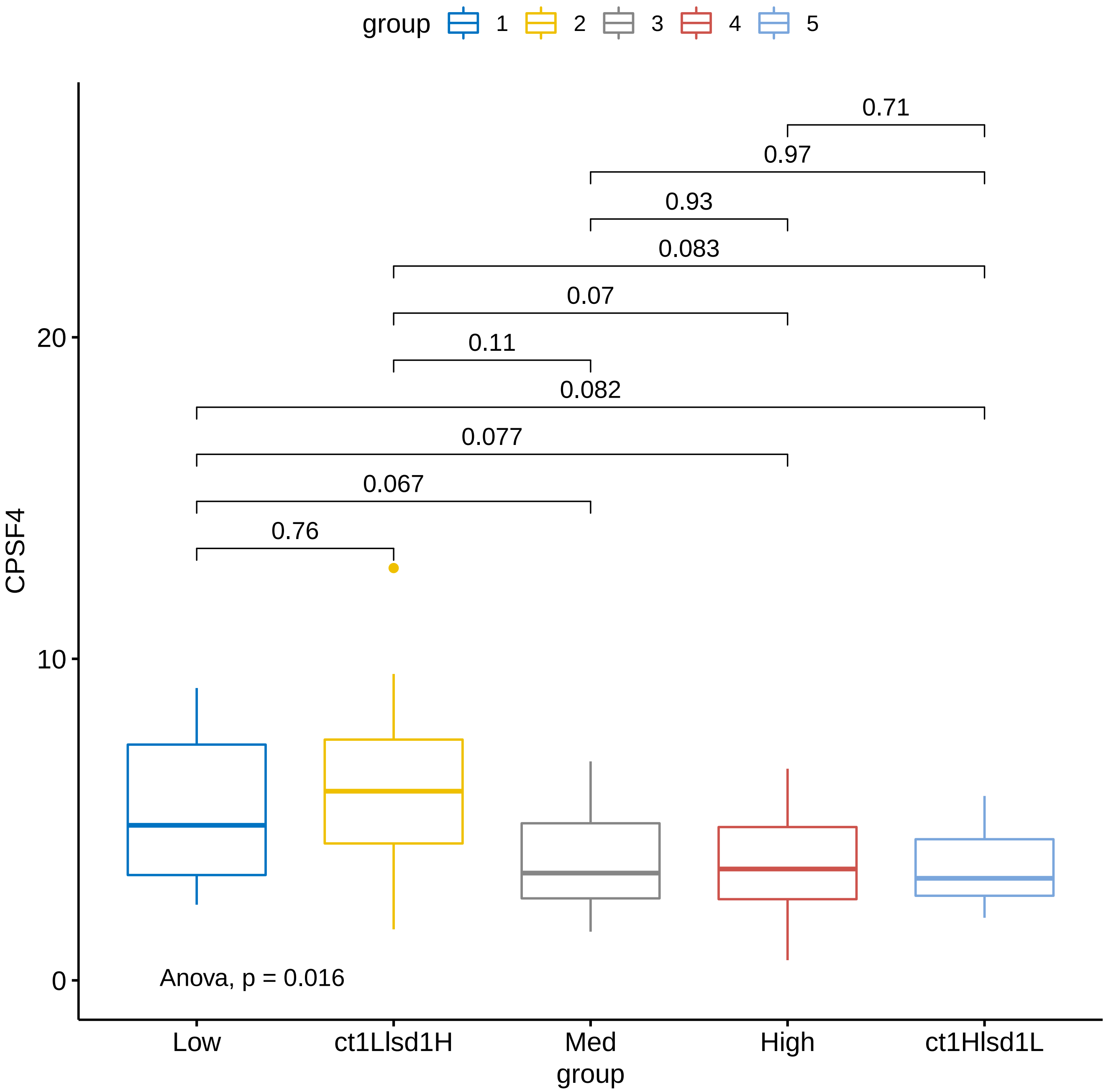
group
1
2
3
4
5
0.71
0.97
0.93
0.083
0.07
0.11
0.082
0.077
0.067
0.76
20
10
0
CPSF4
Anova, p = 0.016
Low
ct1Llsd1H
Med
High
ct1Hlsd1L
group

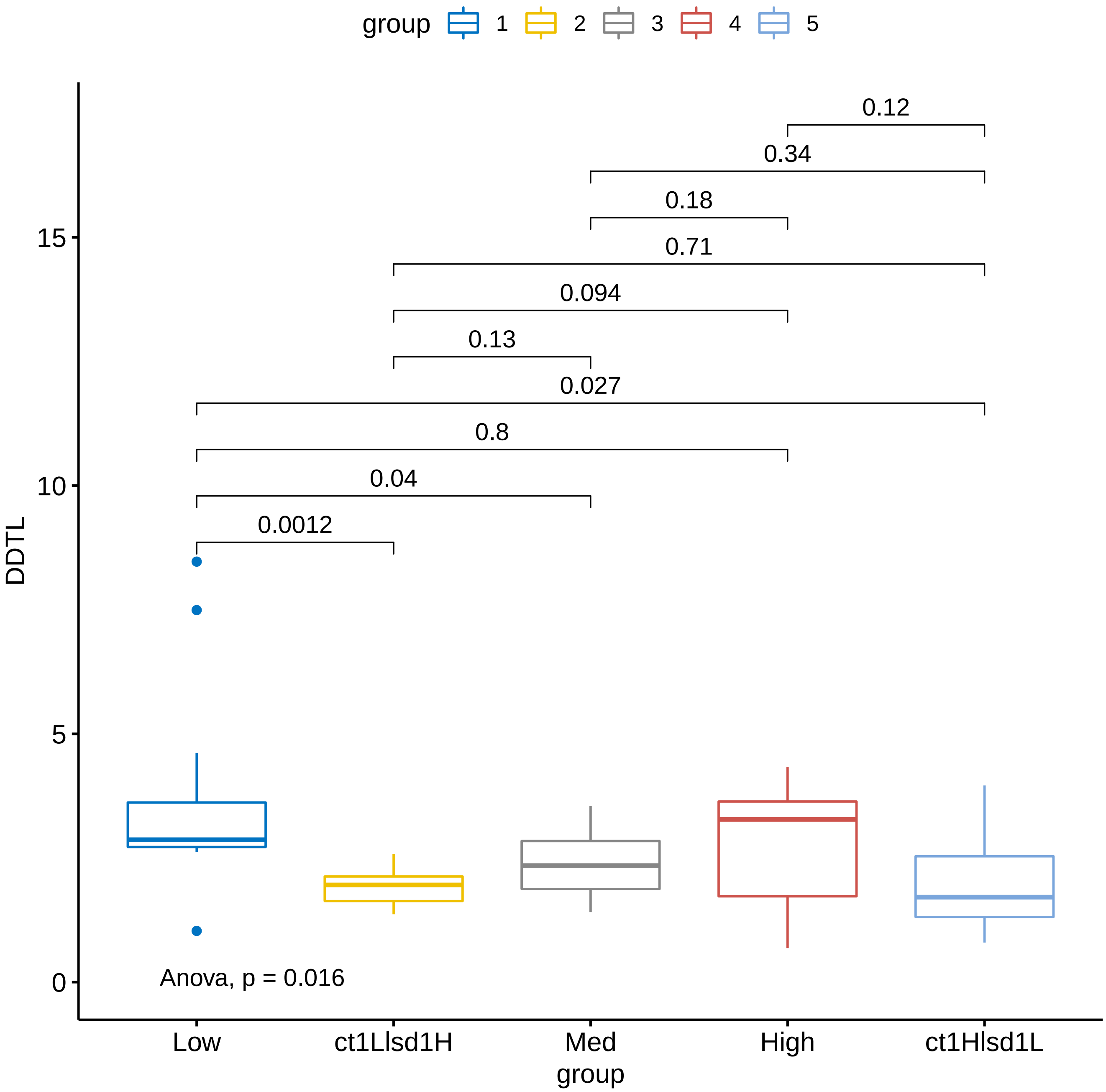

group 1 2 3 4 5
0.12
0.34
0.18
0.71
0.094
0.13
0.027
0.8
0.04
0.0012
15
10
5
0
DDTL
Anova, p = 0.016
Low
ct1Llsd1H
Med
High
ct1Hlsd1L
group

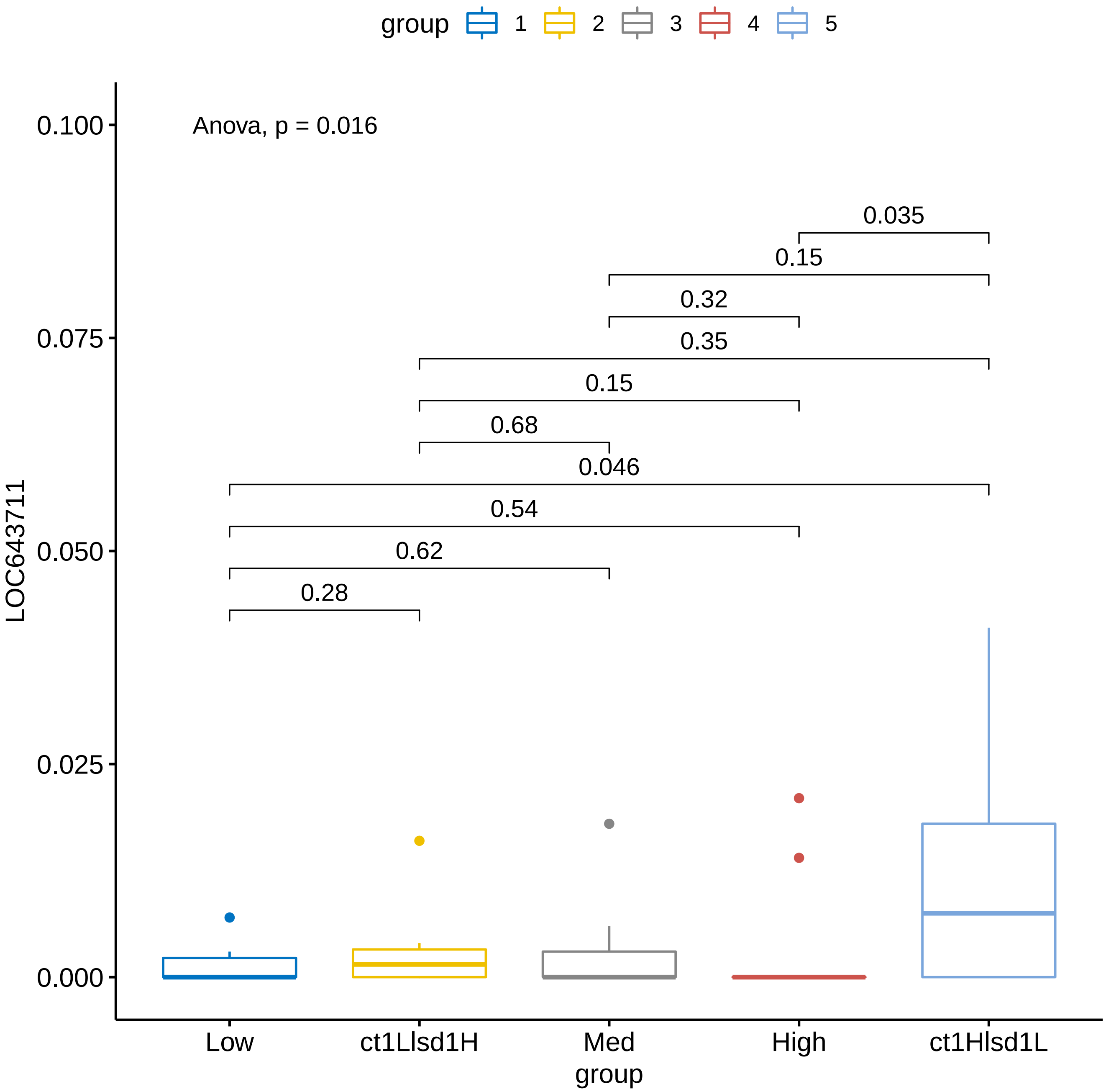

group 1 2 3 4 5
Anova, p = 0.016
0.035
0.15
0.32
0.35
0.15
0.68
0.046
0.54
0.62
0.28
0.100
0.075
0.050
0.025
0.000
LOC643711
Low
ct1Llsd1H
Med
High
ct1Hlsd1L
group

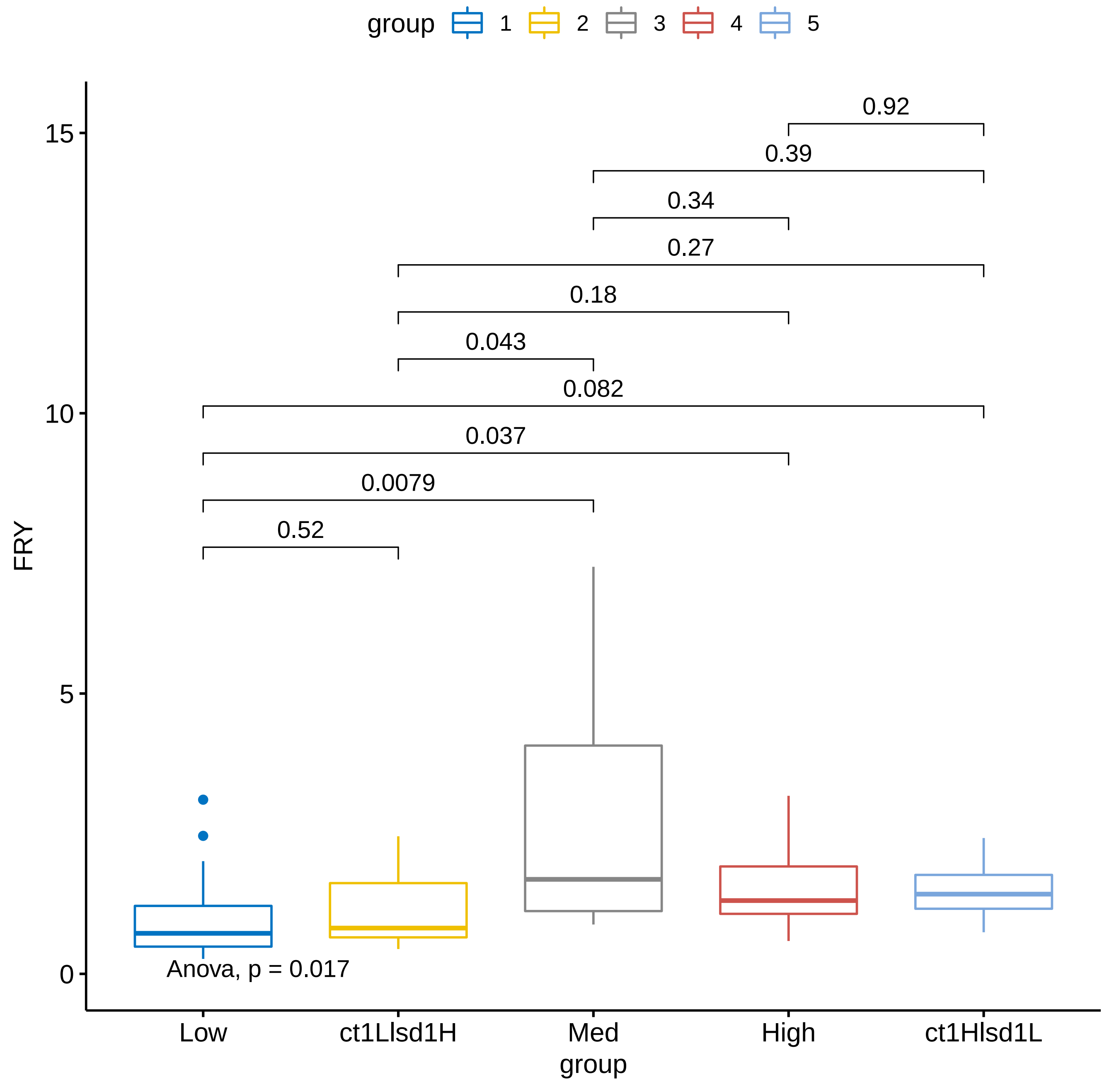

group
1
2
3
4
5
FRY
15
10
5
0
0.92
0.39
0.34
0.27
0.18
0.043
0.082
0.037
0.0079
0.52
Anova, p = 0.017
Low
ct1Llsd1H
Med
High
ct1Hlsd1L
group

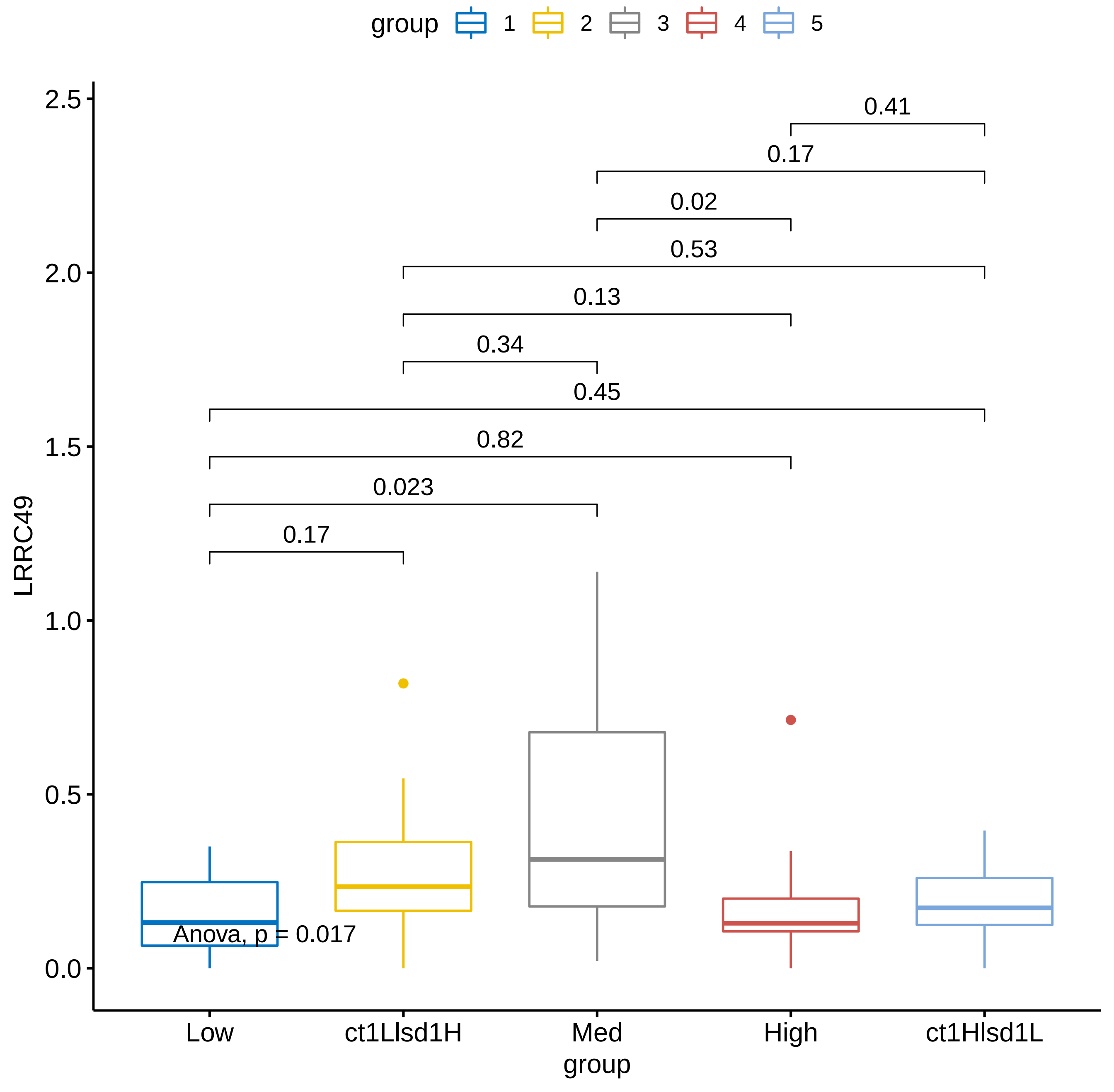

group 1 2 3 4 5
LRRC49
2.5
2.0
1.5
1.0
0.5
0.0
0.41
0.17
0.02
0.53
0.13
0.34
0.45
0.82
0.023
0.17
Anova, p = 0.017
Low
ct1Llsd1H
Med
High
ct1Hlsd1L
group

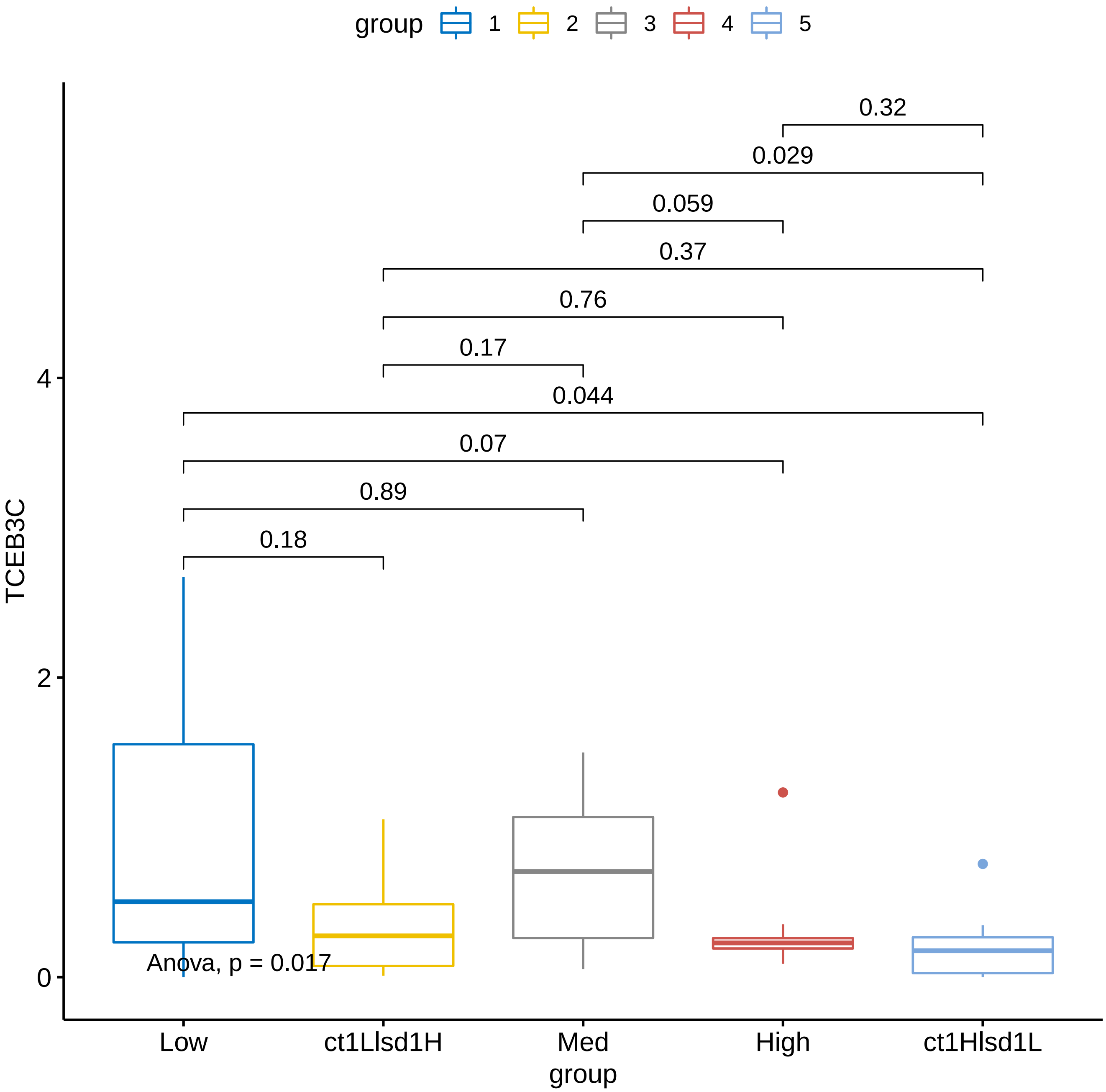
group
1
2
3
4
5
TCEB3C
0
2
4
0.32
0.029
0.059
0.37
0.76
0.17
0.044
0.07
0.89
0.18
Anova, p = 0.017
Low
ct1Llsd1H
Med
High
ct1Hlsd1L
group

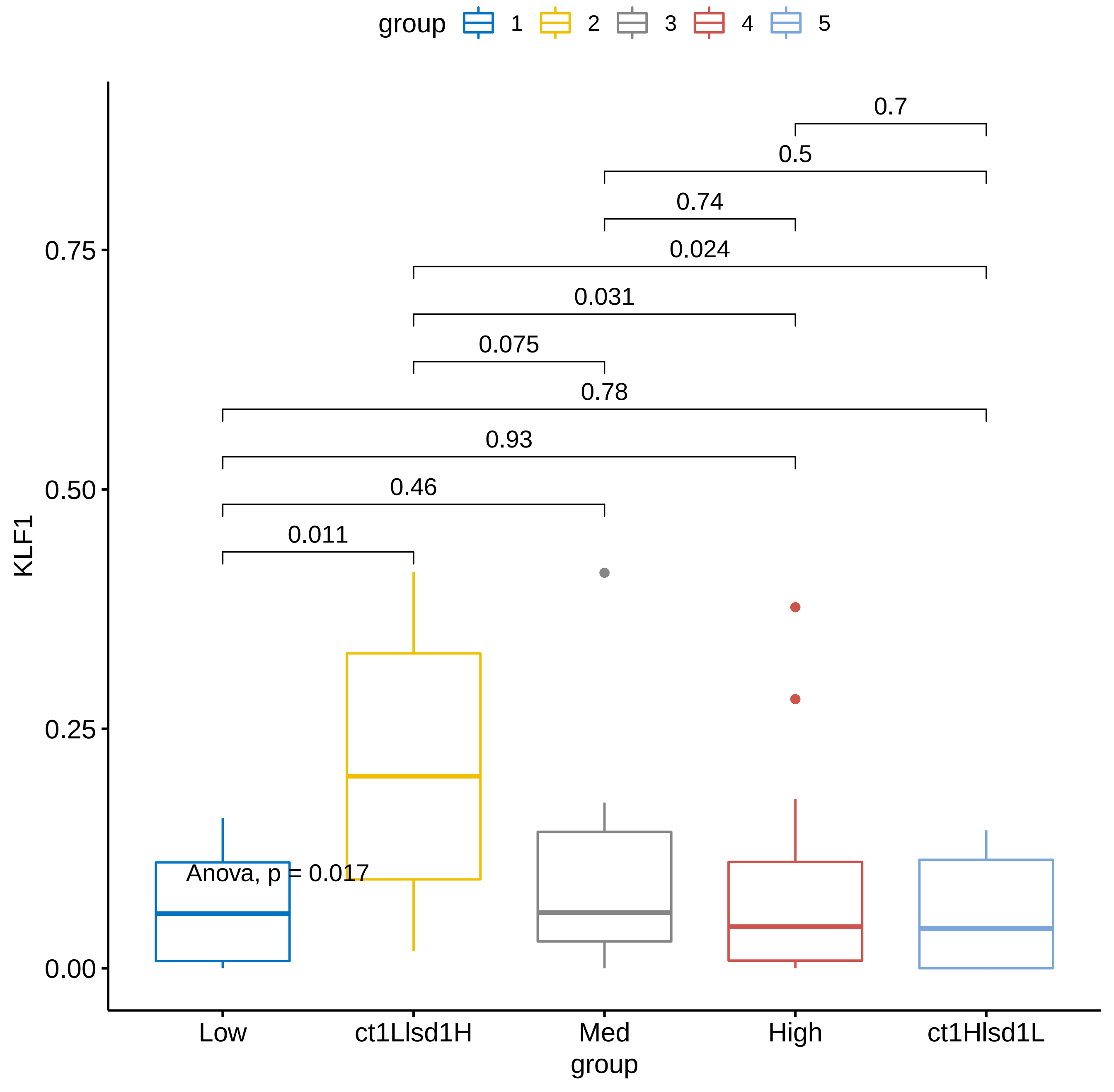

group 1 2 3 4 5
0.7
0.5
0.74
0.024
0.031
0.075
0.78
0.93
0.46
0.011
Anova, p = 0.017
KLF1
0.75
0.50
0.25
0.00
Low
ct1Llsd1H
Med
High
ct1Hlsd1L
group

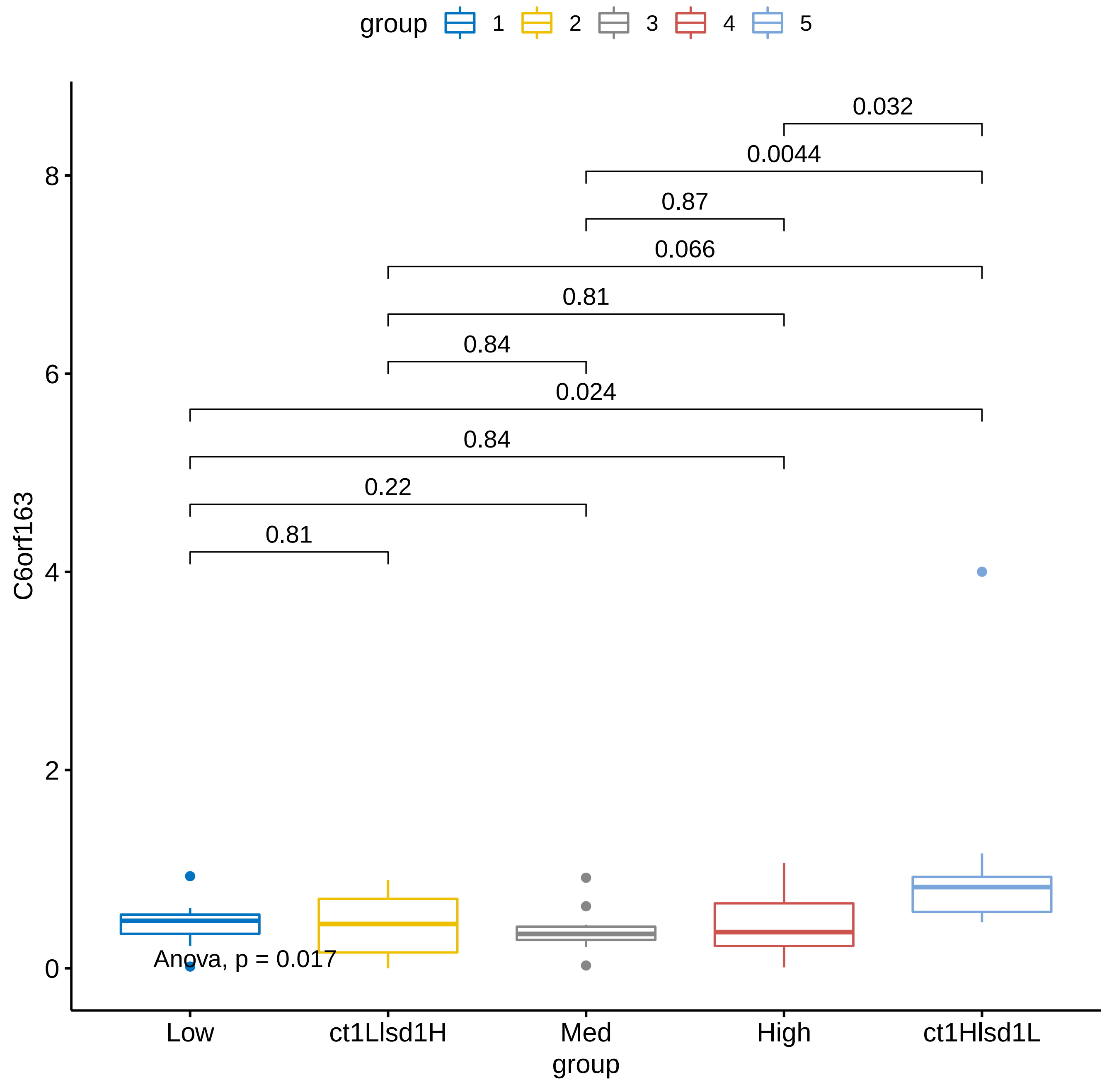
group
1
2
3
4
5
0.032
0.0044
0.87
0.066
0.81
0.84
0.024
0.84
0.22
0.81
Anova, p = 0.017
C6orf163
0
2
4
6
8
Low
ct1Llsd1H
Med
High
ct1Hlsd1L
group

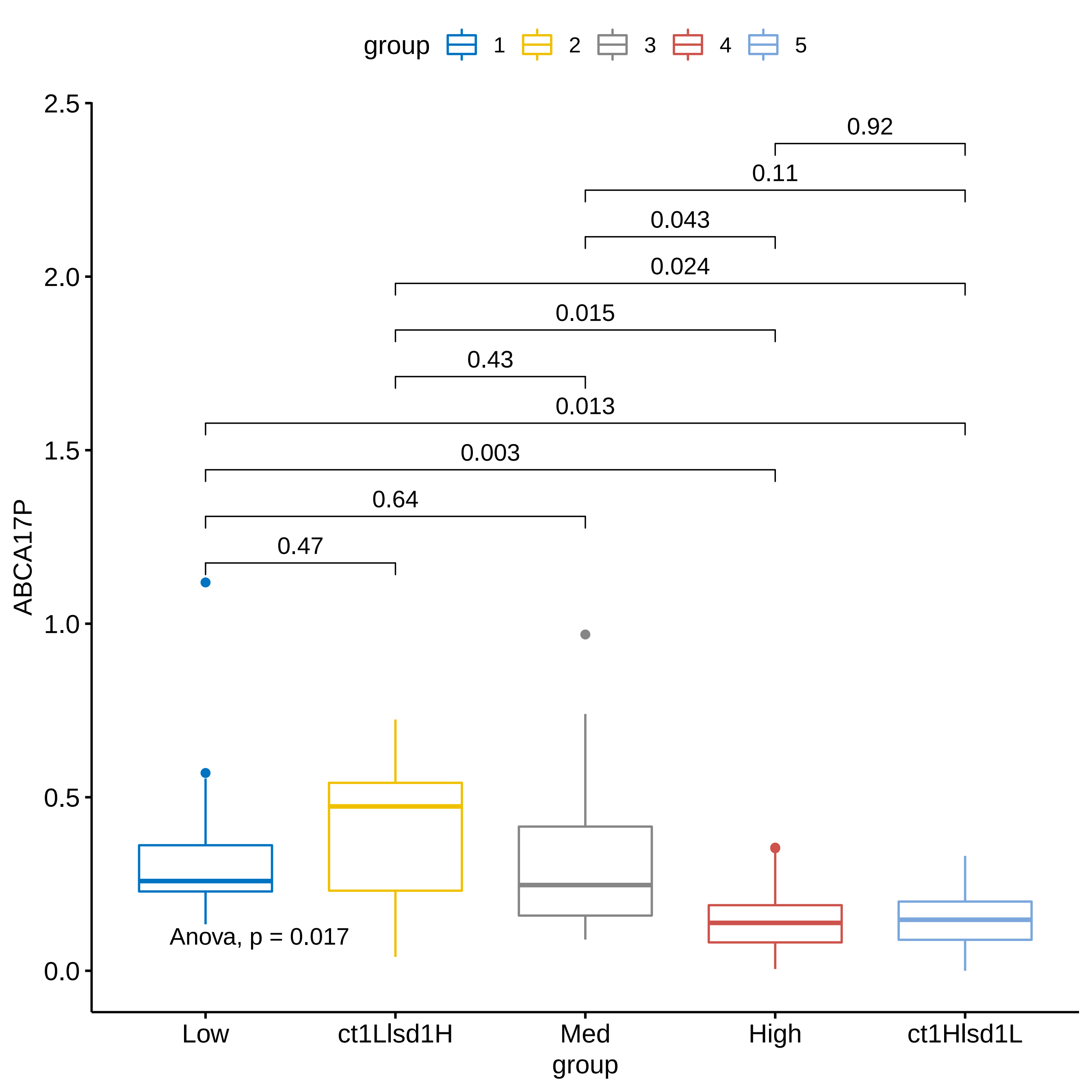

group 1 2 3 4 5
2.5
2.0
1.5
1.0
0.5
0.0
ABCA17P
0.92
0.11
0.043
0.024
0.015
0.43
0.013
0.003
0.64
0.47
Anova, p = 0.017
Low
ct1Llsd1H
Med
High
ct1Hlsd1L
group

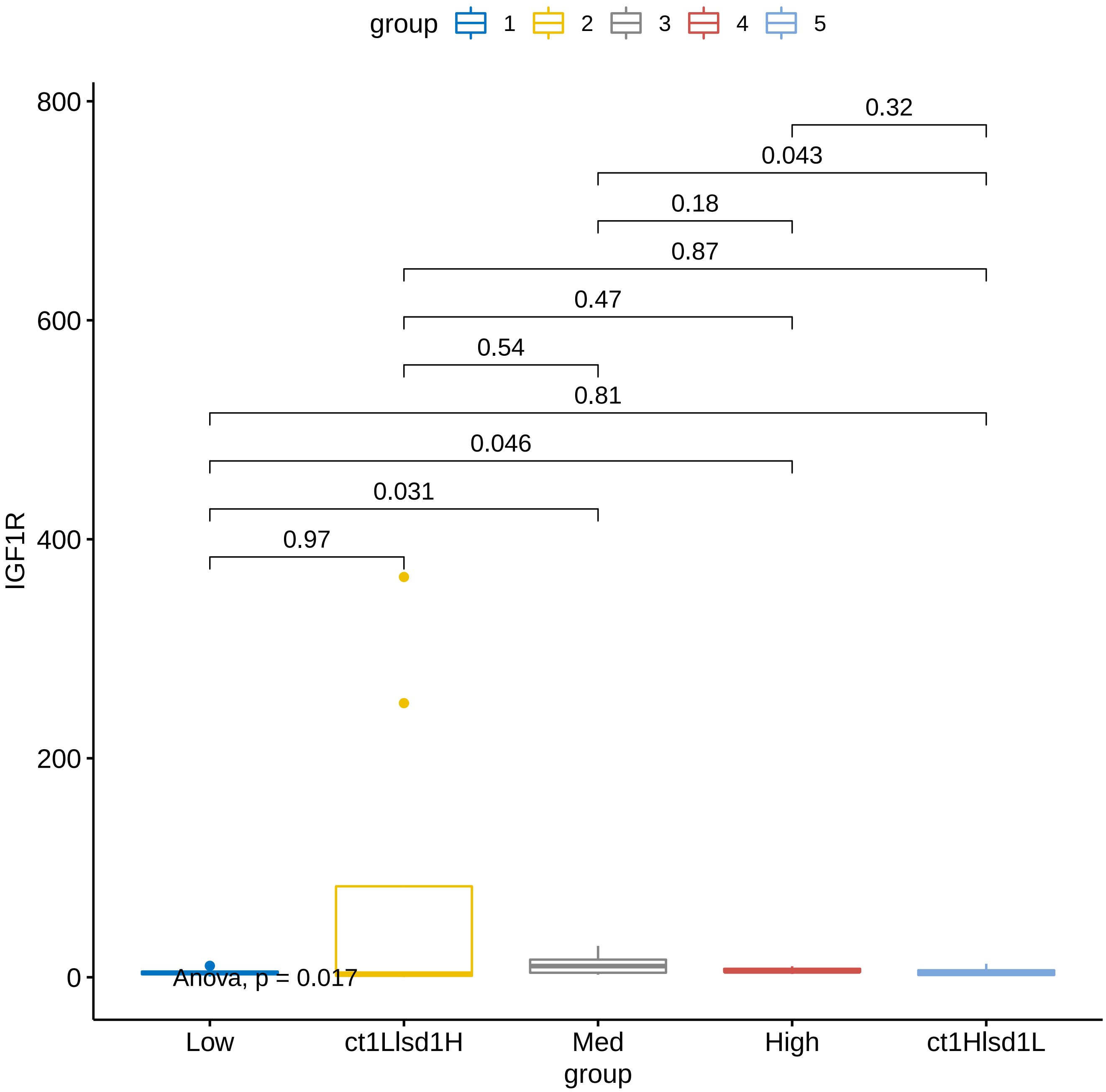

group
1
2
3
4
5
800
600
400
200
0
IGF1R
0.32
0.043
0.18
0.87
0.47
0.54
0.81
0.046
0.031
0.97
Anova, p = 0.017
Low
ct1Llsd1H
Med
High
ct1Hlsd1L
group

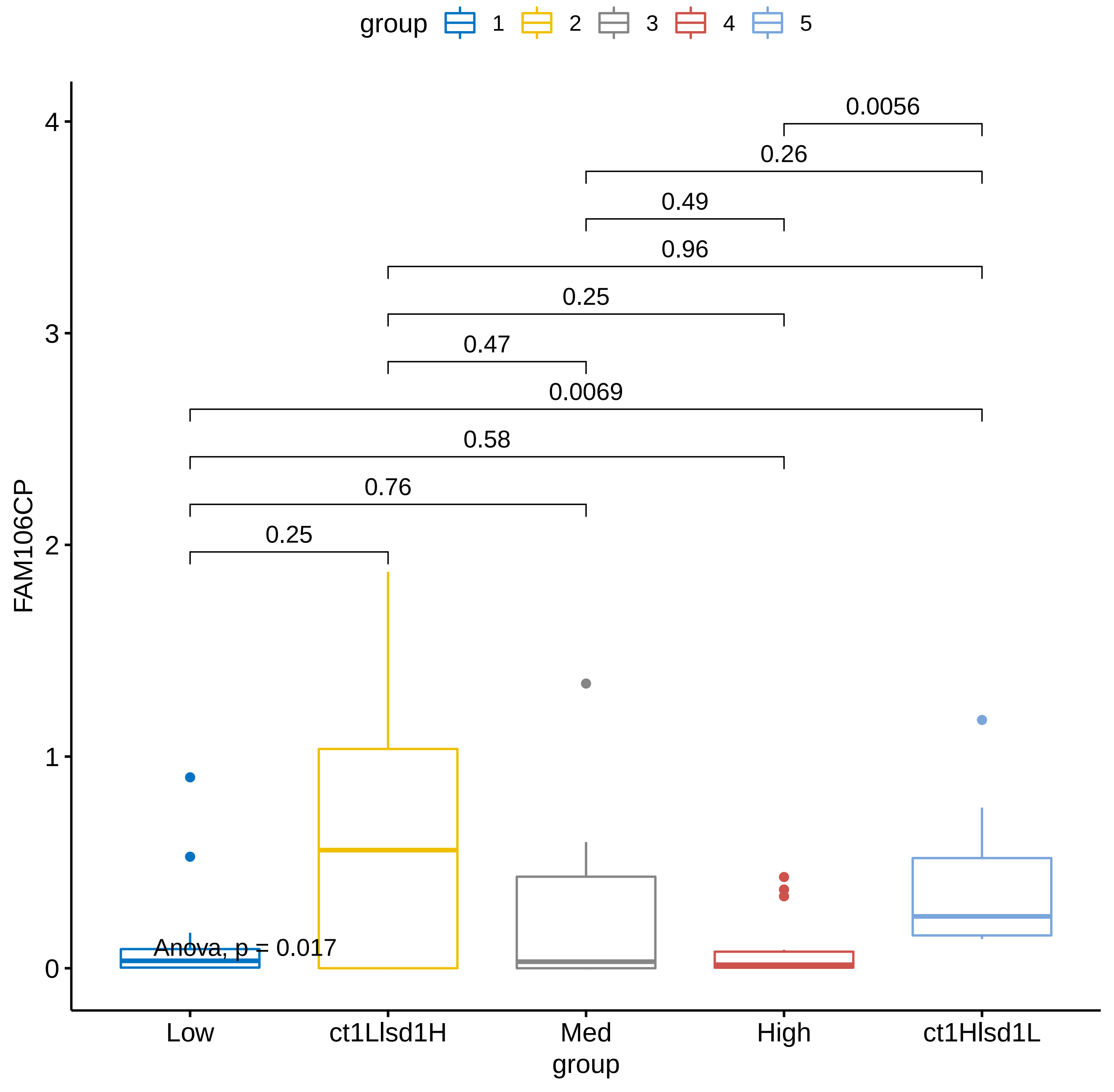

group 1 2 3 4 5
0.0056
0.26
0.49
0.96
0.25
0.47
0.0069
0.58
0.76
0.25
4
3
2
1
0
FAM106CP
Anova, p = 0.017
Low
ct1Llsd1H
Med
High
ct1Hlsd1L
group

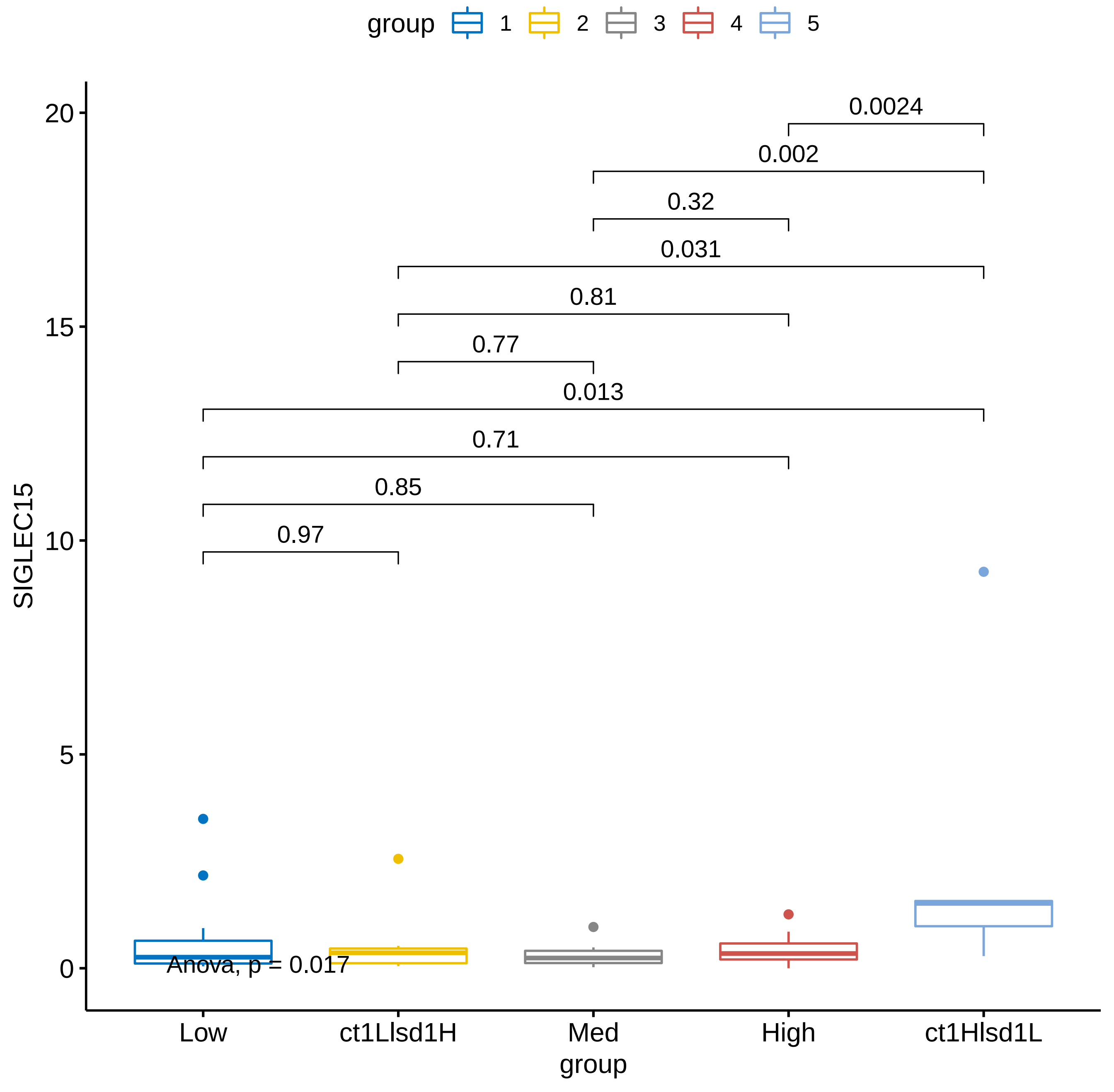

group
1
2
3
4
5
SIGLEC15
20
15
10
5
0
0.0024
0.002
0.32
0.031
0.81
0.77
0.013
0.71
0.85
0.97
Anova, p = 0.017
Low
ct1Llsd1H
Med
High
ct1Hlsd1L
group

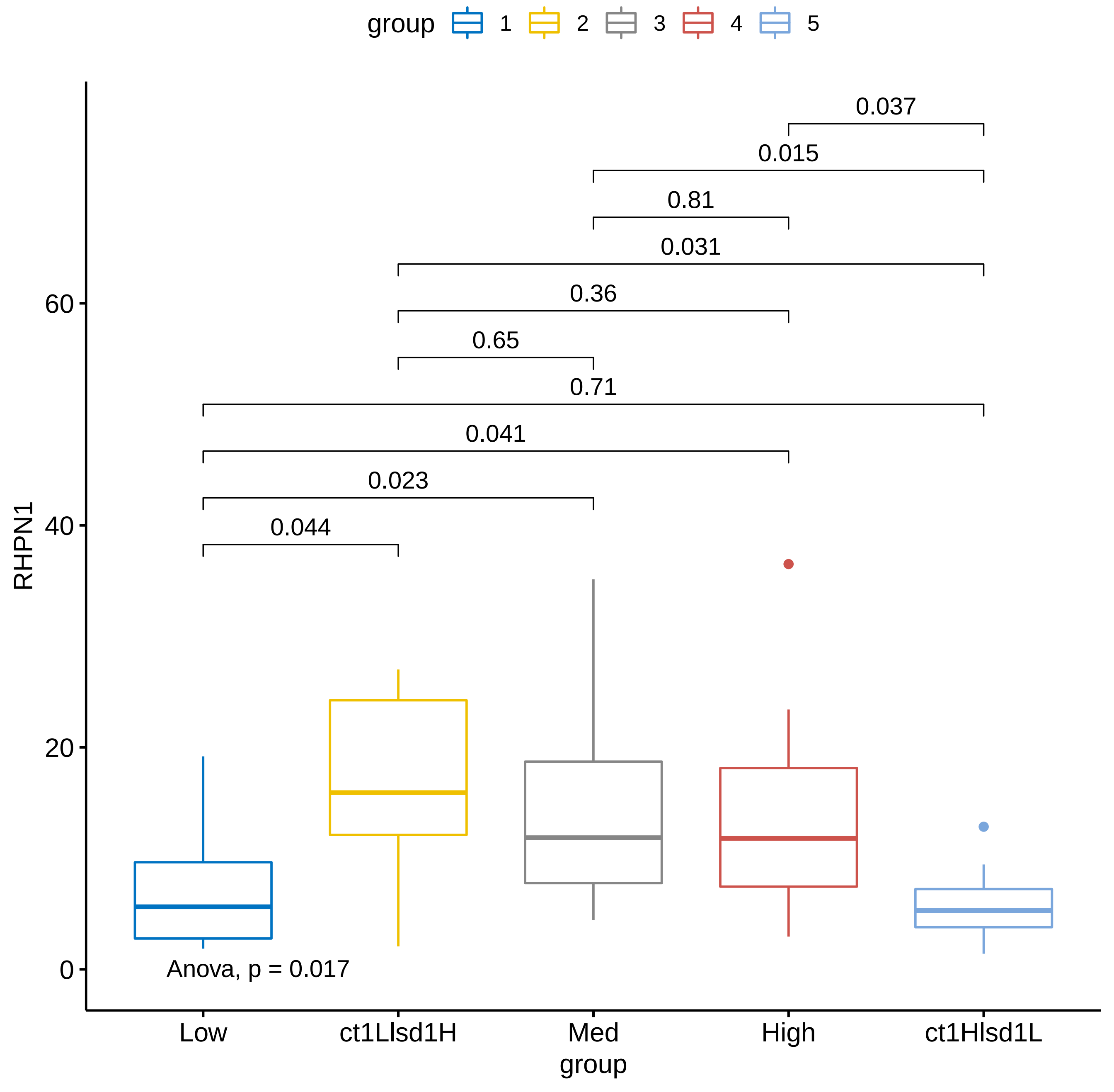

group 1 2 3 4 5
0.037
0.015
0.81
0.031
0.36
0.65
0.71
0.041
0.023
0.044
RHPN1
60
40
20
0
Anova, p = 0.017
Low
ct1Llsd1H
Med
High
ct1Hlsd1L
group

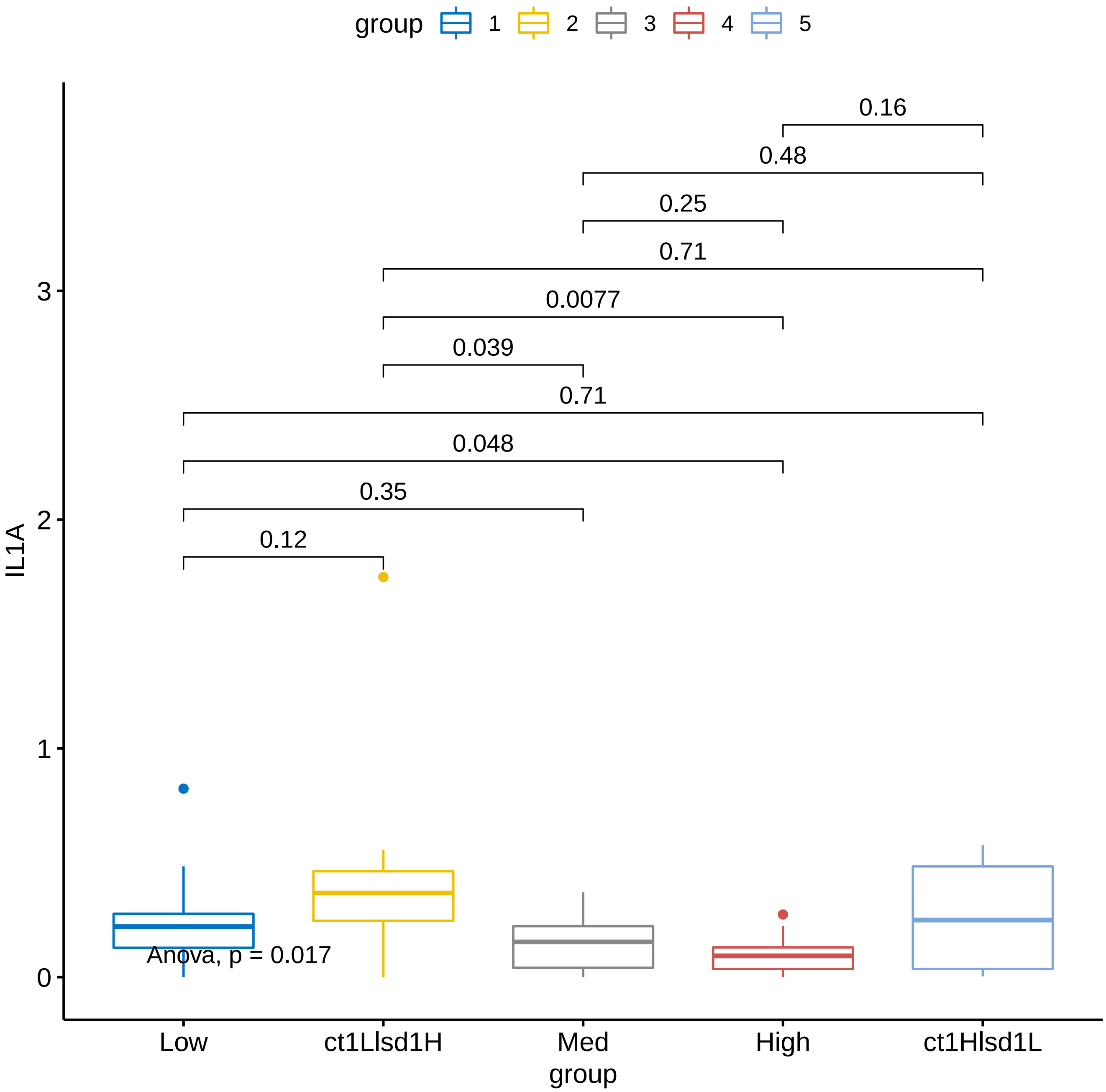
group
1
2
3
4
5
0.16
0.48
0.25
0.71
0.0077
0.039
0.71
0.048
0.35
0.12
Anova, p = 0.017
IL1A
3
2
1
0
Low
ct1Llsd1H
Med
High
ct1Hlsd1L
group

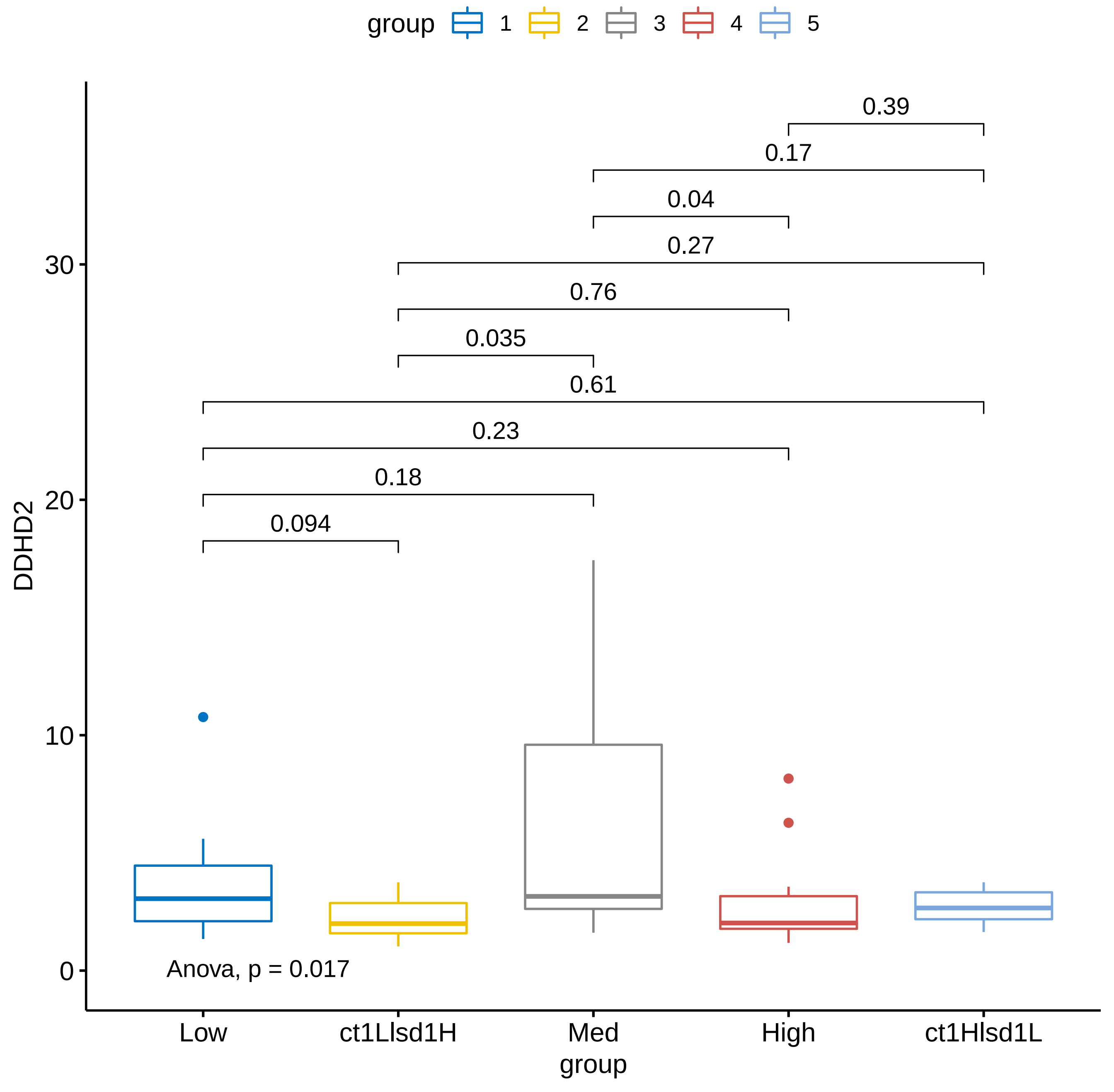

group 1 2 3 4 5
0.39
0.17
0.04
0.27
0.76
0.035
0.61
0.23
0.18
0.094
30
20
10
0
DDHD2
Anova, p = 0.017
Low
ct1Llsd1H
Med
High
ct1Hlsd1L
group

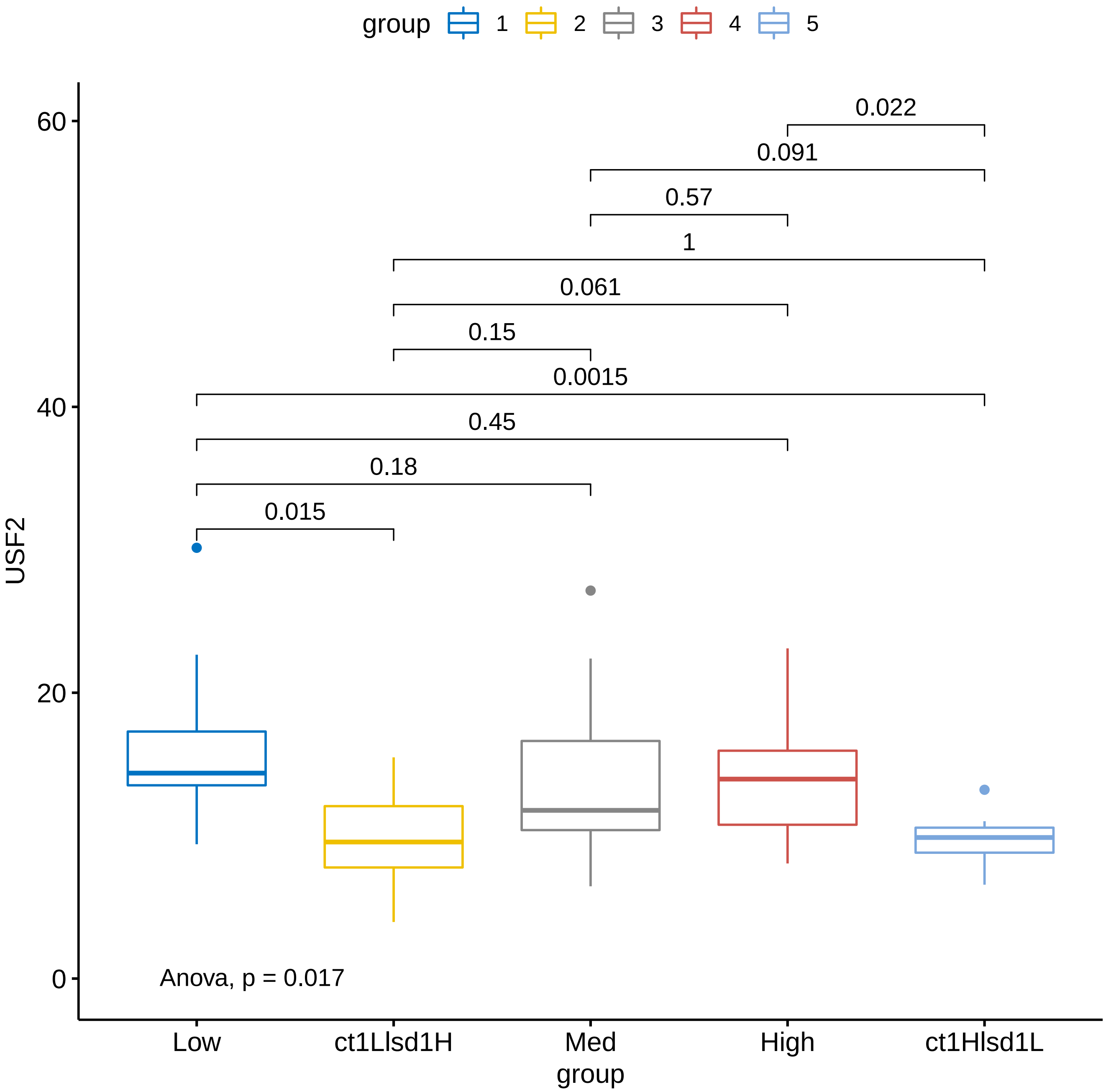

group 1 2 3 4 5
0.022
0.091
0.57
1
0.061
0.15
0.0015
0.45
0.18
0.015
60
40
20
0
USF2
Anova, p = 0.017
Low
ct1Llsd1H
Med
High
ct1Hlsd1L
group

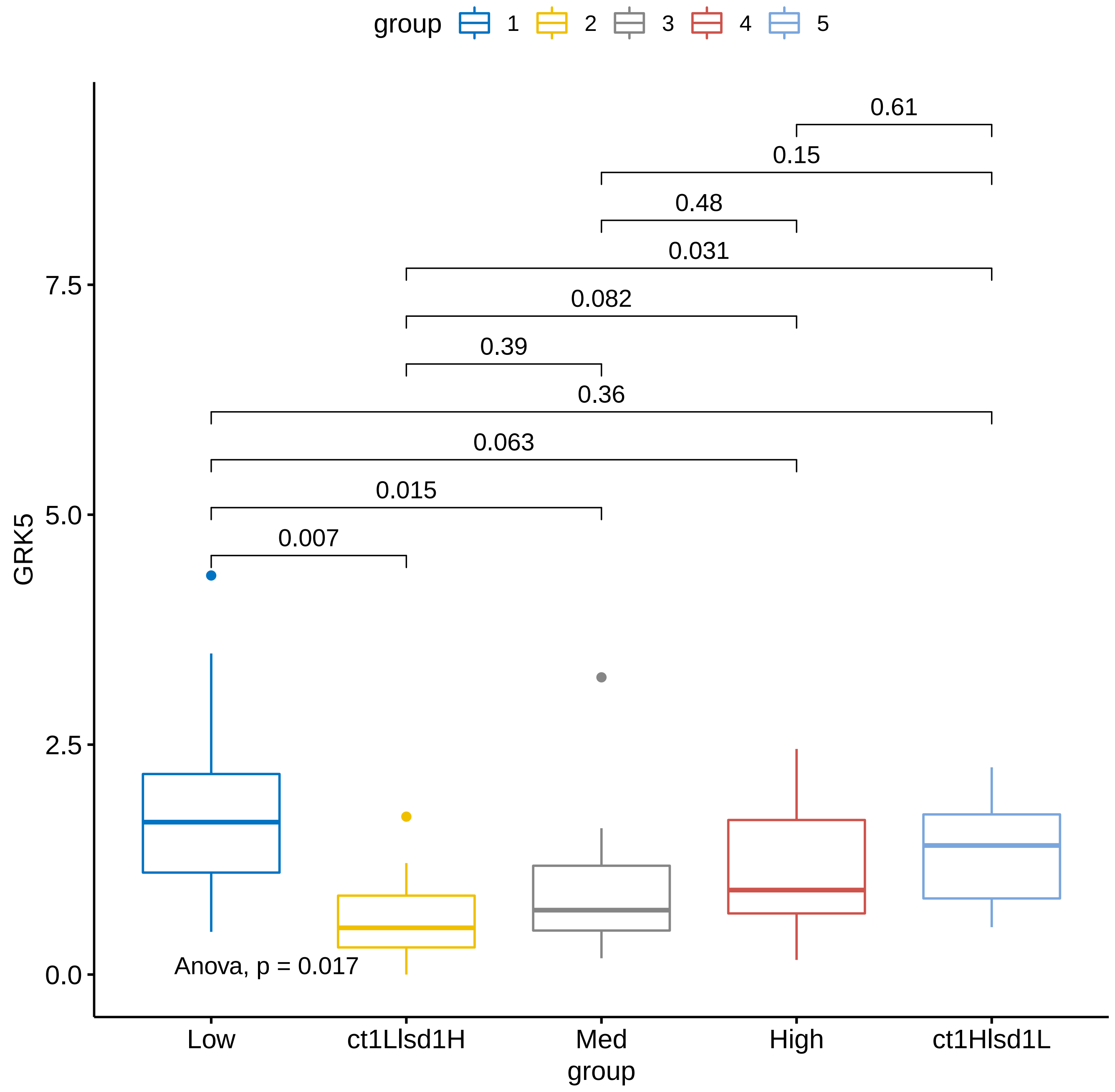
group
1
2
3
4
5
0.61
0.15
0.48
0.031
0.082
0.39
0.36
0.063
0.015
0.007
GRK5
7.5
5.0
2.5
0.0
Anova, p = 0.017
Low
ct1Llsd1H
Med
High
ct1Hlsd1L
group

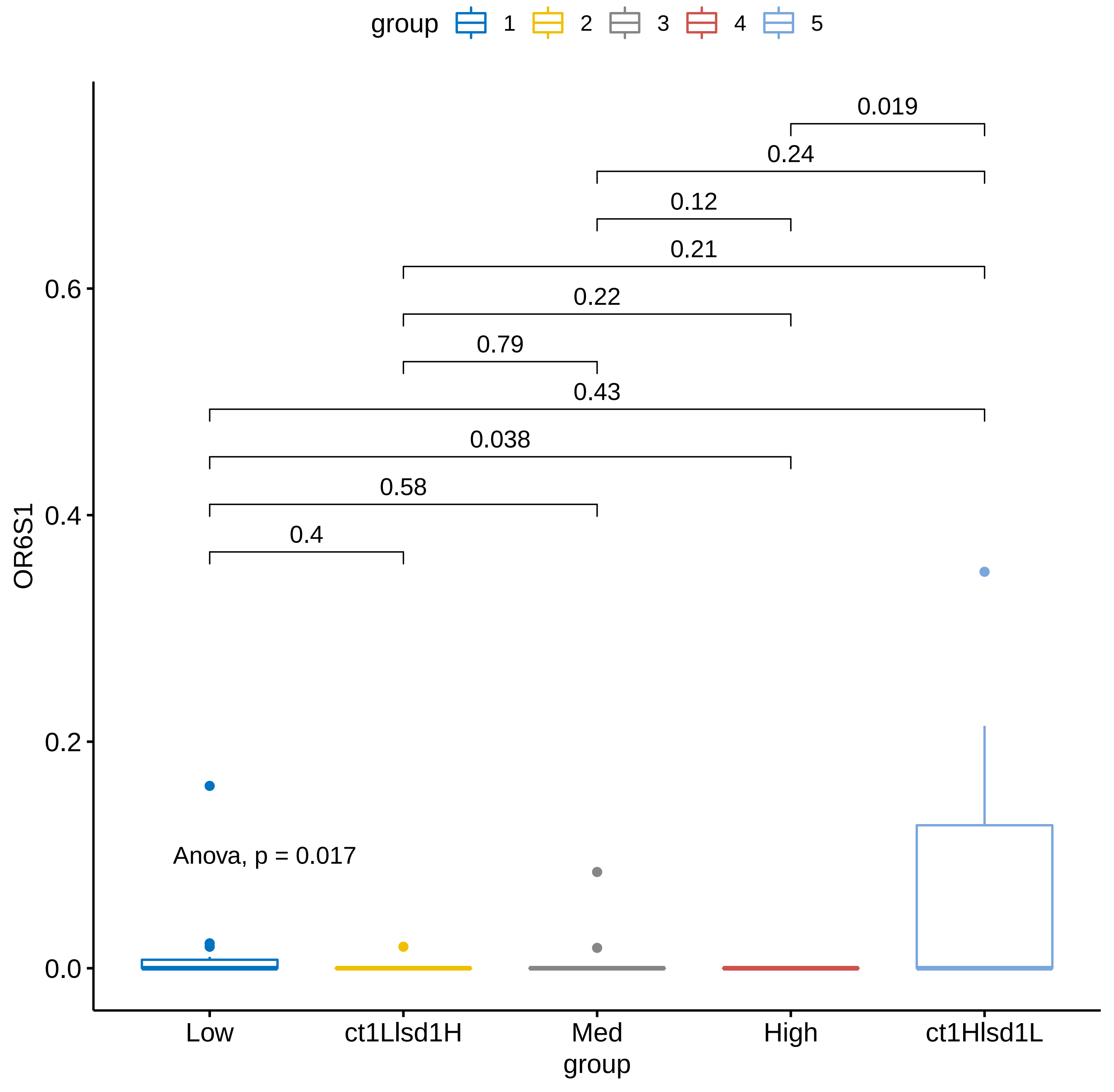

group
1
2
3
4
5
0.019
0.24
0.12
0.21
0.22
0.79
0.43
0.038
0.58
0.4
0.6
0.4
0.2
0.0
OR6S1
Anova, p = 0.017
Low
ct1Llsd1H
Med
High
ct1Hlsd1L
group

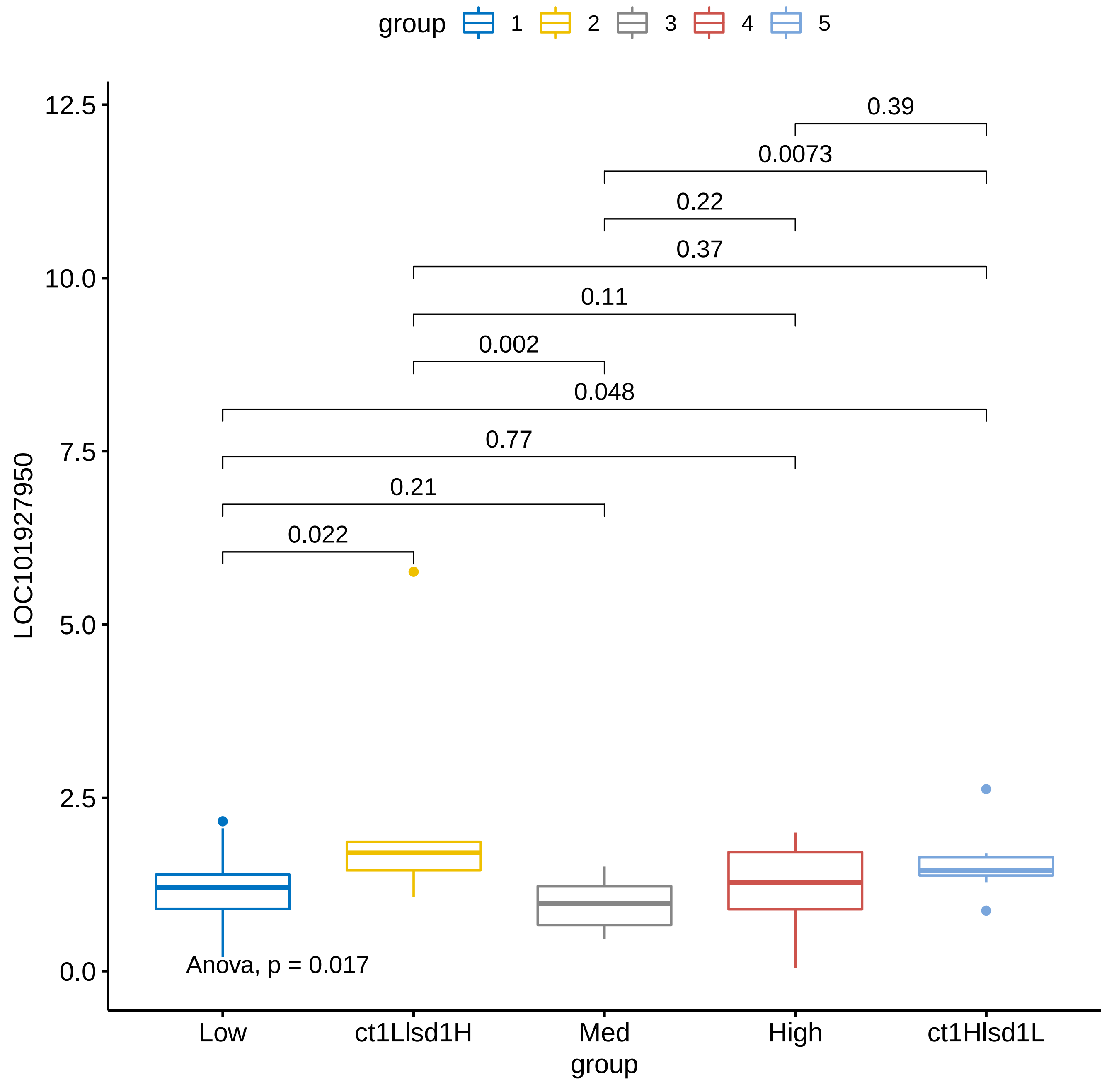

group 1 2 3 4 5
0.39
0.0073
0.22
0.37
0.11
0.002
0.048
0.77
0.21
0.022
Anova, p = 0.017
12.5
10.0
7.5
5.0
2.5
0.0
LOC101927950
Low
ct1Llsd1H
Med
High
ct1Hlsd1L
group

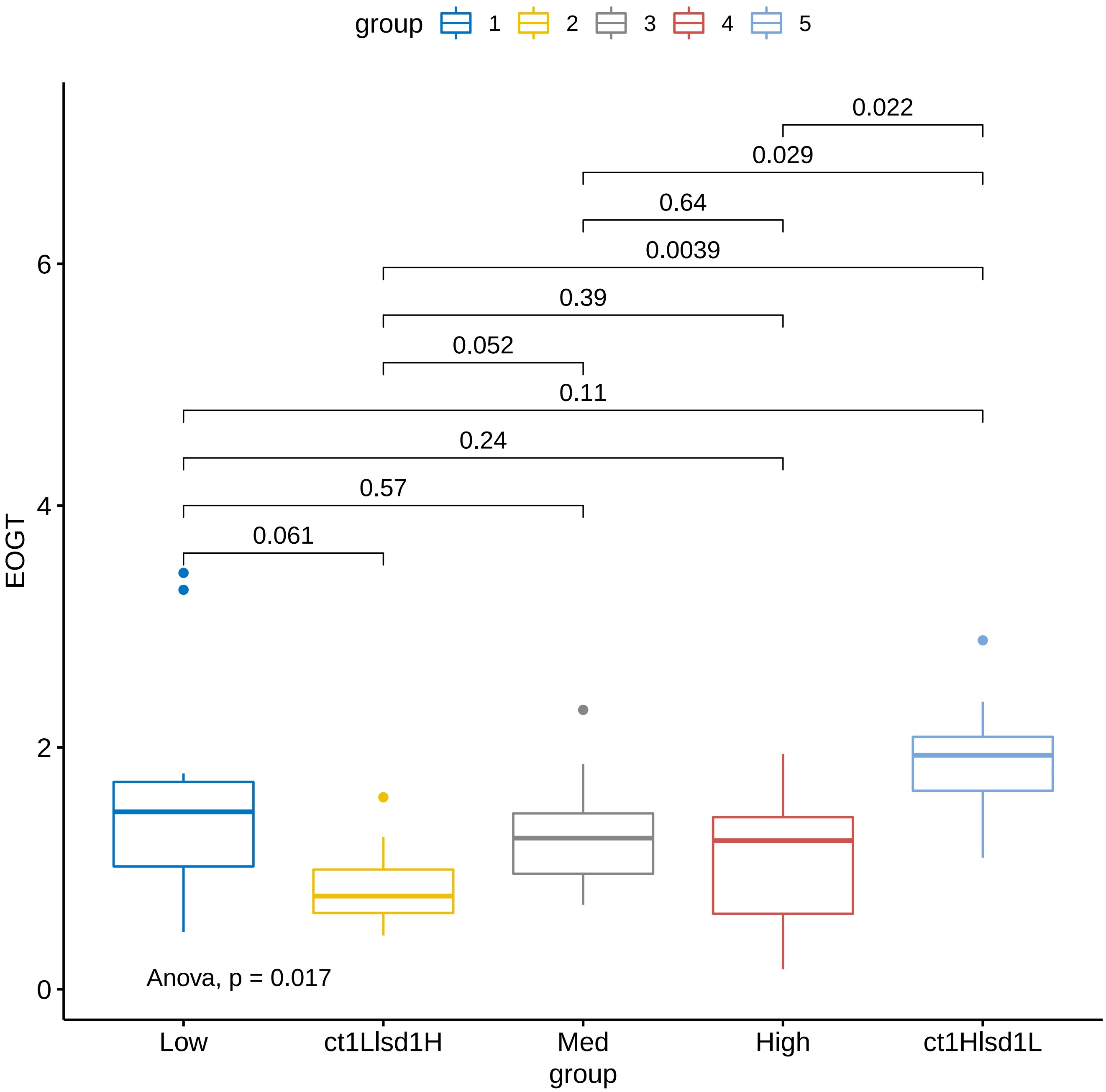

group 1 2 3 4 5
0.022
0.029
0.64
0.0039
0.39
0.052
0.11
0.24
0.57
0.061
EOGT
6
4
2
0
Anova, p = 0.017
Low
ct1Llsd1H
Med
High
ct1Hlsd1L
group

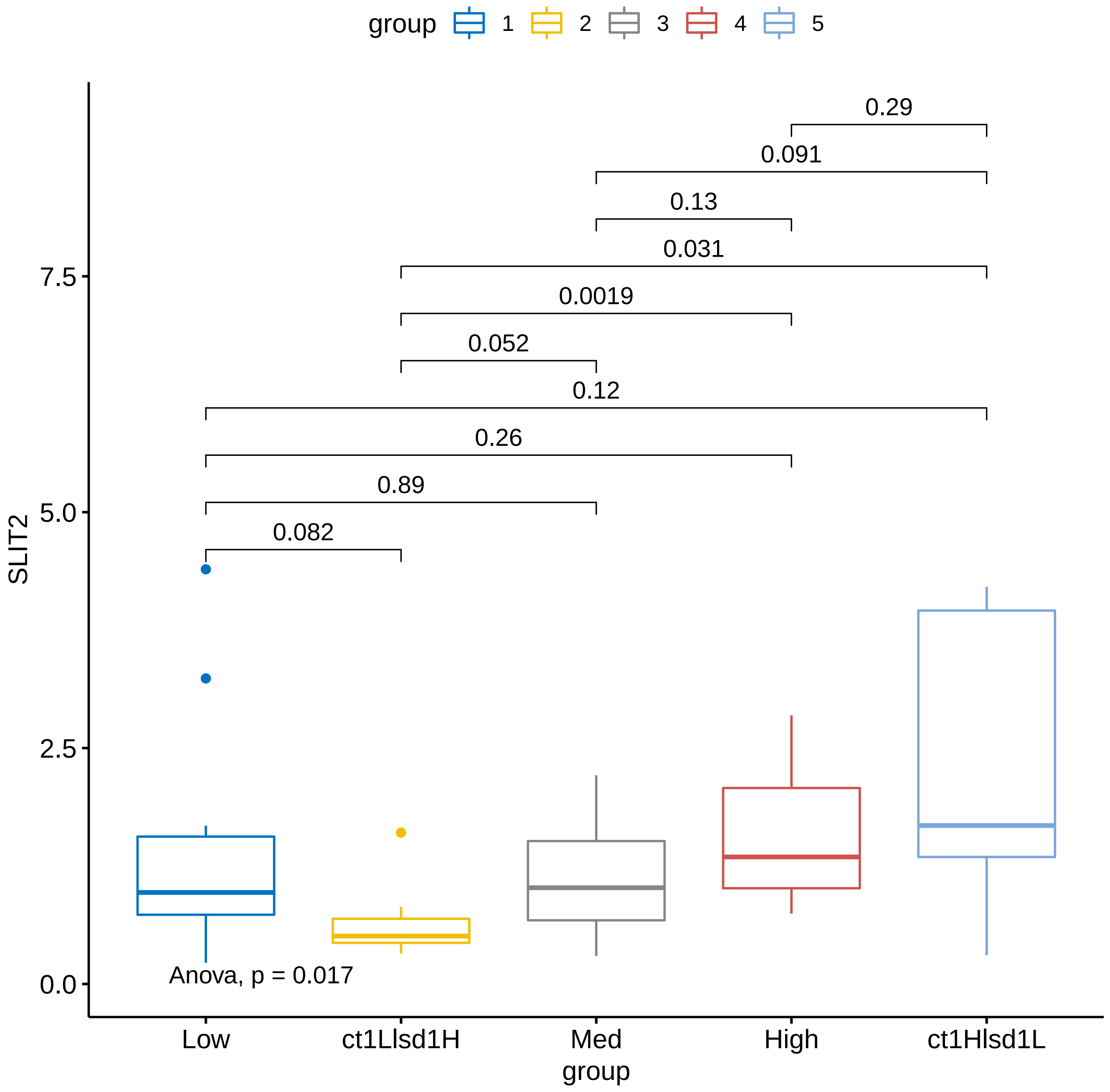
group 1 2 3 4 5
0.29
0.091
0.13
0.031
0.0019
0.052
0.12
0.26
0.89
0.082
7.5
5.0
2.5
0.0
SLIT2
Anova, p = 0.017
Low
ct1Llsd1H
Med
High
ct1Hlsd1L
group

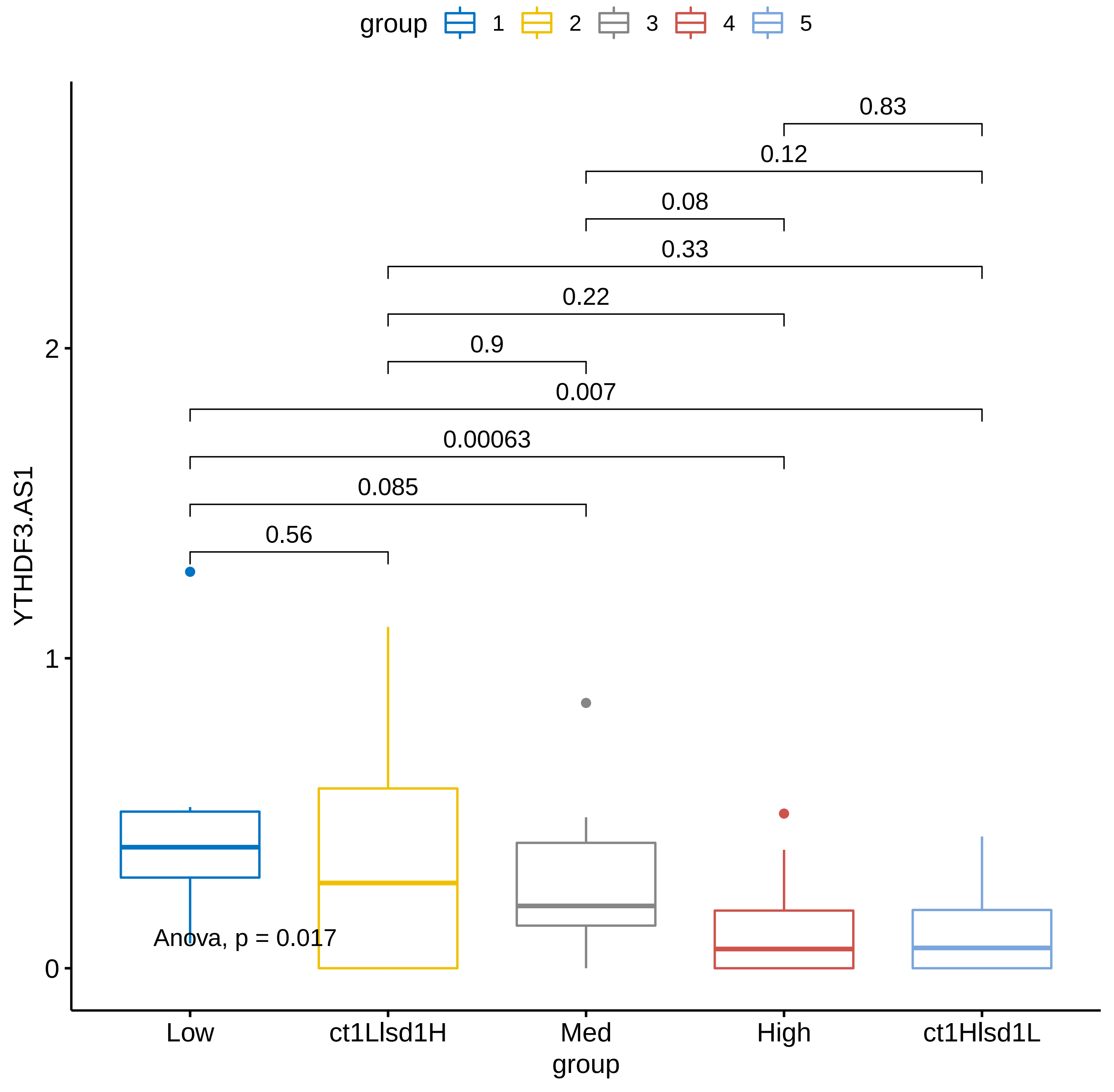

group 1 2 3 4 5
0.83
0.12
0.08
0.33
0.22
0.9
0.007
0.00063
0.085
0.56
YTHDF3.AS1
2
1
0
Anova, p = 0.017
Low
ct1Llsd1H
Med
High
ct1Hlsd1L
group

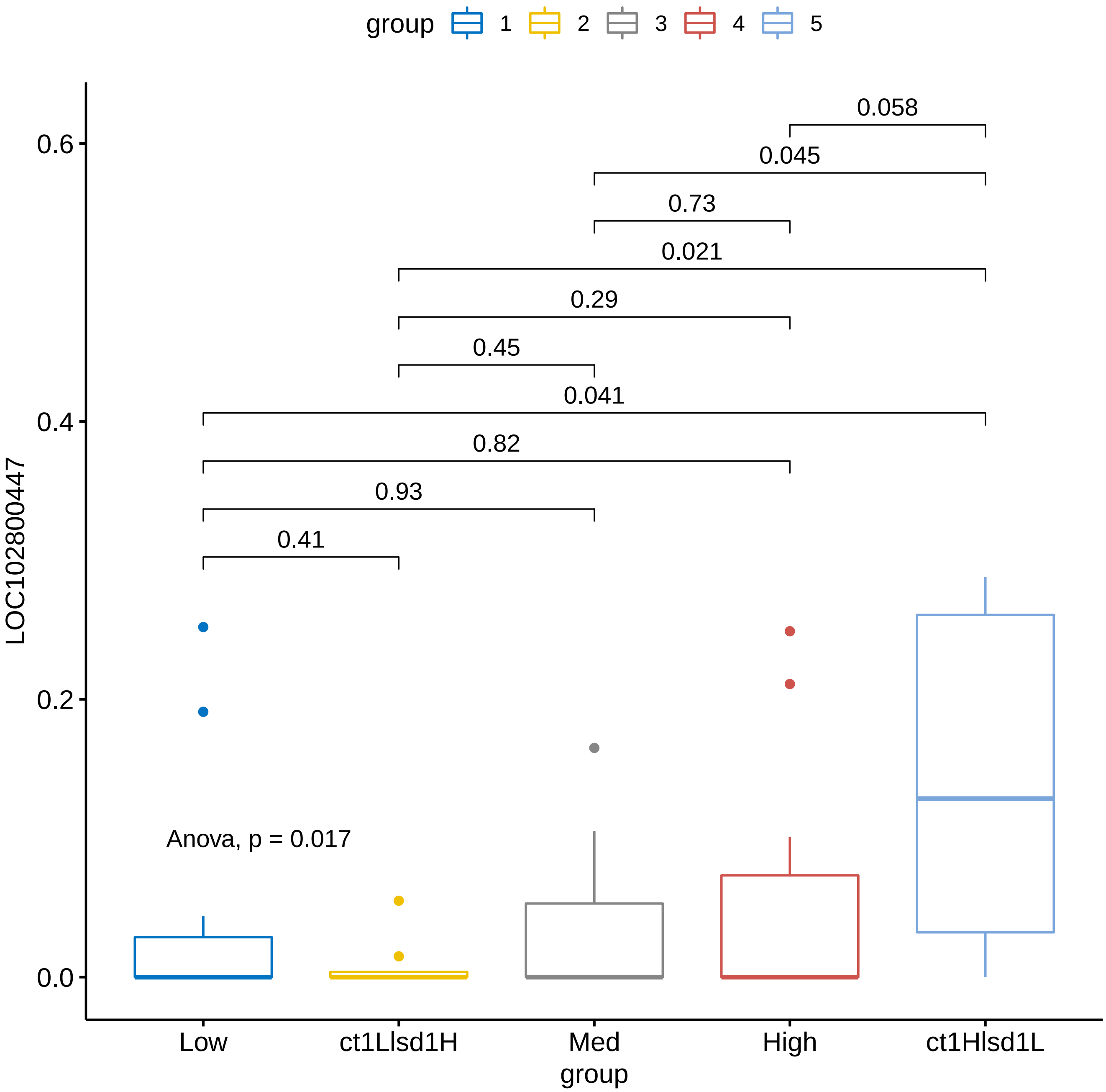

group
1
2
3
4
5
0.058
0.045
0.73
0.021
0.29
0.45
0.041
0.82
0.93
0.41
Anova, p = 0.017
0.6
0.4
0.2
0.0
LOC102800447
Low
ct1Llsd1H
Med
High
ct1Hlsd1L
group

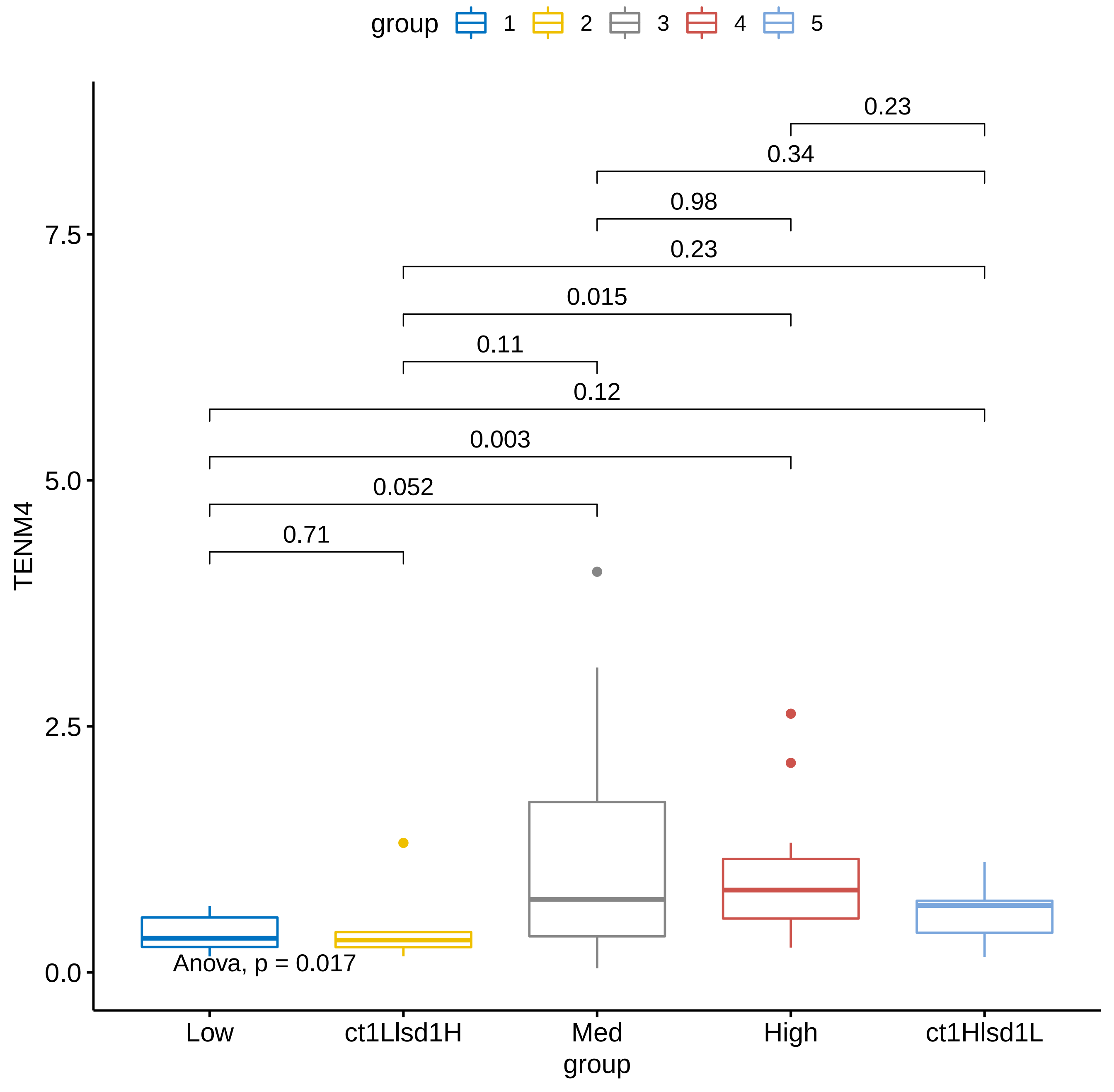

group
1
2
3
4
5
TENM4
7.5
5.0
2.5
0.0
0.23
0.34
0.98
0.23
0.015
0.11
0.12
0.003
0.052
0.71
Anova, p = 0.017
Low
ct1Llsd1H
Med
High
ct1Hlsd1L
group

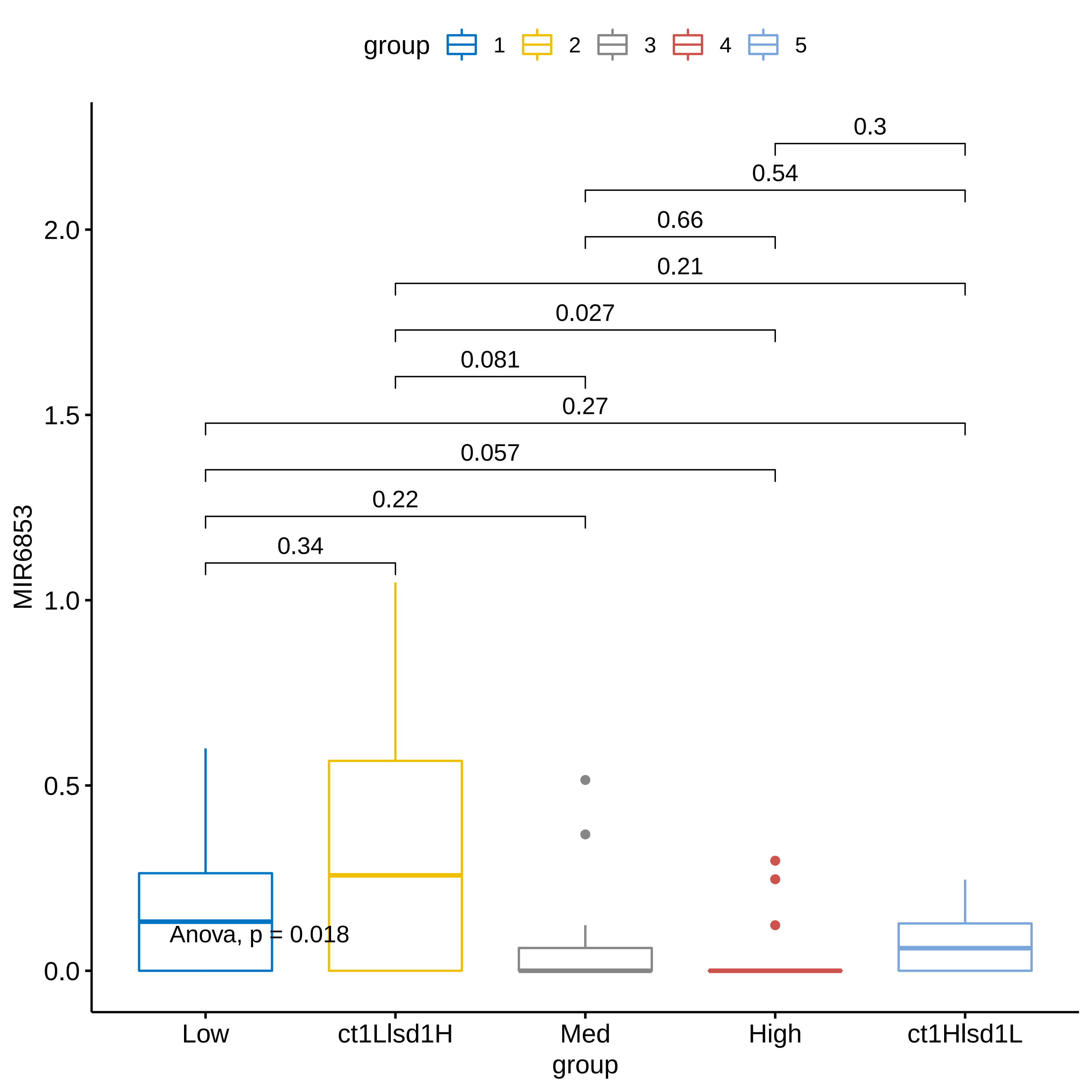
group
1
2
3
4
5
0.3
0.54
0.66
0.21
0.027
0.081
0.27
0.057
0.22
0.34
Anova, p = 0.018
MIR6853
2.0
1.5
1.0
0.5
0.0
Low
ct1Llsd1H
Med
High
ct1Hlsd1L
group

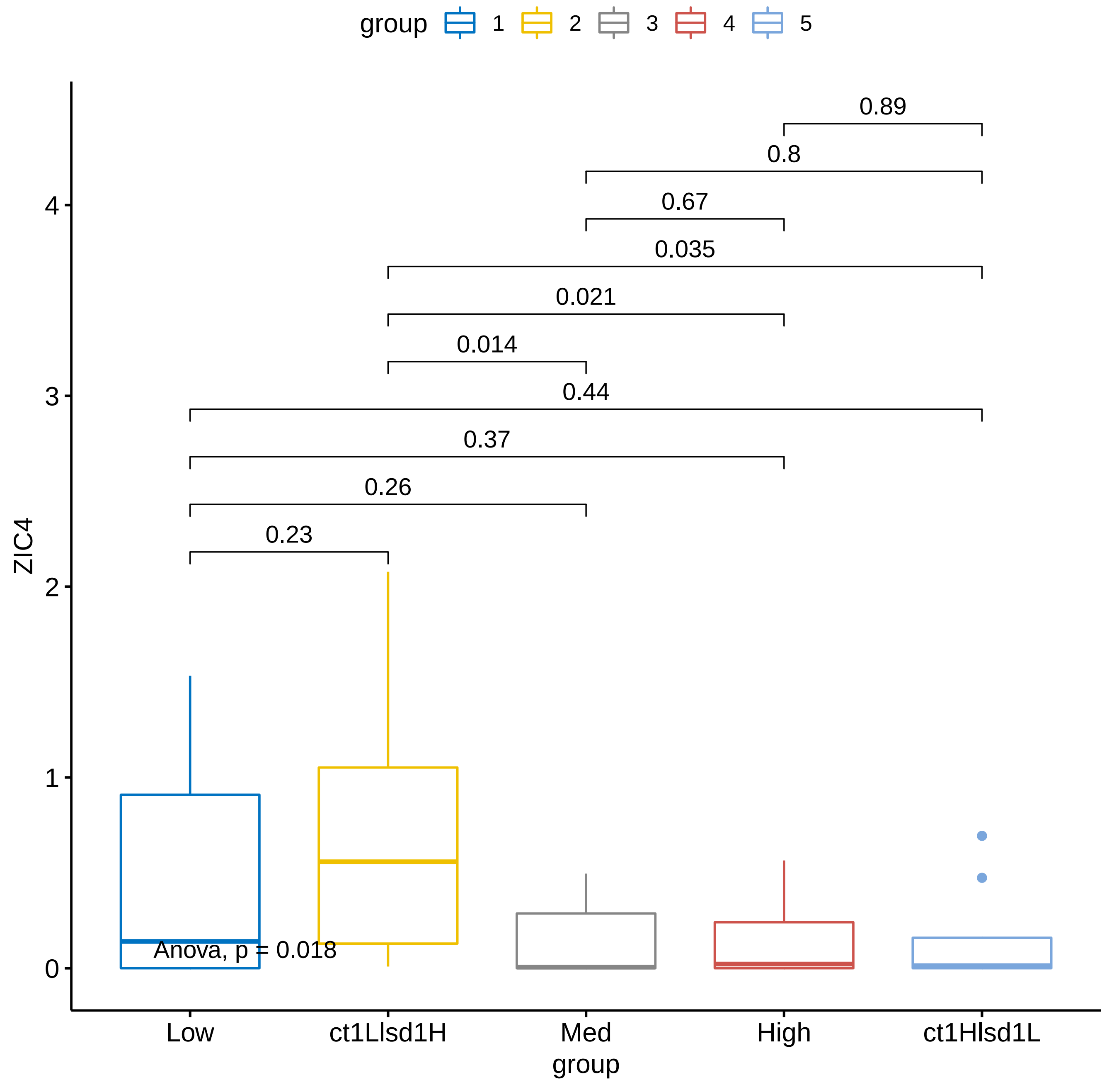

group
1
2
3
4
5
0.89
0.8
0.67
0.035
0.021
0.014
0.44
0.37
0.26
0.23
ZIC4
4
3
2
1
0
Anova, p = 0.018
Low
ct1Llsd1H
Med
High
ct1Hlsd1L
group

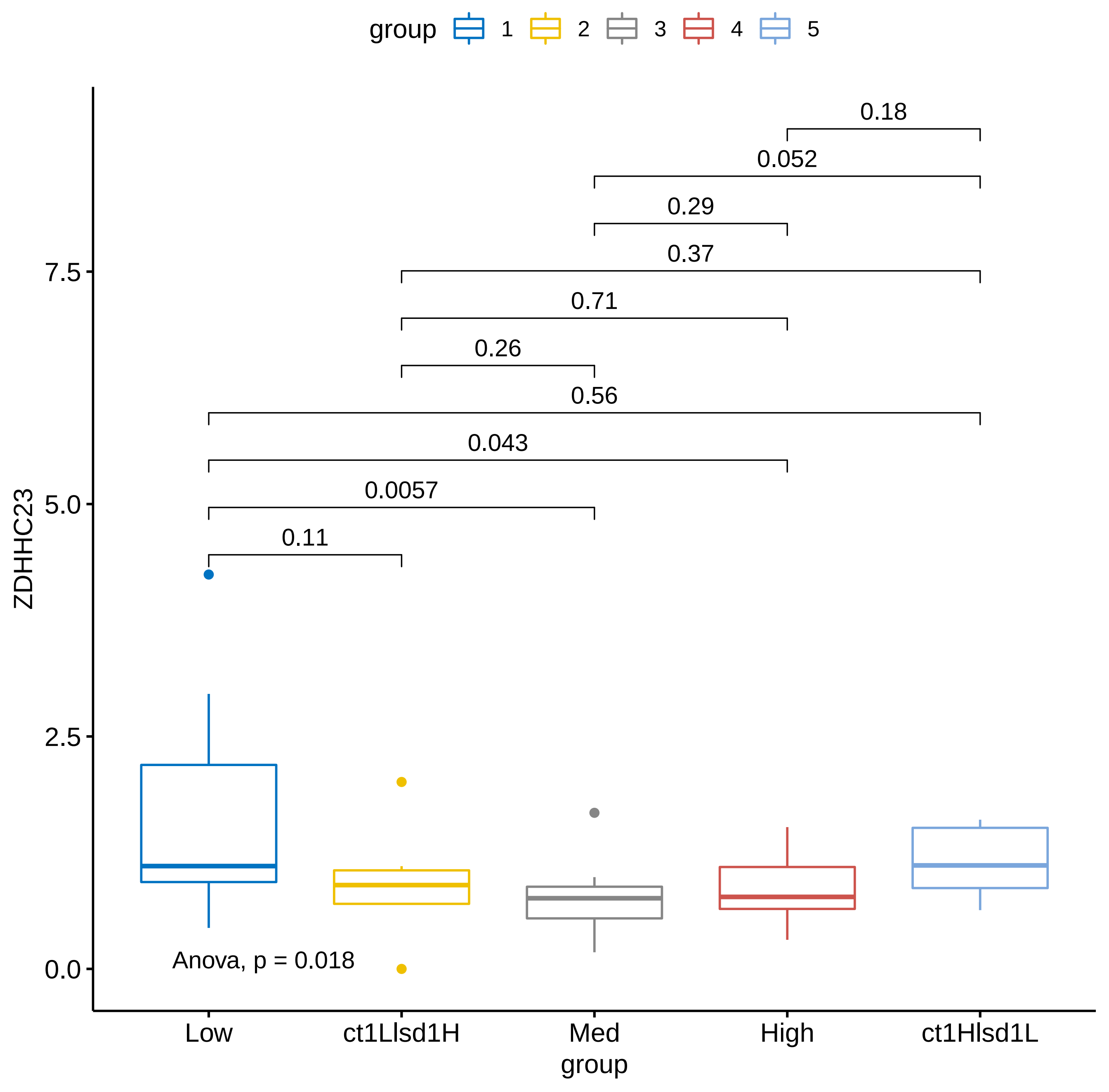

group
1
2
3
4
5
ZDHHC23
7.5
5.0
2.5
0.0
0.18
0.052
0.29
0.37
0.71
0.26
0.56
0.043
0.0057
0.11
Anova, p = 0.018
Low
ct1Llsd1H
Med
High
ct1Hlsd1L
group

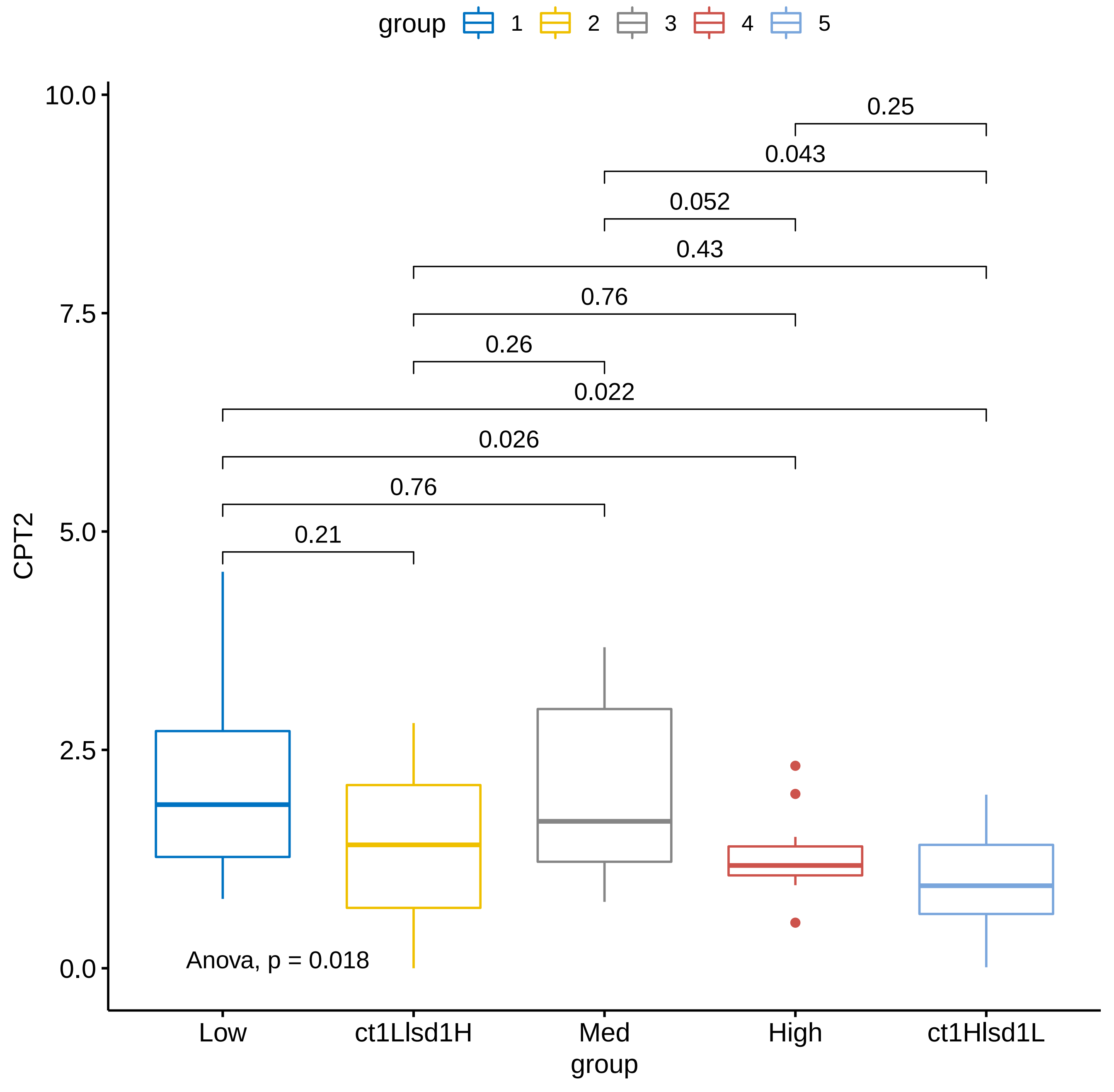

group
1
2
3
4
5
CPT2
10.0
7.5
5.0
2.5
0.0
0.25
0.043
0.052
0.43
0.76
0.26
0.022
0.026
0.76
0.21
Anova, p = 0.018
Low
ct1Llsd1H
Med
High
ct1Hlsd1L
group

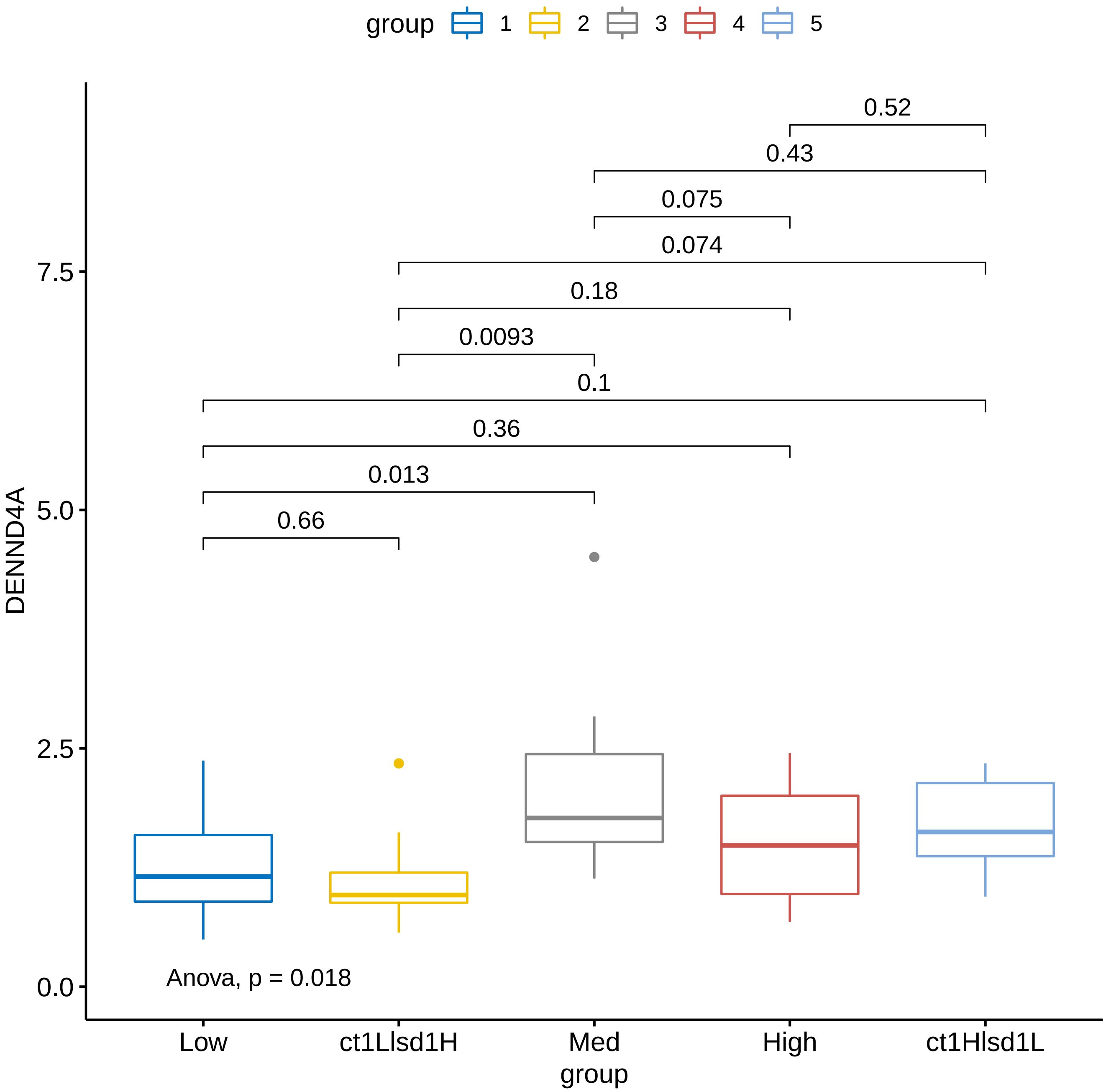

group
1
2
3
4
5
0.52
0.43
0.075
0.074
0.18
0.0093
0.1
0.36
0.013
0.66
DENND4A
7.5
5.0
2.5
0.0
Anova, p = 0.018
Low
ct1Llsd1H
Med
High
ct1Hlsd1L
group

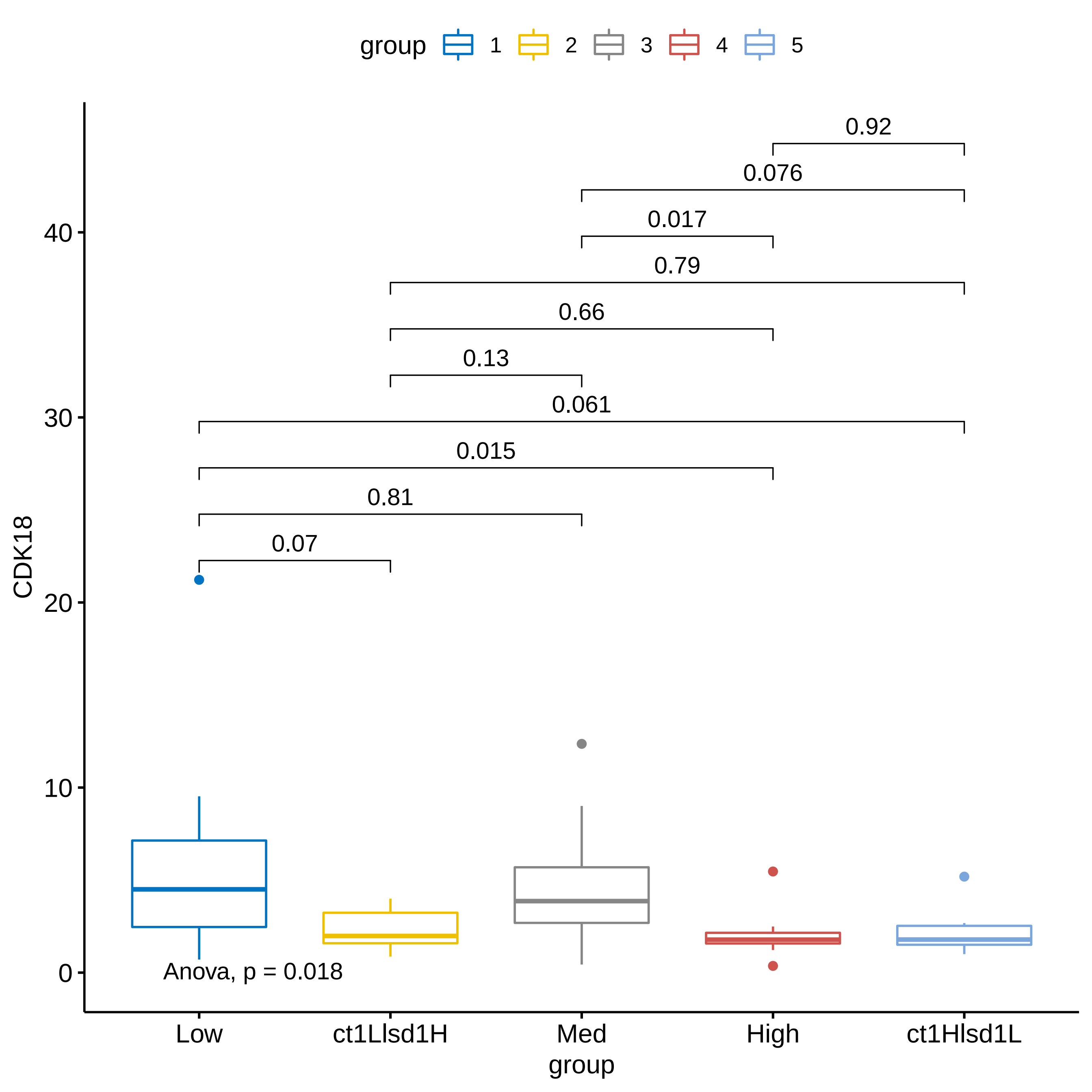
group
1
2
3
4
5
0.92
0.076
0.017
0.79
0.66
0.13
0.061
0.015
0.81
0.07
CDK18
40
30
20
10
0
Anova, p = 0.018
Low
ct1Llsd1H
Med
High
ct1Hlsd1L
group

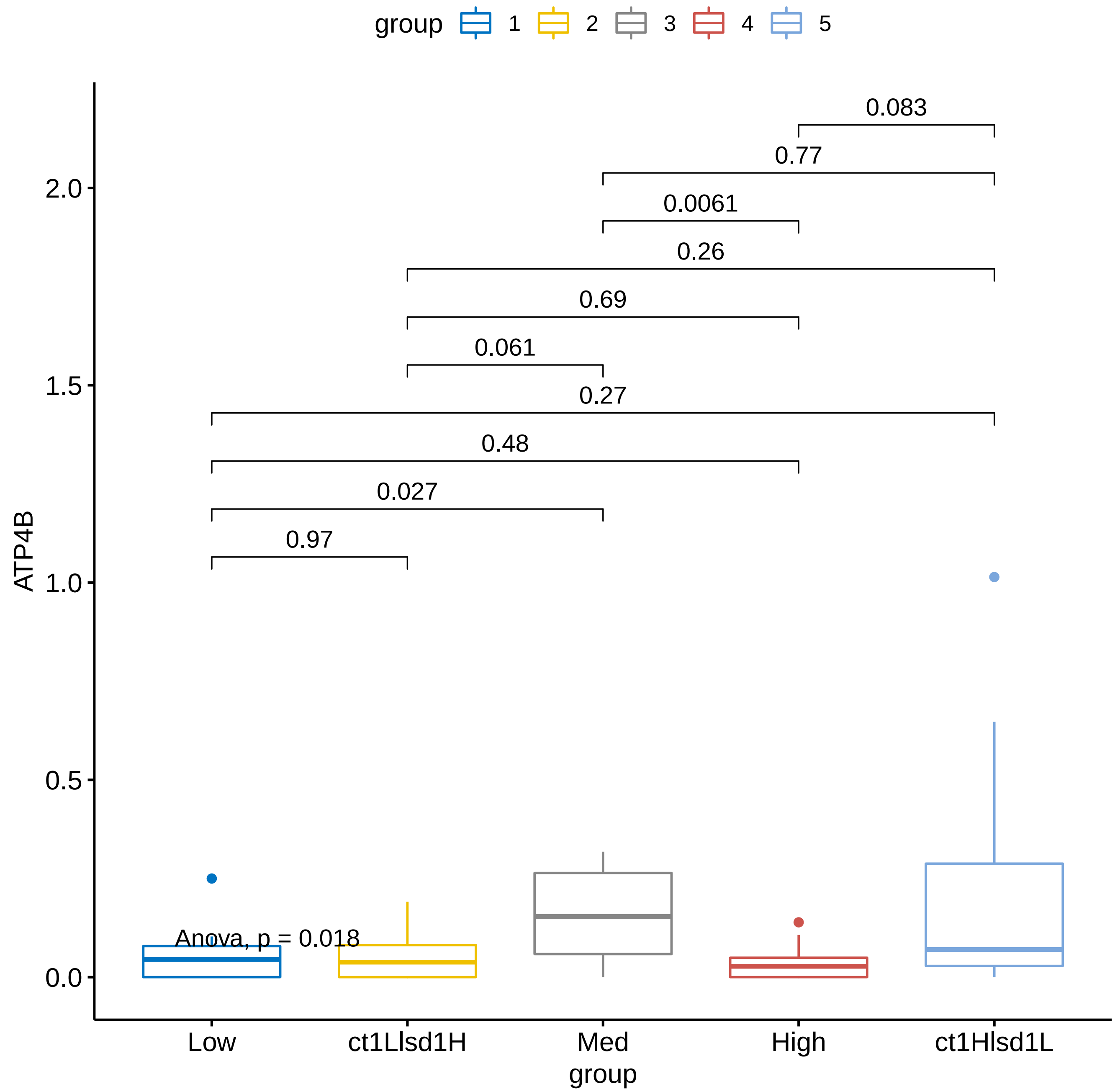

group
1
2
3
4
5
0.083
0.77
0.0061
0.26
0.69
0.061
0.27
0.48
0.027
0.97
Anova, p = 0.018
ATP4B
2.0
1.5
1.0
0.5
0.0
Low
ct1Llsd1H
Med
High
ct1Hlsd1L
group

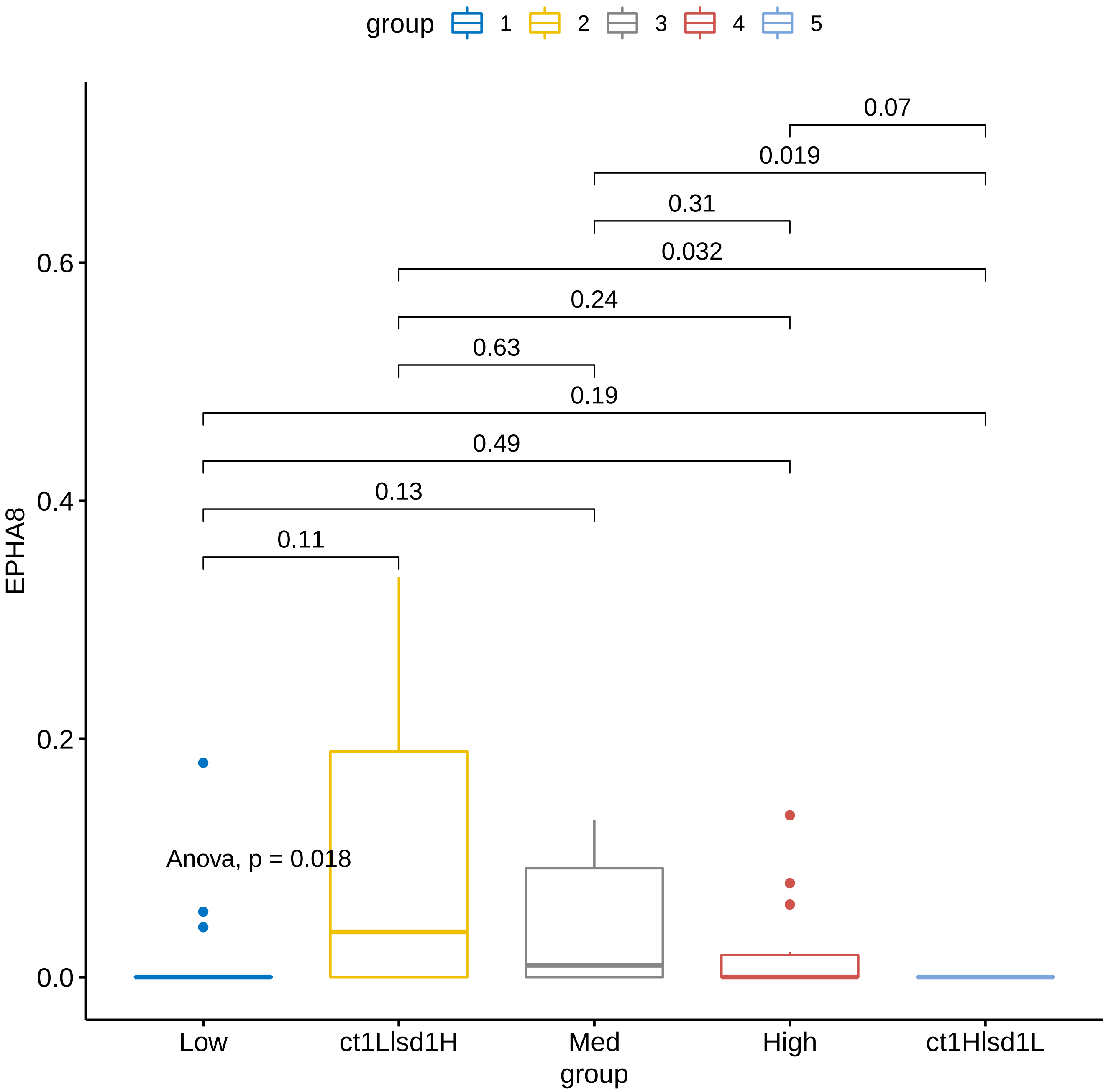

group
1
2
3
4
5
0.07
0.019
0.31
0.032
0.24
0.63
0.19
0.49
0.13
0.11
Anova, p = 0.018
EPHA8
0.0
0.2
0.4
0.6
Low
ct1Llsd1H
Med
High
ct1Hlsd1L
group

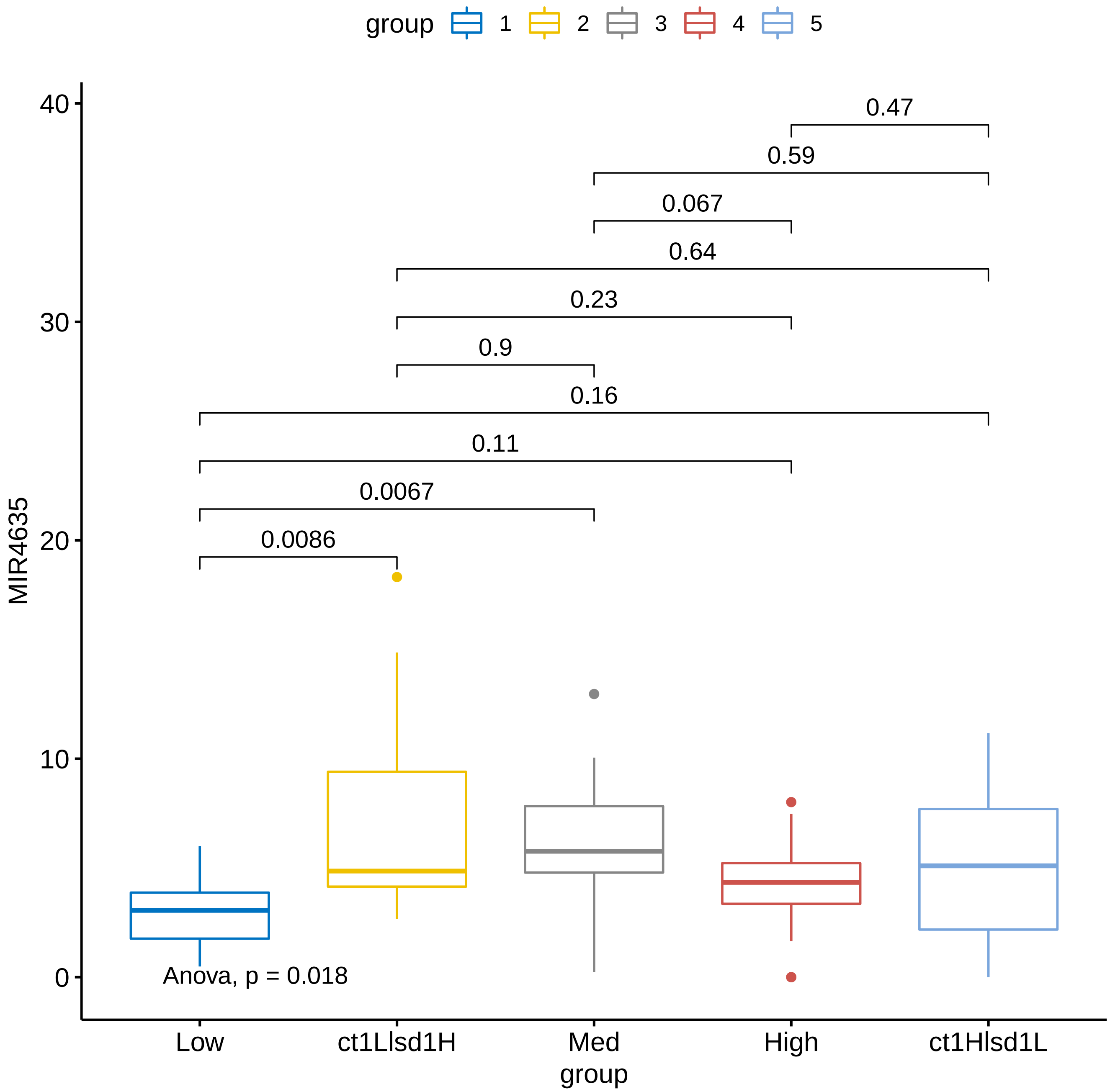

group
1
2
3
4
5
0.47
0.59
0.067
0.64
0.23
0.9
0.16
0.11
0.0067
0.0086
40
30
20
10
0
MIR4635
Anova, p = 0.018
Low
ct1Llsd1H
Med
High
ct1Hlsd1L
group

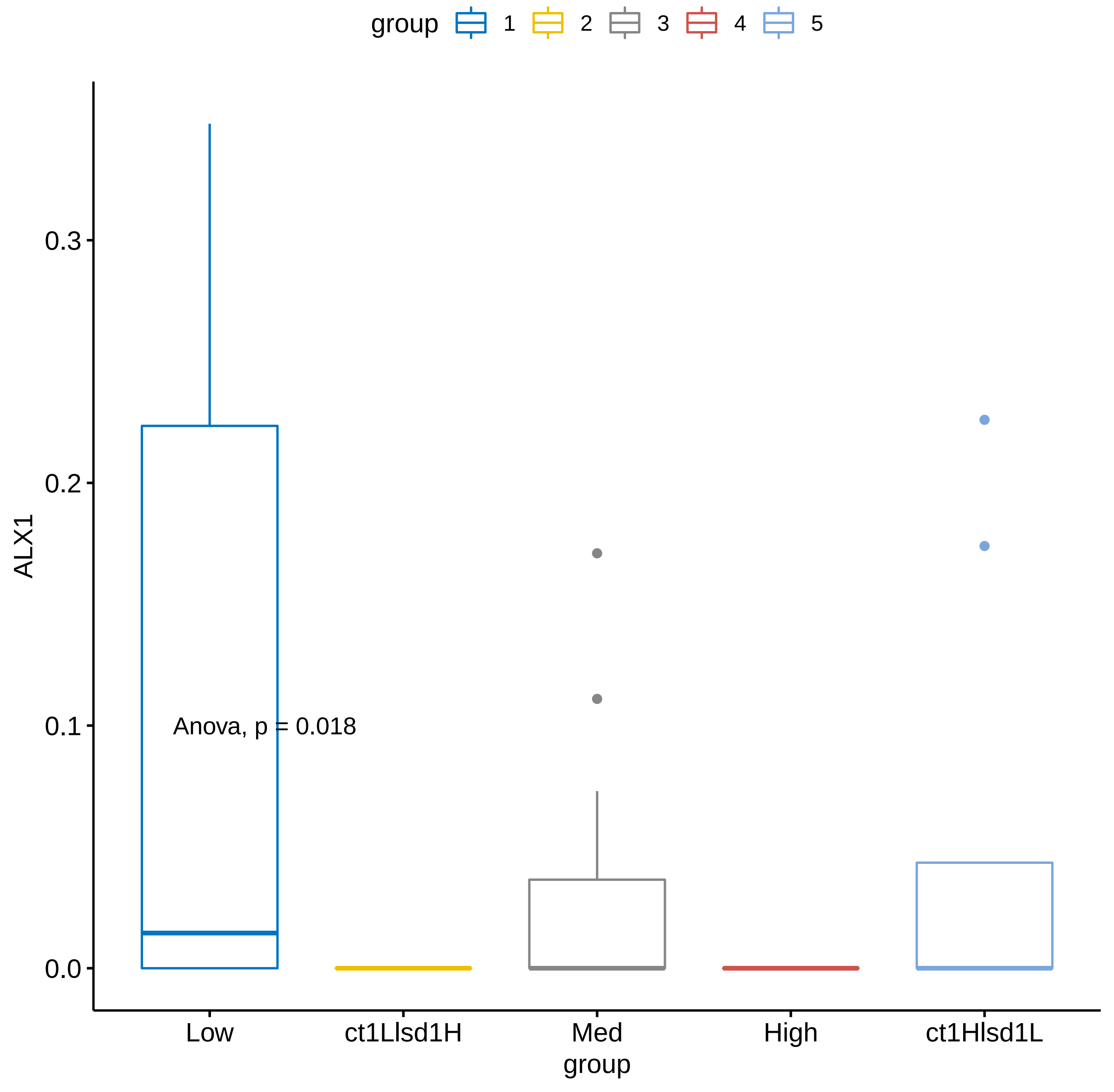

group
1
2
3
4
5
ALX1
0.3
0.2
0.1
0.0
Anova, p = 0.018
Low
ct1Llsd1H
Med
High
ct1Hlsd1L
group

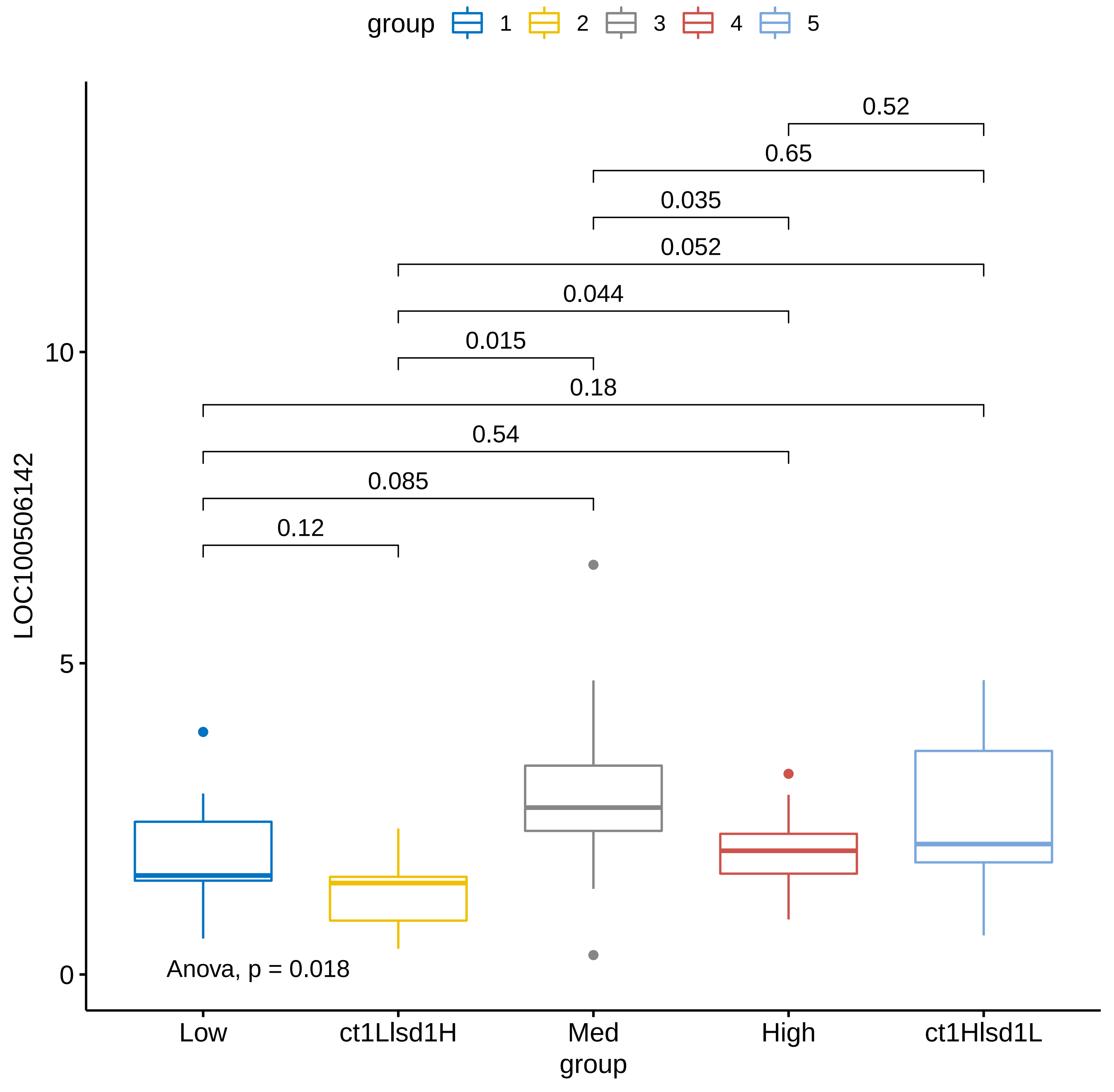

group 1 2 3 4 5
0.52
0.65
0.035
0.052
0.044
0.015
0.18
0.54
0.085
0.12
10
5
0
LOC100506142
Anova, p = 0.018
Low
ct1Llsd1H
Med
High
ct1Hlsd1L
group

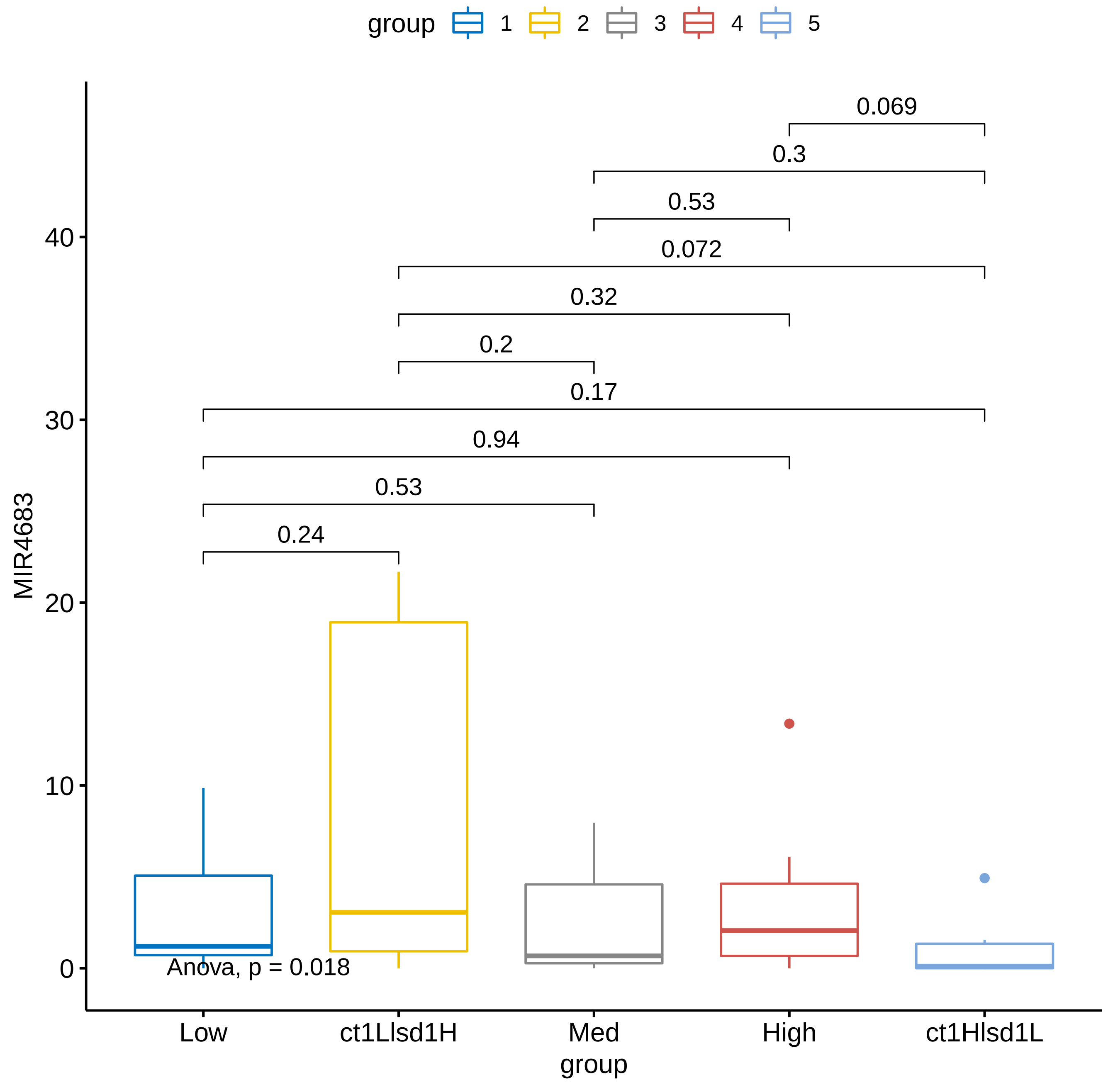

group
1
2
3
4
5
0.069
0.3
0.53
0.072
0.32
0.2
0.17
0.94
0.53
0.24
MIR4683
40
30
20
10
0
Anova, p = 0.018
Low
ct1Llsd1H
Med
High
ct1Hlsd1L
group

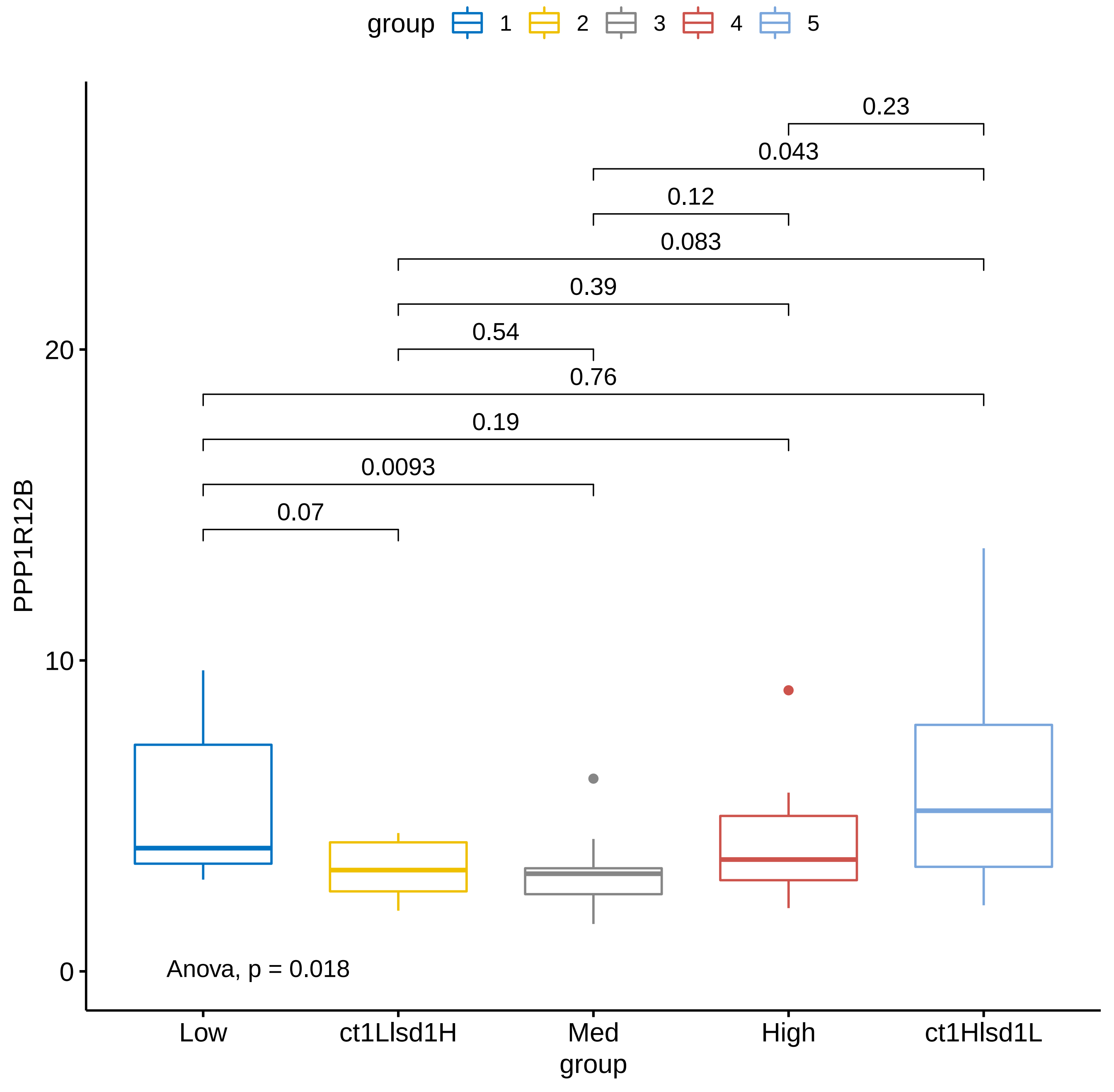

group 1 2 3 4 5
0.23
0.043
0.12
0.083
0.39
0.54
0.76
0.19
0.0093
0.07
PPP1R12B
20
10
0
Anova, p = 0.018
Low
ct1Llsd1H
Med
High
ct1Hlsd1L
group

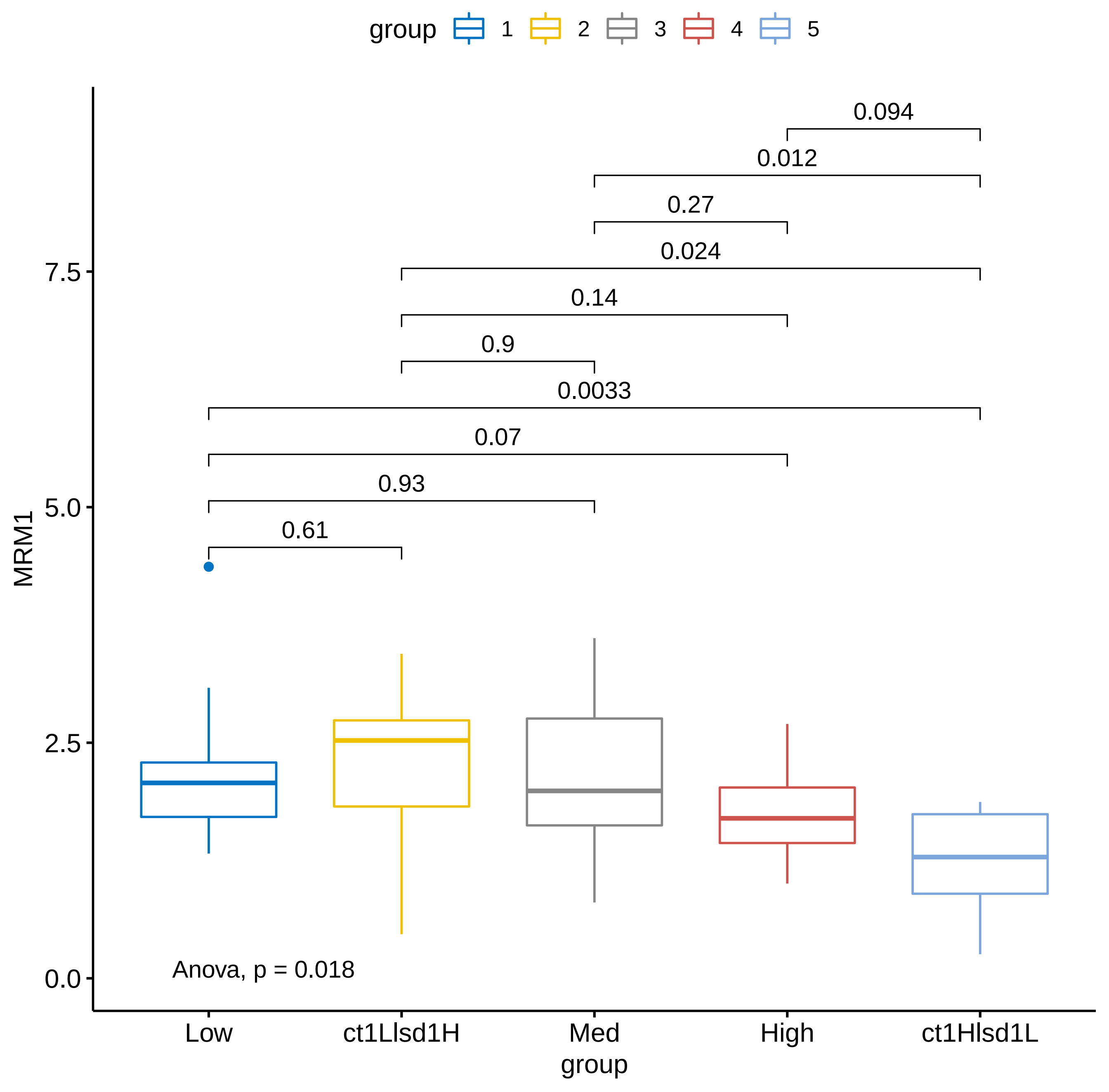
group
1
2
3
4
5
0.094
0.012
0.27
0.024
0.14
0.9
0.0033
0.07
0.93
0.61
7.5
5.0
2.5
0.0
MRM1
Anova, p = 0.018
Low
ct1Llsd1H
Med
High
ct1Hlsd1L
group

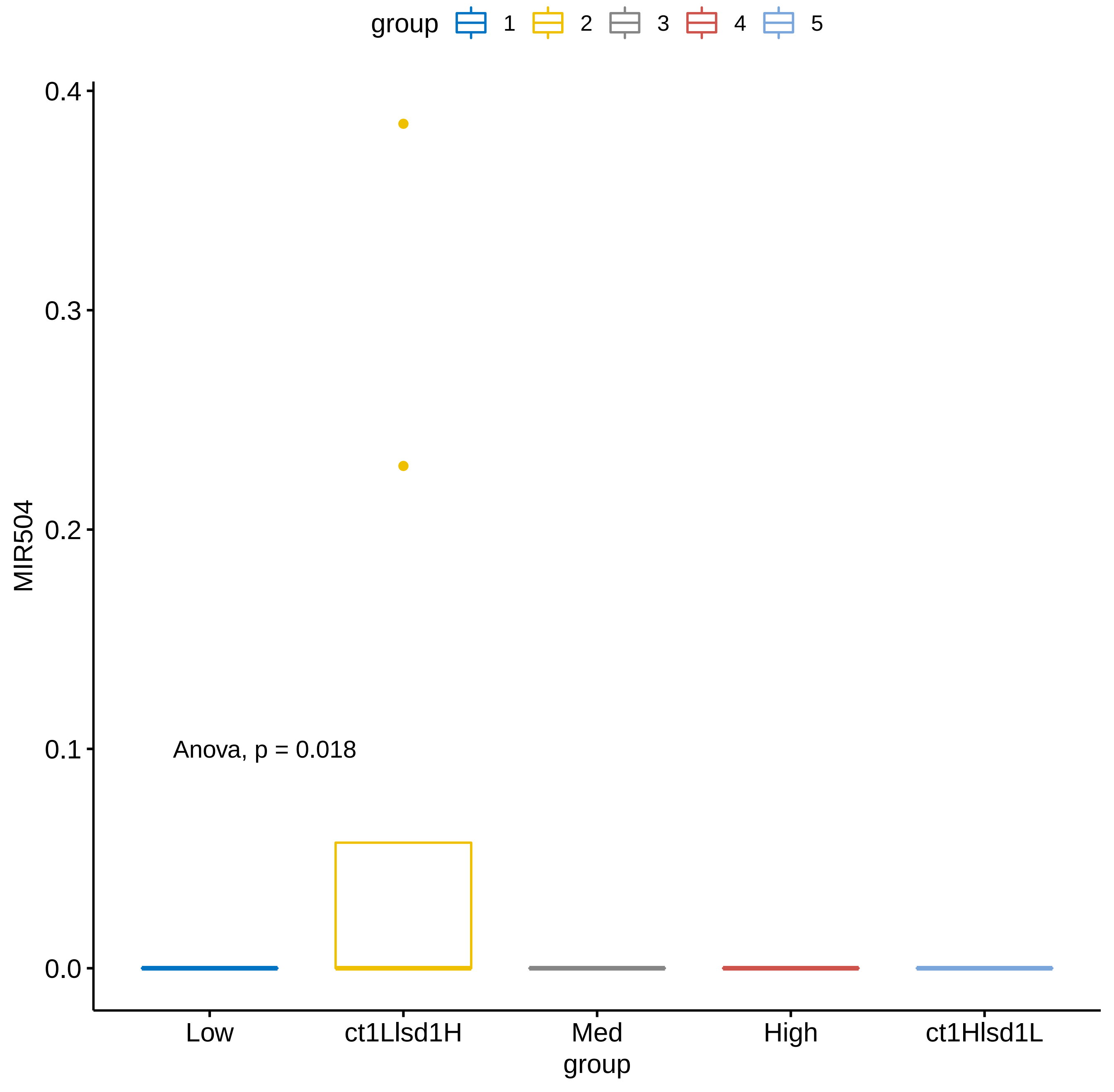

group
1
2
3
4
5
0.4
0.3
0.2
0.1
0.0
MIR504
Anova, p = 0.018
Low
ct1Llsd1H
Med
High
ct1Hlsd1L
group

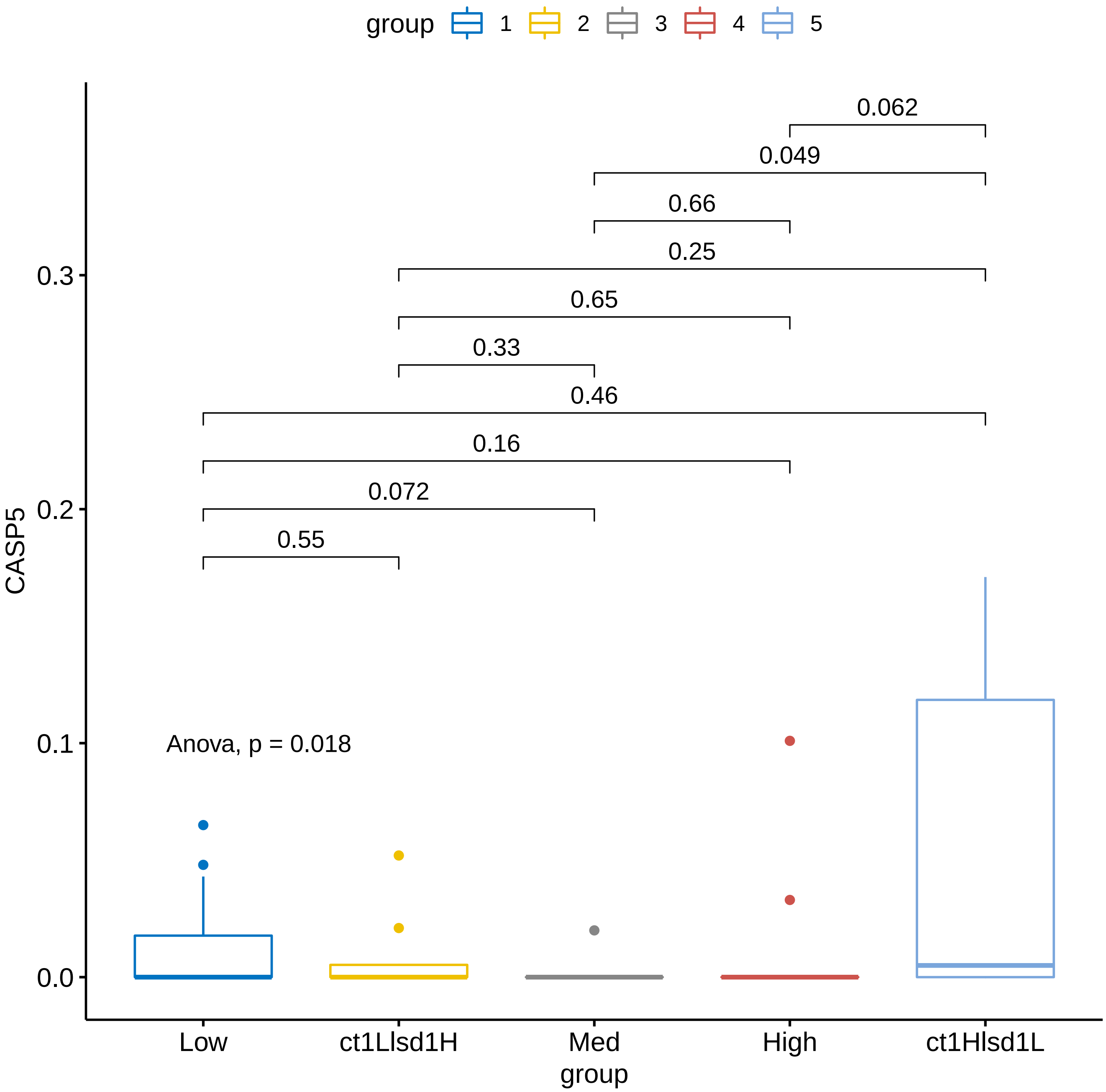

group
1
2
3
4
5
0.062
0.049
0.66
0.25
0.65
0.33
0.46
0.16
0.072
0.55
Anova, p = 0.018
0.3
0.2
0.1
0.0
CASP5
Low
ct1Llsd1H
Med
High
ct1Hlsd1L
group

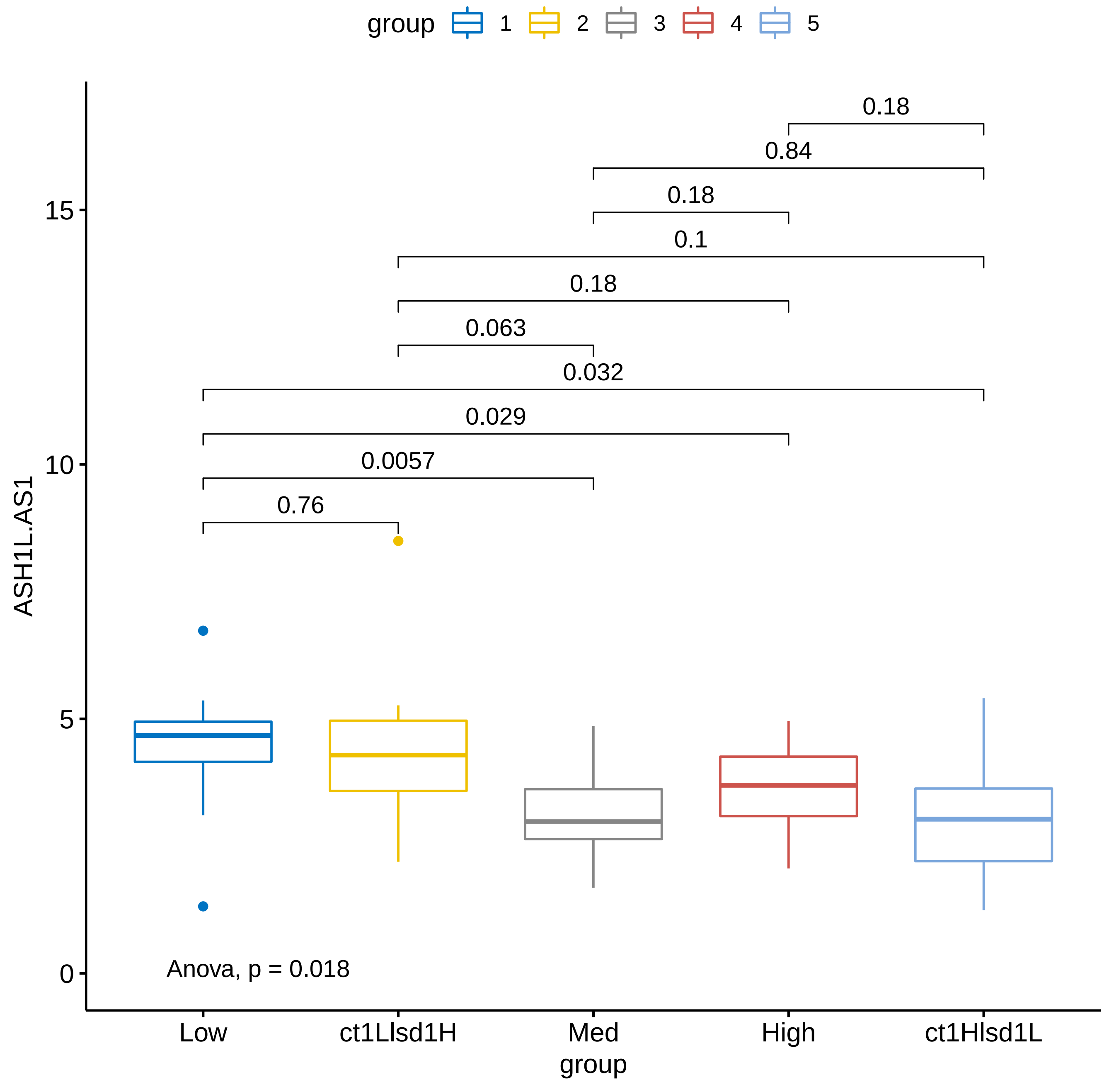

group
1
2
3
4
5
0.18
0.84
0.18
0.1
0.18
0.063
0.032
0.029
0.0057
0.76
15
10
5
0
ASH1L.AS1
Anova, p = 0.018
Low
ct1Llsd1H
Med
High
ct1Hlsd1L
group

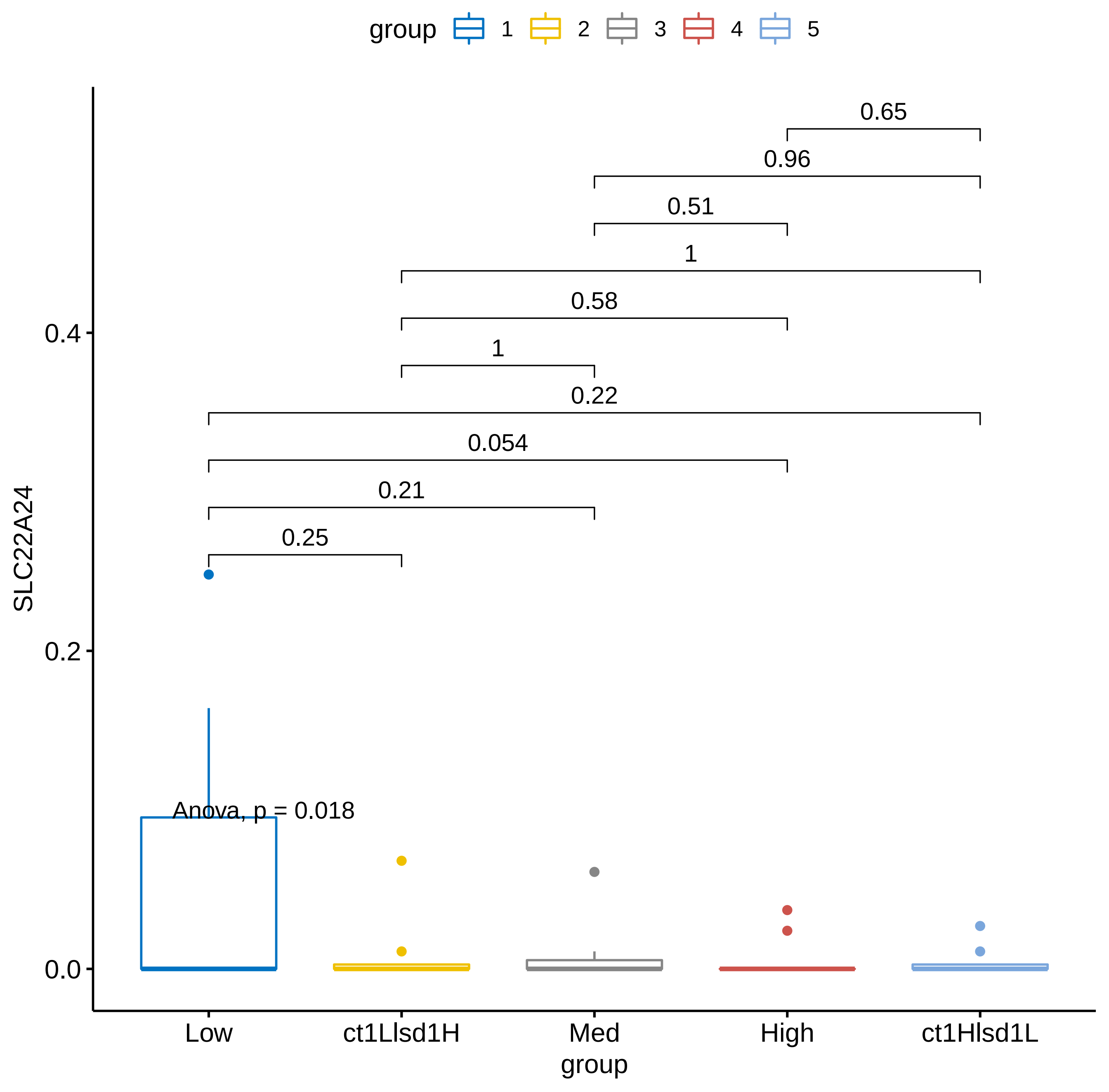
group 1 2 3 4 5
0.65
0.96
0.51
1
0.58
1
0.22
0.054
0.21
0.25
Anova, p = 0.018
SLC22A24
0.4
0.2
0.0
Low
ct1Llsd1H
Med
High
ct1Hlsd1L
group

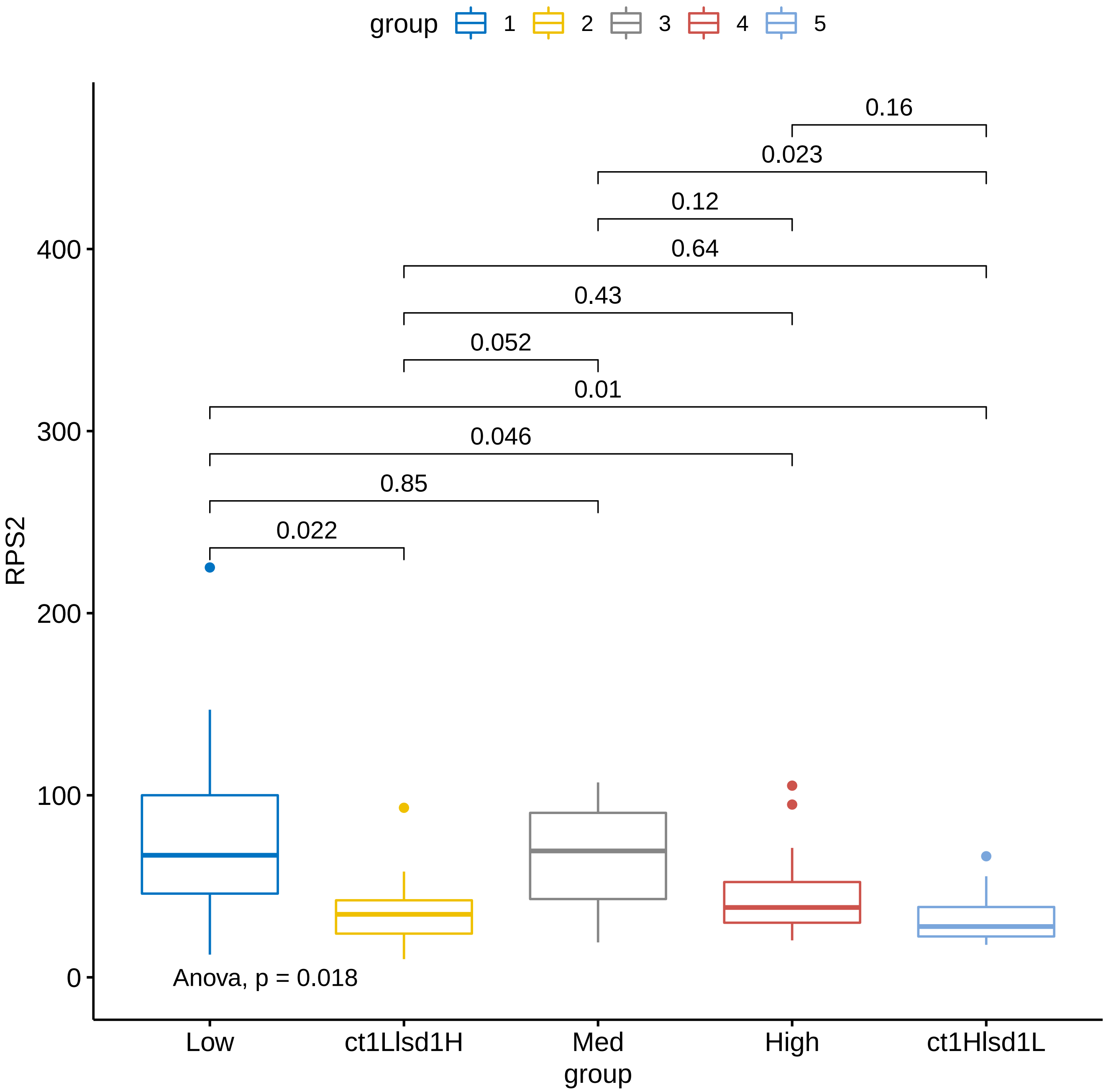

group
1
2
3
4
5
RPS2
0
100
200
300
400
0.16
0.023
0.12
0.64
0.43
0.052
0.01
0.046
0.85
0.022
Anova, p = 0.018
Low
ct1Llsd1H
Med
High
ct1Hlsd1L
group

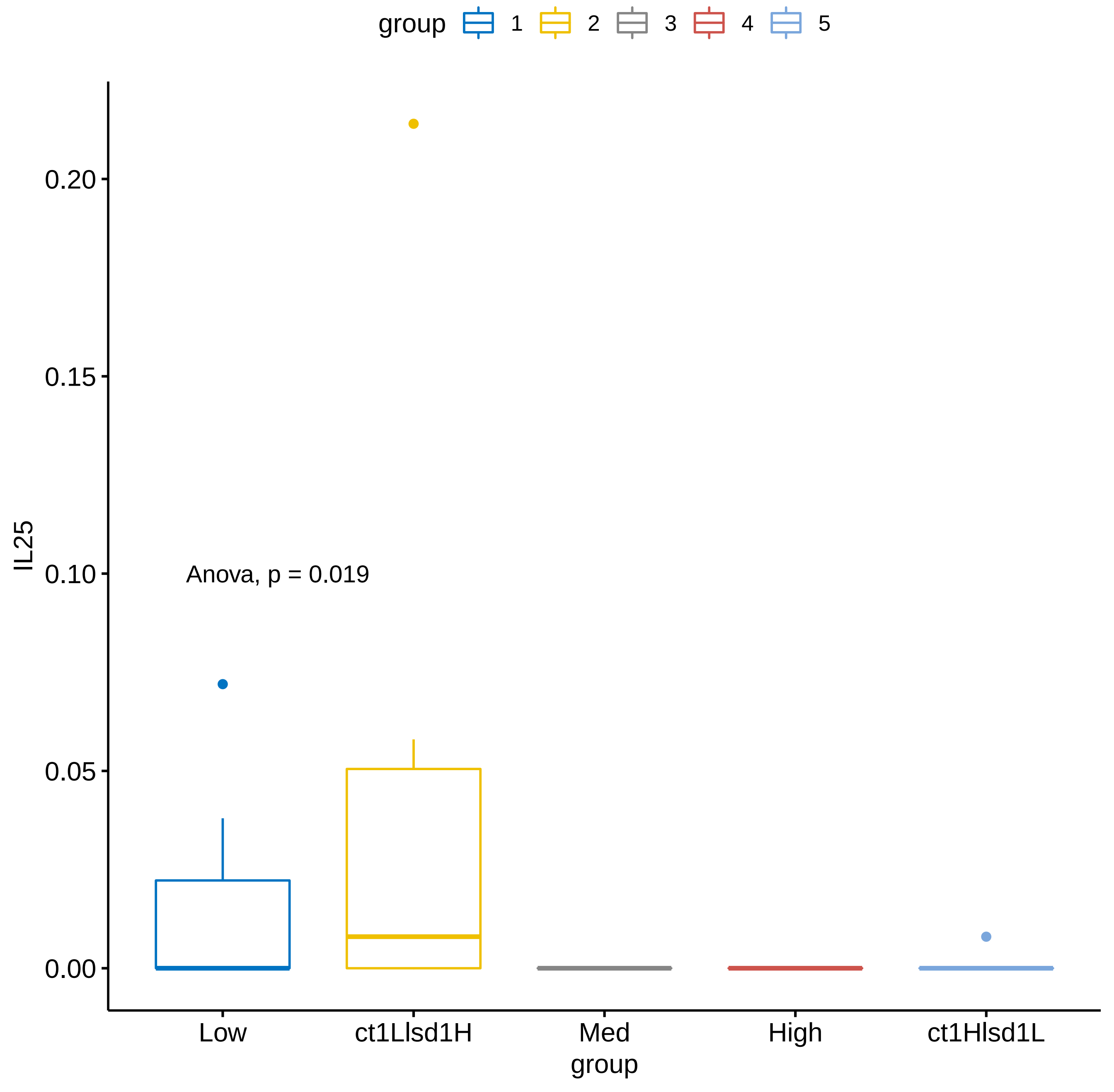
group
1
2
3
4
5
IL25
0.20
0.15
0.10
0.05
0.00
Anova, p = 0.019
Low
ct1Llsd1H
Med
High
ct1Hlsd1L
group

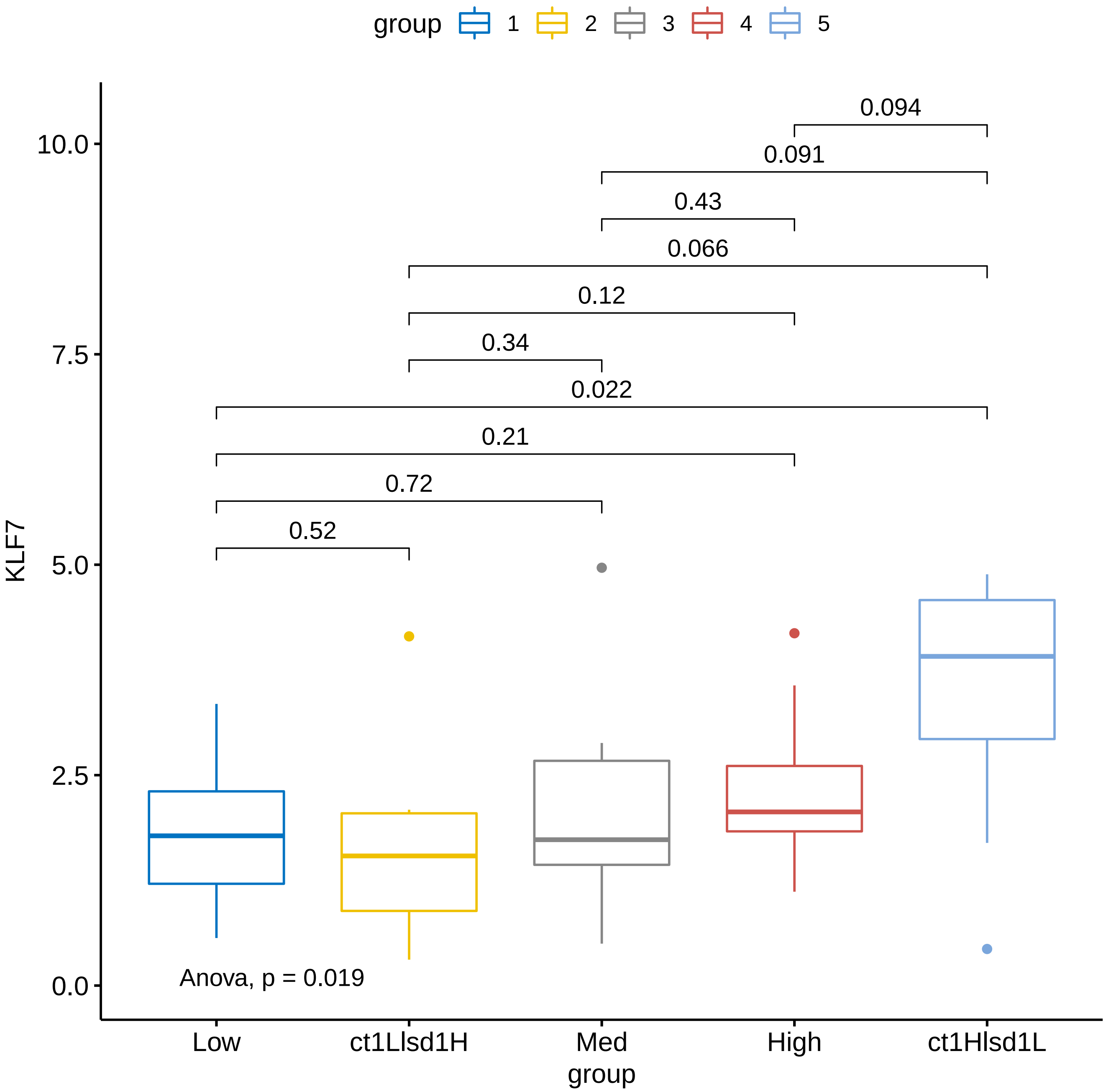

group
1
2
3
4
5
0.094
0.091
0.43
0.066
0.12
0.34
0.022
0.21
0.72
0.52
10.0
7.5
5.0
2.5
0.0
KLF7
Anova, p = 0.019
Low
ct1Llsd1H
Med
High
ct1Hlsd1L
group

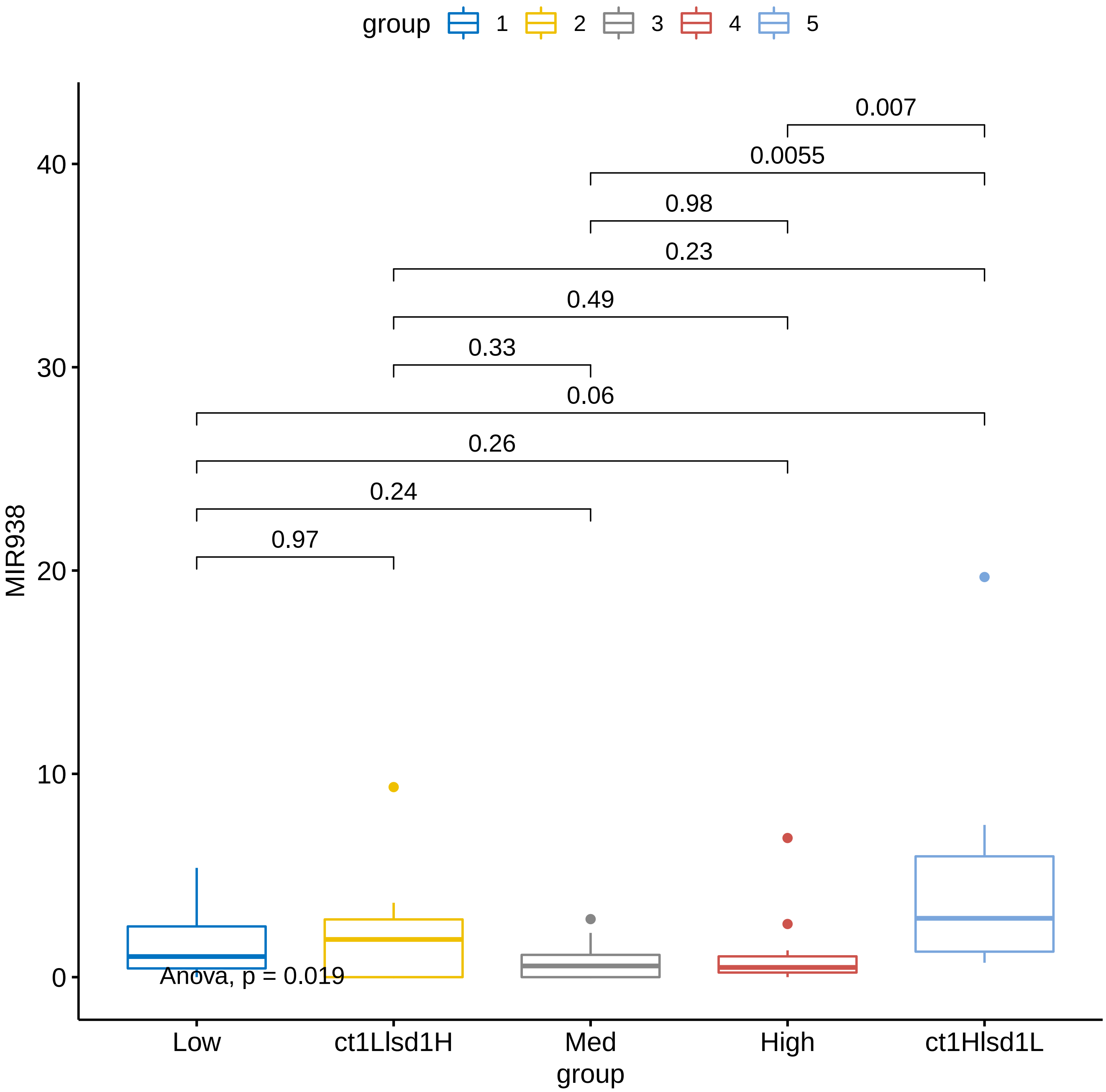
group
1
2
3
4
5
0.007
0.0055
0.98
0.23
0.49
0.33
0.06
0.26
0.24
0.97
40
30
20
10
0
MIR938
Anova, p = 0.019
Low
ct1Llsd1H
Med
High
ct1Hlsd1L
group

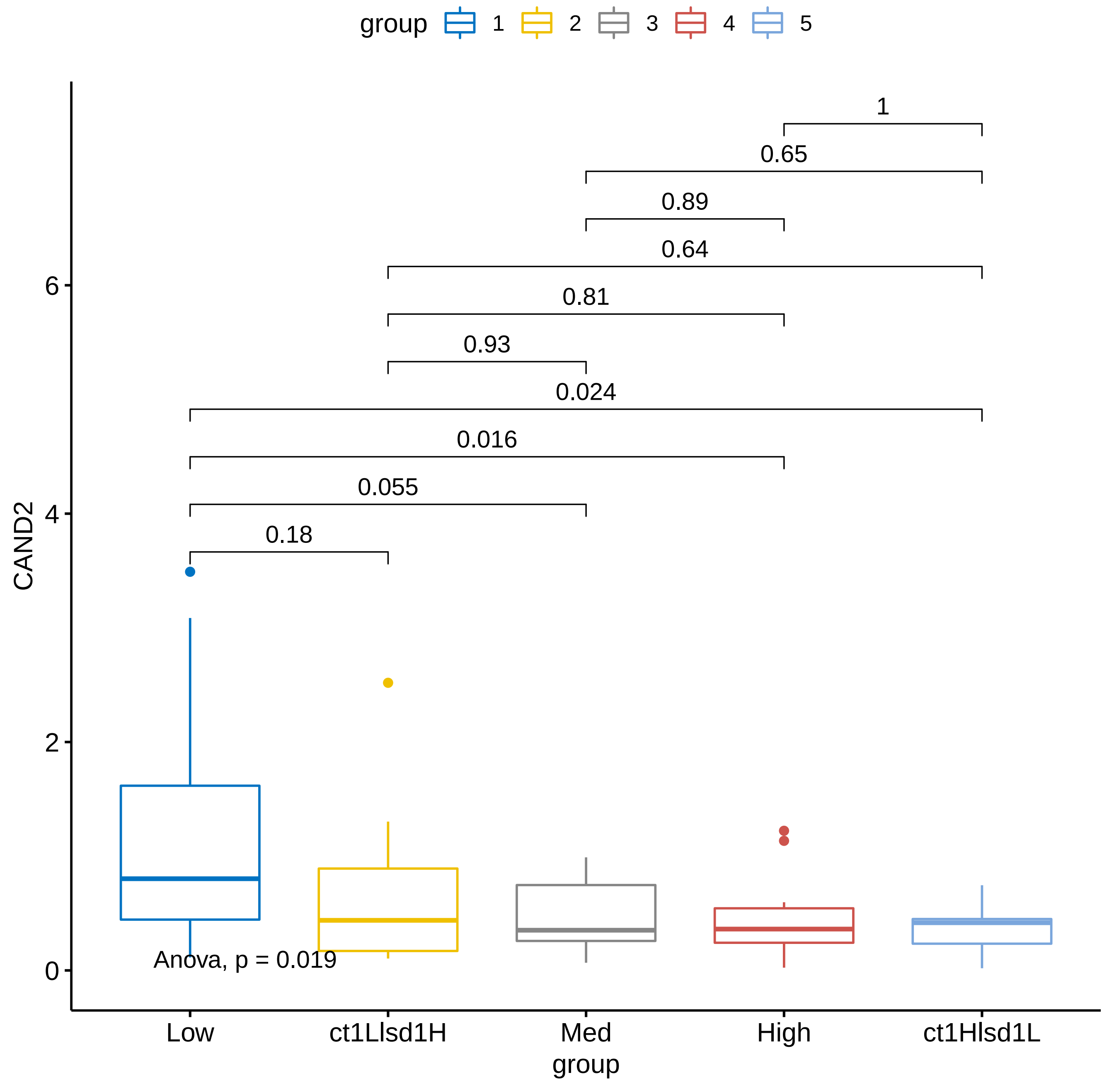

group
1
2
3
4
5
1
0.65
0.89
0.64
0.81
0.93
0.024
0.016
0.055
0.18
CAND2
6
4
2
0
Anova, p = 0.019
Low
ct1Llsd1H
Med
High
ct1Hlsd1L
group

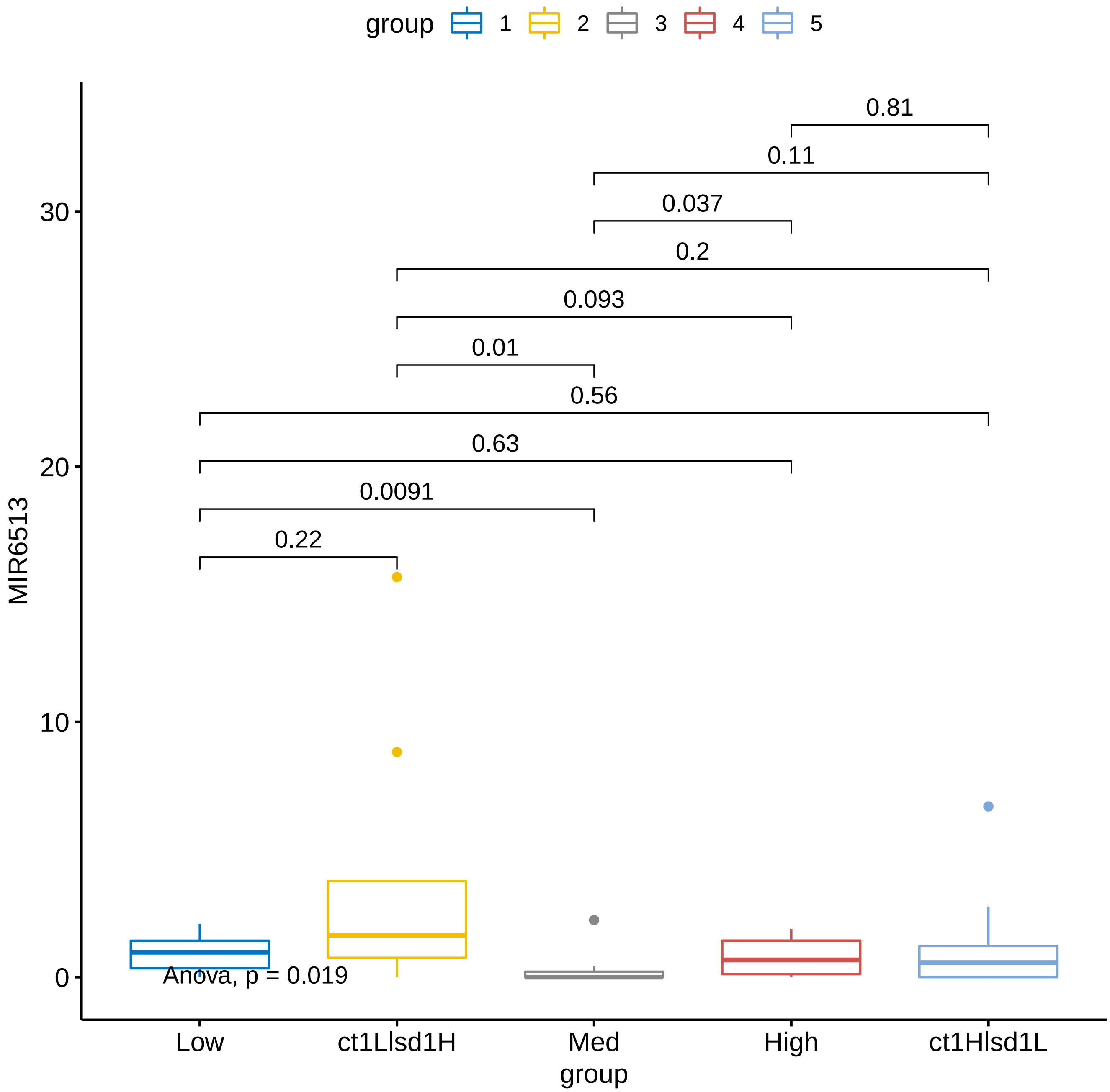

group
1
2
3
4
5
0.81
0.11
0.037
0.2
0.093
0.01
0.56
0.63
0.0091
0.22
30
20
10
0
MIR6513
Anova, p = 0.019
Low
ct1Llsd1H
Med
High
ct1Hlsd1L
group

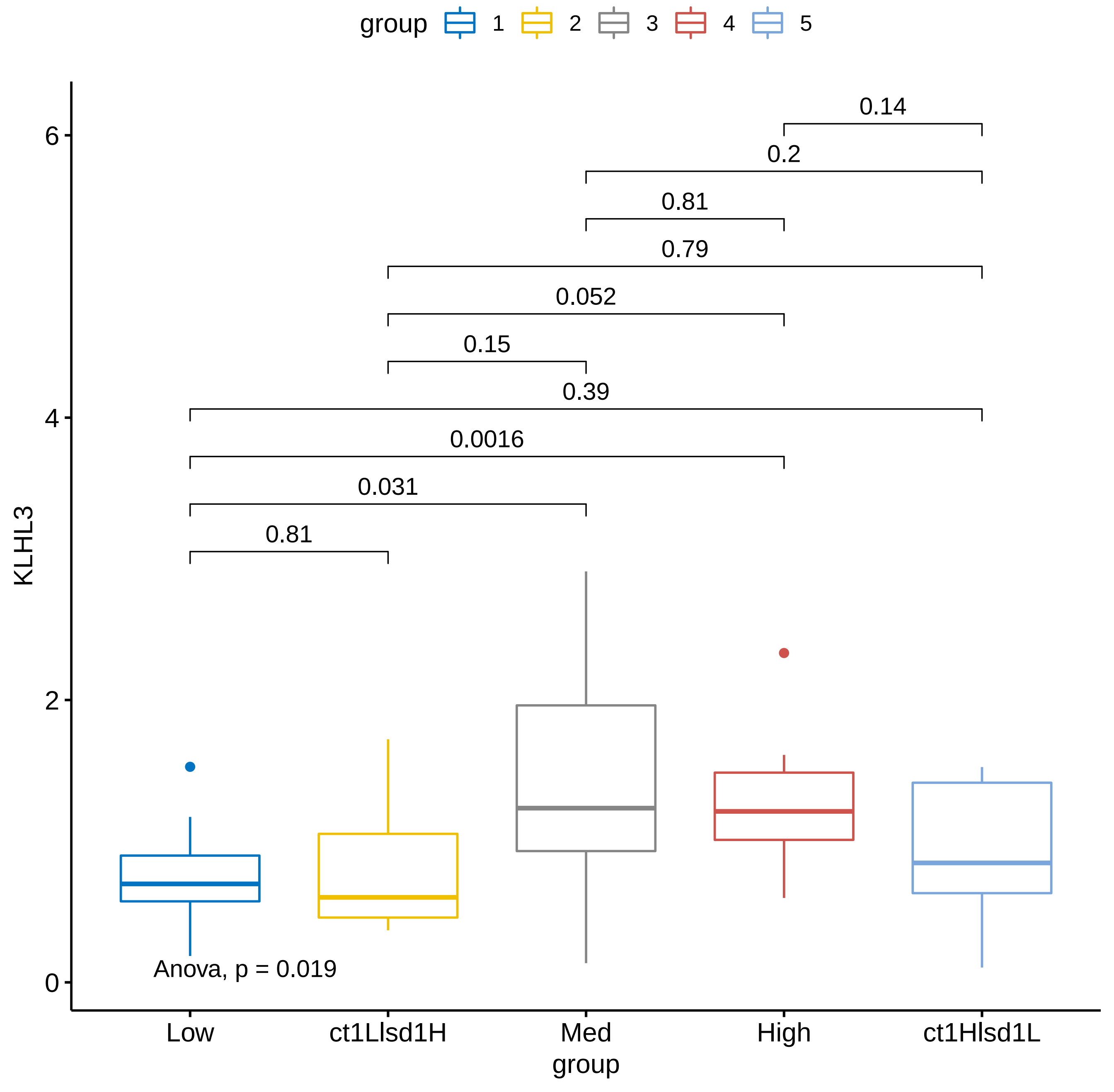
group
1
2
3
4
5
0.14
0.2
0.81
0.79
0.052
0.15
0.39
0.0016
0.031
0.81
KLHL3
6
4
2
0
Anova, p = 0.019
Low
ct1Llsd1H
Med
High
ct1Hlsd1L
group

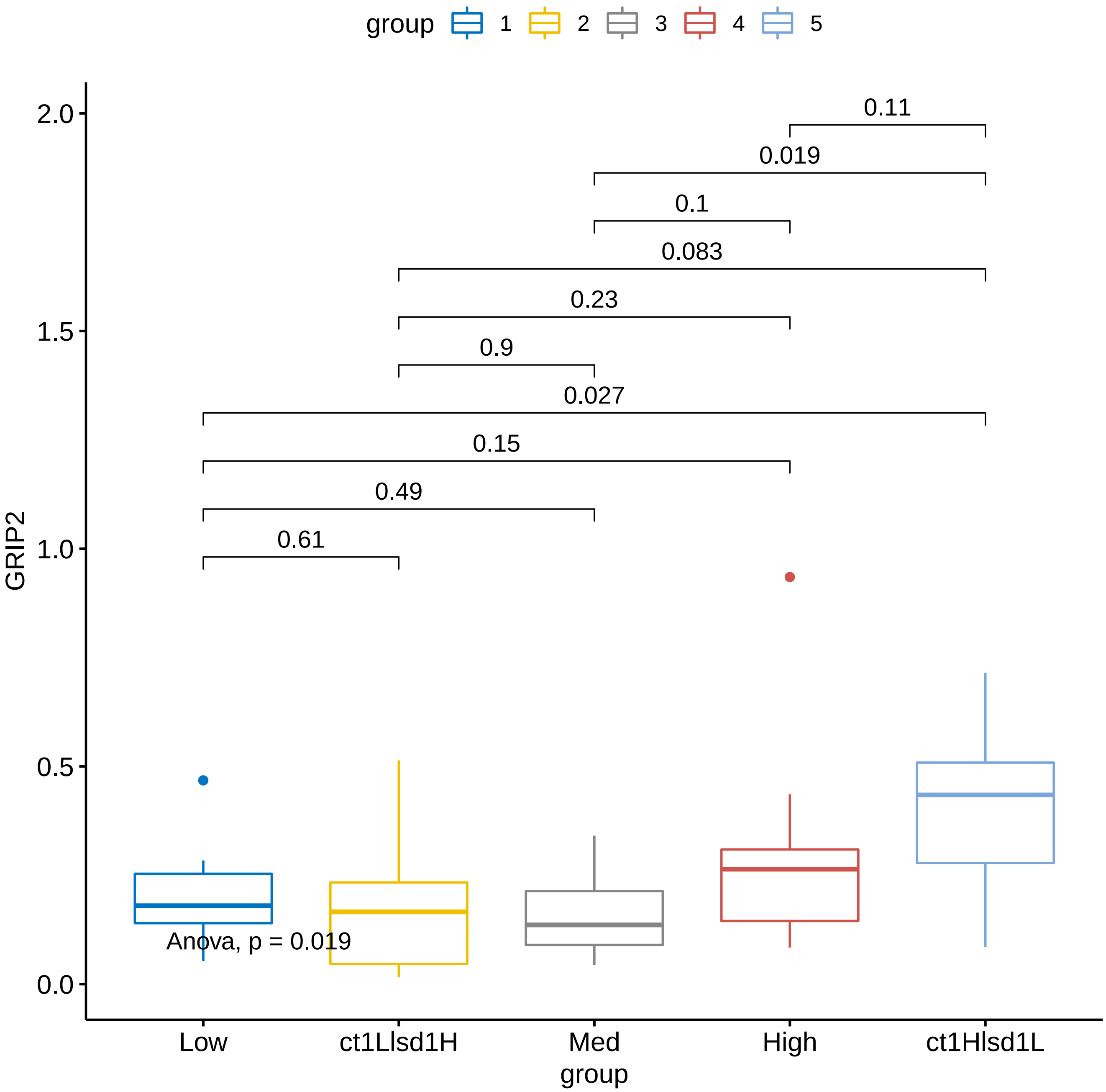

group
1
2
3
4
5
GRIP2
2.0
1.5
1.0
0.5
0.0
0.11
0.019
0.1
0.083
0.23
0.9
0.027
0.15
0.49
0.61
Anova, p = 0.019
Low
ct1Llsd1H
Med
High
ct1Hlsd1L
group

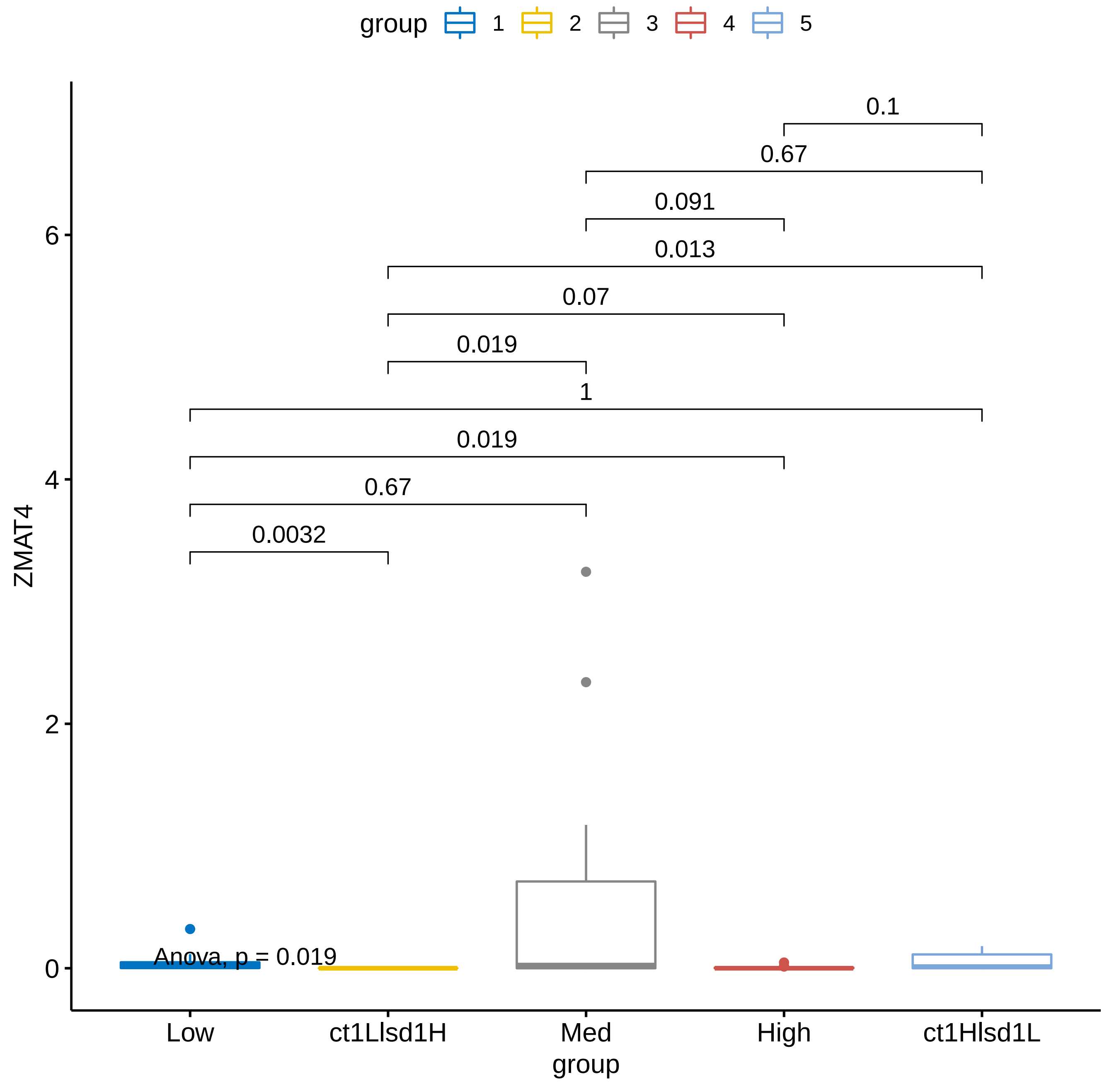

group
1
2
3
4
5
0.1
0.67
0.091
0.013
0.07
0.019
1
0.019
0.67
0.0032
Anova, p = 0.019
ZMAT4
6
4
2
0
Low
ct1Llsd1H
Med
High
ct1Hlsd1L
group

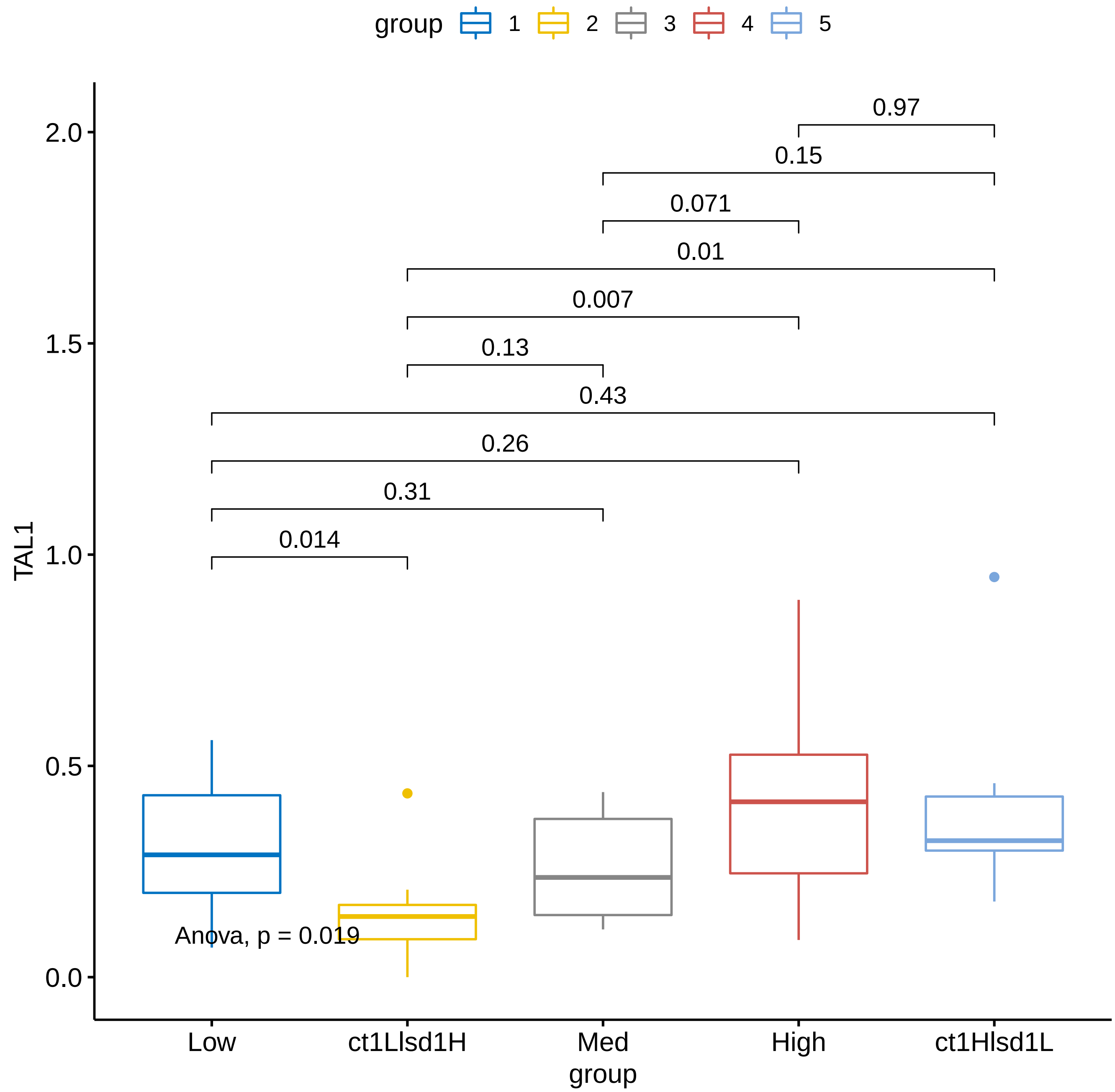

group 1 2 3 4 5
0.97
0.15
0.071
0.01
0.007
0.13
0.43
0.26
0.31
0.014
Anova, p = 0.019
TAL1
2.0
1.5
1.0
0.5
0.0
Low
ct1Llsd1H
Med
High
ct1Hlsd1L
group

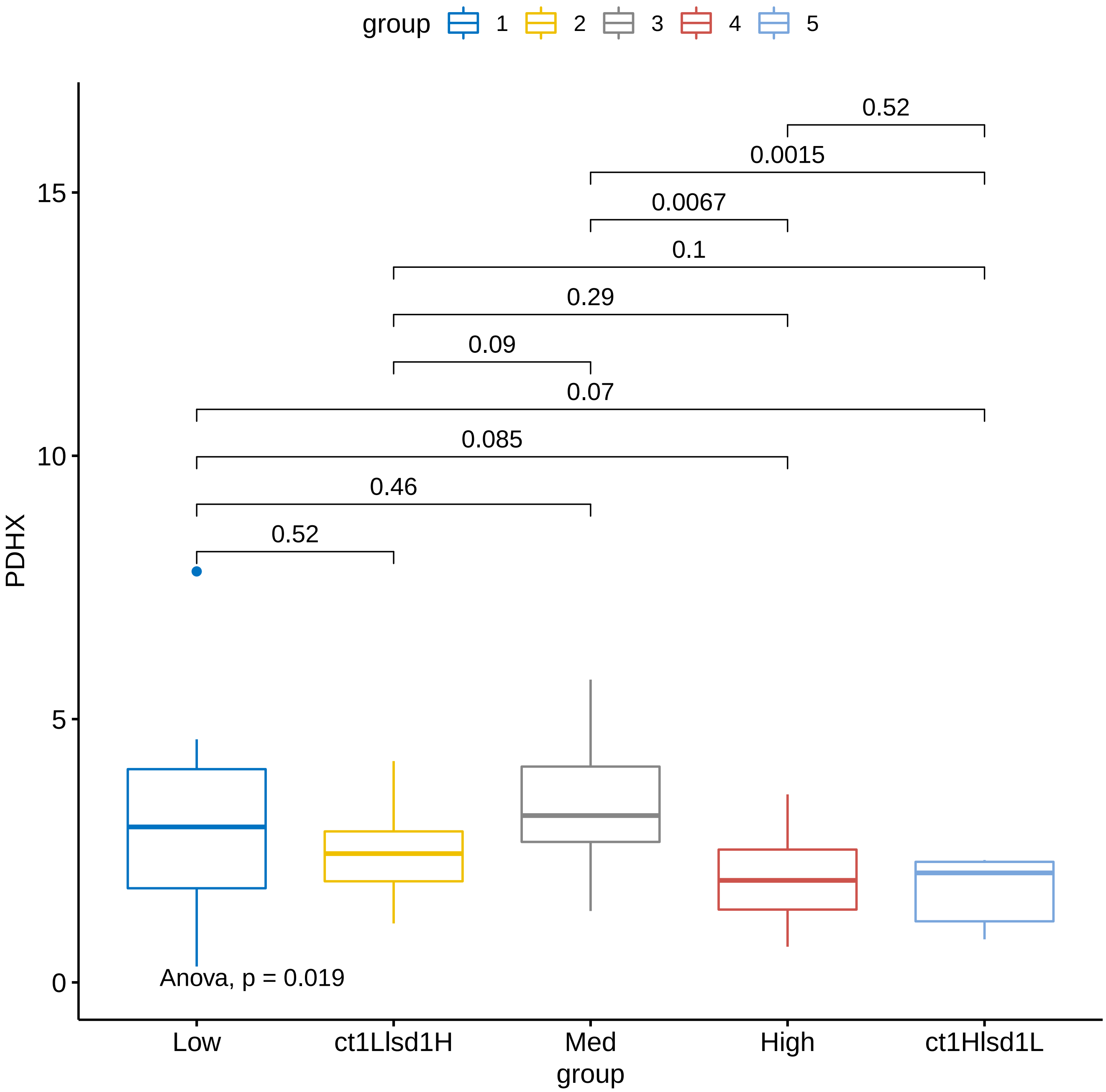
group
1
2
3
4
5
0.52
0.0015
0.0067
0.1
0.29
0.09
0.07
0.085
0.46
0.52
15
10
5
0
PDHX
Anova, p = 0.019
Low
ct1Llsd1H
Med
High
ct1Hlsd1L
group

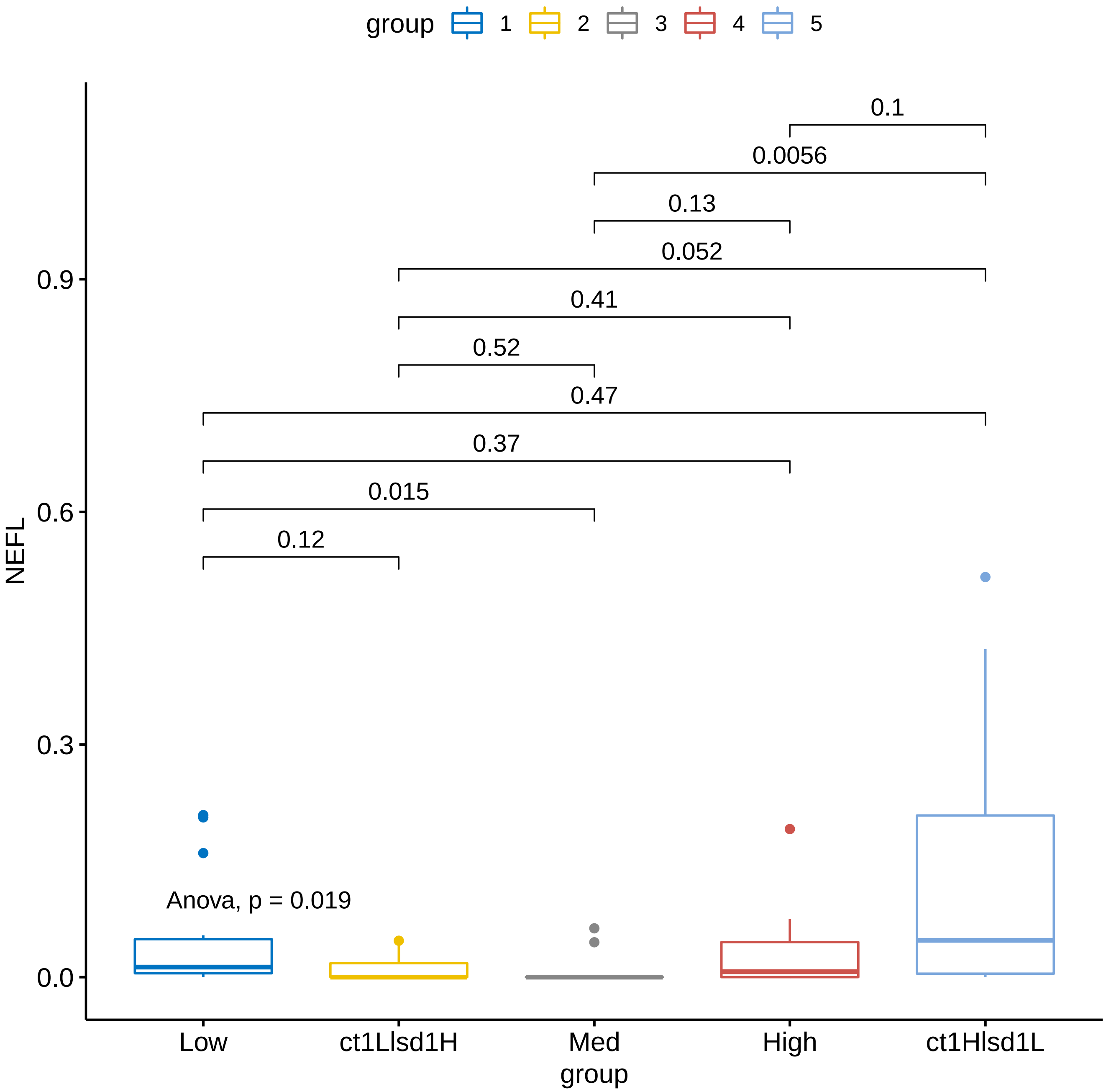

group
1
2
3
4
5
0.1
0.0056
0.13
0.052
0.41
0.52
0.47
0.37
0.015
0.12
Anova, p = 0.019
NEFL
0.9
0.6
0.3
0.0
Low
ct1Llsd1H
Med
High
ct1Hlsd1L
group

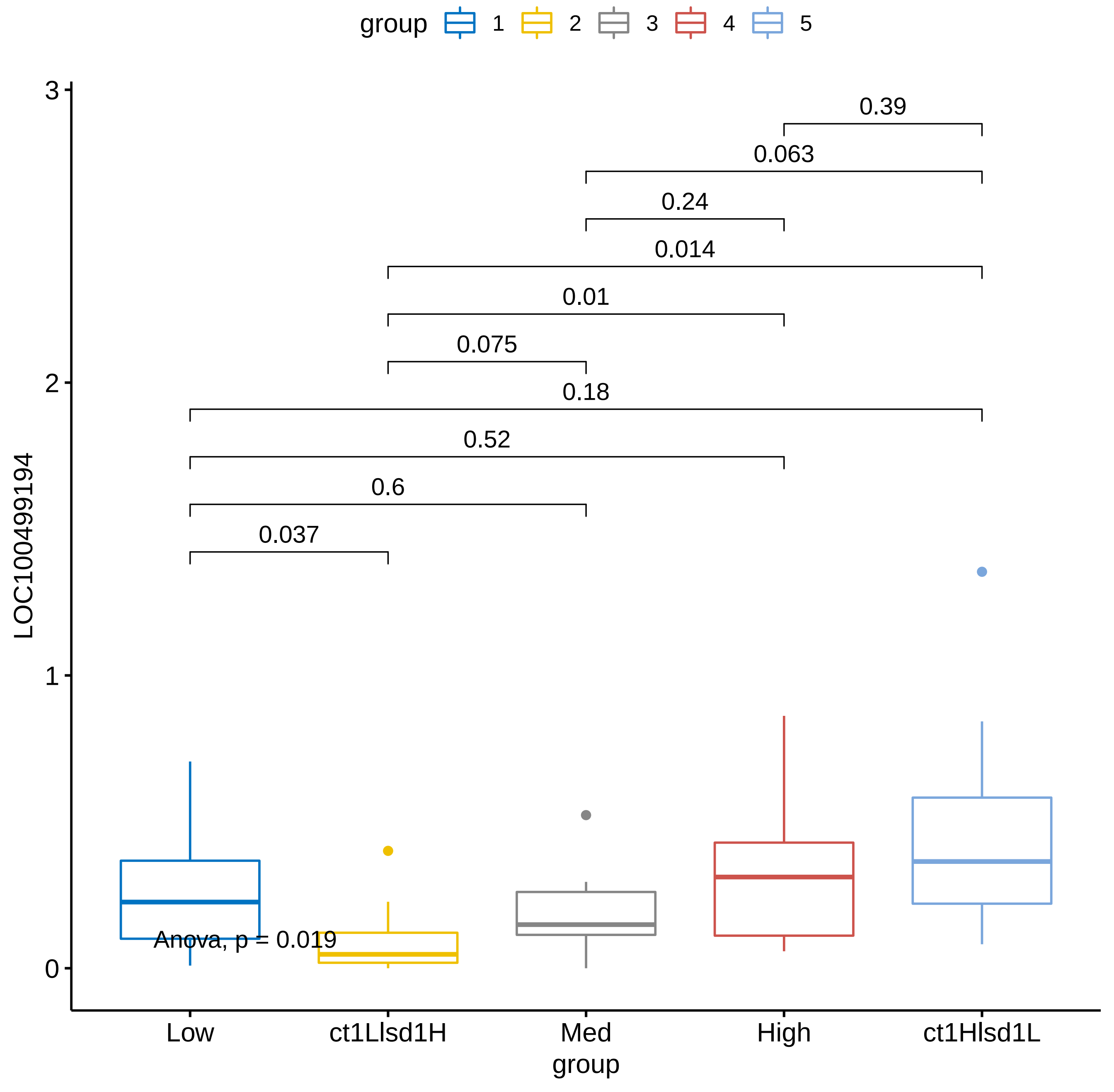
group
1
2
3
4
5
0.39
0.063
0.24
0.014
0.01
0.075
0.18
0.52
0.6
0.037
Anova, p = 0.019
LOC100499194
3
2
1
0
Low
ct1Llsd1H
Med
High
ct1Hlsd1L
group

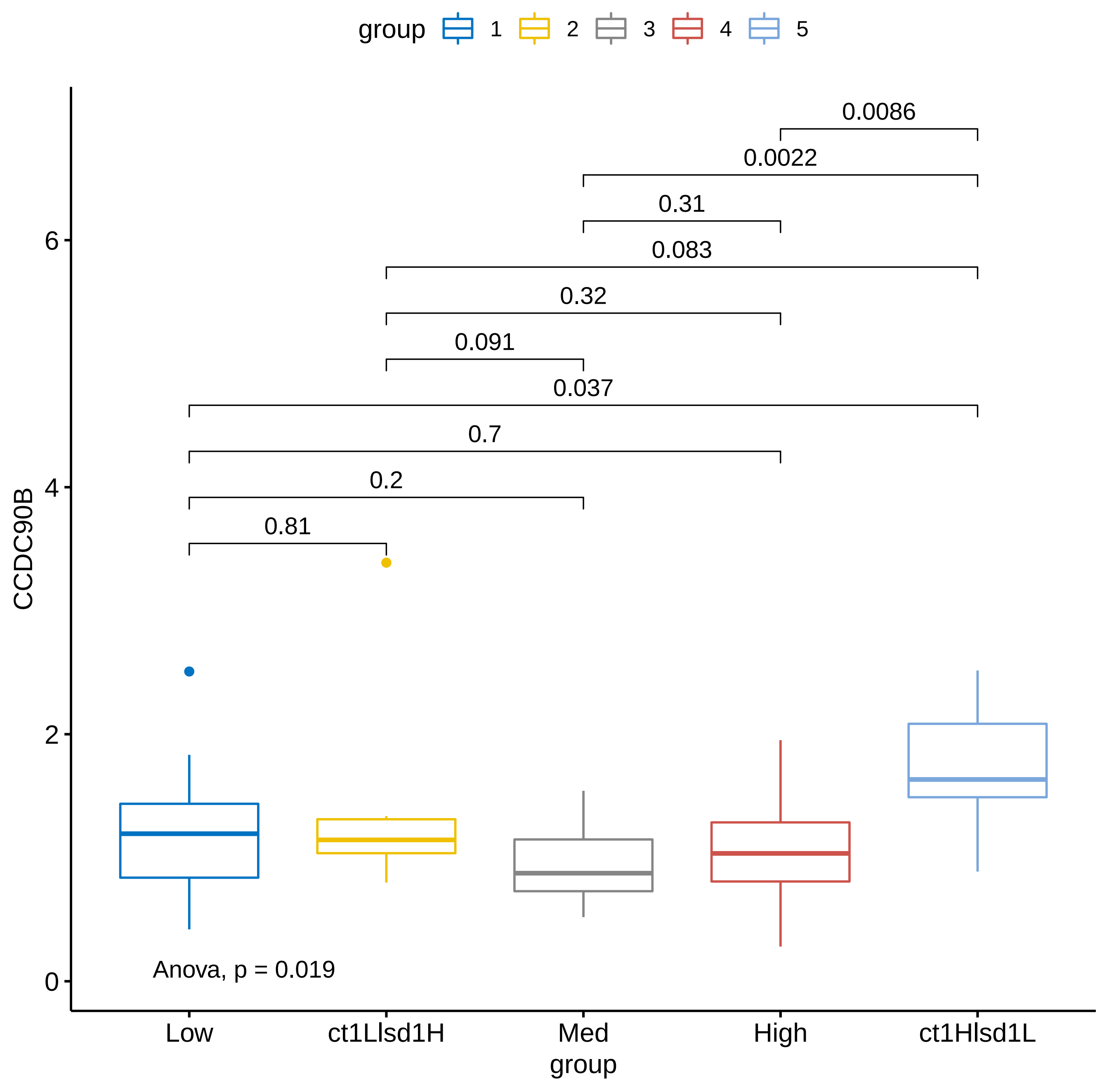

group
1
2
3
4
5
0.0086
0.0022
0.31
0.083
0.32
0.091
0.037
0.7
0.2
0.81
Anova, p = 0.019
CCDC90B
6
4
2
0
Low
ct1Llsd1H
Med
High
ct1Hlsd1L
group

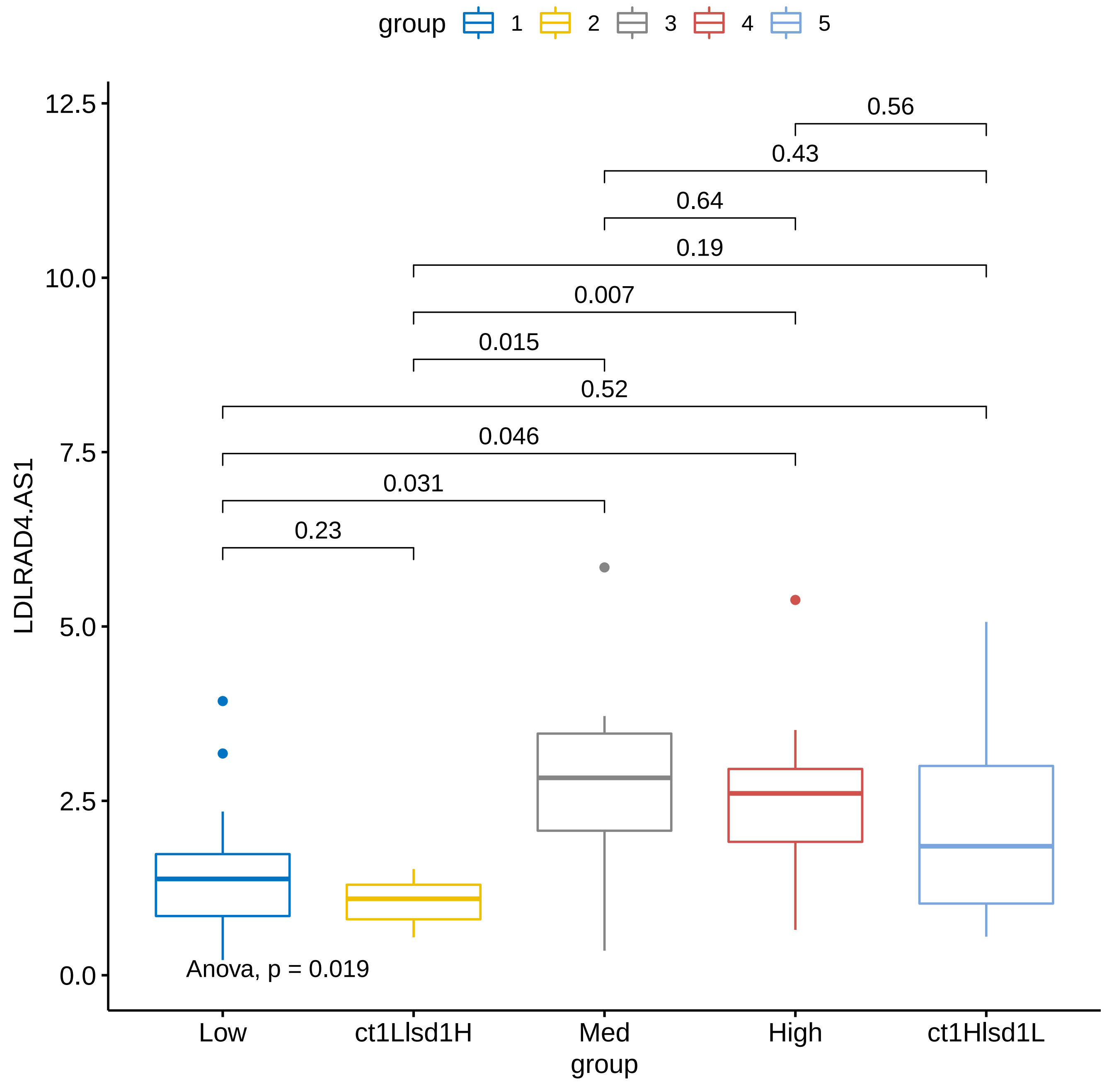
group 1 2 3 4 5
0.56
0.43
0.64
0.19
0.007
0.015
0.52
0.046
0.031
0.23
Anova, p = 0.019
12.5
10.0
7.5
5.0
2.5
0.0
LDLRAD4.AS1
Low
ct1Llsd1H
Med
High
ct1Hlsd1L
group

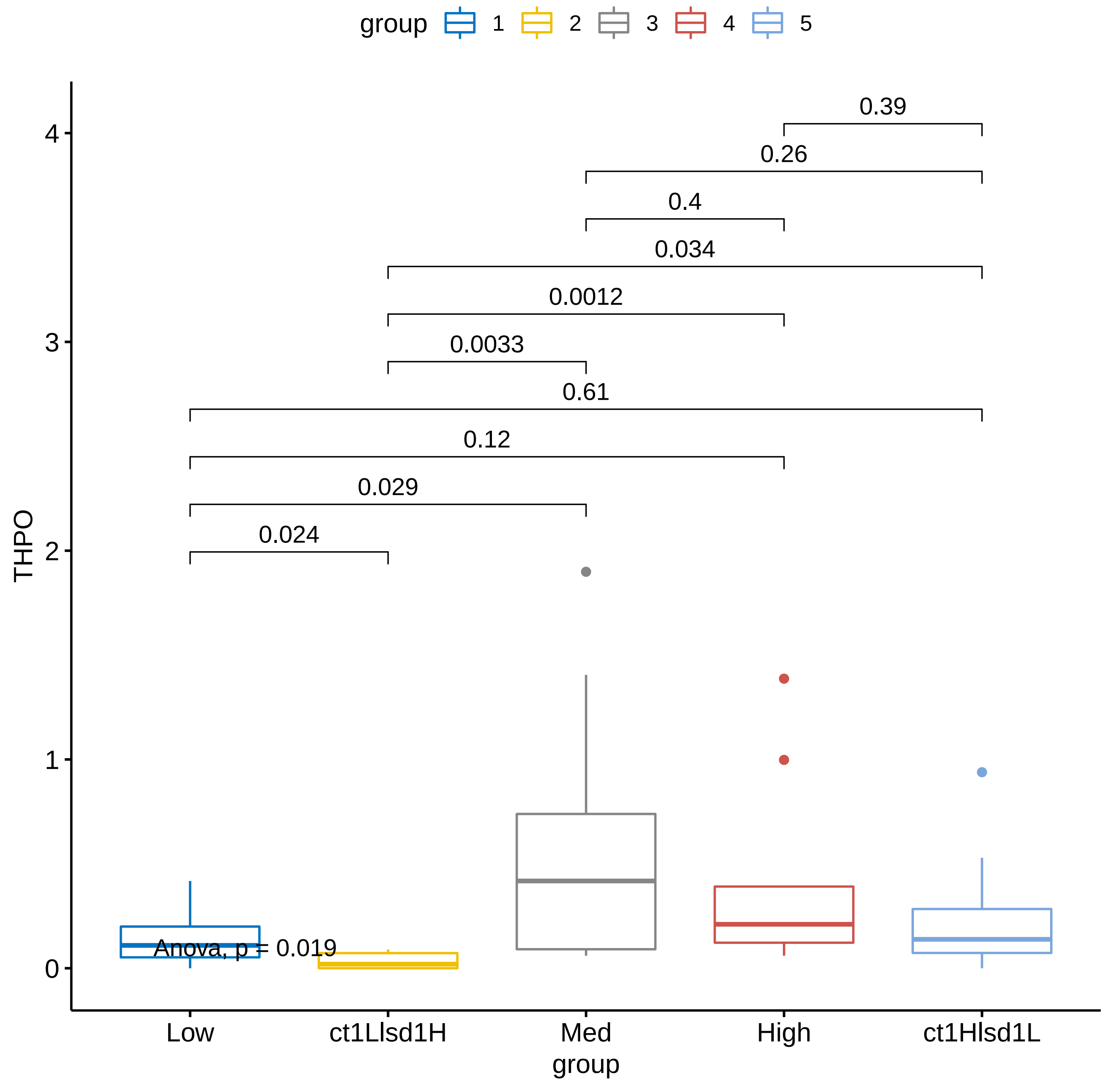

group
1
2
3
4
5
0.39
0.26
0.4
0.034
0.0012
0.0033
0.61
0.12
0.029
0.024
Anova, p = 0.019
THPO
4
3
2
1
0
Low
ct1Llsd1H
Med
High
ct1Hlsd1L
group

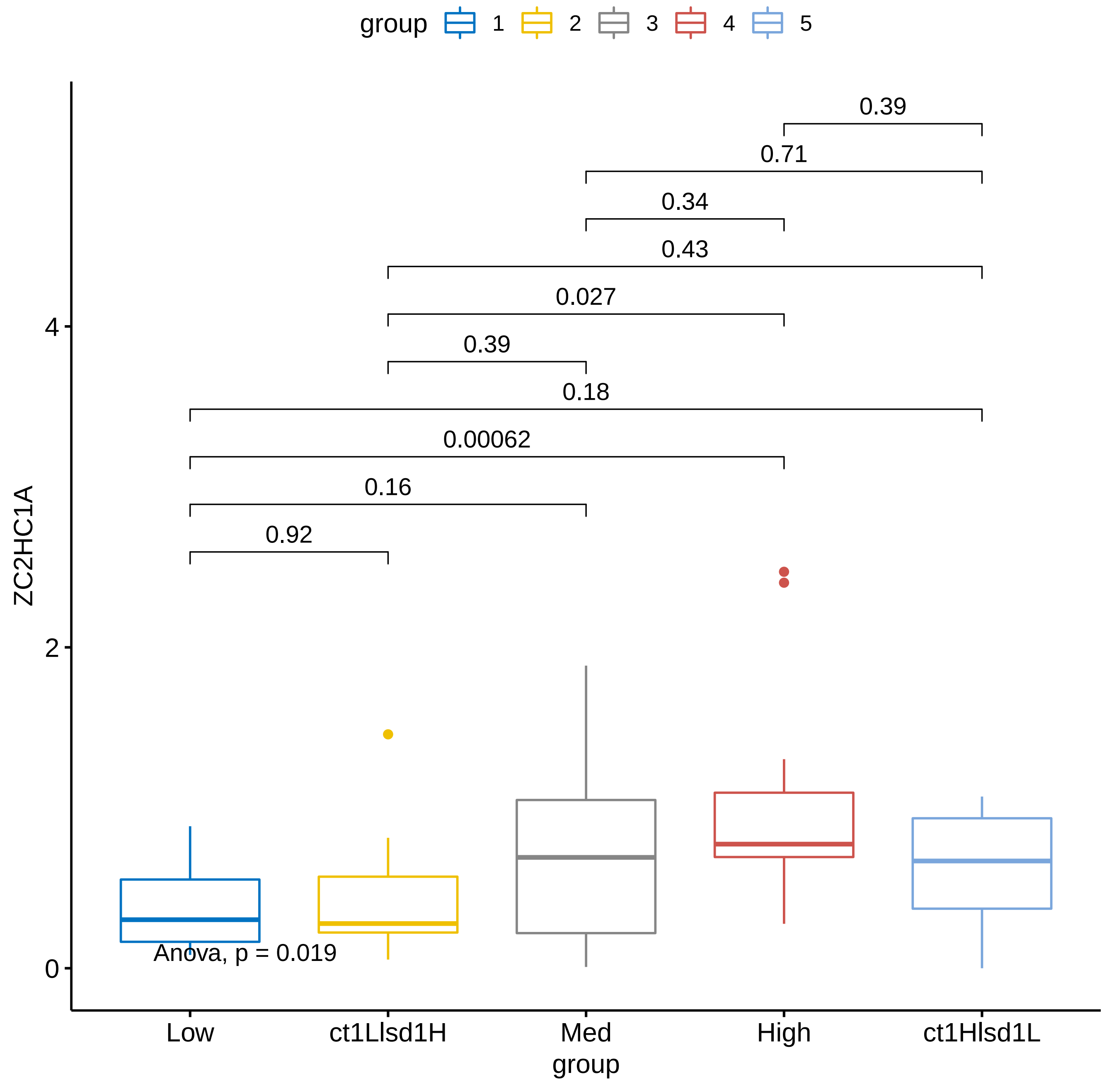
group
1
2
3
4
5
0.39
0.71
0.34
0.43
0.027
0.39
0.18
0.00062
0.16
0.92
Anova, p = 0.019
ZC2HC1A
4
2
0
Low
ct1Llsd1H
Med
High
ct1Hlsd1L
group

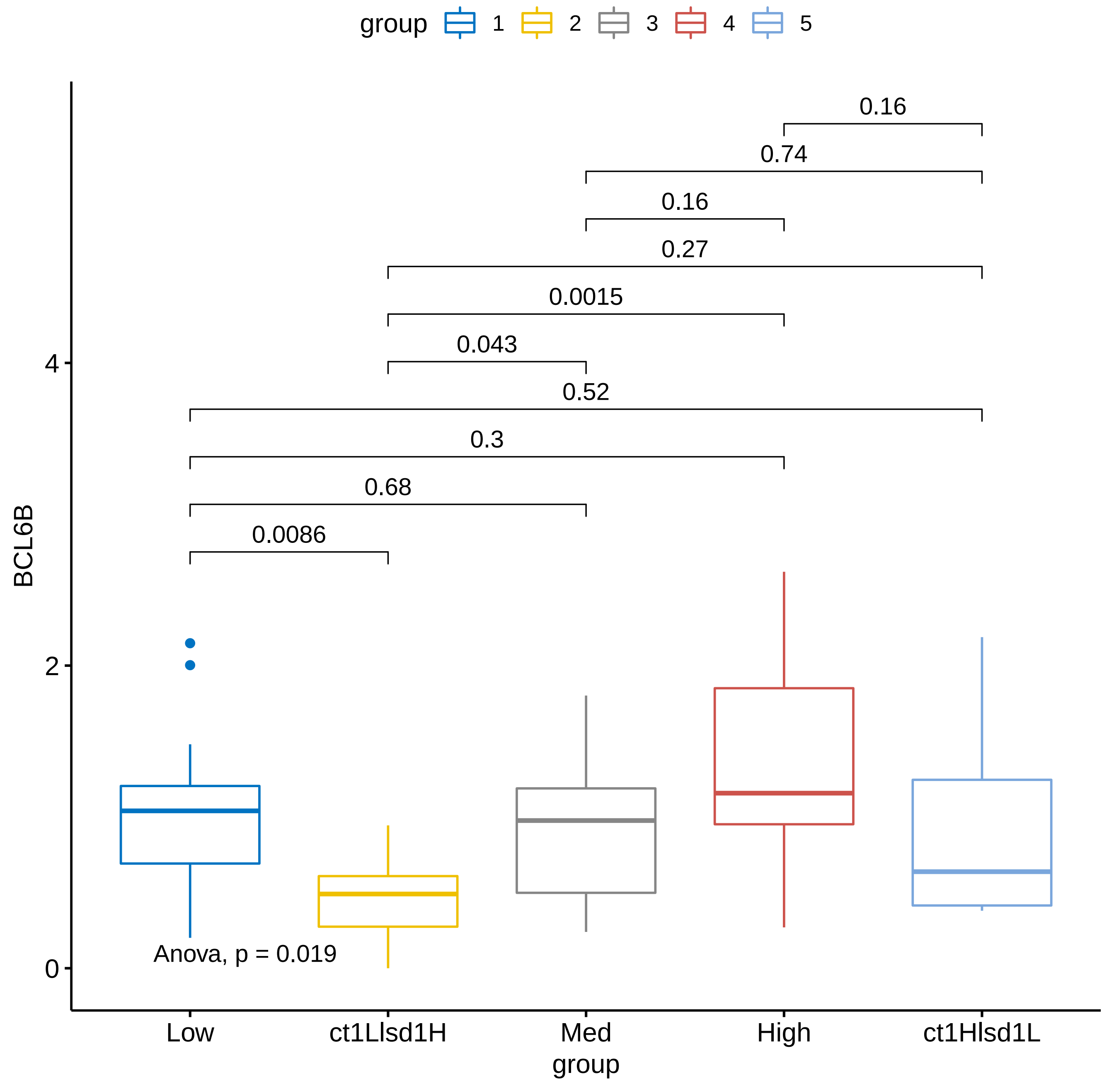
group
1
2
3
4
5
0.16
0.74
0.16
0.27
0.0015
0.043
0.52
0.3
0.68
0.0086
Anova, p = 0.019
BCL6B
4
2
0
Low
ct1Llsd1H
Med
High
ct1Hlsd1L
group

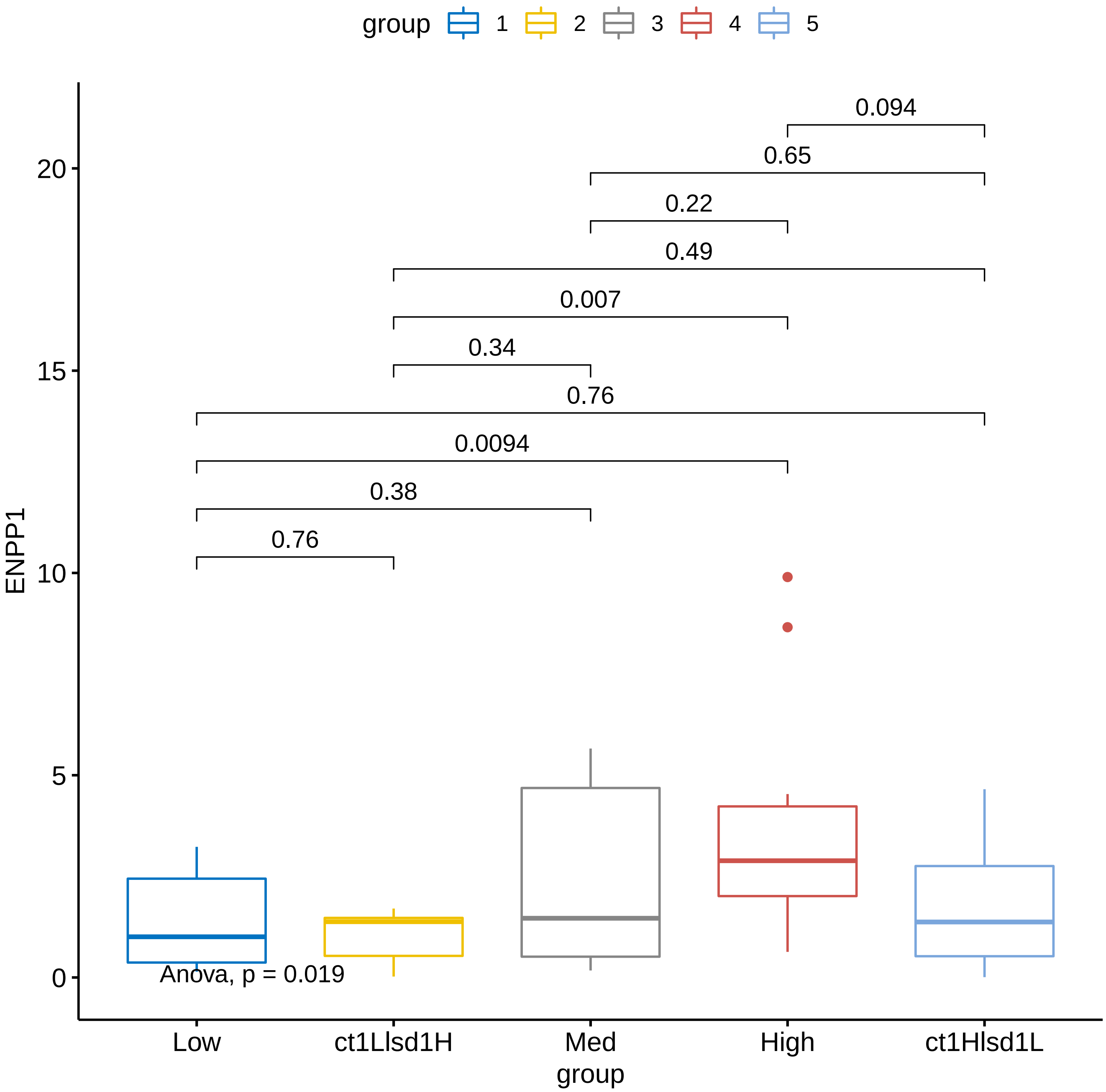

group
1
2
3
4
5
0.094
0.65
0.22
0.49
0.007
0.34
0.76
0.0094
0.38
0.76
Anova, p = 0.019
ENPP1
20
15
10
5
0
Low
ct1Llsd1H
Med
High
ct1Hlsd1L
group

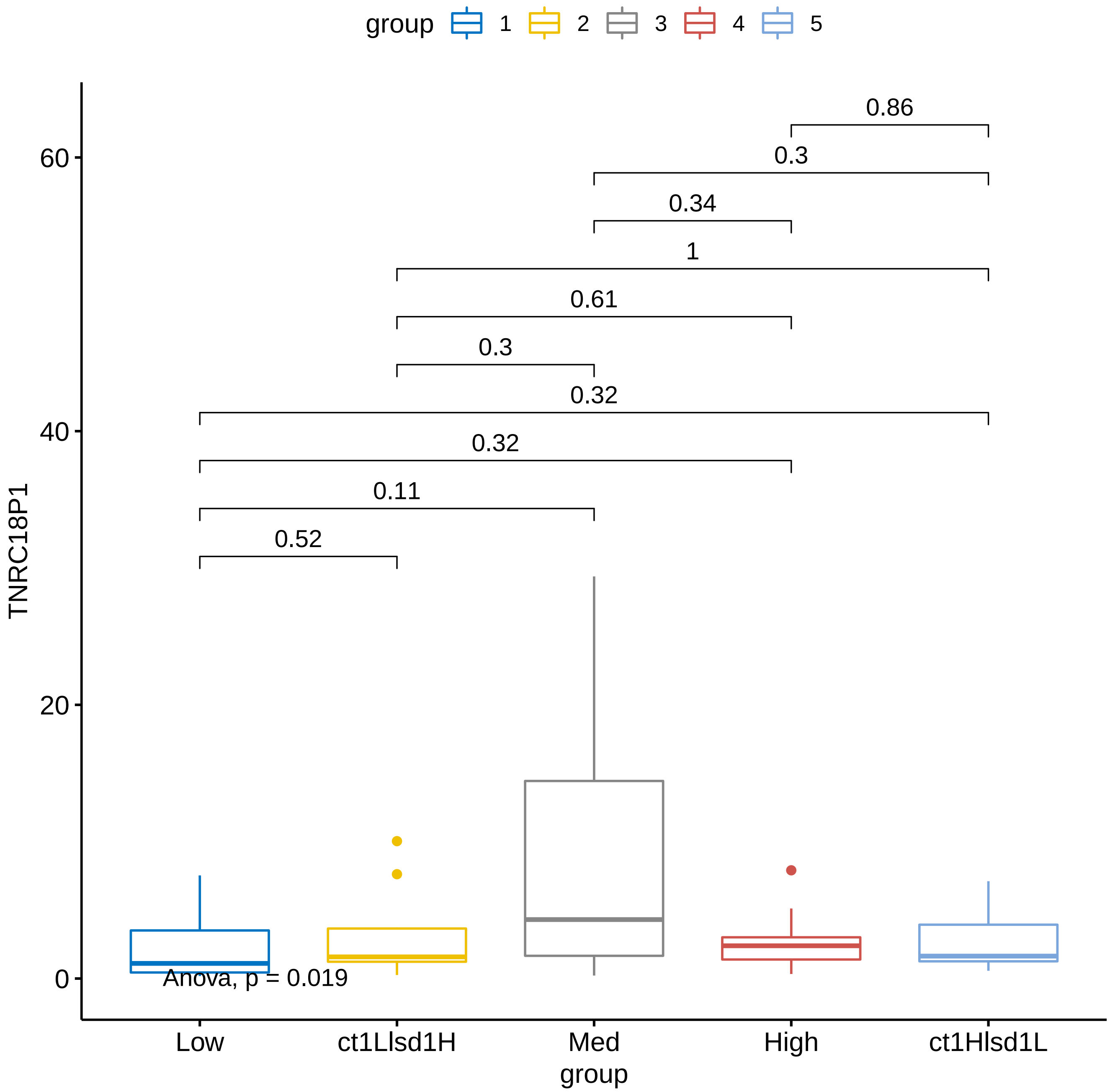

group 1 2 3 4 5
0.86
0.3
0.34
1
0.61
0.3
0.32
0.32
0.11
0.52
60
40
20
0
TNRC18P1
Anova, p = 0.019
Low
ct1Llsd1H
Med
High
ct1Hlsd1L
group

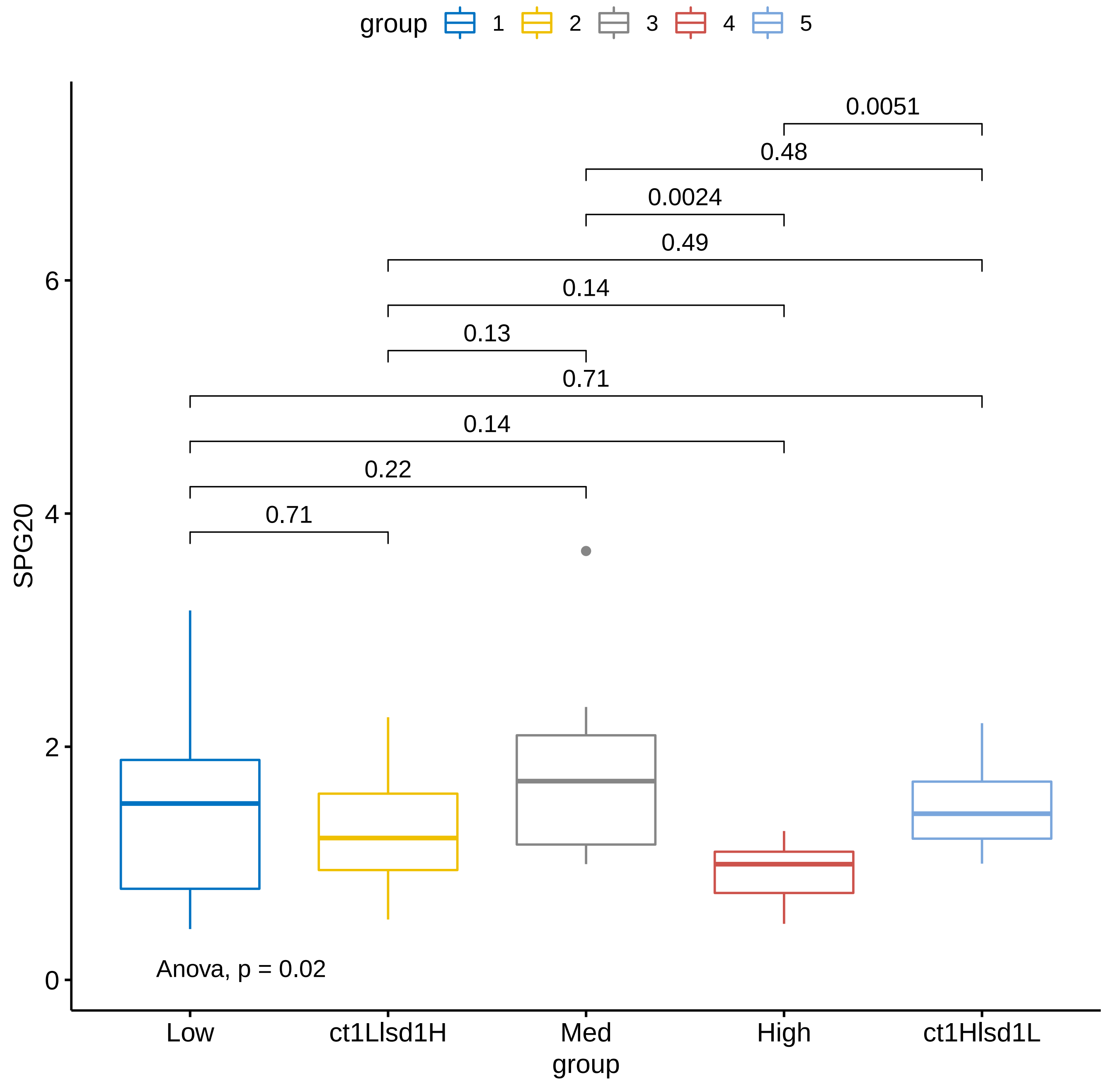

group 1 2 3 4 5
0.0051
0.48
0.0024
0.49
0.14
0.13
0.71
0.14
0.22
0.71
Anova, p = 0.02
SPG20
0
2
4
6
Low
ct1Llsd1H
Med
High
ct1Hlsd1L
group

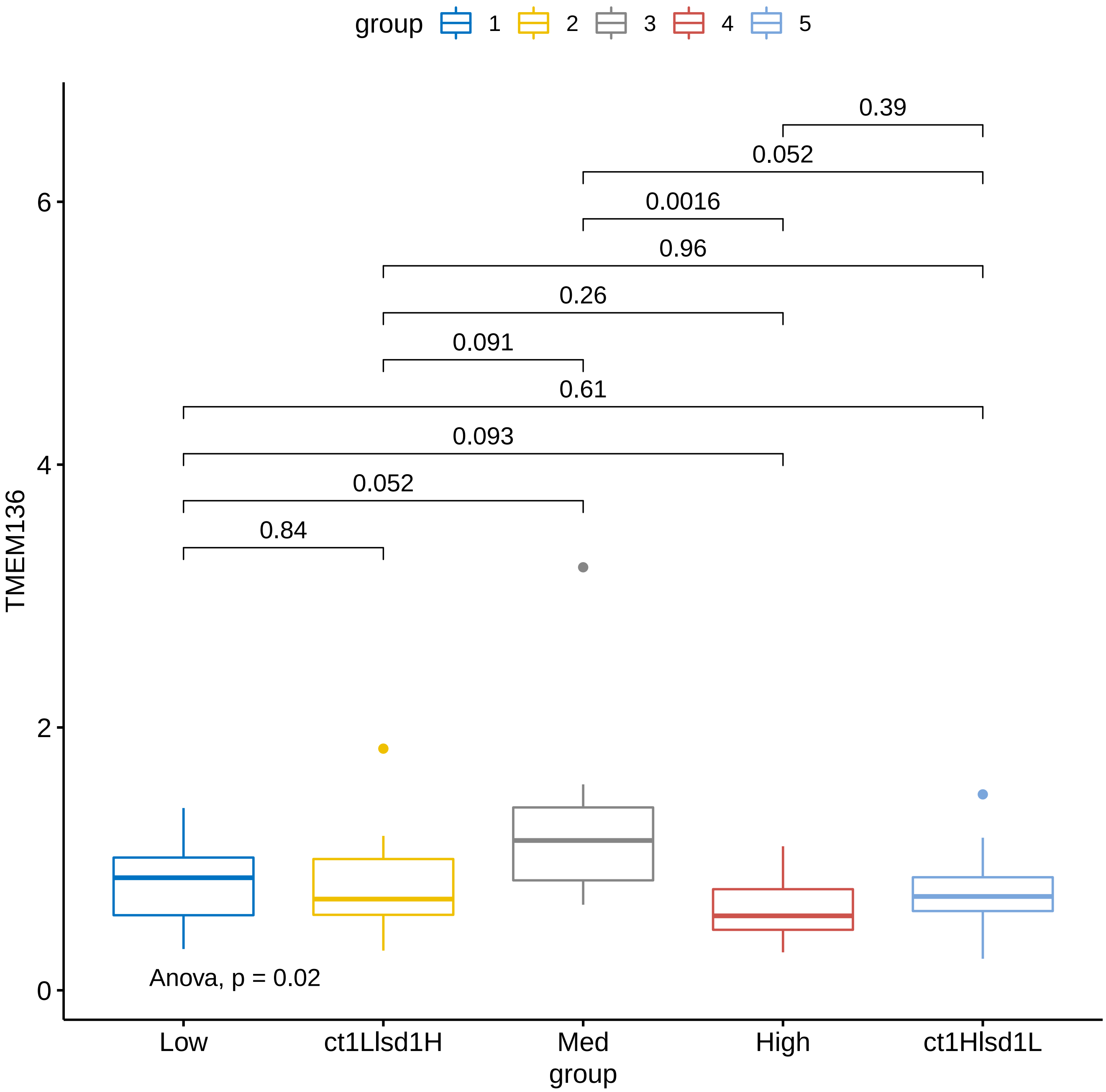

group 1 2 3 4 5
0.39
0.052
0.0016
0.96
0.26
0.091
0.61
0.093
0.052
0.84
Anova, p = 0.02
TMEM136
0
2
4
6
Low
ct1Llsd1H
Med
High
ct1Hlsd1L
group

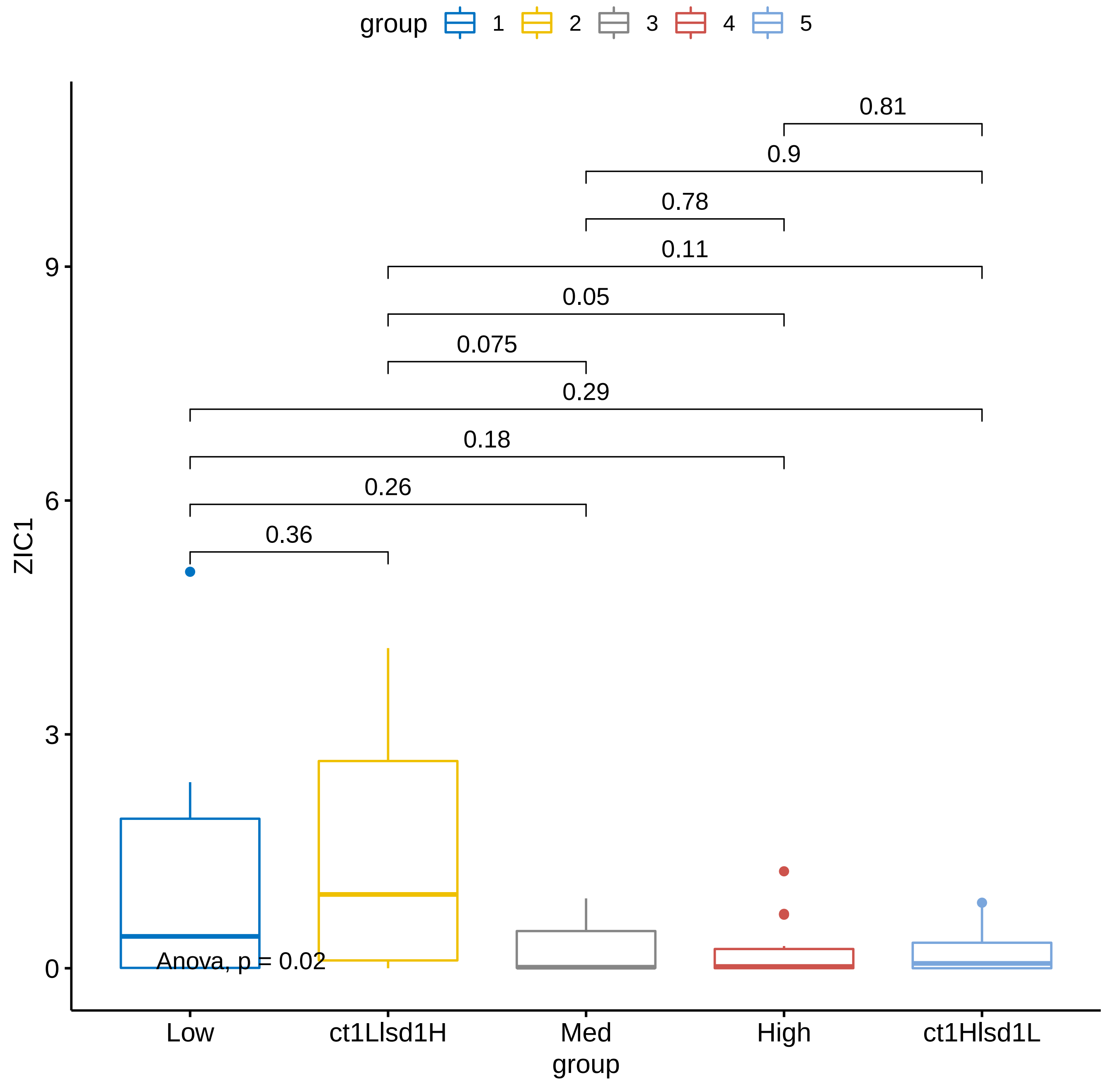
group 1 2 3 4 5
0.81
0.9
0.78
0.11
0.05
0.075
0.29
0.18
0.26
0.36
ZIC1
9
6
3
0
Anova, p = 0.02
Low
ct1Llsd1H
Med
High
ct1Hlsd1L
group

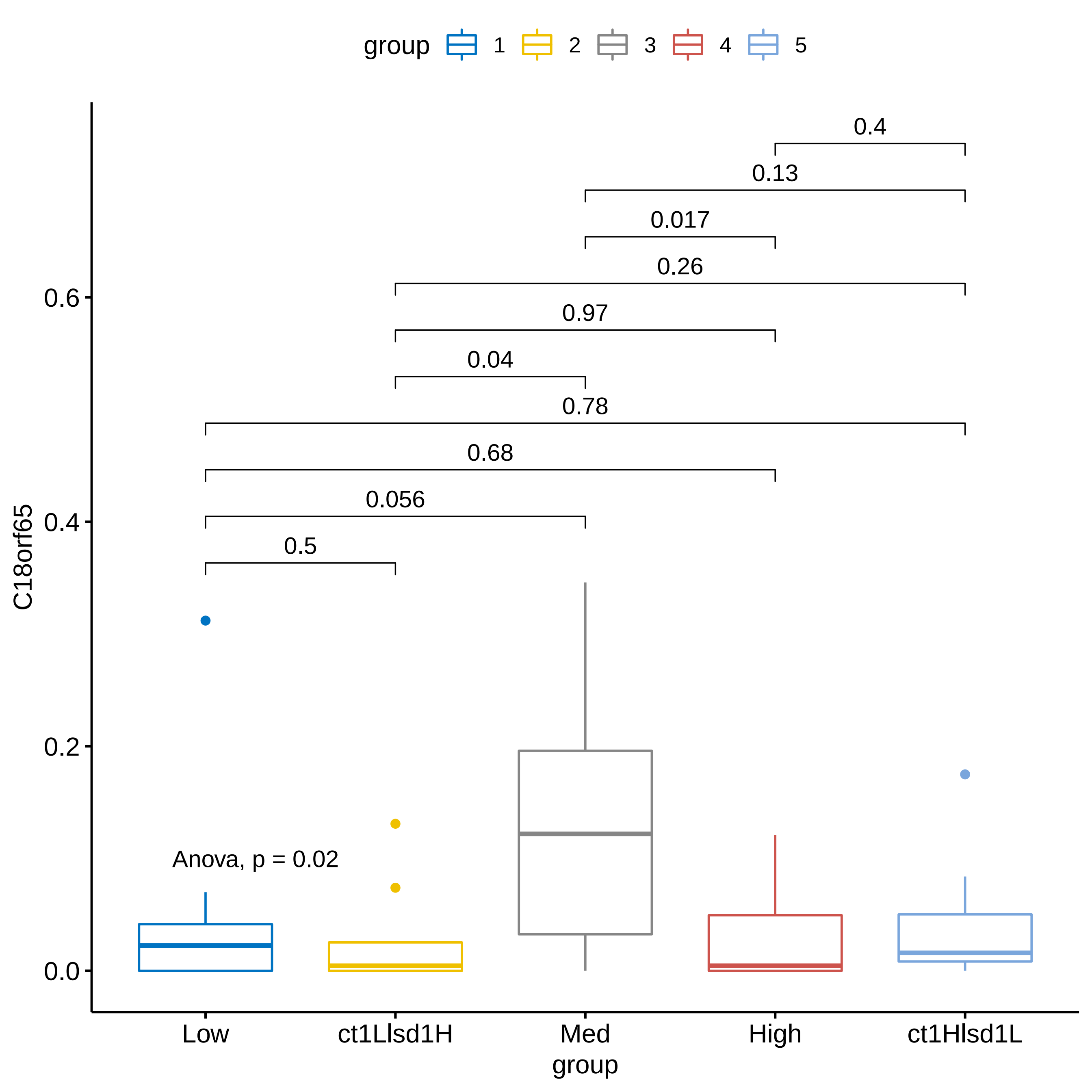
group
1
2
3
4
5
C18orf65
0.0
0.2
0.4
0.6
0.4
0.13
0.017
0.26
0.97
0.04
0.78
0.68
0.056
0.5
Anova, p = 0.02
Low
ct1Llsd1H
Med
High
ct1Hlsd1L
group

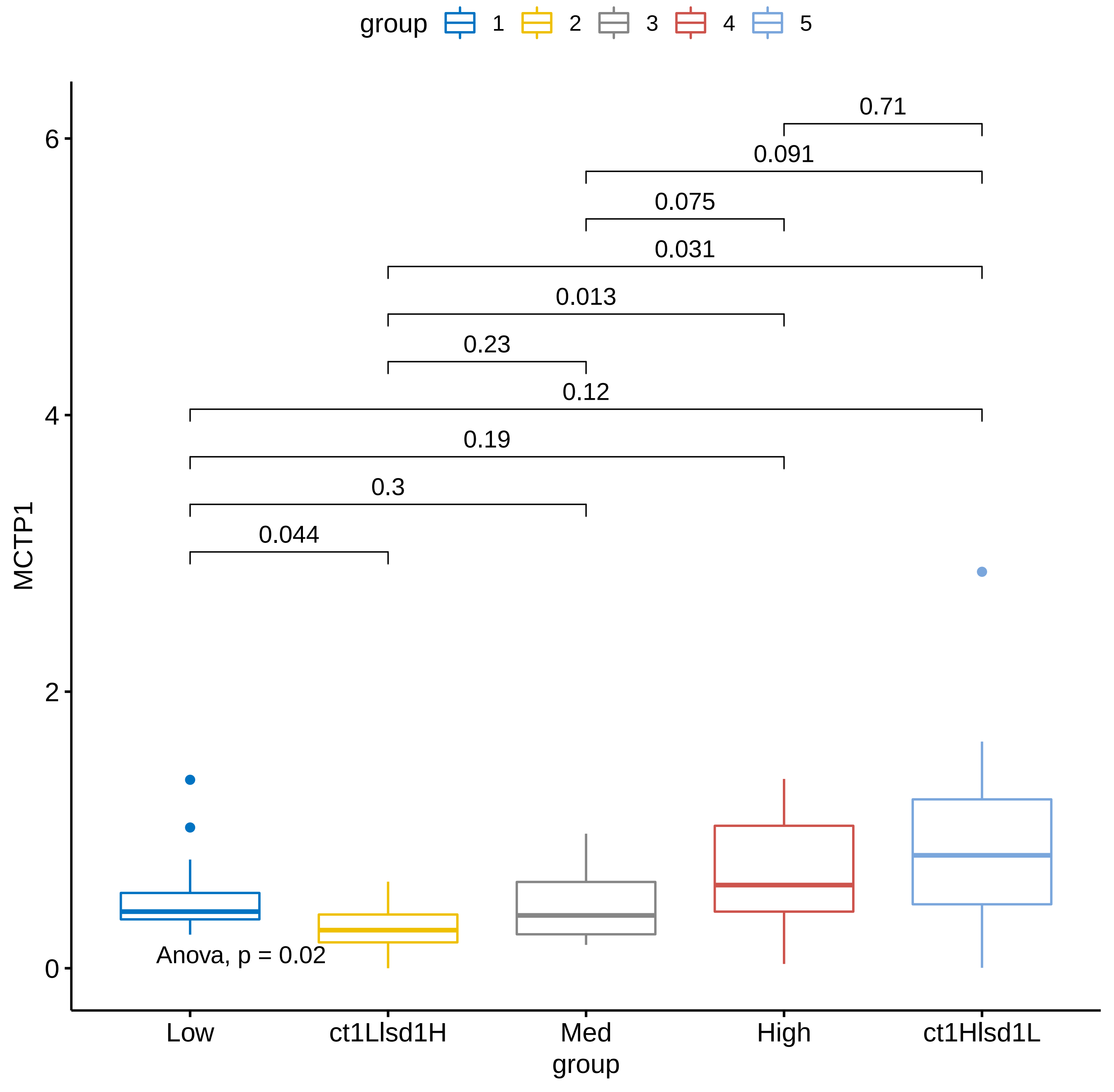

group
1
2
3
4
5
0.71
0.091
0.075
0.031
0.013
0.23
0.12
0.19
0.3
0.044
Anova, p = 0.02
MCTP1
6
4
2
0
Low
ct1Llsd1H
Med
High
ct1Hlsd1L
group

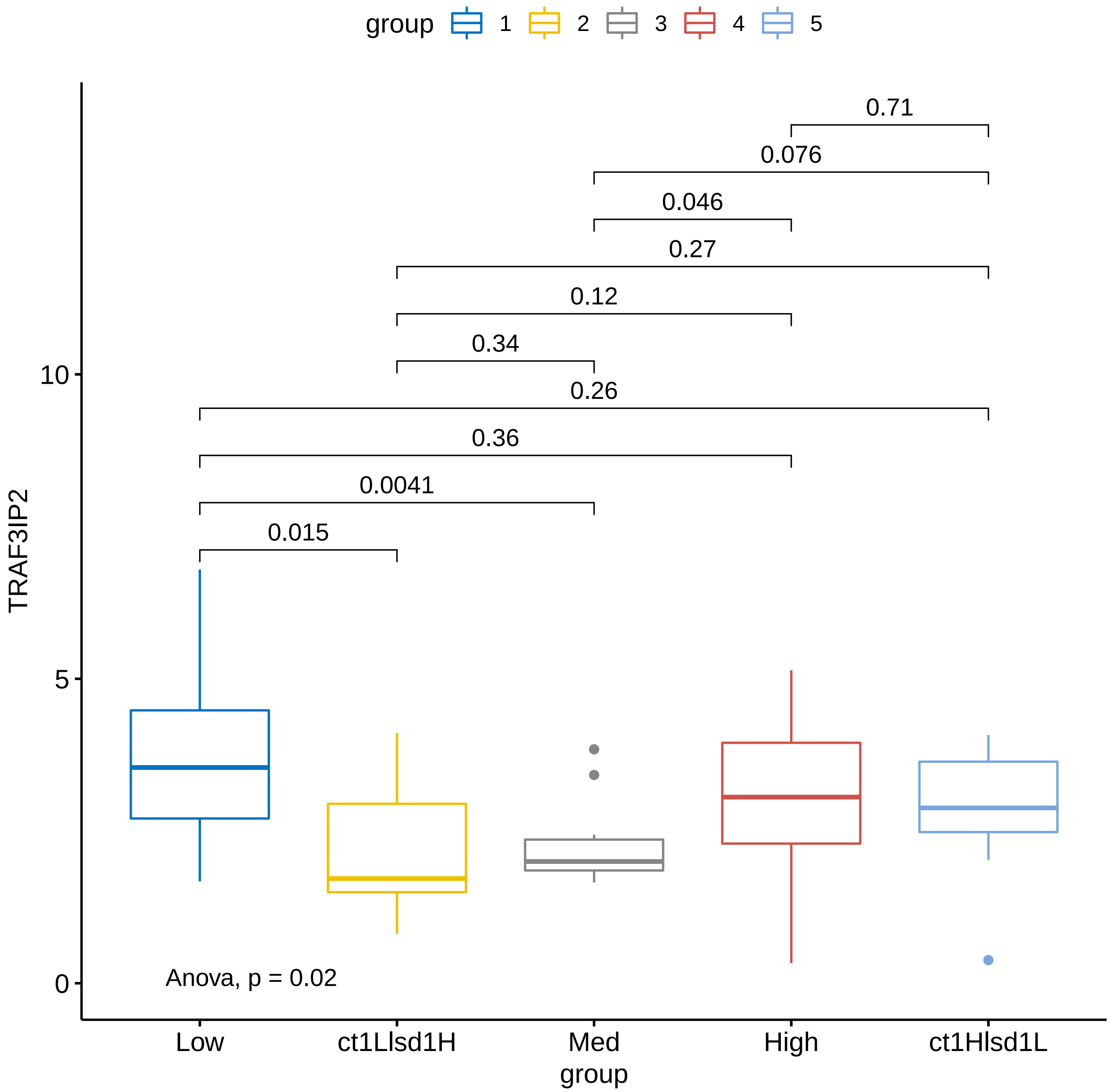

group
1
2
3
4
5
TRAF3IP2
0.71
0.076
0.046
0.27
0.12
0.34
0.26
0.36
0.0041
0.015
10
5
0
Anova, p = 0.02
Low
ct1Llsd1H
Med
High
ct1Hlsd1L
group

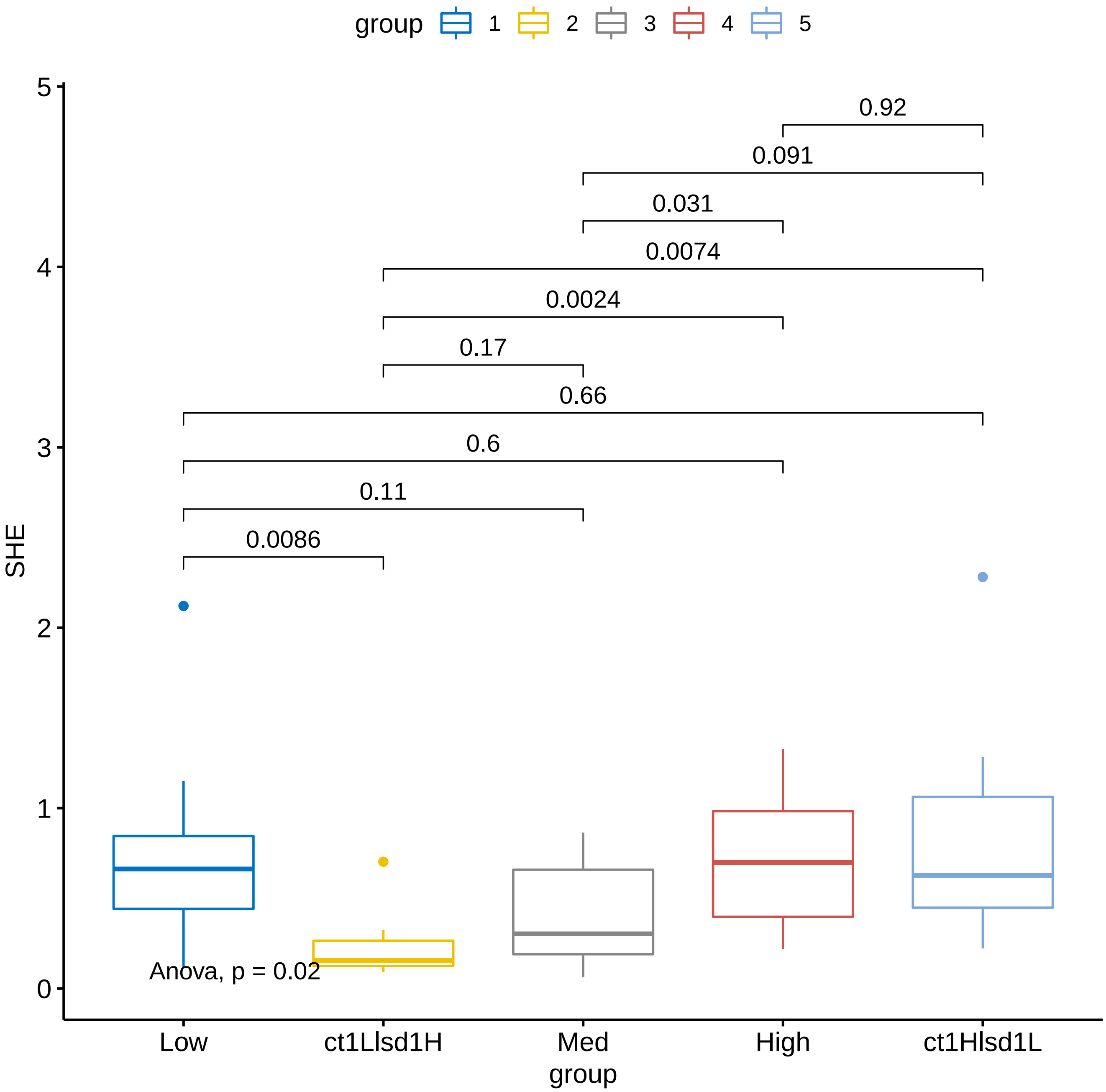
group
1
2
3
4
5
SHE
0.92
0.091
0.031
0.0074
0.0024
0.17
0.66
0.6
0.11
0.0086
Anova, p = 0.02
5
4
3
2
1
0
Low
ct1Llsd1H
Med
High
ct1Hlsd1L
group

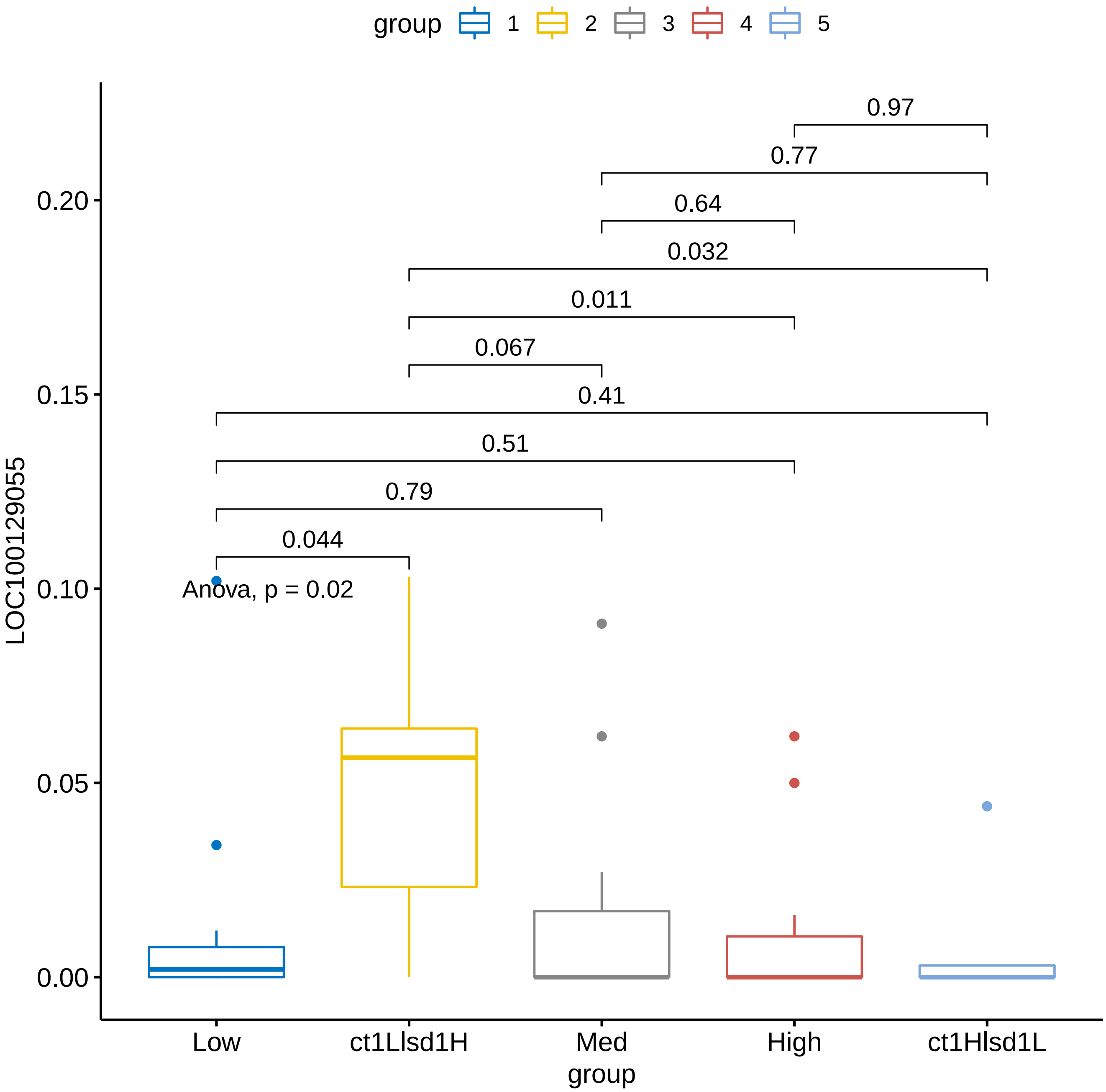

group
1
2
3
4
5
0.97
0.77
0.64
0.032
0.011
0.067
0.41
0.51
0.79
0.044
Anova, p = 0.02
0.20
0.15
0.10
0.05
0.00
LOC100129055
Low
ct1Llsd1H
Med
High
ct1Hlsd1L
group

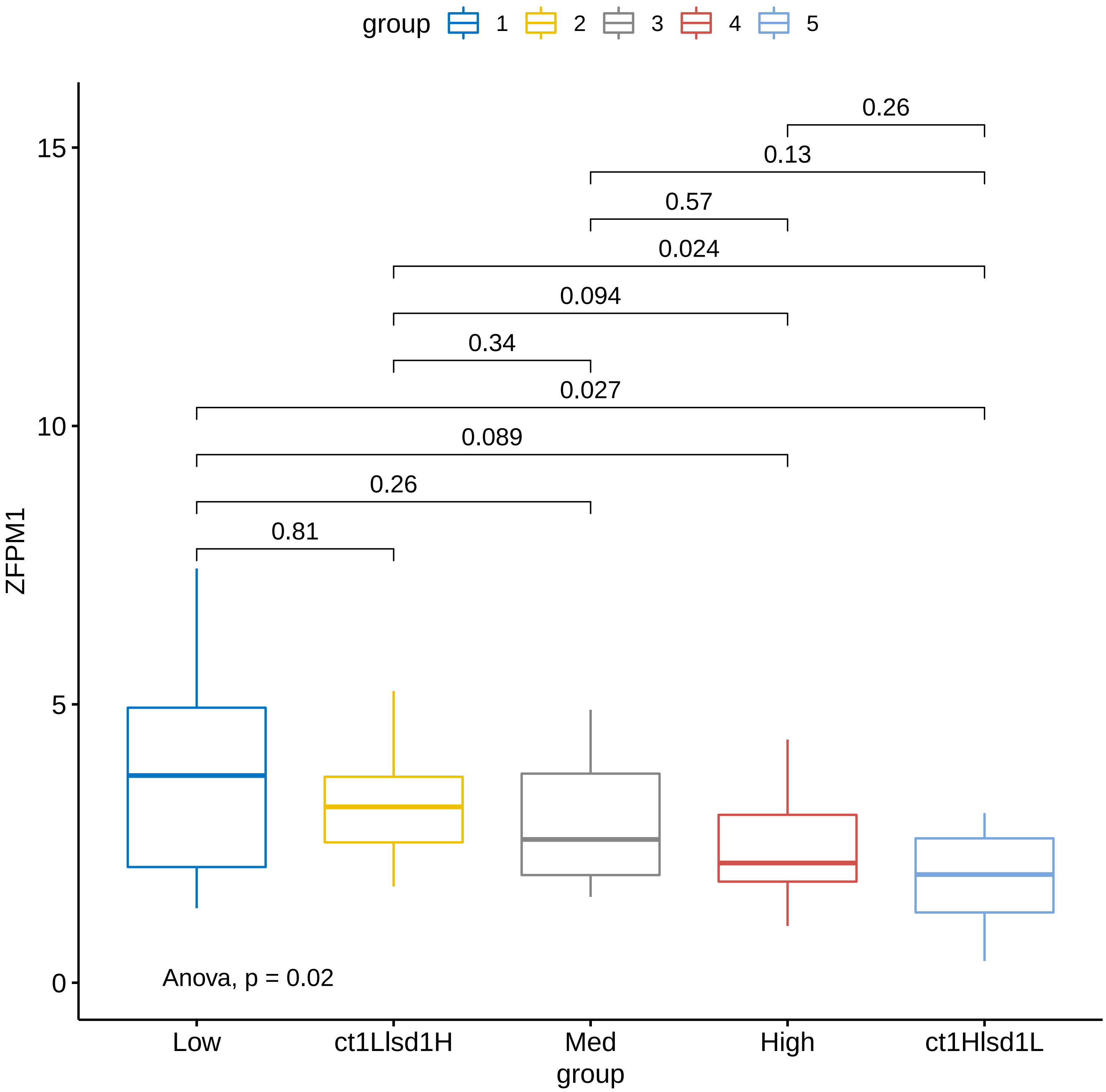
group
1
2
3
4
5
0.26
0.13
0.57
0.024
0.094
0.34
0.027
0.089
0.26
0.81
15
10
5
0
ZFPM1
Anova, p = 0.02
Low
ct1Llsd1H
Med
High
ct1Hlsd1L
group

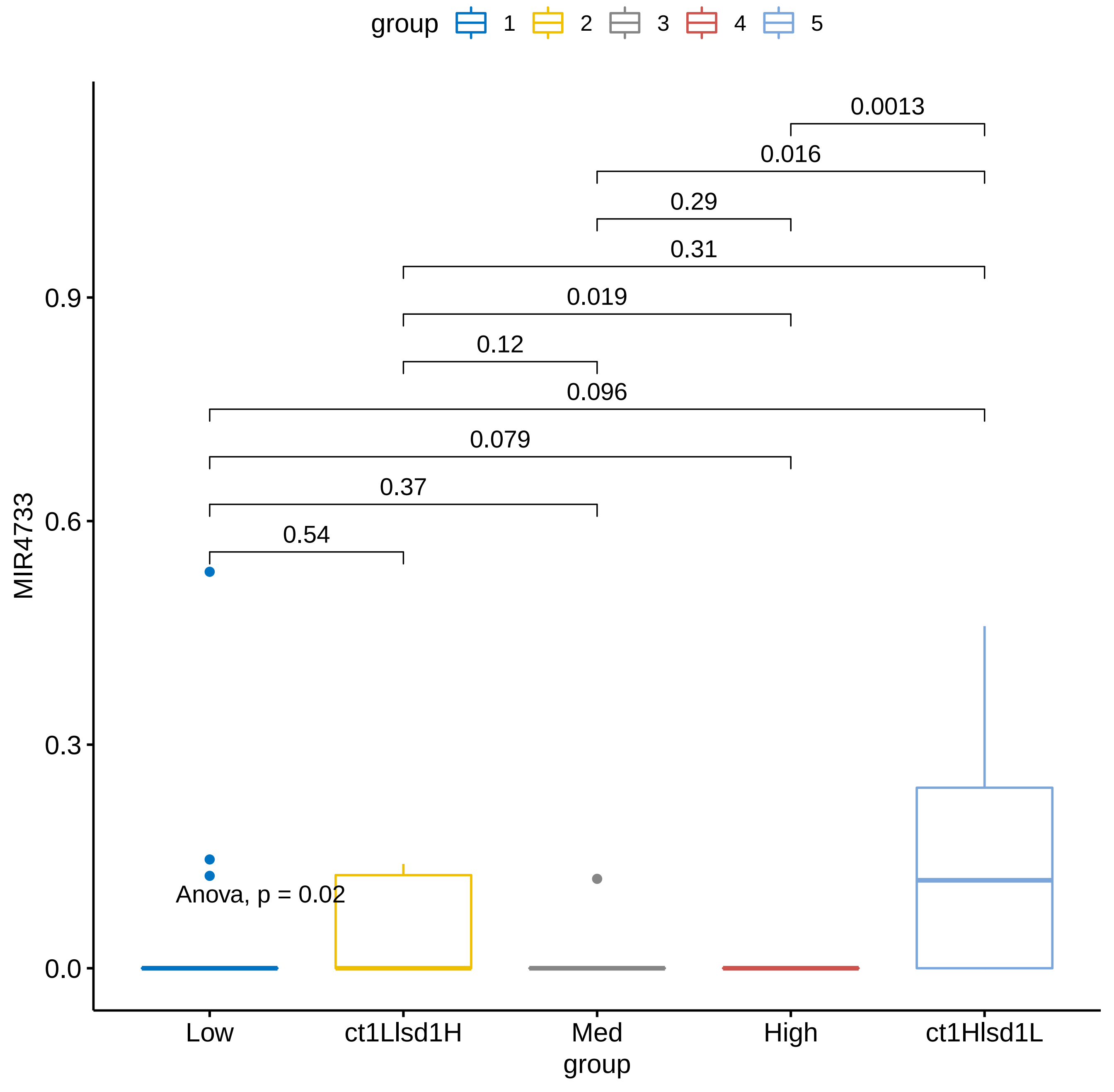

group 1 2 3 4 5
0.0013
0.016
0.29
0.31
0.019
0.12
0.096
0.079
0.37
0.54
Anova, p = 0.02
MIR4733
0.0
0.3
0.6
0.9
Low
ct1Llsd1H
Med
High
ct1Hlsd1L
group

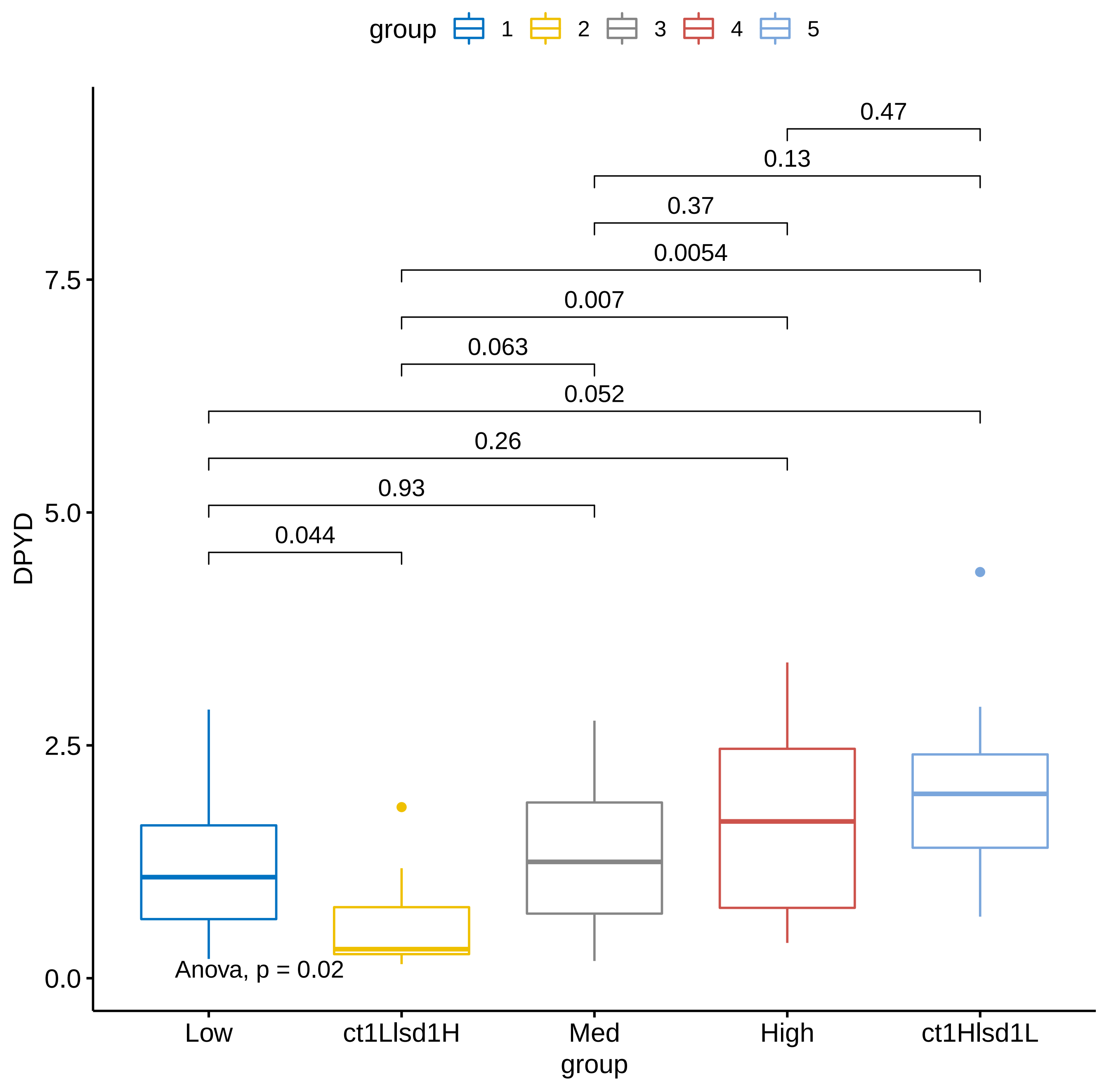
group
1
2
3
4
5
DPYD
7.5
5.0
2.5
0.0
0.47
0.13
0.37
0.0054
0.007
0.063
0.052
0.26
0.93
0.044
Anova, p = 0.02
Low
ct1Llsd1H
Med
High
ct1Hlsd1L
group

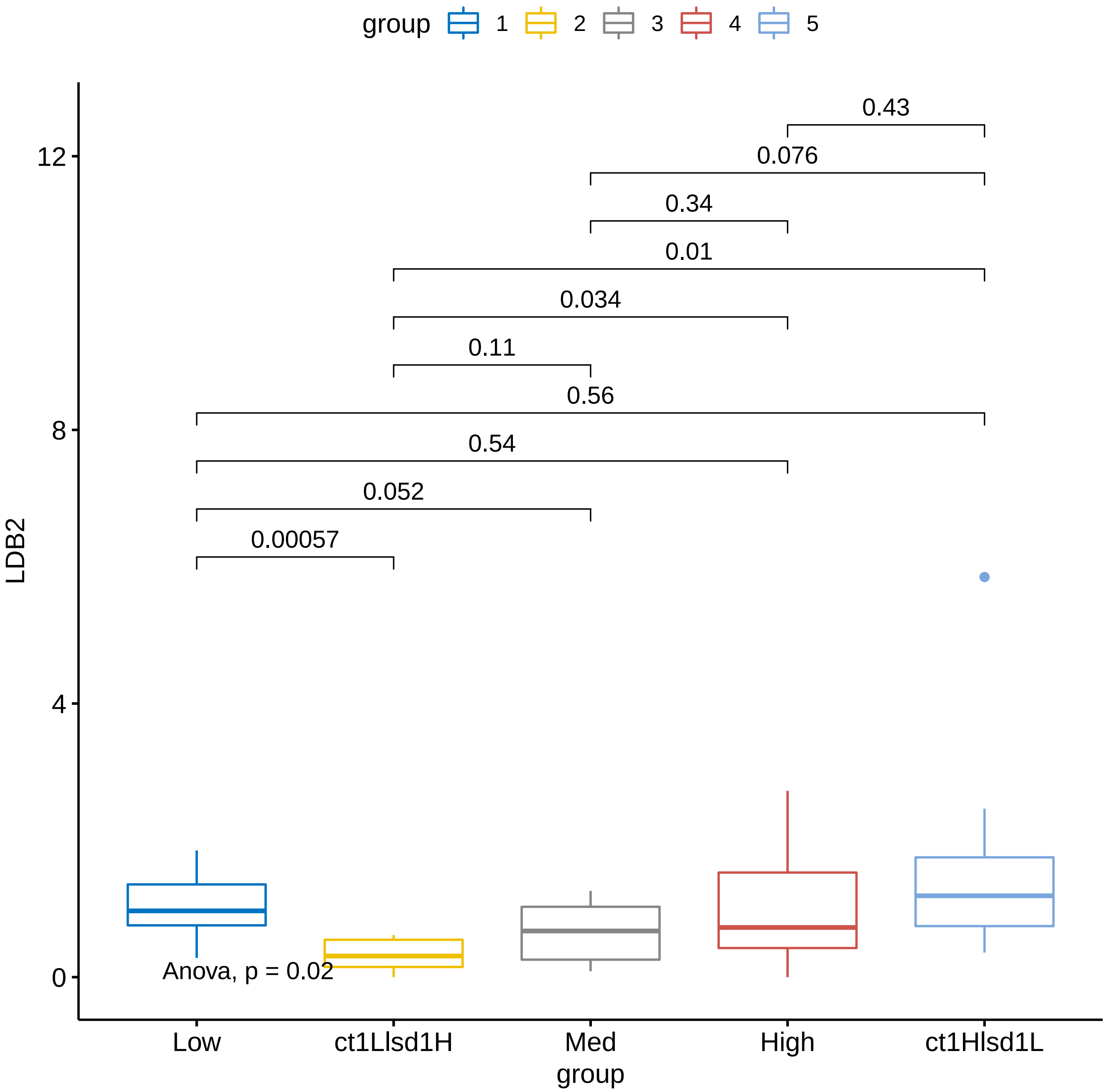

group 1 2 3 4 5
0.43
0.076
0.34
0.01
0.034
0.11
0.56
0.54
0.052
0.00057
12
8
4
0
LDB2
Anova, p = 0.02
Low
ct1Llsd1H
Med
High
ct1Hlsd1L
group

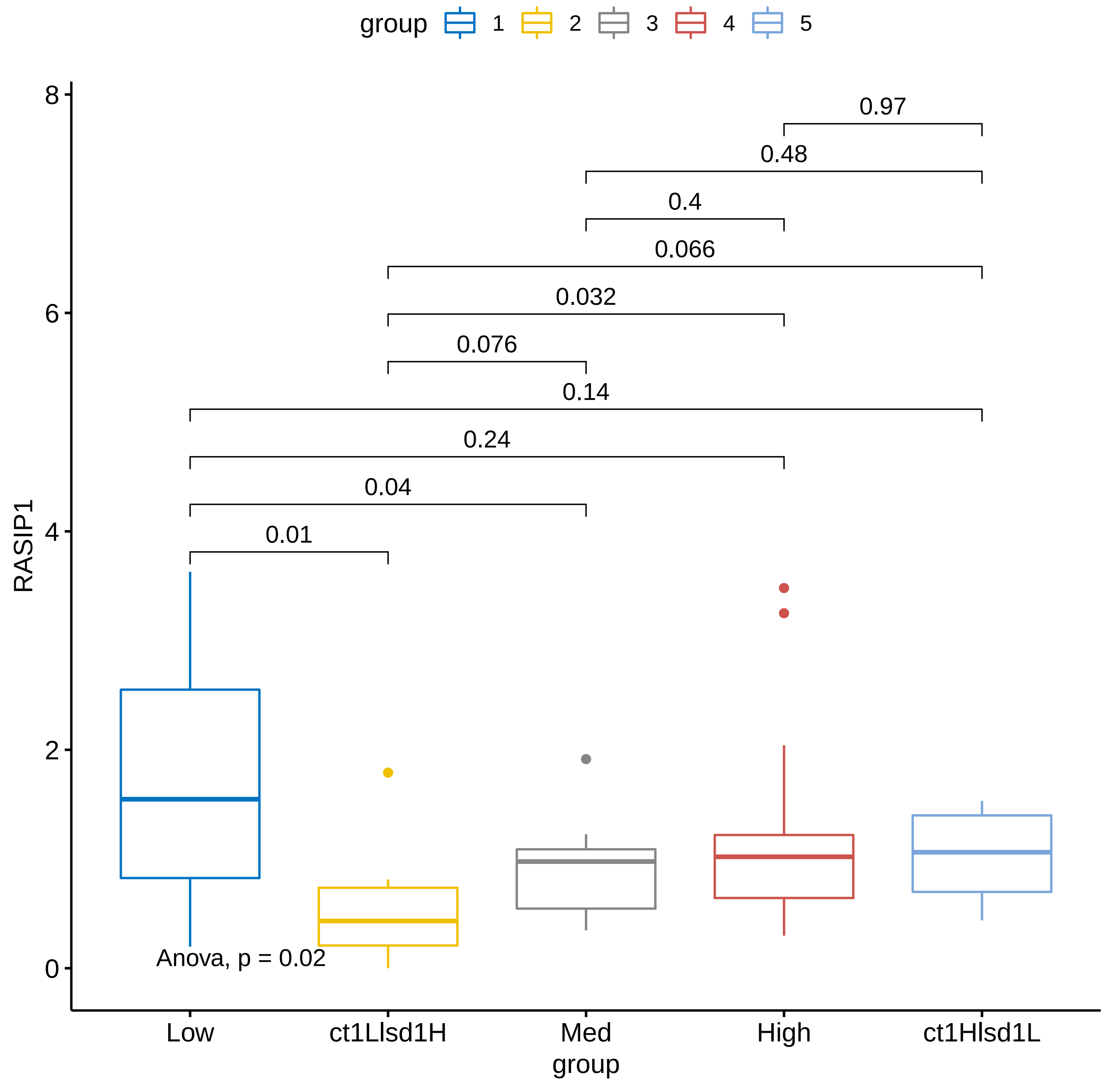

group 1 2 3 4 5
RASIP1
8
6
4
2
0
0.97
0.48
0.4
0.066
0.032
0.076
0.14
0.24
0.04
0.01
Anova, p = 0.02
Low
ct1Llsd1H
Med
High
ct1Hlsd1L
group

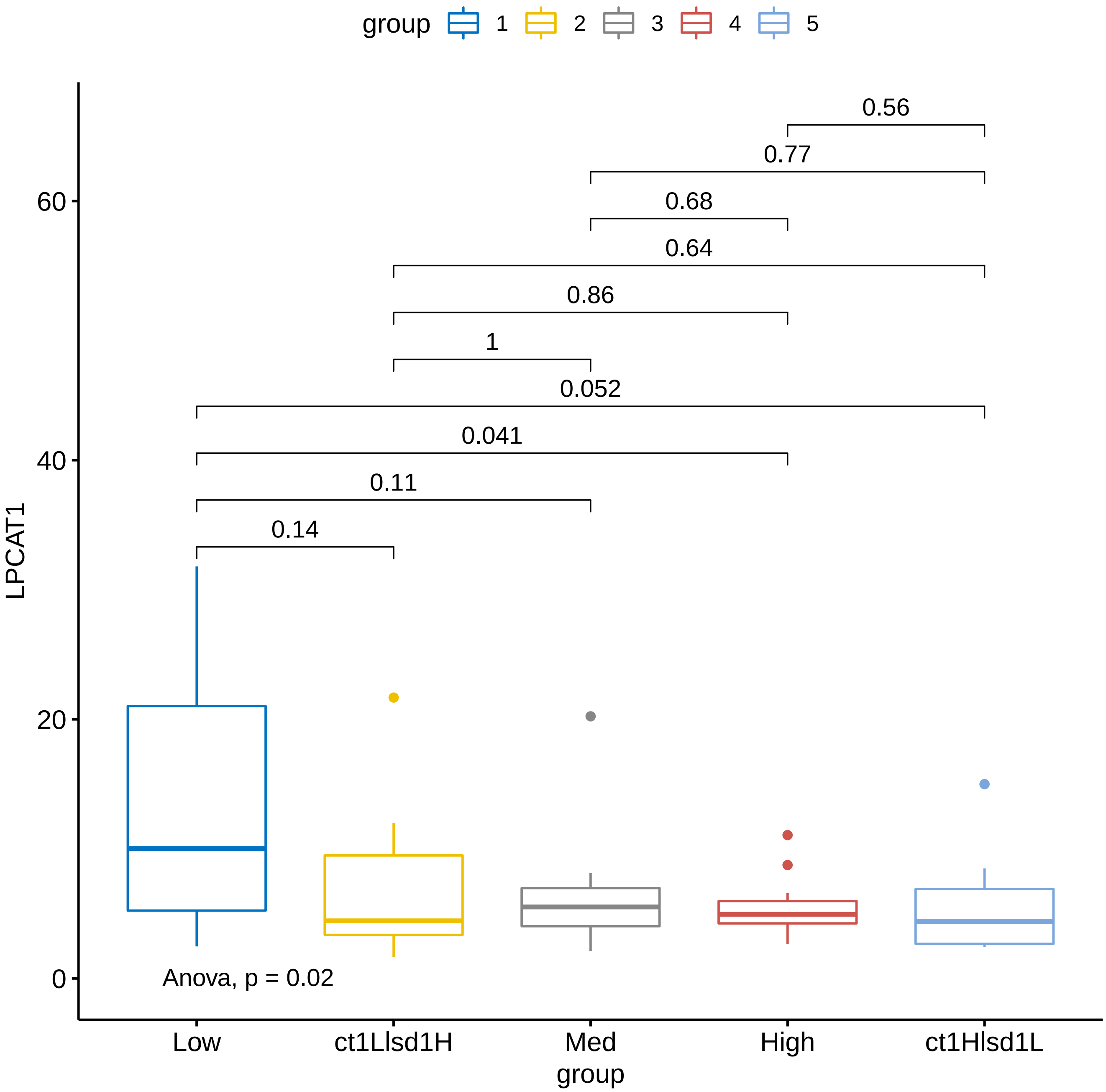
group
1
2
3
4
5
0.56
0.77
0.68
0.64
0.86
1
0.052
0.041
0.11
0.14
60
40
20
0
LPCAT1
Anova, p = 0.02
Low
ct1Llsd1H
Med
High
ct1Hlsd1L
group

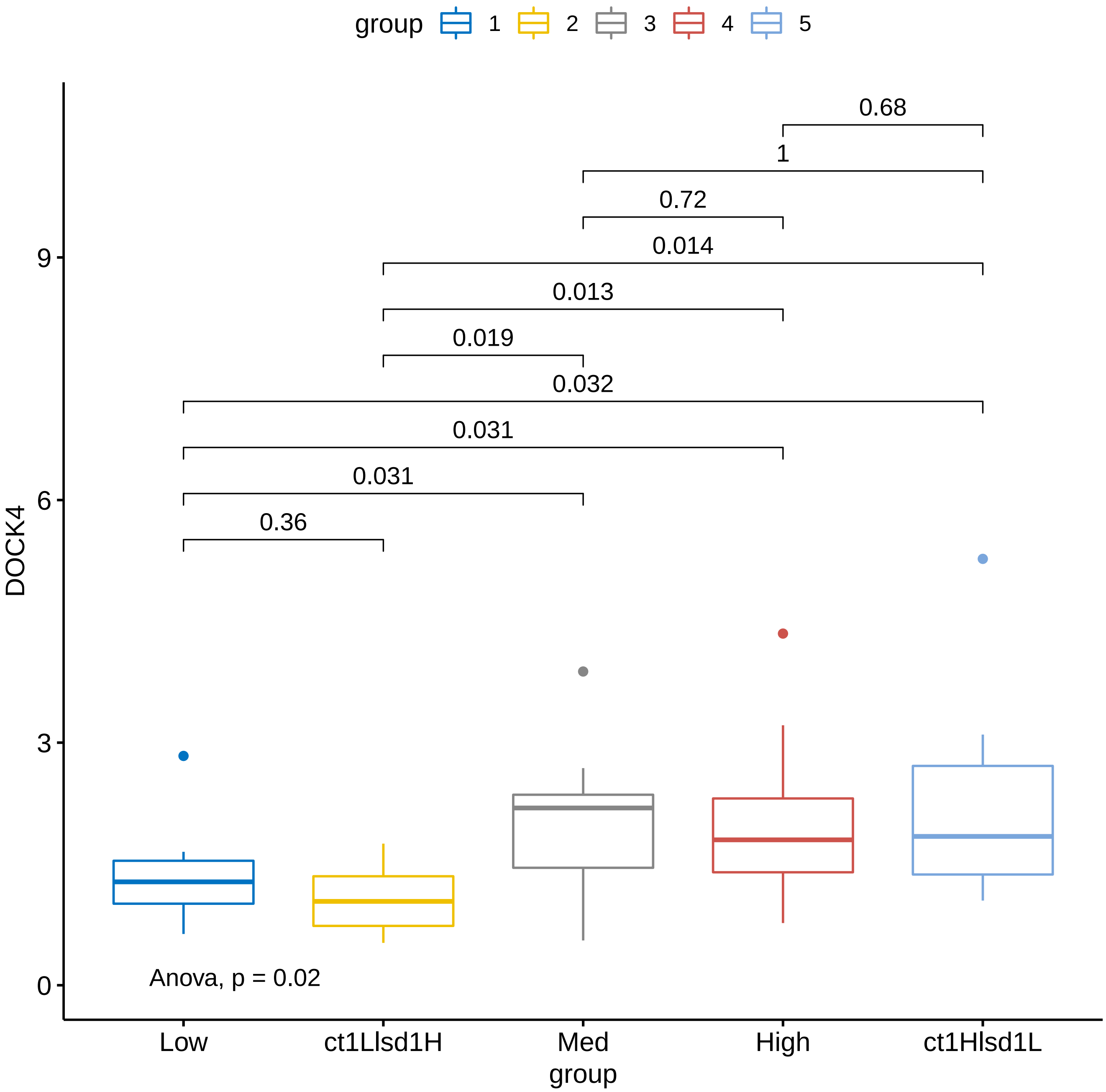

group
1
2
3
4
5
0.68
1
0.72
0.014
0.013
0.019
0.032
0.031
0.031
0.36
Anova, p = 0.02
DOCK4
9
6
3
0
Low
ct1Llsd1H
Med
High
ct1Hlsd1L
group

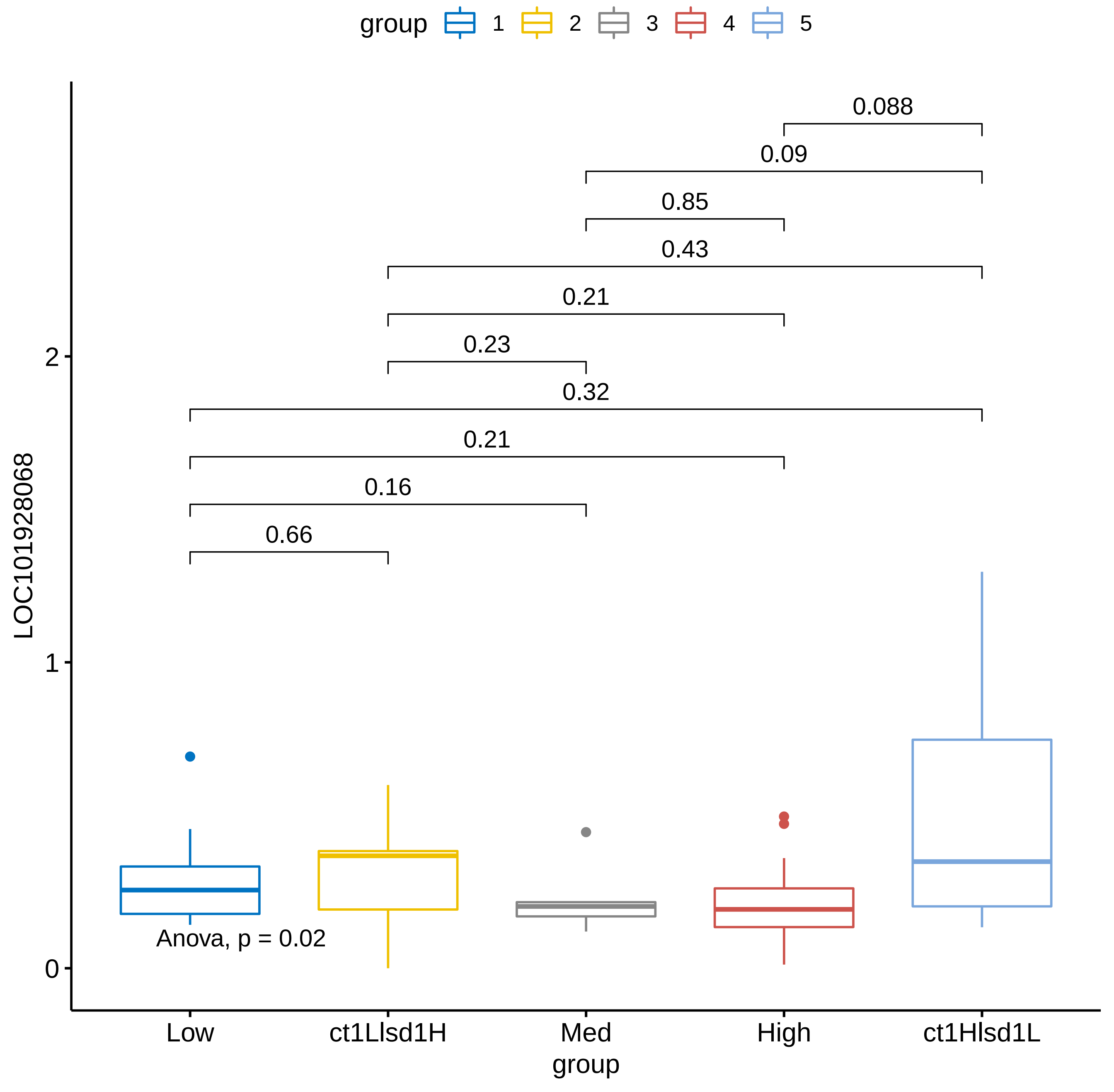
group
1
2
3
4
5
0.088
0.09
0.85
0.43
0.21
0.23
0.32
0.21
0.16
0.66
Anova, p = 0.02
LOC101928068
0
1
2
Low
ct1Llsd1H
Med
High
ct1Hlsd1L
group

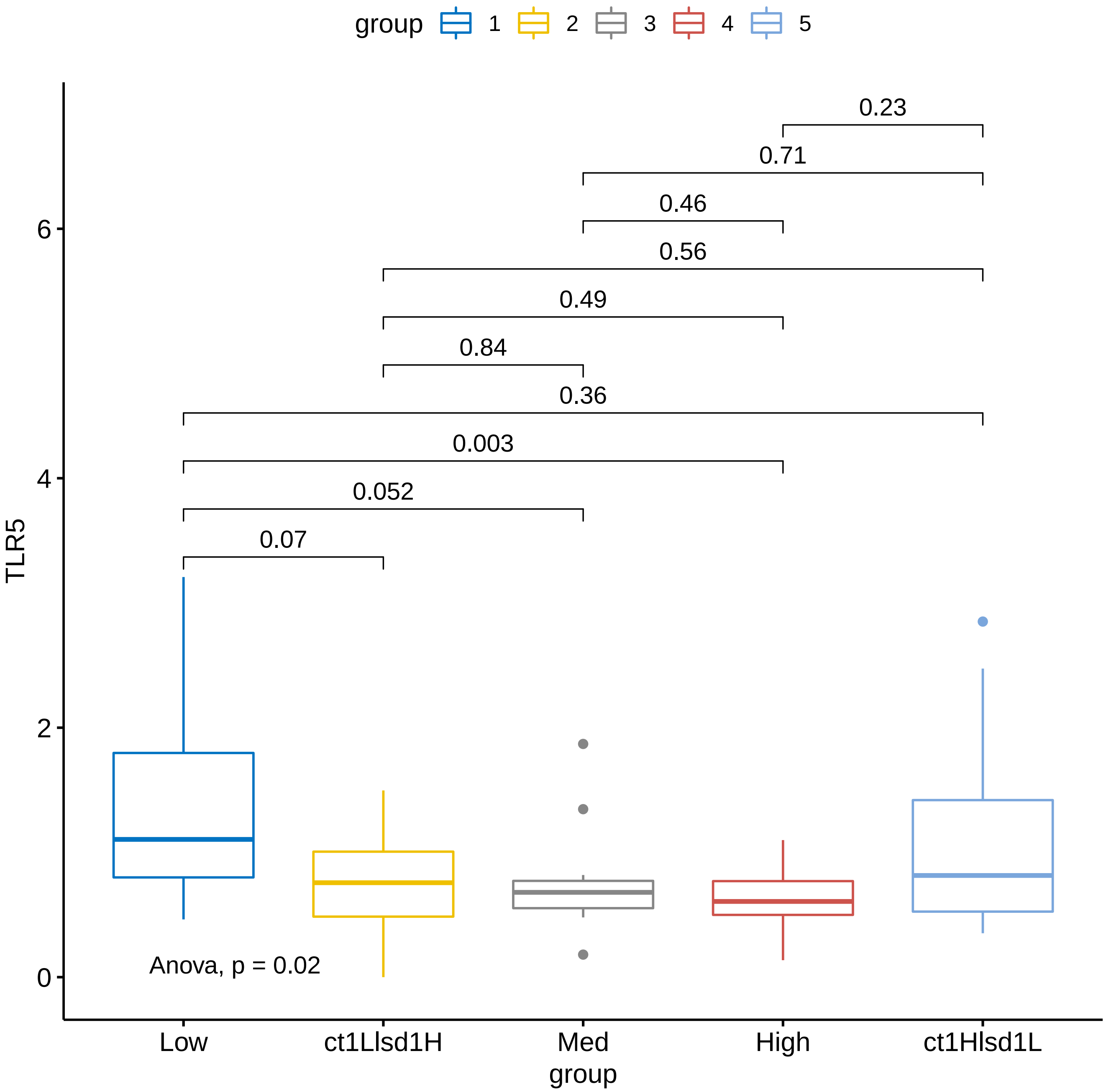
group
1
2
3
4
5
0.23
0.71
0.46
0.56
0.49
0.84
0.36
0.003
0.052
0.07
Anova, p = 0.02
6
4
2
0
TLR5
Low
ct1Llsd1H
Med
High
ct1Hlsd1L
group

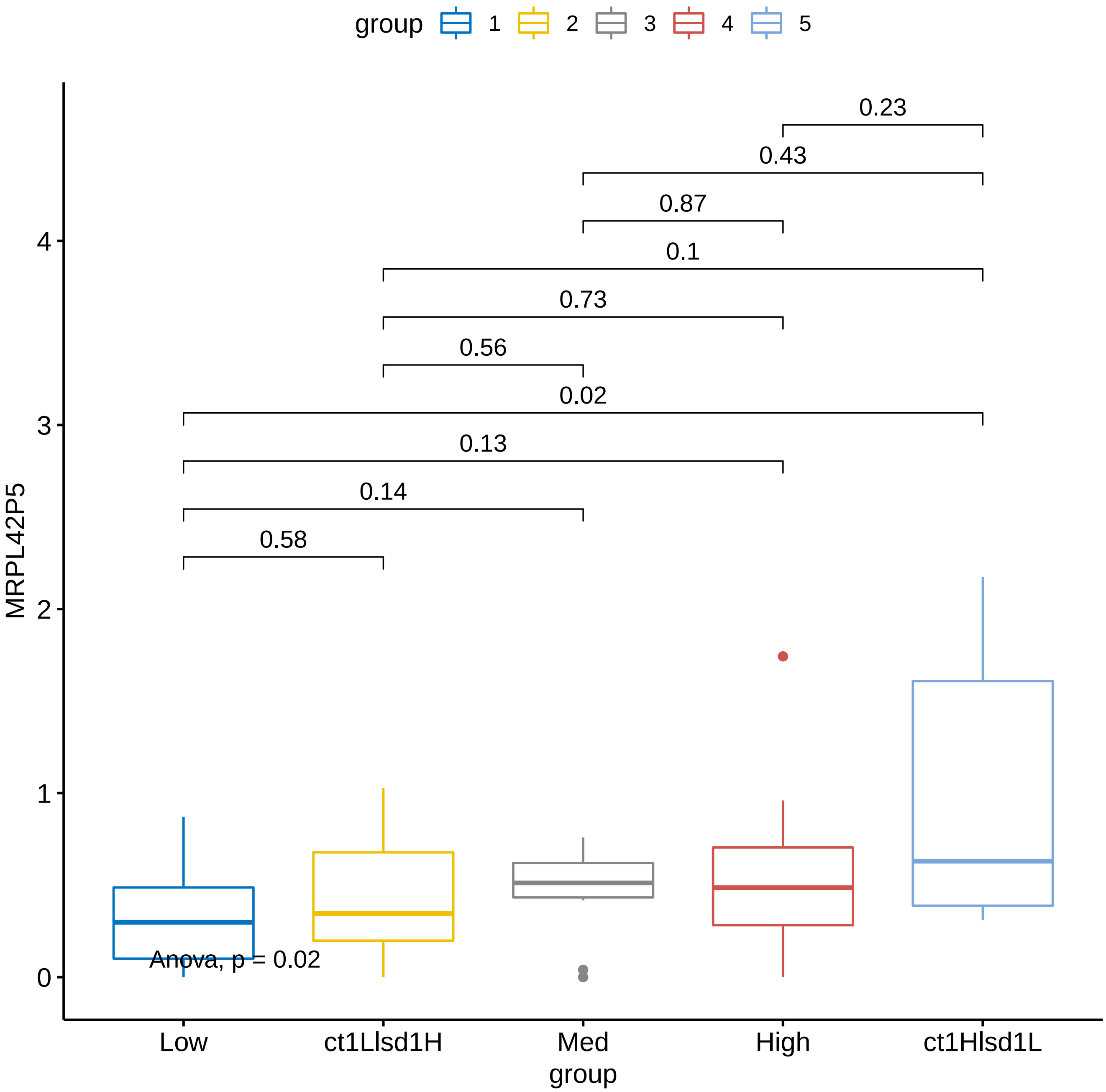

group
1
2
3
4
5
0.23
0.43
0.87
0.1
0.73
0.56
0.02
0.13
0.14
0.58
Anova, p = 0.02
MRPL42P5
0
1
2
3
4
Low
ct1Llsd1H
Med
High
ct1Hlsd1L
group

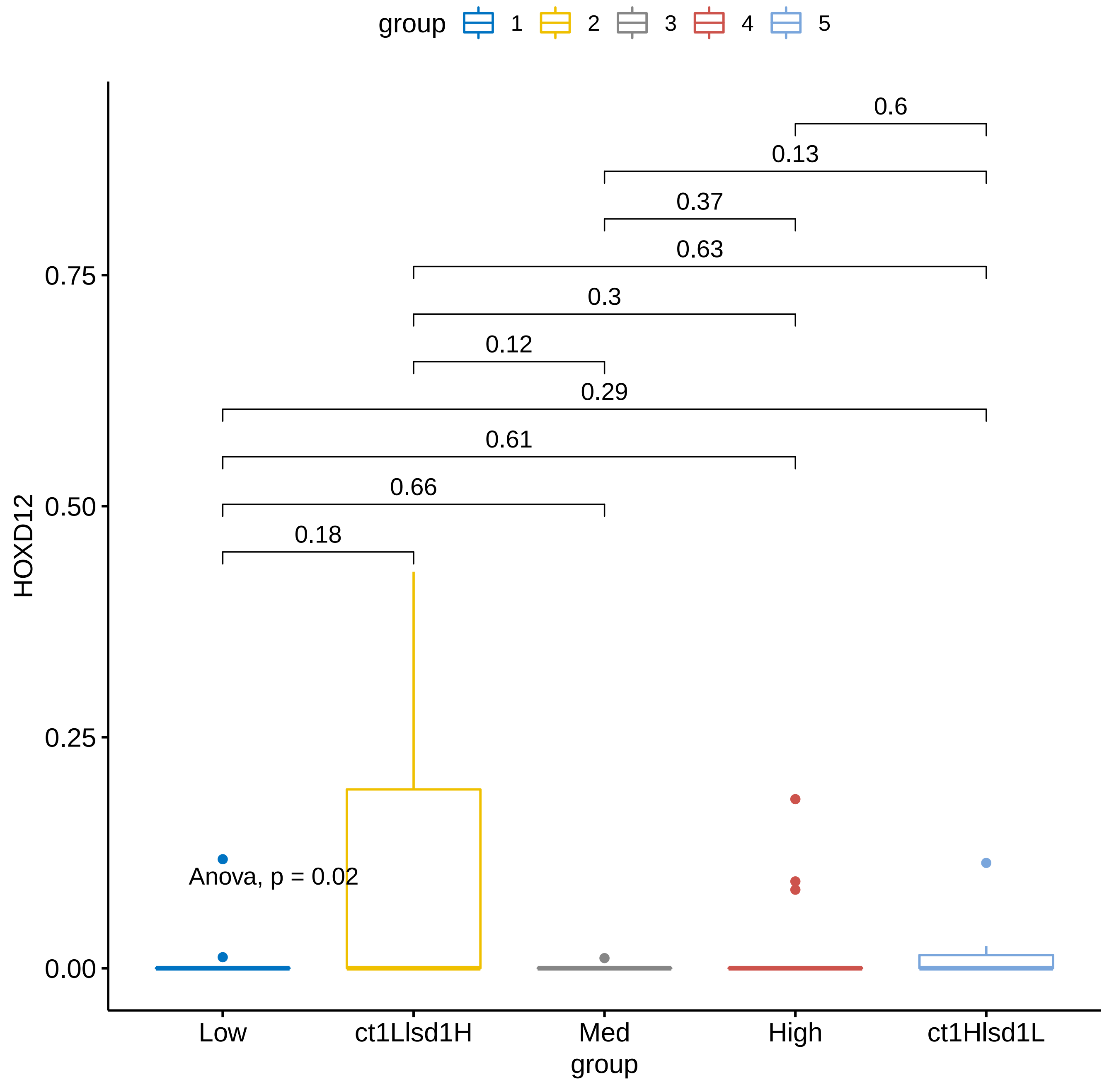

group 1 2 3 4 5
0.6
0.13
0.37
0.63
0.3
0.12
0.29
0.61
0.66
0.18
HOXD12
0.75
0.50
0.25
0.00
Anova, p = 0.02
Low
ct1Llsd1H
Med
High
ct1Hlsd1L
group

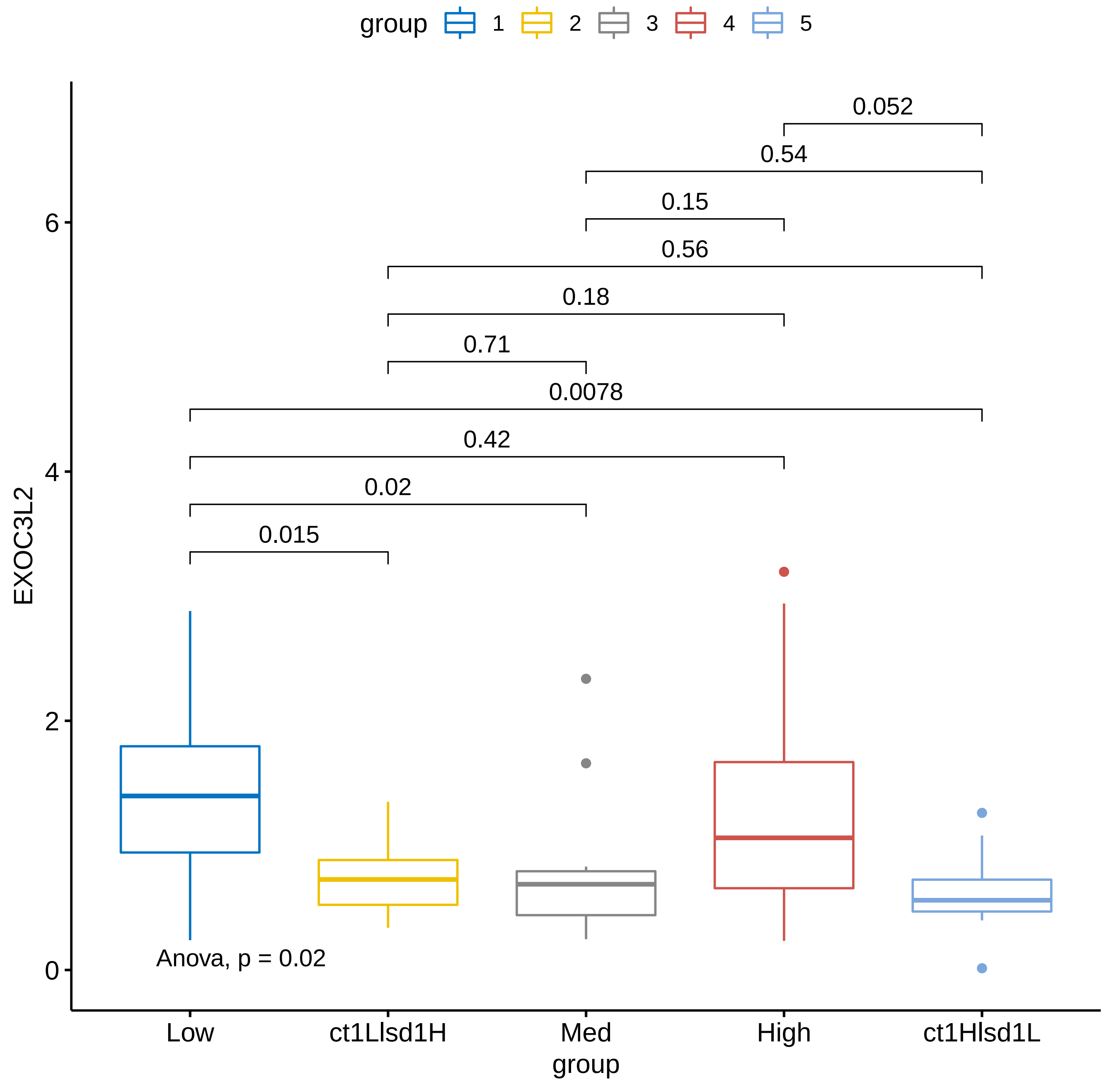

group
1
2
3
4
5
0.052
0.54
0.15
0.56
0.18
0.71
0.0078
0.42
0.02
0.015
Anova, p = 0.02
EXOC3L2
6
4
2
0
Low
ct1Llsd1H
Med
High
ct1Hlsd1L
group

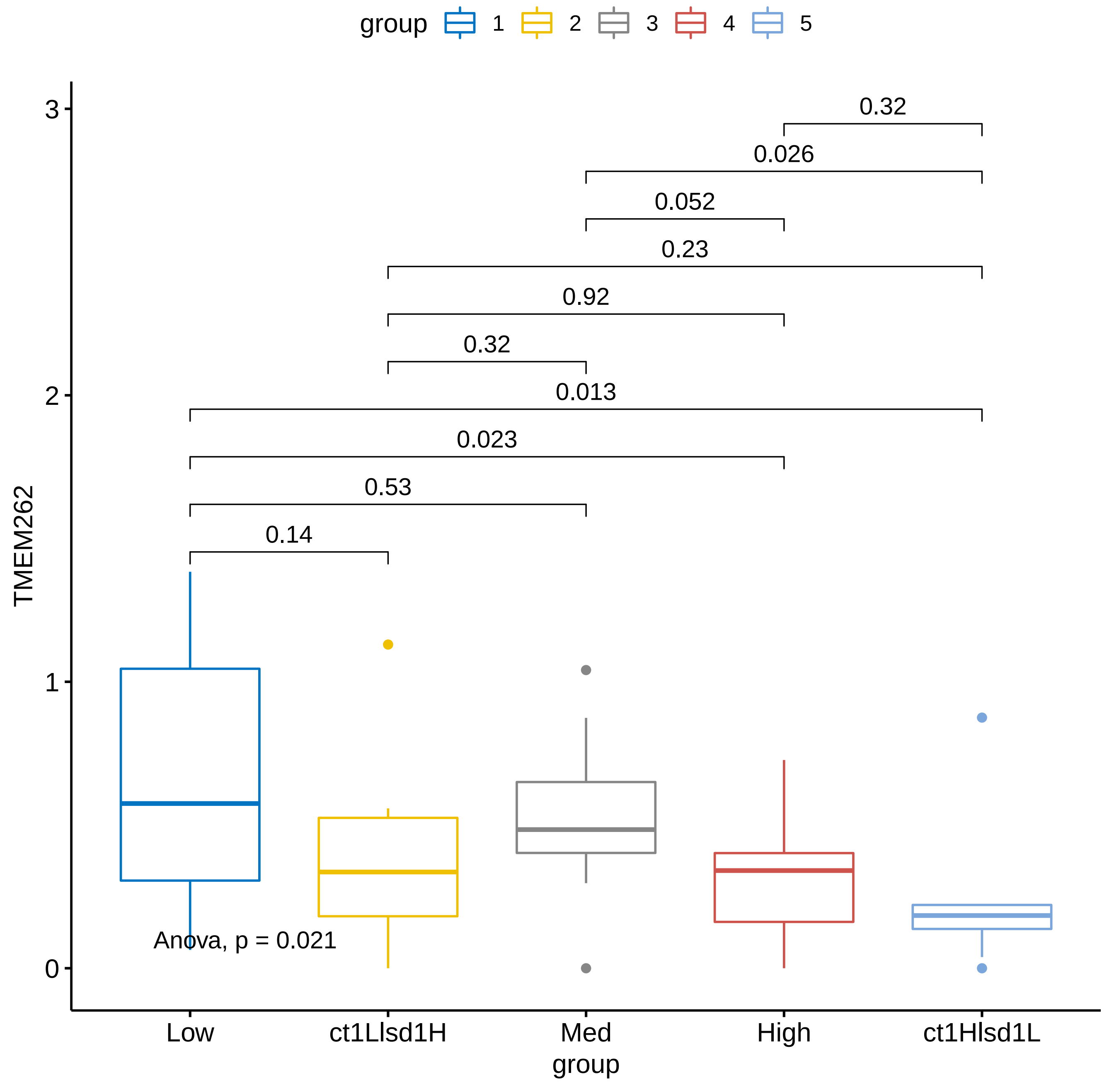
group
1
2
3
4
5
3
2
1
0
TMEM262
0.32
0.026
0.052
0.23
0.92
0.32
0.013
0.023
0.53
0.14
Anova, p = 0.021
Low
ct1Llsd1H
Med
High
ct1Hlsd1L
group

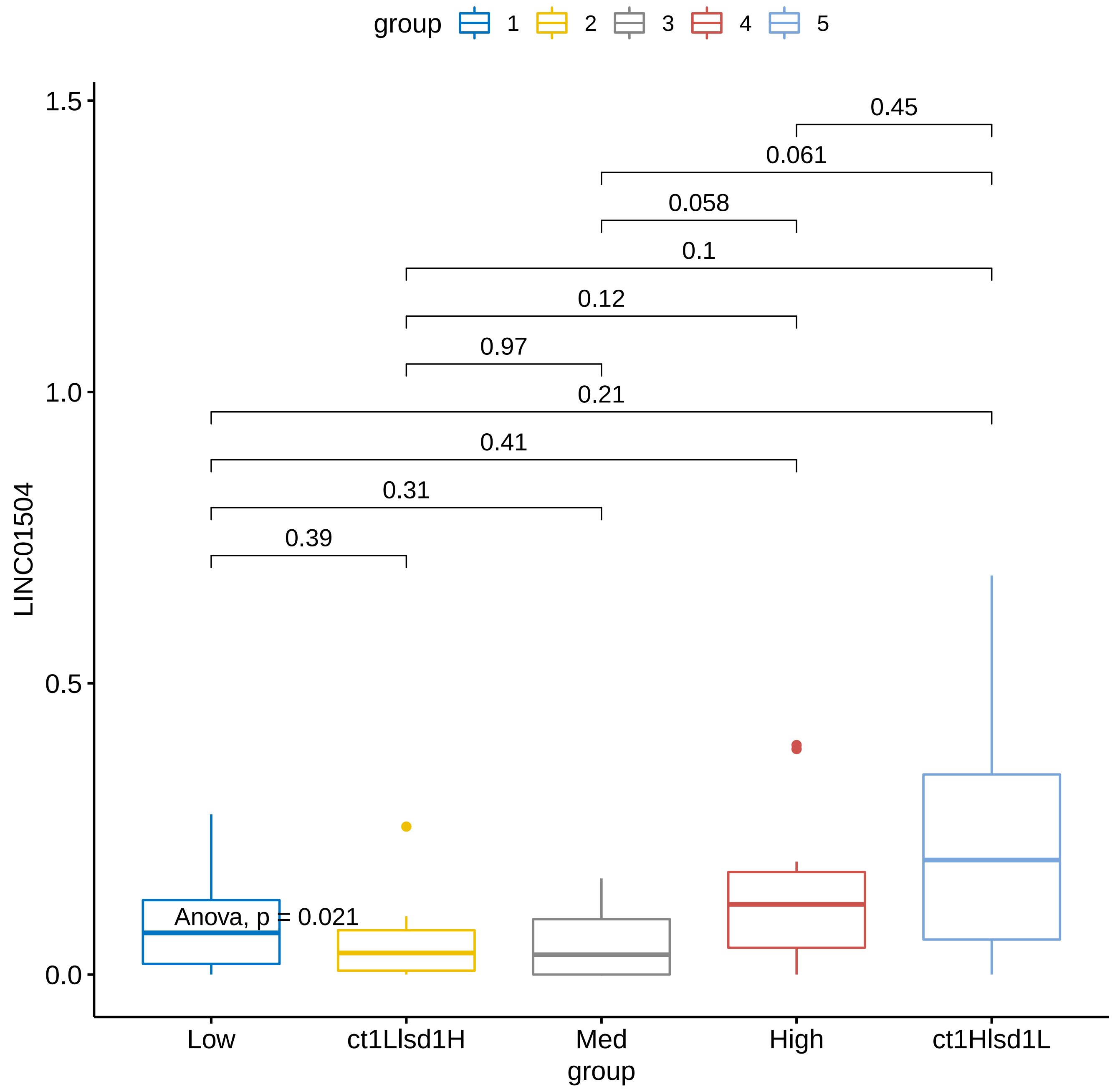

group 1 2 3 4 5
0.45
0.061
0.058
0.1
0.12
0.97
0.21
0.41
0.31
0.39
Anova, p = 0.021
1.5
1.0
0.5
0.0
LINC01504
Low
ct1Llsd1H
Med
High
ct1Hlsd1L
group

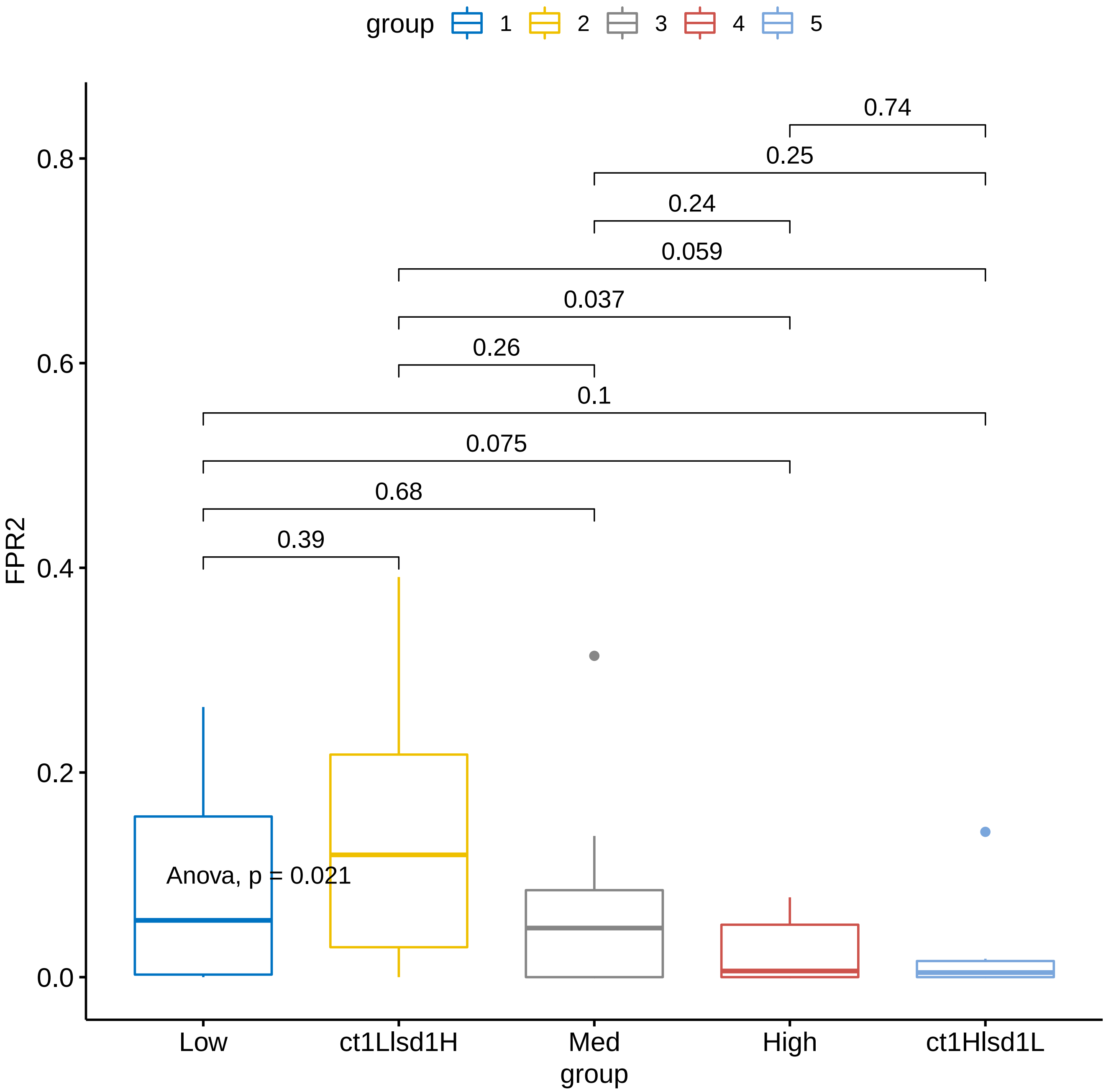

group 1 2 3 4 5
0.74
0.25
0.24
0.059
0.037
0.26
0.1
0.075
0.68
0.39
Anova, p = 0.021
FPR2
0.8
0.6
0.4
0.2
0.0
Low
ct1Llsd1H
Med
High
ct1Hlsd1L
group

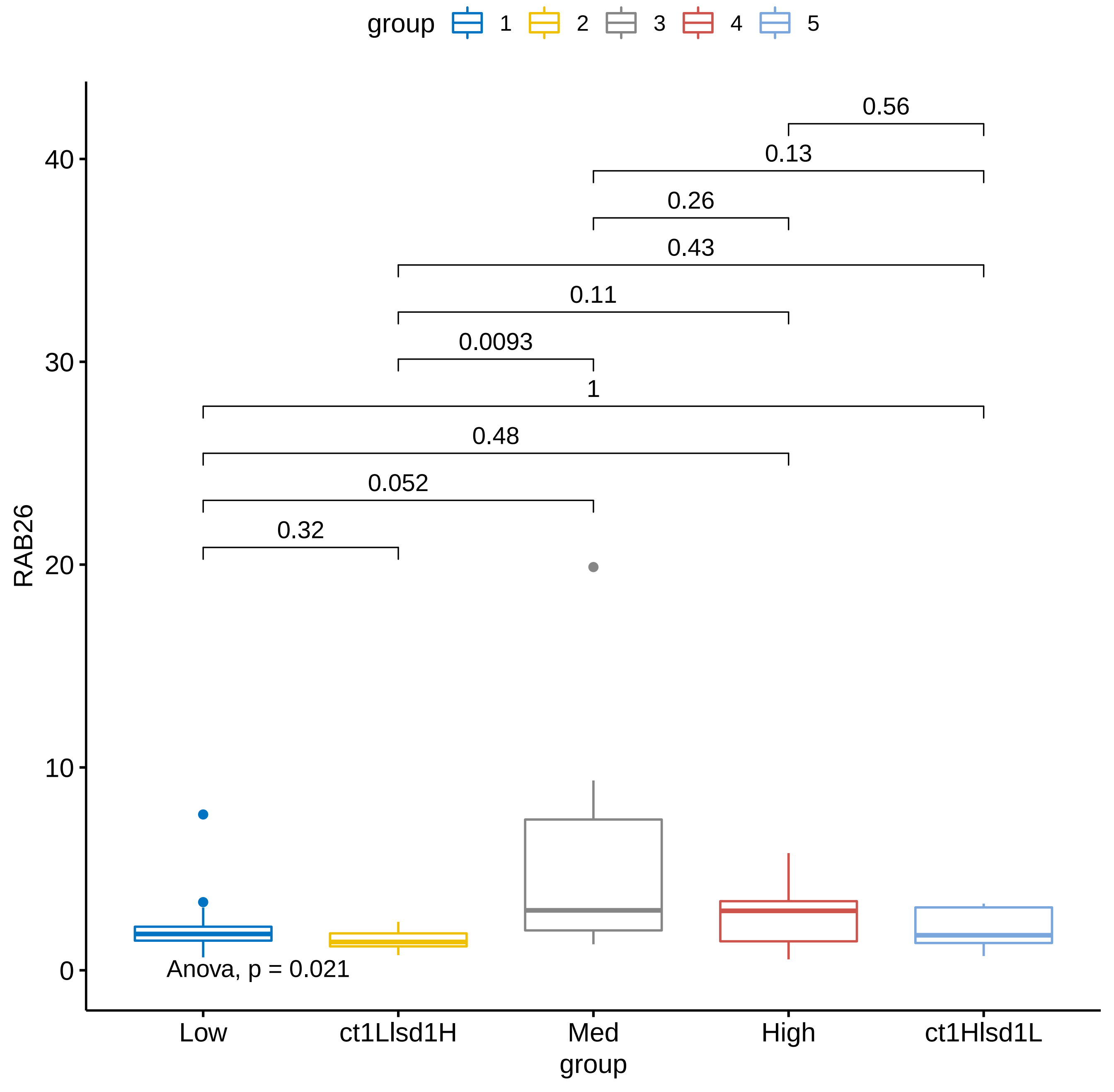

group 1 2 3 4 5
0.56
0.13
0.26
0.43
0.11
0.0093
1
0.48
0.052
0.32
Anova, p = 0.021
RAB26
0
10
20
30
40
Low
ct1Llsd1H
Med
High
ct1Hlsd1L
group

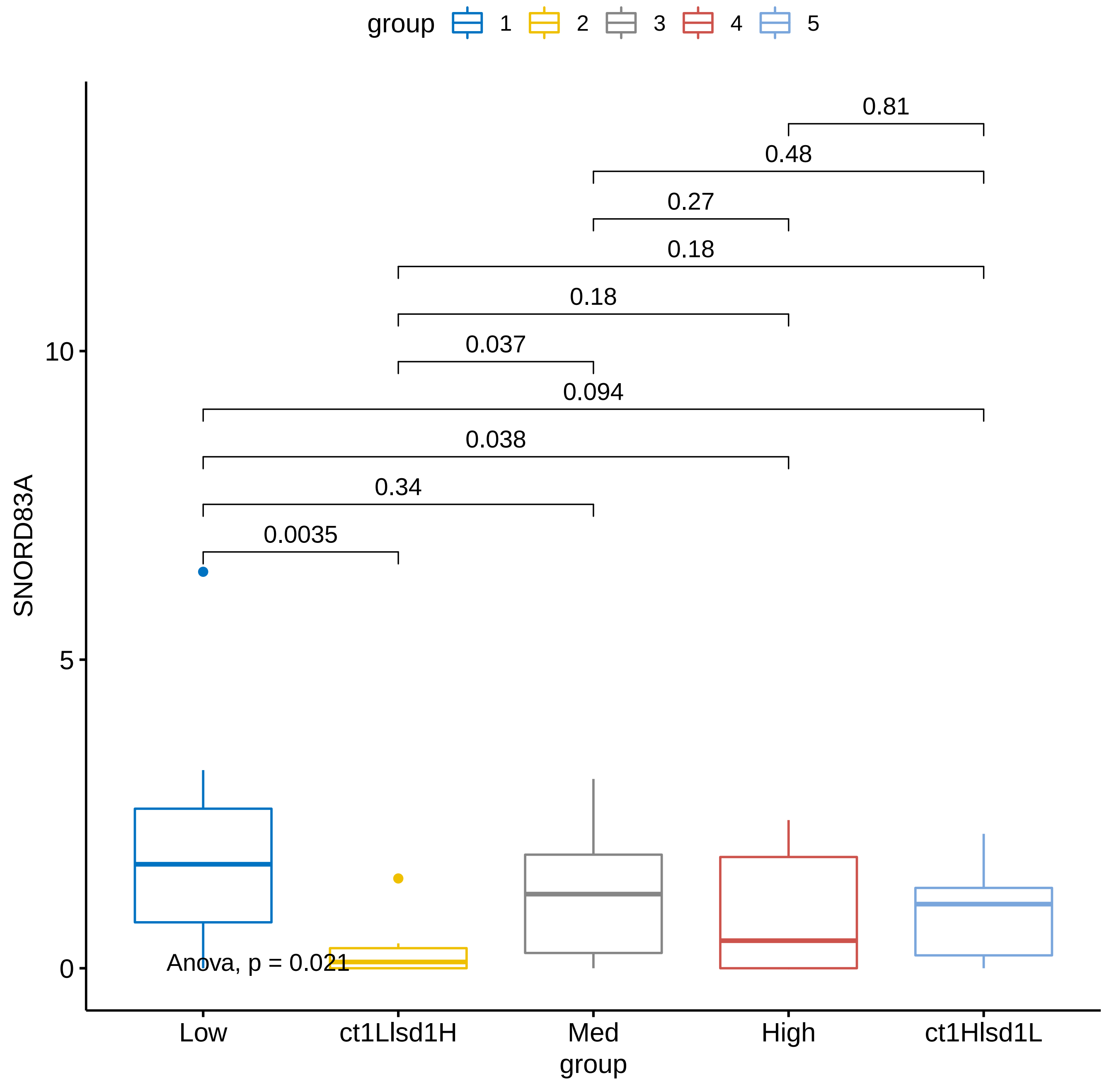

group 1 2 3 4 5
0.81
0.48
0.27
0.18
0.18
0.037
0.094
0.038
0.34
0.0035
Anova, p = 0.021
SNORD83A
10
5
0
Low
ct1Llsd1H
Med
High
ct1Hlsd1L
group

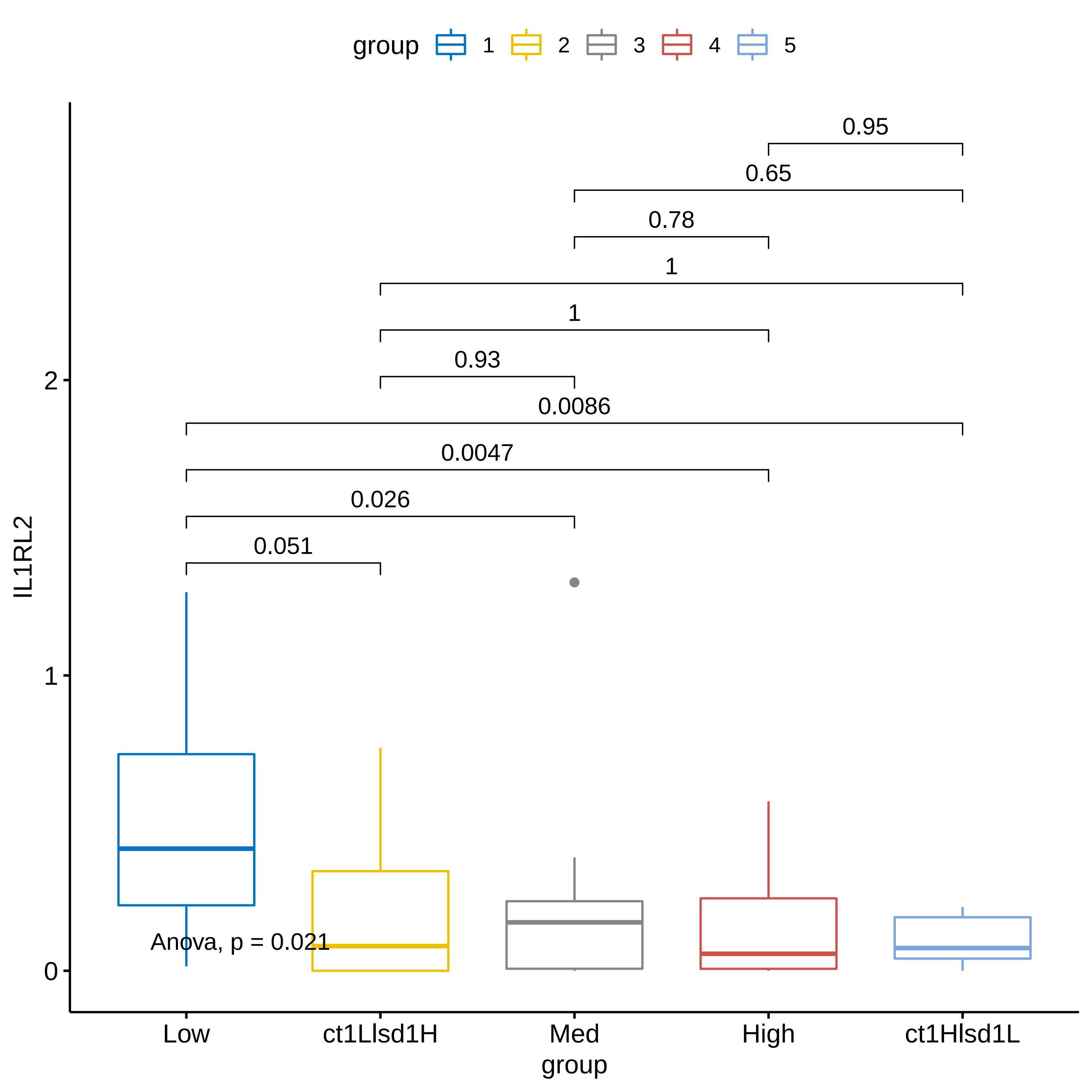

group
1
2
3
4
5
0.95
0.65
0.78
1
1
0.93
0.0086
0.0047
0.026
0.051
Anova, p = 0.021
IL1RL2
2
1
0
Low
ct1Llsd1H
Med
High
ct1Hlsd1L
group

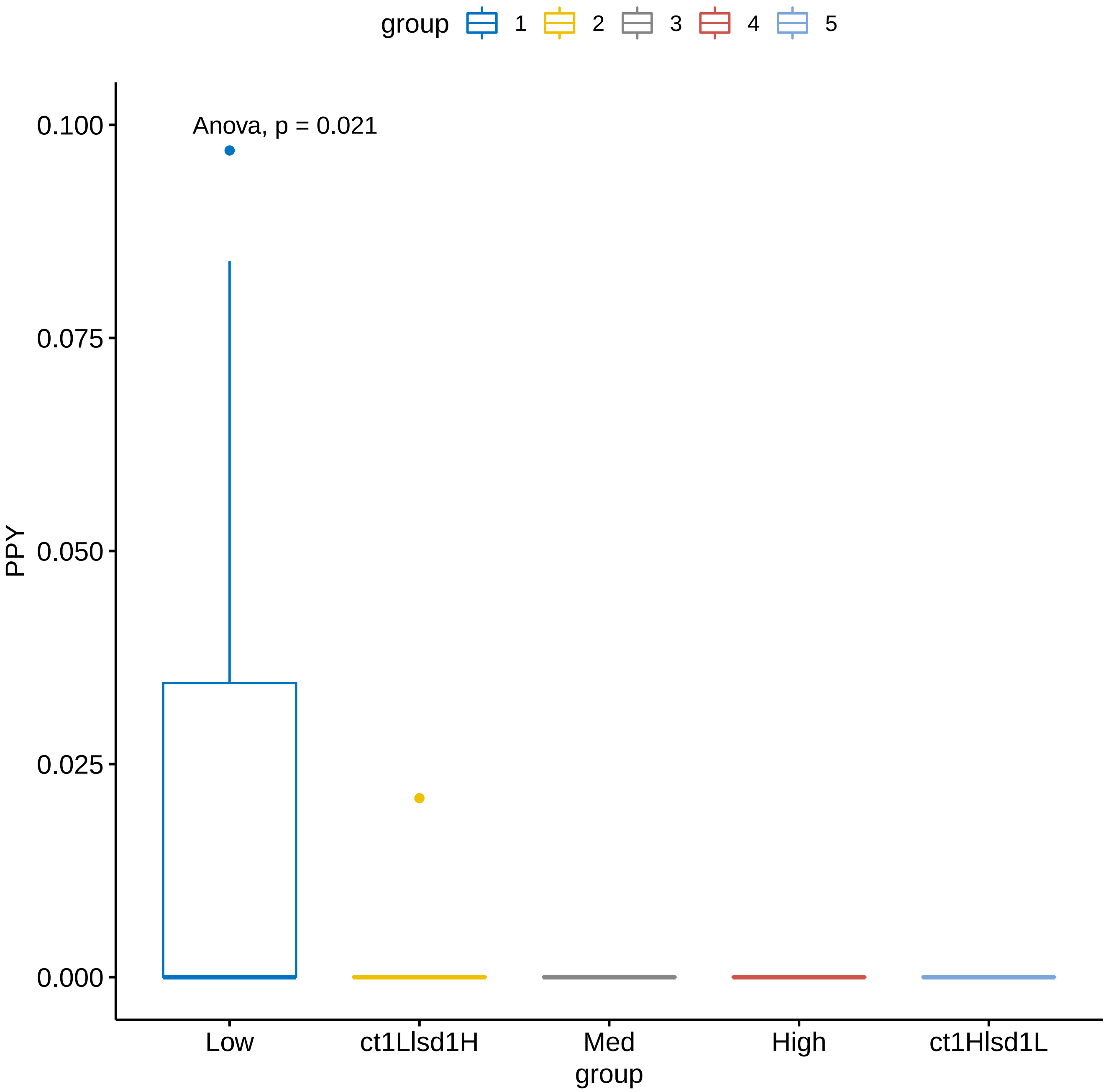

group 1 2 3 4 5
Anova, p = 0.021
PPY
0.100
0.075
0.050
0.025
0.000
Low
ct1Llsd1H
Med
High
ct1Hlsd1L
group

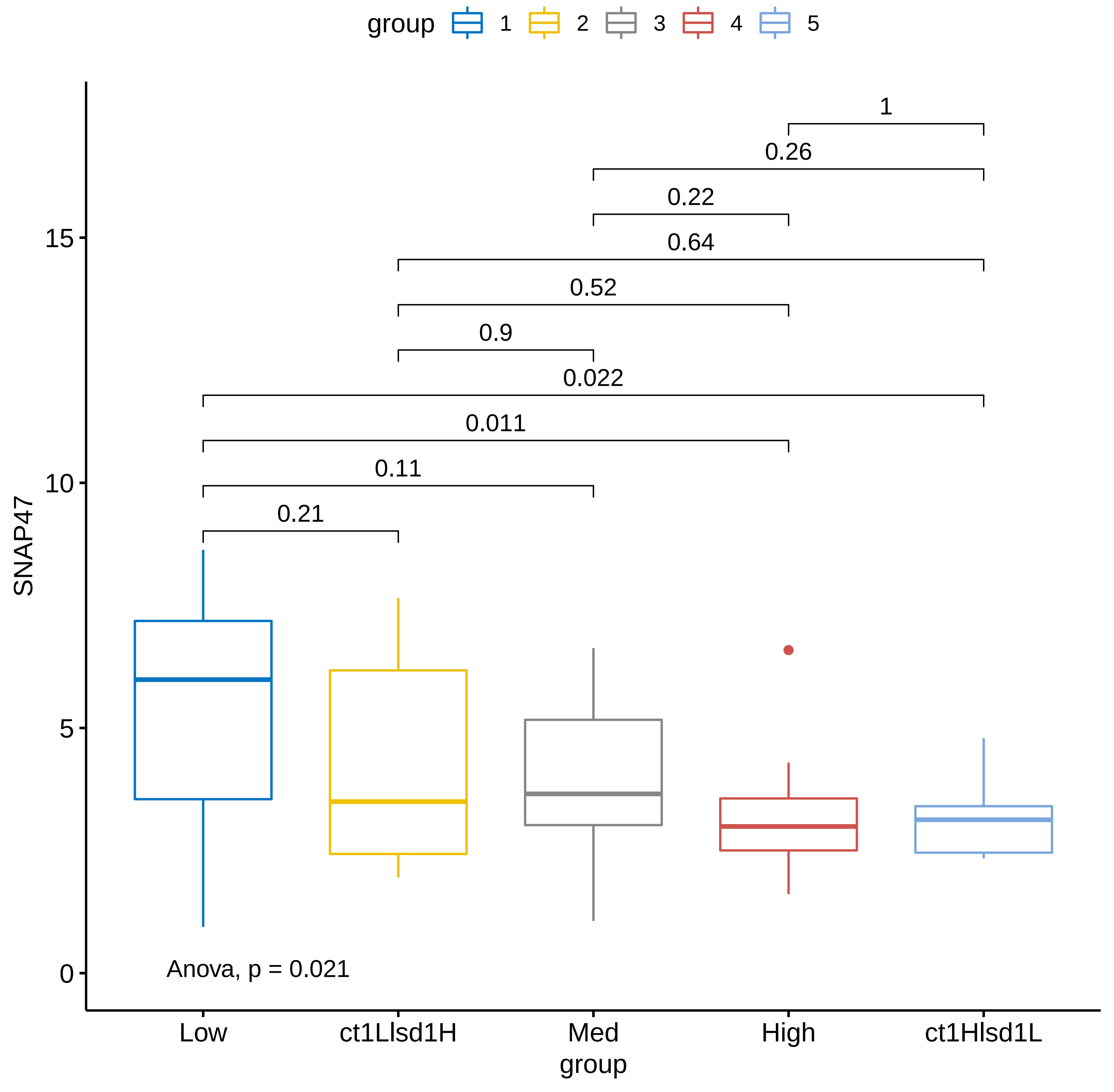

group 1 2 3 4 5
1
0.26
0.22
0.64
0.52
0.9
0.022
0.011
0.11
0.21
15
10
5
0
SNAP47
Anova, p = 0.021
Low
ct1Llsd1H
Med
High
ct1Hlsd1L
group

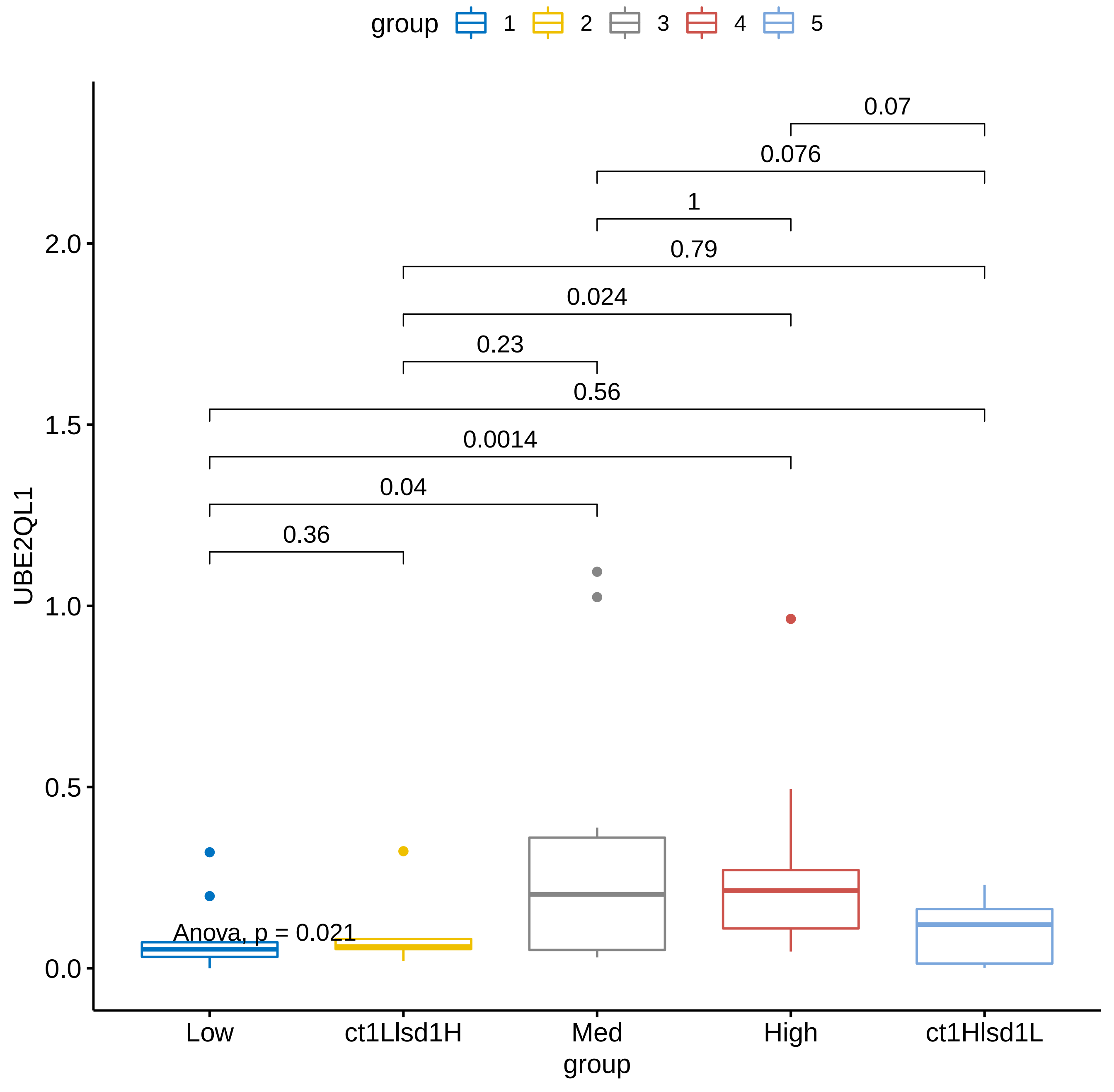

group 1 2 3 4 5
0.07
0.076
1
0.79
0.024
0.23
0.56
0.0014
0.04
0.36
Anova, p = 0.021
UBE2QL1
2.0
1.5
1.0
0.5
0.0
Low
ct1Llsd1H
Med
High
ct1Hlsd1L
group

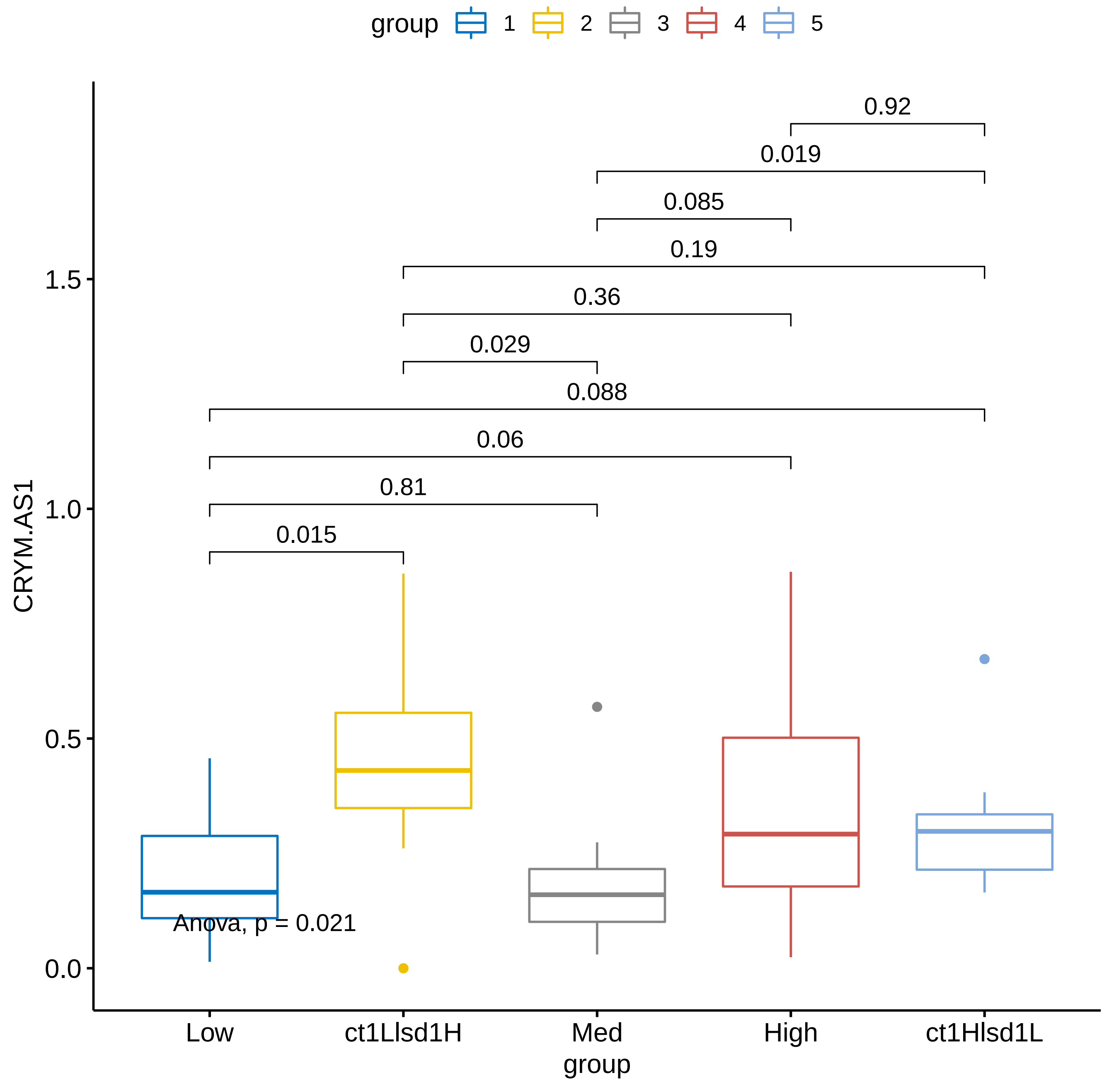
group
1
2
3
4
5
0.92
0.019
0.085
0.19
0.36
0.029
0.088
0.06
0.81
0.015
Anova, p = 0.021
CRYM.AS1
1.5
1.0
0.5
0.0
Low
ct1Llsd1H
Med
High
ct1Hlsd1L
group

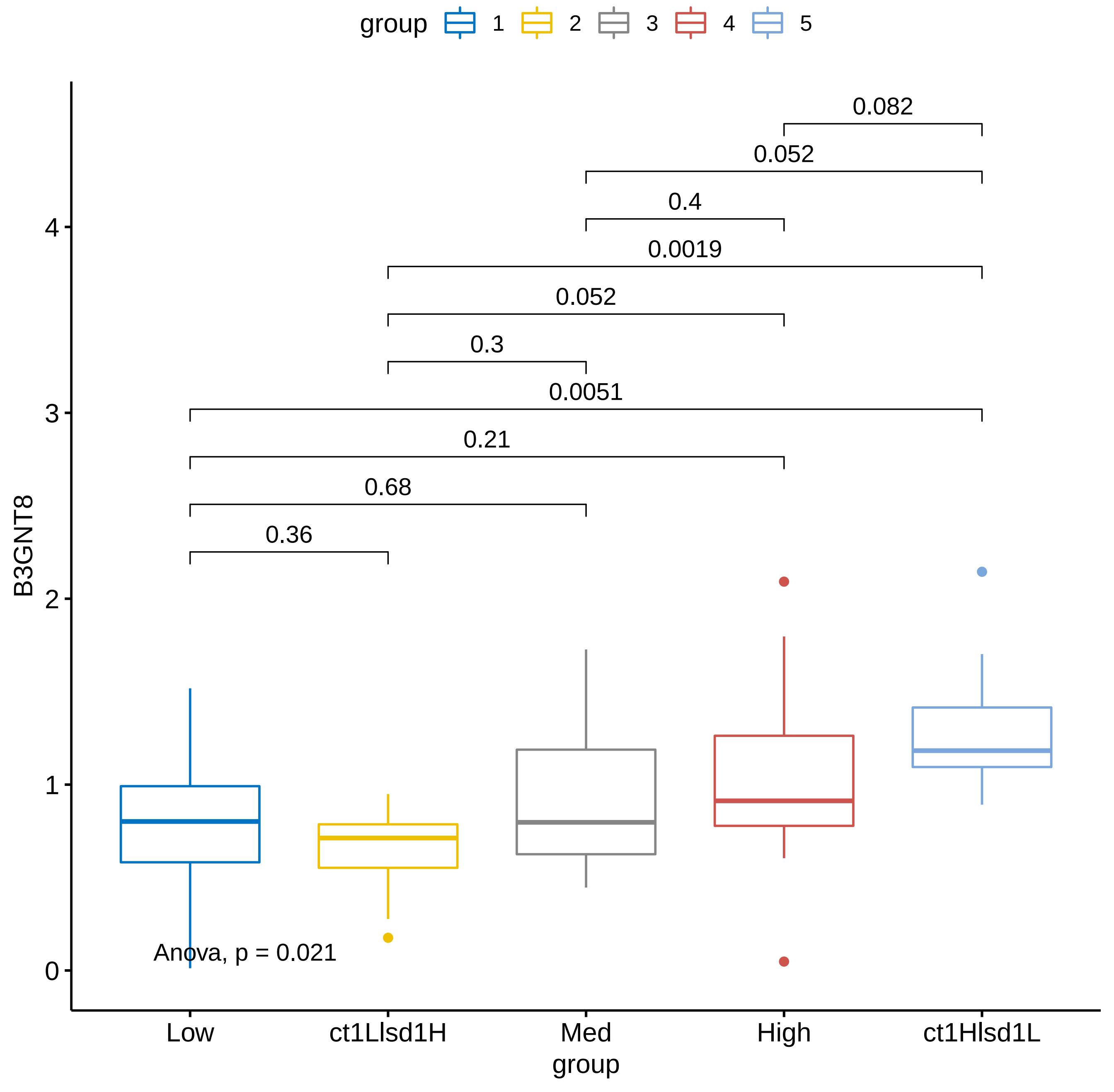
group
1
2
3
4
5
0.082
0.052
0.4
0.0019
0.052
0.3
0.0051
0.21
0.68
0.36
Anova, p = 0.021
B3GNT8
4
3
2
1
0
Low
ct1Llsd1H
Med
High
ct1Hlsd1L
group

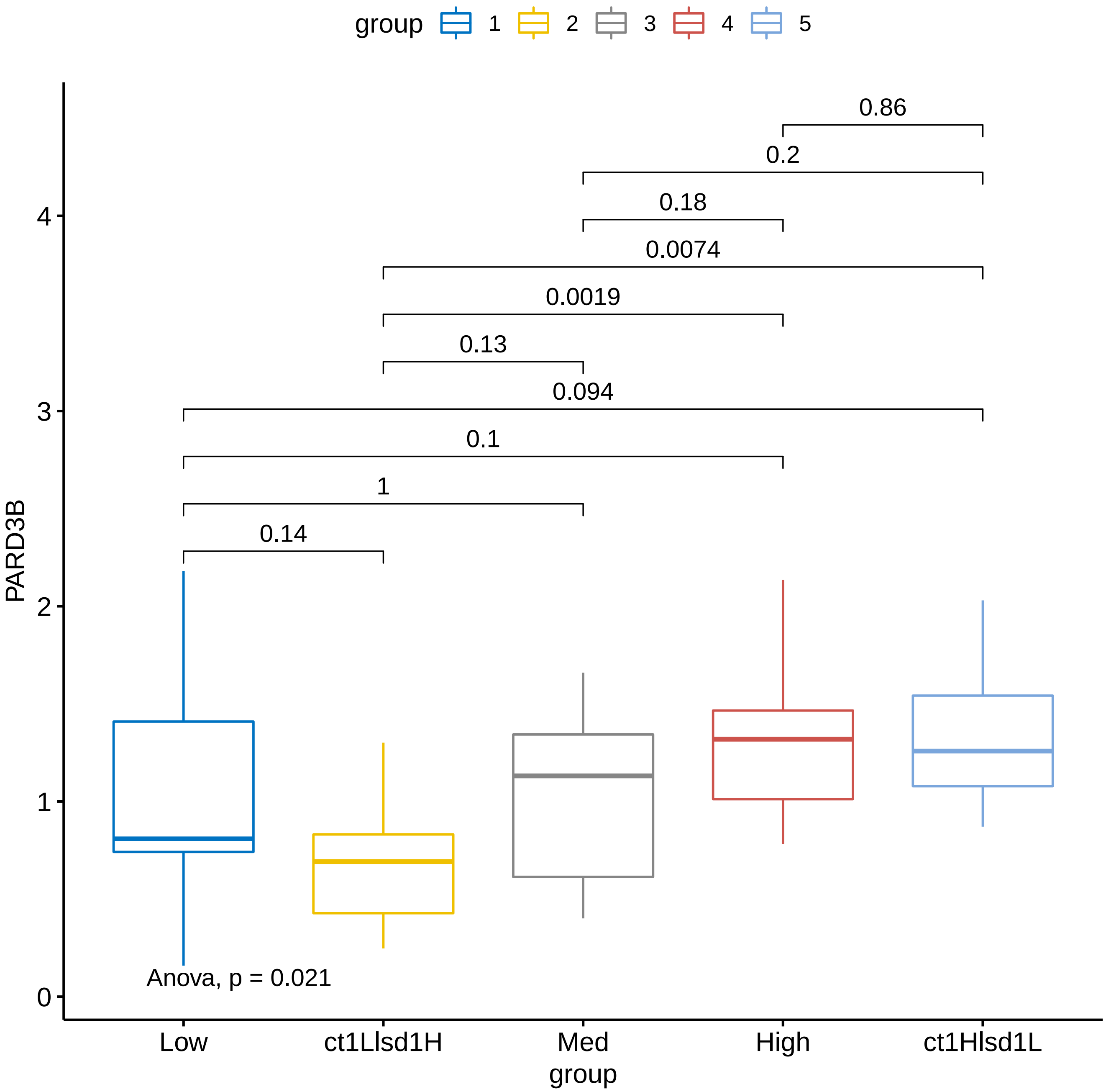

group
1
2
3
4
5
0.86
0.2
0.18
0.0074
0.0019
0.13
0.094
0.1
1
0.14
Anova, p = 0.021
PARD3B
0
1
2
3
4
Low
ct1Llsd1H
Med
High
ct1Hlsd1L
group

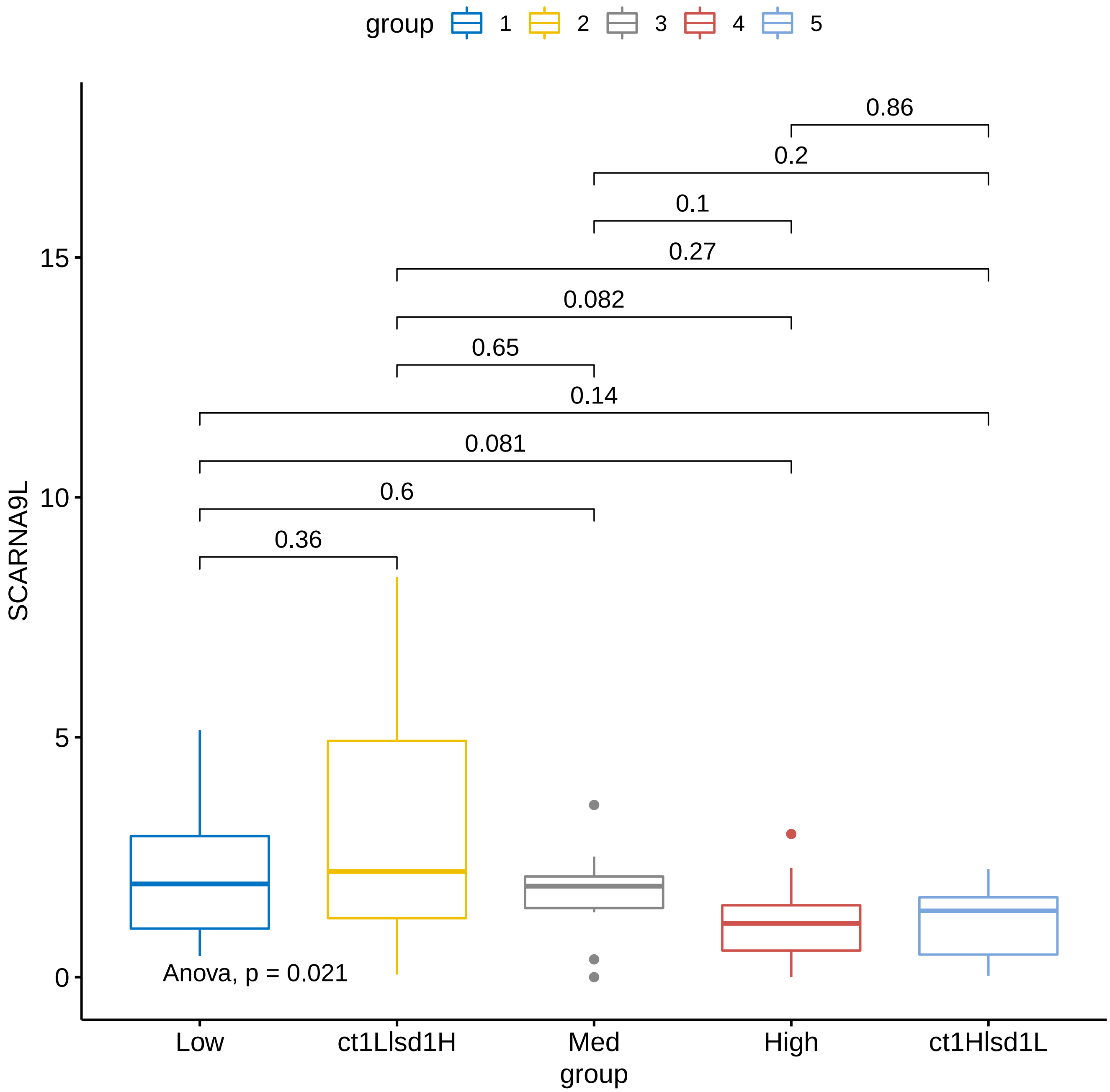

group
1
2
3
4
5
0.86
0.2
0.1
0.27
0.082
0.65
0.14
0.081
0.6
0.36
15
10
5
0
SCARNA9L
Anova, p = 0.021
Low
ct1Llsd1H
Med
High
ct1Hlsd1L
group

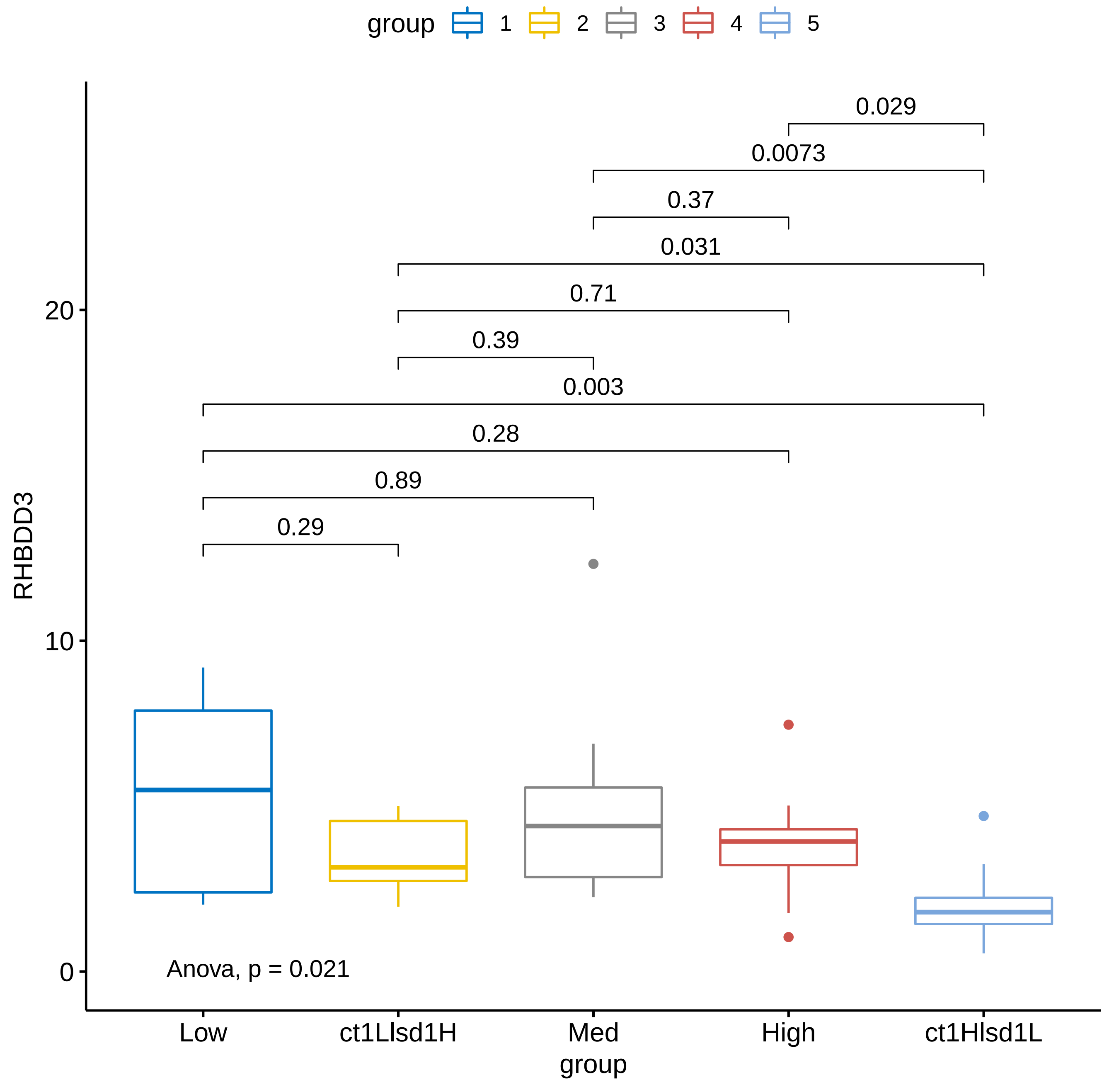
group
1
2
3
4
5
0.029
0.0073
0.37
0.031
0.71
0.39
0.003
0.28
0.89
0.29
20
10
0
RHBDD3
Anova, p = 0.021
Low
ct1Llsd1H
Med
High
ct1Hlsd1L
group

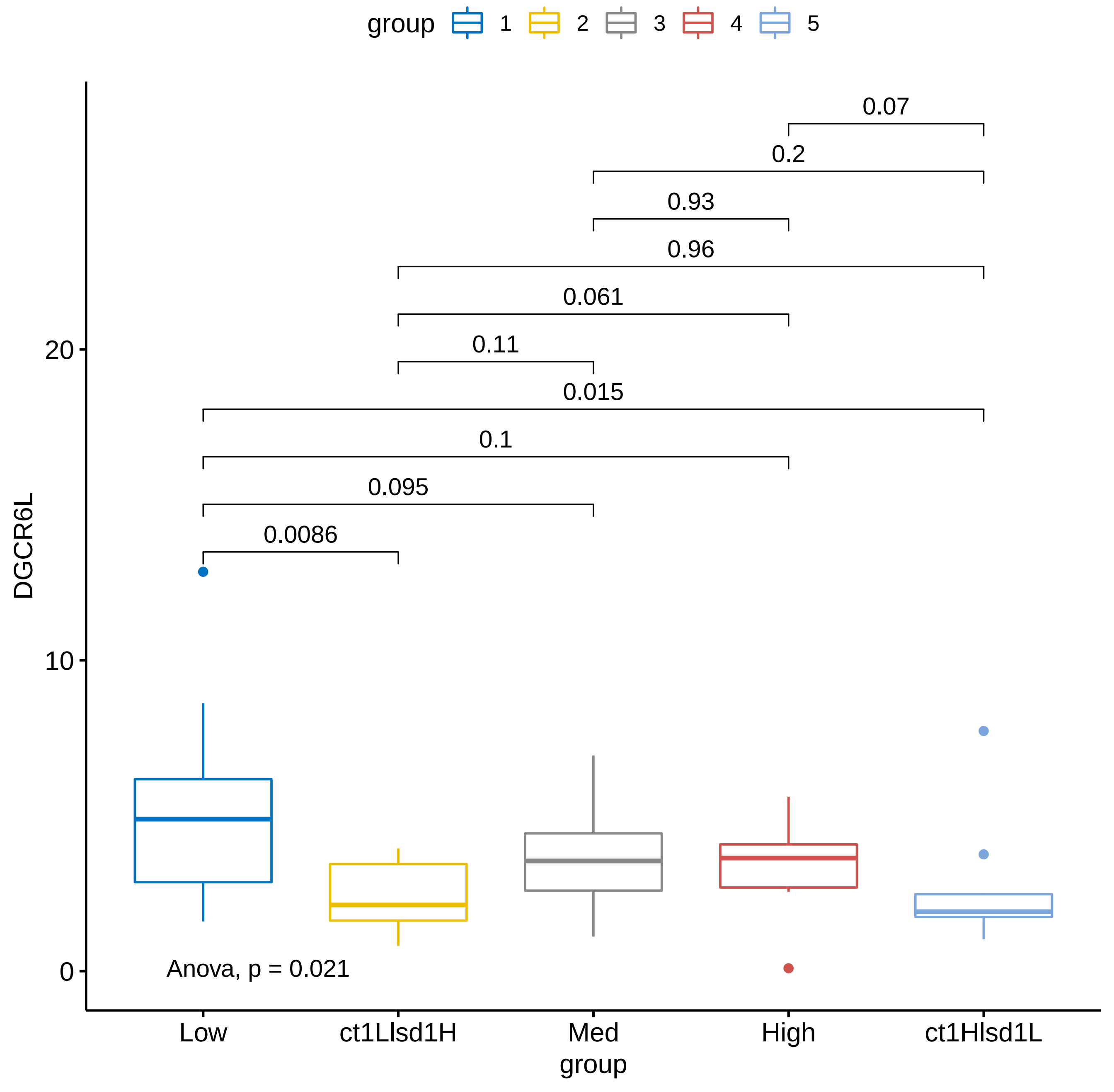

group 1 2 3 4 5
0.07
0.2
0.93
0.96
0.061
0.11
0.015
0.1
0.095
0.0086
DGCR6L
20
10
0
Anova, p = 0.021
Low
ct1Llsd1H
Med
High
ct1Hlsd1L
group

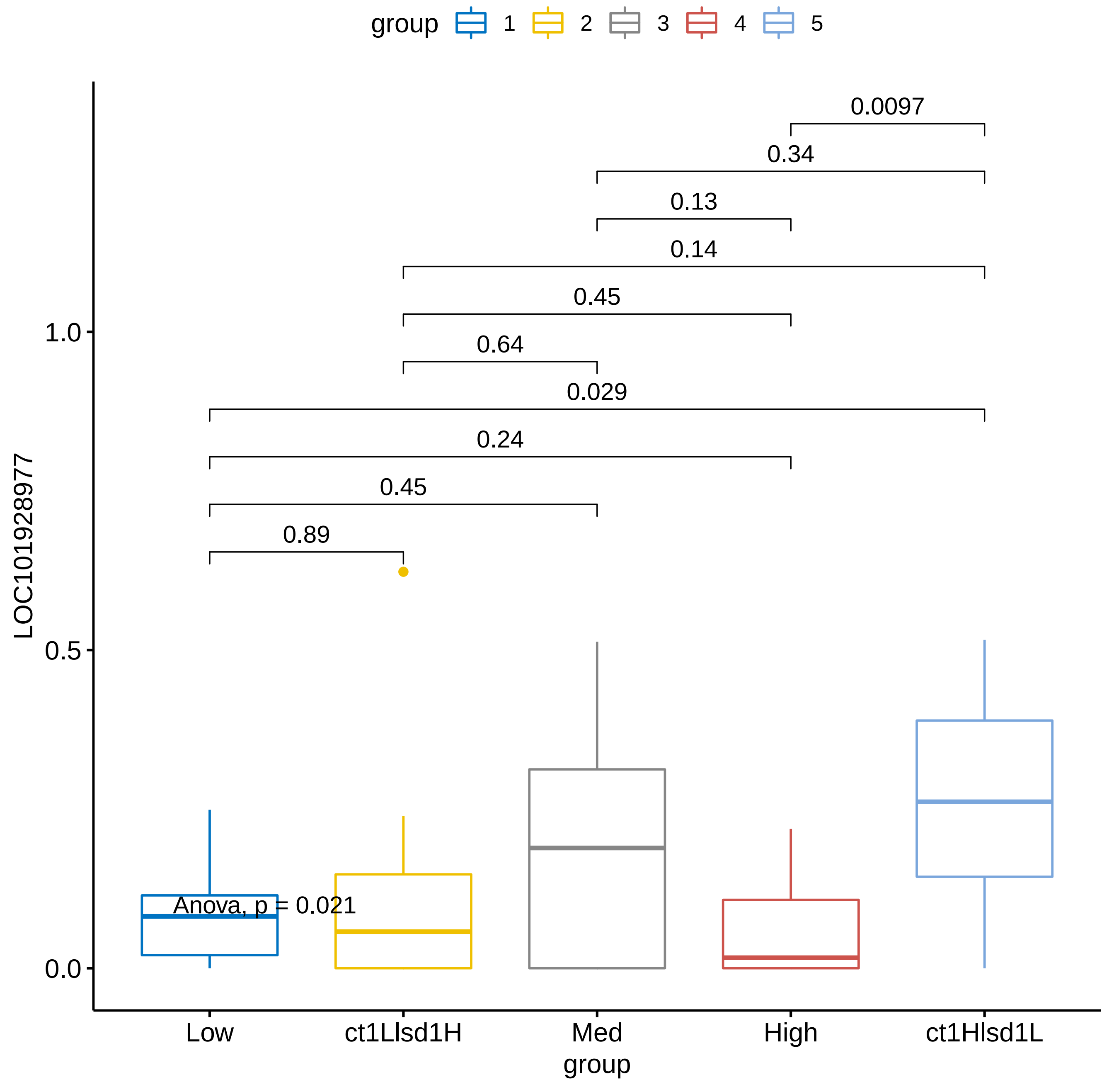

group
1
2
3
4
5
0.0097
0.34
0.13
0.14
0.45
0.64
0.029
0.24
0.45
0.89
1.0
0.5
0.0
LOC101928977
Anova, p = 0.021
Low
ct1Llsd1H
Med
High
ct1Hlsd1L
group

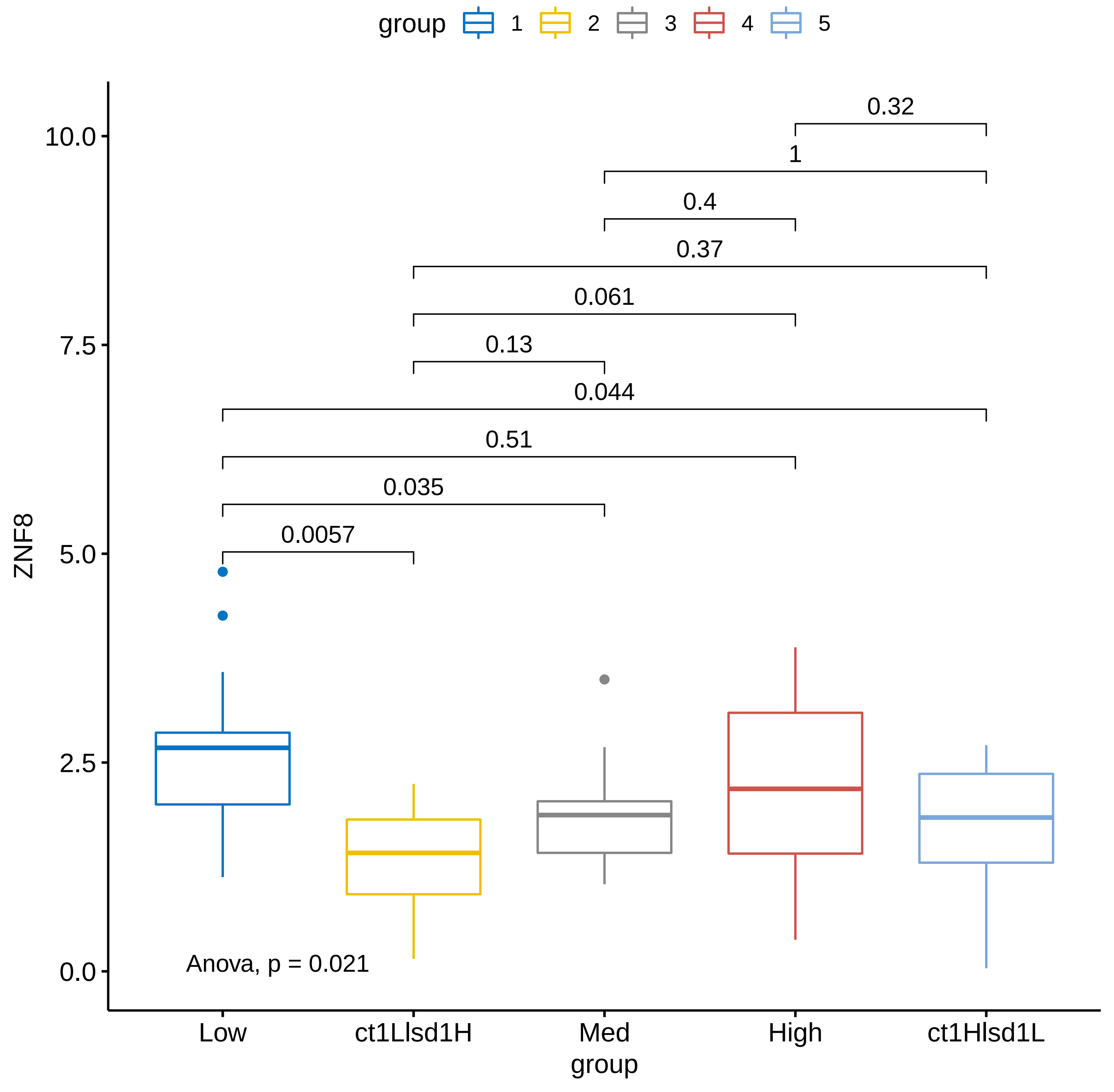

group
1
2
3
4
5
0.32
1
0.4
0.37
0.061
0.13
0.044
0.51
0.035
0.0057
10.0
7.5
5.0
2.5
0.0
ZNF8
Anova, p = 0.021
Low
ct1Llsd1H
Med
High
ct1Hlsd1L
group

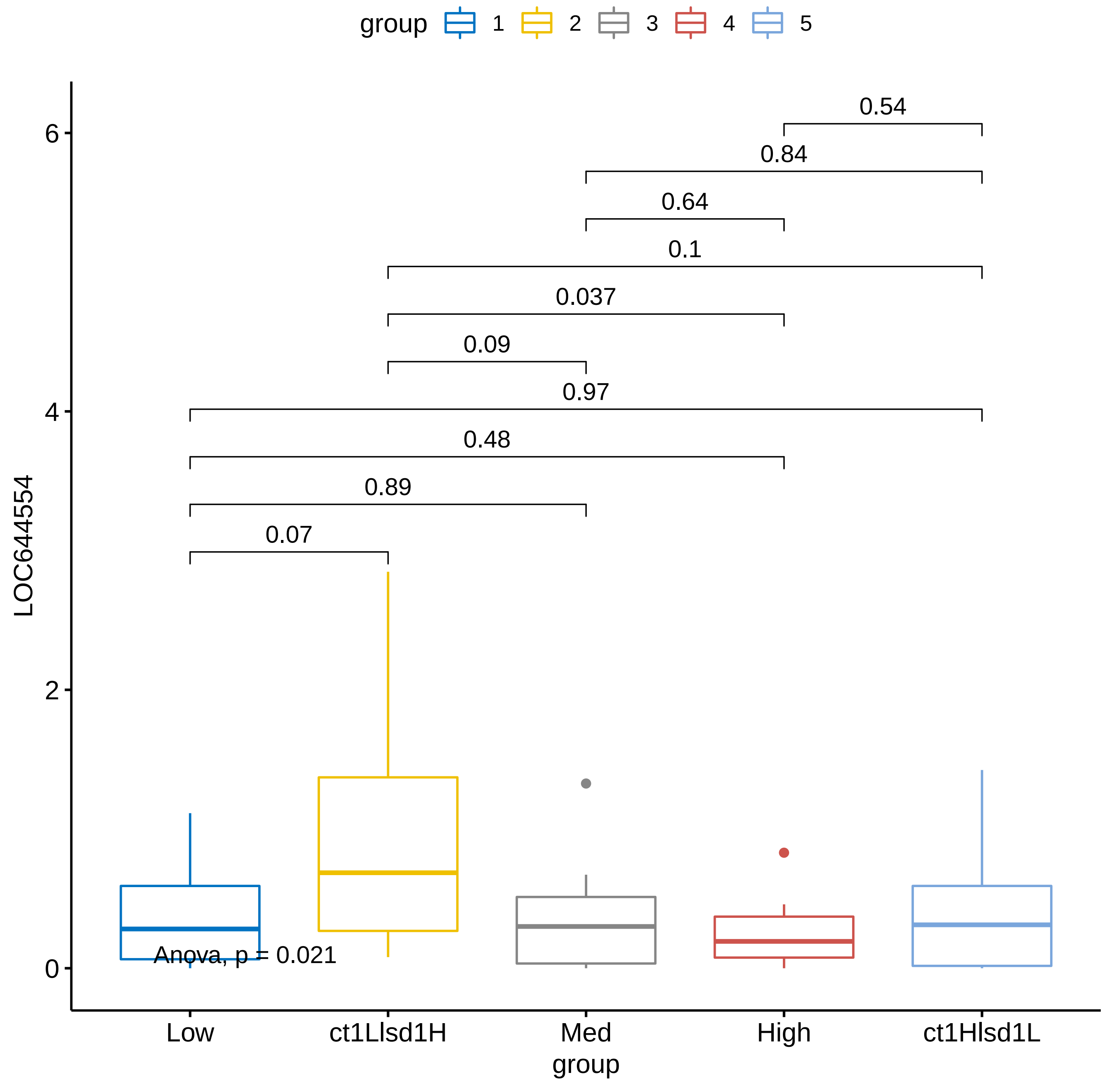

group
1
2
3
4
5
LOC644554
0
2
4
6
0.54
0.84
0.64
0.1
0.037
0.09
0.97
0.48
0.89
0.07
Anova, p = 0.021
Low
ct1Llsd1H
Med
High
ct1Hlsd1L
group

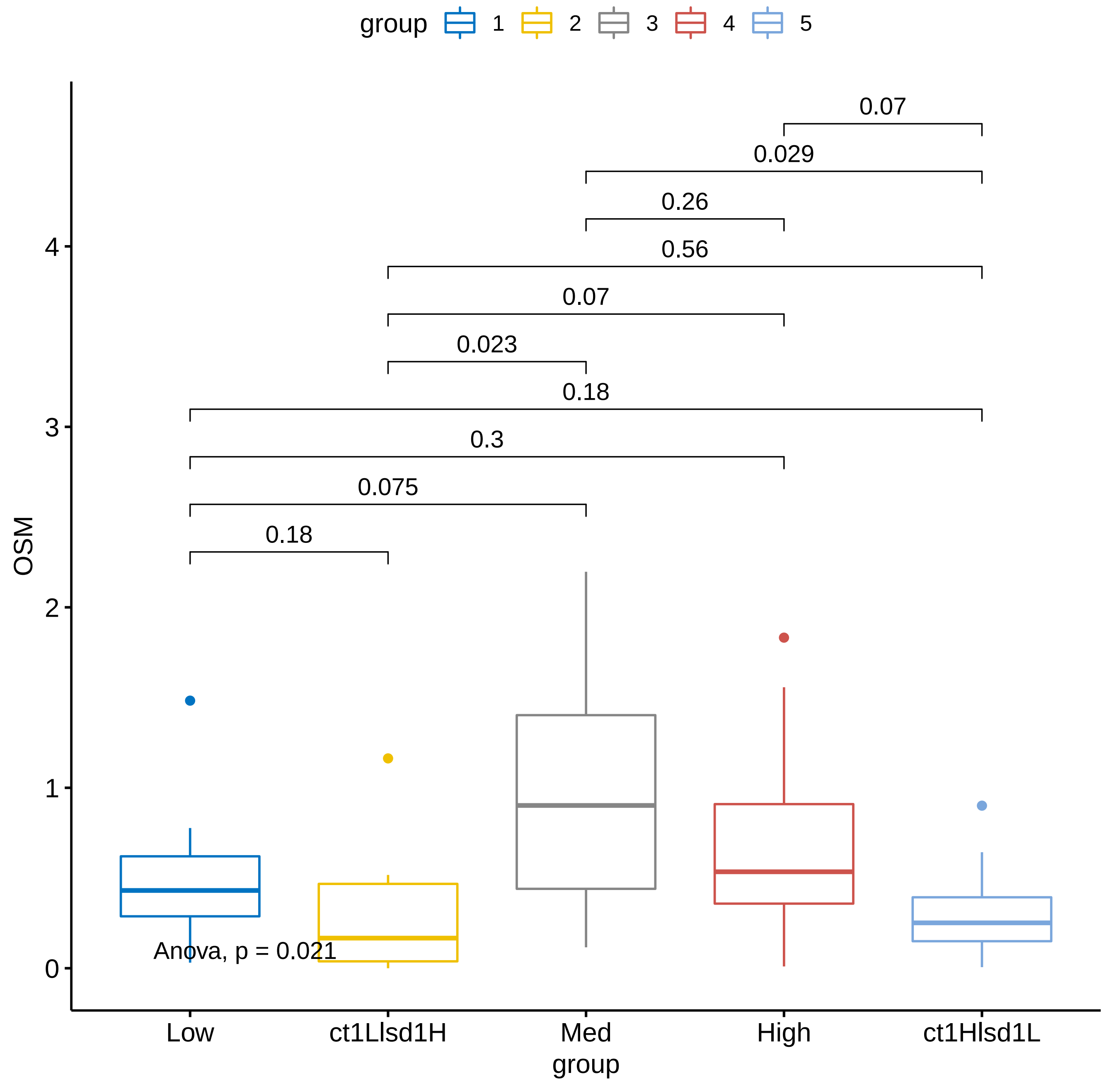

group
1
2
3
4
5
0.07
0.029
0.26
0.56
0.07
0.023
0.18
0.3
0.075
0.18
Anova, p = 0.021
OSM
0
1
2
3
4
Low
ct1Llsd1H
Med
High
ct1Hlsd1L
group

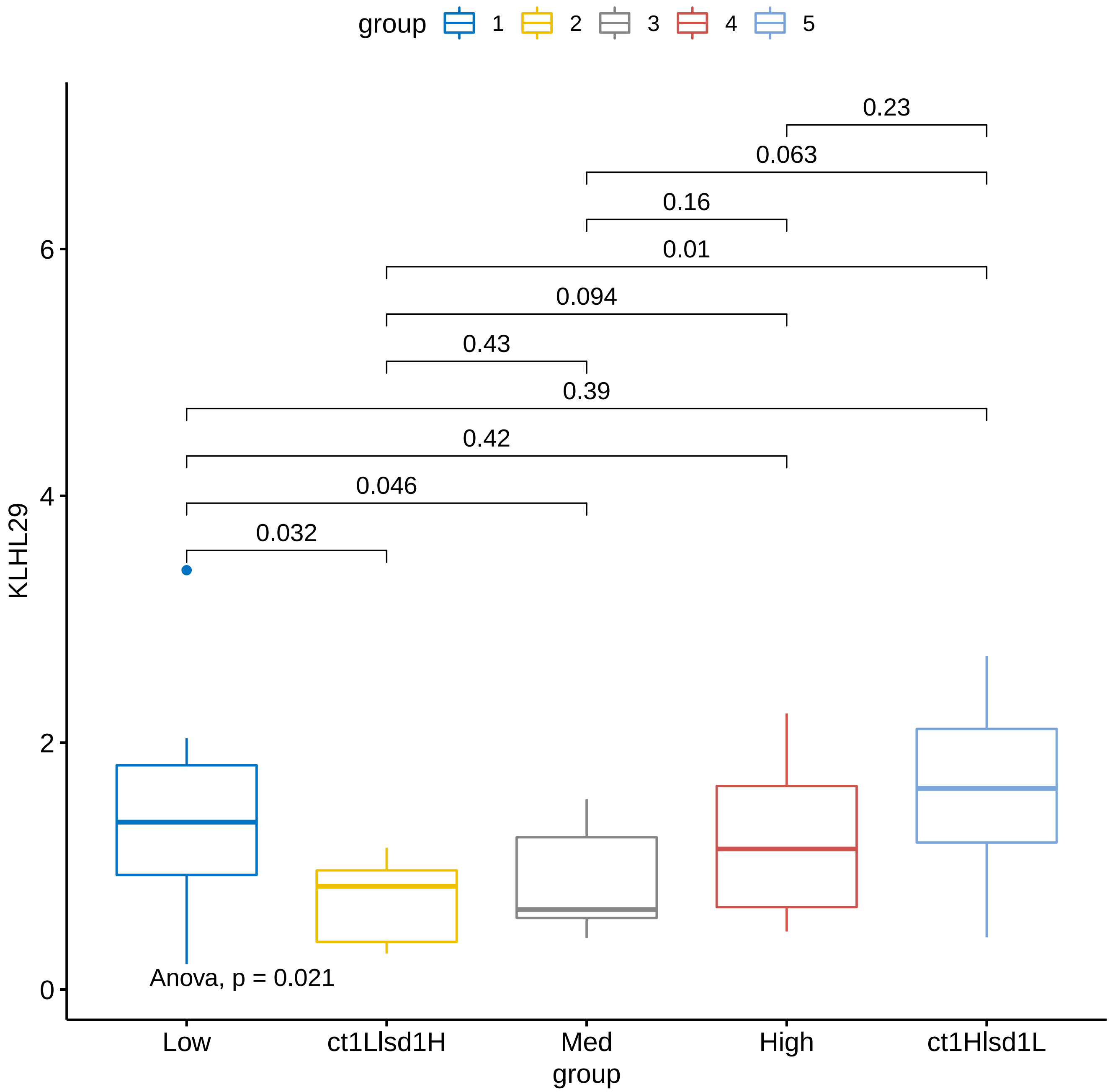

group
1
2
3
4
5
0.23
0.063
0.16
0.01
0.094
0.43
0.39
0.42
0.046
0.032
Anova, p = 0.021
KLHL29
0
2
4
6
Low
ct1Llsd1H
Med
High
ct1Hlsd1L
group

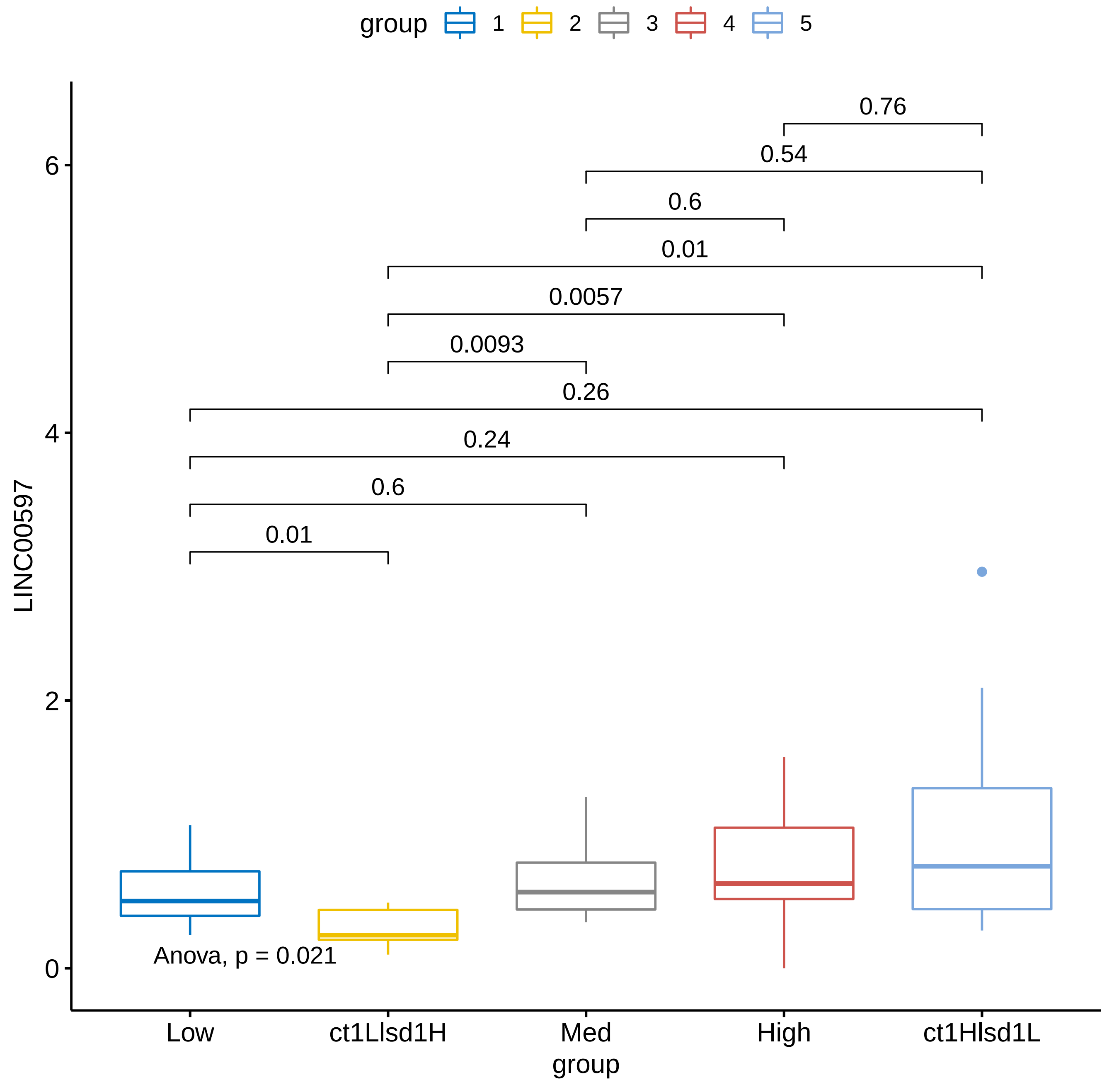
group 1 2 3 4 5
0.76
0.54
0.6
0.01
0.0057
0.0093
0.26
0.24
0.6
0.01
6
4
2
0
LINC00597
Anova, p = 0.021
Low
ct1Llsd1H
Med
High
ct1Hlsd1L
group

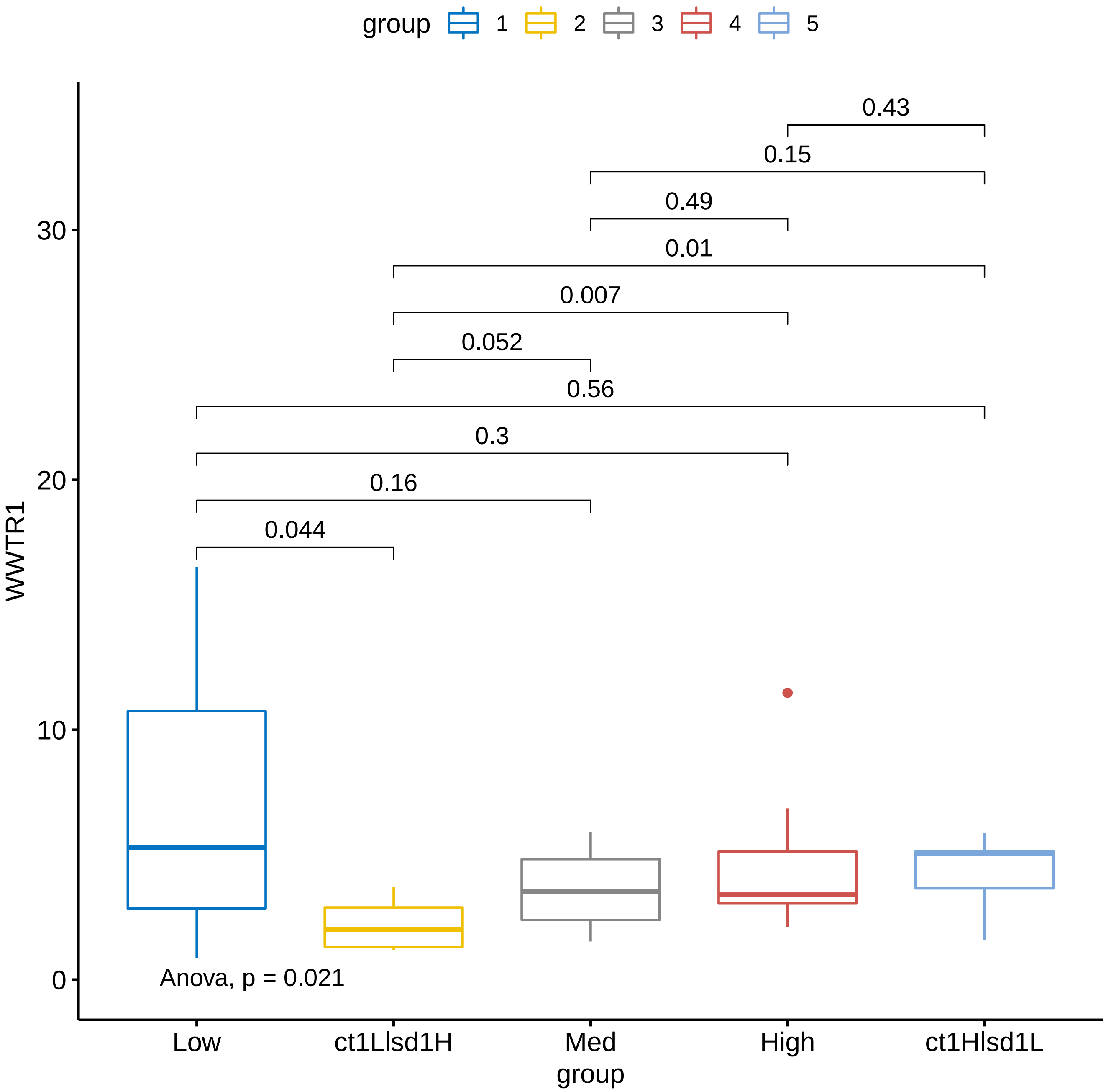

group
1
2
3
4
5
0.43
0.15
0.49
0.01
0.007
0.052
0.56
0.3
0.16
0.044
30
20
10
0
WWTR1
Anova, p = 0.021
Low
ct1Llsd1H
Med
High
ct1Hlsd1L
group

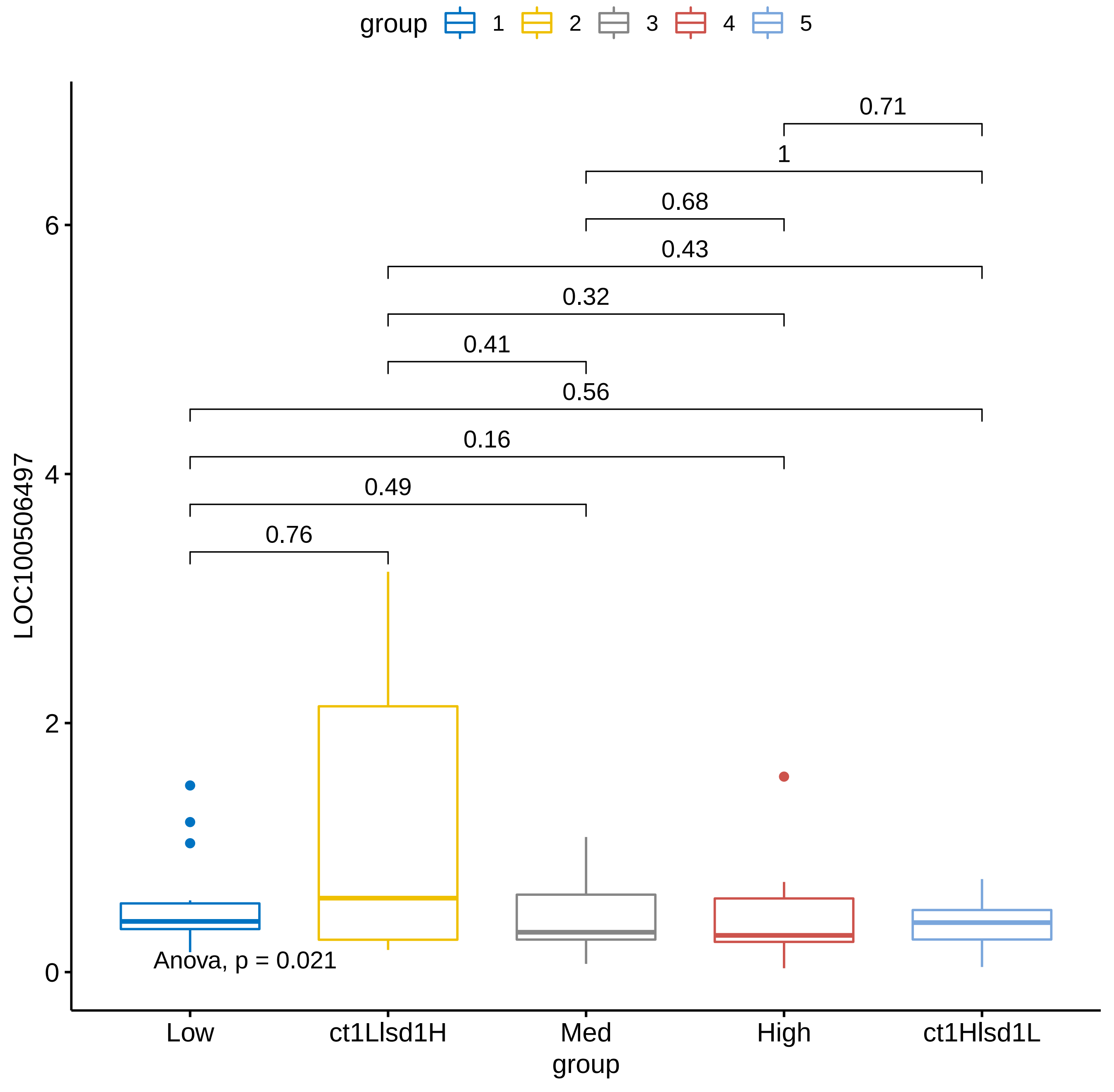

group
1
2
3
4
5
0.71
1
0.68
0.43
0.32
0.41
0.56
0.16
0.49
0.76
Anova, p = 0.021
LOC100506497
0
2
4
6
Low
ct1Llsd1H
Med
High
ct1Hlsd1L
group

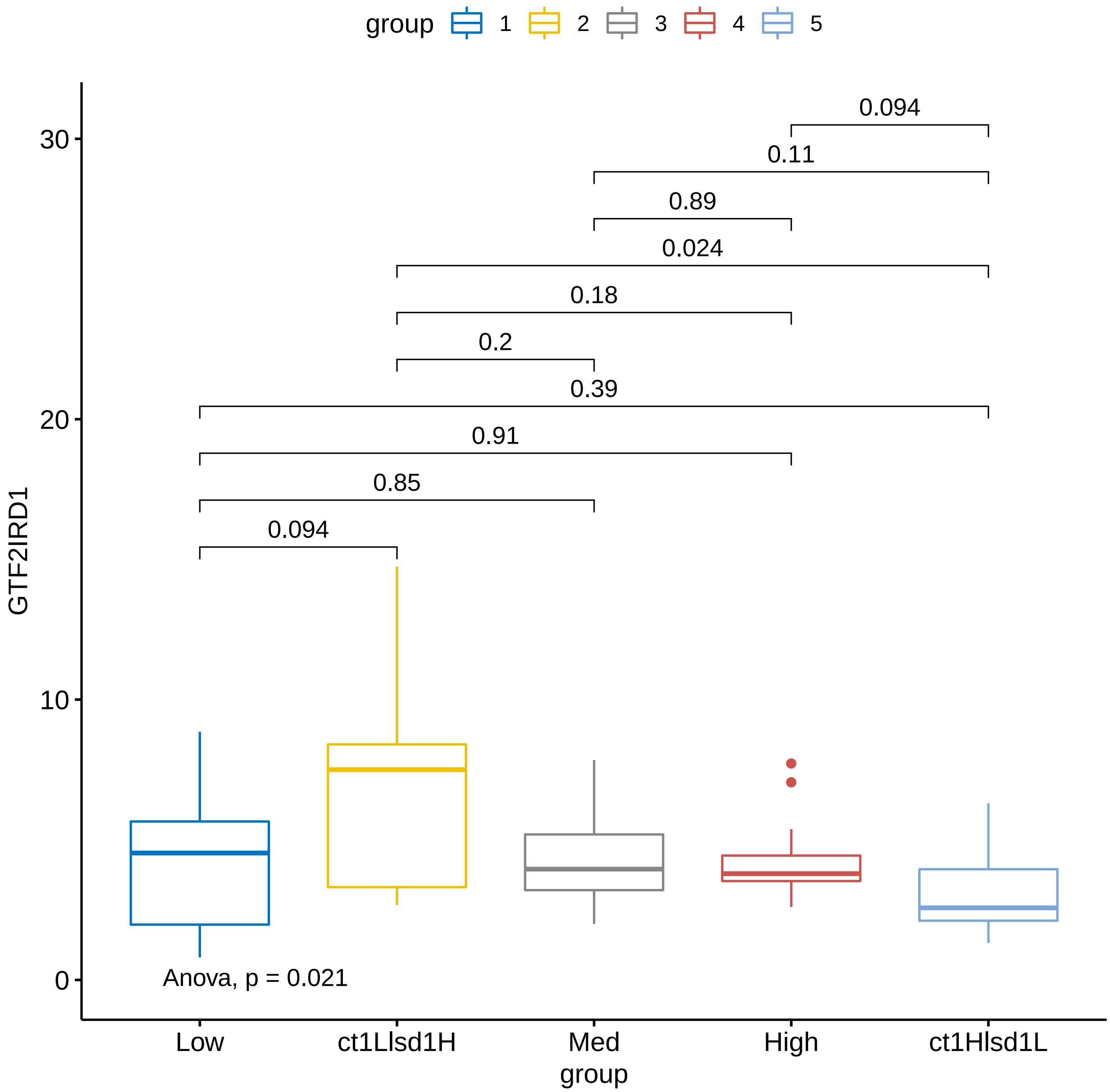
group
1
2
3
4
5
GTF2IRD1
30
20
10
0
0.094
0.11
0.89
0.024
0.18
0.2
0.39
0.91
0.85
0.094
Anova, p = 0.021
Low
ct1Llsd1H
Med
High
ct1Hlsd1L
group

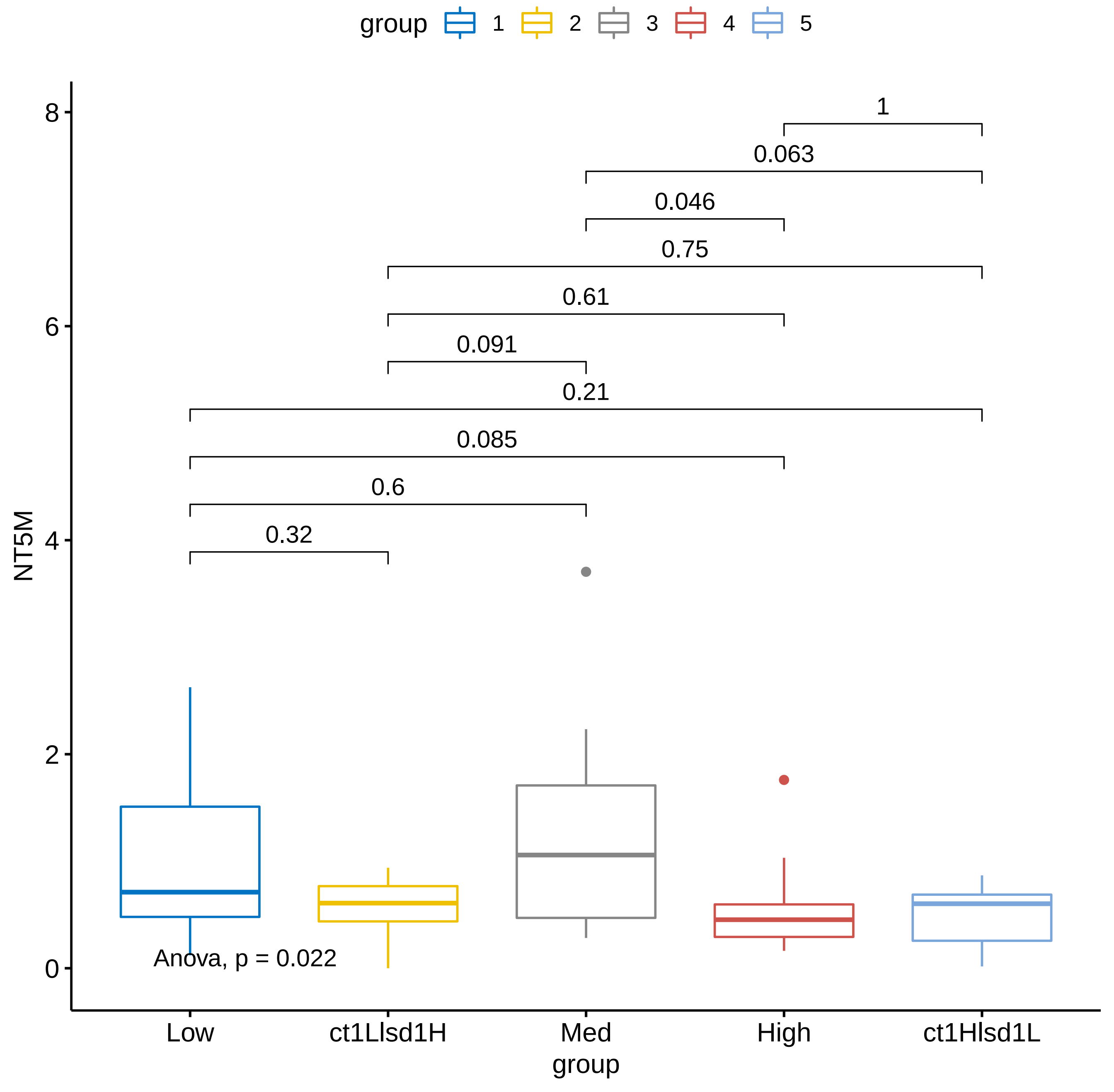

group 1 2 3 4 5
1
0.063
0.046
0.75
0.61
0.091
0.21
0.085
0.6
0.32
Anova, p = 0.022
NT5M
0
2
4
6
8
Low
ct1Llsd1H
Med
High
ct1Hlsd1L
group

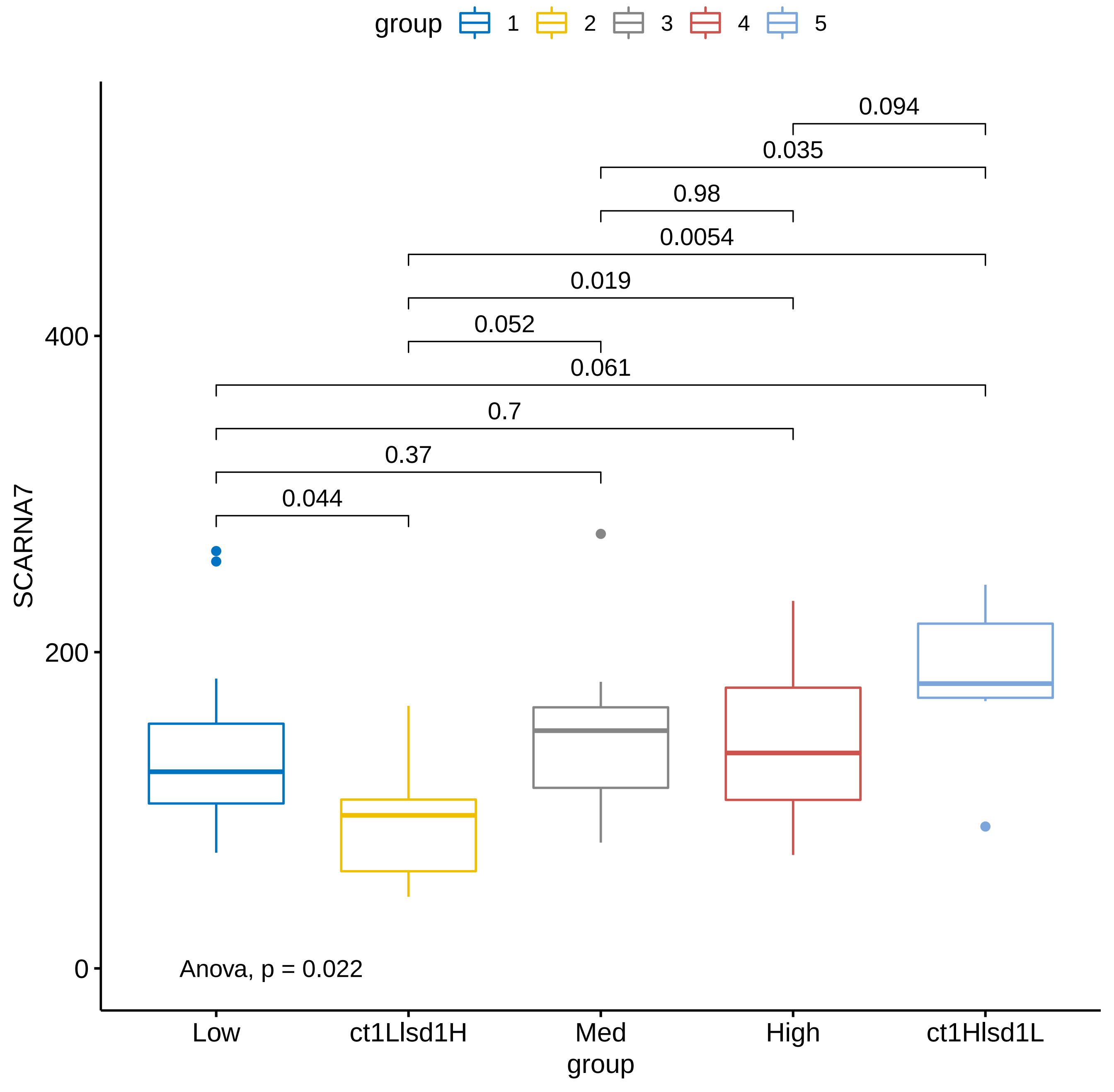

group 1 2 3 4 5
0.094
0.035
0.98
0.0054
0.019
0.052
0.061
0.7
0.37
0.044
SCARNA7
400
200
0
Anova, p = 0.022
Low
ct1Llsd1H
Med
High
ct1Hlsd1L
group

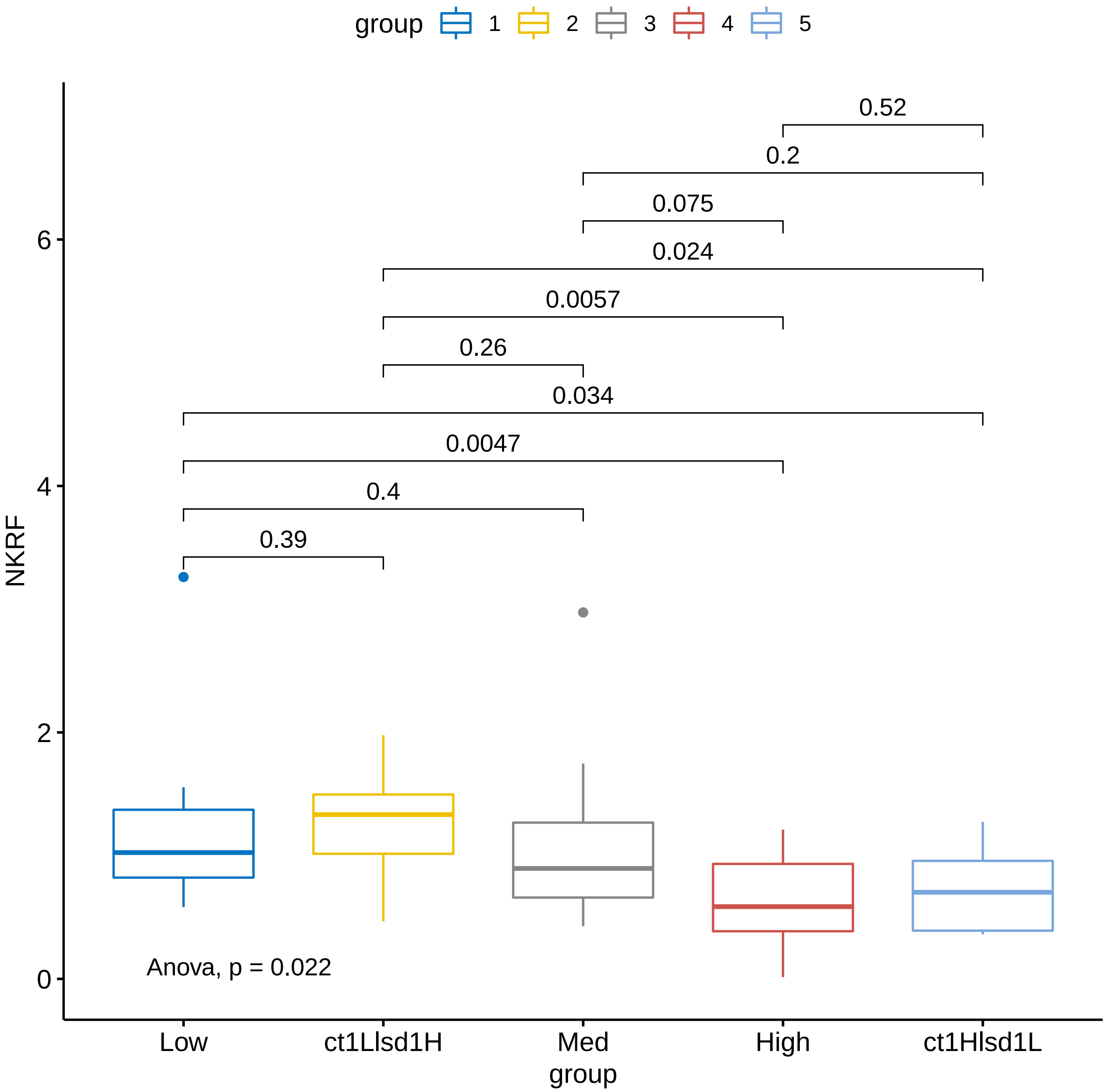
group
1
2
3
4
5
0.52
0.2
0.075
0.024
0.0057
0.26
0.034
0.0047
0.4
0.39
6
4
2
0
NKRF
Anova, p = 0.022
Low
ct1Llsd1H
Med
High
ct1Hlsd1L
group

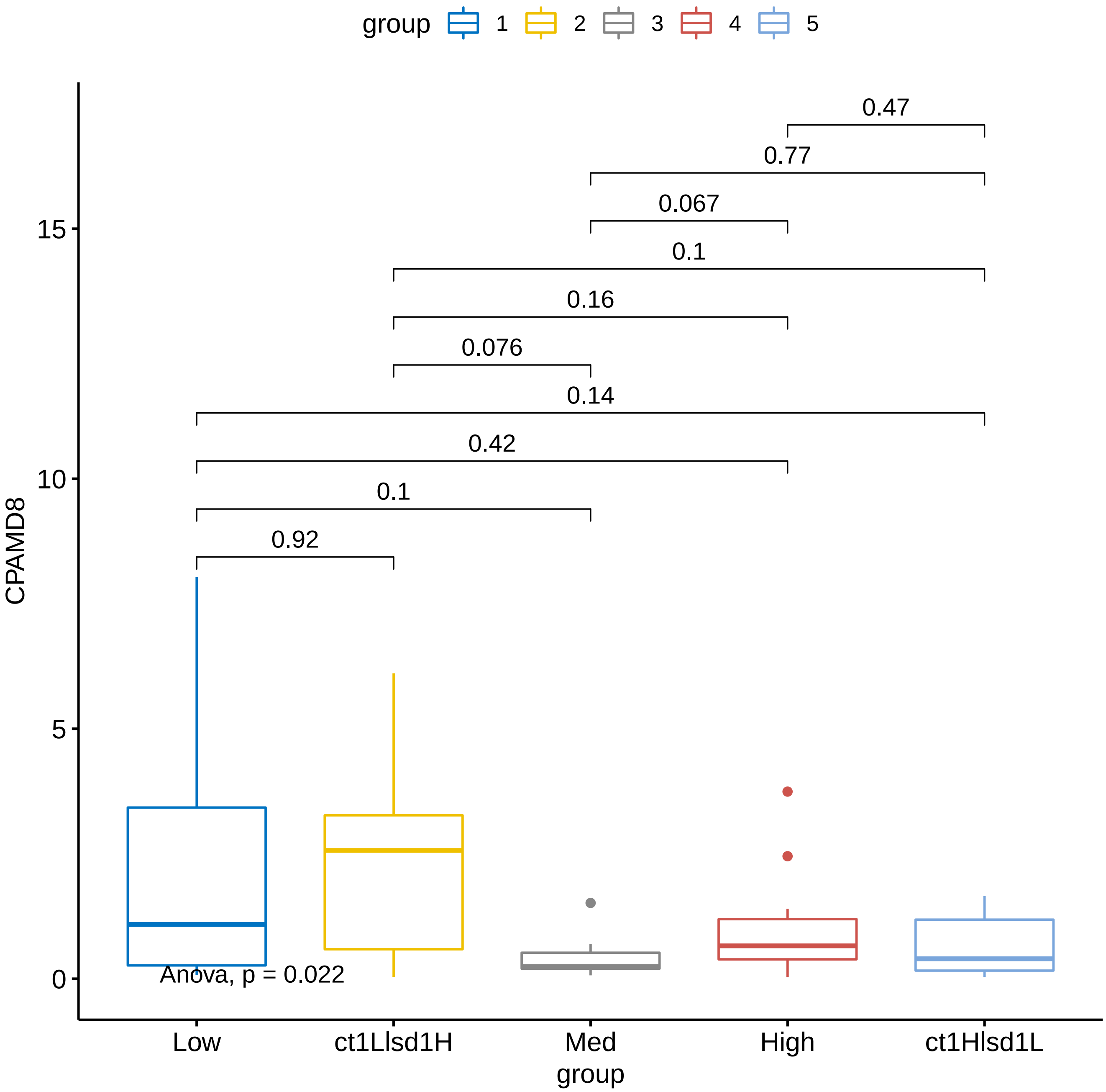

group
1
2
3
4
5
CPAMD8
15
10
5
0
0.47
0.77
0.067
0.1
0.16
0.076
0.14
0.42
0.1
0.92
Anova, p = 0.022
Low
ct1Llsd1H
Med
High
ct1Hlsd1L
group

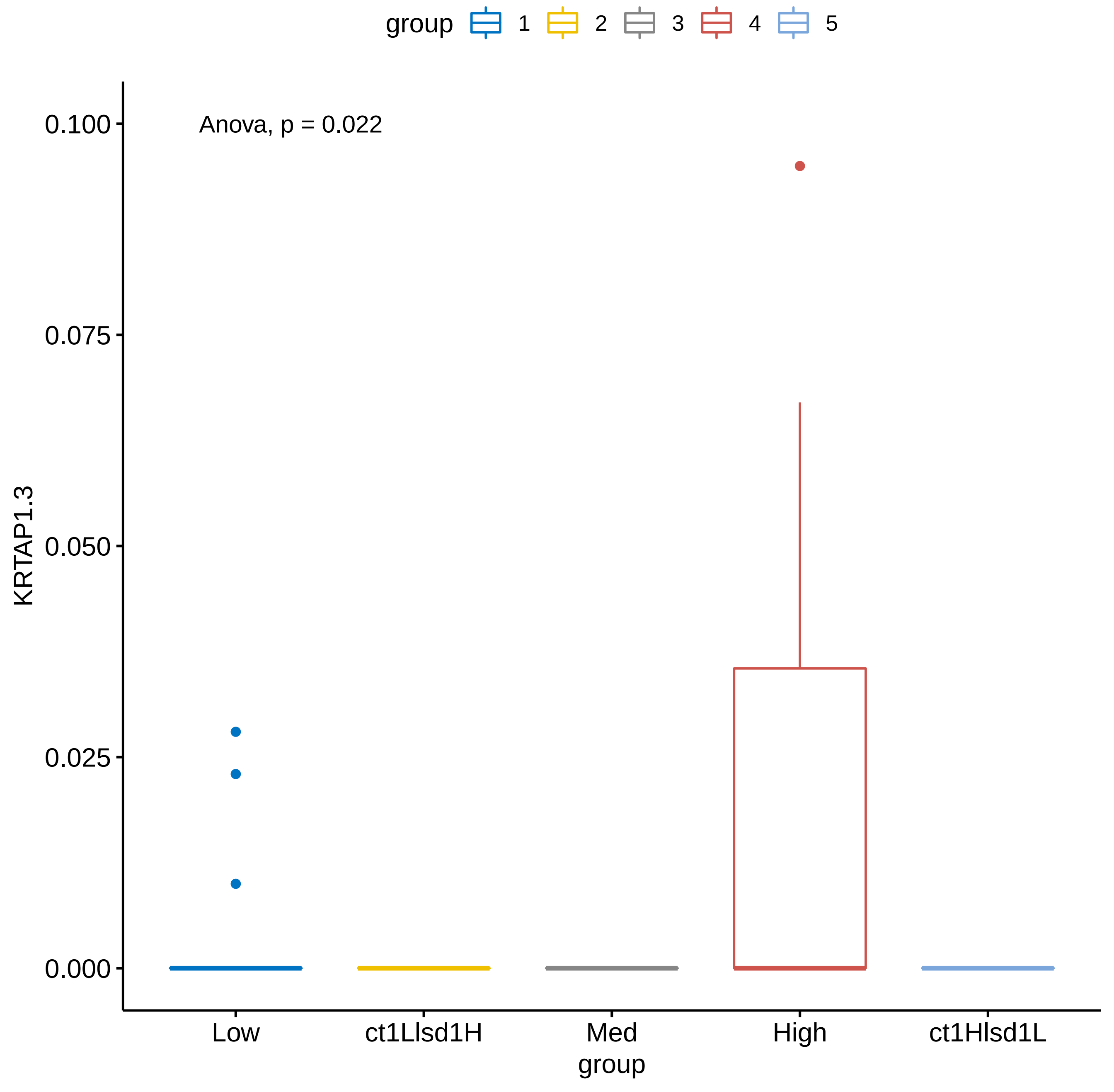

group
1
2
3
4
5
Anova, p = 0.022
KRTAP1.3
0.100
0.075
0.050
0.025
0.000
Low
ct1Llsd1H
Med
High
ct1Hlsd1L
group

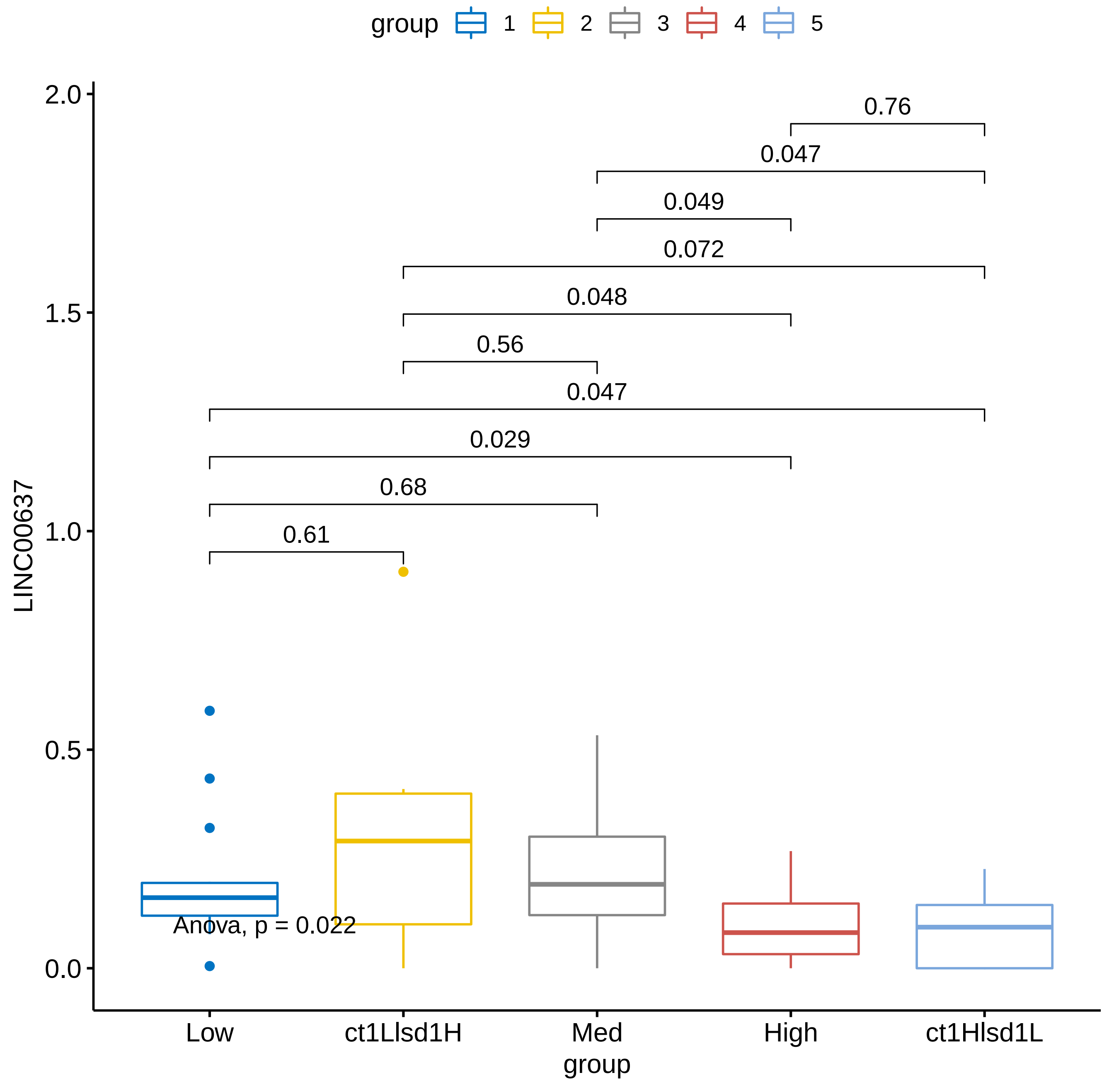

group
1
2
3
4
5
LINC00637
2.0
1.5
1.0
0.5
0.0
0.76
0.047
0.049
0.072
0.048
0.56
0.047
0.029
0.68
0.61
Anova, p = 0.022
Low
ct1Llsd1H
Med
High
ct1Hlsd1L
group

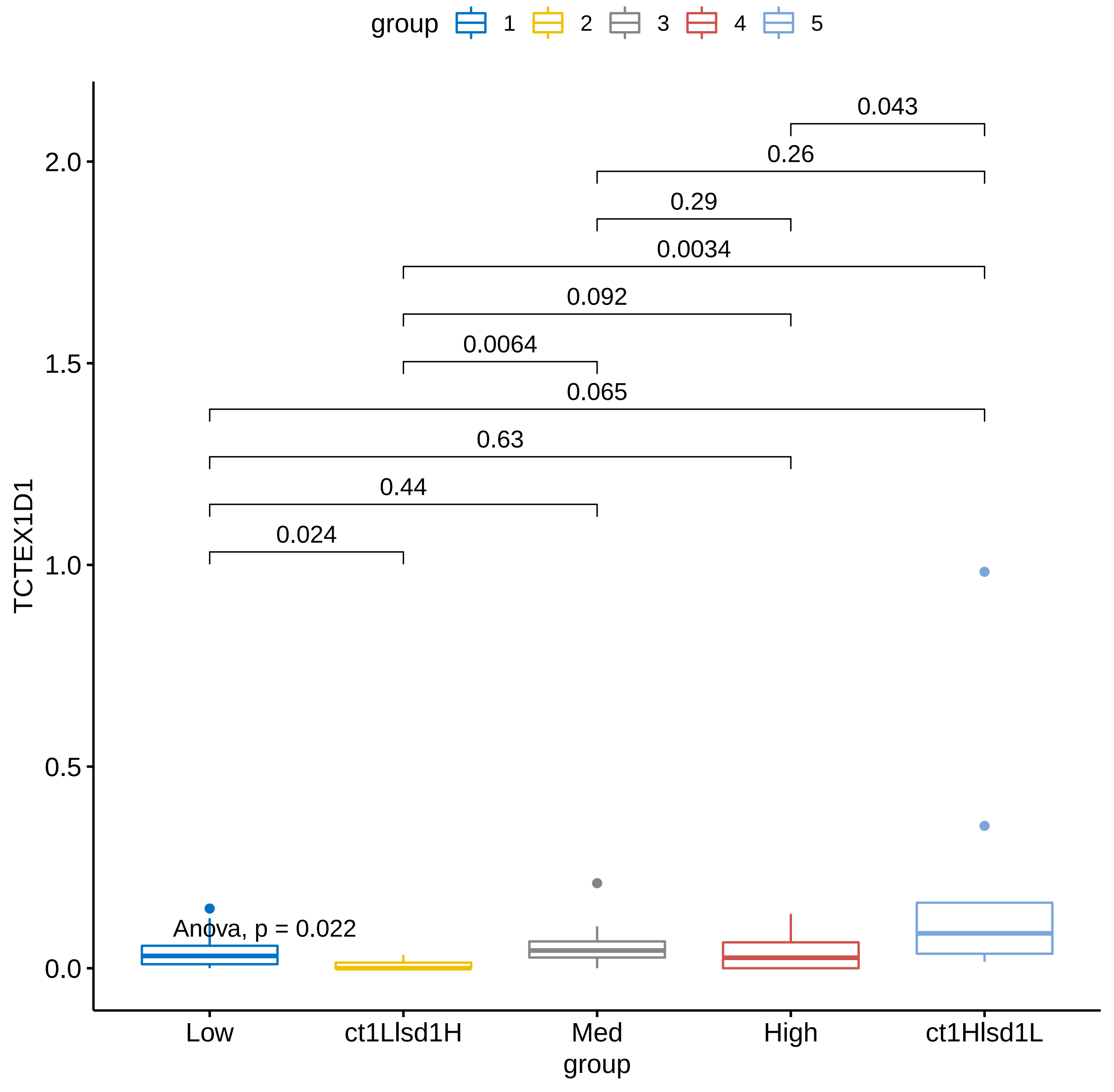
group
1
2
3
4
5
0.043
0.26
0.29
0.0034
0.092
0.0064
0.065
0.63
0.44
0.024
2.0
1.5
1.0
0.5
0.0
TCTEX1D1
Anova, p = 0.022
Low
ct1Llsd1H
Med
High
ct1Hlsd1L
group

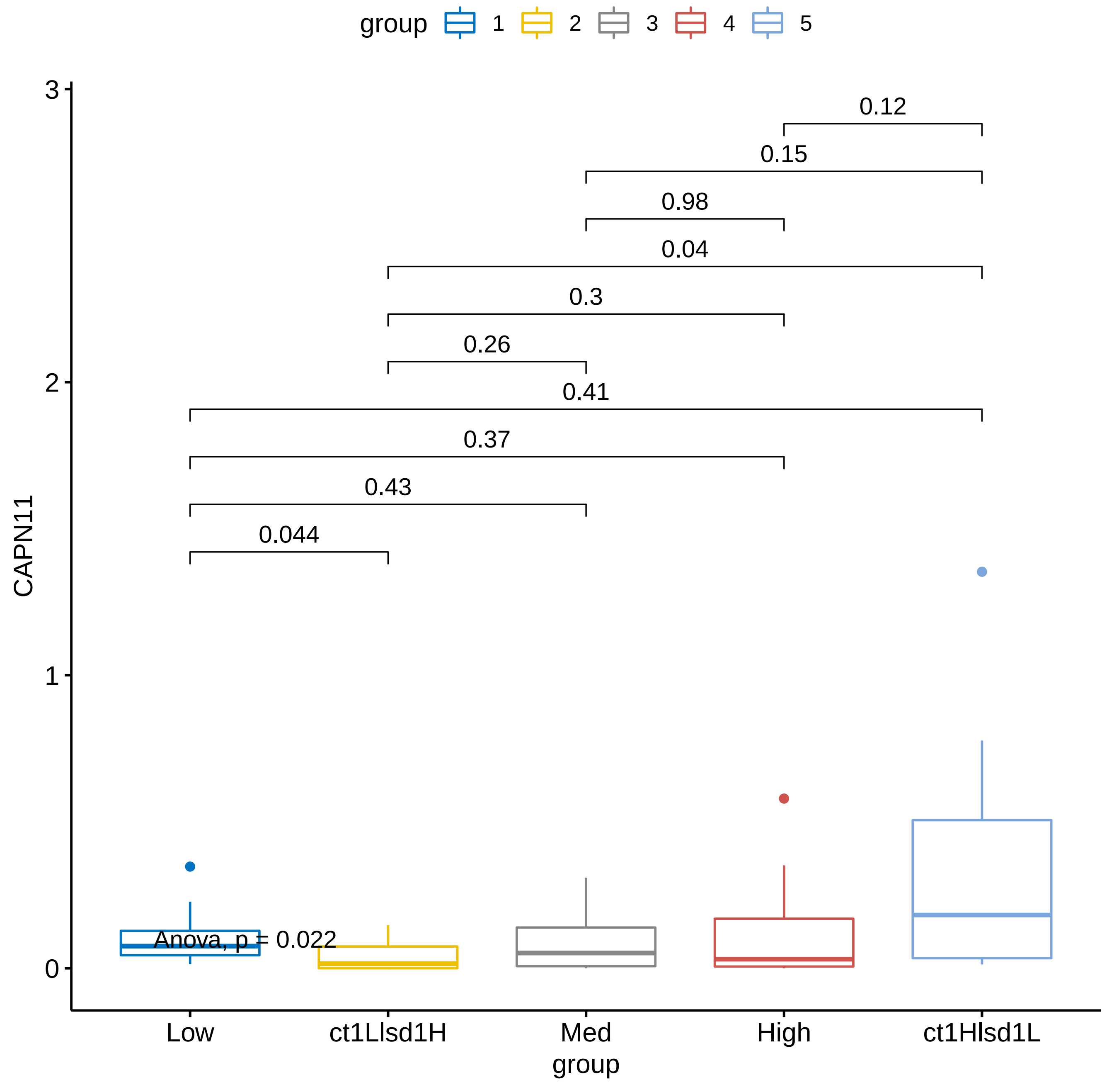

group 1 2 3 4 5
CAPN11
0
1
2
3
0.12
0.15
0.98
0.04
0.3
0.26
0.41
0.37
0.43
0.044
Anova, p = 0.022
Low
ct1Llsd1H
Med
High
ct1Hlsd1L
group

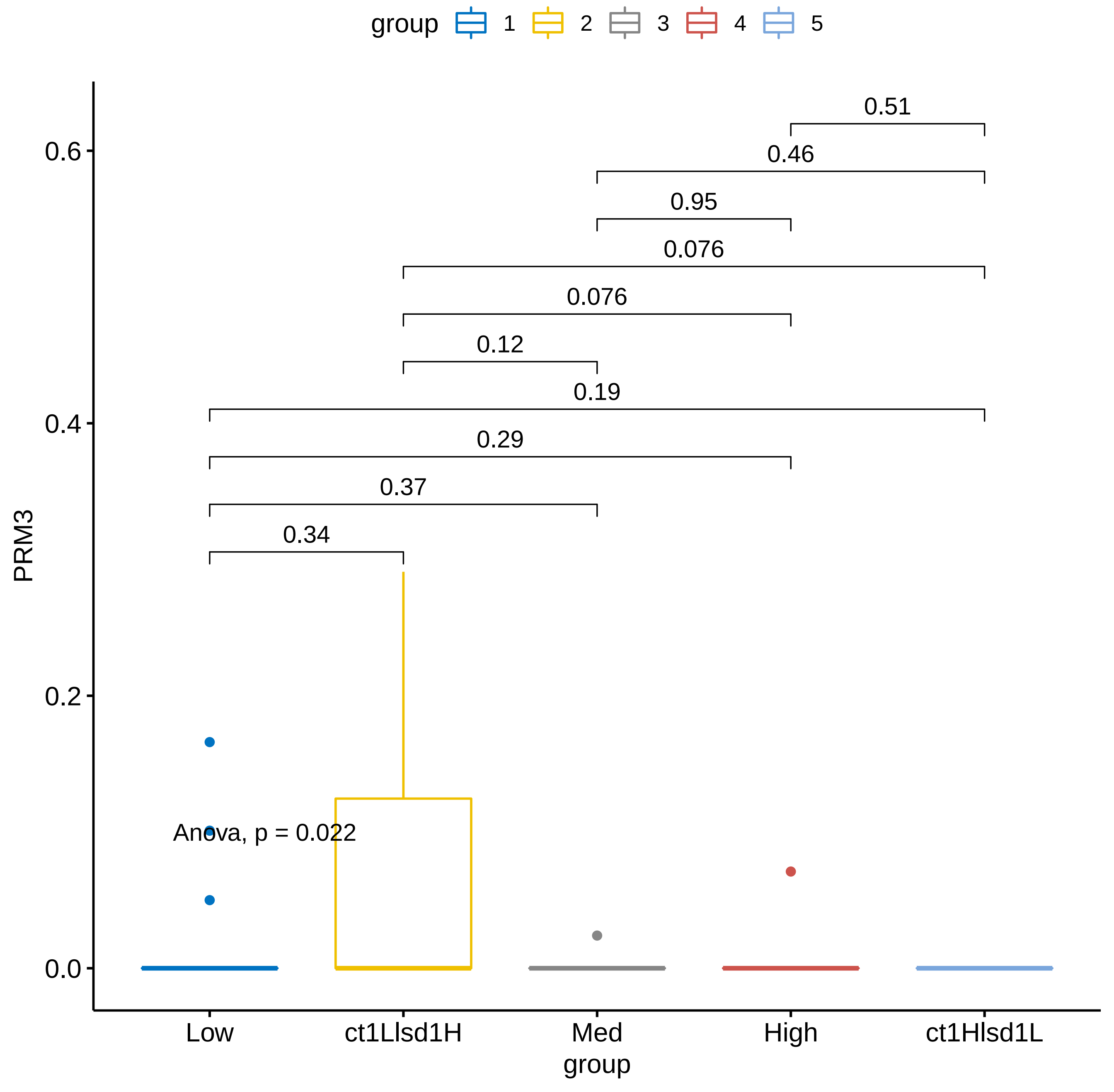

group 1 2 3 4 5
0.51
0.46
0.95
0.076
0.076
0.12
0.19
0.29
0.37
0.34
Anova, p = 0.022
PRM3
0.6
0.4
0.2
0.0
Low
ct1Llsd1H
Med
High
ct1Hlsd1L
group

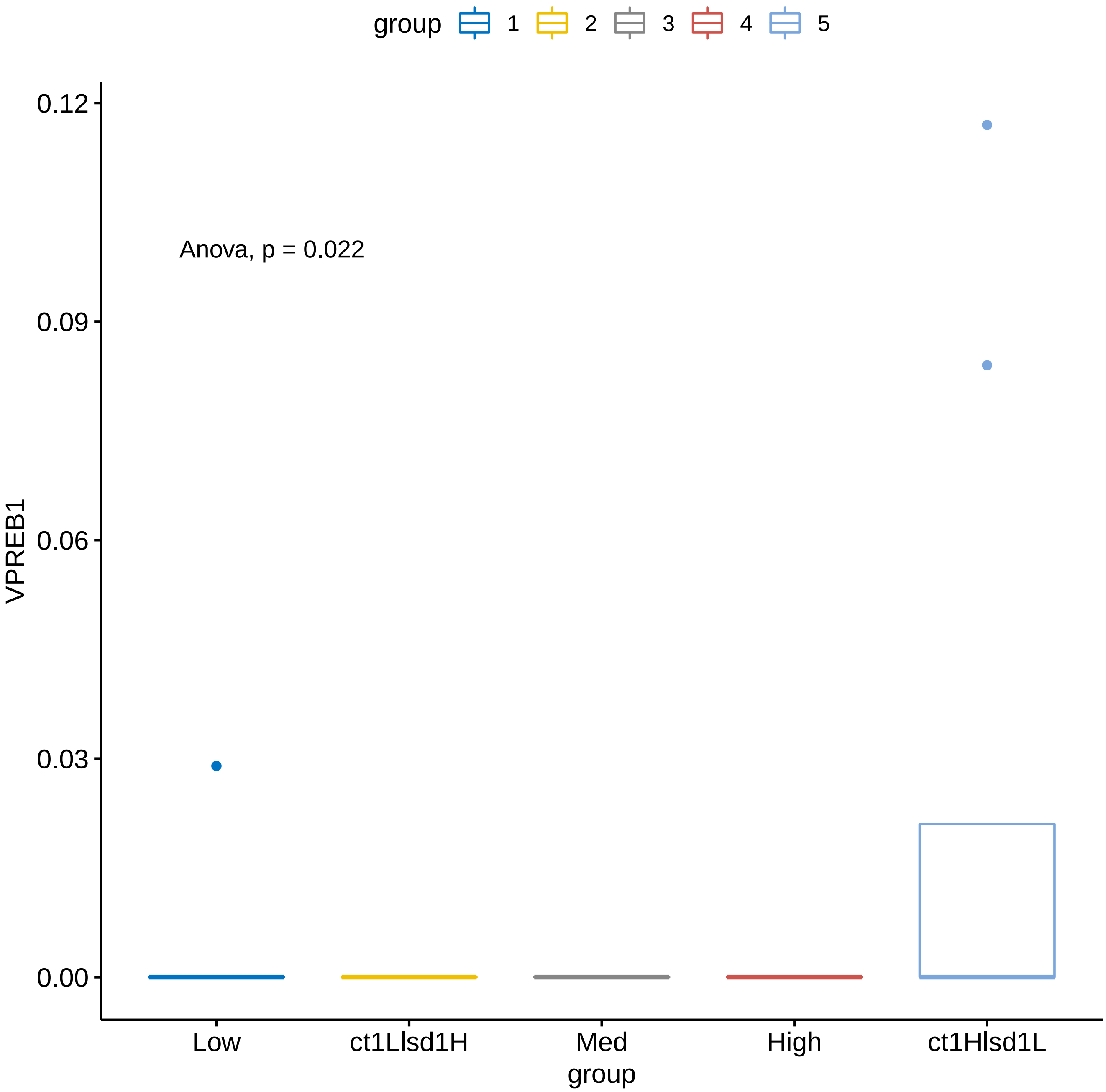

group 1 2 3 4 5
Anova, p = 0.022
0.12
0.09
0.06
0.03
0.00
VPREB1
Low
ct1Llsd1H
Med
High
ct1Hlsd1L
group

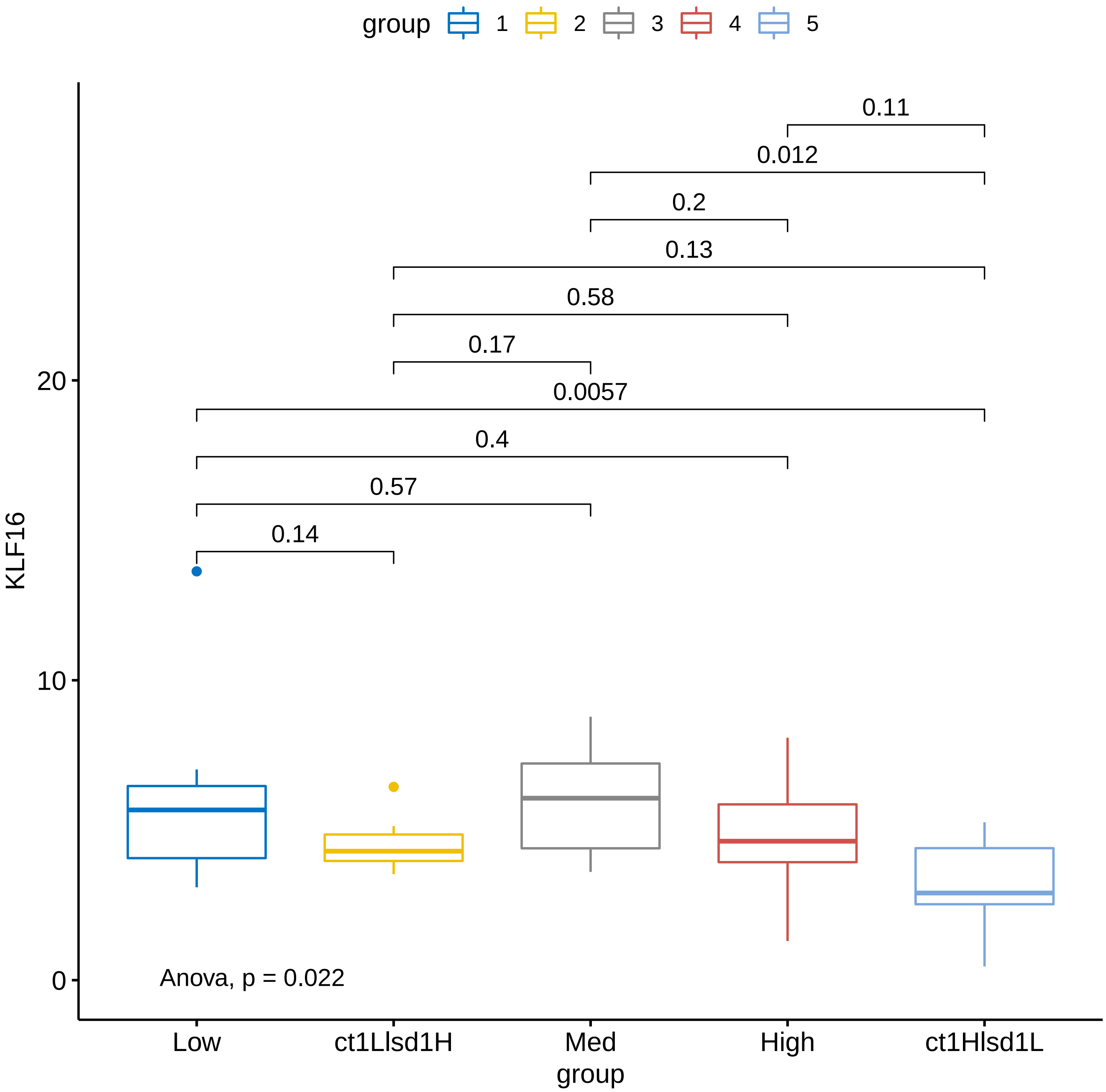

group
1
2
3
4
5
0.11
0.012
0.2
0.13
0.58
0.17
0.0057
0.4
0.57
0.14
KLF16
20
10
0
Anova, p = 0.022
Low
ct1Llsd1H
Med
High
ct1Hlsd1L
group

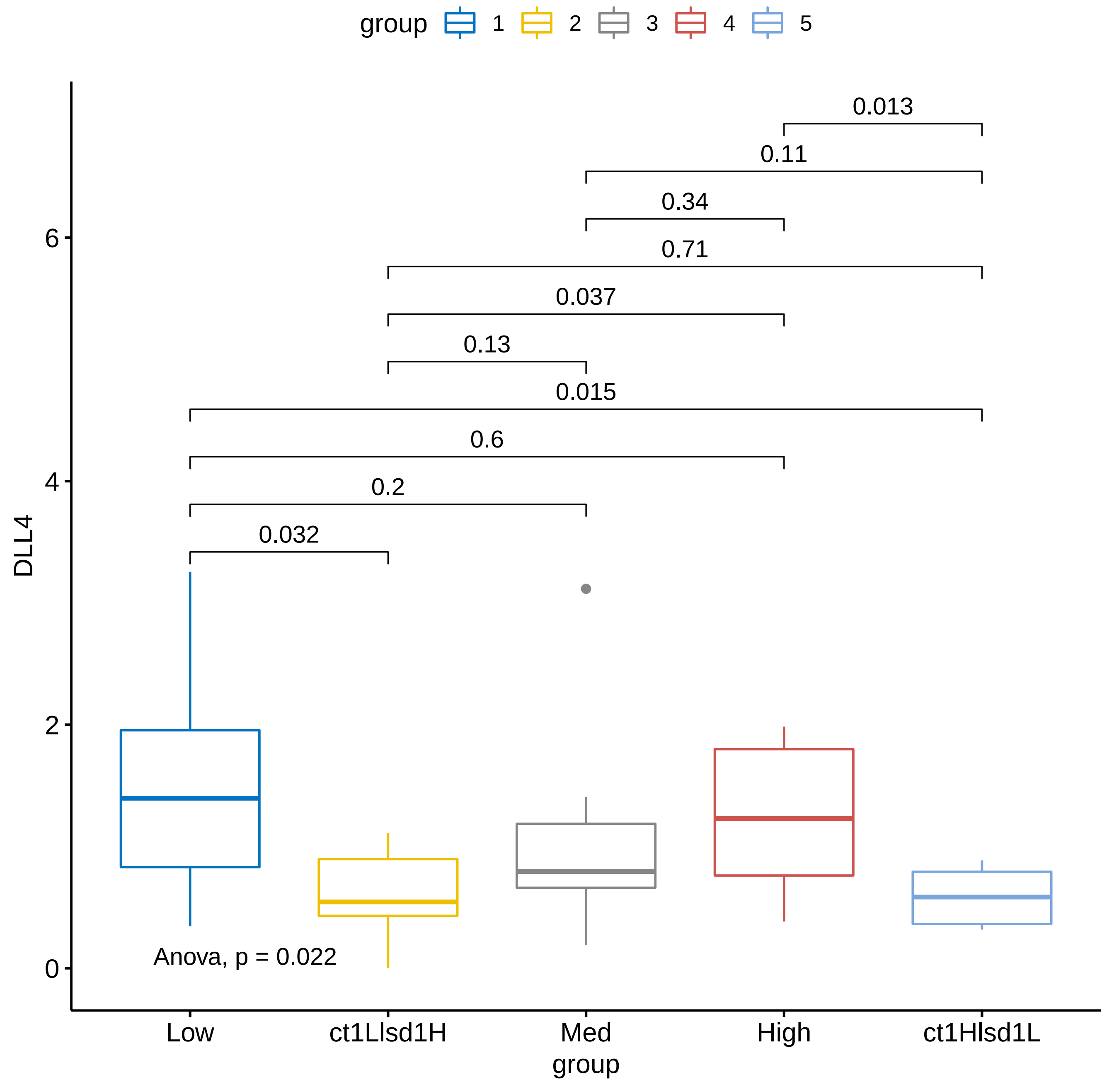

group 1 2 3 4 5
0.013
0.11
0.34
0.71
0.037
0.13
0.015
0.6
0.2
0.032
Anova, p = 0.022
DLL4
0
2
4
6
Low
ct1Llsd1H
Med
High
ct1Hlsd1L
group

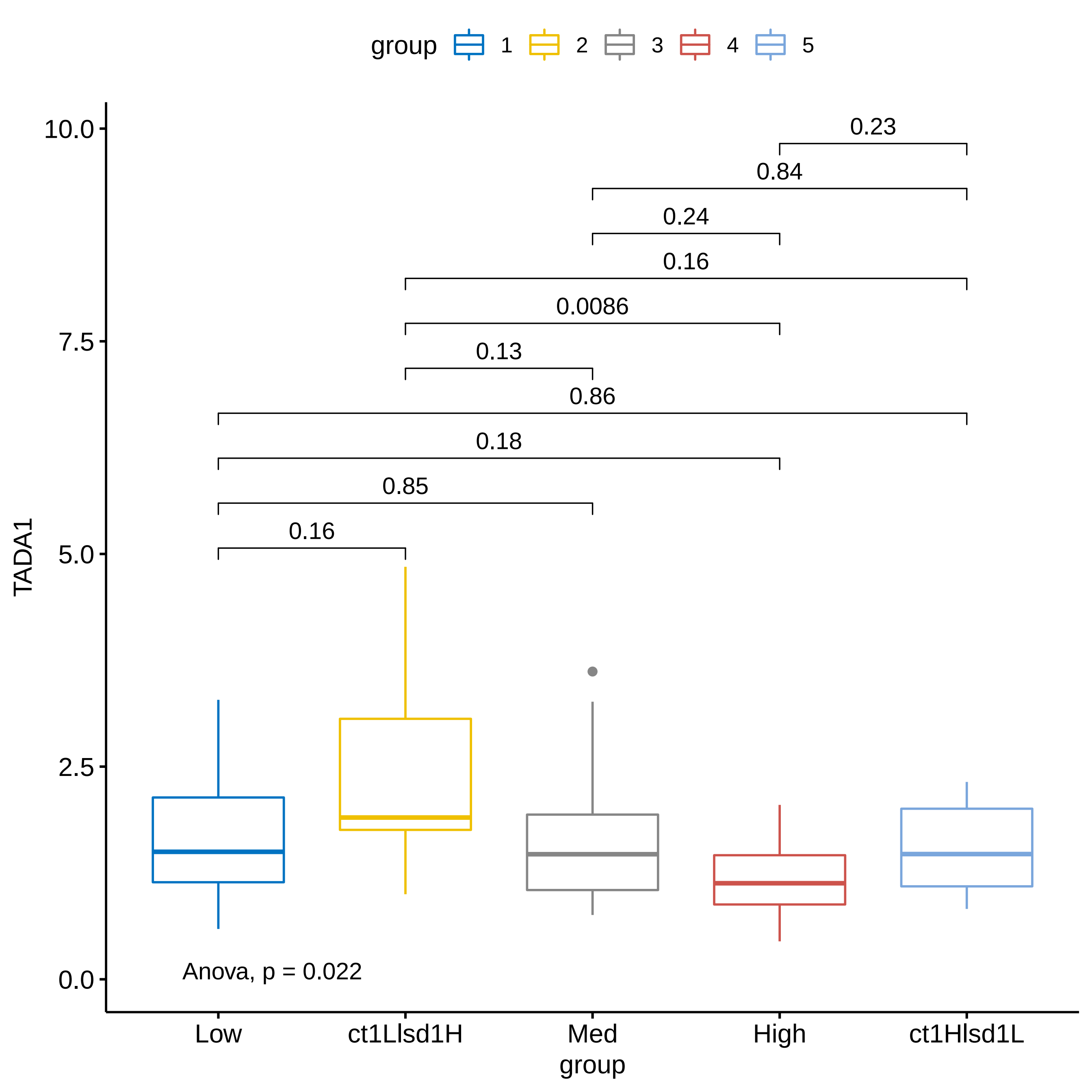
group
1
2
3
4
5
TADA1
10.0
7.5
5.0
2.5
0.0
0.23
0.84
0.24
0.16
0.0086
0.13
0.86
0.18
0.85
0.16
Anova, p = 0.022
Low
ct1Llsd1H
Med
High
ct1Hlsd1L
group

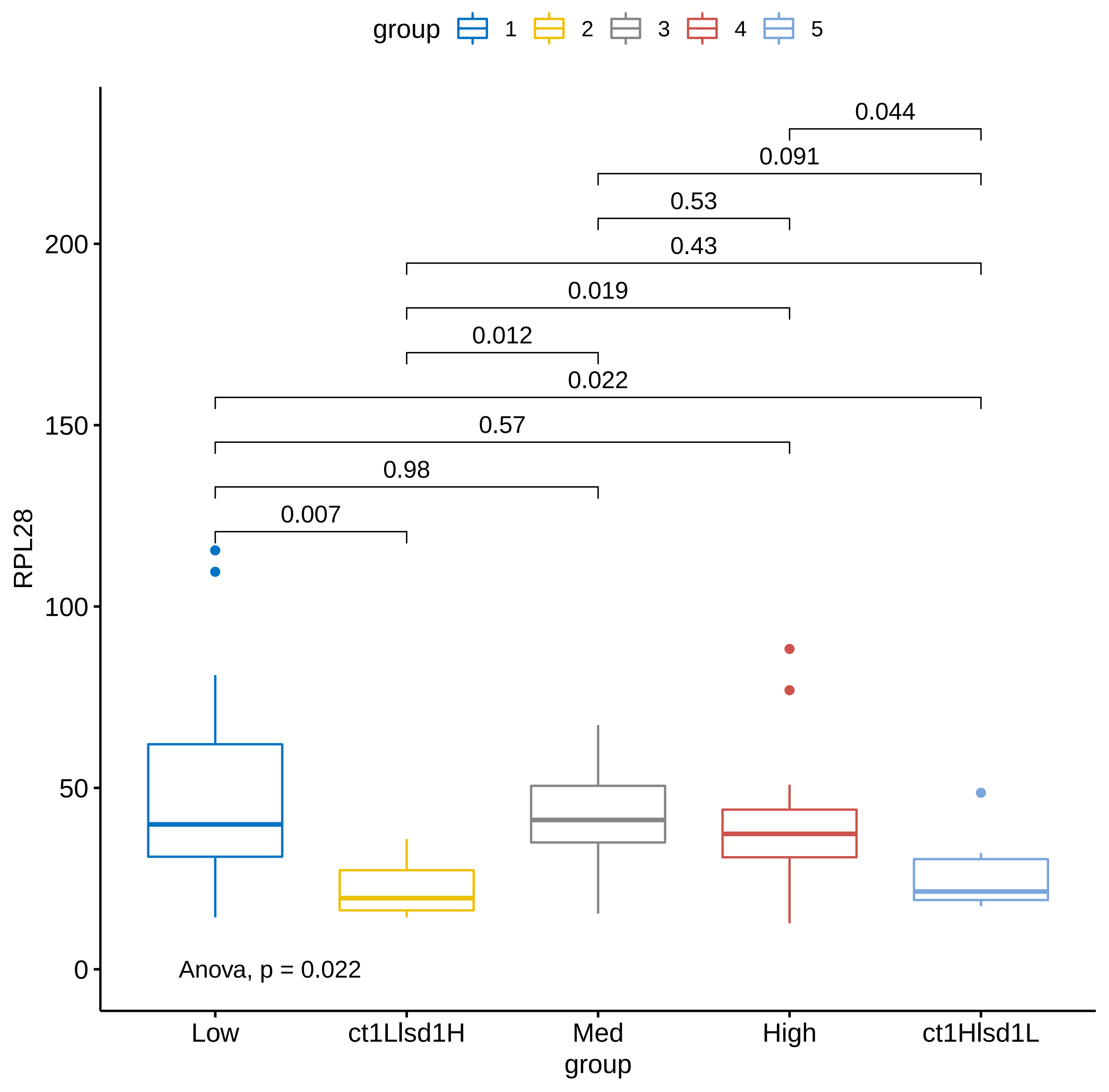
group
1
2
3
4
5
0.044
0.091
0.53
0.43
0.019
0.012
0.022
0.57
0.98
0.007
200
150
100
50
0
RPL28
Anova, p = 0.022
Low
ct1Llsd1H
Med
High
ct1Hlsd1L
group

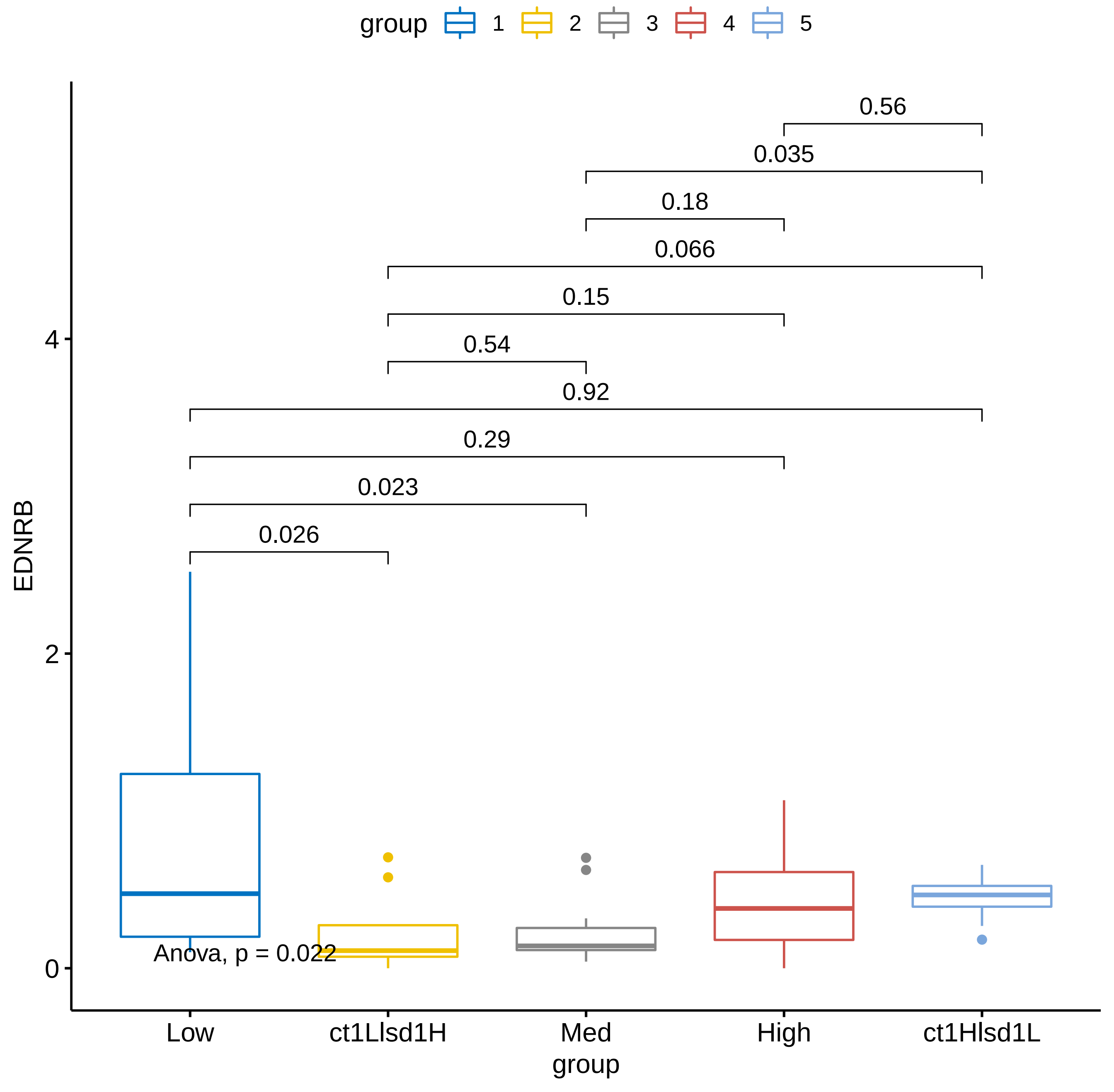

group 1 2 3 4 5
0.56
0.035
0.18
0.066
0.15
0.54
0.92
0.29
0.023
0.026
EDNRB
4
2
0
Anova, p = 0.022
Low
ct1Llsd1H
Med
High
ct1Hlsd1L
group

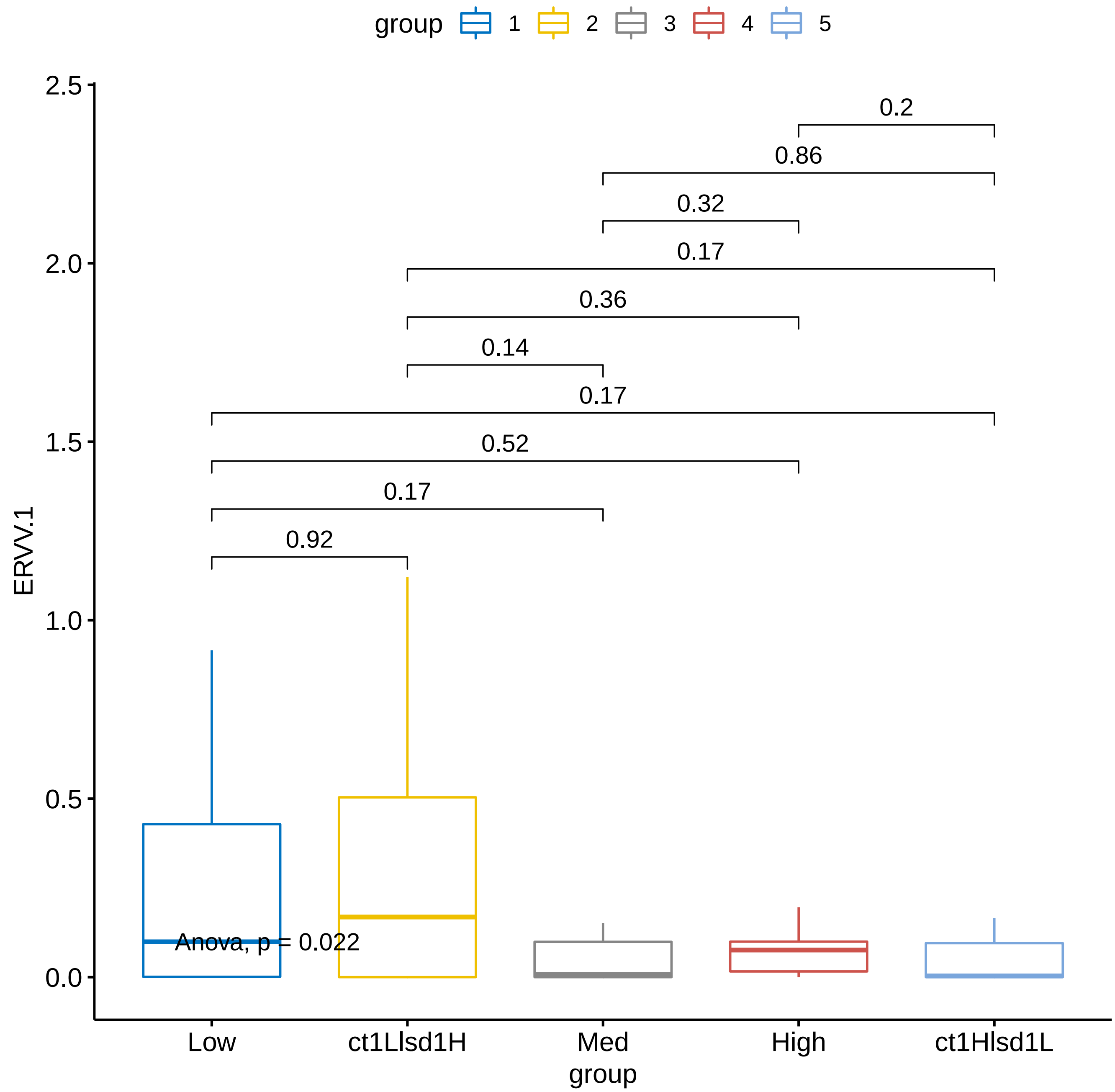

group
1
2
3
4
5
ERVV.1
2.5
2.0
1.5
1.0
0.5
0.0
0.2
0.86
0.32
0.17
0.36
0.14
0.17
0.52
0.17
0.92
Anova, p = 0.022
Low
ct1Llsd1H
Med
High
ct1Hlsd1L
group

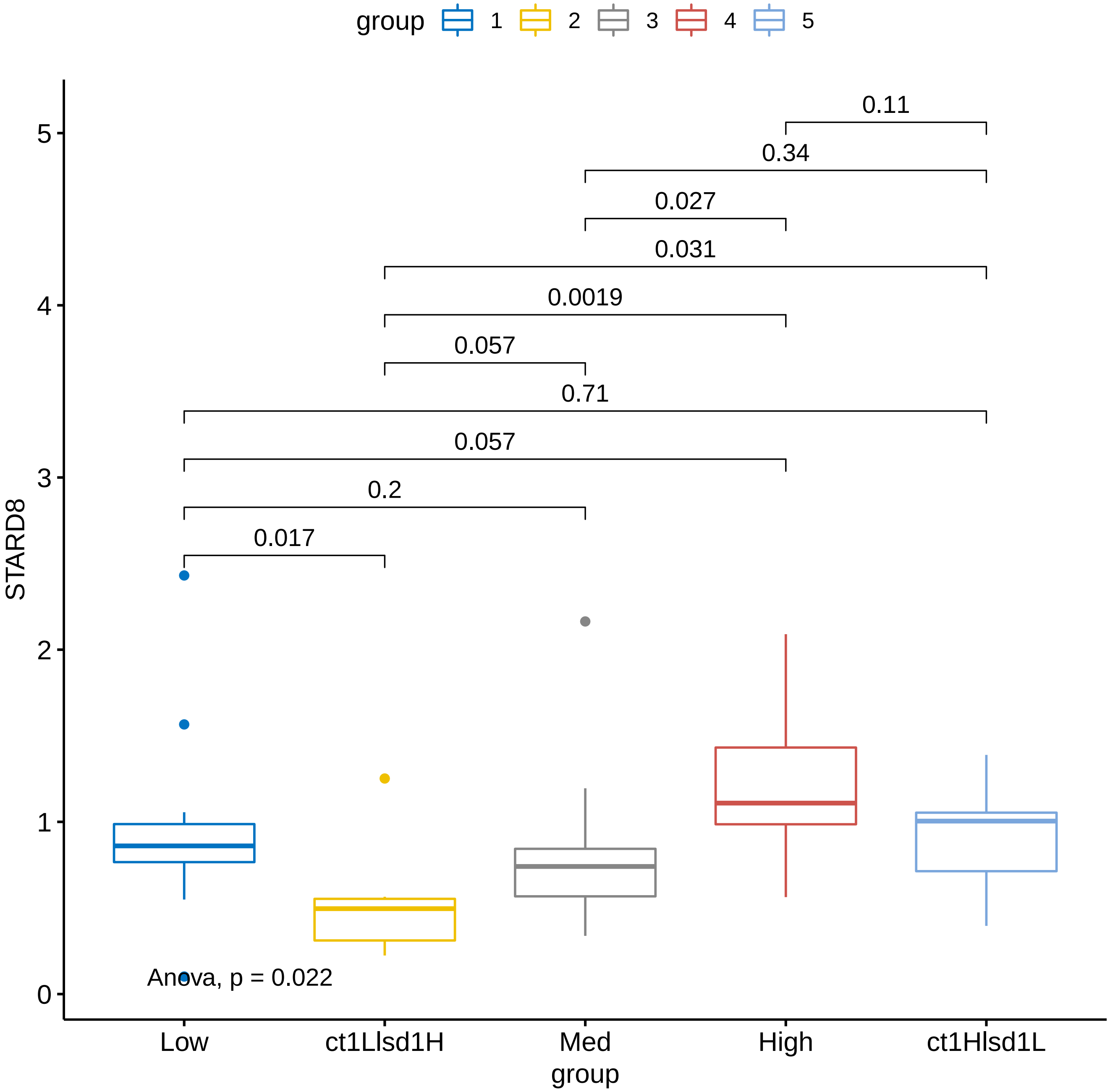

group 1 2 3 4 5
STARD8
0.11
0.34
0.027
0.031
0.0019
0.057
0.71
0.057
0.2
0.017
Anova, p = 0.022
5
4
3
2
1
0
Low
ct1Llsd1H
Med
High
ct1Hlsd1L
group

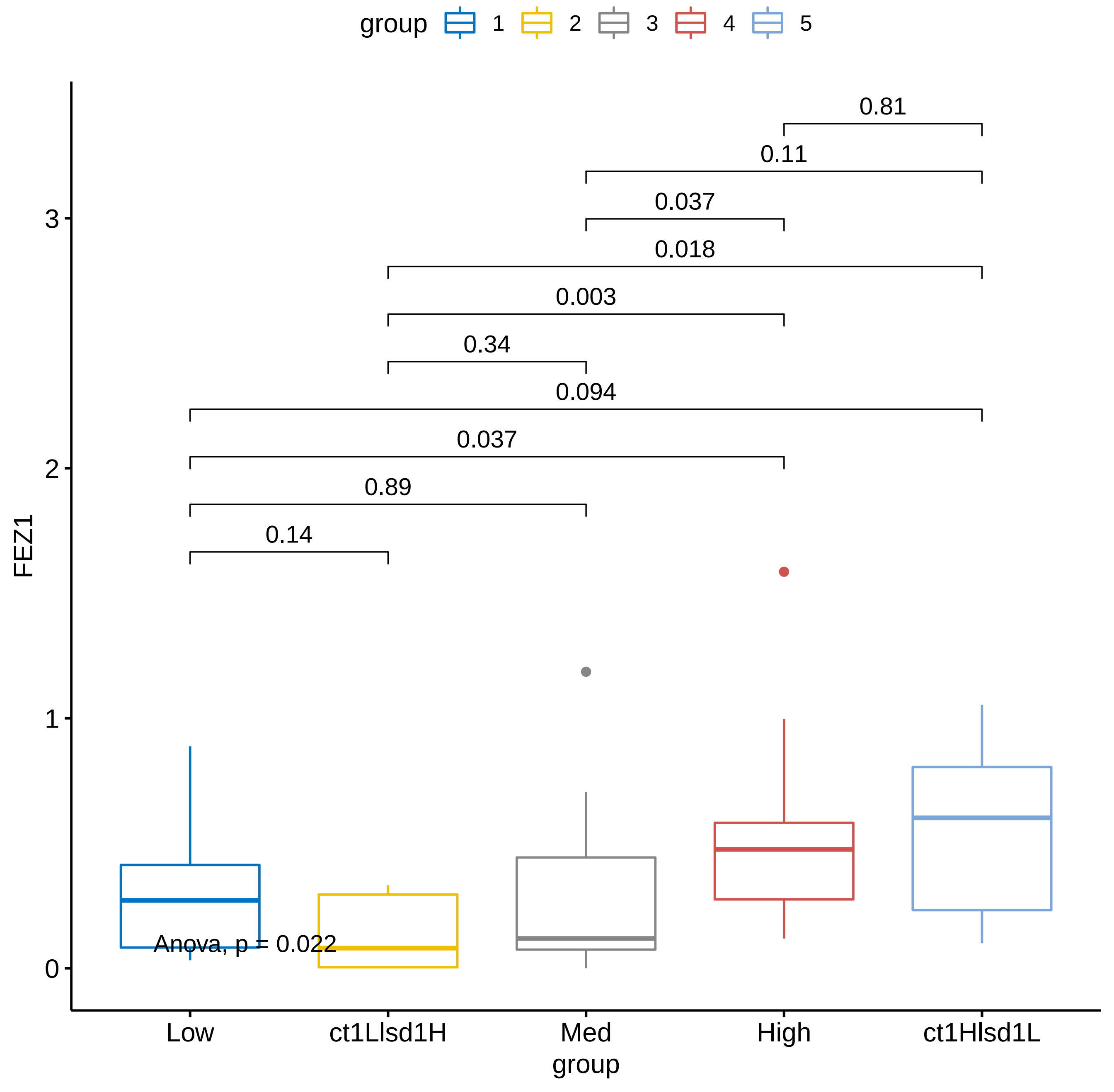

group 1 2 3 4 5
0.81
0.11
0.037
0.018
0.003
0.34
0.094
0.037
0.89
0.14
Anova, p = 0.022
FEZ1
0
1
2
3
Low
ct1Llsd1H
Med
High
ct1Hlsd1L
group

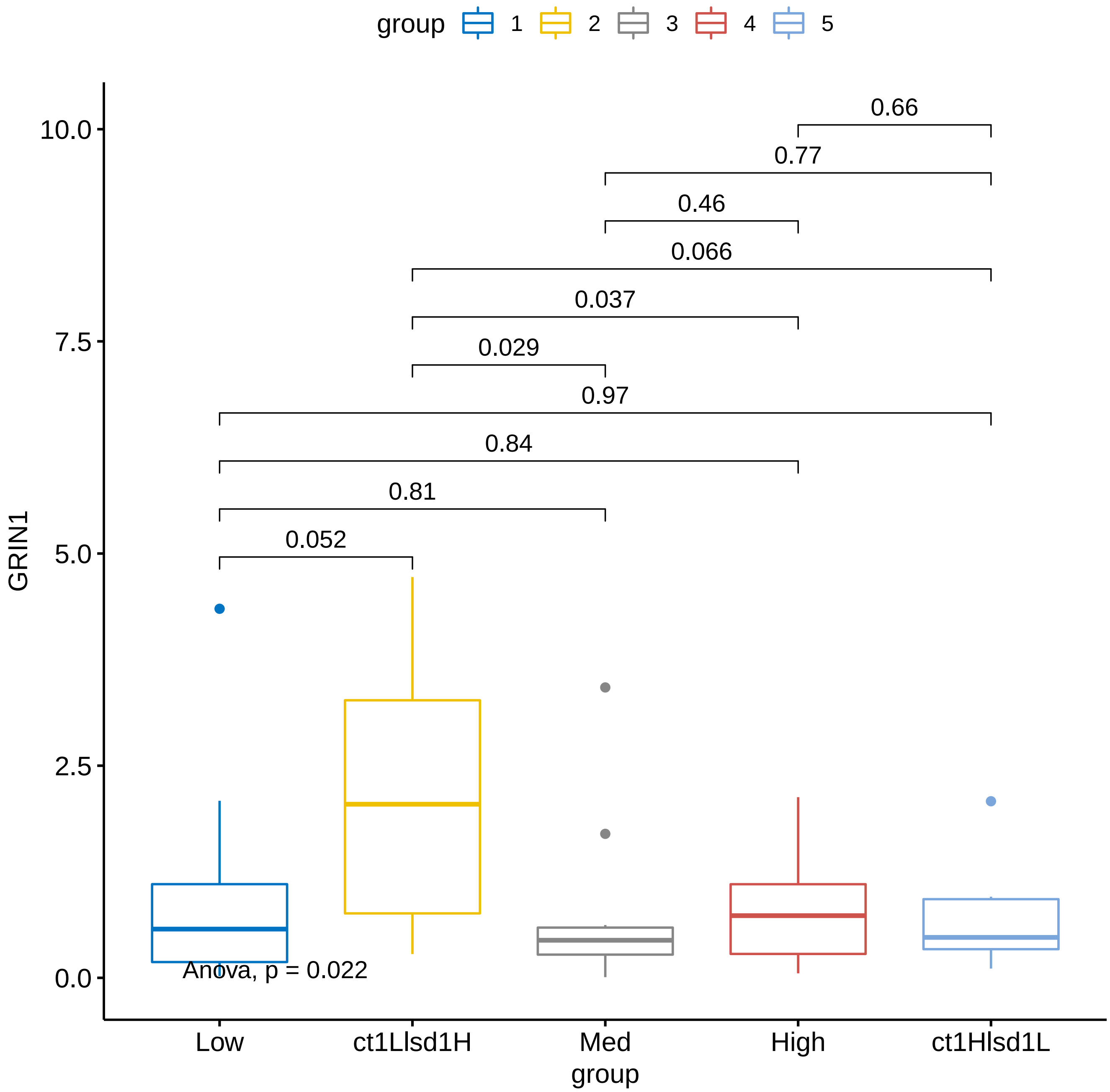

group 1 2 3 4 5
0.66
0.77
0.46
0.066
0.037
0.029
0.97
0.84
0.81
0.052
10.0
7.5
5.0
2.5
0.0
GRIN1
Anova, p = 0.022
Low
ct1Llsd1H
Med
High
ct1Hlsd1L
group

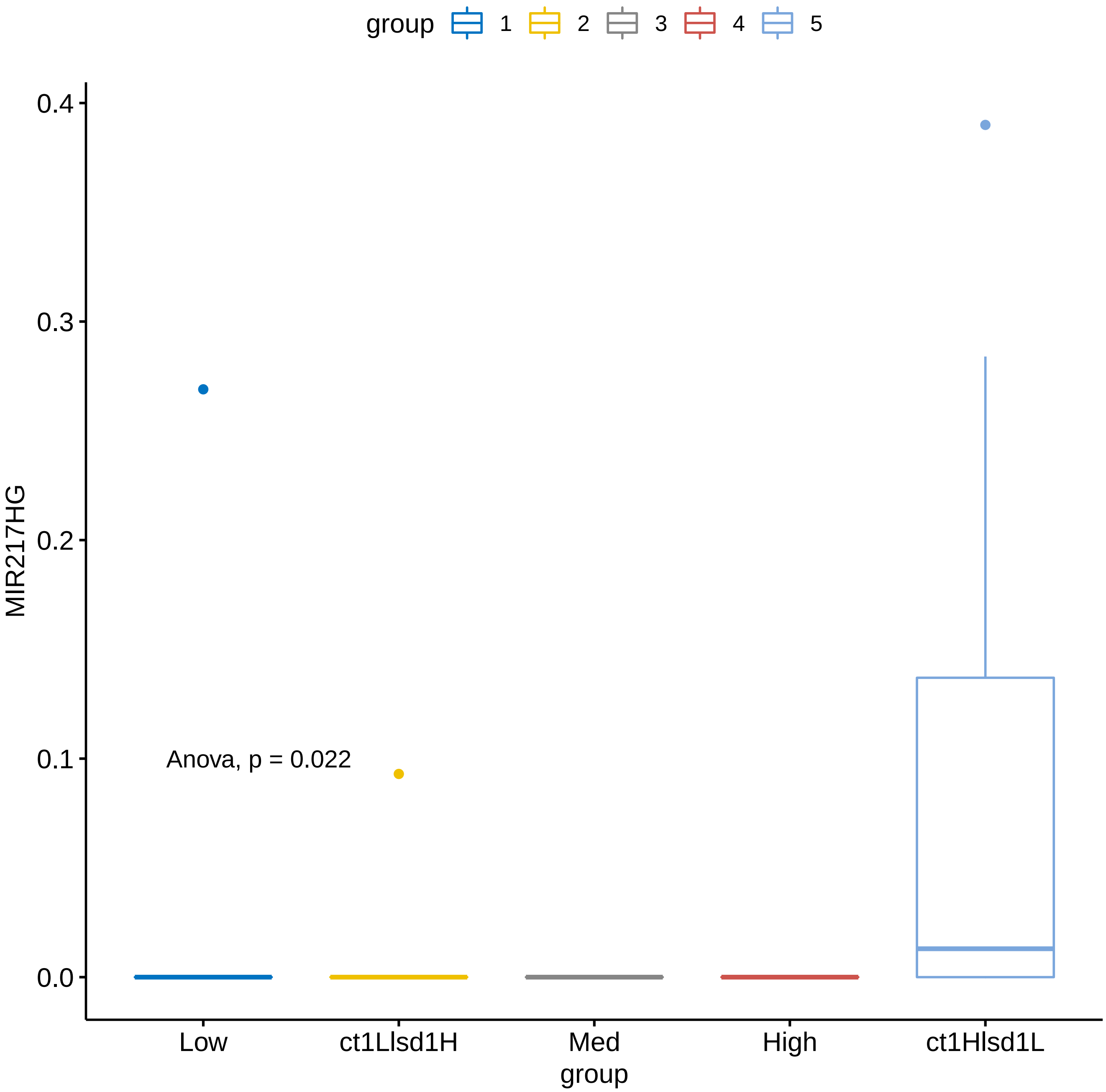

group
1
2
3
4
5
0.4
0.3
0.2
0.1
0.0
MIR217HG
Anova, p = 0.022
Low
ct1Llsd1H
Med
High
ct1Hlsd1L
group

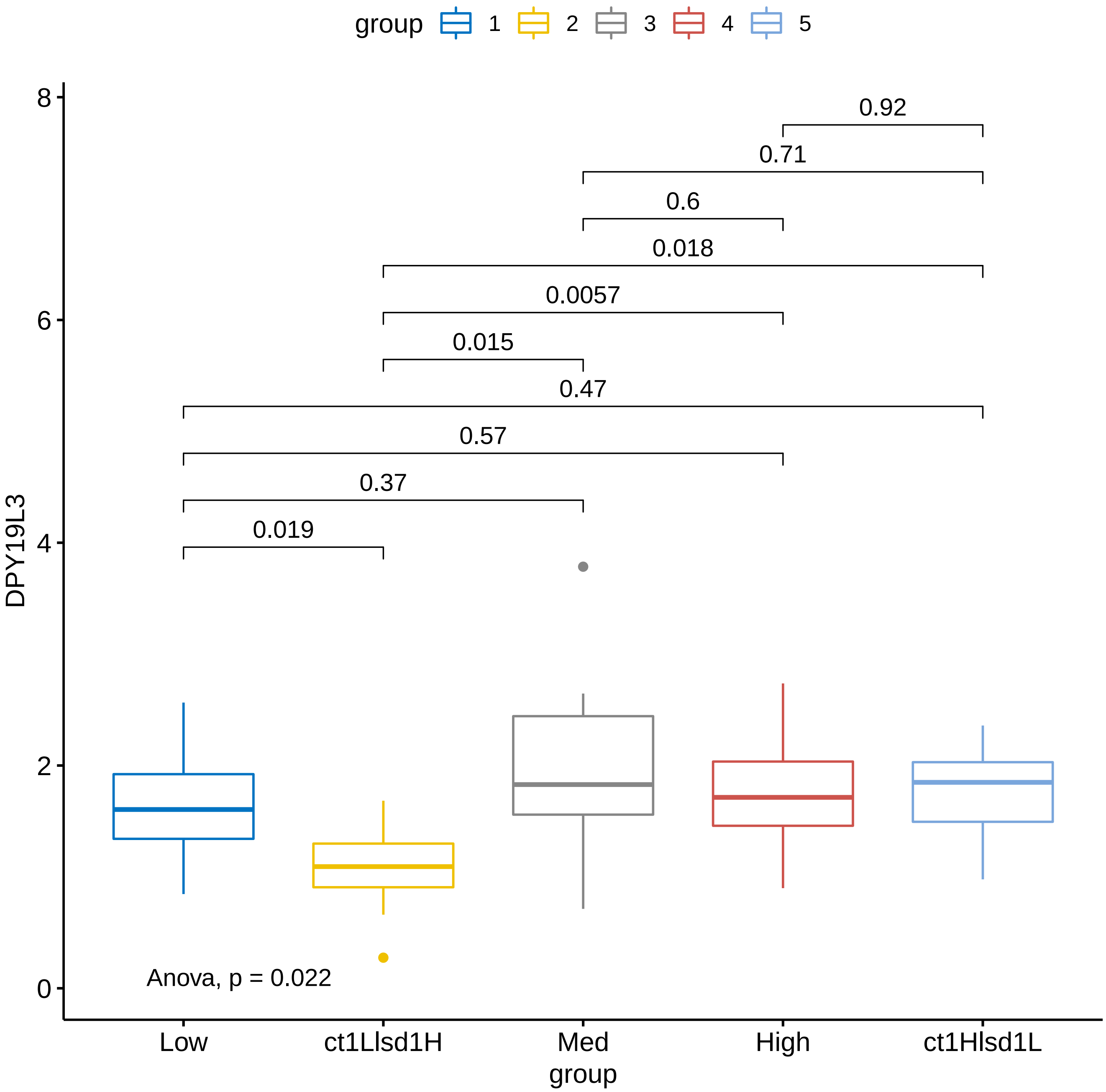
group 1 2 3 4 5
0.92
0.71
0.6
0.018
0.0057
0.015
0.47
0.57
0.37
0.019
DPY19L3
8
6
4
2
0
Anova, p = 0.022
Low
ct1Llsd1H
Med
High
ct1Hlsd1L
group

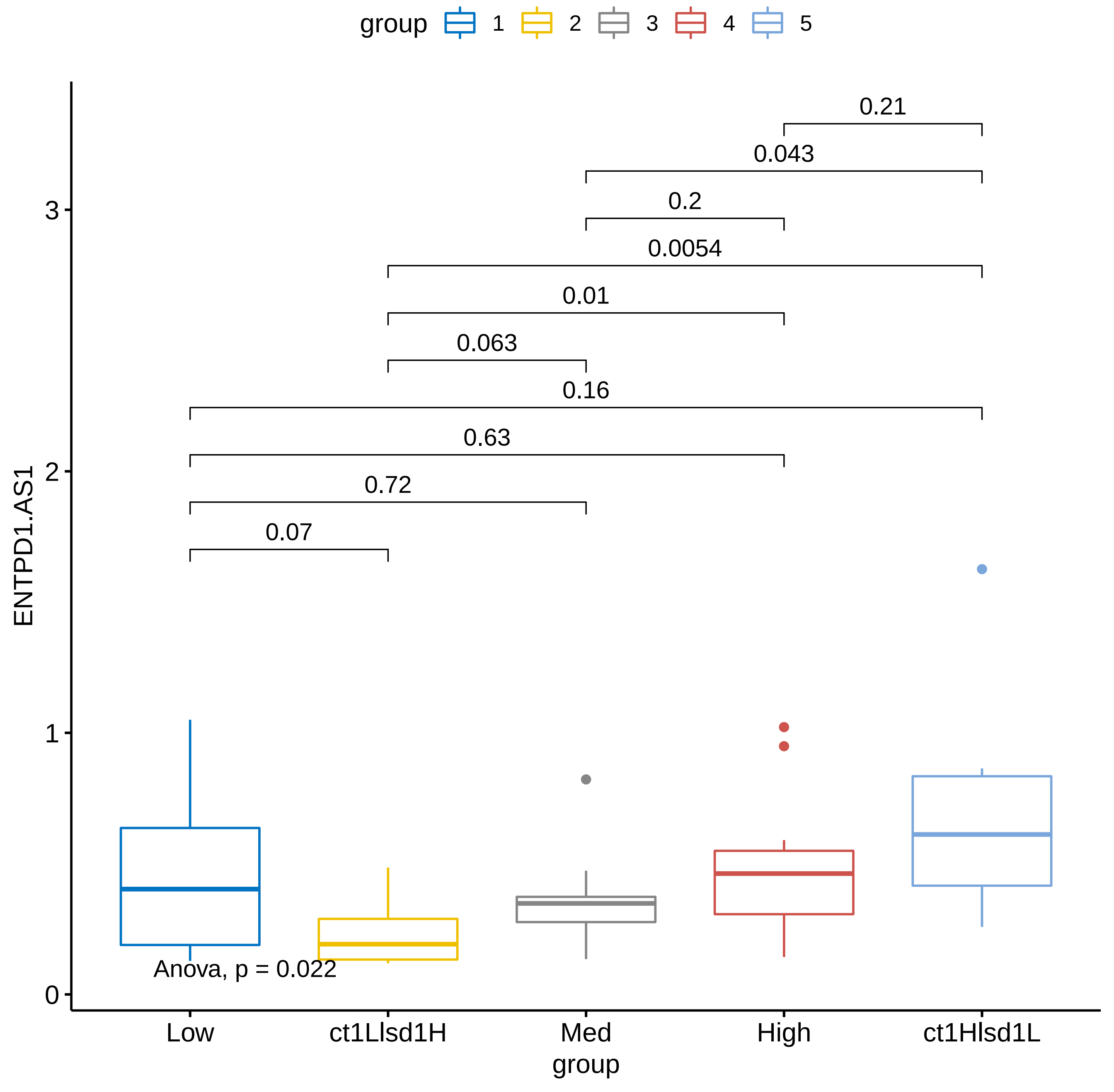

group
1
2
3
4
5
0.21
0.043
0.2
0.0054
0.01
0.063
0.16
0.63
0.72
0.07
Anova, p = 0.022
ENTPD1.AS1
0
1
2
3
Low
ct1Llsd1H
Med
High
ct1Hlsd1L
group

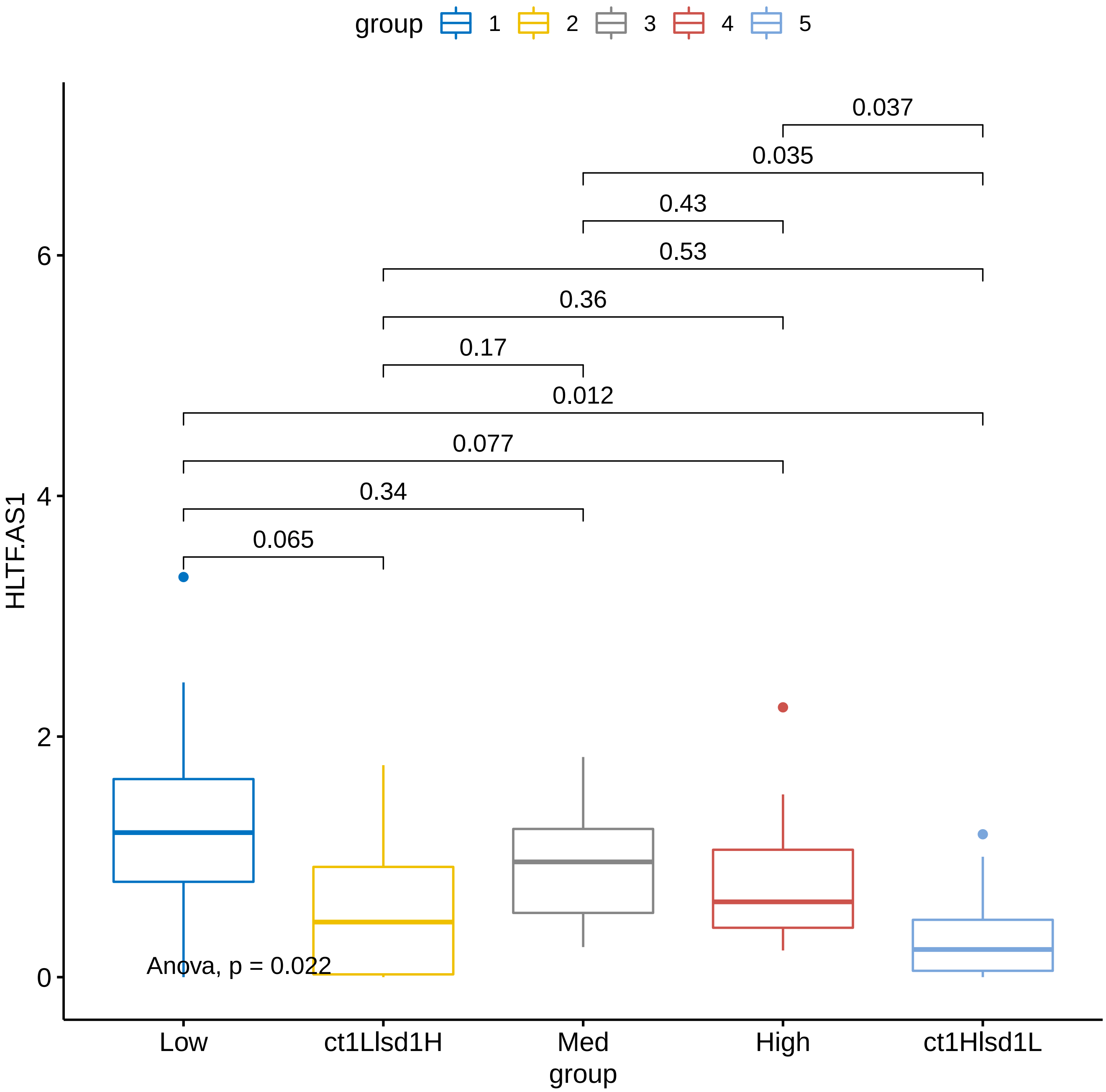

group
1
2
3
4
5
0.037
0.035
0.43
0.53
0.36
0.17
0.012
0.077
0.34
0.065
HLTF.AS1
6
4
2
0
Anova, p = 0.022
Low
ct1Llsd1H
Med
High
ct1Hlsd1L
group

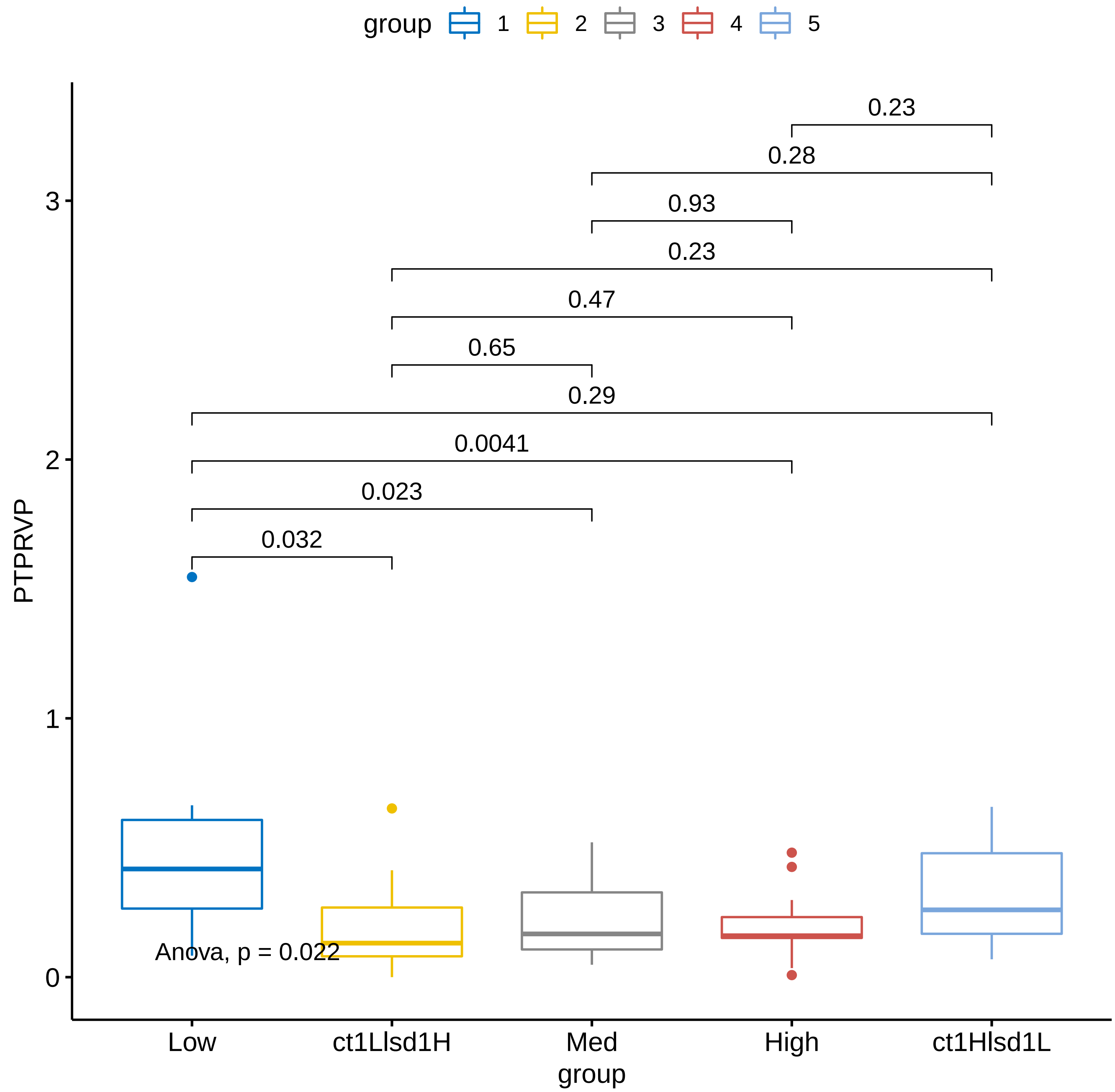

group 1 2 3 4 5
0.23
0.28
0.93
0.23
0.47
0.65
0.29
0.0041
0.023
0.032
Anova, p = 0.022
PTPRVP
3
2
1
0
Low
ct1Llsd1H
Med
High
ct1Hlsd1L
group

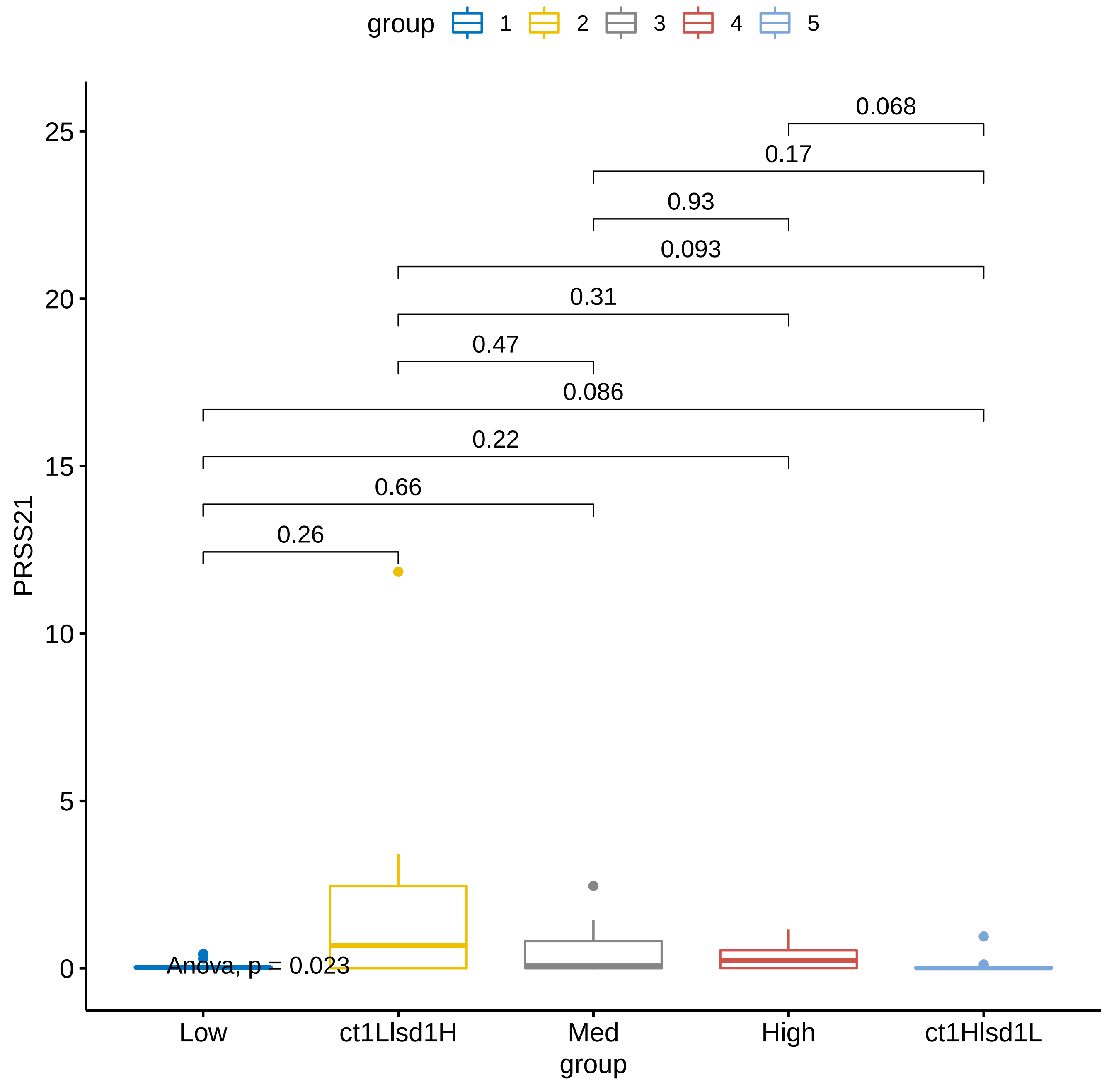
group
1
2
3
4
5
0.068
0.17
0.93
0.093
0.31
0.47
0.086
0.22
0.66
0.26
Anova, p = 0.023
PRSS21
25
20
15
10
5
0
Low
ct1Llsd1H
Med
High
ct1Hlsd1L
group

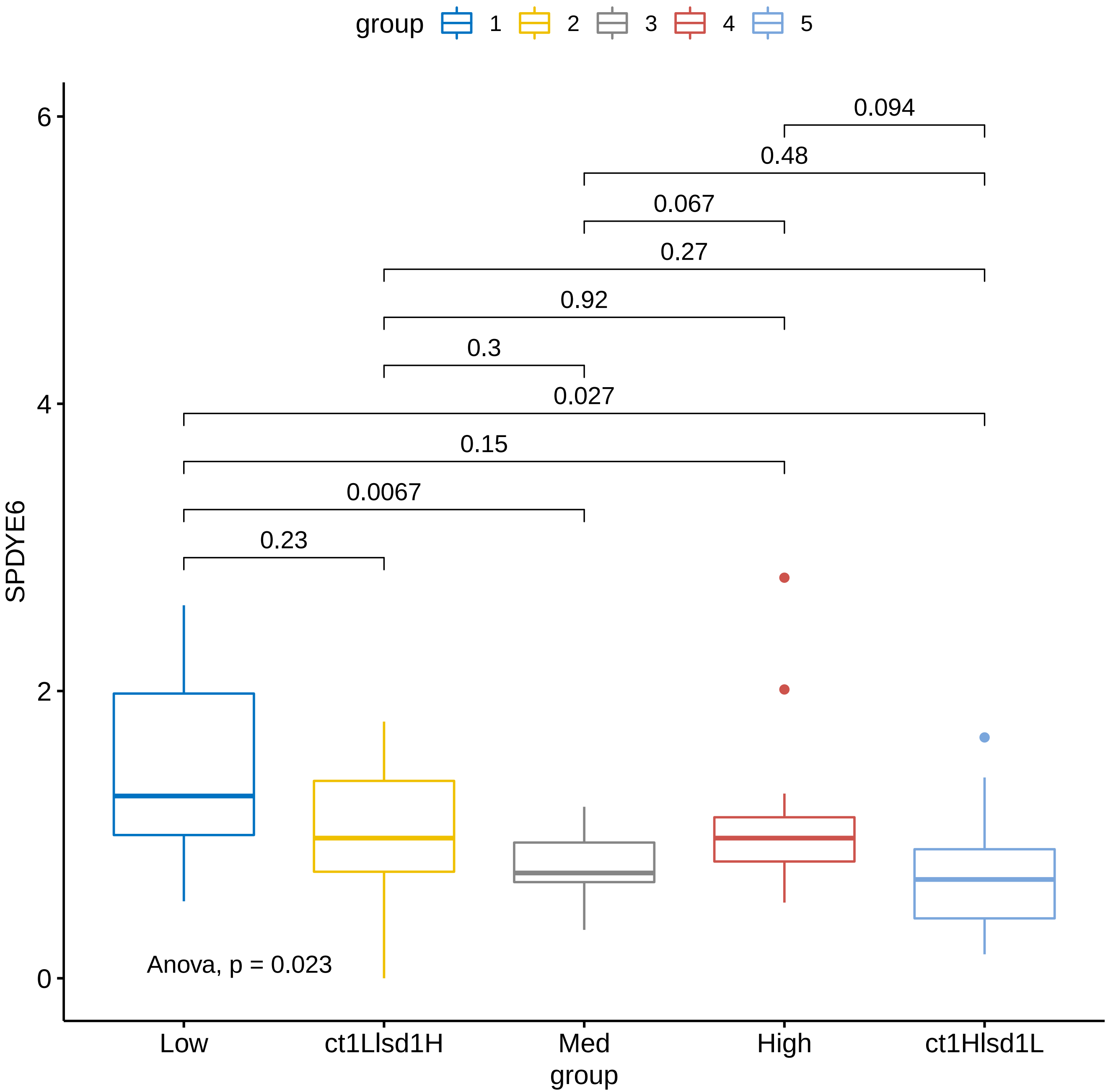

group
1
2
3
4
5
SPDYE6
0.094
0.48
0.067
0.27
0.92
0.3
0.027
0.15
0.0067
0.23
Anova, p = 0.023
6
4
2
0
Low
ct1Llsd1H
Med
High
ct1Hlsd1L
group

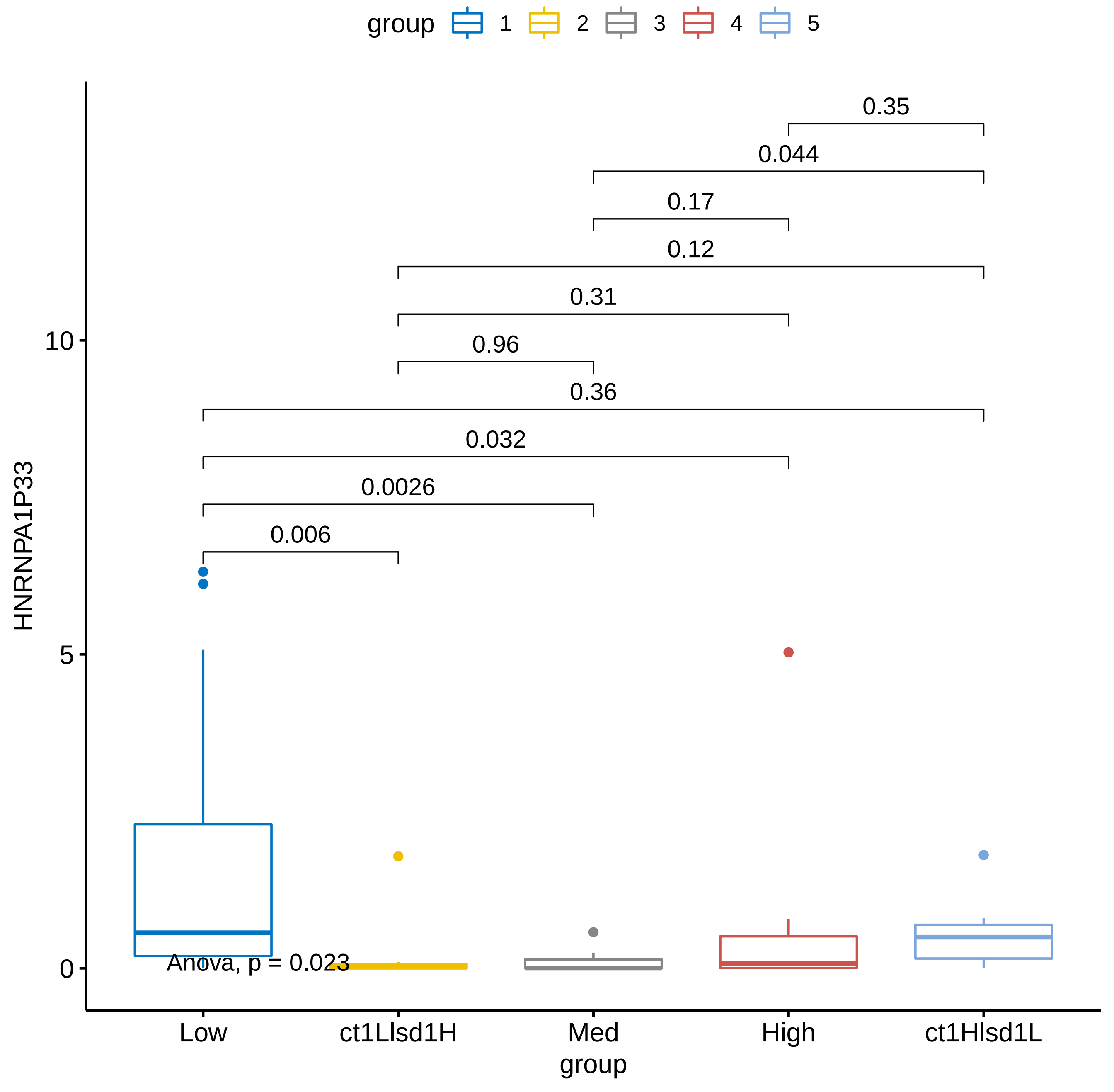

group
1
2
3
4
5
0.35
0.044
0.17
0.12
0.31
0.96
0.36
0.032
0.0026
0.006
10
5
0
HNRNPA1P33
Anova, p = 0.023
Low
ct1Llsd1H
Med
High
ct1Hlsd1L
group

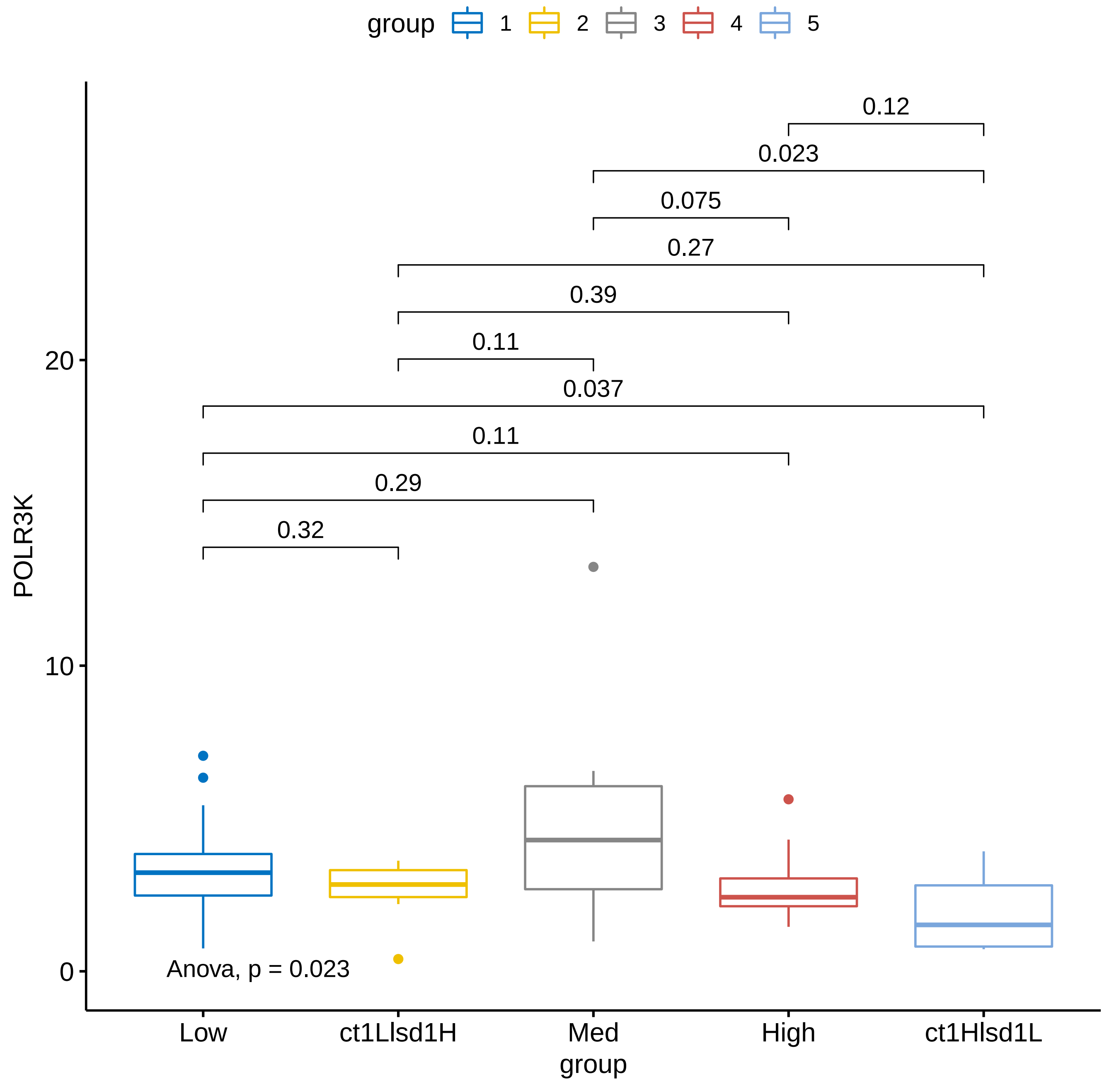

group
1
2
3
4
5
0.12
0.023
0.075
0.27
0.39
0.11
0.037
0.11
0.29
0.32
POLR3K
20
10
0
Anova, p = 0.023
Low
ct1Llsd1H
Med
High
ct1Hlsd1L
group

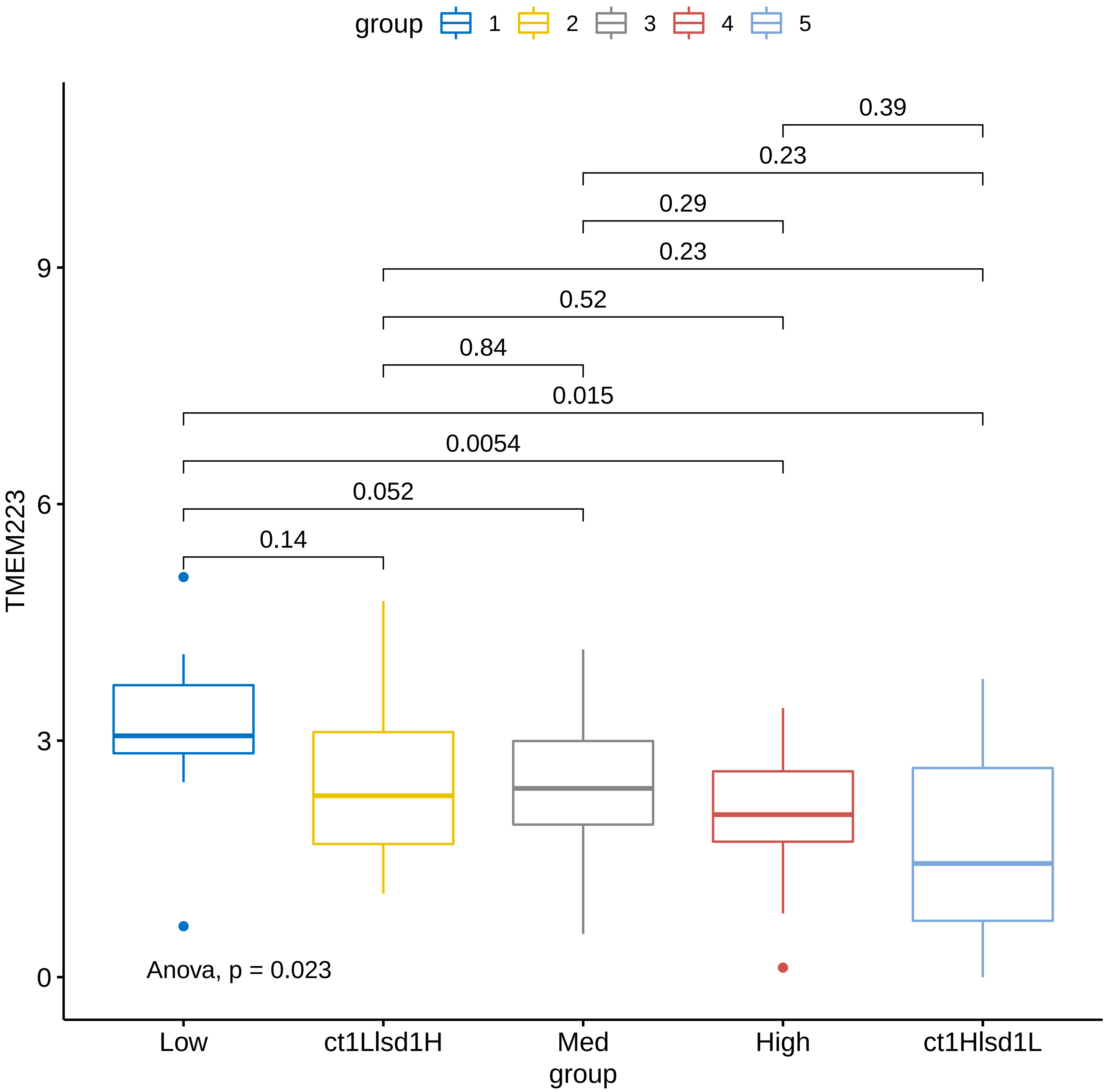

group
1
2
3
4
5
0.39
0.23
0.29
0.23
0.52
0.84
0.015
0.0054
0.052
0.14
9
6
3
0
TMEM223
Anova, p = 0.023
Low
ct1Llsd1H
Med
High
ct1Hlsd1L
group

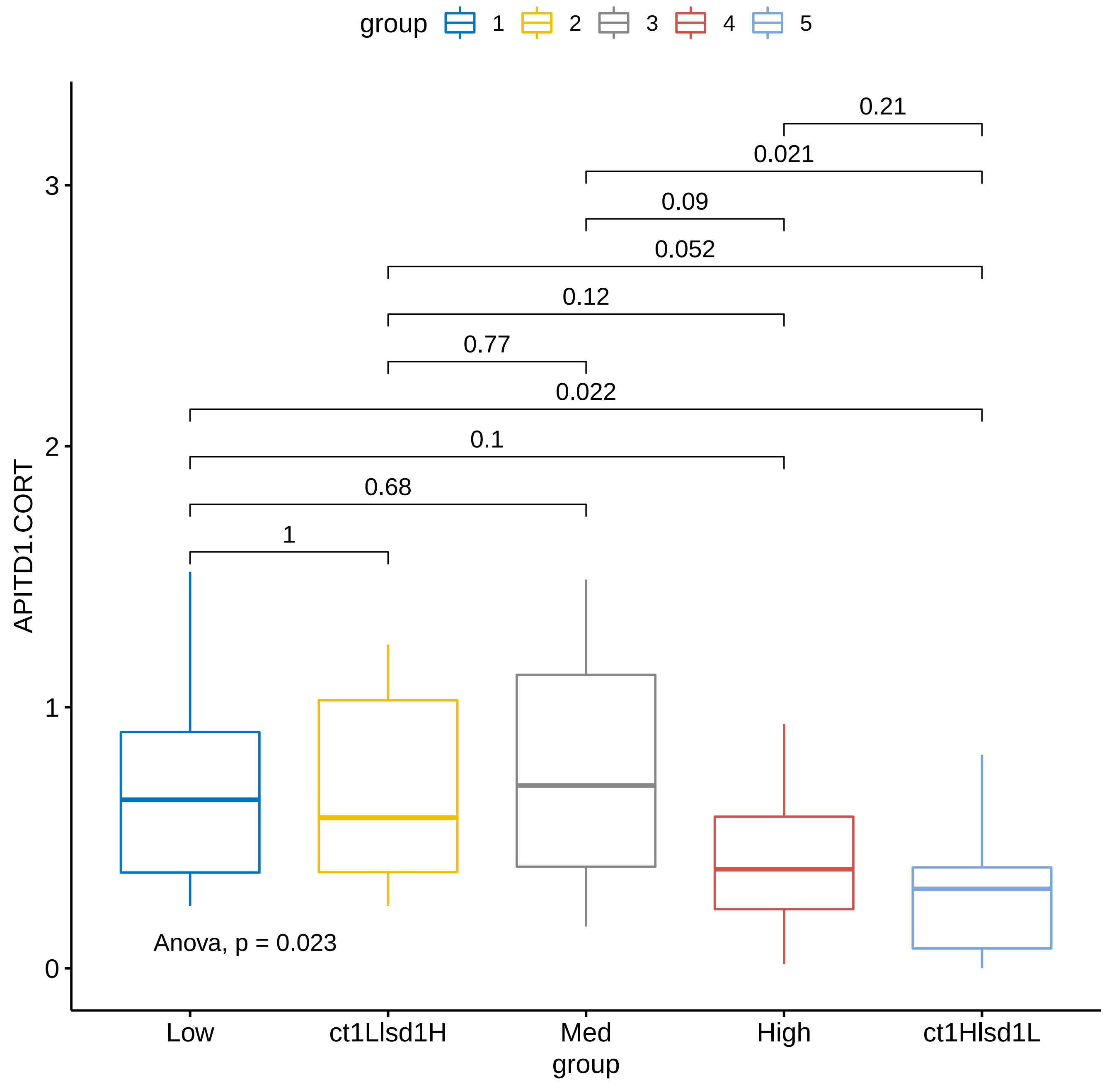

group
1
2
3
4
5
0.21
0.021
0.09
0.052
0.12
0.77
0.022
0.1
0.68
1
Anova, p = 0.023
APITD1.CORT
3
2
1
0
Low
ct1Llsd1H
Med
High
ct1Hlsd1L
group

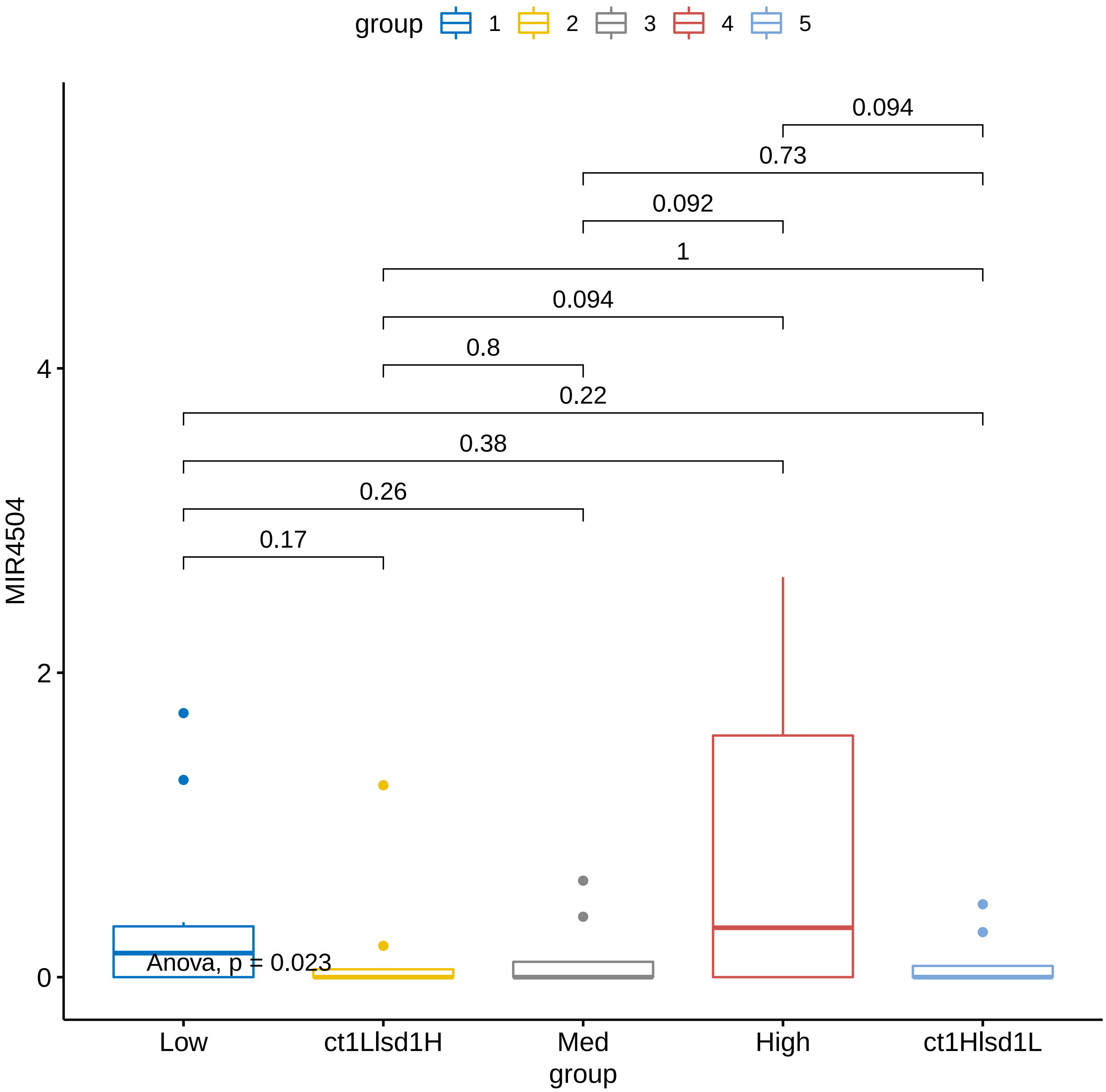

group 1 2 3 4 5
0.094
0.73
0.092
1
0.094
0.8
0.22
0.38
0.26
0.17
Anova, p = 0.023
MIR4504
4
2
0
Low
ct1Llsd1H
Med
High
ct1Hlsd1L
group

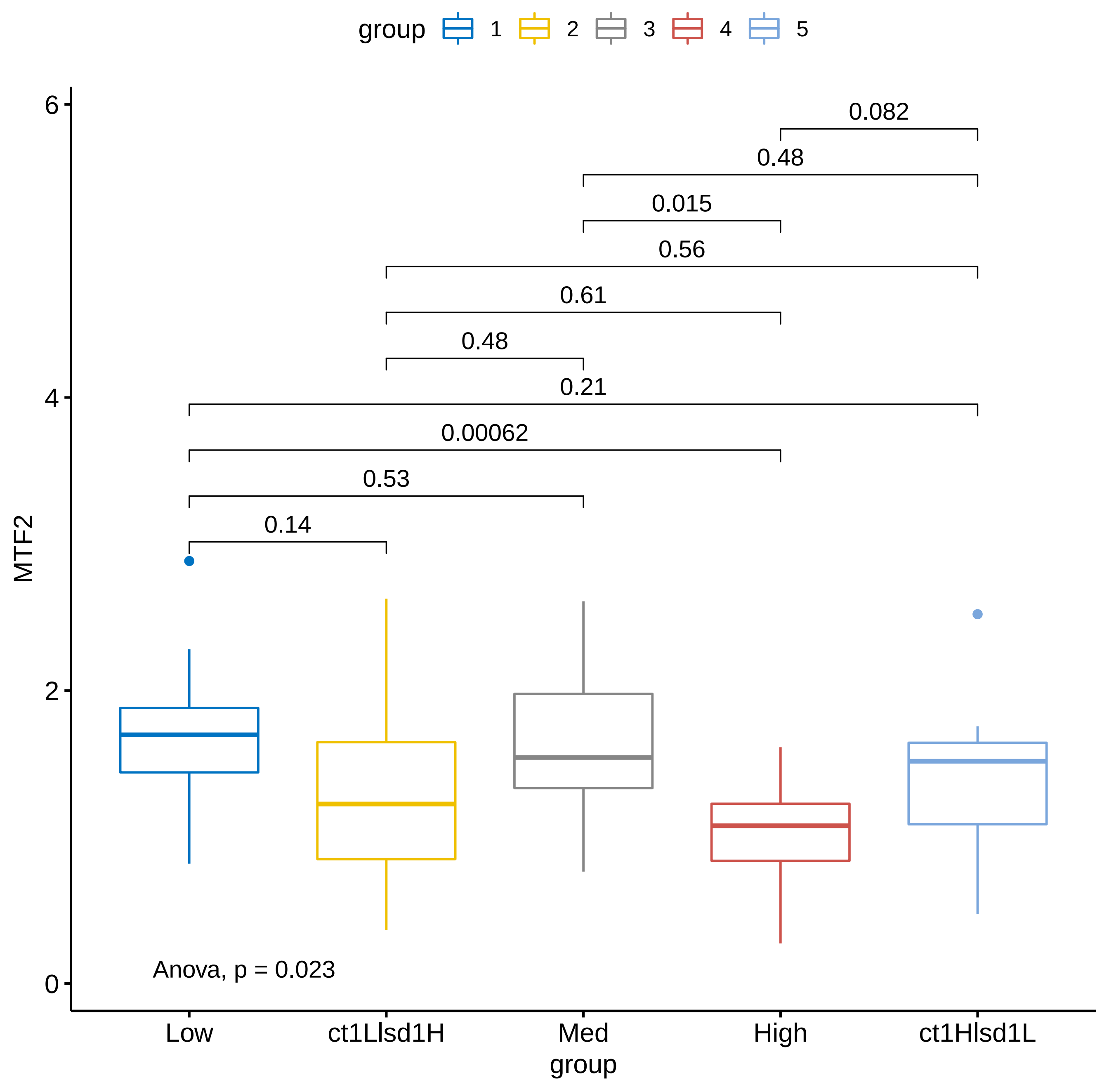

group
1
2
3
4
5
MTF2
0
2
4
6
0.082
0.48
0.015
0.56
0.61
0.48
0.21
0.00062
0.53
0.14
Anova, p = 0.023
Low
ct1Llsd1H
Med
High
ct1Hlsd1L
group

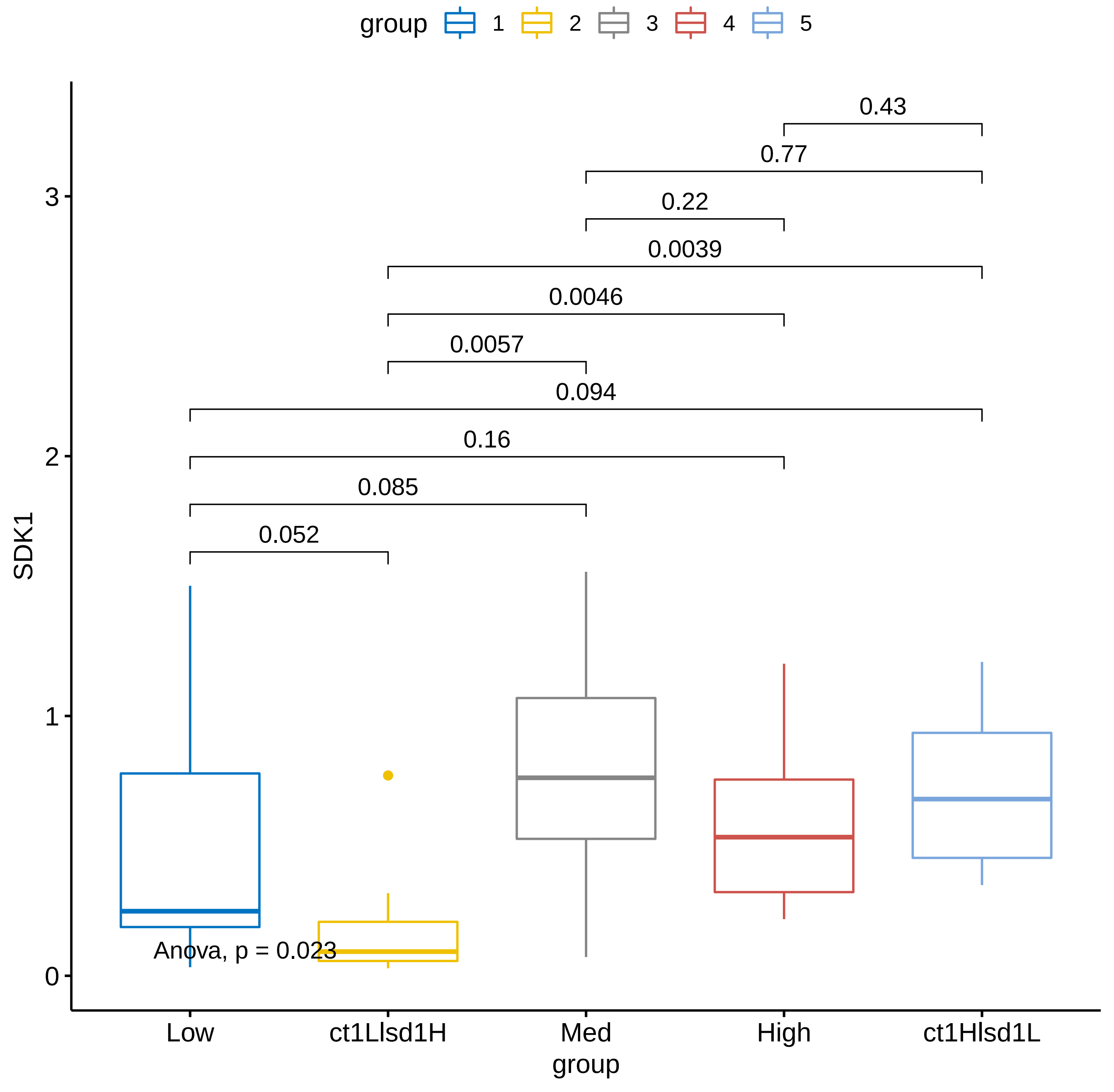
group
1
2
3
4
5
0.43
0.77
0.22
0.0039
0.0046
0.0057
0.094
0.16
0.085
0.052
Anova, p = 0.023
SDK1
3
2
1
0
Low
ct1Llsd1H
Med
High
ct1Hlsd1L
group

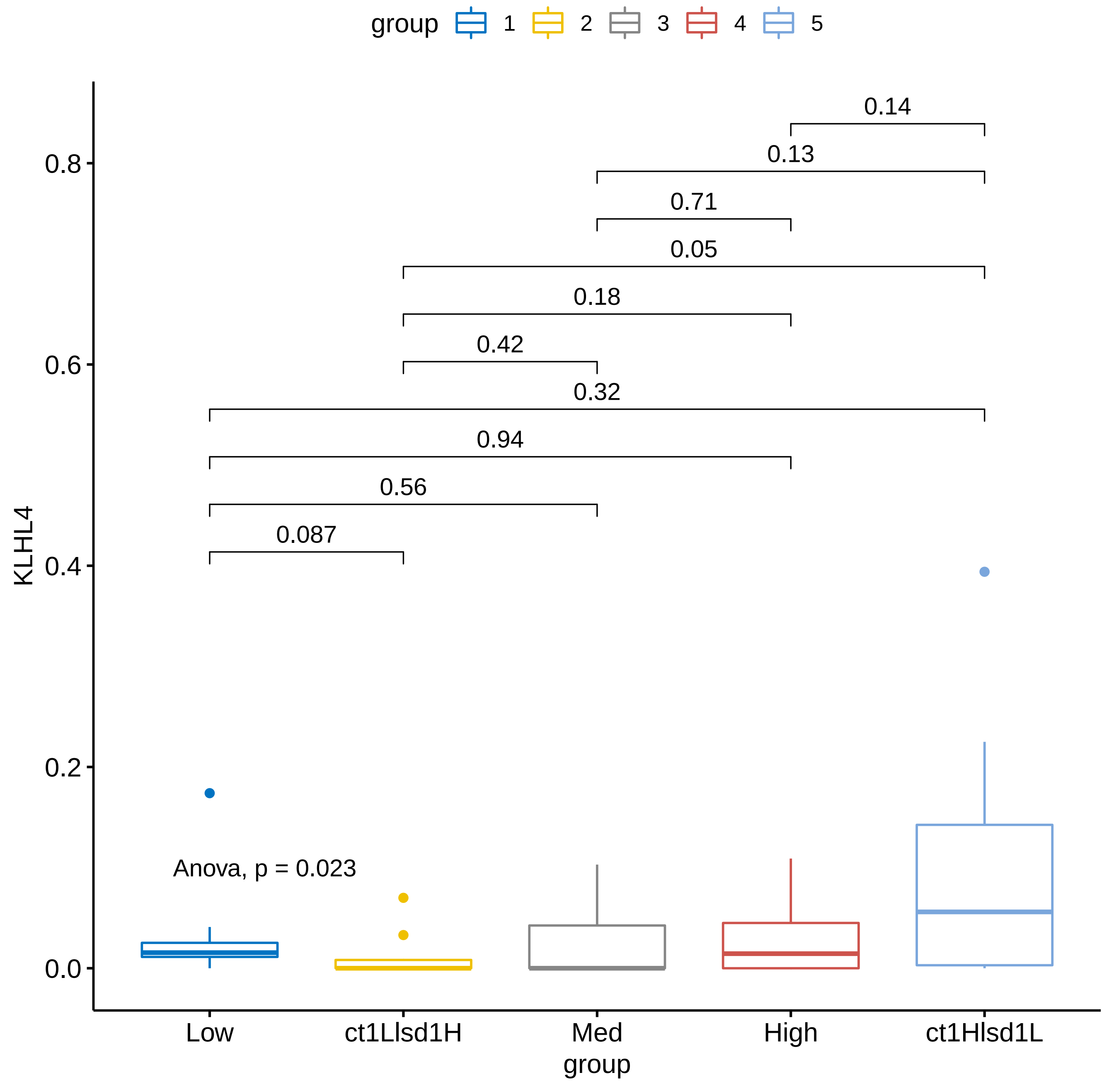

group
1
2
3
4
5
0.14
0.13
0.71
0.05
0.18
0.42
0.32
0.94
0.56
0.087
Anova, p = 0.023
0.8
0.6
0.4
0.2
0.0
KLHL4
Low
ct1Llsd1H
Med
High
ct1Hlsd1L
group

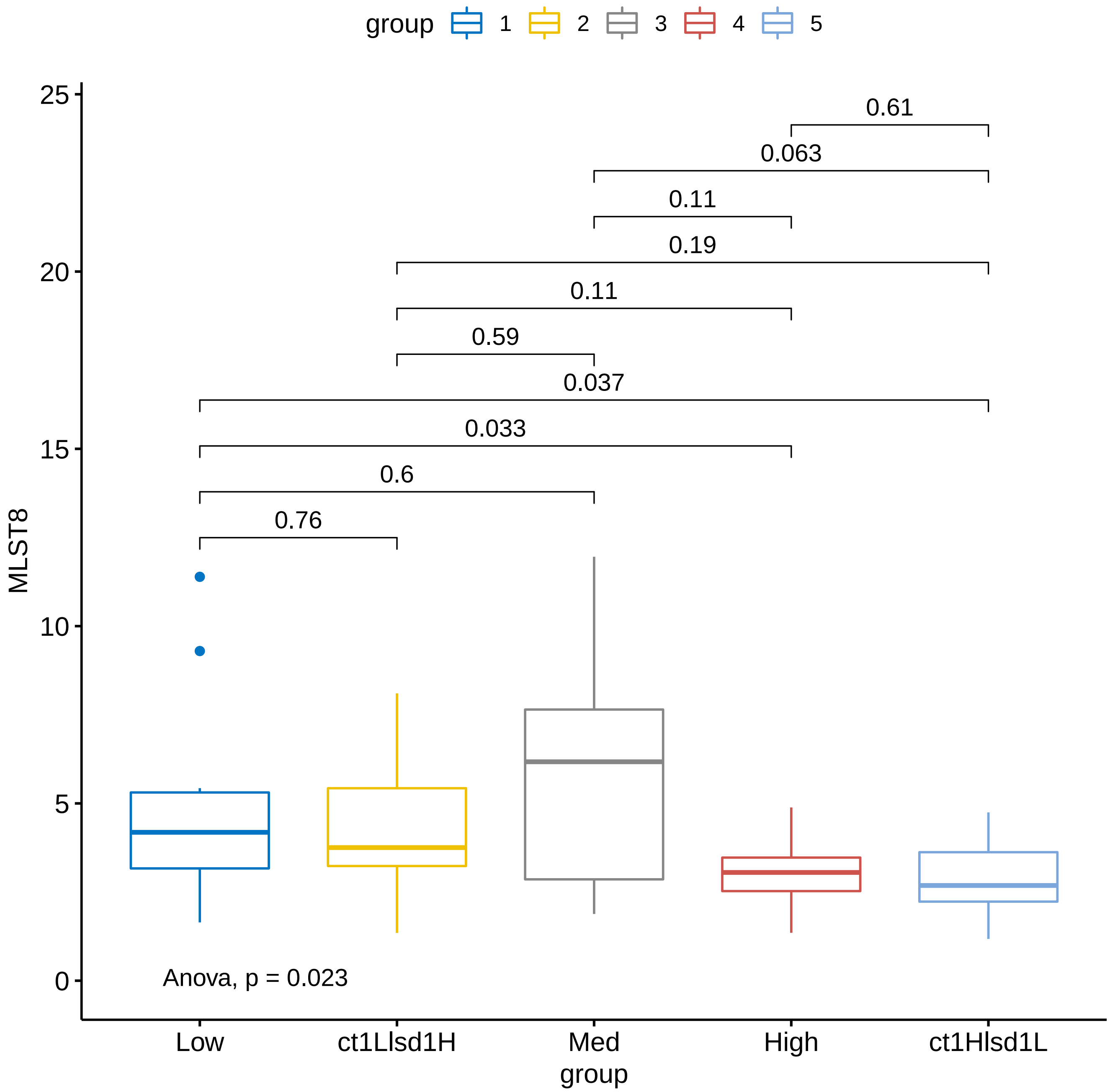
group
1
2
3
4
5
MLST8
25
20
15
10
5
0
0.61
0.063
0.11
0.19
0.11
0.59
0.037
0.033
0.6
0.76
Anova, p = 0.023
Low
ct1Llsd1H
Med
High
ct1Hlsd1L
group

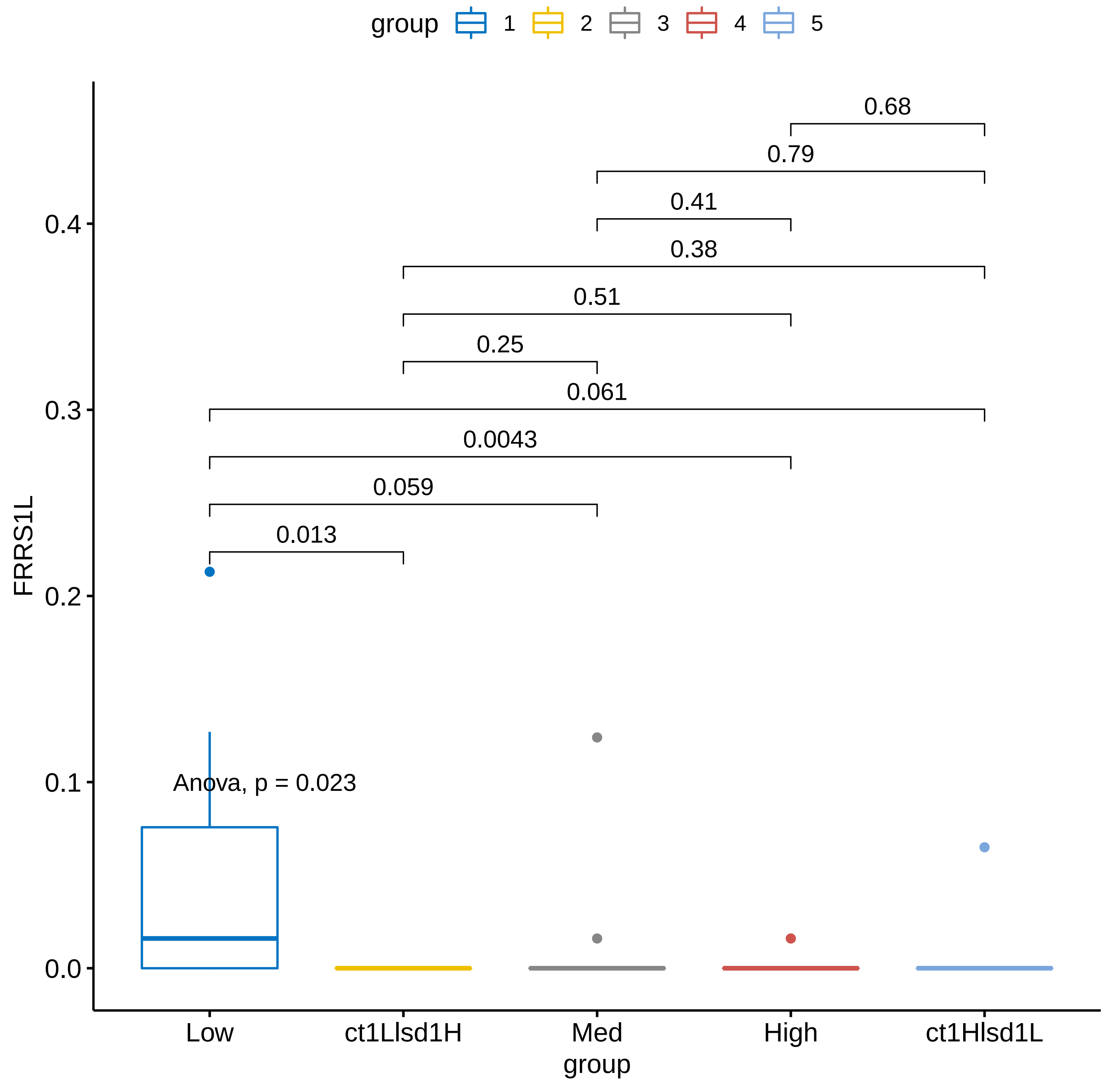
group 1 2 3 4 5
0.68
0.79
0.41
0.38
0.51
0.25
0.061
0.0043
0.059
0.013
Anova, p = 0.023
FRRS1L
0.0
0.1
0.2
0.3
0.4
Low
ct1Llsd1H
Med
High
ct1Hlsd1L
group

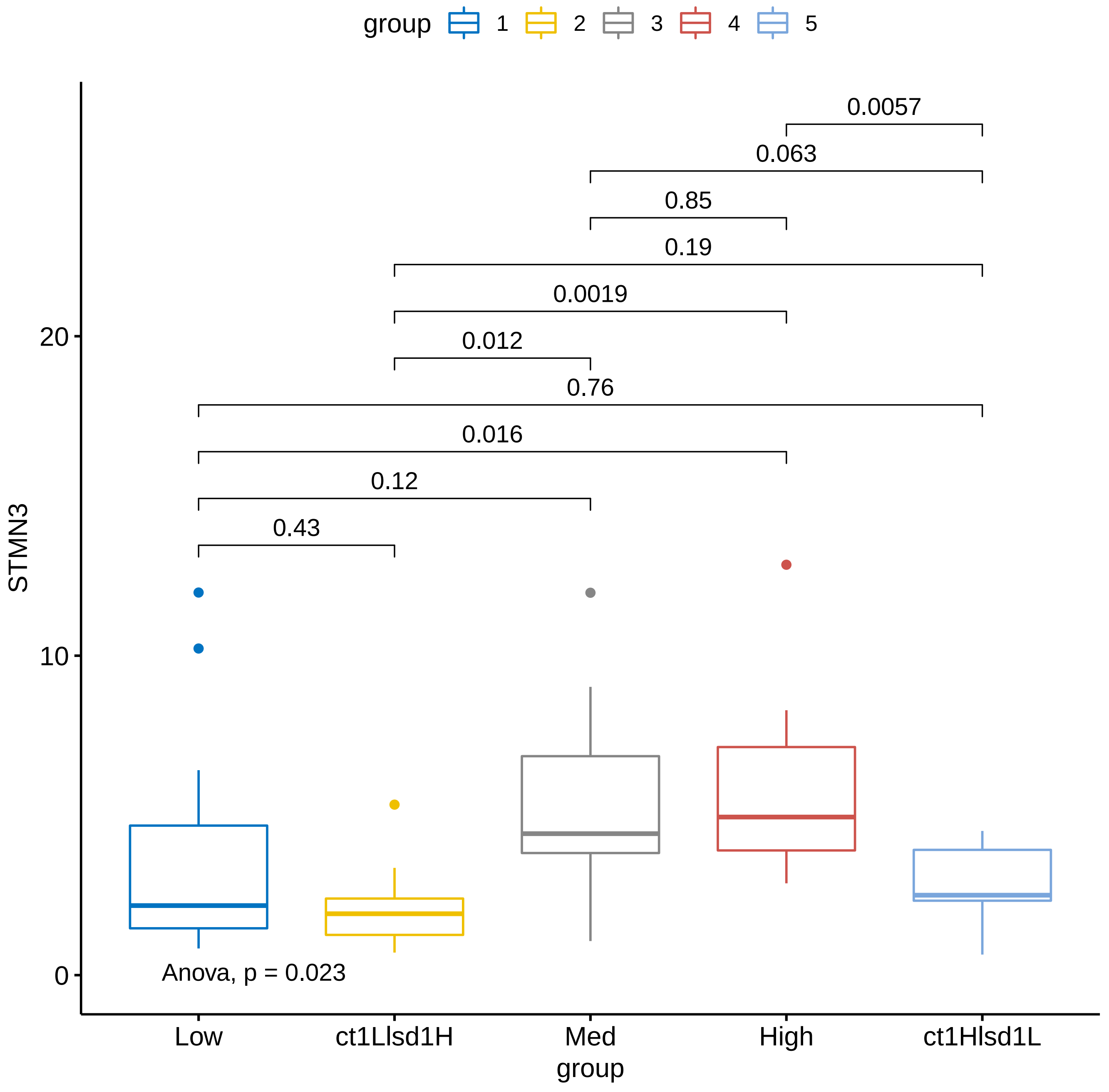
group
1
2
3
4
5
0.0057
0.063
0.85
0.19
0.0019
0.012
0.76
0.016
0.12
0.43
20
10
0
STMN3
Anova, p = 0.023
Low
ct1Llsd1H
Med
High
ct1Hlsd1L
group

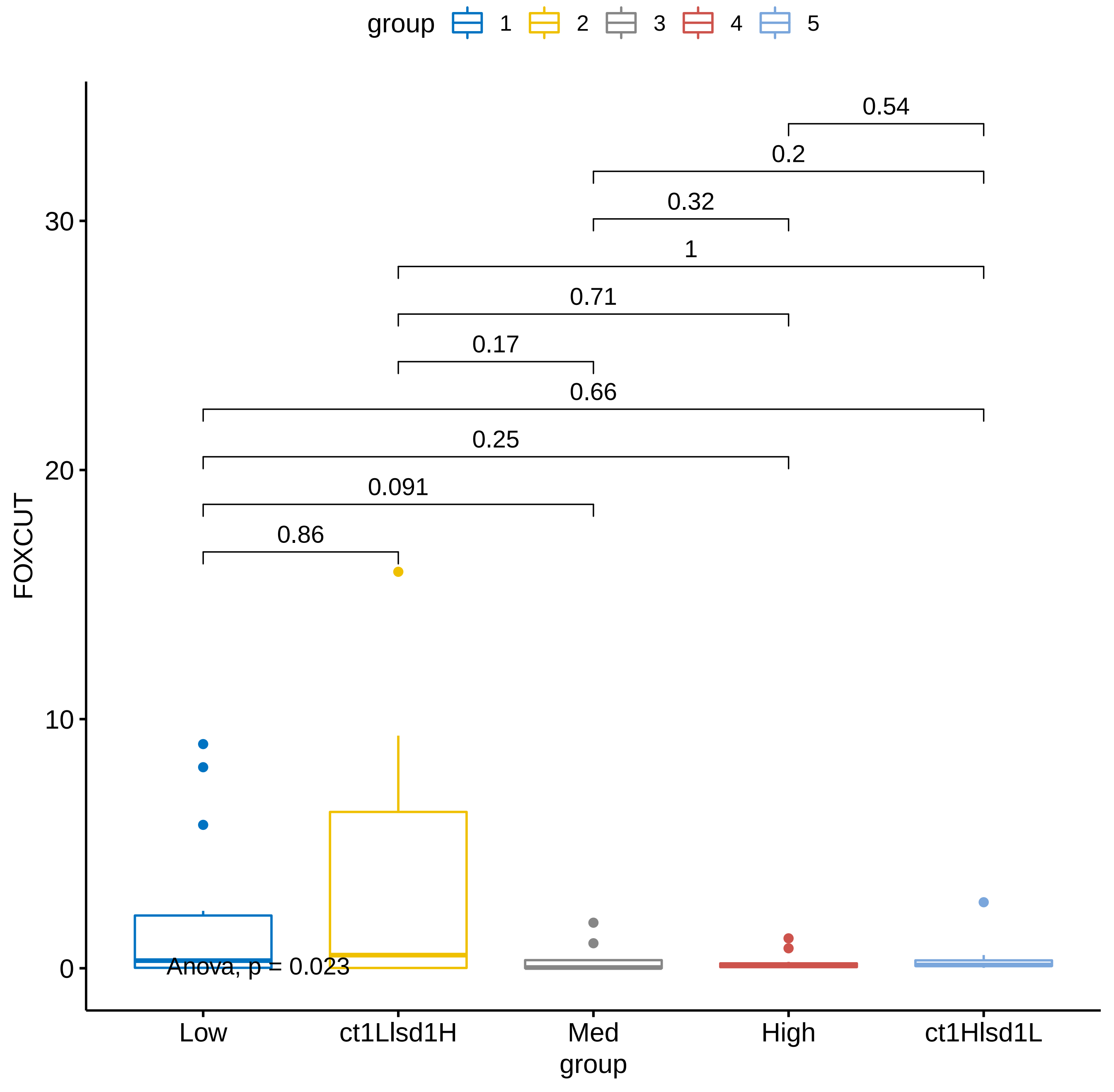

group 1 2 3 4 5
0.54
0.2
0.32
1
0.71
0.17
0.66
0.25
0.091
0.86
30
20
10
0
FOXCUT
Anova, p = 0.023
Low
ct1Llsd1H
Med
High
ct1Hlsd1L
group

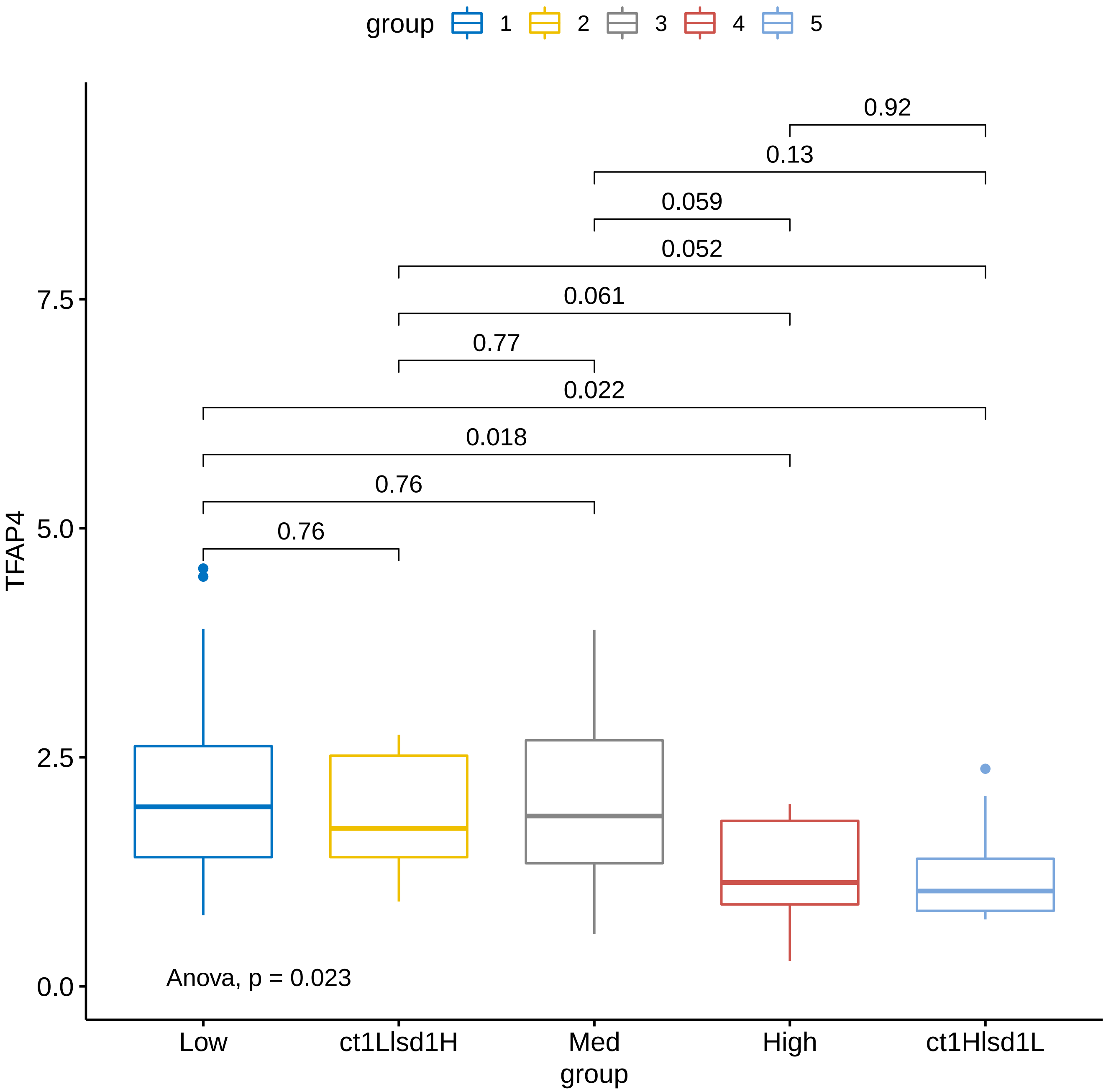

group
1
2
3
4
5
0.92
0.13
0.059
0.052
0.061
0.77
0.022
0.018
0.76
0.76
TFAP4
7.5
5.0
2.5
0.0
Anova, p = 0.023
Low
ct1Llsd1H
Med
High
ct1Hlsd1L
group

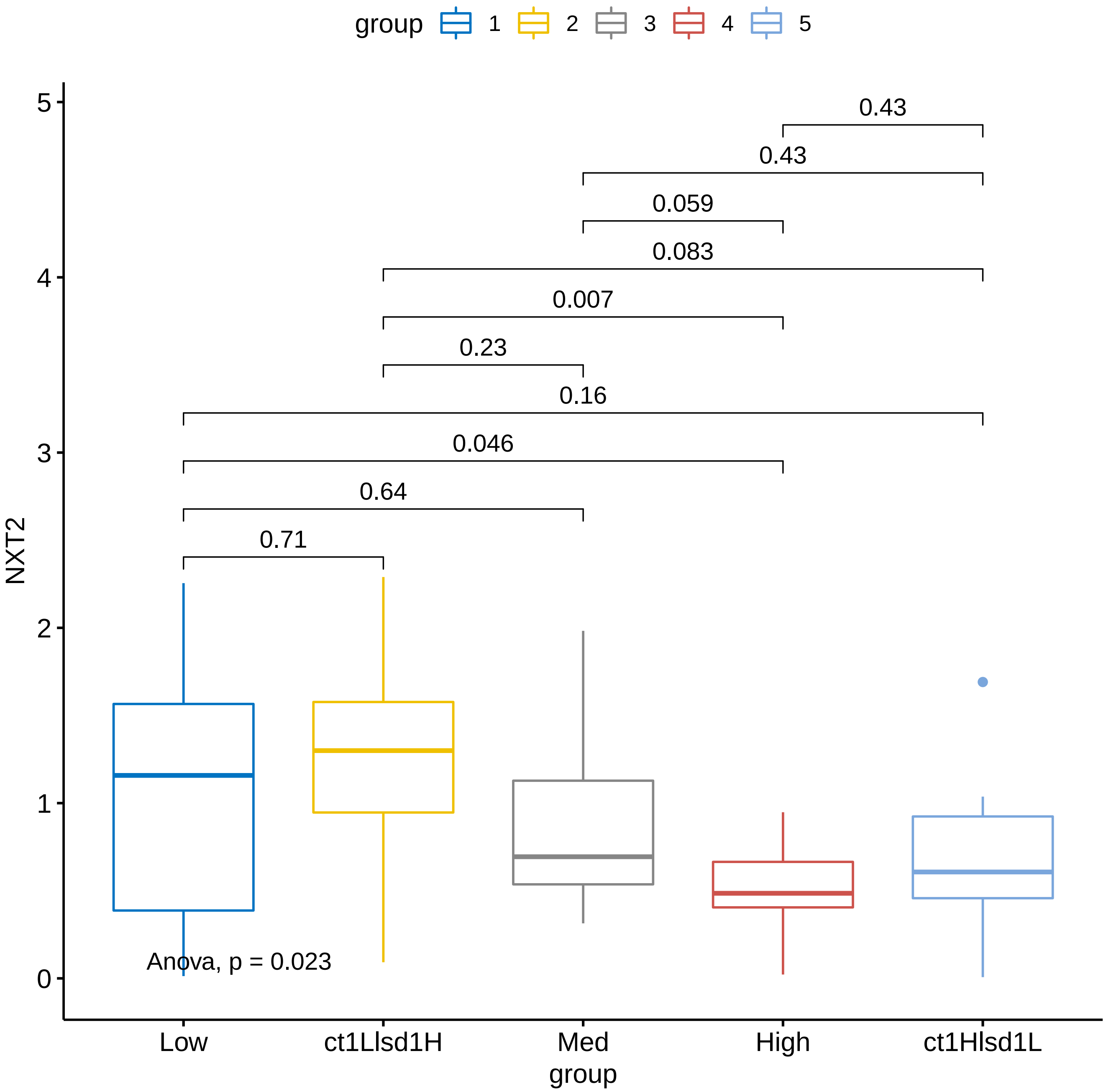

group 1 2 3 4 5
0.43
0.43
0.059
0.083
0.007
0.23
0.16
0.046
0.64
0.71
Anova, p = 0.023
NXT2
0
1
2
3
4
5
Low
ct1Llsd1H
Med
High
ct1Hlsd1L
group

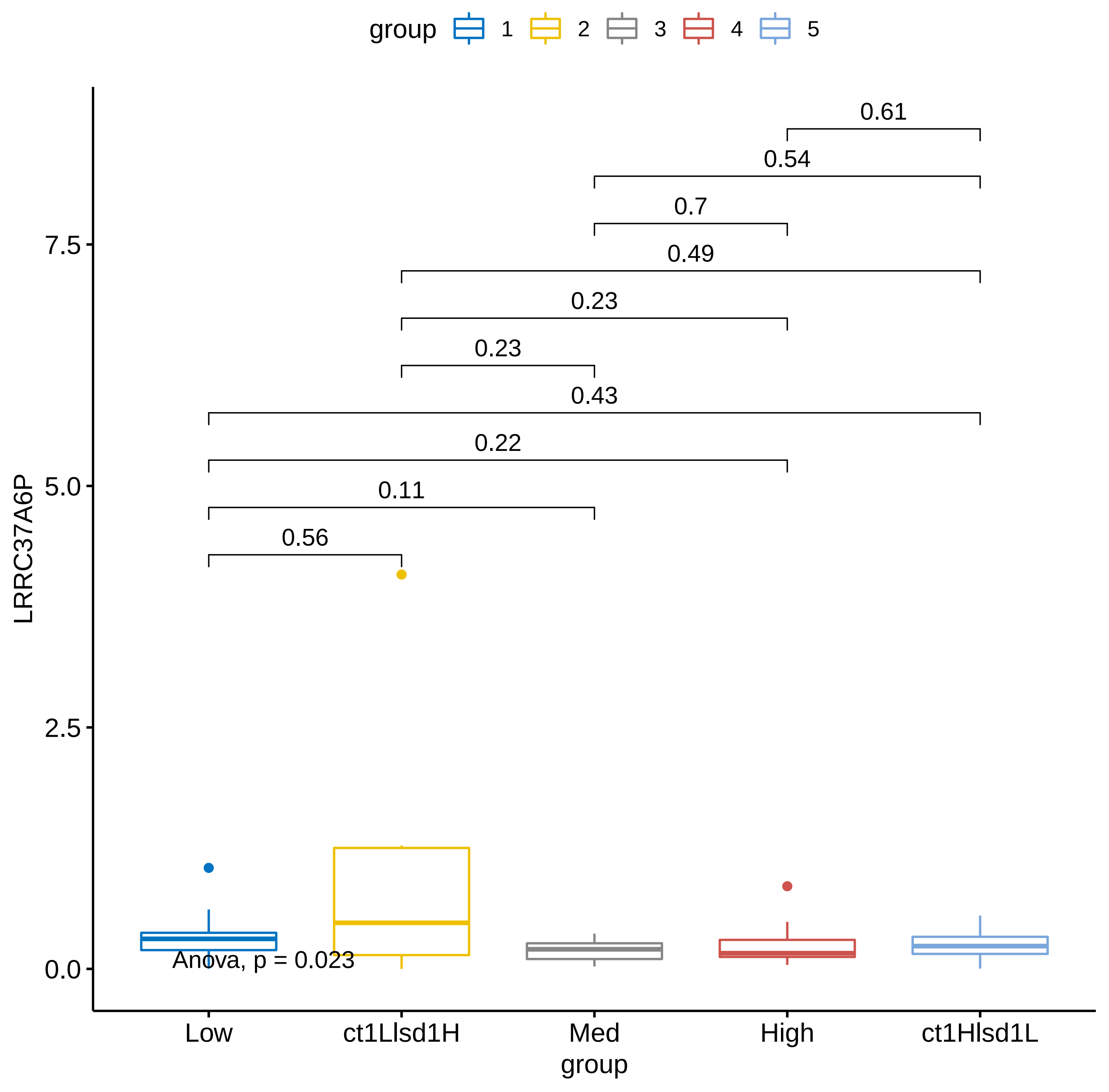

group
1
2
3
4
5
0.61
0.54
0.7
0.49
0.23
0.23
0.43
0.22
0.11
0.56
7.5
5.0
2.5
0.0
LRRC37A6P
Anova, p = 0.023
Low
ct1Llsd1H
Med
High
ct1Hlsd1L
group

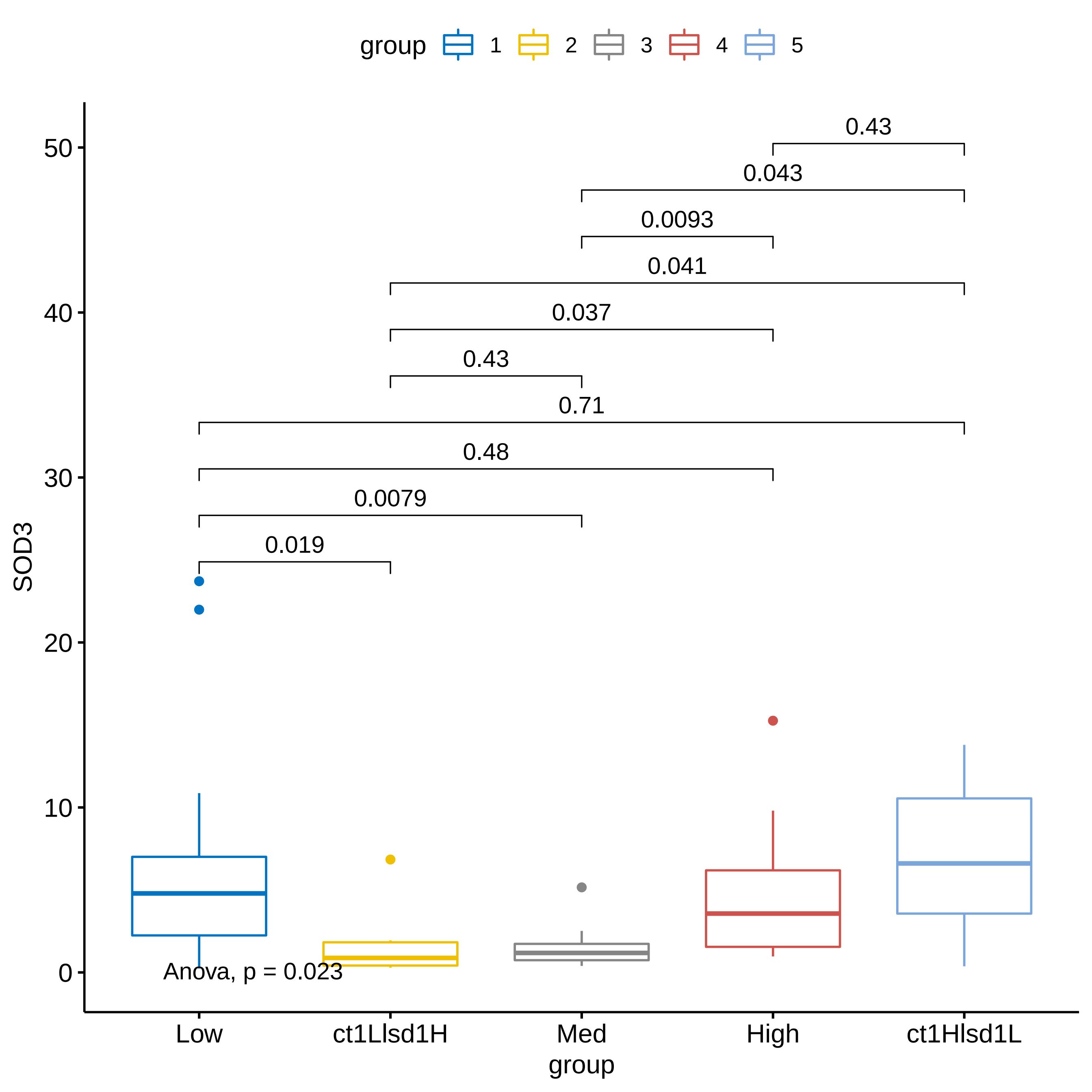

group 1 2 3 4 5
SOD3
50
40
30
20
10
0
0.43
0.043
0.0093
0.041
0.037
0.43
0.71
0.48
0.0079
0.019
Anova, p = 0.023
Low
ct1Llsd1H
Med
High
ct1Hlsd1L
group

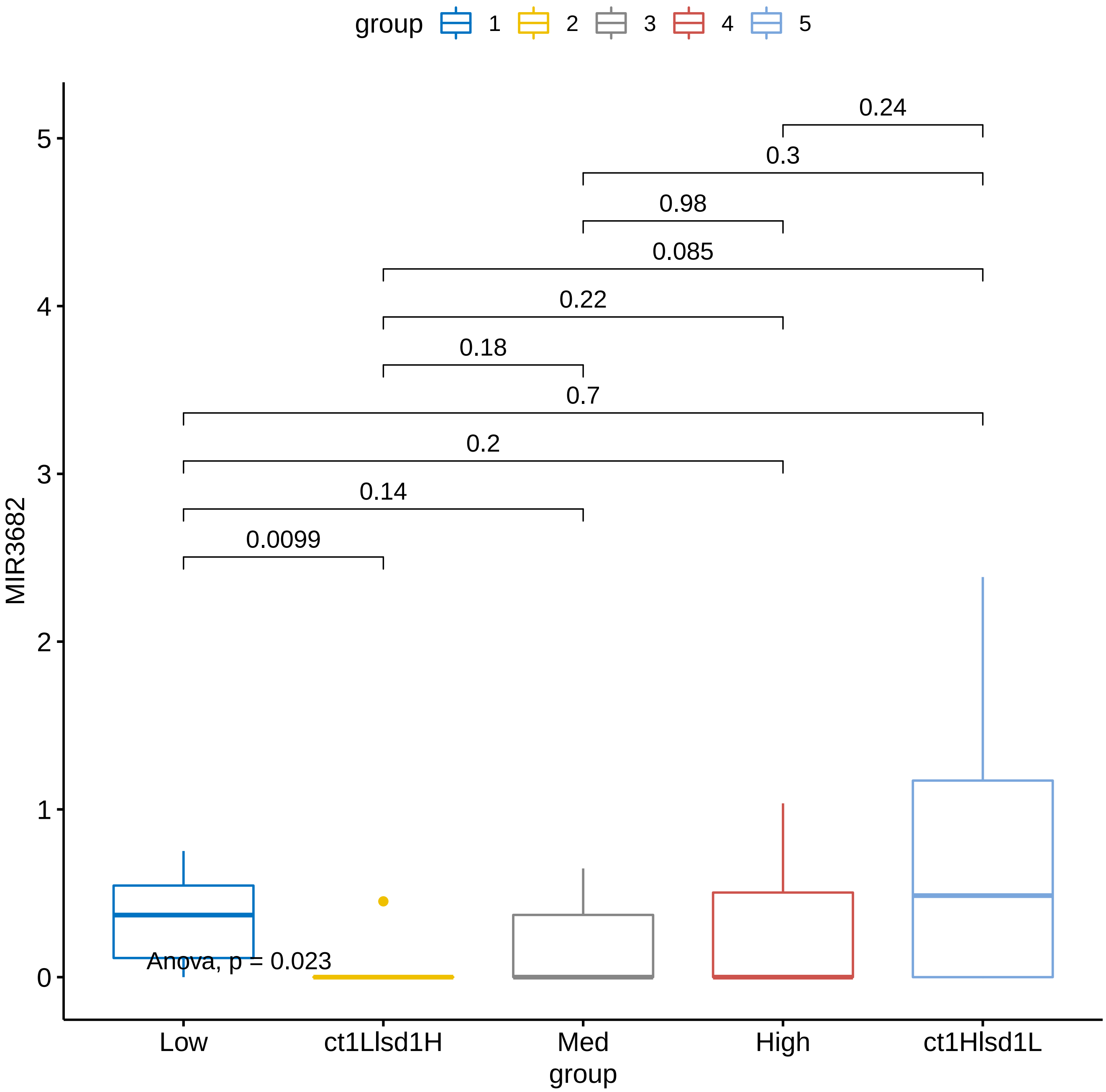

group 1 2 3 4 5
0.24
0.3
0.98
0.085
0.22
0.18
0.7
0.2
0.14
0.0099
Anova, p = 0.023
MIR3682
5
4
3
2
1
0
Low
ct1Llsd1H
Med
High
ct1Hlsd1L
group

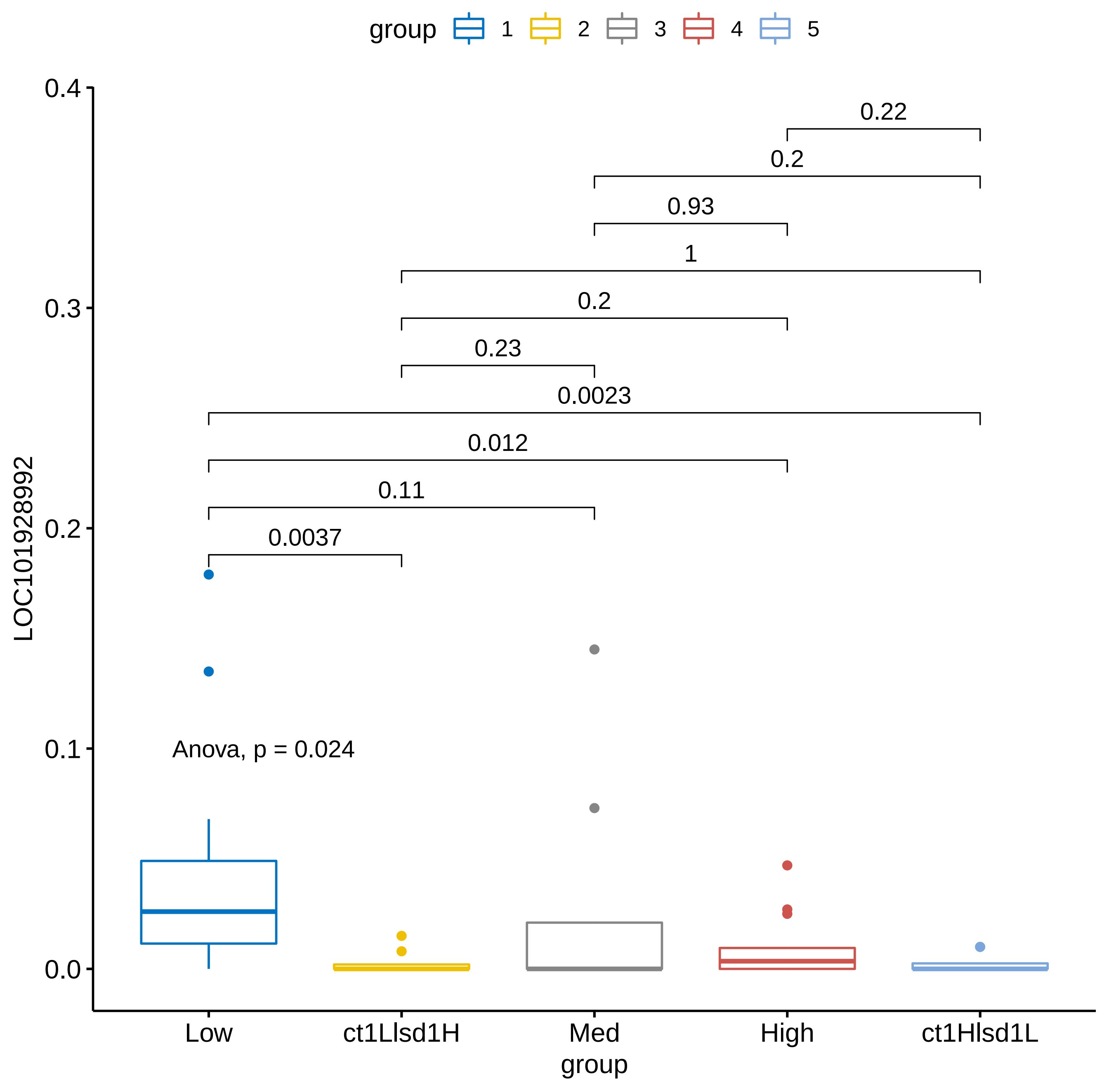
group
1
2
3
4
5
0.4
0.3
0.2
0.1
0.0
LOC101928992
0.22
0.2
0.93
1
0.2
0.23
0.0023
0.012
0.11
0.0037
Anova, p = 0.024
Low
ct1Llsd1H
Med
High
ct1Hlsd1L
group

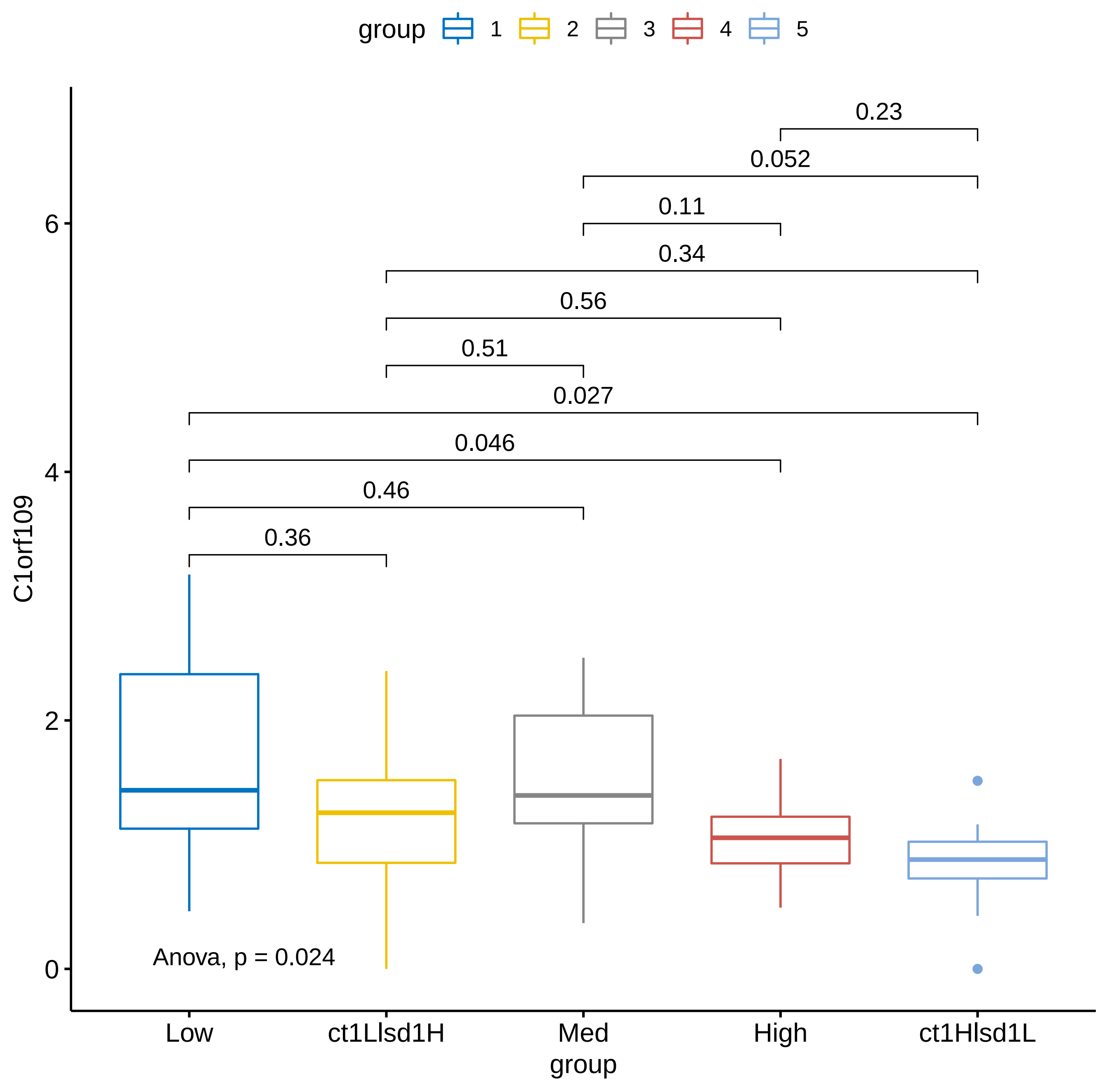

group
1
2
3
4
5
C1orf109
0.23
0.052
0.11
0.34
0.56
0.51
0.027
0.046
0.46
0.36
Anova, p = 0.024
6
4
2
0
Low
ct1Llsd1H
Med
High
ct1Hlsd1L
group

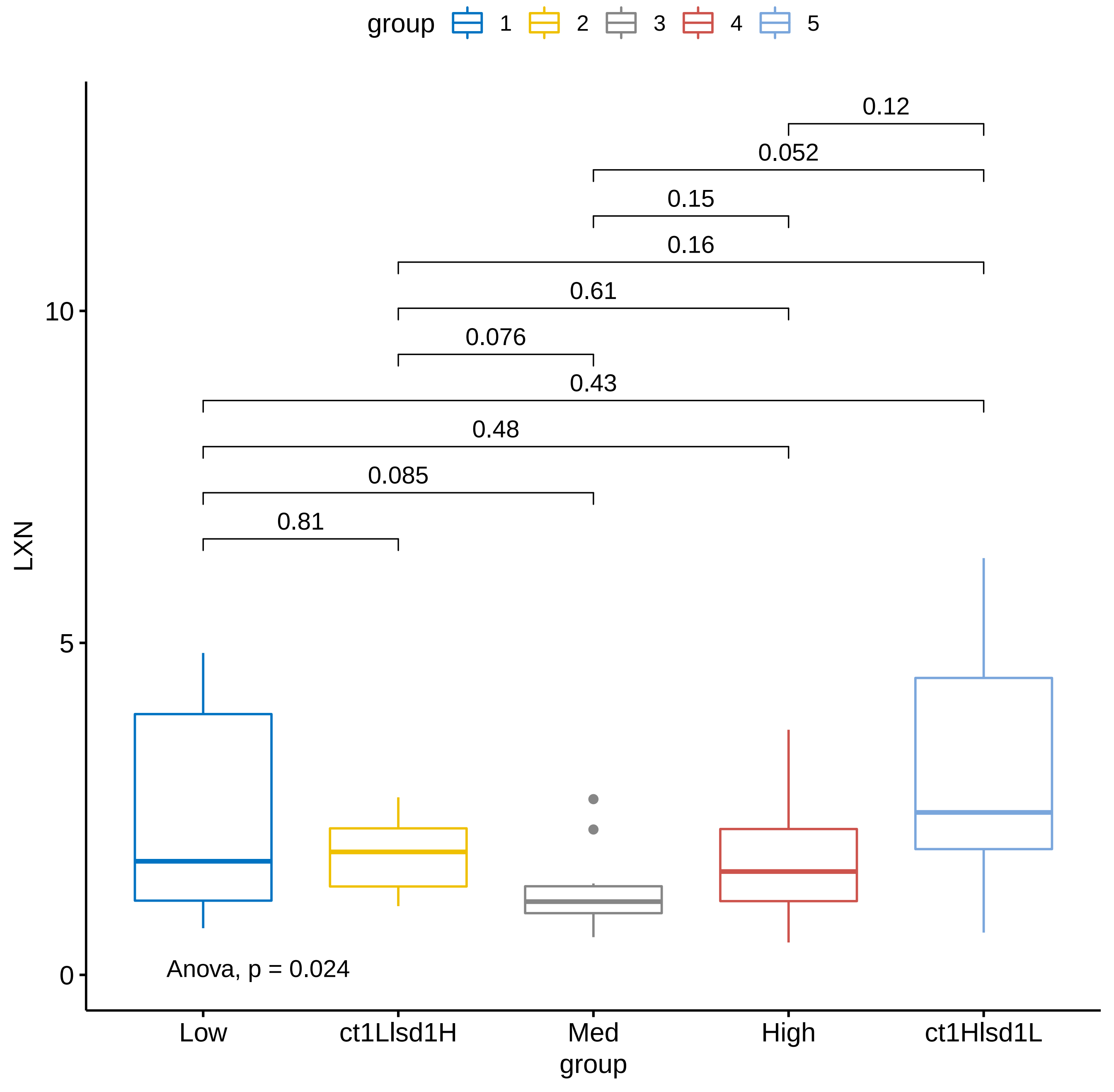

group
1
2
3
4
5
0.12
0.052
0.15
0.16
0.61
0.076
0.43
0.48
0.085
0.81
10
5
0
LXN
Anova, p = 0.024
Low
ct1Llsd1H
Med
High
ct1Hlsd1L
group

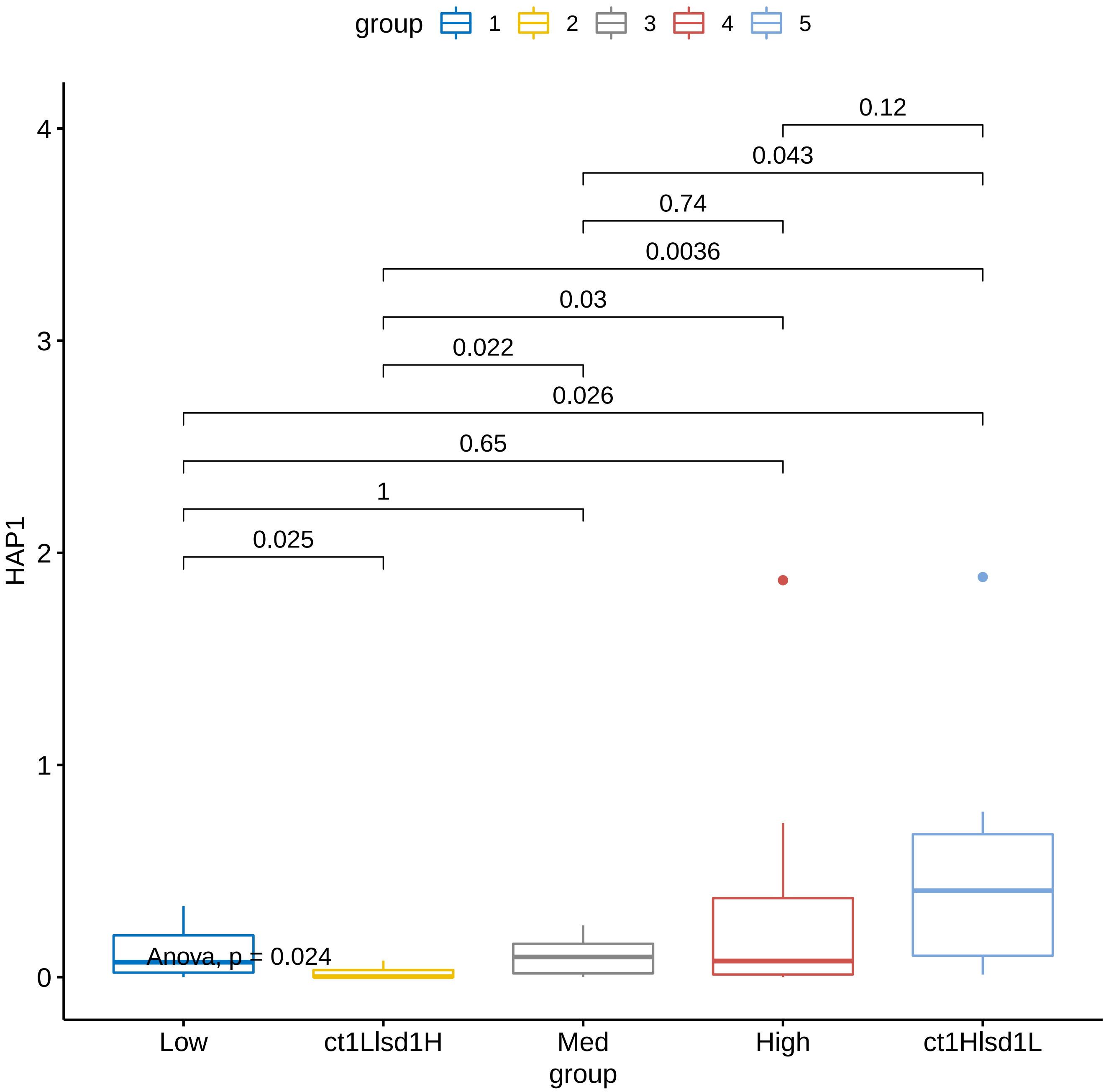
group
1
2
3
4
5
0.12
0.043
0.74
0.0036
0.03
0.022
0.026
0.65
1
0.025
Anova, p = 0.024
HAP1
4
3
2
1
0
Low
ct1Llsd1H
Med
High
ct1Hlsd1L
group

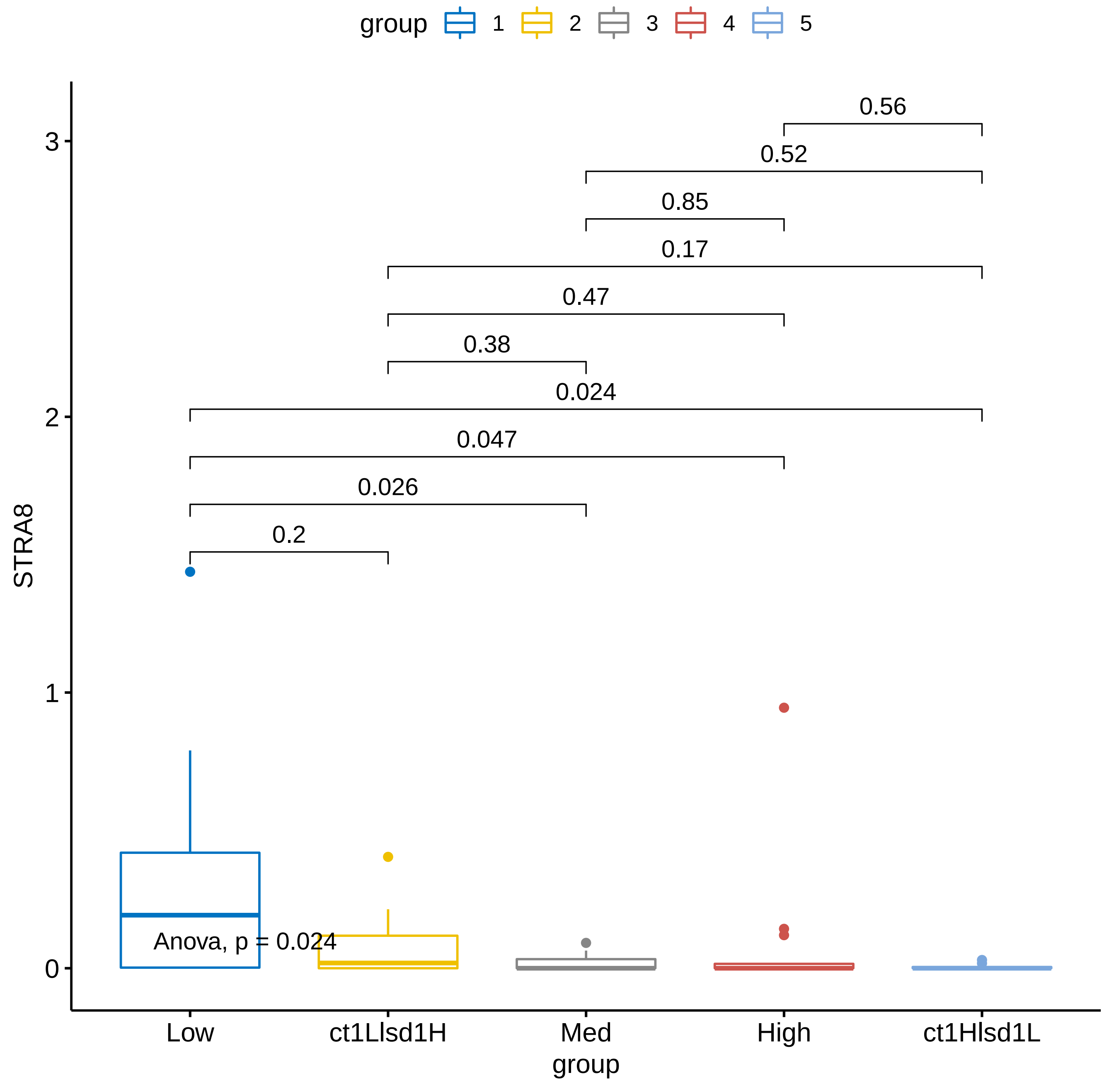

group 1 2 3 4 5
0.56
0.52
0.85
0.17
0.47
0.38
0.024
0.047
0.026
0.2
Anova, p = 0.024
STRA8
3
2
1
0
Low
ct1Llsd1H
Med
High
ct1Hlsd1L
group

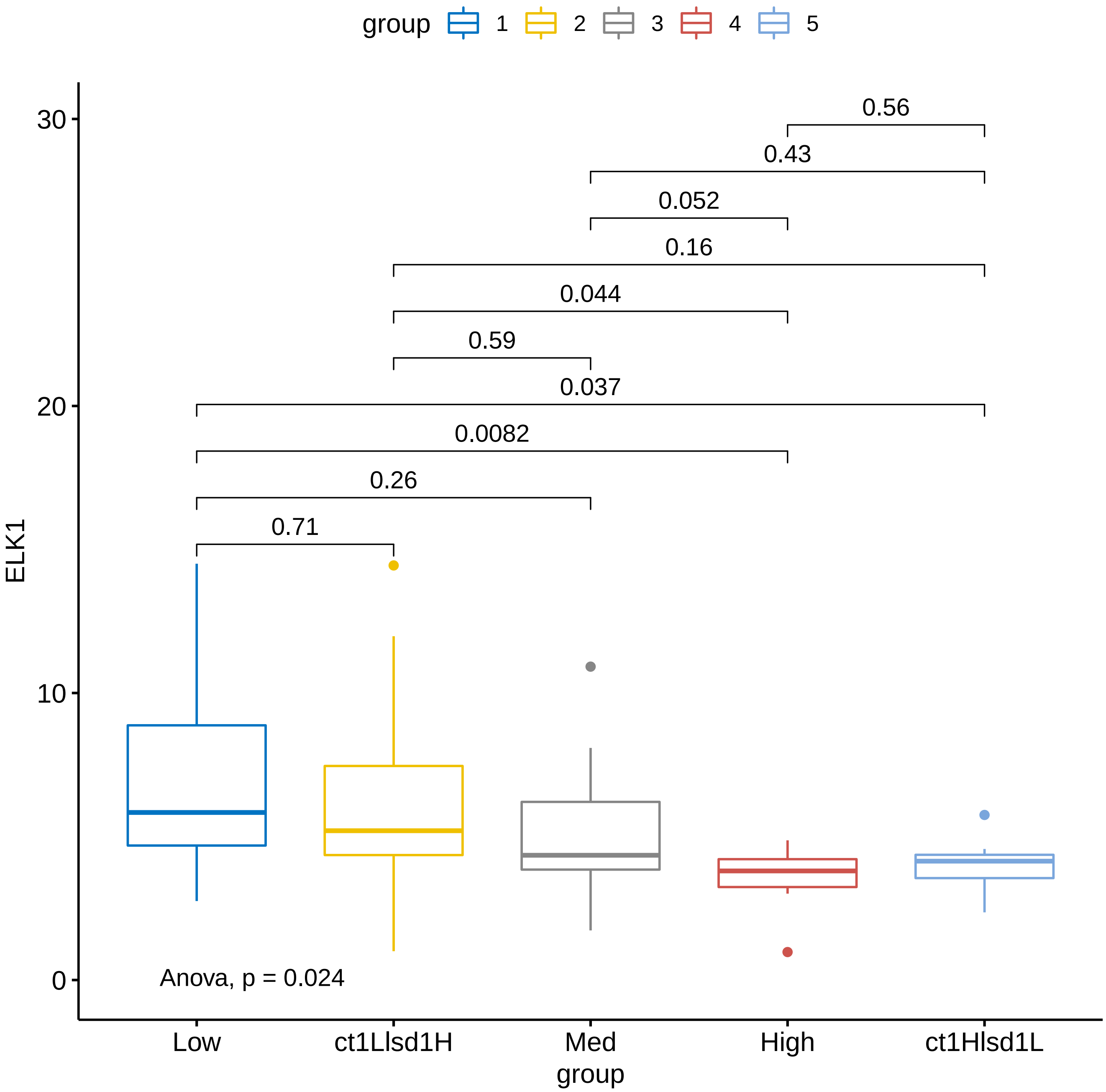

group
1
2
3
4
5
0.56
0.43
0.052
0.16
0.044
0.59
0.037
0.0082
0.26
0.71
30
20
10
0
ELK1
Anova, p = 0.024
Low
ct1Llsd1H
Med
High
ct1Hlsd1L
group

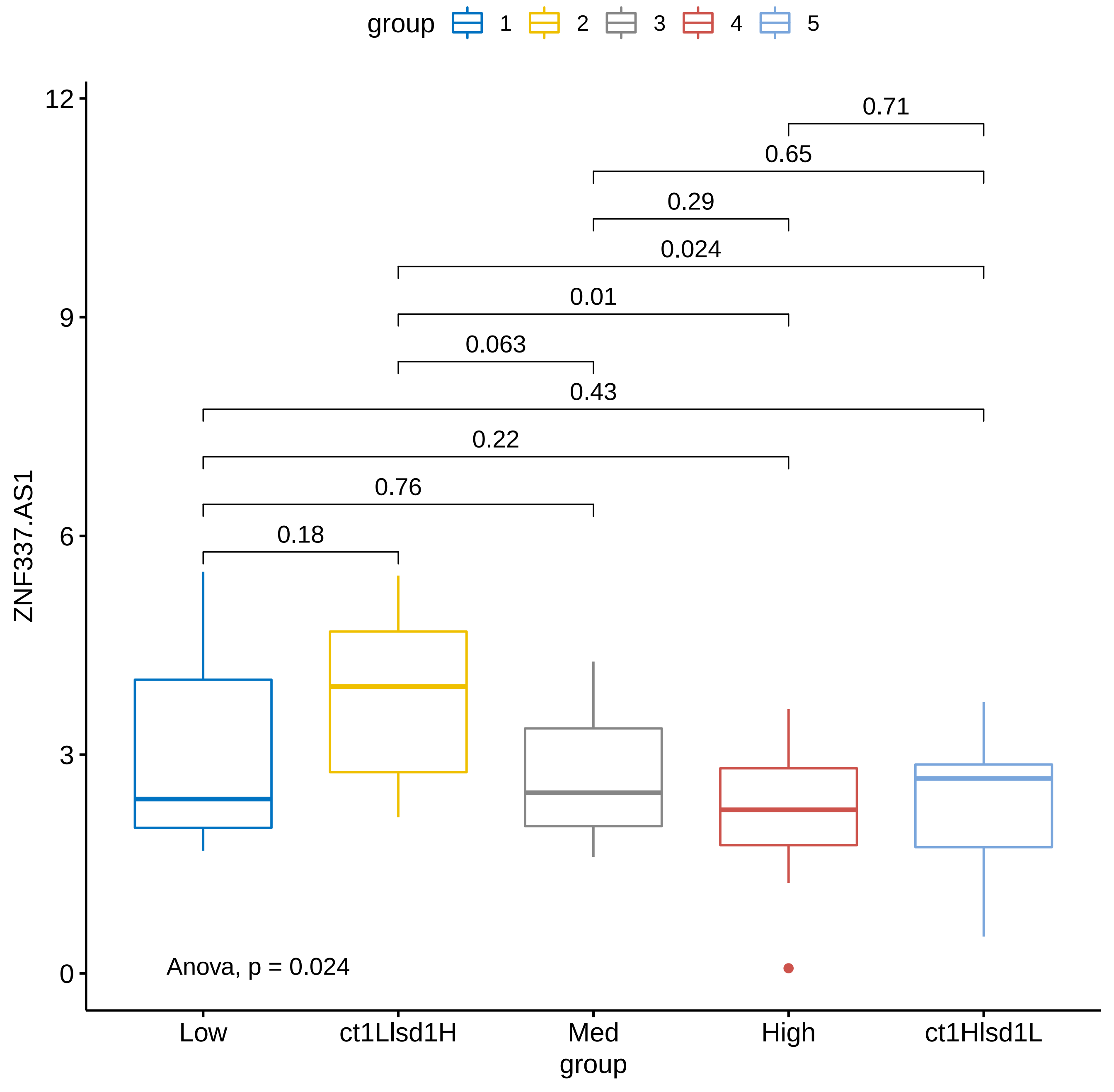
group 1 2 3 4 5
12
9
6
3
0
ZNF337.AS1
0.71
0.65
0.29
0.024
0.01
0.063
0.43
0.22
0.76
0.18
Anova, p = 0.024
Low
ct1Llsd1H
Med
High
ct1Hlsd1L
group

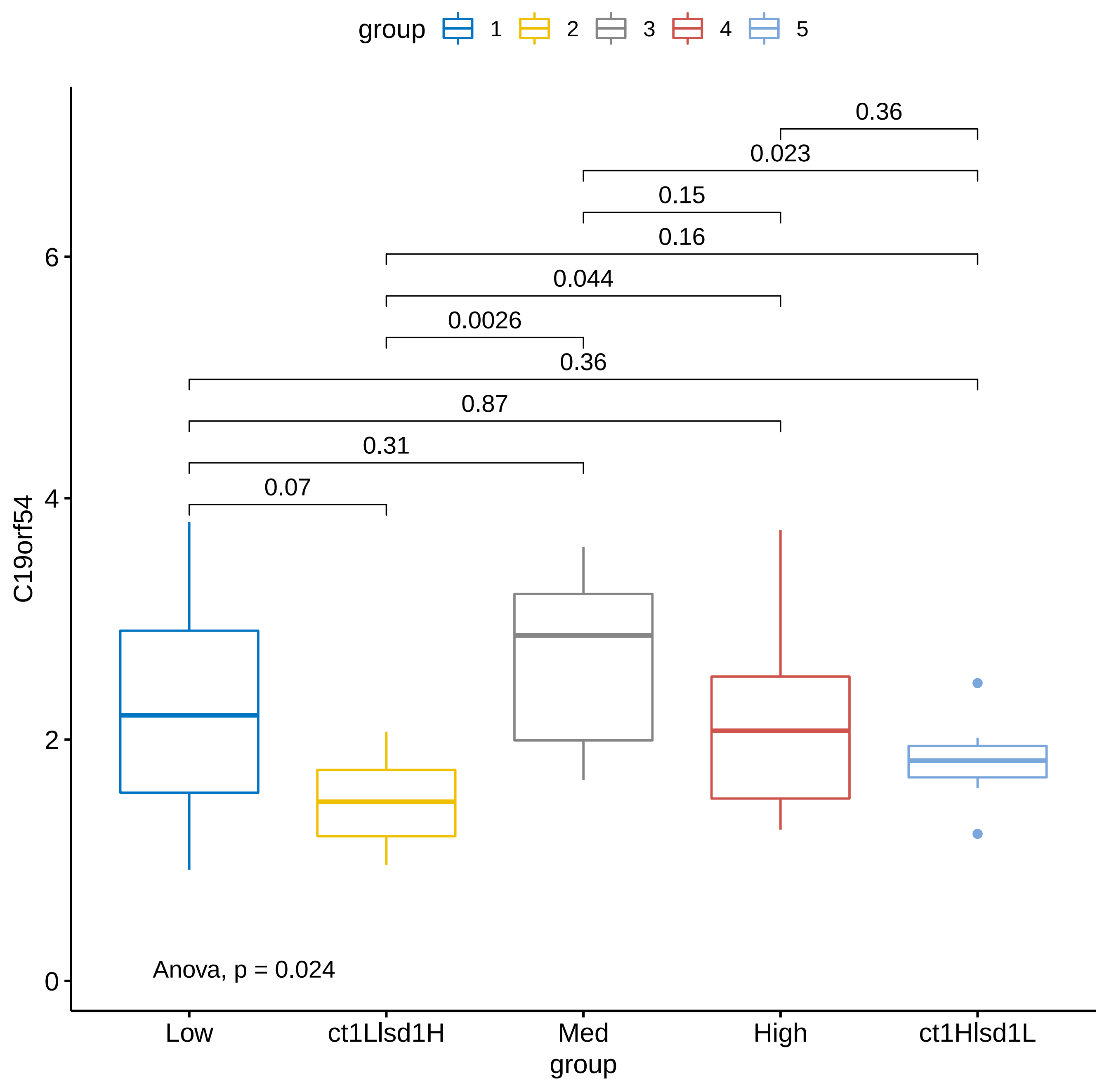

group 1 2 3 4 5
0.36
0.023
0.15
0.16
0.044
0.0026
0.36
0.87
0.31
0.07
6
4
2
0
C19orf54
Anova, p = 0.024
Low
ct1Llsd1H
Med
High
ct1Hlsd1L
group

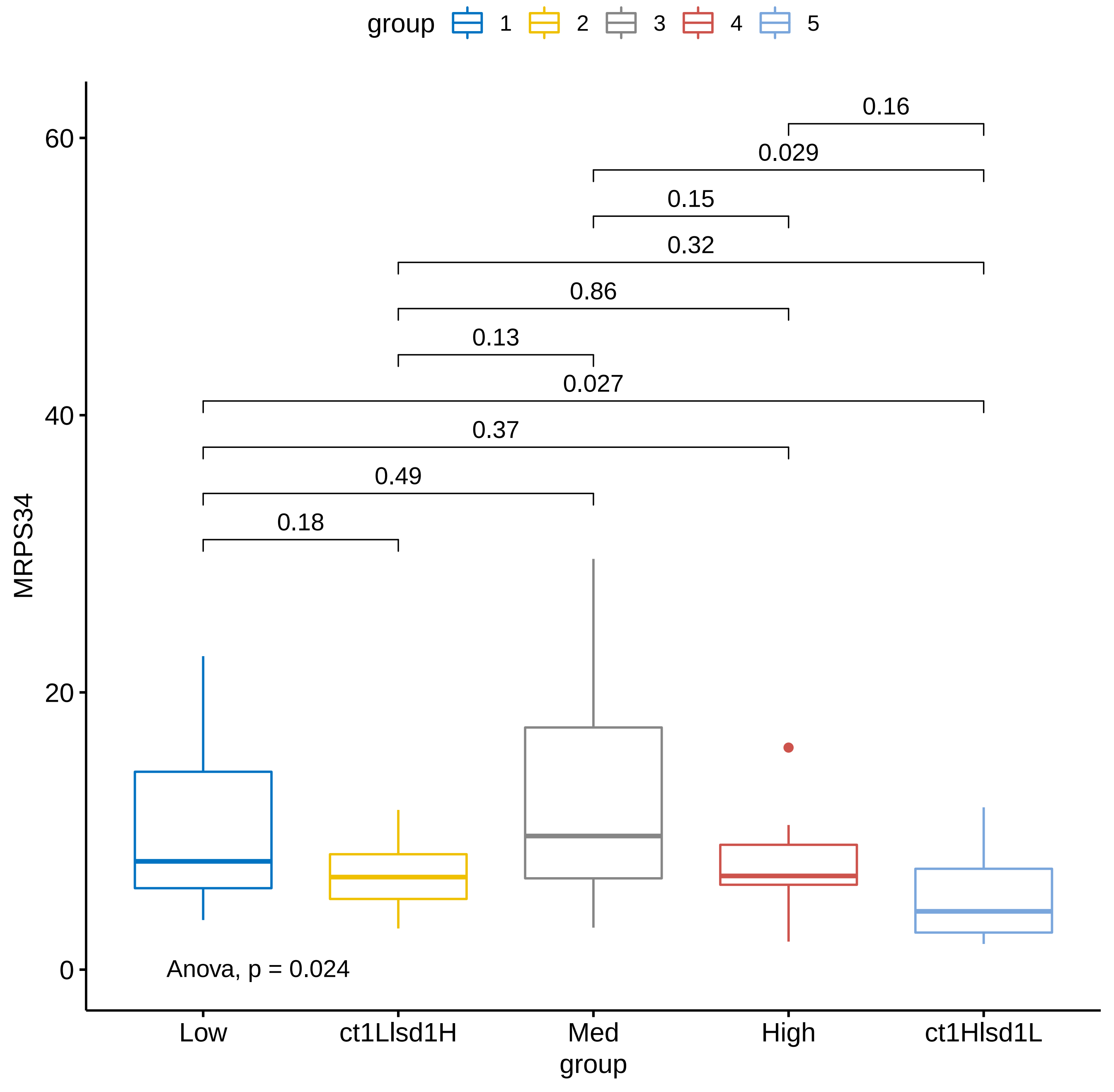

group
1
2
3
4
5
MRPS34
60
40
20
0
0.16
0.029
0.15
0.32
0.86
0.13
0.027
0.37
0.49
0.18
Anova, p = 0.024
Low
ct1Llsd1H
Med
High
ct1Hlsd1L
group

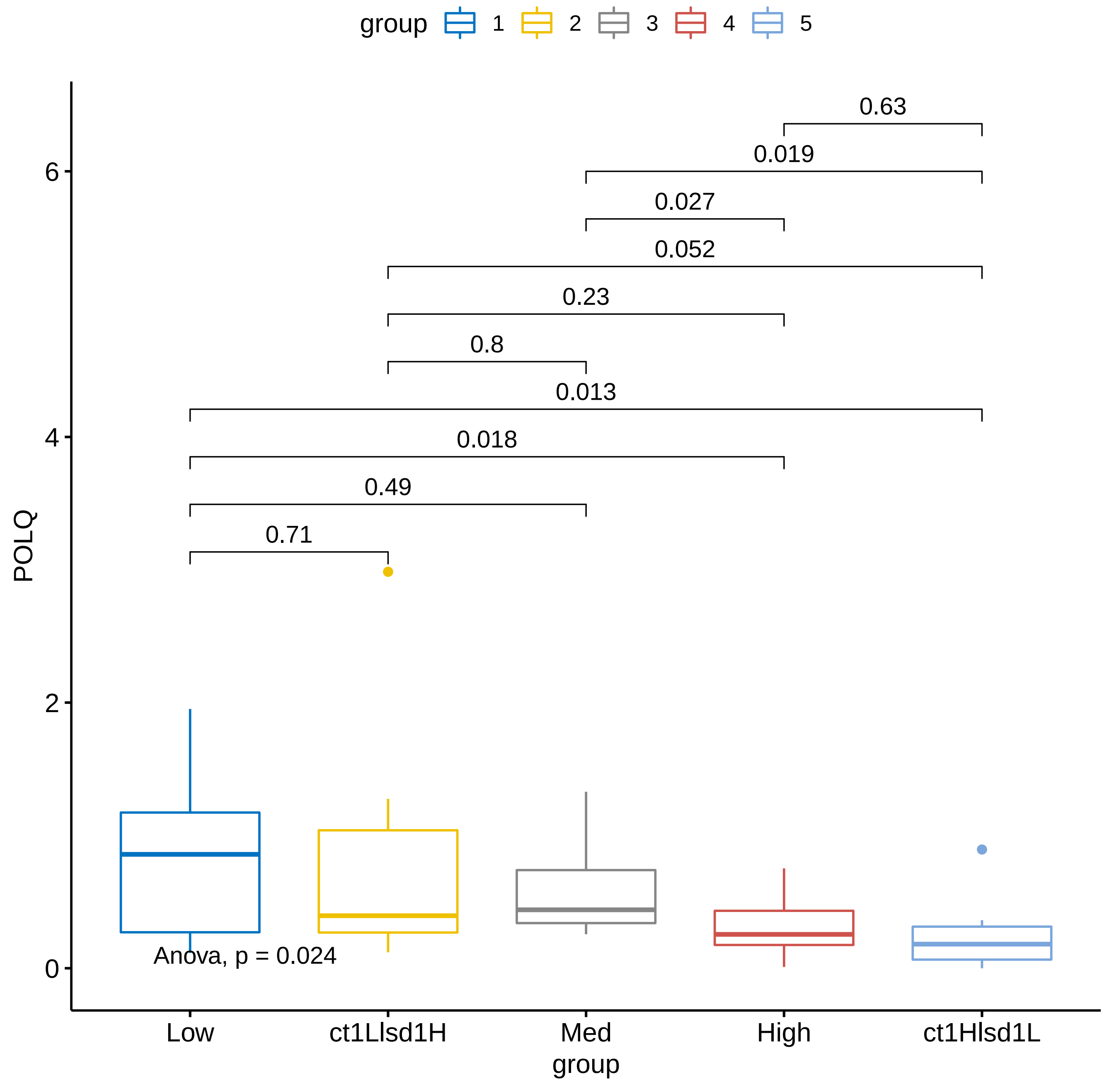

group
1
2
3
4
5
0.63
0.019
0.027
0.052
0.23
0.8
0.013
0.018
0.49
0.71
6
4
2
0
POLQ
Anova, p = 0.024
Low
ct1Llsd1H
Med
High
ct1Hlsd1L
group

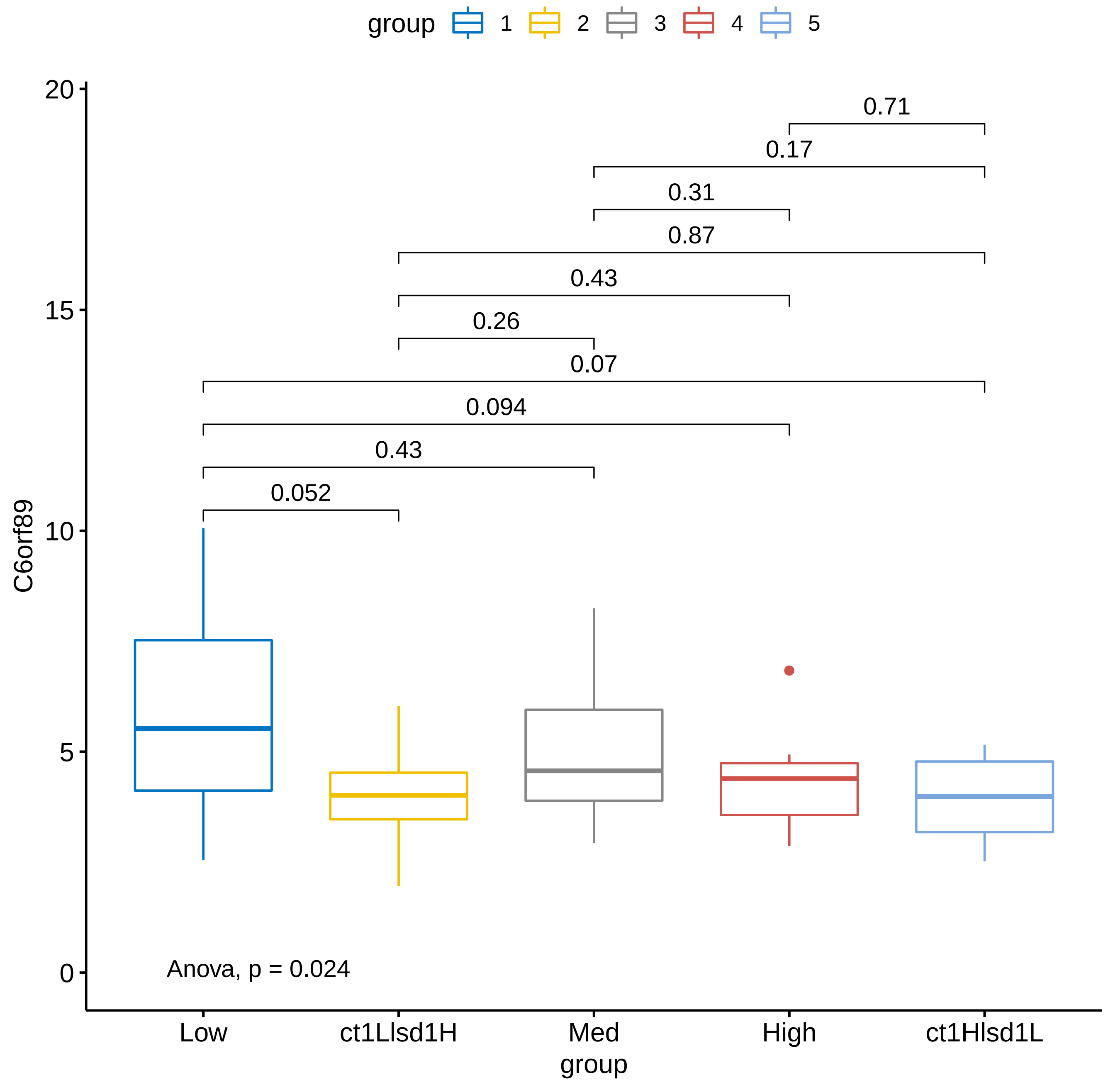
group
1
2
3
4
5
C6orf89
20
15
10
5
0
0.71
0.17
0.31
0.87
0.43
0.26
0.07
0.094
0.43
0.052
Anova, p = 0.024
Low
ct1Llsd1H
Med
High
ct1Hlsd1L
group

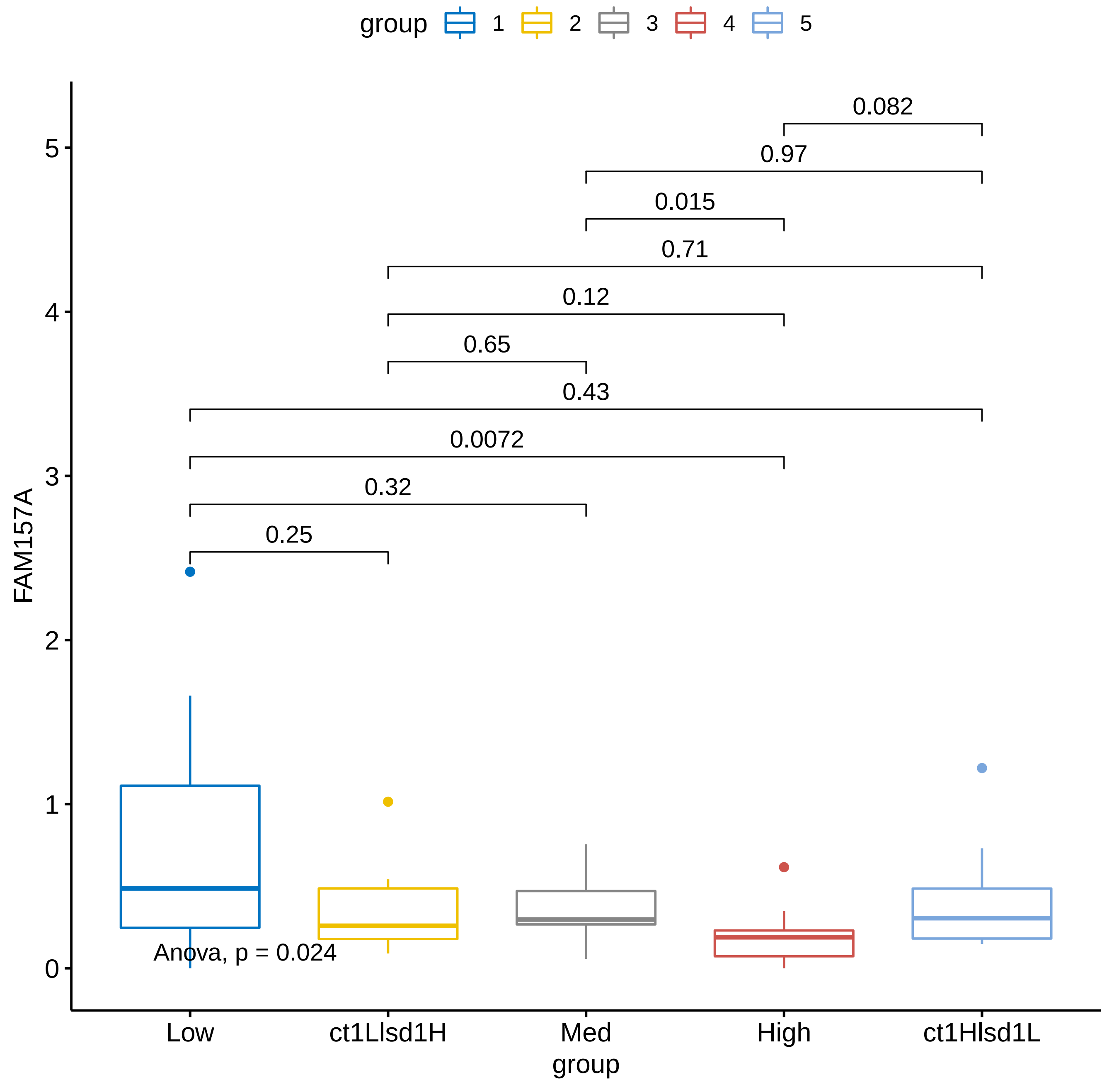

group
1
2
3
4
5
0.082
0.97
0.015
0.71
0.12
0.65
0.43
0.0072
0.32
0.25
Anova, p = 0.024
FAM157A
5
4
3
2
1
0
Low
ct1Llsd1H
Med
High
ct1Hlsd1L
group

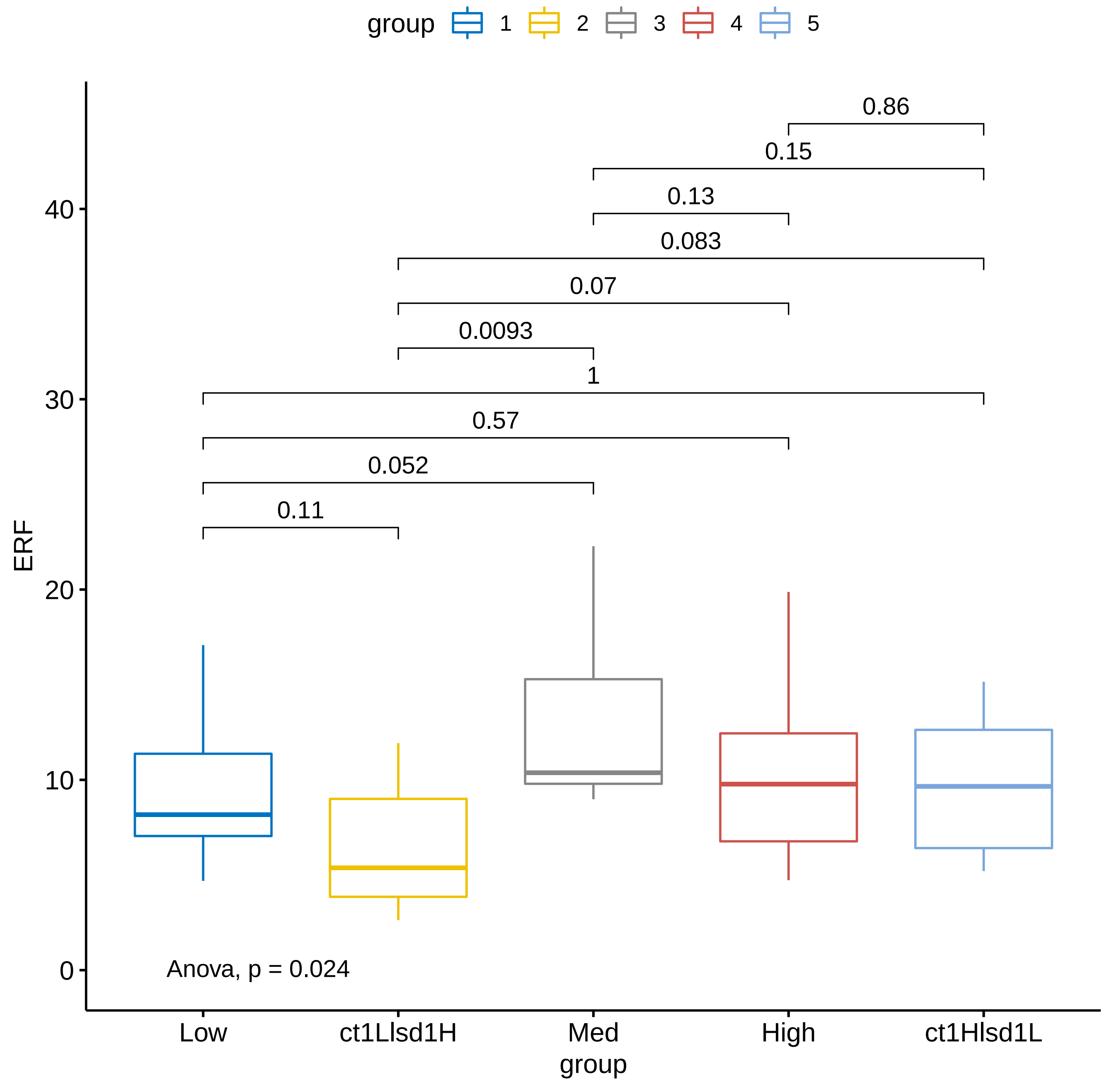

group
1
2
3
4
5
0.86
0.15
0.13
0.083
0.07
0.0093
1
0.57
0.052
0.11
40
30
20
10
0
ERF
Anova, p = 0.024
Low
ct1Llsd1H
Med
High
ct1Hlsd1L
group

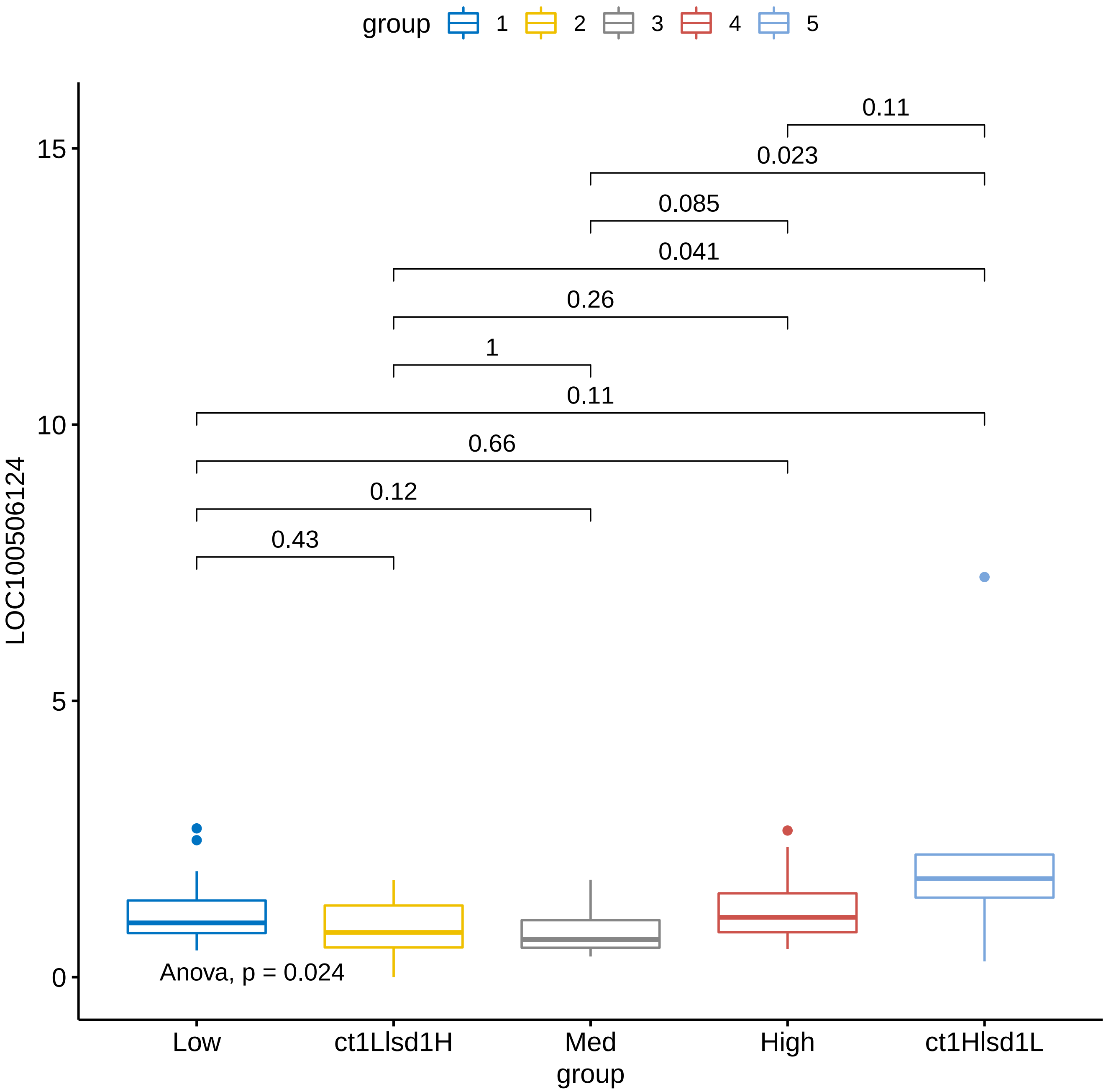

group 1 2 3 4 5
0.11
0.023
0.085
0.041
0.26
1
0.11
0.66
0.12
0.43
Anova, p = 0.024
LOC100506124
15
10
5
0
Low
ct1Llsd1H
Med
High
ct1Hlsd1L
group

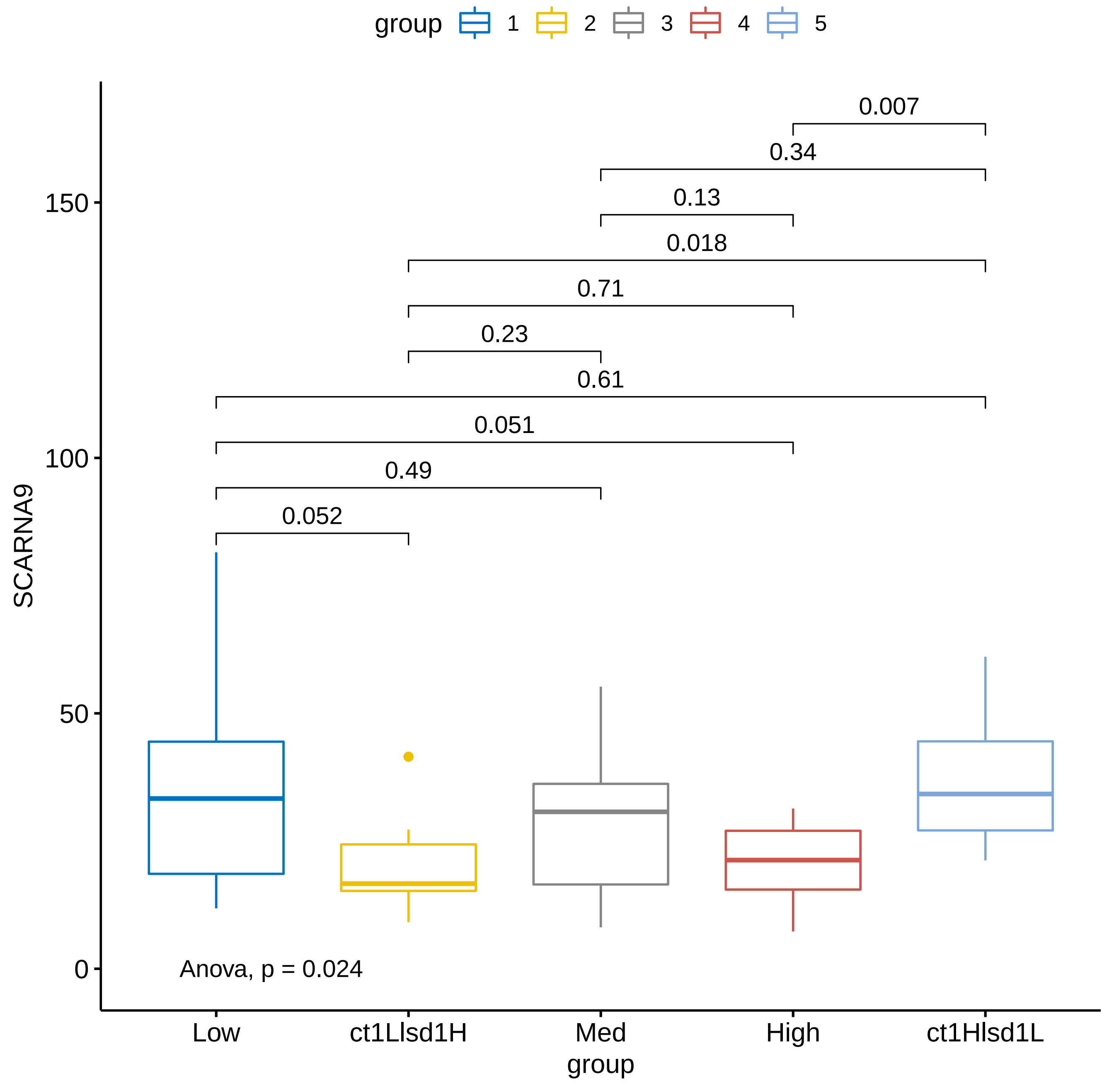

group
1
2
3
4
5
SCARNA9
150
100
50
0
0.007
0.34
0.13
0.018
0.71
0.23
0.61
0.051
0.49
0.052
Anova, p = 0.024
Low
ct1Llsd1H
Med
High
ct1Hlsd1L
group

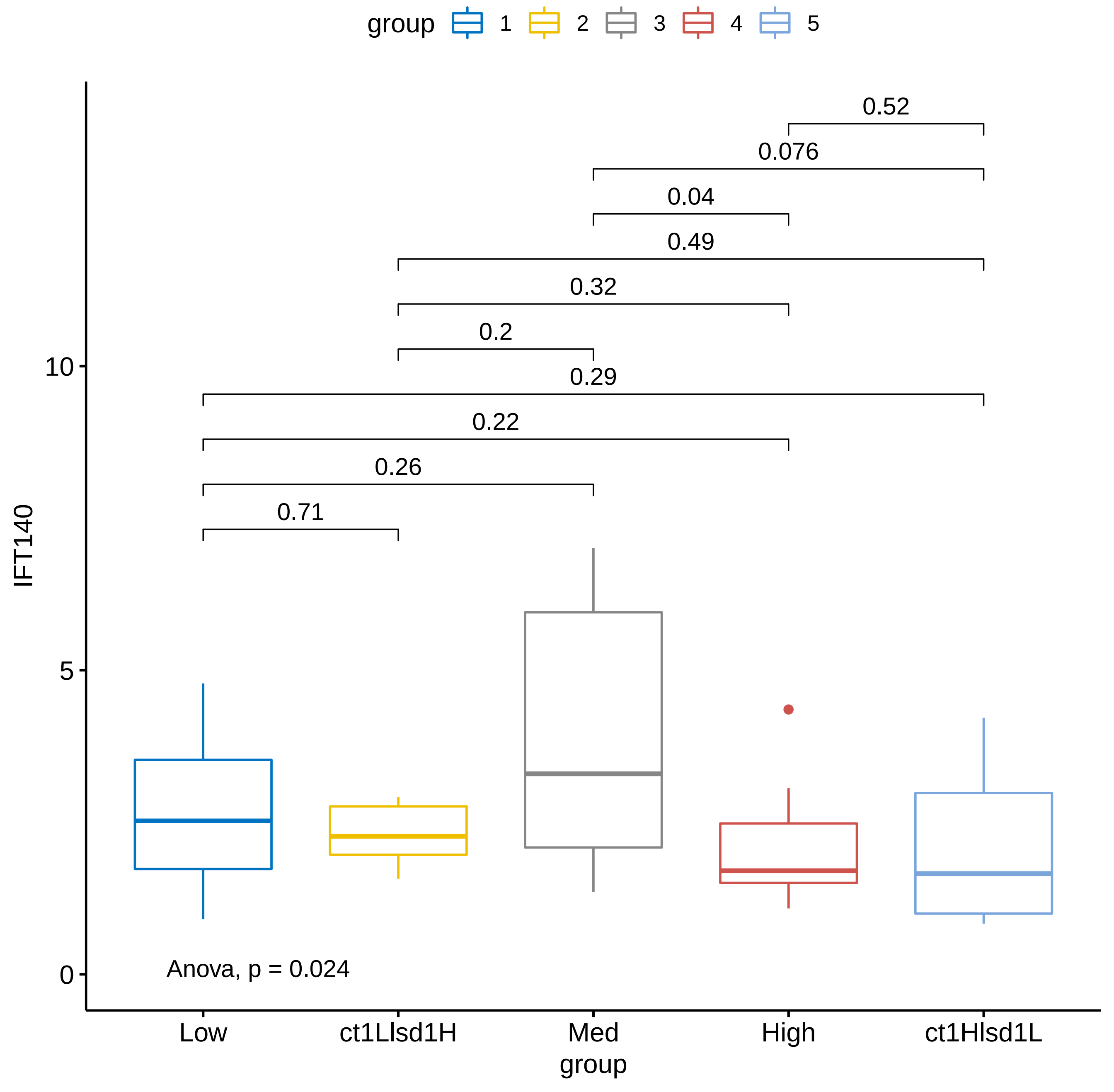

group
1
2
3
4
5
0.52
0.076
0.04
0.49
0.32
0.2
0.29
0.22
0.26
0.71
10
5
0
IFT140
Anova, p = 0.024
Low
ct1Llsd1H
Med
High
ct1Hlsd1L
group

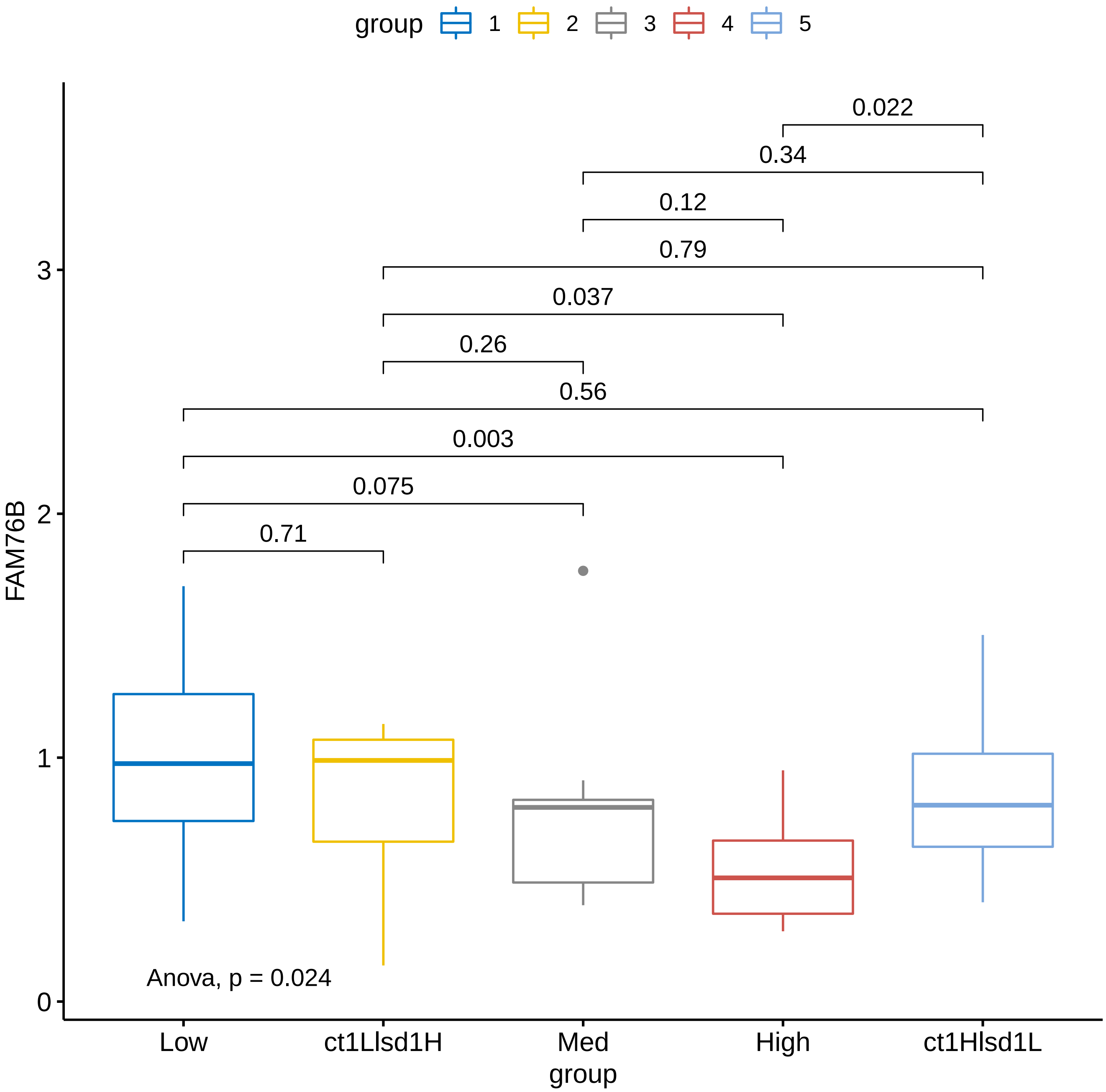

group
1
2
3
4
5
0.022
0.34
0.12
0.79
0.037
0.26
0.56
0.003
0.075
0.71
Anova, p = 0.024
FAM76B
0
1
2
3
Low
ct1Llsd1H
Med
High
ct1Hlsd1L
group

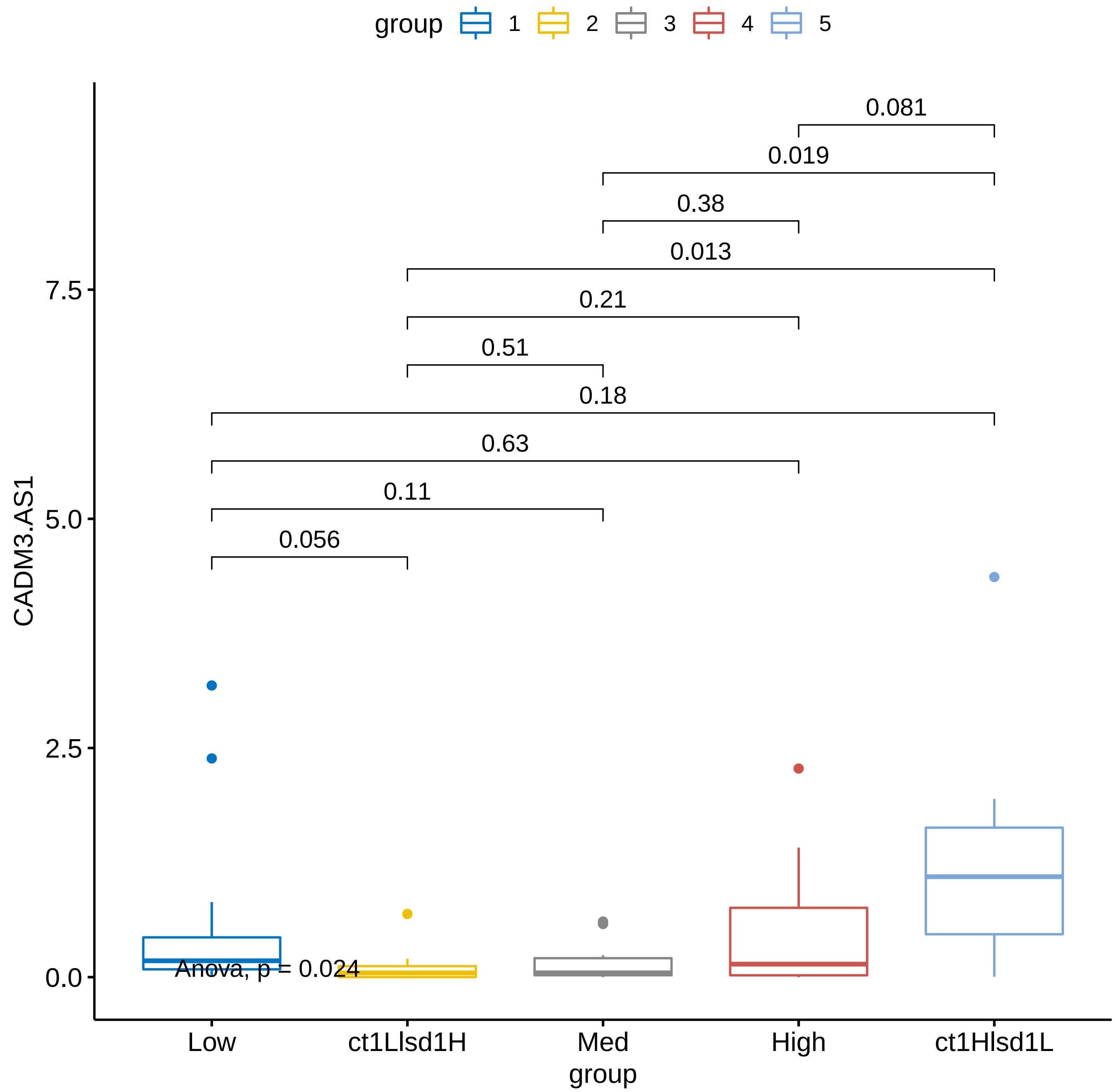

group 1 2 3 4 5
0.081
0.019
0.38
0.013
0.21
0.51
0.18
0.63
0.11
0.056
Anova, p = 0.024
CADM3.AS1
7.5
5.0
2.5
0.0
Low
ct1Llsd1H
Med
High
ct1Hlsd1L
group

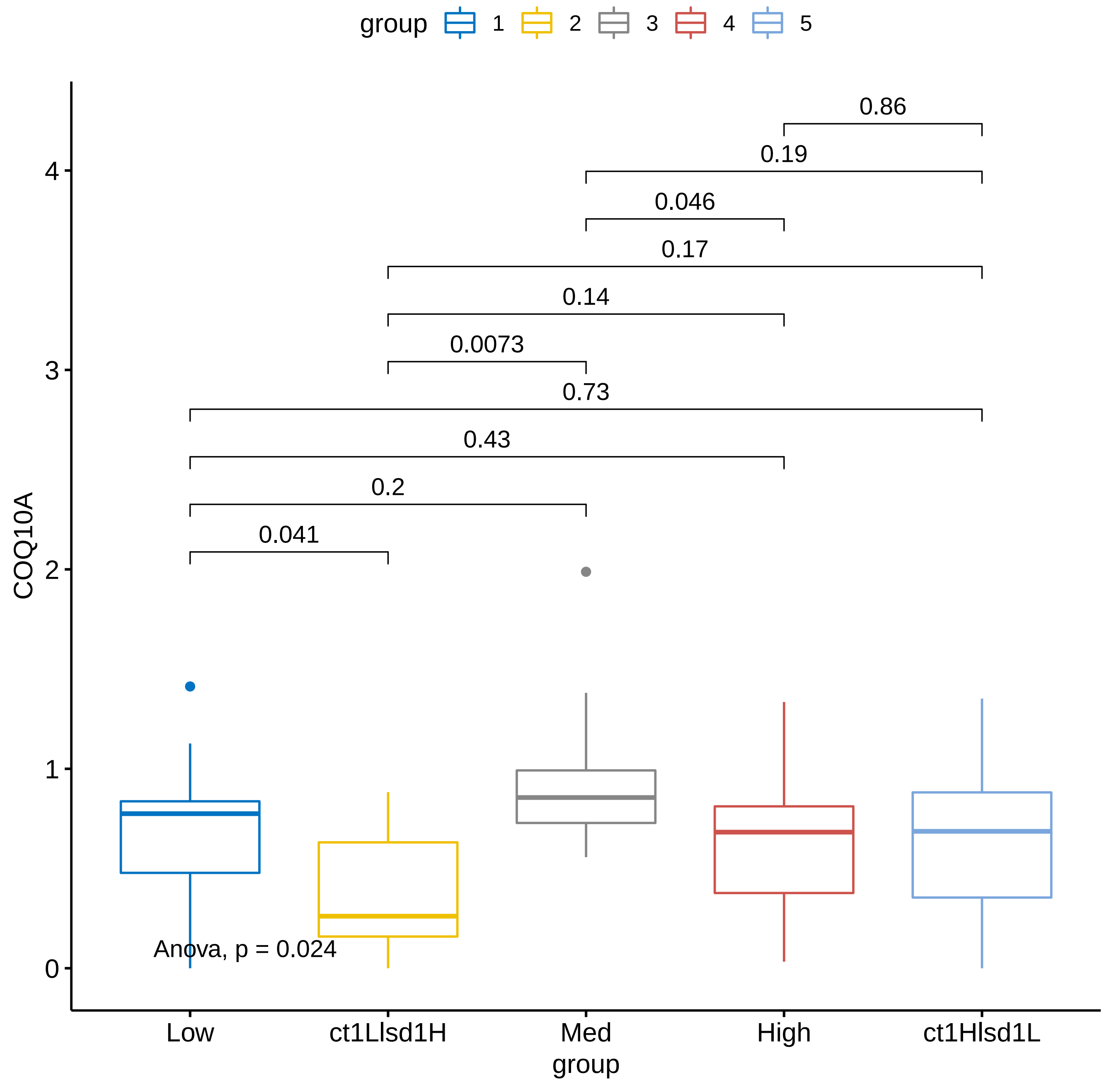

group 1 2 3 4 5
0.86
0.19
0.046
0.17
0.14
0.0073
0.73
0.43
0.2
0.041
COQ10A
4
3
2
1
0
Anova, p = 0.024
Low
ct1Llsd1H
Med
High
ct1Hlsd1L
group

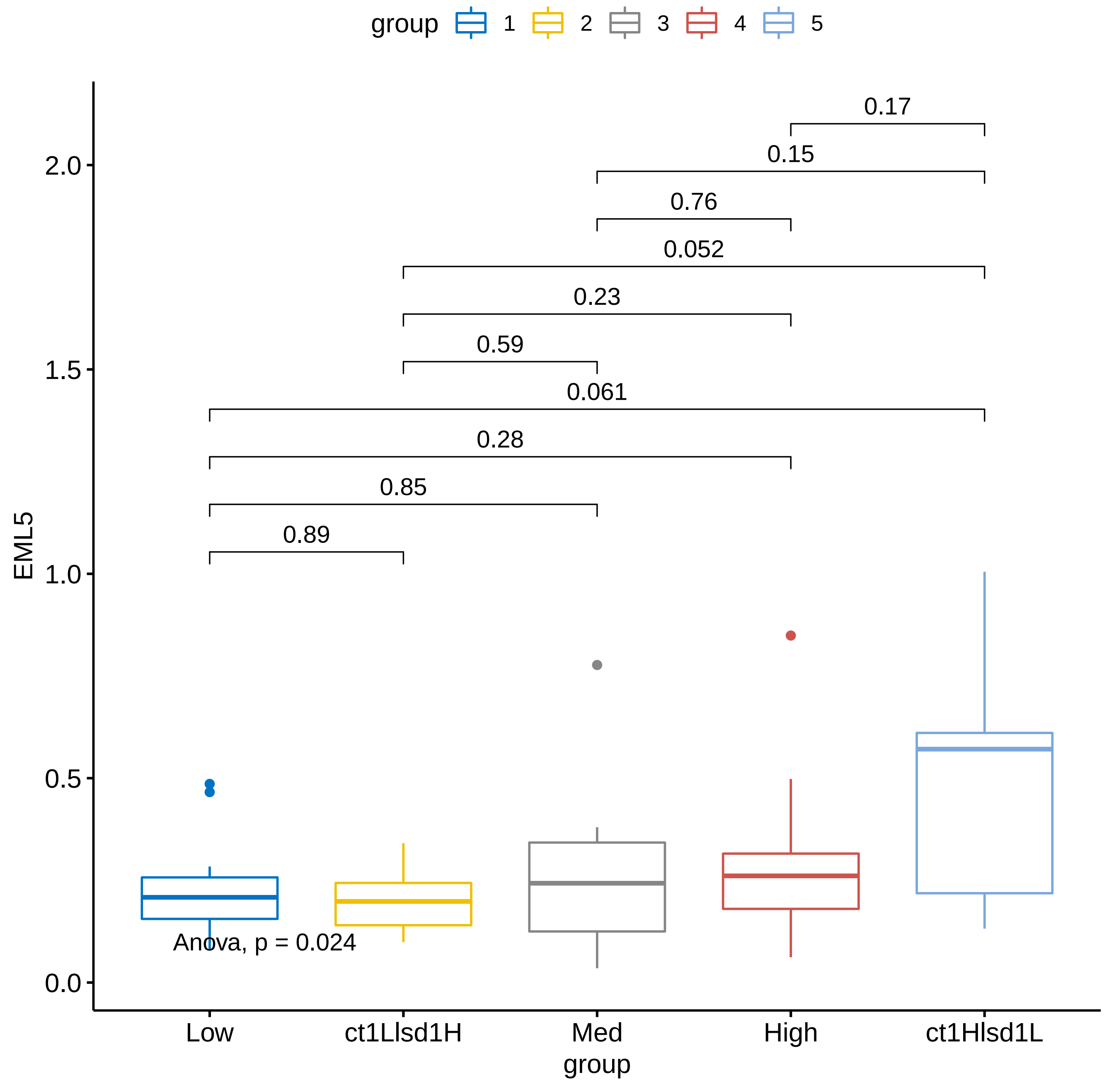
group
1
2
3
4
5
0.17
0.15
0.76
0.052
0.23
0.59
0.061
0.28
0.85
0.89
Anova, p = 0.024
EML5
2.0
1.5
1.0
0.5
0.0
Low
ct1Llsd1H
Med
High
ct1Hlsd1L
group

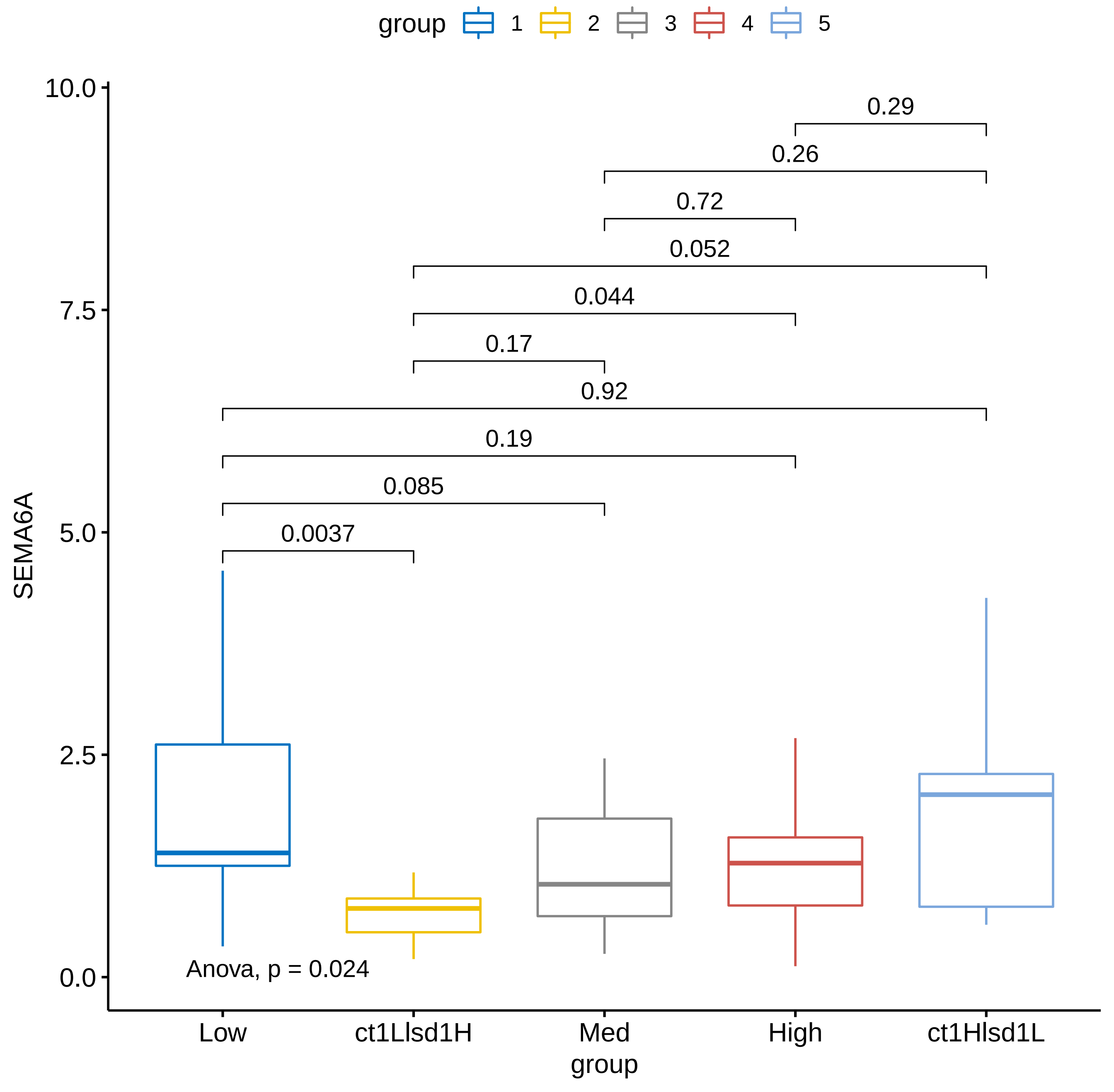

group 1 2 3 4 5
0.29
0.26
0.72
0.052
0.044
0.17
0.92
0.19
0.085
0.0037
Anova, p = 0.024
10.0
7.5
5.0
2.5
0.0
SEMA6A
Low
ct1Llsd1H
Med
High
ct1Hlsd1L
group

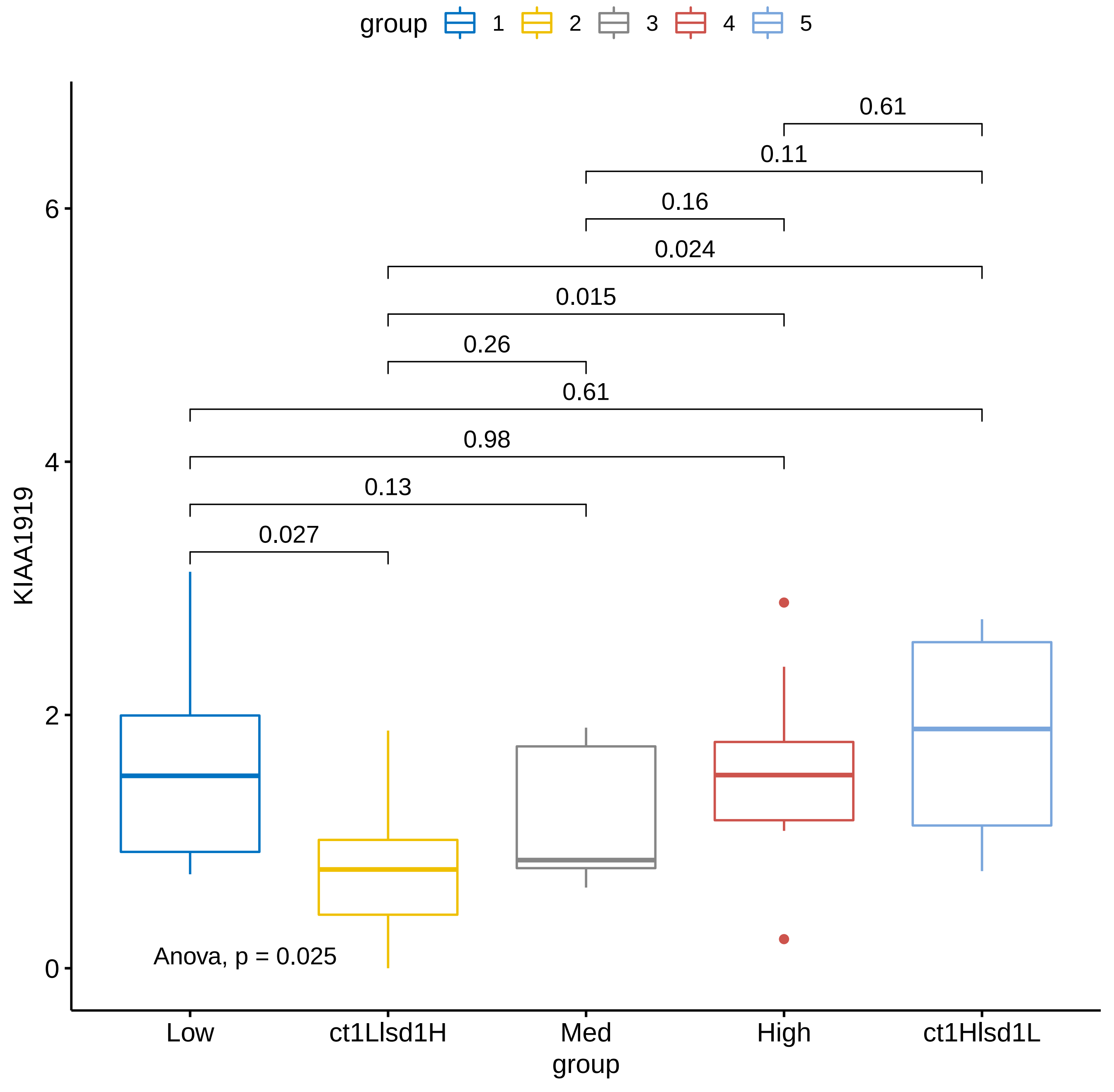

group
1
2
3
4
5
0.61
0.11
0.16
0.024
0.015
0.26
0.61
0.98
0.13
0.027
Anova, p = 0.025
KIAA1919
0
2
4
6
Low
ct1Llsd1H
Med
High
ct1Hlsd1L
group

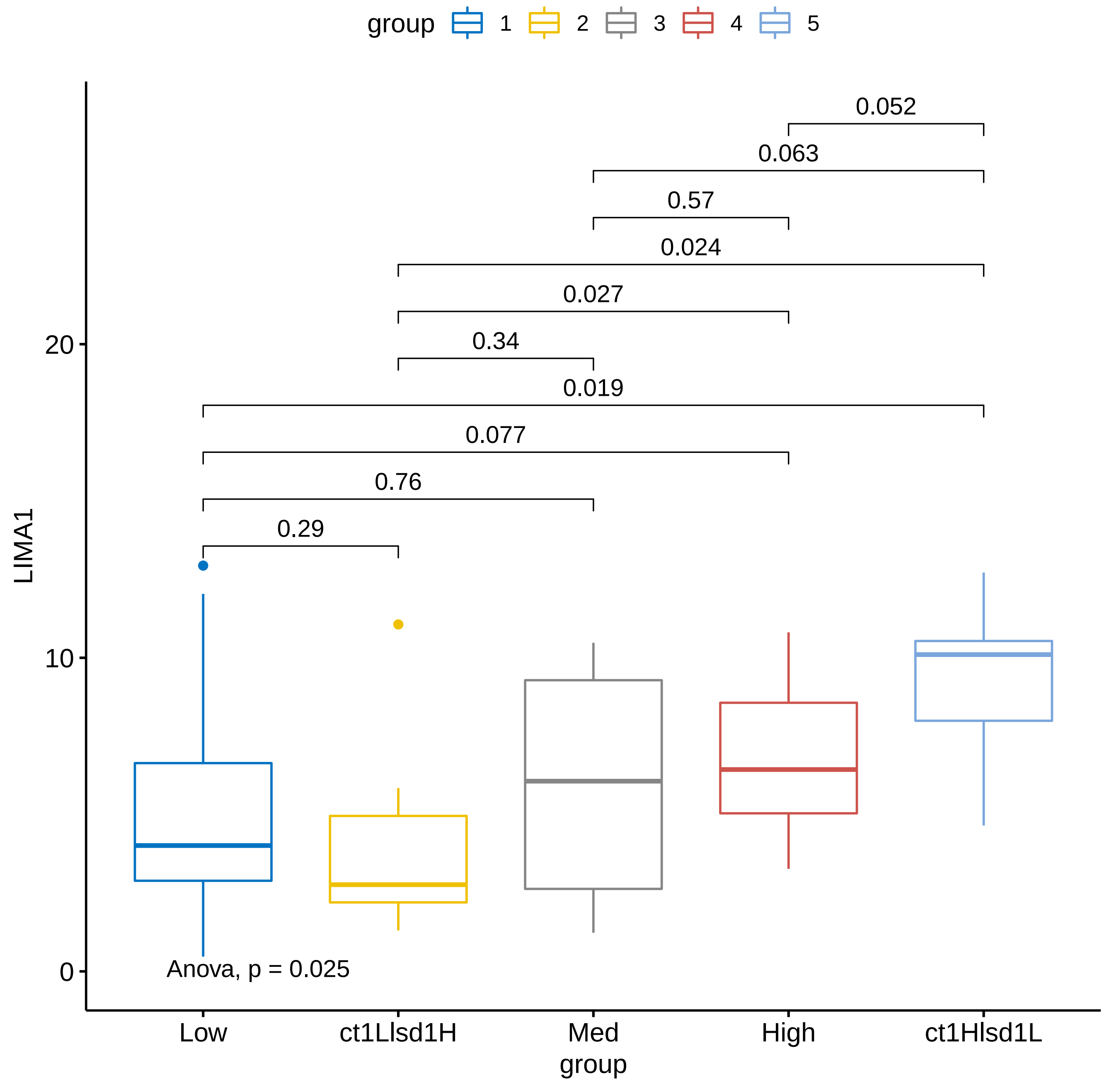

group 1 2 3 4 5
0.052
0.063
0.57
0.024
0.027
0.34
0.019
0.077
0.76
0.29
Anova, p = 0.025
LIMA1
20
10
0
Low
ct1Llsd1H
Med
High
ct1Hlsd1L
group

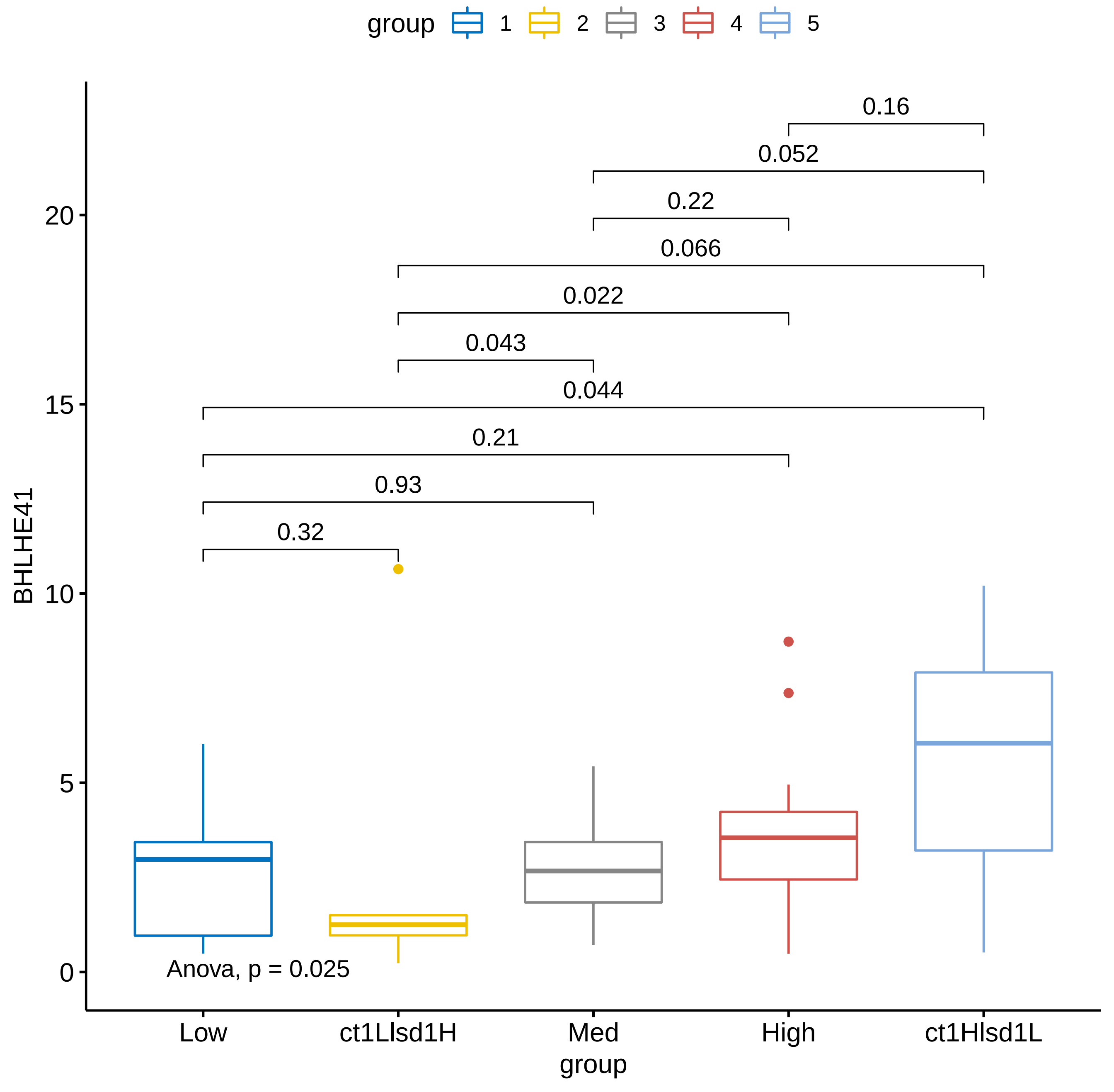

group 1 2 3 4 5
0.16
0.052
0.22
0.066
0.022
0.043
0.044
0.21
0.93
0.32
BHLHE41
20
15
10
5
0
Anova, p = 0.025
Low
ct1Llsd1H
Med
High
ct1Hlsd1L
group

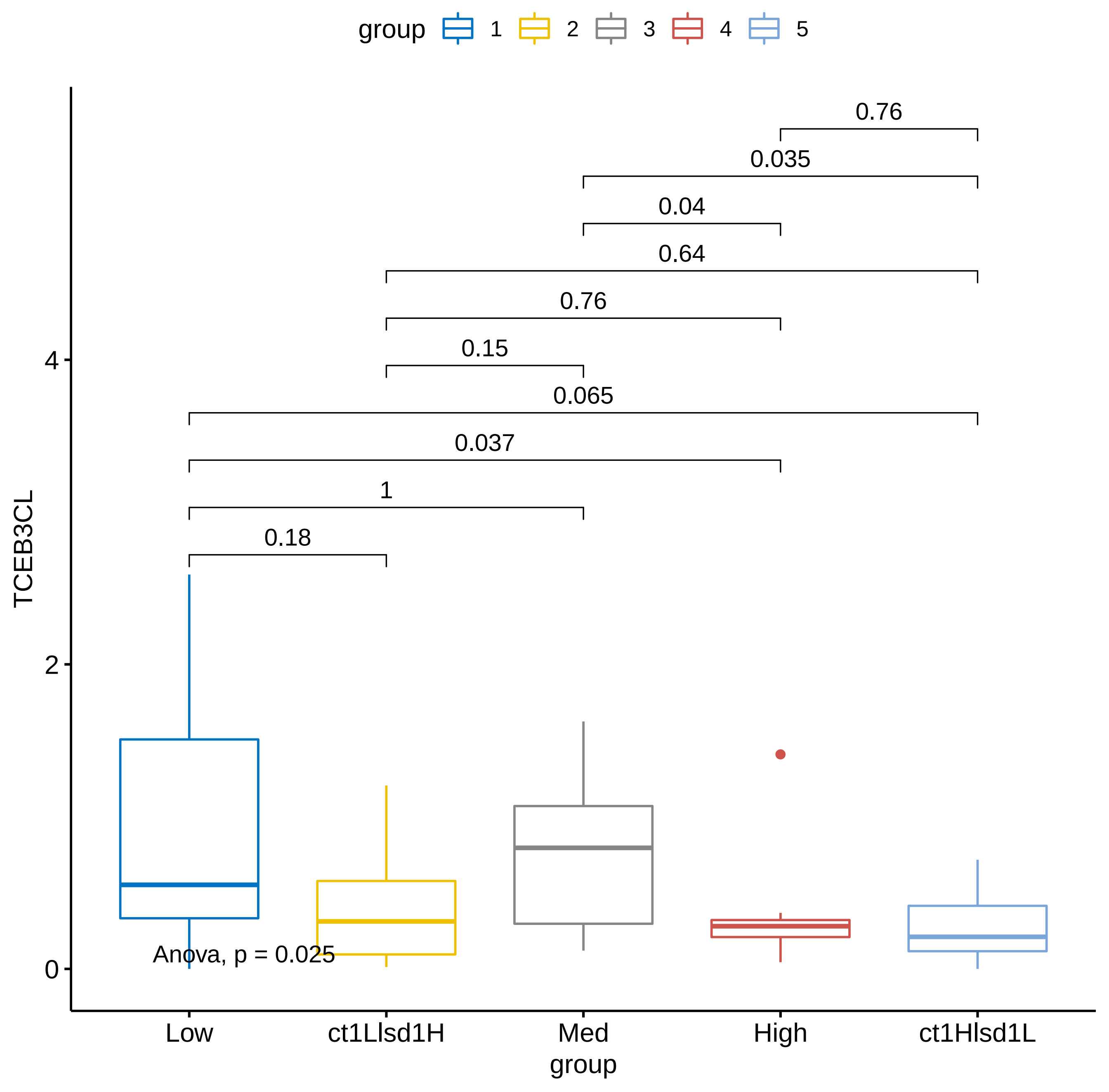

group
1
2
3
4
5
0.76
0.035
0.04
0.64
0.76
0.15
0.065
0.037
1
0.18
4
2
0
TCEB3CL
Anova, p = 0.025
Low
ct1Llsd1H
Med
High
ct1Hlsd1L
group

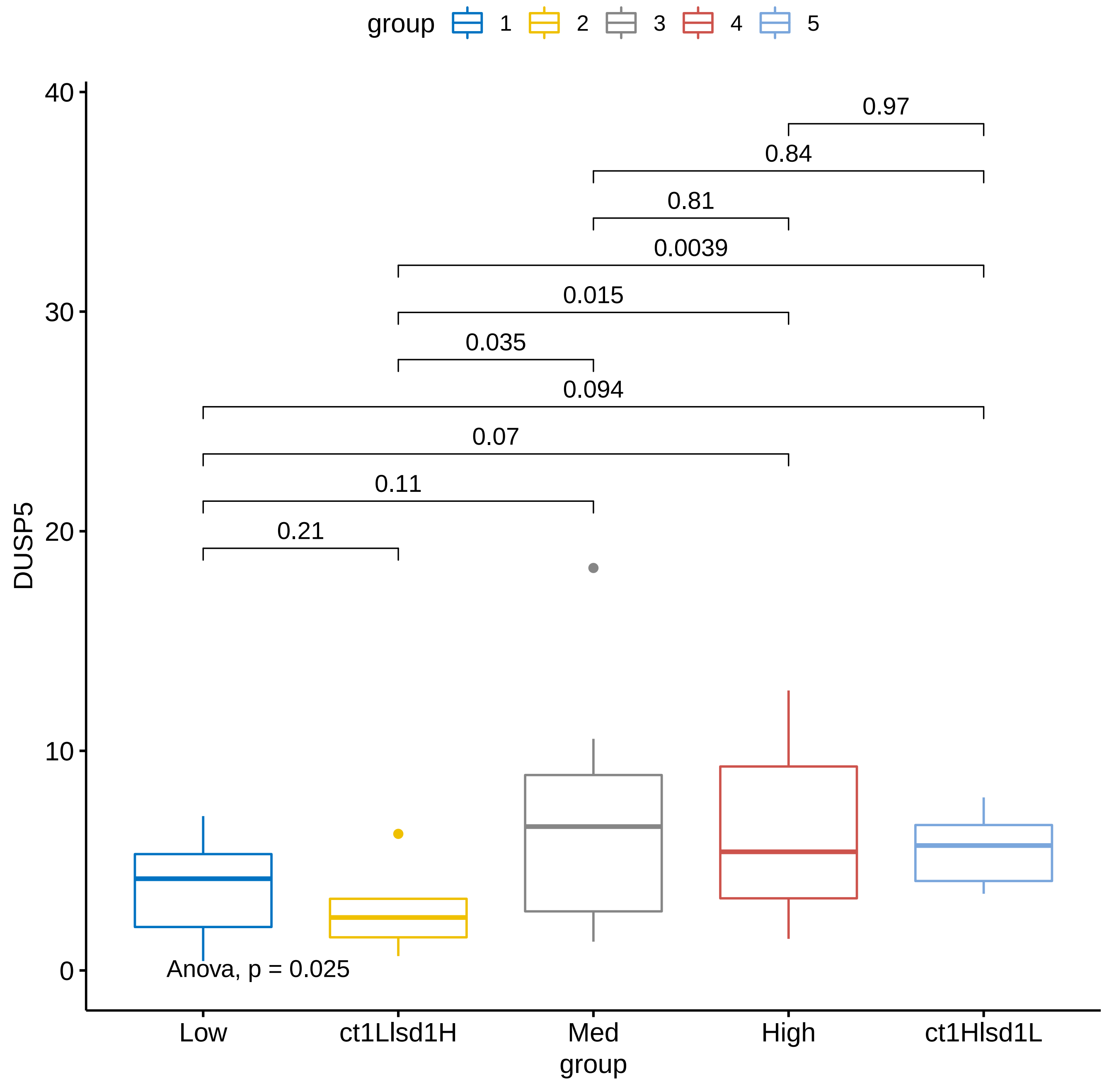

group 1 2 3 4 5
DUSP5
40
30
20
10
0
0.97
0.84
0.81
0.0039
0.015
0.035
0.094
0.07
0.11
0.21
Anova, p = 0.025
Low
ct1Llsd1H
Med
High
ct1Hlsd1L
group

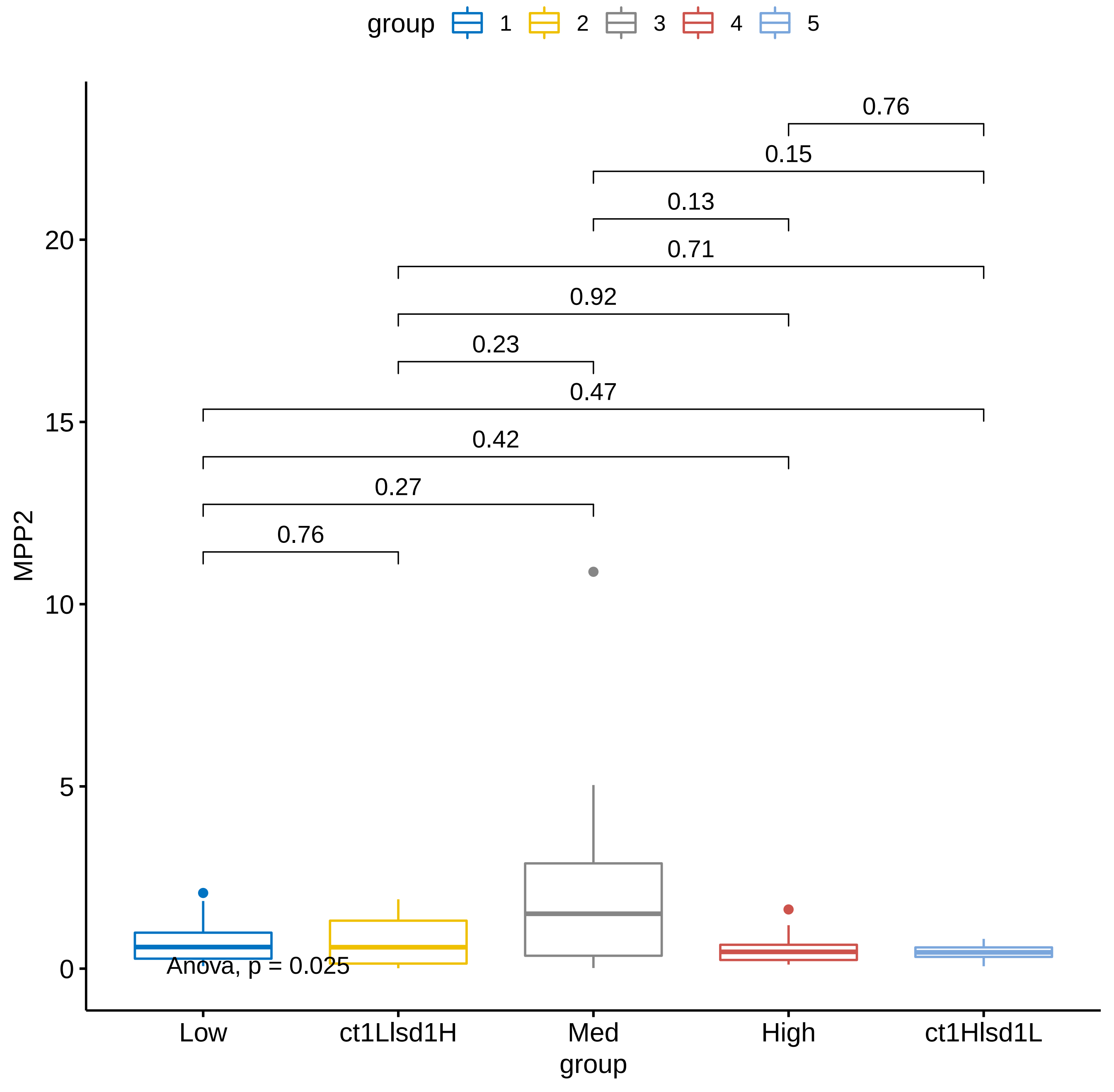

group
1
2
3
4
5
MPP2
20
15
10
5
0
0.76
0.15
0.13
0.71
0.92
0.23
0.47
0.42
0.27
0.76
Anova, p = 0.025
Low
ct1Llsd1H
Med
High
ct1Hlsd1L
group

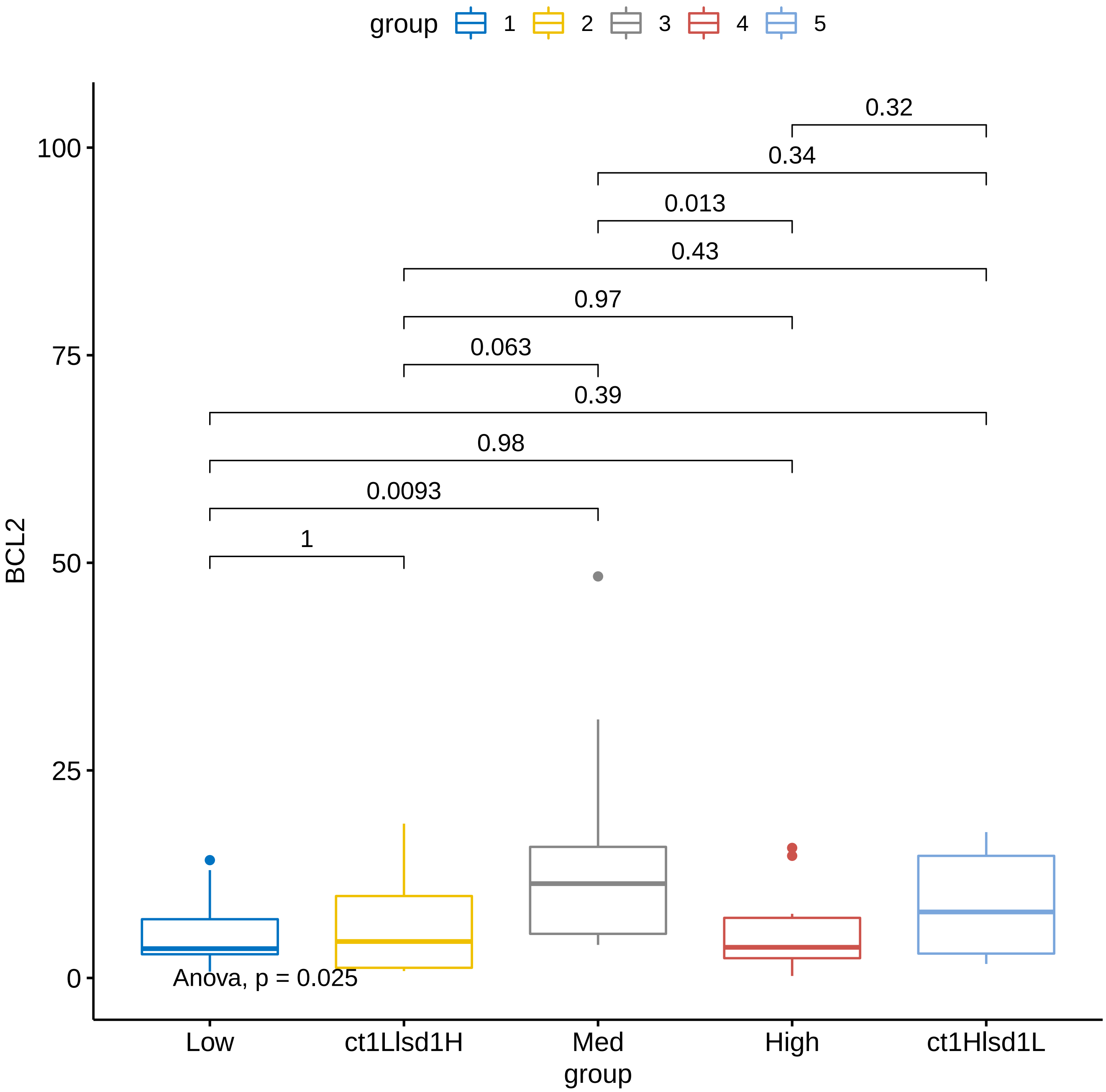

group
1
2
3
4
5
BCL2
100
75
50
25
0
0.32
0.34
0.013
0.43
0.97
0.063
0.39
0.98
0.0093
1
Anova, p = 0.025
Low
ct1Llsd1H
Med
High
ct1Hlsd1L
group

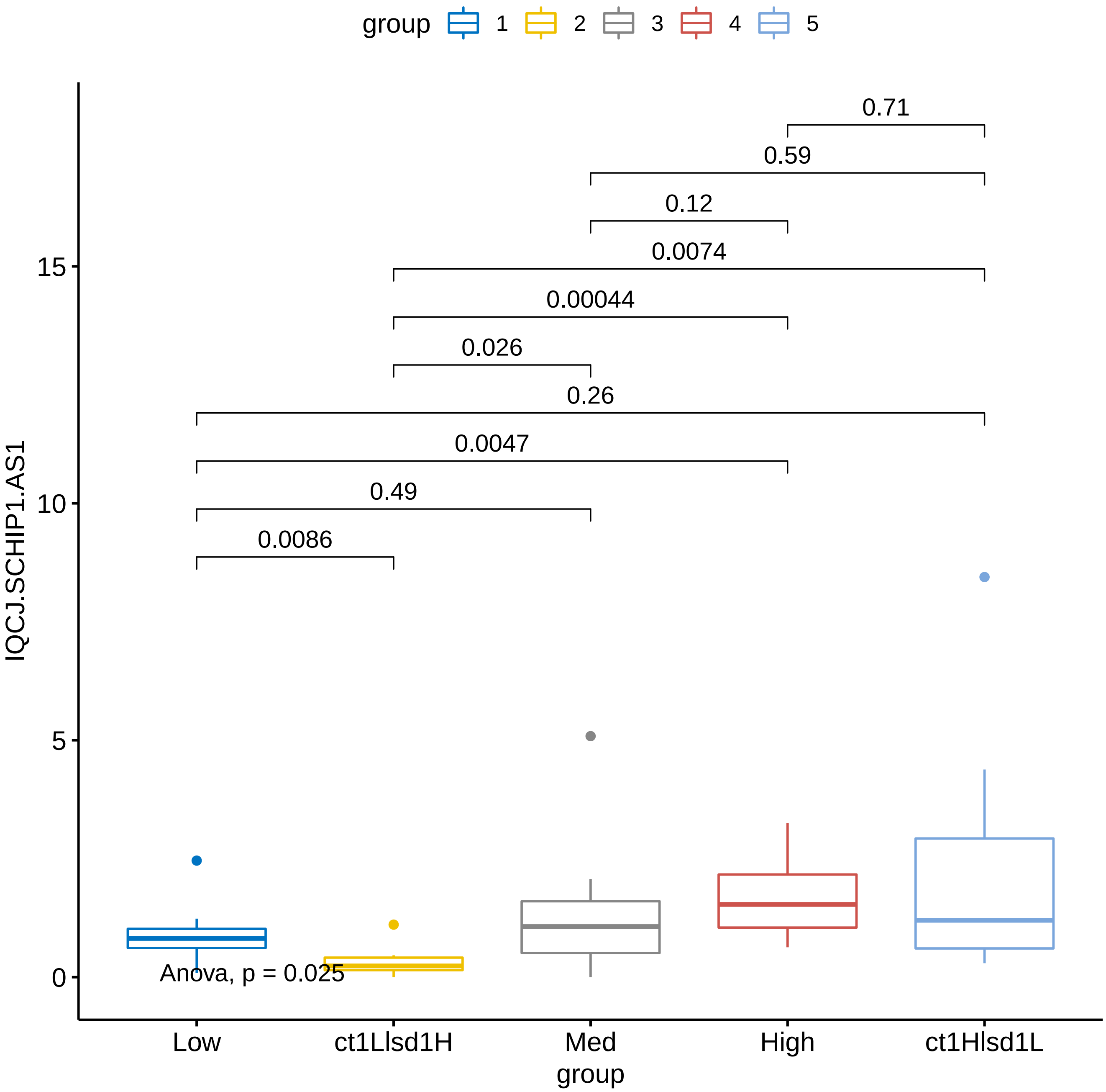
group 1 2 3 4 5
0.71
0.59
0.12
0.0074
0.00044
0.026
0.26
0.0047
0.49
0.0086
Anova, p = 0.025
IQCJ.SCHIP1.AS1
15
10
5
0
Low
ct1Llsd1H
Med
High
ct1Hlsd1L
group

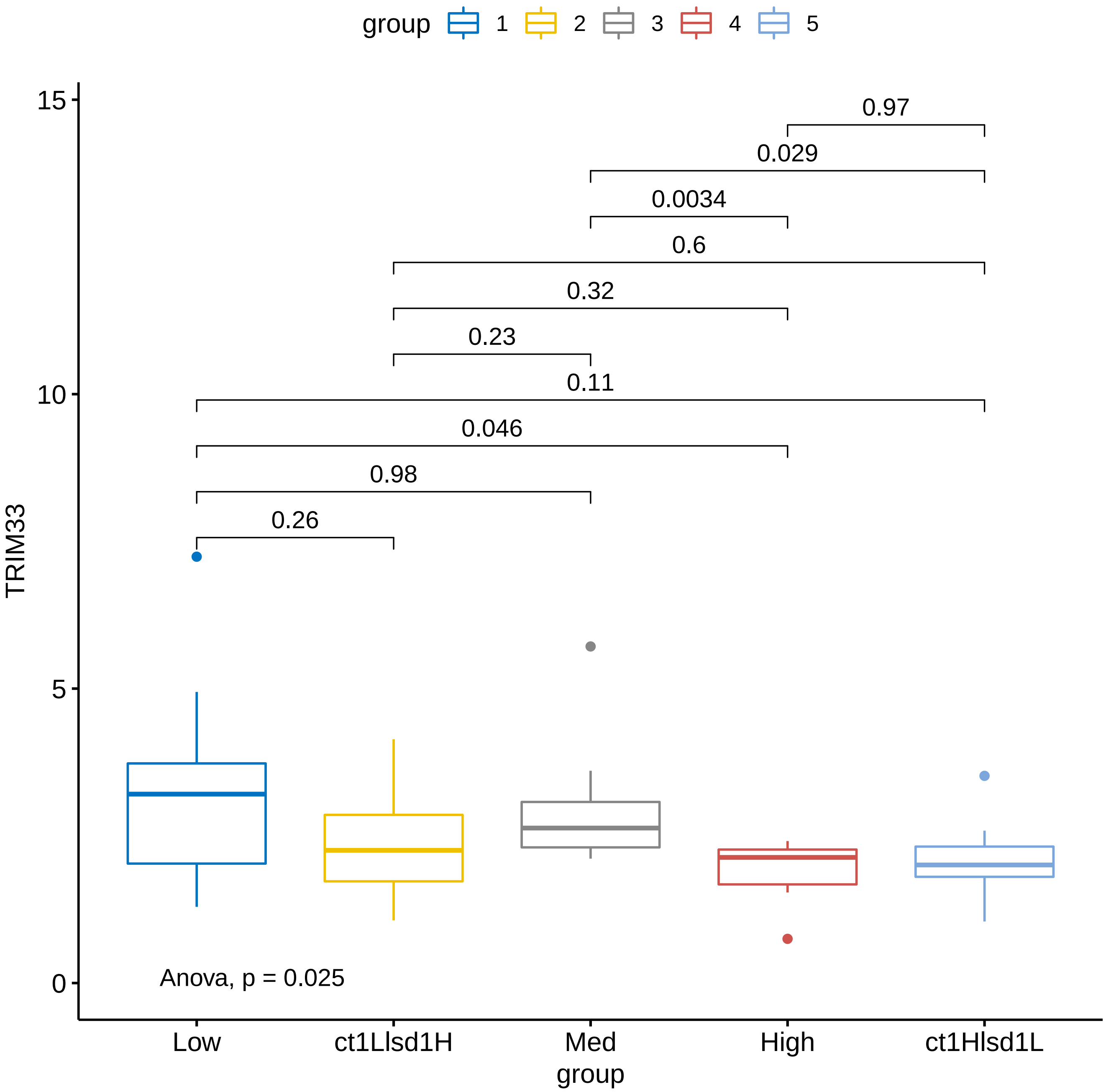
group 1 2 3 4 5
TRIM33
15
10
5
0
0.97
0.029
0.0034
0.6
0.32
0.23
0.11
0.046
0.98
0.26
Anova, p = 0.025
Low
ct1Llsd1H
Med
High
ct1Hlsd1L
group

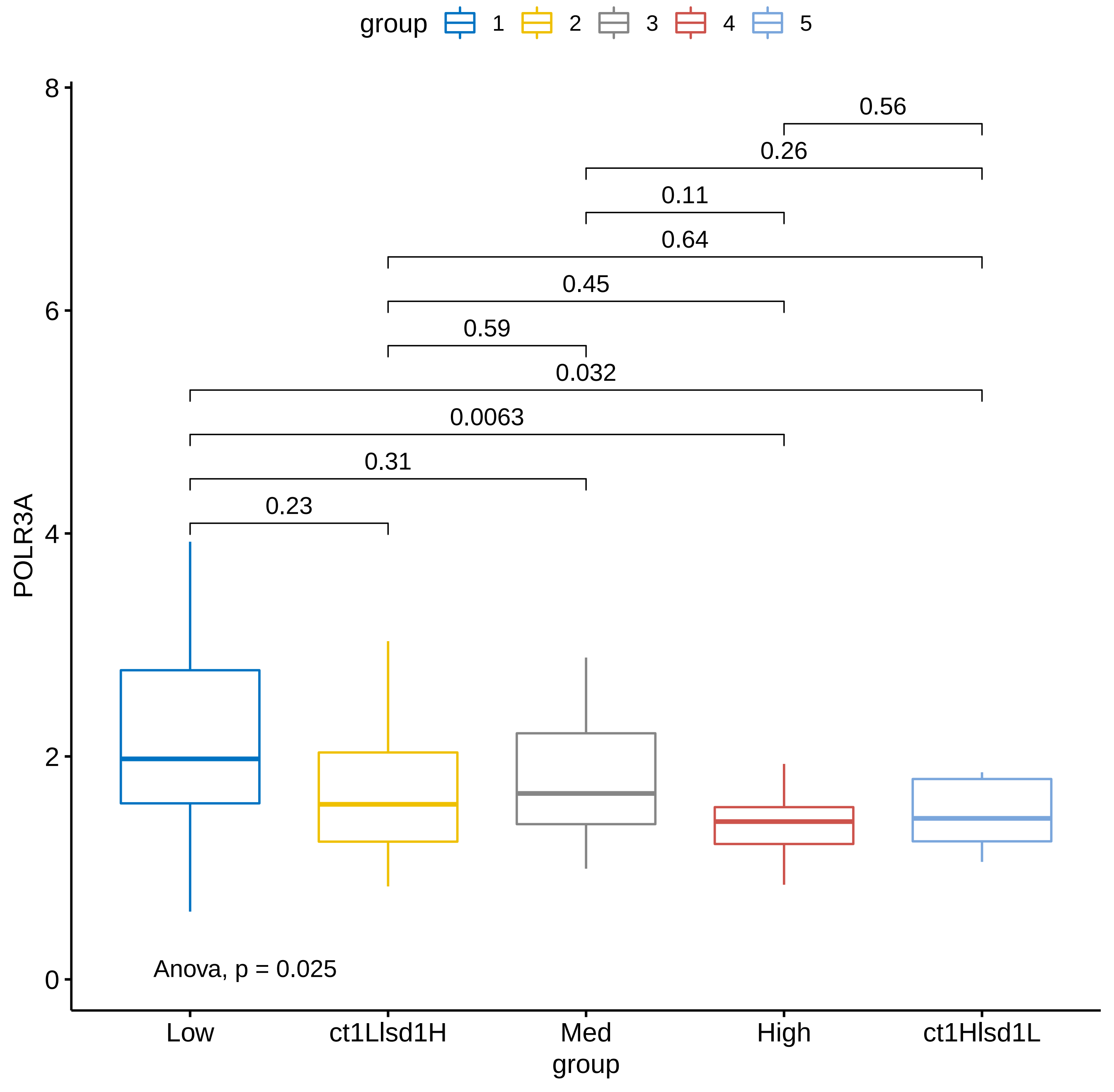

group
1
2
3
4
5
0.56
0.26
0.11
0.64
0.45
0.59
0.032
0.0063
0.31
0.23
Anova, p = 0.025
POLR3A
8
6
4
2
0
Low
ct1Llsd1H
Med
High
ct1Hlsd1L
group

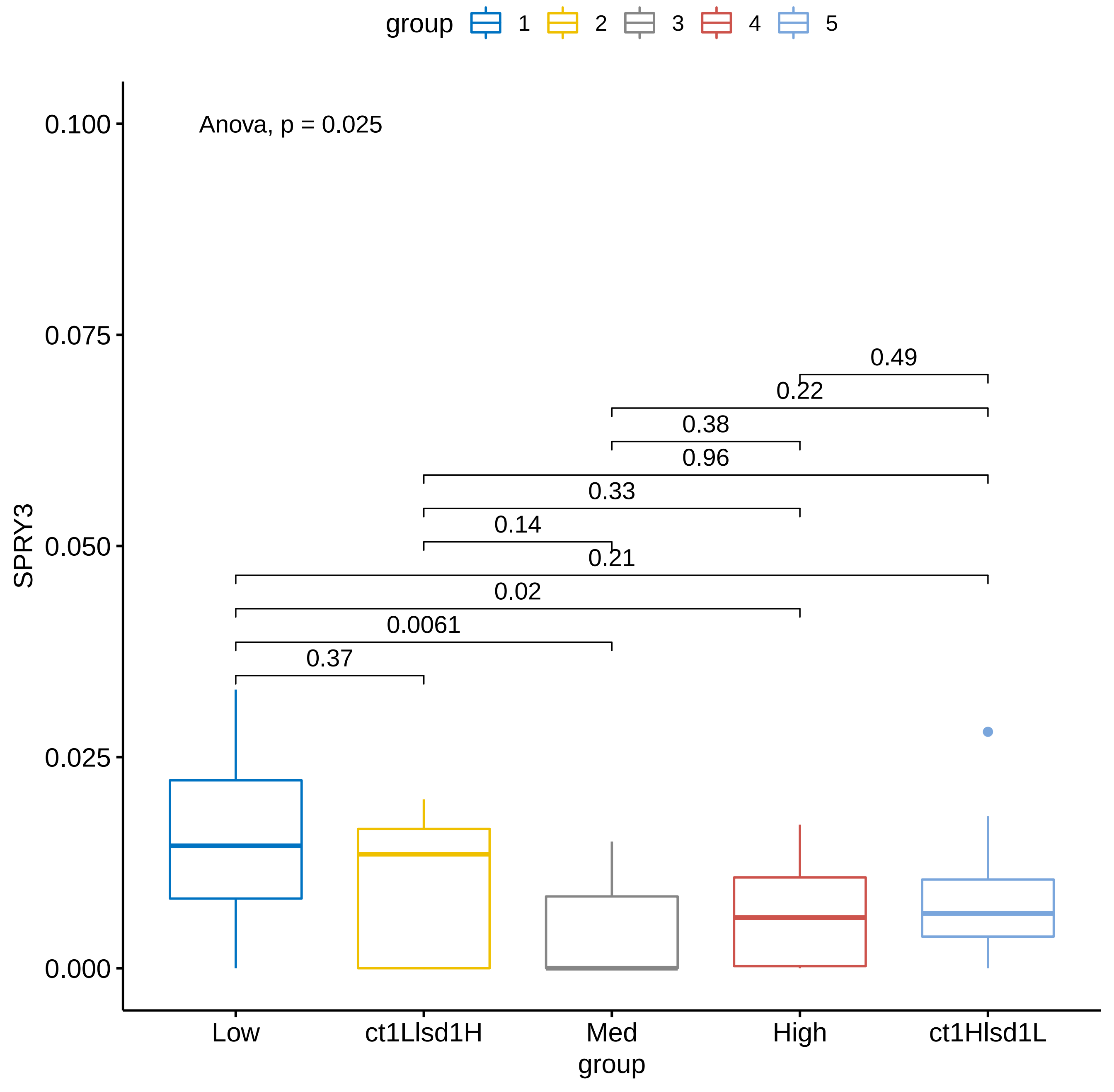

group 1 2 3 4 5
Anova, p = 0.025
0.49
0.22
0.38
0.96
0.33
0.14
0.21
0.02
0.0061
0.37
SPRY3
0.100
0.075
0.050
0.025
0.000
Low
ct1Llsd1H
Med
High
ct1Hlsd1L
group

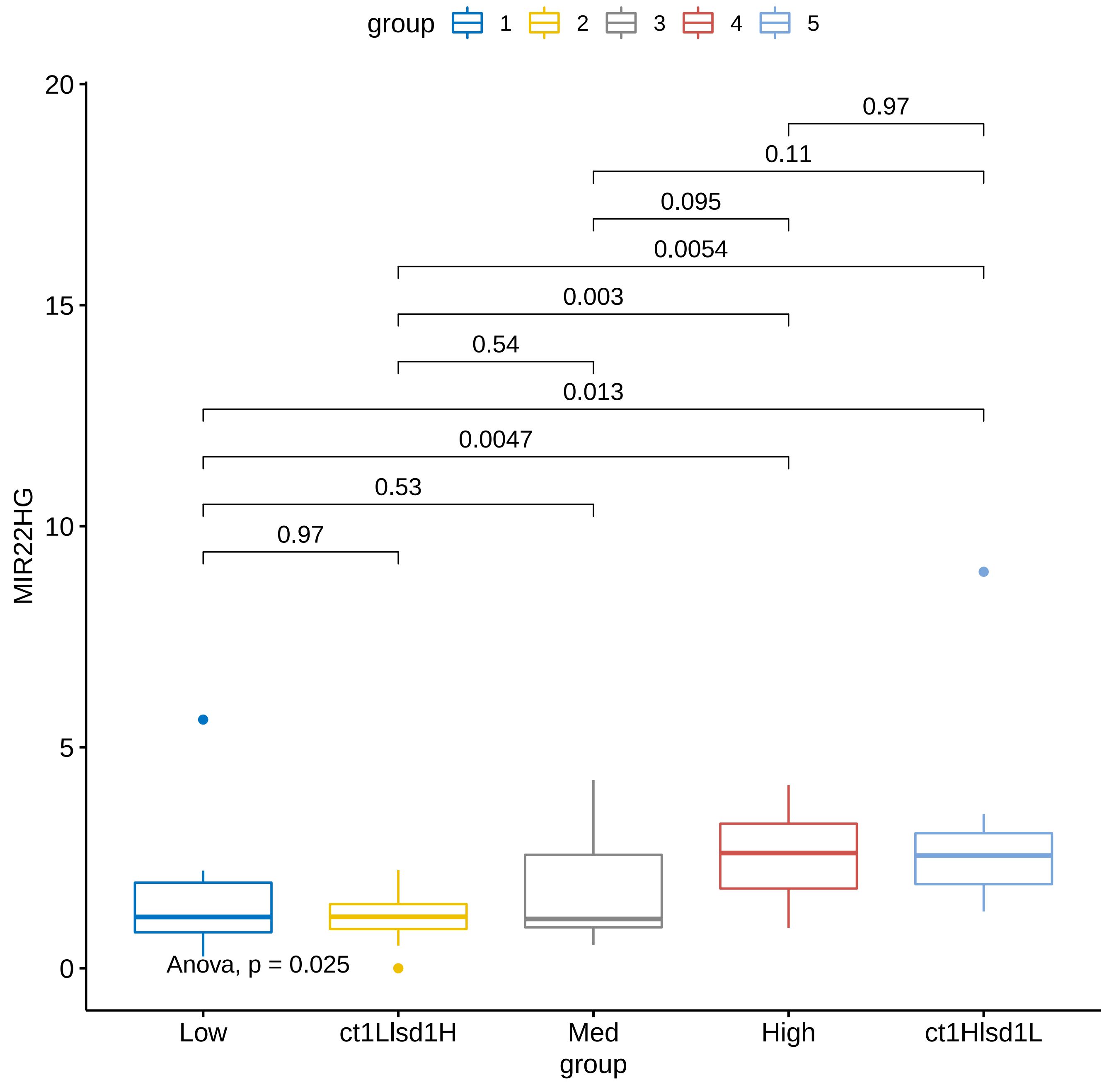

group 1 2 3 4 5
20
15
10
5
0
MIR22HG
0.97
0.11
0.095
0.0054
0.003
0.54
0.013
0.0047
0.53
0.97
Anova, p = 0.025
Low
ct1Llsd1H
Med
High
ct1Hlsd1L
group

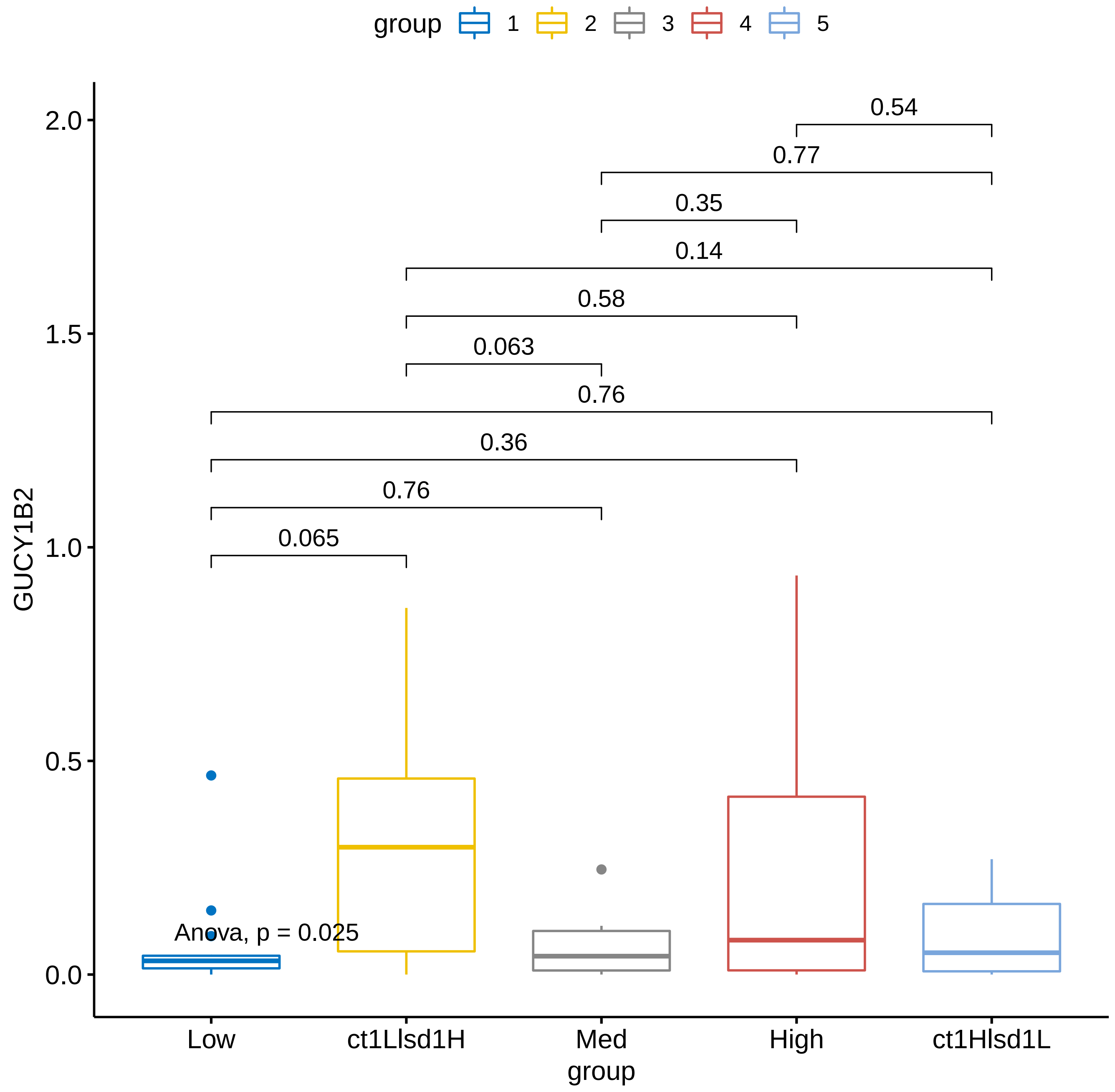

group 1 2 3 4 5
0.54
0.77
0.35
0.14
0.58
0.063
0.76
0.36
0.76
0.065
Anova, p = 0.025
GUCY1B2
2.0
1.5
1.0
0.5
0.0
Low
ct1Llsd1H
Med
High
ct1Hlsd1L
group

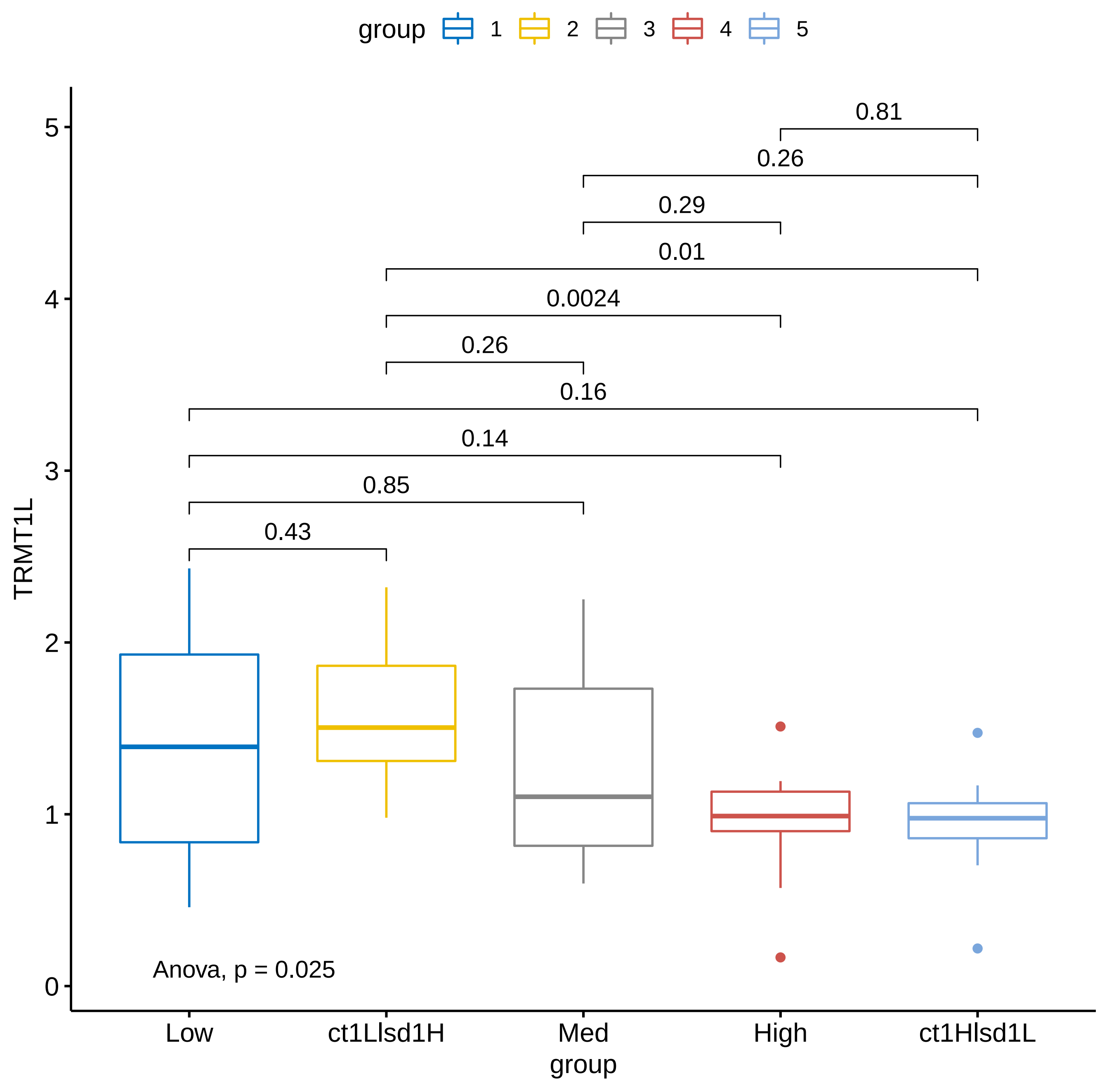
group
1
2
3
4
5
TRMT1L
5
4
3
2
1
0
0.81
0.26
0.29
0.01
0.0024
0.26
0.16
0.14
0.85
0.43
Anova, p = 0.025
Low
ct1Llsd1H
Med
High
ct1Hlsd1L
group

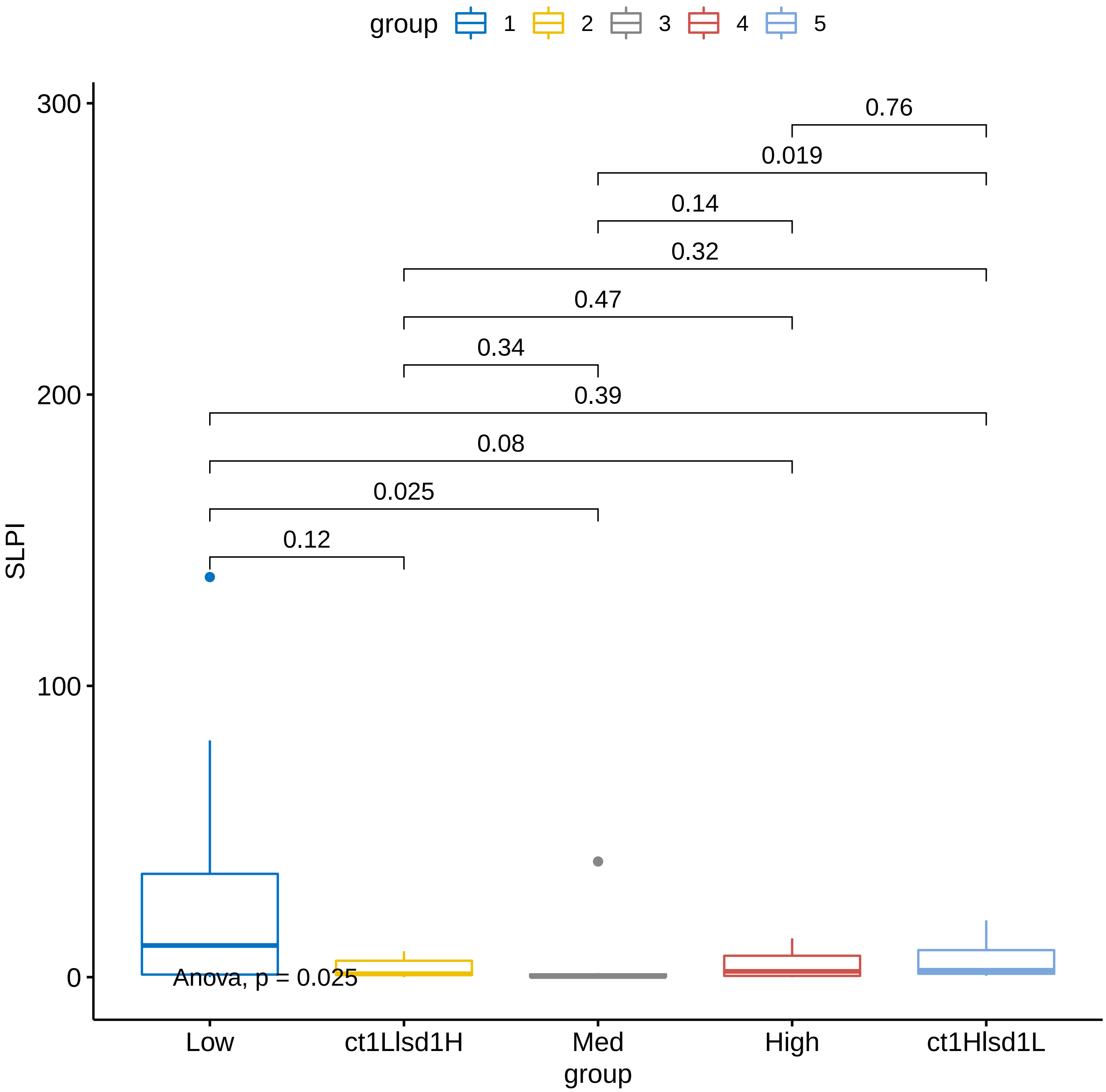
group
1
2
3
4
5
SLPI
300
200
100
0
0.76
0.019
0.14
0.32
0.47
0.34
0.39
0.08
0.025
0.12
Anova, p = 0.025
Low
ct1Llsd1H
Med
High
ct1Hlsd1L
group

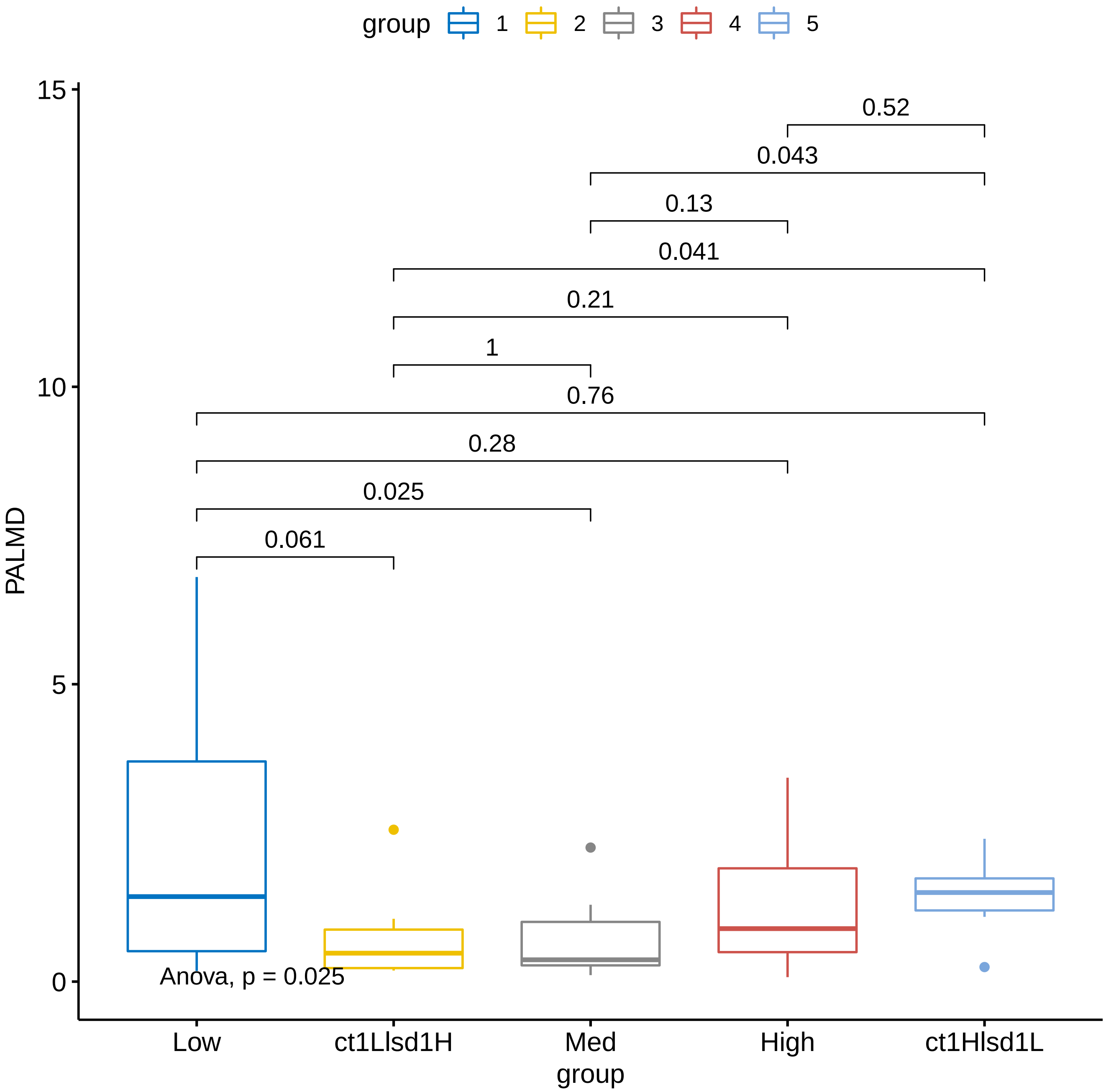

group 1 2 3 4 5
0.52
0.043
0.13
0.041
0.21
1
0.76
0.28
0.025
0.061
15
10
5
0
PALMD
Anova, p = 0.025
Low
ct1Llsd1H
Med
High
ct1Hlsd1L
group

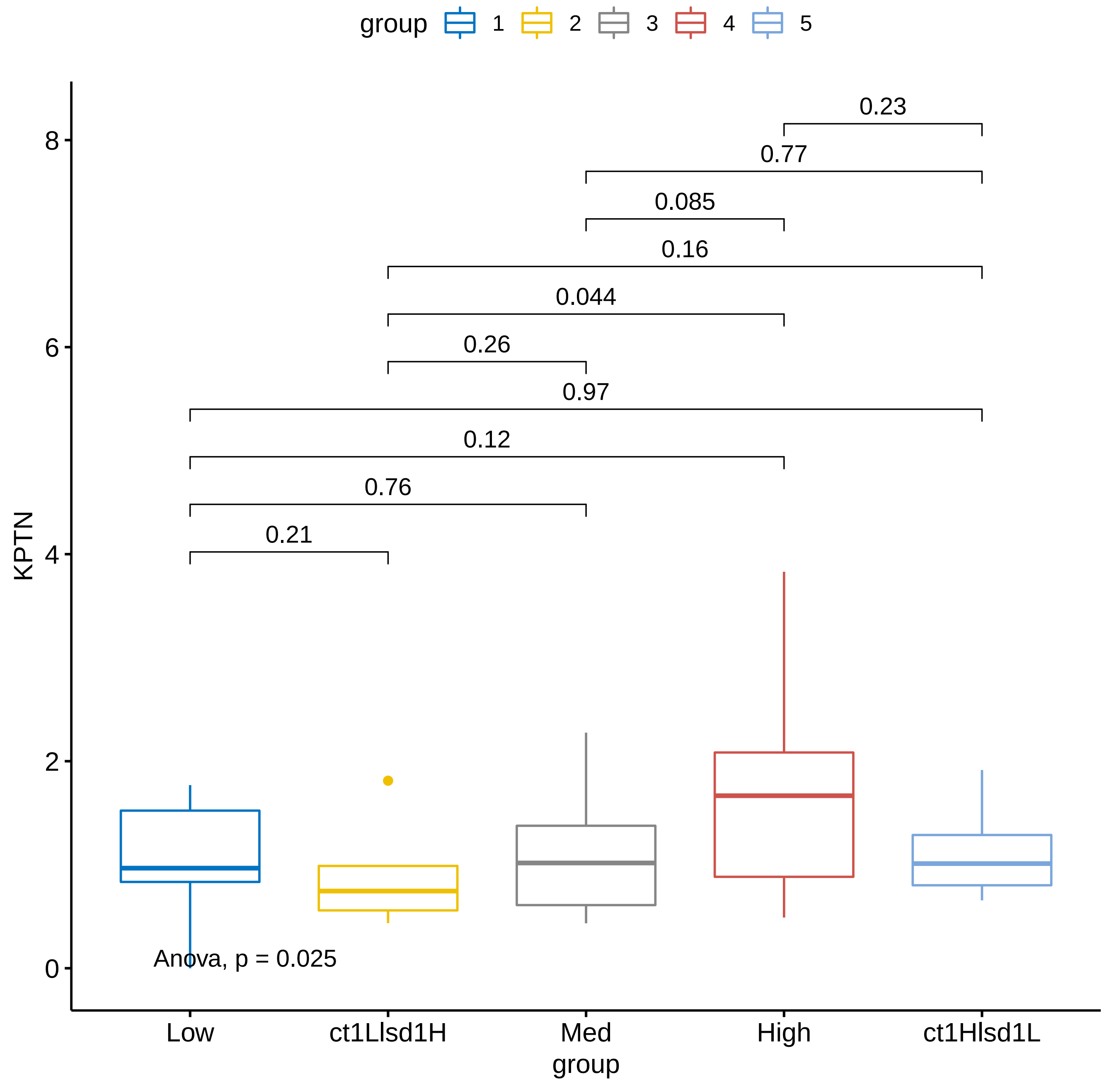
group
1
2
3
4
5
0.23
0.77
0.085
0.16
0.044
0.26
0.97
0.12
0.76
0.21
8
6
4
2
0
KPTN
Anova, p = 0.025
Low
ct1Llsd1H
Med
High
ct1Hlsd1L
group

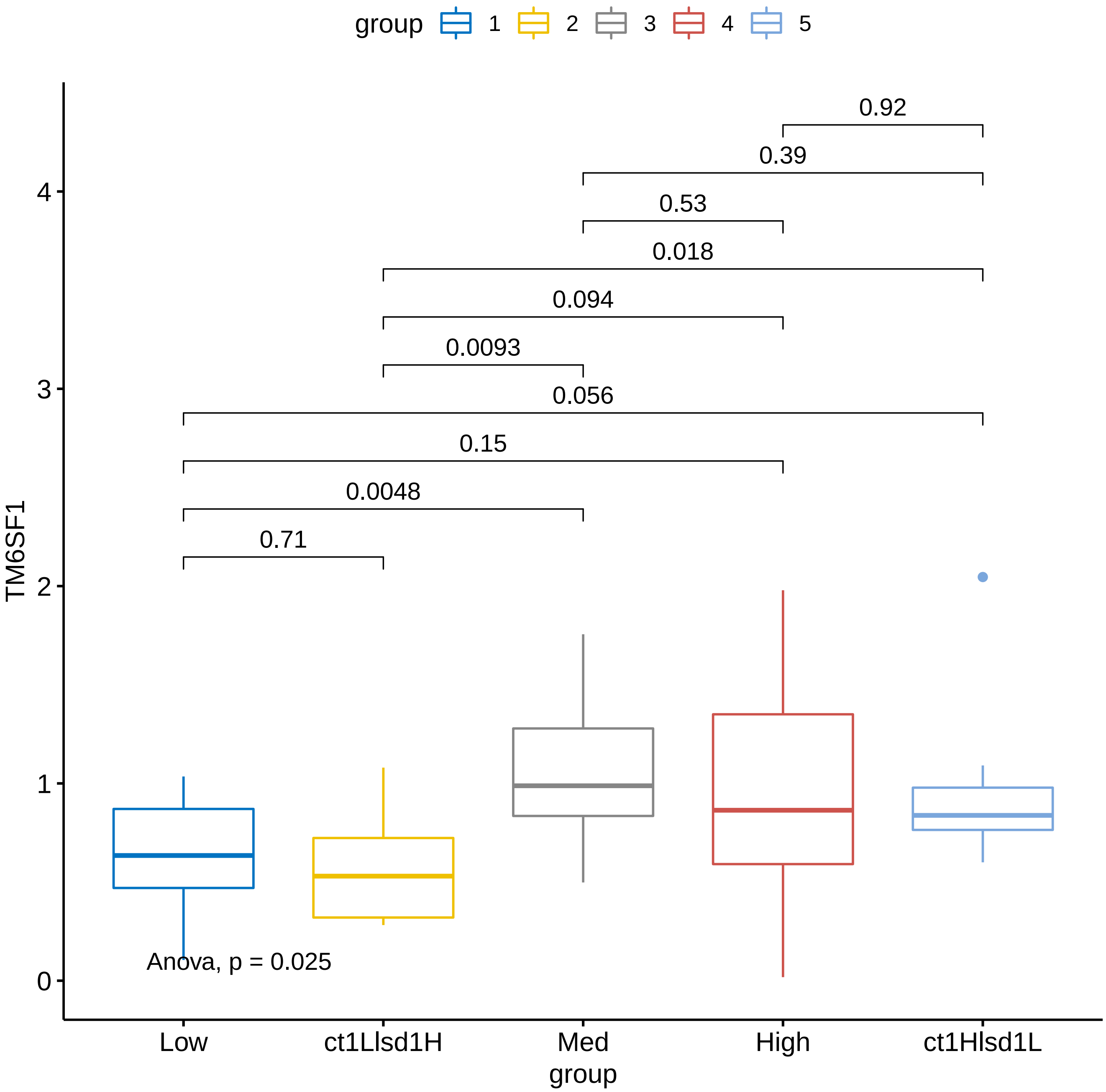

group 1 2 3 4 5
0.92
0.39
0.53
0.018
0.094
0.0093
0.056
0.15
0.0048
0.71
TM6SF1
4
3
2
1
0
Anova, p = 0.025
Low
ct1Llsd1H
Med
High
ct1Hlsd1L
group

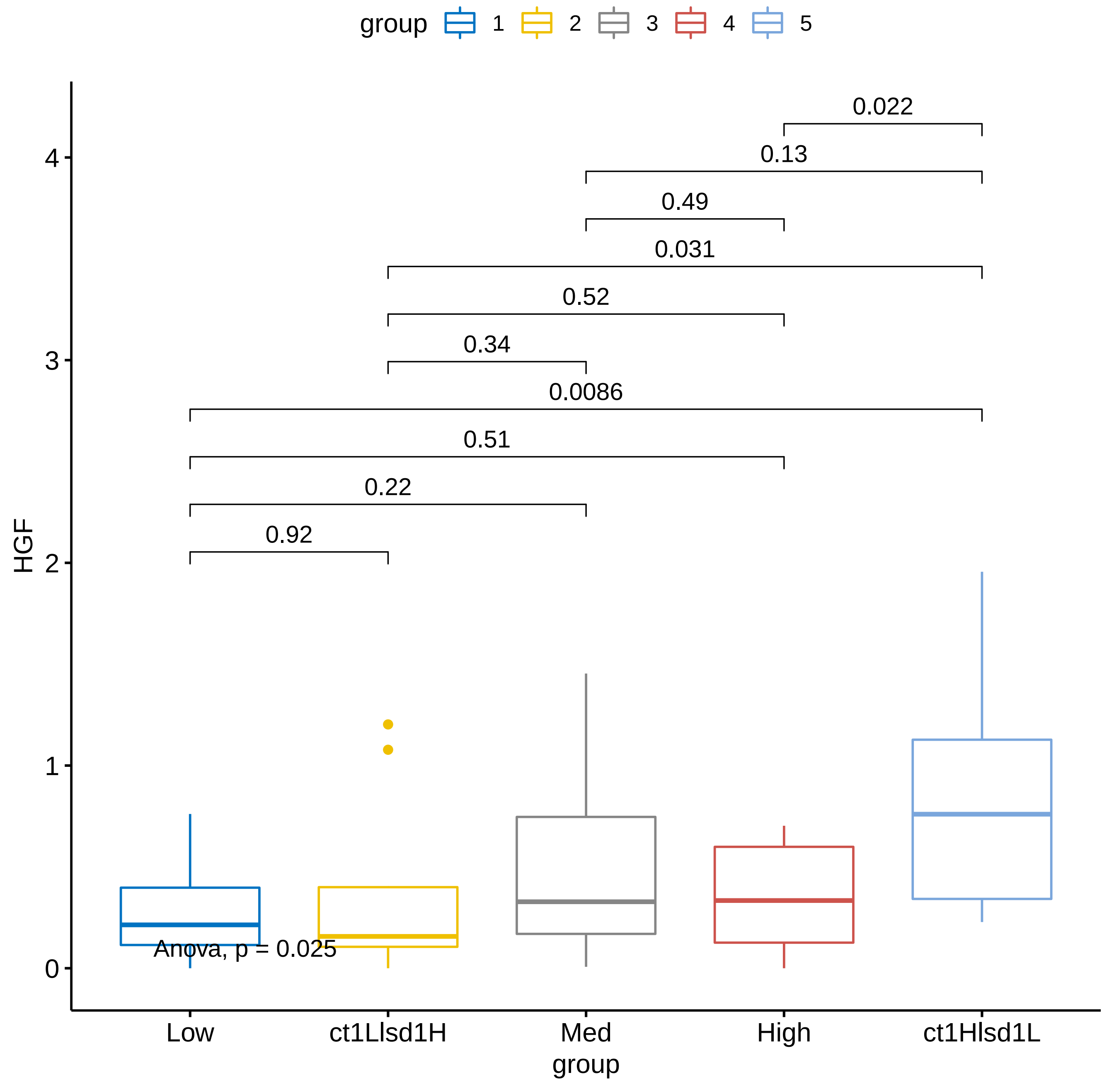

group
1
2
3
4
5
0.022
0.13
0.49
0.031
0.52
0.34
0.0086
0.51
0.22
0.92
Anova, p = 0.025
HGF
4
3
2
1
0
Low
ct1Llsd1H
Med
High
ct1Hlsd1L
group

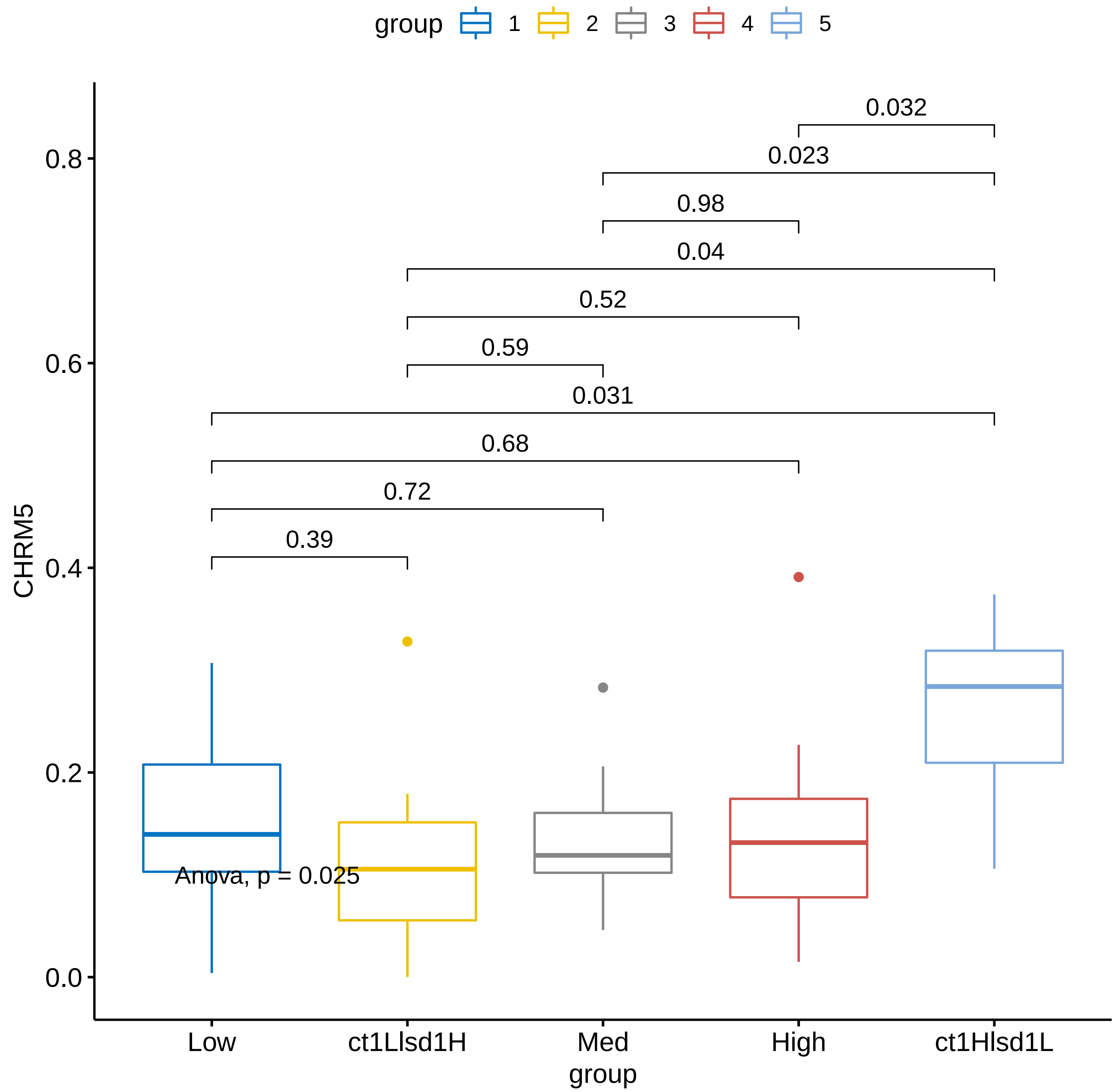

group
1
2
3
4
5
0.032
0.023
0.98
0.04
0.52
0.59
0.031
0.68
0.72
0.39
Anova, p = 0.025
CHRM5
0.0
0.2
0.4
0.6
0.8
Low
ct1Llsd1H
Med
High
ct1Hlsd1L
group

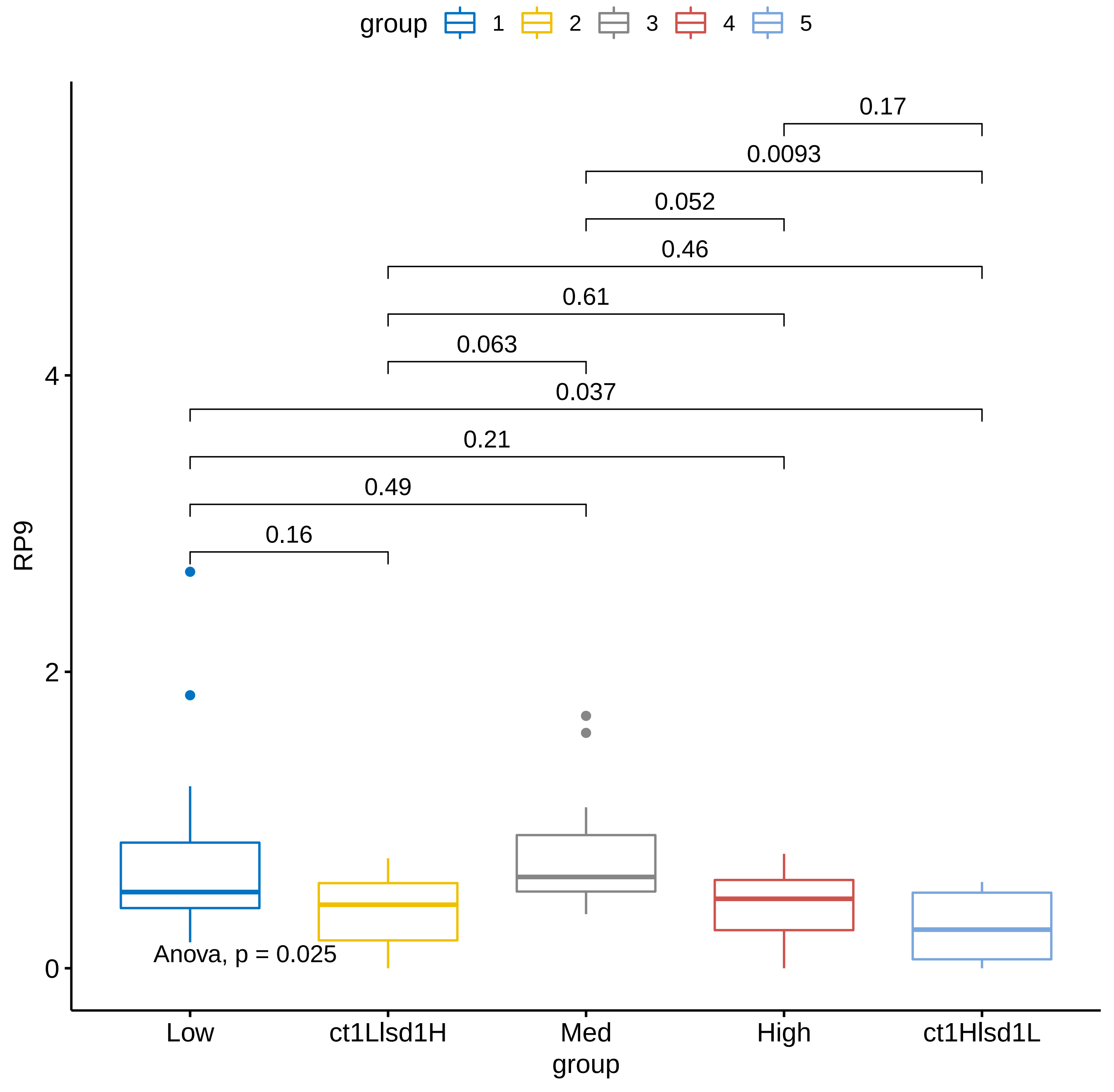

group
1
2
3
4
5
0.17
0.0093
0.052
0.46
0.61
0.063
0.037
0.21
0.49
0.16
RP9
4
2
0
Anova, p = 0.025
Low
ct1Llsd1H
Med
High
ct1Hlsd1L
group

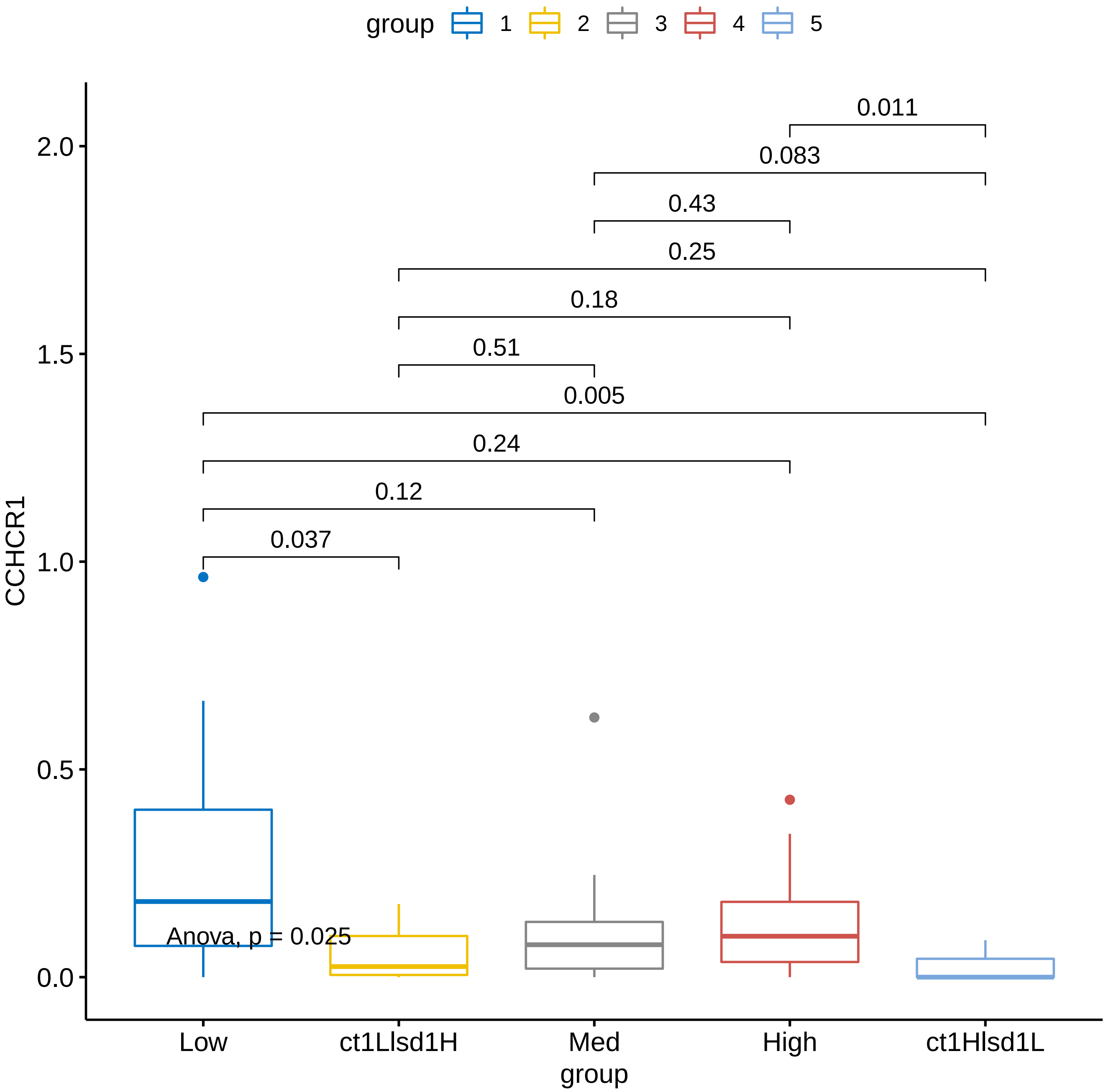
group
1
2
3
4
5
CCHCR1
2.0
1.5
1.0
0.5
0.0
0.011
0.083
0.43
0.25
0.18
0.51
0.005
0.24
0.12
0.037
Anova, p = 0.025
Low
ct1Llsd1H
Med
High
ct1Hlsd1L
group

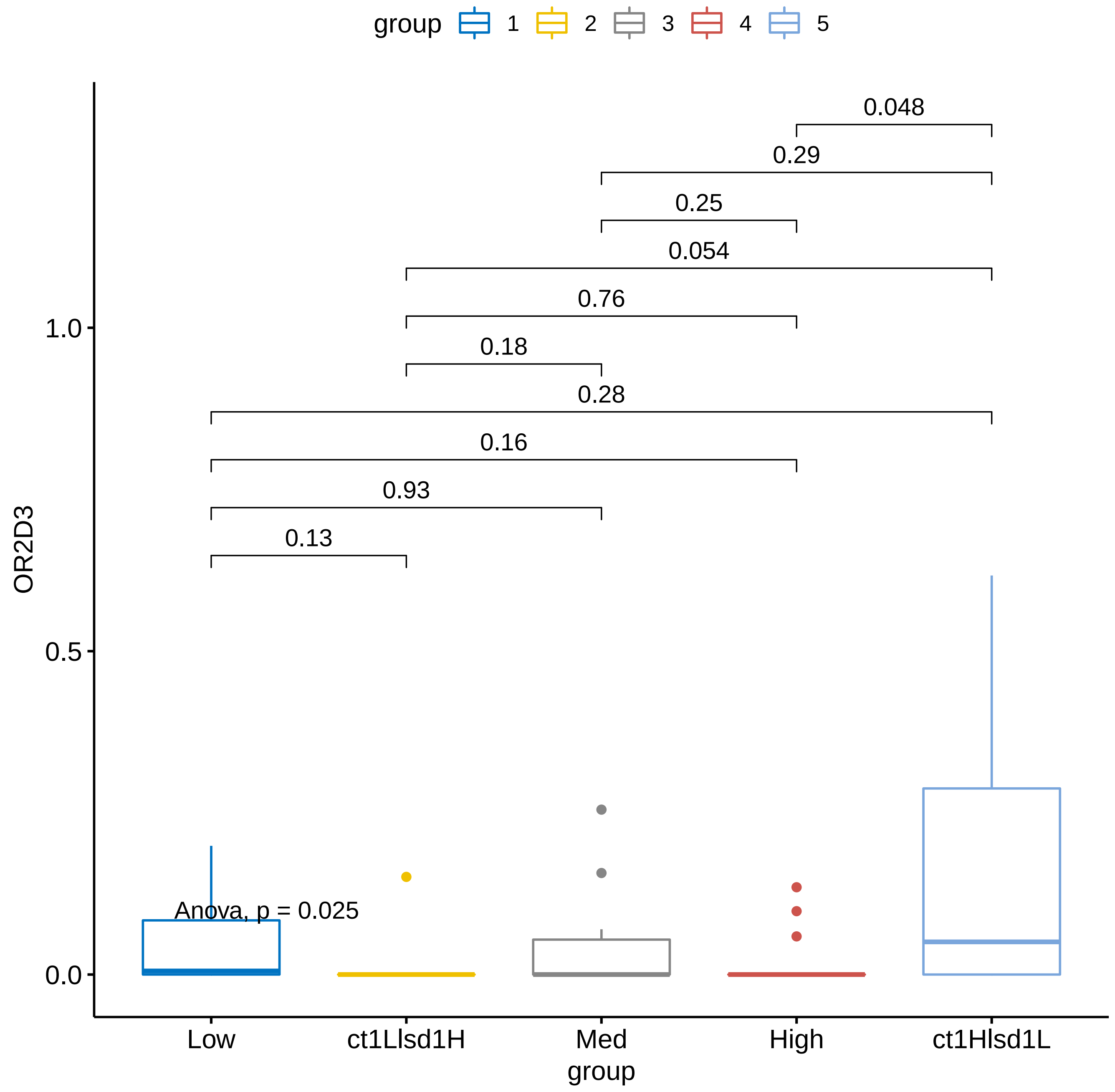
group
1
2
3
4
5
0.048
0.29
0.25
0.054
0.76
0.18
0.28
0.16
0.93
0.13
Anova, p = 0.025
OR2D3
1.0
0.5
0.0
Low
ct1Llsd1H
Med
High
ct1Hlsd1L
group

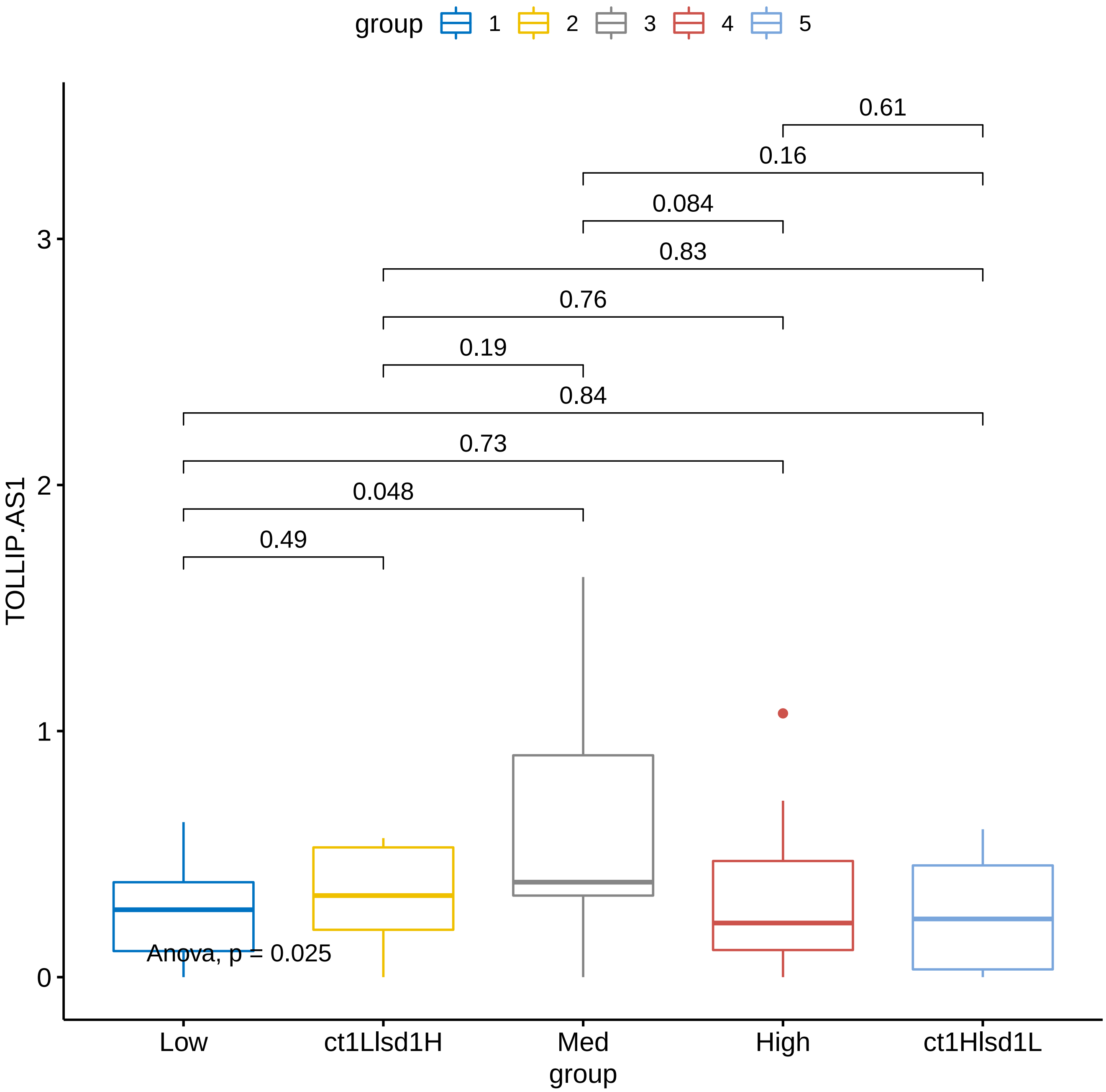
group
1
2
3
4
5
0.61
0.16
0.084
0.83
0.76
0.19
0.84
0.73
0.048
0.49
TOLLIP.AS1
3
2
1
0
Anova, p = 0.025
Low
ct1Llsd1H
Med
High
ct1Hlsd1L
group

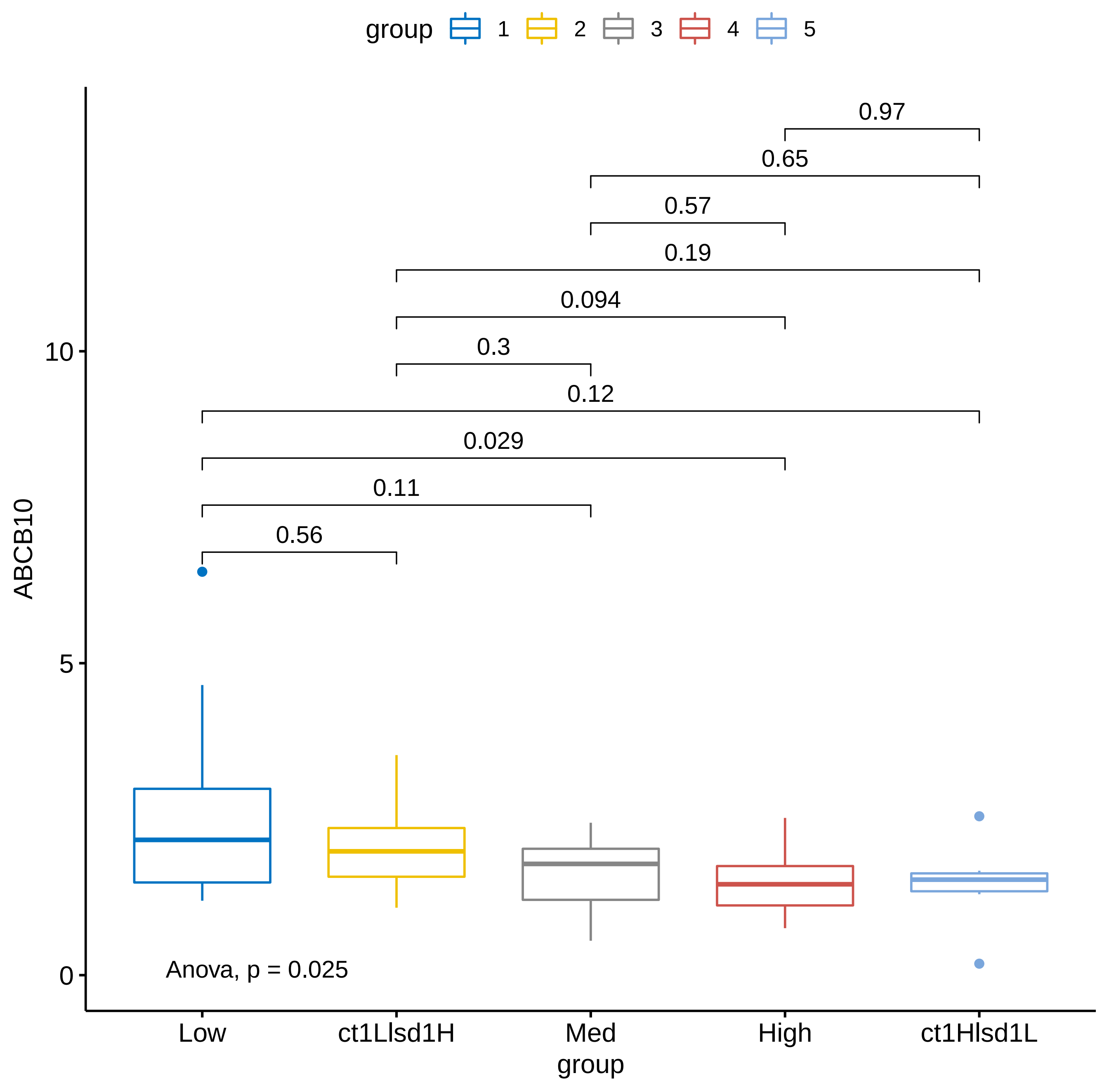

group 1 2 3 4 5
0.97
0.65
0.57
0.19
0.094
0.3
0.12
0.029
0.11
0.56
10
5
0
ABCB10
Anova, p = 0.025
Low
ct1Llsd1H
Med
High
ct1Hlsd1L
group

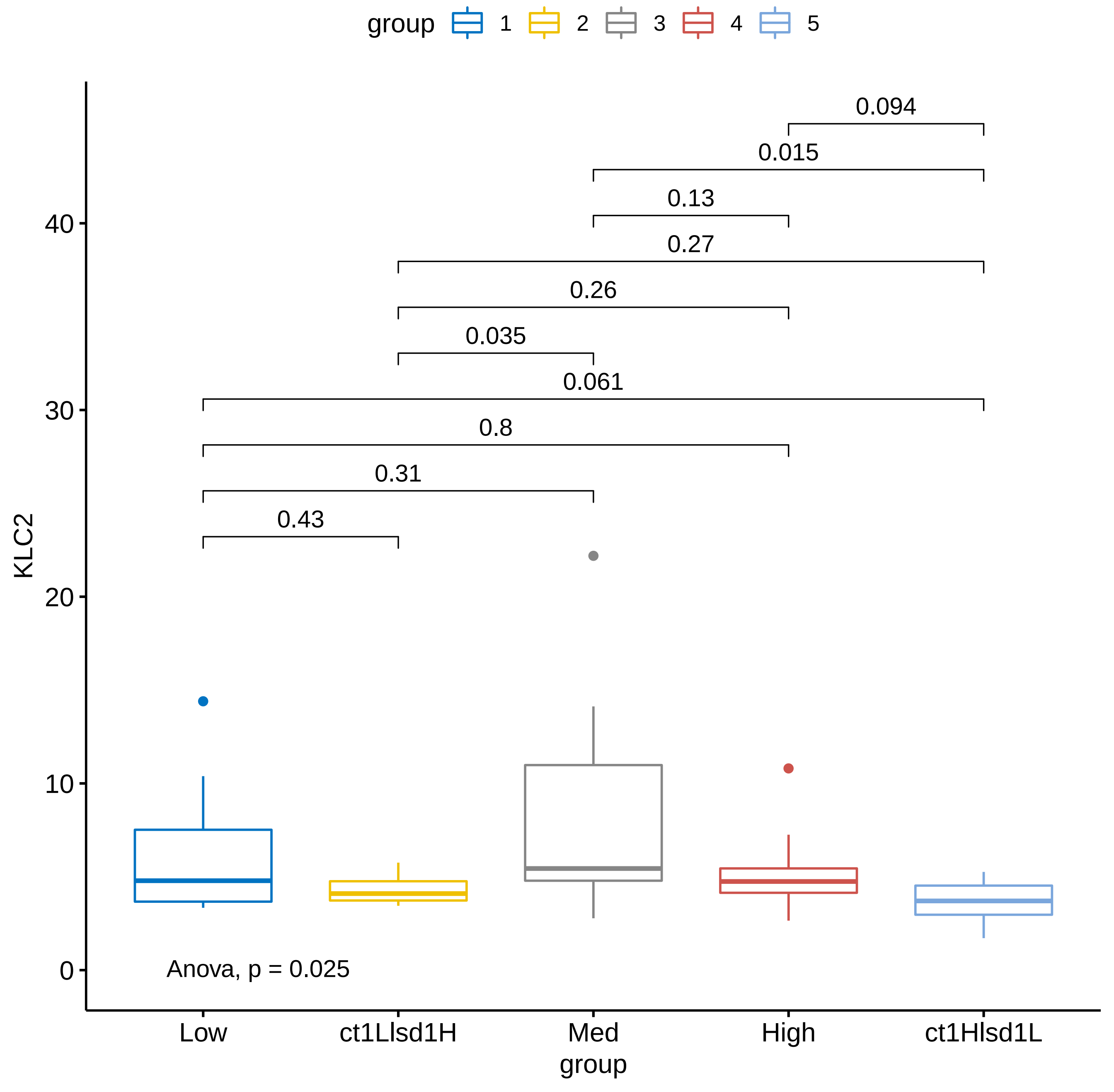

group 1 2 3 4 5
0.094
0.015
0.13
0.27
0.26
0.035
0.061
0.8
0.31
0.43
40
30
20
10
0
KLC2
Anova, p = 0.025
Low
ct1Llsd1H
Med
High
ct1Hlsd1L
group

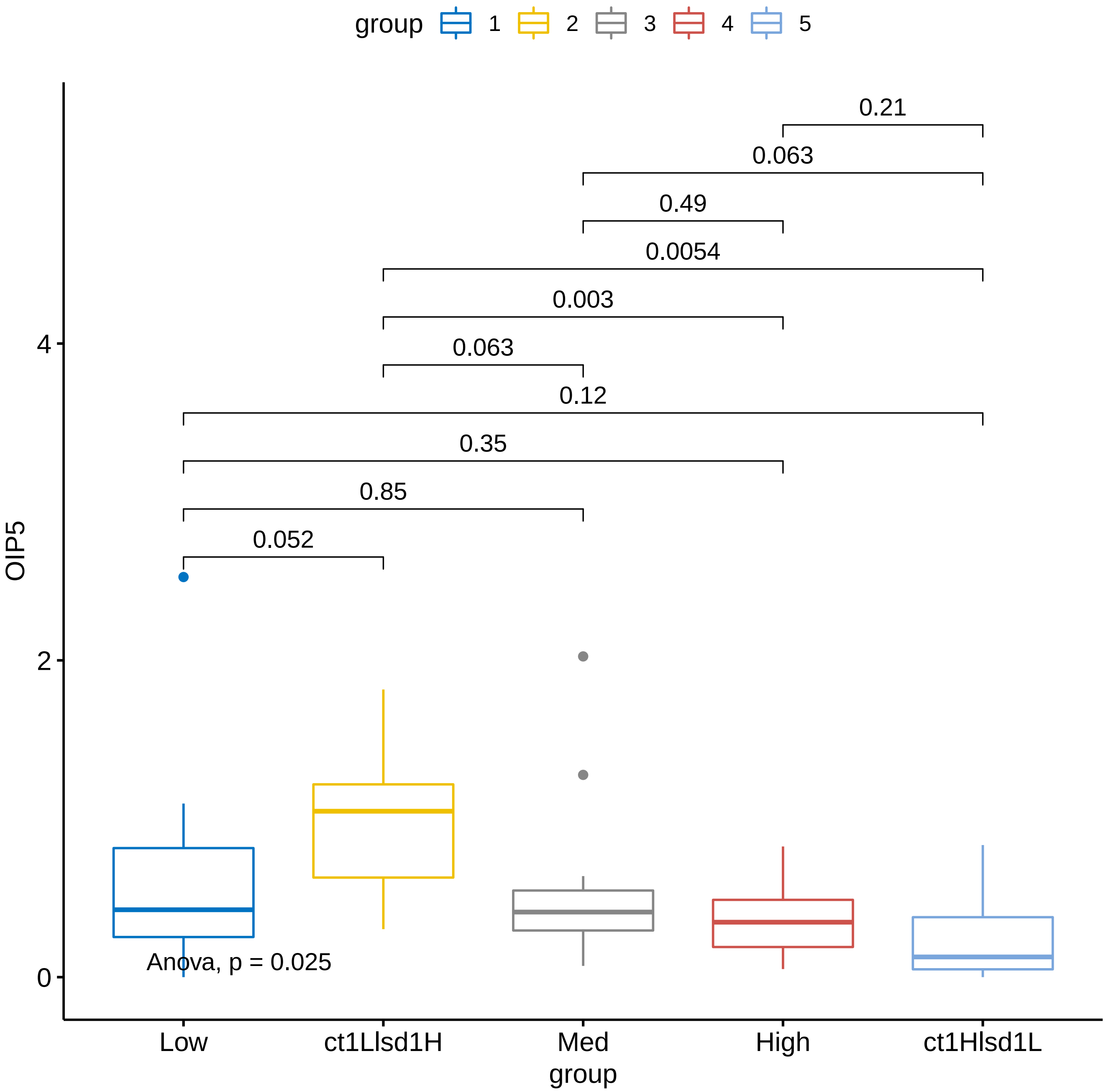

group
1
2
3
4
5
0.21
0.063
0.49
0.0054
0.003
0.063
0.12
0.35
0.85
0.052
OIP5
4
2
0
Anova, p = 0.025
Low
ct1Llsd1H
Med
High
ct1Hlsd1L
group

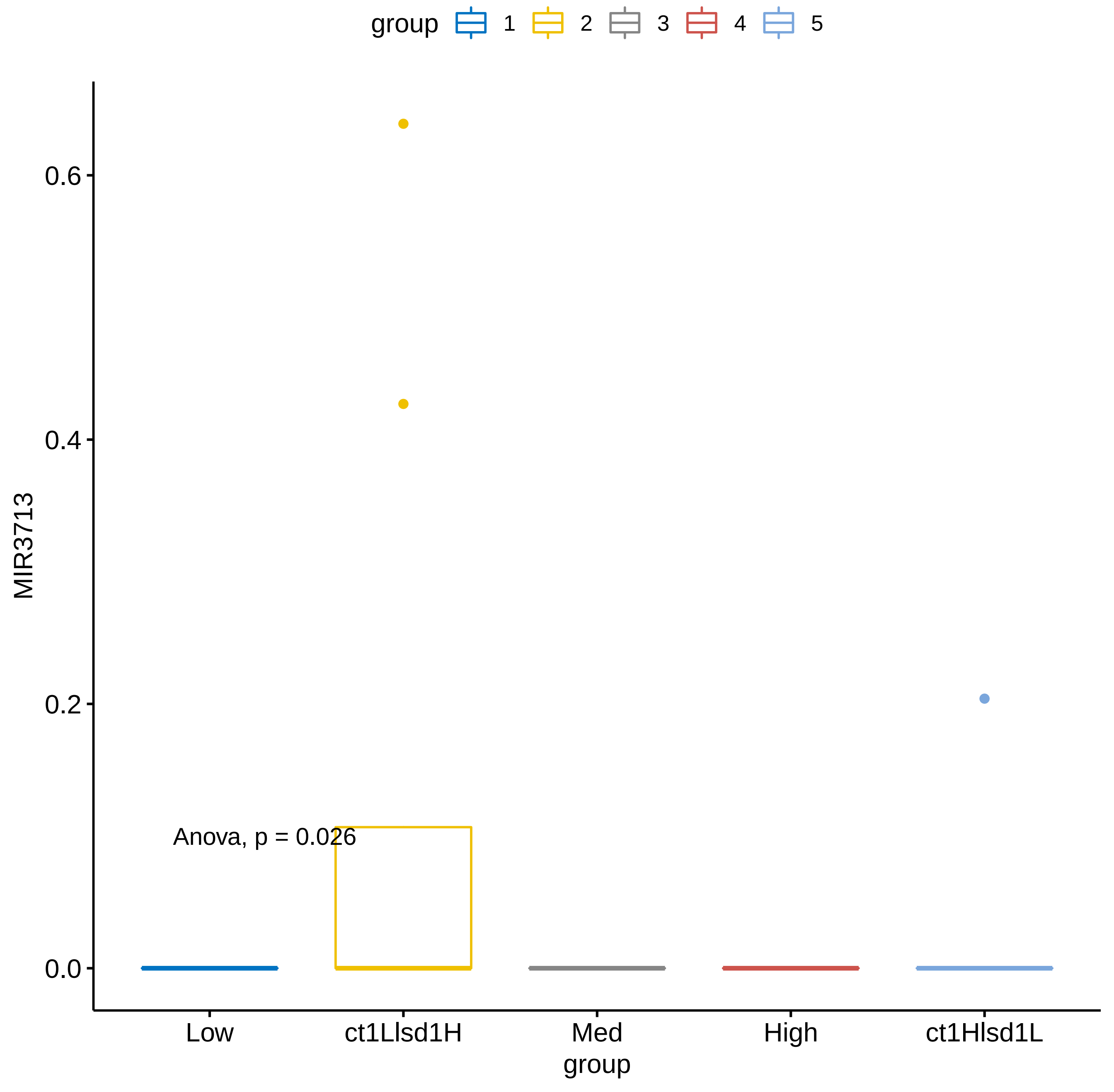
group
1
2
3
4
5
0.6
0.4
0.2
0.0
MIR3713
Anova, p = 0.026
Low
ct1Llsd1H
Med
High
ct1Hlsd1L
group

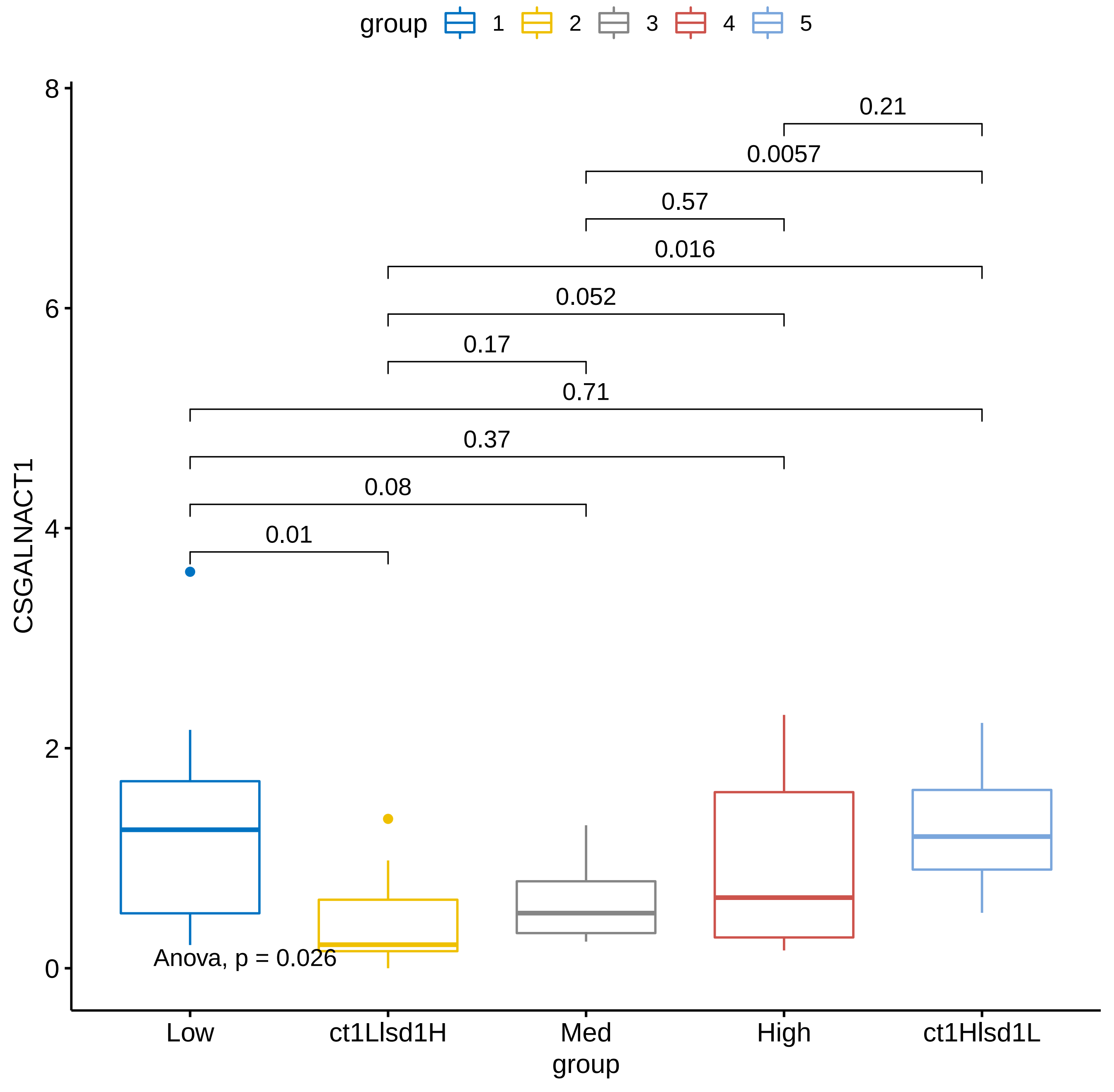

group 1 2 3 4 5
CSGALNACT1
0
2
4
6
8
0.21
0.0057
0.57
0.016
0.052
0.17
0.71
0.37
0.08
0.01
Anova, p = 0.026
Low
ct1Llsd1H
Med
High
ct1Hlsd1L
group

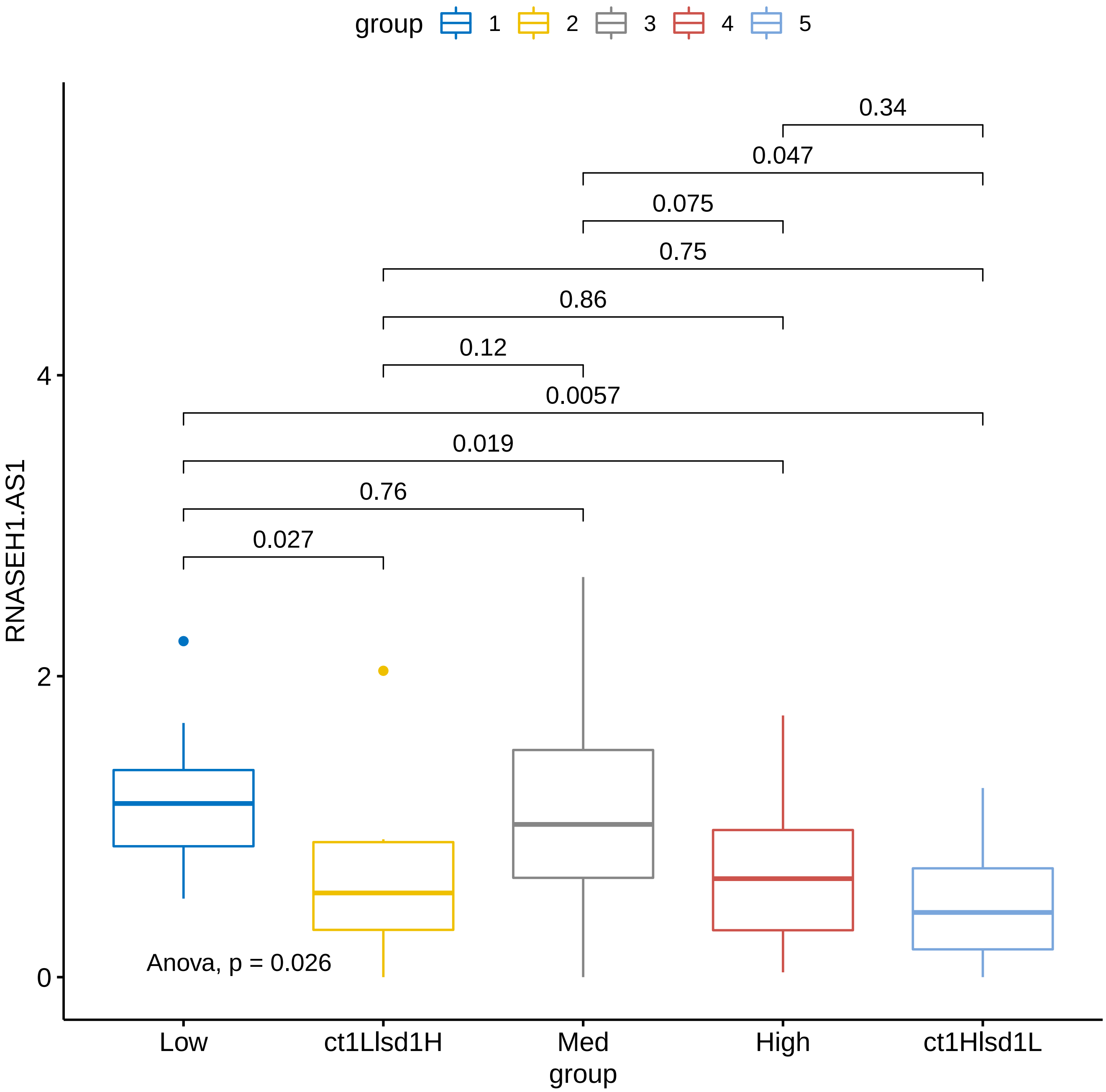

group
1
2
3
4
5
0.34
0.047
0.075
0.75
0.86
0.12
0.0057
0.019
0.76
0.027
Anova, p = 0.026
RNASEH1.AS1
4
2
0
Low
ct1Llsd1H
Med
High
ct1Hlsd1L
group

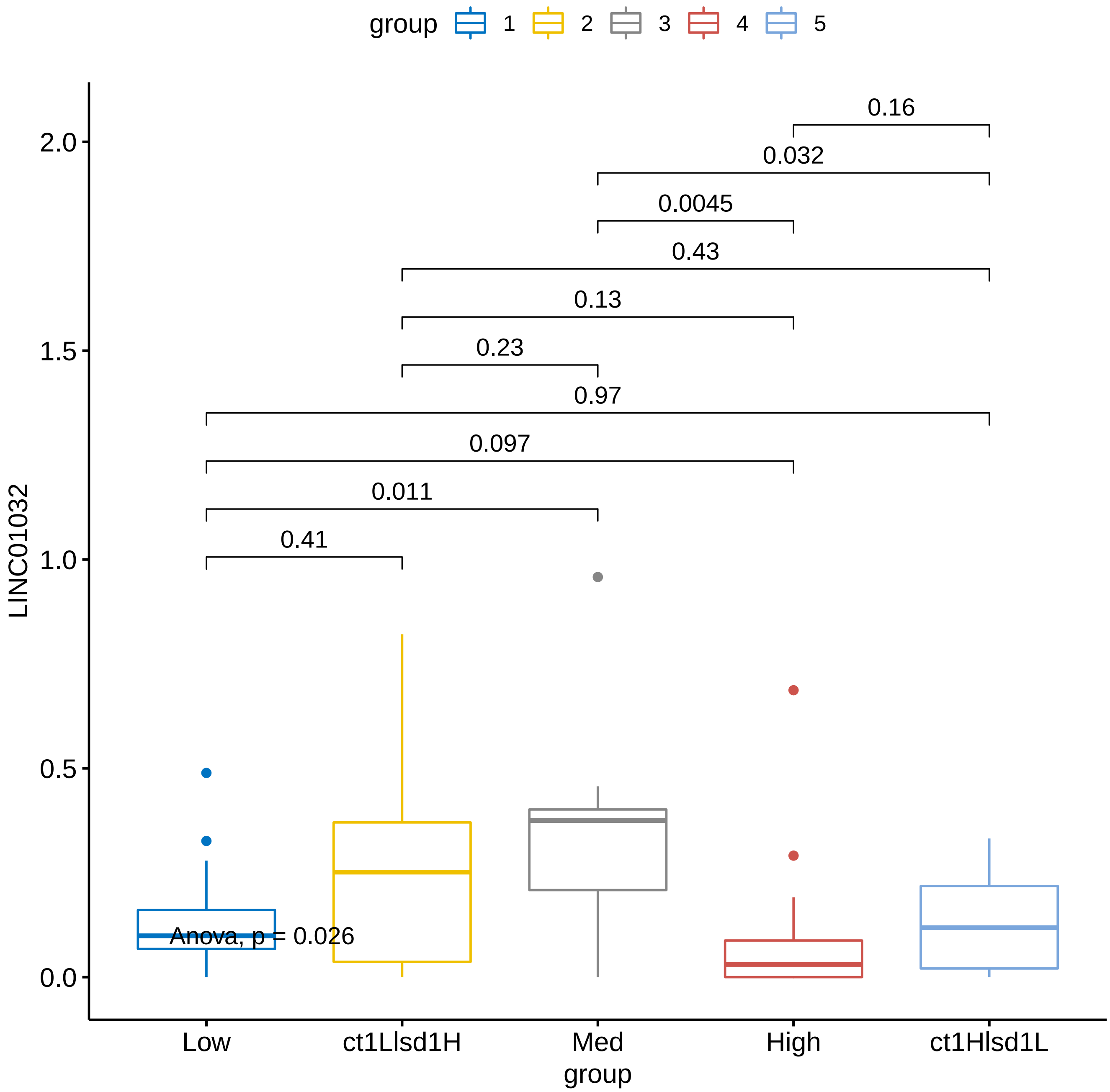
group
1
2
3
4
5
LINC01032
2.0
1.5
1.0
0.5
0.0
0.16
0.032
0.0045
0.43
0.13
0.23
0.97
0.097
0.011
0.41
Anova, p = 0.026
Low
ct1Llsd1H
Med
High
ct1Hlsd1L
group

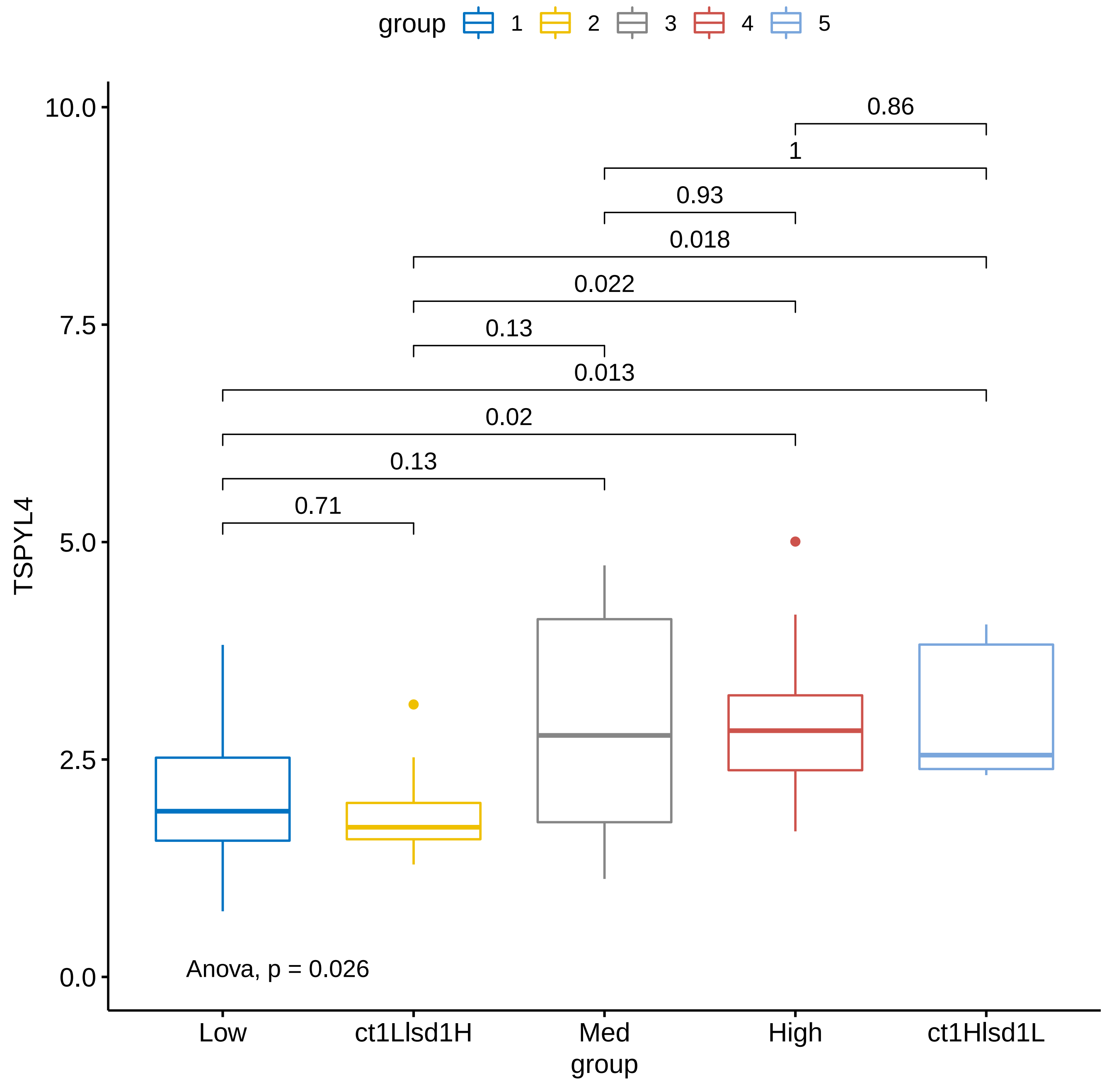

group
1
2
3
4
5
10.0
7.5
5.0
2.5
0.0
TSPYL4
0.86
1
0.93
0.018
0.022
0.13
0.013
0.02
0.13
0.71
Anova, p = 0.026
Low
ct1Llsd1H
Med
High
ct1Hlsd1L
group

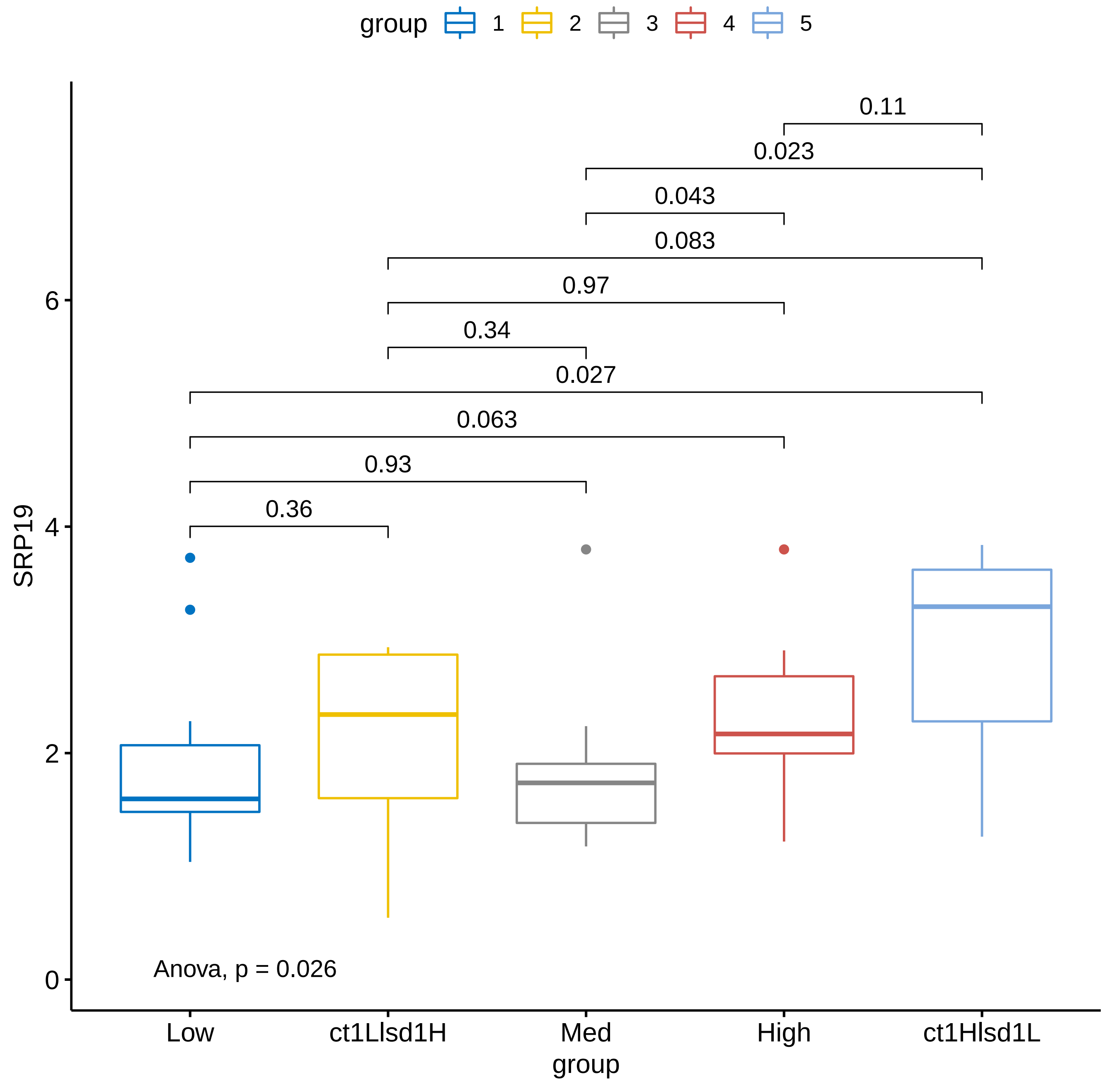

group 1 2 3 4 5
SRP19
0
2
4
6
0.11
0.023
0.043
0.083
0.97
0.34
0.027
0.063
0.93
0.36
Anova, p = 0.026
Low
ct1Llsd1H
Med
High
ct1Hlsd1L
group

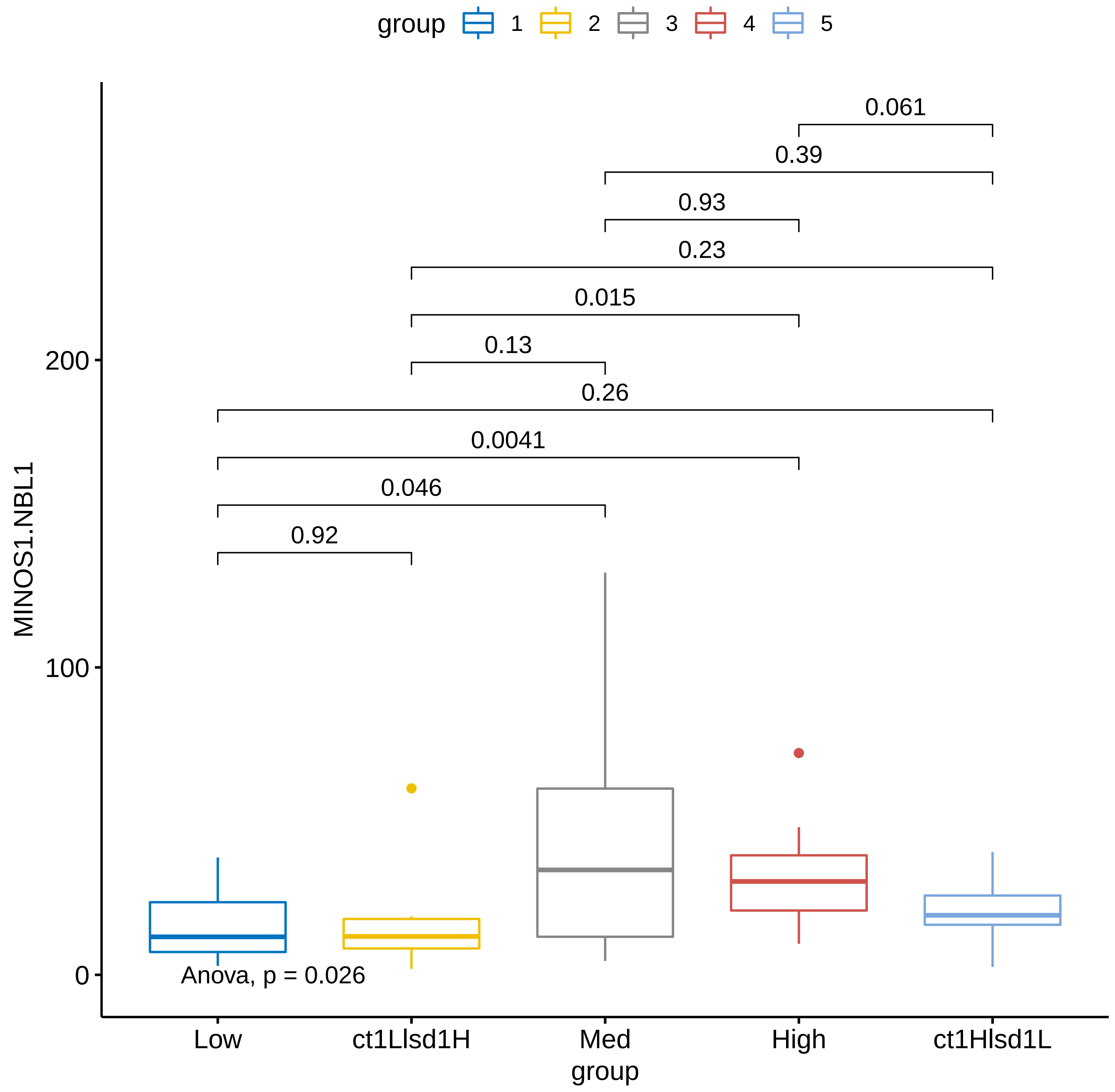
group
1
2
3
4
5
0.061
0.39
0.93
0.23
0.015
0.13
0.26
0.0041
0.046
0.92
Anova, p = 0.026
MINOS1.NBL1
200
100
0
Low
ct1Llsd1H
Med
High
ct1Hlsd1L
group

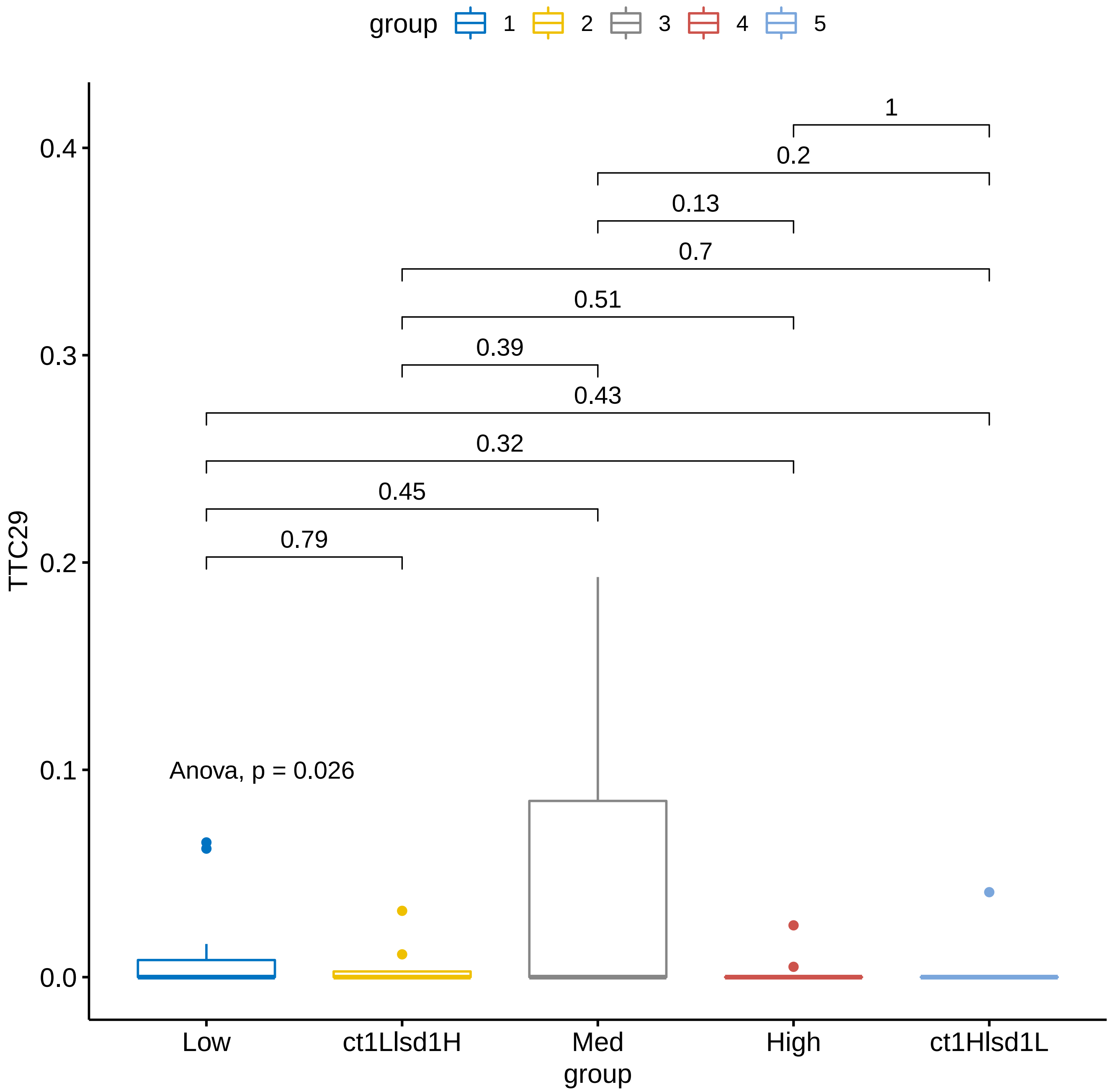

group
1
2
3
4
5
TTC29
0.4
0.3
0.2
0.1
0.0
1
0.2
0.13
0.7
0.51
0.39
0.43
0.32
0.45
0.79
Anova, p = 0.026
Low
ct1Llsd1H
Med
High
ct1Hlsd1L
group

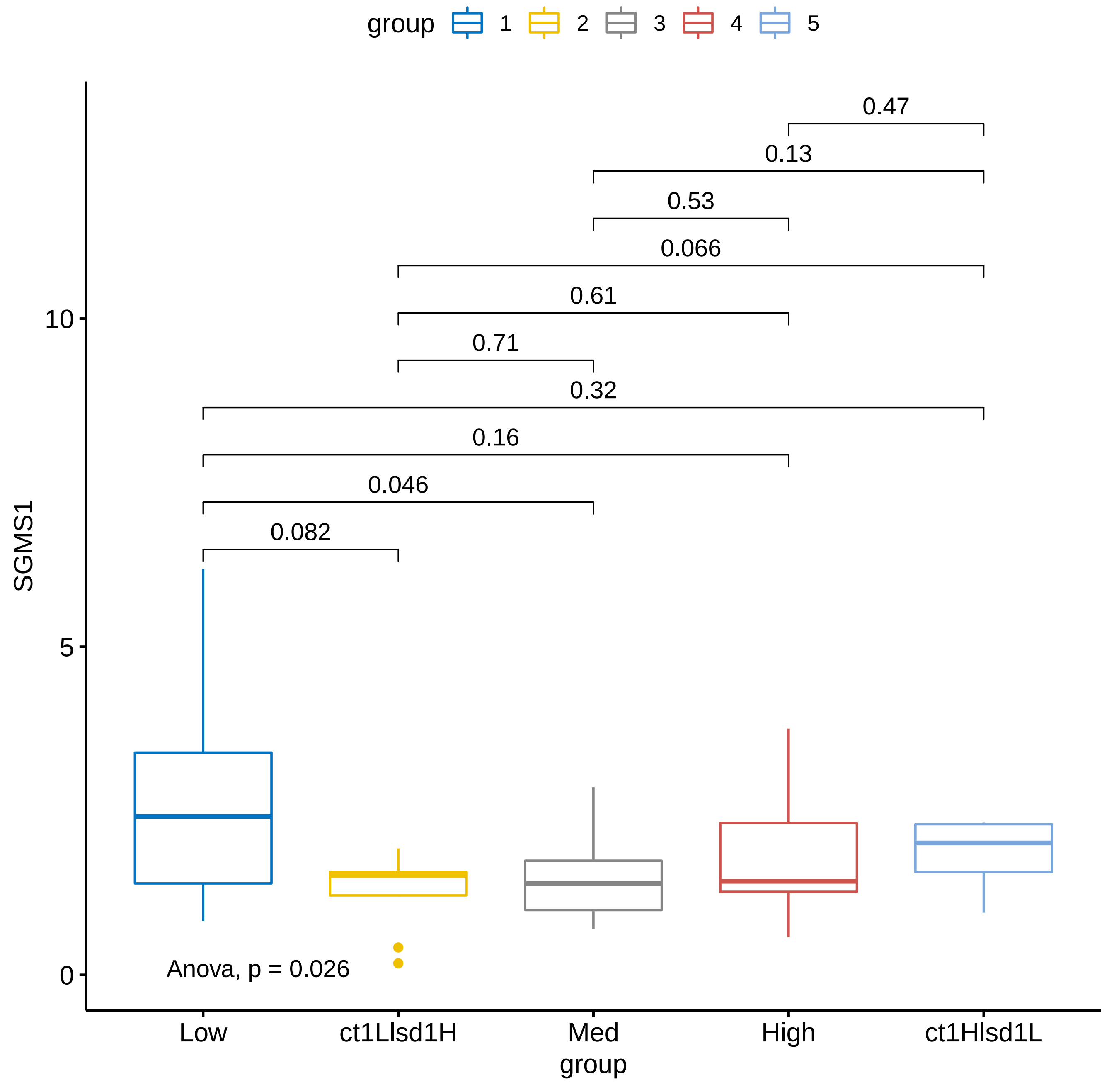

group
1
2
3
4
5
0.47
0.13
0.53
0.066
0.61
0.71
0.32
0.16
0.046
0.082
SGMS1
10
5
0
Anova, p = 0.026
Low
ct1Llsd1H
Med
High
ct1Hlsd1L
group

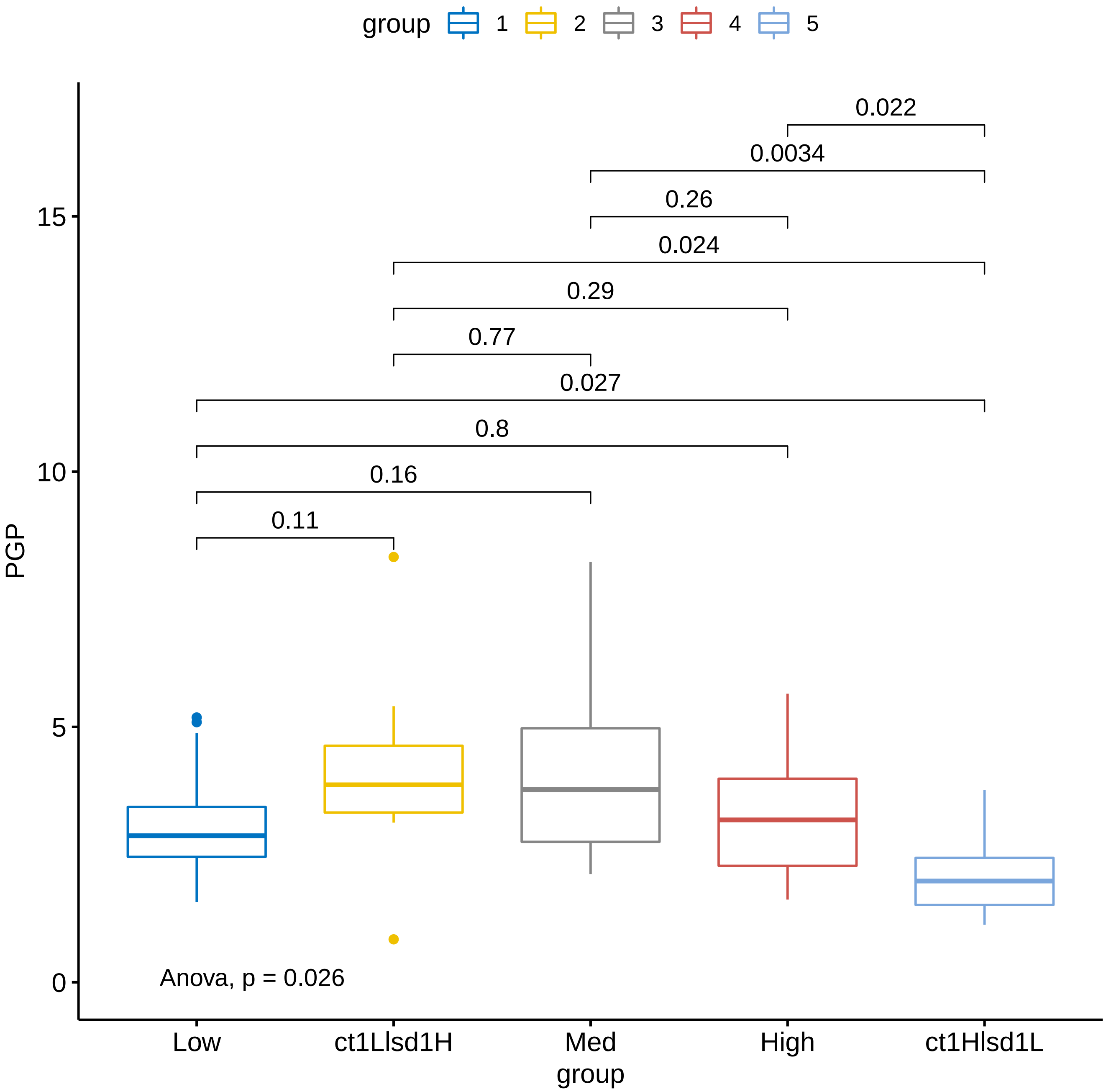
group
1
2
3
4
5
0.022
0.0034
0.26
0.024
0.29
0.77
0.027
0.8
0.16
0.11
15
10
5
0
PGP
Anova, p = 0.026
Low
ct1Llsd1H
Med
High
ct1Hlsd1L
group

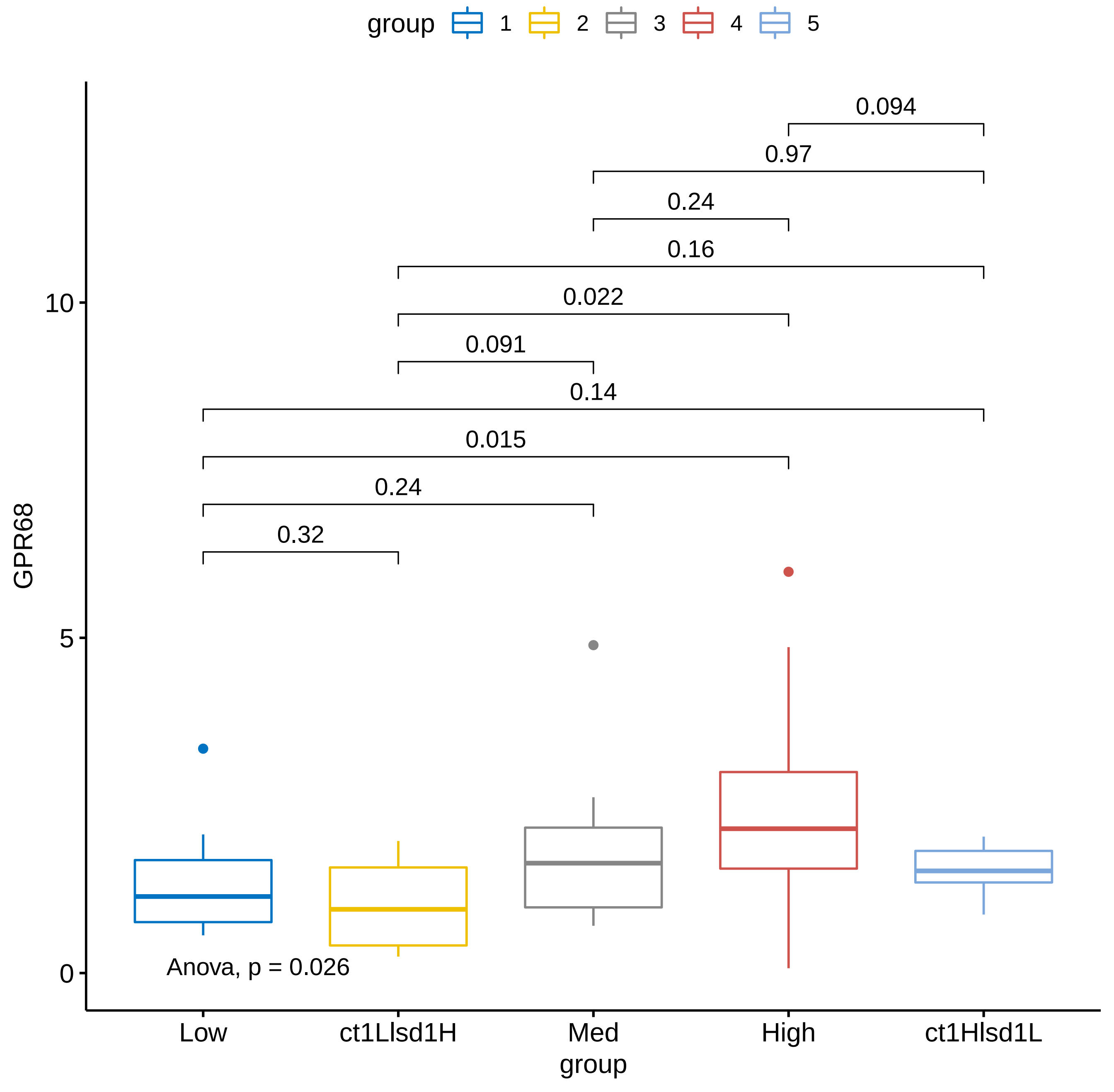

group
1
2
3
4
5
0.094
0.97
0.24
0.16
0.022
0.091
0.14
0.015
0.24
0.32
10
5
0
GPR68
Anova, p = 0.026
Low
ct1Llsd1H
Med
High
ct1Hlsd1L
group

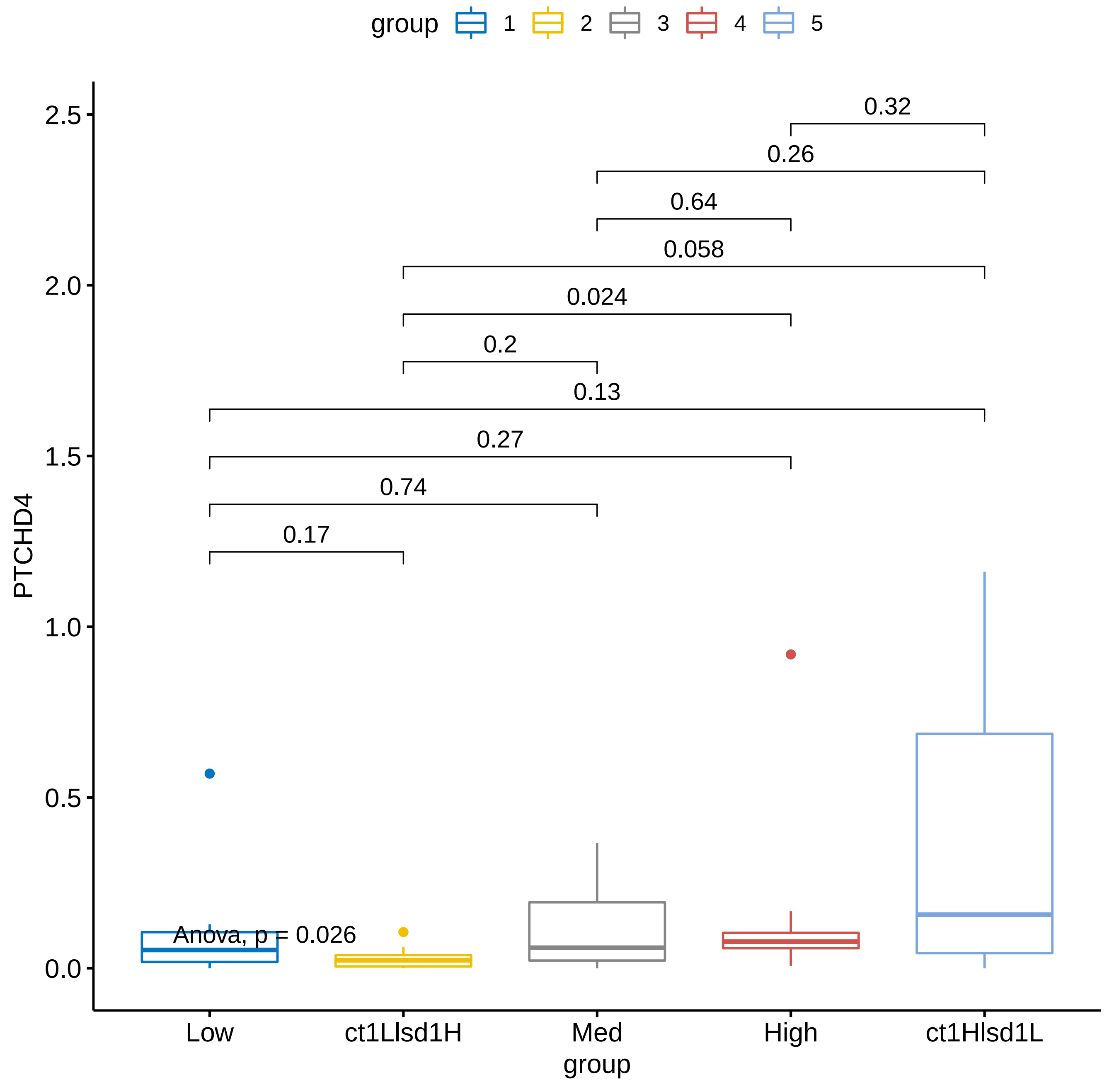

group
1
2
3
4
5
0.32
0.26
0.64
0.058
0.024
0.2
0.13
0.27
0.74
0.17
Anova, p = 0.026
PTCHD4
2.5
2.0
1.5
1.0
0.5
0.0
Low
ct1Llsd1H
Med
High
ct1Hlsd1L
group

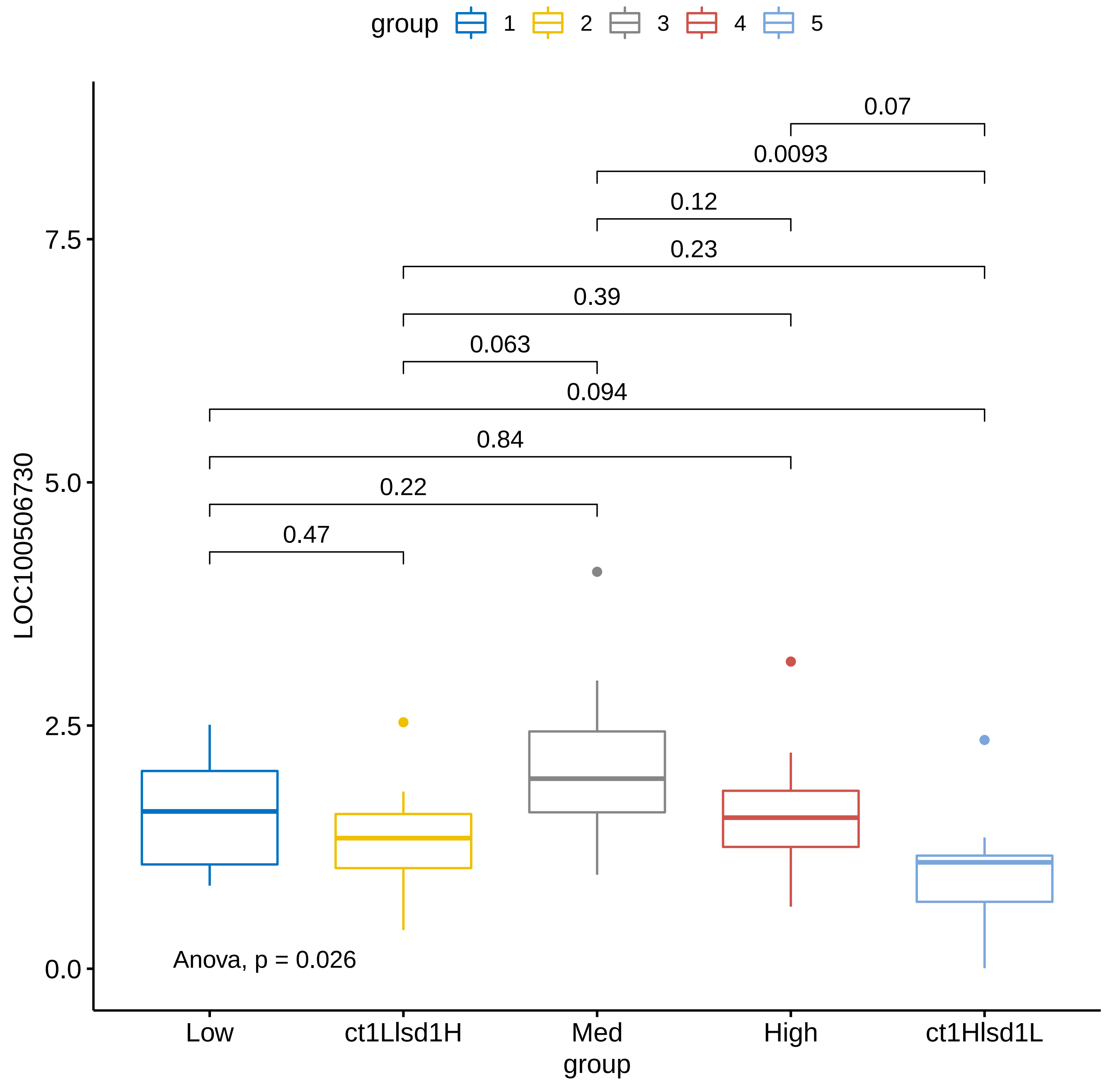

group
1
2
3
4
5
0.07
0.0093
0.12
0.23
0.39
0.063
0.094
0.84
0.22
0.47
7.5
5.0
2.5
0.0
LOC100506730
Anova, p = 0.026
Low
ct1Llsd1H
Med
High
ct1Hlsd1L
group

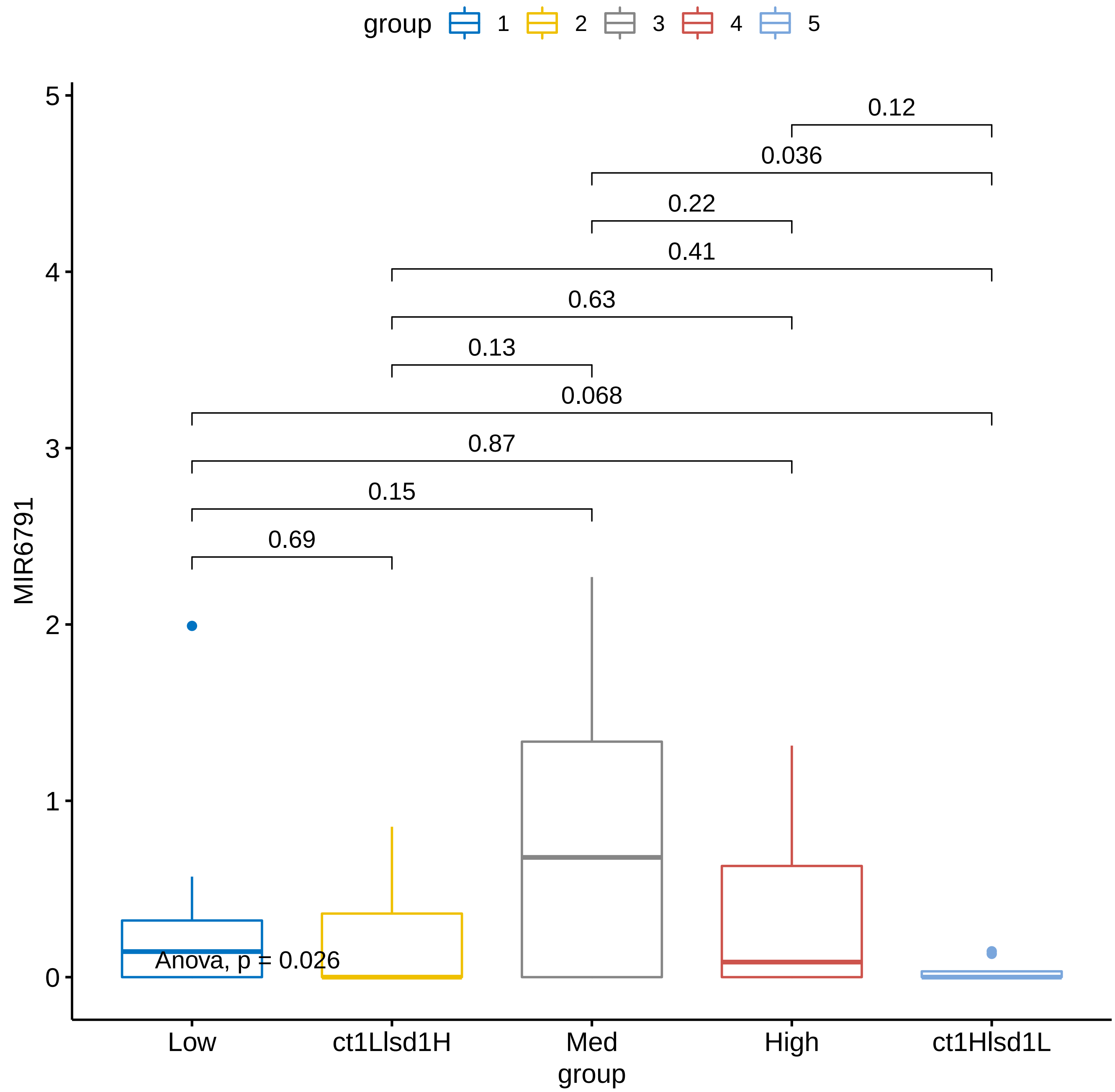

group 1 2 3 4 5
MIR6791
0
1
2
3
4
5
0.12
0.036
0.22
0.41
0.63
0.13
0.068
0.87
0.15
0.69
Anova, p = 0.026
Low
ct1Llsd1H
Med
High
ct1Hlsd1L
group

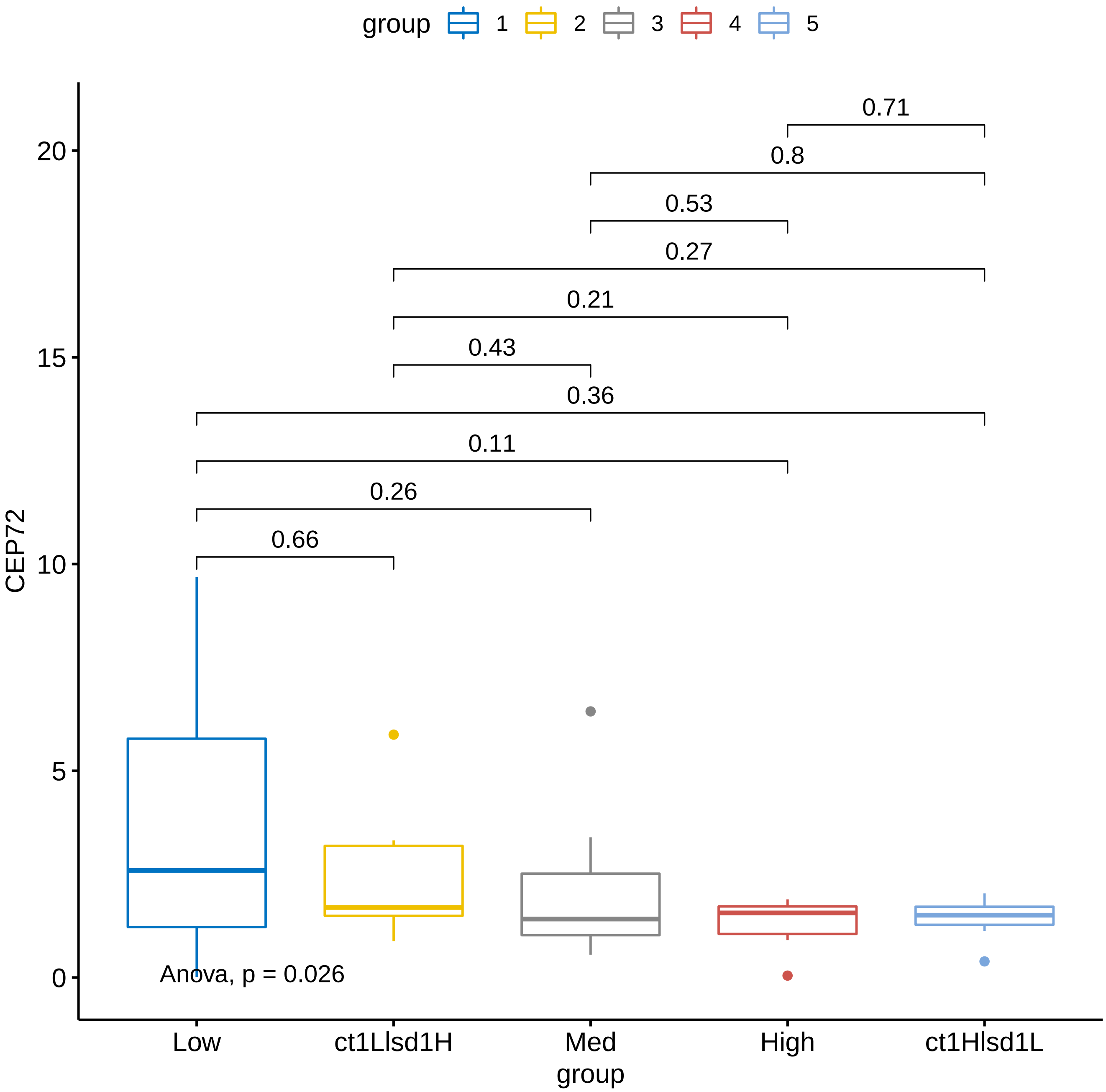
group
1
2
3
4
5
0.71
0.8
0.53
0.27
0.21
0.43
0.36
0.11
0.26
0.66
Anova, p = 0.026
CEP72
0
5
10
15
20
Low
ct1Llsd1H
Med
High
ct1Hlsd1L
group

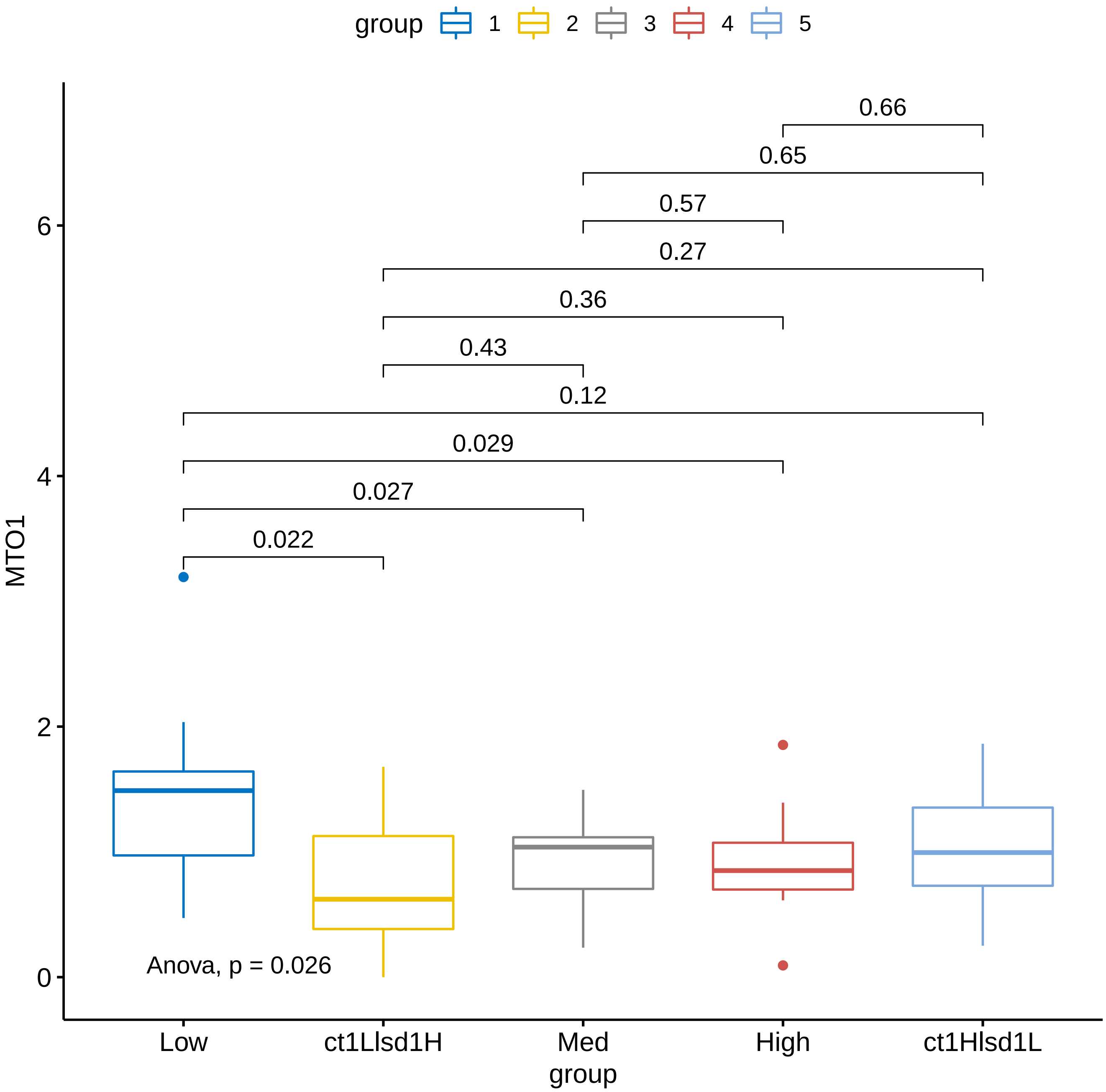
group
1
2
3
4
5
0.66
0.65
0.57
0.27
0.36
0.43
0.12
0.029
0.027
0.022
MTO1
6
4
2
0
Anova, p = 0.026
Low
ct1Llsd1H
Med
High
ct1Hlsd1L
group

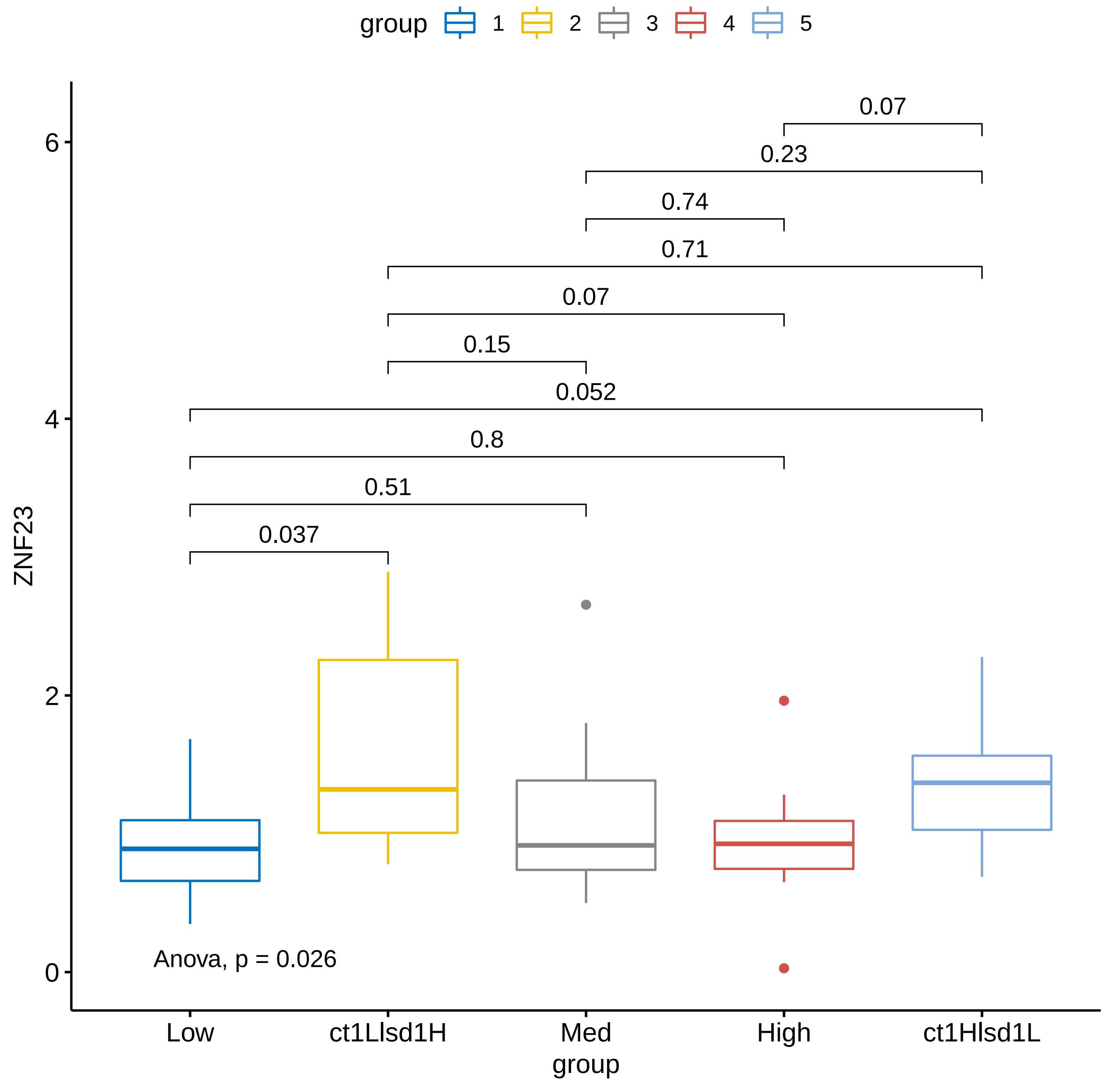
group
1
2
3
4
5
ZNF23
6
4
2
0
0.07
0.23
0.74
0.71
0.07
0.15
0.052
0.8
0.51
0.037
Anova, p = 0.026
Low
ct1Llsd1H
Med
High
ct1Hlsd1L
group

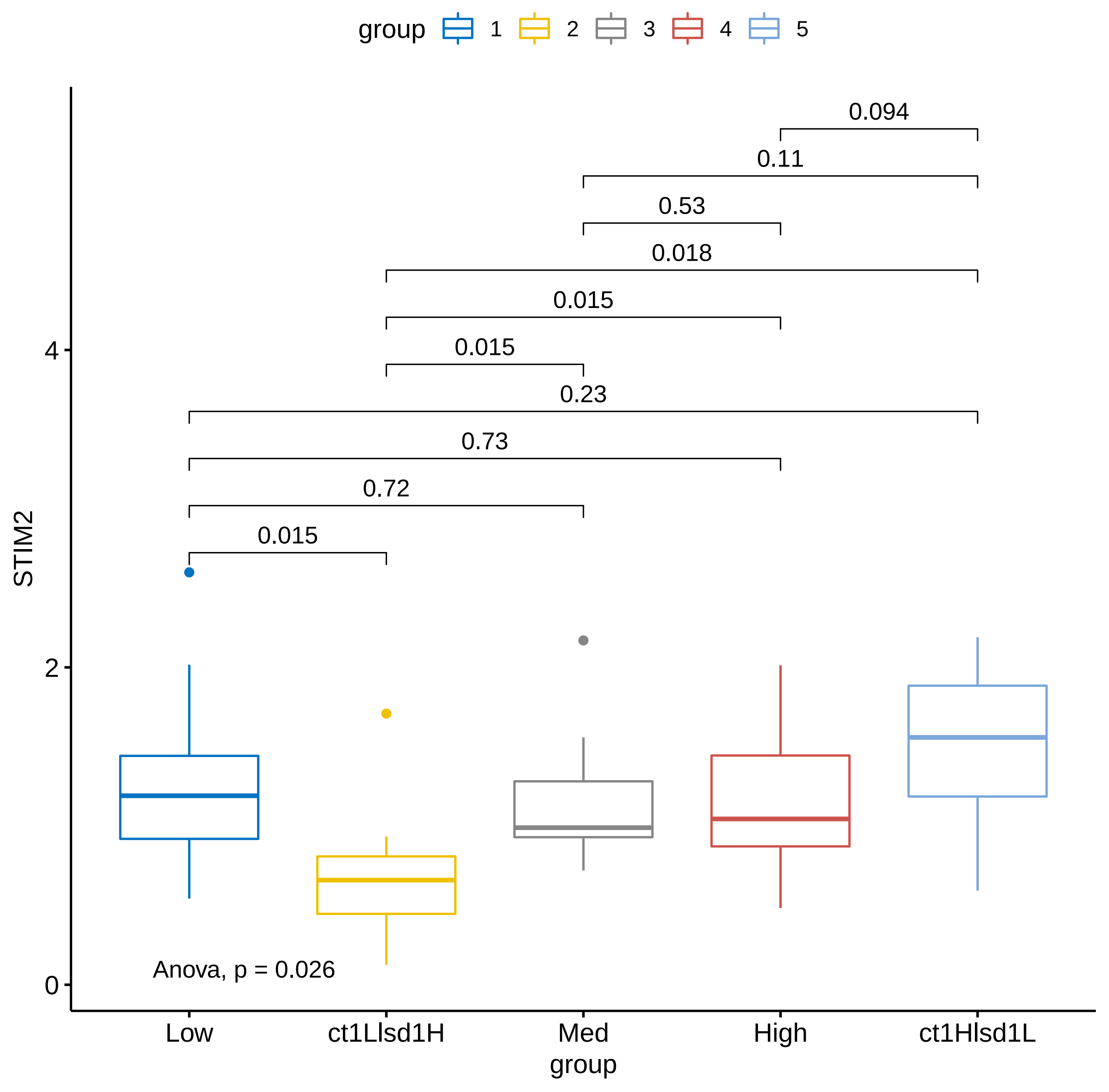

group
1
2
3
4
5
0.094
0.11
0.53
0.018
0.015
0.015
0.23
0.73
0.72
0.015
STIM2
4
2
0
Anova, p = 0.026
Low
ct1Llsd1H
Med
High
ct1Hlsd1L
group

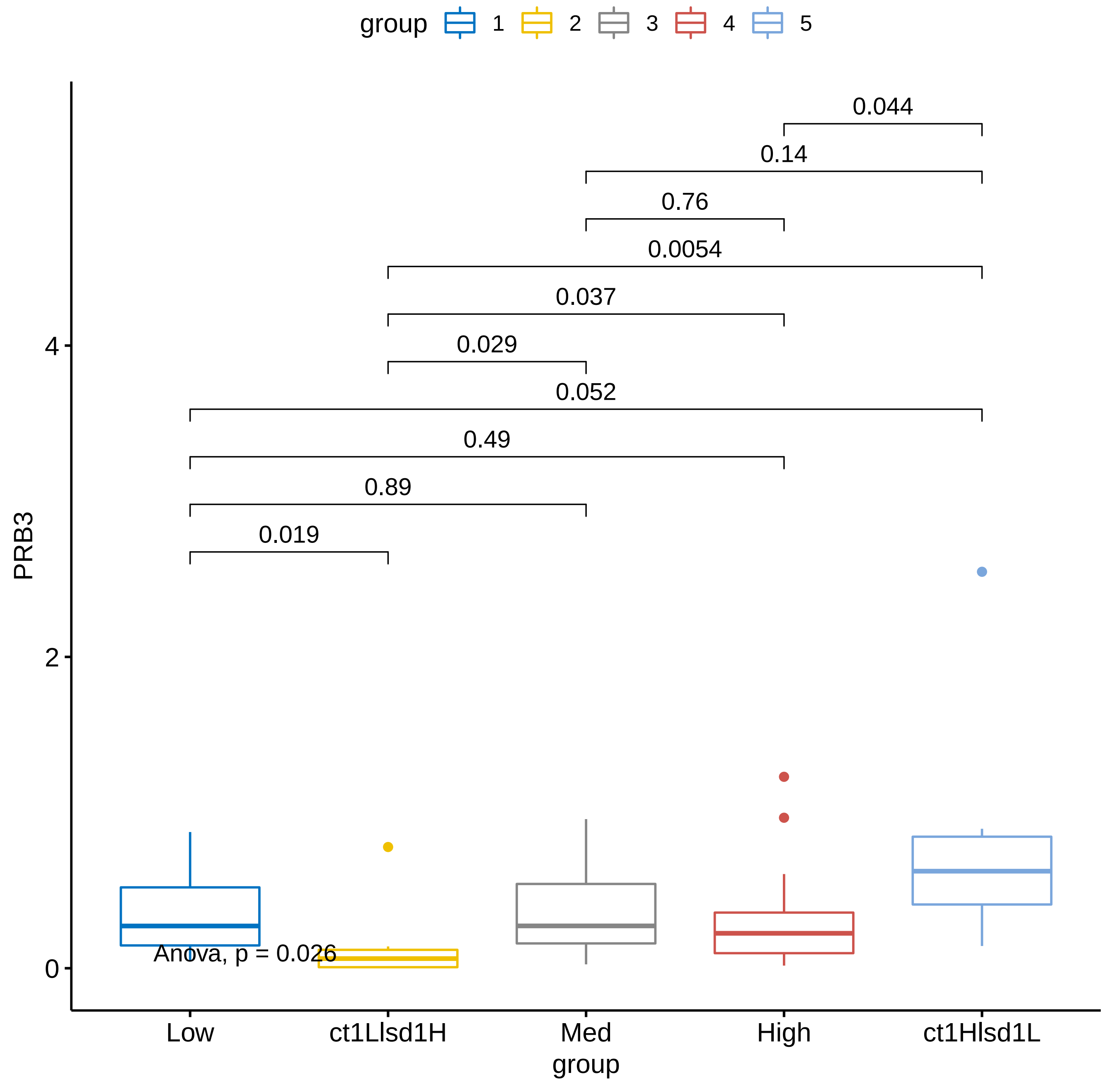
group
1
2
3
4
5
0.044
0.14
0.76
0.0054
0.037
0.029
0.052
0.49
0.89
0.019
PRB3
4
2
0
Anova, p = 0.026
Low
ct1Llsd1H
Med
High
ct1Hlsd1L
group

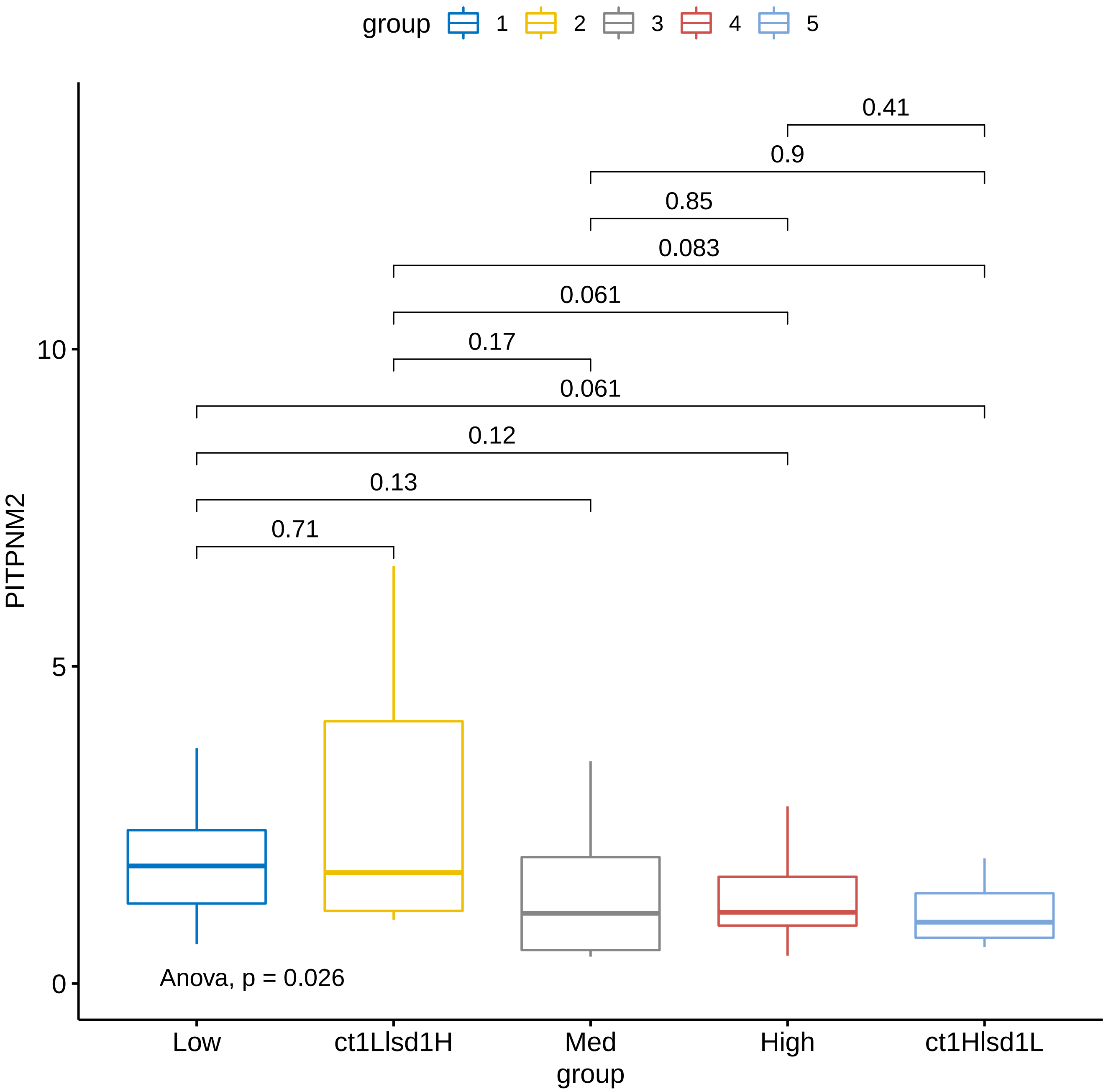

group
1
2
3
4
5
0.41
0.9
0.85
0.083
0.061
0.17
0.061
0.12
0.13
0.71
10
5
0
PITPNM2
Anova, p = 0.026
Low
ct1Llsd1H
Med
High
ct1Hlsd1L
group

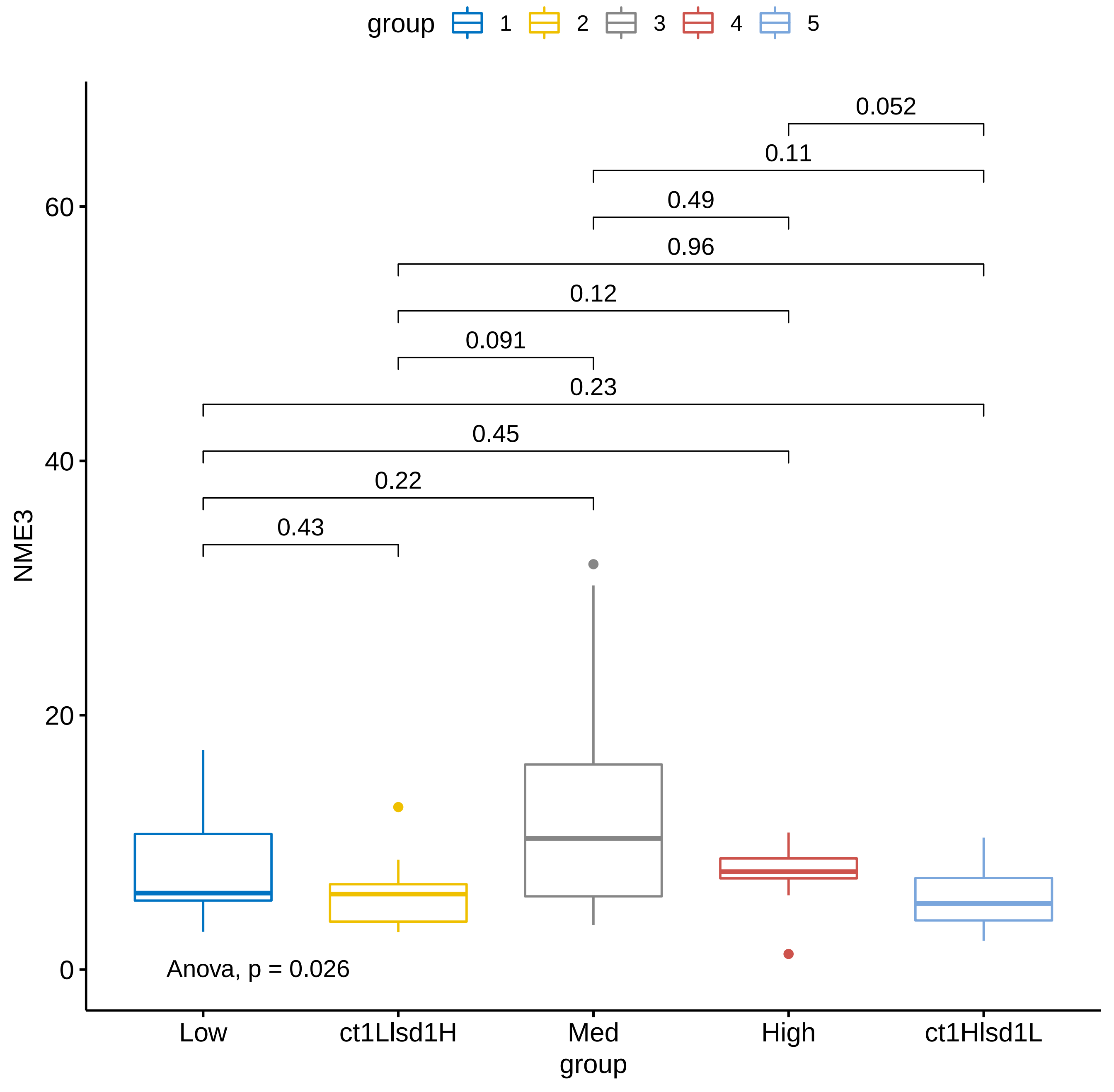
group
1
2
3
4
5
NME3
0
20
40
60
0.052
0.11
0.49
0.96
0.12
0.091
0.23
0.45
0.22
0.43
Anova, p = 0.026
Low
ct1Llsd1H
Med
High
ct1Hlsd1L
group

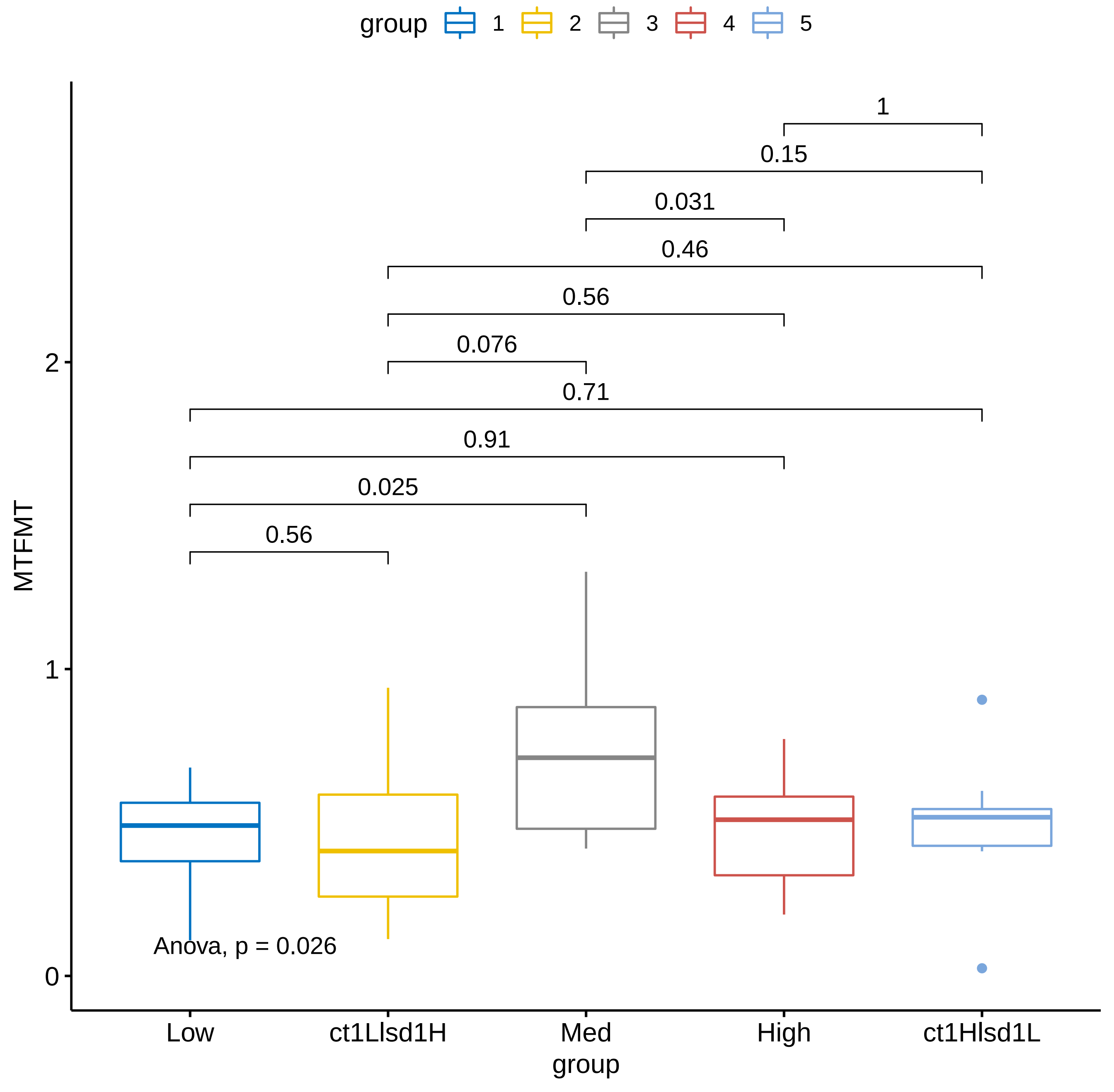
group
1
2
3
4
5
1
0.15
0.031
0.46
0.56
0.076
0.71
0.91
0.025
0.56
Anova, p = 0.026
MTFMT
2
1
0
Low
ct1Llsd1H
Med
High
ct1Hlsd1L
group

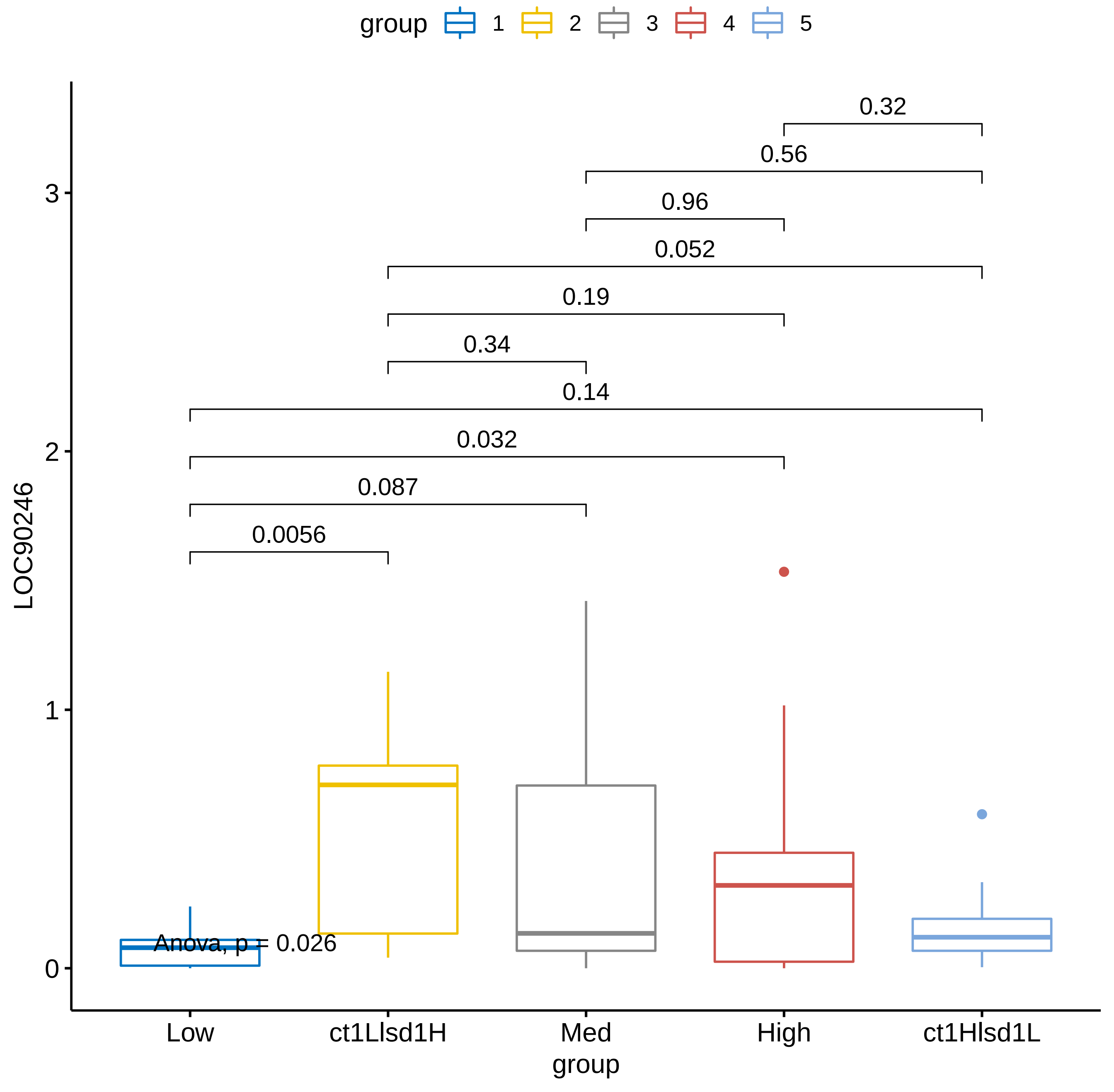

group
1
2
3
4
5
0.32
0.56
0.96
0.052
0.19
0.34
0.14
0.032
0.087
0.0056
Anova, p = 0.026
LOC90246
3
2
1
0
Low
ct1Llsd1H
Med
High
ct1Hlsd1L
group

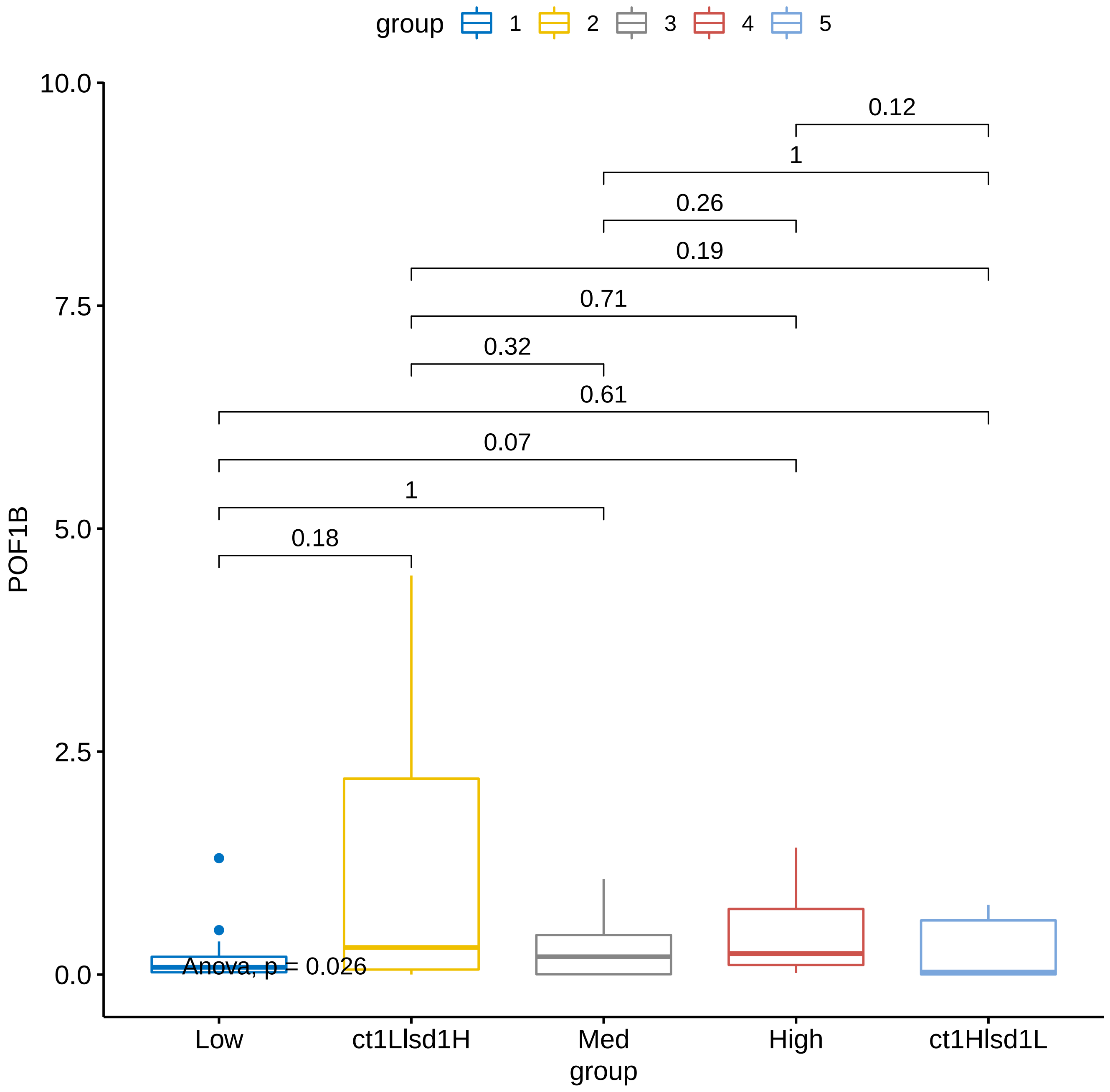
group
1
2
3
4
5
10.0
7.5
5.0
2.5
0.0
POF1B
0.12
1
0.26
0.19
0.71
0.32
0.61
0.07
1
0.18
Anova, p = 0.026
Low
ct1Llsd1H
Med
High
ct1Hlsd1L
group

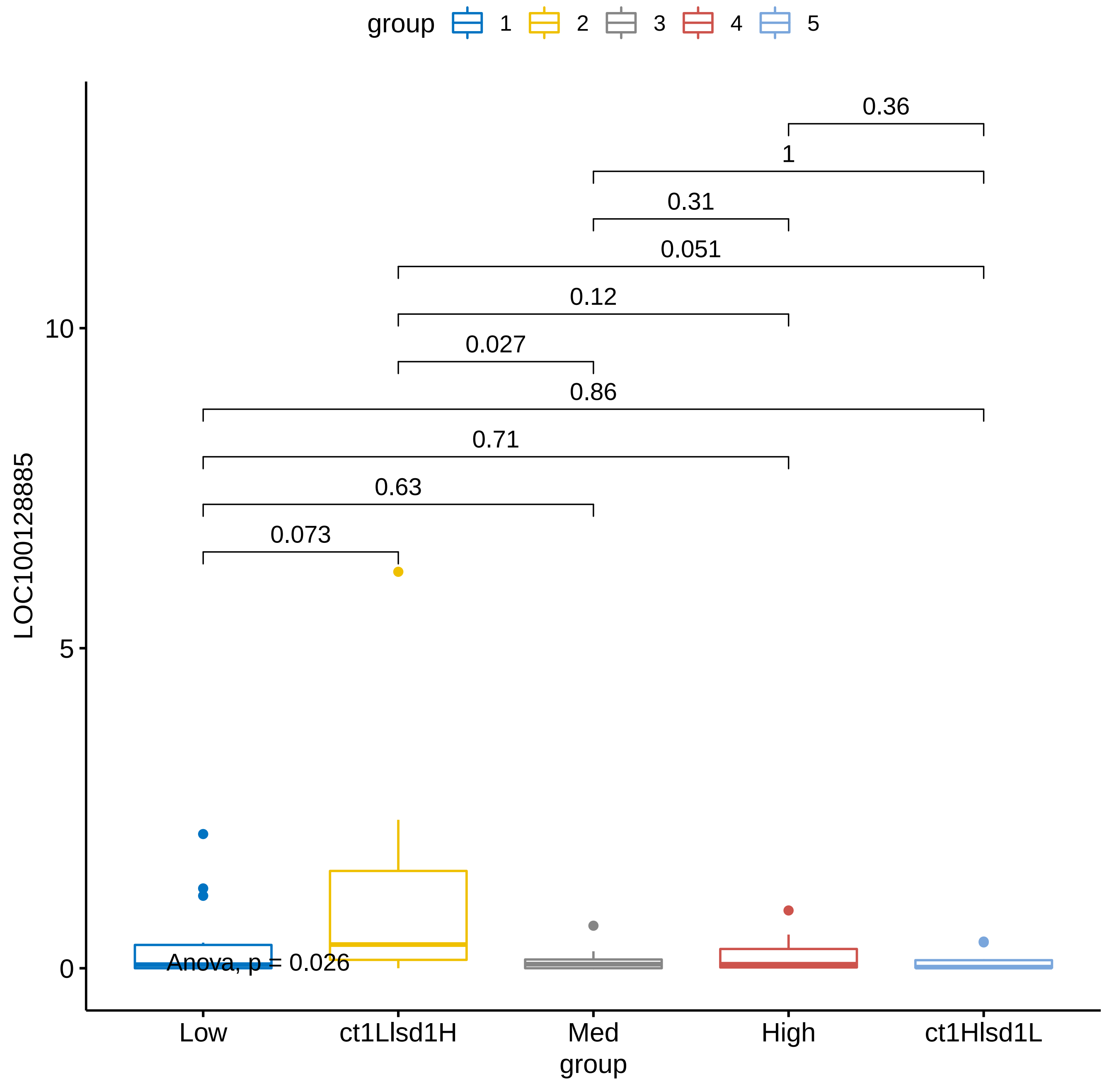

group
1
2
3
4
5
0.36
1
0.31
0.051
0.12
0.027
0.86
0.71
0.63
0.073
Anova, p = 0.026
LOC100128885
10
5
0
Low
ct1Llsd1H
Med
High
ct1Hlsd1L
group

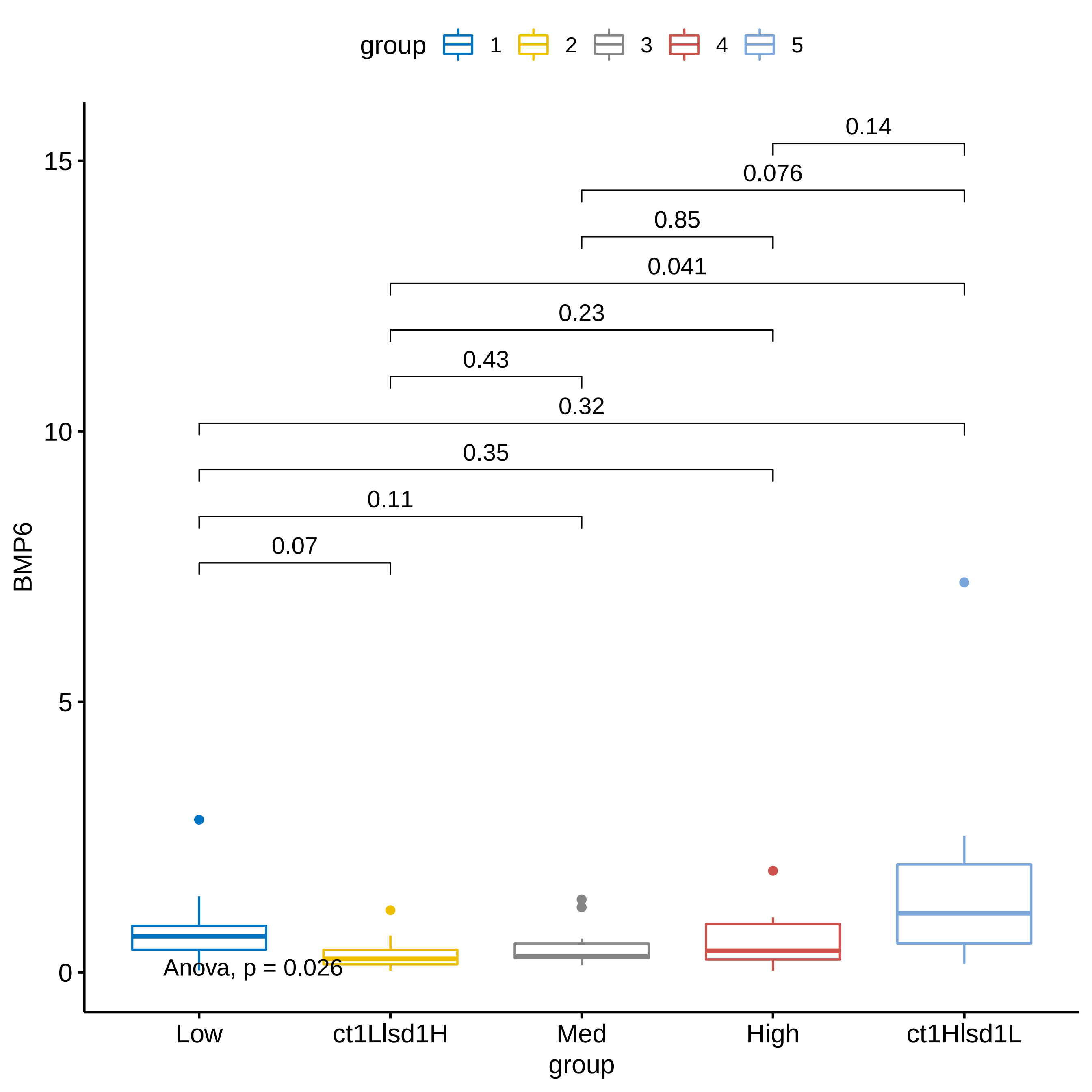
group 1 2 3 4 5
0.14
0.076
0.85
0.041
0.23
0.43
0.32
0.35
0.11
0.07
BMP6
15
10
5
0
Anova, p = 0.026
Low
ct1Llsd1H
Med
High
ct1Hlsd1L
group

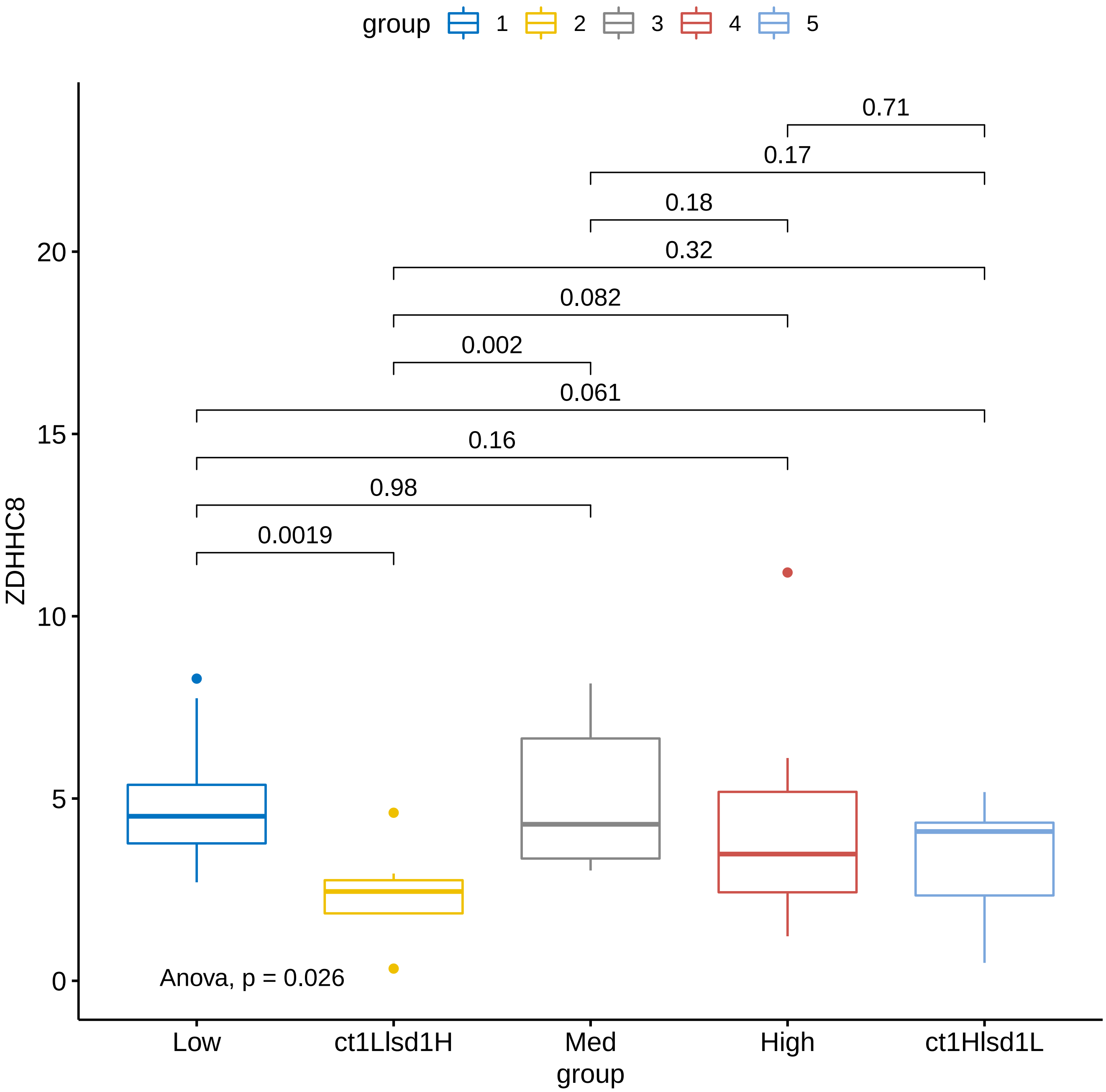

group
1
2
3
4
5
0.71
0.17
0.18
20
0.32
0.082
0.002
0.061
15
0.16
0.98
ZDHHC8
0.0019
10
5
0
Anova, p = 0.026
Low
ct1Llsd1H
Med
High
ct1Hlsd1L
group

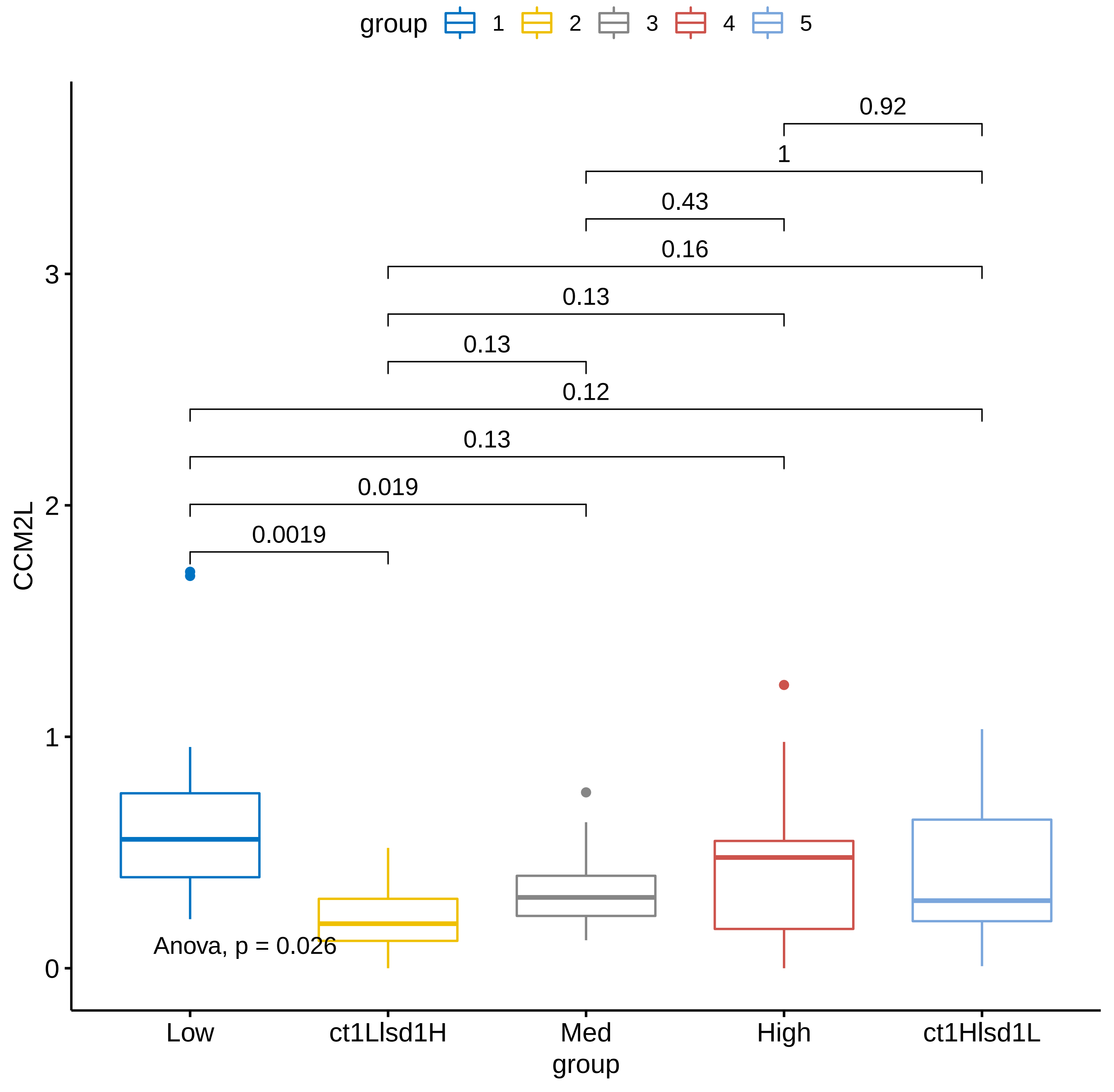
group
1
2
3
4
5
0.92
1
0.43
0.16
0.13
0.13
0.12
0.13
0.019
0.0019
Anova, p = 0.026
CCM2L
3
2
1
0
Low
ct1Llsd1H
Med
High
ct1Hlsd1L
group

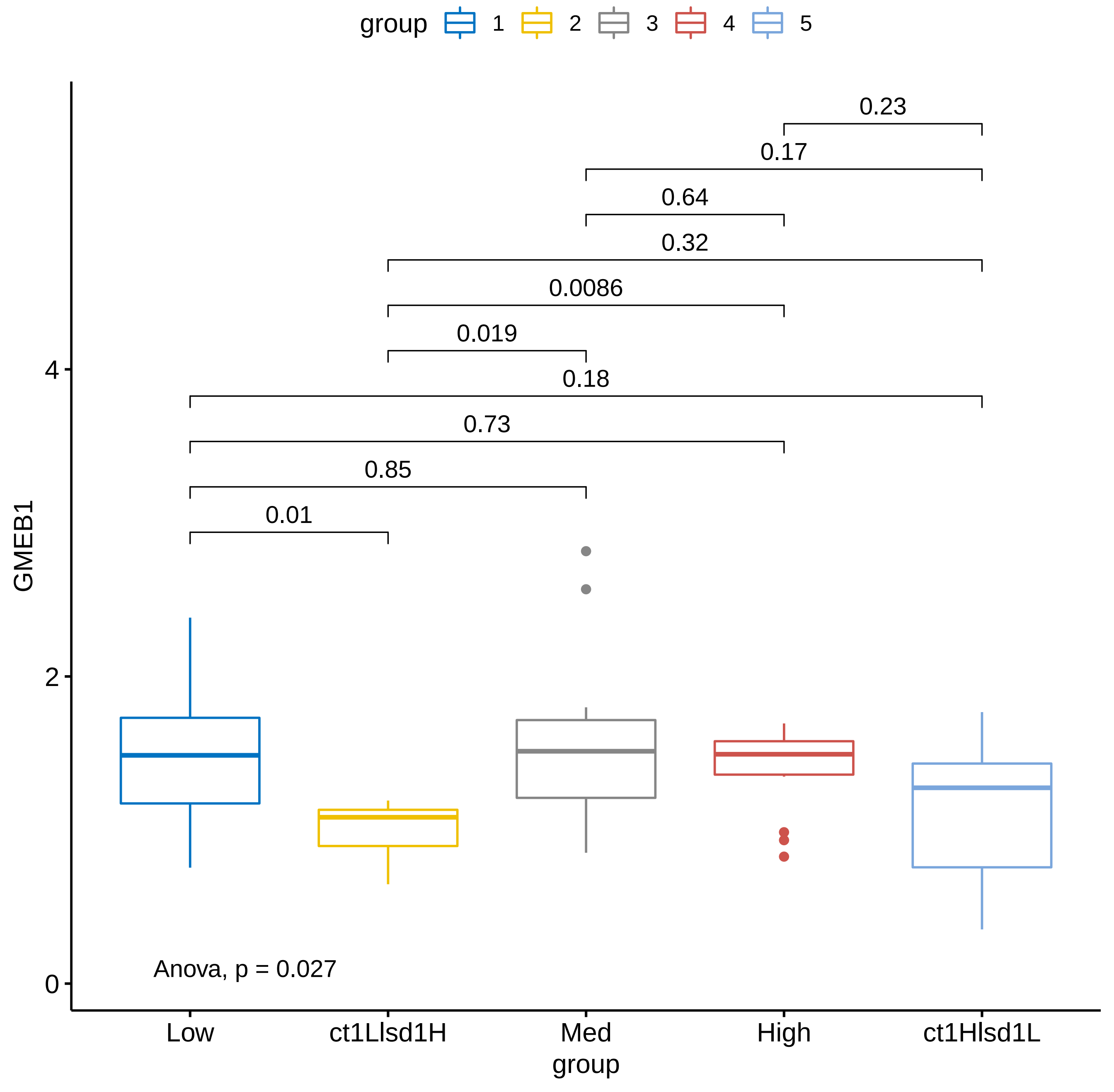

group
1
2
3
4
5
0.23
0.17
0.64
0.32
0.0086
0.019
0.18
0.73
0.85
0.01
4
2
0
GMEB1
Anova, p = 0.027
Low
ct1Llsd1H
Med
High
ct1Hlsd1L
group

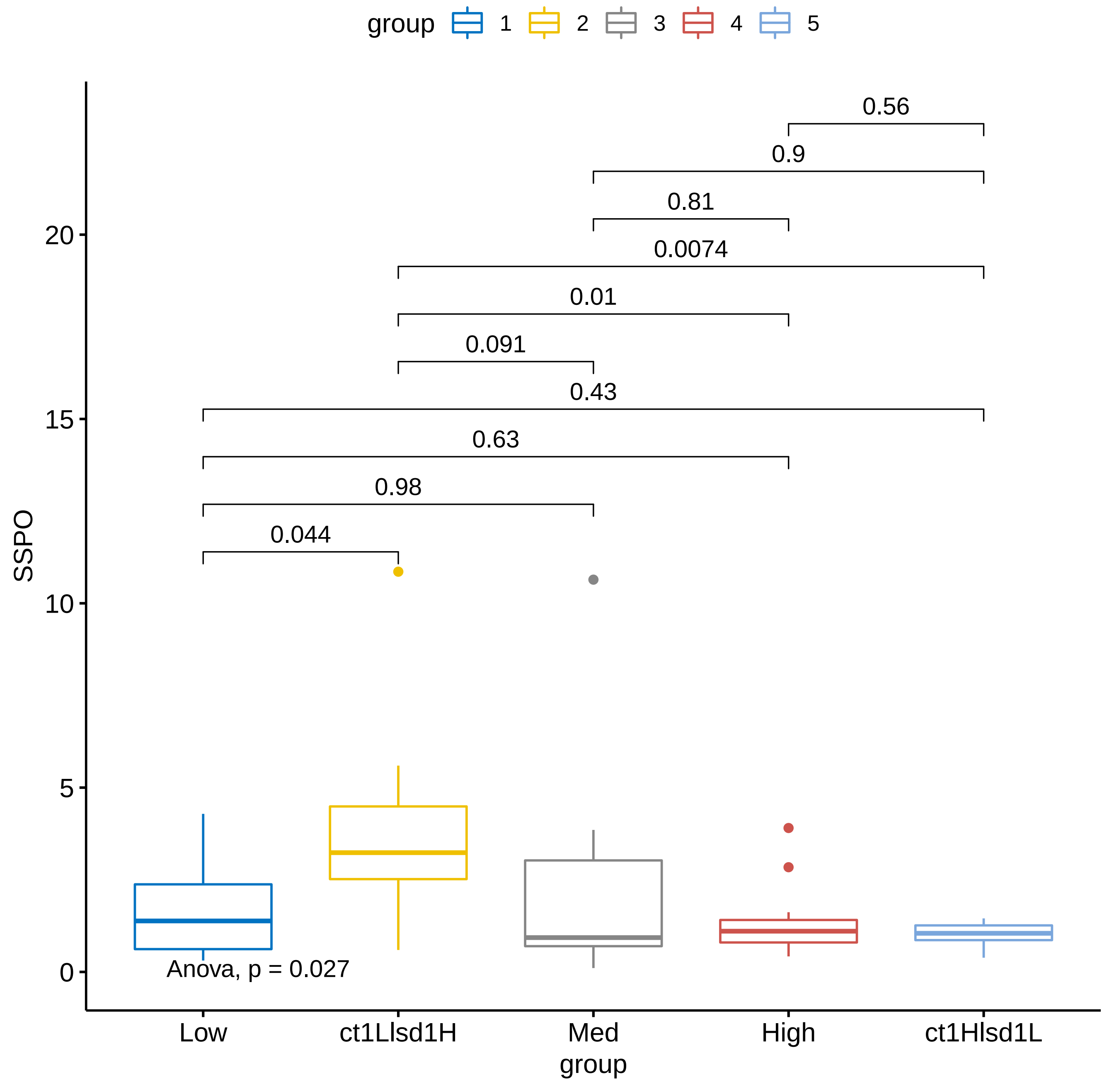

group 1 2 3 4 5
0.56
0.9
0.81
0.0074
0.01
0.091
0.43
0.63
0.98
0.044
SSPO
20
15
10
5
0
Anova, p = 0.027
Low
ct1Llsd1H
Med
High
ct1Hlsd1L
group

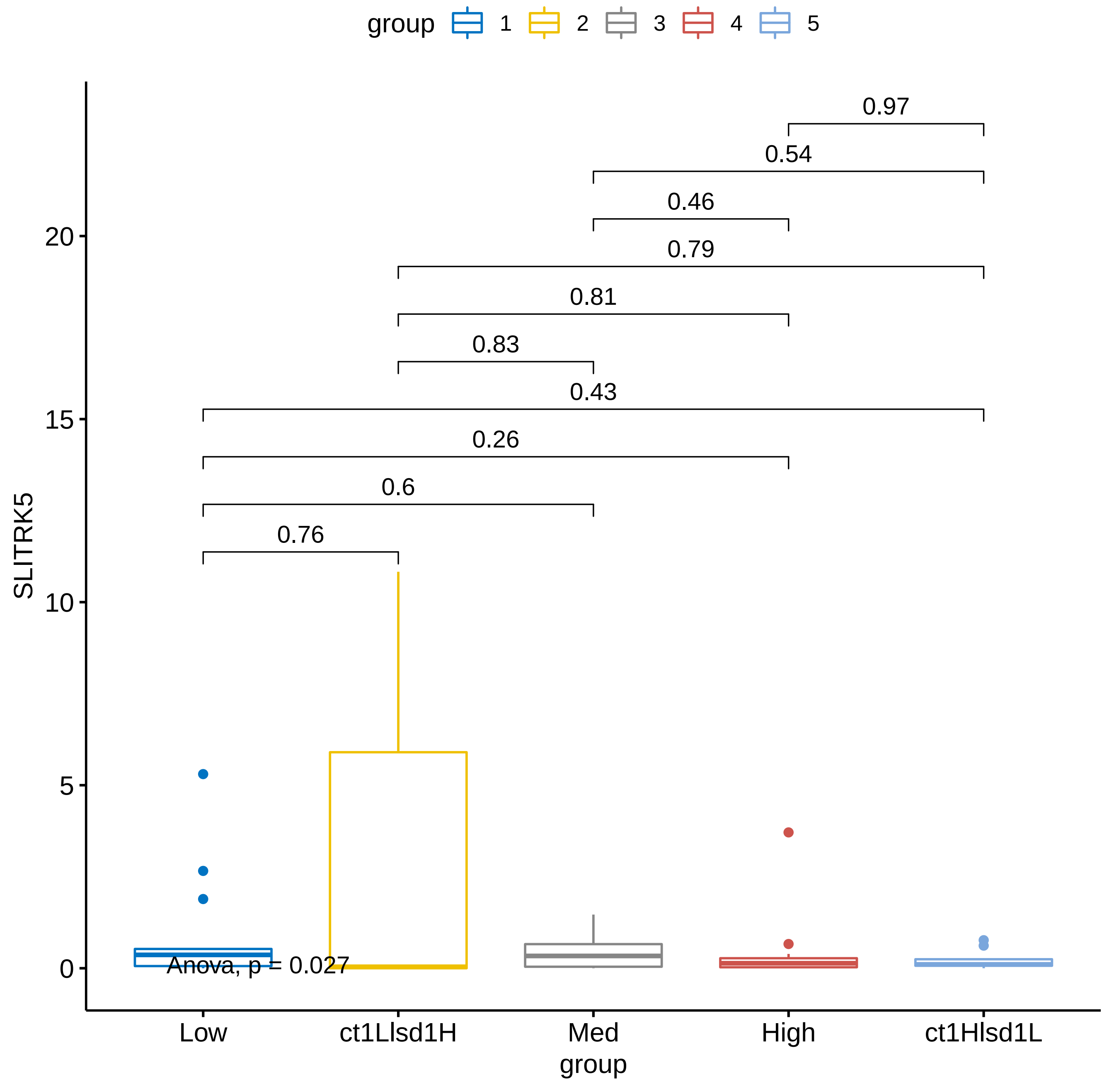
group 1 2 3 4 5
0.97
0.54
0.46
0.79
0.81
0.83
0.43
0.26
0.6
0.76
SLITRK5
20
15
10
5
0
Anova, p = 0.027
Low
ct1Llsd1H
Med
High
ct1Hlsd1L
group

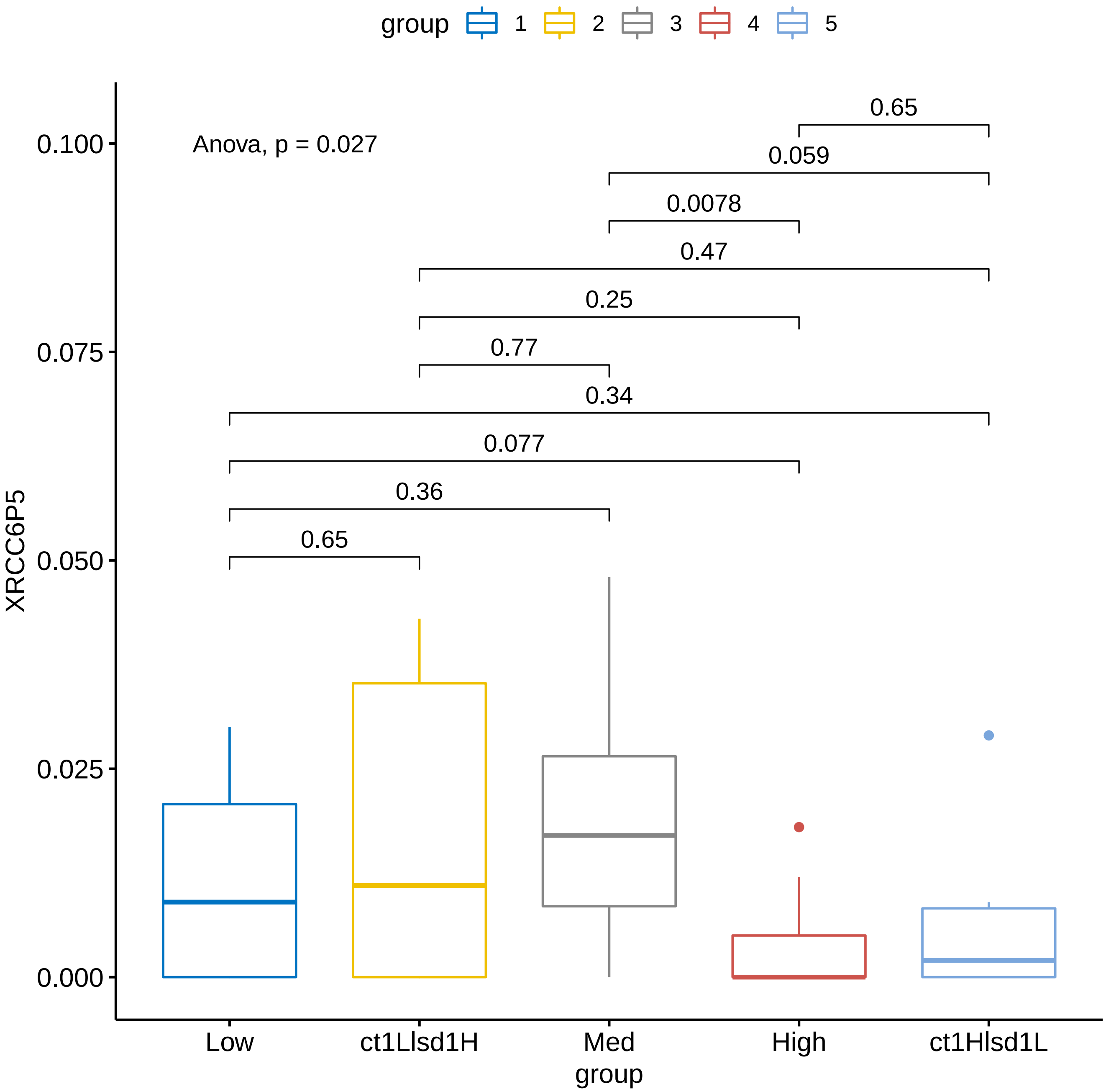

group
1
2
3
4
5
Anova, p = 0.027
0.65
0.059
0.0078
0.47
0.25
0.77
0.34
0.077
0.36
0.65
0.100
0.075
0.050
0.025
0.000
XRCC6P5
Low
ct1Llsd1H
Med
High
ct1Hlsd1L
group

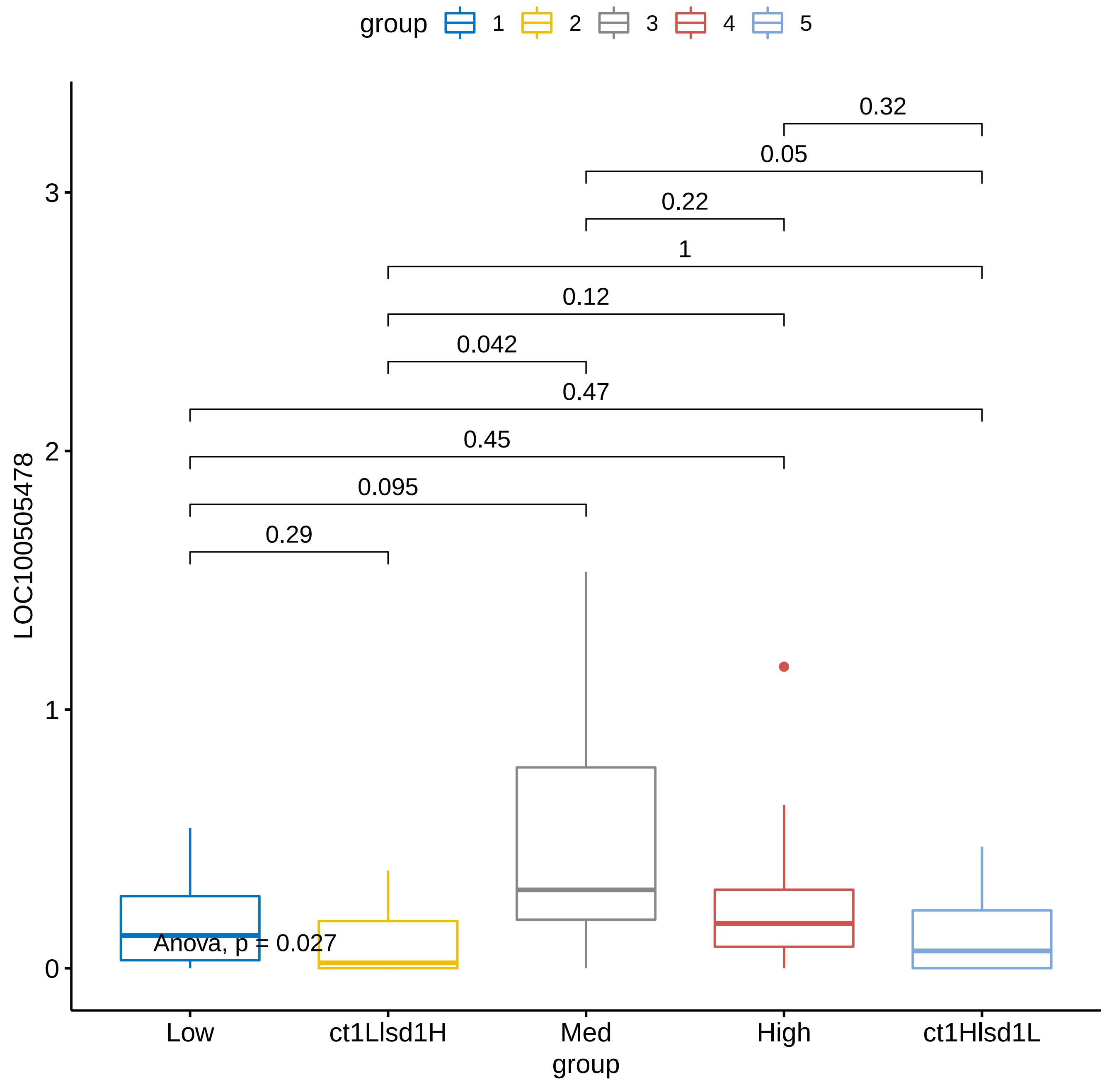

group
1
2
3
4
5
0.32
0.05
0.22
1
0.12
0.042
0.47
0.45
0.095
0.29
Anova, p = 0.027
LOC100505478
3
2
1
0
Low
ct1Llsd1H
Med
High
ct1Hlsd1L
group

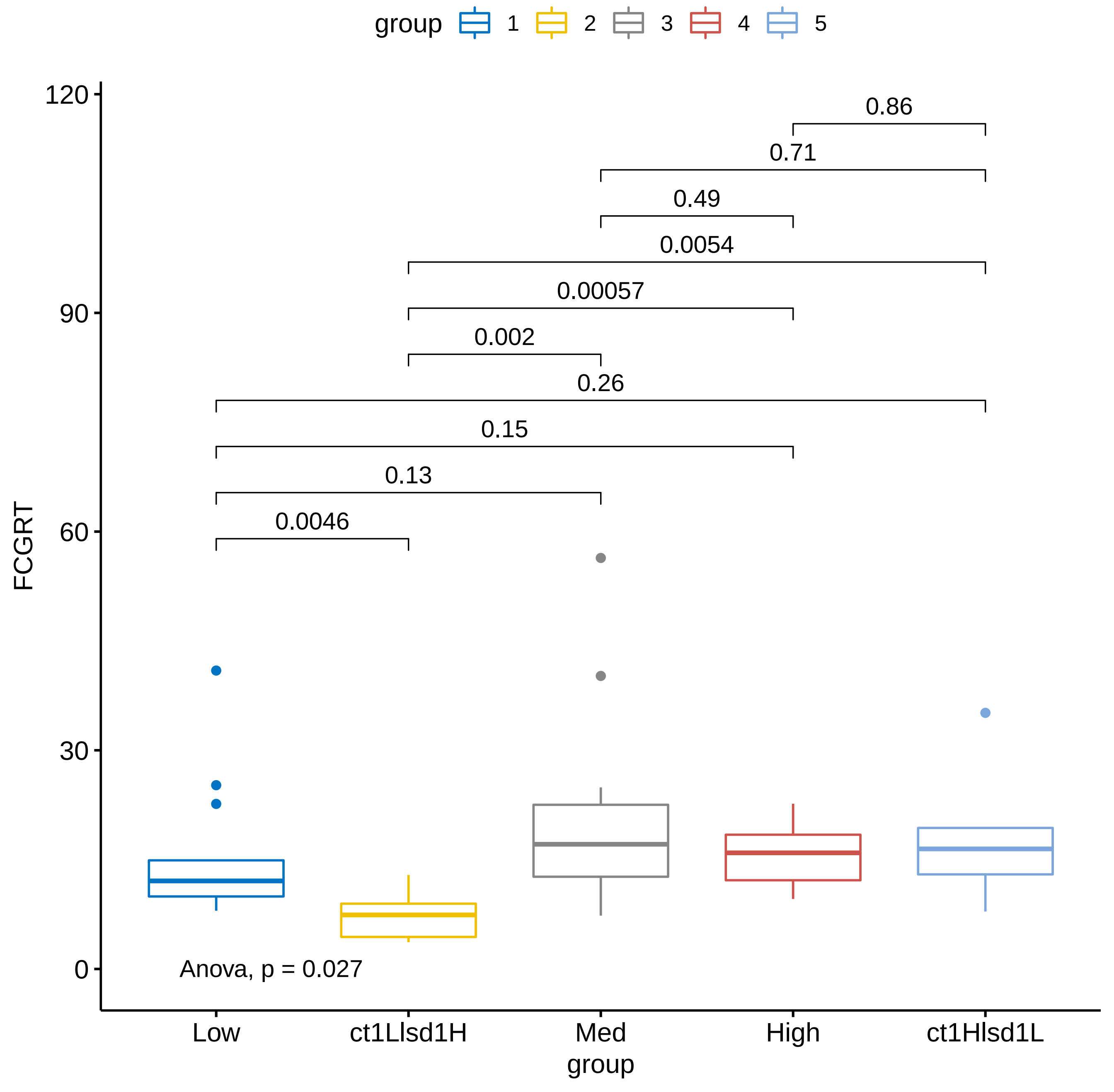

group
1
2
3
4
5
FCGRT
120
90
60
30
0
0.86
0.71
0.49
0.0054
0.00057
0.002
0.26
0.15
0.13
0.0046
Anova, p = 0.027
Low
ct1Llsd1H
Med
High
ct1Hlsd1L
group

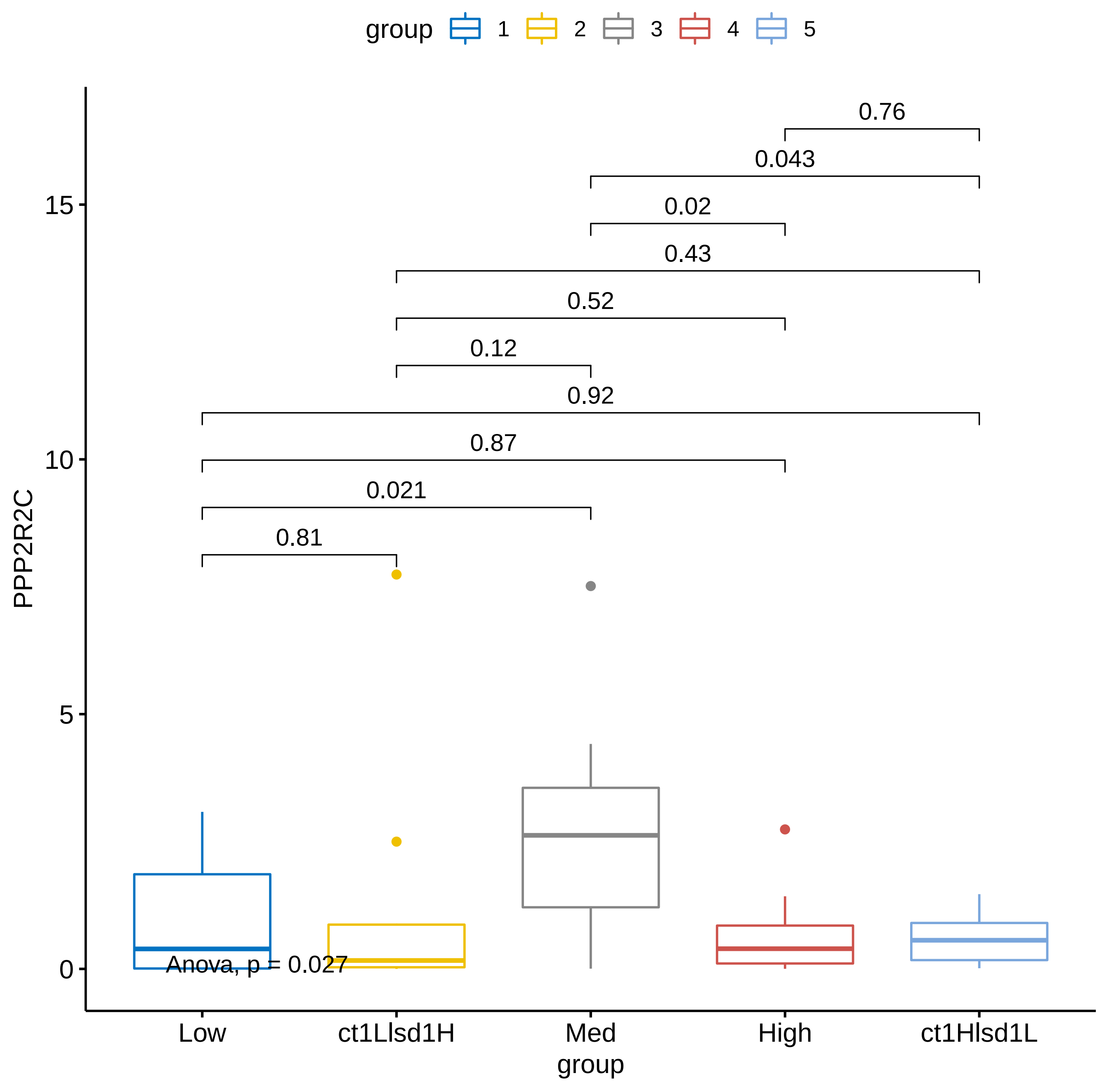

group 1 2 3 4 5
0.76
0.043
0.02
0.43
0.52
0.12
0.92
0.87
0.021
0.81
Anova, p = 0.027
PPP2R2C
15
10
5
0
Low
ct1Llsd1H
Med
High
ct1Hlsd1L
group

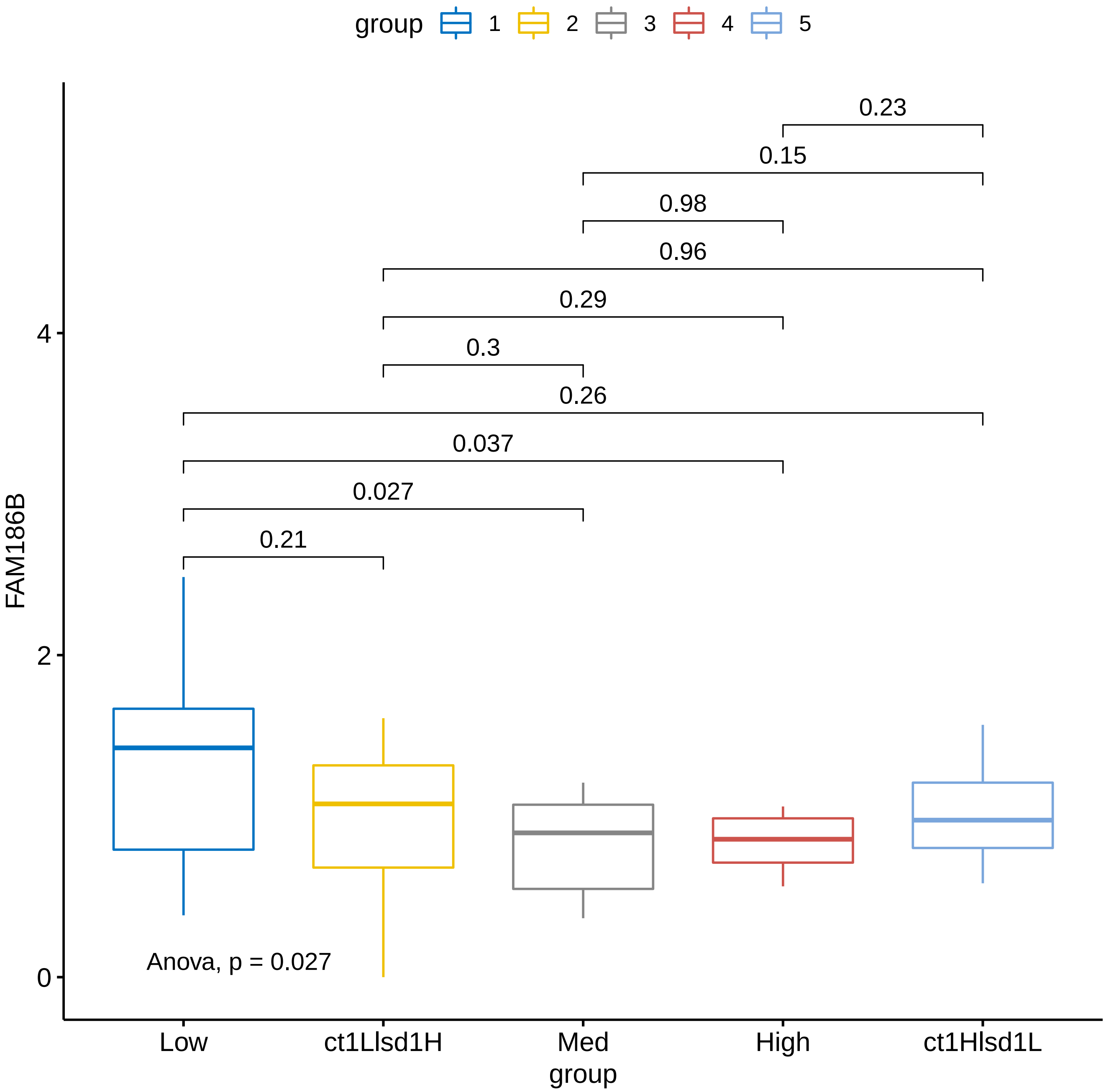

group
1
2
3
4
5
0.23
0.15
0.98
0.96
0.29
0.3
0.26
0.037
0.027
0.21
FAM186B
4
2
0
Anova, p = 0.027
Low
ct1Llsd1H
Med
High
ct1Hlsd1L
group

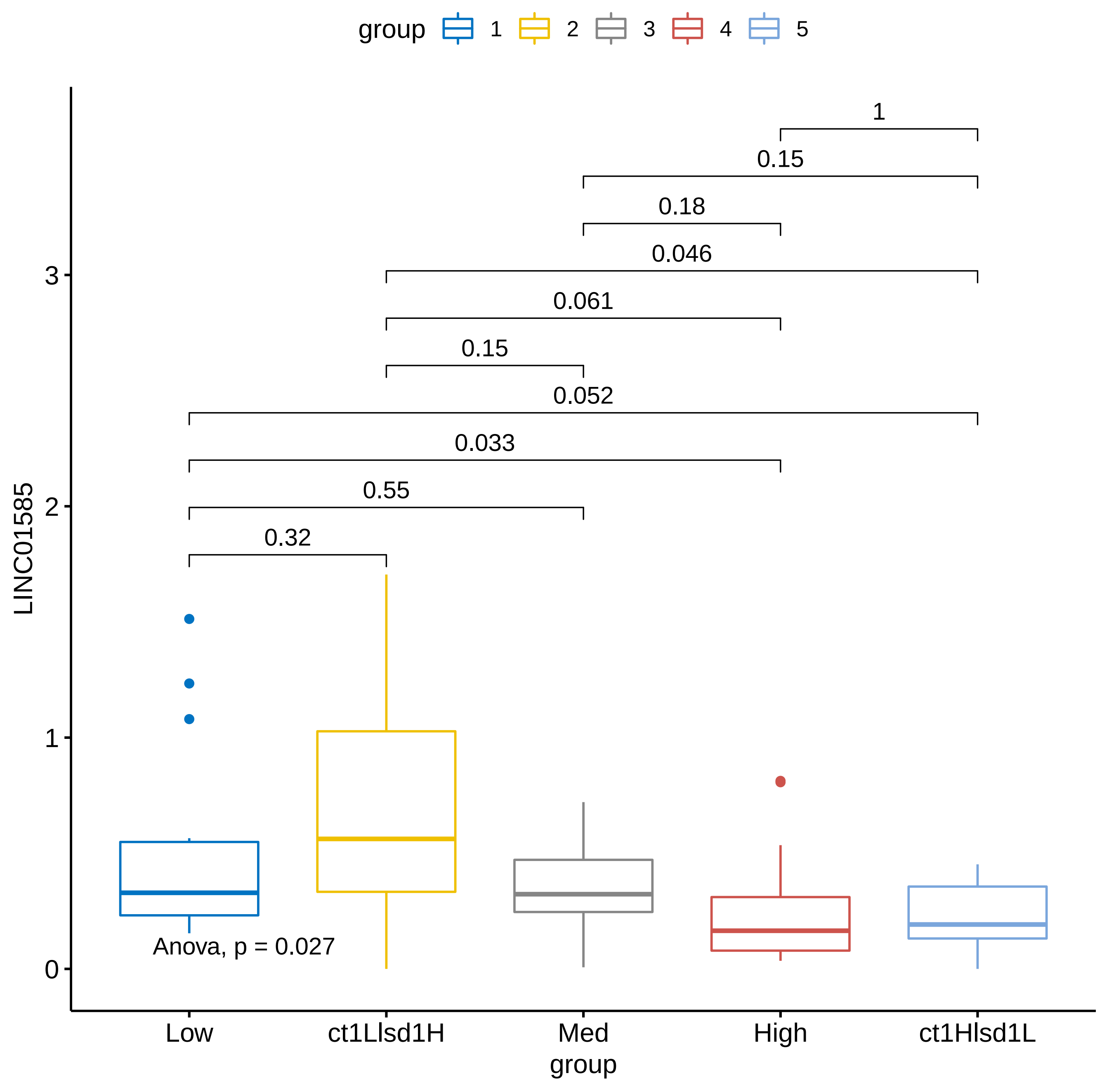
group
1
2
3
4
5
LINC01585
0
1
2
3
1
0.15
0.18
0.046
0.061
0.15
0.052
0.033
0.55
0.32
Anova, p = 0.027
Low
ct1Llsd1H
Med
High
ct1Hlsd1L
group

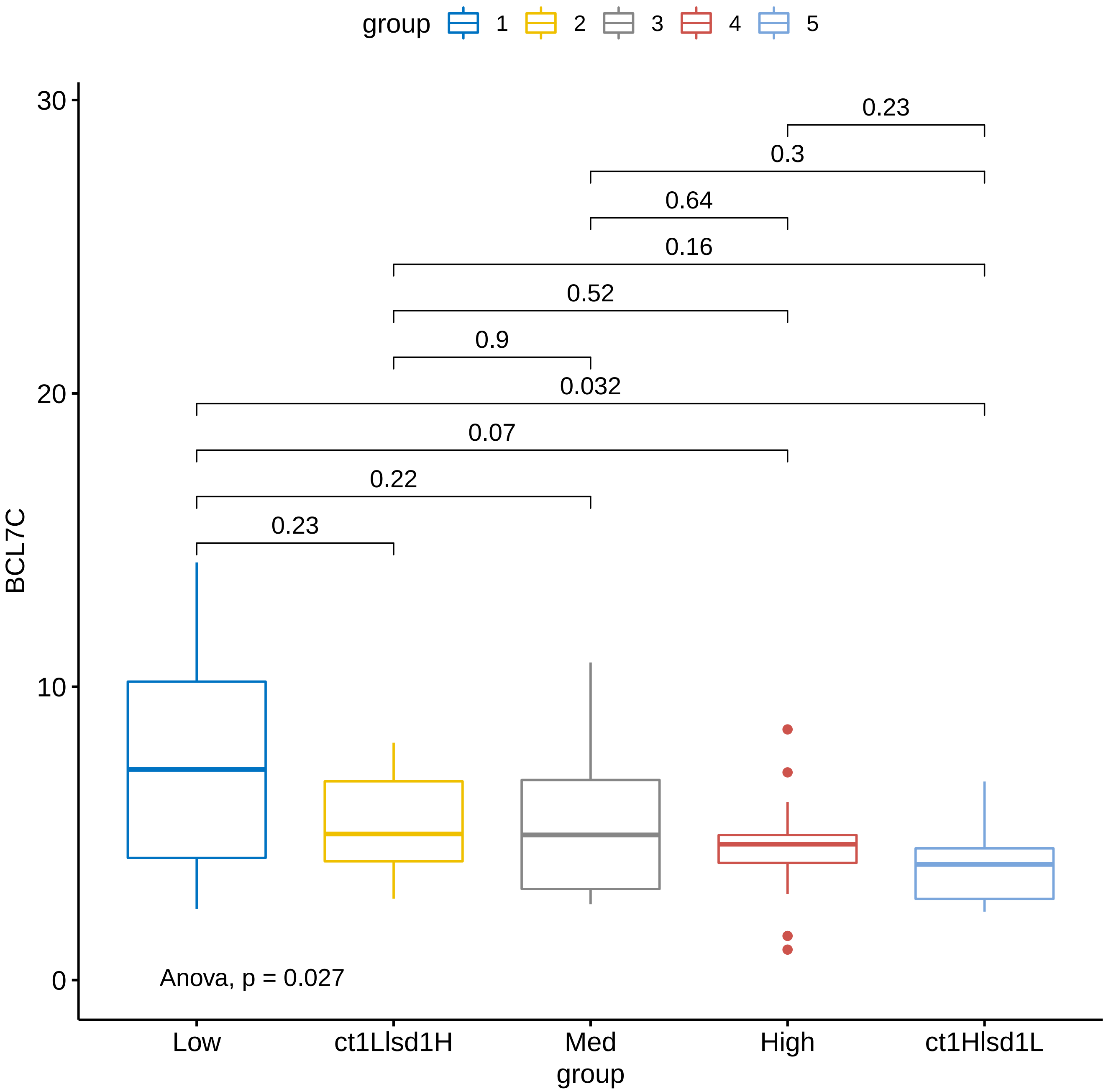

group 1 2 3 4 5
BCL7C
30
20
10
0
0.23
0.3
0.64
0.16
0.52
0.9
0.032
0.07
0.22
0.23
Anova, p = 0.027
Low
ct1Llsd1H
Med
High
ct1Hlsd1L
group

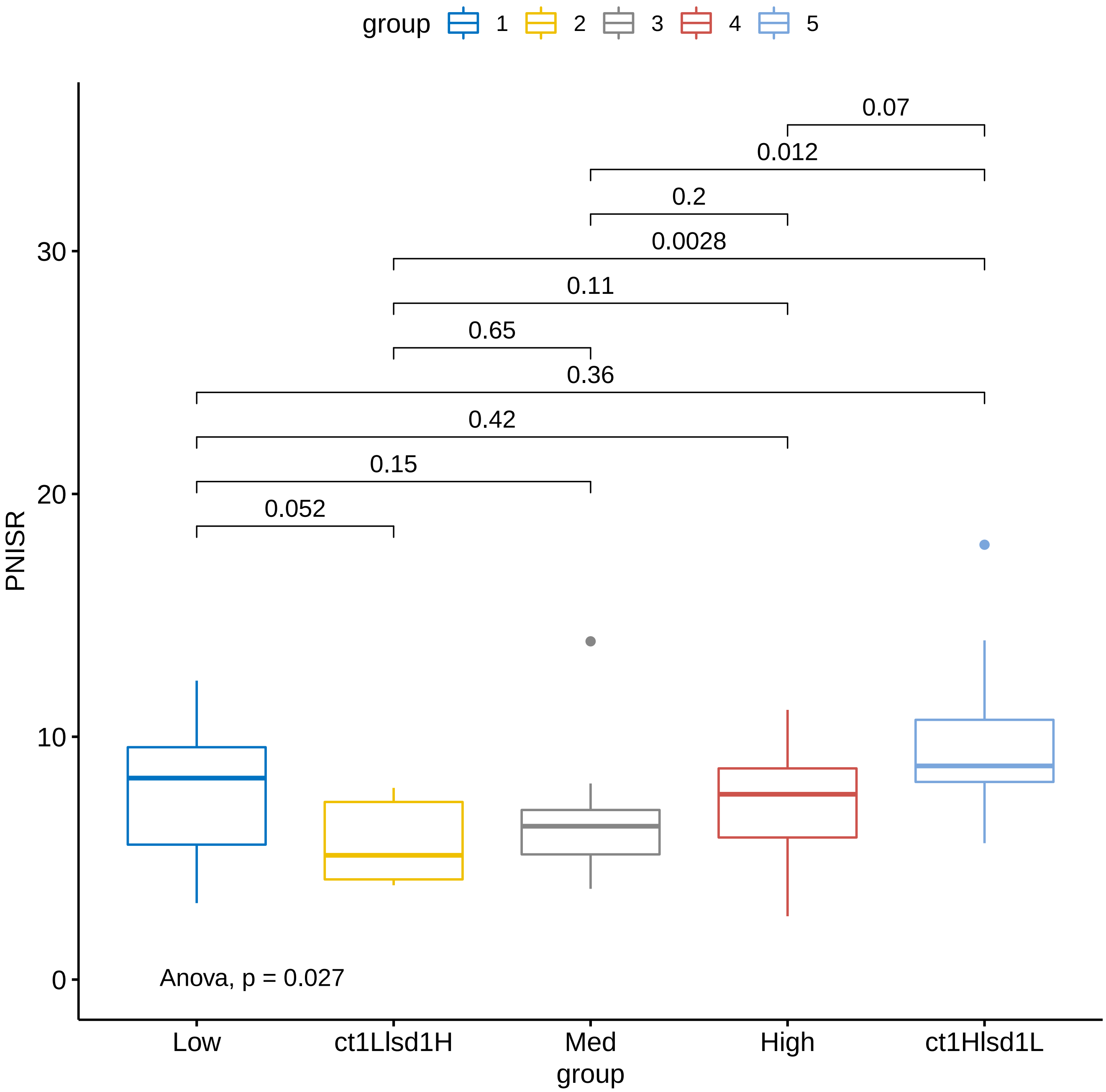
group
1
2
3
4
5
0.07
0.012
0.2
0.0028
0.11
0.65
0.36
0.42
0.15
0.052
30
20
10
0
PNISR
Anova, p = 0.027
Low
ct1Llsd1H
Med
High
ct1Hlsd1L
group

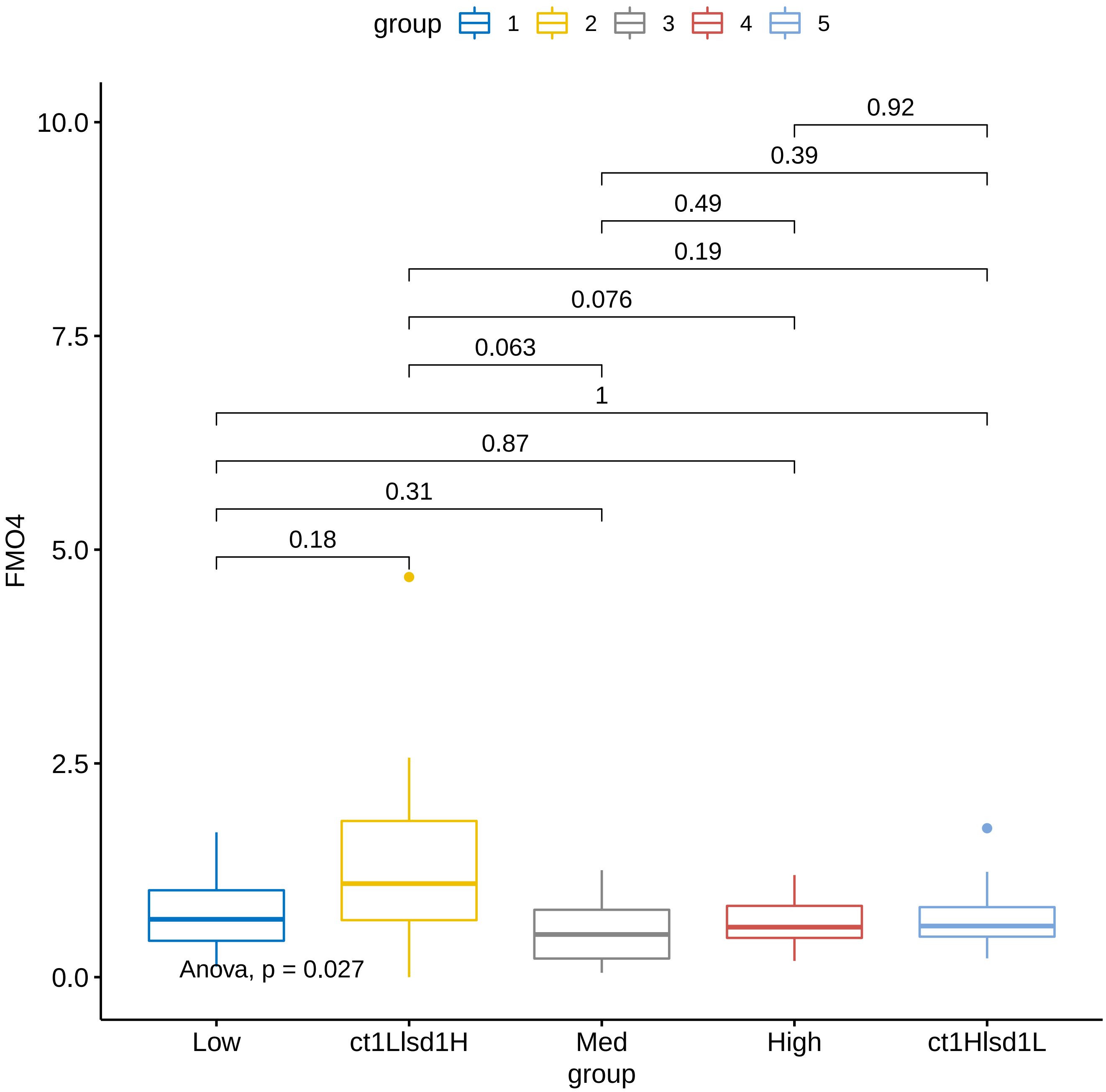

group
1
2
3
4
5
0.92
0.39
0.49
0.19
0.076
0.063
1
0.87
0.31
0.18
Anova, p = 0.027
10.0
7.5
5.0
2.5
0.0
FMO4
Low
ct1Llsd1H
Med
High
ct1Hlsd1L
group

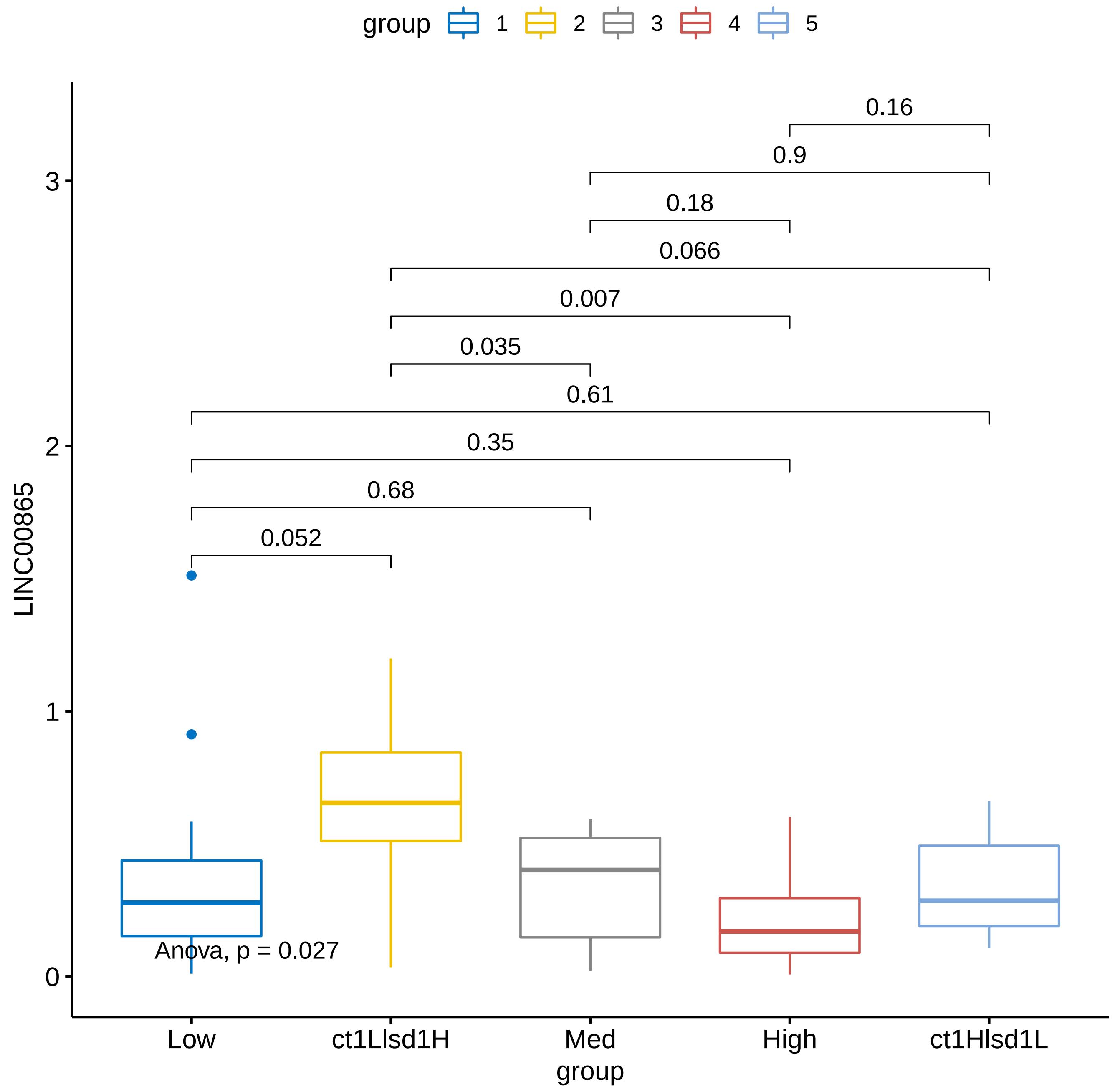

group
1
2
3
4
5
0.16
0.9
0.18
0.066
0.007
0.035
0.61
0.35
0.68
0.052
Anova, p = 0.027
LINC00865
0
1
2
3
Low
ct1Llsd1H
Med
High
ct1Hlsd1L
group

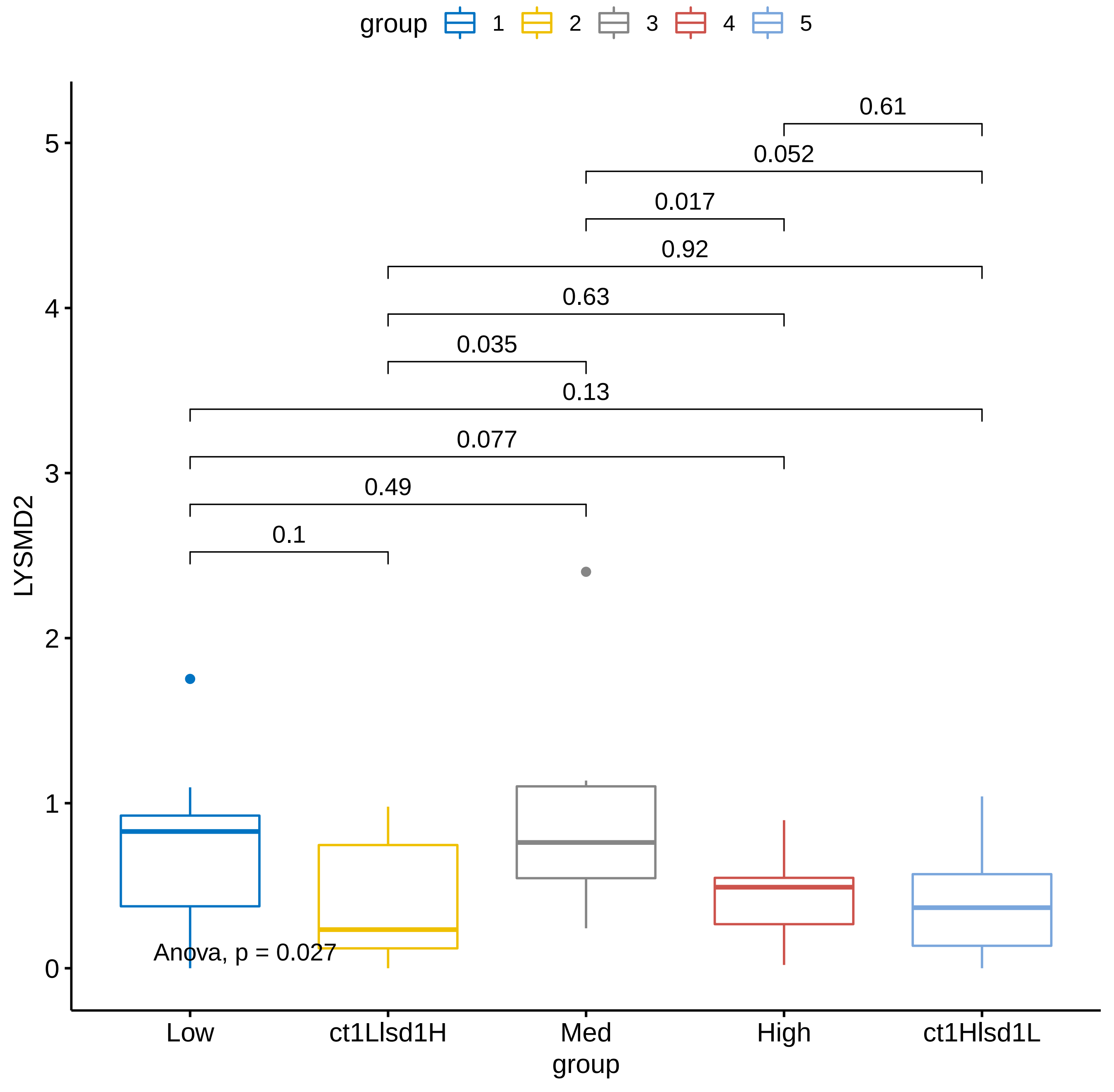

group 1 2 3 4 5
0.61
0.052
0.017
0.92
0.63
0.035
0.13
0.077
0.49
0.1
LYSMD2
5
4
3
2
1
0
Anova, p = 0.027
Low
ct1Llsd1H
Med
High
ct1Hlsd1L
group

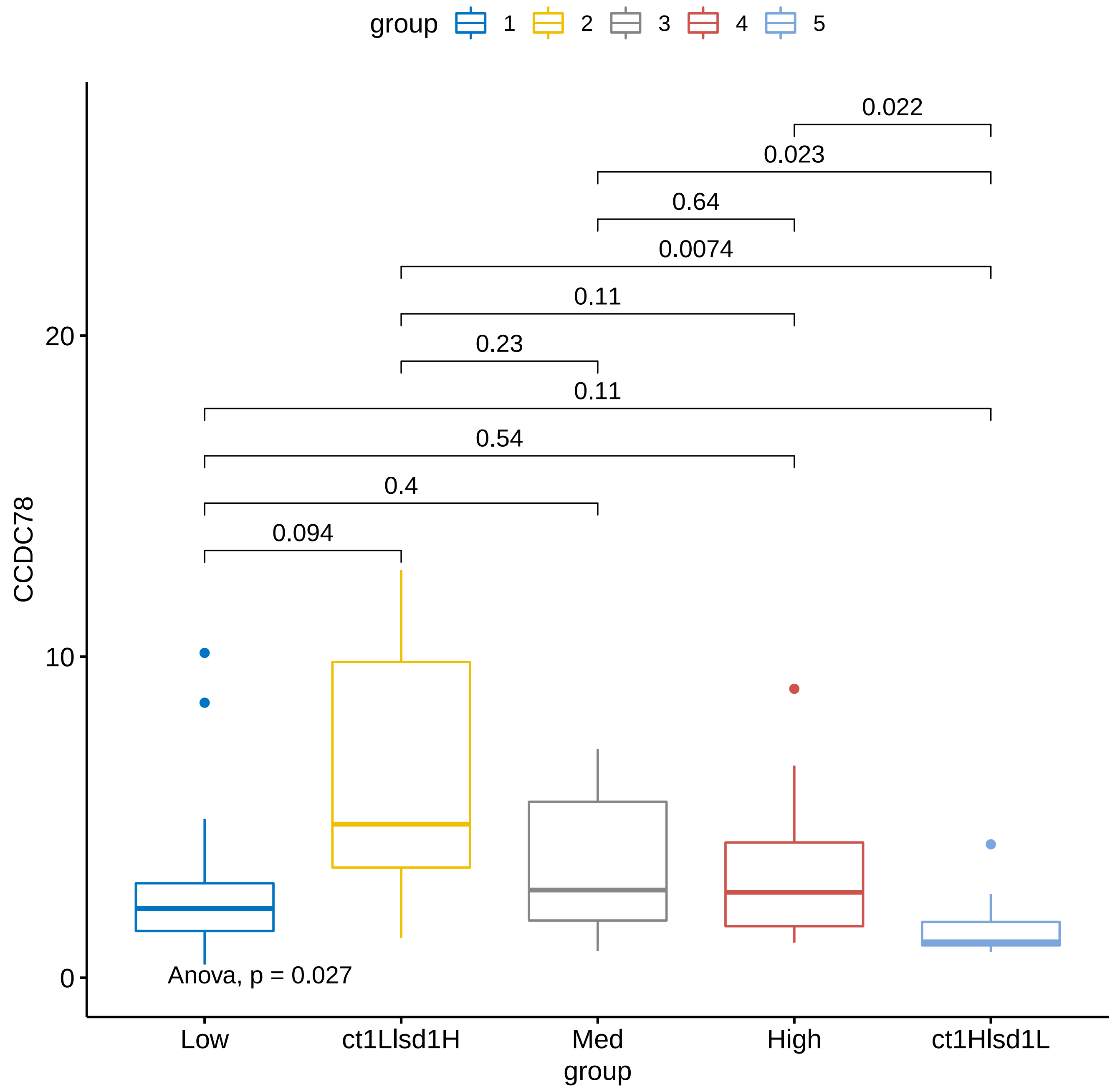

group 1 2 3 4 5
0.022
0.023
0.64
0.0074
0.11
0.23
0.11
0.54
0.4
0.094
Anova, p = 0.027
CCDC78
20
10
0
Low
ct1Llsd1H
Med
High
ct1Hlsd1L
group

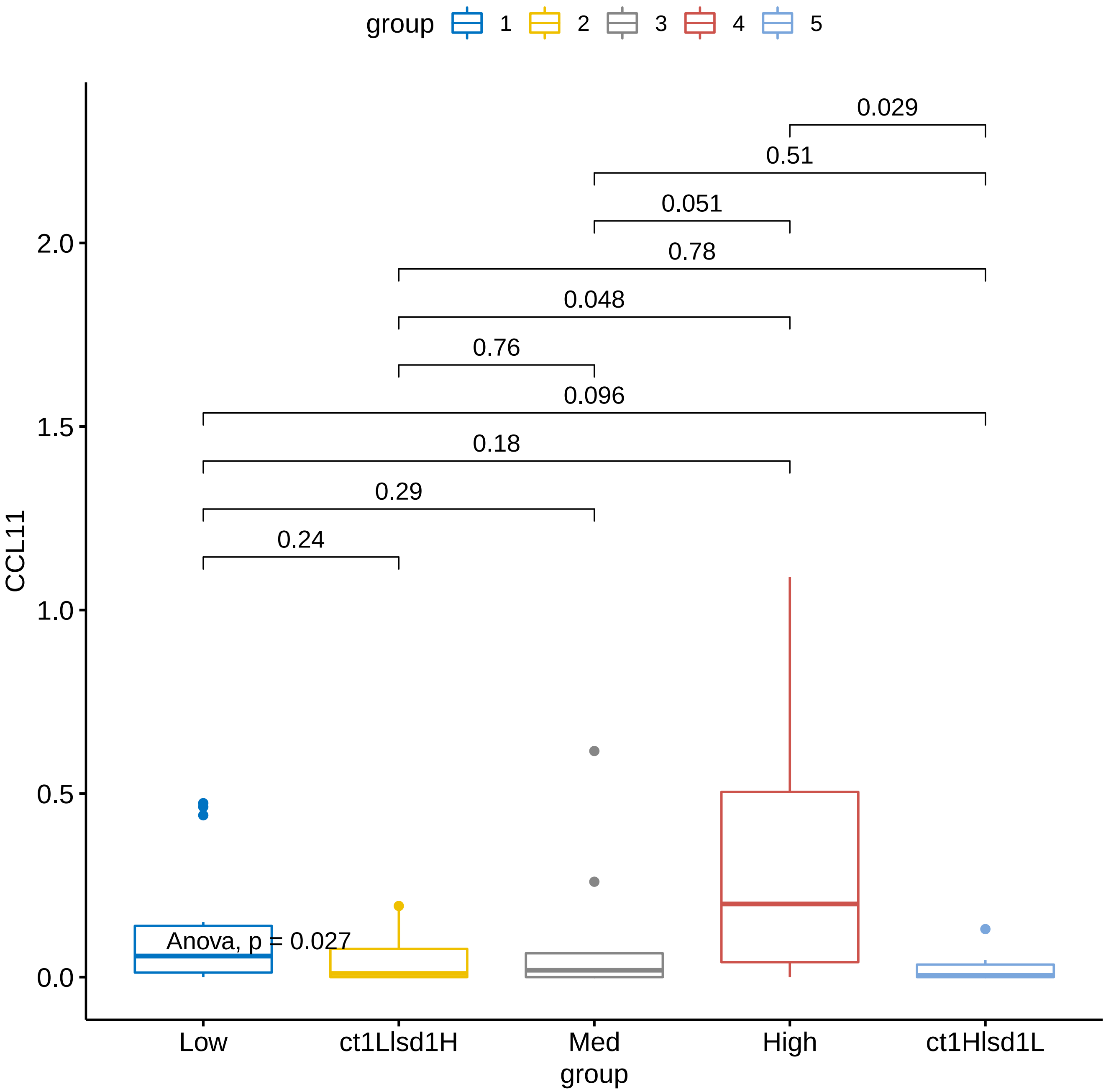

group
1
2
3
4
5
0.029
0.51
0.051
0.78
0.048
0.76
0.096
0.18
0.29
0.24
Anova, p = 0.027
CCL11
2.0
1.5
1.0
0.5
0.0
Low
ct1Llsd1H
Med
High
ct1Hlsd1L
group

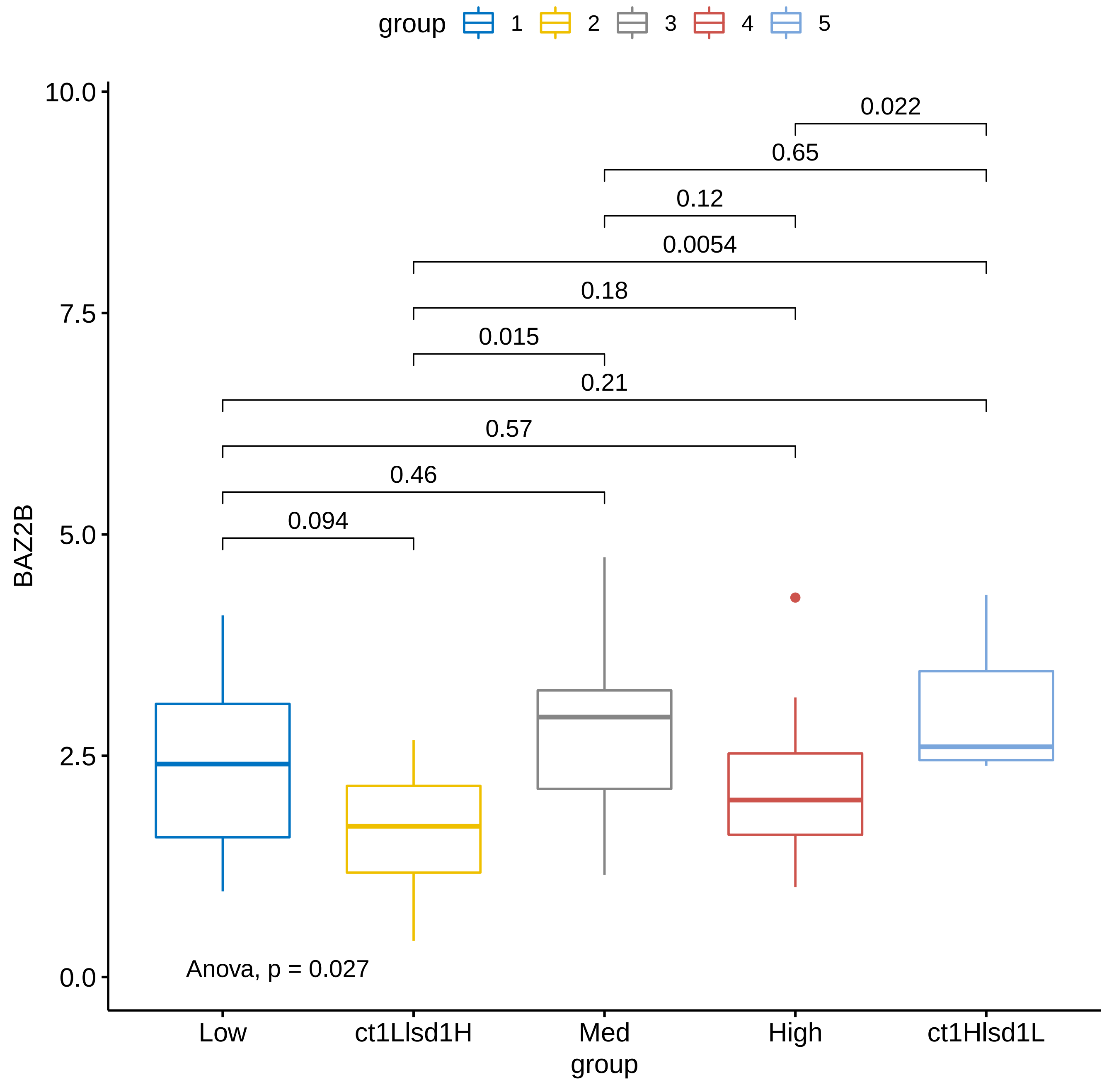
group 1 2 3 4 5
10.0
7.5
5.0
2.5
0.0
BAZ2B
0.022
0.65
0.12
0.0054
0.18
0.015
0.21
0.57
0.46
0.094
Anova, p = 0.027
Low
ct1Llsd1H
Med
High
ct1Hlsd1L
group

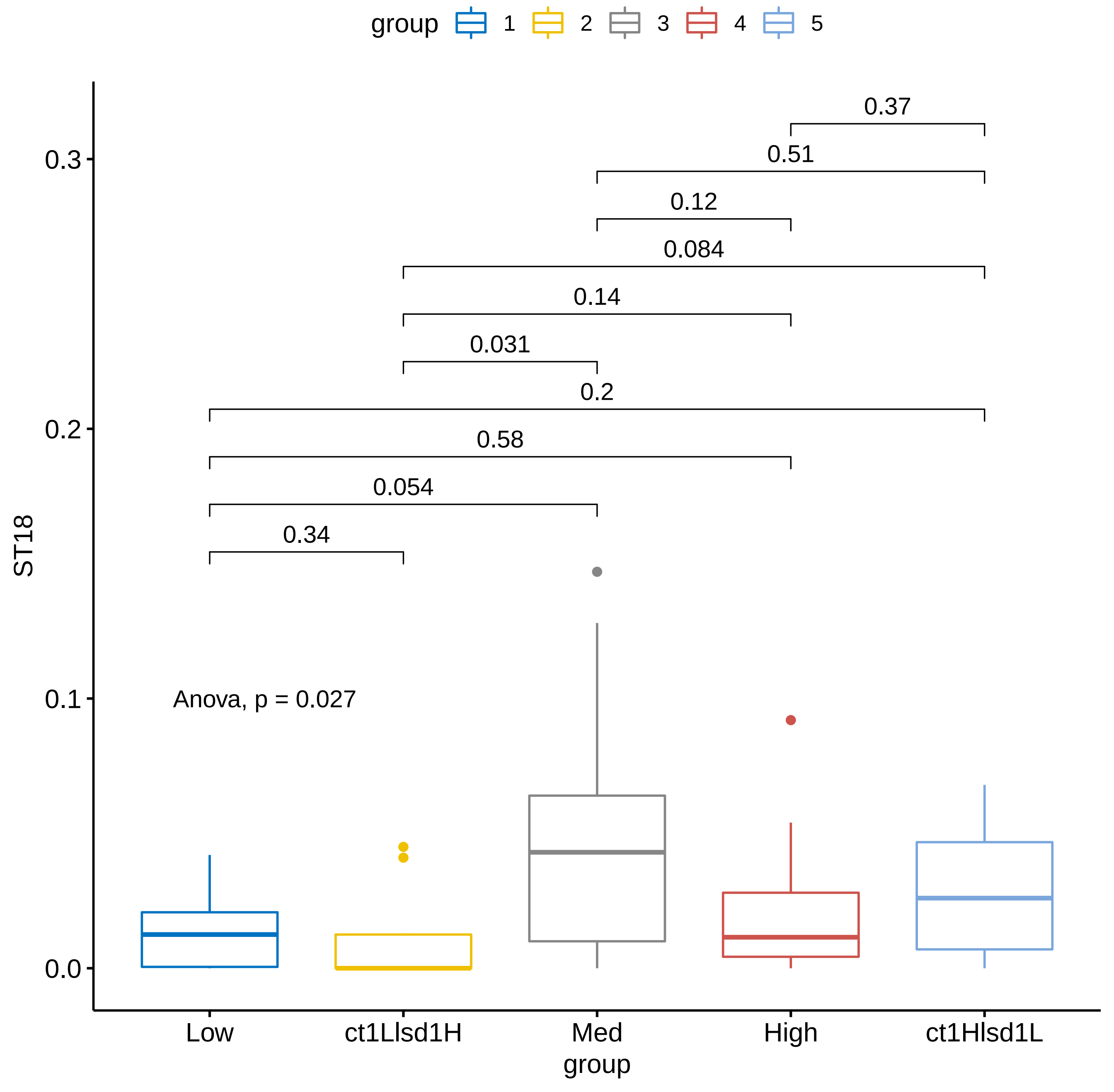

group 1 2 3 4 5
0.37
0.51
0.12
0.084
0.14
0.031
0.2
0.58
0.054
0.34
Anova, p = 0.027
ST18
0.3
0.2
0.1
0.0
Low
ct1Llsd1H
Med
High
ct1Hlsd1L
group

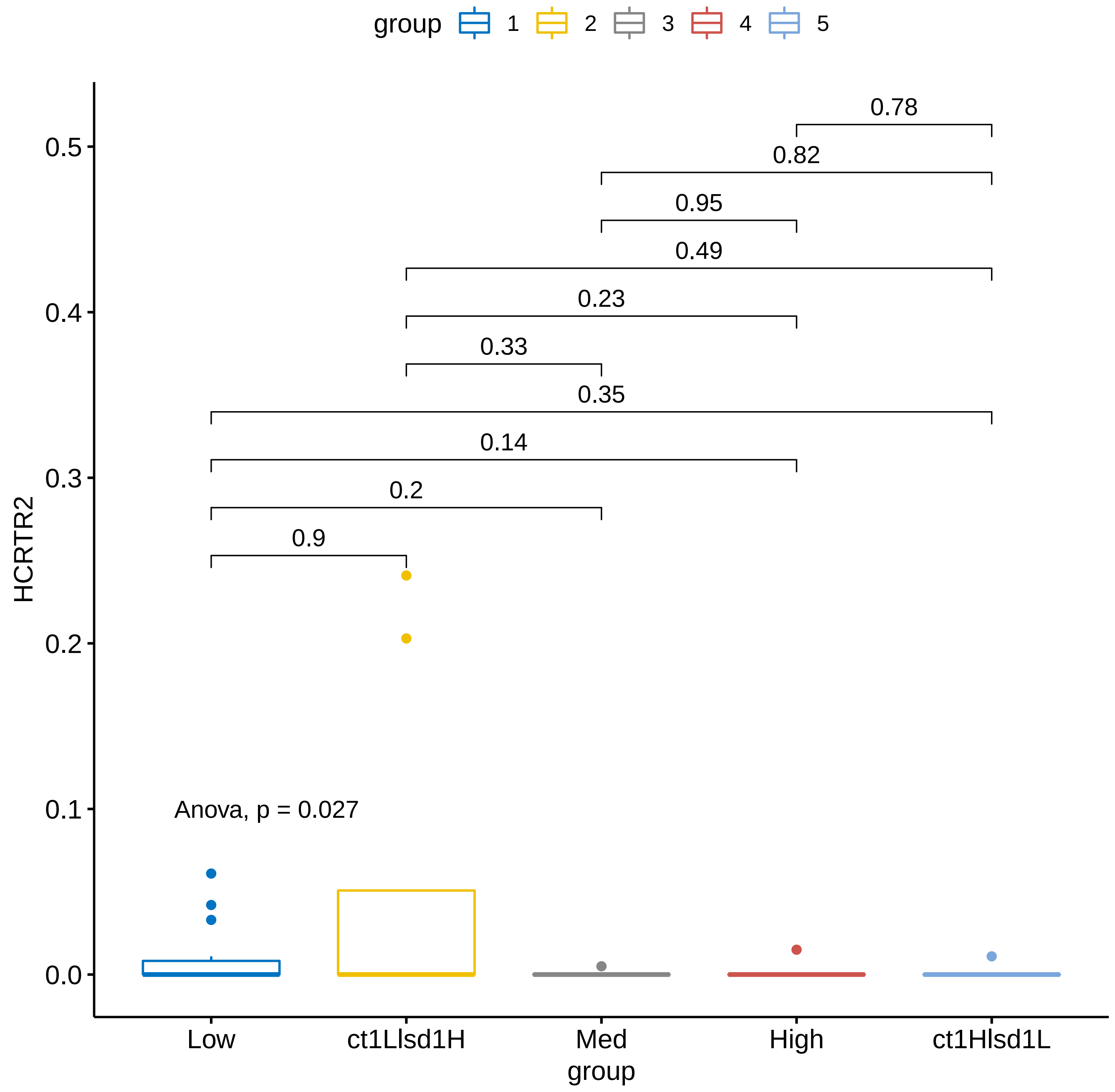

group 1 2 3 4 5
0.78
0.82
0.95
0.49
0.23
0.33
0.35
0.14
0.2
0.9
Anova, p = 0.027
HCRTR2
0.0
0.1
0.2
0.3
0.4
0.5
Low
ct1Llsd1H
Med
High
ct1Hlsd1L
group

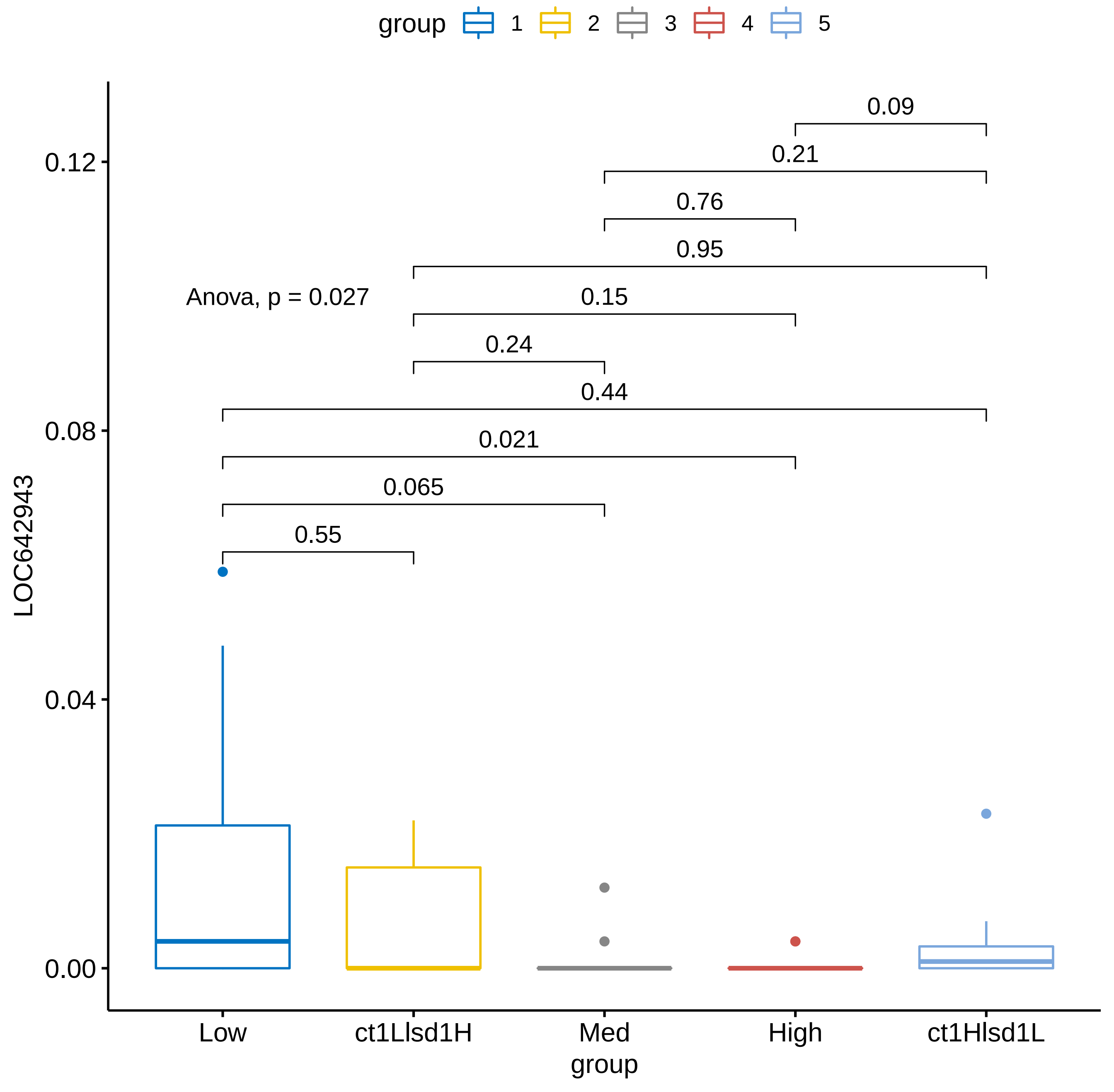

group
1
2
3
4
5
0.09
0.21
0.76
0.95
Anova, p = 0.027
0.15
0.24
0.44
0.021
0.065
0.55
0.12
0.08
0.04
0.00
LOC642943
Low
ct1Llsd1H
Med
High
ct1Hlsd1L
group

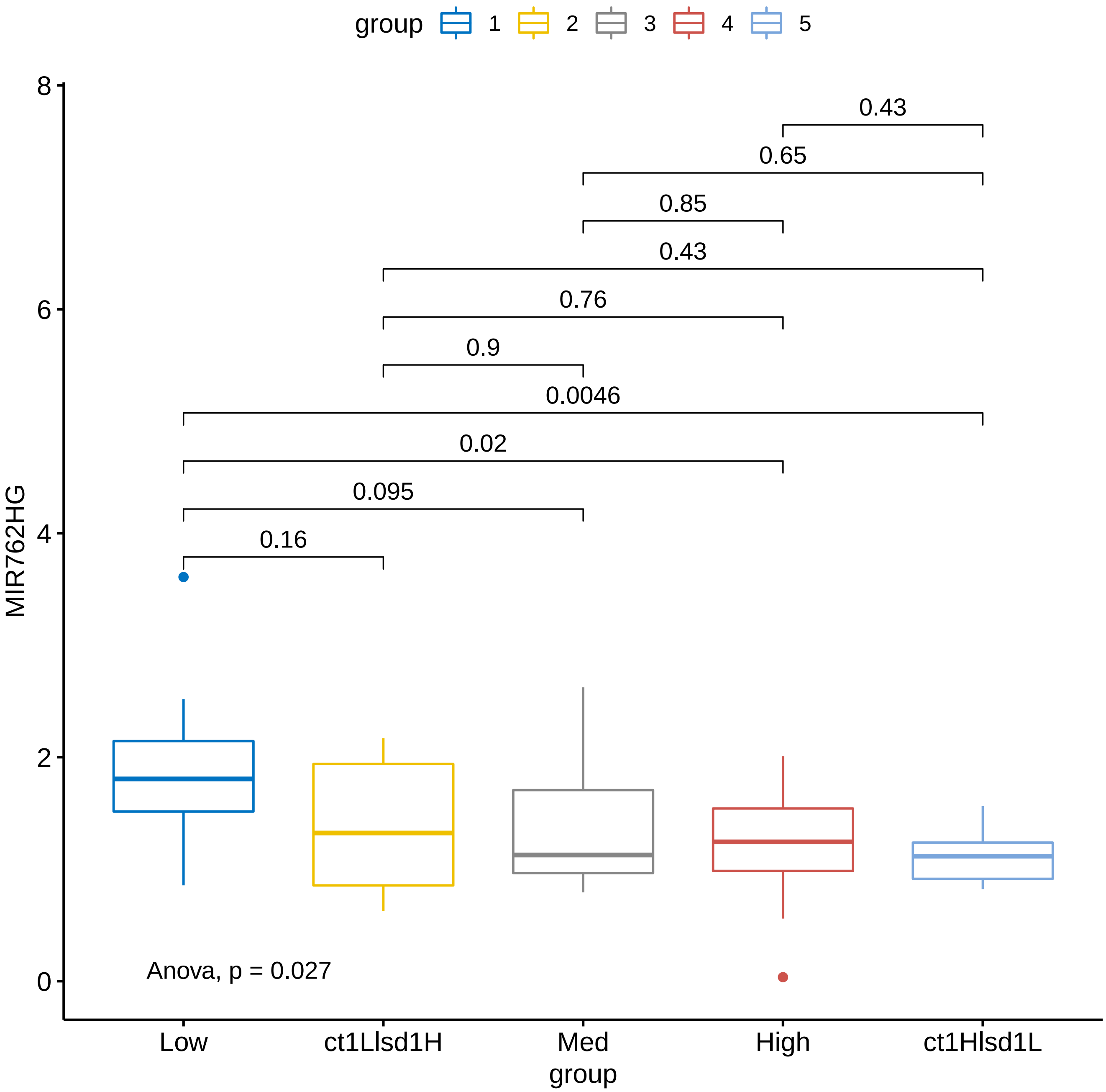

group
1
2
3
4
5
MIR762HG
8
6
4
2
0
0.43
0.65
0.85
0.43
0.76
0.9
0.0046
0.02
0.095
0.16
Anova, p = 0.027
Low
ct1Llsd1H
Med
High
ct1Hlsd1L
group

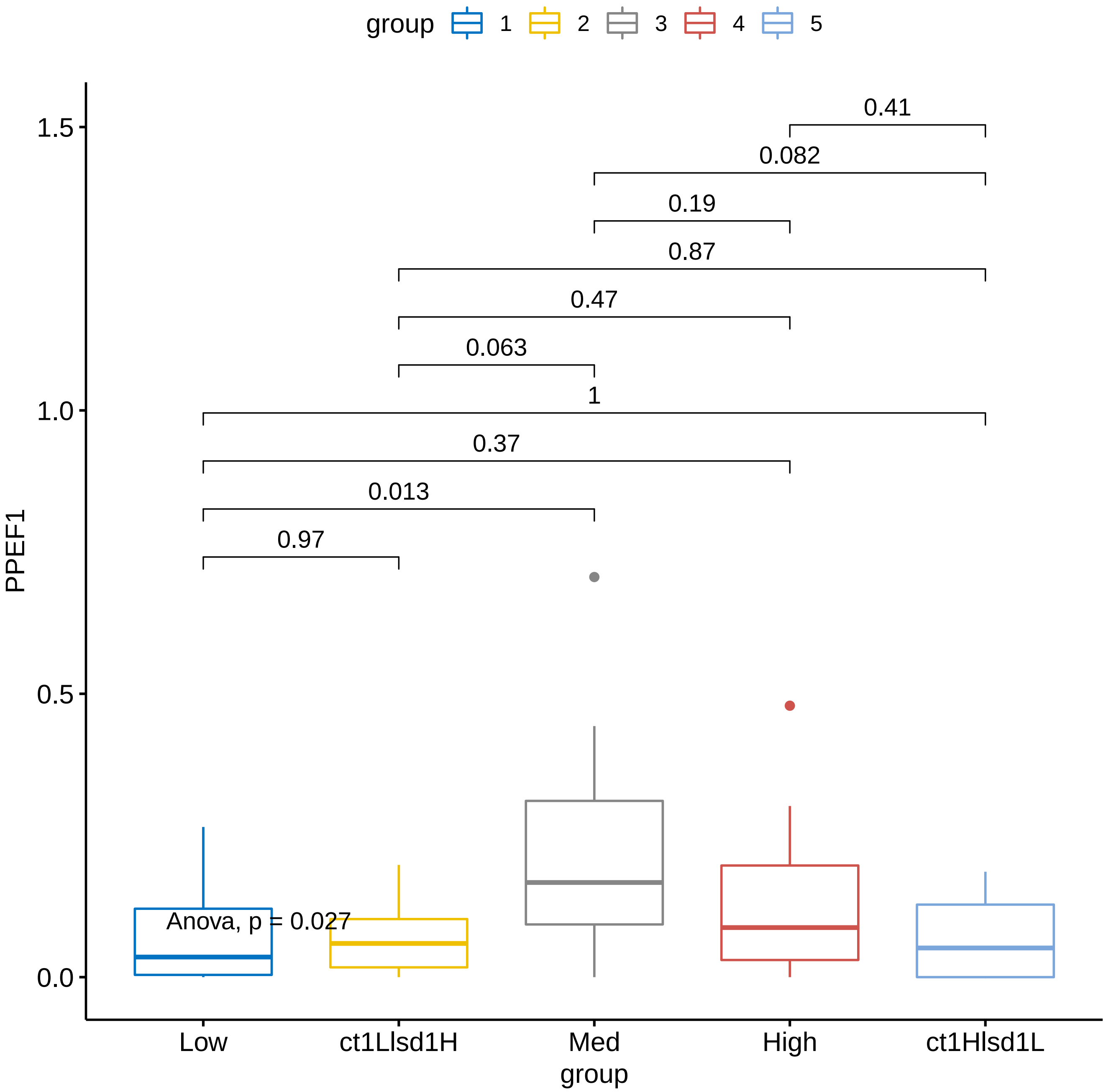

group 1 2 3 4 5
0.41
0.082
0.19
0.87
0.47
0.063
1
0.37
0.013
0.97
Anova, p = 0.027
PPEF1
1.5
1.0
0.5
0.0
Low
ct1Llsd1H
Med
High
ct1Hlsd1L
group

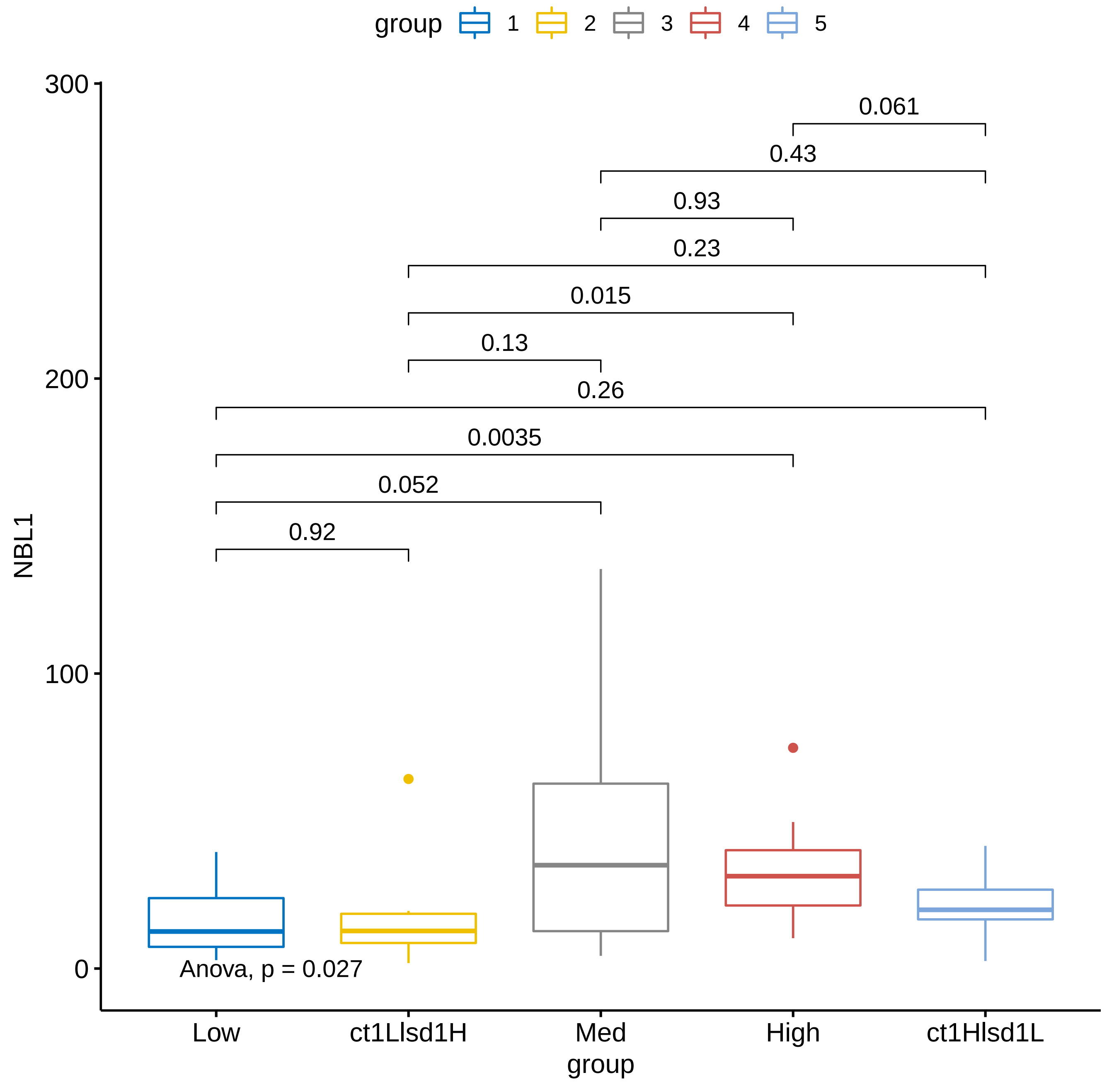
group
1
2
3
4
5
300
200
100
0
NBL1
0.061
0.43
0.93
0.23
0.015
0.13
0.26
0.0035
0.052
0.92
Anova, p = 0.027
Low
ct1Llsd1H
Med
High
ct1Hlsd1L
group

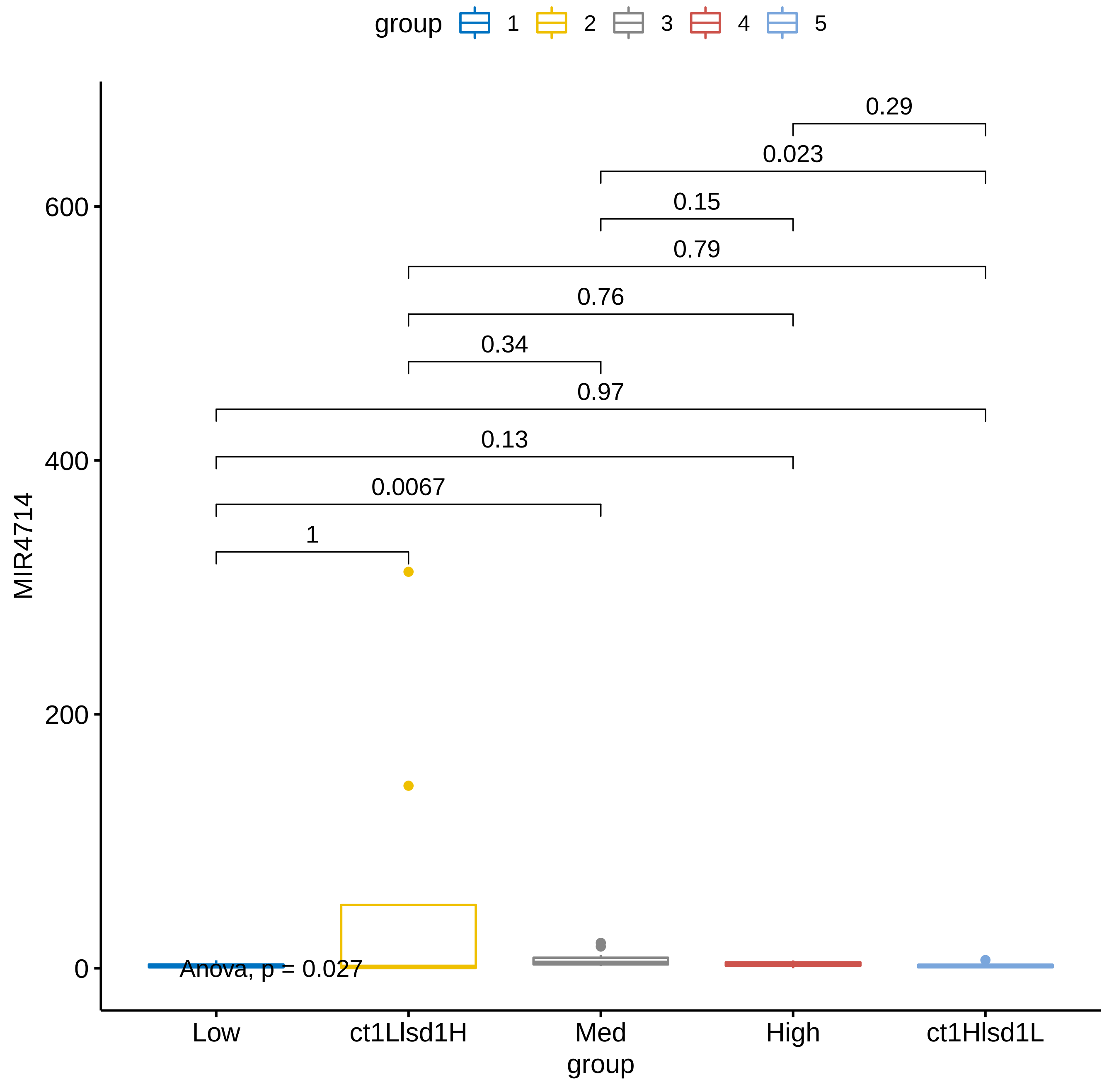
group
1
2
3
4
5
0.29
0.023
0.15
0.79
0.76
0.34
0.97
0.13
0.0067
1
600
400
200
0
MIR4714
Anova, p = 0.027
Low
ct1Llsd1H
Med
High
ct1Hlsd1L
group

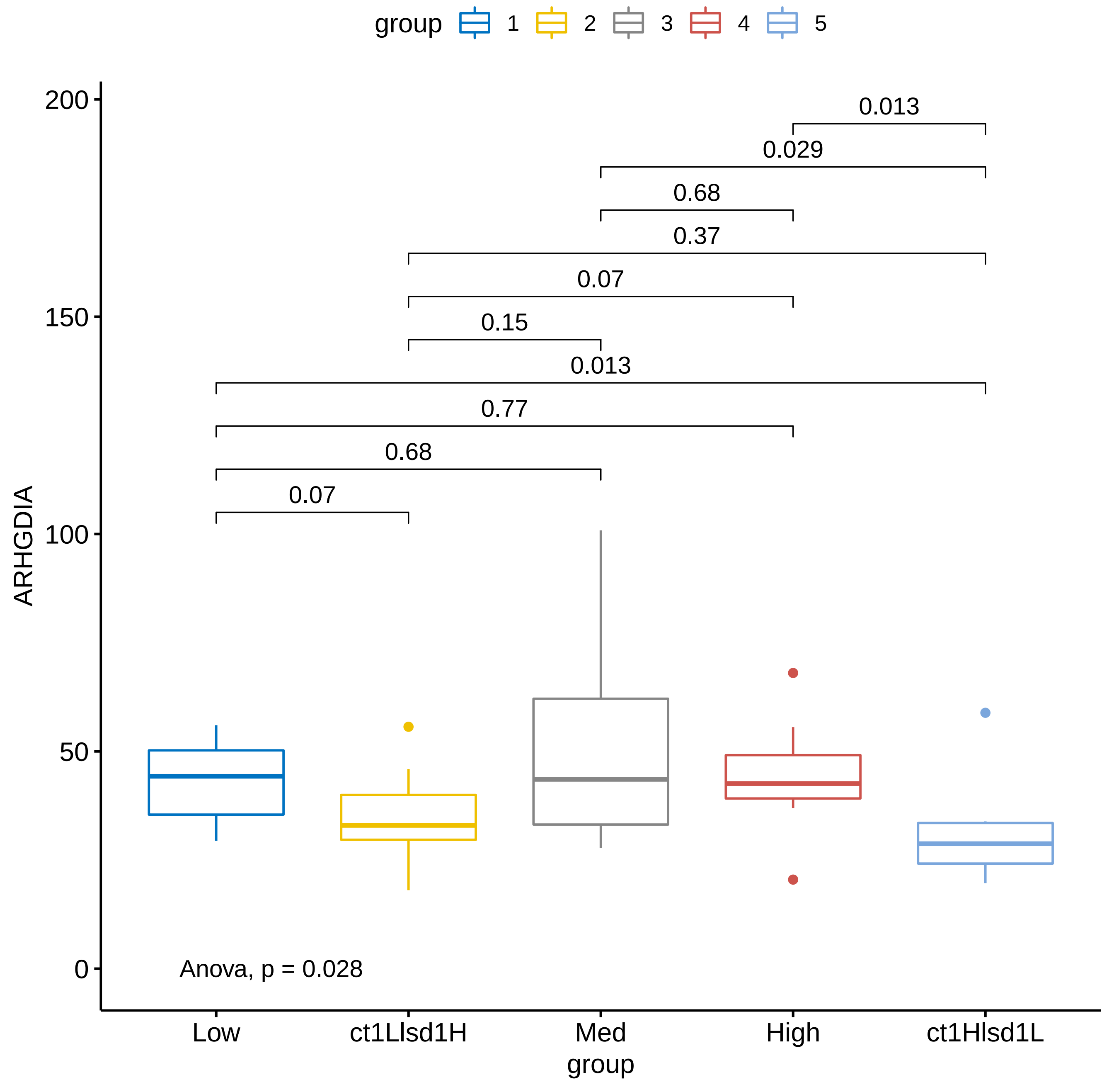

group
1
2
3
4
5
0.013
0.029
0.68
0.37
0.07
0.15
0.013
0.77
0.68
0.07
Anova, p = 0.028
200
150
100
50
0
ARHGDIA
Low
ct1Llsd1H
Med
High
ct1Hlsd1L
group

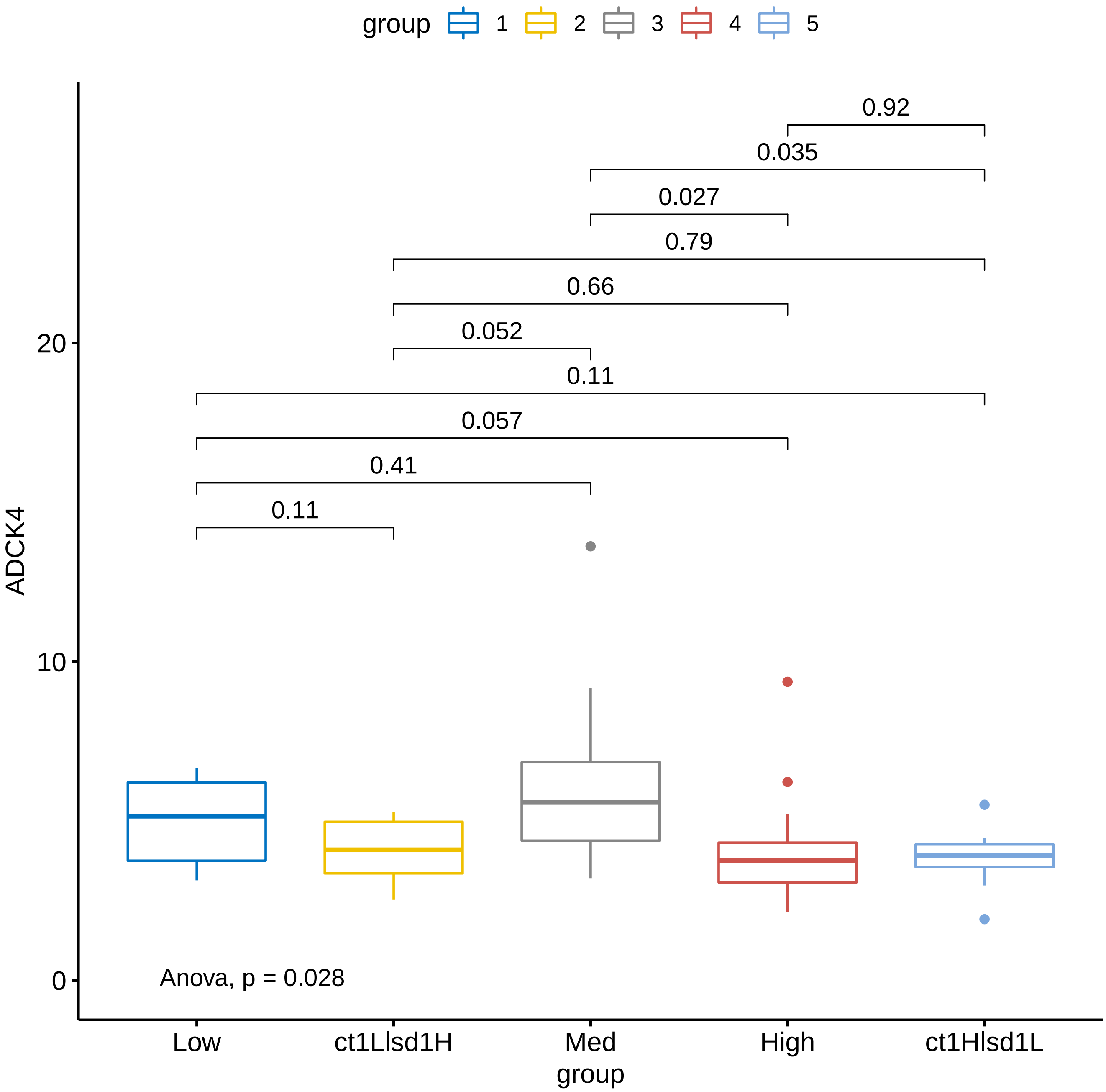
group
1
2
3
4
5
0.92
0.035
0.027
0.79
0.66
0.052
0.11
0.057
0.41
0.11
20
10
0
ADCK4
Anova, p = 0.028
Low
ct1Llsd1H
Med
High
ct1Hlsd1L
group

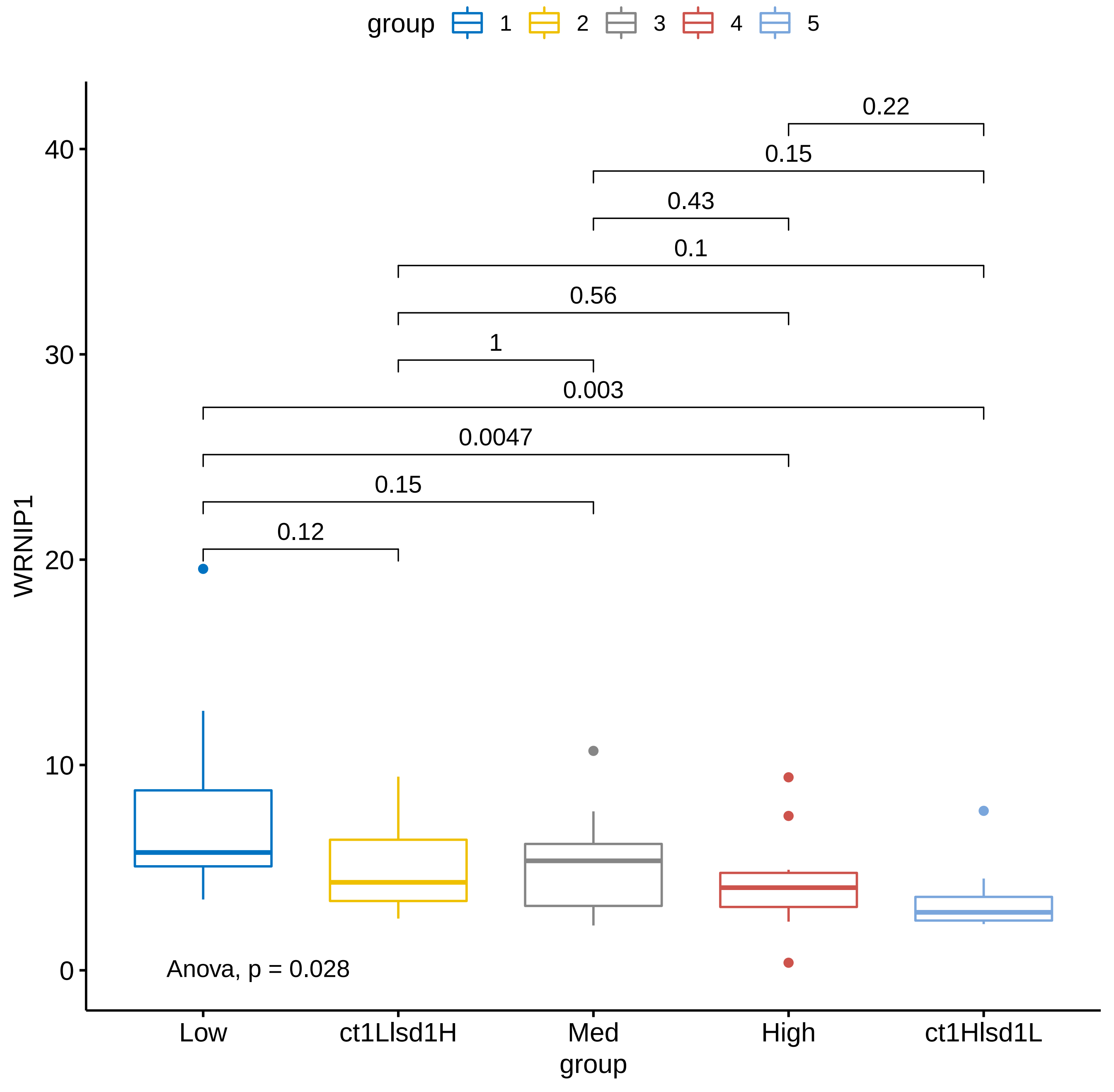

group 1 2 3 4 5
0.22
0.15
0.43
0.1
0.56
1
0.003
0.0047
0.15
0.12
40
30
20
10
0
WRNIP1
Anova, p = 0.028
Low
ct1Llsd1H
Med
High
ct1Hlsd1L
group

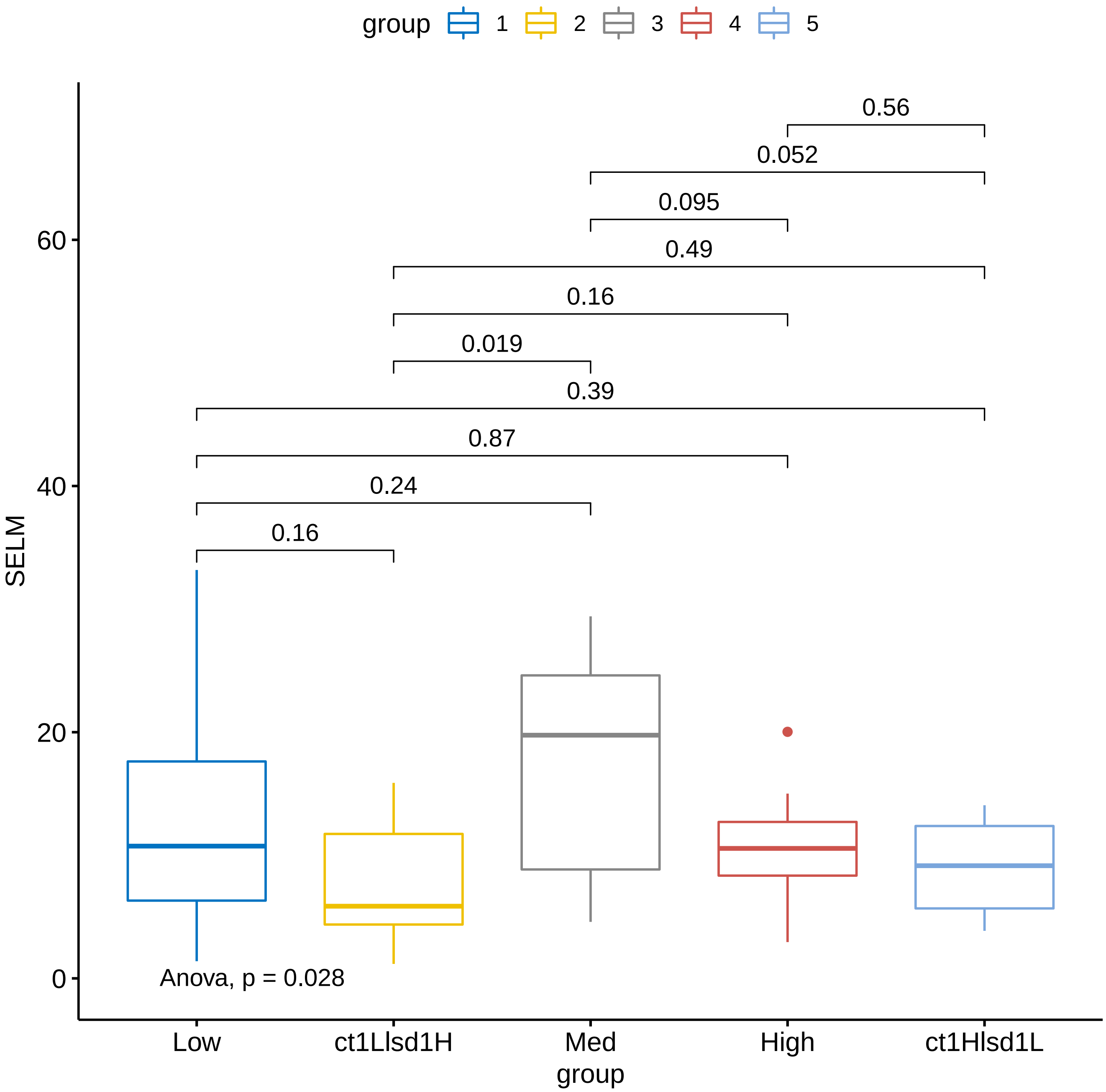
group 1 2 3 4 5
0.56
0.052
0.095
0.49
0.16
0.019
0.39
0.87
0.24
0.16
Anova, p = 0.028
SELM
60
40
20
0
Low
ct1Llsd1H
Med
High
ct1Hlsd1L
group

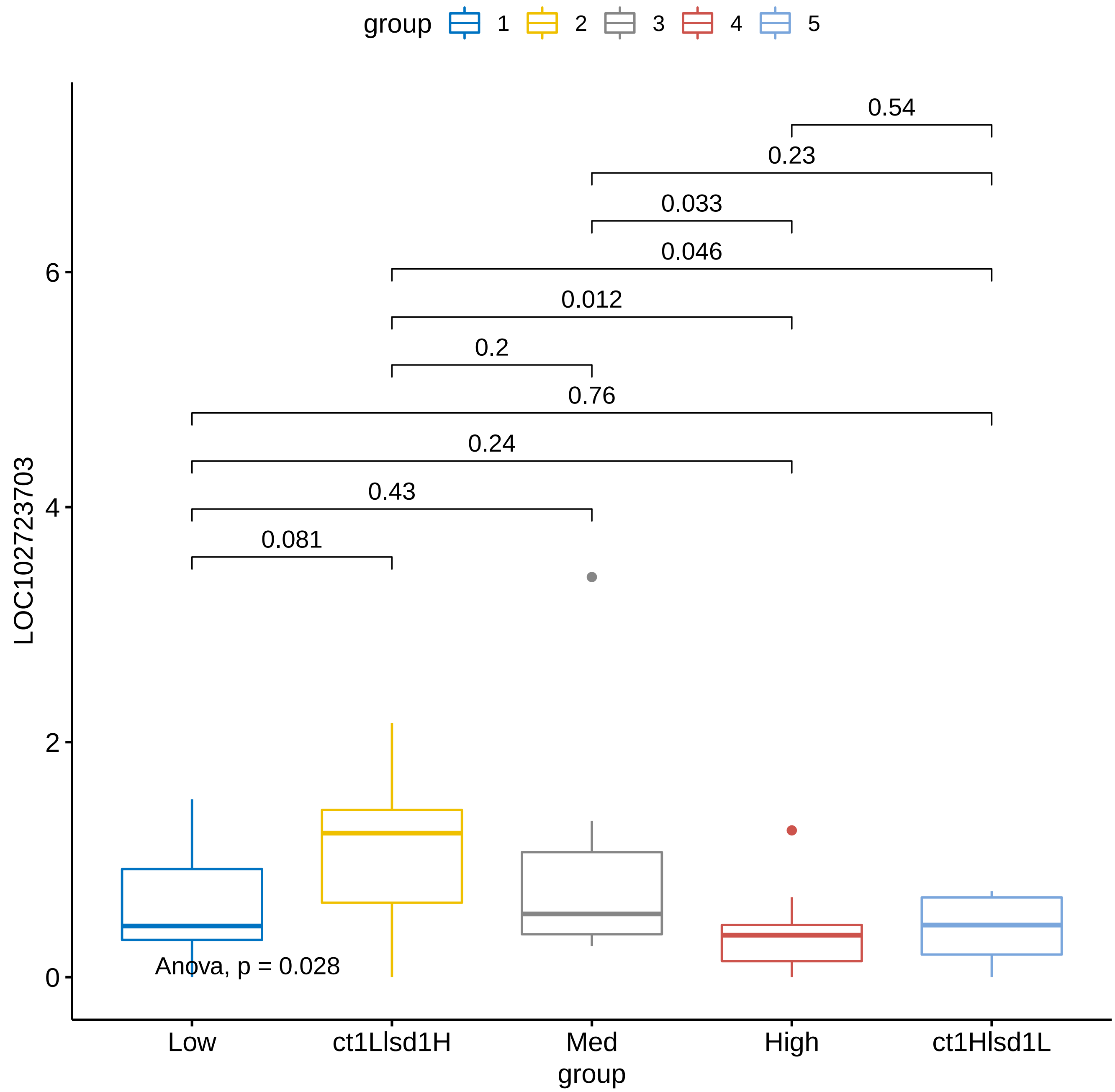

group 1 2 3 4 5
0.54
0.23
0.033
0.046
0.012
0.2
0.76
0.24
0.43
0.081
Anova, p = 0.028
LOC102723703
0
2
4
6
Low
ct1Llsd1H
Med
High
ct1Hlsd1L
group

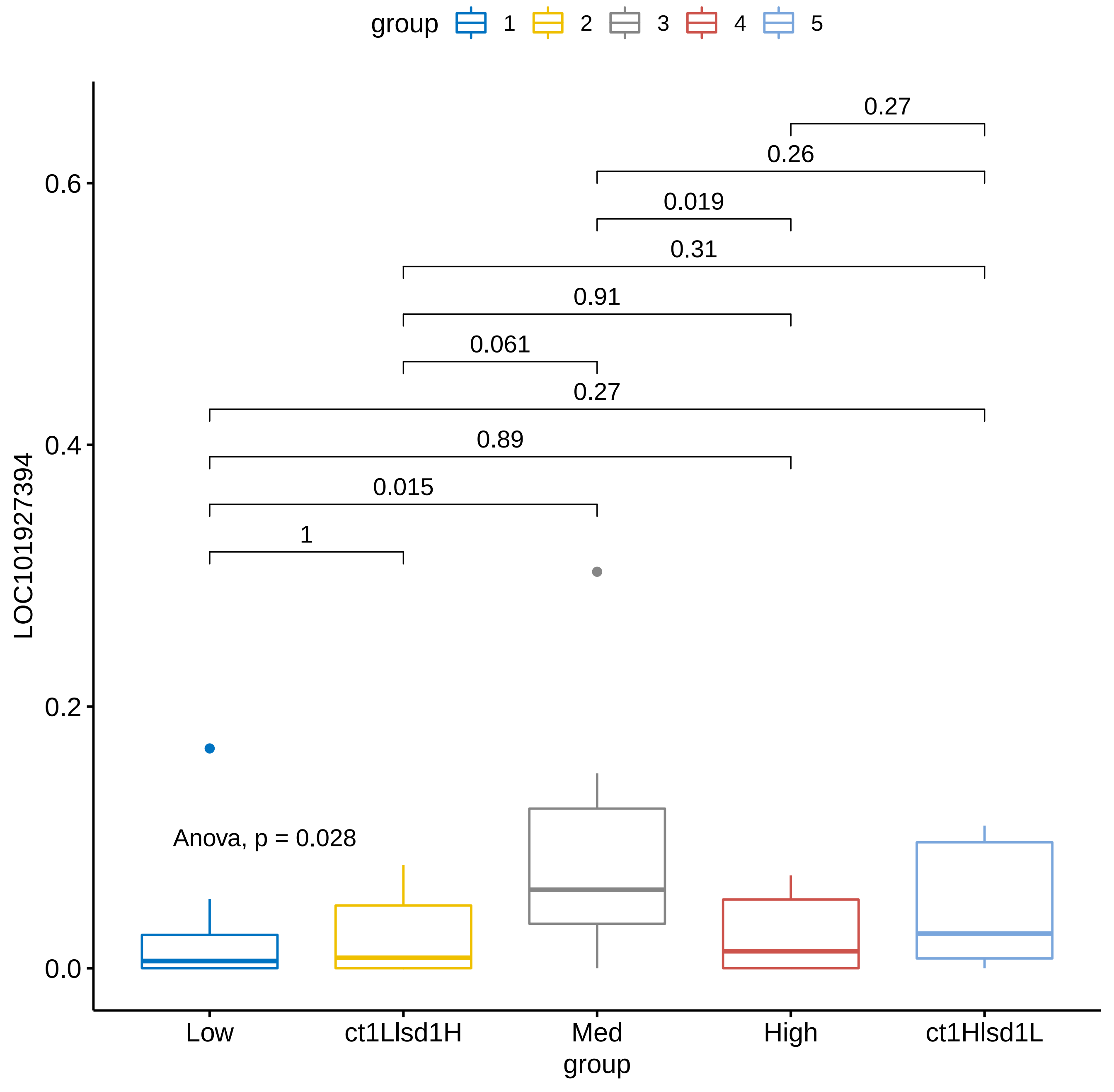
group
1
2
3
4
5
0.27
0.26
0.019
0.31
0.91
0.061
0.27
0.89
0.015
1
Anova, p = 0.028
LOC101927394
0.6
0.4
0.2
0.0
Low
ct1Llsd1H
Med
High
ct1Hlsd1L
group

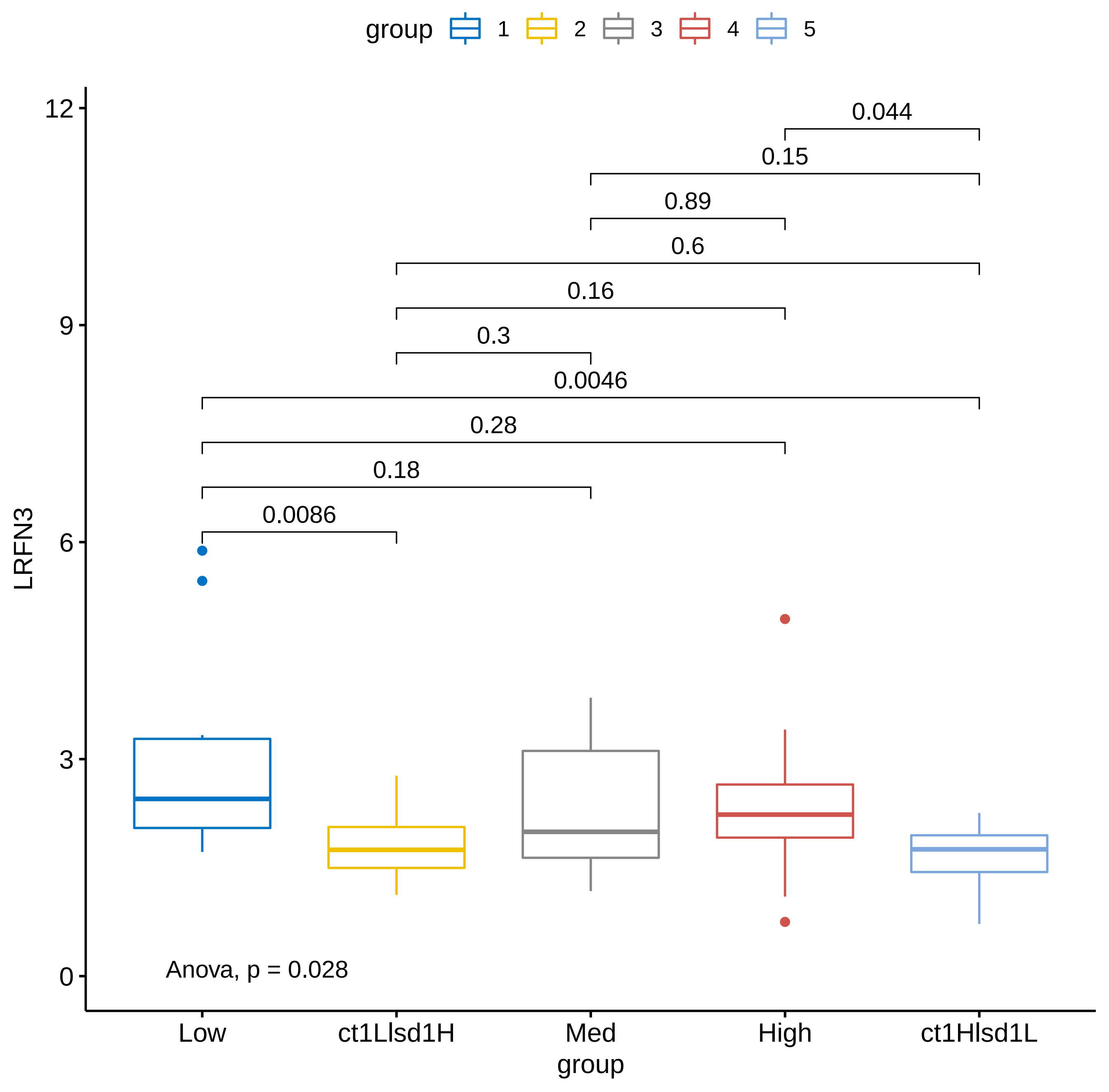

group
1
2
3
4
5
12
9
6
3
0
LRFN3
0.044
0.15
0.89
0.6
0.16
0.3
0.0046
0.28
0.18
0.0086
Anova, p = 0.028
Low
ct1Llsd1H
Med
High
ct1Hlsd1L
group

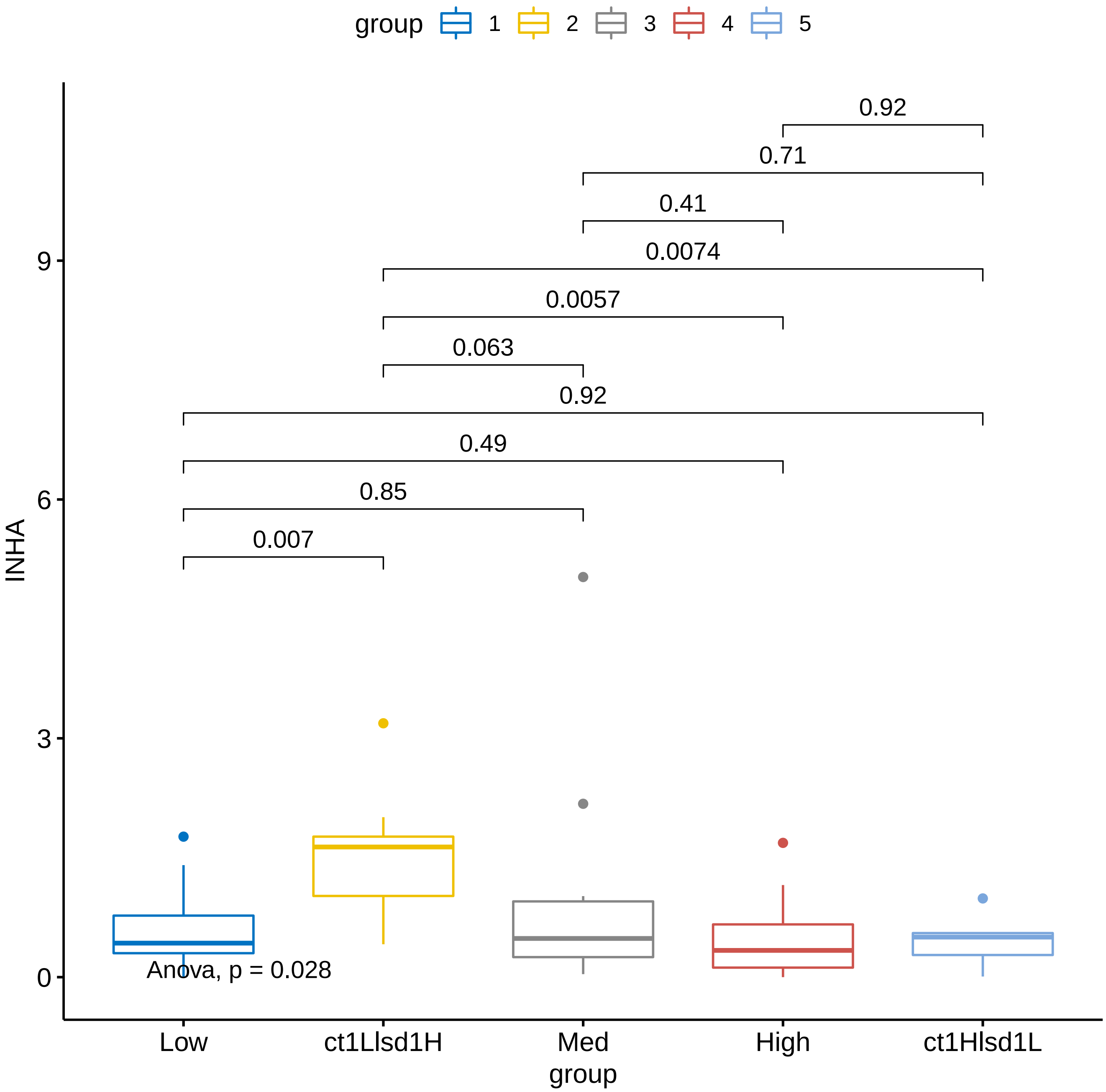

group 1 2 3 4 5
INHA
9
6
3
0
0.92
0.71
0.41
0.0074
0.0057
0.063
0.92
0.49
0.85
0.007
Anova, p = 0.028
Low
ct1Llsd1H
Med
High
ct1Hlsd1L
group

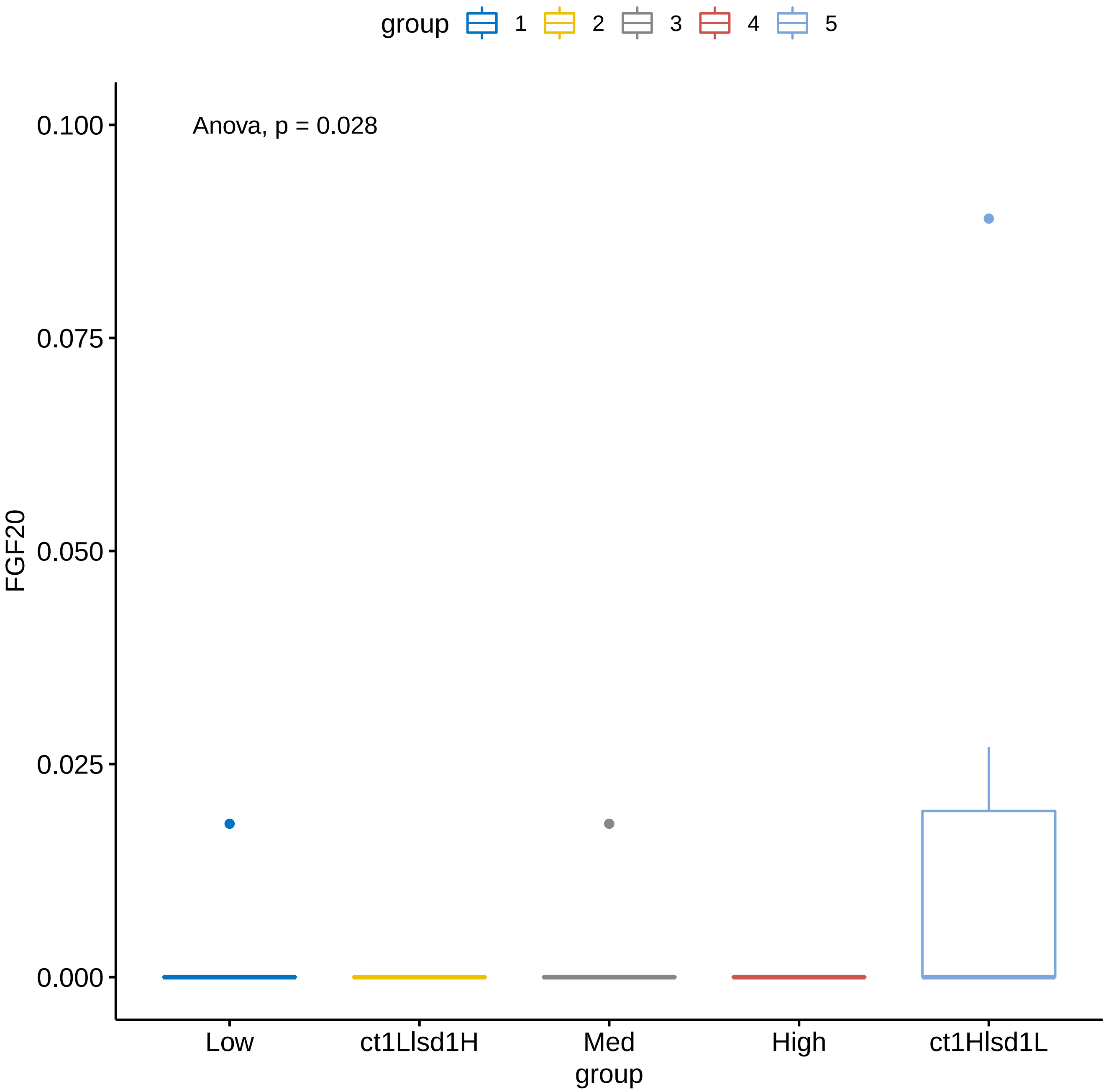

group
1
2
3
4
5
Anova, p = 0.028
0.100
0.075
0.050
0.025
0.000
FGF20
Low
ct1Llsd1H
Med
High
ct1Hlsd1L
group

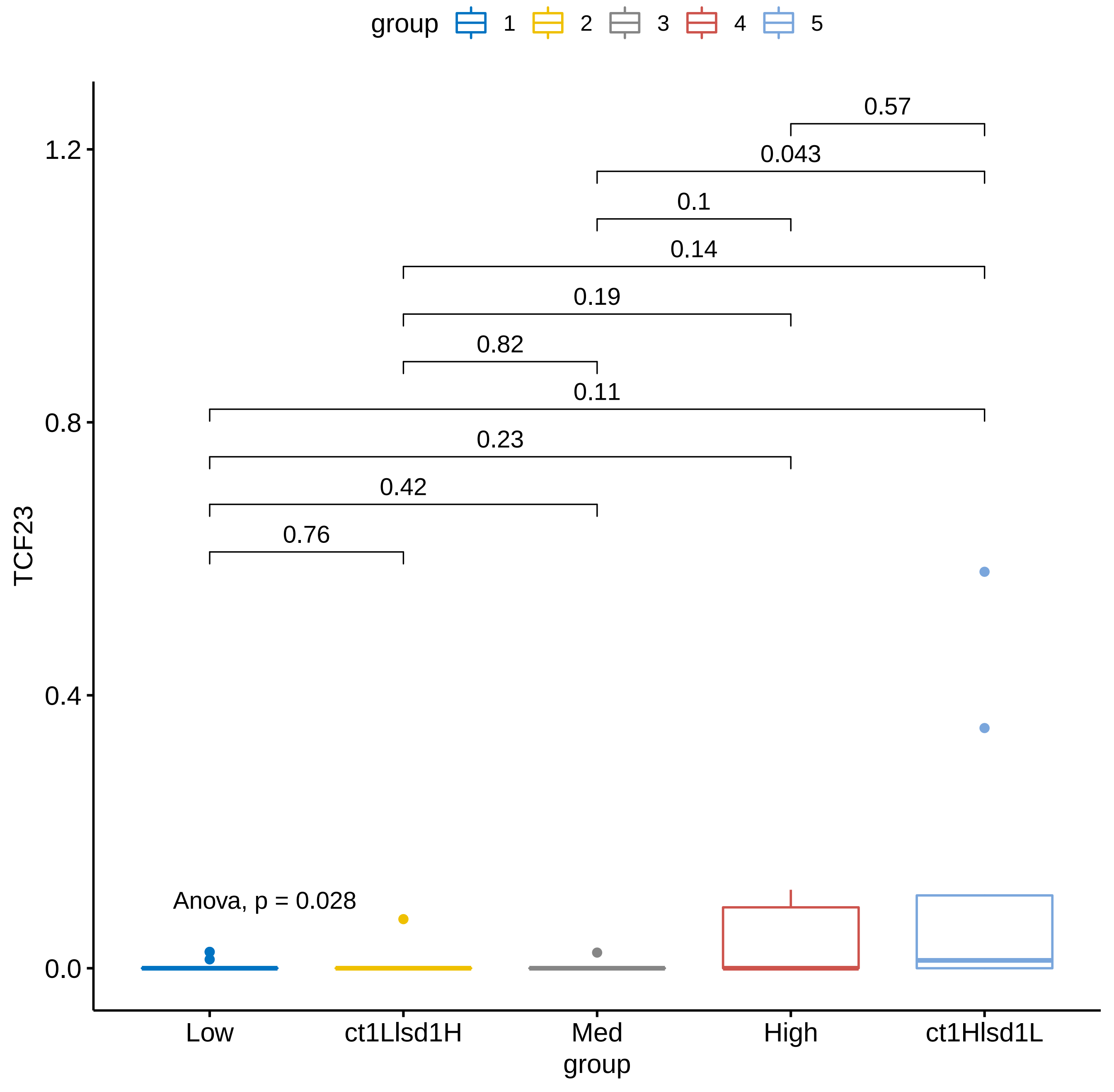

group 1 2 3 4 5
0.57
0.043
0.1
0.14
0.19
0.82
0.11
0.23
0.42
0.76
Anova, p = 0.028
1.2
0.8
0.4
0.0
TCF23
Low
ct1Llsd1H
Med
High
ct1Hlsd1L
group

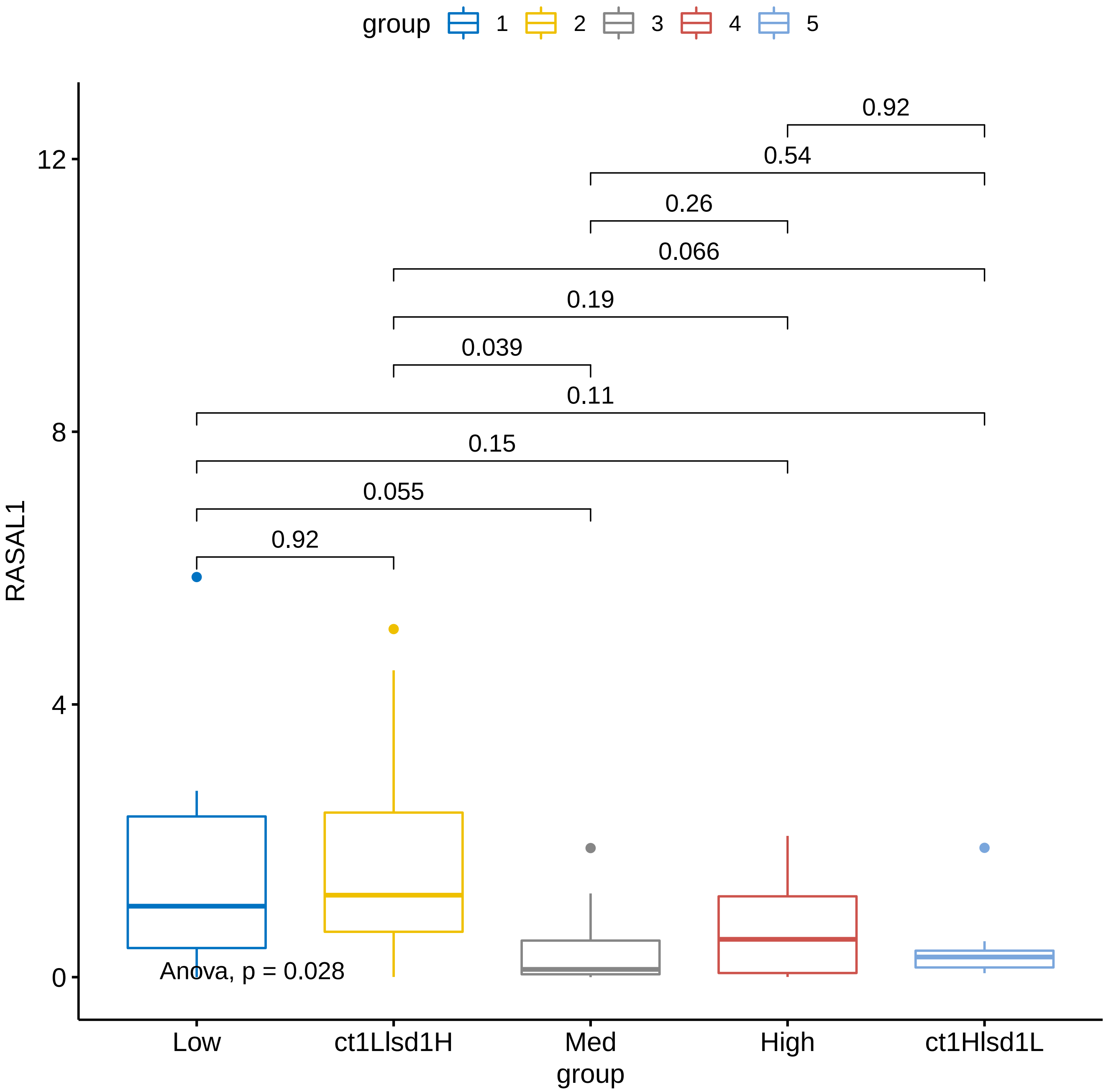
group
1
2
3
4
5
RASAL1
12
8
4
0
0.92
0.54
0.26
0.066
0.19
0.039
0.11
0.15
0.055
0.92
Anova, p = 0.028
Low
ct1Llsd1H
Med
High
ct1Hlsd1L
group

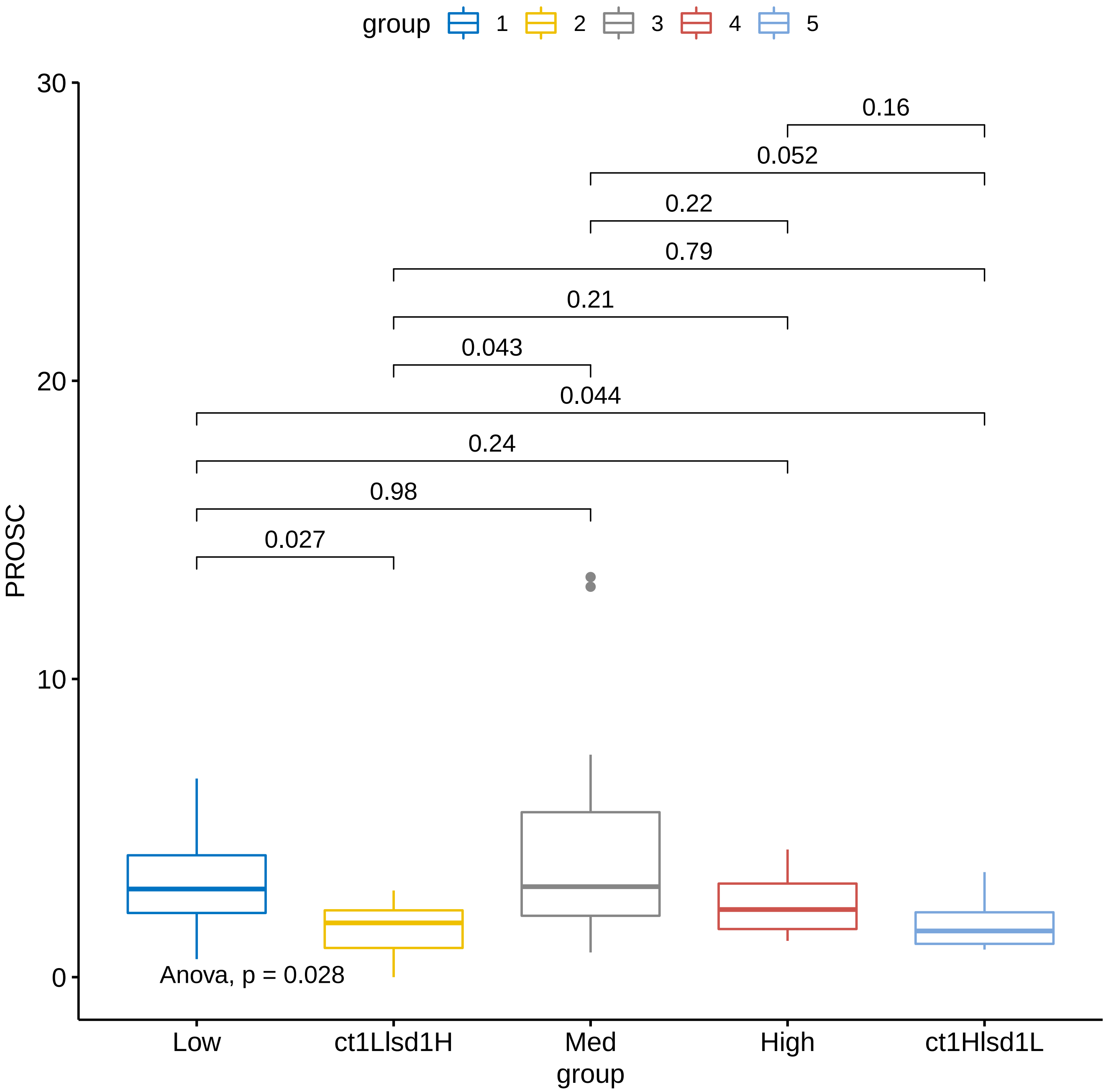

group 1 2 3 4 5
0.16
0.052
0.22
0.79
0.21
0.043
0.044
0.24
0.98
0.027
Anova, p = 0.028
30
20
10
0
PROSC
Low
ct1Llsd1H
Med
High
ct1Hlsd1L
group

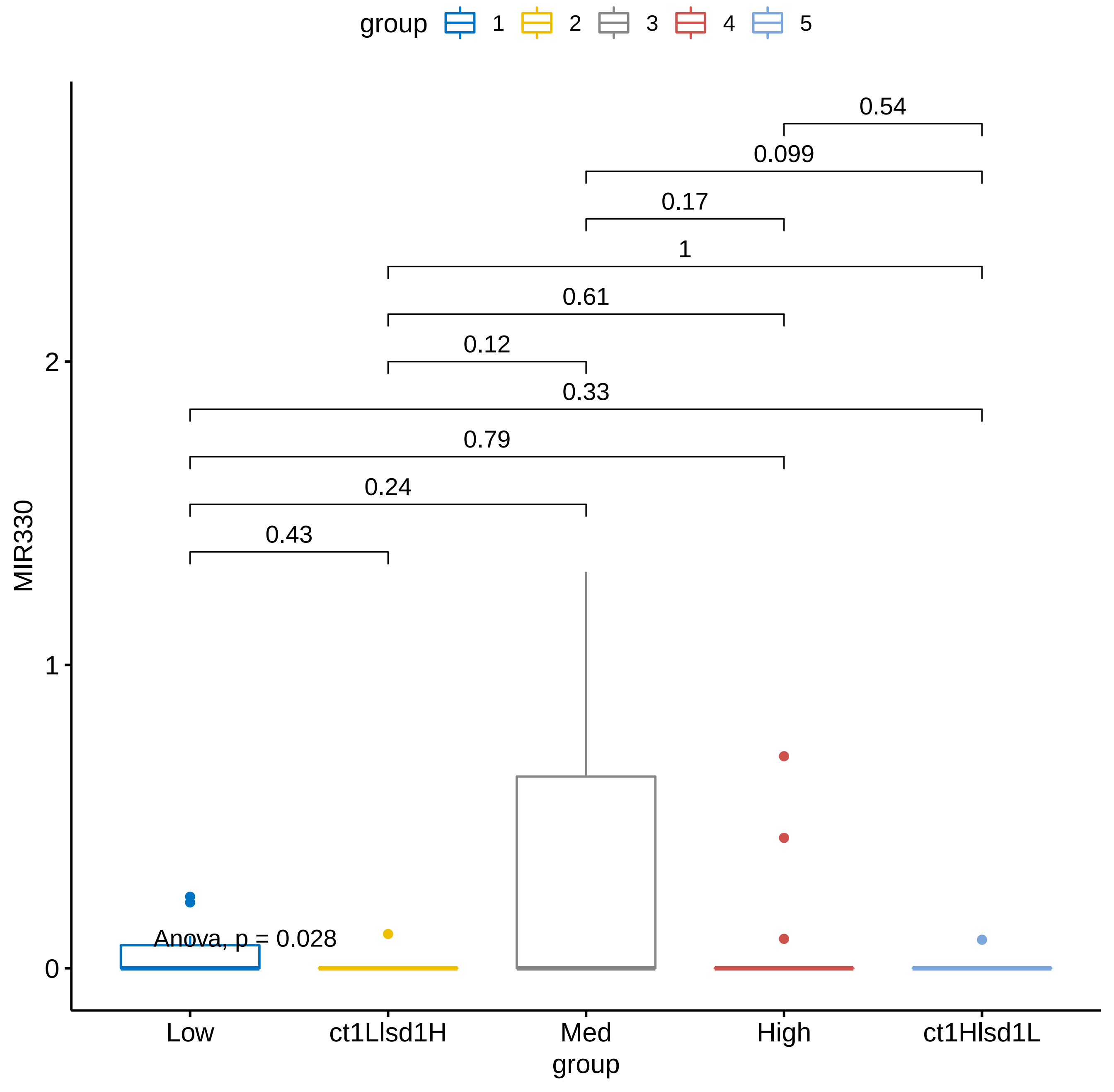

group 1 2 3 4 5
0.54
0.099
0.17
1
0.61
0.12
0.33
0.79
0.24
0.43
Anova, p = 0.028
MIR330
2
1
0
Low
ct1Llsd1H
Med
High
ct1Hlsd1L
group

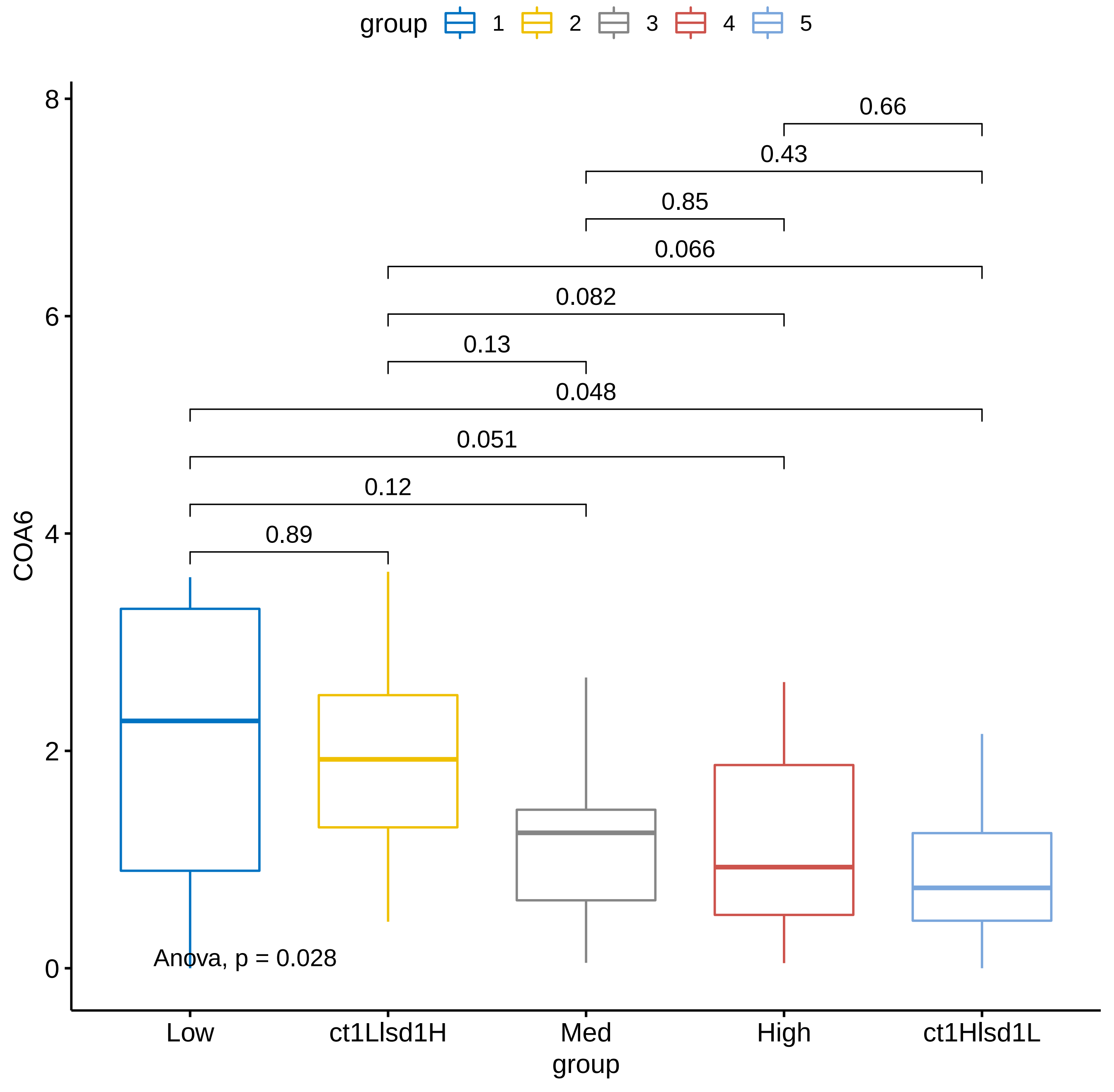

group
1
2
3
4
5
COA6
8
6
4
2
0
0.66
0.43
0.85
0.066
0.082
0.13
0.048
0.051
0.12
0.89
Anova, p = 0.028
Low
ct1Llsd1H
Med
High
ct1Hlsd1L
group

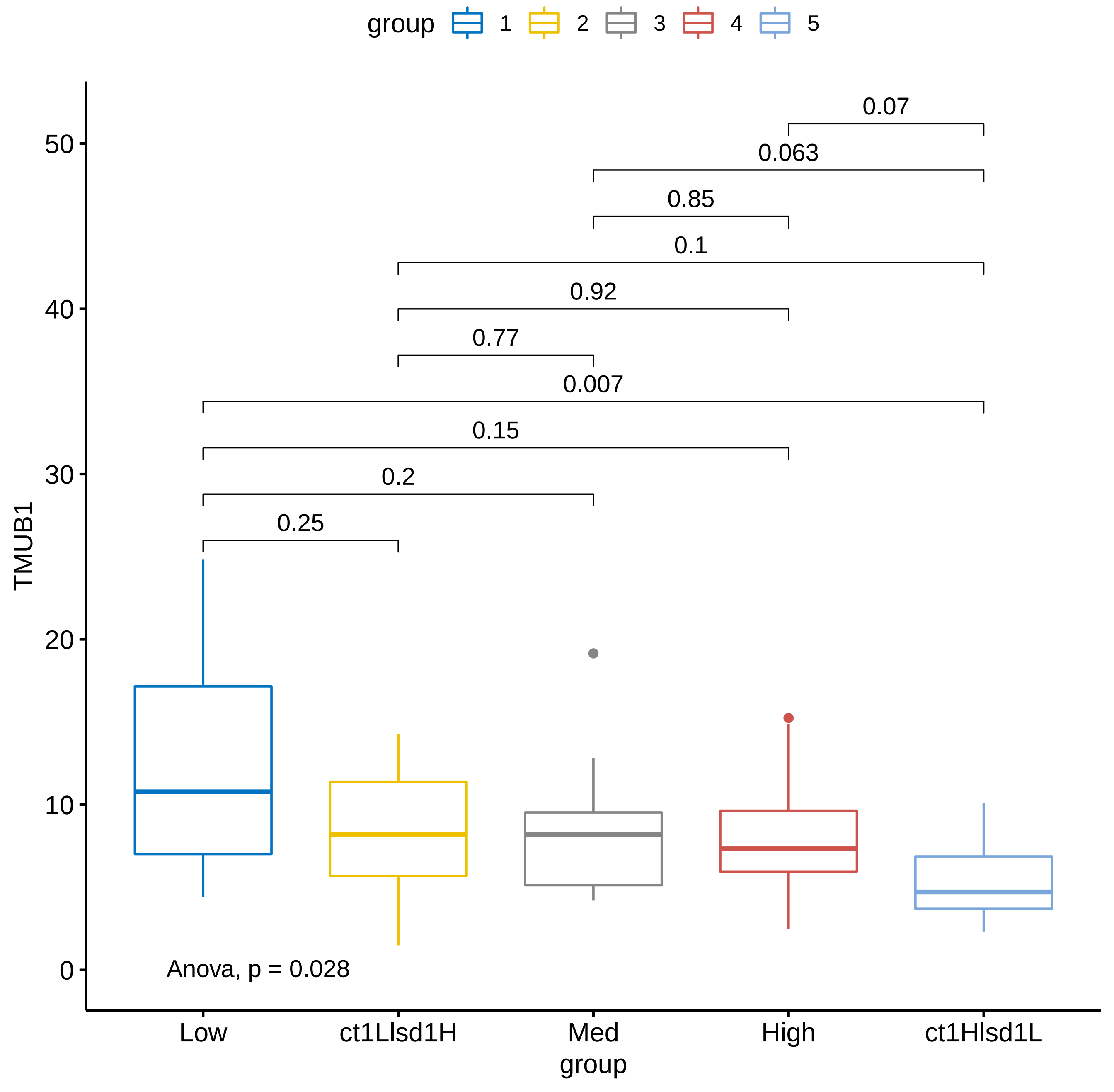

group 1 2 3 4 5
TMUB1
0
10
20
30
40
50
0.07
0.063
0.85
0.1
0.92
0.77
0.007
0.15
0.2
0.25
Anova, p = 0.028
Low
ct1Llsd1H
Med
High
ct1Hlsd1L
group

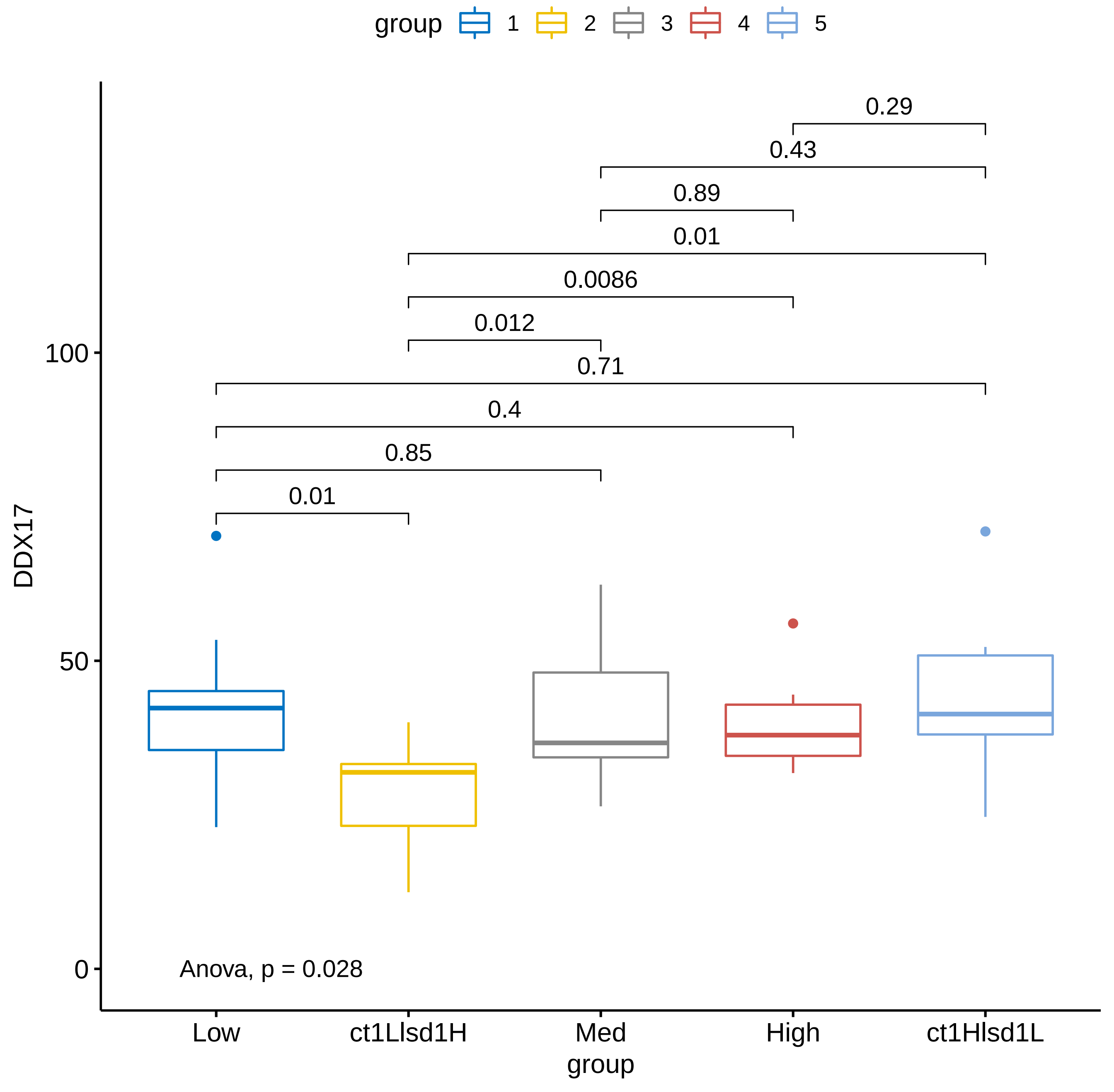

group
1
2
3
4
5
0.29
0.43
0.89
0.01
0.0086
0.012
0.71
0.4
0.85
0.01
100
50
0
DDX17
Anova, p = 0.028
Low
ct1Llsd1H
Med
High
ct1Hlsd1L
group

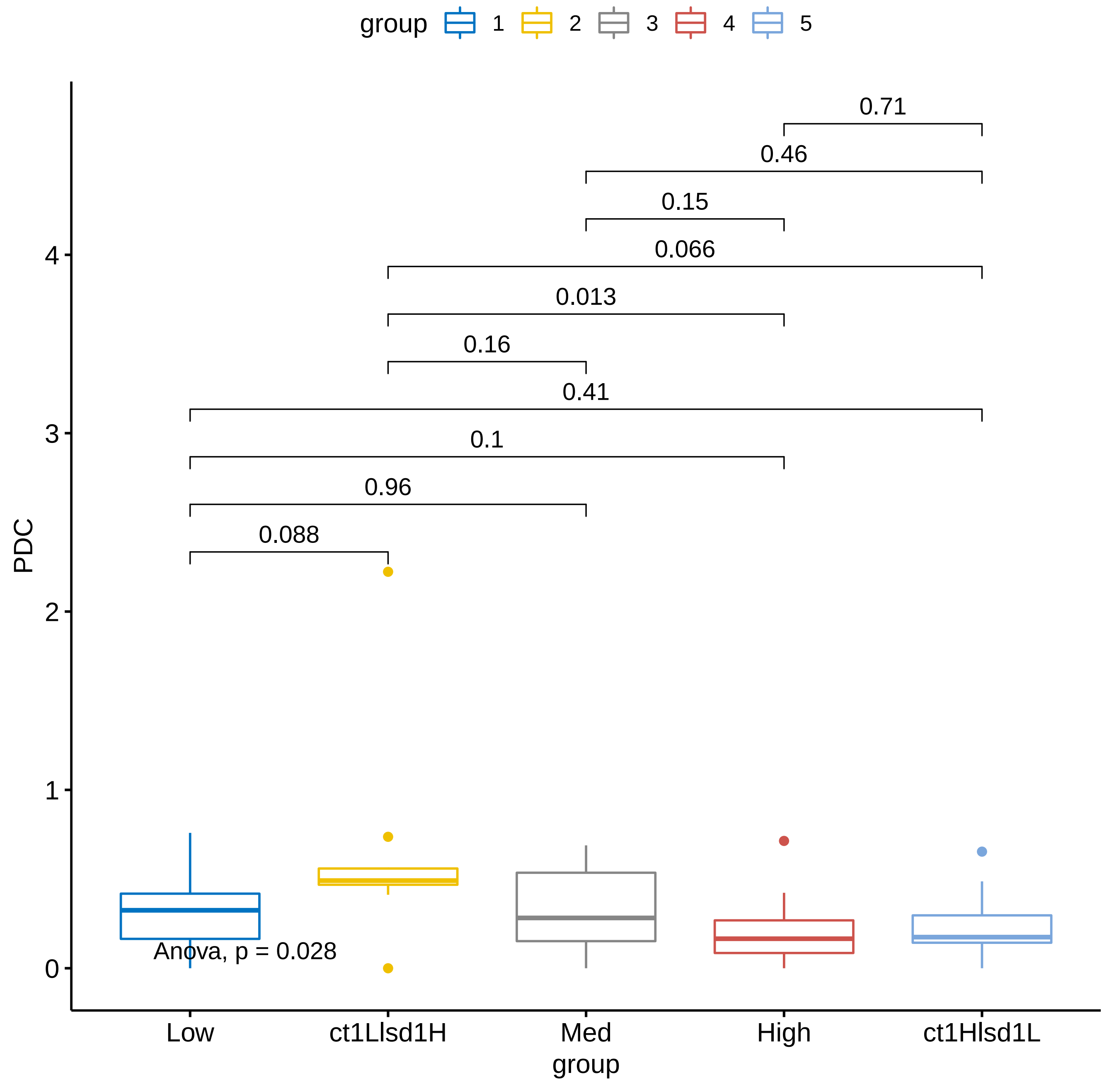

group
1
2
3
4
5
0.71
0.46
0.15
0.066
0.013
0.16
0.41
0.1
0.96
0.088
Anova, p = 0.028
PDC
0
1
2
3
4
Low
ct1Llsd1H
Med
High
ct1Hlsd1L
group

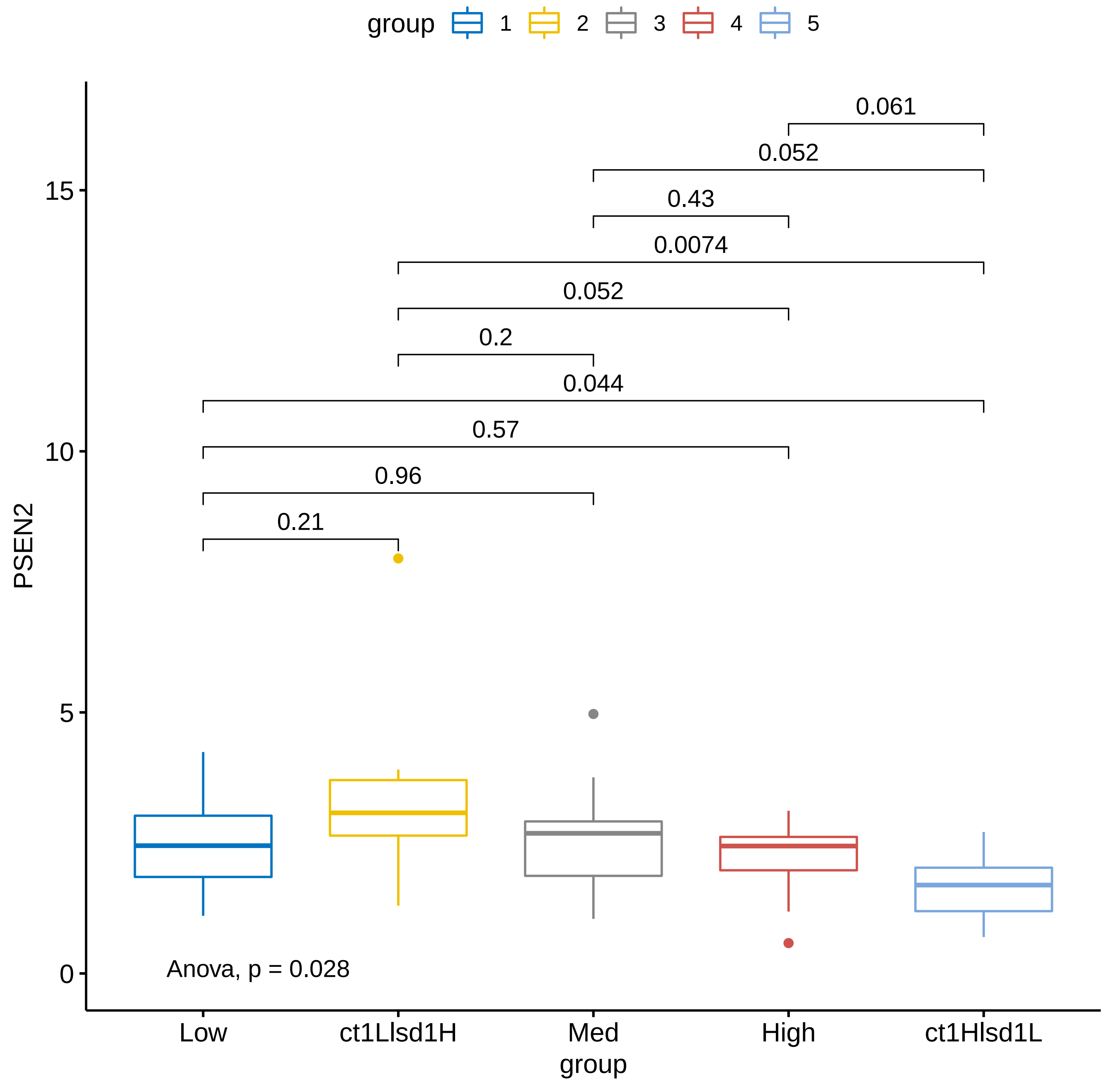
group
1
2
3
4
5
0.061
0.052
0.43
0.0074
0.052
0.2
0.044
0.57
0.96
0.21
PSEN2
15
10
5
0
Anova, p = 0.028
Low
ct1Llsd1H
Med
High
ct1Hlsd1L
group

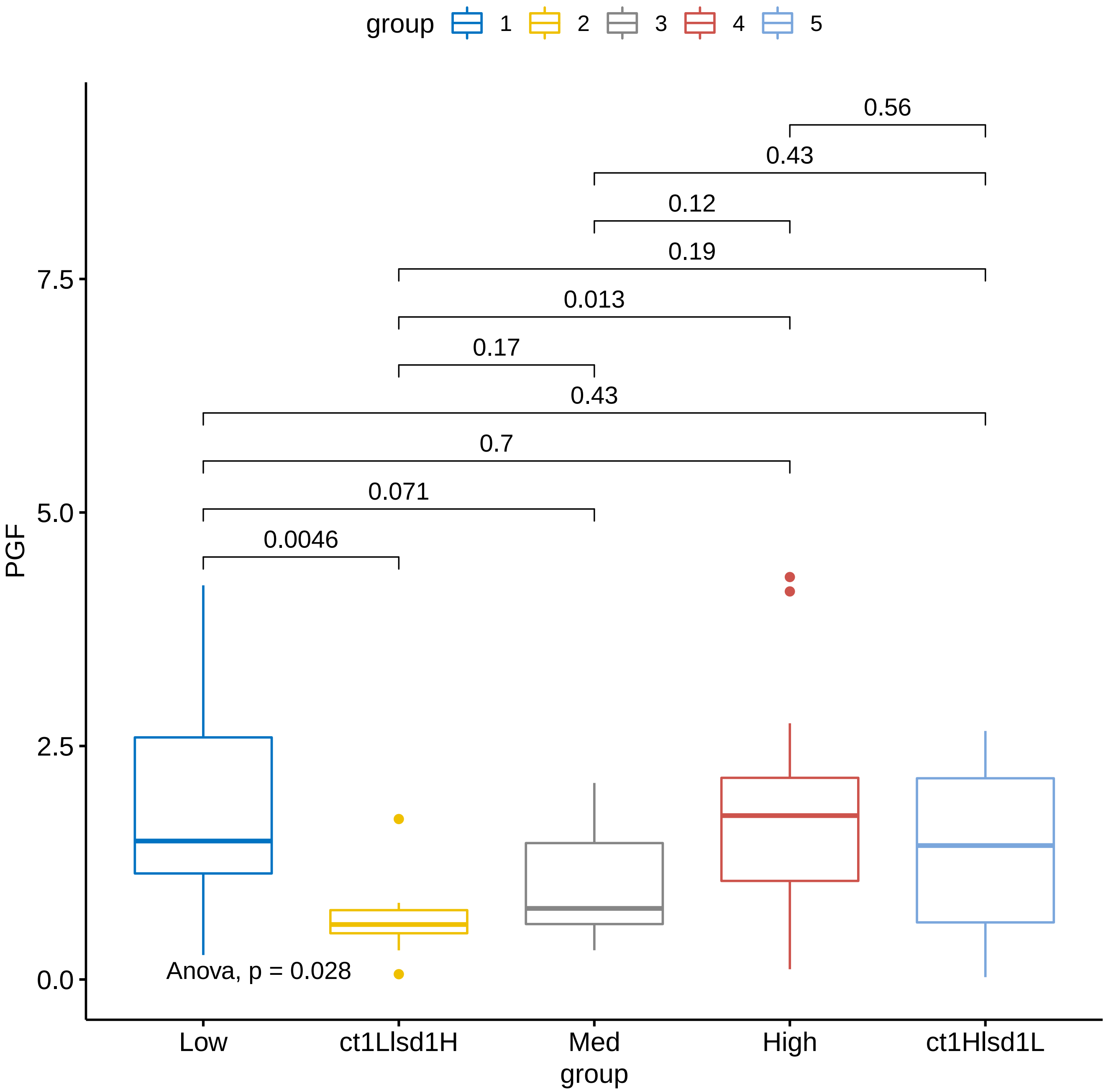

group
1
2
3
4
5
0.56
0.43
0.12
0.19
0.013
0.17
0.43
0.7
0.071
0.0046
Anova, p = 0.028
PGF
7.5
5.0
2.5
0.0
Low
ct1Llsd1H
Med
High
ct1Hlsd1L
group

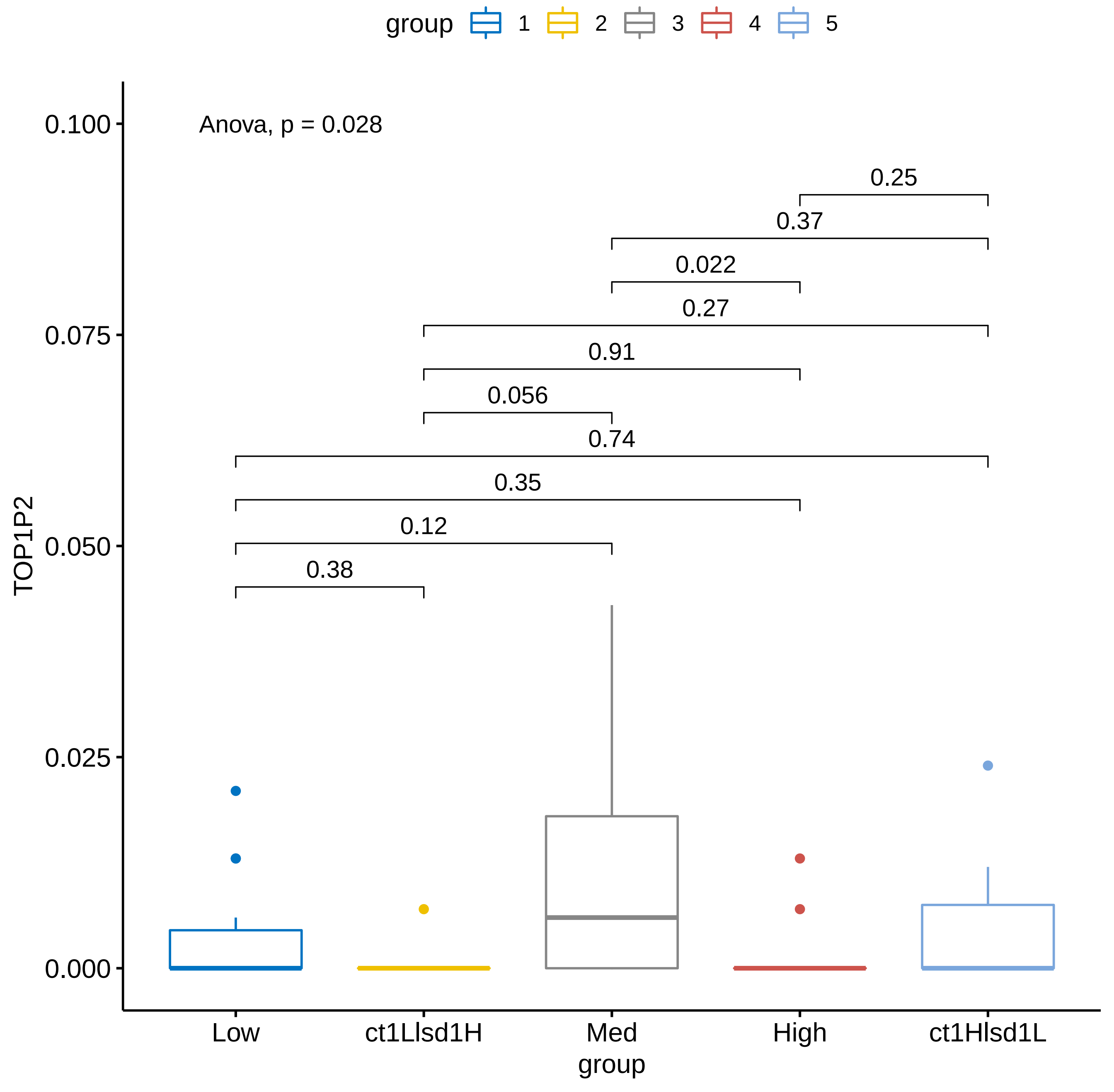

group
1
2
3
4
5
Anova, p = 0.028
0.25
0.37
0.022
0.27
0.91
0.056
0.74
0.35
0.12
0.38
TOP1P2
0.100
0.075
0.050
0.025
0.000
Low
ct1Llsd1H
Med
High
ct1Hlsd1L
group

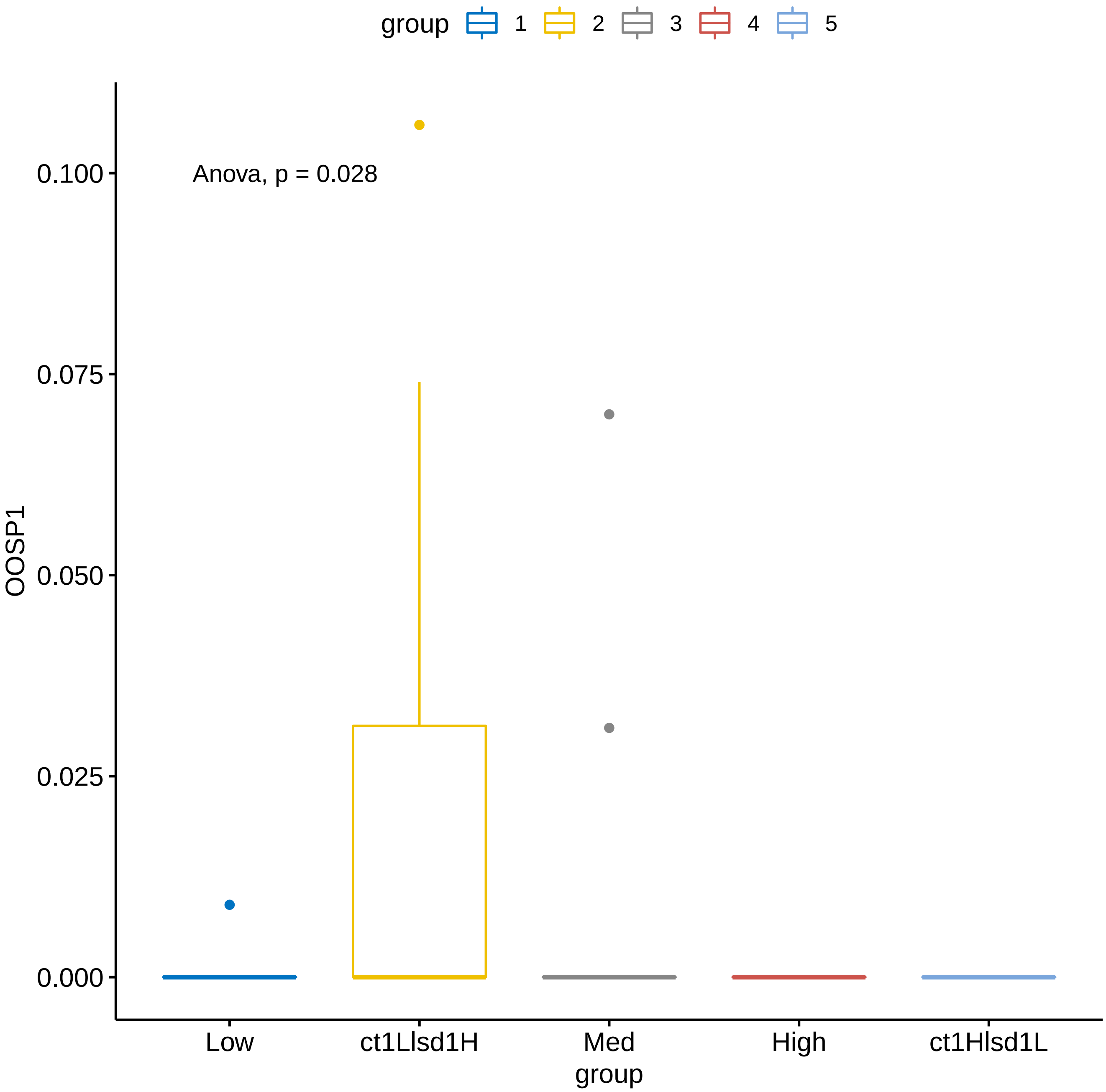

group 1 2 3 4 5
Anova, p = 0.028
OOSP1
0.100
0.075
0.050
0.025
0.000
Low
ct1Llsd1H
Med
High
ct1Hlsd1L
group

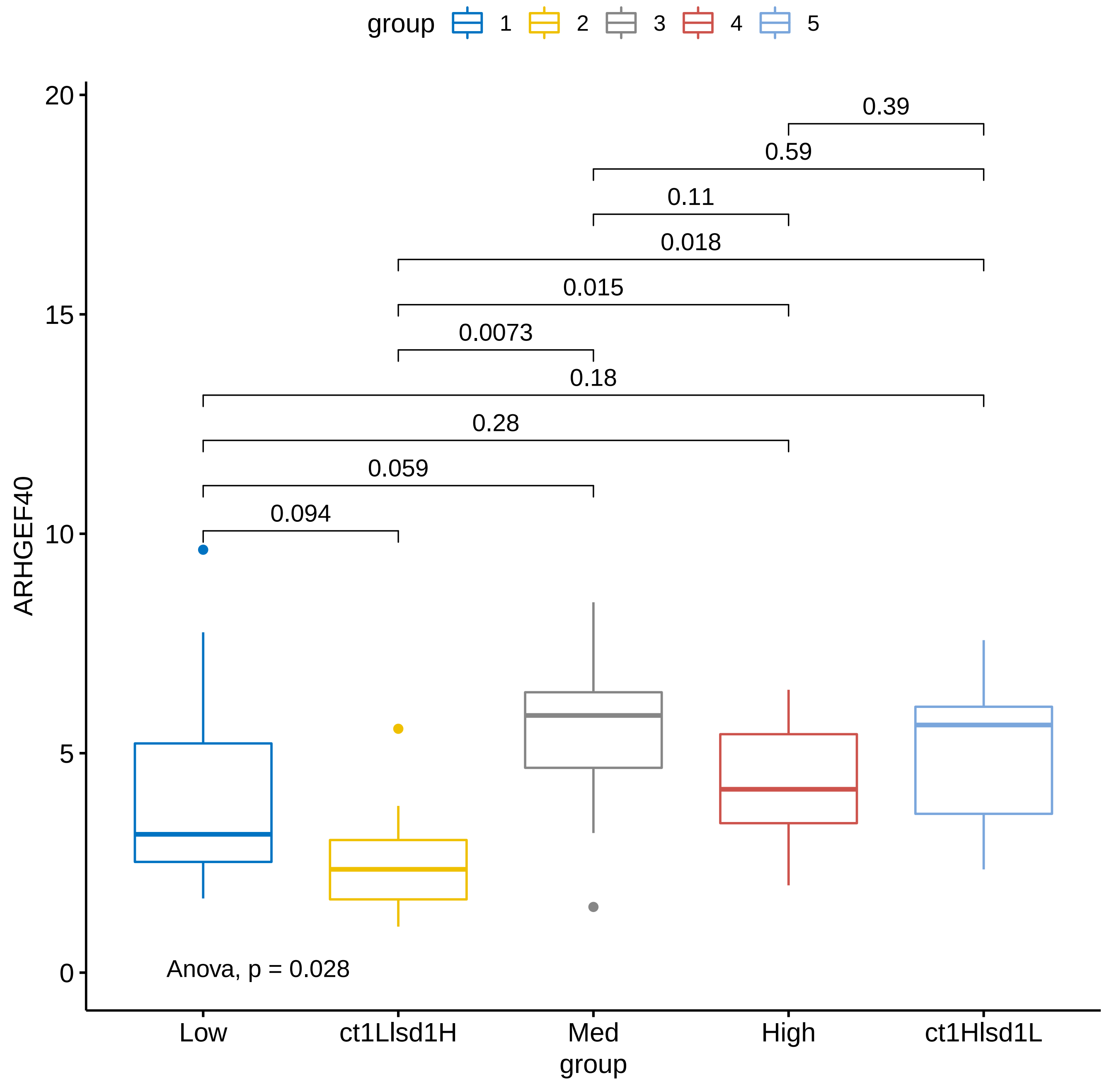

group 1 2 3 4 5
20
15
10
5
0
ARHGEF40
0.39
0.59
0.11
0.018
0.015
0.0073
0.18
0.28
0.059
0.094
Anova, p = 0.028
Low
ct1Llsd1H
Med
High
ct1Hlsd1L
group

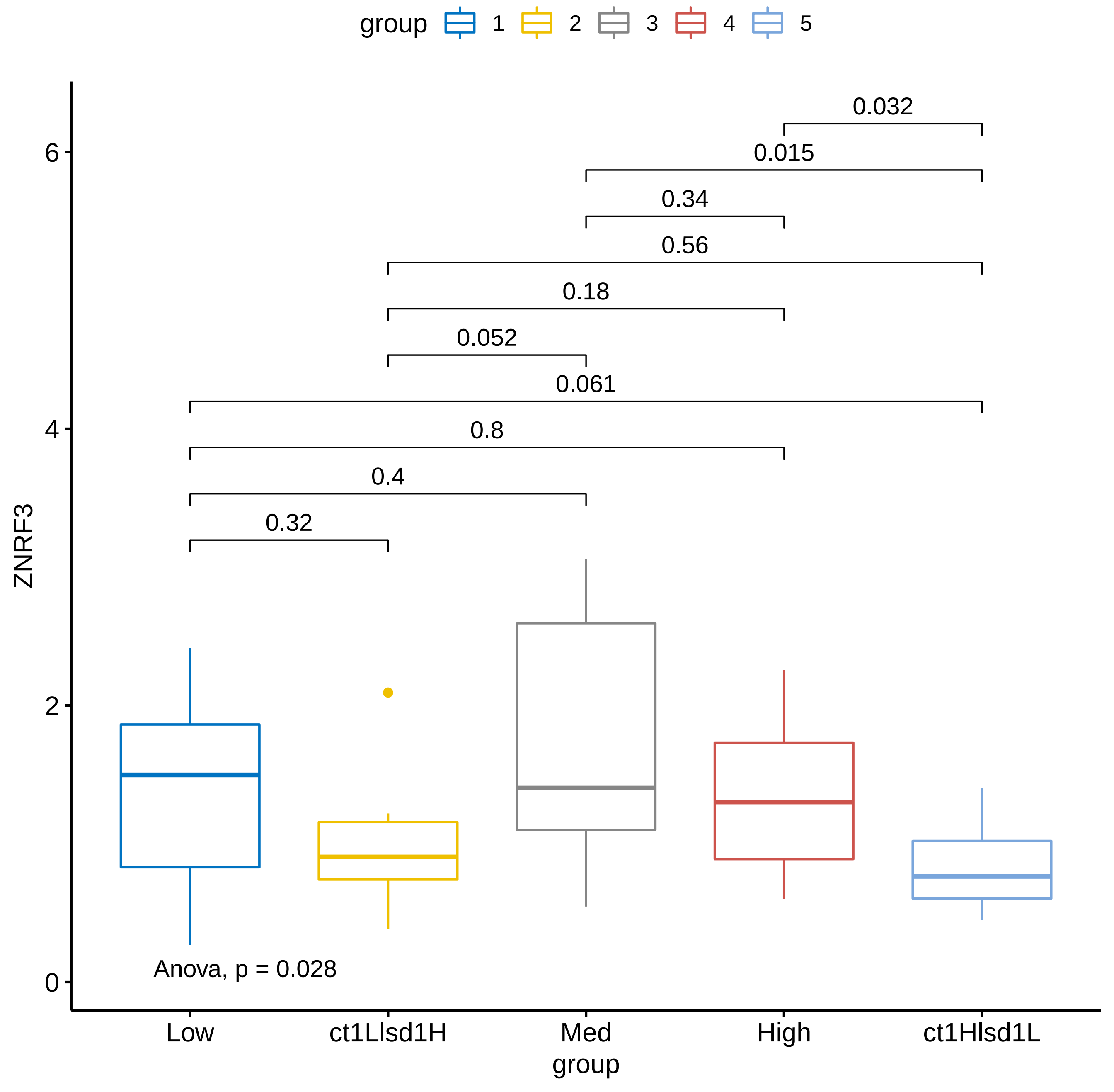

group
1
2
3
4
5
0.032
0.015
0.34
0.56
0.18
0.052
0.061
0.8
0.4
0.32
6
4
2
0
ZNRF3
Anova, p = 0.028
Low
ct1Llsd1H
Med
High
ct1Hlsd1L
group

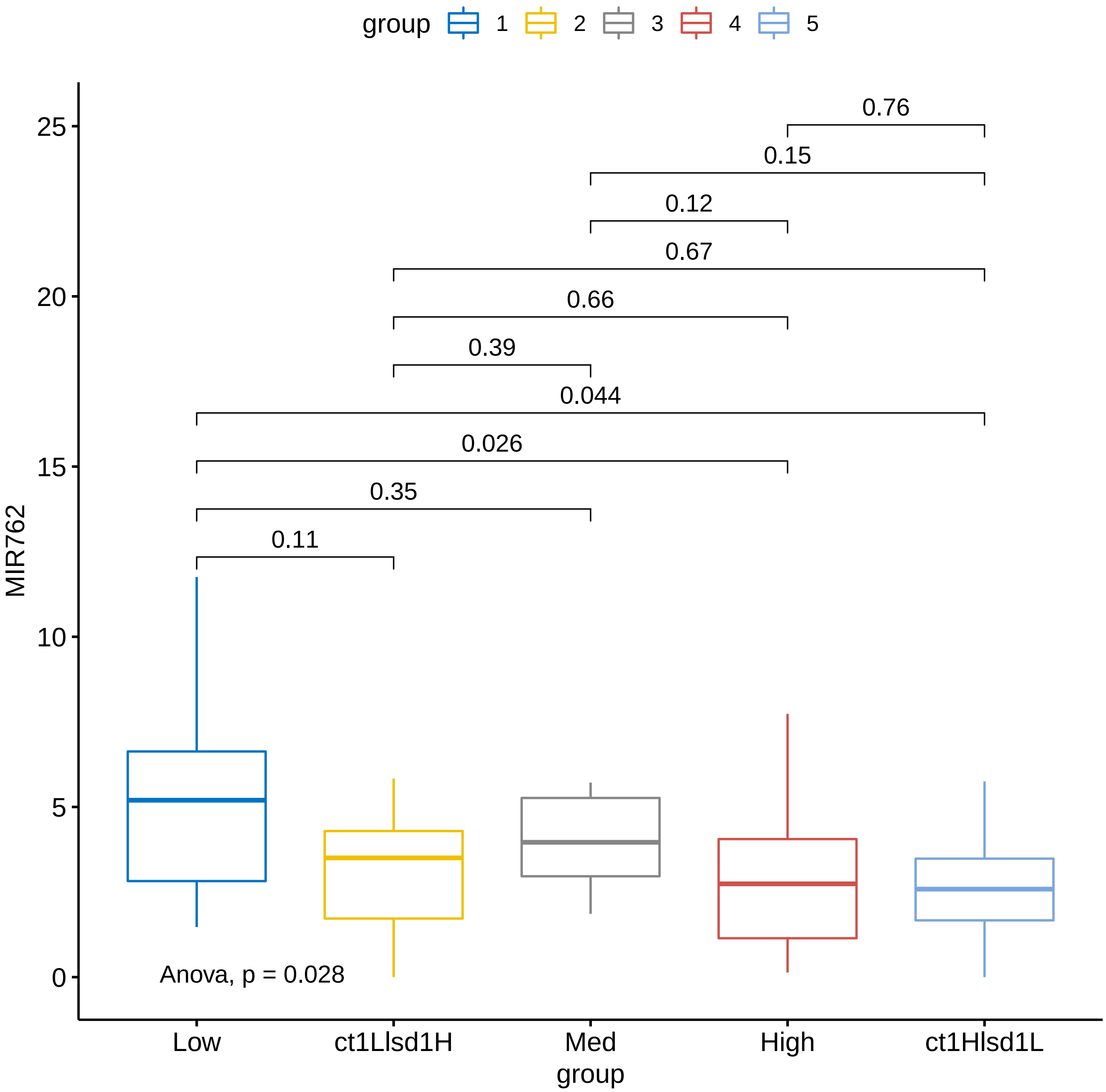

group 1 2 3 4 5
MIR762
25
20
15
10
5
0
0.76
0.15
0.12
0.67
0.66
0.39
0.044
0.026
0.35
0.11
Anova, p = 0.028
Low
ct1Llsd1H
Med
High
ct1Hlsd1L
group

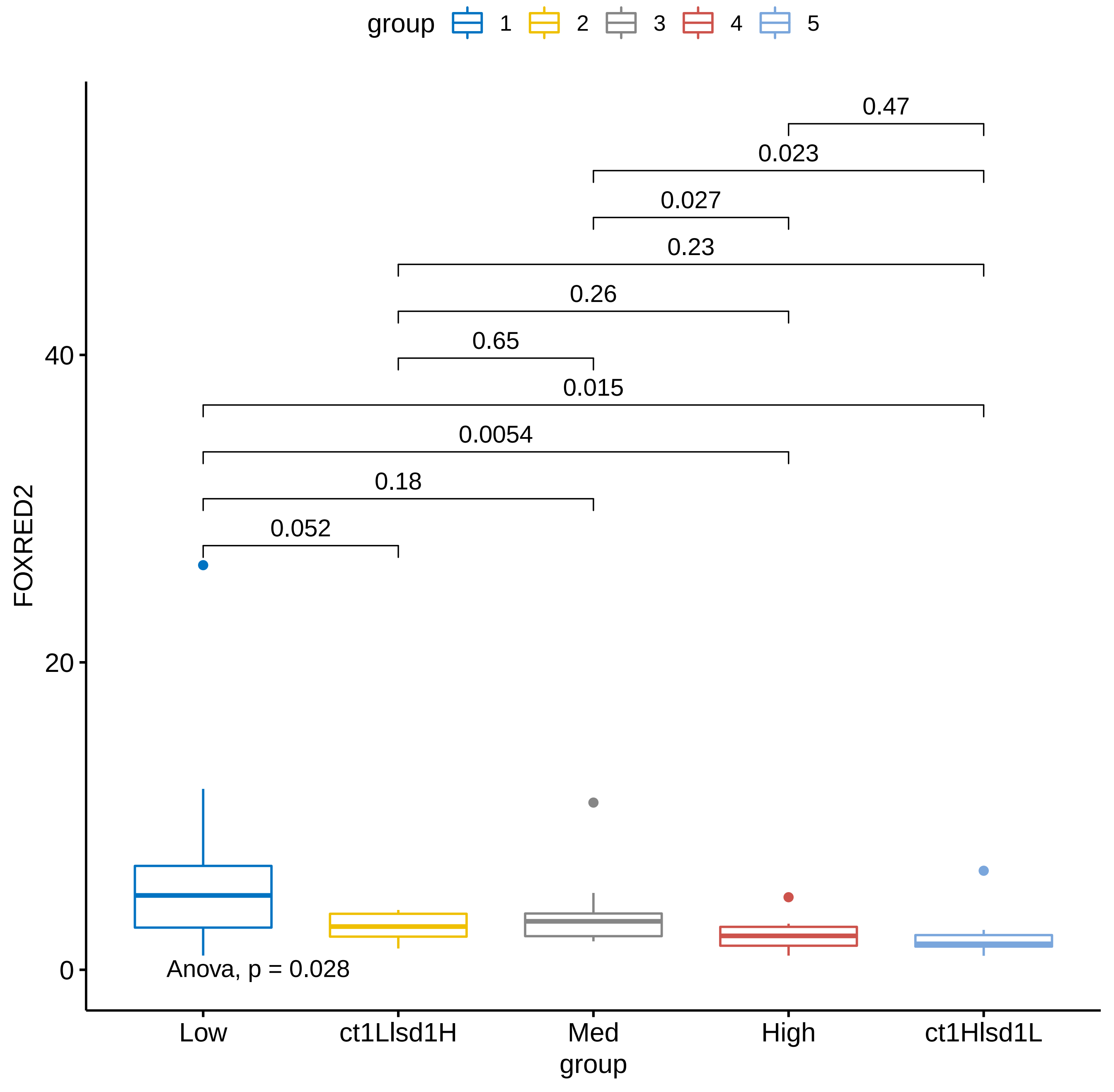

group 1 2 3 4 5
0.47
0.023
0.027
0.23
0.26
0.65
0.015
0.0054
0.18
0.052
FOXRED2
40
20
0
Anova, p = 0.028
Low
ct1Llsd1H
Med
High
ct1Hlsd1L
group

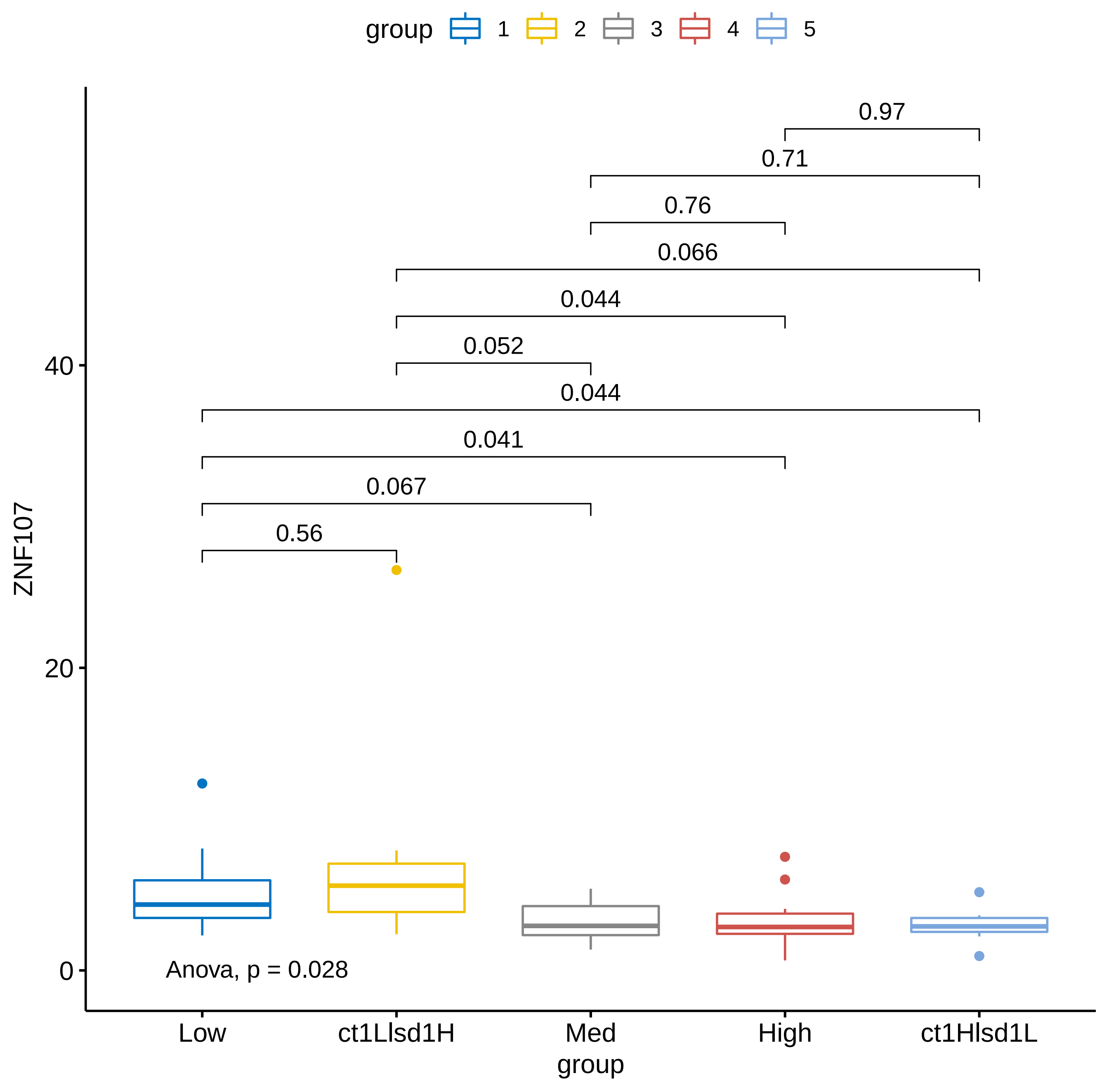
group 1 2 3 4 5
0.97
0.71
0.76
0.066
0.044
0.052
40
0.044
0.041
0.067
0.56
ZNF107
20
0
Anova, p = 0.028
Low
ct1Llsd1H
Med
High
ct1Hlsd1L
group

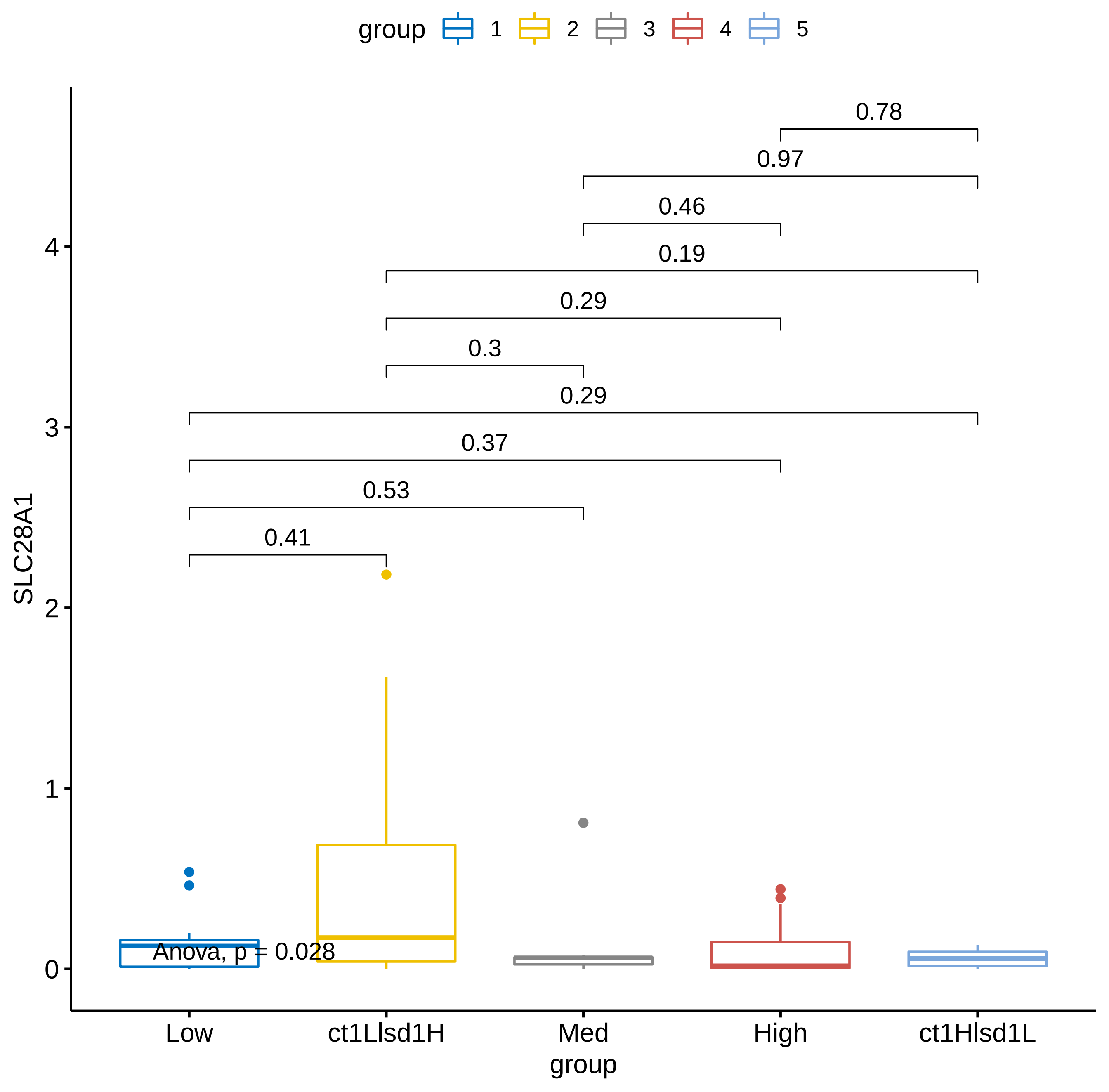
group
1
2
3
4
5
0.78
0.97
0.46
0.19
0.29
0.3
0.29
0.37
0.53
0.41
Anova, p = 0.028
SLC28A1
4
3
2
1
0
Low
ct1Llsd1H
Med
High
ct1Hlsd1L
group

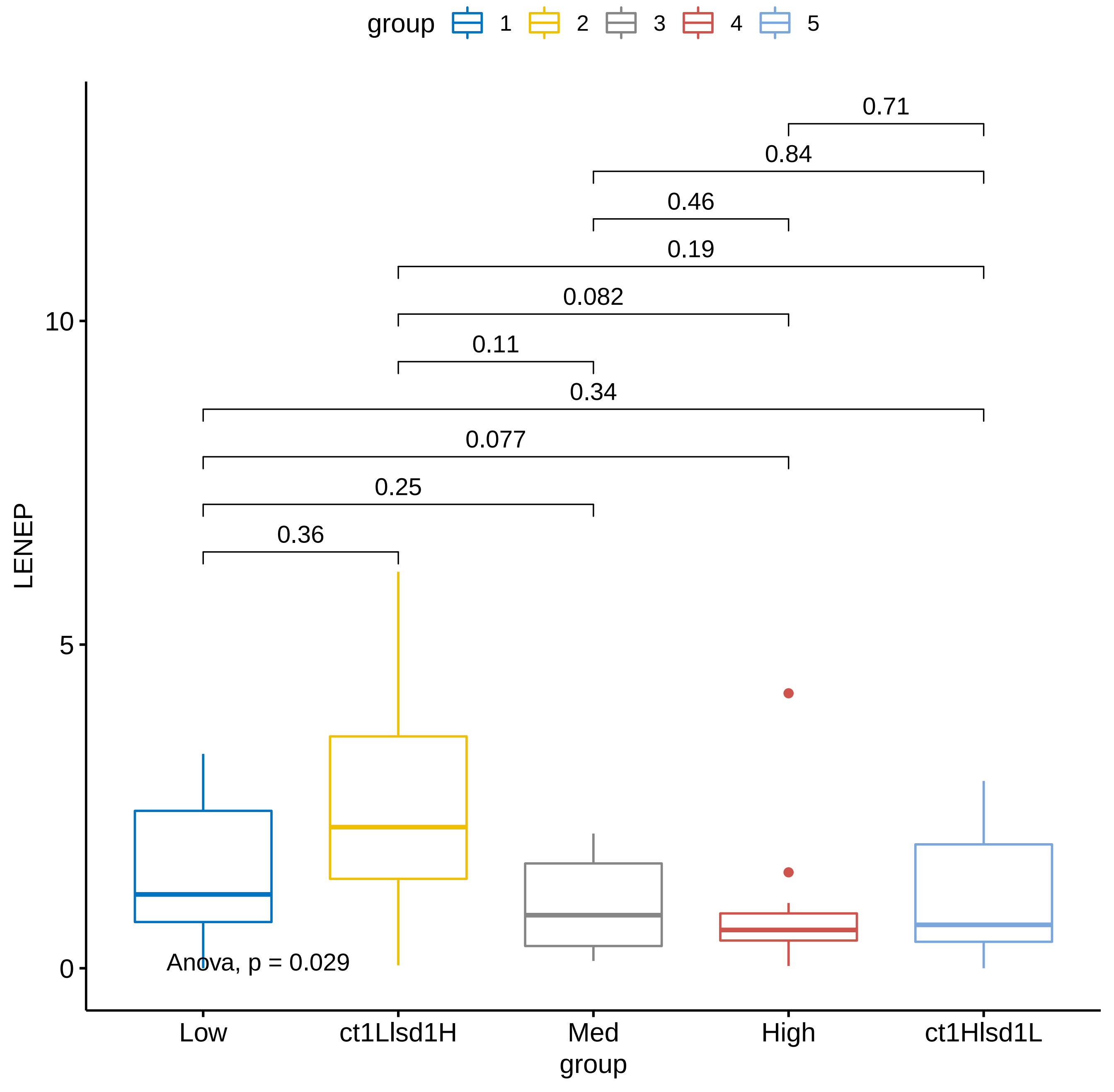
group
1
2
3
4
5
0.71
0.84
0.46
0.19
0.082
0.11
0.34
0.077
0.25
0.36
10
5
0
LENEP
Anova, p = 0.029
Low
ct1Llsd1H
Med
High
ct1Hlsd1L
group

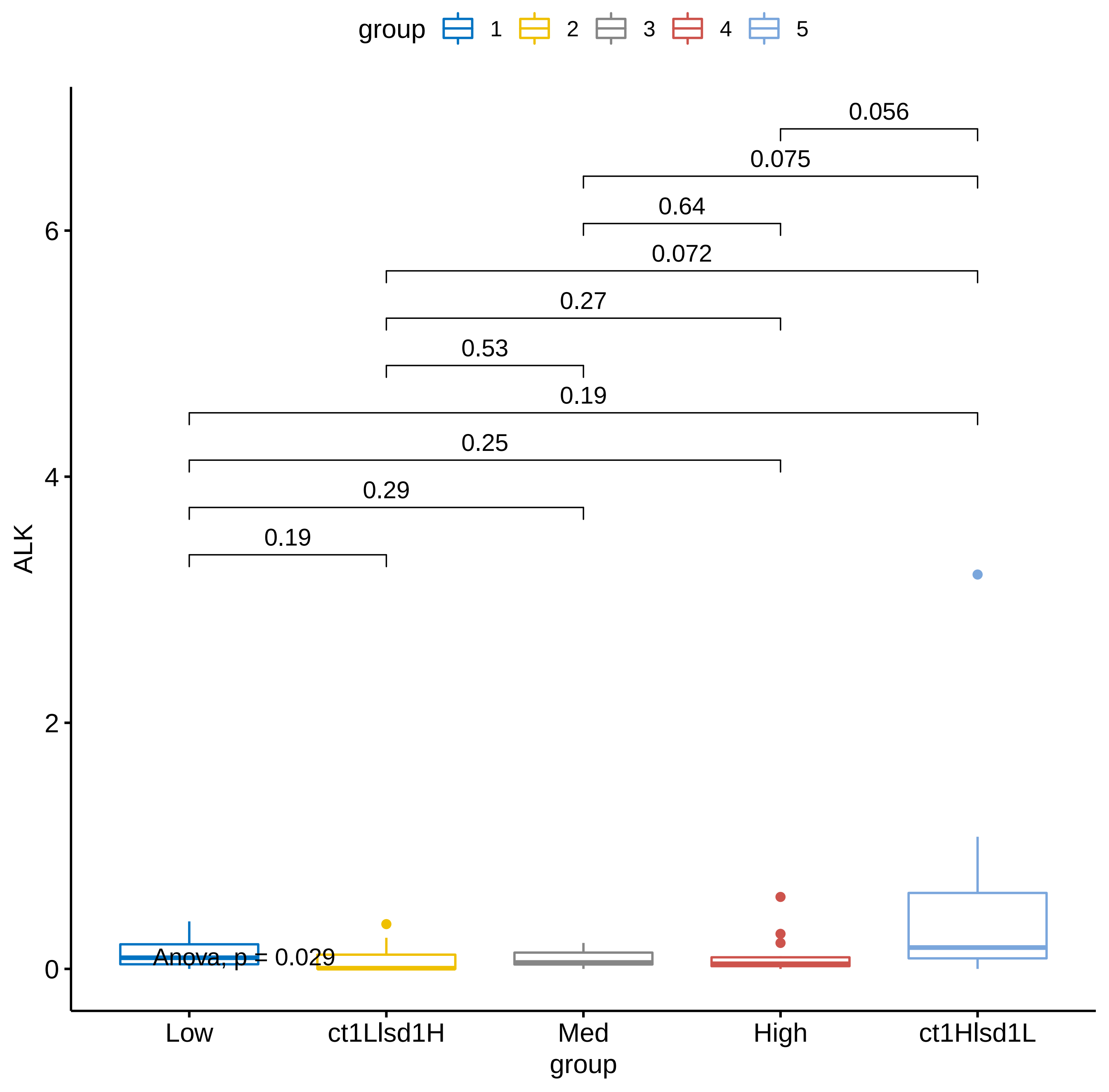

group 1 2 3 4 5
0.056
0.075
0.64
0.072
0.27
0.53
0.19
0.25
0.29
0.19
ALK
6
4
2
0
Anova, p = 0.029
Low
ct1Llsd1H
Med
High
ct1Hlsd1L
group

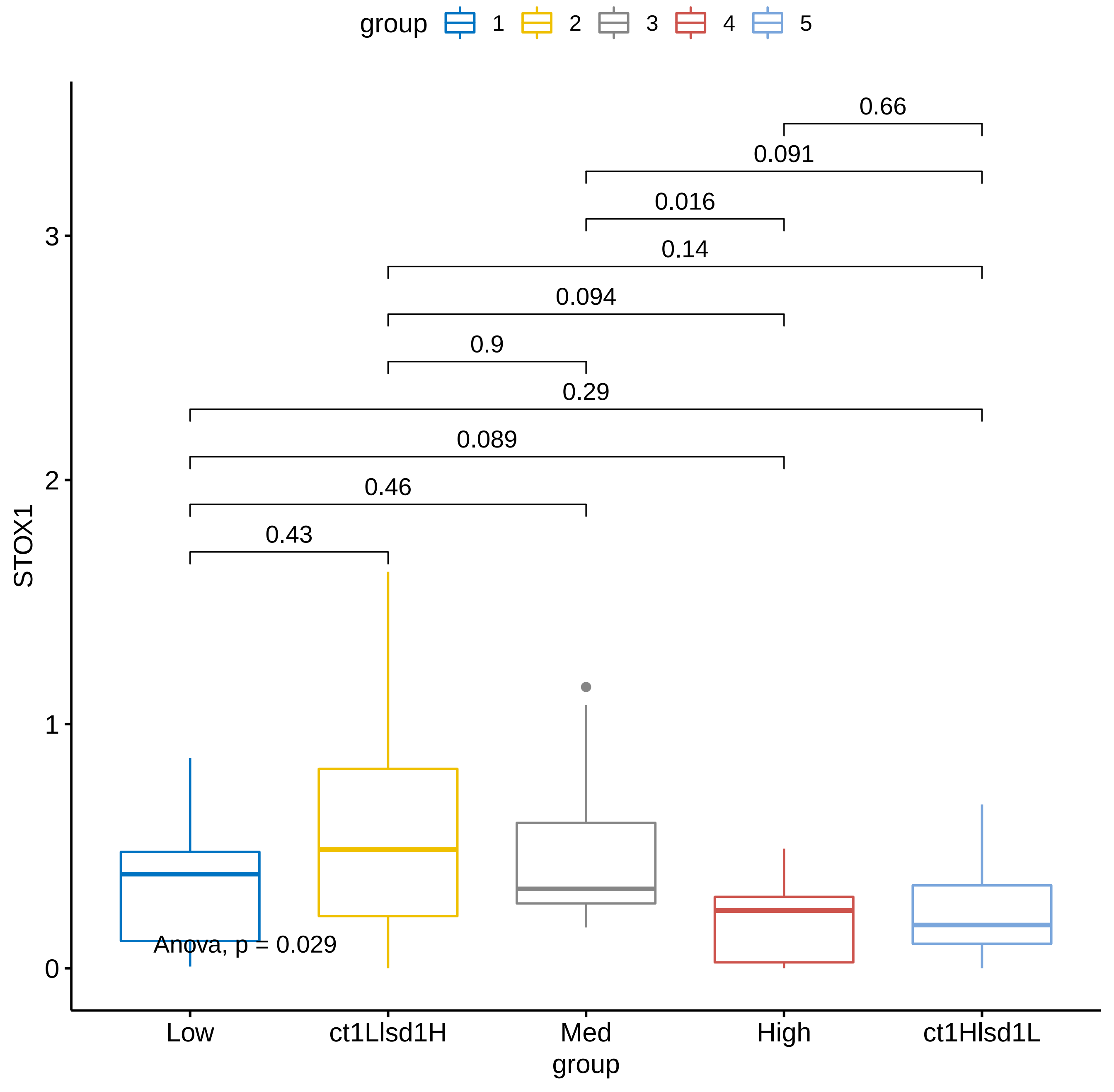

group
1
2
3
4
5
0.66
0.091
0.016
0.14
0.094
0.9
0.29
0.089
0.46
0.43
Anova, p = 0.029
STOX1
3
2
1
0
Low
ct1Llsd1H
Med
High
ct1Hlsd1L
group

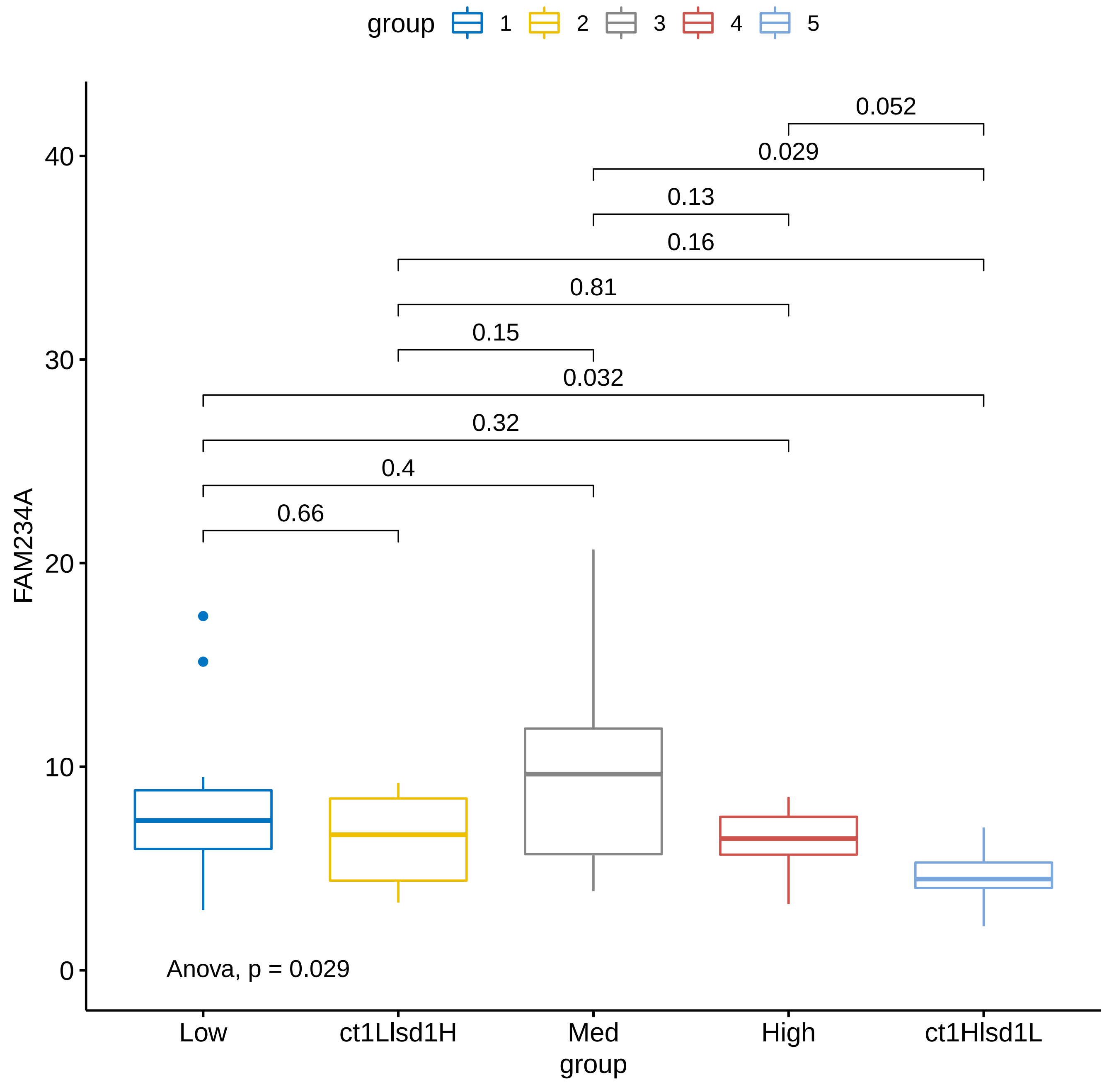
group 1 2 3 4 5
0.052
0.029
0.13
0.16
0.81
0.15
0.032
0.32
0.4
0.66
40
30
20
10
0
FAM234A
Anova, p = 0.029
Low
ct1Llsd1H
Med
High
ct1Hlsd1L
group

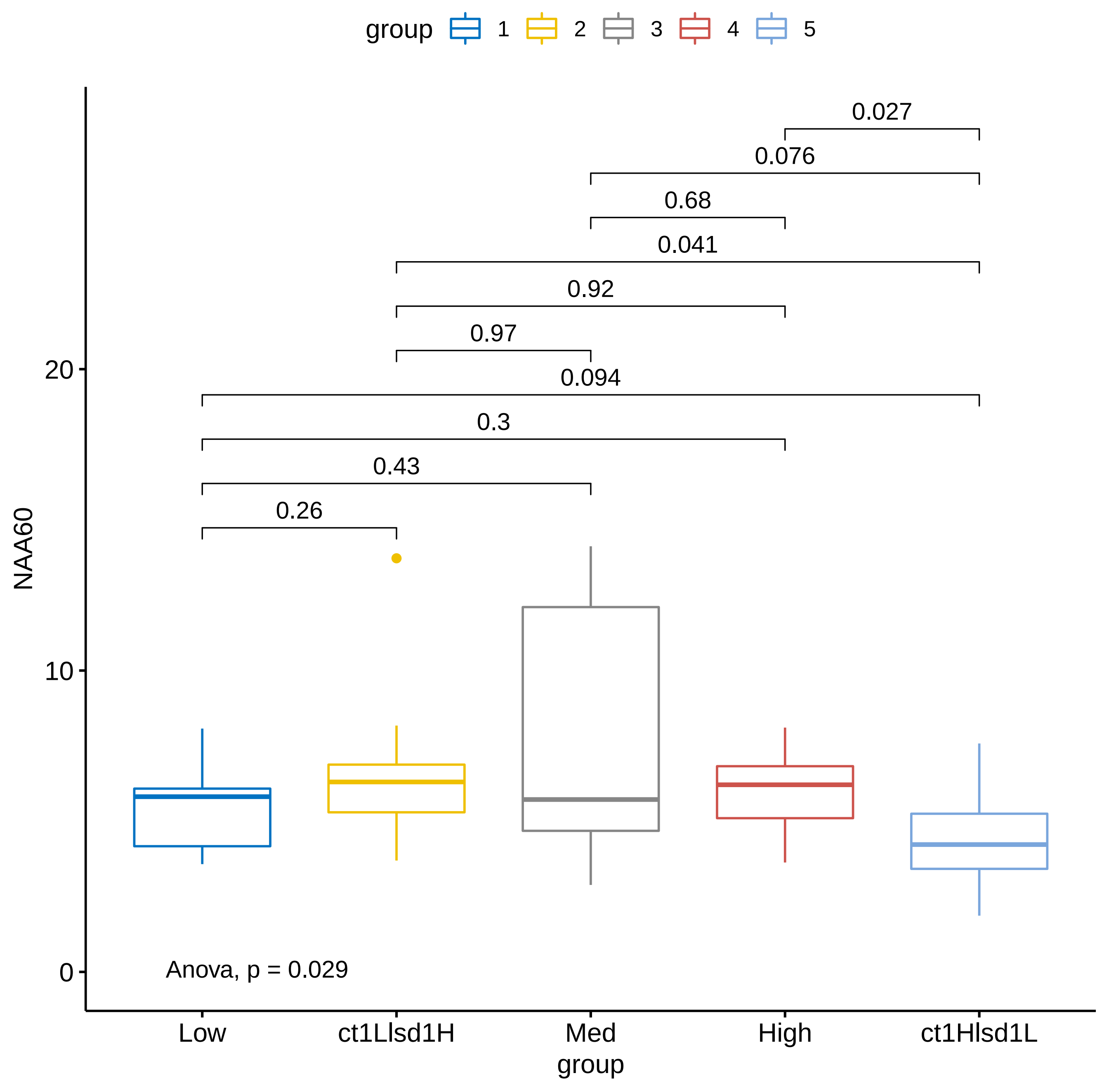

group
1
2
3
4
5
0.027
0.076
0.68
0.041
0.92
0.97
0.094
0.3
0.43
0.26
20
10
0
NAA60
Anova, p = 0.029
Low
ct1Llsd1H
Med
High
ct1Hlsd1L
group

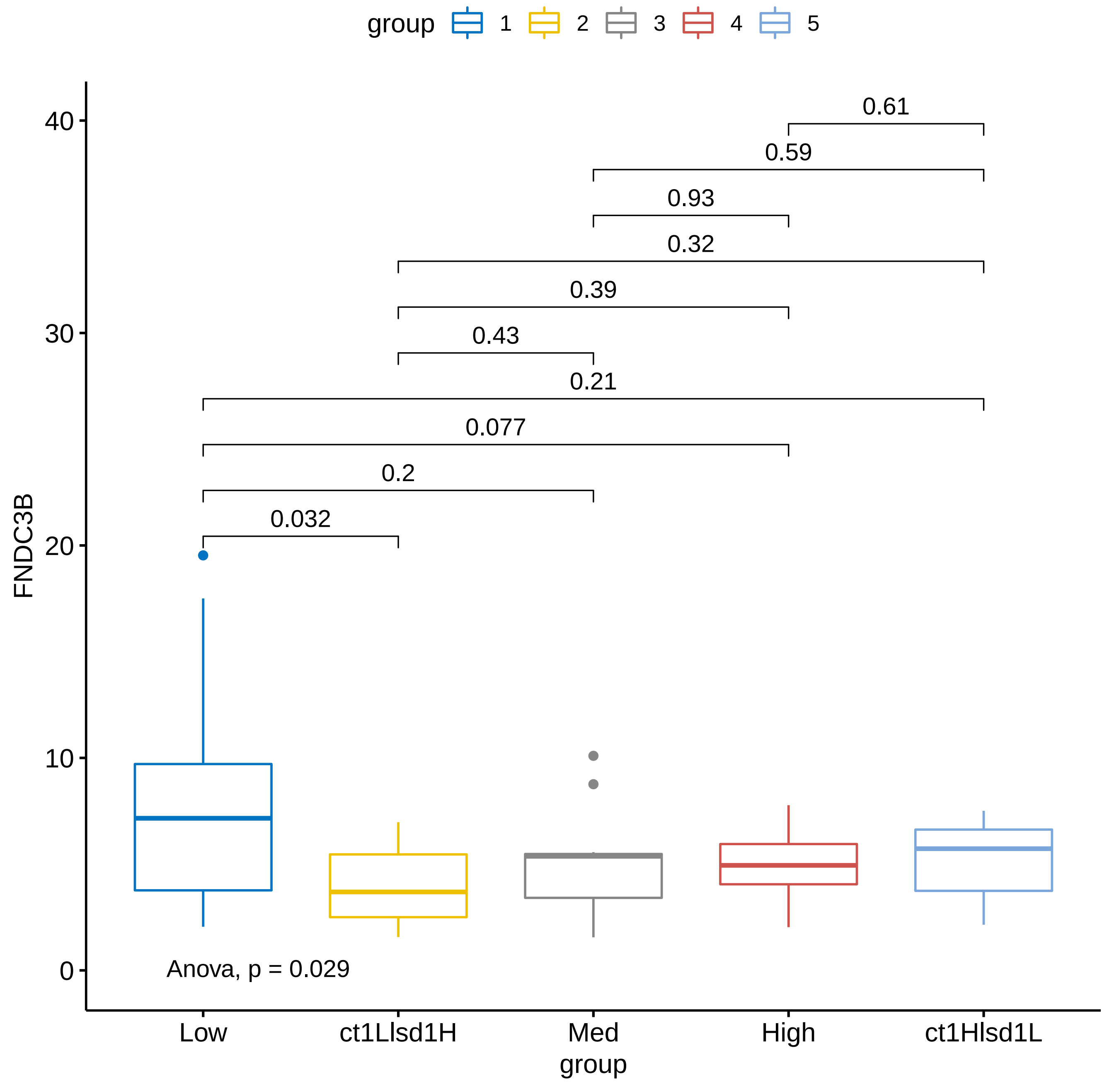

group
1
2
3
4
5
FNDC3B
40
30
20
10
0
0.61
0.59
0.93
0.32
0.39
0.43
0.21
0.077
0.2
0.032
Anova, p = 0.029
Low
ct1Llsd1H
Med
High
ct1Hlsd1L
group

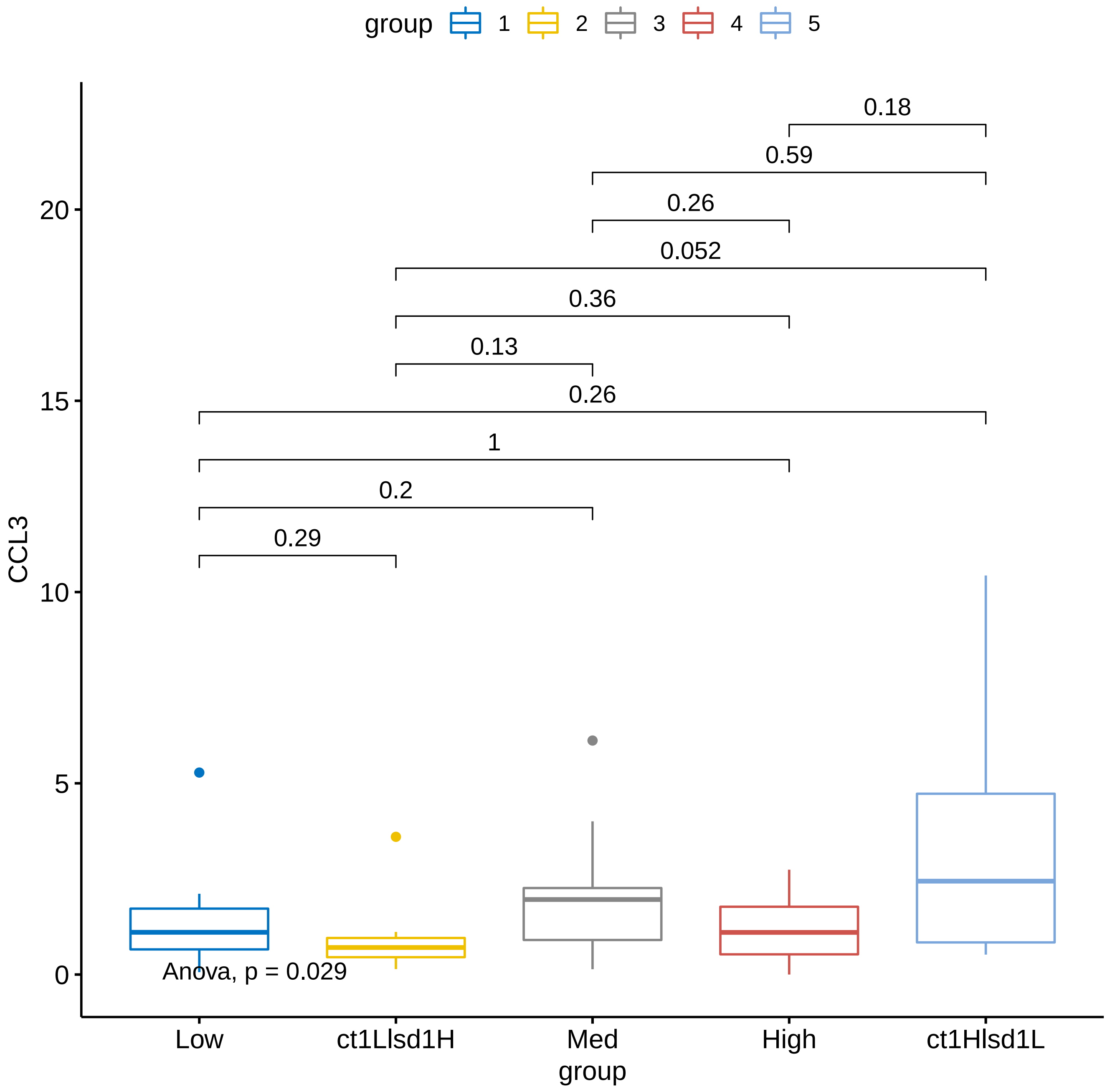
group
1
2
3
4
5
0.18
0.59
0.26
0.052
0.36
0.13
0.26
1
0.2
0.29
CCL3
20
15
10
5
0
Anova, p = 0.029
Low
ct1Llsd1H
Med
High
ct1Hlsd1L
group

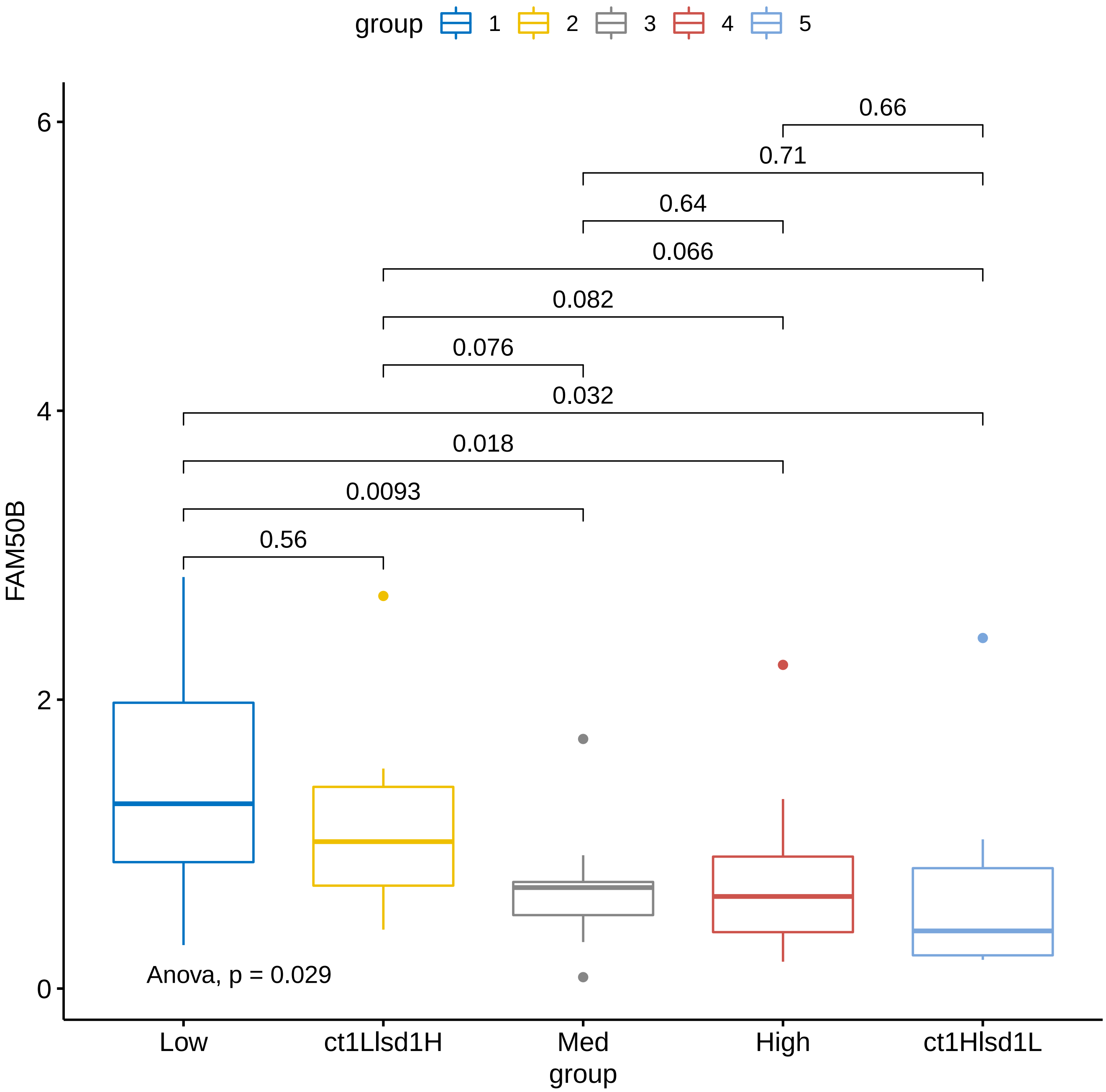
group
1
2
3
4
5
FAM50B
0
2
4
6
0.66
0.71
0.64
0.066
0.082
0.076
0.032
0.018
0.0093
0.56
Anova, p = 0.029
Low
ct1Llsd1H
Med
High
ct1Hlsd1L
group

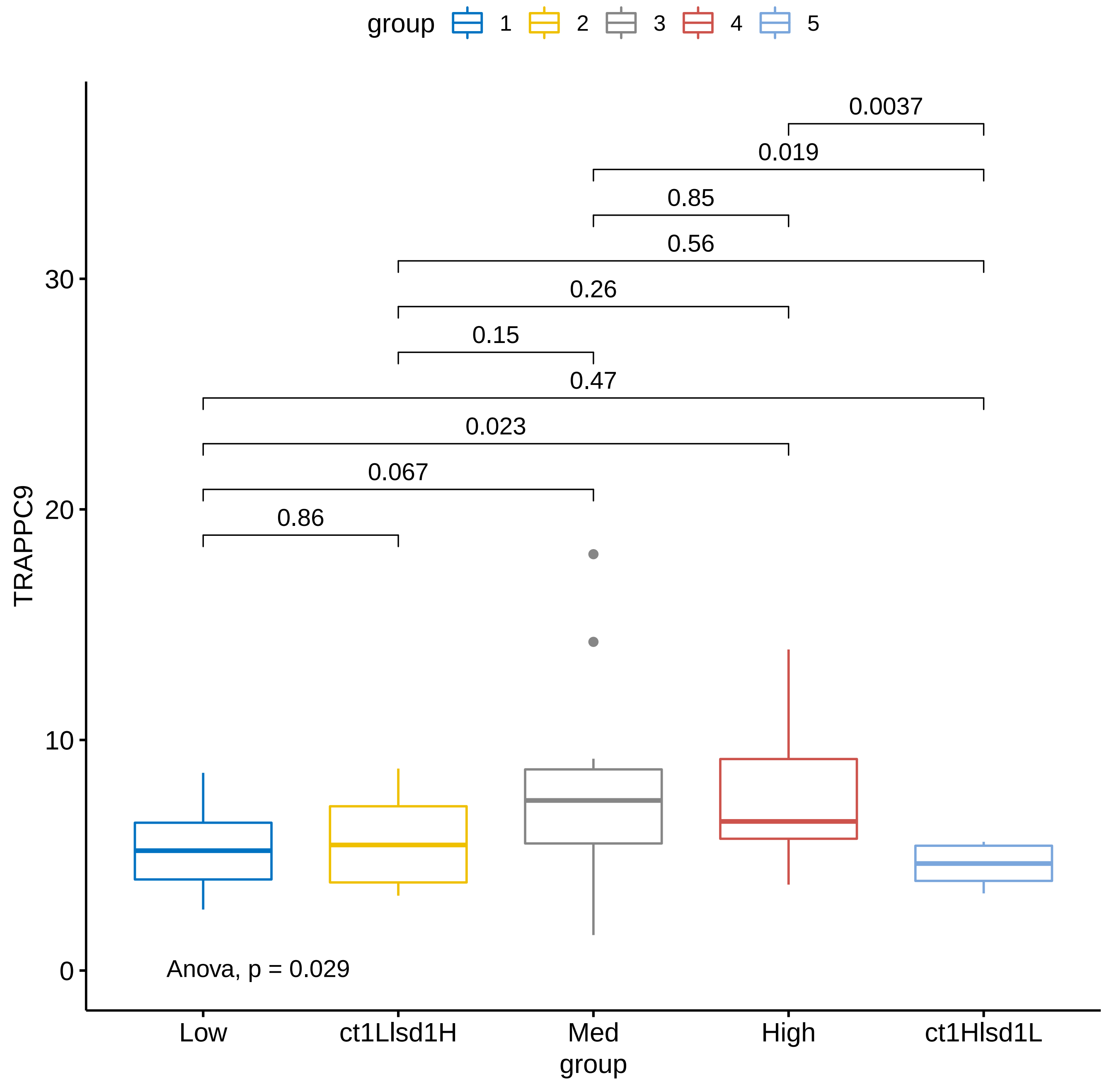
group 1 2 3 4 5
0.0037
0.019
0.85
0.56
0.26
0.15
0.47
0.023
0.067
0.86
30
20
10
0
TRAPPC9
Anova, p = 0.029
Low
ct1Llsd1H
Med
High
ct1Hlsd1L
group

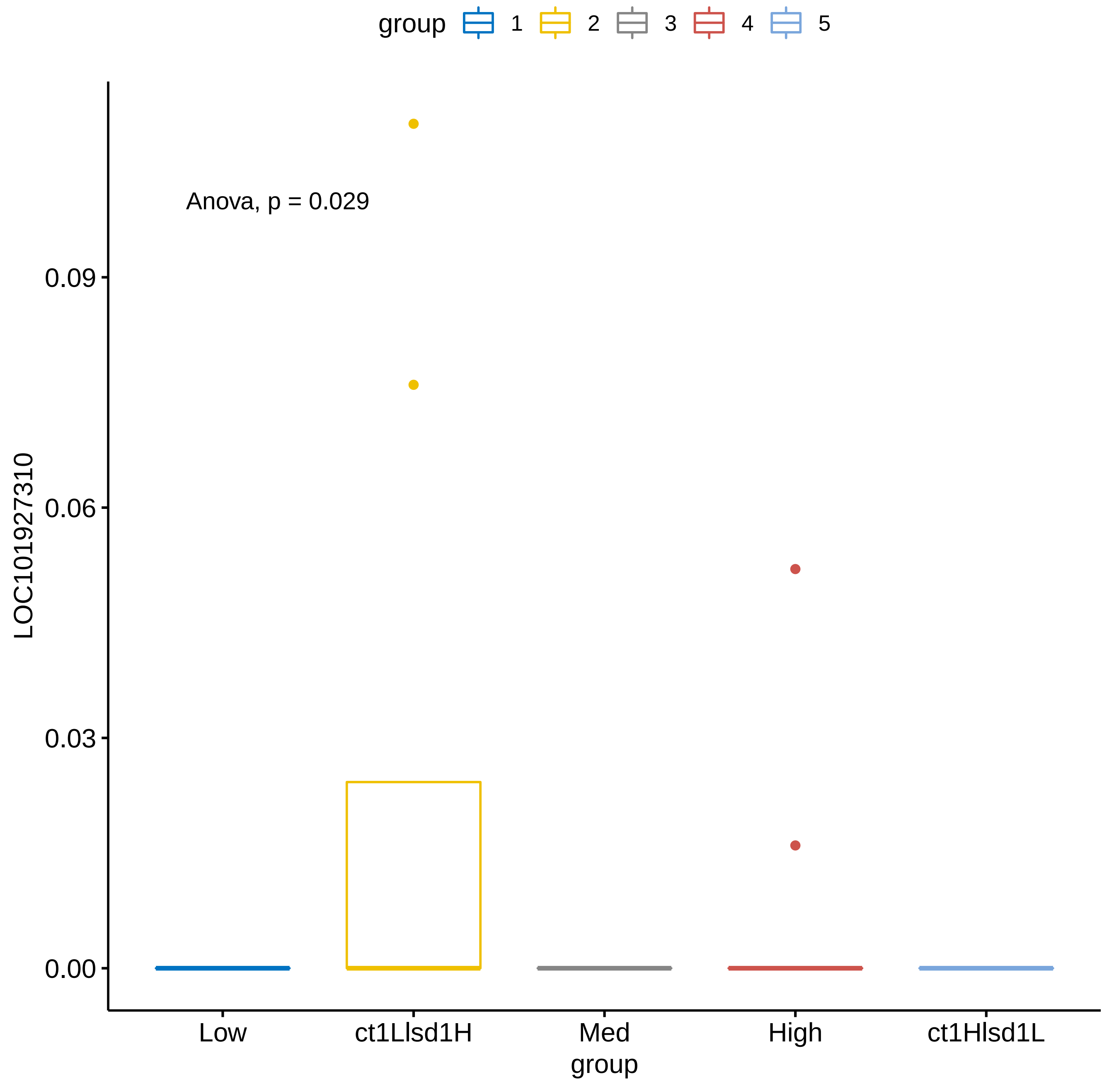
group
1
2
3
4
5
Anova, p = 0.029
LOC101927310
0.09
0.06
0.03
0.00
Low
ct1Llsd1H
Med
High
ct1Hlsd1L
group

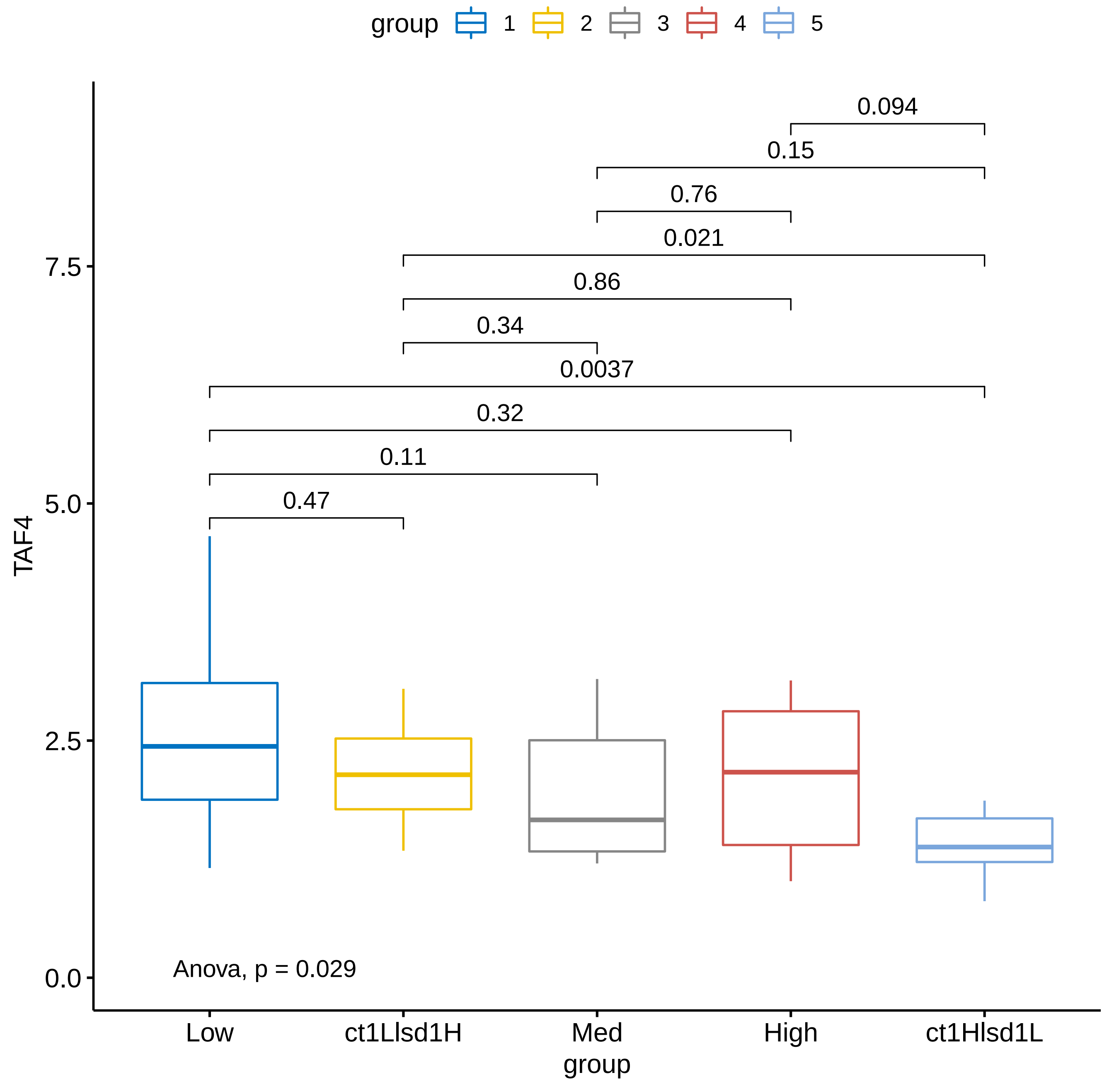

group
1
2
3
4
5
0.094
0.15
0.76
0.021
0.86
0.34
0.0037
0.32
0.11
0.47
7.5
5.0
2.5
0.0
TAF4
Anova, p = 0.029
Low
ct1Llsd1H
Med
High
ct1Hlsd1L
group

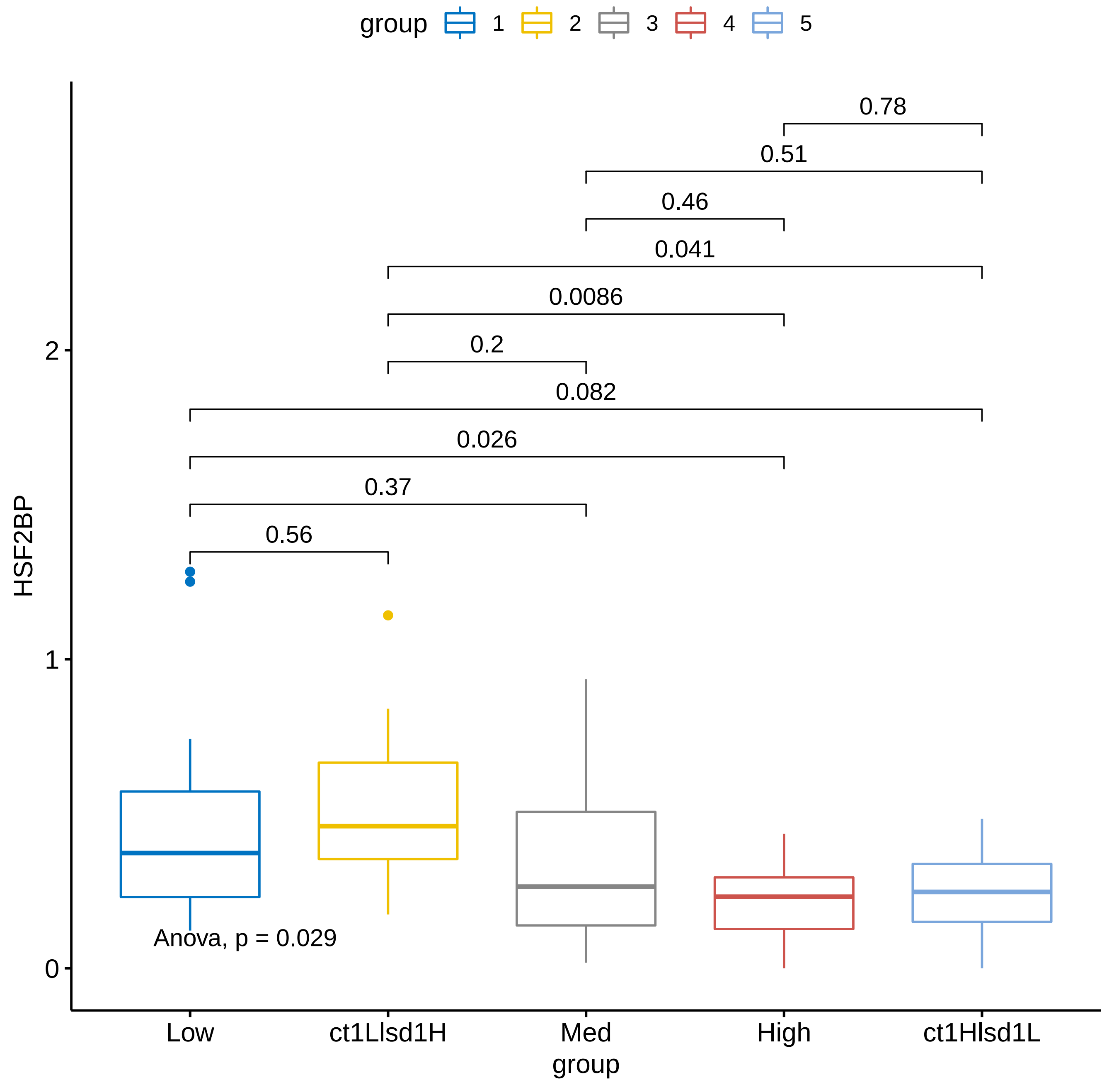

group
1
2
3
4
5
0.78
0.51
0.46
0.041
0.0086
0.2
0.082
0.026
0.37
0.56
Anova, p = 0.029
HSF2BP
2
1
0
Low
ct1Llsd1H
Med
High
ct1Hlsd1L
group

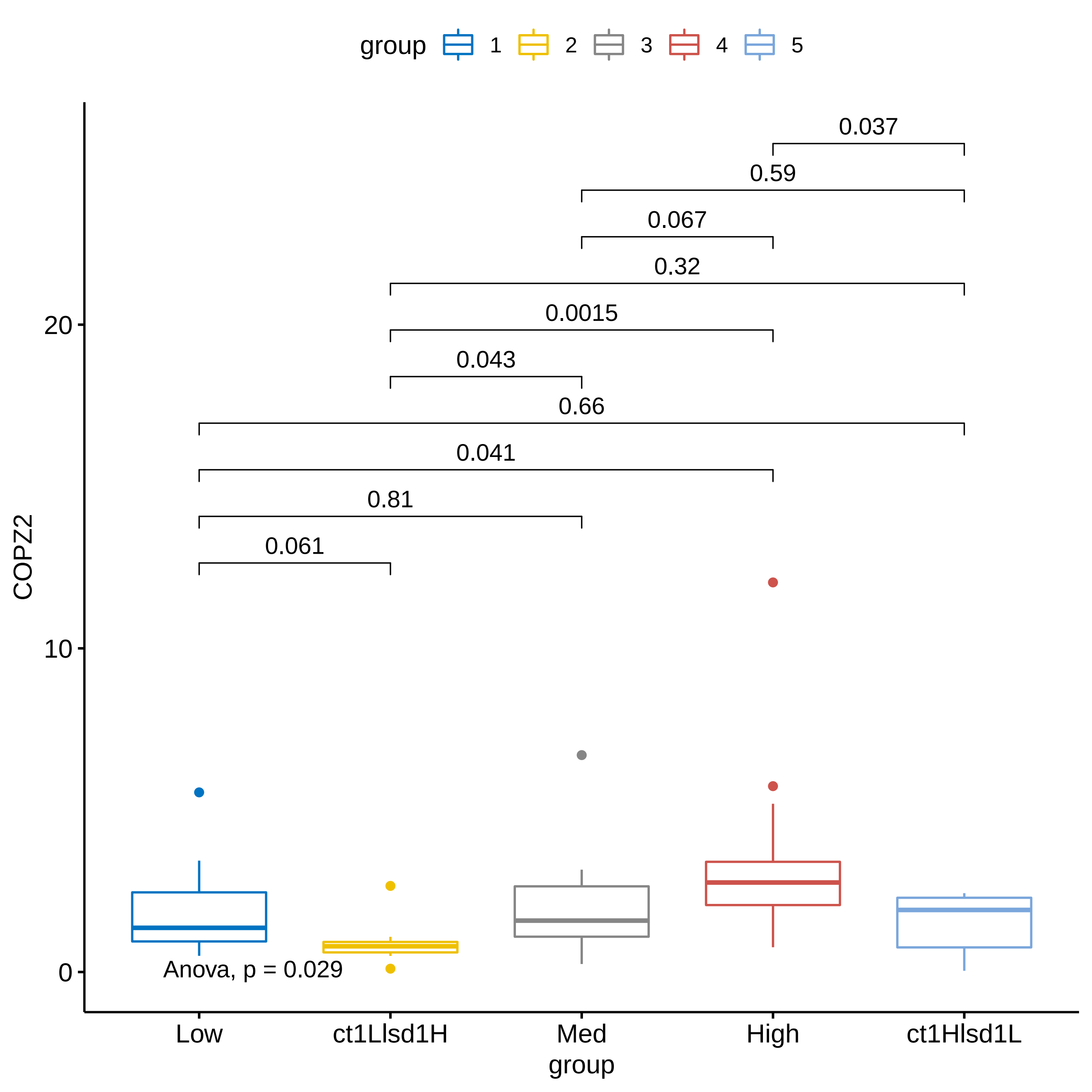

group
1
2
3
4
5
0.037
0.59
0.067
0.32
0.0015
0.043
0.66
0.041
0.81
0.061
20
10
0
COPZ2
Anova, p = 0.029
Low
ct1Llsd1H
Med
High
ct1Hlsd1L
group

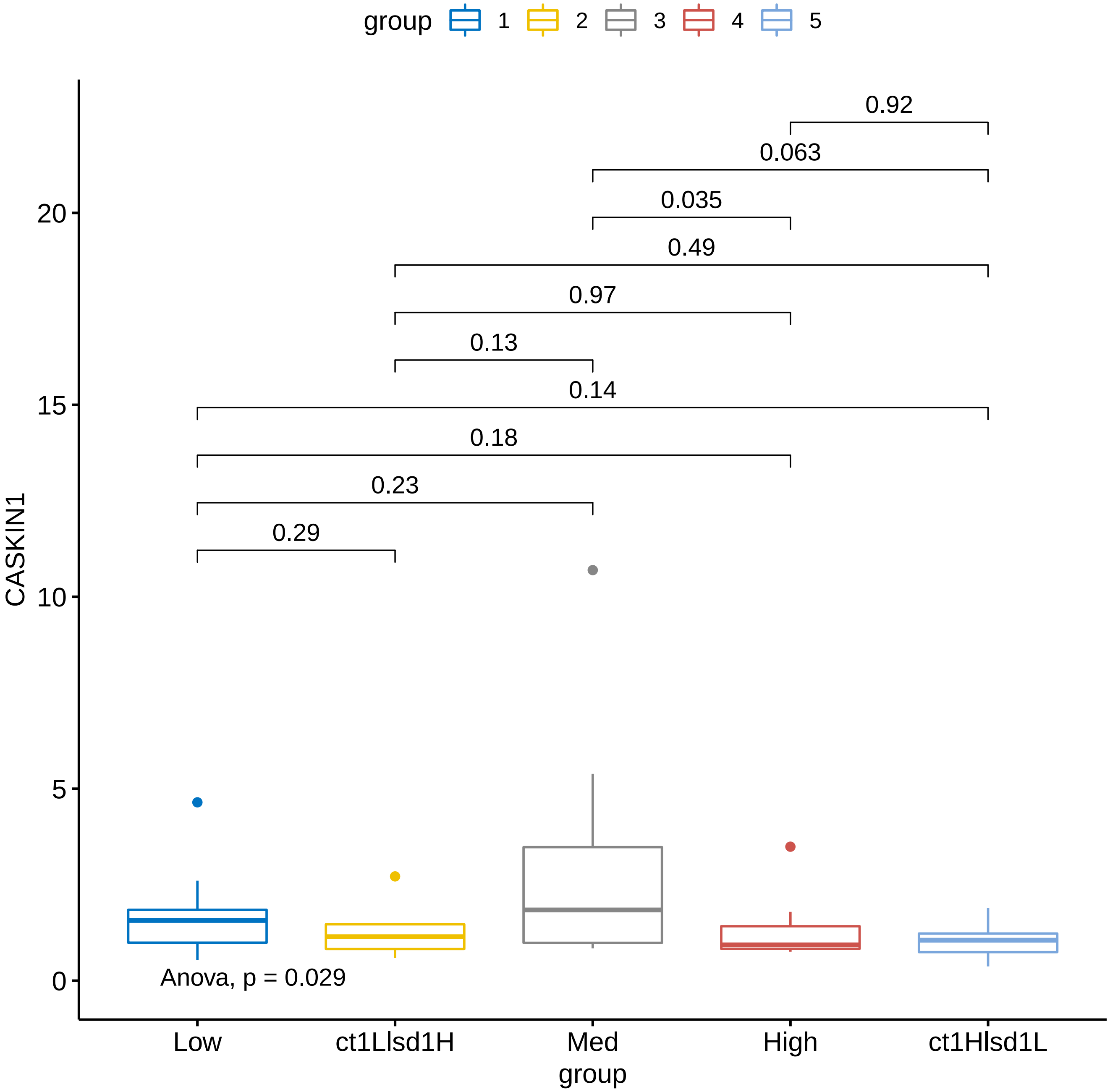

group 1 2 3 4 5
0.92
0.063
0.035
0.49
0.97
0.13
0.14
0.18
0.23
0.29
CASKIN1
20
15
10
5
0
Anova, p = 0.029
Low
ct1Llsd1H
Med
High
ct1Hlsd1L
group

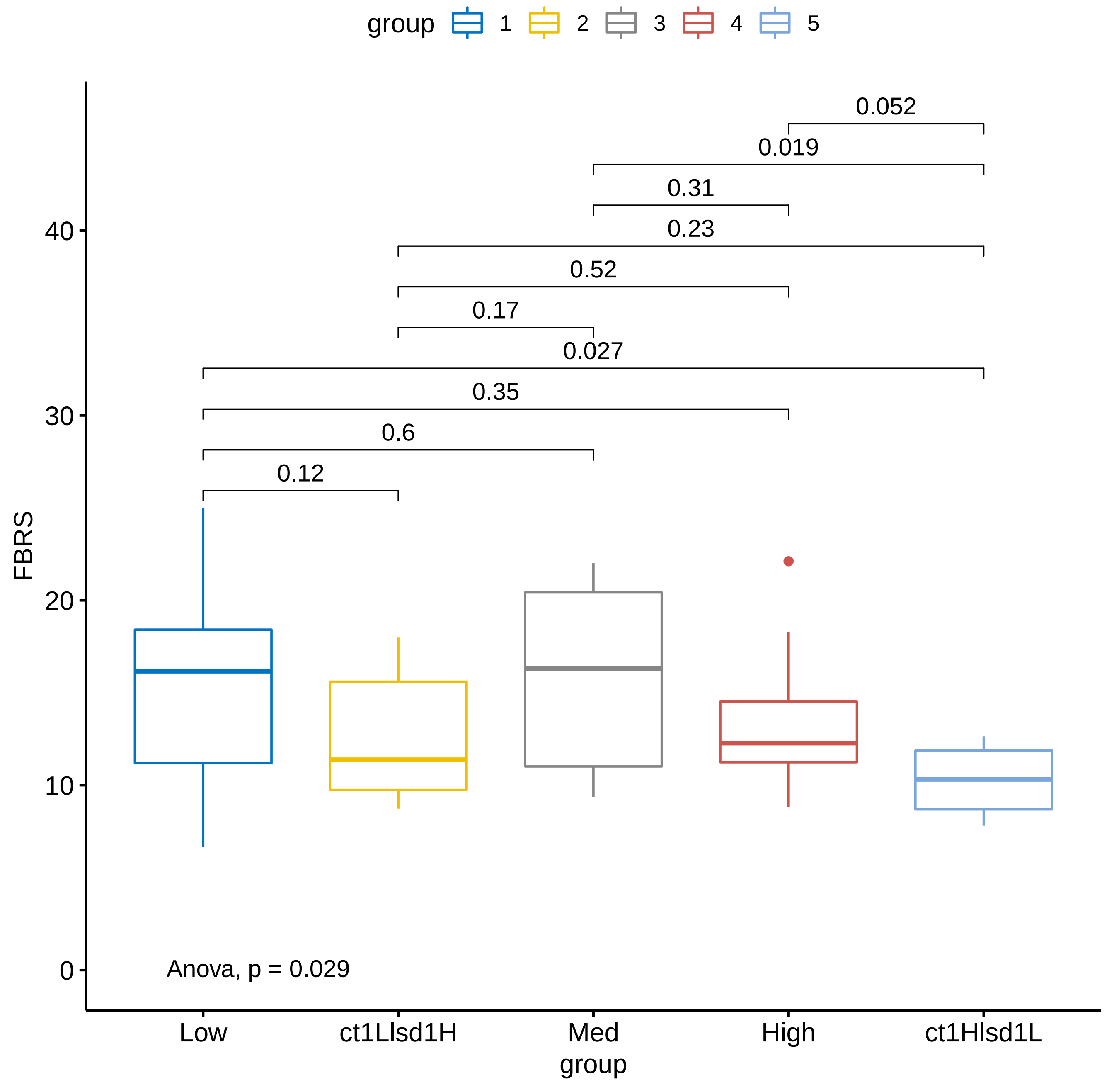

group 1 2 3 4 5
0.052
0.019
0.31
0.23
0.52
0.17
0.027
0.35
0.6
0.12
40
30
20
10
0
FBRS
Anova, p = 0.029
Low
ct1Llsd1H
Med
High
ct1Hlsd1L
group

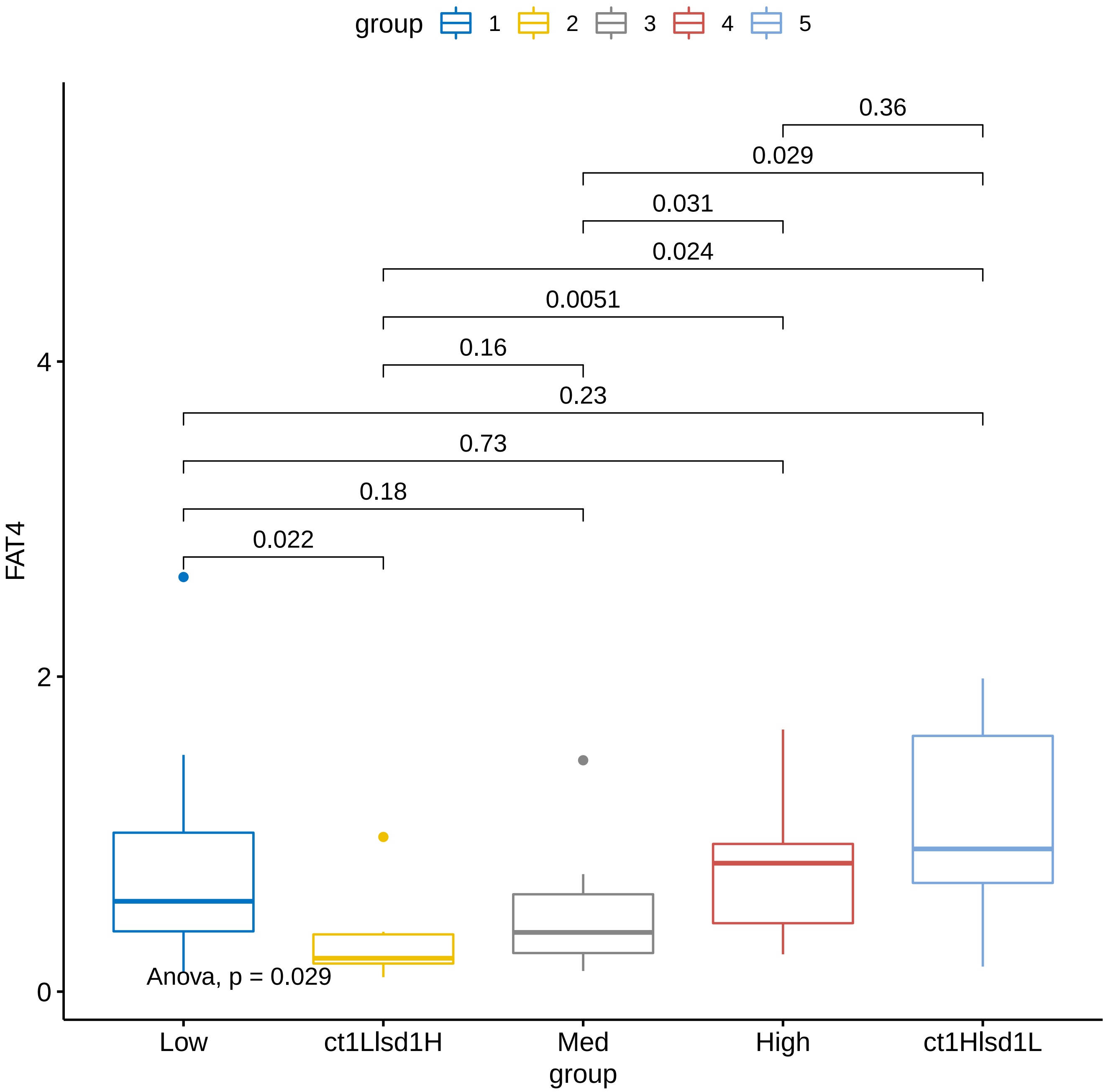

group
1
2
3
4
5
0.36
0.029
0.031
0.024
0.0051
0.16
0.23
0.73
0.18
0.022
4
2
0
FAT4
Anova, p = 0.029
Low
ct1Llsd1H
Med
High
ct1Hlsd1L
group

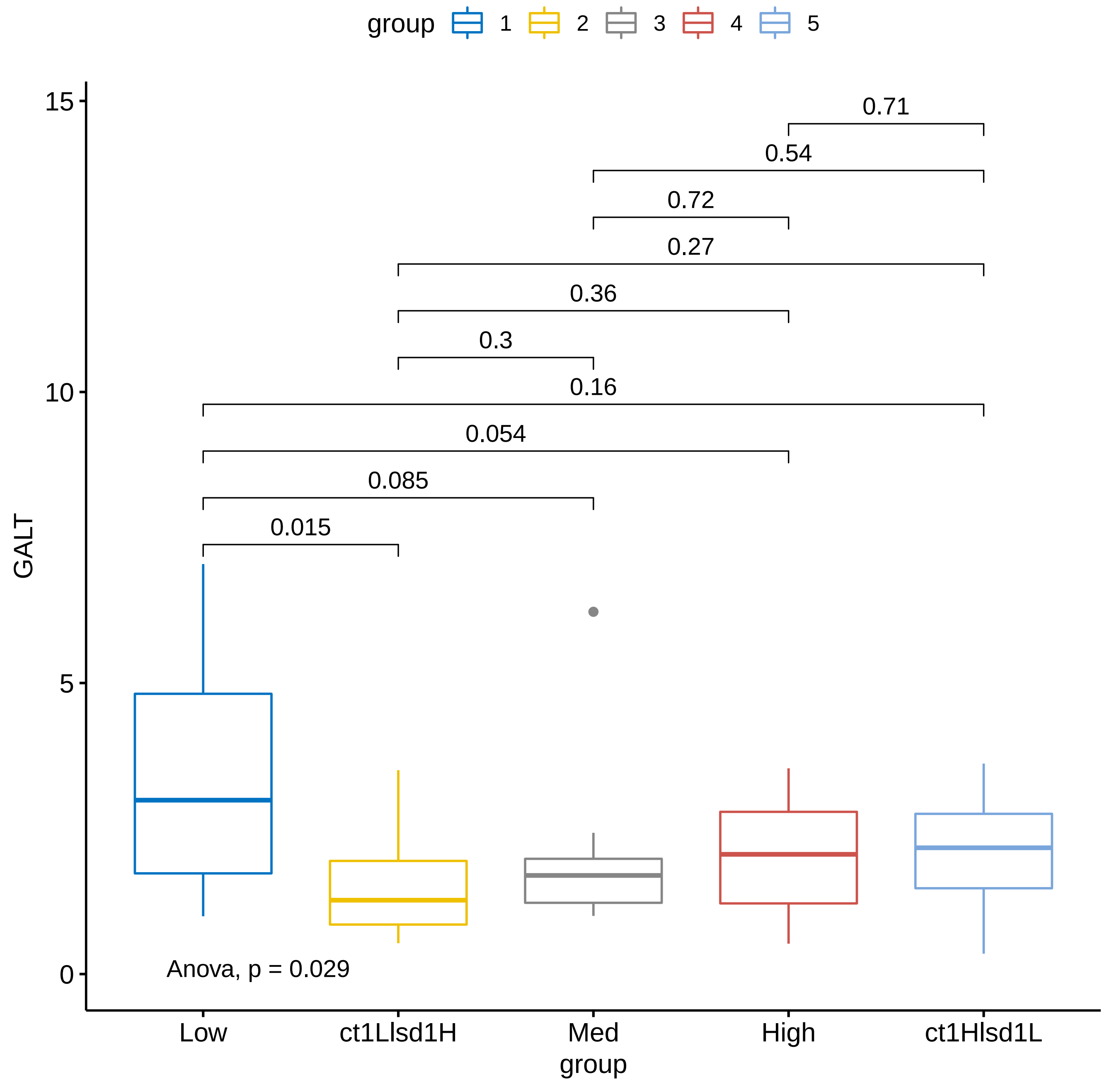

group 1 2 3 4 5
15
10
5
0
GALT
0.71
0.54
0.72
0.27
0.36
0.3
0.16
0.054
0.085
0.015
Anova, p = 0.029
Low
ct1Llsd1H
Med
High
ct1Hlsd1L
group

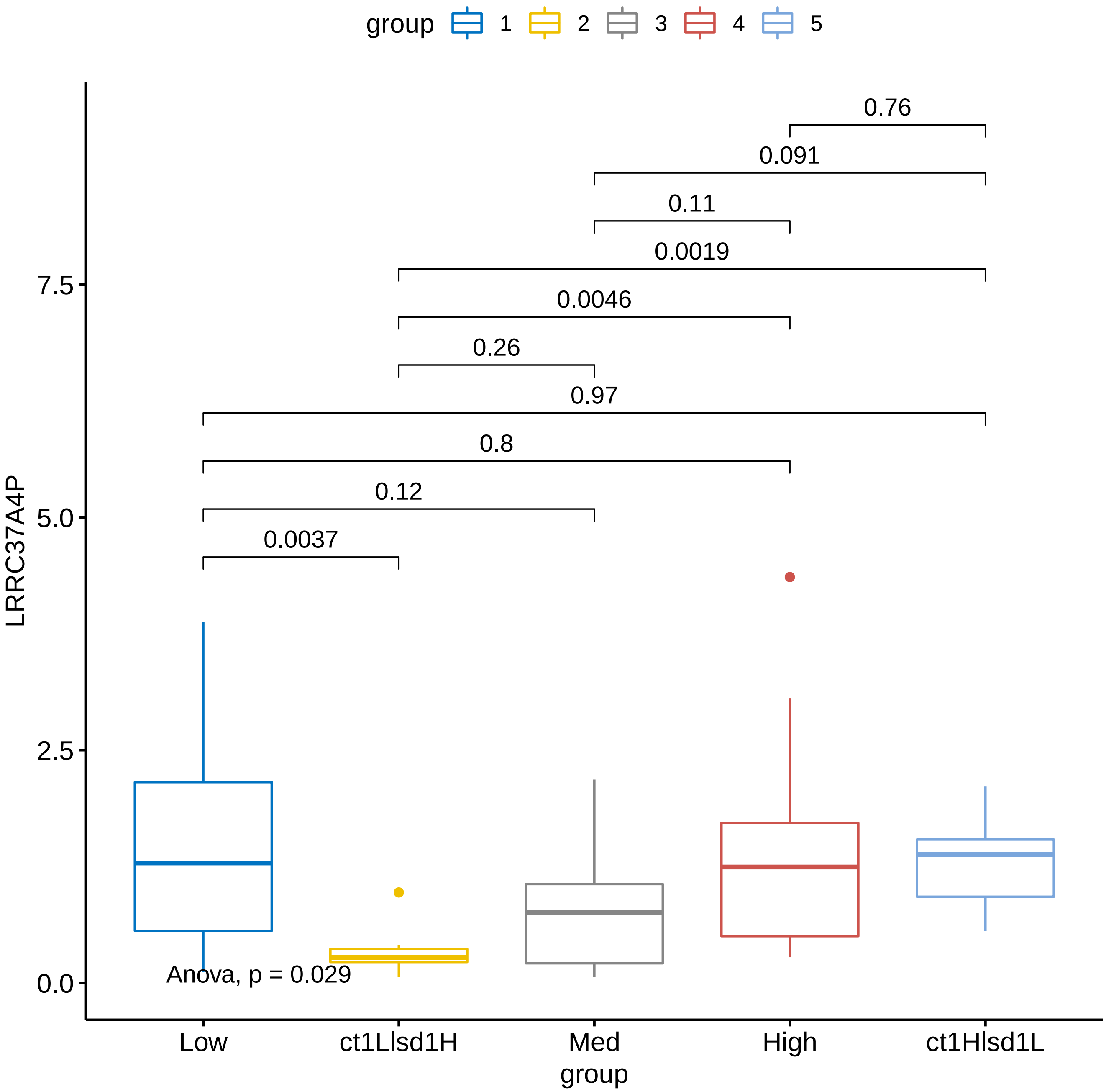
group
1
2
3
4
5
0.76
0.091
0.11
0.0019
0.0046
0.26
0.97
0.8
0.12
0.0037
LRRC37A4P
7.5
5.0
2.5
0.0
Anova, p = 0.029
Low
ct1Llsd1H
Med
High
ct1Hlsd1L
group

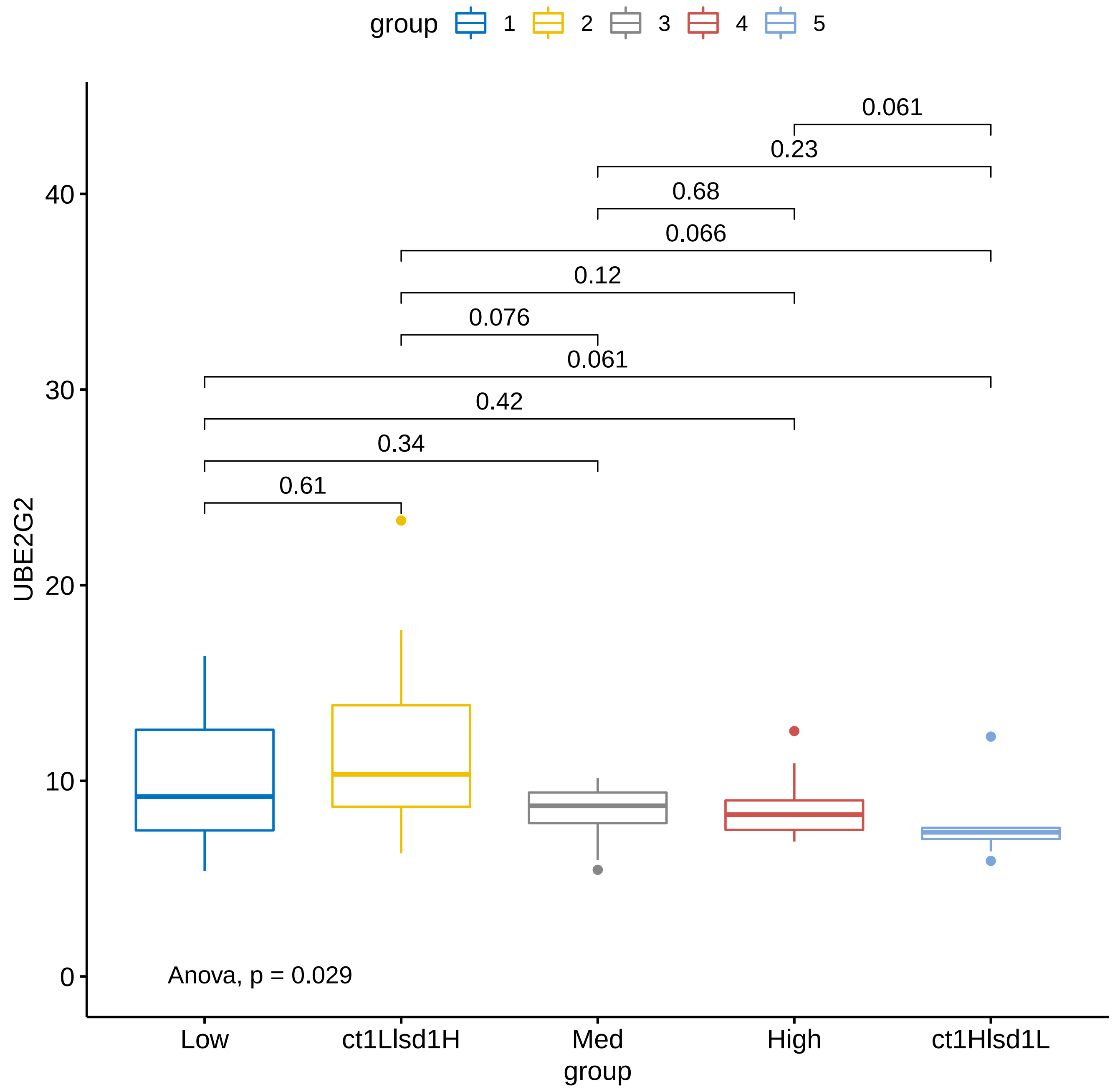

group
1
2
3
4
5
UBE2G2
0
10
20
30
40
0.061
0.23
0.68
0.066
0.12
0.076
0.061
0.42
0.34
0.61
Anova, p = 0.029
Low
ct1Llsd1H
Med
High
ct1Hlsd1L
group

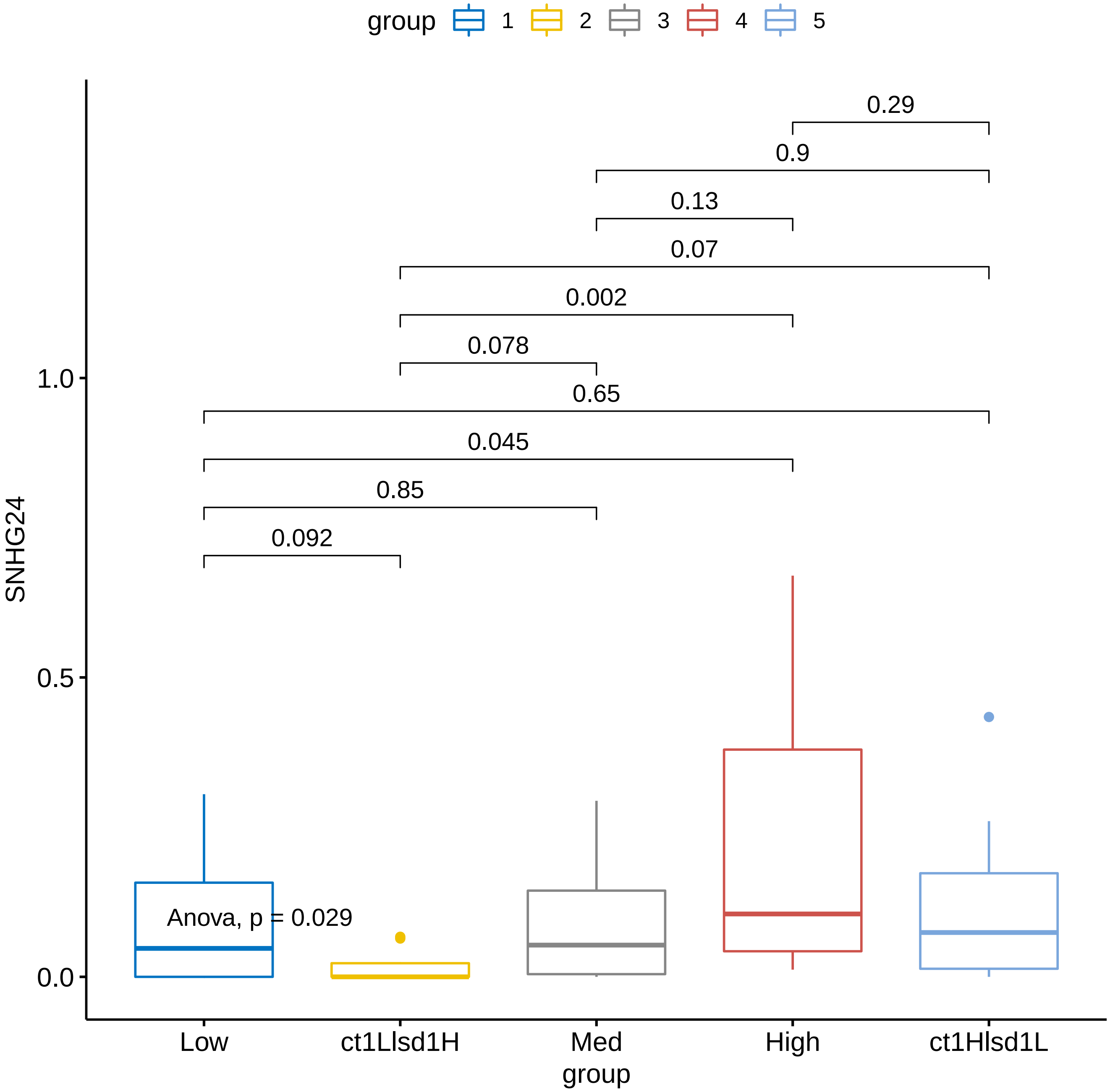

group 1 2 3 4 5
0.29
0.9
0.13
0.07
0.002
0.078
0.65
0.045
0.85
0.092
1.0
0.5
0.0
SNHG24
Anova, p = 0.029
Low
ct1Llsd1H
Med
High
ct1Hlsd1L
group

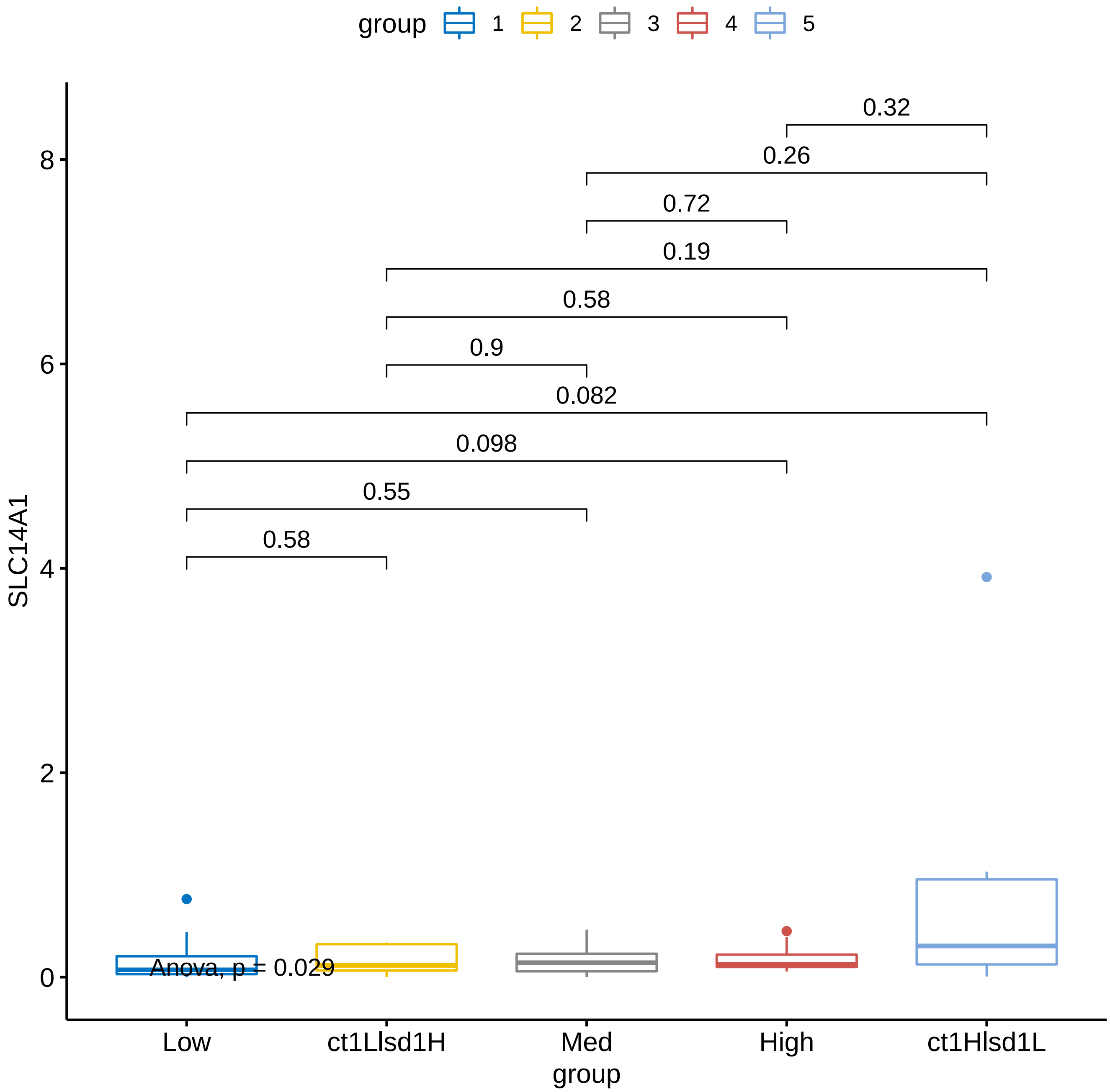

group 1 2 3 4 5
0.32
0.26
0.72
0.19
0.58
0.9
0.082
0.098
0.55
0.58
Anova, p = 0.029
8
6
4
2
0
SLC14A1
Low
ct1Llsd1H
Med
High
ct1Hlsd1L
group

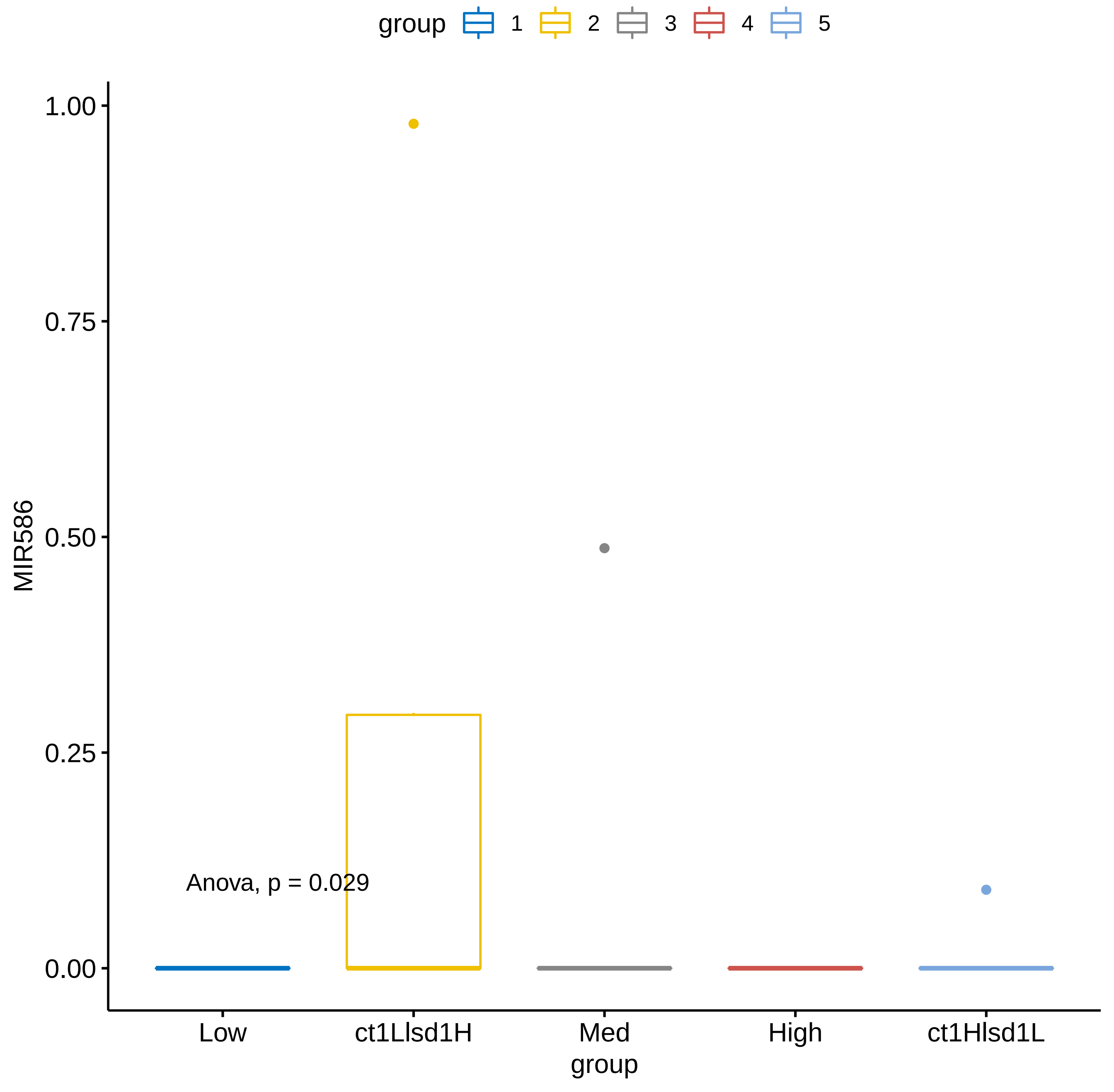
group 1 2 3 4 5
1.00
0.75
0.50
0.25
0.00
MIR586
Anova, p = 0.029
Low
ct1Llsd1H
Med
High
ct1Hlsd1L
group

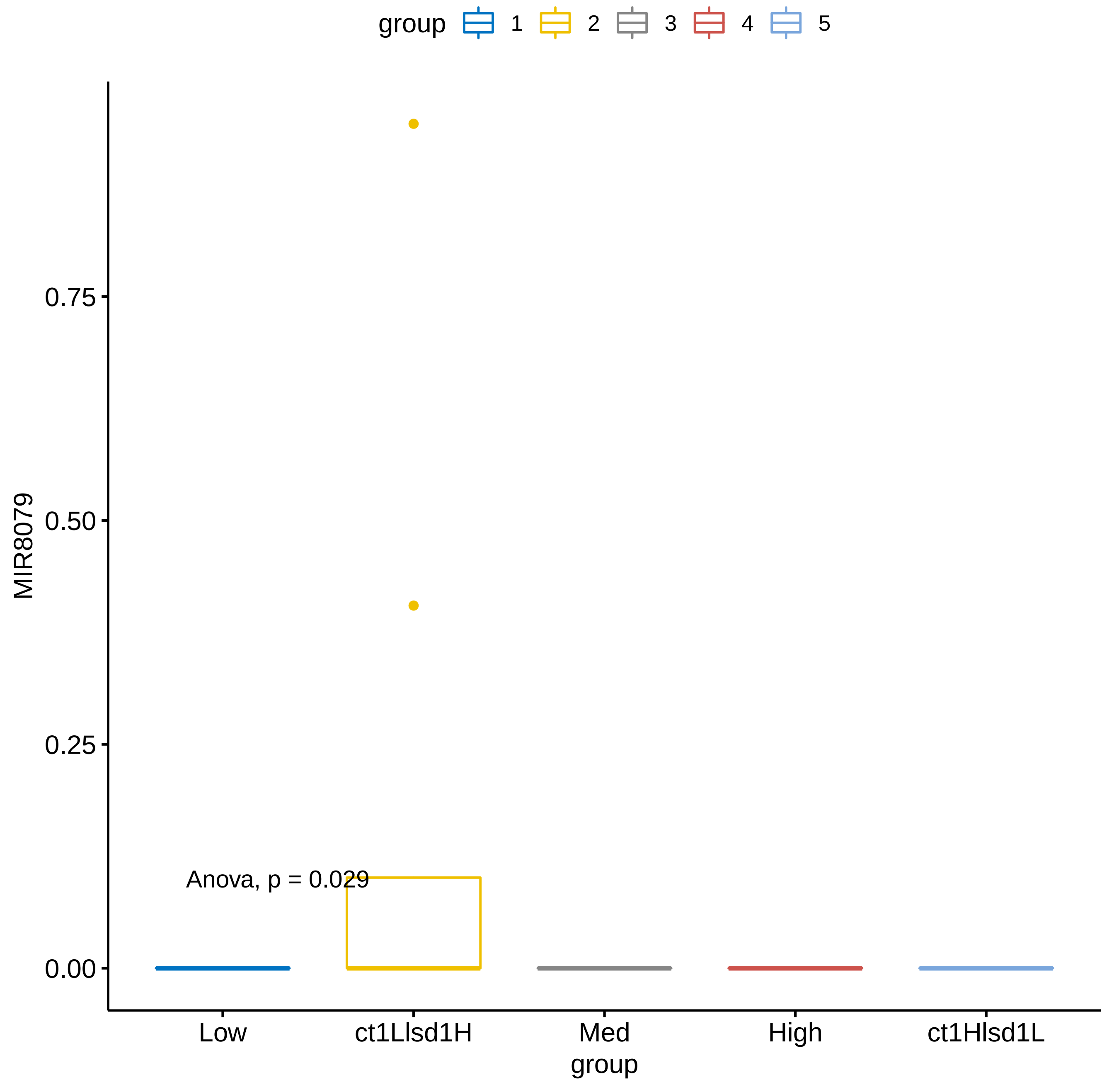

group
1
2
3
4
5
MIR8079
0.75
0.50
0.25
0.00
Anova, p = 0.029
Low
ct1Llsd1H
Med
High
ct1Hlsd1L
group

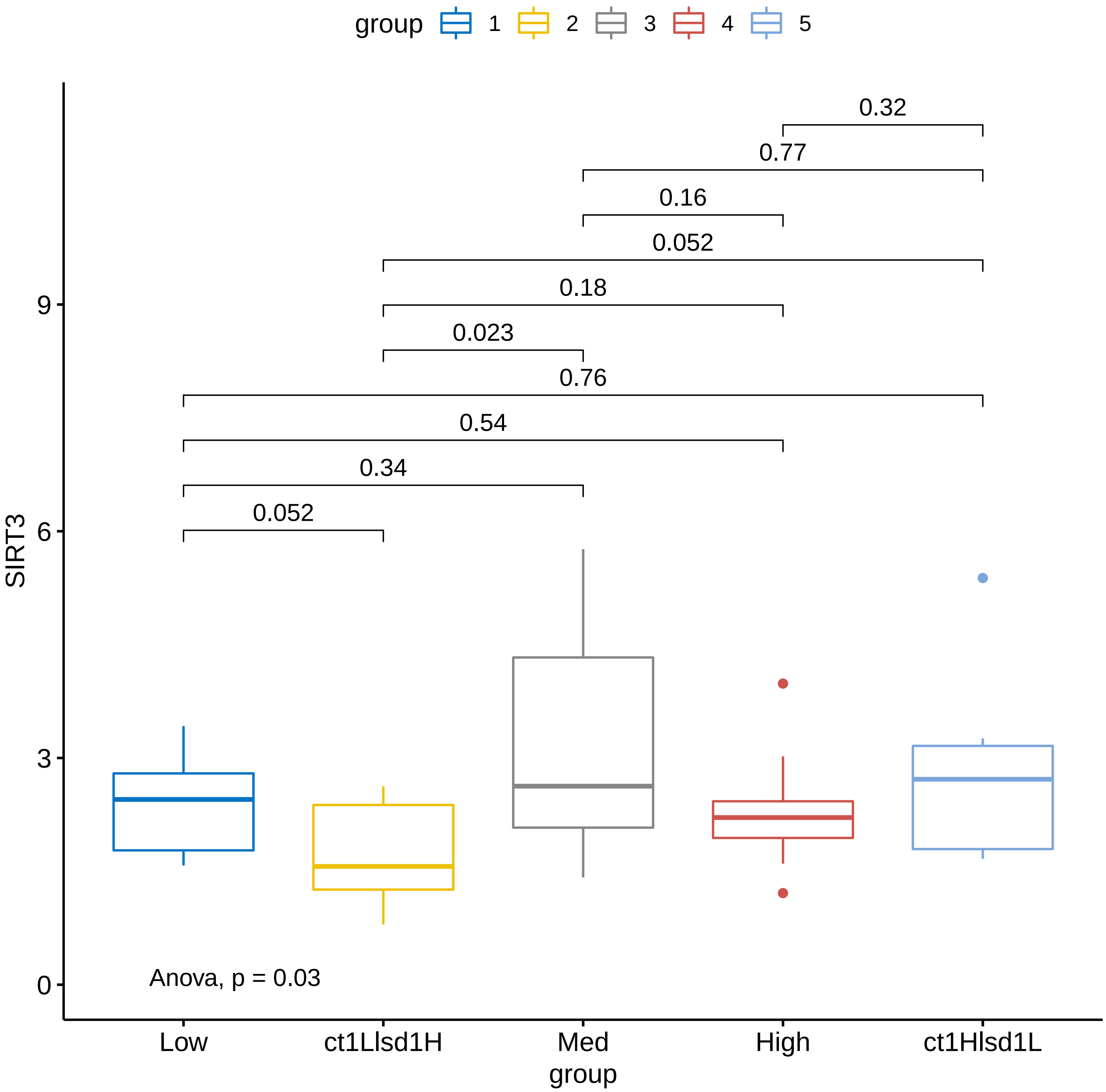
group
1
2
3
4
5
0.32
0.77
0.16
0.052
0.18
0.023
0.76
0.54
0.34
0.052
9
6
3
0
SIRT3
Anova, p = 0.03
Low
ct1Llsd1H
Med
High
ct1Hlsd1L
group

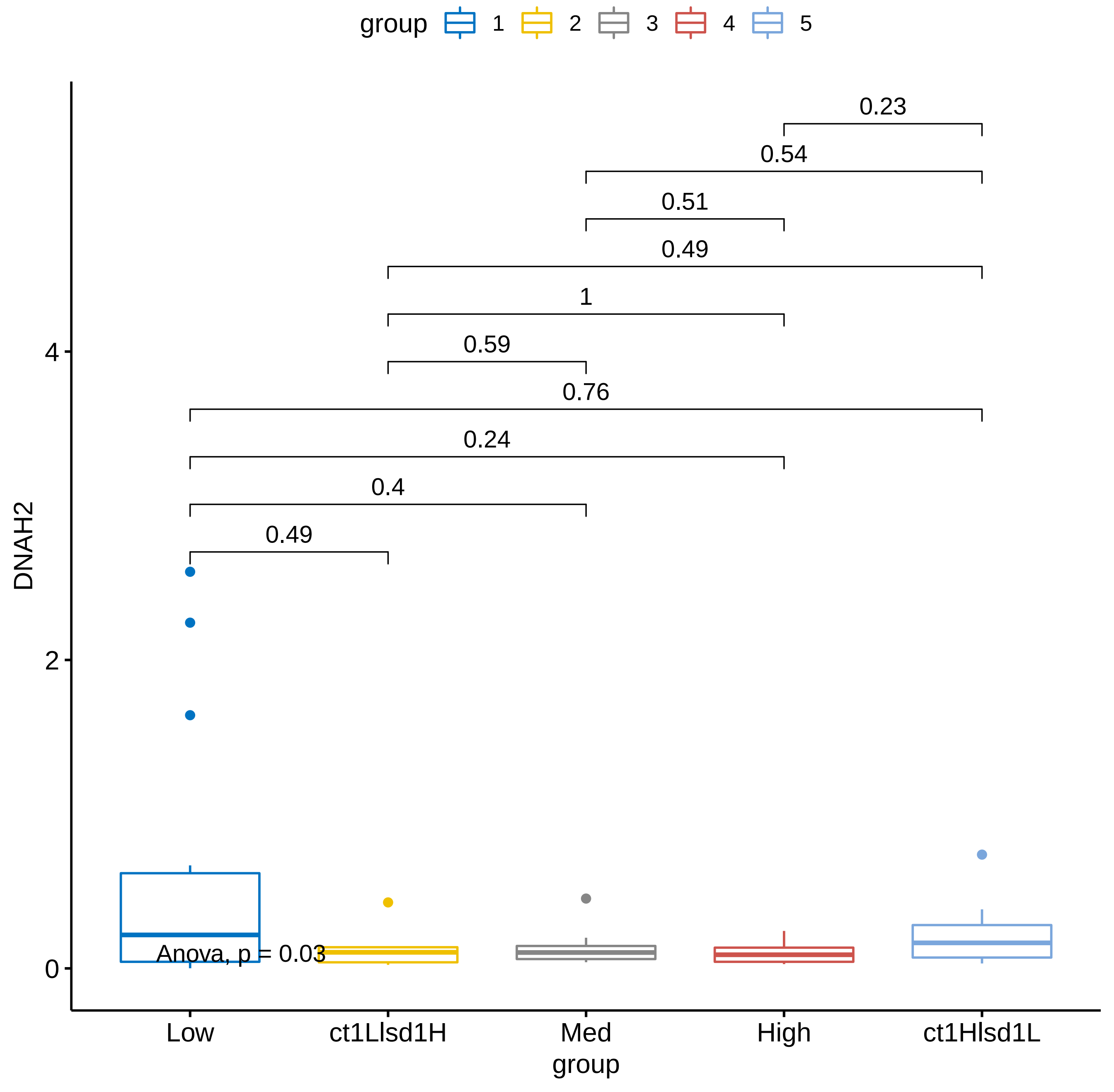
group 1 2 3 4 5
0.23
0.54
0.51
0.49
1
0.59
0.76
0.24
0.4
0.49
Anova, p = 0.03
DNAH2
4
2
0
Low
ct1Llsd1H
Med
High
ct1Hlsd1L
group

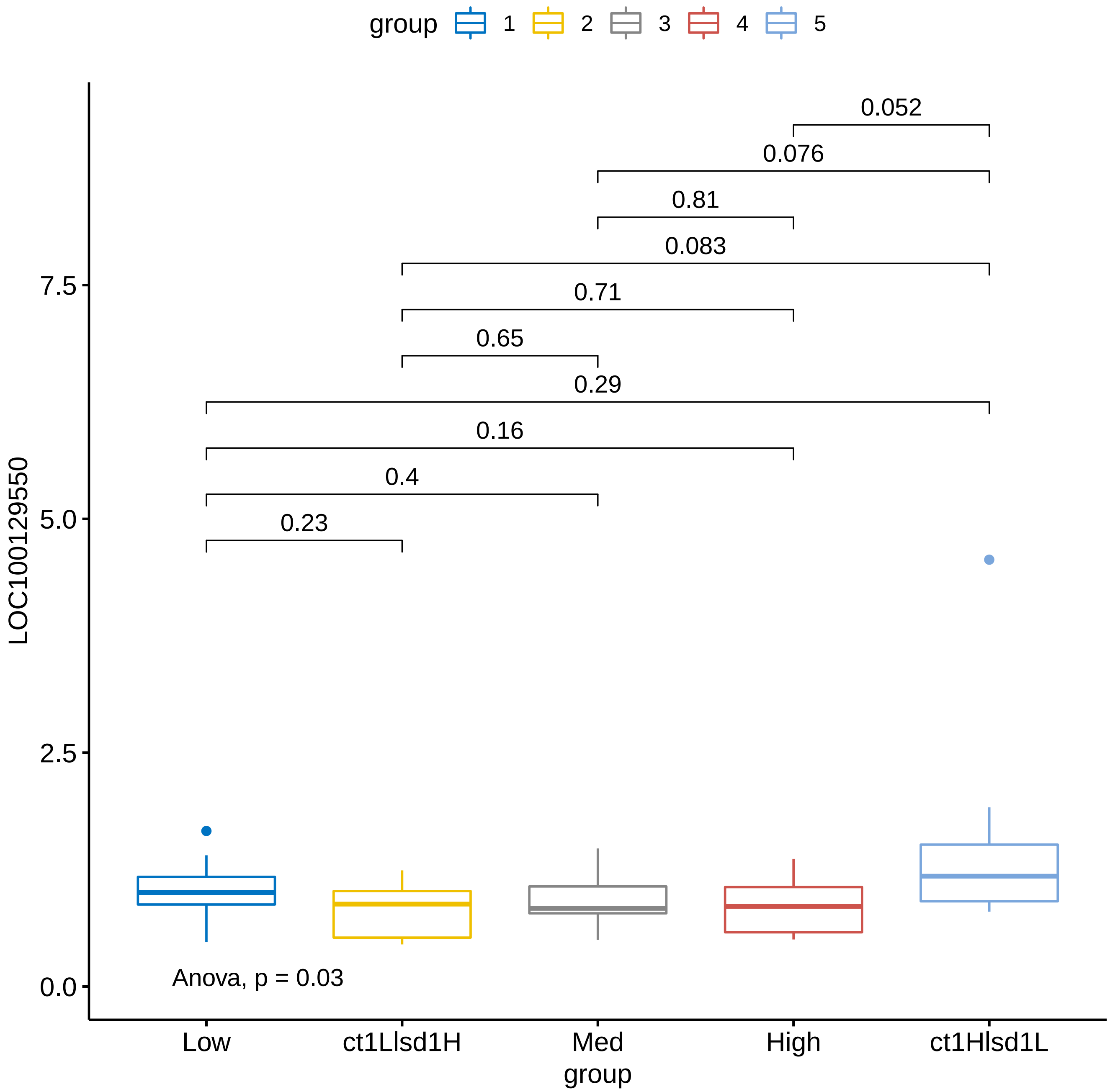
group
1
2
3
4
5
0.052
0.076
0.81
0.083
0.71
0.65
0.29
0.16
0.4
0.23
7.5
5.0
2.5
0.0
LOC100129550
Anova, p = 0.03
Low
ct1Llsd1H
Med
High
ct1Hlsd1L
group

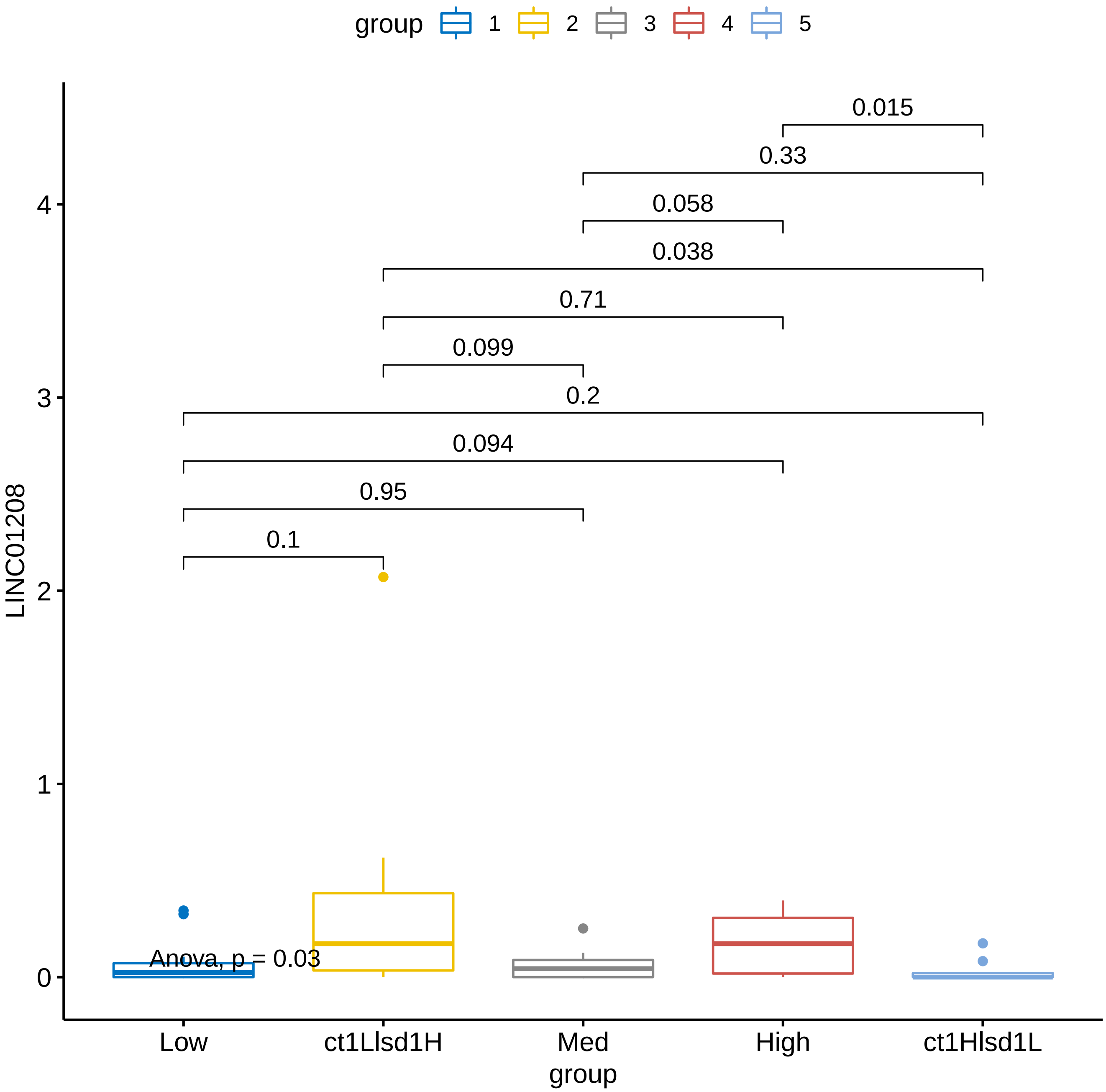
group
1
2
3
4
5
0.015
0.33
0.058
0.038
0.71
0.099
0.2
0.094
0.95
0.1
Anova, p = 0.03
LINC01208
0
1
2
3
4
Low
ct1Llsd1H
Med
High
ct1Hlsd1L
group

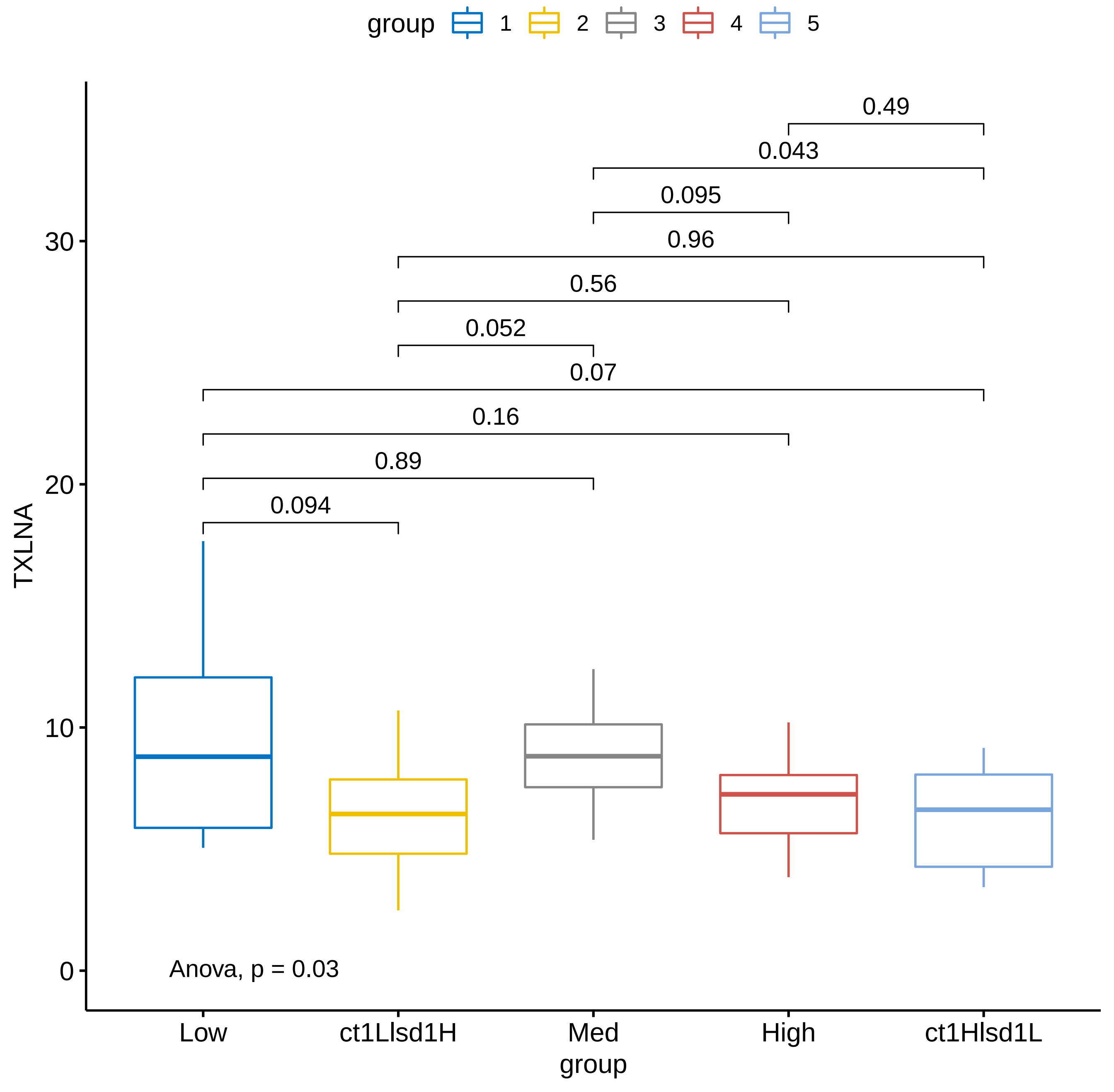
group 1 2 3 4 5
0.49
0.043
0.095
0.96
0.56
0.052
0.07
0.16
0.89
0.094
30
20
10
0
TXLNA
Anova, p = 0.03
Low
ct1Llsd1H
Med
High
ct1Hlsd1L
group

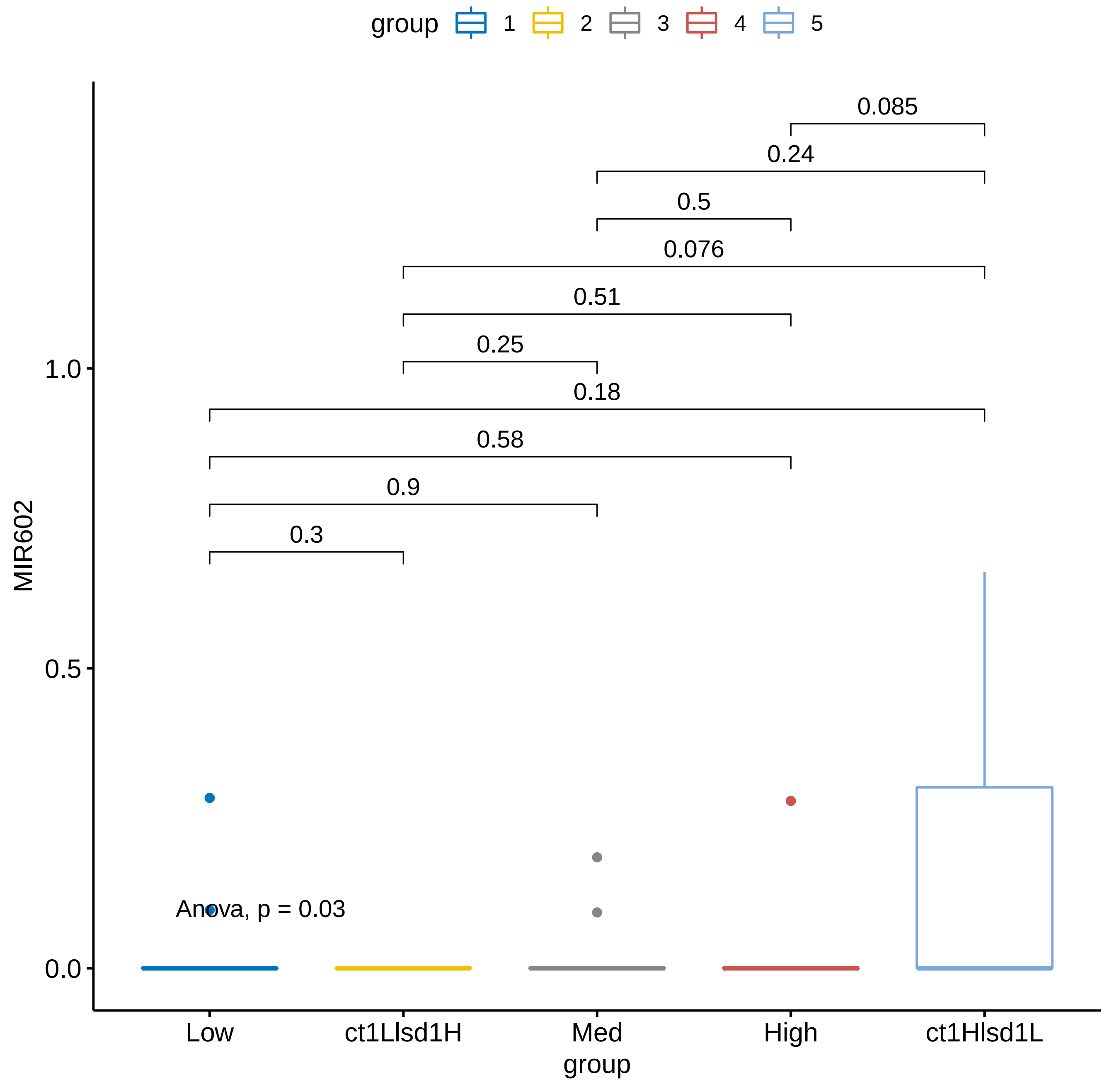
group
1
2
3
4
5
0.085
0.24
0.5
0.076
0.51
0.25
0.18
0.58
0.9
0.3
Anova, p = 0.03
MIR602
1.0
0.5
0.0
Low
ct1Llsd1H
Med
High
ct1Hlsd1L
group

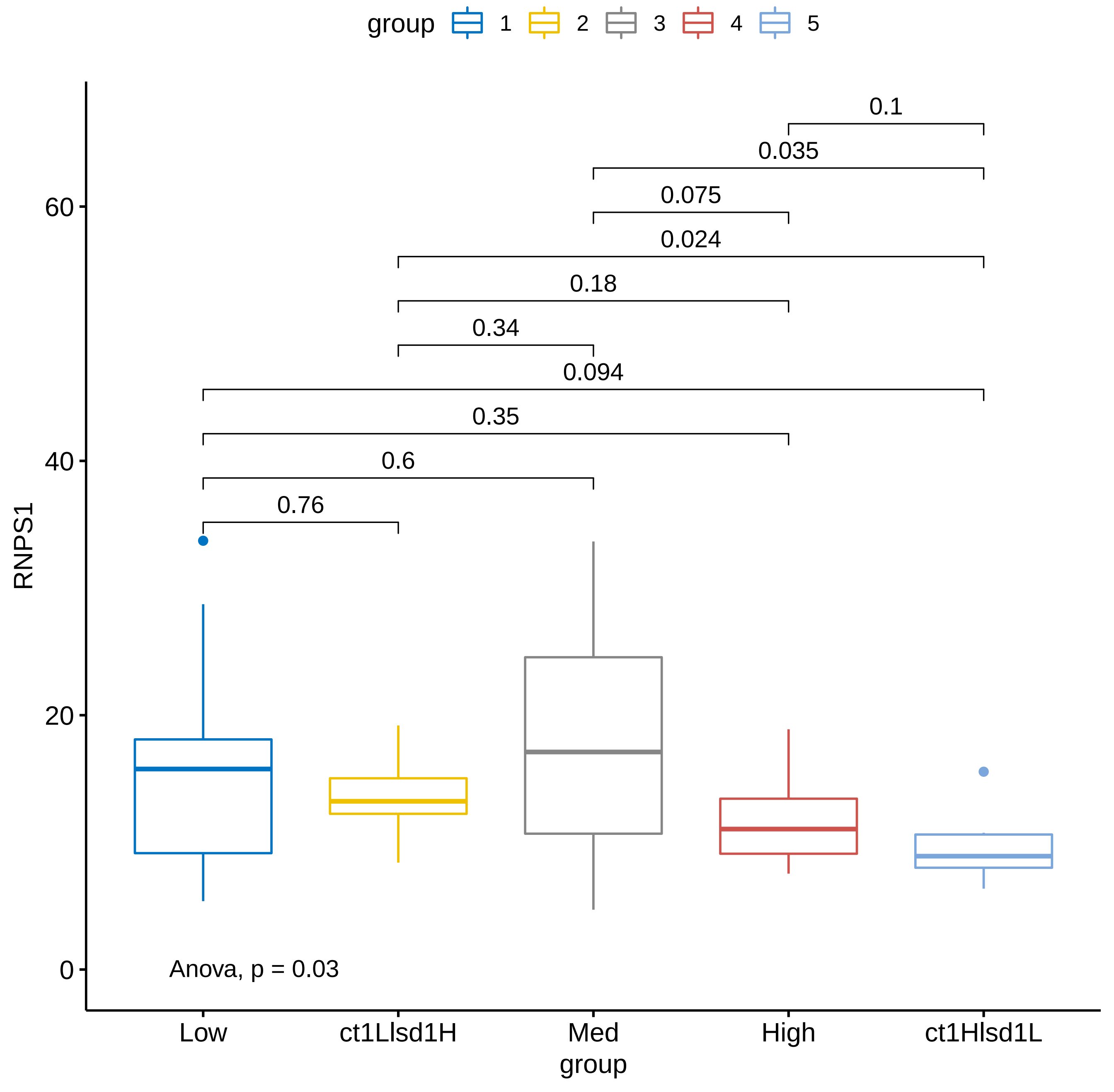

group
1
2
3
4
5
0.1
0.035
0.075
0.024
0.18
0.34
0.094
0.35
0.6
0.76
60
40
20
0
RNPS1
Anova, p = 0.03
Low
ct1Llsd1H
Med
High
ct1Hlsd1L
group

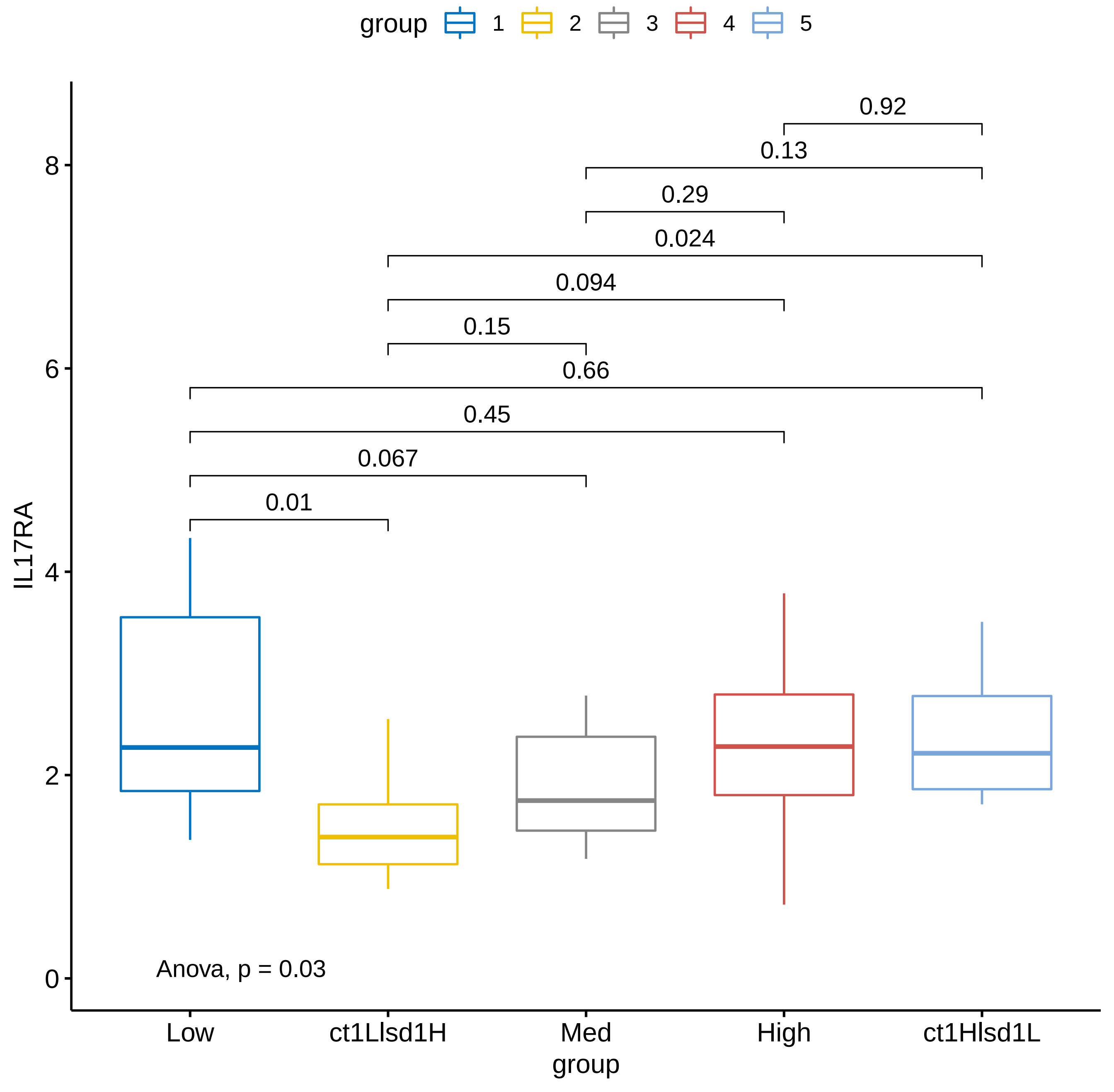

group 1 2 3 4 5
IL17RA
0.92
0.13
0.29
0.024
0.094
0.15
0.66
0.45
0.067
0.01
Anova, p = 0.03
8
6
4
2
0
Low
ct1Llsd1H
Med
High
ct1Hlsd1L
group

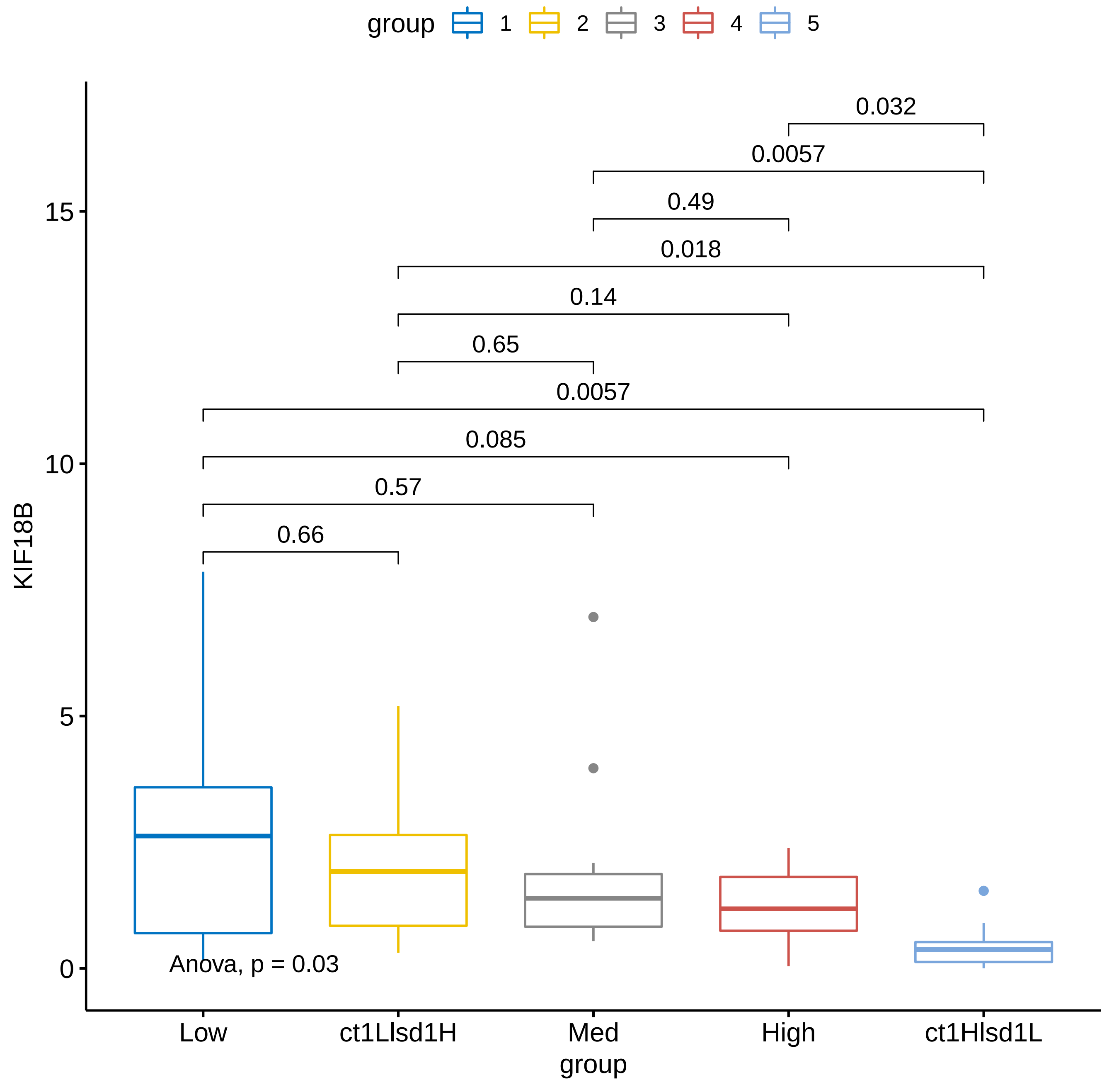

group
1
2
3
4
5
0.032
0.0057
0.49
0.018
0.14
0.65
0.0057
0.085
0.57
0.66
Anova, p = 0.03
15
10
5
0
KIF18B
Low
ct1Llsd1H
Med
High
ct1Hlsd1L
group

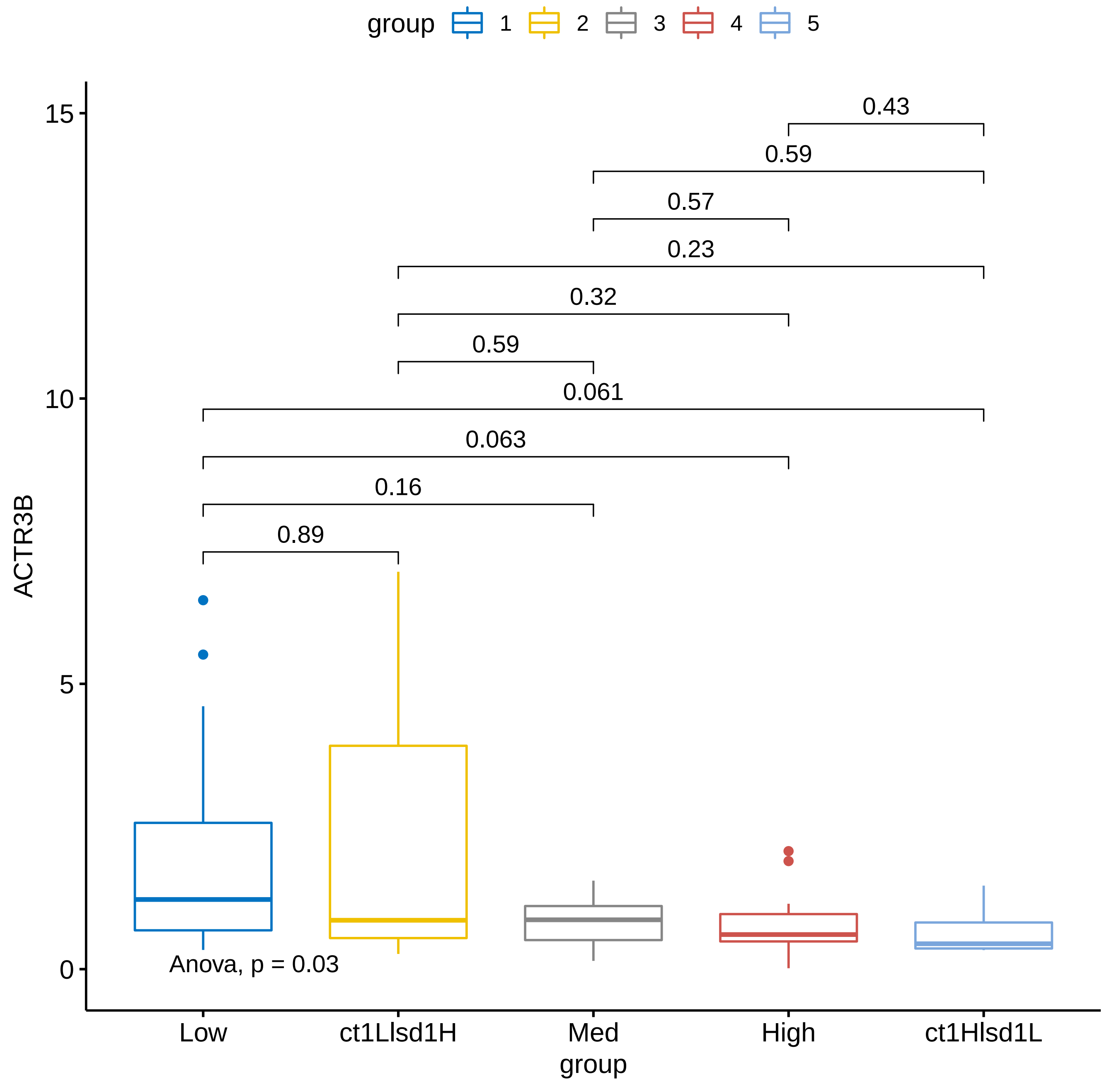
group
1
2
3
4
5
ACTR3B
15
10
5
0
0.43
0.59
0.57
0.23
0.32
0.59
0.061
0.063
0.16
0.89
Anova, p = 0.03
Low
ct1Llsd1H
Med
High
ct1Hlsd1L
group

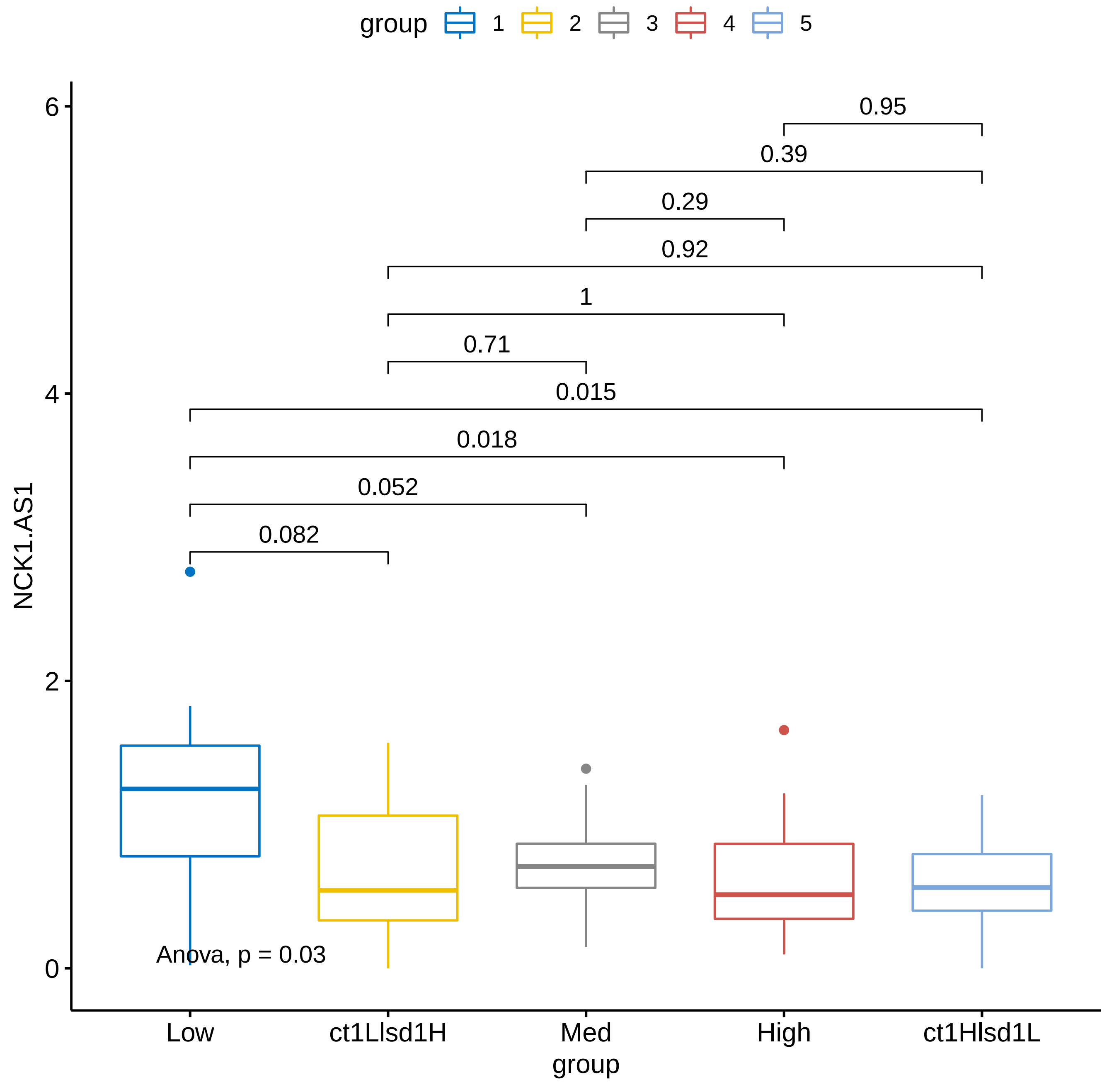
group
1
2
3
4
5
0.95
0.39
0.29
0.92
1
0.71
0.015
0.018
0.052
0.082
Anova, p = 0.03
6
4
2
0
NCK1.AS1
Low
ct1Llsd1H
Med
High
ct1Hlsd1L
group

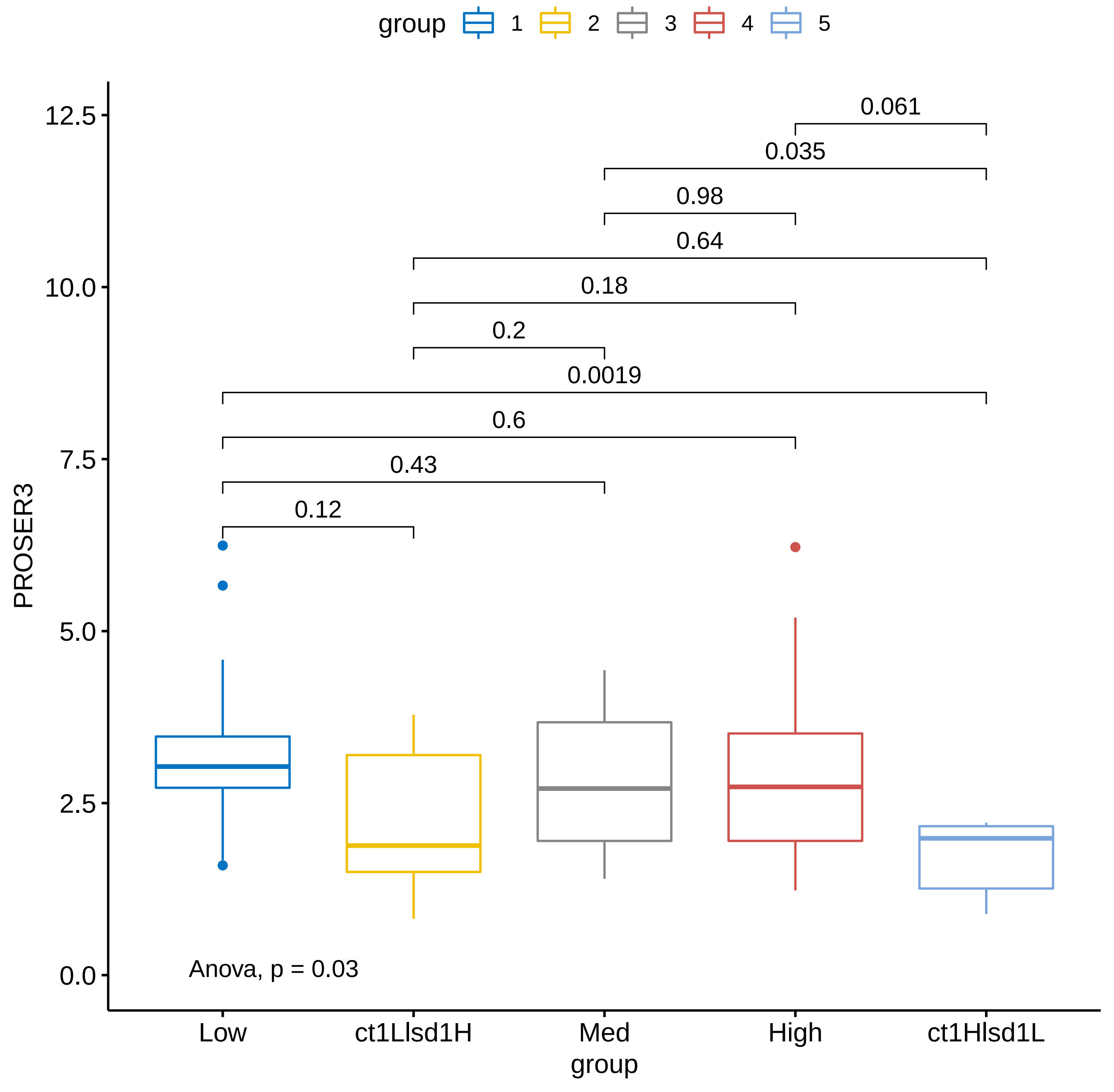

group 1 2 3 4 5
PROSER3
12.5
10.0
7.5
5.0
2.5
0.0
0.061
0.035
0.98
0.64
0.18
0.2
0.0019
0.6
0.43
0.12
Anova, p = 0.03
Low
ct1Llsd1H
Med
High
ct1Hlsd1L
group

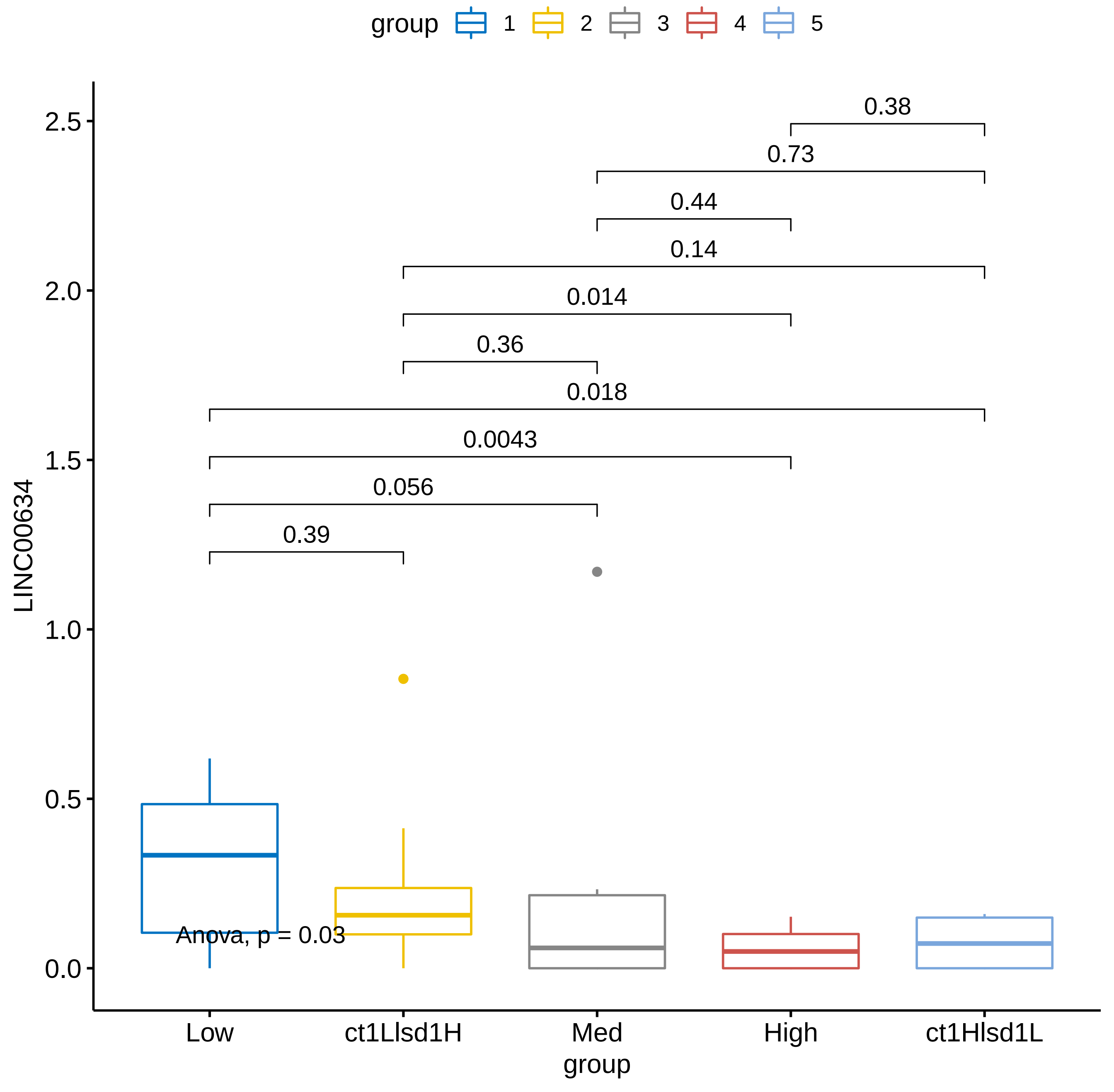

group 1 2 3 4 5
LINC00634
2.5
2.0
1.5
1.0
0.5
0.0
0.38
0.73
0.44
0.14
0.014
0.36
0.018
0.0043
0.056
0.39
Anova, p = 0.03
Low
ct1Llsd1H
Med
High
ct1Hlsd1L
group

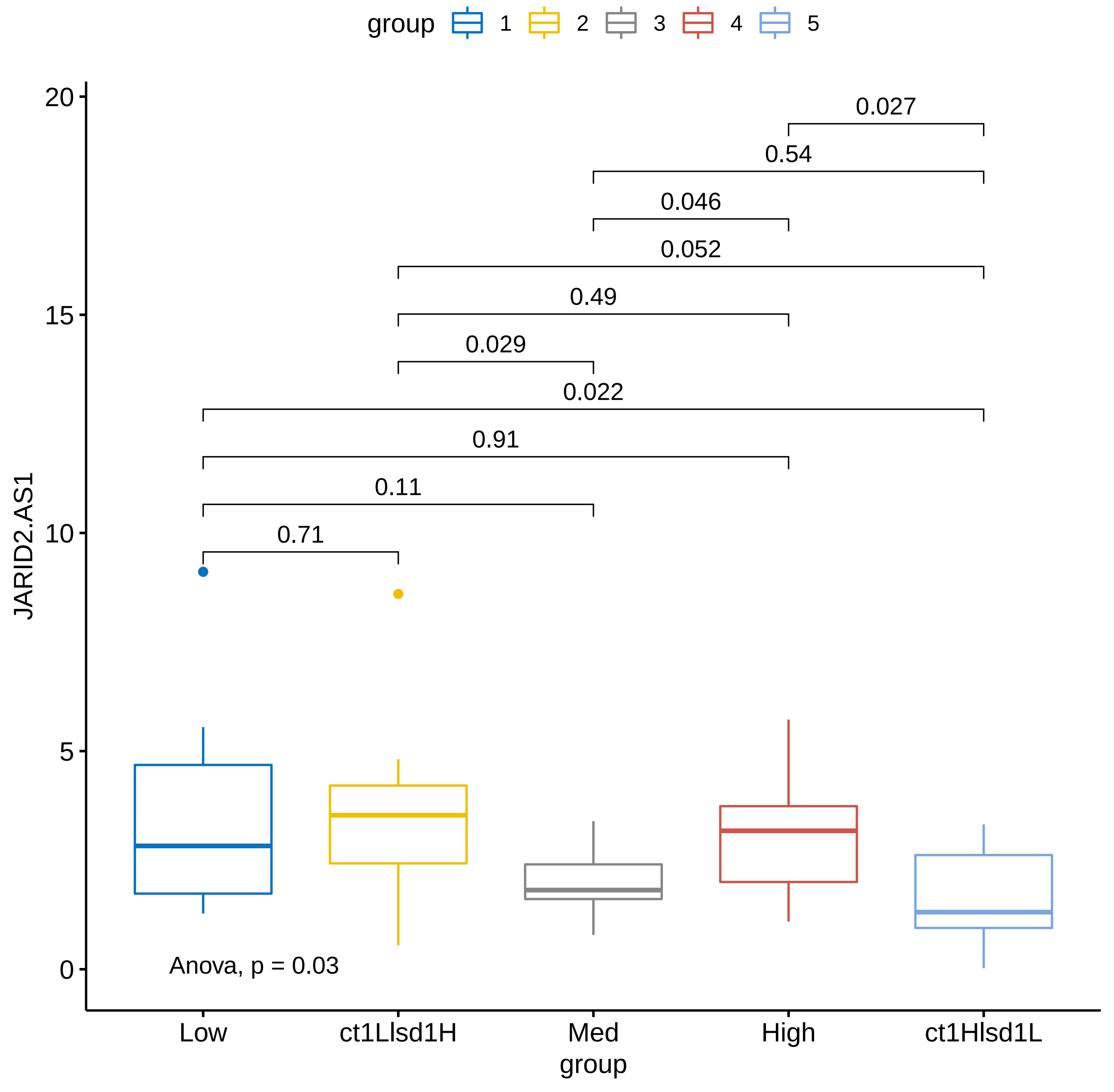

group 1 2 3 4 5
20
15
10
5
0
JARID2.AS1
0.027
0.54
0.046
0.052
0.49
0.029
0.022
0.91
0.11
0.71
Anova, p = 0.03
Low
ct1Llsd1H
Med
High
ct1Hlsd1L
group

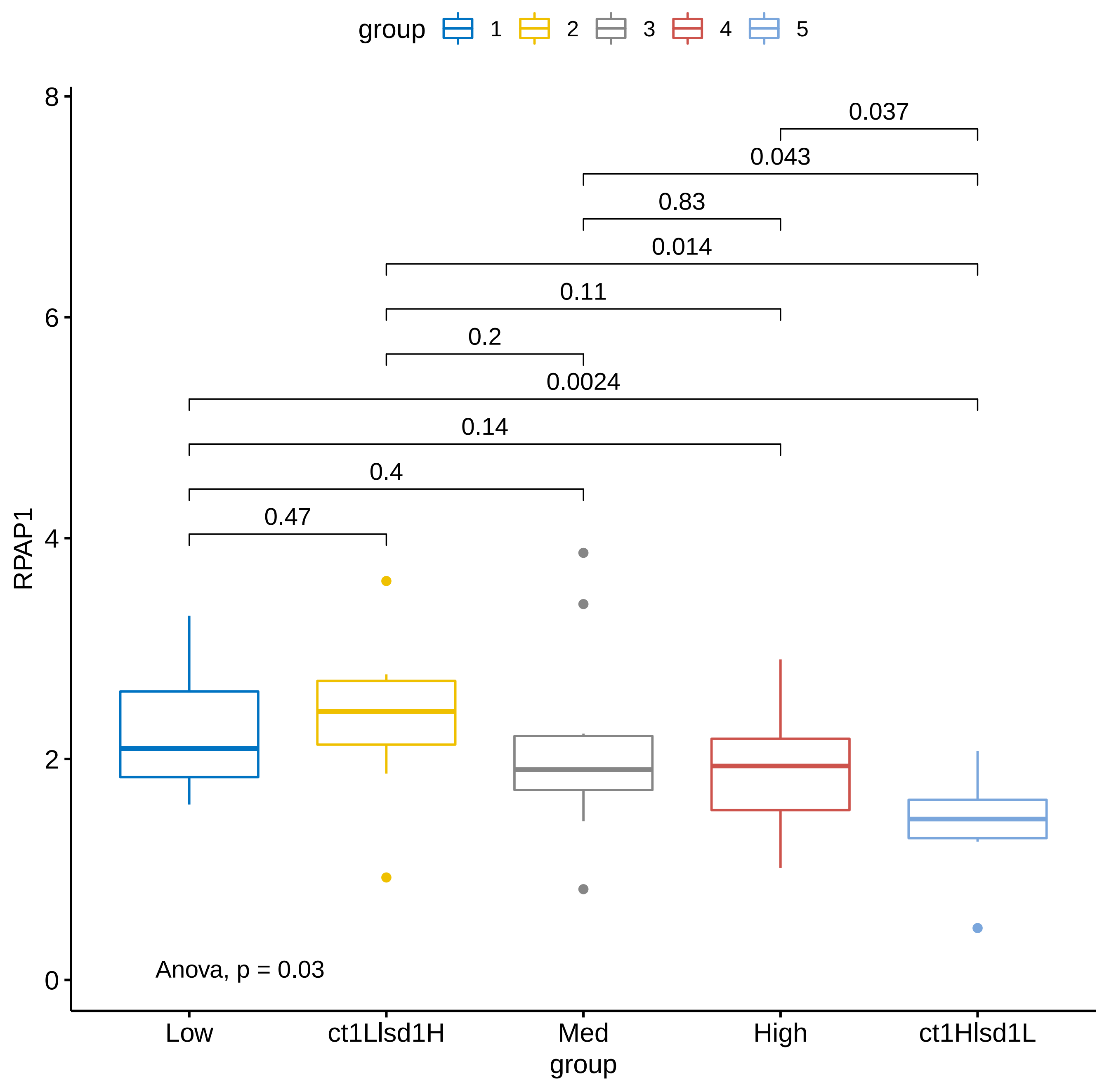

group 1 2 3 4 5
0.037
0.043
0.83
0.014
0.11
0.2
0.0024
0.14
0.4
0.47
RPAP1
8
6
4
2
0
Anova, p = 0.03
Low
ct1Llsd1H
Med
High
ct1Hlsd1L
group

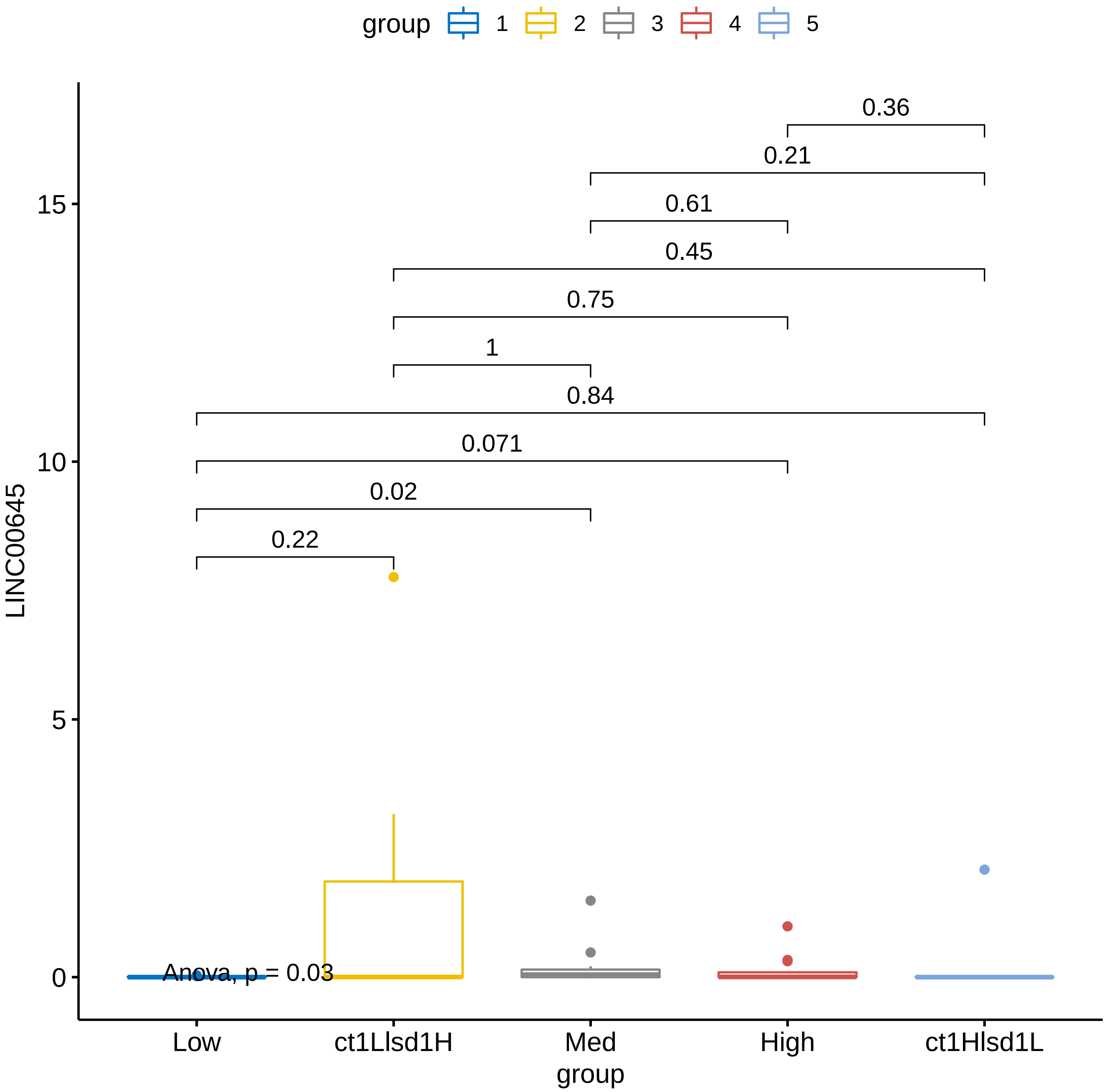

group
1
2
3
4
5
0.36
0.21
0.61
0.45
0.75
1
0.84
0.071
0.02
0.22
15
10
5
0
LINC00645
Anova, p = 0.03
Low
ct1Llsd1H
Med
High
ct1Hlsd1L
group

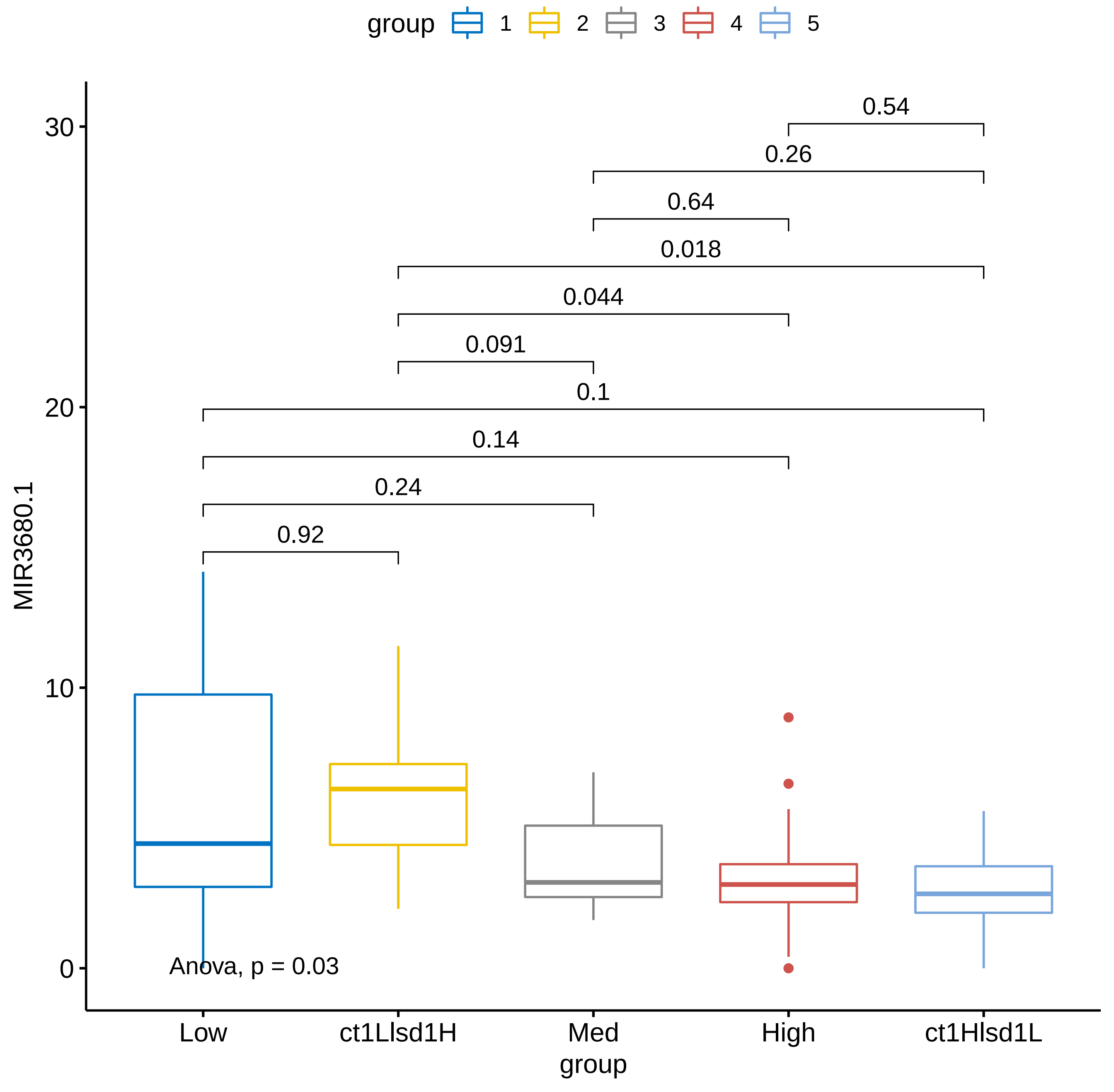
group 1 2 3 4 5
0.54
0.26
0.64
0.018
0.044
0.091
0.1
0.14
0.24
0.92
30
20
10
0
MIR3680.1
Anova, p = 0.03
Low
ct1Llsd1H
Med
High
ct1Hlsd1L
group

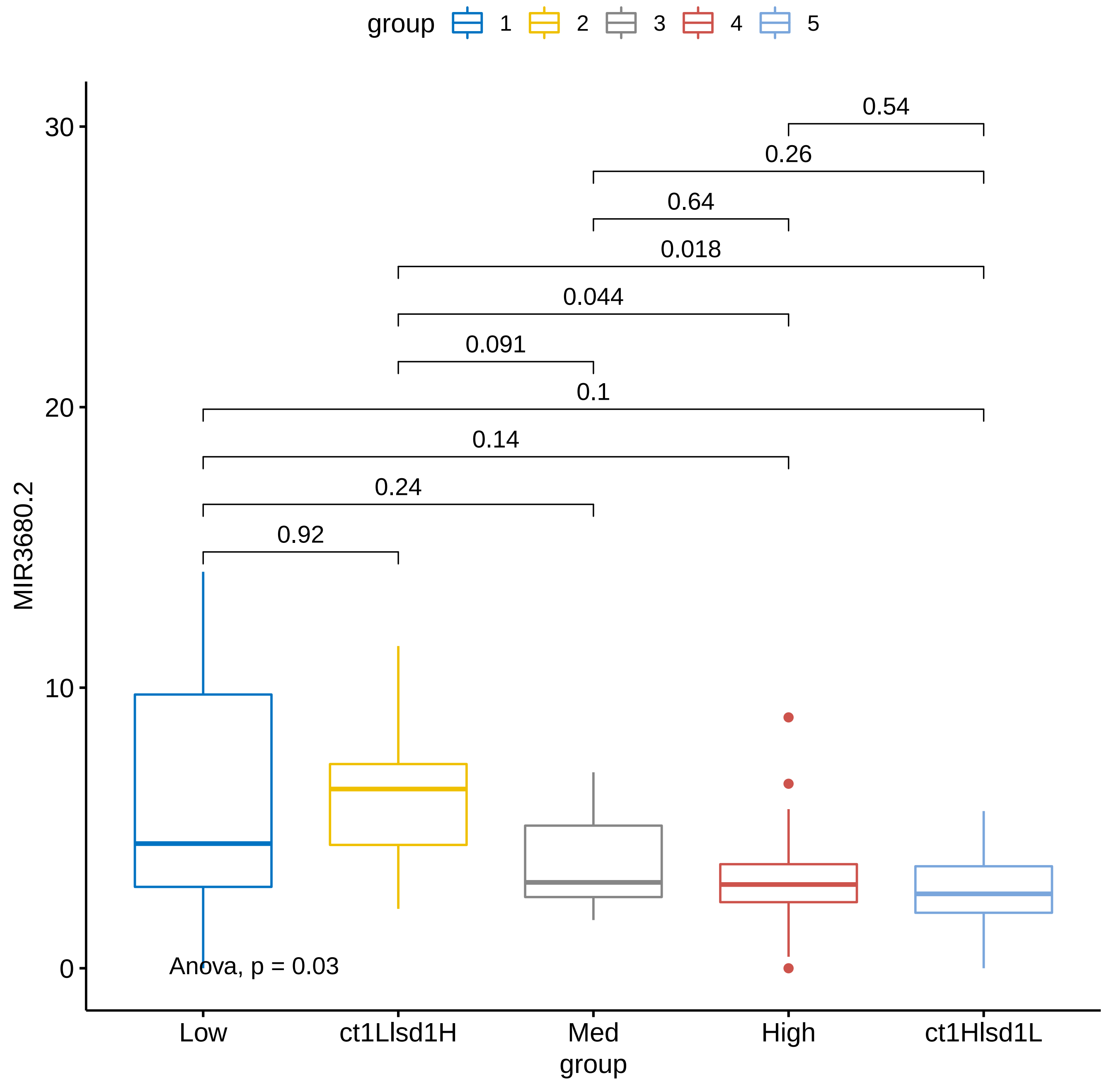

group 1 2 3 4 5
0.54
0.26
0.64
0.018
0.044
0.091
0.1
0.14
0.24
0.92
30
20
10
0
MIR3680.2
Anova, p = 0.03
Low
ct1Llsd1H
Med
High
ct1Hlsd1L
group

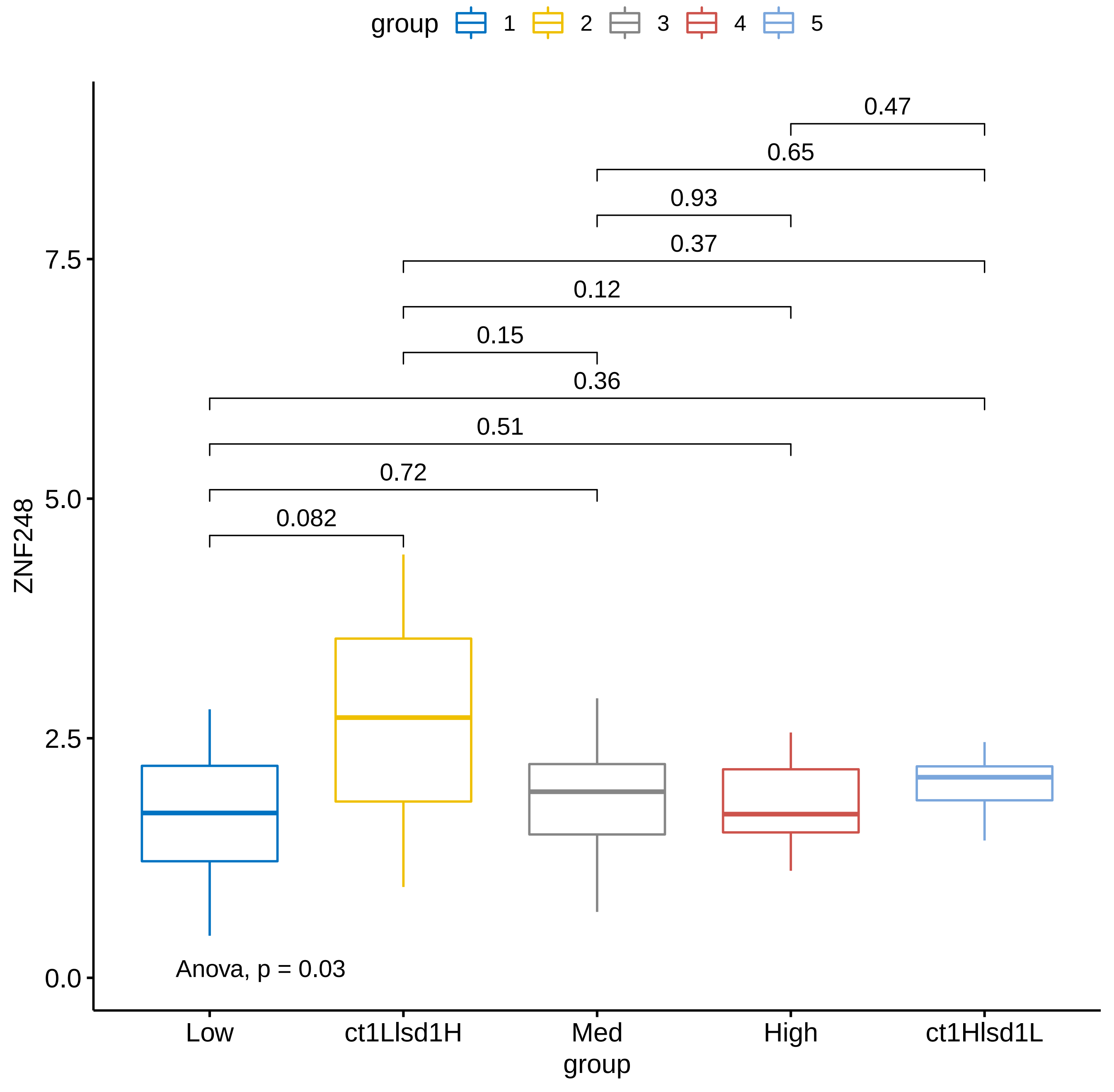
group
1
2
3
4
5
ZNF248
7.5
5.0
2.5
0.0
0.47
0.65
0.93
0.37
0.12
0.15
0.36
0.51
0.72
0.082
Anova, p = 0.03
Low
ct1Llsd1H
Med
High
ct1Hlsd1L
group

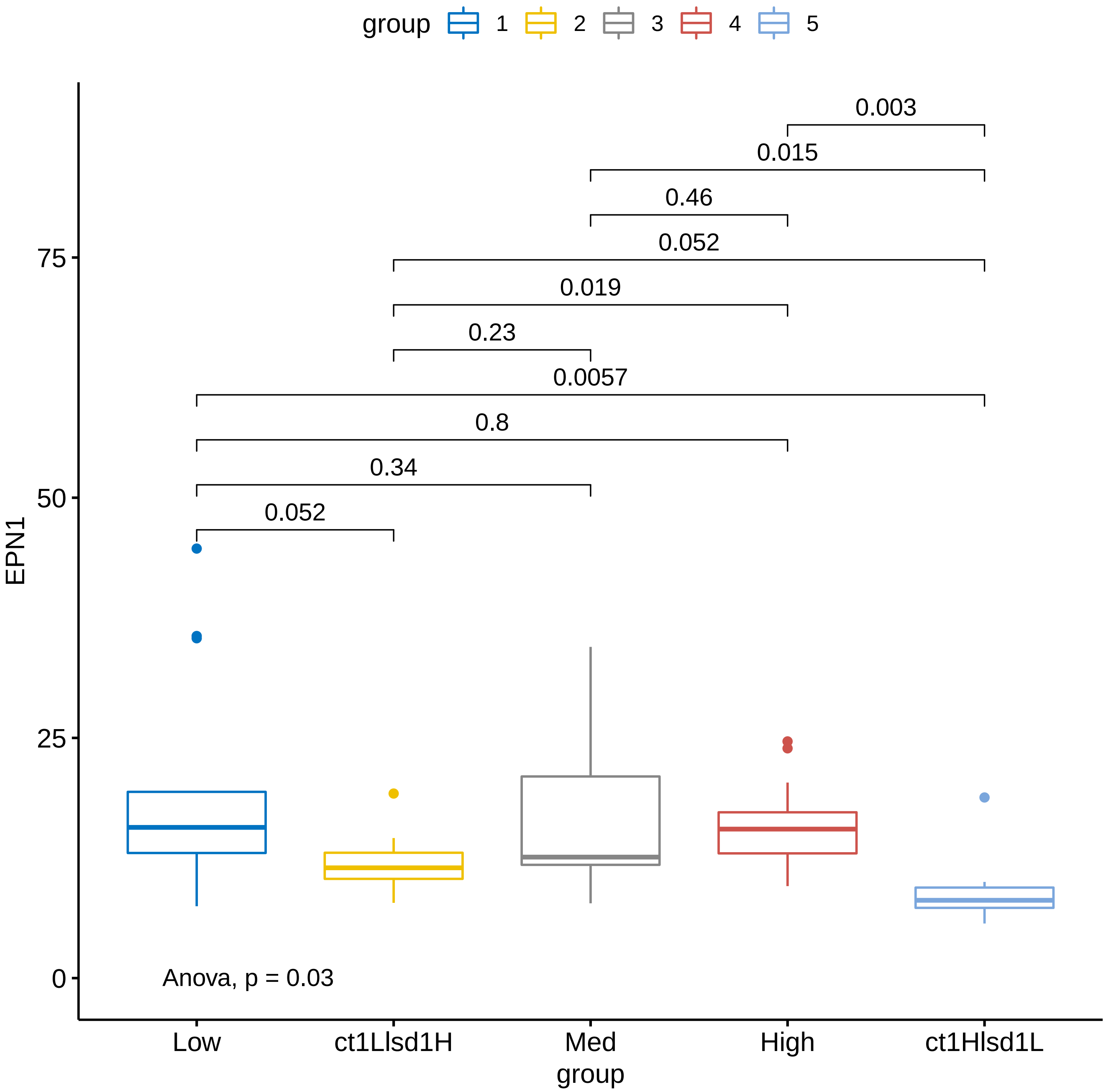

group
1
2
3
4
5
0.003
0.015
0.46
0.052
0.019
0.23
0.0057
0.8
0.34
0.052
75
50
25
0
EPN1
Anova, p = 0.03
Low
ct1Llsd1H
Med
High
ct1Hlsd1L
group

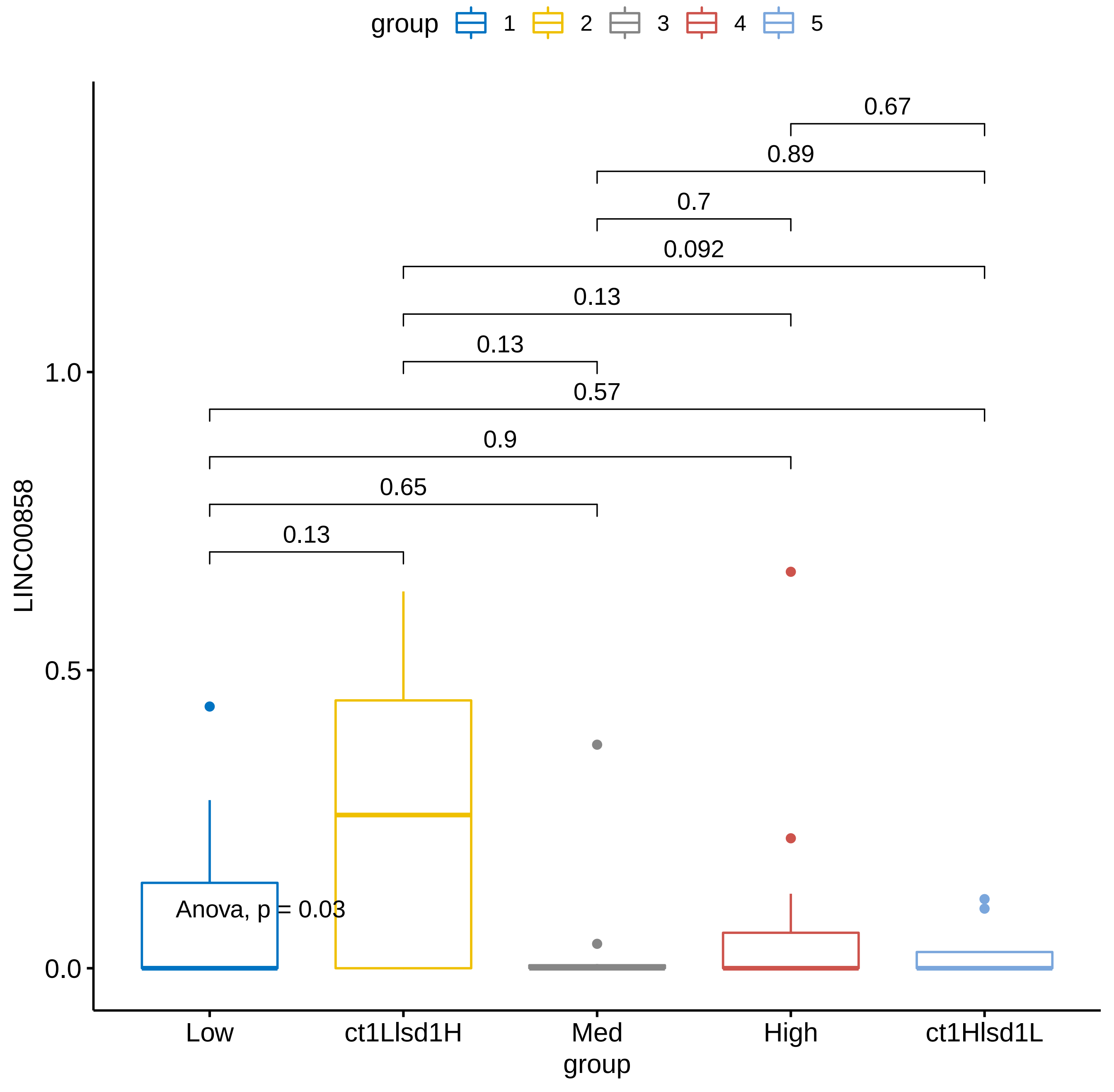

group
1
2
3
4
5
0.67
0.89
0.7
0.092
0.13
0.13
0.57
0.9
0.65
0.13
1.0
0.5
0.0
LINC00858
Anova, p = 0.03
Low
ct1Llsd1H
Med
High
ct1Hlsd1L
group

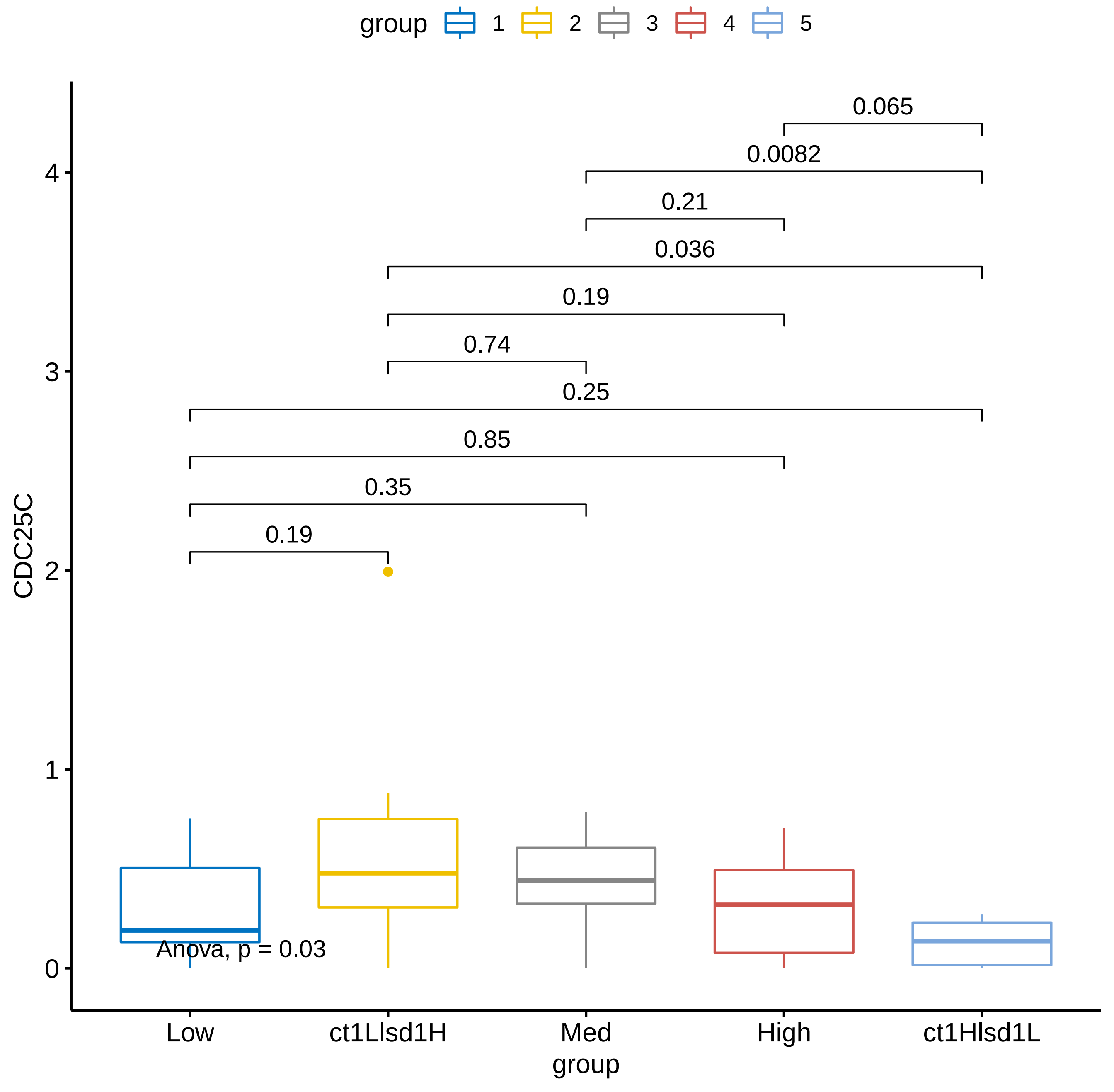

group 1 2 3 4 5
0.065
0.0082
0.21
0.036
0.19
0.74
0.25
0.85
0.35
0.19
Anova, p = 0.03
CDC25C
0
1
2
3
4
Low
ct1Llsd1H
Med
High
ct1Hlsd1L
group

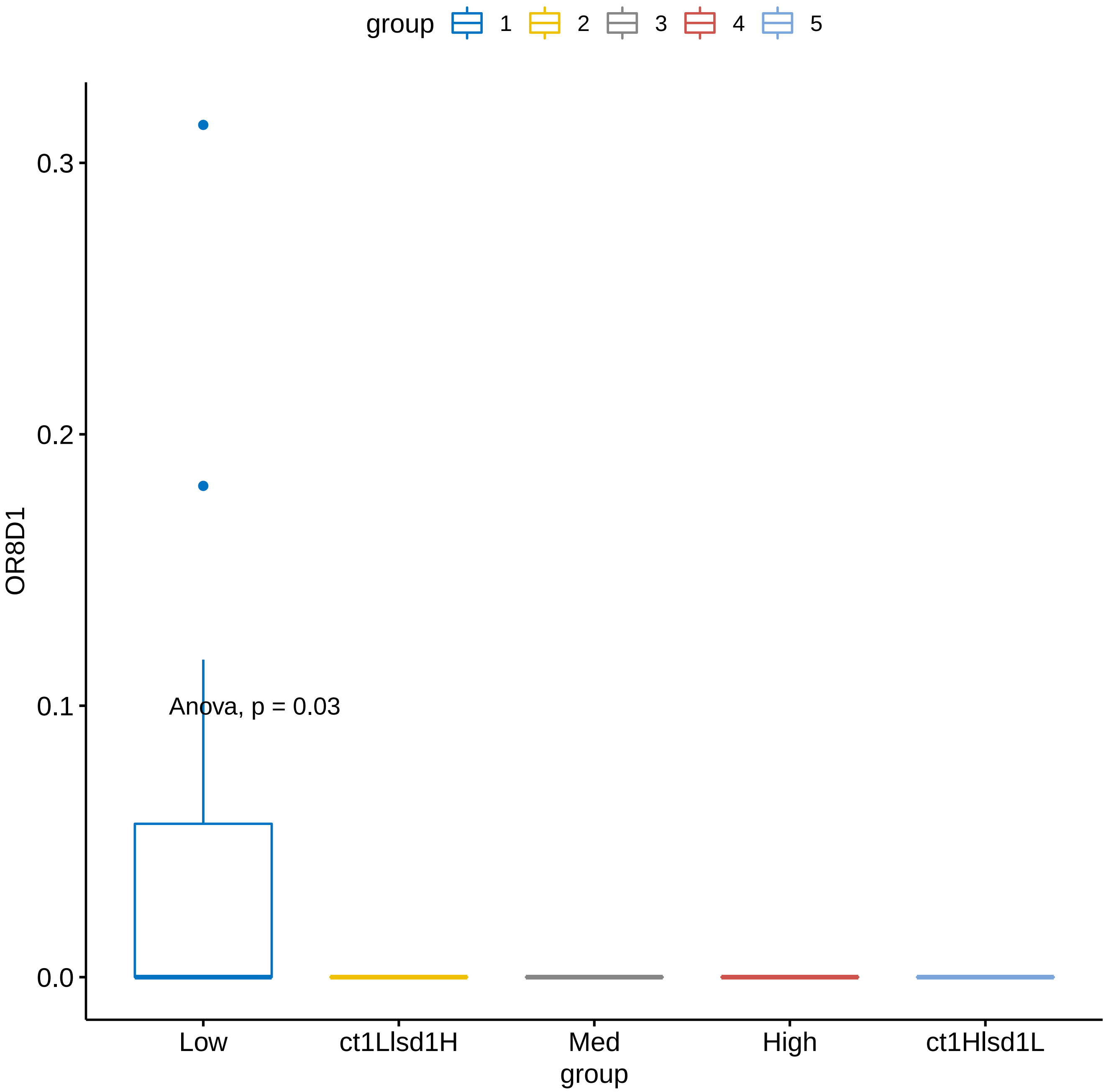
group
1
2
3
4
5
OR8D1
0.3
0.2
0.1
0.0
Anova, p = 0.03
Low
ct1Llsd1H
Med
High
ct1Hlsd1L
group

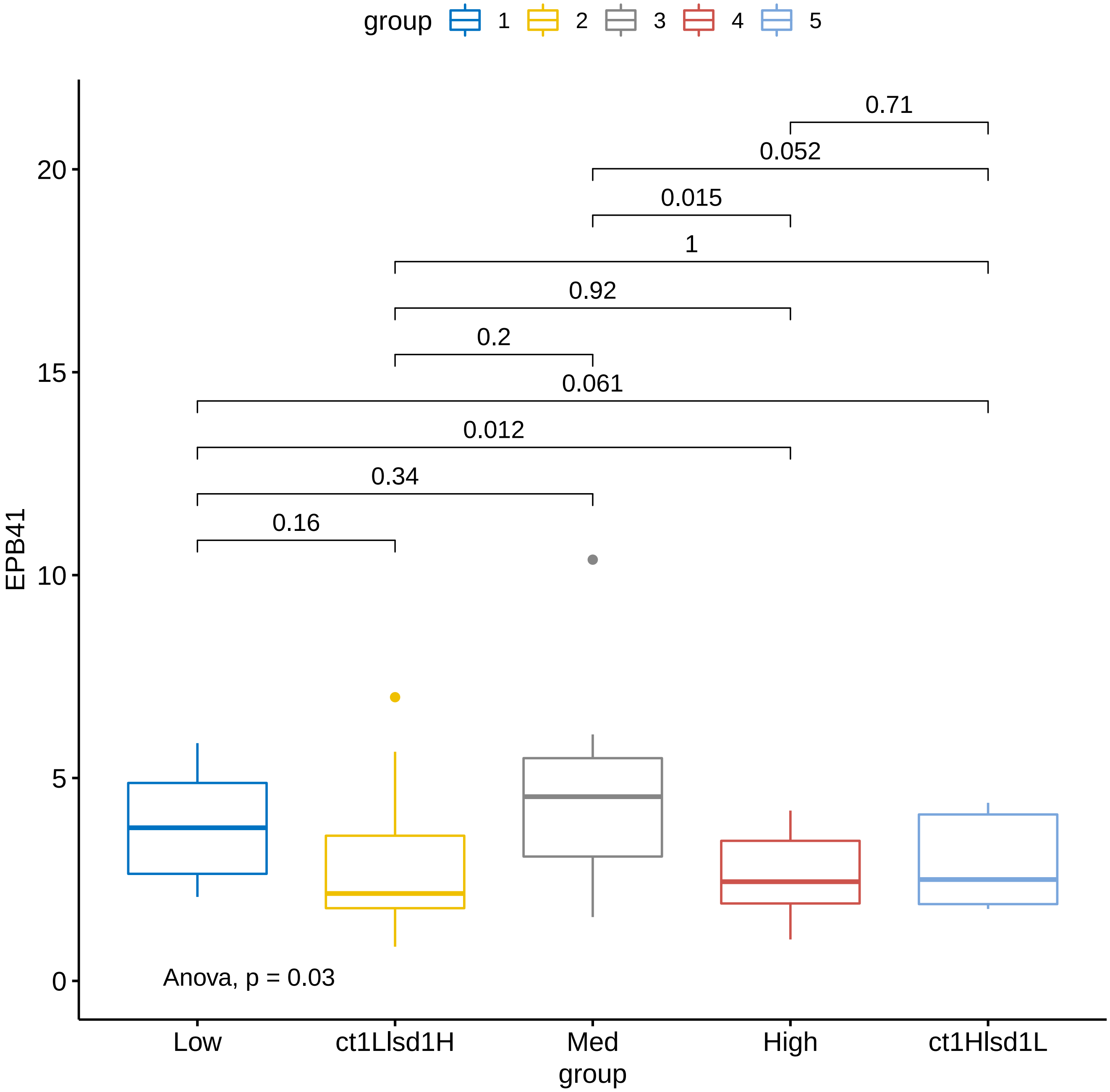

group
1
2
3
4
5
0.71
0.052
0.015
1
0.92
0.2
0.061
0.012
0.34
0.16
EPB41
20
15
10
5
0
Anova, p = 0.03
Low
ct1Llsd1H
Med
High
ct1Hlsd1L
group

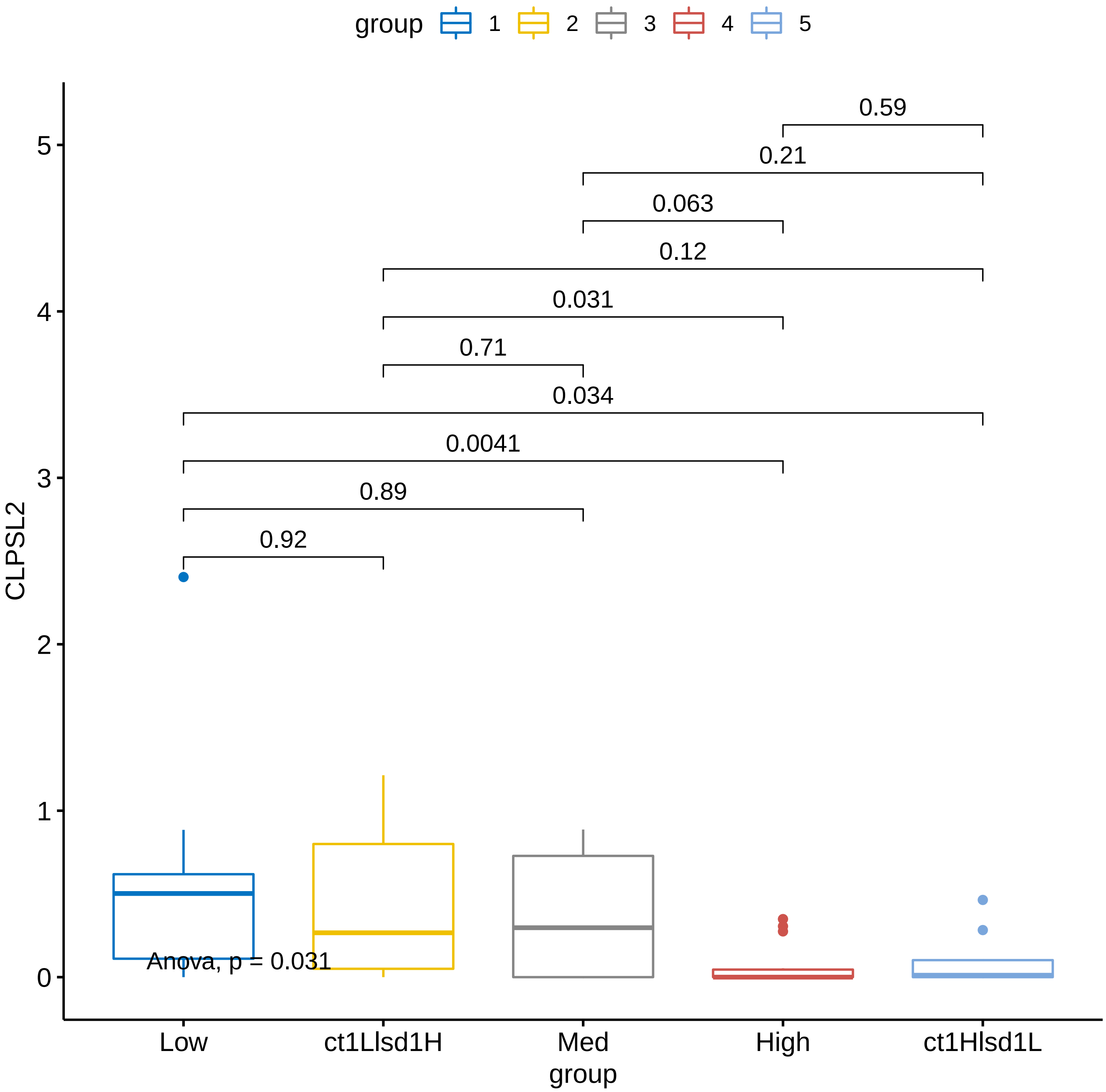

group 1 2 3 4 5
0.59
0.21
0.063
0.12
0.031
0.71
0.034
0.0041
0.89
0.92
Anova, p = 0.031
CLPSL2
5
4
3
2
1
0
Low
ct1Llsd1H
Med
High
ct1Hlsd1L
group

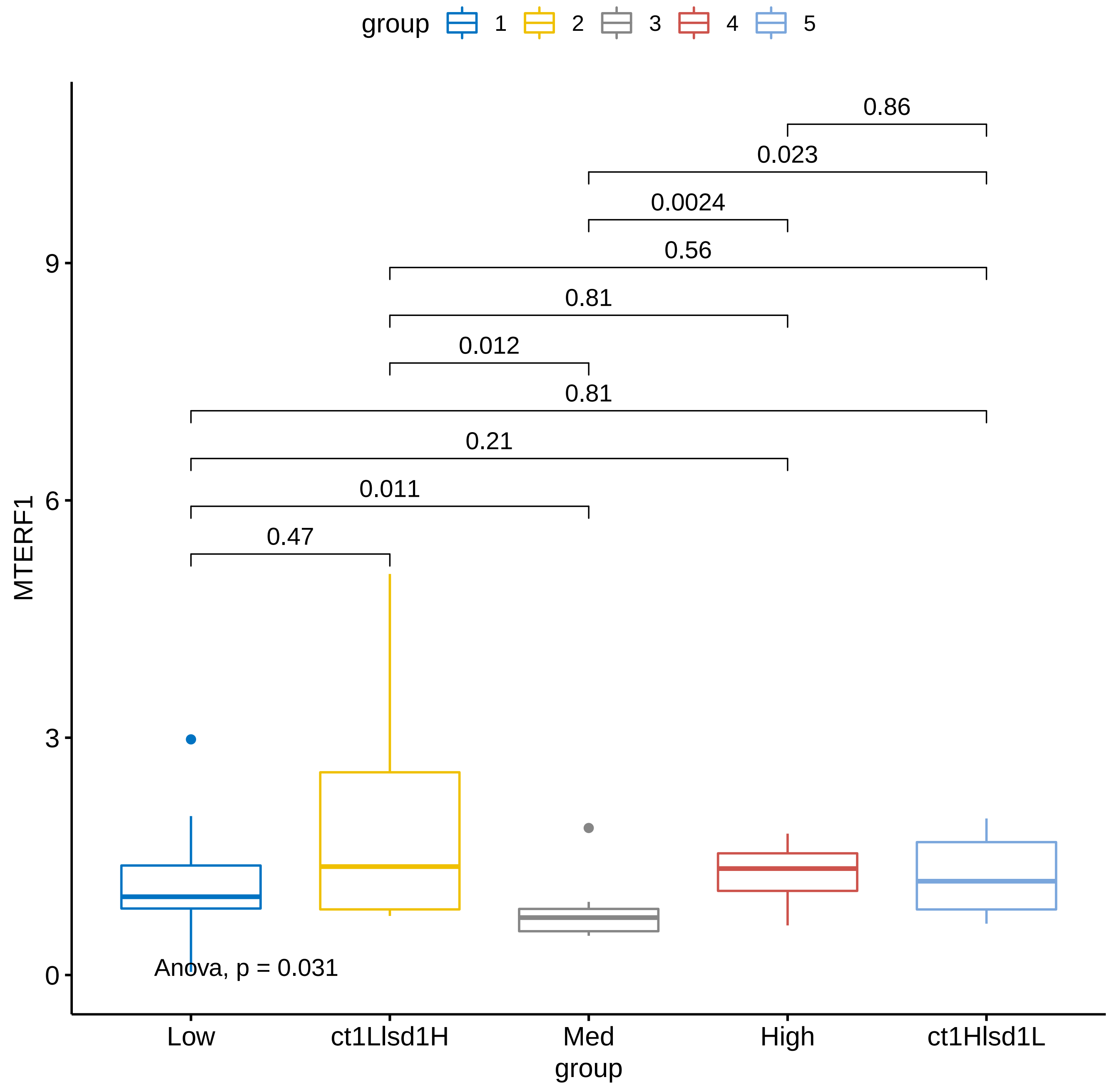

group 1 2 3 4 5
0.86
0.023
0.0024
0.56
0.81
0.012
0.81
0.21
0.011
0.47
MTERF1
9
6
3
0
Anova, p = 0.031
Low
ct1Llsd1H
Med
High
ct1Hlsd1L
group

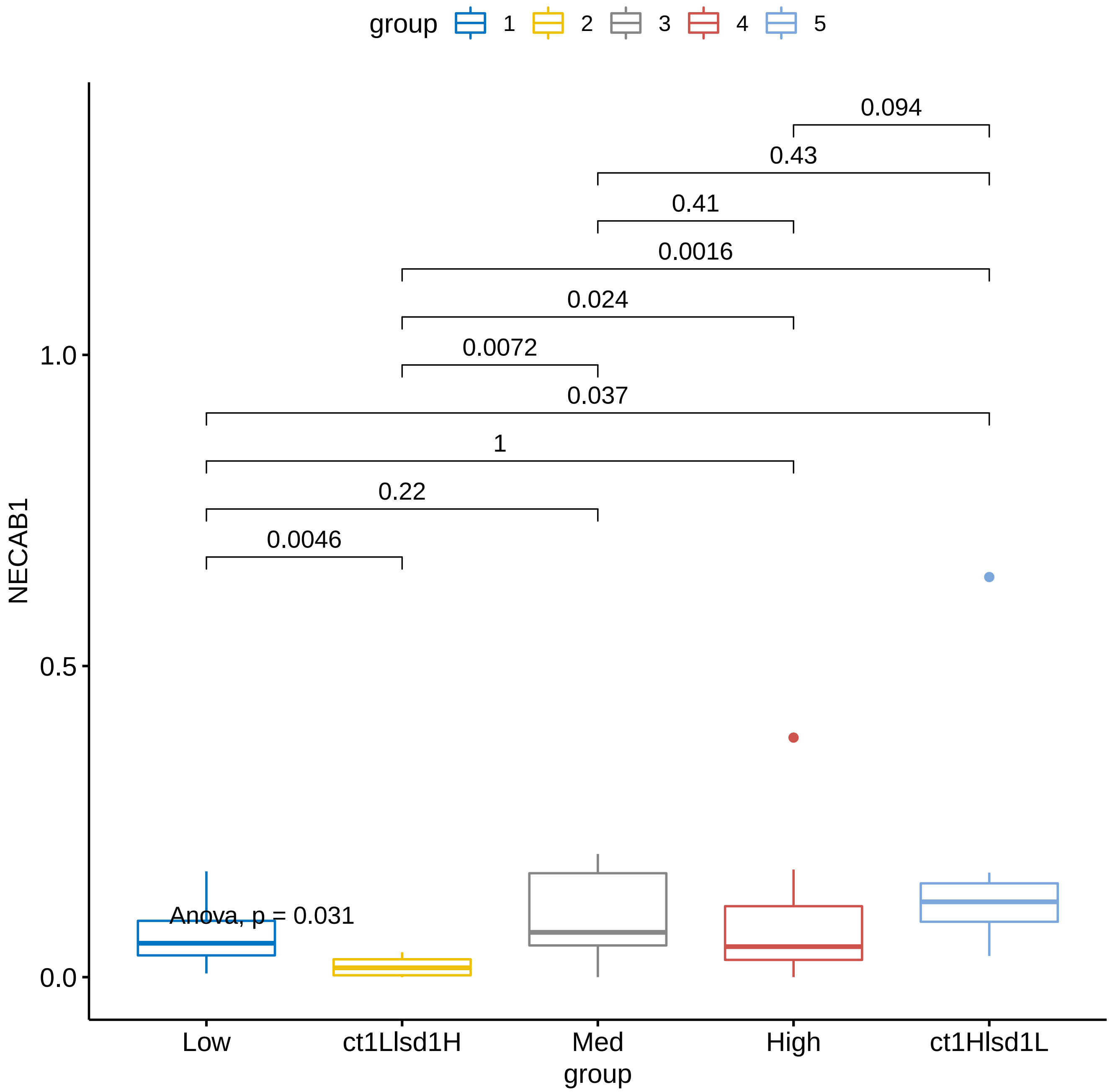

group
1
2
3
4
5
0.094
0.43
0.41
0.0016
0.024
0.0072
0.037
1
0.22
0.0046
1.0
0.5
0.0
NECAB1
Anova, p = 0.031
Low
ct1Llsd1H
Med
High
ct1Hlsd1L
group

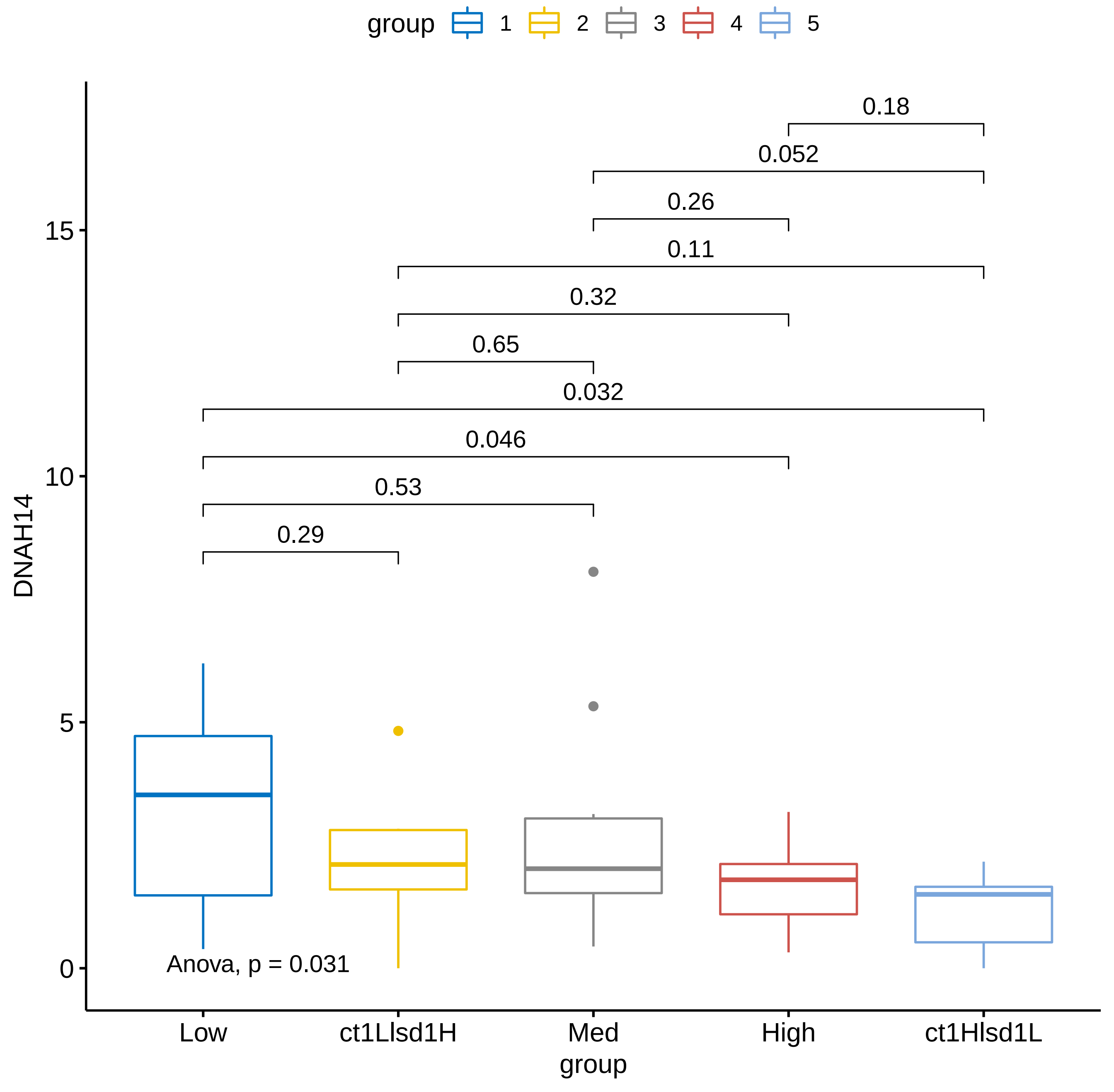

group 1 2 3 4 5
0.18
0.052
0.26
0.11
0.32
0.65
0.032
0.046
0.53
0.29
Anova, p = 0.031
DNAH14
15
10
5
0
Low
ct1Llsd1H
Med
High
ct1Hlsd1L
group

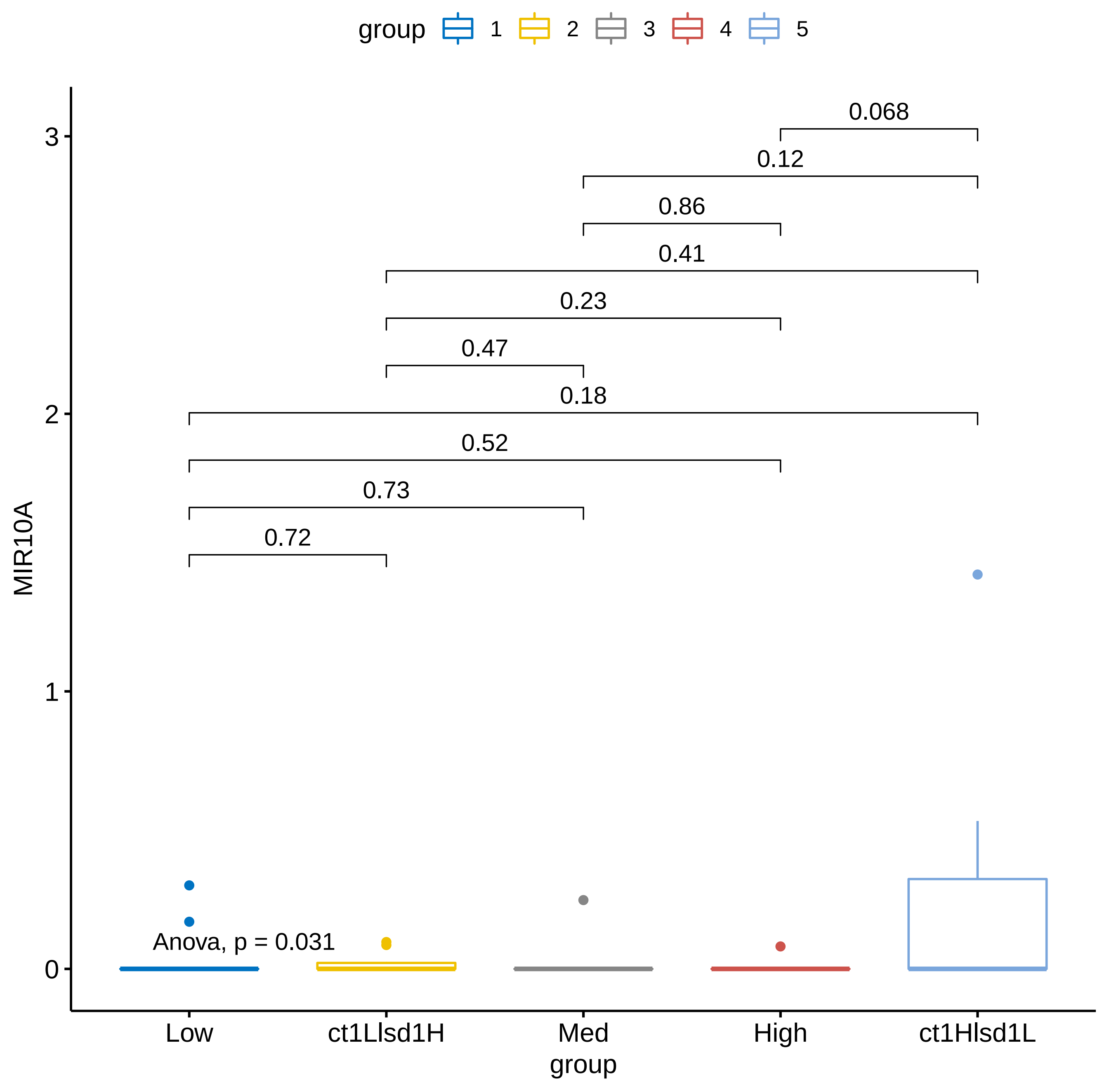
group 1 2 3 4 5
0.068
0.12
0.86
0.41
0.23
0.47
0.18
0.52
0.73
0.72
Anova, p = 0.031
MIR10A
0
1
2
3
Low
ct1Llsd1H
Med
High
ct1Hlsd1L
group

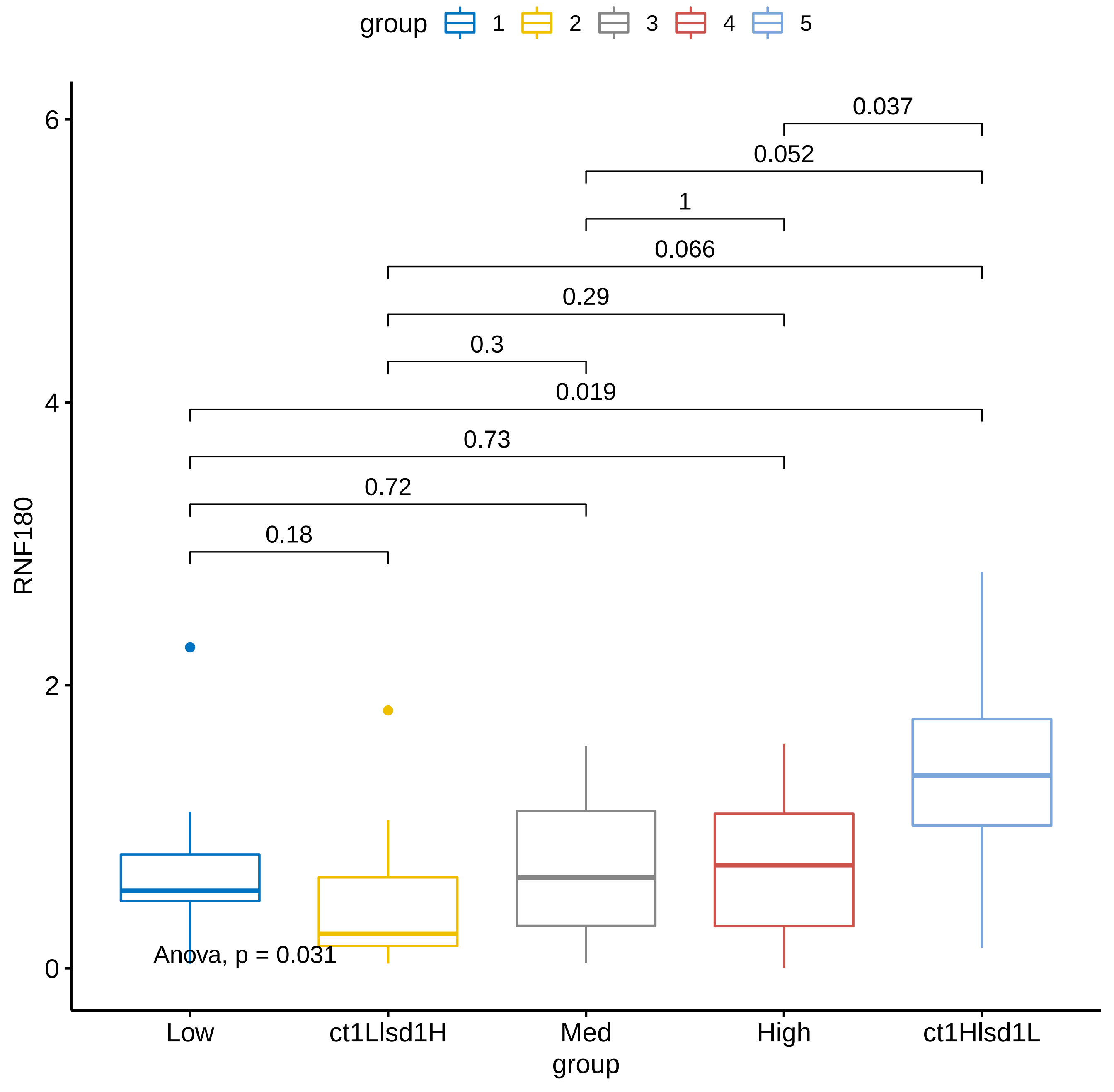

group
1
2
3
4
5
0.037
0.052
1
0.066
0.29
0.3
0.019
0.73
0.72
0.18
6
4
2
0
RNF180
Anova, p = 0.031
Low
ct1Llsd1H
Med
High
ct1Hlsd1L
group

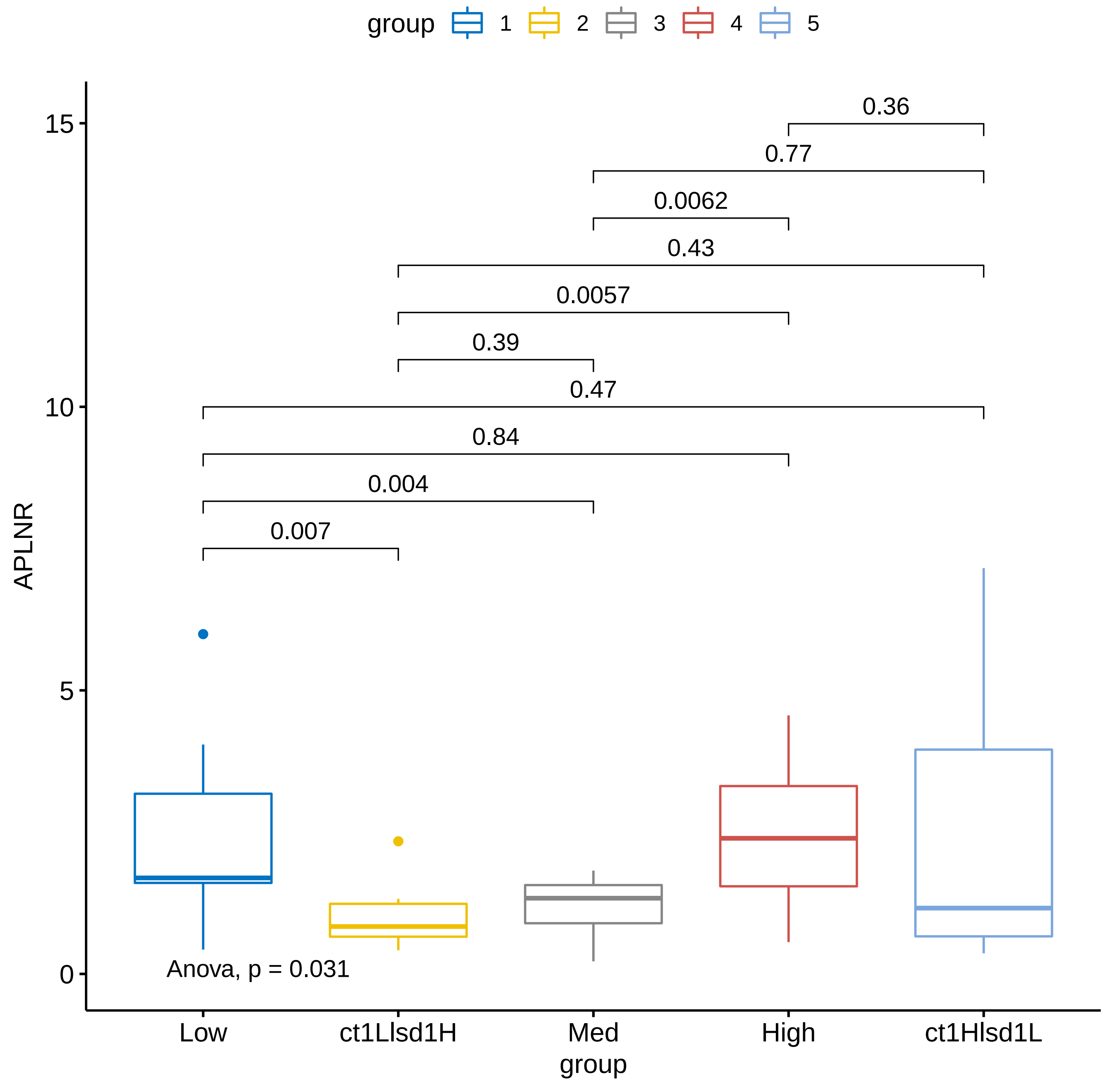
group
1
2
3
4
5
0.36
0.77
0.0062
0.43
0.0057
0.39
0.47
0.84
0.004
0.007
15
10
5
0
APLNR
Anova, p = 0.031
Low
ct1Llsd1H
Med
High
ct1Hlsd1L
group

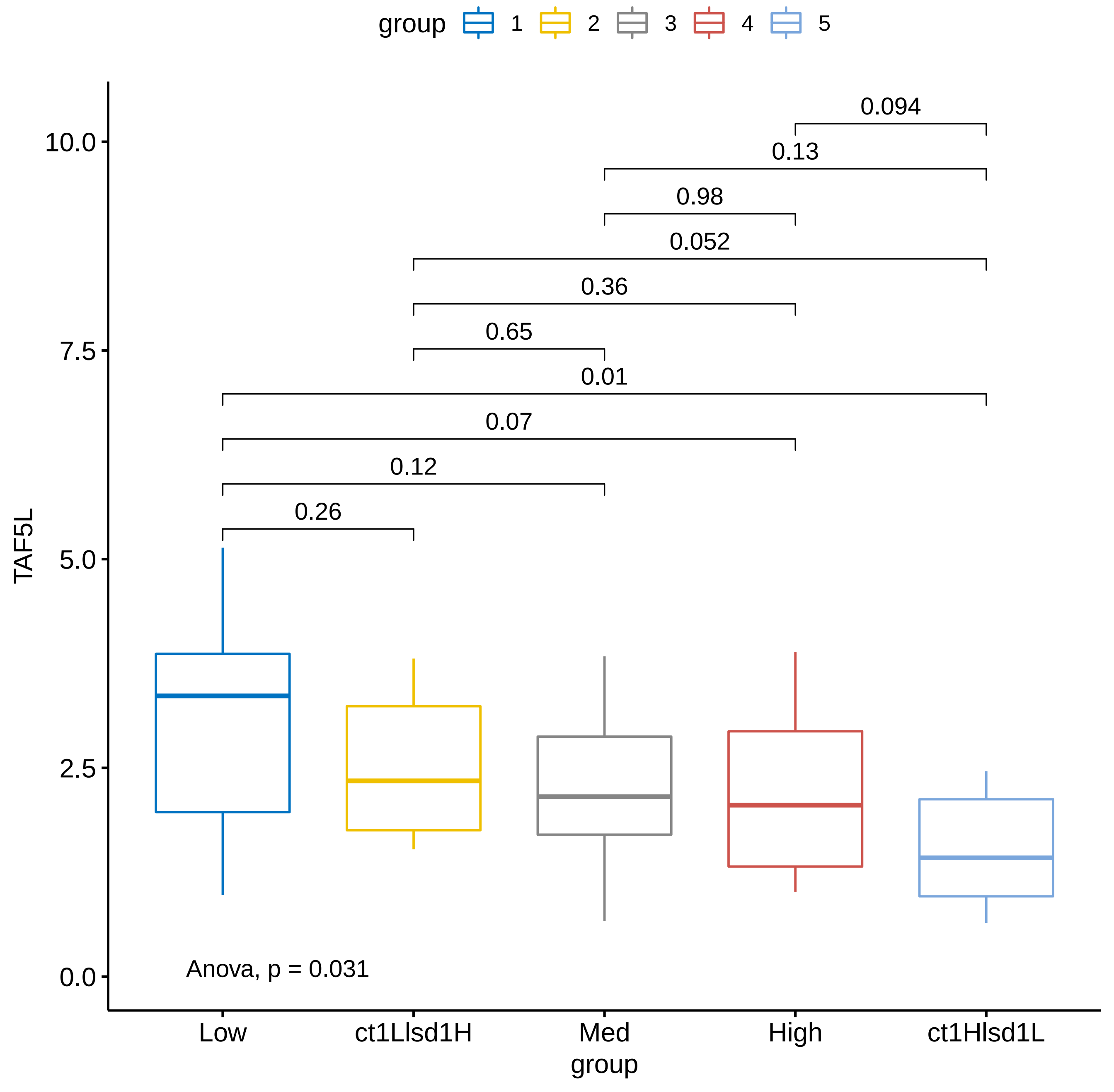

group
1
2
3
4
5
0.094
0.13
0.98
0.052
0.36
0.65
0.01
0.07
0.12
0.26
10.0
7.5
5.0
2.5
0.0
TAF5L
Anova, p = 0.031
Low
ct1Llsd1H
Med
High
ct1Hlsd1L
group

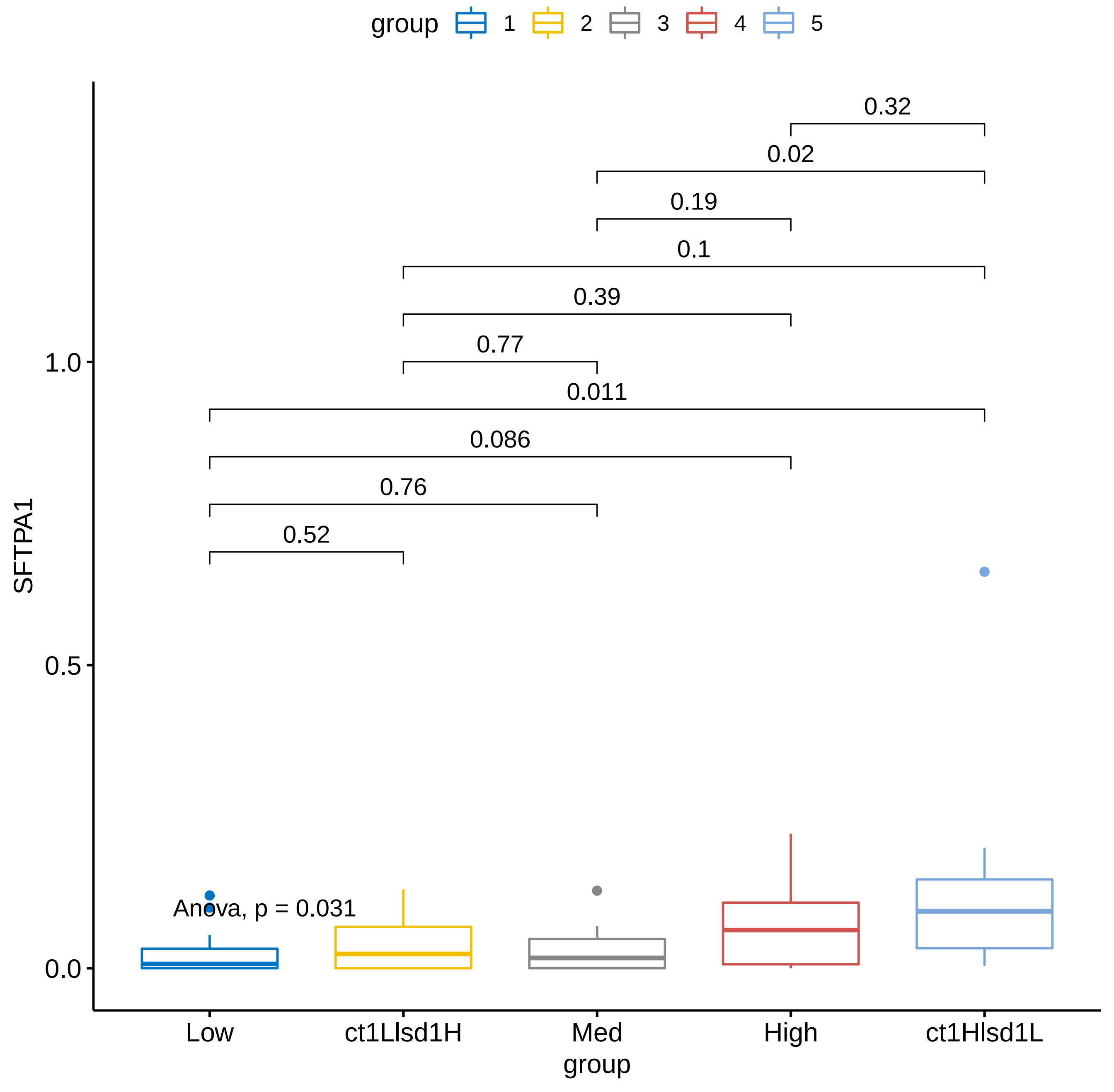

group
1
2
3
4
5
0.32
0.02
0.19
0.1
0.39
0.77
0.011
0.086
0.76
0.52
1.0
0.5
0.0
SFTPA1
Anova, p = 0.031
Low
ct1Llsd1H
Med
High
ct1Hlsd1L
group

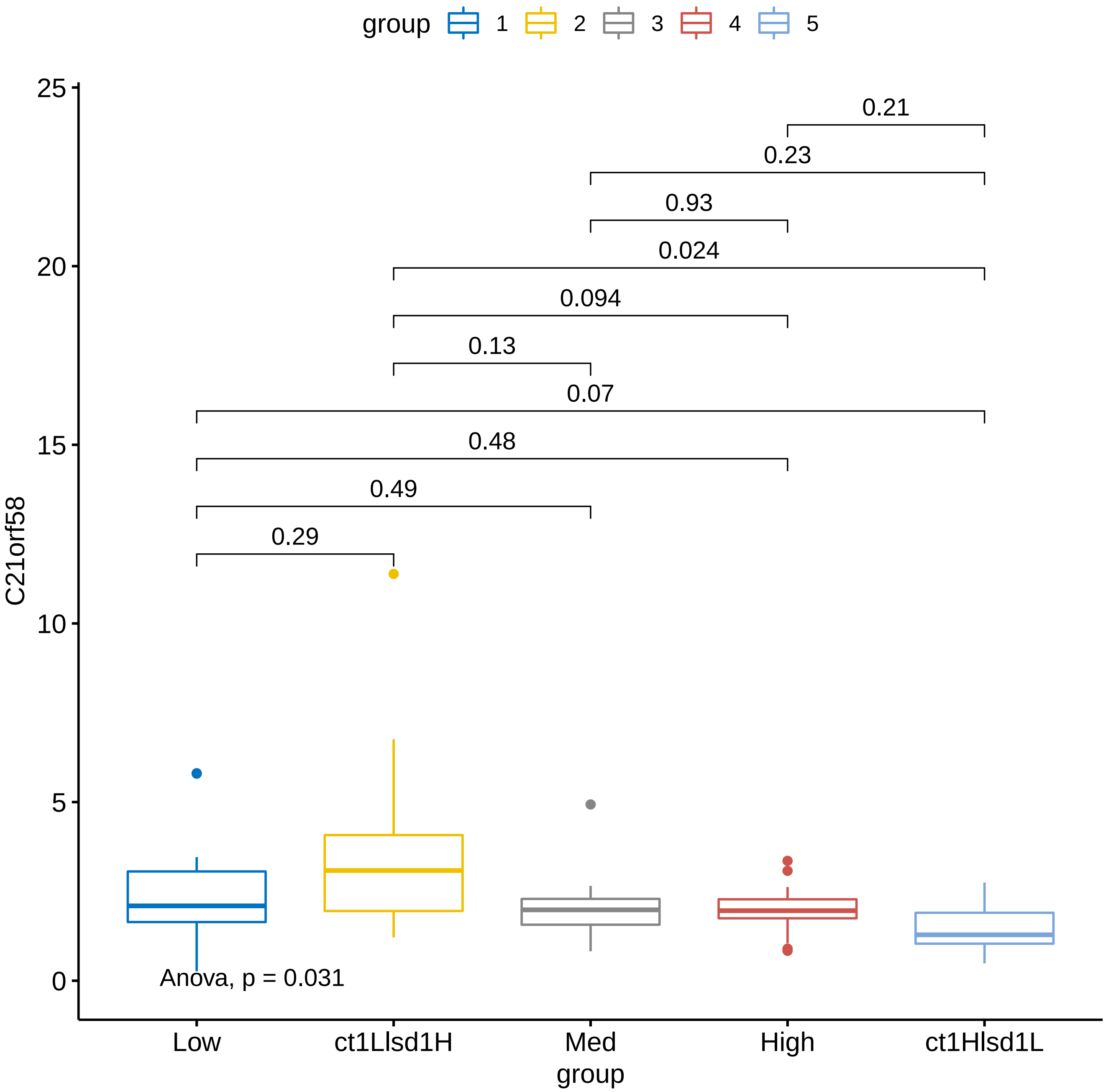

group
1
2
3
4
5
C21orf58
25
20
15
10
5
0
0.21
0.23
0.93
0.024
0.094
0.13
0.07
0.48
0.49
0.29
Anova, p = 0.031
Low
ct1Llsd1H
Med
High
ct1Hlsd1L
group

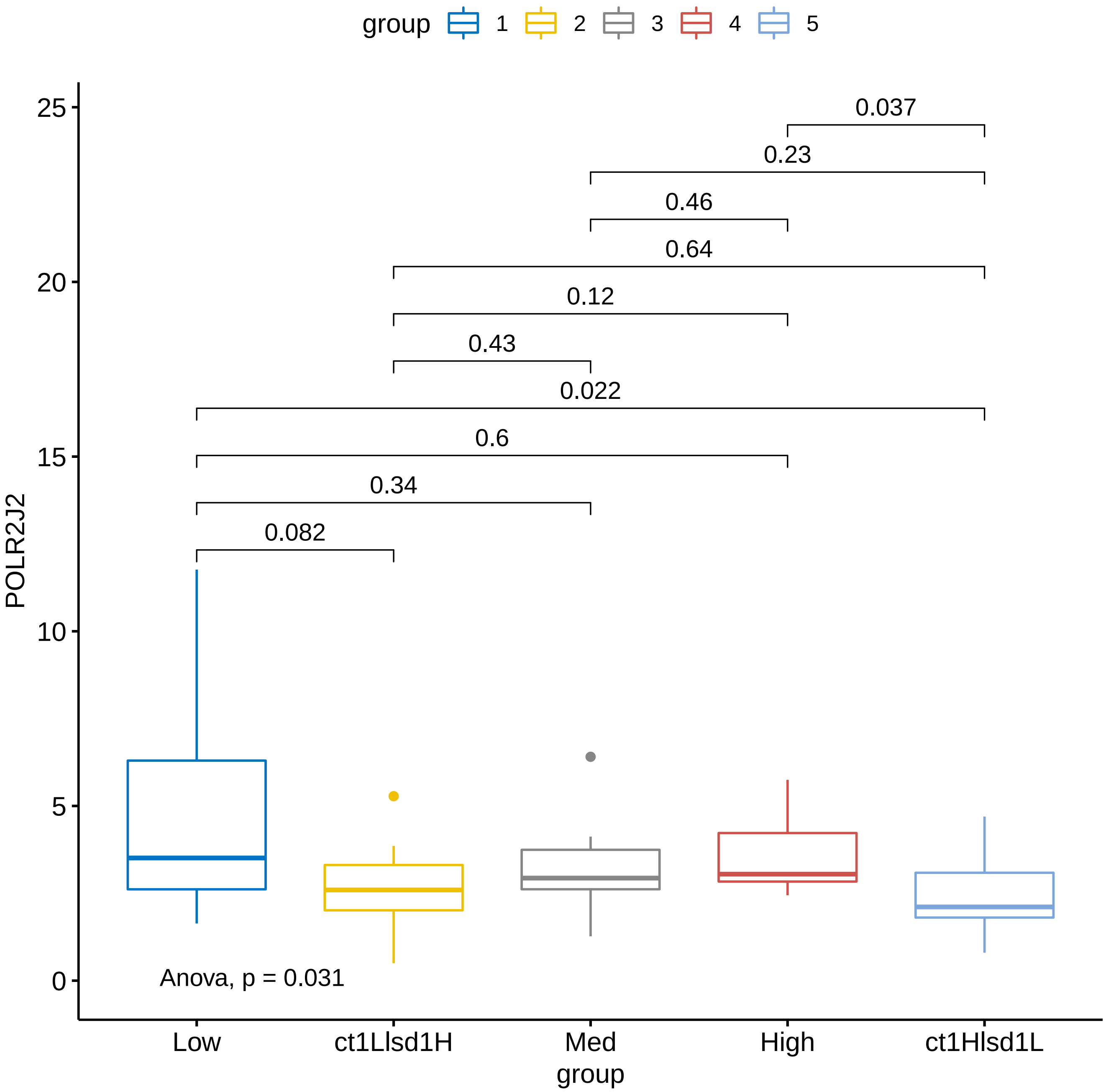

group
1
2
3
4
5
0.037
0.23
0.46
0.64
0.12
0.43
0.022
0.6
0.34
0.082
Anova, p = 0.031
POLR2J2
25
20
15
10
5
0
Low
ct1Llsd1H
Med
High
ct1Hlsd1L
group

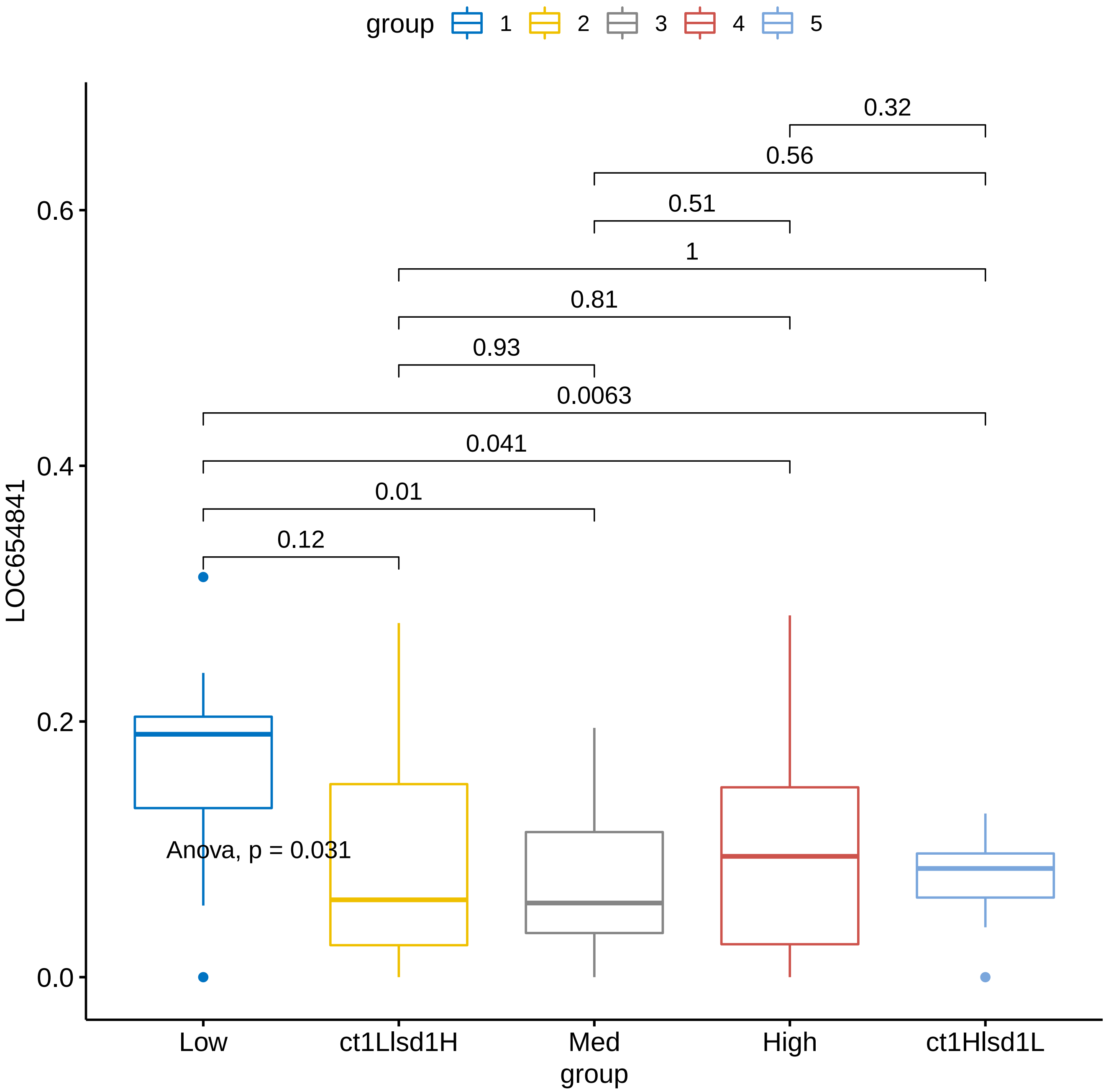
group
1
2
3
4
5
0.32
0.56
0.51
1
0.81
0.93
0.0063
0.041
0.01
0.12
Anova, p = 0.031
0.6
0.4
0.2
0.0
LOC654841
Low
ct1Llsd1H
Med
High
ct1Hlsd1L
group

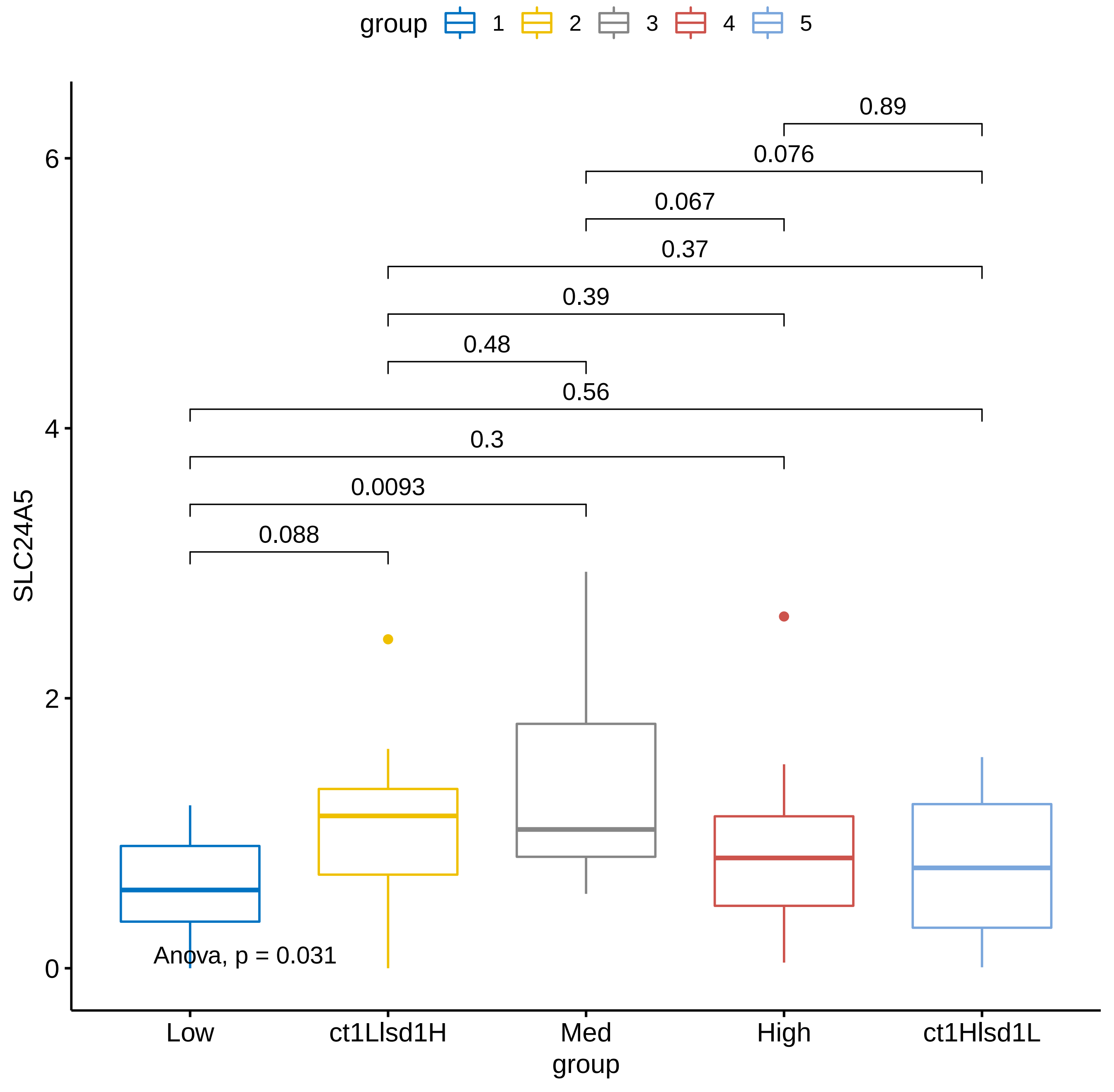

group
1
2
3
4
5
0.89
0.076
0.067
0.37
0.39
0.48
0.56
0.3
0.0093
0.088
Anova, p = 0.031
SLC24A5
6
4
2
0
Low
ct1Llsd1H
Med
High
ct1Hlsd1L
group

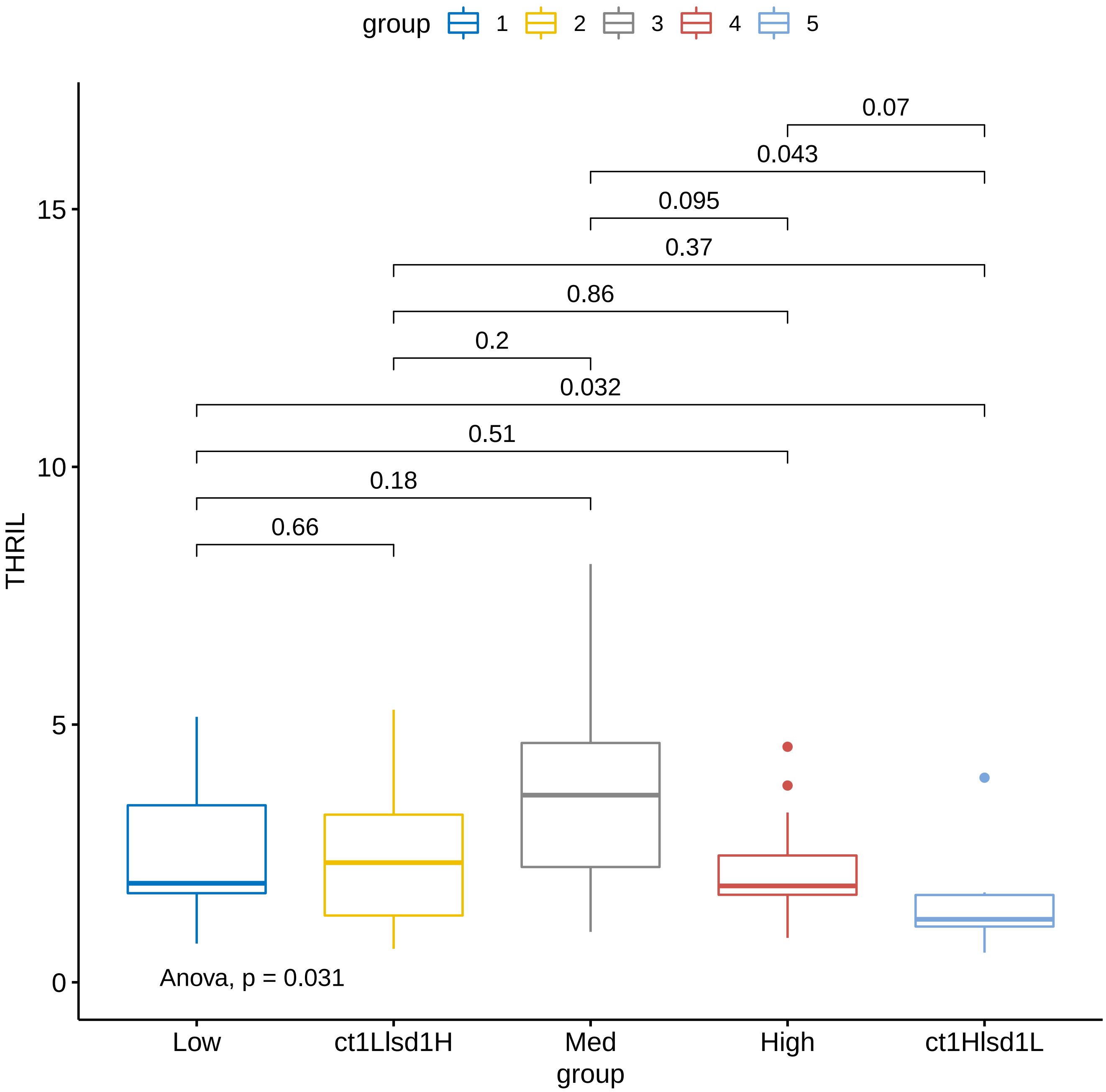
group
1
2
3
4
5
THRIL
15
10
5
0
0.07
0.043
0.095
0.37
0.86
0.2
0.032
0.51
0.18
0.66
Anova, p = 0.031
Low
ct1Llsd1H
Med
High
ct1Hlsd1L
group

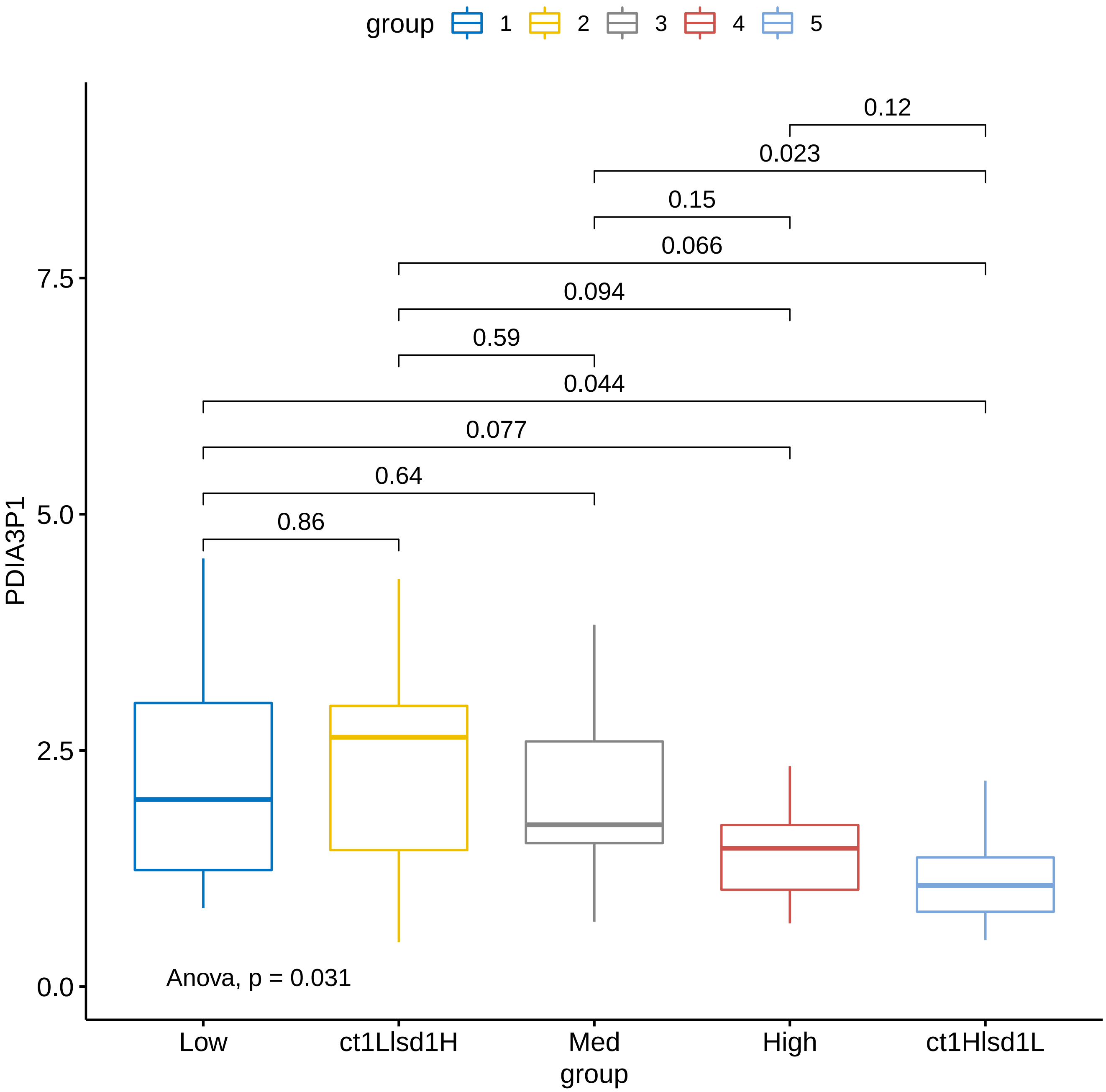

group
1
2
3
4
5
0.12
0.023
0.15
0.066
0.094
0.59
0.044
0.077
0.64
0.86
7.5
5.0
2.5
0.0
PDIA3P1
Anova, p = 0.031
Low
ct1Llsd1H
Med
High
ct1Hlsd1L
group

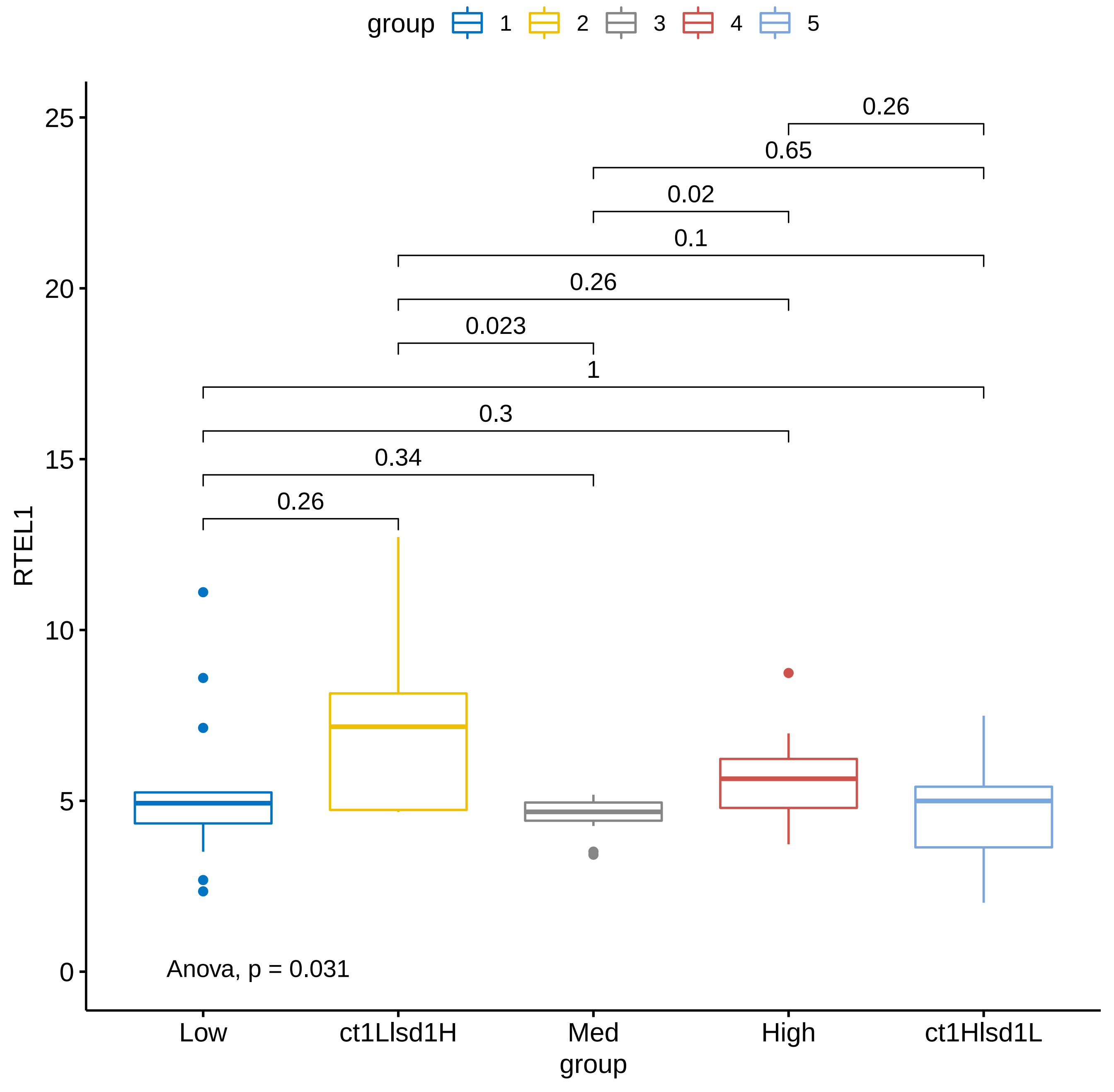

group
1
2
3
4
5
RTEL1
25
20
15
10
5
0
0.26
0.65
0.02
0.1
0.26
0.023
1
0.3
0.34
0.26
Anova, p = 0.031
Low
ct1Llsd1H
Med
High
ct1Hlsd1L
group

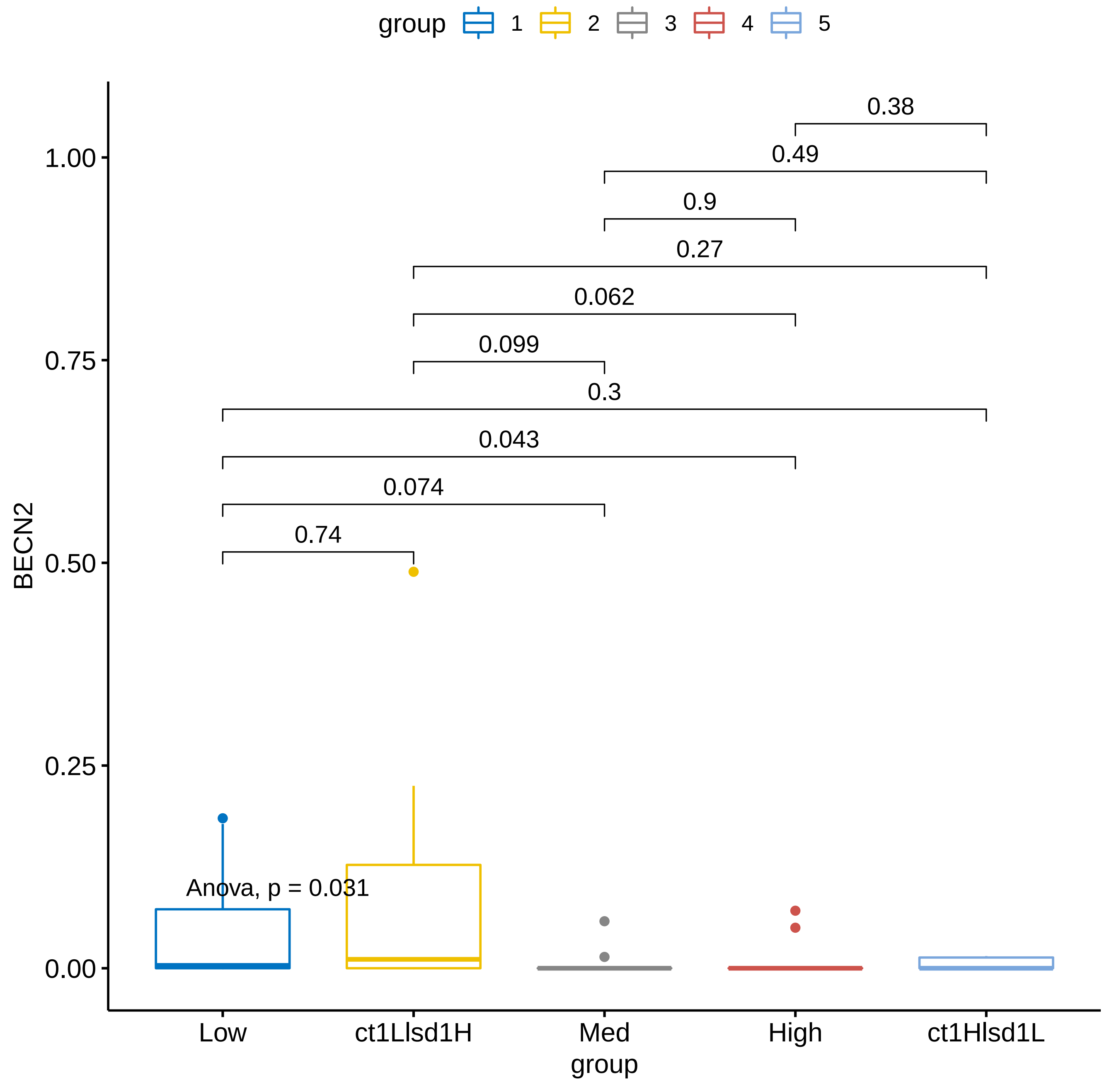
group
1
2
3
4
5
BECN2
1.00
0.75
0.50
0.25
0.00
0.38
0.49
0.9
0.27
0.062
0.099
0.3
0.043
0.074
0.74
Anova, p = 0.031
Low
ct1Llsd1H
Med
High
ct1Hlsd1L
group

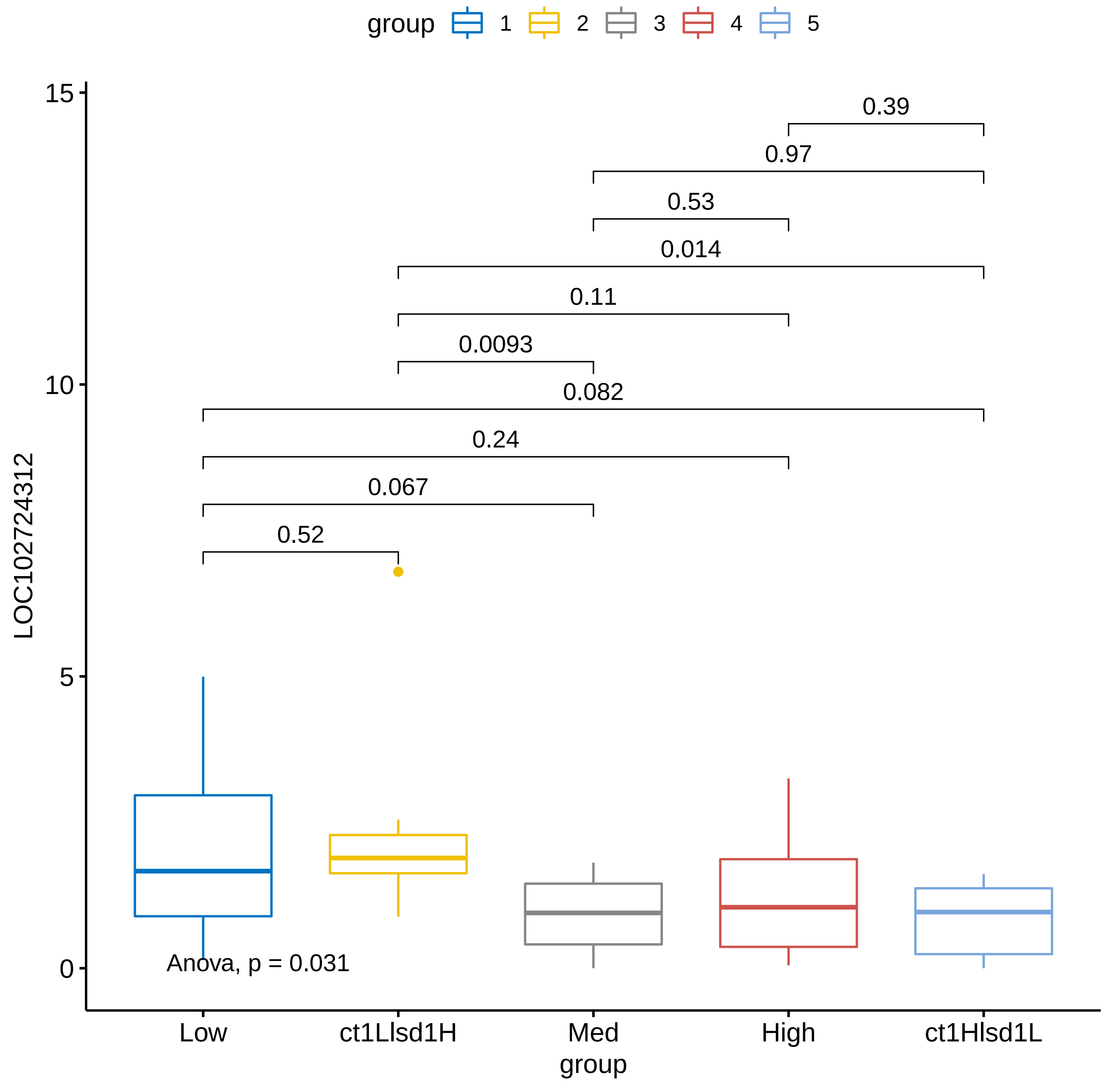
group
1
2
3
4
5
15
10
5
0
LOC102724312
0.39
0.97
0.53
0.014
0.11
0.0093
0.082
0.24
0.067
0.52
Anova, p = 0.031
Low
ct1Llsd1H
Med
High
ct1Hlsd1L
group

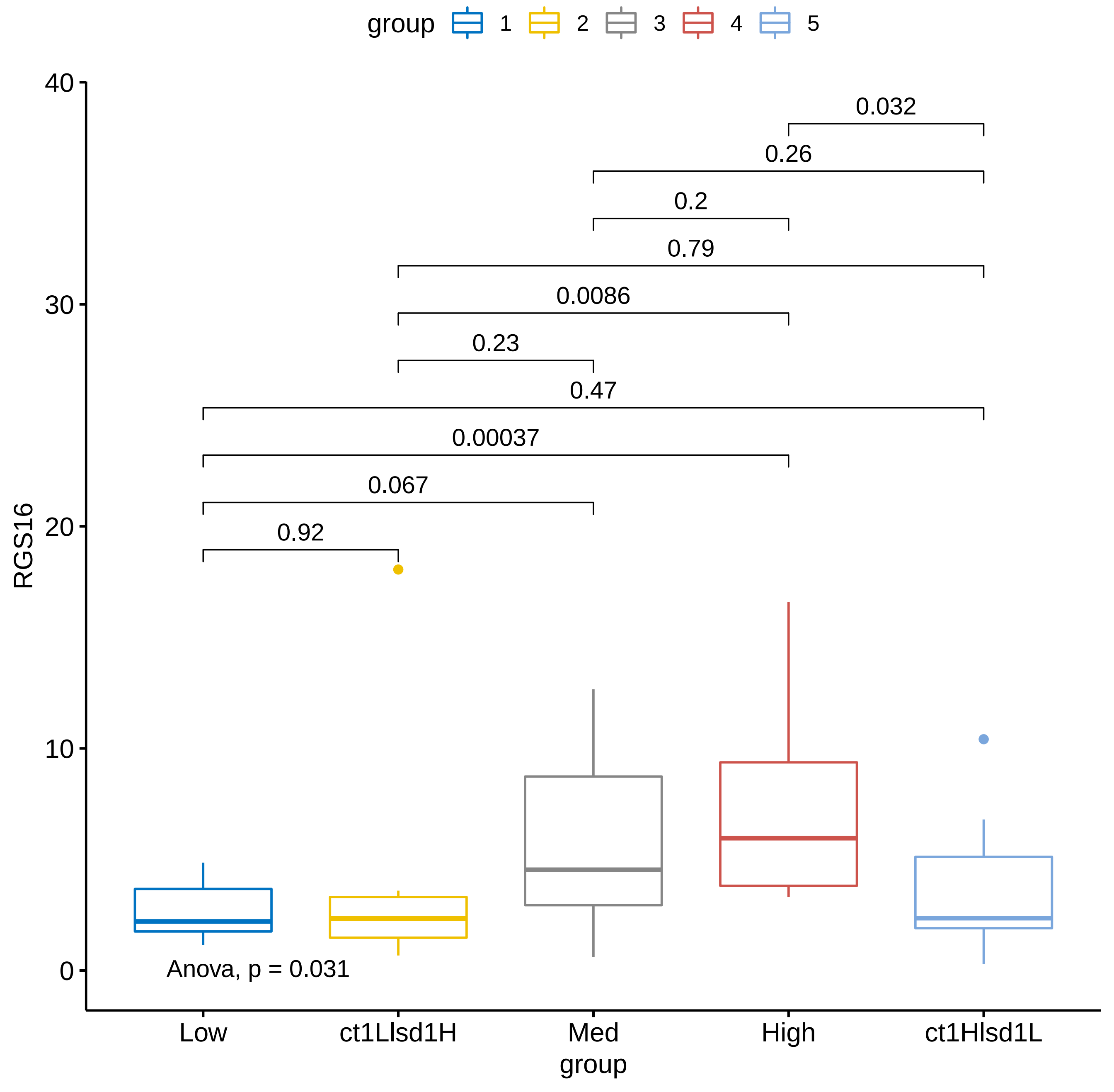

group 1 2 3 4 5
40
30
20
10
0
RGS16
0.032
0.26
0.2
0.79
0.0086
0.23
0.47
0.00037
0.067
0.92
Anova, p = 0.031
Low
ct1Llsd1H
Med
High
ct1Hlsd1L
group

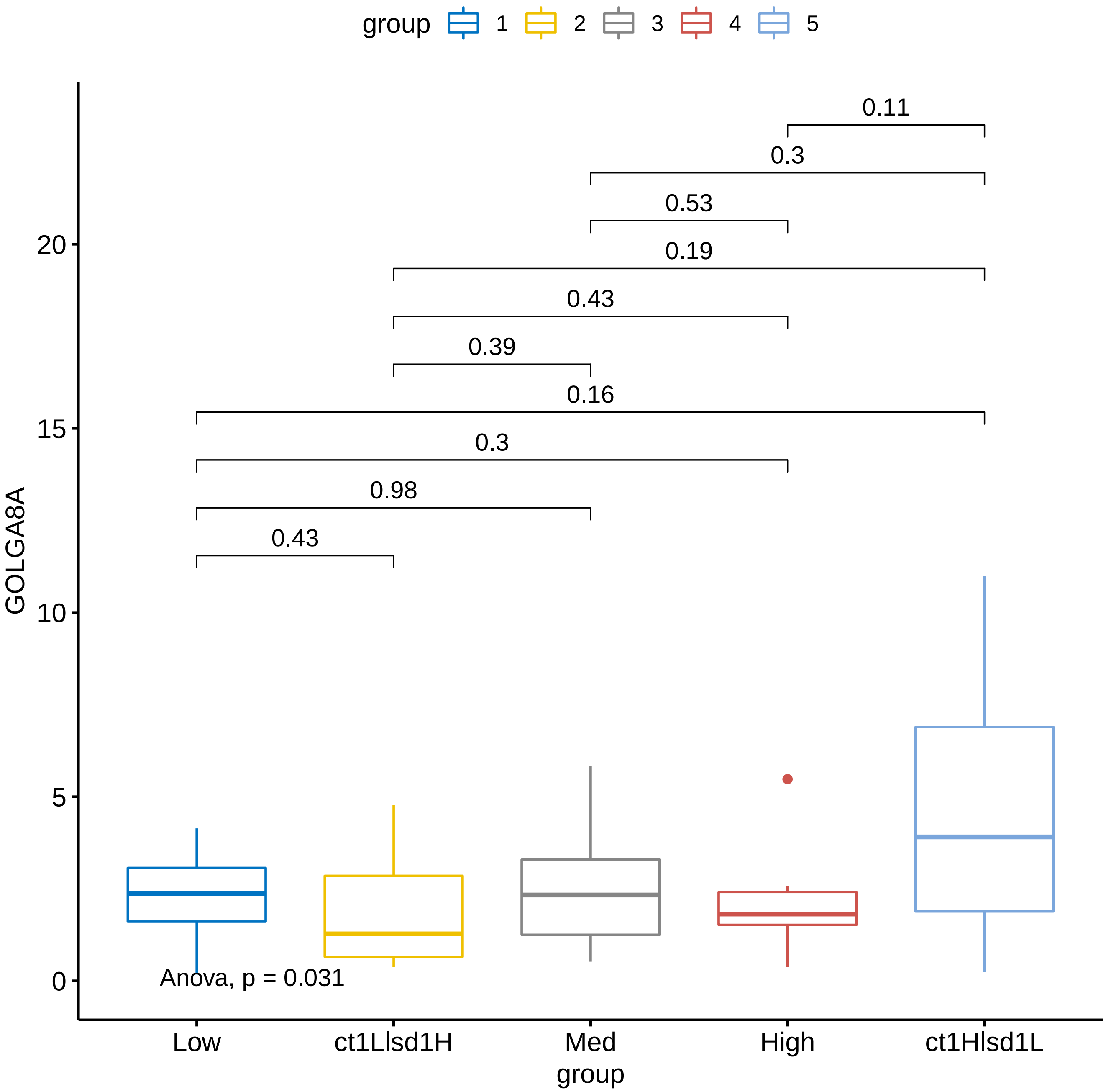

group 1 2 3 4 5
0.11
0.3
0.53
0.19
0.43
0.39
0.16
0.3
0.98
0.43
GOLGA8A
20
15
10
5
0
Anova, p = 0.031
Low
ct1Llsd1H
Med
High
ct1Hlsd1L
group

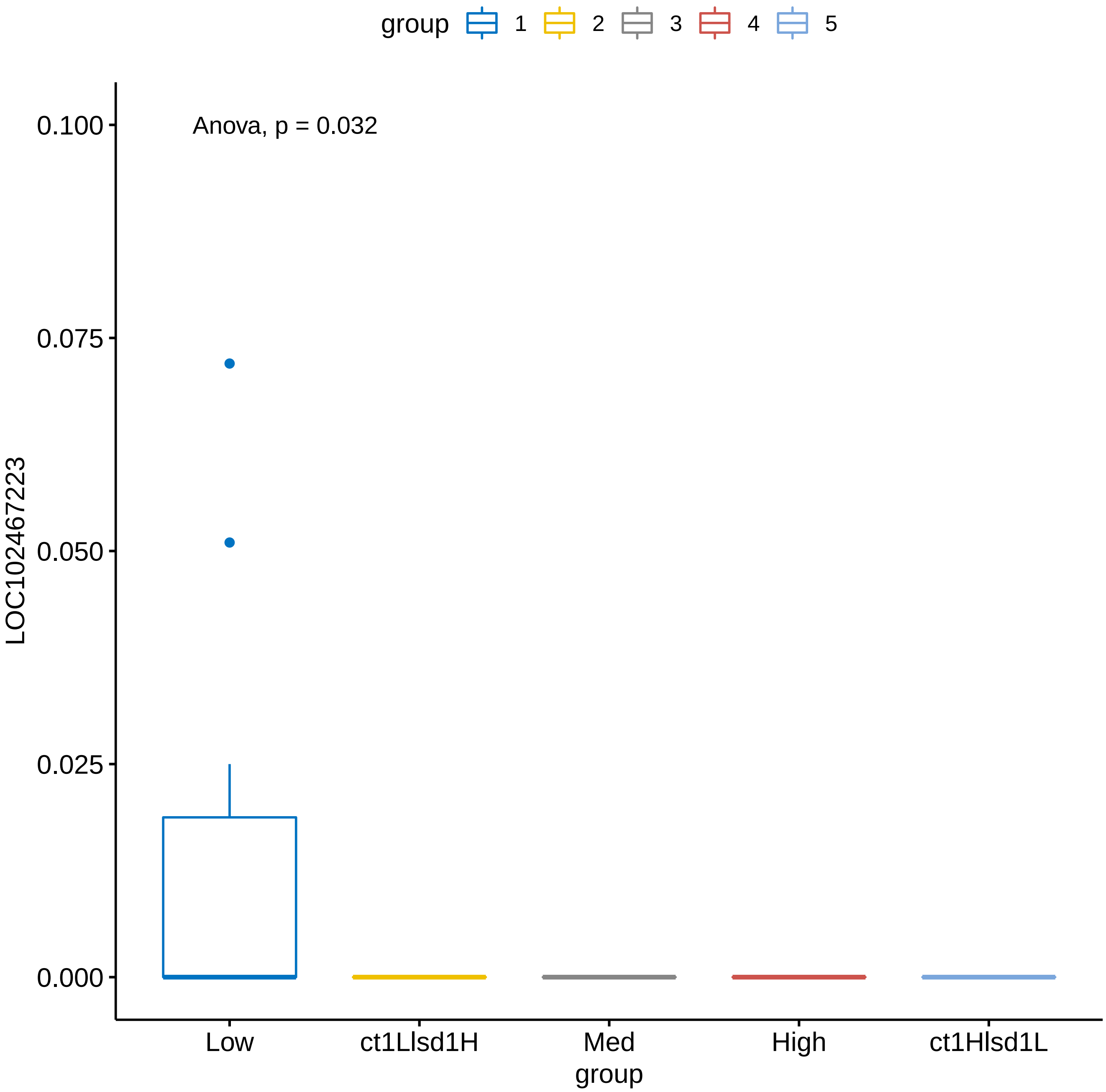
group
1
2
3
4
5
Anova, p = 0.032
0.100
0.075
0.050
0.025
0.000
LOC102467223
Low
ct1Llsd1H
Med
High
ct1Hlsd1L
group

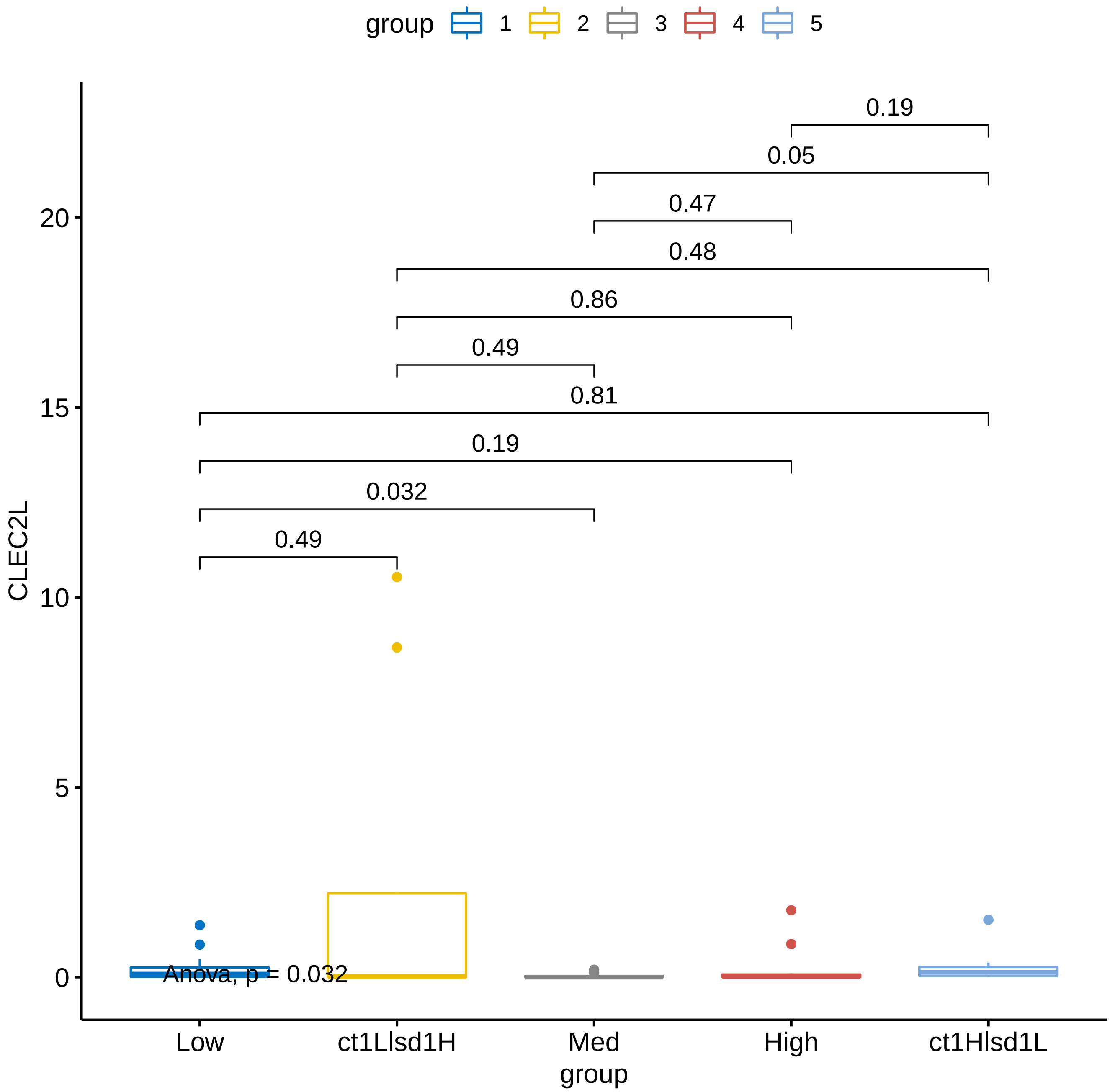
group
1
2
3
4
5
0.19
0.05
0.47
0.48
0.86
0.49
0.81
0.19
0.032
0.49
CLEC2L
20
15
10
5
0
Anova, p = 0.032
Low
ct1Llsd1H
Med
High
ct1Hlsd1L
group

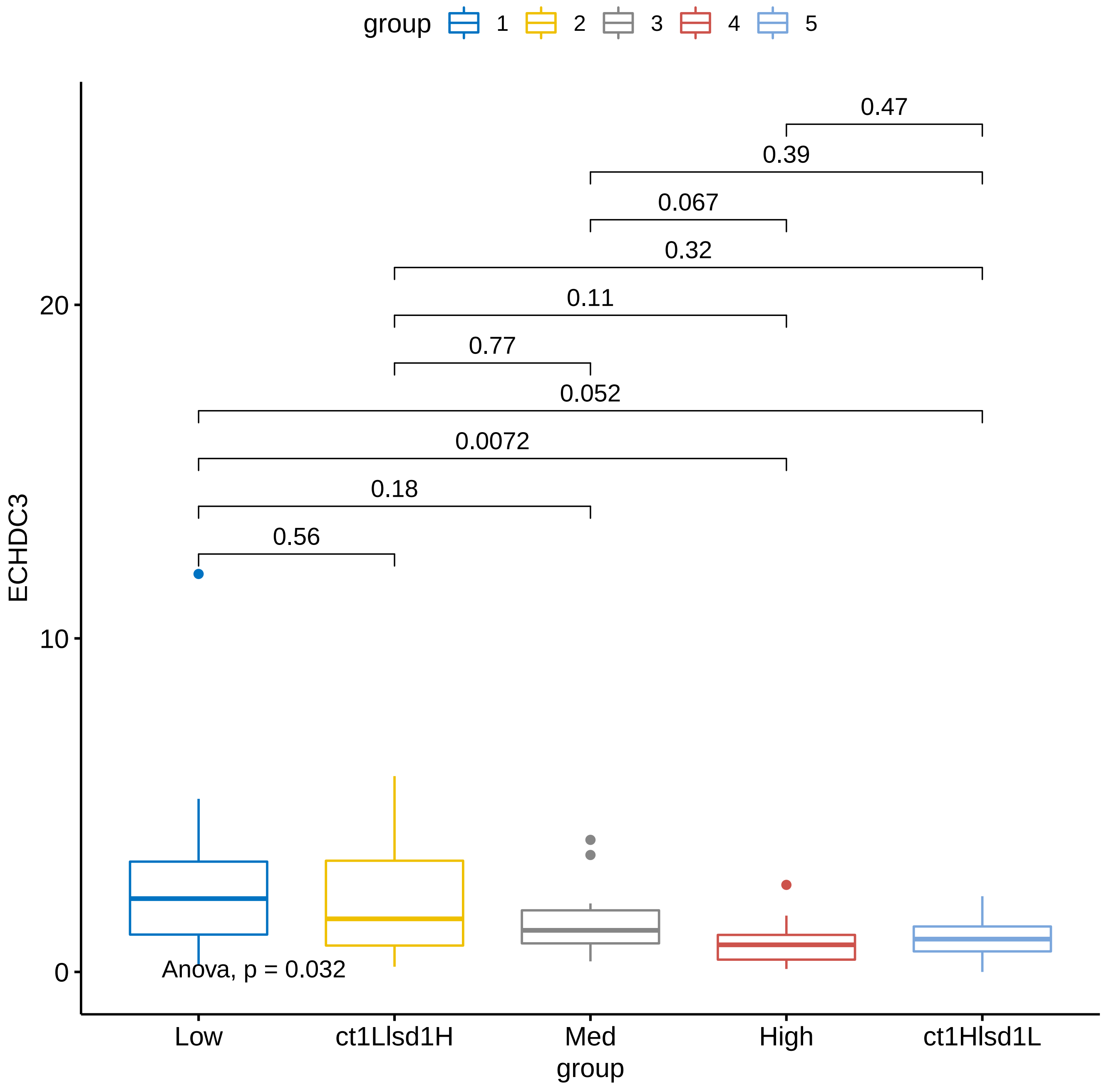

group 1 2 3 4 5
0.47
0.39
0.067
0.32
0.11
0.77
0.052
0.0072
0.18
0.56
20
10
0
ECHDC3
Anova, p = 0.032
Low
ct1Llsd1H
Med
High
ct1Hlsd1L
group

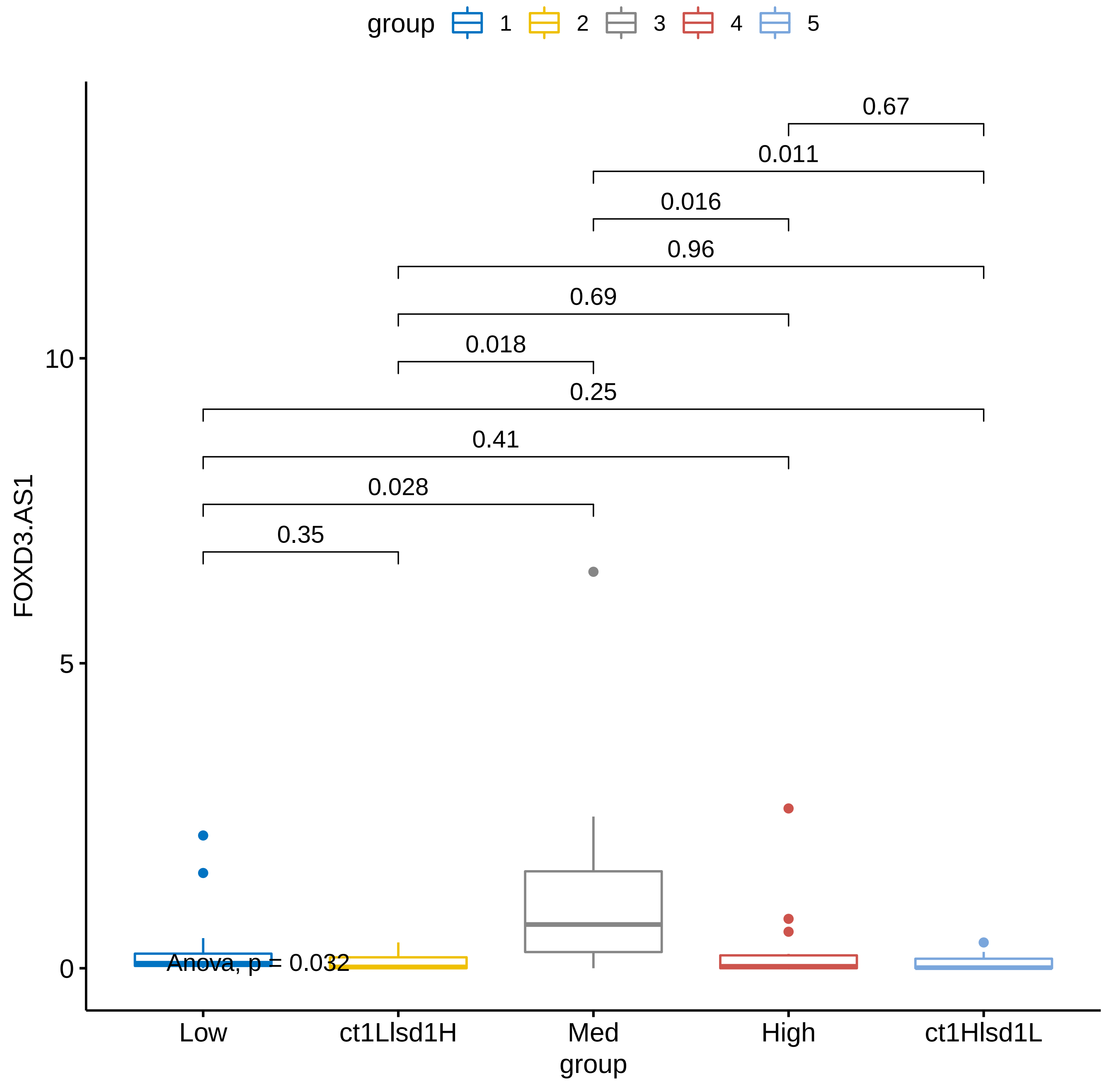

group 1 2 3 4 5
0.67
0.011
0.016
0.96
0.69
0.018
0.25
0.41
0.028
0.35
FOXD3.AS1
10
5
0
Anova, p = 0.032
Low
ct1Llsd1H
Med
High
ct1Hlsd1L
group

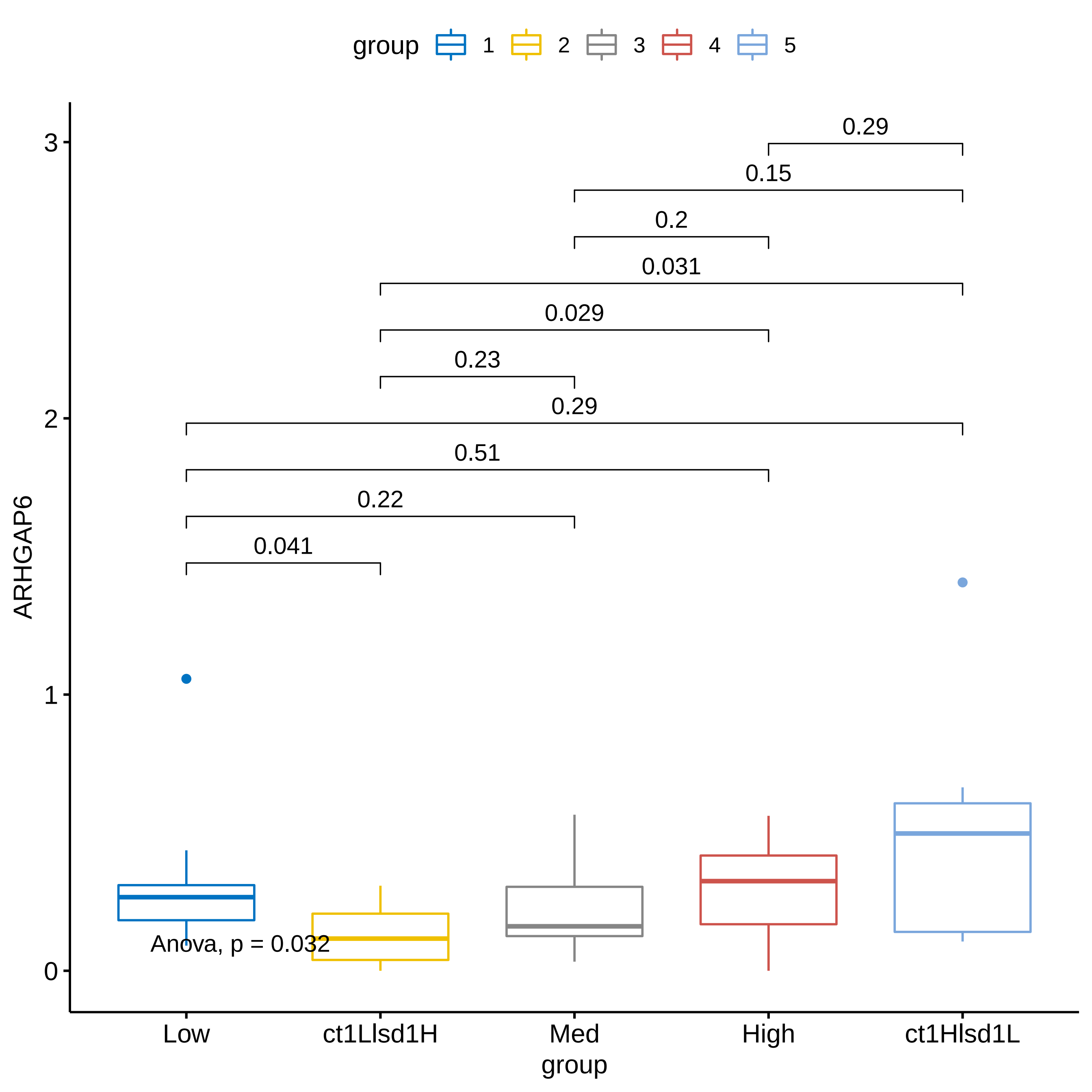

group 1 2 3 4 5
ARHGAP6
0.29
0.15
0.2
0.031
0.029
0.23
0.29
0.51
0.22
0.041
Anova, p = 0.032
3
2
1
0
Low
ct1Llsd1H
Med
High
ct1Hlsd1L
group

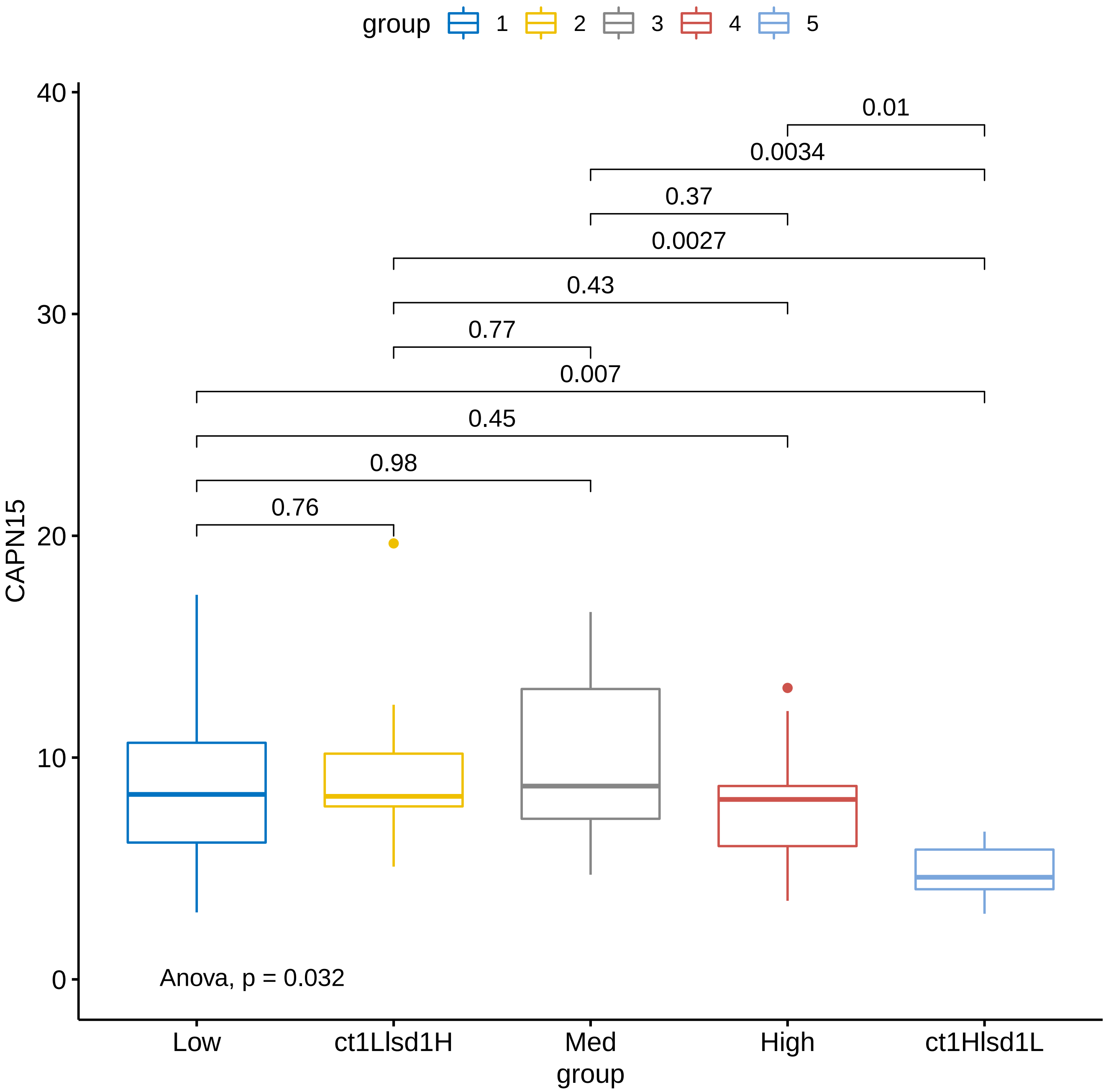
group
1
2
3
4
5
CAPN15
40
30
20
10
0
0.01
0.0034
0.37
0.0027
0.43
0.77
0.007
0.45
0.98
0.76
Anova, p = 0.032
Low
ct1Llsd1H
Med
High
ct1Hlsd1L
group

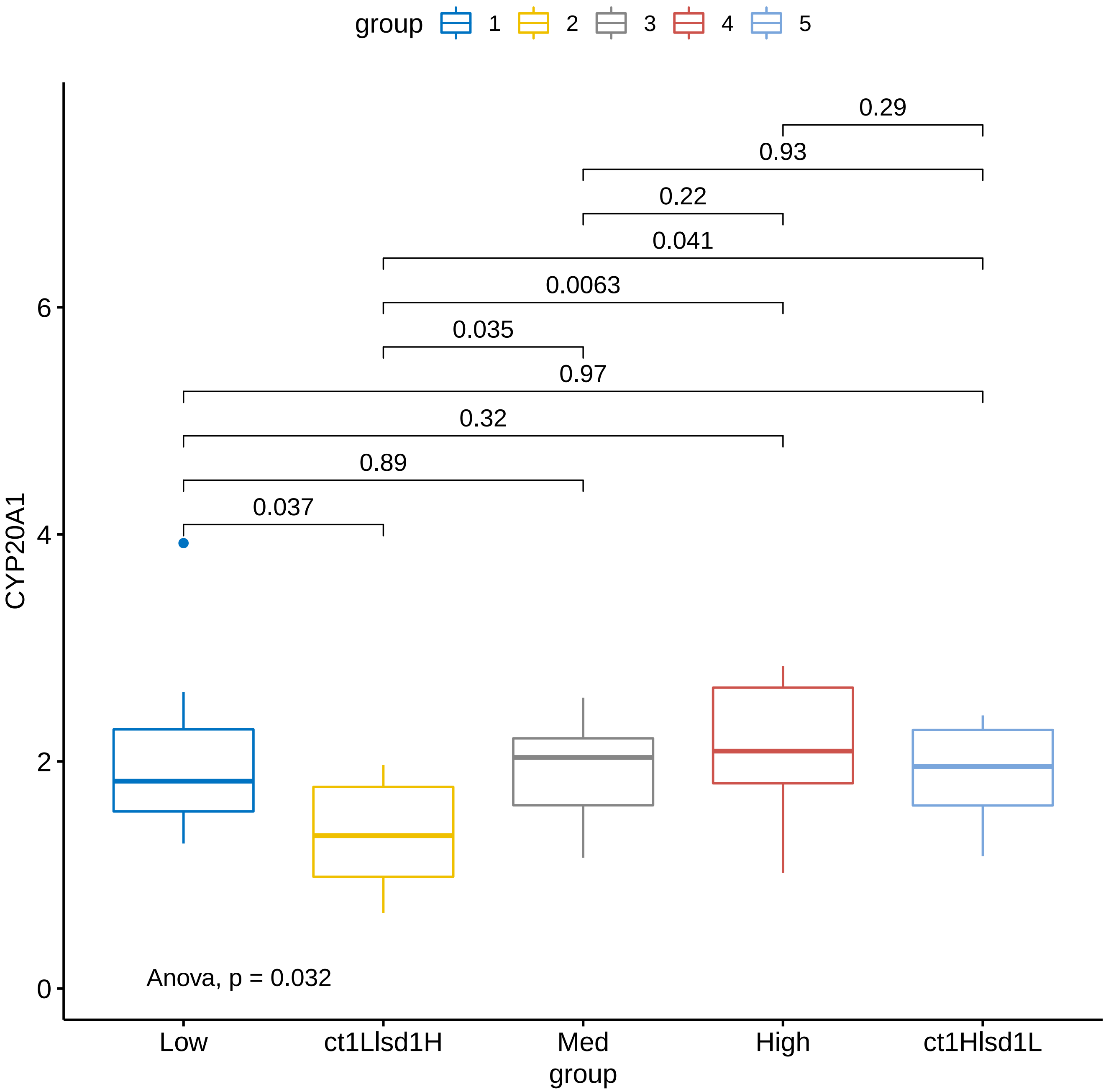
group
1
2
3
4
5
0.29
0.93
0.22
0.041
0.0063
0.035
0.97
0.32
0.89
0.037
CYP20A1
6
4
2
0
Anova, p = 0.032
Low
ct1Llsd1H
Med
High
ct1Hlsd1L
group

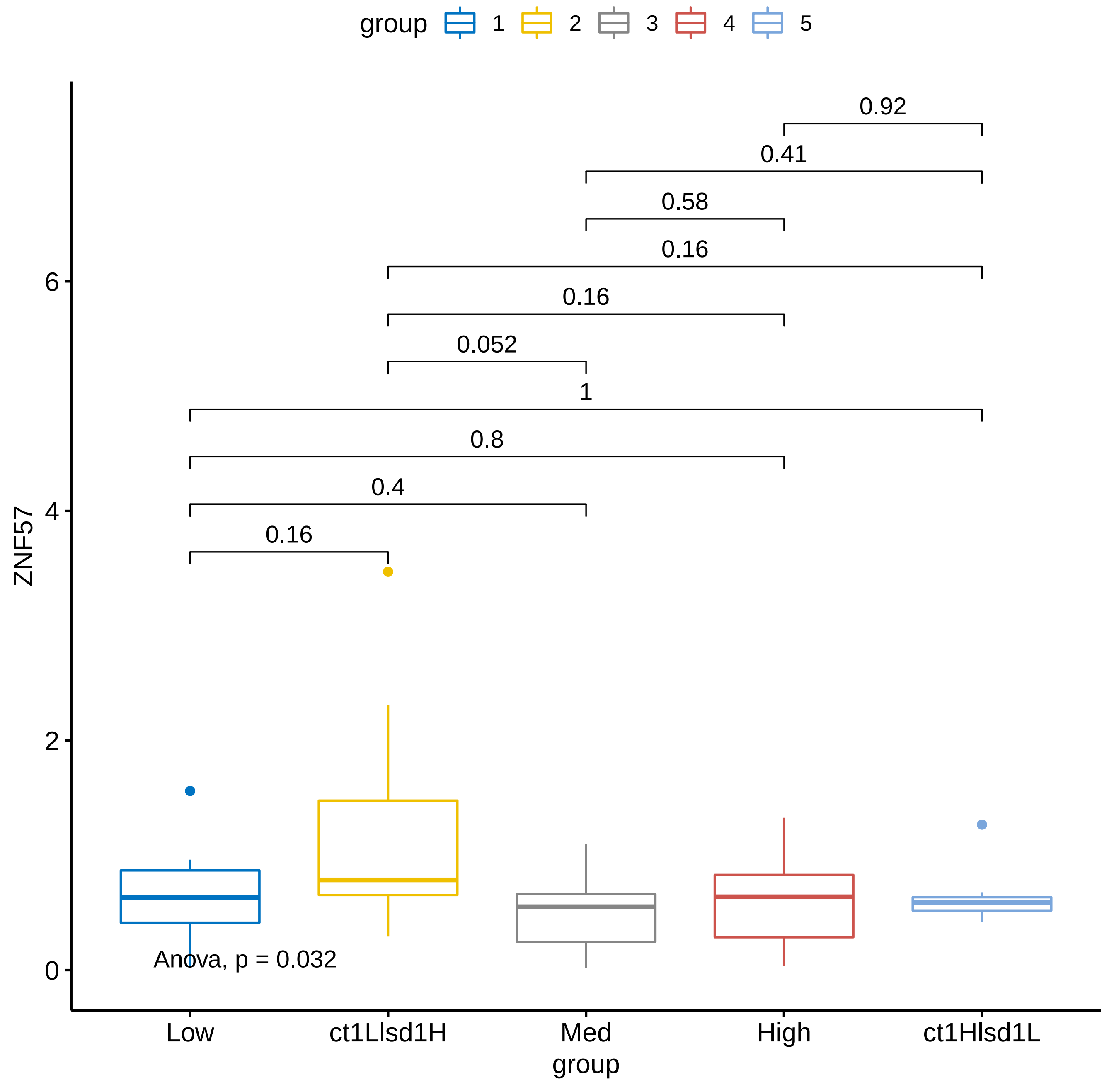

group
1
2
3
4
5
0.92
0.41
0.58
0.16
0.16
0.052
1
0.8
0.4
0.16
Anova, p = 0.032
6
4
2
0
ZNF57
Low
ct1Llsd1H
Med
High
ct1Hlsd1L
group

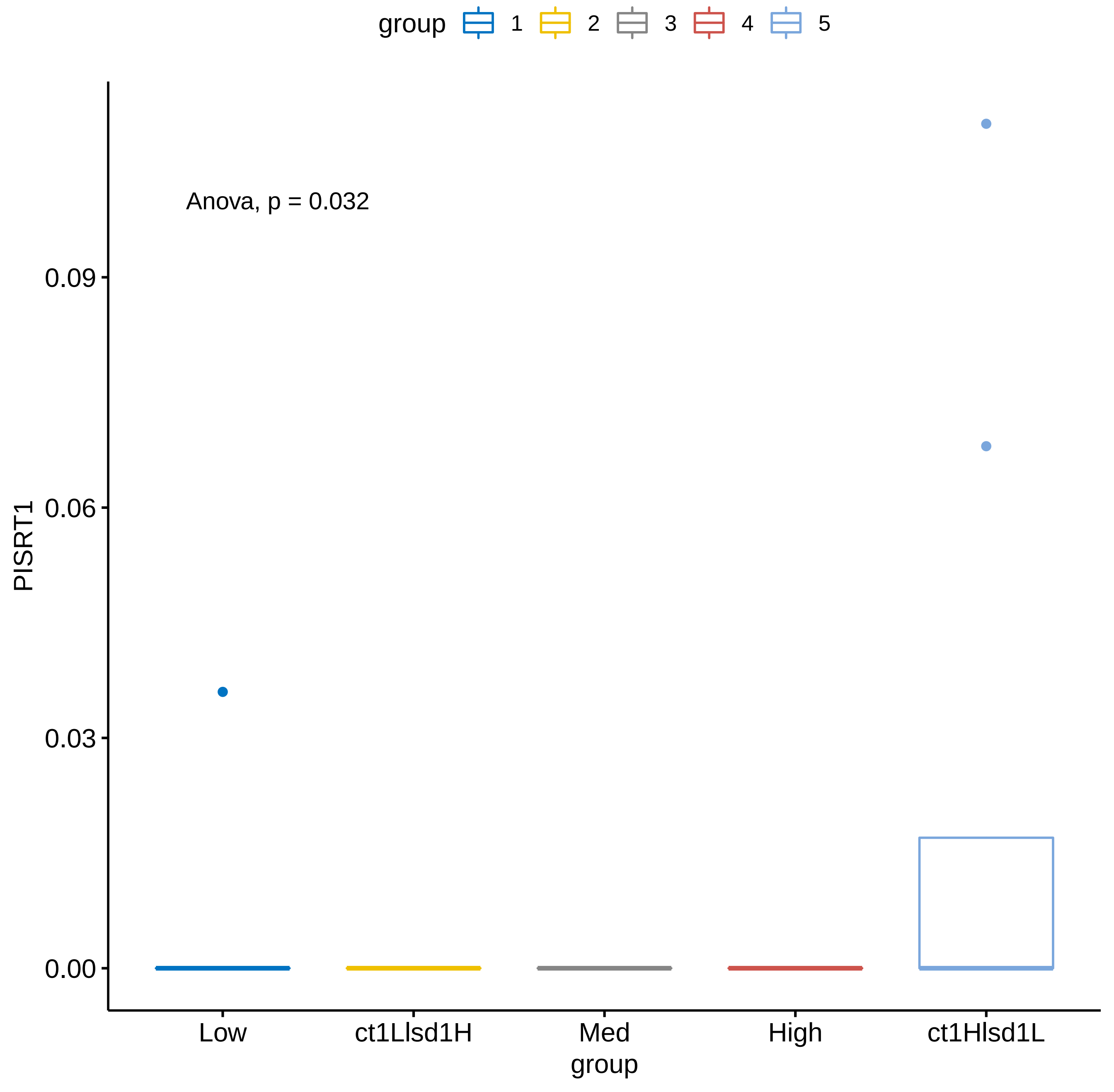

group 1 2 3 4 5
Anova, p = 0.032
PISRT1
0.09
0.06
0.03
0.00
Low
ct1Llsd1H
Med
High
ct1Hlsd1L
group

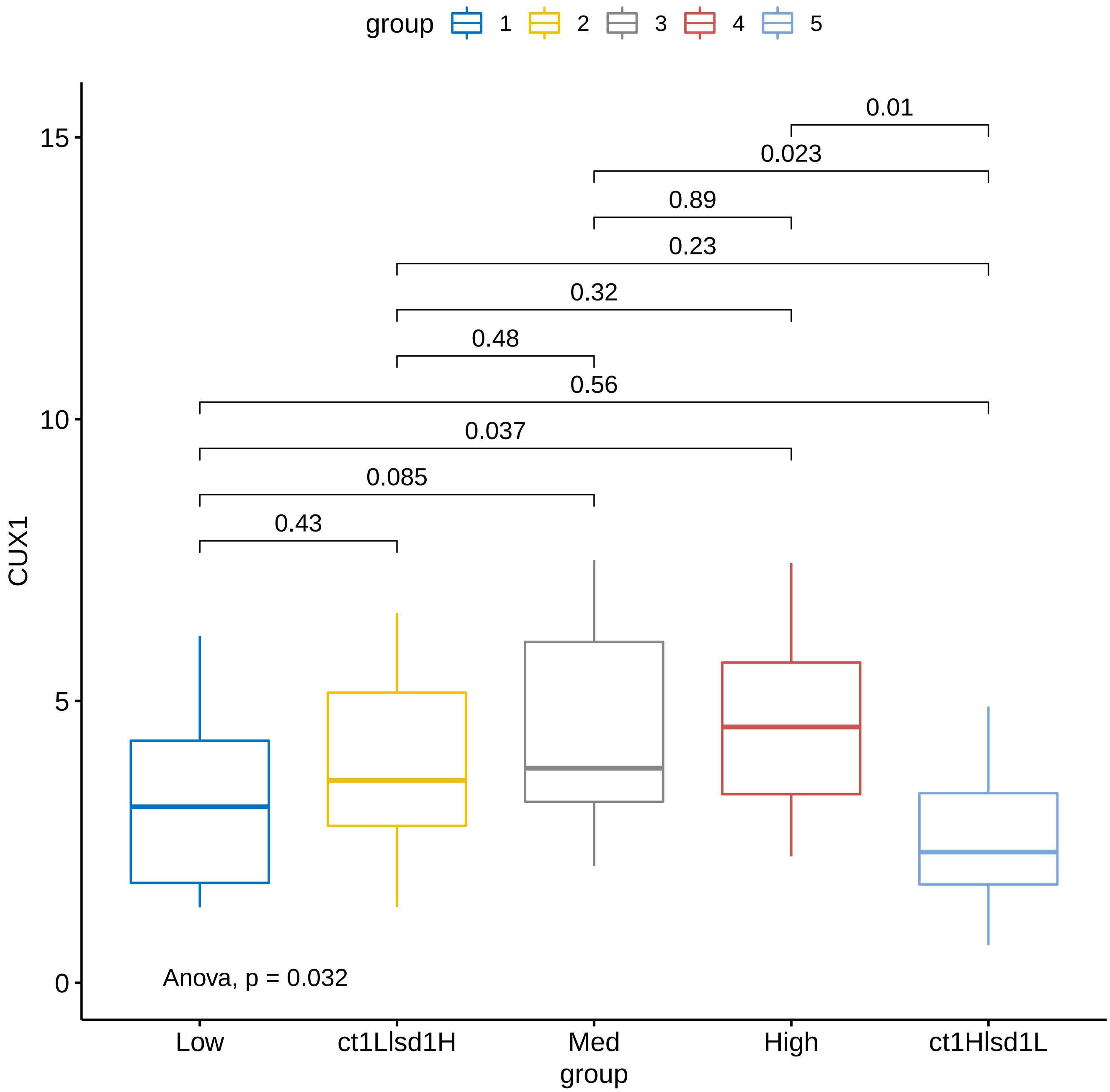

group 1 2 3 4 5
0.01
0.023
0.89
0.23
0.32
0.48
0.56
0.037
0.085
0.43
15
10
5
0
CUX1
Anova, p = 0.032
Low
ct1Llsd1H
Med
High
ct1Hlsd1L
group

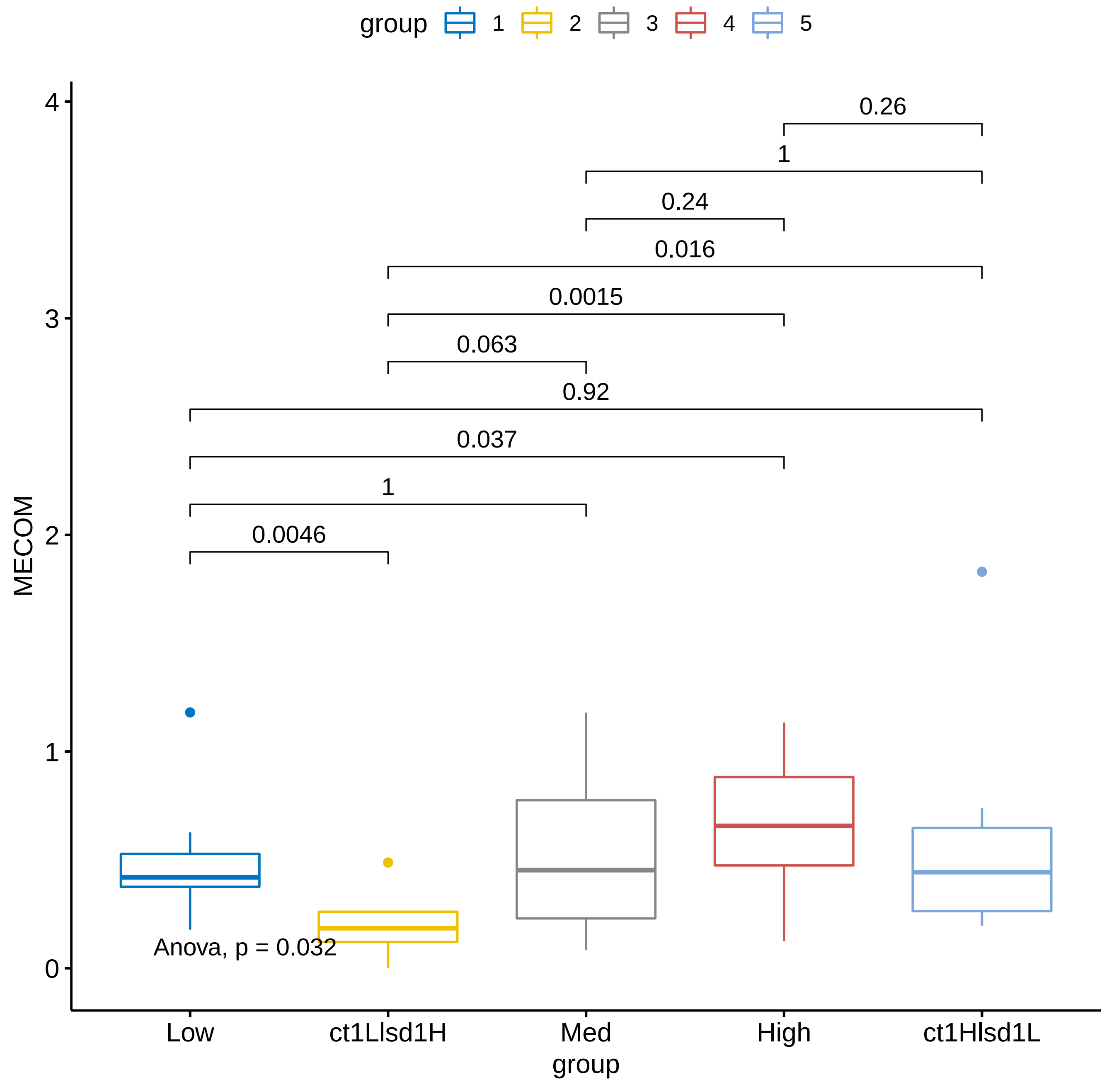

group
1
2
3
4
5
MECOM
4
3
2
1
0
0.26
1
0.24
0.016
0.0015
0.063
0.92
0.037
1
0.0046
Anova, p = 0.032
Low
ct1Llsd1H
Med
High
ct1Hlsd1L
group

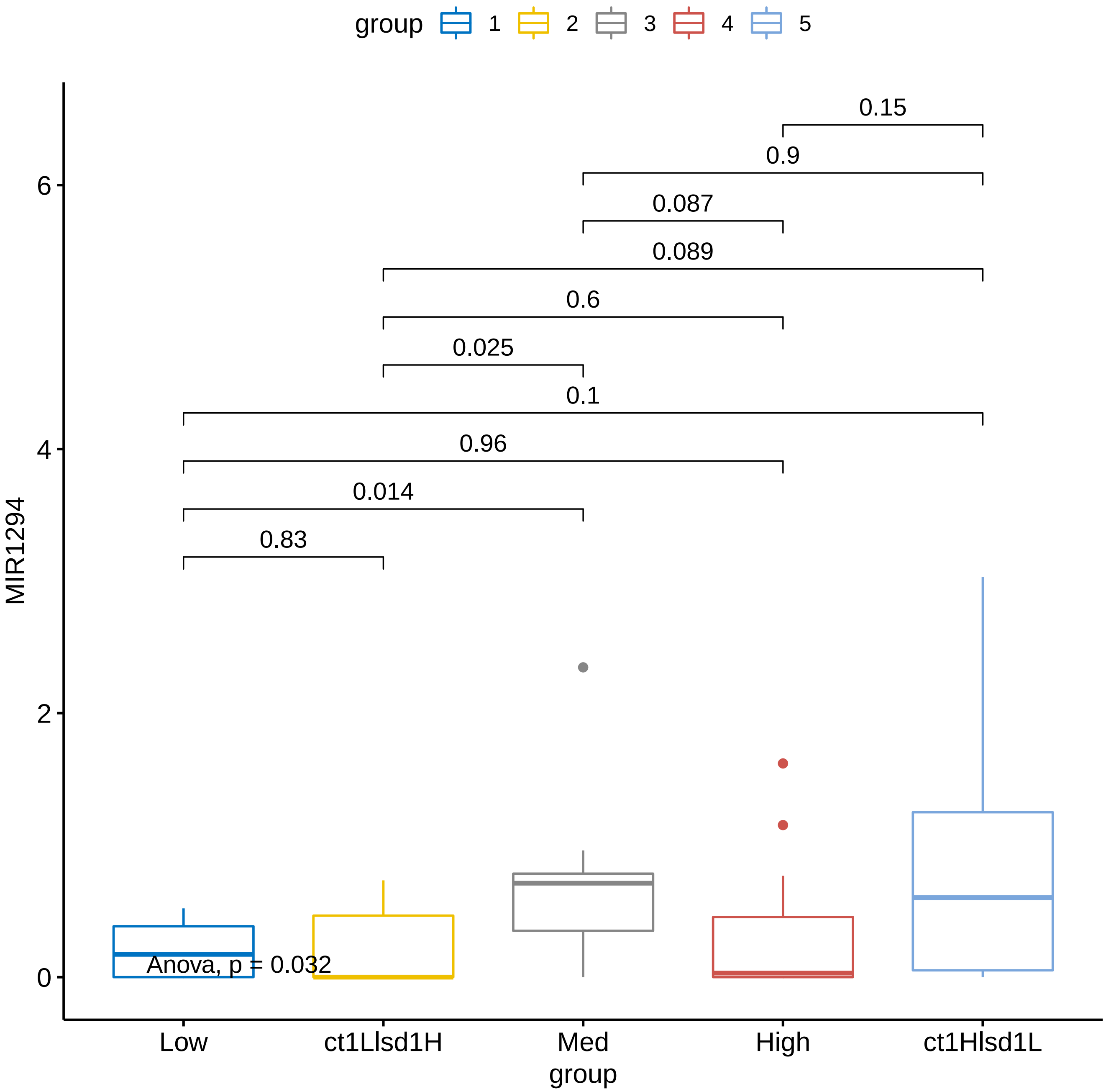

group
1
2
3
4
5
MIR1294
0.15
0.9
0.087
0.089
0.6
0.025
0.1
0.96
0.014
0.83
Anova, p = 0.032
6
4
2
0
Low
ct1Llsd1H
Med
High
ct1Hlsd1L
group

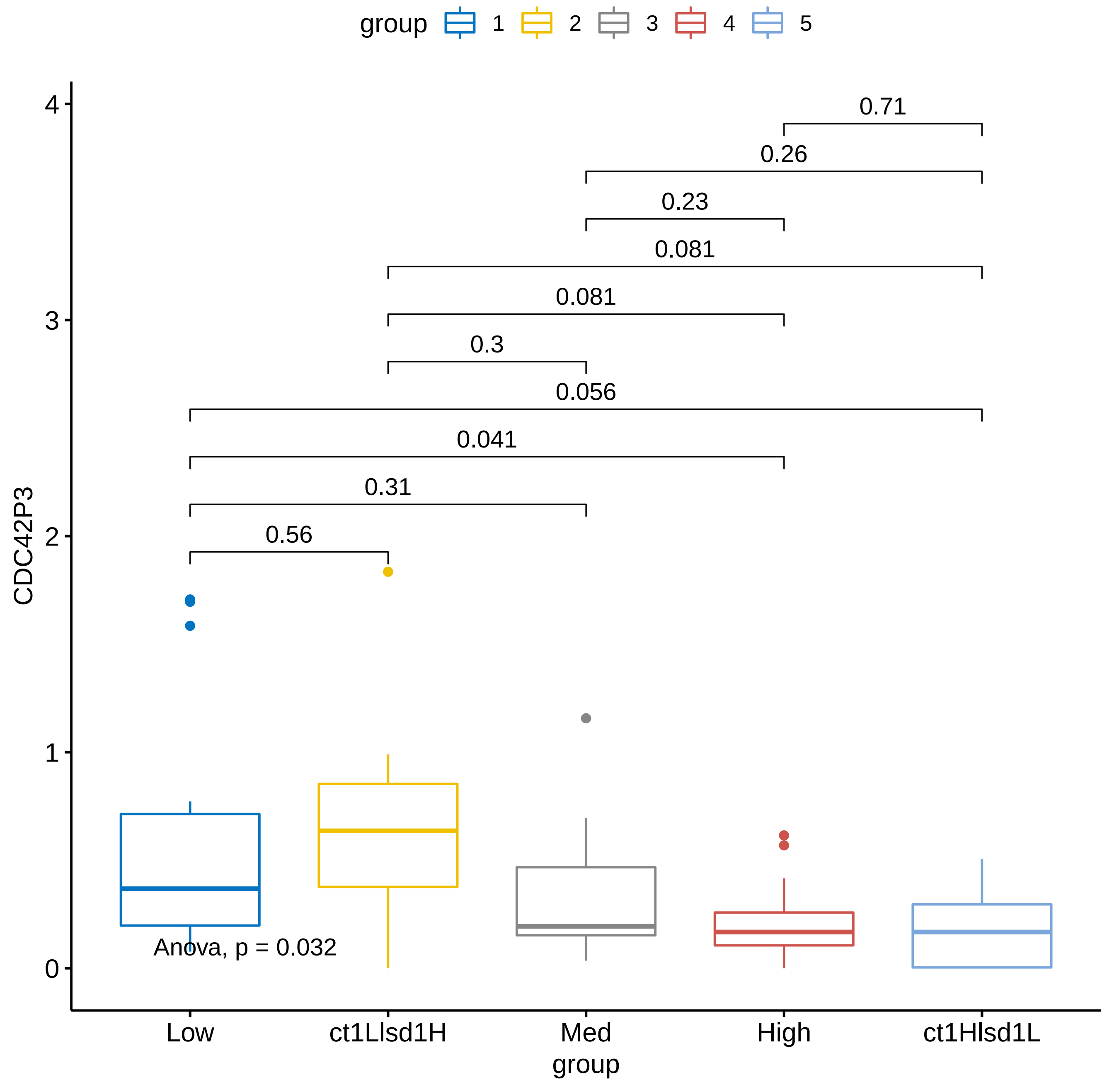
group
1
2
3
4
5
CDC42P3
0.71
0.26
0.23
0.081
0.081
0.3
0.056
0.041
0.31
0.56
Anova, p = 0.032
Low
ct1Llsd1H
Med
High
ct1Hlsd1L
group

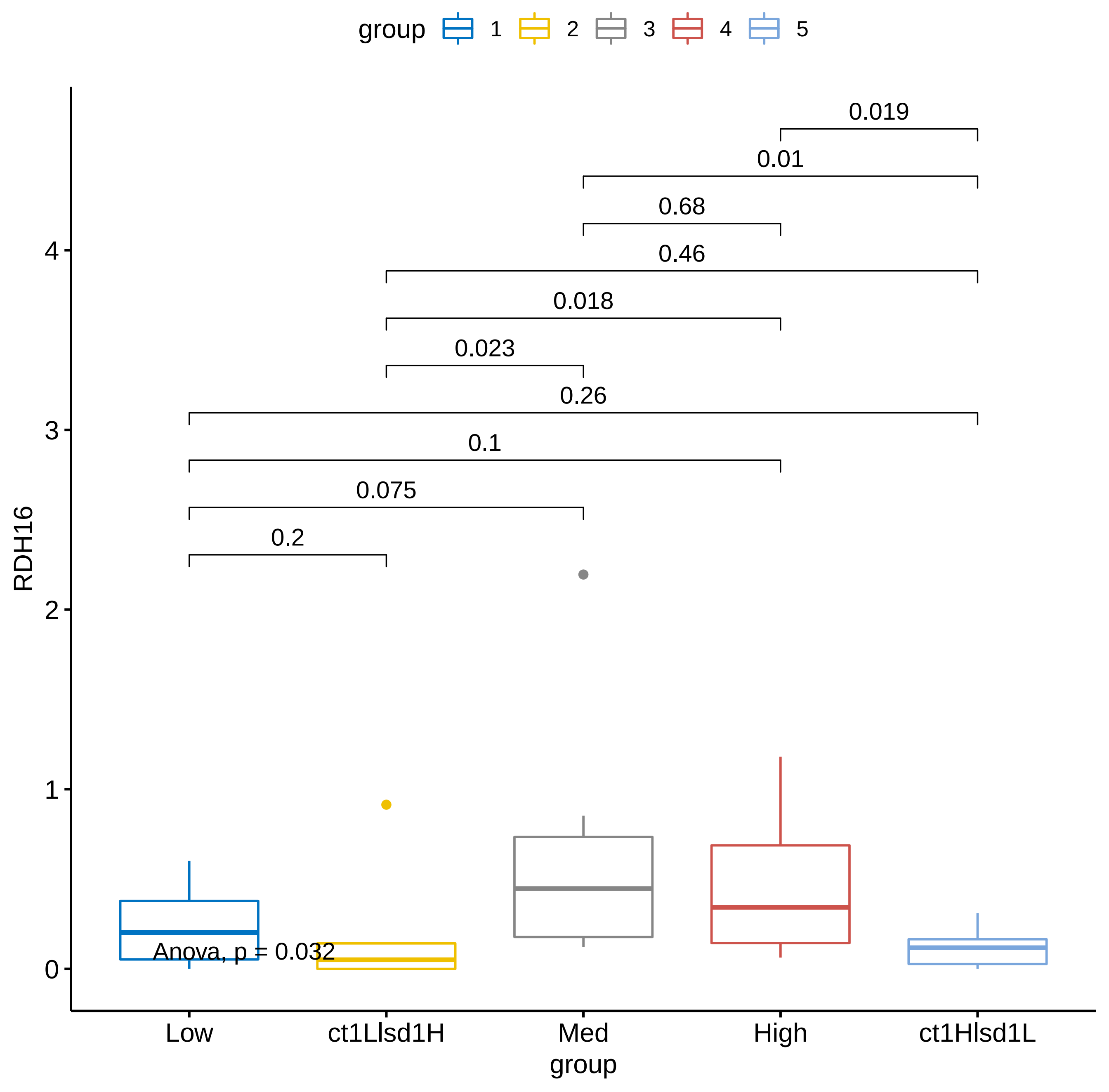

group
1
2
3
4
5
RDH16
0.019
0.01
0.68
0.46
0.018
0.023
0.26
0.1
0.075
0.2
Anova, p = 0.032
0
1
2
3
4
Low
ct1Llsd1H
Med
High
ct1Hlsd1L
group

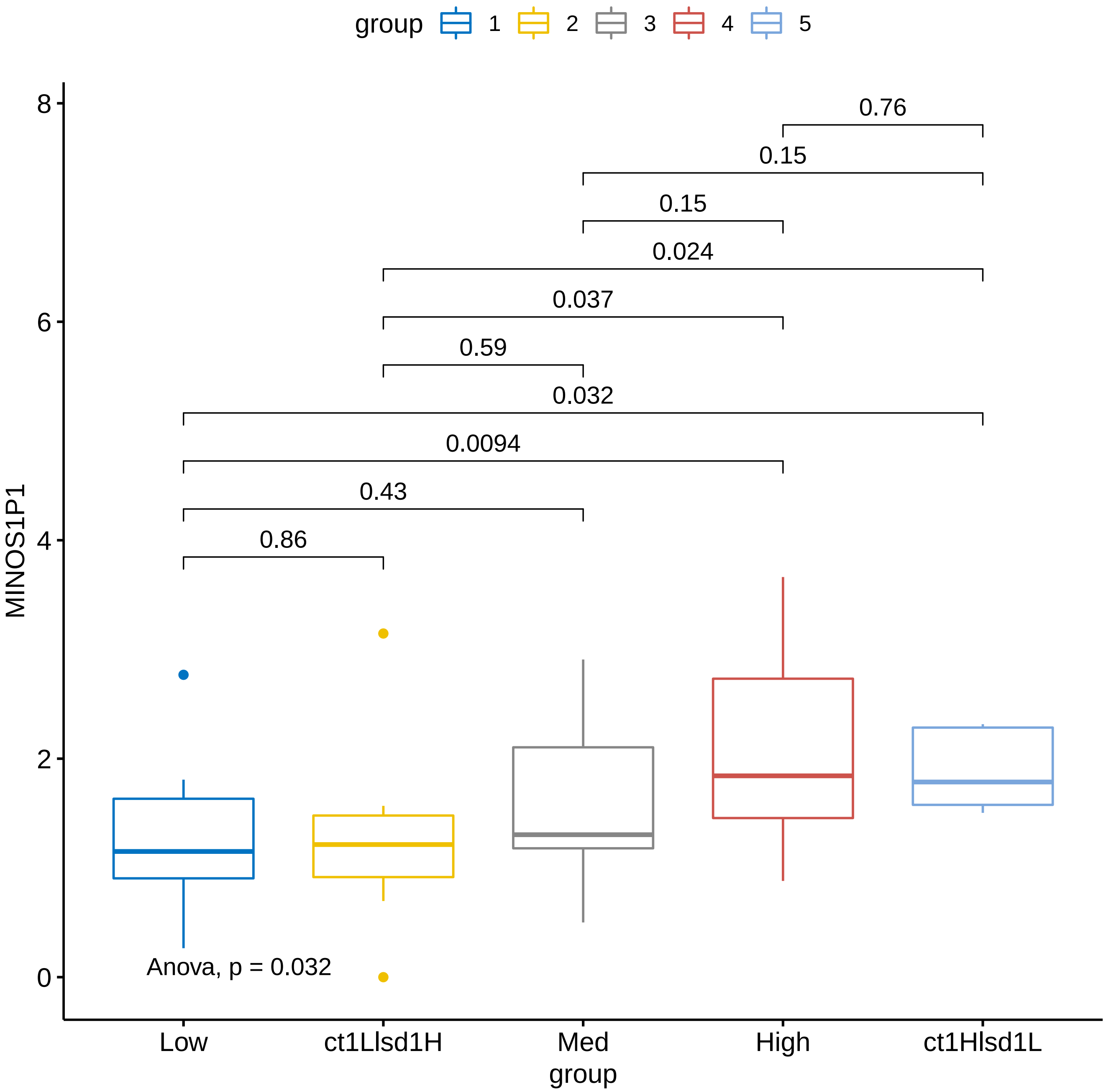

group
1
2
3
4
5
MINOS1P1
0
2
4
6
8
0.76
0.15
0.15
0.024
0.037
0.59
0.032
0.0094
0.43
0.86
Anova, p = 0.032
Low
ct1Llsd1H
Med
High
ct1Hlsd1L
group

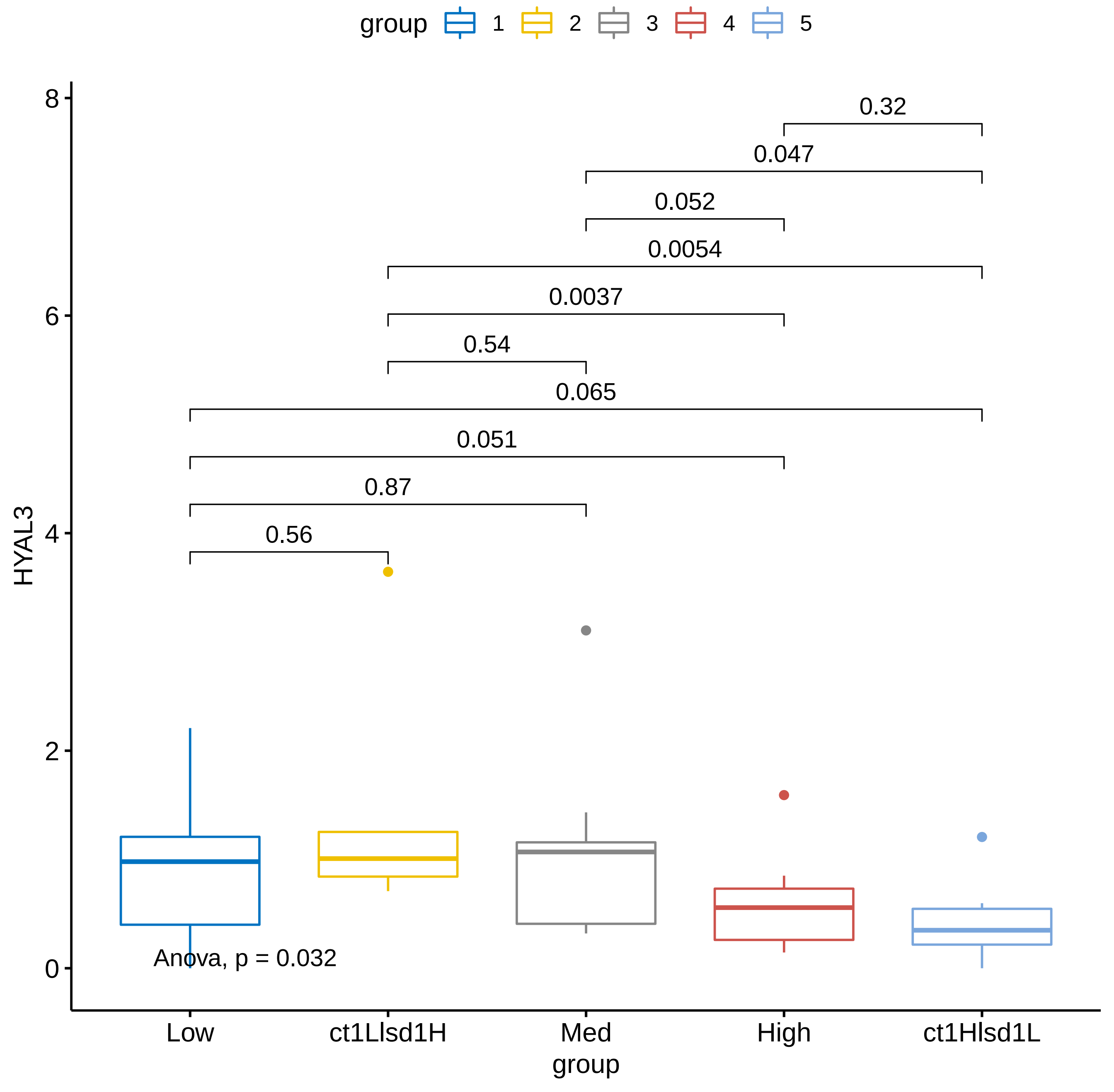
group
1
2
3
4
5
0.32
0.047
0.052
0.0054
0.0037
0.54
0.065
0.051
0.87
0.56
Anova, p = 0.032
8
6
4
2
0
HYAL3
Low
ct1Llsd1H
Med
High
ct1Hlsd1L
group

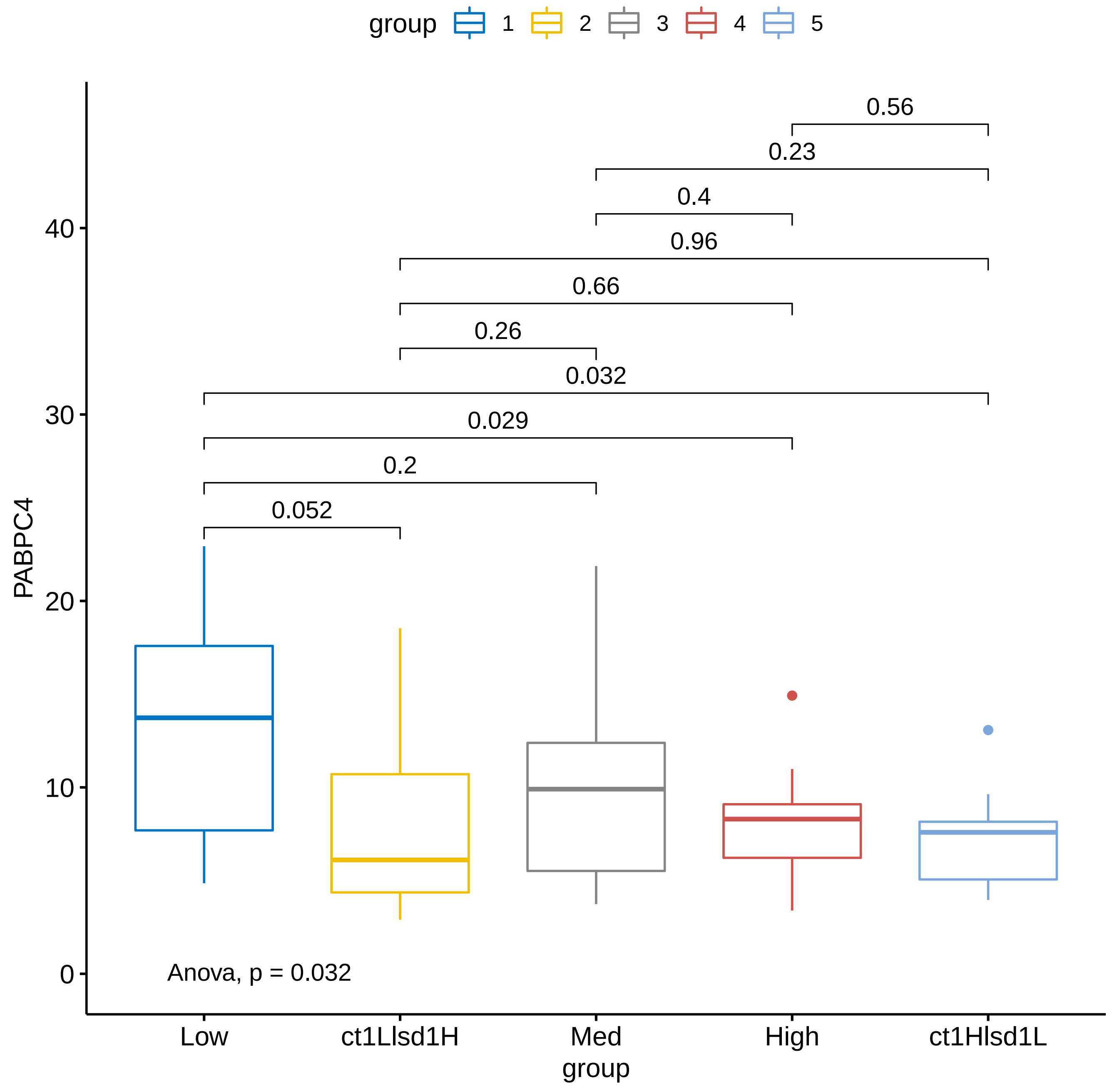

group
1
2
3
4
5
0.56
0.23
0.4
0.96
0.66
0.26
0.032
0.029
0.2
0.052
PABPC4
40
30
20
10
0
Anova, p = 0.032
Low
ct1Llsd1H
Med
High
ct1Hlsd1L
group

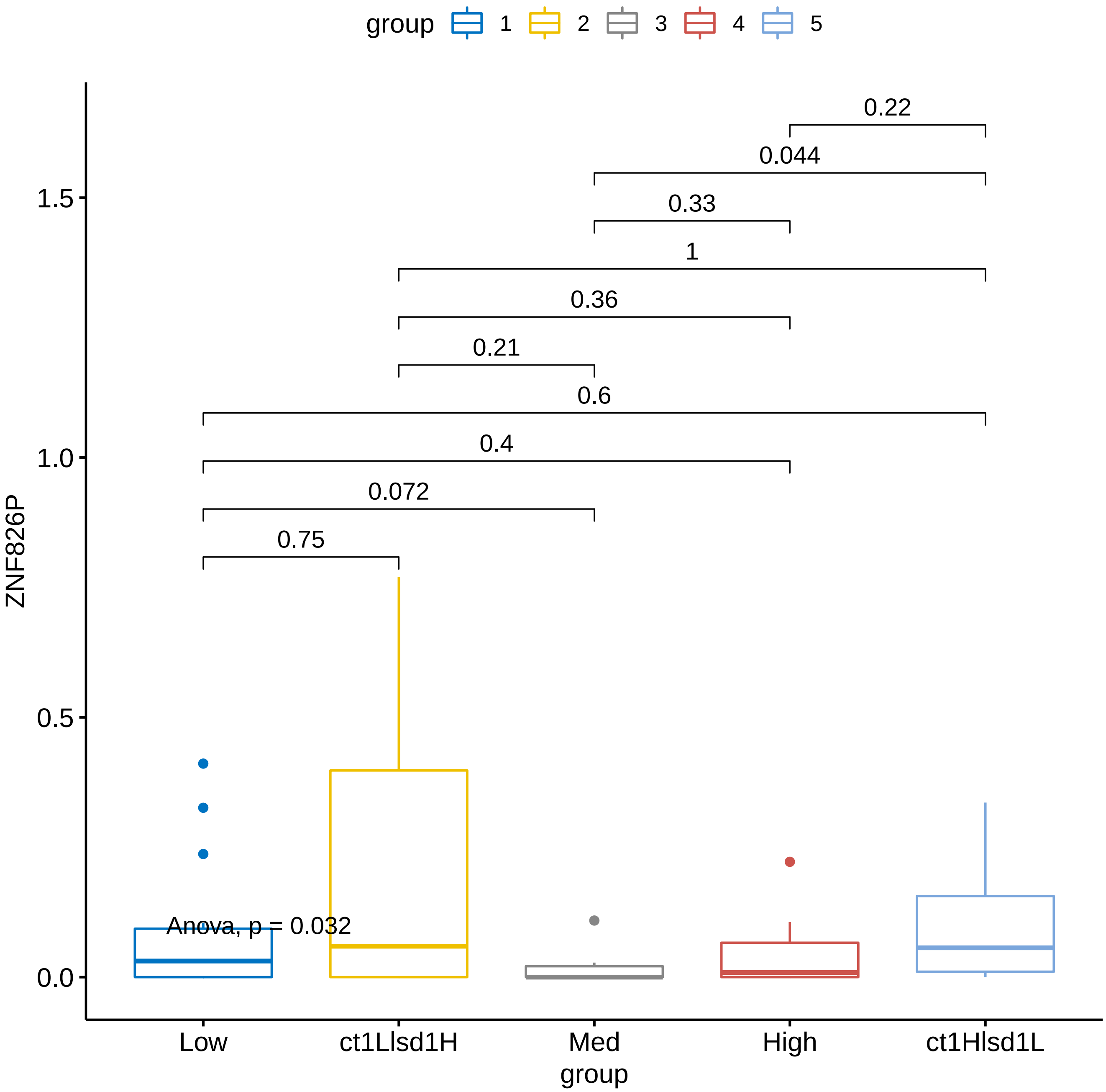

group 1 2 3 4 5
0.22
0.044
0.33
1
0.36
0.21
0.6
0.4
0.072
0.75
Anova, p = 0.032
ZNF826P
1.5
1.0
0.5
0.0
Low
ct1Llsd1H
Med
High
ct1Hlsd1L
group

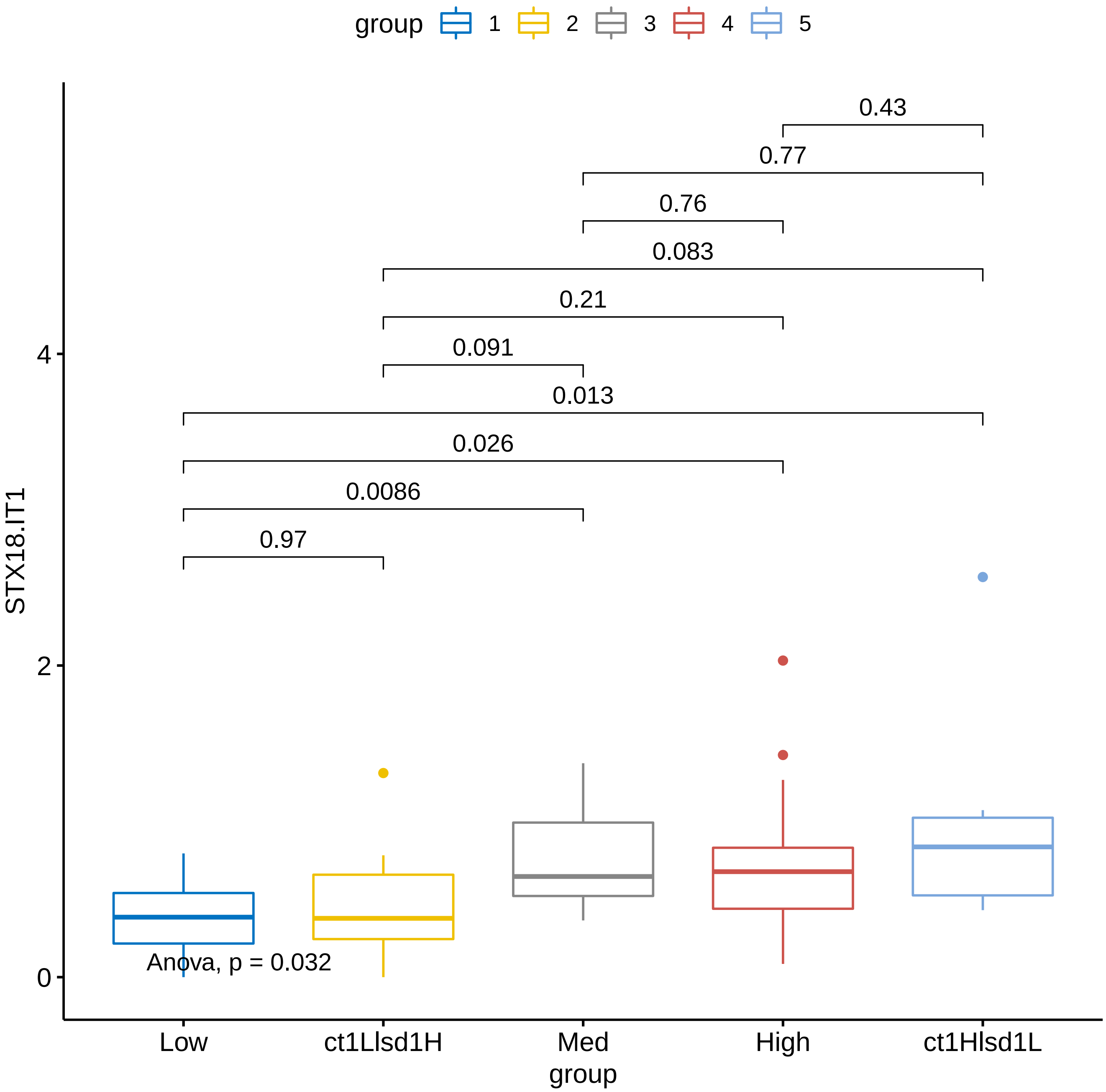
group
1
2
3
4
5
0.43
0.77
0.76
0.083
0.21
0.091
0.013
0.026
0.0086
0.97
Anova, p = 0.032
STX18.IT1
4
2
0
Low
ct1Llsd1H
Med
High
ct1Hlsd1L
group

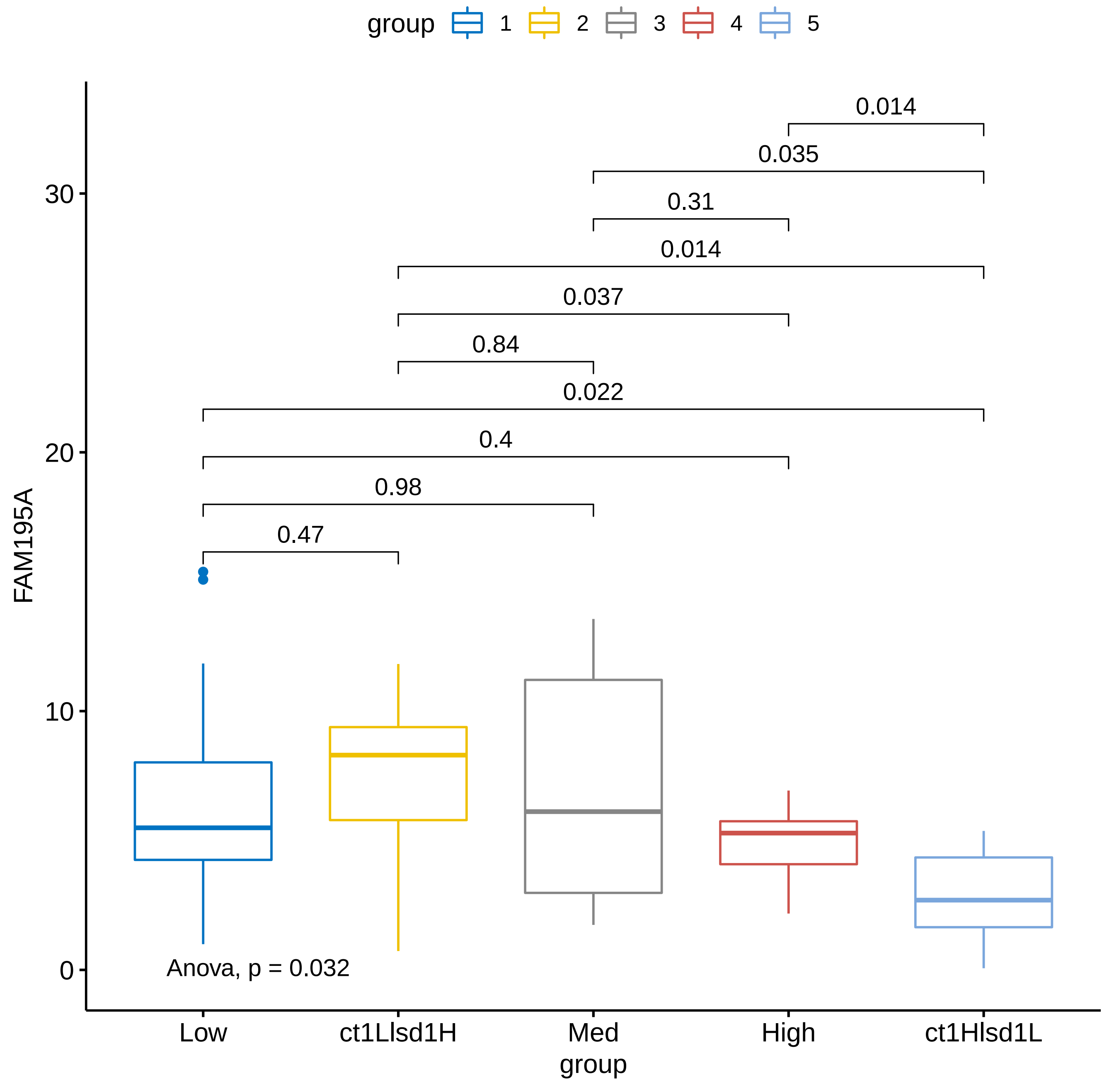

group 1 2 3 4 5
0.014
0.035
0.31
0.014
0.037
0.84
0.022
0.4
0.98
0.47
FAM195A
30
20
10
0
Anova, p = 0.032
Low
ct1Llsd1H
Med
High
ct1Hlsd1L
group

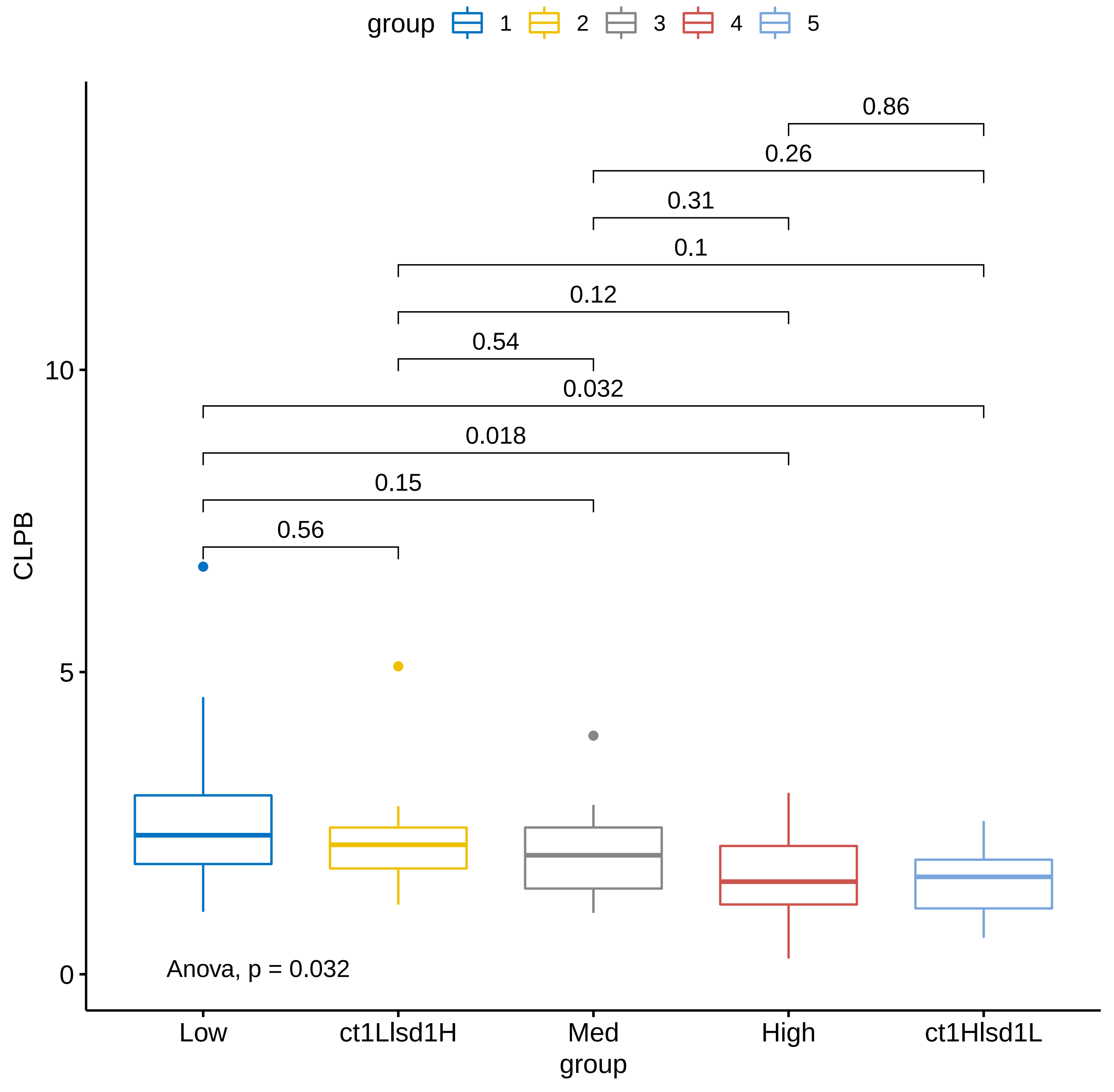

group 1 2 3 4 5
0.86
0.26
0.31
0.1
0.12
0.54
0.032
0.018
0.15
0.56
CLPB
10
5
0
Anova, p = 0.032
Low
ct1Llsd1H
Med
High
ct1Hlsd1L
group

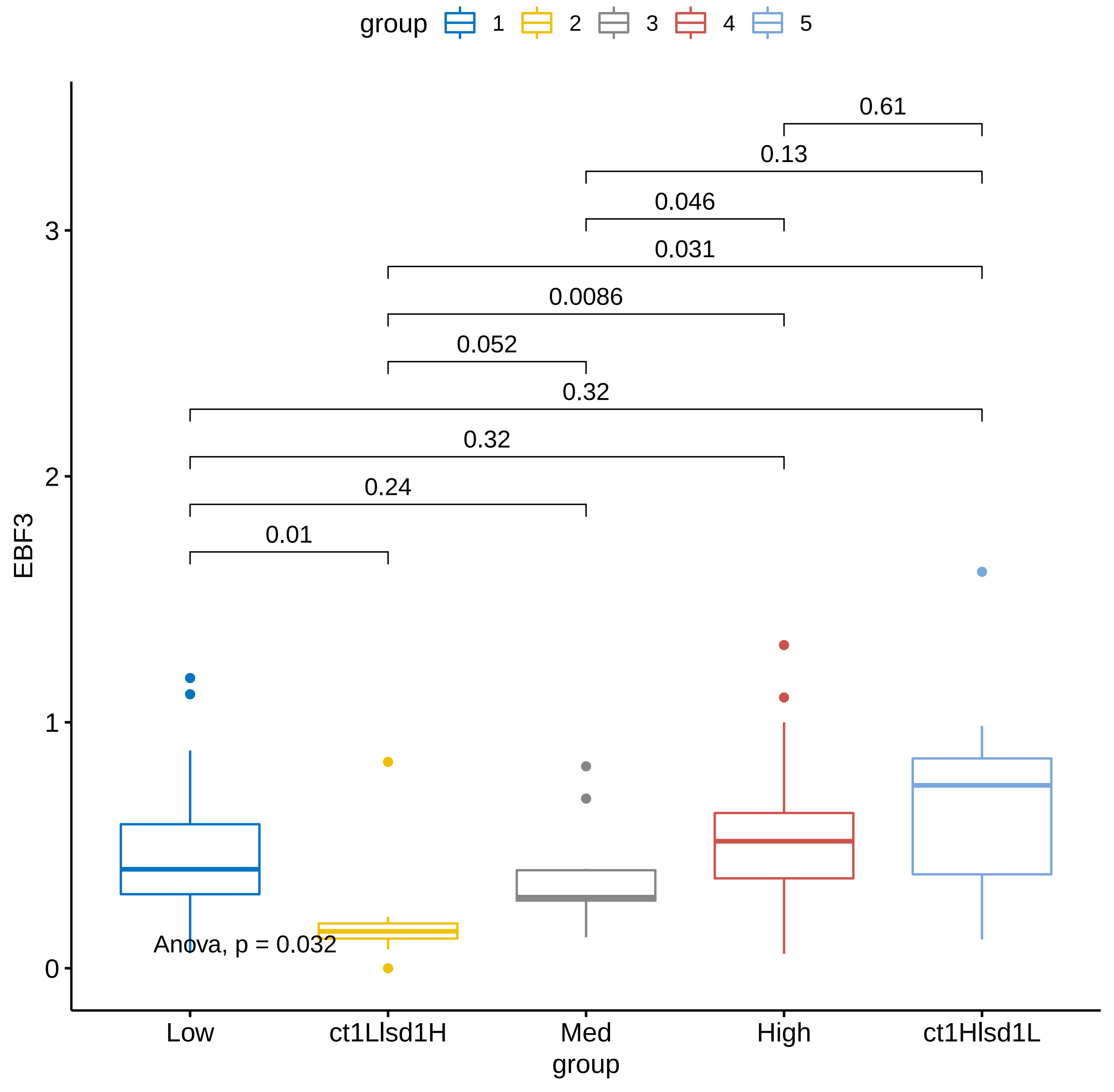

group 1 2 3 4 5
0.61
0.13
0.046
0.031
0.0086
0.052
0.32
0.32
0.24
0.01
EBF3
3
2
1
0
Anova, p = 0.032
Low
ct1Llsd1H
Med
High
ct1Hlsd1L
group

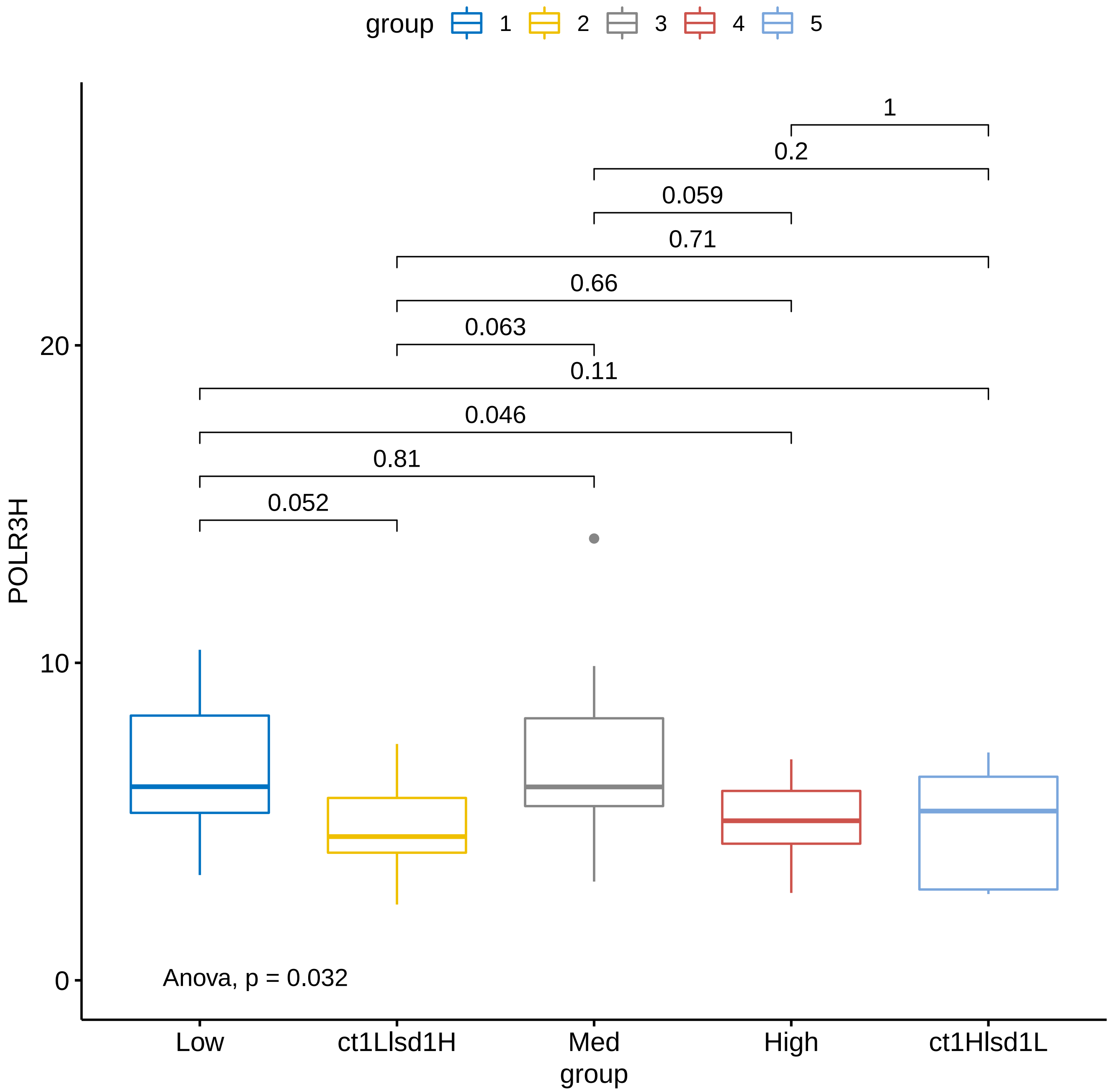

group 1 2 3 4 5
1
0.2
0.059
0.71
0.66
0.063
0.11
0.046
0.81
0.052
POLR3H
20
10
0
Anova, p = 0.032
Low
ct1Llsd1H
Med
High
ct1Hlsd1L
group

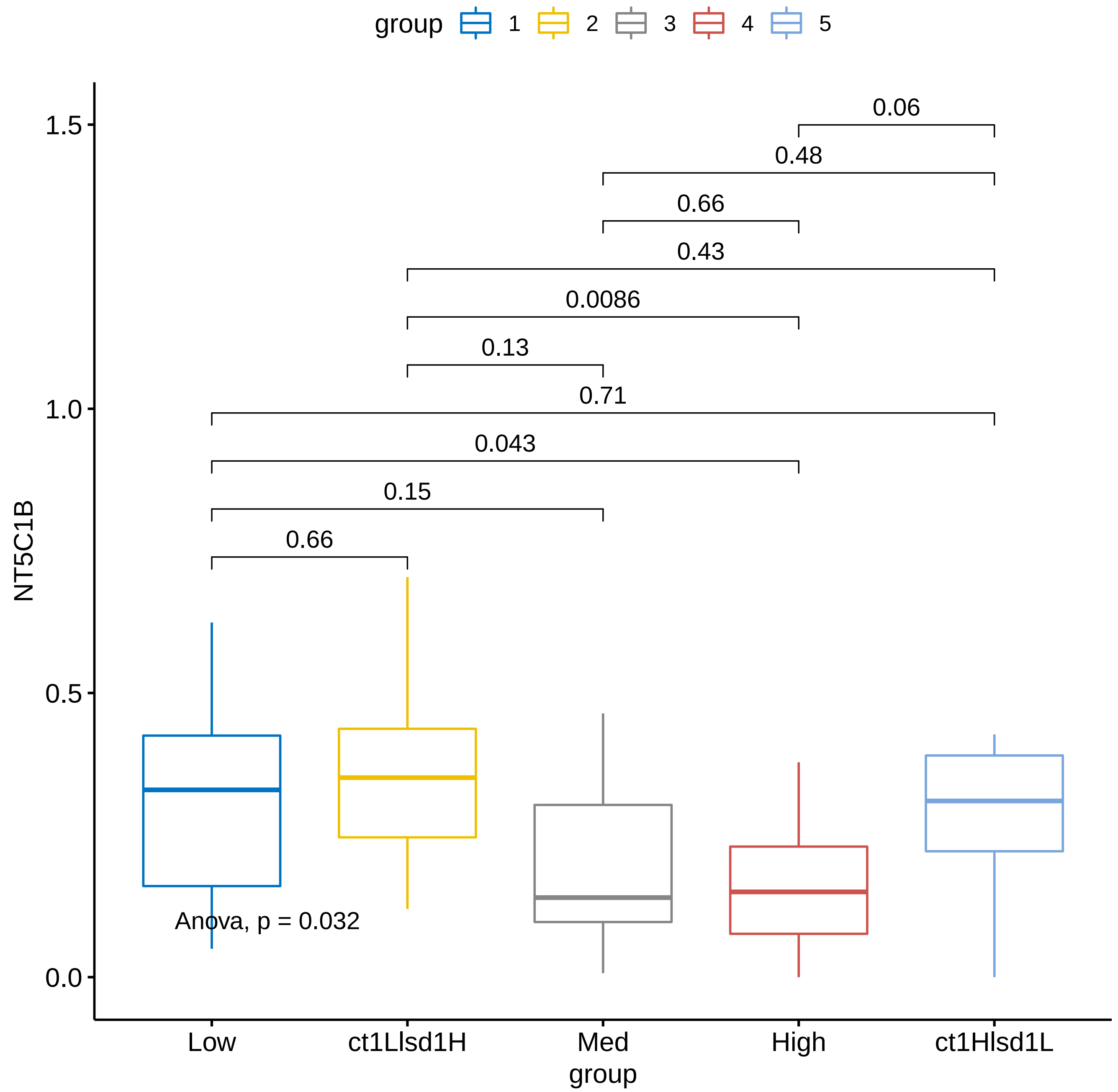

group 1 2 3 4 5
NT5C1B
1.5
1.0
0.5
0.0
0.06
0.48
0.66
0.43
0.0086
0.13
0.71
0.043
0.15
0.66
Anova, p = 0.032
Low
ct1Llsd1H
Med
High
ct1Hlsd1L
group

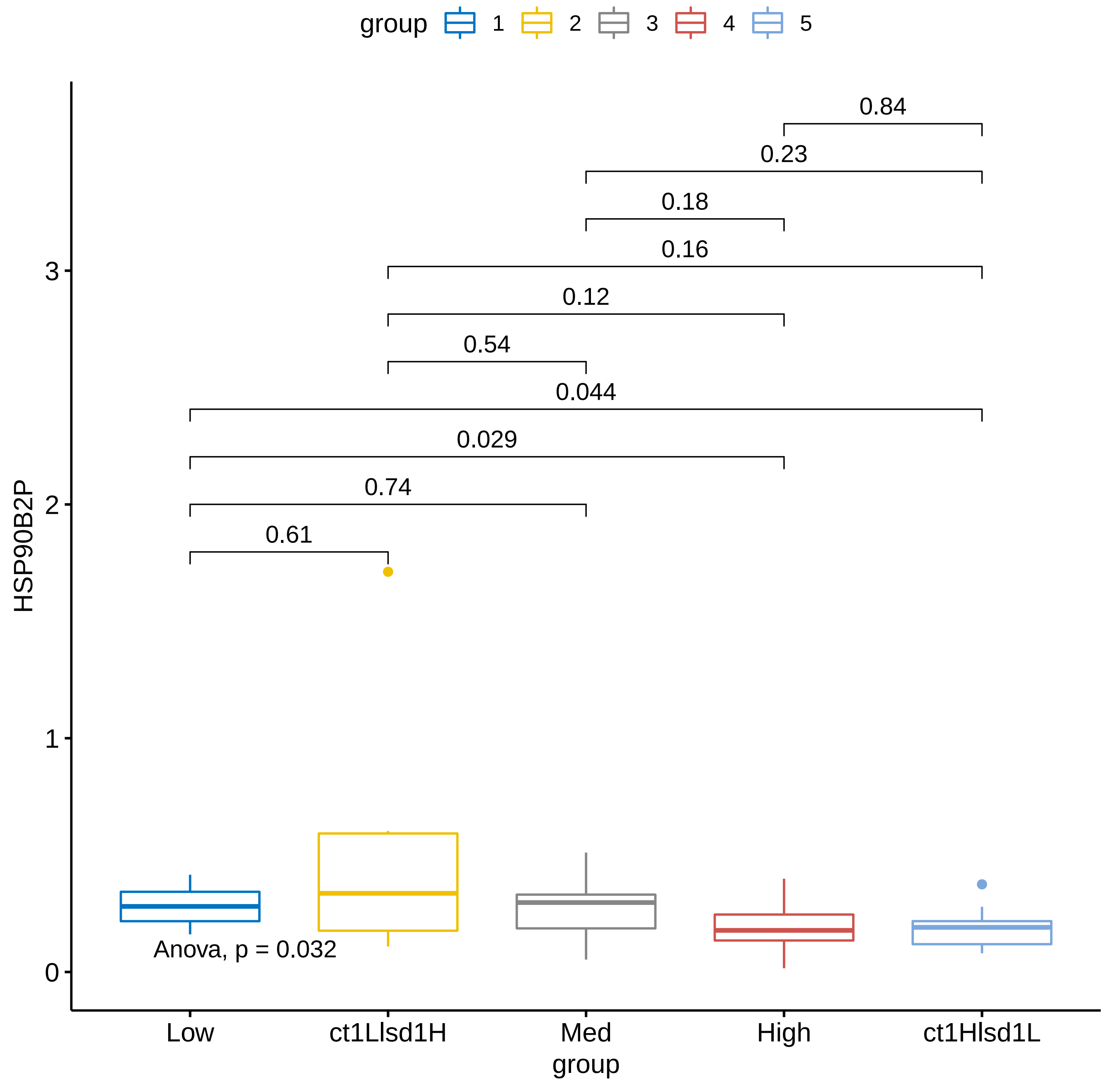

group 1 2 3 4 5
0.84
0.23
0.18
0.16
0.12
0.54
0.044
0.029
0.74
0.61
Anova, p = 0.032
HSP90B2P
0
1
2
3
Low
ct1Llsd1H
Med
High
ct1Hlsd1L
group

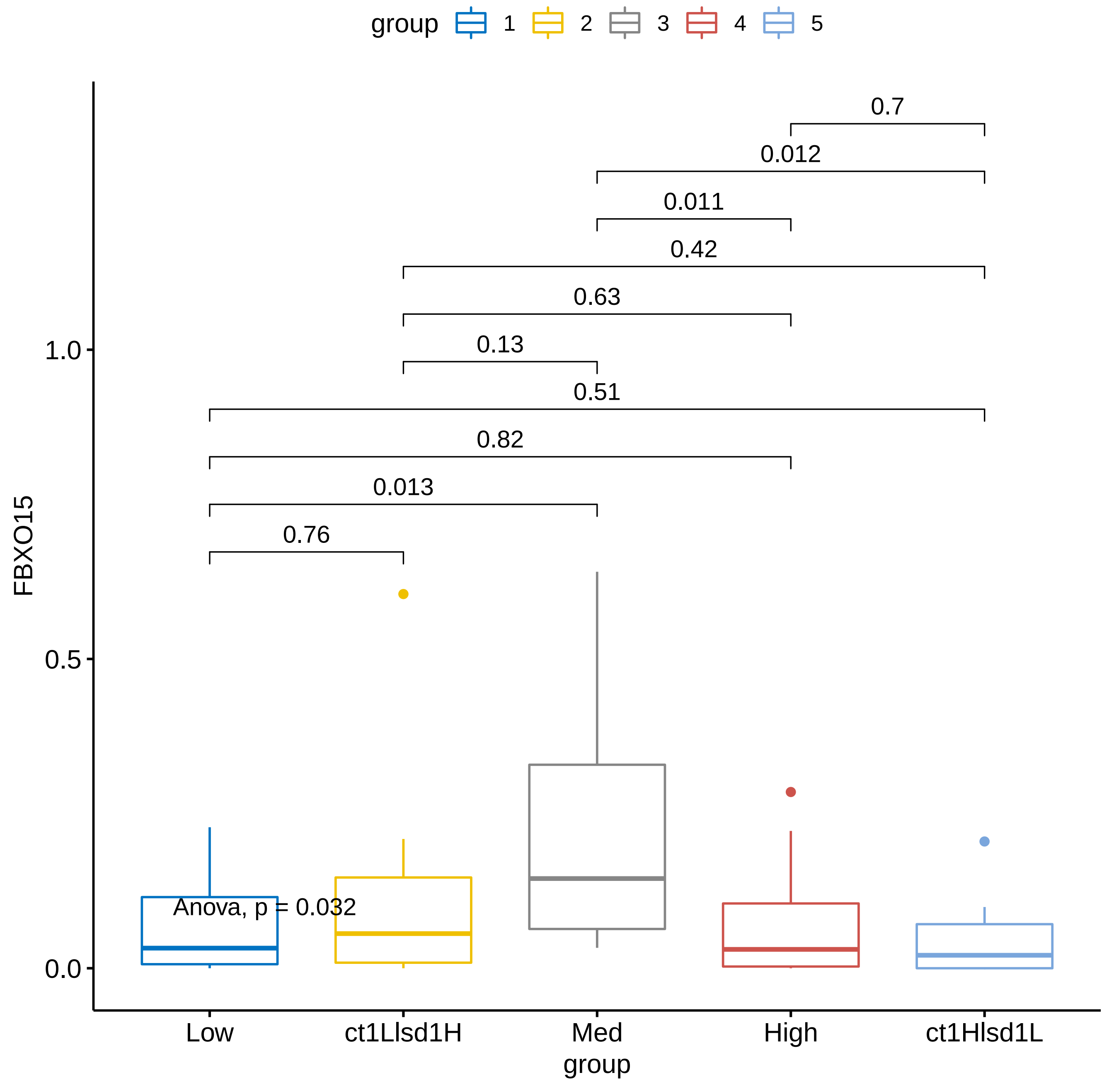

group 1 2 3 4 5
0.7
0.012
0.011
0.42
0.63
0.13
0.51
0.82
0.013
0.76
1.0
0.5
0.0
FBXO15
Anova, p = 0.032
Low
ct1Llsd1H
Med
High
ct1Hlsd1L
group

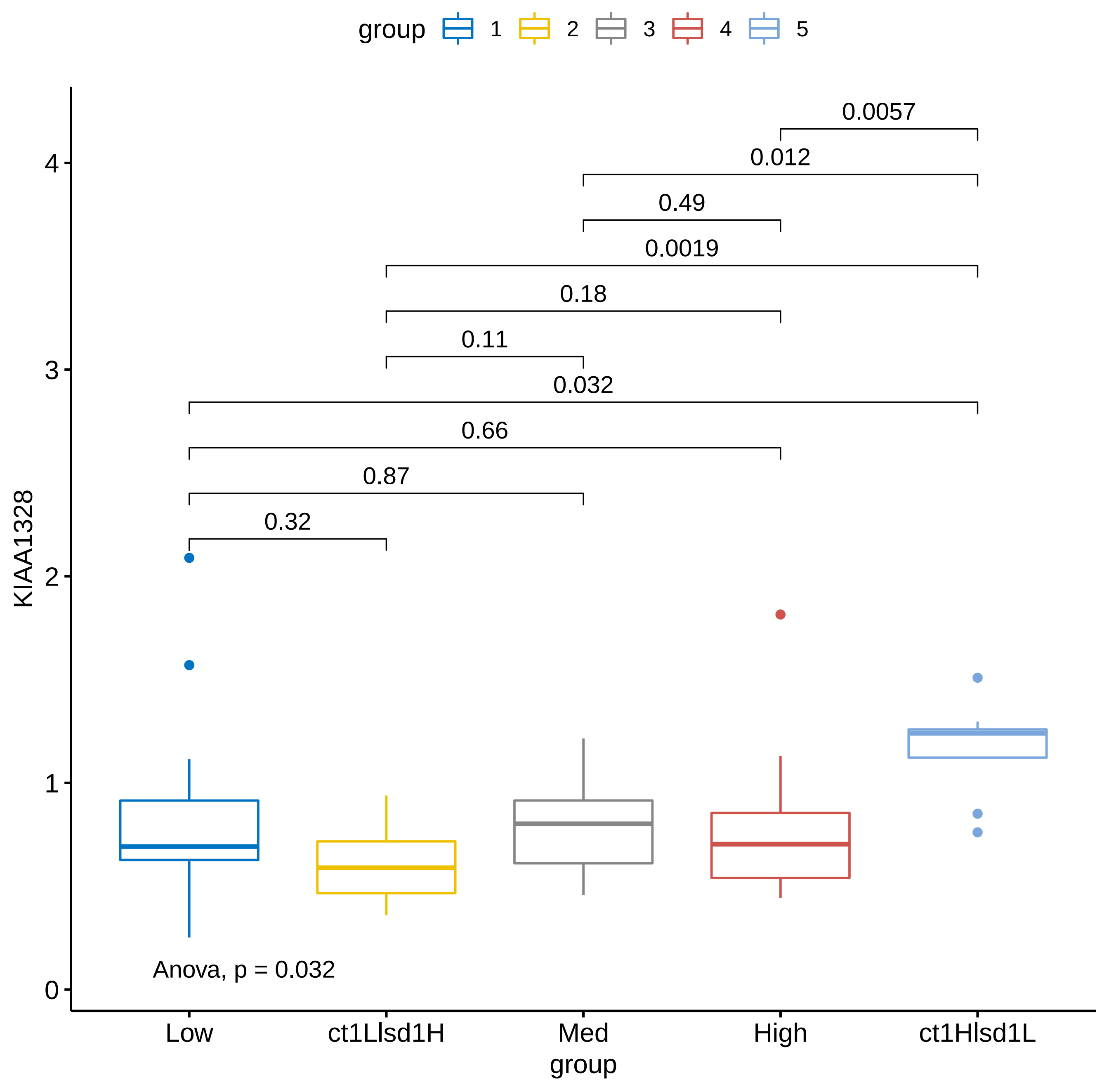

group
1
2
3
4
5
0.0057
0.012
0.49
0.0019
0.18
0.11
0.032
0.66
0.87
0.32
KIAA1328
4
3
2
1
0
Anova, p = 0.032
Low
ct1Llsd1H
Med
High
ct1Hlsd1L
group

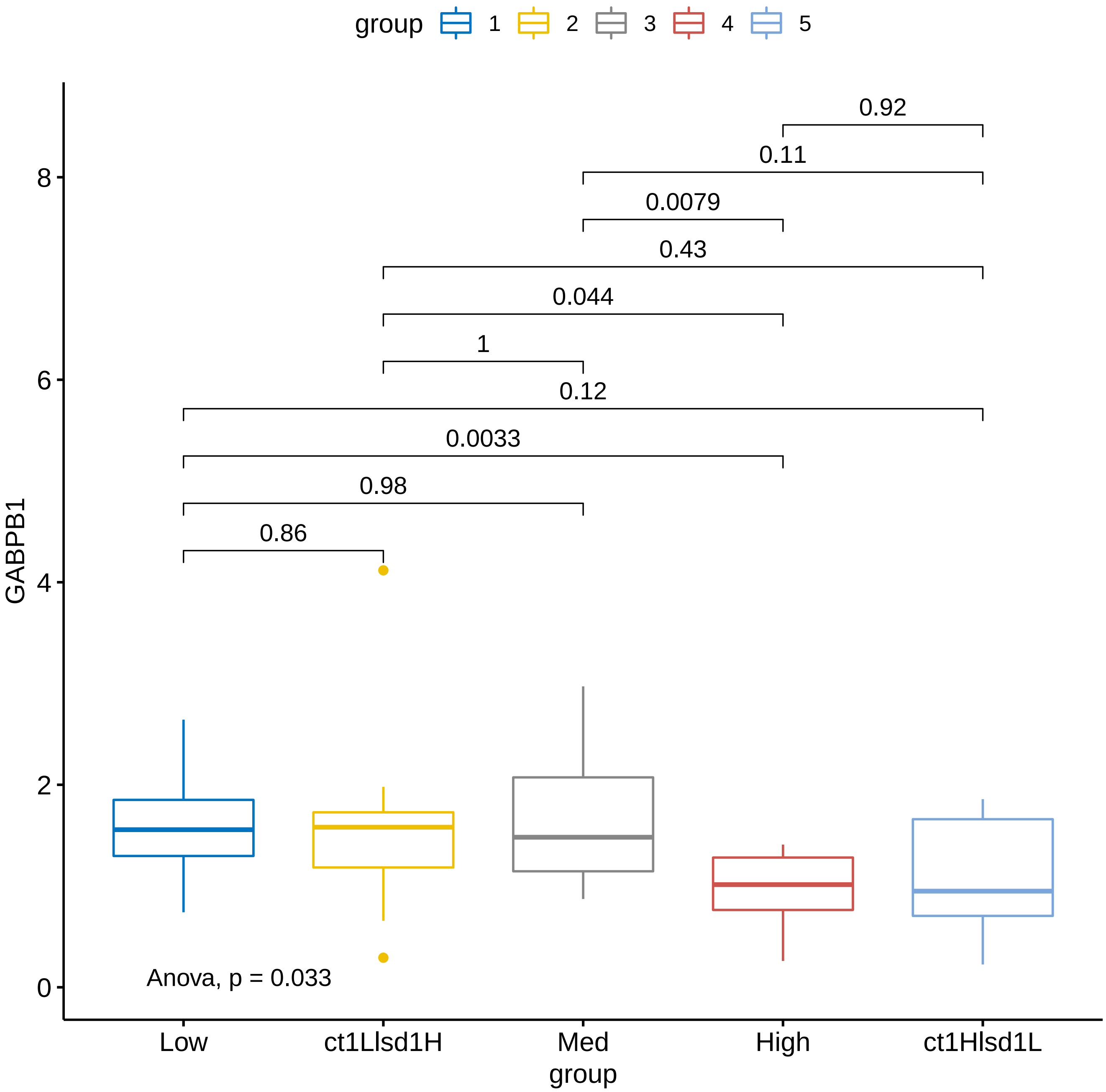
group
1
2
3
4
5
GABPB1
0.92
0.11
0.0079
0.43
0.044
1
0.12
0.0033
0.98
0.86
Anova, p = 0.033
0
2
4
6
8
Low
ct1Llsd1H
Med
High
ct1Hlsd1L
group

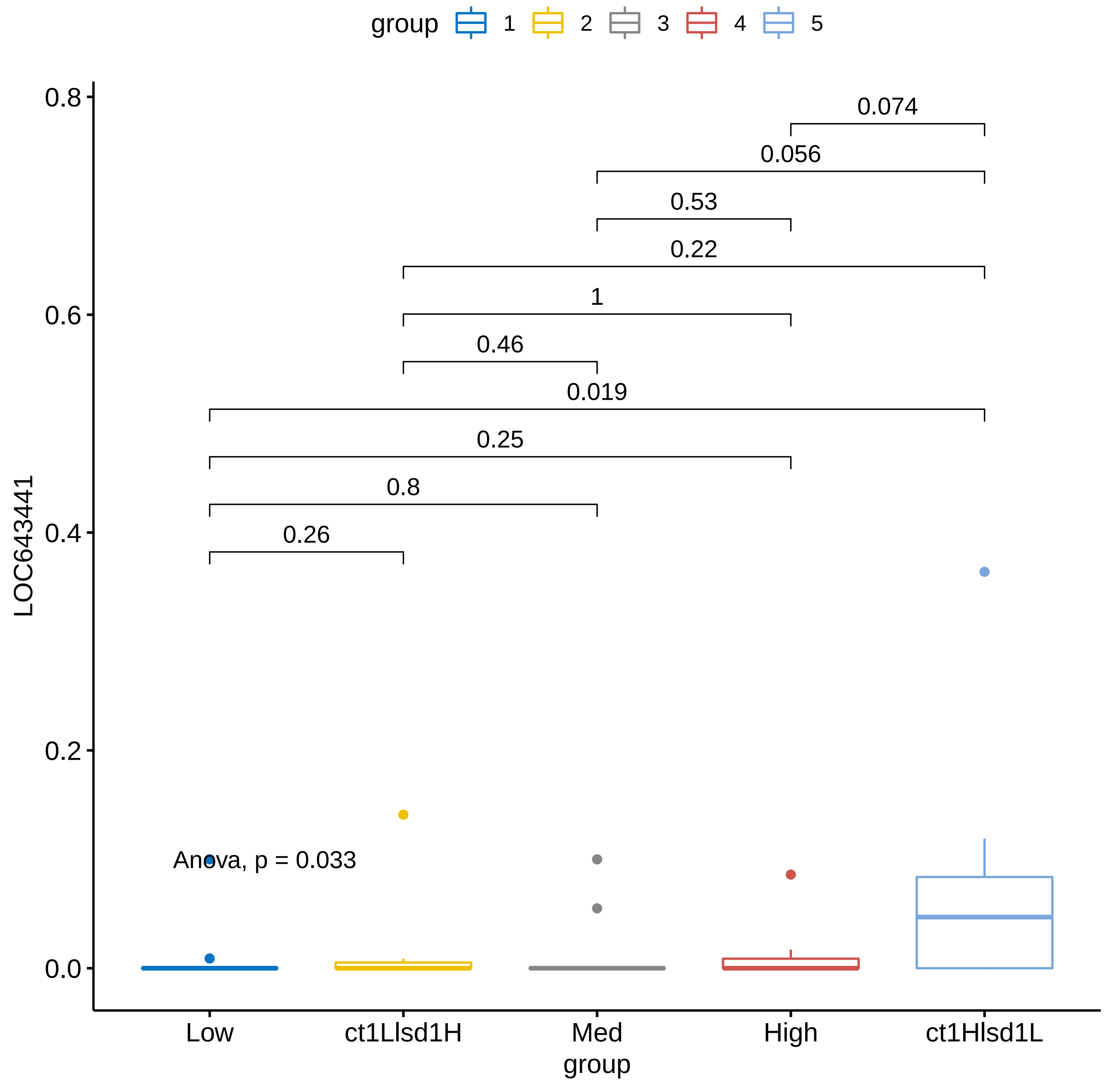
group
1
2
3
4
5
0.074
0.056
0.53
0.22
1
0.46
0.019
0.25
0.8
0.26
Anova, p = 0.033
0.8
0.6
0.4
0.2
0.0
LOC643441
Low
ct1Llsd1H
Med
High
ct1Hlsd1L
group

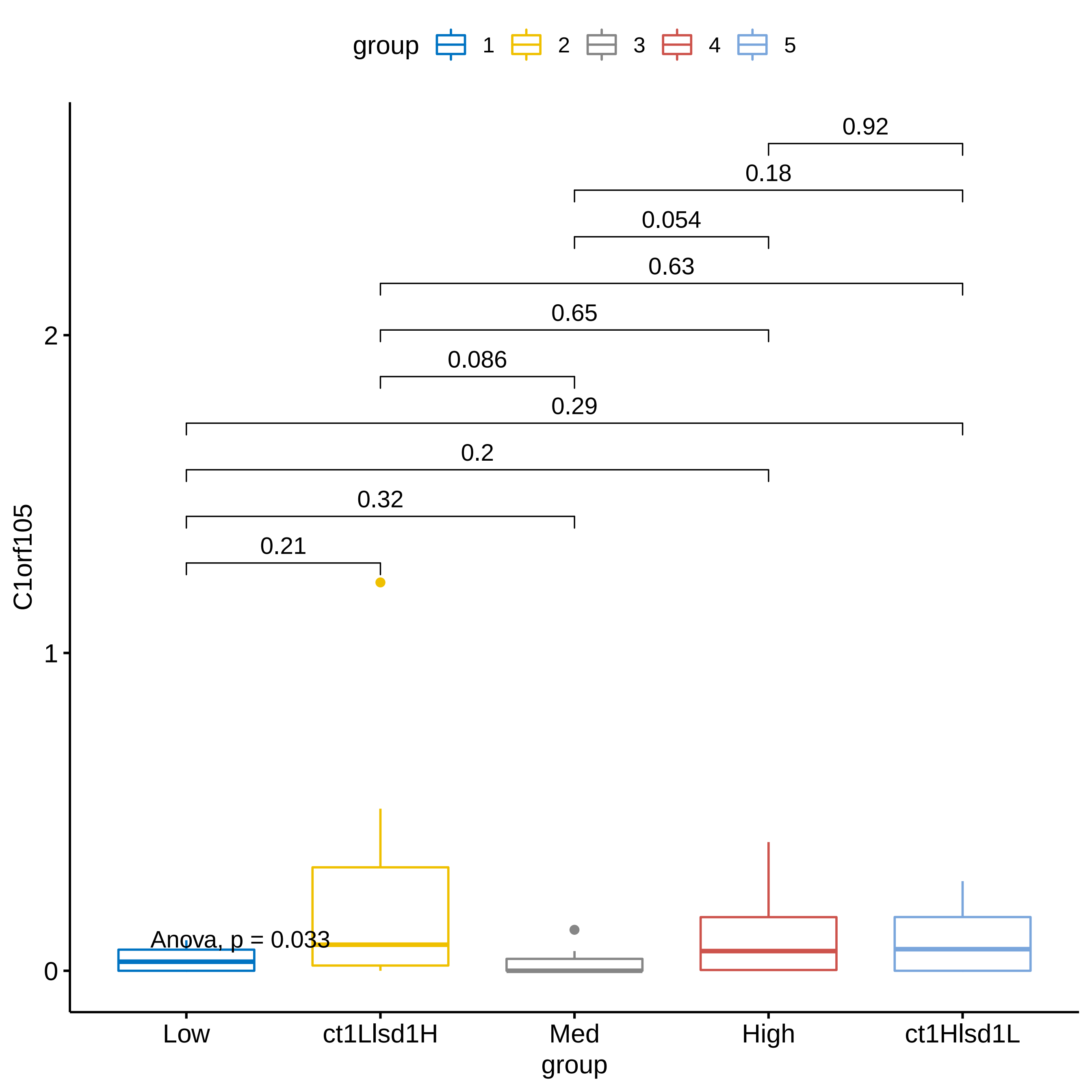
group 1 2 3 4 5
0.92
0.18
0.054
0.63
0.65
0.086
0.29
0.2
0.32
0.21
Anova, p = 0.033
C1orf105
0
1
2
Low
ct1Llsd1H
Med
High
ct1Hlsd1L
group

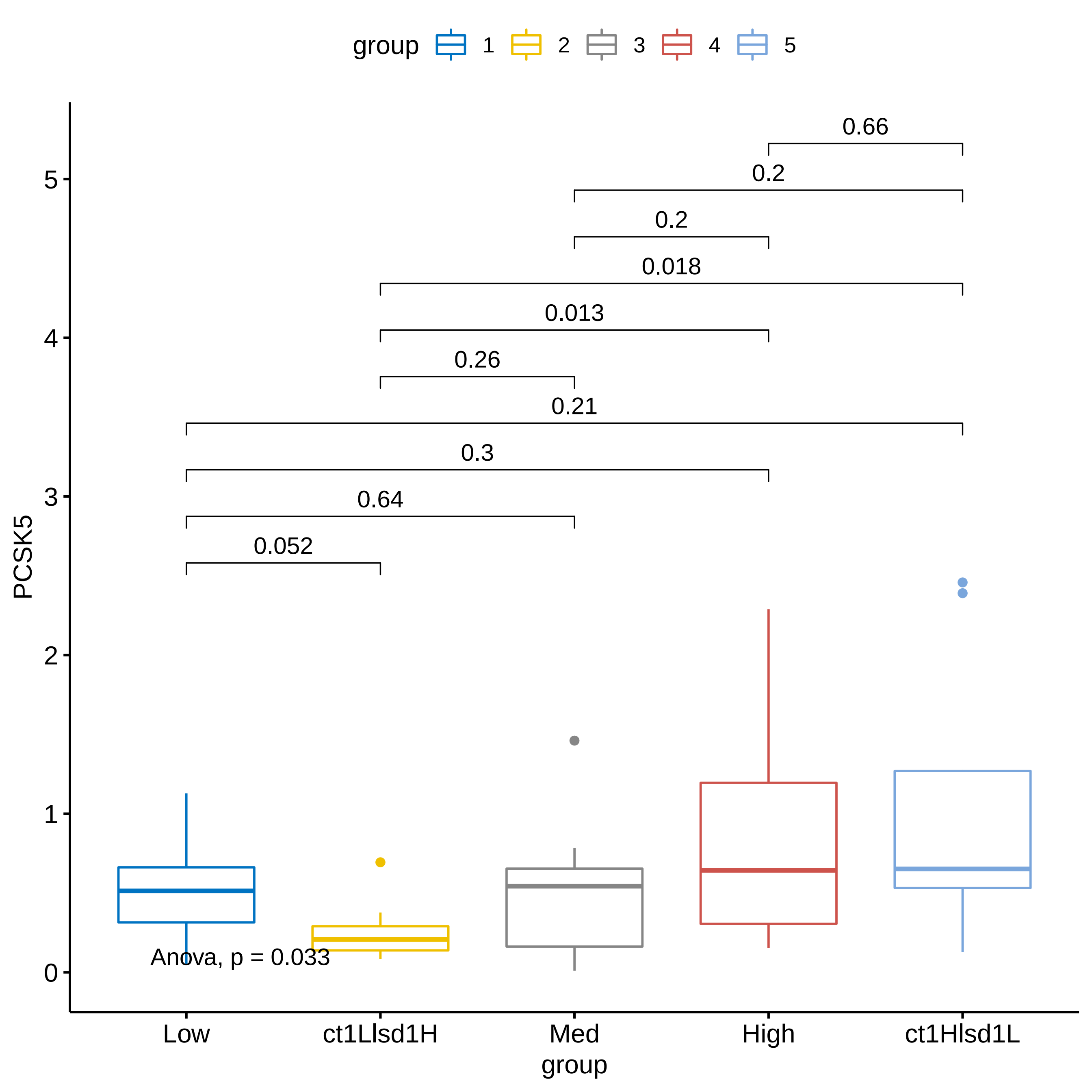

group 1 2 3 4 5
PCSK5
0.66
0.2
0.2
0.018
0.013
0.26
0.21
0.3
0.64
0.052
Anova, p = 0.033
0
1
2
3
4
5
Low
ct1Llsd1H
Med
High
ct1Hlsd1L
group

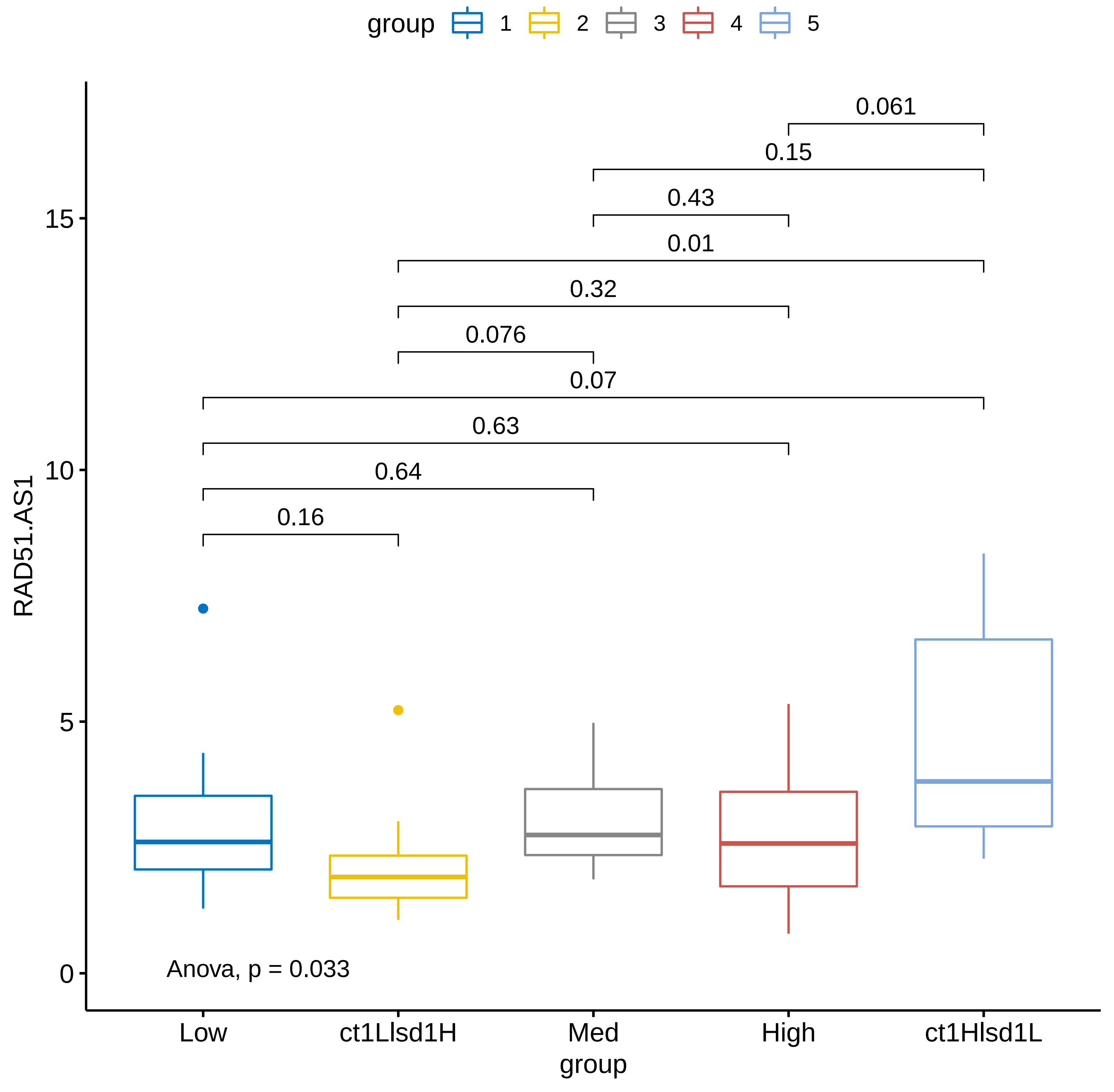
group 1 2 3 4 5
0.061
0.15
0.43
0.01
0.32
0.076
0.07
0.63
0.64
0.16
RAD51.AS1
15
10
5
0
Anova, p = 0.033
Low
ct1Llsd1H
Med
High
ct1Hlsd1L
group

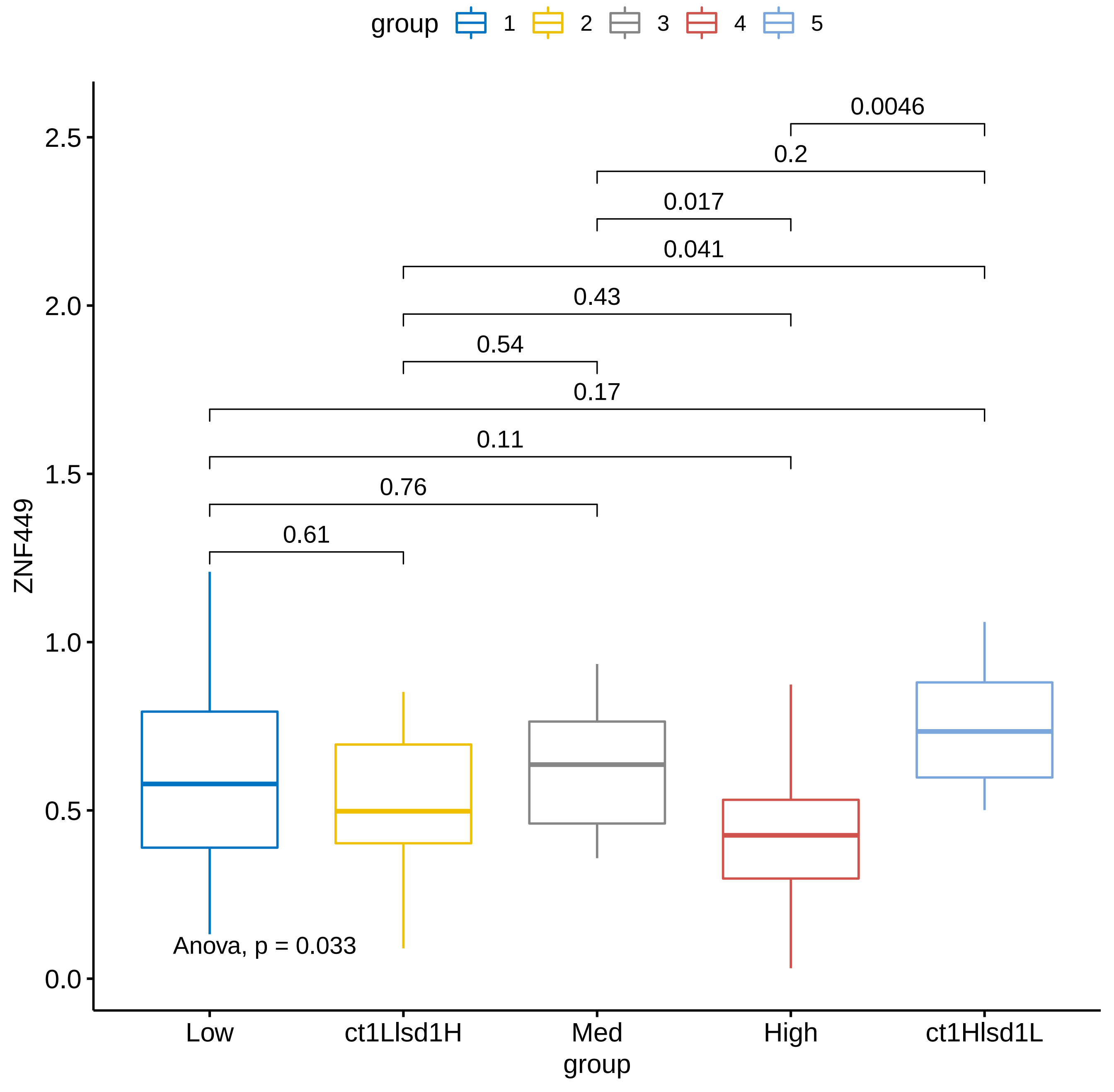

group
1
2
3
4
5
0.0046
0.2
0.017
0.041
0.43
0.54
0.17
0.11
0.76
0.61
Anova, p = 0.033
ZNF449
2.5
2.0
1.5
1.0
0.5
0.0
Low
ct1Llsd1H
Med
High
ct1Hlsd1L
group

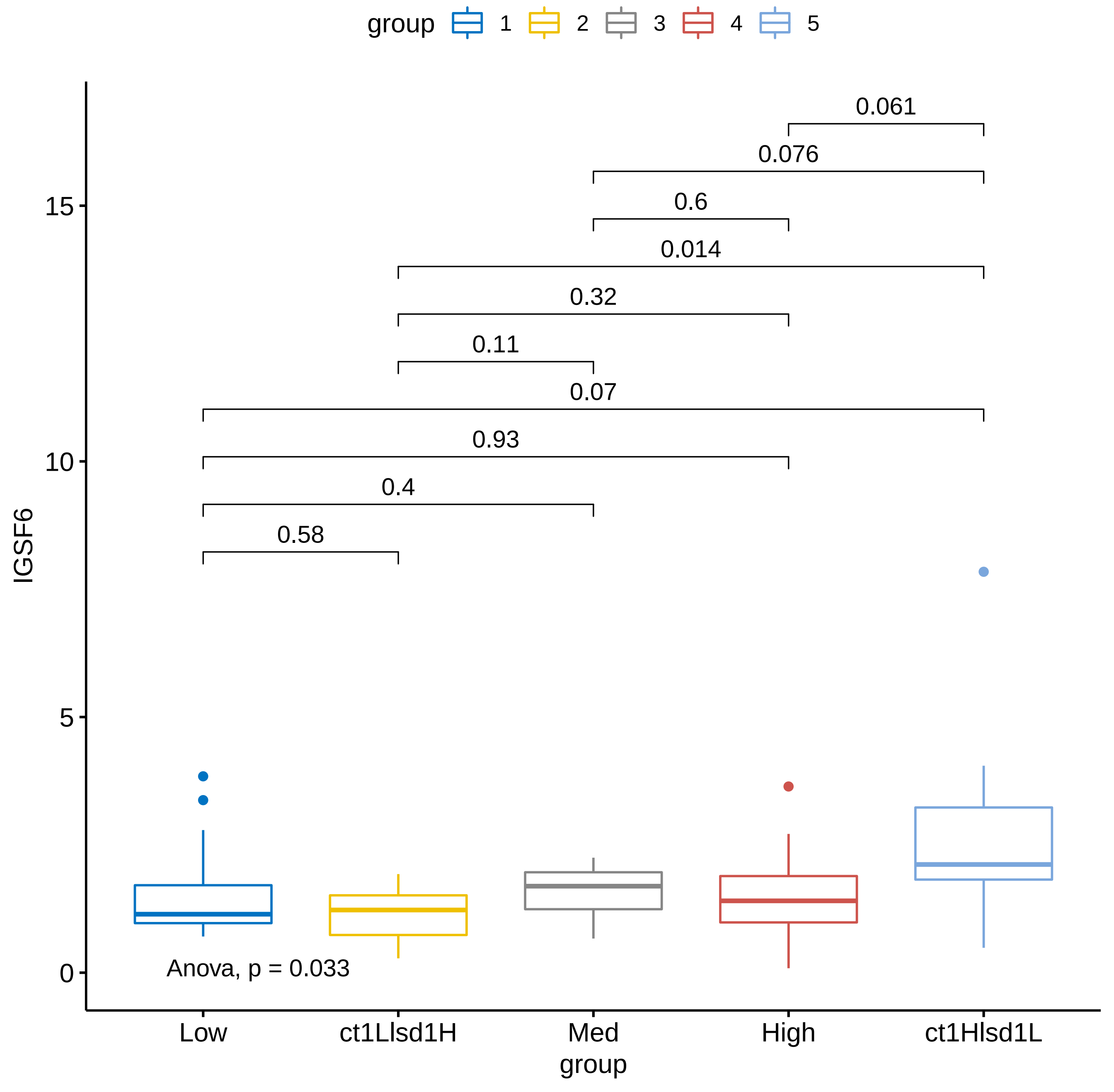

group
1
2
3
4
5
0.061
0.076
0.6
0.014
0.32
0.11
0.07
0.93
0.4
0.58
15
10
5
0
IGSF6
Anova, p = 0.033
Low
ct1Llsd1H
Med
High
ct1Hlsd1L
group

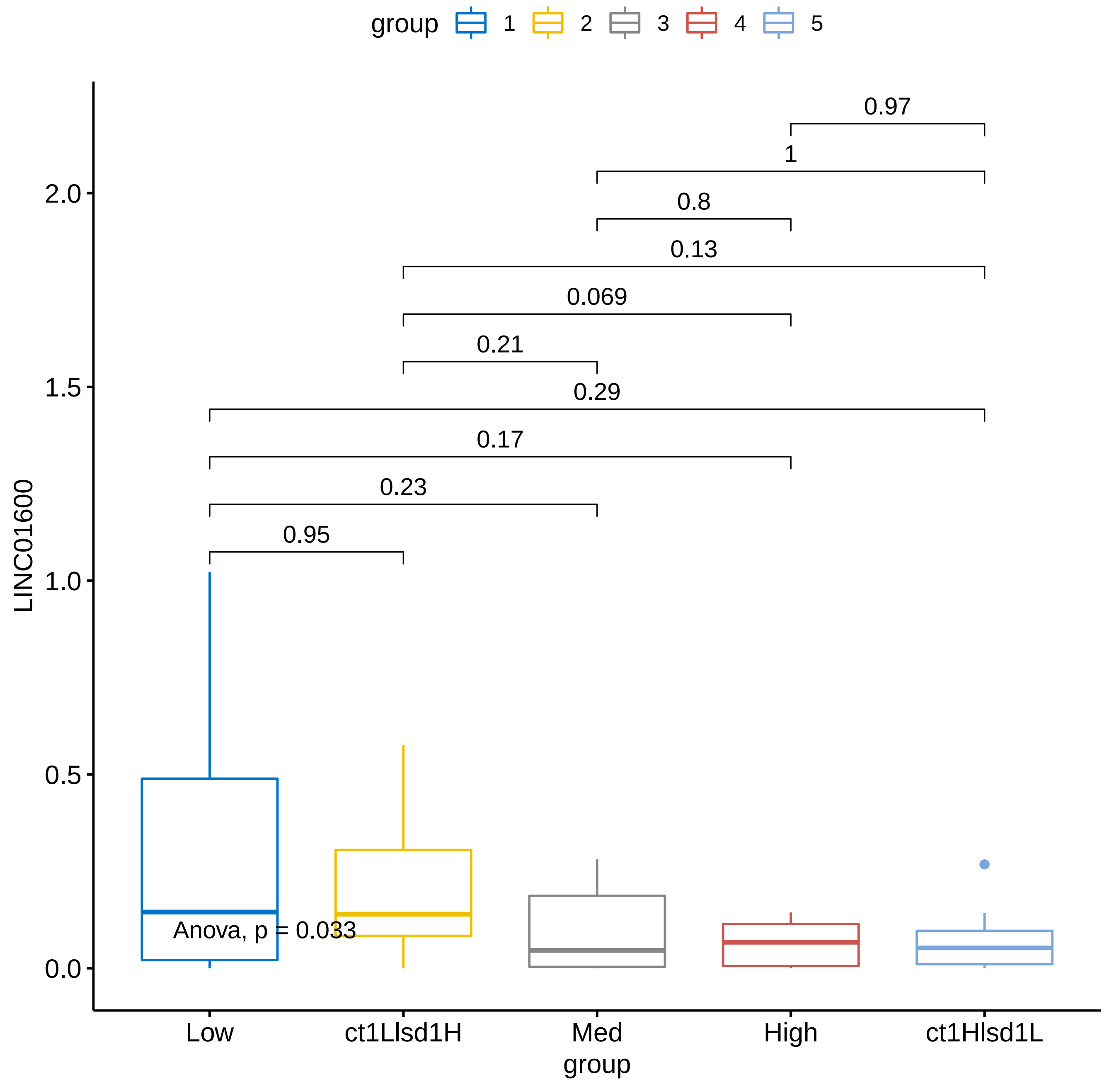

group
1
2
3
4
5
0.97
1
0.8
0.13
0.069
0.21
0.29
0.17
0.23
0.95
2.0
1.5
1.0
0.5
0.0
LINC01600
Anova, p = 0.033
Low
ct1Llsd1H
Med
High
ct1Hlsd1L
group

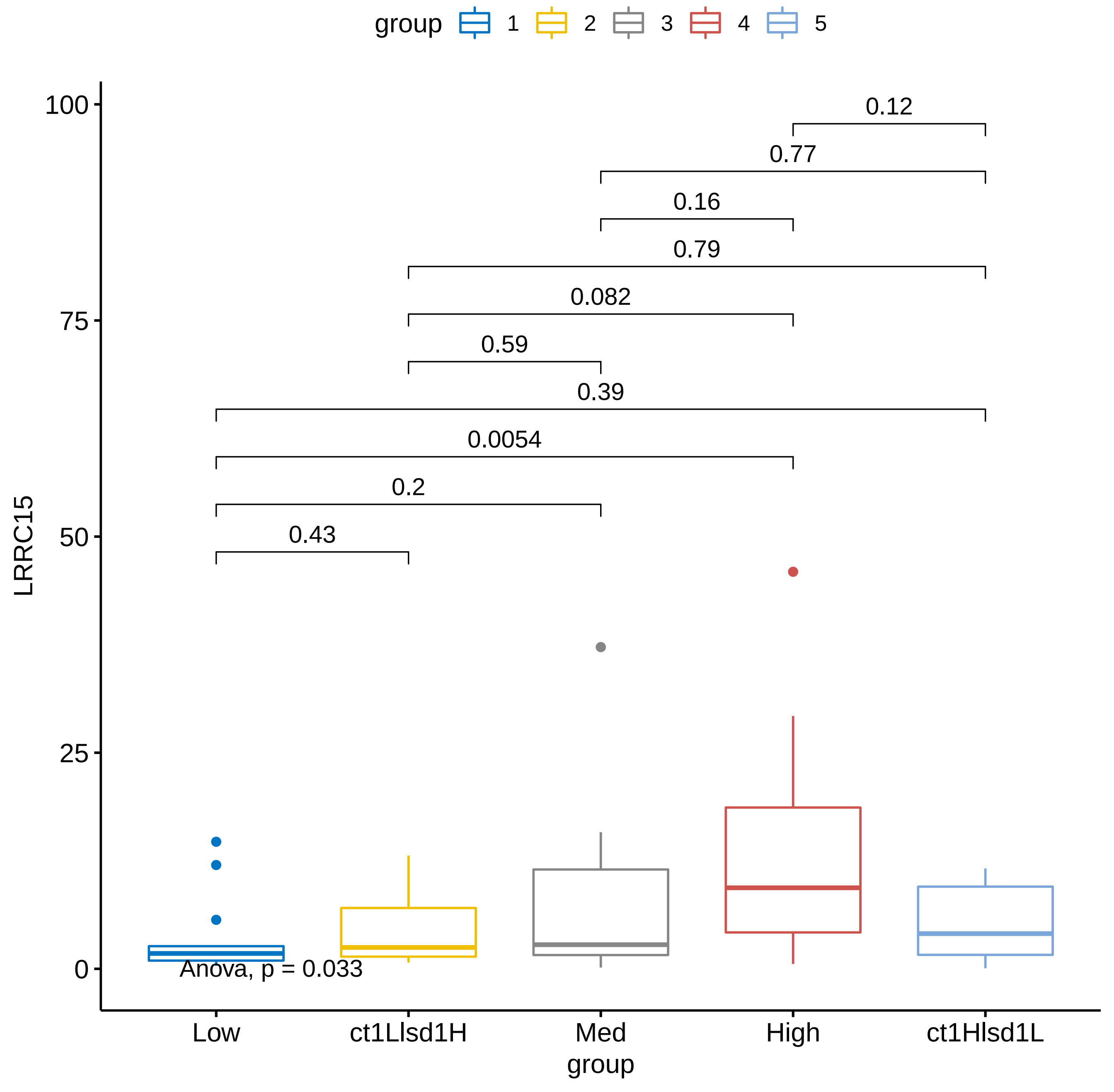

group 1 2 3 4 5
LRRC15
100
75
50
25
0
0.12
0.77
0.16
0.79
0.082
0.59
0.39
0.0054
0.2
0.43
Anova, p = 0.033
Low
ct1Llsd1H
Med
High
ct1Hlsd1L
group

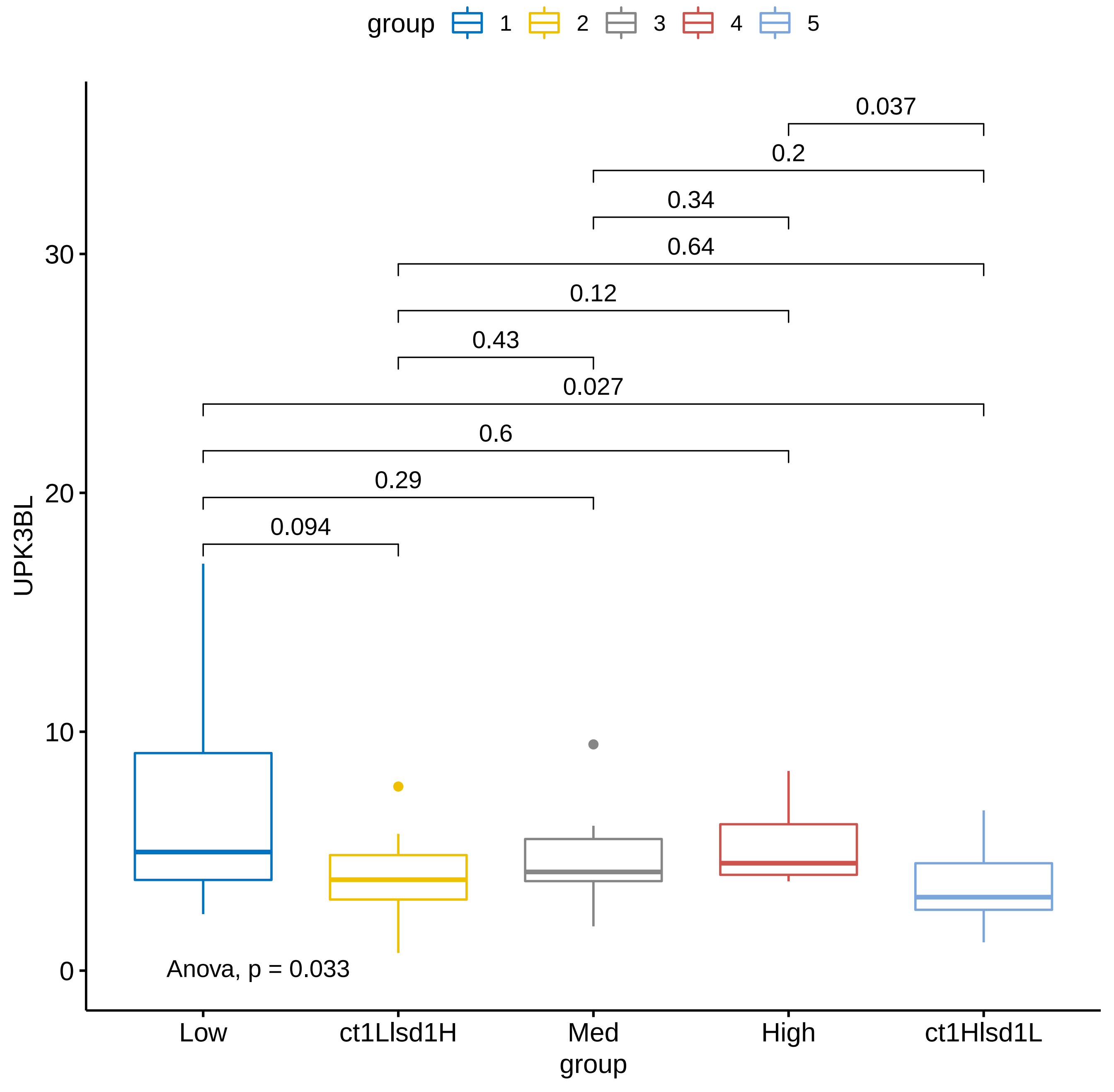
group
1
2
3
4
5
0.037
0.2
0.34
0.64
0.12
0.43
0.027
0.6
0.29
0.094
30
20
10
0
UPK3BL
Anova, p = 0.033
Low
ct1Llsd1H
Med
High
ct1Hlsd1L
group

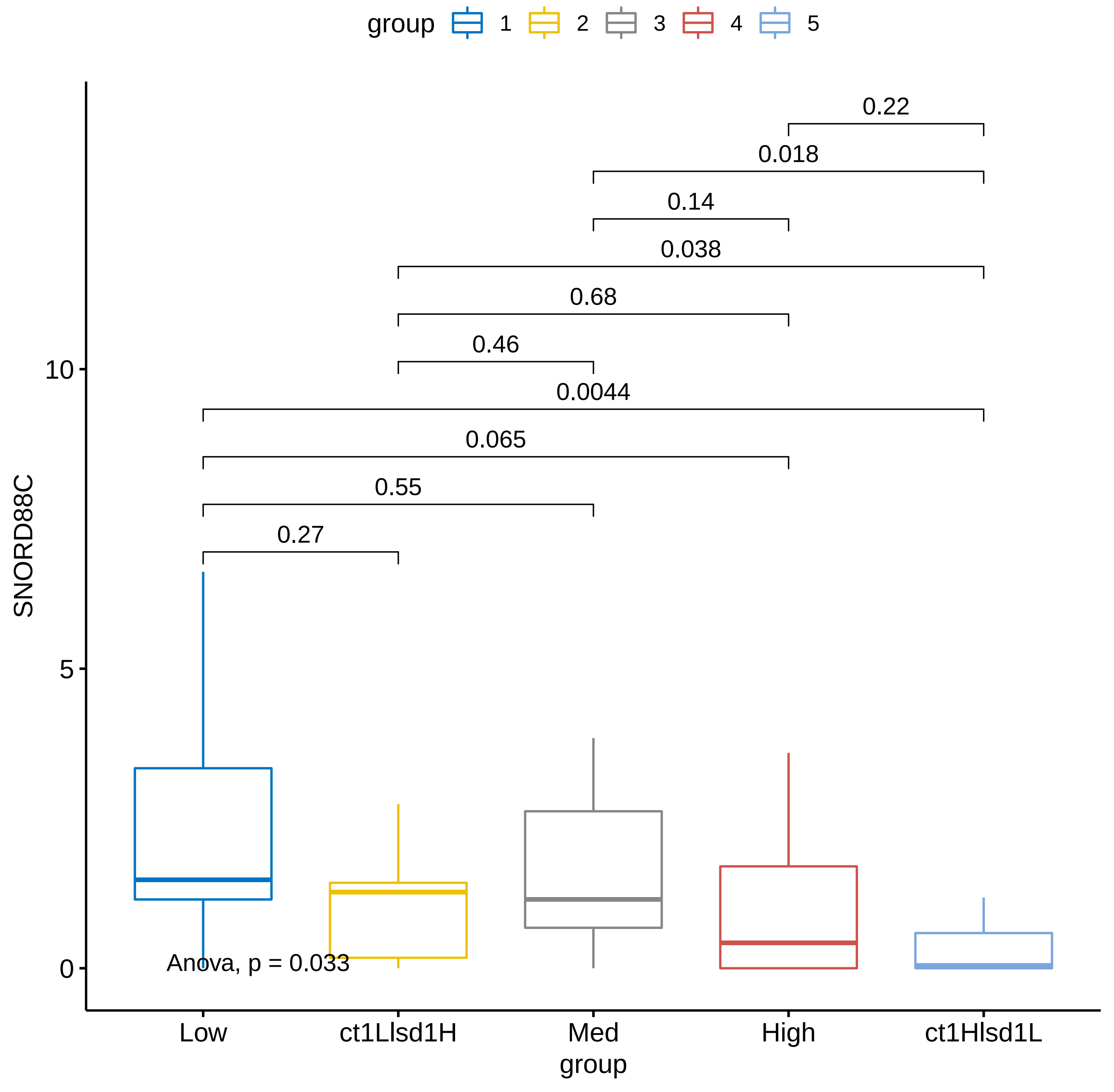

group 1 2 3 4 5
0.22
0.018
0.14
0.038
0.68
0.46
0.0044
0.065
0.55
0.27
SNORD88C
10
5
0
Anova, p = 0.033
Low
ct1Llsd1H
Med
High
ct1Hlsd1L
group

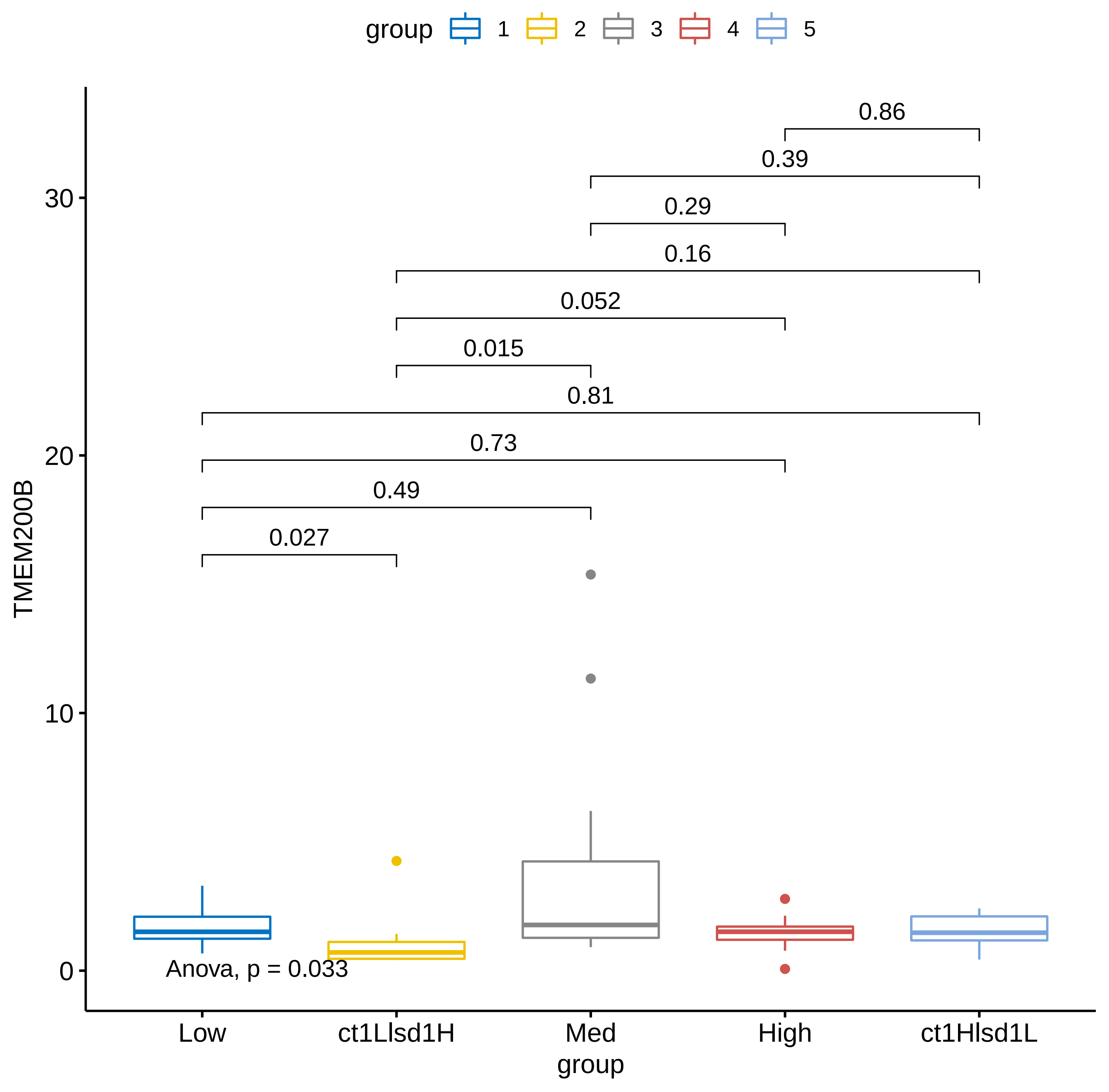
group
1
2
3
4
5
0.86
0.39
0.29
0.16
0.052
0.015
0.81
0.73
0.49
0.027
Anova, p = 0.033
TMEM200B
30
20
10
0
Low
ct1Llsd1H
Med
High
ct1Hlsd1L
group

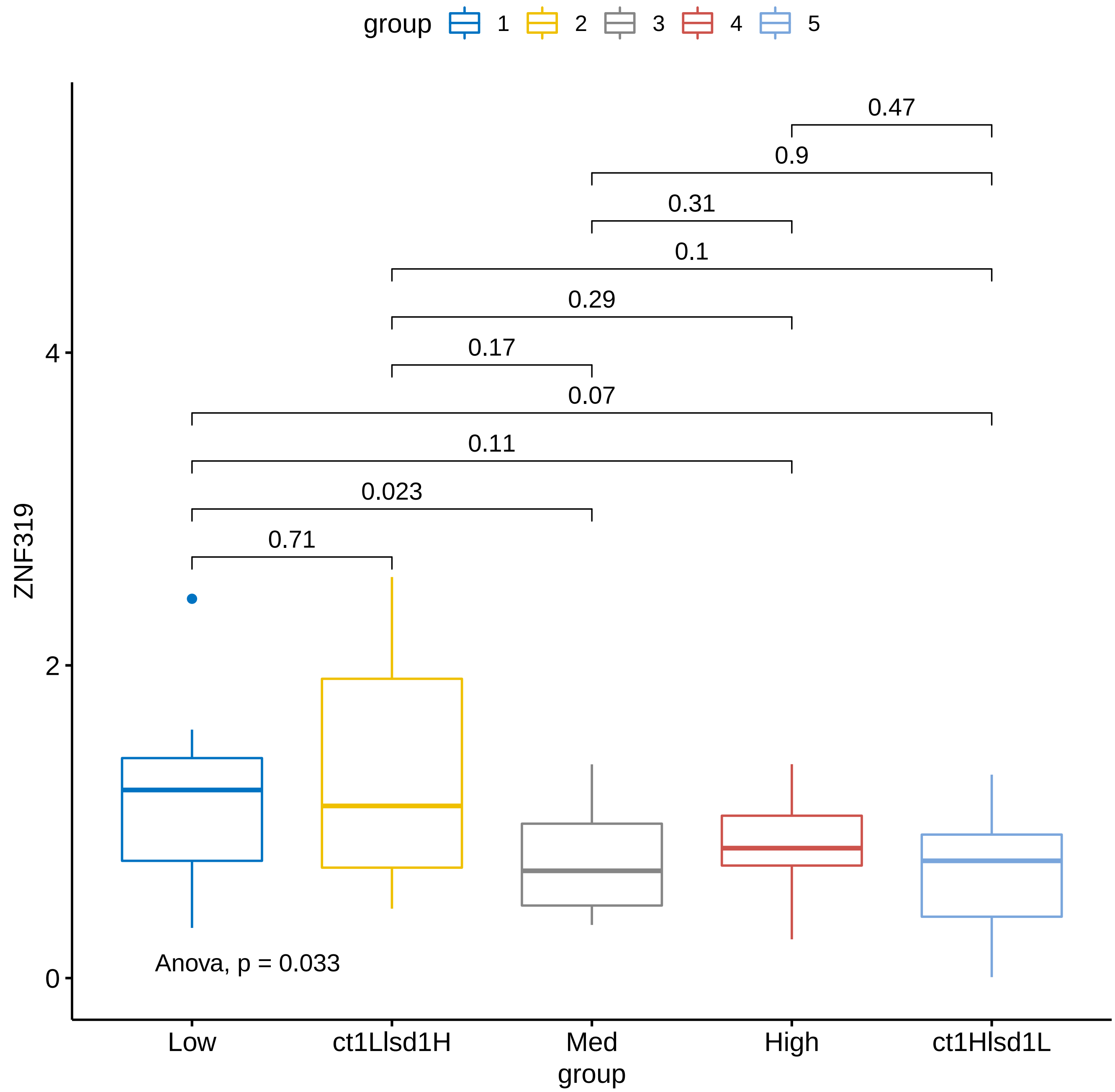

group 1 2 3 4 5
0.47
0.9
0.31
0.1
0.29
0.17
0.07
0.11
0.023
0.71
ZNF319
4
2
0
Anova, p = 0.033
Low
ct1Llsd1H
Med
High
ct1Hlsd1L
group

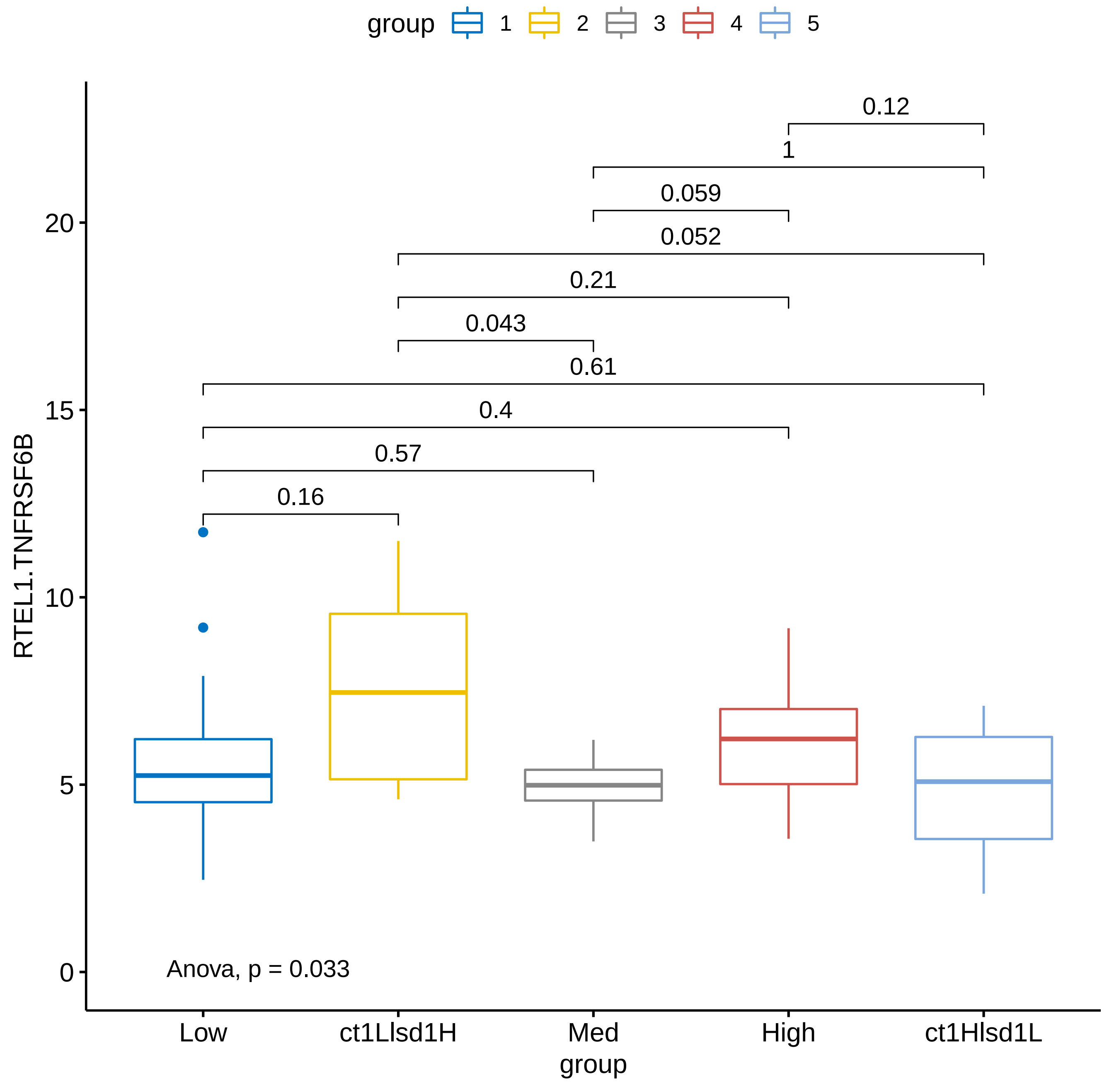

group
1
2
3
4
5
0.12
1
0.059
0.052
0.21
0.043
0.61
0.4
0.57
0.16
20
15
10
5
0
RTEL1.TNFRSF6B
Anova, p = 0.033
Low
ct1Llsd1H
Med
High
ct1Hlsd1L
group

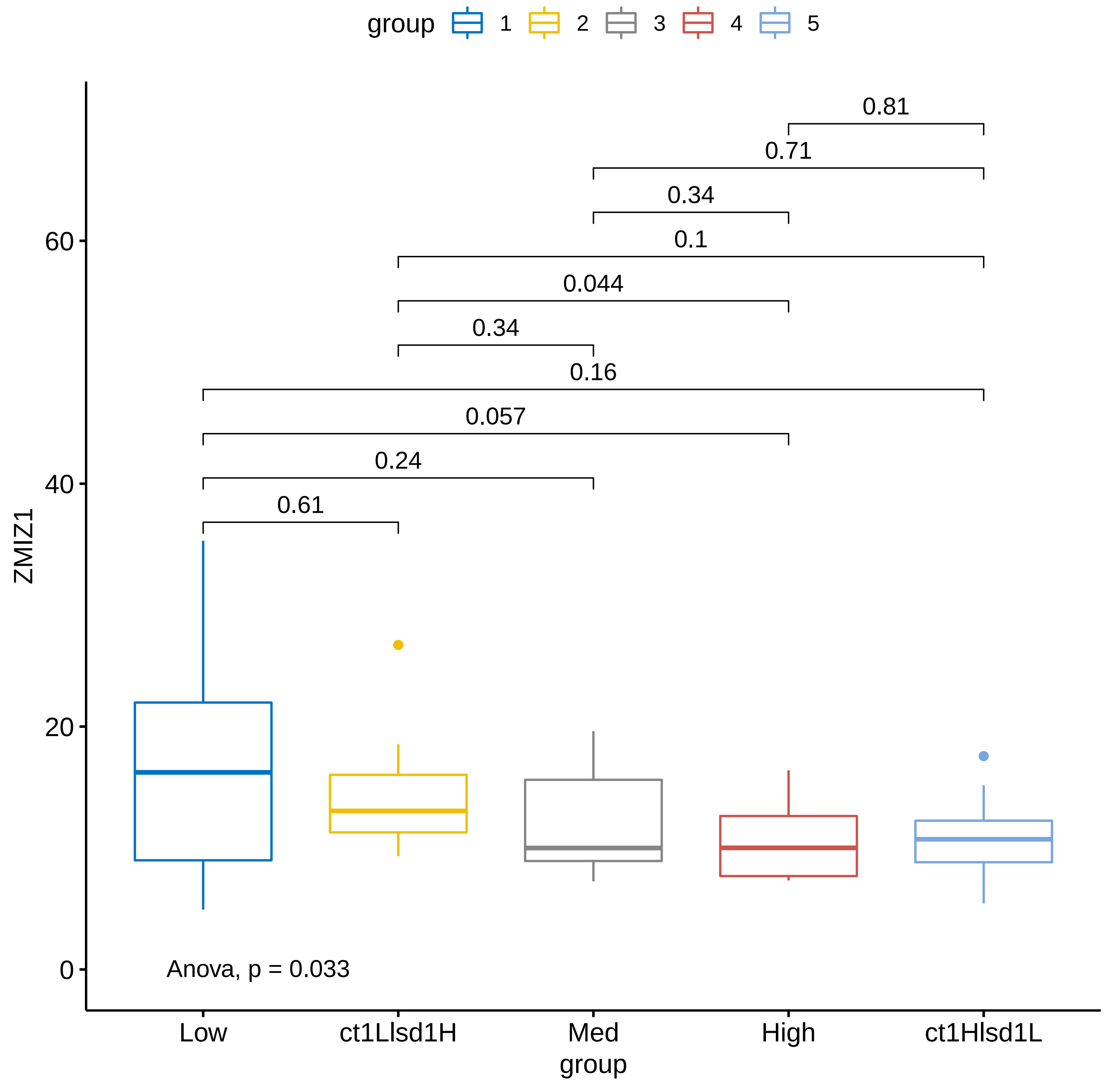
group 1 2 3 4 5
0.81
0.71
0.34
0.1
0.044
0.34
0.16
0.057
0.24
0.61
60
40
20
0
ZMIZ1
Anova, p = 0.033
Low
ct1Llsd1H
Med
High
ct1Hlsd1L
group

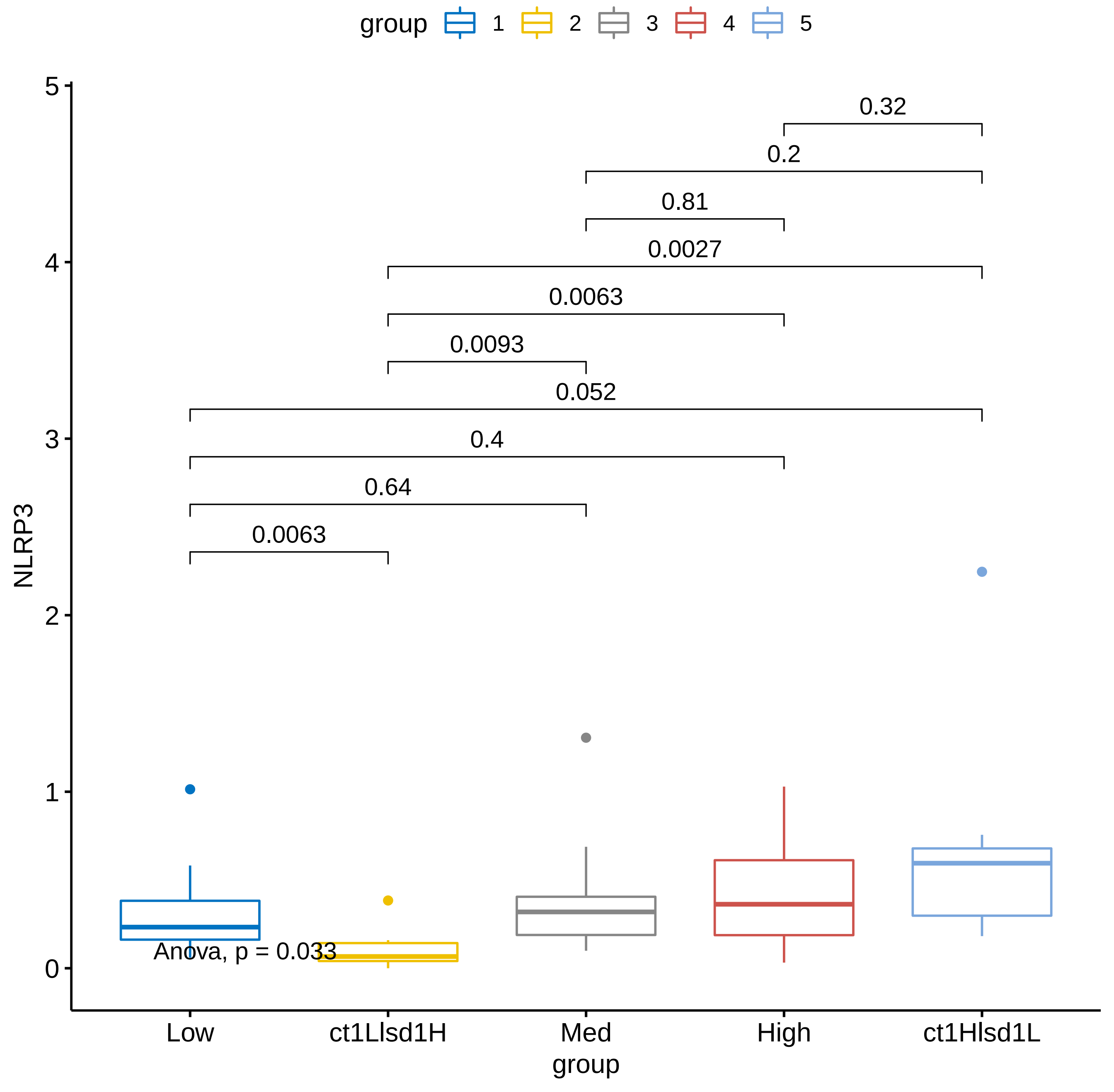

group
1
2
3
4
5
NLRP3
0.32
0.2
0.81
0.0027
0.0063
0.0093
0.052
0.4
0.64
0.0063
Anova, p = 0.033
0
1
2
3
4
5
Low
ct1Llsd1H
Med
High
ct1Hlsd1L
group

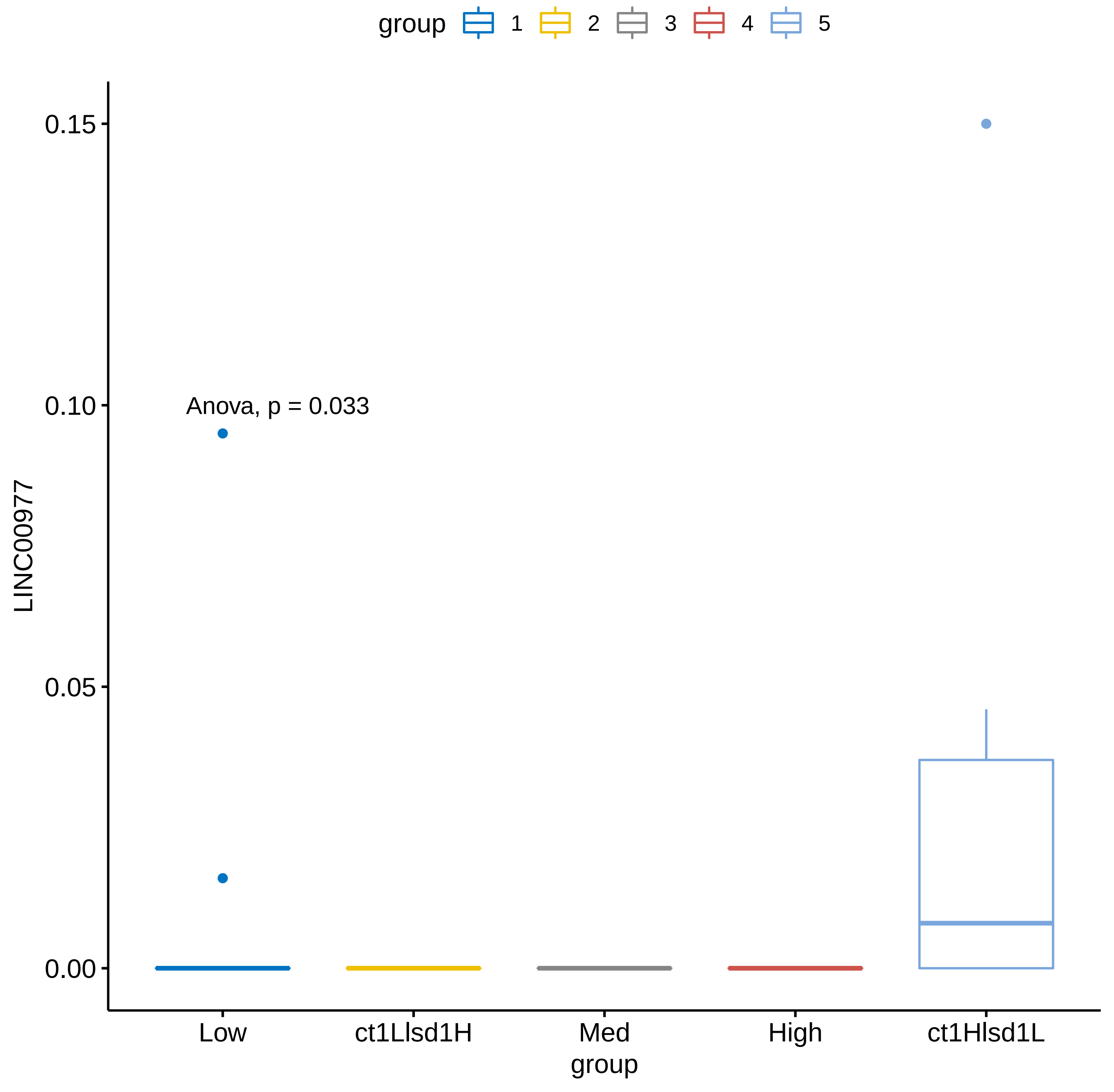
group
1
2
3
4
5
0.15
0.10
0.05
0.00
Anova, p = 0.033
LINC00977
Low
ct1Llsd1H
Med
High
ct1Hlsd1L
group

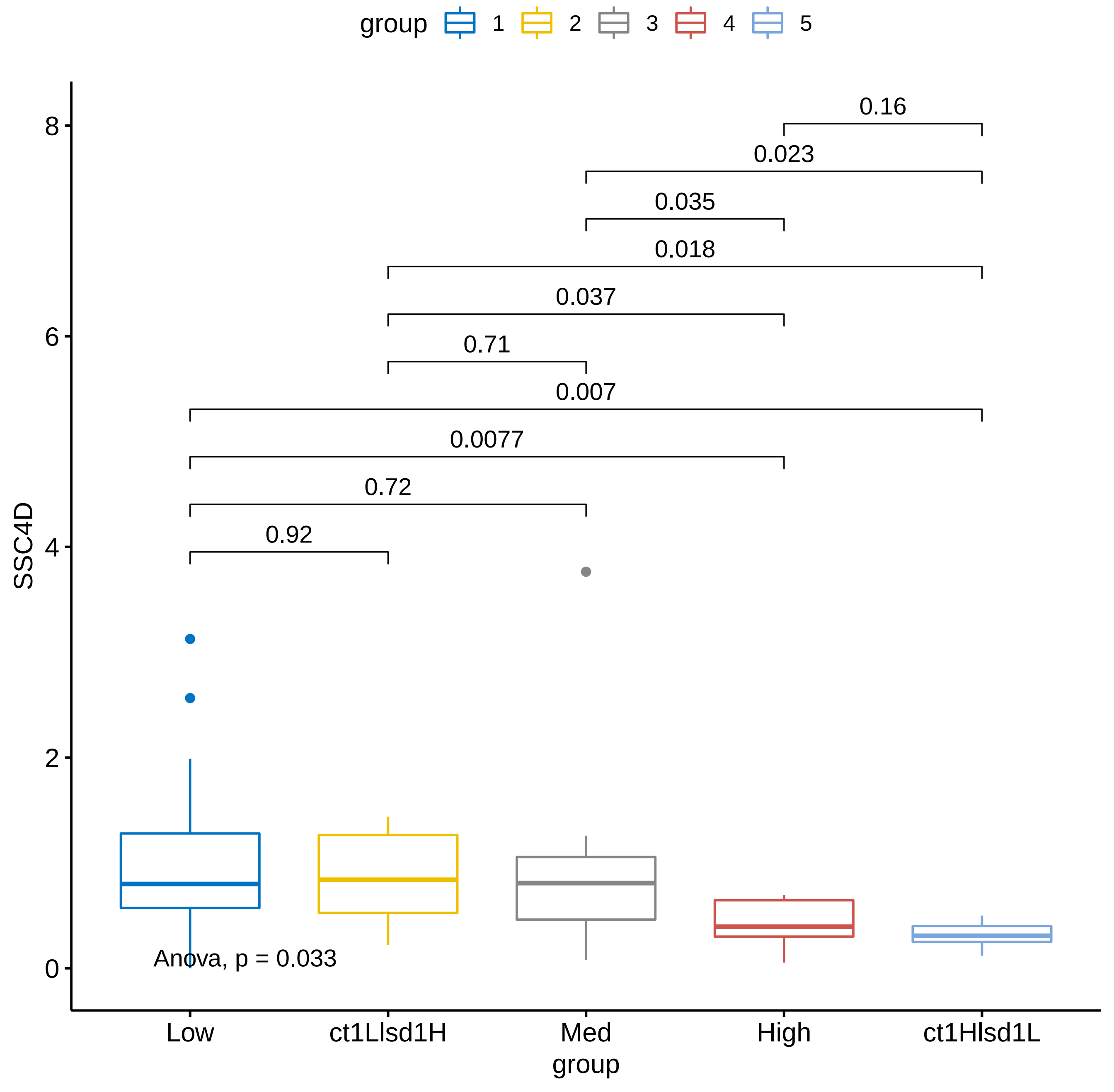

group 1 2 3 4 5
0.16
0.023
0.035
0.018
0.037
0.71
0.007
0.0077
0.72
0.92
Anova, p = 0.033
SSC4D
8
6
4
2
0
Low
ct1Llsd1H
Med
High
ct1Hlsd1L
group

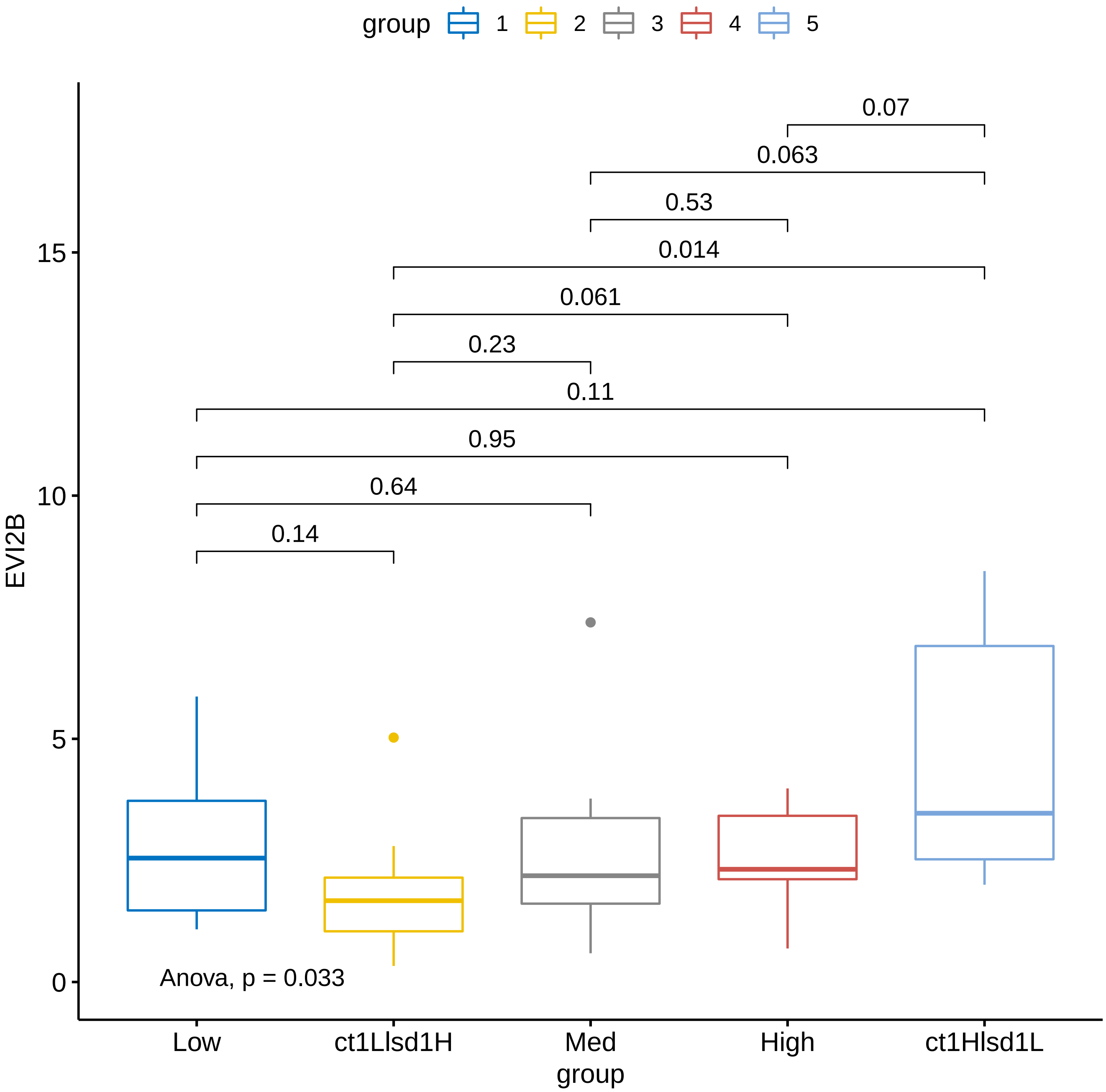

group 1 2 3 4 5
0.07
0.063
0.53
0.014
0.061
0.23
0.11
0.95
0.64
0.14
Anova, p = 0.033
15
10
5
0
EVI2B
Low
ct1Llsd1H
Med
High
ct1Hlsd1L
group

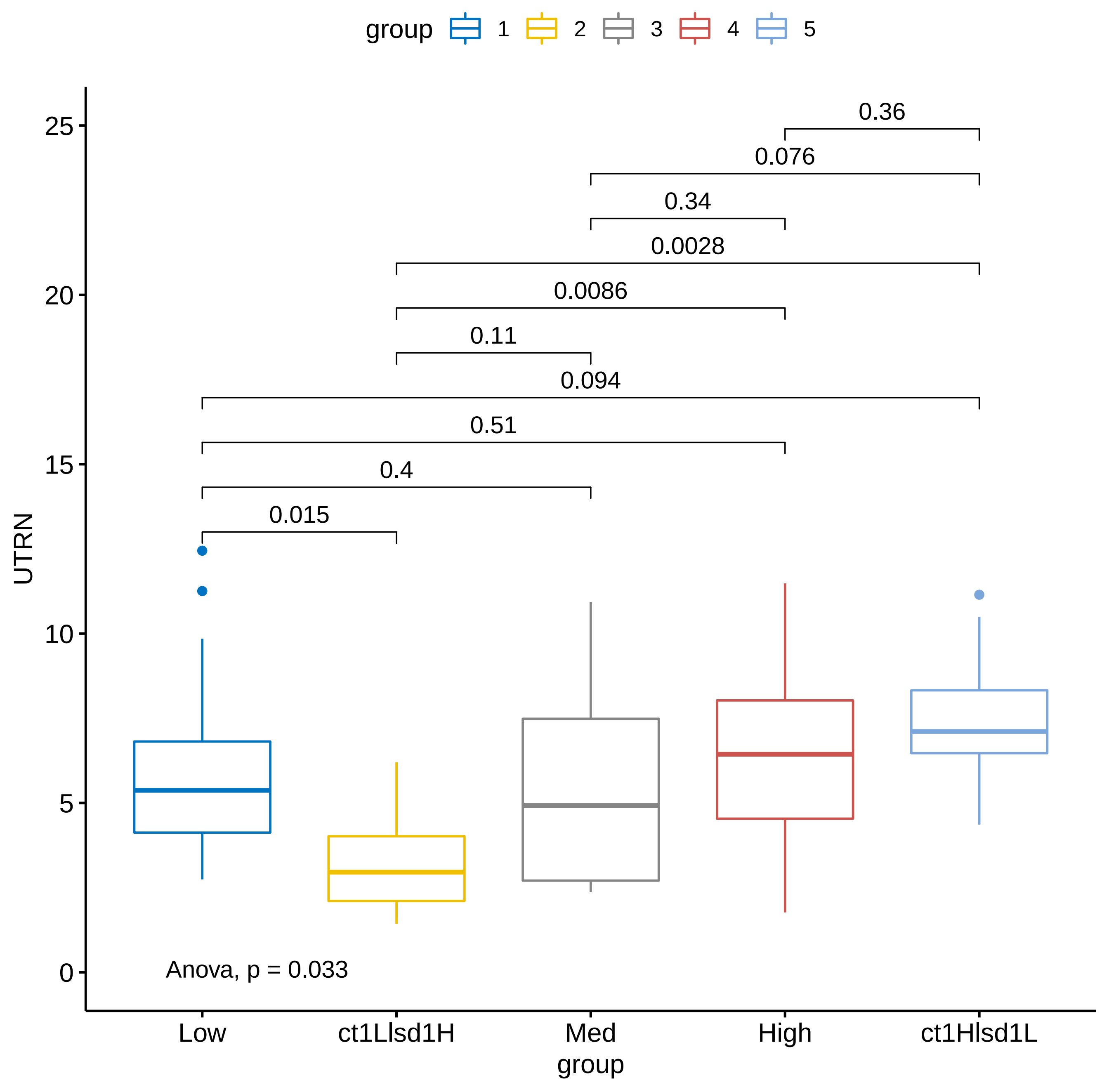
group
1
2
3
4
5
0.36
0.076
0.34
0.0028
0.0086
0.11
0.094
0.51
0.4
0.015
UTRN
25
20
15
10
5
0
Anova, p = 0.033
Low
ct1Llsd1H
Med
High
ct1Hlsd1L
group

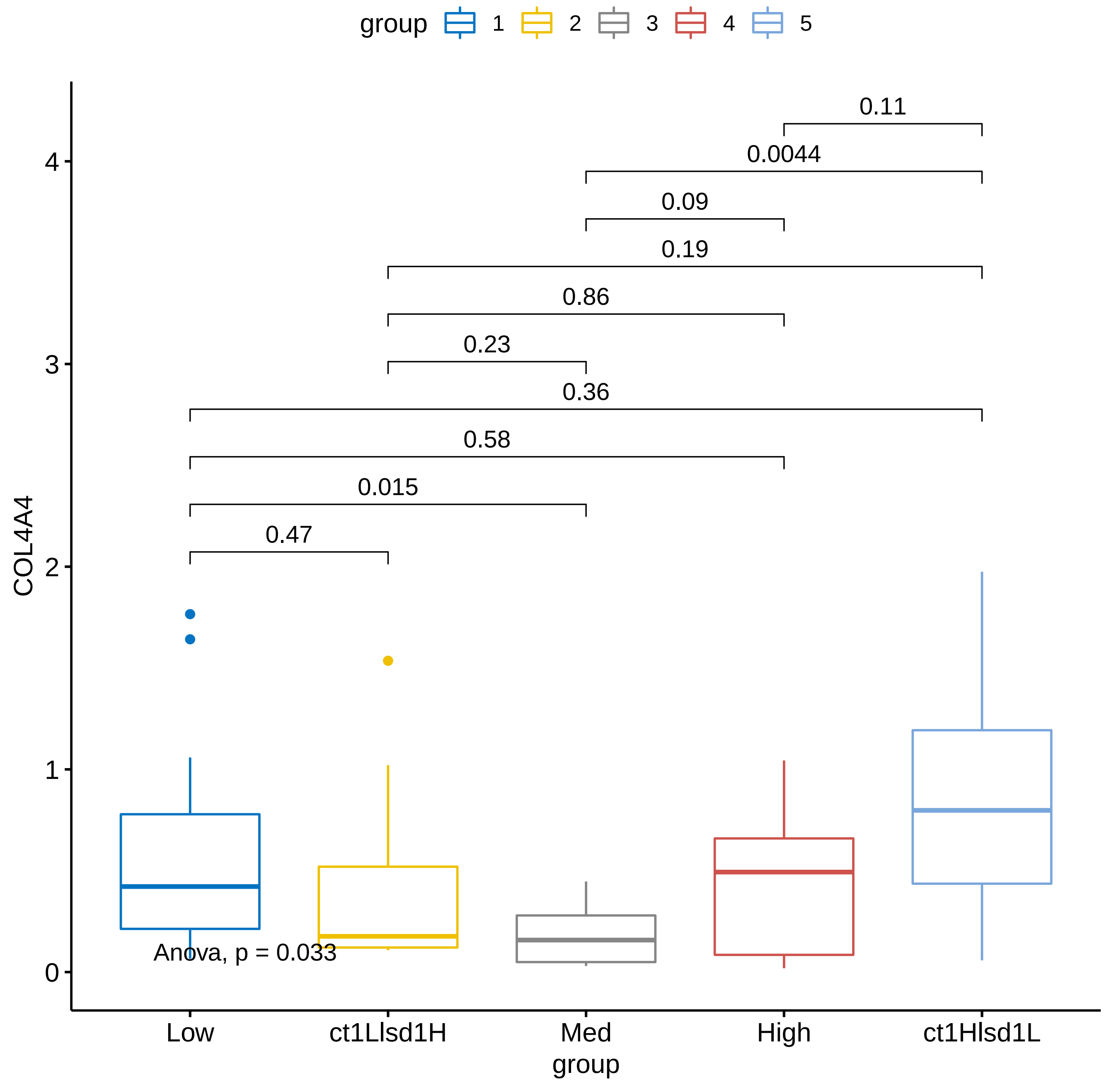
group 1 2 3 4 5
0.11
0.0044
0.09
0.19
0.86
0.23
0.36
0.58
0.015
0.47
COL4A4
4
3
2
1
0
Anova, p = 0.033
Low
ct1Llsd1H
Med
High
ct1Hlsd1L
group

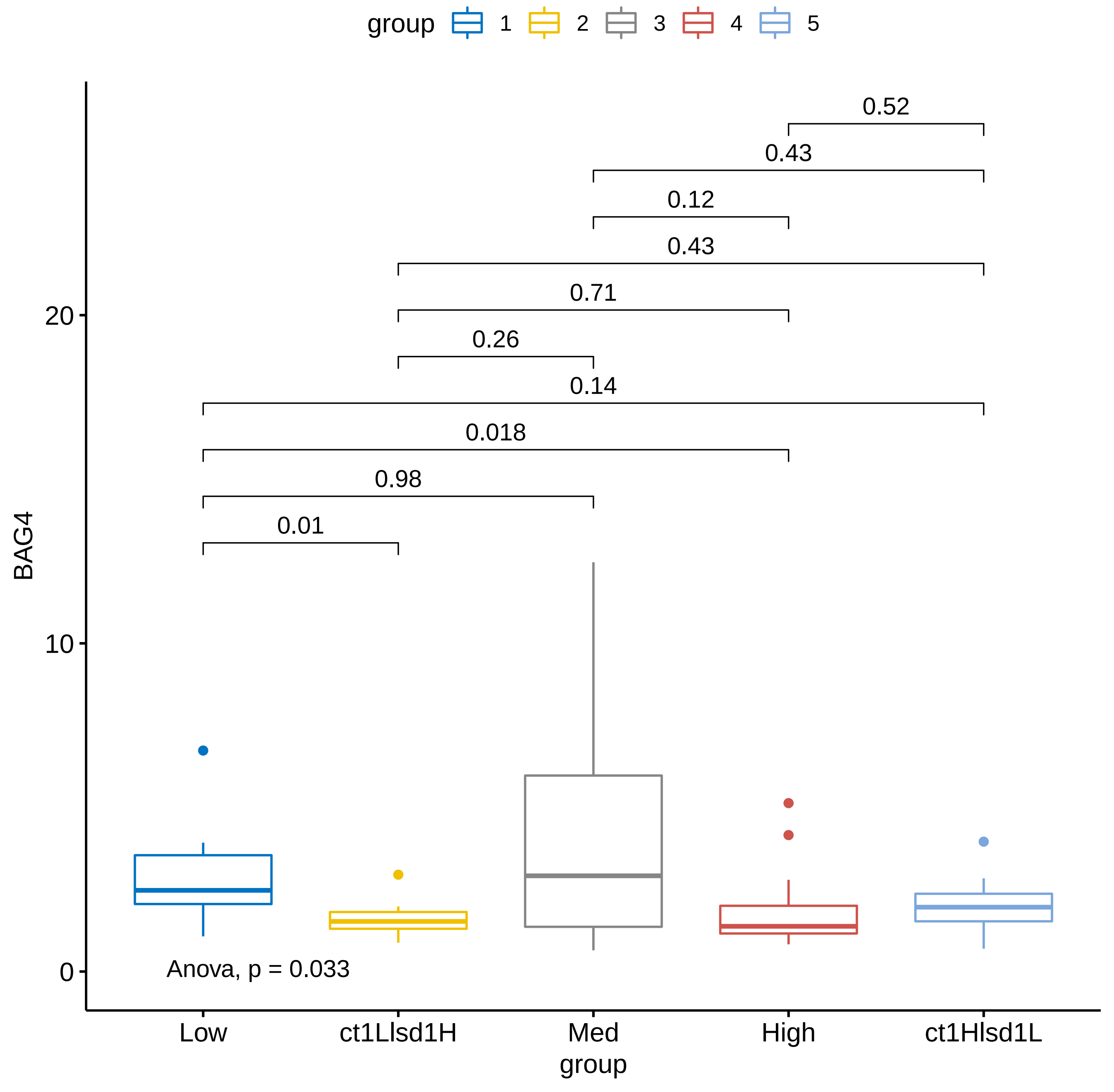

group 1 2 3 4 5
0.52
0.43
0.12
0.43
0.71
0.26
0.14
0.018
0.98
0.01
BAG4
20
10
0
Anova, p = 0.033
Low
ct1Llsd1H
Med
High
ct1Hlsd1L
group

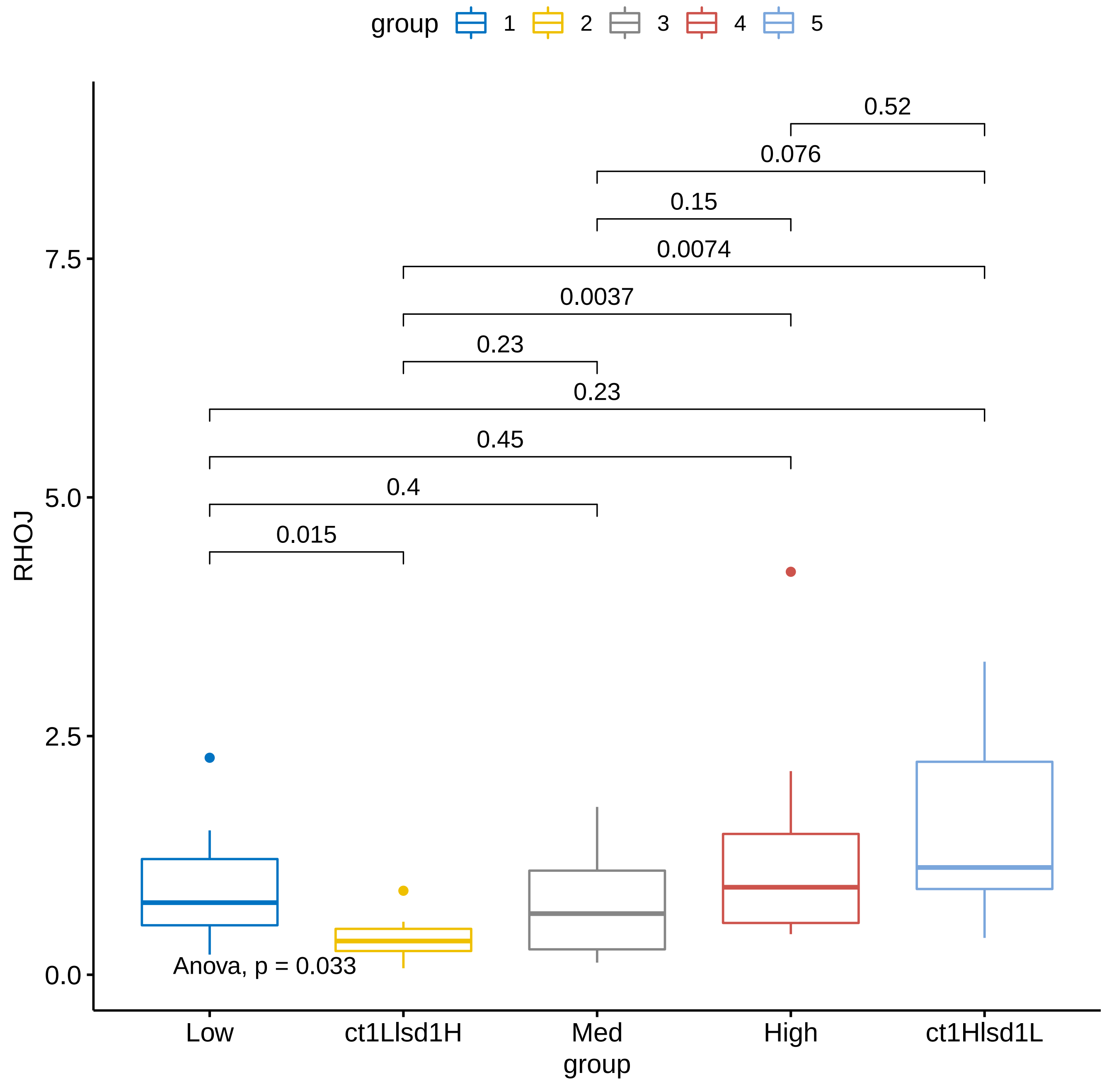

group
1
2
3
4
5
RHOJ
7.5
5.0
2.5
0.0
0.52
0.076
0.15
0.0074
0.0037
0.23
0.23
0.45
0.4
0.015
Anova, p = 0.033
Low
ct1Llsd1H
Med
High
ct1Hlsd1L
group

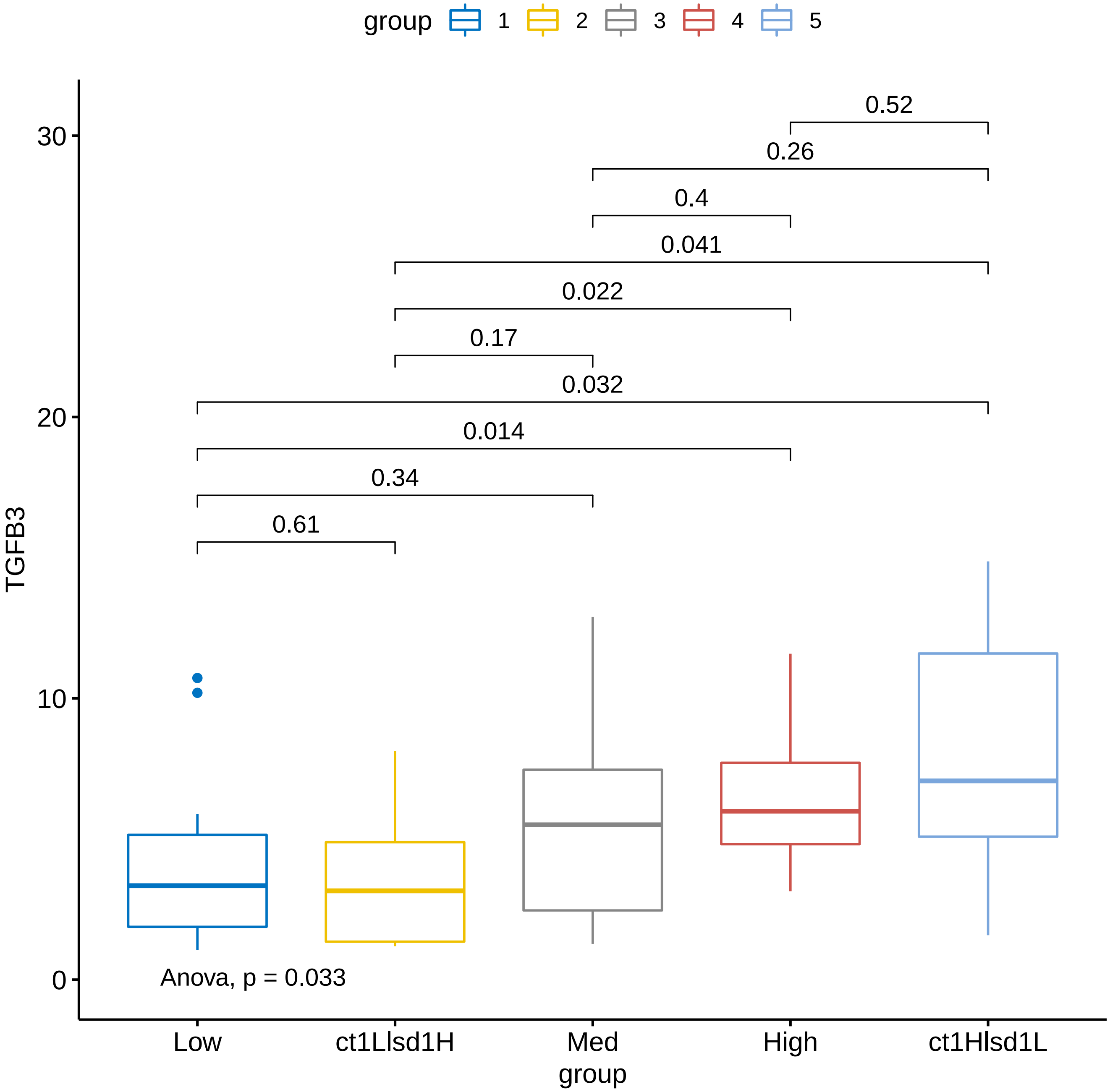
group
1
2
3
4
5
TGFB3
30
20
10
0
0.52
0.26
0.4
0.041
0.022
0.17
0.032
0.014
0.34
0.61
Anova, p = 0.033
Low
ct1Llsd1H
Med
High
ct1Hlsd1L
group

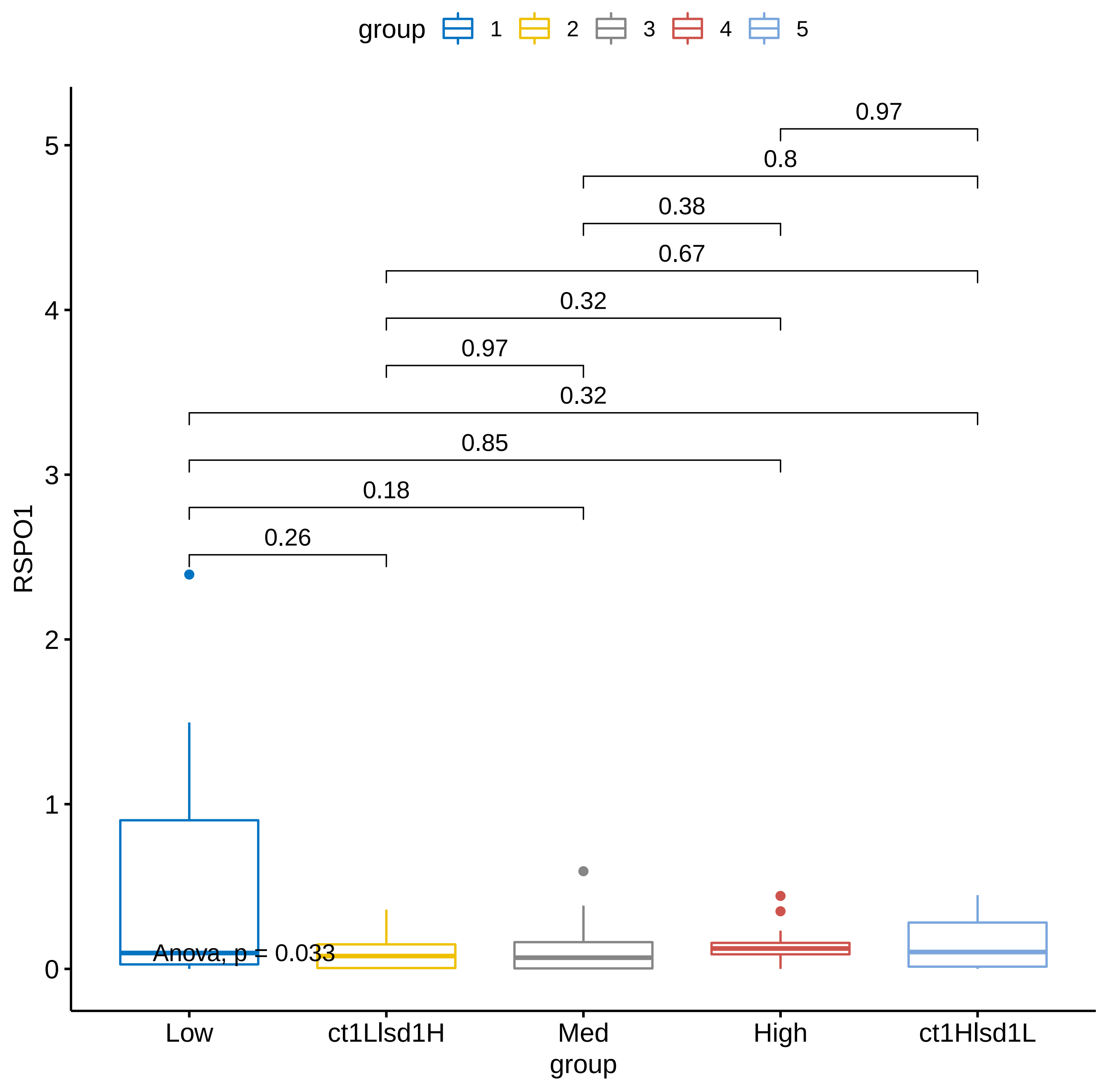

group
1
2
3
4
5
RSPO1
0
1
2
3
4
5
0.97
0.8
0.38
0.67
0.32
0.97
0.32
0.85
0.18
0.26
Anova, p = 0.033
Low
ct1Llsd1H
Med
High
ct1Hlsd1L
group

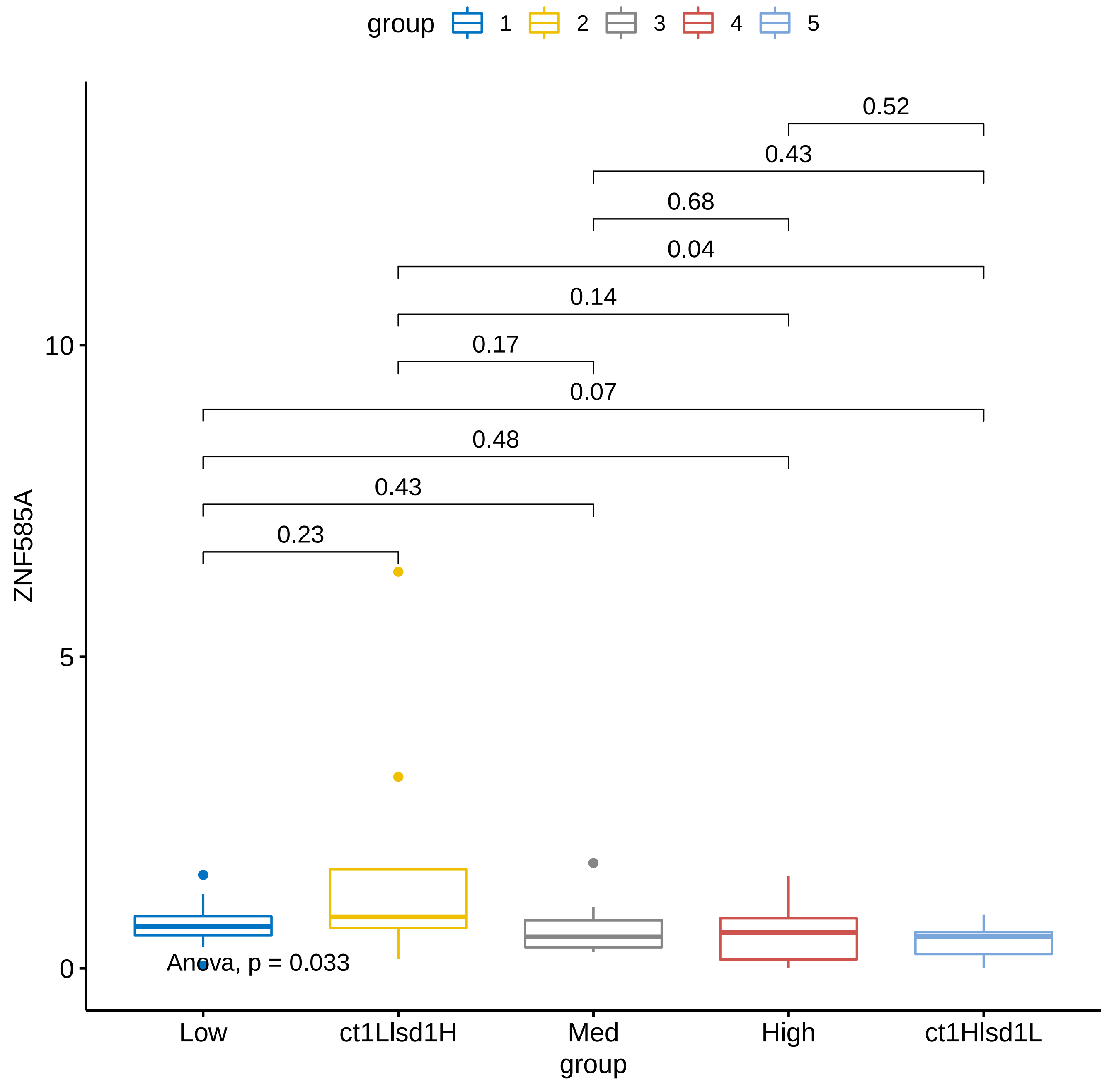

group 1 2 3 4 5
0.52
0.43
0.68
0.04
0.14
0.17
0.07
0.48
0.43
0.23
ZNF585A
10
5
0
Anova, p = 0.033
Low
ct1Llsd1H
Med
High
ct1Hlsd1L
group

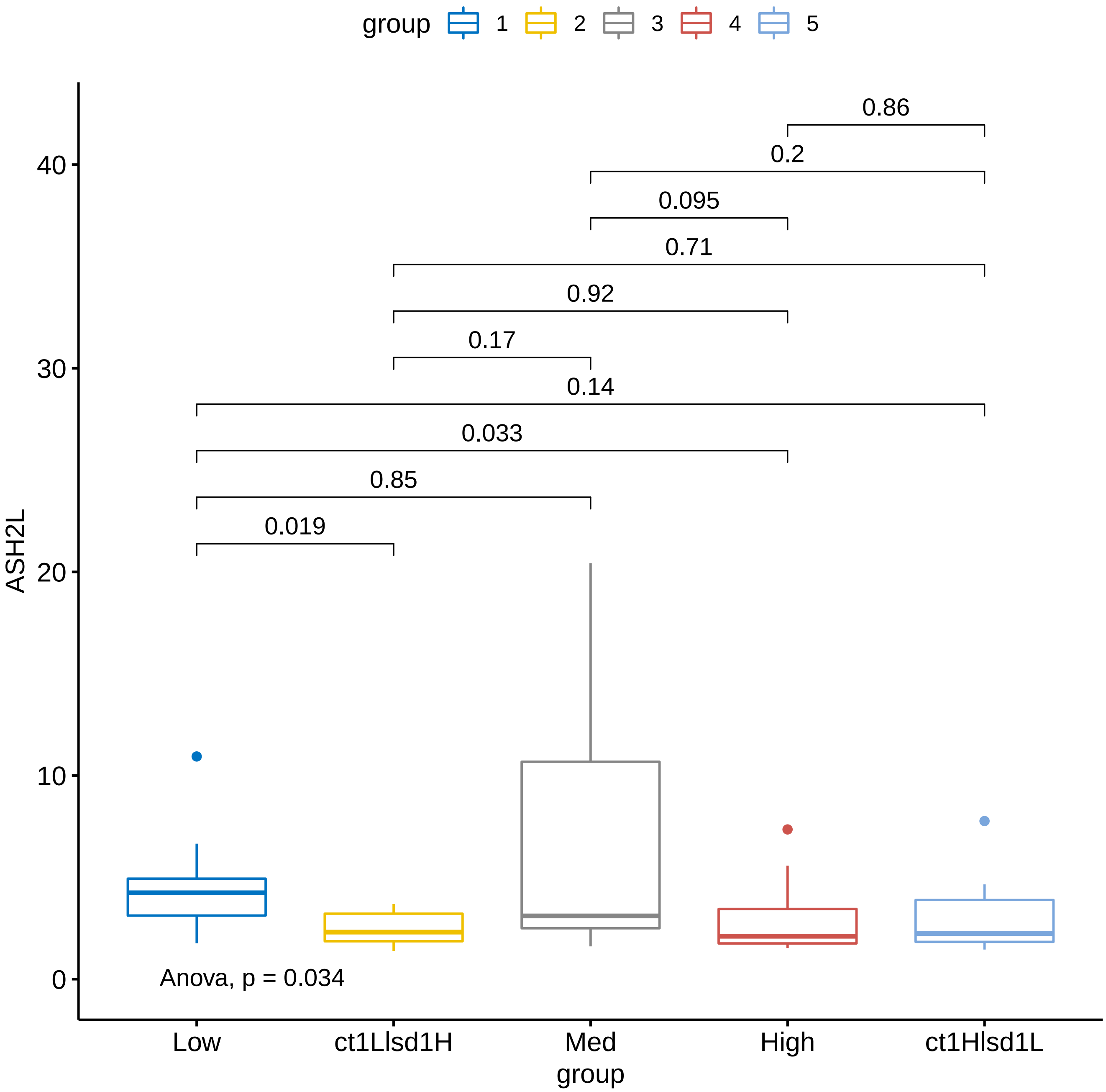
group
1
2
3
4
5
ASH2L
0
10
20
30
40
0.86
0.2
0.095
0.71
0.92
0.17
0.14
0.033
0.85
0.019
Anova, p = 0.034
Low
ct1Llsd1H
Med
High
ct1Hlsd1L
group

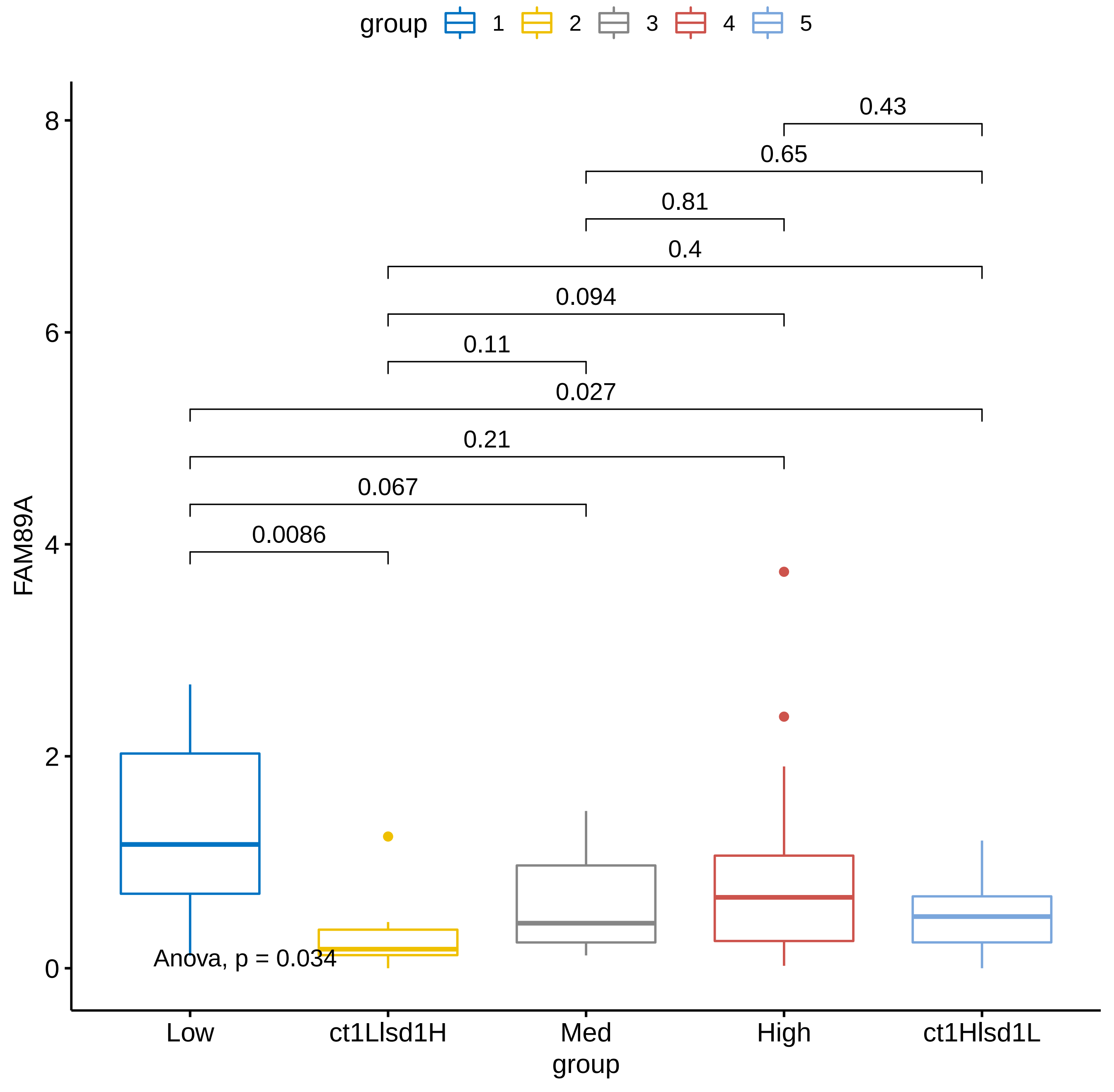
group
1
2
3
4
5
0.43
0.65
0.81
0.4
0.094
0.11
0.027
0.21
0.067
0.0086
Anova, p = 0.034
FAM89A
8
6
4
2
0
Low
ct1Llsd1H
Med
High
ct1Hlsd1L
group

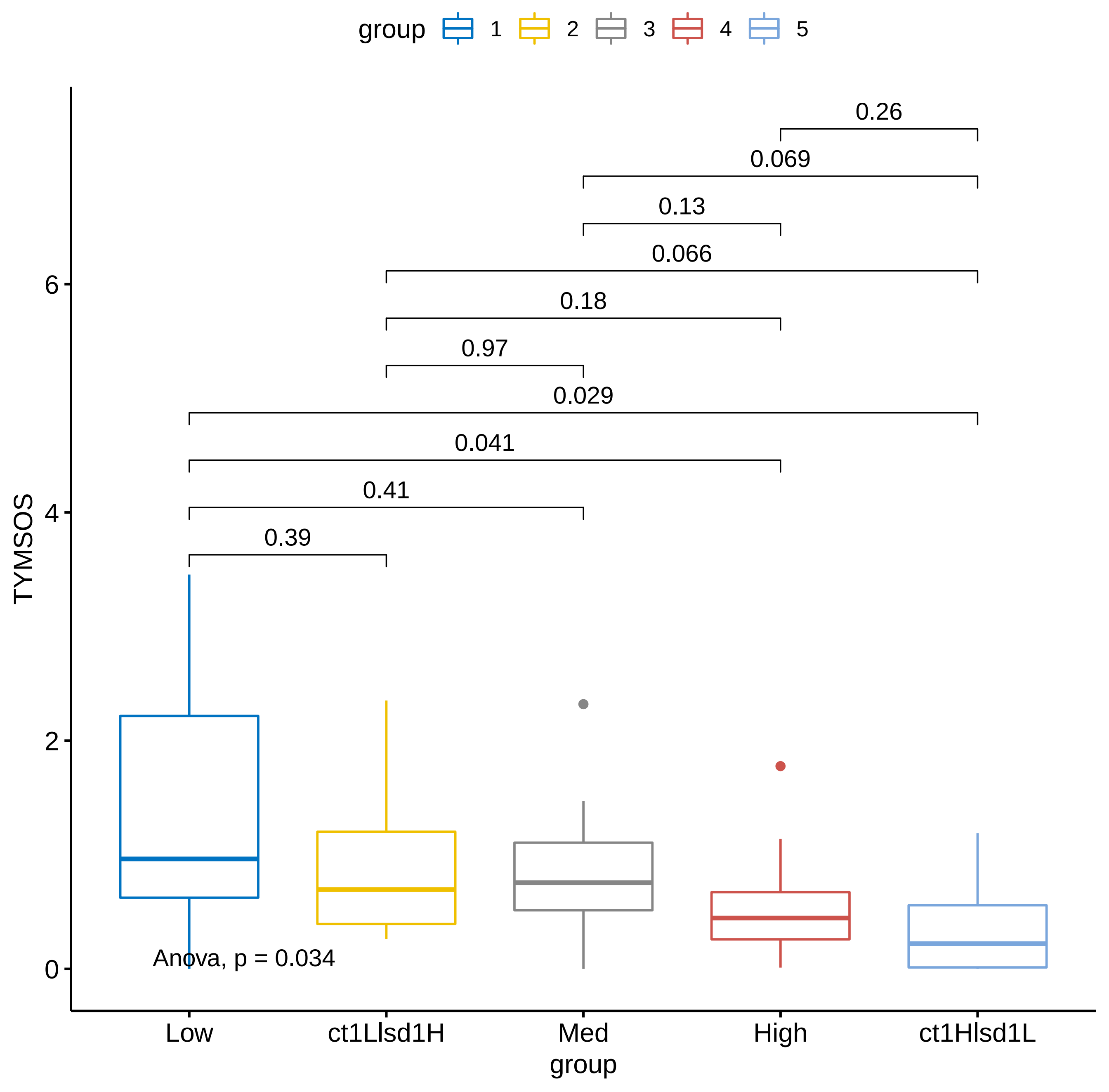
group
1
2
3
4
5
0.26
0.069
0.13
0.066
0.18
0.97
0.029
0.041
0.41
0.39
6
4
2
0
TYMSOS
Anova, p = 0.034
Low
ct1Llsd1H
Med
High
ct1Hlsd1L
group

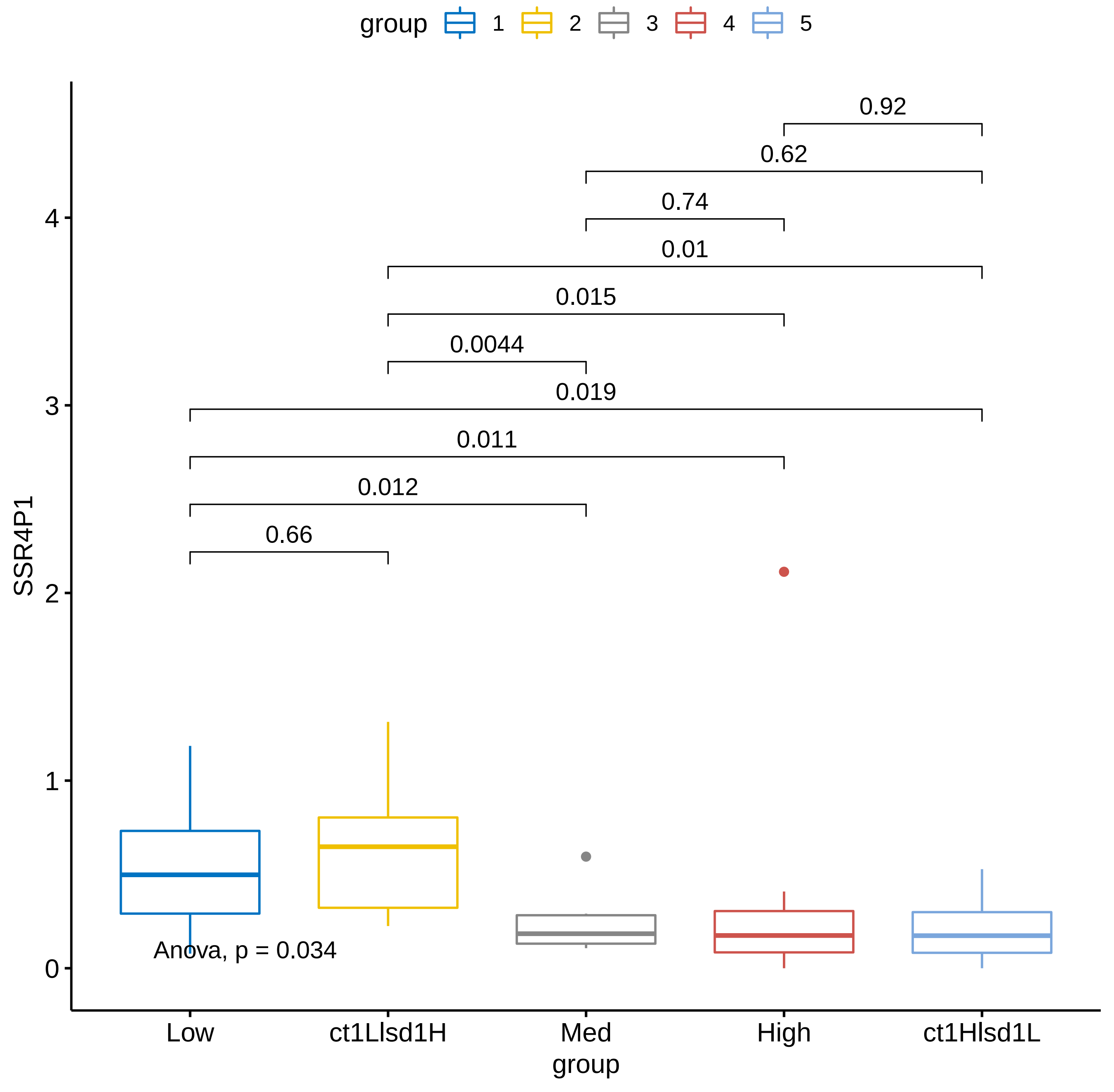

group 1 2 3 4 5
0.92
0.62
0.74
0.01
0.015
0.0044
0.019
0.011
0.012
0.66
Anova, p = 0.034
SSR4P1
0
1
2
3
4
Low
ct1Llsd1H
Med
High
ct1Hlsd1L
group

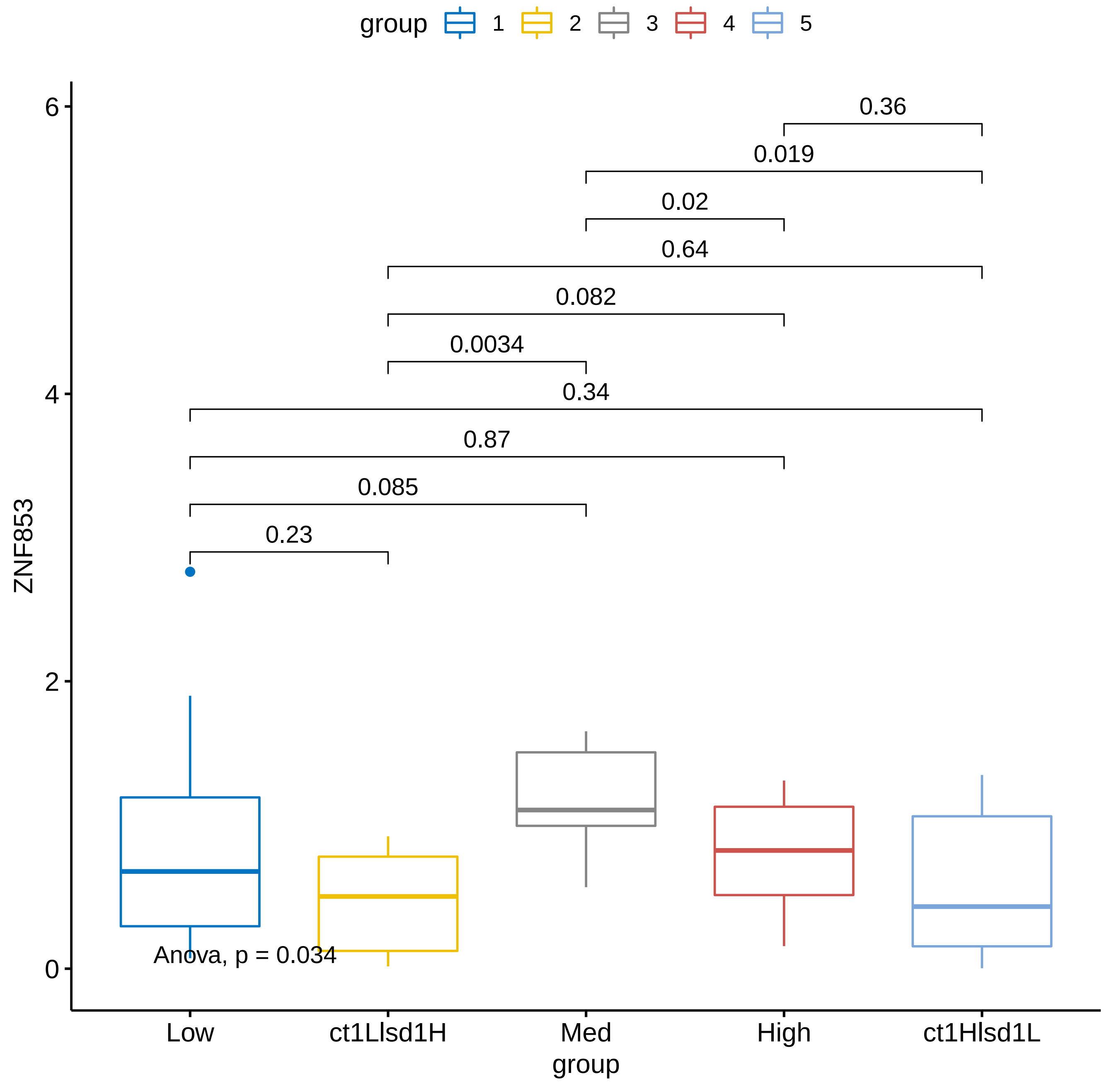

group 1 2 3 4 5
ZNF853
6
4
2
0
0.36
0.019
0.02
0.64
0.082
0.0034
0.34
0.87
0.085
0.23
Anova, p = 0.034
Low
ct1Llsd1H
Med
High
ct1Hlsd1L
group

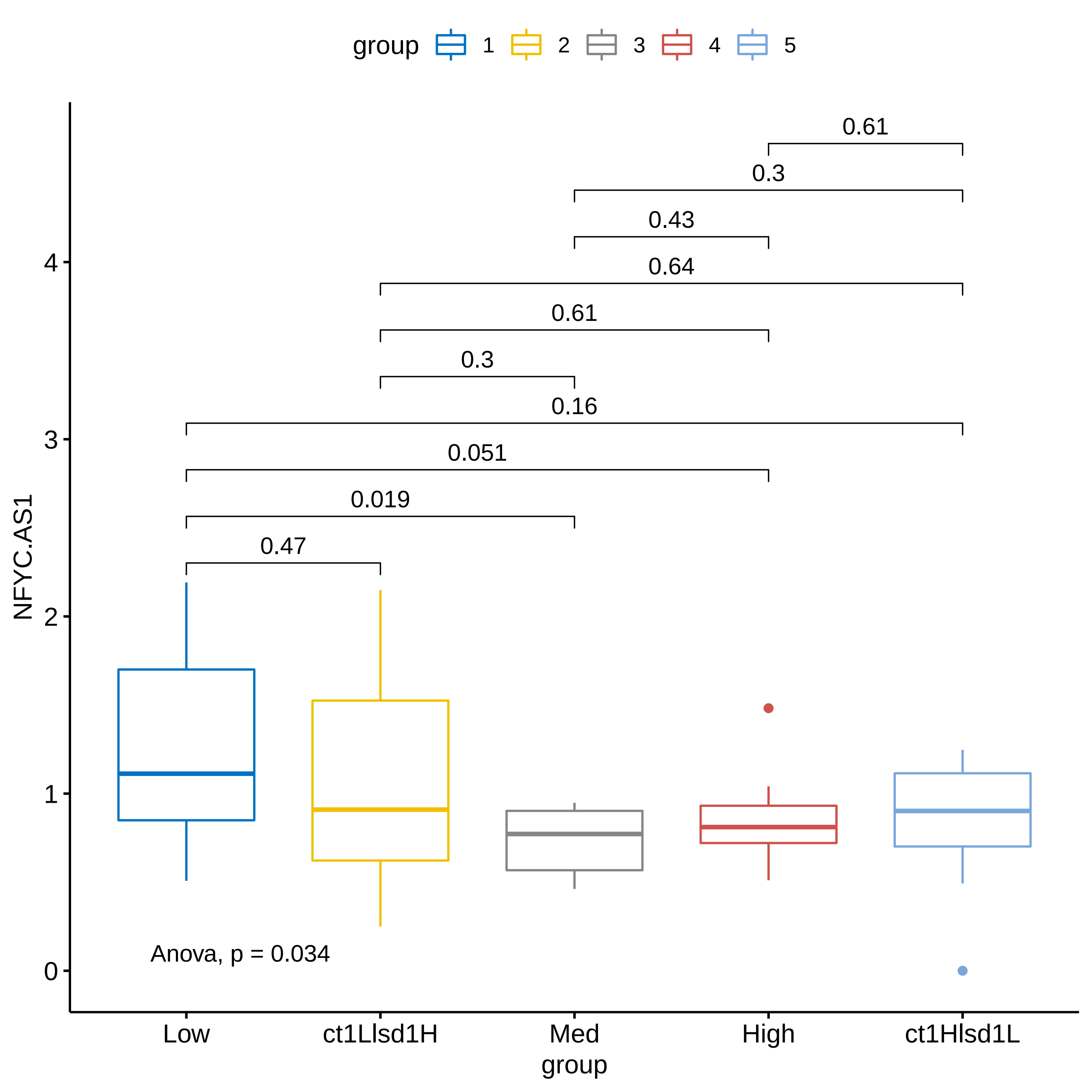

group
1
2
3
4
5
0.61
0.3
0.43
0.64
0.61
0.3
0.16
0.051
0.019
0.47
Anova, p = 0.034
NFYC.AS1
0
1
2
3
4
Low
ct1Llsd1H
Med
High
ct1Hlsd1L
group

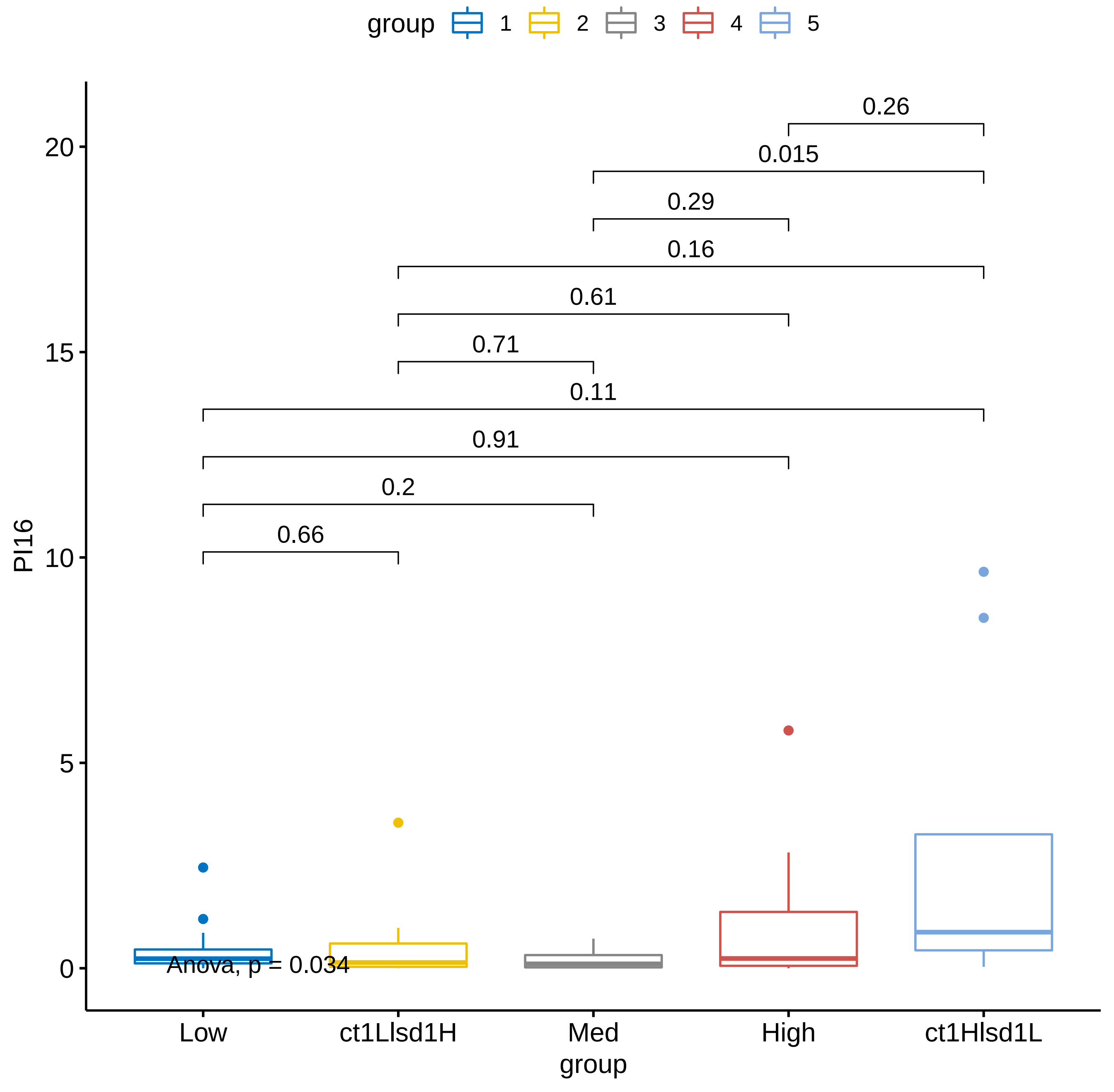

group
1
2
3
4
5
0.26
0.015
0.29
0.16
0.61
0.71
0.11
0.91
0.2
0.66
20
15
10
5
0
PI16
Anova, p = 0.034
Low
ct1Llsd1H
Med
High
ct1Hlsd1L
group

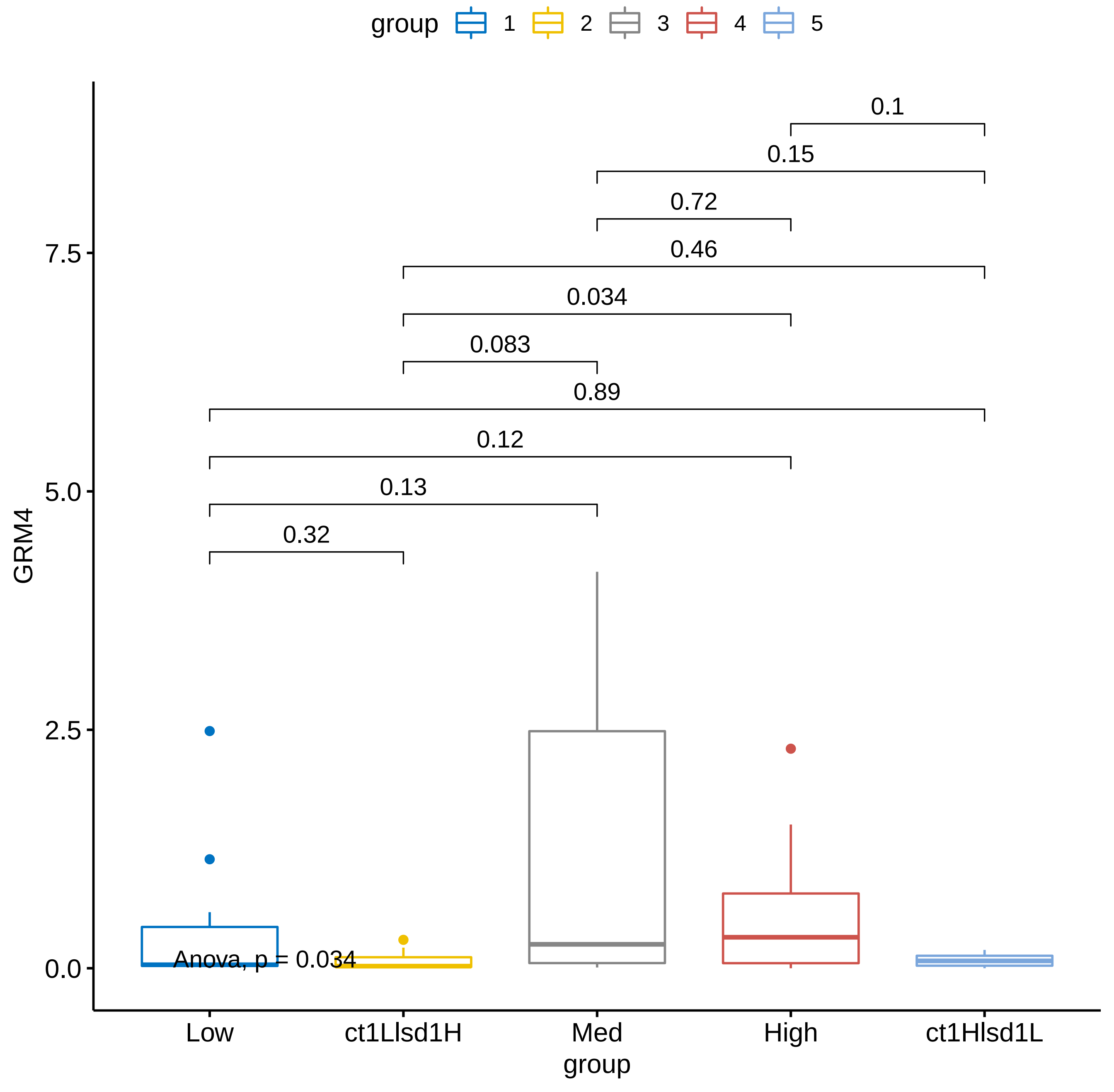

group 1 2 3 4 5
0.1
0.15
0.72
0.46
0.034
0.083
0.89
0.12
0.13
0.32
7.5
5.0
2.5
0.0
GRM4
Anova, p = 0.034
Low
ct1Llsd1H
Med
High
ct1Hlsd1L
group

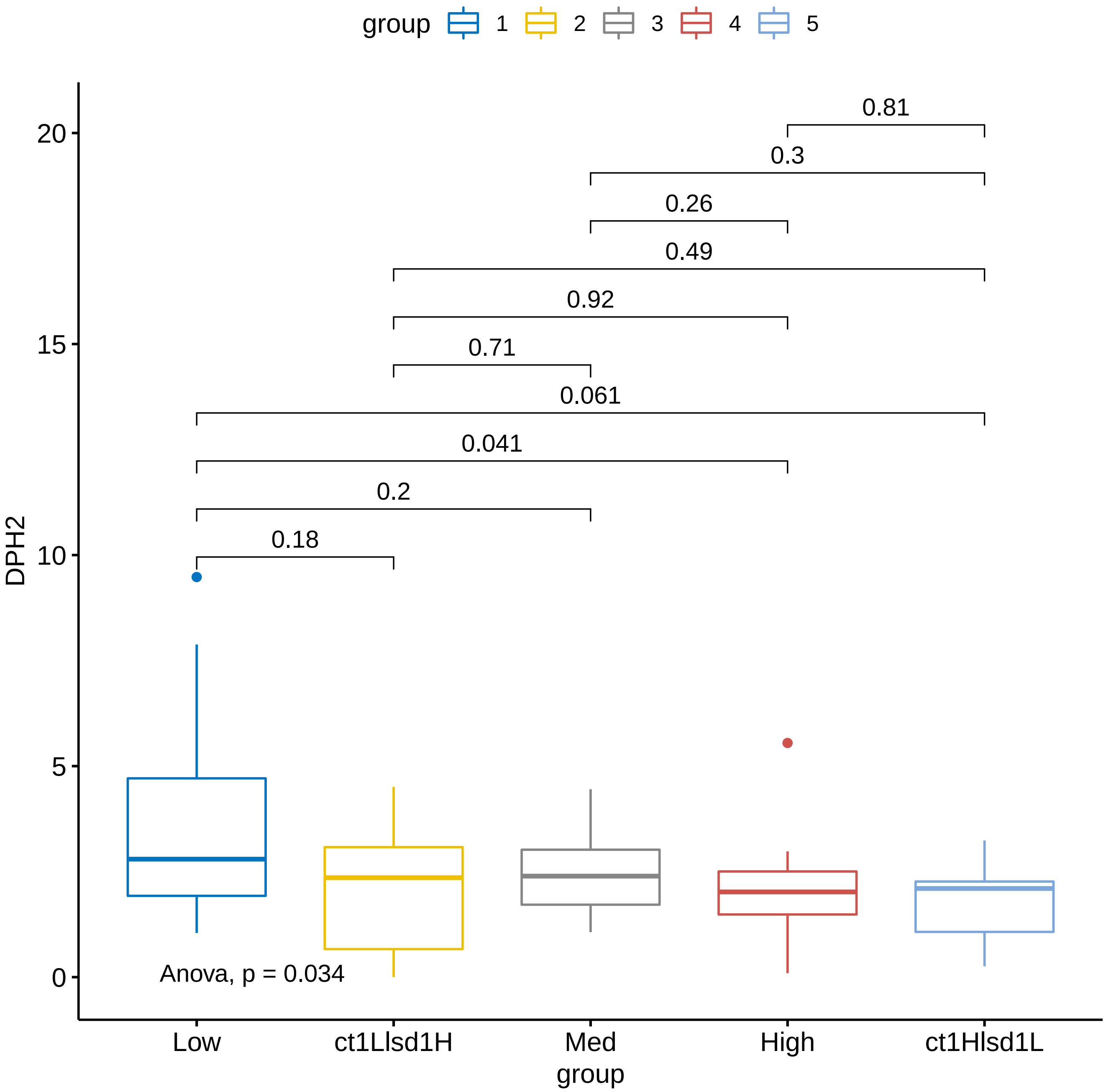

group 1 2 3 4 5
0.81
0.3
0.26
0.49
0.92
0.71
0.061
0.041
0.2
0.18
DPH2
20
15
10
5
0
Anova, p = 0.034
Low
ct1Llsd1H
Med
High
ct1Hlsd1L
group

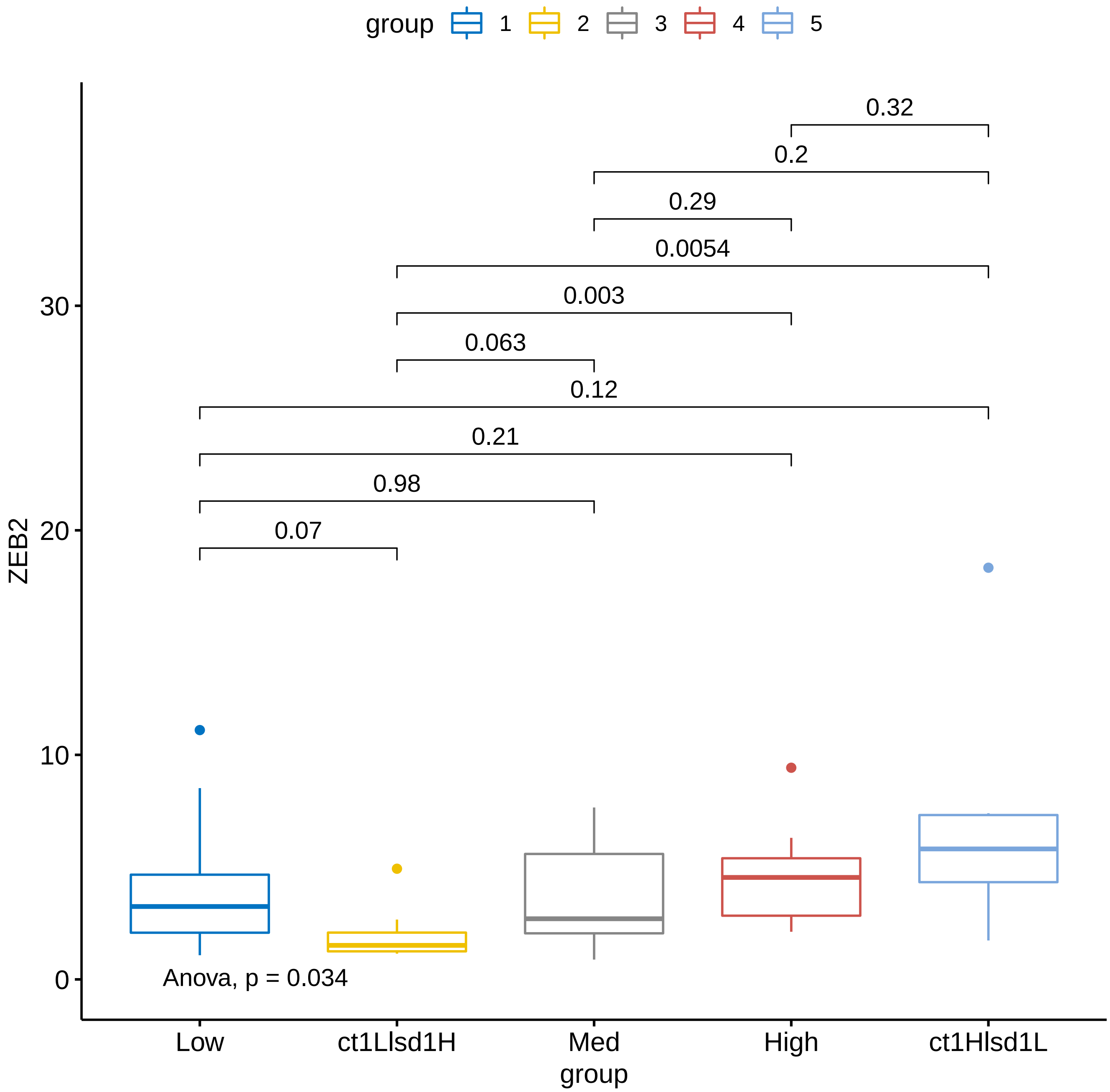

group 1 2 3 4 5
0.32
0.2
0.29
0.0054
0.003
0.063
0.12
0.21
0.98
0.07
30
20
10
0
ZEB2
Anova, p = 0.034
Low
ct1Llsd1H
Med
High
ct1Hlsd1L
group

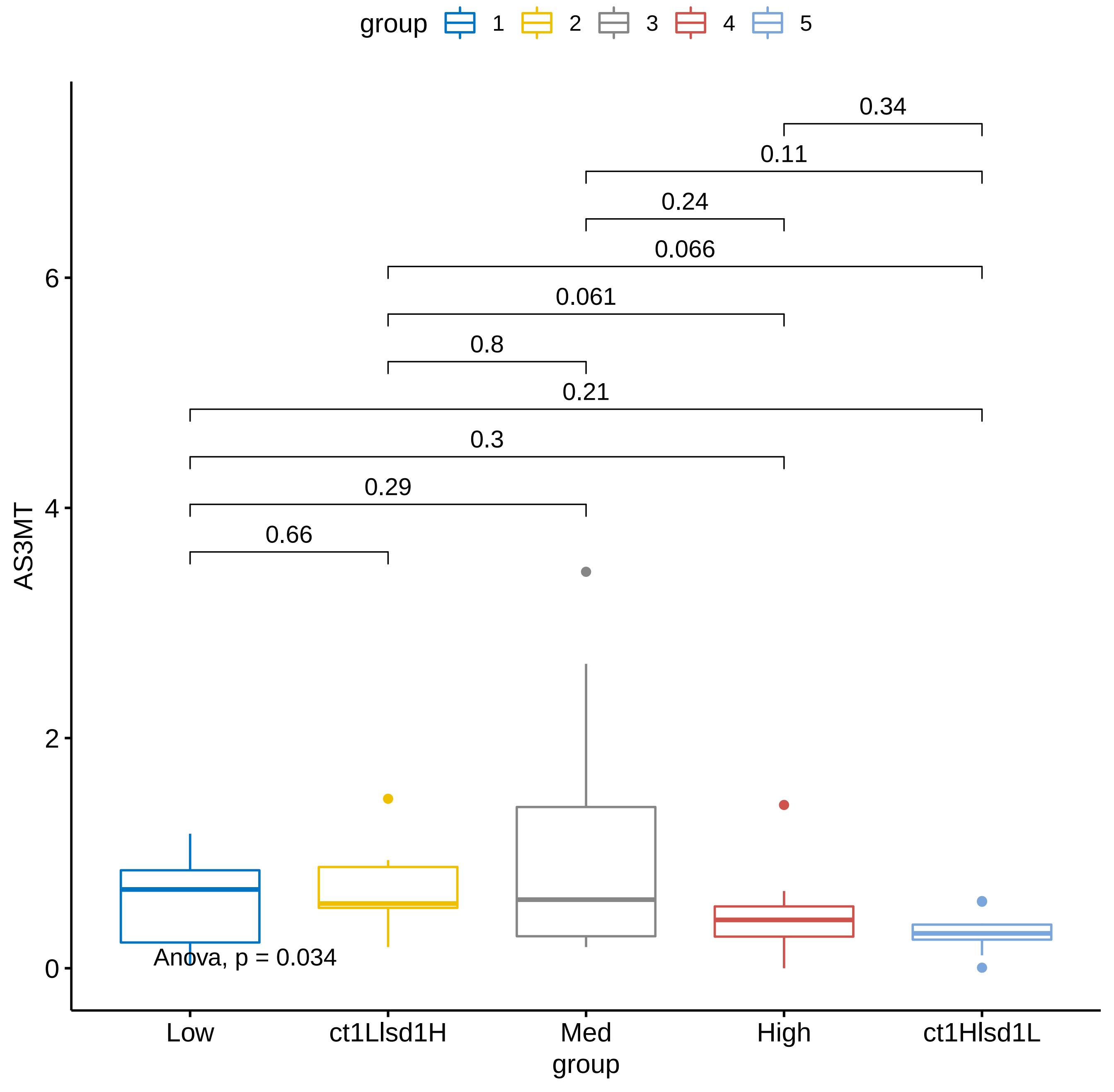

group
1
2
3
4
5
0.34
0.11
0.24
0.066
0.061
0.8
0.21
0.3
0.29
0.66
AS3MT
6
4
2
0
Anova, p = 0.034
Low
ct1Llsd1H
Med
High
ct1Hlsd1L
group

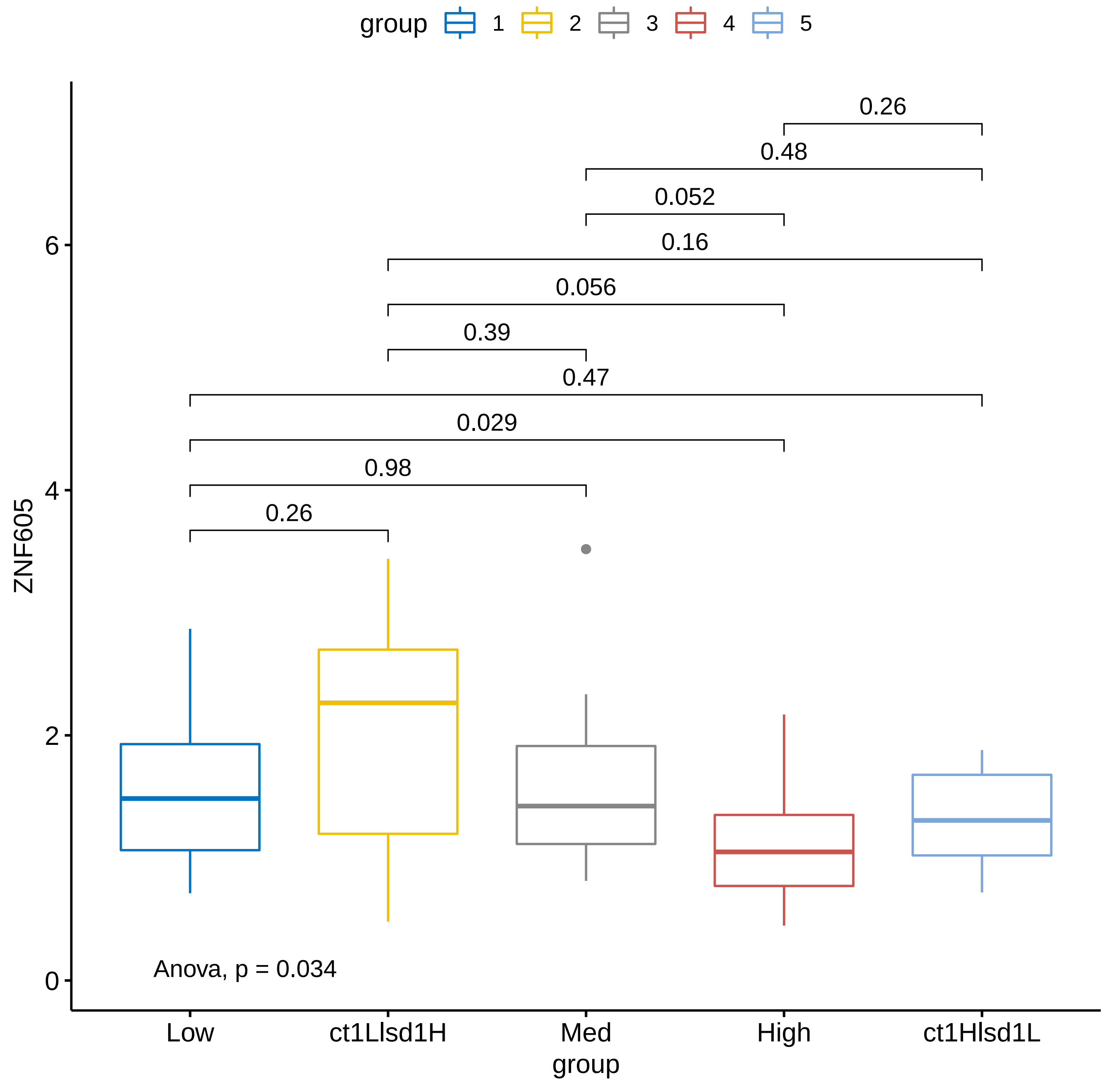
group
1
2
3
4
5
0.26
0.48
0.052
0.16
0.056
0.39
0.47
0.029
0.98
0.26
ZNF605
6
4
2
0
Anova, p = 0.034
Low
ct1Llsd1H
Med
High
ct1Hlsd1L
group

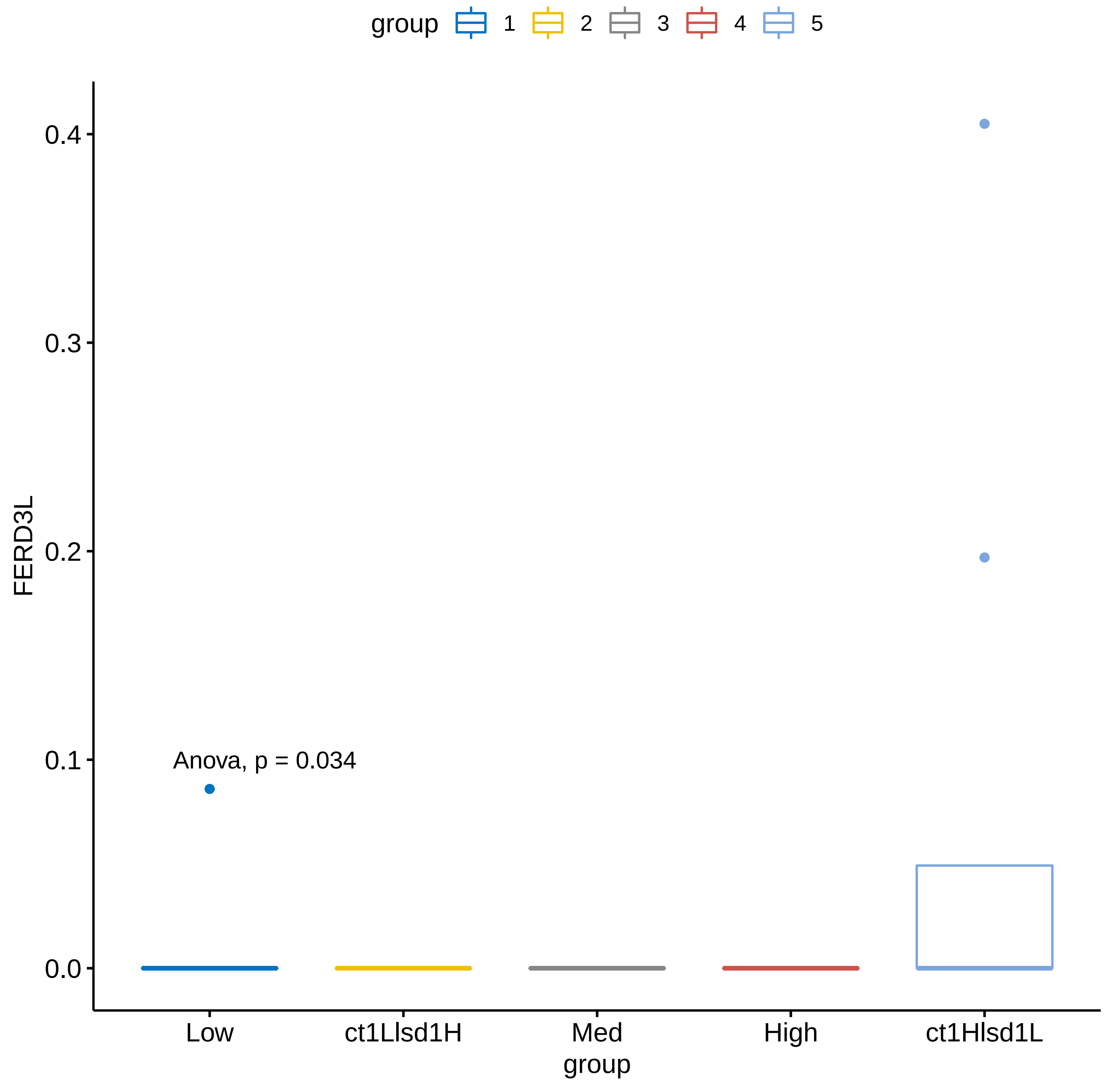

group
1
2
3
4
5
FERD3L
0.4
0.3
0.2
0.1
0.0
Anova, p = 0.034
Low
ct1Llsd1H
Med
High
ct1Hlsd1L
group

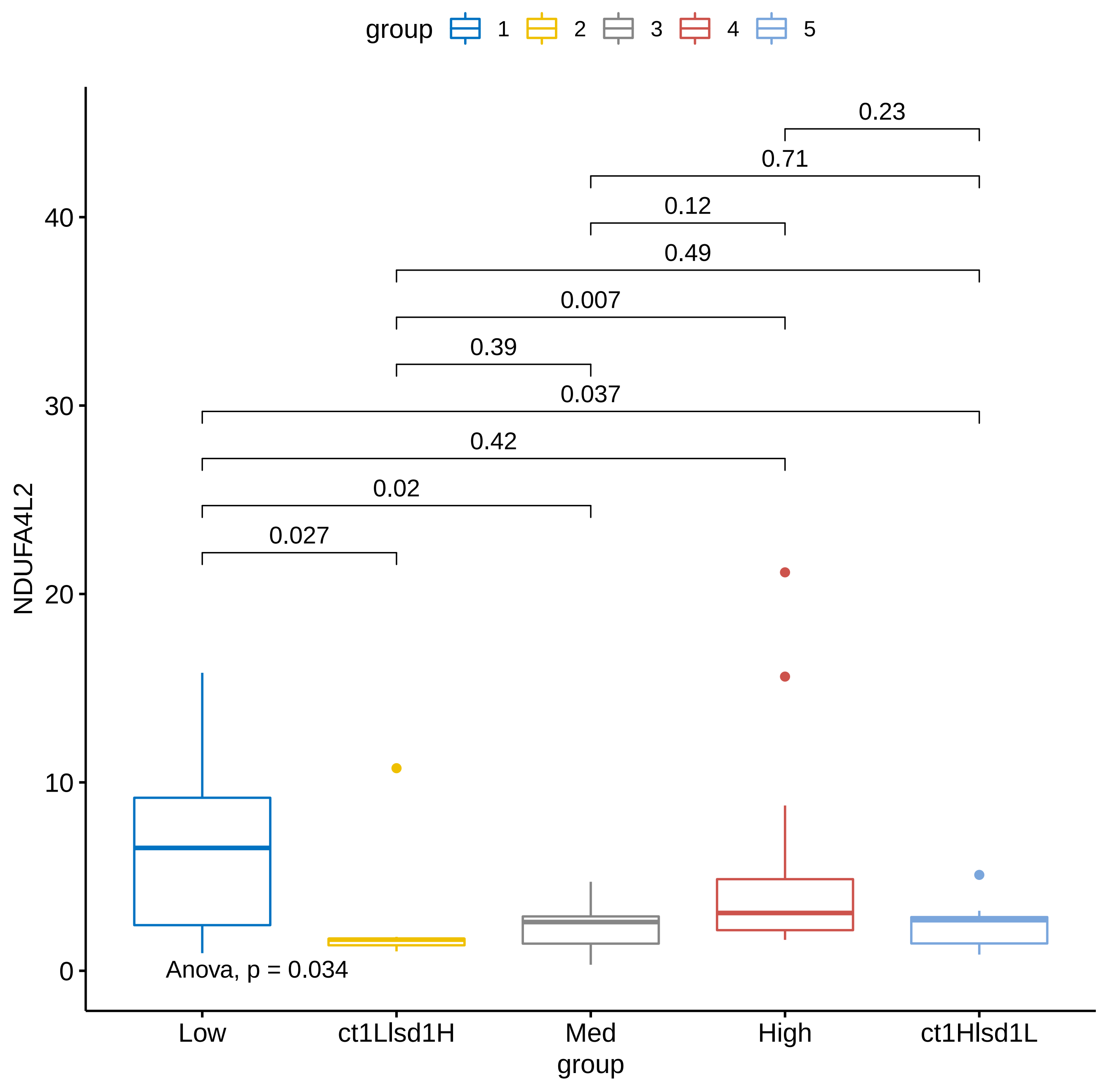
group 1 2 3 4 5
NDUFA4L2
0.23
0.71
0.12
0.49
0.007
0.39
0.037
0.42
0.02
0.027
40
30
20
10
0
Anova, p = 0.034
Low
ct1Llsd1H
Med
High
ct1Hlsd1L
group

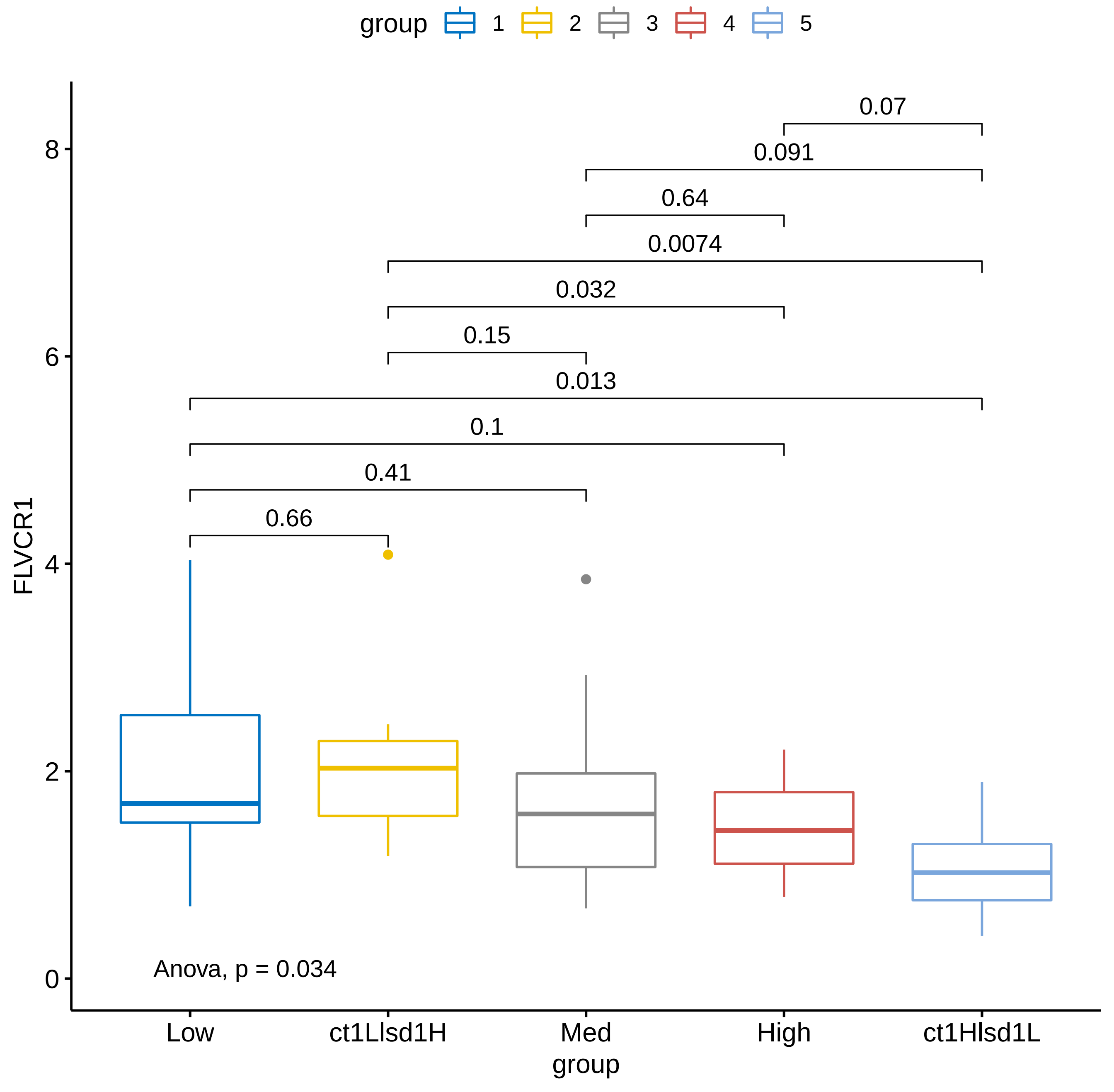
group
1
2
3
4
5
0.07
0.091
0.64
0.0074
0.032
0.15
0.013
0.1
0.41
0.66
FLVCR1
8
6
4
2
0
Anova, p = 0.034
Low
ct1Llsd1H
Med
High
ct1Hlsd1L
group

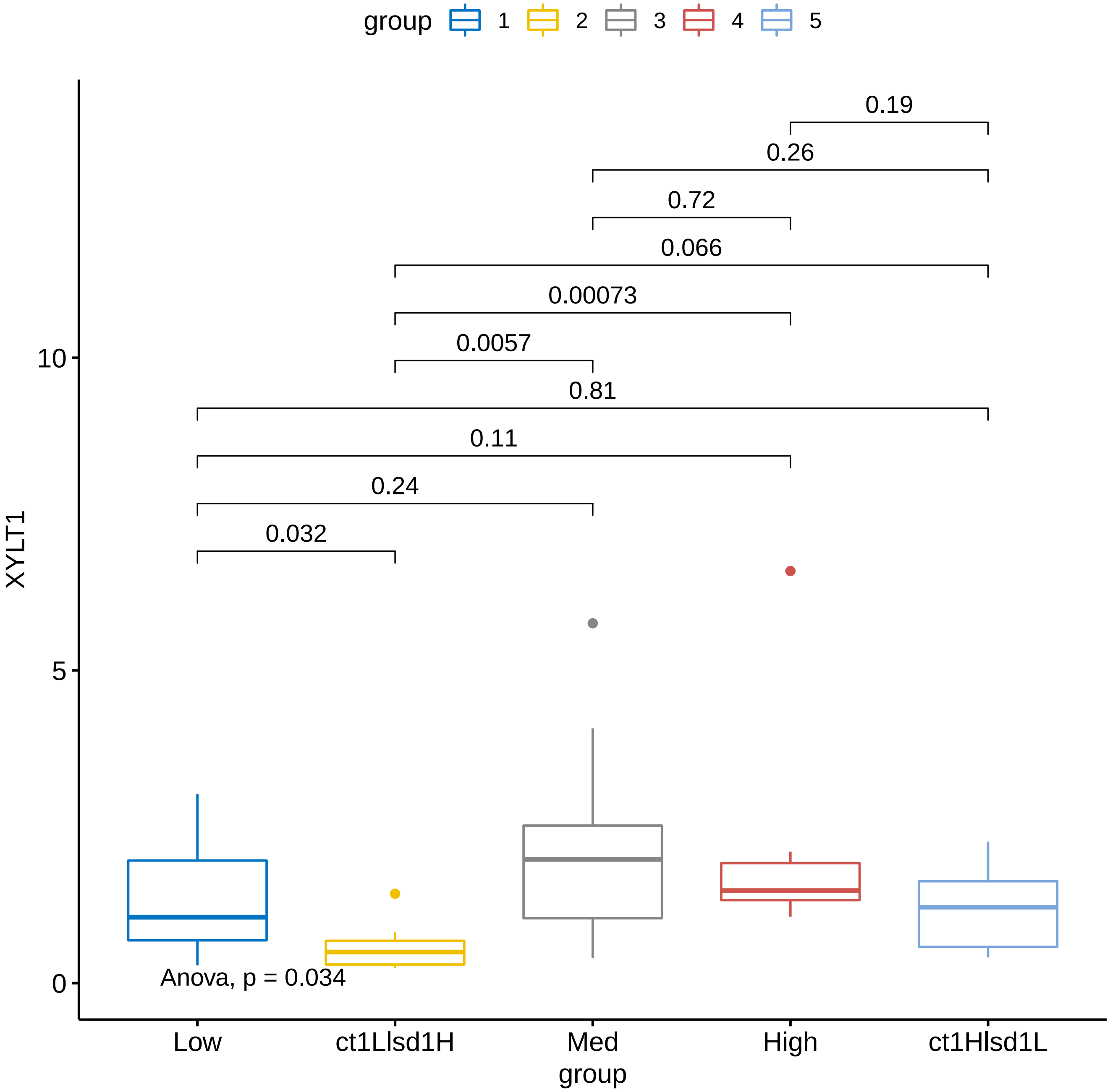
group
1
2
3
4
5
0.19
0.26
0.72
0.066
0.00073
0.0057
0.81
0.11
0.24
0.032
Anova, p = 0.034
XYLT1
10
5
0
Low
ct1Llsd1H
Med
High
ct1Hlsd1L
group

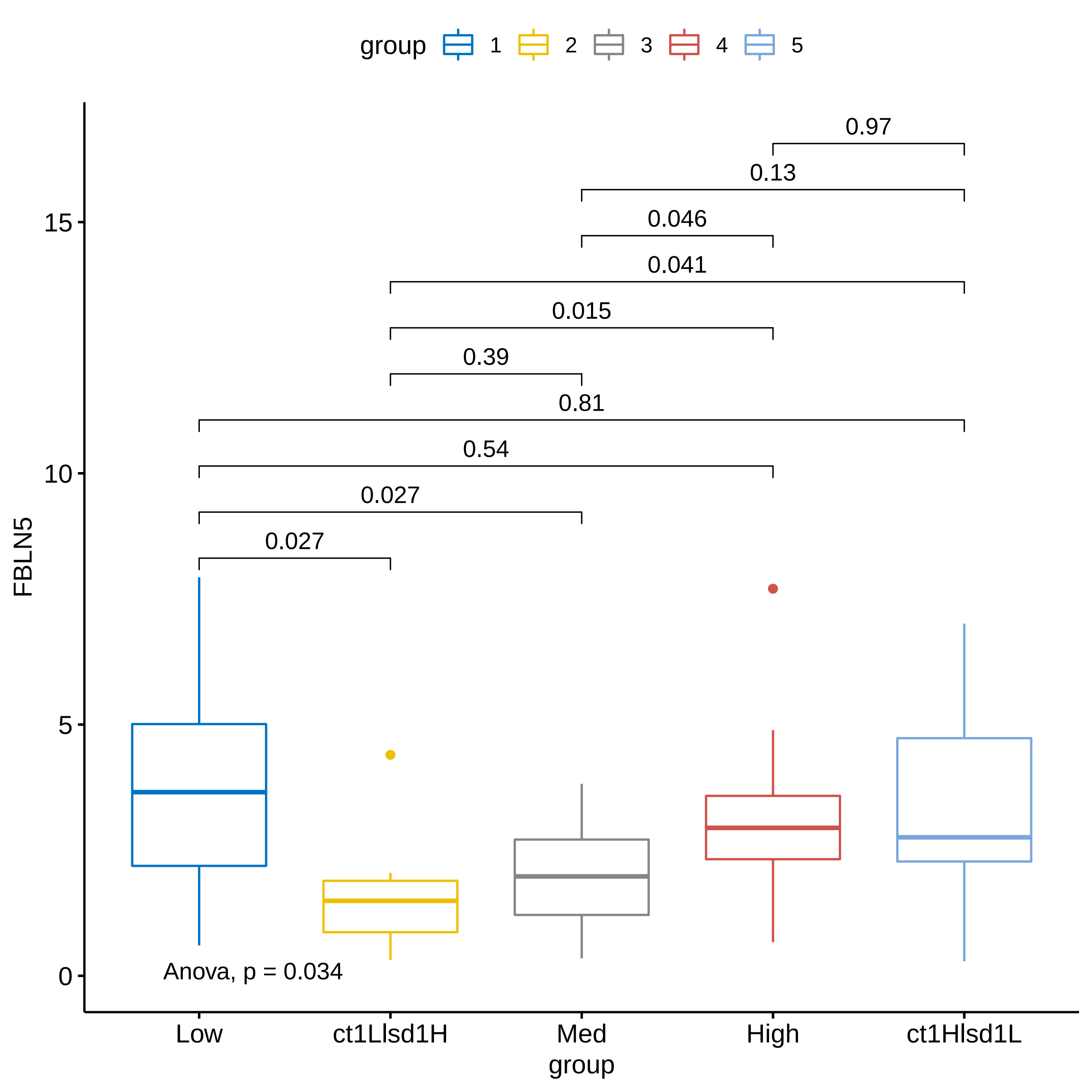

group
1
2
3
4
5
FBLN5
0.97
0.13
0.046
0.041
0.015
0.39
0.81
0.54
0.027
0.027
15
10
5
0
Anova, p = 0.034
Low
ct1Llsd1H
Med
High
ct1Hlsd1L
group

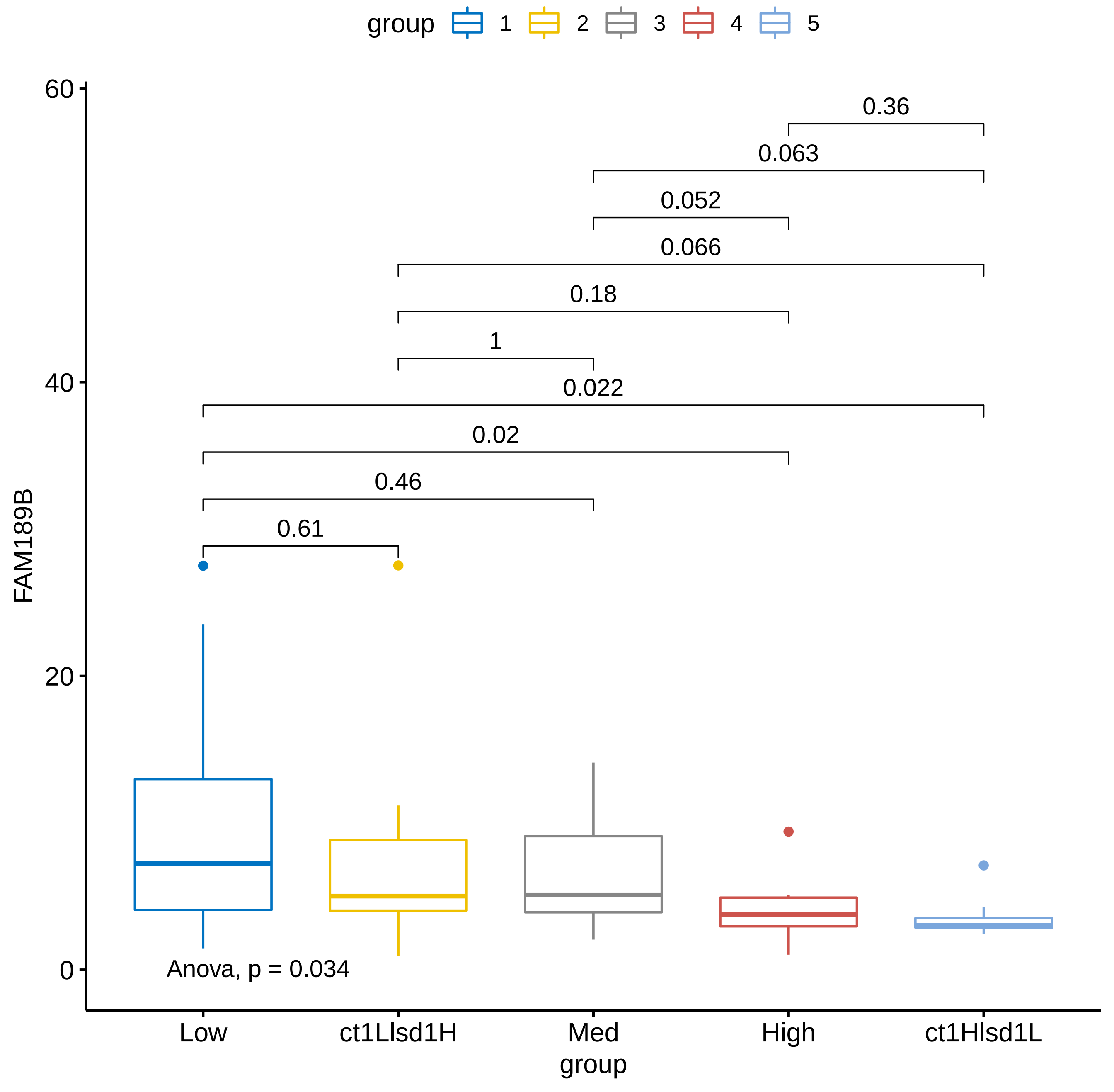
group
1
2
3
4
5
60
40
20
0
FAM189B
0.36
0.063
0.052
0.066
0.18
1
0.022
0.02
0.46
0.61
Anova, p = 0.034
Low
ct1Llsd1H
Med
High
ct1Hlsd1L
group

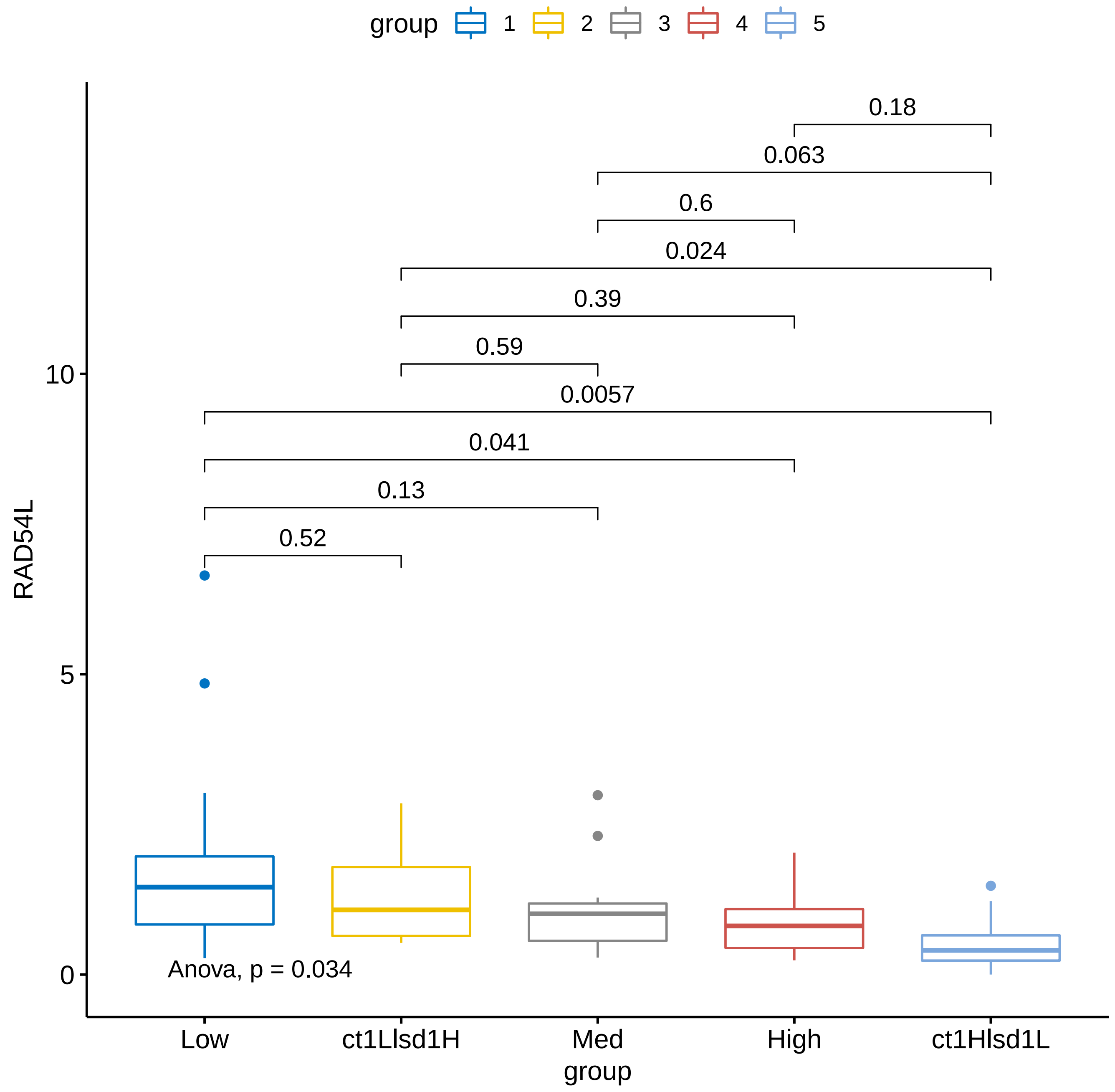

group 1 2 3 4 5
0.18
0.063
0.6
0.024
0.39
0.59
0.0057
0.041
0.13
0.52
RAD54L
10
5
0
Anova, p = 0.034
Low
ct1Llsd1H
Med
High
ct1Hlsd1L
group

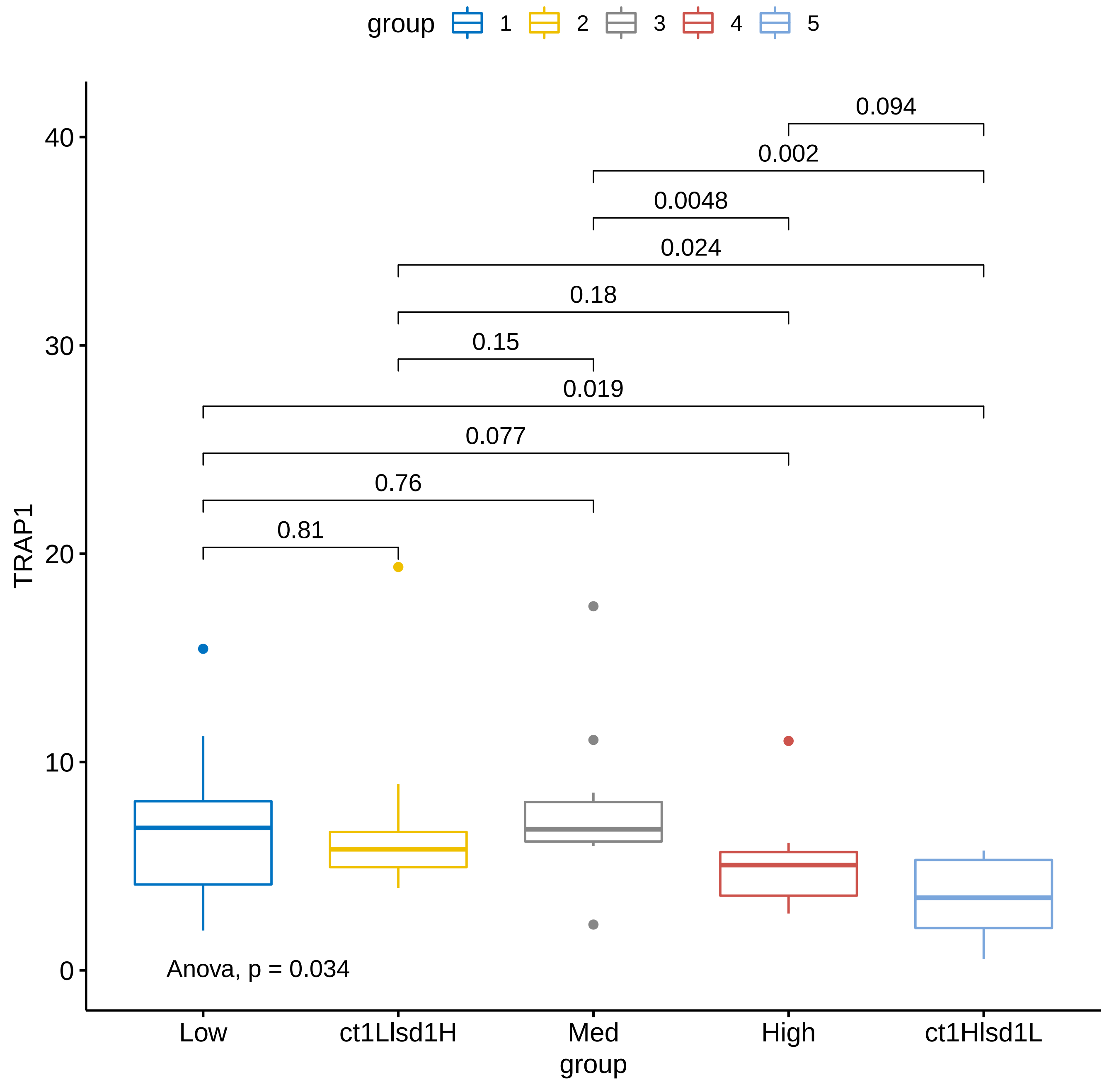

group 1 2 3 4 5
0.094
0.002
0.0048
0.024
0.18
0.15
0.019
0.077
0.76
0.81
TRAP1
40
30
20
10
0
Anova, p = 0.034
Low
ct1Llsd1H
Med
High
ct1Hlsd1L
group

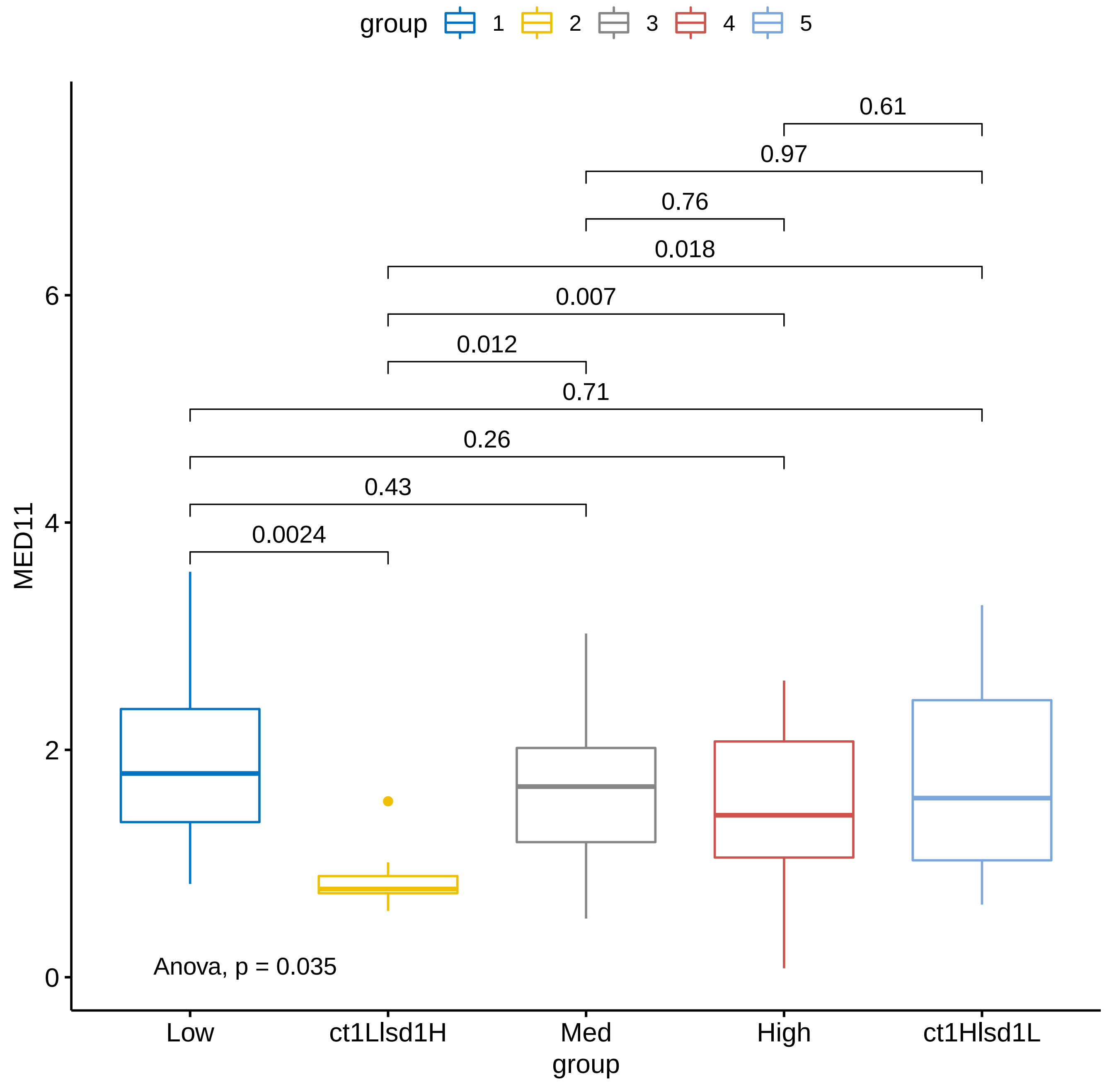
group
1
2
3
4
5
0.61
0.97
0.76
0.018
0.007
0.012
0.71
0.26
0.43
0.0024
MED11
6
4
2
0
Anova, p = 0.035
Low
ct1Llsd1H
Med
High
ct1Hlsd1L
group

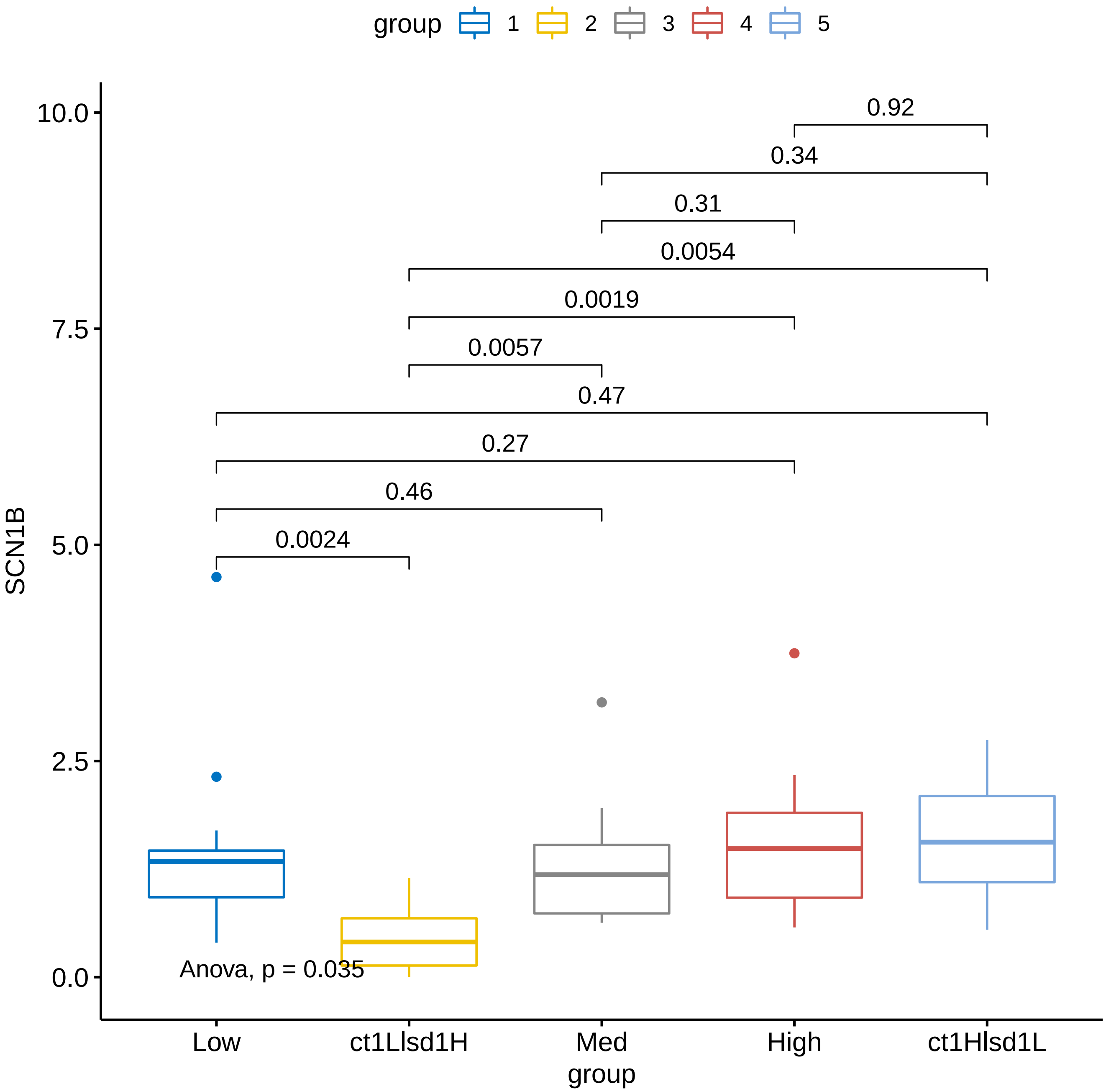
group
1
2
3
4
5
10.0
7.5
5.0
2.5
0.0
SCN1B
0.92
0.34
0.31
0.0054
0.0019
0.0057
0.47
0.27
0.46
0.0024
Anova, p = 0.035
Low
ct1Llsd1H
Med
High
ct1Hlsd1L
group

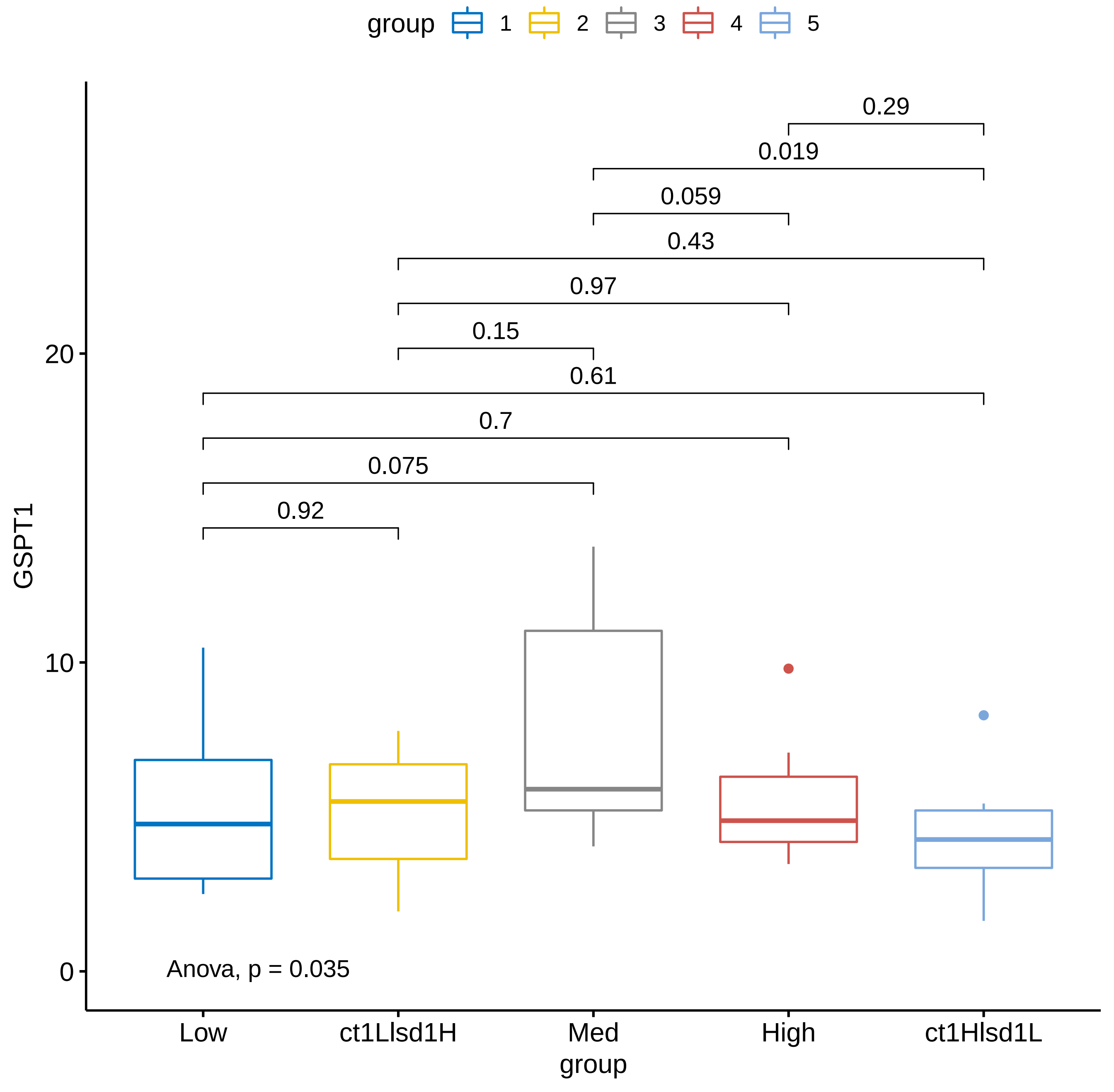
group
1
2
3
4
5
0.29
0.019
0.059
0.43
0.97
0.15
0.61
0.7
0.075
0.92
GSPT1
20
10
0
Anova, p = 0.035
Low
ct1Llsd1H
Med
High
ct1Hlsd1L
group

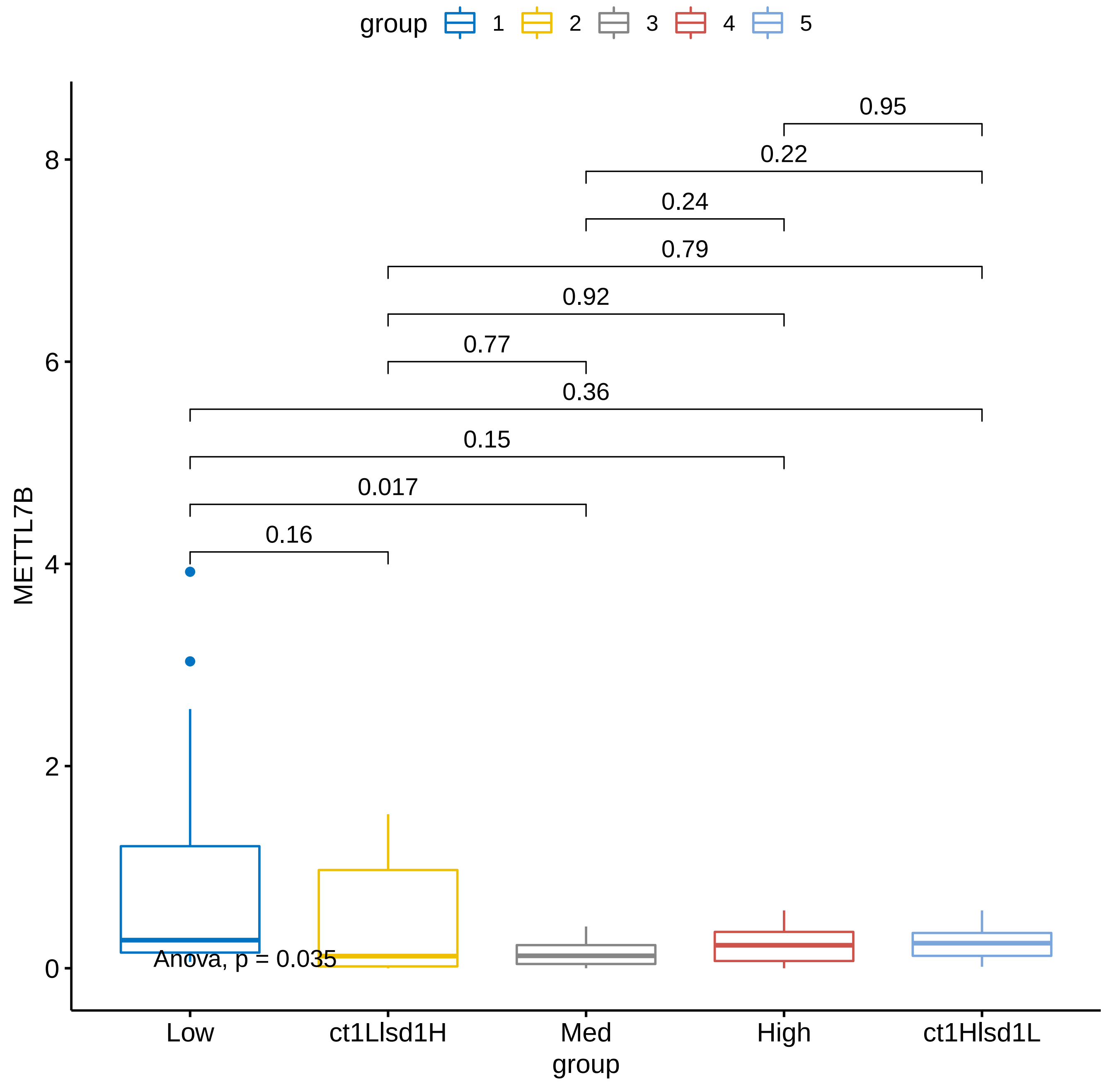

group
1
2
3
4
5
0.95
0.22
0.24
0.79
0.92
0.77
0.36
0.15
0.017
0.16
Anova, p = 0.035
METTL7B
0
2
4
6
8
Low
ct1Llsd1H
Med
High
ct1Hlsd1L
group

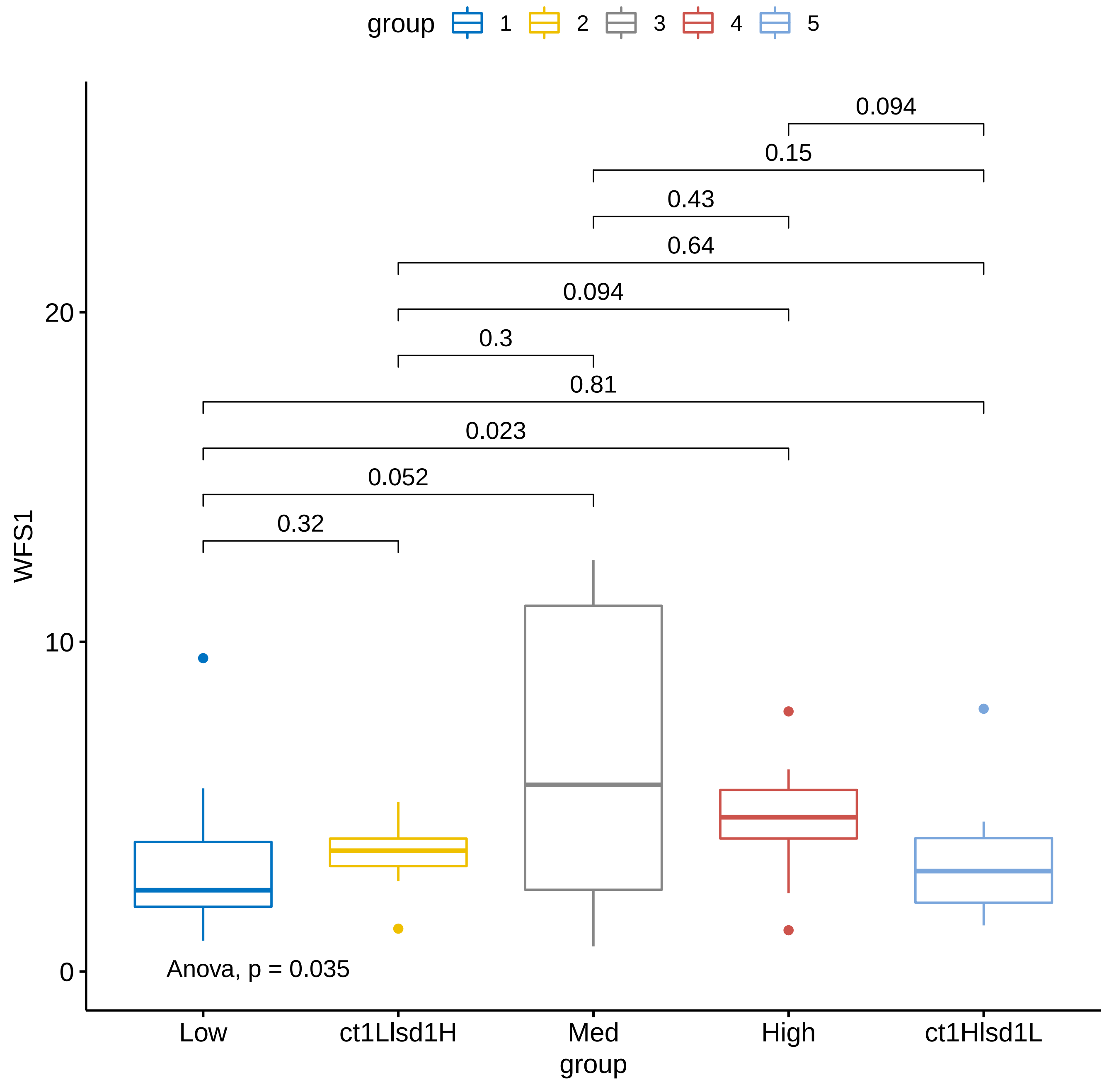

group 1 2 3 4 5
0.094
0.15
0.43
0.64
0.094
0.3
0.81
0.023
0.052
0.32
WFS1
20
10
0
Anova, p = 0.035
Low
ct1Llsd1H
Med
High
ct1Hlsd1L
group

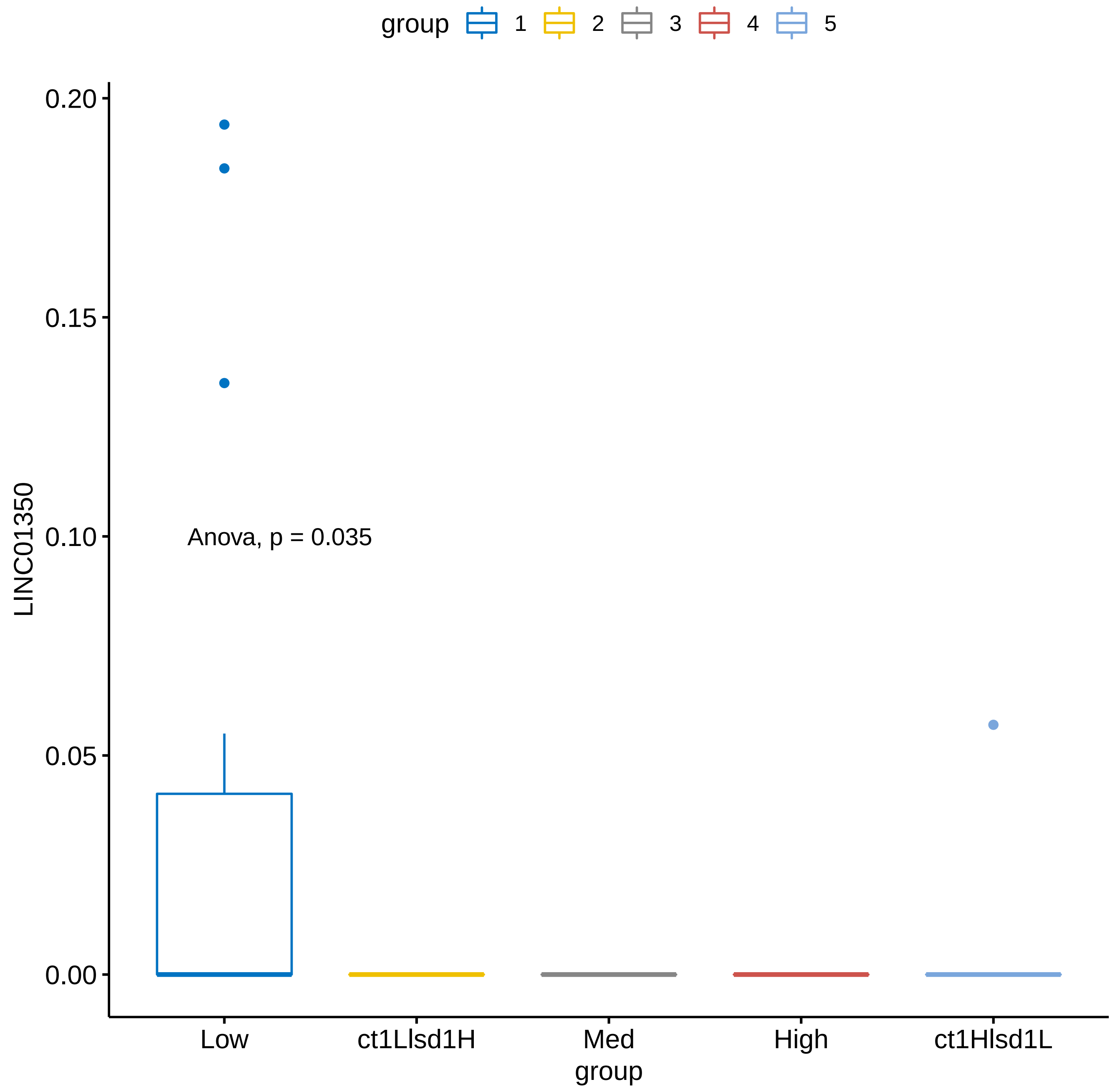

group 1 2 3 4 5
0.20
0.15
0.10
0.05
0.00
LINC01350
Anova, p = 0.035
Low
ct1Llsd1H
Med
High
ct1Hlsd1L
group

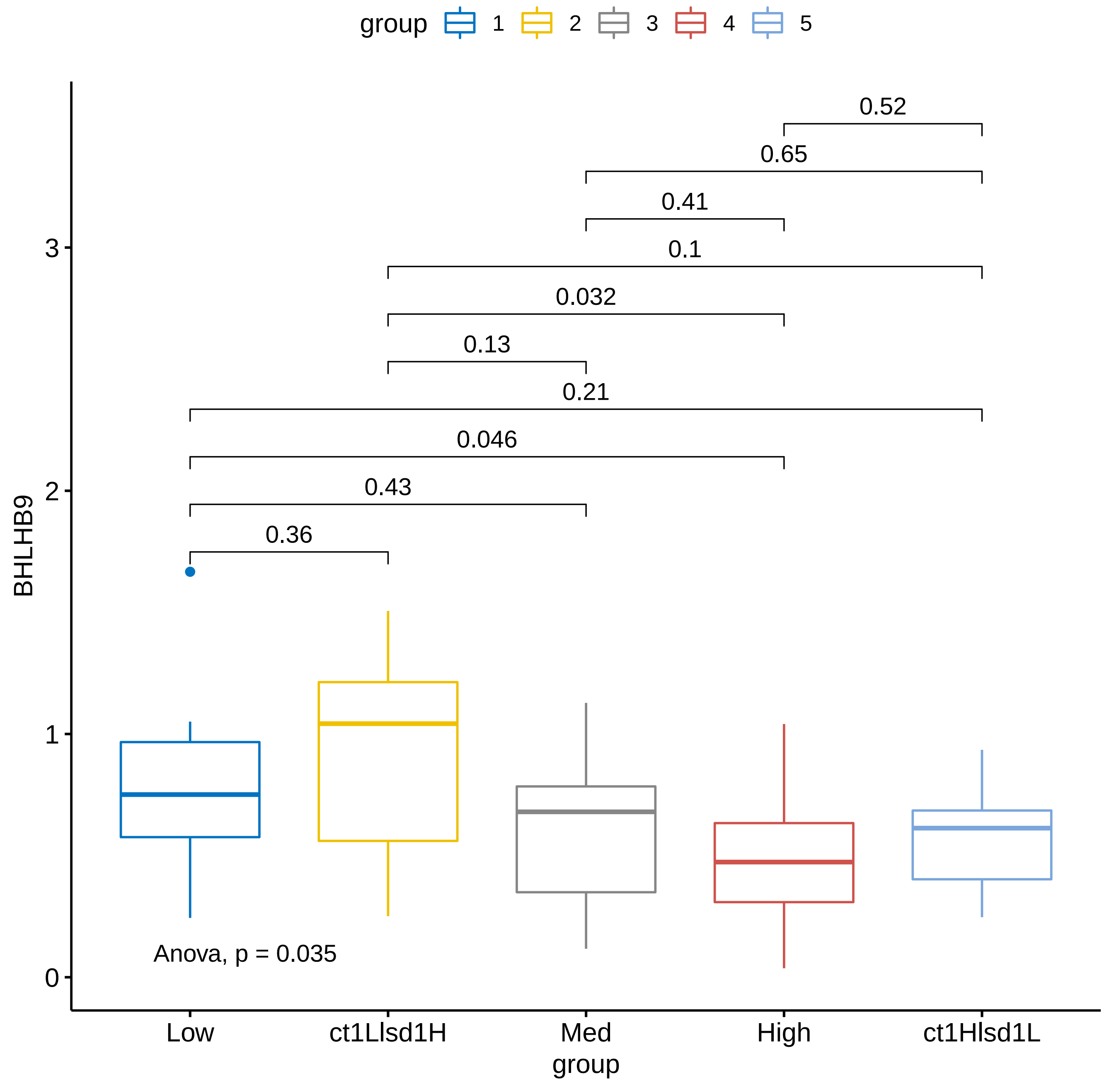

group
1
2
3
4
5
0.52
0.65
0.41
0.1
0.032
0.13
0.21
0.046
0.43
0.36
Anova, p = 0.035
BHLHB9
3
2
1
0
Low
ct1Llsd1H
Med
High
ct1Hlsd1L
group

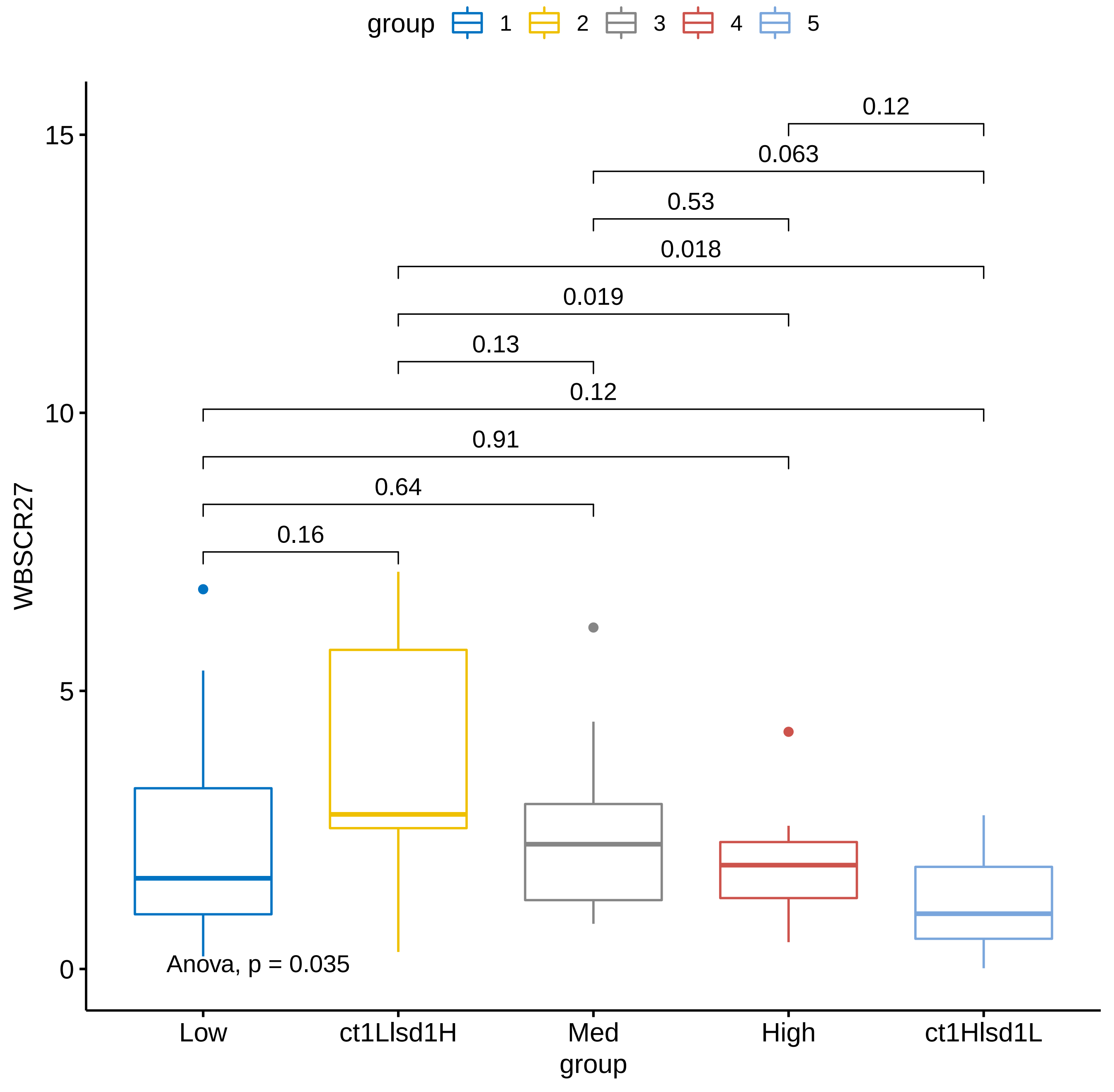

group 1 2 3 4 5
WBSCR27
0.12
0.063
0.53
0.018
0.019
0.13
0.12
0.91
0.64
0.16
Anova, p = 0.035
15
10
5
0
Low
ct1Llsd1H
Med
High
ct1Hlsd1L
group

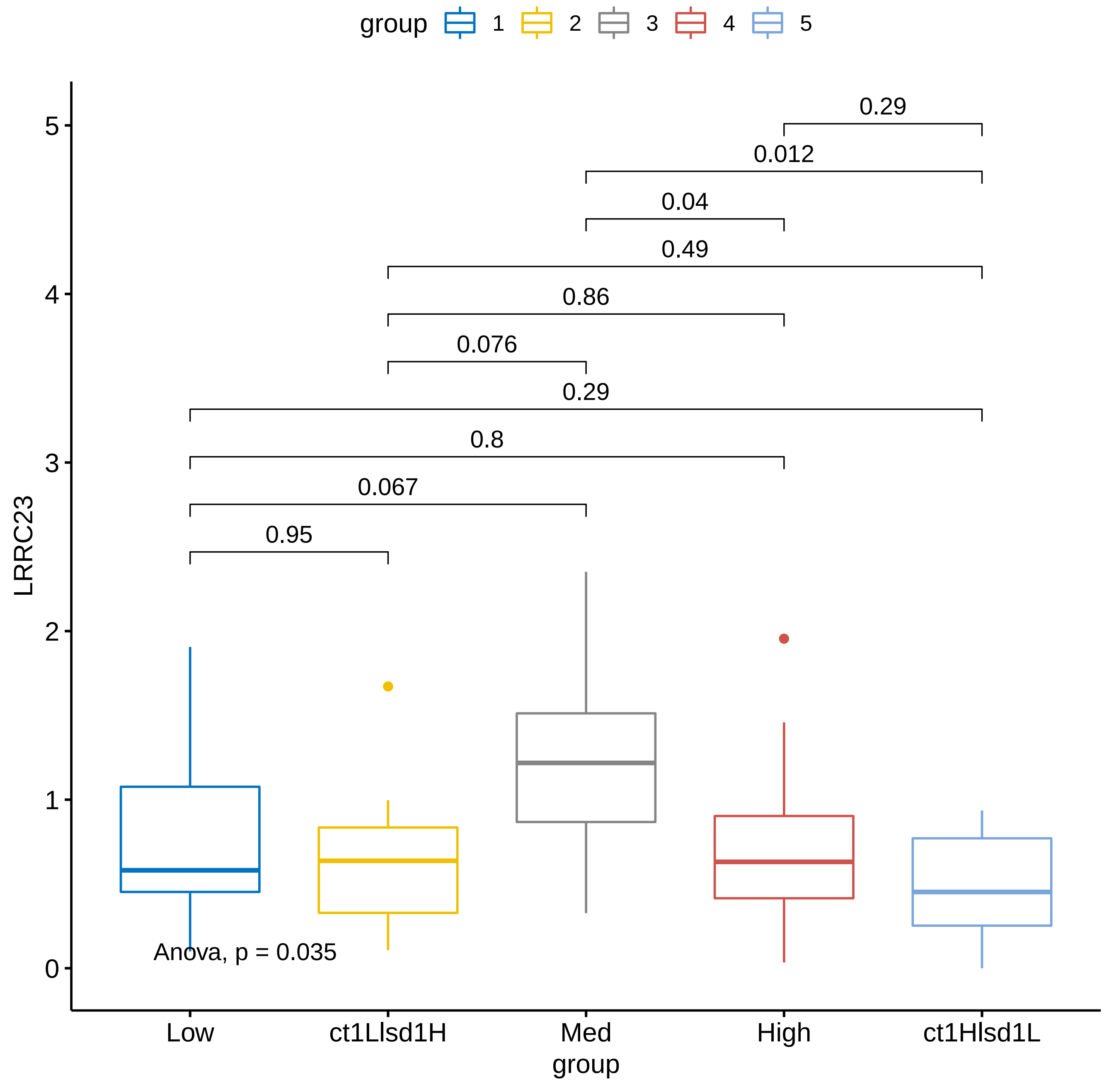

group
1
2
3
4
5
LRRC23
0
1
2
3
4
5
0.29
0.012
0.04
0.49
0.86
0.076
0.29
0.8
0.067
0.95
Anova, p = 0.035
Low
ct1Llsd1H
Med
High
ct1Hlsd1L
group

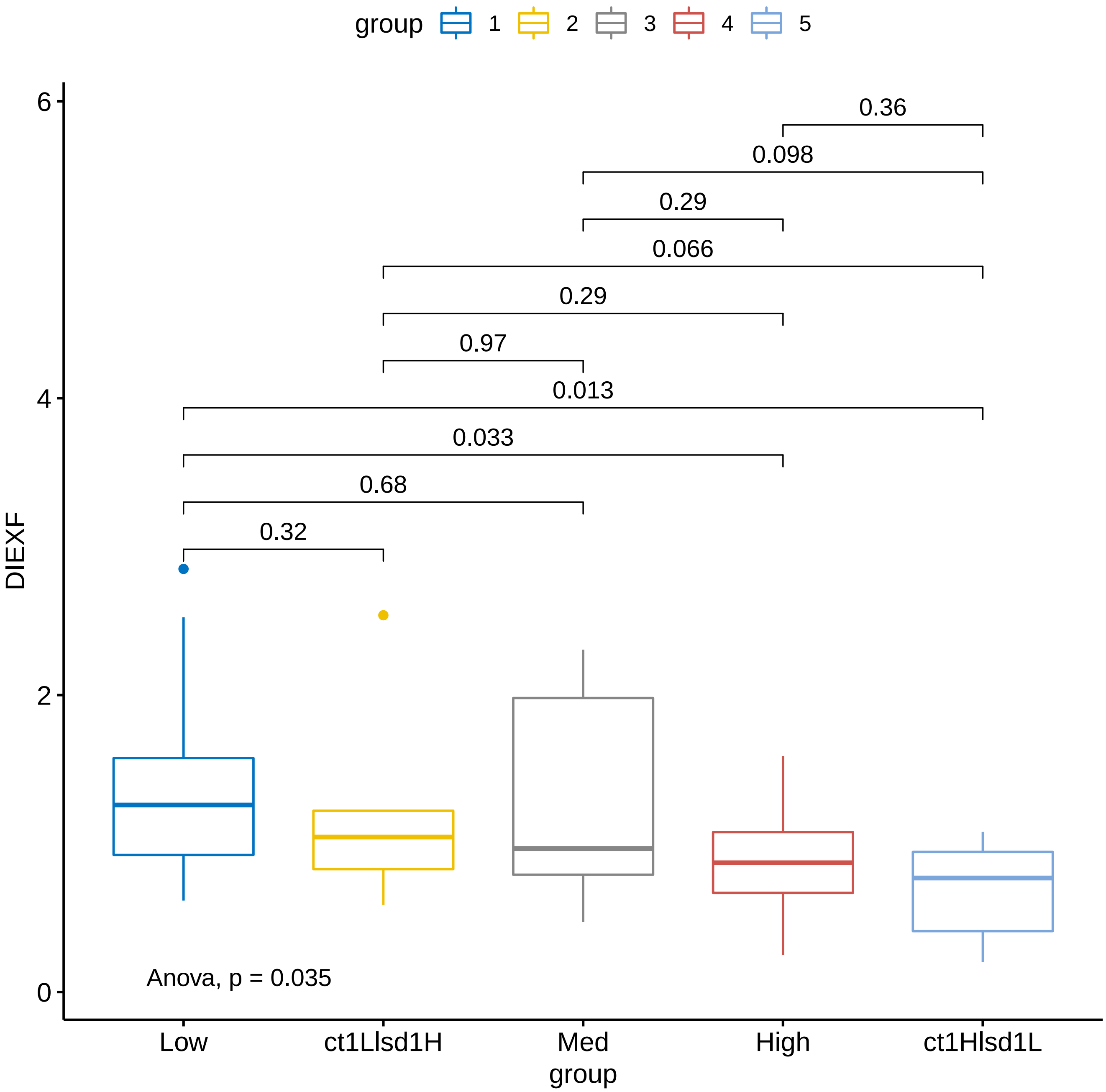
group 1 2 3 4 5
DIEXF
6
4
2
0
0.36
0.098
0.29
0.066
0.29
0.97
0.013
0.033
0.68
0.32
Anova, p = 0.035
Low
ct1Llsd1H
Med
High
ct1Hlsd1L
group

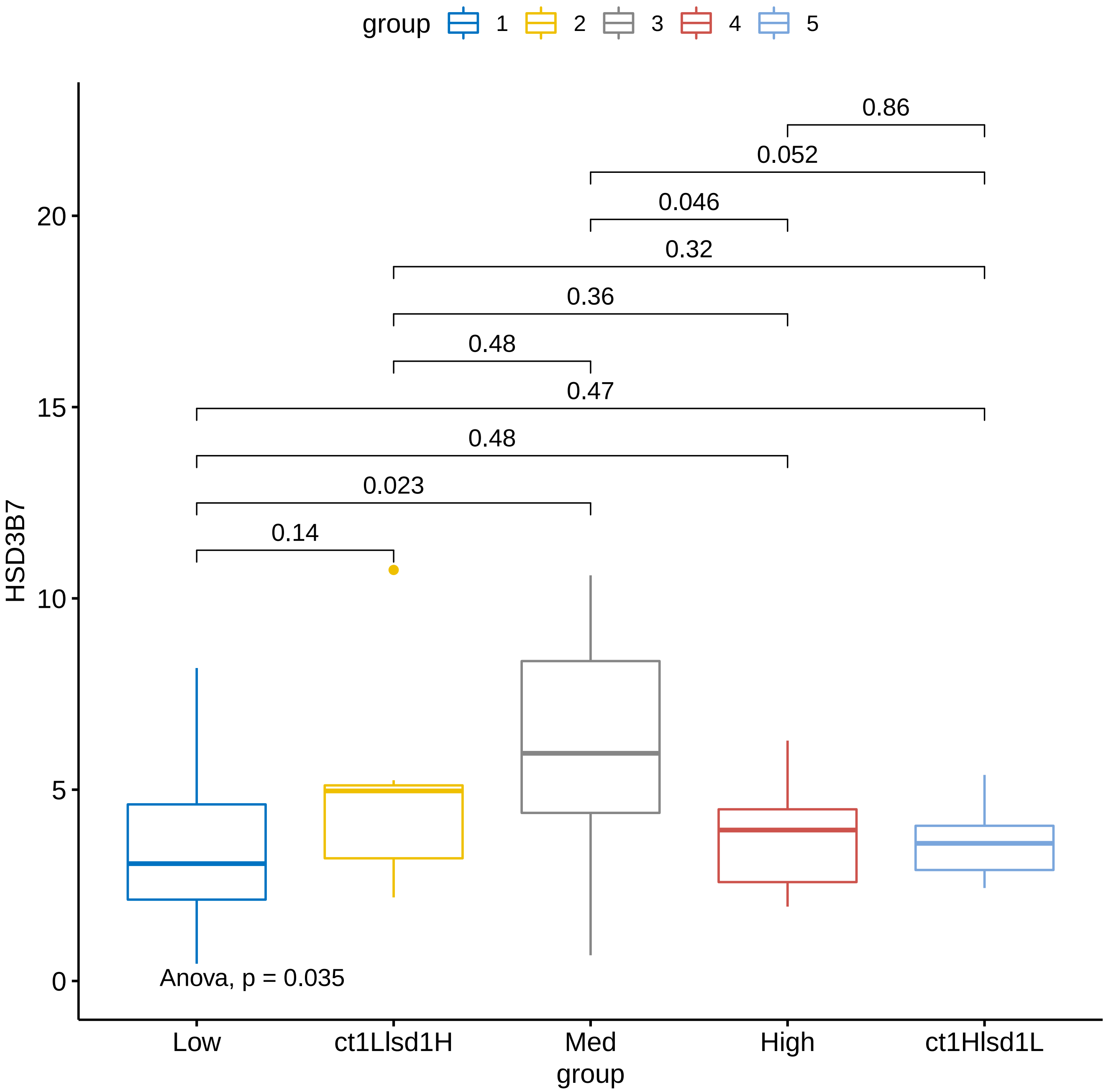

group 1 2 3 4 5
0.86
0.052
0.046
0.32
0.36
0.48
0.47
0.48
0.023
0.14
Anova, p = 0.035
HSD3B7
20
15
10
5
0
Low
ct1Llsd1H
Med
High
ct1Hlsd1L
group

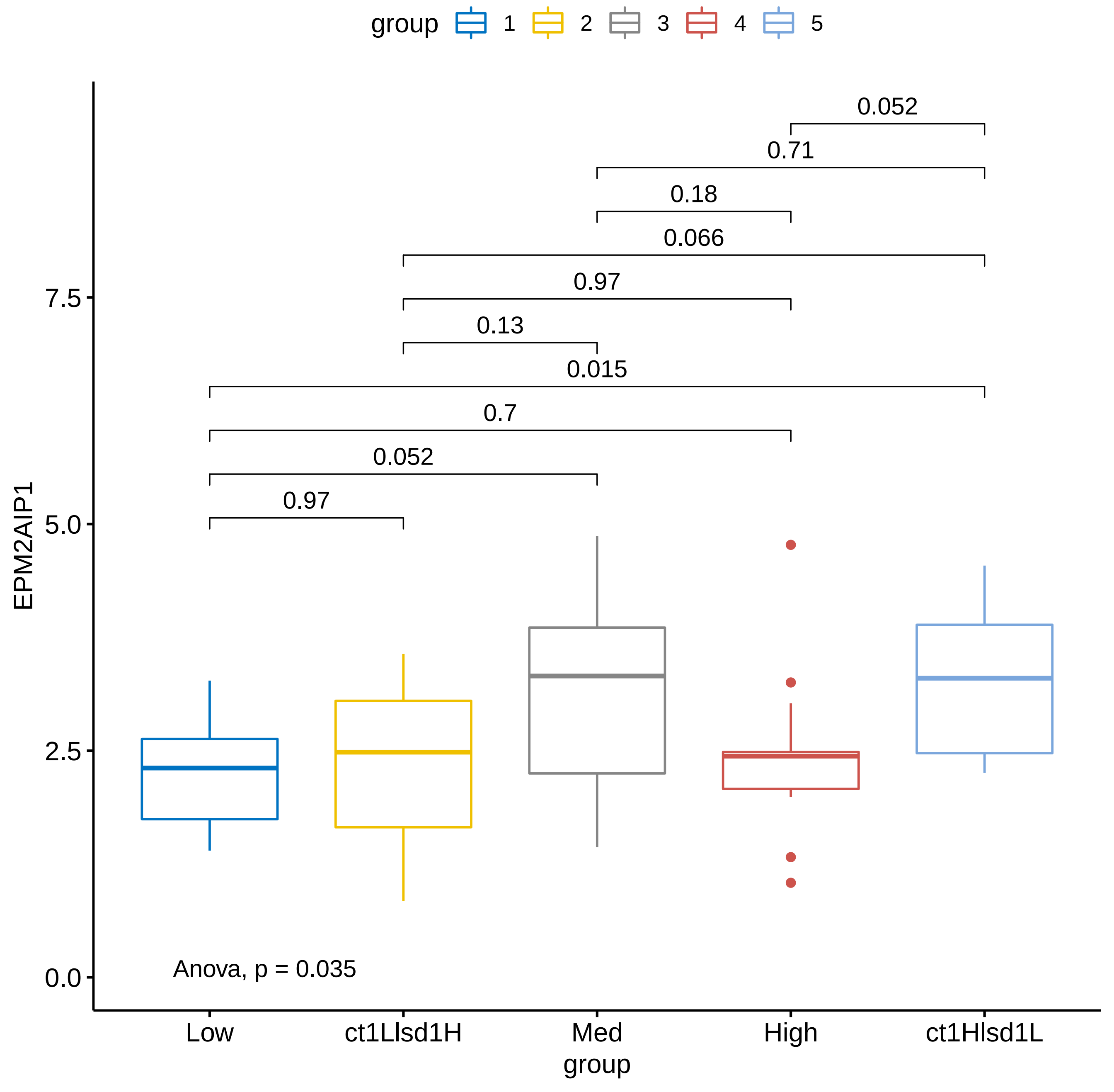

group
1
2
3
4
5
0.052
0.71
0.18
0.066
0.97
0.13
0.015
0.7
0.052
0.97
7.5
5.0
2.5
0.0
EPM2AIP1
Anova, p = 0.035
Low
ct1Llsd1H
Med
High
ct1Hlsd1L
group

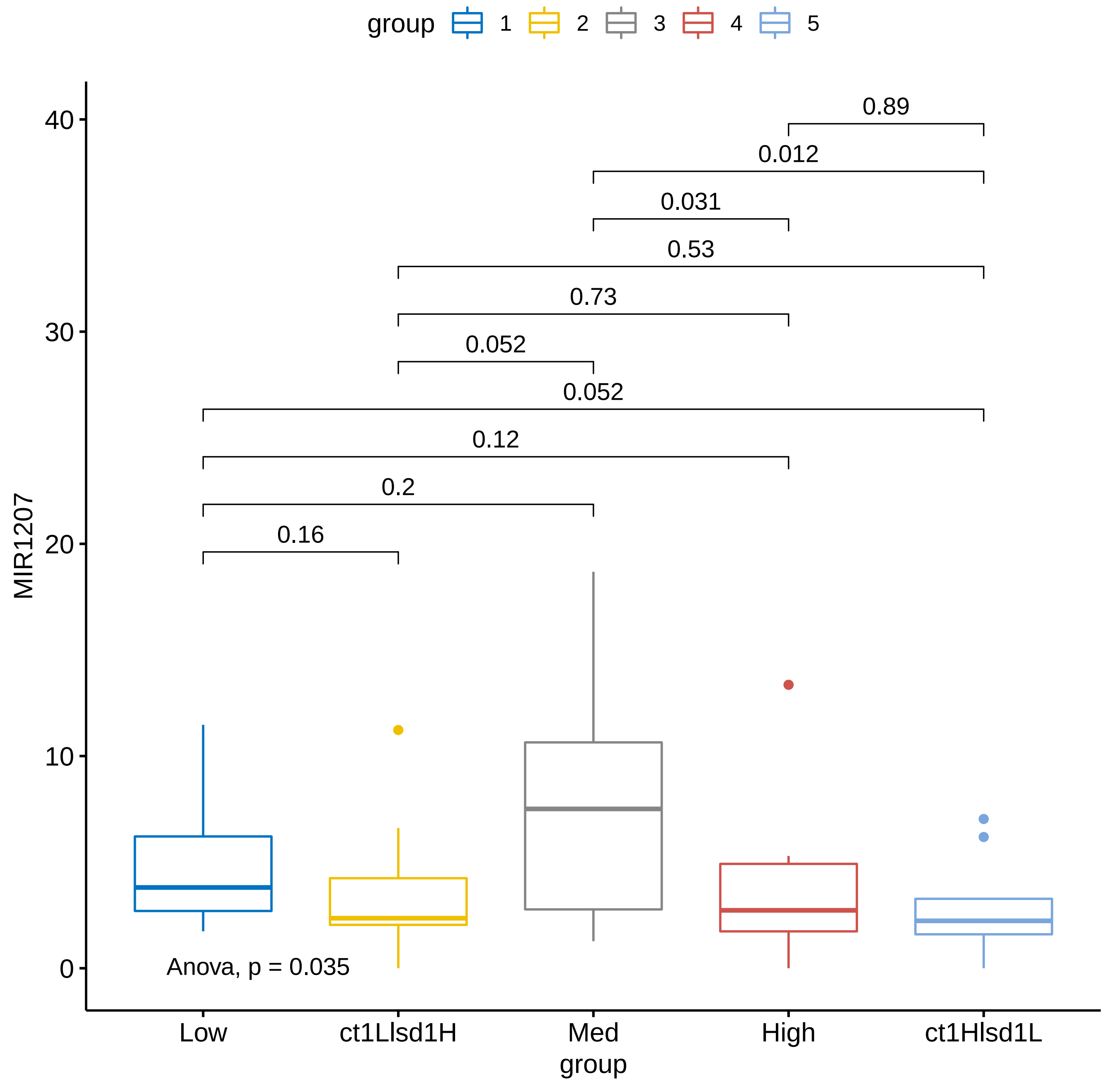

group
1
2
3
4
5
MIR1207
40
30
20
10
0
0.89
0.012
0.031
0.53
0.73
0.052
0.052
0.12
0.2
0.16
Anova, p = 0.035
Low
ct1Llsd1H
Med
High
ct1Hlsd1L
group

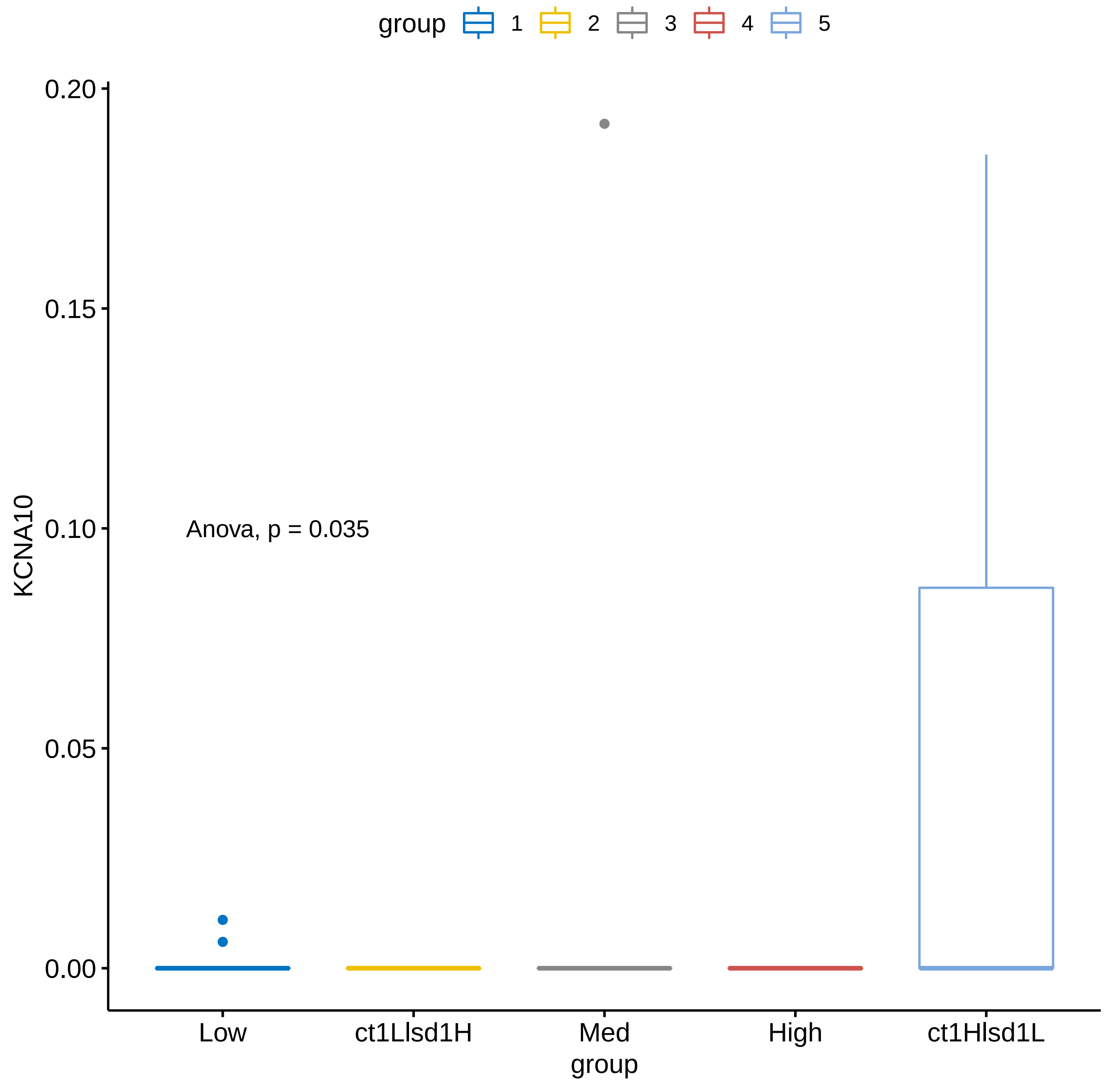

group 1 2 3 4 5
0.20
0.15
0.10
0.05
0.00
KCNA10
Anova, p = 0.035
Low
ct1Llsd1H
Med
High
ct1Hlsd1L
group

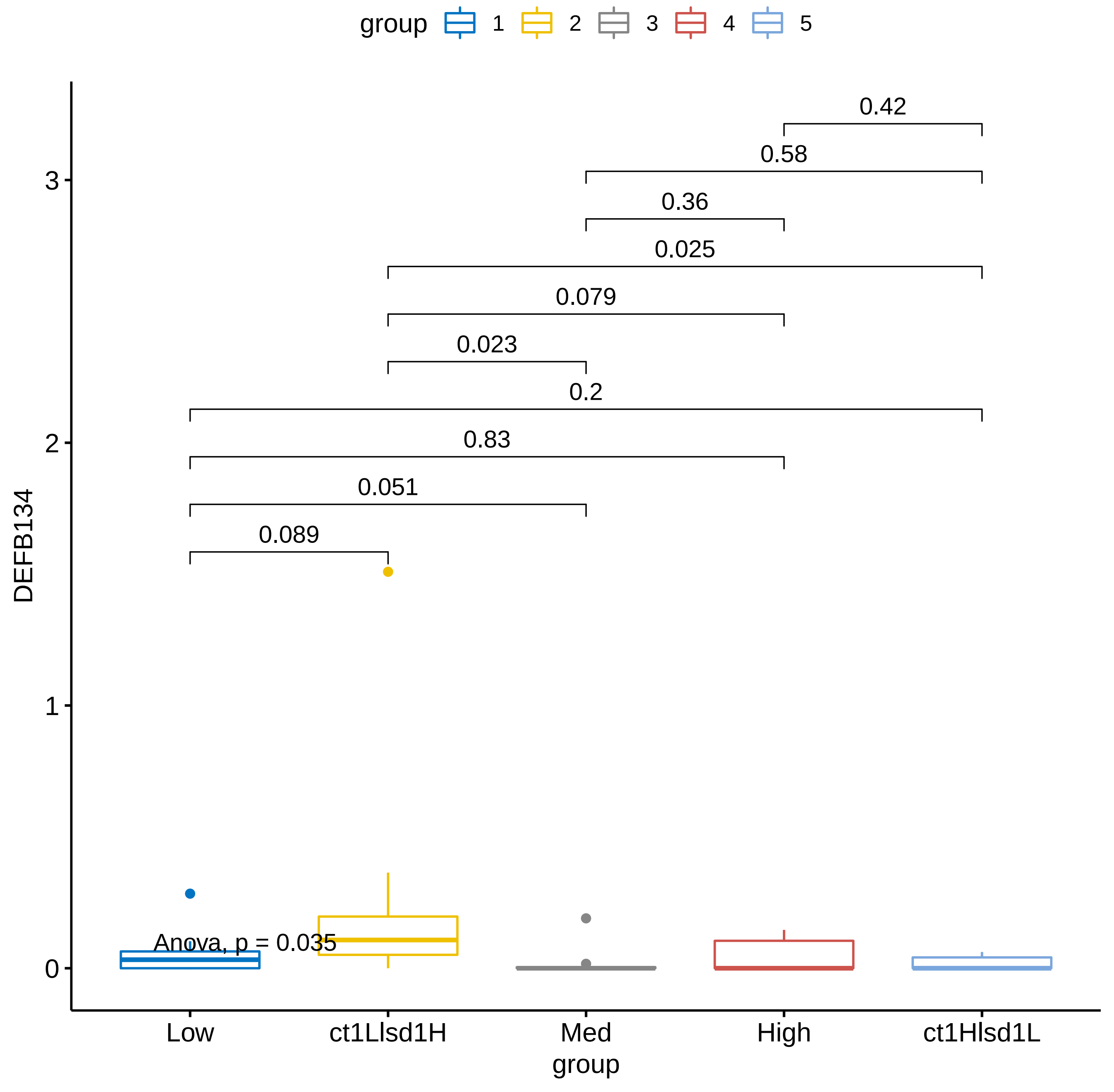

group 1 2 3 4 5
0.42
0.58
0.36
0.025
0.079
0.023
0.2
0.83
0.051
0.089
Anova, p = 0.035
3
2
1
0
DEFB134
Low
ct1Llsd1H
Med
High
ct1Hlsd1L
group

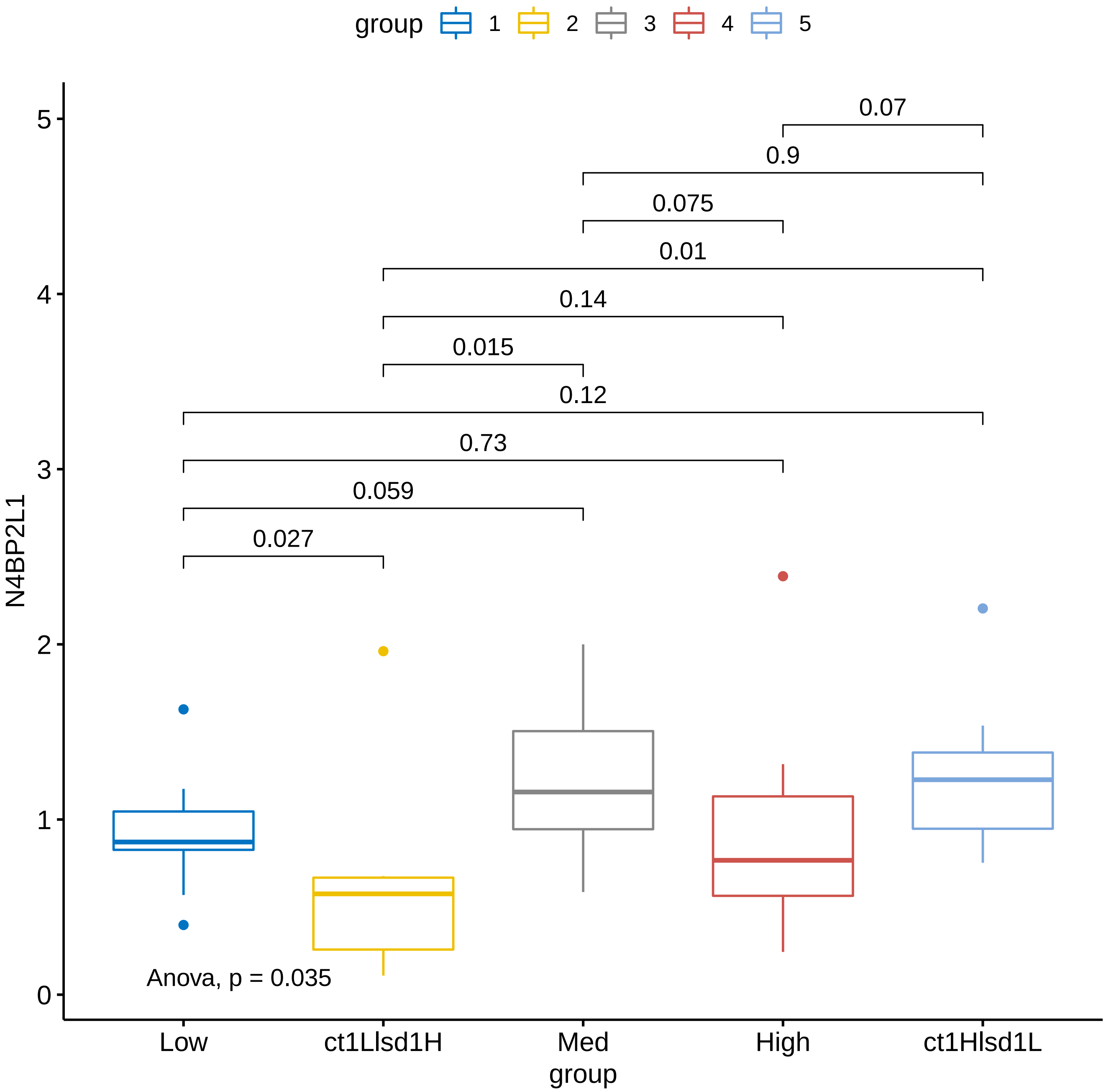
group
1
2
3
4
5
N4BP2L1
0.07
0.9
0.075
0.01
0.14
0.015
0.12
0.73
0.059
0.027
Anova, p = 0.035
0
1
2
3
4
5
Low
ct1Llsd1H
Med
High
ct1Hlsd1L
group

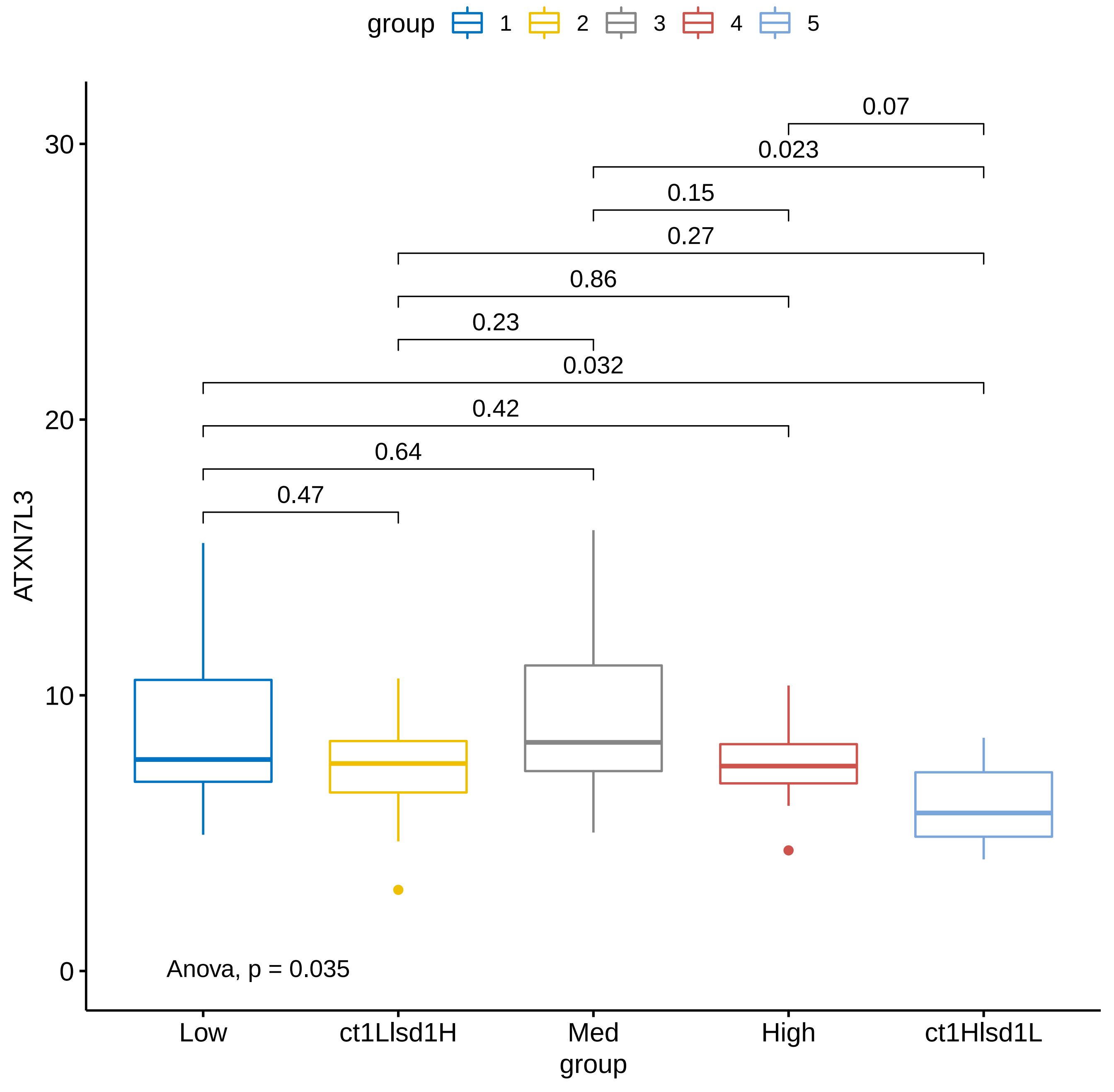
group 1 2 3 4 5
0.07
0.023
0.15
0.27
0.86
0.23
0.032
0.42
0.64
0.47
30
20
10
0
ATXN7L3
Anova, p = 0.035
Low
ct1Llsd1H
Med
High
ct1Hlsd1L
group

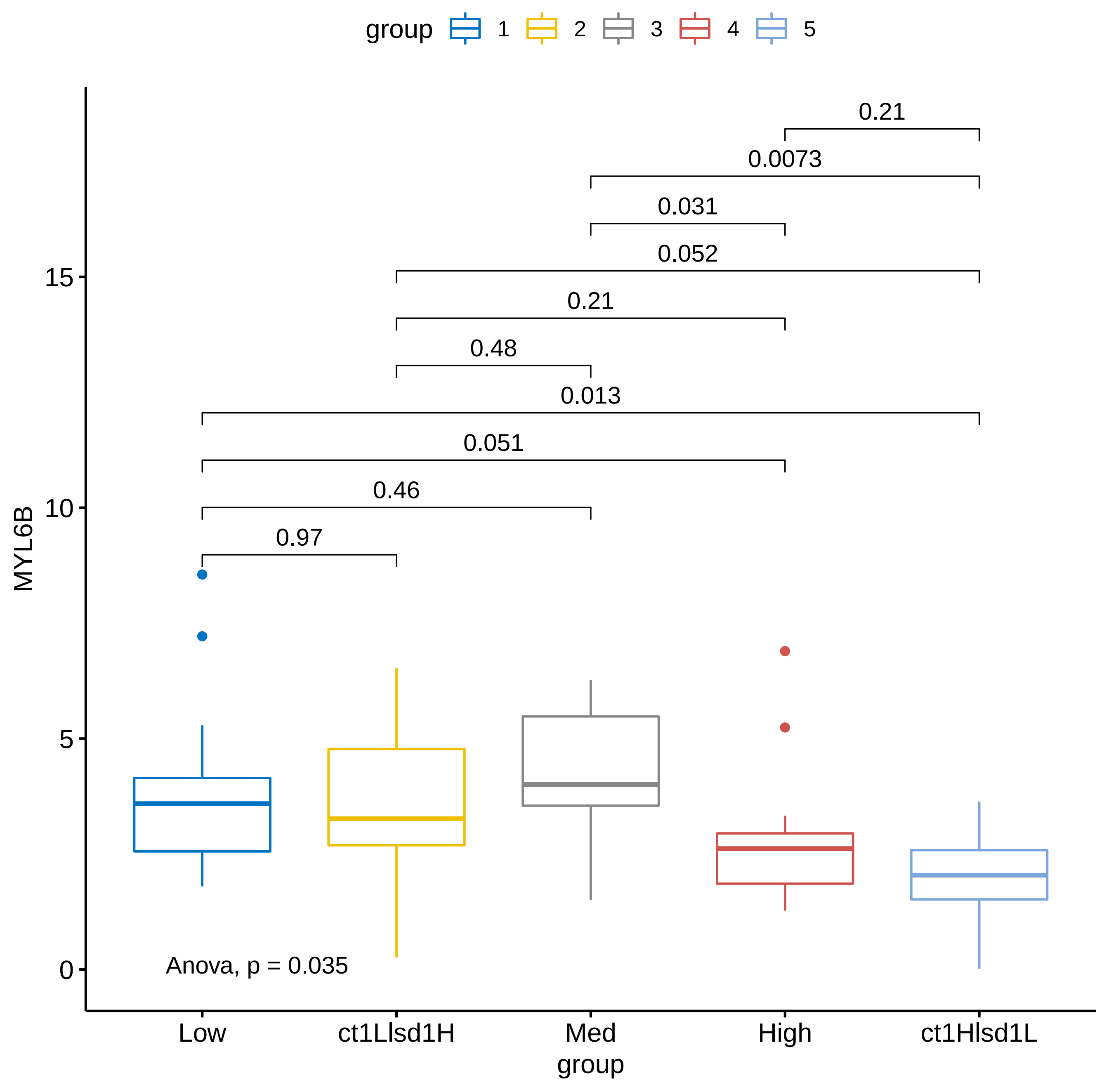

group 1 2 3 4 5
MYL6B
0.21
0.0073
0.031
0.052
0.21
0.48
0.013
0.051
0.46
0.97
Anova, p = 0.035
15
10
5
0
Low
ct1Llsd1H
Med
High
ct1Hlsd1L
group

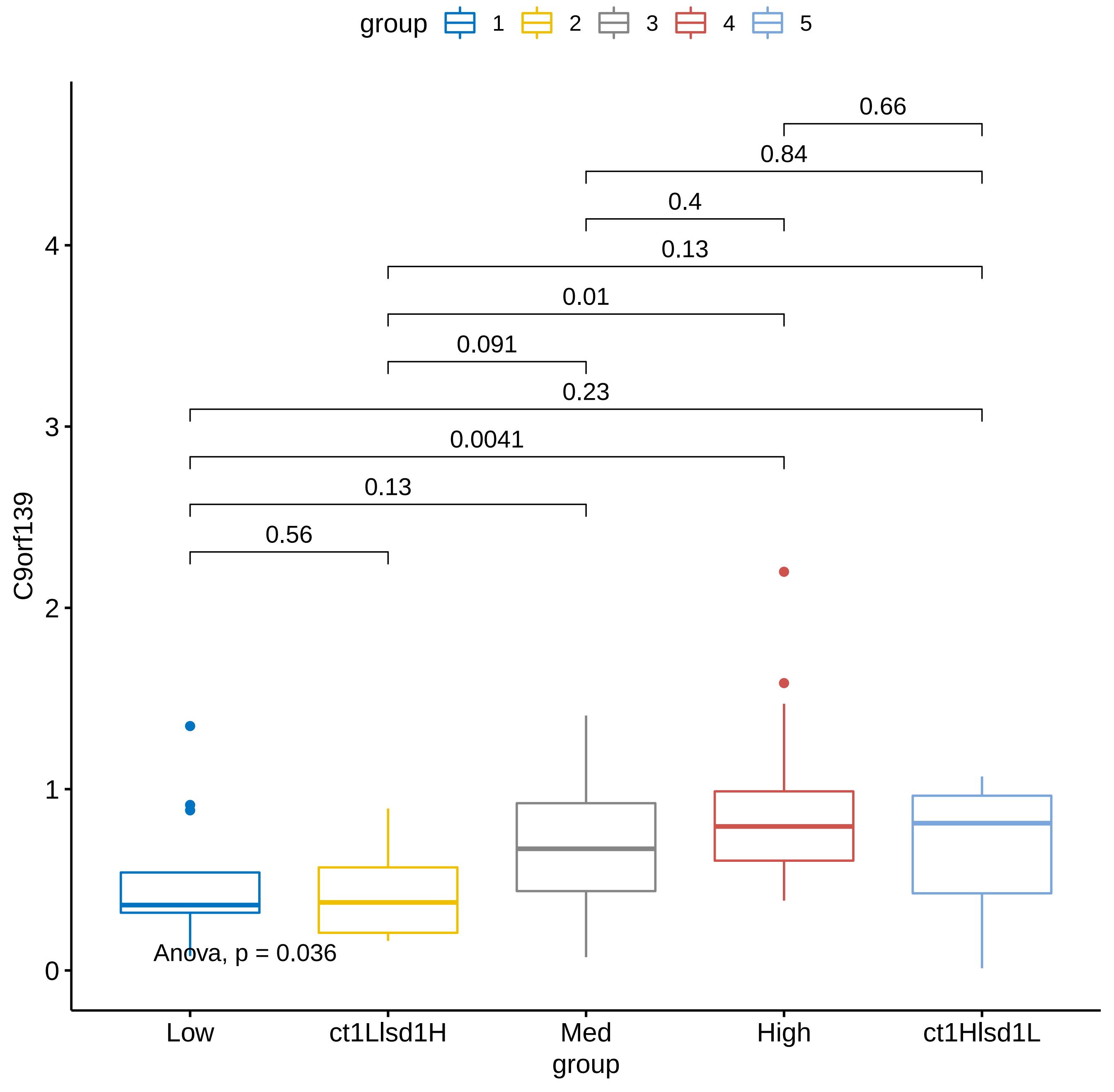

group
1
2
3
4
5
0.66
0.84
0.4
0.13
0.01
0.091
0.23
0.0041
0.13
0.56
Anova, p = 0.036
C9orf139
0
1
2
3
4
Low
ct1Llsd1H
Med
High
ct1Hlsd1L
group

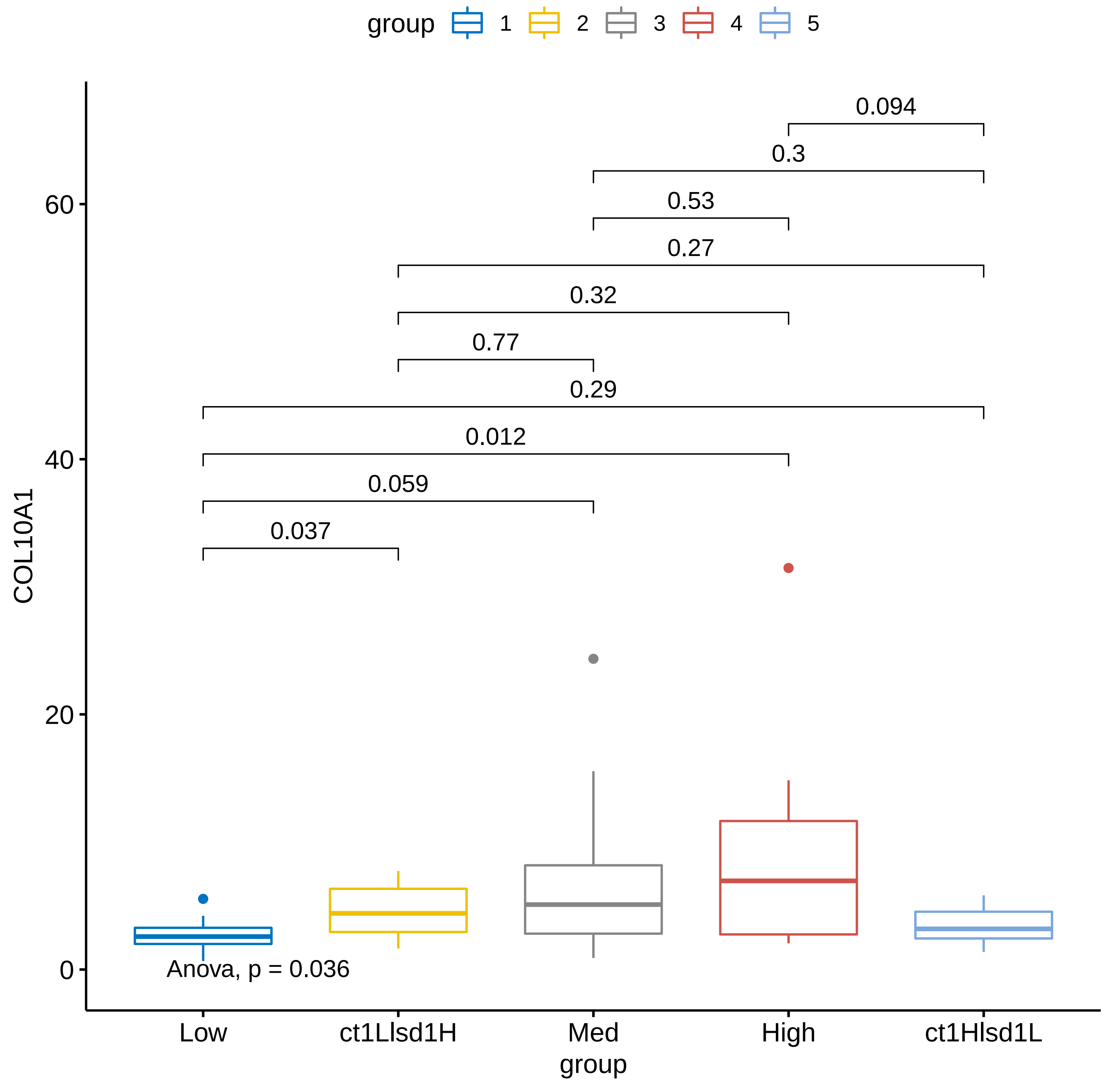

group
1
2
3
4
5
0.094
0.3
0.53
0.27
0.32
0.77
0.29
0.012
0.059
0.037
60
40
20
0
COL10A1
Anova, p = 0.036
Low
ct1Llsd1H
Med
High
ct1Hlsd1L
group

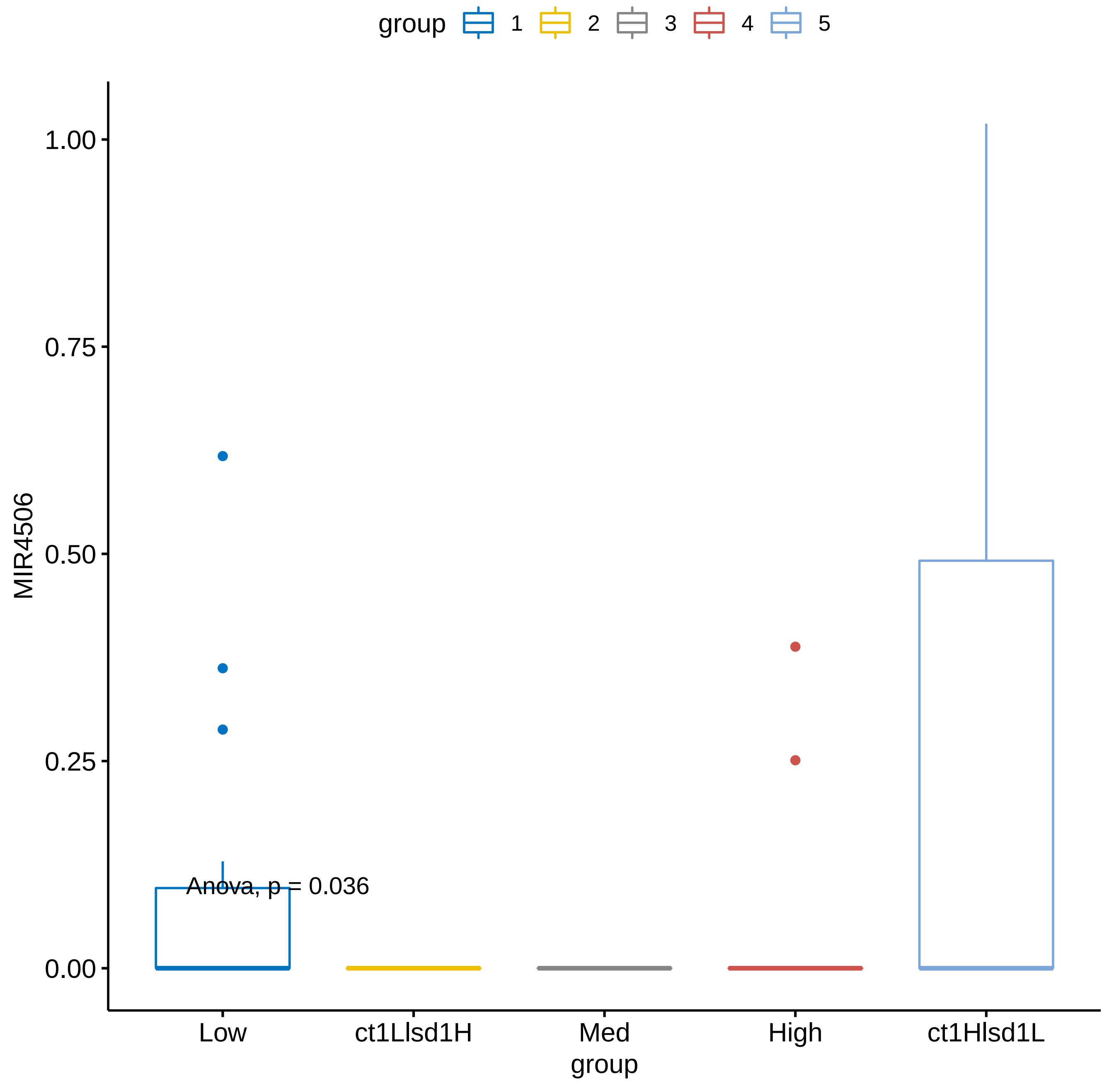

group
1
2
3
4
5
1.00
0.75
0.50
0.25
0.00
MIR4506
Anova, p = 0.036
Low
ct1Llsd1H
Med
High
ct1Hlsd1L
group

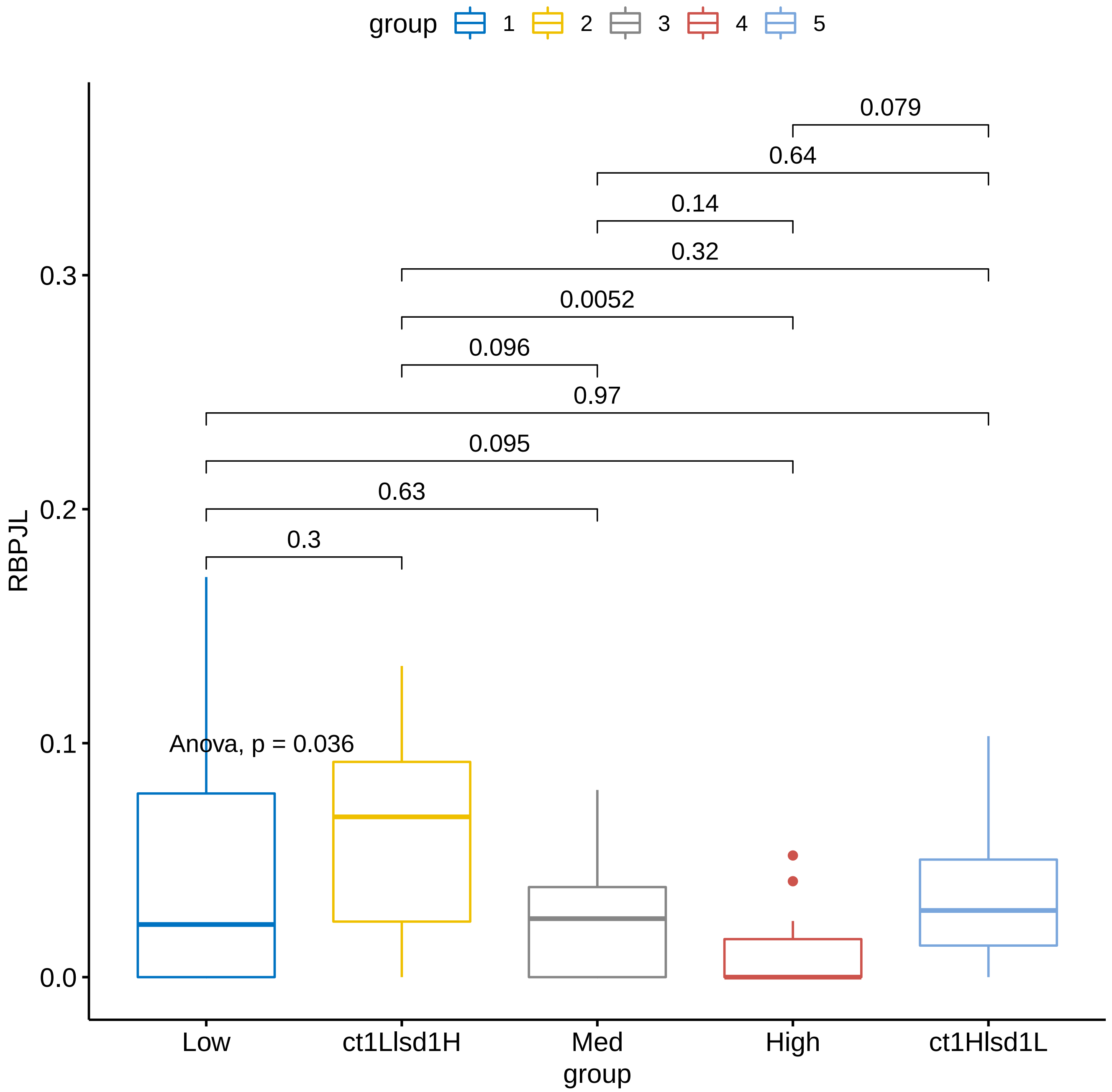

group 1 2 3 4 5
0.079
0.64
0.14
0.32
0.0052
0.096
0.97
0.095
0.63
0.3
Anova, p = 0.036
RBPJL
0.3
0.2
0.1
0.0
Low
ct1Llsd1H
Med
High
ct1Hlsd1L
group

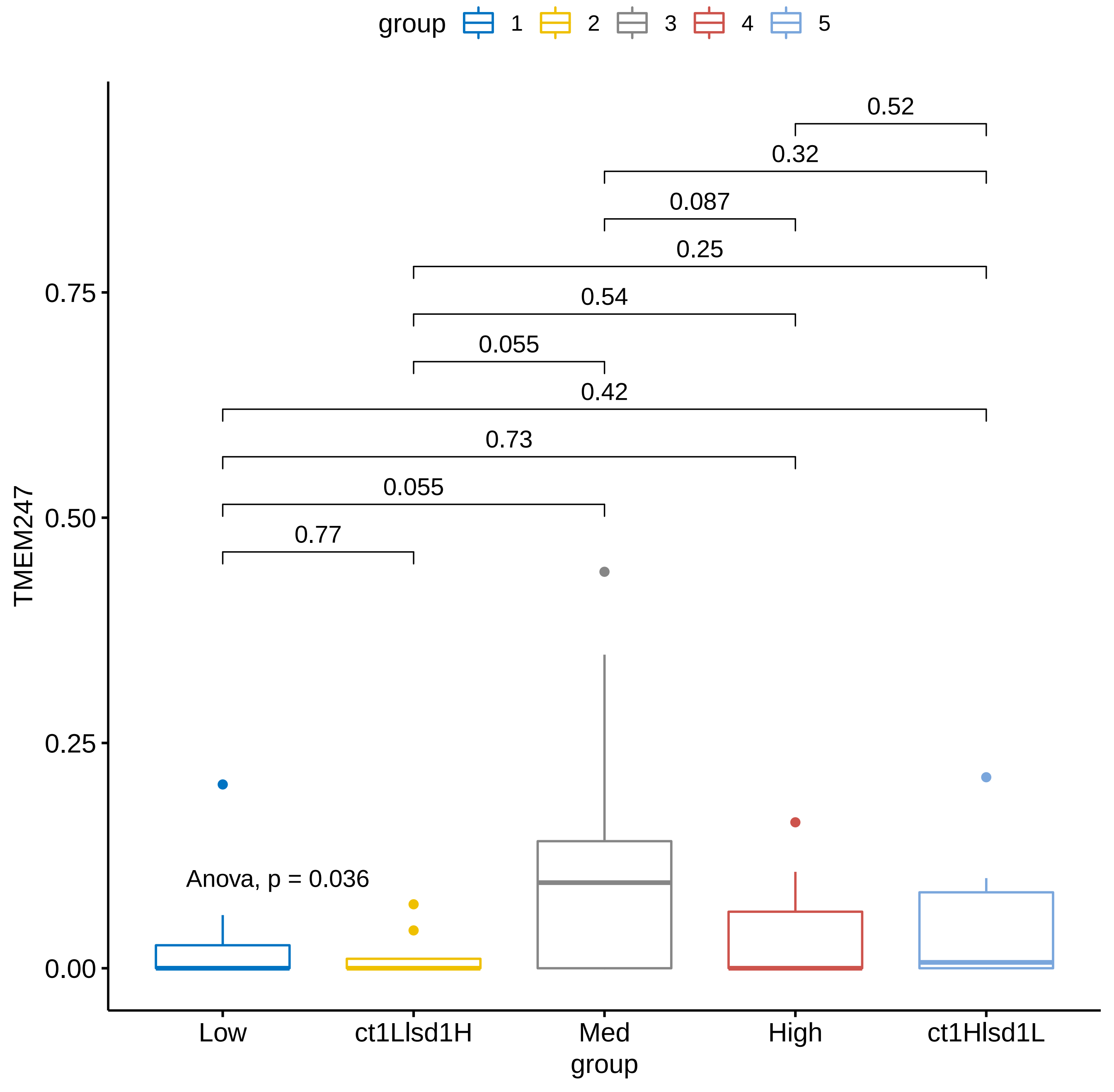

group 1 2 3 4 5
0.52
0.32
0.087
0.25
0.54
0.055
0.42
0.73
0.055
0.77
Anova, p = 0.036
0.75
0.50
0.25
0.00
TMEM247
Low
ct1Llsd1H
Med
High
ct1Hlsd1L
group

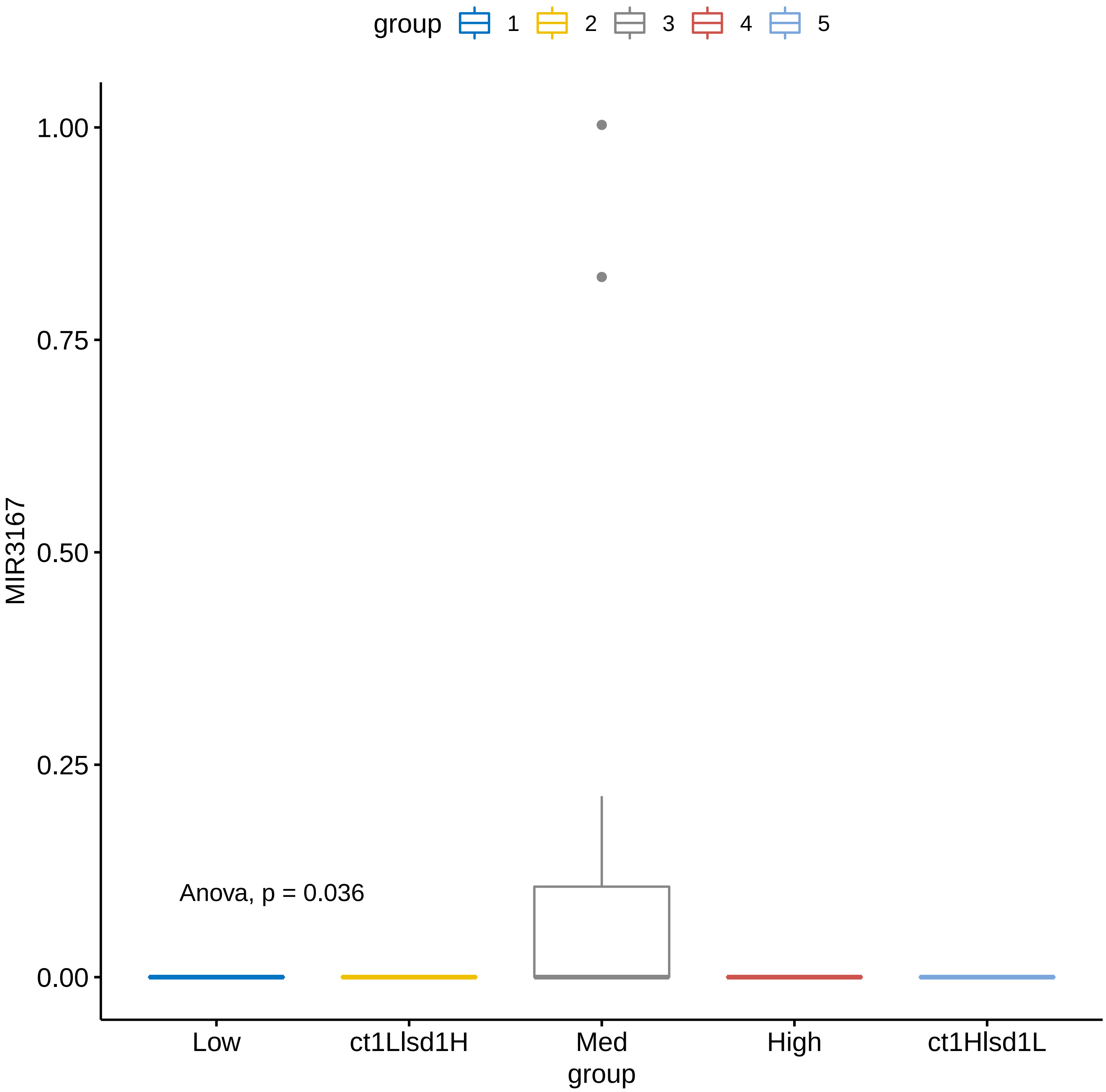

group
1
2
3
4
5
1.00
0.75
0.50
0.25
0.00
MIR3167
Anova, p = 0.036
Low
ct1Llsd1H
Med
High
ct1Hlsd1L
group

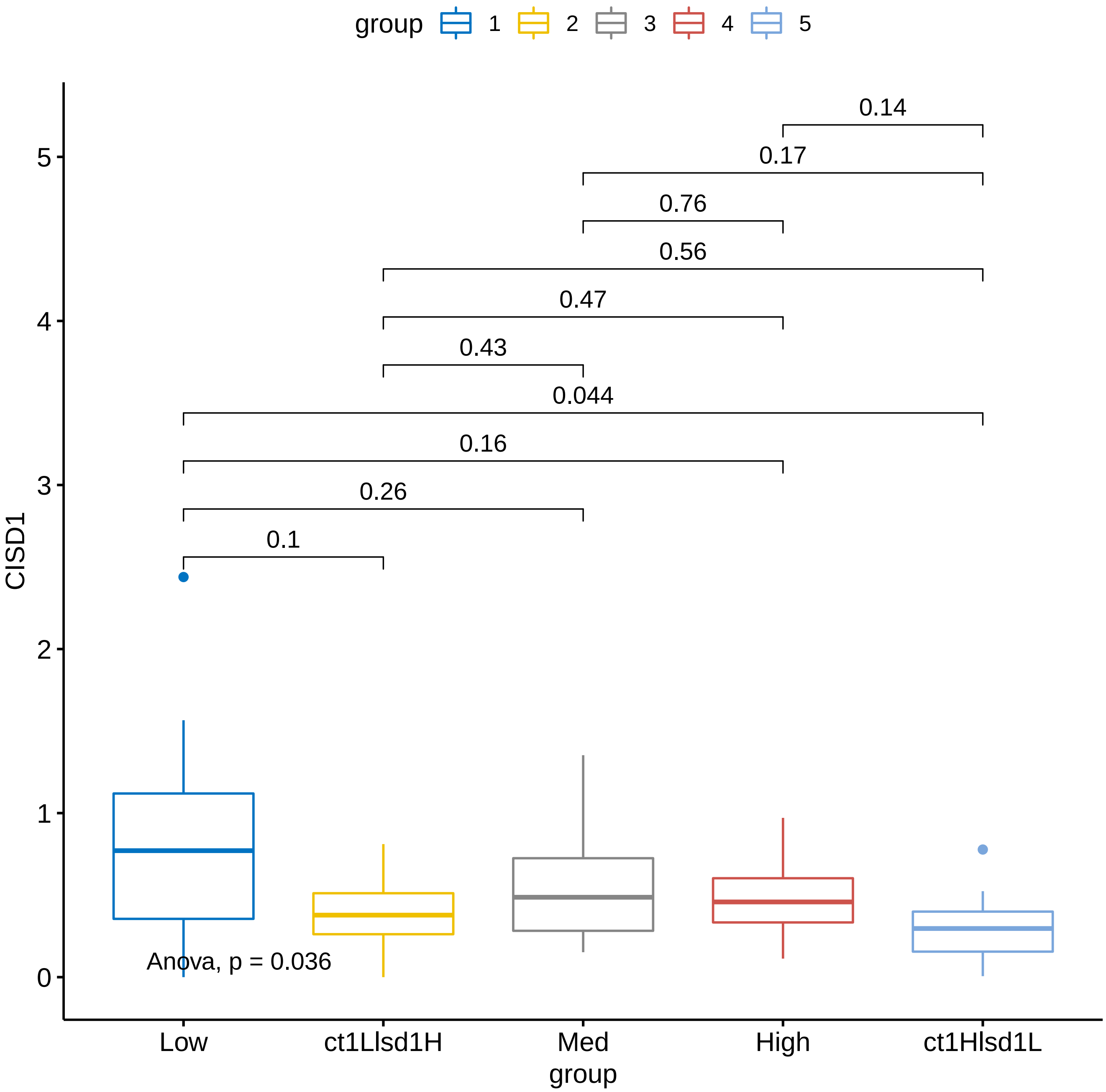

group
1
2
3
4
5
0.14
0.17
0.76
0.56
0.47
0.43
0.044
0.16
0.26
0.1
Anova, p = 0.036
CISD1
0
1
2
3
4
5
Low
ct1Llsd1H
Med
High
ct1Hlsd1L
group

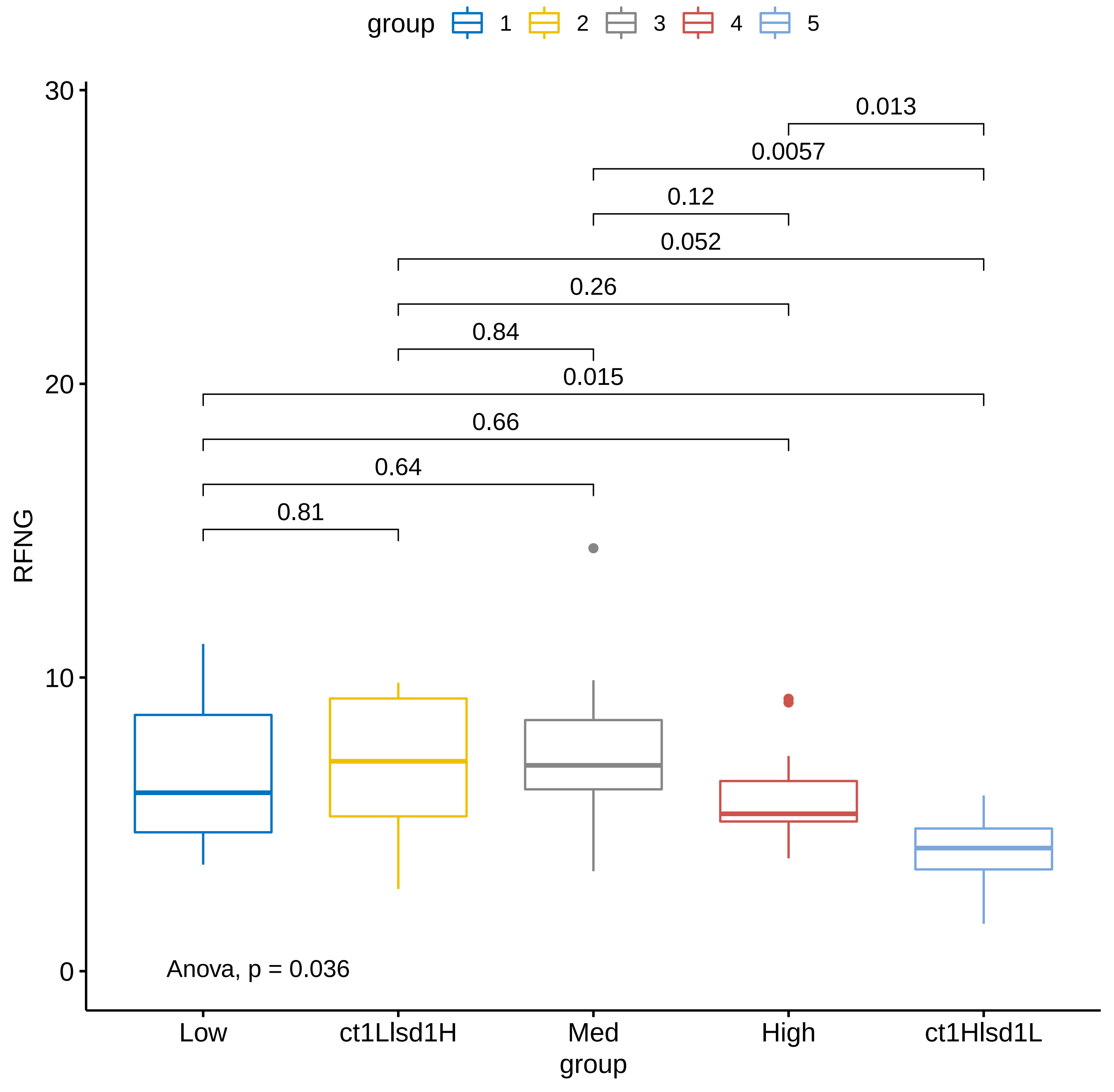
group
1
2
3
4
5
30
20
10
0
RFNG
0.013
0.0057
0.12
0.052
0.26
0.84
0.015
0.66
0.64
0.81
Anova, p = 0.036
Low
ct1Llsd1H
Med
High
ct1Hlsd1L
group

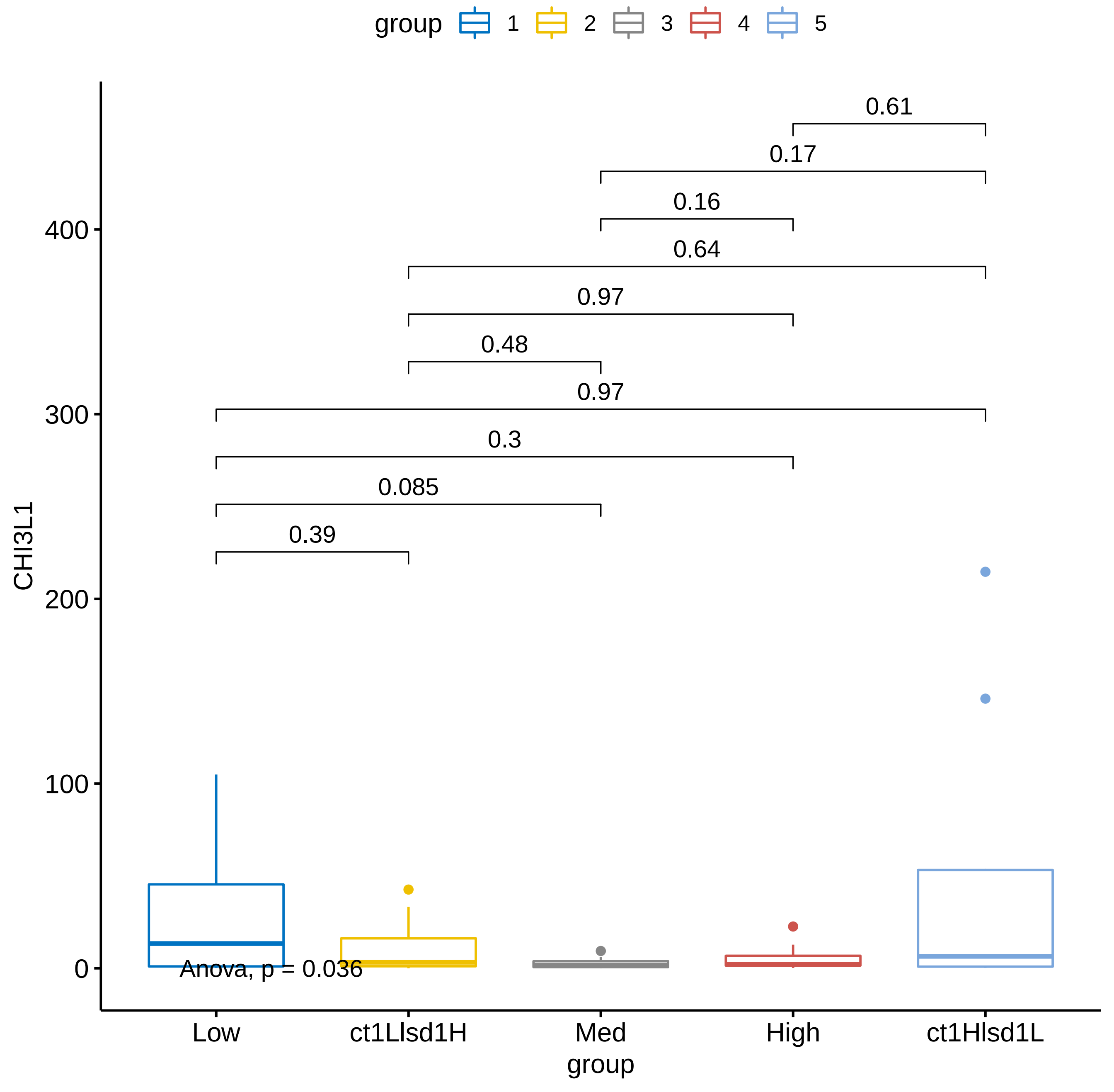

group
1
2
3
4
5
0.61
0.17
0.16
0.64
0.97
0.48
0.97
0.3
0.085
0.39
400
300
200
100
0
CHI3L1
Anova, p = 0.036
Low
ct1Llsd1H
Med
High
ct1Hlsd1L
group

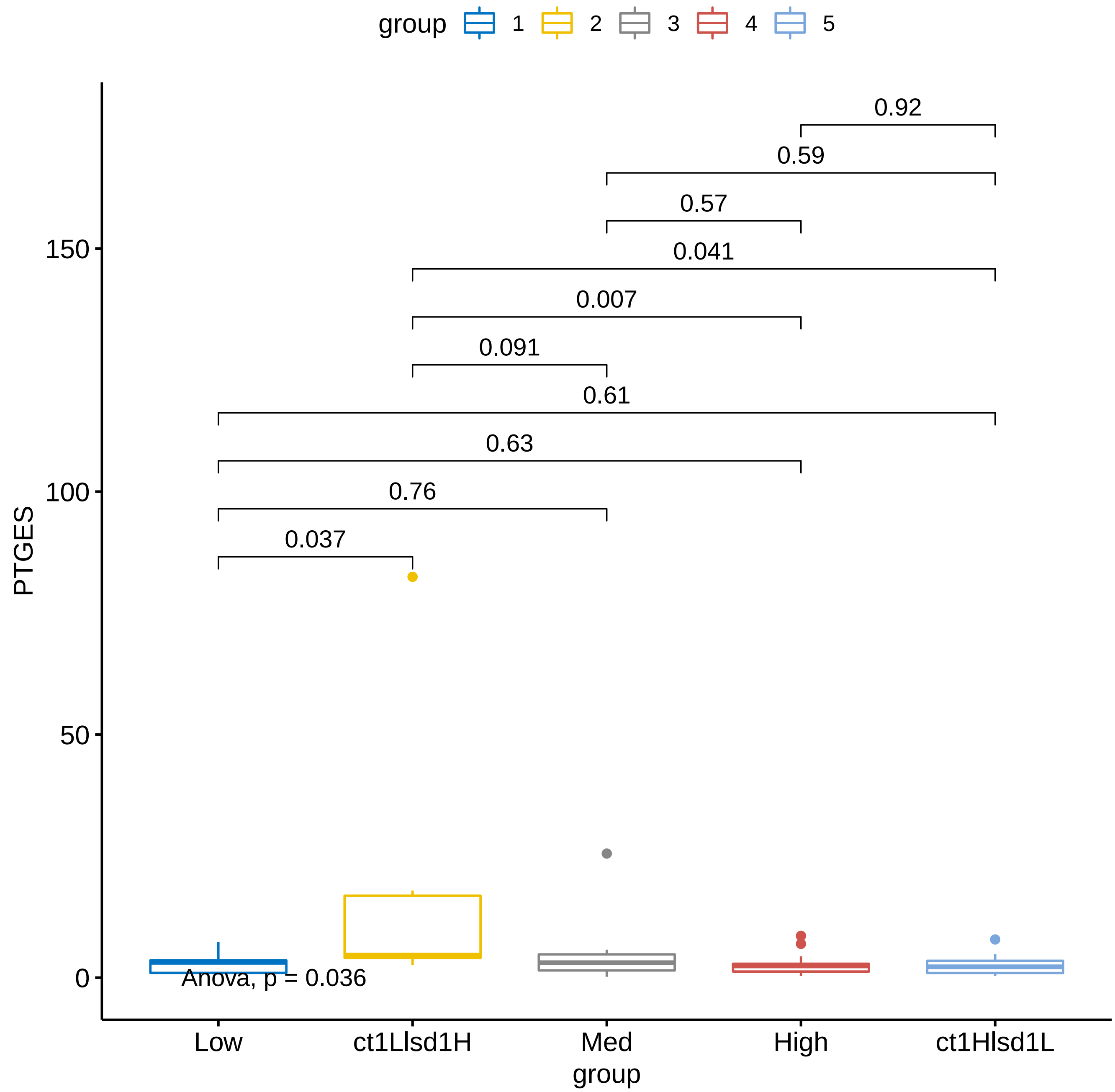

group 1 2 3 4 5
0.92
0.59
0.57
0.041
0.007
0.091
0.61
0.63
0.76
0.037
150
100
50
0
PTGES
Anova, p = 0.036
Low
ct1Llsd1H
Med
High
ct1Hlsd1L
group

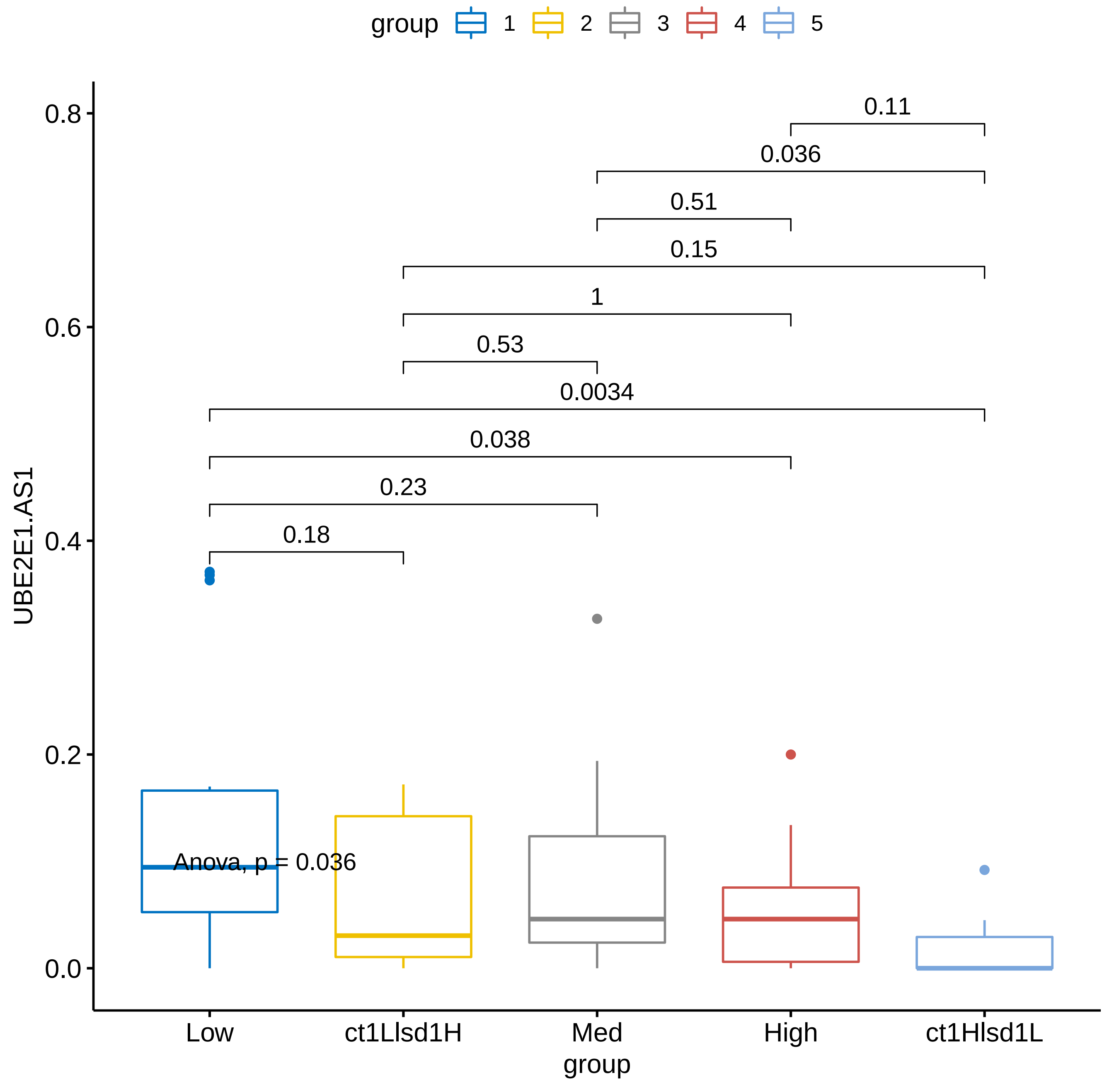

group 1 2 3 4 5
0.11
0.036
0.51
0.15
1
0.53
0.0034
0.038
0.23
0.18
Anova, p = 0.036
UBE2E1.AS1
0.8
0.6
0.4
0.2
0.0
Low
ct1Llsd1H
Med
High
ct1Hlsd1L
group

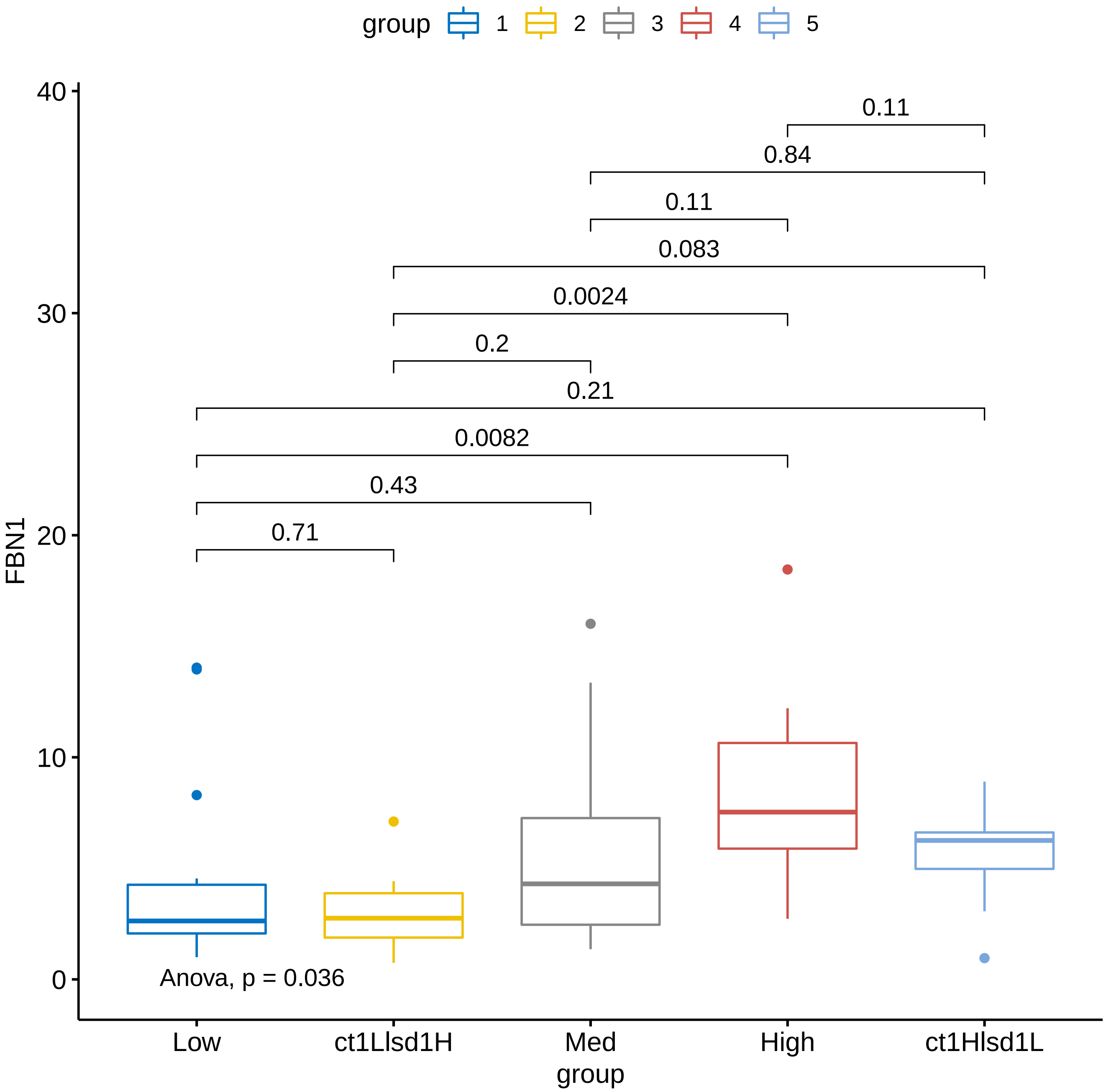

group
1
2
3
4
5
FBN1
40
30
20
10
0
0.11
0.84
0.11
0.083
0.0024
0.2
0.21
0.0082
0.43
0.71
Anova, p = 0.036
Low
ct1Llsd1H
Med
High
ct1Hlsd1L
group

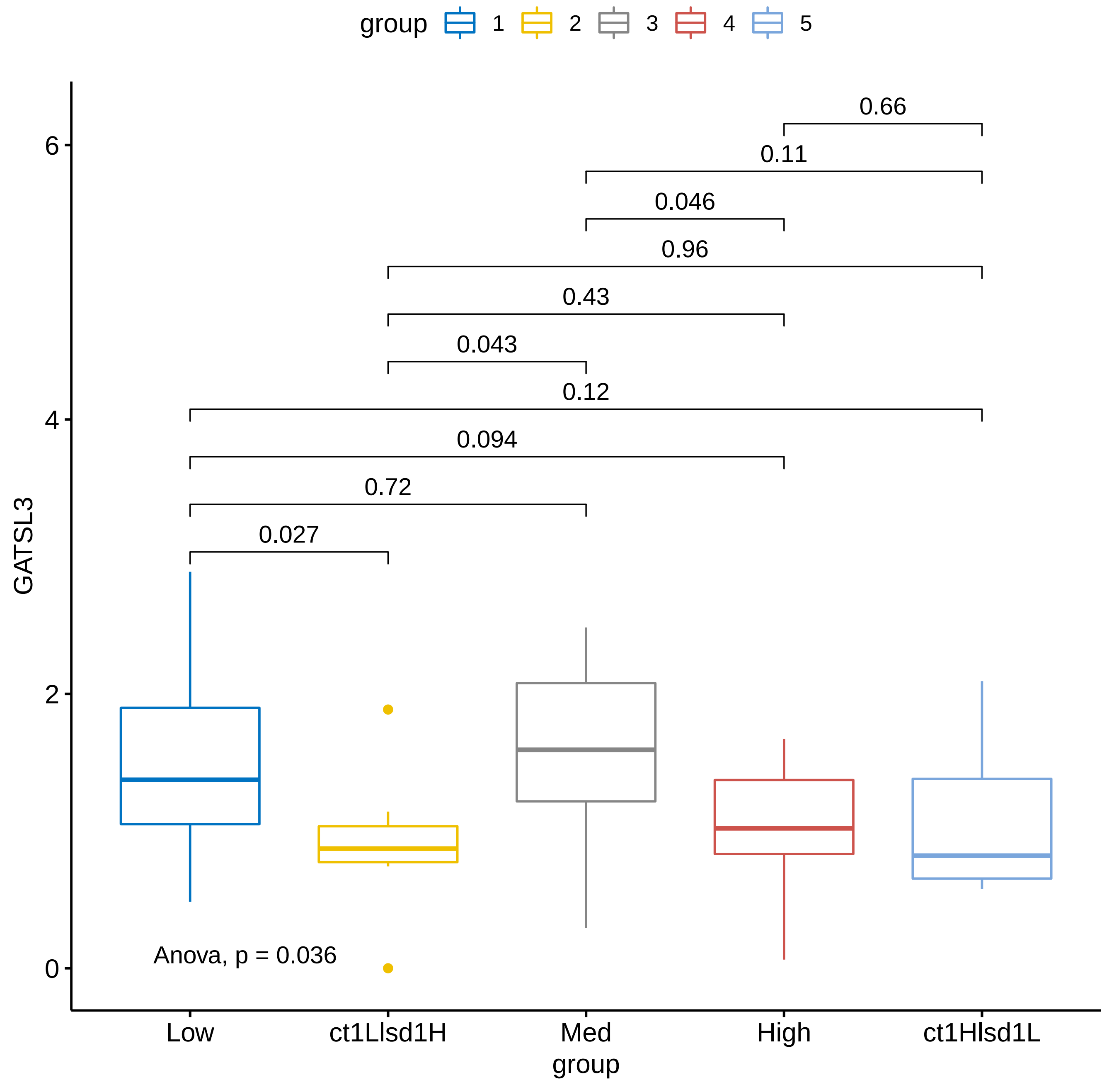
group
1
2
3
4
5
GATSL3
6
4
2
0
0.66
0.11
0.046
0.96
0.43
0.043
0.12
0.094
0.72
0.027
Anova, p = 0.036
Low
ct1Llsd1H
Med
High
ct1Hlsd1L
group

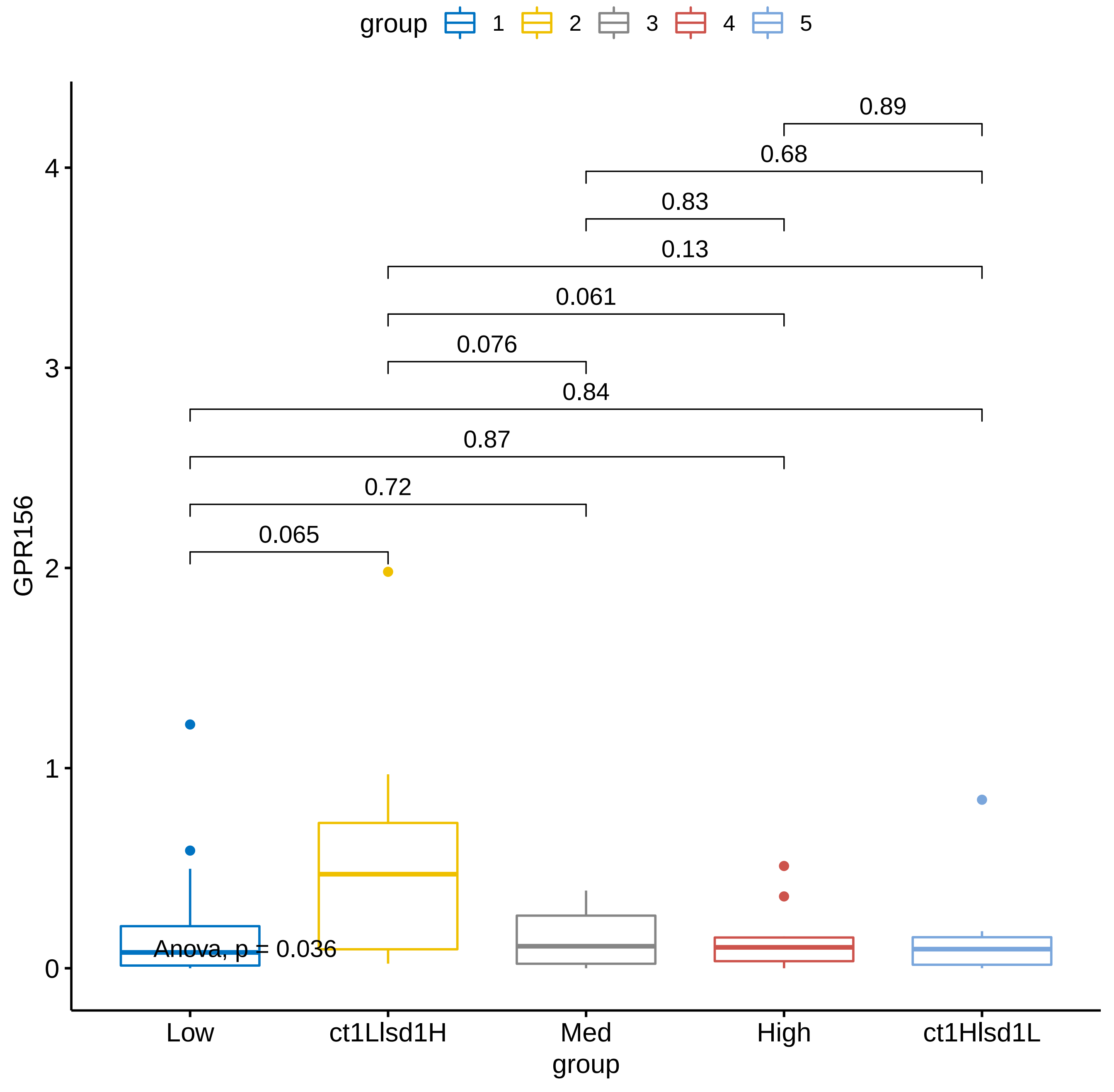
group
1
2
3
4
5
0.89
0.68
0.83
0.13
0.061
0.076
0.84
0.87
0.72
0.065
Anova, p = 0.036
GPR156
4
3
2
1
0
Low
ct1Llsd1H
Med
High
ct1Hlsd1L
group

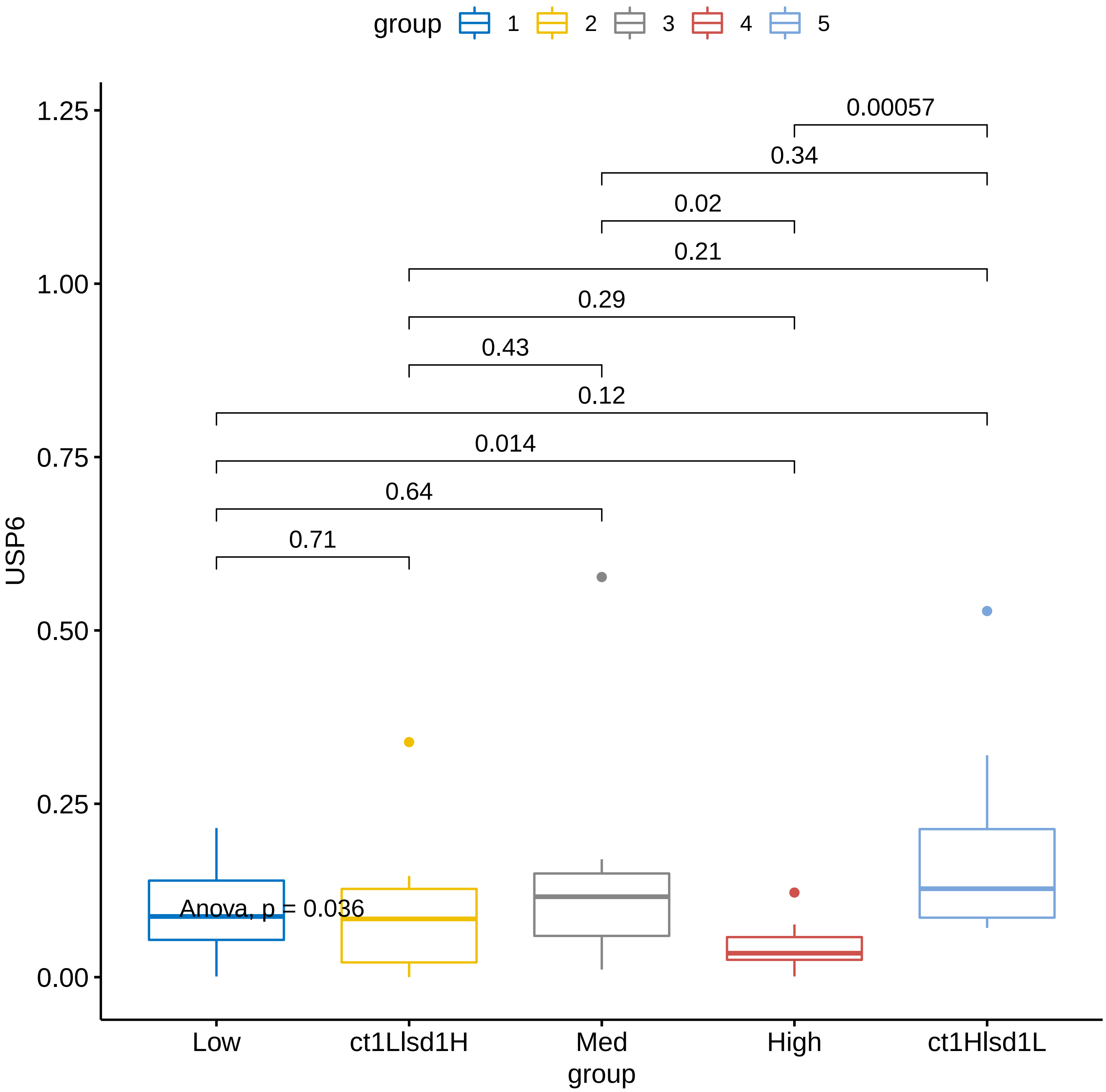
group 1 2 3 4 5
USP6
1.25
1.00
0.75
0.50
0.25
0.00
0.00057
0.34
0.02
0.21
0.29
0.43
0.12
0.014
0.64
0.71
Anova, p = 0.036
Low
ct1Llsd1H
Med
High
ct1Hlsd1L
group

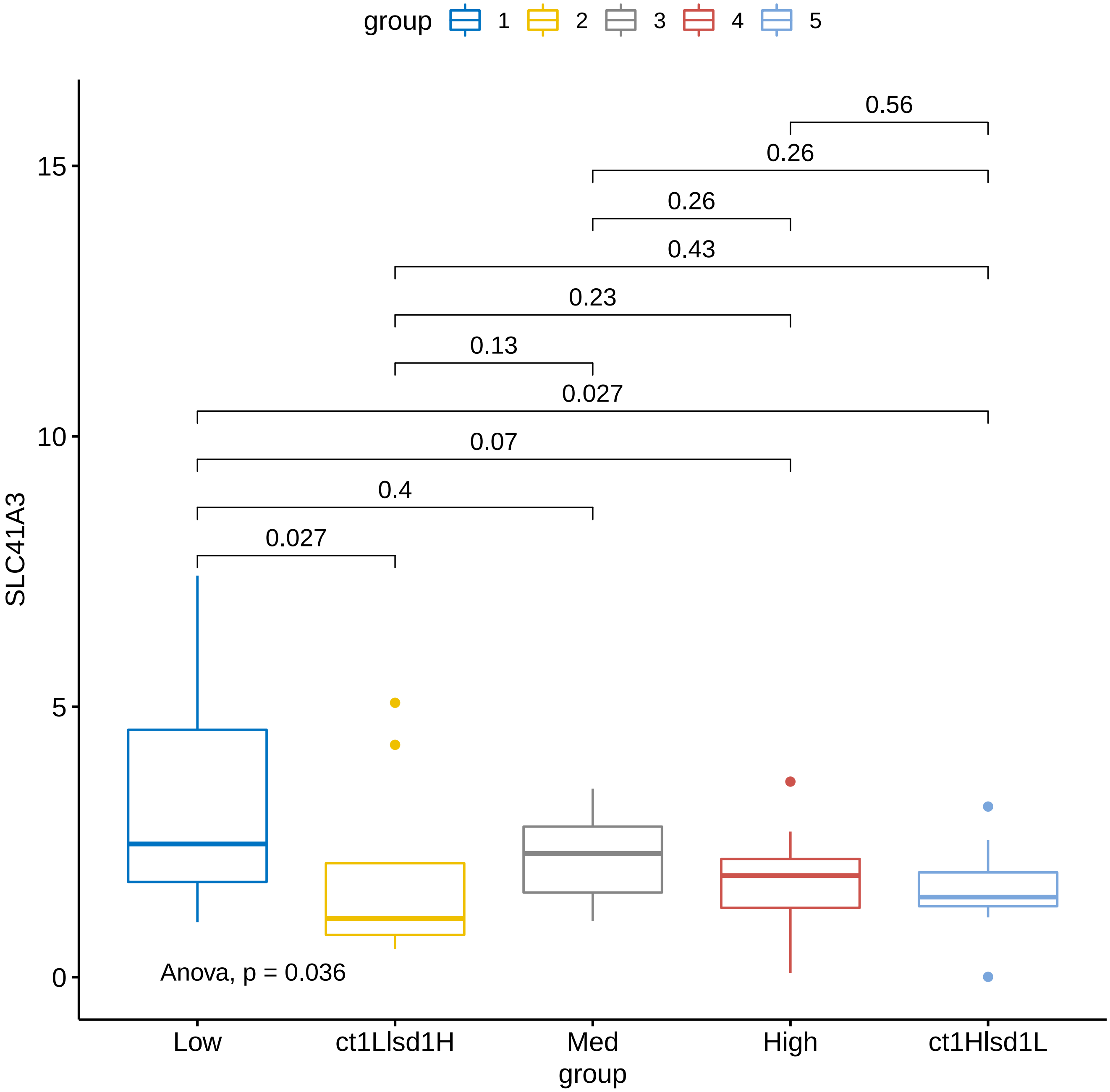

group
1
2
3
4
5
0.56
0.26
0.26
0.43
0.23
0.13
0.027
0.07
0.4
0.027
15
10
5
0
SLC41A3
Anova, p = 0.036
Low
ct1Llsd1H
Med
High
ct1Hlsd1L
group

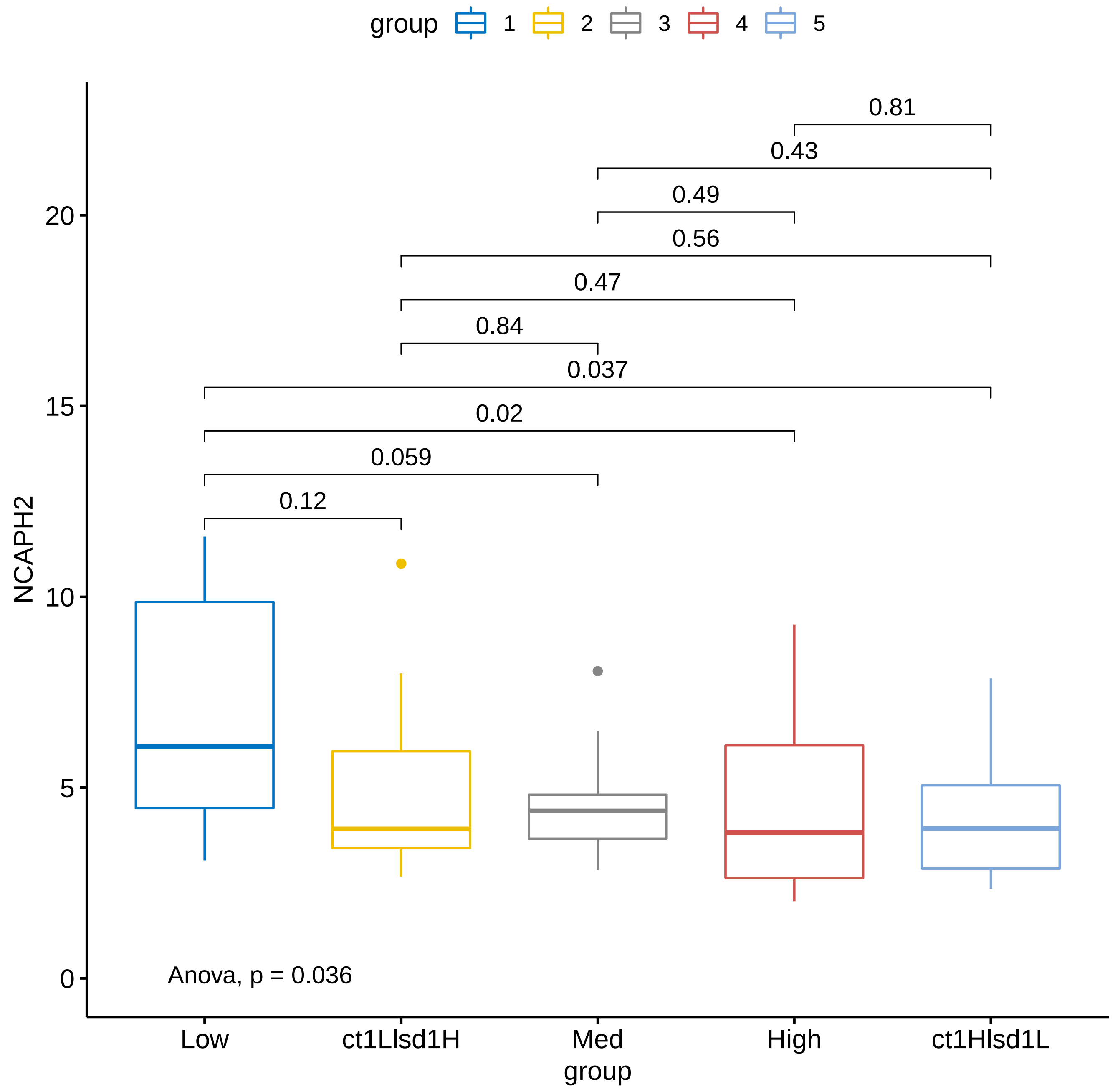

group 1 2 3 4 5
0.81
0.43
0.49
0.56
0.47
0.84
0.037
0.02
0.059
0.12
20
15
10
5
0
NCAPH2
Anova, p = 0.036
Low
ct1Llsd1H
Med
High
ct1Hlsd1L
group

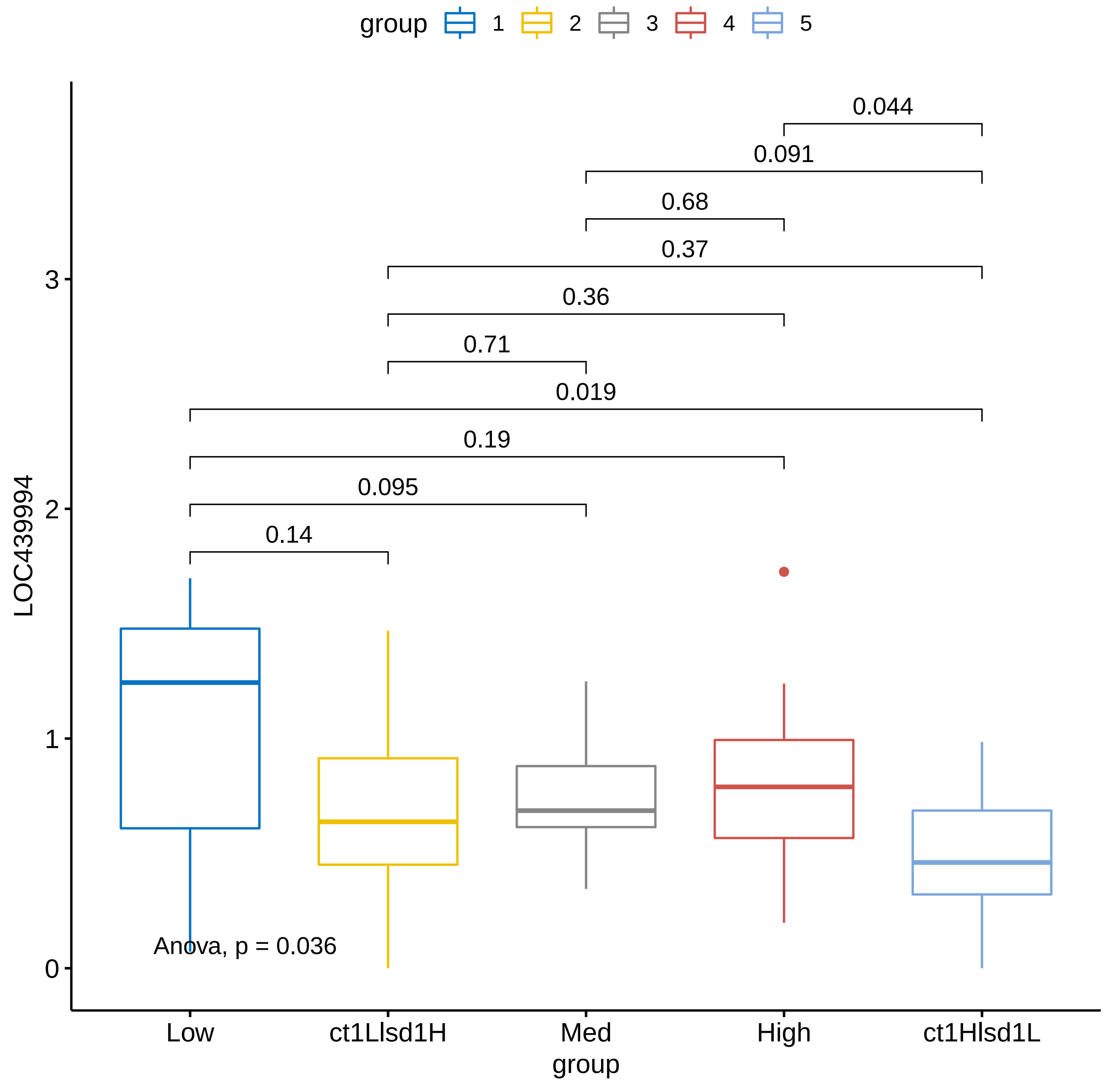
group 1 2 3 4 5
0.044
0.091
0.68
0.37
0.36
0.71
0.019
0.19
0.095
0.14
LOC439994
3
2
1
0
Anova, p = 0.036
Low
ct1Llsd1H
Med
High
ct1Hlsd1L
group

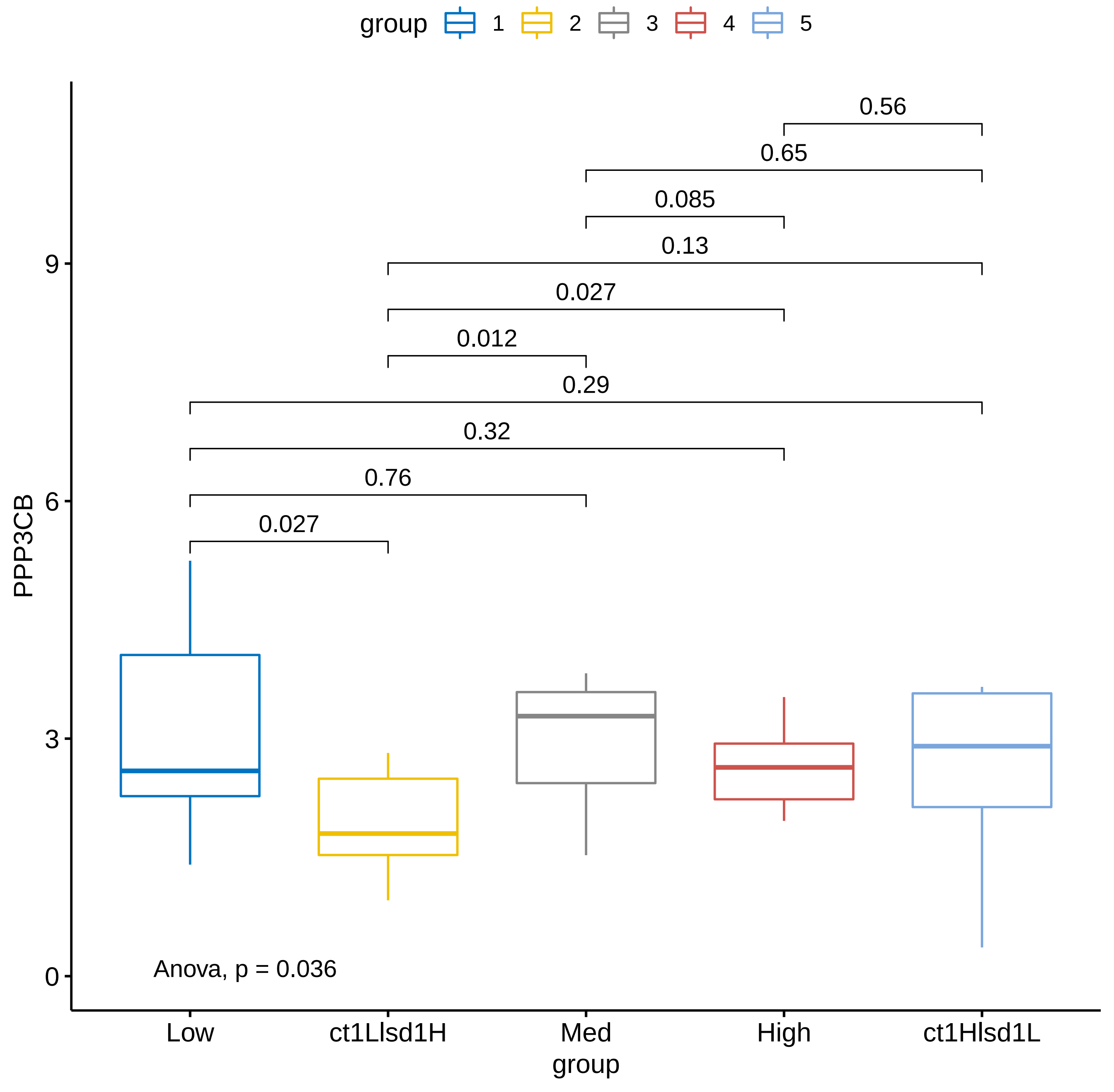

group
1
2
3
4
5
0.56
0.65
0.085
0.13
0.027
0.012
0.29
0.32
0.76
0.027
9
6
3
0
PPP3CB
Anova, p = 0.036
Low
ct1Llsd1H
Med
High
ct1Hlsd1L
group

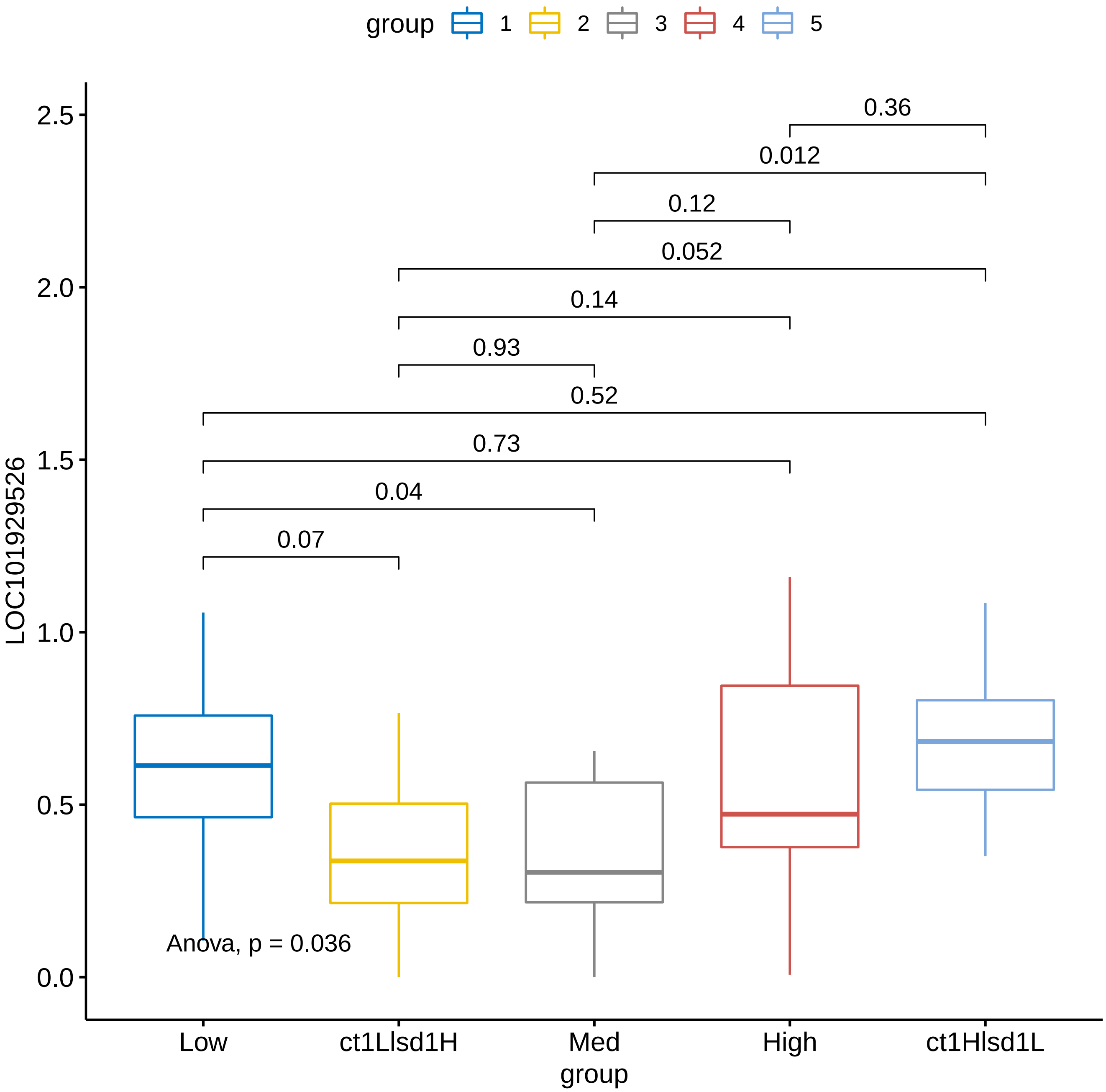
group
1
2
3
4
5
0.36
0.012
0.12
0.052
0.14
0.93
0.52
0.73
0.04
0.07
Anova, p = 0.036
LOC101929526
2.5
2.0
1.5
1.0
0.5
0.0
Low
ct1Llsd1H
Med
High
ct1Hlsd1L
group

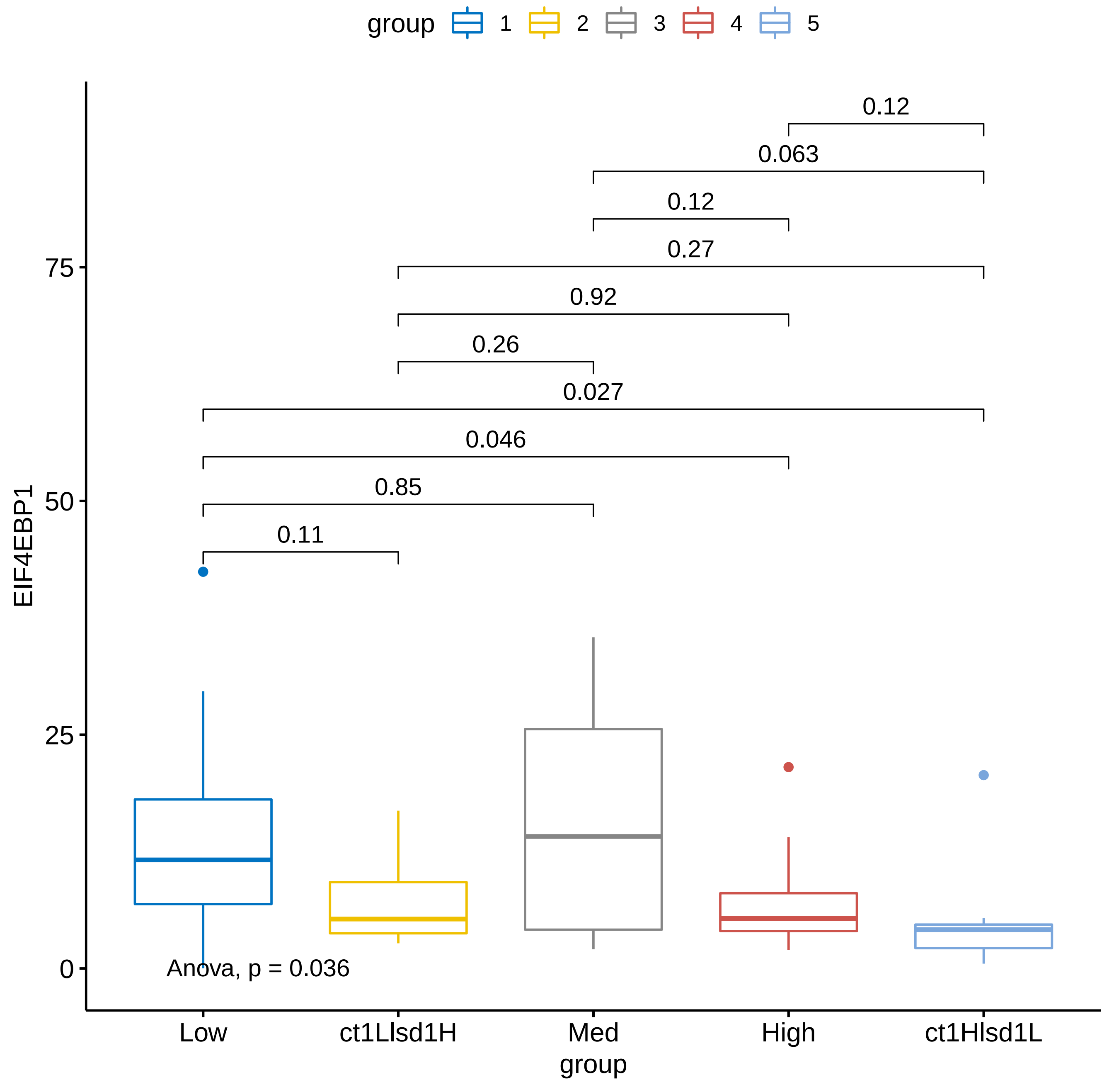

group
1
2
3
4
5
0.12
0.063
0.12
0.27
0.92
0.26
0.027
0.046
0.85
0.11
75
50
25
0
EIF4EBP1
Anova, p = 0.036
Low
ct1Llsd1H
Med
High
ct1Hlsd1L
group

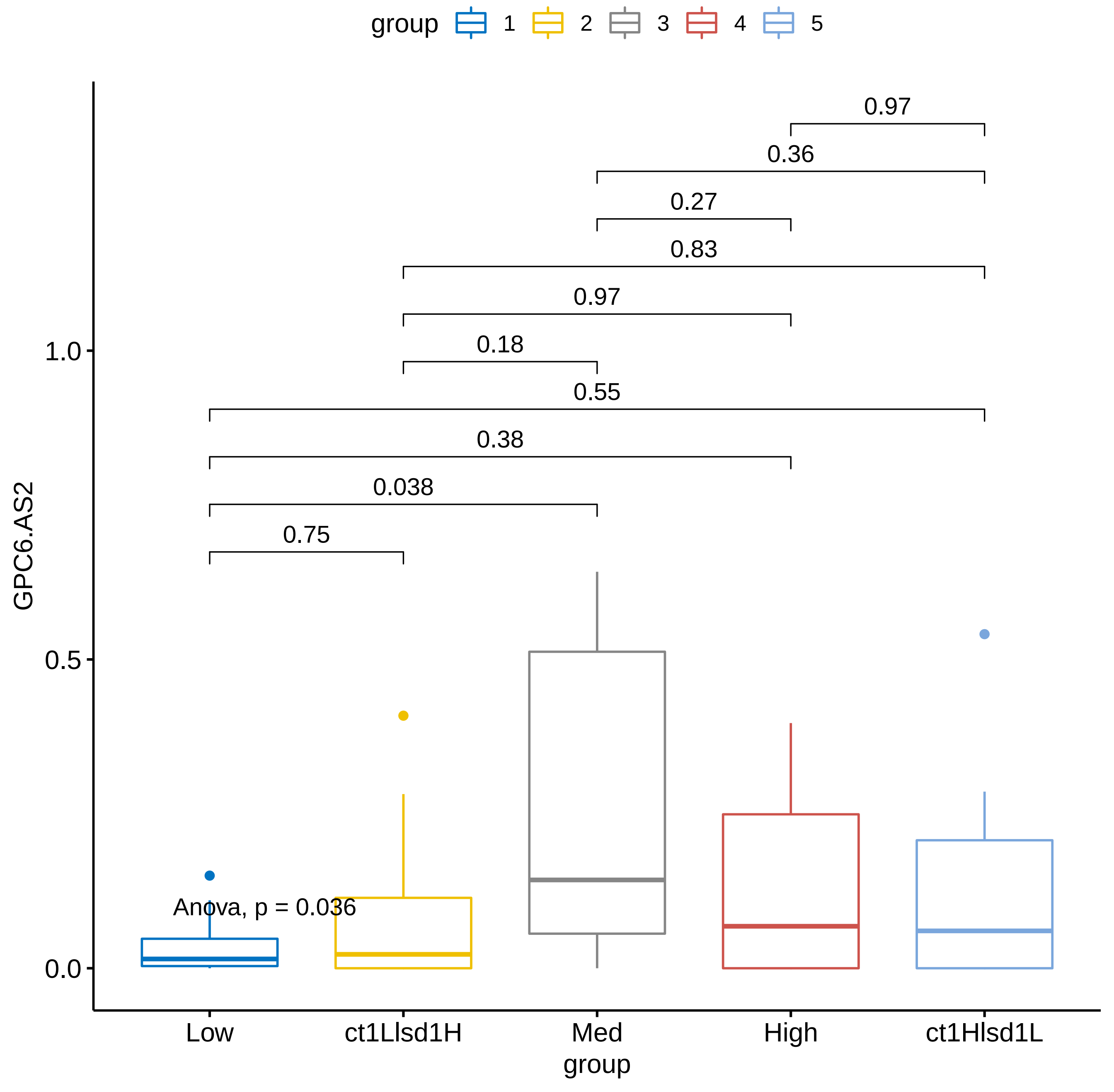
group
1
2
3
4
5
0.97
0.36
0.27
0.83
0.97
0.18
0.55
0.38
0.038
0.75
1.0
0.5
0.0
GPC6.AS2
Anova, p = 0.036
Low
ct1Llsd1H
Med
High
ct1Hlsd1L
group

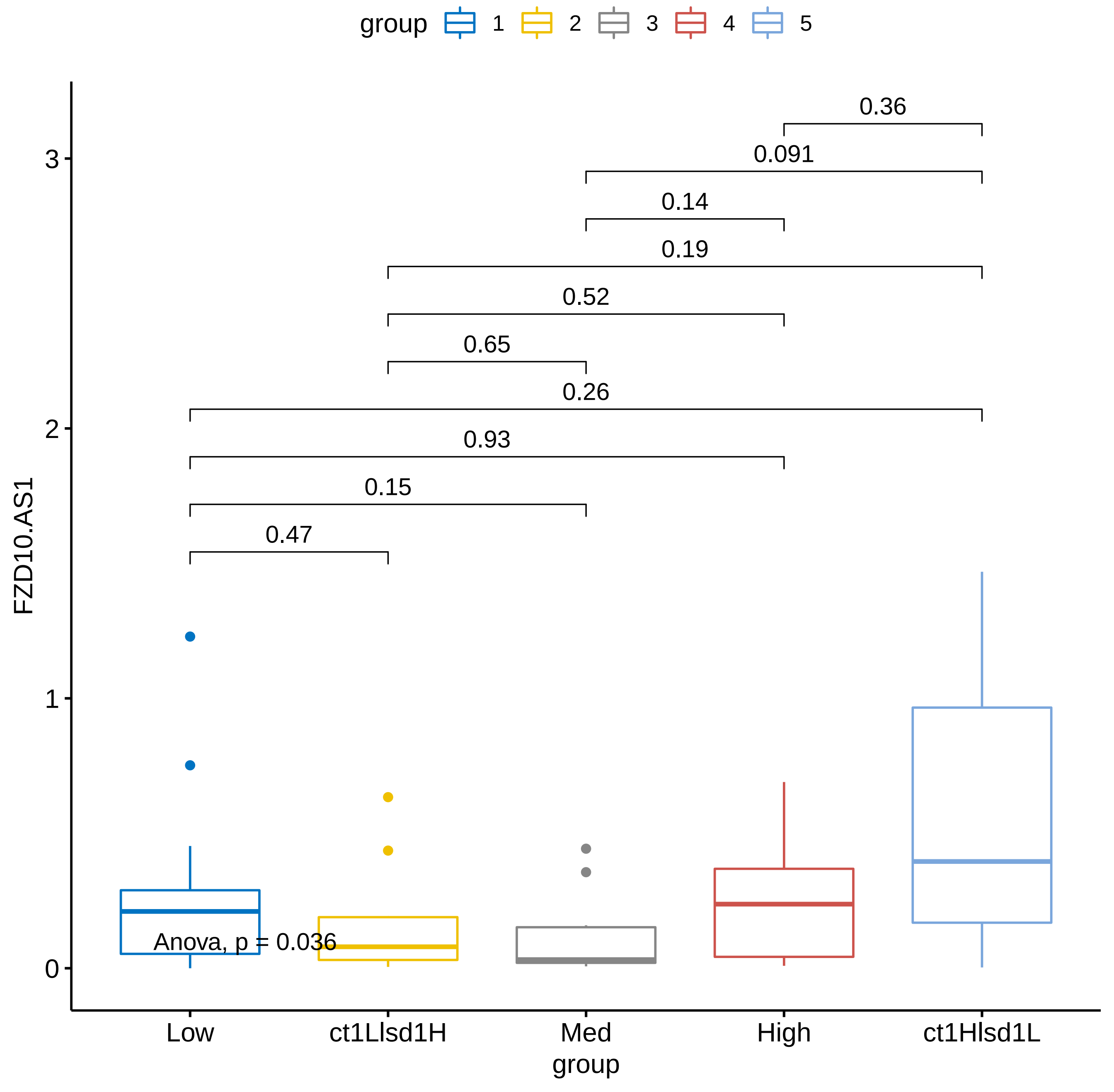

group
1
2
3
4
5
FZD10.AS1
0.36
0.091
0.14
0.19
0.52
0.65
0.26
0.93
0.15
0.47
Anova, p = 0.036
3
2
1
0
Low
ct1Llsd1H
Med
High
ct1Hlsd1L
group

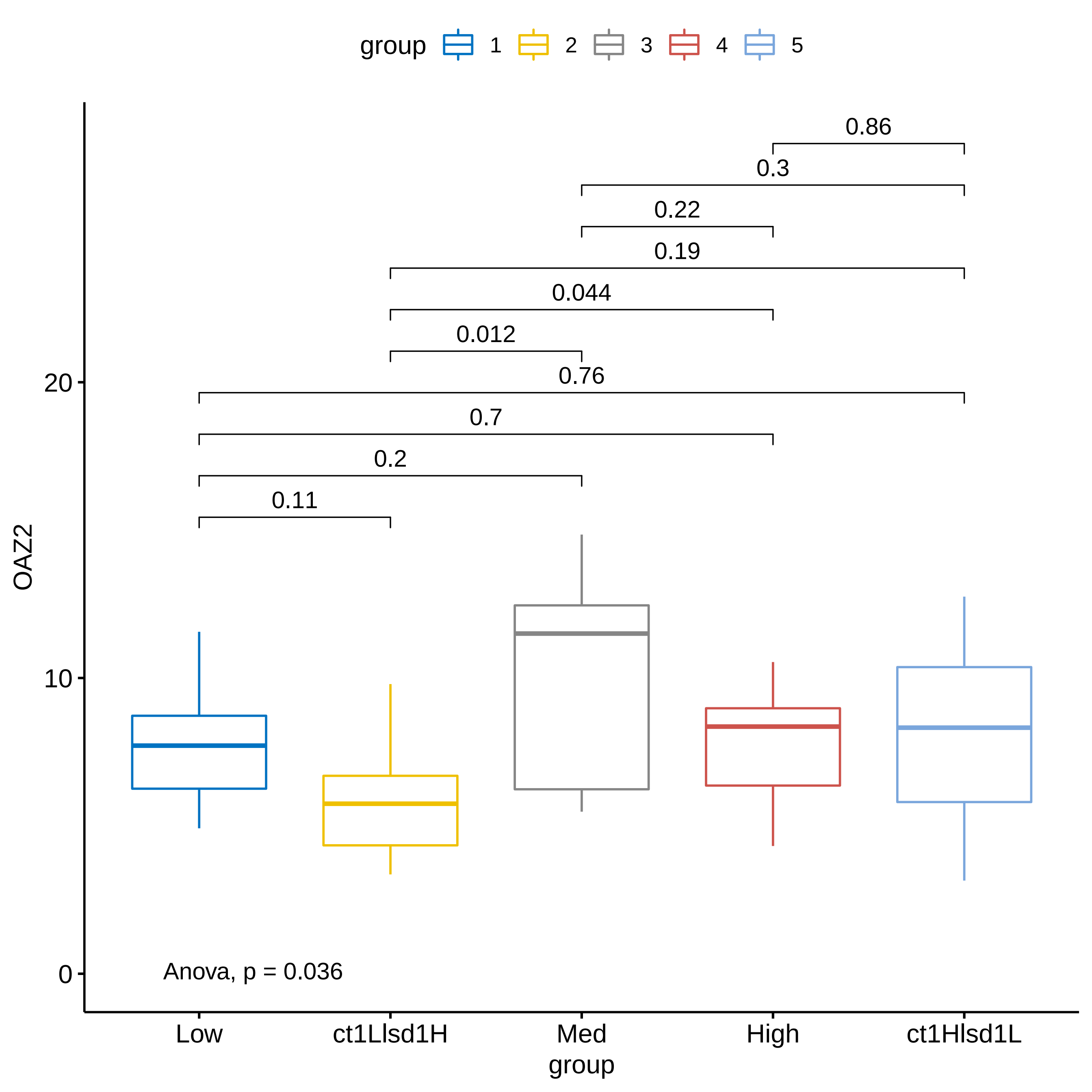
group
1
2
3
4
5
0.86
0.3
0.22
0.19
0.044
0.012
0.76
0.7
0.2
0.11
20
10
0
OAZ2
Anova, p = 0.036
Low
ct1Llsd1H
Med
High
ct1Hlsd1L
group

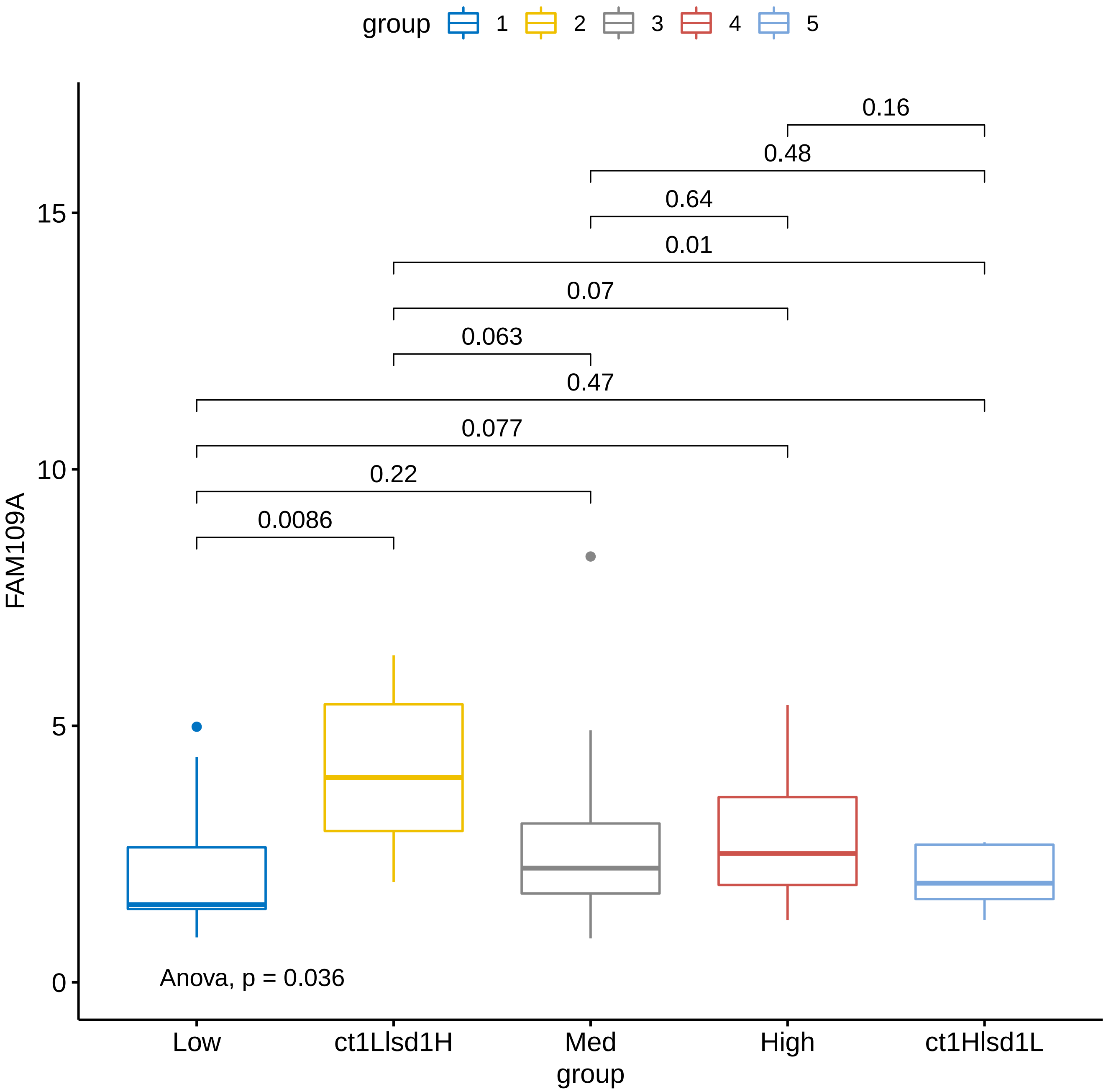

group 1 2 3 4 5
0.16
0.48
0.64
0.01
0.07
0.063
0.47
0.077
0.22
0.0086
FAM109A
15
10
5
0
Anova, p = 0.036
Low
ct1Llsd1H
Med
High
ct1Hlsd1L
group

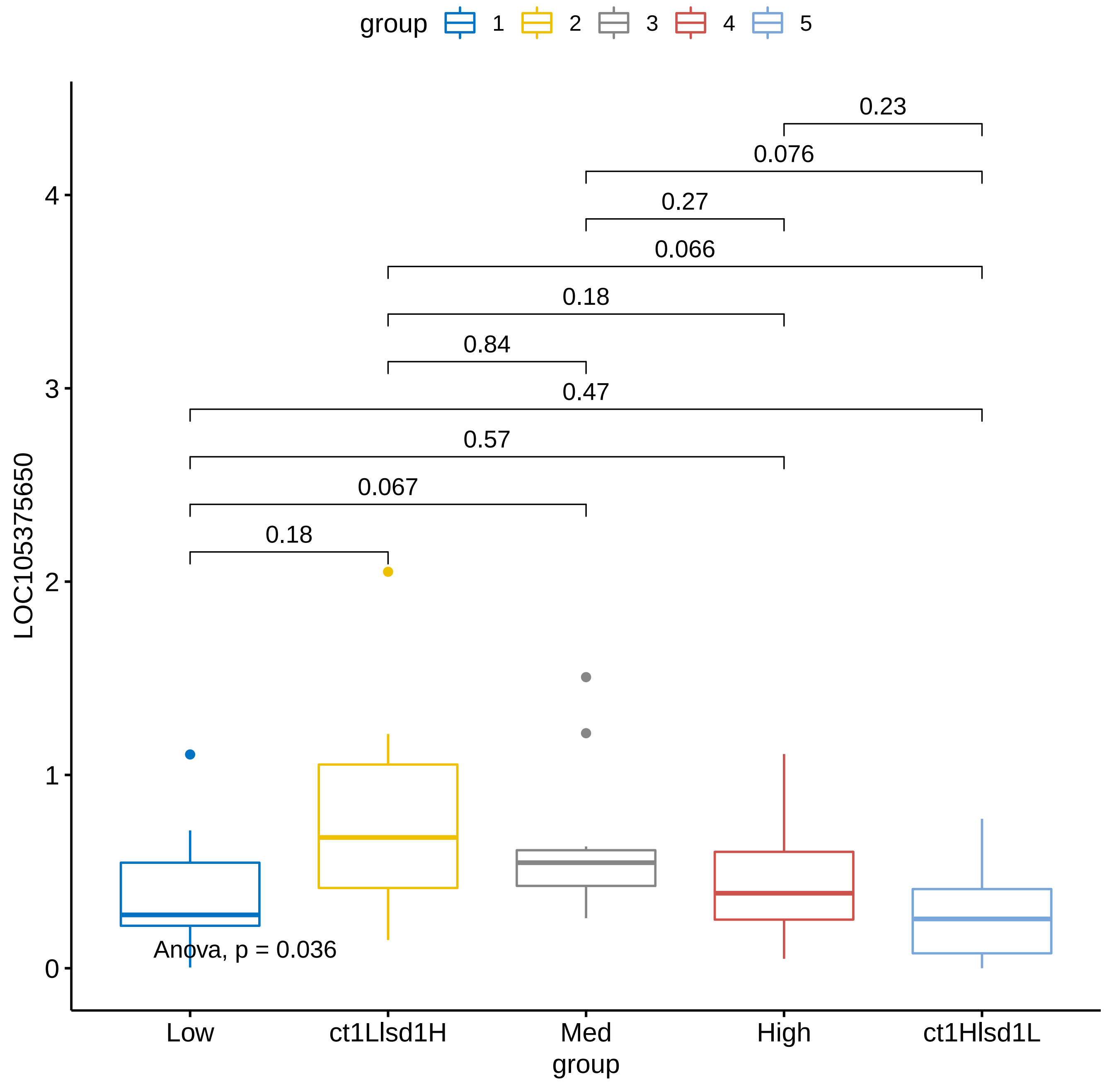

group
1
2
3
4
5
0.23
0.076
0.27
0.066
0.18
0.84
0.47
0.57
0.067
0.18
Anova, p = 0.036
LOC105375650
0
1
2
3
4
Low
ct1Llsd1H
Med
High
ct1Hlsd1L
group

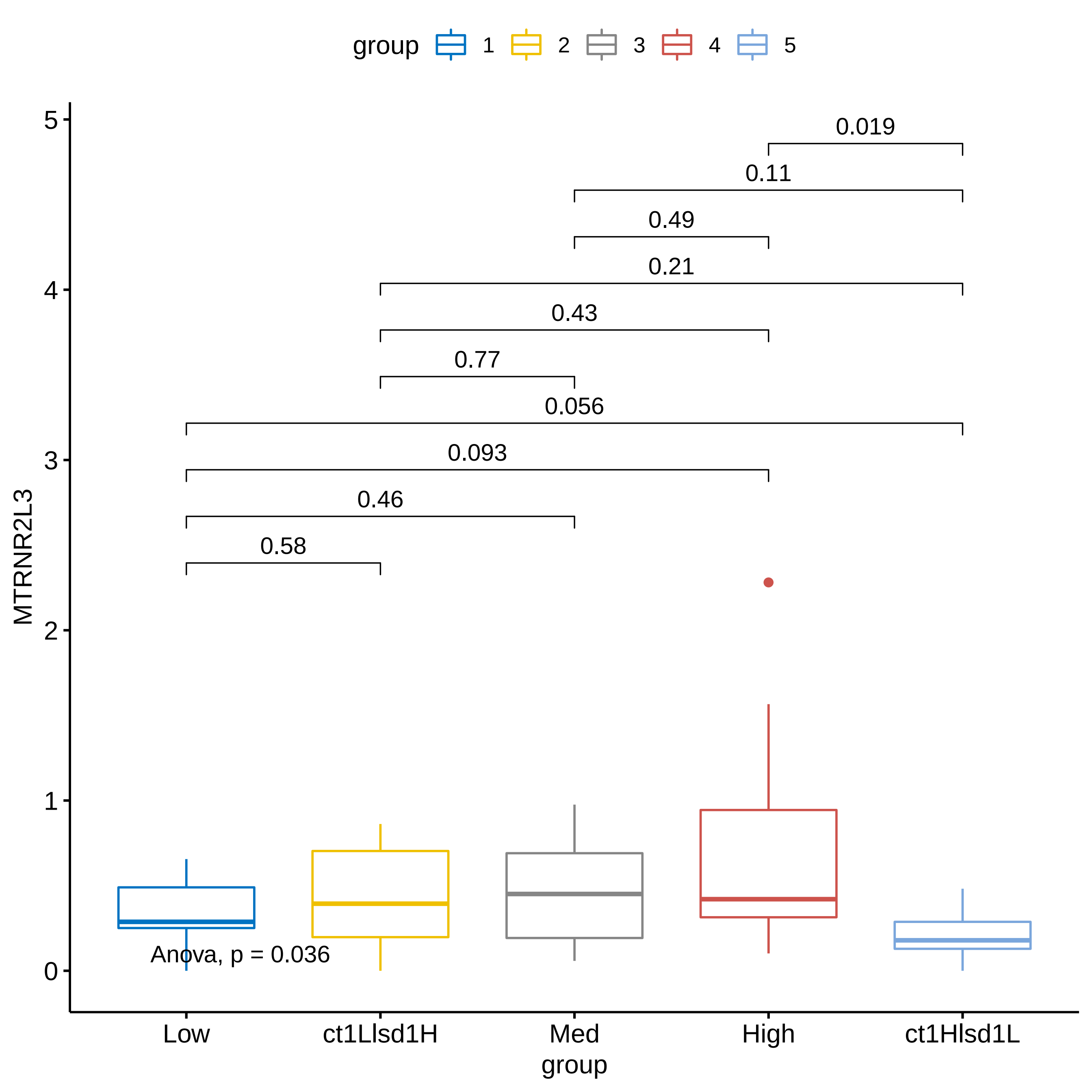
group
1
2
3
4
5
0.019
0.11
0.49
0.21
0.43
0.77
0.056
0.093
0.46
0.58
Anova, p = 0.036
MTRNR2L3
0
1
2
3
4
5
Low
ct1Llsd1H
Med
High
ct1Hlsd1L
group

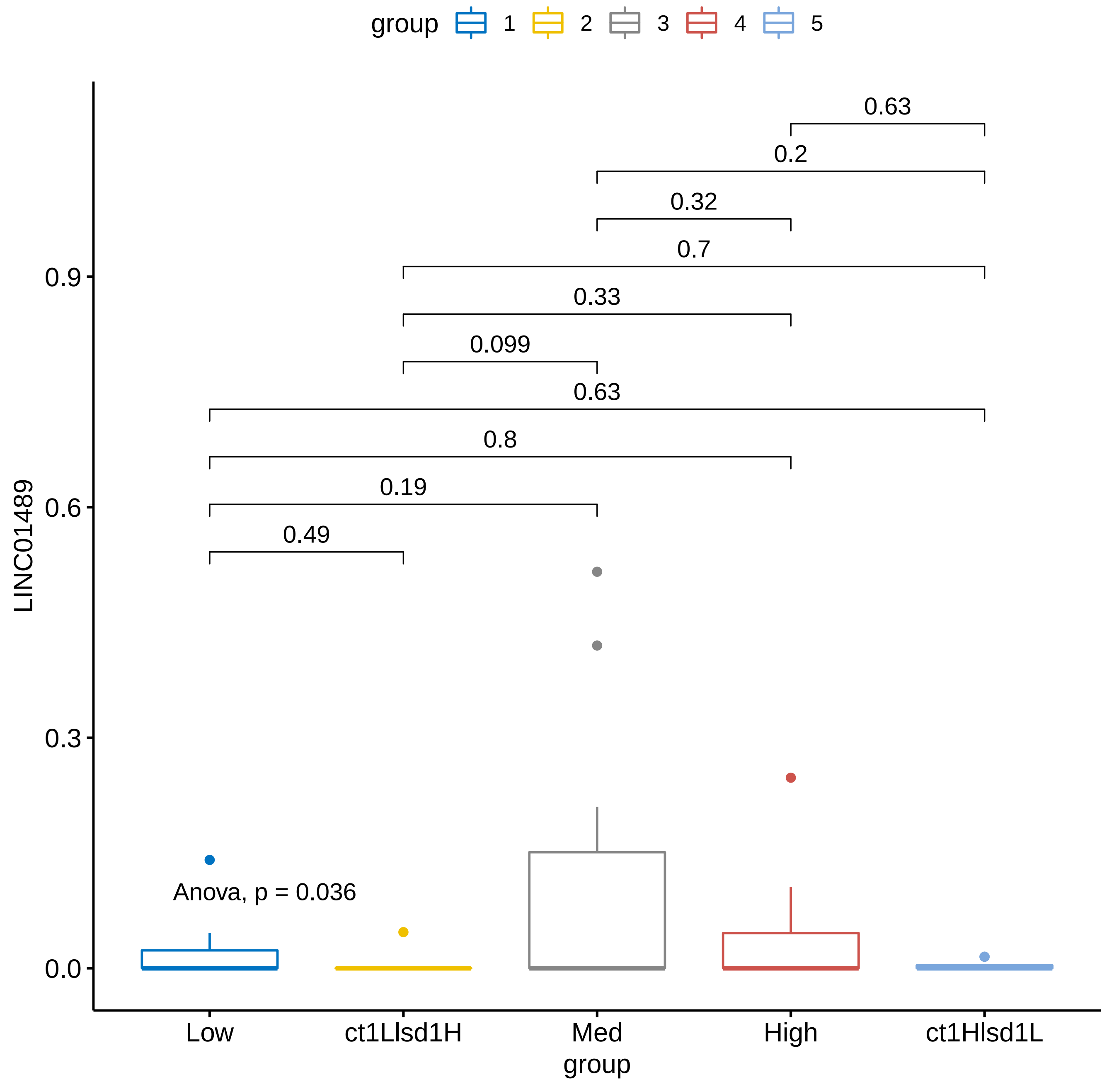

group
1
2
3
4
5
LINC01489
0.0
0.3
0.6
0.9
0.63
0.2
0.32
0.7
0.33
0.099
0.63
0.8
0.19
0.49
Anova, p = 0.036
Low
ct1Llsd1H
Med
High
ct1Hlsd1L
group

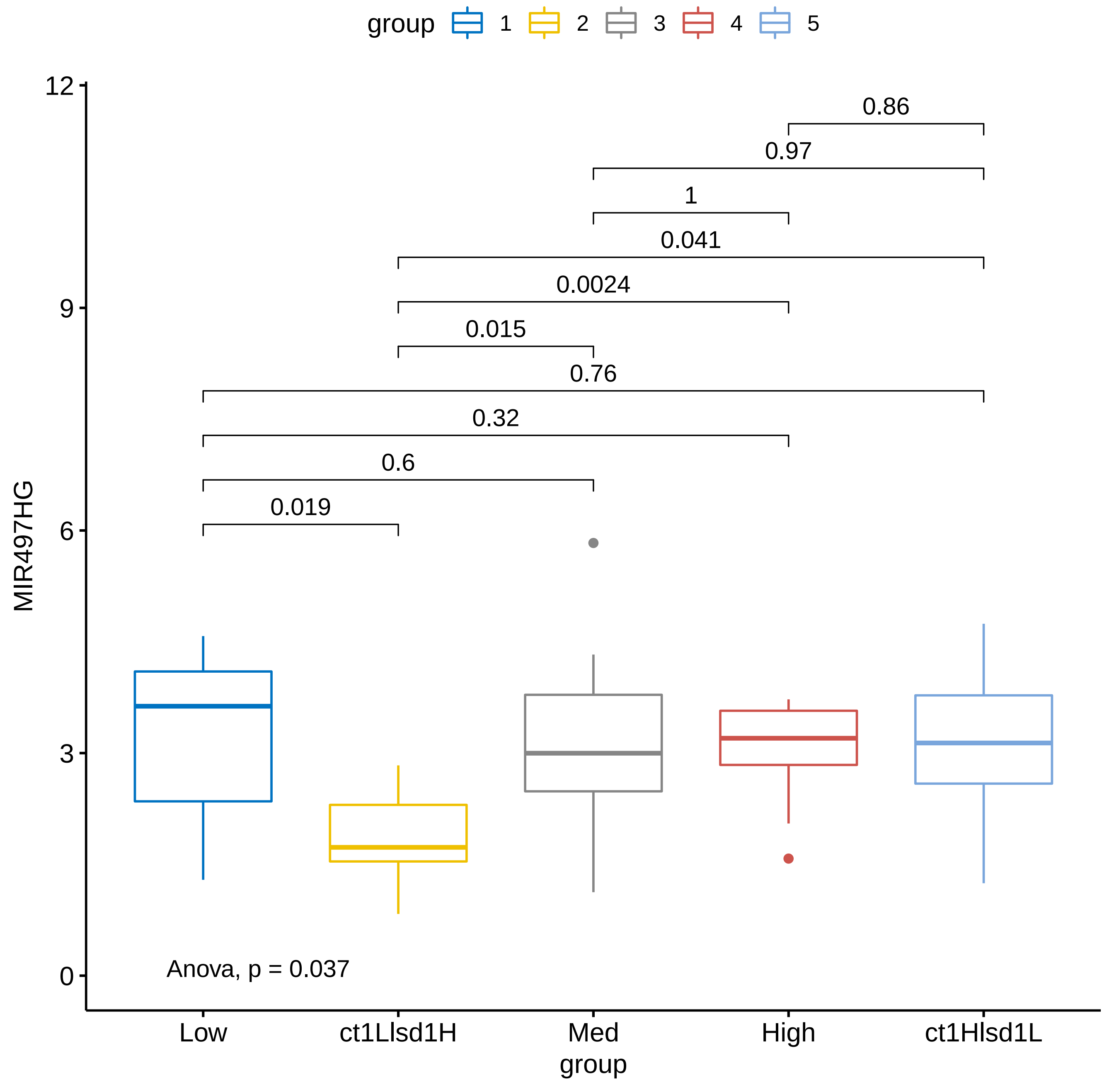

group 1 2 3 4 5
0.86
0.97
1
0.041
0.0024
0.015
0.76
0.32
0.6
0.019
Anova, p = 0.037
MIR497HG
12
9
6
3
0
Low
ct1Llsd1H
Med
High
ct1Hlsd1L
group

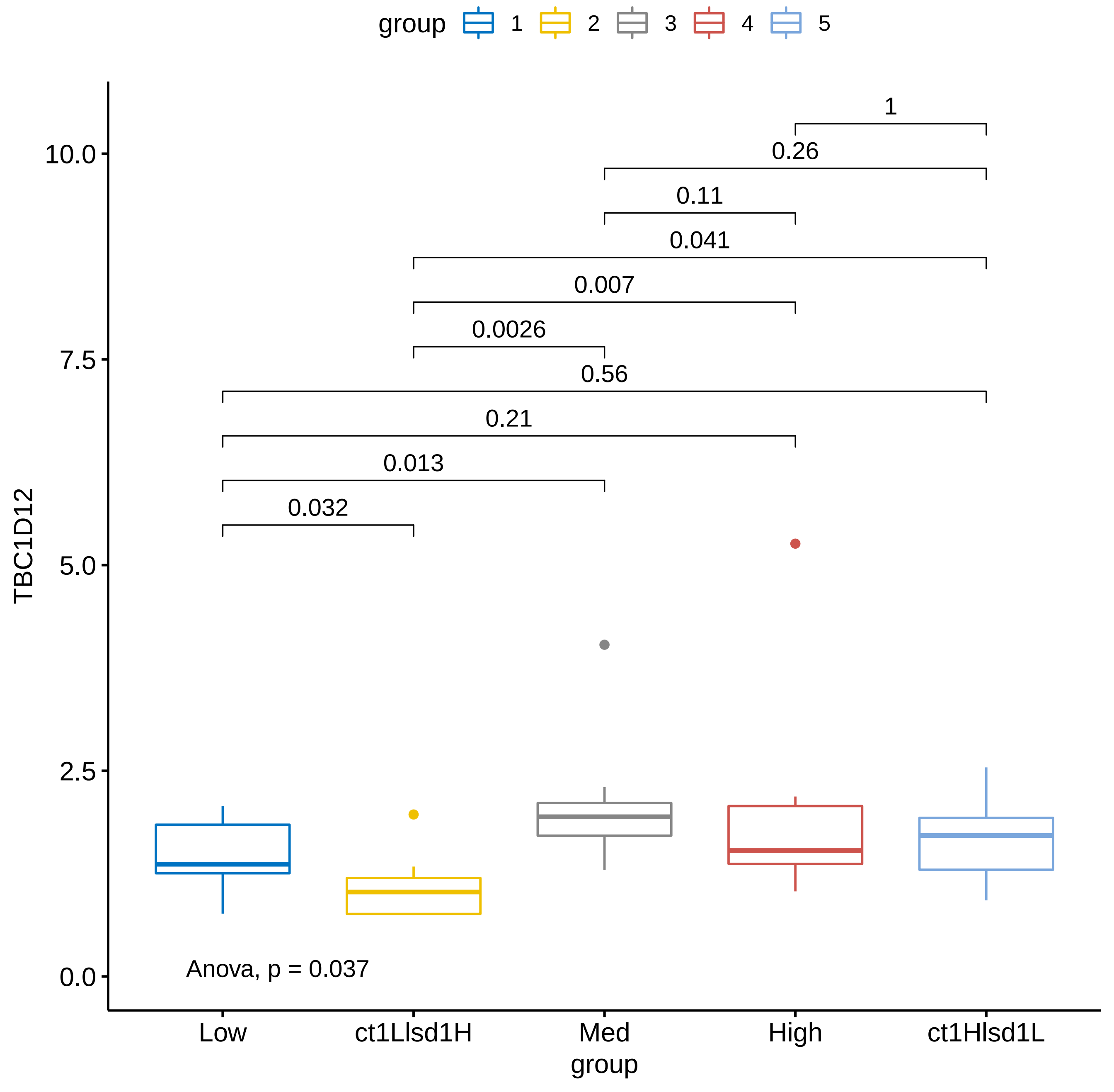

group 1 2 3 4 5
TBC1D12
10.0
7.5
5.0
2.5
0.0
1
0.26
0.11
0.041
0.007
0.0026
0.56
0.21
0.013
0.032
Anova, p = 0.037
Low
ct1Llsd1H
Med
High
ct1Hlsd1L
group

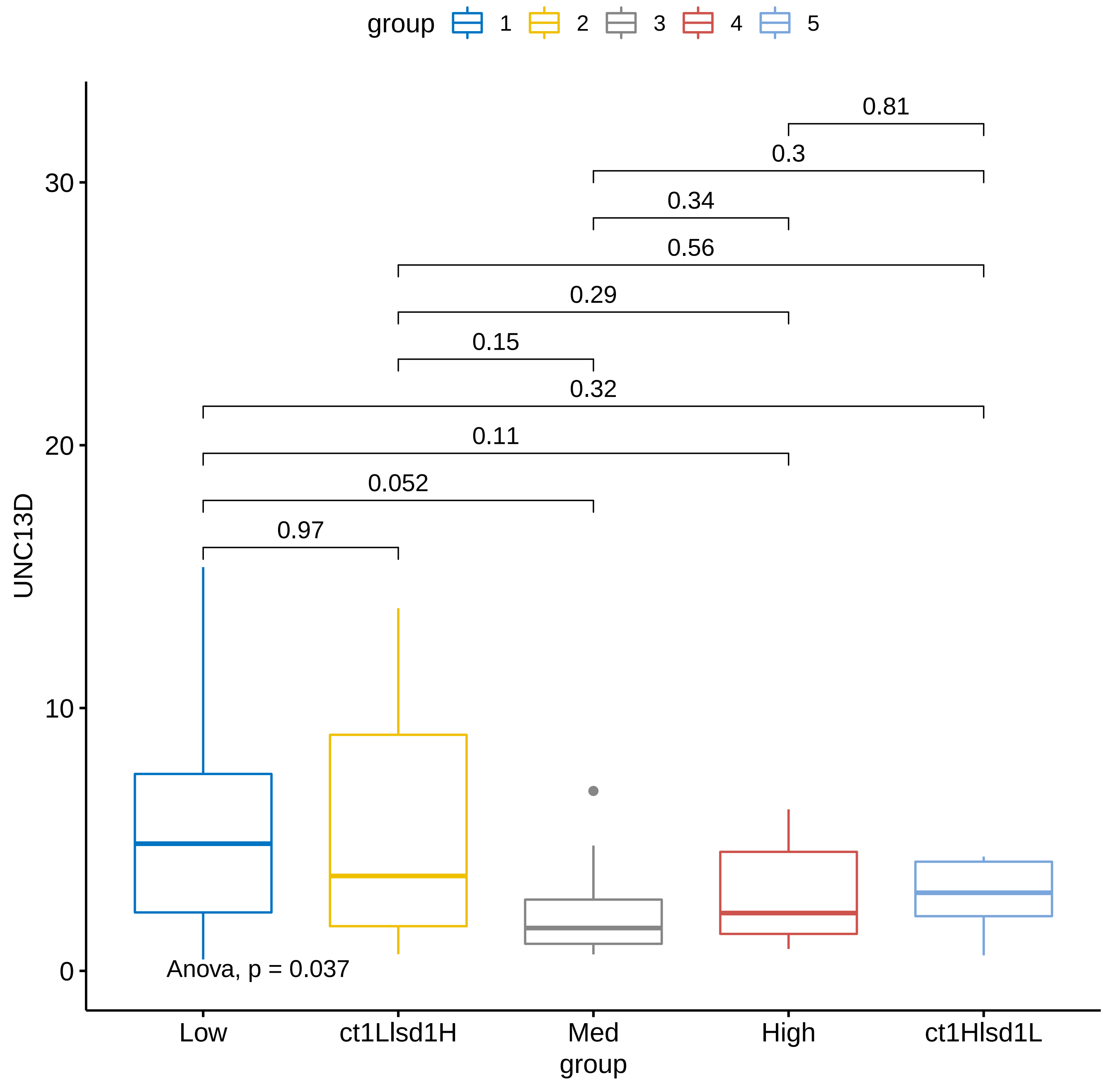
group
1
2
3
4
5
0.81
0.3
0.34
0.56
0.29
0.15
0.32
0.11
0.052
0.97
30
20
10
0
UNC13D
Anova, p = 0.037
Low
ct1Llsd1H
Med
High
ct1Hlsd1L
group

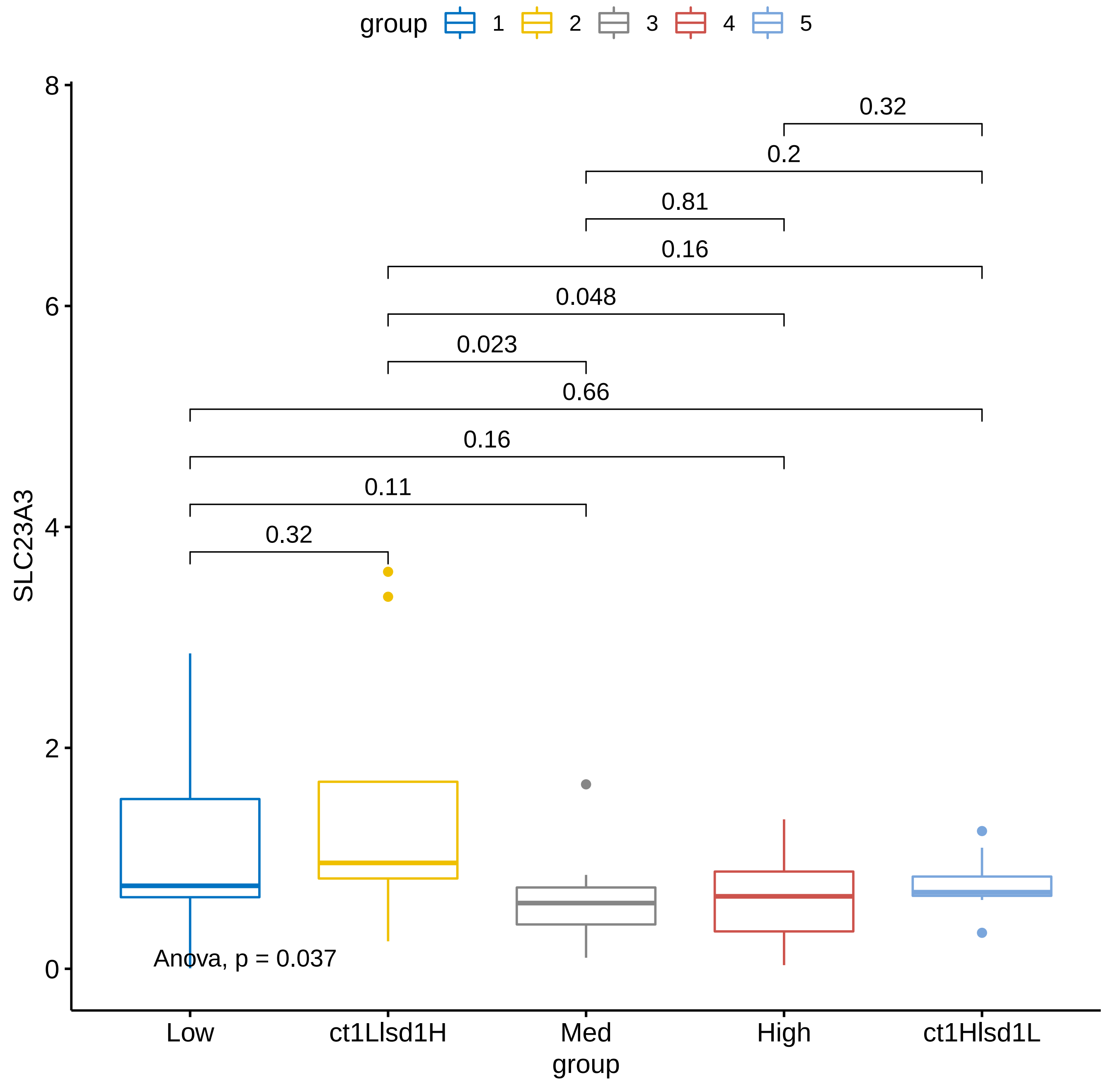

group
1
2
3
4
5
SLC23A3
8
6
4
2
0
0.32
0.2
0.81
0.16
0.048
0.023
0.66
0.16
0.11
0.32
Anova, p = 0.037
Low
ct1Llsd1H
Med
High
ct1Hlsd1L
group

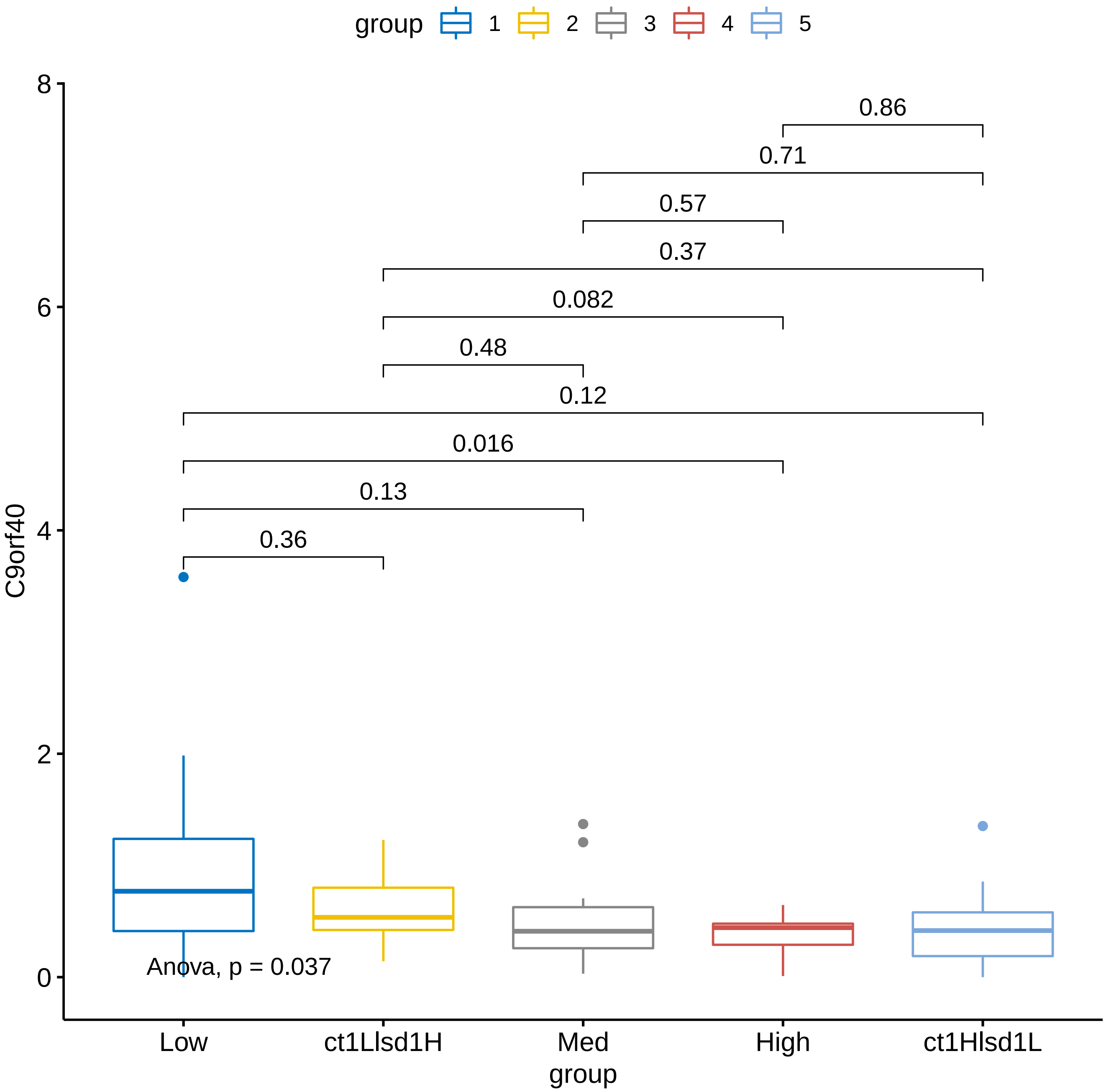

group 1 2 3 4 5
C9orf40
0.86
0.71
0.57
0.37
0.082
0.48
0.12
0.016
0.13
0.36
Anova, p = 0.037
0
2
4
6
8
Low
ct1Llsd1H
Med
High
ct1Hlsd1L
group

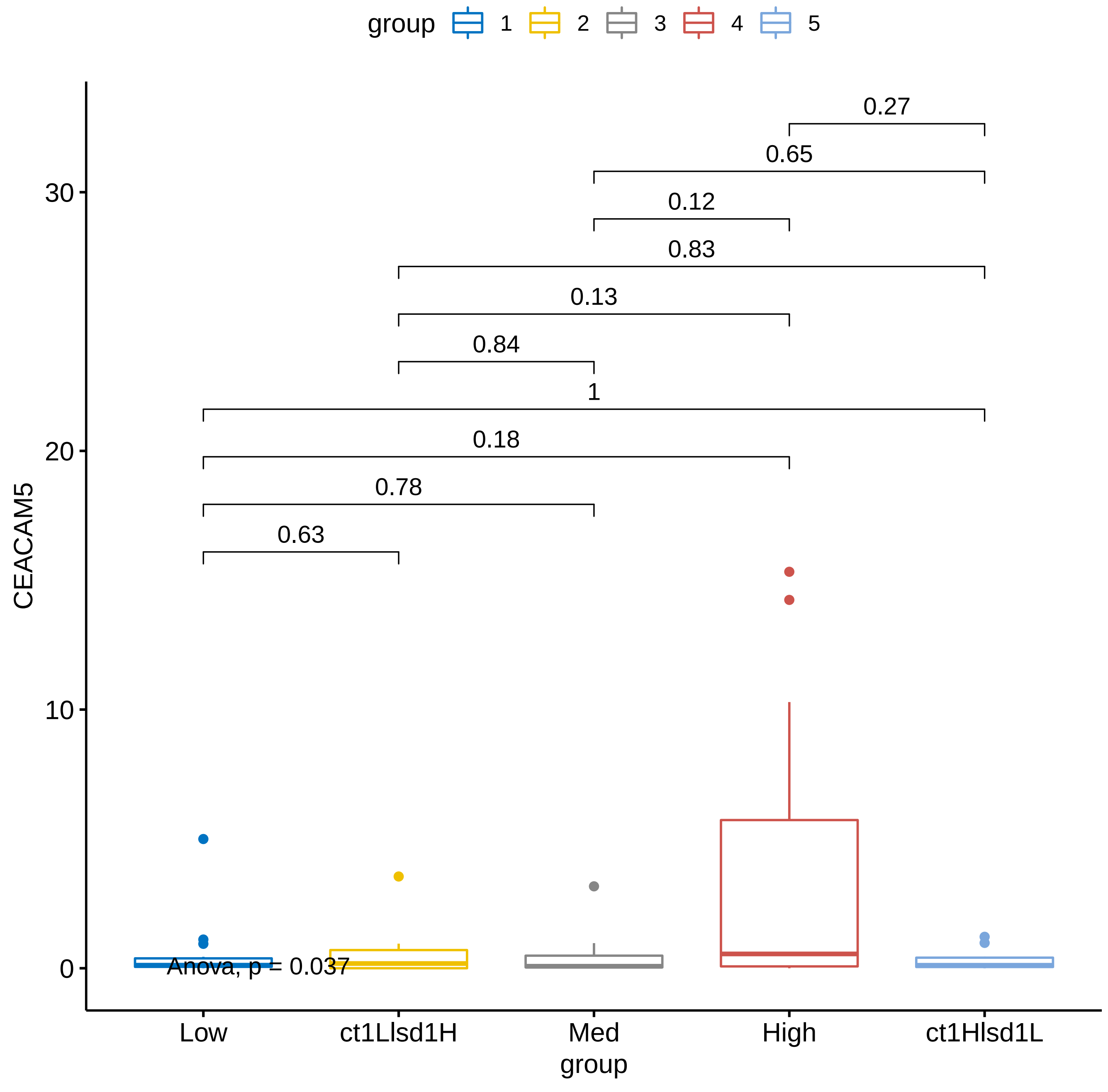

group 1 2 3 4 5
0.27
0.65
0.12
0.83
0.13
0.84
1
0.18
0.78
0.63
30
20
10
0
CEACAM5
Anova, p = 0.037
Low
ct1Llsd1H
Med
High
ct1Hlsd1L
group

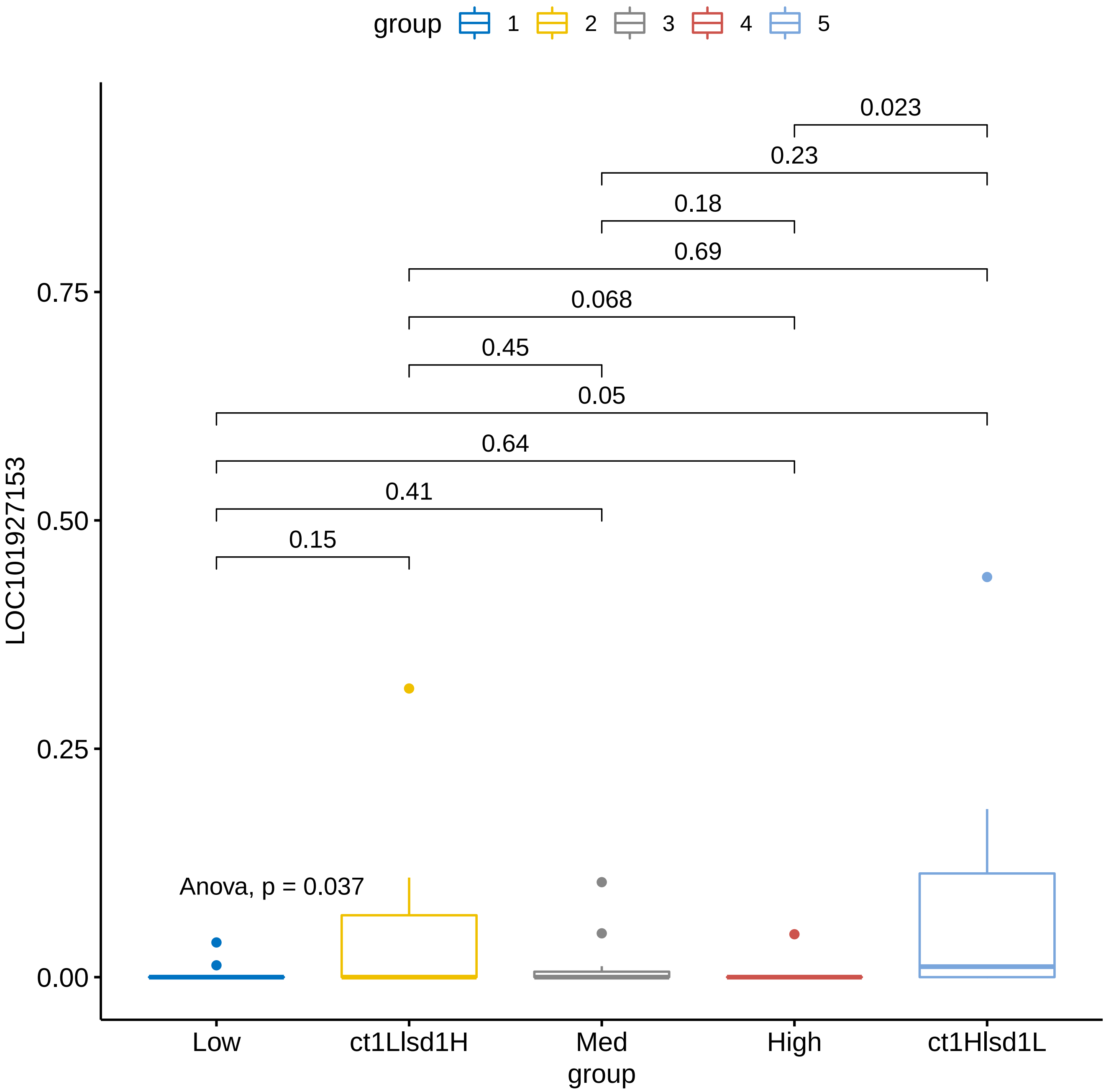
group 1 2 3 4 5
0.023
0.23
0.18
0.69
0.068
0.45
0.05
0.64
0.41
0.15
Anova, p = 0.037
LOC101927153
0.75
0.50
0.25
0.00
Low
ct1Llsd1H
Med
High
ct1Hlsd1L
group

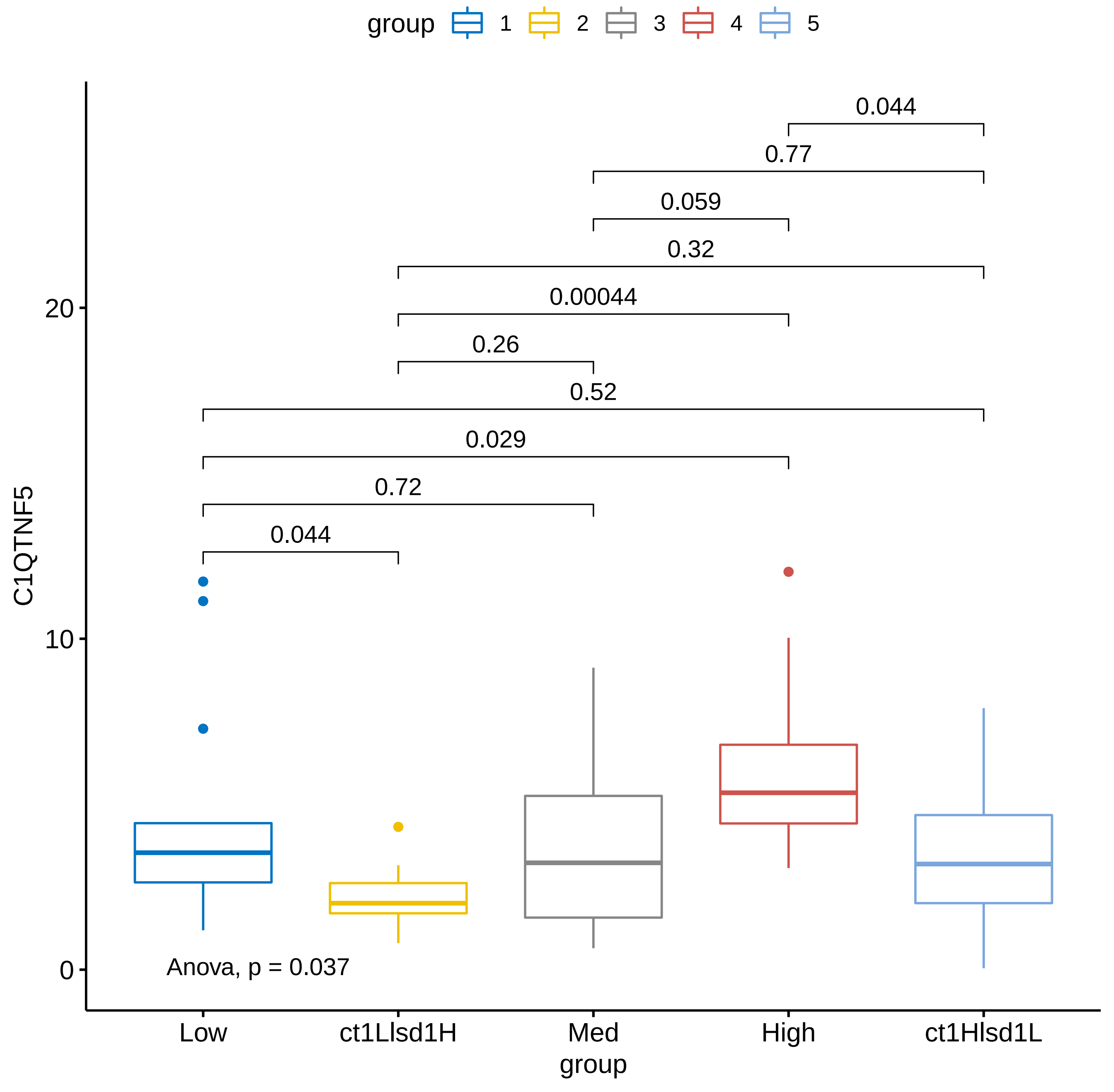

group 1 2 3 4 5
0.044
0.77
0.059
0.32
0.00044
0.26
0.52
0.029
0.72
0.044
C1QTNF5
20
10
0
Anova, p = 0.037
Low
ct1Llsd1H
Med
High
ct1Hlsd1L
group

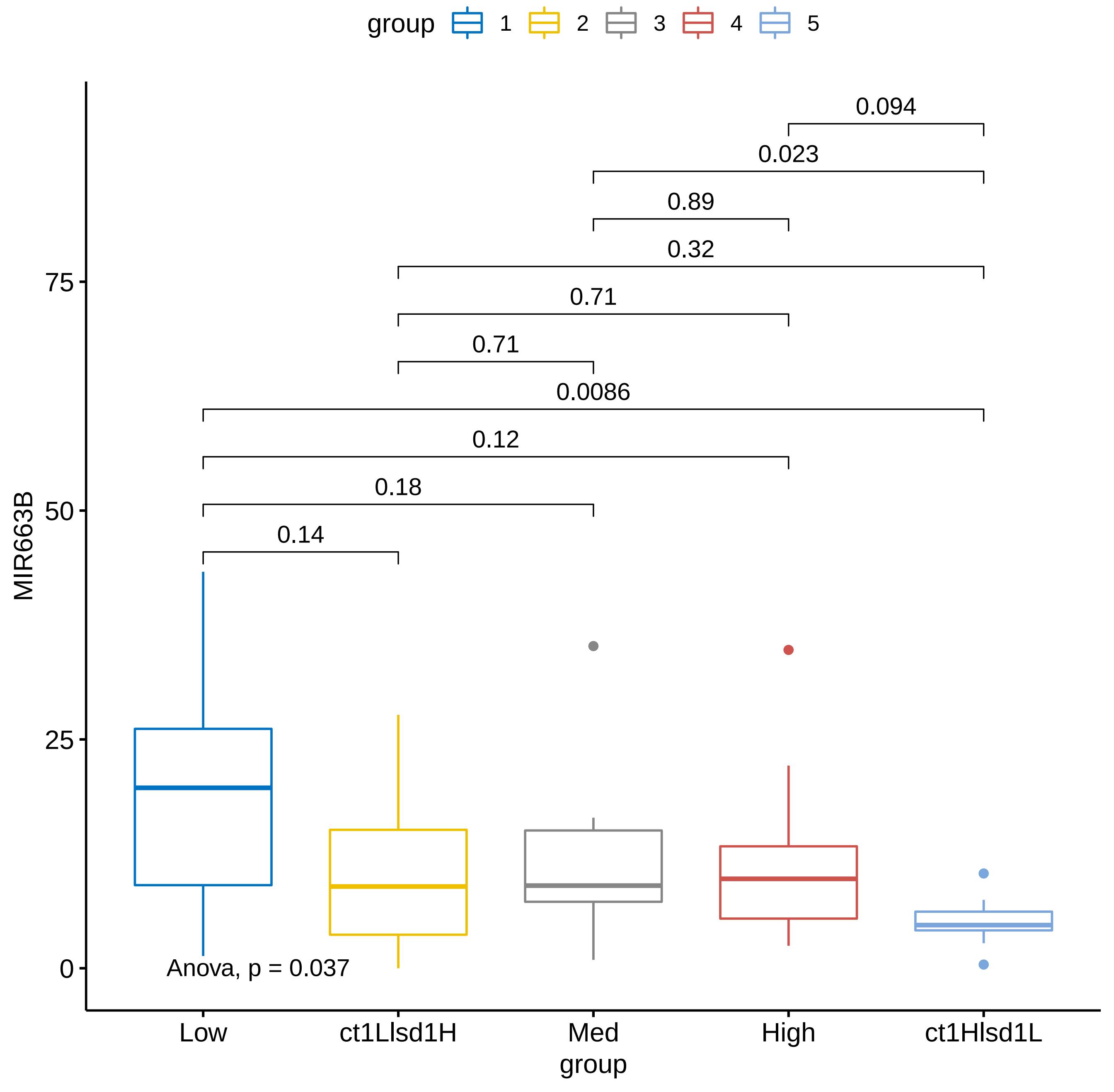

group
1
2
3
4
5
MIR663B
0.094
0.023
0.89
0.32
0.71
0.71
0.0086
0.12
0.18
0.14
75
50
25
0
Anova, p = 0.037
Low
ct1Llsd1H
Med
High
ct1Hlsd1L
group

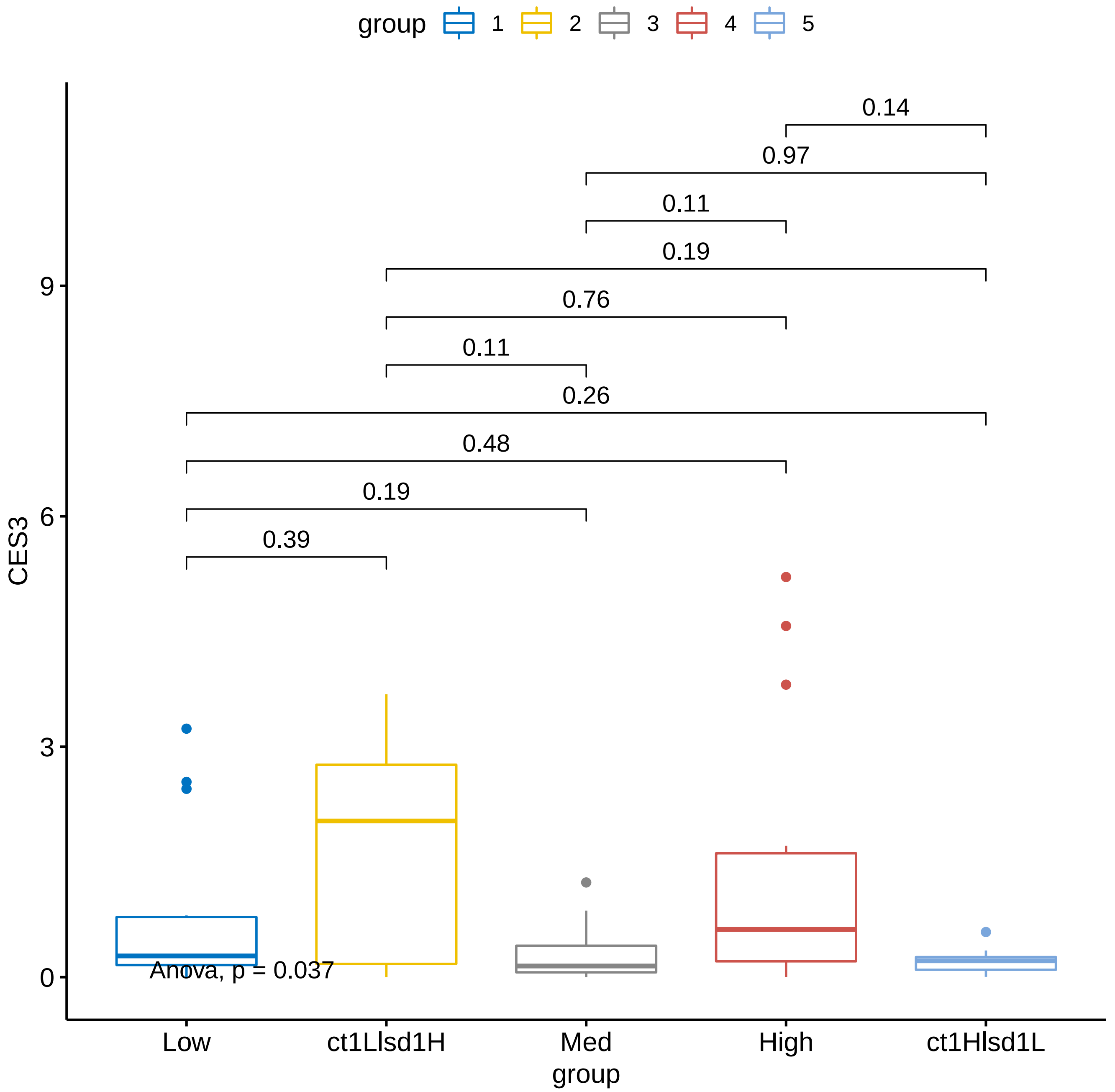

group
1
2
3
4
5
CES3
0
3
6
9
0.14
0.97
0.11
0.19
0.76
0.11
0.26
0.48
0.19
0.39
Anova, p = 0.037
Low
ct1Llsd1H
Med
High
ct1Hlsd1L
group

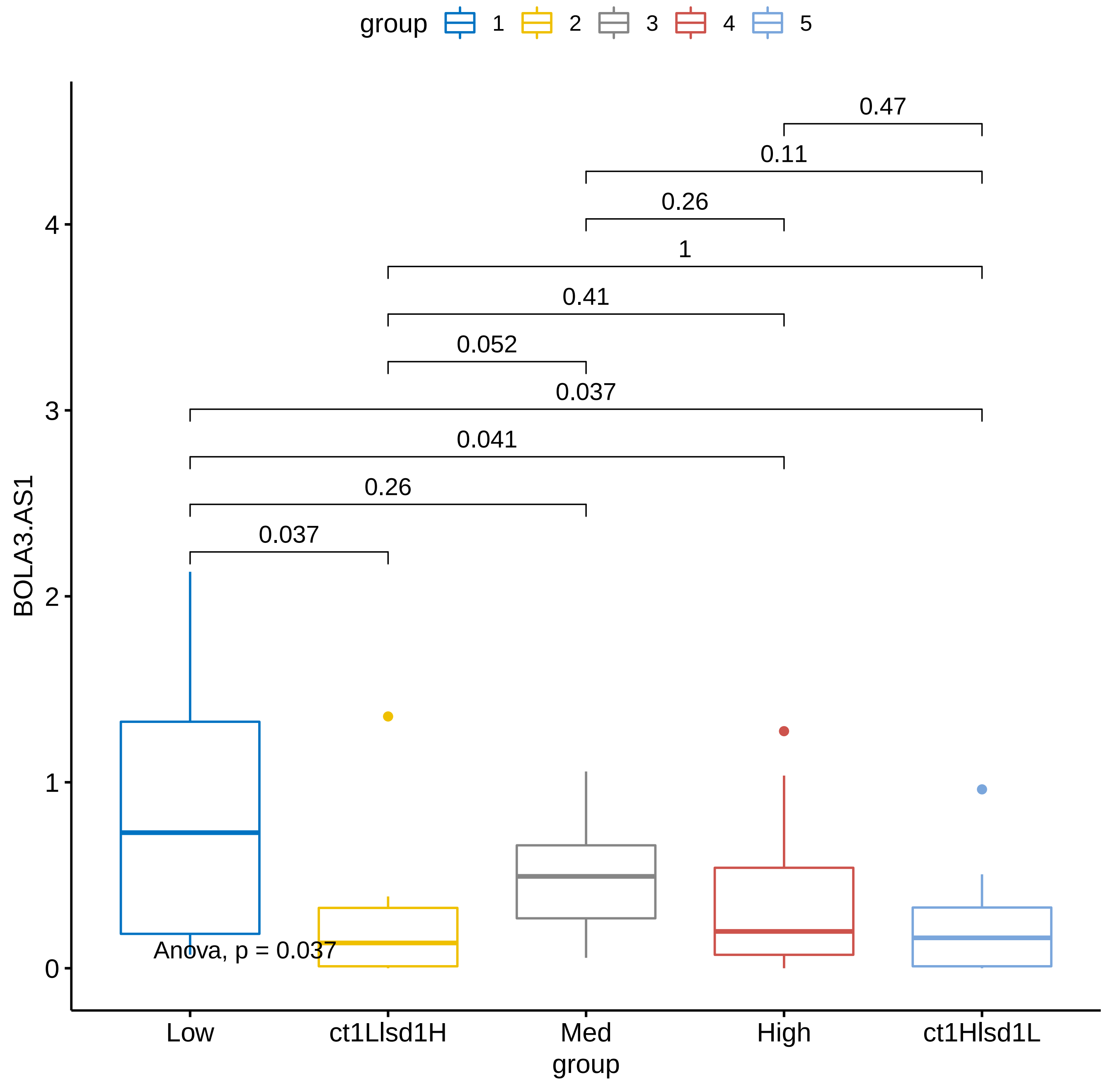

group
1
2
3
4
5
BOLA3.AS1
0
1
2
3
4
0.47
0.11
0.26
1
0.41
0.052
0.037
0.041
0.26
0.037
Anova, p = 0.037
Low
ct1Llsd1H
Med
High
ct1Hlsd1L
group

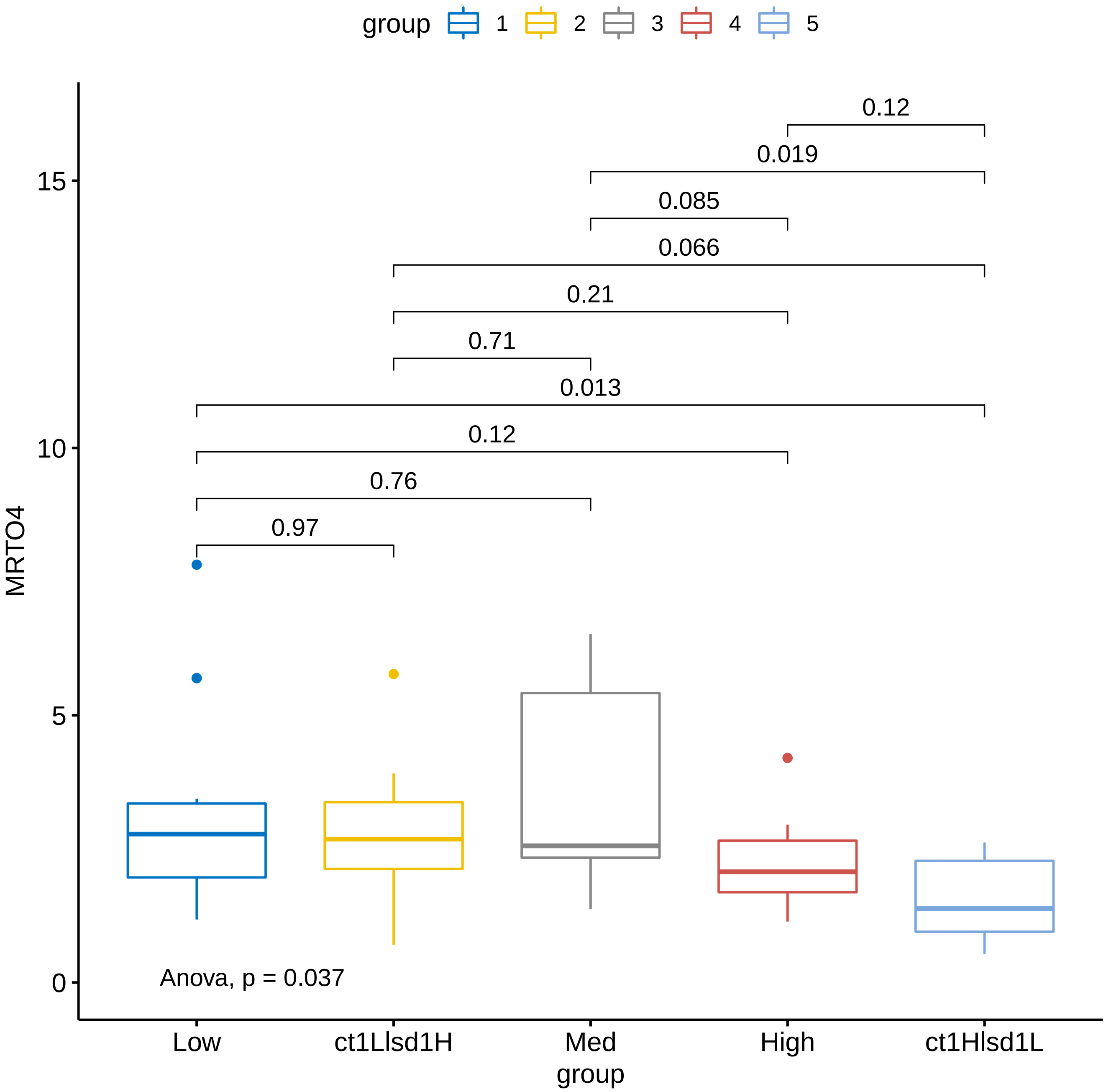
group 1 2 3 4 5
0.12
0.019
0.085
0.066
0.21
0.71
0.013
0.12
0.76
0.97
Anova, p = 0.037
MRTO4
15
10
5
0
Low
ct1Llsd1H
Med
High
ct1Hlsd1L
group

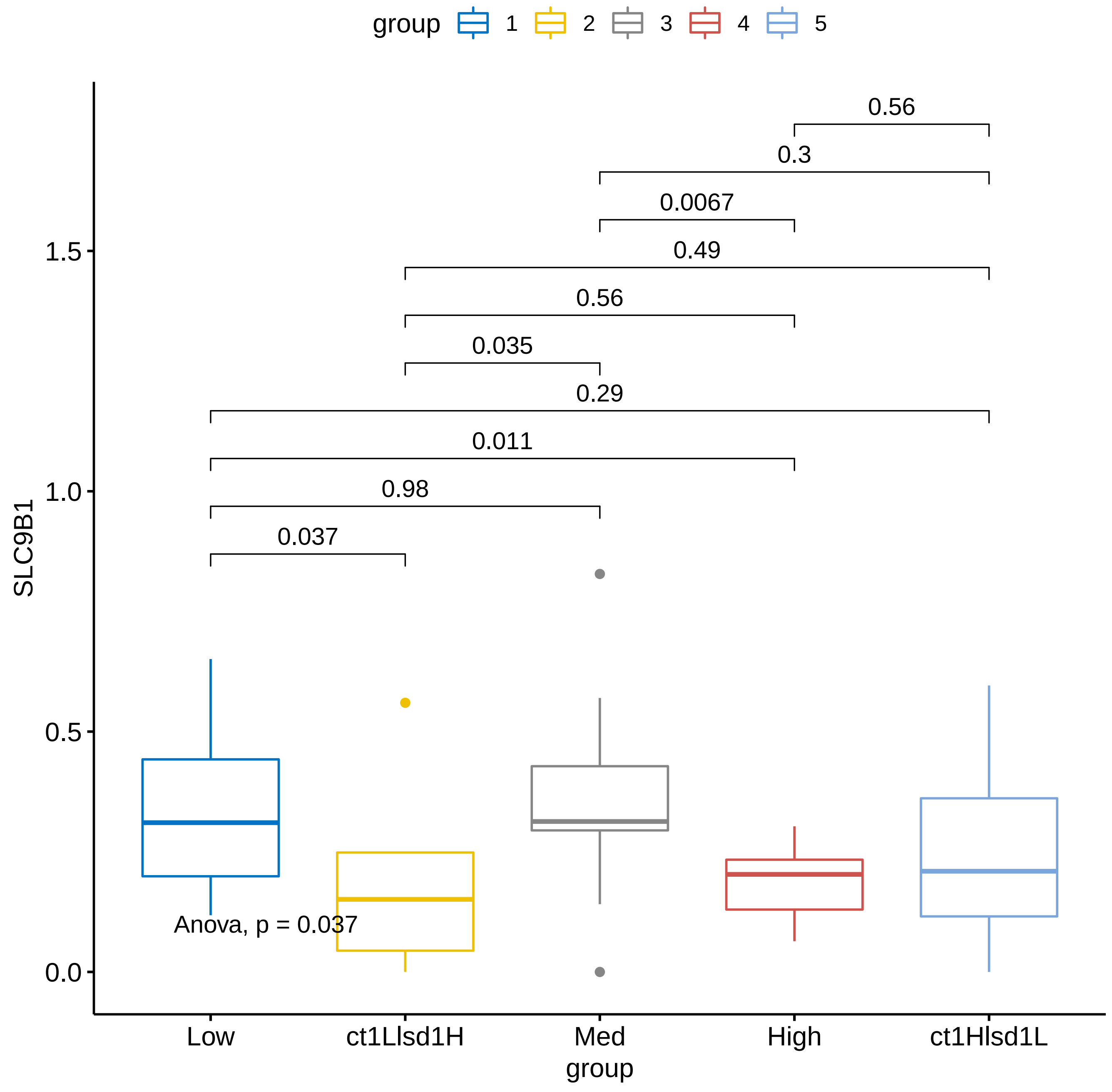

group
1
2
3
4
5
0.56
0.3
0.0067
0.49
0.56
0.035
0.29
0.011
0.98
0.037
1.5
1.0
0.5
0.0
SLC9B1
Anova, p = 0.037
Low
ct1Llsd1H
Med
High
ct1Hlsd1L
group

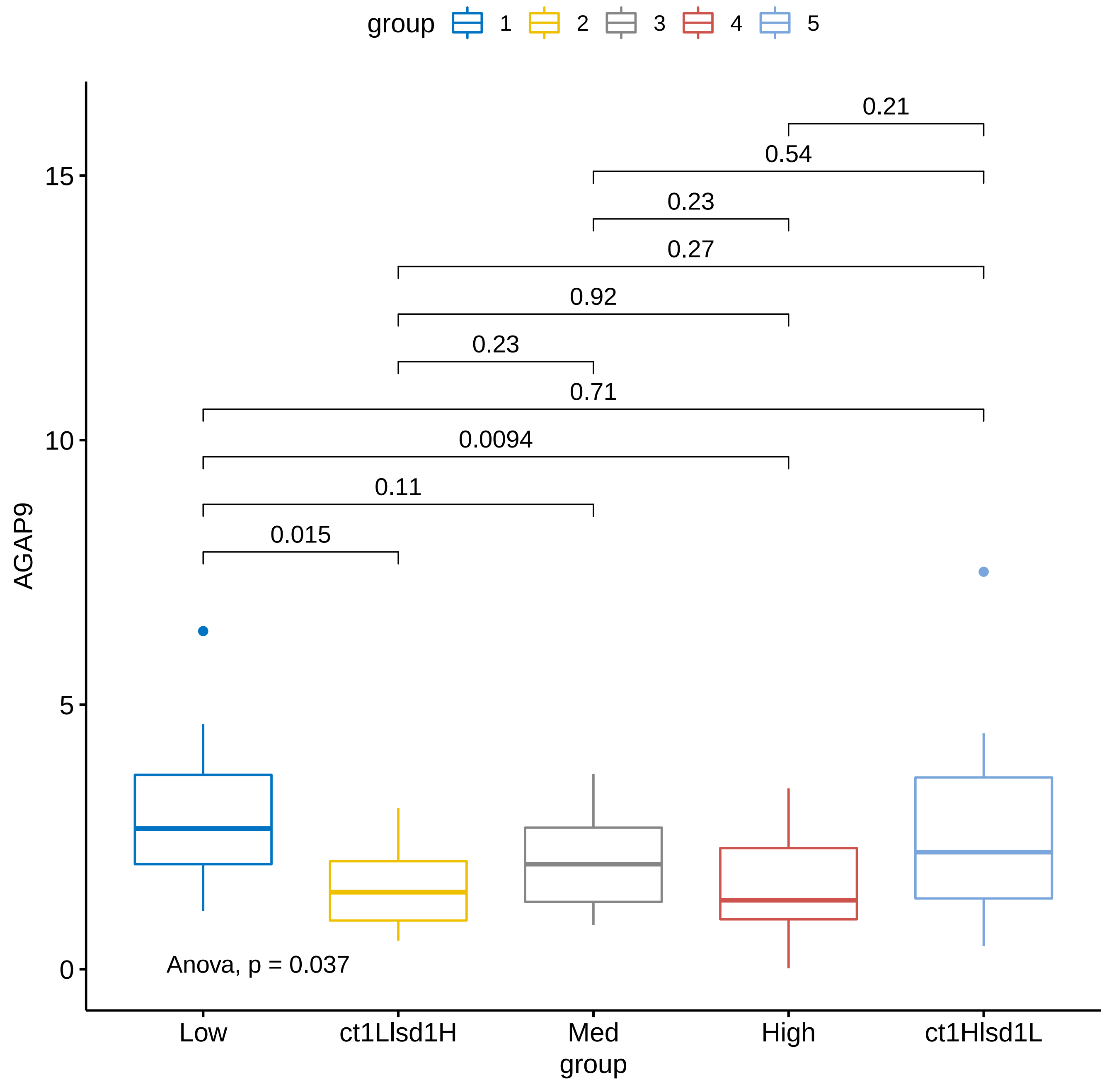

group
1
2
3
4
5
0.21
0.54
0.23
0.27
0.92
0.23
0.71
0.0094
0.11
0.015
15
10
5
0
AGAP9
Anova, p = 0.037
Low
ct1Llsd1H
Med
High
ct1Hlsd1L
group

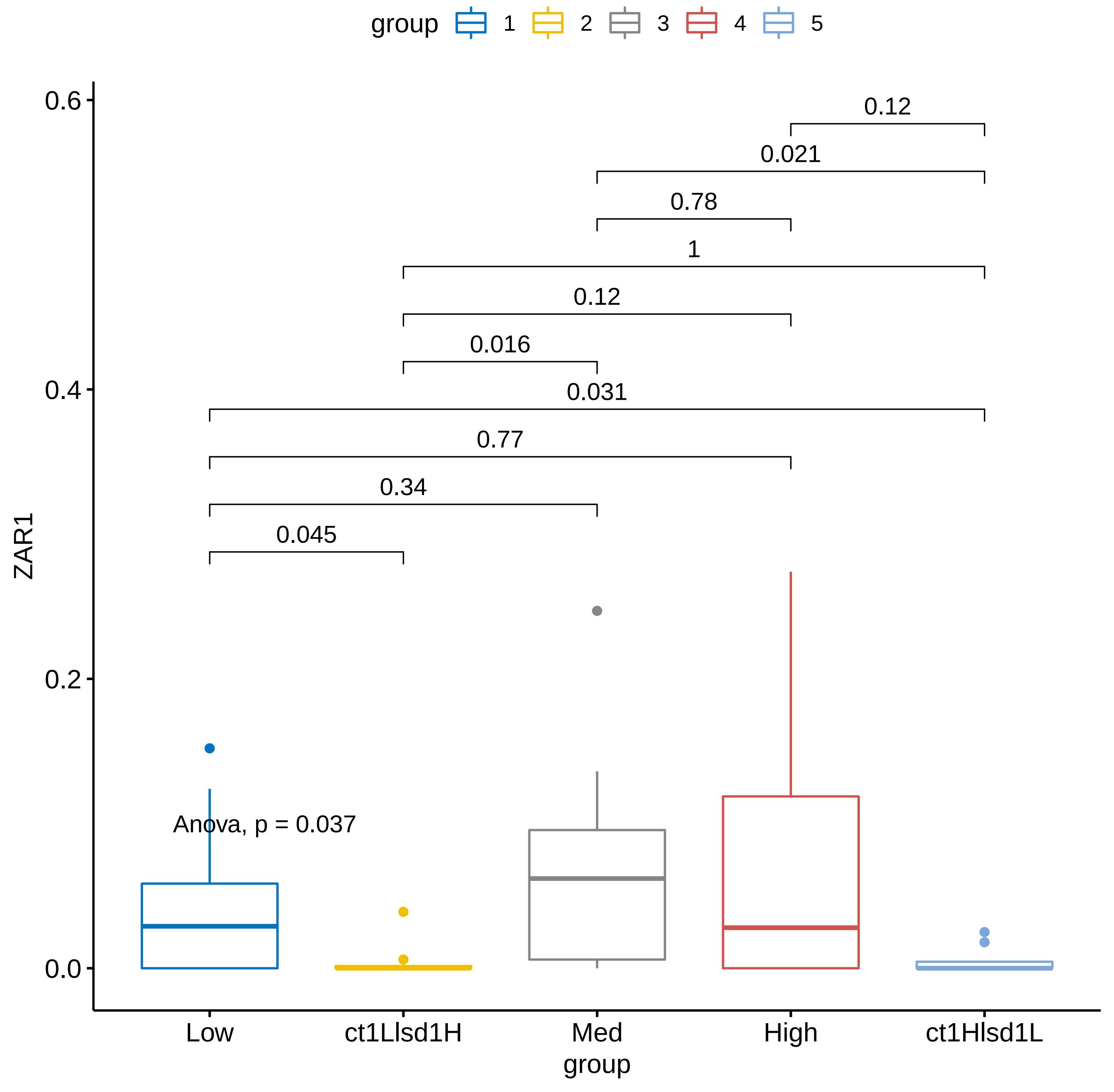
group
1
2
3
4
5
0.6
0.4
0.2
0.0
ZAR1
0.12
0.021
0.78
1
0.12
0.016
0.031
0.77
0.34
0.045
Anova, p = 0.037
Low
ct1Llsd1H
Med
High
ct1Hlsd1L
group

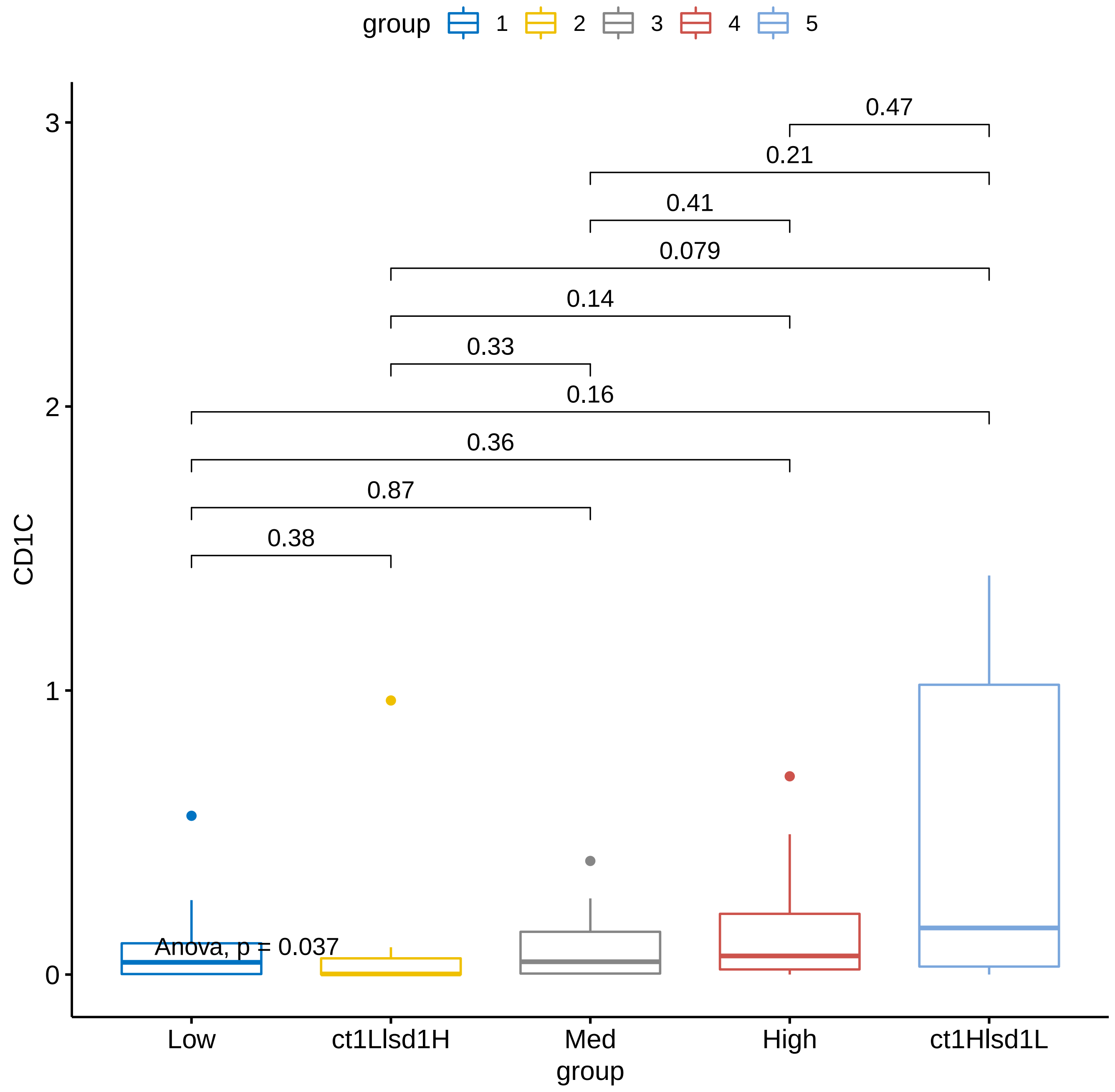

group
1
2
3
4
5
0.47
0.21
0.41
0.079
0.14
0.33
0.16
0.36
0.87
0.38
Anova, p = 0.037
CD1C
3
2
1
0
Low
ct1Llsd1H
Med
High
ct1Hlsd1L
group

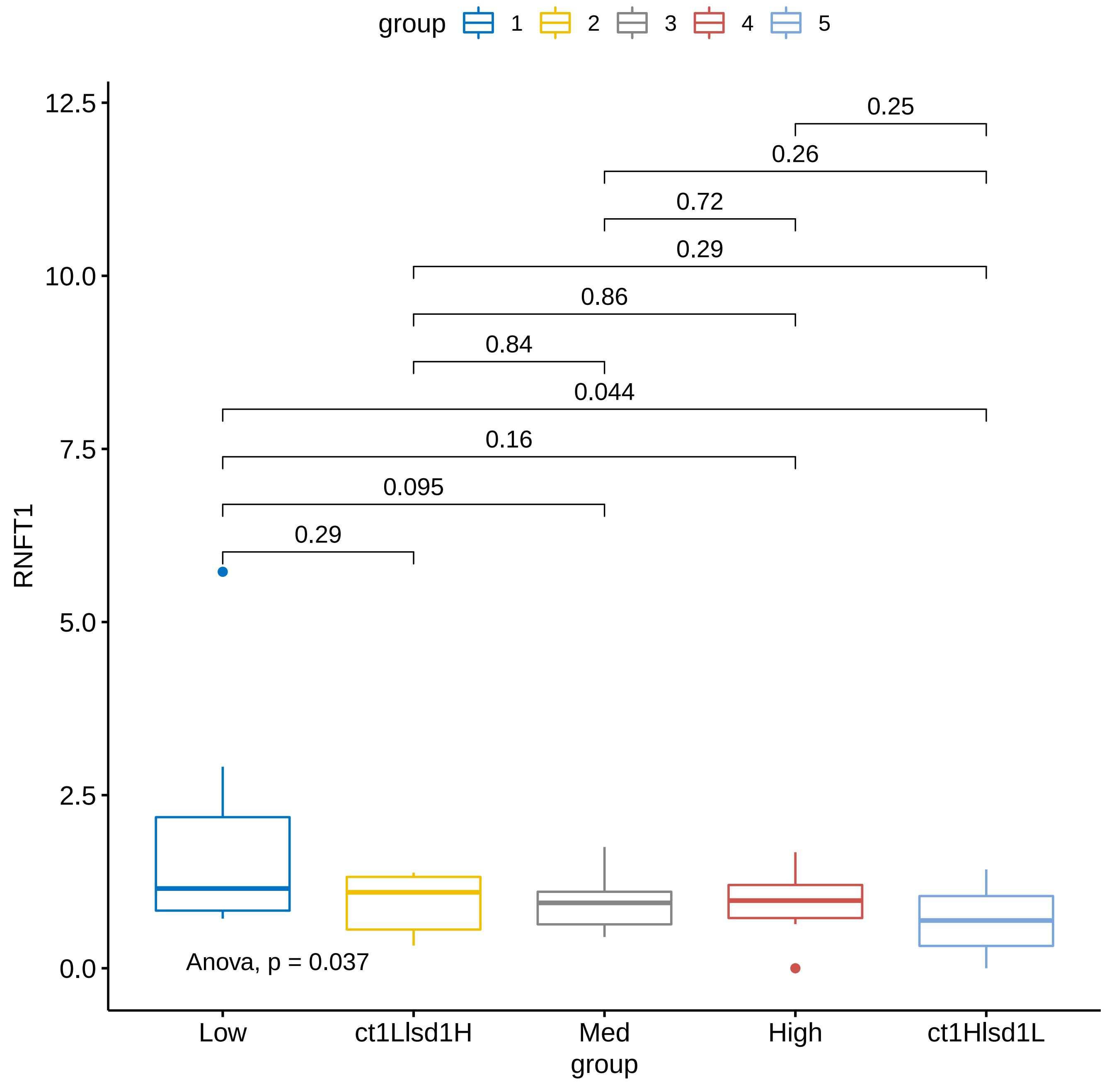

group
1
2
3
4
5
RNFT1
12.5
10.0
7.5
5.0
2.5
0.0
0.25
0.26
0.72
0.29
0.86
0.84
0.044
0.16
0.095
0.29
Anova, p = 0.037
Low
ct1Llsd1H
Med
High
ct1Hlsd1L
group

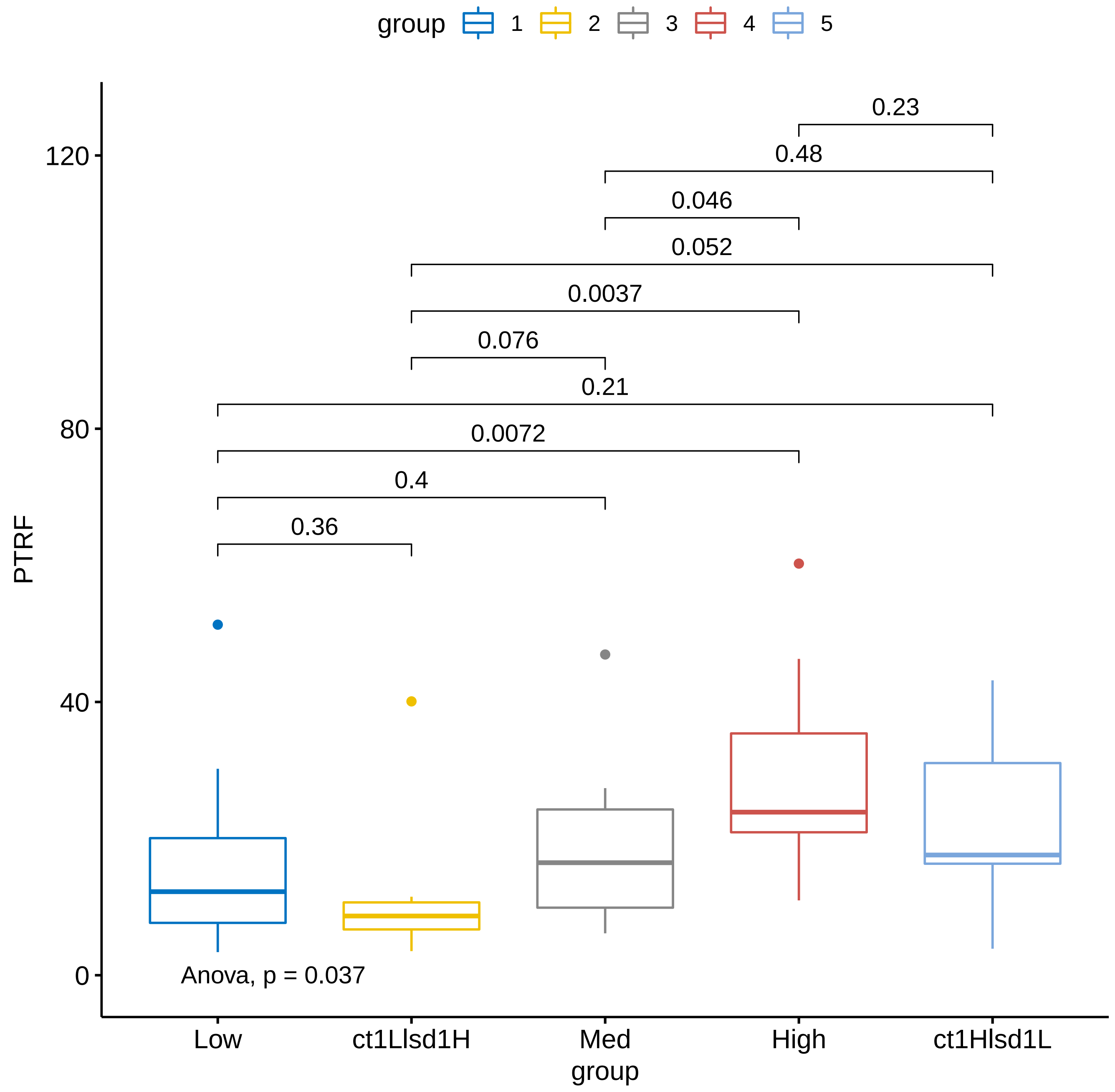

group 1 2 3 4 5
0.23
0.48
0.046
0.052
0.0037
0.076
0.21
0.0072
0.4
0.36
120
80
40
0
PTRF
Anova, p = 0.037
Low
ct1Llsd1H
Med
High
ct1Hlsd1L
group

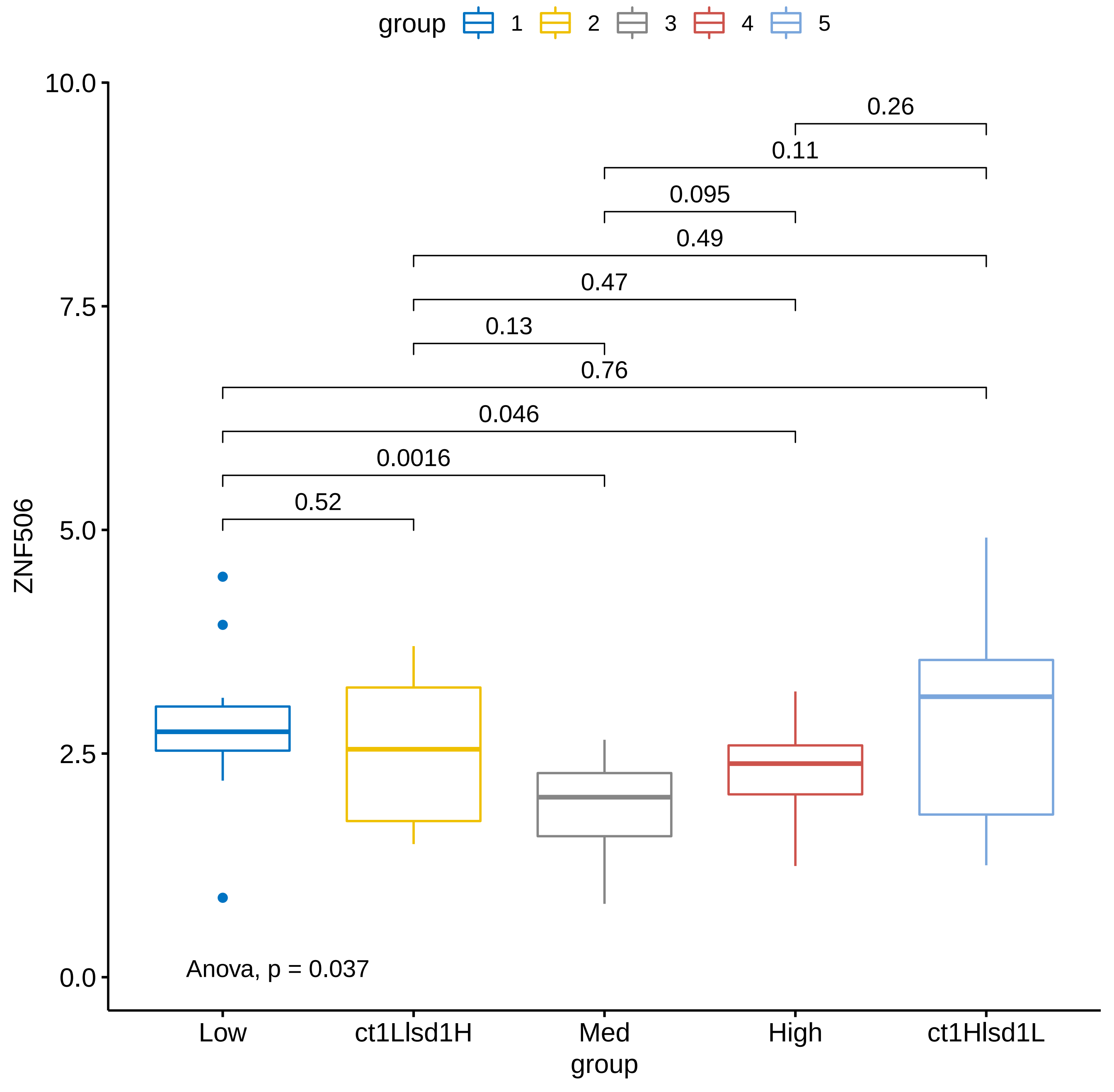
group
1
2
3
4
5
10.0
7.5
5.0
2.5
0.0
ZNF506
0.26
0.11
0.095
0.49
0.47
0.13
0.76
0.046
0.0016
0.52
Anova, p = 0.037
Low
ct1Llsd1H
Med
High
ct1Hlsd1L
group

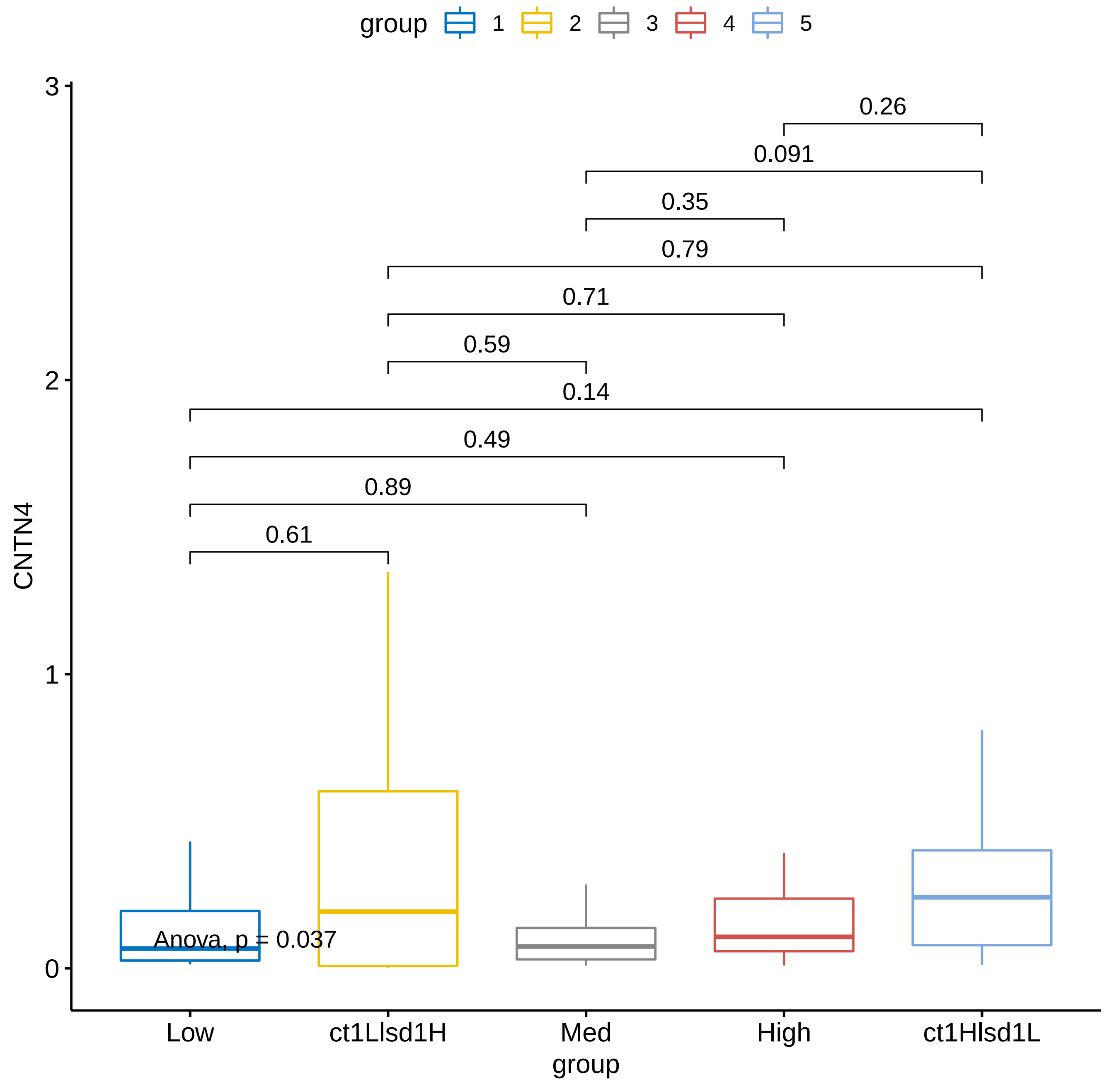
group
1
2
3
4
5
CNTN4
3
2
1
0
0.26
0.091
0.35
0.79
0.71
0.59
0.14
0.49
0.89
0.61
Anova, p = 0.037
Low
ct1Llsd1H
Med
High
ct1Hlsd1L
group

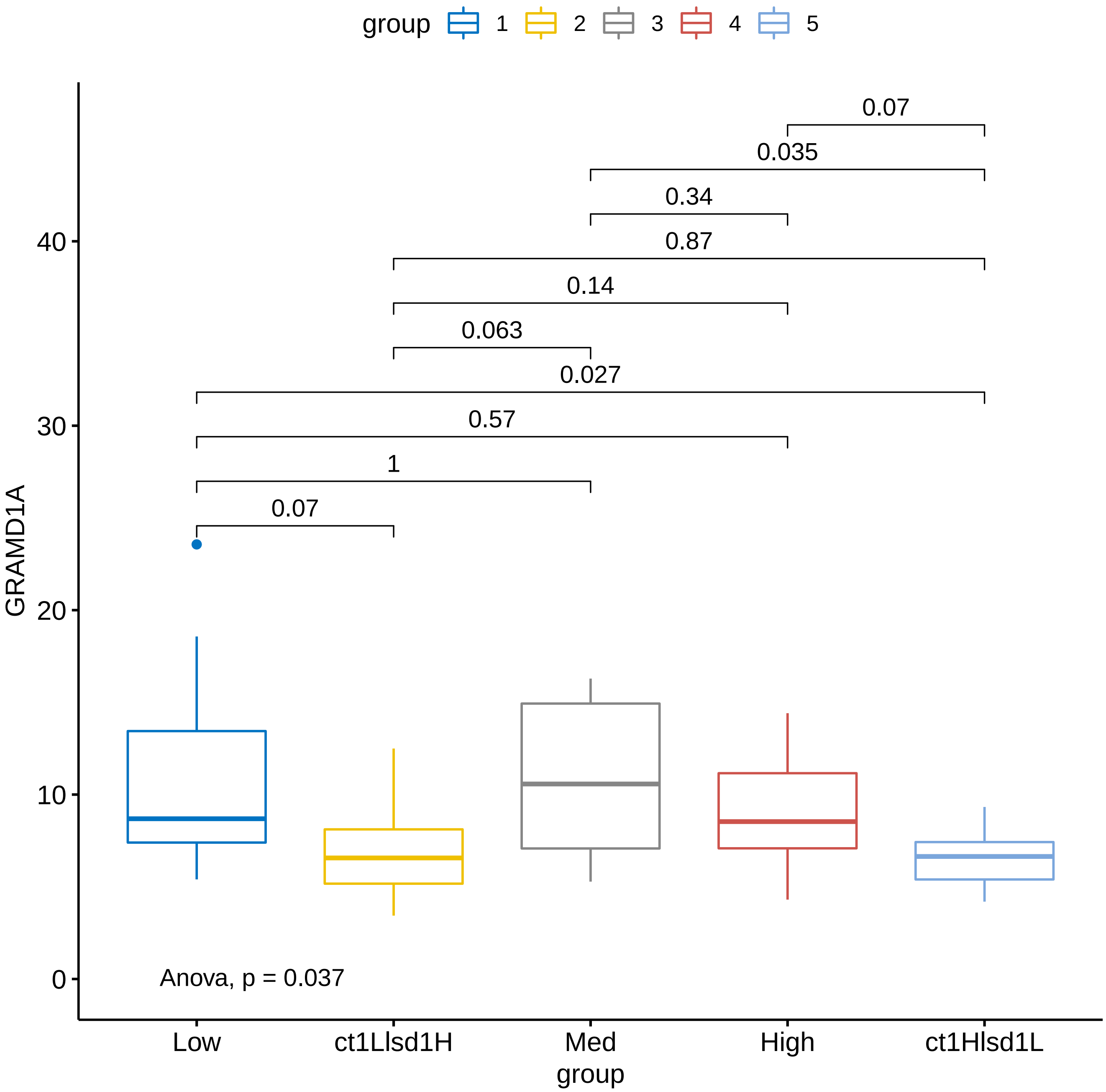
group
1
2
3
4
5
0.07
0.035
0.34
0.87
0.14
0.063
0.027
0.57
1
0.07
40
30
20
10
0
GRAMD1A
Anova, p = 0.037
Low
ct1Llsd1H
Med
High
ct1Hlsd1L
group

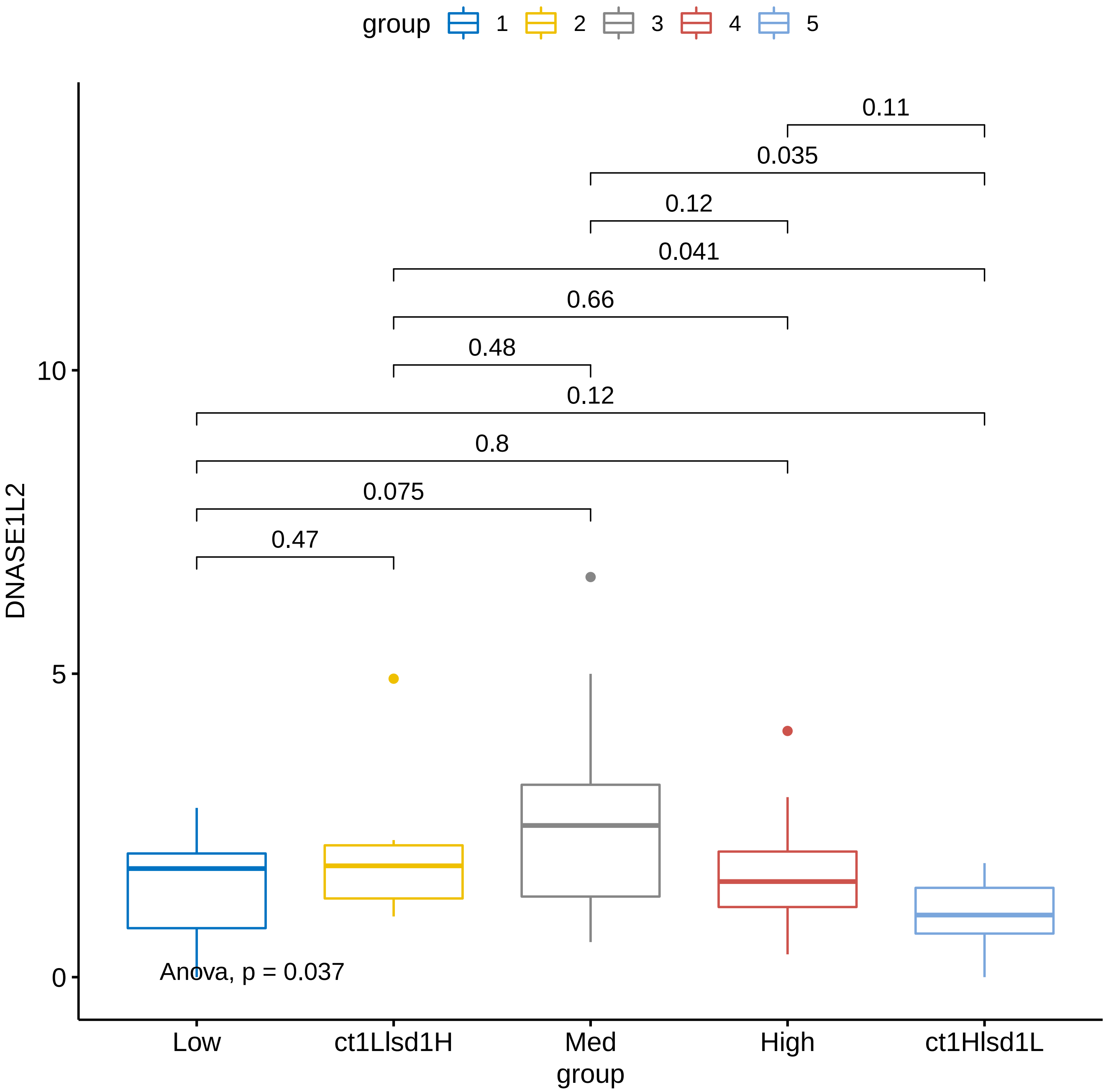
group 1 2 3 4 5
0.11
0.035
0.12
0.041
0.66
0.48
0.12
0.8
0.075
0.47
Anova, p = 0.037
DNASE1L2
10
5
0
Low
ct1Llsd1H
Med
High
ct1Hlsd1L
group

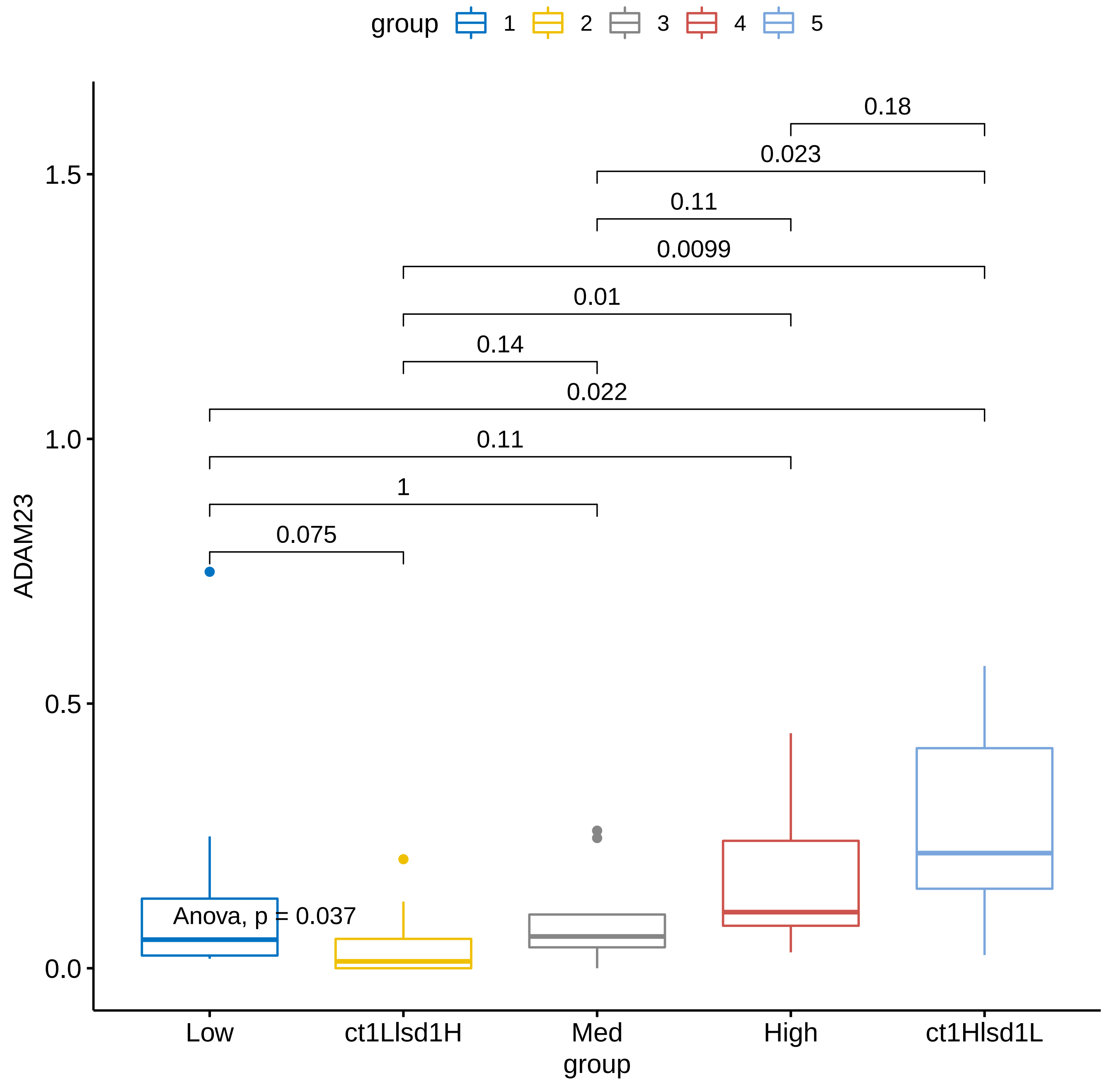

group 1 2 3 4 5
0.18
0.023
1.5
0.11
0.0099
0.01
0.14
0.022
1.0
0.11
1
0.075
ADAM23
0.5
Anova, p = 0.037
0.0
Low
ct1Llsd1H
Med
High
ct1Hlsd1L
group

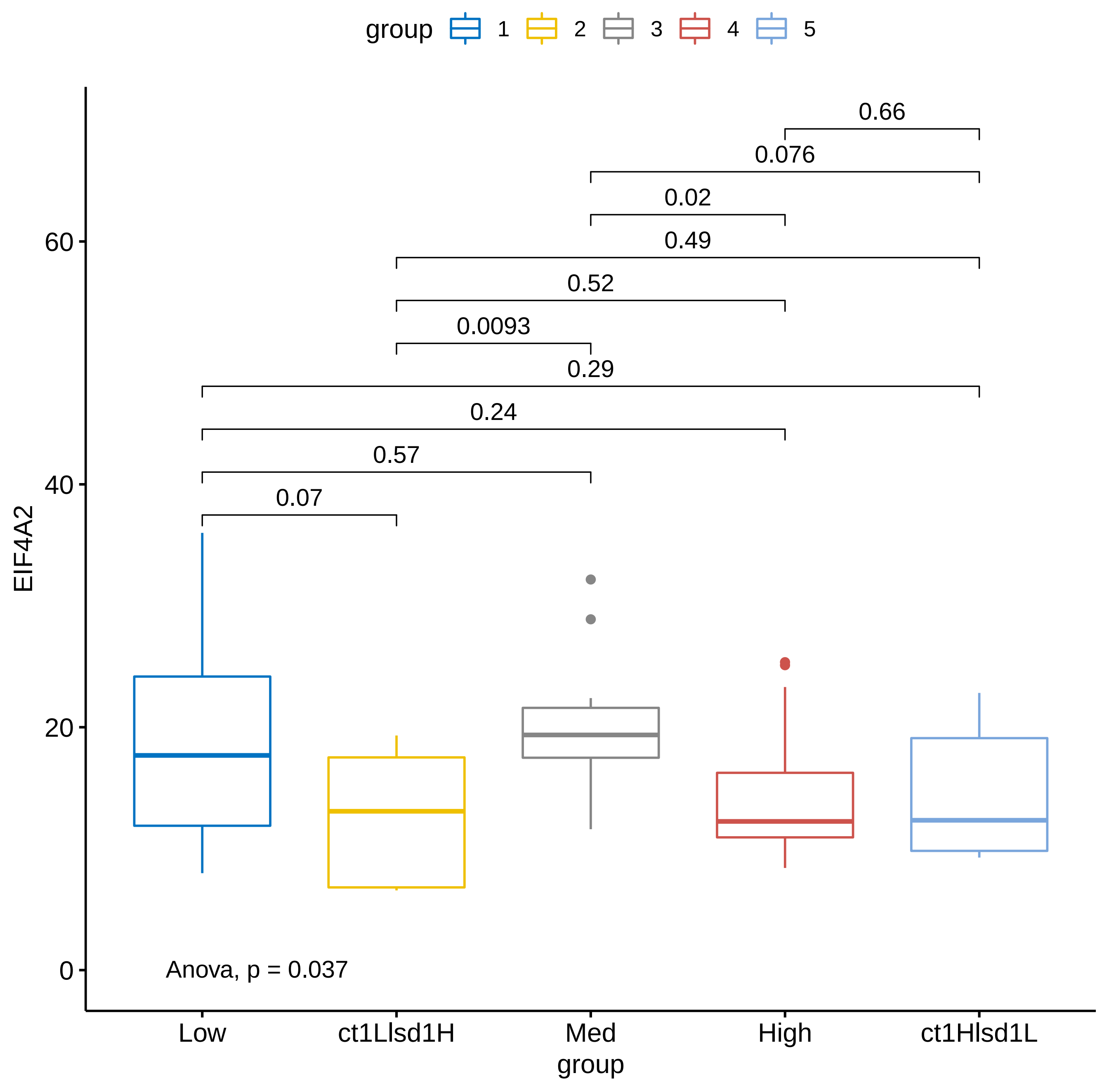

group 1 2 3 4 5
0.66
0.076
0.02
0.49
0.52
0.0093
0.29
0.24
0.57
0.07
60
40
20
0
EIF4A2
Anova, p = 0.037
Low
ct1Llsd1H
Med
High
ct1Hlsd1L
group

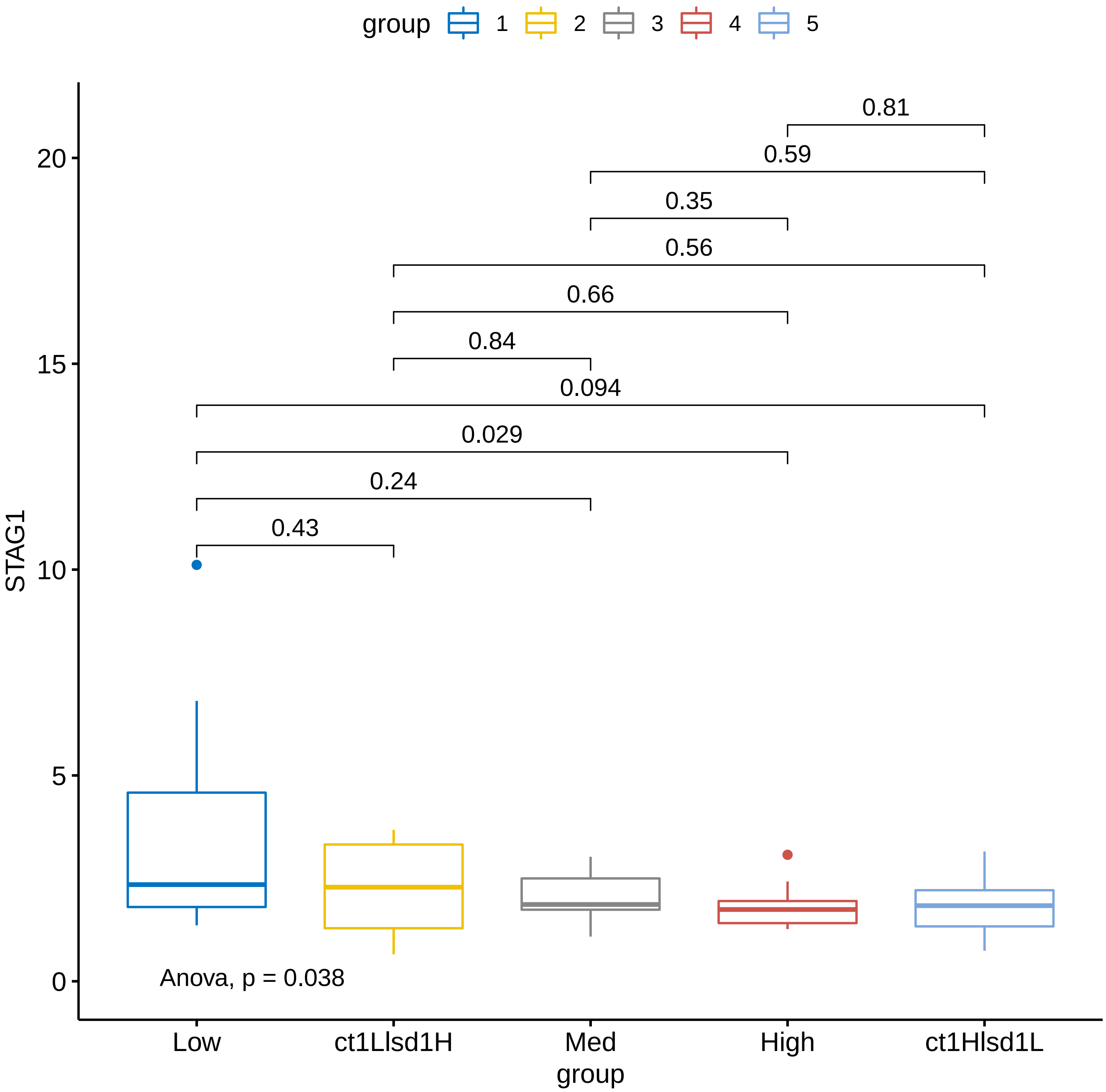

group
1
2
3
4
5
0.81
0.59
0.35
0.56
0.66
0.84
0.094
0.029
0.24
0.43
STAG1
20
15
10
5
0
Anova, p = 0.038
Low
ct1Llsd1H
Med
High
ct1Hlsd1L
group

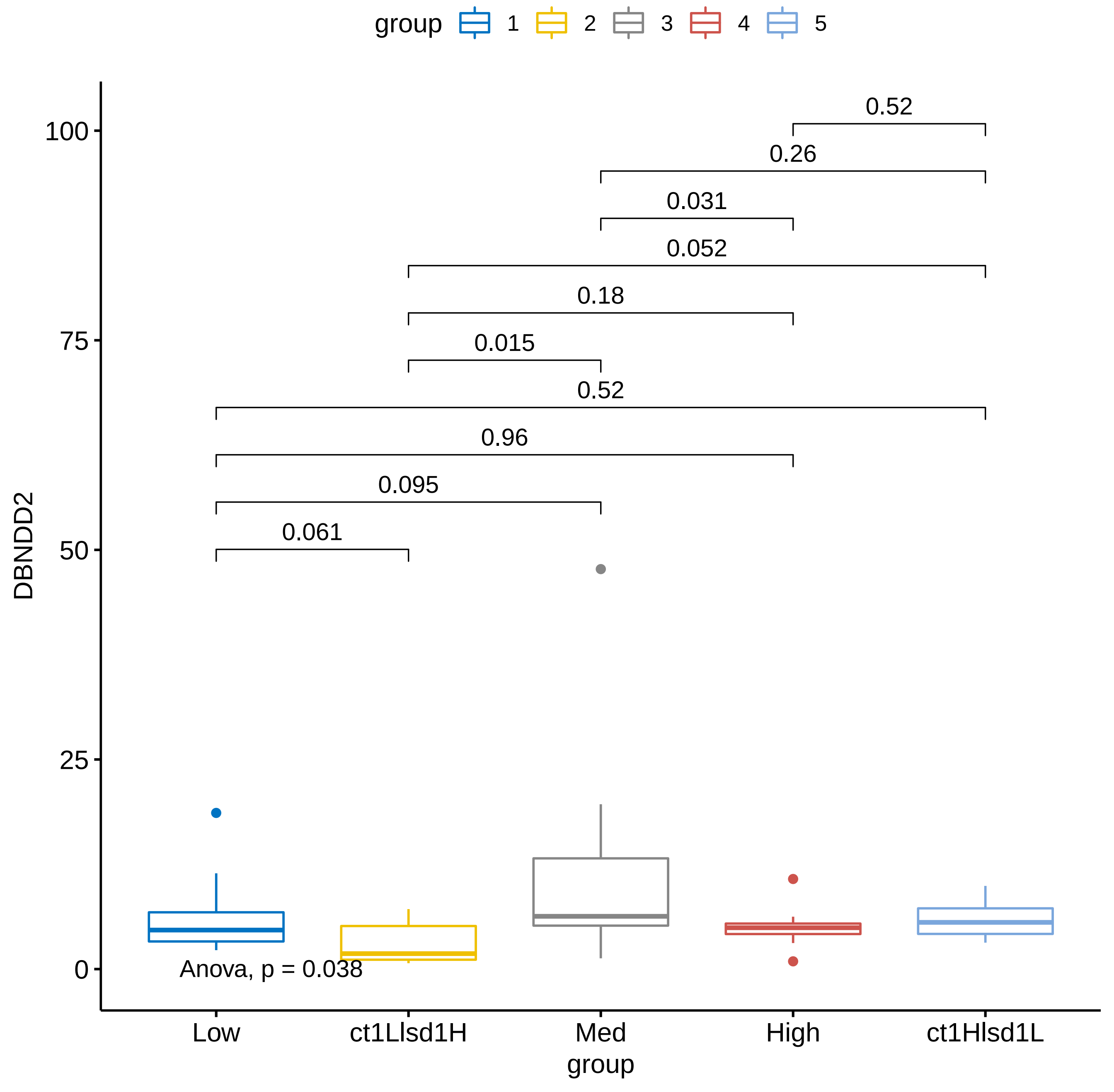
group
1
2
3
4
5
DBNDD2
100
75
50
25
0
0.52
0.26
0.031
0.052
0.18
0.015
0.52
0.96
0.095
0.061
Anova, p = 0.038
Low
ct1Llsd1H
Med
High
ct1Hlsd1L
group

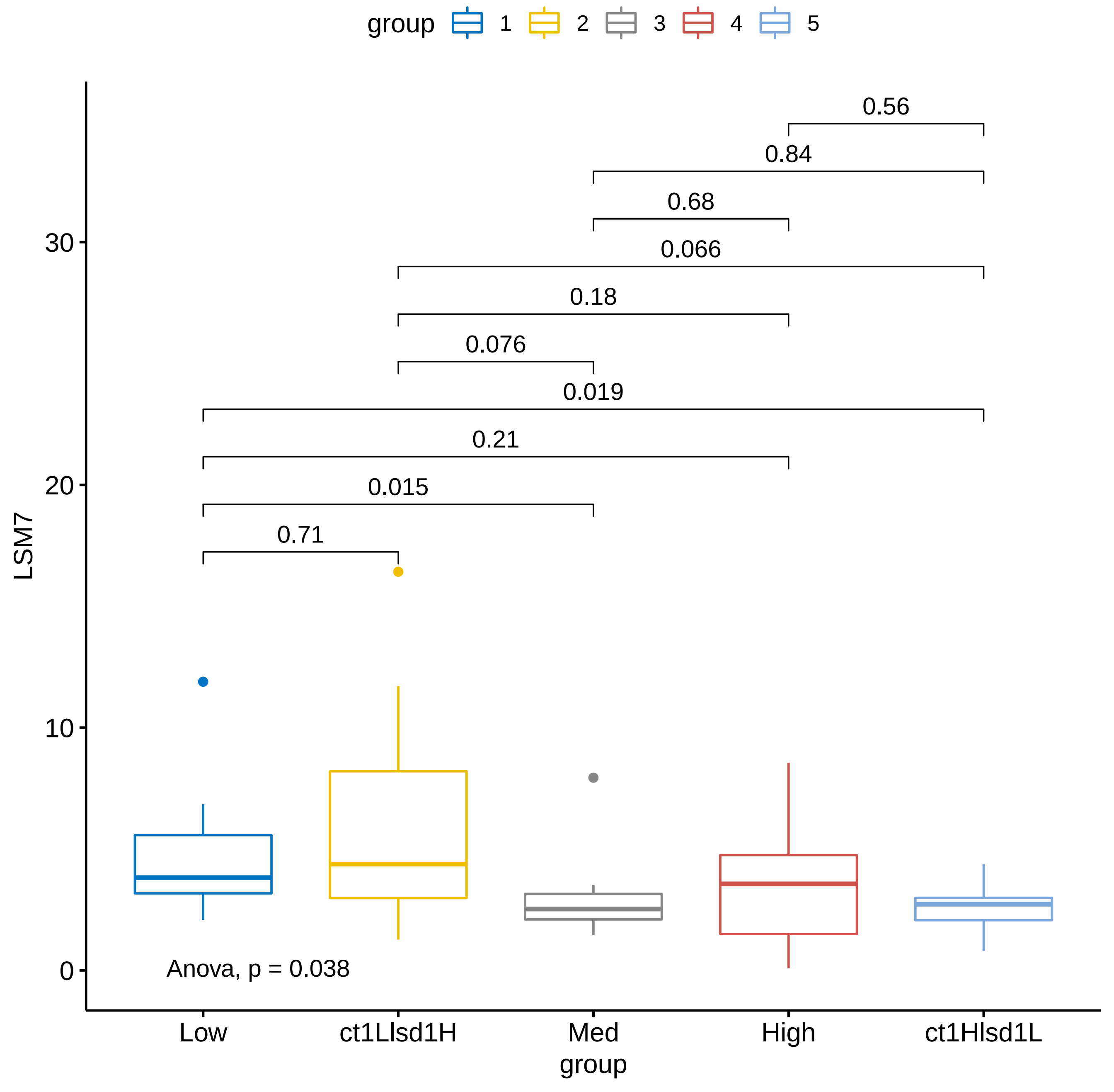

group
1
2
3
4
5
LSM7
30
20
10
0
0.56
0.84
0.68
0.066
0.18
0.076
0.019
0.21
0.015
0.71
Anova, p = 0.038
Low
ct1Llsd1H
Med
High
ct1Hlsd1L
group

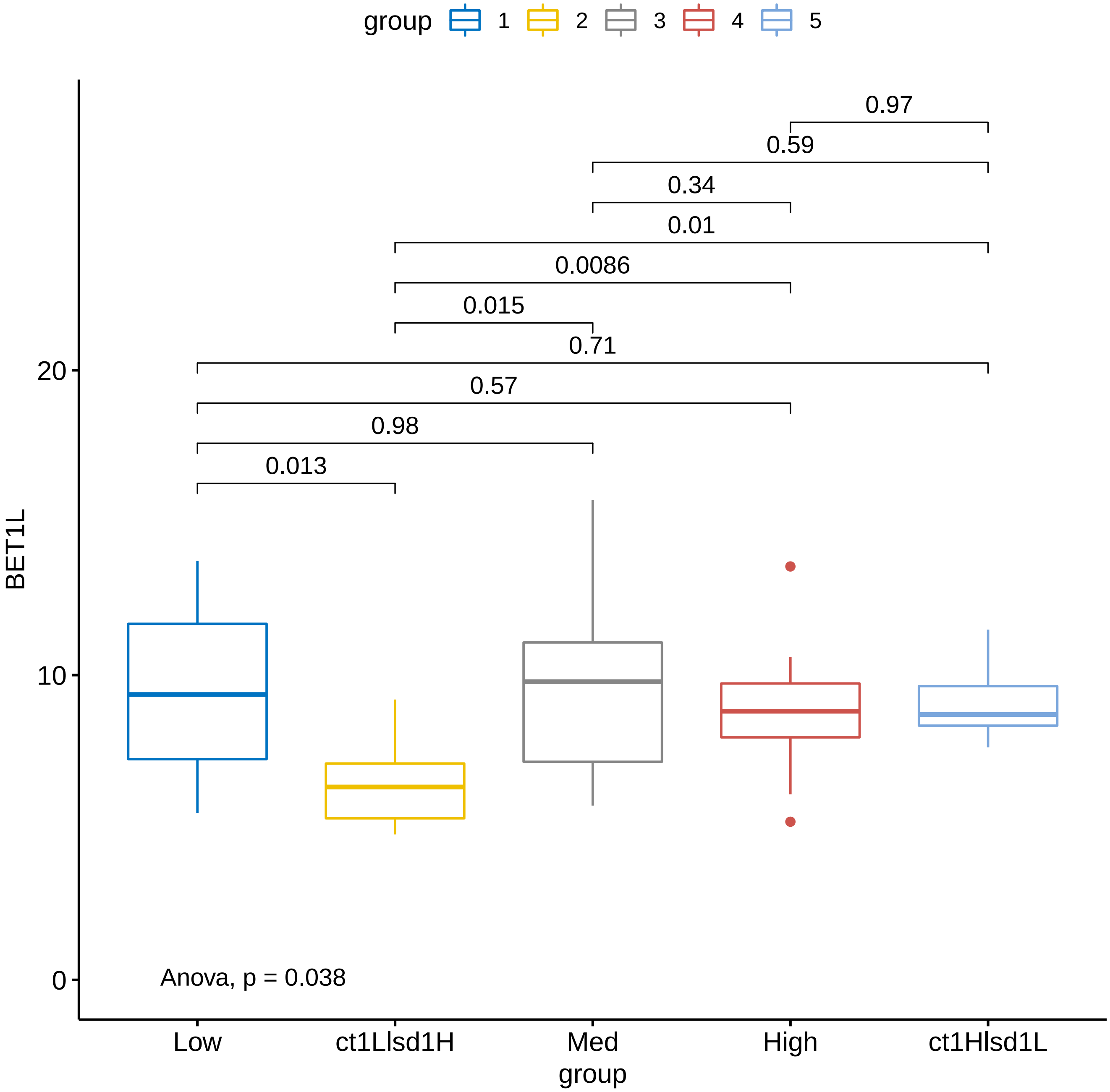

group 1 2 3 4 5
0.97
0.59
0.34
0.01
0.0086
0.015
0.71
0.57
0.98
0.013
BET1L
20
10
0
Anova, p = 0.038
Low
ct1Llsd1H
Med
High
ct1Hlsd1L
group

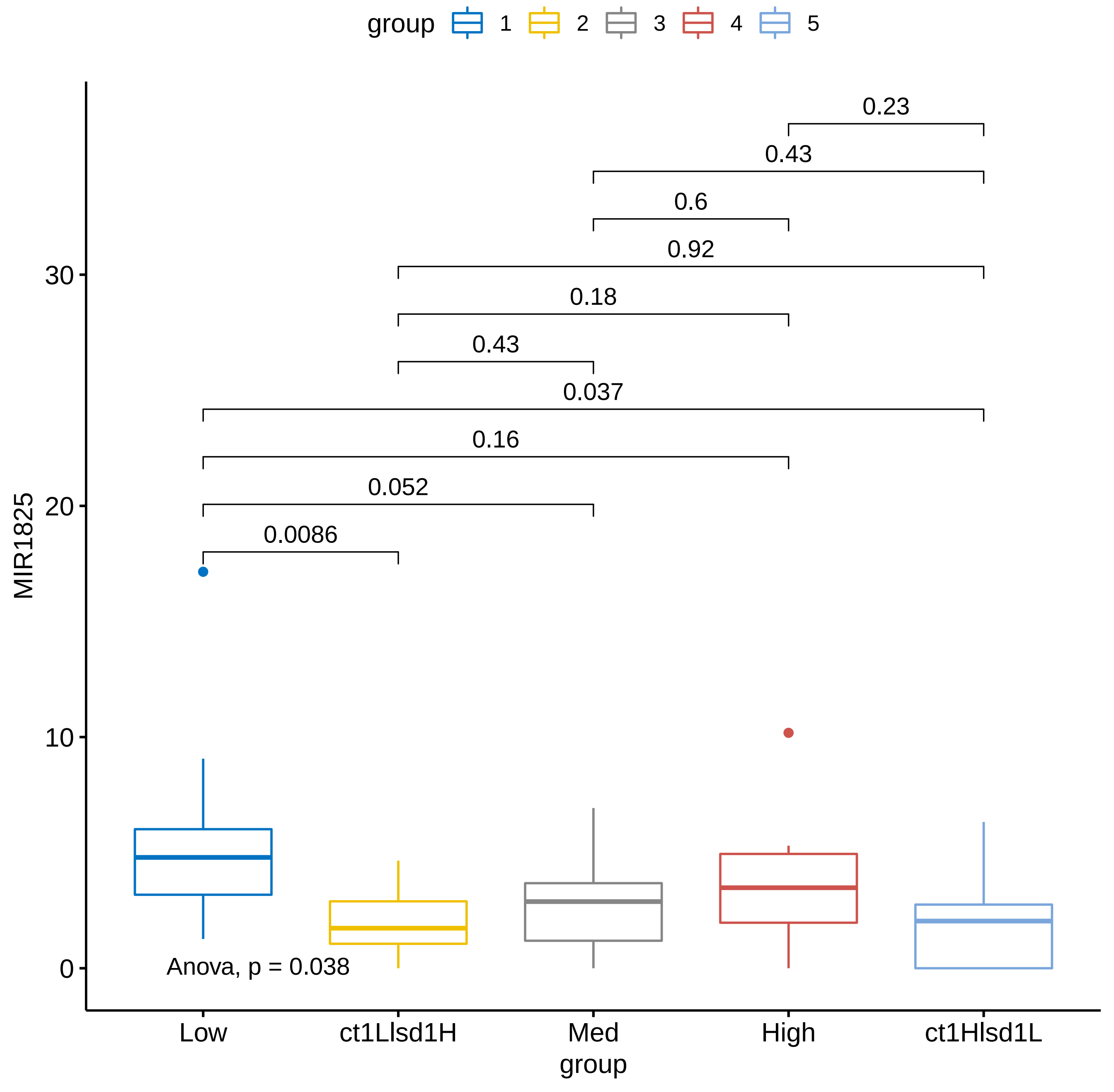
group 1 2 3 4 5
0.23
0.43
0.6
0.92
0.18
0.43
0.037
0.16
0.052
0.0086
Anova, p = 0.038
MIR1825
30
20
10
0
Low
ct1Llsd1H
Med
High
ct1Hlsd1L
group

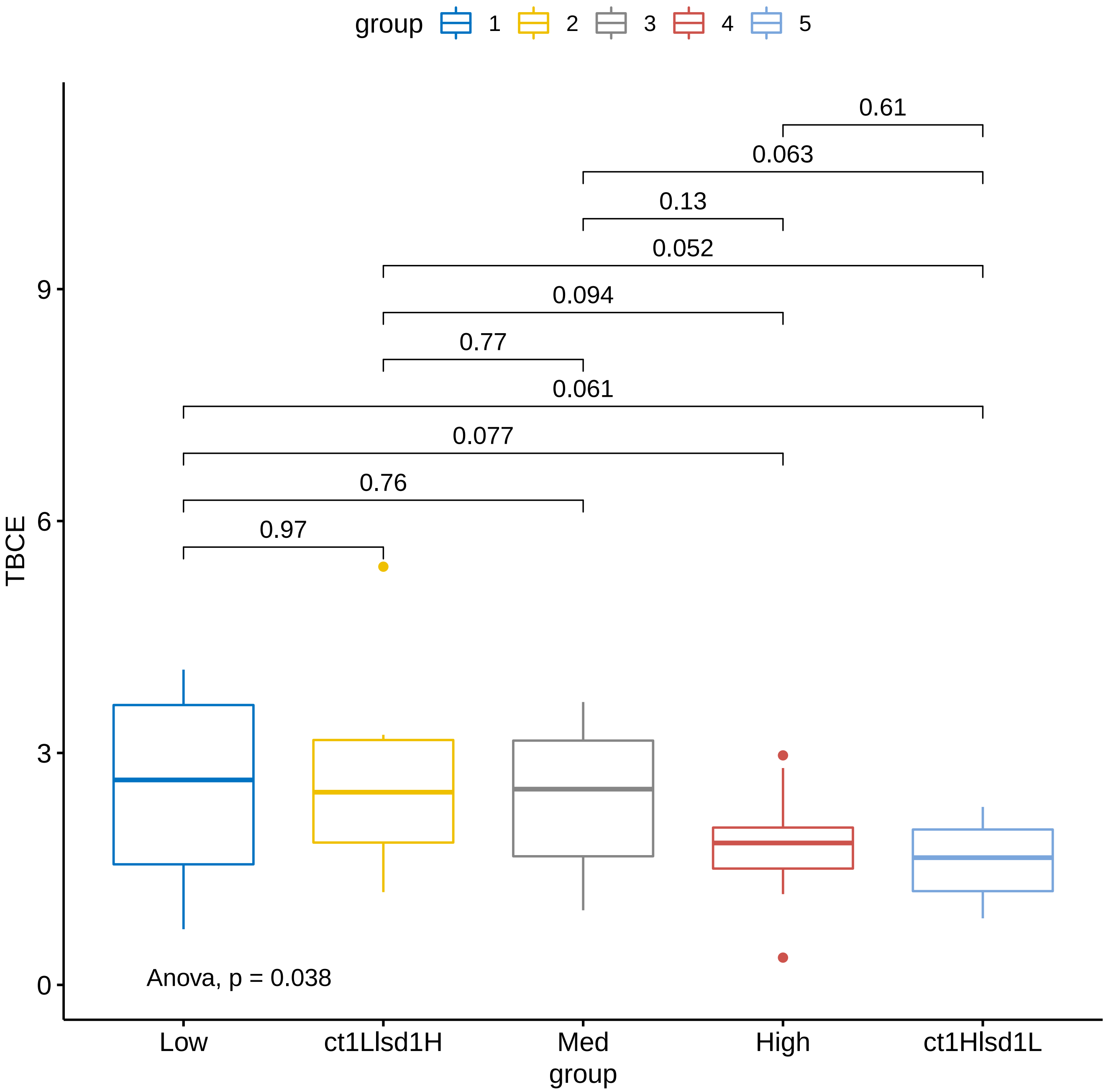

group 1 2 3 4 5
0.61
0.063
0.13
0.052
0.094
0.77
0.061
0.077
0.76
0.97
9
6
3
0
TBCE
Anova, p = 0.038
Low
ct1Llsd1H
Med
High
ct1Hlsd1L
group

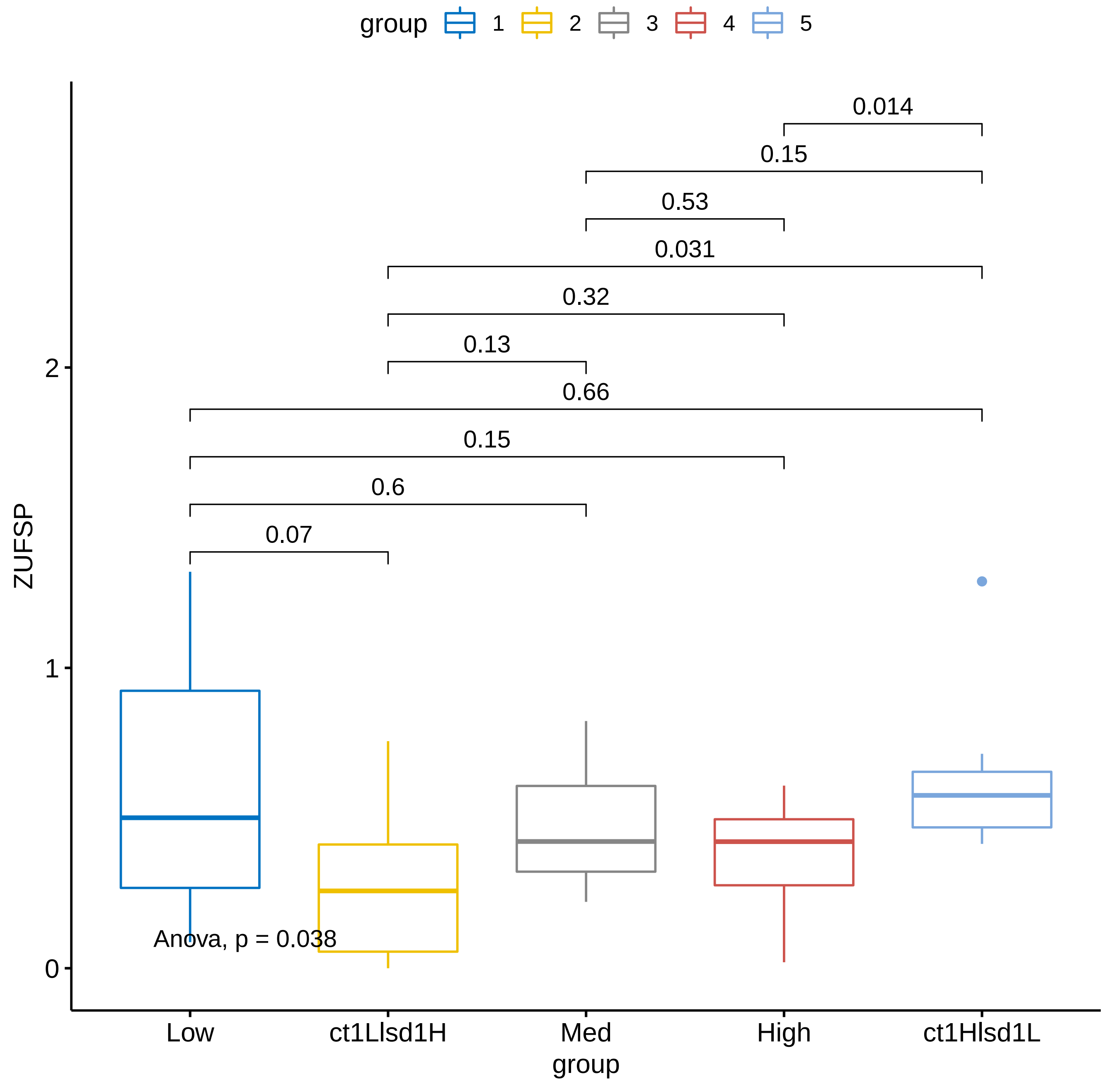

group
1
2
3
4
5
0.014
0.15
0.53
0.031
0.32
0.13
0.66
0.15
0.6
0.07
Anova, p = 0.038
ZUFSP
2
1
0
Low
ct1Llsd1H
Med
High
ct1Hlsd1L
group

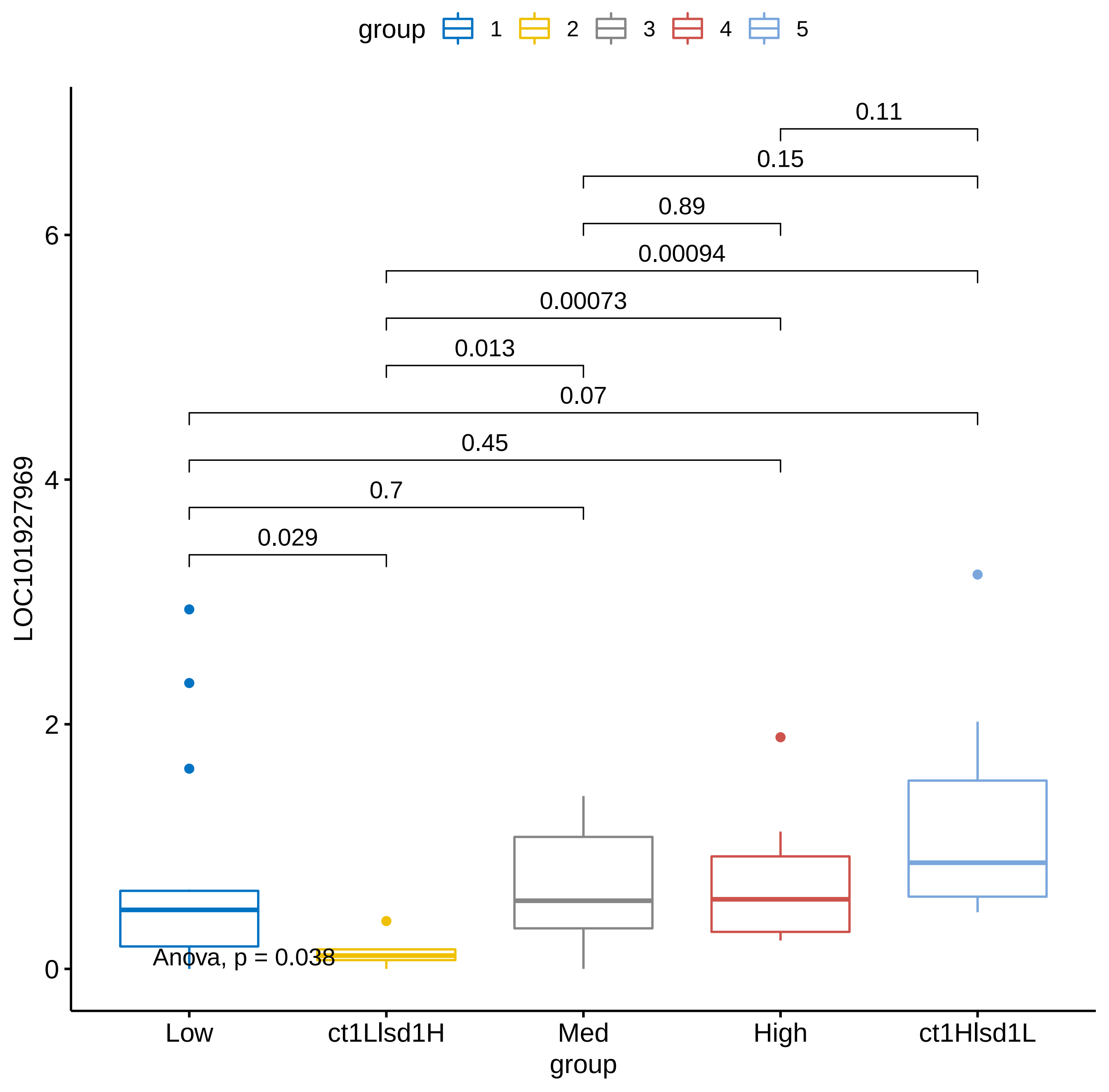

group 1 2 3 4 5
0.11
0.15
0.89
0.00094
0.00073
0.013
0.07
0.45
0.7
0.029
Anova, p = 0.038
LOC101927969
0
2
4
6
Low
ct1Llsd1H
Med
High
ct1Hlsd1L
group

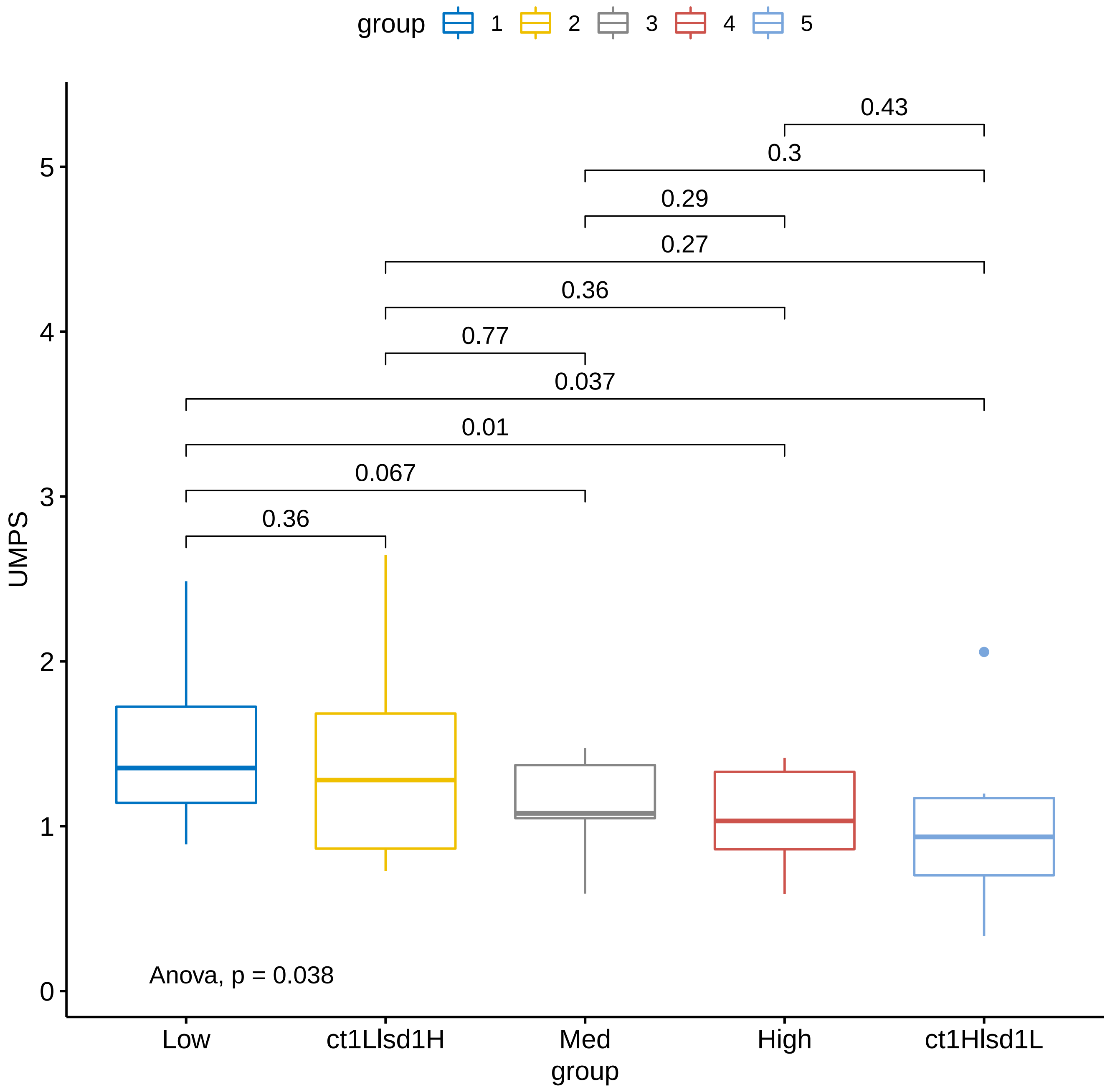

group
1
2
3
4
5
0.43
0.3
0.29
0.27
0.36
0.77
0.037
0.01
0.067
0.36
UMPS
5
4
3
2
1
0
Anova, p = 0.038
Low
ct1Llsd1H
Med
High
ct1Hlsd1L
group

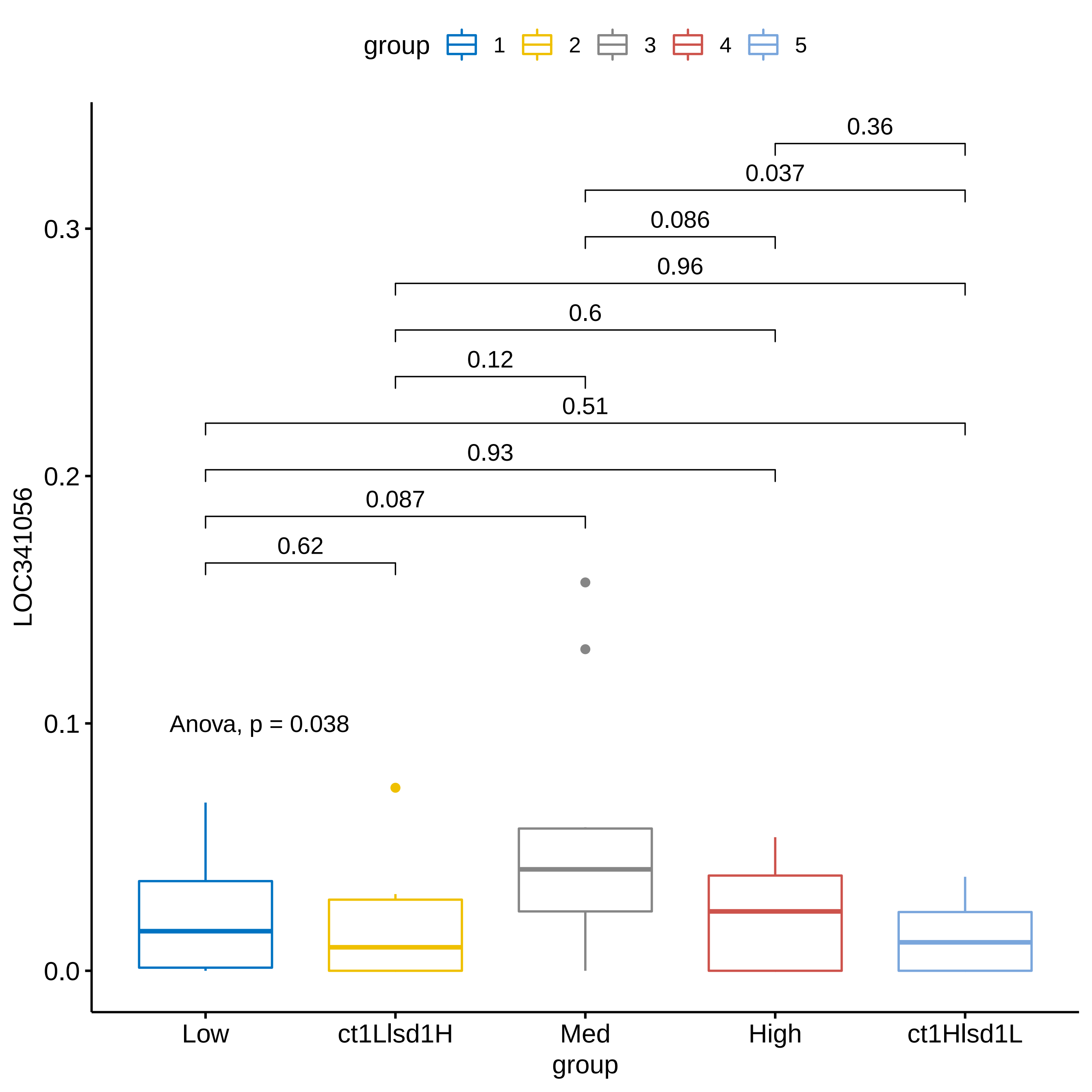
group
1
2
3
4
5
0.36
0.037
0.086
0.96
0.6
0.12
0.51
0.93
0.087
0.62
Anova, p = 0.038
0.3
0.2
0.1
0.0
LOC341056
Low
ct1Llsd1H
Med
High
ct1Hlsd1L
group

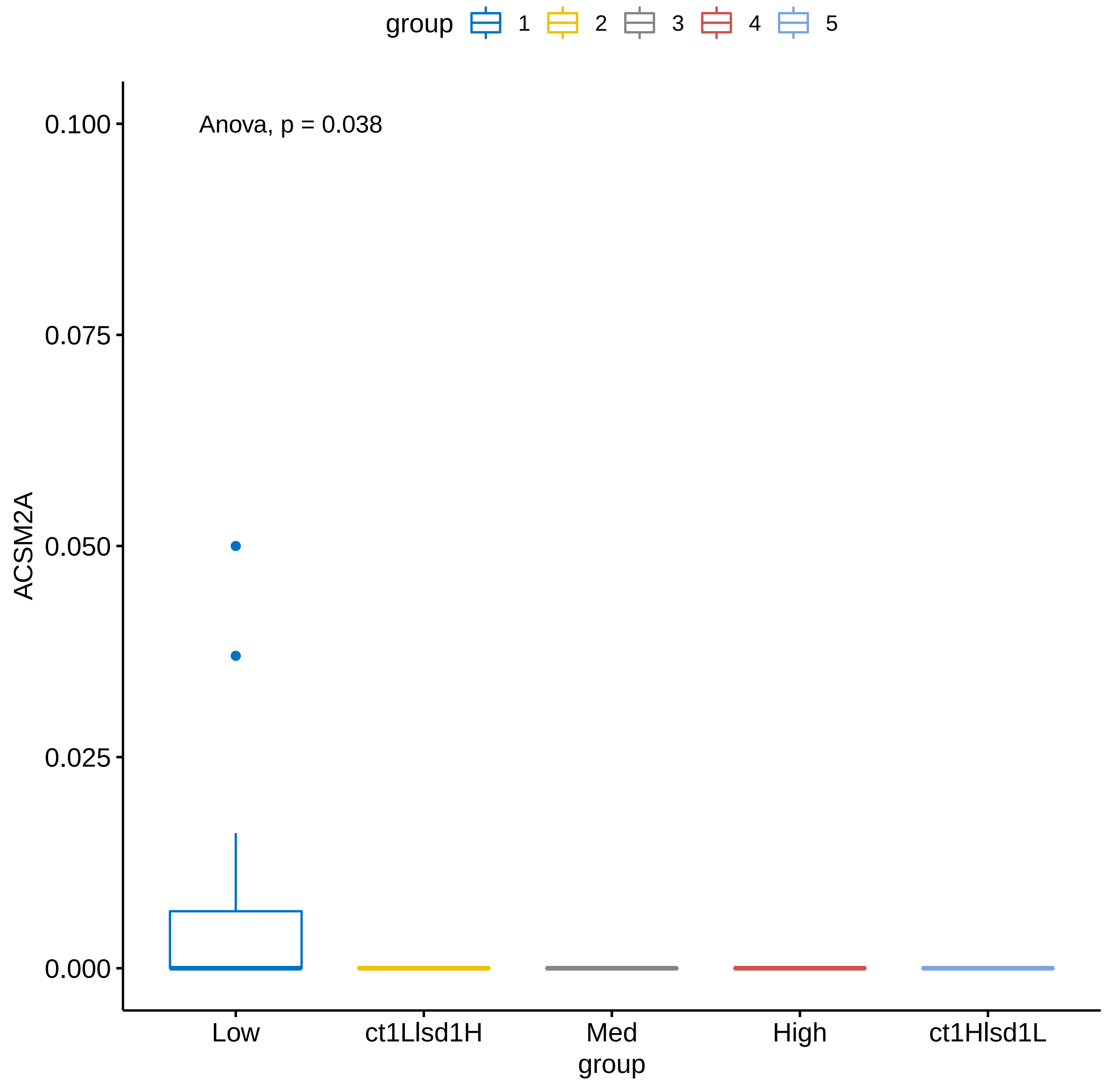

group
1
2
3
4
5
Anova, p = 0.038
ACSM2A
0.100
0.075
0.050
0.025
0.000
Low
ct1Llsd1H
Med
High
ct1Hlsd1L
group

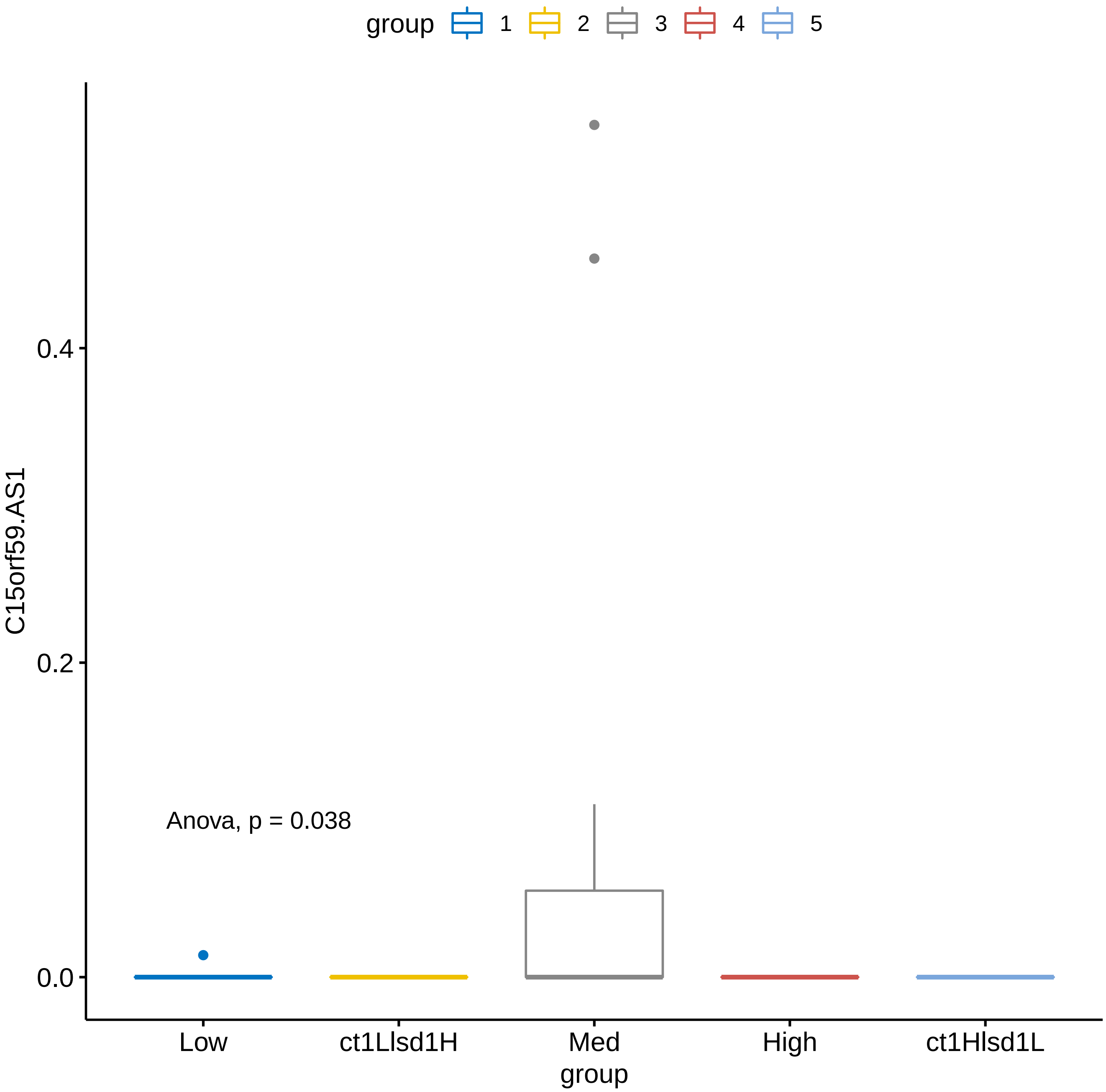

group
1
2
3
4
5
C15orf59.AS1
0.4
0.2
0.0
Anova, p = 0.038
Low
ct1Llsd1H
Med
High
ct1Hlsd1L
group

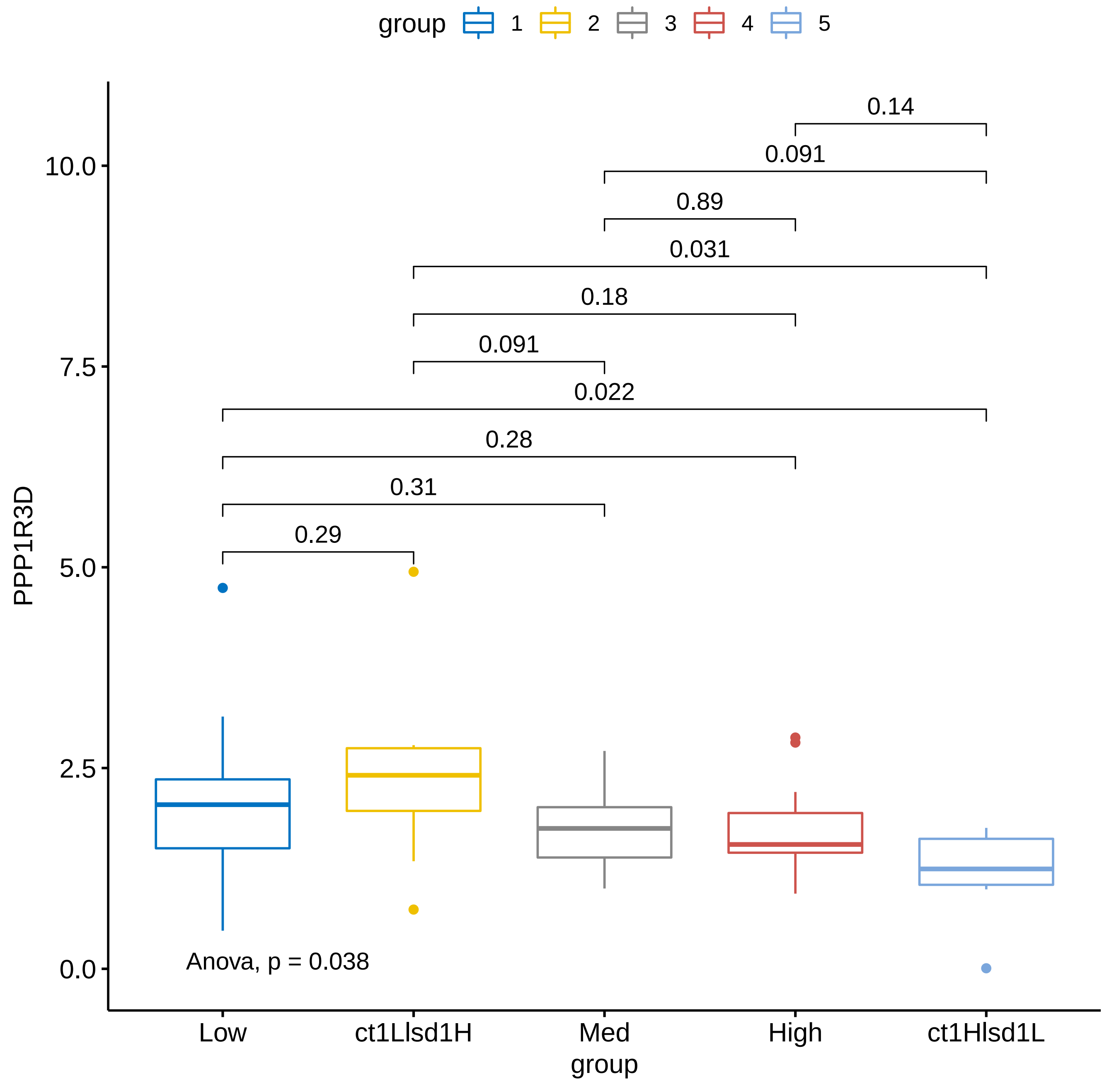

group
1
2
3
4
5
0.14
0.091
0.89
0.031
0.18
0.091
0.022
0.28
0.31
0.29
10.0
7.5
5.0
2.5
0.0
PPP1R3D
Anova, p = 0.038
Low
ct1Llsd1H
Med
High
ct1Hlsd1L
group

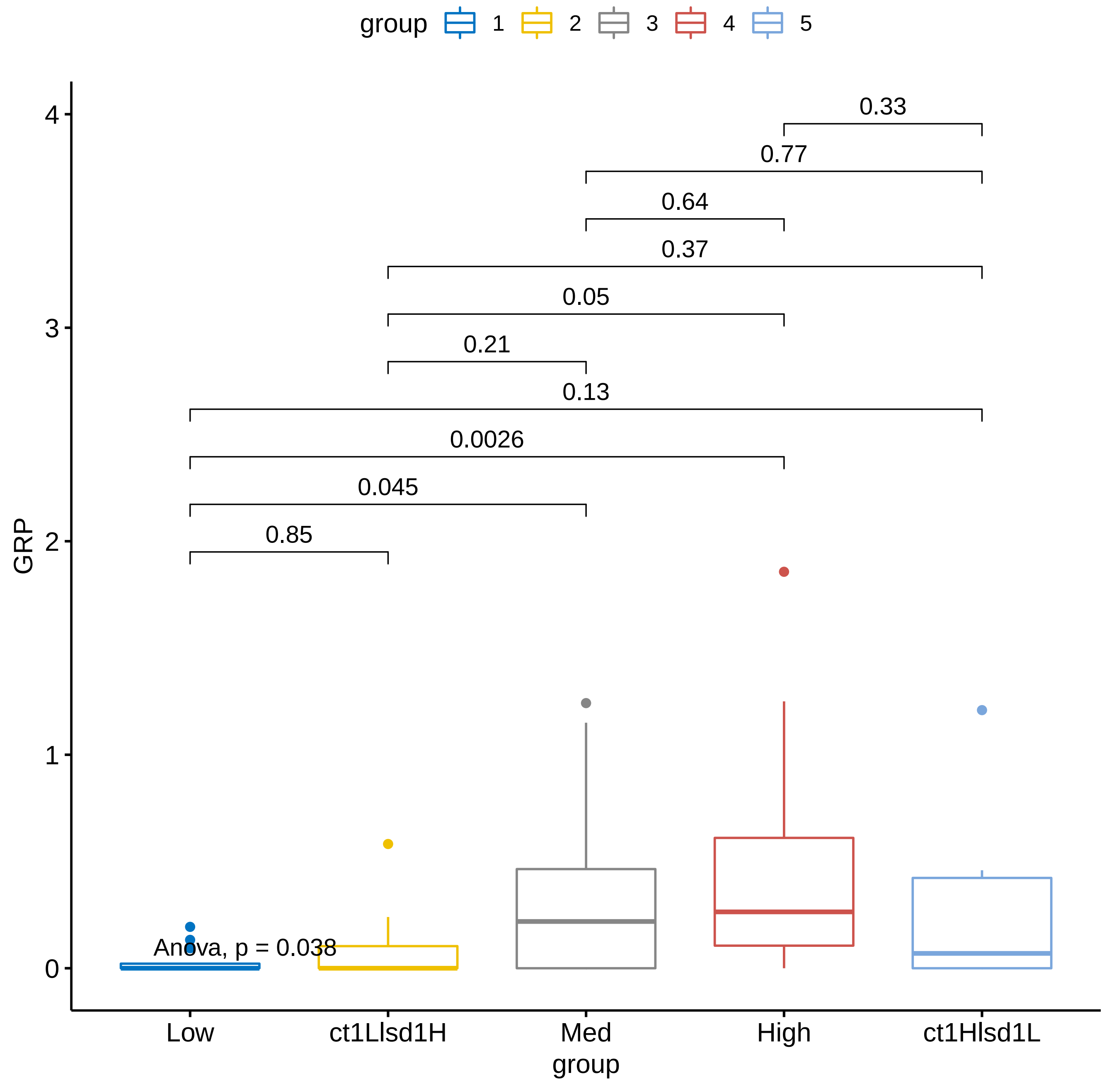

group 1 2 3 4 5
0.33
0.77
0.64
0.37
0.05
0.21
0.13
0.0026
0.045
0.85
Anova, p = 0.038
GRP
0
1
2
3
4
Low
ct1Llsd1H
Med
High
ct1Hlsd1L
group

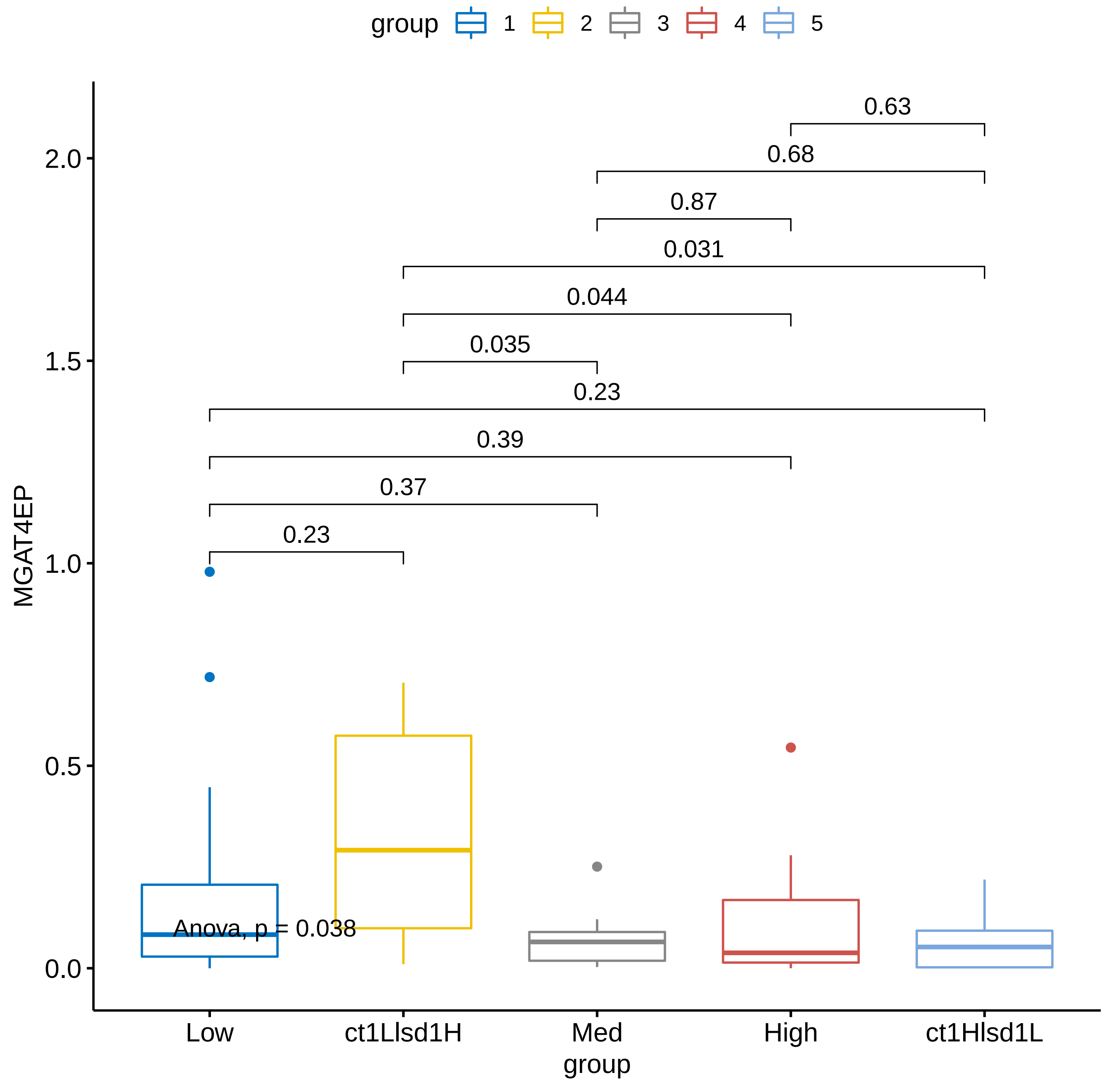
group
1
2
3
4
5
0.63
0.68
0.87
0.031
0.044
0.035
0.23
0.39
0.37
0.23
Anova, p = 0.038
2.0
1.5
1.0
0.5
0.0
MGAT4EP
Low
ct1Llsd1H
Med
High
ct1Hlsd1L
group

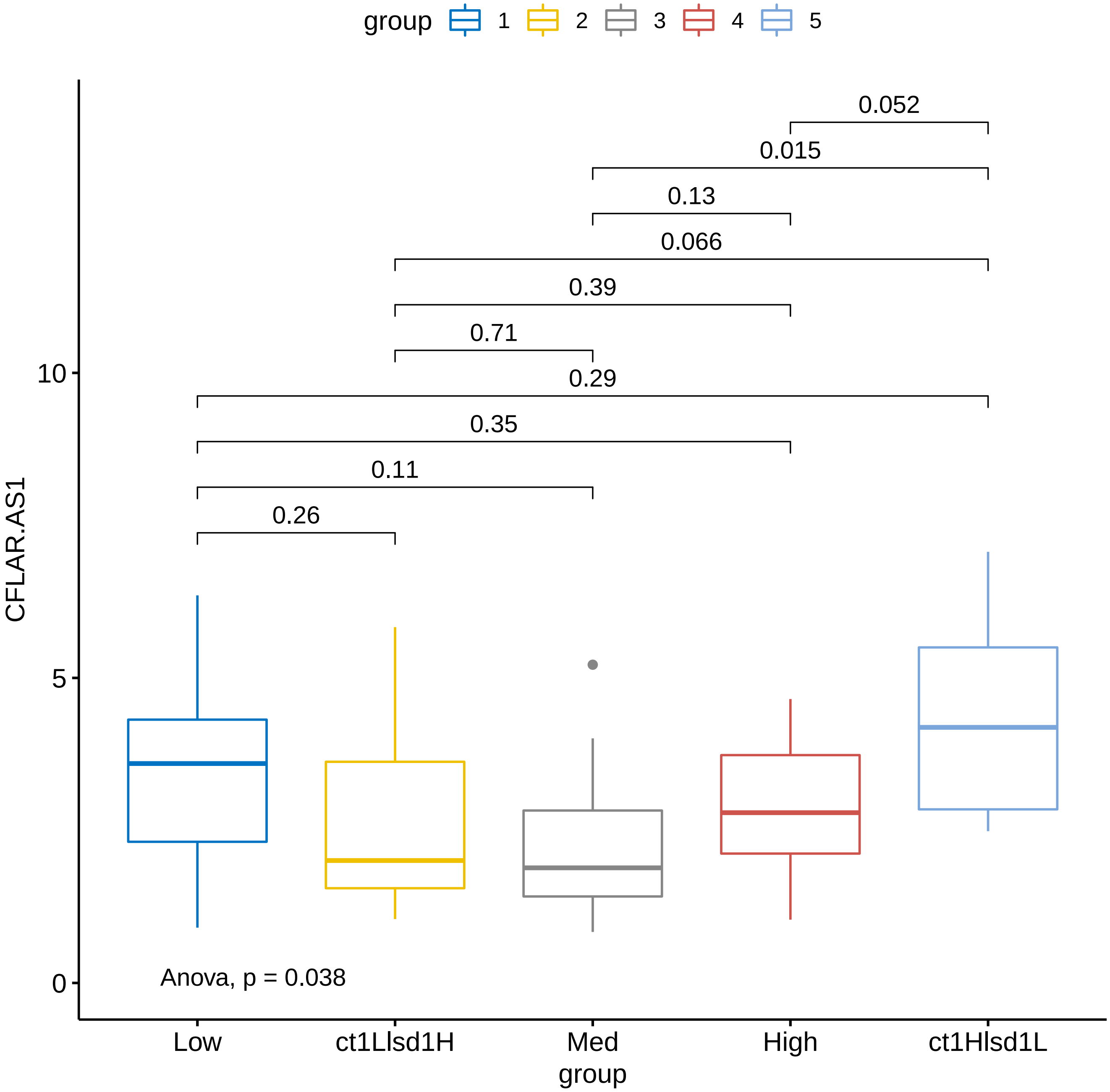
group
1
2
3
4
5
0.052
0.015
0.13
0.066
0.39
0.71
0.29
0.35
0.11
0.26
CFLAR.AS1
10
5
0
Anova, p = 0.038
Low
ct1Llsd1H
Med
High
ct1Hlsd1L
group

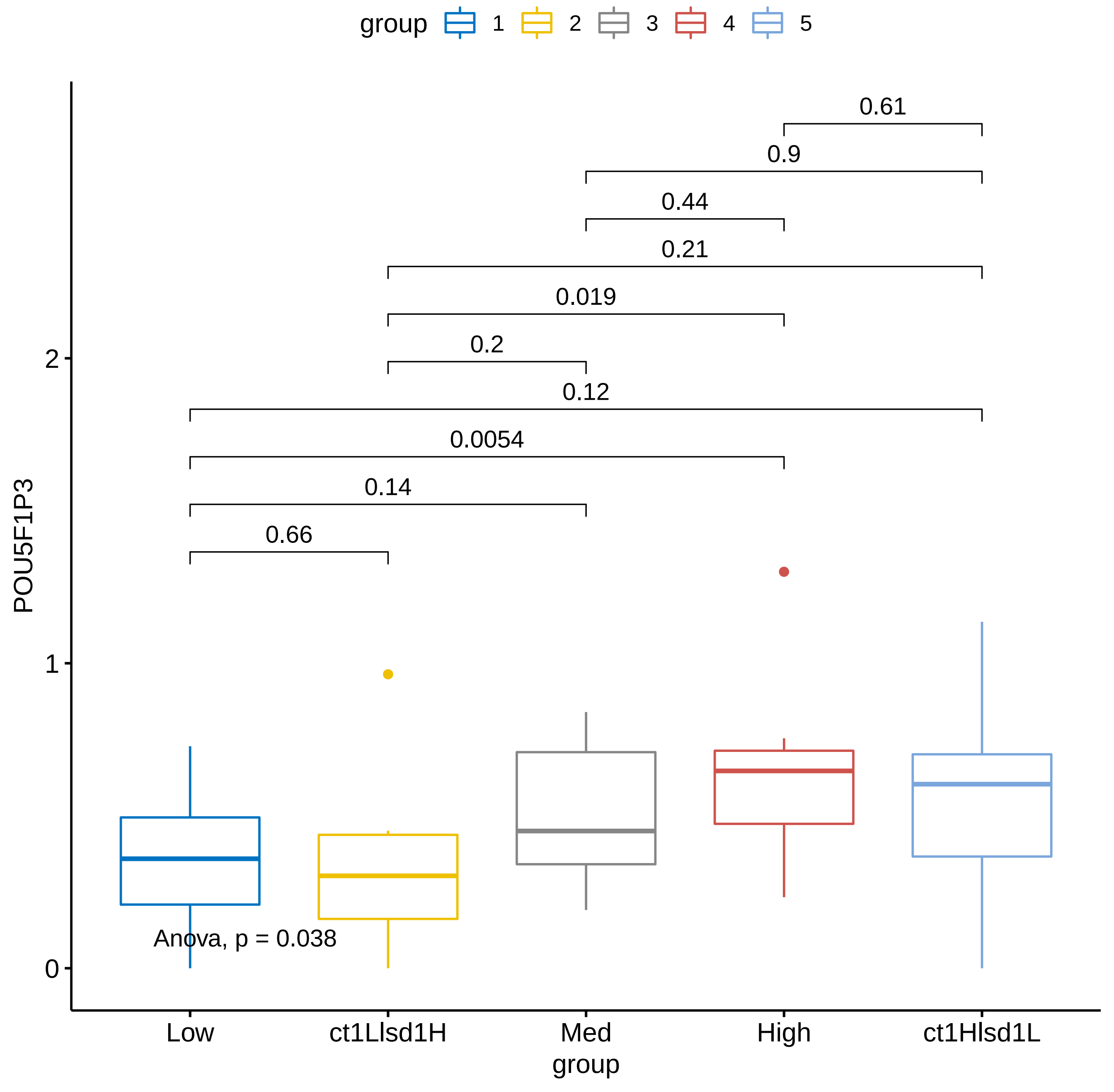

group
1
2
3
4
5
POU5F1P3
0.61
0.9
0.44
0.21
0.019
0.2
0.12
0.0054
0.14
0.66
Anova, p = 0.038
2
1
0
Low
ct1Llsd1H
Med
High
ct1Hlsd1L
group

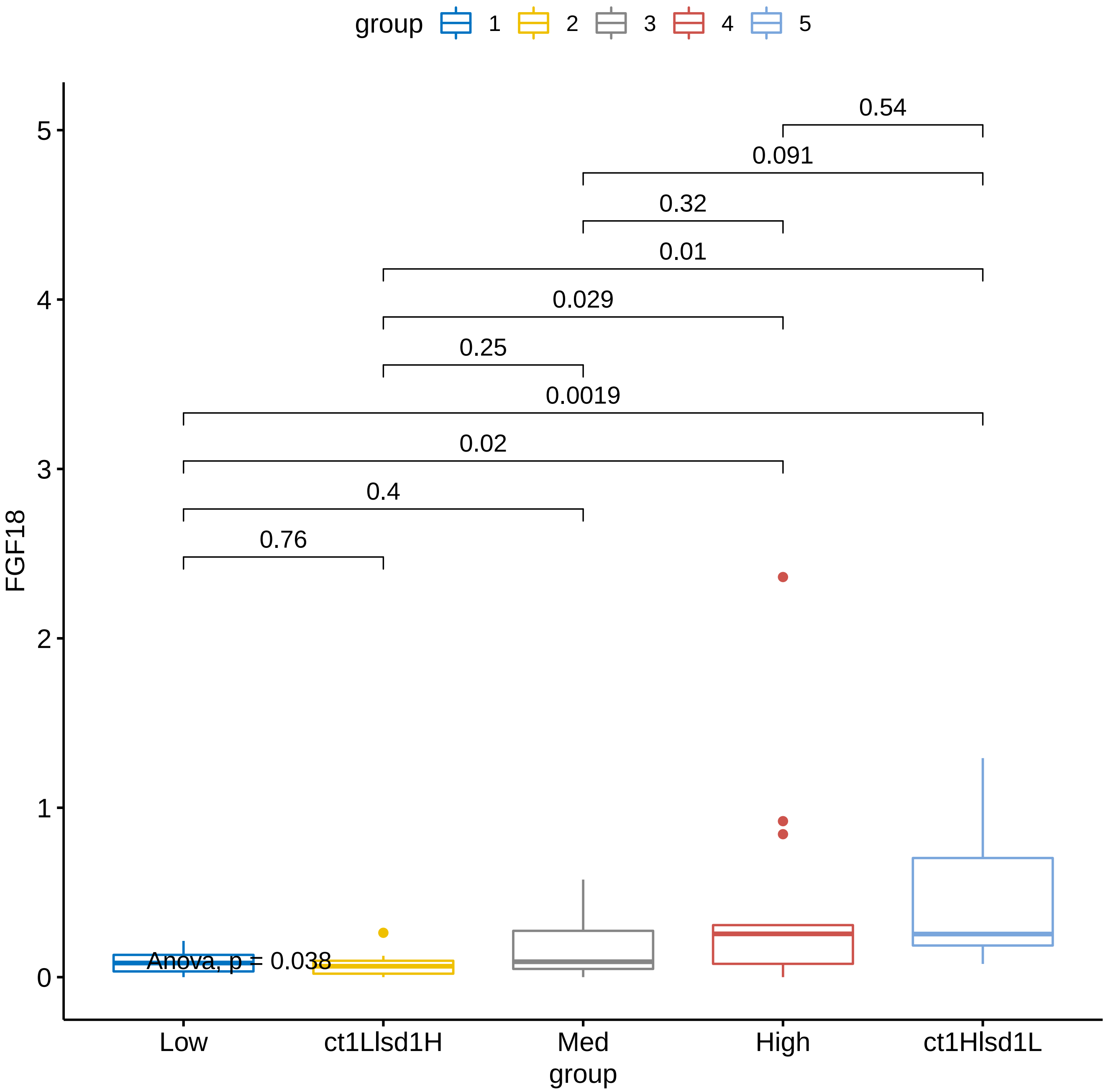
group
1
2
3
4
5
FGF18
0.54
0.091
0.32
0.01
0.029
0.25
0.0019
0.02
0.4
0.76
Anova, p = 0.038
5
4
3
2
1
0
Low
ct1Llsd1H
Med
High
ct1Hlsd1L
group

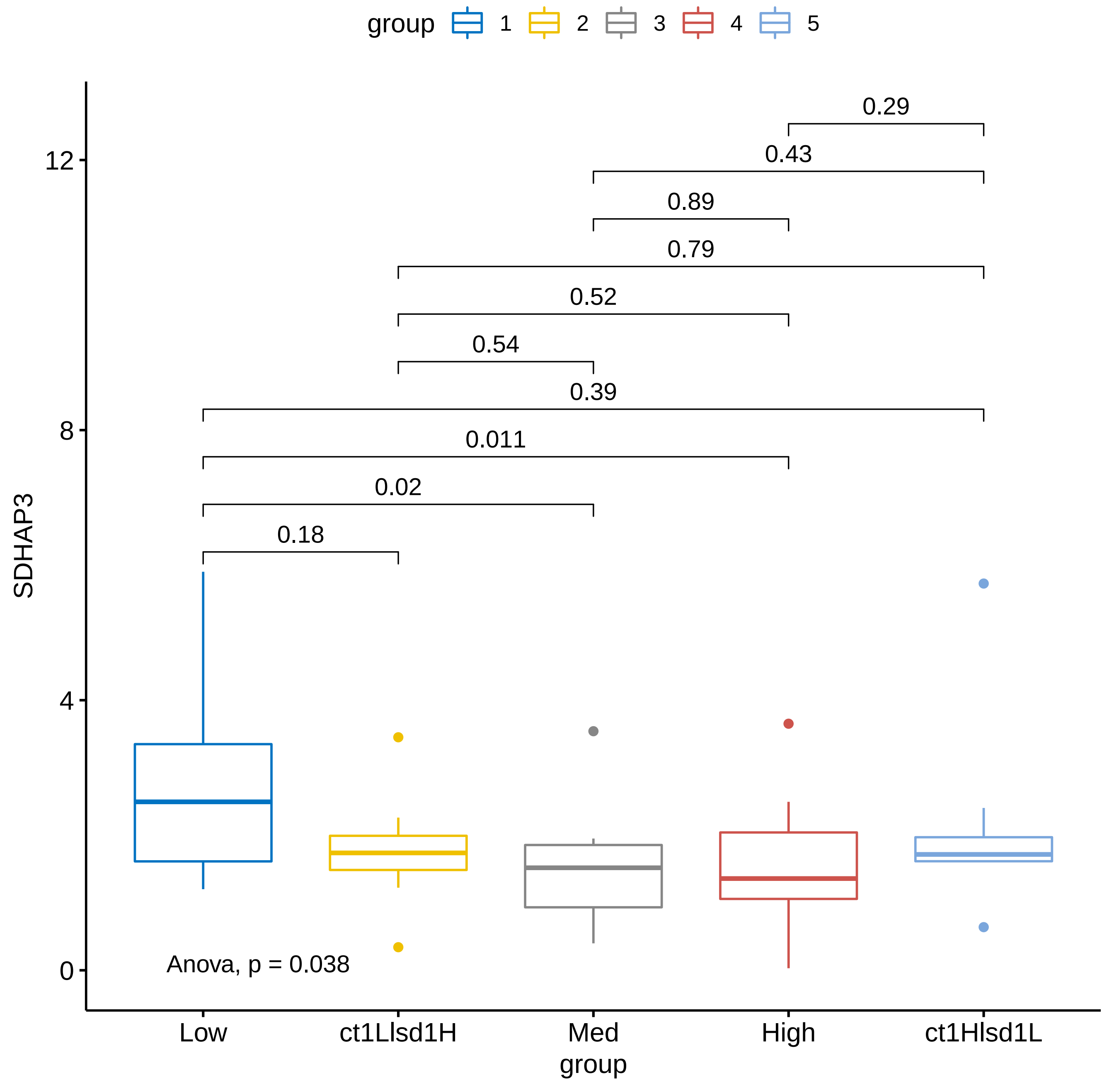

group 1 2 3 4 5
0.29
0.43
0.89
0.79
0.52
0.54
0.39
0.011
0.02
0.18
12
8
4
0
SDHAP3
Anova, p = 0.038
Low
ct1Llsd1H
Med
High
ct1Hlsd1L
group

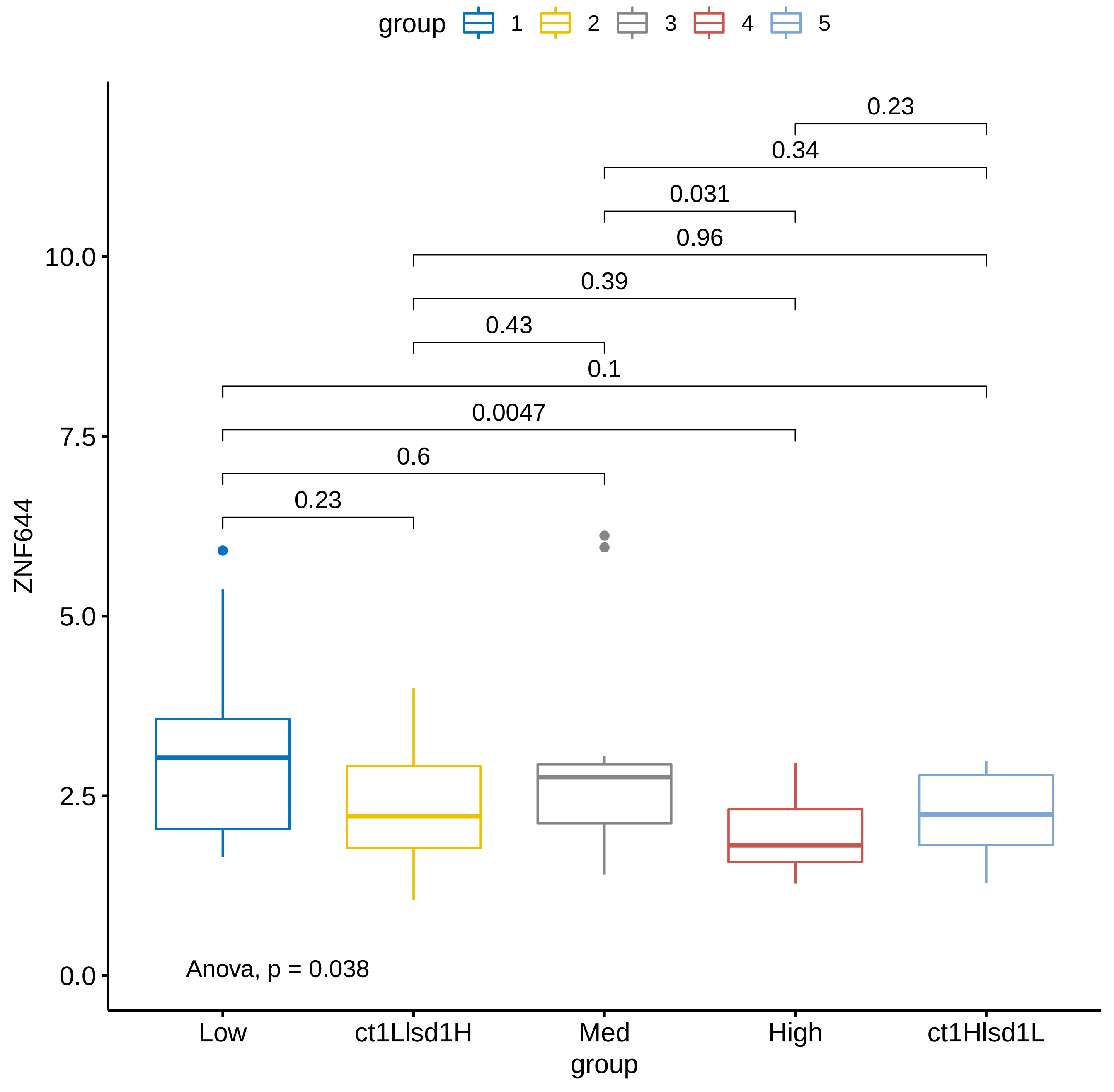

group
1
2
3
4
5
0.23
0.34
0.031
0.96
0.39
0.43
0.1
0.0047
0.6
0.23
ZNF644
10.0
7.5
5.0
2.5
0.0
Anova, p = 0.038
Low
ct1Llsd1H
Med
High
ct1Hlsd1L
group

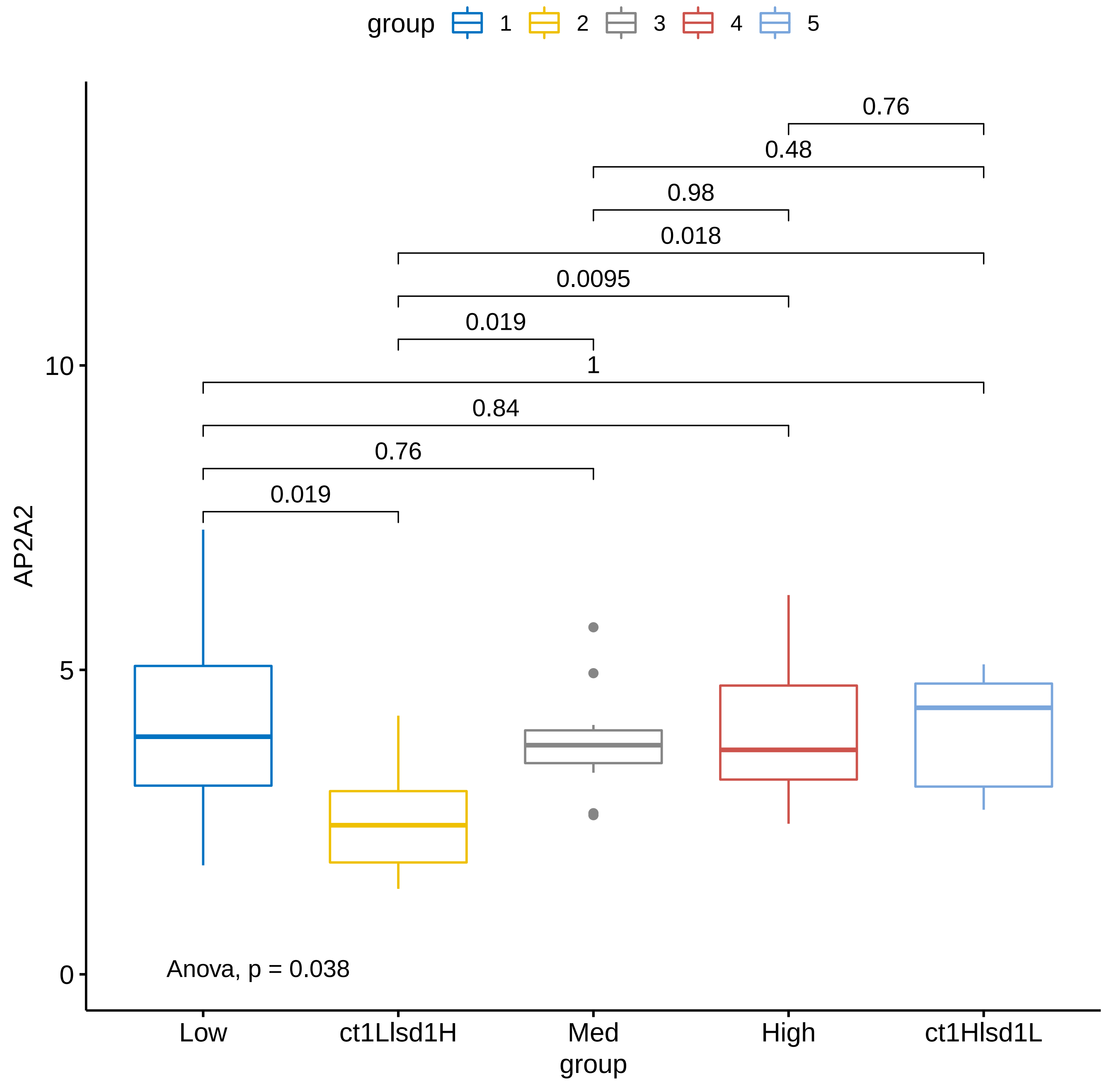

group 1 2 3 4 5
0.76
0.48
0.98
0.018
0.0095
0.019
1
0.84
0.76
0.019
10
5
0
AP2A2
Anova, p = 0.038
Low
ct1Llsd1H
Med
High
ct1Hlsd1L
group

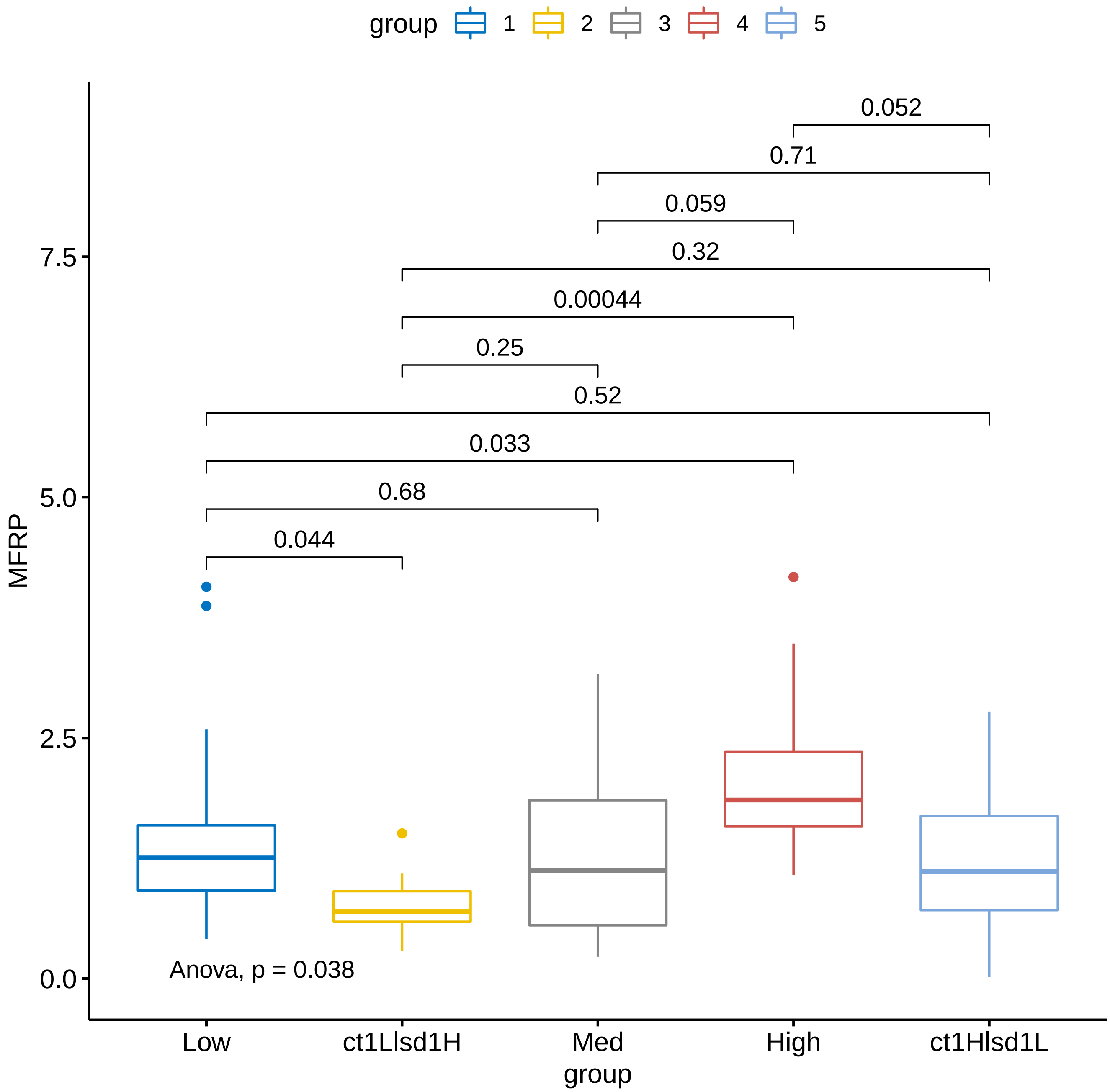
group
1
2
3
4
5
0.052
0.71
0.059
0.32
0.00044
0.25
0.52
0.033
0.68
0.044
MFRP
7.5
5.0
2.5
0.0
Anova, p = 0.038
Low
ct1Llsd1H
Med
High
ct1Hlsd1L
group

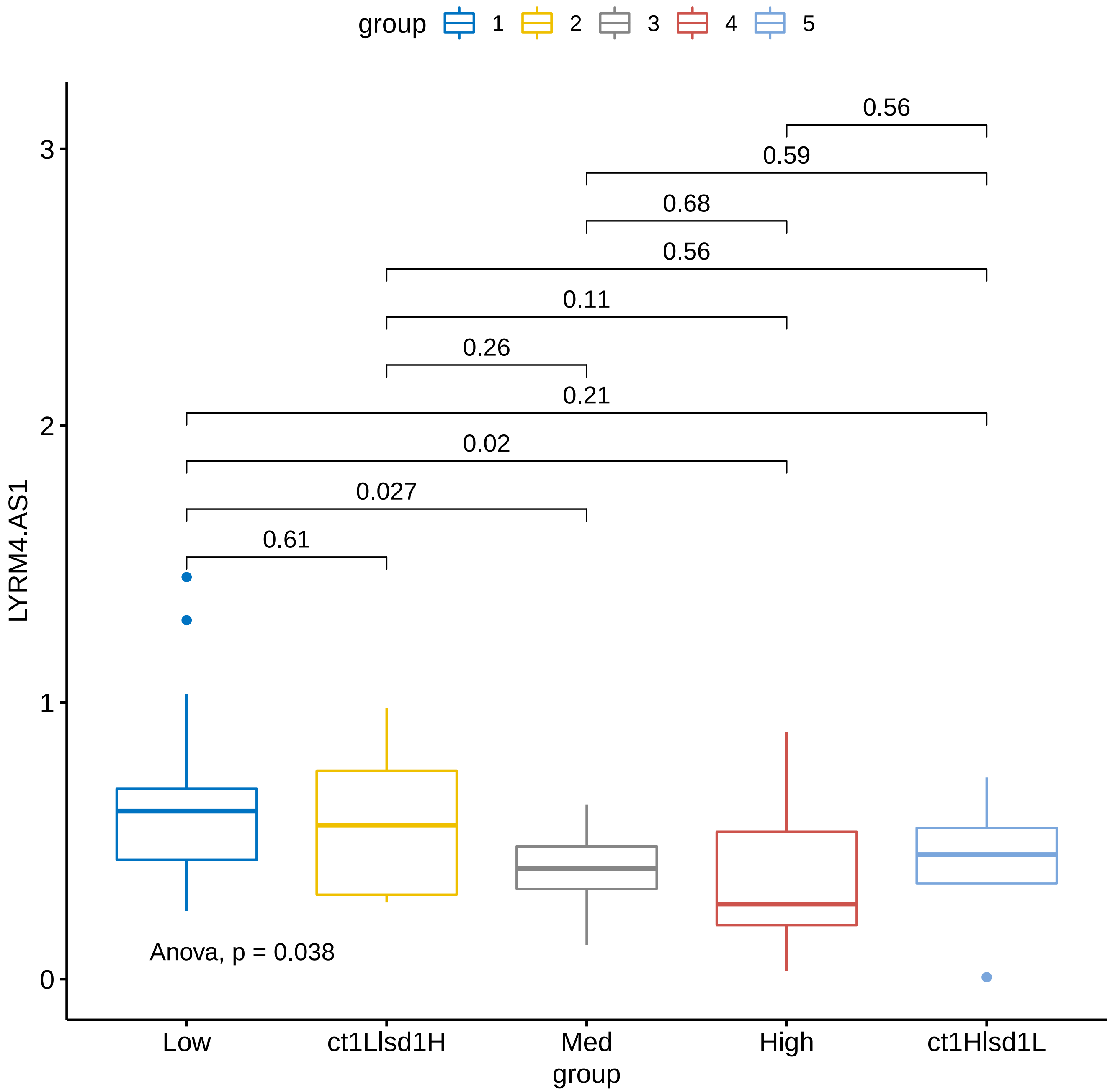

group 1 2 3 4 5
0.56
0.59
0.68
0.56
0.11
0.26
0.21
0.02
0.027
0.61
Anova, p = 0.038
3
2
1
0
LYRM4.AS1
Low
ct1Llsd1H
Med
High
ct1Hlsd1L
group

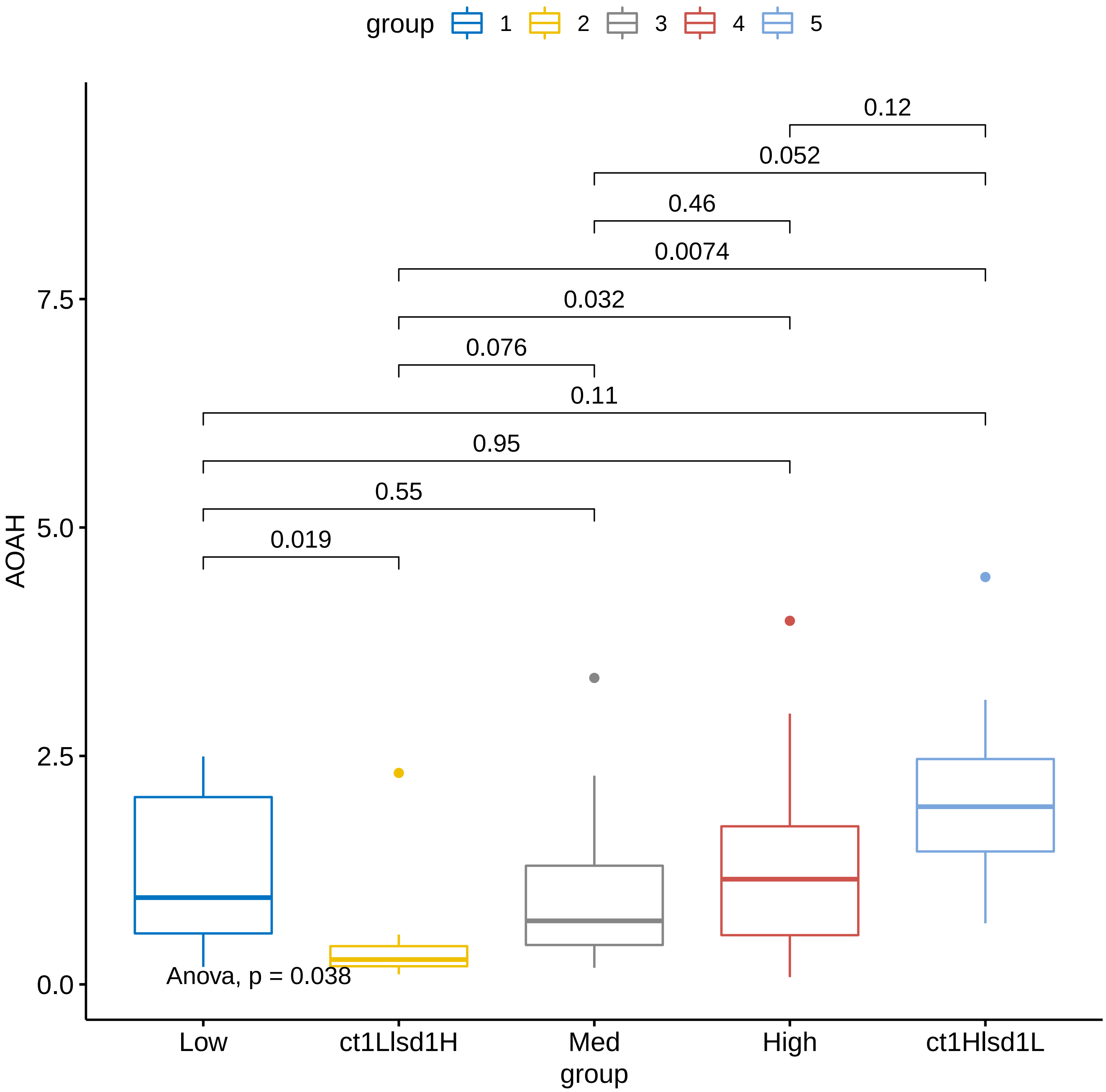

group
1
2
3
4
5
0.12
0.052
0.46
0.0074
0.032
0.076
0.11
0.95
0.55
0.019
Anova, p = 0.038
7.5
5.0
2.5
0.0
AOAH
Low
ct1Llsd1H
Med
High
ct1Hlsd1L
group

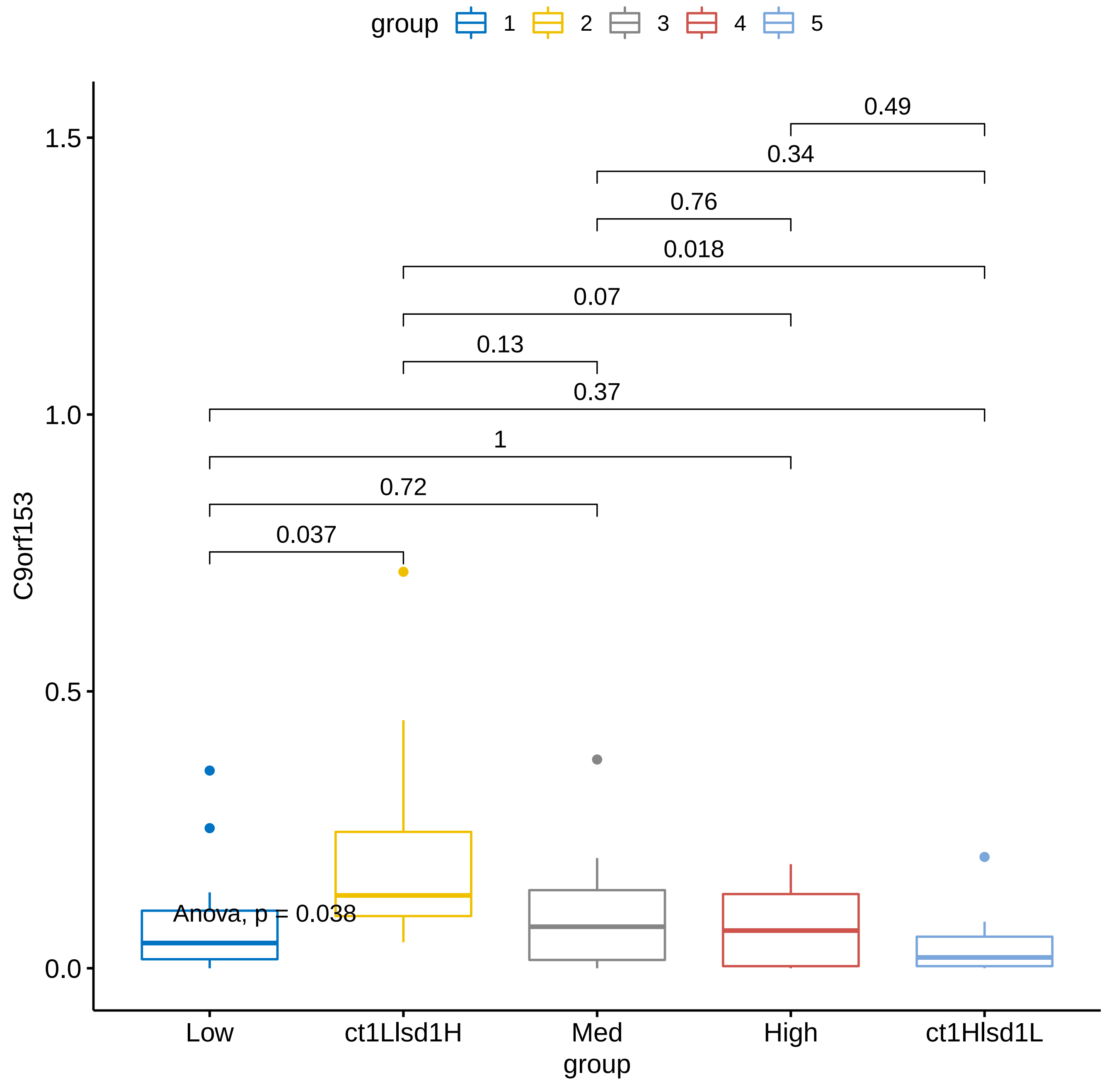
group 1 2 3 4 5
C9orf153
1.5
1.0
0.5
0.0
0.49
0.34
0.76
0.018
0.07
0.13
0.37
1
0.72
0.037
Anova, p = 0.038
Low
ct1Llsd1H
Med
High
ct1Hlsd1L
group

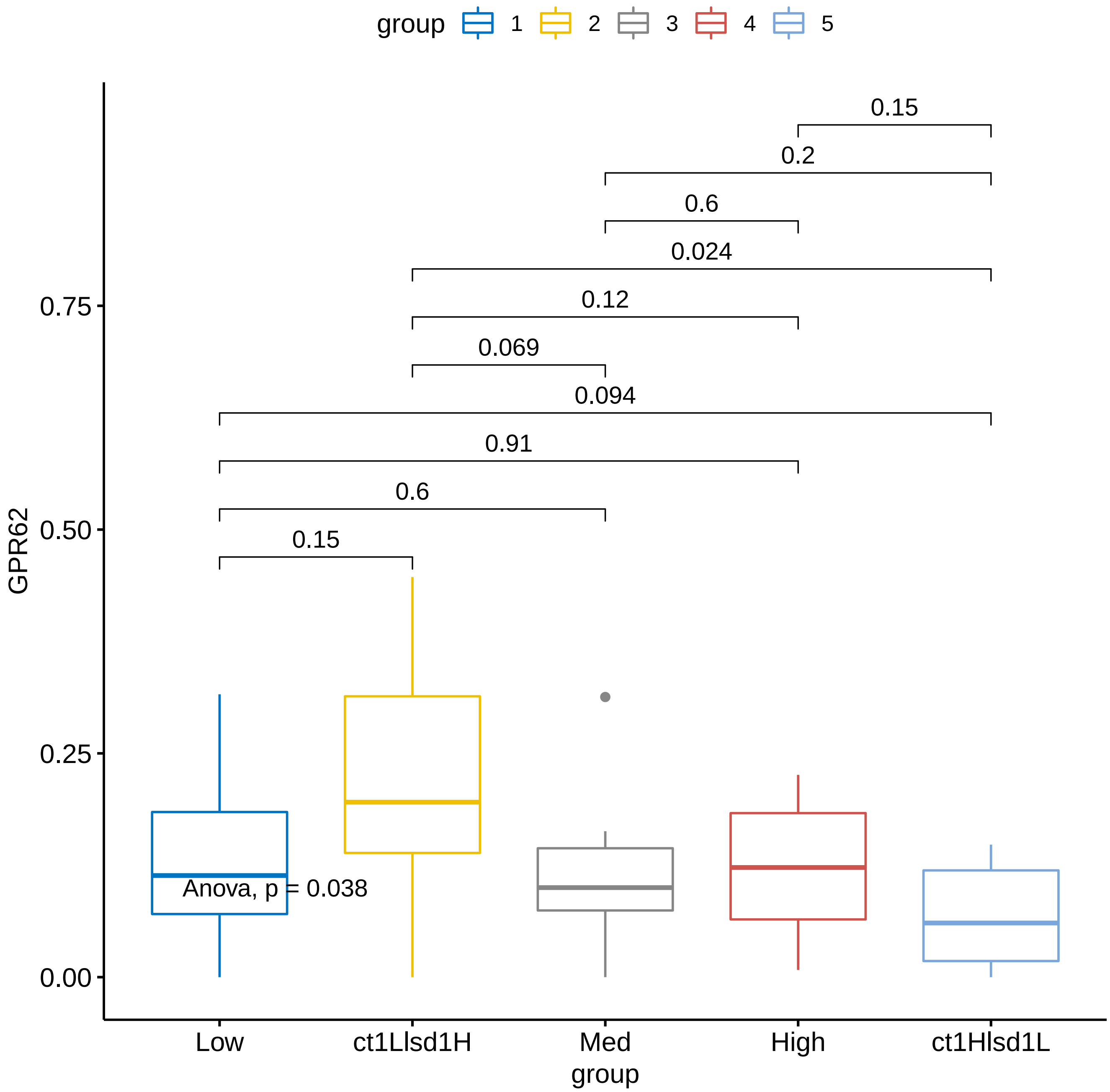

group 1 2 3 4 5
0.15
0.2
0.6
0.024
0.12
0.069
0.094
0.91
0.6
0.15
Anova, p = 0.038
GPR62
0.75
0.50
0.25
0.00
Low
ct1Llsd1H
Med
High
ct1Hlsd1L
group

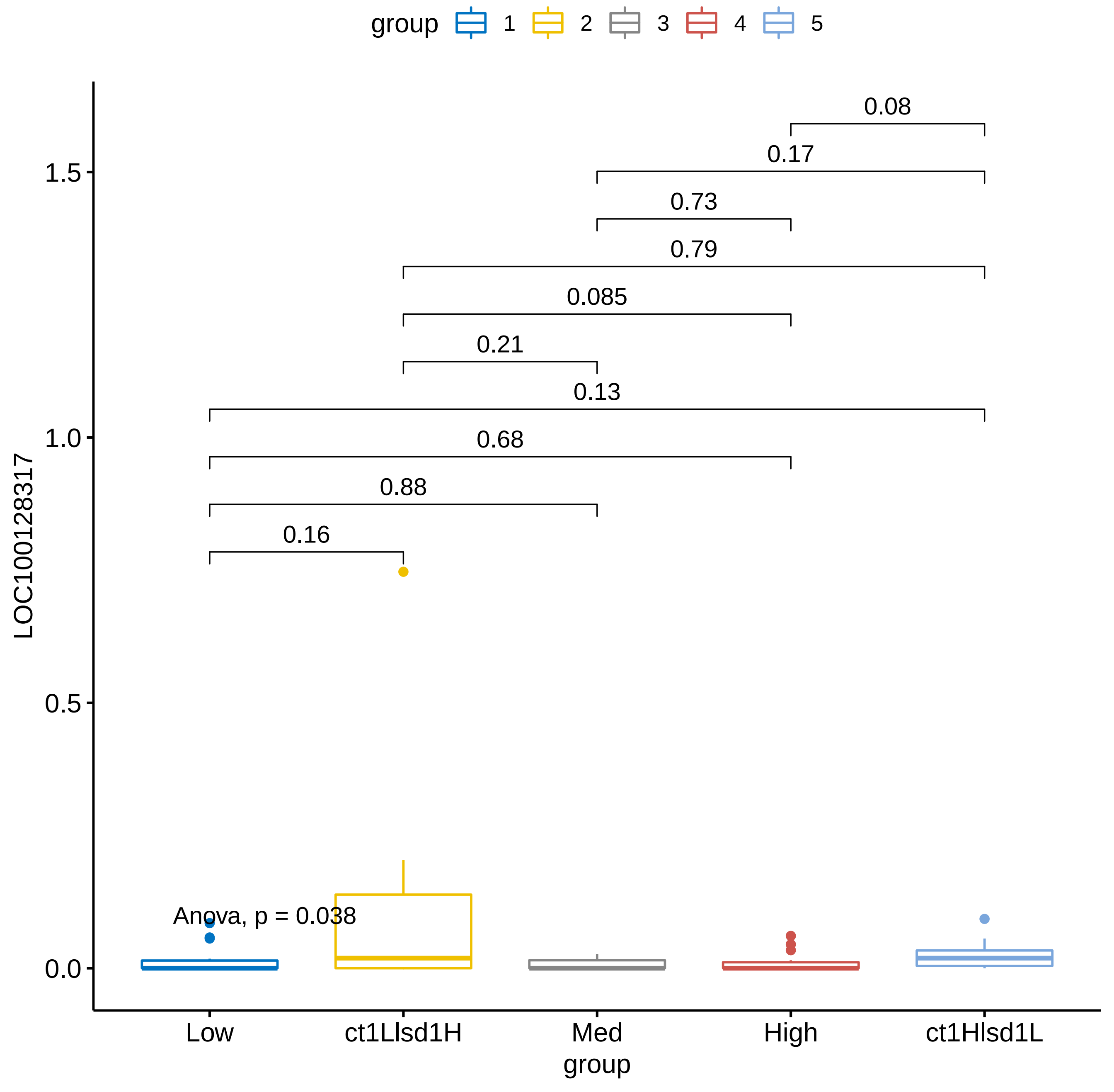

group
1
2
3
4
5
LOC100128317
0.0
0.5
1.0
1.5
0.08
0.17
0.73
0.79
0.085
0.21
0.13
0.68
0.88
0.16
Anova, p = 0.038
Low
ct1Llsd1H
Med
High
ct1Hlsd1L
group

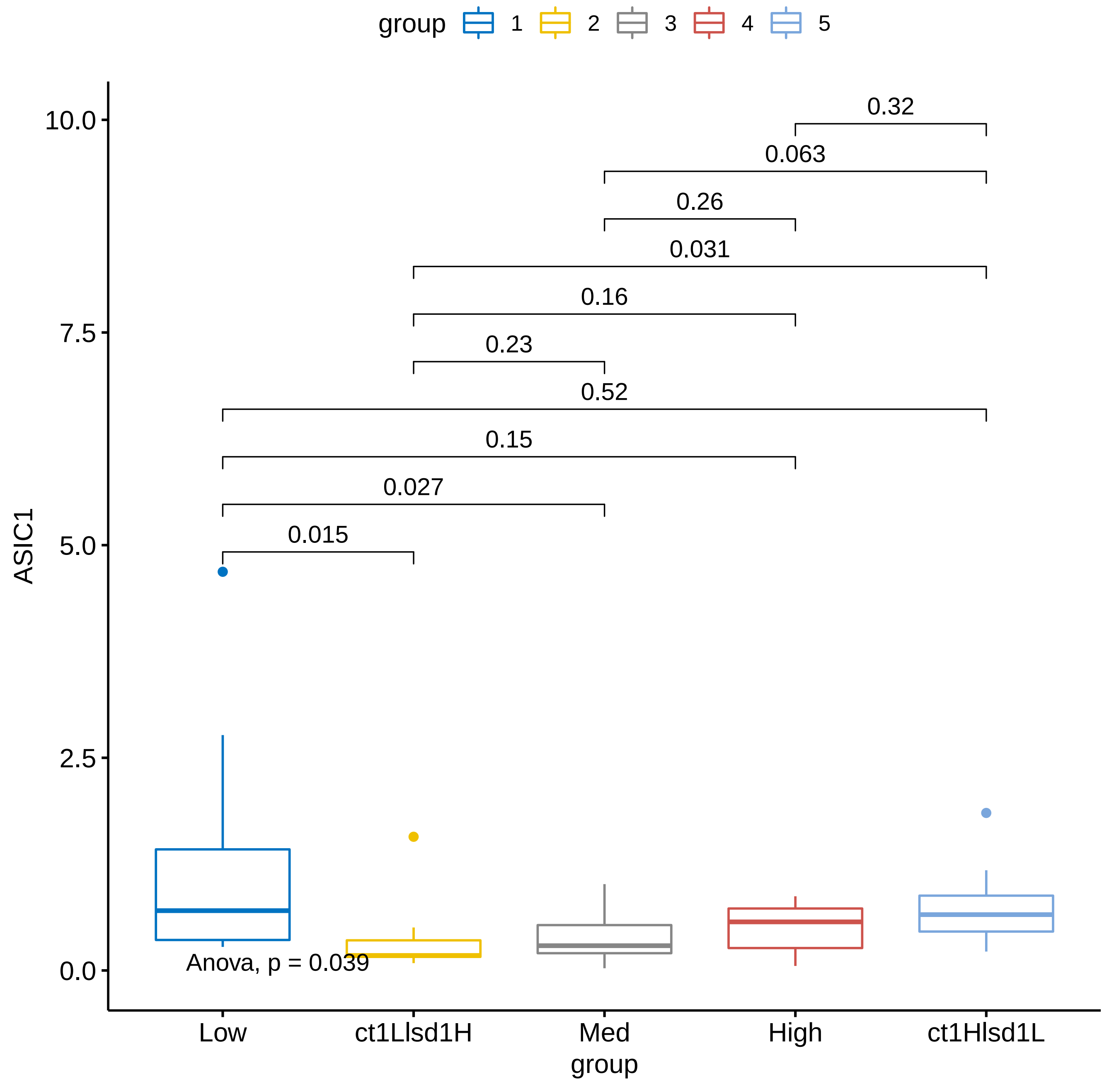

group 1 2 3 4 5
ASIC1
10.0
7.5
5.0
2.5
0.0
0.32
0.063
0.26
0.031
0.16
0.23
0.52
0.15
0.027
0.015
Anova, p = 0.039
Low
ct1Llsd1H
Med
High
ct1Hlsd1L
group

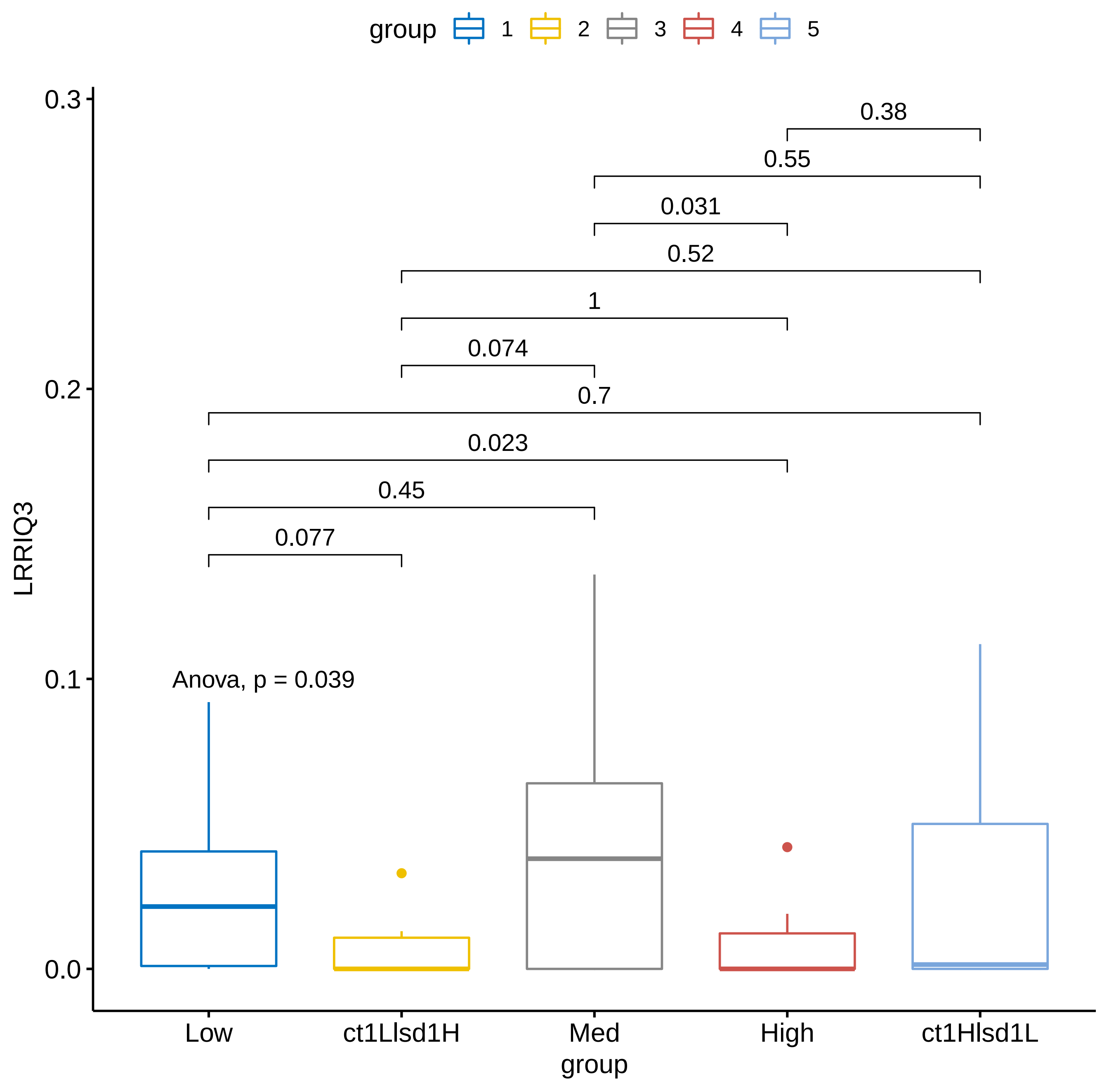
group 1 2 3 4 5
LRRIQ3
0.3
0.2
0.1
0.0
0.38
0.55
0.031
0.52
1
0.074
0.7
0.023
0.45
0.077
Anova, p = 0.039
Low
ct1Llsd1H
Med
High
ct1Hlsd1L
group

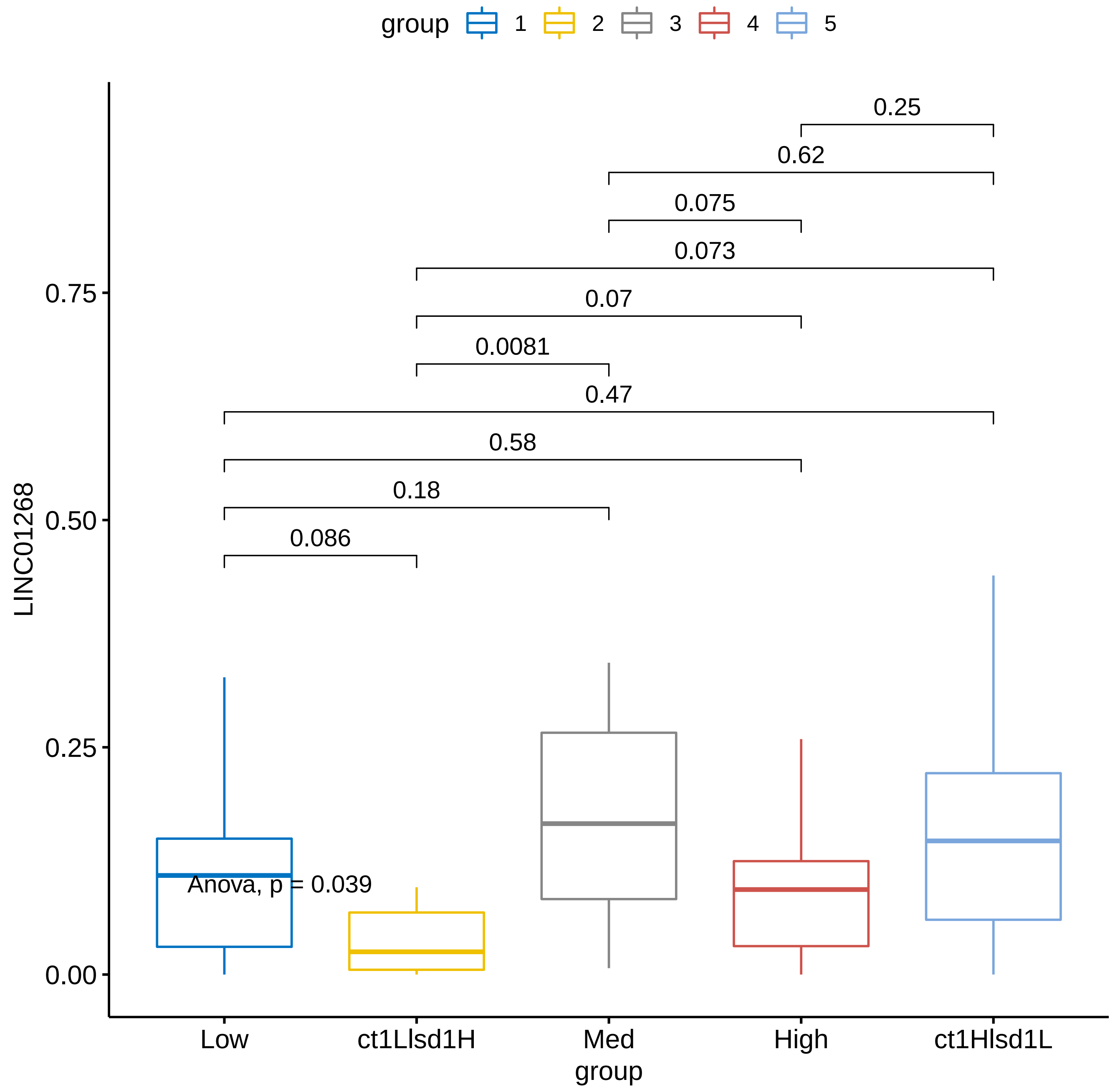
group 1 2 3 4 5
0.25
0.62
0.075
0.073
0.07
0.0081
0.47
0.58
0.18
0.086
Anova, p = 0.039
LINC01268
0.00
0.25
0.50
0.75
Low
ct1Llsd1H
Med
High
ct1Hlsd1L
group

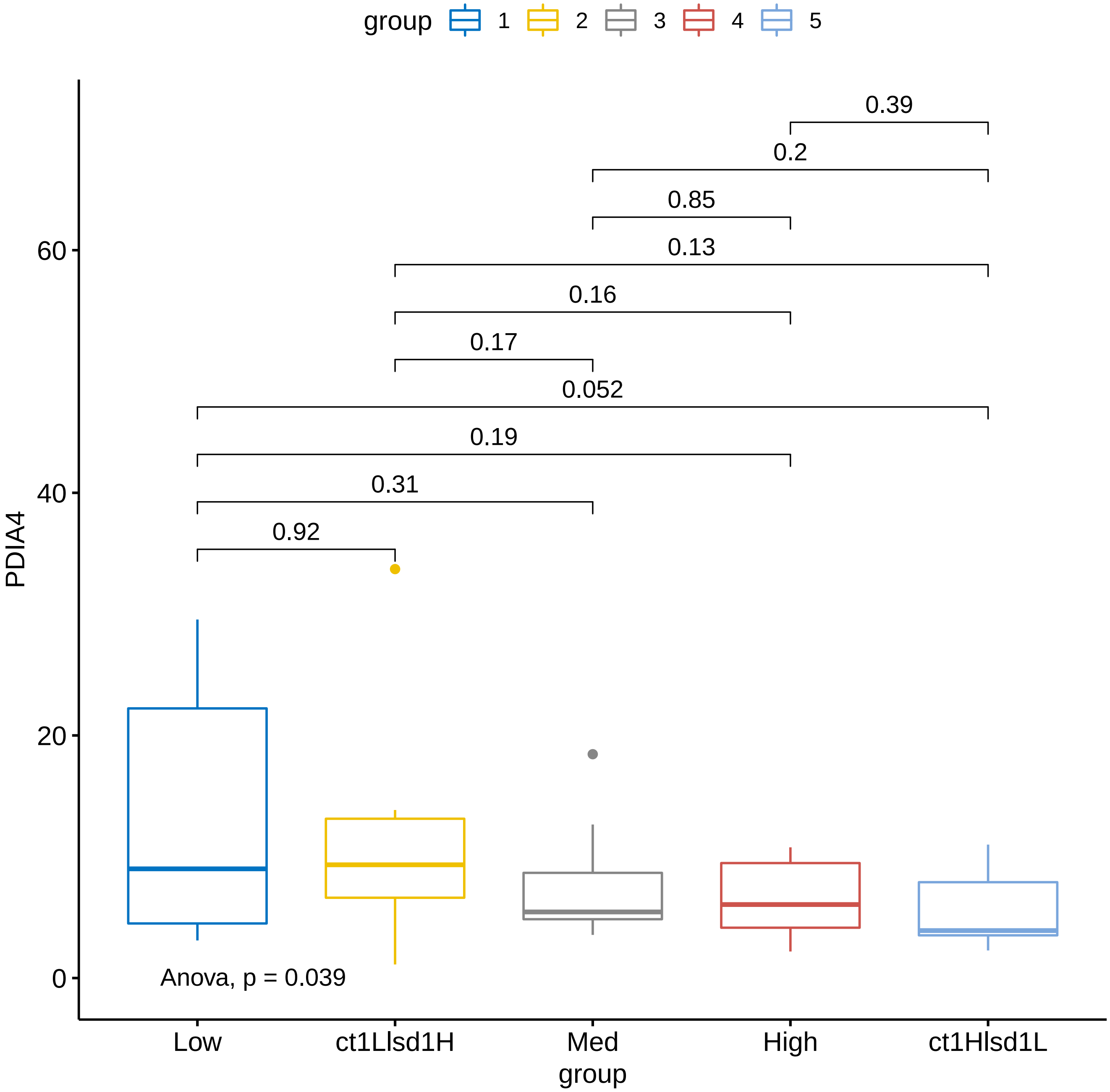

group 1 2 3 4 5
0.39
0.2
0.85
0.13
0.16
0.17
0.052
0.19
0.31
0.92
Anova, p = 0.039
PDIA4
0
20
40
60
Low
ct1Llsd1H
Med
High
ct1Hlsd1L
group

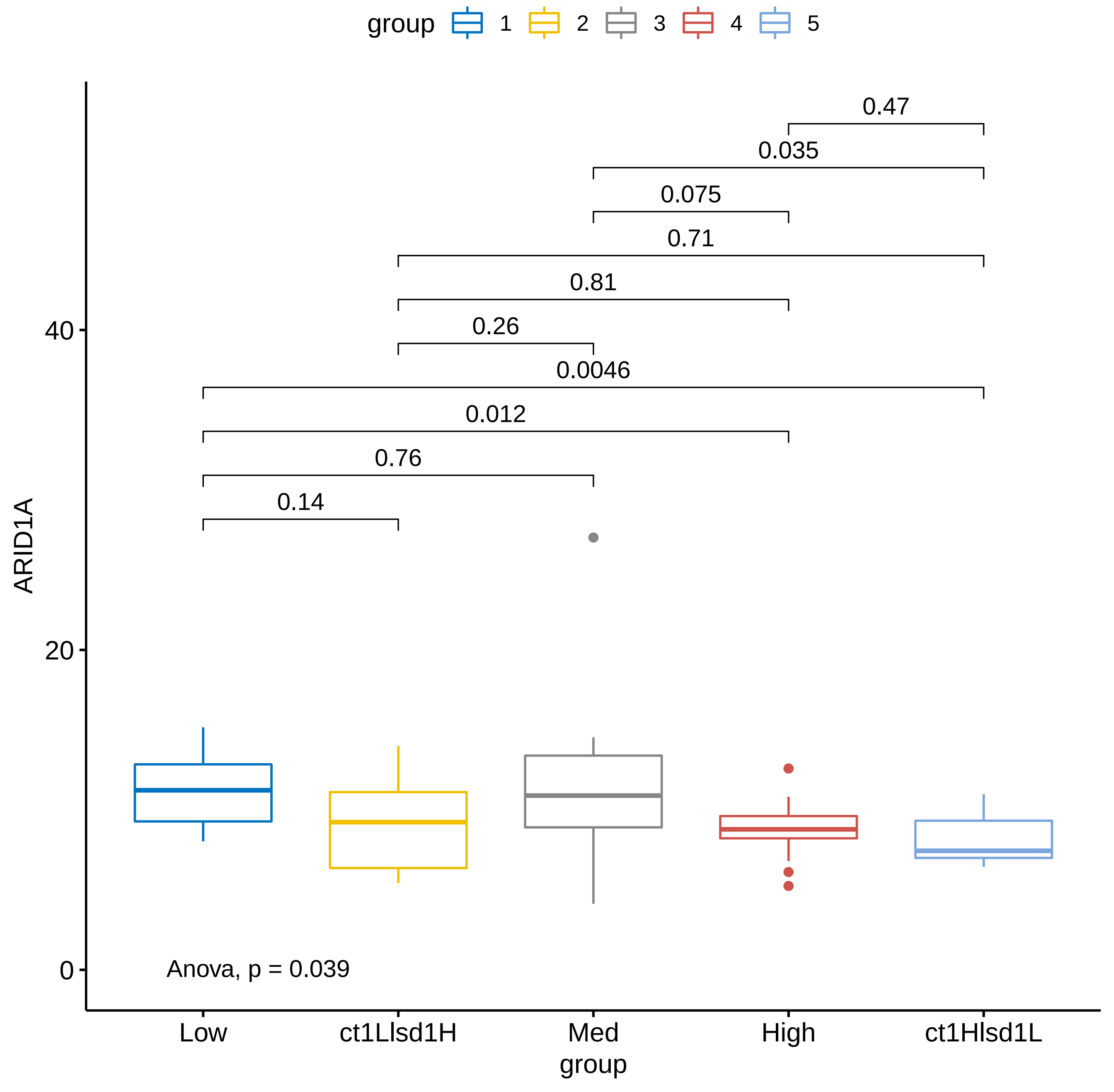
group
1
2
3
4
5
0.47
0.035
0.075
0.71
0.81
0.26
0.0046
0.012
0.76
0.14
ARID1A
40
20
0
Anova, p = 0.039
Low
ct1Llsd1H
Med
High
ct1Hlsd1L
group

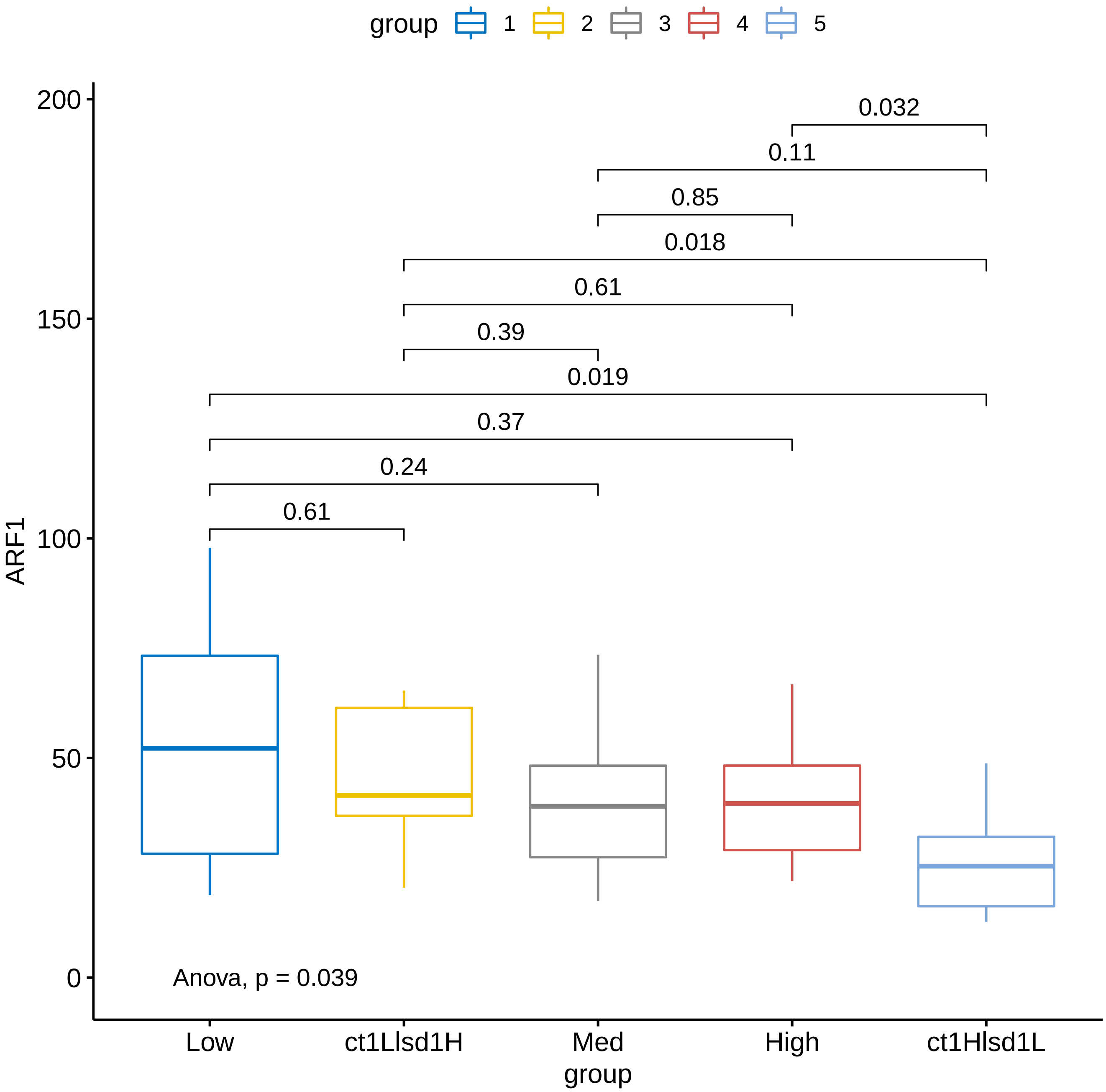
group
1
2
3
4
5
200
150
100
50
0
ARF1
0.032
0.11
0.85
0.018
0.61
0.39
0.019
0.37
0.24
0.61
Anova, p = 0.039
Low
ct1Llsd1H
Med
High
ct1Hlsd1L
group

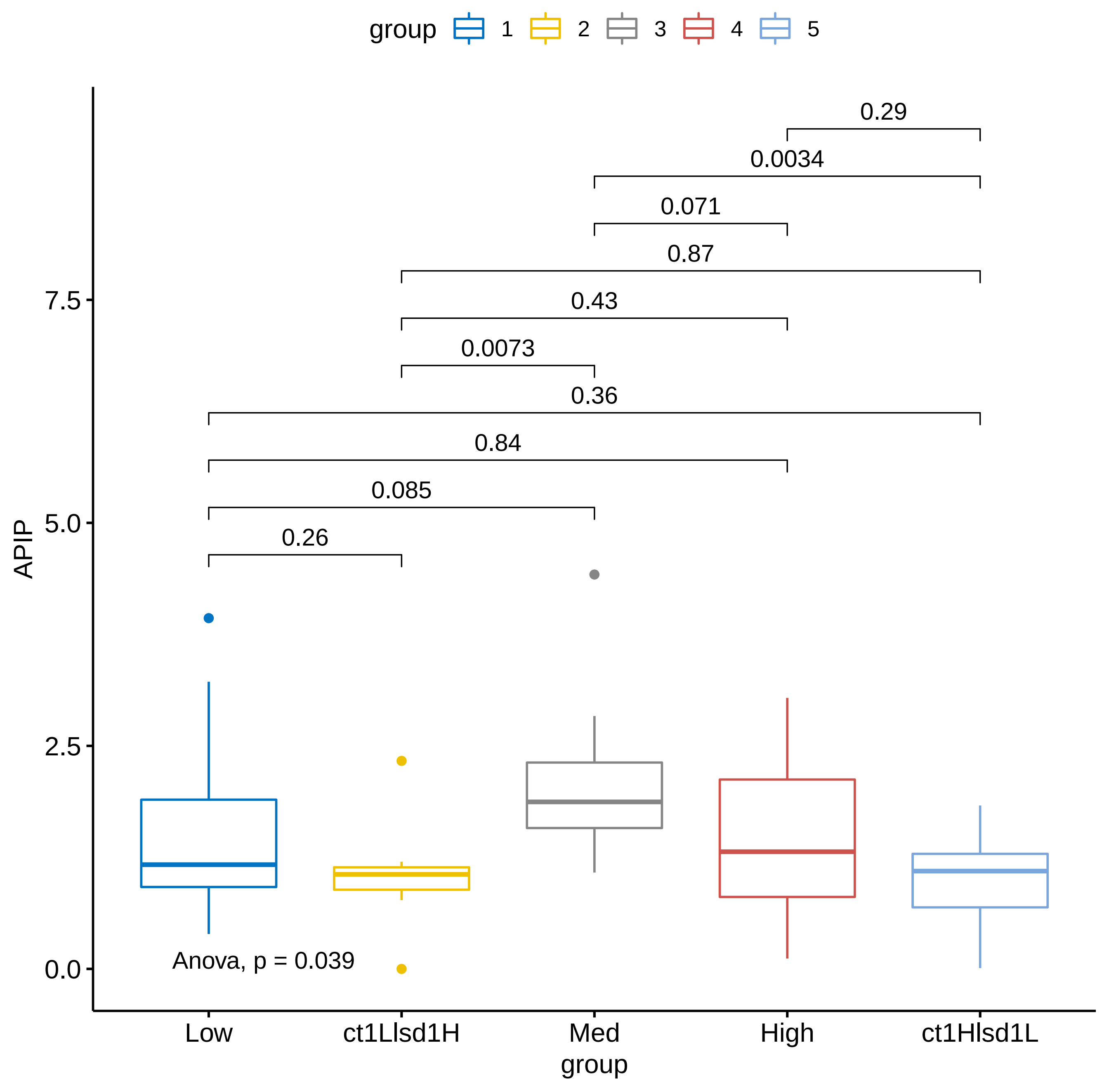
group
1
2
3
4
5
0.29
0.0034
0.071
0.87
0.43
0.0073
0.36
0.84
0.085
0.26
Anova, p = 0.039
APIP
7.5
5.0
2.5
0.0
Low
ct1Llsd1H
Med
High
ct1Hlsd1L
group

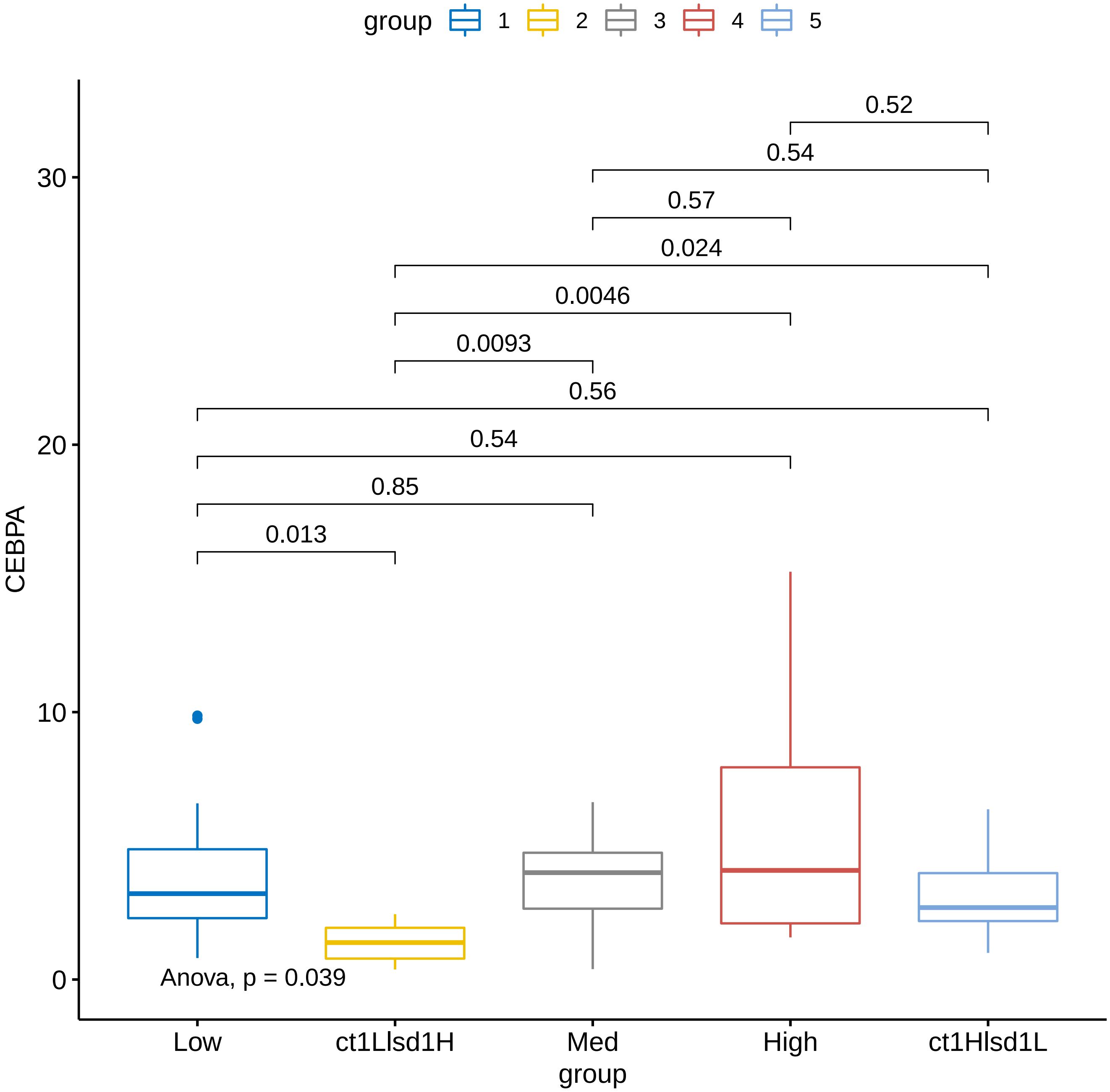
group
1
2
3
4
5
0.52
0.54
0.57
0.024
0.0046
0.0093
0.56
0.54
0.85
0.013
30
20
10
0
CEBPA
Anova, p = 0.039
Low
ct1Llsd1H
Med
High
ct1Hlsd1L
group

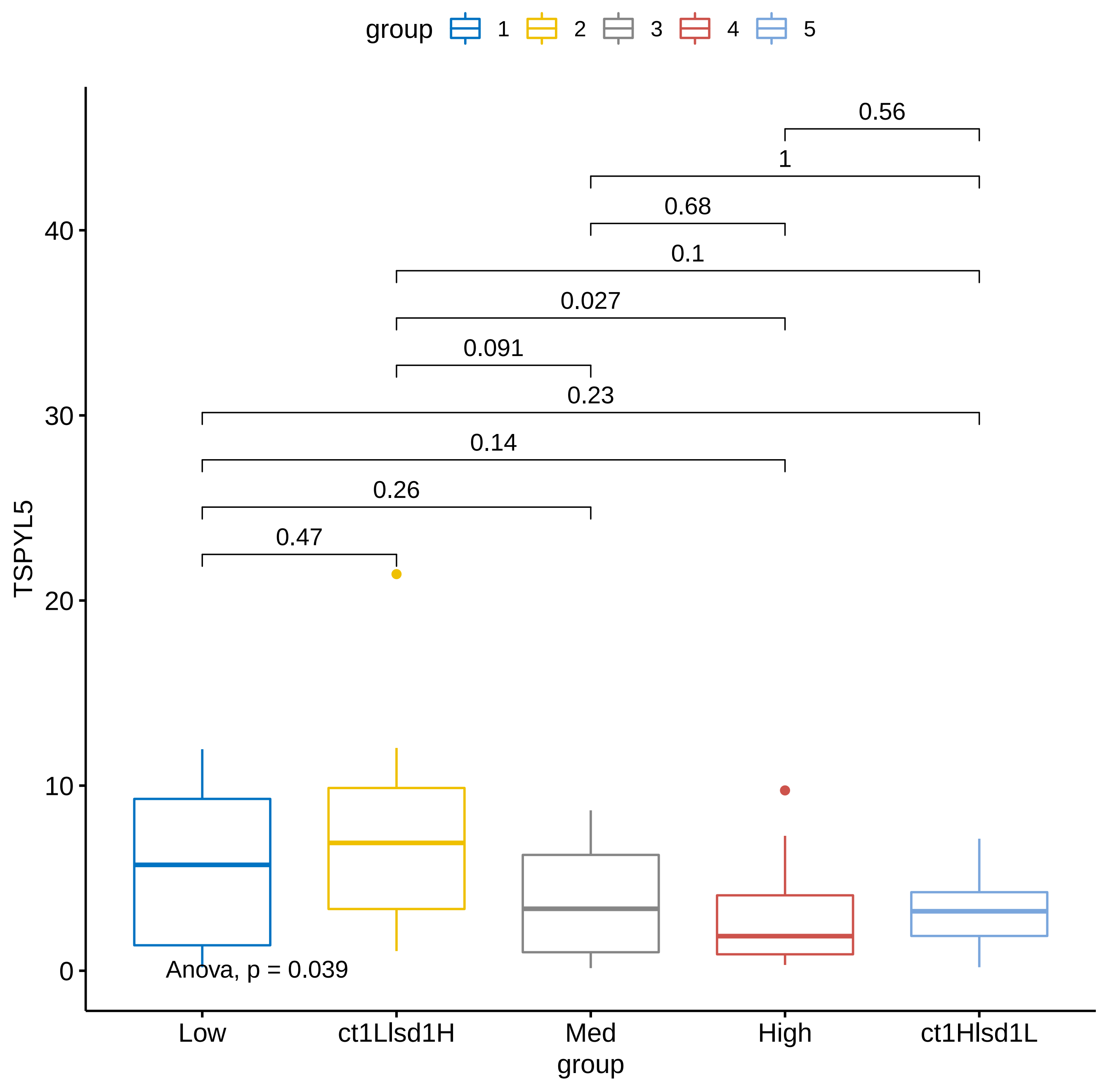
group
1
2
3
4
5
TSPYL5
0.56
1
0.68
0.1
0.027
0.091
0.23
0.14
0.26
0.47
Anova, p = 0.039
0
10
20
30
40
Low
ct1Llsd1H
Med
High
ct1Hlsd1L
group

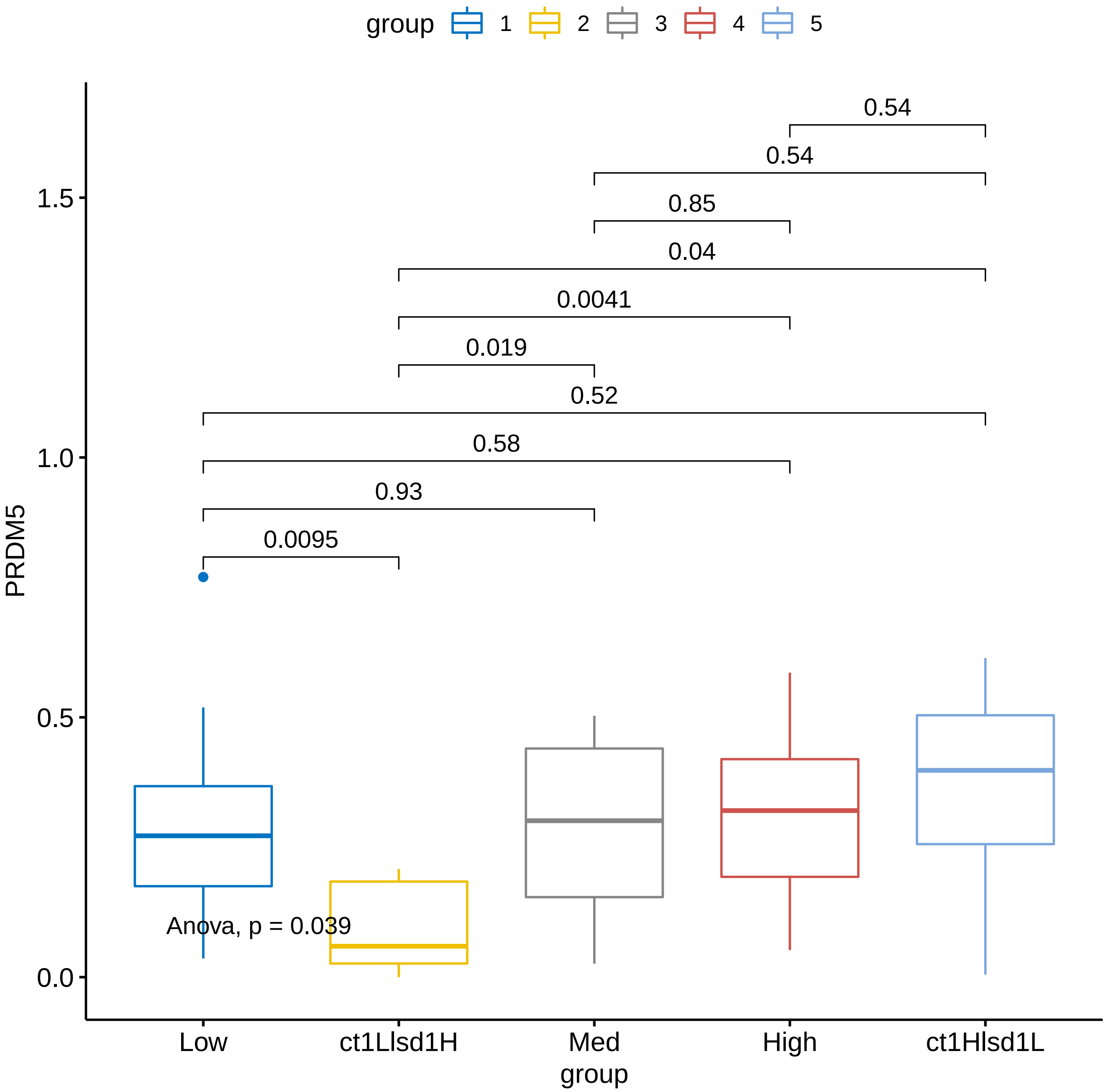

group
1
2
3
4
5
0.54
0.54
0.85
0.04
0.0041
0.019
0.52
0.58
0.93
0.0095
Anova, p = 0.039
PRDM5
1.5
1.0
0.5
0.0
Low
ct1Llsd1H
Med
High
ct1Hlsd1L
group

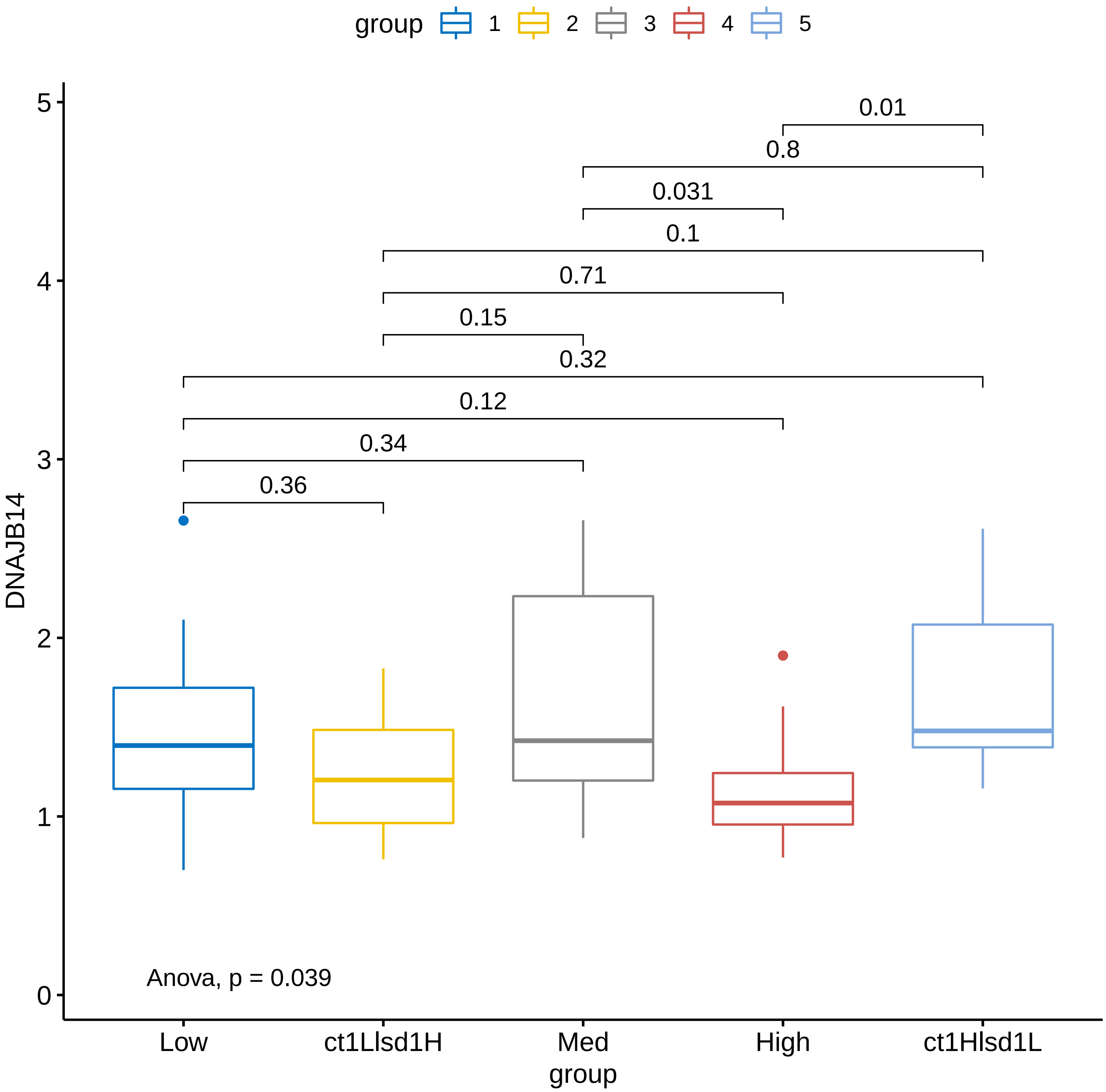
group
1
2
3
4
5
0.01
0.8
0.031
0.1
0.71
0.15
0.32
0.12
0.34
0.36
Anova, p = 0.039
DNAJB14
0
1
2
3
4
5
Low
ct1Llsd1H
Med
High
ct1Hlsd1L
group

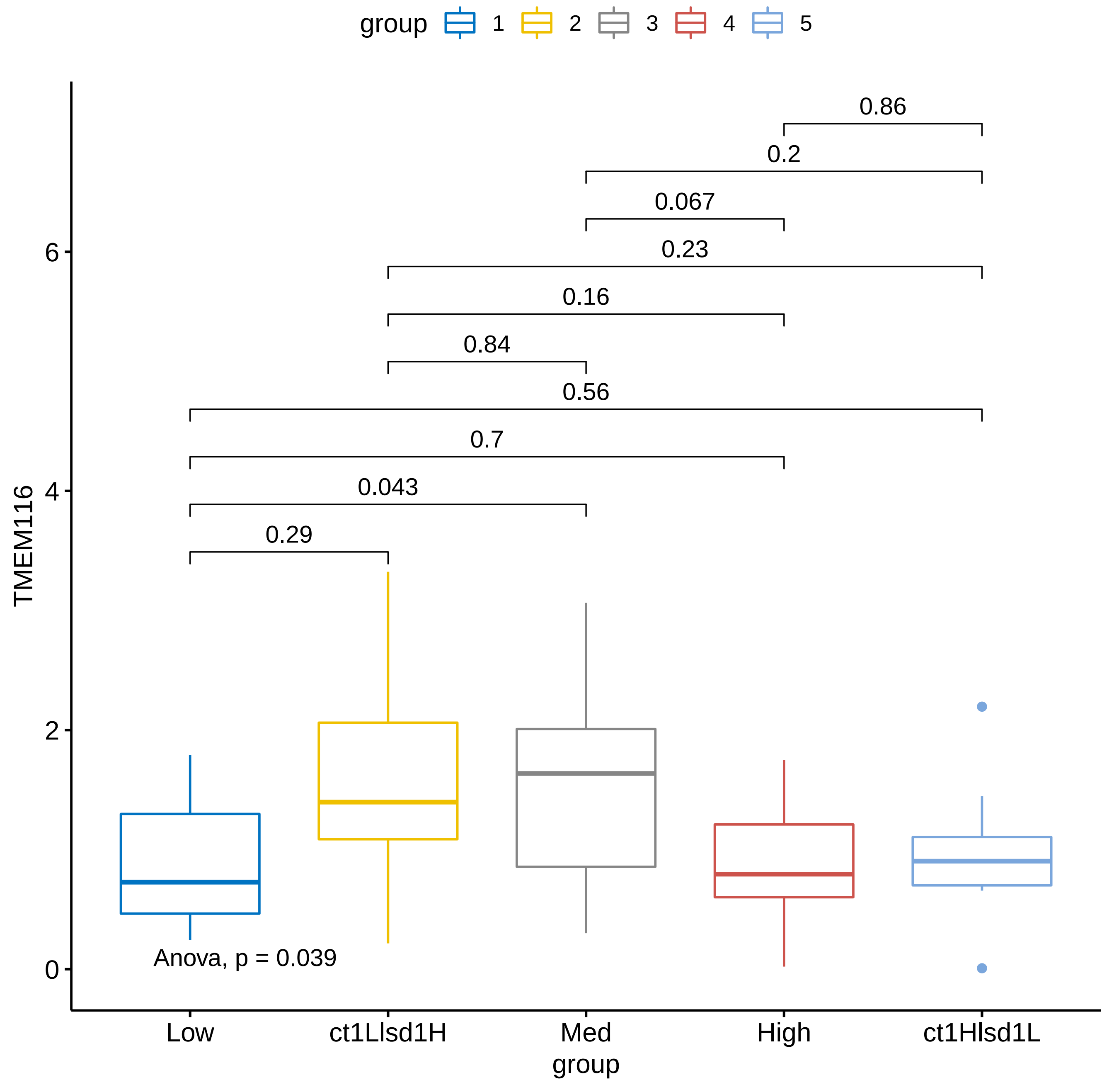

group
1
2
3
4
5
TMEM116
0
2
4
6
0.86
0.2
0.067
0.23
0.16
0.84
0.56
0.7
0.043
0.29
Anova, p = 0.039
Low
ct1Llsd1H
Med
High
ct1Hlsd1L
group

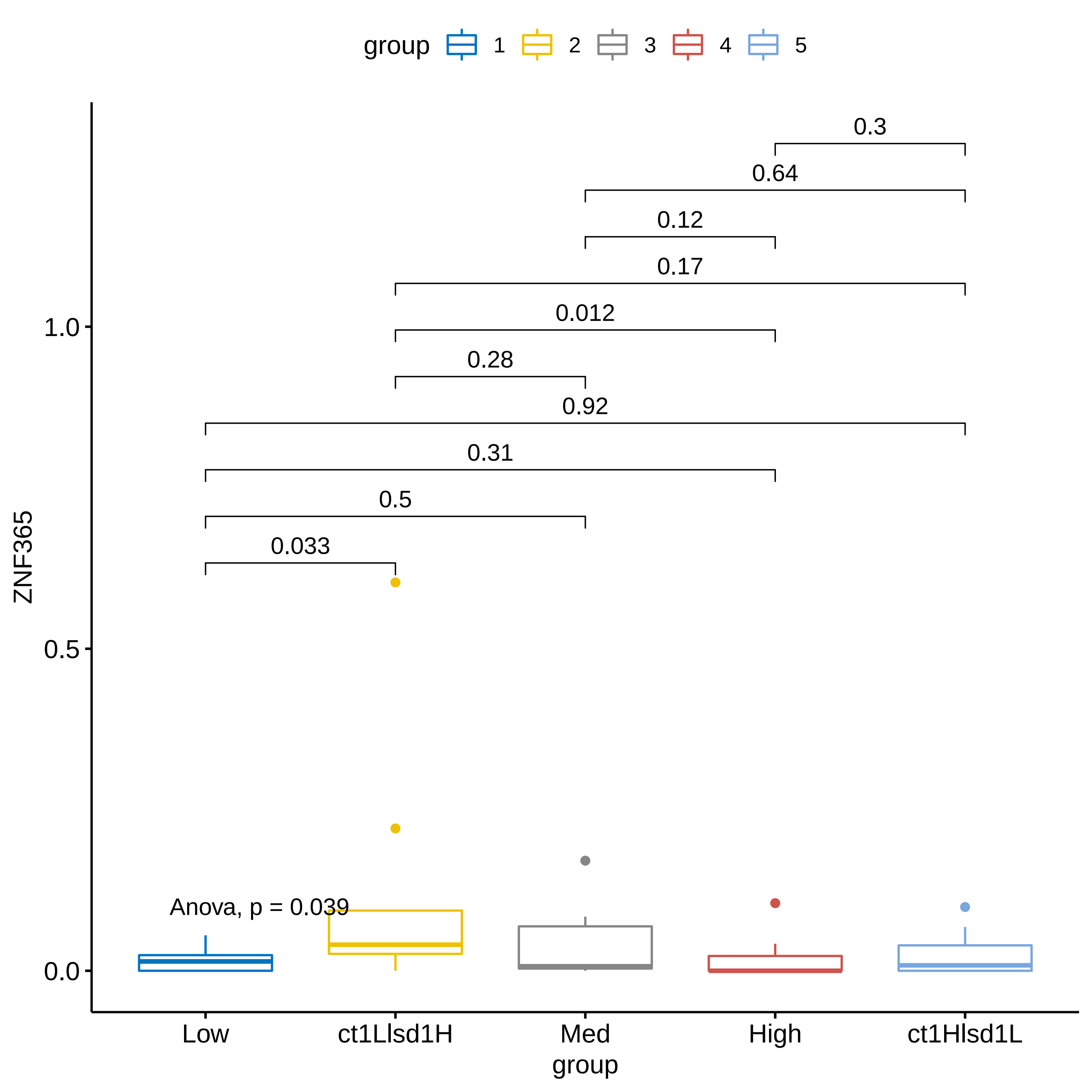
group
1
2
3
4
5
0.3
0.64
0.12
0.17
0.012
0.28
0.92
0.31
0.5
0.033
1.0
0.5
0.0
ZNF365
Anova, p = 0.039
Low
ct1Llsd1H
Med
High
ct1Hlsd1L
group

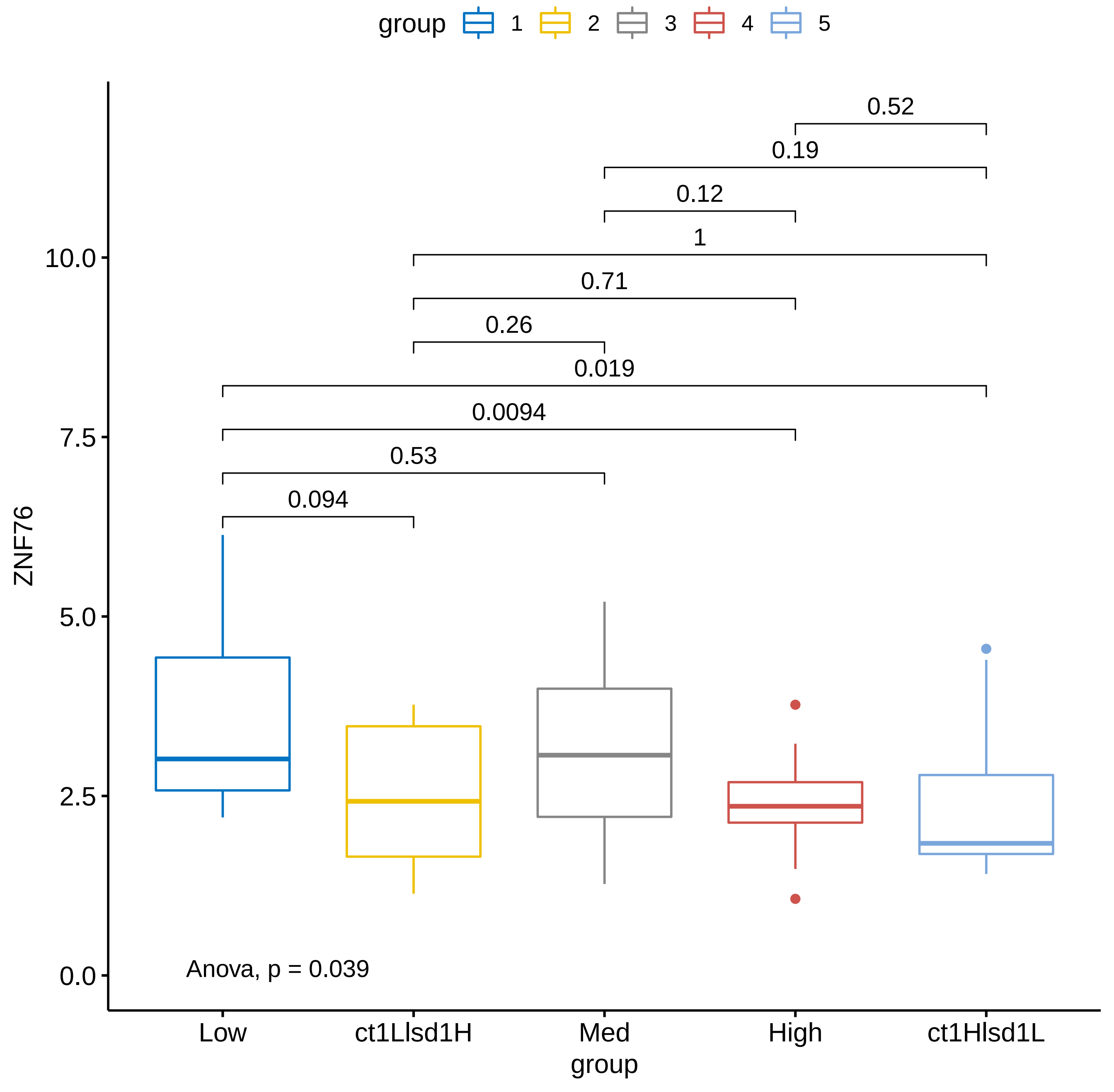
group
1
2
3
4
5
0.52
0.19
0.12
1
0.71
0.26
0.019
0.0094
0.53
0.094
10.0
7.5
5.0
2.5
0.0
ZNF76
Anova, p = 0.039
Low
ct1Llsd1H
Med
High
ct1Hlsd1L
group

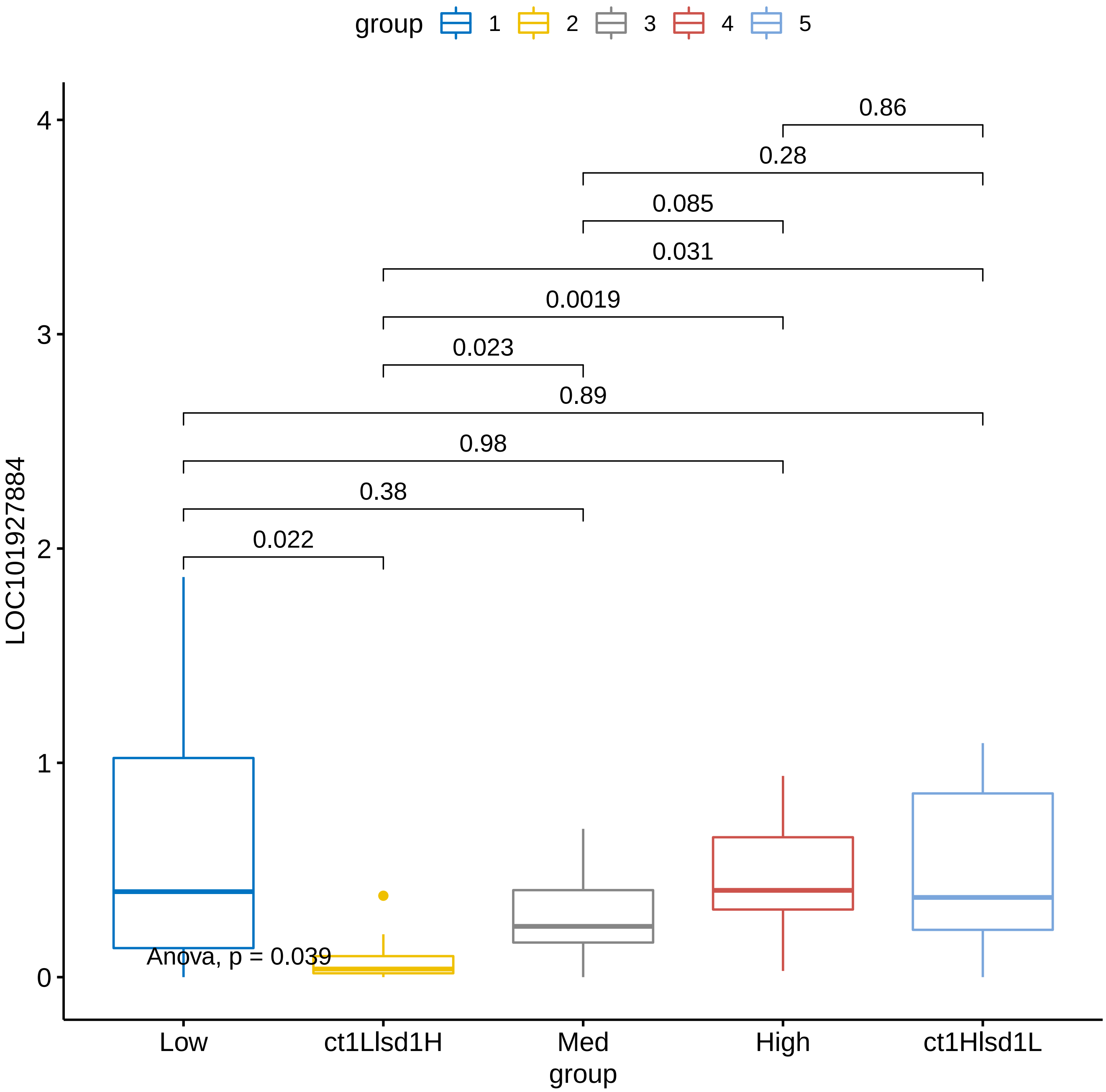

group
1
2
3
4
5
0.86
0.28
0.085
0.031
0.0019
0.023
0.89
0.98
0.38
0.022
Anova, p = 0.039
LOC101927884
4
3
2
1
0
Low
ct1Llsd1H
Med
High
ct1Hlsd1L
group

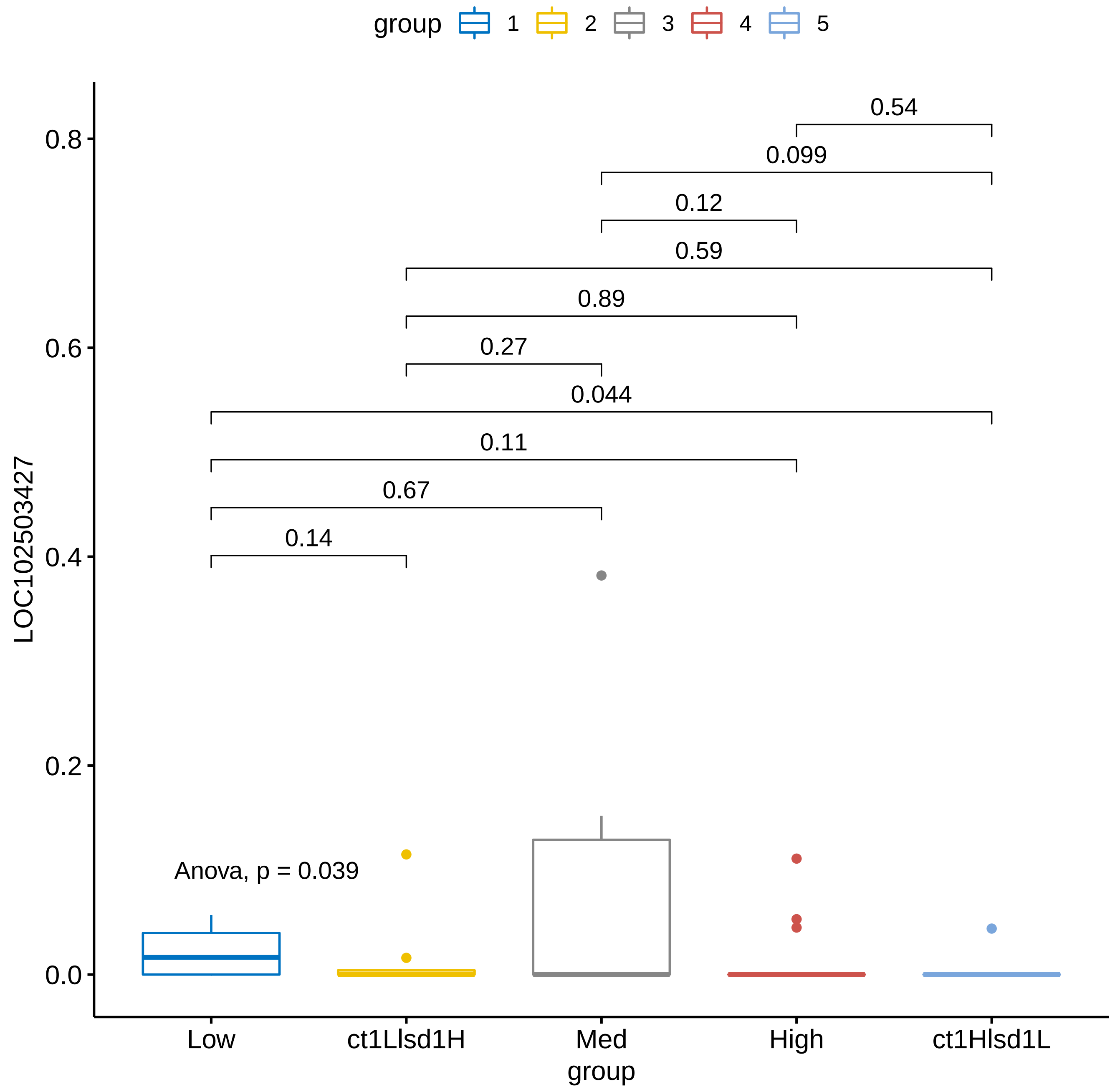

group
1
2
3
4
5
0.54
0.099
0.12
0.59
0.89
0.27
0.044
0.11
0.67
0.14
Anova, p = 0.039
0.8
0.6
0.4
0.2
0.0
LOC102503427
Low
ct1Llsd1H
Med
High
ct1Hlsd1L
group

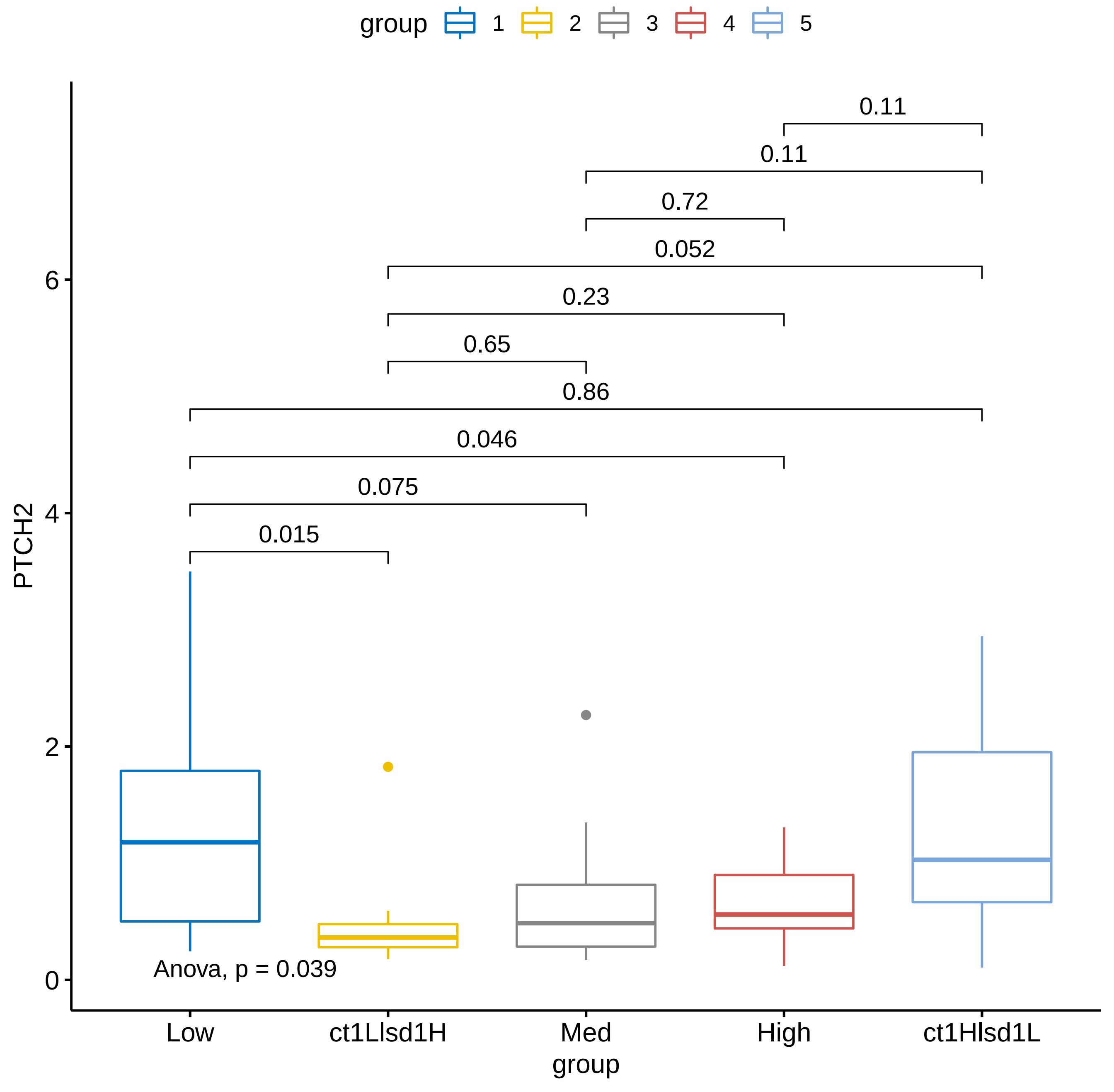

group
1
2
3
4
5
0.11
0.11
0.72
0.052
0.23
0.65
0.86
0.046
0.075
0.015
Anova, p = 0.039
PTCH2
0
2
4
6
Low
ct1Llsd1H
Med
High
ct1Hlsd1L
group

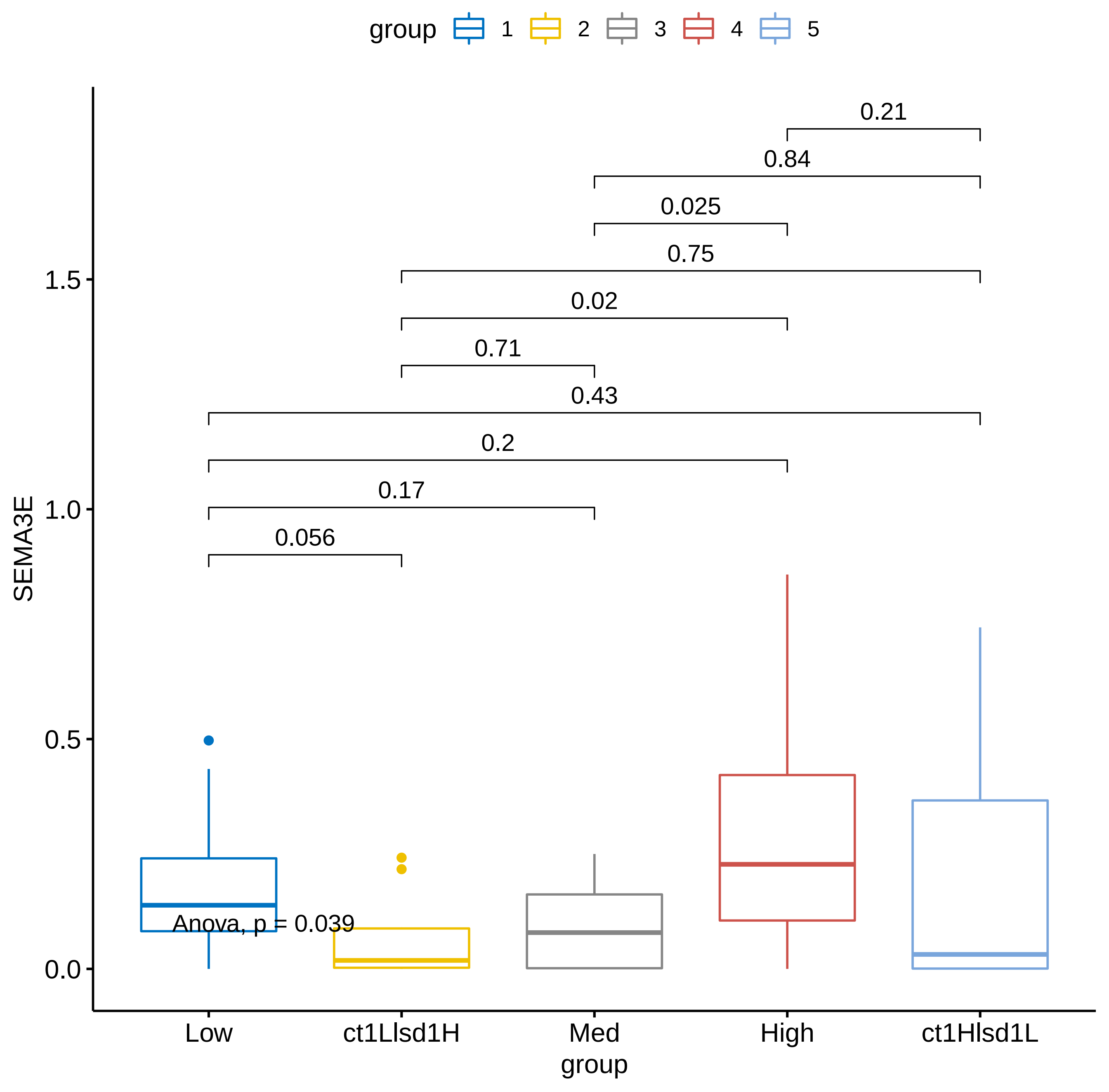

group
1
2
3
4
5
0.21
0.84
0.025
0.75
0.02
0.71
0.43
0.2
0.17
0.056
Anova, p = 0.039
SEMA3E
1.5
1.0
0.5
0.0
Low
ct1Llsd1H
Med
High
ct1Hlsd1L
group

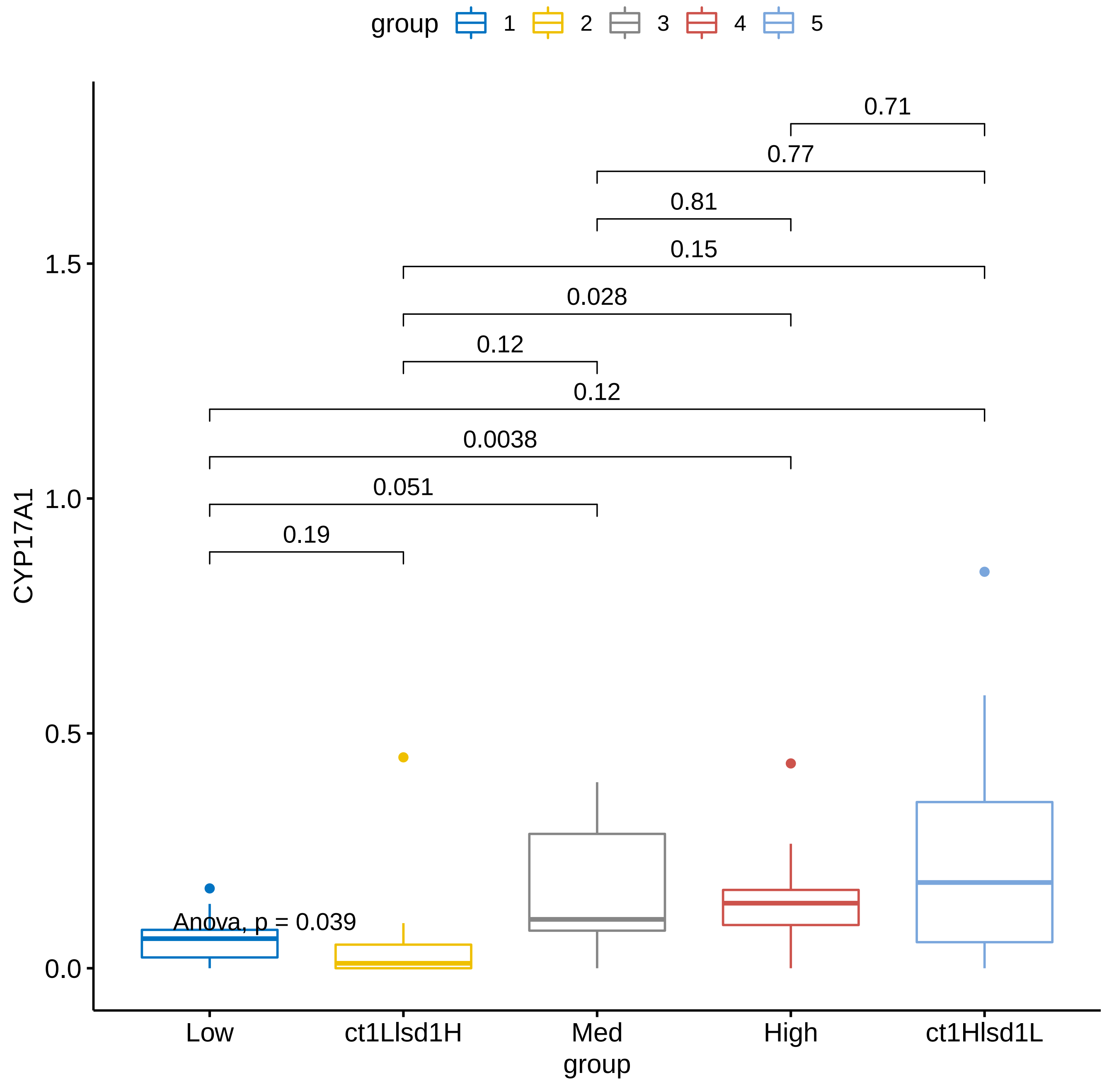
group
1
2
3
4
5
0.71
0.77
0.81
0.15
0.028
0.12
0.12
0.0038
0.051
0.19
Anova, p = 0.039
CYP17A1
1.5
1.0
0.5
0.0
Low
ct1Llsd1H
Med
High
ct1Hlsd1L
group

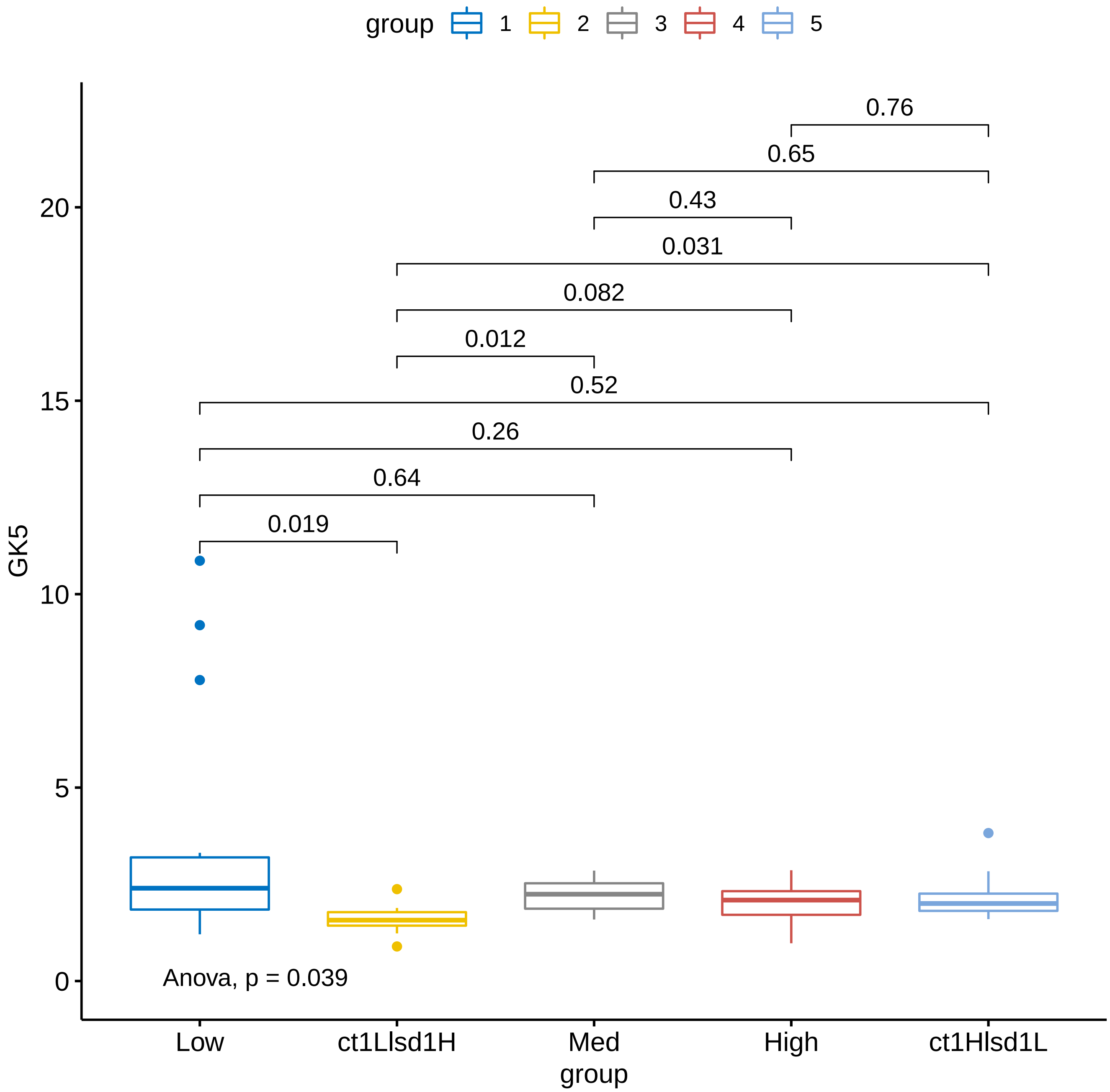
group
1
2
3
4
5
0.76
0.65
0.43
0.031
0.082
0.012
0.52
0.26
0.64
0.019
GK5
20
15
10
5
0
Anova, p = 0.039
Low
ct1Llsd1H
Med
High
ct1Hlsd1L
group

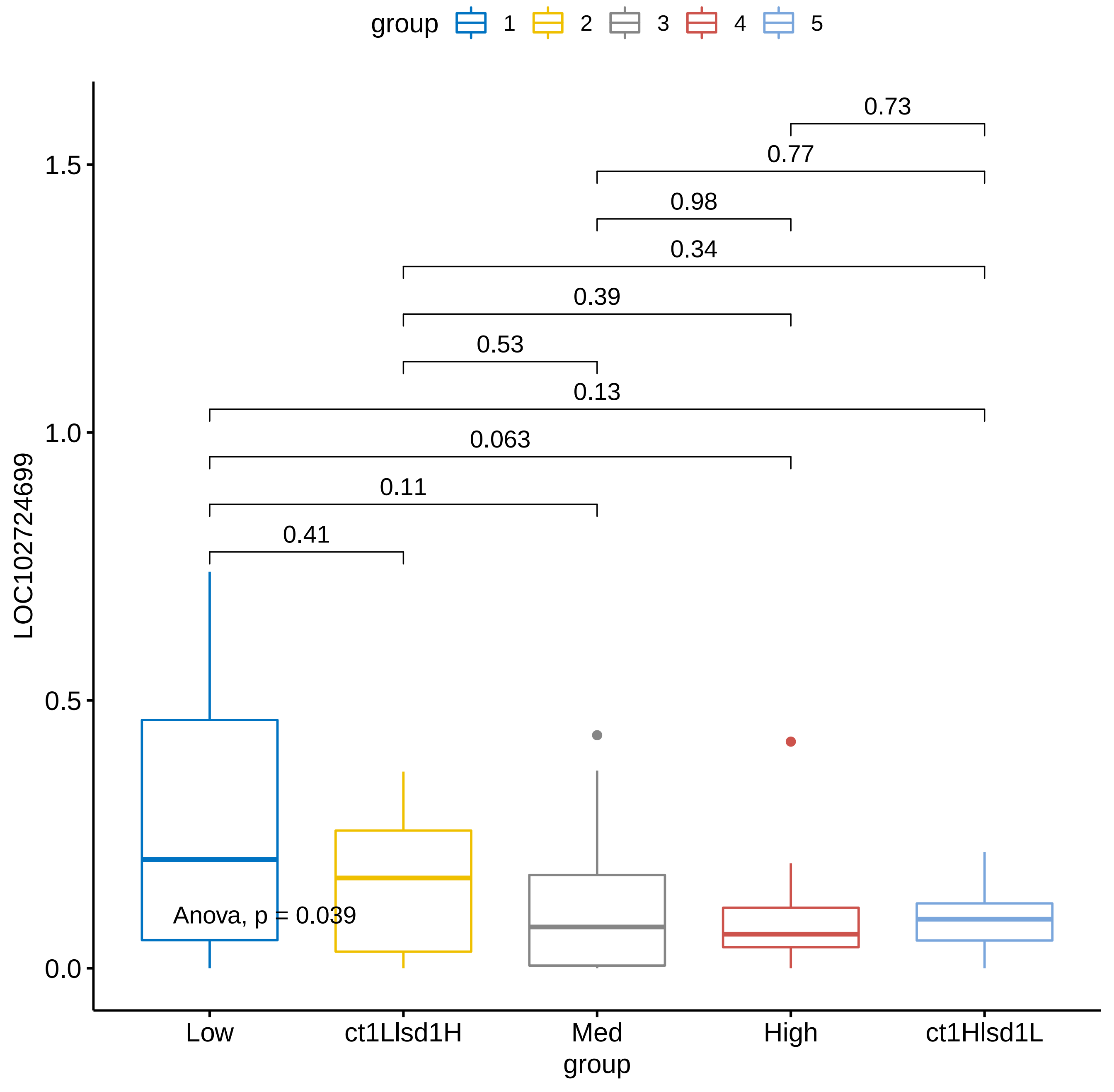
group
1
2
3
4
5
0.73
0.77
0.98
0.34
0.39
0.53
0.13
0.063
0.11
0.41
Anova, p = 0.039
LOC102724699
1.5
1.0
0.5
0.0
Low
ct1Llsd1H
Med
High
ct1Hlsd1L
group

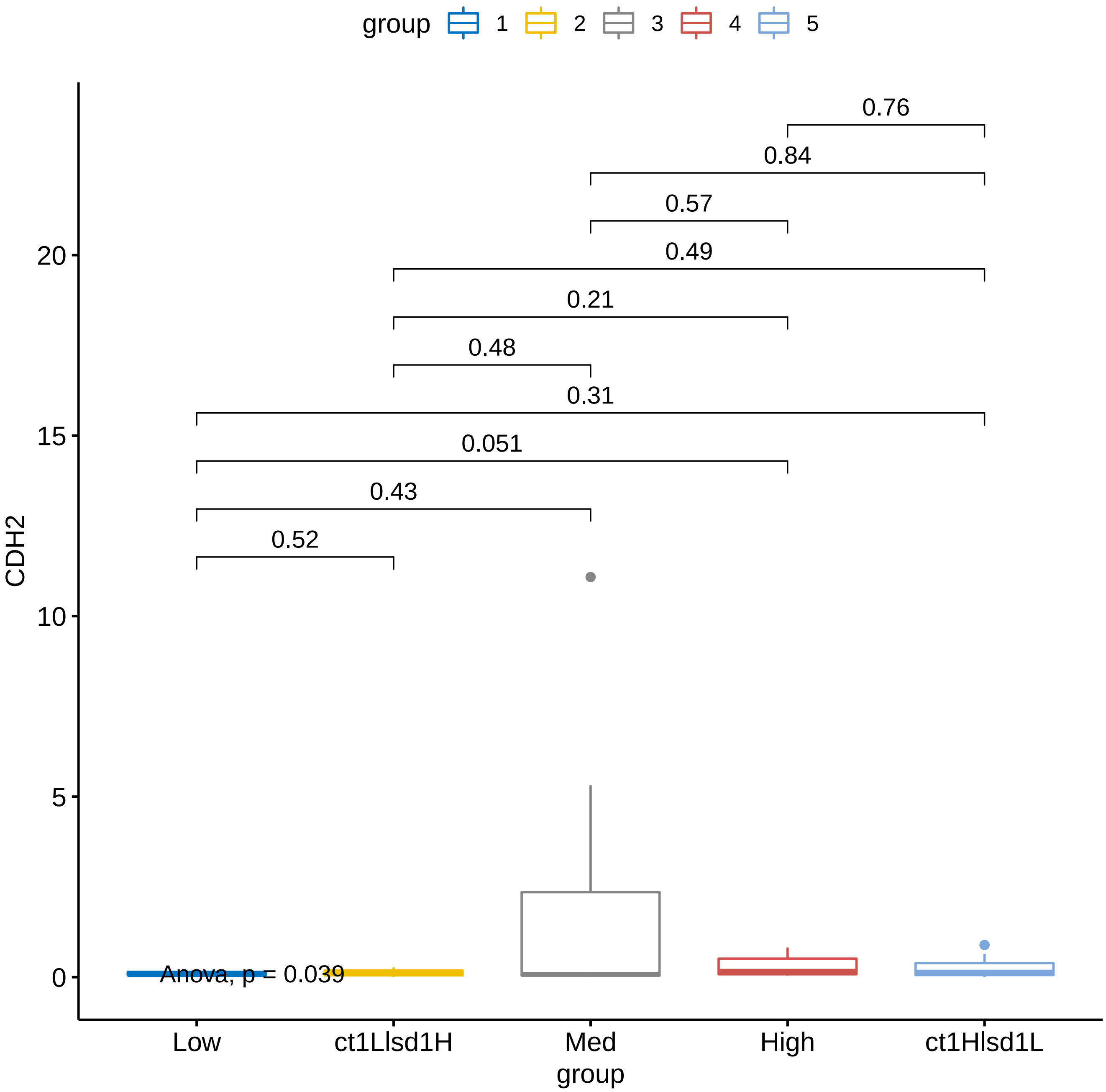

group 1 2 3 4 5
0.76
0.84
0.57
0.49
0.21
0.48
0.31
0.051
0.43
0.52
Anova, p = 0.039
CDH2
20
15
10
5
0
Low
ct1Llsd1H
Med
High
ct1Hlsd1L
group

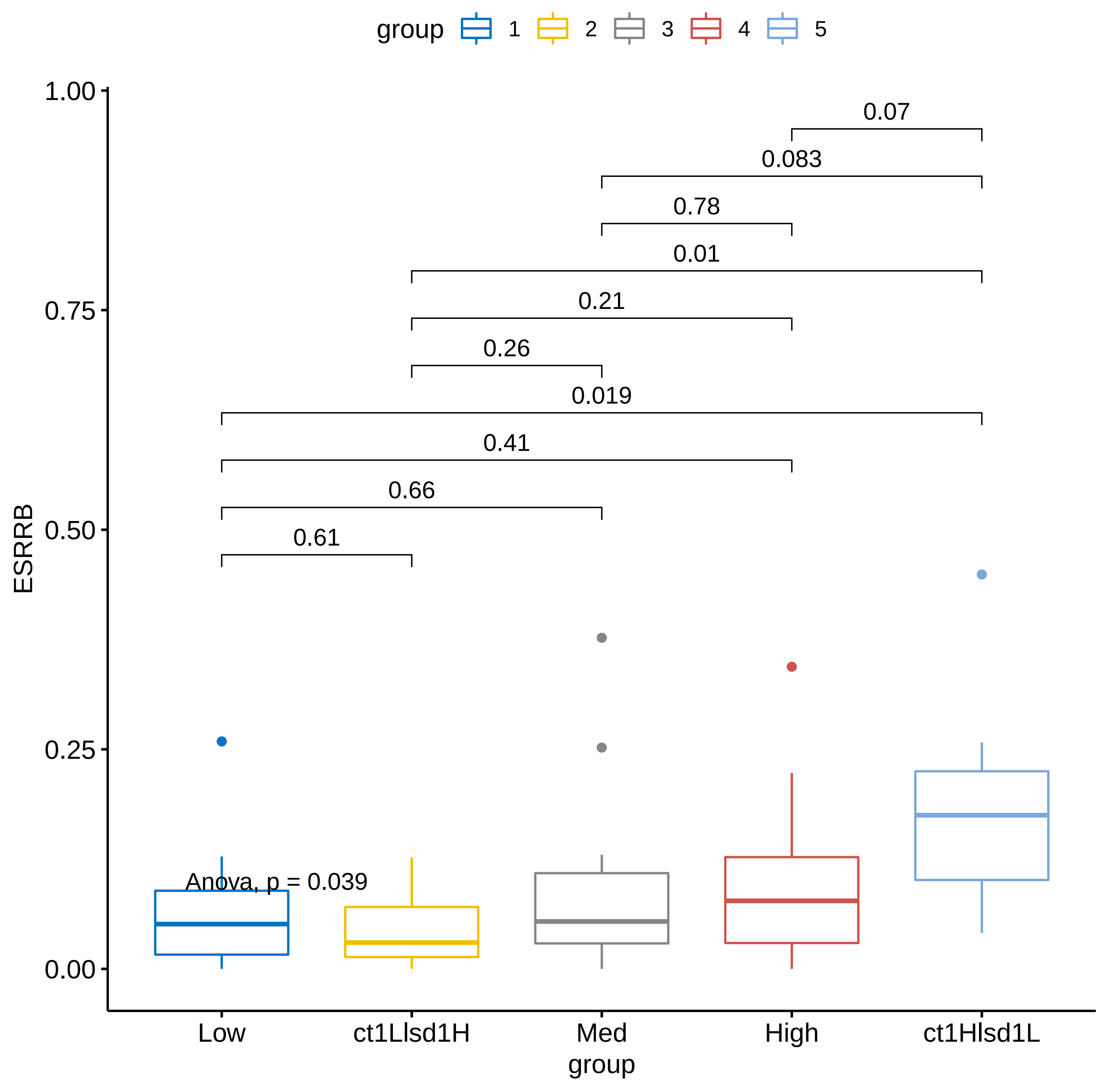

group 1 2 3 4 5
1.00
0.75
0.50
0.25
0.00
ESRRB
0.07
0.083
0.78
0.01
0.21
0.26
0.019
0.41
0.66
0.61
Anova, p = 0.039
Low
ct1Llsd1H
Med
High
ct1Hlsd1L
group

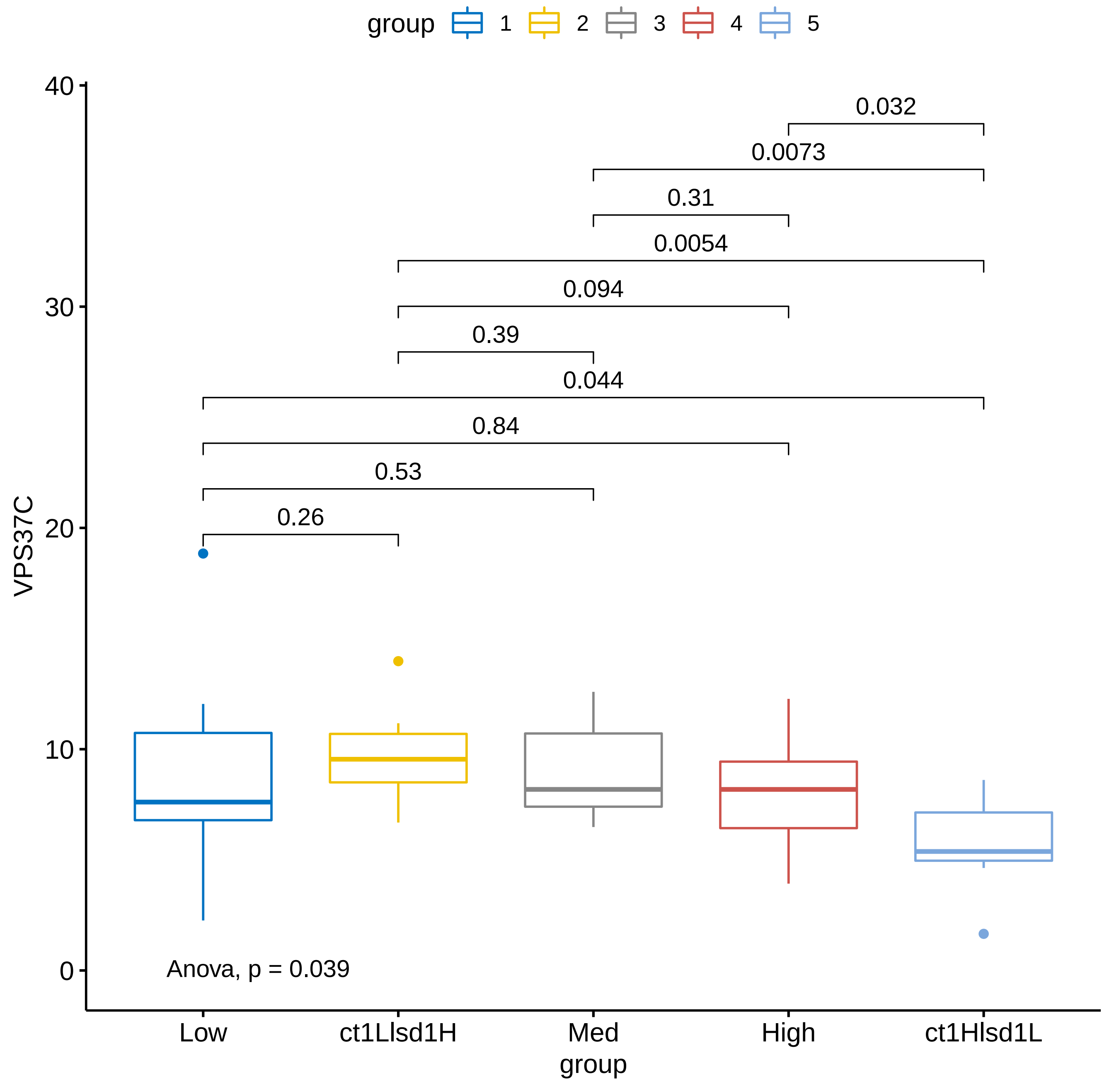

group
1
2
3
4
5
VPS37C
40
30
20
10
0
0.032
0.0073
0.31
0.0054
0.094
0.39
0.044
0.84
0.53
0.26
Anova, p = 0.039
Low
ct1Llsd1H
Med
High
ct1Hlsd1L
group

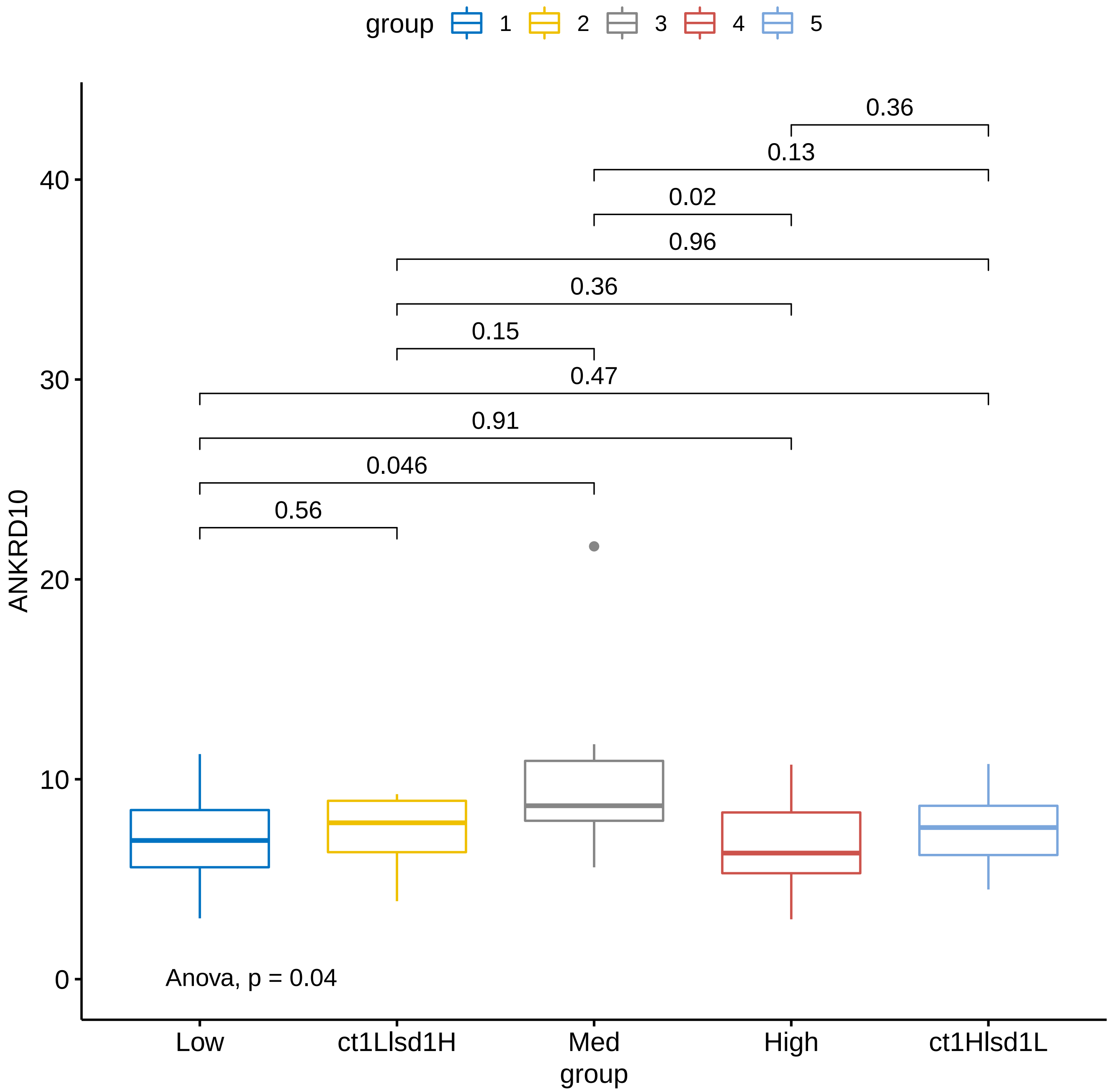

group 1 2 3 4 5
0.36
0.13
0.02
0.96
0.36
0.15
0.47
0.91
0.046
0.56
ANKRD10
40
30
20
10
0
Anova, p = 0.04
Low
ct1Llsd1H
Med
High
ct1Hlsd1L
group

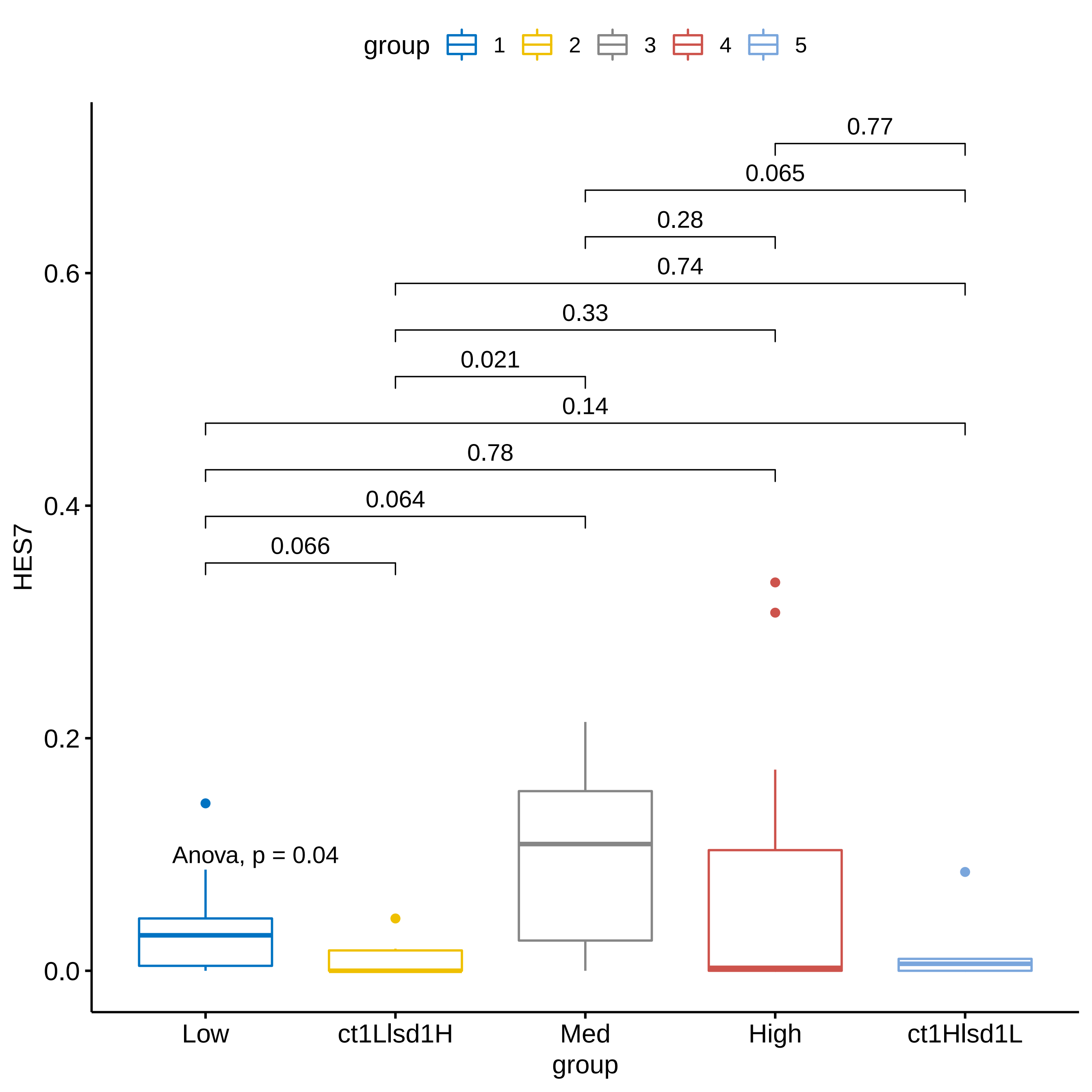

group
1
2
3
4
5
HES7
0.0
0.2
0.4
0.6
0.77
0.065
0.28
0.74
0.33
0.021
0.14
0.78
0.064
0.066
Anova, p = 0.04
Low
ct1Llsd1H
Med
High
ct1Hlsd1L
group

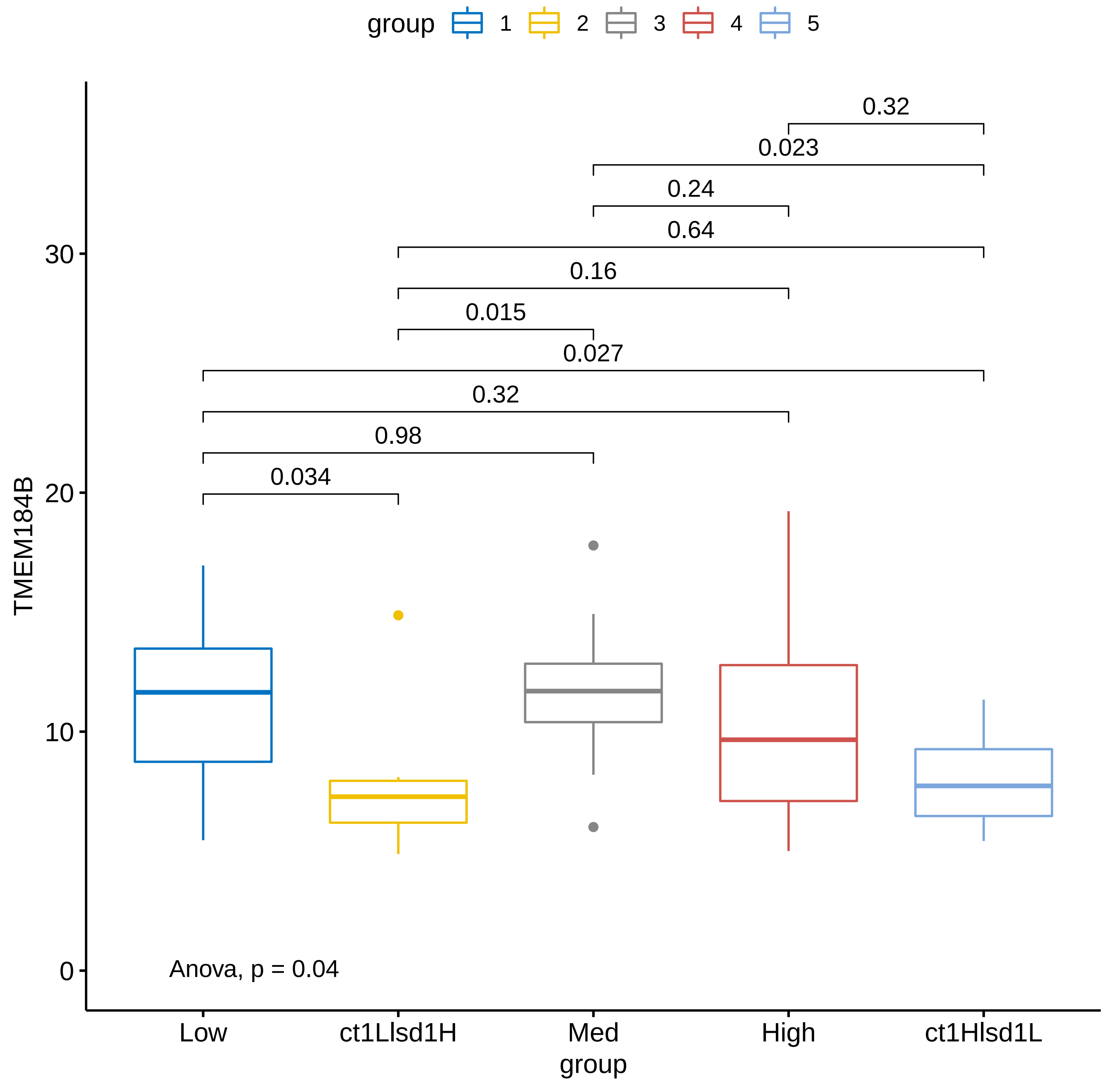

group
1
2
3
4
5
0.32
0.023
0.24
0.64
0.16
0.015
0.027
0.32
0.98
0.034
TMEM184B
30
20
10
0
Anova, p = 0.04
Low
ct1Llsd1H
Med
High
ct1Hlsd1L
group

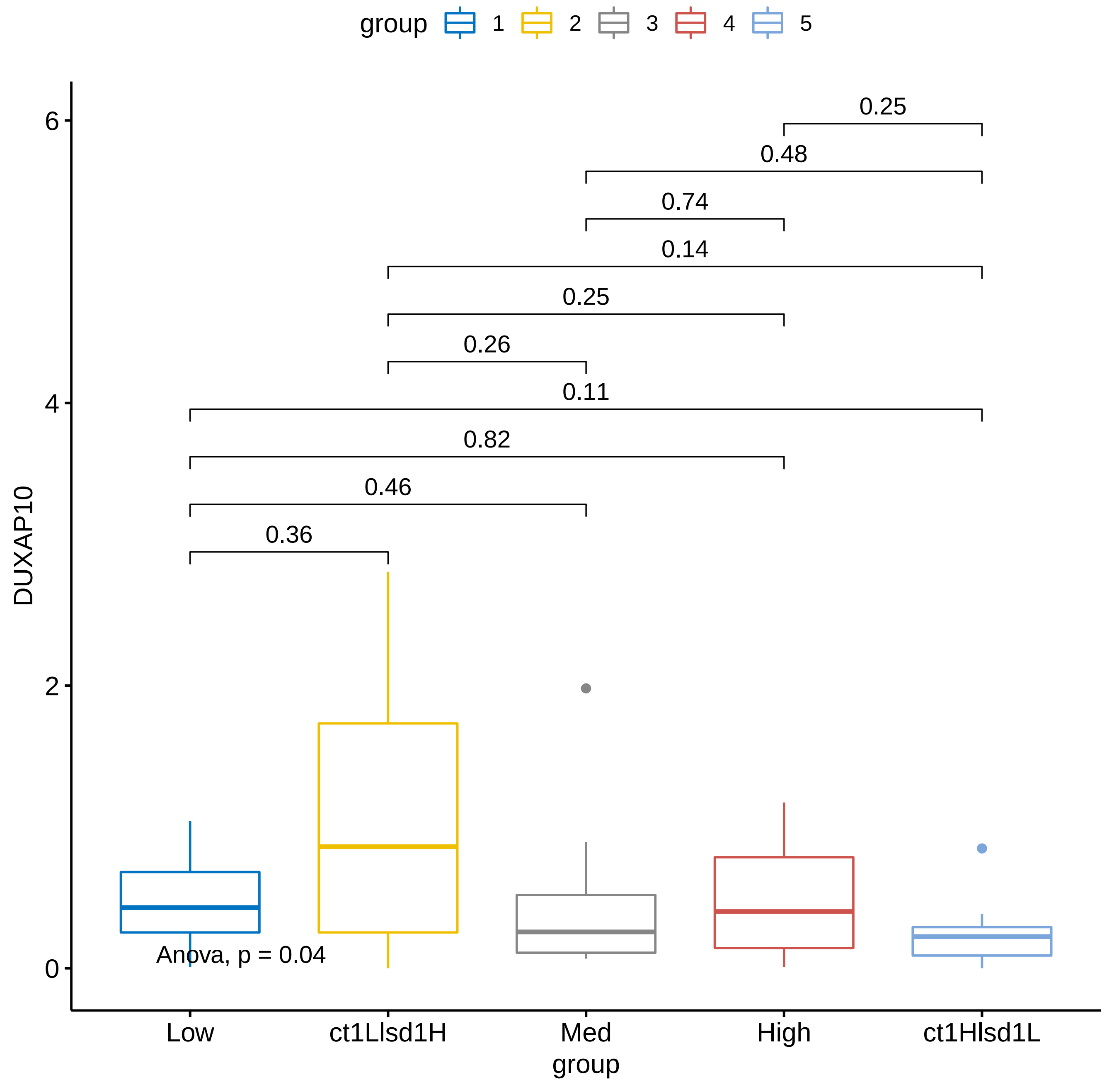
group
1
2
3
4
5
0.25
0.48
0.74
0.14
0.25
0.26
0.11
0.82
0.46
0.36
6
4
2
0
DUXAP10
Anova, p = 0.04
Low
ct1Llsd1H
Med
High
ct1Hlsd1L
group

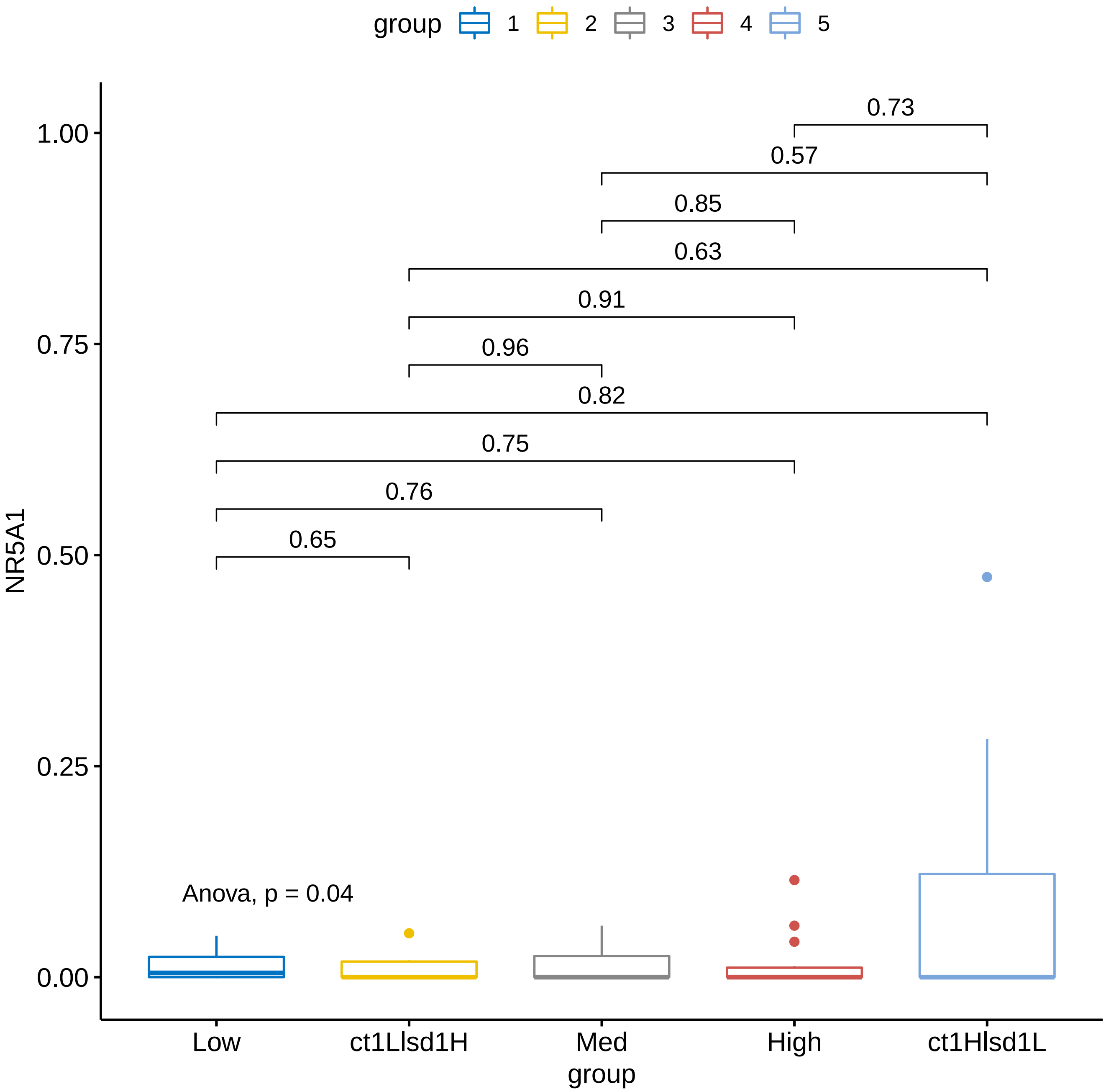

group 1 2 3 4 5
NR5A1
1.00
0.75
0.50
0.25
0.00
0.73
0.57
0.85
0.63
0.91
0.96
0.82
0.75
0.76
0.65
Anova, p = 0.04
Low
ct1Llsd1H
Med
High
ct1Hlsd1L
group

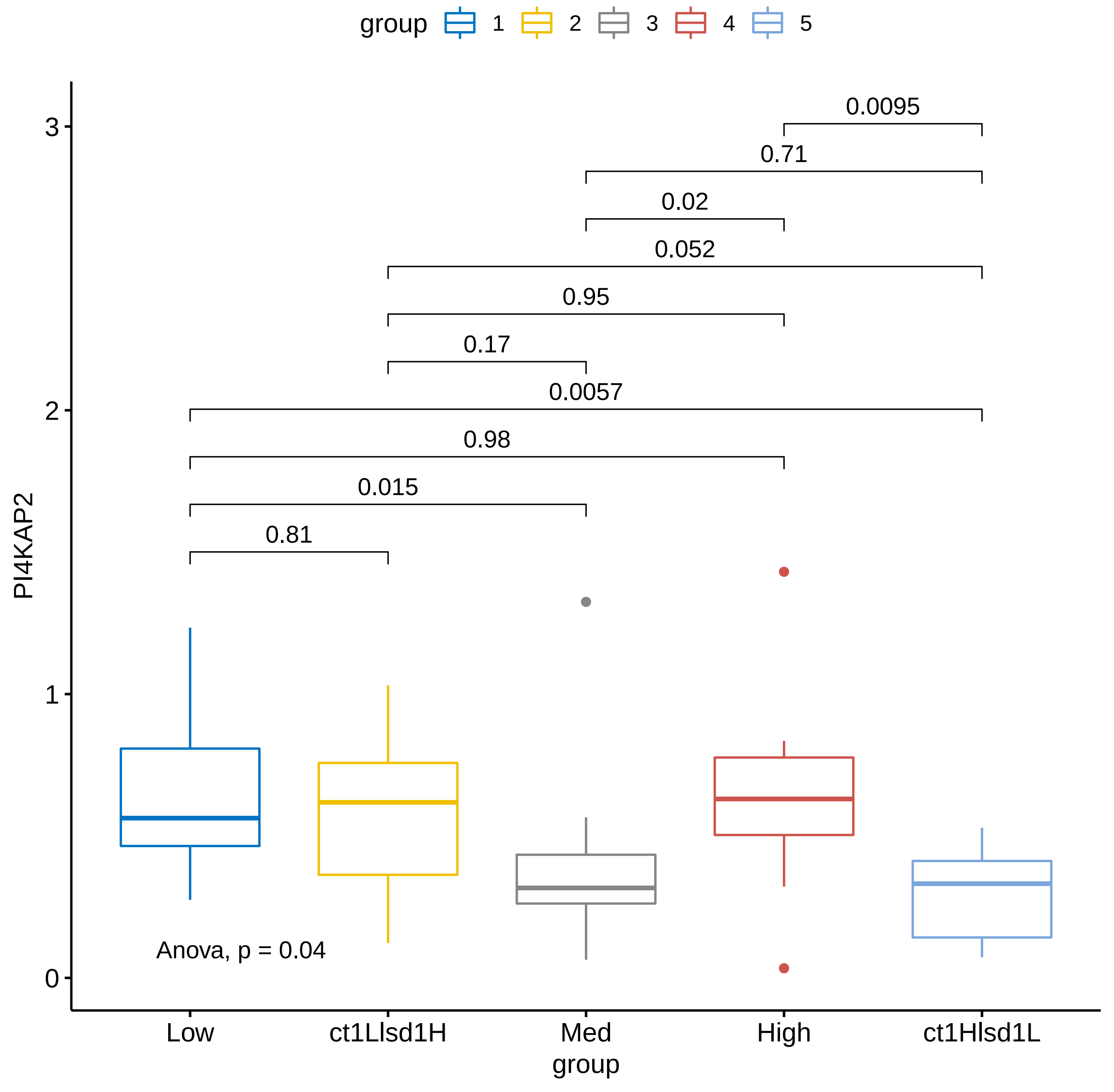

group
1
2
3
4
5
0.0095
0.71
0.02
0.052
0.95
0.17
0.0057
0.98
0.015
0.81
PI4KAP2
3
2
1
0
Anova, p = 0.04
Low
ct1Llsd1H
Med
High
ct1Hlsd1L
group

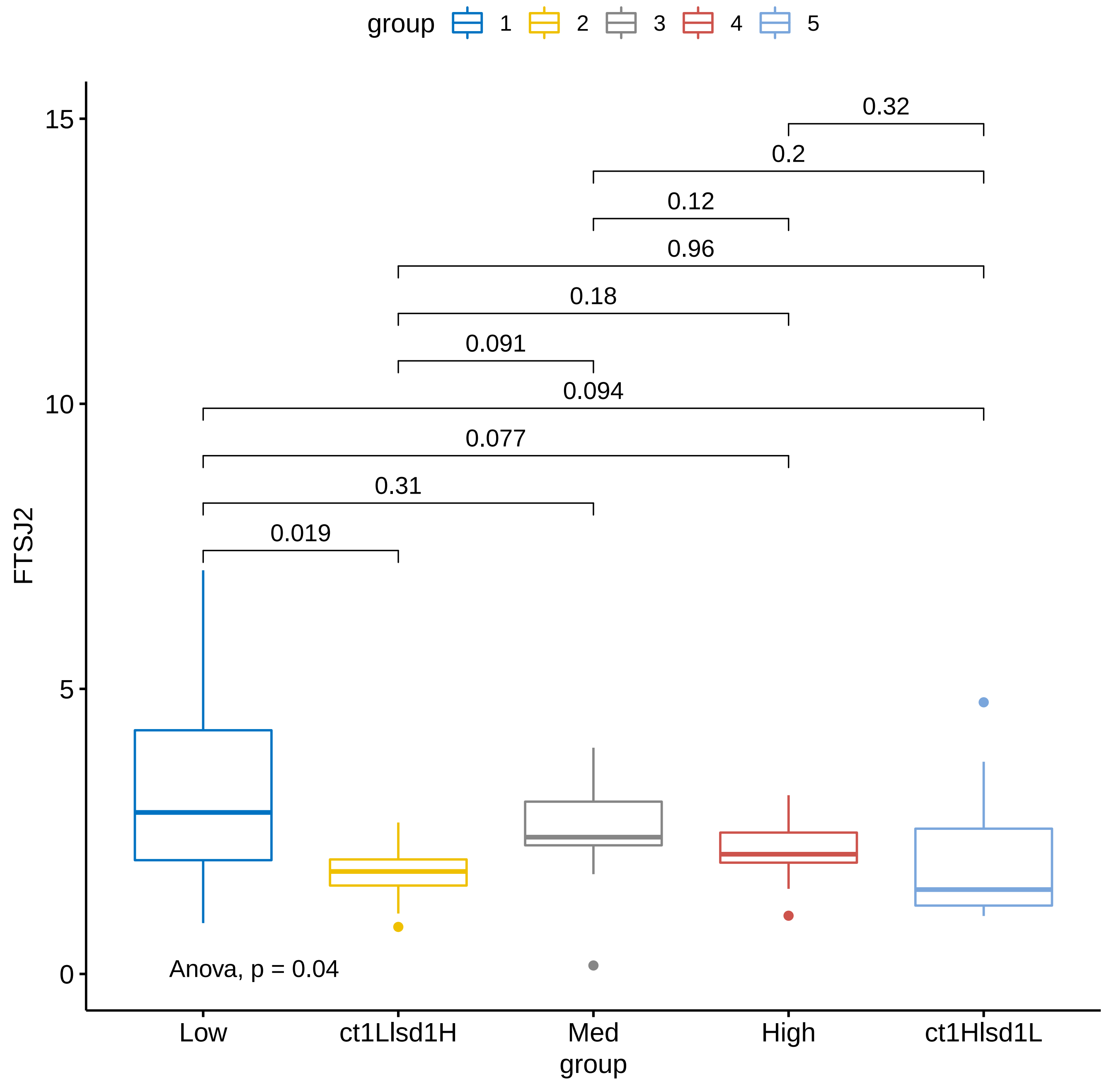

group
1
2
3
4
5
0.32
0.2
0.12
0.96
0.18
0.091
0.094
0.077
0.31
0.019
15
10
5
0
FTSJ2
Anova, p = 0.04
Low
ct1Llsd1H
Med
High
ct1Hlsd1L
group

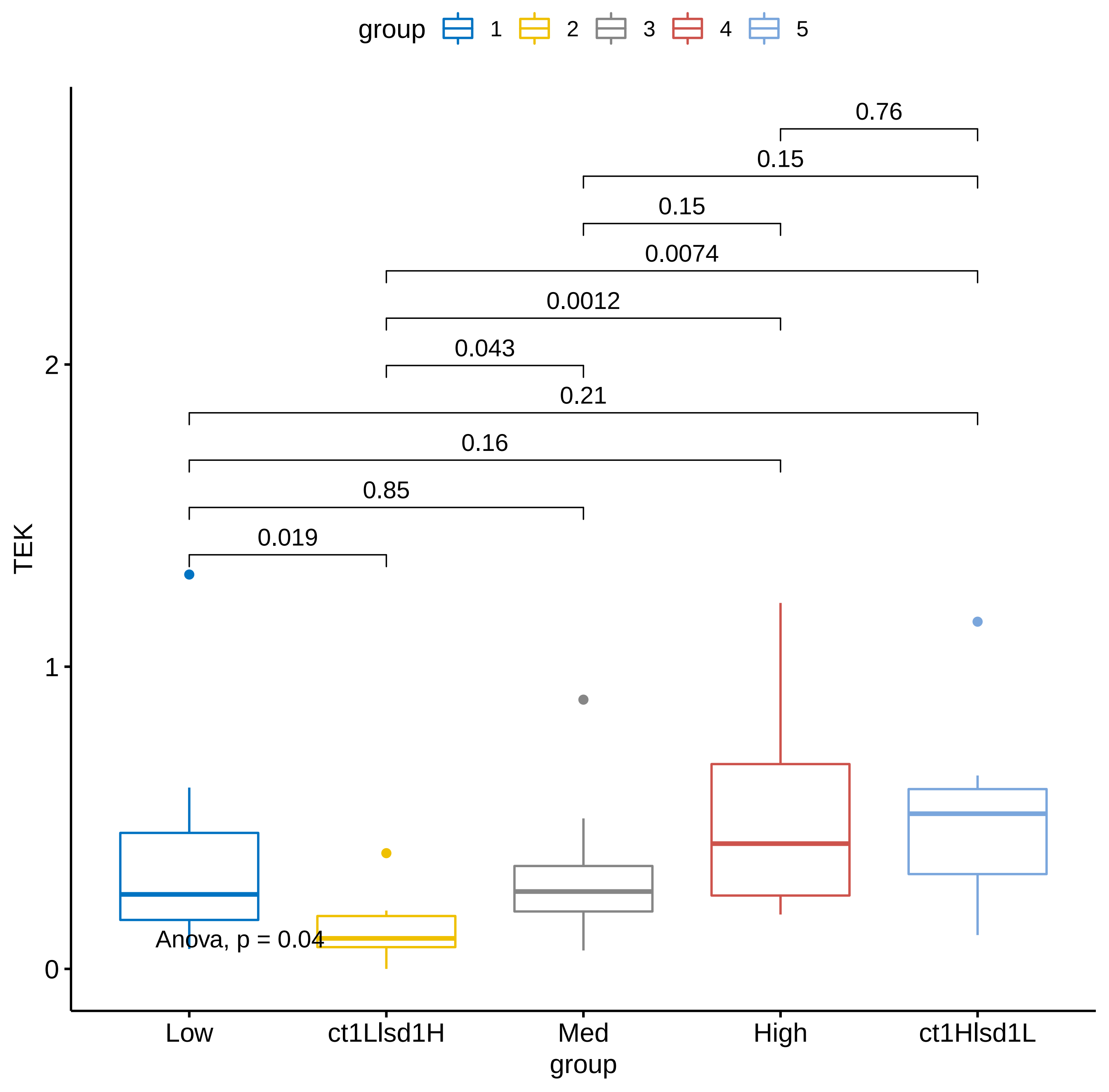

group
1
2
3
4
5
0.76
0.15
0.15
0.0074
0.0012
0.043
0.21
0.16
0.85
0.019
TEK
2
1
0
Anova, p = 0.04
Low
ct1Llsd1H
Med
High
ct1Hlsd1L
group

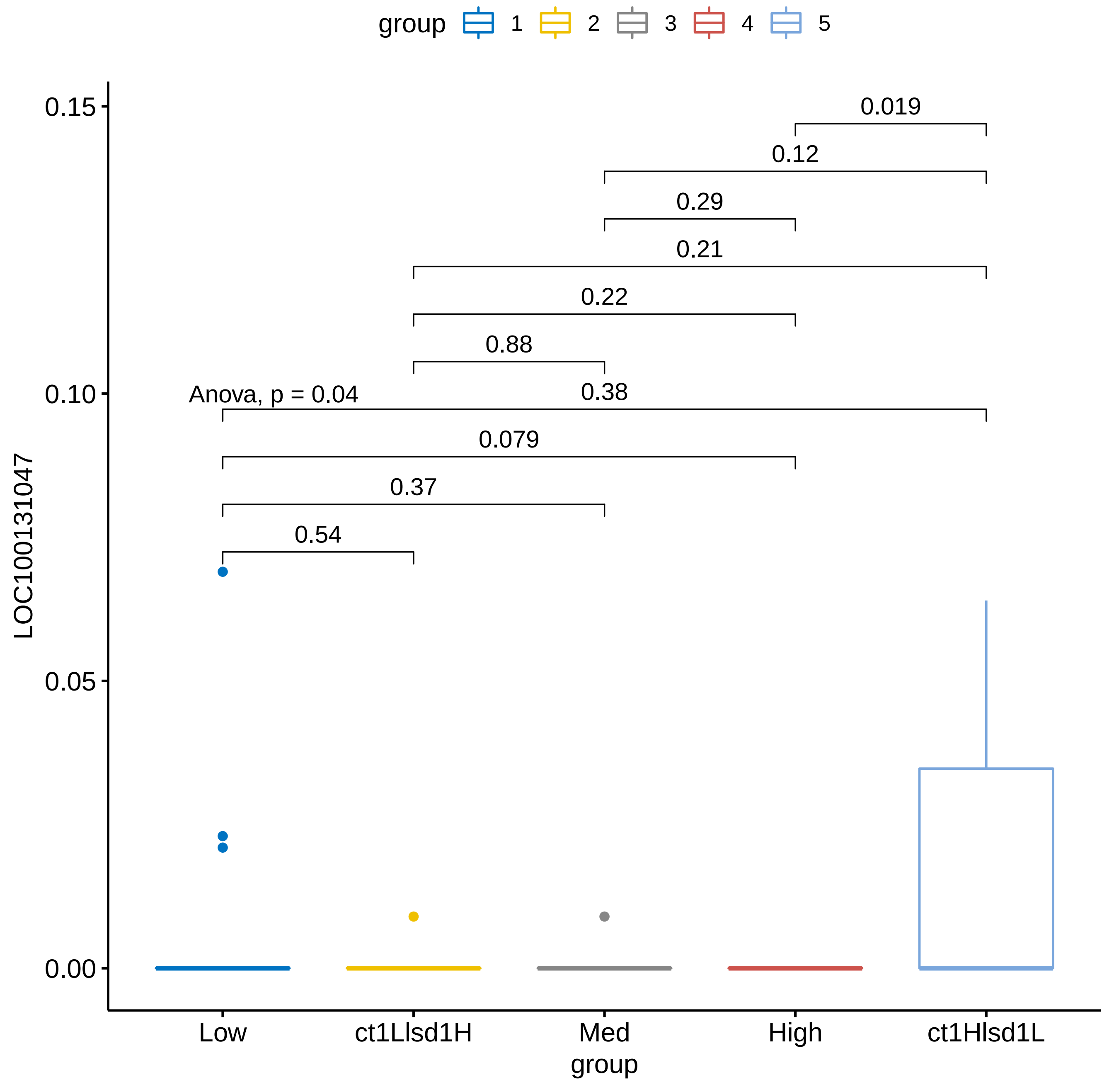

group 1 2 3 4 5
0.019
0.12
0.29
0.21
0.22
0.88
Anova, p = 0.04
0.38
0.079
0.37
0.54
0.15
0.10
0.05
0.00
LOC100131047
Low
ct1Llsd1H
Med
High
ct1Hlsd1L
group

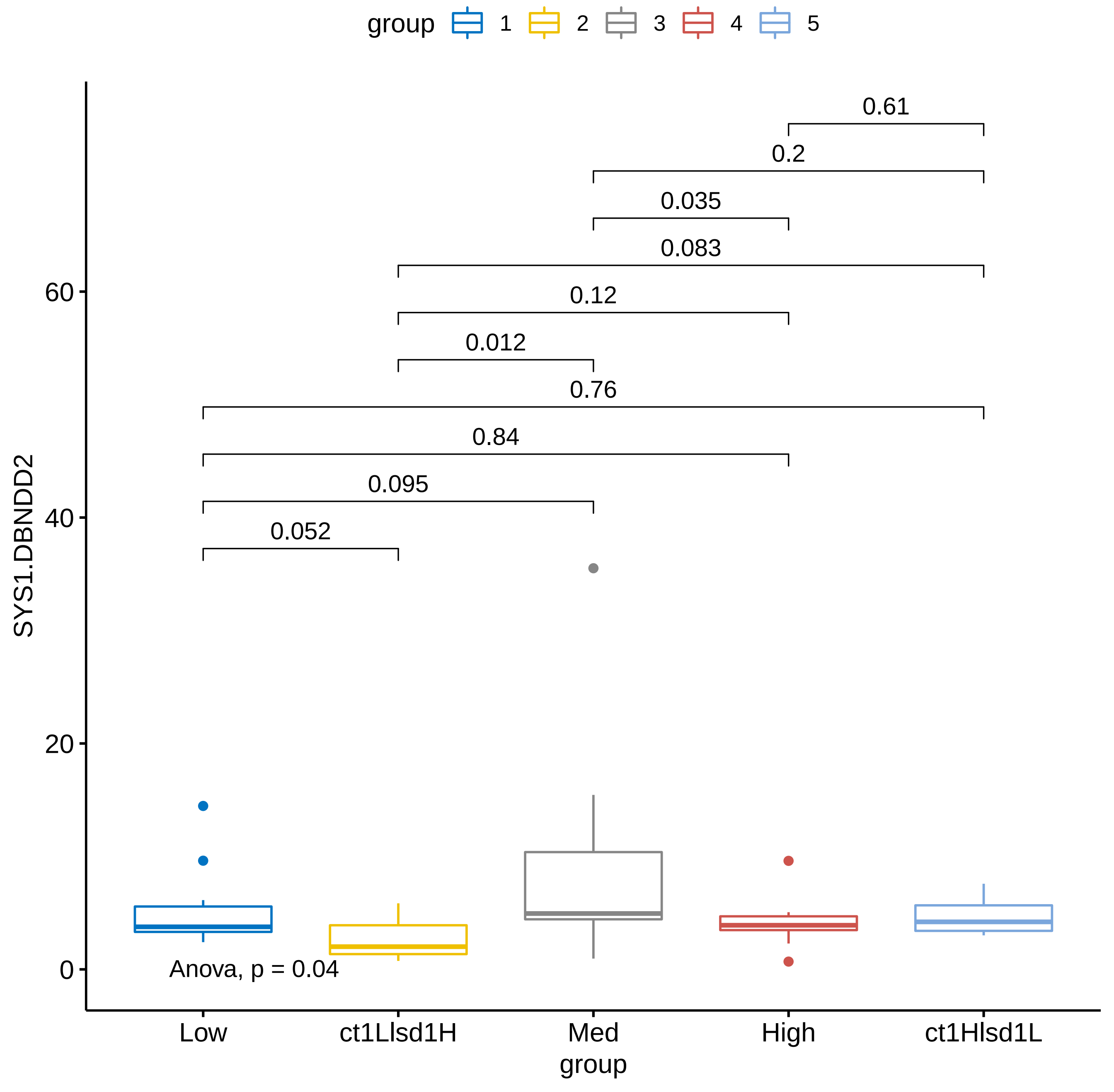
group
1
2
3
4
5
0.61
0.2
0.035
0.083
0.12
0.012
0.76
0.84
0.095
0.052
60
40
20
0
SYS1.DBNDD2
Anova, p = 0.04
Low
ct1Llsd1H
Med
High
ct1Hlsd1L
group

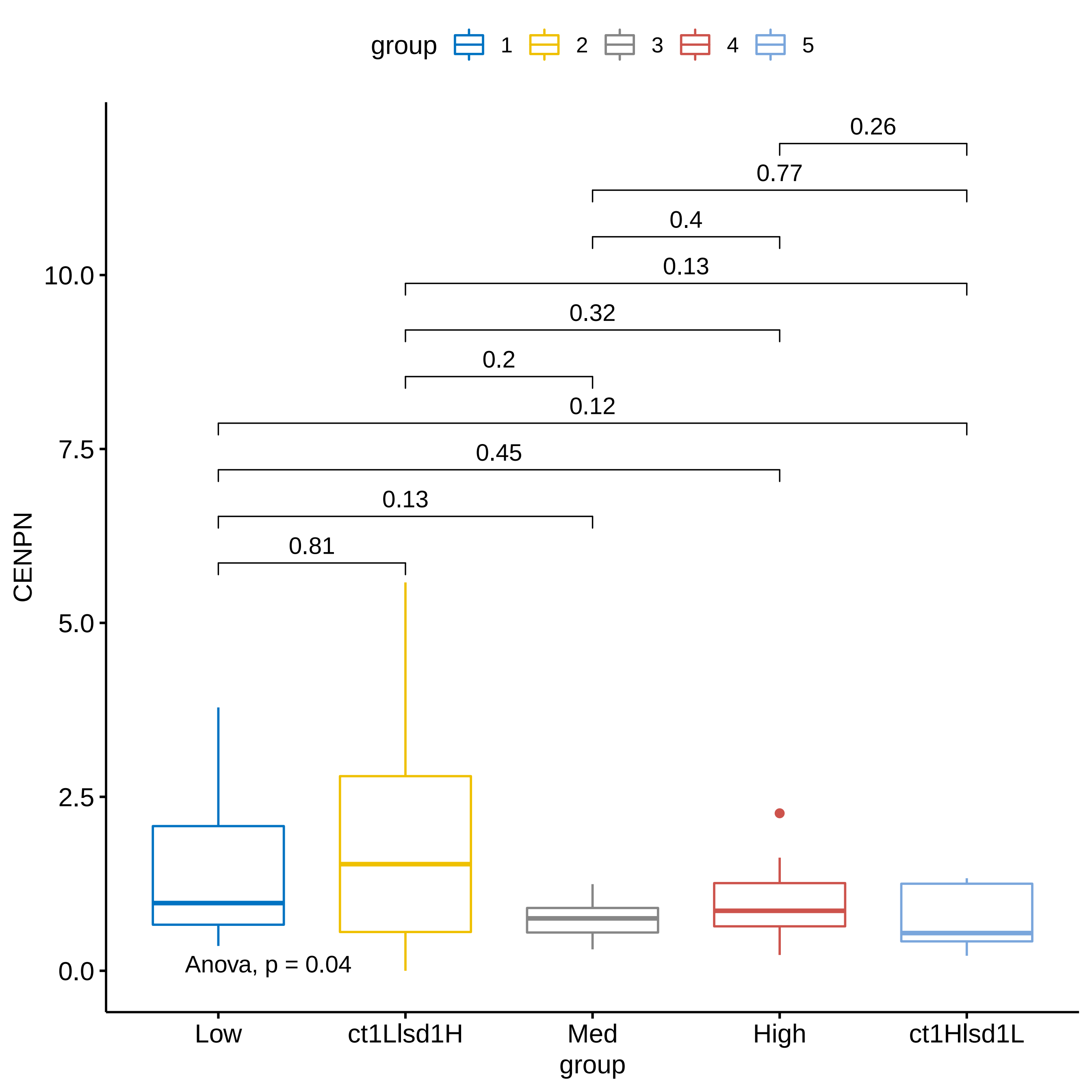

group
1
2
3
4
5
0.26
0.77
0.4
0.13
0.32
0.2
0.12
0.45
0.13
0.81
10.0
7.5
5.0
2.5
0.0
CENPN
Anova, p = 0.04
Low
ct1Llsd1H
Med
High
ct1Hlsd1L
group

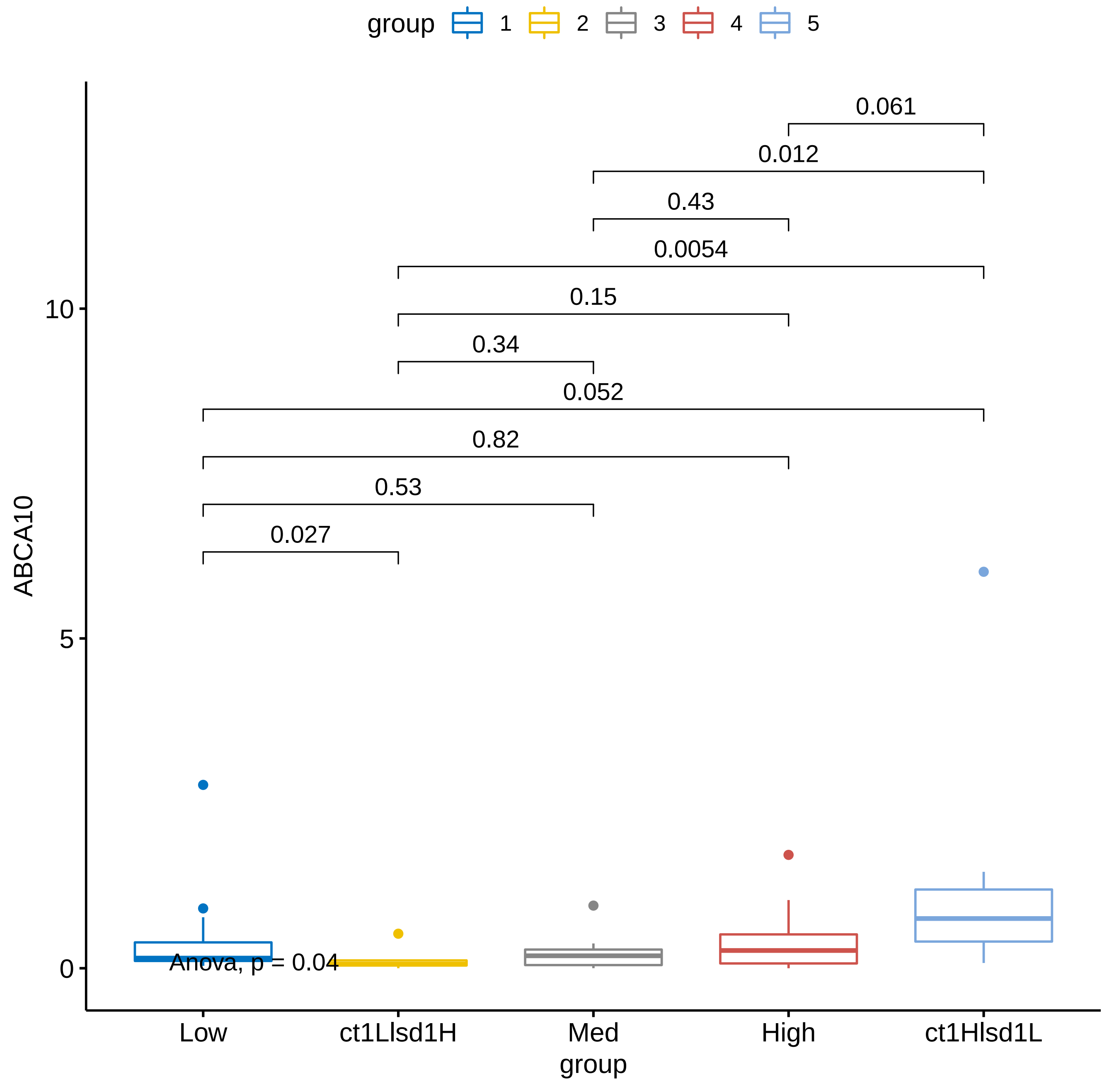
group
1
2
3
4
5
0.061
0.012
0.43
0.0054
0.15
0.34
0.052
0.82
0.53
0.027
10
5
0
ABCA10
Anova, p = 0.04
Low
ct1Llsd1H
Med
High
ct1Hlsd1L
group

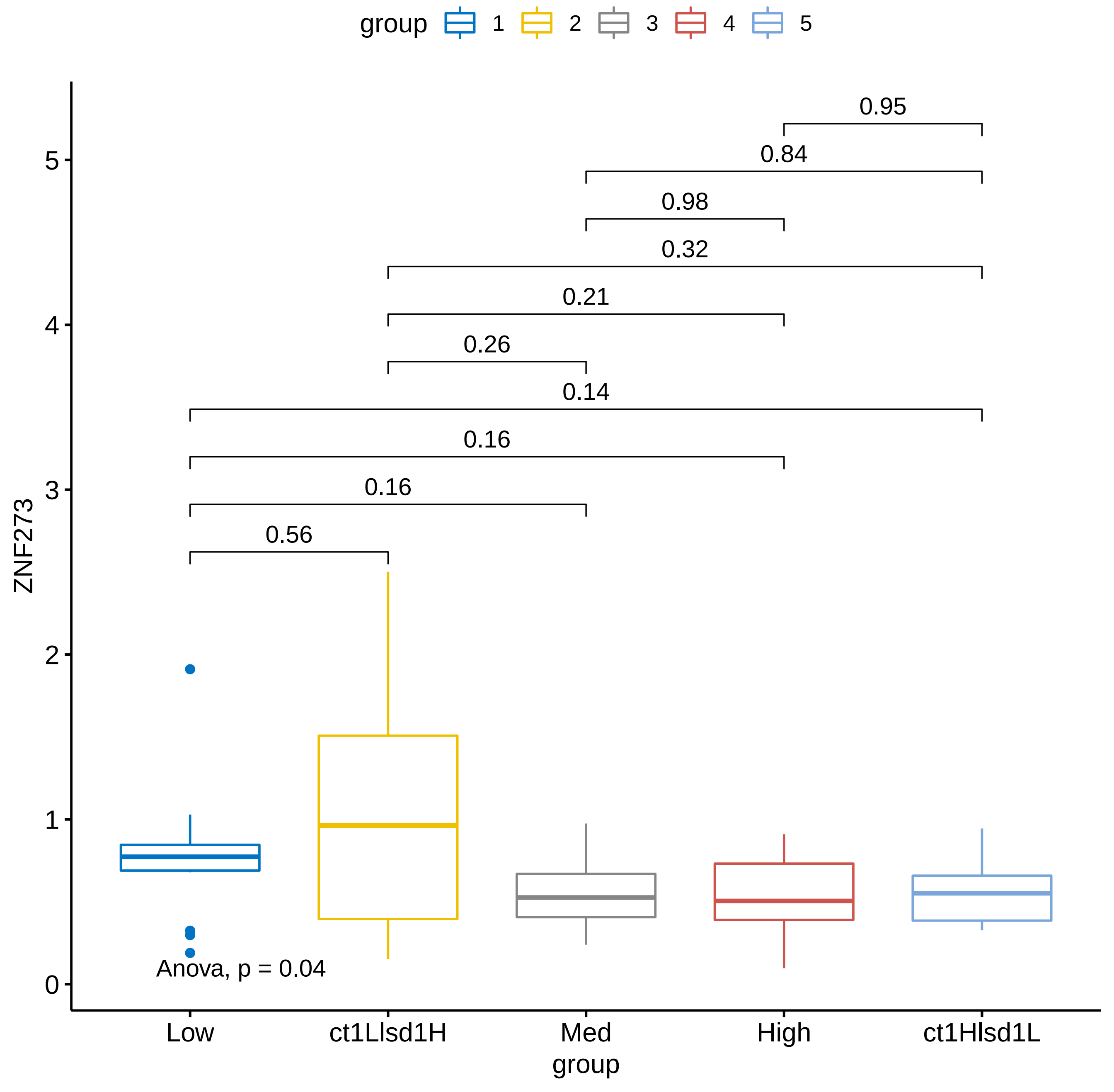
group
1
2
3
4
5
0.95
0.84
0.98
0.32
0.21
0.26
0.14
0.16
0.16
0.56
Anova, p = 0.04
ZNF273
5
4
3
2
1
0
Low
ct1Llsd1H
Med
High
ct1Hlsd1L
group

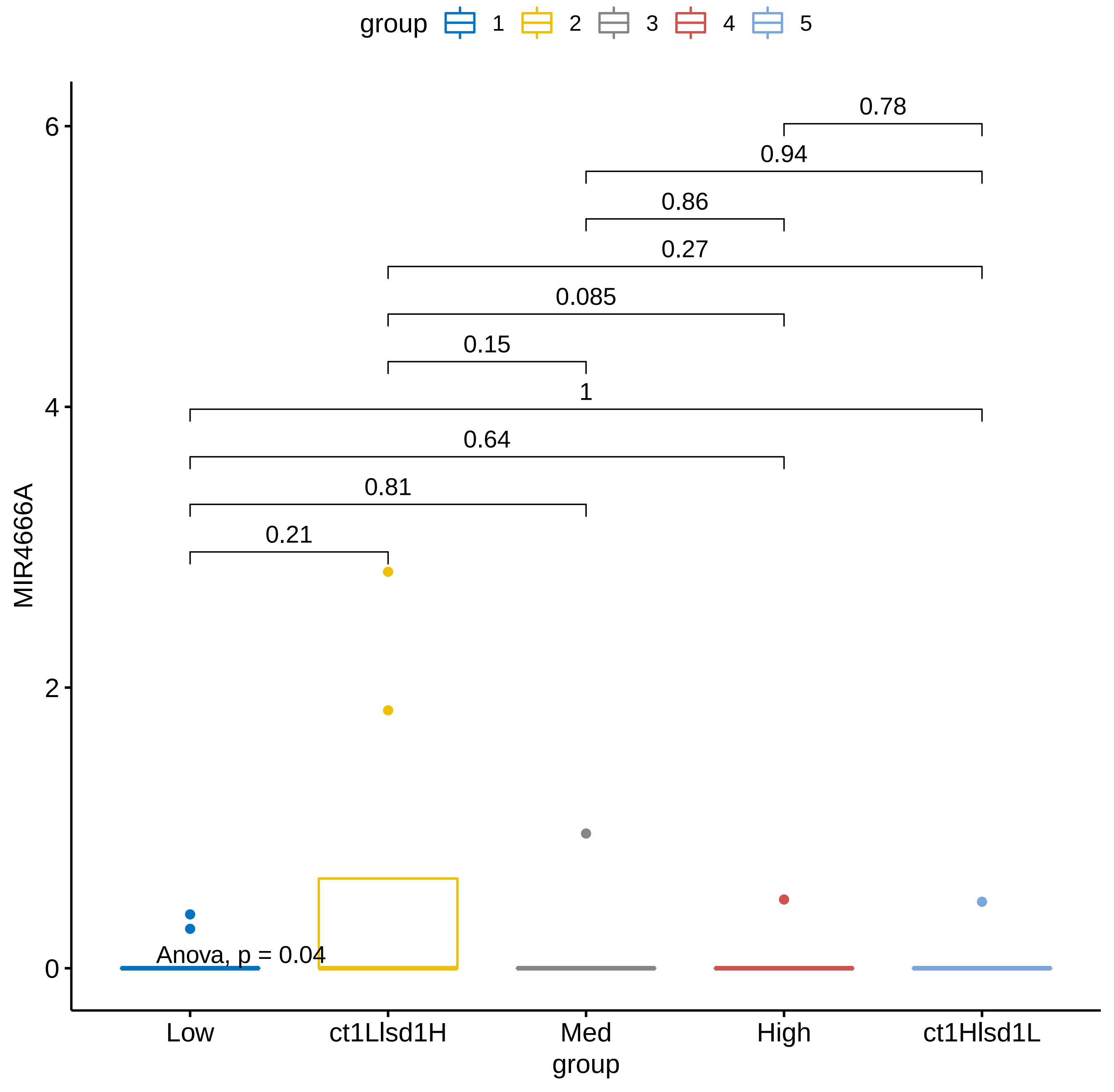

group 1 2 3 4 5
MIR4666A
0.78
0.94
0.86
0.27
0.085
0.15
1
0.64
0.81
0.21
Anova, p = 0.04
6
4
2
0
Low
ct1Llsd1H
Med
High
ct1Hlsd1L
group

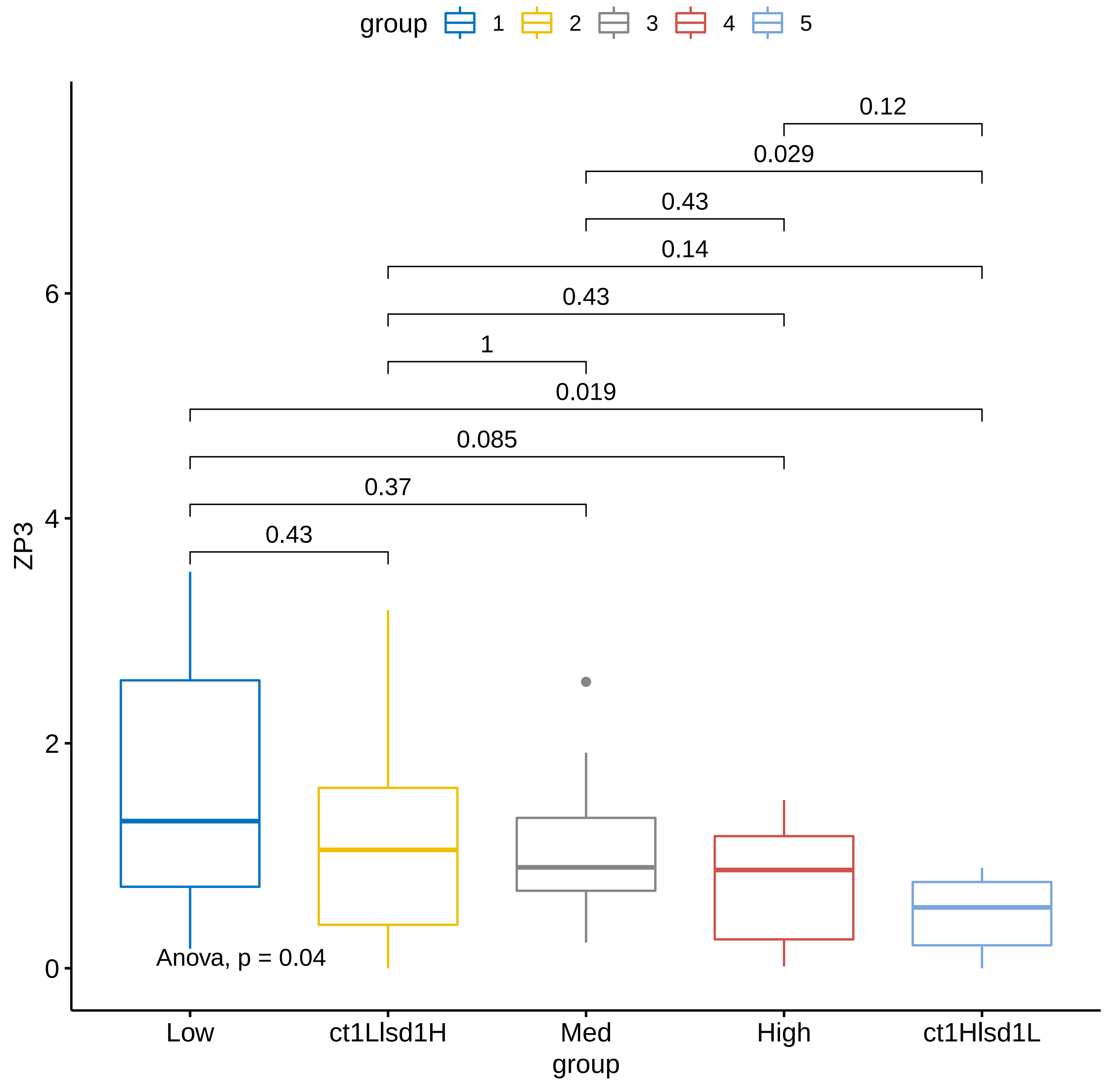
group
1
2
3
4
5
0.12
0.029
0.43
0.14
0.43
1
0.019
0.085
0.37
0.43
Anova, p = 0.04
ZP3
0
2
4
6
Low
ct1Llsd1H
Med
High
ct1Hlsd1L
group

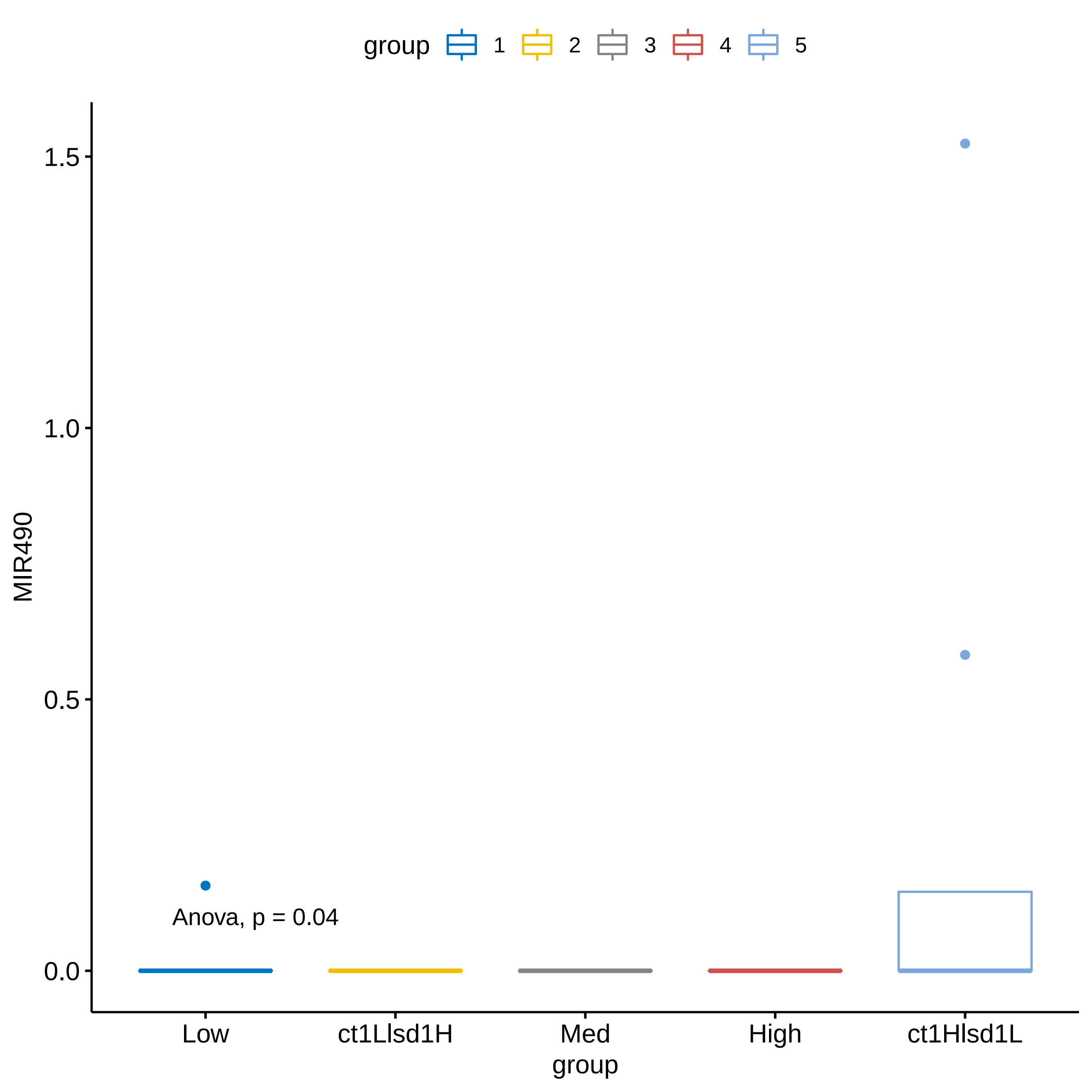

group
1
2
3
4
5
MIR490
1.5
1.0
0.5
0.0
Anova, p = 0.04
Low
ct1Llsd1H
Med
High
ct1Hlsd1L
group

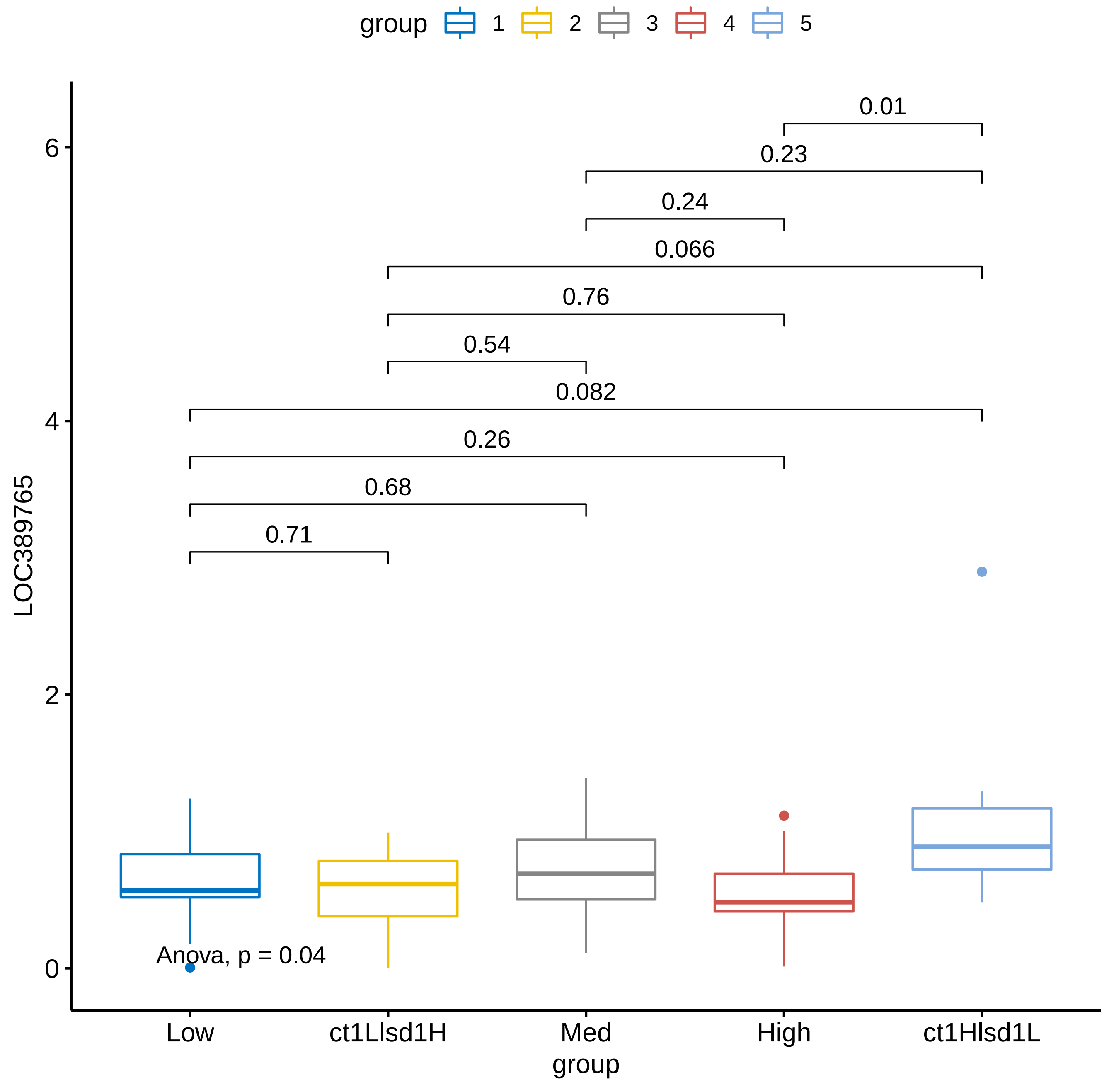

group 1 2 3 4 5
0.01
0.23
0.24
0.066
0.76
0.54
0.082
0.26
0.68
0.71
Anova, p = 0.04
6
4
2
0
LOC389765
Low
ct1Llsd1H
Med
High
ct1Hlsd1L
group

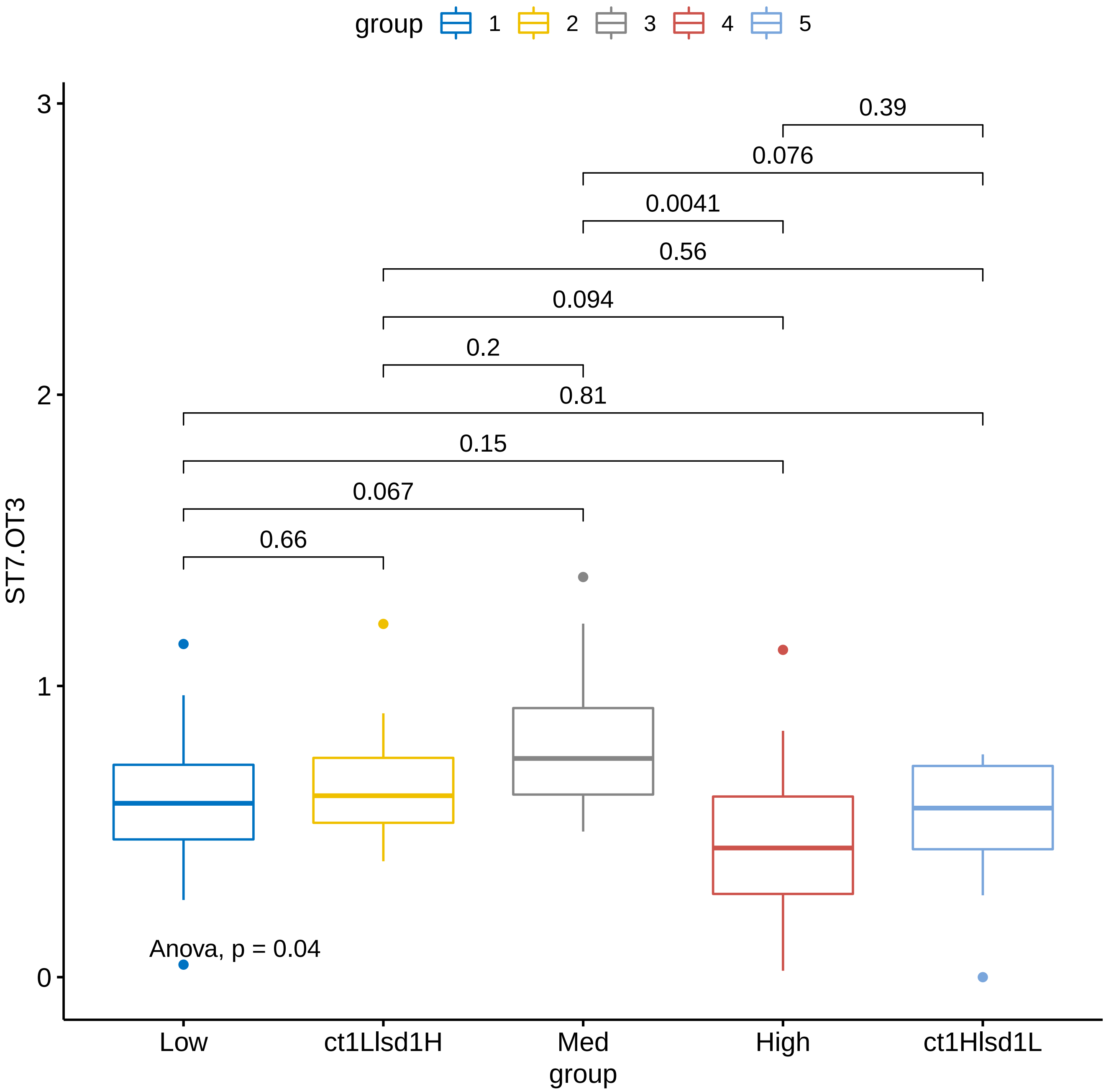

group 1 2 3 4 5
0.39
0.076
0.0041
0.56
0.094
0.2
0.81
0.15
0.067
0.66
Anova, p = 0.04
ST7.OT3
3
2
1
0
Low
ct1Llsd1H
Med
High
ct1Hlsd1L
group

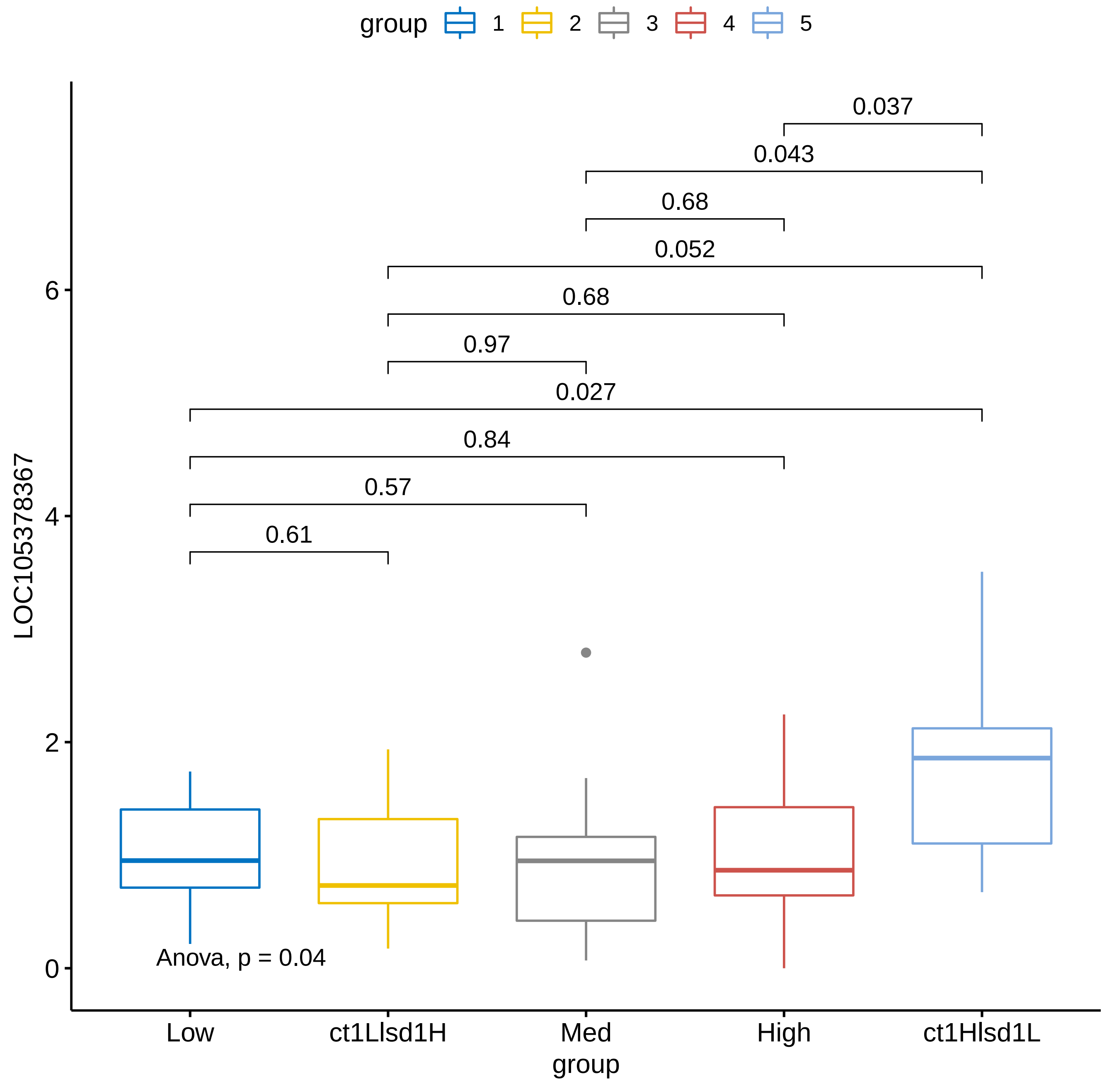

group
1
2
3
4
5
0.037
0.043
0.68
0.052
0.68
0.97
0.027
0.84
0.57
0.61
Anova, p = 0.04
LOC105378367
6
4
2
0
Low
ct1Llsd1H
Med
High
ct1Hlsd1L
group

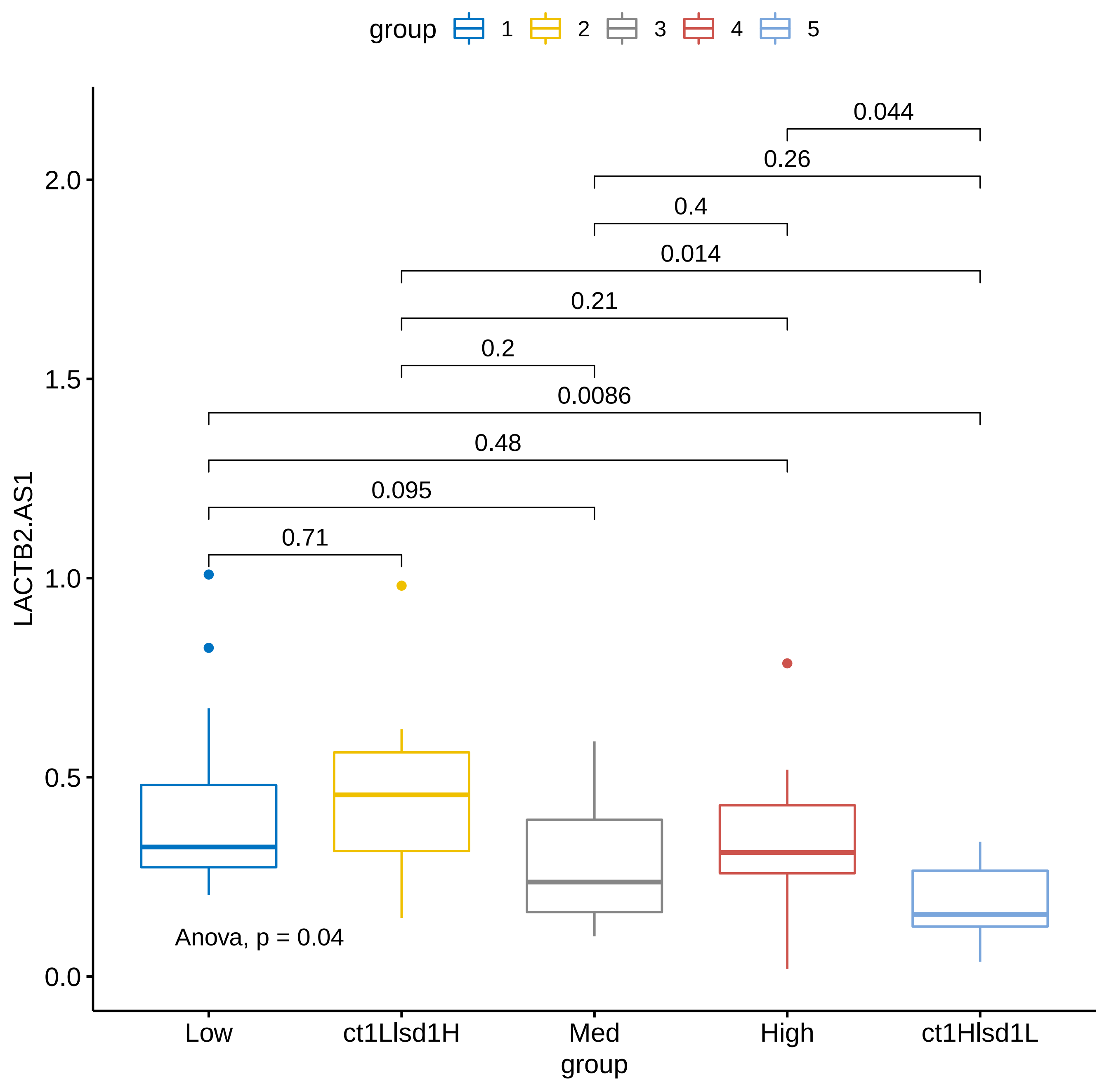

group
1
2
3
4
5
0.044
0.26
0.4
0.014
0.21
0.2
0.0086
0.48
0.095
0.71
2.0
1.5
1.0
0.5
0.0
LACTB2.AS1
Anova, p = 0.04
Low
ct1Llsd1H
Med
High
ct1Hlsd1L
group

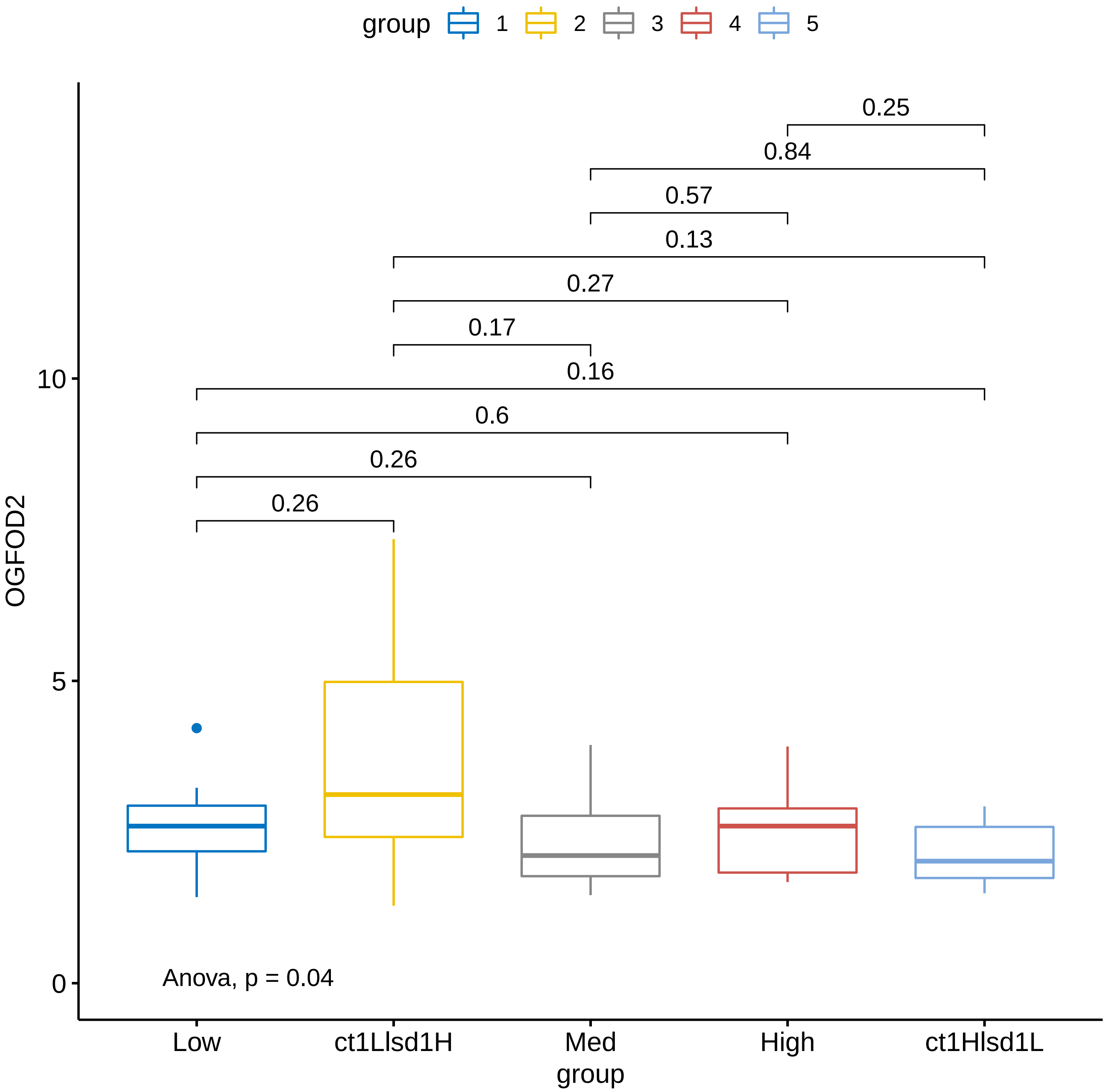

group
1
2
3
4
5
0.25
0.84
0.57
0.13
0.27
0.17
0.16
0.6
0.26
0.26
10
5
0
OGFOD2
Anova, p = 0.04
Low
ct1Llsd1H
Med
High
ct1Hlsd1L
group

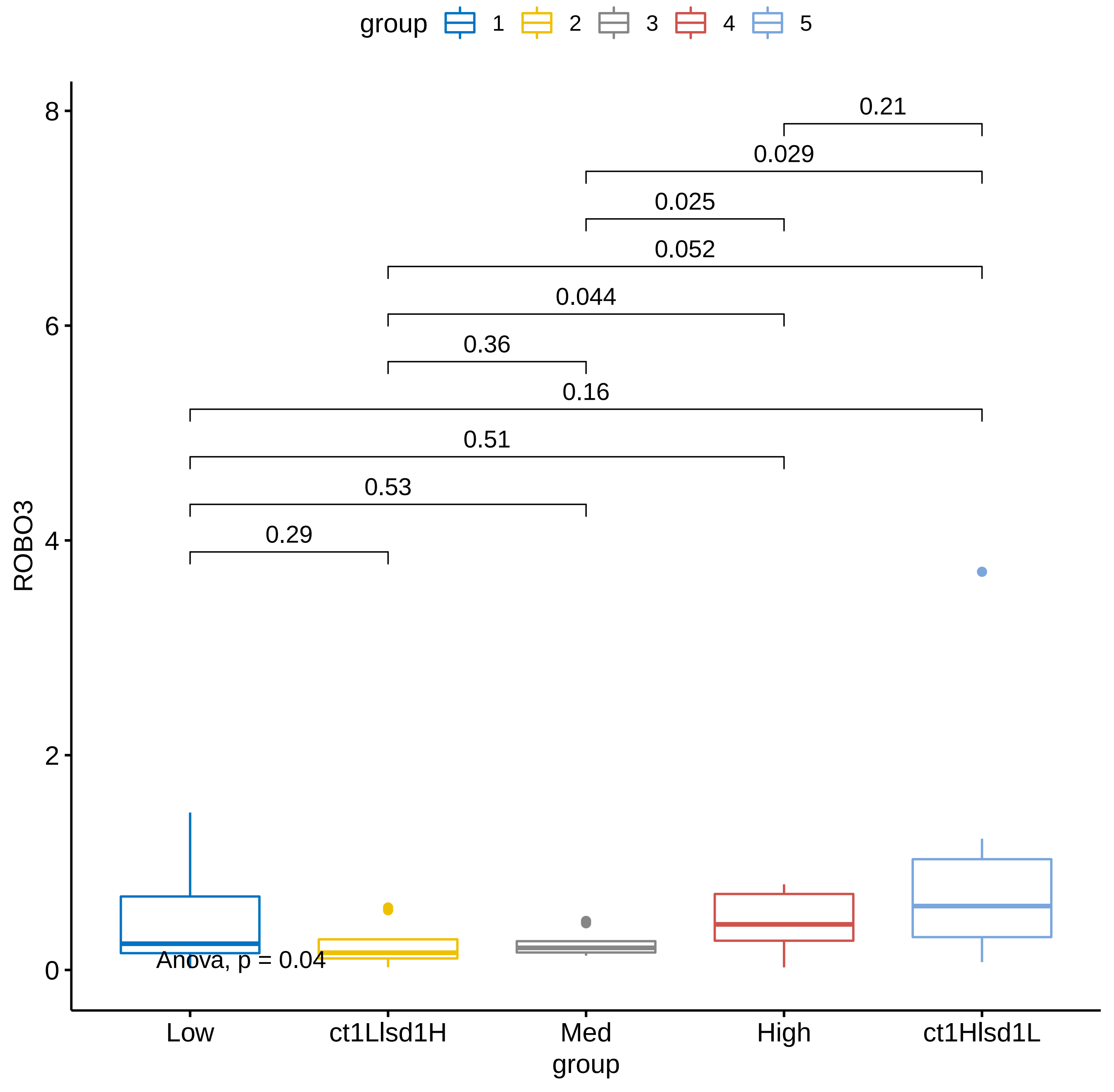

group 1 2 3 4 5
0.21
0.029
0.025
0.052
0.044
0.36
0.16
0.51
0.53
0.29
Anova, p = 0.04
ROBO3
8
6
4
2
0
Low
ct1Llsd1H
Med
High
ct1Hlsd1L
group

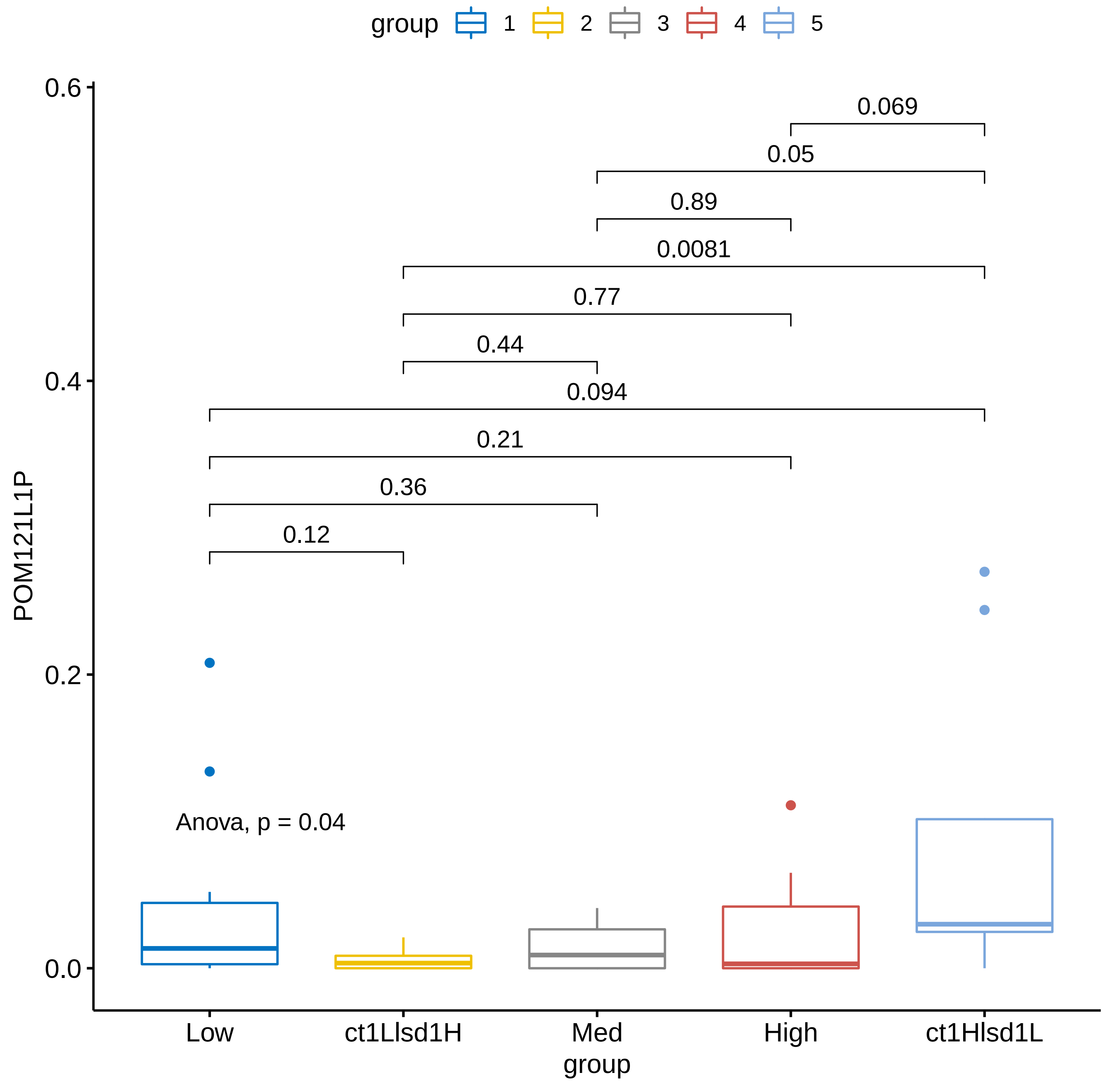
group
1
2
3
4
5
0.6
0.4
0.2
0.0
POM121L1P
0.069
0.05
0.89
0.0081
0.77
0.44
0.094
0.21
0.36
0.12
Anova, p = 0.04
Low
ct1Llsd1H
Med
High
ct1Hlsd1L
group

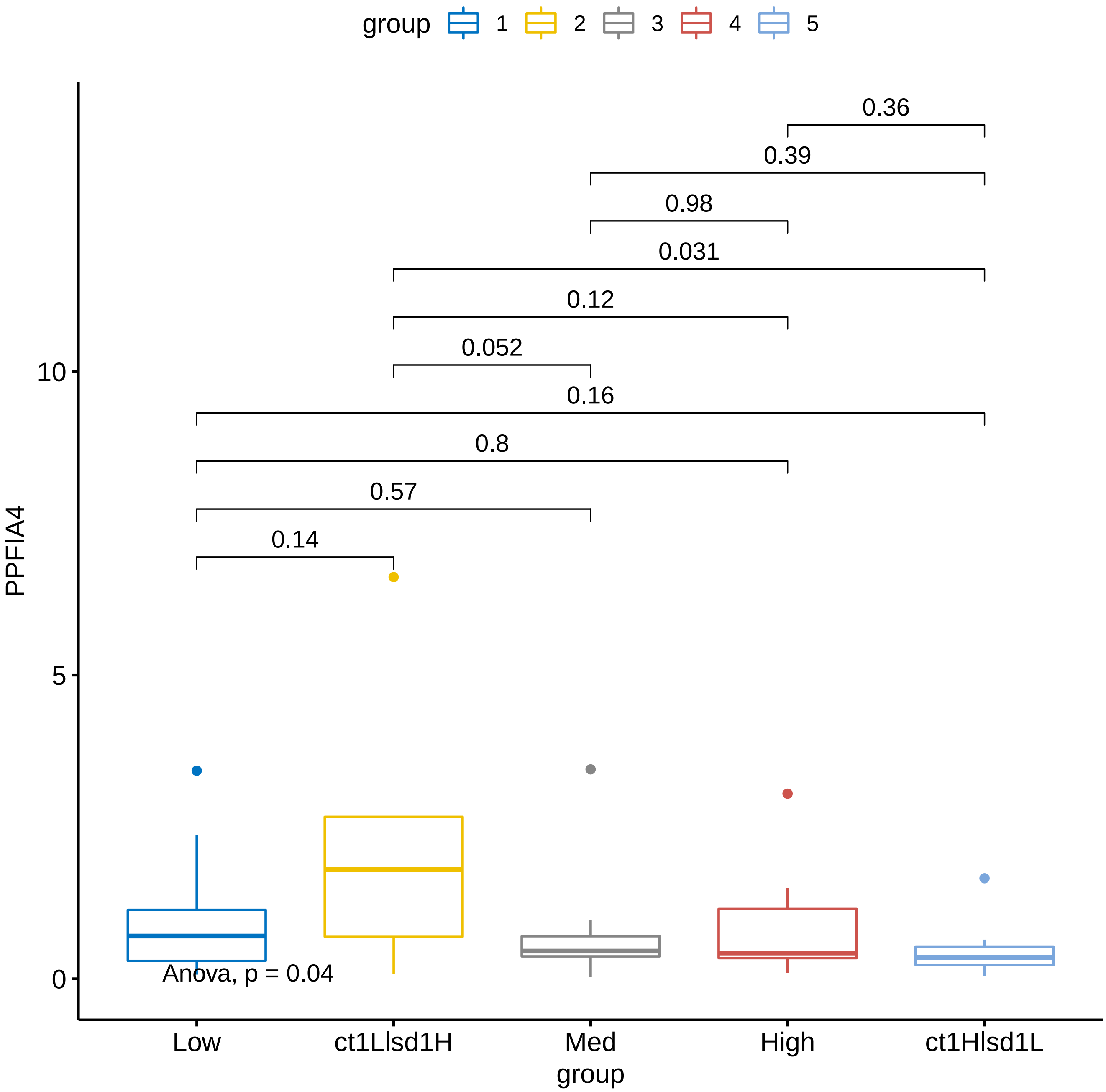
group
1
2
3
4
5
0.36
0.39
0.98
0.031
0.12
0.052
0.16
0.8
0.57
0.14
PPFIA4
10
5
0
Anova, p = 0.04
Low
ct1Llsd1H
Med
High
ct1Hlsd1L
group

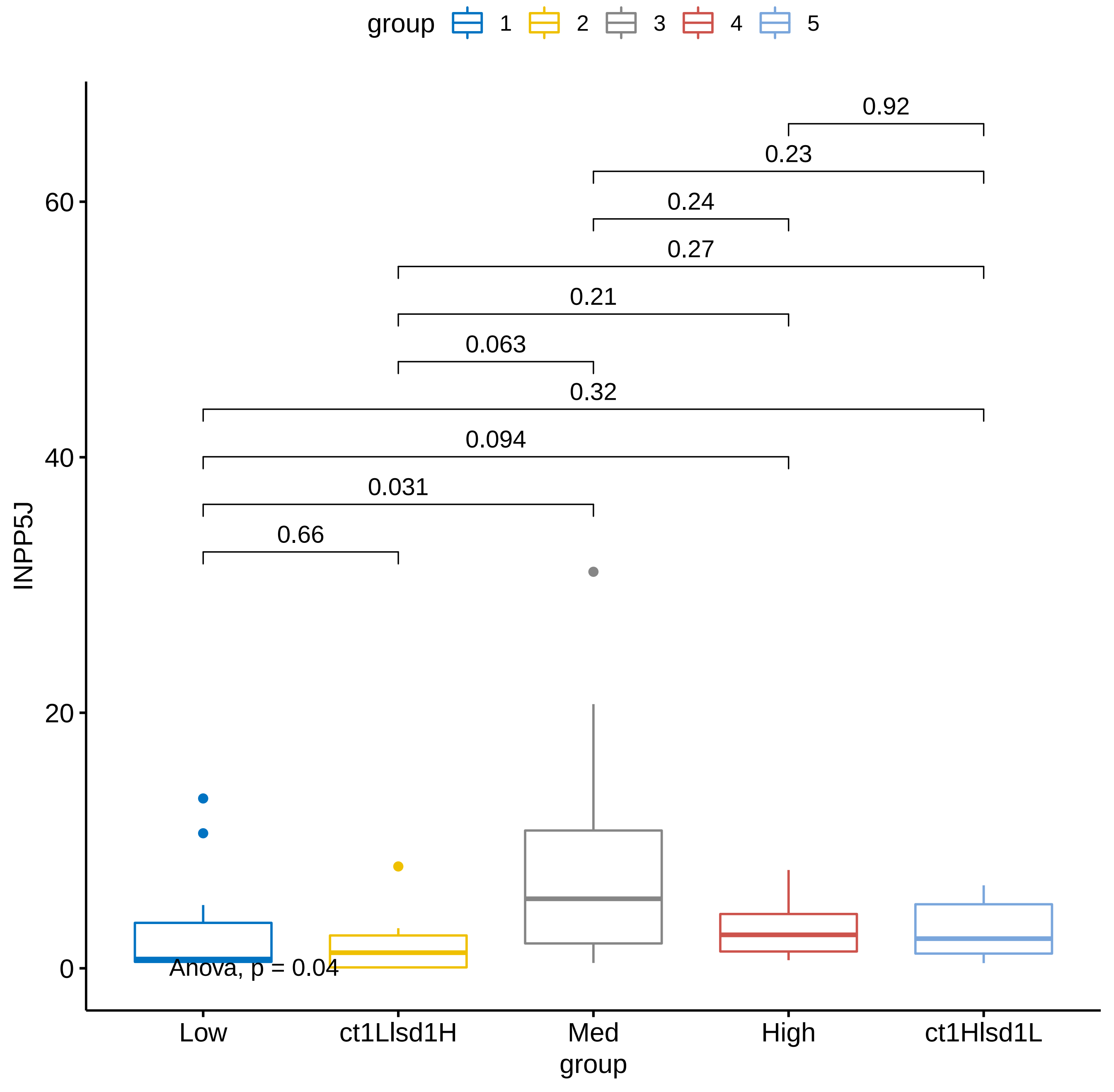
group
1
2
3
4
5
0.92
0.23
0.24
0.27
0.21
0.063
0.32
0.094
0.031
0.66
Anova, p = 0.04
INPP5J
60
40
20
0
Low
ct1Llsd1H
Med
High
ct1Hlsd1L
group

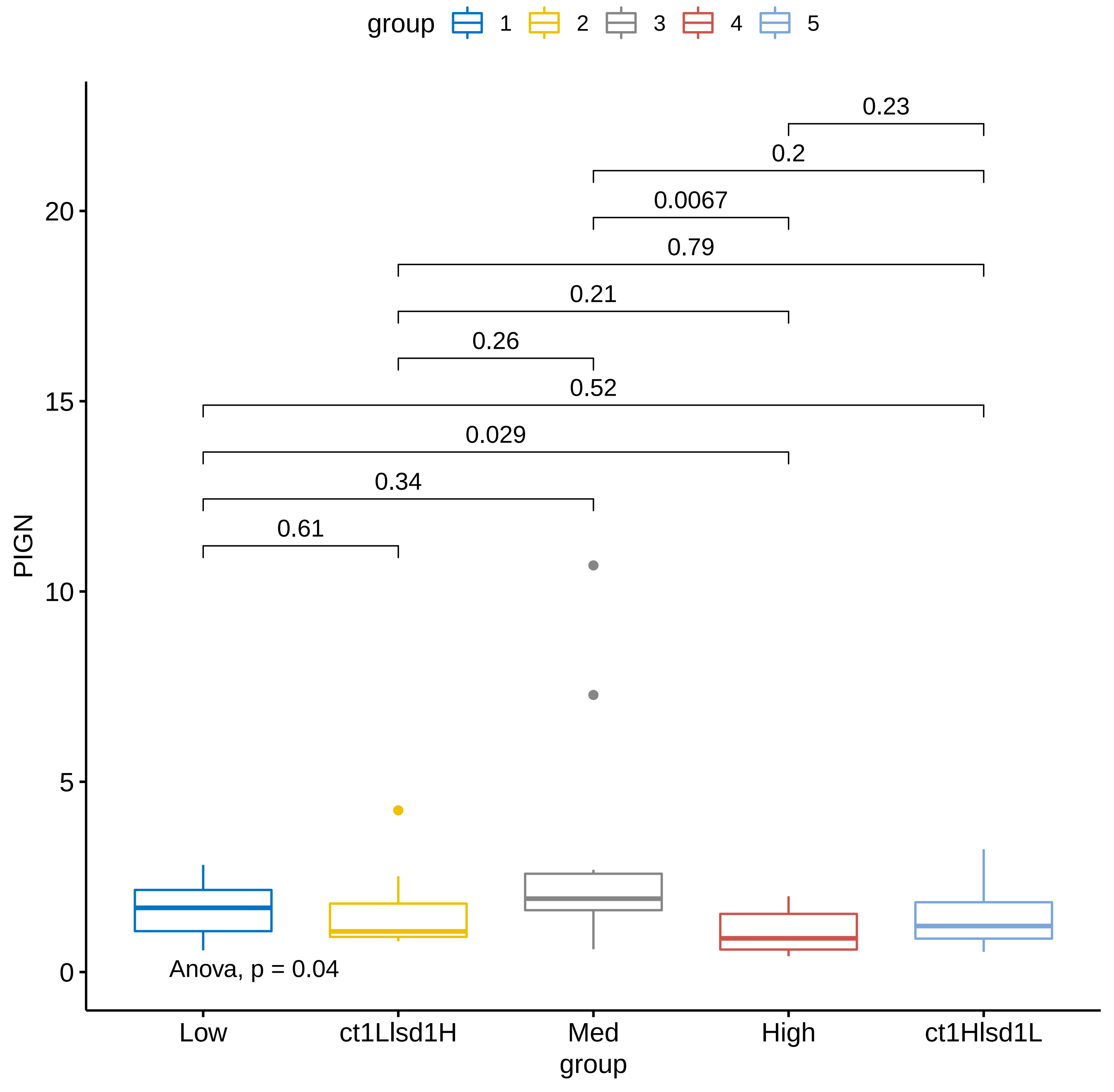
group
1
2
3
4
5
0.23
0.2
0.0067
0.79
0.21
0.26
0.52
0.029
0.34
0.61
20
15
10
5
0
PIGN
Anova, p = 0.04
Low
ct1Llsd1H
Med
High
ct1Hlsd1L
group

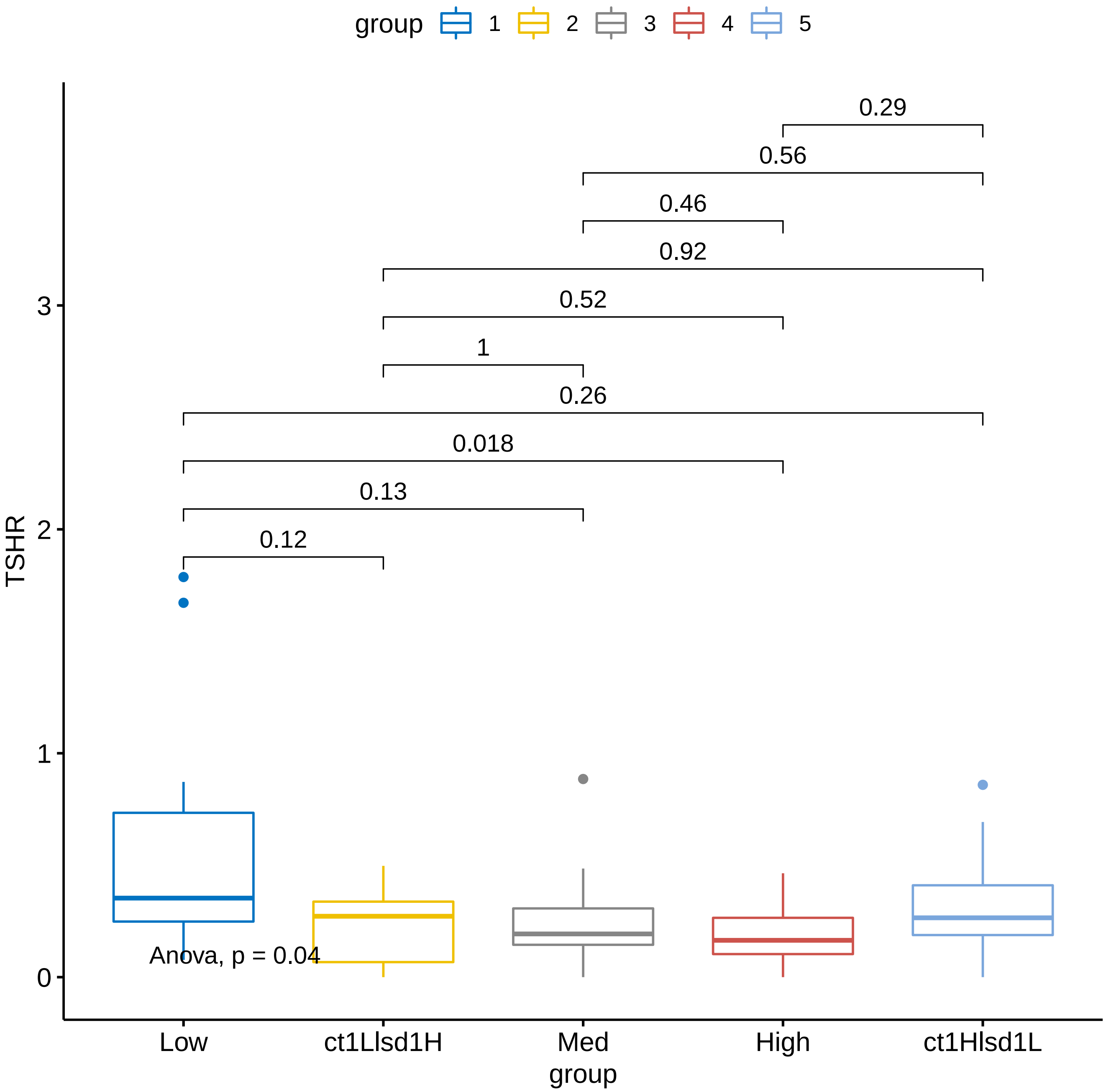
group
1
2
3
4
5
0.29
0.56
0.46
0.92
0.52
1
0.26
0.018
0.13
0.12
3
2
1
0
TSHR
Anova, p = 0.04
Low
ct1Llsd1H
Med
High
ct1Hlsd1L
group

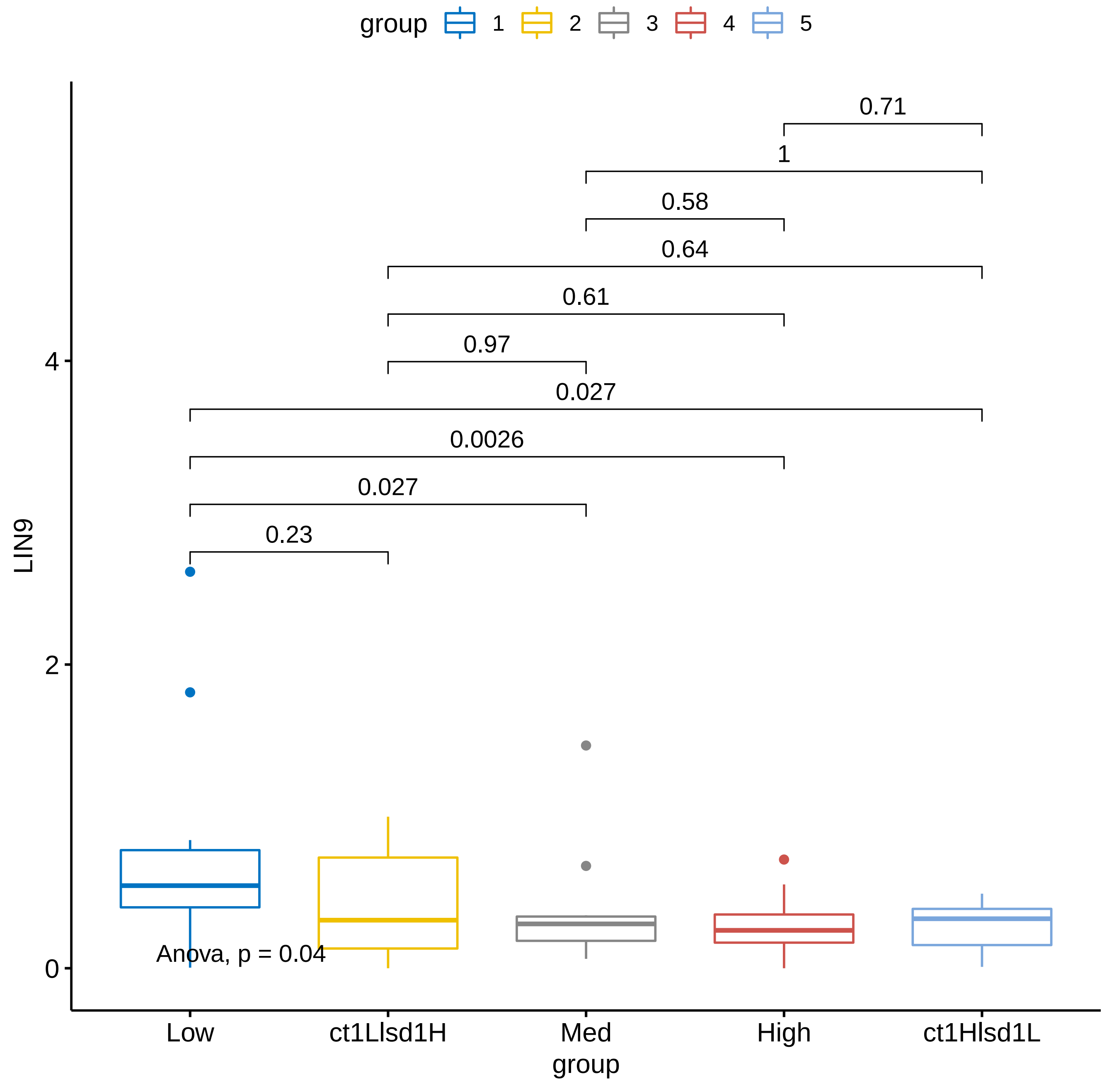

group
1
2
3
4
5
0.71
1
0.58
0.64
0.61
0.97
0.027
0.0026
0.027
0.23
4
2
0
LIN9
Anova, p = 0.04
Low
ct1Llsd1H
Med
High
ct1Hlsd1L
group

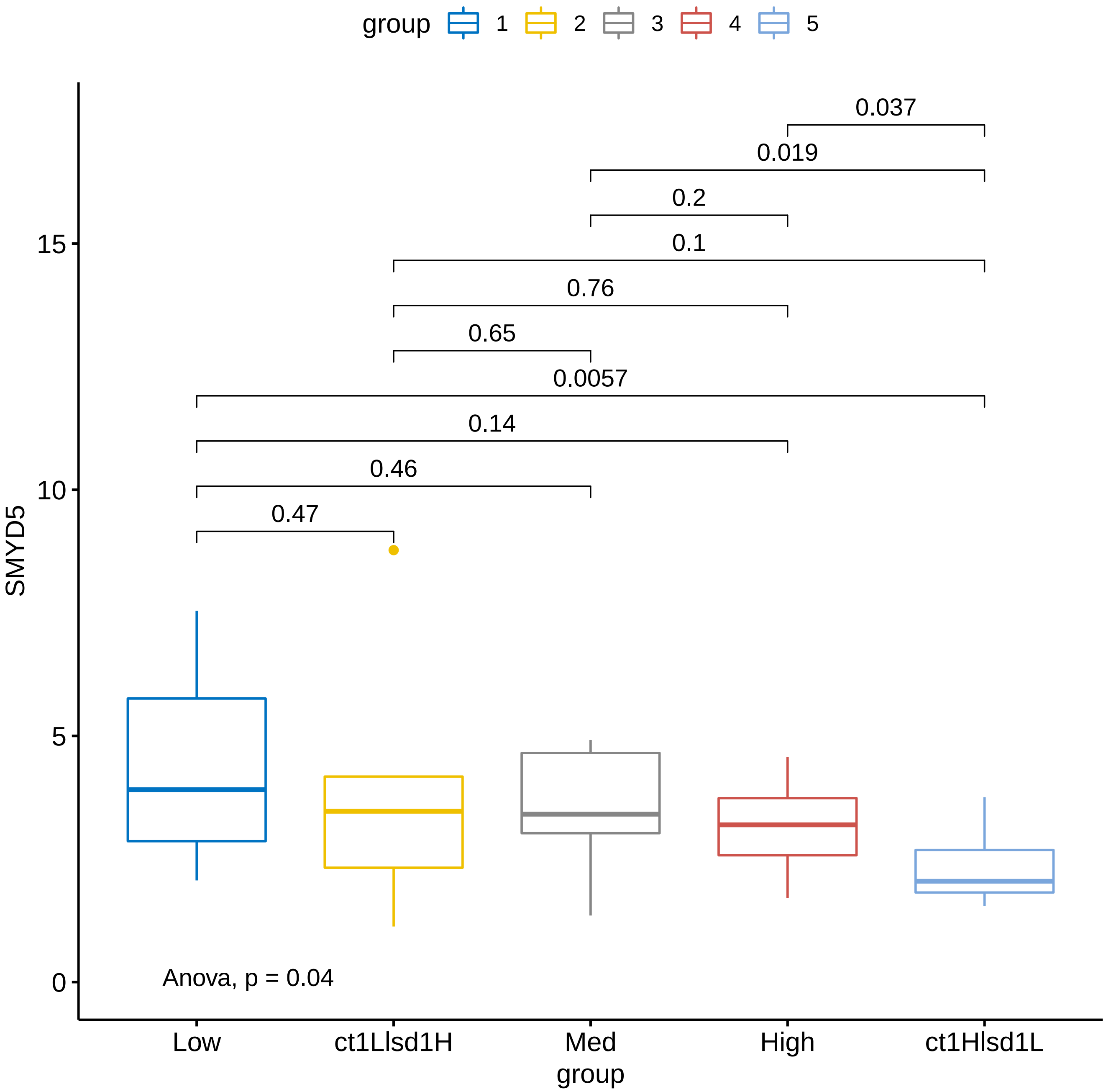

group 1 2 3 4 5
0.037
0.019
0.2
0.1
0.76
0.65
0.0057
0.14
0.46
0.47
15
10
5
0
SMYD5
Anova, p = 0.04
Low
ct1Llsd1H
Med
High
ct1Hlsd1L
group

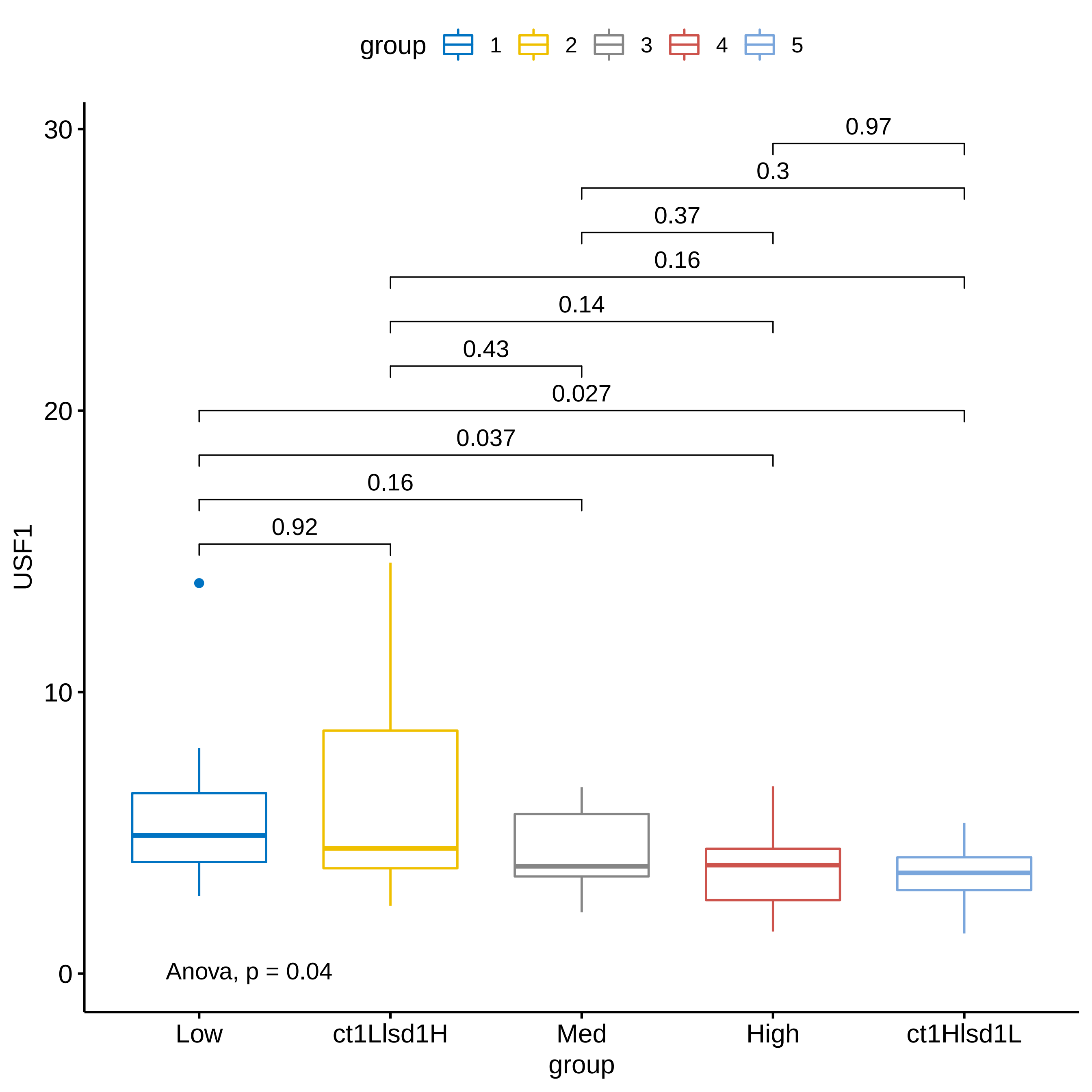

group 1 2 3 4 5
0.97
0.3
0.37
0.16
0.14
0.43
0.027
0.037
0.16
0.92
30
20
10
0
USF1
Anova, p = 0.04
Low
ct1Llsd1H
Med
High
ct1Hlsd1L
group

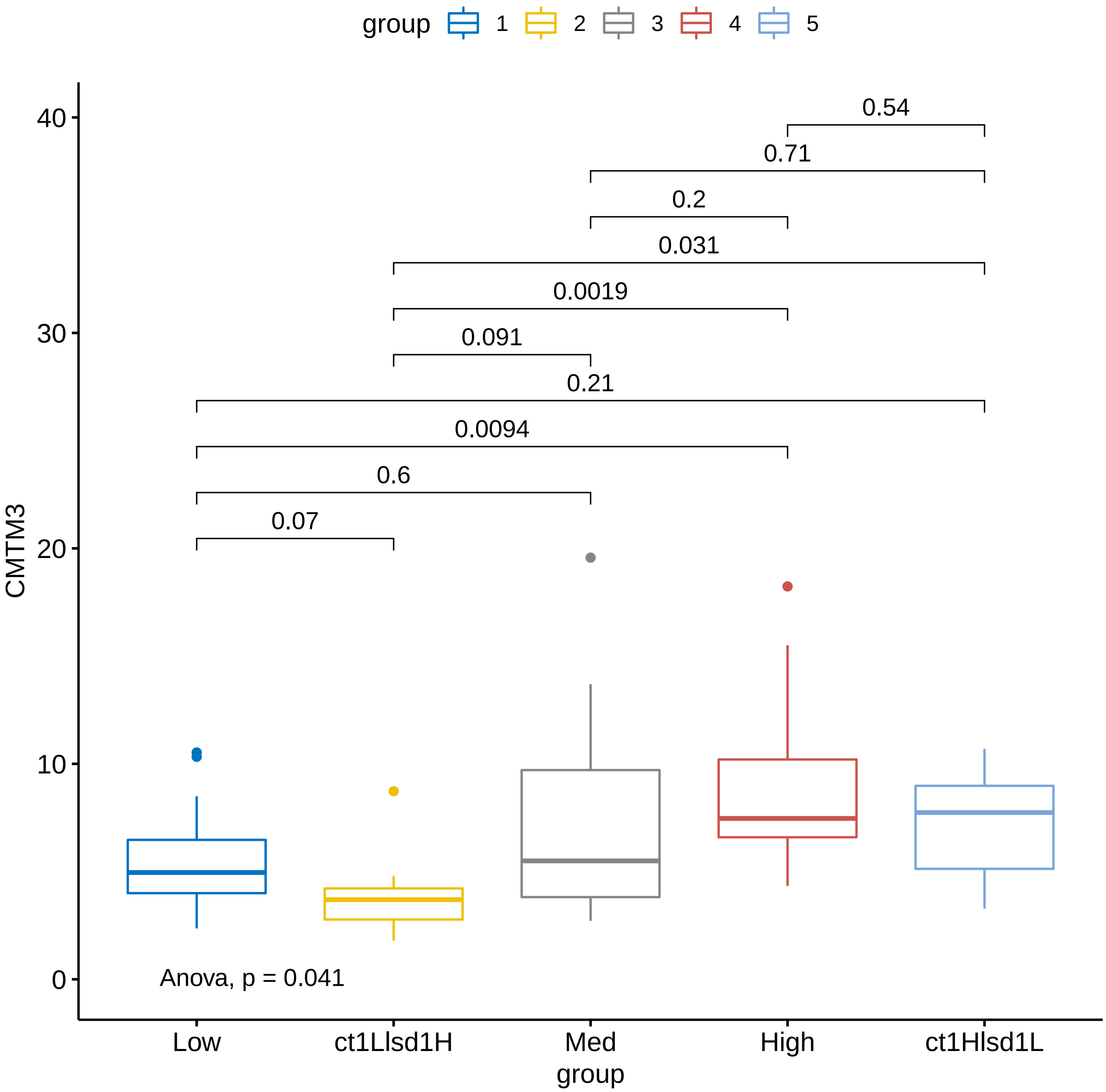

group
1
2
3
4
5
CMTM3
40
30
20
10
0
0.54
0.71
0.2
0.031
0.0019
0.091
0.21
0.0094
0.6
0.07
Anova, p = 0.041
Low
ct1Llsd1H
Med
High
ct1Hlsd1L
group

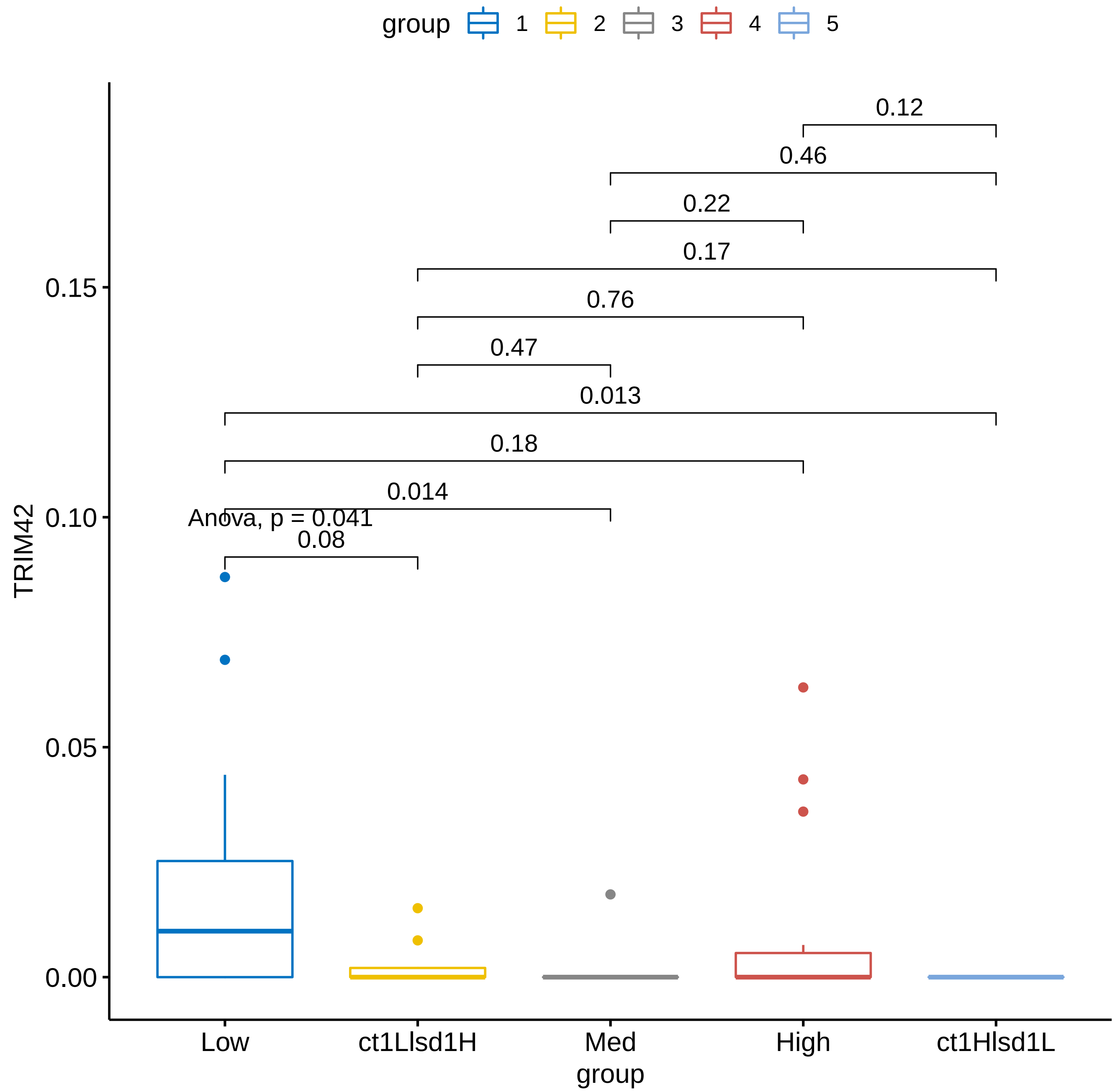

group 1 2 3 4 5
0.12
0.46
0.22
0.17
0.76
0.47
0.013
0.18
0.014
Anova, p = 0.041
0.08
0.15
0.10
0.05
0.00
TRIM42
Low
ct1Llsd1H
Med
High
ct1Hlsd1L
group

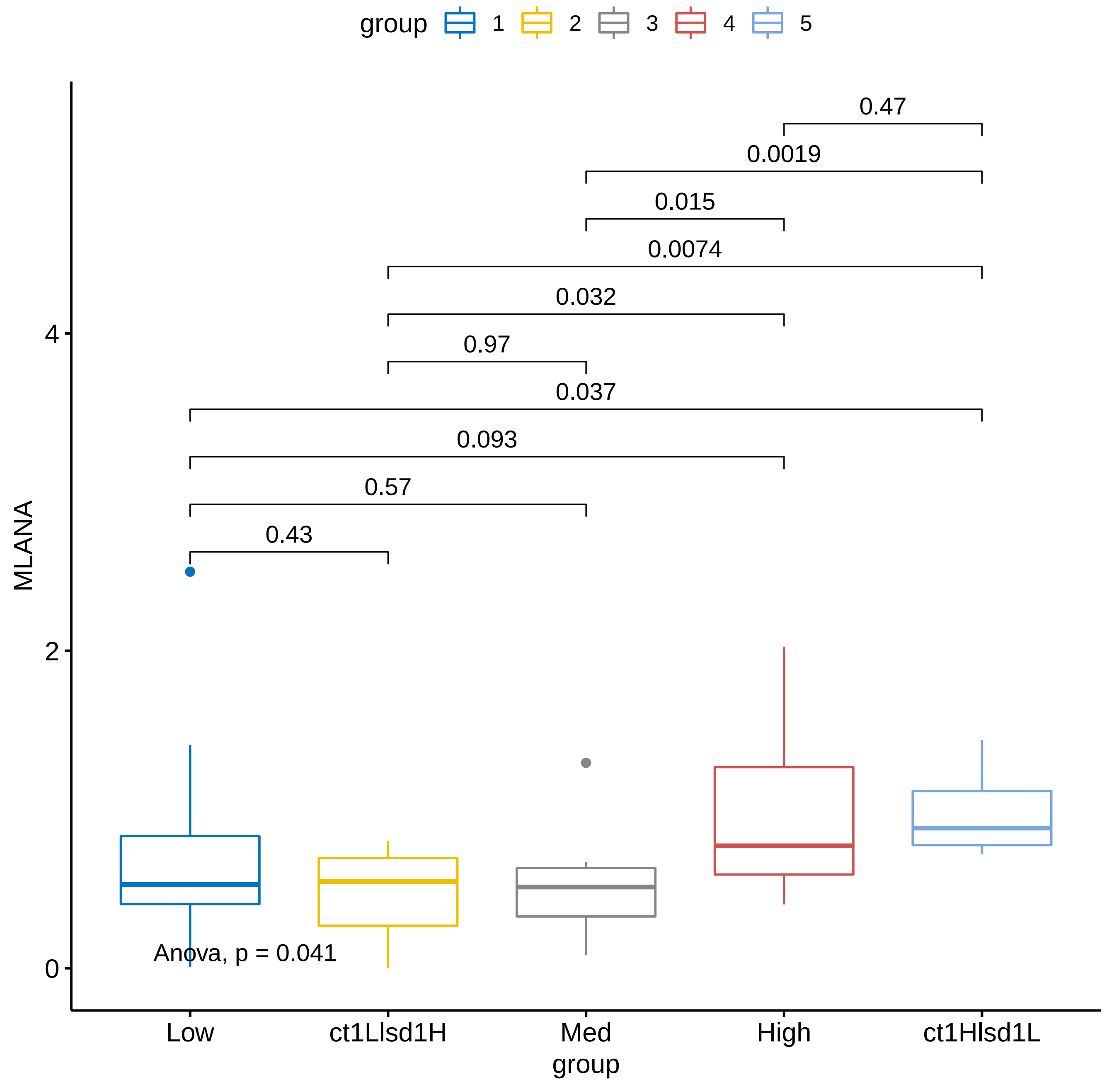

group
1
2
3
4
5
0.47
0.0019
0.015
0.0074
0.032
0.97
0.037
0.093
0.57
0.43
Anova, p = 0.041
MLANA
4
2
0
Low
ct1Llsd1H
Med
High
ct1Hlsd1L
group

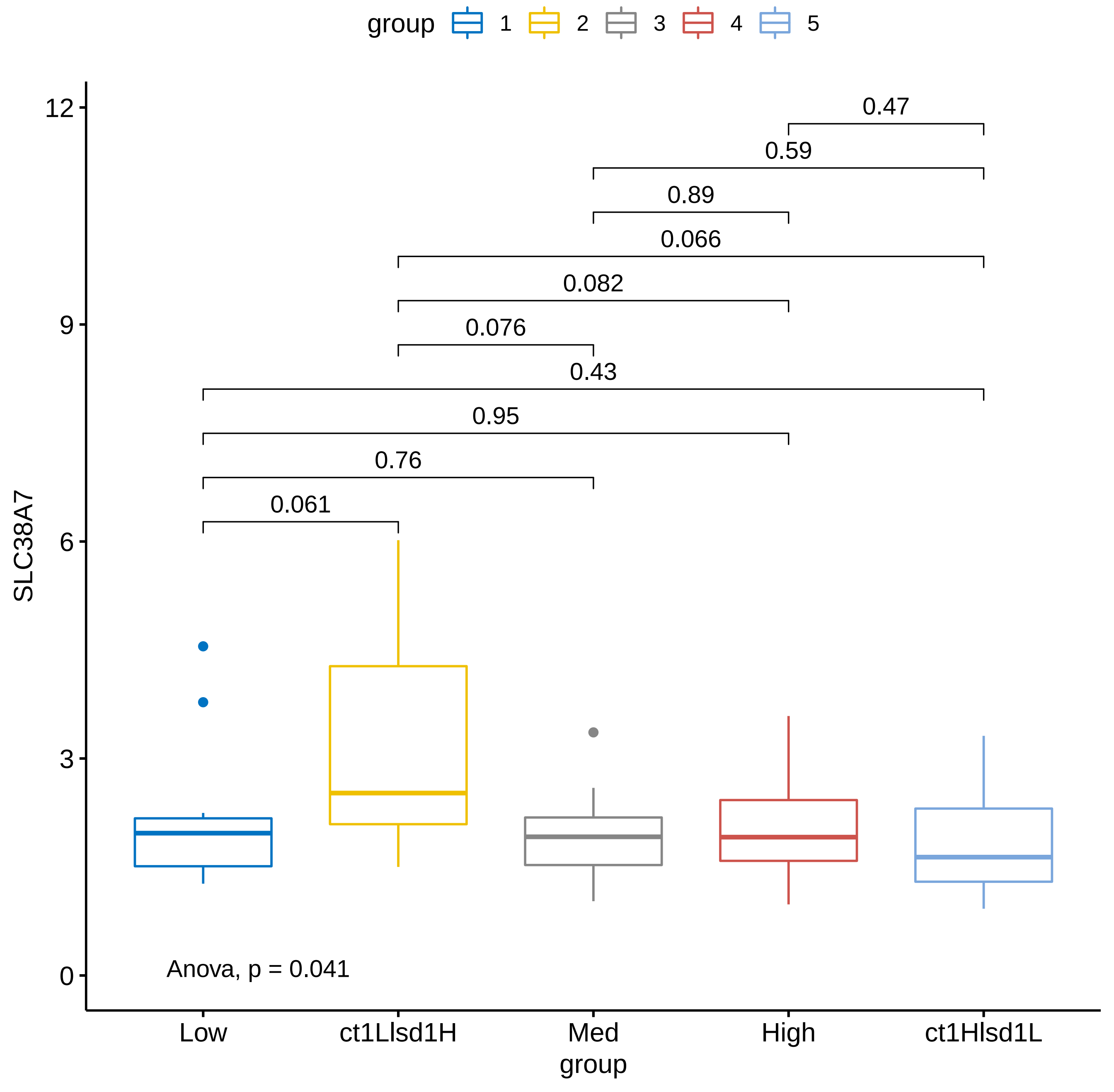

group
1
2
3
4
5
0.47
0.59
0.89
0.066
0.082
0.076
0.43
0.95
0.76
0.061
12
9
6
3
0
SLC38A7
Anova, p = 0.041
Low
ct1Llsd1H
Med
High
ct1Hlsd1L
group

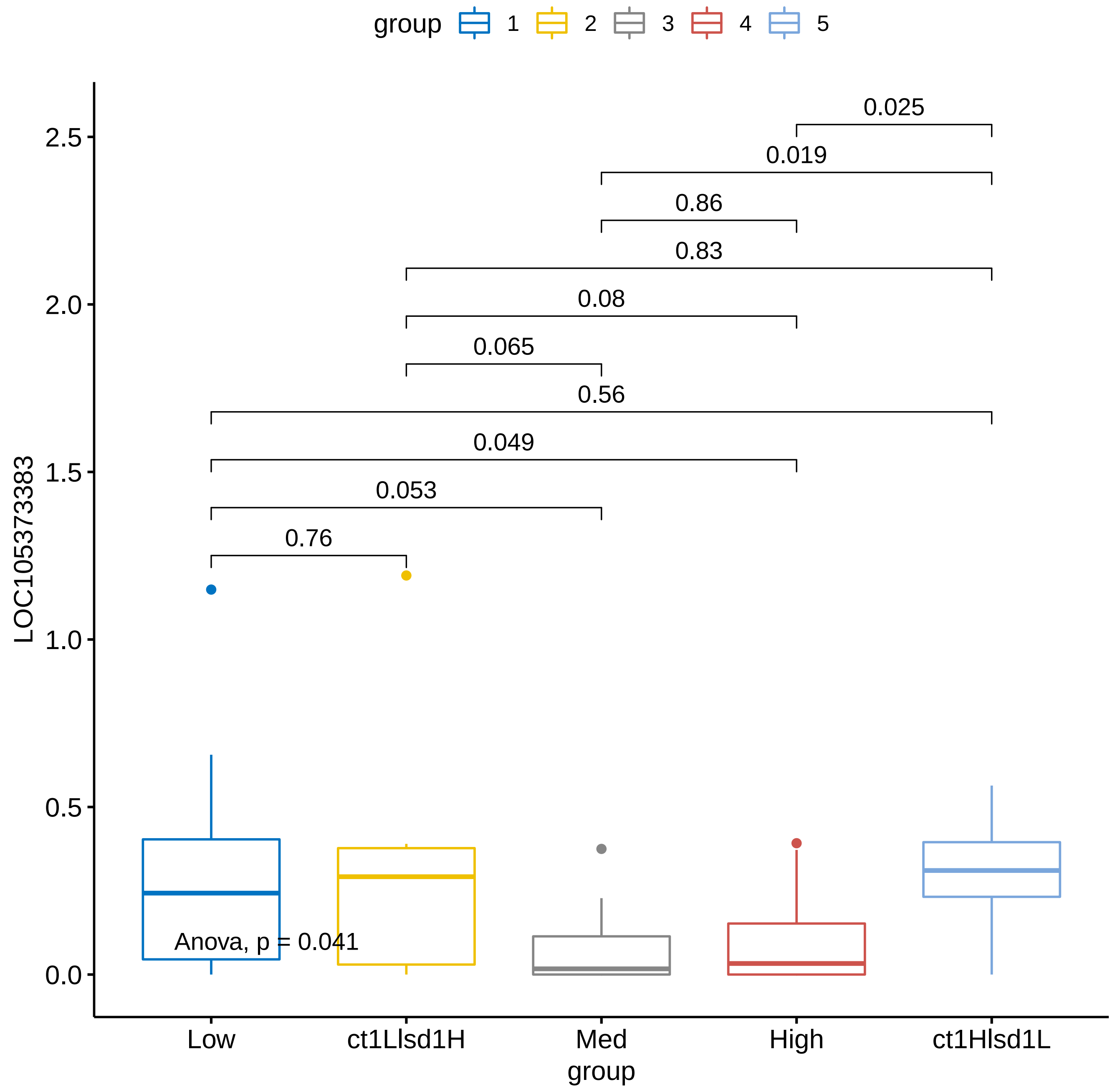

group 1 2 3 4 5
0.025
0.019
0.86
0.83
0.08
0.065
0.56
0.049
0.053
0.76
Anova, p = 0.041
LOC105373383
2.5
2.0
1.5
1.0
0.5
0.0
Low
ct1Llsd1H
Med
High
ct1Hlsd1L
group

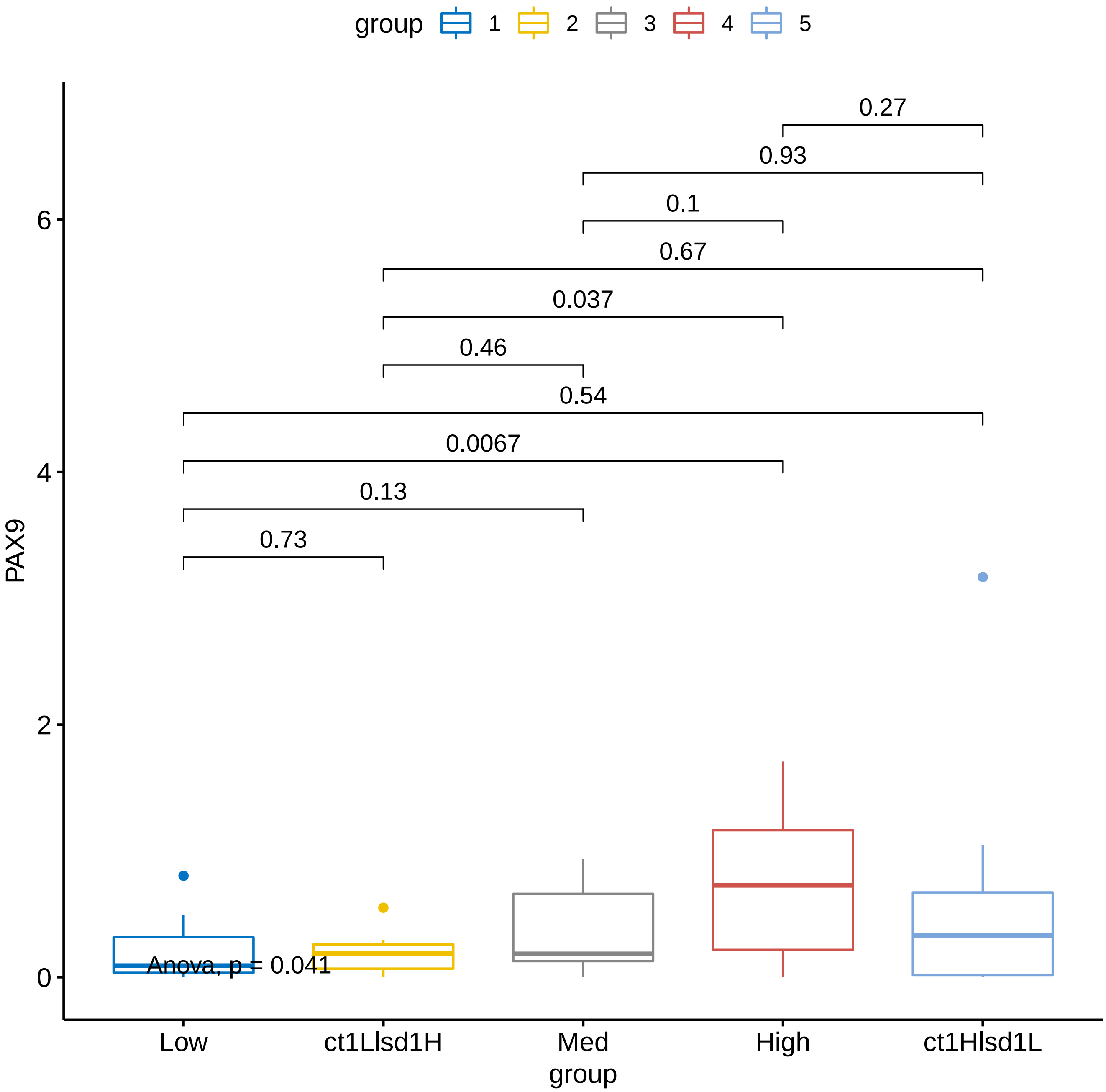

group
1
2
3
4
5
0.27
0.93
0.1
0.67
0.037
0.46
0.54
0.0067
0.13
0.73
Anova, p = 0.041
PAX9
0
2
4
6
Low
ct1Llsd1H
Med
High
ct1Hlsd1L
group

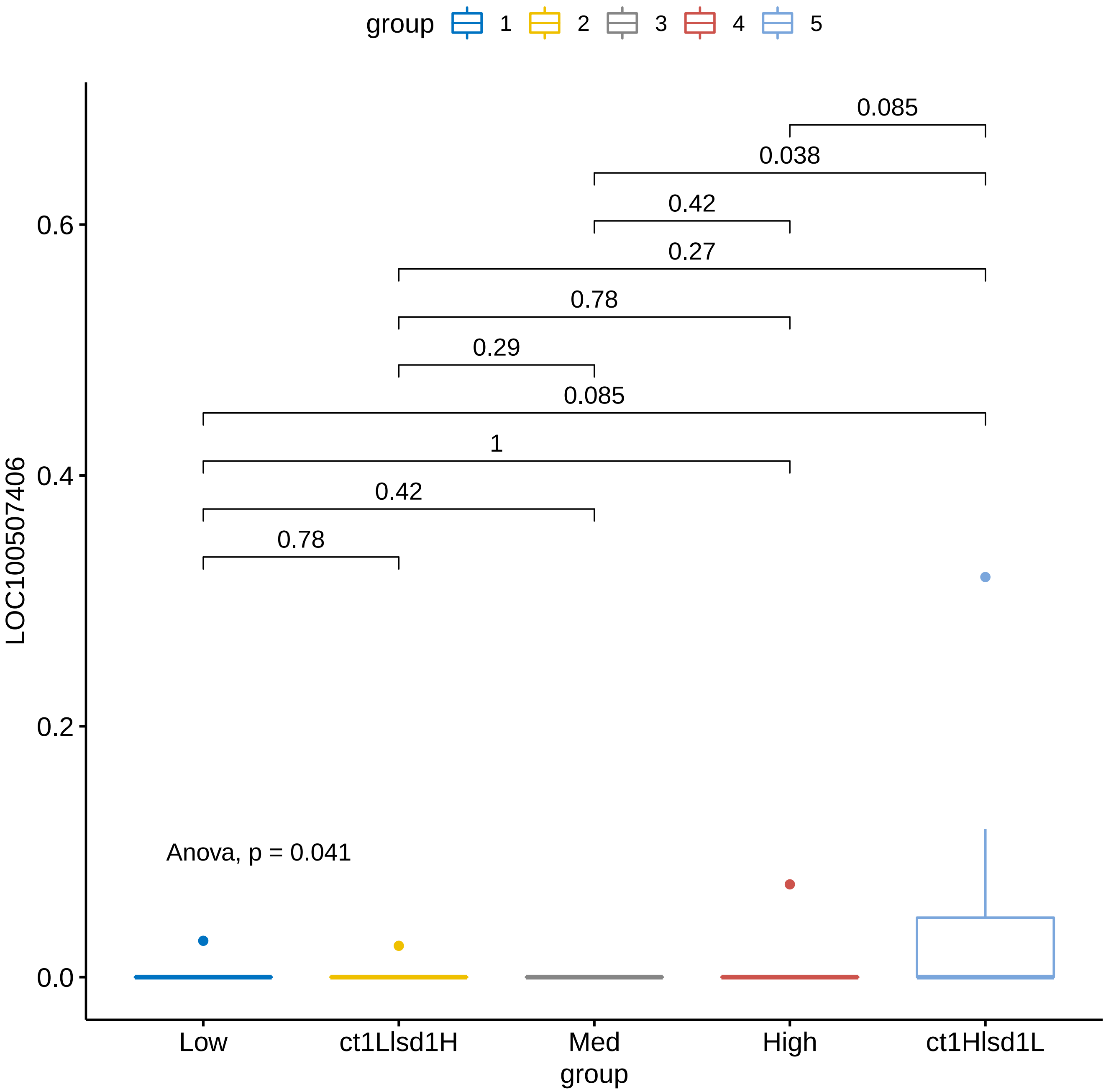
group
1
2
3
4
5
0.085
0.038
0.42
0.27
0.78
0.29
0.085
1
0.42
0.78
Anova, p = 0.041
LOC100507406
0.6
0.4
0.2
0.0
Low
ct1Llsd1H
Med
High
ct1Hlsd1L
group

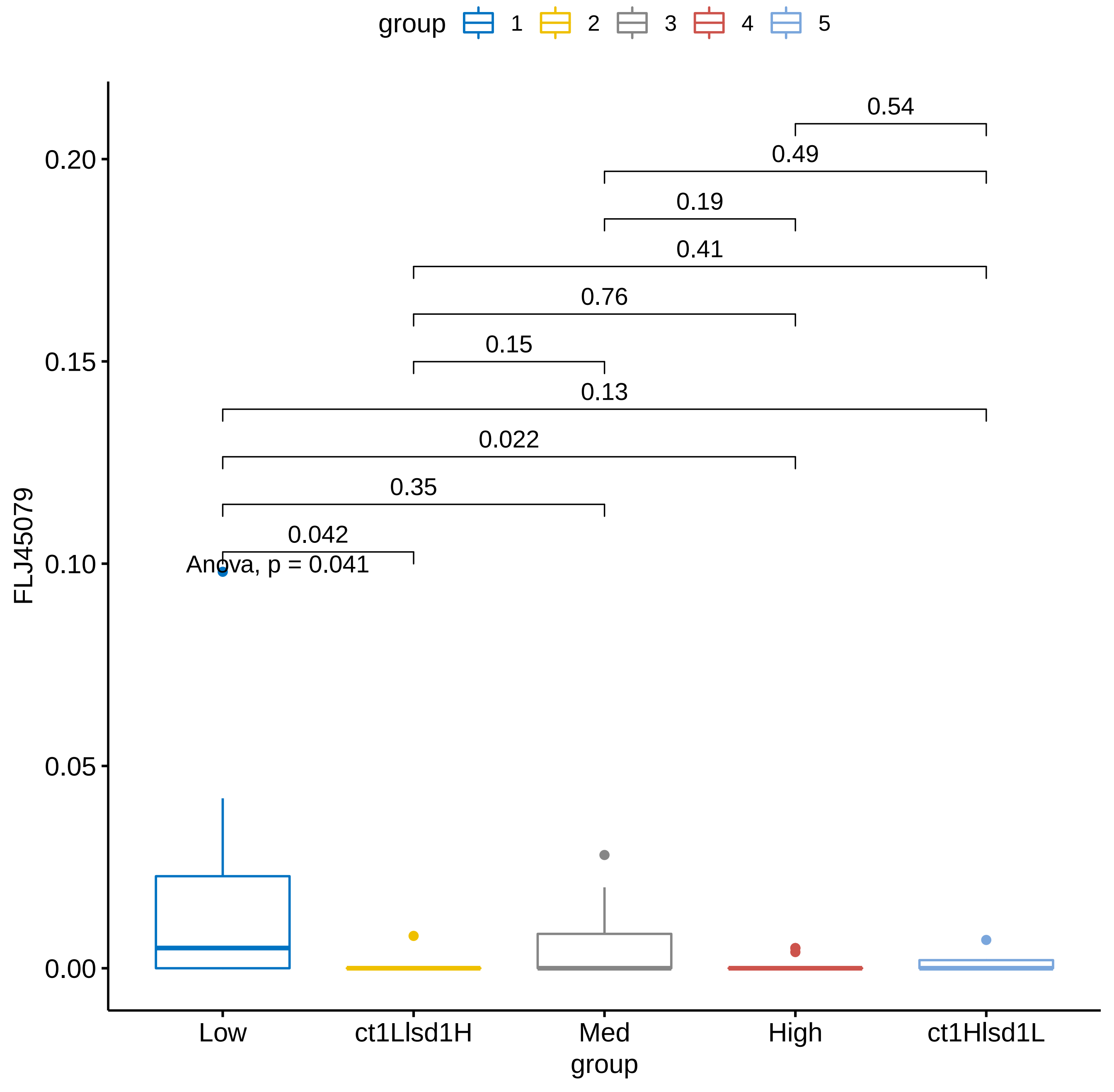
group
1
2
3
4
5
0.54
0.49
0.19
0.41
0.76
0.15
0.13
0.022
0.35
0.042
Anova, p = 0.041
0.20
0.15
0.10
0.05
0.00
FLJ45079
Low
ct1Llsd1H
Med
High
ct1Hlsd1L
group

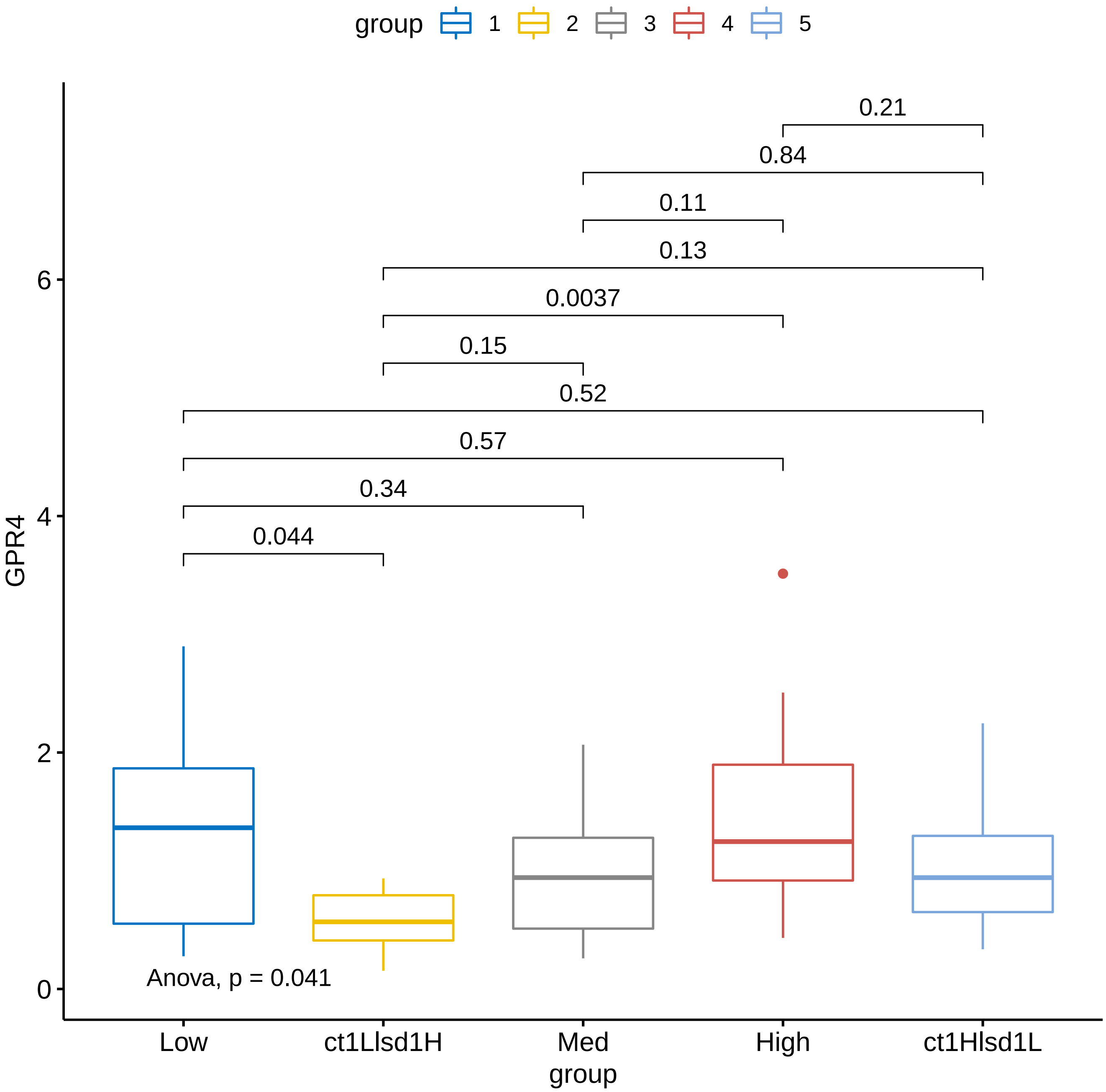

group
1
2
3
4
5
0.21
0.84
0.11
0.13
0.0037
0.15
0.52
0.57
0.34
0.044
6
4
2
0
GPR4
Anova, p = 0.041
Low
ct1Llsd1H
Med
High
ct1Hlsd1L
group

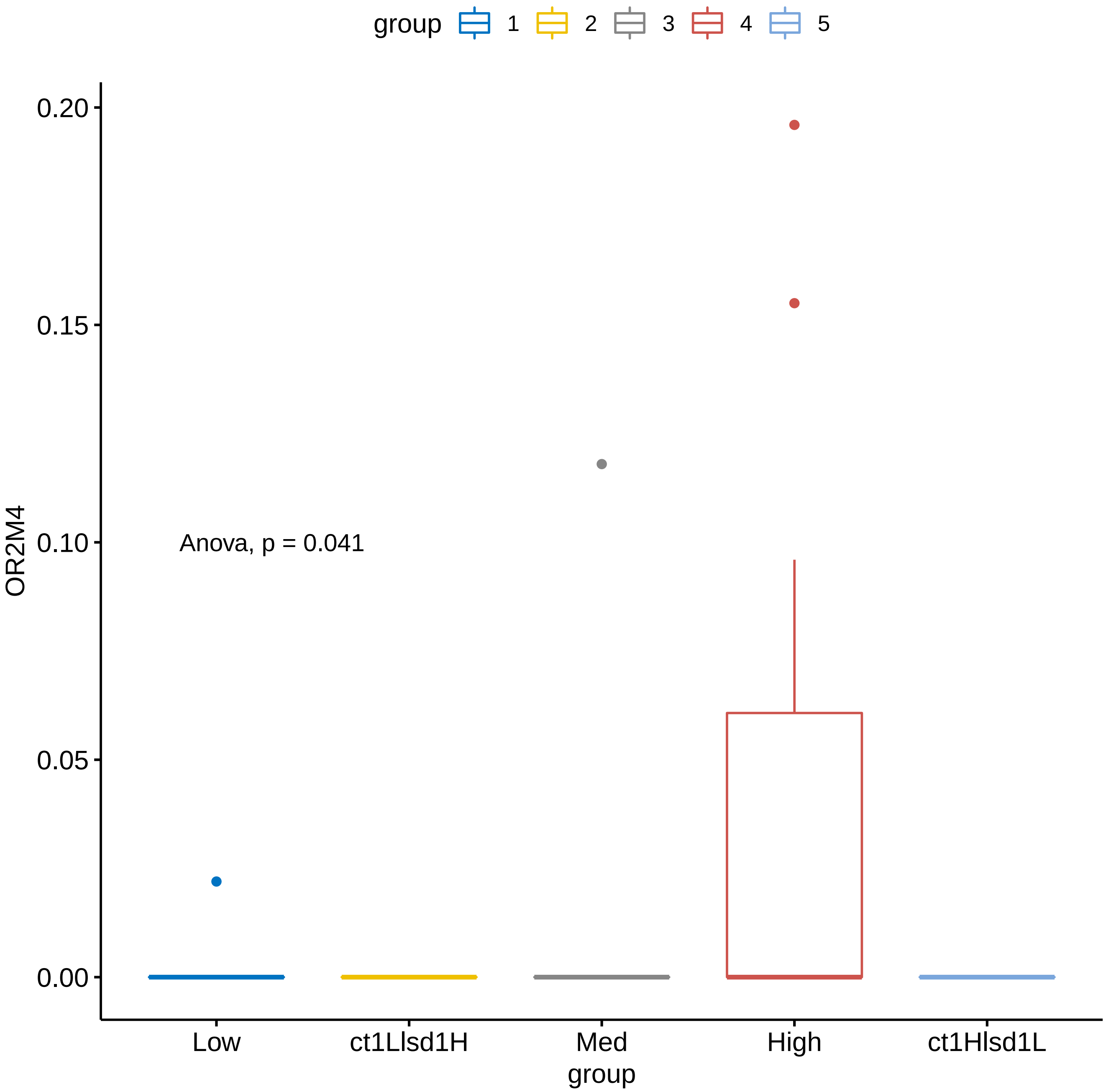

group
1
2
3
4
5
0.20
0.15
0.10
0.05
0.00
OR2M4
Anova, p = 0.041
Low
ct1Llsd1H
Med
High
ct1Hlsd1L
group

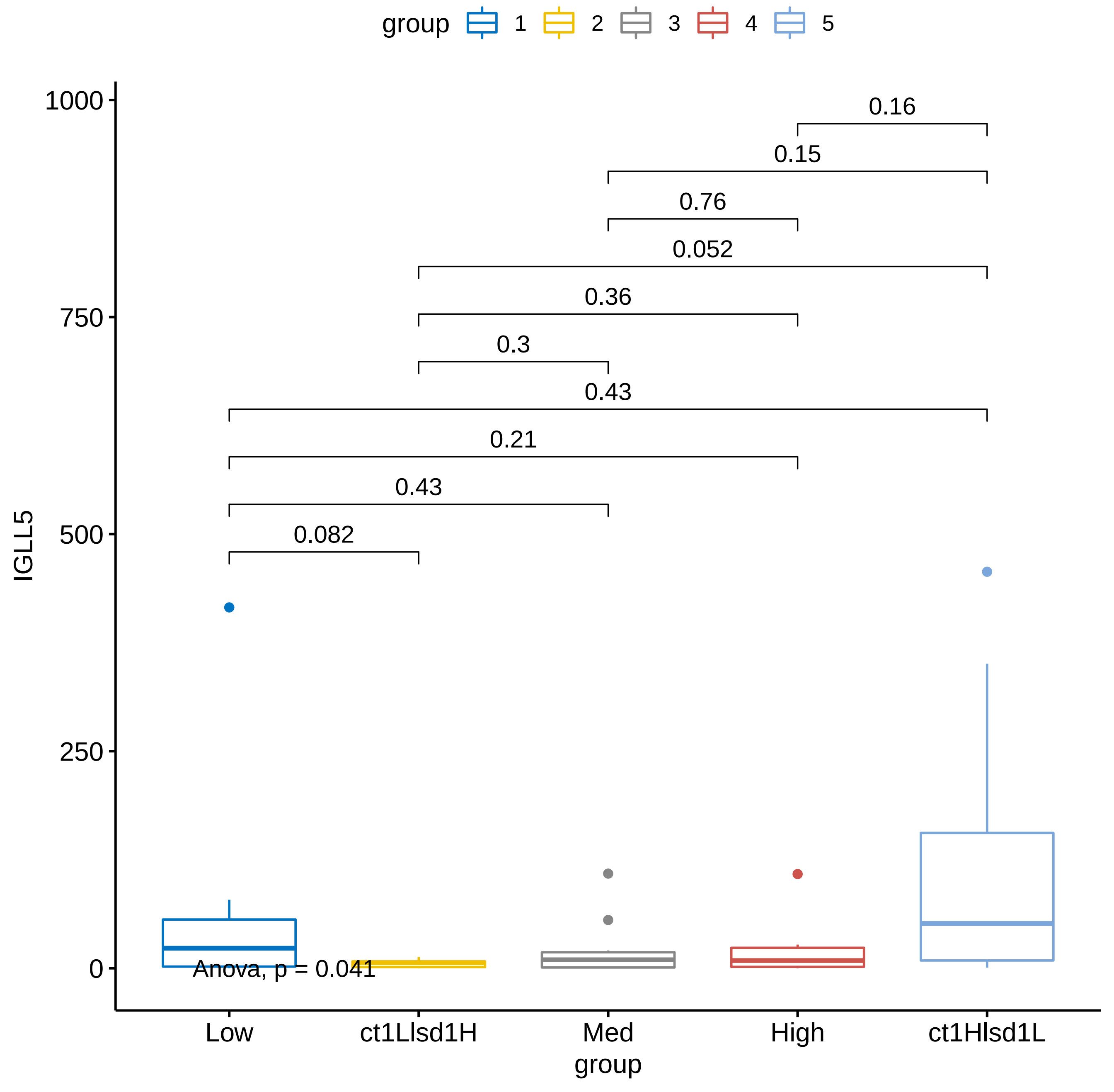

group
1
2
3
4
5
IGLL5
1000
750
500
250
0
0.16
0.15
0.76
0.052
0.36
0.3
0.43
0.21
0.43
0.082
Anova, p = 0.041
Low
ct1Llsd1H
Med
High
ct1Hlsd1L
group

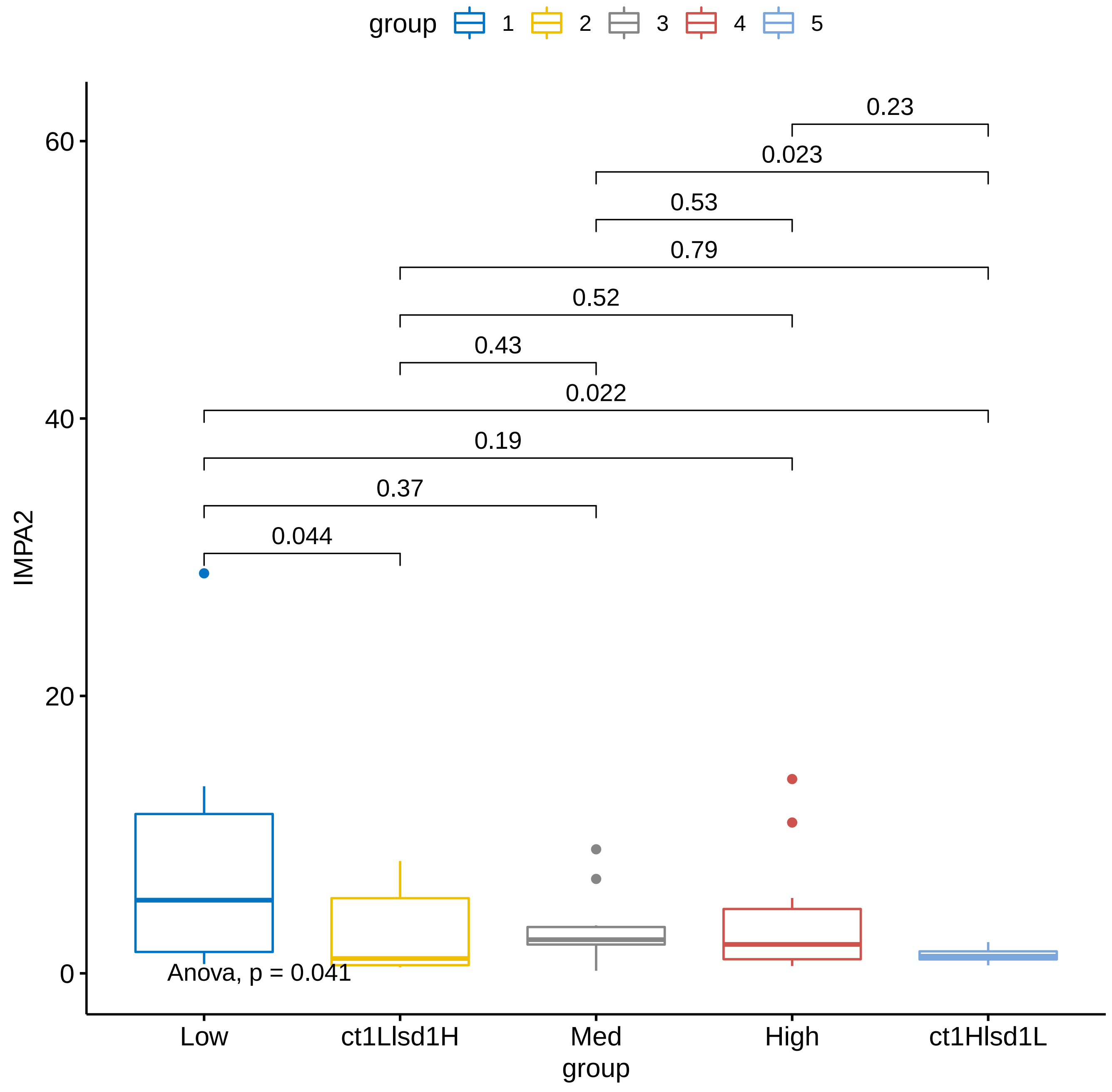

group
1
2
3
4
5
IMPA2
60
40
20
0
0.23
0.023
0.53
0.79
0.52
0.43
0.022
0.19
0.37
0.044
Anova, p = 0.041
Low
ct1Llsd1H
Med
High
ct1Hlsd1L
group

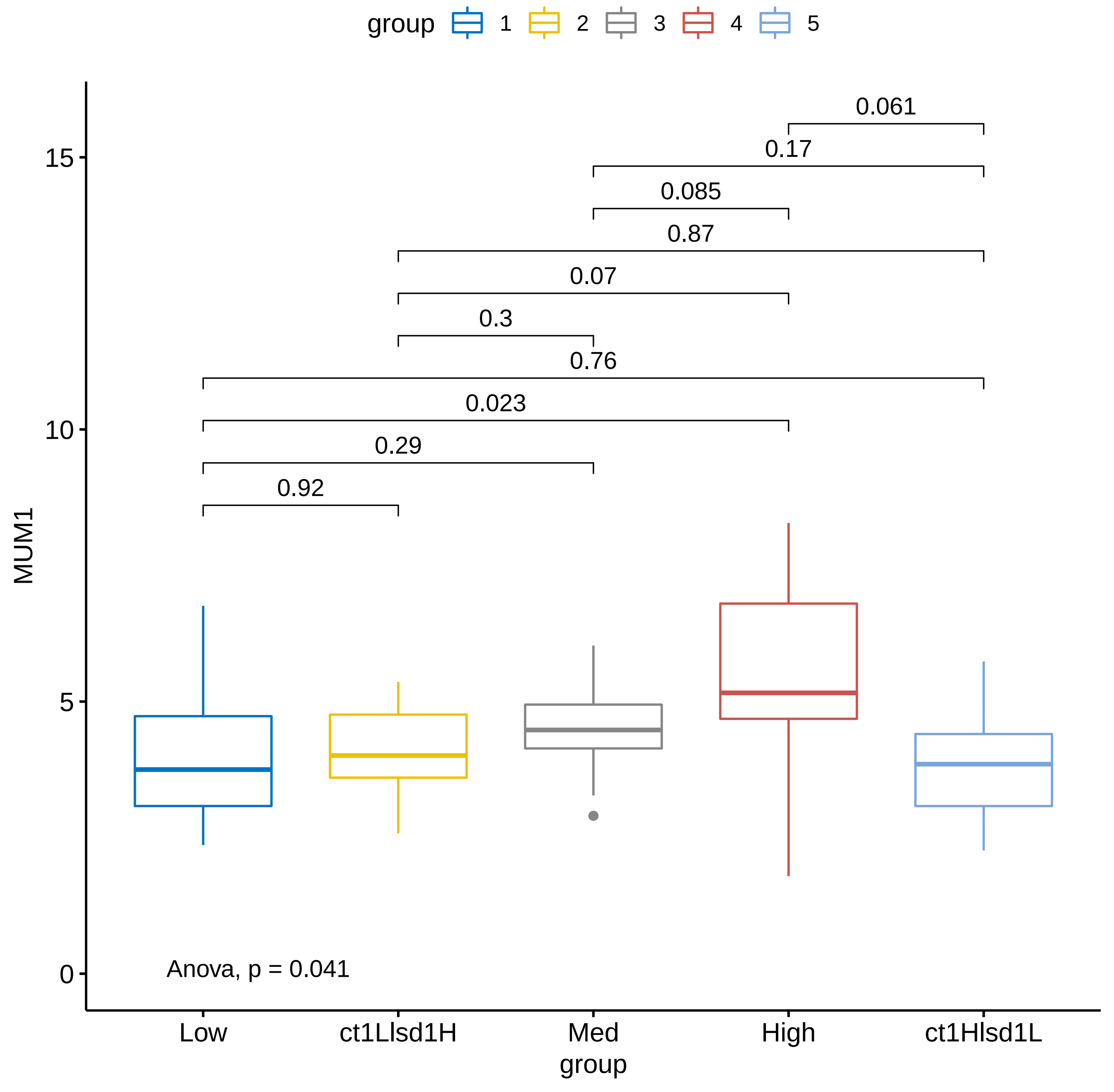
group
1
2
3
4
5
0.061
0.17
0.085
0.87
0.07
0.3
0.76
0.023
0.29
0.92
15
10
5
0
MUM1
Anova, p = 0.041
Low
ct1Llsd1H
Med
High
ct1Hlsd1L
group

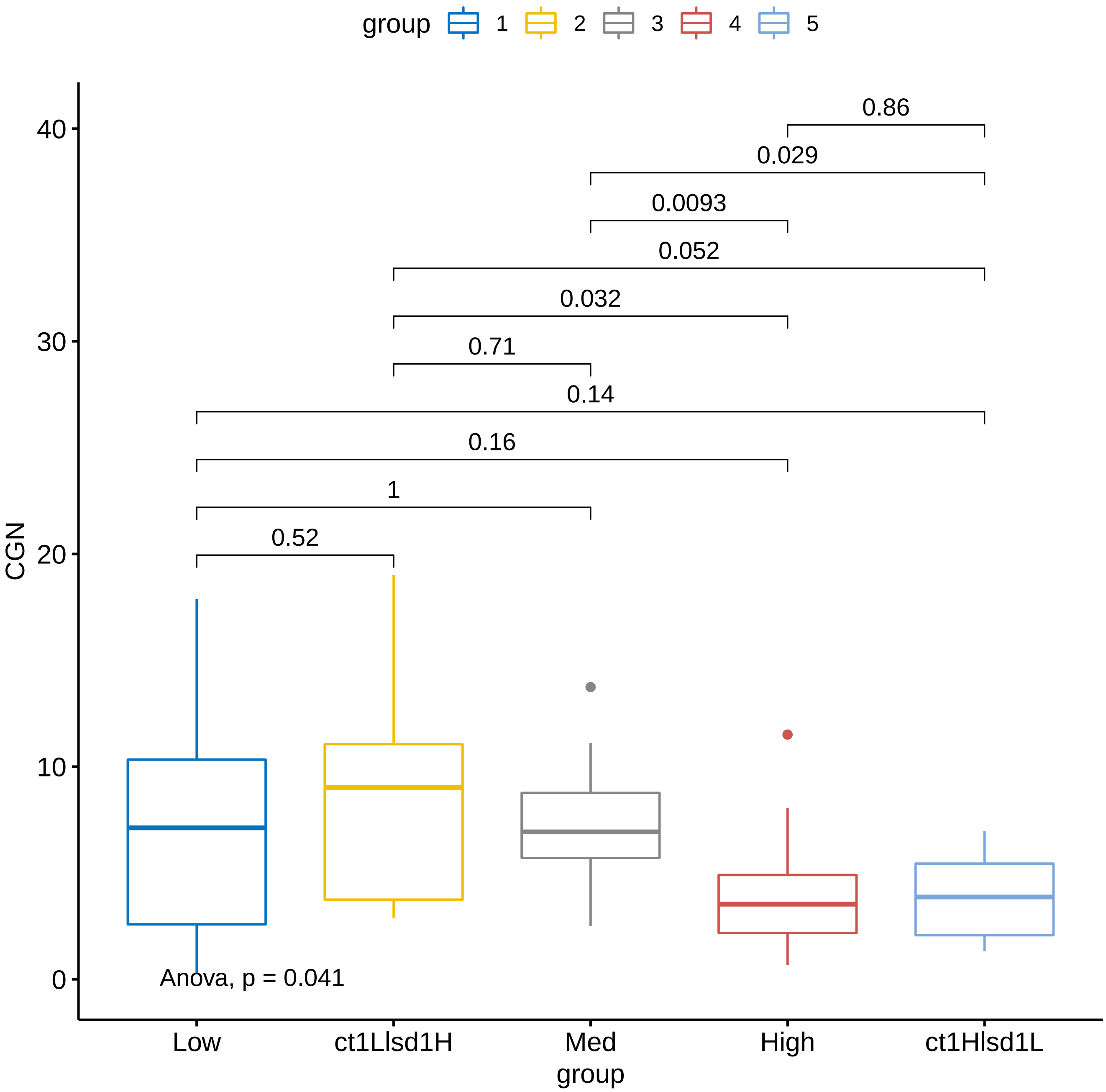

group
1
2
3
4
5
0.86
0.029
0.0093
0.052
0.032
0.71
0.14
0.16
1
0.52
40
30
20
10
0
CGN
Anova, p = 0.041
Low
ct1Llsd1H
Med
High
ct1Hlsd1L
group

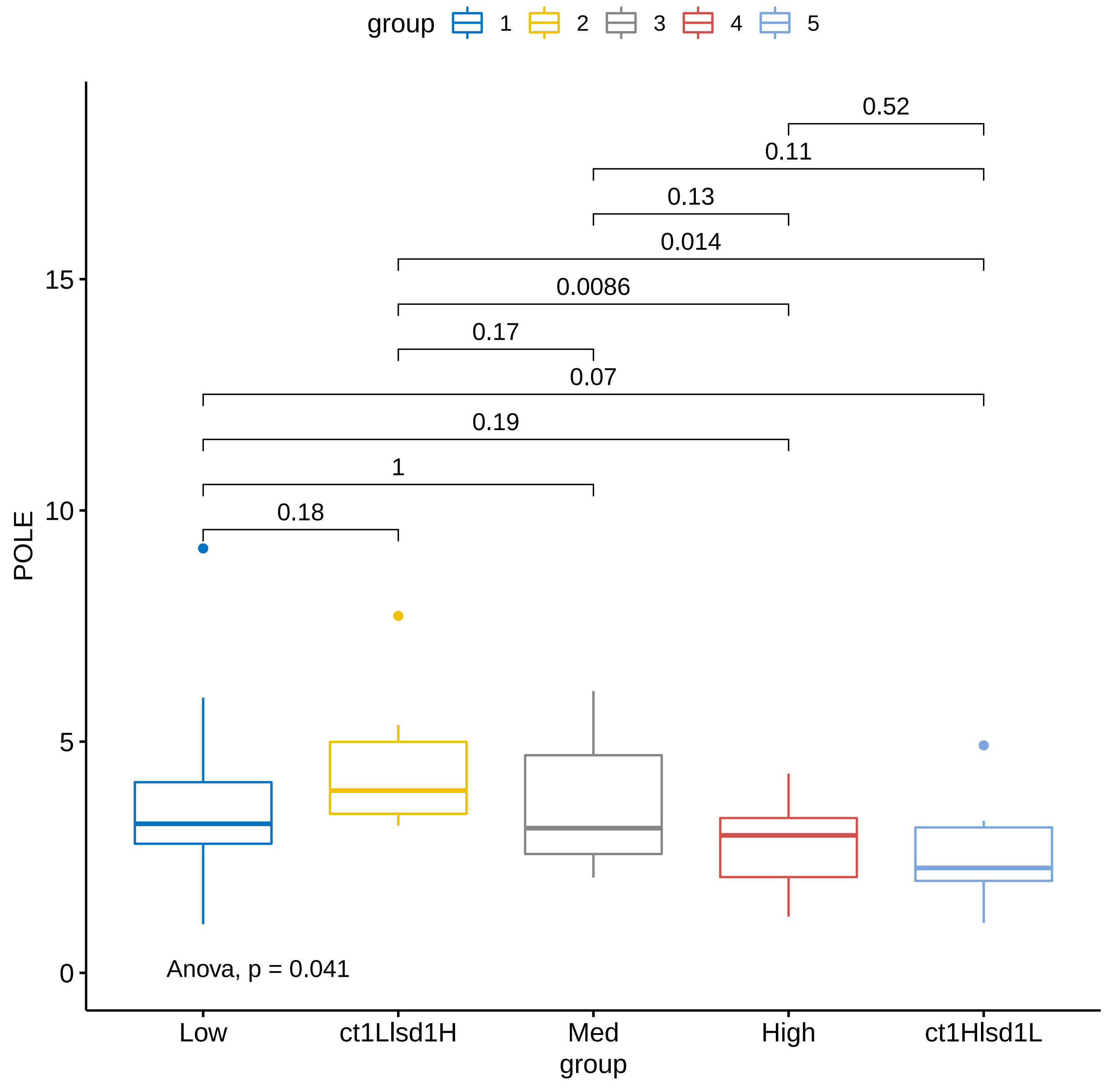

group
1
2
3
4
5
0.52
0.11
0.13
0.014
0.0086
0.17
0.07
0.19
1
0.18
Anova, p = 0.041
POLE
0
5
10
15
Low
ct1Llsd1H
Med
High
ct1Hlsd1L
group

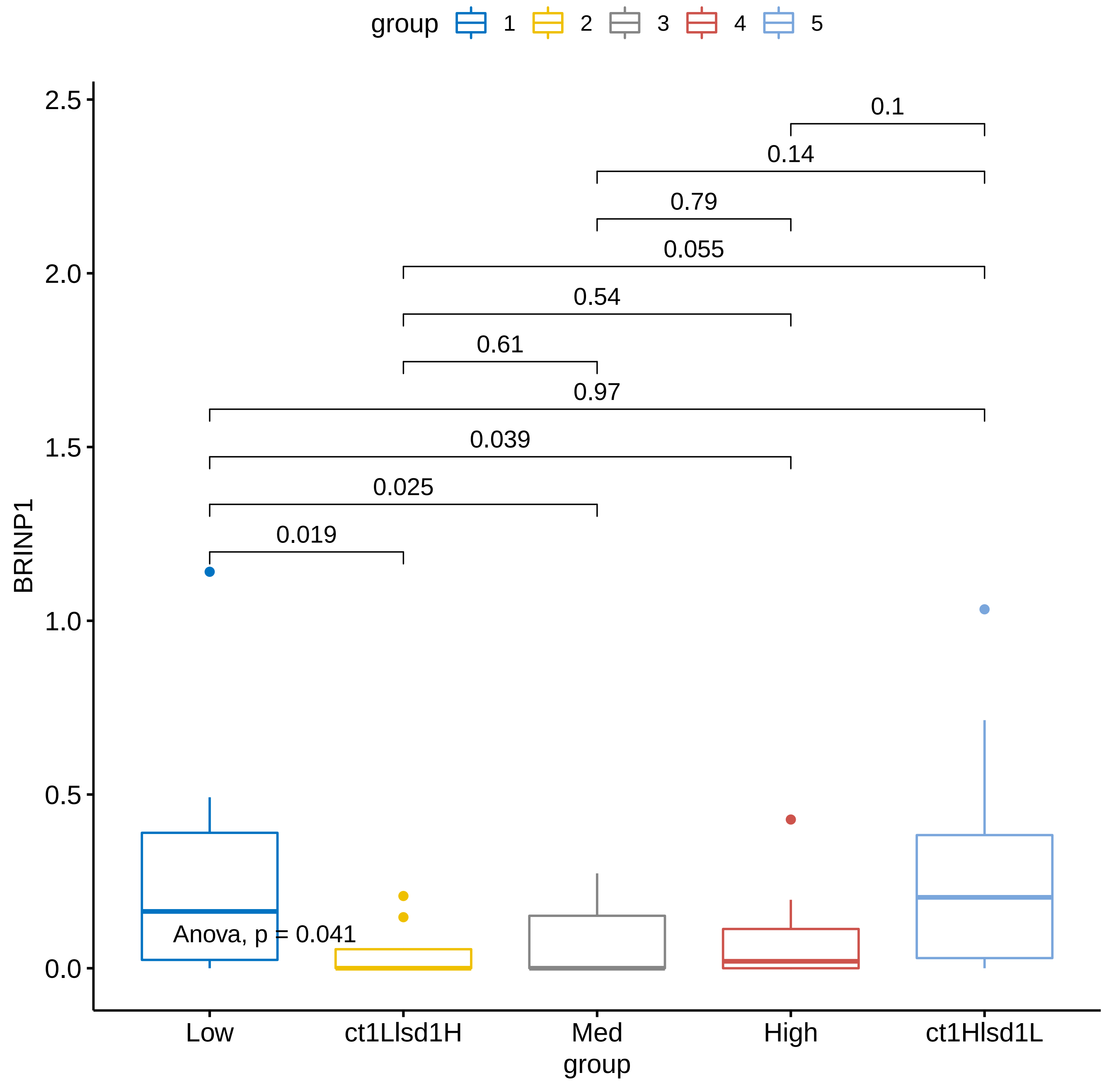
group 1 2 3 4 5
BRINP1
2.5
2.0
1.5
1.0
0.5
0.0
0.1
0.14
0.79
0.055
0.54
0.61
0.97
0.039
0.025
0.019
Anova, p = 0.041
Low
ct1Llsd1H
Med
High
ct1Hlsd1L
group

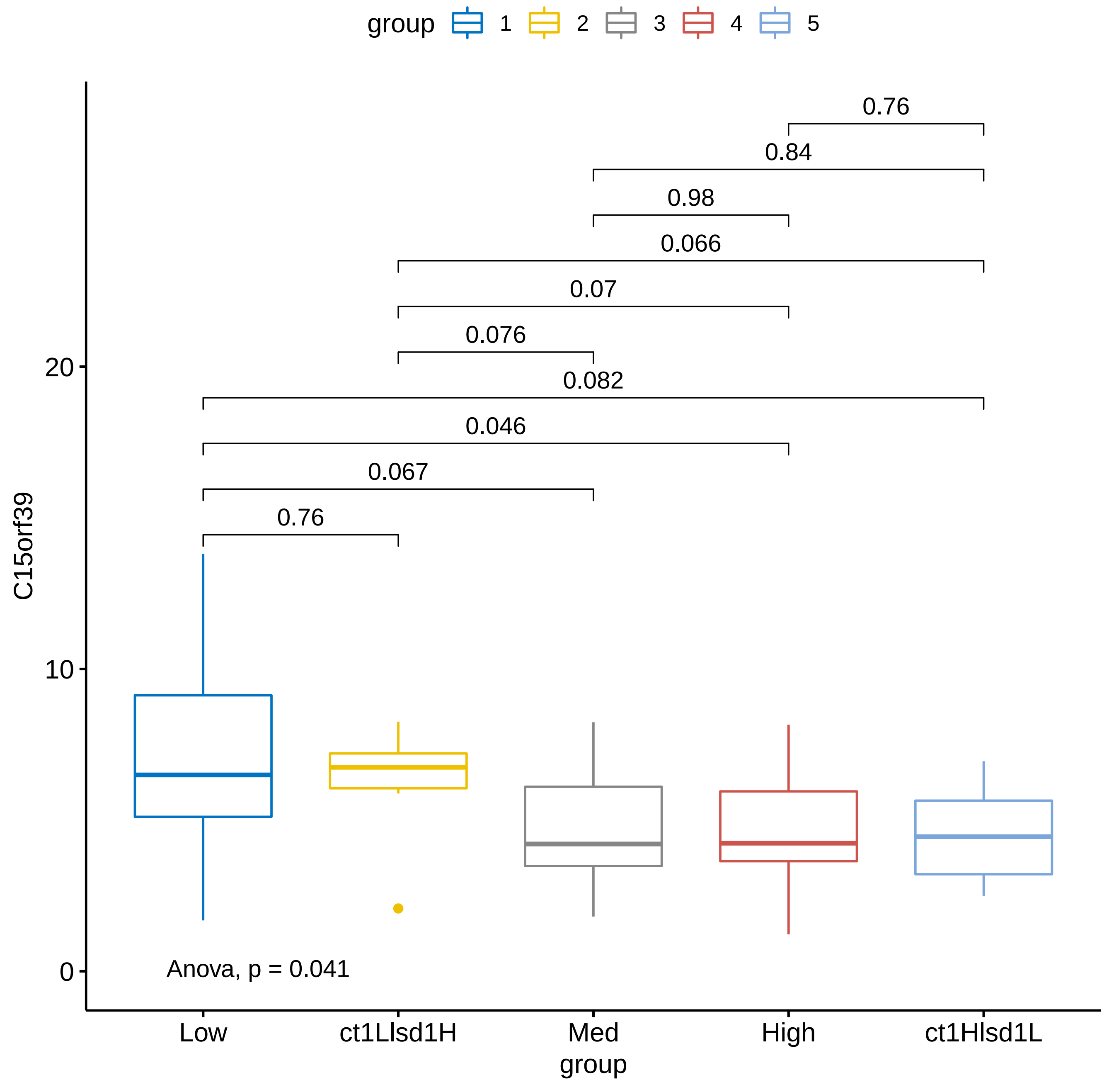

group
1
2
3
4
5
C15orf39
0.76
0.84
0.98
0.066
0.07
0.076
0.082
0.046
0.067
0.76
20
10
0
Anova, p = 0.041
Low
ct1Llsd1H
Med
High
ct1Hlsd1L
group

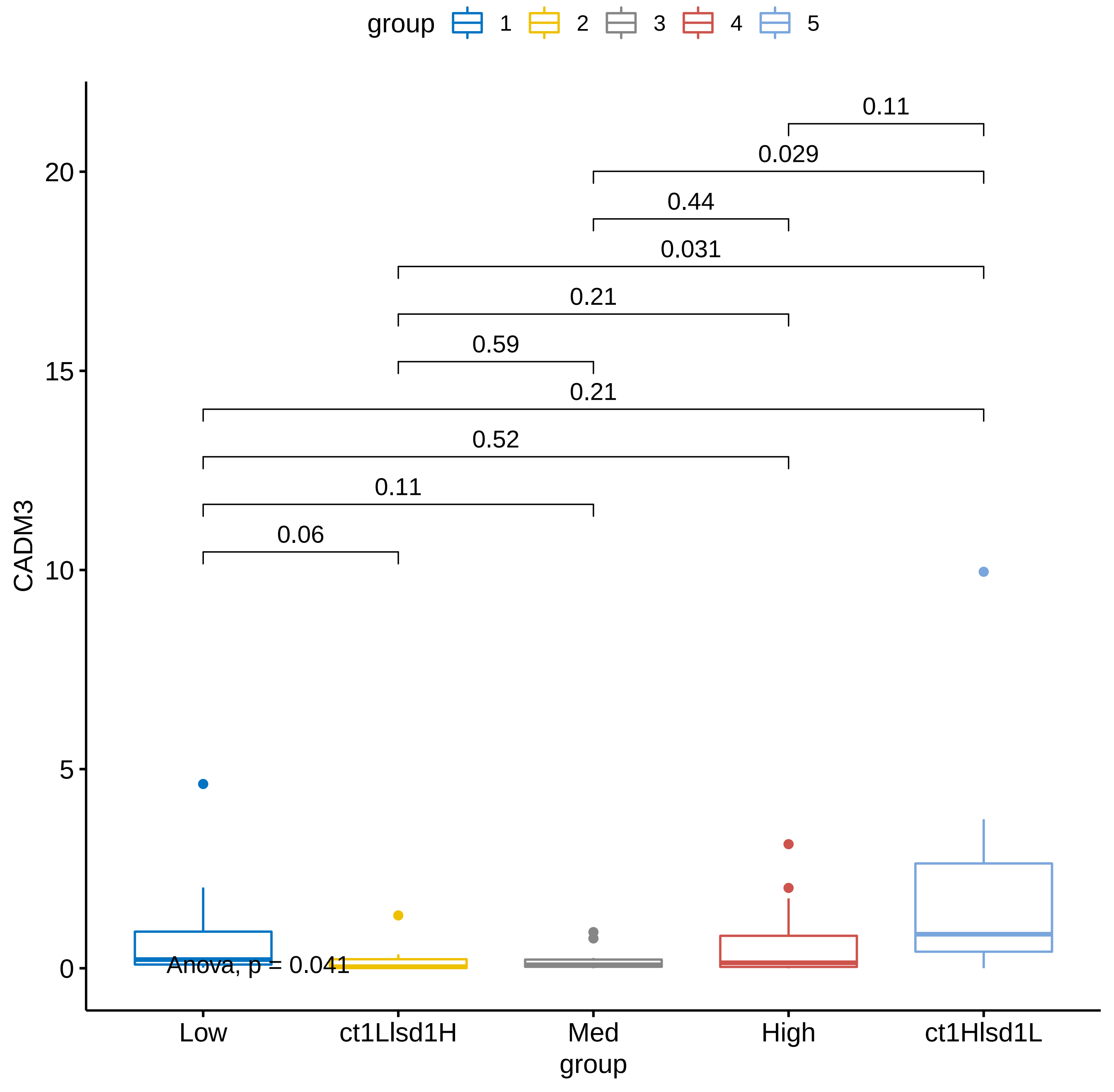
group 1 2 3 4 5
0.11
0.029
0.44
0.031
0.21
0.59
0.21
0.52
0.11
0.06
CADM3
20
15
10
5
0
Anova, p = 0.041
Low
ct1Llsd1H
Med
High
ct1Hlsd1L
group

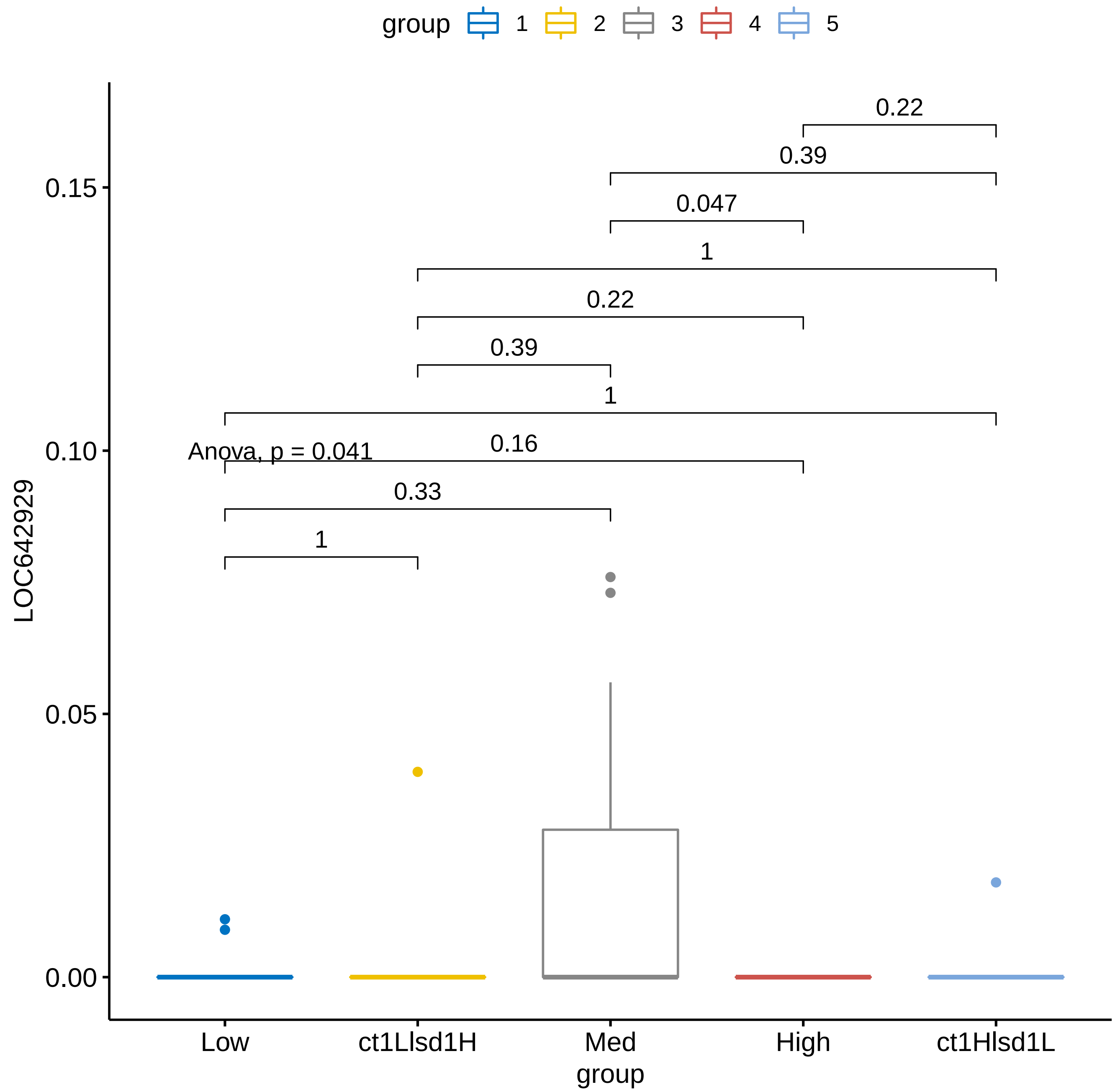

group
1
2
3
4
5
0.22
0.39
0.047
1
0.22
0.39
1
0.16
0.33
1
Anova, p = 0.041
0.15
0.10
0.05
0.00
LOC642929
Low
ct1Llsd1H
Med
High
ct1Hlsd1L
group

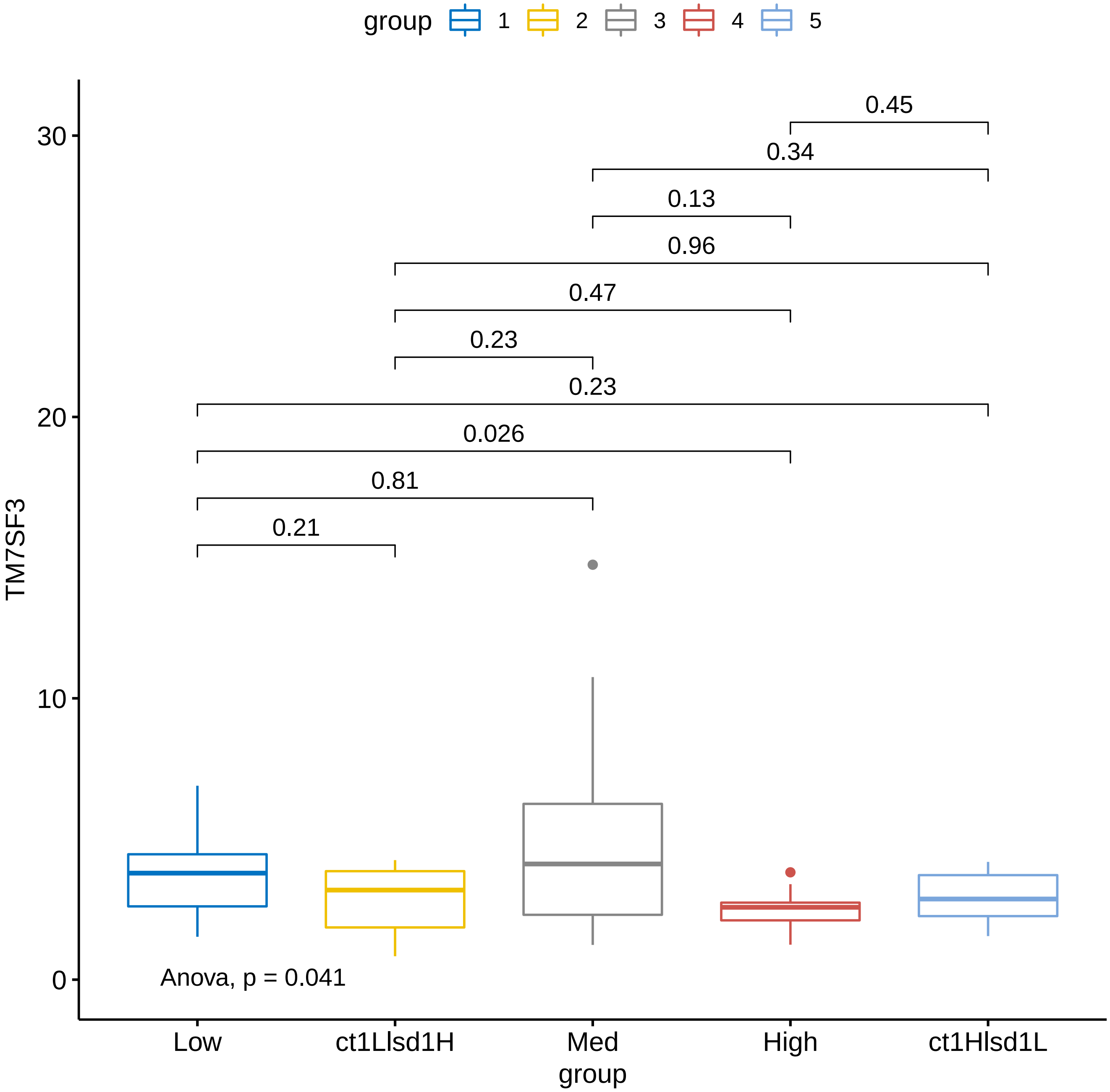
group
1
2
3
4
5
0.45
0.34
0.13
0.96
0.47
0.23
0.23
0.026
0.81
0.21
30
20
10
0
TM7SF3
Anova, p = 0.041
Low
ct1Llsd1H
Med
High
ct1Hlsd1L
group

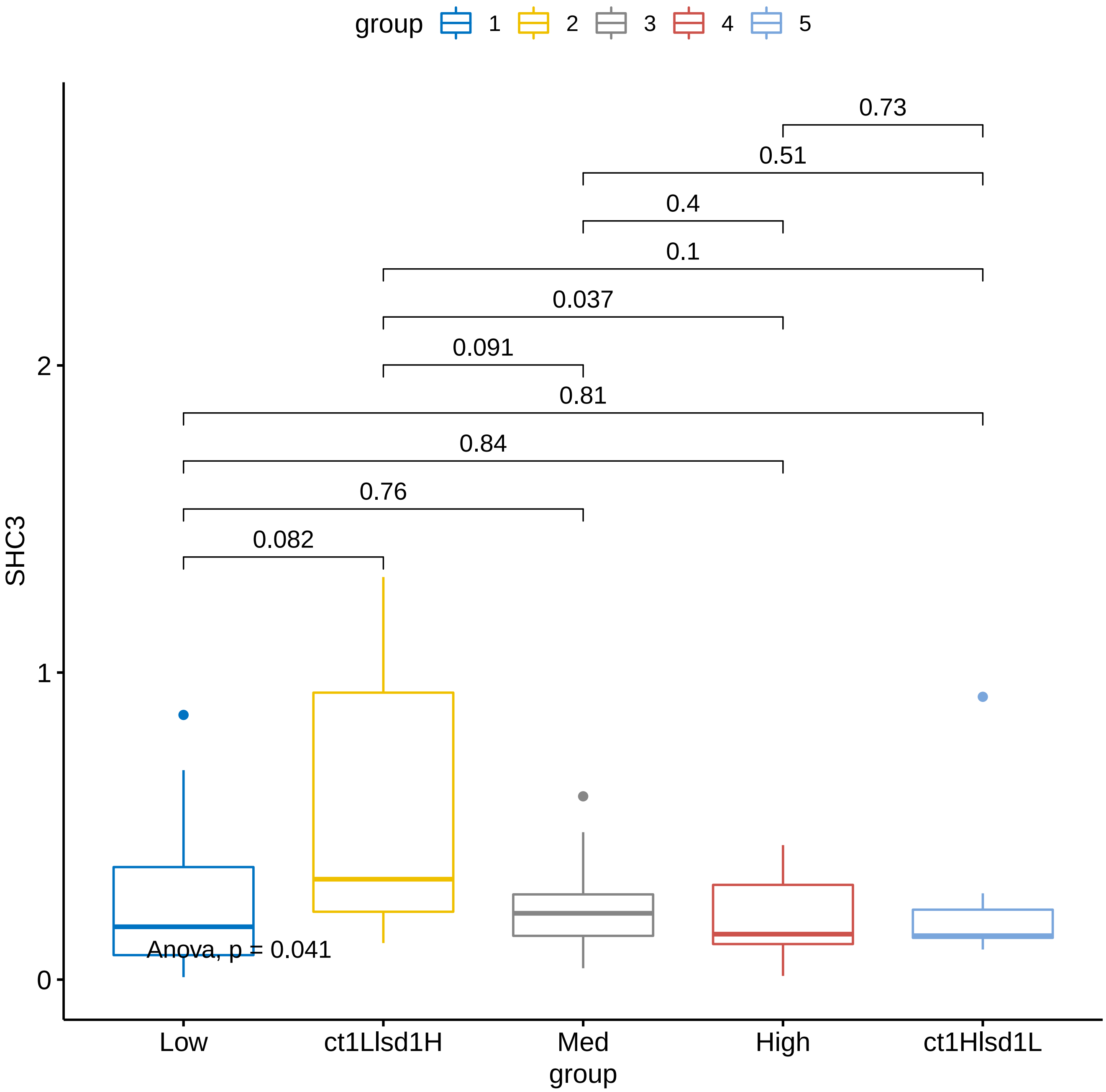

group 1 2 3 4 5
0.73
0.51
0.4
0.1
0.037
0.091
0.81
0.84
0.76
0.082
Anova, p = 0.041
SHC3
2
1
0
Low
ct1Llsd1H
Med
High
ct1Hlsd1L
group

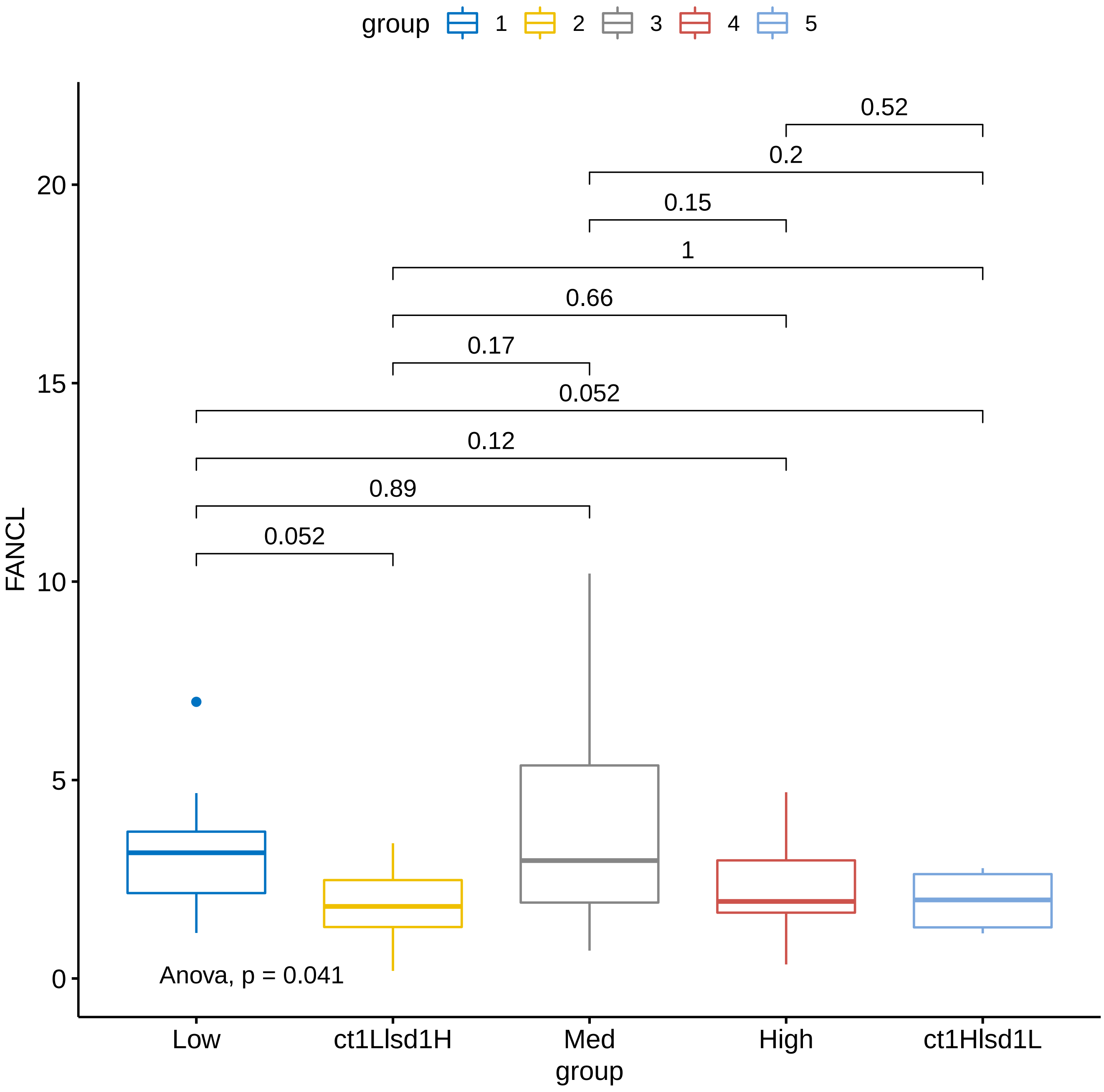

group
1
2
3
4
5
0.52
0.2
0.15
1
0.66
0.17
0.052
0.12
0.89
0.052
20
15
10
5
0
FANCL
Anova, p = 0.041
Low
ct1Llsd1H
Med
High
ct1Hlsd1L
group

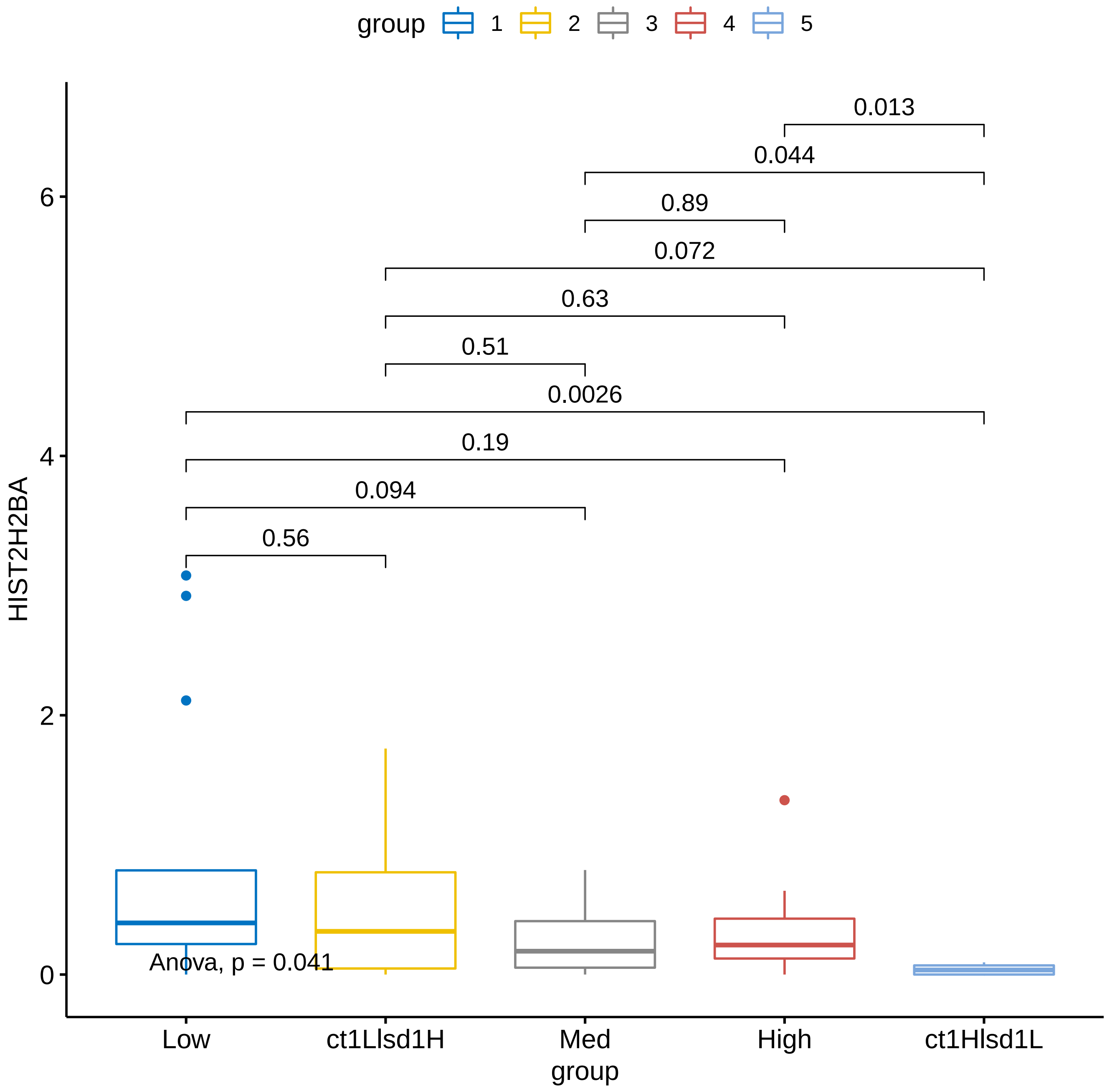
group
1
2
3
4
5
0.013
0.044
0.89
0.072
0.63
0.51
0.0026
0.19
0.094
0.56
6
4
2
0
HIST2H2BA
Anova, p = 0.041
Low
ct1Llsd1H
Med
High
ct1Hlsd1L
group

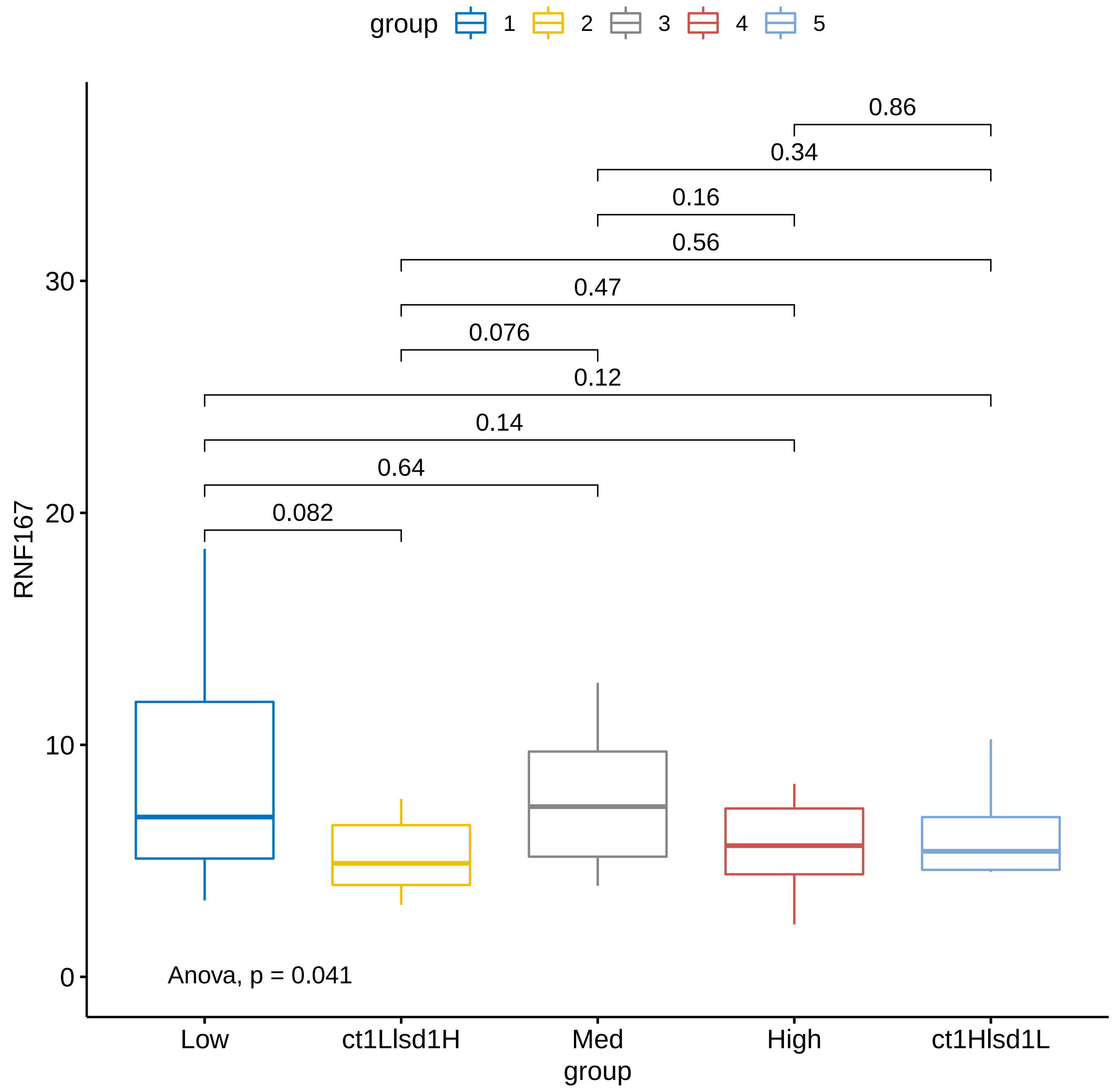
group
1
2
3
4
5
RNF167
0
10
20
30
0.86
0.34
0.16
0.56
0.47
0.076
0.12
0.14
0.64
0.082
Anova, p = 0.041
Low
ct1Llsd1H
Med
High
ct1Hlsd1L
group

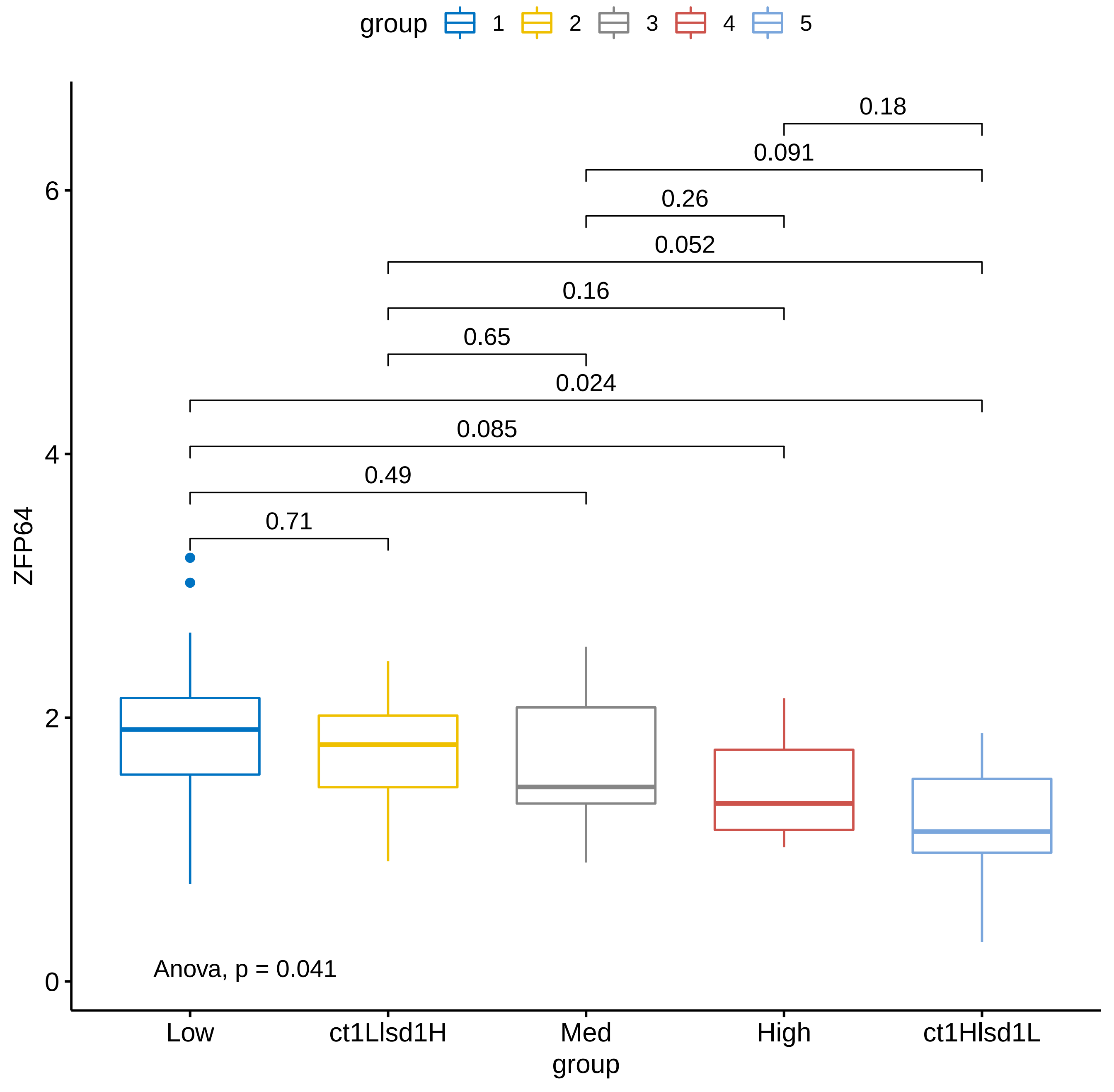

group
1
2
3
4
5
ZFP64
0
2
4
6
0.18
0.091
0.26
0.052
0.16
0.65
0.024
0.085
0.49
0.71
Anova, p = 0.041
Low
ct1Llsd1H
Med
High
ct1Hlsd1L
group

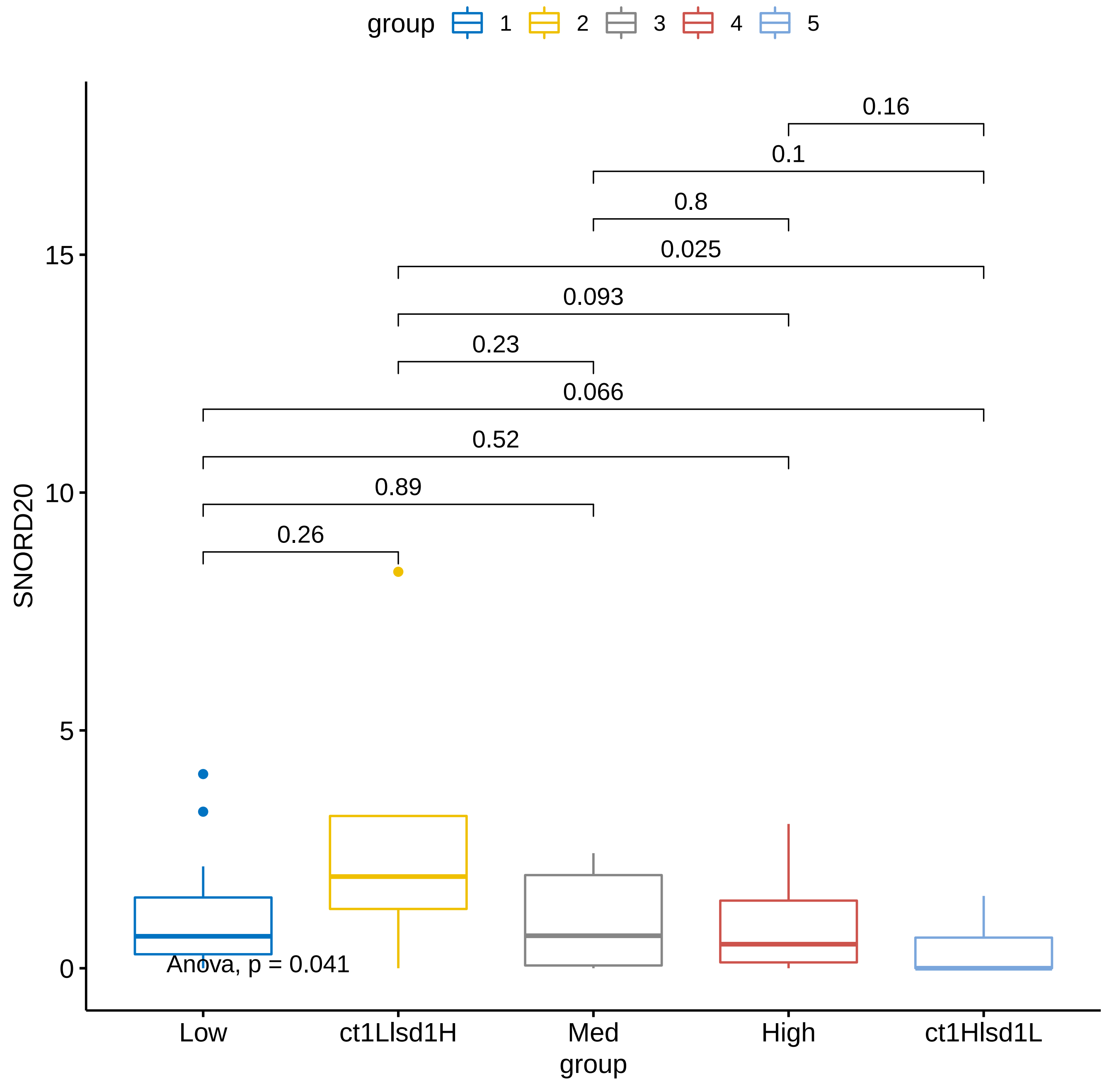

group 1 2 3 4 5
0.16
0.1
0.8
0.025
0.093
0.23
0.066
0.52
0.89
0.26
SNORD20
15
10
5
0
Anova, p = 0.041
Low
ct1Llsd1H
Med
High
ct1Hlsd1L
group

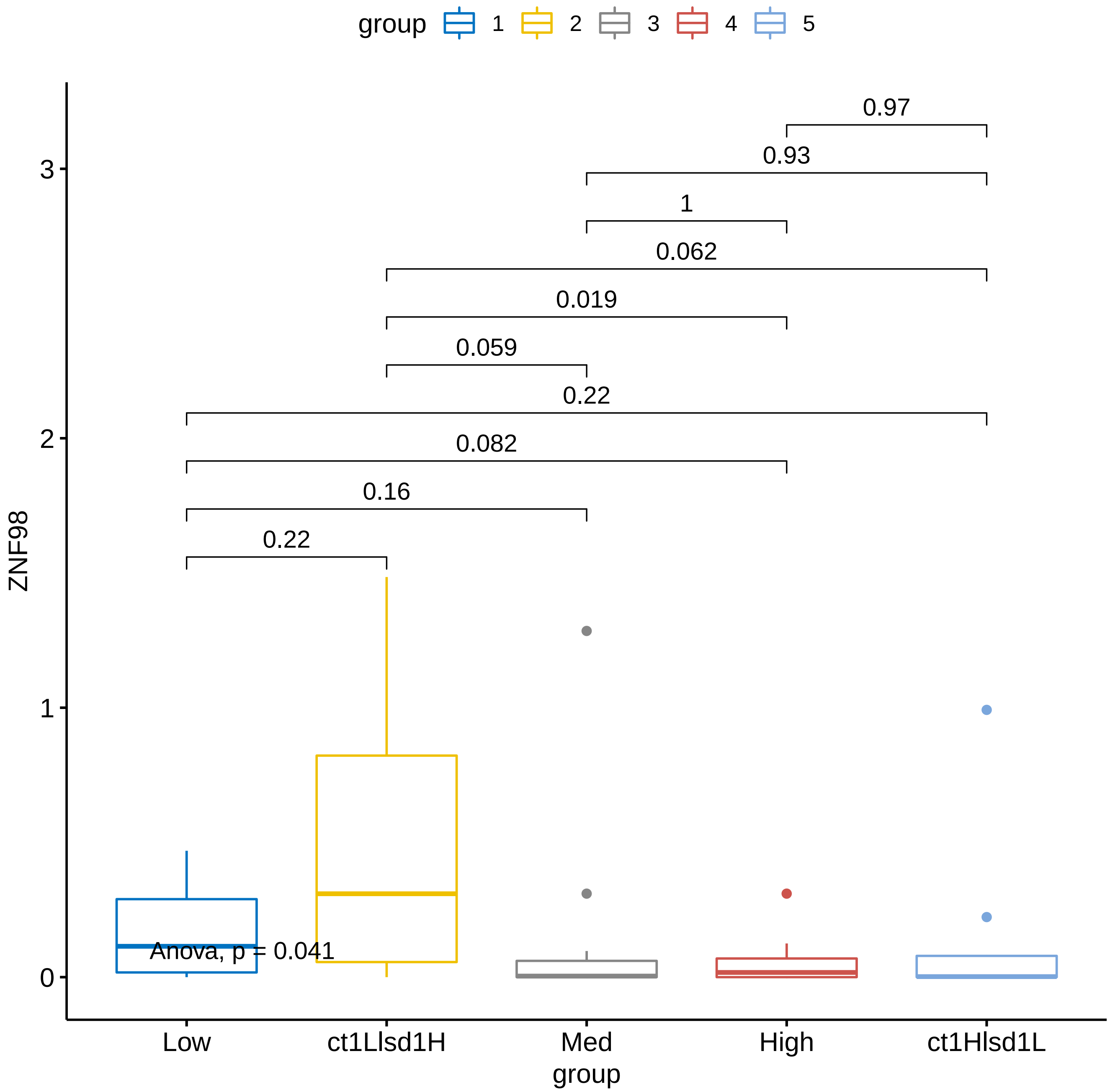
group
1
2
3
4
5
0.97
0.93
1
0.062
0.019
0.059
0.22
0.082
0.16
0.22
Anova, p = 0.041
ZNF98
3
2
1
0
Low
ct1Llsd1H
Med
High
ct1Hlsd1L
group

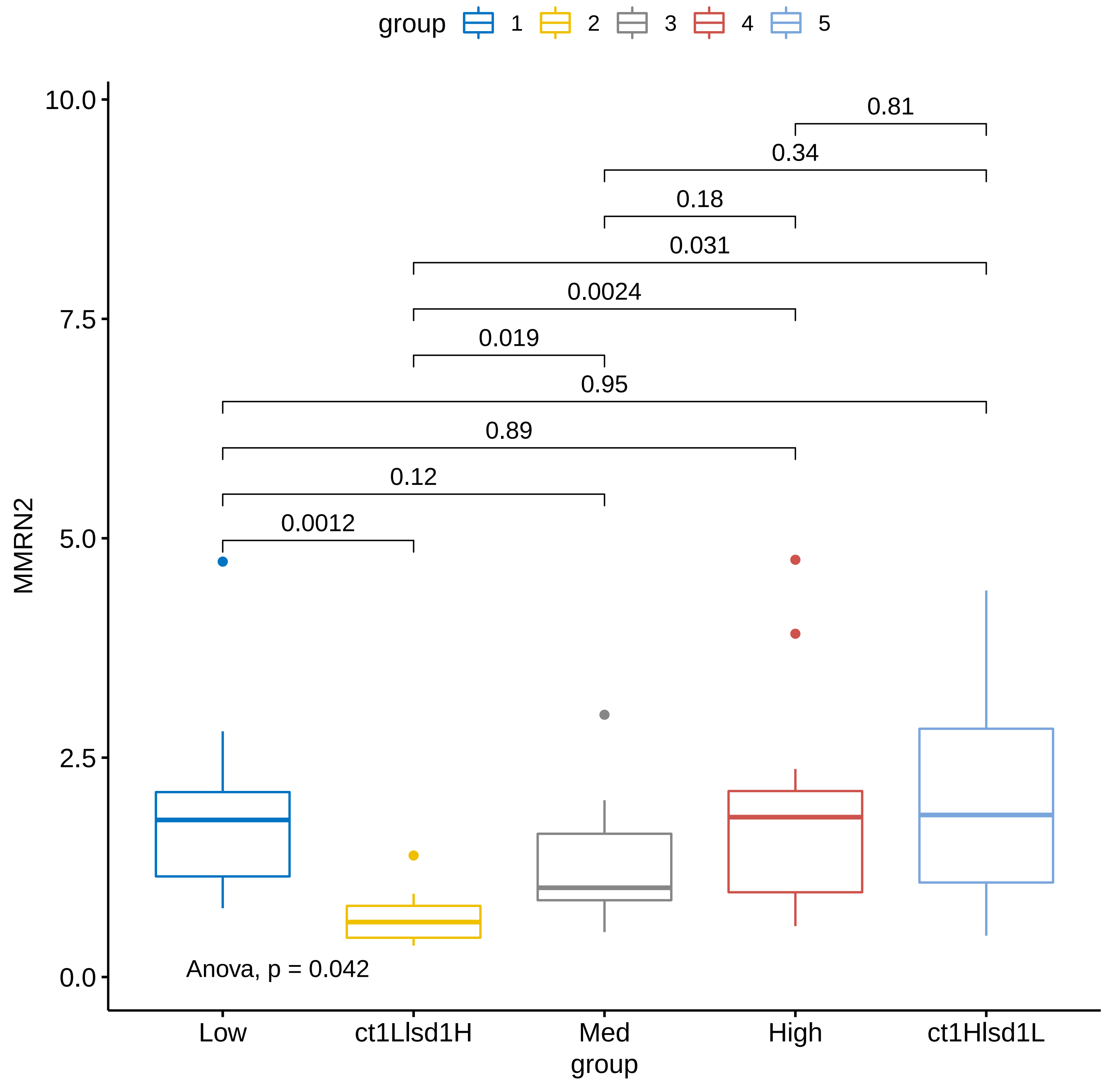

group 1 2 3 4 5
0.81
0.34
0.18
0.031
0.0024
0.019
0.95
0.89
0.12
0.0012
Anova, p = 0.042
10.0
7.5
5.0
2.5
0.0
MMRN2
Low
ct1Llsd1H
Med
High
ct1Hlsd1L
group

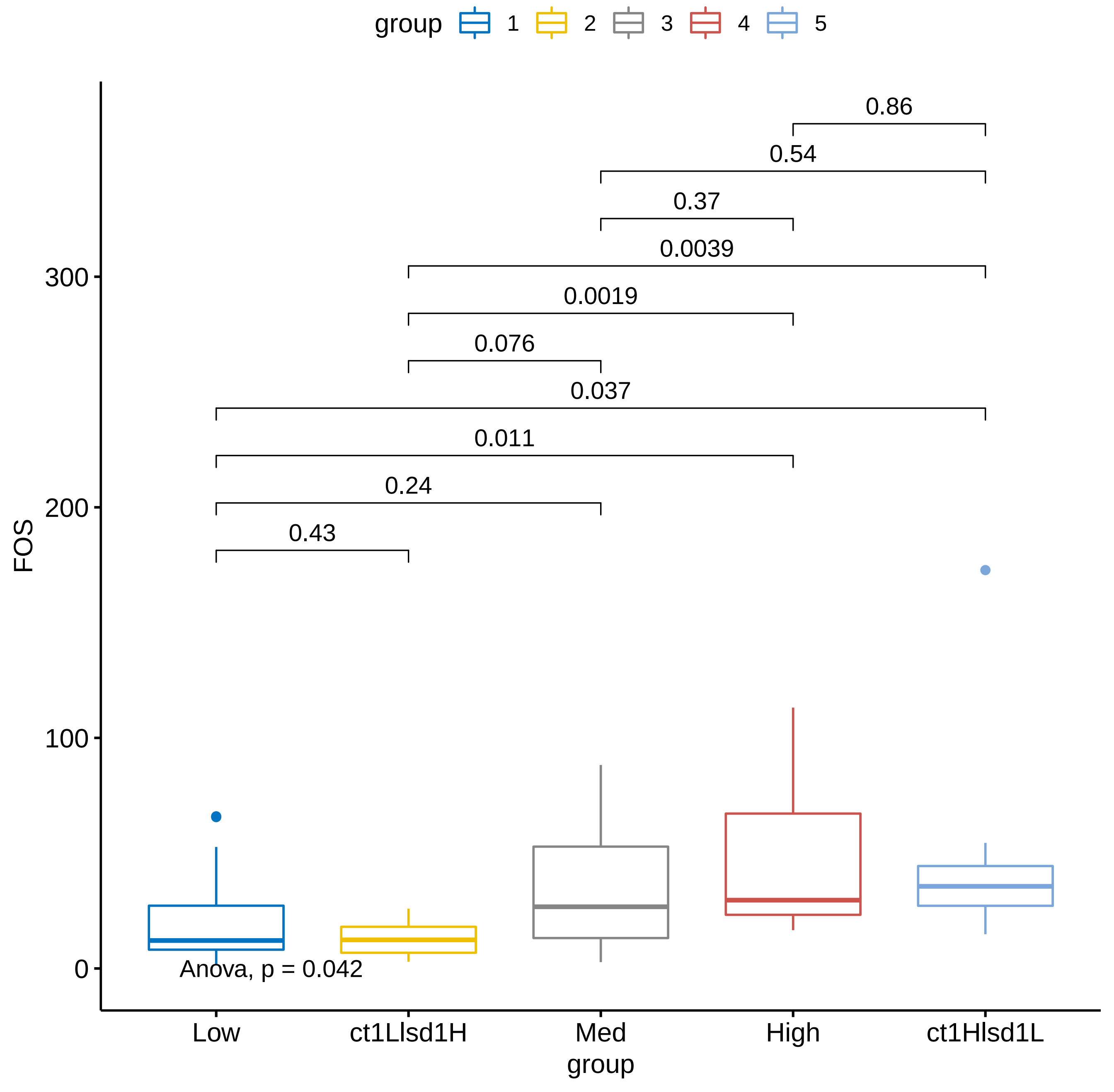
group
1
2
3
4
5
0.86
0.54
0.37
0.0039
0.0019
0.076
0.037
0.011
0.24
0.43
300
200
100
0
FOS
Anova, p = 0.042
Low
ct1Llsd1H
Med
High
ct1Hlsd1L
group

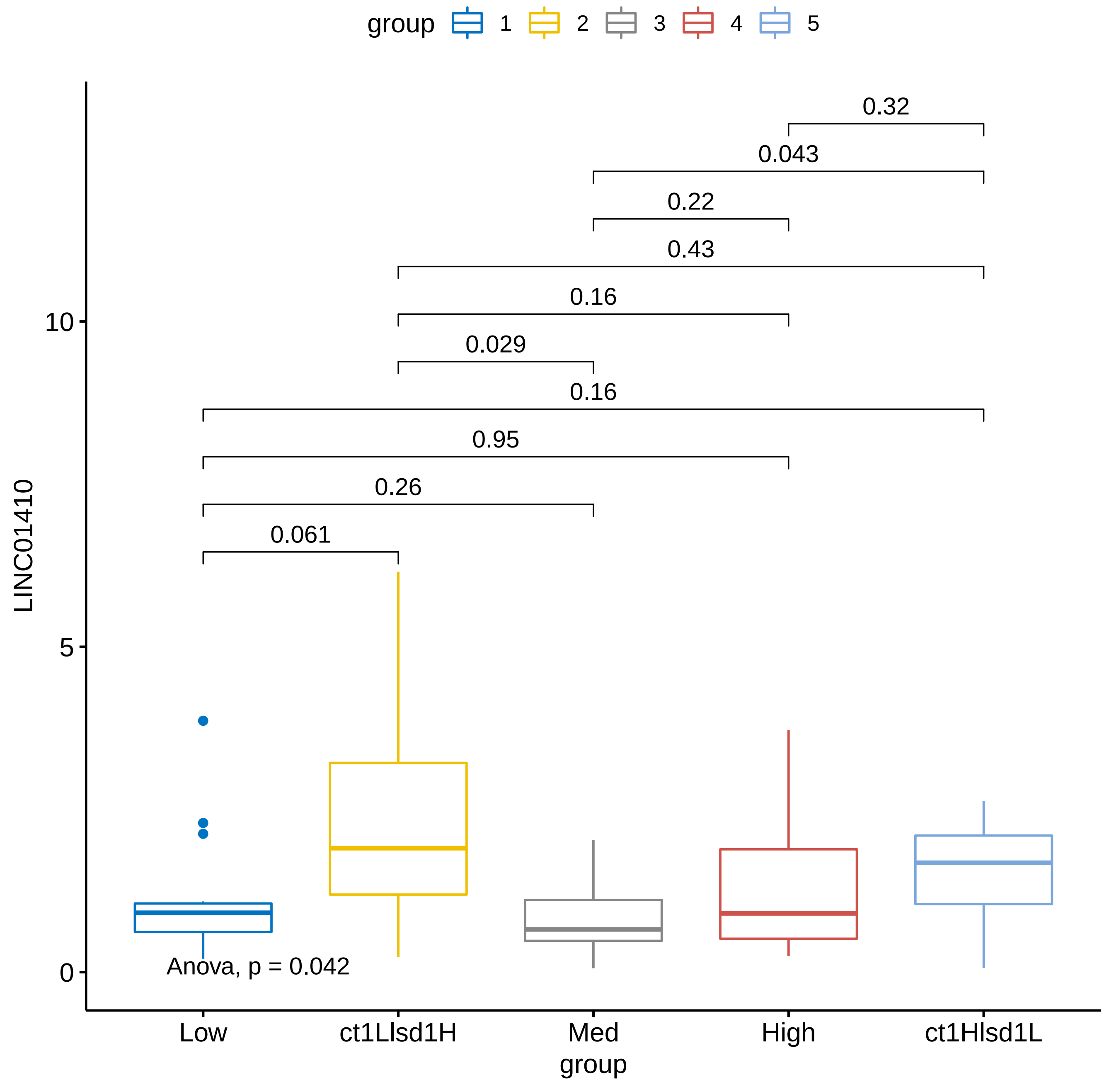

group
1
2
3
4
5
0.32
0.043
0.22
0.43
0.16
0.029
0.16
0.95
0.26
0.061
Anova, p = 0.042
LINC01410
10
5
0
Low
ct1Llsd1H
Med
High
ct1Hlsd1L
group

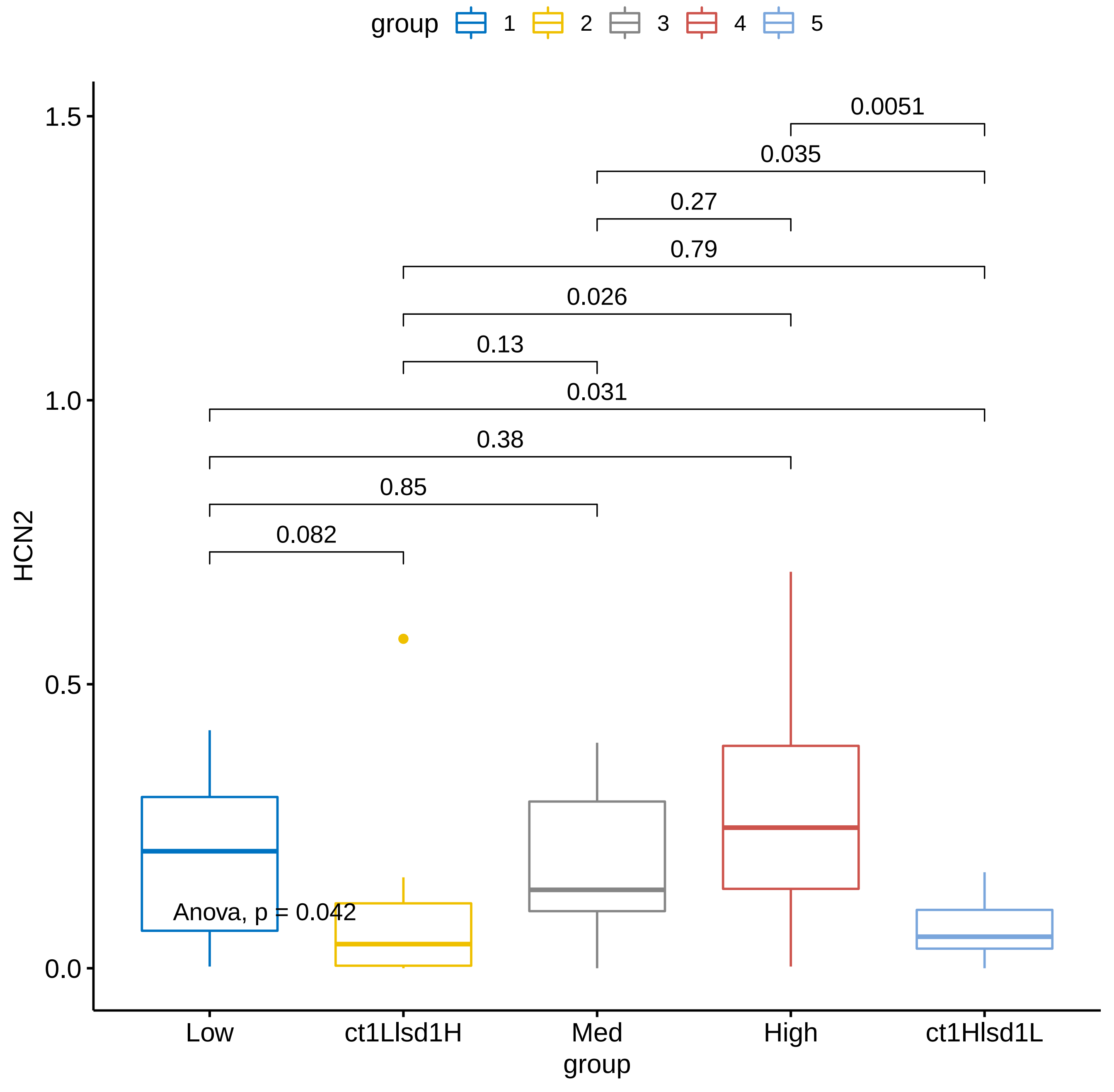
group
1
2
3
4
5
0.0051
0.035
0.27
0.79
0.026
0.13
0.031
0.38
0.85
0.082
Anova, p = 0.042
1.5
1.0
0.5
0.0
HCN2
Low
ct1Llsd1H
Med
High
ct1Hlsd1L
group

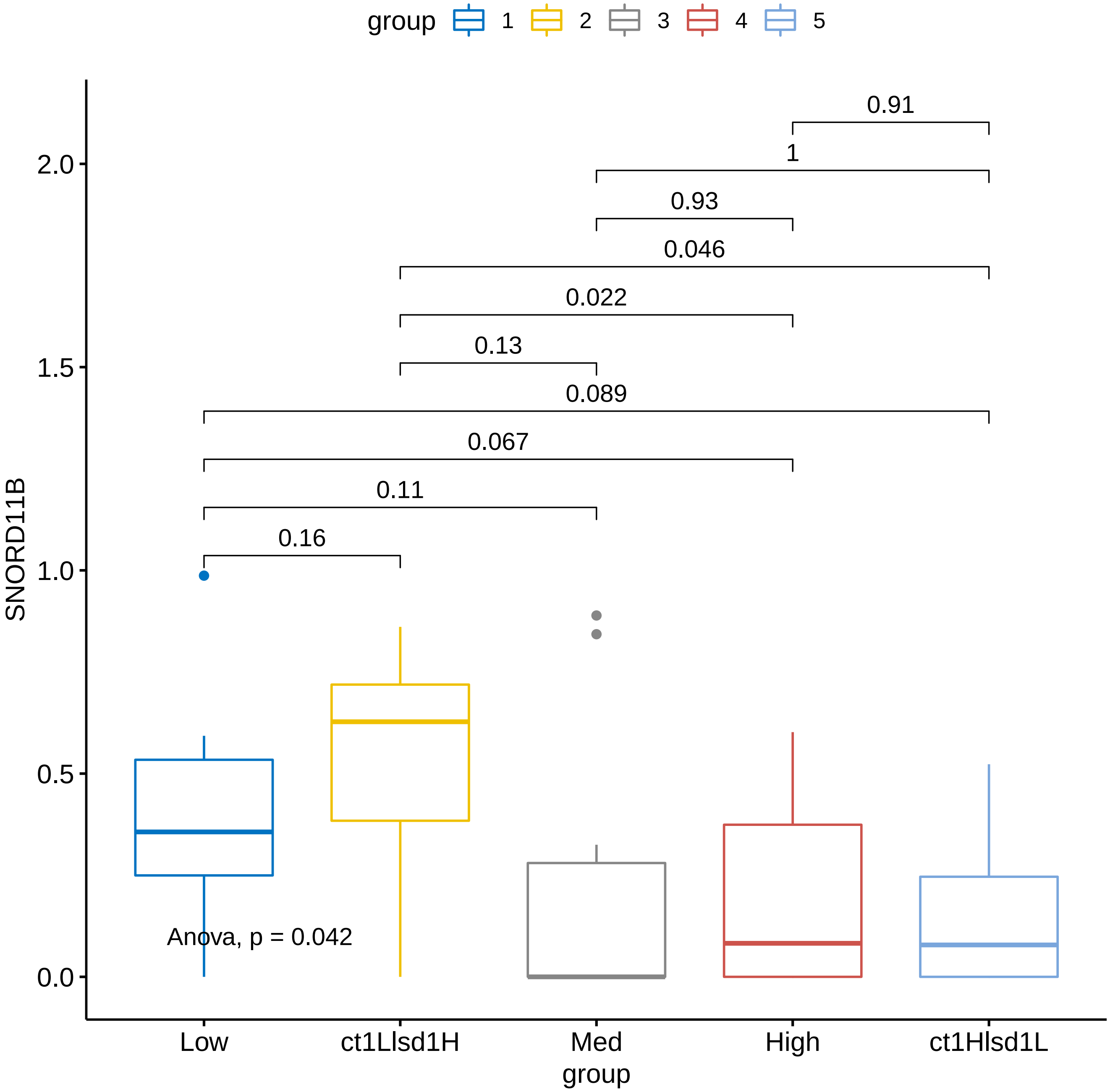
group
1
2
3
4
5
0.91
1
0.93
0.046
0.022
0.13
0.089
0.067
0.11
0.16
2.0
1.5
1.0
0.5
0.0
SNORD11B
Anova, p = 0.042
Low
ct1Llsd1H
Med
High
ct1Hlsd1L
group

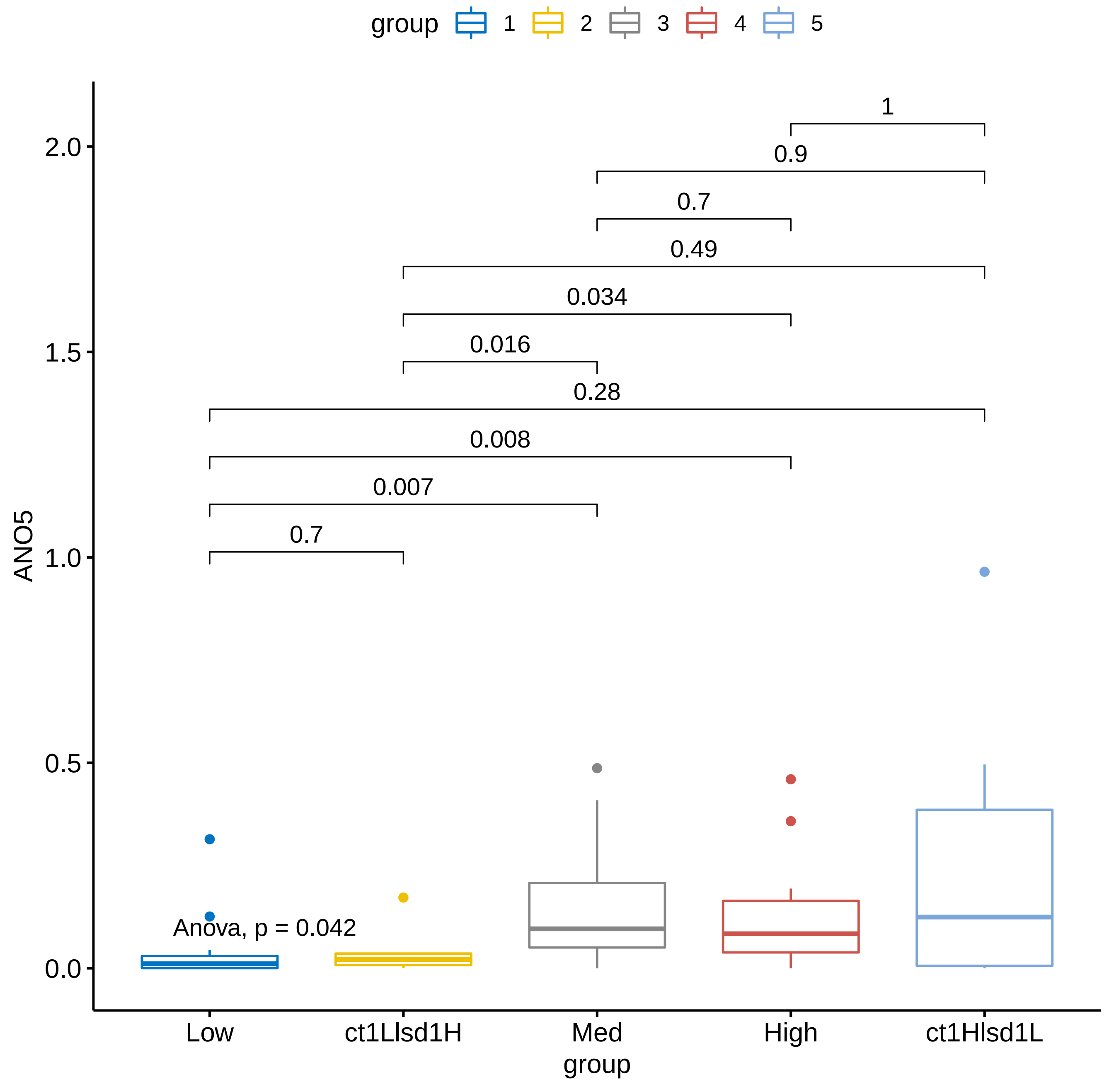
group
1
2
3
4
5
1
0.9
0.7
0.49
0.034
0.016
0.28
0.008
0.007
0.7
2.0
1.5
1.0
0.5
0.0
ANO5
Anova, p = 0.042
Low
ct1Llsd1H
Med
High
ct1Hlsd1L
group

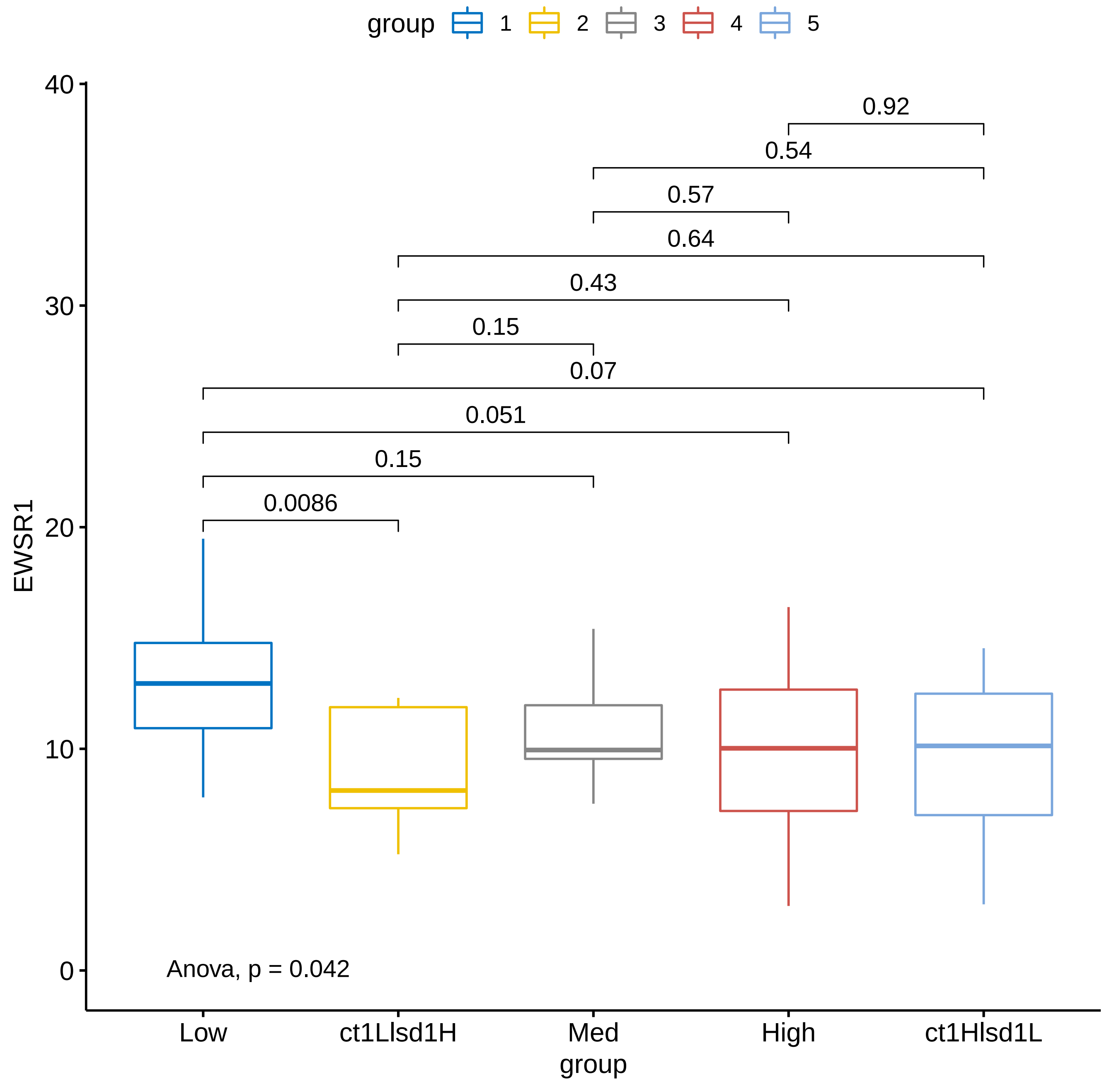
group
1
2
3
4
5
EWSR1
40
30
20
10
0
0.92
0.54
0.57
0.64
0.43
0.15
0.07
0.051
0.15
0.0086
Anova, p = 0.042
Low
ct1Llsd1H
Med
High
ct1Hlsd1L
group

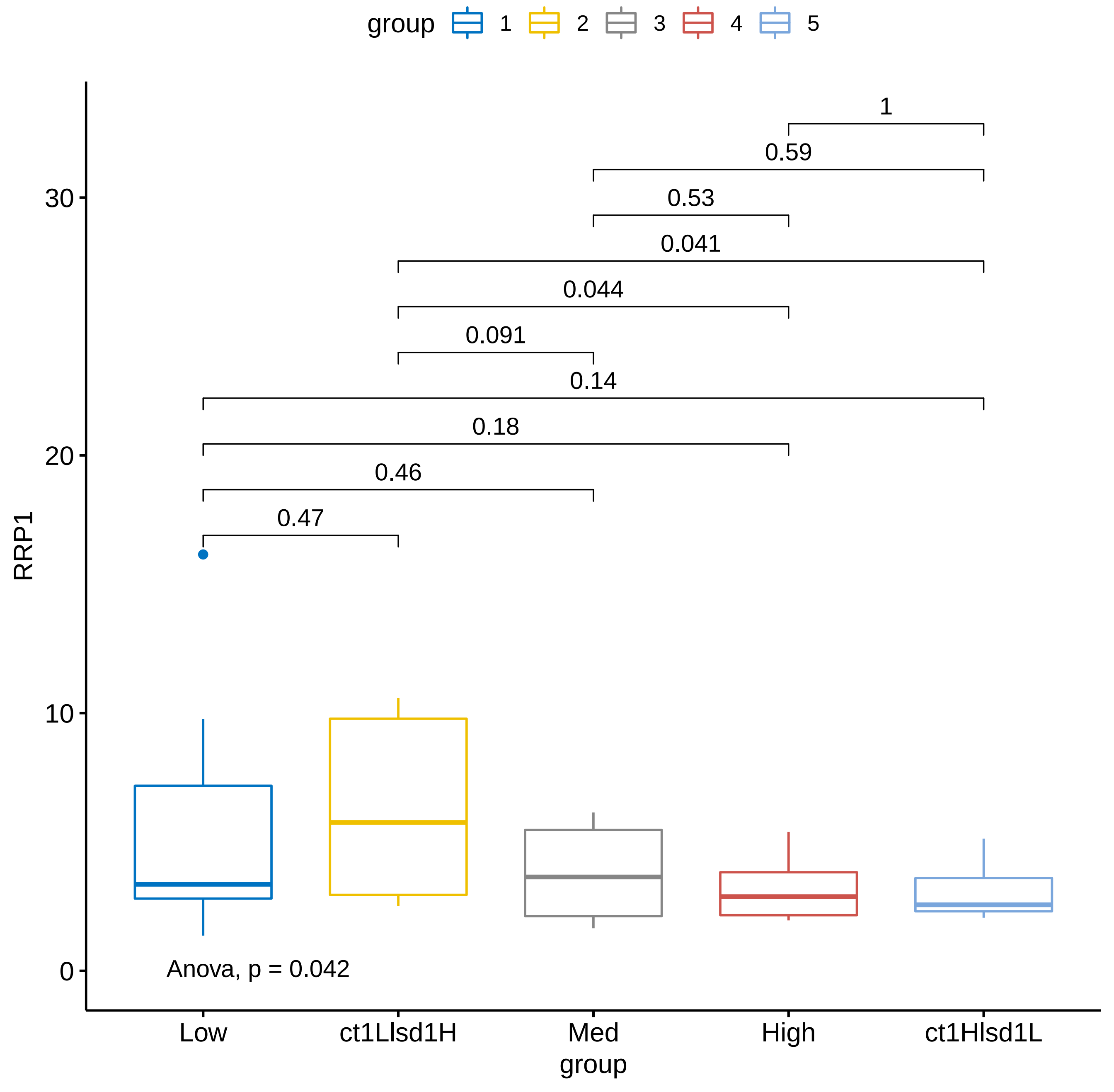

group 1 2 3 4 5
1
0.59
0.53
0.041
0.044
0.091
0.14
0.18
0.46
0.47
30
20
10
0
RRP1
Anova, p = 0.042
Low
ct1Llsd1H
Med
High
ct1Hlsd1L
group

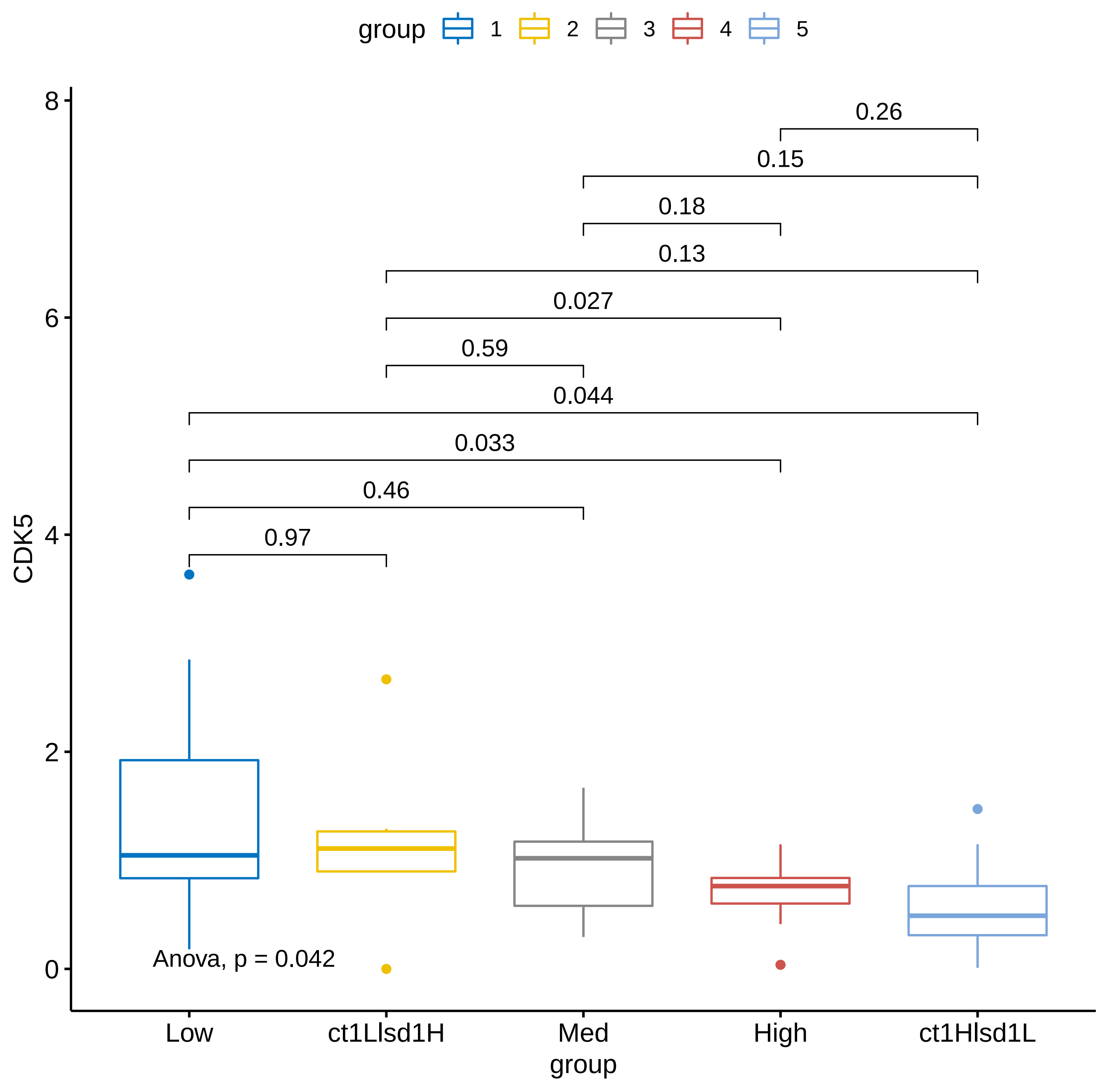

group
1
2
3
4
5
CDK5
8
6
4
2
0
0.26
0.15
0.18
0.13
0.027
0.59
0.044
0.033
0.46
0.97
Anova, p = 0.042
Low
ct1Llsd1H
Med
High
ct1Hlsd1L
group

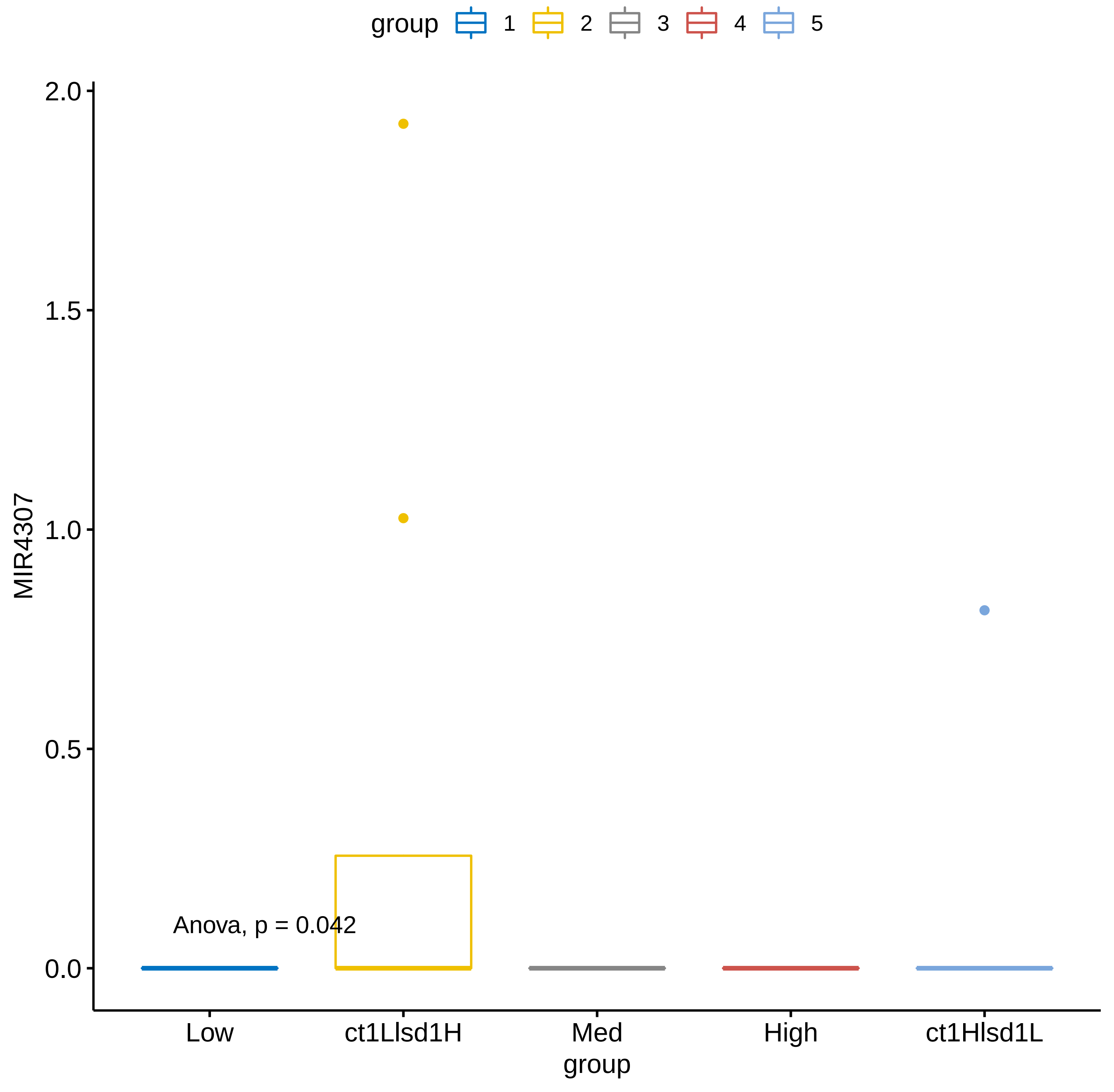

group
1
2
3
4
5
MIR4307
2.0
1.5
1.0
0.5
0.0
Anova, p = 0.042
Low
ct1Llsd1H
Med
High
ct1Hlsd1L
group

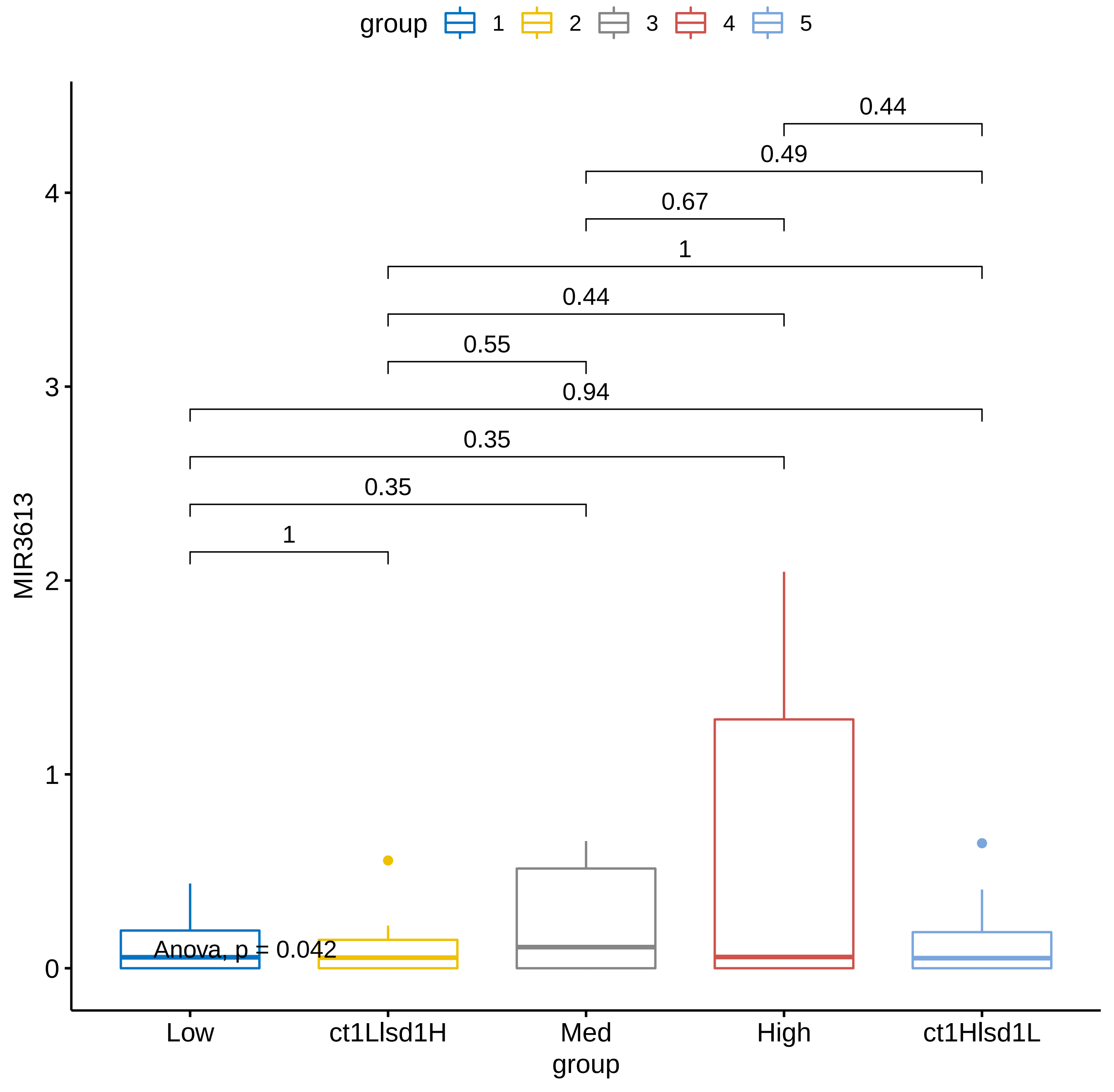

group 1 2 3 4 5
0.44
0.49
0.67
1
0.44
0.55
0.94
0.35
0.35
1
MIR3613
4
3
2
1
0
Anova, p = 0.042
Low
ct1Llsd1H
Med
High
ct1Hlsd1L
group

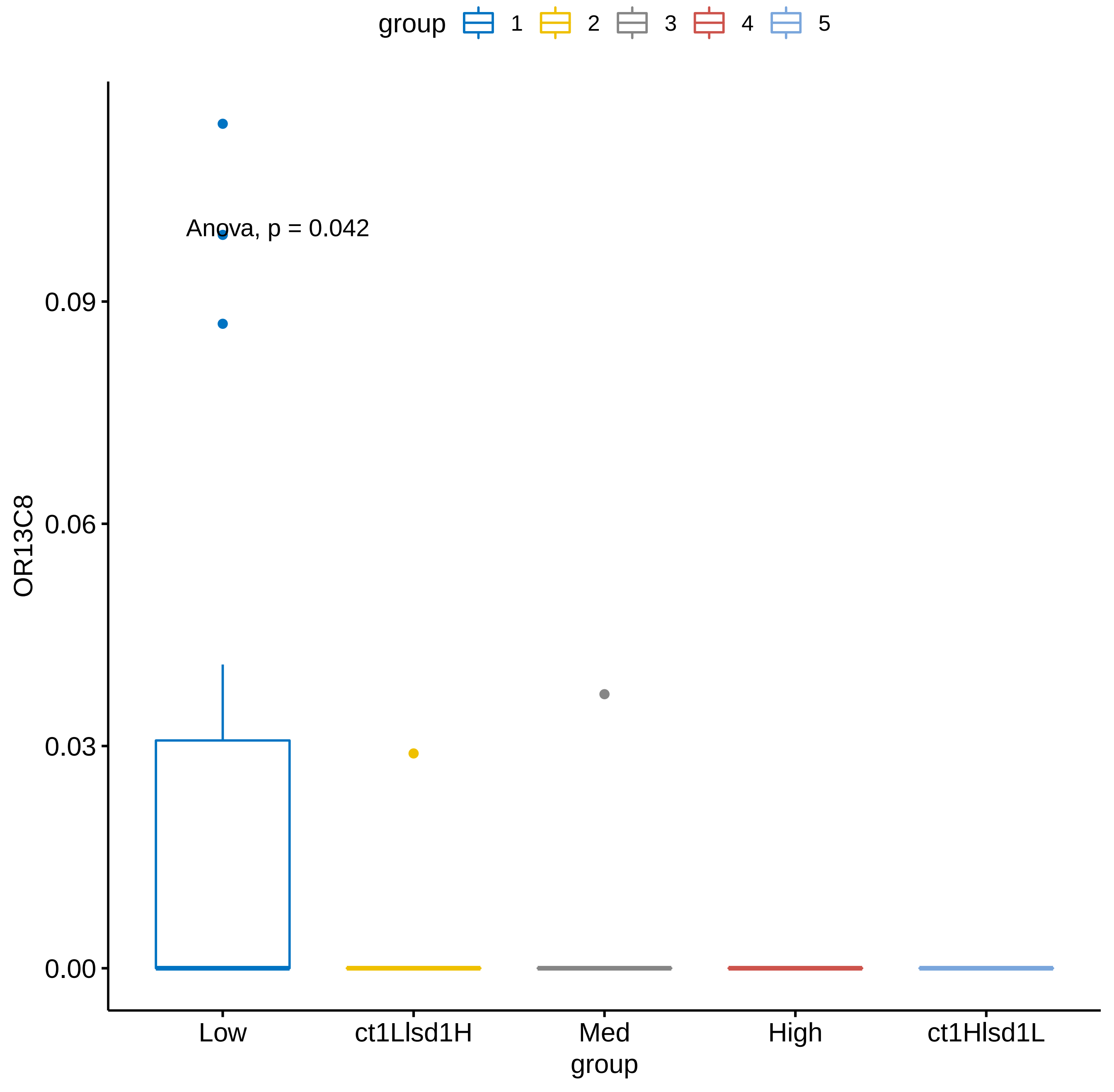

group
1
2
3
4
5
Anova, p = 0.042
OR13C8
0.09
0.06
0.03
0.00
Low
ct1Llsd1H
Med
High
ct1Hlsd1L
group

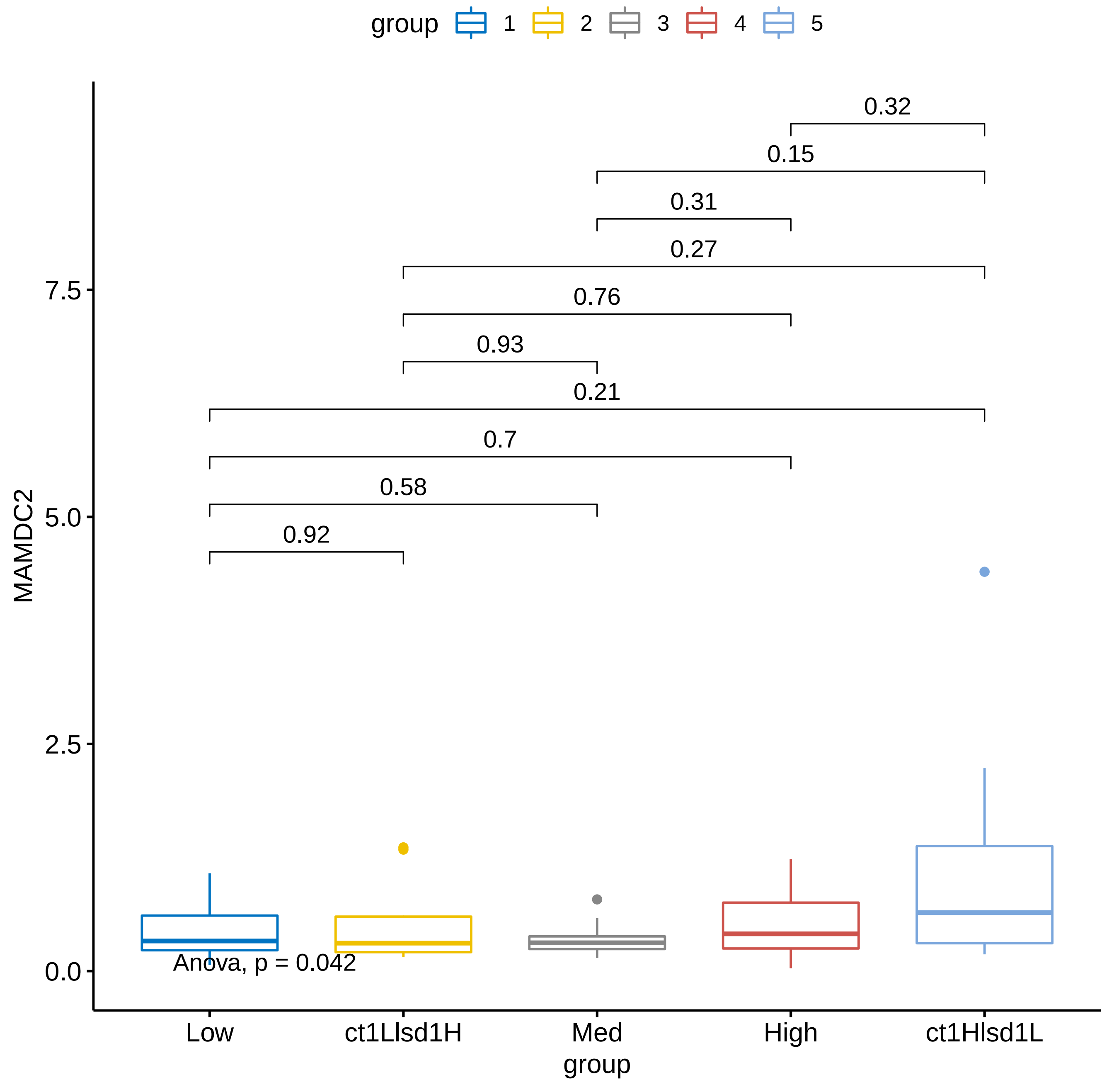

group
1
2
3
4
5
0.32
0.15
0.31
0.27
0.76
0.93
0.21
0.7
0.58
0.92
7.5
5.0
2.5
0.0
MAMDC2
Anova, p = 0.042
Low
ct1Llsd1H
Med
High
ct1Hlsd1L
group

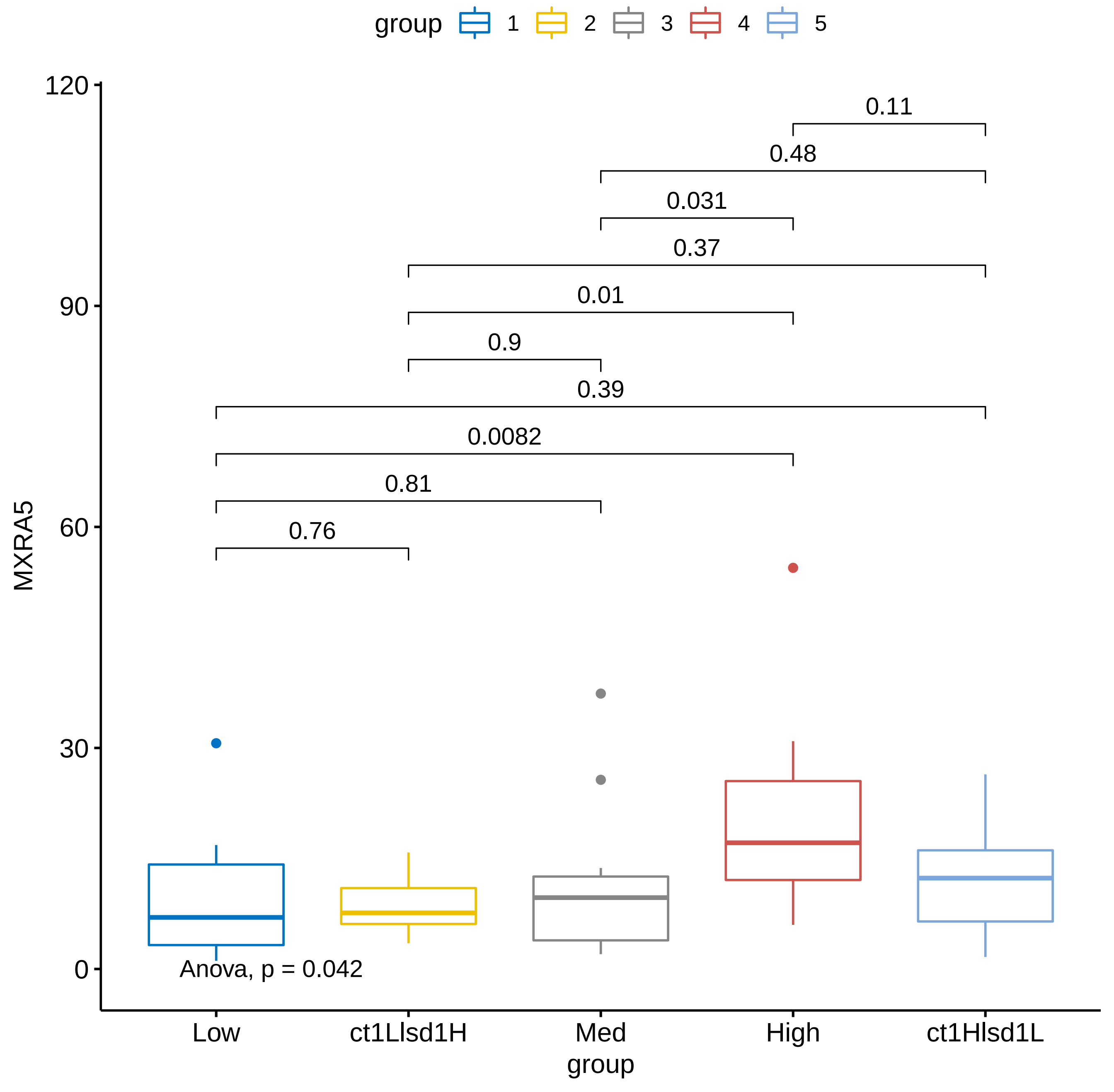

group
1
2
3
4
5
120
90
60
30
0
MXRA5
0.11
0.48
0.031
0.37
0.01
0.9
0.39
0.0082
0.81
0.76
Anova, p = 0.042
Low
ct1Llsd1H
Med
High
ct1Hlsd1L
group

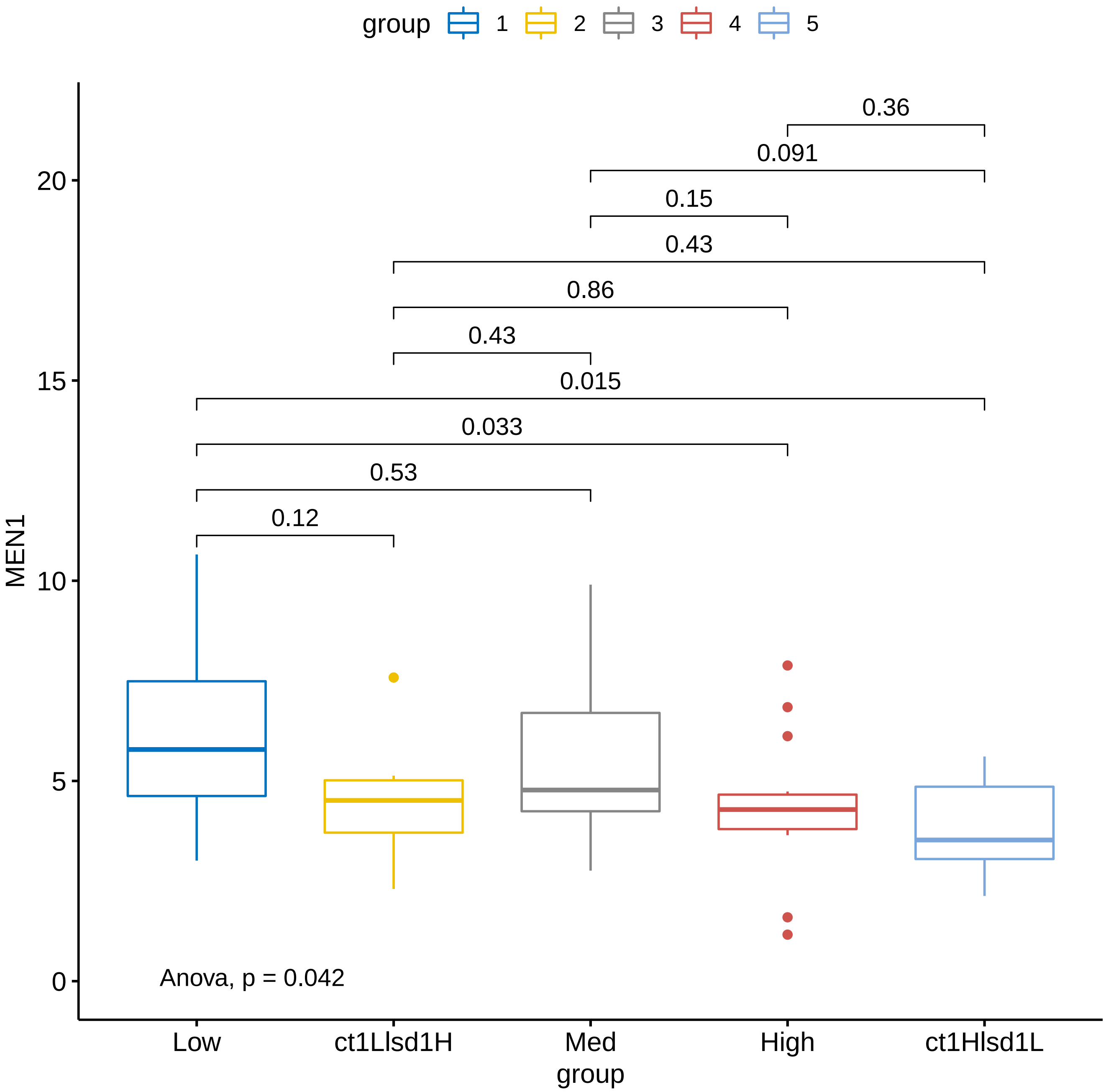
group
1
2
3
4
5
0.36
0.091
0.15
0.43
0.86
0.43
0.015
0.033
0.53
0.12
20
15
10
5
0
MEN1
Anova, p = 0.042
Low
ct1Llsd1H
Med
High
ct1Hlsd1L
group

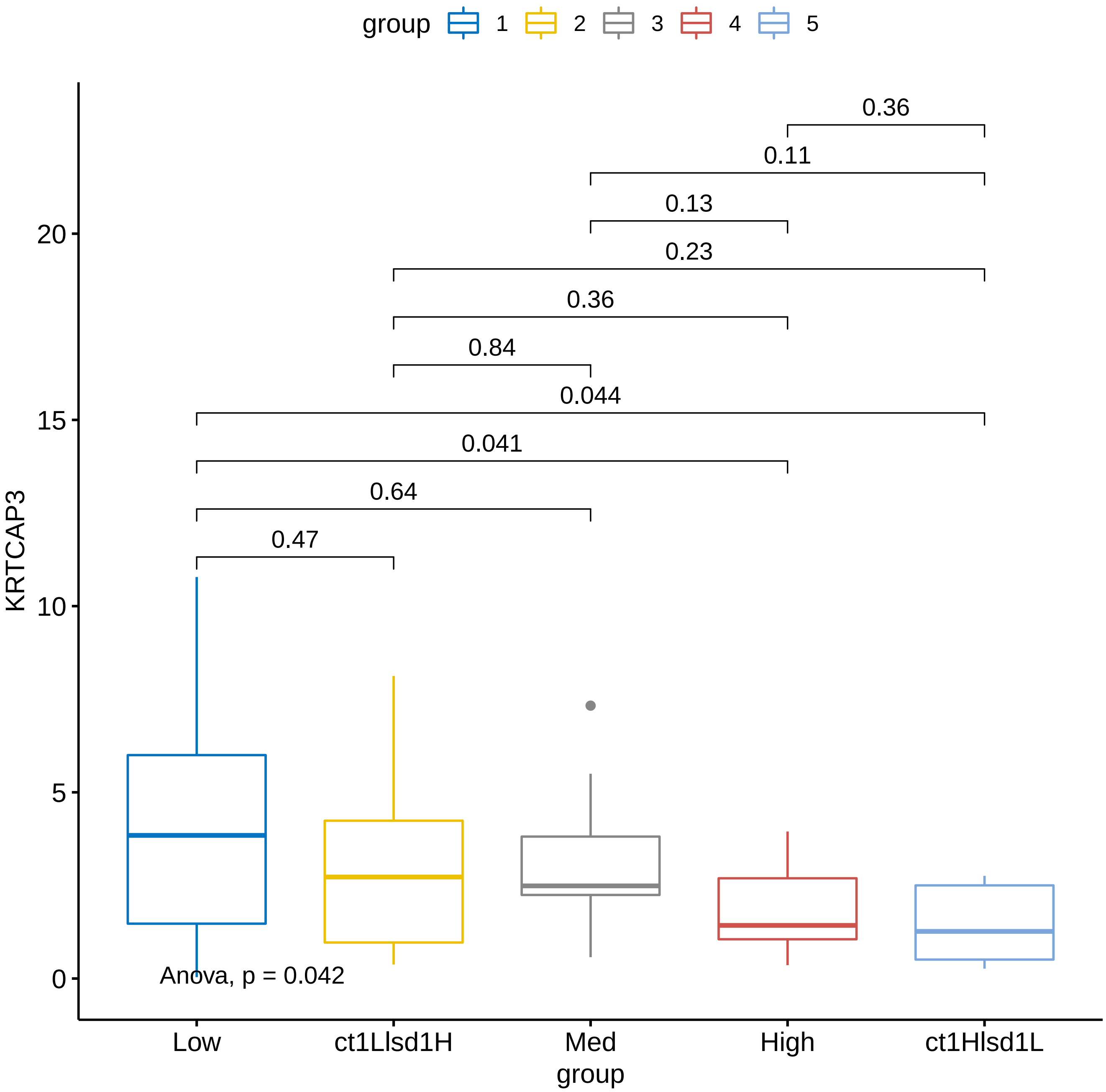

group 1 2 3 4 5
KRTCAP3
0
5
10
15
20
0.36
0.11
0.13
0.23
0.36
0.84
0.044
0.041
0.64
0.47
Anova, p = 0.042
Low
ct1Llsd1H
Med
High
ct1Hlsd1L
group

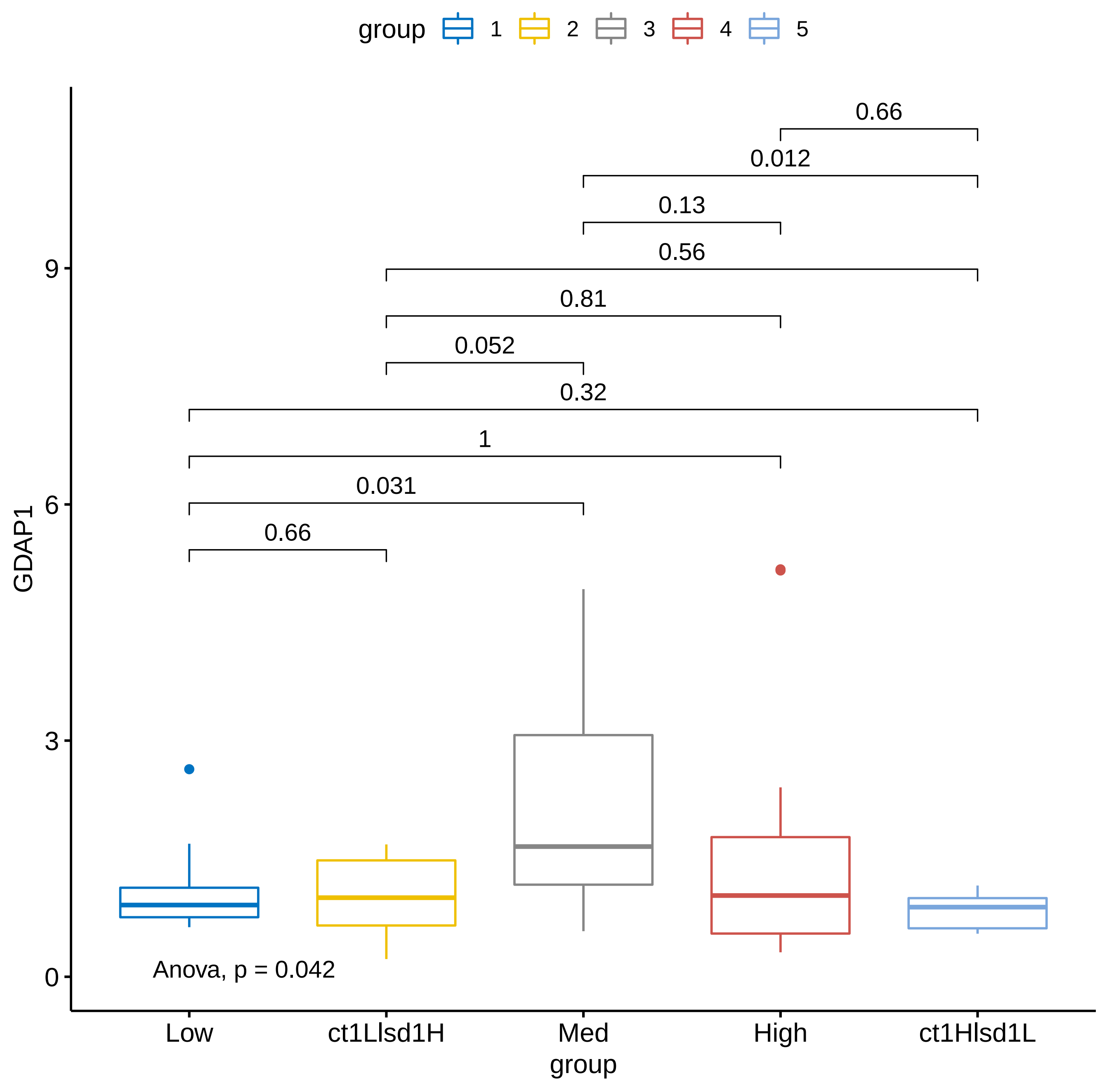
group
1
2
3
4
5
0.66
0.012
0.13
0.56
0.81
0.052
0.32
1
0.031
0.66
9
6
3
0
GDAP1
Anova, p = 0.042
Low
ct1Llsd1H
Med
High
ct1Hlsd1L
group

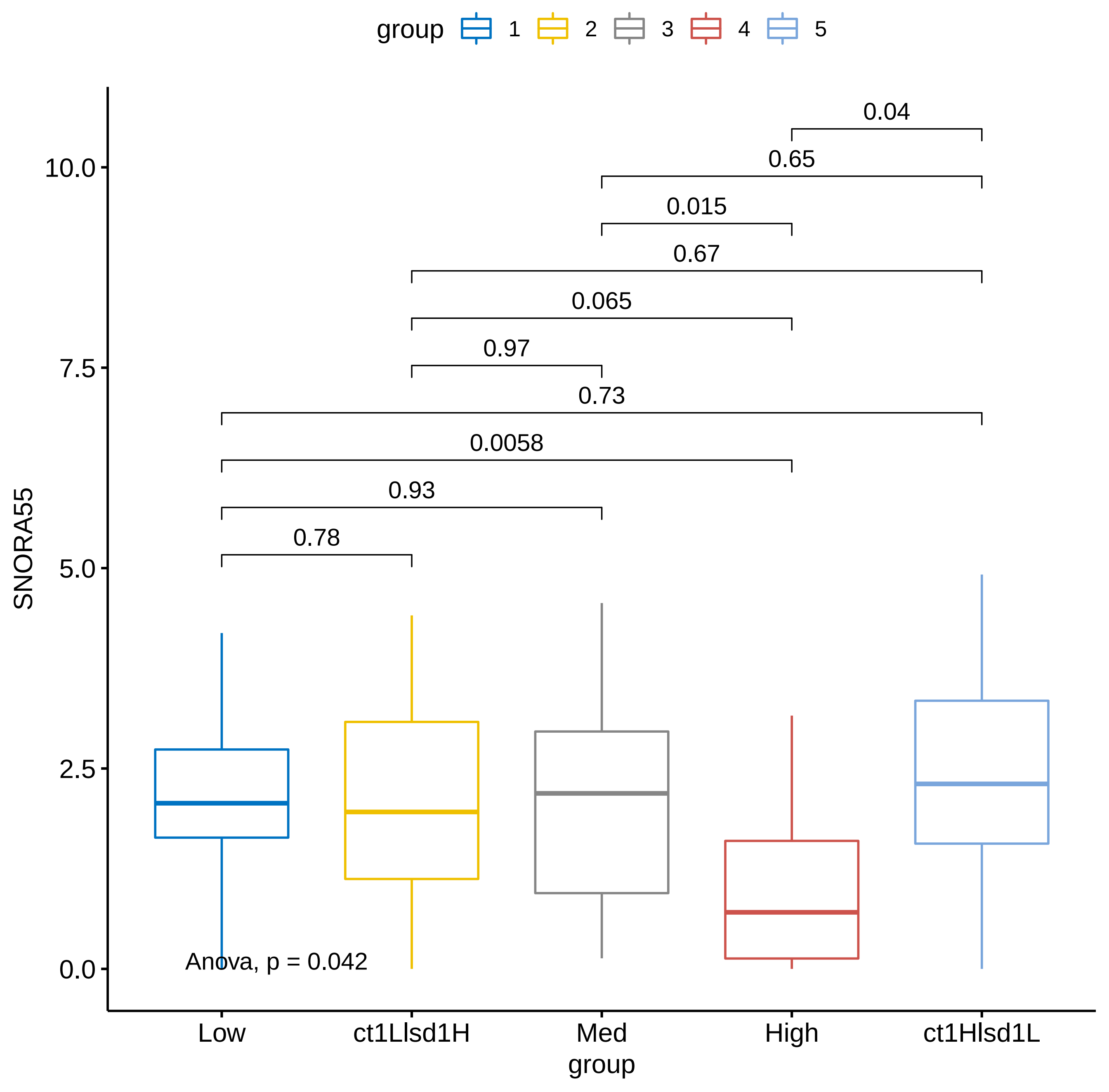

group 1 2 3 4 5
0.04
0.65
0.015
0.67
0.065
0.97
0.73
0.0058
0.93
0.78
10.0
7.5
5.0
2.5
0.0
SNORA55
Anova, p = 0.042
Low
ct1Llsd1H
Med
High
ct1Hlsd1L
group

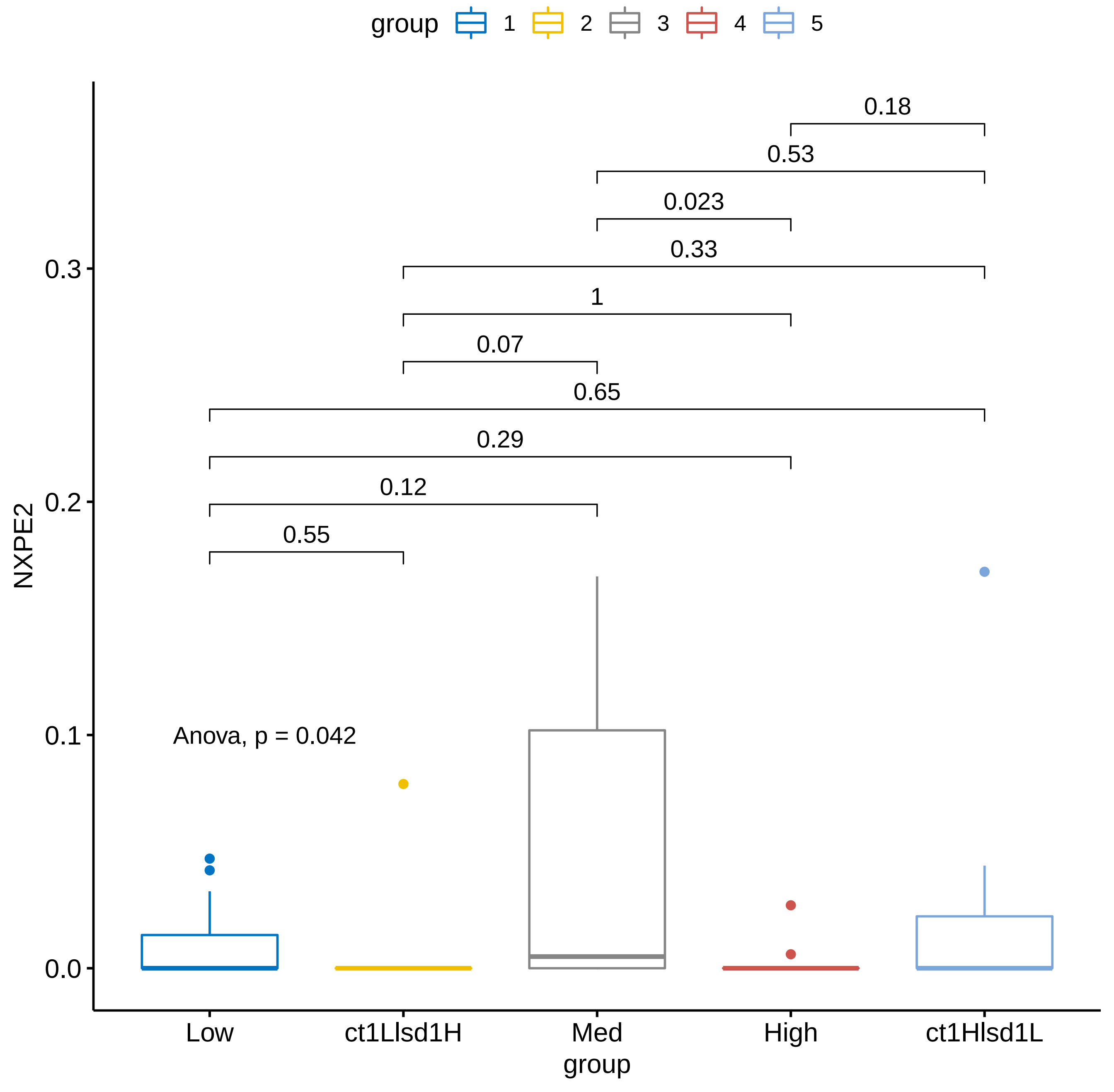
group
1
2
3
4
5
0.18
0.53
0.023
0.33
1
0.07
0.65
0.29
0.12
0.55
Anova, p = 0.042
NXPE2
0.3
0.2
0.1
0.0
Low
ct1Llsd1H
Med
High
ct1Hlsd1L
group

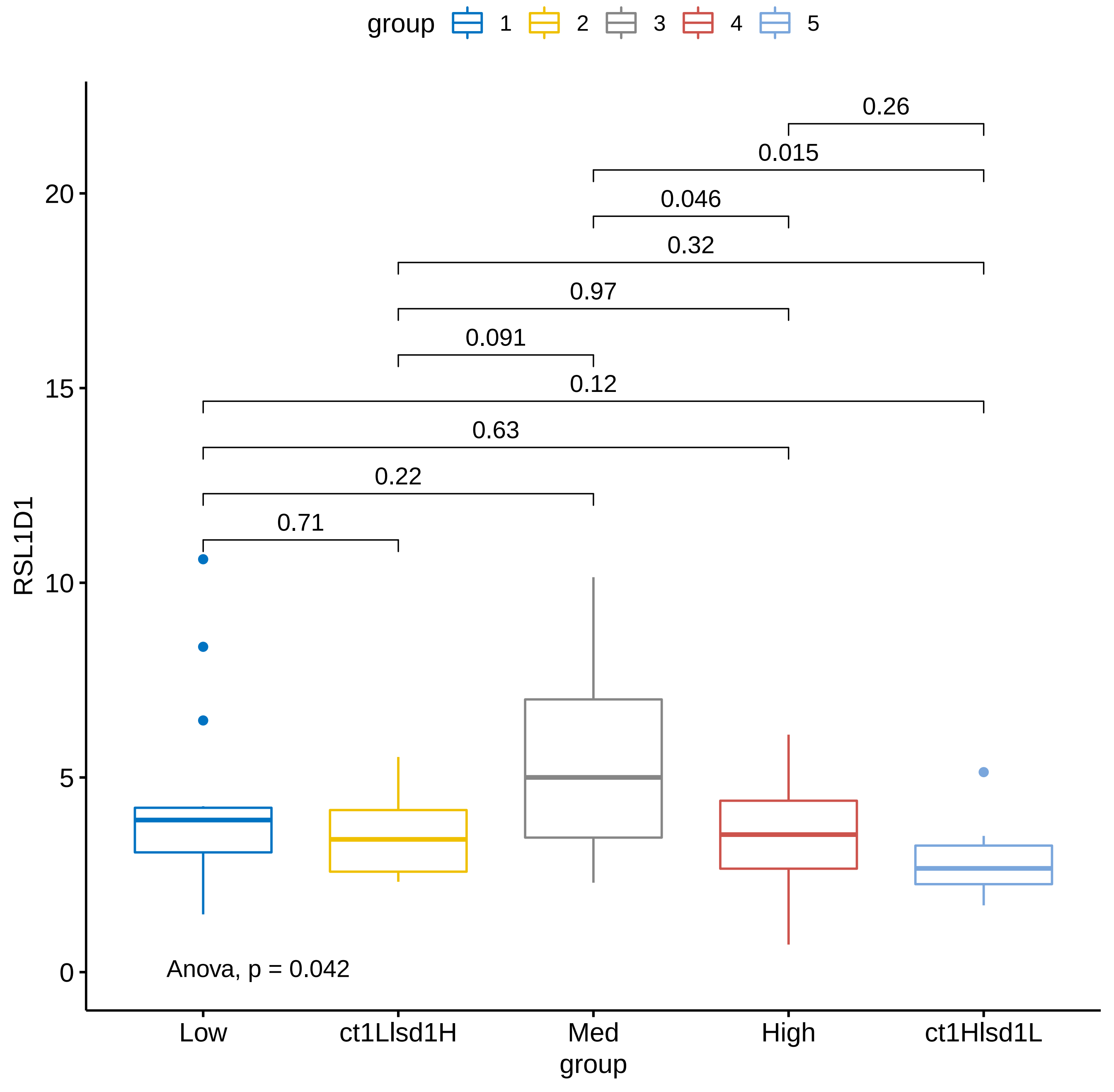
group
1
2
3
4
5
RSL1D1
0.26
0.015
0.046
0.32
0.97
0.091
0.12
0.63
0.22
0.71
20
15
10
5
0
Anova, p = 0.042
Low
ct1Llsd1H
Med
High
ct1Hlsd1L
group

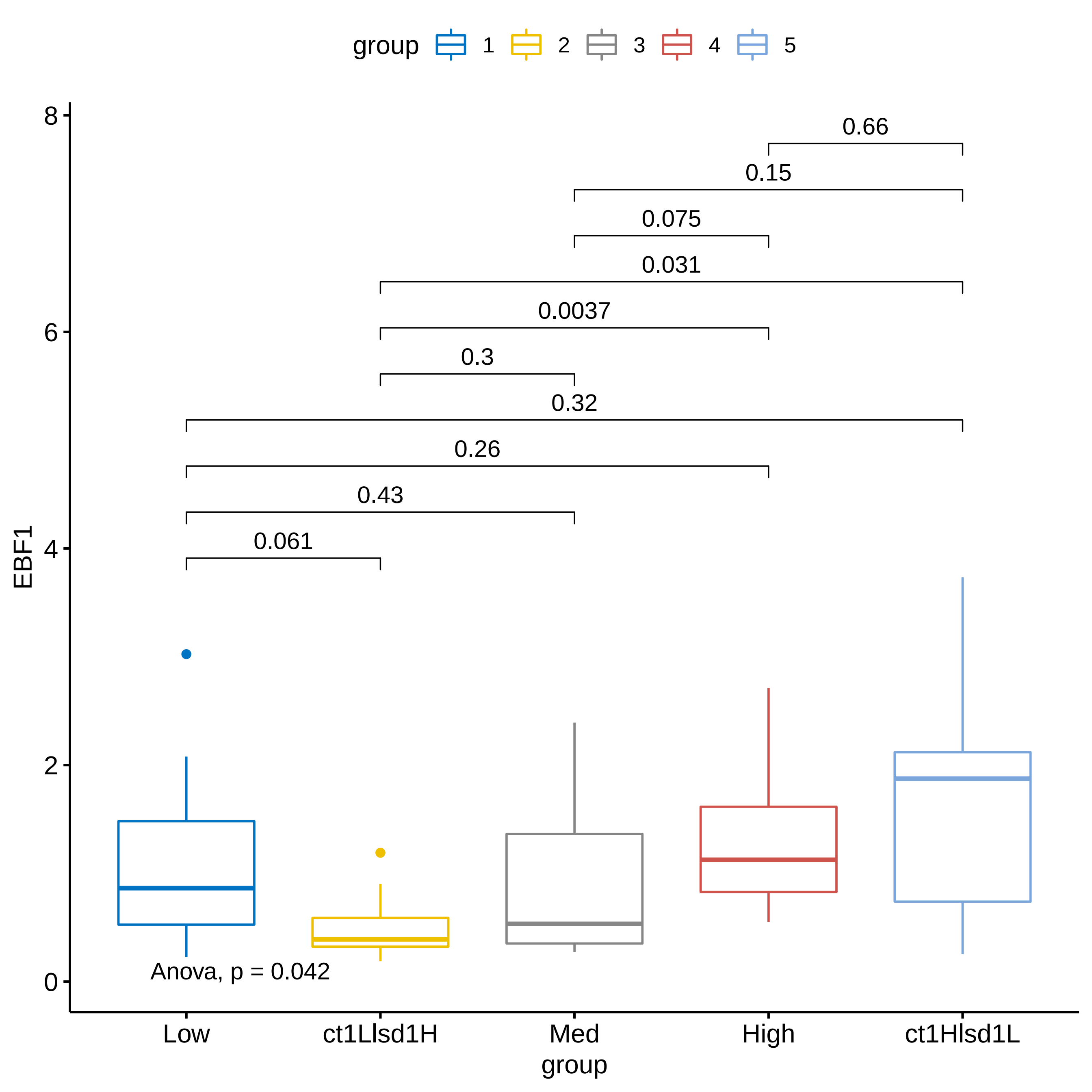

group 1 2 3 4 5
EBF1
0
2
4
6
8
0.66
0.15
0.075
0.031
0.0037
0.3
0.32
0.26
0.43
0.061
Anova, p = 0.042
Low
ct1Llsd1H
Med
High
ct1Hlsd1L
group

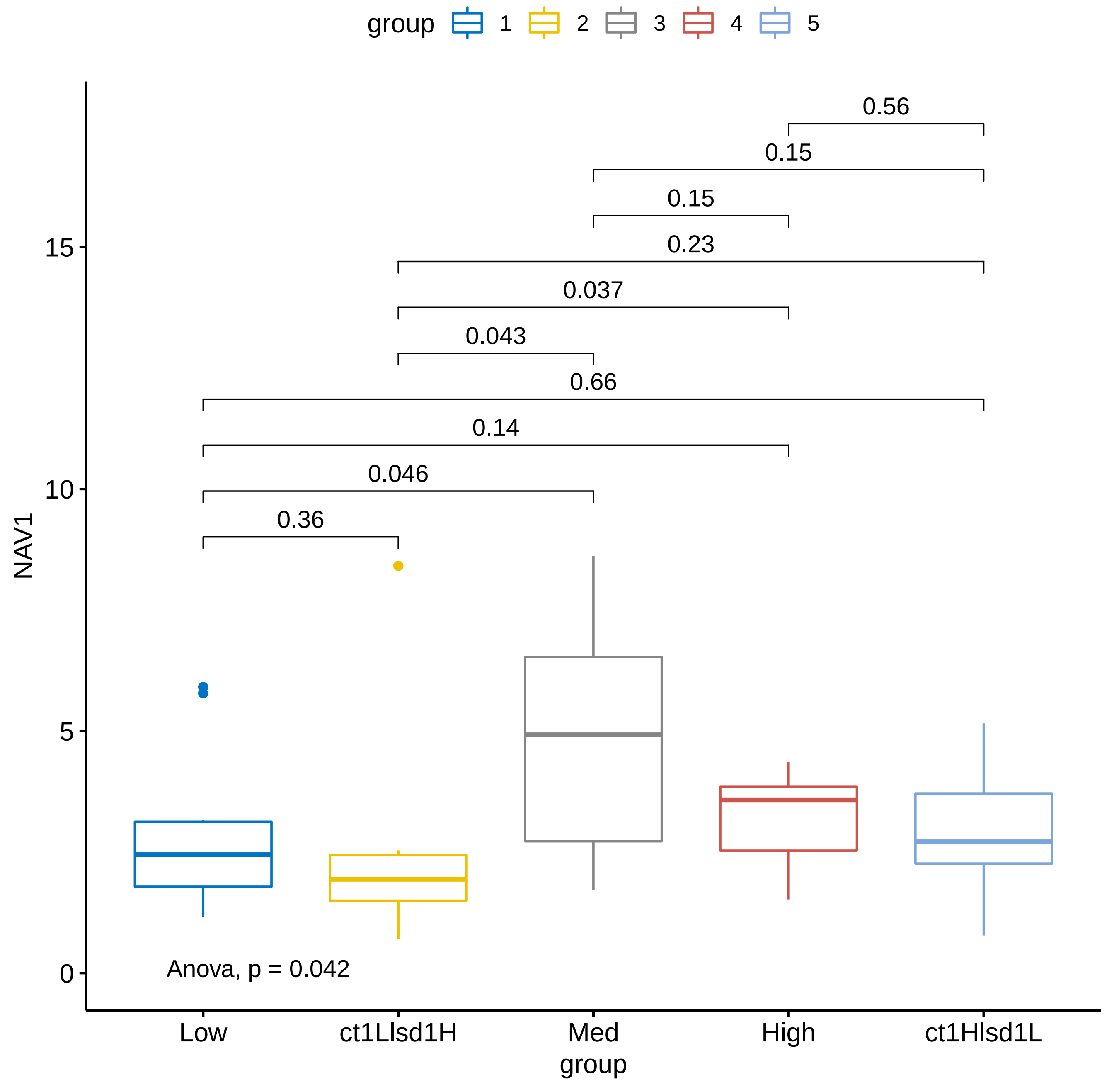

group 1 2 3 4 5
0.56
0.15
0.15
0.23
0.037
0.043
0.66
0.14
0.046
0.36
Anova, p = 0.042
NAV1
0
5
10
15
Low
ct1Llsd1H
Med
High
ct1Hlsd1L
group

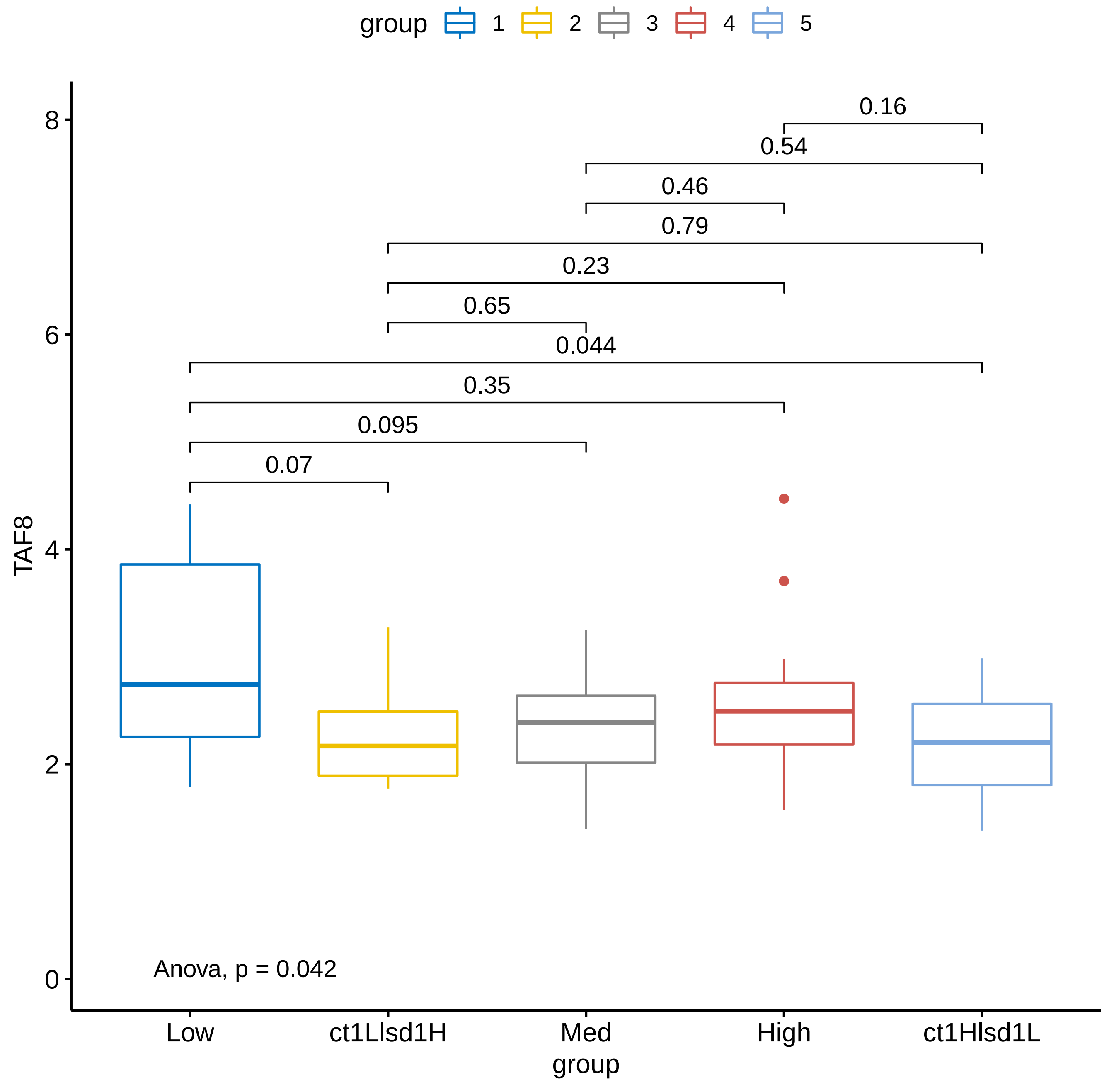

group
1
2
3
4
5
TAF8
8
6
4
2
0
0.16
0.54
0.46
0.79
0.23
0.65
0.044
0.35
0.095
0.07
Anova, p = 0.042
Low
ct1Llsd1H
Med
High
ct1Hlsd1L
group

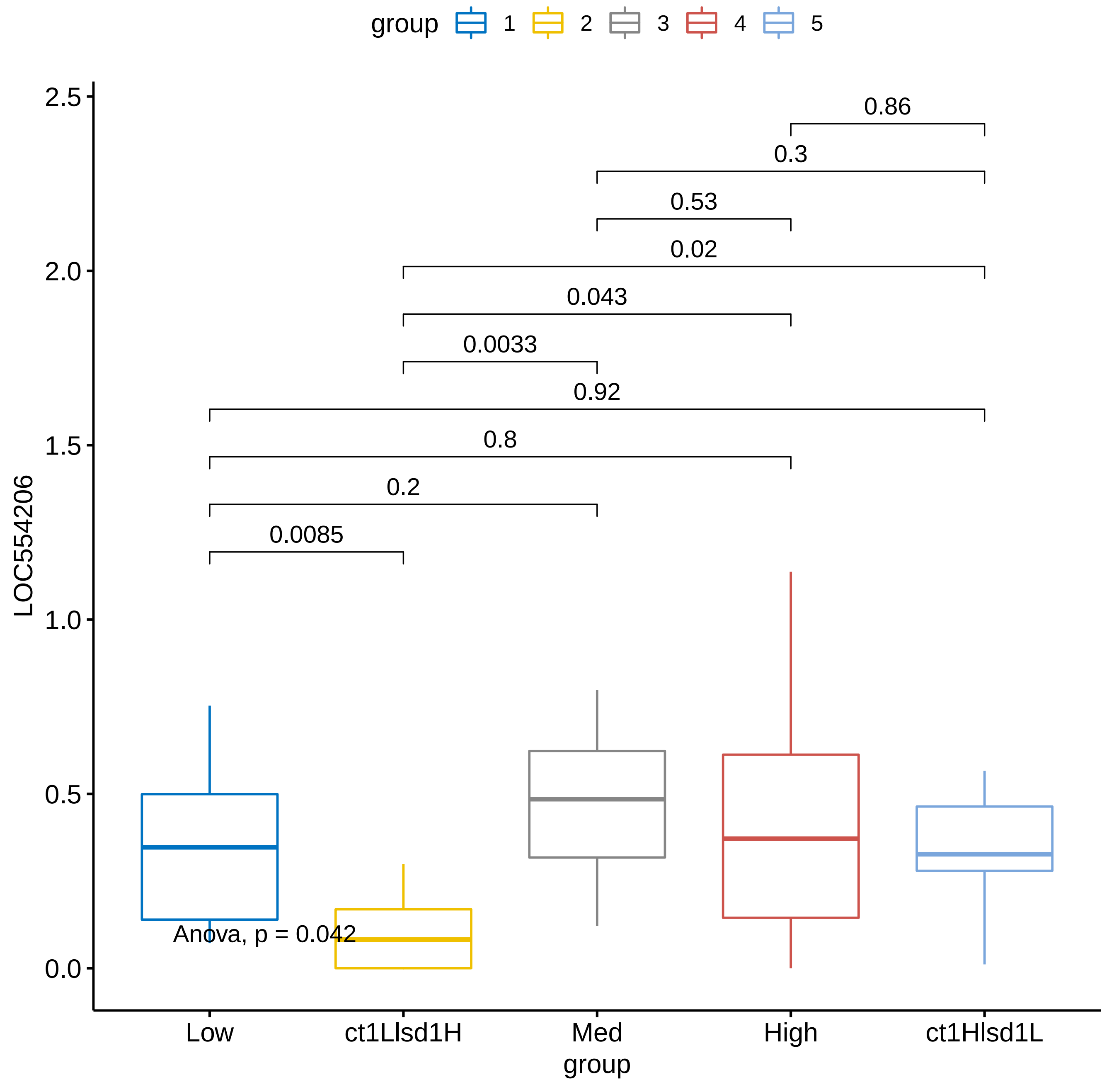
group
1
2
3
4
5
0.86
0.3
0.53
0.02
0.043
0.0033
0.92
0.8
0.2
0.0085
Anova, p = 0.042
2.5
2.0
1.5
1.0
0.5
0.0
LOC554206
Low
ct1Llsd1H
Med
High
ct1Hlsd1L
group

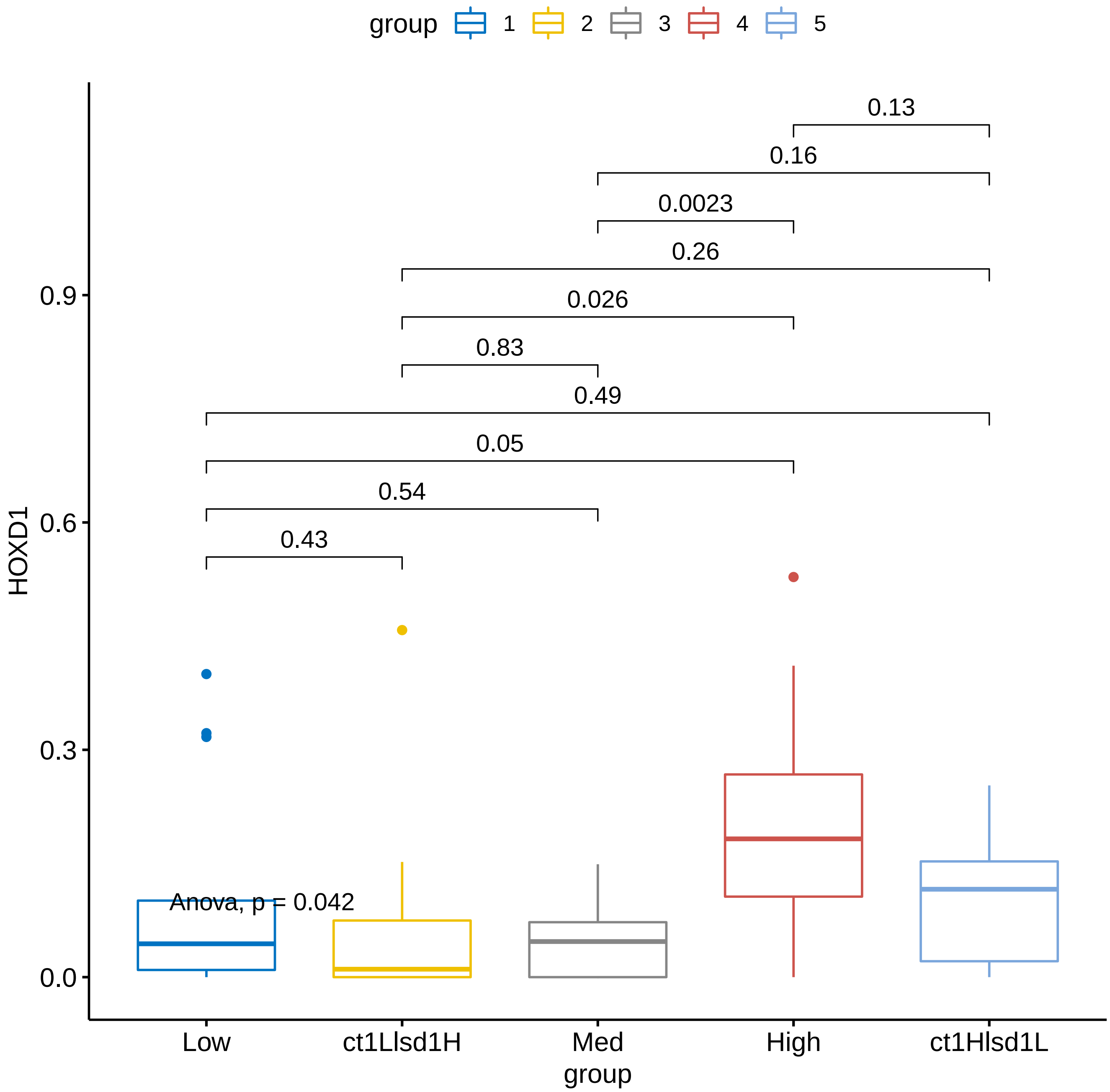

group 1 2 3 4 5
0.13
0.16
0.0023
0.26
0.026
0.83
0.49
0.05
0.54
0.43
Anova, p = 0.042
HOXD1
0.9
0.6
0.3
0.0
Low
ct1Llsd1H
Med
High
ct1Hlsd1L
group

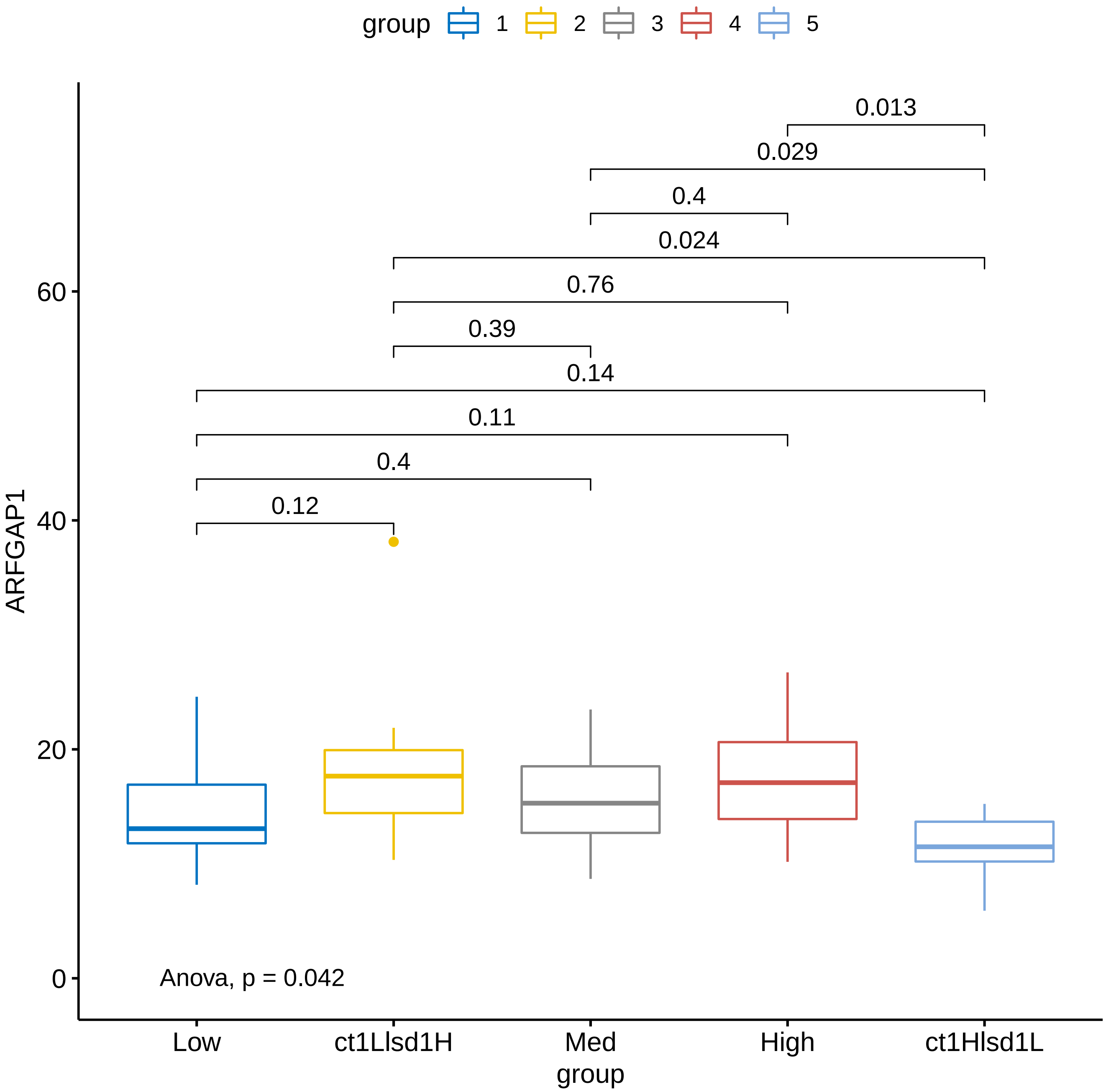
group
1
2
3
4
5
0.013
0.029
0.4
0.024
0.76
0.39
0.14
0.11
0.4
0.12
ARFGAP1
60
40
20
0
Anova, p = 0.042
Low
ct1Llsd1H
Med
High
ct1Hlsd1L
group

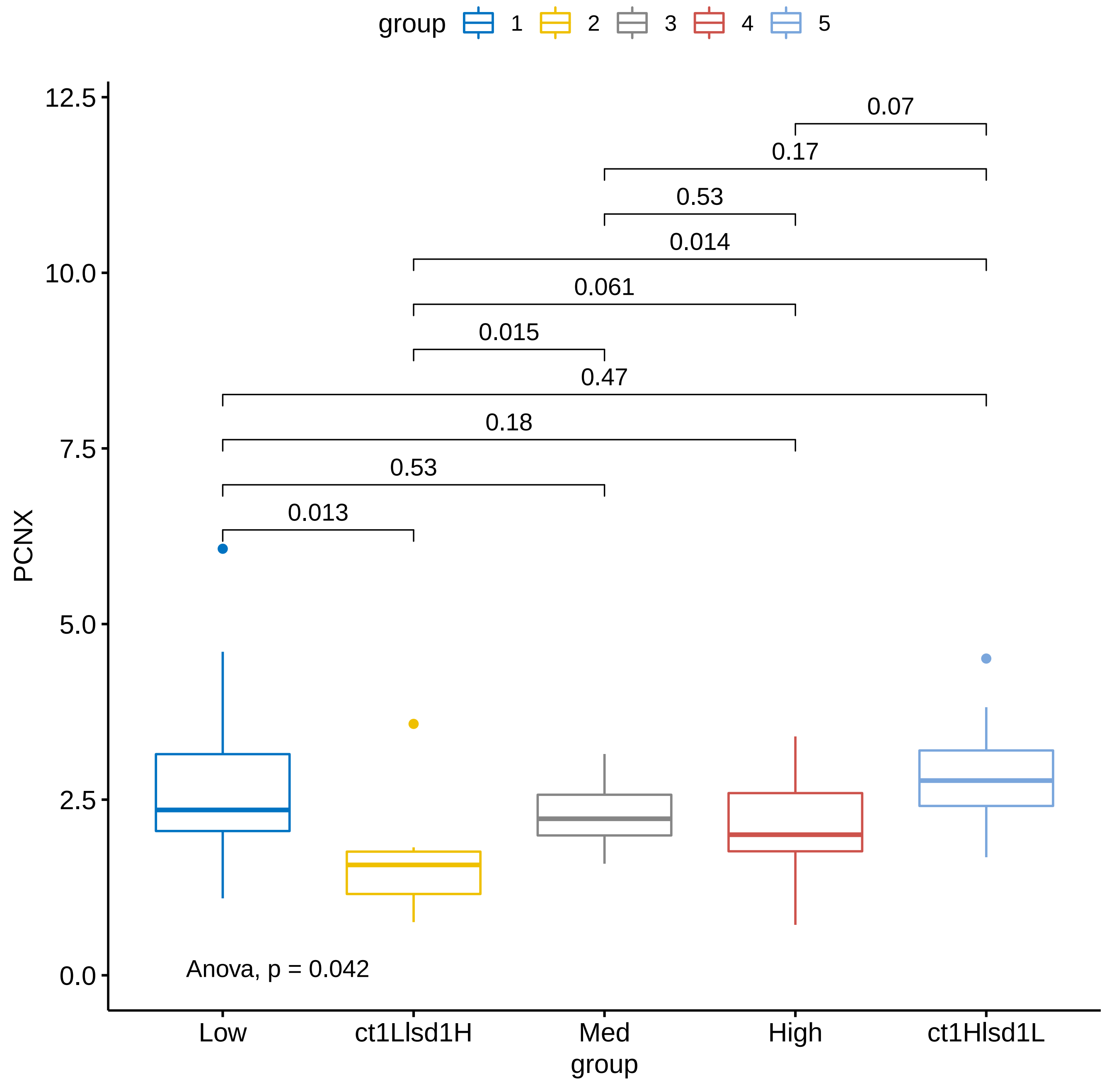

group
1
2
3
4
5
PCNX
12.5
10.0
7.5
5.0
2.5
0.0
0.07
0.17
0.53
0.014
0.061
0.015
0.47
0.18
0.53
0.013
Anova, p = 0.042
Low
ct1Llsd1H
Med
High
ct1Hlsd1L
group

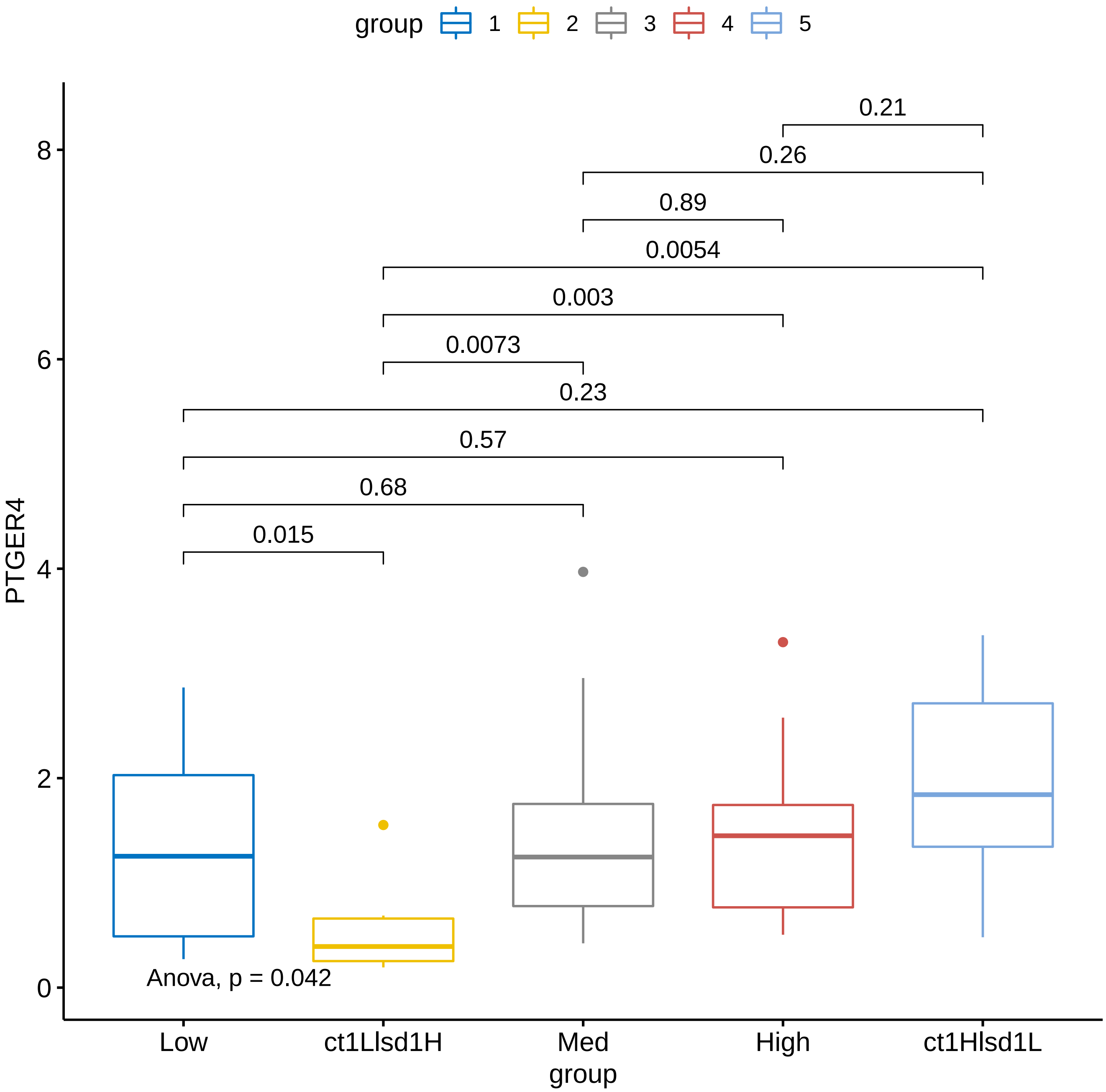

group
1
2
3
4
5
PTGER4
0
2
4
6
8
0.21
0.26
0.89
0.0054
0.003
0.0073
0.23
0.57
0.68
0.015
Anova, p = 0.042
Low
ct1Llsd1H
Med
High
ct1Hlsd1L
group

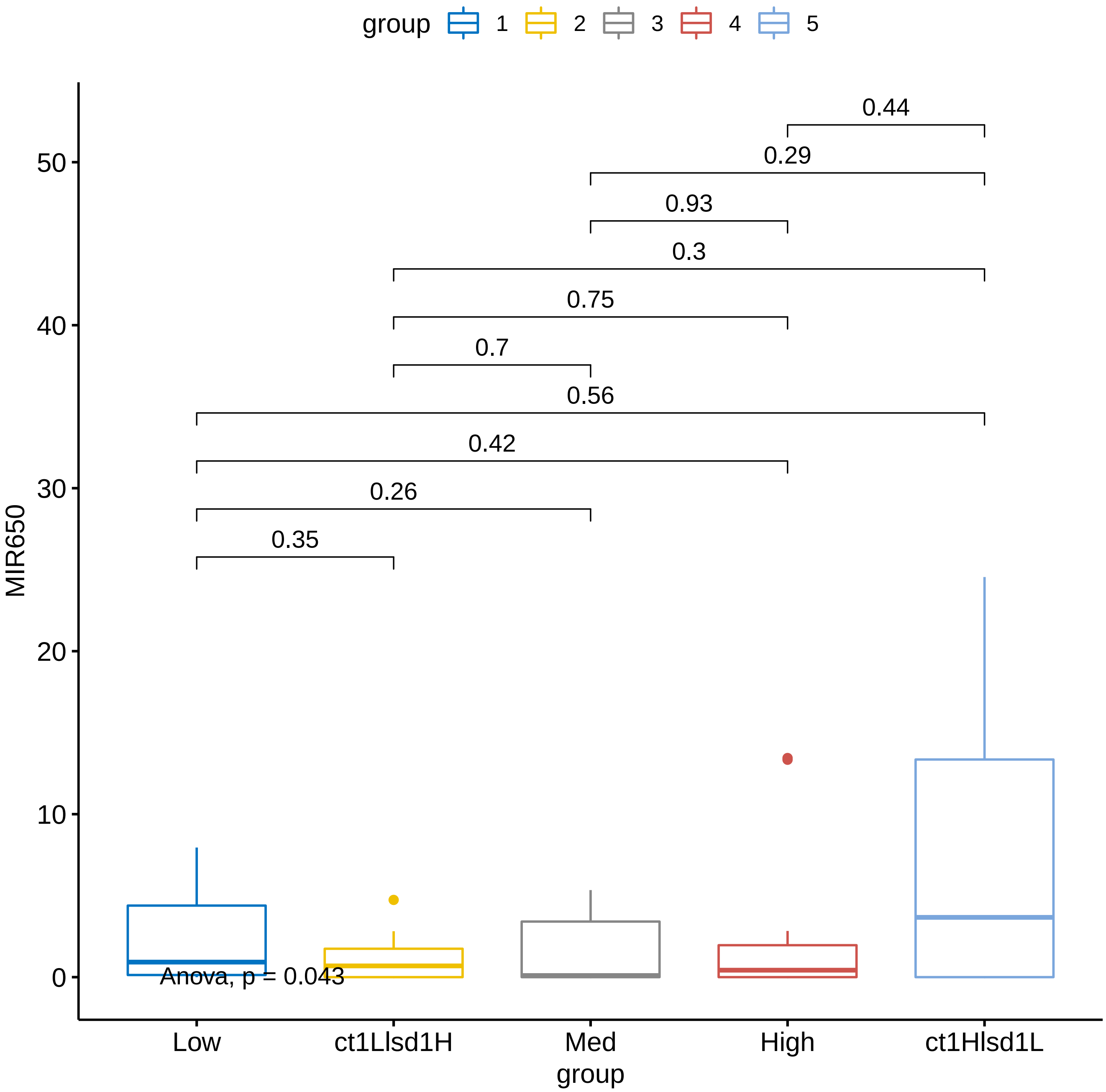

group 1 2 3 4 5
0.44
0.29
0.93
0.3
0.75
0.7
0.56
0.42
0.26
0.35
MIR650
50
40
30
20
10
0
Anova, p = 0.043
Low
ct1Llsd1H
Med
High
ct1Hlsd1L
group

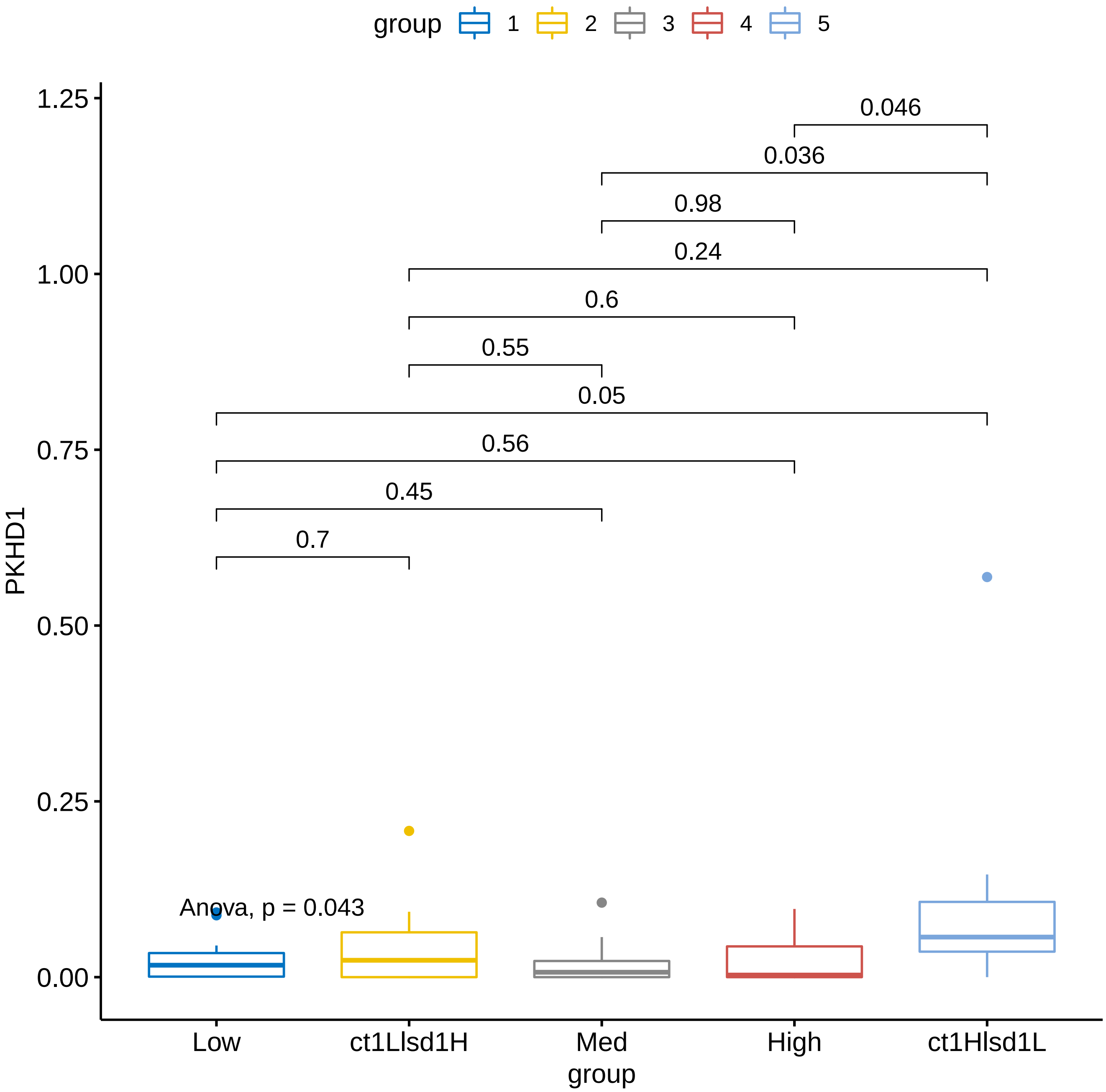

group
1
2
3
4
5
PKHD1
1.25
1.00
0.75
0.50
0.25
0.00
0.046
0.036
0.98
0.24
0.6
0.55
0.05
0.56
0.45
0.7
Anova, p = 0.043
Low
ct1Llsd1H
Med
High
ct1Hlsd1L
group

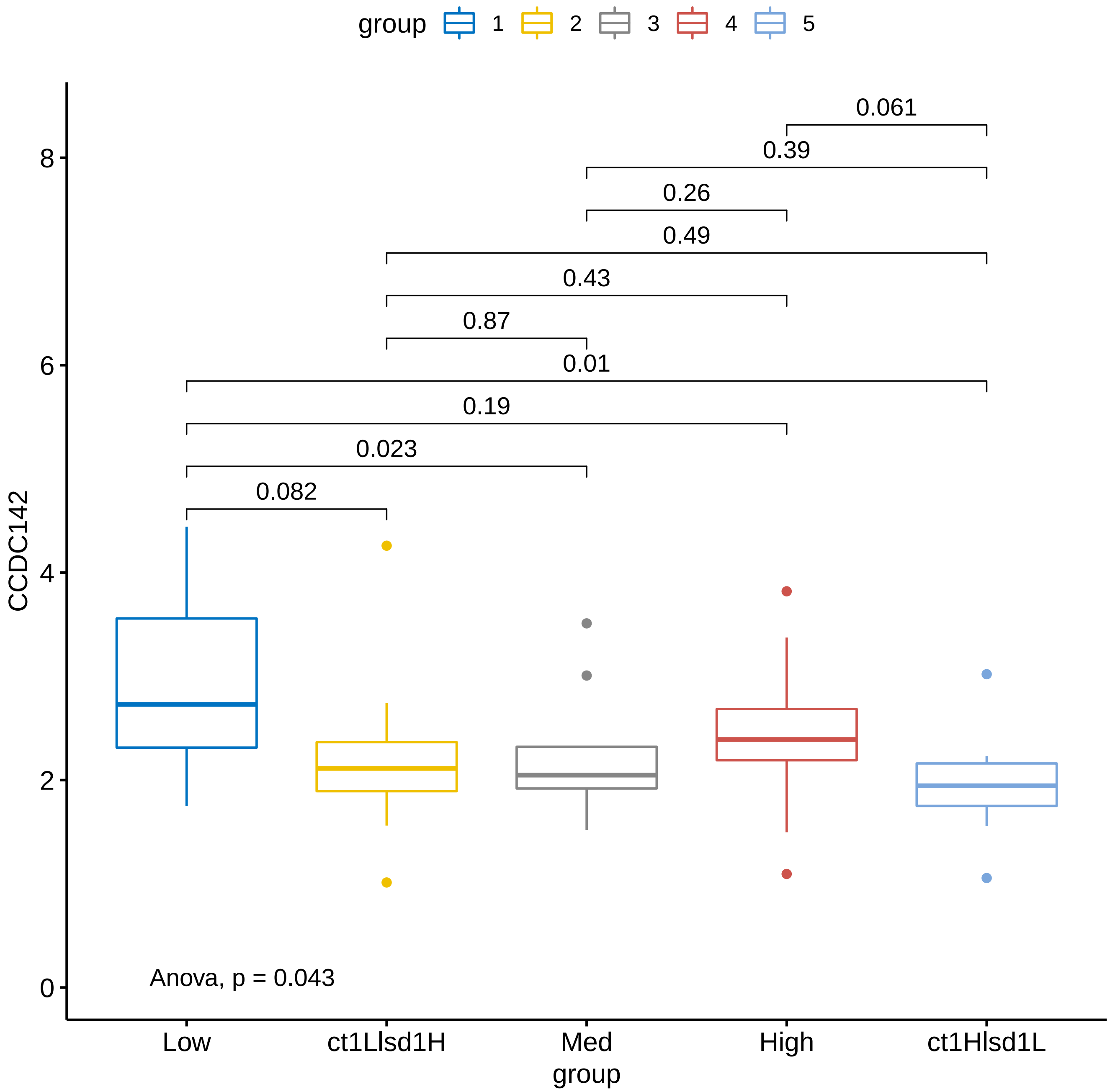

group 1 2 3 4 5
0.061
0.39
0.26
0.49
0.43
0.87
0.01
0.19
0.023
0.082
CCDC142
8
6
4
2
0
Anova, p = 0.043
Low
ct1Llsd1H
Med
High
ct1Hlsd1L
group

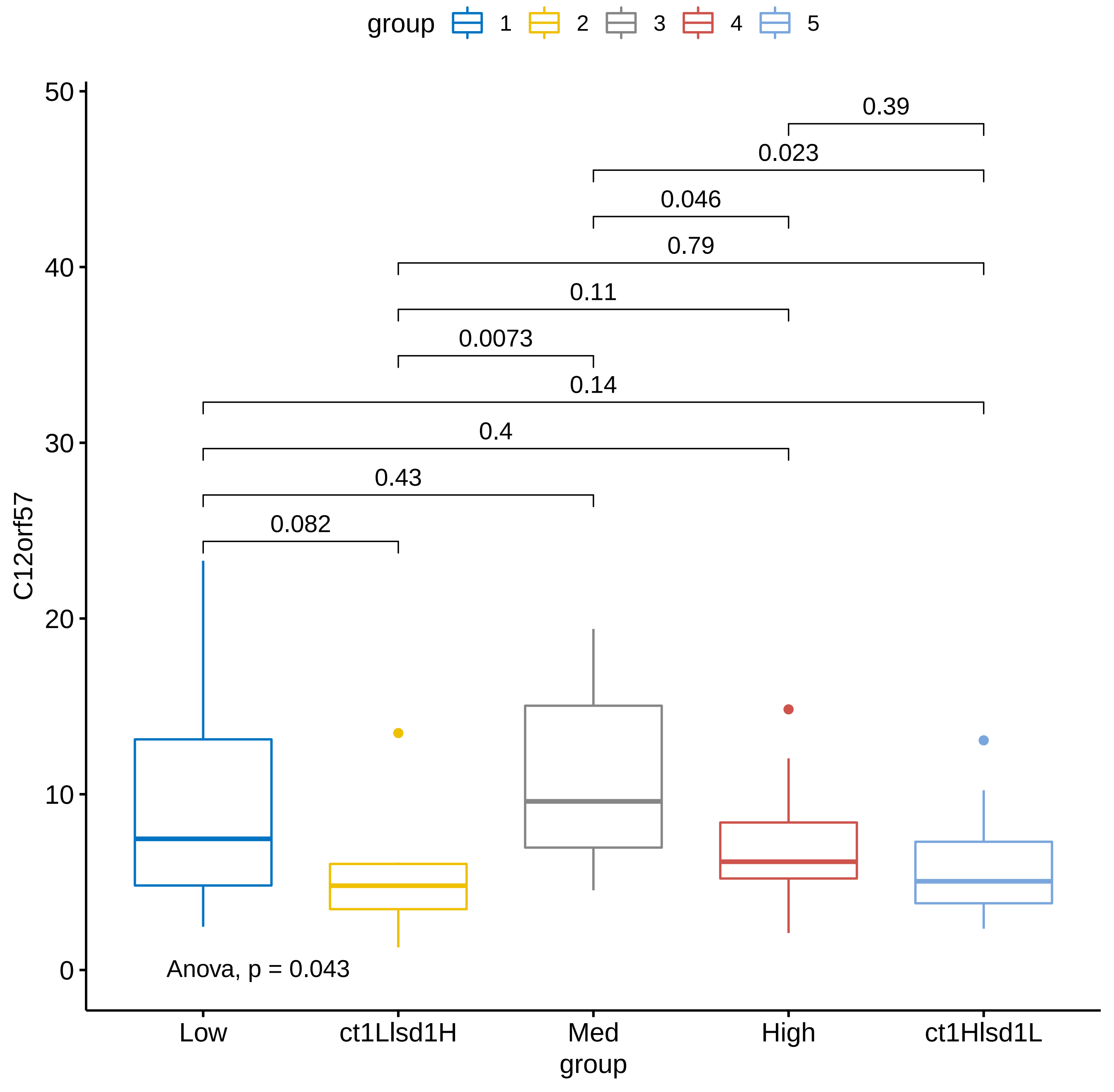

group 1 2 3 4 5
C12orf57
50
40
30
20
10
0
0.39
0.023
0.046
0.79
0.11
0.0073
0.14
0.4
0.43
0.082
Anova, p = 0.043
Low
ct1Llsd1H
Med
High
ct1Hlsd1L
group

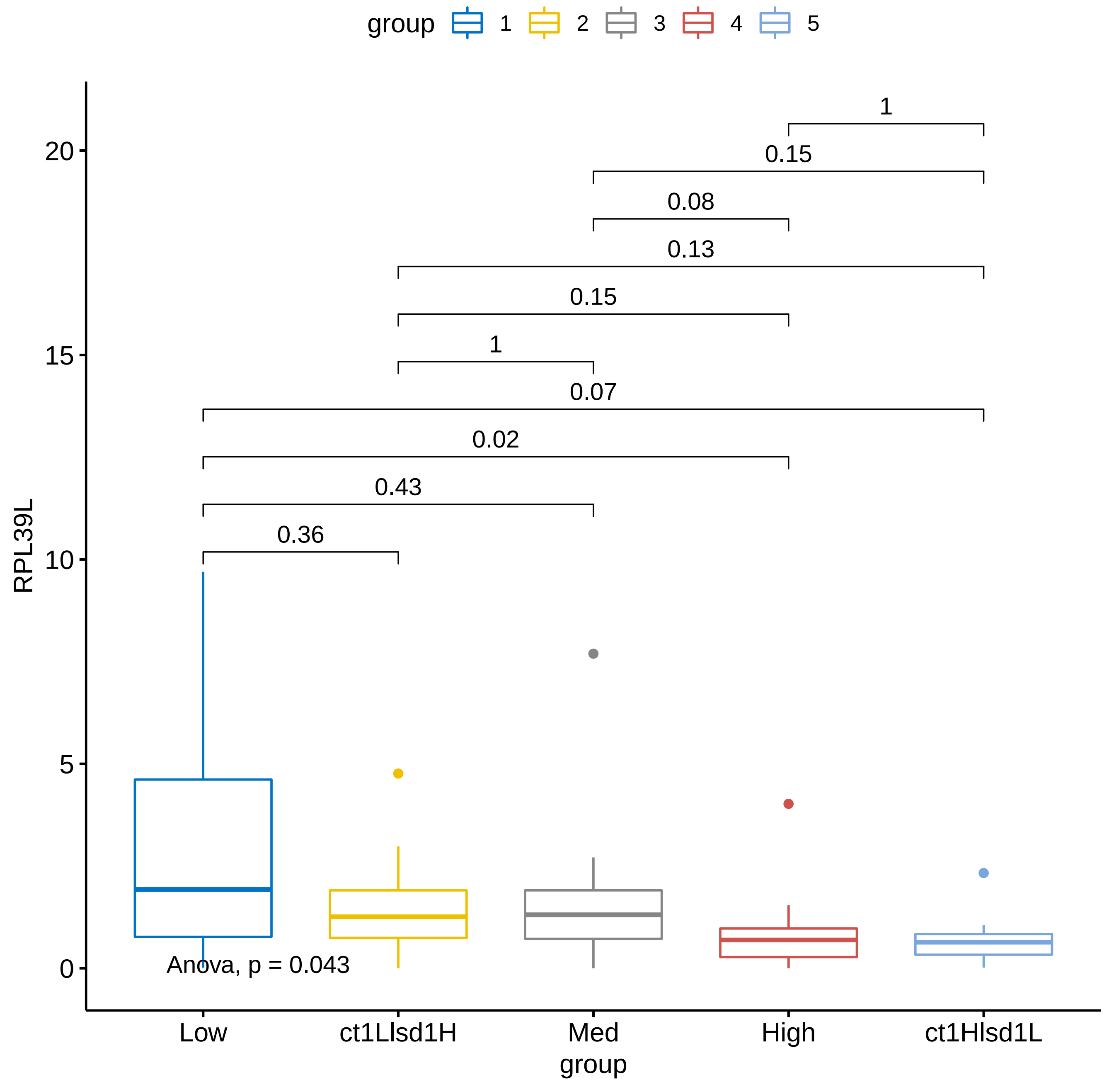
group
1
2
3
4
5
RPL39L
20
15
10
5
0
1
0.15
0.08
0.13
0.15
1
0.07
0.02
0.43
0.36
Anova, p = 0.043
Low
ct1Llsd1H
Med
High
ct1Hlsd1L
group

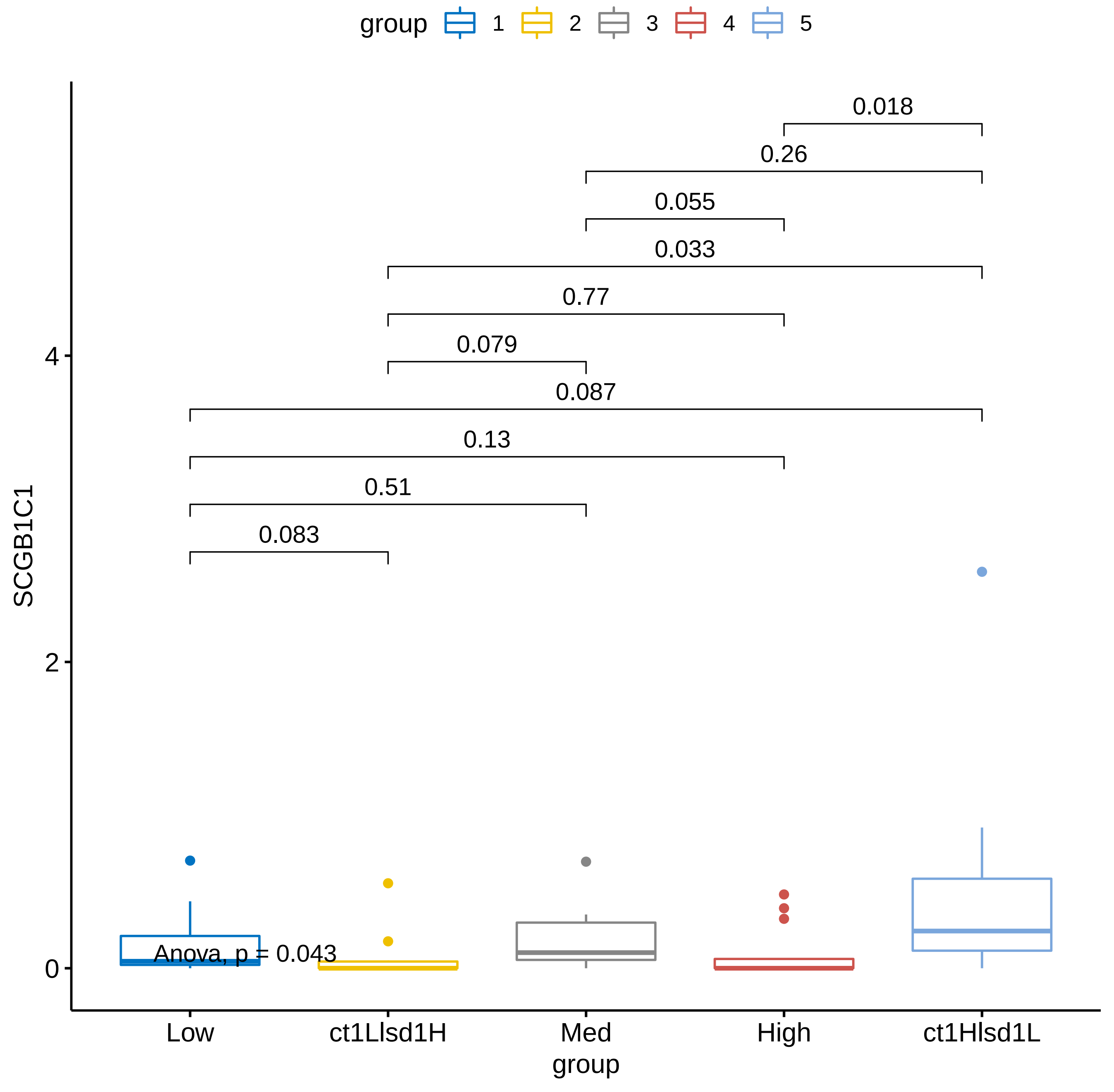
group 1 2 3 4 5
0.018
0.26
0.055
0.033
0.77
0.079
0.087
0.13
0.51
0.083
Anova, p = 0.043
SCGB1C1
0
2
4
Low
ct1Llsd1H
Med
High
ct1Hlsd1L
group

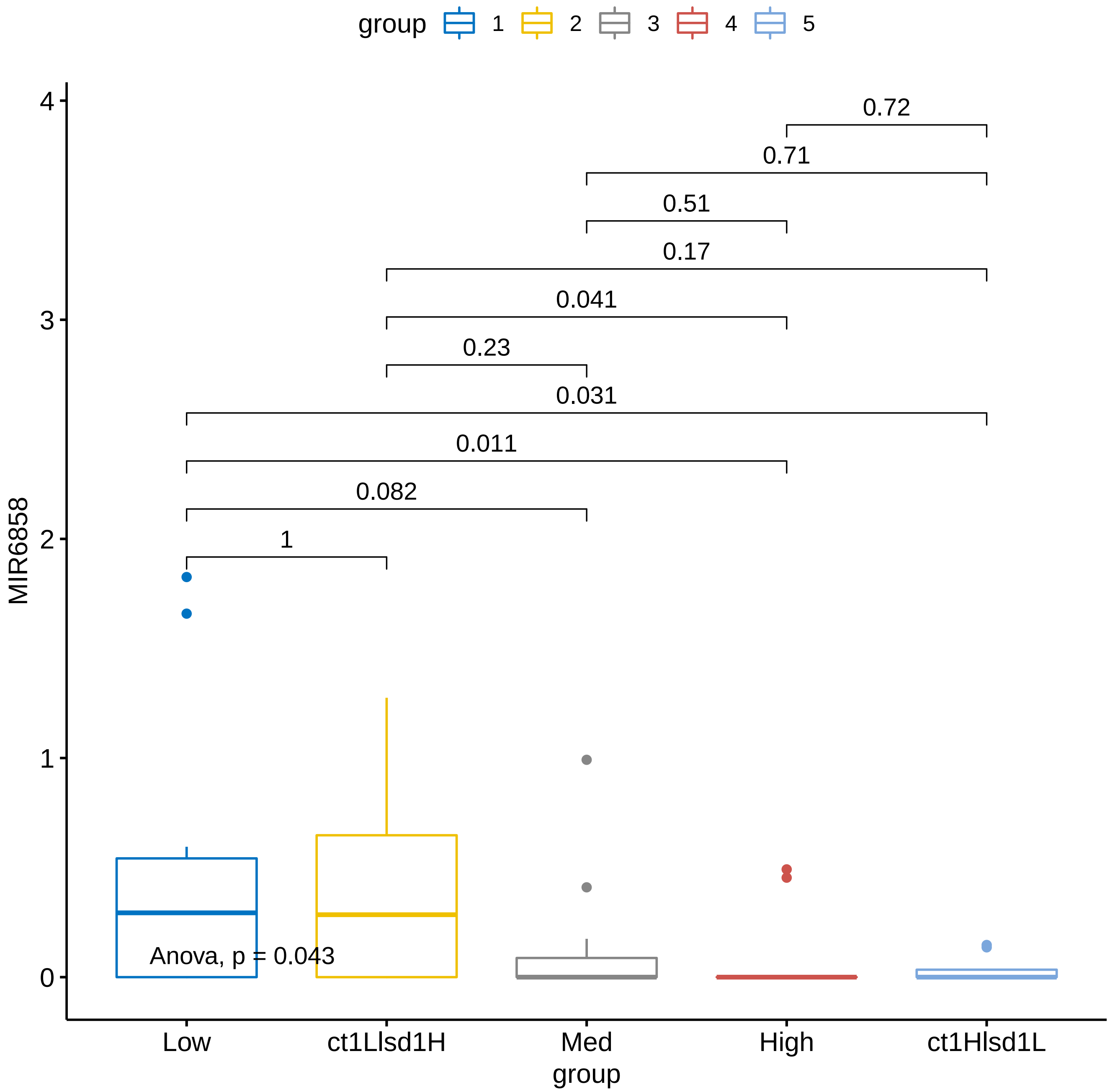

group 1 2 3 4 5
0.72
0.71
0.51
0.17
0.041
0.23
0.031
0.011
0.082
1
Anova, p = 0.043
MIR6858
4
3
2
1
0
Low
ct1Llsd1H
Med
High
ct1Hlsd1L
group

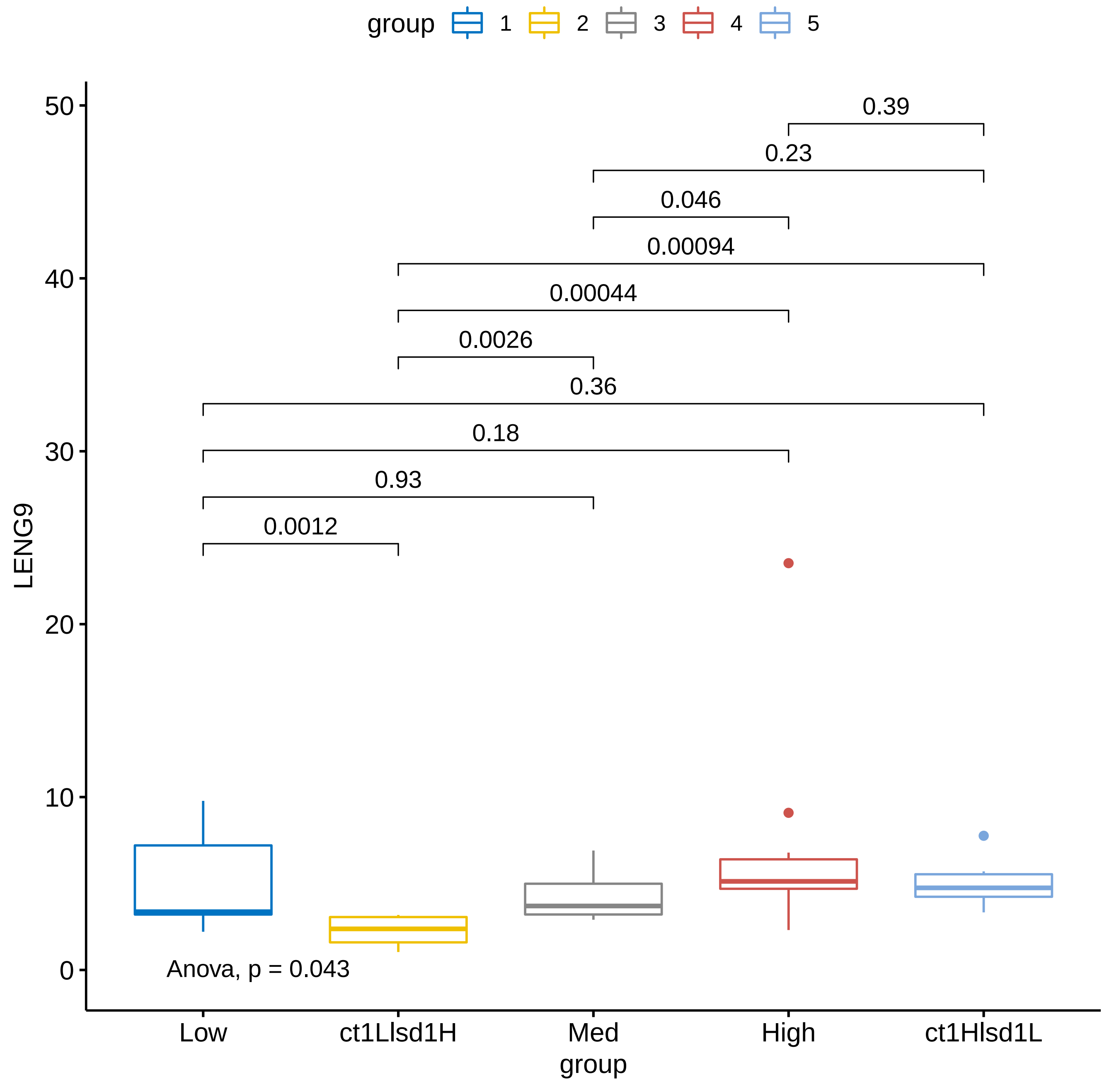
group
1
2
3
4
5
0.39
0.23
0.046
0.00094
0.00044
0.0026
0.36
0.18
0.93
0.0012
Anova, p = 0.043
50
40
30
20
10
0
LENG9
Low
ct1Llsd1H
Med
High
ct1Hlsd1L
group

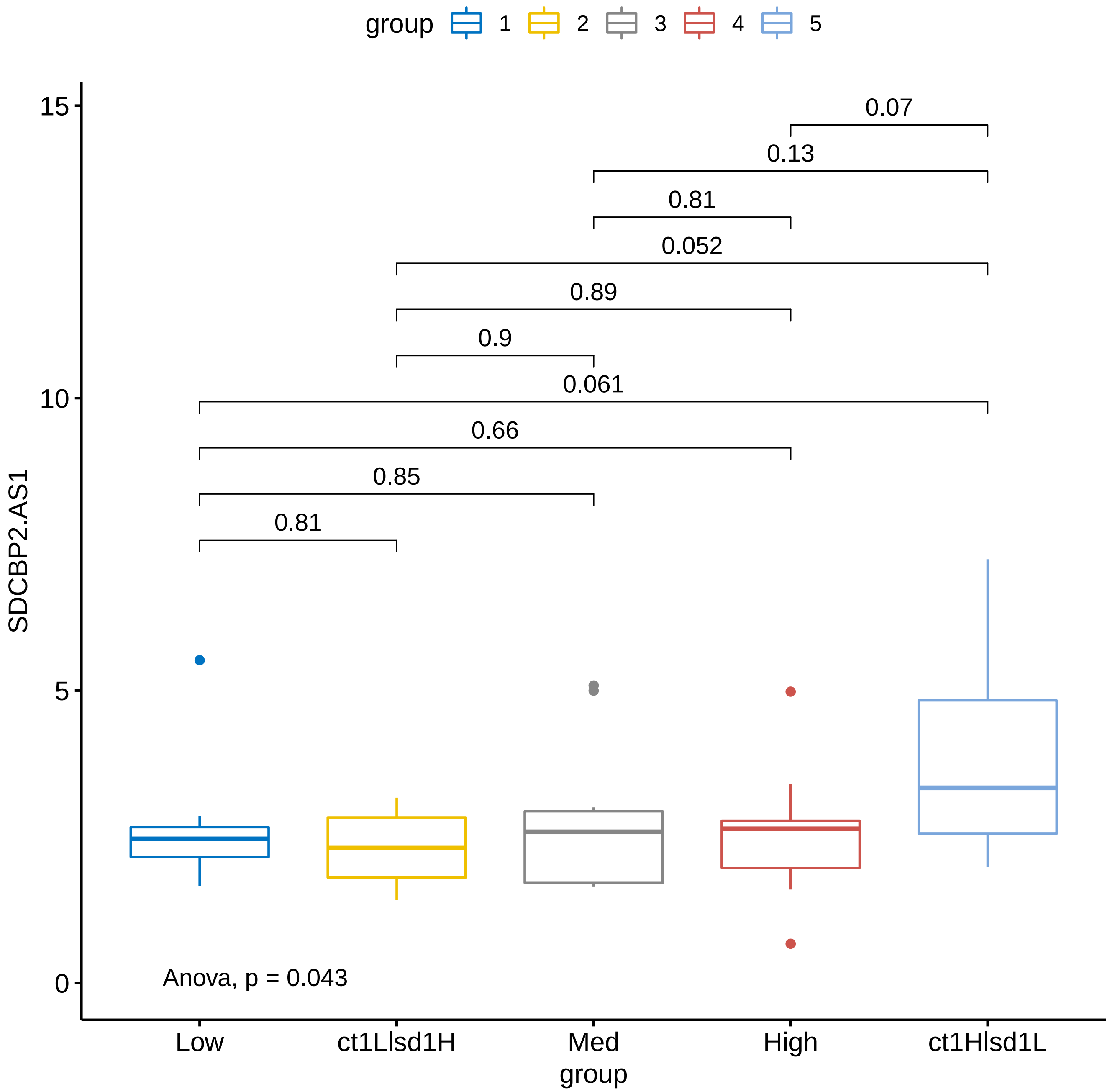
group
1
2
3
4
5
SDCBP2.AS1
15
10
5
0
0.07
0.13
0.81
0.052
0.89
0.9
0.061
0.66
0.85
0.81
Anova, p = 0.043
Low
ct1Llsd1H
Med
High
ct1Hlsd1L
group

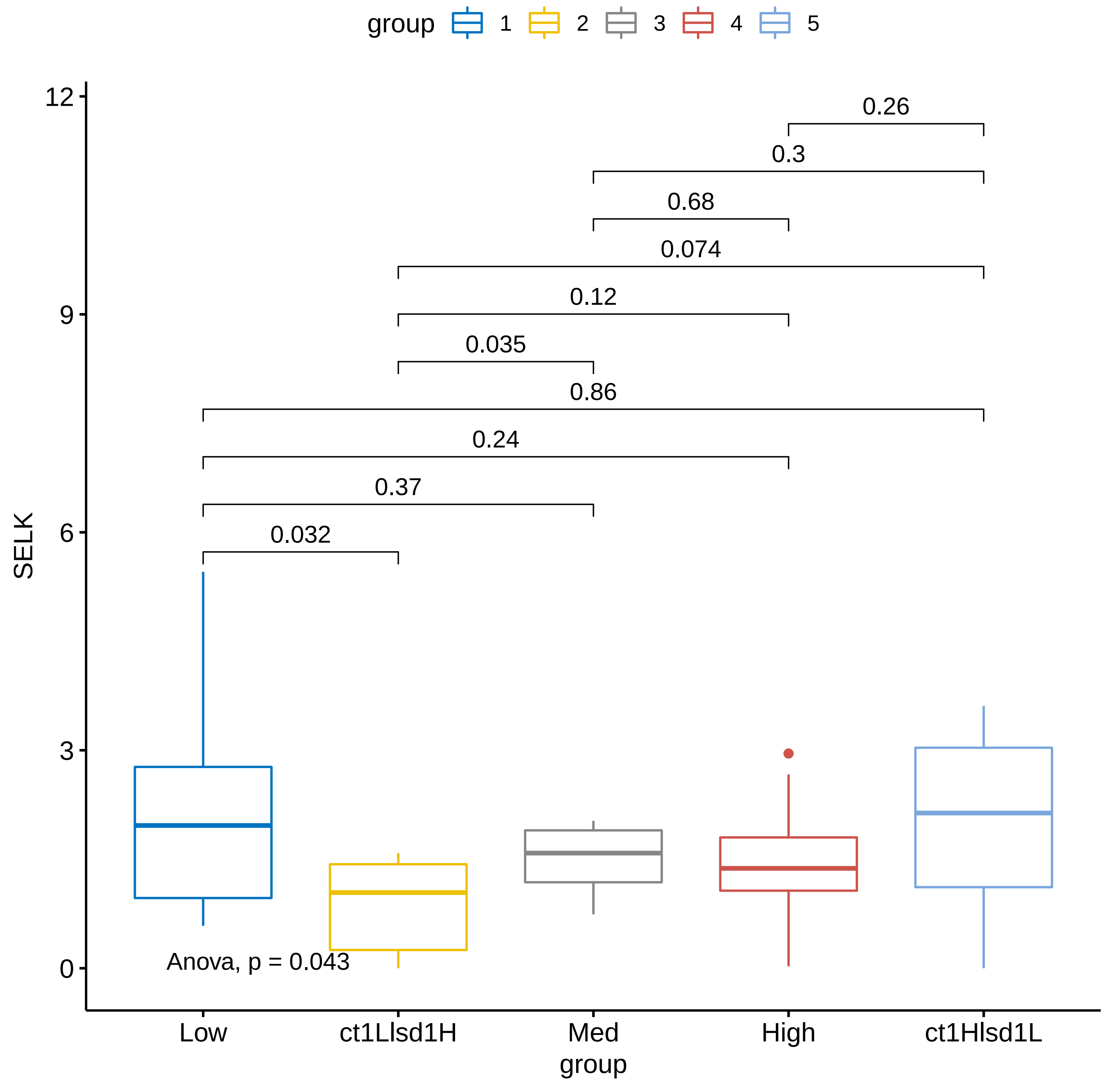

group
1
2
3
4
5
SELK
12
9
6
3
0
0.26
0.3
0.68
0.074
0.12
0.035
0.86
0.24
0.37
0.032
Anova, p = 0.043
Low
ct1Llsd1H
Med
High
ct1Hlsd1L
group

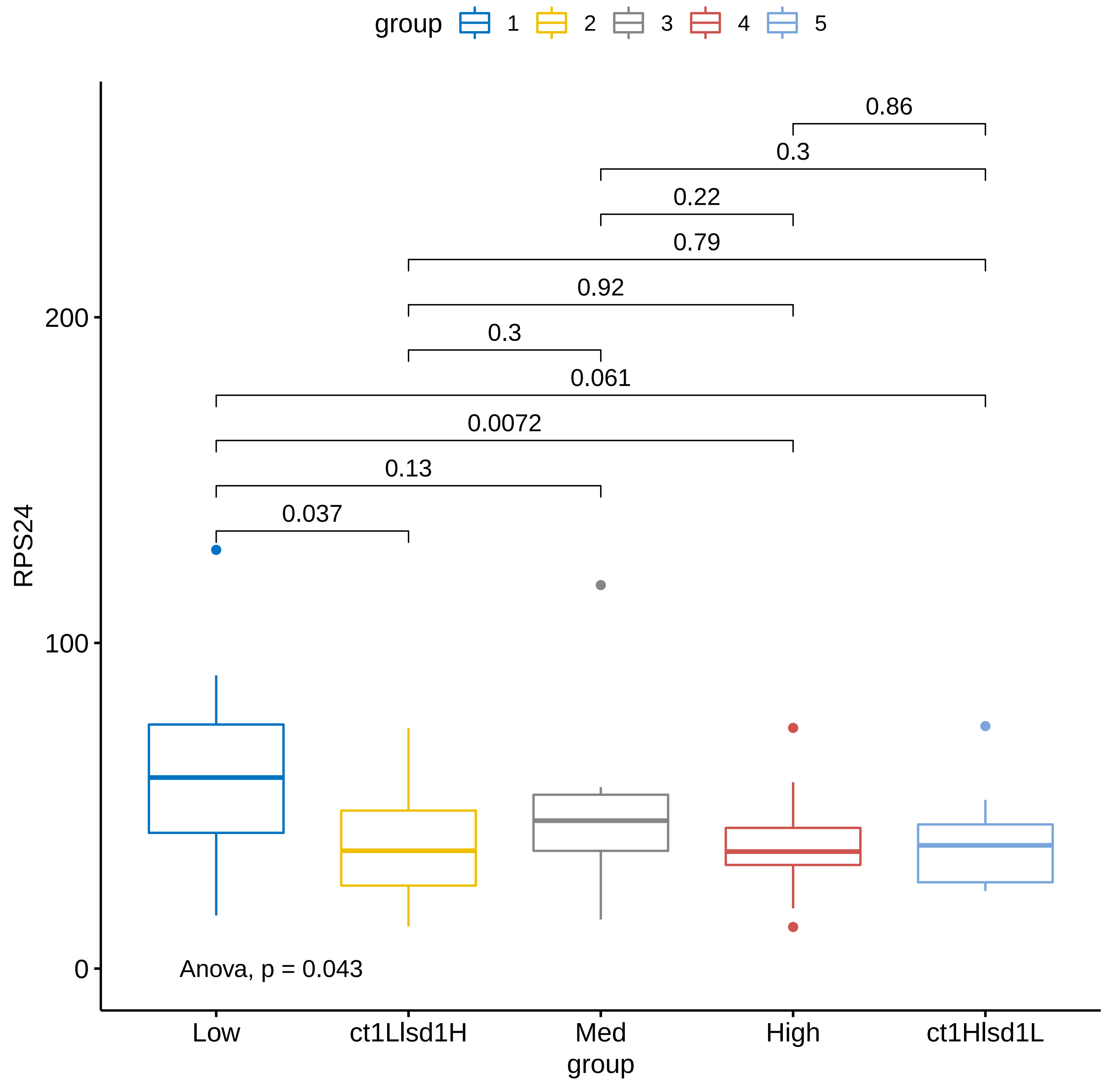

group
1
2
3
4
5
0.86
0.3
0.22
0.79
0.92
0.3
0.061
0.0072
0.13
0.037
200
100
0
RPS24
Anova, p = 0.043
Low
ct1Llsd1H
Med
High
ct1Hlsd1L
group

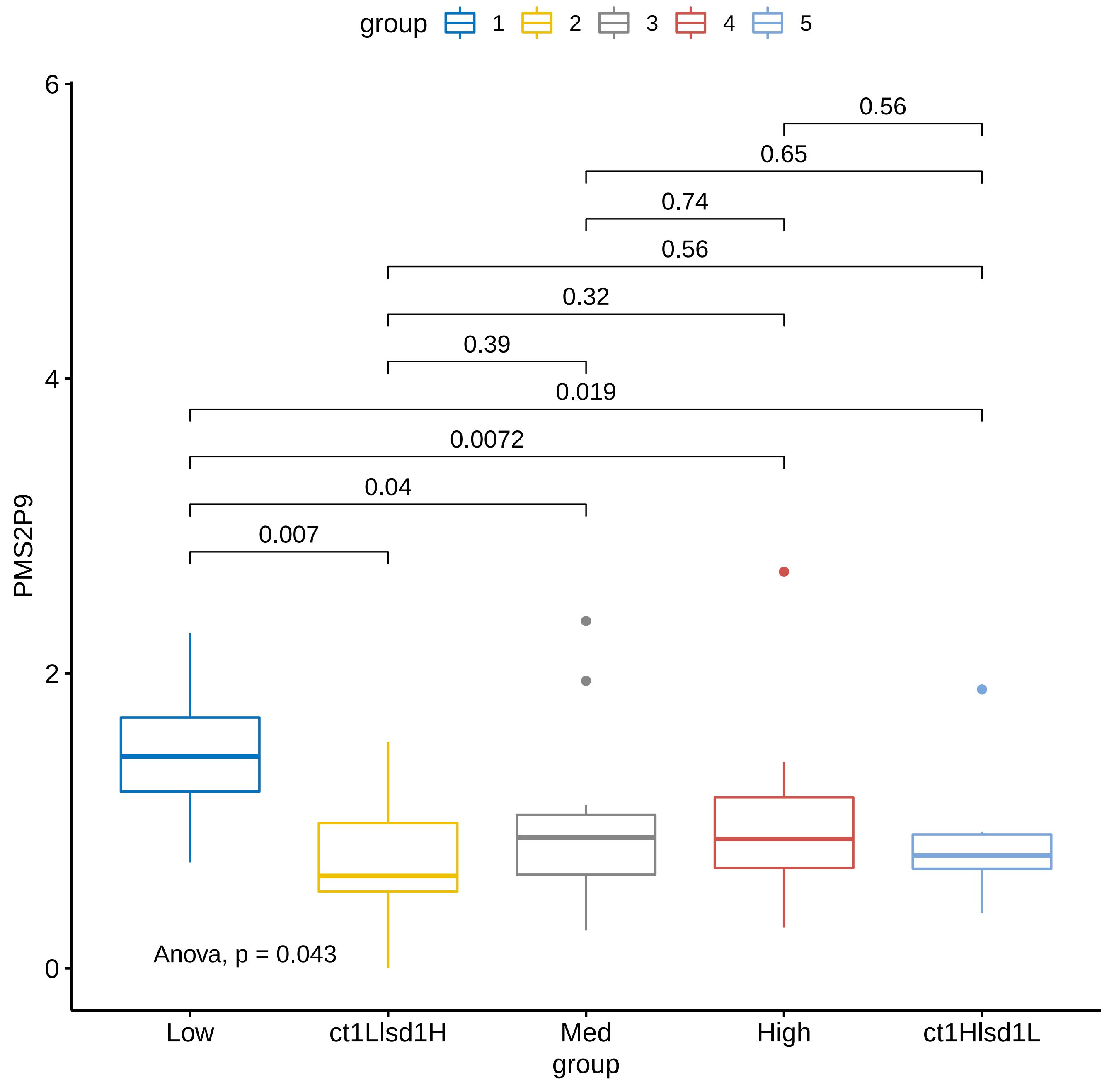

group 1 2 3 4 5
0.56
0.65
0.74
0.56
0.32
0.39
0.019
0.0072
0.04
0.007
Anova, p = 0.043
PMS2P9
6
4
2
0
Low
ct1Llsd1H
Med
High
ct1Hlsd1L
group

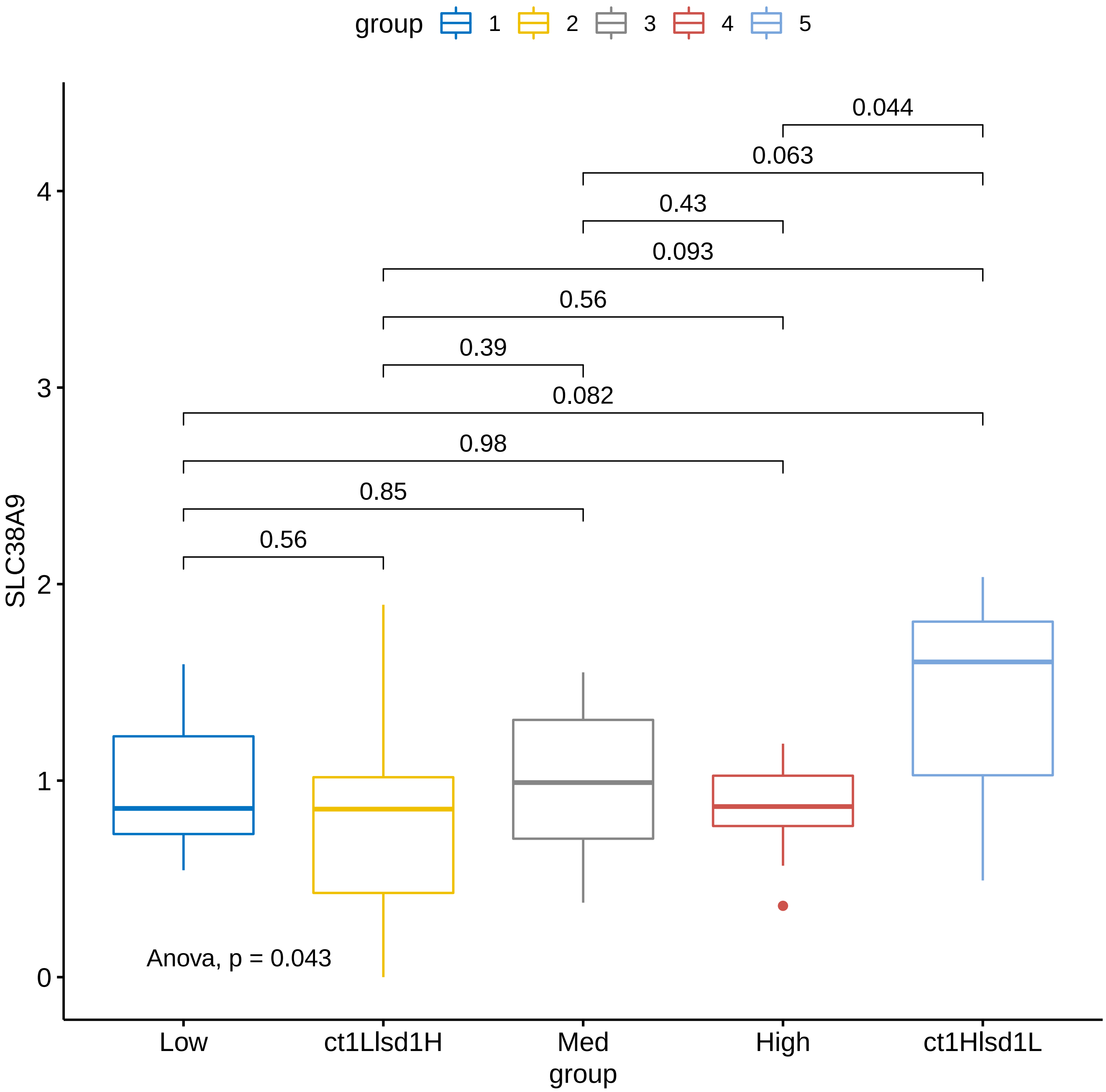

group 1 2 3 4 5
0.044
0.063
0.43
0.093
0.56
0.39
0.082
0.98
0.85
0.56
Anova, p = 0.043
SLC38A9
0
1
2
3
4
Low
ct1Llsd1H
Med
High
ct1Hlsd1L
group

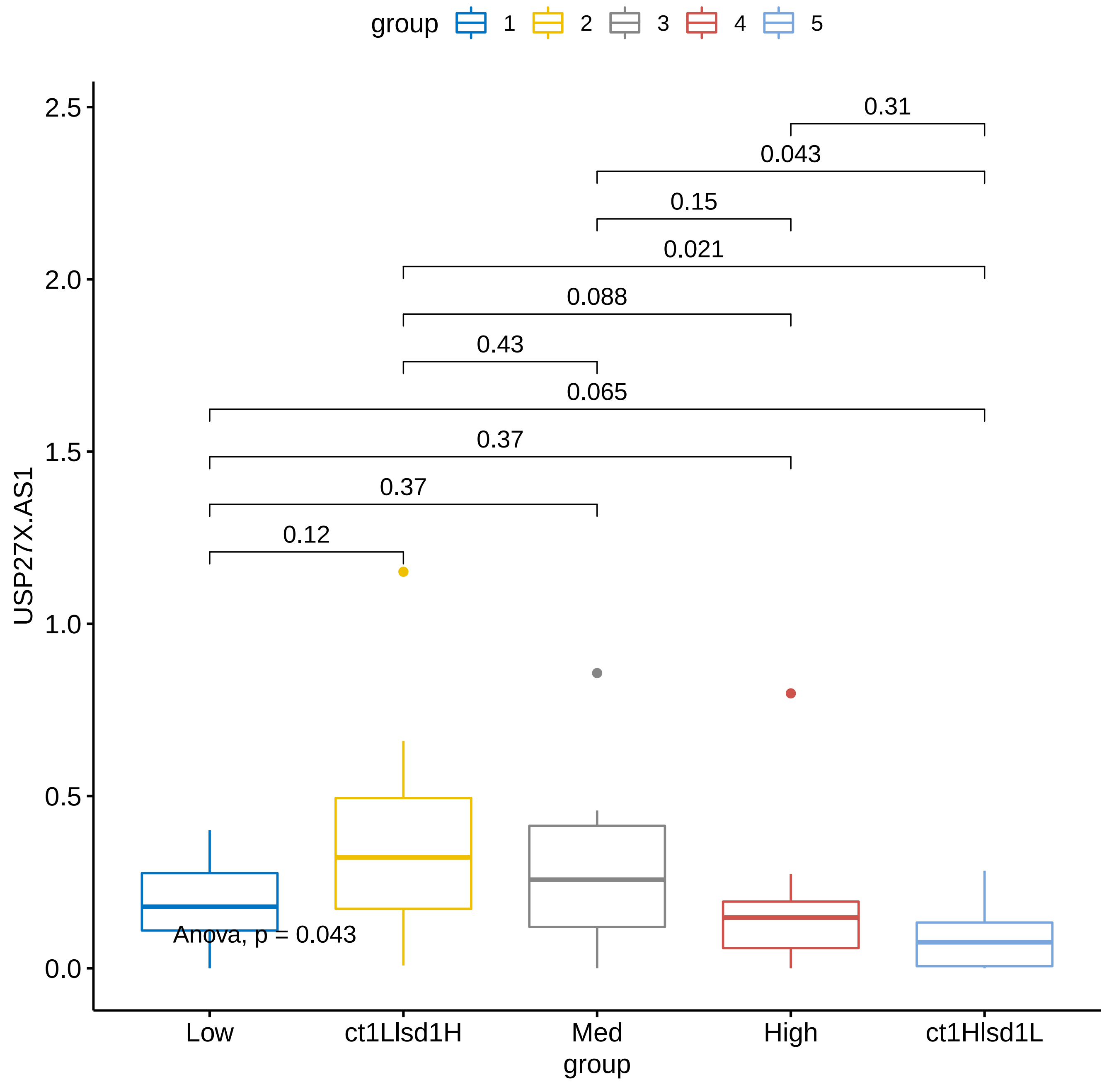

group 1 2 3 4 5
0.31
0.043
0.15
0.021
0.088
0.43
0.065
0.37
0.37
0.12
Anova, p = 0.043
USP27X.AS1
2.5
2.0
1.5
1.0
0.5
0.0
Low
ct1Llsd1H
Med
High
ct1Hlsd1L
group

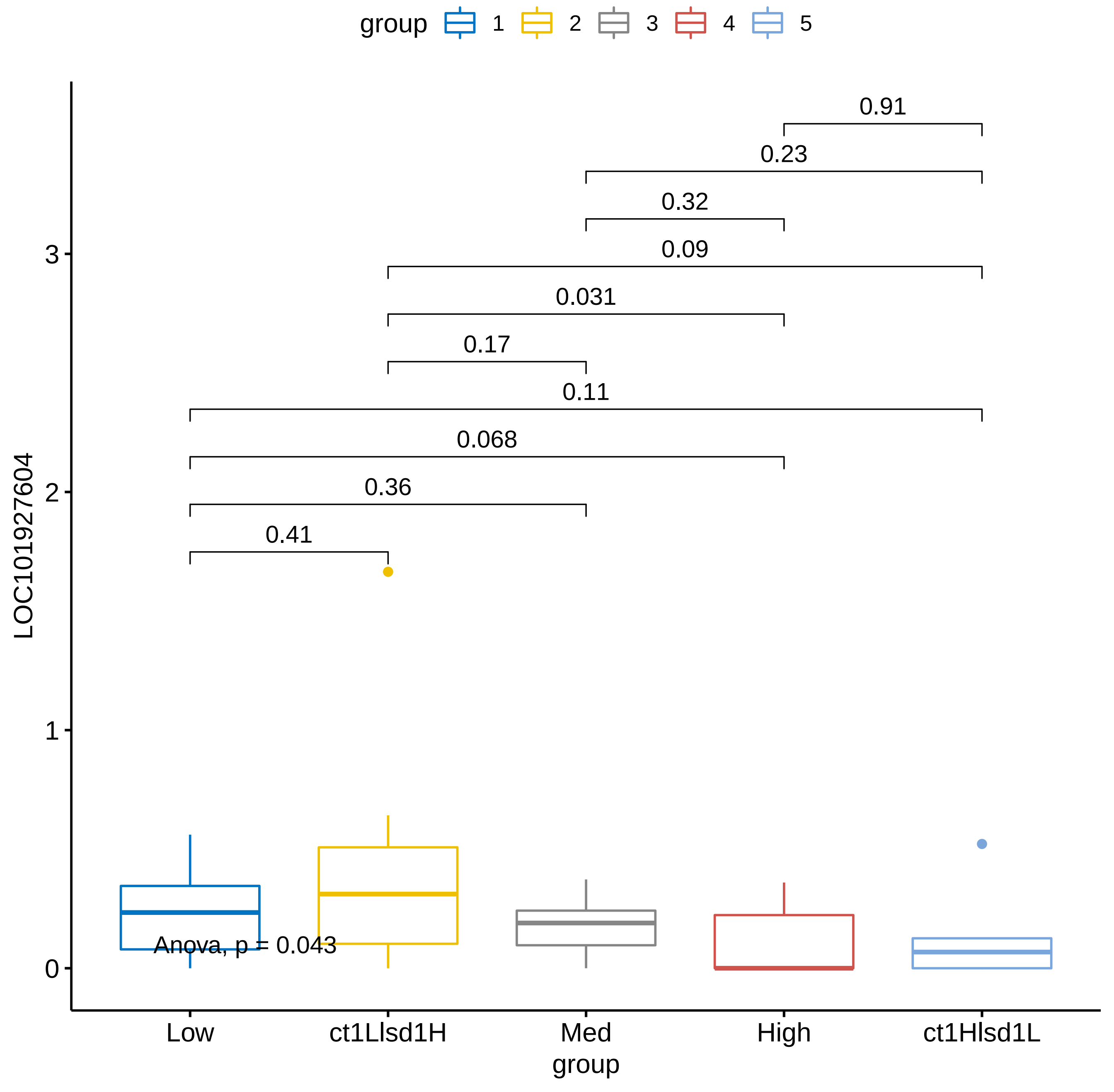

group
1
2
3
4
5
0.91
0.23
0.32
0.09
0.031
0.17
0.11
0.068
0.36
0.41
Anova, p = 0.043
LOC101927604
3
2
1
0
Low
ct1Llsd1H
Med
High
ct1Hlsd1L
group

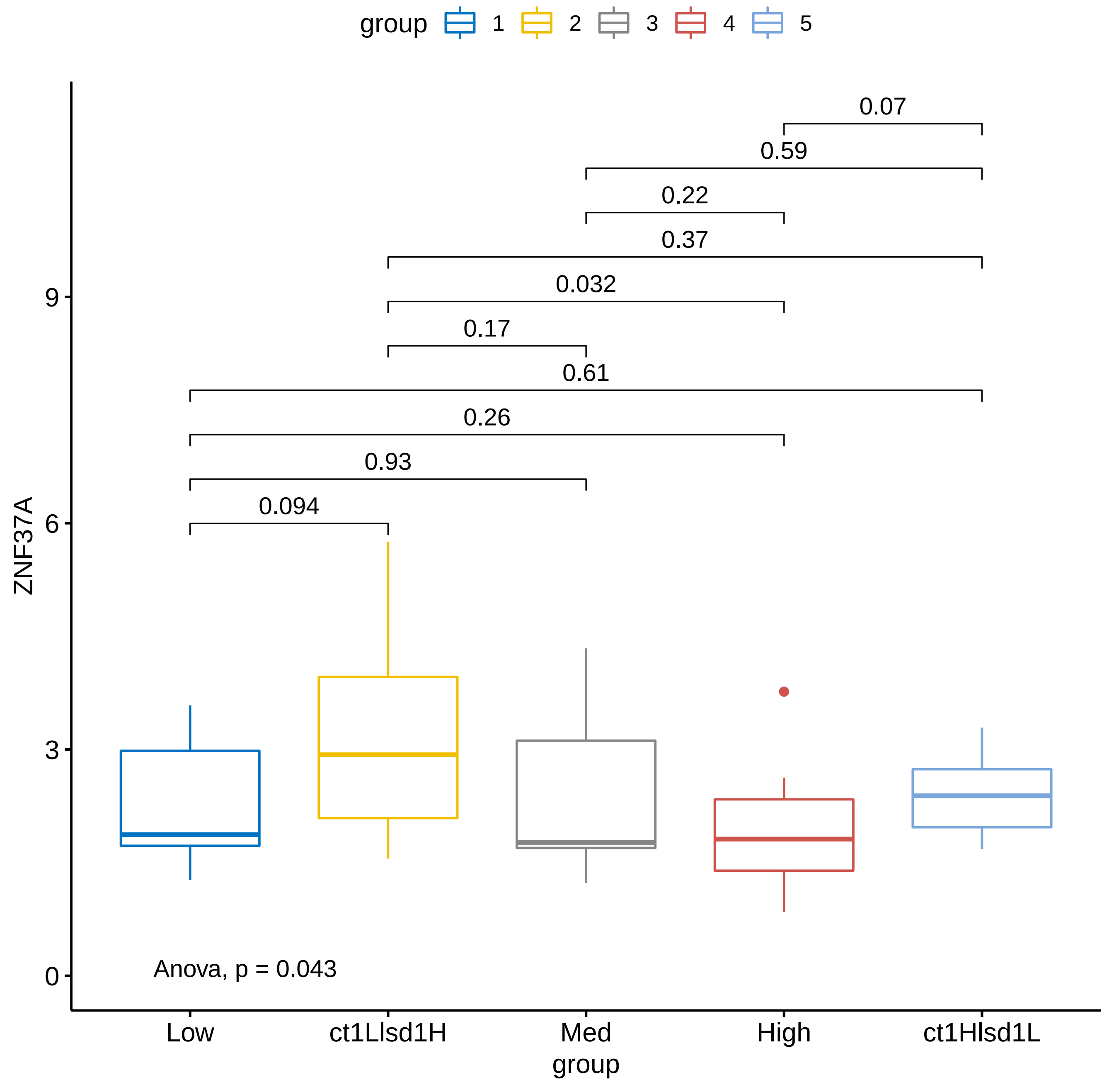
group 1 2 3 4 5
0.07
0.59
0.22
0.37
0.032
0.17
0.61
0.26
0.93
0.094
9
6
3
0
ZNF37A
Anova, p = 0.043
Low
ct1Llsd1H
Med
High
ct1Hlsd1L
group

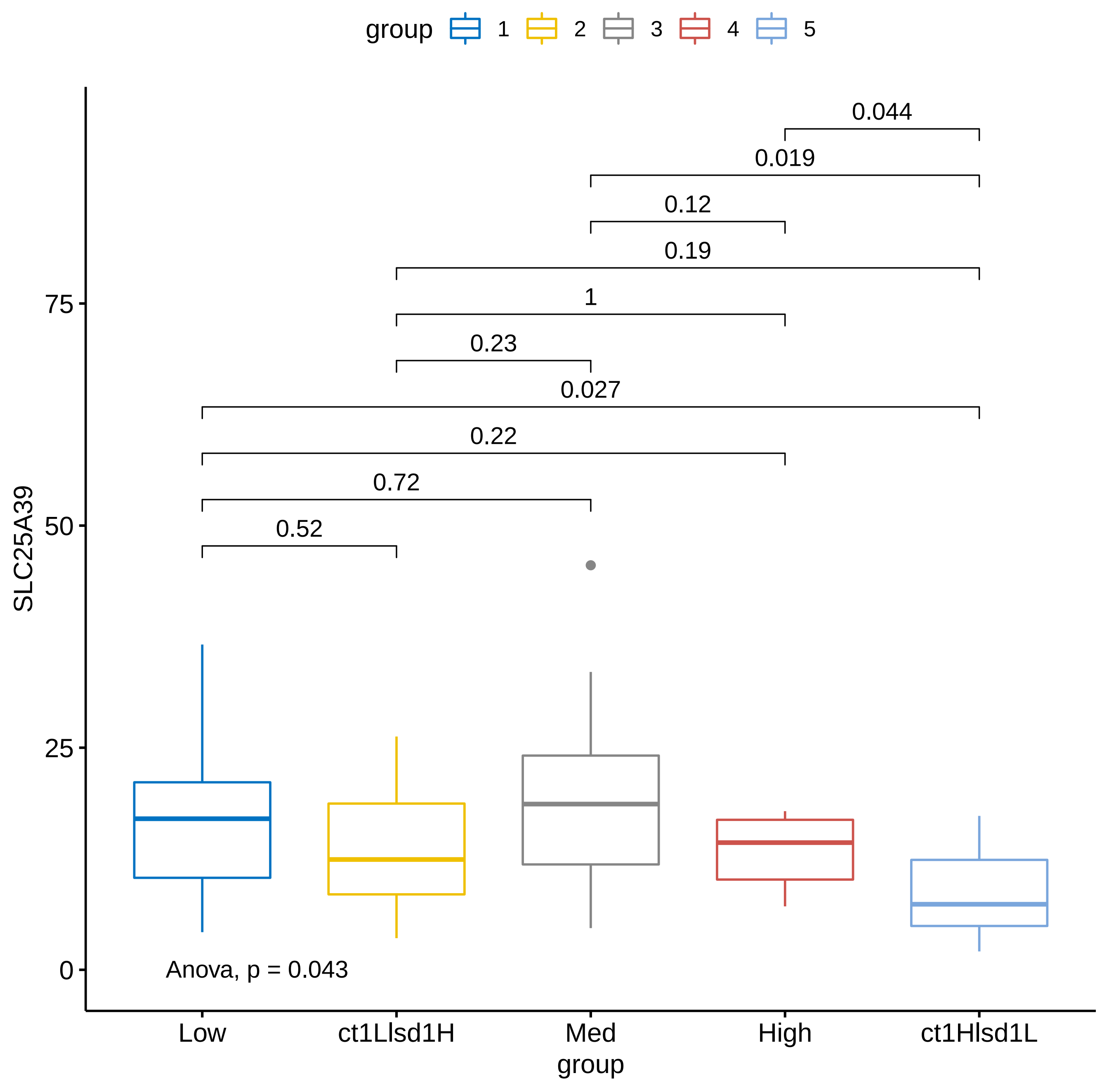
group
1
2
3
4
5
0.044
0.019
0.12
0.19
1
0.23
0.027
0.22
0.72
0.52
75
50
25
0
SLC25A39
Anova, p = 0.043
Low
ct1Llsd1H
Med
High
ct1Hlsd1L
group

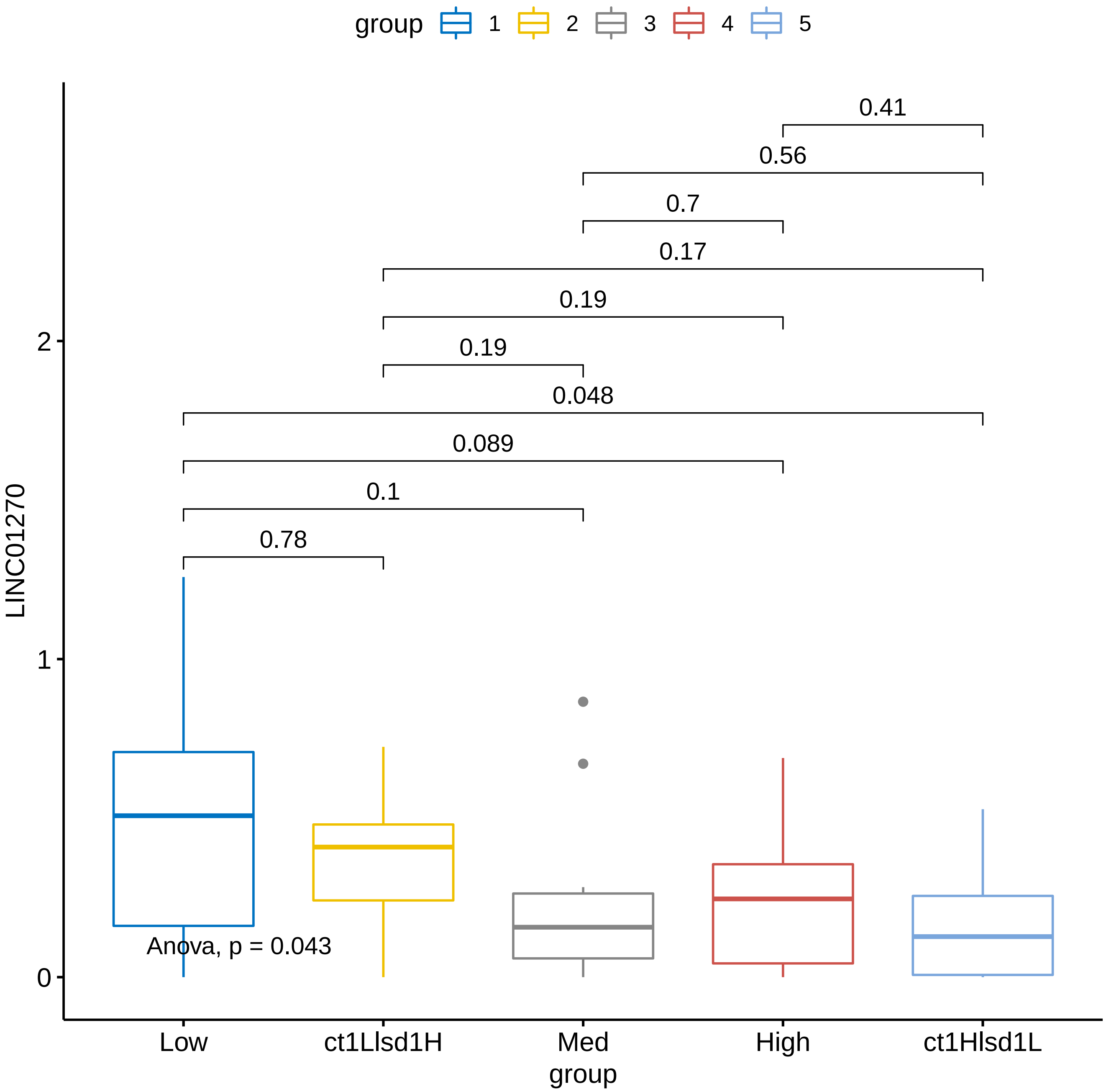
group 1 2 3 4 5
0.41
0.56
0.7
0.17
0.19
0.19
0.048
0.089
0.1
0.78
Anova, p = 0.043
LINC01270
0
1
2
Low
ct1Llsd1H
Med
High
ct1Hlsd1L
group

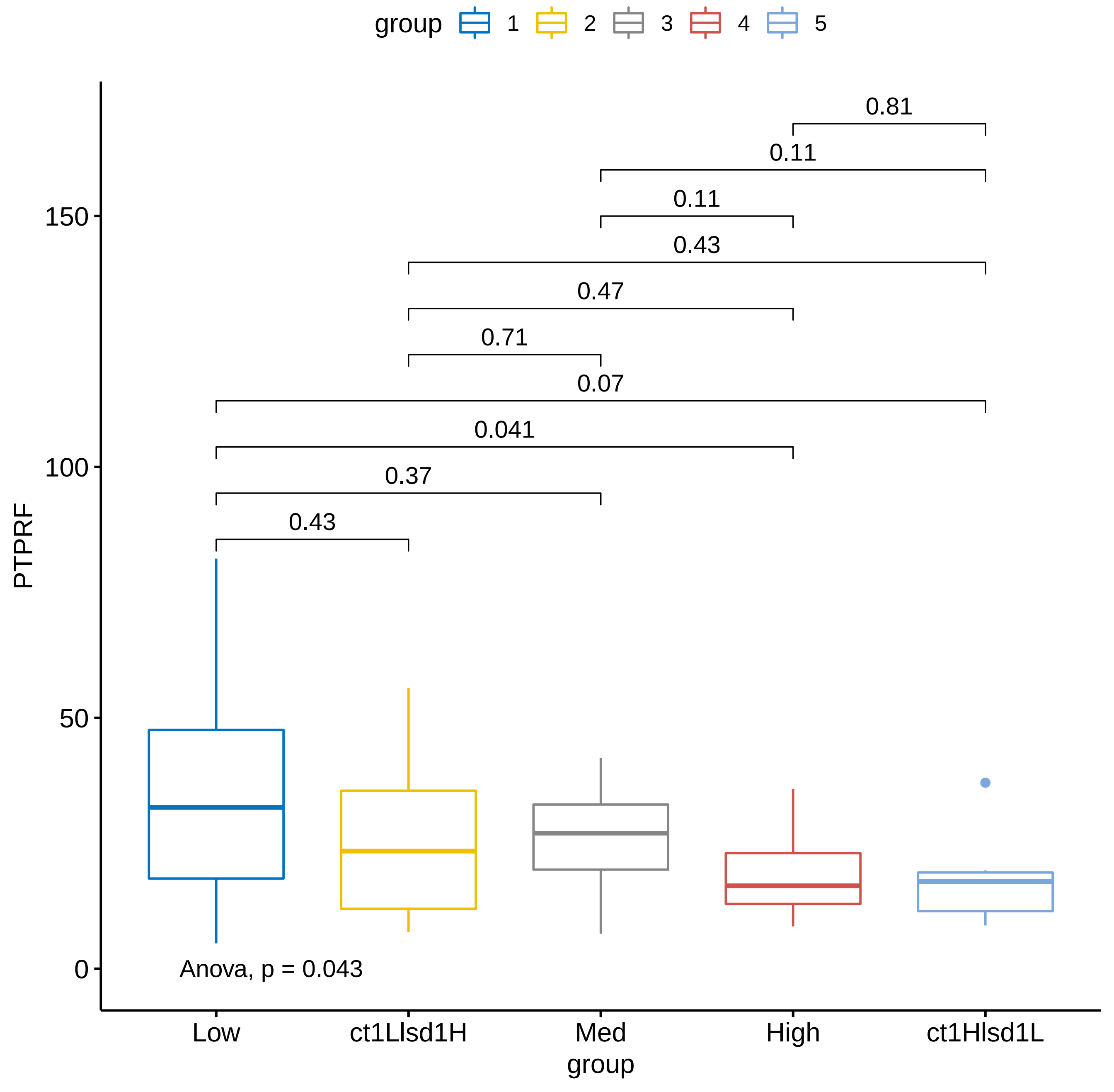

group 1 2 3 4 5
0.81
0.11
0.11
0.43
0.47
0.71
0.07
0.041
0.37
0.43
150
100
50
0
PTPRF
Anova, p = 0.043
Low
ct1Llsd1H
Med
High
ct1Hlsd1L
group

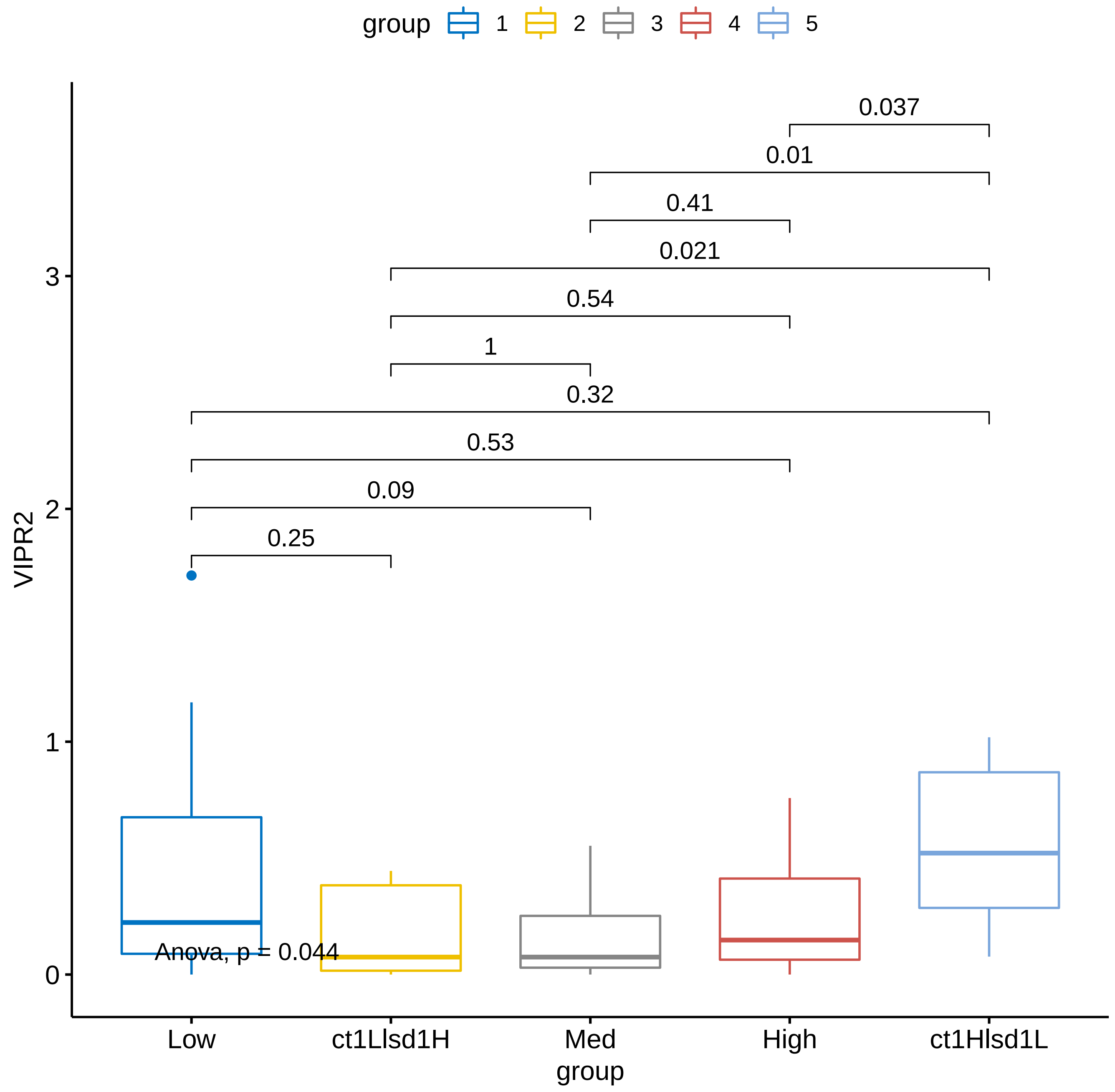

group
1
2
3
4
5
0.037
0.01
0.41
0.021
0.54
1
0.32
0.53
0.09
0.25
Anova, p = 0.044
VIPR2
3
2
1
0
Low
ct1Llsd1H
Med
High
ct1Hlsd1L
group

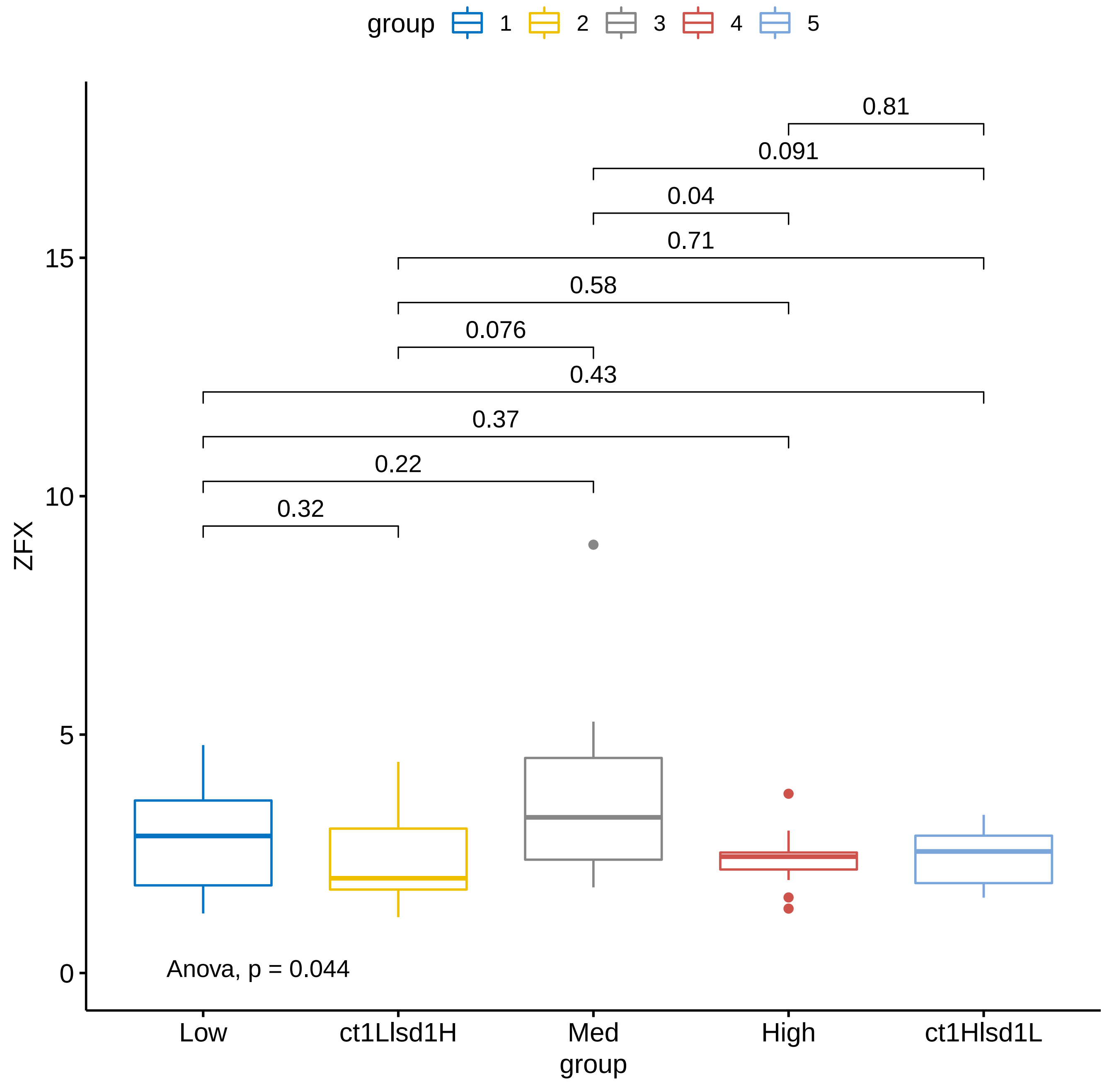

group
1
2
3
4
5
0.81
0.091
0.04
0.71
0.58
0.076
0.43
0.37
0.22
0.32
15
10
5
0
ZFX
Anova, p = 0.044
Low
ct1Llsd1H
Med
High
ct1Hlsd1L
group

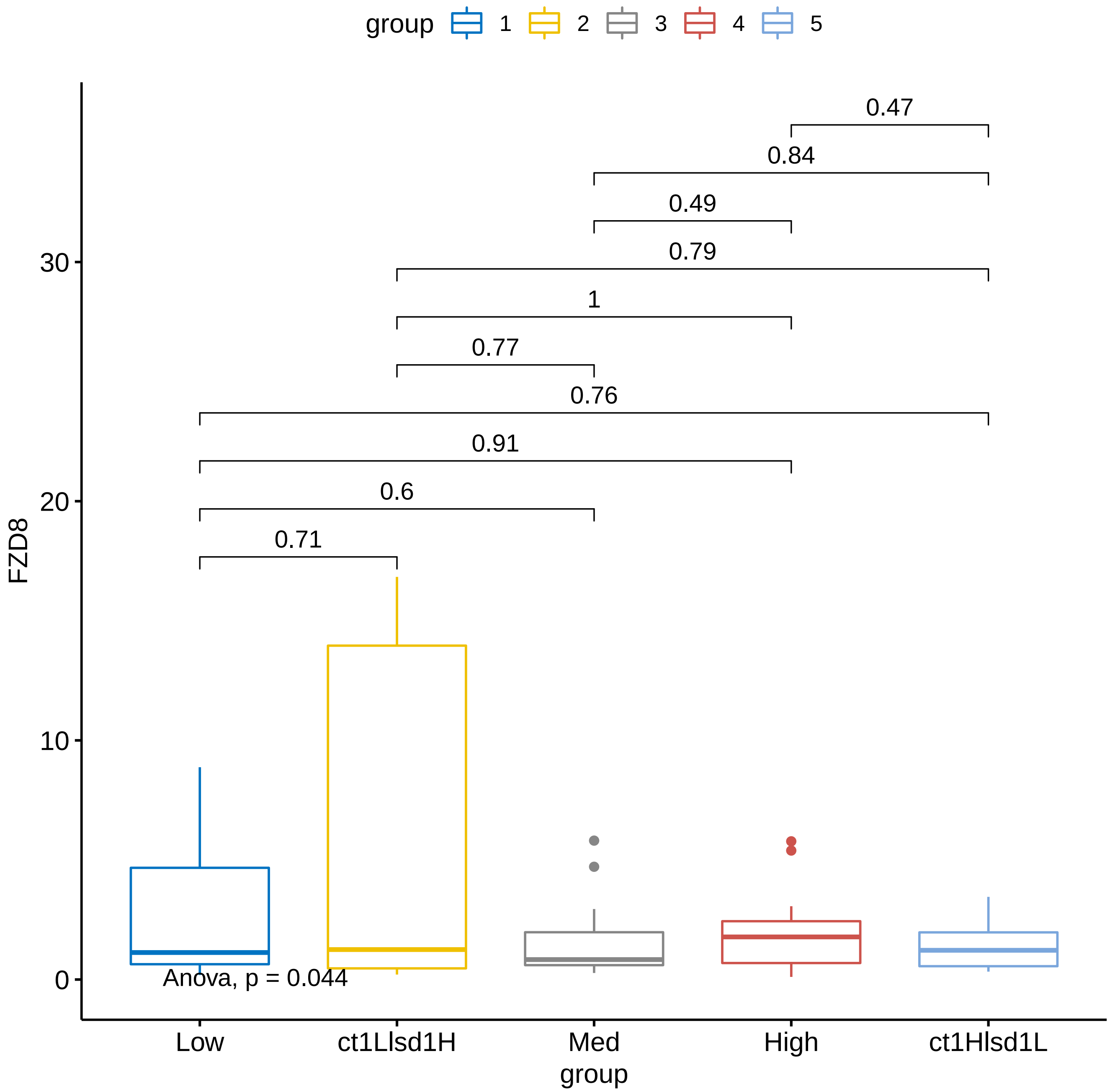
group
1
2
3
4
5
0.47
0.84
0.49
0.79
1
0.77
0.76
0.91
0.6
0.71
30
20
10
0
FZD8
Anova, p = 0.044
Low
ct1Llsd1H
Med
High
ct1Hlsd1L
group

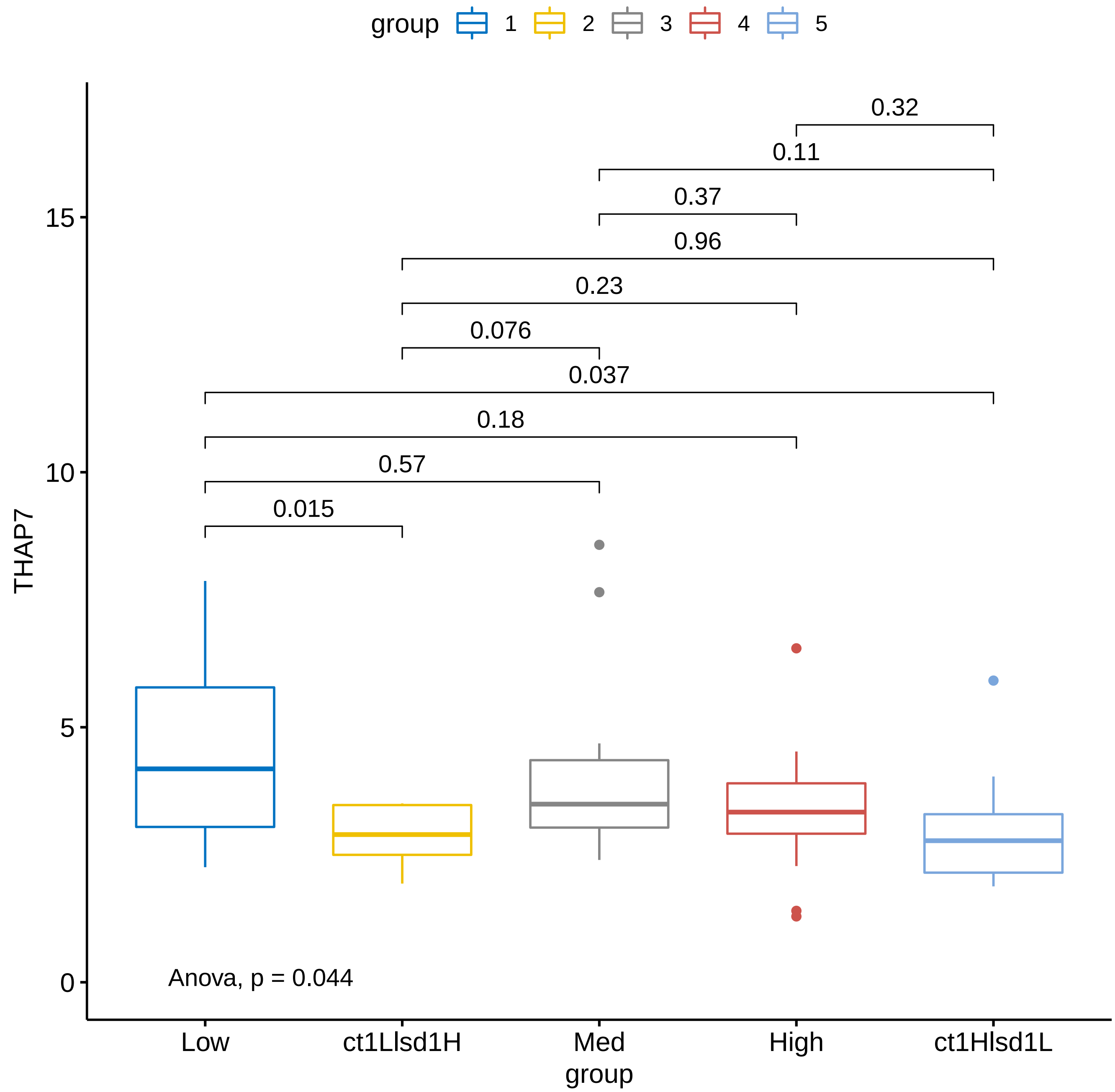

group 1 2 3 4 5
0.32
0.11
0.37
0.96
0.23
0.076
0.037
0.18
0.57
0.015
15
10
5
0
THAP7
Anova, p = 0.044
Low
ct1Llsd1H
Med
High
ct1Hlsd1L
group

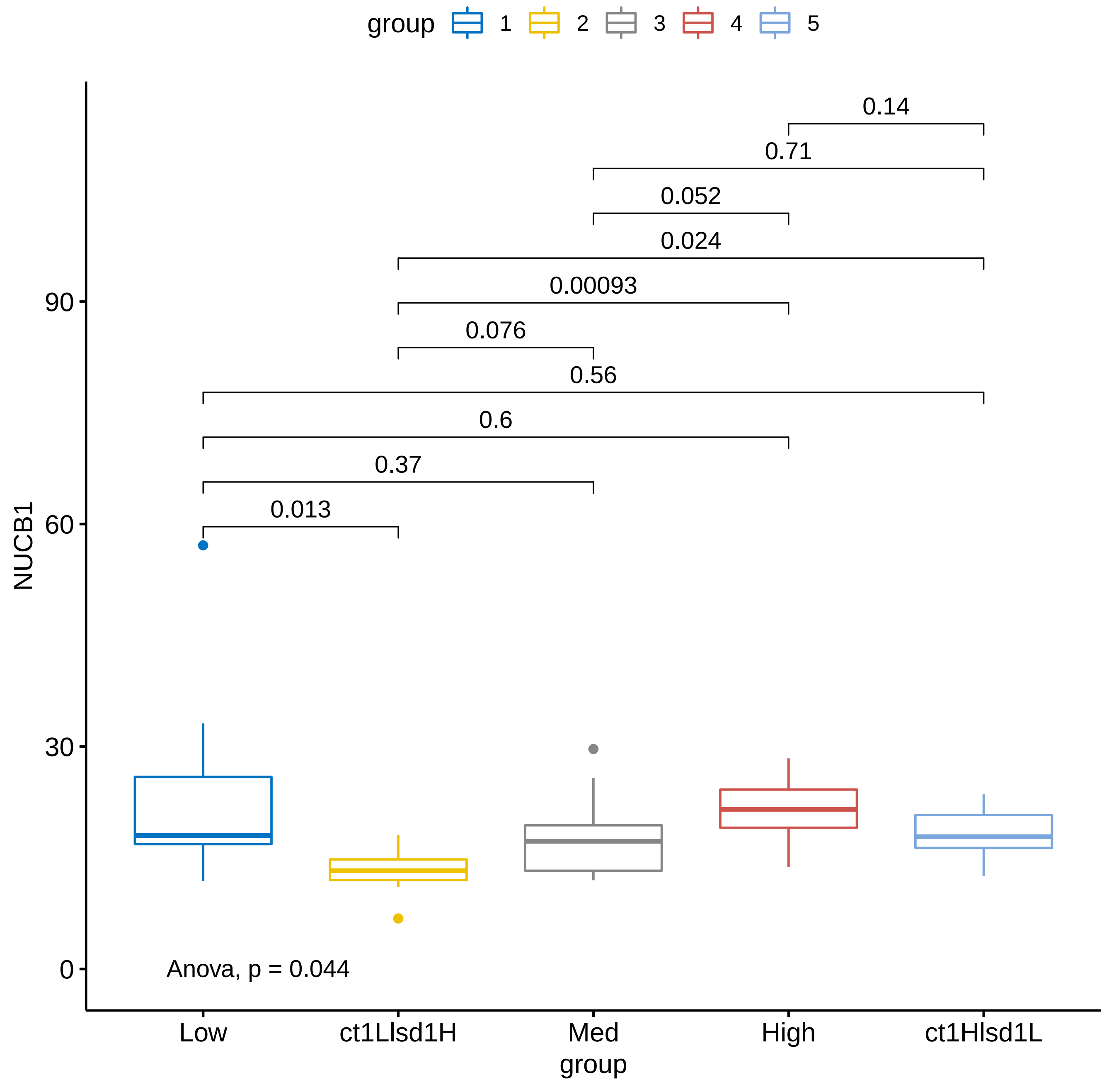
group
1
2
3
4
5
0.14
0.71
0.052
0.024
0.00093
0.076
0.56
0.6
0.37
0.013
90
60
30
0
NUCB1
Anova, p = 0.044
Low
ct1Llsd1H
Med
High
ct1Hlsd1L
group

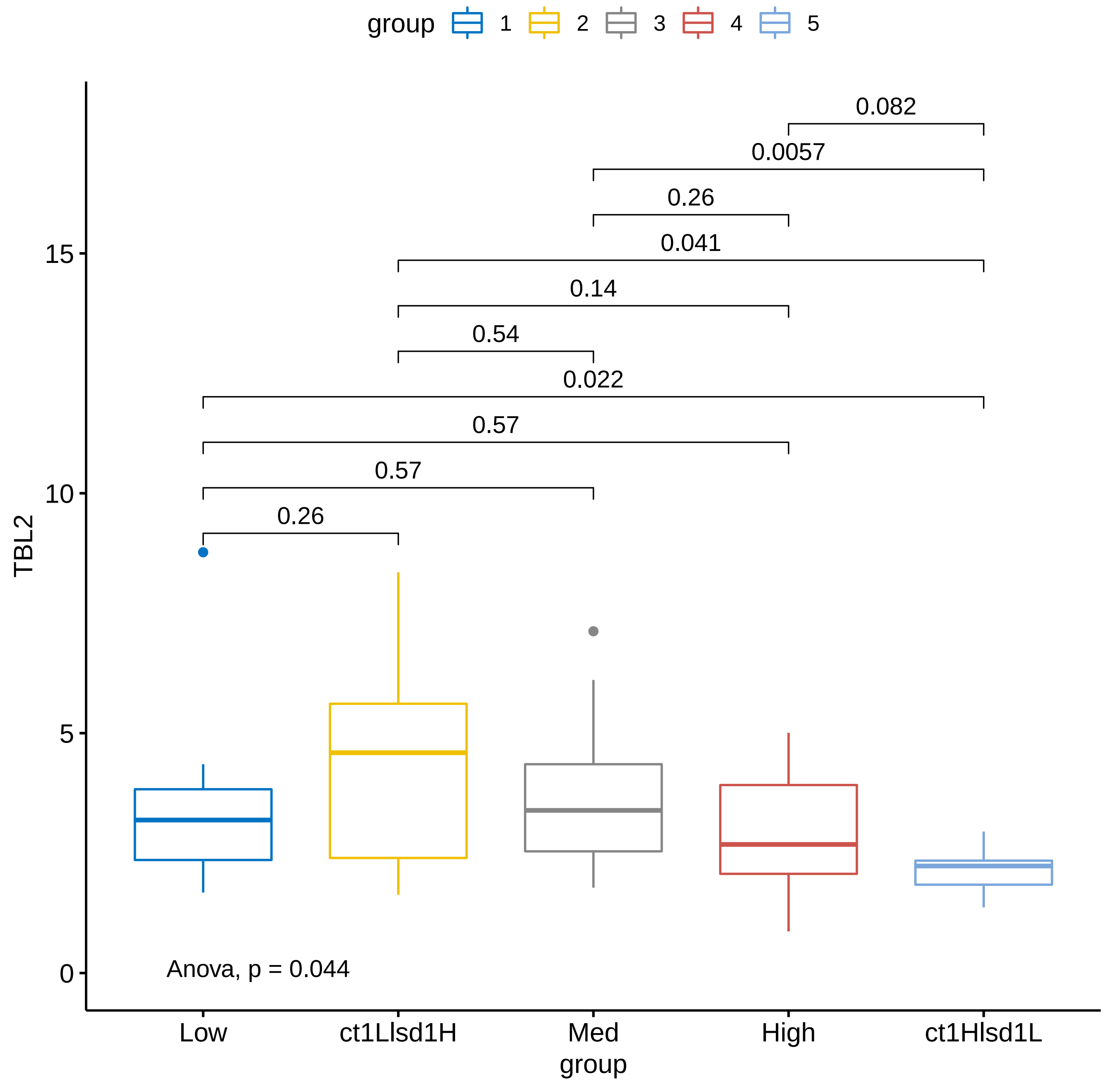

group
1
2
3
4
5
0.082
0.0057
0.26
0.041
0.14
0.54
0.022
0.57
0.57
0.26
15
10
5
0
TBL2
Anova, p = 0.044
Low
ct1Llsd1H
Med
High
ct1Hlsd1L
group

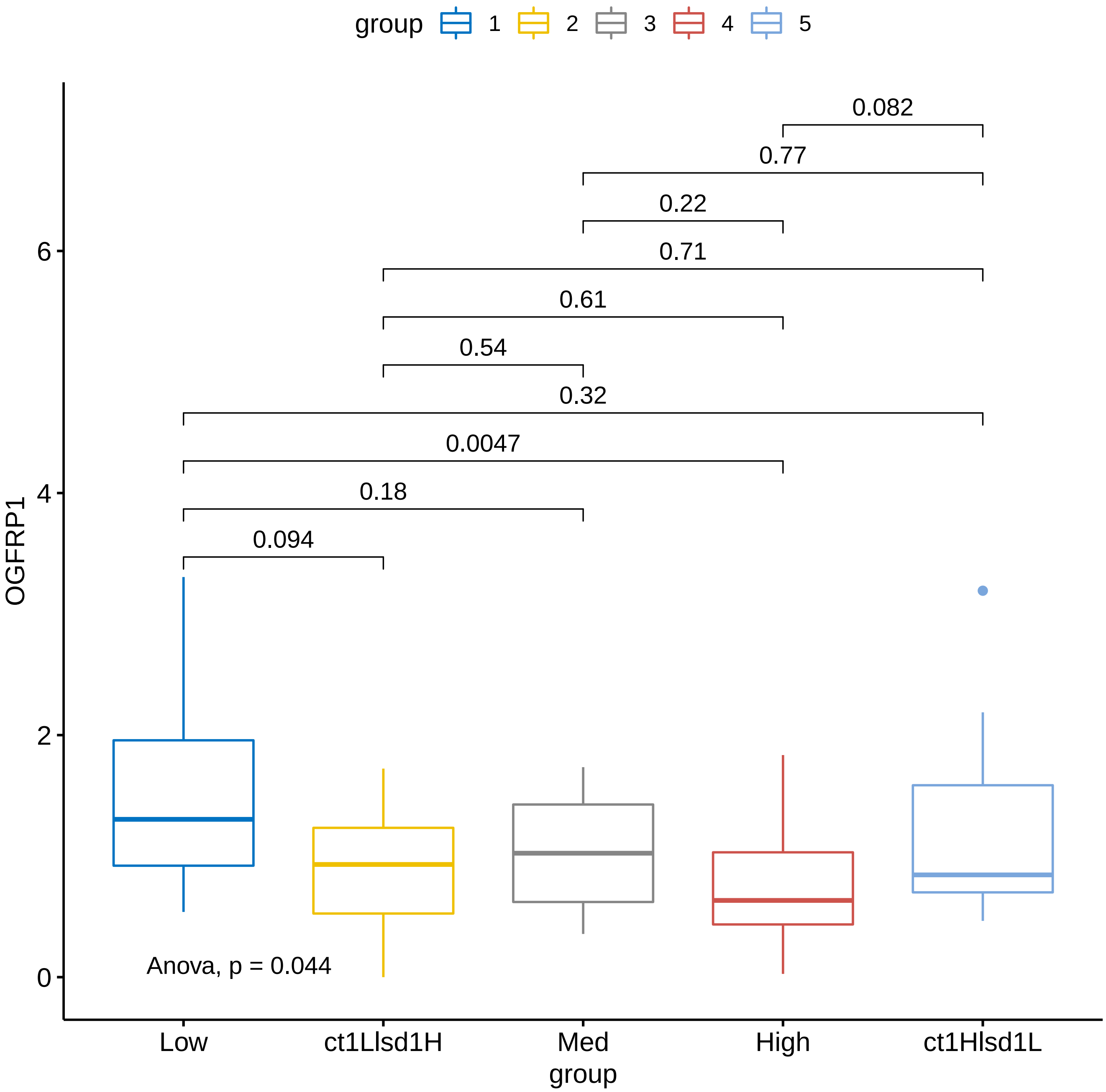
group
1
2
3
4
5
0.082
0.77
0.22
0.71
0.61
0.54
0.32
0.0047
0.18
0.094
Anova, p = 0.044
OGFRP1
6
4
2
0
Low
ct1Llsd1H
Med
High
ct1Hlsd1L
group

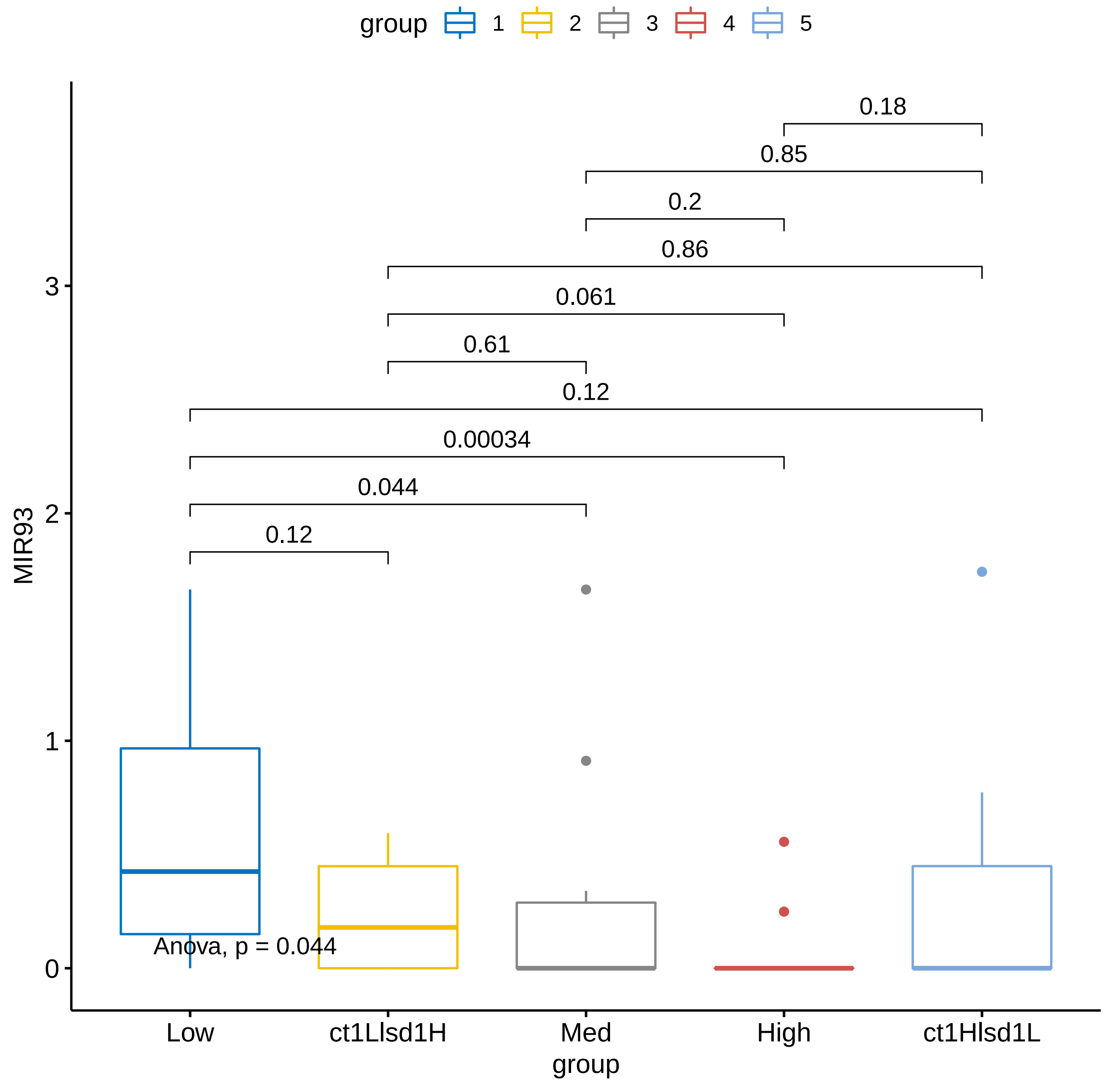
group
1
2
3
4
5
0.18
0.85
0.2
0.86
0.061
0.61
0.12
0.00034
0.044
0.12
Anova, p = 0.044
MIR93
3
2
1
0
Low
ct1Llsd1H
Med
High
ct1Hlsd1L
group

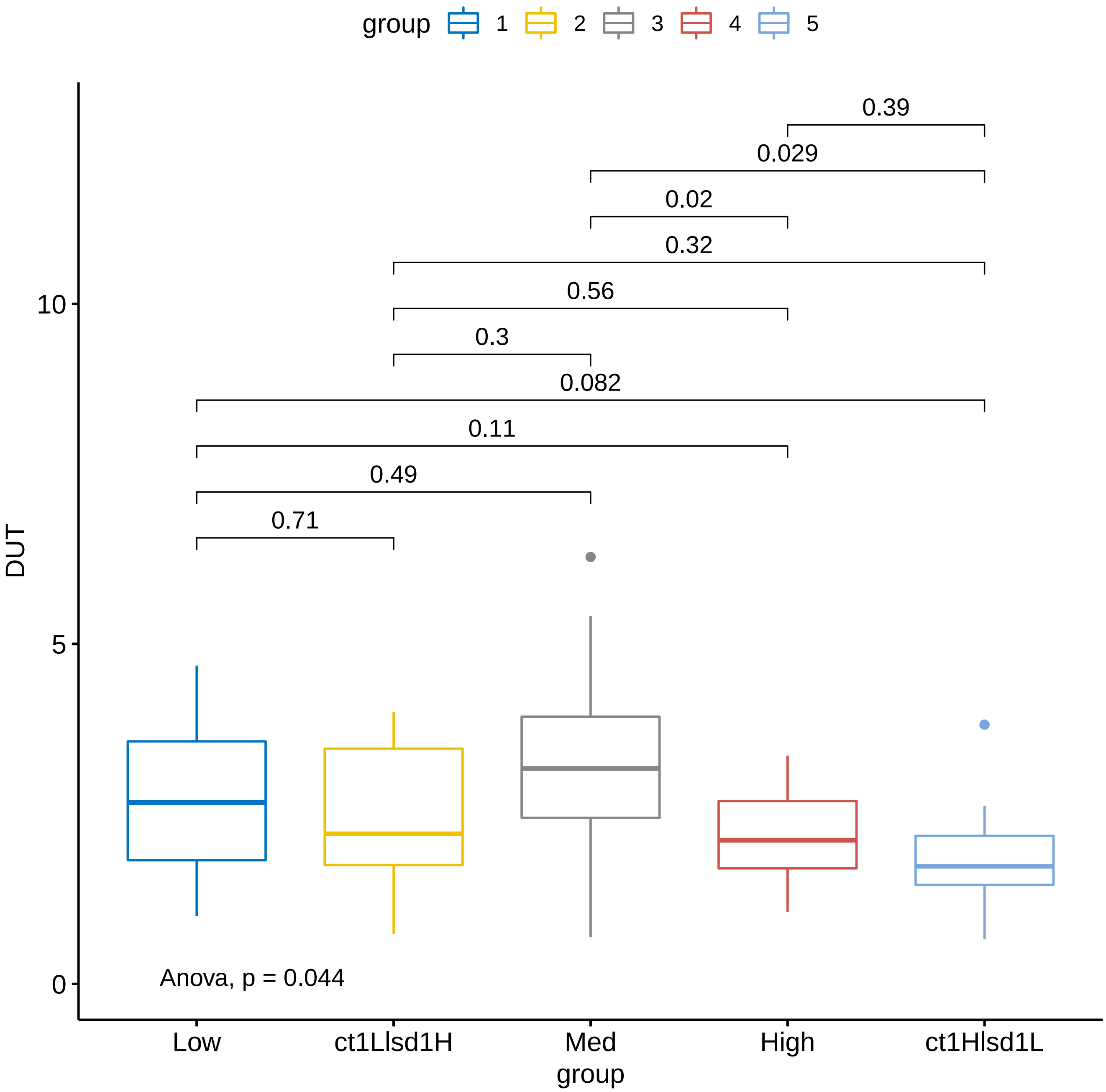

group 1 2 3 4 5
0.39
0.029
0.02
0.32
0.56
0.3
0.082
0.11
0.49
0.71
10
5
0
DUT
Anova, p = 0.044
Low
ct1Llsd1H
Med
High
ct1Hlsd1L
group

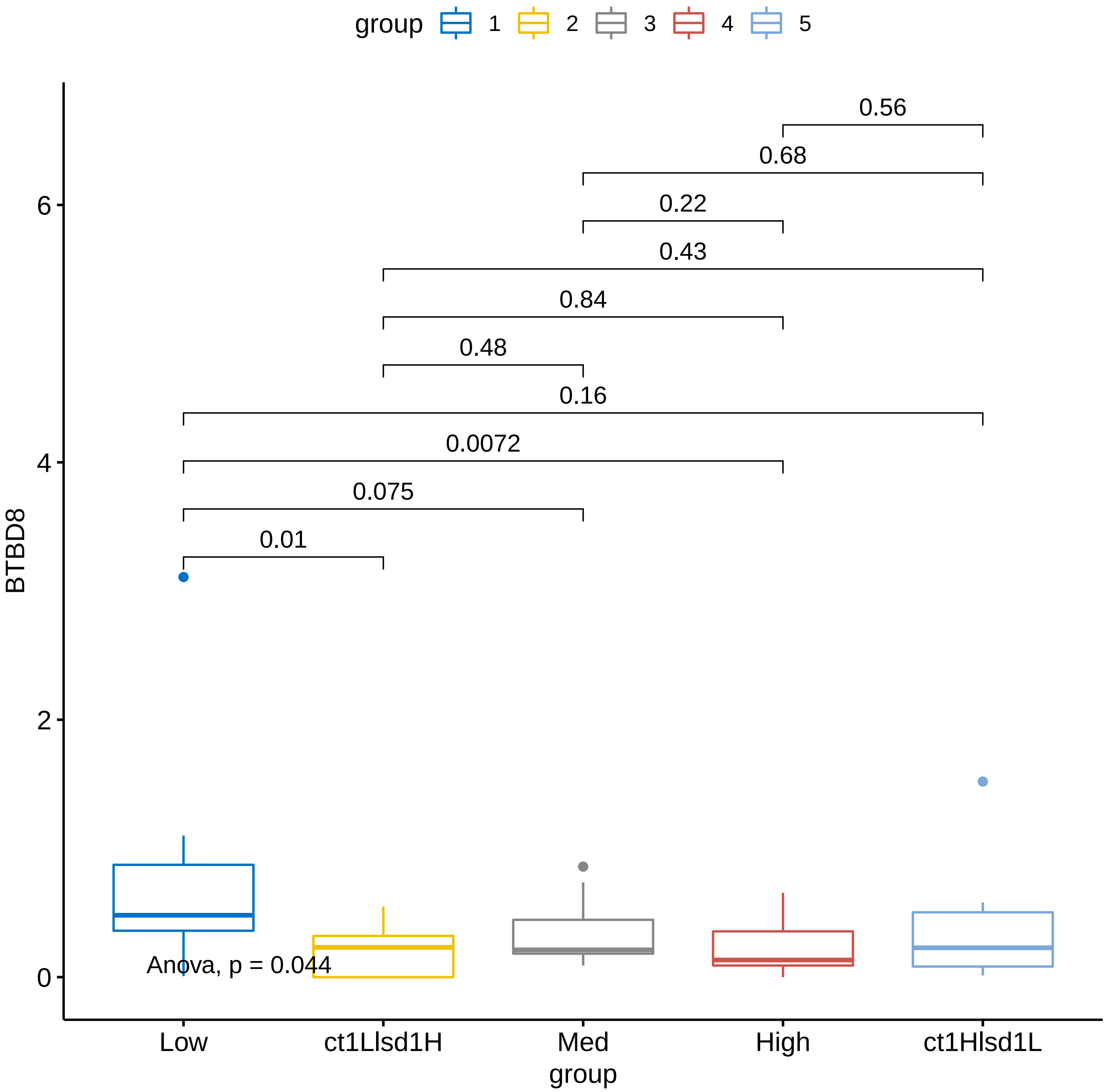

group 1 2 3 4 5
0.56
0.68
0.22
0.43
0.84
0.48
0.16
0.0072
0.075
0.01
6
4
2
0
BTBD8
Anova, p = 0.044
Low
ct1Llsd1H
Med
High
ct1Hlsd1L
group

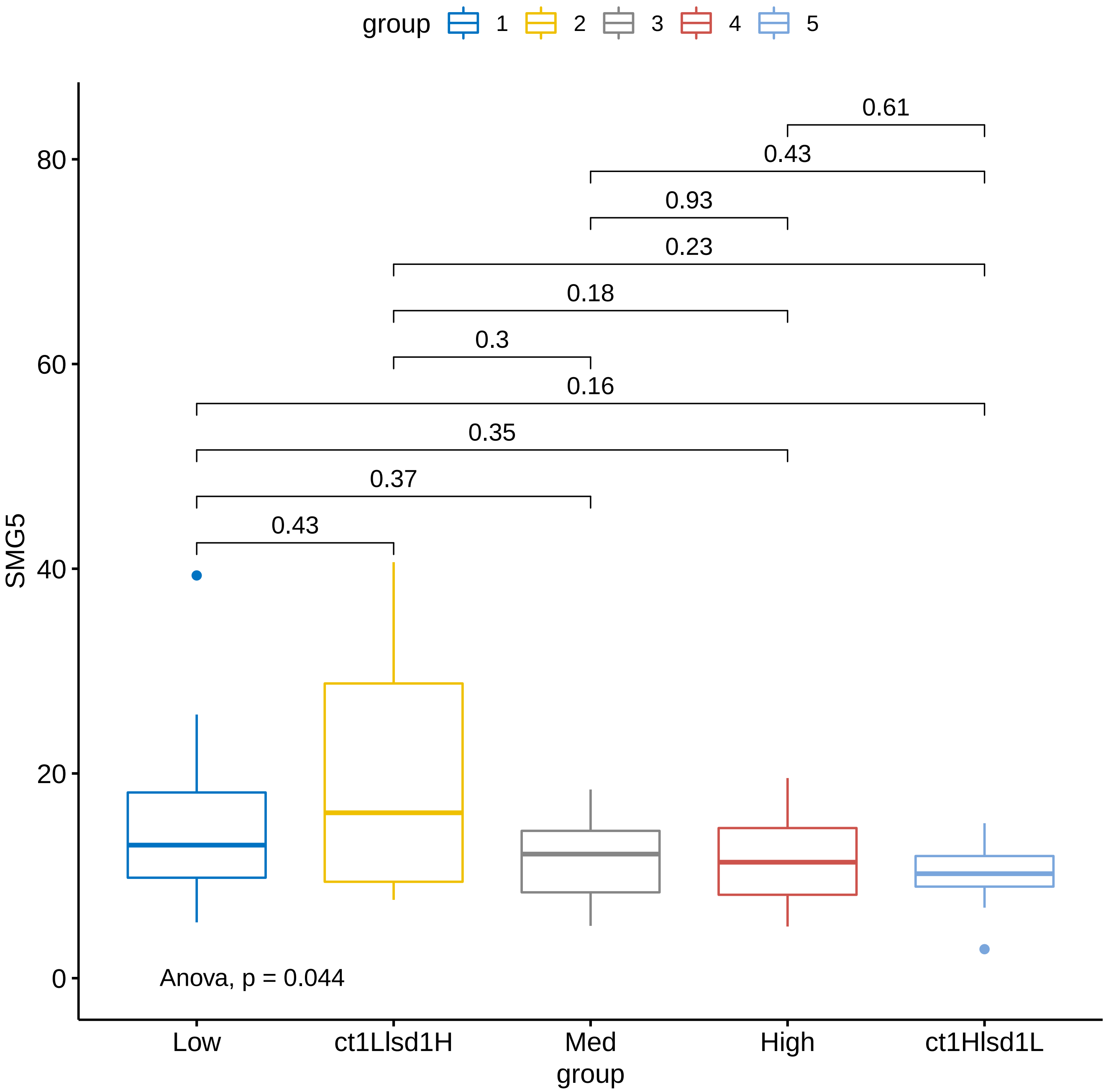

group
1
2
3
4
5
0.61
0.43
0.93
0.23
0.18
0.3
0.16
0.35
0.37
0.43
80
60
40
20
0
SMG5
Anova, p = 0.044
Low
ct1Llsd1H
Med
High
ct1Hlsd1L
group

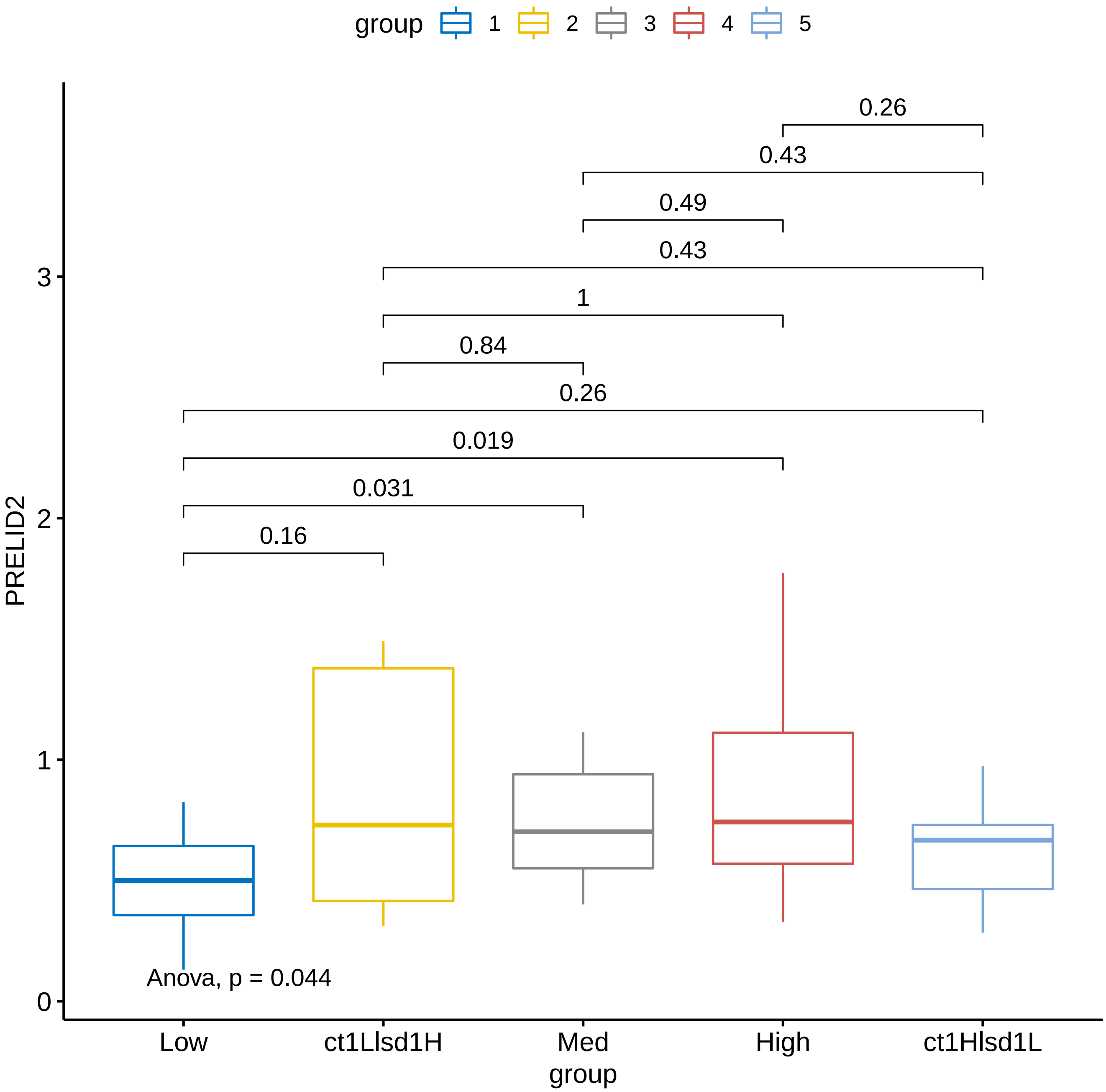
group
1
2
3
4
5
0.26
0.43
0.49
0.43
1
0.84
0.26
0.019
0.031
0.16
PRELID2
3
2
1
0
Anova, p = 0.044
Low
ct1Llsd1H
Med
High
ct1Hlsd1L
group

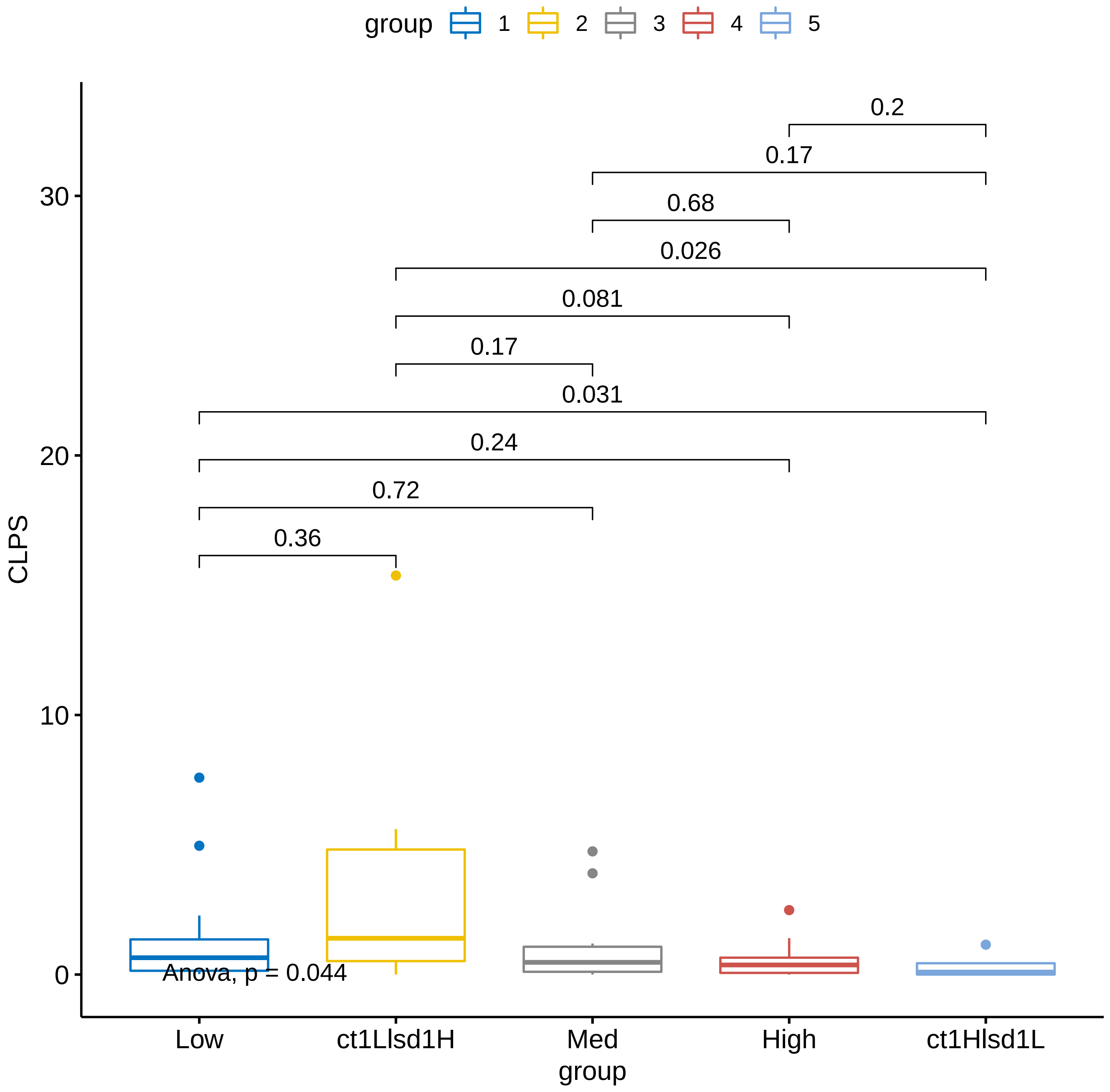

group
1
2
3
4
5
0.2
0.17
0.68
0.026
0.081
0.17
0.031
0.24
0.72
0.36
30
20
10
0
CLPS
Anova, p = 0.044
Low
ct1Llsd1H
Med
High
ct1Hlsd1L
group

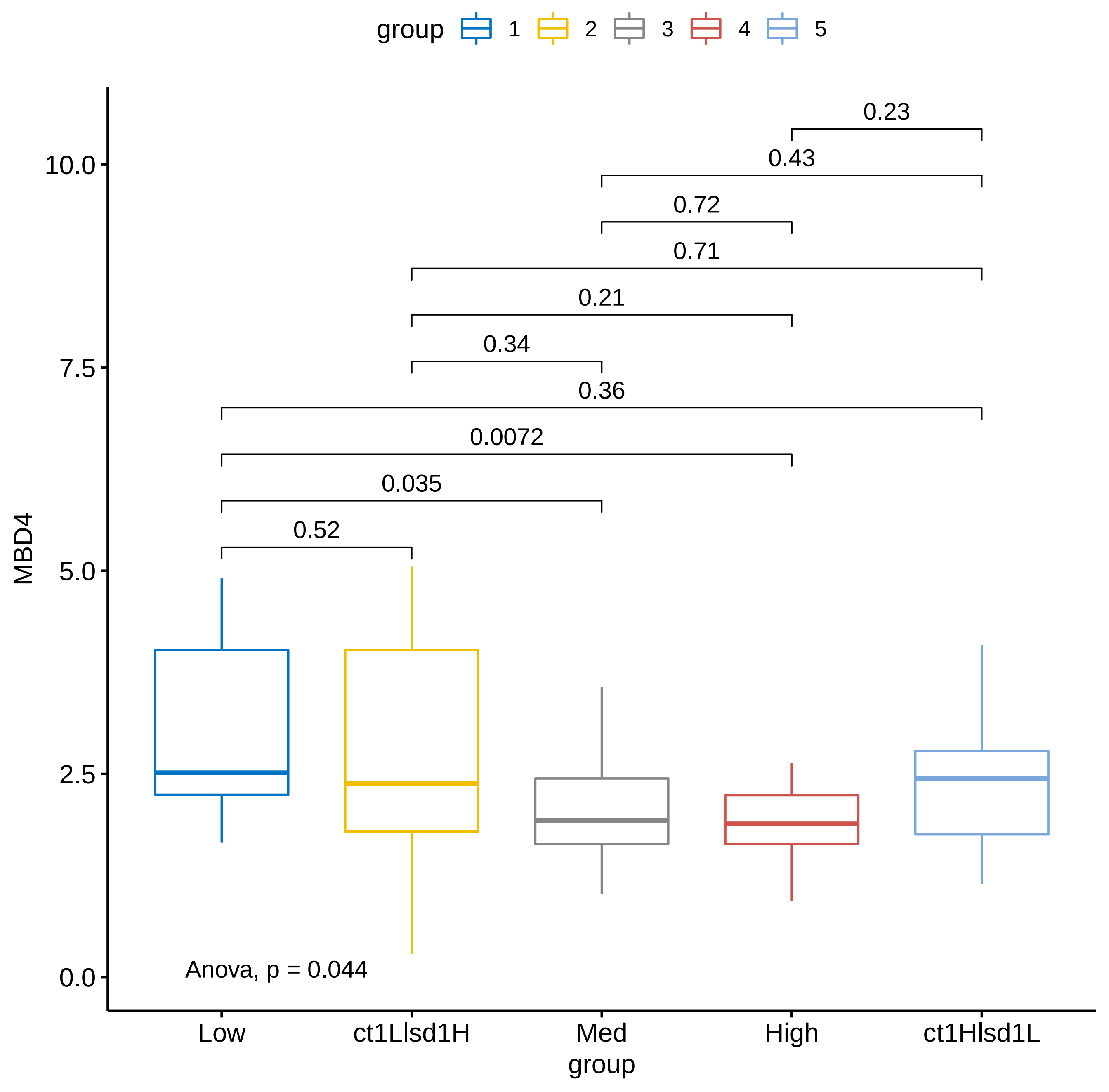

group
1
2
3
4
5
0.23
0.43
0.72
0.71
0.21
0.34
0.36
0.0072
0.035
0.52
10.0
7.5
5.0
2.5
0.0
MBD4
Anova, p = 0.044
Low
ct1Llsd1H
Med
High
ct1Hlsd1L
group

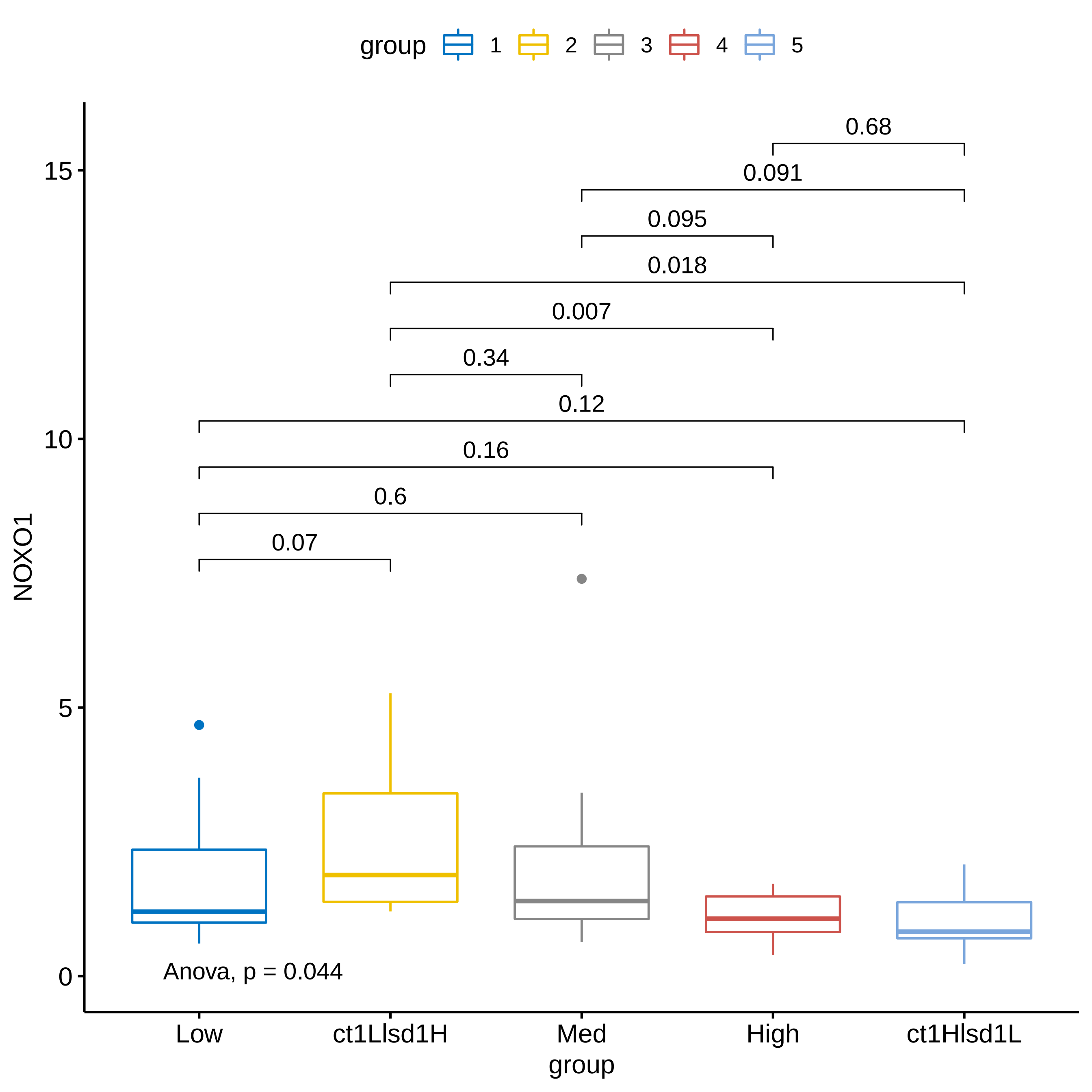
group 1 2 3 4 5
0.68
0.091
0.095
0.018
0.007
0.34
0.12
0.16
0.6
0.07
Anova, p = 0.044
15
10
5
0
NOXO1
Low
ct1Llsd1H
Med
High
ct1Hlsd1L
group

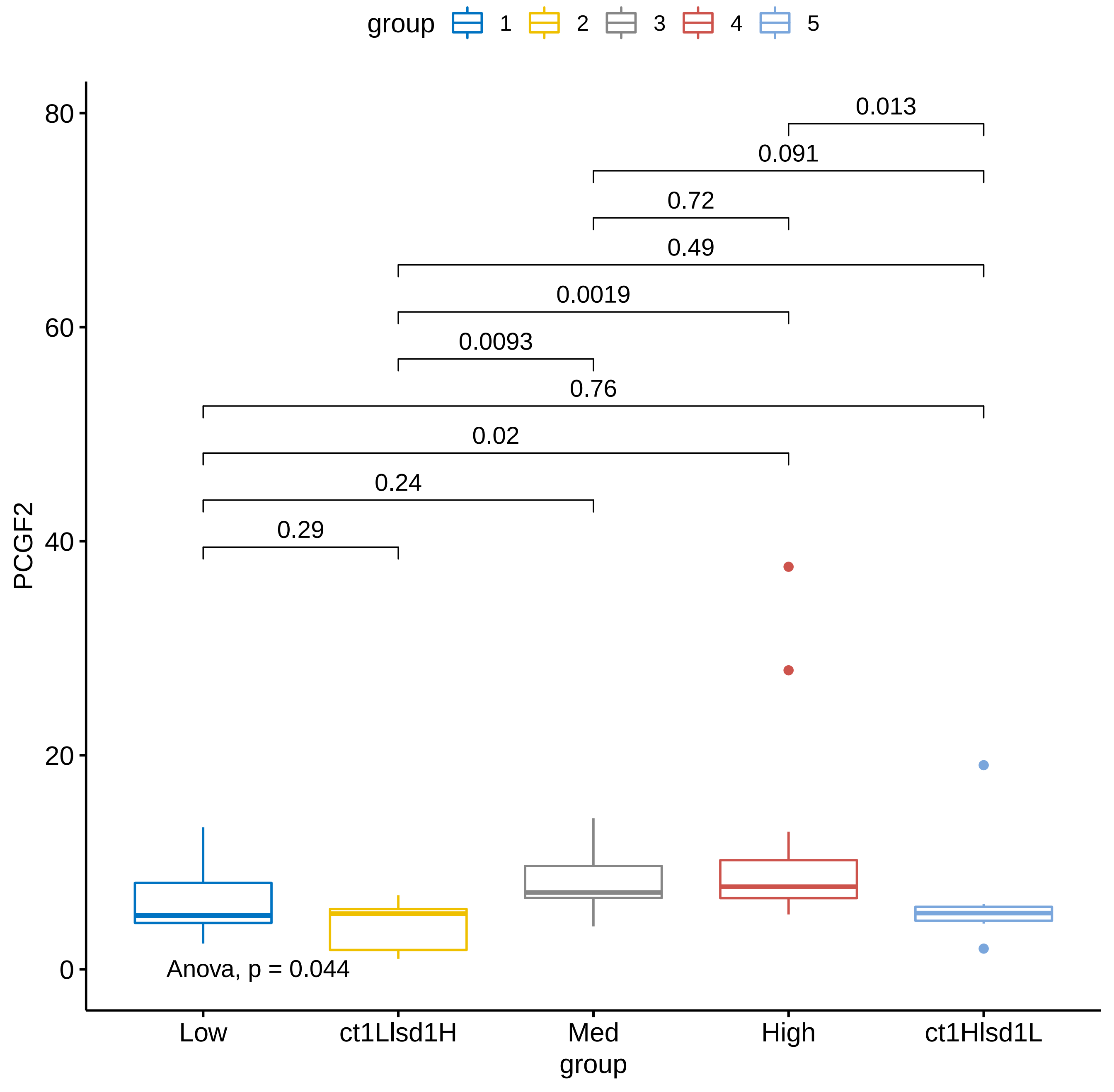
group
1
2
3
4
5
0.013
0.091
0.72
0.49
0.0019
0.0093
0.76
0.02
0.24
0.29
Anova, p = 0.044
PCGF2
0
20
40
60
80
Low
ct1Llsd1H
Med
High
ct1Hlsd1L
group

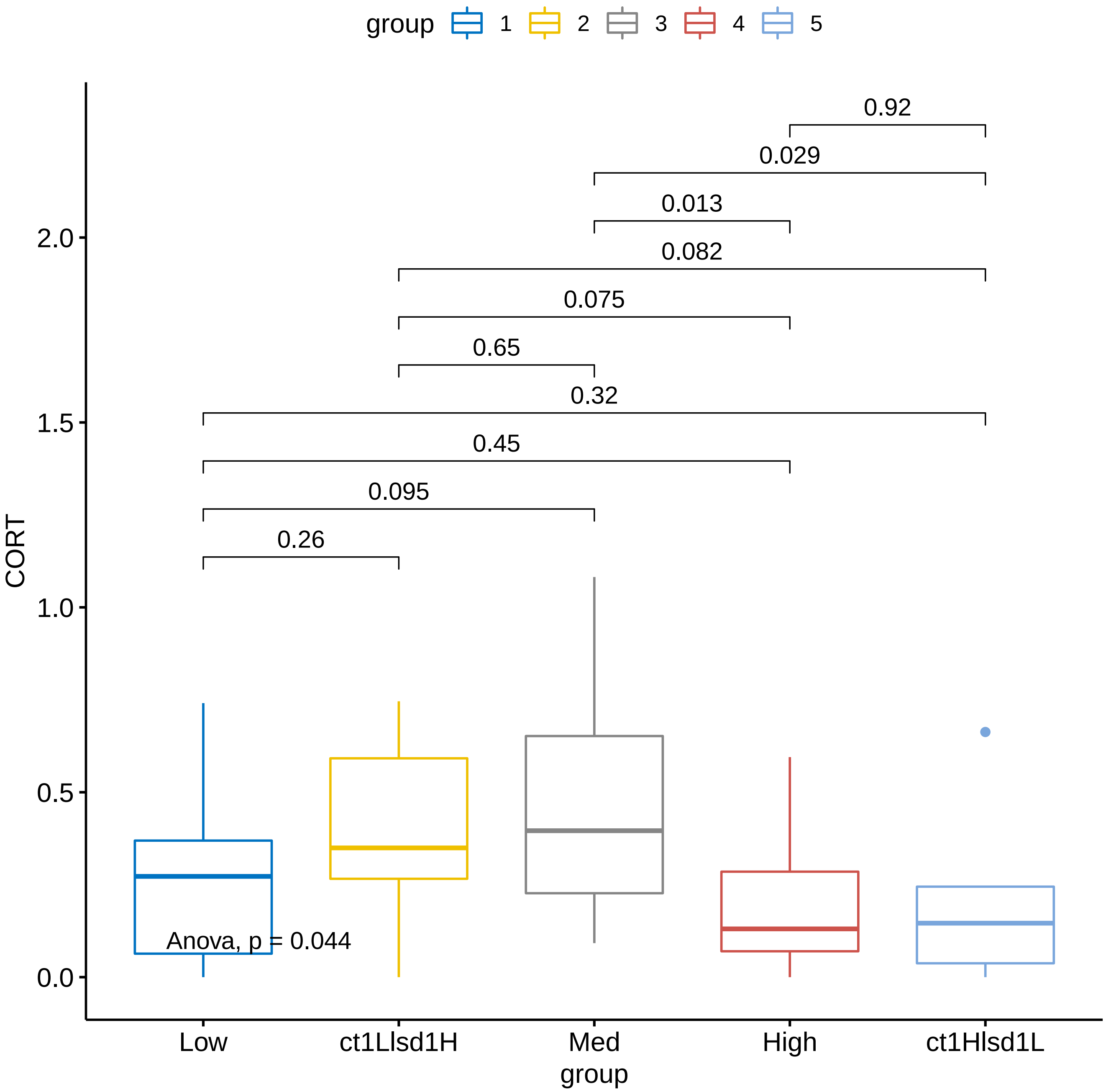

group
1
2
3
4
5
0.92
0.029
0.013
0.082
0.075
0.65
0.32
0.45
0.095
0.26
Anova, p = 0.044
CORT
2.0
1.5
1.0
0.5
0.0
Low
ct1Llsd1H
Med
High
ct1Hlsd1L
group

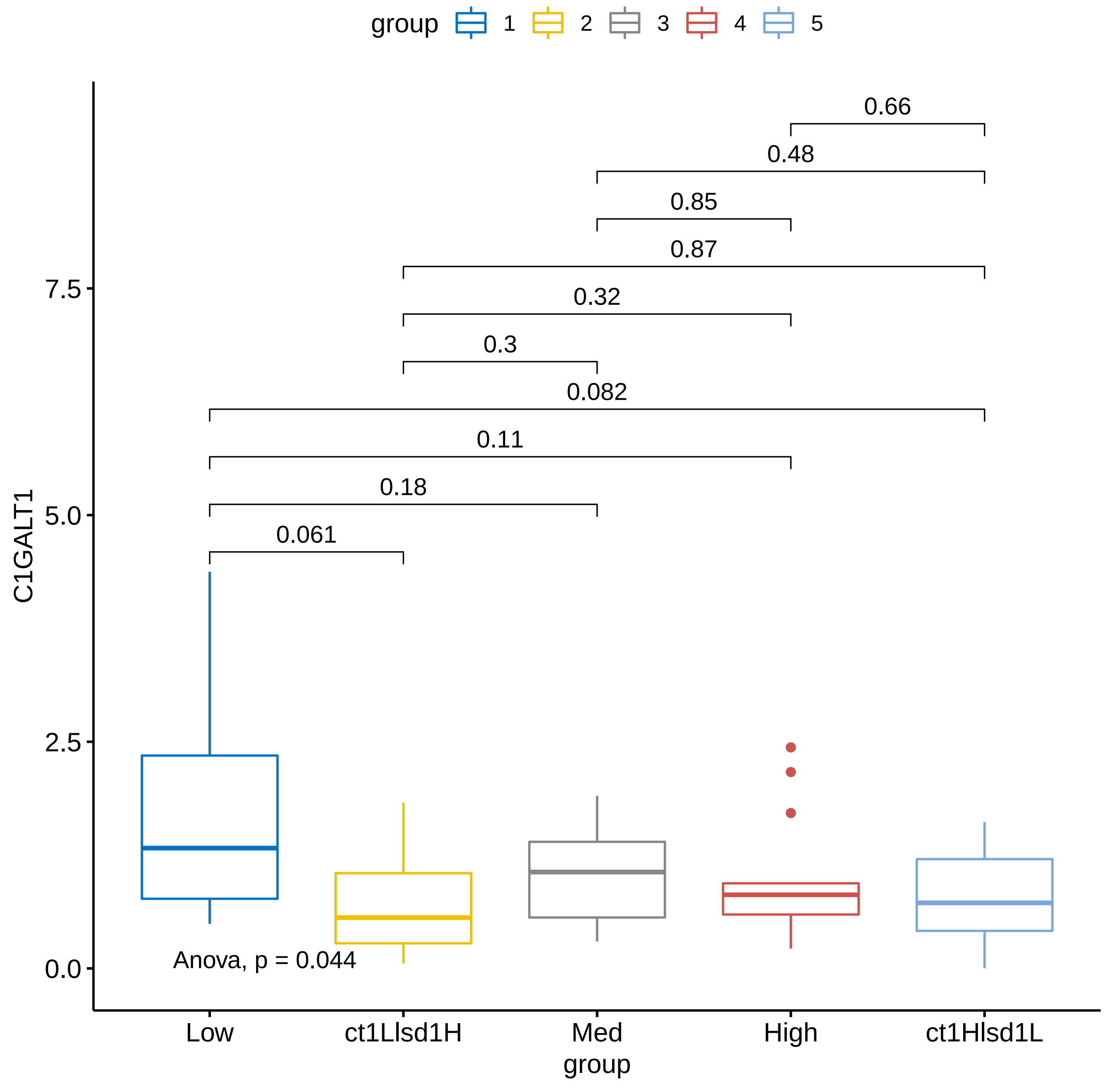

group 1 2 3 4 5
0.66
0.48
0.85
0.87
0.32
0.3
0.082
0.11
0.18
0.061
C1GALT1
7.5
5.0
2.5
0.0
Anova, p = 0.044
Low
ct1Llsd1H
Med
High
ct1Hlsd1L
group

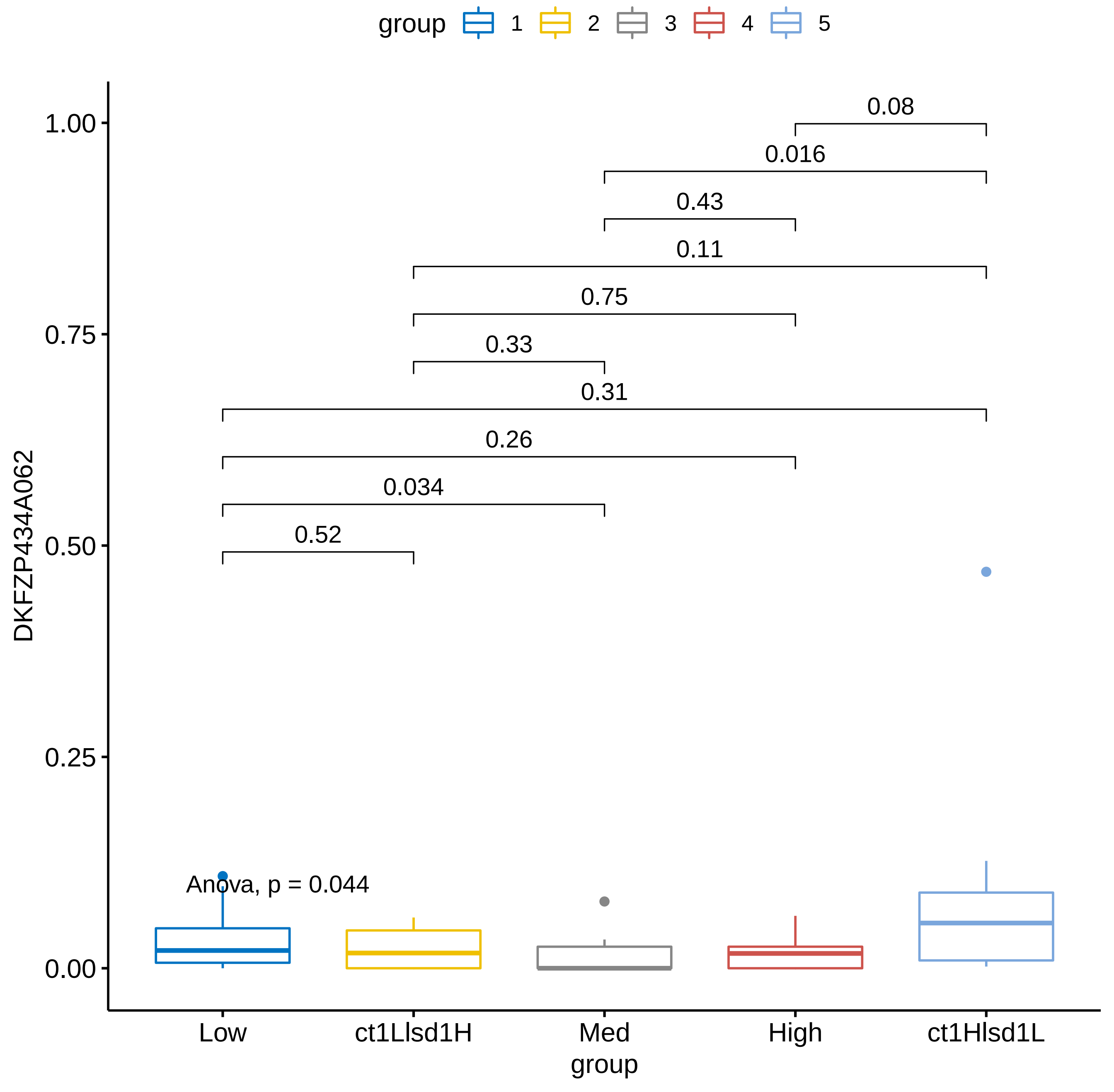

group
1
2
3
4
5
1.00
0.75
0.50
0.25
0.00
DKFZP434A062
0.08
0.016
0.43
0.11
0.75
0.33
0.31
0.26
0.034
0.52
Anova, p = 0.044
Low
ct1Llsd1H
Med
High
ct1Hlsd1L
group

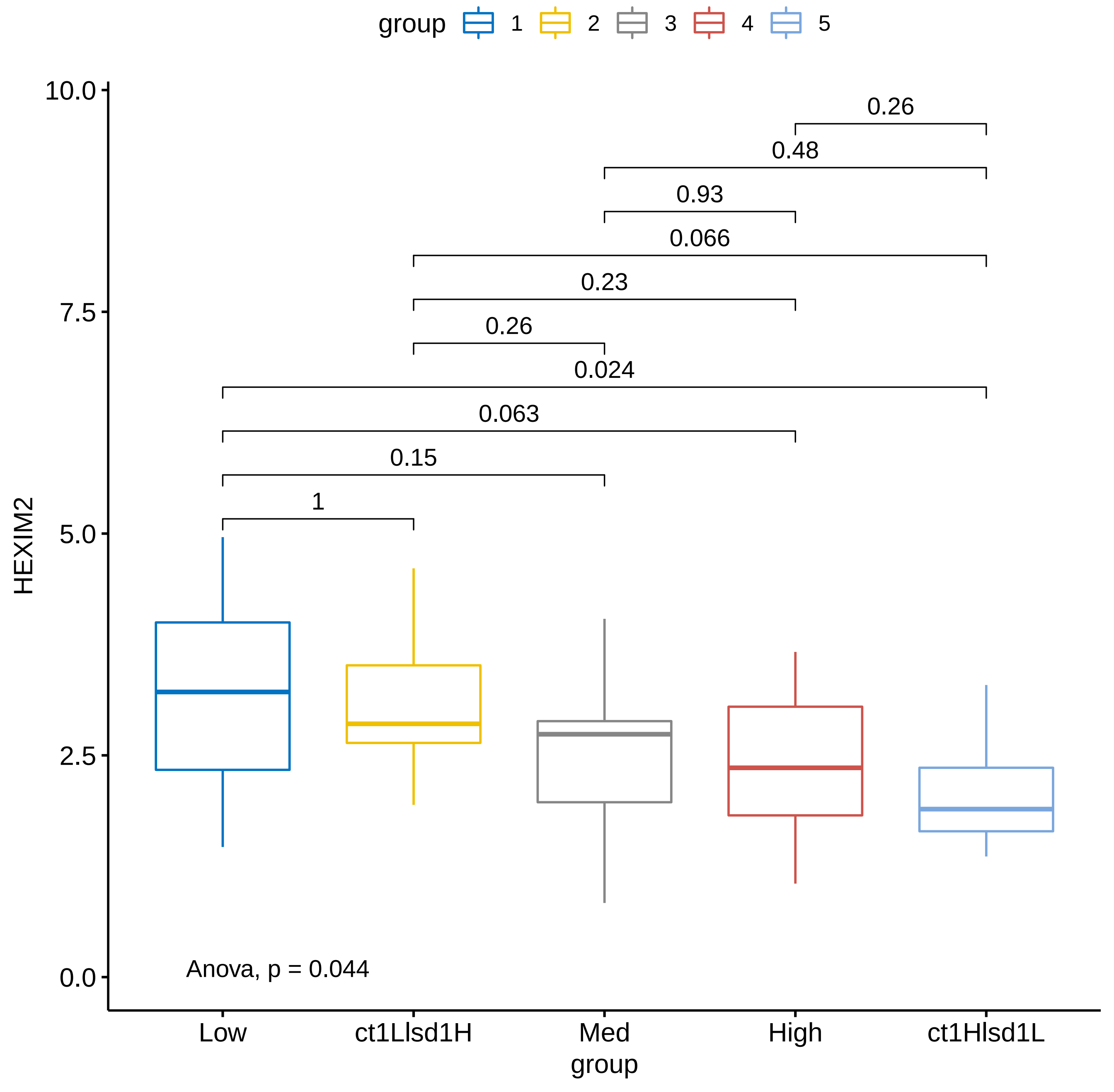

group
1
2
3
4
5
10.0
7.5
5.0
2.5
0.0
HEXIM2
0.26
0.48
0.93
0.066
0.23
0.26
0.024
0.063
0.15
1
Anova, p = 0.044
Low
ct1Llsd1H
Med
High
ct1Hlsd1L
group

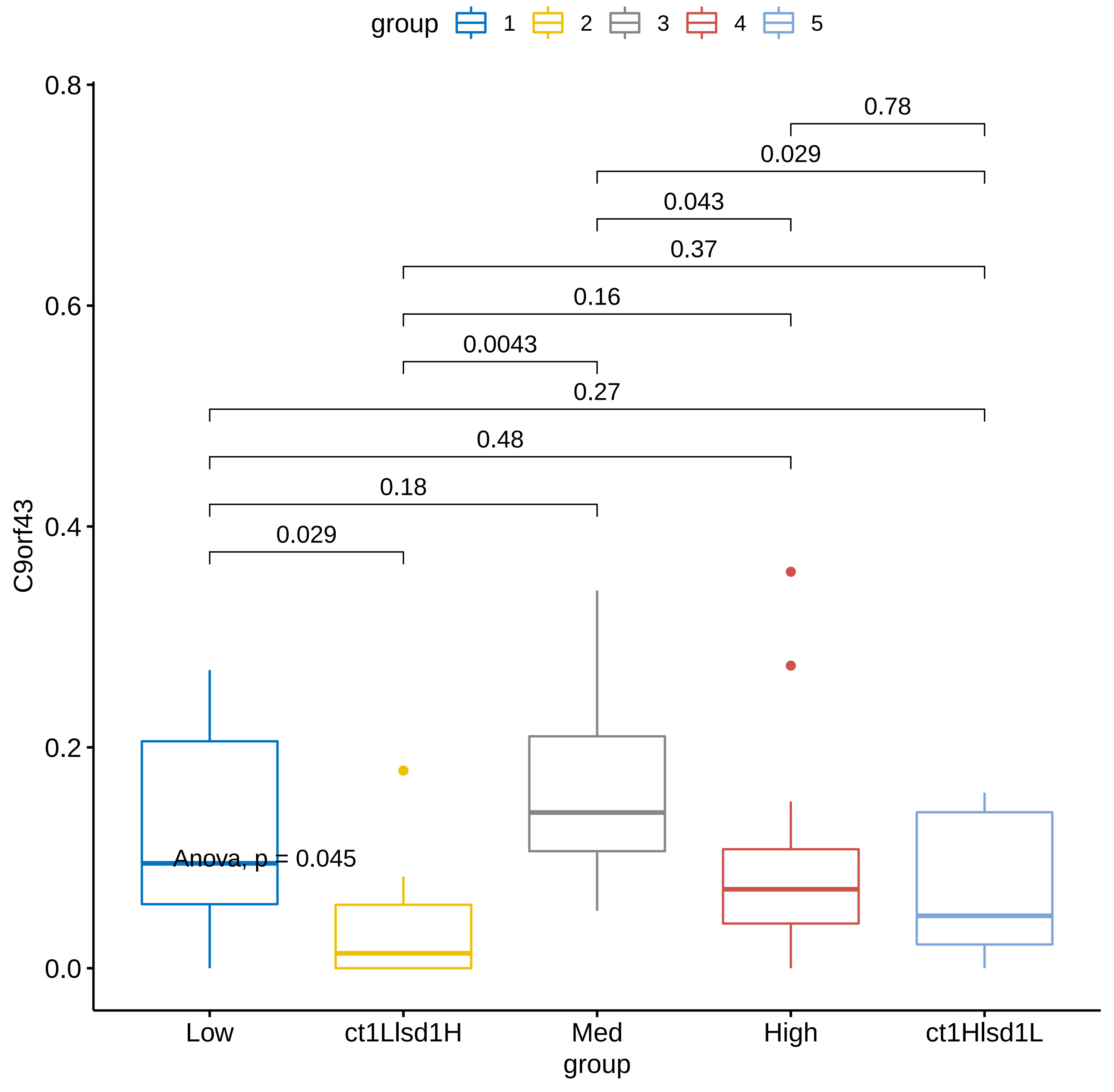

group
1
2
3
4
5
0.8
0.6
0.4
0.2
0.0
C9orf43
0.78
0.029
0.043
0.37
0.16
0.0043
0.27
0.48
0.18
0.029
Anova, p = 0.045
Low
ct1Llsd1H
Med
High
ct1Hlsd1L
group

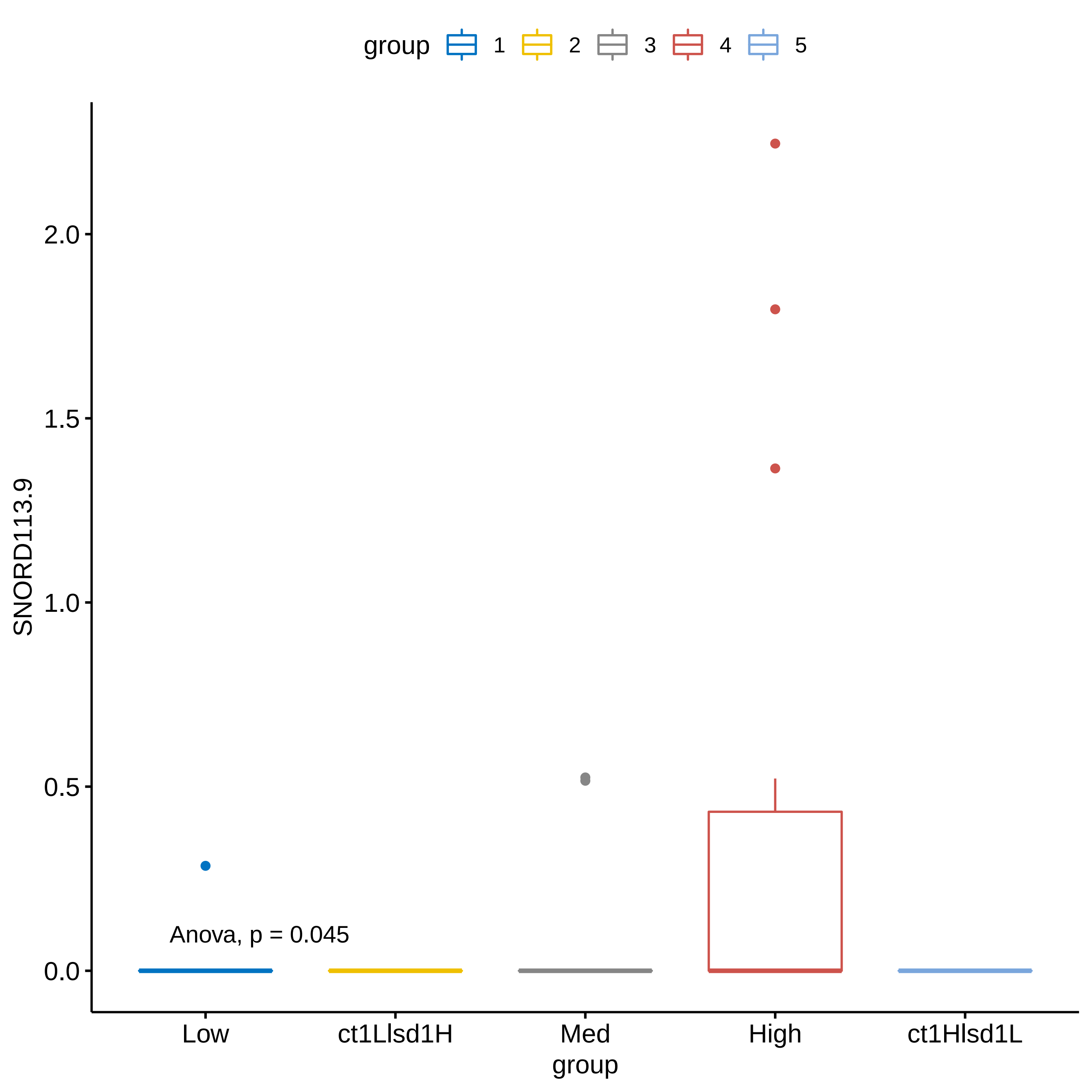
group
1
2
3
4
5
SNORD113.9
2.0
1.5
1.0
0.5
0.0
Anova, p = 0.045
Low
ct1Llsd1H
Med
High
ct1Hlsd1L
group

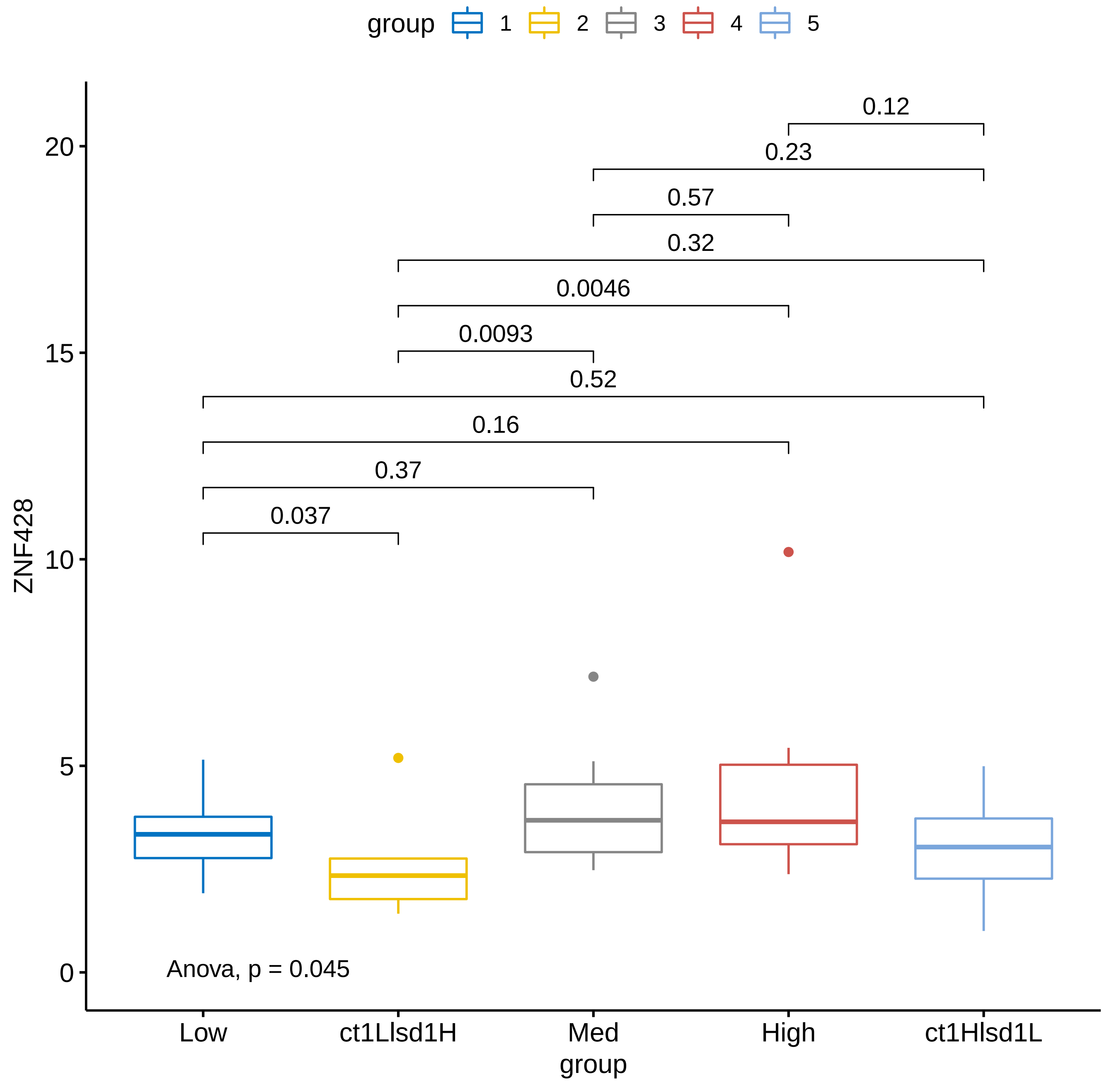
group
1
2
3
4
5
0.12
0.23
0.57
0.32
0.0046
0.0093
0.52
0.16
0.37
0.037
20
15
10
5
0
ZNF428
Anova, p = 0.045
Low
ct1Llsd1H
Med
High
ct1Hlsd1L
group

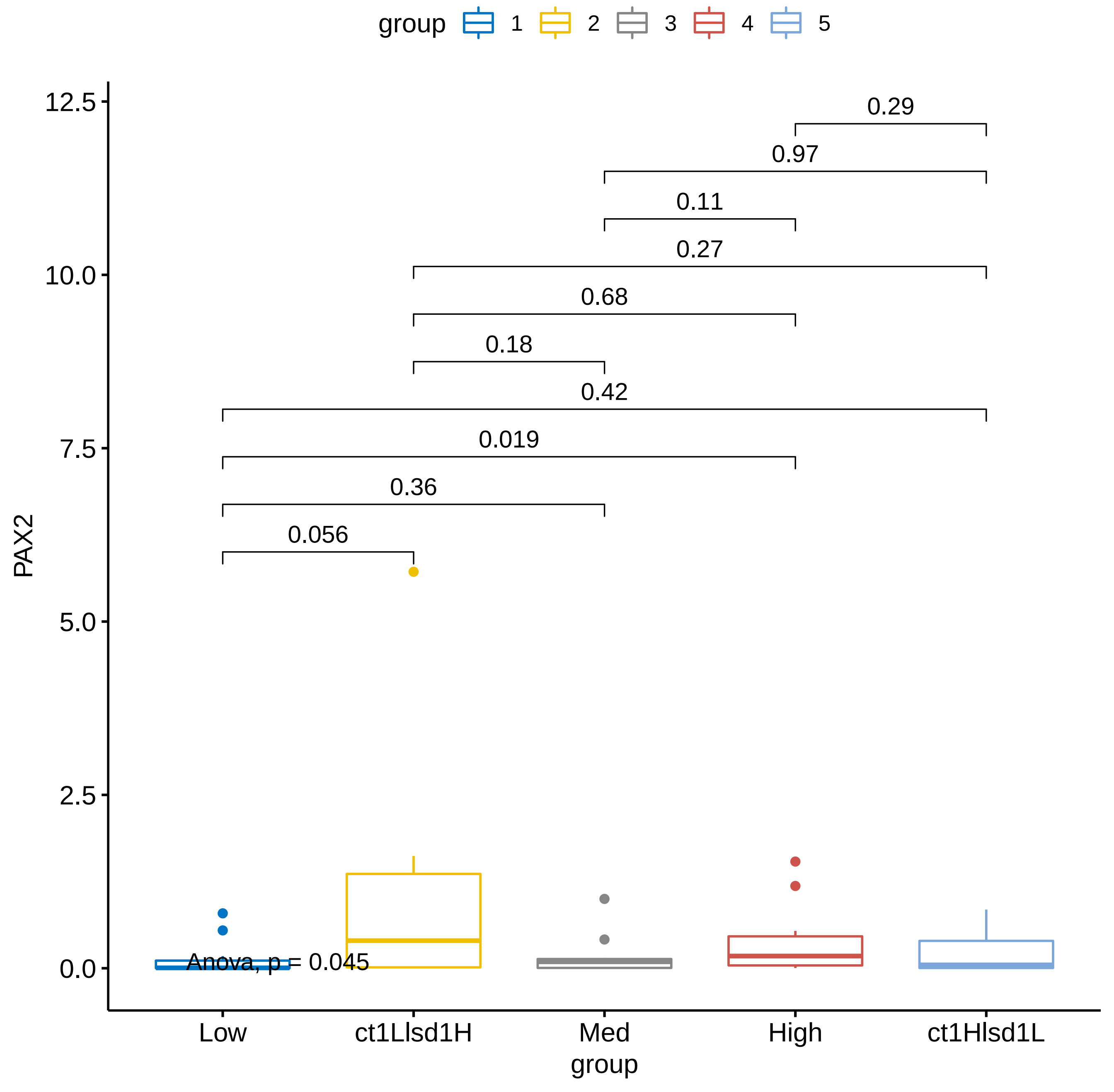

group
1
2
3
4
5
PAX2
12.5
10.0
7.5
5.0
2.5
0.0
0.29
0.97
0.11
0.27
0.68
0.18
0.42
0.019
0.36
0.056
Anova, p = 0.045
Low
ct1Llsd1H
Med
High
ct1Hlsd1L
group

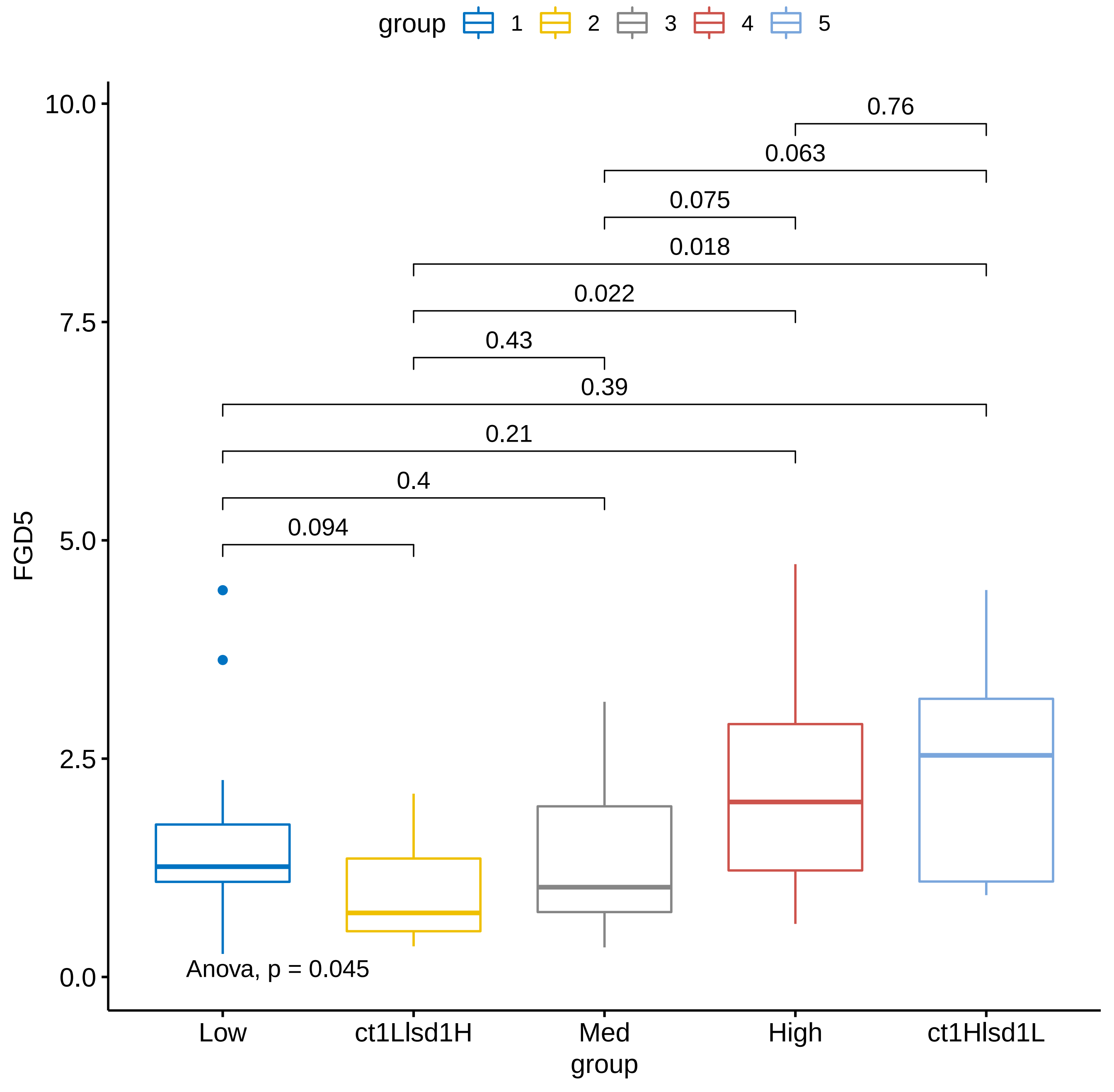
group 1 2 3 4 5
0.76
0.063
0.075
0.018
0.022
0.43
0.39
0.21
0.4
0.094
Anova, p = 0.045
10.0
7.5
5.0
2.5
0.0
FGD5
Low
ct1Llsd1H
Med
High
ct1Hlsd1L
group

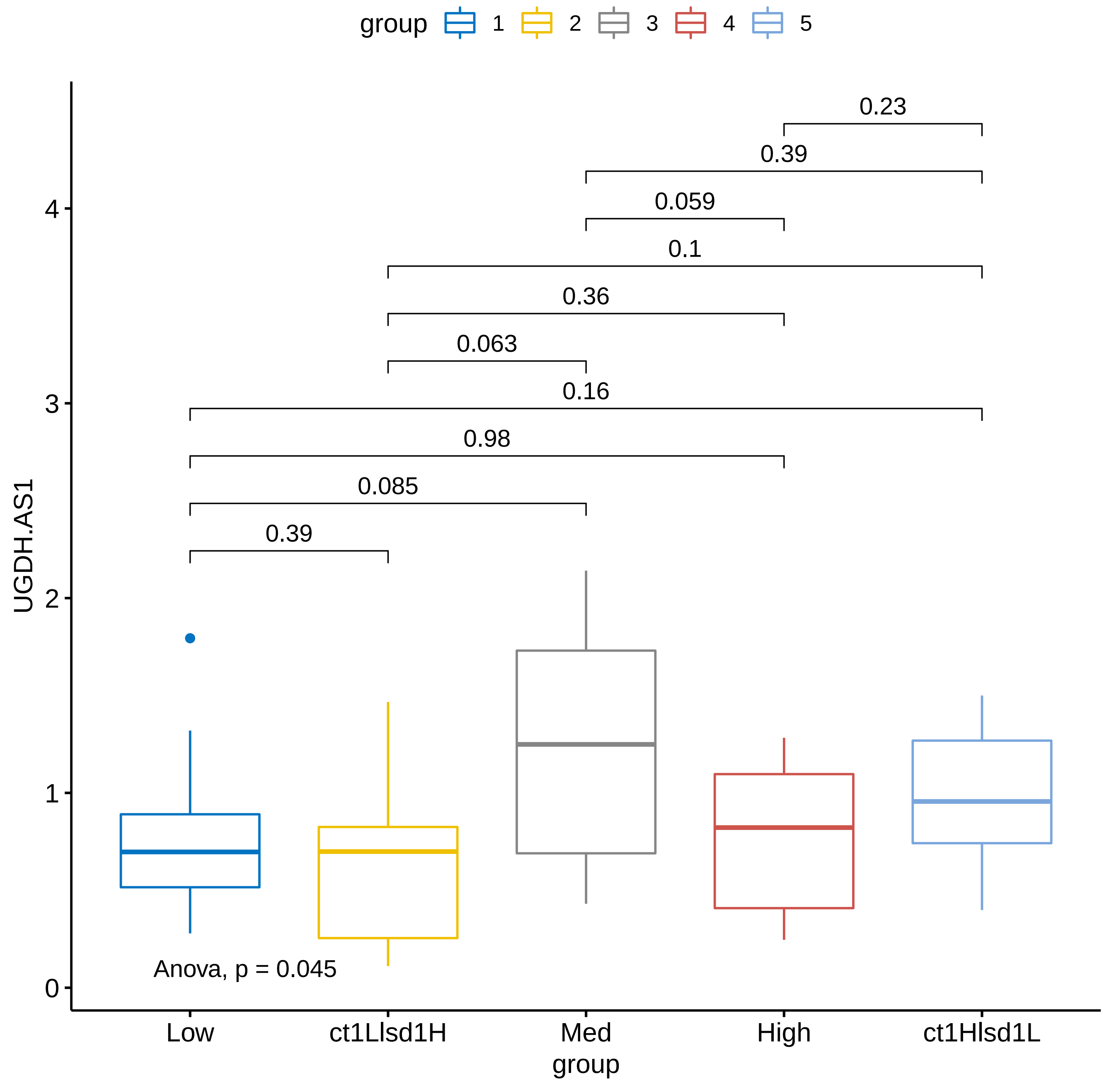

group
1
2
3
4
5
0.23
0.39
0.059
0.1
0.36
0.063
0.16
0.98
0.085
0.39
Anova, p = 0.045
UGDH.AS1
0
1
2
3
4
Low
ct1Llsd1H
Med
High
ct1Hlsd1L
group

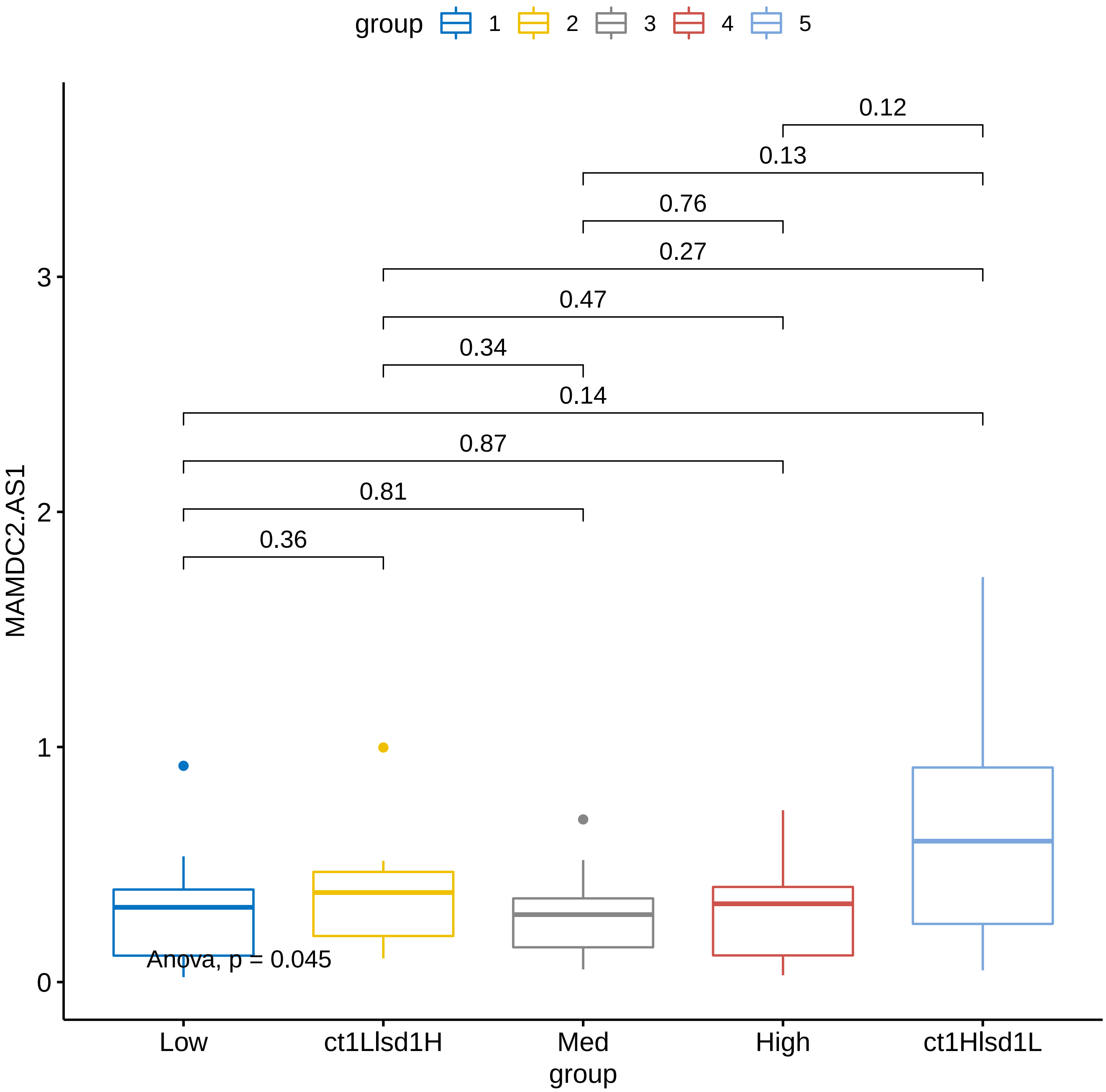
group
1
2
3
4
5
0.12
0.13
0.76
0.27
0.47
0.34
0.14
0.87
0.81
0.36
Anova, p = 0.045
MAMDC2.AS1
0
1
2
3
Low
ct1Llsd1H
Med
High
ct1Hlsd1L
group

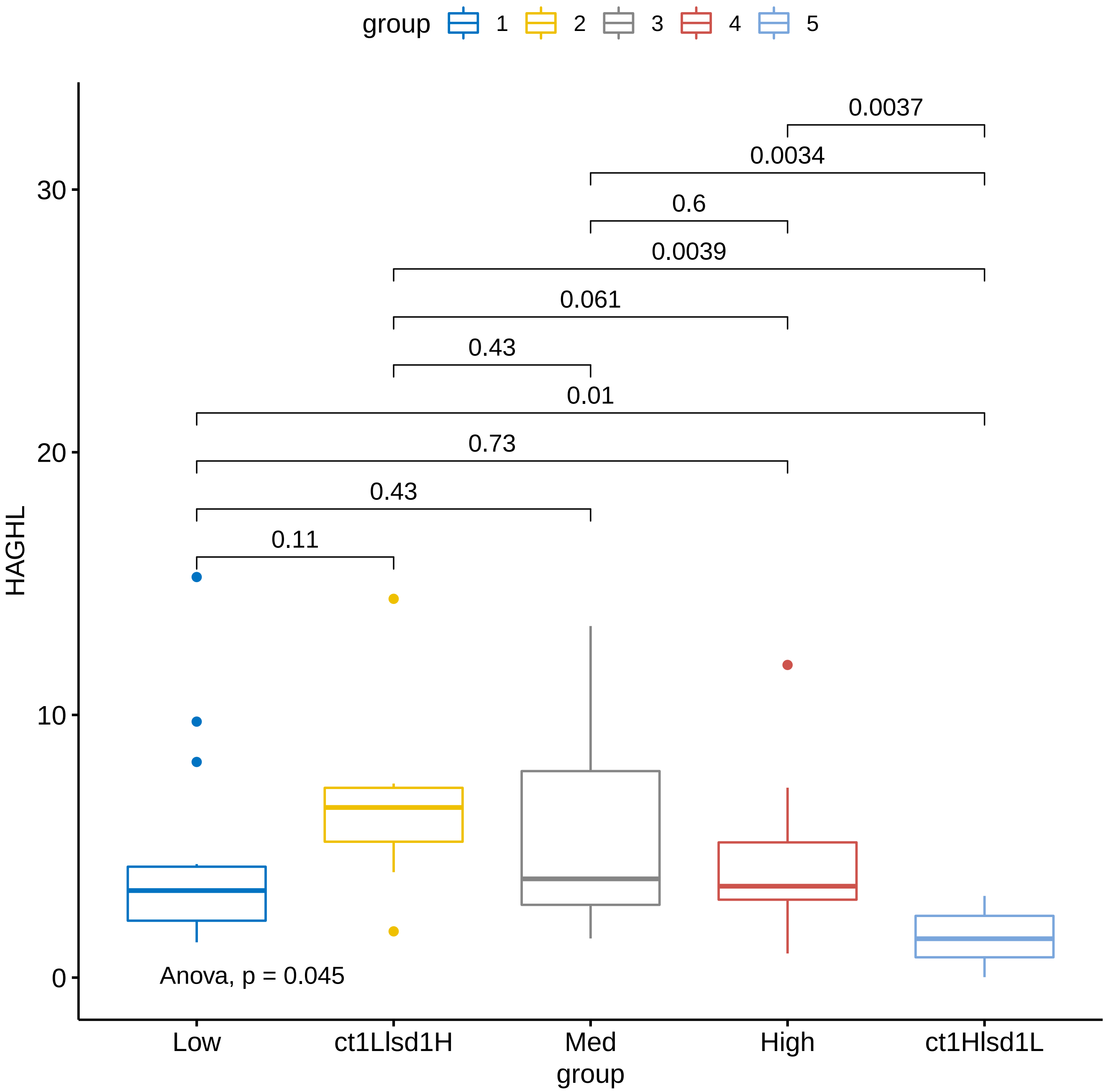

group 1 2 3 4 5
0.0037
0.0034
0.6
0.0039
0.061
0.43
0.01
0.73
0.43
0.11
30
20
10
0
HAGHL
Anova, p = 0.045
Low
ct1Llsd1H
Med
High
ct1Hlsd1L
group

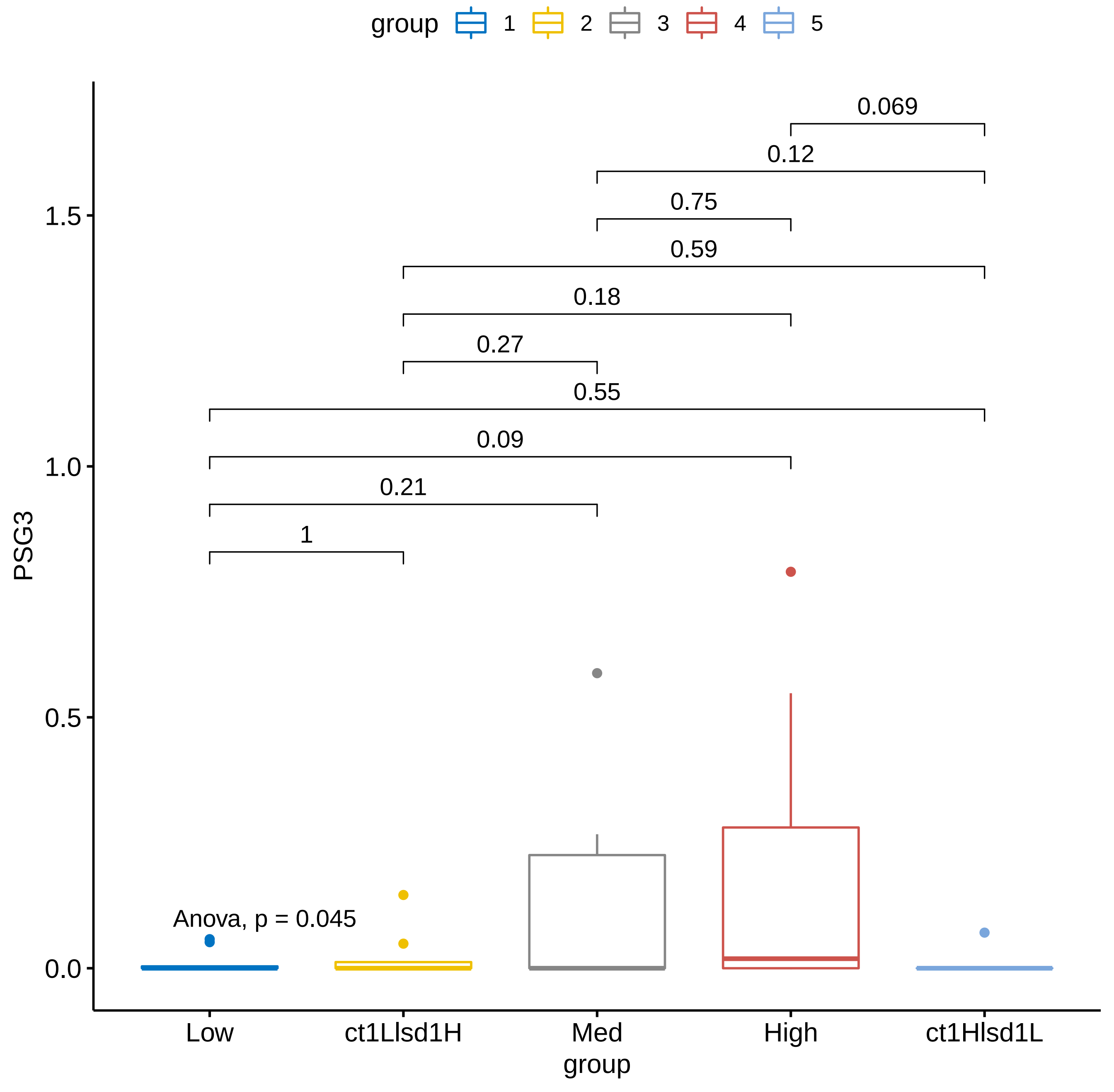
group 1 2 3 4 5
0.069
0.12
0.75
0.59
0.18
0.27
0.55
0.09
0.21
1
Anova, p = 0.045
PSG3
1.5
1.0
0.5
0.0
Low
ct1Llsd1H
Med
High
ct1Hlsd1L
group

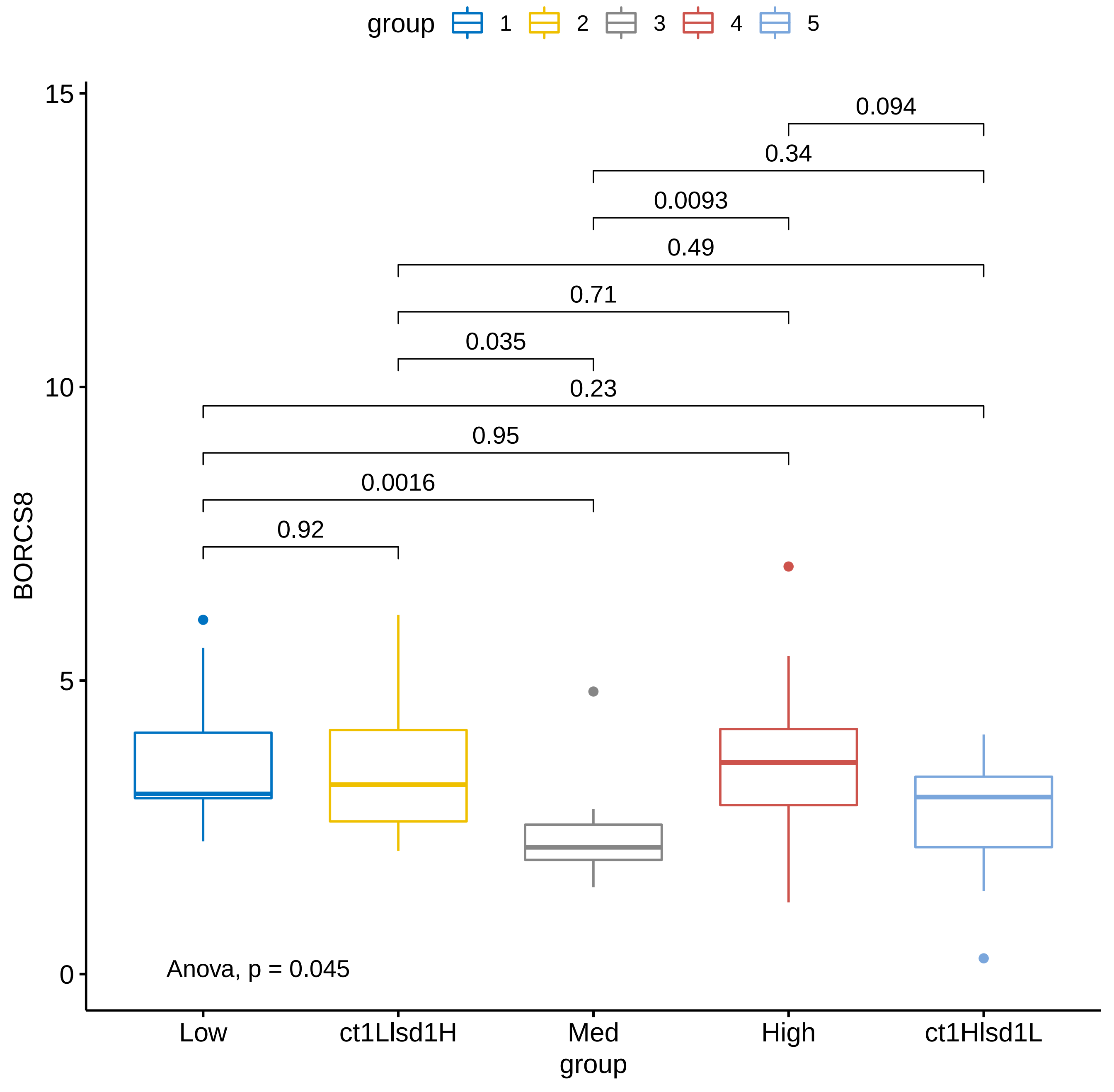

group
1
2
3
4
5
BORCS8
15
10
5
0
0.094
0.34
0.0093
0.49
0.71
0.035
0.23
0.95
0.0016
0.92
Anova, p = 0.045
Low
ct1Llsd1H
Med
High
ct1Hlsd1L
group

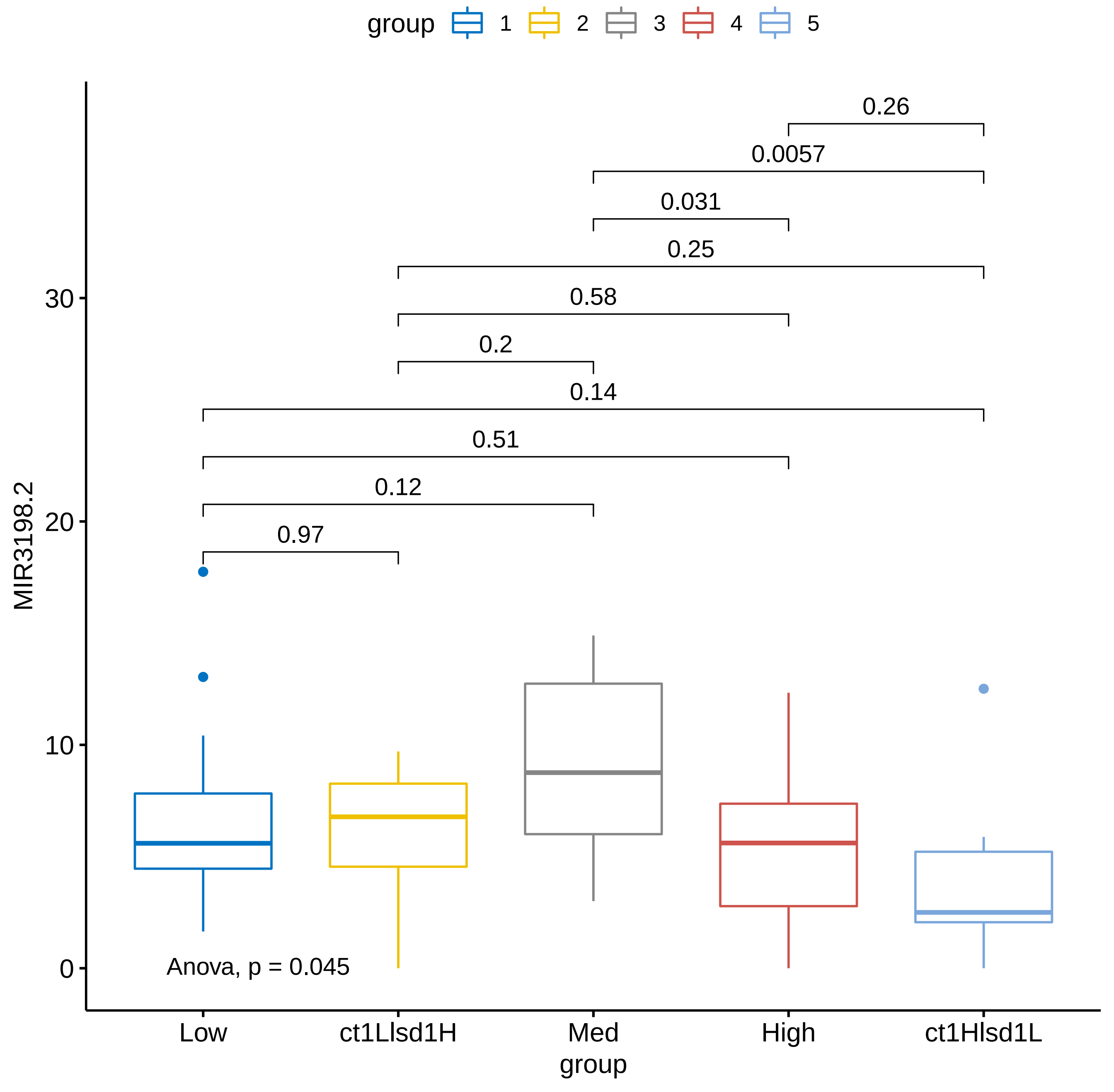

group
1
2
3
4
5
0.26
0.0057
0.031
0.25
0.58
0.2
0.14
0.51
0.12
0.97
30
20
10
0
MIR3198.2
Anova, p = 0.045
Low
ct1Llsd1H
Med
High
ct1Hlsd1L
group

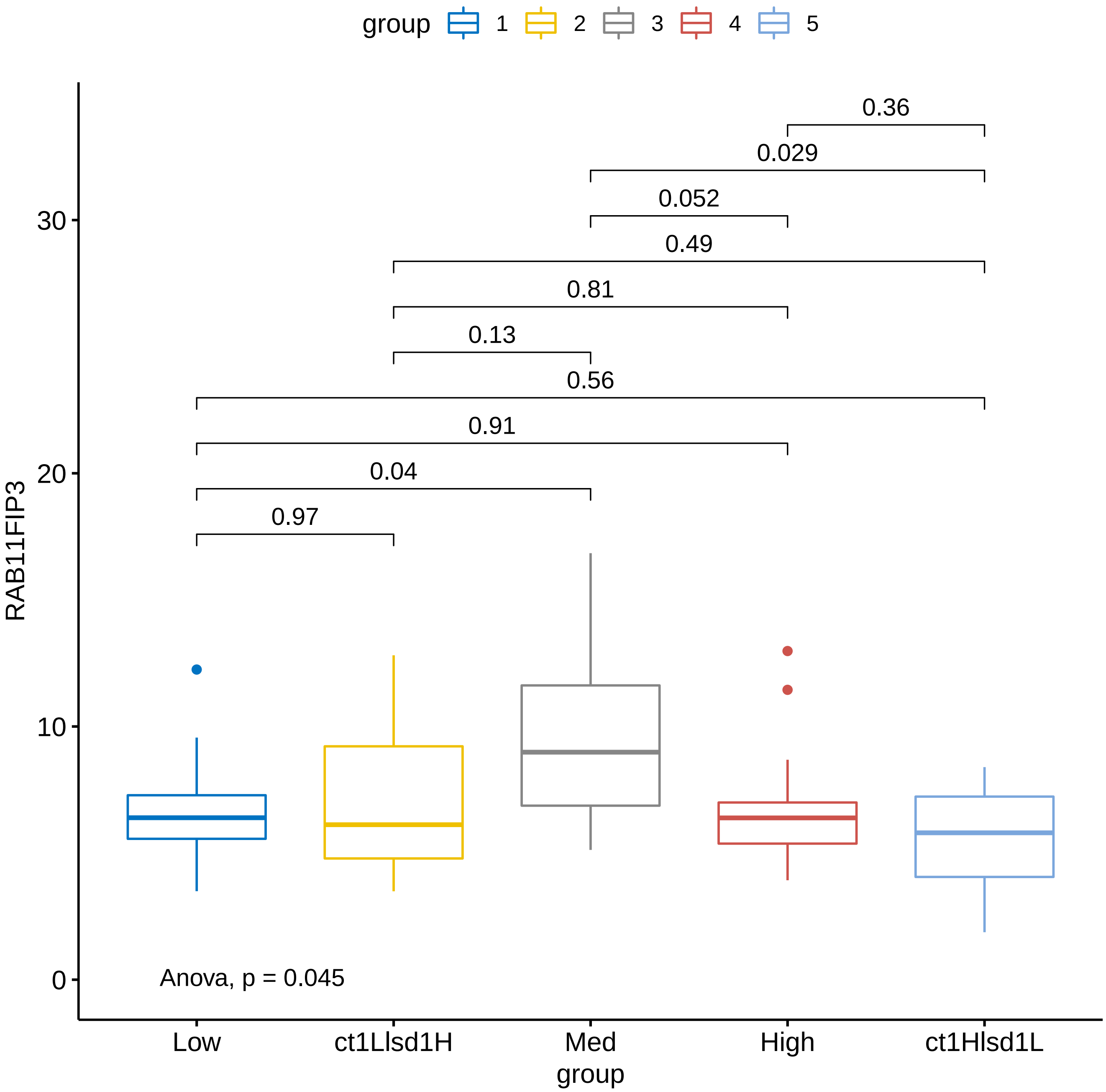
group
1
2
3
4
5
0.36
0.029
0.052
0.49
0.81
0.13
0.56
0.91
0.04
0.97
30
20
10
0
RAB11FIP3
Anova, p = 0.045
Low
ct1Llsd1H
Med
High
ct1Hlsd1L
group

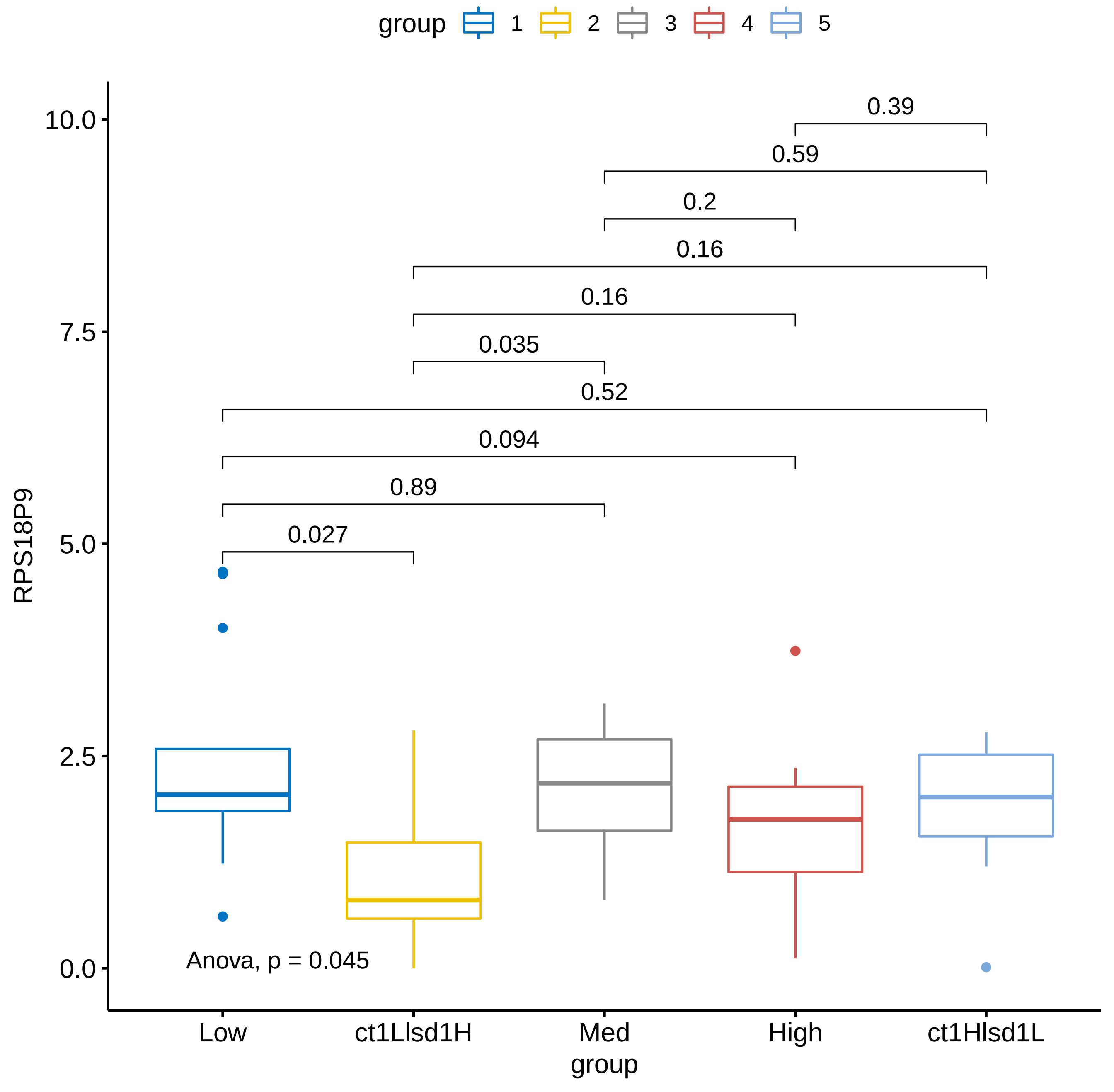

group 1 2 3 4 5
0.39
0.59
0.2
0.16
0.16
0.035
0.52
0.094
0.89
0.027
Anova, p = 0.045
10.0
7.5
5.0
2.5
0.0
RPS18P9
Low
ct1Llsd1H
Med
High
ct1Hlsd1L
group

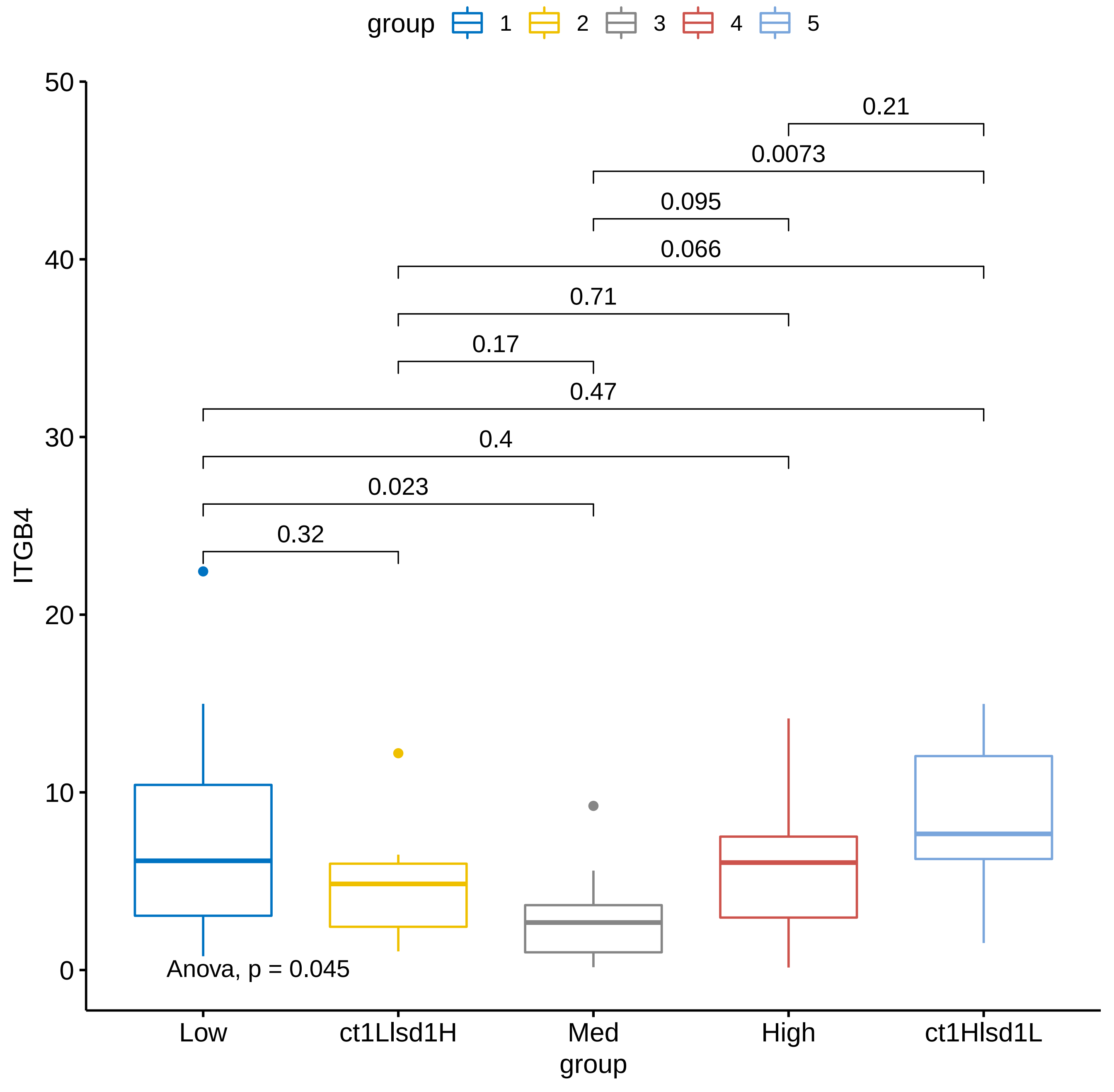

group 1 2 3 4 5
ITGB4
50
40
30
20
10
0
0.21
0.0073
0.095
0.066
0.71
0.17
0.47
0.4
0.023
0.32
Anova, p = 0.045
Low
ct1Llsd1H
Med
High
ct1Hlsd1L
group

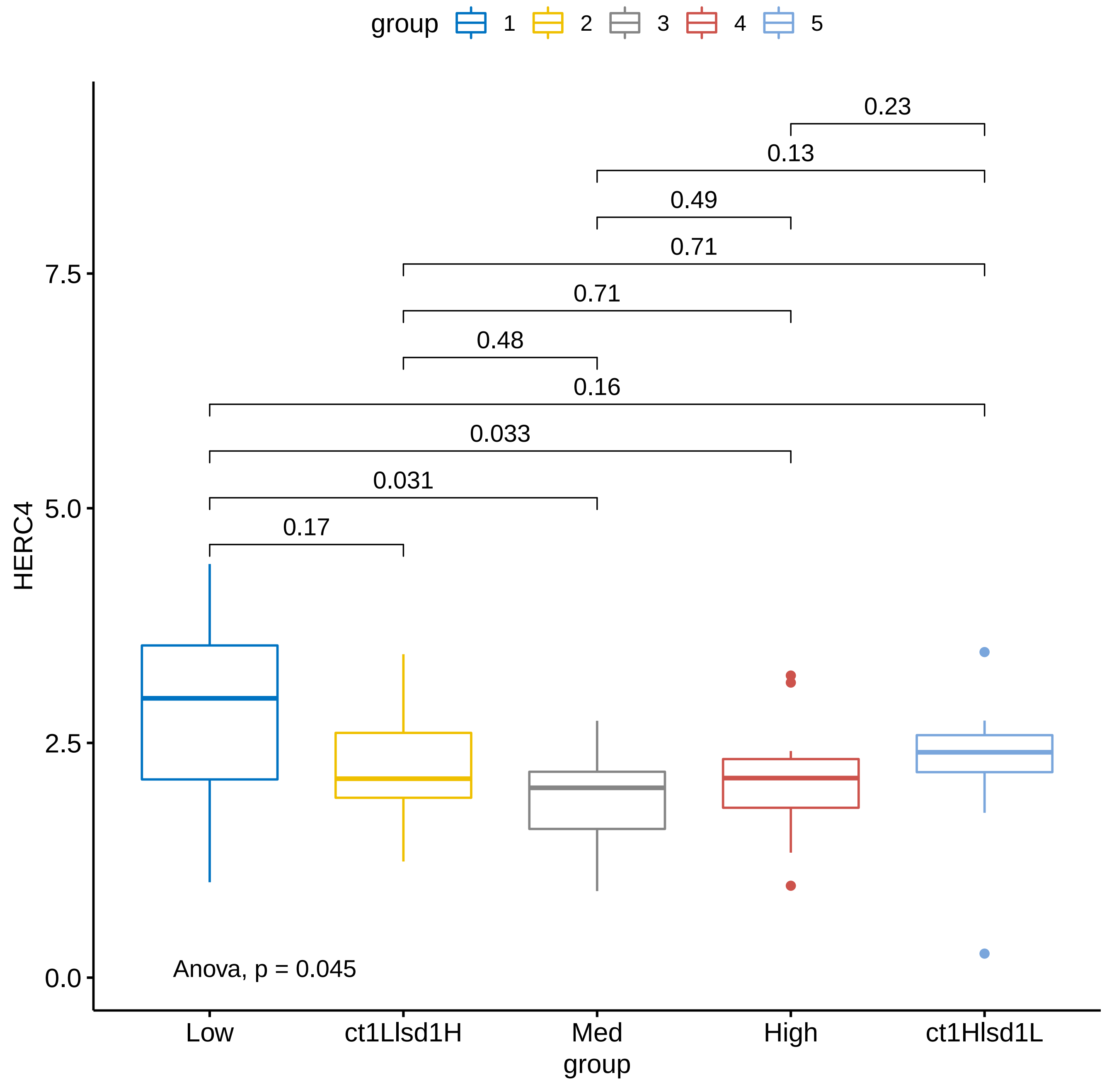
group 1 2 3 4 5
0.23
0.13
0.49
0.71
0.71
0.48
0.16
0.033
0.031
0.17
7.5
5.0
2.5
0.0
HERC4
Anova, p = 0.045
Low
ct1Llsd1H
Med
High
ct1Hlsd1L
group

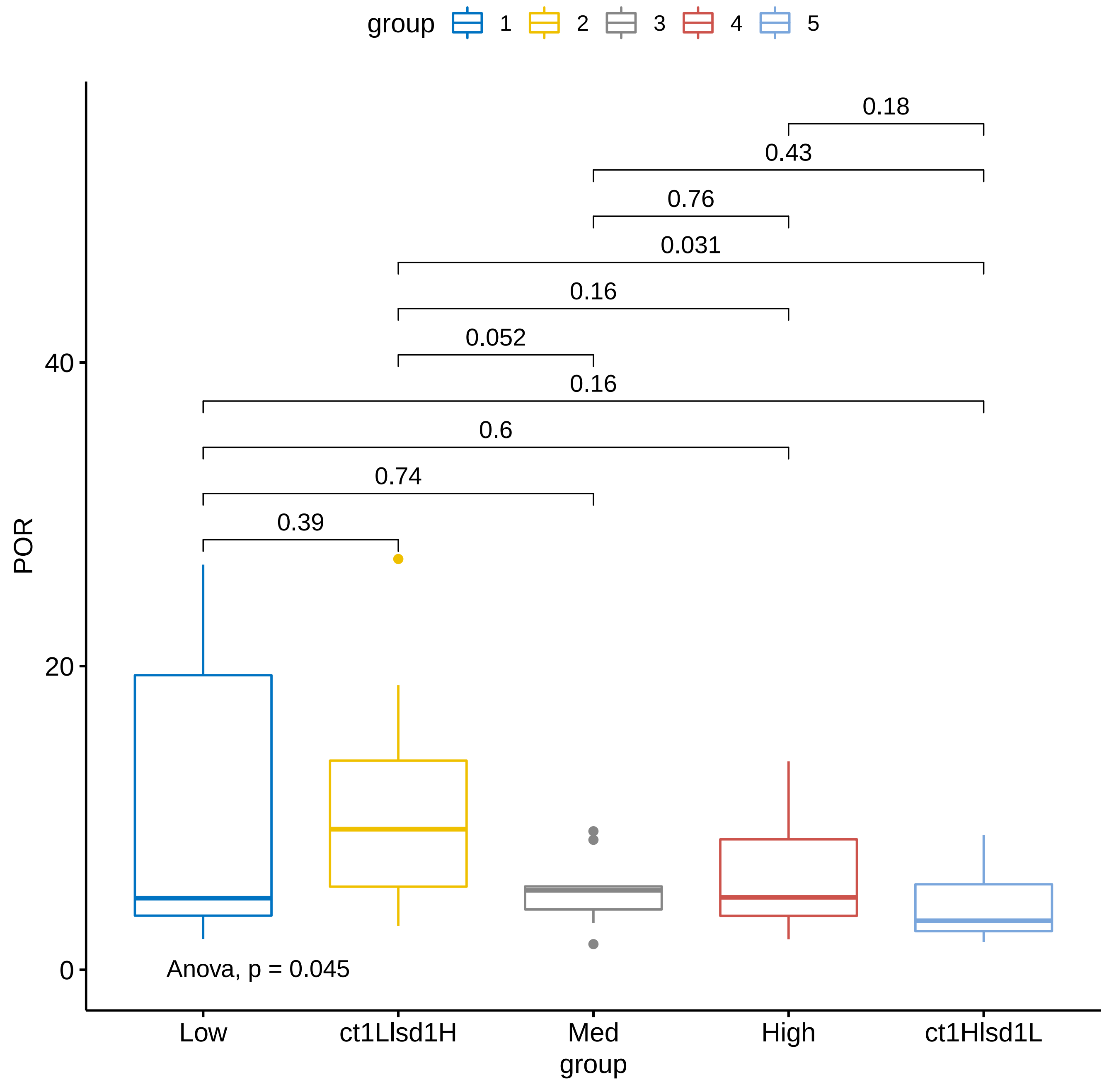

group 1 2 3 4 5
0.18
0.43
0.76
0.031
0.16
0.052
40
0.16
0.6
0.74
0.39
POR
20
0
Anova, p = 0.045
Low
ct1Llsd1H
Med
High
ct1Hlsd1L
group

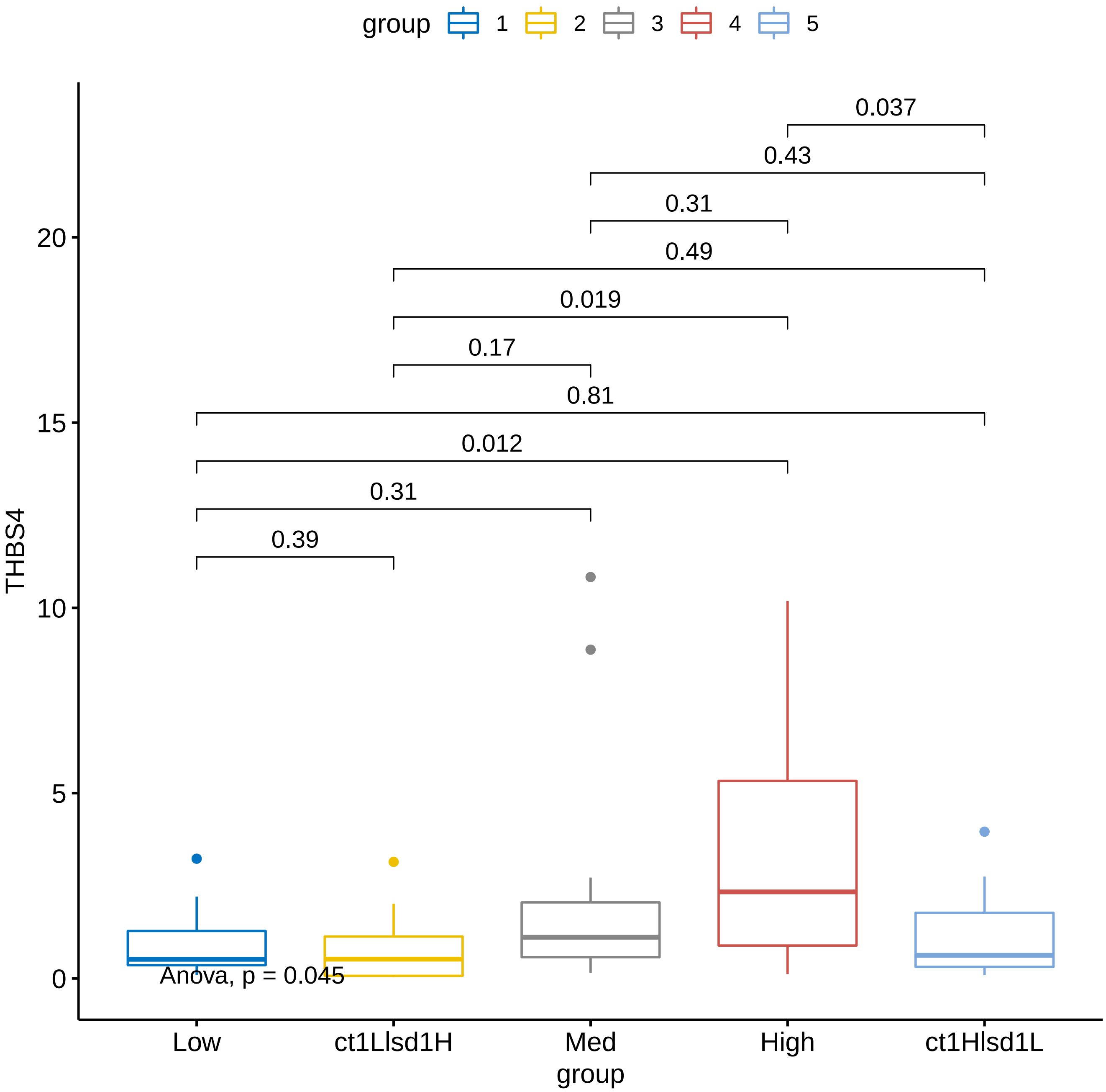
group
1
2
3
4
5
0.037
0.43
0.31
0.49
0.019
0.17
0.81
0.012
0.31
0.39
THBS4
20
15
10
5
0
Anova, p = 0.045
Low
ct1Llsd1H
Med
High
ct1Hlsd1L
group

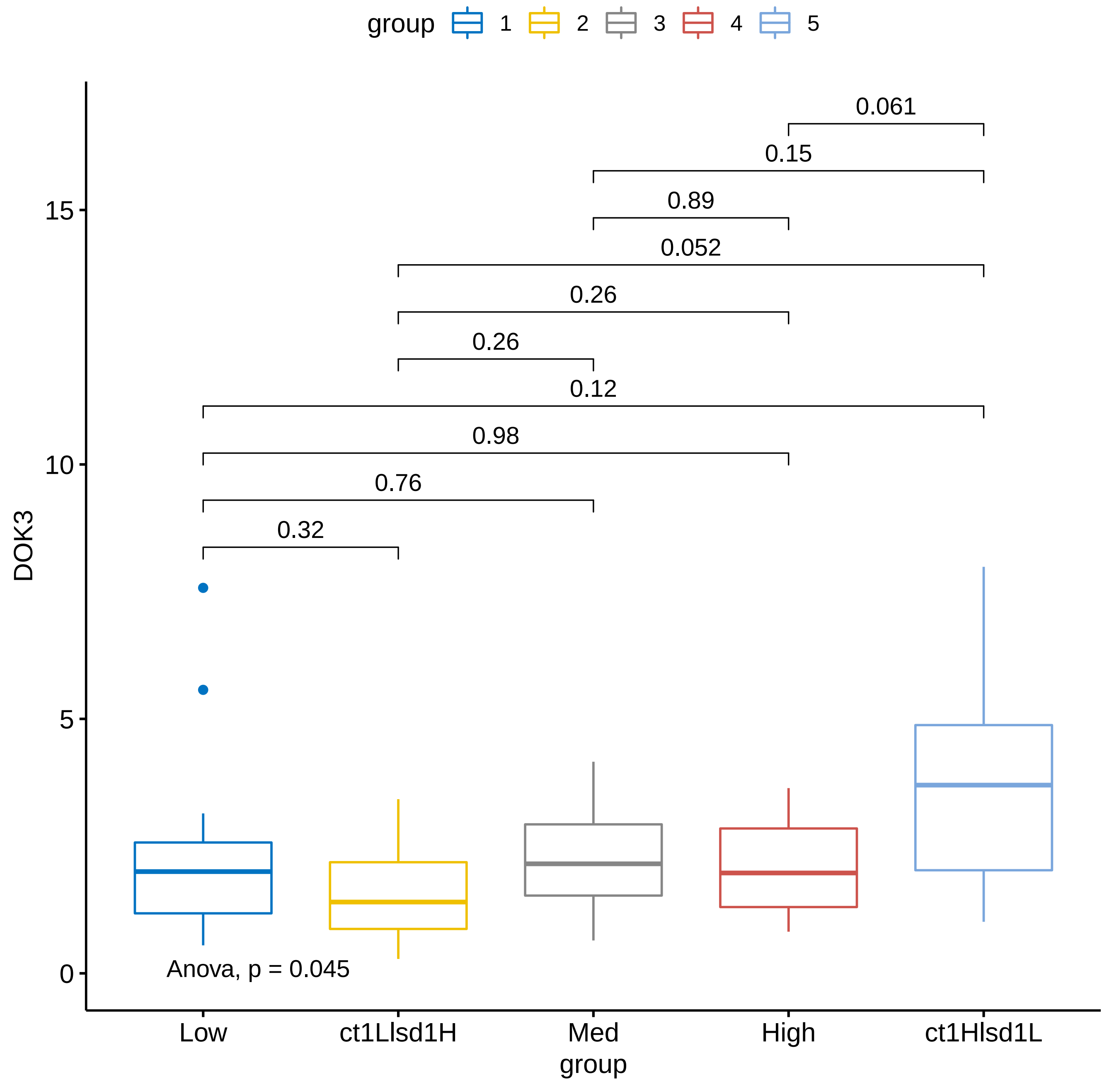
group
1
2
3
4
5
0.061
0.15
0.89
0.052
0.26
0.26
0.12
0.98
0.76
0.32
15
10
5
0
DOK3
Anova, p = 0.045
Low
ct1Llsd1H
Med
High
ct1Hlsd1L
group

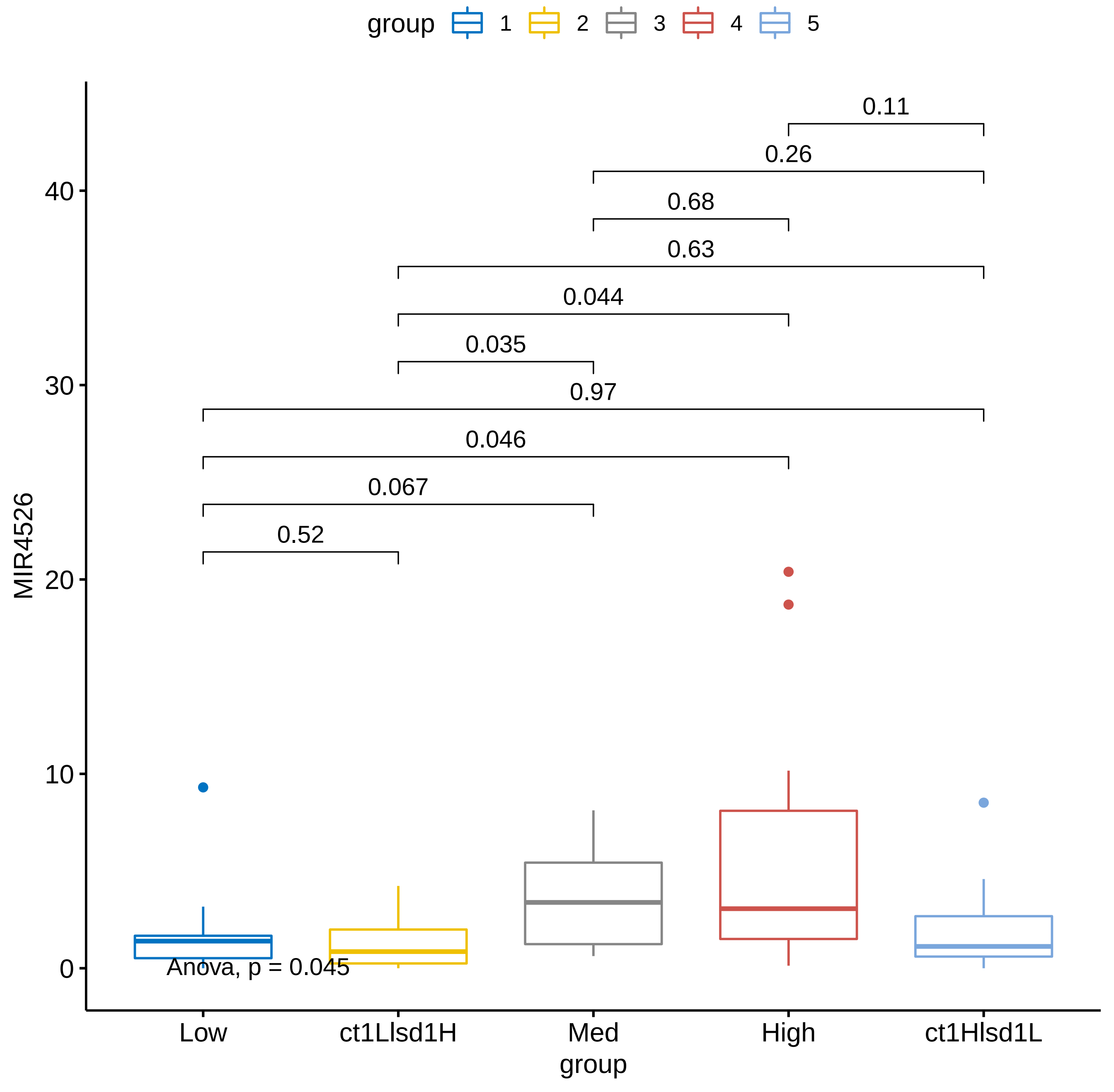

group 1 2 3 4 5
0.11
0.26
0.68
0.63
0.044
0.035
0.97
0.046
0.067
0.52
40
30
20
10
0
MIR4526
Anova, p = 0.045
Low
ct1Llsd1H
Med
High
ct1Hlsd1L
group

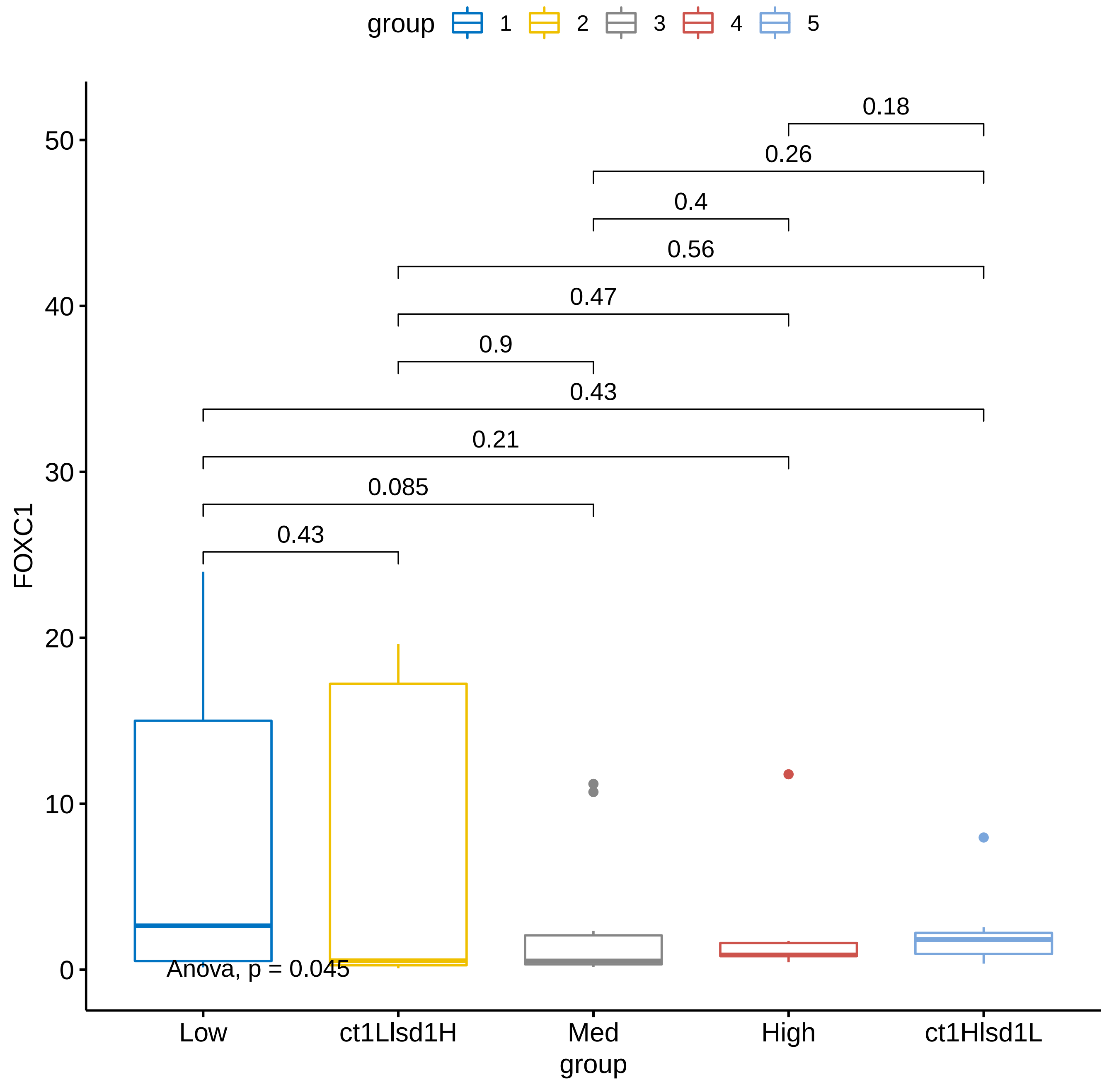

group
1
2
3
4
5
0.18
0.26
0.4
0.56
0.47
0.9
0.43
0.21
0.085
0.43
50
40
30
20
10
0
FOXC1
Anova, p = 0.045
Low
ct1Llsd1H
Med
High
ct1Hlsd1L
group

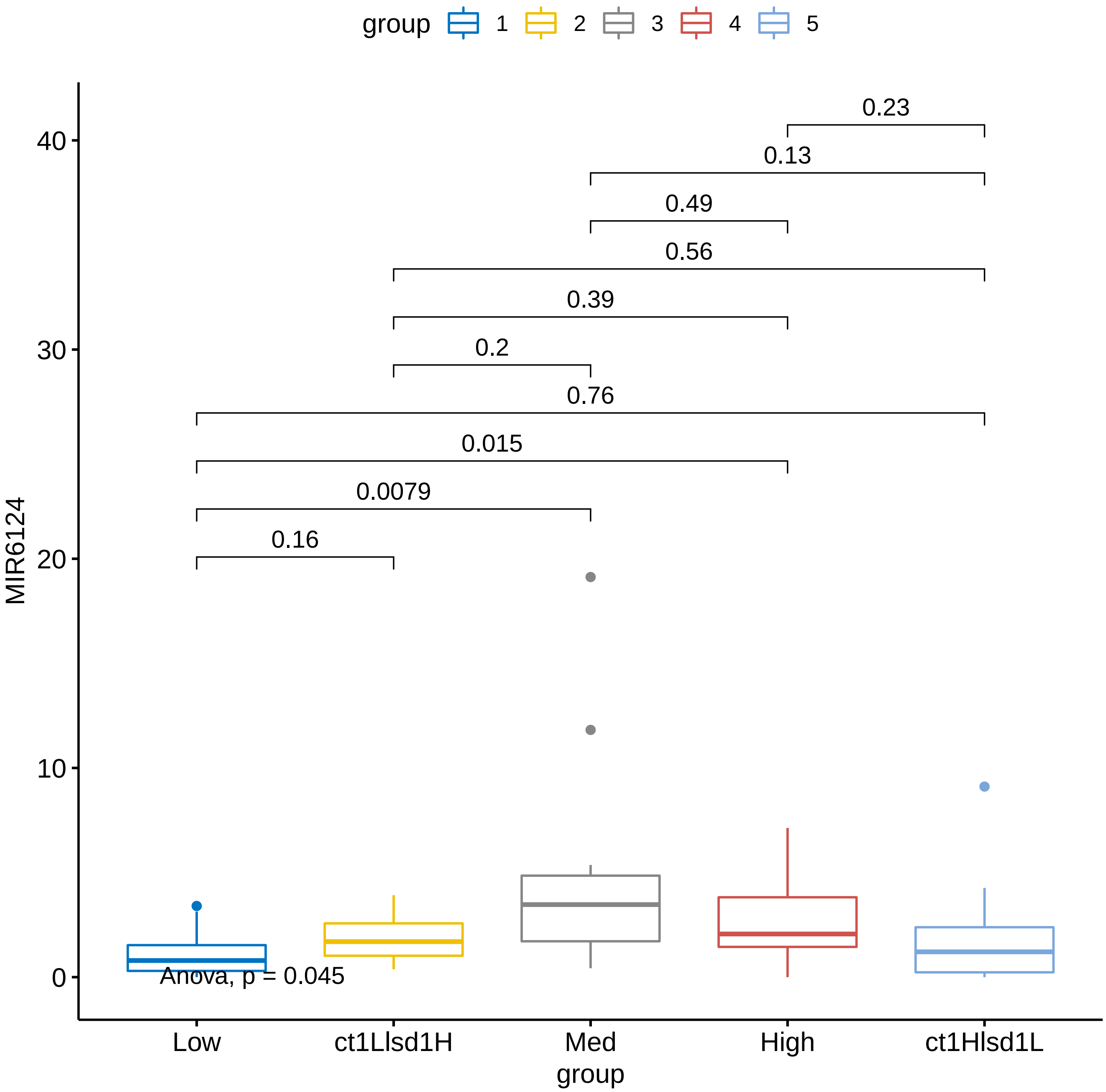

group
1
2
3
4
5
0.23
0.13
0.49
0.56
0.39
0.2
0.76
0.015
0.0079
0.16
MIR6124
40
30
20
10
0
Anova, p = 0.045
Low
ct1Llsd1H
Med
High
ct1Hlsd1L
group

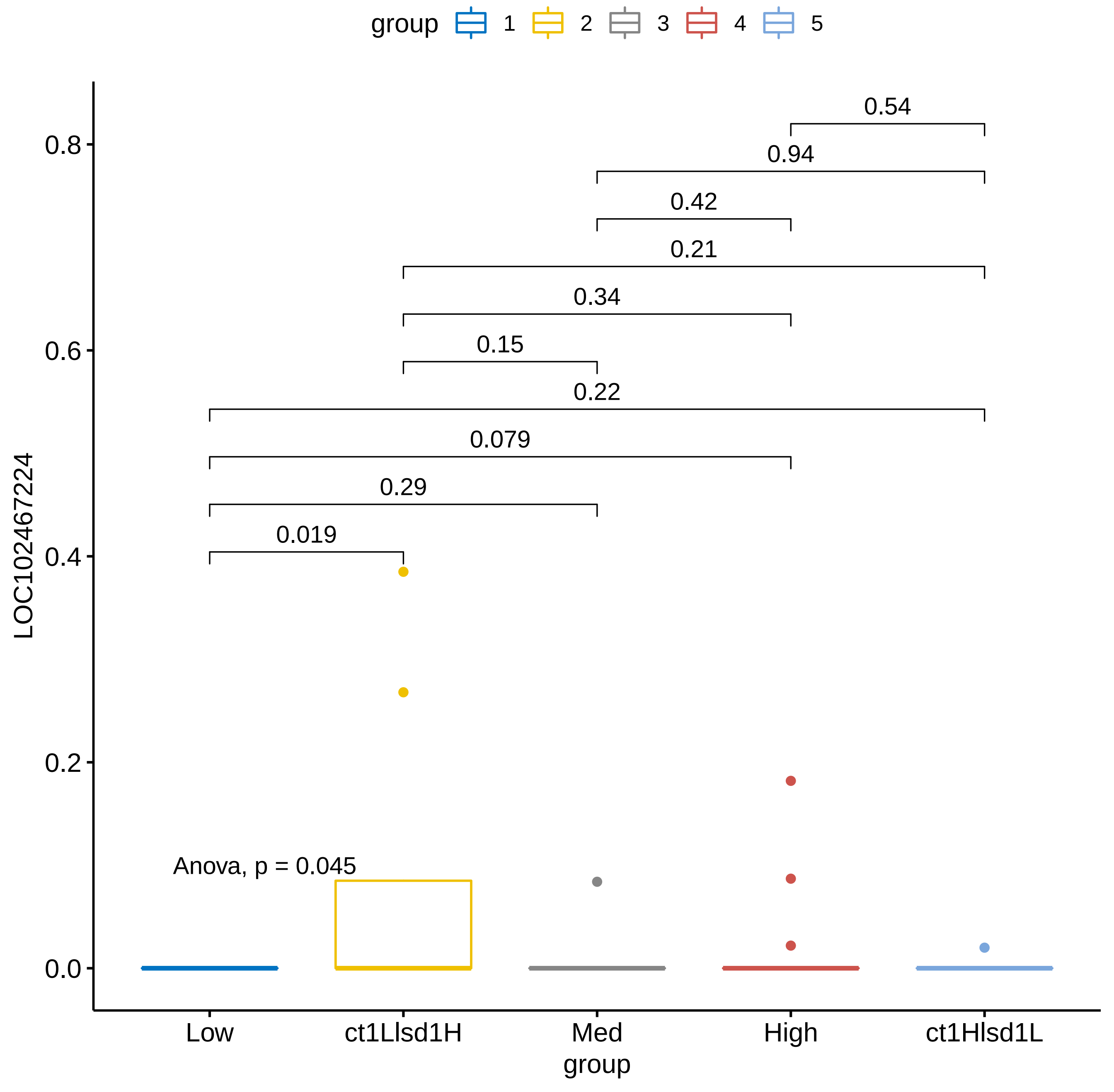

group 1 2 3 4 5
0.54
0.94
0.42
0.21
0.34
0.15
0.22
0.079
0.29
0.019
Anova, p = 0.045
0.8
0.6
0.4
0.2
0.0
LOC102467224
Low
ct1Llsd1H
Med
High
ct1Hlsd1L
group

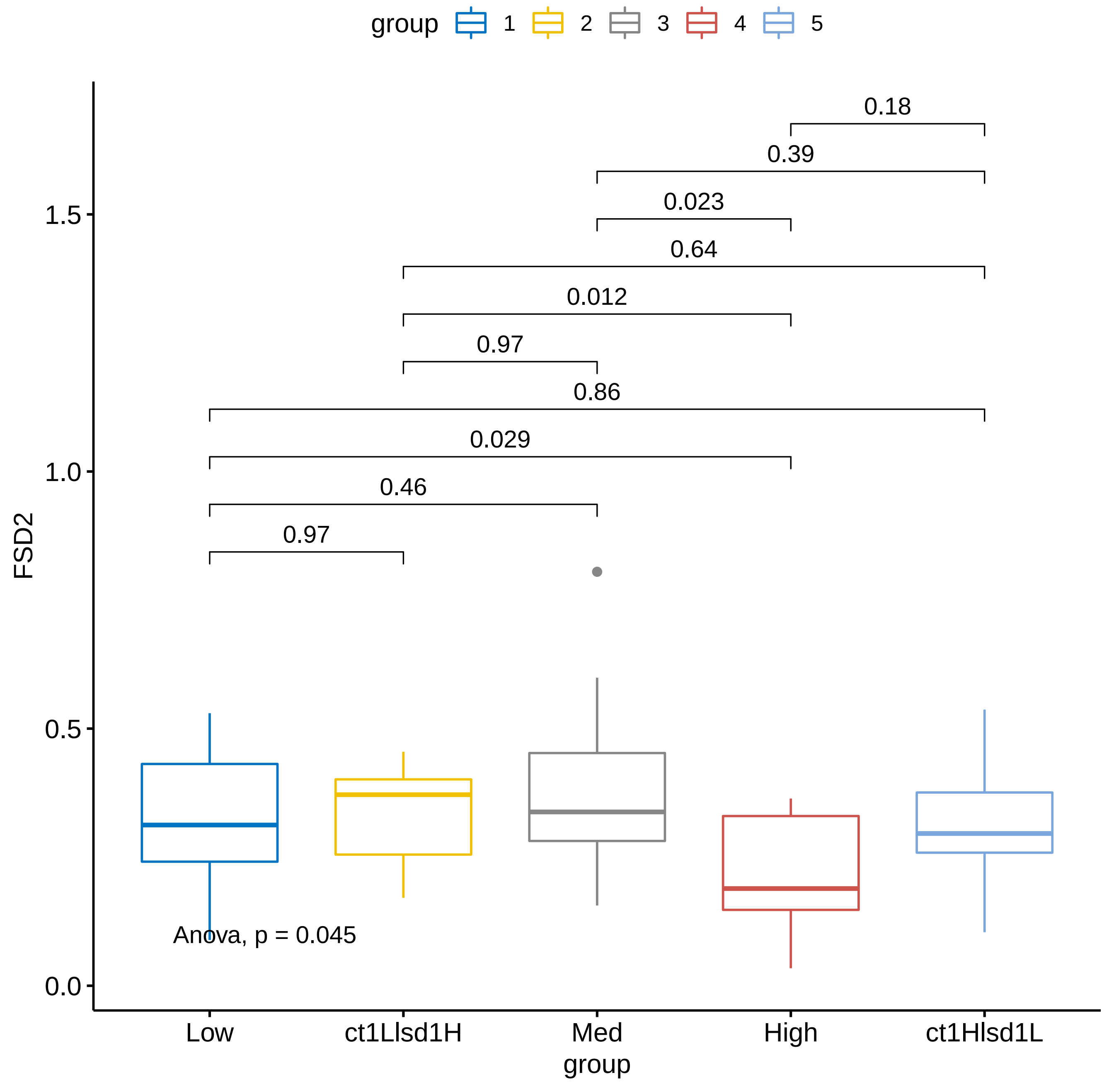
group
1
2
3
4
5
0.18
0.39
0.023
0.64
0.012
0.97
0.86
0.029
0.46
0.97
1.5
1.0
0.5
0.0
FSD2
Anova, p = 0.045
Low
ct1Llsd1H
Med
High
ct1Hlsd1L
group

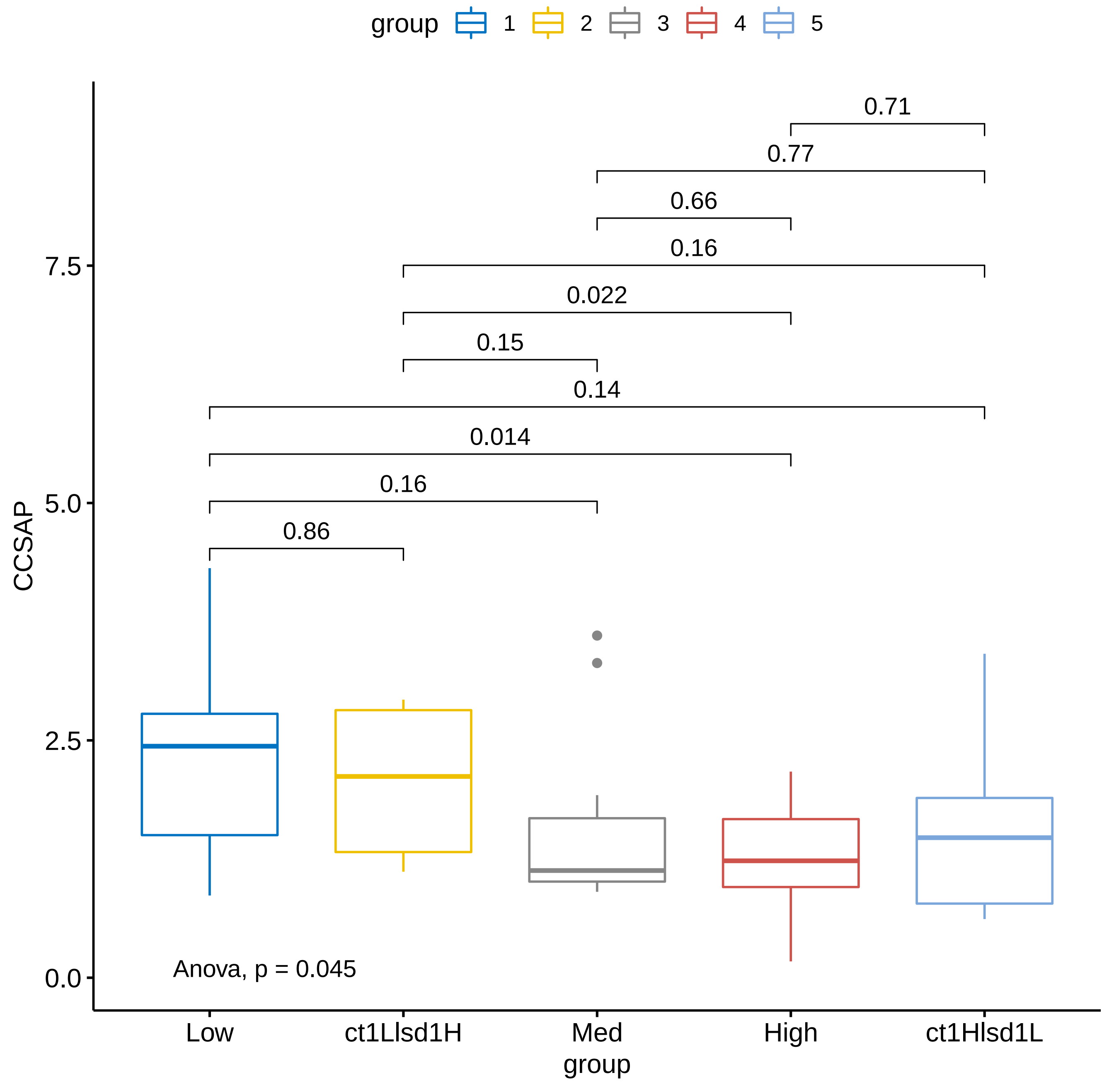

group 1 2 3 4 5
0.71
0.77
0.66
0.16
0.022
0.15
0.14
0.014
0.16
0.86
7.5
5.0
2.5
0.0
CCSAP
Anova, p = 0.045
Low
ct1Llsd1H
Med
High
ct1Hlsd1L
group

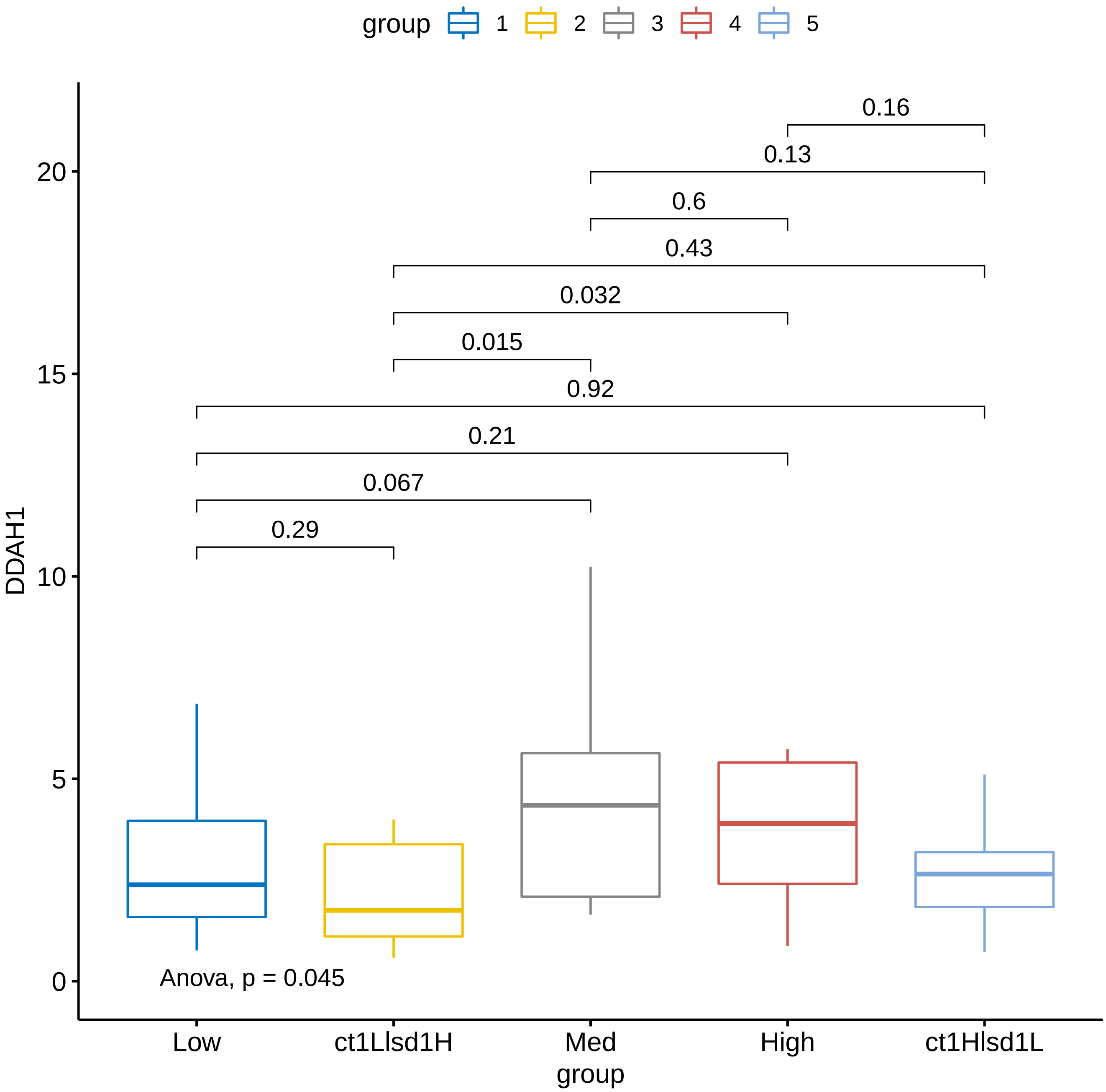
group
1
2
3
4
5
0.16
0.13
0.6
0.43
0.032
0.015
0.92
0.21
0.067
0.29
20
15
10
5
0
DDAH1
Anova, p = 0.045
Low
ct1Llsd1H
Med
High
ct1Hlsd1L
group

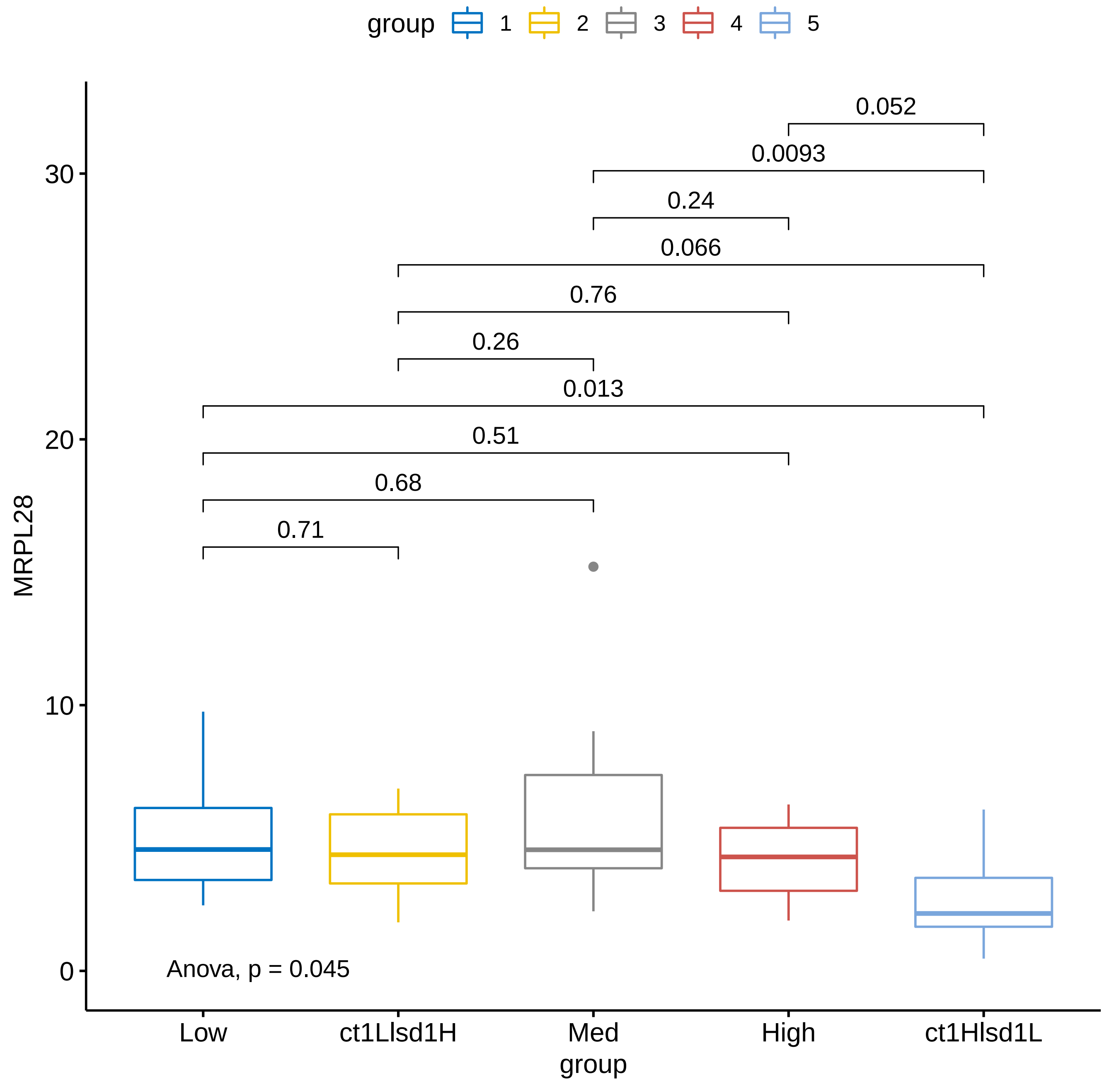

group 1 2 3 4 5
MRPL28
0
10
20
30
0.052
0.0093
0.24
0.066
0.76
0.26
0.013
0.51
0.68
0.71
Anova, p = 0.045
Low
ct1Llsd1H
Med
High
ct1Hlsd1L
group

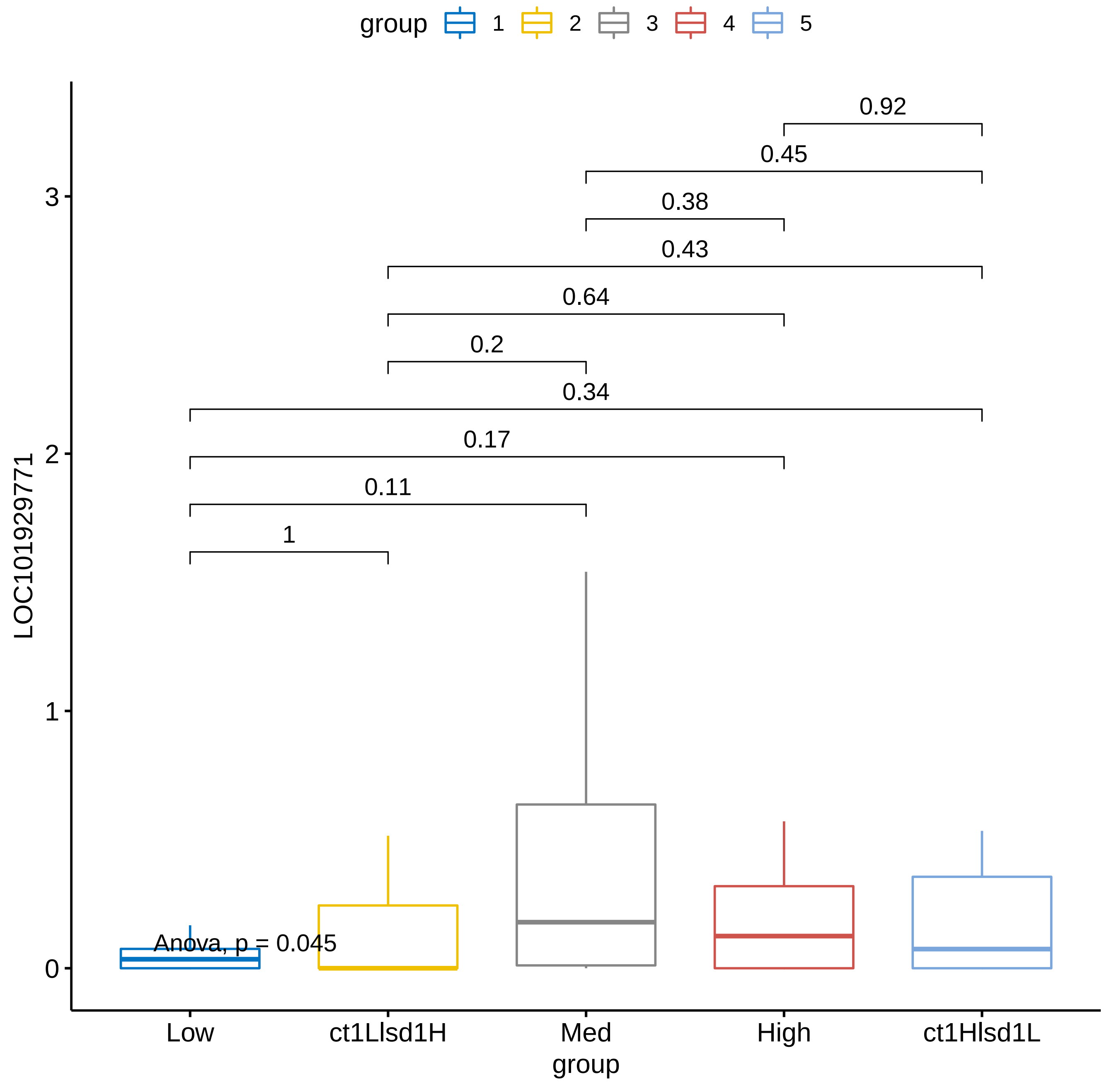
group 1 2 3 4 5
0.92
0.45
0.38
0.43
0.64
0.2
0.34
0.17
0.11
1
Anova, p = 0.045
3
2
1
0
LOC101929771
Low
ct1Llsd1H
Med
High
ct1Hlsd1L
group

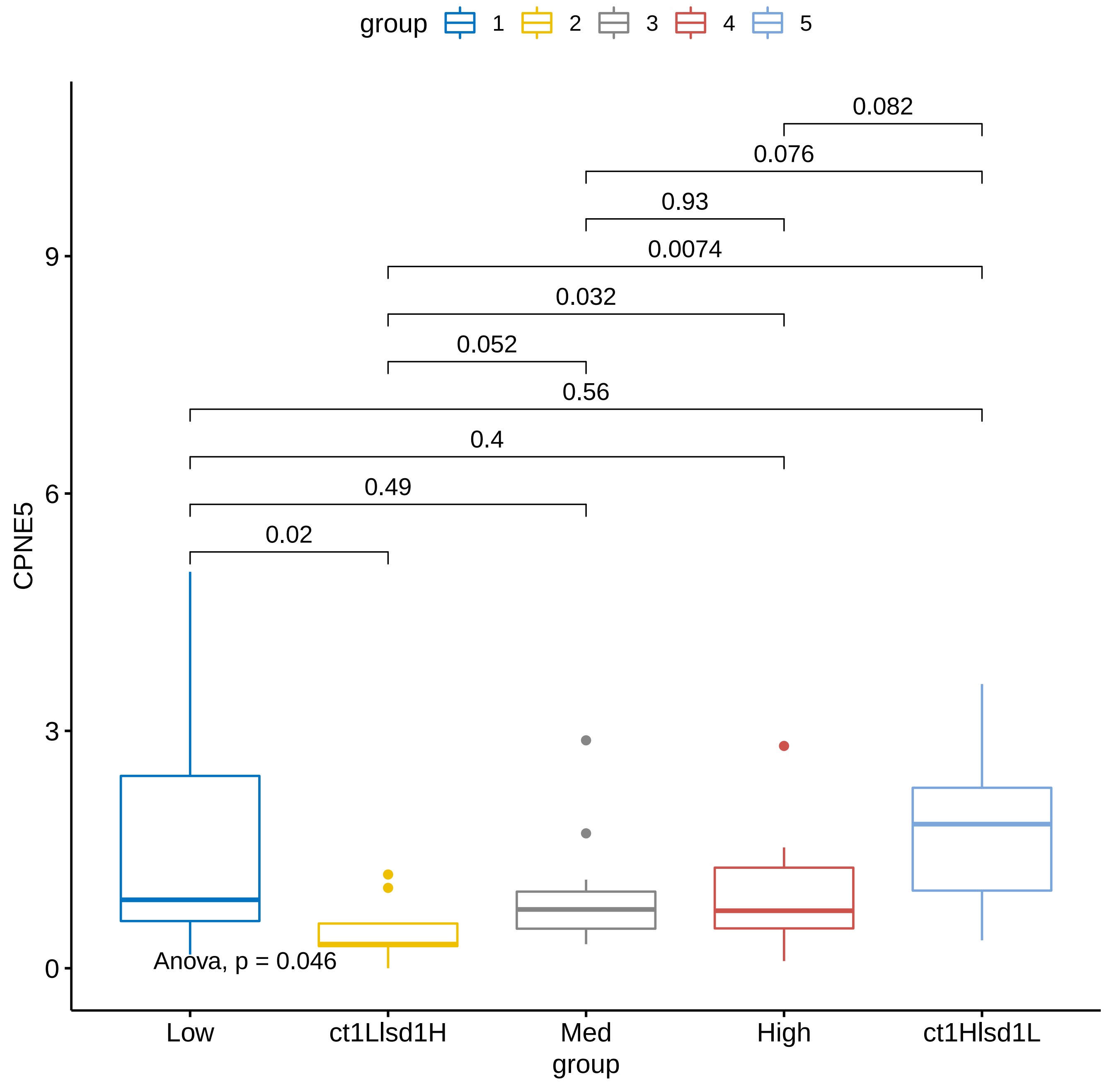
group
1
2
3
4
5
0.082
0.076
0.93
0.0074
0.032
0.052
0.56
0.4
0.49
0.02
CPNE5
9
6
3
0
Anova, p = 0.046
Low
ct1Llsd1H
Med
High
ct1Hlsd1L
group

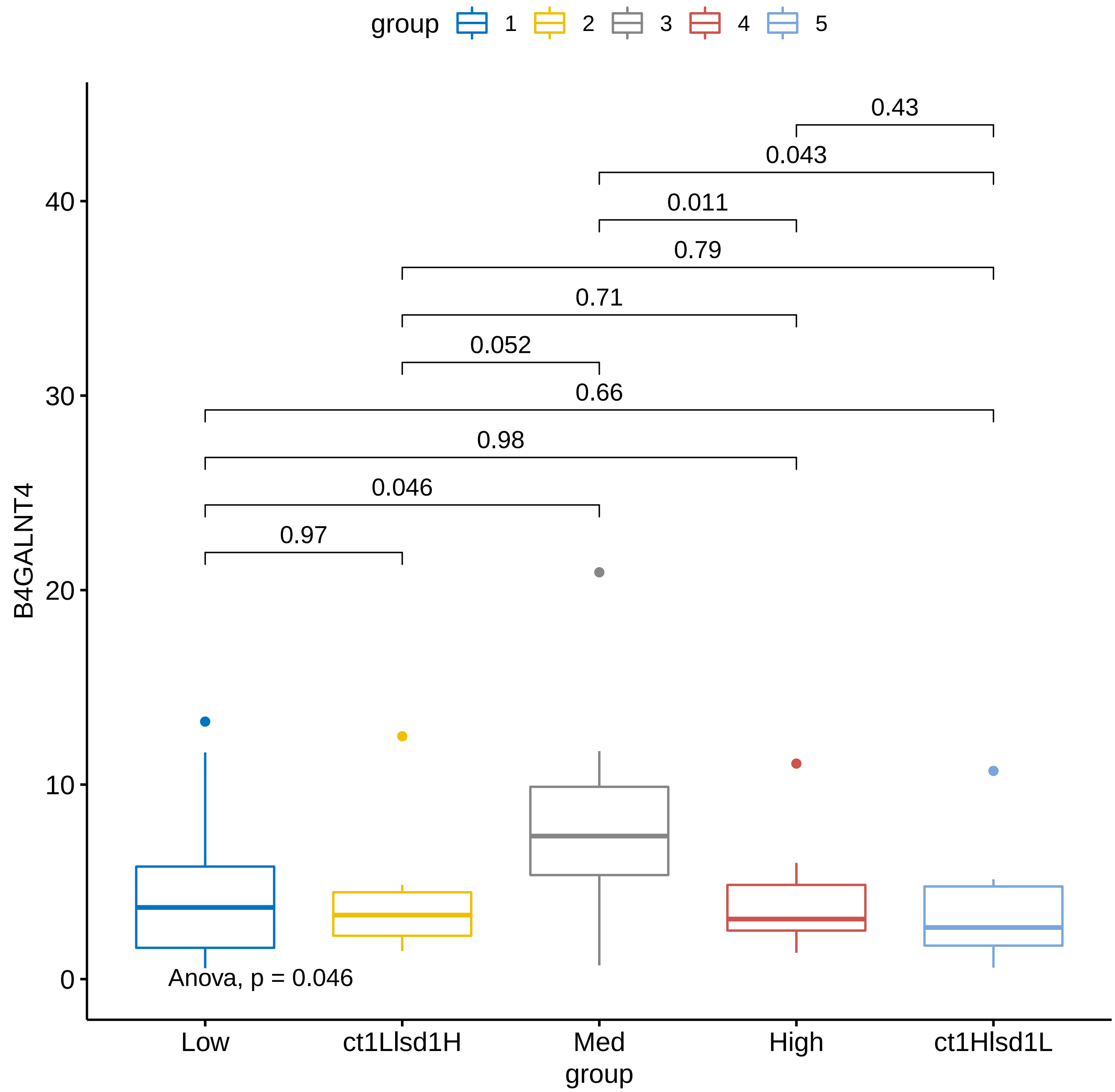
group
1
2
3
4
5
0.43
0.043
0.011
0.79
0.71
0.052
0.66
0.98
0.046
0.97
40
30
20
10
0
B4GALNT4
Anova, p = 0.046
Low
ct1Llsd1H
Med
High
ct1Hlsd1L
group

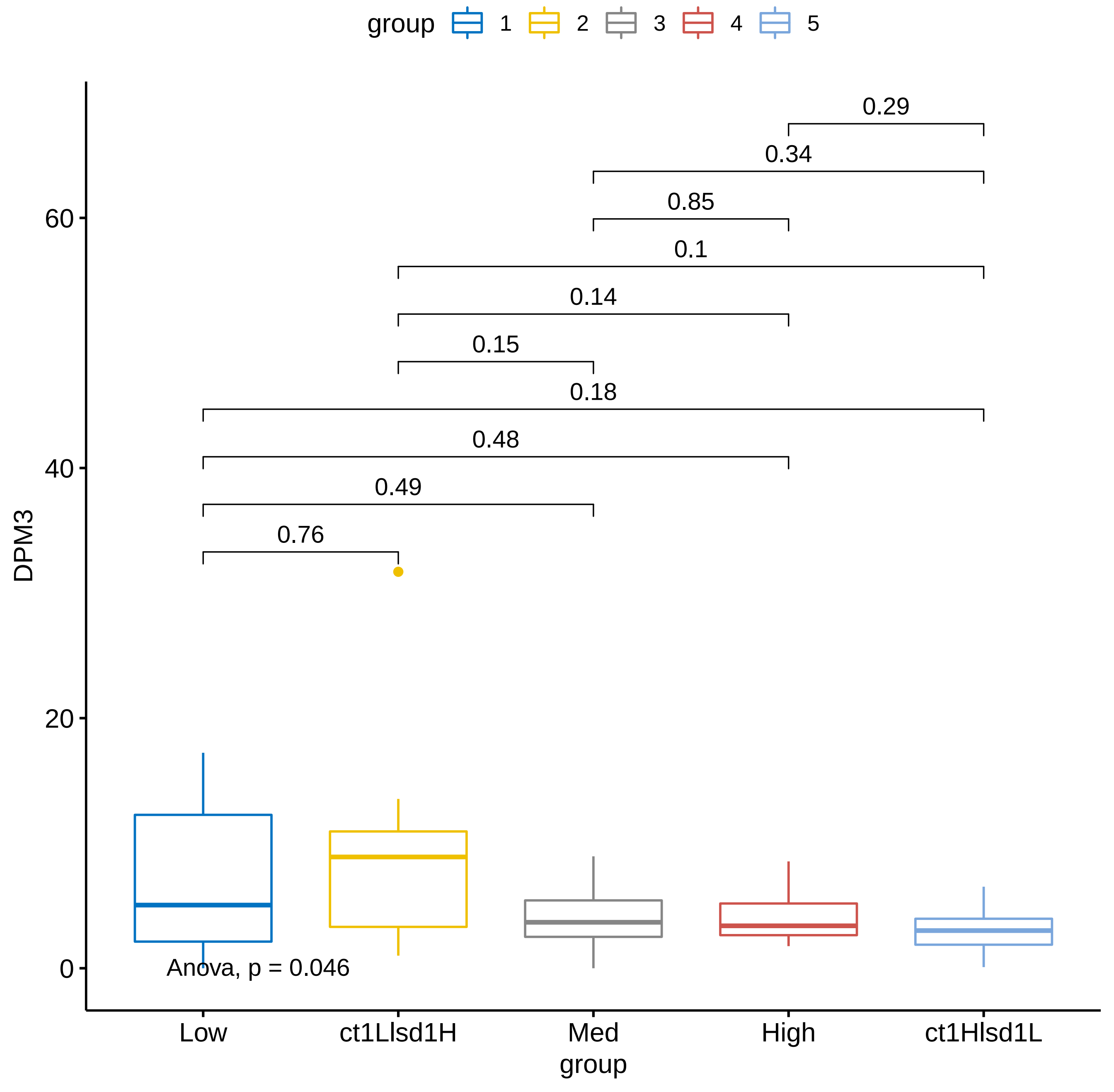
group
1
2
3
4
5
DPM3
60
40
20
0
0.29
0.34
0.85
0.1
0.14
0.15
0.18
0.48
0.49
0.76
Anova, p = 0.046
Low
ct1Llsd1H
Med
High
ct1Hlsd1L
group

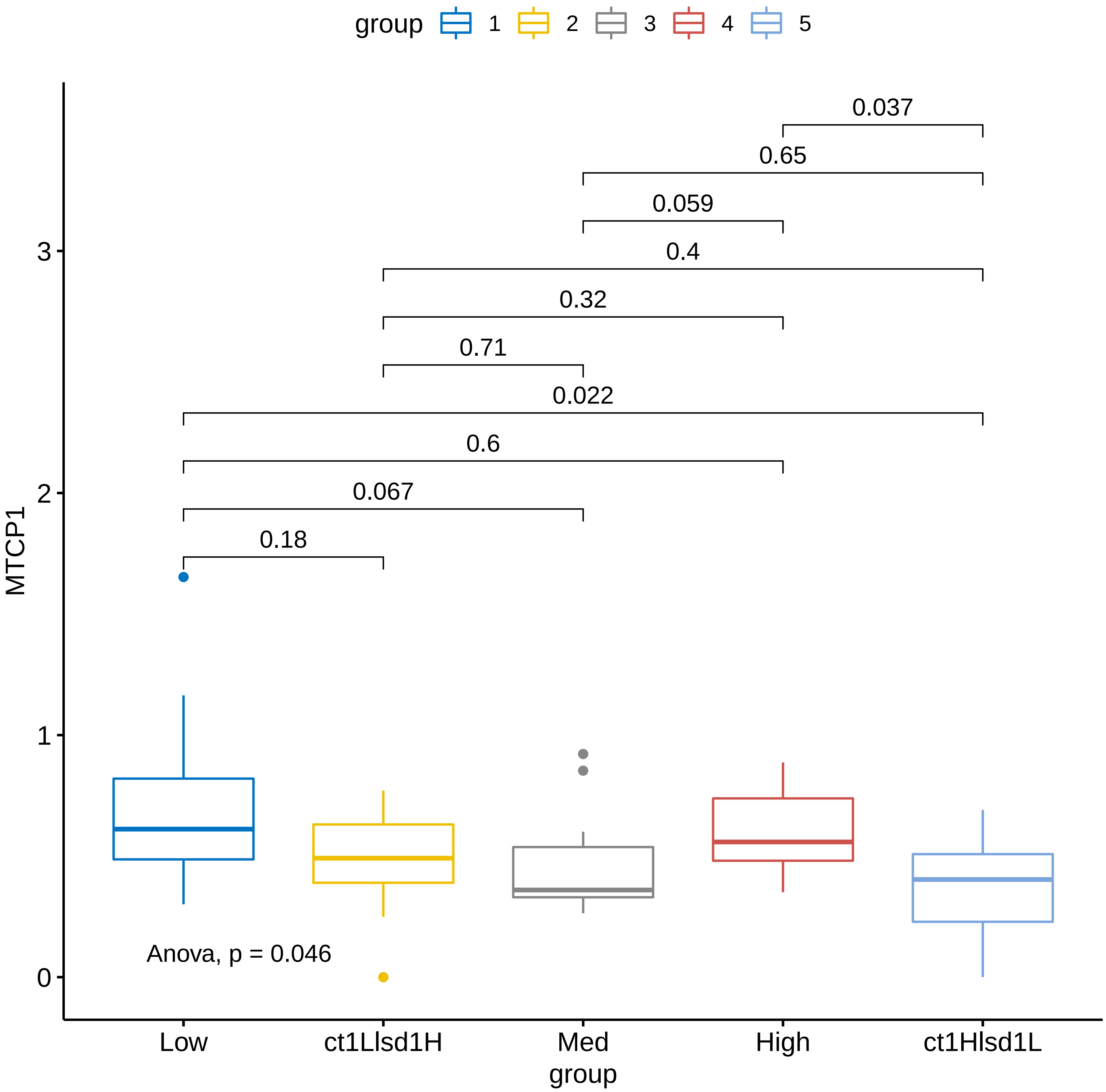

group
1
2
3
4
5
0.037
0.65
0.059
0.4
0.32
0.71
0.022
0.6
0.067
0.18
Anova, p = 0.046
MTCP1
0
1
2
3
Low
ct1Llsd1H
Med
High
ct1Hlsd1L
group

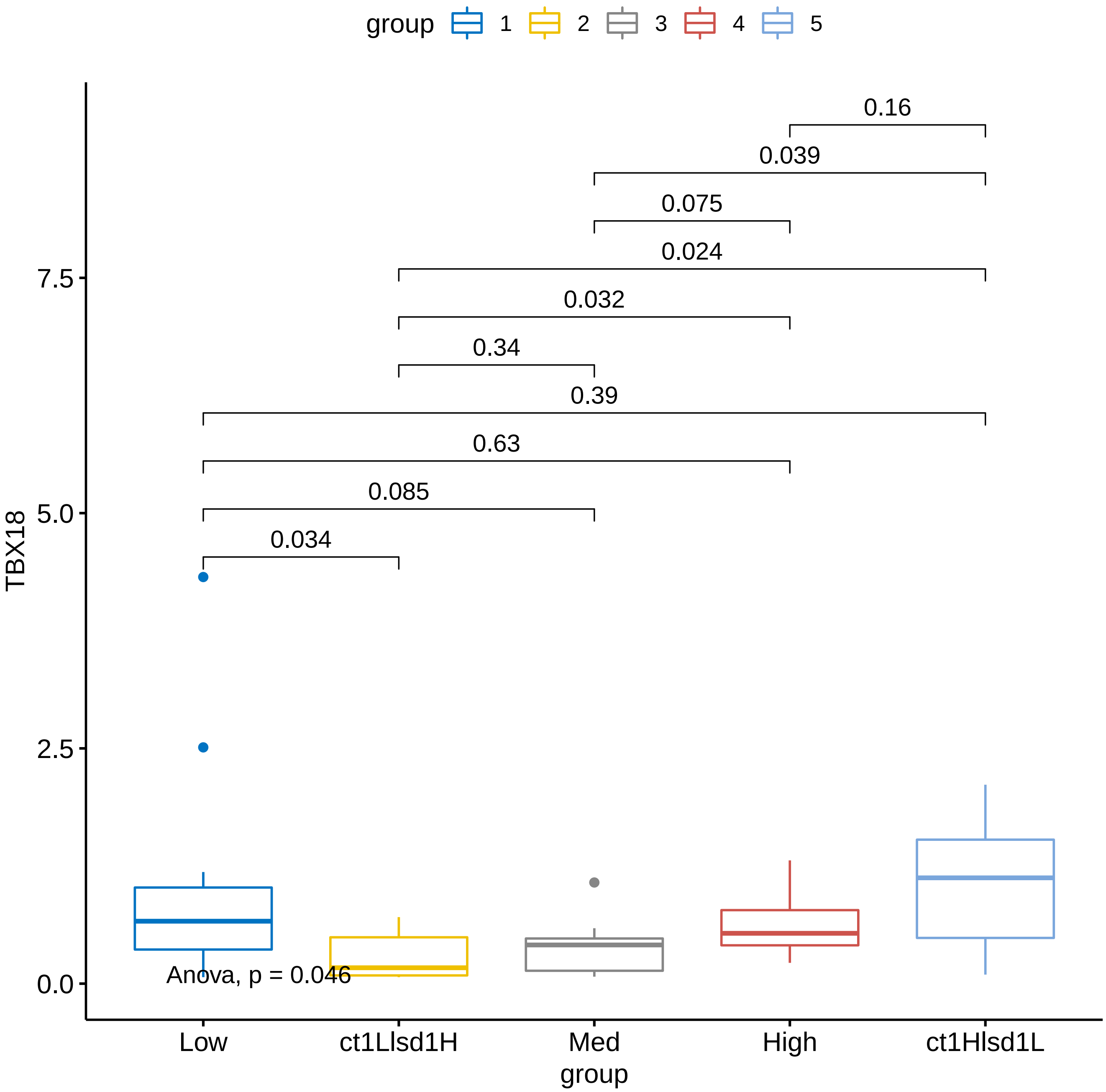
group
1
2
3
4
5
0.16
0.039
0.075
0.024
0.032
0.34
0.39
0.63
0.085
0.034
Anova, p = 0.046
TBX18
7.5
5.0
2.5
0.0
Low
ct1Llsd1H
Med
High
ct1Hlsd1L
group

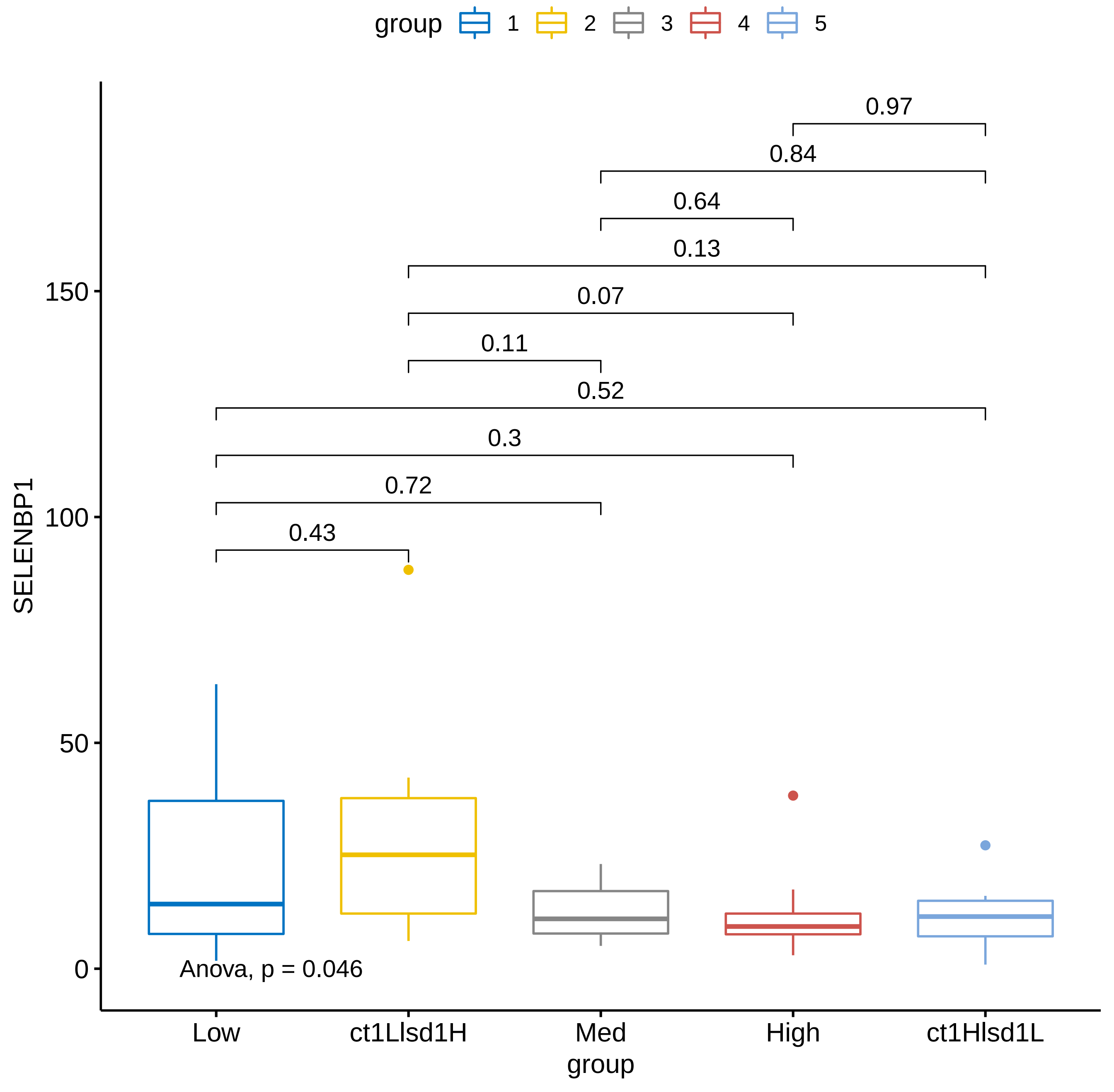
group
1
2
3
4
5
0.97
0.84
0.64
0.13
0.07
0.11
0.52
0.3
0.72
0.43
150
100
50
0
SELENBP1
Anova, p = 0.046
Low
ct1Llsd1H
Med
High
ct1Hlsd1L
group

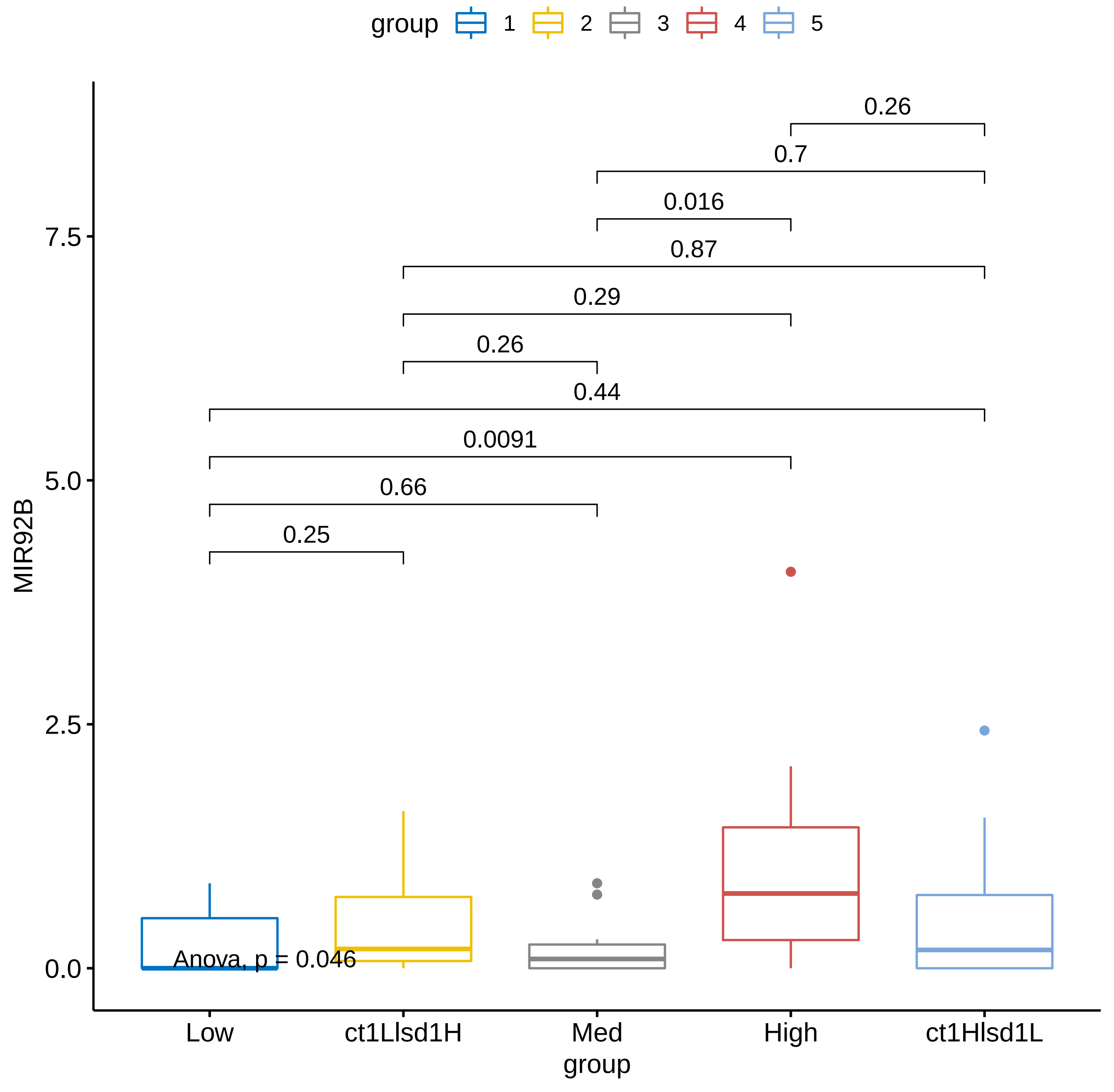

group
1
2
3
4
5
0.26
0.7
0.016
0.87
0.29
0.26
0.44
0.0091
0.66
0.25
Anova, p = 0.046
7.5
5.0
2.5
0.0
MIR92B
Low
ct1Llsd1H
Med
High
ct1Hlsd1L
group

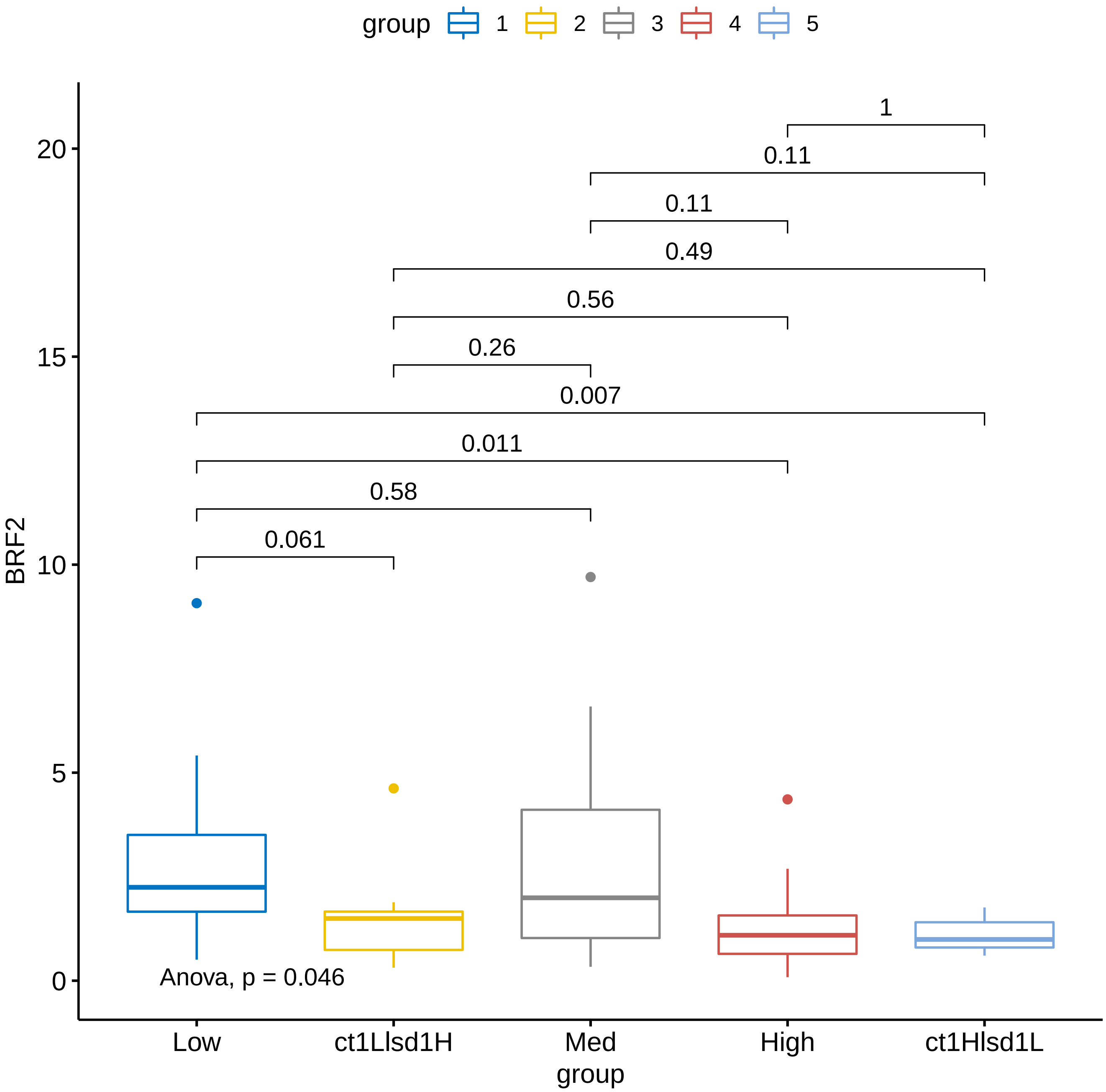
group
1
2
3
4
5
1
0.11
0.11
0.49
0.56
0.26
0.007
0.011
0.58
0.061
Anova, p = 0.046
BRF2
20
15
10
5
0
Low
ct1Llsd1H
Med
High
ct1Hlsd1L
group

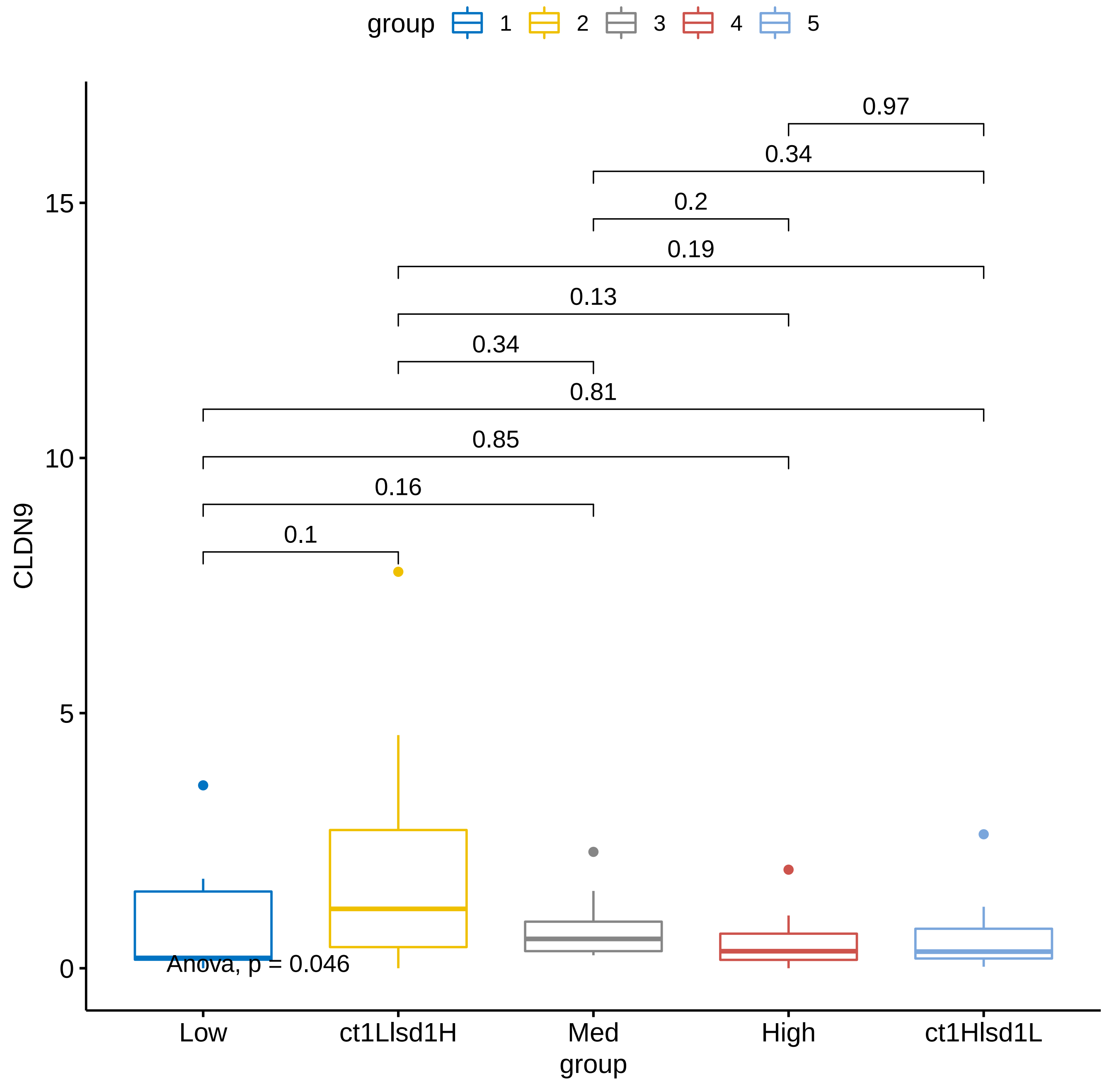
group 1 2 3 4 5
CLDN9
15
10
5
0
0.97
0.34
0.2
0.19
0.13
0.34
0.81
0.85
0.16
0.1
Anova, p = 0.046
Low
ct1Llsd1H
Med
High
ct1Hlsd1L
group

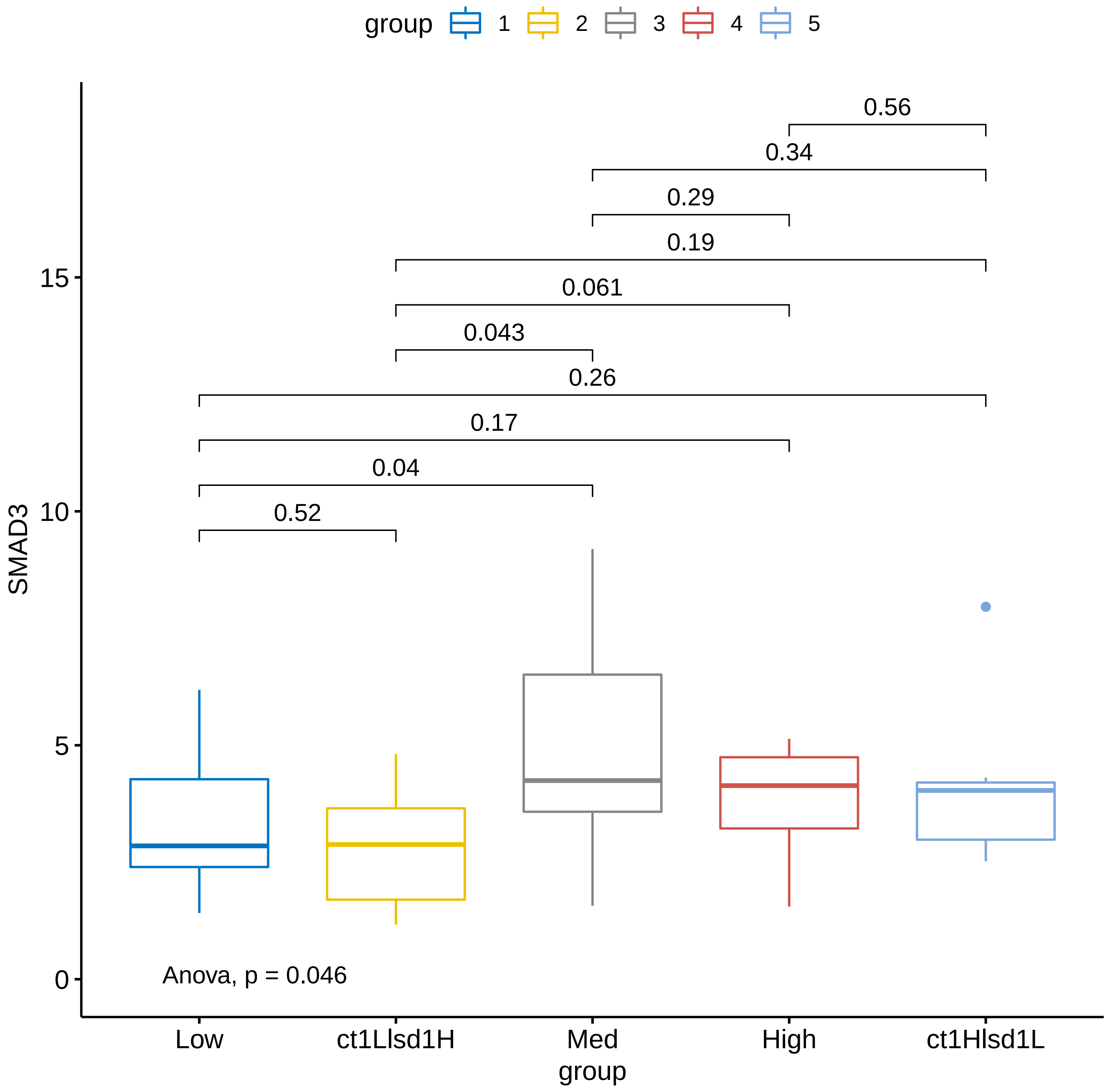

group 1 2 3 4 5
0.56
0.34
0.29
0.19
0.061
0.043
0.26
0.17
0.04
0.52
15
10
5
0
SMAD3
Anova, p = 0.046
Low
ct1Llsd1H
Med
High
ct1Hlsd1L
group

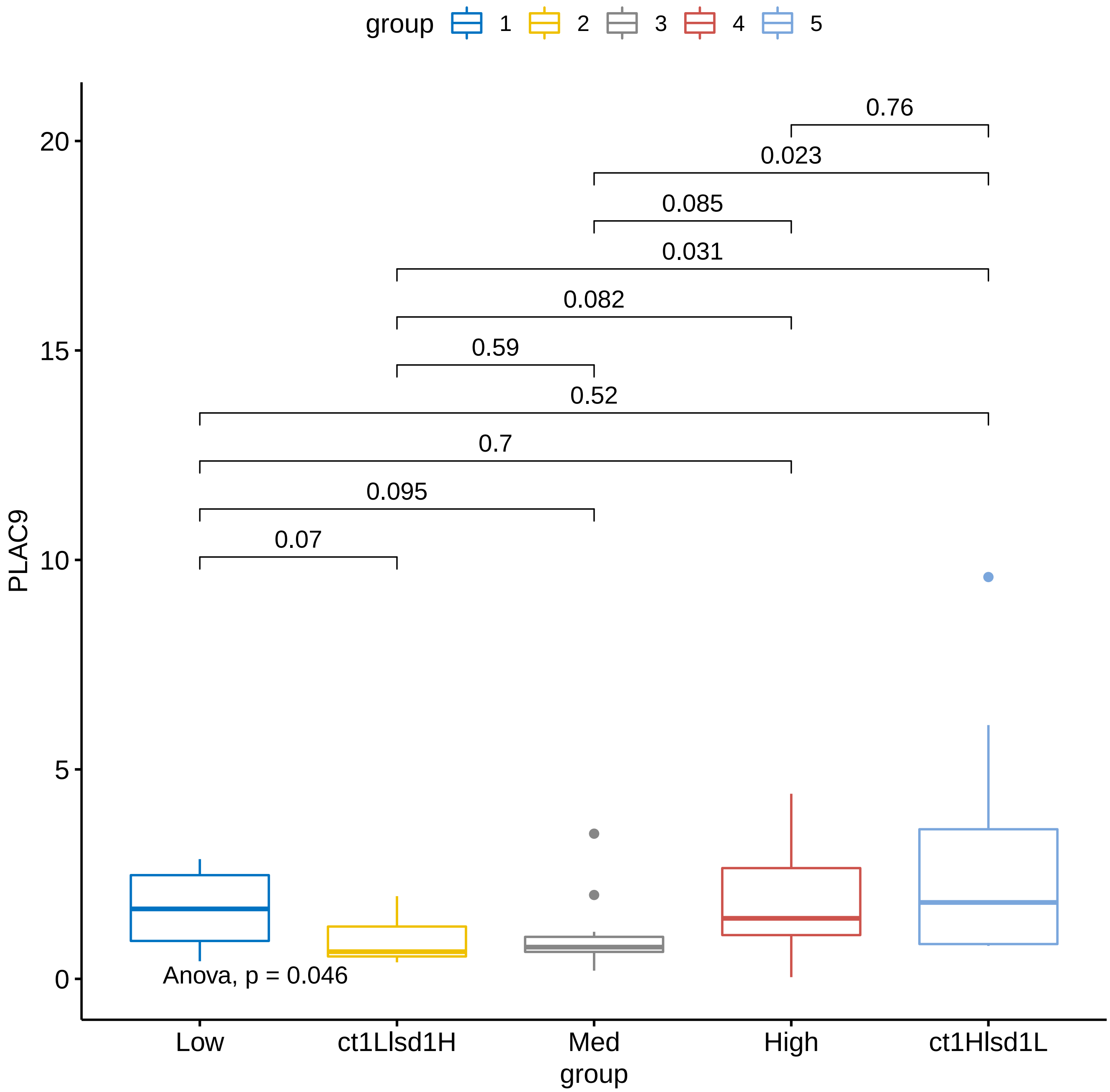
group
1
2
3
4
5
0.76
0.023
0.085
0.031
0.082
0.59
0.52
0.7
0.095
0.07
20
15
10
5
0
PLAC9
Anova, p = 0.046
Low
ct1Llsd1H
Med
High
ct1Hlsd1L
group

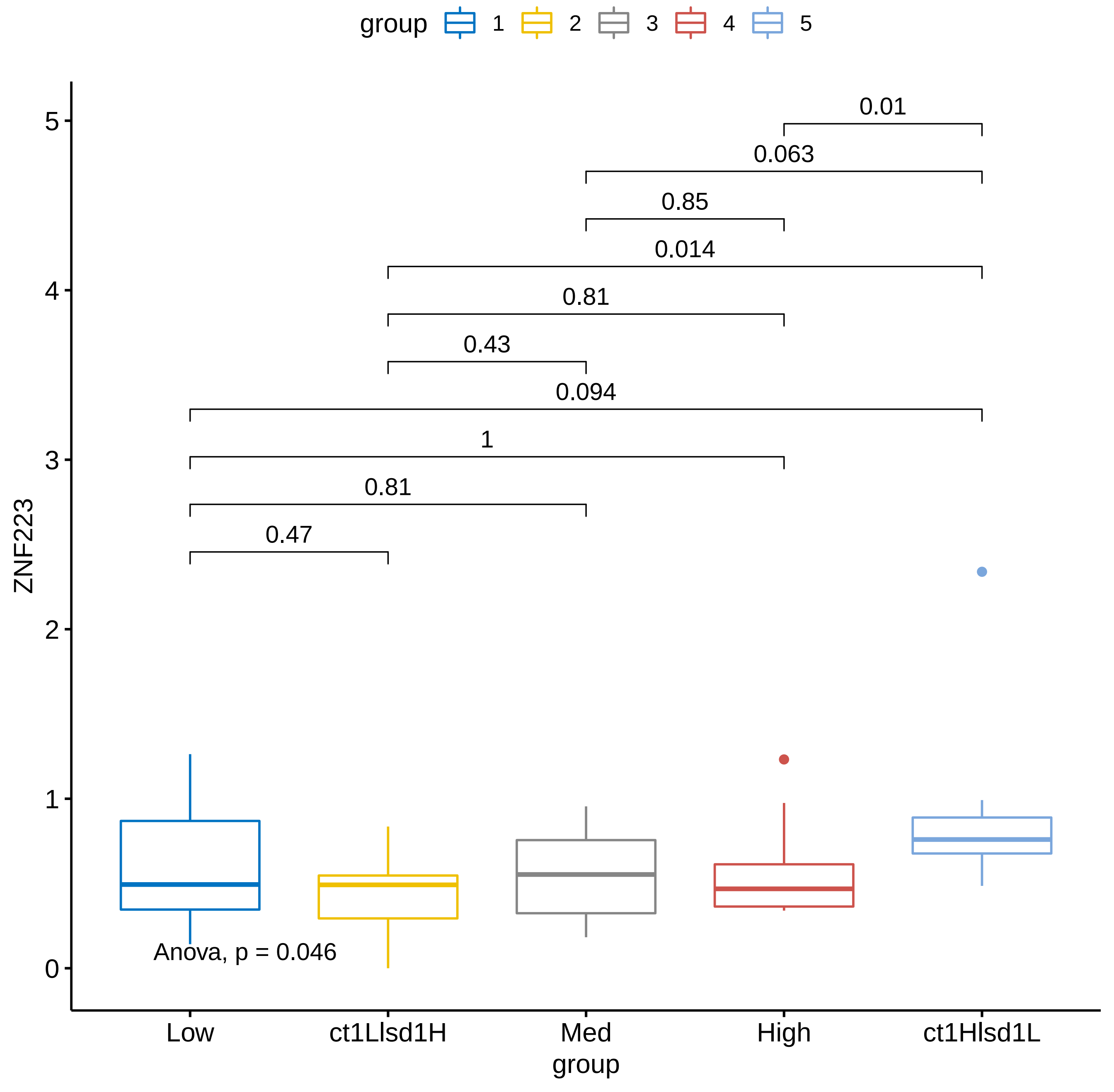

group 1 2 3 4 5
0.01
0.063
0.85
0.014
0.81
0.43
0.094
1
0.81
0.47
Anova, p = 0.046
ZNF223
0
1
2
3
4
5
Low
ct1Llsd1H
Med
High
ct1Hlsd1L
group

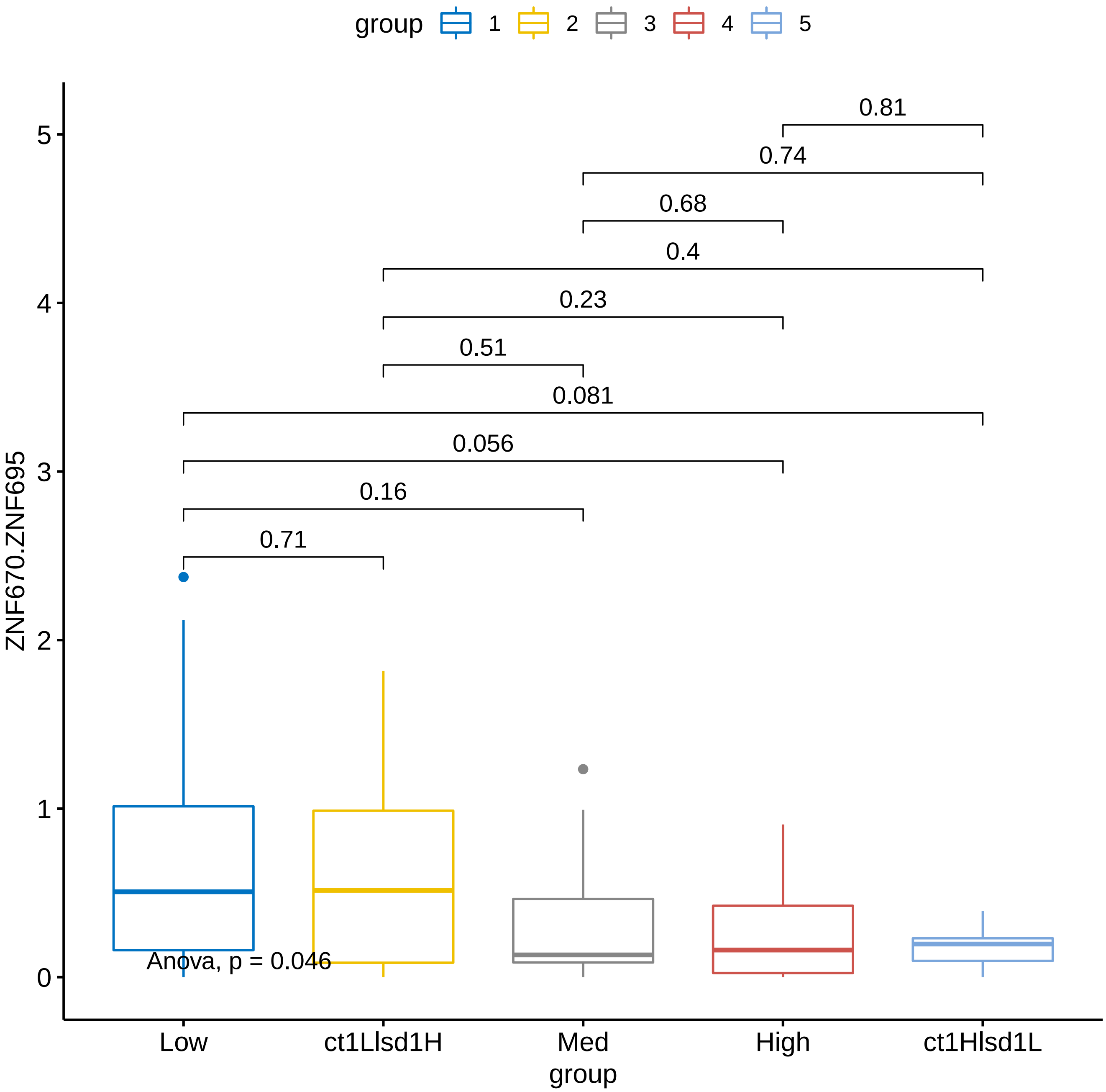
group
1
2
3
4
5
0.81
0.74
0.68
0.4
0.23
0.51
0.081
0.056
0.16
0.71
Anova, p = 0.046
ZNF670.ZNF695
5
4
3
2
1
0
Low
ct1Llsd1H
Med
High
ct1Hlsd1L
group

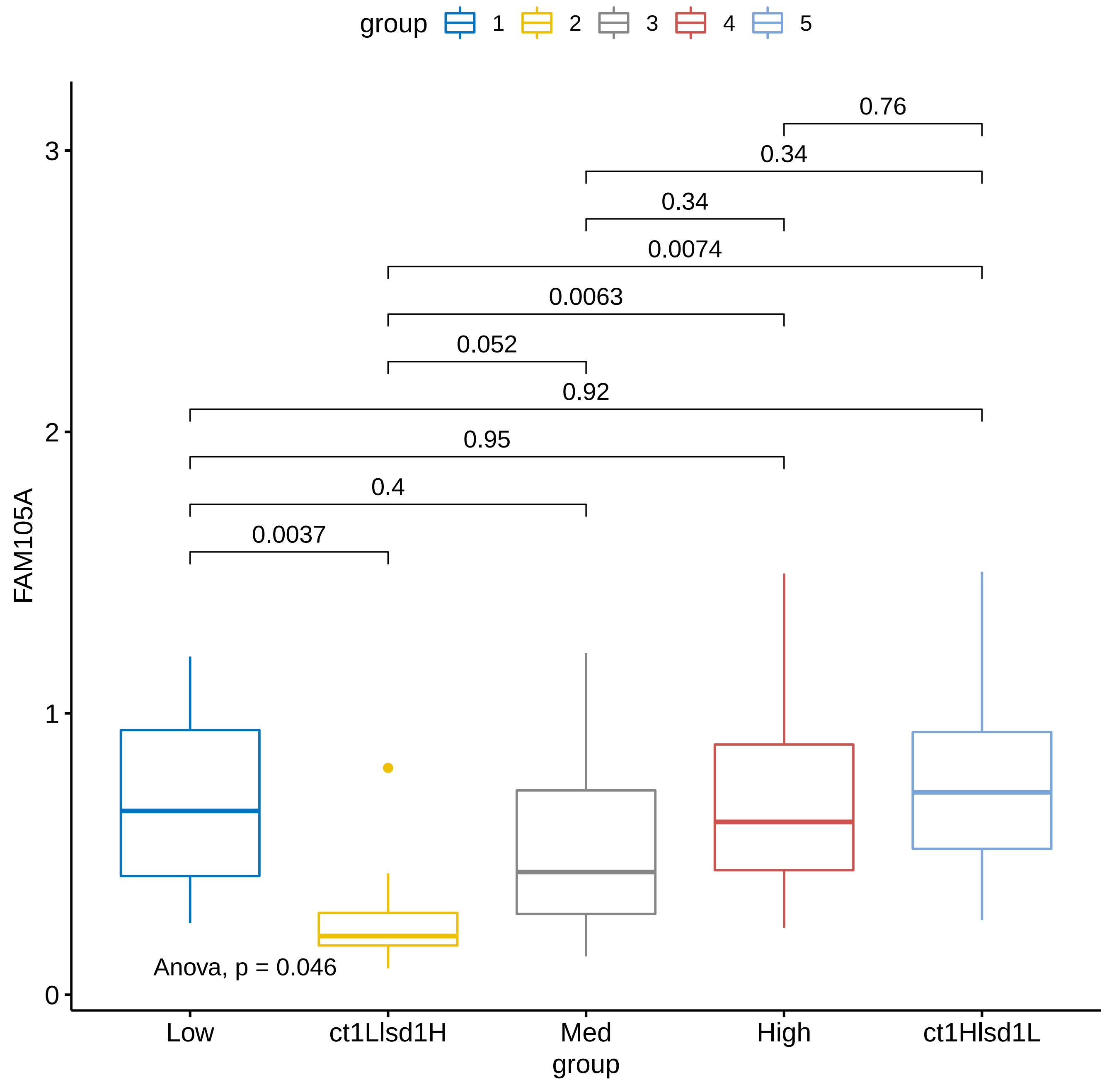

group
1
2
3
4
5
FAM105A
3
2
1
0
0.76
0.34
0.34
0.0074
0.0063
0.052
0.92
0.95
0.4
0.0037
Anova, p = 0.046
Low
ct1Llsd1H
Med
High
ct1Hlsd1L
group

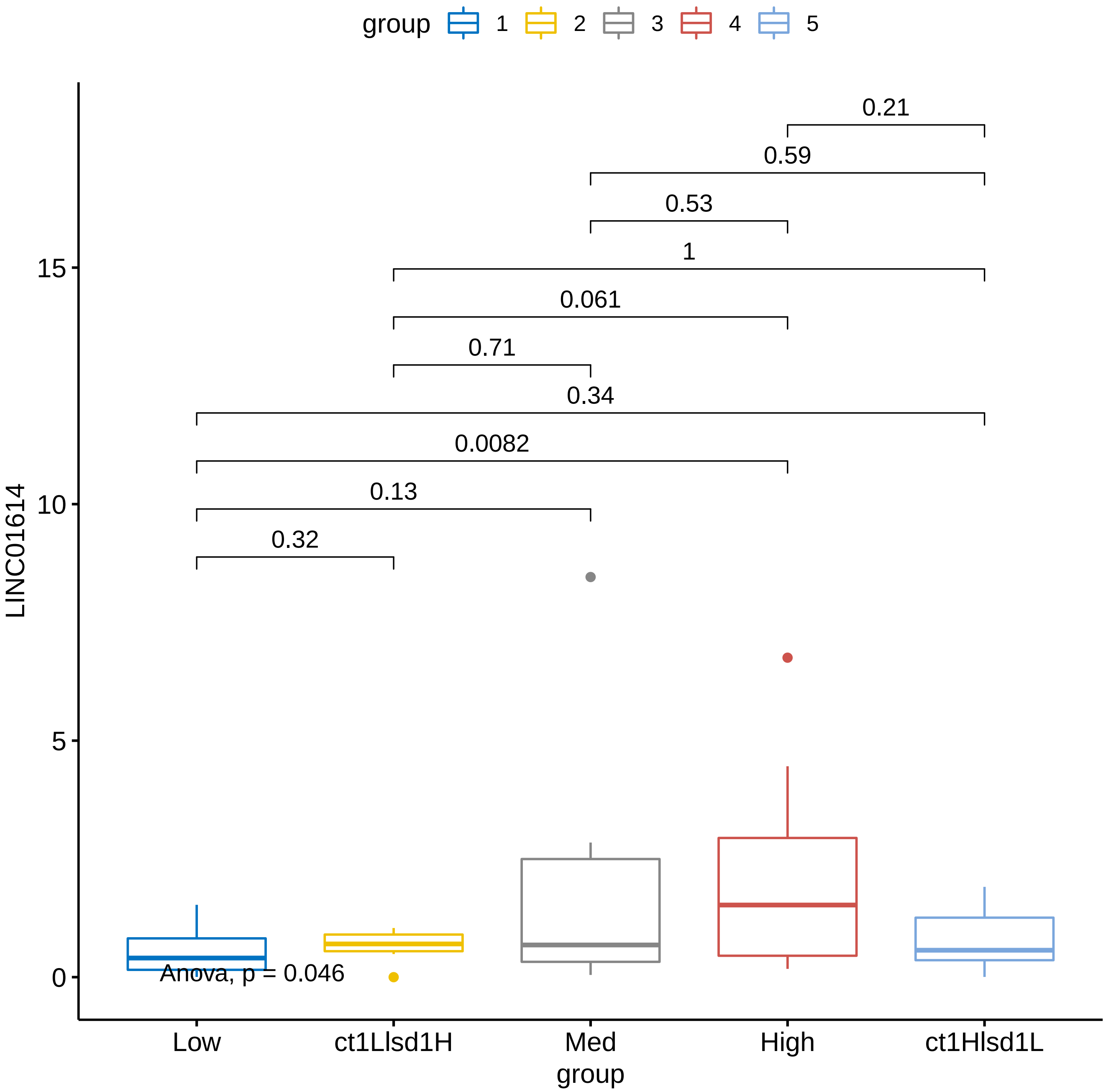
group
1
2
3
4
5
0.21
0.59
0.53
1
0.061
0.71
0.34
0.0082
0.13
0.32
LINC01614
15
10
5
0
Anova, p = 0.046
Low
ct1Llsd1H
Med
High
ct1Hlsd1L
group

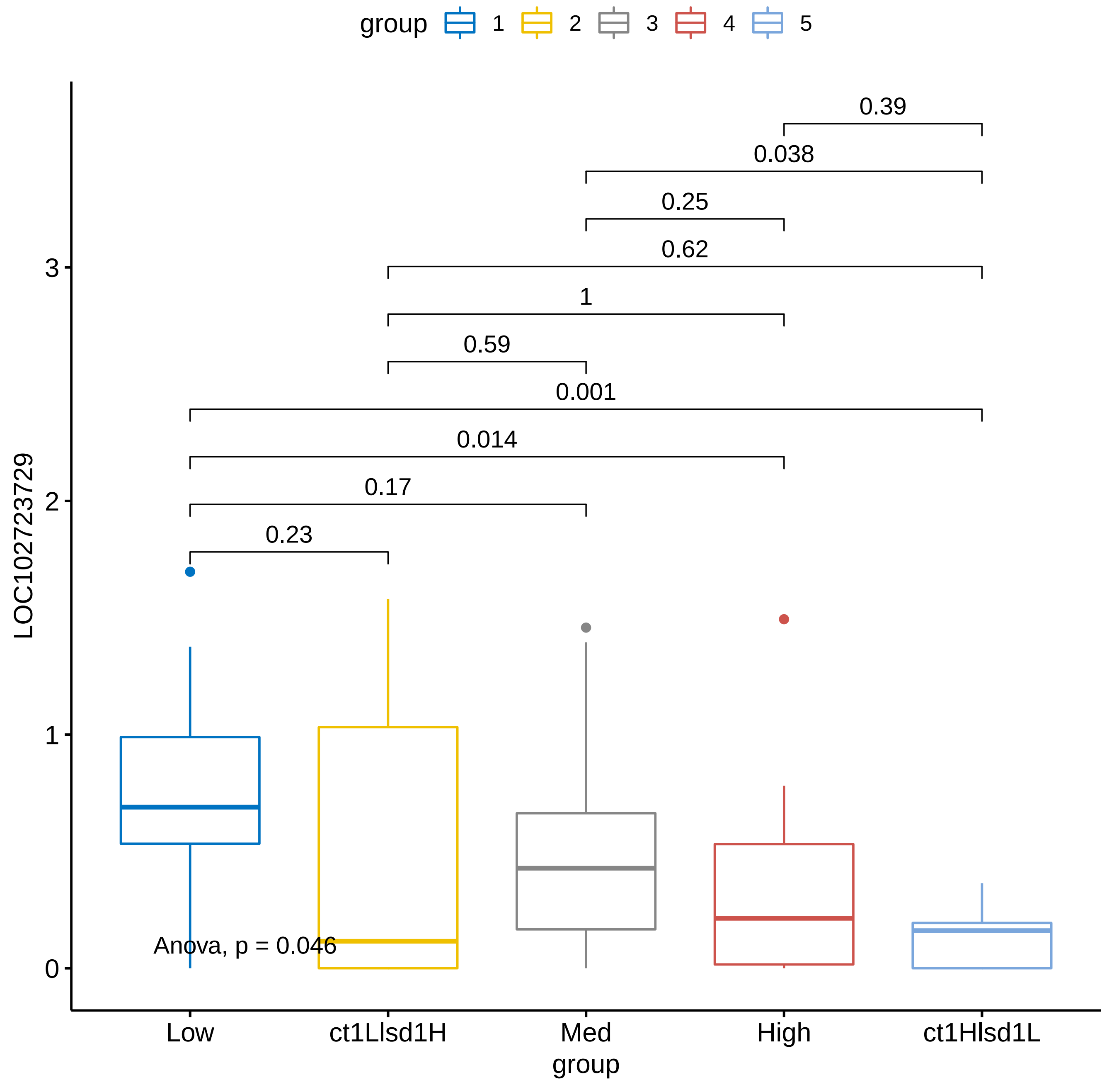

group
1
2
3
4
5
0.39
0.038
0.25
0.62
1
0.59
0.001
0.014
0.17
0.23
Anova, p = 0.046
LOC102723729
0
1
2
3
Low
ct1Llsd1H
Med
High
ct1Hlsd1L
group

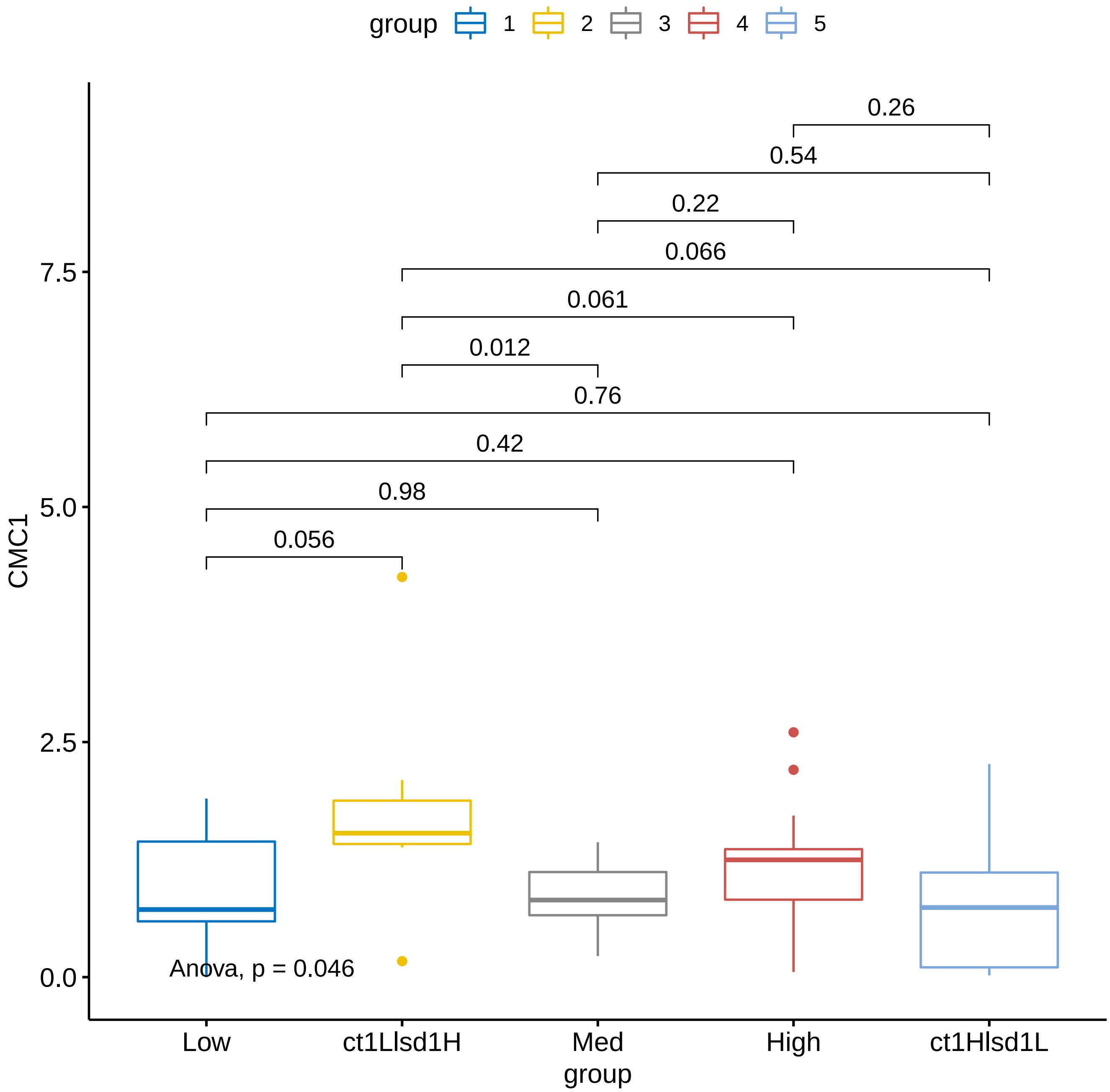

group
1
2
3
4
5
0.26
0.54
0.22
0.066
0.061
0.012
0.76
0.42
0.98
0.056
CMC1
7.5
5.0
2.5
0.0
Anova, p = 0.046
Low
ct1Llsd1H
Med
High
ct1Hlsd1L
group

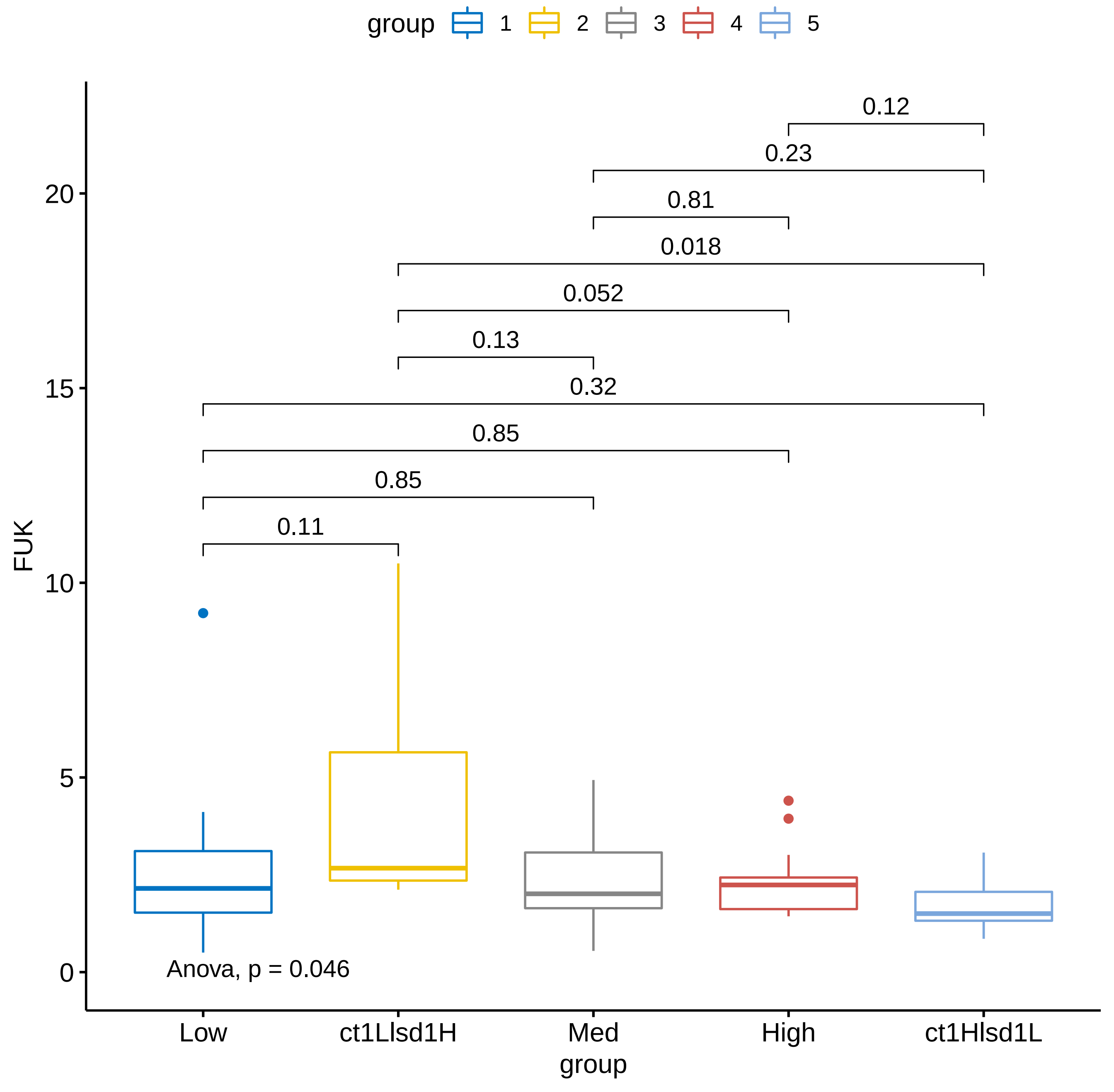
group
1
2
3
4
5
0.12
0.23
0.81
0.018
0.052
0.13
0.32
0.85
0.85
0.11
20
15
10
5
0
FUK
Anova, p = 0.046
Low
ct1Llsd1H
Med
High
ct1Hlsd1L
group

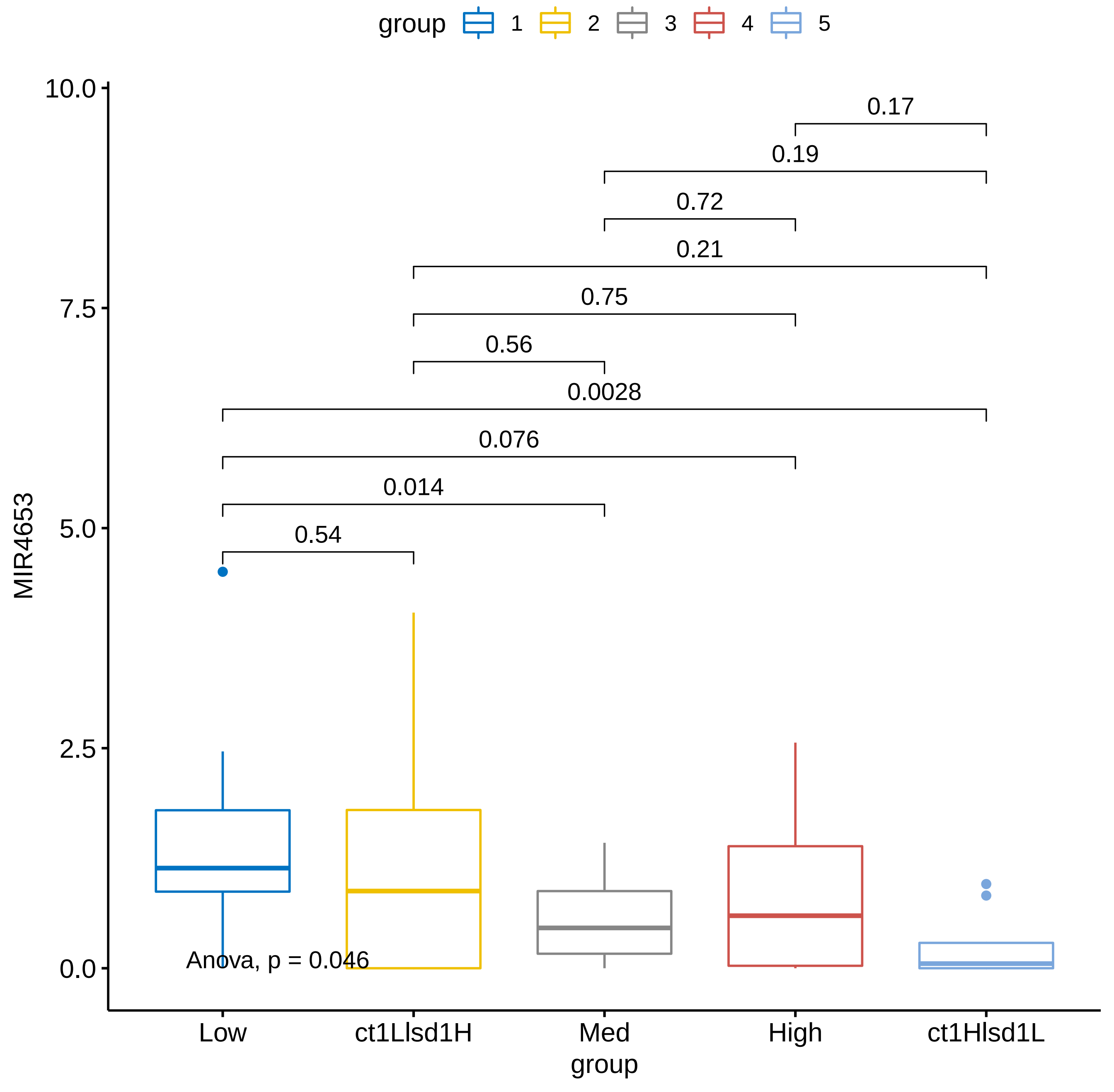

group 1 2 3 4 5
MIR4653
10.0
7.5
5.0
2.5
0.0
0.17
0.19
0.72
0.21
0.75
0.56
0.0028
0.076
0.014
0.54
Anova, p = 0.046
Low
ct1Llsd1H
Med
High
ct1Hlsd1L
group

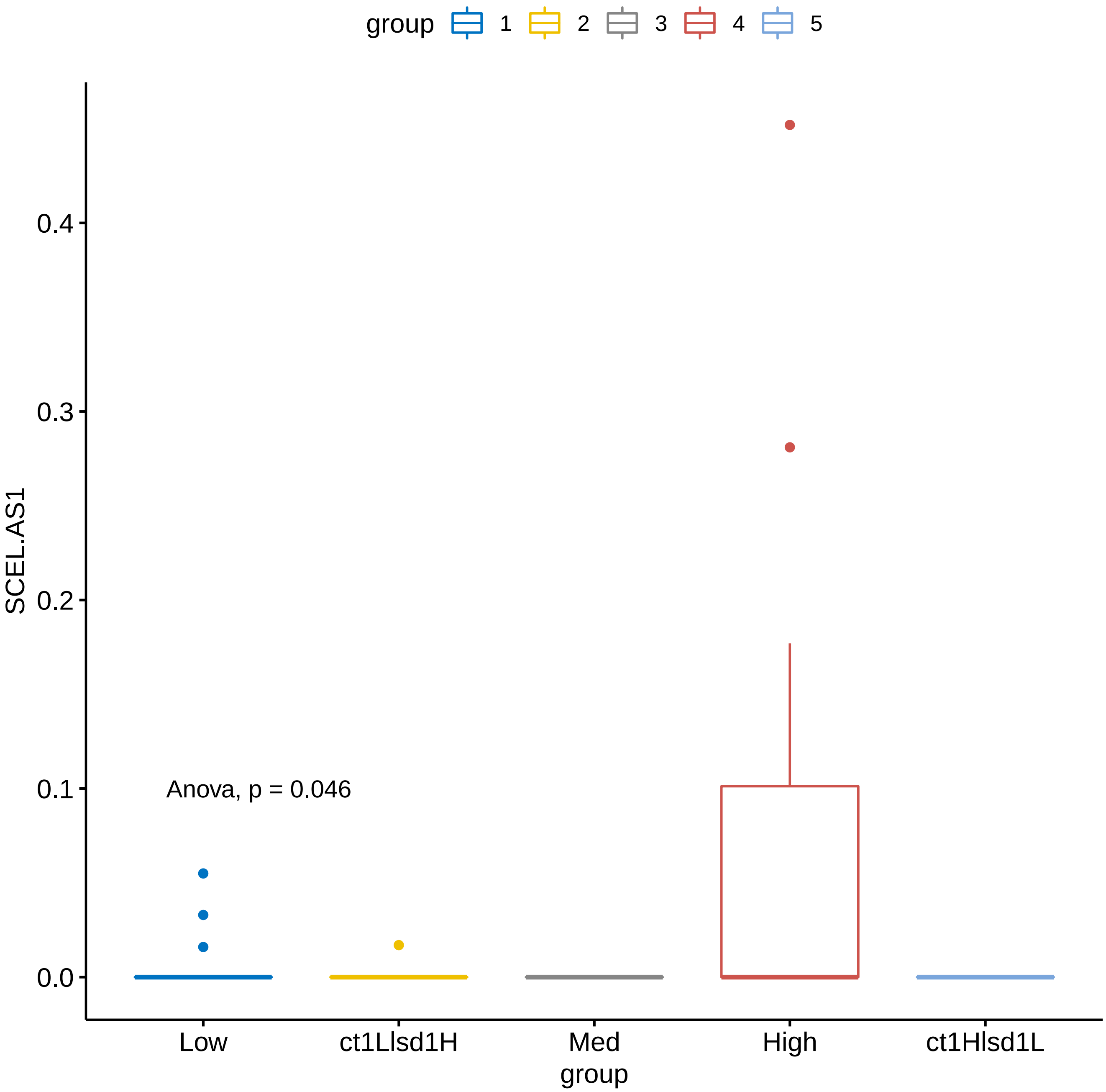
group
1
2
3
4
5
SCEL.AS1
0.4
0.3
0.2
0.1
0.0
Anova, p = 0.046
Low
ct1Llsd1H
Med
High
ct1Hlsd1L
group

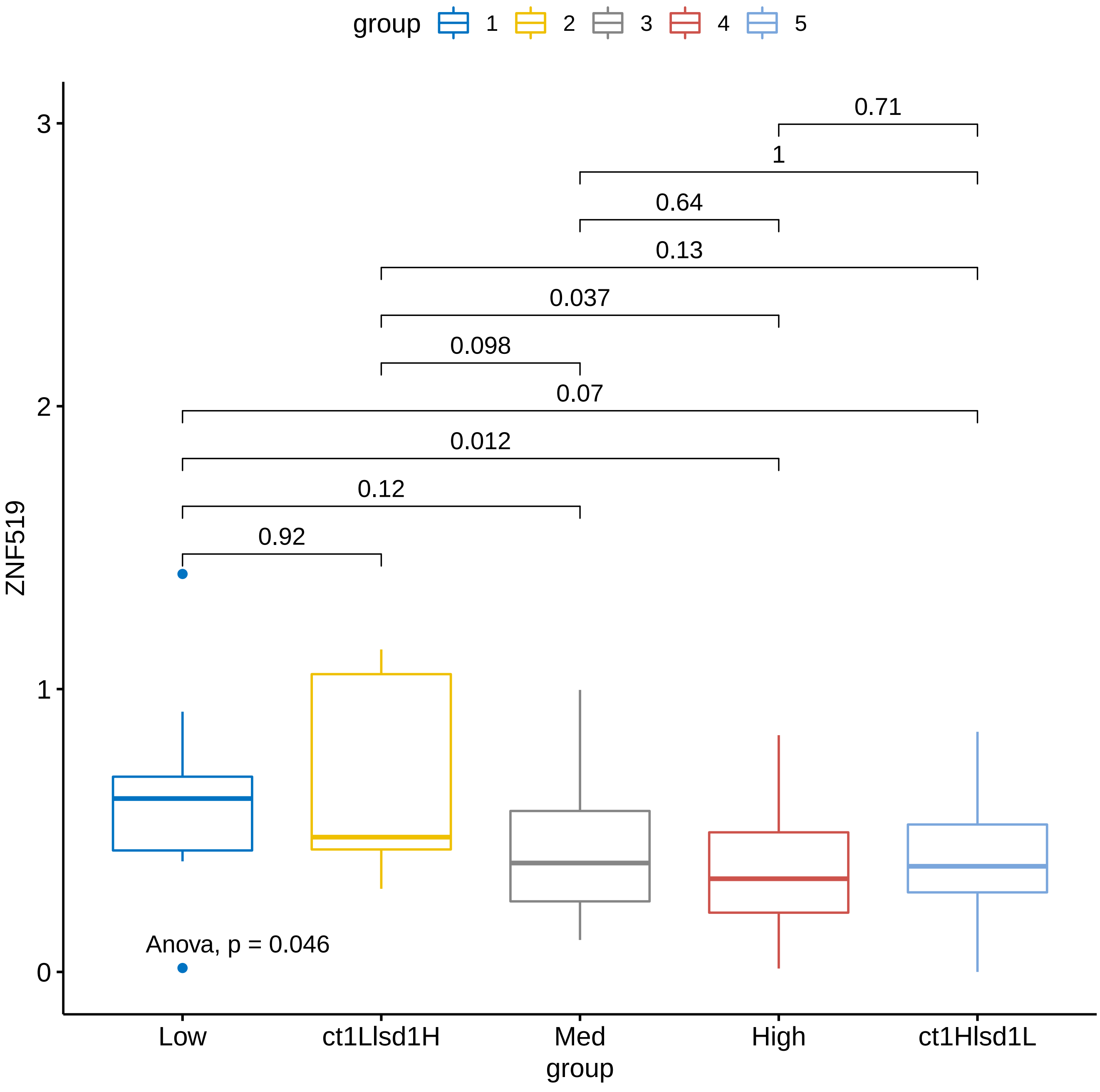
group
1
2
3
4
5
0.71
1
0.64
0.13
0.037
0.098
0.07
0.012
0.12
0.92
Anova, p = 0.046
ZNF519
3
2
1
0
Low
ct1Llsd1H
Med
High
ct1Hlsd1L
group

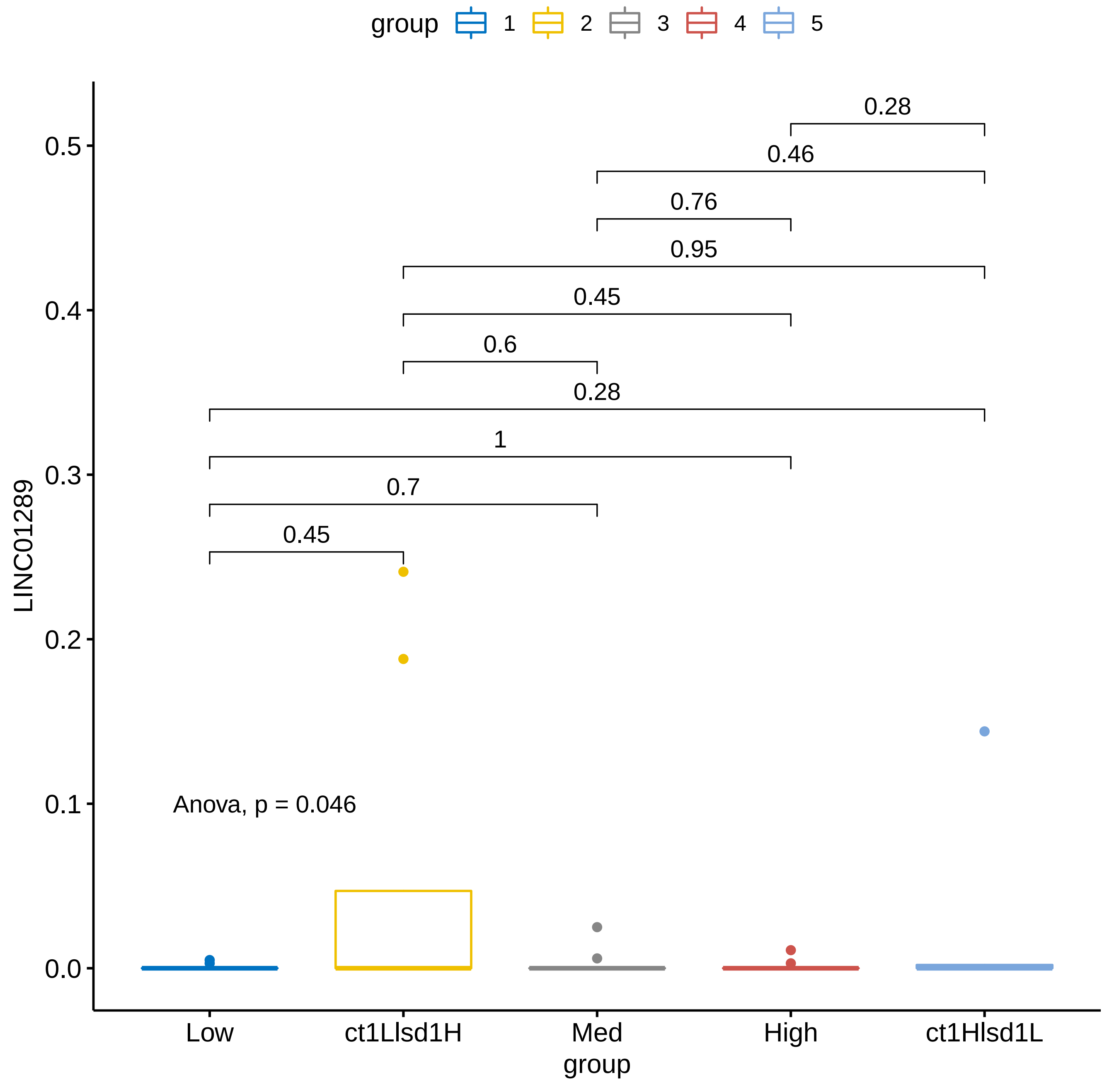
group 1 2 3 4 5
0.28
0.46
0.76
0.95
0.45
0.6
0.28
1
0.7
0.45
Anova, p = 0.046
LINC01289
0.0
0.1
0.2
0.3
0.4
0.5
Low
ct1Llsd1H
Med
High
ct1Hlsd1L
group

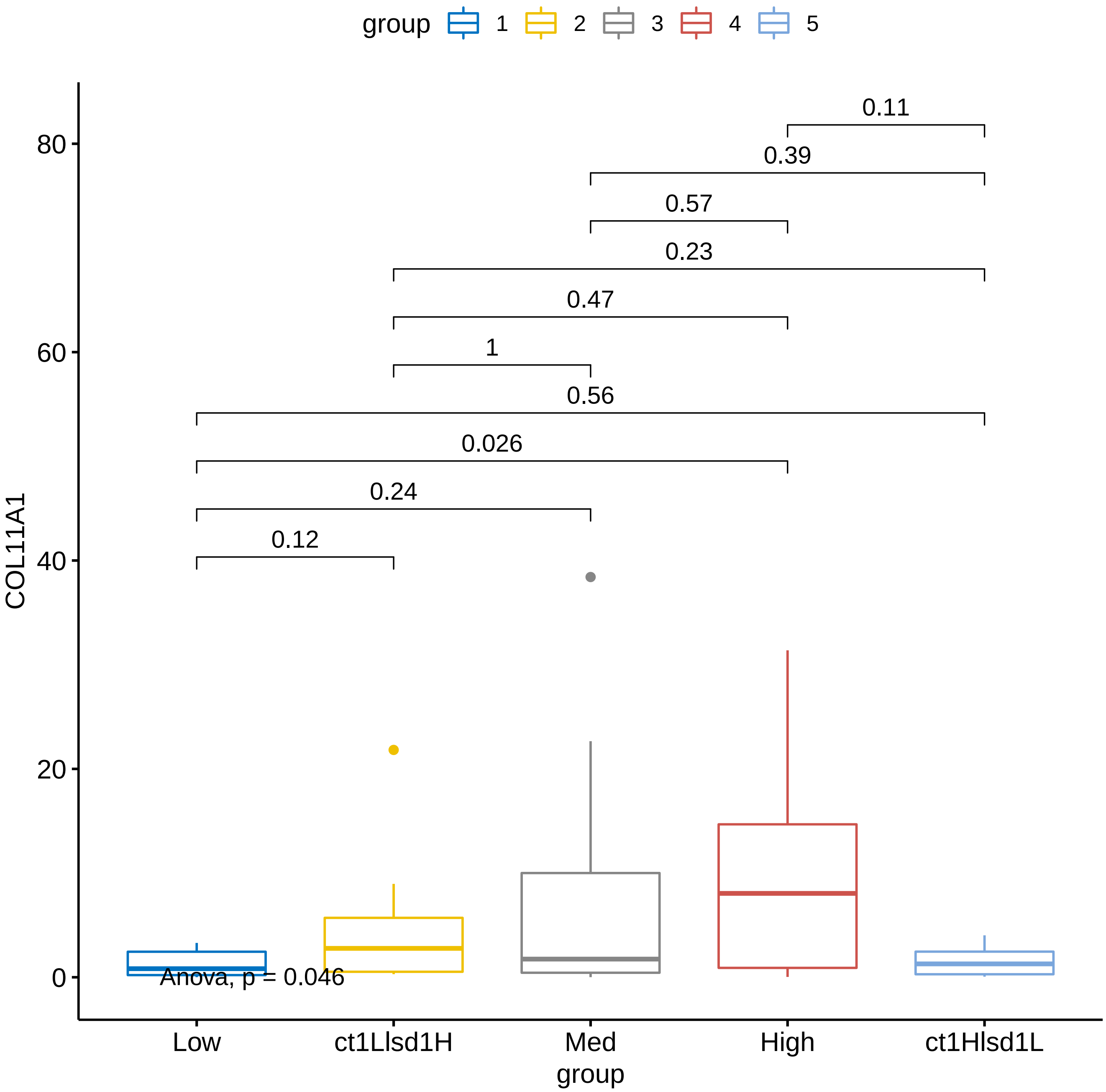

group 1 2 3 4 5
0.11
0.39
0.57
0.23
0.47
1
0.56
0.026
0.24
0.12
Anova, p = 0.046
80
60
40
20
0
COL11A1
Low
ct1Llsd1H
Med
High
ct1Hlsd1L
group

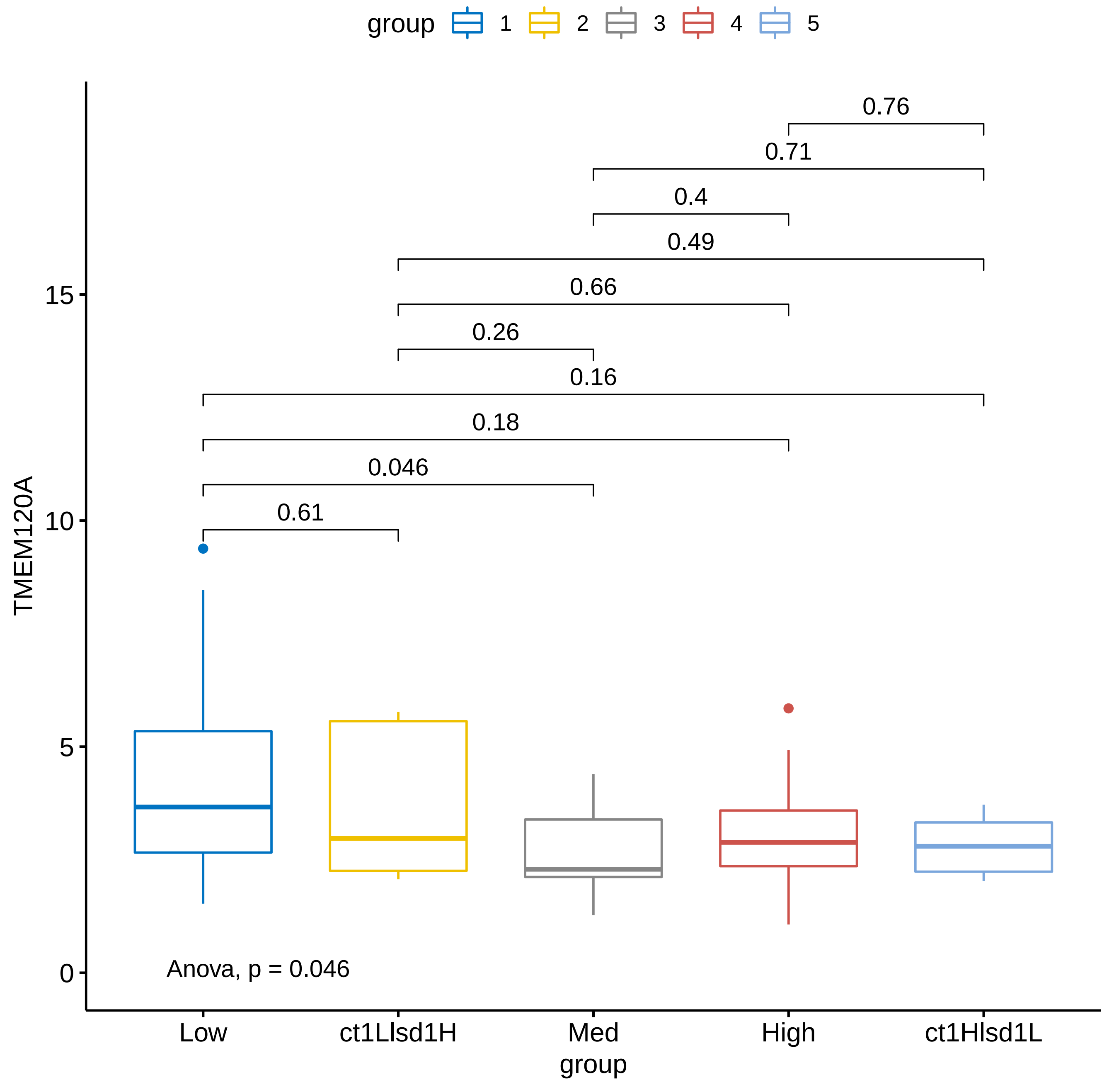

group 1 2 3 4 5
0.76
0.71
0.4
0.49
0.66
0.26
0.16
0.18
0.046
0.61
TMEM120A
15
10
5
0
Anova, p = 0.046
Low
ct1Llsd1H
Med
High
ct1Hlsd1L
group

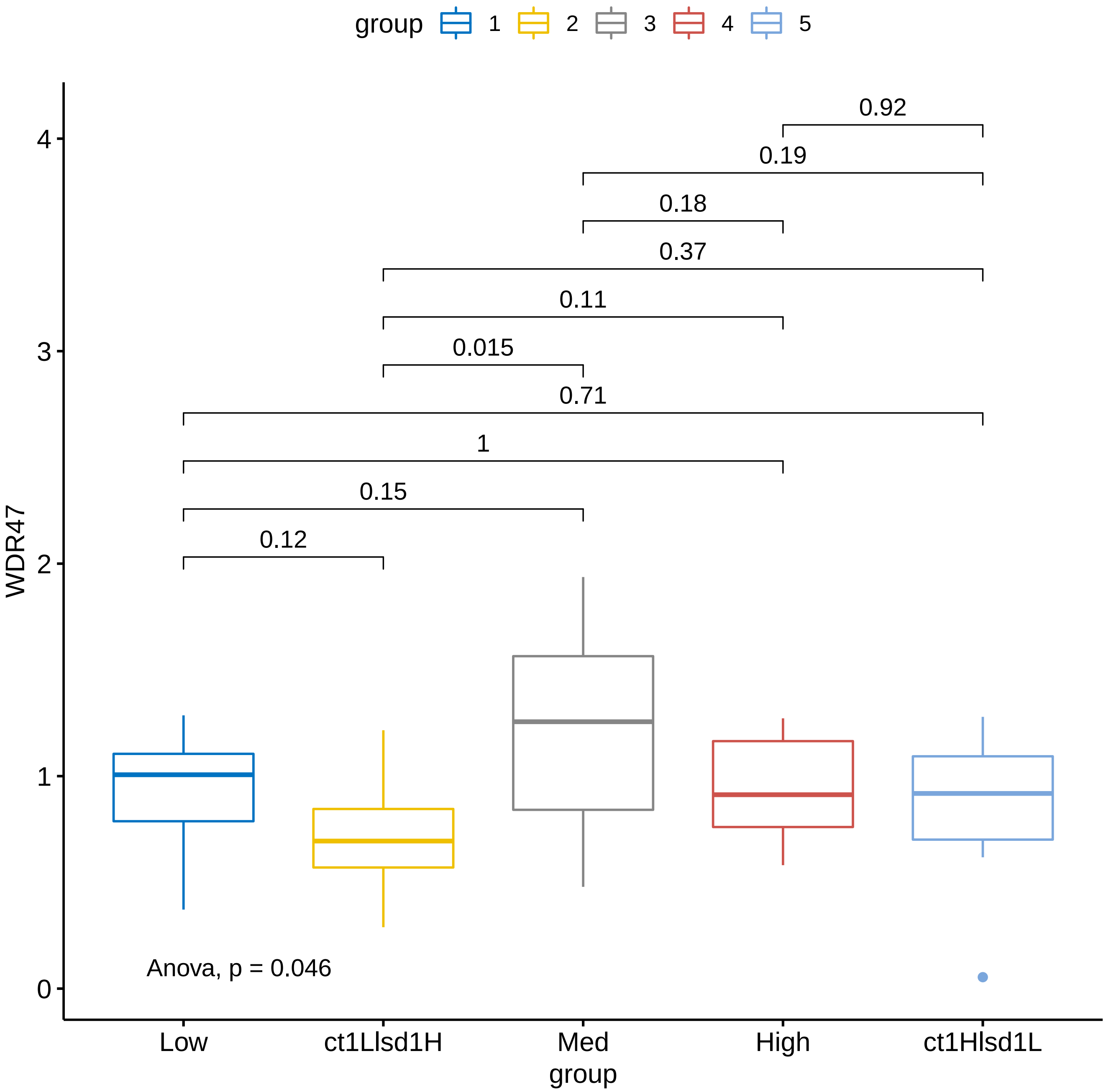
group
1
2
3
4
5
0.92
0.19
0.18
0.37
0.11
0.015
0.71
1
0.15
0.12
Anova, p = 0.046
WDR47
0
1
2
3
4
Low
ct1Llsd1H
Med
High
ct1Hlsd1L
group

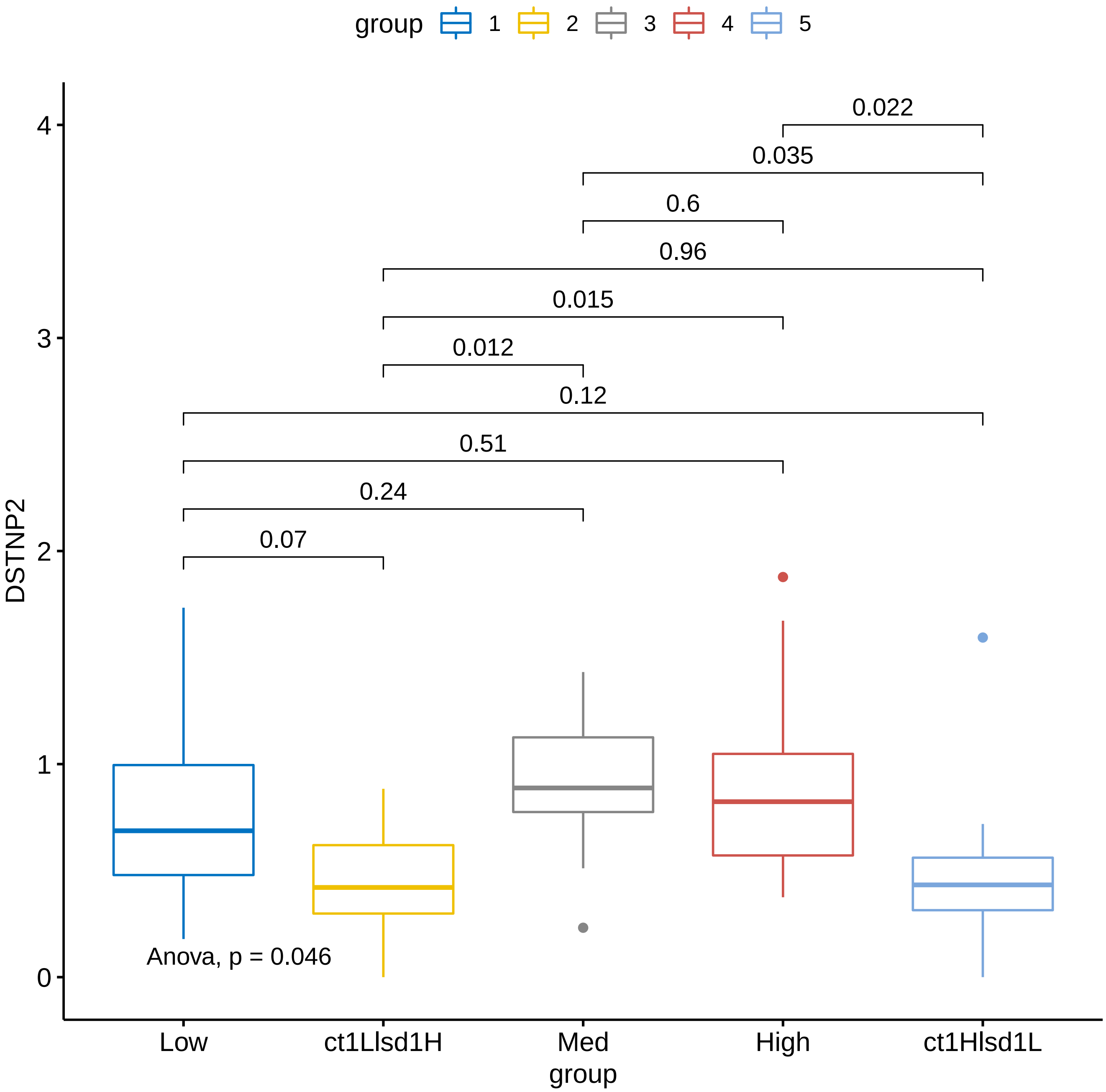
group
1
2
3
4
5
DSTNP2
0.022
0.035
0.6
0.96
0.015
0.012
0.12
0.51
0.24
0.07
Anova, p = 0.046
4
3
2
1
0
Low
ct1Llsd1H
Med
High
ct1Hlsd1L
group

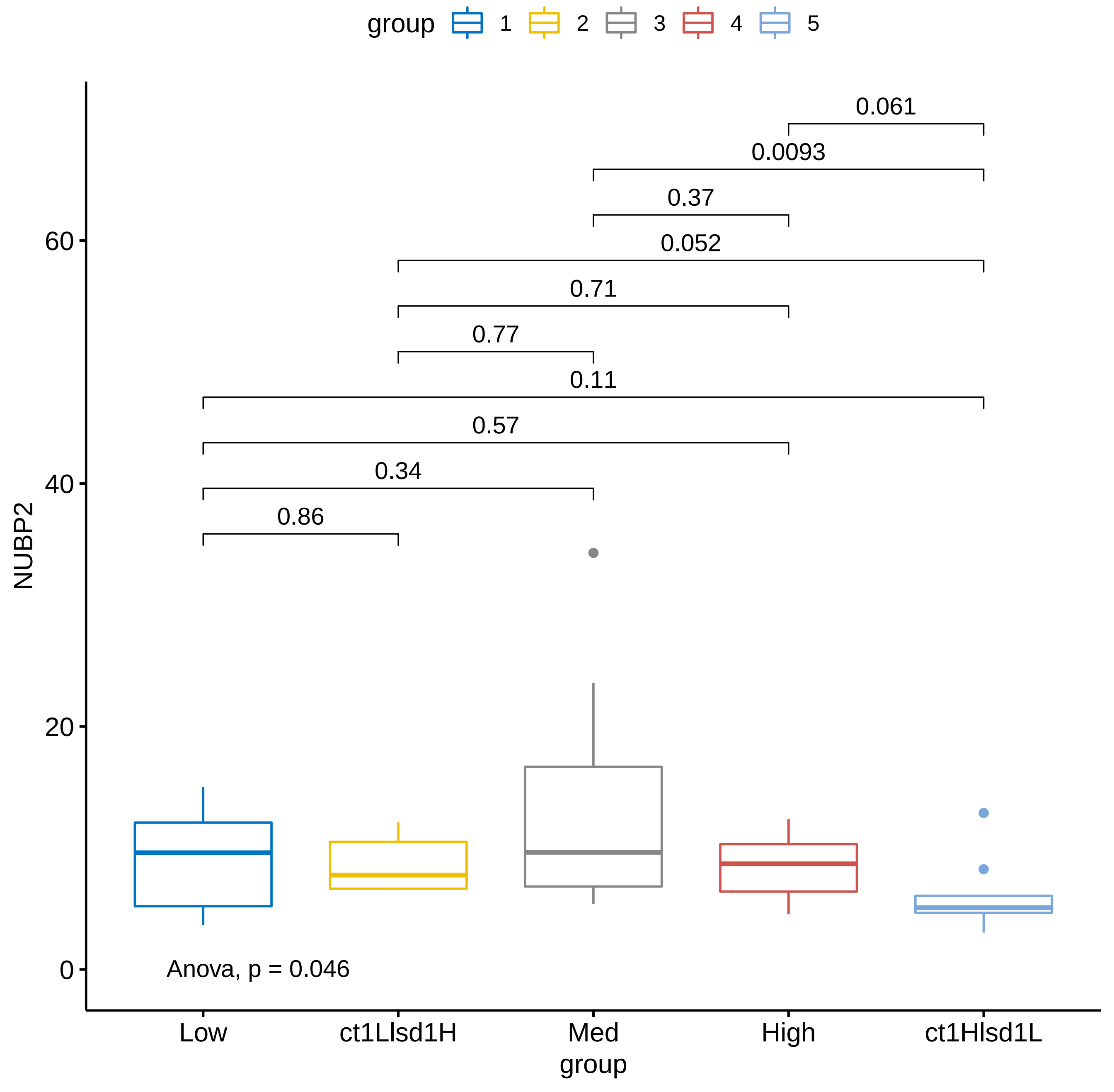
group
1
2
3
4
5
0.061
0.0093
0.37
0.052
0.71
0.77
0.11
0.57
0.34
0.86
NUBP2
60
40
20
0
Anova, p = 0.046
Low
ct1Llsd1H
Med
High
ct1Hlsd1L
group

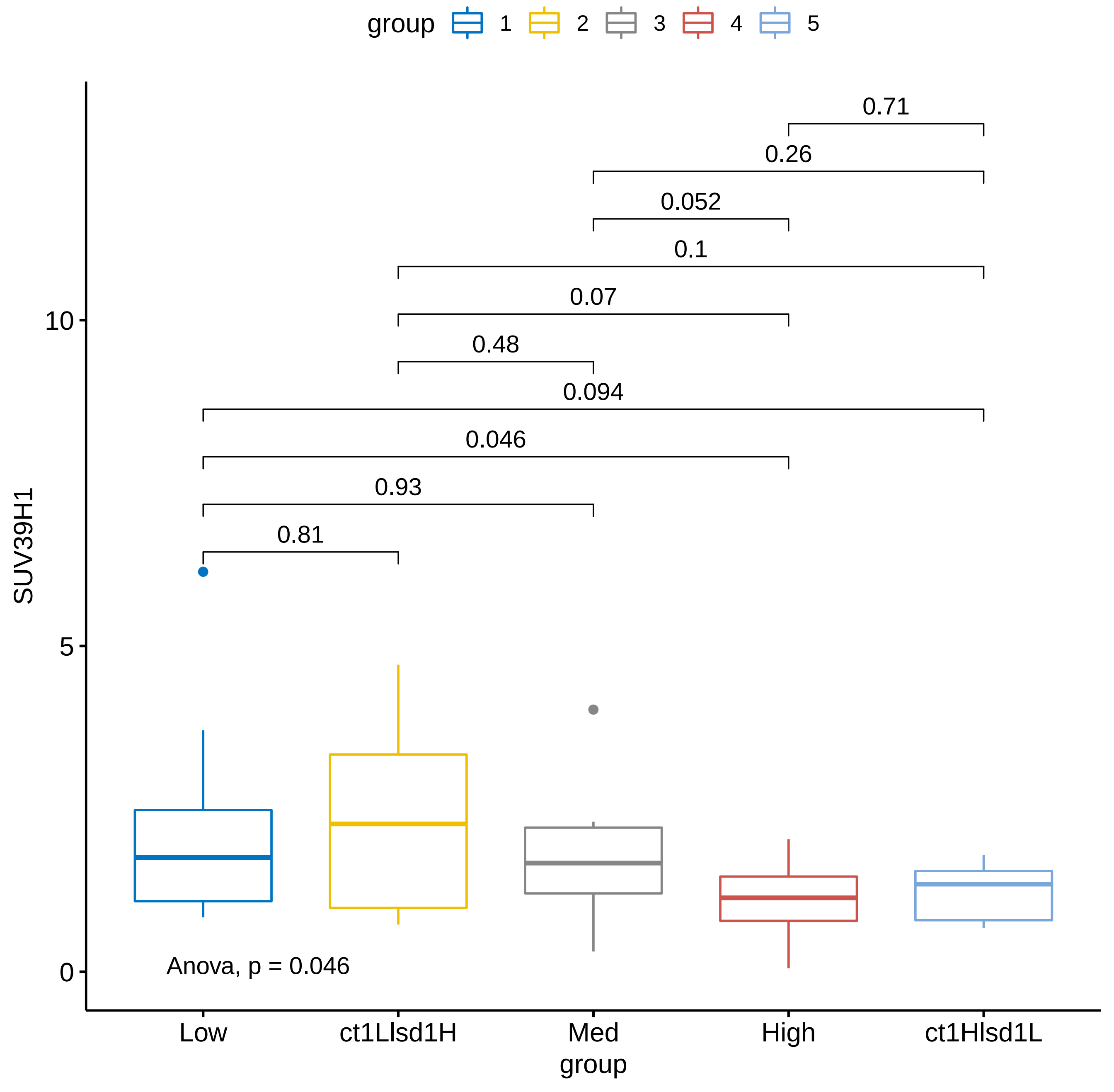
group
1
2
3
4
5
0.71
0.26
0.052
0.1
0.07
0.48
0.094
0.046
0.93
0.81
SUV39H1
10
5
0
Anova, p = 0.046
Low
ct1Llsd1H
Med
High
ct1Hlsd1L
group

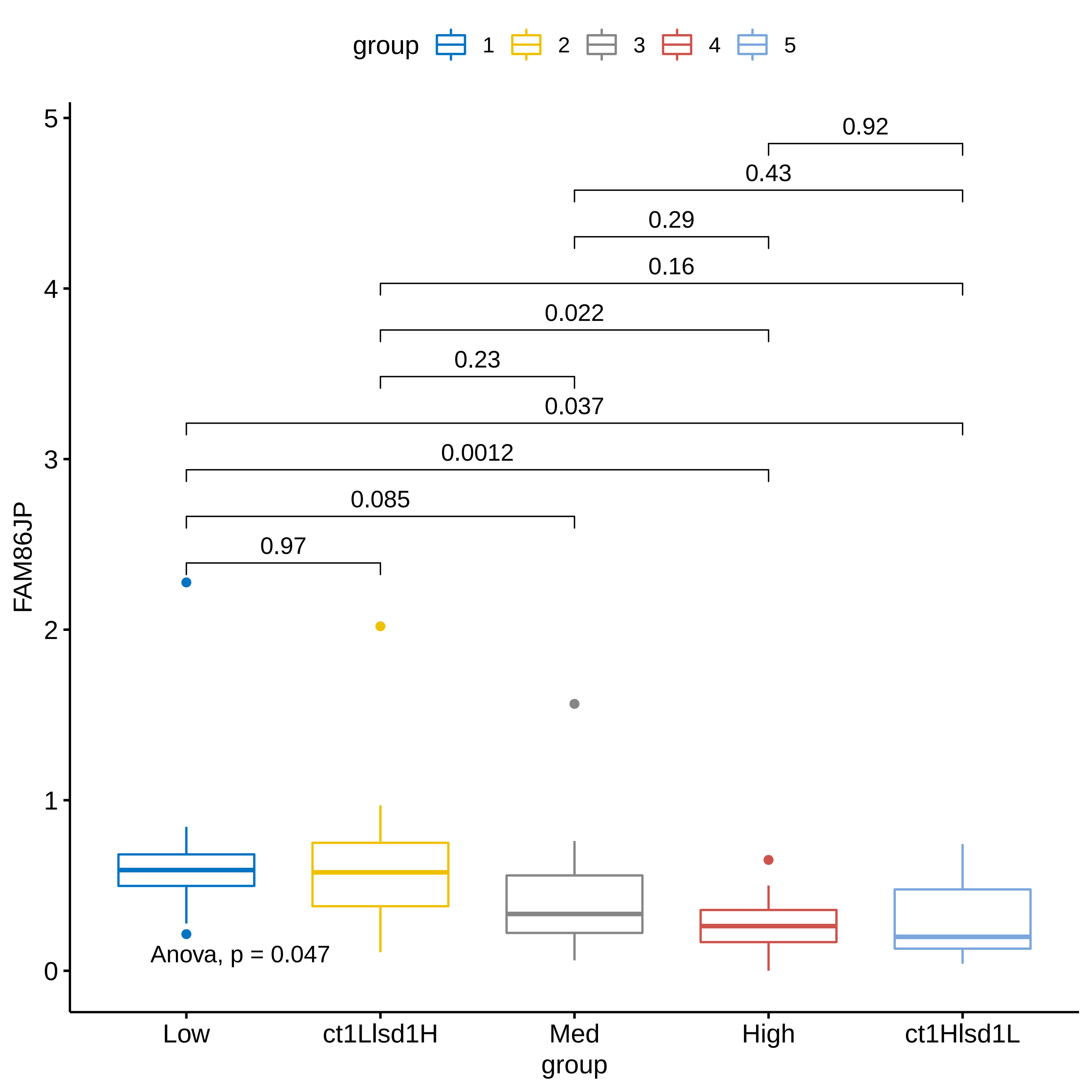

group 1 2 3 4 5
FAM86JP
0.92
0.43
0.29
0.16
0.022
0.23
0.037
0.0012
0.085
0.97
Anova, p = 0.047
0
1
2
3
4
5
Low
ct1Llsd1H
Med
High
ct1Hlsd1L
group

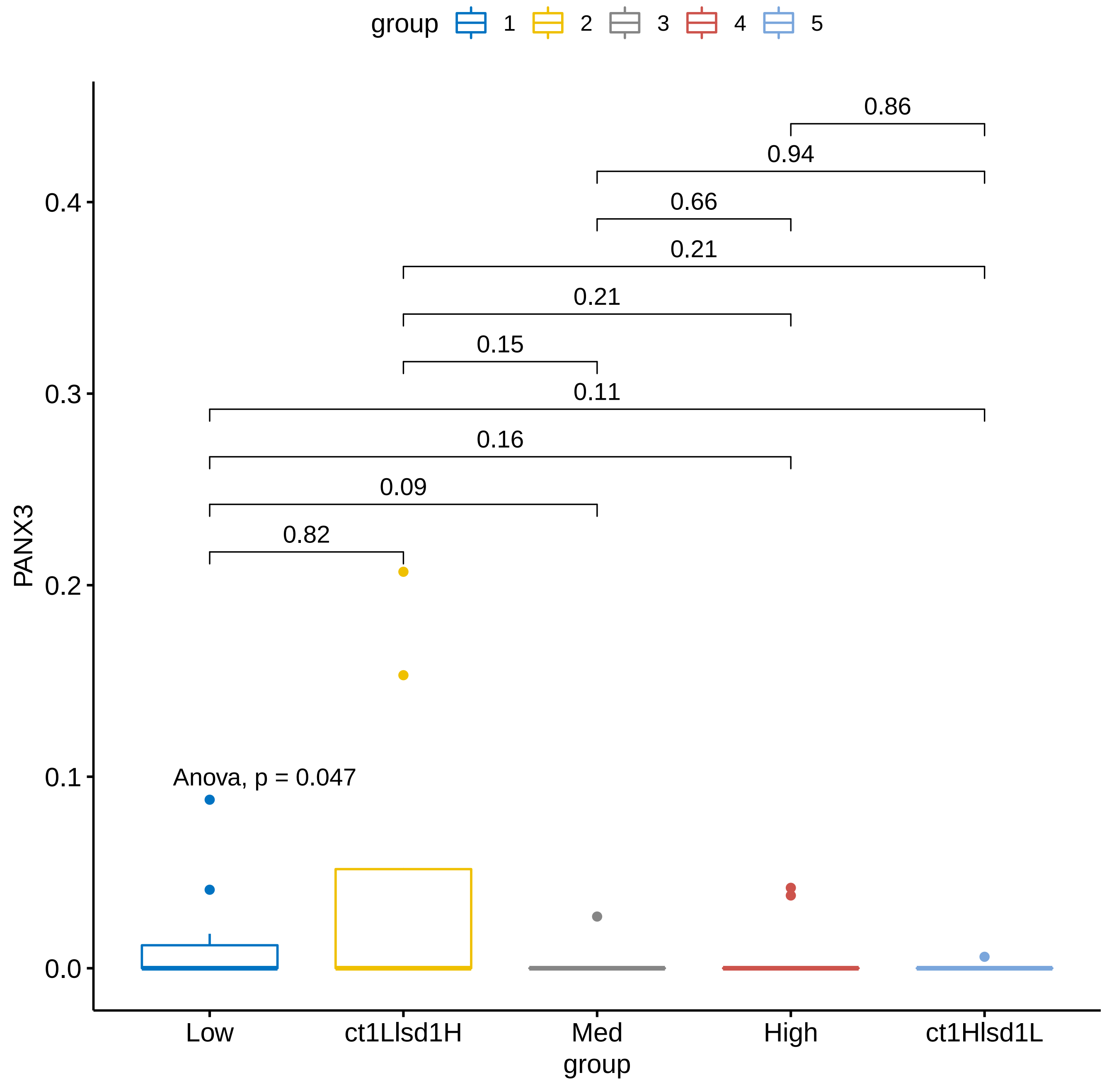
group 1 2 3 4 5
0.86
0.94
0.66
0.21
0.21
0.15
0.11
0.16
0.09
0.82
Anova, p = 0.047
PANX3
0.0
0.1
0.2
0.3
0.4
Low
ct1Llsd1H
Med
High
ct1Hlsd1L
group

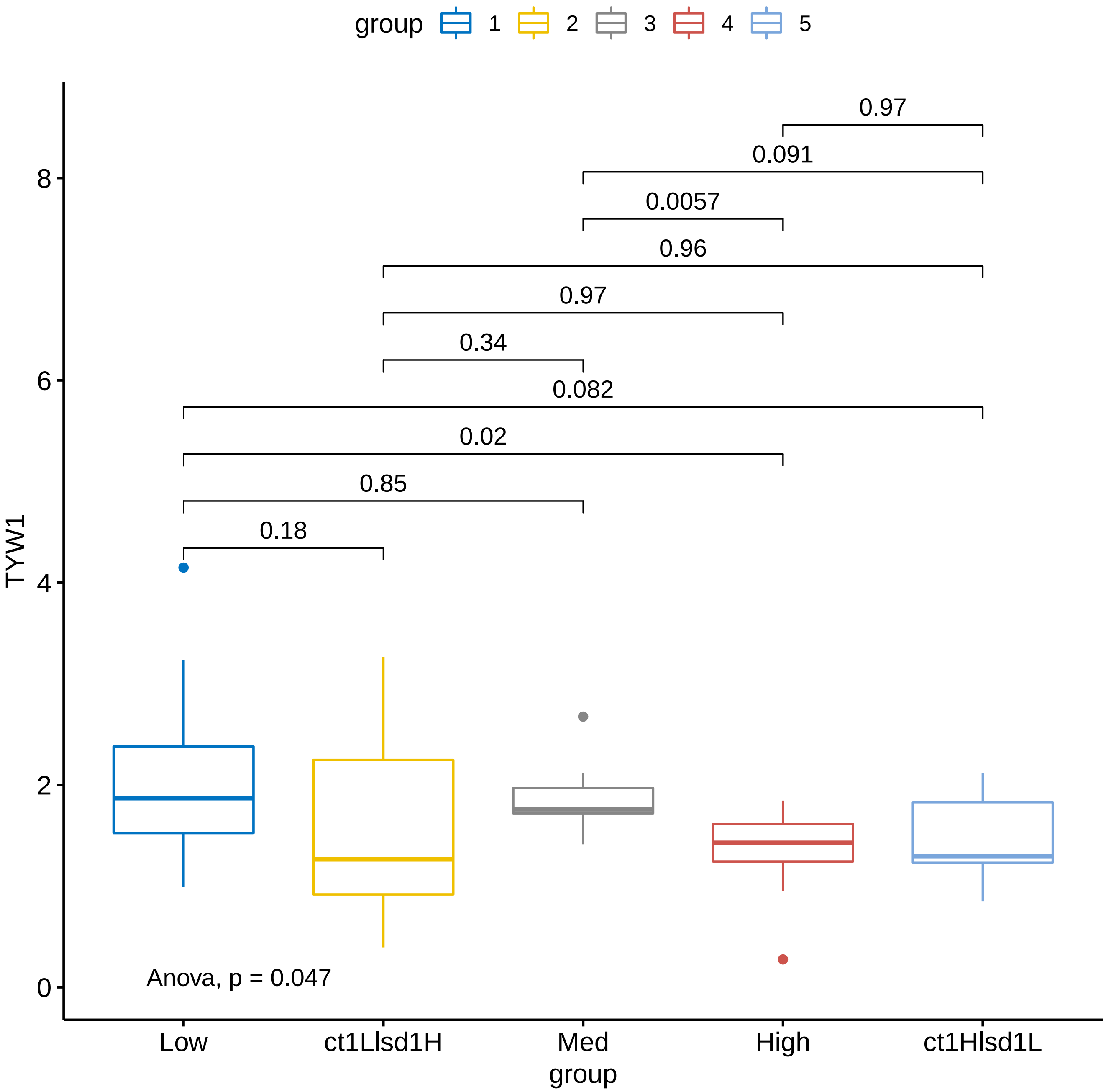

group
1
2
3
4
5
TYW1
0
2
4
6
8
0.97
0.091
0.0057
0.96
0.97
0.34
0.082
0.02
0.85
0.18
Anova, p = 0.047
Low
ct1Llsd1H
Med
High
ct1Hlsd1L
group

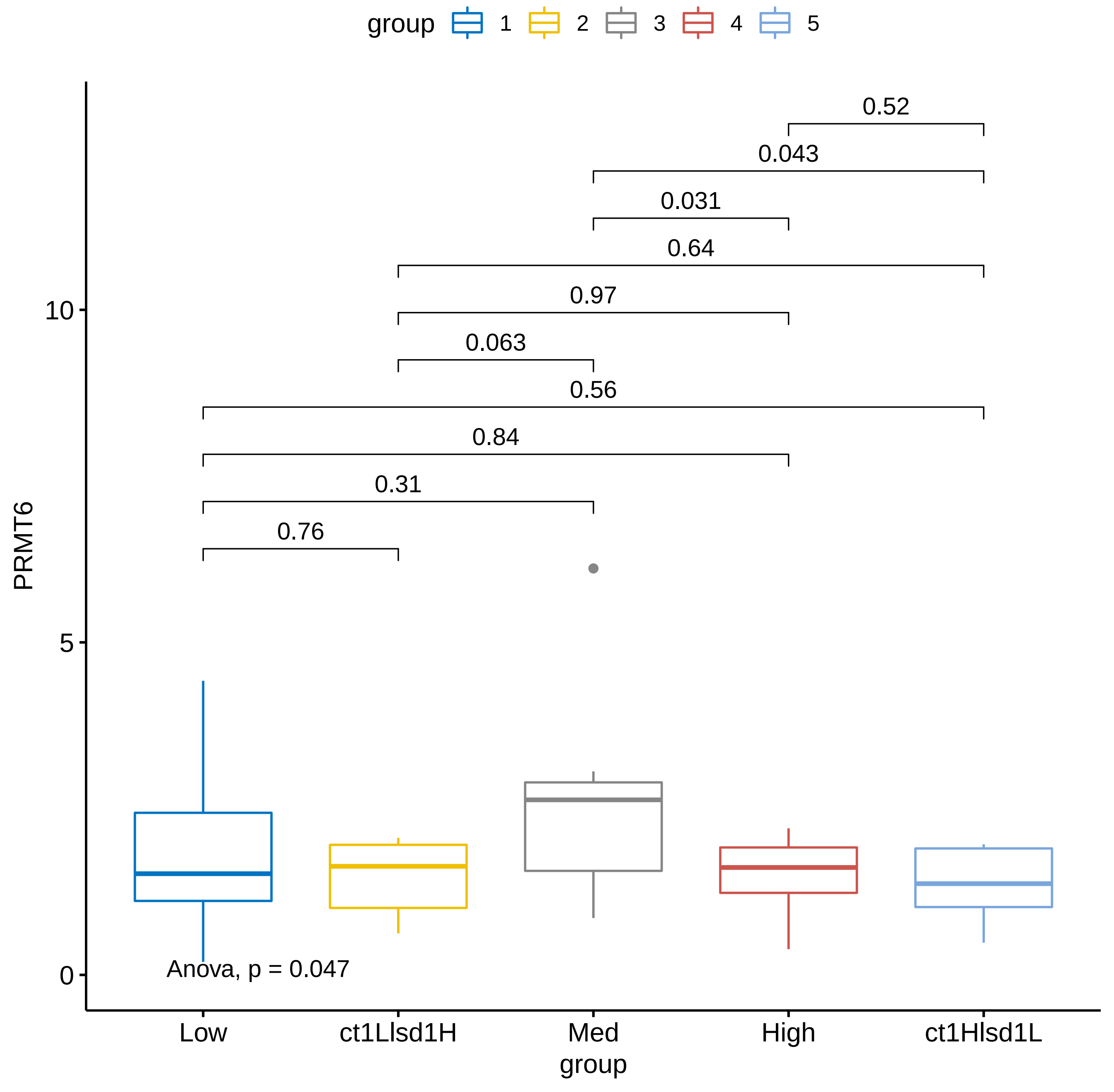

group 1 2 3 4 5
PRMT6
0
5
10
0.52
0.043
0.031
0.64
0.97
0.063
0.56
0.84
0.31
0.76
Anova, p = 0.047
Low
ct1Llsd1H
Med
High
ct1Hlsd1L
group

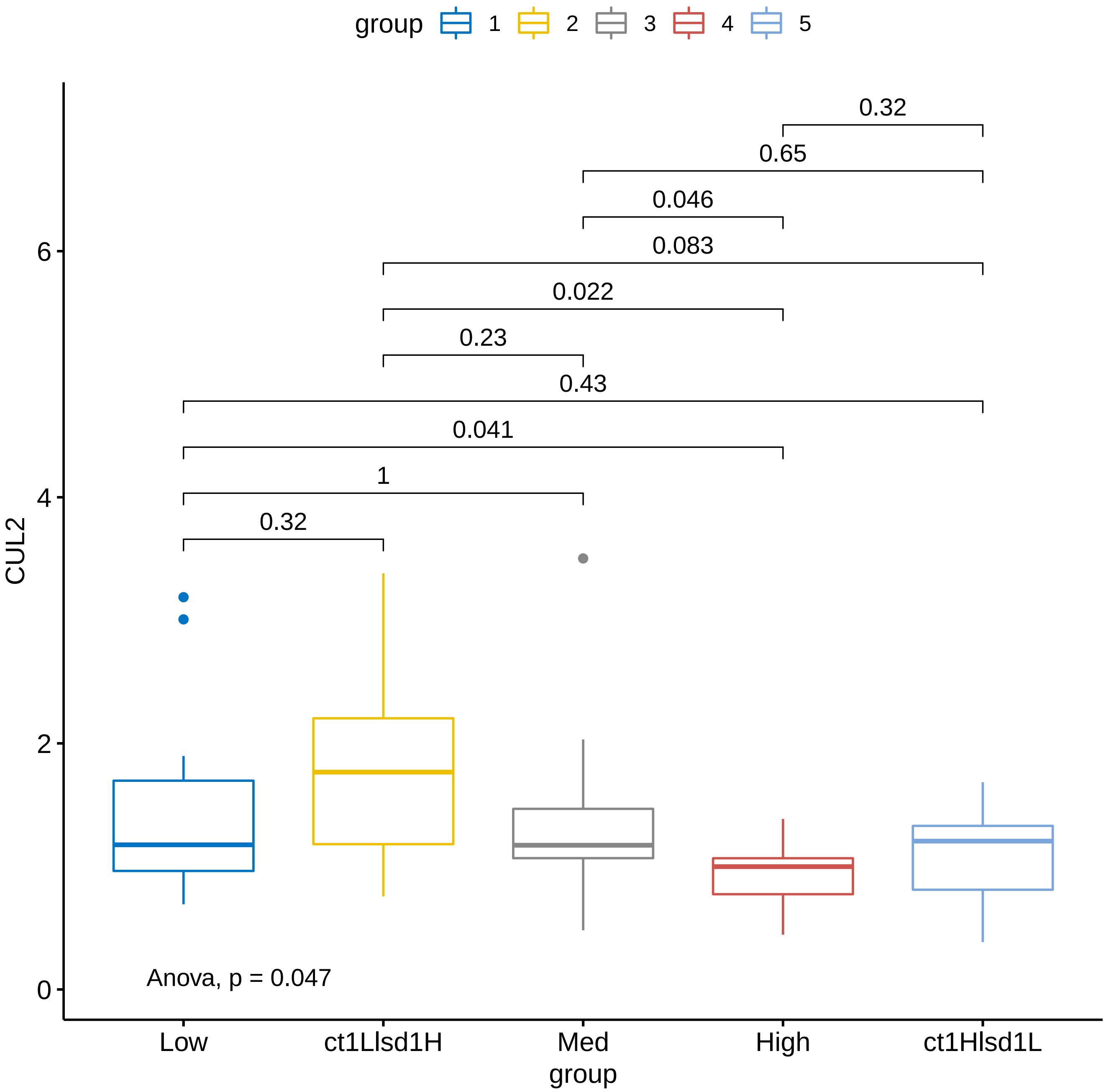

group 1 2 3 4 5
0.32
0.65
0.046
0.083
0.022
0.23
0.43
0.041
1
0.32
6
4
2
0
CUL2
Anova, p = 0.047
Low
ct1Llsd1H
Med
High
ct1Hlsd1L
group

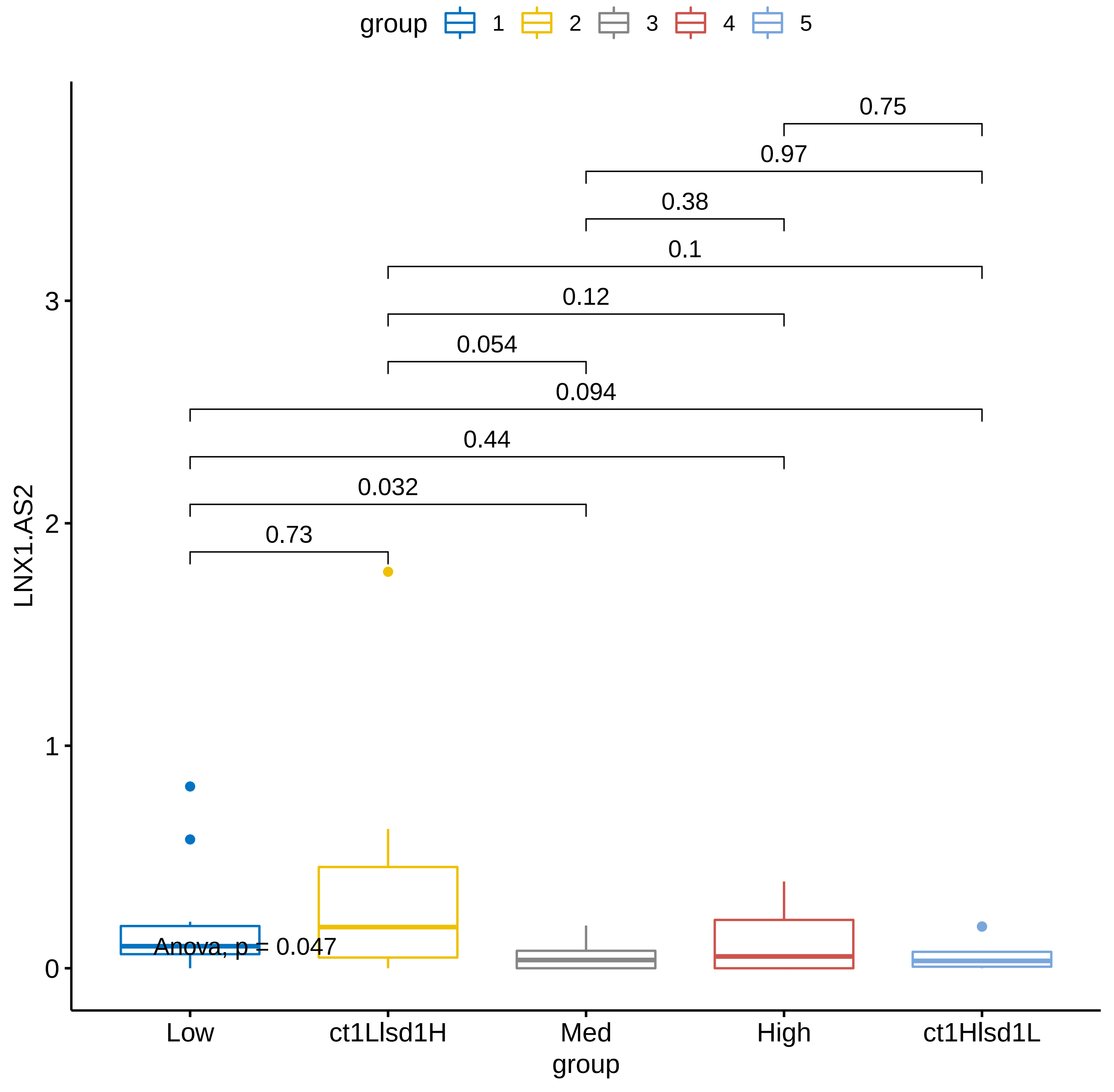
group 1 2 3 4 5
0.75
0.97
0.38
0.1
0.12
0.054
0.094
0.44
0.032
0.73
Anova, p = 0.047
LNX1.AS2
0
1
2
3
Low
ct1Llsd1H
Med
High
ct1Hlsd1L
group

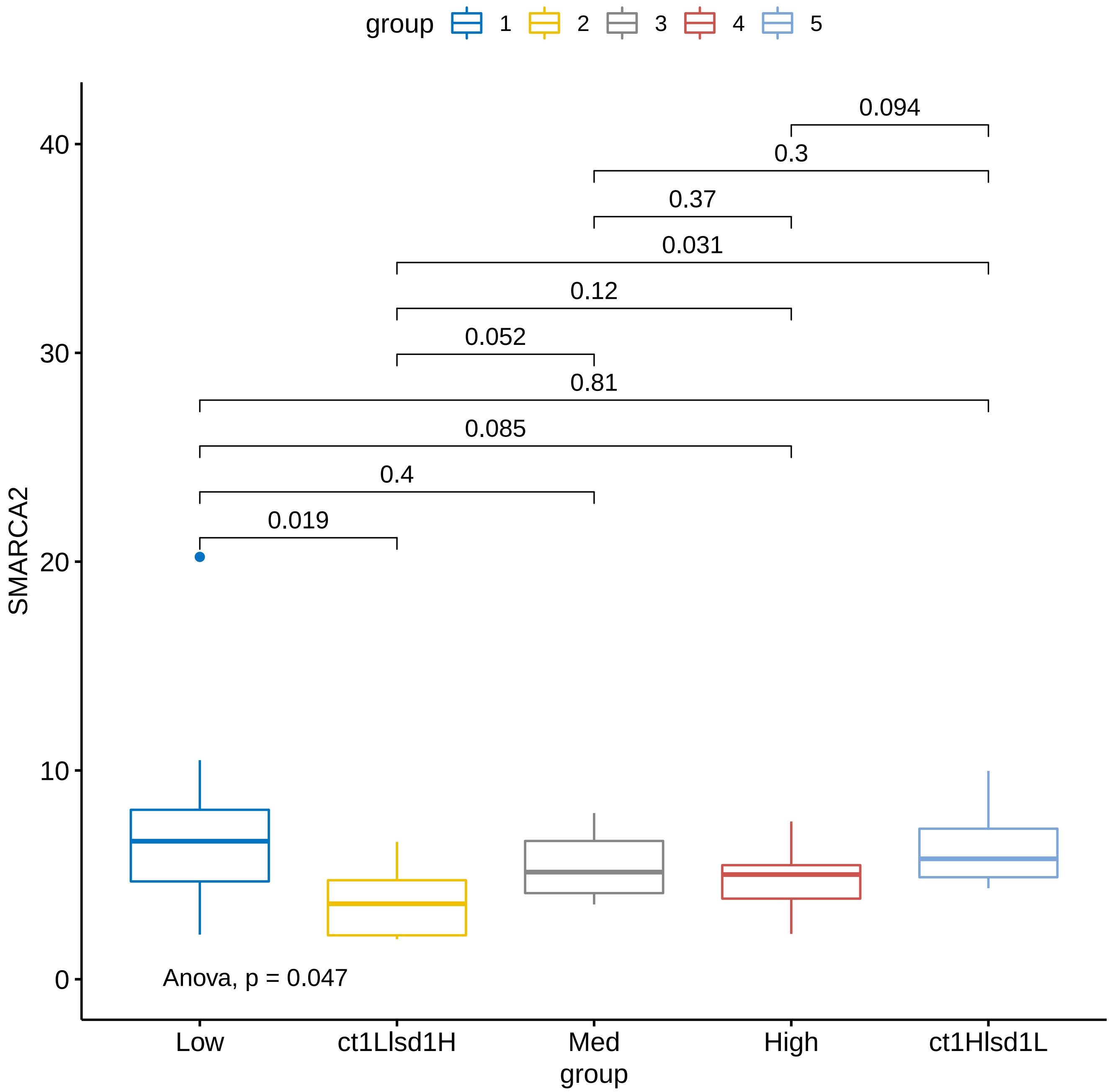

group 1 2 3 4 5
0.094
0.3
0.37
0.031
0.12
0.052
0.81
0.085
0.4
0.019
SMARCA2
40
30
20
10
0
Anova, p = 0.047
Low
ct1Llsd1H
Med
High
ct1Hlsd1L
group

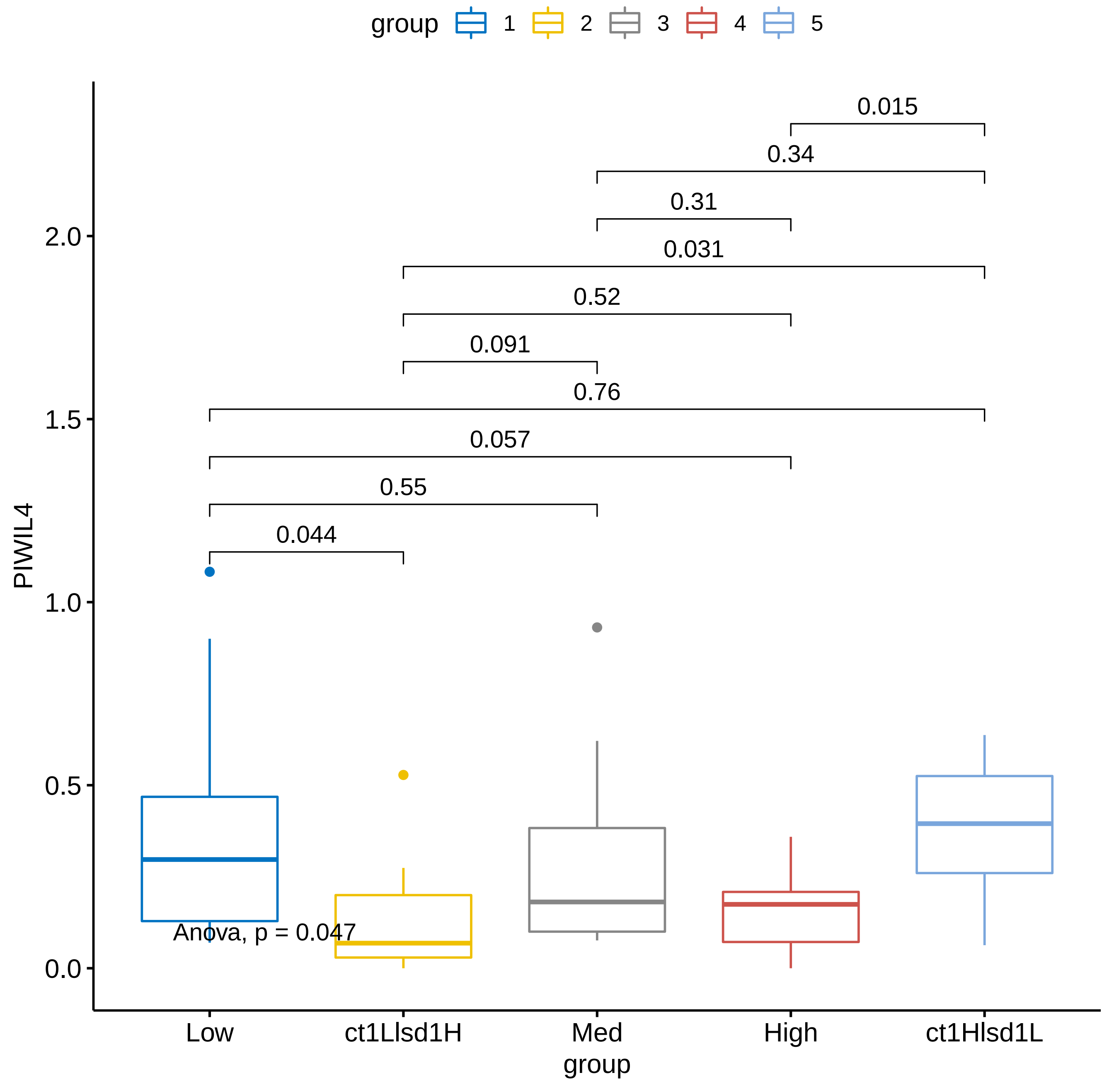
group
1
2
3
4
5
0.015
0.34
0.31
0.031
0.52
0.091
0.76
0.057
0.55
0.044
2.0
1.5
1.0
0.5
0.0
PIWIL4
Anova, p = 0.047
Low
ct1Llsd1H
Med
High
ct1Hlsd1L
group

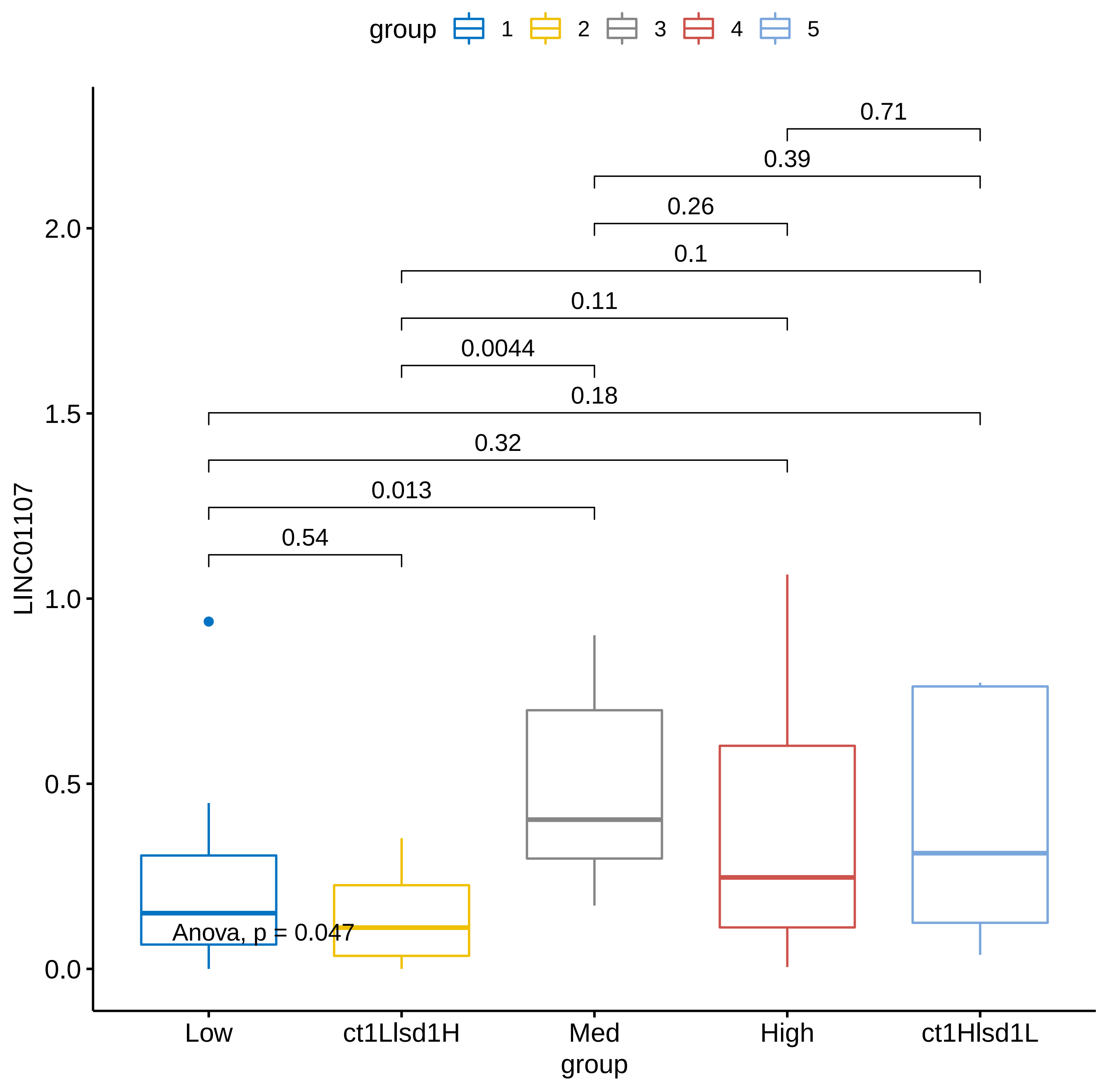
group 1 2 3 4 5
0.71
0.39
0.26
0.1
0.11
0.0044
0.18
0.32
0.013
0.54
Anova, p = 0.047
LINC01107
2.0
1.5
1.0
0.5
0.0
Low
ct1Llsd1H
Med
High
ct1Hlsd1L
group

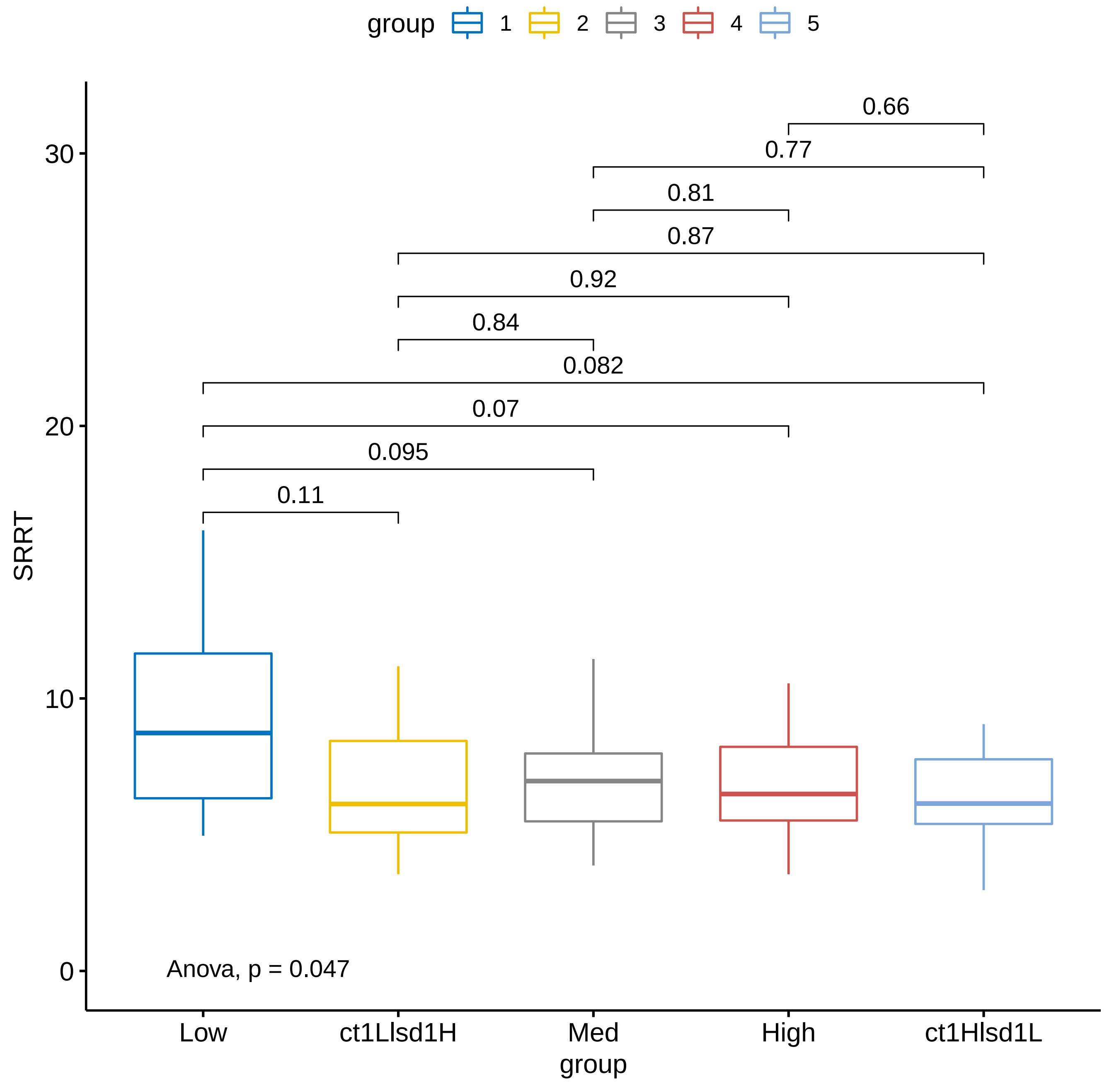

group 1 2 3 4 5
0.66
0.77
0.81
0.87
0.92
0.84
0.082
0.07
0.095
0.11
30
20
10
0
SRRT
Anova, p = 0.047
Low
ct1Llsd1H
Med
High
ct1Hlsd1L
group

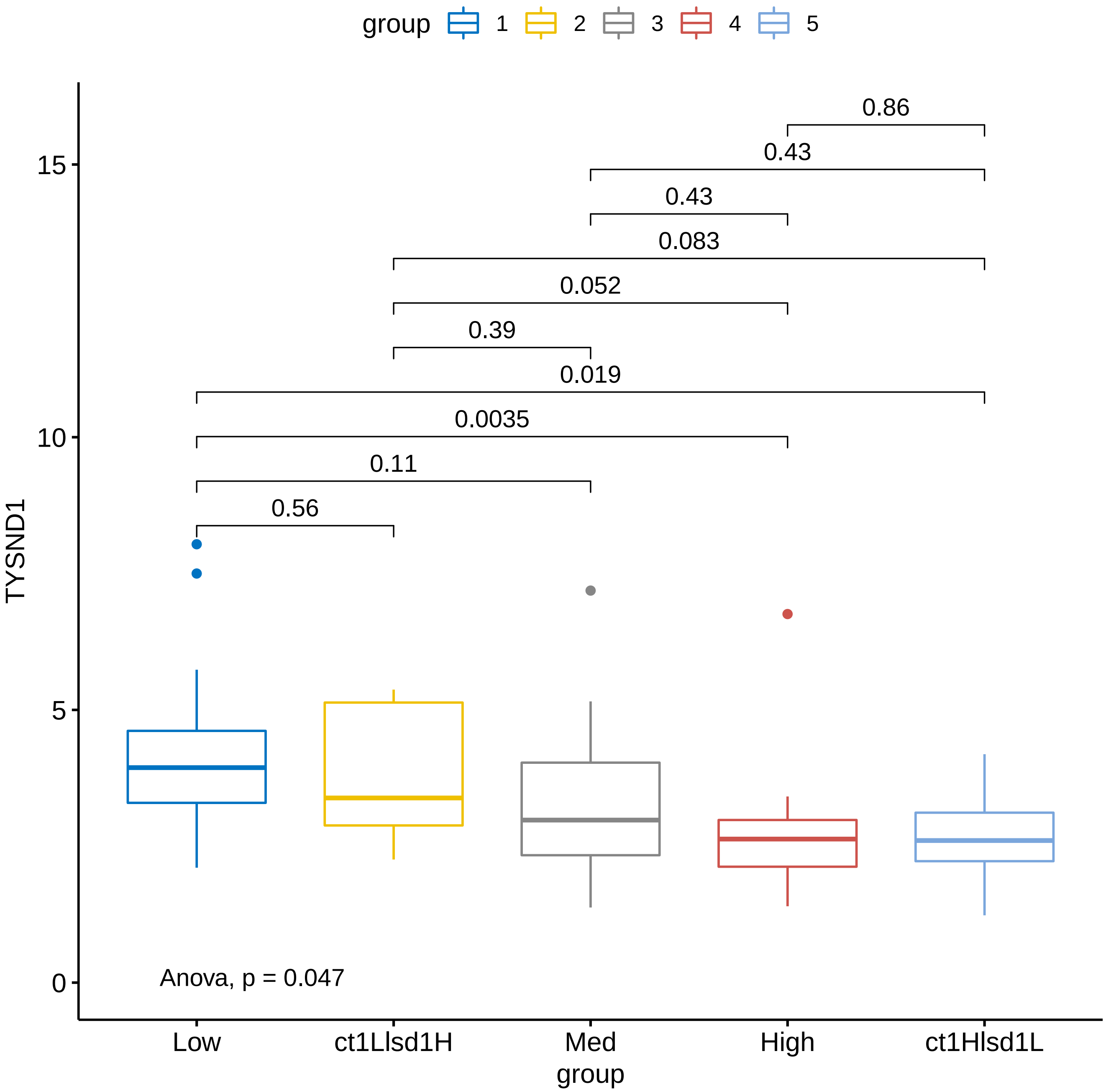
group 1 2 3 4 5
0.86
0.43
0.43
0.083
0.052
0.39
0.019
0.0035
0.11
0.56
15
10
5
0
TYSND1
Anova, p = 0.047
Low
ct1Llsd1H
Med
High
ct1Hlsd1L
group

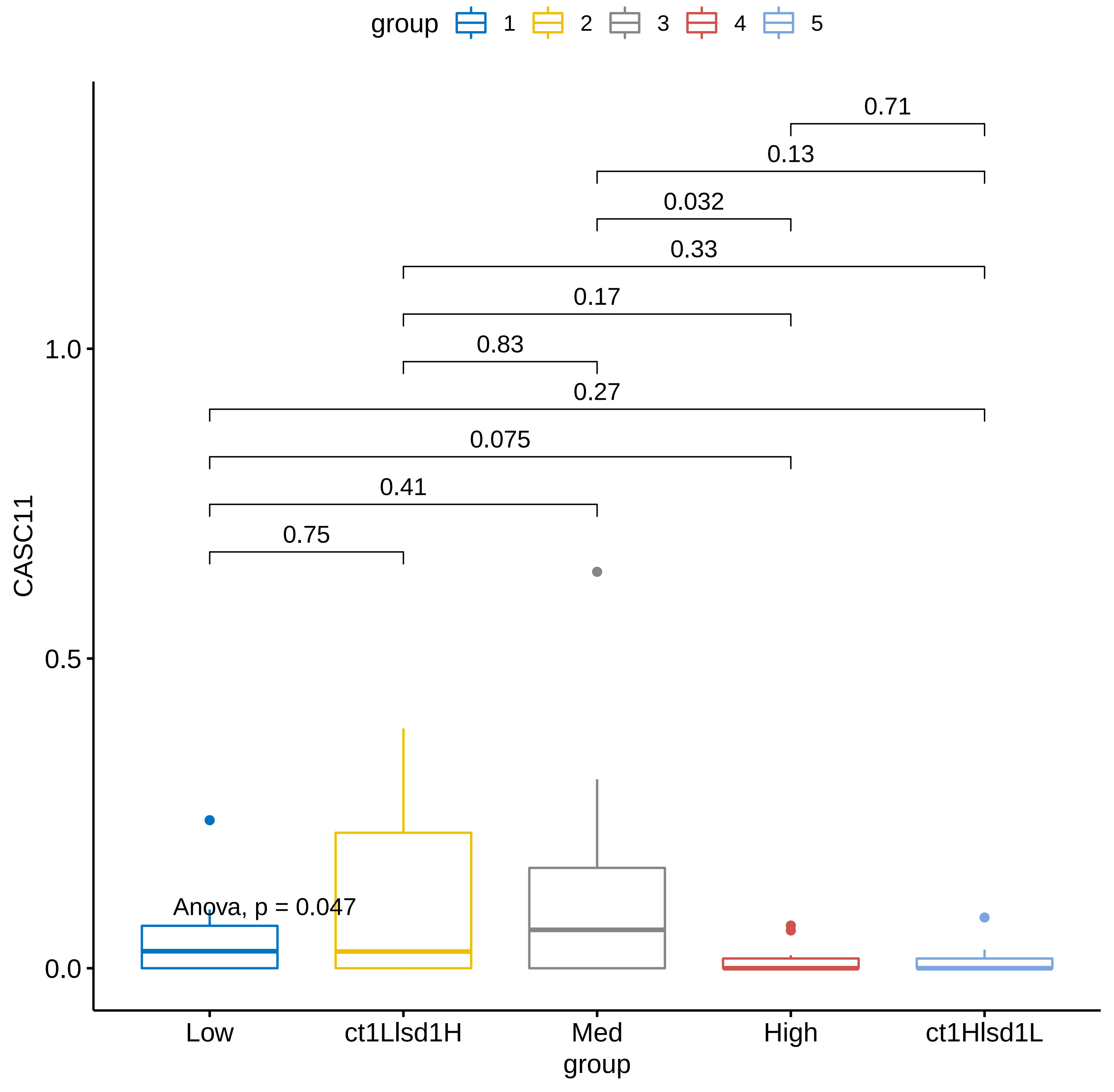
group 1 2 3 4 5
0.71
0.13
0.032
0.33
0.17
0.83
0.27
0.075
0.41
0.75
Anova, p = 0.047
CASC11
1.0
0.5
0.0
Low
ct1Llsd1H
Med
High
ct1Hlsd1L
group

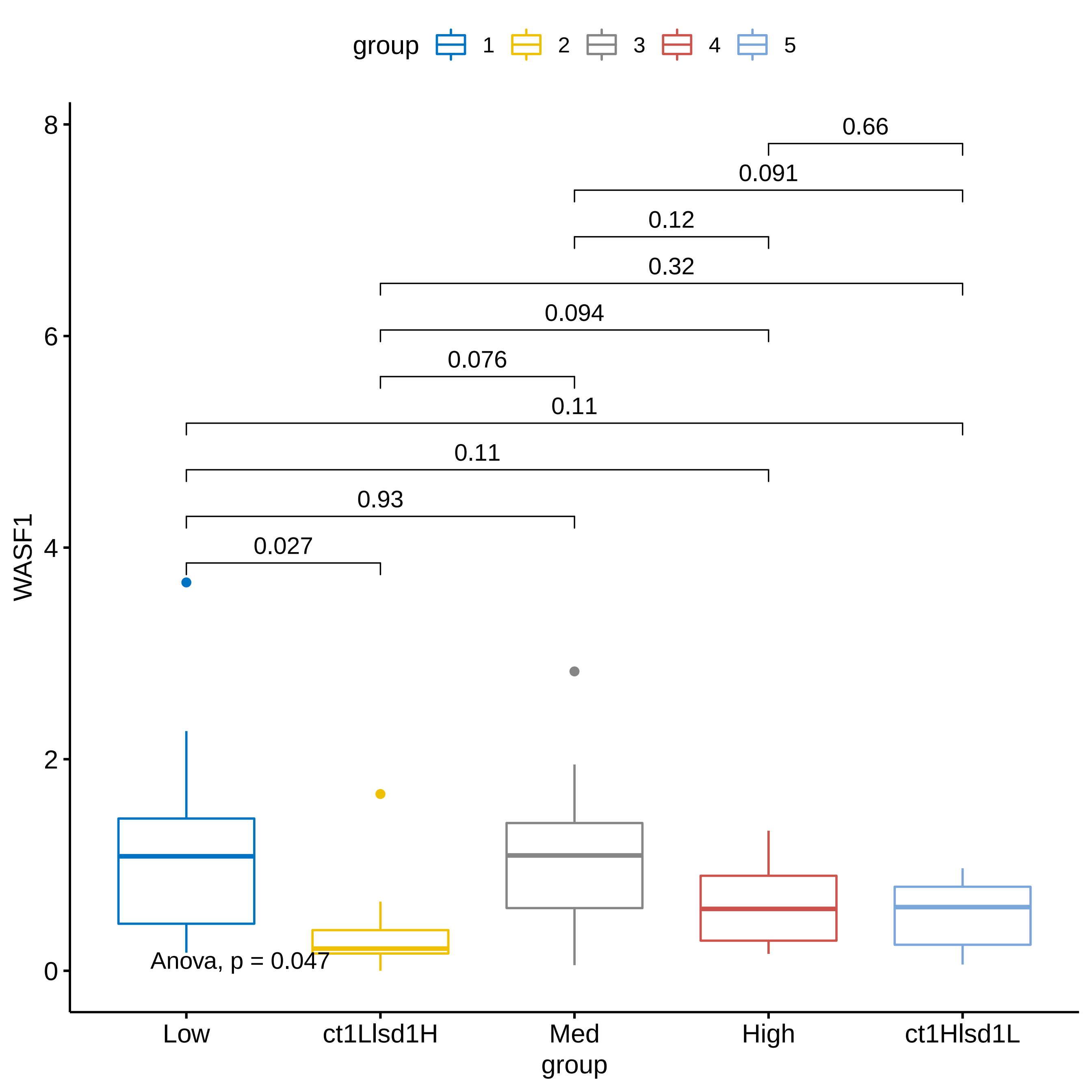
group
1
2
3
4
5
WASF1
0
2
4
6
8
0.66
0.091
0.12
0.32
0.094
0.076
0.11
0.11
0.93
0.027
Anova, p = 0.047
Low
ct1Llsd1H
Med
High
ct1Hlsd1L
group

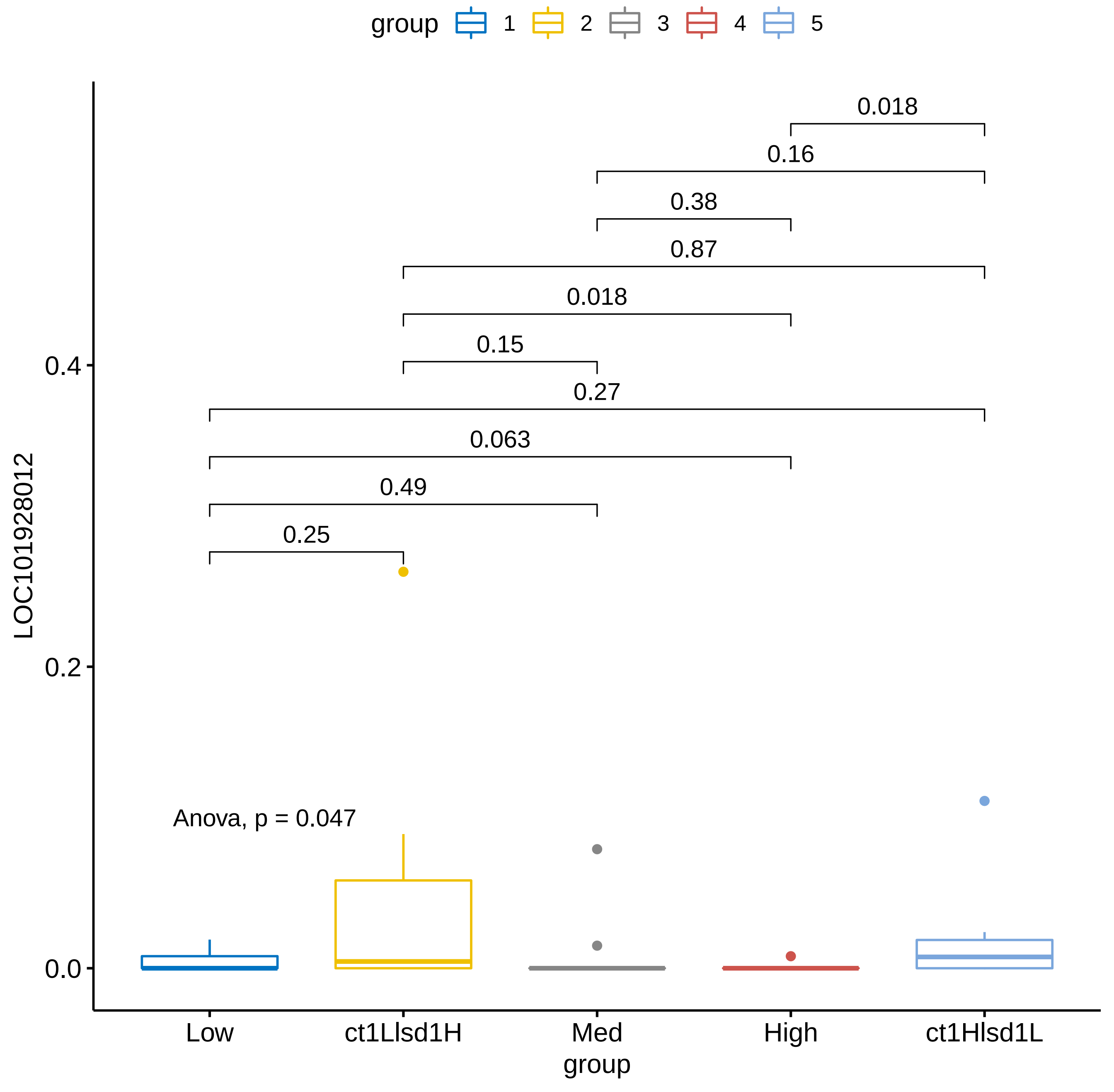

group
1
2
3
4
5
0.018
0.16
0.38
0.87
0.018
0.15
0.27
0.063
0.49
0.25
Anova, p = 0.047
0.4
0.2
0.0
LOC101928012
Low
ct1Llsd1H
Med
High
ct1Hlsd1L
group

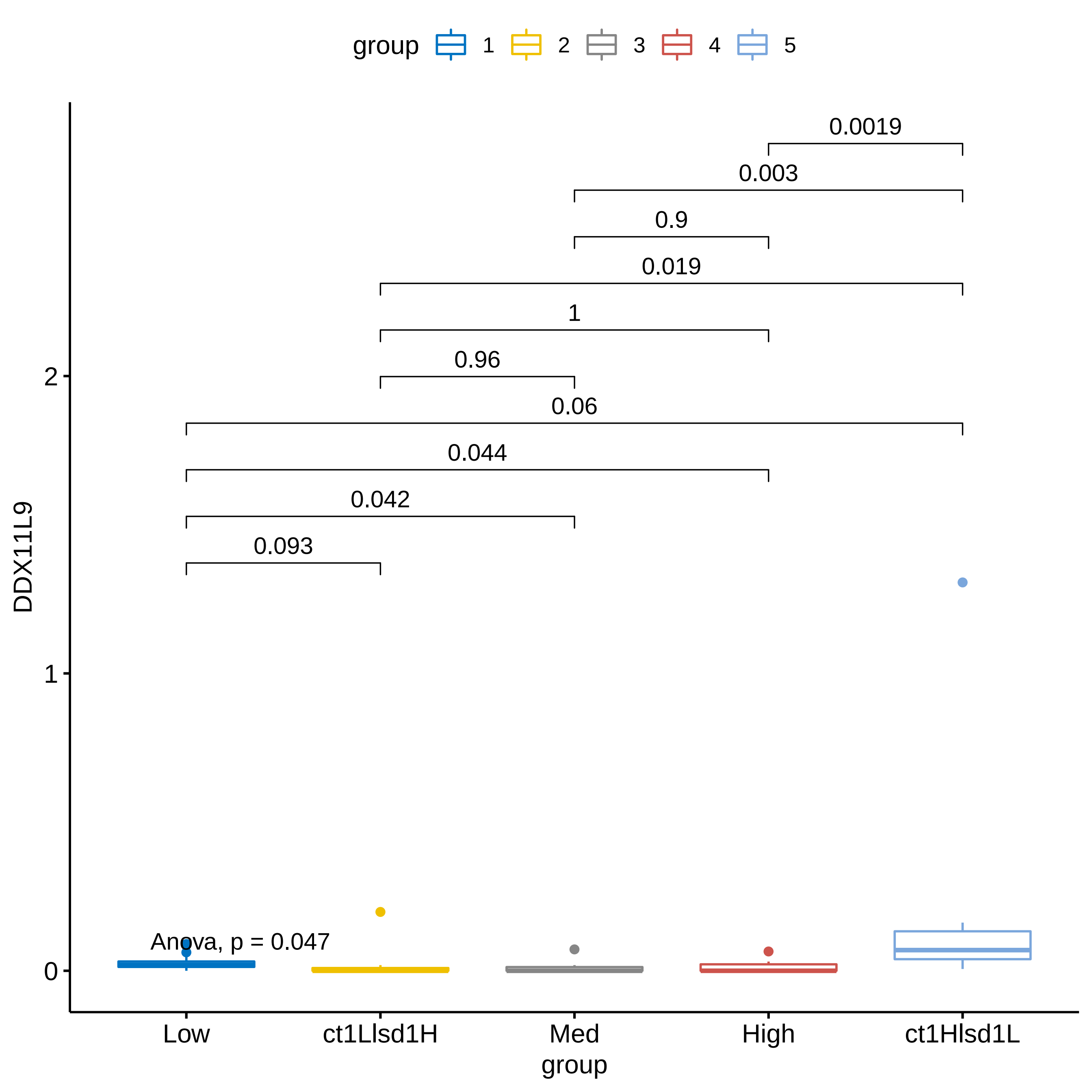

group
1
2
3
4
5
0.0019
0.003
0.9
0.019
1
0.96
0.06
0.044
0.042
0.093
Anova, p = 0.047
DDX11L9
2
1
0
Low
ct1Llsd1H
Med
High
ct1Hlsd1L
group

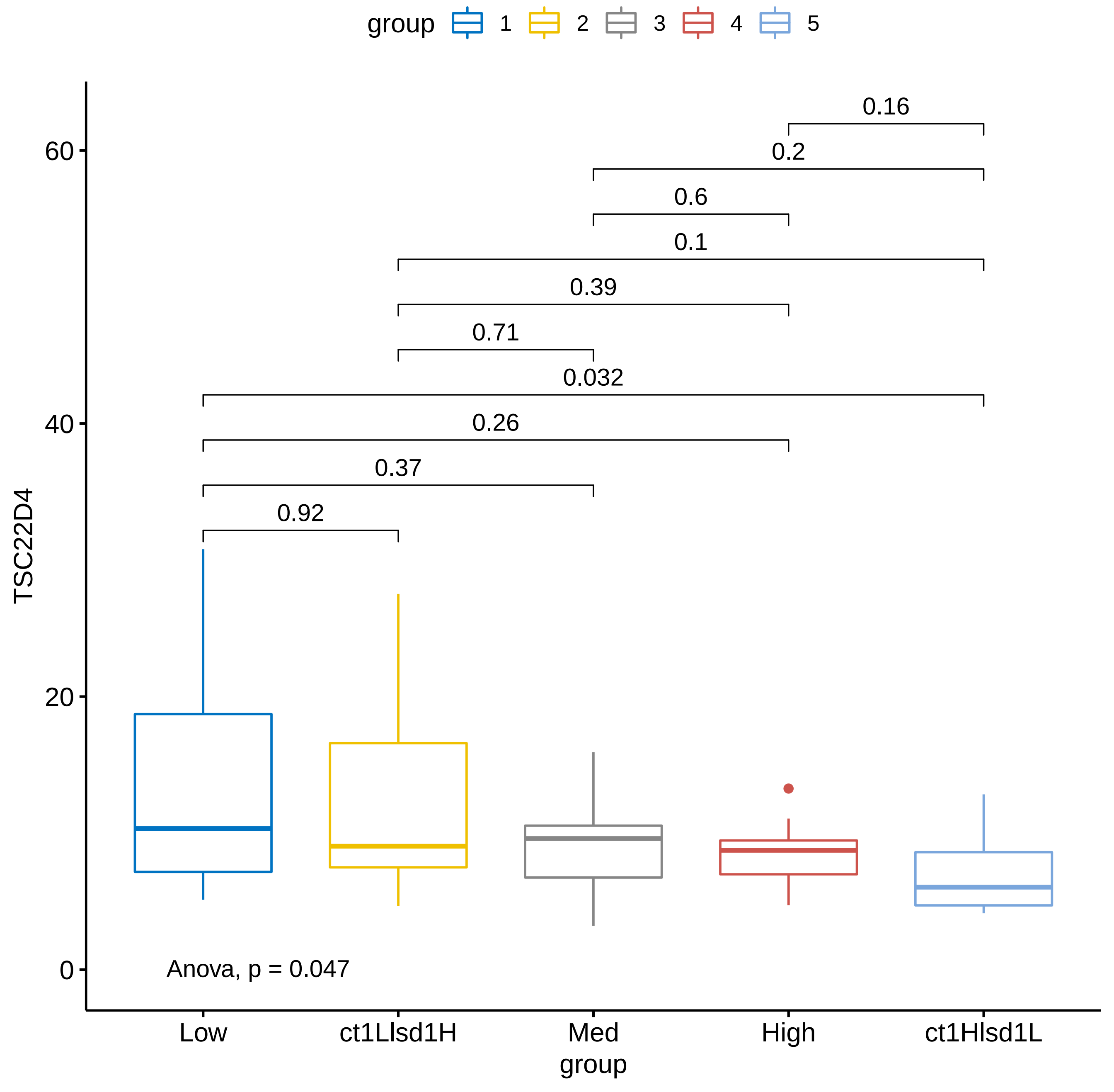
group
1
2
3
4
5
TSC22D4
60
40
20
0
0.16
0.2
0.6
0.1
0.39
0.71
0.032
0.26
0.37
0.92
Anova, p = 0.047
Low
ct1Llsd1H
Med
High
ct1Hlsd1L
group

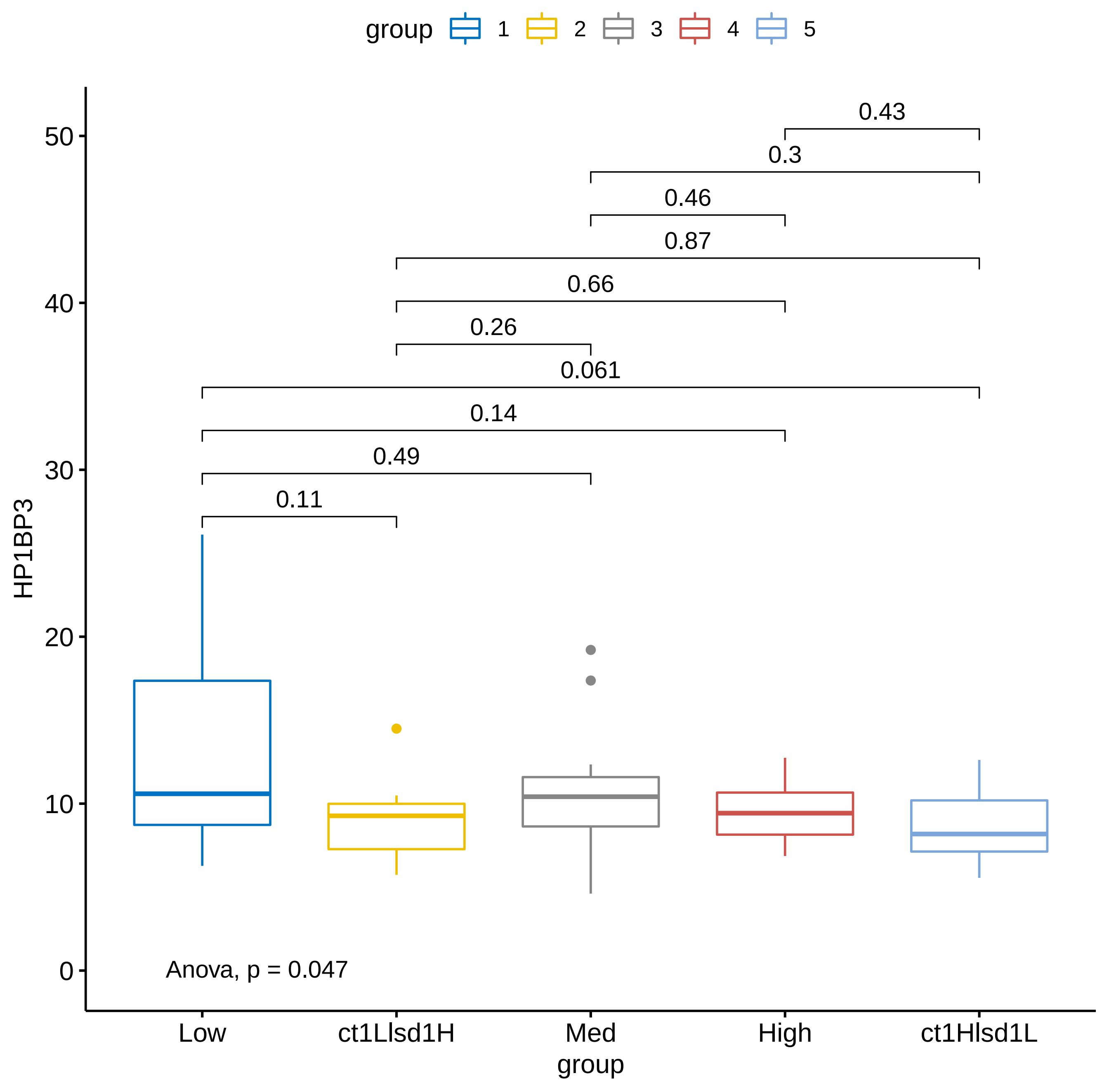
group
1
2
3
4
5
0.43
0.3
0.46
0.87
0.66
0.26
0.061
0.14
0.49
0.11
HP1BP3
50
40
30
20
10
0
Anova, p = 0.047
Low
ct1Llsd1H
Med
High
ct1Hlsd1L
group

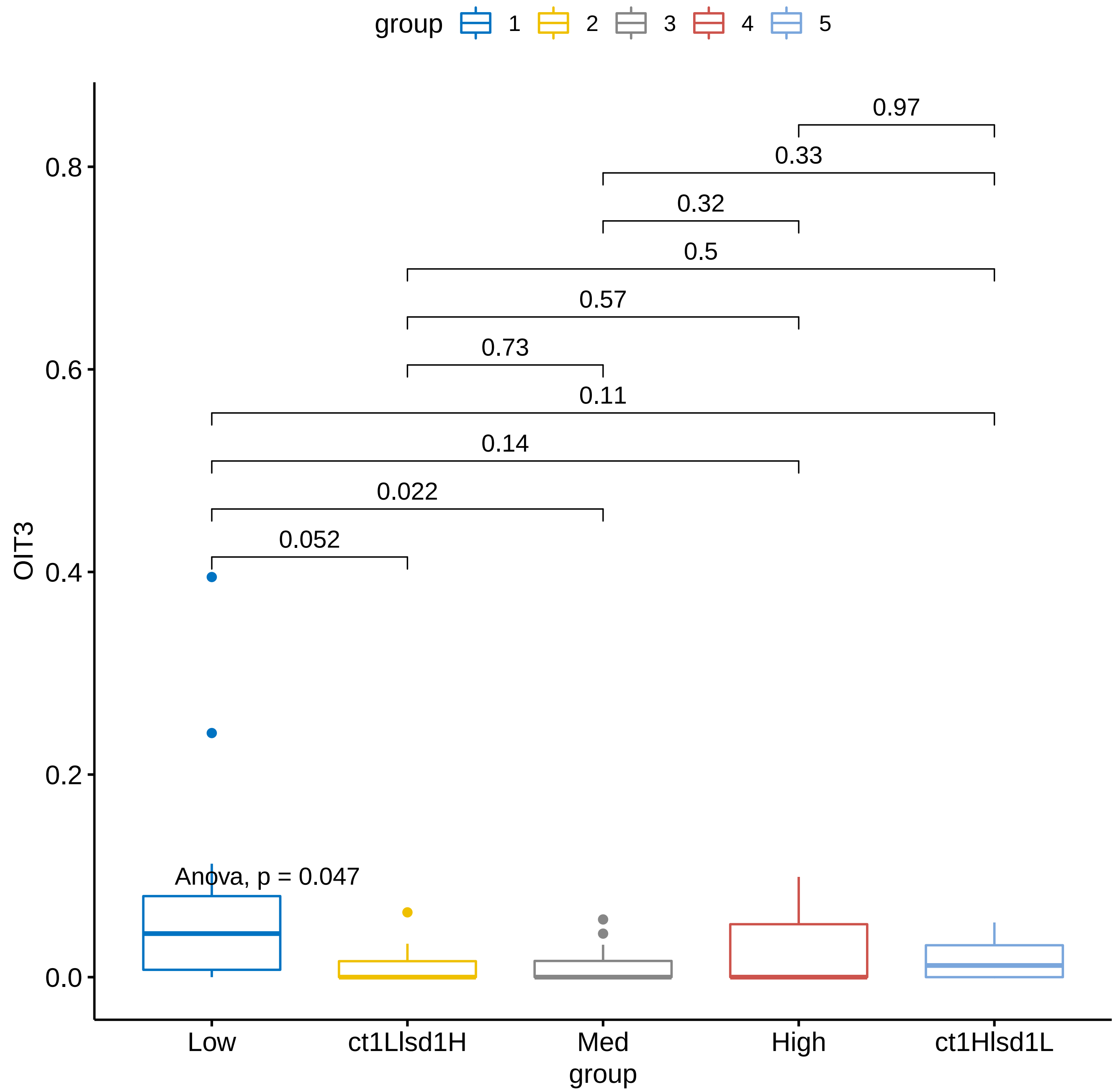

group
1
2
3
4
5
0.97
0.33
0.32
0.5
0.57
0.73
0.11
0.14
0.022
0.052
Anova, p = 0.047
OIT3
0.0
0.2
0.4
0.6
0.8
Low
ct1Llsd1H
Med
High
ct1Hlsd1L
group

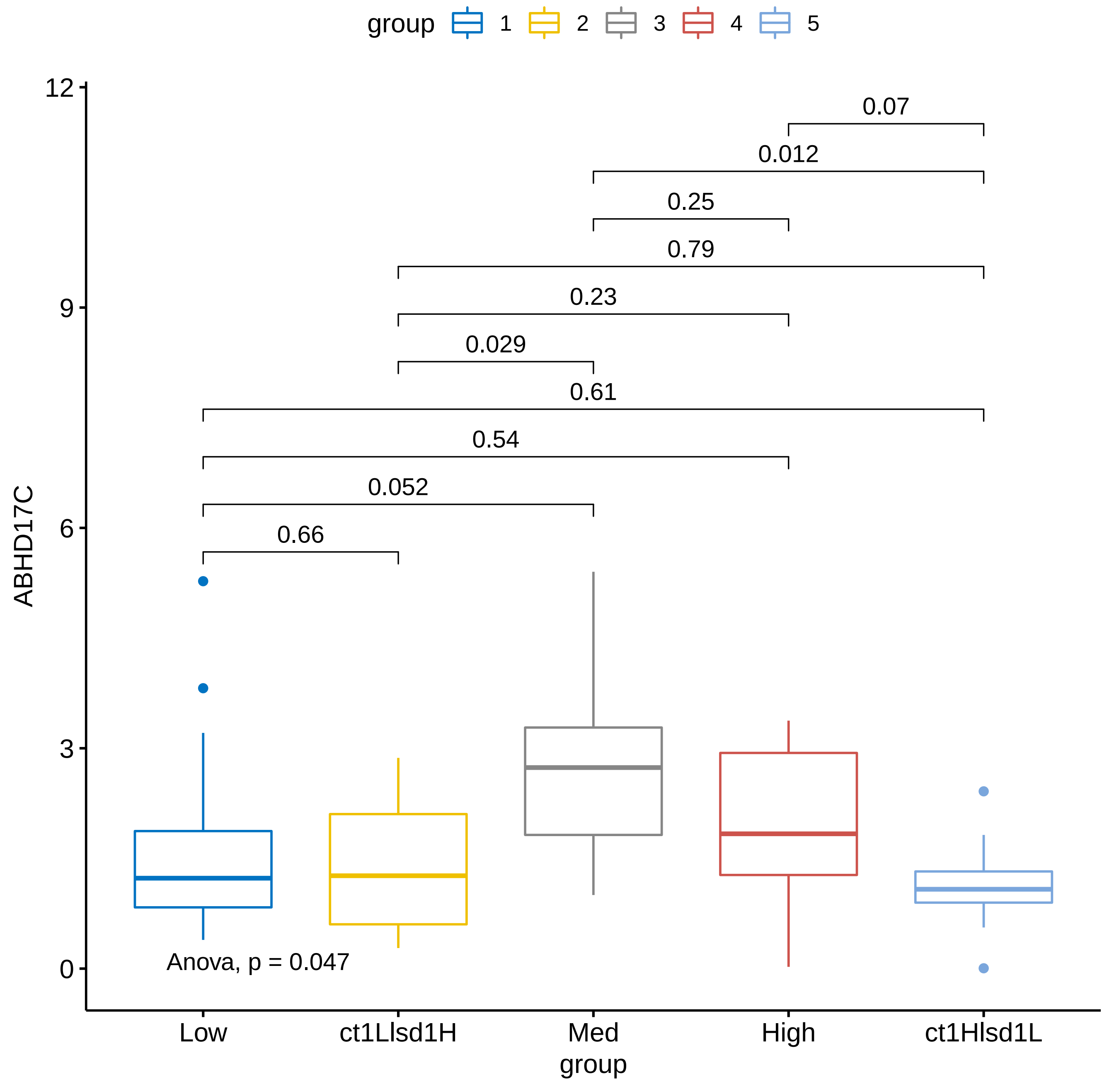
group
1
2
3
4
5
ABHD17C
12
9
6
3
0
0.07
0.012
0.25
0.79
0.23
0.029
0.61
0.54
0.052
0.66
Anova, p = 0.047
Low
ct1Llsd1H
Med
High
ct1Hlsd1L
group

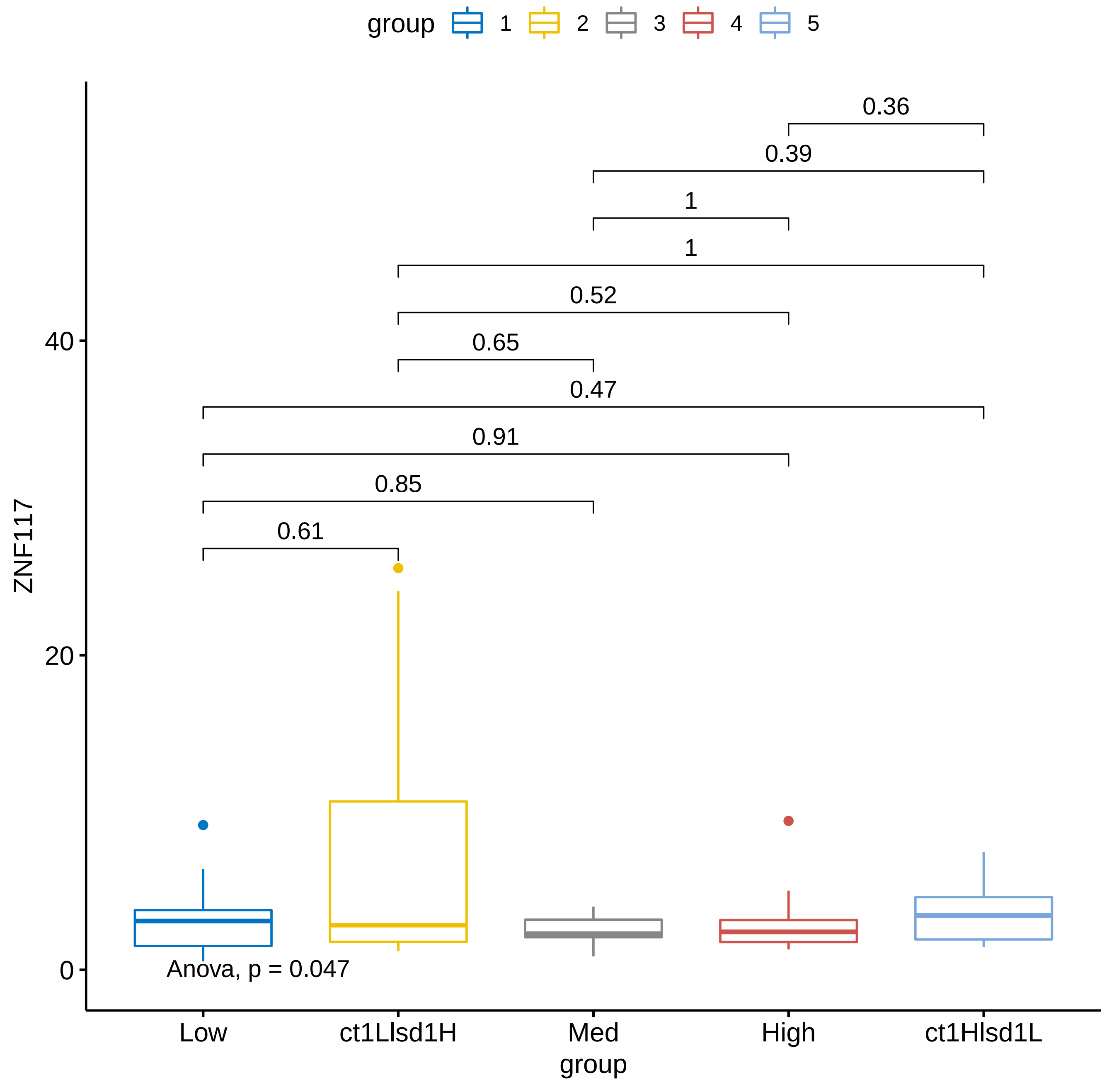

group
1
2
3
4
5
0.36
0.39
1
1
0.52
0.65
0.47
0.91
0.85
0.61
ZNF117
40
20
0
Anova, p = 0.047
Low
ct1Llsd1H
Med
High
ct1Hlsd1L
group

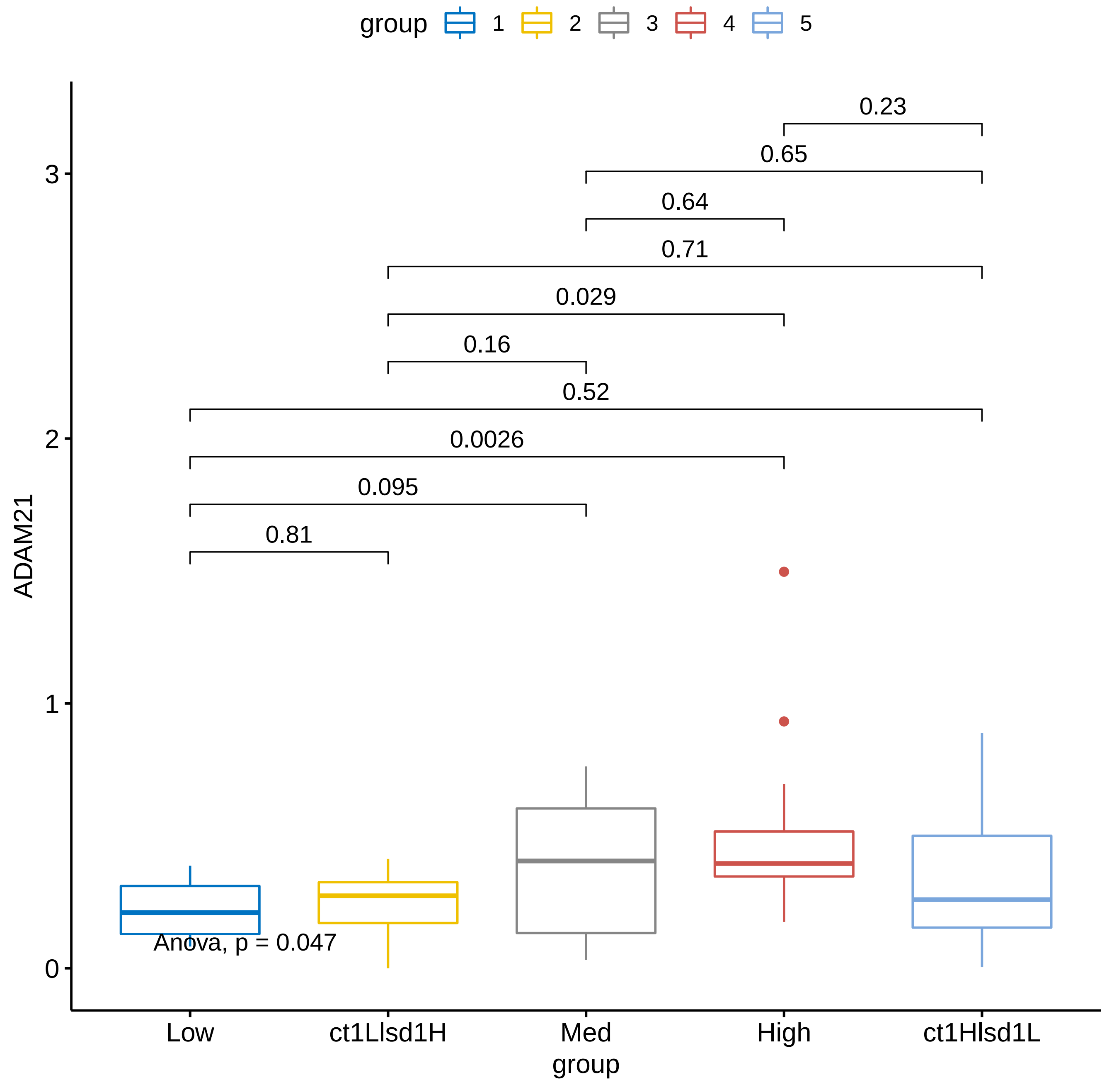

group
1
2
3
4
5
0.23
0.65
0.64
0.71
0.029
0.16
0.52
0.0026
0.095
0.81
Anova, p = 0.047
ADAM21
3
2
1
0
Low
ct1Llsd1H
Med
High
ct1Hlsd1L
group

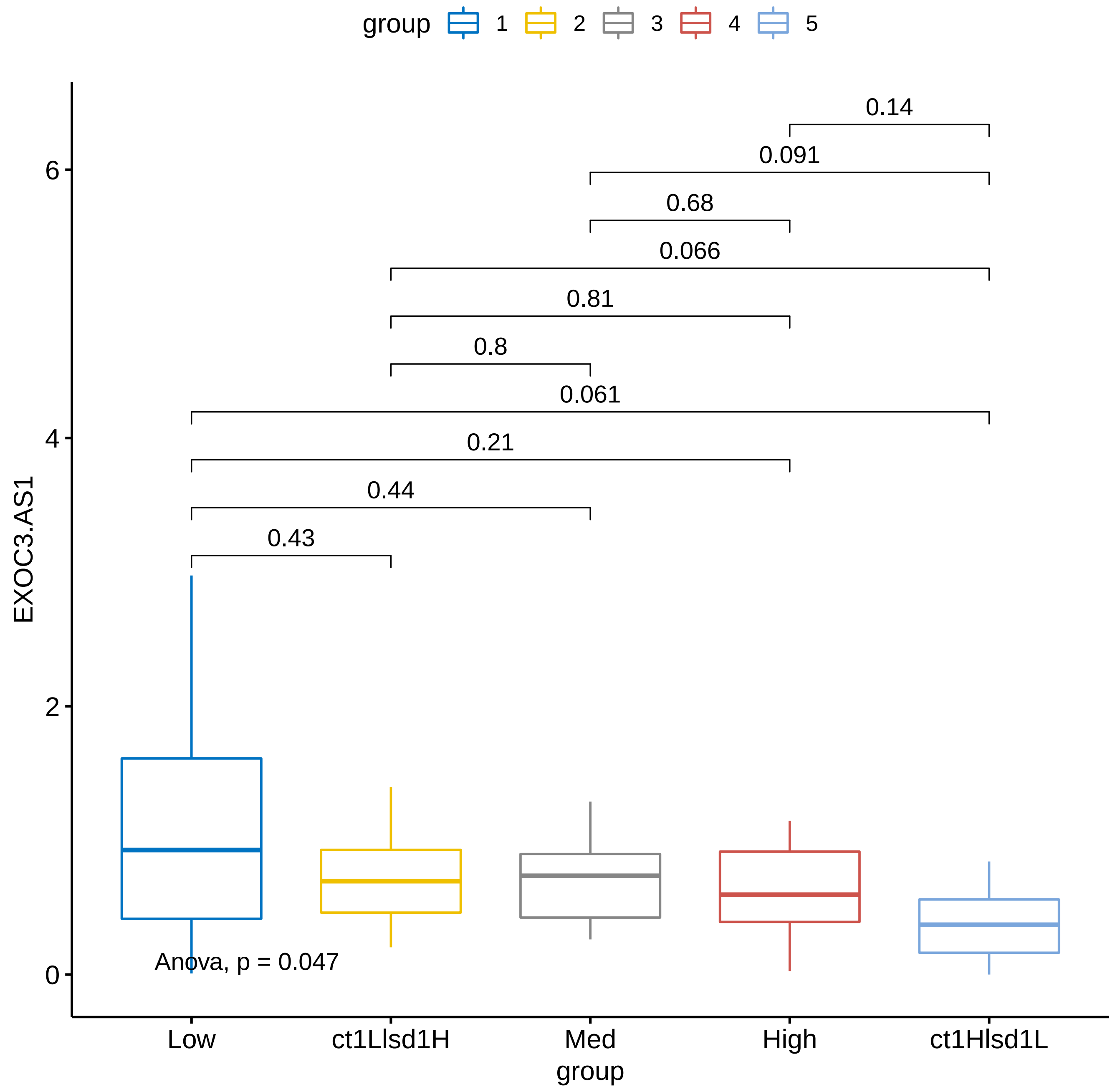
group
1
2
3
4
5
0.14
0.091
0.68
0.066
0.81
0.8
0.061
0.21
0.44
0.43
Anova, p = 0.047
6
4
2
0
EXOC3.AS1
Low
ct1Llsd1H
Med
High
ct1Hlsd1L
group

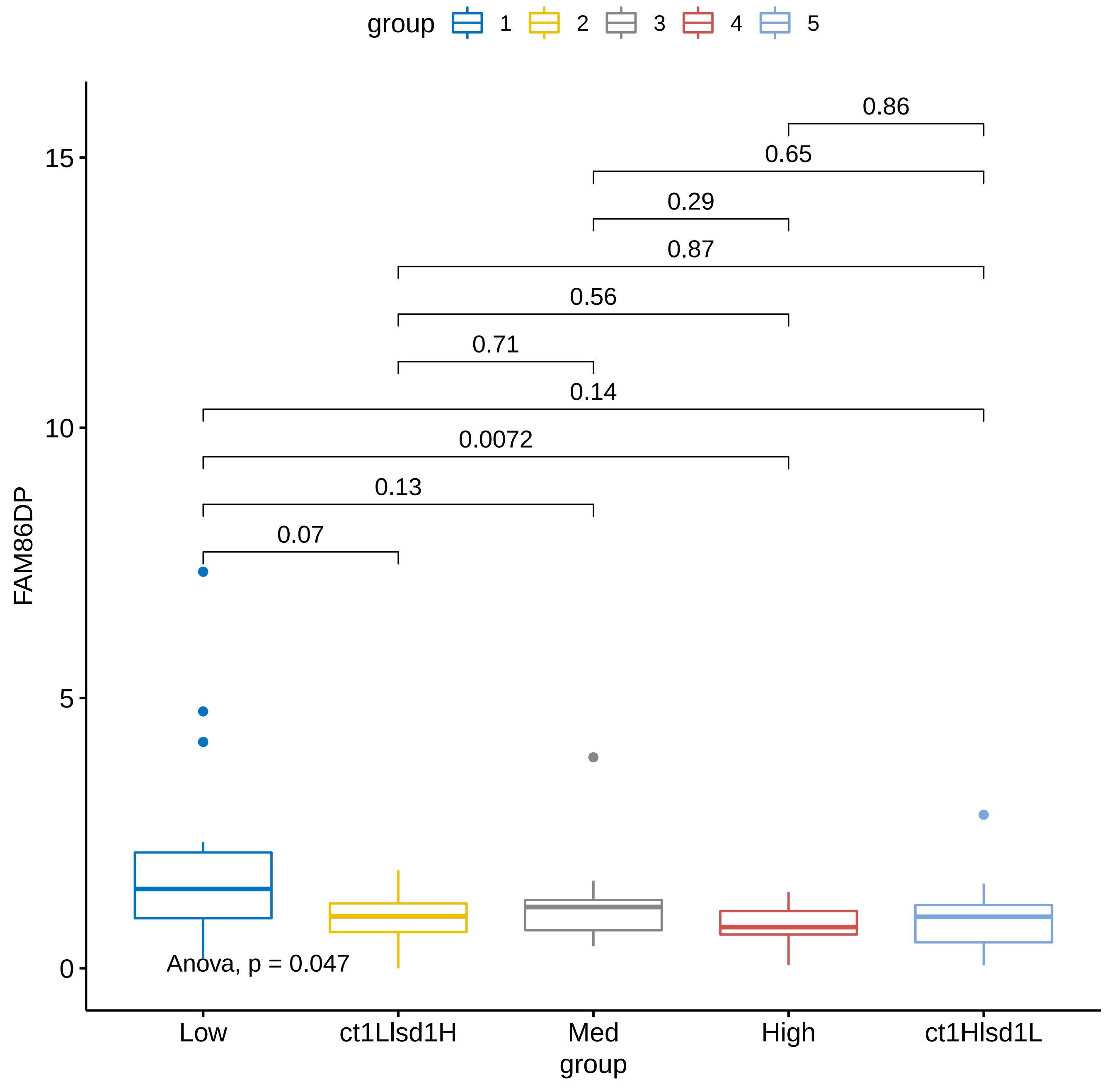
group
1
2
3
4
5
FAM86DP
15
10
5
0
0.86
0.65
0.29
0.87
0.56
0.71
0.14
0.0072
0.13
0.07
Anova, p = 0.047
Low
ct1Llsd1H
Med
High
ct1Hlsd1L
group

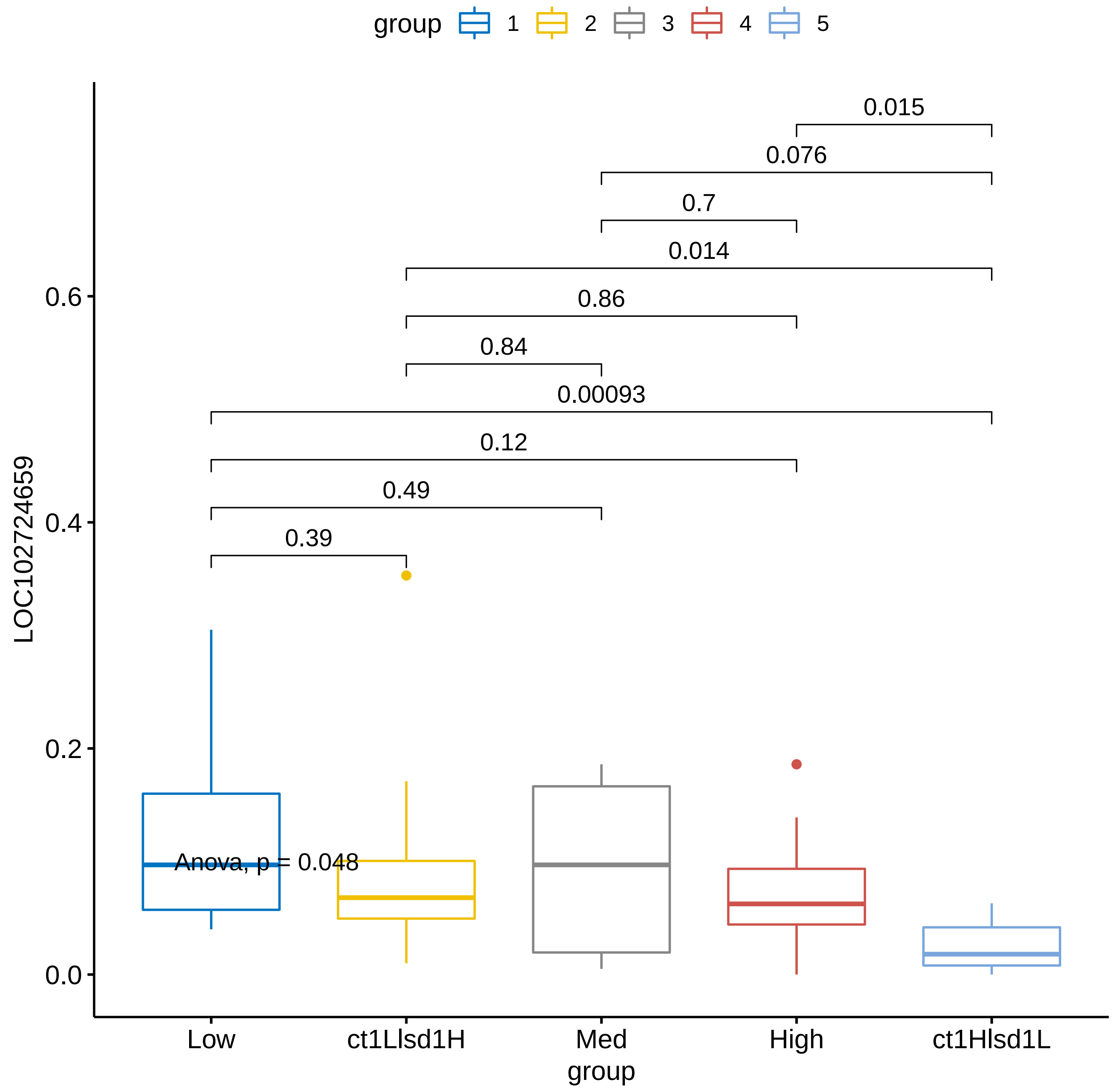

group 1 2 3 4 5
0.015
0.076
0.7
0.014
0.86
0.84
0.00093
0.12
0.49
0.39
Anova, p = 0.048
0.6
0.4
0.2
0.0
LOC102724659
Low
ct1Llsd1H
Med
High
ct1Hlsd1L
group

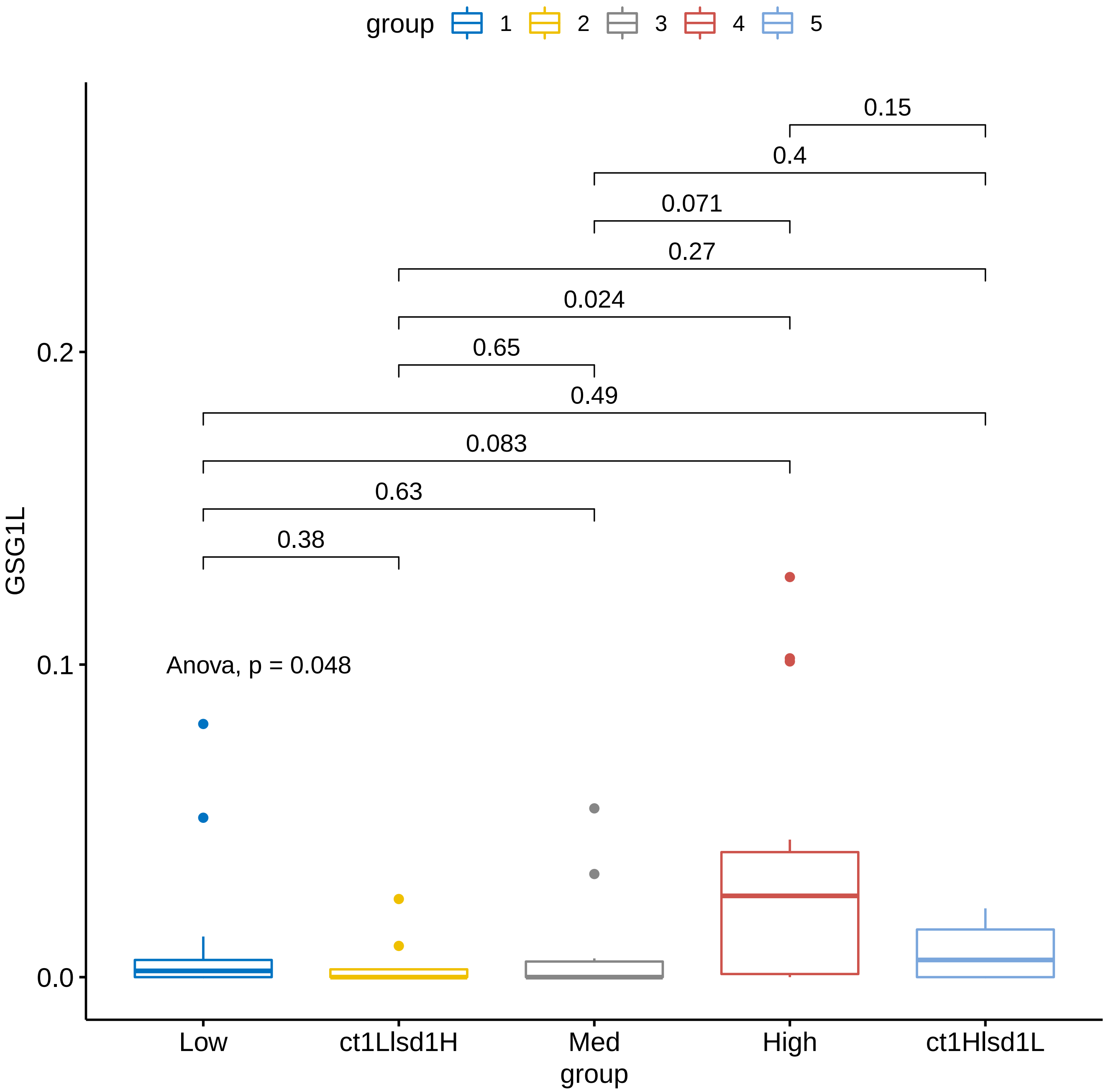

group 1 2 3 4 5
0.15
0.4
0.071
0.27
0.024
0.65
0.49
0.083
0.63
0.38
Anova, p = 0.048
GSG1L
0.2
0.1
0.0
Low
ct1Llsd1H
Med
High
ct1Hlsd1L
group

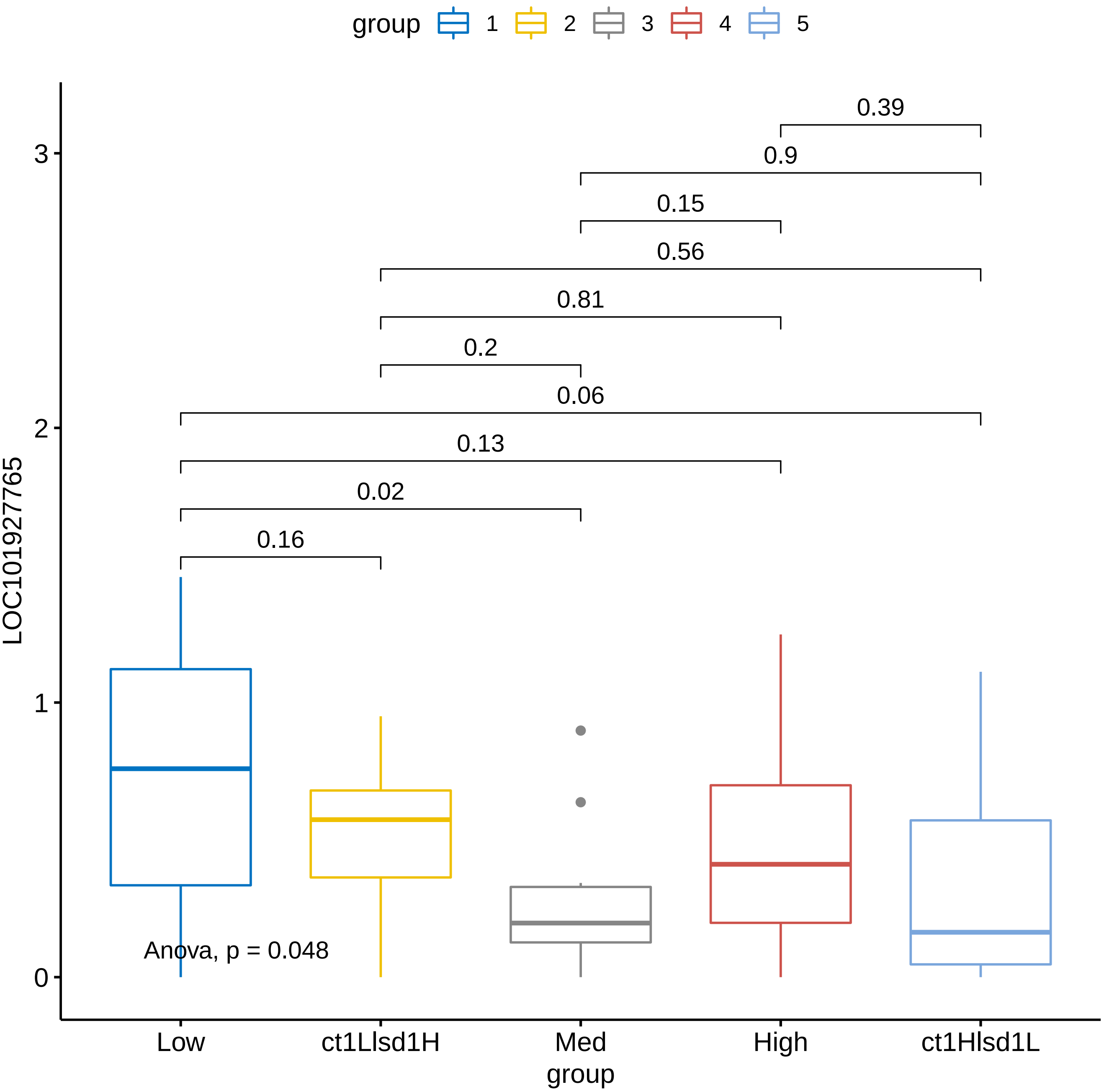
group
1
2
3
4
5
0.39
0.9
0.15
0.56
0.81
0.2
0.06
0.13
0.02
0.16
LOC101927765
3
2
1
0
Anova, p = 0.048
Low
ct1Llsd1H
Med
High
ct1Hlsd1L
group

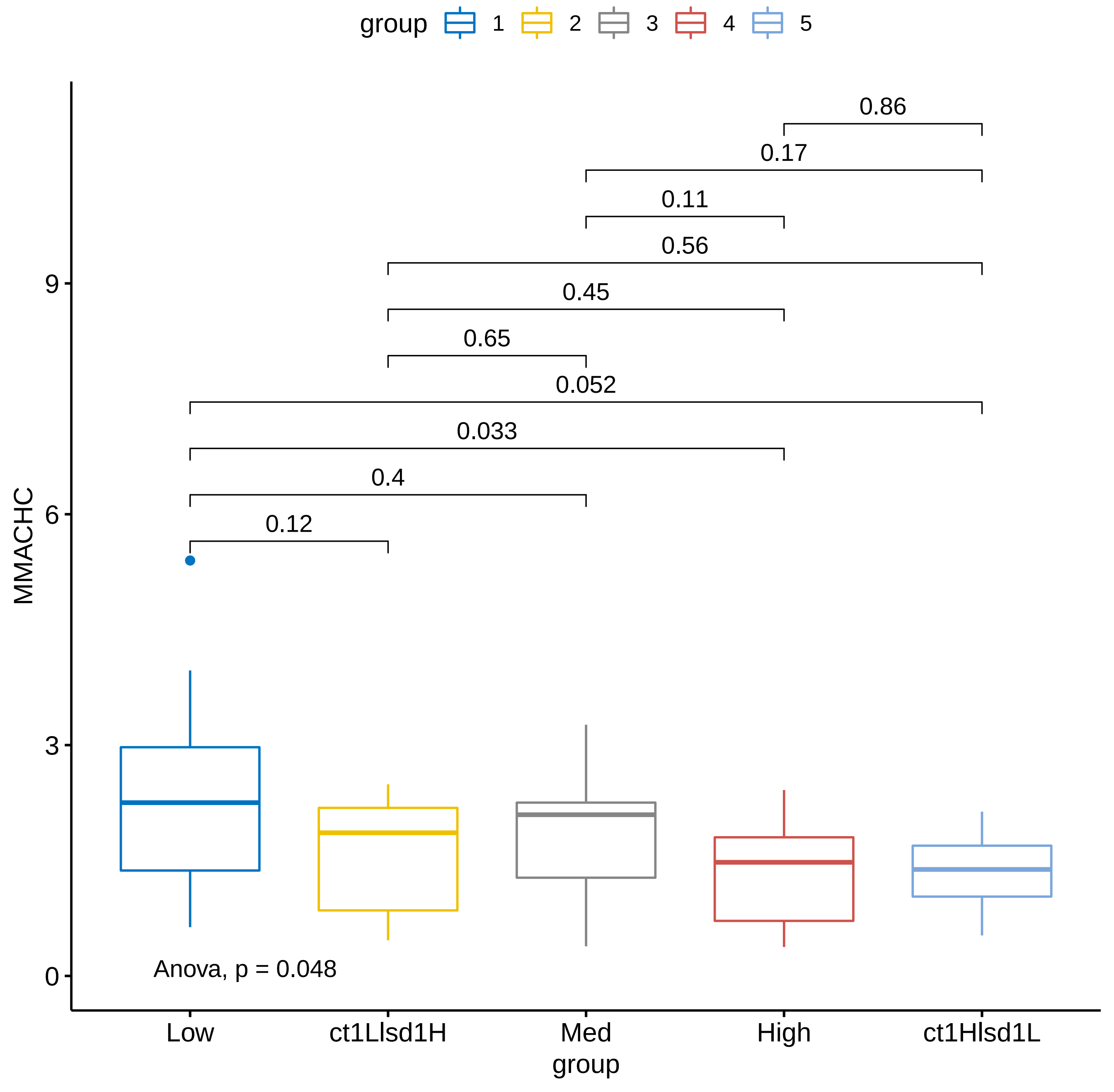
group
1
2
3
4
5
0.86
0.17
0.11
0.56
0.45
0.65
0.052
0.033
0.4
0.12
MMACHC
9
6
3
0
Anova, p = 0.048
Low
ct1Llsd1H
Med
High
ct1Hlsd1L
group

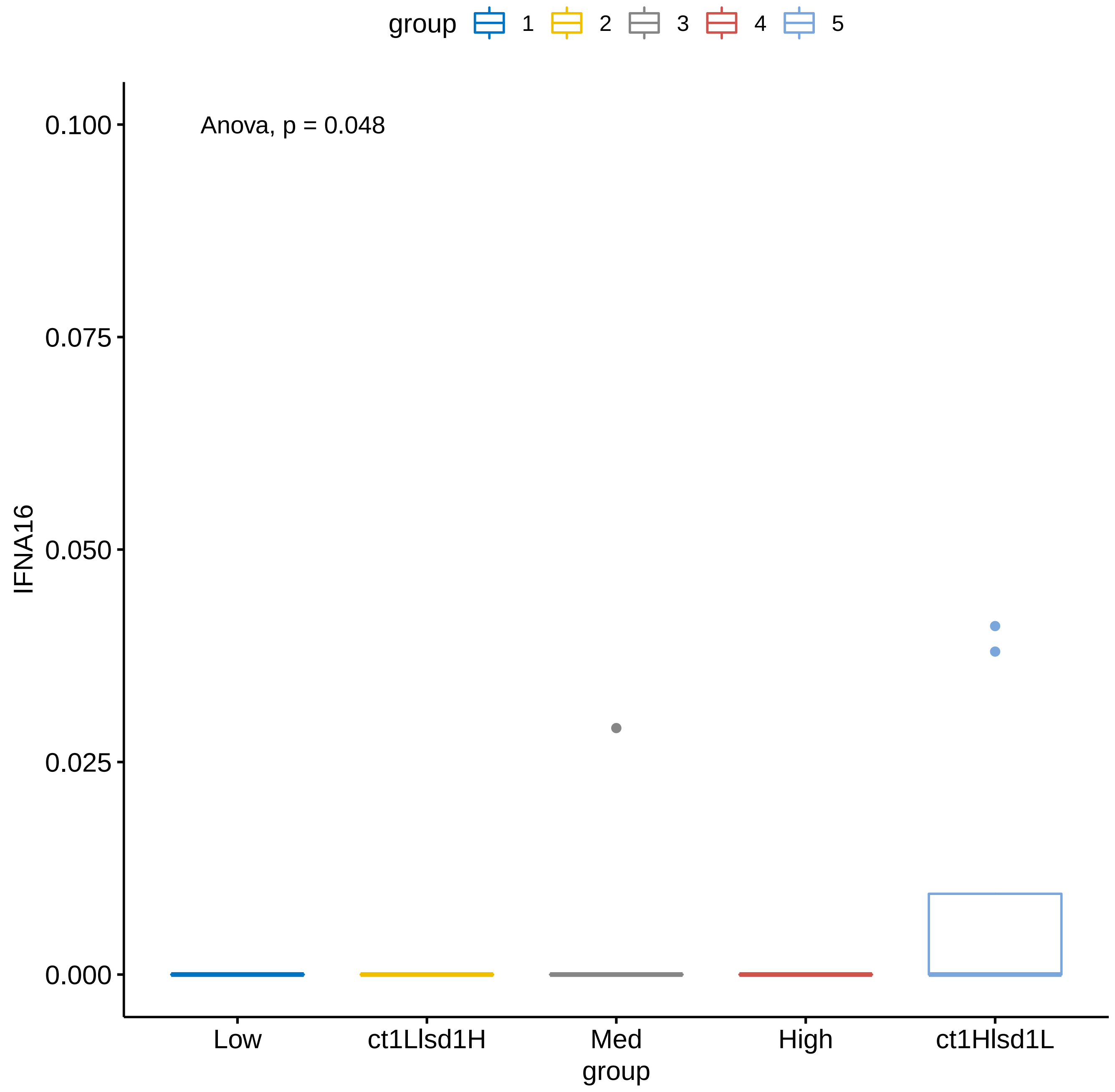

group
1
2
3
4
5
Anova, p = 0.048
IFNA16
0.100
0.075
0.050
0.025
0.000
Low
ct1Llsd1H
Med
High
ct1Hlsd1L
group

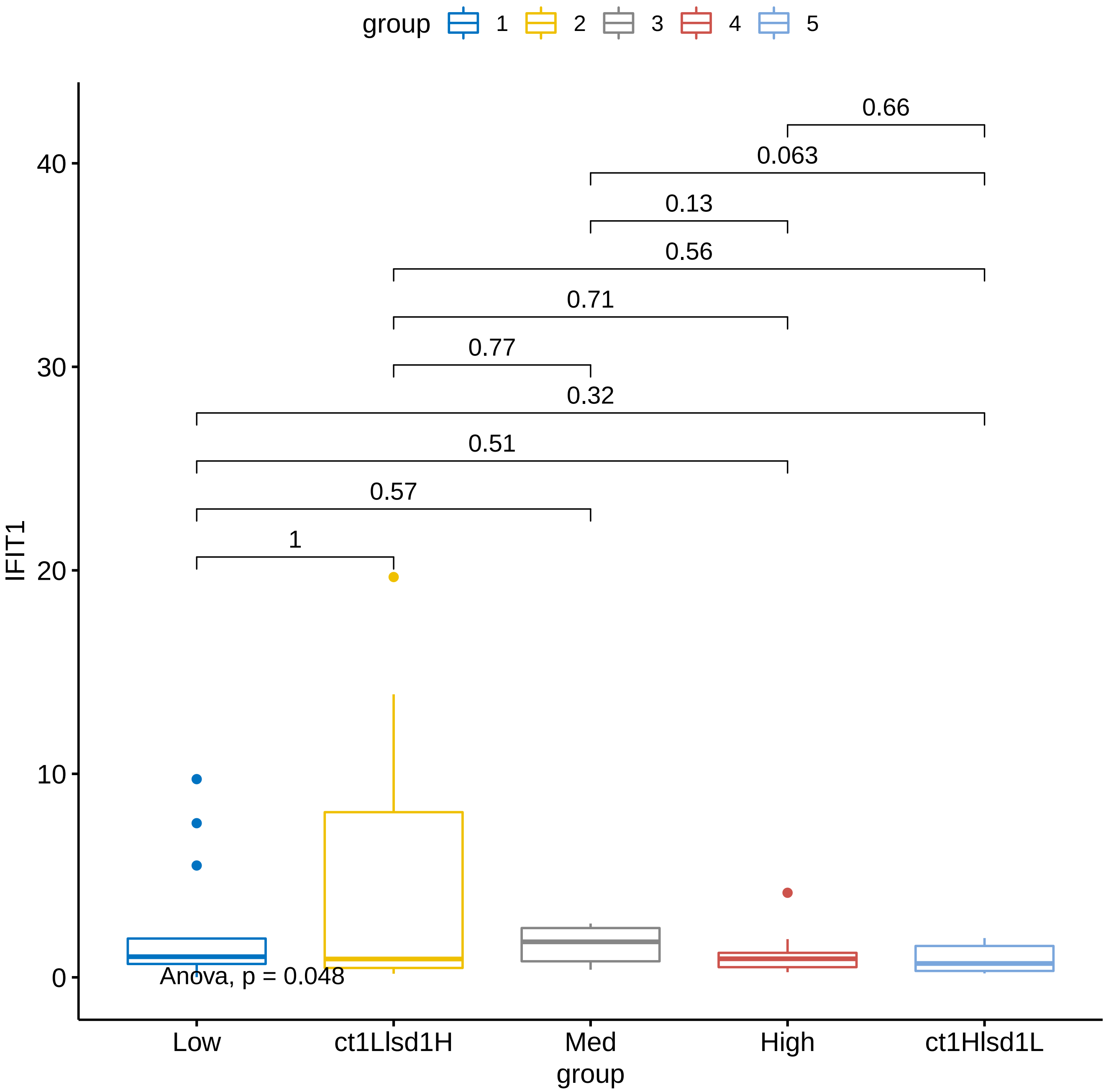

group 1 2 3 4 5
0.66
0.063
0.13
0.56
0.71
0.77
0.32
0.51
0.57
1
Anova, p = 0.048
40
30
20
10
0
IFIT1
Low
ct1Llsd1H
Med
High
ct1Hlsd1L
group

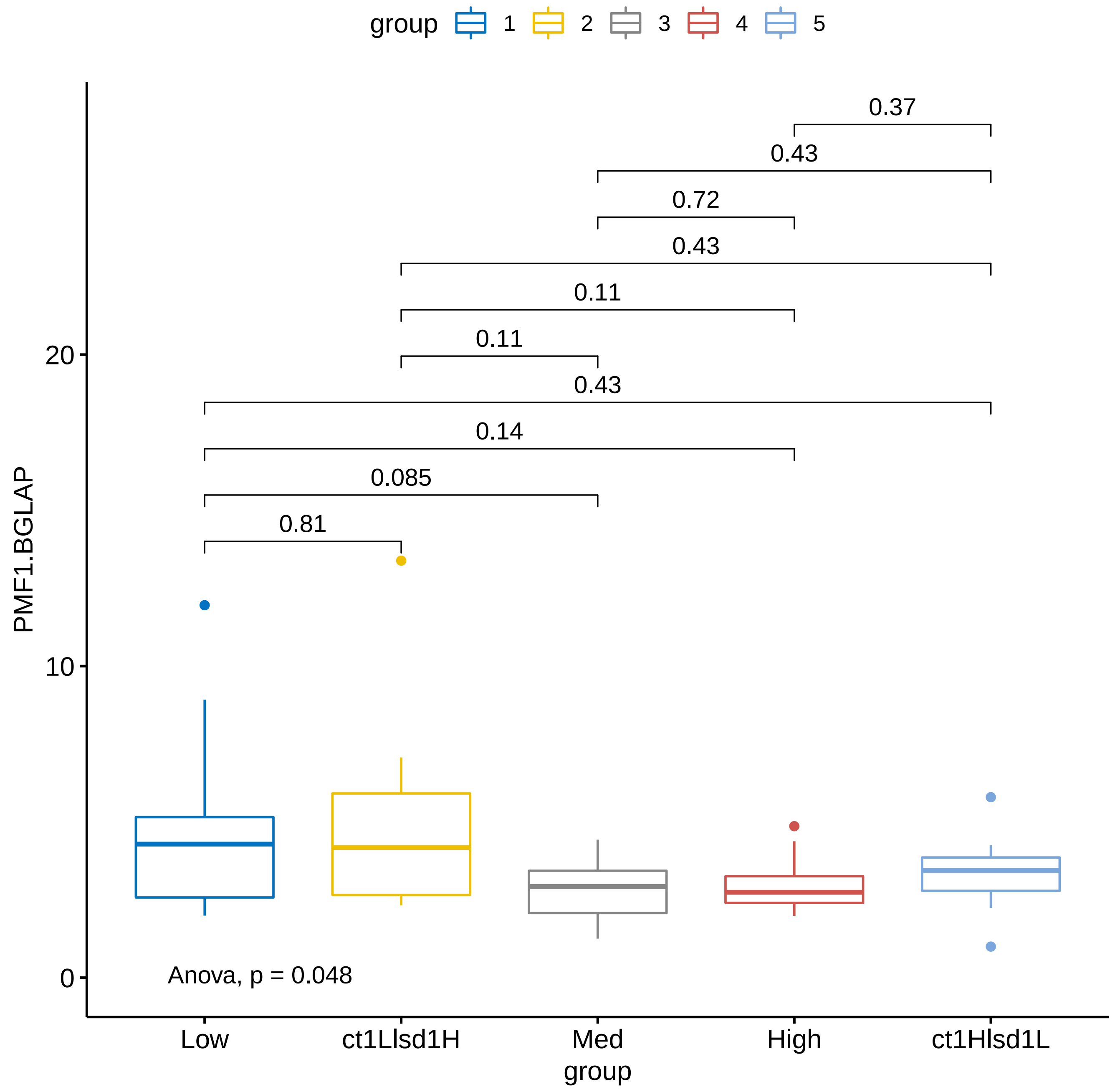

group 1 2 3 4 5
0.37
0.43
0.72
0.43
0.11
0.11
0.43
0.14
0.085
0.81
20
10
0
PMF1.BGLAP
Anova, p = 0.048
Low
ct1Llsd1H
Med
High
ct1Hlsd1L
group

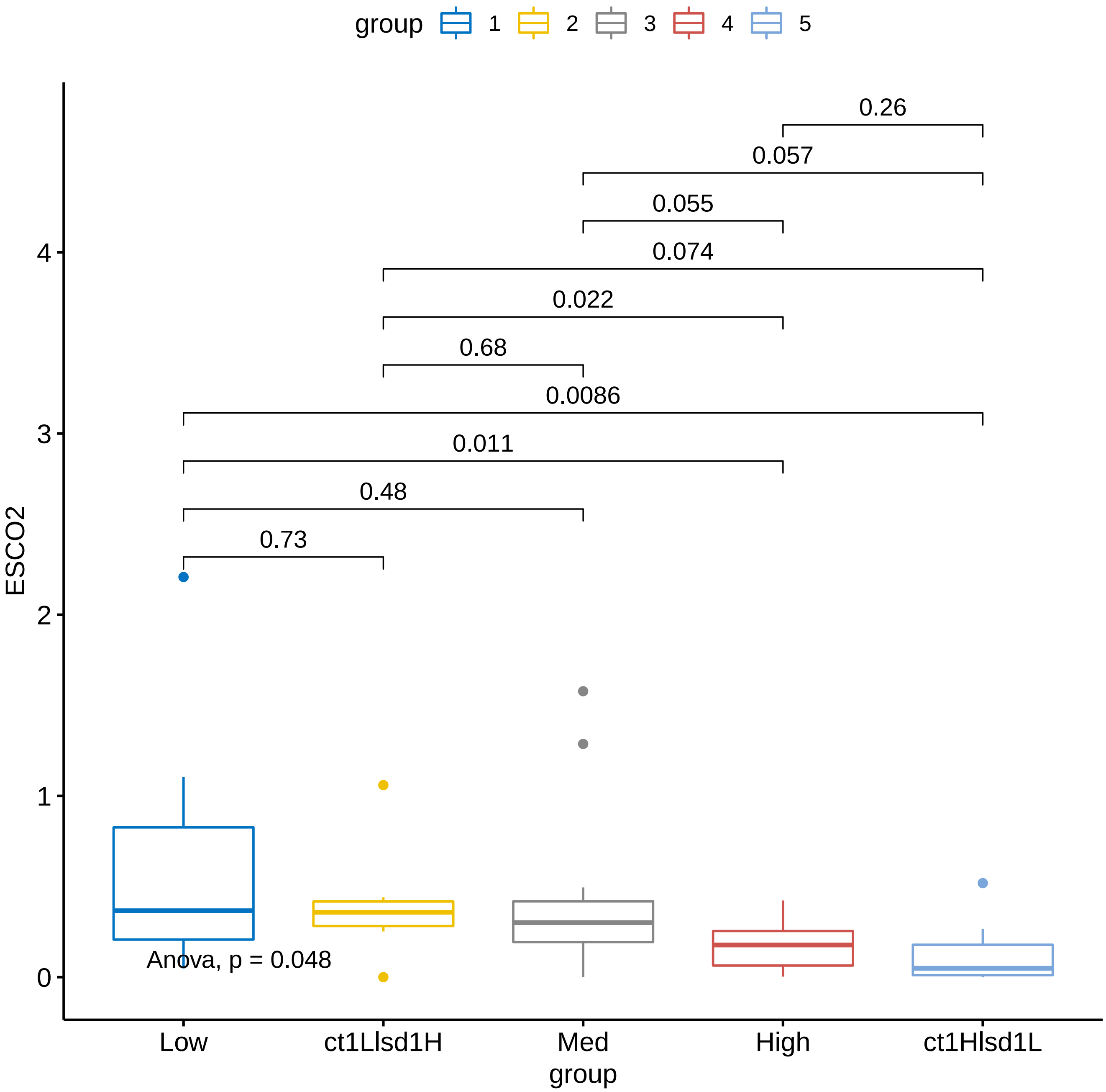

group
1
2
3
4
5
0.26
0.057
0.055
0.074
0.022
0.68
0.0086
0.011
0.48
0.73
Anova, p = 0.048
ESCO2
0
1
2
3
4
Low
ct1Llsd1H
Med
High
ct1Hlsd1L
group

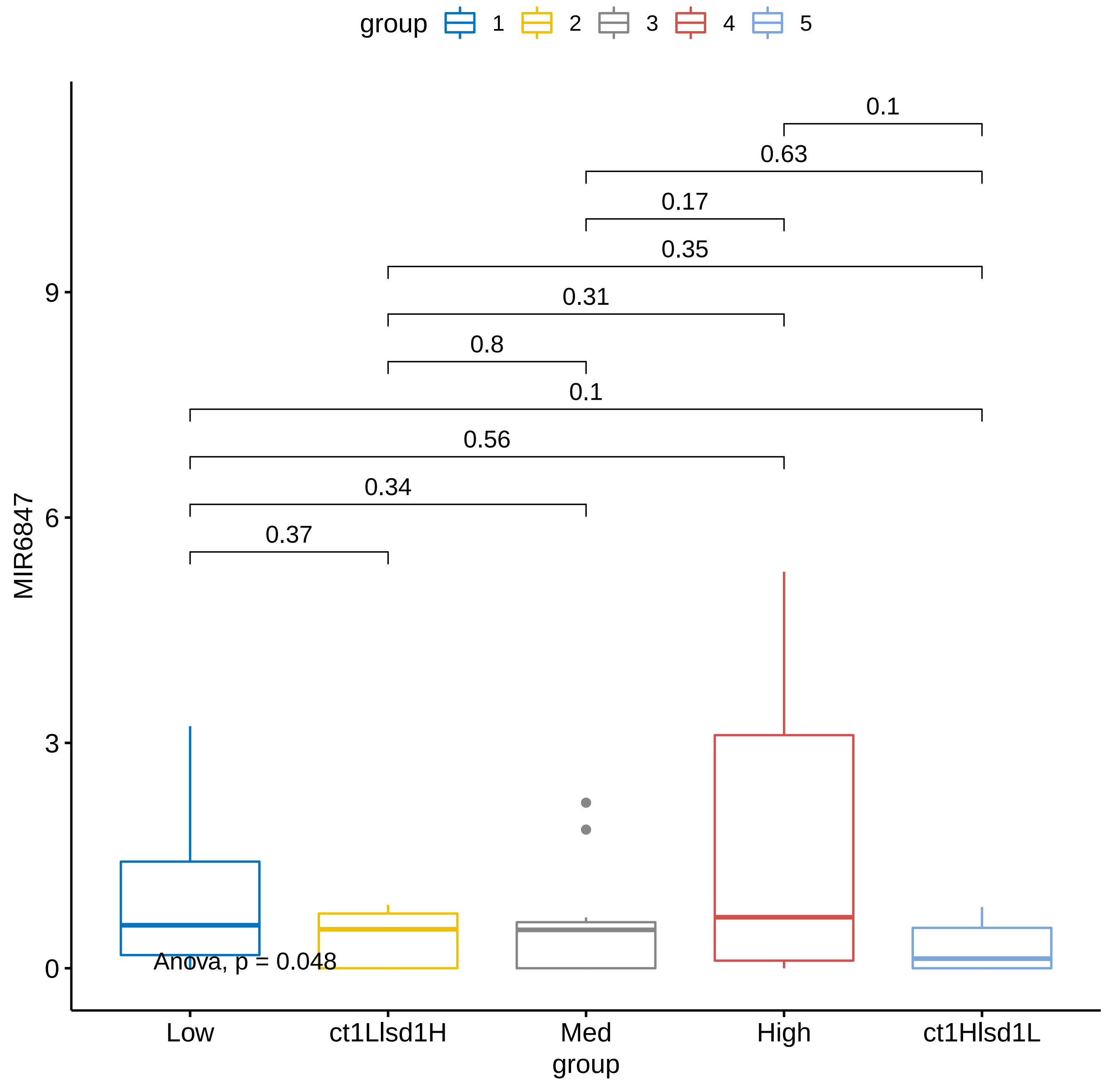

group 1 2 3 4 5
0.1
0.63
0.17
0.35
0.31
0.8
0.1
0.56
0.34
0.37
9
6
3
0
MIR6847
Anova, p = 0.048
Low
ct1Llsd1H
Med
High
ct1Hlsd1L
group

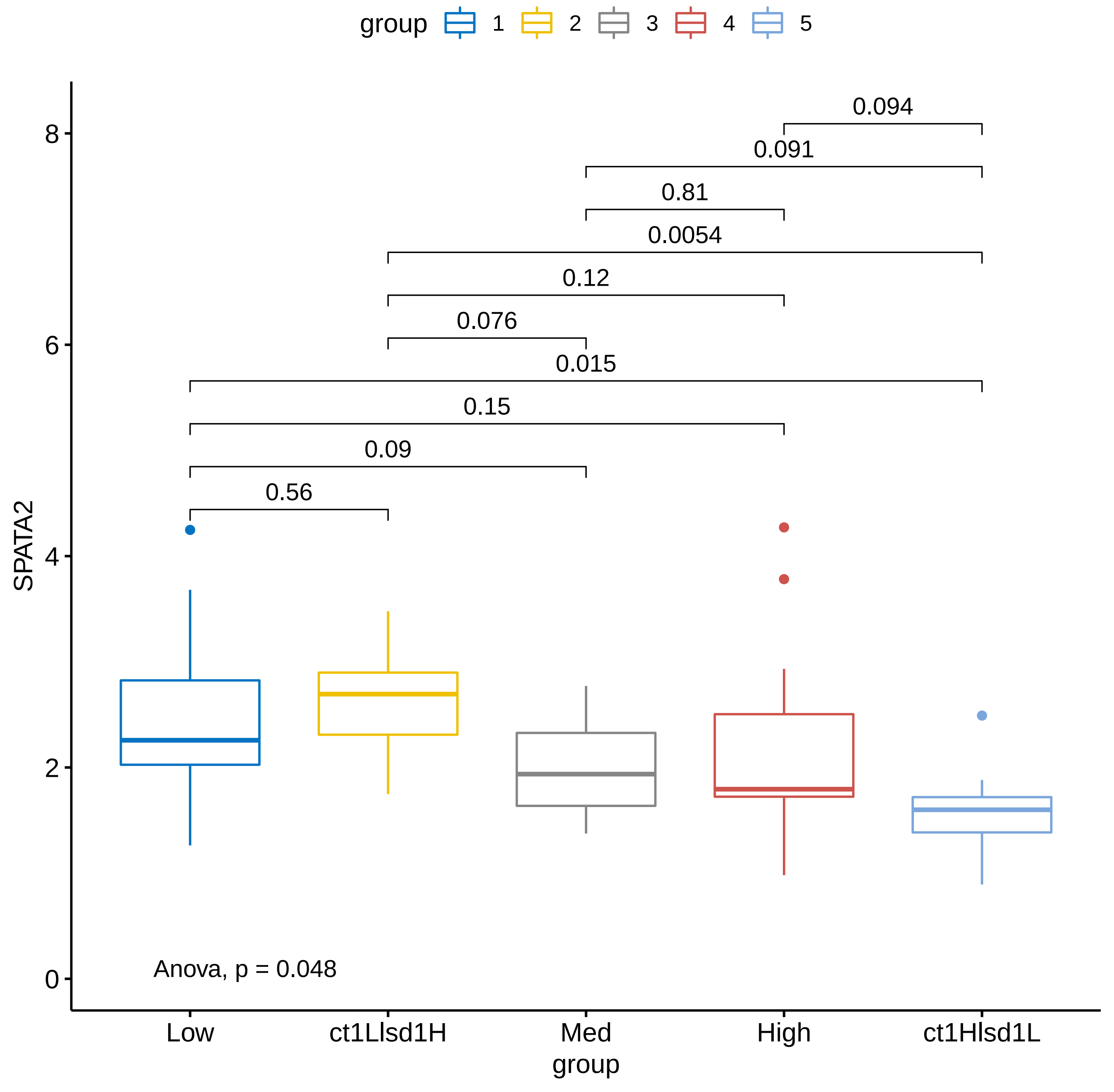
group
1
2
3
4
5
0.094
0.091
0.81
0.0054
0.12
0.076
0.015
0.15
0.09
0.56
SPATA2
8
6
4
2
0
Anova, p = 0.048
Low
ct1Llsd1H
Med
High
ct1Hlsd1L
group

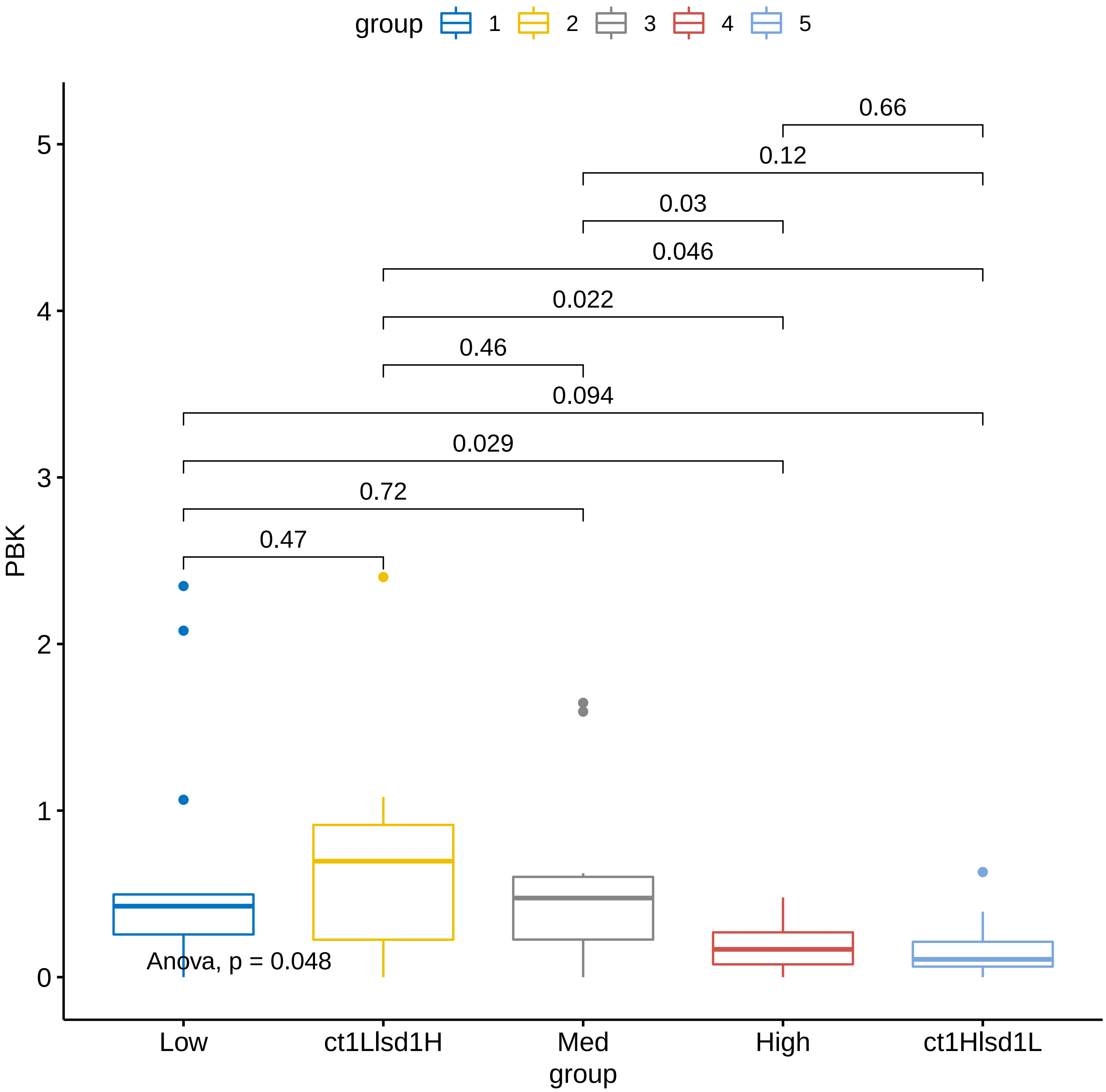
group 1 2 3 4 5
0.66
0.12
0.03
0.046
0.022
0.46
0.094
0.029
0.72
0.47
Anova, p = 0.048
PBK
0
1
2
3
4
5
Low
ct1Llsd1H
Med
High
ct1Hlsd1L
group

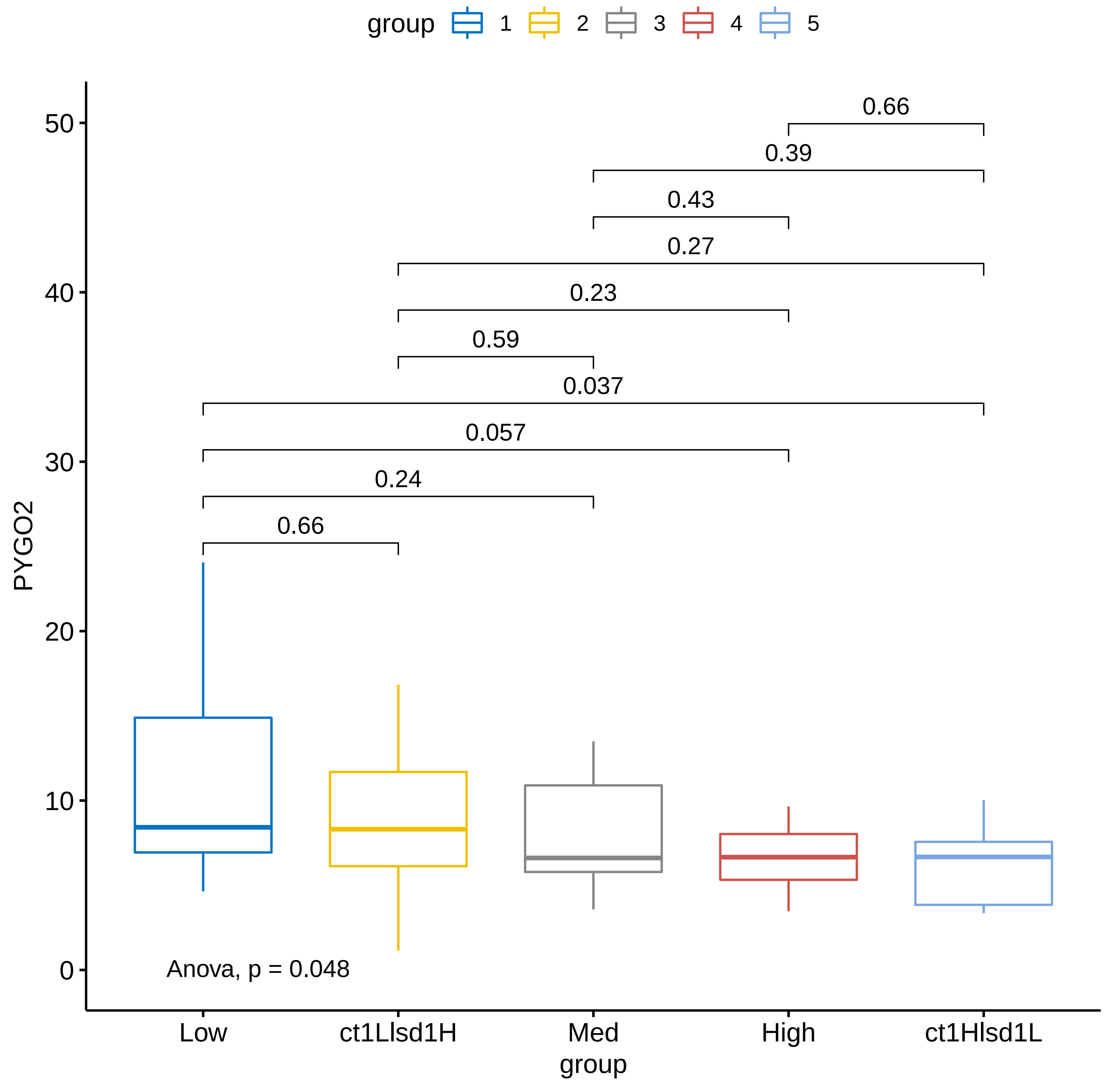

group
1
2
3
4
5
0.66
0.39
0.43
0.27
0.23
0.59
0.037
0.057
0.24
0.66
50
40
30
20
10
0
PYGO2
Anova, p = 0.048
Low
ct1Llsd1H
Med
High
ct1Hlsd1L
group

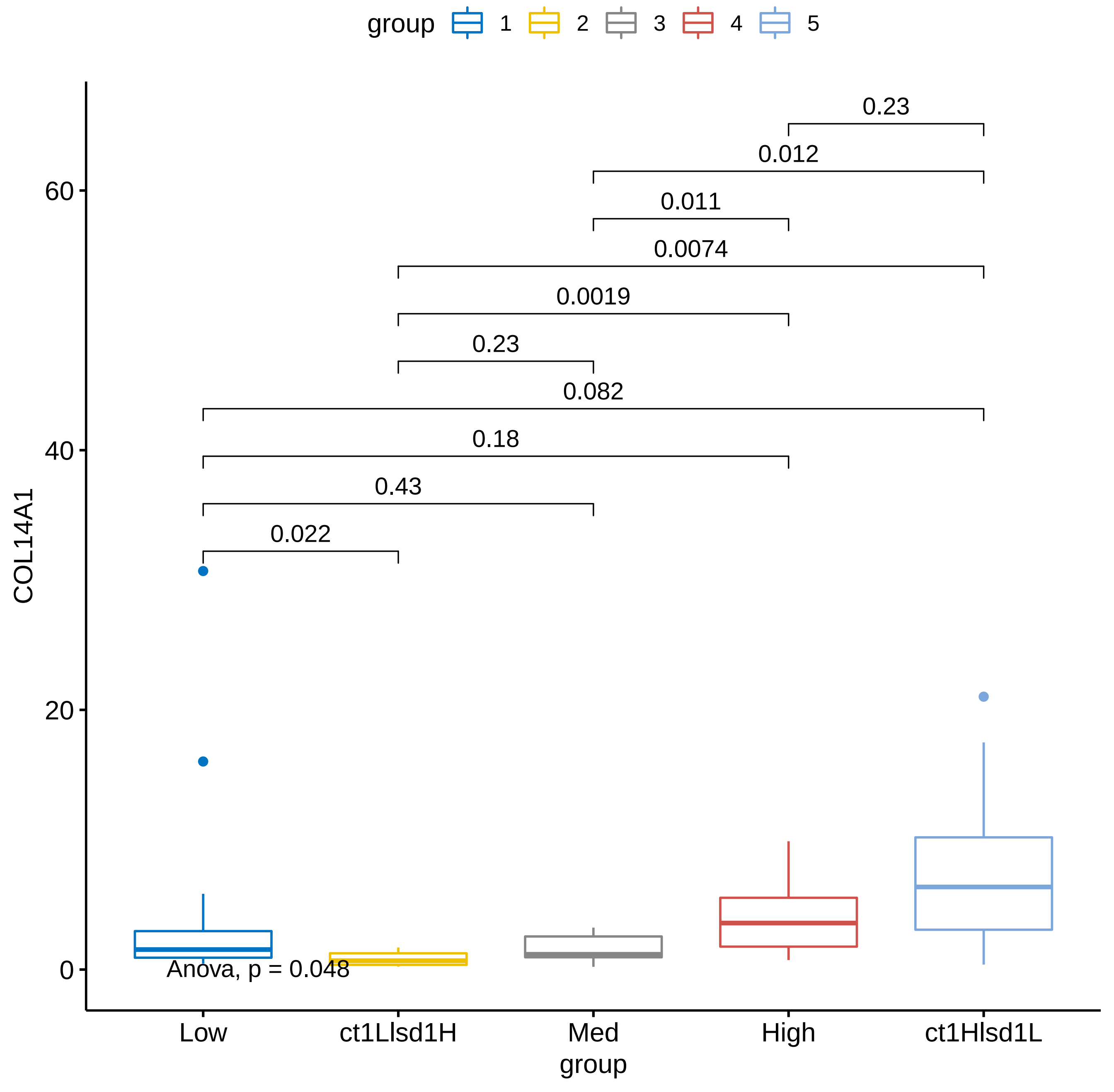
group
1
2
3
4
5
0.23
0.012
0.011
0.0074
0.0019
0.23
0.082
0.18
0.43
0.022
60
40
20
0
COL14A1
Anova, p = 0.048
Low
ct1Llsd1H
Med
High
ct1Hlsd1L
group

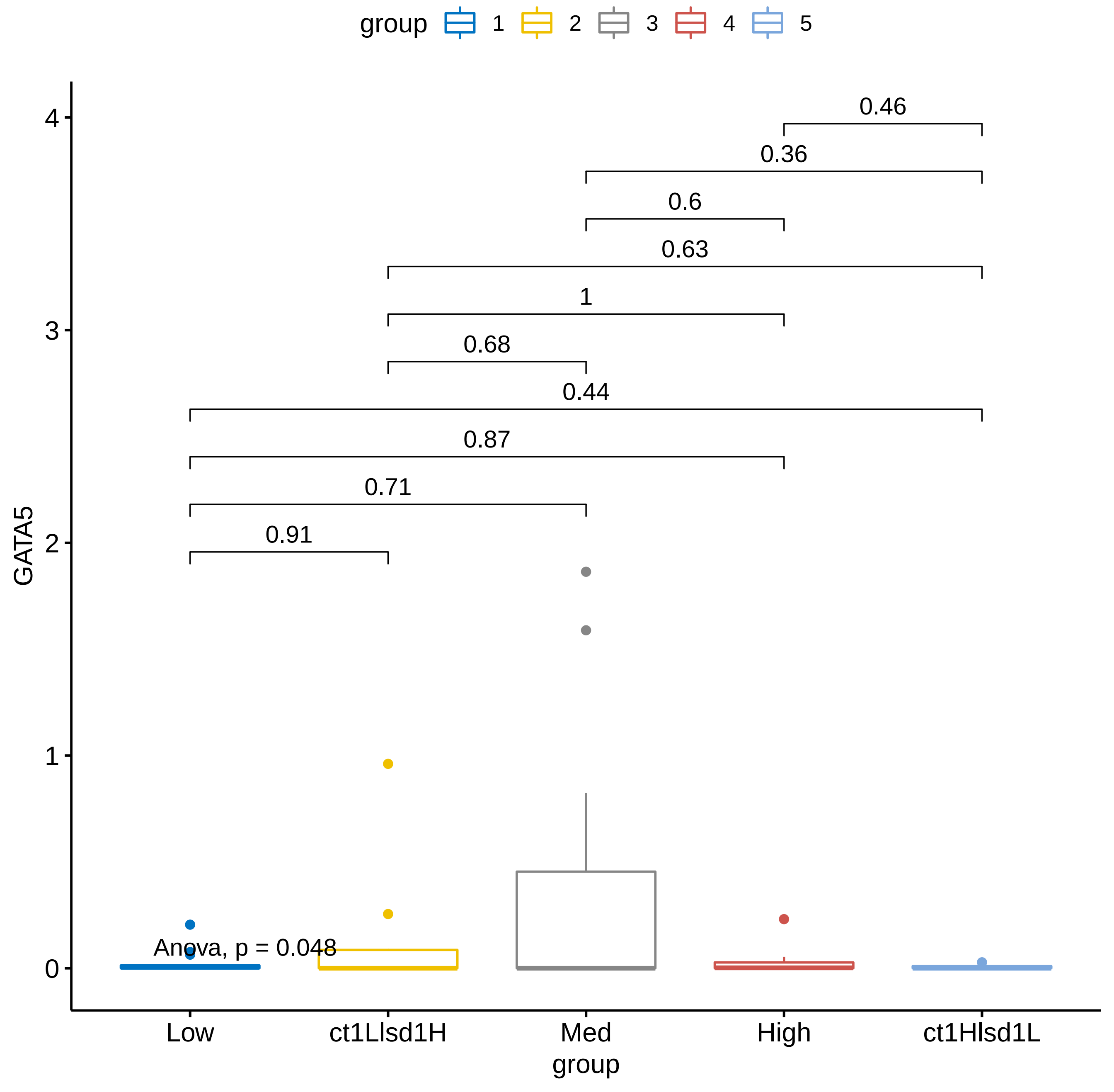

group
1
2
3
4
5
0.46
0.36
0.6
0.63
1
0.68
0.44
0.87
0.71
0.91
Anova, p = 0.048
GATA5
4
3
2
1
0
Low
ct1Llsd1H
Med
High
ct1Hlsd1L
group

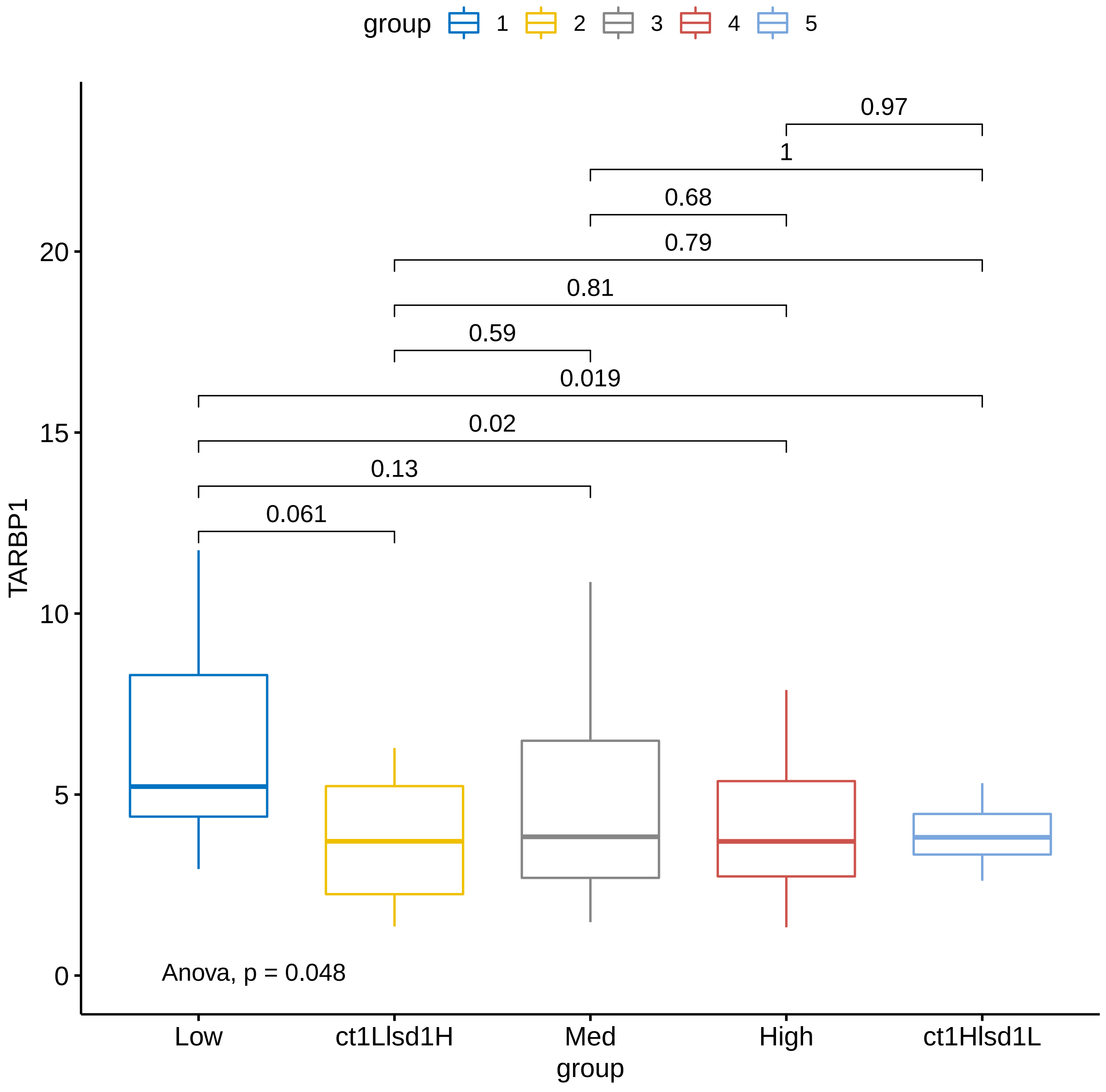
group 1 2 3 4 5
0.97
1
0.68
0.79
0.81
0.59
0.019
0.02
0.13
0.061
TARBP1
20
15
10
5
0
Anova, p = 0.048
Low
ct1Llsd1H
Med
High
ct1Hlsd1L
group

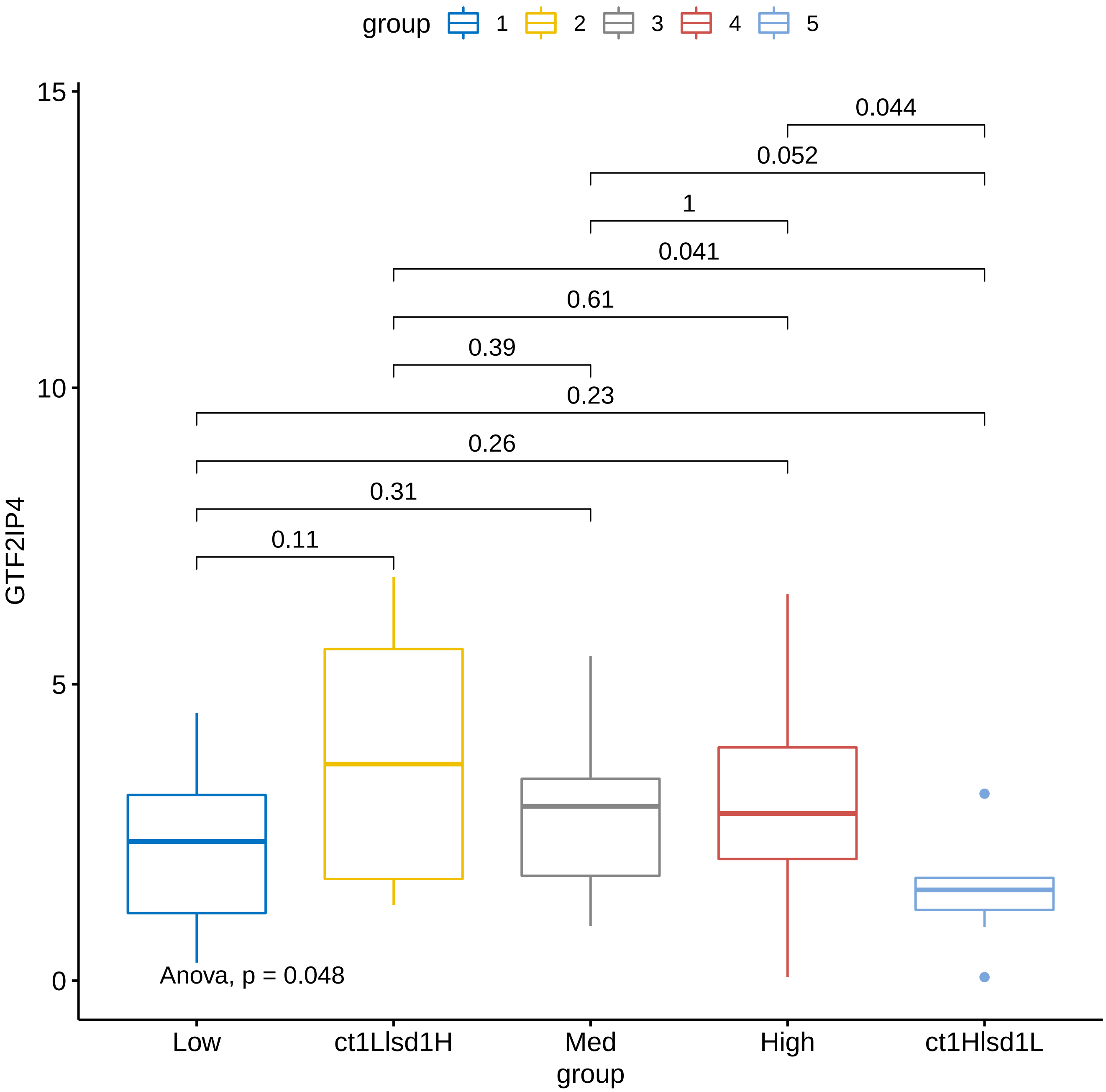
group
1
2
3
4
5
GTF2IP4
15
10
5
0
0.044
0.052
1
0.041
0.61
0.39
0.23
0.26
0.31
0.11
Anova, p = 0.048
Low
ct1Llsd1H
Med
High
ct1Hlsd1L
group

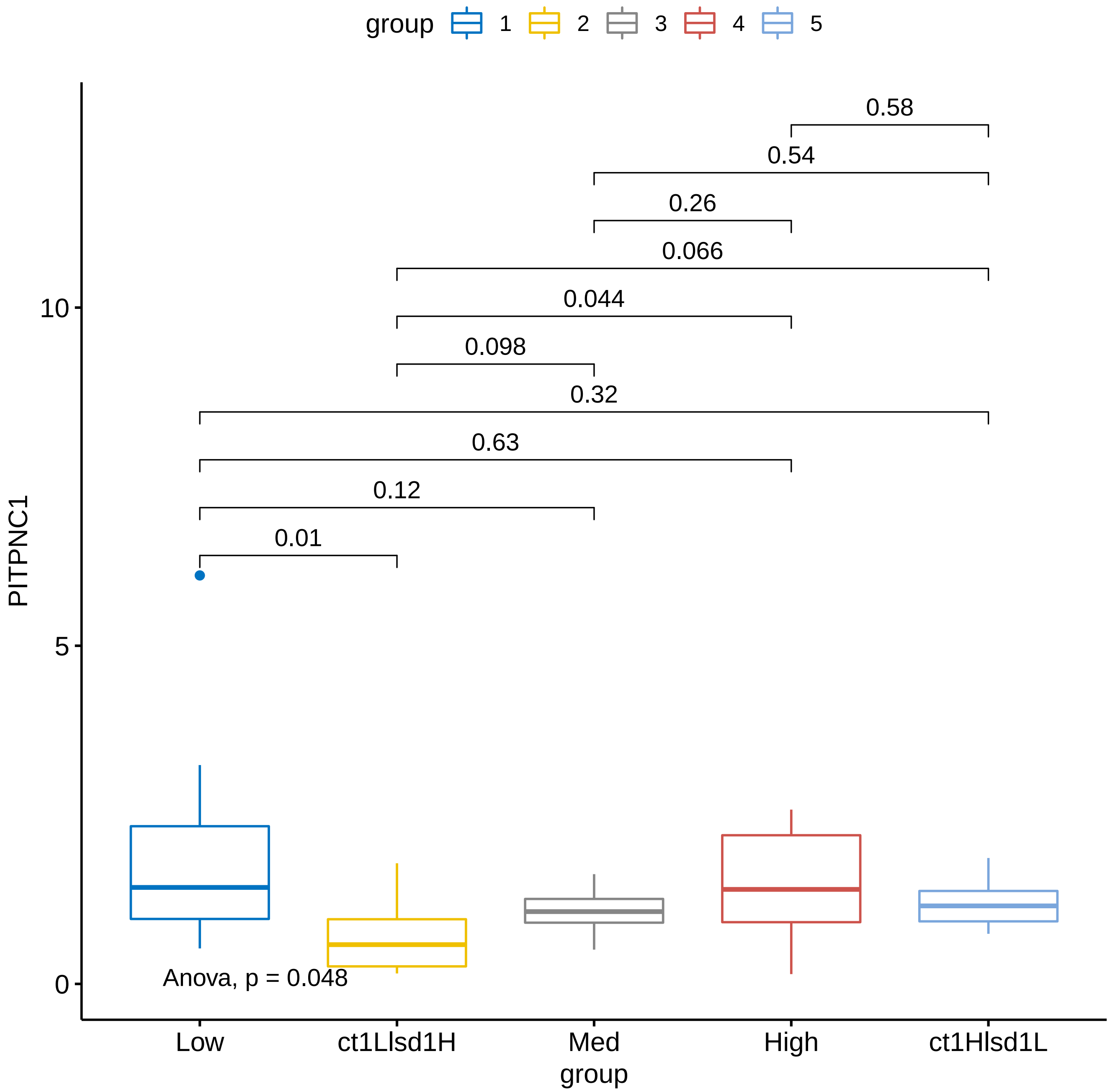
group 1 2 3 4 5
0.58
0.54
0.26
0.066
0.044
0.098
0.32
0.63
0.12
0.01
PITPNC1
10
5
0
Anova, p = 0.048
Low
ct1Llsd1H
Med
High
ct1Hlsd1L
group

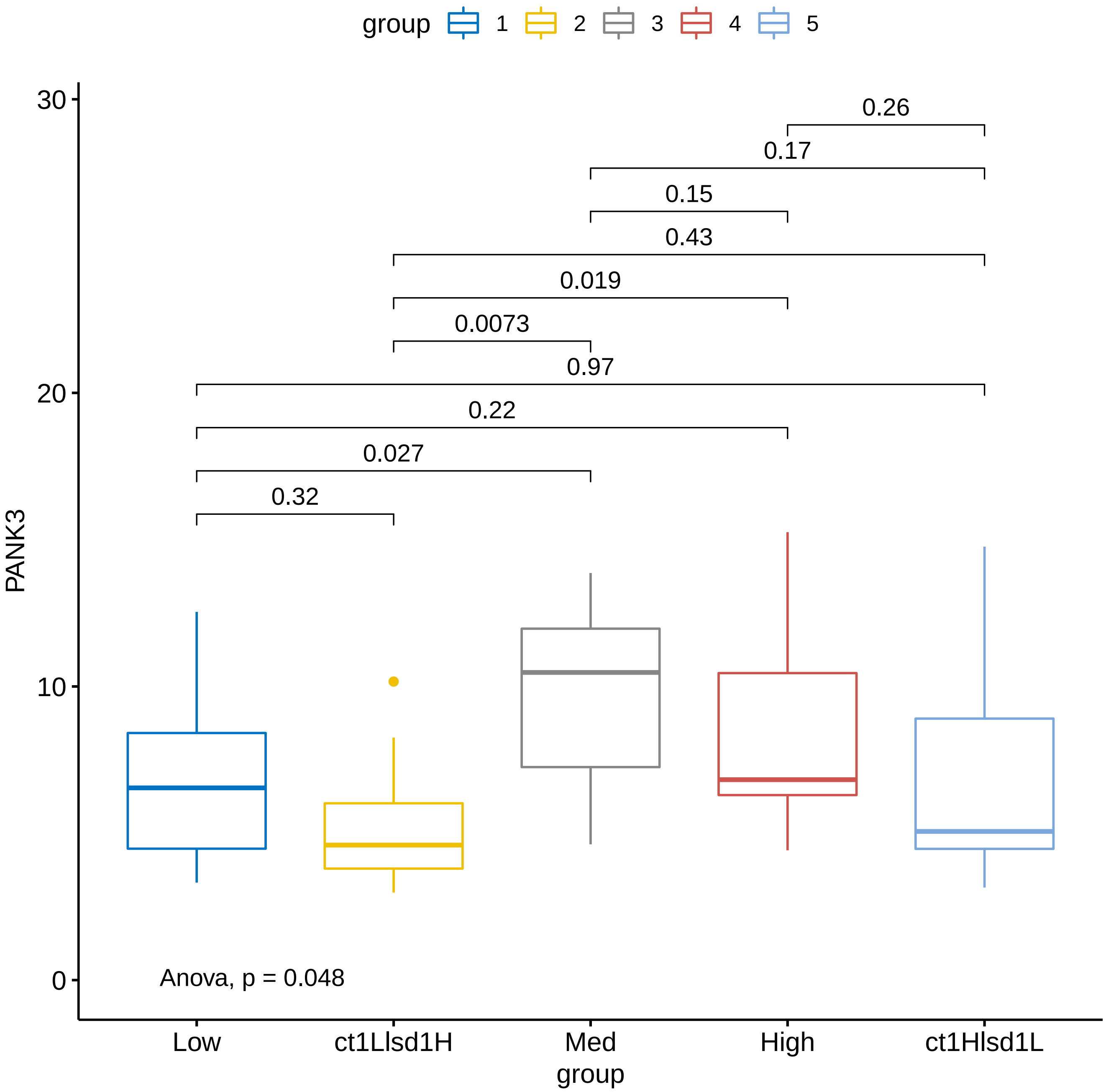

group 1 2 3 4 5
0.26
0.17
0.15
0.43
0.019
0.0073
0.97
0.22
0.027
0.32
30
20
10
0
PANK3
Anova, p = 0.048
Low
ct1Llsd1H
Med
High
ct1Hlsd1L
group

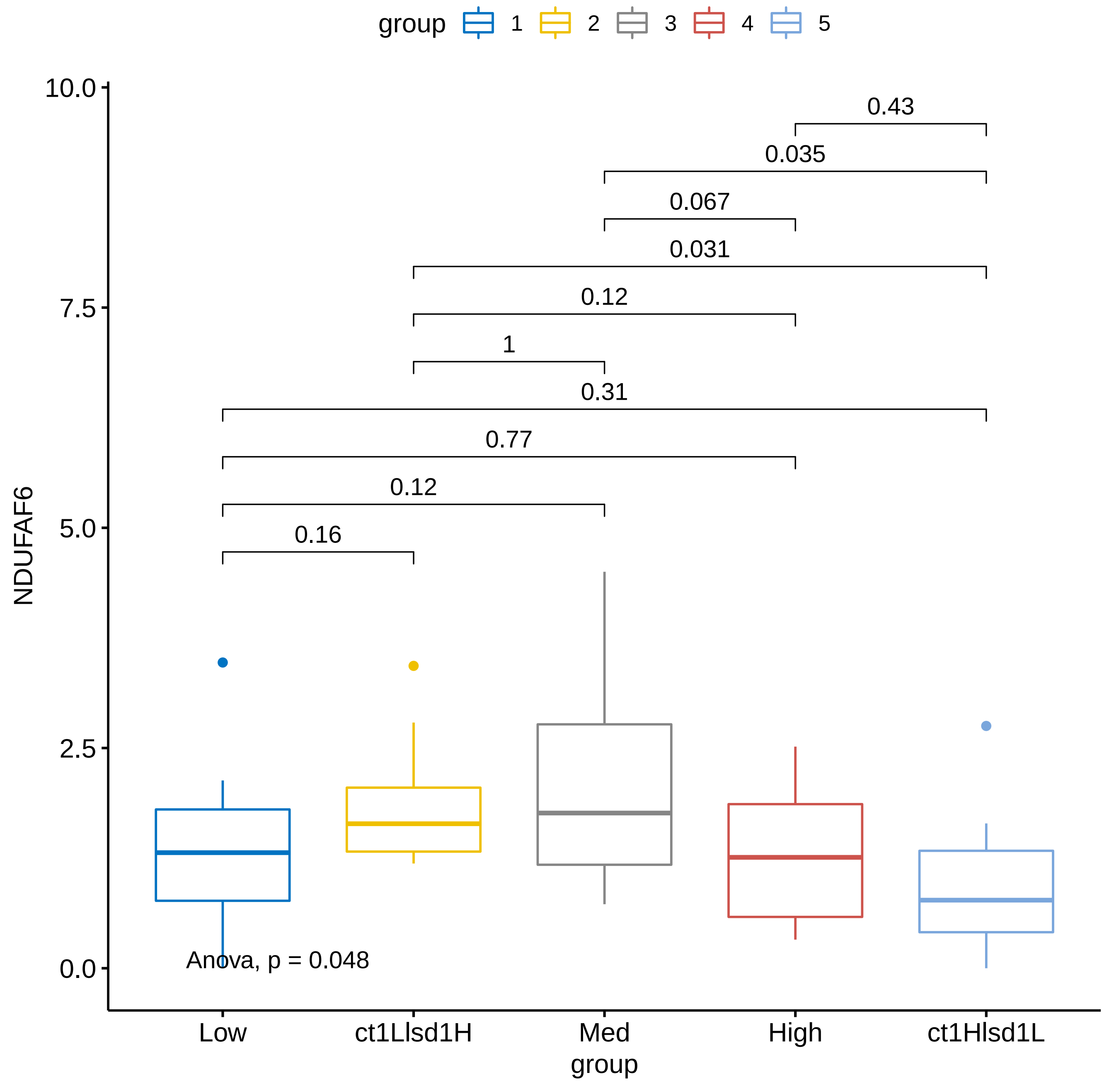
group
1
2
3
4
5
10.0
7.5
5.0
2.5
0.0
NDUFAF6
0.43
0.035
0.067
0.031
0.12
1
0.31
0.77
0.12
0.16
Anova, p = 0.048
Low
ct1Llsd1H
Med
High
ct1Hlsd1L
group

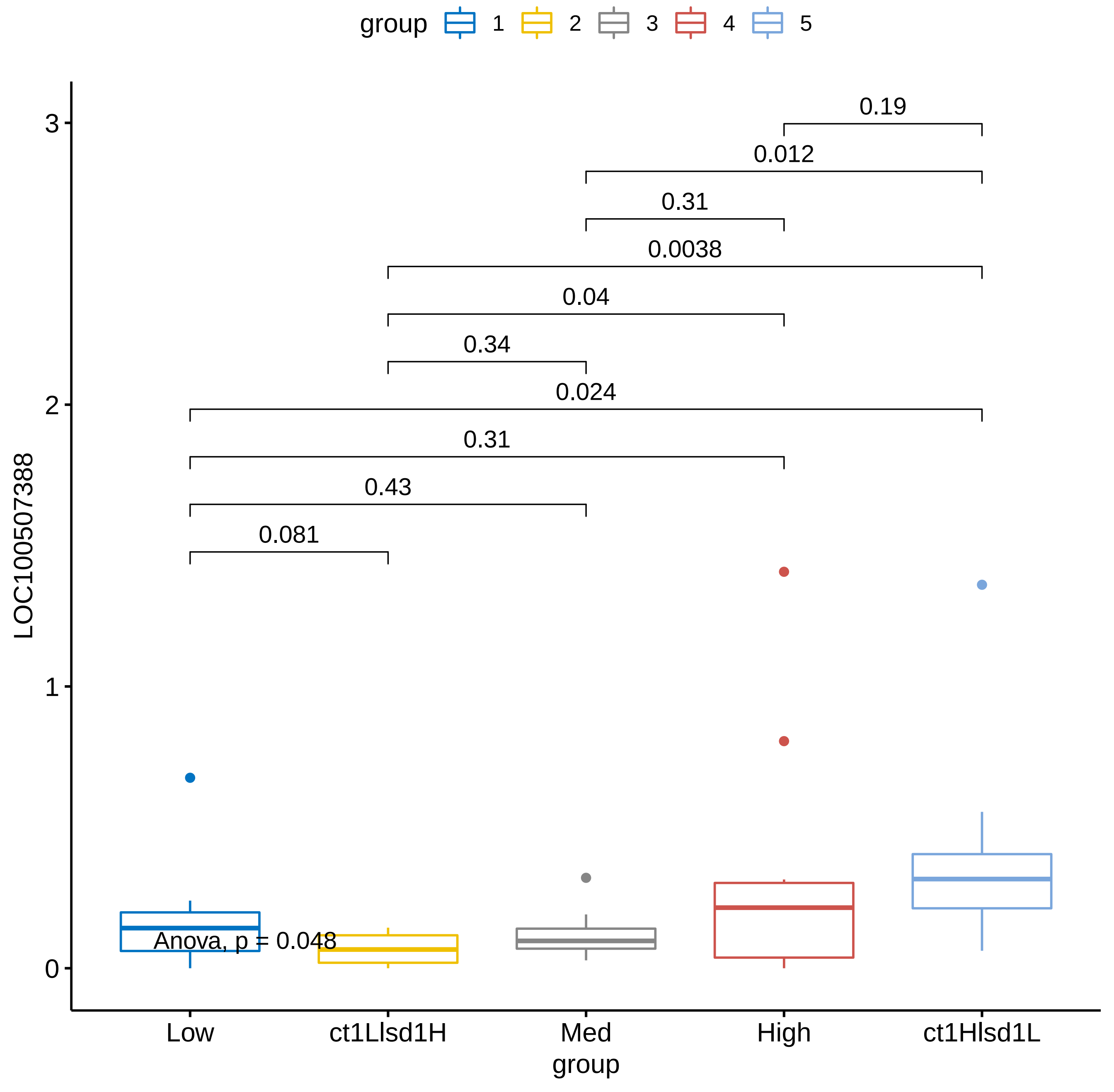
group
1
2
3
4
5
LOC100507388
3
2
1
0
0.19
0.012
0.31
0.0038
0.04
0.34
0.024
0.31
0.43
0.081
Anova, p = 0.048
Low
ct1Llsd1H
Med
High
ct1Hlsd1L
group

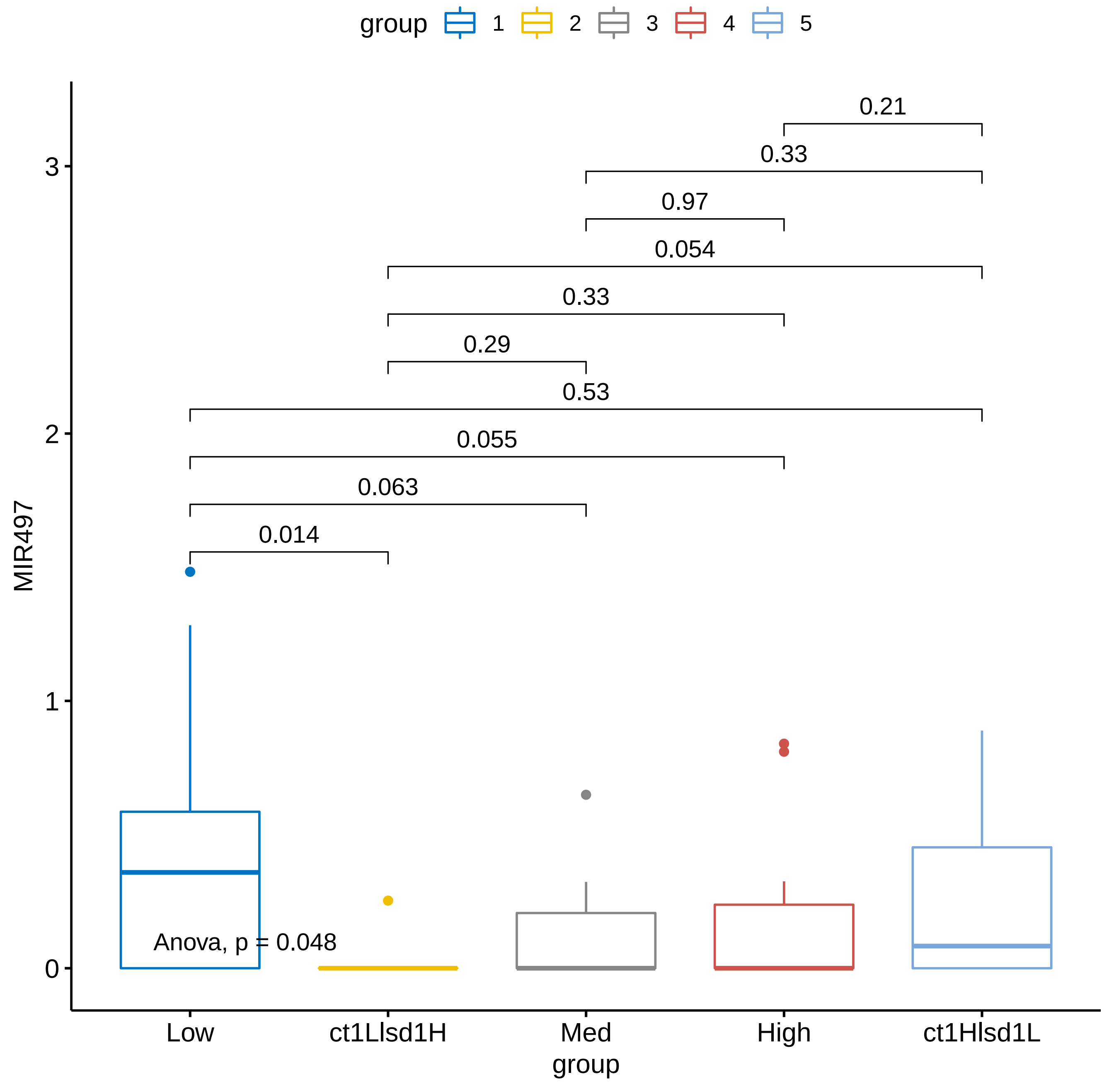
group 1 2 3 4 5
0.21
0.33
0.97
0.054
0.33
0.29
0.53
0.055
0.063
0.014
MIR497
3
2
1
0
Anova, p = 0.048
Low
ct1Llsd1H
Med
High
ct1Hlsd1L
group

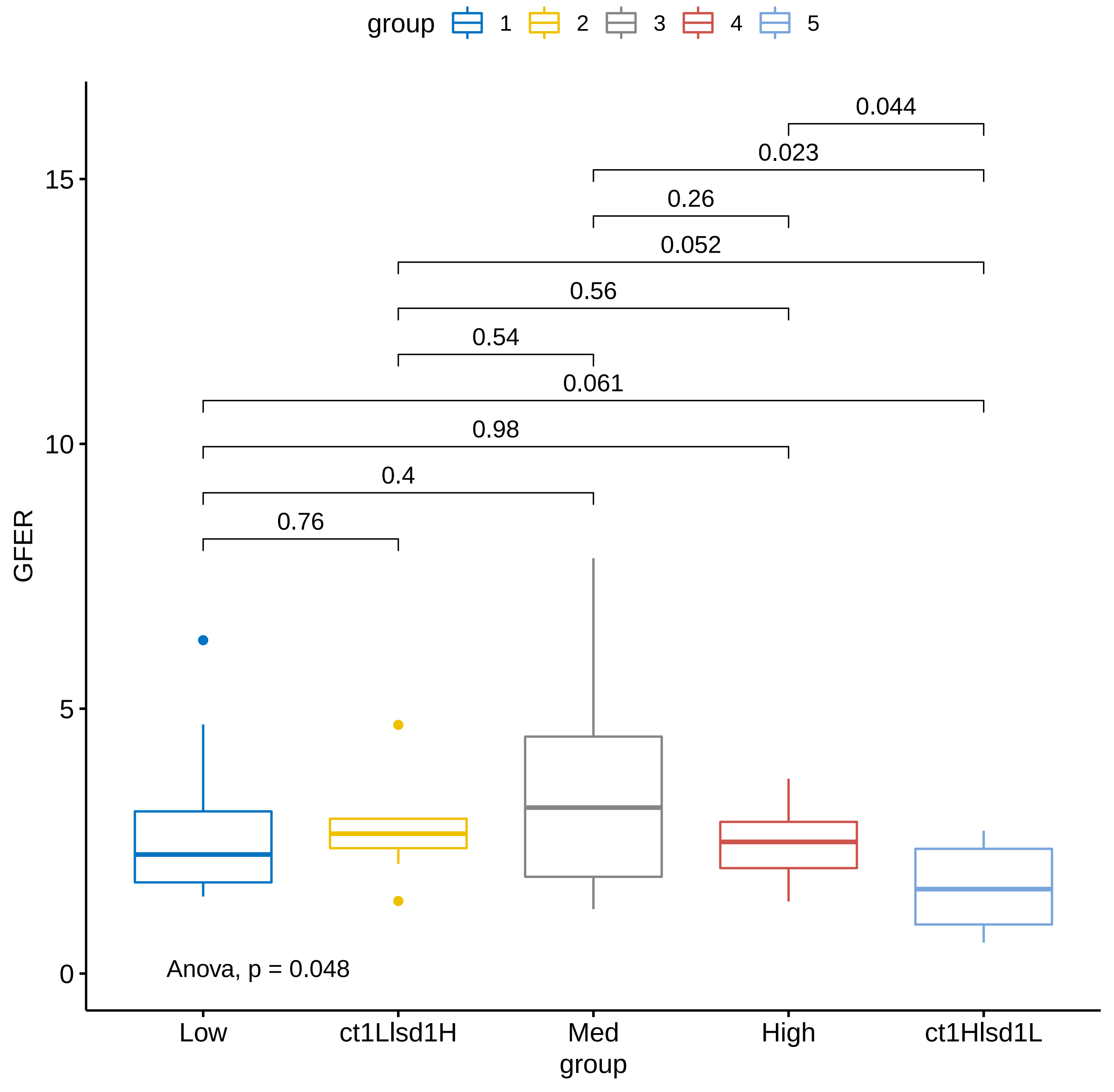

group 1 2 3 4 5
0.044
0.023
0.26
0.052
0.56
0.54
0.061
0.98
0.4
0.76
15
10
5
0
GFER
Anova, p = 0.048
Low
ct1Llsd1H
Med
High
ct1Hlsd1L
group

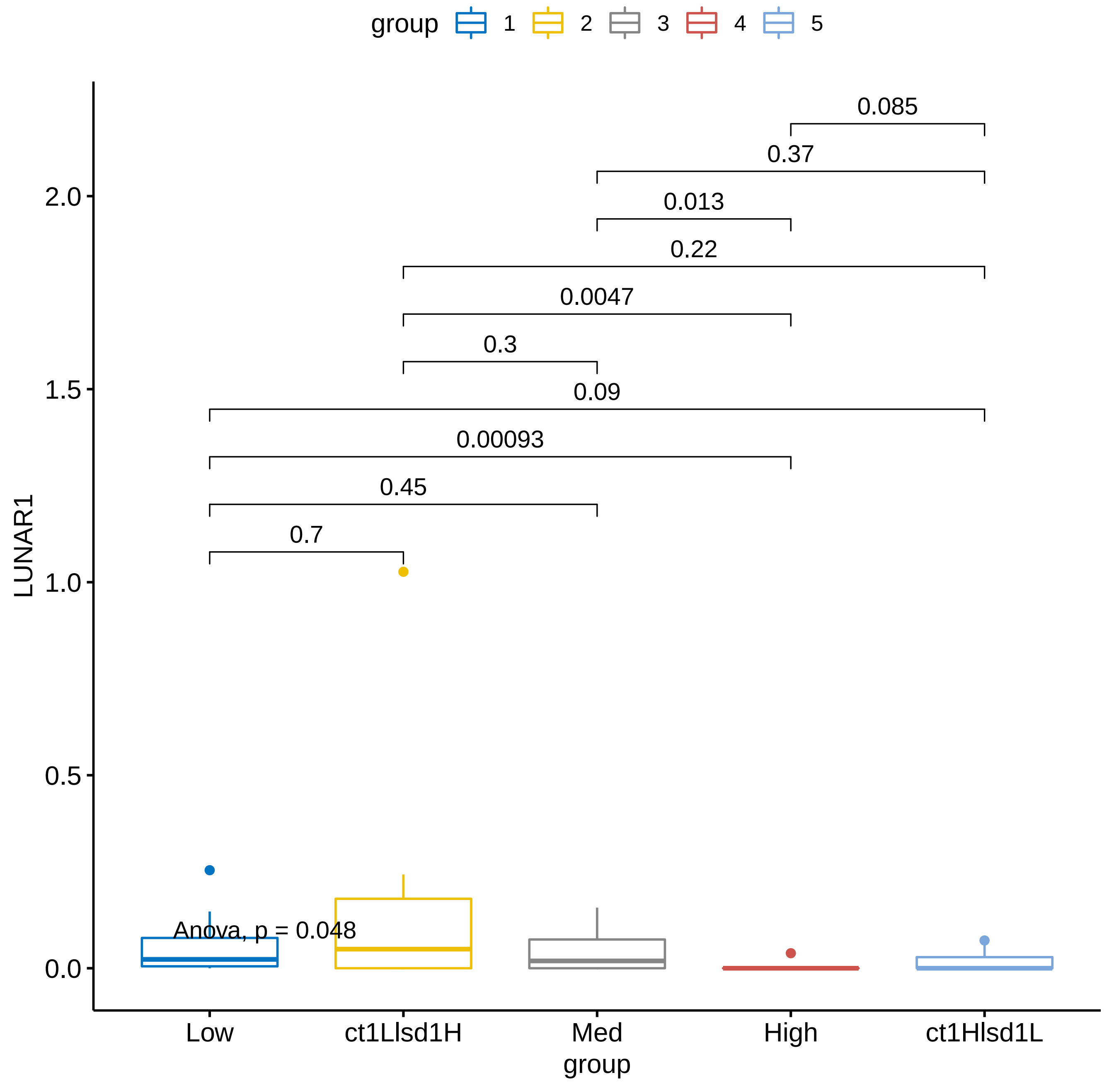
group
1
2
3
4
5
LUNAR1
2.0
1.5
1.0
0.5
0.0
0.085
0.37
0.013
0.22
0.0047
0.3
0.09
0.00093
0.45
0.7
Anova, p = 0.048
Low
ct1Llsd1H
Med
High
ct1Hlsd1L
group

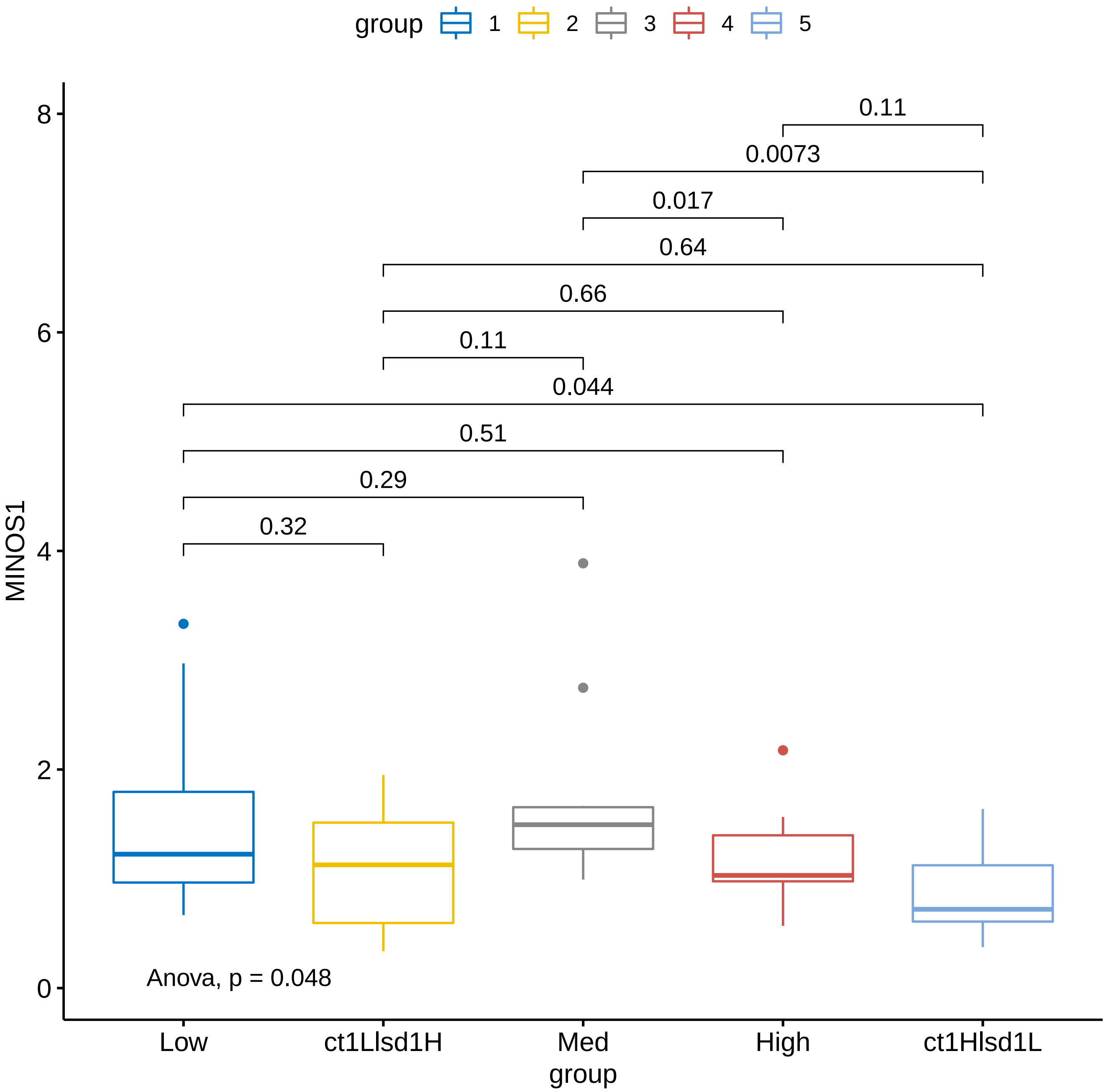

group 1 2 3 4 5
0.11
0.0073
0.017
0.64
0.66
0.11
0.044
0.51
0.29
0.32
MINOS1
8
6
4
2
0
Anova, p = 0.048
Low
ct1Llsd1H
Med
High
ct1Hlsd1L
group

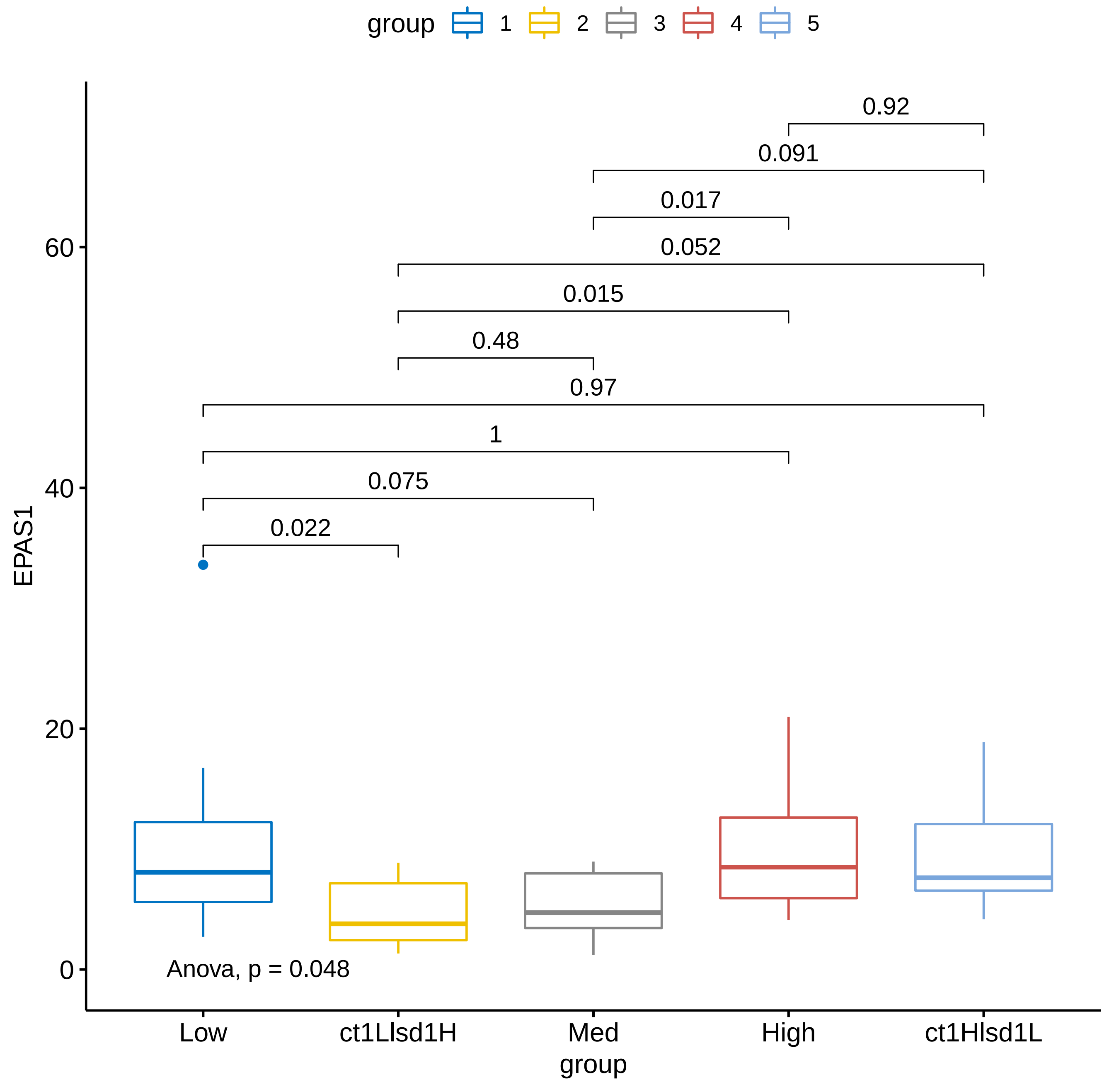

group
1
2
3
4
5
EPAS1
60
40
20
0
0.92
0.091
0.017
0.052
0.015
0.48
0.97
1
0.075
0.022
Anova, p = 0.048
Low
ct1Llsd1H
Med
High
ct1Hlsd1L
group

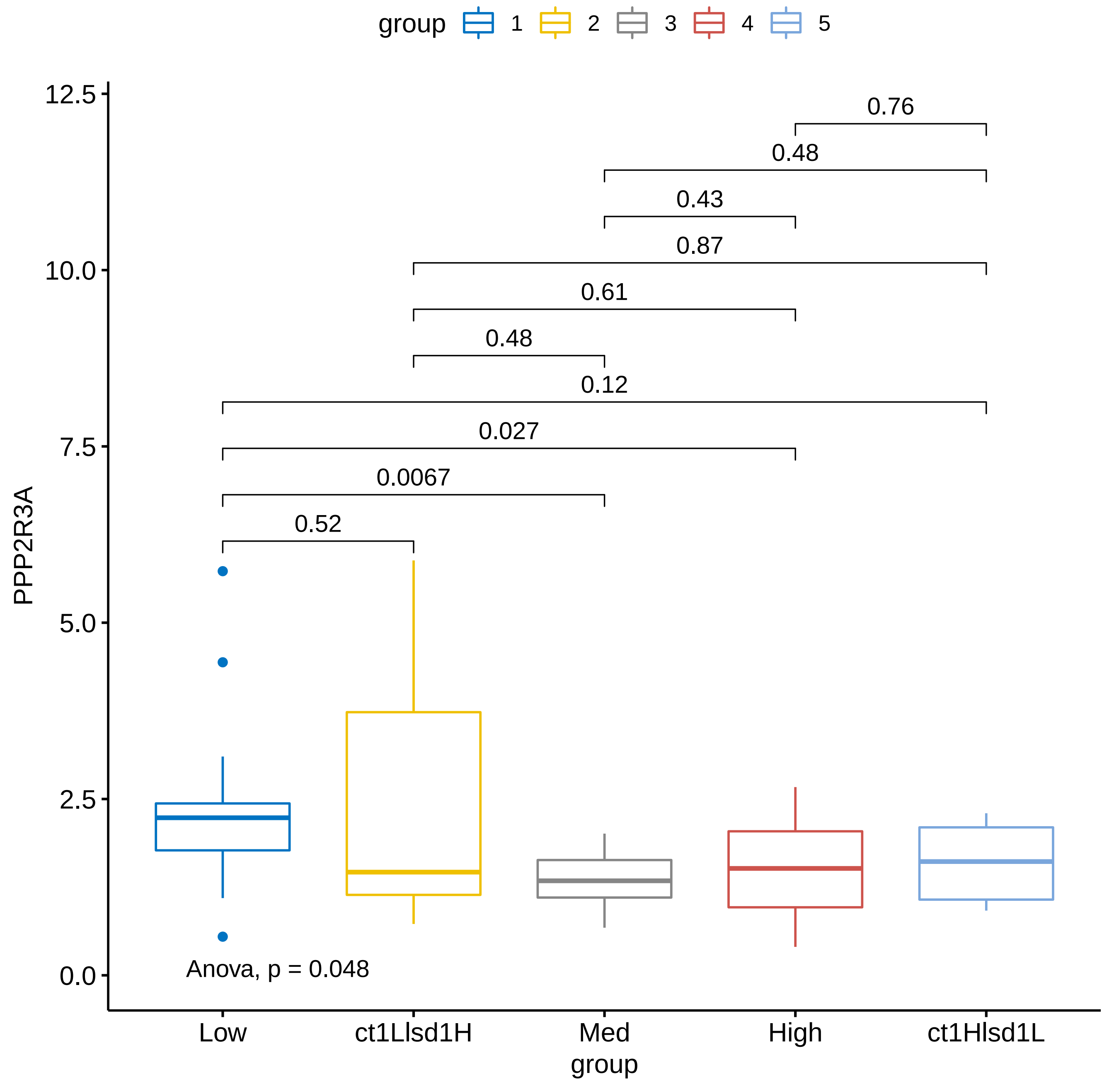
group
1
2
3
4
5
PPP2R3A
12.5
10.0
7.5
5.0
2.5
0.0
0.76
0.48
0.43
0.87
0.61
0.48
0.12
0.027
0.0067
0.52
Anova, p = 0.048
Low
ct1Llsd1H
Med
High
ct1Hlsd1L
group

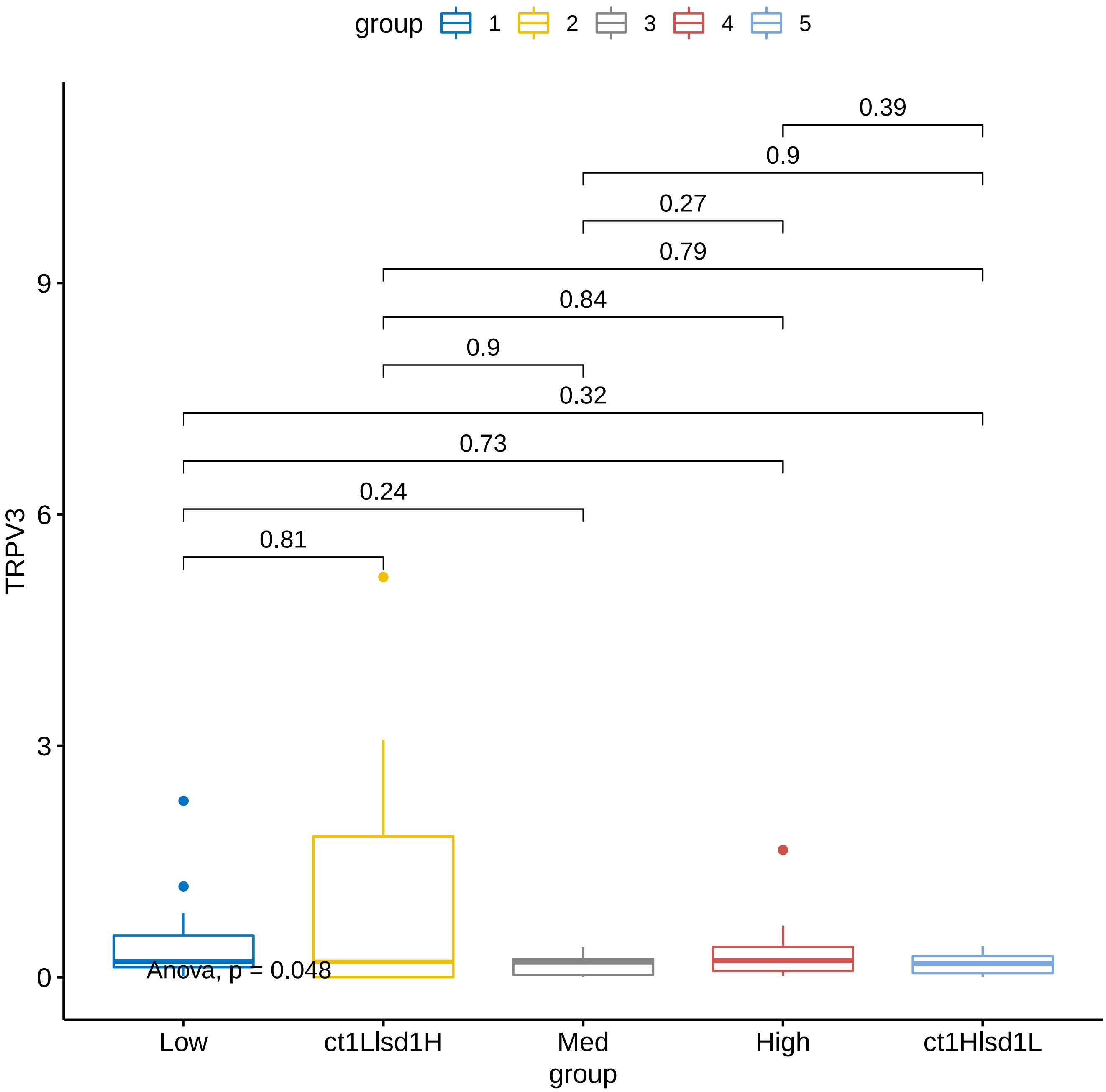

group
1
2
3
4
5
0.39
0.9
0.27
0.79
0.84
0.9
0.32
0.73
0.24
0.81
Anova, p = 0.048
TRPV3
9
6
3
0
Low
ct1Llsd1H
Med
High
ct1Hlsd1L
group

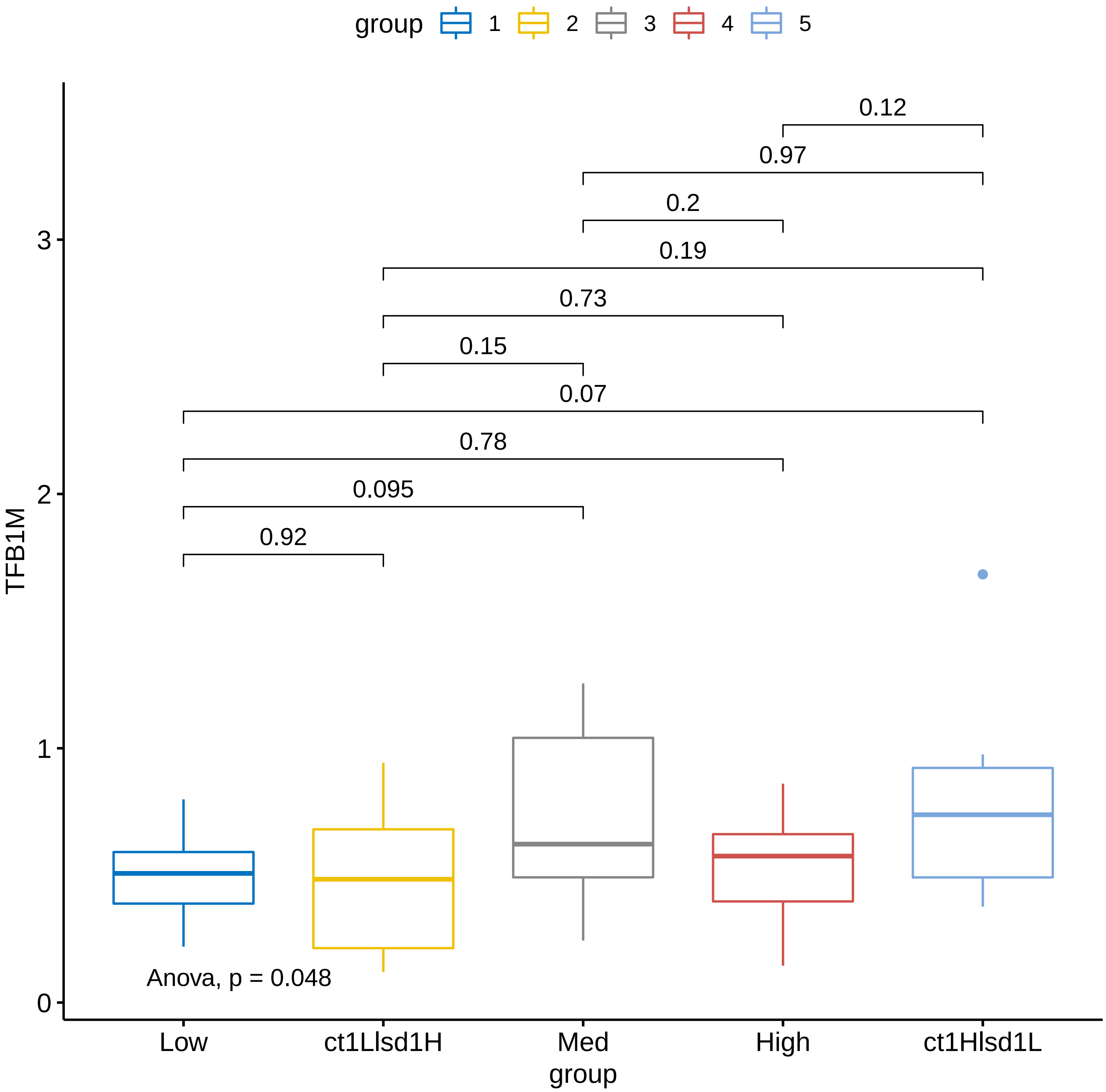
group
1
2
3
4
5
0.12
0.97
0.2
0.19
0.73
0.15
0.07
0.78
0.095
0.92
TFB1M
3
2
1
0
Anova, p = 0.048
Low
ct1Llsd1H
Med
High
ct1Hlsd1L
group

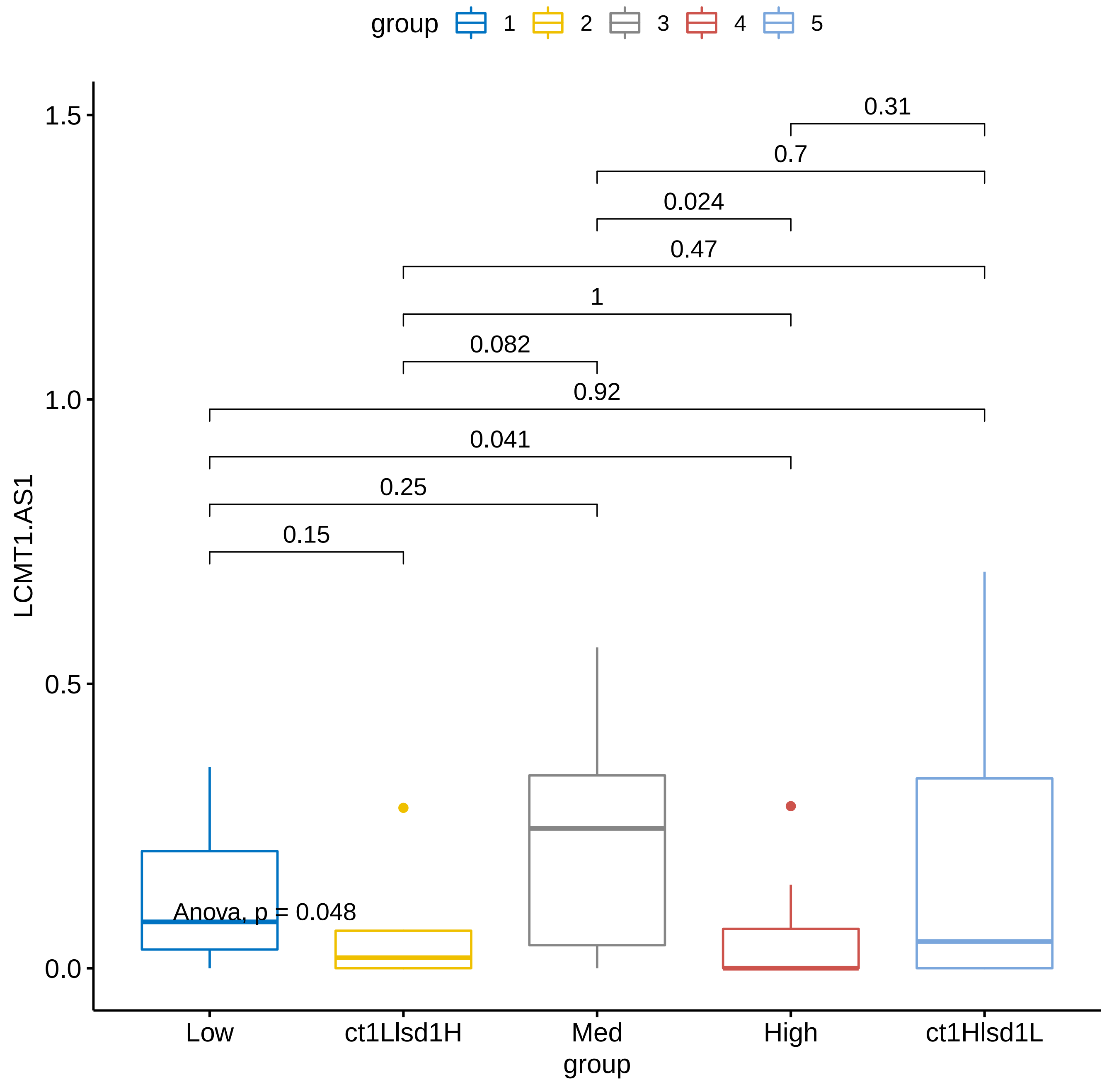

group 1 2 3 4 5
0.31
0.7
0.024
0.47
1
0.082
0.92
0.041
0.25
0.15
1.5
1.0
0.5
0.0
LCMT1.AS1
Anova, p = 0.048
Low
ct1Llsd1H
Med
High
ct1Hlsd1L
group

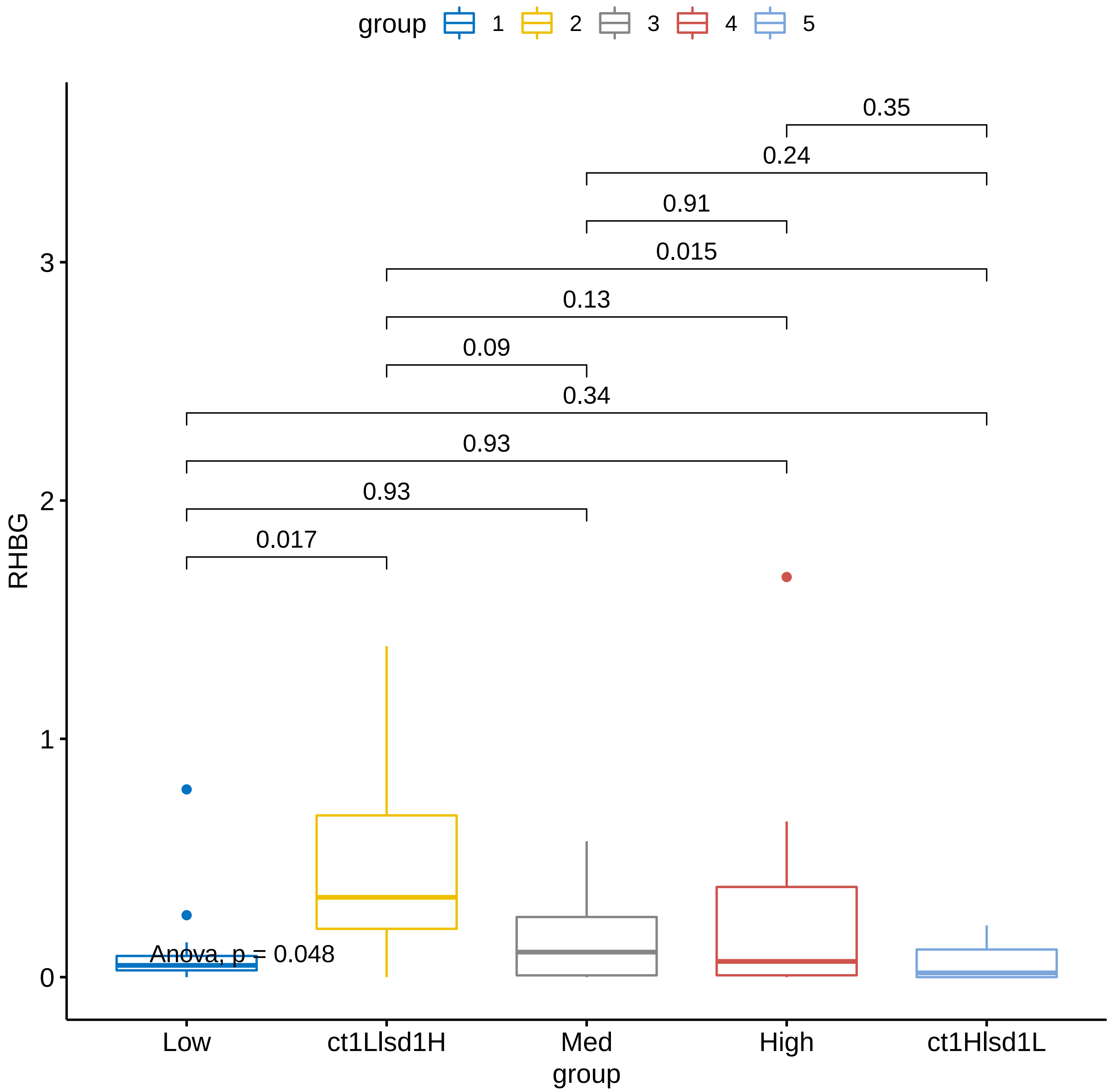

group
1
2
3
4
5
RHBG
0.35
0.24
0.91
0.015
0.13
0.09
0.34
0.93
0.93
0.017
Anova, p = 0.048
3
2
1
0
Low
ct1Llsd1H
Med
High
ct1Hlsd1L
group

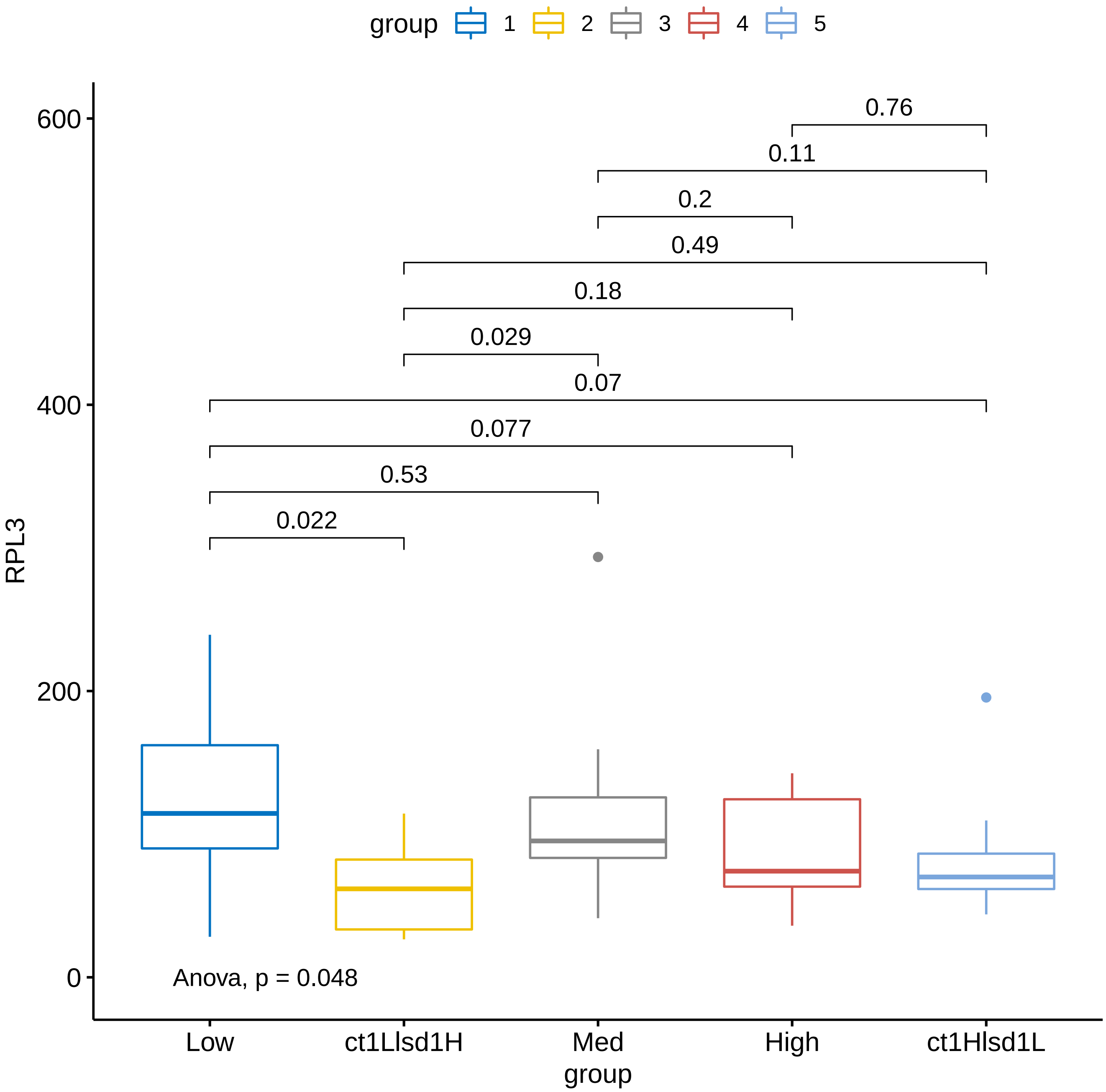
group 1 2 3 4 5
RPL3
600
400
200
0
0.76
0.11
0.2
0.49
0.18
0.029
0.07
0.077
0.53
0.022
Anova, p = 0.048
Low
ct1Llsd1H
Med
High
ct1Hlsd1L
group

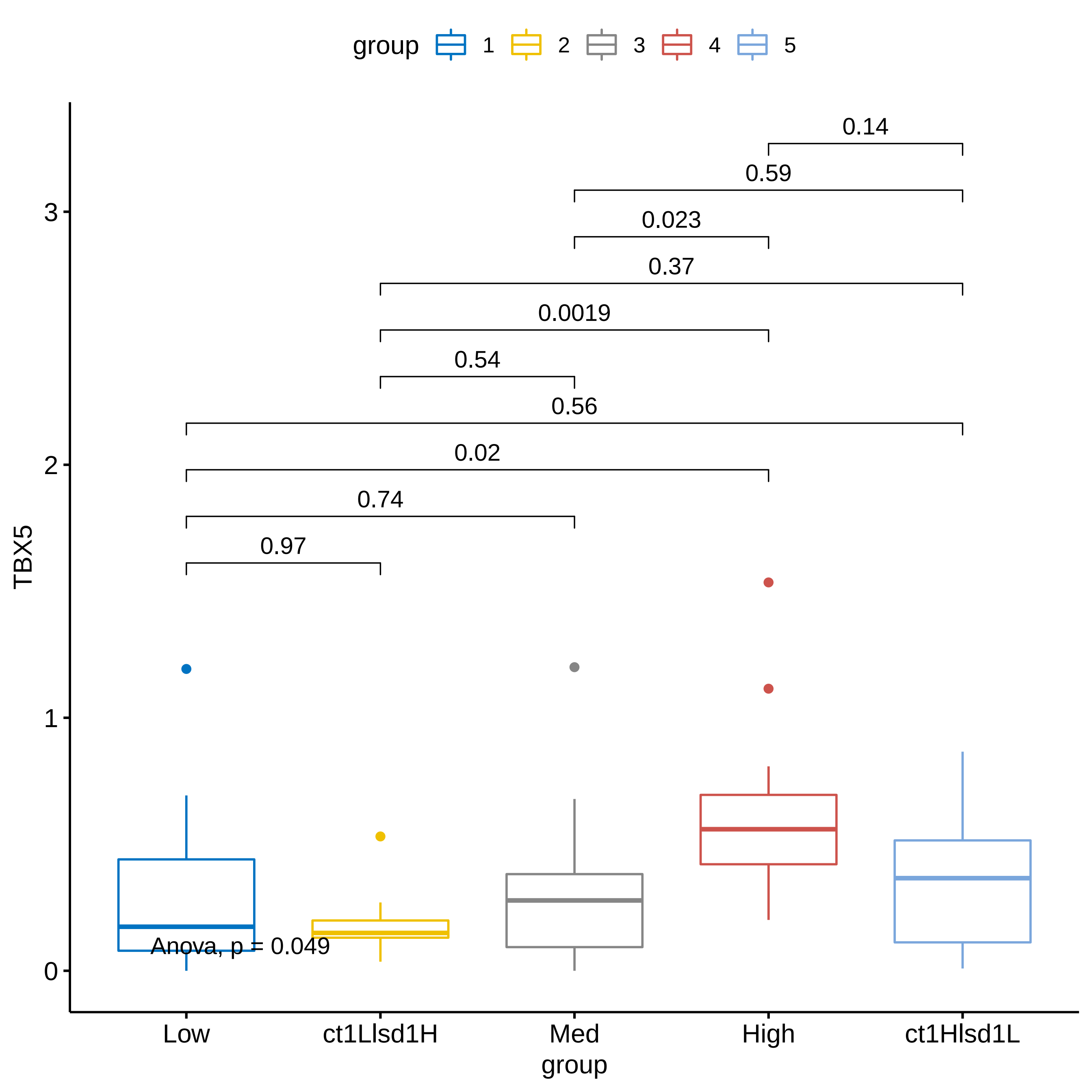
group
1
2
3
4
5
TBX5
0.14
0.59
0.023
0.37
0.0019
0.54
0.56
0.02
0.74
0.97
Anova, p = 0.049
3
2
1
0
Low
ct1Llsd1H
Med
High
ct1Hlsd1L
group

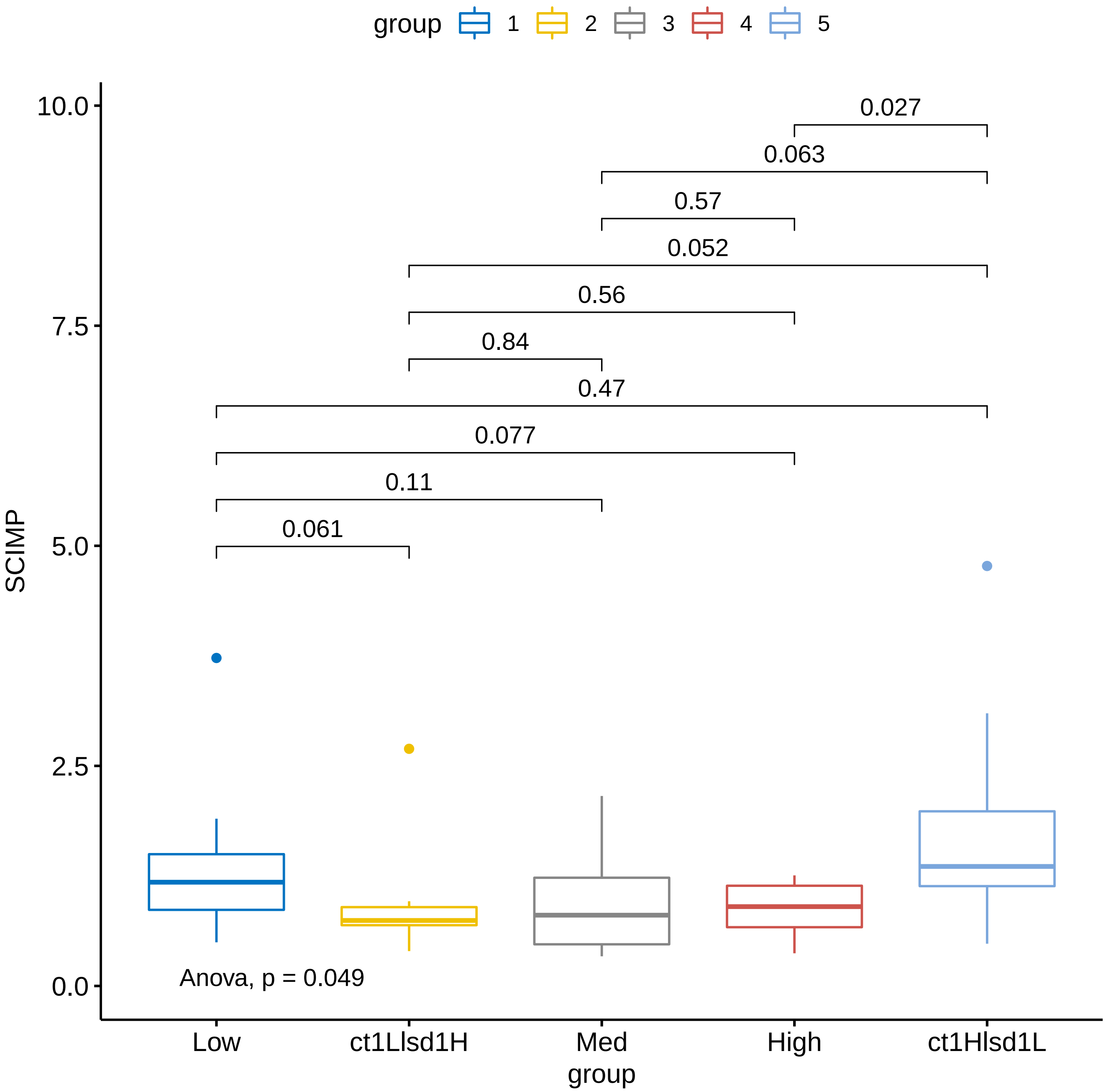
group 1 2 3 4 5
SCIMP
10.0
7.5
5.0
2.5
0.0
0.027
0.063
0.57
0.052
0.56
0.84
0.47
0.077
0.11
0.061
Anova, p = 0.049
Low
ct1Llsd1H
Med
High
ct1Hlsd1L
group

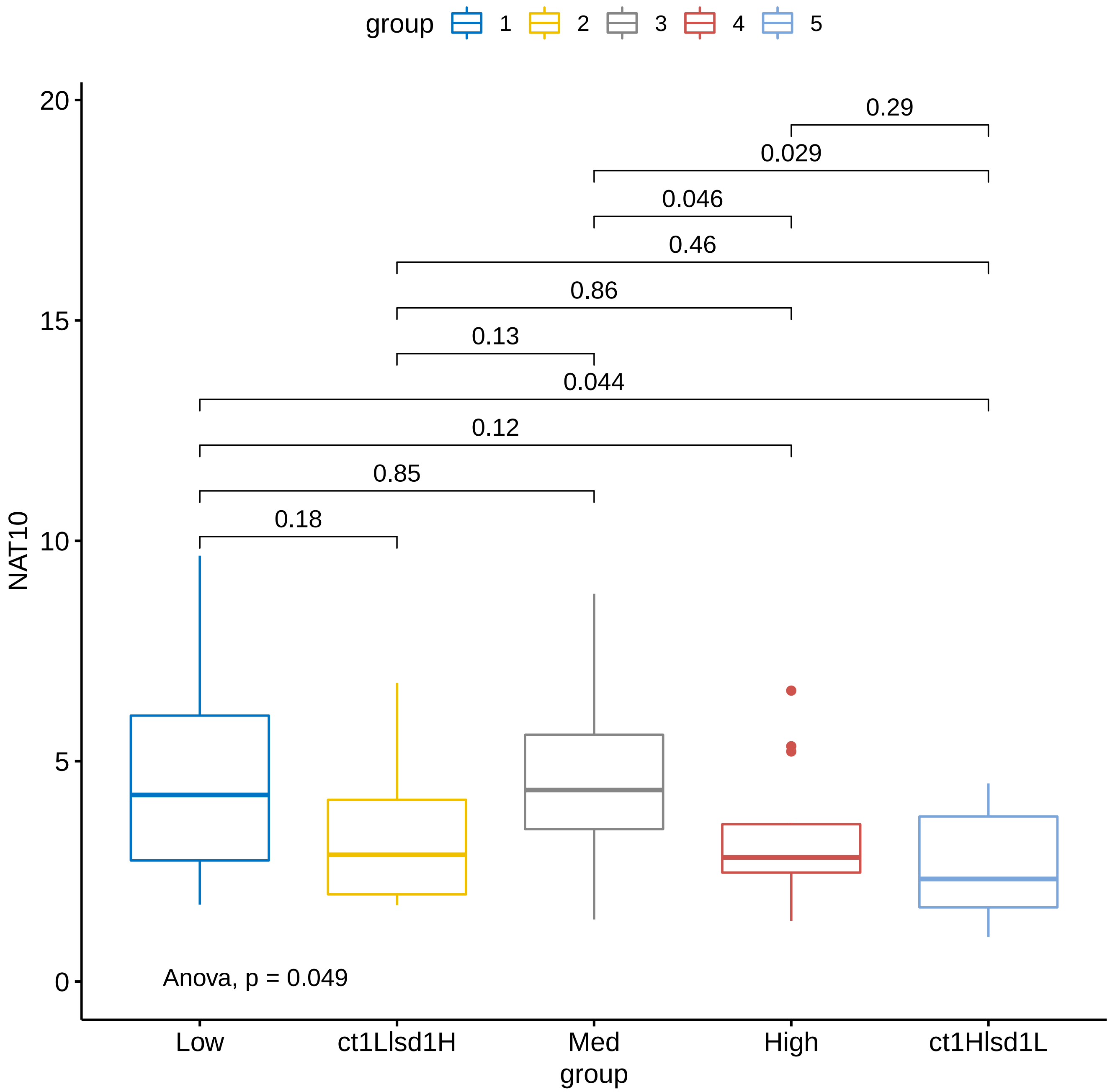

group 1 2 3 4 5
0.29
0.029
0.046
0.46
0.86
0.13
0.044
0.12
0.85
0.18
20
15
10
5
0
NAT10
Anova, p = 0.049
Low
ct1Llsd1H
Med
High
ct1Hlsd1L
group

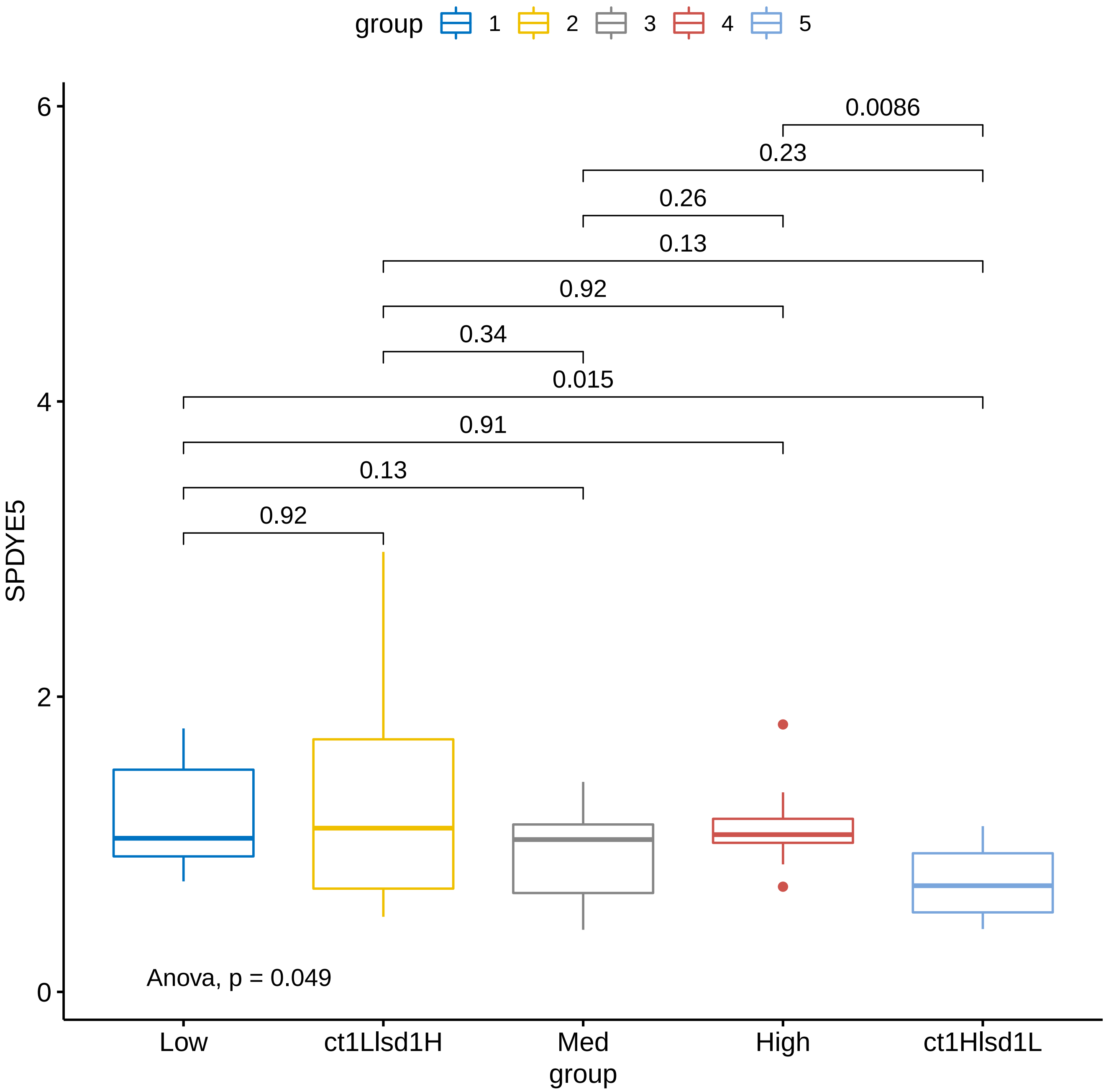
group
1
2
3
4
5
0.0086
0.23
0.26
0.13
0.92
0.34
0.015
0.91
0.13
0.92
6
4
2
0
SPDYE5
Anova, p = 0.049
Low
ct1Llsd1H
Med
High
ct1Hlsd1L
group

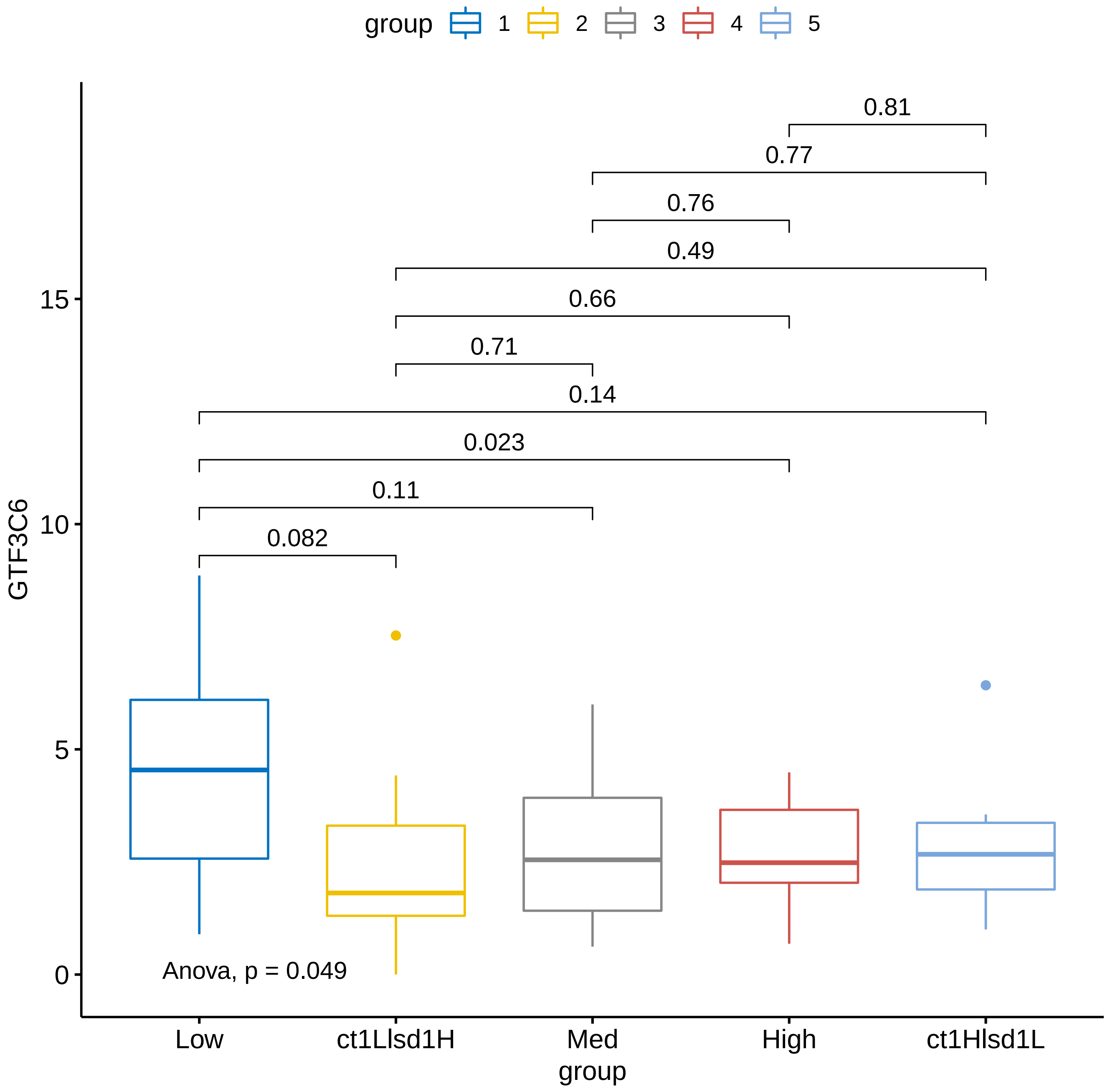

group 1 2 3 4 5
0.81
0.77
0.76
0.49
0.66
0.71
0.14
0.023
0.11
0.082
GTF3C6
15
10
5
0
Anova, p = 0.049
Low
ct1Llsd1H
Med
High
ct1Hlsd1L
group

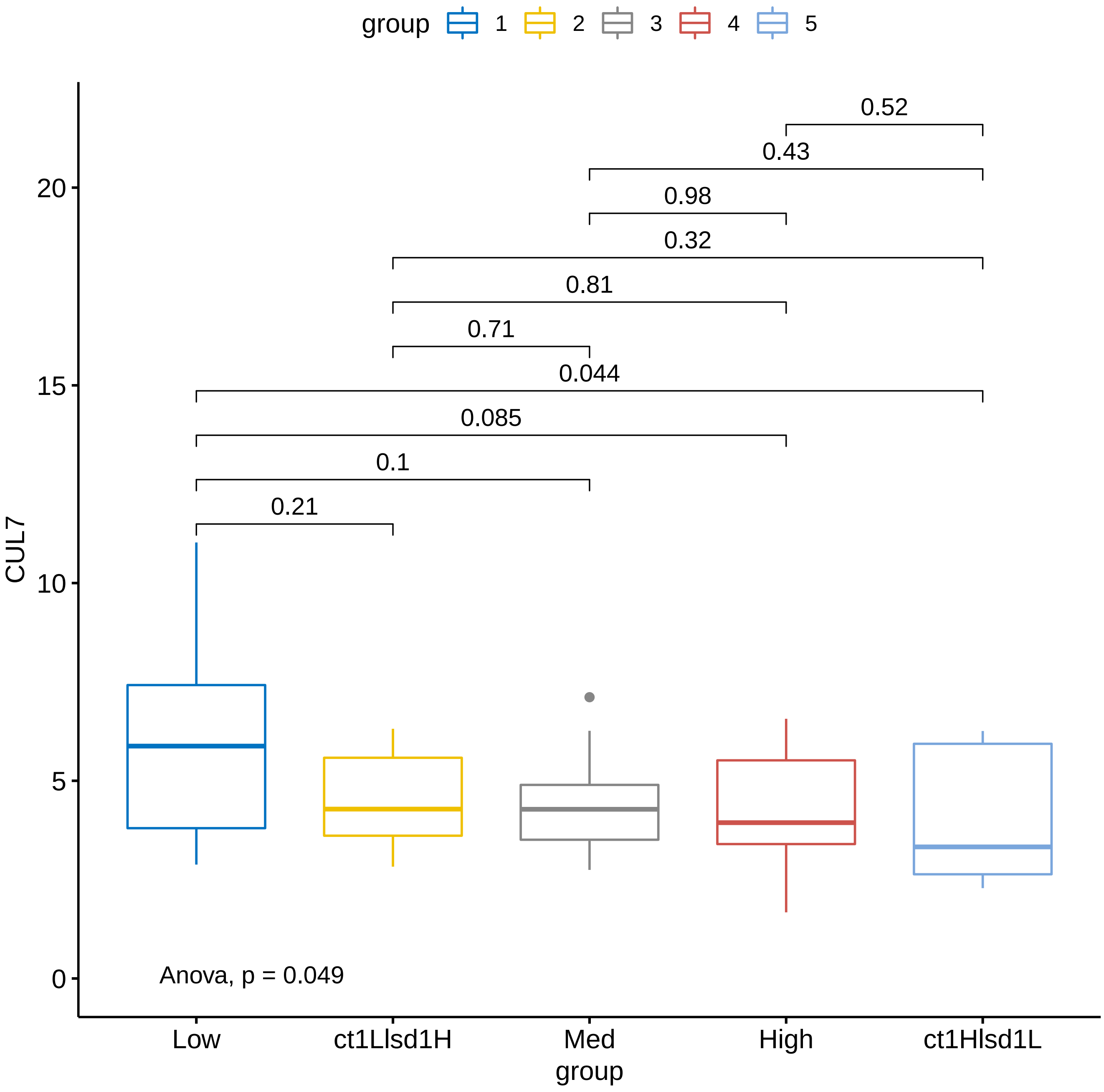

group
1
2
3
4
5
0.52
0.43
0.98
0.32
0.81
0.71
0.044
0.085
0.1
0.21
CUL7
20
15
10
5
0
Anova, p = 0.049
Low
ct1Llsd1H
Med
High
ct1Hlsd1L
group

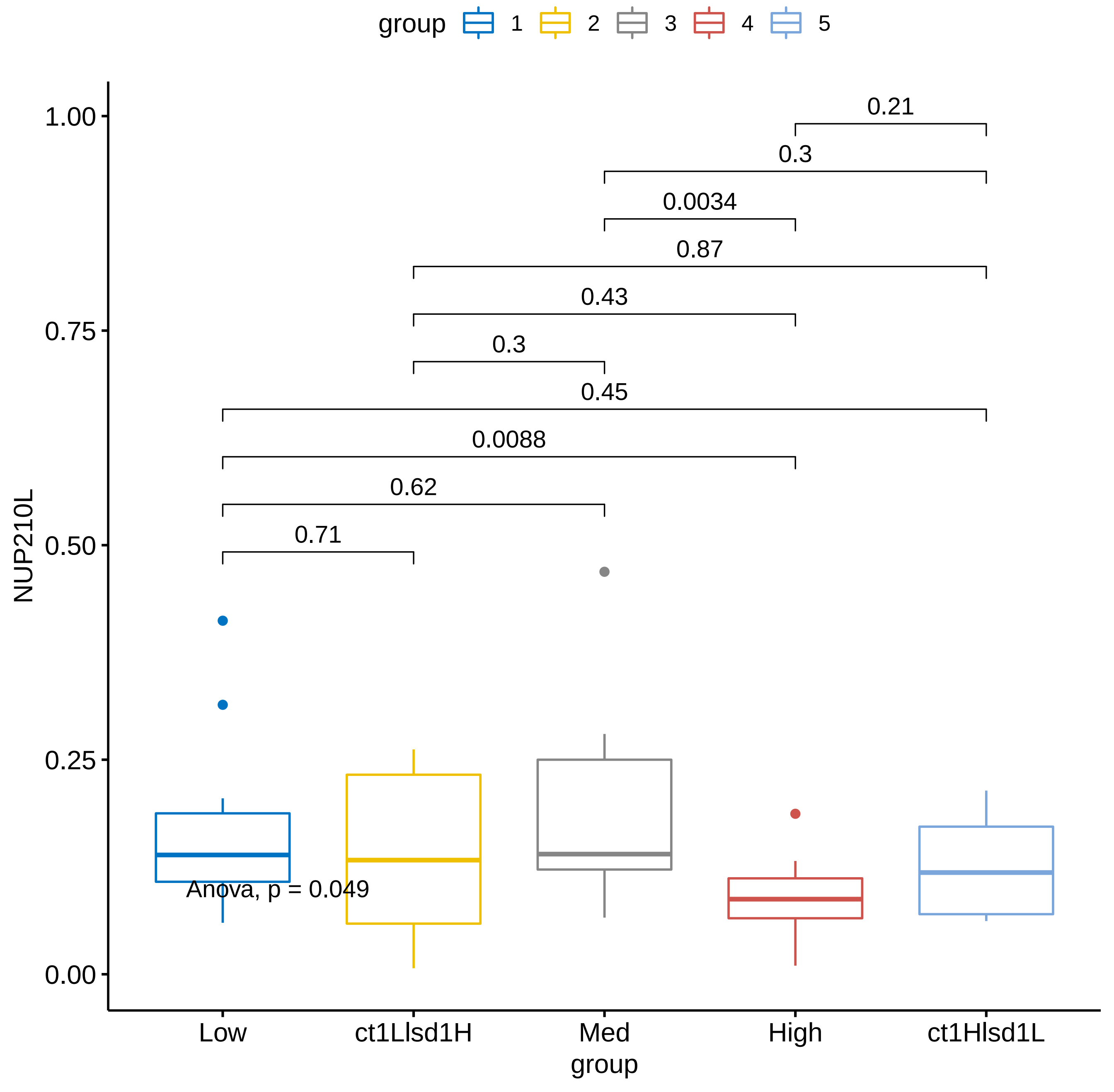

group 1 2 3 4 5
0.21
0.3
0.0034
0.87
0.43
0.3
0.45
0.0088
0.62
0.71
Anova, p = 0.049
1.00
0.75
0.50
0.25
0.00
NUP210L
Low
ct1Llsd1H
Med
High
ct1Hlsd1L
group

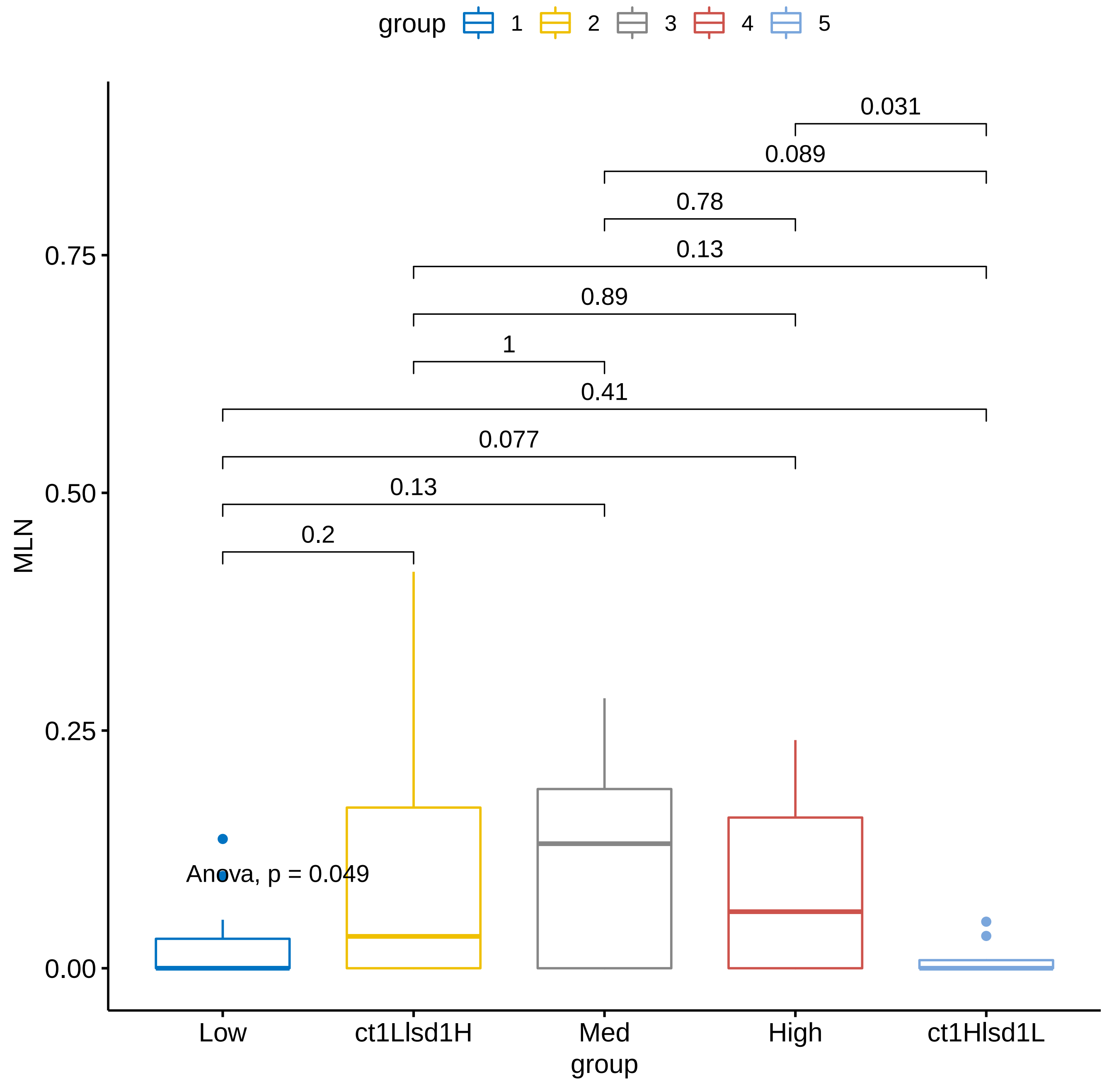
group
1
2
3
4
5
0.031
0.089
0.78
0.13
0.89
1
0.41
0.077
0.13
0.2
Anova, p = 0.049
MLN
0.75
0.50
0.25
0.00
Low
ct1Llsd1H
Med
High
ct1Hlsd1L
group

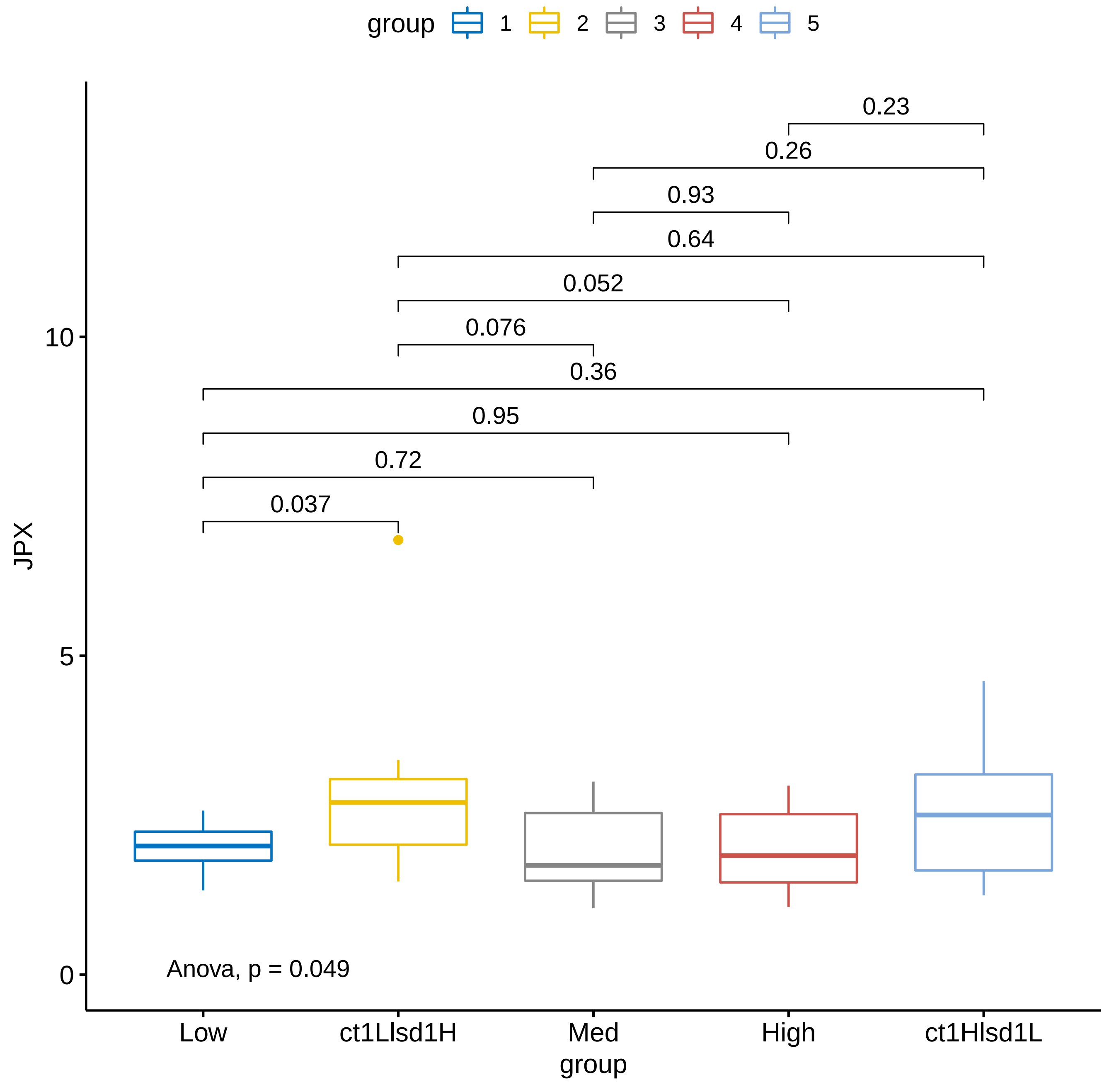

group 1 2 3 4 5
0.23
0.26
0.93
0.64
0.052
0.076
0.36
0.95
0.72
0.037
10
5
0
JPX
Anova, p = 0.049
Low
ct1Llsd1H
Med
High
ct1Hlsd1L
group

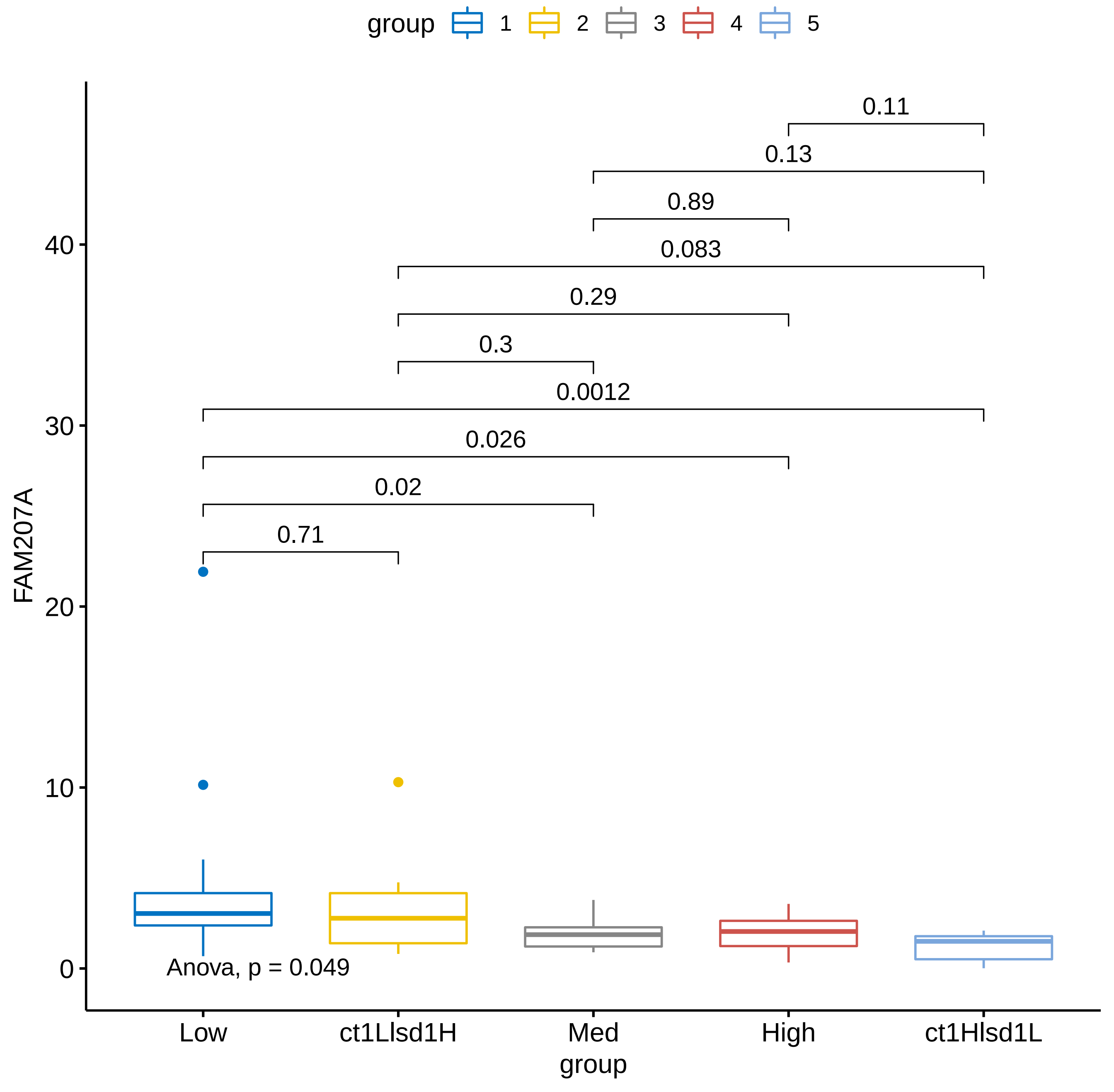

group 1 2 3 4 5
0.11
0.13
0.89
0.083
0.29
0.3
0.0012
0.026
0.02
0.71
FAM207A
40
30
20
10
0
Anova, p = 0.049
Low
ct1Llsd1H
Med
High
ct1Hlsd1L
group

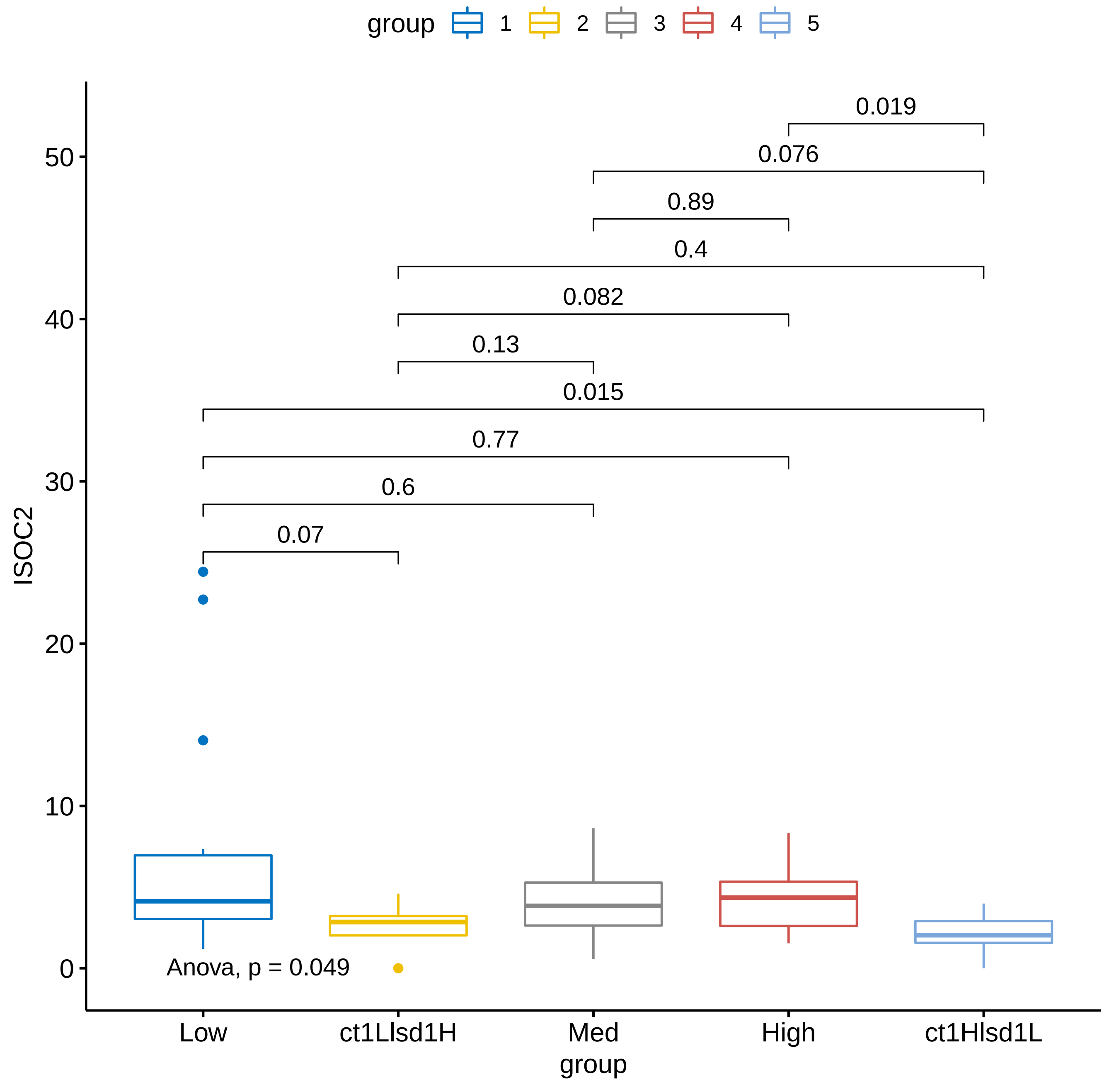
group 1 2 3 4 5
0.019
0.076
0.89
0.4
0.082
0.13
0.015
0.77
0.6
0.07
50
40
30
20
10
0
ISOC2
Anova, p = 0.049
Low
ct1Llsd1H
Med
High
ct1Hlsd1L
group

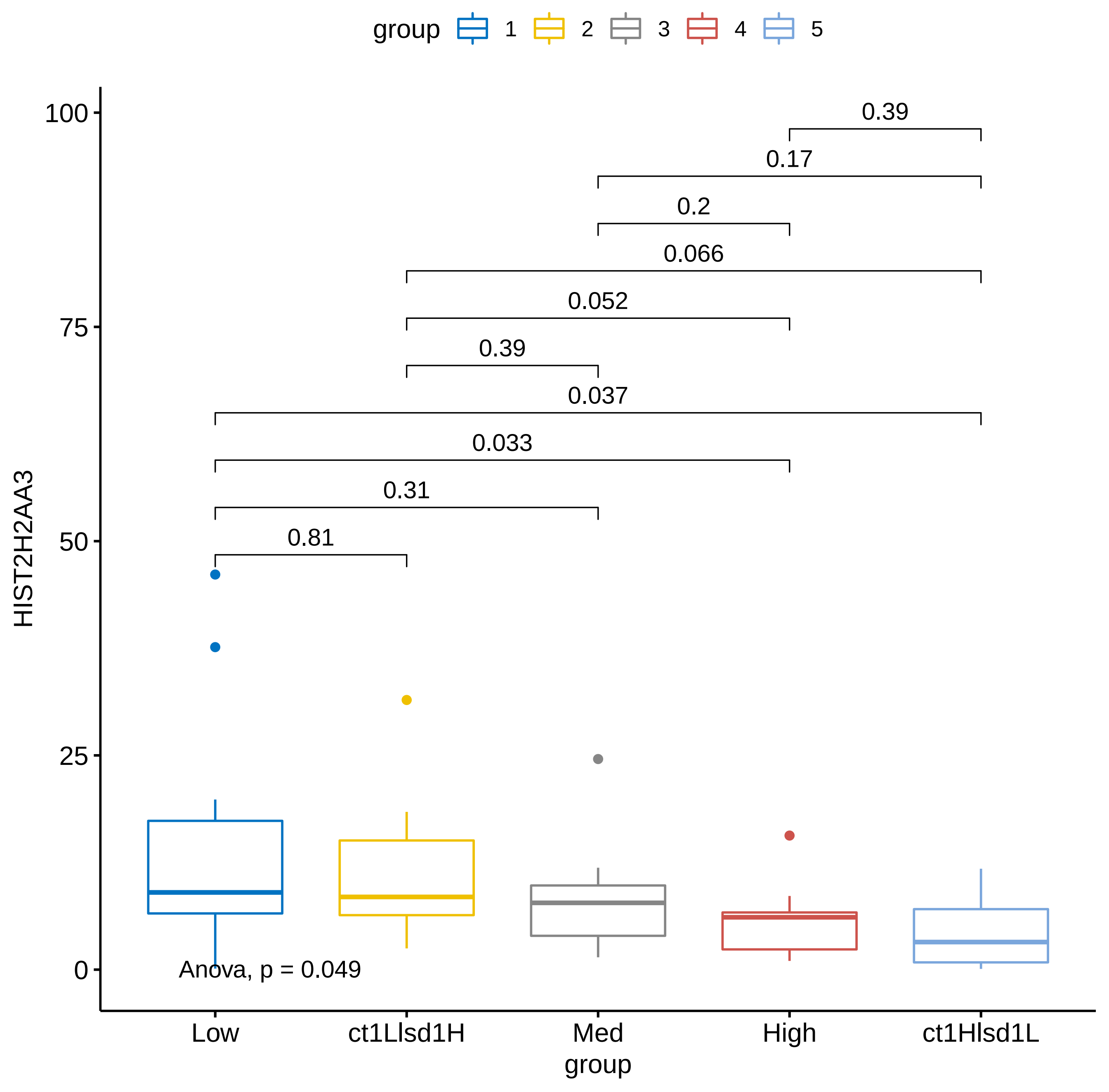

group 1 2 3 4 5
0.39
0.17
0.2
0.066
0.052
0.39
0.037
0.033
0.31
0.81
100
75
50
25
0
HIST2H2AA3
Anova, p = 0.049
Low
ct1Llsd1H
Med
High
ct1Hlsd1L
group

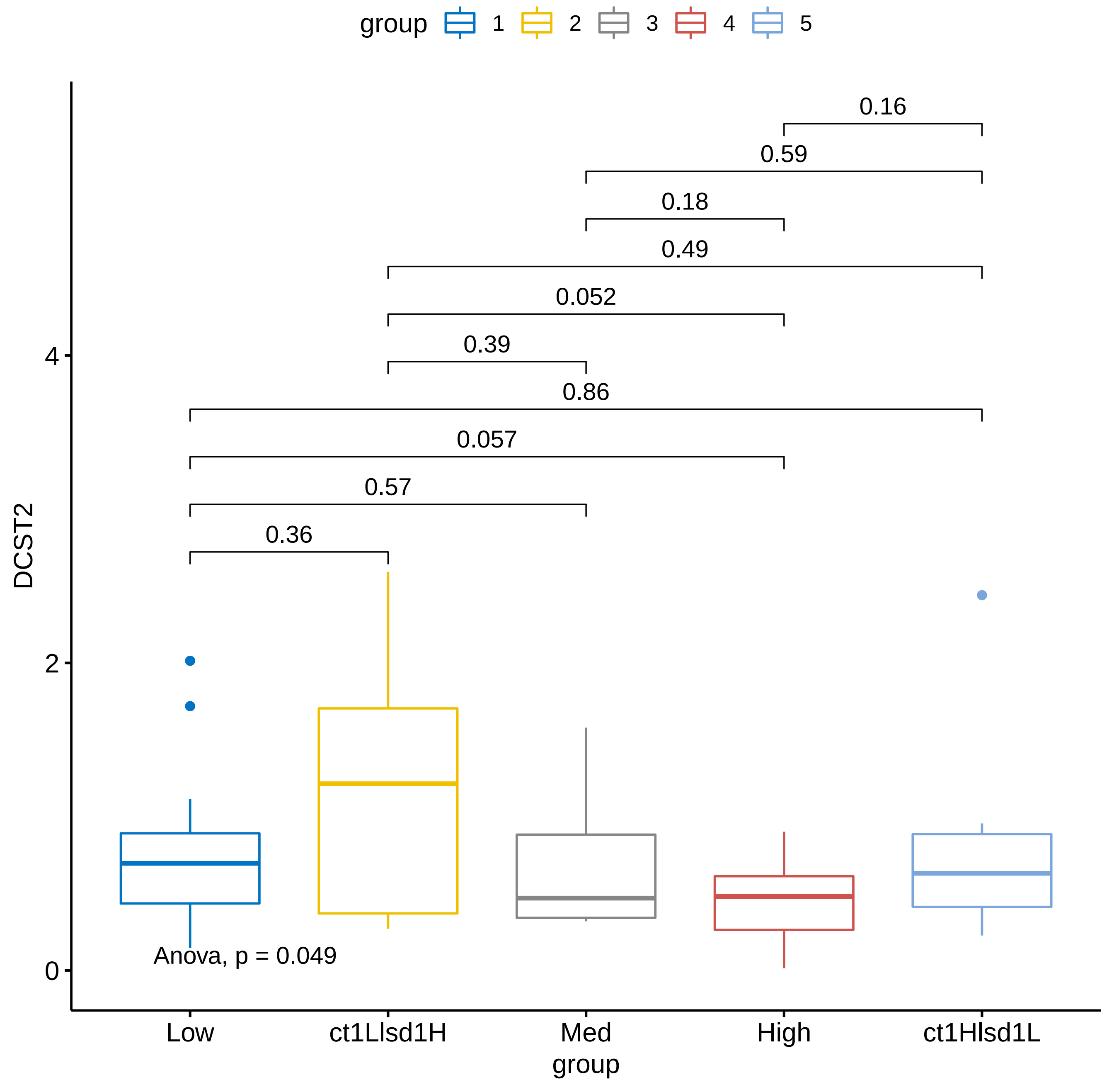

group
1
2
3
4
5
DCST2
0
2
4
0.16
0.59
0.18
0.49
0.052
0.39
0.86
0.057
0.57
0.36
Anova, p = 0.049
Low
ct1Llsd1H
Med
High
ct1Hlsd1L
group

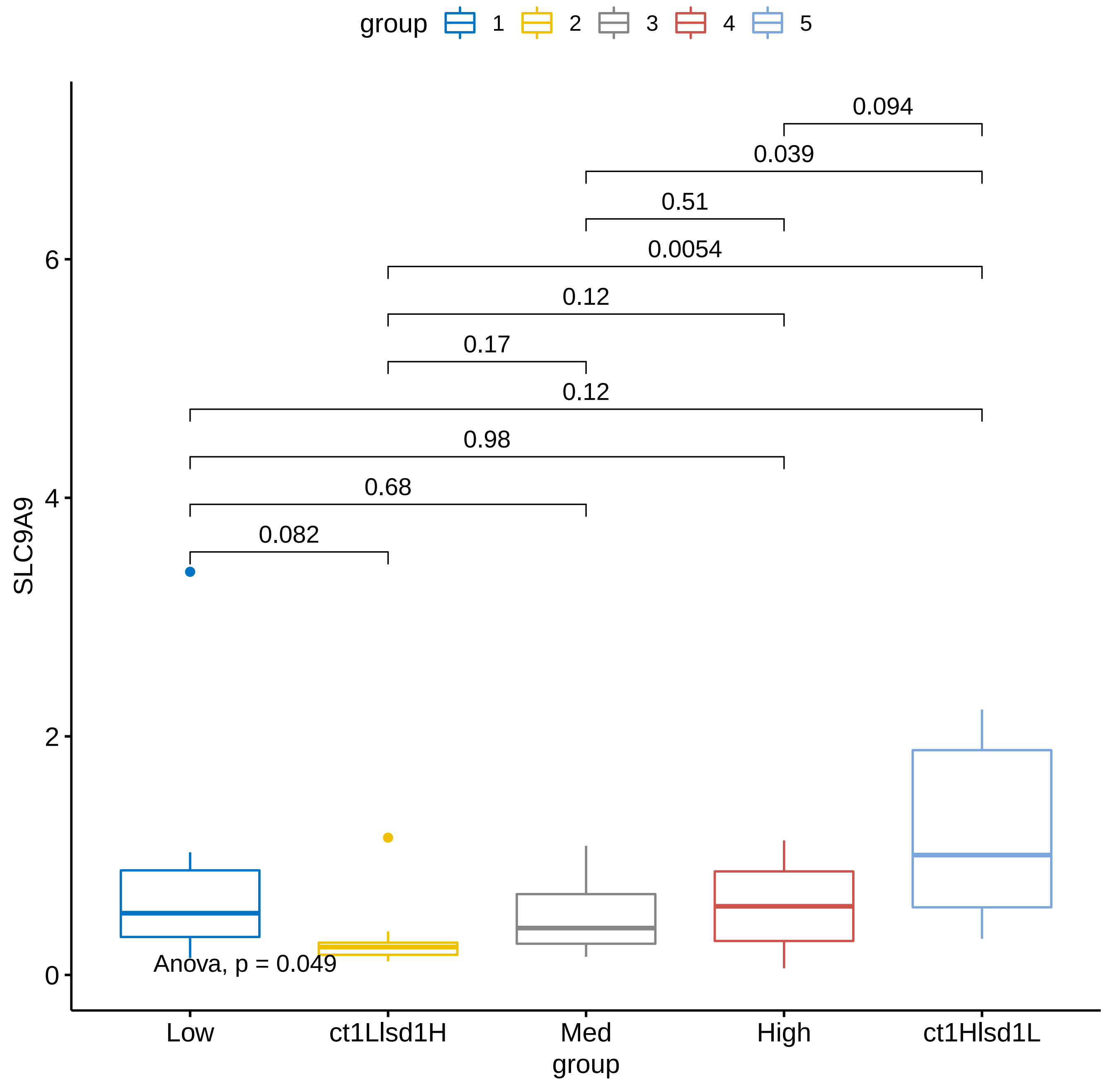

group 1 2 3 4 5
0.094
0.039
0.51
0.0054
0.12
0.17
0.12
0.98
0.68
0.082
Anova, p = 0.049
SLC9A9
0
2
4
6
Low
ct1Llsd1H
Med
High
ct1Hlsd1L
group

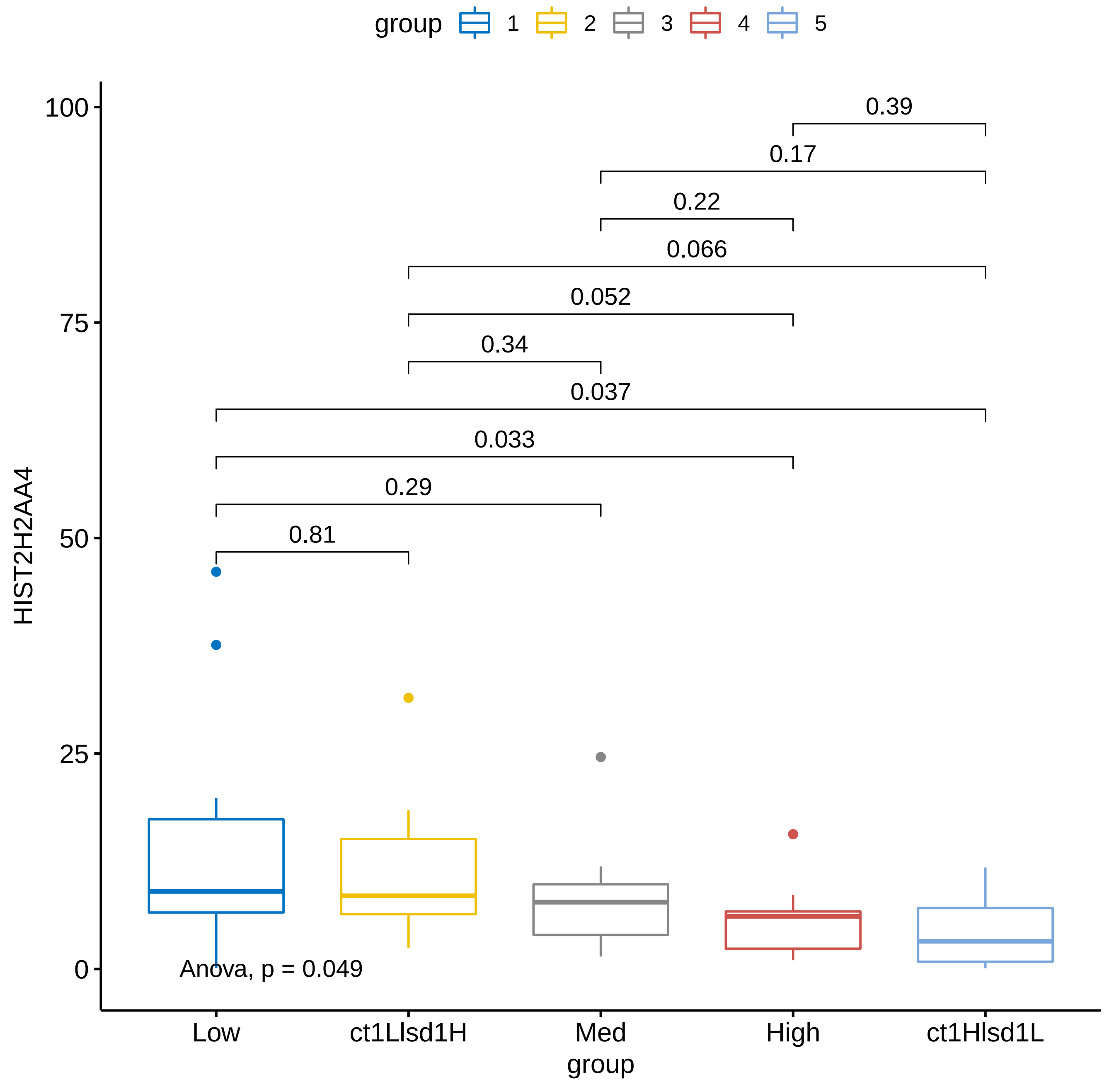

group
1
2
3
4
5
100
75
50
25
0
HIST2H2AA4
0.39
0.17
0.22
0.066
0.052
0.34
0.037
0.033
0.29
0.81
Anova, p = 0.049
Low
ct1Llsd1H
Med
High
ct1Hlsd1L
group

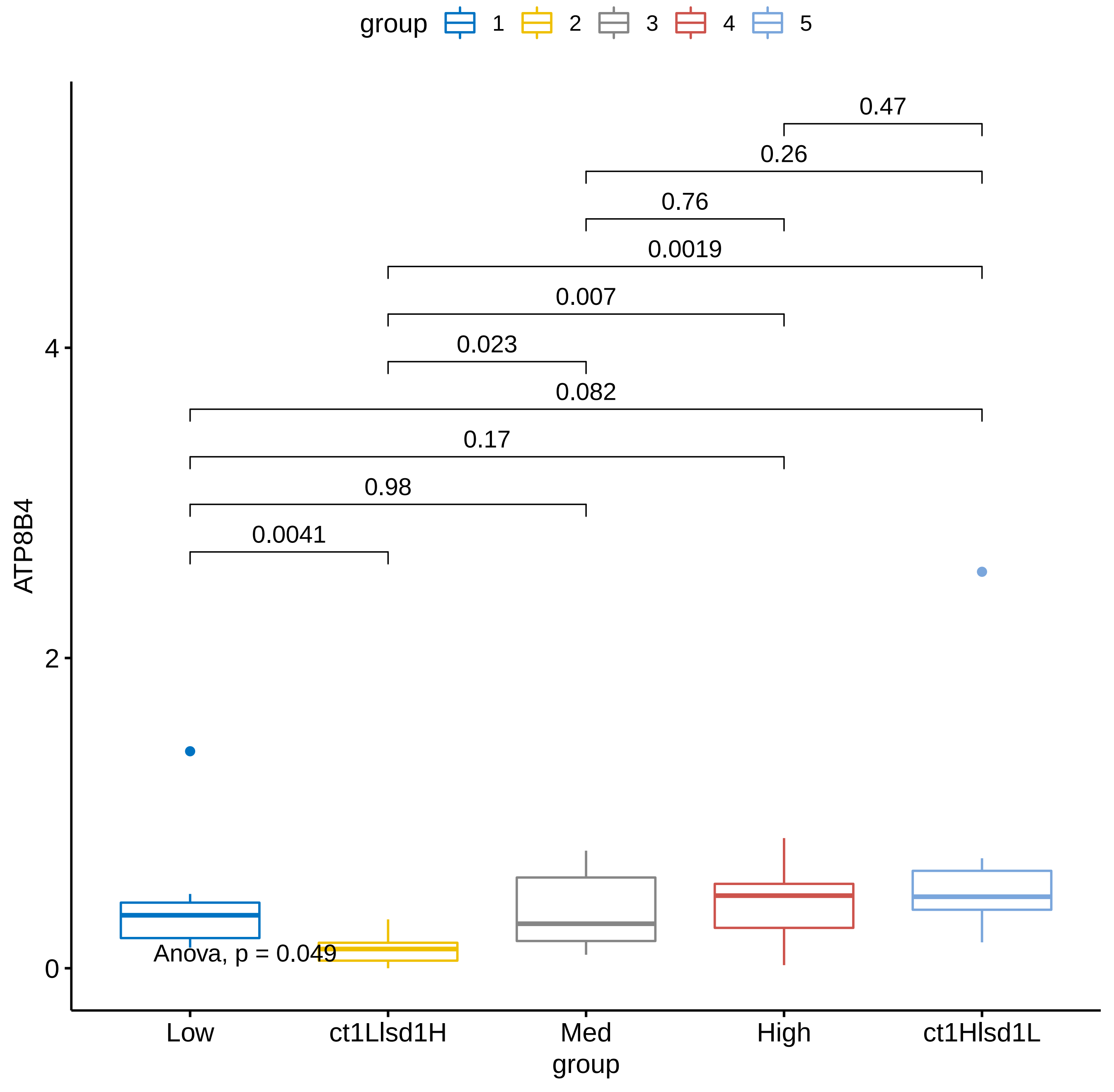
group
1
2
3
4
5
0.47
0.26
0.76
0.0019
0.007
0.023
0.082
0.17
0.98
0.0041
ATP8B4
4
2
0
Anova, p = 0.049
Low
ct1Llsd1H
Med
High
ct1Hlsd1L
group

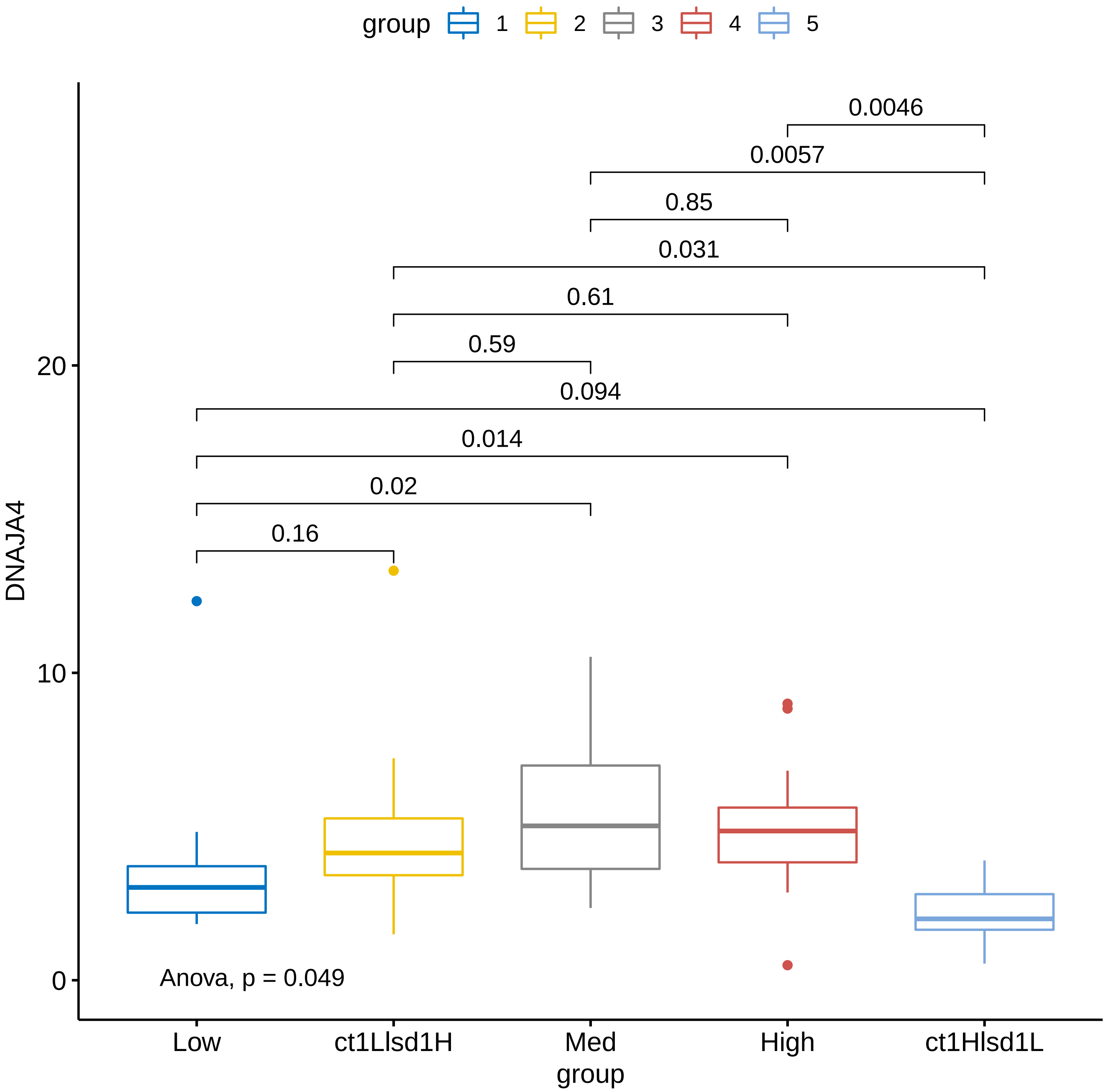

group 1 2 3 4 5
0.0046
0.0057
0.85
0.031
0.61
0.59
0.094
0.014
0.02
0.16
20
10
0
DNAJA4
Anova, p = 0.049
Low
ct1Llsd1H
Med
High
ct1Hlsd1L
group

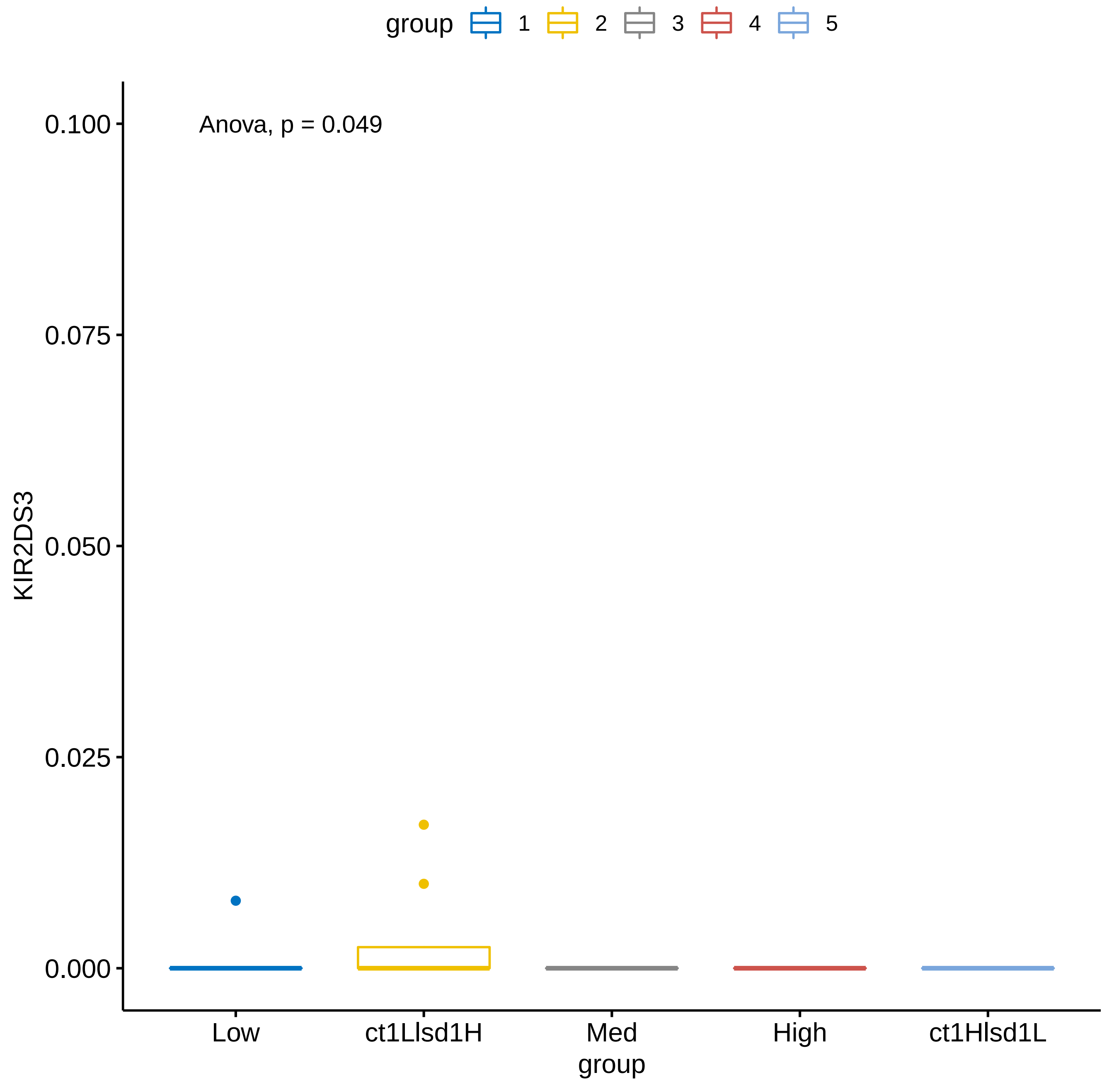

group 1 2 3 4 5
Anova, p = 0.049
KIR2DS3
0.100
0.075
0.050
0.025
0.000
Low
ct1Llsd1H
Med
High
ct1Hlsd1L
group

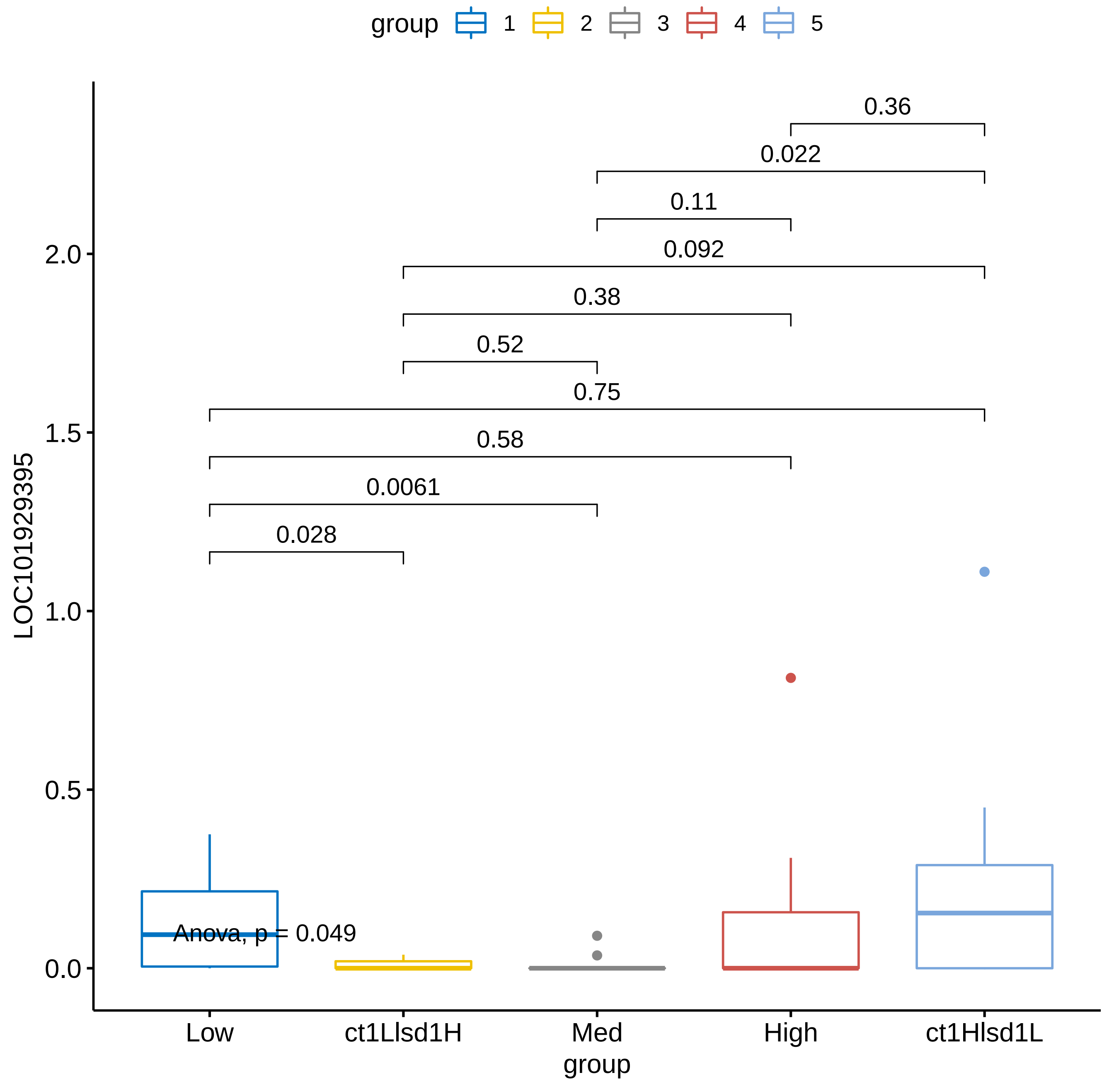

group
1
2
3
4
5
0.36
0.022
0.11
0.092
0.38
0.52
0.75
0.58
0.0061
0.028
Anova, p = 0.049
2.0
1.5
1.0
0.5
0.0
LOC101929395
Low
ct1Llsd1H
Med
High
ct1Hlsd1L
group

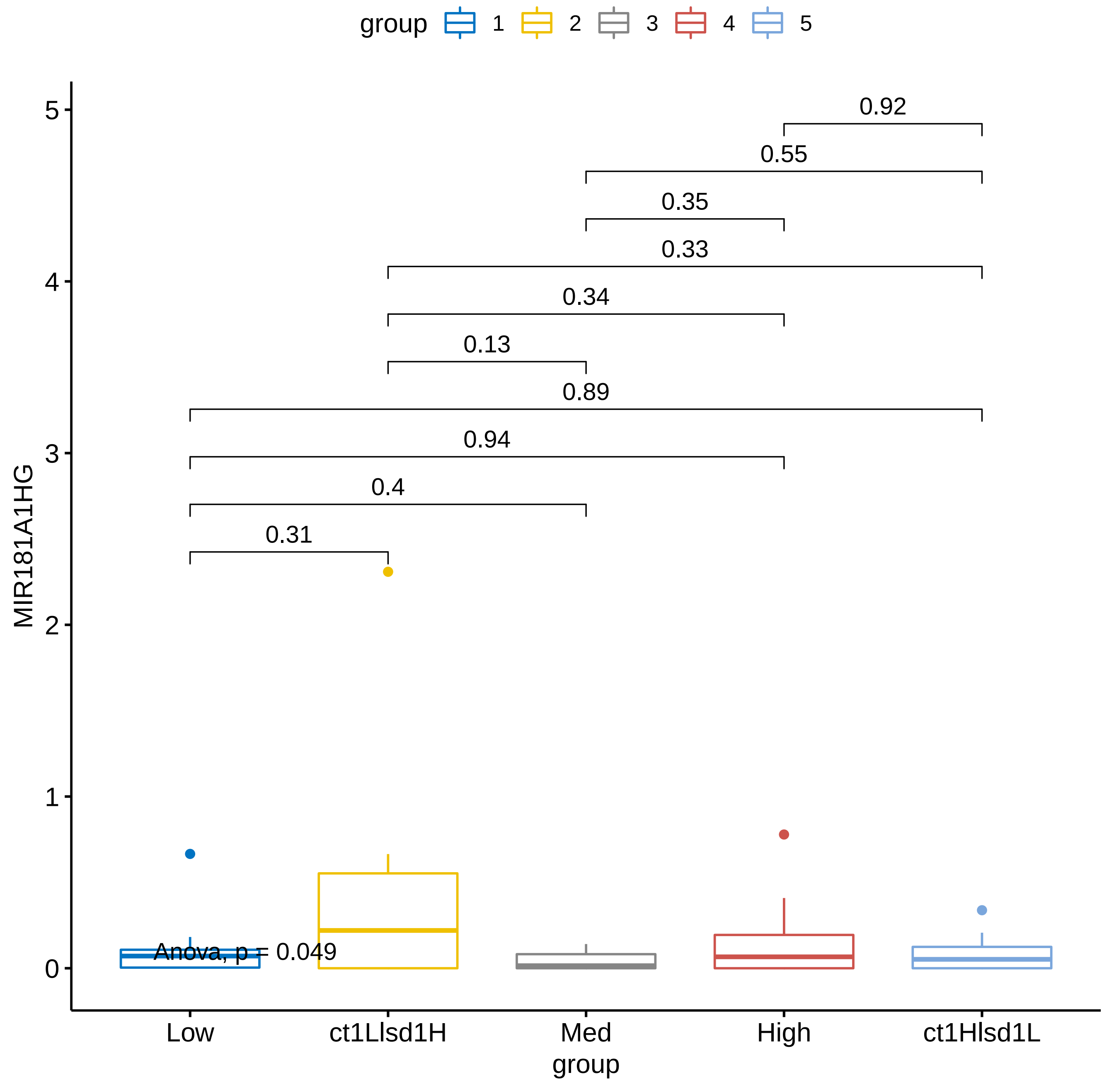
group
1
2
3
4
5
MIR181A1HG
0
1
2
3
4
5
0.92
0.55
0.35
0.33
0.34
0.13
0.89
0.94
0.4
0.31
Anova, p = 0.049
Low
ct1Llsd1H
Med
High
ct1Hlsd1L
group

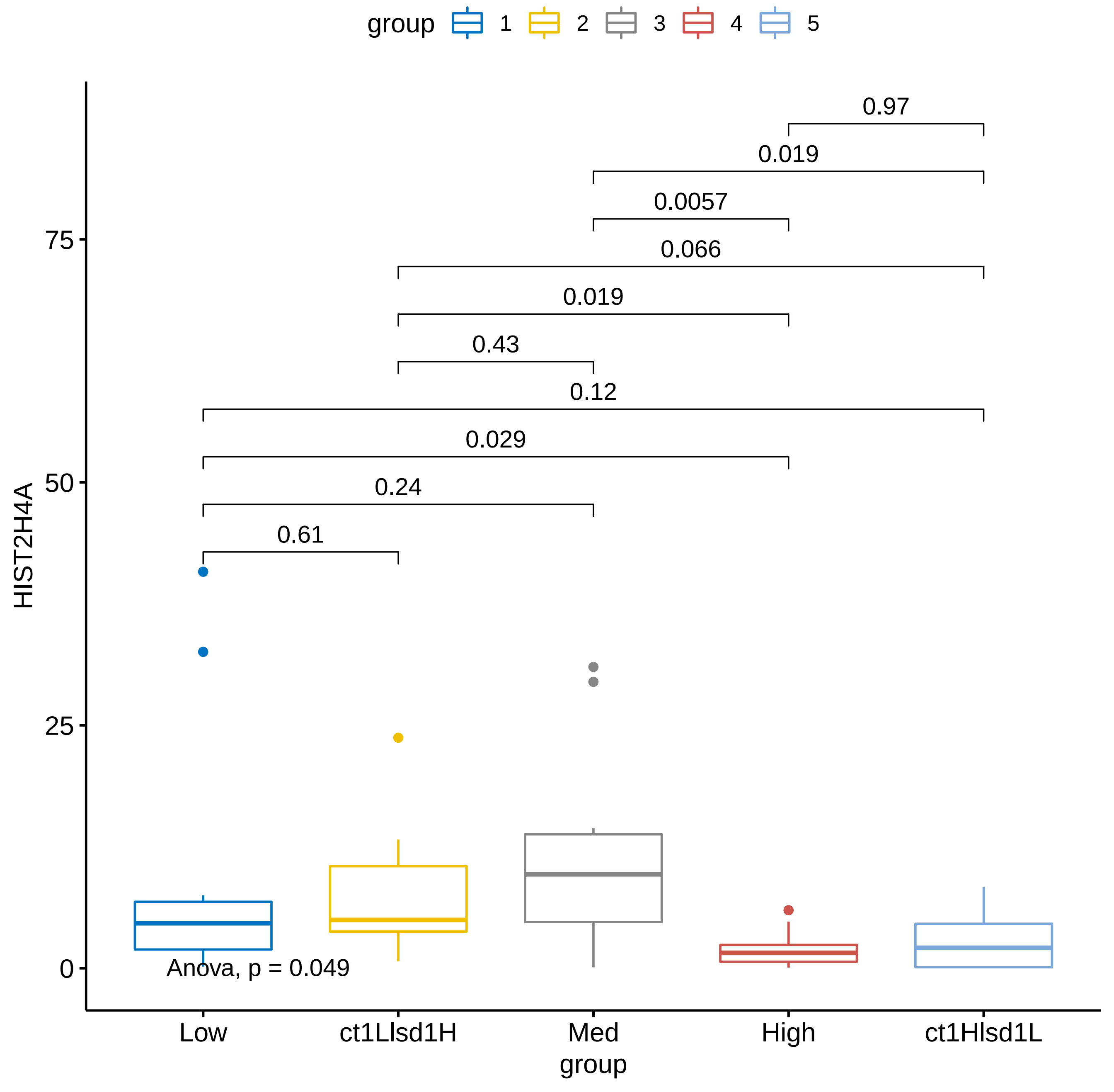

group 1 2 3 4 5
0.97
0.019
0.0057
0.066
0.019
0.43
0.12
0.029
0.24
0.61
75
50
25
0
HIST2H4A
Anova, p = 0.049
Low
ct1Llsd1H
Med
High
ct1Hlsd1L
group

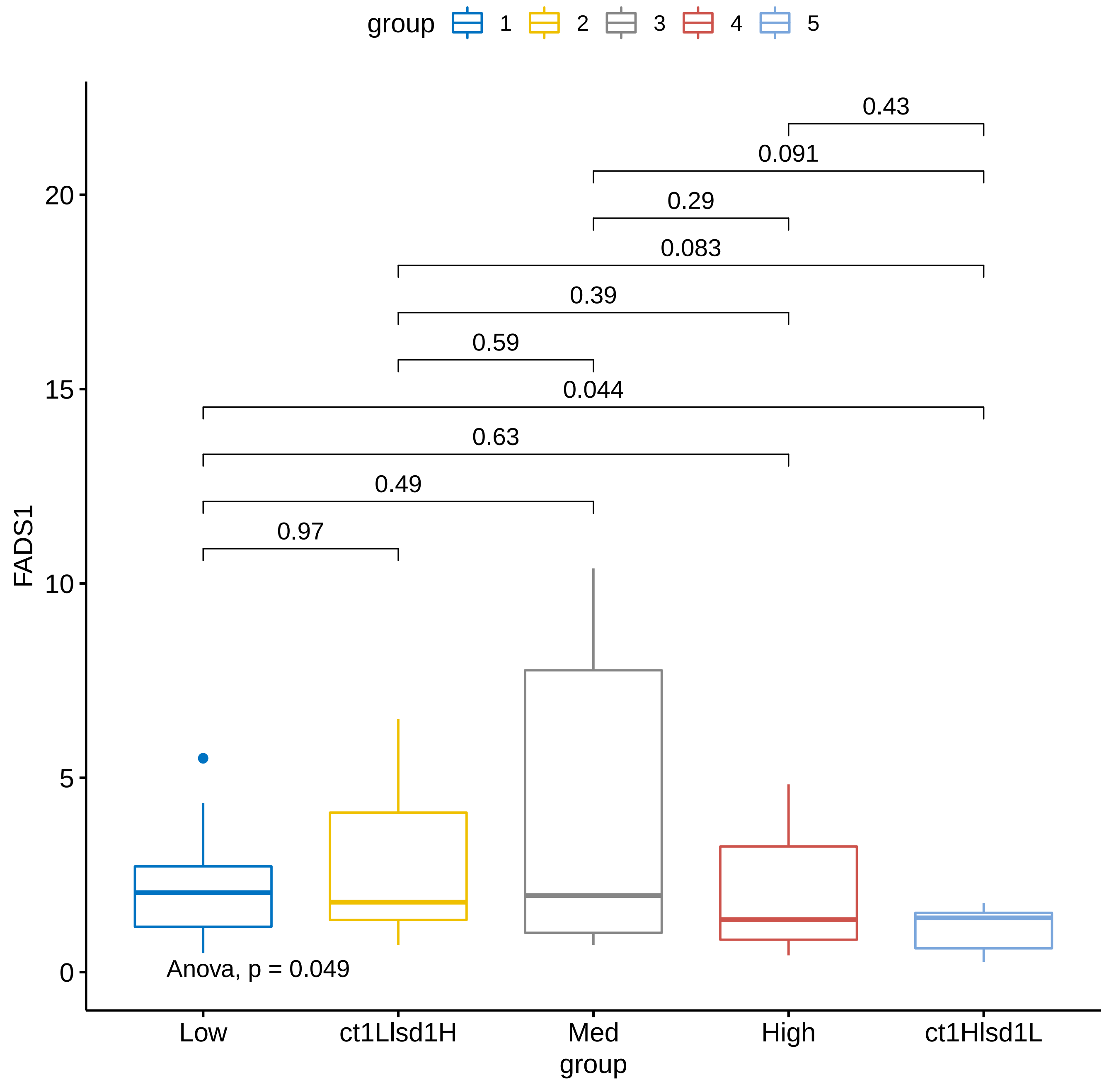
group
1
2
3
4
5
0.43
0.091
0.29
0.083
0.39
0.59
0.044
0.63
0.49
0.97
Anova, p = 0.049
FADS1
0
5
10
15
20
Low
ct1Llsd1H
Med
High
ct1Hlsd1L
group

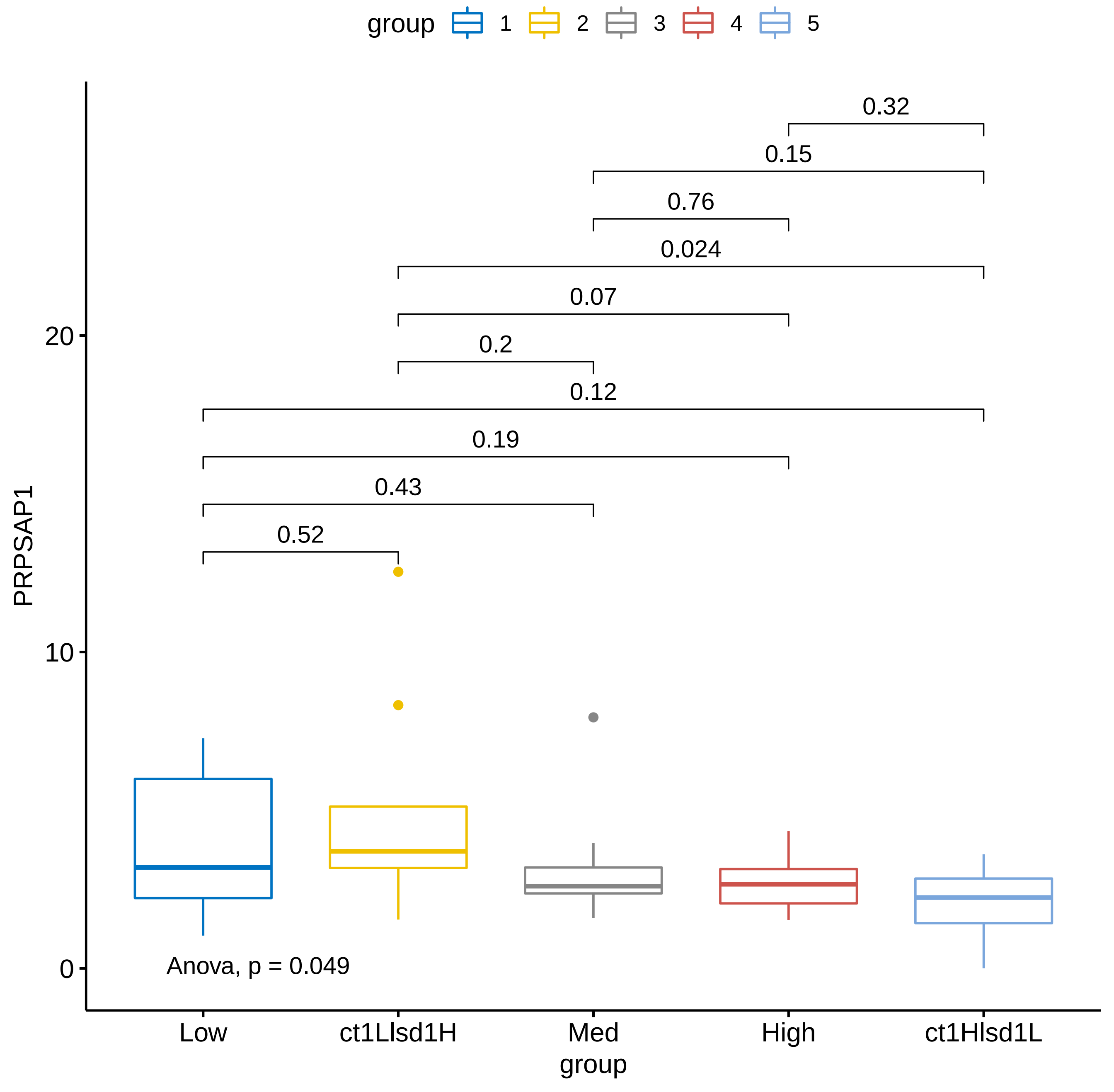
group
1
2
3
4
5
0.32
0.15
0.76
0.024
0.07
0.2
0.12
0.19
0.43
0.52
20
10
0
PRPSAP1
Anova, p = 0.049
Low
ct1Llsd1H
Med
High
ct1Hlsd1L
group

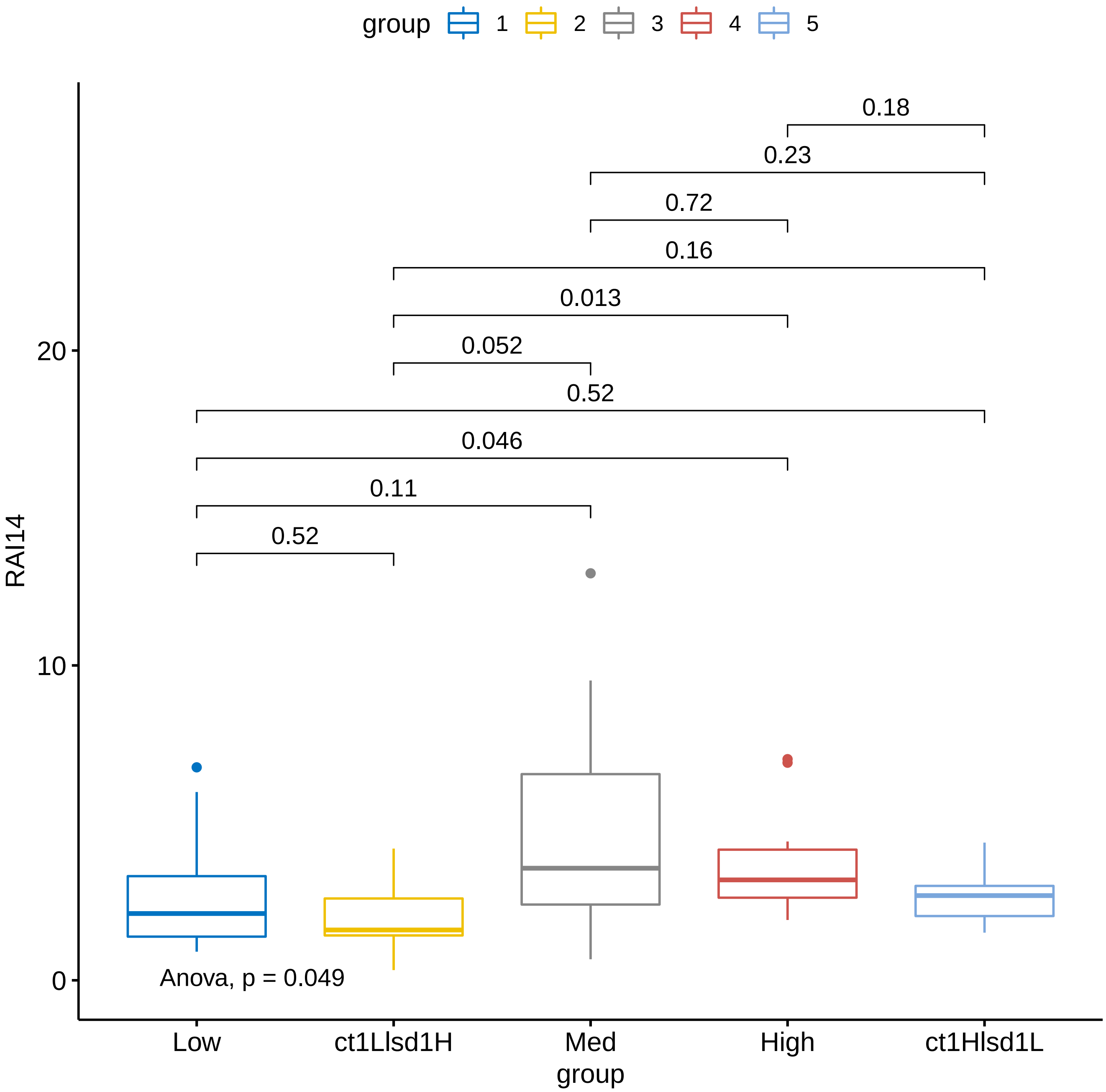
group
1
2
3
4
5
0.18
0.23
0.72
0.16
0.013
0.052
0.52
0.046
0.11
0.52
20
10
0
RAI14
Anova, p = 0.049
Low
ct1Llsd1H
Med
High
ct1Hlsd1L
group

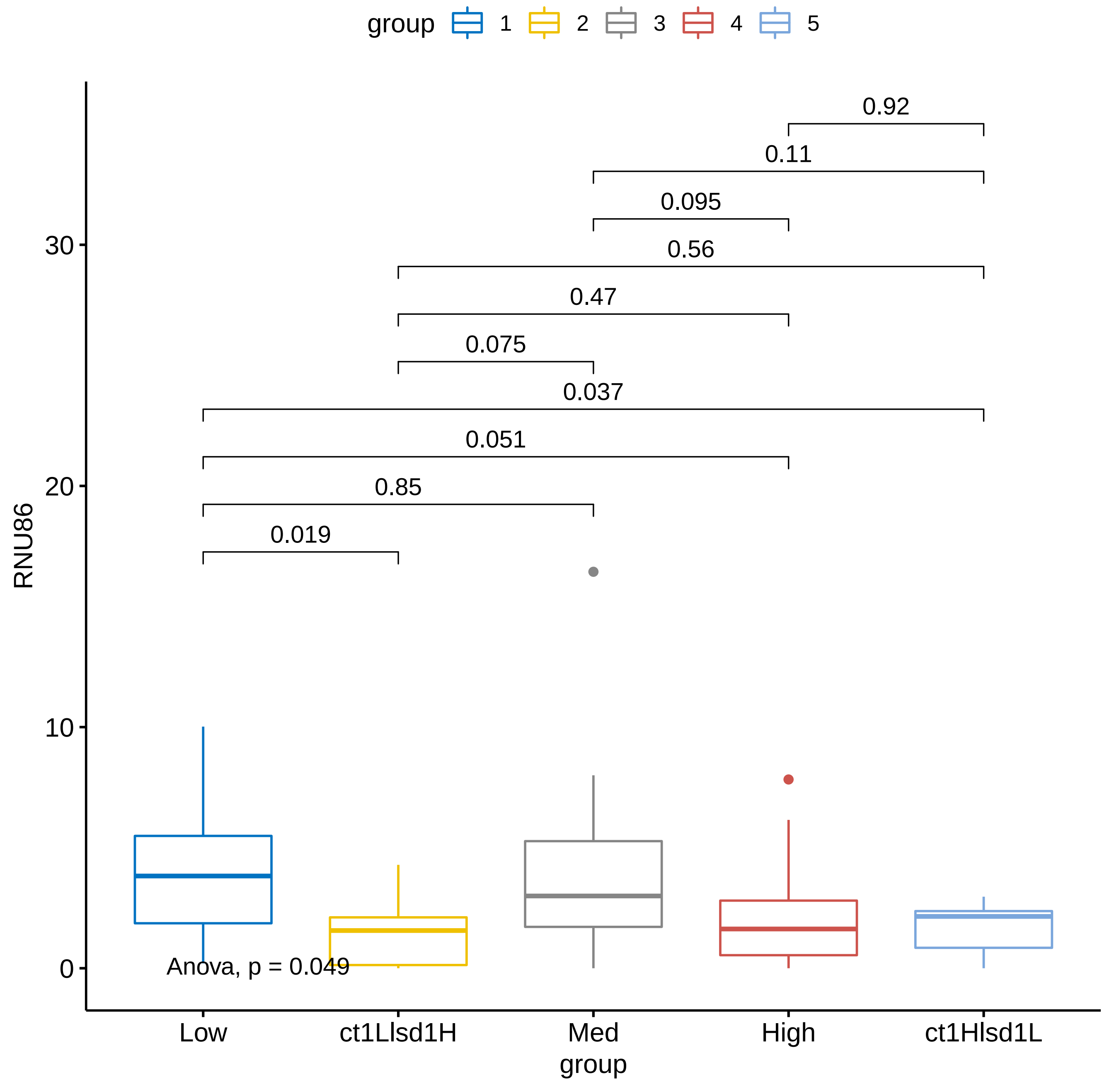
group
1
2
3
4
5
0.92
0.11
0.095
0.56
0.47
0.075
0.037
0.051
0.85
0.019
30
20
10
0
RNU86
Anova, p = 0.049
Low
ct1Llsd1H
Med
High
ct1Hlsd1L
group

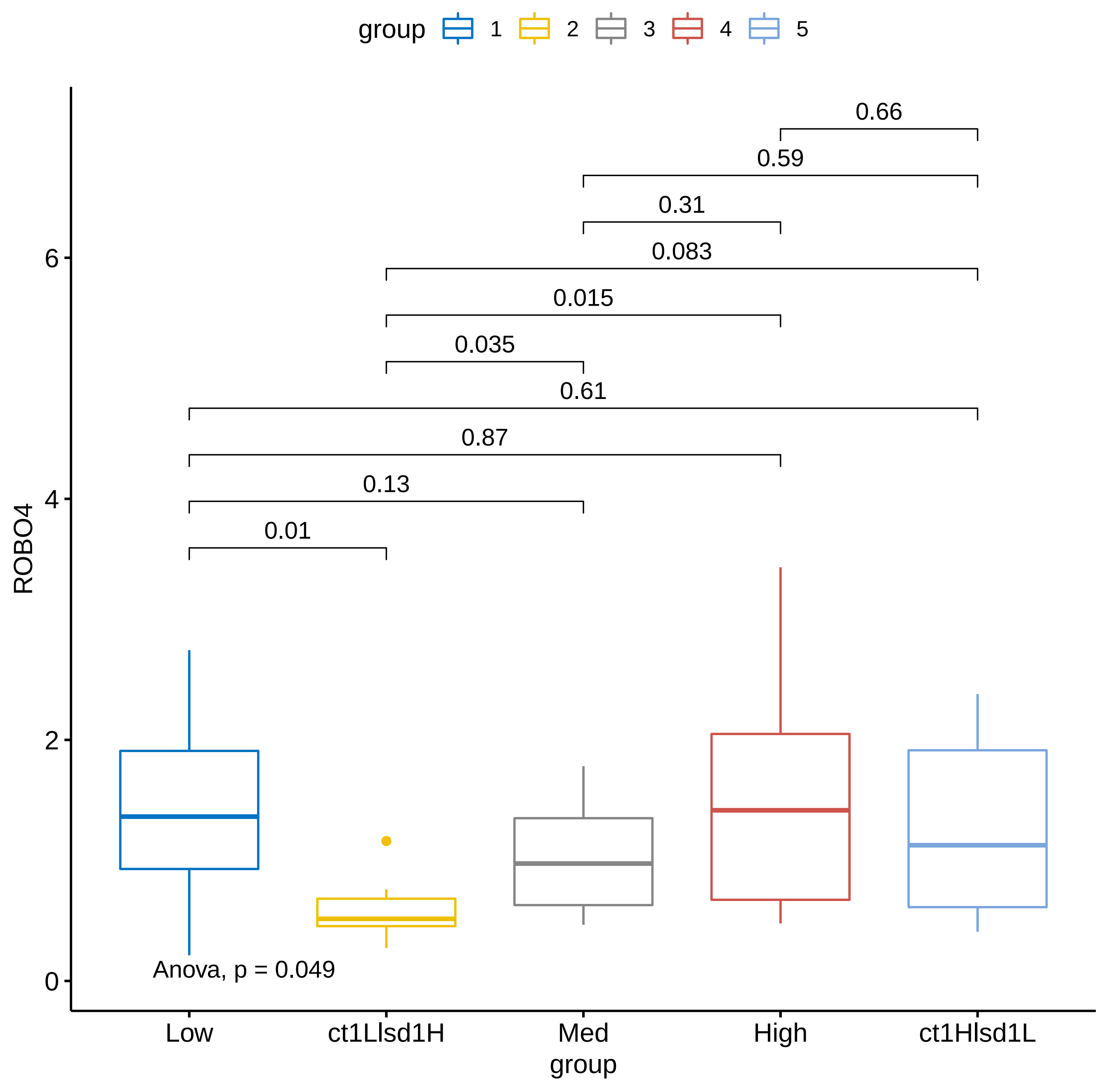

group 1 2 3 4 5
ROBO4
0.66
0.59
0.31
0.083
0.015
0.035
0.61
0.87
0.13
0.01
Anova, p = 0.049
0
2
4
6
Low
ct1Llsd1H
Med
High
ct1Hlsd1L
group

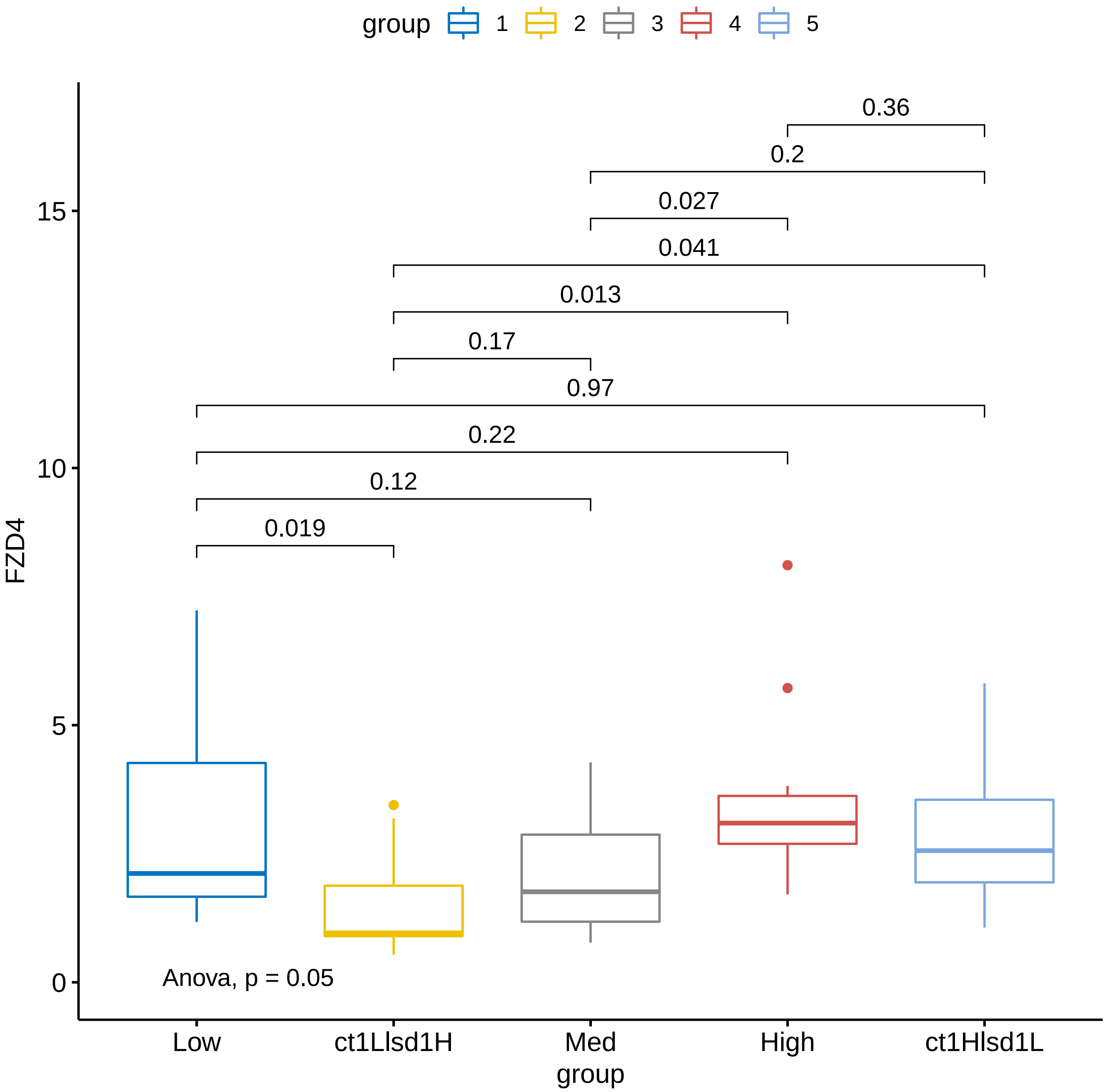
group
1
2
3
4
5
0.36
0.2
0.027
0.041
0.013
0.17
0.97
0.22
0.12
0.019
15
10
5
0
FZD4
Anova, p = 0.05
Low
ct1Llsd1H
Med
High
ct1Hlsd1L
group

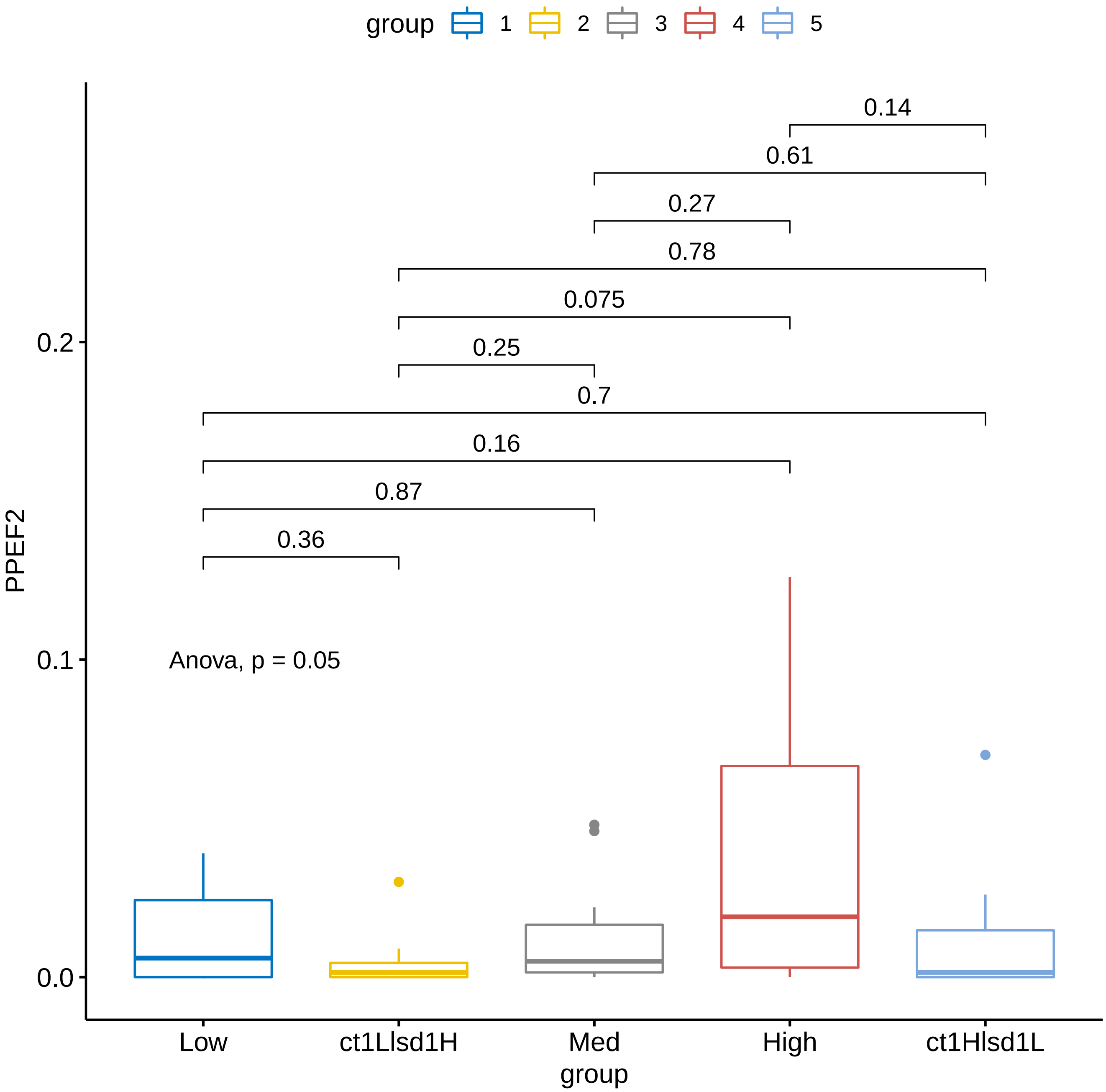

group 1 2 3 4 5
0.14
0.61
0.27
0.78
0.075
0.25
0.7
0.16
0.87
0.36
Anova, p = 0.05
PPEF2
0.2
0.1
0.0
Low
ct1Llsd1H
Med
High
ct1Hlsd1L
group

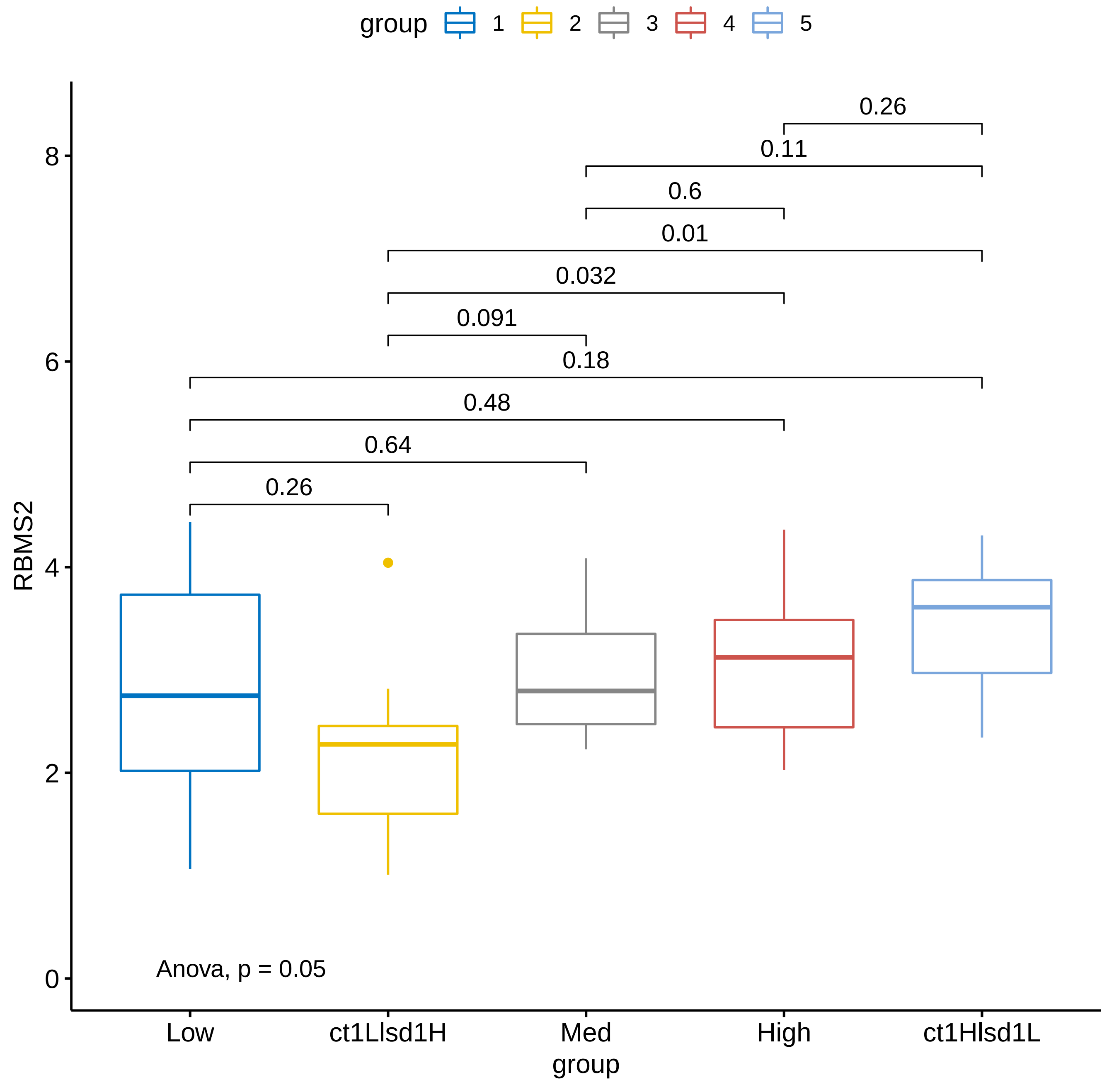
group
1
2
3
4
5
0.26
0.11
0.6
0.01
0.032
0.091
0.18
0.48
0.64
0.26
RBMS2
8
6
4
2
0
Anova, p = 0.05
Low
ct1Llsd1H
Med
High
ct1Hlsd1L
group

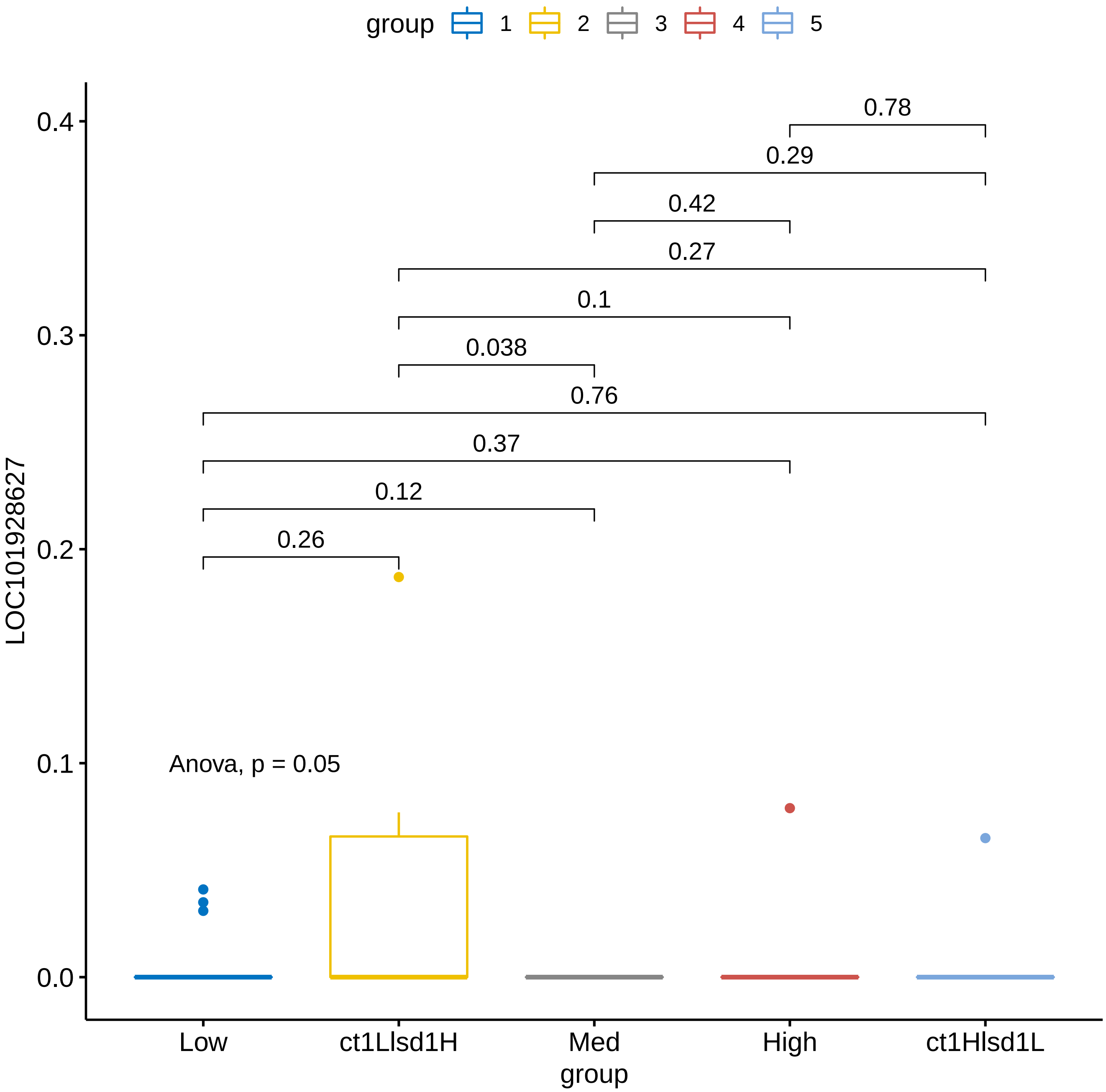
group
1
2
3
4
5
0.78
0.29
0.42
0.27
0.1
0.038
0.76
0.37
0.12
0.26
Anova, p = 0.05
LOC101928627
0.0
0.1
0.2
0.3
0.4
Low
ct1Llsd1H
Med
High
ct1Hlsd1L
group

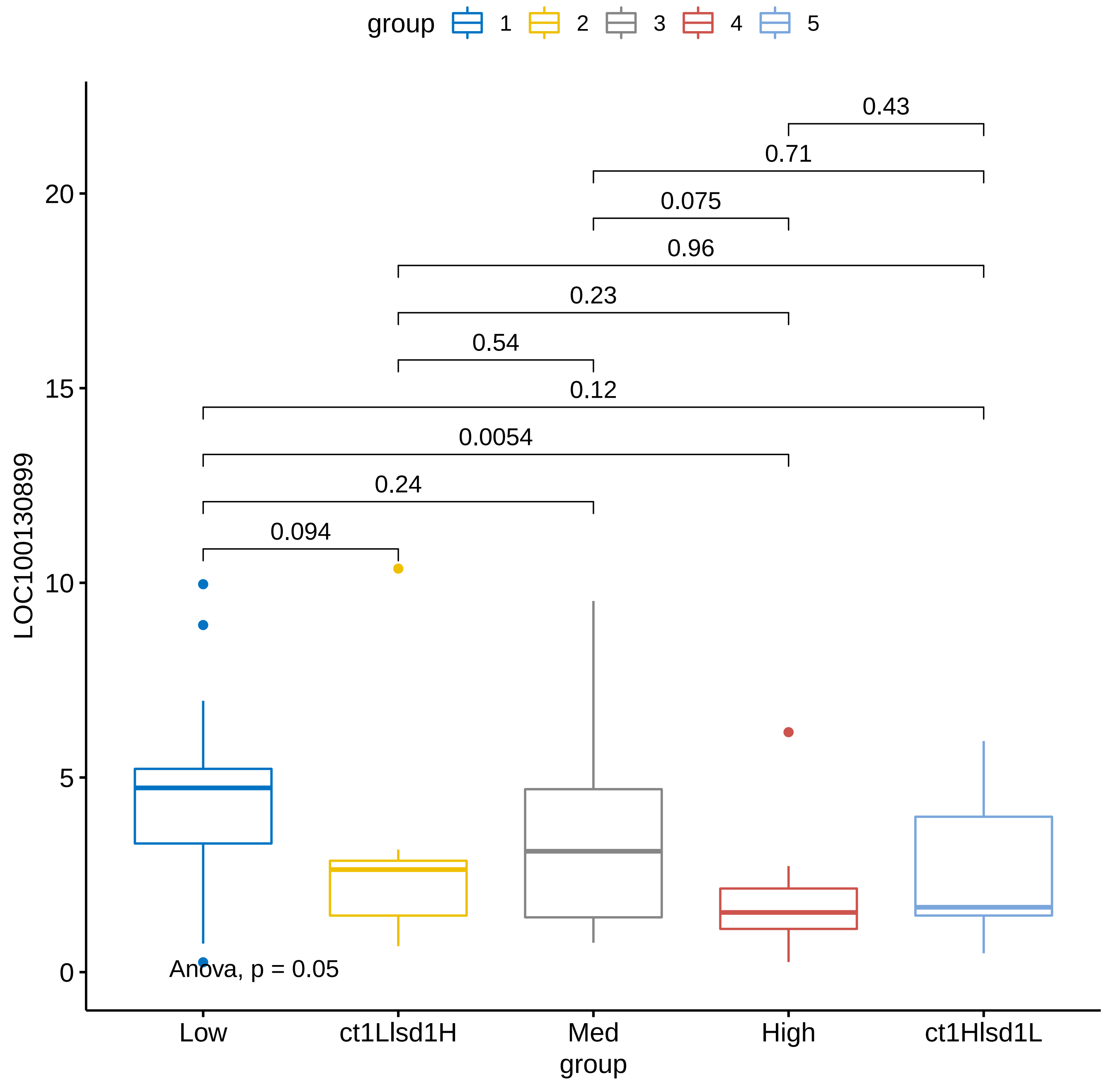
group
1
2
3
4
5
0.43
0.71
0.075
0.96
0.23
0.54
0.12
0.0054
0.24
0.094
20
15
10
5
0
LOC100130899
Anova, p = 0.05
Low
ct1Llsd1H
Med
High
ct1Hlsd1L
group

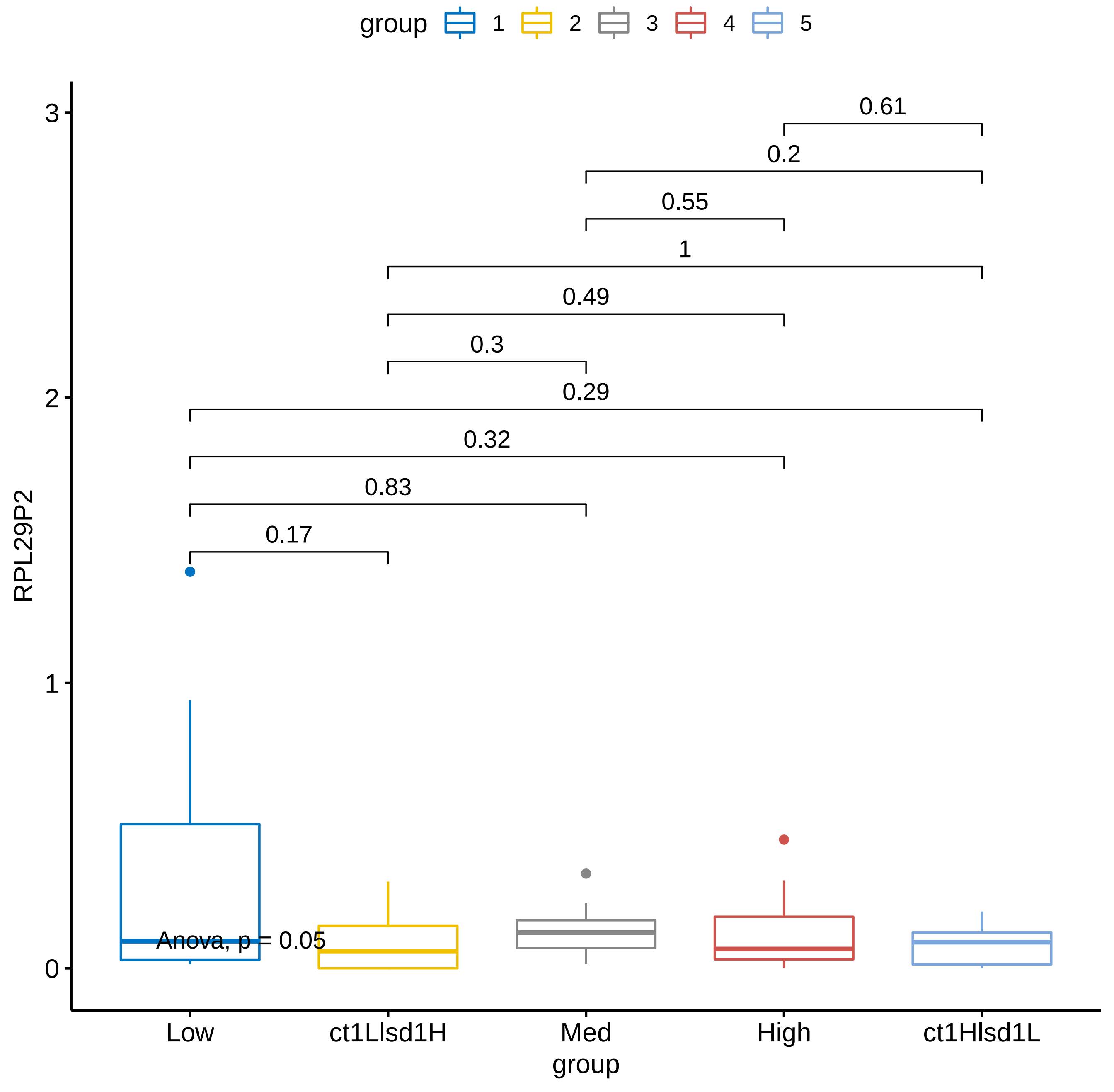

group 1 2 3 4 5
0.61
0.2
0.55
1
0.49
0.3
0.29
0.32
0.83
0.17
Anova, p = 0.05
RPL29P2
3
2
1
0
Low
ct1Llsd1H
Med
High
ct1Hlsd1L
group

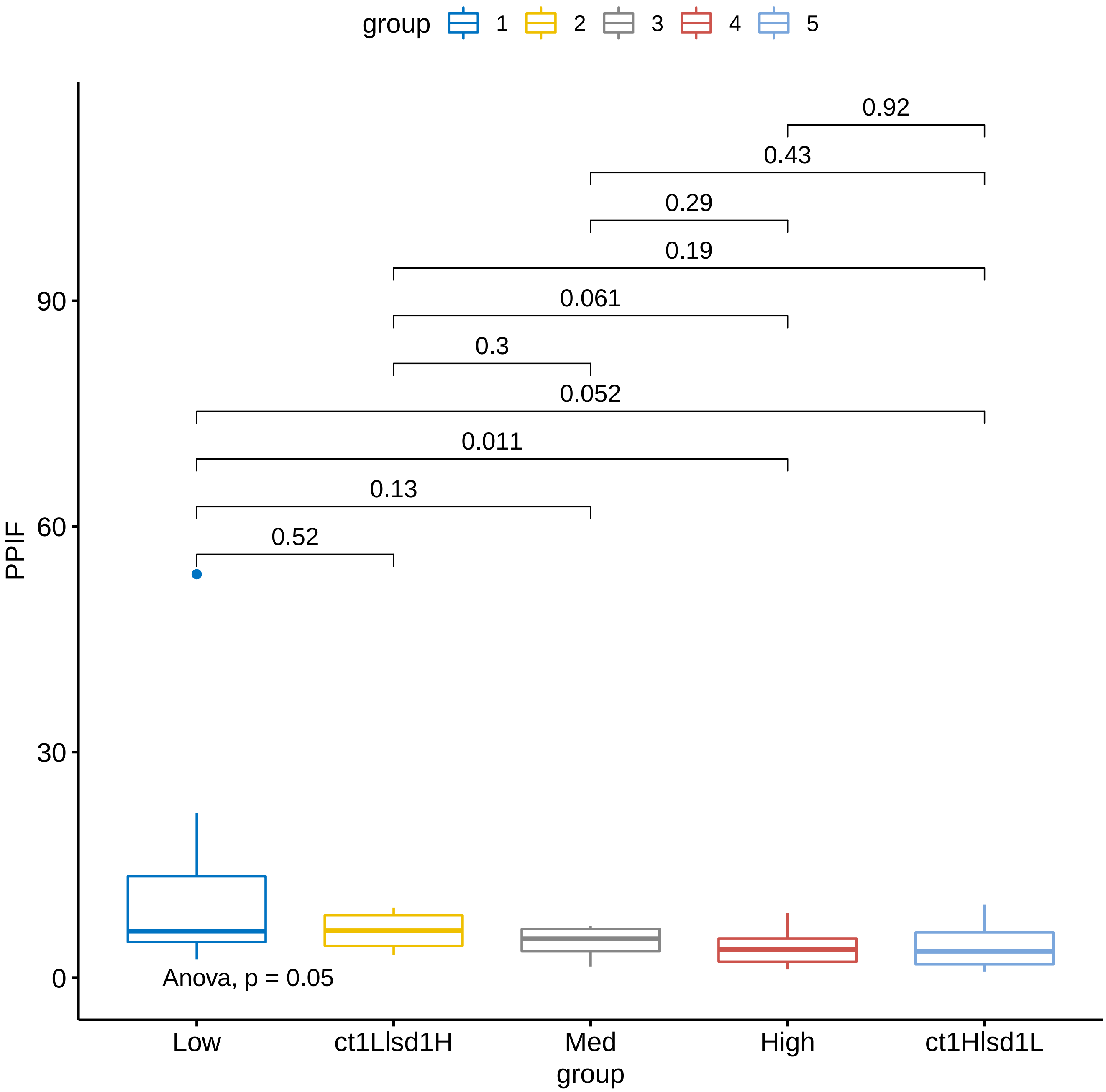

group 1 2 3 4 5
0.92
0.43
0.29
0.19
0.061
0.3
0.052
0.011
0.13
0.52
90
60
30
0
PPIF
Anova, p = 0.05
Low
ct1Llsd1H
Med
High
ct1Hlsd1L
group

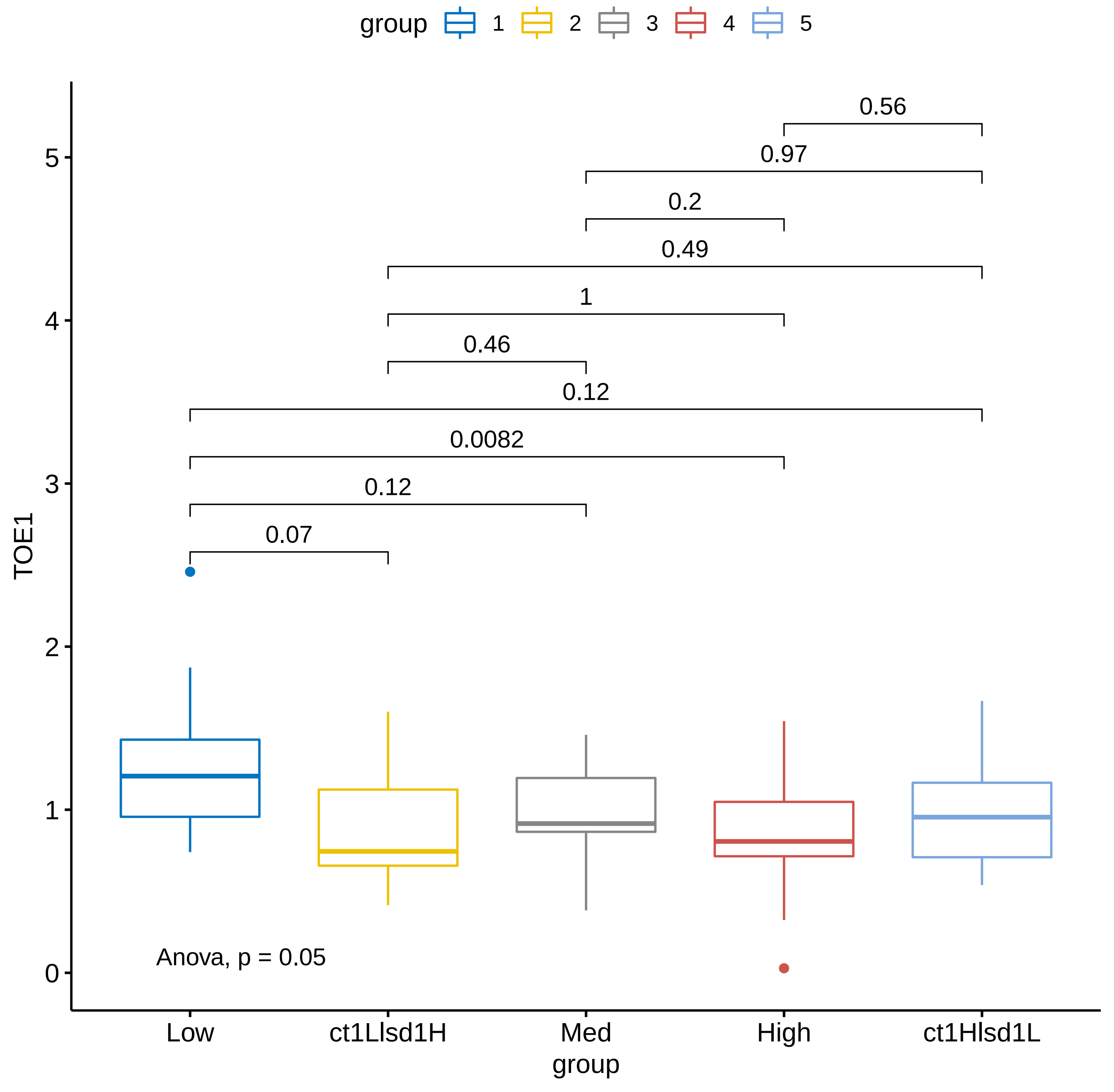
group 1 2 3 4 5
0.56
0.97
0.2
0.49
1
0.46
0.12
0.0082
0.12
0.07
TOE1
5
4
3
2
1
0
Anova, p = 0.05
Low
ct1Llsd1H
Med
High
ct1Hlsd1L
group

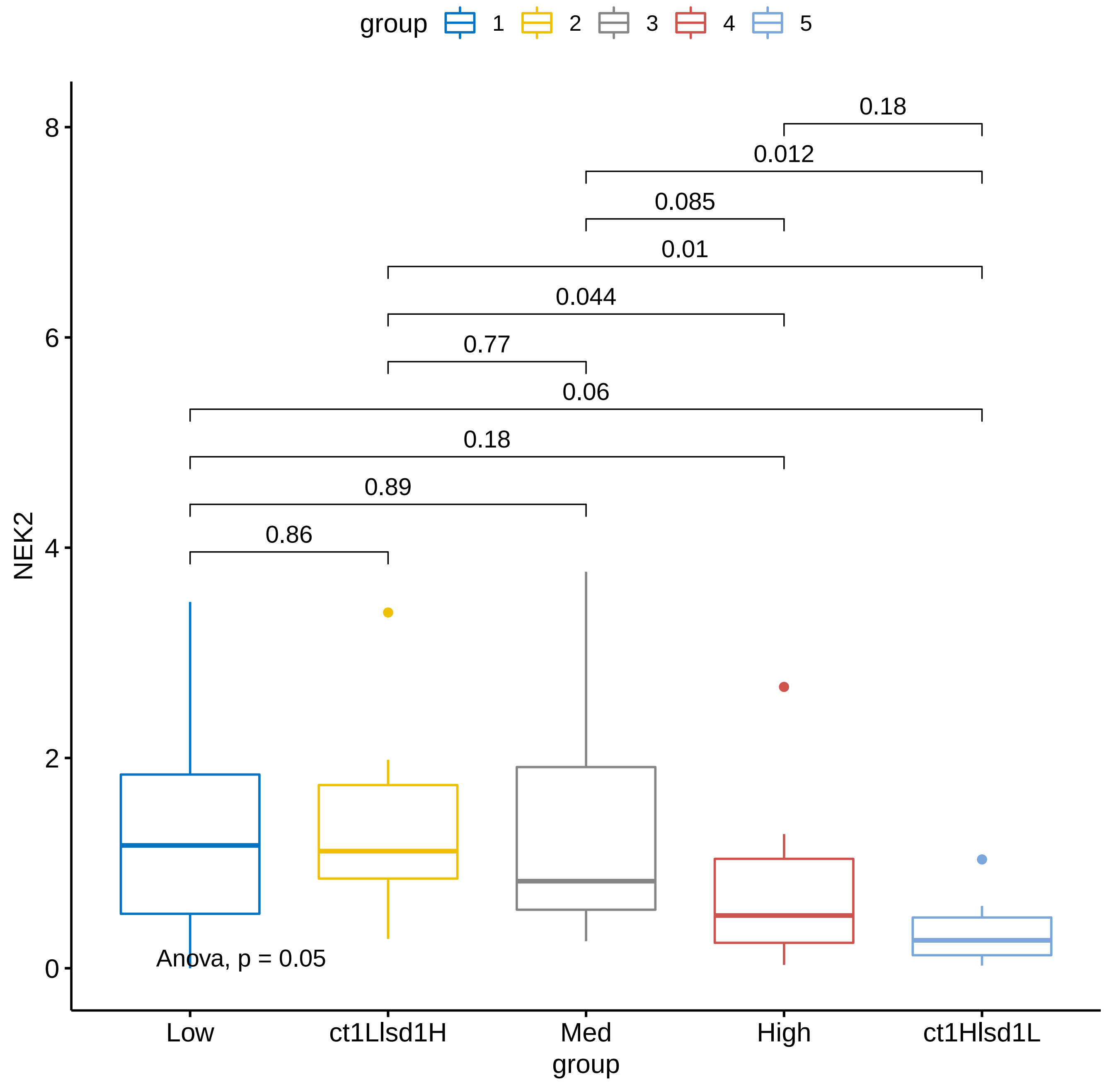

group
1
2
3
4
5
NEK2
8
6
4
2
0
0.18
0.012
0.085
0.01
0.044
0.77
0.06
0.18
0.89
0.86
Anova, p = 0.05
Low
ct1Llsd1H
Med
High
ct1Hlsd1L
group

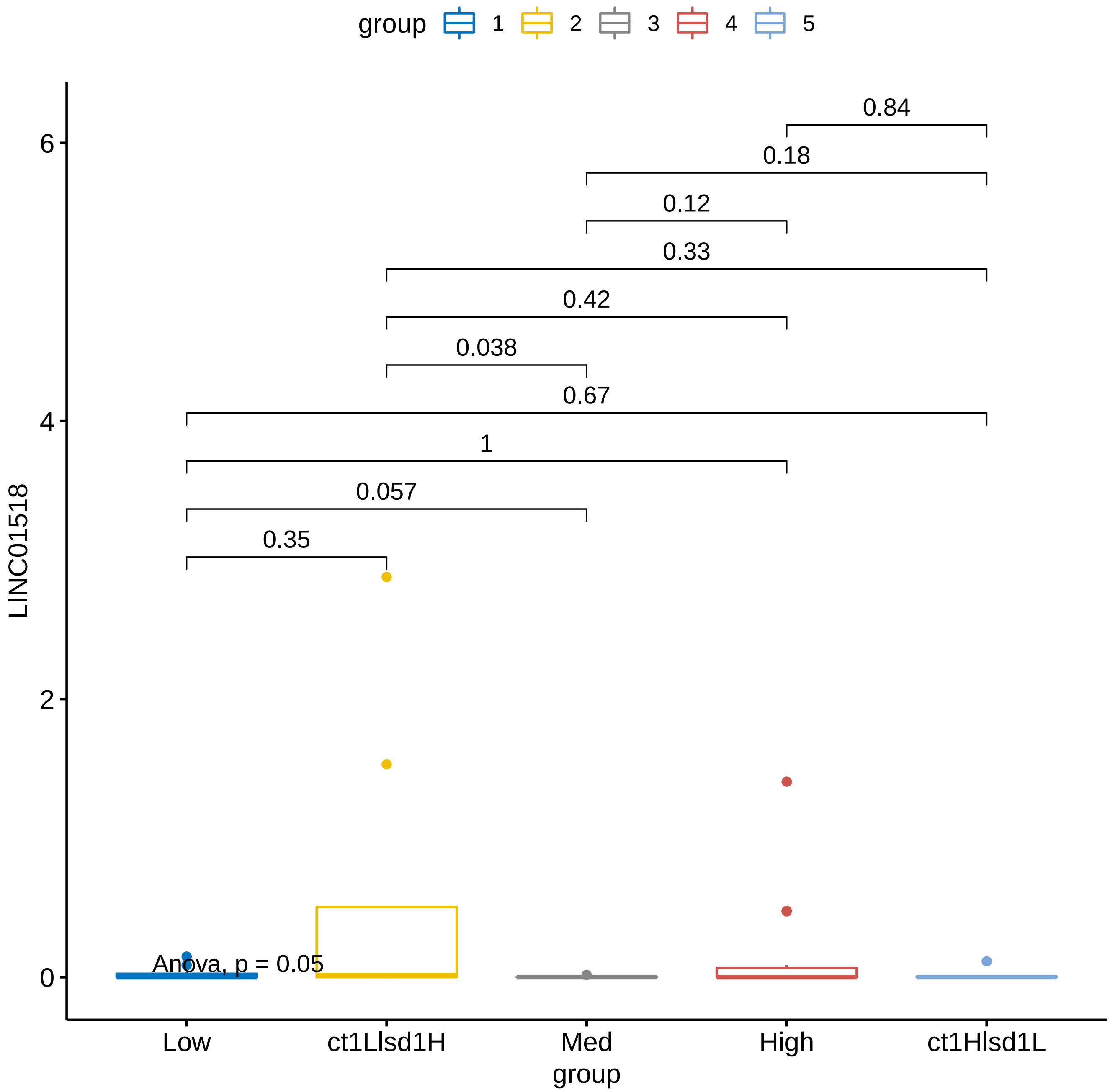
group
1
2
3
4
5
0.84
0.18
0.12
0.33
0.42
0.038
0.67
1
0.057
0.35
Anova, p = 0.05
LINC01518
6
4
2
0
Low
ct1Llsd1H
Med
High
ct1Hlsd1L
group

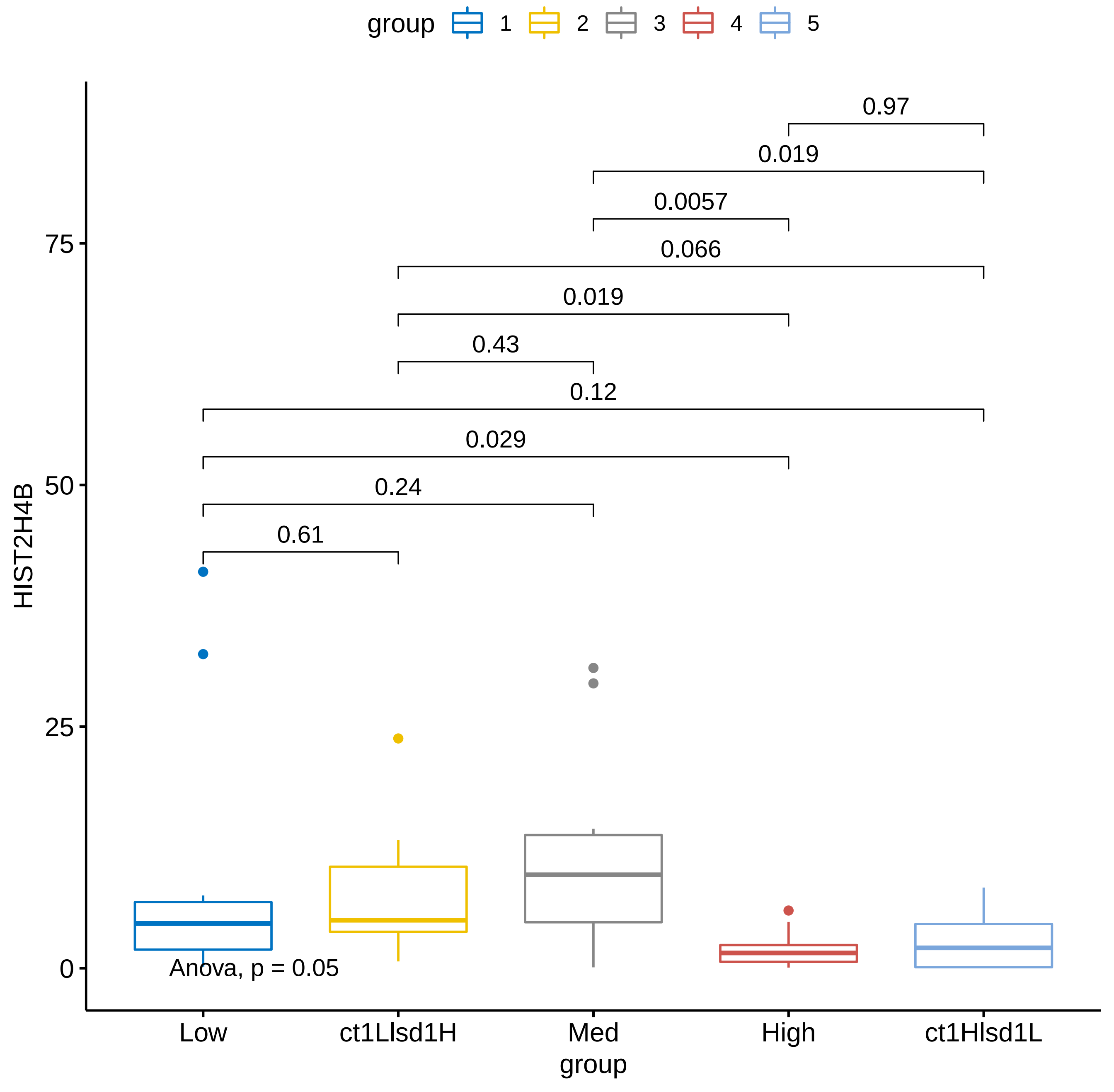

group
1
2
3
4
5
0.97
0.019
0.0057
0.066
0.019
0.43
0.12
0.029
0.24
0.61
75
50
25
0
HIST2H4B
Anova, p = 0.05
Low
ct1Llsd1H
Med
High
ct1Hlsd1L
group

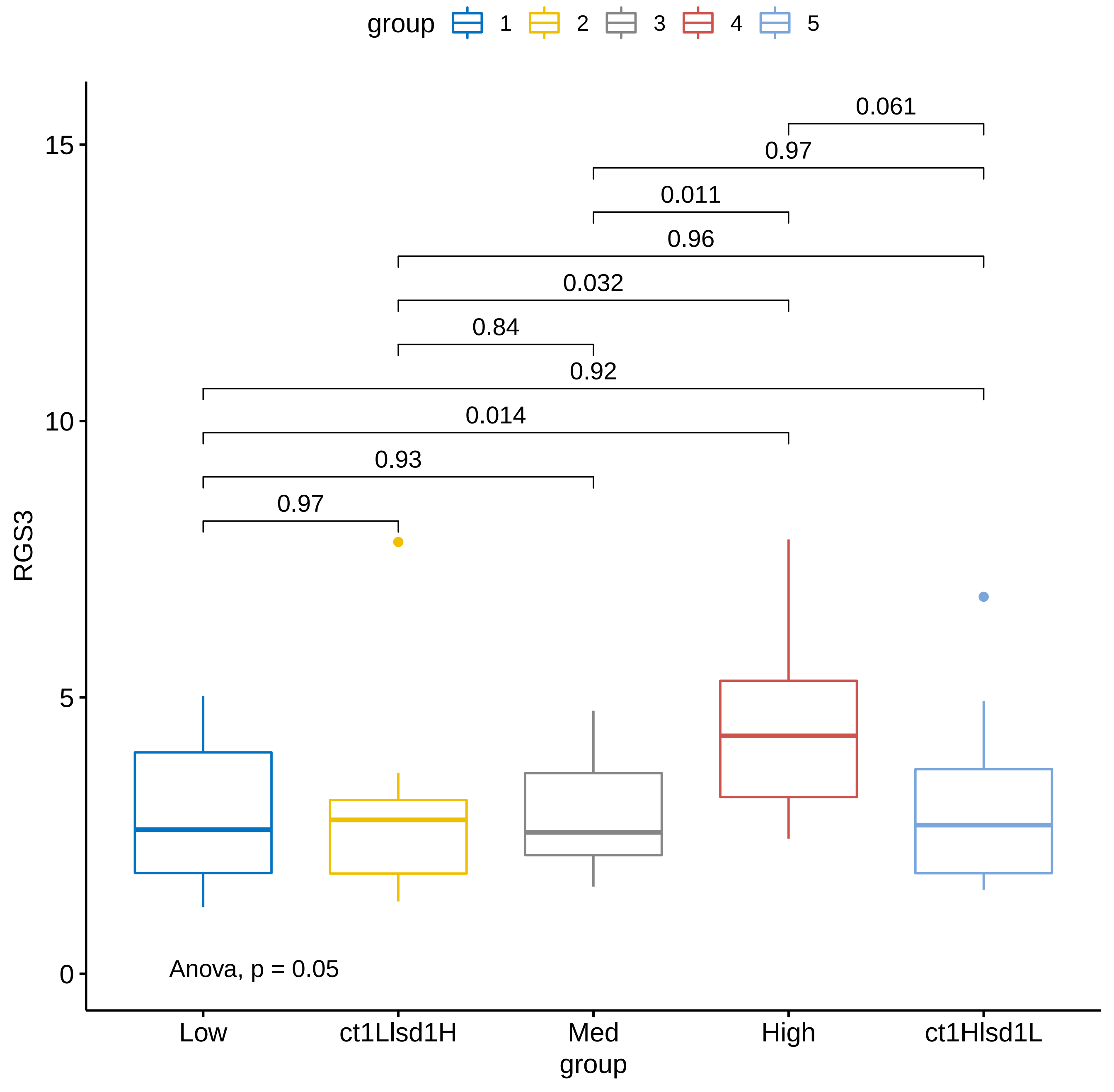

group 1 2 3 4 5
0.061
0.97
0.011
0.96
0.032
0.84
0.92
0.014
0.93
0.97
RGS3
15
10
5
0
Anova, p = 0.05
Low
ct1Llsd1H
Med
High
ct1Hlsd1L
group

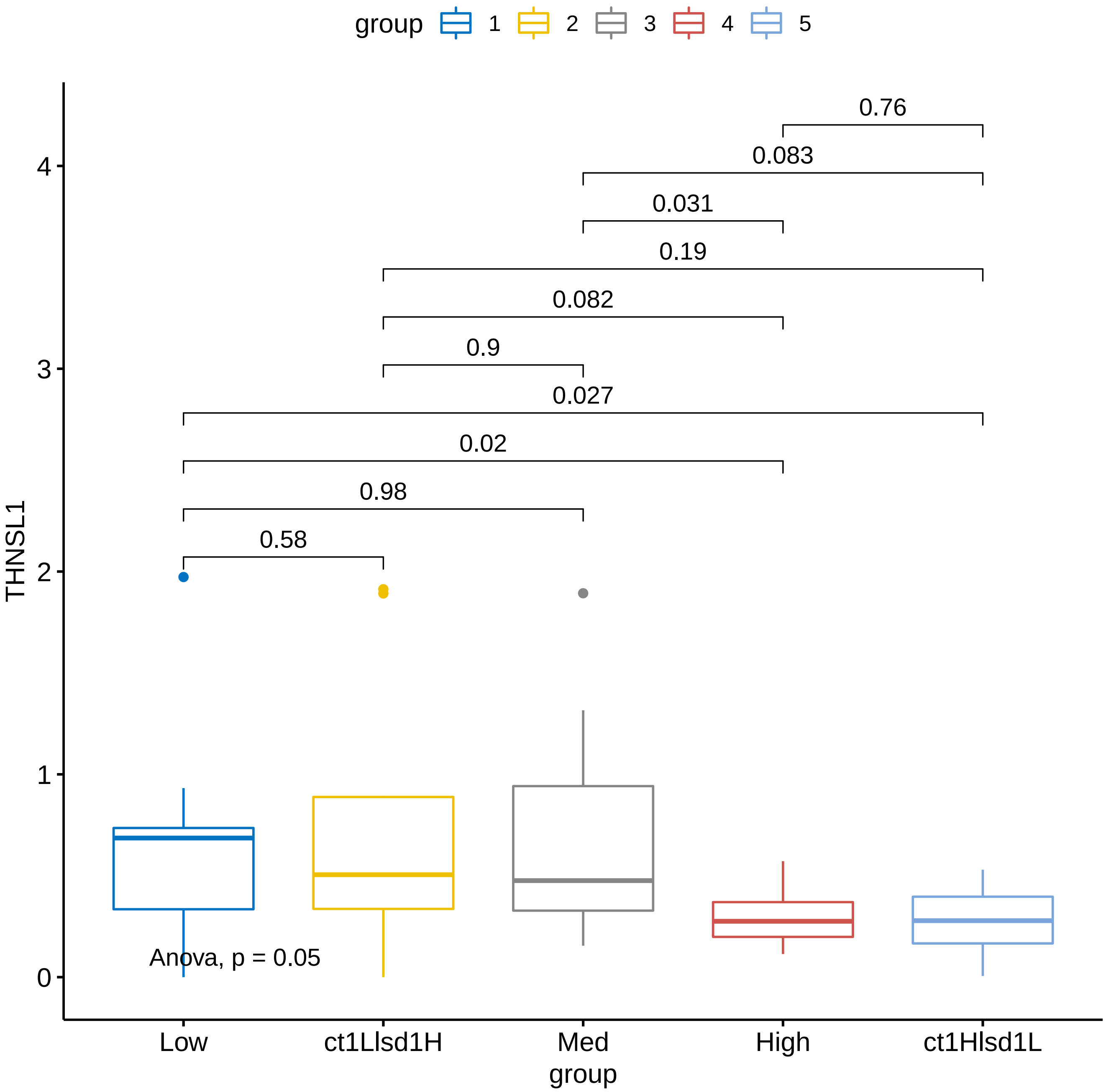
group
1
2
3
4
5
THNSL1
0
1
2
3
4
0.76
0.083
0.031
0.19
0.082
0.9
0.027
0.02
0.98
0.58
Anova, p = 0.05
Low
ct1Llsd1H
Med
High
ct1Hlsd1L
group
